# Diversity, population structure, and linkage disequilibrium among cowpea accessions

**DOI:** 10.1002/tpg2.20113

**Published:** 2021-07-18

**Authors:** Frejus Ariel Kpedetin Sodedji, Symphorien Agbahoungba, Eric Echikintho Agoyi, Médard Konoutan Kafoutchoni, Jaeyoung Choi, Simon‐Pierre Assanvo Nguetta, Achille Ephrem Assogbadjo, Ho‐Youn Kim

**Affiliations:** ^1^ Smart Farm Research Center Korea Institute of Science and Technology (KIST) Gangneung Gangwon 25451 Republic of Korea; ^2^ Non‐timber Forest Products and Orphan Crop Species Unit, Laboratory of Applied Ecology (LEA) University of Abomey–Calavi (UAC) 01 BP: 526 Cotonou Benin; ^3^ West Africa Center of Excellence in Climate Change Biodiversity and Sustainable Agriculture (CEA‐CCBAD), Biosciences Research Unit University Felix Houphouet‐Boigny Abidjan Lagunes 22 BP 461 Côte d'Ivoire

## Abstract

Cowpea [*Vigna unguiculata* (L.) Walp] is a globally important food security crop. However, it is susceptible to pest and disease; hence, constant breeding efforts based on its diversity are required for its improvement. The present study aims to investigate the genetic diversity, population structure, and linkage disequilibrium (LD) among 274 cowpea accessions from different origins. A total of 3,127 single nucleotide polymorphism (SNP) markers generated using diversity array technology (DArT) was used. Population structure, neighbor‐joining clustering, and principal component analyses indicated three subpopulations within the germplasm. Results of STRUCTURE analysis and discriminant analysis of principal components (DAPC) were complementary in assessing the structuration of the diversity among the germplasm, with the grouping of the accessions improved in DAPC. Genetic distances of 0.005–0.44 were observed among accessions. Accessions from western and central Africa, eastern and central Africa, and Asia were predominant and distributed across all subpopulations. The subpopulations had fixation indexes of 0.48–0.56. Analysis of molecular variance revealed that within subpopulation variation accounted for 81% of observed genetic variation in the germplasm. The subpopulations mainly consisted of inbred lines (inbreeding coefficient = 1) with common alleles, although they were from different geographical regions. This reflects considerable seed movement and germplasm exchange between regions. The LD was characterized by low decay for great physical distances between markers. The LD decay distance varied among chromosomes with the average distance of 80–100 kb across the genome. Thus, crop improvement is possible, and the LD will facilitate genome‐wide association studies on quality attributes and critical agronomic traits in cowpea.

## Core Ideas

- Genetic diversity in the cowpea germplasm was low.
- Genetic structuration among cowpea accessions shows potential for crop improvement.
- LD decayed slowly with increasing distance among loci across the cowpea genome.

**Abbreviations**
AMOVA: analysis of molecular variance
DAPC: discriminant analysis of principal components
DArT: diversity array technology
DArT‐seq: diversity array technology sequencing
*F*_IS_
: inbreeding coefficient
*F*_ST_
: fixation index
He: expected heterozygosity
Ho: observed heterozygosity
IITA: International Institute of Tropical Agriculture
LD: linkage disequilibrium
MAF: minor allele frequency
Ne: effective number of alleles
NJ: neighbor‐joining
PIC: polymorphism information content
SNP: single nucleotide polymorphism.

## 1 INTRODUCTION

Diversity in plant genetic resources enables plant breeders to develop improved varieties, with desirable attributes, to cope with ever‐changing environments (Frankham et al., 2002; Govindaraj et al., 2015). Cowpea [*Vigna unguiculata* (L.) Walp] (2*n* = 2*x* = 22) is a globally important grain legume crop (Boukar et al., 2019). The grains, leaves, and pods are the most used parts of the cowpea plant, and their characteristics vary among cultivars (Gonçalves et al., 2016; Herniter et al., 2019). Cowpea contributes to food and nutrition security as well as income generation for millions of households in semi‐arid tropics, including in Asia, Africa, and Latin America (Boukar et al., 2019; Fatokun et al., 2002; Timko & Singh, 2008). Globally, ∼8.9 Tg of cowpea grains were produced in 2019, with most of the production (95.2%) from Africa (FAO, 2020). Although cowpea thrives in drought‐prone environments and on poor soils (Carvalho et al., 2017), it is highly susceptible to pests and diseases, which leads to low yields (25–600 kg ha^−1^) (Boukar et al., 2019; Kamara et al., 2018; Sodedji et al., 2020). These low yields threaten world food security, and, hence, constant breeding efforts are required to explore, create, and use diversity within that species to overcome various biotic and abiotic constraints and satisfy consumer preferences.

The genetic diversity of a given plant population reflects its evolution and potential for improvement. That is, genetic diversity provides information on the patterns and magnitude of population structure, which is driven by the combined effects of evolutionary processes such as recombination, mutation, and genetic drift, demographic history, and natural selection (Andam et al., 2017). Therefore, knowledge of the genetic structure of plant populations can support the formulation of guidelines for appropriate breeding strategies (Hayward & Breese, 1993; Mogga et al., 2018). Notably, investigating the genetic structure of a population requires a thorough analysis of the allelic patterns among individuals within the population to further use the corresponding genetic variation for cultivar development (Eltaher et al., 2018).

To date, a range of molecular and quantitative methods have been developed for easy and effective assessment of genetic diversity (Frankham et al., 2002). Rapid advances in sequencing technologies have provided many possibilities for assessing the organization of natural populations (Jombart et al., 2010). For instance, high‐throughput genotyping using diversity arrays technology (DArT) has emerged as the method of choice for genetic diversity analysis and genomic studies because of its efficiency and low cost (Mogga et al., 2018). It also offers a high‐throughput marker system for genome analysis and has successfully been employed to assess genetic diversity in several legume crops, including cowpea (Fatokun et al., 2018; Qin et al., 2016), common bean (*Phaseolus vulgaris* L.) (Nemli et al., 2017), and pigeonpea (*Cajanus cajan* L. Huth) (Yang et al., 2006).

Previous studies have assessed the population structure of the cultivated cowpea including the USDA cowpea core collection (Qin et al., 2016; Ravelombola et al., 2017; Shi et al., 2016). Two to four subgroups with varying levels of genetic diversity have been identified, depending on the germplasm that was used (Chen et al., 2017; Fatokun et al., 2018; Qin et al., 2016, 2017; Ravelombola et al., 2017; Shi et al., 2016; Xiong et al., 2016). Notably, the reproductive nature of cowpea, which is primarily a self‐pollinating plant (Timko & Singh, 2008), increases the degree of inbreeding, with individuals becoming more homozygous for many alleles; consequently, narrow genetic base and genetic distance have been reported in cowpea (Chen et al., 2017; Wamalwa et al., 2016). Furthermore, the high degree of inbreeding in this species also increases chances of linkage disequilibrium (LD) between loci (Kovi et al., 2015), which is a determining factor in marker–trait association analysis (Laidò et al., 2014).

Linkage disequilibrium can be investigated at haplotype, chromosome, or whole‐genome levels. The whole‐genome LD analysis helps to predict the genotyping efforts needed and the power of association mapping (Balding, 2006; Xu et al., 2012). Generally, slower LD decay is expected in self‐pollinated species like cowpea than in out‐crossing species (Flint‐Garcia et al., 2003), which exhibit a high level of recombination. A rapid LD decay of 5–10 and 7.9–22.7 kb were observed among diverse maize (*Zea mays* L.) germplasm collections (Yan et al., 2009; Badji et al., 2020). In contrast, high and persistence LD level was found in asparagus bean [*V. unguiculata* (L.) Walp. subsp. *unguiculata* Sesquipedalis Group (*V. unguiculata* Sesquipedalis Group)], which varied among chromosomes and decayed at ∼1.88 Mb (∼2 cM) of physical distance (Xu et al., 2012). Similarly, high LD decay distance 2 cM (∼500 kb) was reported in a cowpea collection of 299 accessions (Xu et al., 2017). Noble et al. (2018) reported that LD decayed approximatively at 100 kb in the cultivated germplasm of mungbean [*V. radiata* (L.) R. Wilczek var. *radiata*]. Thus, LD patterns can vary among species and germplasm of same species and should be assessed in a plant germplasm collection designated for long‐term breeding.

In the present study, a cowpea germplasm of 274 cowpea accessions, which will serve as the genetic material for developing a cowpea breeding program in Benin, was acquired from different origins and genotyped using DArT. The objectives were to (a) assess population structure within the germplasm, (b) analyze the genetic diversity among accessions, and (c) assess the extent of LD within the germplasm to support effective breeding decision making.

## 2 MATERIALS AND METHODS

### 2.1 Plant materials

**FIGURE 1:**
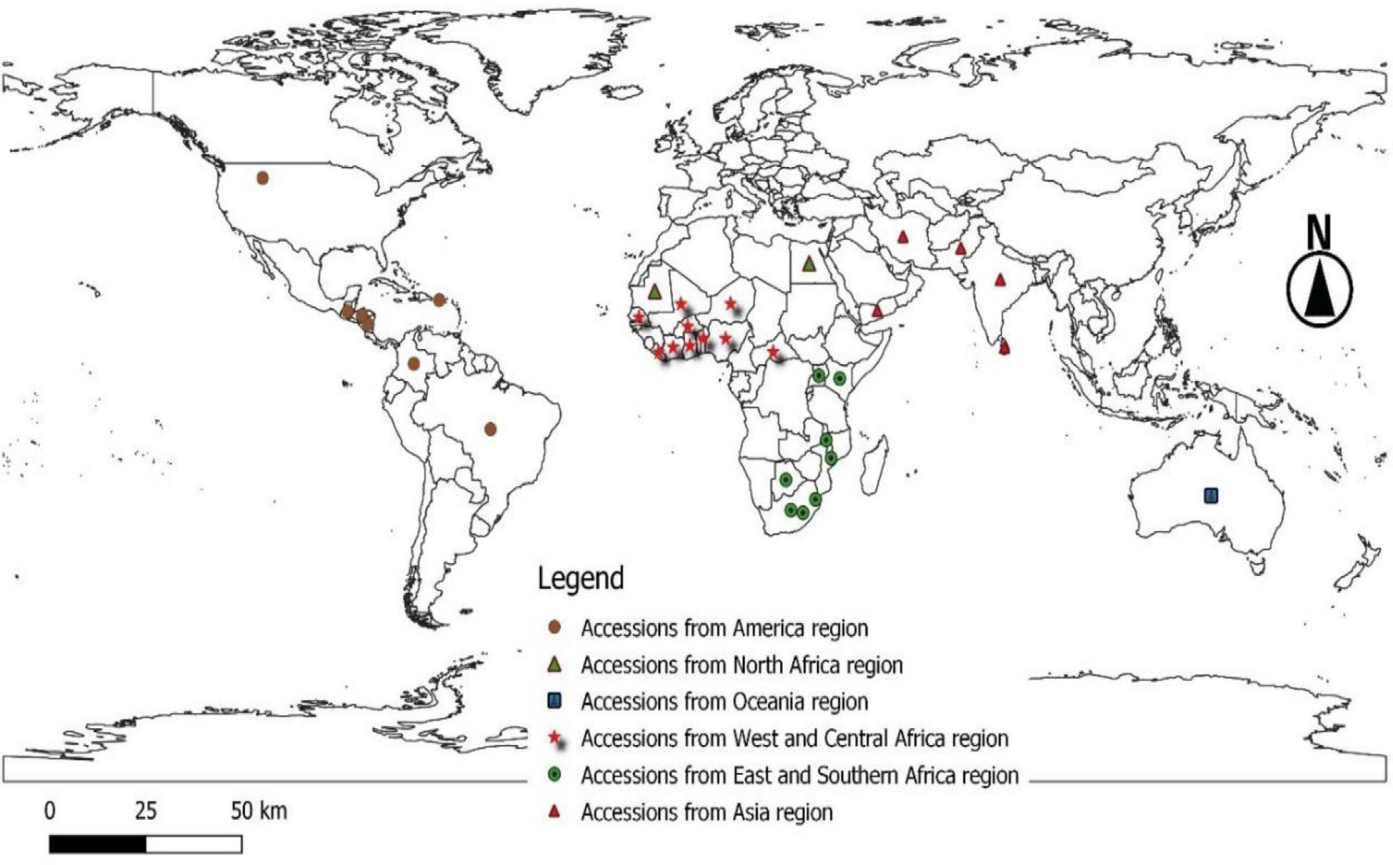
Geographical origins of the accessions. The map was drawn using QGIS version 3.4.4 (https://www.qgis.org/en/site/about/index.html). Each marker on the map represents a single country and accessions from the same regions are depicted with identical markers. A total of 274 accessions from 33 countries and six regions were included in the study

The cowpea germplasm comprised 274 accessions from 33 countries (Figure 1; Supplemental Table S1). Accession seeds were obtained from different sources including the International Institute of Tropical Agriculture (IITA, Nigeria), the Institute of Environment and Agricultural Research (*Institut de l'Environnement et de Recherches Agricoles*—INERA, Burkina‐Faso), the Laboratory of Applied Ecology of the University of Abomey‐Calavi (Benin), the University Naguia Abrogoua (Côte d'Ivoire), the Makerere Regional Center for Crop Improvement (Uganda), and the USDA–ARS. Seeds were sown in plastic bags for leaf tissue sampling, in a greenhouse at the University of Abomey‐Calavi.

### 2.2 DNA extraction and genotyping

Fresh leaf samples were collected from 14‐d‐old plants for each of the 274 cowpea accessions, stored into three 96‐well sample collection plates, and shipped to the Integrated Genotyping Service and Support of the Biosciences eastern and central Africa—ILRI Hub, Kenya, for genotyping. The DNA was extracted from the leaf tissues using the Nucleomag Plant Genomic DNA extraction kit (Macherey‐Nagel), and the quality control was conducted on 0.8% agarose. Genotyping was performed using DArT sequencing (DArT‐seq). We then constructed a genomic DNA library using genomic complexity reduction technology (Kilian et al., 2012). The library was purified and quantified for cluster generation in an automated clonal amplification system (cBOT Illumina). Thereafter, next‐generation sequencing was performed using the sequencer HiSeq 2500 (Illumina). The reads were aligned to the cowpea reference genome *Vigna unguiculata* v1.1 (Lonardi et al., 2019), which is publicly accessible on Phytozome 12 (Goodstein et al., 2012).

**Core Ideas**

- Genetic diversity in the cowpea germplasm was low.
- Genetic structuration among cowpea accessions shows potential for crop improvement.
- LD decayed slowly with increasing distance among loci across the cowpea genome.

### 2.3 Marker diversity analysis

Data quality control and filtering were performed using the R package dartR (Gruber et al., 2018). Single nucleotide polymorphism (SNP) markers with >20% of missing data, with minor allele frequency (MAF) <0.05, or of unknown position were removed. Data were imputed using the expectation maximization algorithm, which recorded the highest simple matching coefficient (SMC = 0.76) among other imputation algorithms, as implemented in the KDCompute pipeline (Diversity Arrays Technology, 2017). The summary statistics of the SNP markers were generated using PowerMarker v3.25 (Liu & Muse, 2005). The computed statistics included allele frequencies, expected heterozygosity (He), observed heterozygosity (Ho), and polymorphism information content (PIC).

### 2.4 Population structure analysis

Filtered SNPs were used to evaluate the population structure within the germplasm, and the structure analysis was conducted with the Bayesian clustering approach in STRUCTURE v2.3.4 (Porras‐Hurtado et al., 2013). The structure analysis was run considering a burn‐in period of 10,000 Markov‐chain Monte Carlo iterations and a 100,000‐run length with an admixture model following the Hardy–Weinberg equilibrium and its correlated allele frequencies. Ten independent runs were performed for the value of each number of clusters (*K*), which ranged from 1 to 11. The structure outputs were analyzed using Structure Harvester (Earl & VonHoldt, 2012) , which enabled the identification of the best *K* value as the distinct peak in the change of likelihood (Δ*K*). The fixation index (*F*_ST_) of each cluster was retrieved and interpreted according to Wright (1984), with an *F*_ST_ value >0.25 indicating high genetic differentiation. The accessions were assigned to their respective clusters based on the coefficient of ancestry values generated by the structure analysis (Supplemental Table S2), with the assumption that an individual is a true member of a given cluster if its coefficient of ancestry in this cluster is >0.52 (Basak et al., 2019).

Discriminant analysis of principal components (DAPC), a multivariate method that uses the sequential means of *K* and model selection to infer and describe clusters in populations of genetically related individuals (Jombart et al., 2010), was performed to confirm the best fitting clusters among the cowpea germplasm. With this approach, the optimum *K* was identified as the minimum number of clusters after which the Bayesian information criterion increases or decreases by a negligible amount (Jombart et al., 2010). The DAPC was conducted using the adegenet package (Jombart, 2008) in R v3.5.0 (R Core Team, 2018).

### 2.5 Genetic relationship and diversity analysis

To examine the phylogenetic relationships among accessions and confirm the number of clusters, an identity‐by‐state distance matrix (Supplemental Table S3) was generated using Tassel v5.2.60 (Bradbury et al., 2007). Moreover, a phylogenetic tree was constructed using the neighbor‐joining (NJ) algorithm in Darwin v6.0.2 (Perrier & Jacquemoud‐Collet, 2016), which was exported to FigTree v1.4.3 (Rambaut, 2016) for annotation. Prior to the analysis, the 274 accessions were grouped according to their geographical origins to describe the composition of the identified clusters (Table 1). Principal component analysis and construction of a scatter plot of the cowpea accessions with the first two principal component axes (PC1 and PC2) were performed using Tassel v5.2.60.

**TABLE 1:** Geographical distribution of the 274 cowpea accessions

Regions	Accessions	Countries of origin
Western and central Africa	113	Benin, Burkina‐Faso, Côte d'Ivoire, Ghana, Liberia, Mali, Niger, Nigeria, Senegal, Central Africa Republic
Eastern and southern Africa	93	Kenya, Malawi, Mozambique, Uganda, Botswana, Lesotho, South Africa, Swaziland
Northern Africa	3	Egypt and Mauritania
Asia	53	India, Siri Lanka, Iran, Pakistan, Yemen
Americas	9	Brazil, Colombia, Guatemala, Honduras, Nicaragua, Puerto Rico, United States
Oceania	3	Australia

The genetic diversity parameters (He, Ho, PIC, and the effective number of alleles [Ne]) of the identified subpopulations were computed through the NJ and DAPC clustering approaches using PowerMarker v3.25 and GenAlEx 6.41 (Peakall & Smouse, 2006). Analysis of molecular variance (AMOVA) was also conducted using GenAlEx 6.41, with the SNP markers and the repartition of the 274 cowpea accessions into different subpopulations based on the clustering analysis. Prior to AMOVA, the marker datasets were numerically coded (A = 1, C = 2, T = 3, and G = 4; see Supplemental Table S4), as suggested in the GenAlEx manual (Blyton & Flanagan, 2006).

### 2.6 LD analysis

The LD analysis was performed between pairs of markers on the same chromosome as well as across the genome using the window size approach (Yan et al., 2009; Xu et al., 2012). The LD values were estimated as square values of correlation coefficient (*r* ^2^) between pairs of SNPs (Yan et al., 2009) in Tassel v5.2.60 (Bradbury et al., 2007). The SNP markers were pruned to identify markers that were in true LD (probability of *r* ^2^ < 0.01), with a sliding window size of 50 markers. Means of *r* ^2^ were calculated between SNPs at different intervals of distance across the genome. Patterns of LD decay was depicted as a plot of variation of *r* ^2^ along the physical genetic distance between pairs of markers in Microsoft Excel (Frye, 2007), and the distance at which LD decays below the threshold of *r* ^2^ = 0.2 (Xu et al., 2017) was identified.

## 3 RESULTS

### 3.1 Profile and diversity of SNP markers

**FIGURE 2:**
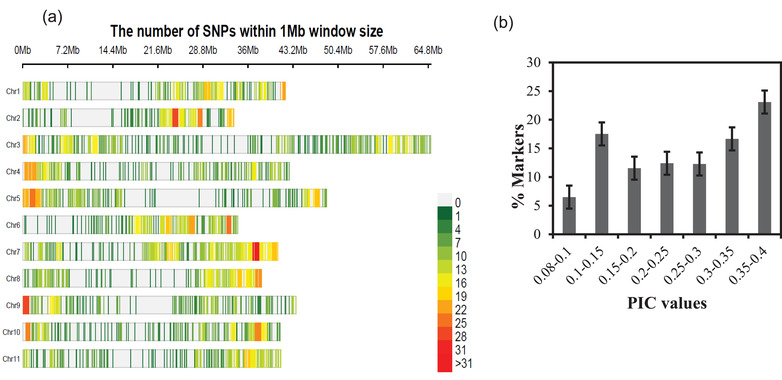
(a) Density of the single nucleotide polymorphism (SNP) markers across the cowpea genome and (b) distribution of their polymorphism information content (PIC) values

A total of 12,689 SNP markers were generated from the DArT‐seq of 274 cowpea accessions of diverse origins (Table 1). A high number of markers (9,562 SNPs) were removed during filtering, while the remaining markers, namely 3,127 SNPs (24.65%) distributed across the 11 cowpea chromosomes (Figure 2), matched the quality criteria used in the diversity analyses. The profiles of the 3,127 SNP markers are presented in Table 2. The markers showed high reproducibility (0.99) with a mean call‐rate value of 0.87. Marker diversity analysis revealed that these markers had an average MAF of 0.22, and the majority (93.51%) had a PIC value above 0.1 with a mean value of 0.24 (Figure 2; Table 2). Furthermore, the mean He of the markers was higher than the mean Ho (He = 0.23, Ho = 0.07).

**TABLE 2:** Quality and diversity of single nucleotide polymorphism (SNP) markers used to investigate genetic diversity and population structure of the cowpea germplasm

Quality and diversity parameters	Mean	Min.	Max.
1‐Markers quality parameters			
Call rate	0.87	0.80	0.98
One ratio, reference allele	0.66	0.05	1.00
One ratio, SNP allele	0.37	0.05	0.99
Reproducibility	0.99	0.91	1.00
2‐Markers diversity			
Major allele frequency	0.78	0.50	0.96
Minor allele frequency	0.22	0.05	0.5
Expected heterozygosity (He)	0.30	0.08	0.5
Observed heterozygosity (Ho)	0.07	0	0.40
Polymorphism information content	0.24	0.08	0.37

### 3.2 Population structure

**FIGURE 3:**
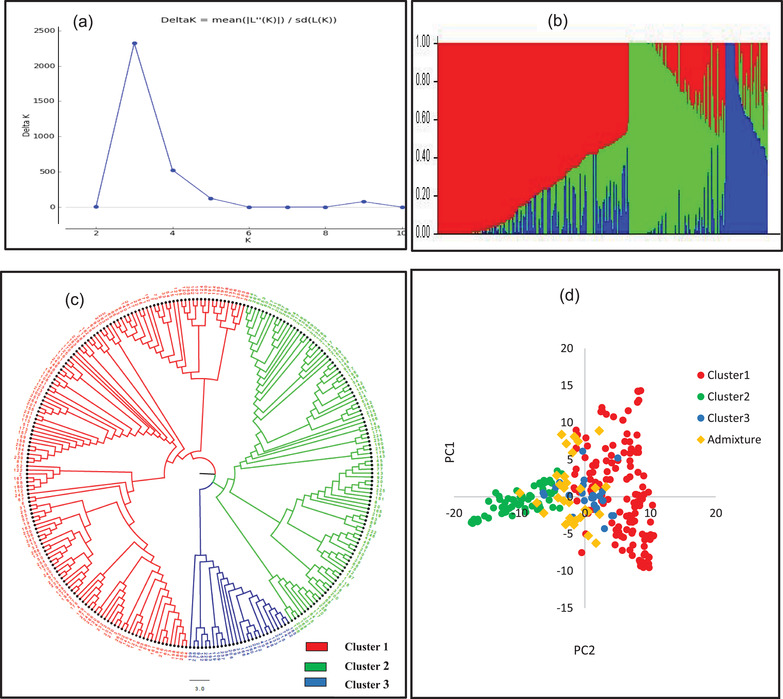
Population structure and phylogenetic relationships among 274 cowpea accessions. (a) Likelihood of Δ*K* showing the best *K* value (*K* = 3); (b) bar plot of accessions assigned into *K* = 3 clusters (Cluster 1, Cluster 2, and Cluster 3 are represented by red, green, and blue colors, respectively); (c) neighbor‐joining phylogenetic tree that groups accessions into three main branches depicting a similitude to the structure analysis; and (d) scatter plot of the 274 cowpea accessions along with PC1 and PC2 that inferred the membership of the accessions to three clusters with some admixed individuals (yellow color) showing similar pattern to the structure analysis

Two different approaches (STRUCTURE and DAPC) were used to identify the optimum *K* within the cowpea germplasm. From the STRUCTURE approach, the cruve of Δ*K* peaked at *K* = 3 (Figure 3a), which indicated that three clusters contributed to the total variation in the diversity panel. Consequently, the 274 cowpea accessions can be grouped into three subpopulations or clusters. The distribution of the cowpea accessions among the different clusters revealed that Cluster 1 had the highest percentage of membership (53.2%), followed by Cluster 2 (31.8%) and Cluster 3 (14.9%). However, the inferred ancestry indicated that some accessions were in admixture (Figure 3b), that is, representing a sum of variation from more than one cluster. Based on the probability value for the assignment of an individual accession to a specific cluster, 31 accessions were categorized into the group of admixed individuals (Supplemental Table S2).

**FIGURE 4:**
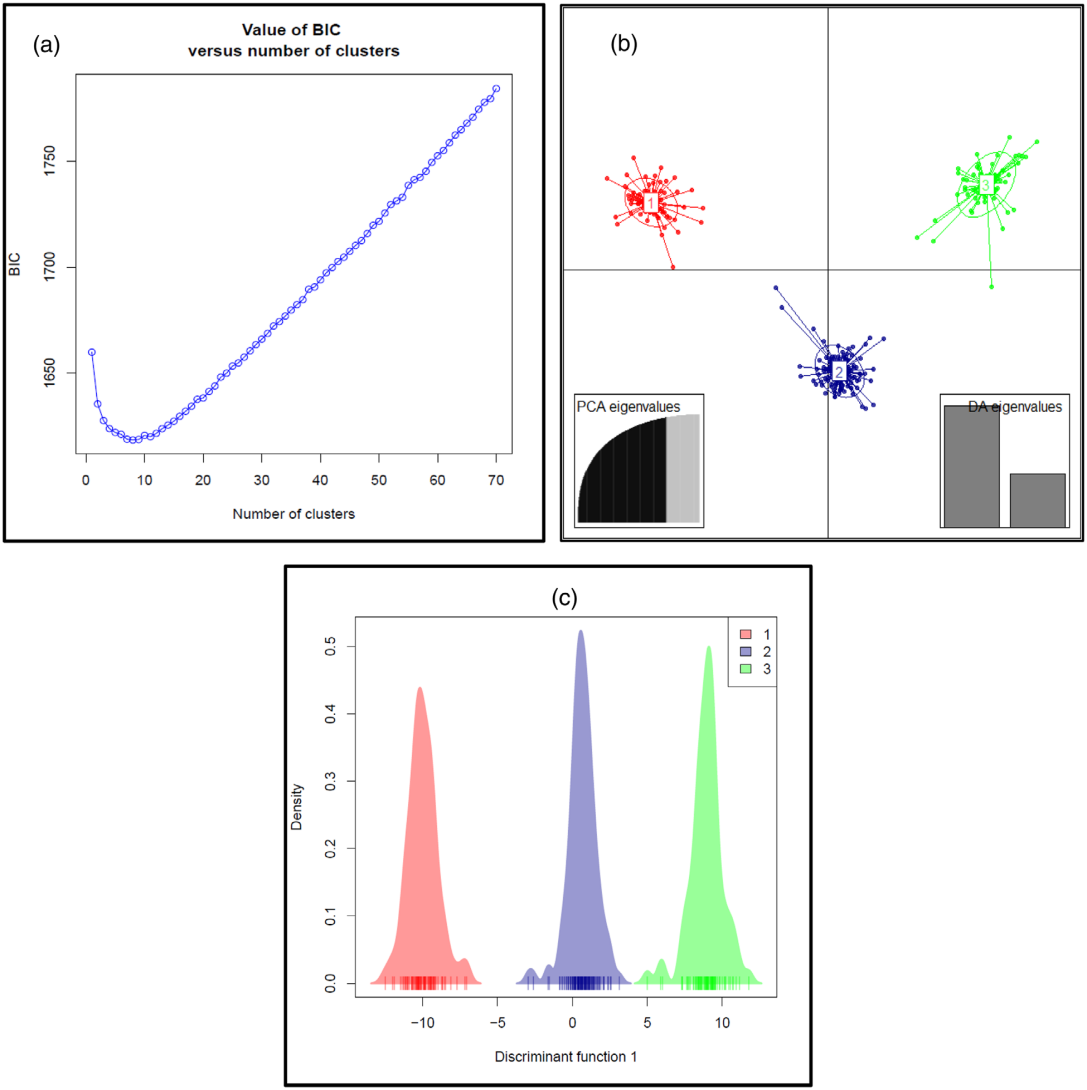
Discriminant analysis of principal components (DAPC). (a) The graph of Bayesian information criterion (BIC) vs. number of clusters indicates the optimum number of clusters (*K* = 3) inferred in the cowpea diversity panel; (b) scatter plot of the 274 cowpea accessions grouped into three clusters using two discriminant functions; and (c) plot of the densities of individuals on the first discriminant function that displays cluster differences

Regarding the DAPC approach, the Bayesian information criterion curve rapidly declined from *K* = 1 to *K* = 3, followed by a prolonged increase (Figure 4a), which suggests that *K* = 3 is the optimum number of clusters. Furthermore, two discriminant functions, which explained 59.09 and 24.92% of the dataset variation, respectively, were detected (Figure 4b). The DAPC biplot (Figure 4b) together with the plot of densities of individuals on the first discriminant function showed a clear separation the 274 accessions into the three clusters with no admixed individuals (Figure 4b, Supplemental S5). Cluster 1 was distant from the two other clusters (Figure 4c). Cluster 2 had the highest membership (117 accessions), followed by Cluster 3 (79 accessions), and Cluster 1 (78 accessions; Supplemental Table S5).

### 3.3 Diversity and genetic relationships among accessions

The grouping of the accessions according to their regions of origin revealed that western and central Africa (113 accessions), eastern and southern Africa (93 accessions), and Asia (53 accessions) were the three dominant geographic origins, whereas the remaining 15 accessions were from northern Africa, the Americas, and Oceania (Table 1). The genetic distance values between pairs of accessions based on marker loci ranged from 0.005 to 0.44 (Supplemental Table S3). The NJ phylogenetic tree depicted three main subroots (Figure 3c), which confirmed the existence of three clusters within the germplasm. Cluster 1 comprised 53.28% of the accessions (146 accessions), while Clusters 2 and 3 included 36.86 (101 accessions) and 9.85% (27 accessions), respectively.

Accessions from all regions, except Oceania, were distributed in two or three subpopulations (Supplemental Table S5). Accessions from western and central Africa and those from eastern and southern Africa were predominant in Clusters 2 and 1, respectively. Regarding within‐country diversity, Nigerian accessions were highly represented in Clusters 1 and 2 (20 and 17 accessions, respectively). The majority of the accessions from Benin belonged to Cluster 2 (27 accessions). Moreover, Ugandan accessions were predominant (76 accessions) among accessions from the eastern and southern Africa region, with the majority (53 accessions) in Cluster 1. Finally, Indian accessions (49 accessions), which were the most predominant accessions in the group from Asia, were distributed in Clusters 1 (28 accessions), 2 (15 accessions), and 3 (6 accessions).

The first two principal component axes of the principal component analysis, which accounted for 23.57% of the variation among accessions, indicated that the 274 accessions could be grouped into three main subpopulations, with some accessions in admixture (Figure 3d). These patterns were consistent with the structure analysis.

### 3.4 Genetic diversity and population differentiation in observed groups

Nei's nucleotide distance among the three inferred subpopulations varied from 0.17 (between Cluster 1 and Cluster 2) to 0.26 (between Cluster 2 and Cluster 3; Table 3), which indicated relatedness between the subpopulations. Notably, Cluster 1 was more related to Cluster 2 than to Cluster 3, whereas Clusters 2 and 3 were the furthest apart. The highest within‐population variation was observed in Cluster 3 (He = 0.32) followed by Cluster 1 (He = 0.26). Cluster 2 contained the highest proportion of genetic variance (*F*_ST_ = 0.56), whereas Clusters 1 and 3 had similar mean population variance values (*F*_ST_ = 0.49 and 0.48, respectively). The NJ clustering revealed that the Ne and PIC values varied across the three subpopulations (Table 4). The highest Ne was recorded in Cluster 3 (Ne = 1.48), whereas the highest PIC mean value was observed in Cluster 2 (PIC = 0.23). The Ho values were generally lower than the He values across subpopulations (Table 4). Cluster 3 had the highest observed heterozygosity (Ho = 0.11), whereas Clusters 1 and 2 had the lowest values for this parameter (Ho = 0.07 and 0.06, respectively).

**TABLE 3:** Genetic variability among (net nucleotide distance) and within (expected heterozygosity) populations, proportion of membership, and mean value of the fixation index (*F*_ST_) observed from the population structure of 274 cowpea cultivars

	Net nucleotide distance				
Population	Cluster 2	Cluster 3	Expected heterozygosity	*F*_ST_	Percentage of Individuals
					%
Cluster 1	0.17	0.21	0.26	0.49	53.2
Cluster 2	–	0.26	0.22	0.56	31.8
Cluster 3	–	–	0.32	0.48	14.9

**TABLE 4:** Diversity parameters of the identified subpopulations in the neighbor‐joining (NJ) clustering and discriminant analysis of principal components (DAPC)

Subpopulations	Subpopulation size	Ne^a^	He^b^	Ho^c^	PIC^d^
NJ clustering					
Cluster 1	146	1.46	0.27	0.07	0.22
Cluster 2	101	1.46	0.28	0.06	0.23
Cluster 3	27	1.48	0.27	0.11	0.22
DAPC clustering					
Cluster 1	78	1.47	0.26	0.05	0.21
Cluster 2	117	1.44	0.29	0.10	0.23
Cluster 3	79	1.48	0.23	0.06	0.19

^a^ Ne, number of effective alleles.

^b^ He, expected heterozygosity.

^c^ Ho, observed heterozygosity.

^d^ PIC, polymorphism information content.

The genetic diversity estimates based on the DAPC clustering method indicated that the Ne values ranged from 1.44 in Cluster 2 to 1.48 in Cluster 3. Cluster 2 had the highest mean PIC value (0.23), whereas the lowest value for this parameter was observed in Cluster 3 (PIC = 0.19). Overall, the He value was higher than the Ho value in all subpopulations (He = 0.23–0.29 and Ho = 0.05 0.10). The highest Ho value was obtained in Cluster 2 (Ho = 0.10).

The AMOVA revealed that the total genetic variation in the germplasm was mainly partitioned into the variation among accessions (81%) and the variation among subpopulations (19%; Table 5). The inbreeding coefficient (*F*_IS_) was remarkably high, implying that the inferred subpopulations were mainly composed of inbred lines.

**TABLE 5:** Analysis of molecular variance (AMOVA) for variation among and within subpopulations in the germplasm collection of 274 cowpea accessions

Source	df	SS	MS	Percentage estimated variance	*F* statistic (*F*_IS_, inbreeding coefficient)
				%	
Among subpopulations	2	33,573.48	16,786.74	19	1 (*p* < .001)
Among accessions	271	212,513.58	784.18	81	
Total	273	246,085.11		100	

### 3.5 LD decay

**FIGURE 5:**
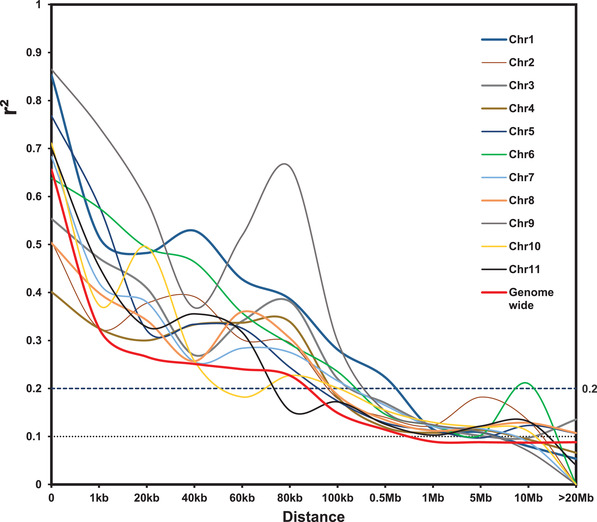
Patterns of linkage disequilibrium (LD) in the cowpea diversity panel based on pairwise correlation between markers on the same chromosome as well as between markers across the 11 cowpea chromosomes. The values on the *y* axis represent the squared correlation coefficient (*r* ^2^), and the *x* axis represents the physical distance. Only significant (*P* < .01) pairwise correlation values between markers are included

The LD analysis revealed that 33,080 (21.36%) of all possible pairs of comparisons (154,876) between SNP markers were in significant LD (*P* < .01; Table 6). These markers were almost evenly distributed across the 11 cowpea chromosomes, with the highest number of SNPs in LD with each other on chromosomes 3 and 7 (Figure 2; Supplemental file S7). The means of *r* ^2^ along intervals of distance between markers in the genome are presented in Table 6. The LD estimates were, overall, low (*r* ^2^ = 0.21). However, a few high LD values were observed over a short physical distance (0–1 kb), but they decayed rapidly, with a slow decline over longer distances between markers across the genome (Table 6, Figure 5). The LD decayed below the threshold of *r* ^2^ = 0.2 at a physical distance of 80–100 kb (Figure 5). For the intrachromosomal analysis, the distance and patterns of decay varied among chromosomes with a large LD block observed on chromosome 6 (Figure 5; Supplemental Table S7). The means of *r* ^2^ did not show similar decline across chromosomes within a distance of 0–100 kb (Figure 5). With few exceptions (chromosomes 6 and 2), a more uniform LD decline was present at higher distance intervals (>100 kb). The LD decays below the critical *r* ^2^ = 0.2 threshold at 80–100 kb in chromosomes 2, 4, 5, and 8; 100–500 kb in chromosomes 3, 6, 7, and 9; and 500 kb–1 Mb in chromosome 1. The shortest distances of LD decay occurred at 40–60 kb in chromosome 10 and at 60–80 kb on chromosome 11.

**TABLE 6:** Summary statistics of the linkage disequilibrium LD (*R*²) among pairs of single nucleotide polymorphism (SNP) markers with maker allele frequency >0.05 across the genome. Only *R*² values with (*P* < .01) were reported

Distance	*N*	Mean (*R*²)	SD (*R*²)	25th percentile	50th percentile	75th percentile	Min. no. of SNP to cover the genome^a^
kb							
0–1	75	0.656	0.398	0.16	0.88	1.00	>640,600
1–20	175	0.325	0.360	0.05	0.12	0.60	640,600–32,030
20–40	166	0.266	0.307	0.05	0.11	0.43	32,030–16,015
40–60	183	0.251	0.303	0.05	0.09	0.39	16,015–10,677
60–80	186	0.240	0.278	0.05	0.11	0.31	10,677–8,006
80–100	188	0.227	0.248	0.06	0.11	0.33	8,006–6,406
100–500	3,550	0.149	0.185	0.05	0.08	0.16	6,406–12,812
500–1,000	3,752	0.114	0.137	0.04	0.07	0.12	12,812–640
1,000–5,000	17,947	0.089	0.085	0.04	0.06	0.10	640–128
5,000–10,000	4,122	0.088	0.081	0.04	0.06	0.10	128–64
10,000–20,000	2,516	0.087	0.079	0.04	0.06	0.10	64–32
>20,000	220	0.088	0.060	0.05	0.07	0.11	32

^a^ Cowpea genome size = 640.6 Mb (Lonardi et al., 2019).

## 4 DISCUSSION

### 4.1 Marker diversity

The present study investigated the genetic diversity and population structure of 274 cowpea accessions from diverse origins with a set of 3,127 SNP markers. The SNP markers selected in this study were informative, suggesting that they were suitable for the reliable fingerprinting and inference of genetic variation within the germplasm. The markers were highly reproducible and scored high call rates, with average MAF and PIC values within the range of the values reported in previous SNP‐based genetic diversity studies conducted on important food crops including cowpea (Fatokun et al., 2018; Xiong et al., 2016), common bean (Nemli et al., 2017), and maize (Adu et al., 2019). Conversely, low heterozygosity was observed for these markers, which is in line with the Ho values that have been reported for the global germplasm collections of cowpea maintained through the Germplasm Resources Information Network of USDA (Xiong et al., 2016) and IITA (Fatokun et al., 2018). In fact, the mean heterozygosity, calculated across several loci is an accurate indicator of the degree of genetic variation within a population (Sbordoni et al., 2012), which implies that the genetic variation in cowpea is low. This reduced genetic diversity can be attributed to the self‐pollinating nature of cowpea and the putative single domestication event of cowpea, which increases the degree of inbreeding with increased homozygosity (Pasquet, 1999; Pasquet, 2000; Timko & Singh, 2008; Badiane et al., 2012; Chen et al., 2017; Farahani et al., 2019).

### 4.2 Population structure and relationships among accessions

Population structure analysis is essential for understanding the genetic diversity and association mapping in a germplasm (Eltaher et al., 2018). In the present study, the cowpea collection was divided into three subgroups, irrespective of the approach used, to infer population structure. Likewise, previous studies on worldwide germplasms of cowpea (298–768 accessions) have also reported the presence of three clusters (Fatokun et al., 2018; Qin et al., 2017; Shi et al., 2016), which suggests that our collection represented, to some extent, the diversity in this crop. Such representativeness of the existing diversity in this germplasm is considerably useful for its use in breeding activities. Conducting crop evaluation for the specific trait of interest with the whole germplasm may be highly informative. Furthermore, the representativeness of the existing diversity in this germplasm can guide genotype selection in a training population for genomic prediction studies. However, this may require high‐density genome‐wide markers (He & Li, 2020).

Differences were observed between results of DAPC and STRUCTURE analyses regarding the separation of accessions into subgroups. The DAPC assigned each of the accessions to a specific group, unlike the STRUCTURE analysis, which indicated the presence of admixed individuals. Similar observations have been reported among a core collection of sweet cherry [*Prunus avium* (L.) L.] (Campoy et, al., 2016), more recently, within Ethiopian cowpea germplasm (Ketama et al., 2020), and among cowpea mutant populations (Diouf et al., 2021). The DAPC was recommended as a good multivariate clustering approach for defining and describing groups of genetically related individuals (Jombart et al., 2010) and was found to be more efficient than STRUCTURE for population structure analysis (Jombart et al., 2010; Diouf et al., 2021). In line with that, the DAPC results effectively showed an improvement in the assignment of the accessions to the three inferred clusters, suggesting that this approach was more appropriate to assess the structuration of genetic diversity among the germplasm (Diouf et al., 2021). In the present study, the majority of accessions within the diversity panel were from Africa, especially the western and central regions, which may substantiate that Africa is the center of diversity and a land with a long tradition of cultivation of cowpea (Lush & Evans, 1981; Timko & Singh, 2008). However, individuals from the same predefined regions were assigned to more than one cluster, which indicates that there is potential for the improvement of this crop across regions. Furthermore, the distribution of accessions across clusters highlights relatedness between accessions from different regions, which can be attributed to the transfer and exchange of seeds between regions through breeding programs, human migration, and farmers exchanging preferred planting materials. Similar observations have been reported in cowpea (Fatokun et al., 2018) and sesame (*Sesamum indicum* L.) (Basak et al., 2019). As for the population structure within each country, accessions from Nigeria, Ghana, Benin, Uganda, and India were predominant in the collection and were distributed in all clusters regardless of the clustering approach that was used. While this may reflect the importance of cowpea in these countries, some of them (e.g., Nigeria, Benin, and Ghana) favor high seed trade and movement (Gomez Carlos, 2004), which would explain the similarity among the accessions from these countries. Beyond geographical proximity, another possible reason is that those materials may have been derived from parents with similar genetic backgrounds. Historically, IITA has the mandate to develop improved lines, which are then widely distributed in its intervention countries for evaluation and release. Therefore, these lines may have been distributed in various countries and used as parents in national breeding programs.

### 4.3 Genetic differentiation and allelic patterns in the subpopulations

Low genetic distances were observed among accessions. The variation range of the genetic distance between pairs of accessions in the germplasm (0.005–0.44) confirmed that some accessions shared multiple alleles. This result corroborates the findings of Fatokun et al. (2018), who have reported a low genetic distance (0.0096–0.462), between pairs of 298 cowpea lines. The low genetic distance may limit the progress in developing superior crop varieties through simple hybridization between accessions. As highlighted in the previous section, the movements of seeds across geographical areas, which promotes gene flow between breeding germplasms, can affect existing genetic boundaries, hence reducing both the genetic distance among individuals and population differentiation.

The high mean *F*_ST_ values observed for the subpopulations identified in the present study suggested a remarkable level of genetic differentiation in the subpopulations, which was confirmed by the large contribution of the variance within subpopulations to the total genetic variation in the germplasm. Previous studies (Fatokun et al., 2018; Kouam et al., 2012) have also reported that the genetic variation in cowpea mainly is due to the within‐subpopulation variation. On the basis of the Ho values, Clusters 3 and 2 were the most diverse. Furthermore, although He was moderately low, it was, overall, higher than Ho for all subpopulations. Fatokun et al. (2018) also observed a similar trend in a mini‐core subset of the world cowpea collection. Notably, a low Ho implies a high proportion of inbred lines within subpopulations (Govindaraj et al., 2015), which was corroborated by the high inbreeding coefficient value obtained in the present study.

### 4.4 Patterns of LD

Linkage disequilibrium is an important parameter in population genetic and genomic studies. In the present study, a low LD characterized the cowpea germplasm collection, with slow LD decay over long physical distances among markers. This result, in line with the findings of Xu et al. (2017), indicates a slower LD decay in cowpea population composed of high number of inbred lines In fact, the self‐pollinating nature of this crop limits the recombination events and delays LD decay (Morrell et al., 2005). The use of techniques such as mutagenesis and crossing between genetically distant individuals can improve the recombination rate and, subsequently, increase genetic diversity within the germplasm as recently reported by Diouf et al. (2021).

The genome‐wide LD decay occurred at 80–100 kb physical distance, suggesting there are possibilities of association mapping analysis and candidate gene selection among the evaluated germplasm. The observed LD decay distance among our cowpea collection is within the range of value reported in mungbean, and shorter than the 500 kb–1.88 Mb reported in some germplasm collections of common and vegetable cowpea (Noble et al., 2018; Xu et al., 2012, 2017). The faster the LD decay is, the better the genetic mapping resolution will be (Hindu et al., 2018). However, considering that the cowpea genome extends over 640.6 Mb (Lonardi et al., 2019), with the observed LD decay distance of 80–100 kb in our germplasm, approximately 6,406–8,006 SNPs markers will be needed to achieve full coverage of the genome. Significant variation was observed in LD patterns among chromosomes, thus agreeing with Yan et al. (2009) that LD decay estimation based on single chromosome may be biased. Nonetheless, LD analysis on chromosome basis help to identify haplotype blocks which is very important in genetic mapping. In the present study, longer LD decay distances were observed on chromosomes 3, 6, 7, 9, and 1, suggesting that these chromosomes could carry more quantitative traits loci. These chromosomes could thus be very useful for association mapping. There have been reports of quantitative trait loci and markers on these cowpea chromosomes that are associated with different traits including pod fiber content (Watcharatpong et al., 2020), perenniality and floral scent (Lo et al., 2020), seed size (Huynh et al., 2018; Lo et al., 2019), and resistance to biotic stresses (e.g., *Fusarium oxysporum* f. sp. *tracheiphilum* race 3 and *Striga gesnerioides* race 1) Ouédraogo et al., 2002; Pottorff et al., 2012). Some accessions in our collection have been reported by other studies to have good attributes for essential traits such as yield and resistance to flower thrips (*Megalurothrips sjostedti*) (Agbahoungba et al., 2017) and bruchids (*Callosobruchus maculatus*) (Kpoviessi et al., 2020). Hence, an in‐depth investigation of LD patterns in the germplasm described herein could help map genomic regions associated with these and other preferred cowpea traits.

## 5 CONCLUSIONS

This study used 3,127 high‐quality DArT‐seq SNP markers to analyze the genetic diversity within a collection of 274 cowpea accessions. An important genetic structuration was observed within the germplasm. Each of the identified subpopulations exhibited a level of genetic diversity that could be leveraged to develop cowpea varieties with desirable attributes. The subpopulations mainly consisted of inbred lines, which shared common alleles although they were from different geographical regions. Our study, by highlighting the presence of structure and LD within the collection, provides valuable insights into the future use of the corresponding germplasm in genome‐wide association studies and its exploitation in cowpea breeding programs.

## AUTHOR CONTRIBUTIONS

Frejus Ariel Kpedetin Sodedji: Conceptualization; Data curation; Formal analysis; Investigation; Methodology; Validation; Writing‐original draft; Writing‐review & editing. Symphorien Agbahoungba: Supervision; Writing‐review & editing. Eric Echikintho Agoyi: Writing‐review & editing. Médard Konoutan Kafoutchoni: Data curation; Writing‐review & editing. Jaeyoung Choi: Formal analysis, Writing‐review & editing. Simon‐Pierre Assanvo Nguetta: Writing‐review & editing. Achille Ephrem Assogbadjo: Writing‐review & editing. Ho‐Youn Kim: Supervision; Writing‐review & editing.

## CONFLICT OF INTEREST

The authors declare no conflict of interest.

## Supporting information

Supplemental Table S1. List of accessions
Supplemental Table S2. Membership of the accessions, as indicated by the structure analysis
Supplemental Table S3. Identity‐by‐state distance matrix.
Supplemental Table S4. Genotype data
Supplemental Table S5. Membership probabilities and clustering of the accessions, as indicated by the discriminant analysis of principal component
Supplemental Table S6. Membership of the accessions, as indicated by the neighbor‐joining clustering
Supplemental Table S7. Summary statistic of the Intra‐chromosomal LD analysis
