# Supplementary material for: Diversity, population structure, and linkage disequilibrium among cowpea accessions
Source: Plant Genome. 2021 Jul 18;14(3):e20113. doi: 10.1002/tpg2.20113 (PMC12807209; doi:10.1002/tpg2.20113)

Table S1 : List of accessions

Identification	Accessions	Regions	Country of origin
4	TVU-16449	Oceania	Australia
101	ALOOMBA	Oceania	Australia
123	MULGRAVE	Oceania	Australia
9	WEWE	West and Central Africa	Benin
12	DEUXTON	West and Central Africa	Benin
14	DESSERT	West and Central Africa	Benin
24	TOLA AKOUMIN	West and Central Africa	Benin
28	TOJOTOHOUN	West and Central Africa	Benin
29	TOLA SINMIN	West and Central Africa	Benin
37	TVU-16408	West and Central Africa	Benin
38	TONTON	West and Central Africa	Benin
45	TVU-16403	West and Central Africa	Benin
78	VIDEGNIKUN	West and Central Africa	Benin
84	TVU-8656	West and Central Africa	Benin
91	YAWAHARI	West and Central Africa	Benin
105	ADJAIKUN	West and Central Africa	Benin
106	AKOUNADO	West and Central Africa	Benin
107	AWLETCHI	West and Central Africa	Benin
111	ANONSIN	West and Central Africa	Benin
115	ATAMARIKO	West and Central Africa	Benin
118	DAMADAMI	West and Central Africa	Benin
127	BLIGOE	West and Central Africa	Benin
137	KAKI GROS GRAIN	West and Central Africa	Benin
140	KAKI PETIT GRAIN	West and Central Africa	Benin
142	DJETOKO	West and Central Africa	Benin
144	ELISABETH	West and Central Africa	Benin
145	KPODJIGUEGUE	West and Central Africa	Benin
147	EYIAWO	West and Central Africa	Benin
149	FLOWOUIWOUI	West and Central Africa	Benin
151	GLESSISSAFFODO	West and Central Africa	Benin
153	MAWUNA	West and Central Africa	Benin
162	KAKI	West and Central Africa	Benin
164	KOMCALLE	West and Central Africa	Benin
168	KPEGNIKOUN	West and Central Africa	Benin
169	KPLOBE	West and Central Africa	Benin
171	KPLOBE ROUGE	West and Central Africa	Benin
177	LAIVI	West and Central Africa	Benin
184	MALANVILLE	West and Central Africa	Benin
196	NONTCHEWAGBEHAMI	West and Central Africa	Benin
224	SÈWÉ	West and Central Africa	Benin
225	SOHOUNGBLO	West and Central Africa	Benin
227	TAWA	West and Central Africa	Benin
233	KPLOBE WEWE	West and Central Africa	Benin
234	TOLA	West and Central Africa	Benin
265	TVU 8834	West and Central Africa	Benin
34	TVU-14253	East and Southern Africa	Bostwana
39	TVU-14272	East and Southern Africa	Bostwana
274	TVU-14759	East and Southern Africa	Bostwana
16	MONTEIRO	America	Brazil
248	TVU 1411	America	Brazil
23	TILIGRE	West and Central Africa	Burkina-Faso
80	TVU-8262	West and Central Africa	Burkina-Faso

For Review Only

174	KVX61-1	West and Central Africa	Burkina-Faso
188	MOUSSA LOCALE	West and Central Africa	Burkina-Faso
267	TVU-10466	West and Central Africa	Burkina-Faso
41	TVU-14633	West and Central Africa	Central Africa
201	TVU-14632	West and Central Africa	Central Africa
268	TVU-10100	America	Colombia
8	TVU-7778	West and Central Africa	Côte d'Ivoire
17	N1-TOUBA	West and Central Africa	Côte d'Ivoire
172	N3-CI	West and Central Africa	Côte d'Ivoire
64	TVU-9492	North Africa	Egypt
79	TVU-9474	North Africa	Egypt
11	AYIYI	West and Central Africa	Ghana
15	KUMASSI	West and Central Africa	Ghana
73	UCR-5272	West and Central Africa	Ghana
110	APAGBAALA	West and Central Africa	Ghana
113	ASETENAPA	West and Central Africa	Ghana
117	IT97K-819-132	West and Central Africa	Ghana
121	EBELATE	West and Central Africa	Ghana
158	NYAPLA	West and Central Africa	Ghana
193	NHYIRA	West and Central Africa	Ghana
210	PADITUYA	West and Central Africa	Ghana
222	SANZI	West and Central Africa	Ghana
239	VIDEZA (WHITE	West and Central Africa	Ghana
247	TONA	West and Central Africa	Ghana
104	51-50	America	Guatemala
1	A-269	America	Honduras
5	TVU-2731	Asia	India
20	P 1120	Asia	India
22	P 721	Asia	India
27	TVU-11577	Asia	India
30	TVU-12897	Asia	India
40	TVU-1429	Asia	India
46	TVU-2736	Asia	India
47	TVU-3234	Asia	India
50	TVU-3279	Asia	India
61	TVU-2971	Asia	India
62	TVU-2785	Asia	India
66	TVU-2933	Asia	India
71	UCR 1340	Asia	India
88	UCR 2576	Asia	India
132	I.C. 2806	Asia	India
133	I.C. 2809	Asia	India
139	I.C. 2916	Asia	India
148	I.C. 2946	Asia	India
160	I.C. 7459	Asia	India
194	P 1115	Asia	India
195	P 1121	Asia	India
197	P 1150	Asia	India
198	P 1159	Asia	India
199	P 1171	Asia	India
200	P 1220	Asia	India
203	P 614	Asia	India
205	P 843	Asia	India
208	P 636	Asia	India
212	P 640	Asia	India

For Review Only

213	P 673	Asia	India
216	P 695	Asia	India
217	PHILIPPINE EARLY	Asia	India
220	PLL 263	Asia	India
223	PLL 282	Asia	India
231	UCR 3326	Asia	India
235	LOBIA 13	Asia	India
246	TVU 2706	Asia	India
250	TVU 2891	Asia	India
252	P 1240	Asia	India
253	TVU 3099	Asia	India
254	TVU 2806	Asia	India
255	TVU 2903	Asia	India
258	TVU 3428	Asia	India
259	P 659	Asia	India
260	P 677	Asia	India
263	P 703	Asia	India
266	P 710	Asia	India
269	TVU-12804	Asia	India
270	TVU-12937	Asia	India
49	TVU-1654	Asia	Iran
89	UCR 707	East and Southern Africa	Kenya
150	KVU 27-1	East and Southern Africa	Kenya
273	TVU-13463	East and Southern Africa	Kenya
31	TVU-15400	East and Southern Africa	Lesotho
69	TVU-6641	West and Central Africa	Liberia
74	TVU-6642	West and Central Africa	Liberia
3	TVU-15143	East and Southern Africa	Malawi
7	TVU-9848	East and Southern Africa	Malawi
70	TVU-9866	East and Southern Africa	Malawi
56	TVU-7642	West and Central Africa	Mali
77	TVU-7625	West and Central Africa	Mali
54	TVU-467	North Africa	Mauritania
90	XINGOVE	East and Southern Africa	Mozambique
122	INIA-40	East and Southern Africa	Mozambique
124	INIA-41	East and Southern Africa	Mozambique
152	MASSAVA-11	East and Southern Africa	Mozambique
245	TIMBAWONE-	East and Southern Africa	Mozambique
10	2925	America	Nicaragua
43	TVU-14971	West and Central Africa	Niger
242	TOUMKALAM	West and Central Africa	Niger
2	IT86D-888	West and Central Africa	Nigeria
13	FUAMPEA 1	West and Central Africa	Nigeria
21	IT97K-449-35	West and Central Africa	Nigeria
25	TVU 17263	West and Central Africa	Nigeria
44	TVU-1509	West and Central Africa	Nigeria
52	TVU-3965	West and Central Africa	Nigeria
53	TVU-43	West and Central Africa	Nigeria
57	TVU-801	West and Central Africa	Nigeria
58	TVU-8461	West and Central Africa	Nigeria
63	TVU-9031	West and Central Africa	Nigeria
65	TVU-972	West and Central Africa	Nigeria
68	TVU-4557	West and Central Africa	Nigeria
82	VITA7	West and Central Africa	Nigeria
85	TVU-946	West and Central Africa	Nigeria

For Review Only

116	IT84S-2049	West and Central Africa	Nigeria
126	AWONLIYIKUN	West and Central Africa	Nigeria
129	IT84D-449	West and Central Africa	Nigeria
130	IT86D-1038	West and Central Africa	Nigeria
134	IT86D-1057	West and Central Africa	Nigeria
135	IT89KD-288	West and Central Africa	Nigeria
136	IT98K-498-1	West and Central Africa	Nigeria
156	IT84E-124	West and Central Africa	Nigeria
165	IT06K-147-1	West and Central Africa	Nigeria
166	IT06K-242-3	West and Central Africa	Nigeria
170	IT07K-188-49	West and Central Africa	Nigeria
178	IT07K-211-1-8	West and Central Africa	Nigeria
179	IT07K-243-1-10	West and Central Africa	Nigeria
183	IT07K318-33	West and Central Africa	Nigeria
185	IT83S-742-2	West and Central Africa	Nigeria
187	IT84S-2246-4	West and Central Africa	Nigeria
202	TVU-15687	West and Central Africa	Nigeria
206	IT87S-1390	West and Central Africa	Nigeria
207	IT90K-76	West and Central Africa	Nigeria
209	IT93K-452-1	West and Central Africa	Nigeria
218	IT97K-499-35	West and Central Africa	Nigeria
221	IT97K-556-6	West and Central Africa	Nigeria
230	IT98K-205-8	West and Central Africa	Nigeria
244	TOUMPIPEAKE	West and Central Africa	Nigeria
257	TVU-1	West and Central Africa	Nigeria
261	TVU 8460	West and Central Africa	Nigeria
159	K522	Asia	Pakistan
33	TVU-1637	America	Puerto Rico
42	TVU-14393	West and Central Africa	Senegal
131	BAMBEY-21	West and Central Africa	Senegal
75	TVU-84	East and Southern Africa	South-Africa
219	POLON LEEMA	Asia	Sri Lanka
32	TVU-15445	East and Southern Africa	Swaziland
6	WC62	East and Southern Africa	Uganda
18	NE15*WC10	East and Southern Africa	Uganda
19	NE48*SECOW5T	East and Southern Africa	Uganda
26	WC29	East and Southern Africa	Uganda
35	WC35B*SUNSHINE	East and Southern Africa	Uganda
36	WC37B	East and Southern Africa	Uganda
48	WC41	East and Southern Africa	Uganda
51	WC46	East and Southern Africa	Uganda
59	WC5	East and Southern Africa	Uganda
67	WC67A	East and Southern Africa	Uganda
72	UCR 5279	East and Southern Africa	Uganda
76	VCI 637	East and Southern Africa	Uganda
81	WC7	East and Southern Africa	Uganda
83	WC35A	East and Southern Africa	Uganda
86	WC30	East and Southern Africa	Uganda
87	WC35B*ALEGI	East and Southern Africa	Uganda
92	VOZZONO BROWN	East and Southern Africa	Uganda
93	WC35B*NE50	East and Southern Africa	Uganda
94	WC35B*SECOW5T	East and Southern Africa	Uganda
95	WC39	East and Southern Africa	Uganda
96	WC66*NE50	East and Southern Africa	Uganda

For Review Only

97	WC66*SECOW5T	East and Southern Africa	Uganda
98	WC66*SUNSHINE	East and Southern Africa	Uganda
99	WC33	East and Southern Africa	Uganda
100	ALEGI	East and Southern Africa	Uganda
102	ACC122*WC66	East and Southern Africa	Uganda
103	ALEGI*SS	East and Southern Africa	Uganda
108	ALEGI*ACC122	East and Southern Africa	Uganda
109	C25	East and Southern Africa	Uganda
112	CIG	East and Southern Africa	Uganda
114	CIT	East and Southern Africa	Uganda
119	MU16	East and Southern Africa	Uganda
120	EBELAT*NE51	East and Southern Africa	Uganda
125	NE19	East and Southern Africa	Uganda
128	F2107T2E	East and Southern Africa	Uganda
138	CJO	East and Southern Africa	Uganda
141	NE20	East and Southern Africa	Uganda
143	EARLY SCARLET	East and Southern Africa	Uganda
146	NE44	East and Southern Africa	Uganda
154	NE45	East and Southern Africa	Uganda
155	NE5	East and Southern Africa	Uganda
157	NE70	East and Southern Africa	Uganda
161	MU9	East and Southern Africa	Uganda
163	MU9KAB	East and Southern Africa	Uganda
167	MUAB	East and Southern Africa	Uganda
173	SECOW 2 W	East and Southern Africa	Uganda
175	SECOW4U	East and Southern Africa	Uganda
176	NE15*NE48	East and Southern Africa	Uganda
180	NE15*WC35B	East and Southern Africa	Uganda
181	SUNSHINE2 SUBMAIN	East and Southern Africa	Uganda
182	NE15*WC36	East and Southern Africa	Uganda
186	NE23	East and Southern Africa	Uganda
189	NE46	East and Southern Africa	Uganda
190	NE50	East and Southern Africa	Uganda
191	NE50*WC10	East and Southern Africa	Uganda
192	NE53	East and Southern Africa	Uganda
211	PAKADHA	East and Southern Africa	Uganda
214	PAKAW	East and Southern Africa	Uganda
215	IT97K-449-39-1-1	East and Southern Africa	Uganda
226	SUNSHINE	East and Southern Africa	Uganda
228	IT97K-569-9	East and Southern Africa	Uganda
229	SECOW 5T*WC36	East and Southern Africa	Uganda
232	SECOW19	East and Southern Africa	Uganda
236	NAMUESSE D	East and Southern Africa	Uganda
237	NE48	East and Southern Africa	Uganda
238	SECOW1T	East and Southern Africa	Uganda
240	SECOW5T	East and Southern Africa	Uganda
241	NE48*SECOW5T	East and Southern Africa	Uganda
243	SECOW5T*NE51	East and Southern Africa	Uganda
249	WARR-20	East and Southern Africa	Uganda
251	WC 10	East and Southern Africa	Uganda
256	WC 36	East and Southern Africa	Uganda
262	WC35B*5T	East and Southern Africa	Uganda
264	WC35B*SS	East and Southern Africa	Uganda
271	TVU-1330	East and Southern Africa	Uganda
272	SEC3B	East and Southern Africa	Uganda

For Review Only

55	TVU-473	America	United States
60	TVU-1727	America	United States
204	TVU-15861	Asia	Yemen

For Review Only

**geopgraphic**
Oceania
Oceania
Oceania
West-Africa
West-Africa
West-Africa
West-Africa
West-Africa
West-Africa
West-Africa
West-Africa
West-Africa
West-Africa
West-Africa
West-Africa
West-Africa
West-Africa
West-Africa
West-Africa
West-Africa
West-Africa
West-Africa
West-Africa
West-Africa
West-Africa
West-Africa
West-Africa
West-Africa
West-Africa
West-Africa
West-Africa
West-Africa
West-Africa
West-Africa
West-Africa
West-Africa
West-Africa
West-Africa
West-Africa
West-Africa
West-Africa
West-Africa
West-Africa
West-Africa
West-Africa
Southern Africa
Southern Africa
Southern Africa
South America
South America
West-Africa
West-Africa

For Review Only

West-Africa
West-Africa
West-Africa
Central Africa
Central Africa
South America
West-Africa
West-Africa
West-Africa
North Africa
North Africa
West-Africa
West-Africa
West-Africa
West-Africa
West-Africa
West-Africa
West-Africa
West-Africa
West-Africa
West-Africa
West-Africa
West-Africa
West-Africa
Central America
North America
South-Asia
South-Asia
South-Asia
South-Asia
South-Asia
South-Asia
South-Asia
South-Asia
South-Asia
South-Asia
South-Asia
South-Asia
South-Asia
South-Asia
South-Asia
South-Asia
South-Asia
South-Asia
South-Asia
South-Asia
South-Asia
South-Asia
South-Asia
South-Asia
South-Asia
South-Asia
South-Asia
South-Asia
South-Asia

For Review Only

South-Asia
South-Asia
South-Asia
South-Asia
South-Asia
South-Asia
South-Asia
South-Asia
South-Asia
South-Asia
South-Asia
South-Asia
South-Asia
South-Asia
South-Asia
South-Asia
South-Asia
South-Asia
South-Asia
South-Asia
South-Asia
East Africa
East Africa
East Africa
Southern Africa
West-Africa
West-Africa
East Africa
East Africa
East Africa
West Africa
West Africa
North Africa
East Africa
East Africa
East Africa
East Africa
East Africa
Central America
West Africa
West Africa
West Africa
West Africa
West Africa
West Africa
West Africa
West Africa
West Africa
West Africa
West Africa
West Africa
West Africa
West Africa
West Africa
West Africa

For Review Only

West Africa
West Africa
West Africa
West Africa
West Africa
West Africa
West Africa
West Africa
West Africa
West Africa
West Africa
West Africa
West Africa
West Africa
West Africa
West Africa
West Africa
West Africa
West Africa
West Africa
West Africa
West Africa
West Africa
West Africa
West Africa
West Africa
South Asia
North America
West Africa
West Africa
Southern Africa
South Asia
Southern Africa
East Africa
East Africa
East Africa
East Africa
East Africa
East Africa
East Africa
East Africa
East Africa
East Africa
East Africa
East Africa
East Africa
East Africa
East Africa
East Africa
East Africa
East Africa
East Africa
East Africa
East Africa

For Review Only

East Africa
East Africa
East Africa
East Africa
East Africa
East Africa
East Africa
East Africa
East Africa
East Africa
East Africa
East Africa
East Africa
East Africa
East Africa
East Africa
East Africa
East Africa
East Africa
East Africa
East Africa
East Africa
East Africa
East Africa
East Africa
East Africa
East Africa
East Africa
East Africa
East Africa
East Africa
East Africa
East Africa
East Africa
East Africa
East Africa
East Africa
East Africa
East Africa
East Africa
East Africa
East Africa
East Africa
East Africa
East Africa
East Africa
East Africa
East Africa
East Africa
East Africa
East Africa
East Africa
East Africa
East Africa
East Africa

For Review Only

North America
North America
Middle East Asia

For Review Only

Table S2: Menbership of the accessions as indicated by the strucure analysis

Structure grouping

	inferred clusters			
Accessions	CLuster1	CLuster2	CLuster3	Structure grouping
1	0.74	0.10	0.16	Cluster1
2	0.52	0.45	0.03	Admixture
3	1.00	0.00	0.00	Cluster1
4	0.63	0.05	0.32	Cluster1
5	0.48	0.41	0.12	Admixture
6	0.94	0.05	0.01	Cluster1
7	0.81	0.16	0.03	Cluster1
8	0.35	0.66	0.00	Cluster2
9	0.66	0.09	0.25	Cluster1
10	0.22	0.78	0.00	Cluster2
11	0.40	0.60	0.00	Cluster2
12	0.77	0.00	0.23	Cluster1
13	0.23	0.16	0.61	Cluster3
14	0.37	0.61	0.02	Cluster2
15	0.40	0.00	0.60	Cluster3
16	0.61	0.29	0.09	Cluster1
17	0.73	0.24	0.04	Cluster1
18	0.65	0.32	0.03	Cluster1
19	1.00	0.00	0.00	Cluster1
20	0.37	0.59	0.04	Cluster2
21	0.51	0.49	0.00	Admixture
22	0.68	0.23	0.08	Cluster1
23	0.15	0.85	0.00	Cluster2
24	0.15	0.81	0.05	Cluster2
25	0.94	0.03	0.03	Cluster1
26	0.97	0.00	0.03	Cluster1
27	0.94	0.04	0.02	Cluster1
28	0.00	0.00	1.00	Cluster3
29	0.58	0.42	0.00	Cluster1
30	0.00	0.99	0.01	Cluster2
31	0.97	0.00	0.03	Cluster1
32	0.95	0.05	0.00	Cluster1
33	0.99	0.01	0.00	Cluster1
34	0.13	0.87	0.00	Cluster2
35	0.16	0.23	0.62	Cluster3
36	0.65	0.36	0.00	Cluster1
37	0.59	0.41	0.00	Cluster1
38	0.52	0.36	0.13	Admixture
39	0.00	0.99	0.01	Cluster2
40	0.32	0.61	0.06	Cluster2
41	0.00	0.97	0.03	Cluster2
42	0.31	0.60	0.09	Cluster2
43	0.25	0.70	0.05	Cluster2
44	0.54	0.40	0.07	Cluster1
45	0.26	0.74	0.00	Cluster2
46	0.80	0.00	0.20	Cluster1
47	0.96	0.00	0.04	Cluster1
48	0.50	0.32	0.17	Admixture

For Review Only

49	0.53	0.40	0.08	Cluster1
50	0.58	0.00	0.42	Cluster1
51	0.99	0.01	0.00	Cluster1
52	0.59	0.18	0.23	Cluster1
53	1.00	0.00	0.00	Cluster1
54	0.98	0.02	0.00	Cluster1
55	0.91	0.03	0.06	Cluster1
56	0.72	0.28	0.00	Cluster1
57	1.00	0.00	0.00	Cluster1
58	0.98	0.00	0.02	Cluster1
59	1.00	0.00	0.00	Cluster1
60	0.00	1.00	0.00	Cluster2
61	0.44	0.55	0.01	Cluster2
62	0.00	0.00	1.00	Cluster3
63	1.00	0.00	0.00	Cluster1
64	1.00	0.00	0.00	Cluster1
65	1.00	0.00	0.00	Cluster1
66	0.58	0.42	0.00	Cluster1
67	0.18	0.00	0.82	Cluster3
68	0.30	0.49	0.21	Admixture
69	0.55	0.28	0.17	Cluster1
70	0.94	0.00	0.06	Cluster1
71	1.00	0.00	0.00	Cluster1
72	0.97	0.02	0.01	Cluster1
73	0.77	0.00	0.23	Cluster1
74	0.17	0.59	0.25	Cluster2
75	0.55	0.27	0.18	Cluster1
76	0.77	0.23	0.00	Cluster1
77	0.26	0.75	0.00	Cluster2
78	0.93	0.07	0.00	Cluster1
79	0.49	0.41	0.09	Admixture
80	0.00	1.00	0.00	Cluster2
81	0.98	0.02	0.00	Cluster1
82	1.00	0.01	0.00	Cluster1
83	0.56	0.31	0.13	Cluster1
84	0.39	0.53	0.08	Cluster2
85	0.00	1.00	0.00	Cluster2
86	1.00	0.00	0.00	Cluster1
87	0.72	0.28	0.00	Cluster1
88	0.26	0.74	0.00	Cluster2
89	0.32	0.65	0.03	Cluster2
90	0.38	0.50	0.13	Admixture
91	0.58	0.42	0.00	Cluster1
92	0.25	0.65	0.11	Cluster2
93	0.98	0.00	0.02	Cluster1
94	0.96	0.00	0.04	Cluster1
95	0.67	0.28	0.05	Cluster1
96	1.00	0.00	0.00	Cluster1
97	0.75	0.15	0.10	Cluster1
98	0.96	0.02	0.02	Cluster1
99	0.00	0.98	0.02	Cluster2
100	0.79	0.13	0.08	Cluster1
101	0.86	0.13	0.01	Cluster1
102	0.48	0.16	0.36	Admixture
103	0.82	0.00	0.18	Cluster1

For Review Only

104	0.00	1.00	0.00	Cluster2
105	0.13	0.87	0.00	Cluster2
106	0.10	0.18	0.72	Cluster3
107	0.77	0.23	0.00	Cluster1
108	0.49	0.16	0.36	Admixture
109	0.25	0.36	0.39	Admixture
110	0.85	0.06	0.09	Cluster1
111	0.03	0.92	0.05	Cluster2
112	0.80	0.00	0.20	Cluster1
113	0.86	0.07	0.07	Cluster1
114	0.99	0.00	0.01	Cluster1
115	0.49	0.19	0.32	Admixture
116	0.42	0.41	0.17	Admixture
117	0.21	0.26	0.54	Cluster3
118	0.54	0.27	0.20	Cluster1
119	0.00	0.00	1.00	Cluster3
120	0.63	0.32	0.05	Cluster1
121	0.58	0.00	0.42	Cluster1
122	0.34	0.13	0.53	Cluster3
123	0.20	0.00	0.80	Cluster3
124	0.73	0.16	0.12	Cluster1
125	0.19	0.00	0.81	Cluster3
126	0.04	0.96	0.00	Cluster2
127	0.61	0.25	0.14	Cluster1
128	0.00	0.00	1.00	Cluster3
129	0.88	0.00	0.12	Cluster1
130	0.88	0.00	0.11	Cluster1
131	0.00	1.00	0.00	Cluster2
132	0.33	0.17	0.50	Admixture
133	0.40	0.57	0.03	Cluster2
134	0.70	0.05	0.25	Cluster1
135	0.77	0.23	0.00	Cluster1
136	0.85	0.00	0.16	Cluster1
137	0.74	0.13	0.13	Cluster1
138	0.00	0.91	0.10	Cluster2
139	0.00	0.00	1.00	Cluster3
140	1.00	0.00	0.00	Cluster1
141	0.34	0.00	0.66	Cluster3
142	0.14	0.86	0.00	Cluster2
143	0.23	0.32	0.45	Admixture
144	0.00	0.99	0.01	Cluster2
145	0.95	0.00	0.05	Cluster1
146	0.26	0.28	0.46	Admixture
147	0.00	0.77	0.23	Cluster2
148	0.26	0.32	0.41	Admixture
149	0.00	1.00	0.00	Cluster2
150	0.92	0.08	0.00	Cluster1
151	0.00	0.50	0.50	Admixture
152	0.52	0.21	0.27	Admixture
153	0.78	0.19	0.03	Cluster1
154	0.99	0.00	0.01	Cluster1
155	1.00	0.00	0.00	Cluster1
156	0.01	0.40	0.59	Cluster3
157	0.76	0.00	0.24	Cluster1
158	0.76	0.00	0.24	Cluster1

For Review Only

159	0.21	0.79	0.00	Cluster2
160	0.53	0.19	0.28	Cluster1
161	0.98	0.01	0.01	Cluster1
162	0.00	0.27	0.73	Cluster3
163	0.98	0.00	0.02	Cluster1
164	0.30	0.68	0.03	Cluster2
165	0.32	0.41	0.27	Admixture
166	0.12	0.34	0.54	Cluster3
167	0.77	0.04	0.19	Cluster1
168	0.49	0.50	0.01	Admixture
169	0.00	0.54	0.46	Cluster2
170	0.00	0.00	1.00	Cluster3
171	0.07	0.42	0.51	Admixture
172	1.00	0.00	0.00	Cluster1
173	0.74	0.00	0.26	Cluster1
174	0.11	0.57	0.32	Cluster2
175	0.70	0.00	0.30	Cluster1
176	0.24	0.14	0.62	Cluster3
177	0.00	0.99	0.02	Cluster2
178	0.33	0.68	0.00	Cluster2
179	0.27	0.15	0.58	Cluster3
180	0.86	0.02	0.13	Cluster1
181	0.84	0.14	0.02	Cluster1
182	0.93	0.00	0.07	Cluster1
183	0.00	0.00	1.00	Cluster3
184	0.01	0.88	0.11	Cluster2
185	0.22	0.68	0.11	Cluster2
186	1.00	0.00	0.00	Cluster1
187	0.50	0.37	0.13	Admixture
188	0.12	0.88	0.00	Cluster2
189	0.68	0.03	0.30	Cluster1
190	0.22	0.13	0.65	Cluster3
191	0.75	0.25	0.00	Cluster1
192	0.93	0.07	0.01	Cluster1
193	0.79	0.21	0.00	Cluster1
194	1.00	0.00	0.00	Cluster1
195	0.86	0.14	0.00	Cluster1
196	0.00	0.93	0.07	Cluster2
197	0.66	0.09	0.25	Cluster1
198	1.00	0.00	0.00	Cluster1
199	0.65	0.13	0.21	Cluster1
200	0.80	0.20	0.01	Cluster1
201	1.00	0.00	0.00	Cluster1
202	0.95	0.05	0.00	Cluster1
203	1.00	0.00	0.00	Cluster1
204	1.00	0.00	0.00	Cluster1
205	0.00	1.00	0.00	Cluster2
206	0.34	0.03	0.62	Cluster3
207	0.33	0.21	0.46	Admixture
208	0.54	0.21	0.25	Cluster1
209	0.33	0.67	0.00	Cluster2
210	0.11	0.89	0.00	Cluster2
211	0.05	0.95	0.00	Cluster2
212	0.56	0.29	0.15	Cluster1
213	0.85	0.05	0.10	Cluster1

For Review Only

214	0.17	0.83	0.00	Cluster2
215	0.18	0.82	0.00	Cluster2
216	1.00	0.00	0.00	Cluster1
217	0.00	1.00	0.00	Cluster2
218	0.58	0.41	0.01	Cluster1
219	0.41	0.50	0.10	Admixture
220	0.83	0.00	0.17	Cluster1
221	0.05	0.26	0.69	Cluster3
222	0.47	0.53	0.00	Cluster2
223	0.84	0.00	0.17	Cluster1
224	0.26	0.60	0.14	Cluster2
225	0.07	0.93	0.00	Cluster2
226	0.02	0.51	0.47	Admixture
227	0.00	1.00	0.00	Cluster2
228	0.00	0.00	1.00	Cluster3
229	0.55	0.39	0.07	Cluster1
230	0.68	0.32	0.00	Cluster1
231	0.89	0.10	0.01	Cluster1
232	0.99	0.01	0.00	Cluster1
233	0.59	0.40	0.01	Cluster1
234	0.15	0.85	0.00	Cluster2
235	0.28	0.54	0.18	Cluster2
236	0.19	0.72	0.09	Cluster2
237	0.55	0.45	0.00	Cluster1
238	0.47	0.48	0.05	Admixture
239	1.00	0.00	0.00	Cluster1
240	0.96	0.04	0.00	Cluster1
241	0.48	0.52	0.00	Admixture
242	0.00	1.00	0.00	Cluster2
243	0.45	0.09	0.46	Admixture
244	0.00	1.00	0.00	Cluster2
245	0.92	0.00	0.09	Cluster1
246	0.46	0.09	0.45	Admixture
247	1.00	0.00	0.00	Cluster1
248	0.02	0.98	0.00	Cluster2
249	0.58	0.25	0.18	Cluster1
250	0.42	0.58	0.00	Cluster2
251	0.81	0.07	0.12	Cluster1
252	0.32	0.68	0.00	Cluster2
253	0.00	1.00	0.00	Cluster2
254	1.00	0.00	0.00	Cluster1
255	1.00	0.00	0.00	Cluster1
256	0.65	0.35	0.00	Cluster1
257	0.00	1.00	0.00	Cluster2
258	0.98	0.01	0.01	Cluster1
259	0.72	0.26	0.02	Cluster1
260	0.45	0.55	0.00	Cluster2
261	1.00	0.00	0.00	Cluster1
262	0.60	0.09	0.31	Cluster1
263	0.66	0.34	0.00	Cluster1
264	0.57	0.38	0.05	Cluster1
265	0.71	0.25	0.04	Cluster1
266	0.51	0.23	0.26	Admixture
267	0.00	1.00	0.00	Cluster2
268	0.99	0.01	0.00	Cluster1

For Review Only

269	0.00	1.00	0.00	Cluster2
270	1.00	0.00	0.00	Cluster1
271	0.99	0.01	0.00	Cluster1
272	0.26	0.74	0.00	Cluster2
273	0.96	0.02	0.02	Cluster1
274	1.00	0.00	0.00	Cluster1

For Review Only

For Review Only

For Review Only

For Review Only

For Review Only

For Review Only

For Review Only

For Review Only

For Review Only

For Review Only

For Review Only

For Review Only

For Review Only

For Review Only

For Review Only

For Review Only

For Review Only

For Review Only

For Review Only

For Review Only

For Review Only

For Review Only

For Review Only

For Review Only

For Review Only

For Review Only

For Review Only

For Review Only

For Review Only

For Review Only

For Review Only

For Review Only

For Review Only

For Review Only

For Review Only

For Review Only

For Review Only

For Review Only

For Review Only

For Review Only

For Review Only

For Review Only

For Review Only

For Review Only

For Review Only

For Review Only

For Review Only

For Review Only

For Review Only

For Review Only

For Review Only

For Review Only

For Review Only

For Review Only

For Review Only

For Review Only

For Review Only

For Review Only

For Review Only

For Review Only

For Review Only

For Review Only

For Review Only

For Review Only

For Review Only

For Review Only

For Review Only

For Review Only

For Review Only

For Review Only

For Review Only

For Review Only

For Review Only

For Review Only

For Review Only

For Review Only

For Review Only

For Review Only

For Review Only

For Review Only

For Review Only

For Review Only

For Review Only

For Review Only

For Review Only

For Review Only

For Review Only

For Review Only

For Review Only

For Review Only

For Review Only

For Review Only

For Review Only

For Review Only

For Review Only

For Review Only

For Review Only

For Review Only

For Review Only

For Review Only

For Review Only

For Review Only

For Review Only

For Review Only

For Review Only

For Review Only

For Review Only

For Review Only

For Review Only

For Review Only

For Review Only

For Review Only

For Review Only

For Review Only

For Review Only

For Review Only

For Review Only

For Review Only

For Review Only

For Review Only

For Review Only

For Review Only

For Review Only

For Review Only

For Review Only

For Review Only

For Review Only

For Review Only

For Review Only

For Review Only

For Review Only

For Review Only

For Review Only

For Review Only

For Review Only

For Review Only

For Review Only

For Review Only

For Review Only

For Review Only

For Review Only

For Review Only

For Review Only

For Review Only

For Review Only

For Review Only

For Review Only

For Review Only

For Review Only

For Review Only

For Review Only

For Review Only

For Review Only

For Review Only

For Review Only

For Review Only

For Review Only

For Review Only

For Review Only

For Review Only

For Review Only

For Review Only

For Review Only

For Review Only

For Review Only

For Review Only

For Review Only

For Review Only

For Review Only

For Review Only

For Review Only

For Review Only

For Review Only

For Review Only

For Review Only

For Review Only

For Review Only

For Review Only

For Review Only

For Review Only

For Review Only

For Review Only

For Review Only

For Review Only

For Review Only

For Review Only

For Review Only

For Review Only

For Review Only

For Review Only

For Review Only

For Review Only

For Review Only

For Review Only

For Review Only

For Review Only

For Review Only

For Review Only

For Review Only

For Review Only

For Review Only

For Review Only

For Review Only

For Review Only

For Review Only

For Review Only

For Review Only

For Review Only

For Review Only

For Review Only

For Review Only

For Review Only

For Review Only

For Review Only

For Review Only

For Review Only

For Review Only

For Review Only

For Review Only

For Review Only

For Review Only

For Review Only

For Review Only

For Review Only

For Review Only

For Review Only

For Review Only

For Review Only

For Review Only

For Review Only

For Review Only

For Review Only

For Review Only

For Review Only

For Review Only

For Review Only

For Review Only

For Review Only

For Review Only

For Review Only

For Review Only

For Review Only

For Review Only

For Review Only

For Review Only

For Review Only

For Review Only

For Review Only

For Review Only

For Review Only

For Review Only

For Review Only

For Review Only

For Review Only

For Review Only

For Review Only

For Review Only

For Review Only

For Review Only

For Review Only

For Review Only

For Review Only

For Review Only

For Review Only

For Review Only

For Review Only

For Review Only

For Review Only

For Review Only

For Review Only

For Review Only

For Review Only

For Review Only

For Review Only

For Review Only

For Review Only

For Review Only

For Review Only

For Review Only

For Review Only

For Review Only

For Review Only

For Review Only

For Review Only

For Review Only

For Review Only

For Review Only

For Review Only

For Review Only

For Review Only

For Review Only

For Review Only

For Review Only

For Review Only

For Review Only

For Review Only

For Review Only

For Review Only

For Review Only

For Review Only

For Review Only

For Review Only

For Review Only

For Review Only

For Review Only

For Review Only

For Review Only

For Review Only

For Review Only

For Review Only

For Review Only

For Review Only

For Review Only

For Review Only

For Review Only

For Review Only

For Review Only

For Review Only

For Review Only

For Review Only

For Review Only

For Review Only

For Review Only

For Review Only

For Review Only

For Review Only

For Review Only

For Review Only

For Review Only

For Review Only

For Review Only

For Review Only

For Review Only

For Review Only

For Review Only

For Review Only

For Review Only

For Review Only

For Review Only

For Review Only

For Review Only

For Review Only

For Review Only

For Review Only

For Review Only

For Review Only

For Review Only

For Review Only

For Review Only

For Review Only

For Review Only

For Review Only

For Review Only

For Review Only

For Review Only

For Review Only

For Review Only

For Review Only

For Review Only

For Review Only

For Review Only

For Review Only

For Review Only

For Review Only

For Review Only

For Review Only

For Review Only

For Review Only

For Review Only

For Review Only

For Review Only

For Review Only

For Review Only

For Review Only

For Review Only

For Review Only

For Review Only

For Review Only

For Review Only

For Review Only

For Review Only

For Review Only

For Review Only

For Review Only

For Review Only

For Review Only

For Review Only

For Review Only

For Review Only

For Review Only

For Review Only

For Review Only

For Review Only

For Review Only

For Review Only

For Review Only

For Review Only

For Review Only

For Review Only

For Review Only

For Review Only

For Review Only

For Review Only

For Review Only

For Review Only

For Review Only

For Review Only

For Review Only

For Review Only

For Review Only

For Review Only

For Review Only

For Review Only

For Review Only

For Review Only

For Review Only

For Review Only

For Review Only

For Review Only

For Review Only

For Review Only

For Review Only

For Review Only

For Review Only

For Review Only

For Review Only

For Review Only

For Review Only

For Review Only

For Review Only

For Review Only

For Review Only

For Review Only

For Review Only

For Review Only

For Review Only

For Review Only

For Review Only

For Review Only

For Review Only

For Review Only

For Review Only

For Review Only

For Review Only

For Review Only

For Review Only

For Review Only

For Review Only

For Review Only

For Review Only

For Review Only

For Review Only

For Review Only

For Review Only

For Review Only

For Review Only

For Review Only

For Review Only

For Review Only

For Review Only

For Review Only

For Review Only

For Review Only

For Review Only

For Review Only

For Review Only

For Review Only

For Review Only

For Review Only

For Review Only

For Review Only

For Review Only

For Review Only

For Review Only

For Review Only

For Review Only

For Review Only

For Review Only

For Review Only

For Review Only

For Review Only

For Review Only

For Review Only

For Review Only

For Review Only

For Review Only

For Review Only

For Review Only

For Review Only

For Review Only

For Review Only

For Review Only

For Review Only

For Review Only

For Review Only

For Review Only

For Review Only

For Review Only

For Review Only

For Review Only

For Review Only

For Review Only

For Review Only

For Review Only

For Review Only

For Review Only

For Review Only

For Review Only

For Review Only

For Review Only

For Review Only

For Review Only

For Review Only

For Review Only

For Review Only

For Review Only

For Review Only

For Review Only

For Review Only

For Review Only

For Review Only

For Review Only

For Review Only

For Review Only

For Review Only

For Review Only

For Review Only

For Review Only

For Review Only

For Review Only

For Review Only

For Review Only

For Review Only

For Review Only

For Review Only

For Review Only

For Review Only

For Review Only

For Review Only

For Review Only

For Review Only

For Review Only

For Review Only

For Review Only

For Review Only

For Review Only

For Review Only

For Review Only

For Review Only

For Review Only

For Review Only

For Review Only

For Review Only

For Review Only

For Review Only

For Review Only

For Review Only

For Review Only

For Review Only

For Review Only

For Review Only

For Review Only

For Review Only

For Review Only

For Review Only

For Review Only

For Review Only

For Review Only

For Review Only

For Review Only

For Review Only

For Review Only

For Review Only

For Review Only

For Review Only

For Review Only

For Review Only

For Review Only

For Review Only

For Review Only

For Review Only

For Review Only

For Review Only

For Review Only

For Review Only

For Review Only

For Review Only

For Review Only

For Review Only

For Review Only

For Review Only

For Review Only

For Review Only

For Review Only

For Review Only

For Review Only

For Review Only

For Review Only

For Review Only

For Review Only

For Review Only

For Review Only

For Review Only

For Review Only

For Review Only

For Review Only

For Review Only

For Review Only

For Review Only

For Review Only

For Review Only

For Review Only

For Review Only

For Review Only

For Review Only

For Review Only

For Review Only

For Review Only

For Review Only

For Review Only

For Review Only

For Review Only

For Review Only

For Review Only

For Review Only

For Review Only

For Review Only

For Review Only

For Review Only

For Review Only

For Review Only

For Review Only

For Review Only

For Review Only

For Review Only

For Review Only

For Review Only

For Review Only

For Review Only

For Review Only

For Review Only

For Review Only

For Review Only

For Review Only

For Review Only

For Review Only

For Review Only

For Review Only

For Review Only

For Review Only

For Review Only

For Review Only

For Review Only

For Review Only

For Review Only

For Review Only

For Review Only

For Review Only

For Review Only

For Review Only

For Review Only

For Review Only

For Review Only

For Review Only

For Review Only

For Review Only

For Review Only

For Review Only

For Review Only

For Review Only

For Review Only

For Review Only

For Review Only

For Review Only

For Review Only

For Review Only

For Review Only

For Review Only

For Review Only

For Review Only

For Review Only

For Review Only

For Review Only

For Review Only

For Review Only

For Review Only

For Review Only

For Review Only

For Review Only

For Review Only

For Review Only

For Review Only

For Review Only

For Review Only

For Review Only

For Review Only

For Review Only

For Review Only

For Review Only

For Review Only

For Review Only

For Review Only

For Review Only

For Review Only

For Review Only

For Review Only

For Review Only

For Review Only

For Review Only

For Review Only

For Review Only

For Review Only

For Review Only

For Review Only

For Review Only

For Review Only

For Review Only

For Review Only

For Review Only

For Review Only

For Review Only

For Review Only

For Review Only

For Review Only

For Review Only

For Review Only

For Review Only

For Review Only

For Review Only

For Review Only

For Review Only

For Review Only

For Review Only

For Review Only

For Review Only

For Review Only

For Review Only

For Review Only

For Review Only

For Review Only

For Review Only

For Review Only

For Review Only

For Review Only

For Review Only

For Review Only

For Review Only

For Review Only

For Review Only

For Review Only

For Review Only

For Review Only

For Review Only

For Review Only

For Review Only

For Review Only

For Review Only

For Review Only

For Review Only

For Review Only

For Review Only

For Review Only

For Review Only

For Review Only

For Review Only

For Review Only

For Review Only

For Review Only

For Review Only

For Review Only

For Review Only

For Review Only

For Review Only

For Review Only

For Review Only

For Review Only

For Review Only

For Review Only

For Review Only

For Review Only

For Review Only

For Review Only

For Review Only

For Review Only

For Review Only

For Review Only

For Review Only

For Review Only

For Review Only

For Review Only

For Review Only

For Review Only

For Review Only

For Review Only

For Review Only

For Review Only

For Review Only

For Review Only

For Review Only

For Review Only

For Review Only

For Review Only

For Review Only

For Review Only

For Review Only

For Review Only

For Review Only

For Review Only

For Review Only

For Review Only

For Review Only

For Review Only

For Review Only

For Review Only

For Review Only

For Review Only

For Review Only

For Review Only

For Review Only

For Review Only

For Review Only

For Review Only

For Review Only

For Review Only

For Review Only

For Review Only

For Review Only

For Review Only

For Review Only

For Review Only

For Review Only

For Review Only

For Review Only

For Review Only

For Review Only

For Review Only

For Review Only

For Review Only

For Review Only

For Review Only

For Review Only

For Review Only

For Review Only

For Review Only

For Review Only

For Review Only

For Review Only

For Review Only

For Review Only

For Review Only

For Review Only

For Review Only

For Review Only

For Review Only

For Review Only

For Review Only

For Review Only

For Review Only

For Review Only

For Review Only

For Review Only

For Review Only

For Review Only

For Review Only

For Review Only

For Review Only

For Review Only

For Review Only

For Review Only

For Review Only

For Review Only

For Review Only

For Review Only

For Review Only

For Review Only

For Review Only

For Review Only

For Review Only

For Review Only

For Review Only

For Review Only

For Review Only

For Review Only

For Review Only

For Review Only

For Review Only

For Review Only

For Review Only

For Review Only

For Review Only

For Review Only

For Review Only

For Review Only

For Review Only

For Review Only

For Review Only

For Review Only

For Review Only

For Review Only

For Review Only

For Review Only

For Review Only

For Review Only

For Review Only

For Review Only

For Review Only

For Review Only

For Review Only

For Review Only

For Review Only

For Review Only

For Review Only

For Review Only

For Review Only

For Review Only

For Review Only

For Review Only

For Review Only

For Review Only

For Review Only

For Review Only

For Review Only

For Review Only

For Review Only

For Review Only

For Review Only

For Review Only

For Review Only

For Review Only

For Review Only

For Review Only

For Review Only

For Review Only

For Review Only

For Review Only

For Review Only

For Review Only

For Review Only

For Review Only

For Review Only

For Review Only

For Review Only

For Review Only

For Review Only

For Review Only

For Review Only

For Review Only

For Review Only

For Review Only

For Review Only

For Review Only

For Review Only

For Review Only

For Review Only

For Review Only

For Review Only

For Review Only

For Review Only

For Review Only

For Review Only

For Review Only

For Review Only

For Review Only

For Review Only

For Review Only

For Review Only

For Review Only

For Review Only

For Review Only

For Review Only

For Review Only

For Review Only

For Review Only

For Review Only

For Review Only

For Review Only

For Review Only

For Review Only

For Review Only

For Review Only

For Review Only

For Review Only

For Review Only

For Review Only

For Review Only

For Review Only

For Review Only

For Review Only

For Review Only

For Review Only

For Review Only

For Review Only

For Review Only

For Review Only

For Review Only

For Review Only

For Review Only

For Review Only

For Review Only

For Review Only

For Review Only

For Review Only

For Review Only

For Review Only

For Review Only

For Review Only

For Review Only

For Review Only

For Review Only

For Review Only

For Review Only

For Review Only

For Review Only

For Review Only

For Review Only

For Review Only

For Review Only

For Review Only

For Review Only

For Review Only

For Review Only

For Review Only

For Review Only

For Review Only

For Review Only

For Review Only

For Review Only

For Review Only

For Review Only

For Review Only

For Review Only

For Review Only

For Review Only

For Review Only

For Review Only

For Review Only

For Review Only

For Review Only

For Review Only

For Review Only

For Review Only

For Review Only

For Review Only

For Review Only

For Review Only

For Review Only

For Review Only

For Review Only

For Review Only

For Review Only

For Review Only

For Review Only

For Review Only

For Review Only

For Review Only

For Review Only

For Review Only

For Review Only

For Review Only

For Review Only

For Review Only

For Review Only

For Review Only

For Review Only

For Review Only

For Review Only

For Review Only

For Review Only

For Review Only

For Review Only

For Review Only

For Review Only

For Review Only

For Review Only

For Review Only

For Review Only

For Review Only

For Review Only

For Review Only

For Review Only

For Review Only

For Review Only

For Review Only

For Review Only

For Review Only

For Review Only

For Review Only

For Review Only

For Review Only

For Review Only

For Review Only

For Review Only

For Review Only

For Review Only

For Review Only

For Review Only

For Review Only

For Review Only

For Review Only

For Review Only

For Review Only

For Review Only

For Review Only

For Review Only

For Review Only

For Review Only

For Review Only

For Review Only

For Review Only

For Review Only

For Review Only

For Review Only

For Review Only

For Review Only

For Review Only

For Review Only

For Review Only

For Review Only

For Review Only

For Review Only

For Review Only

For Review Only

For Review Only

For Review Only

For Review Only

For Review Only

For Review Only

For Review Only

For Review Only

For Review Only

For Review Only

For Review Only

For Review Only

For Review Only

For Review Only

For Review Only

For Review Only

For Review Only

For Review Only

For Review Only

For Review Only

For Review Only

For Review Only

For Review Only

For Review Only

For Review Only

For Review Only

For Review Only

For Review Only

For Review Only

For Review Only

For Review Only

For Review Only

For Review Only

For Review Only

For Review Only

For Review Only

For Review Only

For Review Only

For Review Only

For Review Only

For Review Only

For Review Only

For Review Only

For Review Only

For Review Only

For Review Only

For Review Only

For Review Only

For Review Only

For Review Only

For Review Only

For Review Only

For Review Only

For Review Only

For Review Only

For Review Only

For Review Only

For Review Only

For Review Only

For Review Only

For Review Only

For Review Only

For Review Only

For Review Only

For Review Only

For Review Only

For Review Only

For Review Only

For Review Only

For Review Only

For Review Only

For Review Only

For Review Only

For Review Only

For Review Only

For Review Only

For Review Only

For Review Only

For Review Only

For Review Only

For Review Only

For Review Only

For Review Only

For Review Only

For Review Only

For Review Only

For Review Only

For Review Only

For Review Only

For Review Only

For Review Only

For Review Only

For Review Only

For Review Only

For Review Only

For Review Only

For Review Only

For Review Only

For Review Only

For Review Only

For Review Only

For Review Only

For Review Only

For Review Only

For Review Only

For Review Only

For Review Only

For Review Only

For Review Only

For Review Only

For Review Only

For Review Only

For Review Only

For Review Only

For Review Only

For Review Only

For Review Only

For Review Only

For Review Only

For Review Only

For Review Only

For Review Only

For Review Only

For Review Only

For Review Only

For Review Only

For Review Only

For Review Only

For Review Only

For Review Only

For Review Only

For Review Only

For Review Only

For Review Only

For Review Only

For Review Only

For Review Only

For Review Only

For Review Only

For Review Only

For Review Only

For Review Only

For Review Only

For Review Only

For Review Only

For Review Only

For Review Only

For Review Only

For Review Only

For Review Only

For Review Only

For Review Only

For Review Only

For Review Only

For Review Only

For Review Only

For Review Only

For Review Only

For Review Only

For Review Only

For Review Only

For Review Only

For Review Only

For Review Only

For Review Only

For Review Only

For Review Only

For Review Only

For Review Only

For Review Only

For Review Only

For Review Only

For Review Only

For Review Only

For Review Only

For Review Only

For Review Only

For Review Only

For Review Only

For Review Only

For Review Only

For Review Only

For Review Only

For Review Only

For Review Only

For Review Only

For Review Only

For Review Only

For Review Only

For Review Only

For Review Only

For Review Only

For Review Only

For Review Only

For Review Only

For Review Only

For Review Only

For Review Only

For Review Only

For Review Only

For Review Only

For Review Only

For Review Only

For Review Only

For Review Only

For Review Only

For Review Only

For Review Only

For Review Only

For Review Only

For Review Only

For Review Only

For Review Only

For Review Only

For Review Only

For Review Only

For Review Only

For Review Only

For Review Only

For Review Only

For Review Only

For Review Only

For Review Only

For Review Only

For Review Only

For Review Only

For Review Only

For Review Only

For Review Only

For Review Only

For Review Only

For Review Only

For Review Only

For Review Only

For Review Only

For Review Only

For Review Only

For Review Only

For Review Only

For Review Only

For Review Only

For Review Only

For Review Only

For Review Only

For Review Only

For Review Only

For Review Only

For Review Only

For Review Only

For Review Only

For Review Only

For Review Only

For Review Only

For Review Only

For Review Only

For Review Only

For Review Only

For Review Only

For Review Only

For Review Only

For Review Only

For Review Only

For Review Only

For Review Only

For Review Only

For Review Only

For Review Only

For Review Only

For Review Only

For Review Only

For Review Only

For Review Only

For Review Only

For Review Only

For Review Only

For Review Only

For Review Only

For Review Only

For Review Only

For Review Only

For Review Only

For Review Only

For Review Only

For Review Only

For Review Only

For Review Only

For Review Only

For Review Only

For Review Only

For Review Only

For Review Only

For Review Only

For Review Only

For Review Only

For Review Only

For Review Only

For Review Only

For Review Only

For Review Only

For Review Only

For Review Only

For Review Only

For Review Only

For Review Only

For Review Only

For Review Only

For Review Only

For Review Only

For Review Only

For Review Only

For Review Only

For Review Only

For Review Only

For Review Only

For Review Only

For Review Only

For Review Only

For Review Only

For Review Only

For Review Only

For Review Only

For Review Only

For Review Only

For Review Only

For Review Only

For Review Only

For Review Only

For Review Only

For Review Only

For Review Only

For Review Only

For Review Only

For Review Only

For Review Only

For Review Only

For Review Only

For Review Only

For Review Only

For Review Only

For Review Only

For Review Only

For Review Only

For Review Only

For Review Only

For Review Only

For Review Only

For Review Only

For Review Only

For Review Only

For Review Only

For Review Only

For Review Only

For Review Only

For Review Only

For Review Only

For Review Only

For Review Only

For Review Only

For Review Only

For Review Only

For Review Only

For Review Only

For Review Only

For Review Only

For Review Only

For Review Only

For Review Only

For Review Only

For Review Only

For Review Only

For Review Only

For Review Only

For Review Only

For Review Only

For Review Only

For Review Only

For Review Only

For Review Only

For Review Only

For Review Only

For Review Only

For Review Only

For Review Only

For Review Only

For Review Only

For Review Only

For Review Only

For Review Only

For Review Only

For Review Only

For Review Only

For Review Only

For Review Only

For Review Only

For Review Only

For Review Only

For Review Only

For Review Only

For Review Only

For Review Only

For Review Only

For Review Only

For Review Only

For Review Only

For Review Only

For Review Only

For Review Only

For Review Only

For Review Only

For Review Only

For Review Only

For Review Only

For Review Only

For Review Only

For Review Only

For Review Only

For Review Only

For Review Only

For Review Only

For Review Only

For Review Only

For Review Only

For Review Only

For Review Only

For Review Only

For Review Only

For Review Only

For Review Only

For Review Only

For Review Only

For Review Only

For Review Only

For Review Only

For Review Only

For Review Only

For Review Only

For Review Only

For Review Only

For Review Only

For Review Only

For Review Only

For Review Only

For Review Only

For Review Only

For Review Only

For Review Only

For Review Only

For Review Only

For Review Only

For Review Only

For Review Only

For Review Only

For Review Only

For Review Only

For Review Only

For Review Only

For Review Only

For Review Only

For Review Only

For Review Only

For Review Only

For Review Only

For Review Only

For Review Only

For Review Only

For Review Only

For Review Only

For Review Only

For Review Only

For Review Only

For Review Only

For Review Only

For Review Only

For Review Only

For Review Only

For Review Only

For Review Only

For Review Only

For Review Only

For Review Only

For Review Only

For Review Only

For Review Only

For Review Only

For Review Only

For Review Only

For Review Only

For Review Only

For Review Only

For Review Only

For Review Only

For Review Only

For Review Only

For Review Only

For Review Only

For Review Only

For Review Only

For Review Only

For Review Only

For Review Only

For Review Only

For Review Only

For Review Only

For Review Only

For Review Only

For Review Only

For Review Only

For Review Only

For Review Only

For Review Only

For Review Only

For Review Only

For Review Only

For Review Only

For Review Only

For Review Only

For Review Only

For Review Only

For Review Only

For Review Only

For Review Only

For Review Only

For Review Only

For Review Only

For Review Only

For Review Only

For Review Only

For Review Only

For Review Only

For Review Only

For Review Only

For Review Only

For Review Only

For Review Only

For Review Only

For Review Only

For Review Only

For Review Only

For Review Only

For Review Only

For Review Only

For Review Only

For Review Only

For Review Only

For Review Only

For Review Only

For Review Only

For Review Only

For Review Only

For Review Only

For Review Only

For Review Only

For Review Only

For Review Only

For Review Only

For Review Only

For Review Only

For Review Only

For Review Only

For Review Only

For Review Only

For Review Only

For Review Only

For Review Only

For Review Only

For Review Only

For Review Only

For Review Only

For Review Only

For Review Only

For Review Only

For Review Only

For Review Only

For Review Only

For Review Only

For Review Only

For Review Only

For Review Only

For Review Only

For Review Only

For Review Only

For Review Only

For Review Only

For Review Only

For Review Only

For Review Only

For Review Only

For Review Only

For Review Only

For Review Only

For Review Only

For Review Only

For Review Only

For Review Only

For Review Only

For Review Only

For Review Only

For Review Only

For Review Only

For Review Only

For Review Only

For Review Only

For Review Only

For Review Only

For Review Only

For Review Only

For Review Only

For Review Only

For Review Only

For Review Only

For Review Only

For Review Only

For Review Only

For Review Only

For Review Only

For Review Only

For Review Only

For Review Only

For Review Only

For Review Only

For Review Only

For Review Only

For Review Only

For Review Only

For Review Only

For Review Only

For Review Only

For Review Only

For Review Only

For Review Only

For Review Only

For Review Only

For Review Only

For Review Only

For Review Only

For Review Only

For Review Only

For Review Only

For Review Only

For Review Only

For Review Only

For Review Only

For Review Only

For Review Only

For Review Only

For Review Only

For Review Only

For Review Only

For Review Only

For Review Only

For Review Only

For Review Only

For Review Only

For Review Only

For Review Only

For Review Only

For Review Only

For Review Only

For Review Only

For Review Only

For Review Only

For Review Only

For Review Only

For Review Only

For Review Only

For Review Only

For Review Only

For Review Only

For Review Only

For Review Only

For Review Only

For Review Only

For Review Only

For Review Only

For Review Only

For Review Only

For Review Only

For Review Only

For Review Only

For Review Only

For Review Only

For Review Only

For Review Only

For Review Only

For Review Only

For Review Only

For Review Only

For Review Only

For Review Only

For Review Only

For Review Only

For Review Only

For Review Only

For Review Only

For Review Only

For Review Only

For Review Only

For Review Only

For Review Only

For Review Only

For Review Only

For Review Only

For Review Only

For Review Only

For Review Only

For Review Only

For Review Only

For Review Only

For Review Only

For Review Only

For Review Only

For Review Only

For Review Only

For Review Only

For Review Only

For Review Only

For Review Only

For Review Only

For Review Only

For Review Only

For Review Only

For Review Only

For Review Only

For Review Only

For Review Only

For Review Only

For Review Only

For Review Only

For Review Only

For Review Only

For Review Only

For Review Only

For Review Only

For Review Only

For Review Only

For Review Only

For Review Only

For Review Only

For Review Only

For Review Only

For Review Only

#NAME?

Table S3 : Distance matrix

##IBS_Distance_Matrix.AverageTotalSites=3127.0
##IBS_Distance_Matrix.NumAlleles=3
##Matrix_Type=IBS_Distance_Matrix

	274							
1	0	0.339143	0.28142	0.283658	0.268628	0.231532	0.230892	0.319955
2	0.339143	0	0.31196	0.302207	0.356572	0.306684	0.348257	0.29757
3	0.28142	0.31196	0	0.239687	0.355932	0.260473	0.315318	0.341861
4	0.283658	0.302207	0.239687	0	0.32763	0.254557	0.307163	0.32747
5	0.268628	0.356572	0.355932	0.32763	0	0.323473	0.277103	0.322674
6	0.231532	0.306684	0.260473	0.254557	0.323473	0	0.259354	0.324432
7	0.230892	0.348257	0.315318	0.307163	0.277103	0.259354	0	0.321874
8	0.319955	0.29757	0.341861	0.32747	0.322674	0.324432	0.321874	0
9	0.223377	0.34378	0.319955	0.308123	0.254717	0.255516	0.185162	0.330349
10	0.352255	0.311481	0.372721	0.3441	0.303326	0.358171	0.342981	0.205788
11	0.340902	0.335146	0.350975	0.338823	0.331788	0.345539	0.340582	0.334826
12	0.282699	0.299488	0.070195	0.214743	0.350975	0.255836	0.309082	0.331148
13	0.251359	0.314519	0.314839	0.305564	0.292293	0.273585	0.2496	0.295971
14	0.286217	0.315798	0.32827	0.308443	0.308283	0.322194	0.322993	0.284778
15	0.237448	0.327151	0.268308	0.293412	0.274864	0.249121	0.248961	0.322833
16	0.310042	0.330668	0.267349	0.263831	0.306364	0.308602	0.332587	0.31228
17	0.287016	0.351455	0.32827	0.321234	0.292133	0.303006	0.229453	0.325072
18	0.242405	0.317077	0.306524	0.300608	0.321075	0.258235	0.268788	0.286856
19	0.286856	0.322354	0.244963	0.26495	0.341062	0.251039	0.318356	0.348257
20	0.337544	0.332107	0.367285	0.335945	0.337384	0.34378	0.336585	0.280141
21	0.290054	0.307323	0.34442	0.32843	0.292933	0.315638	0.248481	0.300927
22	0.284618	0.356892	0.327151	0.314679	0.240006	0.293892	0.2181	0.34426
23	0.372562	0.335305	0.397506	0.376559	0.362008	0.389191	0.373201	0.323793
24	0.329389	0.29725	0.354973	0.318516	0.29677	0.332427	0.345859	0.255836
25	0.257755	0.338983	0.267829	0.269428	0.286537	0.236329	0.245283	0.340262
26	0.303965	0.313559	0.231212	0.268948	0.346178	0.274225	0.309562	0.32747
27	0.293252	0.271506	0.217461	0.217621	0.34394	0.252478	0.300128	0.323153
28	0.268948	0.28078	0.257915	0.257755	0.298689	0.263671	0.276143	0.280141
29	0.26511	0.310042	0.329869	0.29645	0.298529	0.272945	0.251679	0.295491
30	0.398625	0.309882	0.431244	0.377359	0.319476	0.398945	0.394148	0.26575
31	0.289255	0.313399	0.235369	0.24992	0.332427	0.244803	0.303006	0.339623
32	0.26559	0.342501	0.251839	0.278702	0.358011	0.258075	0.279341	0.338823
33	0.225136	0.363447	0.290214	0.325072	0.272785	0.257915	0.181484	0.336425
34	0.322194	0.306524	0.363128	0.319955	0.308283	0.321874	0.348257	0.26559
35	0.32779	0.316917	0.322194	0.323633	0.332747	0.320755	0.345219	0.336105
36	0.302526	0.303166	0.274225	0.254237	0.316597	0.293572	0.323953	0.279341
37	0.253758	0.300608	0.313879	0.279501	0.304925	0.283339	0.290534	0.318996
38	0.298849	0.335945	0.285737	0.270387	0.317717	0.314359	0.303006	0.298209
39	0.368084	0.309082	0.397666	0.371922	0.337704	0.388871	0.381036	0.287336
40	0.367605	0.32827	0.347618	0.34314	0.365206	0.345859	0.340742	0.322833
41	0.382635	0.314679	0.409178	0.370163	0.303965	0.388232	0.379117	0.233451
42	0.316917	0.31228	0.34394	0.323313	0.314998	0.32843	0.306524	0.269587
43	0.319635	0.3118	0.362008	0.337864	0.311321	0.330349	0.333866	0.200192
44	0.298369	0.330828	0.354493	0.323313	0.292453	0.333866	0.279181	0.346978
45	0.336904	0.32795	0.3756	0.357052	0.318996	0.340902	0.3433	0.208826
46	0.279821	0.302846	0.108251	0.236808	0.352574	0.257115	0.307163	0.32859
47	0.297889	0.291493	0.226255	0.263351	0.350496	0.275344	0.322514	0.345219
48	0.307643	0.314998	0.291653	0.284778	0.310521	0.291493	0.313559	0.307963
49	0.29741	0.324272	0.349376	0.32891	0.304605	0.32795	0.2496	0.318996
50	0.26511	0.34394	0.273265	0.2819	0.289415	0.275983	0.259354	0.338983

For Review Only

51	0.298849	0.301407	0.216342	0.262072	0.352095	0.278222	0.308922	0.338983
52	0.293732	0.289735	0.286856	0.29693	0.323793	0.288935	0.291653	0.32859
53	0.21842	0.355772	0.295971	0.29693	0.241605	0.248641	0.197314	0.362168
54	0.225296	0.363128	0.290374	0.324912	0.273105	0.258075	0.181324	0.335465
55	0.292773	0.302686	0.240646	0.256796	0.357851	0.23473	0.302047	0.326511
56	0.303006	0.337064	0.329389	0.329549	0.299808	0.314039	0.206588	0.32875
57	0.285257	0.322833	0.245763	0.264631	0.340742	0.25072	0.318036	0.347618
58	0.302846	0.287016	0.23457	0.261593	0.350815	0.277582	0.332747	0.345219
59	0.291014	0.317077	0.23425	0.229453	0.341222	0.26639	0.306524	0.32763
60	0.35913	0.282539	0.404061	0.344899	0.304765	0.376399	0.362008	0.262552
61	0.290534	0.298369	0.338503	0.310681	0.309722	0.303965	0.286377	0.304125
62	0.267029	0.278222	0.243204	0.257115	0.300448	0.255996	0.273745	0.28126
63	0.29757	0.251679	0.205309	0.244803	0.354813	0.251999	0.321714	0.321234
64	0.321714	0.279341	0.215062	0.263192	0.358491	0.275024	0.345859	0.339783
65	0.217781	0.355772	0.29645	0.29645	0.240966	0.248161	0.198113	0.361689
66	0.264311	0.309402	0.32843	0.295651	0.298849	0.272146	0.251999	0.295331
67	0.253758	0.291813	0.264791	0.263831	0.310841	0.17125	0.275664	0.302047
68	0.276143	0.282699	0.338823	0.31244	0.288615	0.305085	0.293572	0.278702
69	0.314359	0.306684	0.259514	0.304125	0.332907	0.310841	0.322993	0.333547
70	0.263351	0.355293	0.241286	0.291653	0.346178	0.263991	0.285737	0.350656
71	0.26543	0.333706	0.289255	0.282379	0.278862	0.257115	0.253598	0.342661
72	0.154461	0.358331	0.292773	0.299488	0.254877	0.246882	0.15686	0.334666
73	0.298369	0.270547	0.21842	0.253118	0.330189	0.255996	0.318516	0.314679
74	0.356732	0.326671	0.360569	0.330349	0.350975	0.339143	0.354333	0.333227
75	0.263511	0.318676	0.326831	0.295971	0.285098	0.274225	0.236649	0.300288
76	0.321714	0.345059	0.284138	0.307483	0.349057	0.28174	0.320755	0.323953
77	0.335785	0.32795	0.3748	0.356412	0.318836	0.340742	0.34394	0.208347
78	0.300768	0.268308	0.214423	0.261273	0.336265	0.257115	0.299009	0.310681
79	0.314839	0.332907	0.340422	0.375919	0.32795	0.341382	0.288935	0.295331
80	0.377998	0.320595	0.399744	0.367605	0.340902	0.386952	0.383914	0.284298
81	0.303166	0.321554	0.237608	0.269587	0.35913	0.28174	0.317557	0.325072
82	0.29645	0.334666	0.282859	0.269587	0.310361	0.288295	0.286217	0.34362
83	0.29741	0.338983	0.327151	0.298049	0.238567	0.305564	0.272785	0.298369
84	0.314839	0.294691	0.317877	0.290054	0.304125	0.299328	0.3118	0.275024
85	0.388391	0.314199	0.420051	0.381836	0.340422	0.394148	0.396866	0.294532
86	0.217781	0.354813	0.29613	0.29629	0.239847	0.248961	0.196994	0.360729
87	0.229933	0.334186	0.297889	0.283179	0.29725	0.267189	0.283818	0.323633
88	0.336745	0.32747	0.3756	0.357371	0.319955	0.341062	0.342341	0.209146
89	0.325392	0.322993	0.351775	0.321075	0.313879	0.333706	0.310042	0.212504
90	0.319476	0.339623	0.366166	0.34394	0.274065	0.334346	0.261433	0.293732
91	0.324592	0.330189	0.300448	0.315798	0.334666	0.317077	0.322514	0.3118
92	0.307323	0.350016	0.355772	0.341062	0.314839	0.322833	0.324432	0.235689
93	0.254077	0.35945	0.285897	0.304765	0.247681	0.278542	0.194755	0.348577
94	0.309082	0.315958	0.216182	0.277263	0.331308	0.28126	0.31308	0.35881
95	0.291973	0.324432	0.303166	0.29725	0.349217	0.308762	0.309242	0.295651
96	0.298529	0.252638	0.205948	0.245283	0.354493	0.252798	0.322194	0.321075
97	0.270547	0.339463	0.294691	0.303166	0.305085	0.285257	0.257275	0.335625
98	0.284138	0.247841	0.258874	0.247681	0.3433	0.255197	0.293252	0.338503
99	0.338823	0.298049	0.365526	0.332107	0.321394	0.349217	0.360409	0.274225
100	0.298049	0.32779	0.256956	0.256156	0.333547	0.279981	0.304125	0.309882
101	0.26639	0.319955	0.248641	0.256636	0.316597	0.275823	0.291813	0.326511
102	0.302047	0.350975	0.318996	0.322833	0.337224	0.307803	0.313559	0.331468
103	0.28174	0.246402	0.236009	0.251999	0.341222	0.23473	0.298529	0.31308
104	0.377199	0.320115	0.40566	0.374001	0.346338	0.396546	0.39127	0.294212
105	0.34394	0.29661	0.383115	0.324752	0.32843	0.35961	0.354653	0.278862

106	0.295811	0.306684	0.298369	0.292613	0.31276	0.310042	0.321394	0.291493
107	0.278542	0.345699	0.299808	0.324272	0.310521	0.291014	0.289575	0.308443
108	0.31228	0.288136	0.289095	0.287656	0.3433	0.29661	0.32763	0.322354
109	0.330029	0.300288	0.32779	0.29693	0.336105	0.310841	0.317717	0.287016
110	0.305884	0.299328	0.254077	0.259994	0.322354	0.275664	0.300288	0.300768
111	0.330349	0.299328	0.360889	0.330508	0.324272	0.330508	0.349856	0.291014
112	0.294212	0.247202	0.205309	0.240006	0.351295	0.255996	0.308123	0.337384
113	0.299009	0.3126	0.229613	0.261273	0.325392	0.271826	0.29757	0.3118
114	0.282859	0.302367	0.20275	0.227694	0.327151	0.255516	0.291334	0.31196
115	0.286217	0.309242	0.266709	0.268148	0.306044	0.244484	0.301567	0.29757
116	0.314679	0.304765	0.291334	0.29709	0.324912	0.307323	0.326031	0.299808
117	0.317557	0.304445	0.29757	0.288136	0.326671	0.316278	0.321394	0.277263
118	0.212824	0.329389	0.322833	0.310521	0.292293	0.251039	0.246242	0.330349
119	0.272945	0.286377	0.26559	0.264311	0.303486	0.267189	0.276463	0.299169
120	0.301887	0.324272	0.306364	0.31276	0.335146	0.299009	0.307483	0.291973
121	0.288775	0.273585	0.225136	0.255037	0.331628	0.241286	0.283978	0.31244
122	0.3126	0.28062	0.287816	0.283339	0.324592	0.290694	0.305724	0.306524
123	0.2819	0.291334	0.259834	0.270387	0.314359	0.283179	0.301567	0.29757
124	0.301087	0.339303	0.293252	0.319316	0.369044	0.291334	0.300288	0.349536
125	0.274225	0.290214	0.228174	0.255676	0.307004	0.261273	0.276303	0.294372
126	0.334986	0.305564	0.368244	0.32859	0.319156	0.337864	0.361529	0.288295
127	0.299968	0.309082	0.3118	0.321075	0.332107	0.295491	0.298209	0.310361
128	0.273265	0.285098	0.255996	0.258874	0.301087	0.260473	0.279661	0.285737
129	0.279981	0.330508	0.276463	0.269587	0.295331	0.269747	0.267988	0.329229
130	0.284938	0.280141	0.170131	0.252958	0.347298	0.2504	0.303166	0.319476
131	0.387912	0.316118	0.405181	0.367605	0.344579	0.386633	0.389351	0.282699
132	0.32779	0.314039	0.288935	0.289894	0.346178	0.31212	0.32779	0.331948
133	0.331788	0.333067	0.342981	0.321075	0.333547	0.330988	0.316118	0.318356
134	0.276943	0.345379	0.293732	0.307004	0.31292	0.285737	0.280461	0.326351
135	0.253758	0.302686	0.295651	0.283978	0.300768	0.275823	0.278702	0.286377
136	0.292293	0.319795	0.223697	0.222897	0.347298	0.251039	0.303166	0.346178
137	0.29709	0.333227	0.304605	0.293892	0.348097	0.276463	0.306524	0.34442
138	0.364087	0.314039	0.395587	0.35945	0.330988	0.37432	0.389351	0.291653
139	0.277422	0.289575	0.272466	0.272466	0.291493	0.278222	0.286697	0.286057
140	0.253438	0.360889	0.285257	0.306524	0.245923	0.280461	0.192517	0.348577
141	0.271186	0.306204	0.257595	0.262552	0.315478	0.270707	0.285577	0.310521
142	0.321075	0.307323	0.361848	0.319955	0.308283	0.320915	0.347618	0.26623
143	0.295651	0.319476	0.333547	0.309722	0.295491	0.304765	0.304285	0.303486
144	0.349376	0.301087	0.378478	0.346338	0.317557	0.356412	0.365206	0.279661
145	0.269428	0.28142	0.233451	0.260633	0.334186	0.240326	0.28142	0.305405
146	0.318836	0.321874	0.31196	0.314199	0.342661	0.318676	0.336904	0.329869
147	0.37576	0.329069	0.402462	0.365046	0.338503	0.388232	0.389511	0.307963
148	0.330828	0.34394	0.335945	0.318516	0.360729	0.320435	0.327151	0.330508
149	0.351935	0.302207	0.376079	0.339942	0.318676	0.350496	0.364087	0.275024
150	0.287496	0.331148	0.263671	0.305245	0.339303	0.285257	0.309882	0.339143
151	0.35977	0.301567	0.382635	0.346338	0.301887	0.363927	0.358491	0.247522
152	0.303965	0.329069	0.288615	0.289095	0.331308	0.293252	0.290214	0.310681
153	0.266709	0.309562	0.287496	0.286697	0.287176	0.258075	0.261753	0.300927
154	0.291493	0.315798	0.223057	0.251039	0.3433	0.276943	0.307643	0.341222
155	0.299328	0.31324	0.219859	0.261273	0.352255	0.275184	0.323633	0.322034
156	0.302686	0.294372	0.331148	0.279341	0.303006	0.308602	0.308123	0.291813
157	0.307643	0.314039	0.213463	0.259034	0.355293	0.267988	0.303486	0.339783
158	0.291014	0.315958	0.252798	0.263351	0.329549	0.28078	0.311001	0.331308
159	0.324912	0.325232	0.37576	0.337384	0.307643	0.357052	0.338024	0.295331
160	0.270387	0.314359	0.270867	0.271986	0.311641	0.263511	0.310681	0.300288

For Review Only

161	0.313879	0.29693	0.227374	0.269907	0.362648	0.277103	0.315798	0.339303
162	0.307163	0.286697	0.331788	0.294212	0.290534	0.314359	0.31292	0.282699
163	0.290214	0.32763	0.236808	0.276303	0.330189	0.271506	0.289894	0.341861
164	0.342661	0.334666	0.370323	0.349856	0.350656	0.358331	0.356892	0.309242
165	0.334826	0.333387	0.338983	0.32891	0.303646	0.336265	0.327151	0.310841
166	0.325232	0.3433	0.340742	0.324752	0.316757	0.332907	0.333706	0.339463
167	0.264791	0.244803	0.252798	0.263991	0.337064	0.257915	0.276303	0.321714
168	0.300128	0.319635	0.333866	0.272146	0.304445	0.306364	0.317237	0.314998
169	0.346178	0.32779	0.362808	0.341861	0.329389	0.353694	0.344899	0.302367
170	0.26607	0.279341	0.253598	0.262392	0.293732	0.26575	0.275344	0.280461
171	0.346019	0.318676	0.349536	0.336585	0.324272	0.337224	0.365206	0.31276
172	0.28142	0.332587	0.28062	0.288136	0.308762	0.273905	0.276943	0.356412
173	0.285737	0.301407	0.213623	0.240646	0.335305	0.263671	0.298529	0.319476
174	0.320915	0.309242	0.340422	0.319316	0.308922	0.317877	0.320435	0.283978
175	0.279181	0.299968	0.229933	0.220019	0.32875	0.243044	0.289255	0.338503
176	0.287976	0.306524	0.314199	0.29693	0.302526	0.286856	0.298529	0.288615
177	0.345699	0.29629	0.365366	0.330988	0.322354	0.350656	0.362008	0.276303
178	0.354653	0.32747	0.366006	0.354973	0.354973	0.360569	0.362968	0.335785
179	0.280301	0.299808	0.2811	0.272306	0.300288	0.272146	0.300608	0.290854
180	0.298849	0.246562	0.243844	0.260793	0.356092	0.268468	0.310521	0.326831
181	0.302047	0.285098	0.244004	0.251359	0.32827	0.271027	0.29677	0.31196
182	0.28078	0.297889	0.204029	0.228814	0.321554	0.257115	0.288775	0.304125
183	0.259834	0.277902	0.254077	0.252319	0.295331	0.254557	0.269268	0.28094
184	0.353854	0.298529	0.377518	0.349057	0.331148	0.35993	0.360249	0.274704
185	0.357052	0.330828	0.374161	0.340742	0.355453	0.351295	0.358331	0.336585
186	0.322194	0.279981	0.215862	0.263351	0.35945	0.275664	0.345539	0.339783
187	0.318996	0.31196	0.347618	0.329389	0.323793	0.305085	0.259834	0.299328
188	0.32811	0.307963	0.363128	0.31196	0.304765	0.323953	0.352414	0.264311
189	0.284938	0.304445	0.226575	0.213303	0.333227	0.245923	0.299968	0.315478
190	0.287016	0.314679	0.308922	0.300927	0.338983	0.284458	0.301087	0.31244
191	0.272306	0.319955	0.29661	0.292613	0.321874	0.287176	0.290054	0.314039
192	0.256316	0.342501	0.267669	0.291174	0.28126	0.251839	0.220019	0.337544
193	0.271027	0.323313	0.257915	0.272466	0.322354	0.268628	0.268788	0.307323
194	0.284778	0.361848	0.271986	0.287816	0.323793	0.28094	0.288775	0.354493
195	0.257755	0.336105	0.292613	0.290214	0.311321	0.29677	0.263032	0.321394
196	0.340422	0.299648	0.367605	0.332107	0.323633	0.351775	0.365366	0.279341
197	0.271986	0.349536	0.304445	0.283339	0.315318	0.293092	0.266549	0.341701
198	0.320915	0.279341	0.215542	0.263192	0.35881	0.274384	0.34442	0.339303
199	0.307483	0.305564	0.257755	0.268948	0.34426	0.268788	0.292773	0.336904
200	0.18804	0.299328	0.285737	0.271027	0.311161	0.248641	0.267669	0.319955
201	0.299648	0.315318	0.213463	0.262392	0.356252	0.278222	0.326511	0.321874
202	0.290214	0.308123	0.248161	0.267029	0.342821	0.276783	0.302367	0.307004
203	0.28062	0.31228	0.008634	0.238567	0.356252	0.261113	0.314839	0.339942
204	0.293732	0.320275	0.228174	0.255197	0.3441	0.280461	0.305564	0.337864
205	0.379437	0.309402	0.402942	0.376079	0.345859	0.39111	0.395107	0.285417
206	0.293252	0.32795	0.270547	0.290054	0.32875	0.293892	0.310201	0.337704
207	0.307323	0.326671	0.284618	0.285737	0.32859	0.326031	0.32859	0.326031
208	0.298849	0.310681	0.290854	0.266549	0.330668	0.294532	0.317877	0.320435
209	0.353694	0.327151	0.364567	0.354493	0.354013	0.35881	0.361689	0.335785
210	0.352574	0.331148	0.372562	0.353374	0.308283	0.375919	0.354333	0.286537
211	0.322034	0.292933	0.367605	0.309242	0.293572	0.329389	0.336105	0.262712
212	0.307004	0.307163	0.295171	0.248481	0.321714	0.308443	0.272945	0.298689
213	0.305724	0.323953	0.255836	0.267988	0.349217	0.270227	0.295171	0.339623
214	0.32811	0.307323	0.381036	0.330828	0.307163	0.349217	0.35993	0.288455
215	0.365046	0.320275	0.387112	0.352095	0.346019	0.362008	0.360889	0.325232

For Review Only

216	0.29741	0.251199	0.204509	0.244803	0.353694	0.251359	0.321714	0.320435
217	0.378638	0.322833	0.40614	0.37464	0.353534	0.396226	0.386792	0.293572
218	0.293732	0.310681	0.288615	0.300288	0.307163	0.295811	0.315478	0.302207
219	0.318836	0.305245	0.339463	0.315638	0.321234	0.325392	0.329709	0.310201
220	0.292453	0.337064	0.17141	0.26623	0.345059	0.269268	0.301407	0.348257
221	0.340102	0.322833	0.354173	0.339303	0.364087	0.341701	0.339143	0.336585
222	0.278382	0.3118	0.340262	0.313719	0.310841	0.31308	0.301567	0.240166
223	0.26495	0.334506	0.257275	0.256636	0.333227	0.252319	0.278062	0.330828
224	0.347458	0.347298	0.355453	0.334986	0.350336	0.336745	0.338503	0.323633
225	0.387752	0.297729	0.400224	0.364087	0.317397	0.383115	0.385513	0.222098
226	0.319156	0.309242	0.32763	0.298049	0.317237	0.317237	0.326351	0.274065
227	0.379917	0.321874	0.414295	0.379277	0.338823	0.393668	0.394627	0.299328
228	0.267509	0.279661	0.261113	0.256796	0.288136	0.256476	0.272146	0.283818
229	0.306364	0.321874	0.31292	0.303166	0.334346	0.301247	0.297729	0.318196
230	0.306684	0.345539	0.302846	0.300608	0.330029	0.305405	0.308283	0.331308
231	0.293732	0.292773	0.241765	0.259994	0.323473	0.268468	0.287656	0.310042
232	0.289095	0.314359	0.098337	0.268308	0.35929	0.271666	0.315158	0.336105
233	0.314519	0.347618	0.337224	0.320595	0.309722	0.338983	0.325232	0.339783
234	0.335785	0.321075	0.35929	0.341062	0.34362	0.356572	0.355133	0.294052
235	0.323313	0.308602	0.360729	0.345859	0.350656	0.3118	0.32795	0.304925
236	0.32875	0.305085	0.377838	0.340102	0.314199	0.341701	0.347937	0.311321
237	0.31228	0.333067	0.294052	0.276783	0.320915	0.313399	0.31324	0.318516
238	0.32779	0.326351	0.317877	0.315478	0.347298	0.308762	0.337384	0.322674
239	0.29741	0.31212	0.23505	0.258395	0.346019	0.268148	0.306844	0.323953
240	0.241765	0.322674	0.283019	0.276783	0.29677	0.260793	0.254397	0.329389
241	0.310841	0.336105	0.331948	0.332587	0.334826	0.322514	0.317877	0.339623
242	0.35897	0.28158	0.403422	0.34426	0.303965	0.3756	0.361848	0.262392
243	0.270387	0.307803	0.271186	0.282859	0.333866	0.269907	0.303326	0.316597
244	0.388871	0.318036	0.417973	0.382795	0.341861	0.395907	0.404061	0.294212
245	0.290534	0.287816	0.195235	0.15558	0.355772	0.240966	0.308123	0.32811
246	0.242725	0.336745	0.315318	0.293252	0.26511	0.278382	0.259354	0.310201
247	0.298369	0.326191	0.246083	0.269907	0.333866	0.255516	0.288935	0.345859
248	0.369044	0.321554	0.388391	0.371442	0.357531	0.376559	0.362648	0.308123
249	0.319795	0.309242	0.257755	0.279661	0.32779	0.291493	0.332267	0.31244
250	0.311481	0.292133	0.341222	0.310201	0.320275	0.306204	0.334826	0.271506
251	0.29725	0.316437	0.253758	0.275664	0.34426	0.2819	0.313719	0.308283
252	0.347937	0.337704	0.369843	0.334506	0.341861	0.338663	0.34362	0.339303
253	0.386313	0.323313	0.416853	0.379597	0.346978	0.39095	0.395267	0.301407
254	0.248321	0.32843	0.236968	0.272785	0.310201	0.248001	0.258075	0.334506
255	0.322194	0.280301	0.216821	0.264471	0.35945	0.275823	0.345539	0.341541
256	0.301727	0.301727	0.274225	0.252159	0.316118	0.291653	0.322034	0.278222
257	0.377518	0.317877	0.404861	0.37496	0.349057	0.400224	0.39111	0.287976
258	0.261593	0.337864	0.28094	0.288935	0.329229	0.274384	0.275504	0.345859
259	0.274225	0.300288	0.284138	0.273745	0.302207	0.270707	0.288295	0.289735
260	0.337224	0.335625	0.34346	0.336105	0.340742	0.337704	0.322354	0.326511
261	0.262712	0.364087	0.286537	0.294052	0.226255	0.269747	0.184682	0.357691
262	0.297889	0.301887	0.208986	0.254717	0.345859	0.28126	0.333067	0.320435
263	0.313399	0.314519	0.295651	0.31228	0.353374	0.304125	0.294691	0.321075
264	0.320115	0.350016	0.290694	0.294372	0.353374	0.321394	0.353694	0.338343
265	0.29693	0.317557	0.289735	0.306684	0.326351	0.29741	0.29757	0.293092
266	0.319955	0.29693	0.260633	0.306684	0.326991	0.304125	0.335785	0.294372
267	0.379917	0.322514	0.416214	0.379597	0.342661	0.386952	0.399424	0.295011
268	0.26623	0.357531	0.278062	0.276783	0.324912	0.268308	0.26607	0.351775
269	0.376079	0.319316	0.408698	0.371602	0.342661	0.395907	0.388551	0.292453
270	0.28078	0.31212	0.006876	0.240486	0.355612	0.261113	0.314839	0.340742

For Review Only

271	0.300448	0.316757	0.222737	0.219699	0.373041	0.25024	0.303326	0.342661
272	0.357531	0.311161	0.348417	0.330828	0.354813	0.354013	0.355612	0.315638
273	0.277422	0.357851	0.289735	0.291973	0.290214	0.291653	0.270547	0.352734
274	0.308602	0.282379	0.21874	0.258555	0.354973	0.258714	0.316597	0.334986

For Review Only

0.223377	0.352255	0.340902	0.282699	0.251359	0.286217	0.237448	0.310042	0.287016	0.242405
0.34378	0.311481	0.335146	0.299488	0.314519	0.315798	0.327151	0.330668	0.351455	0.317077
0.319955	0.372721	0.350975	0.070195	0.314839	0.32827	0.268308	0.267349	0.32827	0.306524
0.308123	0.3441	0.338823	0.214743	0.305564	0.308443	0.293412	0.263831	0.321234	0.300608
0.254717	0.303326	0.331788	0.350975	0.292293	0.308283	0.274864	0.306364	0.292133	0.321075
0.255516	0.358171	0.345539	0.255836	0.273585	0.322194	0.249121	0.308602	0.303006	0.258235
0.185162	0.342981	0.340582	0.309082	0.2496	0.322993	0.248961	0.332587	0.229453	0.268788
0.330349	0.205788	0.334826	0.331148	0.295971	0.284778	0.322833	0.31228	0.325072	0.286856
0	0.347298	0.330189	0.3118	0.259034	0.290374	0.221458	0.321075	0.231212	0.295491
0.347298	0	0.370003	0.361049	0.309882	0.306524	0.353854	0.322833	0.340102	0.300927
0.330189	0.370003	0	0.348097	0.338343	0.29661	0.323793	0.301247	0.345859	0.325392
0.3118	0.361049	0.348097	0	0.310681	0.322833	0.26559	0.267669	0.325871	0.299488
0.259034	0.309882	0.338343	0.310681	0	0.307483	0.254237	0.325552	0.273425	0.268628
0.290374	0.306524	0.29661	0.322833	0.307483	0	0.298209	0.287656	0.325072	0.301887
0.221458	0.353854	0.323793	0.26559	0.254237	0.298209	0	0.305884	0.256316	0.284298
0.321075	0.322833	0.301247	0.267669	0.325552	0.287656	0.305884	0	0.337224	0.321234
0.231212	0.340102	0.345859	0.325871	0.273425	0.325072	0.256316	0.337224	0	0.326351
0.295491	0.300927	0.325392	0.299488	0.268628	0.301887	0.284298	0.321234	0.326351	0
0.31228	0.378158	0.347138	0.23393	0.318996	0.337064	0.275664	0.307963	0.324912	0.32779
0.349217	0.271506	0.354653	0.361529	0.326671	0.322514	0.333547	0.339623	0.329389	0.295491
0.280461	0.306204	0.314998	0.340422	0.254397	0.315798	0.286856	0.353054	0.247522	0.287176
0.232651	0.334506	0.338343	0.320915	0.232491	0.325392	0.24944	0.330029	0.194595	0.310681
0.364567	0.339942	0.272306	0.389351	0.347458	0.294691	0.356572	0.355293	0.381836	0.35945
0.314839	0.271027	0.29661	0.345699	0.298369	0.239367	0.314199	0.277422	0.342181	0.31228
0.24944	0.364407	0.338663	0.268308	0.263032	0.311481	0.243844	0.287496	0.262072	0.271506
0.318996	0.368724	0.354333	0.226895	0.318356	0.313719	0.288615	0.28078	0.349856	0.321234
0.293732	0.353374	0.349696	0.20339	0.294052	0.307323	0.271346	0.282219	0.333866	0.291813
0.26575	0.308762	0.290854	0.251839	0.274065	0.262232	0.248161	0.267988	0.282539	0.275024
0.17125	0.284138	0.329549	0.315958	0.270387	0.315638	0.267189	0.304125	0.273905	0.273585
0.381196	0.228654	0.336265	0.419252	0.314199	0.303806	0.378798	0.356892	0.367445	0.351295
0.302686	0.360569	0.339942	0.227374	0.294851	0.321554	0.262872	0.28094	0.321714	0.302047
0.29725	0.356892	0.350336	0.252638	0.318356	0.333227	0.282379	0.306044	0.310042	0.287016
0.204509	0.37528	0.335625	0.293572	0.259674	0.322034	0.256796	0.333387	0.247841	0.285257
0.314839	0.272146	0.305245	0.354173	0.295491	0.208187	0.321234	0.291493	0.334186	0.308762
0.318356	0.354653	0.29661	0.318036	0.329709	0.307323	0.306524	0.317717	0.369044	0.326831
0.304445	0.299808	0.304765	0.26591	0.308922	0.247841	0.301727	0.231852	0.352574	0.298849
0.283019	0.325712	0.320275	0.316437	0.29613	0.263831	0.284298	0.307323	0.308123	0.301887
0.316118	0.322674	0.299169	0.288775	0.31292	0.291334	0.29741	0.291014	0.327151	0.295011
0.362968	0.287016	0.246242	0.394308	0.319635	0.272306	0.353214	0.299488	0.373681	0.350016
0.348577	0.356252	0.277582	0.339783	0.346338	0.320915	0.321394	0.314679	0.348417	0.332587
0.377678	0.209786	0.32779	0.402303	0.303166	0.295011	0.374001	0.351775	0.366805	0.336585
0.270867	0.251519	0.322993	0.339463	0.292133	0.303806	0.321234	0.324432	0.318676	0.285897
0.324592	0.175088	0.352574	0.348257	0.29693	0.294851	0.335146	0.326511	0.350176	0.28126
0.283499	0.346658	0.319156	0.354013	0.291174	0.299009	0.294372	0.349057	0.269587	0.310521
0.332587	0.182123	0.352574	0.35977	0.330988	0.29757	0.346658	0.340422	0.355772	0.29629
0.317077	0.367925	0.34378	0.129677	0.302846	0.320915	0.264631	0.252478	0.324592	0.290214
0.319476	0.372881	0.348577	0.224336	0.320595	0.314998	0.273905	0.291973	0.327151	0.293252
0.321394	0.349696	0.28094	0.288136	0.307163	0.278702	0.291334	0.294052	0.334666	0.304765
0.251039	0.324752	0.339942	0.342021	0.268468	0.29709	0.290694	0.34314	0.255836	0.313399
0.264791	0.363607	0.332747	0.269108	0.288615	0.327151	0.240166	0.303486	0.267189	0.300608

For Review Only

0.310841	0.362808	0.350176	0.216342	0.316757	0.306684	0.26639	0.290374	0.32763	0.310521
0.294372	0.35929	0.34314	0.277263	0.302846	0.329549	0.294212	0.306364	0.326031	0.323153
0.17173	0.385673	0.344899	0.293892	0.232491	0.321714	0.206908	0.32779	0.170931	0.278062
0.205149	0.37496	0.335465	0.293572	0.258235	0.321075	0.256156	0.333067	0.247362	0.283658
0.292293	0.358011	0.345859	0.230093	0.311321	0.309082	0.264631	0.288295	0.326031	0.278382
0.24944	0.332587	0.338983	0.321554	0.259674	0.305884	0.261113	0.340902	0.180684	0.302367
0.31228	0.377199	0.346498	0.23409	0.318356	0.336105	0.275024	0.307643	0.325072	0.327151
0.318356	0.383275	0.354973	0.221458	0.32811	0.318996	0.276623	0.302526	0.347937	0.309722
0.306844	0.360889	0.341541	0.208507	0.315318	0.309242	0.274864	0.2819	0.339942	0.291174
0.361369	0.251519	0.295971	0.394468	0.314039	0.269907	0.35993	0.321874	0.347777	0.332747
0.277103	0.31244	0.317237	0.332907	0.288295	0.278862	0.288615	0.318836	0.326031	0.293412
0.262552	0.318836	0.290694	0.233131	0.272306	0.270867	0.246402	0.251199	0.285737	0.272785
0.32811	0.37576	0.34362	0.190278	0.320435	0.313559	0.267829	0.285098	0.338663	0.302207
0.32811	0.376239	0.356572	0.205948	0.338823	0.313879	0.282379	0.290534	0.35865	0.319955
0.17157	0.387112	0.34426	0.293572	0.232012	0.321394	0.206428	0.32795	0.170771	0.277422
0.17189	0.283179	0.329229	0.314679	0.270547	0.315478	0.266869	0.304125	0.273265	0.273425
0.267029	0.330828	0.309402	0.261912	0.275024	0.287336	0.248321	0.287176	0.301247	0.263831
0.299009	0.283818	0.313399	0.334346	0.295971	0.274225	0.304925	0.329549	0.320275	0.287176
0.306044	0.35897	0.277582	0.268948	0.321075	0.292453	0.287336	0.315158	0.32875	0.31292
0.285257	0.380876	0.355612	0.25056	0.307323	0.338663	0.236169	0.304765	0.308922	0.283339
0.224177	0.376559	0.332587	0.271666	0.290054	0.332267	0.228814	0.314359	0.23409	0.305405
0.157179	0.371922	0.351775	0.294532	0.252159	0.318356	0.220499	0.324113	0.198273	0.289735
0.303326	0.349057	0.331788	0.211065	0.308283	0.291973	0.259354	0.274704	0.333067	0.295171
0.327151	0.334506	0.274704	0.35913	0.334346	0.316118	0.323953	0.321234	0.352414	0.346978
0.168532	0.313399	0.311161	0.315318	0.26559	0.31276	0.25072	0.310841	0.26543	0.302207
0.316757	0.32795	0.34362	0.28094	0.321075	0.304285	0.304605	0.307803	0.329869	0.304605
0.332267	0.182603	0.351775	0.35913	0.330828	0.29645	0.346019	0.339942	0.354973	0.29677
0.304285	0.349536	0.351935	0.210745	0.302846	0.316917	0.282539	0.274225	0.344579	0.305245
0.309242	0.319795	0.353534	0.340102	0.302207	0.319955	0.322514	0.368404	0.294212	0.331468
0.370483	0.283818	0.274864	0.391589	0.322674	0.273425	0.361209	0.315478	0.373361	0.348097
0.32795	0.366326	0.345059	0.237768	0.322833	0.308123	0.292613	0.294212	0.352734	0.305245
0.282539	0.382315	0.318356	0.272785	0.303646	0.329069	0.264471	0.273905	0.29613	0.311161
0.268628	0.309562	0.326991	0.324432	0.28126	0.305245	0.266709	0.29757	0.270387	0.282379
0.306204	0.28174	0.320115	0.306364	0.299648	0.255836	0.290534	0.267029	0.315958	0.305085
0.383435	0.301887	0.28158	0.412056	0.32779	0.278062	0.365526	0.327151	0.386313	0.35897
0.169971	0.385353	0.342181	0.293572	0.231052	0.320755	0.205788	0.32747	0.169971	0.276623
0.280301	0.338663	0.310361	0.301887	0.277422	0.285577	0.278702	0.291334	0.324592	0.23457
0.332427	0.182123	0.352894	0.36009	0.331308	0.29741	0.346498	0.340582	0.355293	0.29741
0.311481	0.236169	0.322674	0.342821	0.305884	0.287336	0.320915	0.32843	0.326031	0.280141
0.297889	0.29725	0.3441	0.357371	0.255676	0.341382	0.31244	0.356092	0.272785	0.322194
0.334186	0.317077	0.299808	0.29709	0.304445	0.29741	0.321234	0.329549	0.331308	0.32779
0.334026	0.240646	0.339143	0.348897	0.317077	0.28126	0.327151	0.337224	0.356732	0.304925
0.190438	0.391429	0.332907	0.288775	0.260313	0.320275	0.211705	0.314039	0.181804	0.299808
0.295491	0.393828	0.350975	0.225296	0.320915	0.350016	0.274864	0.290854	0.319156	0.338823
0.311641	0.314998	0.379597	0.290374	0.304125	0.306044	0.263831	0.335305	0.321234	0.326671
0.329069	0.37544	0.345219	0.191238	0.322034	0.31292	0.269428	0.287336	0.340422	0.303006
0.267669	0.364407	0.295171	0.299968	0.282539	0.32763	0.276783	0.315638	0.31212	0.316278
0.285257	0.372082	0.340902	0.247362	0.317717	0.321554	0.26511	0.304605	0.31196	0.297729
0.336265	0.278542	0.310681	0.361209	0.302207	0.18804	0.333227	0.301087	0.349057	0.338983
0.303006	0.337704	0.340422	0.244164	0.302207	0.276463	0.277103	0.303006	0.336904	0.297729
0.303326	0.353214	0.326191	0.246083	0.293892	0.272146	0.263032	0.268468	0.331628	0.282699
0.289255	0.355133	0.354653	0.315638	0.345219	0.306684	0.271027	0.32859	0.354013	0.332267
0.29613	0.339303	0.335785	0.222737	0.307323	0.297889	0.253438	0.287656	0.314998	0.298209
0.375919	0.299648	0.21874	0.400863	0.32811	0.287976	0.367765	0.310201	0.378318	0.352255
0.351935	0.273425	0.303486	0.373041	0.326831	0.292773	0.352414	0.322514	0.335465	0.345219

For Review Only

0.29661	0.308443	0.315478	0.290054	0.295491	0.292773	0.286217	0.29725	0.325232	0.31308
0.278702	0.350176	0.354493	0.298209	0.323633	0.29709	0.240006	0.331148	0.334186	0.29629
0.320595	0.373041	0.311161	0.278702	0.333067	0.303646	0.291493	0.306044	0.358171	0.31276
0.327311	0.320435	0.315958	0.317717	0.309882	0.303486	0.318196	0.32779	0.349057	0.315158
0.292133	0.334666	0.335305	0.245923	0.288136	0.291653	0.267189	0.272466	0.336425	0.298049
0.337064	0.294532	0.3126	0.356732	0.295811	0.198913	0.32811	0.299648	0.340102	0.316437
0.310841	0.363927	0.355293	0.195555	0.316757	0.317877	0.278062	0.291813	0.335625	0.304765
0.302686	0.341222	0.345539	0.227534	0.305085	0.307004	0.267669	0.276783	0.335465	0.303326
0.283658	0.345379	0.32811	0.190278	0.310841	0.306364	0.26543	0.135433	0.291813	0.304765
0.298689	0.323473	0.295491	0.26543	0.294372	0.262552	0.26543	0.26575	0.320435	0.288136
0.331468	0.304285	0.32875	0.292453	0.298849	0.279981	0.307483	0.295171	0.351295	0.305884
0.318196	0.295171	0.308602	0.291014	0.299968	0.279341	0.305245	0.280301	0.329069	0.299968
0.251039	0.357211	0.318356	0.320435	0.261113	0.259994	0.266549	0.331628	0.309402	0.244803
0.274225	0.320915	0.297729	0.259354	0.272306	0.276623	0.255676	0.279501	0.292133	0.276463
0.31292	0.29645	0.365366	0.290534	0.310201	0.305245	0.271186	0.336105	0.334506	0.313559
0.288615	0.347138	0.333387	0.217141	0.293732	0.319795	0.257915	0.284298	0.322993	0.295331
0.323473	0.324752	0.339942	0.284138	0.304925	0.29645	0.298209	0.295971	0.325712	0.302047
0.299169	0.332587	0.310042	0.253438	0.289255	0.277582	0.261912	0.277103	0.314519	0.285897
0.299169	0.381036	0.335465	0.290854	0.323633	0.335146	0.254877	0.345859	0.317717	0.310521
0.276303	0.327151	0.293892	0.222258	0.279981	0.272785	0.240966	0.259674	0.298369	0.28078
0.347458	0.294691	0.314679	0.360249	0.299488	0.179725	0.336585	0.300448	0.346978	0.318676
0.285257	0.339303	0.311321	0.303166	0.318356	0.318996	0.26639	0.331308	0.284138	0.320435
0.271506	0.308123	0.294691	0.248481	0.278062	0.269907	0.251519	0.270387	0.292133	0.280301
0.26591	0.372562	0.315318	0.264151	0.293892	0.320755	0.25008	0.259674	0.273265	0.298689
0.299808	0.348417	0.353694	0.192996	0.295971	0.319476	0.26591	0.276463	0.310841	0.293092
0.370643	0.294372	0.28174	0.401343	0.327311	0.267829	0.363927	0.325552	0.379437	0.356892
0.322514	0.350975	0.315158	0.286697	0.332907	0.31276	0.302846	0.299488	0.349376	0.329869
0.29645	0.32827	0.269907	0.339942	0.321714	0.303806	0.290534	0.316917	0.337704	0.310841
0.267988	0.353694	0.336105	0.287816	0.293572	0.333227	0.254237	0.291813	0.294851	0.308922
0.246562	0.302047	0.337224	0.292933	0.287336	0.277103	0.260953	0.29613	0.277263	0.29741
0.303486	0.374161	0.363447	0.195395	0.318516	0.335785	0.26607	0.299808	0.314519	0.301567
0.293732	0.354653	0.336904	0.290854	0.31292	0.329549	0.274065	0.318356	0.307323	0.298209
0.361848	0.287816	0.298049	0.388232	0.320115	0.245283	0.352894	0.31228	0.378318	0.347937
0.275504	0.298849	0.29693	0.26623	0.280461	0.258075	0.26511	0.280141	0.294532	0.283339
0.18724	0.392869	0.333387	0.288615	0.258235	0.321874	0.209786	0.313719	0.179405	0.301887
0.277742	0.337704	0.314998	0.254877	0.278862	0.282219	0.246882	0.267509	0.299009	0.274864
0.313879	0.273265	0.304925	0.352414	0.294851	0.209306	0.319476	0.291493	0.334346	0.307803
0.287336	0.32779	0.333706	0.325712	0.283339	0.283978	0.272306	0.311321	0.3126	0.303965
0.348097	0.285897	0.314998	0.373361	0.303646	0.200192	0.339303	0.310681	0.360249	0.342181
0.289415	0.350975	0.330349	0.221938	0.300768	0.292293	0.241765	0.287976	0.319635	0.29693
0.326831	0.362168	0.26495	0.308443	0.317717	0.292933	0.294691	0.307803	0.348417	0.315318
0.363128	0.331468	0.186281	0.396226	0.342821	0.292773	0.35865	0.311001	0.369364	0.367605
0.308283	0.352894	0.290854	0.32747	0.327311	0.311161	0.306684	0.32763	0.34362	0.3433
0.34378	0.277902	0.31324	0.370803	0.305405	0.191717	0.333547	0.304445	0.349376	0.342021
0.285737	0.373681	0.347777	0.26559	0.31276	0.29677	0.238088	0.318516	0.341861	0.308443
0.351935	0.236009	0.320275	0.37464	0.298849	0.286856	0.348897	0.326991	0.346498	0.316118
0.303486	0.334186	0.324912	0.289894	0.302207	0.272306	0.28142	0.301727	0.333067	0.317557
0.248801	0.338823	0.346498	0.28062	0.276623	0.29677	0.247522	0.307323	0.284298	0.320275
0.322514	0.369683	0.349536	0.217781	0.315478	0.309402	0.273585	0.28174	0.340582	0.298849
0.322194	0.355293	0.353054	0.21826	0.324432	0.318516	0.279181	0.278382	0.345539	0.305245
0.300128	0.299328	0.299488	0.326671	0.278862	0.255037	0.306524	0.29661	0.331948	0.307963
0.31196	0.369524	0.353054	0.211545	0.303965	0.31228	0.271666	0.283658	0.319955	0.31292
0.314839	0.35993	0.345859	0.244643	0.317077	0.290534	0.280461	0.278542	0.355612	0.298529
0.330828	0.298689	0.245283	0.369843	0.317557	0.280461	0.337544	0.309402	0.336425	0.300288
0.291973	0.334666	0.278542	0.275983	0.292933	0.270227	0.266869	0.272785	0.313719	0.287976

For Review Only

0.323633	0.3748	0.35913	0.223377	0.331148	0.310841	0.283019	0.299169	0.349696	0.320275
0.308443	0.289735	0.278222	0.319795	0.282859	0.26639	0.299968	0.292293	0.315958	0.305245
0.29613	0.363128	0.335625	0.23377	0.306364	0.323473	0.244164	0.304925	0.307803	0.310042
0.345699	0.316118	0.287656	0.364247	0.336265	0.290214	0.324592	0.351935	0.353374	0.332587
0.337384	0.294691	0.352414	0.337704	0.325712	0.323313	0.32795	0.32827	0.341222	0.305085
0.3118	0.339783	0.288136	0.340102	0.311641	0.317397	0.307803	0.304765	0.333547	0.325072
0.299169	0.358171	0.349696	0.242565	0.295811	0.310201	0.274225	0.292773	0.300608	0.26511
0.307803	0.330508	0.320595	0.332107	0.316278	0.286217	0.303806	0.295651	0.295651	0.305884
0.337864	0.325392	0.261593	0.361209	0.31196	0.290214	0.320915	0.315958	0.356572	0.331788
0.272306	0.305405	0.301087	0.246562	0.269587	0.257755	0.2504	0.273265	0.29693	0.263511
0.339303	0.336105	0.276463	0.347618	0.330189	0.291174	0.32779	0.309082	0.35993	0.335465
0.273105	0.383115	0.325871	0.275184	0.305245	0.346338	0.257915	0.290214	0.273265	0.31196
0.298369	0.357211	0.331948	0.210106	0.292133	0.302367	0.262072	0.271346	0.318676	0.301087
0.294691	0.306524	0.288455	0.332107	0.305564	0.237768	0.29709	0.282219	0.349057	0.301567
0.29677	0.37432	0.334826	0.216661	0.294052	0.311161	0.259674	0.285897	0.32763	0.290374
0.279981	0.330508	0.329549	0.307323	0.298049	0.277582	0.277742	0.314039	0.316437	0.314359
0.34394	0.284618	0.321394	0.357691	0.299488	0.196514	0.338823	0.307483	0.358491	0.338663
0.345699	0.37544	0.061561	0.362648	0.347618	0.295651	0.332587	0.305085	0.355772	0.341062
0.288935	0.321234	0.28078	0.28158	0.290054	0.25056	0.26575	0.275823	0.308762	0.283339
0.310841	0.369044	0.354013	0.239687	0.322674	0.335945	0.283179	0.299169	0.337544	0.316597
0.29645	0.345059	0.341062	0.241286	0.283019	0.298049	0.264791	0.274384	0.313399	0.284938
0.28078	0.335945	0.325871	0.190918	0.305405	0.302047	0.258555	0.144068	0.289894	0.298689
0.260953	0.311161	0.289095	0.248801	0.271826	0.261433	0.243044	0.270867	0.284138	0.274544
0.340102	0.283658	0.291493	0.373521	0.305245	0.255197	0.335945	0.310841	0.358491	0.332587
0.327151	0.338663	0.277902	0.373841	0.335146	0.309722	0.324592	0.325232	0.348737	0.350496
0.327151	0.376399	0.356572	0.206748	0.338024	0.314039	0.2819	0.290534	0.358171	0.319795
0.230413	0.315318	0.342821	0.339942	0.251519	0.311641	0.28206	0.34346	0.269587	0.291493
0.316917	0.280461	0.275983	0.355453	0.303326	0.209146	0.326991	0.283339	0.337064	0.32843
0.292453	0.342181	0.345379	0.190758	0.307803	0.293572	0.241126	0.285417	0.320915	0.301087
0.289415	0.338503	0.313399	0.301887	0.304605	0.279181	0.269907	0.324912	0.326031	0.280141
0.274704	0.332107	0.362648	0.290534	0.29613	0.302367	0.259194	0.308283	0.316757	0.299328
0.216981	0.364567	0.352574	0.272945	0.274864	0.308283	0.21938	0.306844	0.232651	0.277902
0.279661	0.332107	0.341062	0.257915	0.299009	0.304925	0.272626	0.315958	0.342981	0.291014
0.294851	0.39127	0.341541	0.270227	0.309402	0.337544	0.253598	0.300768	0.304925	0.302207
0.269587	0.351135	0.336585	0.284458	0.299968	0.314359	0.273425	0.302367	0.309082	0.302686
0.344579	0.290854	0.318836	0.35913	0.303486	0.196514	0.338343	0.303006	0.354653	0.345539
0.260313	0.37496	0.34394	0.299808	0.304445	0.314199	0.274384	0.321234	0.290374	0.310841
0.326671	0.3756	0.355932	0.206108	0.337864	0.31324	0.28206	0.289415	0.357691	0.319476
0.305085	0.358171	0.335785	0.255676	0.299808	0.330508	0.292133	0.292933	0.334666	0.304925
0.262072	0.337384	0.332107	0.288775	0.267829	0.29741	0.258075	0.307483	0.282859	0.276623
0.324592	0.354813	0.354333	0.213463	0.326191	0.316757	0.28142	0.280301	0.349057	0.307004
0.303965	0.344739	0.342181	0.243044	0.315798	0.295491	0.255357	0.31292	0.32811	0.315478
0.320595	0.372082	0.350336	0.070675	0.316278	0.326671	0.269268	0.26591	0.32875	0.306524
0.321714	0.369364	0.346658	0.224656	0.314998	0.310042	0.276143	0.286697	0.340902	0.298689
0.37512	0.294372	0.269428	0.398305	0.32843	0.270067	0.367605	0.31228	0.382475	0.361689
0.287336	0.357371	0.302207	0.273425	0.313399	0.307643	0.272306	0.290694	0.317077	0.309402
0.315638	0.334506	0.334346	0.286856	0.315958	0.256636	0.307004	0.283818	0.335305	0.306524
0.302686	0.333706	0.372721	0.290214	0.295331	0.32859	0.288295	0.32875	0.310201	0.333547
0.34426	0.3748	0.060281	0.361209	0.346978	0.294532	0.331148	0.303166	0.354333	0.340422
0.361049	0.303326	0.256636	0.37528	0.29629	0.304285	0.346818	0.321554	0.357052	0.338823
0.327311	0.26591	0.300608	0.354653	0.29645	0.213783	0.320435	0.284778	0.329389	0.323313
0.287816	0.29661	0.311641	0.267509	0.289255	0.280141	0.282859	0.310361	0.261113	0.303326
0.303326	0.358171	0.37432	0.247522	0.319476	0.341062	0.272626	0.319476	0.310681	0.304765
0.341222	0.286377	0.321075	0.373841	0.319476	0.270227	0.336585	0.317877	0.350815	0.346338
0.326511	0.321234	0.277263	0.387912	0.335146	0.308602	0.333067	0.334026	0.354973	0.35977

For Review Only

0.32747	0.37464	0.34362	0.190118	0.320755	0.31276	0.268148	0.285257	0.338823	0.302207
0.372402	0.295011	0.248481	0.400704	0.330508	0.280301	0.363128	0.305405	0.386473	0.361529
0.320595	0.337544	0.289095	0.29757	0.305884	0.262712	0.278862	0.269268	0.337384	0.282219
0.314359	0.294212	0.317877	0.335305	0.316278	0.285737	0.311481	0.306204	0.349376	0.304605
0.303806	0.3748	0.361529	0.191078	0.316917	0.340582	0.263032	0.29741	0.303326	0.3126
0.316118	0.3441	0.316757	0.350336	0.335625	0.32779	0.303326	0.348737	0.330029	0.345539
0.29709	0.28078	0.31276	0.335625	0.304765	0.279181	0.301887	0.316437	0.321714	0.259194
0.283339	0.352894	0.353054	0.247681	0.298849	0.315638	0.261113	0.301247	0.318196	0.279181
0.3441	0.324912	0.302367	0.351295	0.331148	0.302846	0.32779	0.310681	0.353214	0.315798
0.37464	0.178286	0.337384	0.392069	0.319795	0.301887	0.380397	0.355453	0.381516	0.319635
0.325072	0.287816	0.309562	0.321874	0.298209	0.229293	0.313559	0.277902	0.353054	0.316597
0.370163	0.299488	0.274225	0.407579	0.325072	0.272945	0.365046	0.319795	0.384554	0.362488
0.262872	0.303326	0.287176	0.251359	0.270227	0.260473	0.248801	0.26575	0.282859	0.271986
0.311641	0.337544	0.315158	0.309882	0.325712	0.302686	0.292773	0.323473	0.35945	0.310042
0.323793	0.334986	0.279021	0.292613	0.316437	0.298209	0.308602	0.327311	0.320755	0.308602
0.310681	0.34362	0.31196	0.244324	0.280141	0.304445	0.262072	0.266869	0.315958	0.302526
0.326191	0.37448	0.349696	0.136872	0.320275	0.329229	0.269907	0.278542	0.341701	0.29629
0.307803	0.365686	0.239527	0.332267	0.325392	0.293092	0.298369	0.293572	0.348097	0.329869
0.342661	0.29693	0.272945	0.356732	0.294052	0.273905	0.337064	0.29661	0.364407	0.323313
0.315478	0.339783	0.289255	0.349536	0.323153	0.294851	0.318996	0.327311	0.331788	0.316917
0.350016	0.318676	0.279021	0.370963	0.323953	0.286377	0.331468	0.314998	0.334666	0.322354
0.317557	0.329869	0.283339	0.298689	0.322354	0.299009	0.300927	0.303006	0.324272	0.306364
0.345219	0.339942	0.32859	0.308762	0.316917	0.319476	0.31196	0.326511	0.379757	0.323633
0.317877	0.358491	0.337864	0.231372	0.316118	0.305564	0.278382	0.286697	0.34378	0.295811
0.248481	0.366326	0.307004	0.279021	0.275823	0.319795	0.258714	0.291813	0.261912	0.295811
0.275823	0.356252	0.273105	0.333227	0.332907	0.241605	0.268788	0.308443	0.32843	0.310361
0.360729	0.252798	0.29645	0.394148	0.313399	0.268948	0.36009	0.321554	0.347618	0.333067
0.287496	0.333866	0.337224	0.268948	0.286377	0.332107	0.295171	0.319955	0.318036	0.29677
0.384554	0.298689	0.285737	0.411896	0.326671	0.277902	0.371122	0.32859	0.392709	0.363447
0.315638	0.357531	0.351615	0.137832	0.313879	0.325392	0.271506	0.274544	0.318196	0.298209
0.2181	0.333067	0.323473	0.313879	0.267029	0.29725	0.244643	0.309882	0.237128	0.269268
0.294851	0.368244	0.362008	0.240006	0.311641	0.350016	0.260473	0.294851	0.308283	0.306844
0.363128	0.326031	0.247841	0.385993	0.330349	0.263831	0.350336	0.327151	0.373361	0.346178
0.329709	0.331948	0.283019	0.255357	0.314519	0.291174	0.302207	0.28206	0.366805	0.309722
0.309882	0.316597	0.306524	0.334826	0.317077	0.247841	0.308602	0.313879	0.341541	0.303326
0.330828	0.351615	0.335945	0.248481	0.300128	0.305884	0.269428	0.282699	0.349376	0.302526
0.314998	0.339623	0.267669	0.369843	0.338024	0.303646	0.319156	0.32827	0.338184	0.345219
0.381996	0.294851	0.283339	0.410297	0.326671	0.278382	0.37576	0.325232	0.395747	0.358011
0.250879	0.362328	0.323633	0.247522	0.261753	0.329229	0.225456	0.286217	0.263671	0.289575
0.32795	0.377199	0.357211	0.207547	0.338823	0.315158	0.282699	0.291014	0.358171	0.320435
0.303166	0.299648	0.303806	0.26591	0.307643	0.246242	0.300608	0.230732	0.352255	0.299009
0.3756	0.298529	0.242245	0.401343	0.32891	0.283019	0.366326	0.308922	0.385034	0.353214
0.270707	0.369524	0.358491	0.270227	0.313879	0.320115	0.261273	0.324912	0.31276	0.301407
0.292773	0.31324	0.330668	0.284138	0.275823	0.270387	0.26639	0.288935	0.286537	0.283978
0.298369	0.342021	0.288775	0.336904	0.331148	0.270547	0.287336	0.299328	0.355293	0.323793
0.170611	0.389031	0.322993	0.284138	0.24944	0.326991	0.193316	0.308602	0.154301	0.311321
0.32859	0.338823	0.352894	0.216661	0.316757	0.297889	0.294691	0.299328	0.330828	0.299009
0.3126	0.353374	0.29725	0.291973	0.303965	0.298849	0.299968	0.339143	0.304125	0.331308
0.346338	0.364567	0.306844	0.287016	0.332747	0.284938	0.305564	0.285737	0.369044	0.32747
0.311481	0.316278	0.355932	0.287816	0.304605	0.274864	0.272626	0.317877	0.351615	0.285577
0.332587	0.304285	0.358011	0.26607	0.314679	0.315638	0.309722	0.321075	0.341541	0.297729
0.383914	0.300448	0.286537	0.408539	0.325871	0.28094	0.368724	0.325712	0.396546	0.356252
0.253918	0.382635	0.32875	0.270227	0.305724	0.331948	0.257595	0.283499	0.277742	0.306364
0.373361	0.29741	0.240166	0.401823	0.324752	0.279181	0.368564	0.307163	0.380556	0.356732
0.319795	0.371602	0.351615	0.070515	0.315638	0.32875	0.269268	0.267349	0.32875	0.306844

0.31292	0.379277	0.356092	0.189159	0.316757	0.339783	0.270867	0.31228	0.325392	0.314199
0.336745	0.335465	0.26511	0.344739	0.339463	0.299328	0.323153	0.302846	0.365526	0.333227
0.258235	0.384554	0.233611	0.28126	0.304605	0.332747	0.259994	0.29661	0.268308	0.322034
0.313399	0.373201	0.364407	0.211065	0.3126	0.32875	0.270067	0.298689	0.34314	0.313559

For Review Only

0.286856	0.337544	0.290054	0.284618	0.372562	0.329389	0.257755	0.303965	0.293252	0.268948
0.322354	0.332107	0.307323	0.356892	0.335305	0.29725	0.338983	0.313559	0.271506	0.28078
0.244963	0.367285	0.34442	0.327151	0.397506	0.354973	0.267829	0.231212	0.217461	0.257915
0.26495	0.335945	0.32843	0.314679	0.376559	0.318516	0.269428	0.268948	0.217621	0.257755
0.341062	0.337384	0.292933	0.240006	0.362008	0.29677	0.286537	0.346178	0.34394	0.298689
0.251039	0.34378	0.315638	0.293892	0.389191	0.332427	0.236329	0.274225	0.252478	0.263671
0.318356	0.336585	0.248481	0.2181	0.373201	0.345859	0.245283	0.309562	0.300128	0.276143
0.348257	0.280141	0.300927	0.34426	0.323793	0.255836	0.340262	0.32747	0.323153	0.280141
0.31228	0.349217	0.280461	0.232651	0.364567	0.314839	0.24944	0.318996	0.293732	0.26575
0.378158	0.271506	0.306204	0.334506	0.339942	0.271027	0.364407	0.368724	0.353374	0.308762
0.347138	0.354653	0.314998	0.338343	0.272306	0.29661	0.338663	0.354333	0.349696	0.290854
0.23393	0.361529	0.340422	0.320915	0.389351	0.345699	0.268308	0.226895	0.20339	0.251839
0.318996	0.326671	0.254397	0.232491	0.347458	0.298369	0.263032	0.318356	0.294052	0.274065
0.337064	0.322514	0.315798	0.325392	0.294691	0.239367	0.311481	0.313719	0.307323	0.262232
0.275664	0.333547	0.286856	0.24944	0.356572	0.314199	0.243844	0.288615	0.271346	0.248161
0.307963	0.339623	0.353054	0.330029	0.355293	0.277422	0.287496	0.28078	0.282219	0.267988
0.324912	0.329389	0.247522	0.194595	0.381836	0.342181	0.262072	0.349856	0.333866	0.282539
0.32779	0.295491	0.287176	0.310681	0.35945	0.31228	0.271506	0.321234	0.291813	0.275024
0	0.370643	0.354493	0.330029	0.40582	0.349696	0.252798	0.278382	0.256316	0.273265
0.370643	0	0.288455	0.349536	0.356252	0.301567	0.357691	0.367285	0.34426	0.308602
0.354493	0.288455	0	0.226895	0.32827	0.29613	0.308123	0.353054	0.330029	0.289894
0.330029	0.349536	0.226895	0	0.377678	0.32795	0.264631	0.330349	0.318036	0.279821
0.40582	0.356252	0.32827	0.377678	0	0.273425	0.377199	0.39111	0.358491	0.31292
0.349696	0.301567	0.29613	0.32795	0.273425	0	0.336745	0.335945	0.307483	0.263831
0.252798	0.357691	0.308123	0.264631	0.377199	0.336745	0	0.290534	0.251839	0.251519
0.278382	0.367285	0.353054	0.330349	0.39111	0.335945	0.290534	0	0.215062	0.253598
0.256316	0.34426	0.330029	0.318036	0.358491	0.307483	0.251839	0.215062	0	0.247681
0.273265	0.308602	0.289894	0.279821	0.31292	0.263831	0.251519	0.253598	0.247681	0
0.324912	0.29629	0.240166	0.270547	0.338343	0.280461	0.269907	0.325552	0.292933	0.263991
0.42149	0.254077	0.292773	0.338983	0.291014	0.23505	0.408379	0.409818	0.389031	0.32811
0.213943	0.370643	0.341222	0.310681	0.377199	0.327311	0.206268	0.248961	0.205309	0.259354
0.254077	0.358331	0.340902	0.308602	0.398145	0.352414	0.291813	0.303965	0.274544	0.275823
0.300448	0.383754	0.301887	0.239367	0.383914	0.353054	0.236489	0.311001	0.303326	0.280301
0.364087	0.313879	0.305245	0.321075	0.278702	0.195875	0.339942	0.341222	0.316118	0.270067
0.329869	0.346338	0.336425	0.346178	0.307004	0.304285	0.322354	0.302846	0.303326	0.291493
0.277422	0.315638	0.344899	0.349856	0.336105	0.213463	0.29693	0.26591	0.247841	0.2496
0.313719	0.314839	0.302686	0.325871	0.331948	0.283978	0.298849	0.329229	0.286377	0.26559
0.333387	0.342341	0.322833	0.330988	0.335785	0.31292	0.298689	0.313559	0.295971	0.274704
0.409498	0.329709	0.301567	0.362008	0.194436	0.222578	0.383115	0.380876	0.360889	0.29693
0.367605	0.34442	0.346019	0.371442	0.252798	0.29709	0.344579	0.347298	0.348737	0.29645
0.418932	0.260473	0.275024	0.338184	0.2819	0.242725	0.398785	0.394627	0.382795	0.315318
0.360889	0.264471	0.229933	0.336745	0.329869	0.279341	0.3441	0.348257	0.321714	0.286217
0.361848	0.256636	0.306364	0.342181	0.340422	0.262552	0.346658	0.362968	0.330988	0.293572
0.35929	0.31276	0.261912	0.251039	0.35865	0.315478	0.301567	0.368084	0.339942	0.299488
0.386473	0.269747	0.336904	0.362648	0.342981	0.279501	0.351615	0.372402	0.345059	0.305085
0.24928	0.351135	0.331148	0.320275	0.39095	0.336105	0.262072	0.18804	0.212344	0.247042
0.261273	0.352414	0.342181	0.323313	0.399584	0.336105	0.276623	0.184842	0.225136	0.256316
0.318996	0.346978	0.324912	0.318036	0.320275	0.274225	0.284618	0.238887	0.288295	0.251999
0.352734	0.305245	0.270547	0.246562	0.371922	0.32891	0.331148	0.366166	0.345379	0.292933
0.276303	0.352255	0.323633	0.257115	0.371762	0.331468	0.252478	0.29757	0.283978	0.260953

For Review Only

0.261593	0.362328	0.330508	0.321874	0.387592	0.335625	0.259354	0.157659	0.228814	0.247522
0.290534	0.367765	0.333067	0.324592	0.353854	0.32891	0.290534	0.29757	0.263991	0.271826
0.278222	0.353854	0.275024	0.181004	0.397825	0.355612	0.141509	0.304125	0.291973	0.267988
0.300448	0.382955	0.301567	0.239687	0.383275	0.351935	0.236649	0.311321	0.303166	0.279501
0.215062	0.356092	0.342501	0.322354	0.389191	0.330988	0.239847	0.277902	0.225616	0.258395
0.32827	0.322354	0.244004	0.174129	0.367605	0.342981	0.26559	0.342821	0.325552	0.283658
0.011353	0.369364	0.351775	0.329389	0.40566	0.348897	0.253278	0.278702	0.256796	0.273265
0.255037	0.353854	0.345859	0.338663	0.396866	0.330029	0.292613	0.183882	0.202111	0.261593
0.269747	0.335305	0.342181	0.332267	0.39079	0.32891	0.294372	0.119763	0.194755	0.248321
0.402782	0.282379	0.293092	0.351135	0.255676	0.204829	0.381836	0.385833	0.35945	0.297889
0.341062	0.311001	0.298529	0.295011	0.309082	0.252638	0.313879	0.342821	0.291493	0.270067
0.263991	0.318356	0.294691	0.286057	0.316118	0.269587	0.24976	0.257595	0.242085	0.216342
0.240006	0.358011	0.342341	0.331788	0.393348	0.32747	0.276623	0.245123	0.215062	0.256476
0.250879	0.357371	0.365366	0.351135	0.388711	0.316597	0.283339	0.255516	0.217461	0.268308
0.277103	0.353854	0.273905	0.180524	0.396706	0.356732	0.14055	0.303006	0.290854	0.267829
0.323793	0.29613	0.240646	0.269747	0.337544	0.279821	0.270067	0.324272	0.291493	0.263511
0.268948	0.329549	0.309882	0.29741	0.338663	0.292133	0.253598	0.258075	0.254237	0.240806
0.35913	0.304765	0.287976	0.329709	0.302207	0.269108	0.320755	0.336585	0.326991	0.283658
0.309562	0.338663	0.321075	0.340102	0.298209	0.317877	0.302207	0.304925	0.292613	0.268148
0.272306	0.362168	0.332587	0.306684	0.409498	0.348737	0.270707	0.275823	0.289095	0.266549
0.263032	0.35913	0.305245	0.206428	0.386952	0.344739	0.238088	0.305245	0.278702	0.262872
0.293412	0.35993	0.283179	0.21938	0.398945	0.344579	0.23505	0.298209	0.291653	0.271826
0.251679	0.332267	0.336585	0.325871	0.363767	0.291334	0.267349	0.255197	0.211225	0.251359
0.35881	0.313399	0.320275	0.358171	0.254077	0.278382	0.34442	0.349057	0.336585	0.287496
0.318676	0.305245	0.235369	0.24992	0.339623	0.300927	0.278222	0.321394	0.302686	0.268308
0.301087	0.340902	0.321075	0.322674	0.369524	0.310201	0.275664	0.287656	0.275184	0.28158
0.386792	0.269268	0.337064	0.362328	0.342661	0.278542	0.351295	0.372242	0.34394	0.304925
0.237448	0.350016	0.348897	0.331628	0.388391	0.320115	0.271506	0.24928	0.222098	0.261113
0.357691	0.333866	0.314199	0.292613	0.371122	0.332267	0.341861	0.362808	0.361209	0.31228
0.412056	0.323633	0.315318	0.371282	0.207867	0.226575	0.392389	0.382955	0.366485	0.302526
0.288615	0.356252	0.345699	0.332427	0.398465	0.340262	0.304925	0.060122	0.232971	0.254877
0.257115	0.37496	0.322354	0.293092	0.381676	0.329709	0.252958	0.298369	0.268148	0.26623
0.330508	0.320115	0.299328	0.252159	0.354333	0.295491	0.263991	0.330828	0.311321	0.279981
0.31324	0.316118	0.306044	0.318996	0.310042	0.183722	0.302686	0.301247	0.279021	0.252319
0.414934	0.319476	0.32875	0.380397	0.217141	0.227374	0.404061	0.393668	0.37544	0.307323
0.276943	0.354013	0.273425	0.180045	0.395427	0.354813	0.14055	0.303806	0.291014	0.267509
0.310841	0.316917	0.311161	0.306844	0.354333	0.294052	0.270547	0.302207	0.302526	0.258075
0.386153	0.270067	0.338184	0.362328	0.342821	0.279341	0.350975	0.372242	0.344899	0.305085
0.357052	0.275344	0.28174	0.32827	0.313879	0.254557	0.330189	0.354813	0.322354	0.287176
0.35977	0.31228	0.222098	0.237448	0.337544	0.316757	0.284778	0.37448	0.348417	0.305085
0.315958	0.346498	0.310201	0.324752	0.313559	0.308602	0.319476	0.301247	0.295171	0.284618
0.366965	0.292933	0.3126	0.35865	0.340422	0.277263	0.336105	0.368084	0.35945	0.298209
0.278062	0.361369	0.264151	0.17173	0.393188	0.349856	0.217781	0.295971	0.287976	0.271346
0.252478	0.382795	0.353694	0.317877	0.409818	0.353534	0.26591	0.293412	0.243684	0.273265
0.291334	0.338983	0.32747	0.332907	0.358331	0.32811	0.299009	0.304925	0.292293	0.279501
0.242245	0.358171	0.344579	0.332107	0.393508	0.327151	0.277422	0.246242	0.215702	0.257275
0.300927	0.371762	0.314998	0.285737	0.361209	0.307004	0.277742	0.309402	0.292773	0.280301
0.24928	0.350336	0.330508	0.306364	0.371602	0.34346	0.263991	0.259834	0.23473	0.259674
0.372721	0.308602	0.314839	0.34426	0.26543	0.192517	0.364087	0.355293	0.330029	0.276303
0.279341	0.330349	0.334346	0.332747	0.362008	0.272785	0.299648	0.18708	0.240806	0.247681
0.278862	0.333067	0.330029	0.317557	0.364567	0.283019	0.294212	0.192677	0.232811	0.24928
0.305724	0.366485	0.355453	0.34378	0.331148	0.332747	0.315958	0.325871	0.298849	0.289255
0.261433	0.34346	0.320595	0.316437	0.367765	0.295651	0.277582	0.242245	0.197474	0.2496
0.42117	0.337384	0.311481	0.368244	0.193796	0.229453	0.395267	0.392549	0.370963	0.305884
0.382315	0.304605	0.303326	0.348897	0.276463	0.238727	0.377359	0.379917	0.336745	0.299009

For Review Only

0.294851	0.307643	0.29757	0.322354	0.301407	0.263032	0.303646	0.310681	0.284458	0.258235
0.297889	0.381196	0.342341	0.323793	0.365206	0.32843	0.294851	0.322674	0.297889	0.275184
0.313719	0.341382	0.330189	0.348577	0.324592	0.308602	0.326831	0.194755	0.252798	0.261433
0.337704	0.323633	0.323793	0.351135	0.319156	0.290694	0.332427	0.322993	0.28094	0.290694
0.287656	0.336105	0.329869	0.326671	0.361689	0.28174	0.268468	0.167253	0.213303	0.244324
0.371122	0.3126	0.304285	0.331788	0.280461	0.209946	0.354013	0.349057	0.335945	0.274864
0.235209	0.365846	0.346338	0.322034	0.39063	0.32795	0.275344	0.2189	0.174288	0.251519
0.270867	0.341861	0.32763	0.313399	0.35977	0.292933	0.274384	0.149344	0.239847	0.242725
0.241605	0.355932	0.34362	0.298849	0.385353	0.327151	0.242565	0.262072	0.232811	0.246083
0.264631	0.318196	0.306044	0.326831	0.327311	0.236968	0.263192	0.279181	0.236649	0.244004
0.326991	0.329709	0.326671	0.336265	0.346178	0.285577	0.32875	0.258395	0.267988	0.273745
0.307483	0.303006	0.307323	0.321234	0.329549	0.252159	0.307803	0.270387	0.288455	0.257435
0.325552	0.340102	0.300288	0.298689	0.339303	0.29741	0.274225	0.32891	0.308762	0.271506
0.284618	0.314199	0.294851	0.290214	0.3126	0.275823	0.272146	0.254877	0.263671	0.233291
0.294052	0.354493	0.32747	0.330189	0.341701	0.307483	0.31292	0.299328	0.276463	0.28142
0.239847	0.361848	0.332427	0.314519	0.379117	0.314519	0.255996	0.237768	0.211705	0.246722
0.290214	0.340742	0.317397	0.335146	0.34346	0.29613	0.274544	0.293732	0.270067	0.273105
0.280141	0.316757	0.304605	0.314039	0.336745	0.28142	0.279981	0.196514	0.247362	0.235849
0.298849	0.355453	0.333547	0.325072	0.369204	0.342981	0.302207	0.294691	0.318356	0.280141
0.254397	0.318516	0.29725	0.289735	0.332907	0.285897	0.259354	0.226575	0.236169	0.221458
0.373841	0.309402	0.310681	0.3433	0.283818	0.200032	0.35977	0.354493	0.336904	0.277742
0.295331	0.347618	0.308283	0.287656	0.329869	0.334346	0.28078	0.324752	0.310841	0.269268
0.270387	0.306044	0.287496	0.288136	0.319316	0.271666	0.256956	0.238407	0.243364	0.217781
0.255516	0.369044	0.322034	0.28126	0.370803	0.321394	0.232811	0.29677	0.273265	0.264791
0.244164	0.358331	0.316597	0.299488	0.365366	0.31212	0.260953	0.267829	0.222897	0.252638
0.414455	0.313399	0.318196	0.367925	0.210106	0.220659	0.397346	0.384714	0.367445	0.301887
0.317877	0.32875	0.324752	0.348097	0.314519	0.307323	0.324113	0.209786	0.275024	0.26591
0.348097	0.330189	0.313879	0.3433	0.228174	0.273105	0.316118	0.330029	0.305245	0.279181
0.285737	0.355932	0.326831	0.294212	0.364567	0.320915	0.262232	0.29645	0.287496	0.275823
0.275504	0.333067	0.305245	0.298049	0.347937	0.26623	0.260793	0.304125	0.255357	0.242565
0.240326	0.371122	0.338184	0.290214	0.407579	0.354013	0.263991	0.267988	0.222897	0.258714
0.26607	0.333547	0.335465	0.3126	0.378478	0.34394	0.275664	0.310201	0.299488	0.275664
0.402143	0.316917	0.321075	0.365046	0.235529	0.223217	0.386952	0.382315	0.362968	0.300128
0.282379	0.315958	0.289095	0.289255	0.303166	0.263032	0.268628	0.268148	0.256316	0.23457
0.279021	0.362008	0.263032	0.169012	0.394148	0.352095	0.216501	0.295651	0.289415	0.271666
0.28142	0.319795	0.307643	0.300927	0.336585	0.287496	0.255357	0.195715	0.233611	0.233291
0.362008	0.31324	0.304765	0.319955	0.278542	0.196674	0.337544	0.337864	0.313559	0.269108
0.339143	0.332427	0.291334	0.285417	0.311001	0.258395	0.308762	0.323313	0.302367	0.273425
0.383914	0.310681	0.309562	0.346818	0.279181	0.200192	0.373361	0.365046	0.342341	0.288136
0.23441	0.346658	0.326831	0.315158	0.384234	0.323793	0.279821	0.248481	0.21858	0.255676
0.339783	0.34394	0.333227	0.339783	0.29661	0.289575	0.308922	0.258075	0.309402	0.26543
0.403422	0.331948	0.325552	0.370323	0.231532	0.264311	0.388871	0.391909	0.372242	0.311481
0.339463	0.350656	0.334346	0.350656	0.266709	0.298209	0.334666	0.298049	0.311641	0.286057
0.380397	0.305724	0.309882	0.344739	0.276943	0.188519	0.365366	0.360409	0.338663	0.28158
0.271186	0.368404	0.342021	0.334026	0.387912	0.332907	0.286377	0.276463	0.263991	0.264311
0.384234	0.277902	0.300448	0.331148	0.304605	0.241925	0.365846	0.3748	0.353054	0.305724
0.3118	0.350176	0.306684	0.324752	0.325871	0.288775	0.300448	0.239527	0.277902	0.262392
0.28094	0.346019	0.3126	0.278382	0.362968	0.308123	0.271986	0.279661	0.263671	0.259034
0.270387	0.349057	0.332427	0.326511	0.394308	0.340742	0.284938	0.092261	0.214423	0.246083
0.276463	0.338343	0.345699	0.340902	0.396706	0.325871	0.284138	0.152542	0.20275	0.251679
0.334026	0.316437	0.301407	0.314359	0.285577	0.233451	0.31308	0.329869	0.295811	0.26591
0.269907	0.35945	0.331468	0.321394	0.392229	0.336105	0.272306	0.098337	0.212824	0.25056
0.283019	0.34378	0.346658	0.335785	0.376559	0.310361	0.287176	0.121202	0.221458	0.253438
0.373201	0.307163	0.286377	0.322354	0.274065	0.253438	0.363927	0.345699	0.357851	0.293572
0.282539	0.341062	0.325232	0.31276	0.331628	0.268788	0.275983	0.284778	0.285897	0.25072

For Review Only

0.270867	0.370803	0.361529	0.339783	0.394787	0.319156	0.282219	0.215702	0.231212	0.26607
0.334186	0.300128	0.283179	0.305405	0.264151	0.244963	0.31276	0.319635	0.302207	0.264151
0.255197	0.367605	0.324272	0.29661	0.396226	0.34378	0.272146	0.240326	0.270387	0.262072
0.372562	0.349376	0.322354	0.3756	0.261593	0.283339	0.36009	0.346818	0.352255	0.299169
0.357691	0.299328	0.324432	0.330189	0.362488	0.307004	0.330029	0.348097	0.325072	0.303326
0.336904	0.348577	0.325072	0.327311	0.275184	0.276303	0.3126	0.335146	0.322674	0.289575
0.258395	0.339143	0.334666	0.306524	0.394308	0.334826	0.257275	0.262072	0.254877	0.263032
0.325072	0.3126	0.325232	0.325232	0.336904	0.283978	0.318996	0.32795	0.333706	0.271666
0.373201	0.342981	0.322993	0.348897	0.247362	0.267189	0.339783	0.356892	0.339143	0.290694
0.279021	0.298369	0.286057	0.286217	0.322514	0.255197	0.267509	0.230253	0.239687	0.221938
0.35977	0.340742	0.329389	0.345699	0.262072	0.272466	0.354653	0.353694	0.315958	0.29661
0.246882	0.362968	0.32827	0.270547	0.394148	0.338663	0.248321	0.300288	0.276623	0.26527
0.259514	0.340422	0.31292	0.307323	0.361689	0.307323	0.269268	0.139431	0.208666	0.240646
0.349217	0.314679	0.310521	0.333227	0.267349	0.176207	0.313879	0.326831	0.297729	0.262392
0.259354	0.346818	0.316757	0.306044	0.368084	0.316437	0.254557	0.236489	0.199872	0.245123
0.311641	0.332747	0.319476	0.315798	0.334506	0.286537	0.295811	0.303646	0.286697	0.271666
0.3756	0.305405	0.310841	0.349376	0.26623	0.200512	0.365686	0.36009	0.331308	0.277103
0.361529	0.361209	0.333067	0.363767	0.254877	0.308123	0.361209	0.372881	0.371282	0.302526
0.288775	0.323313	0.310042	0.298049	0.319635	0.259994	0.283339	0.280461	0.279181	0.241765
0.245283	0.363288	0.34362	0.336105	0.389511	0.333387	0.26511	0.258714	0.244164	0.270547
0.274384	0.341701	0.311161	0.293732	0.341222	0.271346	0.270547	0.190278	0.226255	0.237608
0.244803	0.350656	0.339303	0.29693	0.378478	0.321554	0.241765	0.260153	0.230253	0.242405
0.267669	0.307323	0.282699	0.279341	0.308123	0.261753	0.256476	0.256156	0.24928	0.21858
0.377359	0.300288	0.31292	0.355293	0.241445	0.185162	0.35993	0.354813	0.335305	0.28062
0.367925	0.308443	0.322194	0.356572	0.249121	0.283339	0.352734	0.357052	0.342501	0.293892
0.25056	0.35881	0.365526	0.351135	0.388871	0.317557	0.282699	0.256476	0.2181	0.268468
0.347937	0.301407	0.230253	0.269587	0.346818	0.284618	0.290374	0.345699	0.31324	0.28206
0.355612	0.314039	0.305245	0.318196	0.279821	0.200192	0.338983	0.339942	0.320595	0.268148
0.251839	0.358331	0.329389	0.315478	0.364407	0.303806	0.258075	0.247202	0.20387	0.24992
0.302367	0.335465	0.319156	0.325392	0.321874	0.283978	0.290374	0.305245	0.302207	0.267988
0.310841	0.332427	0.334826	0.305724	0.335625	0.31276	0.28174	0.268148	0.253278	0.261273
0.288295	0.353374	0.290214	0.233611	0.380077	0.327151	0.237768	0.298369	0.285098	0.260633
0.277103	0.357691	0.307643	0.316917	0.361689	0.303326	0.274065	0.268148	0.263192	0.266549
0.25024	0.369683	0.334346	0.291334	0.388391	0.352574	0.273105	0.283339	0.284618	0.274704
0.300768	0.350176	0.317397	0.289415	0.347618	0.313879	0.274704	0.295811	0.268628	0.268948
0.376079	0.306844	0.314519	0.352894	0.26591	0.205149	0.361529	0.357531	0.329549	0.277422
0.294532	0.342181	0.318996	0.286856	0.377678	0.319156	0.274384	0.311001	0.292933	0.271986
0.25024	0.357851	0.365206	0.350656	0.389031	0.316597	0.282859	0.255516	0.217301	0.267829
0.295331	0.338503	0.318036	0.333387	0.347458	0.318516	0.275184	0.238887	0.220179	0.268948
0.282699	0.338343	0.299328	0.285897	0.364886	0.314998	0.259354	0.31308	0.288775	0.261273
0.274225	0.341222	0.351935	0.3433	0.398145	0.325232	0.287176	0.163895	0.201311	0.251679
0.252798	0.342501	0.330668	0.325552	0.363927	0.31324	0.251519	0.266549	0.246882	0.262392
0.245443	0.366326	0.34442	0.327311	0.397346	0.353374	0.269747	0.229613	0.216182	0.257435
0.276943	0.348257	0.332107	0.324912	0.394308	0.341541	0.287976	0.09402	0.216661	0.247202
0.409658	0.322354	0.317557	0.379757	0.209626	0.229613	0.391589	0.382155	0.367125	0.304605
0.290054	0.329549	0.329389	0.313879	0.332907	0.316278	0.288935	0.269747	0.278222	0.257595
0.314679	0.315958	0.352255	0.32875	0.362968	0.286377	0.321234	0.291493	0.273265	0.272626
0.283339	0.361689	0.316118	0.310361	0.356572	0.307963	0.28174	0.337704	0.284778	0.277742
0.36009	0.361049	0.330988	0.362168	0.252958	0.308602	0.35961	0.371282	0.370803	0.302047
0.389351	0.340102	0.307163	0.340422	0.245123	0.260953	0.355133	0.382475	0.363288	0.299488
0.356252	0.298689	0.291653	0.326031	0.270227	0.160217	0.340262	0.341861	0.308922	0.26639
0.304925	0.324272	0.287336	0.270707	0.322674	0.2819	0.289735	0.319795	0.271506	0.261433
0.277582	0.361049	0.332107	0.299808	0.396226	0.351455	0.270387	0.258714	0.262552	0.263831
0.369843	0.286537	0.322993	0.35929	0.292453	0.233451	0.364247	0.377838	0.338503	0.289894
0.381676	0.321714	0.318516	0.352095	0.228654	0.258874	0.357211	0.370003	0.356412	0.290534

For Review Only

0.240166	0.357052	0.342021	0.332107	0.392229	0.327311	0.275983	0.245603	0.214902	0.255996
0.413975	0.336585	0.316757	0.370643	0.185002	0.227854	0.395107	0.388232	0.35929	0.307323
0.324432	0.344899	0.315958	0.32827	0.318036	0.257115	0.29677	0.315958	0.305724	0.266549
0.345219	0.271986	0.332587	0.332587	0.330668	0.283658	0.324113	0.341382	0.317557	0.286697
0.257435	0.361049	0.344739	0.307323	0.403582	0.367285	0.268308	0.267189	0.26575	0.261753
0.341222	0.336904	0.324912	0.3433	0.286217	0.317397	0.336745	0.352894	0.335945	0.294212
0.338983	0.254557	0.298369	0.336105	0.330828	0.271346	0.309562	0.341861	0.315318	0.280461
0.28126	0.368084	0.331148	0.300128	0.404221	0.3441	0.26591	0.164375	0.241925	0.248961
0.376399	0.331788	0.302047	0.323473	0.291174	0.295171	0.353854	0.326671	0.32875	0.29757
0.40678	0.26511	0.309082	0.35897	0.311321	0.25072	0.396866	0.393508	0.372721	0.321874
0.34346	0.311161	0.317077	0.324432	0.301247	0.213943	0.326671	0.299488	0.295171	0.267509
0.417493	0.32891	0.322674	0.374161	0.208507	0.228174	0.400704	0.393348	0.371282	0.307004
0.270067	0.305564	0.276783	0.274384	0.308443	0.256956	0.256316	0.254397	0.245923	0.216182
0.326511	0.34346	0.305564	0.345059	0.308123	0.302846	0.311321	0.311481	0.307803	0.275823
0.304125	0.350656	0.320435	0.306204	0.31244	0.316917	0.307483	0.300448	0.287336	0.278862
0.26527	0.342821	0.307803	0.298529	0.352574	0.275184	0.258395	0.191877	0.247841	0.248801
0.258714	0.366645	0.338823	0.333227	0.401023	0.345379	0.275504	0.231692	0.233291	0.268628
0.337224	0.341062	0.355453	0.330349	0.293572	0.307483	0.305564	0.334666	0.330029	0.292933
0.379757	0.331308	0.315798	0.338343	0.232651	0.252159	0.361209	0.349057	0.326351	0.282859
0.347618	0.329389	0.304445	0.32875	0.29677	0.277582	0.333227	0.361049	0.346978	0.292133
0.366006	0.337544	0.322354	0.338983	0.274384	0.26559	0.342661	0.358331	0.354813	0.292293
0.339143	0.349856	0.318036	0.334506	0.327311	0.314679	0.303166	0.32827	0.310681	0.279501
0.338823	0.332587	0.323793	0.354333	0.304445	0.291653	0.329869	0.307483	0.292933	0.293412
0.276463	0.350496	0.339942	0.325871	0.392709	0.336105	0.294532	0.071954	0.229933	0.244484
0.254397	0.352894	0.292773	0.252958	0.367125	0.324432	0.247522	0.314199	0.278862	0.261593
0.338823	0.351615	0.341062	0.350336	0.275024	0.295651	0.303166	0.327151	0.324272	0.271027
0.402143	0.283339	0.292773	0.350656	0.255996	0.204669	0.381836	0.386473	0.35945	0.297729
0.284298	0.341701	0.316118	0.31324	0.37528	0.318676	0.271506	0.29629	0.267988	0.275823
0.416374	0.32843	0.329389	0.381356	0.206748	0.232012	0.404541	0.397186	0.37512	0.314679
0.226575	0.363128	0.340422	0.313879	0.394468	0.338823	0.263991	0.221778	0.183722	0.25056
0.309402	0.303006	0.285257	0.254077	0.357371	0.298689	0.222098	0.320115	0.291334	0.269907
0.260153	0.365046	0.333067	0.287016	0.396067	0.345059	0.260793	0.278062	0.248481	0.256956
0.40582	0.351455	0.325871	0.365846	0.189319	0.23473	0.381676	0.381036	0.372402	0.295971
0.287656	0.345859	0.347618	0.340422	0.338184	0.286697	0.293572	0.214423	0.270867	0.2504
0.344579	0.31292	0.320595	0.352414	0.294691	0.260153	0.323633	0.325712	0.309882	0.274544
0.28142	0.337224	0.321874	0.326191	0.369044	0.300768	0.284298	0.157339	0.232491	0.251519
0.364247	0.318196	0.324432	0.347138	0.241286	0.278862	0.350815	0.353534	0.350975	0.287176
0.417813	0.32843	0.330828	0.383754	0.217781	0.232171	0.398625	0.393508	0.382635	0.31212
0.264631	0.348097	0.299968	0.253278	0.393828	0.32843	0.251199	0.284458	0.270867	0.258874
0.25056	0.35897	0.365526	0.350975	0.389671	0.318196	0.282859	0.256156	0.21874	0.268628
0.276783	0.314839	0.34346	0.348897	0.334986	0.213623	0.295491	0.26495	0.246722	0.248801
0.418932	0.337064	0.315158	0.370803	0.204029	0.223217	0.397985	0.395107	0.370483	0.309402
0.272146	0.37448	0.333706	0.304765	0.385034	0.34314	0.285257	0.286377	0.268308	0.275184
0.29645	0.310201	0.28206	0.28206	0.334026	0.270387	0.261273	0.288935	0.244164	0.253438
0.34314	0.340902	0.347618	0.361209	0.257115	0.278062	0.309242	0.320115	0.29725	0.28062
0.28142	0.362008	0.253118	0.128238	0.392229	0.354013	0.188999	0.307643	0.291653	0.26511
0.274384	0.341062	0.351135	0.338823	0.357211	0.306044	0.273105	0.267509	0.248481	0.264151
0.317557	0.361369	0.314839	0.313879	0.320755	0.31228	0.305724	0.31308	0.272306	0.28206
0.340422	0.340102	0.344739	0.353214	0.320435	0.292453	0.331148	0.240006	0.267349	0.277103
0.316917	0.3441	0.320115	0.334026	0.353854	0.315318	0.279981	0.288775	0.267189	0.275024
0.313559	0.339783	0.337864	0.331148	0.387592	0.321714	0.304925	0.277902	0.279821	0.286377
0.414934	0.326191	0.332267	0.385993	0.21826	0.227374	0.400544	0.396866	0.371602	0.311321
0.24976	0.3748	0.332427	0.284298	0.399264	0.355612	0.245283	0.303965	0.280301	0.26639
0.417173	0.329869	0.315798	0.368884	0.204029	0.232651	0.397825	0.396866	0.373521	0.306844
0.245603	0.366965	0.345859	0.32747	0.397186	0.354173	0.267988	0.230253	0.21842	0.257755

For Review Only

0.247362	0.366485	0.321554	0.315158	0.404701	0.35993	0.272785	0.240326	0.224816	0.26575
0.377678	0.32795	0.345379	0.392549	0.255836	0.272466	0.350336	0.355293	0.341062	0.287336
0.253758	0.381356	0.32763	0.26623	0.37512	0.342181	0.246402	0.309882	0.288455	0.269428
0.236489	0.373201	0.349376	0.333706	0.393508	0.341382	0.255197	0.236808	0.205788	0.254237

For Review Only

0.26511	0.398625	0.289255	0.26559	0.225136	0.322194	0.32779	0.302526	0.253758	0.298849
0.310042	0.309882	0.313399	0.342501	0.363447	0.306524	0.316917	0.303166	0.300608	0.335945
0.329869	0.431244	0.235369	0.251839	0.290214	0.363128	0.322194	0.274225	0.313879	0.285737
0.29645	0.377359	0.24992	0.278702	0.325072	0.319955	0.323633	0.254237	0.279501	0.270387
0.298529	0.319476	0.332427	0.358011	0.272785	0.308283	0.332747	0.316597	0.304925	0.317717
0.272945	0.398945	0.244803	0.258075	0.257915	0.321874	0.320755	0.293572	0.283339	0.314359
0.251679	0.394148	0.303006	0.279341	0.181484	0.348257	0.345219	0.323953	0.290534	0.303006
0.295491	0.26575	0.339623	0.338823	0.336425	0.26559	0.336105	0.279341	0.318996	0.298209
0.17125	0.381196	0.302686	0.29725	0.204509	0.314839	0.318356	0.304445	0.283019	0.316118
0.284138	0.228654	0.360569	0.356892	0.37528	0.272146	0.354653	0.299808	0.325712	0.322674
0.329549	0.336265	0.339942	0.350336	0.335625	0.305245	0.29661	0.304765	0.320275	0.299169
0.315958	0.419252	0.227374	0.252638	0.293572	0.354173	0.318036	0.26591	0.316437	0.288775
0.270387	0.314199	0.294851	0.318356	0.259674	0.295491	0.329709	0.308922	0.29613	0.31292
0.315638	0.303806	0.321554	0.333227	0.322034	0.208187	0.307323	0.247841	0.263831	0.291334
0.267189	0.378798	0.262872	0.282379	0.256796	0.321234	0.306524	0.301727	0.284298	0.29741
0.304125	0.356892	0.28094	0.306044	0.333387	0.291493	0.317717	0.231852	0.307323	0.291014
0.273905	0.367445	0.321714	0.310042	0.247841	0.334186	0.369044	0.352574	0.308123	0.327151
0.273585	0.351295	0.302047	0.287016	0.285257	0.308762	0.326831	0.298849	0.301887	0.295011
0.324912	0.42149	0.213943	0.254077	0.300448	0.364087	0.329869	0.277422	0.313719	0.333387
0.29629	0.254077	0.370643	0.358331	0.383754	0.313879	0.346338	0.315638	0.314839	0.342341
0.240166	0.292773	0.341222	0.340902	0.301887	0.305245	0.336425	0.344899	0.302686	0.322833
0.270547	0.338983	0.310681	0.308602	0.239367	0.321075	0.346178	0.349856	0.325871	0.330988
0.338343	0.291014	0.377199	0.398145	0.383914	0.278702	0.307004	0.336105	0.331948	0.335785
0.280461	0.23505	0.327311	0.352414	0.353054	0.195875	0.304285	0.213463	0.283978	0.31292
0.269907	0.408379	0.206268	0.291813	0.236489	0.339942	0.322354	0.29693	0.298849	0.298689
0.325552	0.409818	0.248961	0.303965	0.311001	0.341222	0.302846	0.26591	0.329229	0.313559
0.292933	0.389031	0.205309	0.274544	0.303326	0.316118	0.303326	0.247841	0.286377	0.295971
0.263991	0.32811	0.259354	0.275823	0.280301	0.270067	0.291493	0.2496	0.26559	0.274704
0	0.315798	0.306524	0.31324	0.295171	0.267988	0.322034	0.285737	0.303806	0.322833
0.315798	0	0.401343	0.420691	0.417333	0.261113	0.360409	0.329709	0.340902	0.342341
0.306524	0.401343	0	0.262072	0.289735	0.333387	0.326671	0.267349	0.320755	0.322514
0.31324	0.420691	0.262072	0	0.20323	0.354973	0.350656	0.301727	0.314519	0.326031
0.295171	0.417333	0.289735	0.20323	0	0.339783	0.335625	0.331948	0.289894	0.306684
0.267988	0.261113	0.333387	0.354973	0.339783	0	0.318676	0.247841	0.287976	0.323793
0.322034	0.360409	0.326671	0.350656	0.335625	0.318676	0	0.311321	0.306524	0.324752
0.285737	0.329709	0.267349	0.301727	0.331948	0.247841	0.311321	0	0.277422	0.283339
0.303806	0.340902	0.320755	0.314519	0.289894	0.287976	0.306524	0.277422	0	0.256476
0.322833	0.342341	0.322514	0.326031	0.306684	0.323793	0.324752	0.283339	0.256476	0
0.315318	0.252638	0.387592	0.400224	0.388711	0.230892	0.305245	0.305085	0.325871	0.326351
0.326191	0.337544	0.349536	0.361529	0.366006	0.330828	0.290054	0.316917	0.314839	0.258395
0.307643	0.141509	0.399264	0.404541	0.411896	0.259834	0.347138	0.320115	0.342821	0.326031
0.151423	0.266709	0.350656	0.345539	0.337224	0.287016	0.330189	0.306524	0.295811	0.32747
0.269108	0.24992	0.355133	0.350496	0.350496	0.270387	0.334186	0.288136	0.314039	0.319156
0.330189	0.319156	0.339143	0.355133	0.300448	0.342341	0.335785	0.336265	0.269907	0.308602
0.301567	0.257435	0.365686	0.360249	0.35945	0.287976	0.348417	0.298369	0.331788	0.314359
0.320275	0.416693	0.237288	0.263991	0.295491	0.353534	0.317397	0.255836	0.303965	0.283179
0.315478	0.414615	0.255037	0.29645	0.302846	0.3441	0.235849	0.267029	0.322833	0.322993
0.31212	0.347618	0.290054	0.324912	0.320435	0.295811	0.284138	0.269108	0.323473	0.292133
0.280301	0.301087	0.347138	0.342341	0.291973	0.332907	0.363447	0.322034	0.300448	0.307483
0.300608	0.409498	0.269268	0.268788	0.261753	0.337544	0.322194	0.302047	0.285257	0.29629

For Review Only

0.308762	0.411417	0.246882	0.277582	0.298849	0.349536	0.290854	0.267988	0.322034	0.310361
0.298209	0.387592	0.282699	0.301407	0.309082	0.324912	0.331148	0.307004	0.332587	0.323473
0.23409	0.425488	0.263192	0.291973	0.190758	0.350176	0.3441	0.325232	0.301407	0.31308
0.294052	0.416374	0.289255	0.20243	0.008794	0.338823	0.335785	0.331468	0.289575	0.306044
0.308602	0.417013	0.223217	0.260473	0.288935	0.349376	0.314519	0.28174	0.310201	0.31292
0.306044	0.326191	0.303646	0.320755	0.254077	0.34394	0.37464	0.334986	0.306684	0.322833
0.325712	0.419731	0.214263	0.253438	0.300608	0.363447	0.330189	0.276943	0.31244	0.332747
0.318356	0.4063	0.23489	0.283179	0.320435	0.352095	0.282699	0.252478	0.335945	0.32859
0.310042	0.399584	0.241126	0.275823	0.305405	0.334346	0.303806	0.270547	0.308762	0.299169
0.29629	0.175728	0.386952	0.397346	0.389831	0.225935	0.330349	0.292133	0.304605	0.309082
0.279341	0.306524	0.319316	0.324752	0.287656	0.248321	0.306044	0.277263	0.206908	0.317717
0.267349	0.335945	0.247042	0.28062	0.271027	0.275024	0.293252	0.245443	0.272146	0.271826
0.323793	0.410937	0.23473	0.278702	0.311481	0.341062	0.295651	0.248641	0.324272	0.3118
0.324752	0.401503	0.23425	0.291493	0.331468	0.338503	0.28142	0.235689	0.325552	0.322194
0.23377	0.425807	0.263032	0.292453	0.190118	0.349057	0.34442	0.326031	0.301727	0.31212
0.005117	0.315958	0.305884	0.31228	0.295331	0.268148	0.321234	0.285098	0.303006	0.322993
0.284778	0.364407	0.255996	0.276943	0.271346	0.293092	0.298369	0.268468	0.270227	0.288455
0.286217	0.303166	0.345699	0.345379	0.300927	0.275344	0.301567	0.307163	0.230732	0.292133
0.319635	0.371282	0.31244	0.329709	0.329069	0.322194	0.287816	0.316917	0.302686	0.31276
0.298849	0.43828	0.254717	0.252798	0.267988	0.362488	0.323633	0.319635	0.306524	0.320755
0.28078	0.414615	0.227055	0.273425	0.263351	0.351135	0.329389	0.298849	0.315478	0.335465
0.253438	0.42181	0.277902	0.288935	0.176847	0.337064	0.34442	0.32779	0.294212	0.318036
0.29613	0.371282	0.231212	0.28142	0.3126	0.307643	0.275823	0.226735	0.29757	0.303006
0.299009	0.313879	0.347937	0.351135	0.364407	0.292933	0.286377	0.316597	0.290214	0.315318
0.113847	0.341701	0.304285	0.31212	0.259354	0.289735	0.31196	0.304605	0.298849	0.308922
0.292133	0.357851	0.283019	0.277582	0.294851	0.307483	0.335785	0.293572	0.309242	0.334986
0.302207	0.257755	0.366006	0.35993	0.35961	0.287496	0.347458	0.29741	0.330988	0.314199
0.304765	0.391749	0.245923	0.292773	0.315798	0.333067	0.31276	0.245123	0.309242	0.317717
0.326351	0.324272	0.368724	0.321234	0.275664	0.340422	0.348577	0.345059	0.326991	0.342821
0.317717	0.239047	0.395267	0.399264	0.401023	0.235849	0.315958	0.303806	0.334186	0.336105
0.32795	0.408379	0.268308	0.307323	0.317717	0.348257	0.302047	0.275184	0.325712	0.313879
0.311161	0.409978	0.23489	0.26559	0.273105	0.338823	0.349536	0.286856	0.307963	0.318356
0.295011	0.336265	0.304285	0.319476	0.278542	0.302526	0.333067	0.292453	0.274065	0.279821
0.284618	0.290694	0.304765	0.315798	0.319635	0.21826	0.3118	0.199712	0.285257	0.295171
0.32779	0.239687	0.399744	0.413335	0.40662	0.227215	0.320435	0.309562	0.342821	0.348737
0.232171	0.424368	0.262232	0.292133	0.188999	0.348097	0.34378	0.325712	0.301247	0.31212
0.283658	0.361848	0.303166	0.283179	0.263351	0.284138	0.311161	0.289095	0.261113	0.276783
0.301407	0.258555	0.365366	0.35929	0.35929	0.288136	0.348417	0.29741	0.331308	0.314519
0.270707	0.257275	0.340742	0.340902	0.358171	0.277742	0.321234	0.291493	0.300927	0.305405
0.29693	0.269747	0.351615	0.37528	0.305405	0.304605	0.355133	0.34314	0.331788	0.325232
0.337064	0.340742	0.308922	0.316118	0.322514	0.306044	0.318036	0.302207	0.301887	0.315798
0.326351	0.257275	0.363288	0.361529	0.354013	0.298369	0.336904	0.298049	0.308283	0.306844
0.26575	0.430125	0.269428	0.286697	0.18724	0.348737	0.340582	0.324432	0.301727	0.308283
0.330508	0.439239	0.23441	0.26495	0.301727	0.363128	0.345699	0.253598	0.322034	0.331308
0.329229	0.361049	0.307803	0.326351	0.331308	0.350176	0.331788	0.316597	0.308602	0.318676
0.326351	0.410457	0.236169	0.279821	0.31228	0.340902	0.29693	0.24976	0.323633	0.31212
0.309562	0.387752	0.287816	0.302526	0.26495	0.313559	0.334186	0.278862	0.303646	0.308762
0.302686	0.415414	0.254557	0.248001	0.290214	0.334506	0.315158	0.28062	0.29677	0.292453
0.293732	0.252478	0.360249	0.371122	0.362968	0.14071	0.315318	0.258075	0.274225	0.316278
0.301407	0.35945	0.272945	0.311481	0.320915	0.303166	0.3118	0.228494	0.28206	0.303806
0.314199	0.369524	0.268948	0.292133	0.291174	0.31196	0.300288	0.225776	0.289415	0.299648
0.323953	0.382475	0.310201	0.317557	0.319635	0.347618	0.31228	0.298049	0.299968	0.306044
0.288295	0.381356	0.225296	0.277263	0.307323	0.311001	0.306204	0.248481	0.300608	0.300927
0.316278	0.259194	0.396386	0.4055	0.399105	0.241765	0.313559	0.304765	0.331788	0.323953
0.295331	0.238887	0.367445	0.377039	0.386633	0.255836	0.330668	0.293732	0.299648	0.318676

For Review Only

0.283499	0.323313	0.307643	0.311481	0.31244	0.275983	0.303806	0.284138	0.287496	0.307803
0.318996	0.393188	0.300288	0.307483	0.287176	0.342981	0.316597	0.304125	0.29613	0.289255
0.314519	0.364886	0.292933	0.319476	0.334826	0.326511	0.286217	0.286537	0.320435	0.310042
0.320115	0.310201	0.323313	0.330349	0.342181	0.324912	0.319156	0.289255	0.289894	0.259514
0.290054	0.377518	0.24976	0.302367	0.304925	0.292293	0.303646	0.153822	0.285257	0.287176
0.289735	0.264791	0.356252	0.377518	0.350975	0.177646	0.308283	0.275983	0.283818	0.304605
0.3126	0.413495	0.230253	0.261753	0.293572	0.338343	0.31324	0.264151	0.300448	0.29741
0.309402	0.368564	0.2496	0.293892	0.294372	0.301407	0.293732	0.23473	0.301247	0.292133
0.292933	0.408698	0.227055	0.262392	0.287976	0.32859	0.322514	0.270547	0.307004	0.29757
0.302047	0.339143	0.244324	0.28174	0.307323	0.26511	0.309242	0.21938	0.273745	0.286537
0.316437	0.318196	0.31244	0.346178	0.337064	0.287816	0.321554	0.267829	0.307963	0.307803
0.276143	0.304125	0.301247	0.319156	0.32795	0.260633	0.304605	0.191557	0.295331	0.302047
0.286377	0.369044	0.310201	0.298369	0.254877	0.292773	0.318836	0.298209	0.26495	0.284298
0.271986	0.335785	0.271027	0.289415	0.28078	0.293732	0.294212	0.268468	0.271186	0.246242
0.320435	0.346178	0.295811	0.333866	0.331948	0.321394	0.32875	0.29741	0.304445	0.317077
0.299328	0.398465	0.247042	0.274225	0.299169	0.331468	0.310361	0.259034	0.311321	0.316917
0.3118	0.353214	0.269268	0.31324	0.324592	0.307004	0.332907	0.261273	0.300927	0.311321
0.295491	0.354013	0.269268	0.290374	0.292293	0.291014	0.274065	0.260313	0.28126	0.289415
0.307323	0.394468	0.310201	0.29677	0.304925	0.362168	0.327151	0.342661	0.327311	0.316597
0.284778	0.353214	0.25072	0.271826	0.273585	0.294691	0.28078	0.248961	0.28094	0.276623
0.299009	0.257915	0.356412	0.373681	0.361848	0.161976	0.309402	0.277422	0.271506	0.3118
0.300288	0.382795	0.299328	0.301087	0.314039	0.336425	0.334506	0.32763	0.326031	0.314679
0.268628	0.331468	0.258235	0.287496	0.283179	0.275983	0.28078	0.24928	0.274704	0.28126
0.291014	0.407419	0.229773	0.259994	0.248001	0.336585	0.329869	0.290694	0.303326	0.293412
0.29693	0.394148	0.227215	0.262232	0.300768	0.31308	0.3126	0.253758	0.291813	0.303646
0.309562	0.231372	0.394787	0.403902	0.401023	0.227534	0.317877	0.309882	0.334986	0.334506
0.305245	0.354173	0.29661	0.323953	0.332747	0.315638	0.287656	0.287016	0.300927	0.304925
0.299488	0.330508	0.330508	0.333547	0.329549	0.284938	0.285577	0.298689	0.276623	0.286856
0.284618	0.399264	0.275504	0.276303	0.279181	0.330189	0.334346	0.285897	0.286057	0.302367
0.261593	0.358491	0.262552	0.274225	0.267189	0.268948	0.319795	0.246562	0.199712	0.289735
0.310681	0.428686	0.232171	0.258395	0.295811	0.354653	0.337384	0.285257	0.327151	0.318036
0.294691	0.4063	0.279981	0.288615	0.29757	0.347777	0.335146	0.308283	0.313559	0.309242
0.309882	0.243204	0.389351	0.403262	0.394308	0.211705	0.310521	0.291653	0.309242	0.332267
0.272306	0.324912	0.280461	0.286856	0.29677	0.276623	0.292773	0.250879	0.2811	0.273585
0.267029	0.432683	0.269747	0.288935	0.185801	0.351935	0.341861	0.327311	0.303646	0.307963
0.279181	0.366805	0.251199	0.291334	0.283818	0.298049	0.28206	0.256316	0.277742	0.274065
0.267029	0.261593	0.332267	0.352894	0.338983	0.011832	0.318996	0.248961	0.288775	0.322514
0.28078	0.322034	0.314519	0.329229	0.298849	0.237448	0.308762	0.285098	0.300448	0.321394
0.29613	0.251199	0.363927	0.389671	0.374001	0.164695	0.31212	0.277582	0.279661	0.318836
0.303806	0.40662	0.236329	0.257755	0.295331	0.331308	0.316597	0.273585	0.309882	0.291493
0.315158	0.350656	0.320595	0.337224	0.339463	0.299808	0.290694	0.302846	0.326191	0.306044
0.336425	0.277742	0.396546	0.396866	0.382315	0.268948	0.309722	0.317557	0.34378	0.323153
0.29709	0.357691	0.331628	0.334186	0.337384	0.324272	0.305085	0.314519	0.303486	0.307323
0.295491	0.244004	0.362808	0.380237	0.373361	0.153822	0.313879	0.262072	0.276463	0.32811
0.303326	0.416374	0.252638	0.292933	0.300288	0.348097	0.317237	0.284138	0.303326	0.32779
0.298369	0.195875	0.368244	0.378957	0.383754	0.256316	0.338663	0.295171	0.320275	0.321554
0.305884	0.350496	0.315158	0.331948	0.300927	0.300128	0.307963	0.280141	0.272306	0.288615
0.284458	0.366485	0.271986	0.300448	0.261113	0.314839	0.315798	0.285417	0.260153	0.291653
0.325871	0.411577	0.253758	0.291174	0.302686	0.349856	0.295491	0.263671	0.321874	0.301567
0.322194	0.408858	0.244484	0.288136	0.319795	0.349376	0.297889	0.257595	0.323313	0.305884
0.278062	0.279341	0.319156	0.331468	0.310681	0.246722	0.29725	0.261912	0.226095	0.280141
0.31276	0.409018	0.245923	0.298209	0.301407	0.348897	0.304285	0.267509	0.323633	0.306524
0.316597	0.394627	0.259034	0.299009	0.304925	0.323153	0.298209	0.221138	0.310841	0.302846
0.309402	0.283818	0.369204	0.369204	0.341861	0.263032	0.291813	0.304445	0.291653	0.290694
0.294372	0.353054	0.271186	0.280301	0.290054	0.270227	0.294691	0.26591	0.260473	0.290534

For Review Only

0.329389	0.40678	0.245123	0.295651	0.314679	0.348897	0.299808	0.232491	0.323953	0.322354
0.285897	0.26559	0.31228	0.332907	0.329869	0.259834	0.299009	0.270387	0.278382	0.278382
0.307483	0.417493	0.254237	0.272785	0.268788	0.353854	0.306364	0.29613	0.302846	0.306844
0.345379	0.303486	0.362168	0.382955	0.37576	0.319955	0.31212	0.321714	0.301087	0.297889
0.333387	0.308762	0.354013	0.352255	0.330668	0.331308	0.353854	0.298689	0.308123	0.317557
0.288935	0.337864	0.32859	0.330029	0.329069	0.291014	0.294372	0.314359	0.304765	0.307323
0.304125	0.408698	0.267988	0.291973	0.287816	0.326031	0.326511	0.268948	0.31324	0.304925
0.301407	0.326351	0.323793	0.313399	0.311481	0.287816	0.308443	0.314039	0.276943	0.279341
0.323793	0.304125	0.357851	0.377039	0.346019	0.279341	0.292773	0.311001	0.321394	0.308283
0.269428	0.333706	0.262232	0.283818	0.278382	0.270547	0.28206	0.240486	0.278542	0.280301
0.32763	0.324912	0.358331	0.363607	0.366805	0.274704	0.304925	0.306844	0.321234	0.329229
0.308123	0.423089	0.241925	0.259514	0.256316	0.346818	0.34442	0.29709	0.304925	0.335785
0.306684	0.381196	0.242245	0.279501	0.284778	0.322514	0.288455	0.253758	0.301087	0.291973
0.290214	0.284938	0.321554	0.337864	0.326511	0.243044	0.275024	0.245283	0.288935	0.28078
0.314199	0.397346	0.229613	0.278702	0.293732	0.319795	0.300608	0.263671	0.279341	0.291174
0.292453	0.349856	0.29725	0.318836	0.295011	0.288295	0.31244	0.28078	0.261912	0.302047
0.293092	0.24944	0.362328	0.372721	0.365846	0.155261	0.307163	0.258235	0.274704	0.324752
0.350176	0.339783	0.363927	0.370483	0.350815	0.317557	0.287656	0.318036	0.337544	0.301407
0.286697	0.32859	0.271346	0.291813	0.292933	0.255676	0.294851	0.262072	0.263511	0.288136
0.31196	0.413495	0.24928	0.286537	0.300448	0.342341	0.323313	0.267988	0.307323	0.320915
0.29757	0.368884	0.255676	0.280301	0.298369	0.300927	0.294851	0.231532	0.294212	0.301407
0.288295	0.400863	0.225935	0.264791	0.287176	0.323473	0.316917	0.264631	0.300288	0.288615
0.26543	0.329549	0.253278	0.276943	0.273905	0.271027	0.28174	0.242405	0.267349	0.273265
0.299968	0.240646	0.361369	0.376399	0.373841	0.223057	0.306524	0.255676	0.301247	0.325072
0.305245	0.31244	0.361049	0.356732	0.366166	0.287976	0.290374	0.315318	0.284298	0.314359
0.324113	0.401183	0.23457	0.291653	0.331148	0.338823	0.282219	0.236489	0.325712	0.322674
0.217301	0.300768	0.321714	0.341062	0.28174	0.295331	0.338503	0.315318	0.29757	0.32843
0.276783	0.268148	0.340262	0.365526	0.347298	0.04765	0.31324	0.241605	0.279821	0.321874
0.292453	0.382155	0.230253	0.278702	0.299328	0.317237	0.310521	0.259674	0.29725	0.287816
0.283499	0.340582	0.304765	0.309082	0.299648	0.259354	0.308762	0.295011	0.294052	0.304125
0.294212	0.365526	0.294691	0.301247	0.291174	0.313719	0.324592	0.29709	0.268308	0.292933
0.259674	0.410457	0.271826	0.272626	0.241765	0.331148	0.322354	0.311321	0.287496	0.314039
0.290534	0.385513	0.270707	0.309562	0.283499	0.310201	0.309402	0.270387	0.261753	0.284618
0.322674	0.425807	0.248481	0.270867	0.279181	0.363767	0.338983	0.311321	0.320915	0.331148
0.282699	0.392709	0.263991	0.270867	0.264311	0.319795	0.32891	0.285897	0.267829	0.305085
0.291174	0.250879	0.362648	0.376879	0.368564	0.159098	0.307004	0.264791	0.276143	0.325072
0.282219	0.393028	0.282699	0.29693	0.259354	0.319476	0.330189	0.291174	0.263351	0.29725
0.322833	0.400863	0.23377	0.290374	0.330349	0.338343	0.28206	0.235849	0.325232	0.321714
0.306364	0.384554	0.251199	0.29741	0.311641	0.337064	0.306684	0.238088	0.294691	0.29725
0.293892	0.380237	0.283179	0.267669	0.24944	0.313399	0.325072	0.290214	0.141989	0.292293
0.326191	0.408379	0.242405	0.290854	0.321874	0.349696	0.29741	0.260313	0.322514	0.306364
0.309882	0.39095	0.261593	0.294532	0.298369	0.317717	0.322194	0.260313	0.309402	0.29709
0.330189	0.429485	0.23441	0.251359	0.289575	0.361848	0.321234	0.274065	0.31324	0.284938
0.325712	0.409018	0.256476	0.29661	0.299169	0.349217	0.290854	0.278062	0.316437	0.300288
0.318036	0.240966	0.387752	0.398785	0.396546	0.223217	0.309082	0.31292	0.334026	0.339303
0.29661	0.384554	0.292453	0.293412	0.29677	0.324752	0.291813	0.276303	0.295651	0.285098
0.324272	0.355293	0.300288	0.318356	0.333387	0.287176	0.322514	0.242885	0.295011	0.300448
0.294851	0.363767	0.28126	0.302526	0.306524	0.31196	0.32891	0.3118	0.284298	0.325871
0.349057	0.341222	0.362808	0.368884	0.348737	0.316917	0.286856	0.316597	0.337064	0.300448
0.325552	0.275024	0.383275	0.393028	0.350016	0.258235	0.314359	0.315478	0.319795	0.31244
0.276943	0.236808	0.339463	0.35993	0.348097	0.171091	0.310361	0.235689	0.266869	0.31324
0.289894	0.31324	0.283339	0.31308	0.308443	0.300927	0.330189	0.298049	0.288295	0.301247
0.319795	0.412856	0.268628	0.273905	0.280461	0.346658	0.3441	0.311641	0.32859	0.309082
0.301727	0.246083	0.367445	0.373521	0.358331	0.238727	0.317397	0.286057	0.245443	0.295811
0.297729	0.283499	0.37448	0.379757	0.369524	0.276303	0.288455	0.314998	0.283978	0.32843

For Review Only

0.323633	0.411097	0.235689	0.278382	0.310681	0.340422	0.295171	0.248961	0.324113	0.310841
0.316917	0.261912	0.389671	0.4055	0.404541	0.235369	0.308283	0.298369	0.324592	0.329069
0.302047	0.336745	0.286377	0.314519	0.322034	0.252159	0.302367	0.291973	0.28174	0.285577
0.298209	0.294212	0.323953	0.316597	0.335945	0.299808	0.322674	0.303646	0.295651	0.319635
0.330029	0.434282	0.269907	0.262072	0.284298	0.369204	0.34426	0.300128	0.311641	0.313399
0.317237	0.34314	0.338503	0.348737	0.352414	0.32827	0.319955	0.336105	0.297889	0.325232
0.286856	0.294372	0.340262	0.32795	0.311641	0.290534	0.306364	0.304125	0.278222	0.295811
0.29725	0.415254	0.255836	0.26511	0.279021	0.340102	0.319955	0.28094	0.29693	0.304925
0.309722	0.32811	0.358331	0.349536	0.344739	0.287816	0.284458	0.318836	0.324752	0.319635
0.307963	0.178286	0.397026	0.397026	0.414135	0.267189	0.341222	0.316118	0.330189	0.331148
0.290854	0.274065	0.316278	0.338983	0.341541	0.226255	0.315158	0.242405	0.280461	0.302526
0.320115	0.232811	0.396386	0.411737	0.407099	0.23393	0.323793	0.308443	0.340422	0.339783
0.260633	0.324912	0.257595	0.28062	0.280461	0.269268	0.285257	0.241765	0.273105	0.275024
0.299169	0.348737	0.331148	0.340262	0.310042	0.303326	0.295971	0.299808	0.263192	0.290694
0.332587	0.363767	0.303326	0.307004	0.305884	0.323953	0.313399	0.29693	0.302367	0.316917
0.29709	0.35881	0.245603	0.286537	0.291334	0.299009	0.291014	0.243524	0.278222	0.288295
0.332107	0.418292	0.244164	0.262712	0.286377	0.349856	0.32795	0.273105	0.32875	0.291014
0.346658	0.366006	0.319955	0.32843	0.31276	0.32763	0.294851	0.298529	0.31292	0.316757
0.314679	0.277263	0.364727	0.37544	0.354813	0.255996	0.307483	0.286697	0.295011	0.285897
0.300448	0.318676	0.347777	0.351135	0.350815	0.276623	0.320115	0.304765	0.323153	0.330029
0.326831	0.300448	0.368084	0.371762	0.350176	0.270547	0.306044	0.317877	0.335625	0.32811
0.319795	0.337224	0.332267	0.335465	0.316597	0.325232	0.31292	0.297889	0.261433	0.067317
0.313399	0.341701	0.317237	0.339303	0.366805	0.317077	0.315798	0.316597	0.335465	0.334186
0.321714	0.400704	0.257595	0.29677	0.311481	0.337864	0.291174	0.263671	0.311321	0.304125
0.299808	0.401983	0.258235	0.268948	0.25024	0.322514	0.336105	0.279021	0.271027	0.302047
0.304925	0.357371	0.323633	0.319635	0.322993	0.323633	0.261273	0.297729	0.305245	0.277742
0.295331	0.175408	0.385673	0.397825	0.389191	0.224816	0.330988	0.291813	0.303486	0.310201
0.273105	0.368724	0.267349	0.291334	0.305245	0.322514	0.339143	0.285737	0.319476	0.330029
0.325072	0.237608	0.396226	0.411257	0.411896	0.230413	0.321075	0.313719	0.346978	0.353214
0.305405	0.410457	0.21842	0.258874	0.311001	0.351615	0.32891	0.258555	0.330349	0.303166
0.25072	0.358011	0.29661	0.299488	0.237128	0.305085	0.319635	0.305405	0.275344	0.294691
0.311481	0.418292	0.238567	0.259994	0.280141	0.342661	0.339303	0.299648	0.306044	0.322993
0.329869	0.270227	0.389511	0.397186	0.371442	0.255836	0.310841	0.303166	0.325392	0.316278
0.31228	0.346338	0.283019	0.318836	0.316278	0.304925	0.282219	0.229133	0.307643	0.304765
0.303965	0.321714	0.331628	0.334506	0.322674	0.248641	0.299488	0.270387	0.253278	0.299169
0.309882	0.372242	0.258555	0.29709	0.310841	0.322674	0.29613	0.255357	0.308283	0.295651
0.307004	0.31196	0.364886	0.34426	0.356412	0.298689	0.278862	0.320595	0.272626	0.31212
0.318996	0.248961	0.397026	0.405341	0.410617	0.223537	0.325072	0.314519	0.336425	0.34362
0.274384	0.413815	0.248161	0.24976	0.253598	0.32763	0.332107	0.295011	0.28158	0.311321
0.324752	0.401823	0.23473	0.292453	0.330668	0.339623	0.283179	0.237768	0.325712	0.322674
0.284458	0.329229	0.26591	0.300927	0.330189	0.246402	0.311001	0.008315	0.276143	0.282379
0.324272	0.260633	0.393188	0.409338	0.400064	0.236808	0.308443	0.311481	0.333866	0.331628
0.280301	0.423409	0.251359	0.261273	0.263991	0.334986	0.339783	0.295971	0.276303	0.314519
0.279501	0.339783	0.288615	0.292133	0.272306	0.259514	0.305884	0.255516	0.256156	0.290534
0.318836	0.346978	0.313879	0.350016	0.32811	0.306044	0.263192	0.29629	0.323473	0.287176
0.262712	0.431404	0.264791	0.287656	0.195715	0.349376	0.336425	0.323153	0.303326	0.307803
0.32827	0.372721	0.268148	0.302207	0.319476	0.322354	0.305724	0.264631	0.315798	0.307163
0.317877	0.366645	0.292613	0.318996	0.300448	0.313879	0.339783	0.300608	0.298369	0.29677
0.337544	0.356572	0.300448	0.32875	0.354013	0.325552	0.301567	0.278702	0.34314	0.299169
0.322034	0.363927	0.285257	0.323953	0.305884	0.318836	0.314679	0.301407	0.320275	0.311481
0.330988	0.331628	0.289735	0.317877	0.338503	0.335305	0.330508	0.289575	0.341701	0.313559
0.331628	0.242725	0.394947	0.409018	0.408858	0.227374	0.316757	0.310042	0.339623	0.348737
0.298689	0.43732	0.251679	0.235529	0.263671	0.351935	0.346178	0.293892	0.298689	0.302846
0.319955	0.25024	0.398625	0.409338	0.402143	0.230892	0.310042	0.31212	0.330508	0.32859
0.330988	0.429965	0.235849	0.251839	0.289415	0.363767	0.321714	0.275024	0.314359	0.284938

For Review Only

0.31212	0.427087	0.253118	0.293252	0.325232	0.364247	0.336745	0.278702	0.340422	0.311161
0.313399	0.326511	0.365846	0.369044	0.367445	0.31212	0.274225	0.306524	0.282539	0.252958
0.31276	0.430285	0.257115	0.268628	0.245763	0.356092	0.337704	0.293412	0.308123	0.303006
0.313399	0.416054	0.21842	0.29629	0.318836	0.34346	0.324272	0.260793	0.304605	0.325712

For Review Only

0.368084	0.367605	0.382635	0.316917	0.319635	0.298369	0.336904	0.279821	0.297889	0.307643
0.309082	0.32827	0.314679	0.31228	0.3118	0.330828	0.32795	0.302846	0.291493	0.314998
0.397666	0.347618	0.409178	0.34394	0.362008	0.354493	0.3756	0.108251	0.226255	0.291653
0.371922	0.34314	0.370163	0.323313	0.337864	0.323313	0.357052	0.236808	0.263351	0.284778
0.337704	0.365206	0.303965	0.314998	0.311321	0.292453	0.318996	0.352574	0.350496	0.310521
0.388871	0.345859	0.388232	0.32843	0.330349	0.333866	0.340902	0.257115	0.275344	0.291493
0.381036	0.340742	0.379117	0.306524	0.333866	0.279181	0.3433	0.307163	0.322514	0.313559
0.287336	0.322833	0.233451	0.269587	0.200192	0.346978	0.208826	0.32859	0.345219	0.307963
0.362968	0.348577	0.377678	0.270867	0.324592	0.283499	0.332587	0.317077	0.319476	0.321394
0.287016	0.356252	0.209786	0.251519	0.175088	0.346658	0.182123	0.367925	0.372881	0.349696
0.246242	0.277582	0.32779	0.322993	0.352574	0.319156	0.352574	0.34378	0.348577	0.28094
0.394308	0.339783	0.402303	0.339463	0.348257	0.354013	0.35977	0.129677	0.224336	0.288136
0.319635	0.346338	0.303166	0.292133	0.29693	0.291174	0.330988	0.302846	0.320595	0.307163
0.272306	0.320915	0.295011	0.303806	0.294851	0.299009	0.29757	0.320915	0.314998	0.278702
0.353214	0.321394	0.374001	0.321234	0.335146	0.294372	0.346658	0.264631	0.273905	0.291334
0.299488	0.314679	0.351775	0.324432	0.326511	0.349057	0.340422	0.252478	0.291973	0.294052
0.373681	0.348417	0.366805	0.318676	0.350176	0.269587	0.355772	0.324592	0.327151	0.334666
0.350016	0.332587	0.336585	0.285897	0.28126	0.310521	0.29629	0.290214	0.293252	0.304765
0.409498	0.367605	0.418932	0.360889	0.361848	0.35929	0.386473	0.24928	0.261273	0.318996
0.329709	0.34442	0.260473	0.264471	0.256636	0.31276	0.269747	0.351135	0.352414	0.346978
0.301567	0.346019	0.275024	0.229933	0.306364	0.261912	0.336904	0.331148	0.342181	0.324912
0.362008	0.371442	0.338184	0.336745	0.342181	0.251039	0.362648	0.320275	0.323313	0.318036
0.194436	0.252798	0.2819	0.329869	0.340422	0.35865	0.342981	0.39095	0.399584	0.320275
0.222578	0.29709	0.242725	0.279341	0.262552	0.315478	0.279501	0.336105	0.336105	0.274225
0.383115	0.344579	0.398785	0.3441	0.346658	0.301567	0.351615	0.262072	0.276623	0.284618
0.380876	0.347298	0.394627	0.348257	0.362968	0.368084	0.372402	0.18804	0.184842	0.238887
0.360889	0.348737	0.382795	0.321714	0.330988	0.339942	0.345059	0.212344	0.225136	0.288295
0.29693	0.29645	0.315318	0.286217	0.293572	0.299488	0.305085	0.247042	0.256316	0.251999
0.315318	0.326191	0.307643	0.151423	0.269108	0.330189	0.301567	0.320275	0.315478	0.31212
0.252638	0.337544	0.141509	0.266709	0.24992	0.319156	0.257435	0.416693	0.414615	0.347618
0.387592	0.349536	0.399264	0.350656	0.355133	0.339143	0.365686	0.237288	0.255037	0.290054
0.400224	0.361529	0.404541	0.345539	0.350496	0.355133	0.360249	0.263991	0.29645	0.324912
0.388711	0.366006	0.411896	0.337224	0.350496	0.300448	0.35945	0.295491	0.302846	0.320435
0.230892	0.330828	0.259834	0.287016	0.270387	0.342341	0.287976	0.353534	0.3441	0.295811
0.305245	0.290054	0.347138	0.330189	0.334186	0.335785	0.348417	0.317397	0.235849	0.284138
0.305085	0.316917	0.320115	0.306524	0.288136	0.336265	0.298369	0.255836	0.267029	0.269108
0.325871	0.314839	0.342821	0.295811	0.314039	0.269907	0.331788	0.303965	0.322833	0.323473
0.326351	0.258395	0.326031	0.32747	0.319156	0.308602	0.314359	0.283179	0.322993	0.292133
0	0.251999	0.236329	0.291653	0.29741	0.347937	0.314839	0.392229	0.383435	0.302846
0.251999	0	0.329389	0.326831	0.348097	0.341861	0.342821	0.340422	0.346338	0.279981
0.236329	0.329389	0	0.24976	0.222098	0.329549	0.233291	0.400704	0.398945	0.333547
0.291653	0.326831	0.24976	0	0.257755	0.318836	0.272945	0.334506	0.336745	0.319476
0.29741	0.348097	0.222098	0.257755	0	0.34362	0.10905	0.352734	0.358011	0.323153
0.347937	0.341861	0.329549	0.318836	0.34362	0	0.337544	0.346818	0.341382	0.332747
0.314839	0.342821	0.233291	0.272945	0.10905	0.337544	0	0.363447	0.371282	0.319316
0.392229	0.340422	0.400704	0.334506	0.352734	0.346818	0.363447	0	0.228973	0.26575
0.383435	0.346338	0.398945	0.336745	0.358011	0.341382	0.371282	0.228973	0	0.282699
0.302846	0.279981	0.333547	0.319476	0.323153	0.332747	0.319316	0.26575	0.282699	0
0.348737	0.348897	0.304285	0.308602	0.308602	0.206268	0.318036	0.352414	0.350016	0.338503
0.38999	0.325392	0.401183	0.350496	0.352574	0.309722	0.356732	0.269587	0.292613	0.292613

For Review Only

0.380876	0.348897	0.396866	0.346019	0.350176	0.348737	0.365046	0.197634	0.180684	0.269428
0.333067	0.333547	0.377838	0.330508	0.342661	0.356892	0.353854	0.283179	0.303646	0.300768
0.409658	0.35961	0.420051	0.356412	0.364567	0.253438	0.374161	0.293732	0.291813	0.31324
0.388391	0.366326	0.410297	0.335945	0.349536	0.299648	0.357851	0.295011	0.302846	0.319795
0.393348	0.349696	0.411896	0.3441	0.354653	0.339463	0.362968	0.233131	0.251679	0.295171
0.362648	0.361529	0.335465	0.333387	0.334026	0.226095	0.345059	0.321234	0.331148	0.341541
0.408858	0.367925	0.416693	0.35961	0.361209	0.358331	0.385353	0.24944	0.261593	0.319795
0.389831	0.354013	0.393828	0.334986	0.367445	0.357691	0.378957	0.217461	0.125999	0.291973
0.384394	0.334186	0.392229	0.331628	0.350975	0.347138	0.355453	0.180365	0.197634	0.269907
0.195395	0.300768	0.193636	0.28142	0.25072	0.309722	0.269907	0.392869	0.394468	0.306684
0.285098	0.338823	0.305405	0.298689	0.300608	0.293412	0.316917	0.325871	0.336425	0.318676
0.308283	0.294532	0.329069	0.295811	0.299808	0.303646	0.311161	0.237768	0.255037	0.26527
0.384394	0.358331	0.398145	0.34442	0.349696	0.346338	0.36009	0.20387	0.165494	0.300288
0.380876	0.367125	0.394947	0.341382	0.354493	0.358491	0.362168	0.225616	0.132555	0.3126
0.408858	0.35881	0.420211	0.357371	0.364886	0.252638	0.37448	0.293252	0.290694	0.31276
0.315478	0.326351	0.308123	0.151743	0.268788	0.329549	0.301087	0.318996	0.314359	0.311161
0.323153	0.306044	0.347777	0.3126	0.314519	0.306684	0.323313	0.255357	0.273105	0.271666
0.299169	0.315318	0.292773	0.280301	0.251519	0.295971	0.271186	0.324432	0.334666	0.303965
0.304285	0.304605	0.35897	0.314679	0.346338	0.316437	0.345379	0.270387	0.2819	0.285257
0.420851	0.362008	0.433323	0.347618	0.376719	0.354493	0.385513	0.235209	0.255357	0.306684
0.408379	0.367125	0.409178	0.346658	0.360569	0.280141	0.373361	0.28174	0.287336	0.304445
0.400704	0.354813	0.408858	0.331468	0.337704	0.287816	0.345059	0.286697	0.303646	0.302686
0.347777	0.339942	0.367445	0.318196	0.330668	0.332427	0.342981	0.227694	0.15686	0.29693
0.227055	0.229933	0.307004	0.29709	0.326831	0.34442	0.341382	0.350815	0.347458	0.293252
0.331788	0.336265	0.338343	0.222098	0.294372	0.292613	0.329389	0.318836	0.317717	0.307643
0.357211	0.367925	0.346658	0.303006	0.320435	0.344899	0.334986	0.276463	0.303326	0.332907
0.313879	0.342181	0.232811	0.273105	0.10873	0.336745	0.007675	0.362648	0.370483	0.319156
0.37464	0.362968	0.388711	0.321554	0.326671	0.353054	0.344899	0.216821	0.244803	0.298529
0.345539	0.361049	0.315318	0.341701	0.302686	0.269428	0.31212	0.341062	0.348417	0.350496
0.129357	0.269268	0.244164	0.304285	0.29725	0.357531	0.316437	0.393508	0.395907	0.306684
0.380397	0.346658	0.393668	0.346019	0.360889	0.362008	0.3748	0.195235	0.181484	0.248961
0.392069	0.347937	0.410617	0.349057	0.367125	0.306524	0.385034	0.275983	0.3118	0.306524
0.345219	0.340742	0.331628	0.305724	0.305405	0.282219	0.306684	0.317077	0.329389	0.305564
0.270547	0.313399	0.287656	0.295011	0.278702	0.32795	0.292773	0.303326	0.302846	0.267189
0.131116	0.26591	0.237928	0.304445	0.300288	0.366166	0.323793	0.407099	0.404381	0.313399
0.407899	0.35865	0.419092	0.355772	0.364087	0.252958	0.373841	0.293092	0.291334	0.3126
0.344739	0.3441	0.348097	0.303166	0.320275	0.298529	0.338663	0.293412	0.294212	0.298209
0.314519	0.342341	0.23425	0.273905	0.10905	0.338024	0.007835	0.363607	0.371442	0.319476
0.293092	0.330029	0.247202	0.247042	0.2504	0.311161	0.243524	0.339783	0.349696	0.305245
0.32747	0.37496	0.26559	0.314039	0.314519	0.268468	0.323633	0.353374	0.364087	0.321234
0.316118	0.347777	0.335305	0.317237	0.311161	0.333706	0.310201	0.286057	0.334826	0.31244
0.318356	0.337544	0.247681	0.285098	0.205469	0.315798	0.162936	0.351775	0.362808	0.3126
0.40646	0.362488	0.424528	0.353214	0.370643	0.248001	0.386633	0.283339	0.297889	0.316917
0.416374	0.365046	0.42229	0.371122	0.382475	0.347777	0.400064	0.235209	0.277103	0.332107
0.36009	0.342981	0.365526	0.3433	0.304925	0.333866	0.31292	0.29725	0.315478	0.326991
0.384874	0.35993	0.397985	0.346178	0.350656	0.345539	0.35977	0.205788	0.167093	0.300768
0.356892	0.366166	0.383595	0.348577	0.355453	0.309242	0.360889	0.287976	0.306844	0.300768
0.391909	0.333227	0.409978	0.342981	0.363288	0.335465	0.378798	0.254077	0.232491	0.305564
0.212504	0.306044	0.252319	0.286217	0.278062	0.337864	0.291493	0.364087	0.356892	0.290534
0.34346	0.331308	0.356092	0.311001	0.334026	0.330189	0.334826	0.217461	0.241765	0.261273
0.350975	0.322674	0.361529	0.324113	0.344899	0.318036	0.350176	0.230572	0.207867	0.237768
0.35945	0.329389	0.373041	0.348577	0.337224	0.332427	0.344739	0.313399	0.302526	0.32763
0.348577	0.333067	0.366645	0.311321	0.317877	0.339942	0.333227	0.241126	0.207067	0.290534
0.087144	0.255197	0.241925	0.293252	0.305085	0.350336	0.319476	0.401503	0.391749	0.304125
0.227055	0.309242	0.25024	0.29629	0.289095	0.324272	0.298209	0.37464	0.379437	0.333227

For Review Only

0.282539	0.311161	0.315318	0.29645	0.306524	0.330668	0.330349	0.29677	0.309242	0.29677
0.376239	0.356572	0.379757	0.339143	0.318996	0.329389	0.327151	0.303646	0.279981	0.315798
0.313399	0.274544	0.355772	0.32875	0.346338	0.350016	0.350975	0.264631	0.240966	0.258395
0.309242	0.287176	0.306204	0.29677	0.31244	0.323953	0.319476	0.316917	0.326191	0.308123
0.357211	0.342341	0.367285	0.318036	0.317077	0.338823	0.32875	0.199232	0.230093	0.250879
0.224017	0.295491	0.257275	0.291174	0.278542	0.331148	0.29645	0.351775	0.338024	0.287976
0.386633	0.34378	0.40662	0.340422	0.350176	0.355293	0.370323	0.204669	0.209626	0.293892
0.355293	0.325232	0.355453	0.330988	0.332587	0.340262	0.346178	0.197793	0.228014	0.261753
0.380876	0.334186	0.395107	0.329229	0.336105	0.339143	0.342821	0.216022	0.252319	0.301567
0.298209	0.313719	0.325072	0.297729	0.306044	0.304765	0.306844	0.267988	0.270227	0.257275
0.285897	0.326991	0.305405	0.315158	0.298849	0.333706	0.308922	0.288455	0.279021	0.24944
0.282859	0.316278	0.295811	0.291493	0.300448	0.331948	0.321234	0.279981	0.271826	0.267509
0.329389	0.322833	0.357851	0.323633	0.331948	0.293572	0.344579	0.318516	0.314359	0.307323
0.307483	0.277742	0.331628	0.298209	0.303006	0.294691	0.316278	0.252478	0.268468	0.262232
0.34378	0.331948	0.347138	0.335945	0.302367	0.350815	0.289415	0.29709	0.304445	0.298849
0.365046	0.342021	0.387752	0.333706	0.32779	0.345539	0.342181	0.224656	0.235209	0.295491
0.332907	0.336265	0.345539	0.326351	0.310521	0.340102	0.321394	0.286217	0.293892	0.298049
0.311481	0.29693	0.336105	0.305884	0.319476	0.321554	0.333706	0.232491	0.208347	0.257915
0.397346	0.330029	0.389671	0.342661	0.366326	0.342821	0.382315	0.291014	0.290694	0.311481
0.319156	0.307004	0.340742	0.308762	0.306524	0.303965	0.323953	0.220819	0.225456	0.264791
0.229453	0.308922	0.253918	0.293092	0.277582	0.32875	0.292773	0.35977	0.342501	0.294052
0.349217	0.315478	0.381516	0.347937	0.32843	0.318836	0.318676	0.309882	0.318676	0.311161
0.29693	0.297729	0.323313	0.295331	0.298209	0.300608	0.308283	0.242085	0.240966	0.257595
0.391429	0.331468	0.405341	0.345859	0.355932	0.320755	0.376239	0.270707	0.295811	0.301887
0.364727	0.351775	0.378318	0.330988	0.323793	0.334986	0.344739	0.211545	0.235369	0.302207
0.144228	0.2811	0.236329	0.293892	0.297889	0.358491	0.315158	0.397346	0.400544	0.302047
0.307004	0.28206	0.341382	0.319635	0.342181	0.344899	0.352574	0.26527	0.259834	0.276783
0.258555	0.225616	0.324113	0.302207	0.317877	0.332427	0.32827	0.329069	0.32779	0.29677
0.375919	0.335785	0.393828	0.329389	0.353694	0.331148	0.371442	0.28174	0.291174	0.325871
0.32827	0.325871	0.355453	0.286377	0.29693	0.318516	0.322194	0.292293	0.303486	0.311481
0.413655	0.371602	0.411417	0.353694	0.366645	0.350656	0.380237	0.228494	0.240166	0.300128
0.389351	0.344899	0.39047	0.336904	0.342341	0.325712	0.353374	0.293892	0.304285	0.323633
0.166294	0.28142	0.243364	0.290054	0.293892	0.346338	0.307963	0.391429	0.386792	0.311001
0.290854	0.29757	0.314679	0.299169	0.289415	0.306684	0.301407	0.266869	0.270547	0.270227
0.407419	0.362968	0.426607	0.354333	0.373201	0.246083	0.388391	0.282379	0.298529	0.319635
0.329229	0.295971	0.354493	0.309722	0.321394	0.3126	0.337384	0.23377	0.211385	0.252478
0.232171	0.329229	0.260473	0.286377	0.269268	0.341701	0.289255	0.351775	0.342021	0.293732
0.290054	0.32747	0.316597	0.311161	0.311481	0.315958	0.320595	0.321234	0.317717	0.291493
0.224177	0.303326	0.252159	0.287976	0.285098	0.336745	0.295651	0.368724	0.360889	0.300128
0.377199	0.334346	0.39015	0.336425	0.336265	0.346498	0.350975	0.227694	0.251199	0.293732
0.288455	0.285257	0.337224	0.334826	0.334506	0.344899	0.338503	0.290374	0.29741	0.235849
0.160857	0.271666	0.28126	0.322354	0.325072	0.353694	0.335465	0.394308	0.391589	0.305564
0.279981	0.240806	0.346019	0.322674	0.34362	0.364886	0.357691	0.32811	0.336425	0.300768
0.21906	0.300768	0.24976	0.284778	0.277422	0.335785	0.29629	0.366485	0.365046	0.298049
0.380077	0.367285	0.411737	0.339623	0.356412	0.34426	0.370803	0.259834	0.268628	0.300288
0.261753	0.323473	0.179565	0.266869	0.254397	0.318836	0.253758	0.374001	0.37512	0.315478
0.329069	0.330349	0.347298	0.311001	0.332747	0.320275	0.340102	0.261113	0.293892	0.276783
0.35929	0.321554	0.370643	0.334186	0.327311	0.310521	0.335945	0.277582	0.288615	0.300448
0.385833	0.342661	0.394308	0.34426	0.366006	0.348577	0.370643	0.176047	0.162296	0.243684
0.382315	0.345379	0.391589	0.335945	0.341062	0.350496	0.354493	0.205628	0.177167	0.273745
0.256316	0.31244	0.279501	0.285737	0.284778	0.309562	0.31292	0.323313	0.323953	0.294851
0.382475	0.342501	0.392069	0.336745	0.35865	0.34426	0.370483	0.17221	0.18708	0.255357
0.371922	0.339623	0.381836	0.336745	0.349856	0.355133	0.355932	0.208027	0.1882	0.242405
0.221938	0.275823	0.269907	0.291973	0.285737	0.317077	0.301247	0.357851	0.335305	0.304285
0.319156	0.315798	0.347618	0.315158	0.326511	0.32763	0.336904	0.263671	0.291813	0.269268

For Review Only

0.385833	0.371122	0.396067	0.351935	0.351615	0.358171	0.362488	0.221298	0.178286	0.287976
0.244643	0.282699	0.26559	0.286377	0.28174	0.299968	0.290054	0.321394	0.321554	0.28206
0.393028	0.351935	0.403902	0.348257	0.356412	0.341382	0.367285	0.243364	0.223857	0.286377
0.26511	0.259194	0.299648	0.310681	0.316437	0.329229	0.29629	0.357851	0.351775	0.290534
0.338823	0.337864	0.301727	0.322993	0.269747	0.307803	0.232171	0.333547	0.342501	0.334506
0.26543	0.284298	0.32747	0.310681	0.326831	0.347138	0.351455	0.332587	0.334666	0.310042
0.382795	0.342021	0.401183	0.334026	0.337224	0.340262	0.355293	0.243204	0.235689	0.309402
0.291813	0.262712	0.323633	0.303166	0.306524	0.321075	0.32795	0.326671	0.315158	0.294851
0.204509	0.238567	0.295011	0.31212	0.309882	0.335945	0.329229	0.35897	0.356252	0.288295
0.29757	0.303486	0.314998	0.284938	0.295011	0.303006	0.309722	0.239047	0.235209	0.251679
0.238088	0.289894	0.317077	0.318996	0.330668	0.336265	0.34394	0.345219	0.354973	0.283019
0.404061	0.360729	0.417173	0.356732	0.371122	0.317397	0.381516	0.271346	0.294691	0.321874
0.354013	0.315478	0.370483	0.322354	0.34442	0.326991	0.351295	0.182123	0.190438	0.235689
0.224017	0.251999	0.279181	0.287016	0.288935	0.310841	0.298049	0.329869	0.317237	0.271027
0.366326	0.330668	0.388391	0.342661	0.354493	0.308762	0.365846	0.206588	0.244643	0.28094
0.335305	0.323793	0.349057	0.316917	0.316437	0.314998	0.323633	0.29709	0.31308	0.295651
0.220019	0.302846	0.25056	0.287336	0.279661	0.342501	0.292293	0.363288	0.354173	0.29661
0.223697	0.258395	0.330828	0.340422	0.352255	0.333227	0.355293	0.362168	0.366326	0.276943
0.298849	0.308443	0.321075	0.298689	0.307963	0.319476	0.325552	0.267509	0.292133	0.260953
0.384714	0.354813	0.404861	0.339942	0.352095	0.352414	0.368084	0.245923	0.254397	0.307963
0.338983	0.31292	0.362488	0.325871	0.32747	0.325712	0.345219	0.220179	0.224177	0.255836
0.37496	0.32747	0.388232	0.321714	0.329069	0.335146	0.335305	0.216342	0.251039	0.294212
0.294052	0.294532	0.317397	0.292133	0.294851	0.294691	0.302846	0.245603	0.252319	0.259514
0.180844	0.28078	0.243364	0.291973	0.28142	0.331948	0.29661	0.368404	0.367605	0.295811
0.233451	0.239847	0.303326	0.297889	0.32891	0.34362	0.34442	0.356572	0.360889	0.31212
0.380237	0.366965	0.394947	0.341382	0.354653	0.358331	0.362008	0.226255	0.133515	0.31244
0.340262	0.348737	0.298209	0.231052	0.306364	0.261273	0.318356	0.332587	0.340582	0.336425
0.232651	0.323473	0.261433	0.287656	0.272626	0.338343	0.290854	0.356732	0.342821	0.303326
0.369524	0.335945	0.369843	0.326671	0.326671	0.339623	0.332587	0.209306	0.238248	0.267509
0.331948	0.326511	0.340742	0.318676	0.316437	0.334986	0.336904	0.302846	0.293092	0.29661
0.340742	0.318036	0.361049	0.322354	0.329069	0.341222	0.334826	0.271506	0.289575	0.304765
0.384714	0.342501	0.399744	0.324912	0.350016	0.289095	0.364886	0.272306	0.28126	0.31212
0.356412	0.342501	0.363607	0.31244	0.323473	0.32779	0.348577	0.264151	0.277902	0.303806
0.414934	0.352095	0.425328	0.360249	0.383595	0.326191	0.399584	0.275504	0.269428	0.311481
0.369843	0.34346	0.391749	0.317237	0.348737	0.318836	0.360729	0.293732	0.289575	0.32747
0.216342	0.299328	0.252798	0.289255	0.285417	0.341541	0.300768	0.360729	0.352894	0.29645
0.379277	0.348737	0.388072	0.332427	0.349856	0.298049	0.363607	0.29693	0.295491	0.309082
0.380077	0.366326	0.393988	0.340262	0.353694	0.358171	0.361049	0.225776	0.132715	0.31276
0.348737	0.295651	0.374001	0.333227	0.346498	0.341382	0.356412	0.251359	0.251199	0.297889
0.357371	0.346498	0.379437	0.322833	0.327151	0.284778	0.345859	0.276143	0.316917	0.315638
0.379437	0.345059	0.391589	0.339143	0.339463	0.352574	0.353214	0.205628	0.177486	0.276143
0.368244	0.364886	0.384554	0.3433	0.325552	0.339143	0.350815	0.247042	0.261113	0.291973
0.396546	0.347937	0.407899	0.34394	0.360409	0.353054	0.373201	0.10889	0.225935	0.291174
0.384234	0.340262	0.395747	0.338983	0.362488	0.349536	0.370483	0.191078	0.165014	0.247042
0.127119	0.275983	0.240486	0.300288	0.304445	0.363128	0.320115	0.398145	0.39079	0.311321
0.333866	0.292773	0.371602	0.320595	0.345859	0.319795	0.356092	0.266709	0.26591	0.284618
0.329869	0.345379	0.352255	0.323313	0.321874	0.335945	0.342181	0.28094	0.292773	0.307643
0.340582	0.368884	0.361049	0.324592	0.335945	0.323153	0.362808	0.307004	0.29693	0.346178
0.221618	0.256956	0.331308	0.339942	0.351295	0.333227	0.354973	0.361369	0.365366	0.275344
0.185321	0.282379	0.267189	0.310361	0.301247	0.32891	0.323793	0.372562	0.384394	0.29629
0.21858	0.295491	0.239207	0.269108	0.268628	0.316757	0.279981	0.352255	0.348257	0.293092
0.332427	0.338983	0.318676	0.299968	0.295811	0.294372	0.3118	0.299169	0.304285	0.307803
0.398465	0.364247	0.403742	0.349696	0.356252	0.356732	0.371122	0.256636	0.275024	0.303006
0.257595	0.29629	0.261912	0.298369	0.285417	0.306364	0.300128	0.372562	0.376879	0.310361
0.20243	0.255676	0.279341	0.299648	0.320435	0.338024	0.334666	0.371922	0.372562	0.306684

For Review Only

0.383275	0.358011	0.397506	0.3441	0.350016	0.345699	0.35897	0.204669	0.165654	0.300448
0.107931	0.258395	0.260153	0.293412	0.307643	0.365206	0.32747	0.394947	0.392229	0.317077
0.309722	0.310042	0.335945	0.320115	0.341222	0.322034	0.347298	0.285417	0.317557	0.271506
0.315958	0.315158	0.279501	0.290694	0.280461	0.311641	0.301887	0.332107	0.325871	0.293892
0.417173	0.356252	0.420851	0.355612	0.372721	0.337864	0.389031	0.215862	0.251839	0.305724
0.29677	0.274544	0.340102	0.326511	0.336265	0.335625	0.339783	0.34426	0.346978	0.331788
0.315478	0.314839	0.284938	0.269587	0.247841	0.304125	0.245443	0.330828	0.311161	0.300288
0.398305	0.362488	0.40614	0.337544	0.348897	0.352574	0.357851	0.233291	0.221298	0.262392
0.253758	0.274704	0.325392	0.305724	0.322354	0.356572	0.346658	0.336105	0.340902	0.311001
0.261593	0.329869	0.175568	0.263351	0.196514	0.336745	0.216981	0.392709	0.388871	0.342341
0.252638	0.311481	0.269268	0.291493	0.287976	0.311321	0.298849	0.311001	0.313719	0.268468
0.12424	0.277263	0.239527	0.298369	0.310521	0.35929	0.320595	0.404861	0.40566	0.320115
0.287656	0.29613	0.310841	0.287176	0.291174	0.295491	0.303166	0.25072	0.2504	0.255357
0.325552	0.31308	0.333706	0.288935	0.32827	0.333866	0.341382	0.29613	0.309402	0.290854
0.337864	0.342501	0.354173	0.339623	0.327151	0.333067	0.329549	0.287656	0.314359	0.304125
0.338823	0.323633	0.356892	0.322034	0.334186	0.310361	0.350815	0.215062	0.206108	0.262712
0.39079	0.344739	0.40614	0.348257	0.368244	0.354493	0.369524	0.160217	0.227215	0.297729
0.335146	0.308762	0.354813	0.353374	0.346818	0.31276	0.348897	0.321075	0.345859	0.29677
0.145667	0.28174	0.263671	0.29709	0.295811	0.332107	0.319156	0.345539	0.356092	0.306844
0.273105	0.289095	0.324113	0.32747	0.327151	0.320755	0.336745	0.361369	0.351775	0.300927
0.221618	0.262072	0.289575	0.321394	0.326671	0.347937	0.334026	0.370163	0.345539	0.282219
0.320115	0.262872	0.323473	0.326831	0.324912	0.318356	0.322354	0.291493	0.332907	0.280141
0.28158	0.284938	0.333547	0.308443	0.347777	0.362008	0.355293	0.311161	0.325552	0.315638
0.377359	0.339463	0.384714	0.340262	0.350815	0.352574	0.35945	0.189319	0.168052	0.23409
0.383914	0.347937	0.399744	0.348737	0.349057	0.305085	0.361369	0.278702	0.293892	0.302367
0.255197	0.212184	0.341861	0.321234	0.335465	0.317557	0.342181	0.32763	0.314998	0.278222
0.195715	0.300608	0.193796	0.280461	0.252319	0.309402	0.271186	0.392869	0.395107	0.307163
0.356572	0.363288	0.367285	0.304925	0.326351	0.334506	0.333387	0.28174	0.290374	0.324272
0.134474	0.277422	0.237448	0.301407	0.309402	0.367605	0.32875	0.407899	0.40662	0.315798
0.399744	0.344899	0.398945	0.341701	0.352095	0.349856	0.35945	0.179885	0.228973	0.282699
0.349376	0.330349	0.354333	0.304285	0.305564	0.271506	0.310521	0.310521	0.300768	0.301567
0.403262	0.365686	0.407259	0.349536	0.366645	0.342181	0.379597	0.256316	0.266549	0.305884
0.133195	0.244963	0.269428	0.316757	0.316757	0.348257	0.330828	0.379437	0.377838	0.288455
0.314359	0.31324	0.332587	0.320595	0.323473	0.354013	0.331628	0.241445	0.252958	0.222897
0.299968	0.314039	0.314998	0.29709	0.291973	0.309882	0.294851	0.319476	0.337544	0.288615
0.347618	0.329389	0.367925	0.332587	0.334986	0.341062	0.350975	0.205788	0.211864	0.255516
0.244164	0.23505	0.303646	0.31196	0.32827	0.329069	0.341062	0.357052	0.353374	0.304765
0.134794	0.269428	0.251679	0.300927	0.307004	0.361848	0.321075	0.407419	0.404861	0.315958
0.39079	0.374001	0.410617	0.338983	0.353374	0.31228	0.366326	0.244803	0.282699	0.318196
0.381516	0.367285	0.395427	0.341701	0.355293	0.358331	0.362808	0.227055	0.133515	0.314199
0.303486	0.315798	0.319795	0.305724	0.288455	0.335625	0.29757	0.255516	0.266869	0.267988
0.059322	0.232651	0.246083	0.301887	0.301247	0.358331	0.321234	0.404701	0.396226	0.303965
0.398465	0.351615	0.418772	0.342341	0.353534	0.34426	0.369524	0.278222	0.292933	0.308922
0.319795	0.339143	0.333067	0.299968	0.298209	0.302686	0.315478	0.271826	0.264791	0.301887
0.247042	0.211545	0.336425	0.325232	0.319635	0.341861	0.335625	0.333227	0.324272	0.293252
0.408858	0.357851	0.42197	0.35977	0.380876	0.23425	0.386153	0.282699	0.299009	0.307163
0.347458	0.357052	0.370003	0.342501	0.318356	0.340422	0.339143	0.226095	0.231052	0.303806
0.333547	0.32795	0.363607	0.333547	0.339303	0.333866	0.338663	0.284138	0.323633	0.306364
0.286377	0.295491	0.347138	0.341382	0.340422	0.347777	0.349376	0.275823	0.257275	0.266869
0.355612	0.352734	0.353374	0.320115	0.294372	0.337224	0.319635	0.273905	0.291174	0.29741
0.362968	0.349856	0.3118	0.323313	0.284458	0.339623	0.26527	0.271027	0.271186	0.304765
0.133834	0.270387	0.240966	0.310201	0.303646	0.368564	0.32843	0.405341	0.403902	0.316917
0.412536	0.339463	0.424209	0.347777	0.37576	0.316437	0.376079	0.272306	0.292133	0.319156
0.082028	0.248641	0.246083	0.298529	0.299648	0.355293	0.323953	0.404701	0.397346	0.299169
0.397186	0.346818	0.408858	0.345539	0.361049	0.354653	0.37448	0.10889	0.226095	0.291334

For Review Only

0.416054	0.365846	0.42165	0.345379	0.366006	0.344739	0.367445	0.196194	0.244963	0.292293
0.212344	0.130796	0.313559	0.301567	0.310681	0.334826	0.32859	0.346178	0.354813	0.289095
0.409658	0.342821	0.42245	0.366006	0.378798	0.309242	0.383435	0.279821	0.307163	0.316118
0.385833	0.366645	0.400544	0.340582	0.35865	0.352414	0.373521	0.221938	0.229613	0.306364

For Review Only

0.29741	0.26511	0.298849	0.293732	0.21842	0.225296	0.292773	0.303006	0.285257	0.302846
0.324272	0.34394	0.301407	0.289735	0.355772	0.363128	0.302686	0.337064	0.322833	0.287016
0.349376	0.273265	0.216342	0.286856	0.295971	0.290374	0.240646	0.329389	0.245763	0.23457
0.32891	0.2819	0.262072	0.29693	0.29693	0.324912	0.256796	0.329549	0.264631	0.261593
0.304605	0.289415	0.352095	0.323793	0.241605	0.273105	0.357851	0.299808	0.340742	0.350815
0.32795	0.275983	0.278222	0.288935	0.248641	0.258075	0.23473	0.314039	0.25072	0.277582
0.2496	0.259354	0.308922	0.291653	0.197314	0.181324	0.302047	0.206588	0.318036	0.332747
0.318996	0.338983	0.338983	0.32859	0.362168	0.335465	0.326511	0.32875	0.347618	0.345219
0.251039	0.264791	0.310841	0.294372	0.17173	0.205149	0.292293	0.24944	0.31228	0.318356
0.324752	0.363607	0.362808	0.35929	0.385673	0.37496	0.358011	0.332587	0.377199	0.383275
0.339942	0.332747	0.350176	0.34314	0.344899	0.335465	0.345859	0.338983	0.346498	0.354973
0.342021	0.269108	0.216342	0.277263	0.293892	0.293572	0.230093	0.321554	0.23409	0.221458
0.268468	0.288615	0.316757	0.302846	0.232491	0.258235	0.311321	0.259674	0.318356	0.32811
0.29709	0.327151	0.306684	0.329549	0.321714	0.321075	0.309082	0.305884	0.336105	0.318996
0.290694	0.240166	0.26639	0.294212	0.206908	0.256156	0.264631	0.261113	0.275024	0.276623
0.34314	0.303486	0.290374	0.306364	0.32779	0.333067	0.288295	0.340902	0.307643	0.302526
0.255836	0.267189	0.32763	0.326031	0.170931	0.247362	0.326031	0.180684	0.325072	0.347937
0.313399	0.300608	0.310521	0.323153	0.278062	0.283658	0.278382	0.302367	0.327151	0.309722
0.352734	0.276303	0.261593	0.290534	0.278222	0.300448	0.215062	0.32827	0.011353	0.255037
0.305245	0.352255	0.362328	0.367765	0.353854	0.382955	0.356092	0.322354	0.369364	0.353854
0.270547	0.323633	0.330508	0.333067	0.275024	0.301567	0.342501	0.244004	0.351775	0.345859
0.246562	0.257115	0.321874	0.324592	0.181004	0.239687	0.322354	0.174129	0.329389	0.338663
0.371922	0.371762	0.387592	0.353854	0.397825	0.383275	0.389191	0.367605	0.40566	0.396866
0.32891	0.331468	0.335625	0.32891	0.355612	0.351935	0.330988	0.342981	0.348897	0.330029
0.331148	0.252478	0.259354	0.290534	0.141509	0.236649	0.239847	0.26559	0.253278	0.292613
0.366166	0.29757	0.157659	0.29757	0.304125	0.311321	0.277902	0.342821	0.278702	0.183882
0.345379	0.283978	0.228814	0.263991	0.291973	0.303166	0.225616	0.325552	0.256796	0.202111
0.292933	0.260953	0.247522	0.271826	0.267988	0.279501	0.258395	0.283658	0.273265	0.261593
0.280301	0.300608	0.308762	0.298209	0.23409	0.294052	0.308602	0.306044	0.325712	0.318356
0.301087	0.409498	0.411417	0.387592	0.425488	0.416374	0.417013	0.326191	0.419731	0.4063
0.347138	0.269268	0.246882	0.282699	0.263192	0.289255	0.223217	0.303646	0.214263	0.23489
0.342341	0.268788	0.277582	0.301407	0.291973	0.20243	0.260473	0.320755	0.253438	0.283179
0.291973	0.261753	0.298849	0.309082	0.190758	0.008794	0.288935	0.254077	0.300608	0.320435
0.332907	0.337544	0.349536	0.324912	0.350176	0.338823	0.349376	0.34394	0.363447	0.352095
0.363447	0.322194	0.290854	0.331148	0.3441	0.335785	0.314519	0.37464	0.330189	0.282699
0.322034	0.302047	0.267988	0.307004	0.325232	0.331468	0.28174	0.334986	0.276943	0.252478
0.300448	0.285257	0.322034	0.332587	0.301407	0.289575	0.310201	0.306684	0.31244	0.335945
0.307483	0.29629	0.310361	0.323473	0.31308	0.306044	0.31292	0.322833	0.332747	0.32859
0.348737	0.38999	0.380876	0.333067	0.409658	0.388391	0.393348	0.362648	0.408858	0.389831
0.348897	0.325392	0.348897	0.333547	0.35961	0.366326	0.349696	0.361529	0.367925	0.354013
0.304285	0.401183	0.396866	0.377838	0.420051	0.410297	0.411896	0.335465	0.416693	0.393828
0.308602	0.350496	0.346019	0.330508	0.356412	0.335945	0.3441	0.333387	0.35961	0.334986
0.308602	0.352574	0.350176	0.342661	0.364567	0.349536	0.354653	0.334026	0.361209	0.367445
0.206268	0.309722	0.348737	0.356892	0.253438	0.299648	0.339463	0.226095	0.358331	0.357691
0.318036	0.356732	0.365046	0.353854	0.374161	0.357851	0.362968	0.345059	0.385353	0.378957
0.352414	0.269587	0.197634	0.283179	0.293732	0.295011	0.233131	0.321234	0.24944	0.217461
0.350016	0.292613	0.180684	0.303646	0.291813	0.302846	0.251679	0.331148	0.261593	0.125999
0.338503	0.292613	0.269428	0.300768	0.31324	0.319795	0.295171	0.341541	0.319795	0.291973
0	0.329549	0.352734	0.337544	0.268628	0.292133	0.335305	0.217141	0.353374	0.351455
0.329549	0	0.283179	0.300128	0.232171	0.261113	0.277422	0.276463	0.274864	0.311321

For Review Only

0.352734	0.283179	0	0.295491	0.279821	0.298209	0.251199	0.338983	0.262552	0.175248
0.337544	0.300128	0.295491	0	0.282859	0.309562	0.276303	0.319795	0.291174	0.28158
0.268628	0.232171	0.279821	0.282859	0	0.191078	0.280461	0.190278	0.278222	0.313879
0.292133	0.261113	0.298209	0.309562	0.191078	0	0.288775	0.254237	0.300448	0.320595
0.335305	0.277422	0.251199	0.276303	0.280461	0.288775	0	0.32827	0.215542	0.250879
0.217141	0.276463	0.338983	0.319795	0.190278	0.254237	0.32827	0	0.32779	0.344579
0.353374	0.274864	0.262552	0.291174	0.278222	0.300448	0.215542	0.32779	0	0.255197
0.351455	0.311321	0.175248	0.28158	0.313879	0.320595	0.250879	0.344579	0.255197	0
0.357531	0.283818	0.182763	0.287496	0.295171	0.306364	0.251359	0.332267	0.268788	0.168052
0.307803	0.370163	0.378638	0.354653	0.400704	0.388871	0.387592	0.347138	0.402782	0.391749
0.310042	0.295491	0.326031	0.334826	0.307004	0.286856	0.330508	0.303965	0.339942	0.333387
0.294691	0.256636	0.252798	0.278222	0.263831	0.270547	0.255516	0.282859	0.264471	0.258714
0.349057	0.286856	0.227694	0.268468	0.316757	0.311321	0.227694	0.333866	0.239687	0.160058
0.356572	0.307163	0.232971	0.294851	0.329869	0.331148	0.245123	0.345539	0.251039	0.110489
0.268948	0.232491	0.279821	0.283339	0.008475	0.190598	0.28062	0.189319	0.277582	0.31228
0.279821	0.299488	0.307643	0.298209	0.23377	0.294212	0.307803	0.305724	0.324592	0.317237
0.311641	0.271986	0.269587	0.2811	0.267829	0.271027	0.254237	0.298209	0.269108	0.271186
0.318036	0.309882	0.329069	0.327311	0.332427	0.300288	0.338184	0.325232	0.35881	0.342821
0.340742	0.309082	0.261912	0.304765	0.320755	0.329229	0.303486	0.334346	0.309722	0.283019
0.341222	0.204349	0.256796	0.321394	0.272146	0.266869	0.25056	0.318516	0.272466	0.267988
0.283339	0.220019	0.29613	0.284618	0.179405	0.263991	0.259194	0.233131	0.262232	0.285098
0.264791	0.245283	0.307323	0.291813	0.149504	0.177007	0.287976	0.237448	0.293572	0.303486
0.330668	0.291334	0.237128	0.278062	0.304445	0.31212	0.240966	0.326831	0.251839	0.142309
0.347937	0.330189	0.354333	0.329389	0.370323	0.364087	0.348897	0.358011	0.35865	0.351295
0.257595	0.28206	0.314039	0.307803	0.237928	0.258714	0.302207	0.282699	0.318036	0.321234
0.341701	0.304605	0.271186	0.324432	0.336585	0.294851	0.284298	0.335945	0.301087	0.285577
0.317717	0.356572	0.364886	0.353694	0.37432	0.358491	0.362488	0.345219	0.385993	0.379117
0.338184	0.302367	0.237768	0.235849	0.301887	0.316118	0.240486	0.324912	0.237928	0.225616
0.256796	0.335945	0.350496	0.361049	0.299328	0.275024	0.353534	0.283499	0.357211	0.367765
0.346338	0.387112	0.386313	0.337864	0.418932	0.400384	0.382795	0.367285	0.410937	0.392869
0.371602	0.298849	0.154301	0.305564	0.31212	0.318196	0.272466	0.348097	0.288295	0.180205
0.325392	0.255357	0.288136	0.287176	0.248161	0.272306	0.269747	0.278862	0.258075	0.292613
0.309082	0.28126	0.324912	0.3118	0.239527	0.278702	0.317077	0.240006	0.330189	0.330349
0.321394	0.303326	0.29677	0.311001	0.324592	0.318676	0.306204	0.332107	0.31324	0.29677
0.357371	0.399584	0.398945	0.339942	0.428526	0.40598	0.396866	0.380237	0.415574	0.404541
0.267988	0.231852	0.279981	0.28206	0.008475	0.189319	0.280301	0.189159	0.276943	0.31228
0.3118	0.272466	0.302686	0.31308	0.270707	0.262552	0.302526	0.329549	0.309242	0.307963
0.318356	0.356412	0.365046	0.353214	0.373521	0.357851	0.362808	0.34426	0.385513	0.379597
0.324113	0.32811	0.346178	0.332267	0.350016	0.357531	0.333866	0.337864	0.356572	0.349536
0.237128	0.335465	0.366805	0.345219	0.260473	0.304765	0.347298	0.214263	0.360249	0.370003
0.338024	0.317557	0.307643	0.333387	0.338184	0.322514	0.325392	0.307963	0.315638	0.338823
0.309722	0.341222	0.354973	0.366965	0.362168	0.353054	0.349057	0.321075	0.366485	0.378638
0.24944	0.235369	0.288455	0.301727	0.131276	0.1882	0.291174	0.173969	0.277902	0.305884
0.356092	0.264791	0.275024	0.305884	0.276143	0.301887	0.26623	0.322674	0.253918	0.259354
0.334986	0.272146	0.237448	0.303806	0.294532	0.330668	0.293892	0.319795	0.292293	0.319795
0.348897	0.287816	0.228334	0.269268	0.317717	0.31244	0.228654	0.332747	0.241925	0.162136
0.307163	0.277582	0.307004	0.323793	0.268308	0.264471	0.306684	0.292933	0.300288	0.317557
0.330988	0.253278	0.246402	0.288455	0.279821	0.289735	0.246722	0.306364	0.24944	0.222578
0.32763	0.354493	0.366006	0.322993	0.3756	0.362168	0.362808	0.347618	0.371922	0.361529
0.351775	0.289095	0.224336	0.307963	0.307643	0.321075	0.262072	0.326031	0.278702	0.222897
0.334506	0.271826	0.237928	0.313399	0.293732	0.291174	0.273585	0.31308	0.277422	0.205309
0.326511	0.29661	0.329709	0.331788	0.319316	0.318516	0.289894	0.323633	0.306844	0.308283
0.332907	0.286537	0.243044	0.26559	0.295491	0.306844	0.232331	0.316278	0.261593	0.20243
0.354493	0.392389	0.393508	0.345379	0.418292	0.398305	0.397506	0.368404	0.420211	0.393508
0.318196	0.362648	0.363767	0.350496	0.39047	0.385993	0.384074	0.338663	0.381996	0.373201

For Review Only

0.319156	0.29613	0.301407	0.299808	0.311481	0.311481	0.295011	0.322354	0.294691	0.305405
0.322993	0.288295	0.319156	0.330349	0.304605	0.286697	0.290534	0.331628	0.298689	0.299169
0.346818	0.306844	0.227215	0.2811	0.333387	0.334986	0.29757	0.34346	0.31324	0.217461
0.330508	0.32795	0.337064	0.321394	0.347458	0.342021	0.319795	0.334186	0.337224	0.316597
0.326991	0.286856	0.193156	0.291174	0.292293	0.304925	0.256796	0.325552	0.287336	0.217141
0.322833	0.344739	0.355932	0.330828	0.364407	0.349217	0.352734	0.338343	0.370323	0.35929
0.353054	0.284618	0.201791	0.270067	0.299328	0.293732	0.222578	0.332107	0.236009	0.198113
0.341382	0.278382	0.202111	0.29629	0.299488	0.294052	0.259034	0.322034	0.270547	0.224336
0.320755	0.257435	0.253758	0.277902	0.26543	0.287336	0.243044	0.286697	0.242565	0.260793
0.325552	0.287176	0.280461	0.298689	0.300288	0.306844	0.259514	0.308283	0.264471	0.262552
0.318036	0.322833	0.282859	0.338024	0.338343	0.336585	0.323473	0.338184	0.326031	0.295491
0.319476	0.302686	0.264311	0.316917	0.329389	0.327311	0.301567	0.32795	0.306844	0.290054
0.301407	0.288136	0.307163	0.313879	0.244324	0.253438	0.308922	0.300128	0.324432	0.321714
0.295971	0.26607	0.260473	0.28078	0.268788	0.280301	0.274384	0.289575	0.284138	0.271826
0.347777	0.275823	0.285417	0.291813	0.319476	0.330668	0.292293	0.314839	0.294691	0.313559
0.331468	0.283339	0.231052	0.196994	0.2811	0.299808	0.23377	0.314359	0.240166	0.216661
0.329069	0.302207	0.294532	0.298209	0.304445	0.325072	0.28142	0.316278	0.289735	0.286377
0.329869	0.279021	0.199073	0.295811	0.292933	0.291813	0.26639	0.31244	0.280301	0.216022
0.324432	0.273105	0.301727	0.331788	0.306364	0.304285	0.288935	0.325072	0.298849	0.295811
0.309562	0.263831	0.223217	0.274704	0.272785	0.272945	0.2504	0.292133	0.252958	0.221458
0.329229	0.350975	0.362328	0.327151	0.372402	0.360569	0.354973	0.348257	0.373521	0.357211
0.313719	0.28158	0.290854	0.3126	0.26575	0.31308	0.282699	0.285897	0.29645	0.326511
0.29709	0.26543	0.233451	0.279181	0.274384	0.282859	0.24928	0.290054	0.270067	0.239847
0.320435	0.248001	0.279021	0.289415	0.225456	0.247681	0.26591	0.269108	0.255996	0.287176
0.344579	0.26639	0.25008	0.276303	0.289575	0.300608	0.247522	0.31212	0.244164	0.247042
0.34442	0.394627	0.391589	0.347937	0.418452	0.400384	0.392069	0.370003	0.414295	0.396386
0.346498	0.311641	0.248481	0.314039	0.340582	0.332747	0.301567	0.341861	0.318196	0.257275
0.350496	0.304605	0.332747	0.330189	0.330189	0.329389	0.32843	0.32763	0.347138	0.330508
0.318516	0.262392	0.303326	0.292453	0.247202	0.278062	0.283019	0.28126	0.285417	0.29661
0.31308	0.247042	0.294532	0.277103	0.262552	0.267349	0.284138	0.280141	0.275504	0.301727
0.340102	0.260633	0.239527	0.275823	0.271666	0.295971	0.247362	0.304285	0.240166	0.230572
0.316437	0.259834	0.305085	0.323793	0.269268	0.29757	0.28126	0.297729	0.26575	0.294851
0.333706	0.383754	0.384874	0.335625	0.411737	0.393668	0.380556	0.362648	0.401503	0.389671
0.303806	0.278542	0.264471	0.273425	0.278062	0.295811	0.26591	0.288455	0.28126	0.267829
0.247362	0.23457	0.288935	0.301887	0.127279	0.18692	0.291973	0.170611	0.279021	0.308762
0.319795	0.263671	0.20259	0.288295	0.262392	0.283339	0.275184	0.295011	0.28142	0.211225
0.333706	0.337384	0.345859	0.325392	0.348417	0.338024	0.346978	0.34346	0.361369	0.350176
0.322354	0.298049	0.325552	0.316118	0.290854	0.297889	0.320275	0.301887	0.339303	0.317717
0.337704	0.35897	0.370003	0.337864	0.386952	0.372562	0.369843	0.35865	0.383754	0.368724
0.333706	0.274544	0.244803	0.26575	0.282539	0.295651	0.228174	0.315798	0.23457	0.226735
0.35993	0.315638	0.278062	0.306684	0.326031	0.338823	0.314839	0.355453	0.339463	0.300927
0.344899	0.379437	0.393828	0.34314	0.407099	0.381996	0.384394	0.373521	0.404061	0.391589
0.351455	0.310681	0.324113	0.318516	0.346818	0.336904	0.334026	0.340102	0.338823	0.323793
0.32843	0.35945	0.370163	0.335305	0.379117	0.372402	0.367285	0.349217	0.380077	0.370803
0.34442	0.255357	0.262552	0.298529	0.304445	0.299488	0.252319	0.334666	0.271986	0.267829
0.313719	0.371922	0.370483	0.365686	0.382155	0.382795	0.379597	0.32875	0.383435	0.371602
0.344579	0.288615	0.253118	0.308443	0.31228	0.300448	0.294532	0.334346	0.310521	0.292933
0.299009	0.270067	0.273585	0.310681	0.25056	0.260633	0.273585	0.29693	0.28078	0.300608
0.35897	0.286856	0.119923	0.294691	0.293252	0.302686	0.258395	0.32795	0.269747	0.144068
0.361848	0.298849	0.175887	0.290214	0.310201	0.320275	0.252478	0.335785	0.276143	0.162296
0.305564	0.31308	0.32763	0.315638	0.329869	0.310681	0.322993	0.318836	0.333387	0.330668
0.350336	0.300927	0.151743	0.291334	0.282699	0.301247	0.260313	0.319795	0.269907	0.17221
0.358491	0.290854	0.162136	0.297889	0.301407	0.305245	0.272146	0.341541	0.282539	0.168212
0.31196	0.342501	0.361209	0.354333	0.367125	0.340422	0.366965	0.332747	0.371922	0.344899
0.34346	0.242885	0.276943	0.311641	0.295651	0.290214	0.274225	0.333866	0.28158	0.309562

For Review Only

0.366006	0.306044	0.20339	0.300768	0.322993	0.313719	0.245123	0.340102	0.271186	0.17173
0.299328	0.308443	0.321394	0.308762	0.325072	0.32875	0.325392	0.307323	0.333866	0.319476
0.330189	0.257115	0.204029	0.3126	0.264151	0.268308	0.255197	0.313879	0.255676	0.244324
0.355453	0.330668	0.331148	0.351615	0.378798	0.37448	0.358331	0.340582	0.372562	0.361848
0.311641	0.333706	0.347138	0.363927	0.341541	0.329709	0.342981	0.333866	0.354973	0.360729
0.34442	0.300927	0.342661	0.329229	0.330508	0.329069	0.337384	0.349376	0.336265	0.341382
0.338663	0.278542	0.255996	0.277422	0.253438	0.288136	0.254717	0.294212	0.259034	0.240166
0.302367	0.298689	0.309242	0.301887	0.318676	0.3118	0.32747	0.332747	0.323313	0.319156
0.350176	0.346978	0.361529	0.32843	0.366326	0.346658	0.356412	0.340902	0.372721	0.370323
0.300927	0.26623	0.238567	0.277263	0.270547	0.278542	0.253278	0.29725	0.278702	0.238248
0.364727	0.34314	0.356412	0.339303	0.363128	0.367125	0.349696	0.369683	0.35993	0.35945
0.326831	0.240006	0.282699	0.301407	0.228973	0.256476	0.269907	0.272785	0.247202	0.284298
0.338343	0.275024	0.165494	0.294052	0.283179	0.284298	0.252638	0.311641	0.259034	0.185801
0.323473	0.323793	0.324113	0.31324	0.348577	0.326671	0.313559	0.340422	0.349057	0.307643
0.337704	0.26511	0.228494	0.292773	0.28142	0.292613	0.245443	0.311321	0.259514	0.230413
0.321394	0.292293	0.304125	0.319156	0.295651	0.294691	0.300927	0.322354	0.311161	0.321234
0.32891	0.358171	0.364407	0.320915	0.387272	0.364567	0.360889	0.354493	0.37496	0.355772
0.342501	0.350336	0.366166	0.346498	0.366965	0.350815	0.352414	0.353214	0.362328	0.37528
0.326191	0.248641	0.278542	0.308283	0.301567	0.292453	0.277902	0.318676	0.287816	0.302686
0.349217	0.292933	0.241286	0.276623	0.306524	0.300927	0.248481	0.340102	0.245923	0.231692
0.338184	0.274384	0.205948	0.292453	0.284458	0.297729	0.269907	0.313399	0.274065	0.223537
0.317237	0.254077	0.251519	0.276303	0.263351	0.286057	0.241925	0.284938	0.245603	0.260153
0.289735	0.257595	0.256316	0.277582	0.26527	0.273425	0.254557	0.282859	0.267349	0.261753
0.330189	0.362648	0.352414	0.324592	0.378318	0.373041	0.352414	0.345379	0.376879	0.364407
0.342341	0.339303	0.365046	0.331788	0.370803	0.366006	0.357371	0.350016	0.368564	0.364727
0.356412	0.307163	0.233451	0.295331	0.329229	0.330508	0.245123	0.344579	0.250879	0.112088
0.262872	0.303965	0.339303	0.316118	0.2504	0.28094	0.330349	0.252478	0.346818	0.326671
0.329229	0.332267	0.349217	0.326991	0.349536	0.346338	0.350336	0.341382	0.354813	0.357052
0.334506	0.263351	0.240966	0.287976	0.297889	0.298529	0.238407	0.308602	0.251519	0.2189
0.334026	0.290694	0.305884	0.322354	0.300608	0.298849	0.301727	0.325712	0.302047	0.300927
0.334026	0.240006	0.288455	0.305884	0.290214	0.290534	0.291174	0.313559	0.310201	0.283019
0.267189	0.215862	0.286217	0.279021	0.188359	0.242085	0.278542	0.216022	0.288136	0.29693
0.333387	0.289894	0.267988	0.318356	0.306364	0.283339	0.264791	0.326671	0.276783	0.278222
0.347618	0.247362	0.2819	0.306844	0.267988	0.278542	0.271506	0.303326	0.25072	0.272785
0.307963	0.257915	0.303326	0.289894	0.268148	0.263192	0.292453	0.291813	0.299808	0.302846
0.331468	0.35961	0.365206	0.317717	0.383115	0.367605	0.363767	0.350975	0.3756	0.354813
0.279181	0.263831	0.307643	0.32795	0.260953	0.258555	0.293572	0.284938	0.295011	0.303965
0.355133	0.306204	0.232331	0.295011	0.32891	0.329709	0.244484	0.344579	0.2504	0.110649
0.334186	0.303166	0.256796	0.302846	0.302686	0.3118	0.280301	0.334666	0.295011	0.235369
0.306524	0.257435	0.298689	0.326511	0.237928	0.2496	0.290214	0.300448	0.28206	0.330029
0.361369	0.300608	0.178446	0.294052	0.315158	0.322194	0.252958	0.337544	0.274065	0.170611
0.354333	0.288455	0.206108	0.305405	0.286856	0.298049	0.257755	0.330349	0.251999	0.251039
0.348417	0.272945	0.216501	0.286217	0.29709	0.289735	0.240486	0.329389	0.245923	0.23393
0.362648	0.287976	0.128078	0.299328	0.29741	0.299328	0.263671	0.32843	0.276303	0.148865
0.360729	0.395267	0.385034	0.341701	0.42197	0.396226	0.386473	0.378318	0.409818	0.394468
0.326671	0.273425	0.269428	0.301887	0.291014	0.29629	0.276143	0.319635	0.288615	0.261113
0.317717	0.310521	0.298209	0.315478	0.330189	0.332587	0.291813	0.324113	0.314359	0.29629
0.31308	0.285737	0.305085	0.305884	0.305405	0.306524	0.304285	0.326991	0.283658	0.289575
0.342021	0.349696	0.365846	0.346178	0.365526	0.348737	0.352734	0.352414	0.361049	0.37448
0.340742	0.361209	0.378957	0.335785	0.373201	0.349856	0.376879	0.336904	0.389671	0.397985
0.322034	0.334826	0.349376	0.330668	0.361848	0.347138	0.349057	0.337544	0.355453	0.347777
0.283499	0.288775	0.305405	0.308443	0.289255	0.308602	0.29645	0.220979	0.304125	0.305085
0.348737	0.267829	0.259514	0.292613	0.278702	0.28078	0.26591	0.303166	0.277902	0.271506
0.300288	0.345859	0.367445	0.348417	0.374161	0.357531	0.364727	0.368724	0.368724	0.370803
0.338823	0.348737	0.353214	0.345219	0.378478	0.369044	0.367765	0.363128	0.381996	0.373521

For Review Only

0.349376	0.286537	0.228014	0.267988	0.316278	0.310521	0.227694	0.333227	0.239687	0.161657
0.356572	0.398625	0.387272	0.340422	0.420531	0.404381	0.39031	0.376879	0.413975	0.395267
0.35897	0.271666	0.297889	0.325232	0.31244	0.321874	0.300288	0.34362	0.324113	0.329549
0.325072	0.324592	0.339783	0.334666	0.338024	0.334826	0.332427	0.324912	0.3441	0.326191
0.34442	0.249121	0.248961	0.306044	0.273265	0.283978	0.261273	0.29709	0.257755	0.275664
0.339143	0.317717	0.330988	0.32827	0.330668	0.351935	0.326511	0.319476	0.342181	0.356732
0.295651	0.31276	0.342181	0.32891	0.324272	0.310841	0.318036	0.327151	0.337544	0.320915
0.337544	0.2496	0.208507	0.295331	0.271666	0.279021	0.262392	0.305884	0.28158	0.224976
0.365686	0.346338	0.316917	0.352095	0.371442	0.344739	0.337064	0.35881	0.376239	0.342021
0.336904	0.391909	0.385513	0.379757	0.42181	0.413176	0.388711	0.351615	0.404861	0.400064
0.31212	0.322674	0.313879	0.321075	0.345859	0.340902	0.32763	0.331148	0.342661	0.319476
0.348417	0.391909	0.396546	0.345539	0.423729	0.40614	0.397186	0.371442	0.417013	0.398625
0.286856	0.263032	0.250879	0.273425	0.26559	0.279821	0.259514	0.28078	0.270387	0.254557
0.355293	0.302367	0.29661	0.342981	0.350815	0.309402	0.3118	0.362008	0.325072	0.317717
0.340582	0.303006	0.294691	0.32859	0.31324	0.306044	0.303646	0.301407	0.304125	0.325871
0.329549	0.282379	0.194436	0.295811	0.275983	0.290854	0.260793	0.299009	0.26527	0.21938
0.354813	0.290374	0.221938	0.302686	0.309082	0.286856	0.251839	0.331148	0.259834	0.221298
0.320755	0.309402	0.333227	0.338983	0.307963	0.31244	0.321714	0.333866	0.336745	0.340102
0.324432	0.364087	0.351135	0.334826	0.383275	0.354813	0.355772	0.345379	0.379277	0.354813
0.320595	0.329229	0.349057	0.323473	0.323953	0.350496	0.342821	0.326671	0.348097	0.353214
0.361848	0.339303	0.354973	0.339463	0.355772	0.350656	0.347458	0.356412	0.365846	0.367445
0.330189	0.301087	0.313879	0.330508	0.321075	0.316437	0.325552	0.336904	0.338823	0.341861
0.372402	0.34378	0.317877	0.334986	0.372562	0.367125	0.298849	0.384874	0.338184	0.313719
0.363767	0.286217	0.150144	0.300927	0.300128	0.311481	0.26511	0.337384	0.276623	0.175887
0.304285	0.23457	0.277263	0.294212	0.211385	0.25024	0.273425	0.255357	0.253918	0.298849
0.321714	0.318036	0.307163	0.329069	0.315318	0.323153	0.317877	0.333067	0.338184	0.318676
0.307483	0.370323	0.378798	0.355133	0.400224	0.388072	0.386952	0.346338	0.401823	0.391429
0.325232	0.303166	0.294851	0.298049	0.271027	0.305724	0.284458	0.319156	0.283499	0.285737
0.361848	0.399424	0.400704	0.34378	0.432044	0.411257	0.39111	0.382155	0.416693	0.405181
0.346338	0.271346	0.21938	0.266549	0.286537	0.310681	0.224177	0.317717	0.226415	0.202271
0.283179	0.259514	0.313879	0.304285	0.155421	0.237288	0.299169	0.263831	0.308602	0.310681
0.341222	0.24976	0.261273	0.275024	0.267988	0.279661	0.254397	0.29709	0.259834	0.262712
0.366805	0.370963	0.371442	0.362968	0.400224	0.370803	0.382635	0.35865	0.40582	0.395267
0.367125	0.295171	0.211545	0.318676	0.322993	0.315798	0.283019	0.351935	0.287816	0.246242
0.333227	0.316597	0.335625	0.317557	0.345539	0.322354	0.329069	0.342981	0.34426	0.337544
0.361209	0.295811	0.188359	0.299009	0.317237	0.311001	0.274225	0.329549	0.28094	0.195875
0.334186	0.326351	0.356412	0.330668	0.368084	0.356252	0.348097	0.339783	0.364407	0.362008
0.364087	0.401023	0.399424	0.340582	0.425488	0.410138	0.388551	0.385193	0.418132	0.40566
0.297889	0.200352	0.266549	0.299328	0.217781	0.252958	0.262392	0.256796	0.264311	0.290534
0.356092	0.307963	0.233611	0.295651	0.329069	0.330029	0.246083	0.344899	0.25072	0.111129
0.320275	0.301407	0.267349	0.303965	0.324113	0.329549	0.279821	0.334026	0.276463	0.251679
0.353854	0.398465	0.39031	0.340902	0.42245	0.400064	0.397825	0.381356	0.418292	0.396546
0.320755	0.248321	0.29693	0.298689	0.274544	0.263671	0.272945	0.29693	0.271826	0.283339
0.315638	0.271986	0.293412	0.308602	0.274704	0.271186	0.271506	0.284778	0.29645	0.288775
0.351775	0.320275	0.324432	0.319635	0.342181	0.32843	0.318676	0.340422	0.342501	0.320435
0.252159	0.211225	0.287336	0.295331	0.088264	0.196194	0.285417	0.134314	0.28094	0.316118
0.346338	0.300768	0.230253	0.309242	0.306204	0.318836	0.256636	0.332107	0.273745	0.225616
0.322993	0.298049	0.300608	0.310521	0.300608	0.301087	0.322034	0.278222	0.318036	0.32779
0.367605	0.322354	0.278062	0.325392	0.357691	0.353534	0.309242	0.355772	0.338983	0.248321
0.32843	0.321714	0.251199	0.314359	0.315478	0.305405	0.289575	0.324752	0.317397	0.278062
0.331788	0.321075	0.277263	0.322833	0.333387	0.338343	0.288295	0.334826	0.313719	0.275983
0.364567	0.398465	0.400704	0.340902	0.430604	0.408858	0.39063	0.388711	0.414775	0.4055
0.314199	0.21874	0.290694	0.255676	0.224177	0.263671	0.262392	0.274225	0.2504	0.289735
0.348097	0.393828	0.397186	0.334186	0.420691	0.401023	0.393828	0.370803	0.416214	0.400384
0.349856	0.273425	0.216022	0.287816	0.295971	0.289735	0.241445	0.330189	0.246562	0.23457

For Review Only

0.344899	0.283019	0.228494	0.283499	0.285257	0.324432	0.251359	0.317877	0.247362	0.224017
0.345059	0.349217	0.351135	0.324912	0.376719	0.367605	0.356092	0.367925	0.377039	0.352095
0.316917	0.235689	0.298209	0.290534	0.21874	0.246242	0.271027	0.263351	0.254077	0.309882
0.350815	0.302526	0.244324	0.275504	0.295971	0.319635	0.21842	0.333866	0.236329	0.217301

For Review Only

0.291014	0.35913	0.290534	0.267029	0.29757	0.321714	0.217781	0.264311	0.253758	0.276143
0.317077	0.282539	0.298369	0.278222	0.251679	0.279341	0.355772	0.309402	0.291813	0.282699
0.23425	0.404061	0.338503	0.243204	0.205309	0.215062	0.29645	0.32843	0.264791	0.338823
0.229453	0.344899	0.310681	0.257115	0.244803	0.263192	0.29645	0.295651	0.263831	0.31244
0.341222	0.304765	0.309722	0.300448	0.354813	0.358491	0.240966	0.298849	0.310841	0.288615
0.26639	0.376399	0.303965	0.255996	0.251999	0.275024	0.248161	0.272146	0.17125	0.305085
0.306524	0.362008	0.286377	0.273745	0.321714	0.345859	0.198113	0.251999	0.275664	0.293572
0.32763	0.262552	0.304125	0.28126	0.321234	0.339783	0.361689	0.295331	0.302047	0.278702
0.306844	0.361369	0.277103	0.262552	0.32811	0.32811	0.17157	0.17189	0.267029	0.299009
0.360889	0.251519	0.31244	0.318836	0.37576	0.376239	0.387112	0.283179	0.330828	0.283818
0.341541	0.295971	0.317237	0.290694	0.34362	0.356572	0.34426	0.329229	0.309402	0.313399
0.208507	0.394468	0.332907	0.233131	0.190278	0.205948	0.293572	0.314679	0.261912	0.334346
0.315318	0.314039	0.288295	0.272306	0.320435	0.338823	0.232012	0.270547	0.275024	0.295971
0.309242	0.269907	0.278862	0.270867	0.313559	0.313879	0.321394	0.315478	0.287336	0.274225
0.274864	0.35993	0.288615	0.246402	0.267829	0.282379	0.206428	0.266869	0.248321	0.304925
0.2819	0.321874	0.318836	0.251199	0.285098	0.290534	0.32795	0.304125	0.287176	0.329549
0.339942	0.347777	0.326031	0.285737	0.338663	0.35865	0.170771	0.273265	0.301247	0.320275
0.291174	0.332747	0.293412	0.272785	0.302207	0.319955	0.277422	0.273425	0.263831	0.287176
0.269747	0.402782	0.341062	0.263991	0.240006	0.250879	0.277103	0.323793	0.268948	0.35913
0.335305	0.282379	0.311001	0.318356	0.358011	0.357371	0.353854	0.29613	0.329549	0.304765
0.342181	0.293092	0.298529	0.294691	0.342341	0.365366	0.273905	0.240646	0.309882	0.287976
0.332267	0.351135	0.295011	0.286057	0.331788	0.351135	0.180524	0.269747	0.29741	0.329709
0.39079	0.255676	0.309082	0.316118	0.393348	0.388711	0.396706	0.337544	0.338663	0.302207
0.32891	0.204829	0.252638	0.269587	0.32747	0.316597	0.356732	0.279821	0.292133	0.269108
0.294372	0.381836	0.313879	0.24976	0.276623	0.283339	0.14055	0.270067	0.253598	0.320755
0.119763	0.385833	0.342821	0.257595	0.245123	0.255516	0.303006	0.324272	0.258075	0.336585
0.194755	0.35945	0.291493	0.242085	0.215062	0.217461	0.290854	0.291493	0.254237	0.326991
0.248321	0.297889	0.270067	0.216342	0.256476	0.268308	0.267829	0.263511	0.240806	0.283658
0.310042	0.29629	0.279341	0.267349	0.323793	0.324752	0.23377	0.005117	0.284778	0.286217
0.399584	0.175728	0.306524	0.335945	0.410937	0.401503	0.425807	0.315958	0.364407	0.303166
0.241126	0.386952	0.319316	0.247042	0.23473	0.23425	0.263032	0.305884	0.255996	0.345699
0.275823	0.397346	0.324752	0.28062	0.278702	0.291493	0.292453	0.31228	0.276943	0.345379
0.305405	0.389831	0.287656	0.271027	0.311481	0.331468	0.190118	0.295331	0.271346	0.300927
0.334346	0.225935	0.248321	0.275024	0.341062	0.338503	0.349057	0.268148	0.293092	0.275344
0.303806	0.330349	0.306044	0.293252	0.295651	0.28142	0.34442	0.321234	0.298369	0.301567
0.270547	0.292133	0.277263	0.245443	0.248641	0.235689	0.326031	0.285098	0.268468	0.307163
0.308762	0.304605	0.206908	0.272146	0.324272	0.325552	0.301727	0.303006	0.270227	0.230732
0.299169	0.309082	0.317717	0.271826	0.3118	0.322194	0.31212	0.322993	0.288455	0.292133
0.384394	0.195395	0.285098	0.308283	0.384394	0.380876	0.408858	0.315478	0.323153	0.299169
0.334186	0.300768	0.338823	0.294532	0.358331	0.367125	0.35881	0.326351	0.306044	0.315318
0.392229	0.193636	0.305405	0.329069	0.398145	0.394947	0.420211	0.308123	0.347777	0.292773
0.331628	0.28142	0.298689	0.295811	0.34442	0.341382	0.357371	0.151743	0.3126	0.280301
0.350975	0.25072	0.300608	0.299808	0.349696	0.354493	0.364886	0.268788	0.314519	0.251519
0.347138	0.309722	0.293412	0.303646	0.346338	0.358491	0.252638	0.329549	0.306684	0.295971
0.355453	0.269907	0.316917	0.311161	0.36009	0.362168	0.37448	0.301087	0.323313	0.271186
0.180365	0.392869	0.325871	0.237768	0.20387	0.225616	0.293252	0.318996	0.255357	0.324432
0.197634	0.394468	0.336425	0.255037	0.165494	0.132555	0.290694	0.314359	0.273105	0.334666
0.269907	0.306684	0.318676	0.26527	0.300288	0.3126	0.31276	0.311161	0.271666	0.303965
0.357531	0.307803	0.310042	0.294691	0.349057	0.356572	0.268948	0.279821	0.311641	0.318036
0.283818	0.370163	0.295491	0.256636	0.286856	0.307163	0.232491	0.299488	0.271986	0.309882

For Review Only

0.182763	0.378638	0.326031	0.252798	0.227694	0.232971	0.279821	0.307643	0.269587	0.329069
0.287496	0.354653	0.334826	0.278222	0.268468	0.294851	0.283339	0.298209	0.2811	0.327311
0.295171	0.400704	0.307004	0.263831	0.316757	0.329869	0.008475	0.23377	0.267829	0.332427
0.306364	0.388871	0.286856	0.270547	0.311321	0.331148	0.190598	0.294212	0.271027	0.300288
0.251359	0.387592	0.330508	0.255516	0.227694	0.245123	0.28062	0.307803	0.254237	0.338184
0.332267	0.347138	0.303965	0.282859	0.333866	0.345539	0.189319	0.305724	0.298209	0.325232
0.268788	0.402782	0.339942	0.264471	0.239687	0.251039	0.277582	0.324592	0.269108	0.35881
0.168052	0.391749	0.333387	0.258714	0.160058	0.110489	0.31228	0.317237	0.271186	0.342821
0	0.380397	0.330029	0.252798	0.224816	0.242245	0.294212	0.309242	0.264631	0.331788
0.380397	0	0.247681	0.306204	0.383435	0.382635	0.400544	0.295491	0.330029	0.286057
0.330029	0.247681	0	0.275983	0.329389	0.326831	0.306364	0.278542	0.276783	0.255197
0.252798	0.306204	0.275983	0	0.245603	0.259514	0.263351	0.266869	0.243204	0.283179
0.224816	0.383435	0.329389	0.245603	0	0.080588	0.315958	0.322993	0.262712	0.331628
0.242245	0.382635	0.326831	0.259514	0.080588	0	0.32891	0.323793	0.279181	0.339942
0.294212	0.400544	0.306364	0.263351	0.315958	0.32891	0	0.23377	0.267189	0.330828
0.309242	0.295491	0.278542	0.266869	0.322993	0.323793	0.23377	0	0.283818	0.285737
0.264631	0.330029	0.276783	0.243204	0.262712	0.279181	0.267189	0.283818	0	0.285257
0.331788	0.286057	0.255197	0.283179	0.331628	0.339942	0.330828	0.285737	0.285257	0
0.2819	0.340422	0.309402	0.26543	0.288136	0.292933	0.320435	0.319476	0.279981	0.307323
0.251679	0.411577	0.318356	0.262392	0.270867	0.291174	0.271826	0.298209	0.277582	0.321714
0.280461	0.400863	0.315318	0.260793	0.277422	0.28126	0.179405	0.280461	0.271027	0.34442
0.286856	0.389671	0.292453	0.26575	0.310521	0.332427	0.148705	0.253438	0.26495	0.308762
0.23457	0.351295	0.302207	0.243684	0.108411	0.058043	0.303965	0.295011	0.257435	0.313559
0.339463	0.284618	0.292293	0.294851	0.349376	0.345059	0.369524	0.298849	0.302207	0.301567
0.31228	0.329549	0.294851	0.266709	0.316757	0.329229	0.237768	0.114327	0.276623	0.290854
0.286217	0.348417	0.309562	0.286057	0.276623	0.270067	0.335146	0.291014	0.279181	0.319795
0.354973	0.269428	0.317397	0.311001	0.35961	0.361848	0.37432	0.301727	0.322674	0.270867
0.241605	0.365526	0.324113	0.24928	0.185162	0.206908	0.301567	0.303806	0.271186	0.32795
0.361689	0.300448	0.322674	0.309722	0.355772	0.37448	0.299488	0.326351	0.324432	0.314998
0.385353	0.194116	0.293412	0.308762	0.388391	0.389511	0.418612	0.317717	0.330988	0.307004
0.098017	0.386153	0.346658	0.263511	0.248001	0.26639	0.311481	0.326991	0.267349	0.337384
0.291174	0.385673	0.326671	0.254237	0.272785	0.29629	0.248001	0.311001	0.273745	0.339143
0.309242	0.333067	0.291653	0.28206	0.32811	0.340742	0.238887	0.295011	0.290214	0.291334
0.29661	0.245923	0.257595	0.259034	0.289415	0.289735	0.323953	0.284778	0.271666	0.275344
0.398305	0.185321	0.298849	0.317397	0.400384	0.401023	0.428206	0.32795	0.341382	0.31244
0.294372	0.399105	0.305245	0.262552	0.315638	0.32891	0.007355	0.232171	0.267349	0.330029
0.295491	0.332267	0.255357	0.271506	0.318676	0.323953	0.270867	0.282859	0.264631	0.273905
0.355932	0.269907	0.317077	0.311321	0.360409	0.362648	0.373681	0.300768	0.323153	0.271027
0.330988	0.258874	0.279021	0.294212	0.337384	0.349057	0.349856	0.270387	0.306684	0.284138
0.369843	0.288136	0.310681	0.303806	0.365206	0.378158	0.259674	0.29645	0.322514	0.318996
0.307643	0.330668	0.286537	0.28142	0.3126	0.331628	0.337544	0.336904	0.305085	0.308922
0.356412	0.255516	0.302367	0.304605	0.350815	0.364247	0.363288	0.325712	0.316118	0.289735
0.290534	0.40662	0.308602	0.26559	0.307963	0.325871	0.129997	0.26591	0.282699	0.323953
0.2819	0.407419	0.329709	0.266869	0.235529	0.248801	0.275983	0.330029	0.285577	0.356572
0.31276	0.336585	0.318196	0.270867	0.315158	0.321234	0.294212	0.32843	0.289894	0.326031
0.225935	0.382315	0.330189	0.246722	0.008475	0.082347	0.316757	0.325552	0.263511	0.332107
0.31292	0.348577	0.276783	0.272945	0.299169	0.3126	0.267669	0.308762	0.28062	0.309882
0.244004	0.389031	0.320595	0.255676	0.216182	0.244164	0.278382	0.301567	0.259034	0.323313
0.352095	0.217941	0.251839	0.287016	0.355772	0.349856	0.376399	0.293892	0.305724	0.288615
0.132235	0.335305	0.307323	0.255197	0.254397	0.255996	0.307803	0.300608	0.270707	0.310201
0.176847	0.3433	0.308443	0.254717	0.257115	0.258395	0.293092	0.31324	0.256636	0.309562
0.31292	0.356892	0.323313	0.286856	0.299968	0.292293	0.318356	0.323953	0.298209	0.335625
0.235849	0.353054	0.306204	0.241605	0.169811	0.18724	0.295331	0.287976	0.239847	0.323313
0.39031	0.192837	0.292453	0.315958	0.389031	0.389031	0.417333	0.315958	0.334026	0.307004
0.366485	0.143428	0.238727	0.308602	0.361848	0.365526	0.39015	0.294532	0.316437	0.309562

For Review Only

0.291813	0.278222	0.278542	0.258395	0.303806	0.307483	0.310361	0.283499	0.283339	0.290694
0.31228	0.364407	0.310681	0.278222	0.290694	0.298049	0.304445	0.319156	0.290854	0.31196
0.198753	0.337704	0.321554	0.270707	0.283179	0.292773	0.331468	0.313879	0.267189	0.319156
0.308283	0.303486	0.315958	0.294212	0.307483	0.321394	0.346818	0.320275	0.292773	0.31196
0.181324	0.350656	0.292773	0.240646	0.243204	0.248641	0.291653	0.289095	0.268788	0.302846
0.345059	0.225616	0.266869	0.287496	0.353694	0.353054	0.364247	0.289095	0.303166	0.283499
0.20355	0.382955	0.326351	0.24928	0.175568	0.205149	0.298849	0.31196	0.263032	0.339143
0.185801	0.34426	0.304765	0.245443	0.247042	0.253118	0.299808	0.308762	0.256156	0.3126
0.2504	0.380077	0.32891	0.204669	0.231852	0.244803	0.26527	0.292133	0.270227	0.329069
0.257115	0.29661	0.287176	0.239687	0.255676	0.261912	0.300448	0.300927	0.240006	0.295491
0.26575	0.290374	0.300608	0.283019	0.301087	0.307323	0.337544	0.315158	0.284138	0.294052
0.275823	0.264791	0.268308	0.264631	0.290854	0.290214	0.32875	0.275823	0.283658	0.291493
0.300448	0.325552	0.264631	0.277582	0.324272	0.333387	0.243684	0.286537	0.262552	0.26559
0.250879	0.305724	0.289415	0.233131	0.259834	0.276623	0.267829	0.271666	0.243524	0.28142
0.31292	0.324113	0.31196	0.269747	0.307963	0.308922	0.318996	0.319476	0.28078	0.323153
0.224496	0.367285	0.320915	0.246242	0.196994	0.21874	0.28094	0.298529	0.247681	0.323153
0.285577	0.319156	0.314998	0.274065	0.266709	0.279181	0.302686	0.311001	0.278542	0.310042
0.18772	0.321554	0.290534	0.242405	0.252478	0.262712	0.292293	0.294851	0.253118	0.294372
0.29645	0.339143	0.313399	0.278542	0.31244	0.327311	0.305724	0.306684	0.28142	0.322194
0.221778	0.325392	0.285098	0.226895	0.216821	0.232171	0.272306	0.283818	0.243204	0.29757
0.342181	0.216342	0.258714	0.288775	0.352095	0.348257	0.372082	0.298209	0.307323	0.279661
0.319156	0.346178	0.336585	0.271826	0.31324	0.330349	0.26527	0.299968	0.290054	0.325232
0.238727	0.299009	0.2819	0.224017	0.248001	0.256316	0.273425	0.267988	0.240966	0.287176
0.287976	0.383754	0.307323	0.245443	0.270387	0.292293	0.224177	0.291174	0.270867	0.332907
0.263831	0.363927	0.299808	0.245443	0.21826	0.228334	0.289415	0.295971	0.267988	0.311641
0.387112	0.191877	0.294052	0.31196	0.396226	0.391589	0.417333	0.309562	0.338663	0.308922
0.215222	0.325072	0.306684	0.268948	0.285257	0.291973	0.339463	0.304765	0.278222	0.315638
0.314359	0.299648	0.287016	0.277422	0.336105	0.331308	0.329869	0.298849	0.294851	0.285737
0.288935	0.374001	0.309562	0.267669	0.285098	0.29677	0.247202	0.284618	0.277103	0.327151
0.294052	0.324752	0.259674	0.251839	0.292453	0.294212	0.262872	0.260793	0.263192	0.277103
0.23393	0.402942	0.324272	0.248001	0.215382	0.235209	0.271186	0.309882	0.272785	0.357052
0.292293	0.373521	0.331788	0.277422	0.294212	0.302047	0.268308	0.294052	0.28126	0.342181
0.381676	0.199712	0.287496	0.309242	0.388551	0.381356	0.411257	0.309402	0.32827	0.293412
0.268308	0.2811	0.280461	0.237608	0.262072	0.270867	0.277582	0.271506	0.254717	0.285577
0.288935	0.410138	0.311481	0.26623	0.309242	0.32747	0.125999	0.267029	0.284938	0.323793
0.20243	0.337704	0.294532	0.237928	0.255516	0.26495	0.261912	0.278702	0.242725	0.298529
0.330988	0.226895	0.24976	0.274065	0.339942	0.337704	0.347138	0.267189	0.292773	0.275504
0.308283	0.295971	0.279501	0.275344	0.323473	0.330508	0.289735	0.280301	0.286217	0.294052
0.35961	0.210905	0.248321	0.294851	0.362968	0.354813	0.386633	0.295491	0.315478	0.278382
0.231052	0.372881	0.329549	0.25024	0.192677	0.228014	0.28158	0.303166	0.254877	0.319795
0.26559	0.308283	0.319316	0.274065	0.318836	0.330828	0.324752	0.314998	0.292133	0.293572
0.387432	0.233291	0.319476	0.319476	0.389671	0.391589	0.40614	0.336265	0.337704	0.322833
0.306684	0.326671	0.307323	0.289735	0.330029	0.333706	0.346338	0.29709	0.288775	0.315958
0.36009	0.200032	0.244803	0.287976	0.361848	0.356412	0.379117	0.295011	0.314839	0.283179
0.271186	0.37528	0.307963	0.263192	0.253918	0.267669	0.304605	0.302686	0.28126	0.315798
0.363447	0.217941	0.300128	0.310042	0.377518	0.371602	0.381676	0.298689	0.329389	0.288455
0.255197	0.323953	0.293092	0.26591	0.29629	0.307163	0.31276	0.304765	0.269268	0.289575
0.280301	0.326511	0.278542	0.257755	0.271027	0.291653	0.250879	0.283818	0.26511	0.289575
0.103454	0.389031	0.341062	0.256316	0.232331	0.245283	0.293092	0.325072	0.259354	0.338343
0.147905	0.384874	0.345059	0.251999	0.204829	0.21874	0.309242	0.321874	0.269108	0.344579
0.316437	0.226895	0.232331	0.268948	0.333067	0.322354	0.329549	0.277742	0.276783	0.252478
0.137512	0.384394	0.340102	0.251839	0.227694	0.242565	0.28174	0.311481	0.260793	0.332107
0.12536	0.366006	0.320755	0.258395	0.251999	0.260473	0.300608	0.316278	0.263991	0.322674
0.333387	0.216022	0.275344	0.294052	0.369843	0.368564	0.367125	0.308762	0.310042	0.295171
0.283179	0.319635	0.2811	0.244324	0.278702	0.299009	0.294851	0.293892	0.258395	0.261273

For Review Only

0.219699	0.37432	0.331948	0.26591	0.15702	0.116246	0.323473	0.32843	0.280301	0.333866
0.31228	0.189639	0.26495	0.263831	0.319635	0.321714	0.325232	0.285417	0.283658	0.276943
0.227055	0.395107	0.325392	0.261273	0.247681	0.273265	0.264311	0.307004	0.271826	0.329229
0.350016	0.287336	0.317557	0.299009	0.362968	0.364407	0.378318	0.345539	0.308443	0.306044
0.330668	0.308123	0.326511	0.31276	0.344579	0.354173	0.341541	0.332267	0.317237	0.299648
0.332907	0.299968	0.29645	0.285257	0.34314	0.346498	0.329709	0.288935	0.298849	0.309402
0.251839	0.389671	0.341222	0.259194	0.211864	0.243204	0.251999	0.303965	0.26511	0.32859
0.307323	0.268468	0.295331	0.282539	0.336585	0.3433	0.318516	0.300608	0.284618	0.298049
0.361209	0.261753	0.293892	0.297729	0.35945	0.364727	0.365366	0.323793	0.308602	0.305405
0.221938	0.299488	0.277742	0.222737	0.244004	0.254397	0.269907	0.268628	0.244963	0.278862
0.348417	0.286377	0.308283	0.29677	0.355293	0.357531	0.362808	0.327311	0.310361	0.319316
0.282379	0.399584	0.323313	0.260953	0.271027	0.283339	0.228654	0.307323	0.273425	0.346978
0.150943	0.35993	0.319316	0.241765	0.227854	0.239047	0.282219	0.305724	0.248641	0.314839
0.310841	0.243204	0.262552	0.26623	0.320435	0.31228	0.347937	0.290214	0.283499	0.272945
0.204189	0.372082	0.293092	0.240486	0.225935	0.240806	0.279821	0.314199	0.242725	0.307323
0.299968	0.319316	0.278062	0.270707	0.299808	0.317397	0.295971	0.291973	0.279501	0.28206
0.355453	0.20355	0.251999	0.284458	0.353374	0.34314	0.387752	0.292773	0.314839	0.282219
0.361369	0.290534	0.331468	0.305724	0.356252	0.371762	0.366645	0.349376	0.319316	0.326831
0.28206	0.301727	0.266869	0.241605	0.2811	0.292613	0.301567	0.286537	0.257755	0.267509
0.254717	0.384074	0.321714	0.262552	0.201311	0.226415	0.305405	0.31196	0.273105	0.330668
0.20323	0.333547	0.285897	0.244963	0.24928	0.254237	0.285257	0.29645	0.259514	0.313559
0.24928	0.372562	0.322354	0.201471	0.232491	0.246562	0.263351	0.287336	0.26639	0.324592
0.251679	0.29741	0.267509	0.21954	0.245763	0.260153	0.264471	0.264631	0.238727	0.272785
0.354493	0.208986	0.278062	0.292293	0.361689	0.362488	0.378318	0.299968	0.311161	0.298369
0.351455	0.279981	0.285417	0.298849	0.362968	0.361369	0.369683	0.304765	0.307963	0.305884
0.242885	0.382155	0.326671	0.260473	0.082187	0.005916	0.32811	0.323313	0.279341	0.340902
0.32827	0.308762	0.277422	0.2811	0.331788	0.330828	0.25024	0.216501	0.305724	0.283658
0.338503	0.229613	0.260633	0.273745	0.348737	0.344899	0.348897	0.276943	0.295971	0.275024
0.216182	0.360409	0.303486	0.246083	0.212024	0.208666	0.29725	0.291653	0.257115	0.31324
0.298369	0.301727	0.275983	0.264311	0.309562	0.314839	0.299968	0.283179	0.277103	0.284458
0.258874	0.32827	0.271986	0.274065	0.286537	0.292453	0.288455	0.293572	0.263032	0.300927
0.286377	0.381996	0.292133	0.259354	0.286377	0.297729	0.18804	0.259194	0.256636	0.307643
0.271826	0.349057	0.267189	0.26623	0.273585	0.28142	0.306364	0.290214	0.268308	0.26527
0.270867	0.403102	0.342821	0.264631	0.274864	0.300128	0.267669	0.322194	0.275983	0.339942
0.289255	0.365526	0.283658	0.268628	0.286697	0.29645	0.267669	0.28206	0.272146	0.311481
0.352734	0.208666	0.260473	0.285257	0.352574	0.34362	0.383115	0.290854	0.311321	0.288136
0.292453	0.35865	0.28142	0.272785	0.298529	0.306524	0.260473	0.282219	0.279981	0.291973
0.242245	0.381516	0.325552	0.259354	0.080748	0.005437	0.32795	0.321874	0.278542	0.340102
0.237448	0.356252	0.303646	0.267189	0.267988	0.262712	0.302207	0.305724	0.267029	0.330189
0.300288	0.342661	0.232012	0.257115	0.307483	0.329229	0.237928	0.292773	0.257115	0.253118
0.159578	0.386313	0.3441	0.251039	0.204989	0.217941	0.314199	0.325712	0.270547	0.345219
0.264471	0.367925	0.319635	0.257595	0.213463	0.229933	0.284618	0.310042	0.271826	0.3118
0.232012	0.402143	0.336105	0.242885	0.204989	0.214583	0.29757	0.32875	0.26575	0.338184
0.087944	0.388711	0.337704	0.257755	0.239207	0.254237	0.29709	0.324752	0.26607	0.332427
0.388391	0.186121	0.294212	0.311321	0.389671	0.387592	0.420211	0.317717	0.335785	0.309082
0.255676	0.353694	0.316437	0.263831	0.287176	0.288615	0.290694	0.29613	0.267829	0.314519
0.279981	0.322194	0.304125	0.272785	0.295011	0.289415	0.330029	0.323473	0.300128	0.325712
0.332907	0.325712	0.302367	0.279661	0.278542	0.276783	0.304285	0.293892	0.287656	0.327311
0.360729	0.291334	0.330508	0.304125	0.355133	0.370643	0.365366	0.347937	0.316757	0.326031
0.385034	0.221938	0.295171	0.307323	0.386633	0.393988	0.372082	0.325552	0.323473	0.313559
0.333387	0.186441	0.251519	0.270067	0.338823	0.337864	0.362488	0.276623	0.29661	0.270547
0.285257	0.306844	0.302047	0.260953	0.287656	0.29613	0.289415	0.288775	0.294532	0.291493
0.255996	0.383115	0.324592	0.266709	0.263192	0.273265	0.278062	0.319476	0.28142	0.350496
0.366645	0.167893	0.252638	0.302367	0.368884	0.366645	0.373521	0.300608	0.307643	0.261593
0.365046	0.2504	0.269108	0.300128	0.37496	0.367925	0.377039	0.29757	0.310521	0.280141

For Review Only

0.225616	0.381836	0.329069	0.244963	0.007035	0.081068	0.315478	0.322833	0.262392	0.331788
0.391589	0.201951	0.29613	0.316118	0.393188	0.388072	0.419252	0.315798	0.337864	0.310361
0.307803	0.298529	0.284458	0.262072	0.298209	0.319955	0.3118	0.301727	0.283658	0.277263
0.322194	0.291334	0.303646	0.290854	0.338503	0.329549	0.337224	0.29757	0.302686	0.285737
0.260953	0.411737	0.337224	0.257755	0.257755	0.276463	0.272785	0.329549	0.282699	0.34394
0.34426	0.321874	0.317877	0.301247	0.355772	0.354813	0.330029	0.316597	0.309882	0.316597
0.320755	0.279821	0.293252	0.284938	0.333866	0.339143	0.323793	0.287176	0.294372	0.269907
0.196994	0.389671	0.311001	0.25072	0.277422	0.294372	0.271666	0.29709	0.257435	0.314998
0.325392	0.284938	0.300448	0.305085	0.360569	0.364886	0.371442	0.309402	0.314039	0.324592
0.380716	0.200192	0.303166	0.32843	0.394627	0.388711	0.42229	0.307803	0.347298	0.275664
0.307004	0.240806	0.274704	0.276943	0.320115	0.322833	0.345379	0.290374	0.293412	0.285737
0.397985	0.193956	0.301087	0.315798	0.399105	0.394947	0.42245	0.320115	0.339463	0.31244
0.253758	0.289735	0.268628	0.21826	0.251839	0.257115	0.264791	0.259994	0.235689	0.279981
0.31324	0.314679	0.278862	0.282699	0.314839	0.319635	0.350496	0.298529	0.283179	0.273105
0.280141	0.353694	0.299328	0.277902	0.311161	0.332107	0.31276	0.332587	0.298209	0.323633
0.209466	0.341062	0.293252	0.241286	0.235369	0.248481	0.276303	0.29629	0.260793	0.293572
0.238727	0.395267	0.335785	0.255037	0.205309	0.21826	0.308443	0.331468	0.268788	0.337704
0.325392	0.330349	0.297729	0.290214	0.32827	0.341382	0.307483	0.345379	0.303646	0.331308
0.345059	0.23441	0.274225	0.29645	0.357211	0.357851	0.381676	0.314359	0.299009	0.29757
0.357851	0.272945	0.287496	0.288455	0.354013	0.362488	0.323313	0.301087	0.302367	0.303965
0.356732	0.253118	0.323473	0.301407	0.357211	0.37432	0.354333	0.326831	0.307483	0.290694
0.311481	0.305724	0.318676	0.279021	0.320435	0.330508	0.320755	0.319955	0.295811	0.295491
0.305564	0.299968	0.322674	0.298049	0.299648	0.308922	0.372721	0.313719	0.295331	0.331308
0.113687	0.377838	0.334346	0.253598	0.242885	0.261113	0.299808	0.320915	0.257435	0.326031
0.301247	0.379597	0.283339	0.253278	0.282219	0.303166	0.210585	0.299009	0.270547	0.307323
0.324752	0.316437	0.300448	0.282859	0.318516	0.317237	0.314679	0.305085	0.284938	0.291813
0.380397	0.007035	0.246562	0.305884	0.382795	0.381996	0.400064	0.294532	0.329709	0.285577
0.294372	0.350975	0.301407	0.270707	0.279501	0.290534	0.270387	0.272306	0.284938	0.330029
0.400544	0.190278	0.307483	0.322674	0.399264	0.399264	0.431724	0.324272	0.341222	0.31196
0.165814	0.387912	0.338503	0.240326	0.18676	0.209466	0.285737	0.304445	0.255037	0.336265
0.29661	0.335945	0.289735	0.268148	0.326991	0.327311	0.153822	0.2504	0.274704	0.291493
0.26559	0.389191	0.309402	0.253758	0.25072	0.259354	0.267669	0.311001	0.273905	0.346338
0.386633	0.214263	0.287336	0.303166	0.382475	0.385833	0.399264	0.329389	0.324912	0.304125
0.221458	0.318356	0.29757	0.258075	0.28142	0.277902	0.323633	0.311321	0.272466	0.31292
0.320275	0.29693	0.275344	0.279021	0.323633	0.335785	0.345059	0.303166	0.282699	0.26575
0.163096	0.353214	0.316917	0.261113	0.235369	0.255037	0.315318	0.309882	0.262392	0.315798
0.341701	0.277103	0.285417	0.291493	0.357052	0.354813	0.367445	0.306524	0.298209	0.298529
0.398625	0.210425	0.304605	0.325232	0.400224	0.395747	0.424209	0.318676	0.335785	0.311161
0.273585	0.395907	0.293412	0.246882	0.26607	0.289575	0.217141	0.273745	0.264311	0.309242
0.243524	0.383435	0.327311	0.260953	0.081548	0.005756	0.32827	0.323793	0.279821	0.341382
0.269747	0.292613	0.276623	0.244324	0.247841	0.235529	0.324912	0.284138	0.266869	0.307163
0.397506	0.199232	0.291174	0.318196	0.395267	0.393668	0.42213	0.324272	0.332107	0.306684
0.272146	0.396386	0.292133	0.274864	0.271346	0.294691	0.273745	0.280141	0.269747	0.326031
0.28126	0.314039	0.288295	0.258235	0.26511	0.271506	0.273105	0.279341	0.26527	0.286856
0.321394	0.315958	0.289255	0.284618	0.317077	0.316917	0.341861	0.318516	0.295811	0.307963
0.300448	0.415254	0.309562	0.258714	0.301727	0.323473	0.087304	0.262712	0.272945	0.334666
0.253598	0.337064	0.320115	0.263351	0.217621	0.202111	0.305884	0.327311	0.275184	0.310841
0.302686	0.352734	0.298529	0.271826	0.311161	0.329549	0.299968	0.317397	0.294532	0.314359
0.221458	0.315638	0.347618	0.28142	0.279501	0.292773	0.355932	0.337224	0.292933	0.345379
0.292453	0.345219	0.293732	0.276303	0.286377	0.285737	0.315158	0.322514	0.293252	0.305564
0.293252	0.338024	0.324432	0.285257	0.270387	0.288295	0.333706	0.330508	0.305405	0.313879
0.398625	0.190918	0.303806	0.319635	0.396067	0.396386	0.429965	0.331308	0.335465	0.309882
0.284938	0.412696	0.314519	0.267349	0.284458	0.289735	0.224177	0.29757	0.275184	0.342981
0.39047	0.195875	0.295491	0.316757	0.392389	0.392549	0.420371	0.319955	0.334346	0.308922
0.232971	0.403422	0.336904	0.243044	0.205469	0.215062	0.29661	0.329549	0.264631	0.337544

For Review Only

0.210425	0.409018	0.339942	0.257755	0.21938	0.230732	0.283499	0.311481	0.270387	0.346658
0.341222	0.278702	0.317237	0.292133	0.361529	0.363128	0.377039	0.31308	0.307163	0.287656
0.295651	0.403262	0.321234	0.267509	0.282859	0.306044	0.21858	0.31212	0.283019	0.335785
0.229933	0.391589	0.325392	0.257435	0.194755	0.199552	0.295331	0.313399	0.262872	0.337064

For Review Only

0.314359	0.263351	0.26543	0.154461	0.298369	0.356732	0.263511	0.321714	0.335785	0.300768
0.306684	0.355293	0.333706	0.358331	0.270547	0.326671	0.318676	0.345059	0.32795	0.268308
0.259514	0.241286	0.289255	0.292773	0.21842	0.360569	0.326831	0.284138	0.3748	0.214423
0.304125	0.291653	0.282379	0.299488	0.253118	0.330349	0.295971	0.307483	0.356412	0.261273
0.332907	0.346178	0.278862	0.254877	0.330189	0.350975	0.285098	0.349057	0.318836	0.336265
0.310841	0.263991	0.257115	0.246882	0.255996	0.339143	0.274225	0.28174	0.340742	0.257115
0.322993	0.285737	0.253598	0.15686	0.318516	0.354333	0.236649	0.320755	0.34394	0.299009
0.333547	0.350656	0.342661	0.334666	0.314679	0.333227	0.300288	0.323953	0.208347	0.310681
0.306044	0.285257	0.224177	0.157179	0.303326	0.327151	0.168532	0.316757	0.332267	0.304285
0.35897	0.380876	0.376559	0.371922	0.349057	0.334506	0.313399	0.32795	0.182603	0.349536
0.277582	0.355612	0.332587	0.351775	0.331788	0.274704	0.311161	0.34362	0.351775	0.351935
0.268948	0.25056	0.271666	0.294532	0.211065	0.35913	0.315318	0.28094	0.35913	0.210745
0.321075	0.307323	0.290054	0.252159	0.308283	0.334346	0.26559	0.321075	0.330828	0.302846
0.292453	0.338663	0.332267	0.318356	0.291973	0.316118	0.31276	0.304285	0.29645	0.316917
0.287336	0.236169	0.228814	0.220499	0.259354	0.323953	0.25072	0.304605	0.346019	0.282539
0.315158	0.304765	0.314359	0.324113	0.274704	0.321234	0.310841	0.307803	0.339942	0.274225
0.32875	0.308922	0.23409	0.198273	0.333067	0.352414	0.26543	0.329869	0.354973	0.344579
0.31292	0.283339	0.305405	0.289735	0.295171	0.346978	0.302207	0.304605	0.29677	0.305245
0.309562	0.272306	0.263032	0.293412	0.251679	0.35881	0.318676	0.301087	0.386792	0.237448
0.338663	0.362168	0.35913	0.35993	0.332267	0.313399	0.305245	0.340902	0.269268	0.350016
0.321075	0.332587	0.305245	0.283179	0.336585	0.320275	0.235369	0.321075	0.337064	0.348897
0.340102	0.306684	0.206428	0.21938	0.325871	0.358171	0.24992	0.322674	0.362328	0.331628
0.298209	0.409498	0.386952	0.398945	0.363767	0.254077	0.339623	0.369524	0.342661	0.388391
0.317877	0.348737	0.344739	0.344579	0.291334	0.278382	0.300927	0.310201	0.278542	0.320115
0.302207	0.270707	0.238088	0.23505	0.267349	0.34442	0.278222	0.275664	0.351295	0.271506
0.304925	0.275823	0.305245	0.298209	0.255197	0.349057	0.321394	0.287656	0.372242	0.24928
0.292613	0.289095	0.278702	0.291653	0.211225	0.336585	0.302686	0.275184	0.34394	0.222098
0.268148	0.266549	0.262872	0.271826	0.251359	0.287496	0.268308	0.28158	0.304925	0.261113
0.319635	0.298849	0.28078	0.253438	0.29613	0.299009	0.113847	0.292133	0.302207	0.304765
0.371282	0.43828	0.414615	0.42181	0.371282	0.313879	0.341701	0.357851	0.257755	0.391749
0.31244	0.254717	0.227055	0.277902	0.231212	0.347937	0.304285	0.283019	0.366006	0.245923
0.329709	0.252798	0.273425	0.288935	0.28142	0.351135	0.31212	0.277582	0.35993	0.292773
0.329069	0.267988	0.263351	0.176847	0.3126	0.364407	0.259354	0.294851	0.35961	0.315798
0.322194	0.362488	0.351135	0.337064	0.307643	0.292933	0.289735	0.307483	0.287496	0.333067
0.287816	0.323633	0.329389	0.34442	0.275823	0.286377	0.31196	0.335785	0.347458	0.31276
0.316917	0.319635	0.298849	0.32779	0.226735	0.316597	0.304605	0.293572	0.29741	0.245123
0.302686	0.306524	0.315478	0.294212	0.29757	0.290214	0.298849	0.309242	0.330988	0.309242
0.31276	0.320755	0.335465	0.318036	0.303006	0.315318	0.308922	0.334986	0.314199	0.317717
0.304285	0.420851	0.408379	0.400704	0.347777	0.227055	0.331788	0.357211	0.313879	0.37464
0.304605	0.362008	0.367125	0.354813	0.339942	0.229933	0.336265	0.367925	0.342181	0.362968
0.35897	0.433323	0.409178	0.408858	0.367445	0.307004	0.338343	0.346658	0.232811	0.388711
0.314679	0.347618	0.346658	0.331468	0.318196	0.29709	0.222098	0.303006	0.273105	0.321554
0.346338	0.376719	0.360569	0.337704	0.330668	0.326831	0.294372	0.320435	0.10873	0.326671
0.316437	0.354493	0.280141	0.287816	0.332427	0.34442	0.292613	0.344899	0.336745	0.353054
0.345379	0.385513	0.373361	0.345059	0.342981	0.341382	0.329389	0.334986	0.007675	0.344899
0.270387	0.235209	0.28174	0.286697	0.227694	0.350815	0.318836	0.276463	0.362648	0.216821
0.2819	0.255357	0.287336	0.303646	0.15686	0.347458	0.317717	0.303326	0.370483	0.244803
0.285257	0.306684	0.304445	0.302686	0.29693	0.293252	0.307643	0.332907	0.319156	0.298529
0.340742	0.341222	0.283339	0.264791	0.330668	0.347937	0.257595	0.341701	0.317717	0.338184
0.309082	0.204349	0.220019	0.245283	0.291334	0.330189	0.28206	0.304605	0.356572	0.302367

For Review Only

0.261912	0.256796	0.29613	0.307323	0.237128	0.354333	0.314039	0.271186	0.364886	0.237768
0.304765	0.321394	0.284618	0.291813	0.278062	0.329389	0.307803	0.324432	0.353694	0.235849
0.320755	0.272146	0.179405	0.149504	0.304445	0.370323	0.237928	0.336585	0.37432	0.301887
0.329229	0.266869	0.263991	0.177007	0.31212	0.364087	0.258714	0.294851	0.358491	0.316118
0.303486	0.25056	0.259194	0.287976	0.240966	0.348897	0.302207	0.284298	0.362488	0.240486
0.334346	0.318516	0.233131	0.237448	0.326831	0.358011	0.282699	0.335945	0.345219	0.324912
0.309722	0.272466	0.262232	0.293572	0.251839	0.35865	0.318036	0.301087	0.385993	0.237928
0.283019	0.267988	0.285098	0.303486	0.142309	0.351295	0.321234	0.285577	0.379117	0.225616
0.2819	0.251679	0.280461	0.286856	0.23457	0.339463	0.31228	0.286217	0.354973	0.241605
0.340422	0.411577	0.400863	0.389671	0.351295	0.284618	0.329549	0.348417	0.269428	0.365526
0.309402	0.318356	0.315318	0.292453	0.302207	0.292293	0.294851	0.309562	0.317397	0.324113
0.26543	0.262392	0.260793	0.26575	0.243684	0.294851	0.266709	0.286057	0.311001	0.24928
0.288136	0.270867	0.277422	0.310521	0.108411	0.349376	0.316757	0.276623	0.35961	0.185162
0.292933	0.291174	0.28126	0.332427	0.058043	0.345059	0.329229	0.270067	0.361848	0.206908
0.320435	0.271826	0.179405	0.148705	0.303965	0.369524	0.237768	0.335146	0.37432	0.301567
0.319476	0.298209	0.280461	0.253438	0.295011	0.298849	0.114327	0.291014	0.301727	0.303806
0.279981	0.277582	0.271027	0.26495	0.257435	0.302207	0.276623	0.279181	0.322674	0.271186
0.307323	0.321714	0.34442	0.308762	0.313559	0.301567	0.290854	0.319795	0.270867	0.32795
0	0.308602	0.305085	0.327151	0.276463	0.279501	0.305085	0.322993	0.345219	0.292933
0.308602	0	0.264791	0.262232	0.278222	0.349536	0.298689	0.305724	0.384874	0.305085
0.305085	0.264791	0	0.232491	0.268948	0.355932	0.249121	0.316757	0.373521	0.288455
0.327151	0.262232	0.232491	0	0.308283	0.363607	0.223217	0.320435	0.345219	0.307483
0.276463	0.278222	0.268948	0.308283	0	0.322514	0.29757	0.263511	0.342981	0.205469
0.279501	0.349536	0.355932	0.363607	0.322514	0	0.293572	0.329389	0.341222	0.349376
0.305085	0.298689	0.249121	0.223217	0.29757	0.293572	0	0.295651	0.329709	0.314679
0.322993	0.305724	0.316757	0.320435	0.263511	0.329389	0.295651	0	0.334826	0.284778
0.345219	0.384874	0.373521	0.345219	0.342981	0.341222	0.329709	0.334826	0	0.345059
0.292933	0.305085	0.288455	0.307483	0.205469	0.349376	0.314679	0.284778	0.345059	0
0.339143	0.346019	0.323633	0.311001	0.349217	0.366965	0.334826	0.362648	0.31244	0.350336
0.325392	0.420851	0.409498	0.4055	0.354973	0.252958	0.337864	0.362968	0.315798	0.376879
0.293572	0.263511	0.315318	0.307323	0.26543	0.348257	0.32763	0.300608	0.37464	0.269268
0.309882	0.277902	0.235529	0.26623	0.270387	0.345379	0.292293	0.298529	0.384874	0.276623
0.329709	0.327151	0.270387	0.270387	0.314679	0.34426	0.289255	0.334986	0.306684	0.29661
0.321874	0.318356	0.319316	0.311481	0.266549	0.310521	0.290854	0.279181	0.292293	0.286856
0.342021	0.431884	0.420051	0.417333	0.366326	0.252958	0.347777	0.3748	0.322993	0.382635
0.319476	0.270867	0.178286	0.148705	0.303326	0.368564	0.236329	0.333067	0.373841	0.301567
0.31228	0.263511	0.304445	0.258395	0.303486	0.32763	0.286697	0.300288	0.338503	0.316118
0.346019	0.385353	0.373201	0.345219	0.34346	0.340582	0.329709	0.334826	0.008794	0.344579
0.336265	0.357211	0.337064	0.336745	0.322354	0.324912	0.294372	0.325232	0.243684	0.310841
0.35913	0.364886	0.305245	0.289894	0.352095	0.349856	0.274704	0.370963	0.323633	0.352574
0.270067	0.333706	0.333067	0.332587	0.311161	0.307803	0.331948	0.324272	0.310361	0.290054
0.329069	0.367445	0.366006	0.342821	0.344739	0.332427	0.332747	0.339942	0.162936	0.348097
0.314199	0.259034	0.193796	0.167253	0.306204	0.371122	0.241605	0.315478	0.386313	0.302686
0.31244	0.286217	0.251999	0.290534	0.239527	0.362168	0.309562	0.291973	0.399424	0.256476
0.295331	0.298209	0.317077	0.308283	0.305085	0.367125	0.319316	0.32827	0.31228	0.319955
0.289735	0.272785	0.277742	0.311161	0.110489	0.350496	0.318036	0.277902	0.35897	0.186121
0.322833	0.276303	0.284938	0.269108	0.292773	0.347937	0.305245	0.335465	0.360409	0.292613
0.309402	0.253278	0.269907	0.275983	0.237608	0.337704	0.287176	0.279501	0.378158	0.248801
0.321075	0.383435	0.37576	0.358331	0.317877	0.286057	0.310201	0.331948	0.290534	0.345539
0.268788	0.268628	0.295491	0.306204	0.244324	0.314519	0.320275	0.303965	0.334986	0.247202
0.307963	0.268948	0.29709	0.290054	0.253758	0.331948	0.314199	0.304605	0.349696	0.278062
0.322833	0.286217	0.293572	0.304445	0.283179	0.277742	0.307483	0.336425	0.34442	0.305724
0.276623	0.28126	0.26575	0.291973	0.185002	0.333387	0.292613	0.276943	0.332907	0.217461
0.32875	0.426767	0.418612	0.407099	0.358171	0.237288	0.340582	0.364247	0.318356	0.385034
0.336265	0.393988	0.386473	0.376239	0.339623	0.294532	0.320435	0.339143	0.297729	0.358491

For Review Only

0.29741	0.304285	0.305245	0.308762	0.286377	0.291174	0.288615	0.290534	0.330029	0.301887
0.324113	0.24976	0.277103	0.290054	0.2811	0.354333	0.316278	0.342981	0.326351	0.295491
0.288615	0.299169	0.323473	0.314998	0.278702	0.285897	0.309722	0.317557	0.350176	0.299488
0.318516	0.352095	0.329709	0.339942	0.301887	0.293572	0.322993	0.335625	0.318836	0.315318
0.291174	0.270227	0.286537	0.304605	0.236489	0.321714	0.295651	0.287816	0.32843	0.226575
0.325871	0.369683	0.372402	0.354333	0.320115	0.286377	0.307323	0.333227	0.295491	0.356092
0.288455	0.284618	0.276303	0.298689	0.207067	0.345219	0.3118	0.270707	0.369843	0.196994
0.289095	0.267029	0.292933	0.29645	0.245603	0.322034	0.306844	0.273265	0.346498	0.238407
0.290374	0.25008	0.244484	0.268948	0.238248	0.348257	0.294532	0.307483	0.342661	0.228174
0.295331	0.284778	0.303806	0.291973	0.245603	0.293732	0.299169	0.279501	0.306524	0.256956
0.316278	0.336265	0.352734	0.341382	0.293252	0.31276	0.313559	0.333866	0.308922	0.309562
0.305085	0.322993	0.324432	0.339783	0.271186	0.287656	0.287656	0.289894	0.320275	0.287496
0.320115	0.291014	0.299808	0.257275	0.309082	0.32875	0.291973	0.308762	0.3441	0.323473
0.279021	0.269587	0.28174	0.276303	0.257115	0.290854	0.263671	0.29725	0.316118	0.275024
0.304925	0.29677	0.295491	0.320435	0.295491	0.363128	0.319476	0.331148	0.289575	0.307963
0.277582	0.279661	0.264471	0.287336	0.210425	0.337224	0.299488	0.264311	0.342181	0.134474
0.308602	0.322354	0.303646	0.306684	0.262712	0.330988	0.316437	0.303326	0.321394	0.274544
0.26591	0.269428	0.300448	0.292453	0.248321	0.304925	0.294691	0.288295	0.332907	0.272306
0.300128	0.188679	0.292133	0.301087	0.309882	0.338503	0.295171	0.317397	0.381676	0.337384
0.262552	0.266709	0.273745	0.276303	0.222418	0.307803	0.278382	0.274384	0.323473	0.246882
0.326991	0.37464	0.380556	0.364567	0.317237	0.289894	0.319476	0.326351	0.291493	0.353694
0.290694	0.295011	0.258235	0.283978	0.306684	0.340262	0.284618	0.336265	0.319476	0.307643
0.270707	0.272466	0.269108	0.274544	0.241765	0.284778	0.269108	0.278062	0.307963	0.258075
0.302686	0.254557	0.220019	0.251199	0.271346	0.340102	0.277422	0.311481	0.376079	0.284298
0.286537	0.2504	0.279341	0.292613	0.223057	0.340422	0.308602	0.272306	0.34426	0.230413
0.32811	0.42181	0.407739	0.412216	0.356412	0.253438	0.32875	0.35977	0.315158	0.379917
0.290054	0.301407	0.324912	0.326351	0.28078	0.204349	0.295971	0.302367	0.352734	0.29757
0.286856	0.326511	0.323313	0.331948	0.307643	0.193476	0.290694	0.321394	0.32763	0.322514
0.323313	0.26639	0.260633	0.268308	0.279181	0.344579	0.279661	0.283179	0.371122	0.298049
0.309562	0.271506	0.269268	0.258075	0.270707	0.310521	0.263991	0.295331	0.321554	0.2811
0.305245	0.252958	0.238887	0.285098	0.231852	0.374161	0.315318	0.286856	0.380876	0.246562
0.335465	0.247681	0.267029	0.29613	0.290534	0.346818	0.292773	0.309882	0.352574	0.310361
0.333706	0.42165	0.404061	0.398625	0.350496	0.271666	0.331788	0.360889	0.307323	0.373361
0.284938	0.289255	0.275983	0.286377	0.254237	0.286697	0.274384	0.286856	0.301087	0.256476
0.313559	0.259834	0.192197	0.165174	0.307323	0.372082	0.241126	0.319316	0.387752	0.303965
0.271506	0.24928	0.280461	0.272785	0.247681	0.302846	0.274704	0.290854	0.337064	0.272785
0.321075	0.361369	0.351775	0.336425	0.307004	0.291653	0.290694	0.307483	0.288615	0.331148
0.321874	0.298209	0.293252	0.282859	0.304925	0.322674	0.28062	0.333067	0.320115	0.325712
0.331628	0.386473	0.382315	0.370323	0.325712	0.290534	0.316118	0.340422	0.294851	0.357052
0.295331	0.249121	0.260793	0.279181	0.220179	0.352574	0.293732	0.279501	0.350496	0.207547
0.267988	0.316757	0.337384	0.327151	0.310361	0.2819	0.319156	0.32795	0.338024	0.315638
0.318836	0.419252	0.391909	0.397666	0.35929	0.260313	0.330189	0.369044	0.334986	0.39063
0.297889	0.32859	0.333387	0.330349	0.308762	0.217141	0.299808	0.302047	0.357691	0.347618
0.336904	0.389671	0.3756	0.368404	0.326831	0.290214	0.317717	0.339942	0.295171	0.350176
0.297889	0.17237	0.28158	0.302686	0.258075	0.342821	0.310042	0.310042	0.370163	0.263192
0.34442	0.395907	0.384234	0.385993	0.34378	0.315158	0.324272	0.352414	0.253278	0.358171
0.282379	0.283179	0.323953	0.314359	0.290374	0.320115	0.293412	0.294372	0.340102	0.29645
0.324912	0.26511	0.297889	0.245443	0.271346	0.345699	0.284618	0.314839	0.336904	0.28078
0.28062	0.25072	0.303166	0.294052	0.245123	0.350656	0.325552	0.29661	0.370163	0.252958
0.271346	0.259354	0.299328	0.305564	0.21906	0.335146	0.325552	0.294372	0.353694	0.241286
0.299169	0.332107	0.331628	0.322194	0.294691	0.269108	0.285257	0.320915	0.31276	0.319795
0.293412	0.275983	0.301887	0.290694	0.243204	0.34314	0.314839	0.282539	0.370483	0.232811
0.29677	0.256316	0.303965	0.303166	0.251999	0.337704	0.320915	0.300288	0.355133	0.26543
0.300288	0.362008	0.365686	0.336745	0.340422	0.256956	0.307323	0.347298	0.300927	0.36009
0.287816	0.217141	0.278862	0.288295	0.280301	0.295011	0.291174	0.292933	0.336265	0.287816

For Review Only

0.304285	0.292453	0.295651	0.326991	0.148065	0.356252	0.336904	0.279021	0.363128	0.232491
0.291334	0.337224	0.318196	0.324432	0.294372	0.272146	0.295491	0.326191	0.290374	0.31292
0.275184	0.207547	0.26607	0.279501	0.262392	0.349217	0.305724	0.310361	0.366965	0.271346
0.263032	0.361209	0.372562	0.373681	0.341382	0.262072	0.334026	0.365206	0.29629	0.357851
0.357531	0.35929	0.355293	0.347937	0.333547	0.336105	0.336745	0.331628	0.231852	0.354333
0.3118	0.307004	0.322034	0.335305	0.320115	0.252319	0.293092	0.316917	0.350656	0.340902
0.301407	0.275664	0.286377	0.267669	0.237928	0.358171	0.319316	0.314039	0.354813	0.231372
0.295171	0.304125	0.323473	0.301727	0.315798	0.274225	0.308922	0.320275	0.32747	0.335785
0.306684	0.375919	0.371122	0.360729	0.338343	0.246882	0.322514	0.357052	0.32843	0.353214
0.276143	0.268628	0.276303	0.269907	0.239047	0.290854	0.270387	0.275344	0.308922	0.252638
0.292133	0.370483	0.356252	0.35961	0.329709	0.289415	0.325392	0.35993	0.342981	0.34314
0.319156	0.258075	0.220659	0.258395	0.267829	0.352574	0.292453	0.301887	0.380876	0.279181
0.283818	0.261273	0.283978	0.280461	0.235689	0.315638	0.302047	0.277263	0.350815	0.237128
0.288295	0.34314	0.336585	0.329869	0.284298	0.258714	0.29645	0.301087	0.29757	0.311001
0.272146	0.263192	0.267029	0.282539	0.230892	0.314839	0.295971	0.288615	0.365846	0.240806
0.302846	0.288775	0.311321	0.287016	0.293572	0.32827	0.289894	0.321394	0.324113	0.302686
0.327311	0.387592	0.380077	0.363927	0.314998	0.288615	0.31196	0.333547	0.291334	0.341222
0.288775	0.384394	0.355932	0.367125	0.347777	0.274225	0.324432	0.36009	0.354173	0.370643
0.286057	0.242885	0.274864	0.291334	0.273905	0.287496	0.282859	0.286377	0.325552	0.295651
0.304285	0.300128	0.283019	0.301407	0.228814	0.347458	0.297889	0.256316	0.367925	0.197793
0.285098	0.272146	0.275823	0.295171	0.247202	0.332747	0.305085	0.301087	0.345219	0.251999
0.285098	0.246882	0.240806	0.26591	0.236968	0.339623	0.288775	0.304605	0.335305	0.228654
0.279981	0.264631	0.26575	0.26527	0.245123	0.287336	0.26639	0.285737	0.302686	0.264631
0.32843	0.39127	0.39127	0.372721	0.329389	0.267669	0.318036	0.337064	0.29613	0.346818
0.29725	0.358331	0.355932	0.369204	0.335465	0.084426	0.298209	0.330029	0.34394	0.349057
0.293412	0.290694	0.28078	0.332107	0.059002	0.345379	0.329229	0.270387	0.362008	0.207867
0.325712	0.326671	0.274384	0.275344	0.309242	0.336265	0.235849	0.307643	0.318996	0.310681
0.311321	0.372082	0.34426	0.340422	0.314359	0.284458	0.285417	0.311001	0.290374	0.329389
0.298529	0.26559	0.259674	0.287976	0.211864	0.329869	0.29725	0.282539	0.332107	0.226575
0.299488	0.247681	0.306364	0.302846	0.29725	0.322034	0.291653	0.307643	0.336904	0.323153
0.305564	0.246402	0.284938	0.271986	0.279501	0.316437	0.298209	0.291174	0.334986	0.295491
0.322993	0.224656	0.206908	0.211385	0.284138	0.338184	0.242565	0.298369	0.364567	0.295491
0.29613	0.285737	0.303486	0.278702	0.270227	0.337384	0.28142	0.261912	0.348417	0.257595
0.301727	0.212024	0.248001	0.273745	0.275983	0.347777	0.311161	0.316597	0.399105	0.299488
0.332267	0.266869	0.256316	0.267988	0.28126	0.337224	0.277263	0.284298	0.35945	0.303166
0.325232	0.388232	0.376239	0.362168	0.314679	0.285098	0.31292	0.336425	0.299648	0.342181
0.324752	0.26511	0.267829	0.241765	0.290214	0.335785	0.26575	0.289095	0.363128	0.303326
0.292773	0.290374	0.28126	0.331148	0.058203	0.344739	0.32811	0.270067	0.361209	0.207707
0.313559	0.31228	0.301407	0.304925	0.25072	0.300288	0.298849	0.300608	0.356092	0.264631
0.299648	0.263511	0.278862	0.225456	0.302047	0.331308	0.28142	0.306844	0.344899	0.300927
0.270867	0.262872	0.299009	0.308443	0.216342	0.335305	0.327311	0.293412	0.352574	0.241925
0.275184	0.29645	0.285257	0.292453	0.223217	0.346818	0.295491	0.268148	0.351135	0.227694
0.259994	0.240166	0.290054	0.292933	0.21858	0.360569	0.326351	0.284138	0.372562	0.213783
0.277103	0.24976	0.301407	0.29677	0.251039	0.345059	0.324912	0.304925	0.370163	0.253758
0.325072	0.42165	0.412536	0.409338	0.356732	0.254557	0.332107	0.365366	0.319795	0.382475
0.279981	0.276143	0.280141	0.293252	0.278222	0.282699	0.2819	0.298369	0.355293	0.291813
0.313719	0.31292	0.319476	0.326991	0.275024	0.330349	0.323793	0.32795	0.341222	0.280461
0.333387	0.301887	0.303166	0.307163	0.26559	0.338343	0.292773	0.303486	0.362008	0.301727
0.287976	0.383435	0.354813	0.365206	0.346178	0.272785	0.323153	0.35913	0.353854	0.370323
0.335785	0.404861	0.388391	0.37576	0.362808	0.274704	0.337384	0.380397	0.322993	0.371922
0.321394	0.368724	0.349696	0.346338	0.307004	0.288935	0.298049	0.313559	0.279821	0.329549
0.299328	0.314998	0.278862	0.292293	0.283978	0.321874	0.283339	0.299488	0.3118	0.29693
0.323473	0.26623	0.263511	0.287816	0.26495	0.362968	0.320915	0.29741	0.371602	0.279021
0.336105	0.373361	0.377998	0.364407	0.334826	0.287656	0.306684	0.342021	0.300288	0.354493
0.285737	0.366485	0.364407	0.372721	0.339143	0.113687	0.295971	0.334346	0.334826	0.361848

For Review Only

0.287336	0.272146	0.277422	0.310201	0.10953	0.349217	0.316437	0.276463	0.358491	0.185321
0.329229	0.427886	0.415574	0.40678	0.355612	0.250879	0.336904	0.368084	0.326191	0.377518
0.309082	0.247841	0.31308	0.318036	0.301407	0.321714	0.311001	0.321554	0.346818	0.3118
0.321075	0.319955	0.337384	0.338184	0.31308	0.307163	0.304605	0.301247	0.301727	0.329389
0.29645	0.232331	0.257115	0.2819	0.266709	0.35993	0.320755	0.306684	0.388391	0.276303
0.304445	0.336105	0.329869	0.339942	0.333547	0.206108	0.307803	0.338983	0.339783	0.350496
0.314679	0.31196	0.331788	0.29677	0.3126	0.314679	0.278702	0.313399	0.245603	0.32811
0.309562	0.225616	0.260793	0.26639	0.284458	0.356092	0.286057	0.290854	0.357851	0.288775
0.324752	0.346658	0.35913	0.351295	0.340102	0.283179	0.314839	0.341861	0.345699	0.367605
0.35945	0.417813	0.404861	0.412696	0.364567	0.314998	0.334986	0.346019	0.216981	0.3748
0.325712	0.345219	0.3441	0.339942	0.300448	0.302367	0.306364	0.317557	0.298209	0.311481
0.333067	0.432044	0.413335	0.413335	0.35993	0.260153	0.339783	0.367605	0.320115	0.386952
0.271346	0.267829	0.258235	0.271506	0.242405	0.28206	0.263991	0.2819	0.302846	0.254877
0.294212	0.302526	0.326511	0.326831	0.302686	0.306844	0.285897	0.302047	0.340742	0.310042
0.269108	0.316597	0.316757	0.314199	0.3126	0.305564	0.334826	0.313719	0.329229	0.304925
0.26575	0.264471	0.299488	0.299169	0.233131	0.310201	0.303166	0.289894	0.350656	0.23393
0.262552	0.253598	0.301887	0.298049	0.220979	0.35913	0.32891	0.284618	0.368724	0.224336
0.299808	0.329869	0.303006	0.314359	0.323153	0.295651	0.318356	0.341222	0.348897	0.333227
0.300288	0.383435	0.389671	0.363447	0.327311	0.254717	0.324912	0.34362	0.317397	0.347298
0.313879	0.352734	0.323473	0.335785	0.334666	0.286697	0.295811	0.365366	0.336904	0.349217
0.306044	0.377039	0.372082	0.354973	0.348737	0.253758	0.325712	0.377199	0.333866	0.365526
0.300128	0.319635	0.332587	0.330668	0.313719	0.301727	0.302686	0.331948	0.321874	0.330828
0.321234	0.348417	0.350336	0.35929	0.295651	0.317237	0.318036	0.259034	0.354813	0.304445
0.28094	0.256636	0.300608	0.29693	0.257755	0.342341	0.319635	0.294691	0.35961	0.264471
0.295011	0.264311	0.221938	0.23425	0.279021	0.339463	0.277902	0.31228	0.361049	0.283019
0.261912	0.317717	0.319156	0.323153	0.295331	0.244484	0.299328	0.31308	0.341541	0.331148
0.340102	0.411896	0.400224	0.389511	0.350496	0.284458	0.329069	0.349057	0.271027	0.364727
0.314199	0.309882	0.290214	0.29709	0.272306	0.34346	0.292133	0.3126	0.332747	0.253438
0.342181	0.429325	0.425488	0.42261	0.364727	0.261433	0.349696	0.370963	0.32795	0.387272
0.293732	0.280141	0.252319	0.29661	0.214423	0.364567	0.3126	0.297889	0.35945	0.212024
0.311481	0.287816	0.248641	0.212664	0.298369	0.32811	0.246402	0.307483	0.310361	0.303326
0.311001	0.244324	0.236808	0.278062	0.248001	0.356092	0.31292	0.286537	0.380556	0.260313
0.317077	0.403422	0.410297	0.385673	0.355772	0.255037	0.345219	0.37576	0.329869	0.377678
0.28062	0.31196	0.319156	0.331948	0.269587	0.314519	0.322674	0.308283	0.331468	0.267988
0.271027	0.329069	0.322194	0.330828	0.308123	0.304285	0.291653	0.321234	0.294532	0.324113
0.294372	0.26607	0.313879	0.310361	0.243844	0.325871	0.307803	0.28094	0.351135	0.269428
0.288295	0.350815	0.345859	0.353534	0.330029	0.09338	0.290854	0.318516	0.340742	0.351615
0.34378	0.433003	0.416214	0.415894	0.362328	0.258075	0.337864	0.368564	0.320595	0.383754
0.308762	0.172689	0.237768	0.233291	0.274864	0.349696	0.269428	0.303166	0.366166	0.276943
0.293892	0.291653	0.28158	0.331948	0.059002	0.346498	0.329549	0.270707	0.362808	0.208507
0.315478	0.318356	0.29725	0.326511	0.226095	0.314199	0.303326	0.292613	0.29661	0.244803
0.320275	0.426127	0.416693	0.407099	0.35977	0.237768	0.341222	0.367765	0.320275	0.386633
0.335146	0.235209	0.252958	0.253598	0.275823	0.34346	0.269428	0.31228	0.368884	0.293252
0.314679	0.274225	0.301727	0.278862	0.251359	0.310201	0.285577	0.290694	0.314359	0.272945
0.289575	0.330828	0.337224	0.338663	0.295171	0.23393	0.298689	0.325712	0.335146	0.319635
0.301887	0.263192	0.157179	0.158778	0.302686	0.364727	0.21922	0.320595	0.385993	0.294212
0.270227	0.293252	0.307483	0.315798	0.205309	0.347618	0.32875	0.293092	0.338823	0.240326
0.28062	0.31212	0.321234	0.29741	0.308762	0.315478	0.311641	0.31228	0.338663	0.308602
0.302526	0.308922	0.353694	0.340262	0.276303	0.297729	0.339623	0.323153	0.348097	0.315958
0.293092	0.288935	0.29645	0.313879	0.275344	0.35913	0.311161	0.323793	0.319316	0.287816
0.31212	0.323473	0.322674	0.324592	0.279821	0.373681	0.34362	0.323793	0.264151	0.278862
0.346818	0.428686	0.424848	0.417013	0.361848	0.256796	0.349696	0.371442	0.326991	0.382795
0.314998	0.243364	0.21938	0.243364	0.28142	0.341382	0.283499	0.299808	0.376079	0.290054
0.321714	0.428846	0.415574	0.40694	0.358331	0.241765	0.341541	0.370483	0.323313	0.383754
0.258714	0.241126	0.290534	0.292133	0.2181	0.360889	0.32747	0.285417	0.373681	0.215542

For Review Only

0.280141	0.277263	0.26527	0.295651	0.232491	0.364886	0.307163	0.305405	0.367445	0.237608
0.294691	0.377518	0.376719	0.369204	0.332907	0.221618	0.316278	0.361209	0.327311	0.355133
0.310681	0.266549	0.227215	0.262872	0.292453	0.335945	0.286377	0.309082	0.382795	0.299648
0.291014	0.290054	0.276783	0.306364	0.199392	0.353854	0.318196	0.26623	0.373201	0.185321

For Review Only

0.314839	0.377998	0.303166	0.29645	0.29741	0.314839	0.388391	0.217781	0.229933	0.336745
0.332907	0.320595	0.321554	0.334666	0.338983	0.294691	0.314199	0.354813	0.334186	0.32747
0.340422	0.399744	0.237608	0.282859	0.327151	0.317877	0.420051	0.29613	0.297889	0.3756
0.375919	0.367605	0.269587	0.269587	0.298049	0.290054	0.381836	0.29629	0.283179	0.357371
0.32795	0.340902	0.35913	0.310361	0.238567	0.304125	0.340422	0.239847	0.29725	0.319955
0.341382	0.386952	0.28174	0.288295	0.305564	0.299328	0.394148	0.248961	0.267189	0.341062
0.288935	0.383914	0.317557	0.286217	0.272785	0.3118	0.396866	0.196994	0.283818	0.342341
0.295331	0.284298	0.325072	0.34362	0.298369	0.275024	0.294532	0.360729	0.323633	0.209146
0.309242	0.370483	0.32795	0.282539	0.268628	0.306204	0.383435	0.169971	0.280301	0.332427
0.319795	0.283818	0.366326	0.382315	0.309562	0.28174	0.301887	0.385353	0.338663	0.182123
0.353534	0.274864	0.345059	0.318356	0.326991	0.320115	0.28158	0.342181	0.310361	0.352894
0.340102	0.391589	0.237768	0.272785	0.324432	0.306364	0.412056	0.293572	0.301887	0.36009
0.302207	0.322674	0.322833	0.303646	0.28126	0.299648	0.32779	0.231052	0.277422	0.331308
0.319955	0.273425	0.308123	0.329069	0.305245	0.255836	0.278062	0.320755	0.285577	0.29741
0.322514	0.361209	0.292613	0.264471	0.266709	0.290534	0.365526	0.205788	0.278702	0.346498
0.368404	0.315478	0.294212	0.273905	0.29757	0.267029	0.327151	0.32747	0.291334	0.340582
0.294212	0.373361	0.352734	0.29613	0.270387	0.315958	0.386313	0.169971	0.324592	0.355293
0.331468	0.348097	0.305245	0.311161	0.282379	0.305085	0.35897	0.276623	0.23457	0.29741
0.357691	0.412056	0.288615	0.257115	0.330508	0.31324	0.414934	0.276943	0.310841	0.386153
0.333866	0.323633	0.356252	0.37496	0.320115	0.316118	0.319476	0.354013	0.316917	0.270067
0.314199	0.315318	0.345699	0.322354	0.299328	0.306044	0.32875	0.273425	0.311161	0.338184
0.292613	0.371282	0.332427	0.293092	0.252159	0.318996	0.380397	0.180045	0.306844	0.362328
0.371122	0.207867	0.398465	0.381676	0.354333	0.310042	0.217141	0.395427	0.354333	0.342821
0.332267	0.226575	0.340262	0.329709	0.295491	0.183722	0.227374	0.354813	0.294052	0.279341
0.341861	0.392389	0.304925	0.252958	0.263991	0.302686	0.404061	0.14055	0.270547	0.350975
0.362808	0.382955	0.060122	0.298369	0.330828	0.301247	0.393668	0.303806	0.302207	0.372242
0.361209	0.366485	0.232971	0.268148	0.311321	0.279021	0.37544	0.291014	0.302526	0.344899
0.31228	0.302526	0.254877	0.26623	0.279981	0.252319	0.307323	0.267509	0.258075	0.305085
0.326351	0.317717	0.32795	0.311161	0.295011	0.284618	0.32779	0.232171	0.283658	0.301407
0.324272	0.239047	0.408379	0.409978	0.336265	0.290694	0.239687	0.424368	0.361848	0.258555
0.368724	0.395267	0.268308	0.23489	0.304285	0.304765	0.399744	0.262232	0.303166	0.365366
0.321234	0.399264	0.307323	0.26559	0.319476	0.315798	0.413335	0.292133	0.283179	0.35929
0.275664	0.401023	0.317717	0.273105	0.278542	0.319635	0.40662	0.188999	0.263351	0.35929
0.340422	0.235849	0.348257	0.338823	0.302526	0.21826	0.227215	0.348097	0.284138	0.288136
0.348577	0.315958	0.302047	0.349536	0.333067	0.3118	0.320435	0.34378	0.311161	0.348417
0.345059	0.303806	0.275184	0.286856	0.292453	0.199712	0.309562	0.325712	0.289095	0.29741
0.326991	0.334186	0.325712	0.307963	0.274065	0.285257	0.342821	0.301247	0.261113	0.331308
0.342821	0.336105	0.313879	0.318356	0.279821	0.295171	0.348737	0.31212	0.276783	0.314519
0.345539	0.129357	0.380397	0.392069	0.345219	0.270547	0.131116	0.407899	0.344739	0.314519
0.361049	0.269268	0.346658	0.347937	0.340742	0.313399	0.26591	0.35865	0.3441	0.342341
0.315318	0.244164	0.393668	0.410617	0.331628	0.287656	0.237928	0.419092	0.348097	0.23425
0.341701	0.304285	0.346019	0.349057	0.305724	0.295011	0.304445	0.355772	0.303166	0.273905
0.302686	0.29725	0.360889	0.367125	0.305405	0.278702	0.300288	0.364087	0.320275	0.10905
0.269428	0.357531	0.362008	0.306524	0.282219	0.32795	0.366166	0.252958	0.298529	0.338024
0.31212	0.316437	0.3748	0.385034	0.306684	0.292773	0.323793	0.373841	0.338663	0.007835
0.341062	0.393508	0.195235	0.275983	0.317077	0.303326	0.407099	0.293092	0.293412	0.363607
0.348417	0.395907	0.181484	0.3118	0.329389	0.302846	0.404381	0.291334	0.294212	0.371442
0.350496	0.306684	0.248961	0.306524	0.305564	0.267189	0.313399	0.3126	0.298209	0.319476
0.256796	0.346338	0.371602	0.325392	0.309082	0.321394	0.357371	0.267988	0.3118	0.318356
0.335945	0.387112	0.298849	0.255357	0.28126	0.303326	0.399584	0.231852	0.272466	0.356412

For Review Only

0.350496	0.386313	0.154301	0.288136	0.324912	0.29677	0.398945	0.279981	0.302686	0.365046
0.361049	0.337864	0.305564	0.287176	0.3118	0.311001	0.339942	0.28206	0.31308	0.353214
0.299328	0.418932	0.31212	0.248161	0.239527	0.324592	0.428526	0.008475	0.270707	0.373521
0.275024	0.400384	0.318196	0.272306	0.278702	0.318676	0.40598	0.189319	0.262552	0.357851
0.353534	0.382795	0.272466	0.269747	0.317077	0.306204	0.396866	0.280301	0.302526	0.362808
0.283499	0.367285	0.348097	0.278862	0.240006	0.332107	0.380237	0.189159	0.329549	0.34426
0.357211	0.410937	0.288295	0.258075	0.330189	0.31324	0.415574	0.276943	0.309242	0.385513
0.367765	0.392869	0.180205	0.292613	0.330349	0.29677	0.404541	0.31228	0.307963	0.379597
0.361689	0.385353	0.098017	0.291174	0.309242	0.29661	0.398305	0.294372	0.295491	0.355932
0.300448	0.194116	0.386153	0.385673	0.333067	0.245923	0.185321	0.399105	0.332267	0.269907
0.322674	0.293412	0.346658	0.326671	0.291653	0.257595	0.298849	0.305245	0.255357	0.317077
0.309722	0.308762	0.263511	0.254237	0.28206	0.259034	0.317397	0.262552	0.271506	0.311321
0.355772	0.388391	0.248001	0.272785	0.32811	0.289415	0.400384	0.315638	0.318676	0.360409
0.37448	0.389511	0.26639	0.29629	0.340742	0.289735	0.401023	0.32891	0.323953	0.362648
0.299488	0.418612	0.311481	0.248001	0.238887	0.323953	0.428206	0.007355	0.270867	0.373681
0.326351	0.317717	0.326991	0.311001	0.295011	0.284778	0.32795	0.232171	0.282859	0.300768
0.324432	0.330988	0.267349	0.273745	0.290214	0.271666	0.341382	0.267349	0.264631	0.323153
0.314998	0.307004	0.337384	0.339143	0.291334	0.275344	0.31244	0.330029	0.273905	0.271027
0.339143	0.325392	0.293572	0.309882	0.329709	0.321874	0.342021	0.319476	0.31228	0.346019
0.346019	0.420851	0.263511	0.277902	0.327151	0.318356	0.431884	0.270867	0.263511	0.385353
0.323633	0.409498	0.315318	0.235529	0.270387	0.319316	0.420051	0.178286	0.304445	0.373201
0.311001	0.4055	0.307323	0.26623	0.270387	0.311481	0.417333	0.148705	0.258395	0.345219
0.349217	0.354973	0.26543	0.270387	0.314679	0.266549	0.366326	0.303326	0.303486	0.34346
0.366965	0.252958	0.348257	0.345379	0.34426	0.310521	0.252958	0.368564	0.32763	0.340582
0.334826	0.337864	0.32763	0.292293	0.289255	0.290854	0.347777	0.236329	0.286697	0.329709
0.362648	0.362968	0.300608	0.298529	0.334986	0.279181	0.3748	0.333067	0.300288	0.334826
0.31244	0.315798	0.37464	0.384874	0.306684	0.292293	0.322993	0.373841	0.338503	0.008794
0.350336	0.376879	0.269268	0.276623	0.29661	0.286856	0.382635	0.301567	0.316118	0.344579
0	0.351295	0.35929	0.342661	0.324752	0.338503	0.342341	0.299009	0.336904	0.311641
0.351295	0	0.385673	0.392549	0.346658	0.271826	0.102494	0.417493	0.346818	0.315958
0.35929	0.385673	0	0.309722	0.333706	0.3126	0.396866	0.3118	0.298849	0.3748
0.342661	0.392549	0.309722	0	0.279181	0.301247	0.399264	0.247681	0.306684	0.385034
0.324752	0.346658	0.333706	0.279181	0	0.290694	0.353694	0.238727	0.294532	0.306844
0.338503	0.271826	0.3126	0.301247	0.290694	0	0.275344	0.322833	0.278382	0.292933
0.342341	0.102494	0.396866	0.399264	0.353694	0.275344	0	0.427087	0.361049	0.323153
0.299009	0.417493	0.3118	0.247681	0.238727	0.322833	0.427087	0	0.269747	0.373201
0.336904	0.346818	0.298849	0.306684	0.294532	0.278382	0.361049	0.269747	0	0.339463
0.311641	0.315958	0.3748	0.385034	0.306844	0.292933	0.323153	0.373201	0.339463	0
0.319795	0.291334	0.353054	0.333547	0.191238	0.270067	0.298209	0.350016	0.313879	0.244484
0.282379	0.324752	0.378638	0.337704	0.299009	0.309082	0.330189	0.259514	0.331148	0.324113
0.321394	0.325712	0.324752	0.303646	0.315798	0.299009	0.338184	0.336585	0.317077	0.310201
0.295811	0.298049	0.364886	0.376879	0.315318	0.305245	0.308123	0.363128	0.316437	0.163256
0.303486	0.415574	0.304925	0.242085	0.240806	0.321874	0.426287	0.129837	0.267029	0.386313
0.369204	0.417653	0.310201	0.166134	0.318836	0.309402	0.424528	0.276463	0.324912	0.398945
0.317557	0.363128	0.306524	0.318036	0.337384	0.31244	0.368404	0.294691	0.322354	0.313399
0.355612	0.388232	0.249121	0.272626	0.32779	0.290054	0.400224	0.316437	0.320115	0.35993
0.319316	0.368244	0.317877	0.262232	0.298369	0.298849	0.377199	0.267029	0.275823	0.360569
0.360249	0.393508	0.268948	0.278222	0.316597	0.295491	0.40582	0.278542	0.28126	0.378638
0.34394	0.215382	0.354333	0.354013	0.320275	0.223057	0.210745	0.37448	0.306684	0.291174
0.342821	0.349217	0.162456	0.304925	0.300128	0.259514	0.356252	0.307323	0.302047	0.335465
0.350016	0.35865	0.170771	0.29629	0.295811	0.260953	0.367765	0.293732	0.268788	0.350815
0.341541	0.364407	0.32891	0.31292	0.331788	0.324113	0.361369	0.318036	0.324432	0.34394
0.353054	0.358331	0.253918	0.26575	0.304125	0.255516	0.365046	0.295651	0.303006	0.333387
0.346658	0.133834	0.389351	0.400863	0.342501	0.2819	0.145827	0.415894	0.349376	0.318836
0.336745	0.231212	0.377838	0.383914	0.3433	0.259514	0.238887	0.389511	0.327151	0.298529

For Review Only

0.321394	0.279181	0.31324	0.305884	0.308443	0.257915	0.283818	0.308922	0.291653	0.330508
0.310042	0.3756	0.319795	0.315638	0.321874	0.31196	0.375919	0.303646	0.29709	0.327311
0.370643	0.320435	0.18724	0.317077	0.333706	0.293892	0.323153	0.331788	0.308123	0.350815
0.338024	0.323153	0.331148	0.333067	0.316118	0.297889	0.325232	0.346338	0.323153	0.318676
0.35993	0.357211	0.18772	0.285417	0.294052	0.252958	0.365846	0.291493	0.292293	0.32875
0.329869	0.225616	0.342821	0.355612	0.316917	0.245443	0.212344	0.363128	0.303166	0.295811
0.352095	0.39079	0.224976	0.279661	0.322674	0.295331	0.403422	0.298369	0.297729	0.370163
0.334346	0.352414	0.17221	0.283499	0.306204	0.233131	0.35881	0.298849	0.290854	0.346658
0.347618	0.386633	0.276623	0.215702	0.291174	0.295651	0.397825	0.264631	0.298369	0.34314
0.340422	0.305405	0.282859	0.269268	0.293092	0.229613	0.314359	0.300288	0.283658	0.307483
0.355453	0.313879	0.247042	0.337224	0.331948	0.289894	0.317717	0.338024	0.314359	0.309242
0.330668	0.287496	0.262872	0.315318	0.321874	0.237928	0.285257	0.32811	0.294851	0.320435
0.324432	0.334506	0.323153	0.316597	0.302047	0.280301	0.337384	0.243044	0.216981	0.345539
0.31292	0.318516	0.259194	0.28078	0.282379	0.274225	0.324432	0.267669	0.278222	0.315958
0.330668	0.346818	0.316597	0.329229	0.335146	0.302047	0.351935	0.318996	0.339303	0.289894
0.34442	0.366166	0.251199	0.268628	0.307803	0.283499	0.373041	0.279981	0.302526	0.342021
0.351135	0.329709	0.300288	0.310841	0.3126	0.274225	0.3433	0.302846	0.307323	0.320915
0.319635	0.319635	0.18676	0.29741	0.29613	0.283179	0.32827	0.292933	0.282699	0.333866
0.340422	0.388232	0.292773	0.325552	0.356252	0.32827	0.389351	0.304925	0.292933	0.381996
0.314199	0.324432	0.219859	0.271666	0.288615	0.263511	0.334506	0.272146	0.26527	0.323953
0.335305	0.226735	0.350496	0.360409	0.315958	0.238727	0.214263	0.370963	0.301407	0.291813
0.329389	0.355133	0.336265	0.279821	0.301407	0.306524	0.351935	0.26495	0.345539	0.319955
0.307004	0.302526	0.239367	0.276783	0.288136	0.257435	0.3118	0.273585	0.275823	0.308283
0.352095	0.39127	0.311001	0.131116	0.267189	0.292453	0.399105	0.224177	0.287016	0.376399
0.347138	0.367285	0.270067	0.262392	0.307483	0.28206	0.378957	0.289415	0.286537	0.34426
0.351455	0.10953	0.383275	0.398145	0.351295	0.272945	0.128238	0.416693	0.349536	0.314839
0.362968	0.319316	0.216501	0.315158	0.334986	0.298849	0.323473	0.339783	0.311641	0.352095
0.374001	0.291014	0.336105	0.335146	0.310521	0.2819	0.287976	0.329229	0.3126	0.32747
0.337704	0.380876	0.307963	0.271666	0.287336	0.302846	0.39047	0.247042	0.288455	0.371762
0.329549	0.339783	0.300768	0.252478	0.277103	0.247681	0.341062	0.261912	0.260313	0.321075
0.354173	0.412216	0.271027	0.252798	0.318196	0.305564	0.419891	0.270387	0.311481	0.381036
0.333706	0.391749	0.306364	0.284938	0.32827	0.314998	0.395587	0.266709	0.283019	0.352574
0.358171	0.141829	0.39015	0.384234	0.34378	0.256796	0.150464	0.410617	0.342981	0.307963
0.315638	0.290854	0.270707	0.286697	0.293412	0.257595	0.299808	0.276783	0.2819	0.301567
0.302686	0.418772	0.304125	0.240806	0.240646	0.326191	0.429325	0.125839	0.267829	0.388072
0.341861	0.335785	0.180844	0.273425	0.285417	0.269428	0.346818	0.261113	0.263032	0.337704
0.339463	0.236968	0.344899	0.337384	0.303006	0.21906	0.229293	0.346178	0.284298	0.289255
0.32747	0.2819	0.32763	0.306364	0.288615	0.267349	0.294851	0.289255	0.289415	0.320595
0.351615	0.221938	0.366166	0.365686	0.326991	0.243364	0.213783	0.385513	0.320435	0.295011
0.337544	0.380237	0.247522	0.246722	0.300448	0.291653	0.39127	0.282539	0.304765	0.351295
0.349057	0.290054	0.251199	0.333067	0.325072	0.299488	0.29693	0.324912	0.298849	0.338663
0.357851	0.165494	0.39063	0.37576	0.353694	0.295331	0.170291	0.404221	0.352894	0.335625
0.377518	0.302047	0.301087	0.309562	0.34314	0.308443	0.313879	0.345379	0.329549	0.356892
0.34346	0.208027	0.360409	0.35913	0.320275	0.227055	0.199872	0.377838	0.321234	0.295811
0.341382	0.381036	0.270867	0.275184	0.330668	0.294372	0.387912	0.303166	0.286377	0.370643
0.309242	0.261912	0.376079	0.377998	0.313559	0.28126	0.253118	0.380397	0.336425	0.253918
0.349217	0.336904	0.23393	0.318676	0.31324	0.284618	0.342821	0.31244	0.284298	0.340422
0.315638	0.351935	0.283978	0.28174	0.294052	0.274065	0.364567	0.25024	0.284138	0.336425
0.361689	0.388551	0.074832	0.29725	0.319316	0.300448	0.403422	0.293412	0.294691	0.370643
0.350176	0.384394	0.144867	0.276623	0.317877	0.291973	0.397026	0.309562	0.309882	0.354333
0.326991	0.251359	0.32843	0.32747	0.297889	0.261593	0.255676	0.32859	0.274225	0.3126
0.361848	0.382795	0.10905	0.294691	0.324592	0.301567	0.396546	0.28206	0.304925	0.370483
0.35977	0.371282	0.111129	0.303006	0.31196	0.278862	0.382155	0.300608	0.290694	0.355772
0.320435	0.251359	0.336904	0.372562	0.32795	0.294372	0.243844	0.365846	0.311001	0.301727
0.339783	0.316917	0.289575	0.282379	0.287016	0.256796	0.329709	0.293412	0.247362	0.336425

For Review Only

0.353054	0.386153	0.212184	0.307643	0.34426	0.284618	0.395907	0.324432	0.322034	0.362808
0.310361	0.259194	0.324432	0.316917	0.300608	0.259194	0.254557	0.324113	0.303326	0.290694
0.316437	0.397346	0.223377	0.26639	0.313879	0.314519	0.409658	0.264151	0.305085	0.367605
0.349856	0.285897	0.346978	0.366965	0.339143	0.309722	0.284138	0.377518	0.349696	0.29741
0.334026	0.339463	0.34442	0.366326	0.320275	0.299968	0.347777	0.340742	0.305724	0.232651
0.358331	0.291973	0.34426	0.332587	0.329869	0.291813	0.294052	0.32827	0.297889	0.350975
0.332587	0.392229	0.259194	0.287176	0.305245	0.298369	0.402462	0.251839	0.279181	0.354813
0.335146	0.288455	0.331148	0.334986	0.309402	0.283658	0.29693	0.317877	0.257115	0.32811
0.345859	0.20387	0.35945	0.356892	0.329069	0.289894	0.201791	0.364727	0.329549	0.329549
0.313399	0.305085	0.228174	0.274704	0.285257	0.247522	0.3126	0.269747	0.262872	0.309722
0.355133	0.256156	0.355293	0.334666	0.345379	0.293412	0.256796	0.362488	0.326671	0.34426
0.333866	0.413176	0.31308	0.161657	0.304925	0.303486	0.420691	0.228174	0.29629	0.380876
0.347777	0.356572	0.139271	0.279661	0.302047	0.286217	0.370643	0.282539	0.284458	0.351615
0.339942	0.236968	0.331148	0.336585	0.29757	0.221298	0.248161	0.346498	0.295491	0.298049
0.357851	0.3748	0.23489	0.226575	0.292133	0.286537	0.381036	0.280141	0.285417	0.366645
0.332587	0.332267	0.307323	0.299328	0.304285	0.274065	0.339783	0.295171	0.292613	0.324432
0.352894	0.209946	0.363288	0.355772	0.322674	0.224816	0.205788	0.386792	0.315638	0.292133
0.35865	0.254397	0.362488	0.339143	0.340582	0.329389	0.264151	0.365366	0.324752	0.355293
0.332267	0.301247	0.284298	0.279661	0.291813	0.254557	0.307004	0.300927	0.254717	0.325712
0.363767	0.38999	0.274225	0.292933	0.32795	0.295651	0.398145	0.304285	0.306044	0.367765
0.355612	0.339463	0.183722	0.277103	0.299488	0.230253	0.341861	0.284778	0.275983	0.345699
0.342661	0.379117	0.273905	0.214263	0.285737	0.290054	0.39095	0.262552	0.29725	0.335625
0.311481	0.299009	0.262552	0.271826	0.28174	0.257915	0.308762	0.264311	0.266709	0.302686
0.320915	0.165174	0.357211	0.35977	0.313399	0.229773	0.154781	0.377678	0.322674	0.29661
0.360729	0.258714	0.357851	0.357531	0.341861	0.308443	0.24928	0.368884	0.322674	0.342821
0.37448	0.388871	0.267189	0.29645	0.339942	0.290054	0.401183	0.32843	0.324272	0.362488
0.319955	0.34426	0.355932	0.311321	0.292293	0.293412	0.346338	0.249121	0.29725	0.318996
0.340742	0.239207	0.340902	0.336425	0.29725	0.228814	0.226415	0.347937	0.287976	0.291174
0.344739	0.368244	0.256636	0.263032	0.301727	0.278222	0.376399	0.29709	0.303646	0.332587
0.337064	0.334986	0.304285	0.323953	0.319955	0.2811	0.334666	0.299169	0.271346	0.336904
0.349057	0.345859	0.278862	0.306844	0.323633	0.295811	0.354333	0.287656	0.284618	0.334986
0.321714	0.388072	0.301887	0.255197	0.267669	0.301567	0.395747	0.186441	0.277902	0.364247
0.339623	0.357531	0.269268	0.301407	0.287016	0.28206	0.364087	0.304925	0.261593	0.348897
0.338983	0.414135	0.274864	0.130157	0.316757	0.320595	0.419731	0.267349	0.29693	0.399264
0.351135	0.37448	0.3126	0.270227	0.29629	0.293092	0.388551	0.266869	0.263032	0.360249
0.348737	0.213144	0.36009	0.353854	0.324912	0.230093	0.210425	0.382315	0.318516	0.300448
0.330988	0.380077	0.311481	0.278382	0.29629	0.293252	0.39111	0.259034	0.259194	0.362968
0.373361	0.388391	0.26639	0.29613	0.340742	0.289095	0.400384	0.32795	0.322674	0.361529
0.373361	0.356572	0.249121	0.300768	0.320595	0.29677	0.366645	0.302846	0.304285	0.355932
0.323793	0.369364	0.314199	0.270547	0.285417	0.298369	0.382315	0.237928	0.236169	0.345539
0.350815	0.383595	0.15574	0.277902	0.317557	0.293252	0.396706	0.314679	0.31244	0.353054
0.334506	0.37544	0.271826	0.28158	0.310201	0.276783	0.385513	0.285257	0.302207	0.351455
0.339143	0.398945	0.236329	0.282539	0.32827	0.316437	0.419572	0.29725	0.29693	0.373521
0.364247	0.387592	0.064918	0.29693	0.315158	0.3118	0.402782	0.29741	0.295011	0.370803
0.349057	0.121362	0.383914	0.394468	0.353854	0.279981	0.127598	0.419412	0.339463	0.320115
0.341062	0.3433	0.26495	0.283818	0.301727	0.292133	0.35865	0.290214	0.290854	0.355932
0.341541	0.315958	0.294851	0.318516	0.31244	0.283818	0.32779	0.329549	0.302846	0.341861
0.340742	0.340582	0.344739	0.302526	0.336265	0.29693	0.355133	0.303965	0.284778	0.362648
0.358011	0.252958	0.361848	0.338503	0.340582	0.329229	0.264151	0.363927	0.323953	0.354973
0.320915	0.158139	0.376239	0.366485	0.324912	0.291014	0.152702	0.371442	0.326671	0.324272
0.325712	0.215542	0.344739	0.330189	0.295491	0.206108	0.213463	0.361689	0.303646	0.279821
0.337064	0.335465	0.333547	0.287976	0.284778	0.302047	0.344739	0.288455	0.31228	0.31228
0.351295	0.393188	0.268628	0.280141	0.321075	0.302686	0.403102	0.277263	0.304605	0.371602
0.322833	0.254077	0.382475	0.361689	0.321874	0.246083	0.251519	0.372562	0.295171	0.300448
0.354813	0.224017	0.367925	0.35945	0.34346	0.290534	0.221778	0.376239	0.327151	0.333706

For Review Only

0.355932	0.387272	0.248641	0.272626	0.326991	0.289894	0.400064	0.315158	0.318516	0.35929
0.35929	0.135593	0.38999	0.394308	0.347618	0.285257	0.145987	0.418452	0.352894	0.326831
0.353374	0.305085	0.313879	0.292933	0.287176	0.269108	0.315958	0.311001	0.259354	0.347458
0.330668	0.325232	0.332107	0.346818	0.324912	0.304605	0.323313	0.336105	0.292933	0.302047
0.337544	0.414295	0.261912	0.23489	0.316118	0.323633	0.426607	0.272466	0.300768	0.389191
0.358171	0.321075	0.353214	0.332587	0.339463	0.322354	0.313399	0.329389	0.342821	0.339303
0.316437	0.320115	0.338663	0.329069	0.280141	0.280301	0.318196	0.323633	0.279501	0.246722
0.362488	0.399105	0.158459	0.286537	0.310201	0.301407	0.413495	0.271027	0.279661	0.357531
0.370803	0.251839	0.315638	0.371602	0.348417	0.303326	0.26495	0.370323	0.310201	0.346658
0.323153	0.259354	0.389351	0.402942	0.32891	0.290534	0.26543	0.420531	0.364727	0.217621
0.325712	0.252638	0.300288	0.337864	0.303166	0.241286	0.253598	0.345059	0.302526	0.298369
0.360729	0.096418	0.399264	0.401183	0.356252	0.273585	0.116565	0.42133	0.351135	0.320275
0.307483	0.295171	0.258395	0.268308	0.283339	0.2496	0.305884	0.264311	0.26591	0.303166
0.350815	0.329389	0.311001	0.331948	0.304445	0.294212	0.325232	0.350016	0.275024	0.341541
0.324113	0.346818	0.301407	0.298689	0.315638	0.316917	0.362008	0.311481	0.308283	0.329549
0.326671	0.336585	0.196834	0.256156	0.283339	0.256476	0.346178	0.275504	0.284458	0.350656
0.331948	0.39111	0.245443	0.285897	0.331628	0.320275	0.409818	0.308443	0.305724	0.369683
0.342501	0.325072	0.335146	0.283818	0.305884	0.313879	0.338024	0.307163	0.294212	0.349536
0.32891	0.194755	0.340742	0.369843	0.32891	0.273905	0.208666	0.381036	0.305085	0.318516
0.322034	0.257115	0.354653	0.331788	0.335785	0.289255	0.266869	0.322514	0.321075	0.336904
0.341382	0.217141	0.351935	0.360889	0.32779	0.289255	0.204669	0.354493	0.330668	0.334506
0.352095	0.334666	0.329069	0.323793	0.289575	0.303646	0.346338	0.319316	0.301247	0.322194
0.348577	0.295651	0.31196	0.352574	0.337864	0.289575	0.29645	0.370483	0.347458	0.354973
0.357531	0.382315	0.052446	0.307803	0.322034	0.300448	0.391909	0.299808	0.292933	0.35977
0.329549	0.385833	0.314039	0.21906	0.258874	0.310361	0.399584	0.209306	0.280461	0.361689
0.357851	0.274225	0.329709	0.326351	0.324592	0.290694	0.292773	0.313399	0.305564	0.341701
0.300128	0.194116	0.386792	0.384714	0.332587	0.245603	0.185641	0.398625	0.332907	0.271346
0.35993	0.357531	0.314679	0.302367	0.309882	0.306684	0.372881	0.269587	0.295491	0.333067
0.348417	0.101535	0.401503	0.400704	0.353534	0.277582	0.074033	0.430445	0.35961	0.32811
0.355612	0.394627	0.231532	0.252638	0.317717	0.298529	0.407739	0.285737	0.324592	0.35977
0.322993	0.362328	0.326351	0.277103	0.244324	0.289255	0.367445	0.153502	0.259194	0.310841
0.348417	0.398945	0.285257	0.233131	0.307483	0.303166	0.404381	0.266709	0.305884	0.380556
0.337544	0.147586	0.37576	0.395427	0.34426	0.279501	0.146946	0.398305	0.336745	0.330029
0.34346	0.324752	0.204829	0.314519	0.303326	0.286537	0.331148	0.323313	0.291493	0.331148
0.338983	0.29725	0.333547	0.318996	0.31196	0.26527	0.303326	0.34426	0.302526	0.295491
0.356892	0.356732	0.157819	0.305085	0.304605	0.272785	0.362648	0.316437	0.291653	0.351135
0.360569	0.255676	0.351775	0.341382	0.345059	0.310681	0.262392	0.366805	0.329549	0.339783
0.35993	0.105053	0.397186	0.401023	0.357211	0.28078	0.110169	0.423729	0.346658	0.320915
0.32795	0.399105	0.283339	0.248161	0.270067	0.299648	0.407899	0.217141	0.246242	0.365686
0.37464	0.39063	0.267349	0.29645	0.340742	0.290854	0.402143	0.32827	0.324912	0.363288
0.34442	0.302846	0.274065	0.285417	0.292133	0.199712	0.308443	0.324592	0.289095	0.29661
0.352574	0.133994	0.393188	0.402942	0.348257	0.276943	0.135114	0.420851	0.348737	0.320915
0.347618	0.403742	0.29725	0.25072	0.311161	0.309082	0.414934	0.272945	0.292133	0.369044
0.318676	0.321554	0.287016	0.283499	0.287816	0.24976	0.330508	0.273425	0.258235	0.315318
0.370963	0.279181	0.335305	0.336105	0.322993	0.286217	0.282379	0.341222	0.313879	0.335146
0.301727	0.418452	0.317237	0.224976	0.205948	0.320755	0.432683	0.087144	0.293092	0.385833
0.337224	0.34426	0.267349	0.31228	0.313879	0.290534	0.365686	0.305564	0.286377	0.339623
0.336585	0.346178	0.321394	0.301567	0.324752	0.302686	0.360889	0.299169	0.314519	0.337864
0.372082	0.289255	0.237288	0.331468	0.332427	0.293412	0.306844	0.356092	0.322674	0.349856
0.323153	0.361049	0.286697	0.317877	0.335305	0.29661	0.364247	0.314359	0.295011	0.320435
0.321554	0.346978	0.287496	0.317077	0.308283	0.309242	0.356572	0.333227	0.330508	0.264631
0.349057	0.101695	0.400224	0.400704	0.353214	0.272306	0.071954	0.429005	0.357052	0.32779
0.348097	0.420851	0.307643	0.231052	0.2819	0.315958	0.430764	0.223697	0.282539	0.375919
0.348737	0.131436	0.393348	0.399264	0.345539	0.283658	0.134474	0.418132	0.350975	0.323633
0.340102	0.399424	0.235849	0.283179	0.32747	0.317397	0.420051	0.29613	0.298209	0.37464

For Review Only

0.356412	0.414615	0.24992	0.283339	0.329869	0.316917	0.428846	0.283818	0.321874	0.367765
0.364407	0.239847	0.356412	0.357851	0.323793	0.301247	0.246242	0.376399	0.323633	0.32811
0.353374	0.411417	0.309242	0.208507	0.284618	0.324912	0.417973	0.217621	0.279341	0.383115
0.362488	0.393188	0.253598	0.28142	0.325072	0.303006	0.399584	0.294372	0.318356	0.373041

For Review Only

0.325392	0.319476	0.324592	0.307323	0.254077	0.309082	0.291973	0.298529	0.270547	0.284138
0.322993	0.339623	0.330189	0.350016	0.35945	0.315958	0.324432	0.252638	0.339463	0.247841
0.351775	0.366166	0.300448	0.355772	0.285897	0.216182	0.303166	0.205948	0.294691	0.258874
0.321075	0.34394	0.315798	0.341062	0.304765	0.277263	0.29725	0.245283	0.303166	0.247681
0.313879	0.274065	0.334666	0.314839	0.247681	0.331308	0.349217	0.354493	0.305085	0.3433
0.333706	0.334346	0.317077	0.322833	0.278542	0.28126	0.308762	0.252798	0.285257	0.255197
0.310042	0.261433	0.322514	0.324432	0.194755	0.31308	0.309242	0.322194	0.257275	0.293252
0.212504	0.293732	0.3118	0.235689	0.348577	0.35881	0.295651	0.321075	0.335625	0.338503
0.311481	0.297889	0.334186	0.334026	0.190438	0.295491	0.311641	0.329069	0.267669	0.285257
0.236169	0.29725	0.317077	0.240646	0.391429	0.393828	0.314998	0.37544	0.364407	0.372082
0.322674	0.3441	0.299808	0.339143	0.332907	0.350975	0.379597	0.345219	0.295171	0.340902
0.342821	0.357371	0.29709	0.348897	0.288775	0.225296	0.290374	0.191238	0.299968	0.247362
0.305884	0.255676	0.304445	0.317077	0.260313	0.320915	0.304125	0.322034	0.282539	0.317717
0.287336	0.341382	0.29741	0.28126	0.320275	0.350016	0.306044	0.31292	0.32763	0.321554
0.320915	0.31244	0.321234	0.327151	0.211705	0.274864	0.263831	0.269428	0.276783	0.26511
0.32843	0.356092	0.329549	0.337224	0.314039	0.290854	0.335305	0.287336	0.315638	0.304605
0.326031	0.272785	0.331308	0.356732	0.181804	0.319156	0.321234	0.340422	0.31212	0.31196
0.280141	0.322194	0.32779	0.304925	0.299808	0.338823	0.326671	0.303006	0.316278	0.297729
0.357052	0.35977	0.315958	0.366965	0.278062	0.252478	0.291334	0.242245	0.300927	0.24928
0.275344	0.31228	0.346498	0.292933	0.361369	0.382795	0.338983	0.358171	0.371762	0.350336
0.28174	0.222098	0.310201	0.3126	0.264151	0.353694	0.32747	0.344579	0.314998	0.330508
0.32827	0.237448	0.324752	0.35865	0.17173	0.317877	0.332907	0.332107	0.285737	0.306364
0.313879	0.337544	0.313559	0.340422	0.393188	0.409818	0.358331	0.393508	0.361209	0.371602
0.254557	0.316757	0.308602	0.277263	0.349856	0.353534	0.32811	0.327151	0.307004	0.34346
0.330189	0.284778	0.319476	0.336105	0.217781	0.26591	0.299009	0.277422	0.277742	0.263991
0.354813	0.37448	0.301247	0.368084	0.295971	0.293412	0.304925	0.246242	0.309402	0.259834
0.322354	0.348417	0.295171	0.35945	0.287976	0.243684	0.292293	0.215702	0.292773	0.23473
0.287176	0.305085	0.284618	0.298209	0.271346	0.273265	0.279501	0.257275	0.280301	0.259674
0.270707	0.29693	0.337064	0.326351	0.26575	0.330508	0.329229	0.326351	0.309562	0.302686
0.257275	0.269747	0.340742	0.257275	0.430125	0.439239	0.361049	0.410457	0.387752	0.415414
0.340742	0.351615	0.308922	0.363288	0.269428	0.23441	0.307803	0.236169	0.287816	0.254557
0.340902	0.37528	0.316118	0.361529	0.286697	0.26495	0.326351	0.279821	0.302526	0.248001
0.358171	0.305405	0.322514	0.354013	0.18724	0.301727	0.331308	0.31228	0.26495	0.290214
0.277742	0.304605	0.306044	0.298369	0.348737	0.363128	0.350176	0.340902	0.313559	0.334506
0.321234	0.355133	0.318036	0.336904	0.340582	0.345699	0.331788	0.29693	0.334186	0.315158
0.291493	0.34314	0.302207	0.298049	0.324432	0.253598	0.316597	0.24976	0.278862	0.28062
0.300927	0.331788	0.301887	0.308283	0.301727	0.322034	0.308602	0.323633	0.303646	0.29677
0.305405	0.325232	0.315798	0.306844	0.308283	0.331308	0.318676	0.31212	0.308762	0.292453
0.293092	0.32747	0.316118	0.318356	0.40646	0.416374	0.36009	0.384874	0.356892	0.391909
0.330029	0.37496	0.347777	0.337544	0.362488	0.365046	0.342981	0.35993	0.366166	0.333227
0.247202	0.26559	0.335305	0.247681	0.424528	0.42229	0.365526	0.397985	0.383595	0.409978
0.247042	0.314039	0.317237	0.285098	0.353214	0.371122	0.3433	0.346178	0.348577	0.342981
0.2504	0.314519	0.311161	0.205469	0.370643	0.382475	0.304925	0.350656	0.355453	0.363288
0.311161	0.268468	0.333706	0.315798	0.248001	0.347777	0.333866	0.345539	0.309242	0.335465
0.243524	0.323633	0.310201	0.162936	0.386633	0.400064	0.31292	0.35977	0.360889	0.378798
0.339783	0.353374	0.286057	0.351775	0.283339	0.235209	0.29725	0.205788	0.287976	0.254077
0.349696	0.364087	0.334826	0.362808	0.297889	0.277103	0.315478	0.167093	0.306844	0.232491
0.305245	0.321234	0.31244	0.3126	0.316917	0.332107	0.326991	0.300768	0.300768	0.305564
0.324113	0.237128	0.338024	0.309722	0.24944	0.356092	0.334986	0.348897	0.307163	0.330988
0.32811	0.335465	0.317557	0.341222	0.235369	0.264791	0.272146	0.287816	0.277582	0.253278

For Review Only

0.346178	0.366805	0.307643	0.354973	0.288455	0.275024	0.237448	0.228334	0.307004	0.246402
0.332267	0.345219	0.333387	0.366965	0.301727	0.305884	0.303806	0.269268	0.323793	0.288455
0.350016	0.260473	0.338184	0.362168	0.131276	0.276143	0.294532	0.317717	0.268308	0.279821
0.357531	0.304765	0.322514	0.353054	0.1882	0.301887	0.330668	0.31244	0.264471	0.289735
0.333866	0.347298	0.325392	0.349057	0.291174	0.26623	0.293892	0.228654	0.306684	0.246722
0.337864	0.214263	0.307963	0.321075	0.173969	0.322674	0.319795	0.332747	0.292933	0.306364
0.356572	0.360249	0.315638	0.366485	0.277902	0.253918	0.292293	0.241925	0.300288	0.24944
0.349536	0.370003	0.338823	0.378638	0.305884	0.259354	0.319795	0.162136	0.317557	0.222578
0.330988	0.369843	0.307643	0.356412	0.290534	0.2819	0.31276	0.225935	0.31292	0.244004
0.258874	0.288136	0.330668	0.255516	0.40662	0.407419	0.336585	0.382315	0.348577	0.389031
0.279021	0.310681	0.286537	0.302367	0.308602	0.329709	0.318196	0.330189	0.276783	0.320595
0.294212	0.303806	0.28142	0.304605	0.26559	0.266869	0.270867	0.246722	0.272945	0.255676
0.337384	0.365206	0.3126	0.350815	0.307963	0.235529	0.315158	0.008475	0.299169	0.216182
0.349057	0.378158	0.331628	0.364247	0.325871	0.248801	0.321234	0.082347	0.3126	0.244164
0.349856	0.259674	0.337544	0.363288	0.129997	0.275983	0.294212	0.316757	0.267669	0.278382
0.270387	0.29645	0.336904	0.325712	0.26591	0.330029	0.32843	0.325552	0.308762	0.301567
0.306684	0.322514	0.305085	0.316118	0.282699	0.285577	0.289894	0.263511	0.28062	0.259034
0.284138	0.318996	0.308922	0.289735	0.323953	0.356572	0.326031	0.332107	0.309882	0.323313
0.336265	0.35913	0.270067	0.329069	0.314199	0.31244	0.295331	0.289735	0.322833	0.309402
0.357211	0.364886	0.333706	0.367445	0.259034	0.286217	0.298209	0.272785	0.276303	0.253278
0.337064	0.305245	0.333067	0.366006	0.193796	0.251999	0.317077	0.277742	0.284938	0.269907
0.336745	0.289894	0.332587	0.342821	0.167253	0.290534	0.308283	0.311161	0.269108	0.275983
0.322354	0.352095	0.311161	0.344739	0.306204	0.239527	0.305085	0.110489	0.292773	0.237608
0.324912	0.349856	0.307803	0.332427	0.371122	0.362168	0.367125	0.350496	0.347937	0.337704
0.294372	0.274704	0.331948	0.332747	0.241605	0.309562	0.319316	0.318036	0.305245	0.287176
0.325232	0.370963	0.324272	0.339942	0.315478	0.291973	0.32827	0.277902	0.335465	0.279501
0.243684	0.323633	0.310361	0.162936	0.386313	0.399424	0.31228	0.35897	0.360409	0.378158
0.310841	0.352574	0.290054	0.348097	0.302686	0.256476	0.319955	0.186121	0.292613	0.248801
0.319795	0.282379	0.321394	0.295811	0.303486	0.369204	0.317557	0.355612	0.319316	0.360249
0.291334	0.324752	0.325712	0.298049	0.415574	0.417653	0.363128	0.388232	0.368244	0.393508
0.353054	0.378638	0.324752	0.364886	0.304925	0.310201	0.306524	0.249121	0.317877	0.268948
0.333547	0.337704	0.303646	0.376879	0.242085	0.166134	0.318036	0.272626	0.262232	0.278222
0.191238	0.299009	0.315798	0.315318	0.240806	0.318836	0.337384	0.32779	0.298369	0.316597
0.270067	0.309082	0.299009	0.305245	0.321874	0.309402	0.31244	0.290054	0.298849	0.295491
0.298209	0.330189	0.338184	0.308123	0.426287	0.424528	0.368404	0.400224	0.377199	0.40582
0.350016	0.259514	0.336585	0.363128	0.129837	0.276463	0.294691	0.316437	0.267029	0.278542
0.313879	0.331148	0.317077	0.316437	0.267029	0.324912	0.322354	0.320115	0.275823	0.28126
0.244484	0.324113	0.310201	0.163256	0.386313	0.398945	0.313399	0.35993	0.360569	0.378638
0	0.297729	0.320115	0.255357	0.349057	0.356892	0.315318	0.337704	0.329229	0.342341
0.297729	0	0.334026	0.293412	0.289894	0.372721	0.335785	0.363927	0.318516	0.346978
0.320115	0.334026	0	0.306364	0.319476	0.317557	0.315158	0.314039	0.291653	0.336585
0.255357	0.293412	0.306364	0	0.366326	0.388551	0.302846	0.350336	0.342181	0.369683
0.349057	0.289894	0.319476	0.366326	0	0.271506	0.307643	0.308123	0.25056	0.26559
0.356892	0.372721	0.317557	0.388551	0.271506	0	0.319635	0.236009	0.294532	0.26527
0.315318	0.335785	0.315158	0.302846	0.307643	0.319635	0	0.315958	0.331788	0.308283
0.337704	0.363927	0.314039	0.350336	0.308123	0.236009	0.315958	0	0.300927	0.216501
0.329229	0.318516	0.291653	0.342181	0.25056	0.294532	0.331788	0.300927	0	0.299488
0.342341	0.346978	0.336585	0.369683	0.26559	0.26527	0.308283	0.216501	0.299488	0
0.2811	0.319476	0.309722	0.291973	0.378318	0.379917	0.339942	0.356732	0.338503	0.35913
0.309882	0.35961	0.308283	0.315798	0.306044	0.302846	0.303646	0.255996	0.298049	0.268628
0.326511	0.355453	0.316437	0.32747	0.282379	0.295811	0.307004	0.258395	0.298529	0.258874
0.347298	0.351455	0.32747	0.317237	0.323153	0.313879	0.341701	0.300128	0.322514	0.305085
0.321874	0.356252	0.306844	0.342181	0.300927	0.230892	0.295811	0.170611	0.287816	0.227854
0.290374	0.334666	0.330668	0.314839	0.412376	0.423249	0.3748	0.389031	0.367125	0.401023
0.286856	0.298049	0.331948	0.286856	0.388551	0.392229	0.331468	0.361848	0.330189	0.365366

For Review Only

0.288455	0.313879	0.299808	0.318836	0.317397	0.318836	0.311641	0.304605	0.316597	0.298369
0.333387	0.348897	0.326351	0.31292	0.309402	0.320275	0.309722	0.290854	0.292453	0.295491
0.335785	0.366006	0.329229	0.348737	0.320435	0.31292	0.319156	0.284458	0.331468	0.264471
0.302526	0.342341	0.322674	0.309562	0.344899	0.327151	0.336585	0.308443	0.345219	0.310042
0.314039	0.335625	0.292293	0.332907	0.280301	0.258714	0.302846	0.244324	0.269268	0.26511
0.29677	0.313719	0.332747	0.286377	0.35961	0.388232	0.34394	0.354493	0.342821	0.346658
0.338663	0.369044	0.307963	0.365526	0.29693	0.252319	0.300608	0.176047	0.305085	0.191398
0.320435	0.342021	0.288775	0.338983	0.277422	0.272146	0.302526	0.248321	0.287976	0.258874
0.331468	0.342021	0.307643	0.346978	0.255357	0.237288	0.292133	0.233131	0.293572	0.252638
0.302367	0.334026	0.292133	0.298529	0.300448	0.273905	0.315478	0.256316	0.285257	0.274544
0.309722	0.339143	0.337064	0.301407	0.342501	0.345219	0.302686	0.302367	0.309722	0.318516
0.29661	0.316757	0.298849	0.308602	0.334026	0.318356	0.330988	0.292133	0.294851	0.306364
0.315318	0.310681	0.338503	0.321714	0.285737	0.347777	0.303326	0.325232	0.285098	0.295491
0.302686	0.31212	0.292773	0.305085	0.275344	0.299648	0.284298	0.261113	0.288455	0.261433
0.295491	0.314839	0.313719	0.291653	0.32891	0.326511	0.145507	0.308443	0.334506	0.305245
0.329869	0.348417	0.295971	0.3441	0.286217	0.257435	0.299328	0.198433	0.287976	0.240326
0.315158	0.320435	0.309402	0.317077	0.319476	0.29757	0.310201	0.267349	0.318356	0.279341
0.306844	0.333706	0.295331	0.321554	0.286697	0.304925	0.285098	0.253598	0.293572	0.256316
0.348417	0.352414	0.368884	0.353054	0.305245	0.32763	0.294851	0.31308	0.321394	0.272466
0.307004	0.317717	0.282219	0.31228	0.263831	0.270227	0.278542	0.21874	0.28078	0.247681
0.285897	0.316278	0.329069	0.283179	0.370963	0.389511	0.341861	0.352894	0.348097	0.35881
0.334666	0.318196	0.31212	0.324113	0.264791	0.304605	0.268468	0.313719	0.325232	0.293412
0.298689	0.305245	0.285257	0.298689	0.276303	0.279181	0.282219	0.248801	0.286377	0.255836
0.326031	0.323313	0.301407	0.367125	0.223857	0.21826	0.31244	0.271506	0.267509	0.261113
0.327151	0.347458	0.31292	0.339783	0.286537	0.209786	0.288615	0.220339	0.284618	0.240006
0.289575	0.326831	0.330189	0.314199	0.413655	0.42117	0.369843	0.396226	0.369843	0.396067
0.336265	0.353694	0.307323	0.336265	0.325072	0.317717	0.34426	0.285897	0.326031	0.285417
0.323793	0.349217	0.287816	0.317557	0.322194	0.338823	0.34362	0.337704	0.32747	0.308762
0.331308	0.330508	0.331788	0.350975	0.239847	0.291653	0.307163	0.285897	0.289735	0.258075
0.29757	0.331788	0.292453	0.322514	0.261912	0.284298	0.2811	0.293412	0.275024	0.272306
0.341062	0.352894	0.31292	0.371922	0.276143	0.25008	0.286377	0.216501	0.298369	0.236489
0.354493	0.336265	0.353374	0.350336	0.288136	0.302367	0.303806	0.29645	0.315318	0.272626
0.303006	0.321714	0.322034	0.300608	0.412696	0.4055	0.353534	0.387912	0.368084	0.39063
0.29693	0.301407	0.292933	0.295011	0.283339	0.29661	0.285577	0.262872	0.293252	0.267349
0.351615	0.290214	0.318196	0.367605	0.01583	0.273905	0.307483	0.309562	0.248321	0.26623
0.31196	0.337864	0.305564	0.325392	0.264471	0.286377	0.291014	0.257275	0.287176	0.251039
0.278382	0.303806	0.306844	0.299328	0.348577	0.362488	0.350016	0.339623	0.31324	0.333866
0.304125	0.302207	0.326191	0.325712	0.285897	0.337704	0.319955	0.324912	0.304125	0.320435
0.284778	0.313719	0.325232	0.288455	0.384714	0.393508	0.340422	0.363607	0.342021	0.360569
0.317557	0.358011	0.321554	0.351775	0.278862	0.25072	0.29645	0.193476	0.287816	0.232971
0.324432	0.347618	0.316917	0.324752	0.326351	0.344739	0.326031	0.319795	0.316437	0.318836
0.319635	0.333706	0.32891	0.329709	0.397026	0.399424	0.373201	0.39015	0.35881	0.386952
0.346178	0.377039	0.302846	0.354173	0.332907	0.331148	0.331628	0.332427	0.332907	0.319795
0.283179	0.31212	0.319955	0.283818	0.379597	0.382315	0.336904	0.362168	0.340582	0.362808
0.352574	0.369524	0.31276	0.351455	0.295171	0.287336	0.294052	0.255836	0.259674	0.28094
0.254237	0.283978	0.333866	0.253278	0.392709	0.394947	0.346818	0.378158	0.355293	0.3756
0.304765	0.344899	0.314998	0.322674	0.299488	0.317877	0.282859	0.29645	0.292613	0.295651
0.317557	0.313879	0.300608	0.317717	0.248321	0.283818	0.290534	0.272306	0.257275	0.259994
0.354973	0.368564	0.314998	0.357691	0.284618	0.290694	0.301567	0.233611	0.298849	0.244803
0.339623	0.372082	0.31276	0.356092	0.307323	0.272945	0.31308	0.204989	0.326831	0.238887
0.282219	0.295491	0.285897	0.298049	0.339303	0.345379	0.319795	0.333547	0.302846	0.324113
0.355772	0.370163	0.307004	0.364087	0.276303	0.2819	0.301887	0.228973	0.298849	0.263511
0.336265	0.371442	0.314199	0.347458	0.301567	0.295971	0.31196	0.253278	0.298209	0.260953
0.314519	0.31212	0.321234	0.295171	0.360889	0.393828	0.361689	0.371762	0.349376	0.344899
0.298689	0.350496	0.290054	0.317077	0.285417	0.293572	0.322674	0.279821	0.279181	0.275823

FOR REVIEW ONLY

0.355932	0.373361	0.335945	0.346338	0.321554	0.267988	0.317077	0.158778	0.302207	0.258395
0.28142	0.297729	0.298049	0.285257	0.32811	0.341541	0.307643	0.319476	0.305724	0.316757
0.357052	0.349856	0.308123	0.347138	0.26543	0.287176	0.292773	0.247042	0.283179	0.251839
0.309882	0.34346	0.289894	0.28078	0.377678	0.385673	0.252958	0.363927	0.35865	0.358171
0.317877	0.324592	0.34394	0.278222	0.346338	0.377518	0.331468	0.345219	0.333706	0.337704
0.323633	0.329709	0.320115	0.3433	0.333067	0.339143	0.351295	0.344739	0.320115	0.322993
0.346658	0.347777	0.317717	0.346658	0.268468	0.284458	0.321554	0.212984	0.292933	0.189958
0.322514	0.344899	0.323953	0.332907	0.313719	0.348737	0.320915	0.337064	0.349536	0.306844
0.314839	0.329709	0.329389	0.320755	0.361529	0.379277	0.340582	0.360409	0.334826	0.361049
0.289575	0.305724	0.28126	0.305724	0.274384	0.286537	0.282859	0.244324	0.278862	0.259354
0.316437	0.351935	0.307643	0.340102	0.355133	0.345859	0.342021	0.355612	0.338823	0.354333
0.356732	0.338184	0.301567	0.378957	0.229453	0.208826	0.319635	0.271506	0.244963	0.261593
0.335785	0.351775	0.29677	0.341222	0.275184	0.282379	0.301727	0.229453	0.288295	0.247681
0.274065	0.324912	0.307004	0.293572	0.338823	0.34378	0.326991	0.320915	0.309402	0.329389
0.332107	0.339942	0.290214	0.346019	0.279341	0.23505	0.282699	0.227534	0.269587	0.245923
0.306524	0.336265	0.298689	0.316757	0.288935	0.309242	0.307963	0.300608	0.288295	0.292293
0.284618	0.318196	0.314998	0.287016	0.383754	0.369683	0.338983	0.354013	0.347618	0.355133
0.334666	0.356572	0.311161	0.342501	0.35993	0.364567	0.368564	0.356892	0.323313	0.357851
0.294691	0.325392	0.288775	0.306204	0.292613	0.29613	0.307323	0.28126	0.2819	0.272785
0.335625	0.372562	0.32779	0.365206	0.302526	0.255357	0.309882	0.200831	0.313399	0.195075
0.321554	0.349856	0.305564	0.339623	0.282219	0.271506	0.280141	0.251519	0.283339	0.254397
0.325392	0.337704	0.299009	0.339143	0.253118	0.236649	0.284778	0.23393	0.291174	0.248321
0.288455	0.300608	0.283019	0.295651	0.26607	0.279821	0.273425	0.246722	0.270227	0.242245
0.263192	0.321075	0.318516	0.303326	0.37528	0.378318	0.333706	0.362808	0.339783	0.368884
0.32875	0.351775	0.305884	0.330988	0.369524	0.367285	0.370803	0.364087	0.342021	0.344899
0.348577	0.378318	0.333067	0.364247	0.325232	0.248641	0.321394	0.084426	0.31244	0.244643
0.272306	0.291174	0.325871	0.307803	0.257915	0.332267	0.324752	0.333547	0.29709	0.320755
0.287816	0.304285	0.313879	0.295011	0.349696	0.360889	0.347298	0.348417	0.315478	0.334986
0.320435	0.340902	0.294691	0.326831	0.292293	0.264631	0.290054	0.213783	0.29757	0.247841
0.309722	0.333227	0.336585	0.318356	0.307163	0.335146	0.306524	0.310201	0.298049	0.284458
0.319795	0.348897	0.325232	0.349536	0.292773	0.301887	0.267988	0.288935	0.29757	0.263511
0.331148	0.304765	0.322034	0.342661	0.175887	0.269907	0.283499	0.286697	0.292133	0.26495
0.302686	0.334506	0.314998	0.325712	0.291014	0.294691	0.31228	0.274544	0.288935	0.266869
0.347298	0.355133	0.318996	0.384394	0.256156	0.222098	0.315478	0.275024	0.273745	0.259674
0.322354	0.32891	0.322993	0.354333	0.257915	0.294052	0.310681	0.287336	0.29613	0.254557
0.287816	0.318356	0.316757	0.291174	0.380716	0.370963	0.338024	0.352734	0.347458	0.352894
0.330349	0.311161	0.332107	0.34346	0.248481	0.301247	0.322194	0.299488	0.277582	0.254877
0.348417	0.377678	0.330668	0.363288	0.325072	0.248161	0.320275	0.082987	0.31228	0.244004
0.339143	0.362968	0.324113	0.351455	0.307803	0.255197	0.32747	0.269747	0.316278	0.275504
0.317397	0.326511	0.291973	0.321075	0.26527	0.287496	0.280461	0.308123	0.276463	0.287016
0.338663	0.373841	0.310841	0.358171	0.308123	0.263671	0.305405	0.205469	0.326351	0.241605
0.311321	0.3441	0.290694	0.338343	0.293572	0.255357	0.260473	0.213623	0.302207	0.263192
0.350016	0.365686	0.301087	0.355293	0.285417	0.216821	0.301567	0.205628	0.294532	0.258874
0.350336	0.368884	0.317877	0.358011	0.284138	0.300128	0.31276	0.240326	0.303166	0.248961
0.293892	0.322674	0.323953	0.305245	0.417653	0.422929	0.365846	0.388551	0.371282	0.394148
0.324752	0.349696	0.324752	0.353214	0.275983	0.294212	0.323153	0.287816	0.298049	0.260473
0.320115	0.341861	0.311641	0.333387	0.317237	0.31276	0.331948	0.295811	0.306844	0.286217
0.339143	0.310521	0.335305	0.357211	0.310361	0.292453	0.294212	0.280141	0.321234	0.290534
0.333387	0.357052	0.309082	0.342341	0.358171	0.362488	0.368724	0.355932	0.321714	0.356892
0.304285	0.284298	0.341382	0.308602	0.366485	0.396067	0.347458	0.387112	0.334186	0.376879
0.255836	0.300768	0.304445	0.278702	0.353214	0.362808	0.318036	0.339623	0.308123	0.346658
0.285577	0.291653	0.280141	0.29757	0.271186	0.318036	0.282859	0.287816	0.324113	0.276143
0.342661	0.362968	0.308283	0.36009	0.286057	0.287816	0.29693	0.263511	0.307643	0.254877
0.2819	0.31196	0.340582	0.299968	0.380397	0.378957	0.34314	0.368084	0.334986	0.353694
0.319156	0.337224	0.297889	0.327311	0.381516	0.380237	0.35993	0.376239	0.342501	0.364567

For Review Only

0.336265	0.364407	0.311641	0.350336	0.307963	0.235209	0.315158	0.008954	0.299648	0.215862
0.293252	0.336585	0.323793	0.322993	0.413975	0.419092	0.368244	0.393028	0.368084	0.401663
0.294212	0.340742	0.303965	0.319635	0.304125	0.31212	0.332747	0.298049	0.300768	0.303806
0.299328	0.354493	0.342021	0.329869	0.34426	0.351135	0.331628	0.340582	0.342821	0.285577
0.355453	0.354653	0.303806	0.366805	0.268948	0.248641	0.302207	0.258395	0.288295	0.262392
0.34378	0.347777	0.315318	0.326511	0.334986	0.34394	0.306204	0.356732	0.355453	0.330508
0.240006	0.315478	0.333866	0.272785	0.321554	0.361049	0.310201	0.333387	0.34394	0.290854
0.34442	0.34362	0.322034	0.340262	0.271666	0.288935	0.279821	0.278062	0.299968	0.258395
0.329389	0.347138	0.310521	0.326671	0.362328	0.385513	0.347777	0.361848	0.376239	0.339143
0.232651	0.29741	0.332107	0.251519	0.431244	0.425008	0.335146	0.393828	0.384234	0.391749
0.284138	0.317877	0.305884	0.286057	0.339143	0.344899	0.323313	0.320435	0.306524	0.331628
0.29757	0.32763	0.333866	0.3118	0.417653	0.427247	0.367605	0.398145	0.370483	0.400384
0.291493	0.299648	0.284458	0.29645	0.26607	0.278382	0.276303	0.252798	0.272146	0.258714
0.302047	0.34378	0.310042	0.319795	0.336585	0.338024	0.32827	0.315958	0.336265	0.298849
0.338983	0.338343	0.082187	0.318996	0.299009	0.320275	0.31196	0.31212	0.293572	0.315958
0.322034	0.330828	0.282699	0.326351	0.272466	0.278542	0.29677	0.236808	0.279981	0.254237
0.34442	0.364567	0.309722	0.349856	0.284938	0.229773	0.29725	0.205628	0.300128	0.261593
0.335945	0.353534	0.257915	0.3433	0.294212	0.319795	0.334186	0.32875	0.269747	0.318996
0.294212	0.319476	0.302047	0.318836	0.369204	0.392389	0.337384	0.358491	0.339783	0.35961
0.319476	0.313879	0.314679	0.314839	0.347618	0.363128	0.346178	0.355293	0.308922	0.355293
0.315318	0.317237	0.347138	0.329709	0.369044	0.387272	0.344899	0.357211	0.348417	0.348897
0.311641	0.330349	0.314199	0.308123	0.322354	0.334506	0.329869	0.320915	0.319795	0.310681
0.303965	0.352095	0.342661	0.3441	0.367125	0.341861	0.333067	0.300448	0.352255	0.326991
0.344739	0.370483	0.319635	0.348257	0.300927	0.289894	0.292613	0.243364	0.301887	0.259514
0.323793	0.308123	0.285417	0.346498	0.221618	0.248641	0.295011	0.28206	0.223217	0.277103
0.321394	0.382635	0.320275	0.336425	0.322514	0.340902	0.309402	0.319476	0.332267	0.324912
0.259994	0.287496	0.330349	0.256476	0.4063	0.4063	0.338343	0.381836	0.348737	0.389191
0.310042	0.330988	0.310201	0.332907	0.307323	0.301407	0.322514	0.279821	0.303806	0.286217
0.291493	0.338983	0.345539	0.306684	0.429005	0.422929	0.371602	0.399264	0.37544	0.40598
0.332747	0.350016	0.299009	0.351295	0.301887	0.243364	0.287016	0.1882	0.31292	0.239527
0.295971	0.305245	0.324752	0.335945	0.212184	0.310361	0.309562	0.327311	0.298209	0.274544
0.336585	0.349856	0.31212	0.366166	0.268148	0.251999	0.300608	0.251519	0.287016	0.244963
0.307004	0.344899	0.318836	0.301887	0.404861	0.415734	0.35881	0.381996	0.351775	0.39079
0.319476	0.352414	0.302047	0.325712	0.322354	0.293412	0.313399	0.2811	0.304765	0.293252
0.292453	0.349856	0.293892	0.303806	0.333866	0.336904	0.322194	0.324113	0.320915	0.324912
0.316597	0.350975	0.315958	0.337064	0.299169	0.305245	0.303806	0.235849	0.303486	0.261433
0.332587	0.34346	0.297889	0.326351	0.360889	0.360249	0.361689	0.357691	0.354653	0.336105
0.306524	0.331468	0.341861	0.310201	0.426447	0.425807	0.373201	0.399584	0.383754	0.402462
0.330349	0.330349	0.306204	0.346658	0.201311	0.267988	0.303965	0.267829	0.229933	0.256316
0.350496	0.379117	0.333067	0.364886	0.325552	0.248641	0.322194	0.083786	0.31276	0.244963
0.291813	0.342021	0.301567	0.29709	0.323953	0.252638	0.316917	0.248801	0.278062	0.279181
0.29677	0.336745	0.333067	0.324912	0.417813	0.428526	0.370643	0.395427	0.368884	0.405021
0.346019	0.356252	0.308762	0.361049	0.26527	0.269747	0.306044	0.272945	0.280301	0.259834
0.316118	0.300608	0.289735	0.321714	0.269268	0.304925	0.302526	0.26639	0.269108	0.26559
0.325072	0.367765	0.321874	0.342021	0.335945	0.342021	0.321234	0.318356	0.316118	0.334026
0.344899	0.251519	0.321394	0.369044	0.072434	0.257595	0.308762	0.301567	0.242085	0.262232
0.327311	0.353854	0.294212	0.329869	0.320915	0.288136	0.303806	0.217781	0.309402	0.2811
0.345699	0.339143	0.15606	0.327311	0.290214	0.306204	0.295171	0.3118	0.288775	0.325072
0.32827	0.385193	0.320915	0.332107	0.347777	0.331948	0.330508	0.28062	0.350975	0.300288
0.308762	0.32795	0.308283	0.311001	0.304765	0.3118	0.245603	0.287496	0.302526	0.291334
0.301567	0.336425	0.303806	0.247362	0.329229	0.298689	0.330029	0.272146	0.330029	0.307803
0.294372	0.339623	0.338184	0.308443	0.428526	0.426767	0.369364	0.395907	0.373681	0.40614
0.34346	0.351775	0.32875	0.367925	0.227215	0.257115	0.300128	0.285897	0.274065	0.252478
0.299808	0.331628	0.325552	0.317877	0.416214	0.426767	0.366645	0.392709	0.37512	0.4063
0.351455	0.366326	0.300768	0.355133	0.286217	0.216342	0.303326	0.205948	0.295491	0.259674

For Review Only

0.354013	0.346338	0.287816	0.342661	0.295011	0.248641	0.274065	0.220339	0.308283	0.254877
0.308283	0.373681	0.348737	0.333067	0.362008	0.364567	0.339143	0.362008	0.365846	0.353854
0.35881	0.338024	0.287336	0.364407	0.205948	0.245763	0.316278	0.285098	0.211705	0.282699
0.336585	0.356732	0.305405	0.363607	0.307004	0.241925	0.316278	0.196035	0.319955	0.248321

For Review Only

0.338823	0.298049	0.26639	0.302047	0.28174	0.377199	0.34394	0.295811	0.278542	0.31228
0.298049	0.32779	0.319955	0.350975	0.246402	0.320115	0.29661	0.306684	0.345699	0.288136
0.365526	0.256956	0.248641	0.318996	0.236009	0.40566	0.383115	0.298369	0.299808	0.289095
0.332107	0.256156	0.256636	0.322833	0.251999	0.374001	0.324752	0.292613	0.324272	0.287656
0.321394	0.333547	0.316597	0.337224	0.341222	0.346338	0.32843	0.31276	0.310521	0.3433
0.349217	0.279981	0.275823	0.307803	0.23473	0.396546	0.35961	0.310042	0.291014	0.29661
0.360409	0.304125	0.291813	0.313559	0.298529	0.39127	0.354653	0.321394	0.289575	0.32763
0.274225	0.309882	0.326511	0.331468	0.31308	0.294212	0.278862	0.291493	0.308443	0.322354
0.336265	0.303006	0.303326	0.289255	0.29613	0.375919	0.351935	0.29661	0.278702	0.320595
0.278542	0.337704	0.353214	0.355133	0.339303	0.299648	0.273425	0.308443	0.350176	0.373041
0.310681	0.340422	0.326191	0.354653	0.335785	0.21874	0.303486	0.315478	0.354493	0.311161
0.361209	0.244164	0.246083	0.315638	0.222737	0.400863	0.373041	0.290054	0.298209	0.278702
0.302207	0.302207	0.293892	0.345219	0.307323	0.32811	0.326831	0.295491	0.323633	0.333067
0.18804	0.276463	0.272146	0.306684	0.297889	0.287976	0.292773	0.292773	0.29709	0.303646
0.333227	0.277103	0.263032	0.271027	0.253438	0.367765	0.352414	0.286217	0.240006	0.291493
0.301087	0.303006	0.268468	0.32859	0.287656	0.310201	0.322514	0.29725	0.331148	0.306044
0.349057	0.336904	0.331628	0.354013	0.314998	0.378318	0.335465	0.325232	0.334186	0.358171
0.338983	0.297729	0.282699	0.332267	0.298209	0.352255	0.345219	0.31308	0.29629	0.31276
0.372721	0.279341	0.278862	0.305724	0.261433	0.42117	0.382315	0.294851	0.297889	0.313719
0.308602	0.330349	0.333067	0.366485	0.34346	0.337384	0.304605	0.307643	0.381196	0.341382
0.314839	0.334346	0.330029	0.355453	0.320595	0.311481	0.303326	0.29757	0.342341	0.330189
0.34426	0.332747	0.317557	0.34378	0.316437	0.368244	0.348897	0.322354	0.323793	0.348577
0.26543	0.362008	0.364567	0.331148	0.367765	0.193796	0.276463	0.301407	0.365206	0.324592
0.192517	0.272785	0.283019	0.332747	0.295651	0.229453	0.238727	0.263032	0.32843	0.308602
0.364087	0.299648	0.294212	0.315958	0.277582	0.395267	0.377359	0.303646	0.294851	0.326831
0.355293	0.18708	0.192677	0.325871	0.242245	0.392549	0.379917	0.310681	0.322674	0.194755
0.330029	0.240806	0.232811	0.298849	0.197474	0.370963	0.336745	0.284458	0.297889	0.252798
0.276303	0.247681	0.24928	0.289255	0.2496	0.305884	0.299009	0.258235	0.275184	0.261433
0.293732	0.301407	0.314199	0.323953	0.288295	0.316278	0.295331	0.283499	0.318996	0.314519
0.252478	0.35945	0.369524	0.382475	0.381356	0.259194	0.238887	0.323313	0.393188	0.364886
0.360249	0.272945	0.268948	0.310201	0.225296	0.396386	0.367445	0.307643	0.300288	0.292933
0.371122	0.311481	0.292133	0.317557	0.277263	0.4055	0.377039	0.311481	0.307483	0.319476
0.362968	0.320915	0.291174	0.319635	0.307323	0.399105	0.386633	0.31244	0.287176	0.334826
0.14071	0.303166	0.31196	0.347618	0.311001	0.241765	0.255836	0.275983	0.342981	0.326511
0.315318	0.3118	0.300288	0.31228	0.306204	0.313559	0.330668	0.303806	0.316597	0.286217
0.258075	0.228494	0.225776	0.298049	0.248481	0.304765	0.293732	0.284138	0.304125	0.286537
0.274225	0.28206	0.289415	0.299968	0.300608	0.331788	0.299648	0.287496	0.29613	0.320435
0.316278	0.303806	0.299648	0.306044	0.300927	0.323953	0.318676	0.307803	0.289255	0.310042
0.212504	0.34346	0.350975	0.35945	0.348577	0.087144	0.227055	0.282539	0.376239	0.313399
0.306044	0.331308	0.322674	0.329389	0.333067	0.255197	0.309242	0.311161	0.356572	0.274544
0.252319	0.356092	0.361529	0.373041	0.366645	0.241925	0.25024	0.315318	0.379757	0.355772
0.286217	0.311001	0.324113	0.348577	0.311321	0.293252	0.29629	0.29645	0.339143	0.32875
0.278062	0.334026	0.344899	0.337224	0.317877	0.305085	0.289095	0.306524	0.318996	0.346338
0.337864	0.330189	0.318036	0.332427	0.339942	0.350336	0.324272	0.330668	0.329389	0.350016
0.291493	0.334826	0.350176	0.344739	0.333227	0.319476	0.298209	0.330349	0.327151	0.350975
0.364087	0.217461	0.230572	0.313399	0.241126	0.401503	0.37464	0.29677	0.303646	0.264631
0.356892	0.241765	0.207867	0.302526	0.207067	0.391749	0.379437	0.309242	0.279981	0.240966
0.290534	0.261273	0.237768	0.32763	0.290534	0.304125	0.333227	0.29677	0.315798	0.258395
0.32763	0.351775	0.334506	0.326511	0.332907	0.354493	0.318196	0.319156	0.322993	0.346818
0.354493	0.289095	0.271826	0.29661	0.286537	0.392389	0.362648	0.29613	0.288295	0.306844

For Review Only

0.366006	0.224336	0.237928	0.329709	0.243044	0.393508	0.363767	0.301407	0.319156	0.227215
0.322993	0.307963	0.313399	0.331788	0.26559	0.345379	0.350496	0.299808	0.330349	0.2811
0.3756	0.307643	0.293732	0.319316	0.295491	0.418292	0.39047	0.311481	0.304605	0.333387
0.362168	0.321075	0.291174	0.318516	0.306844	0.398305	0.385993	0.311481	0.286697	0.334986
0.362808	0.262072	0.273585	0.289894	0.232331	0.397506	0.384074	0.295011	0.290534	0.29757
0.347618	0.326031	0.31308	0.323633	0.316278	0.368404	0.338663	0.322354	0.331628	0.34346
0.371922	0.278702	0.277422	0.306844	0.261593	0.420211	0.381996	0.294691	0.298689	0.31324
0.361529	0.222897	0.205309	0.308283	0.20243	0.393508	0.373201	0.305405	0.299169	0.217461
0.352095	0.132235	0.176847	0.31292	0.235849	0.39031	0.366485	0.291813	0.31228	0.198753
0.217941	0.335305	0.3433	0.356892	0.353054	0.192837	0.143428	0.278222	0.364407	0.337704
0.251839	0.307323	0.308443	0.323313	0.306204	0.292453	0.238727	0.278542	0.310681	0.321554
0.287016	0.255197	0.254717	0.286856	0.241605	0.315958	0.308602	0.258395	0.278222	0.270707
0.355772	0.254397	0.257115	0.299968	0.169811	0.389031	0.361848	0.303806	0.290694	0.283179
0.349856	0.255996	0.258395	0.292293	0.18724	0.389031	0.365526	0.307483	0.298049	0.292773
0.376399	0.307803	0.293092	0.318356	0.295331	0.417333	0.39015	0.310361	0.304445	0.331468
0.293892	0.300608	0.31324	0.323953	0.287976	0.315958	0.294532	0.283499	0.319156	0.313879
0.305724	0.270707	0.256636	0.298209	0.239847	0.334026	0.316437	0.283339	0.290854	0.267189
0.288615	0.310201	0.309562	0.335625	0.323313	0.307004	0.309562	0.290694	0.31196	0.319156
0.321075	0.268788	0.307963	0.322833	0.276623	0.32875	0.336265	0.29741	0.324113	0.288615
0.383435	0.268628	0.268948	0.286217	0.28126	0.426767	0.393988	0.304285	0.24976	0.299169
0.37576	0.295491	0.29709	0.293572	0.26575	0.418612	0.386473	0.305245	0.277103	0.323473
0.358331	0.306204	0.290054	0.304445	0.291973	0.407099	0.376239	0.308762	0.290054	0.314998
0.317877	0.244324	0.253758	0.283179	0.185002	0.358171	0.339623	0.286377	0.2811	0.278702
0.286057	0.314519	0.331948	0.277742	0.333387	0.237288	0.294532	0.291174	0.354333	0.285897
0.310201	0.320275	0.314199	0.307483	0.292613	0.340582	0.320435	0.288615	0.316278	0.309722
0.331948	0.303965	0.304605	0.336425	0.276943	0.364247	0.339143	0.290534	0.342981	0.317557
0.290534	0.334986	0.349696	0.34442	0.332907	0.318356	0.297729	0.330029	0.326351	0.350176
0.345539	0.247202	0.278062	0.305724	0.217461	0.385034	0.358491	0.301887	0.295491	0.299488
0.34394	0.342821	0.350016	0.341541	0.353054	0.346658	0.336745	0.321394	0.310042	0.370643
0.215382	0.349217	0.35865	0.364407	0.358331	0.133834	0.231212	0.279181	0.3756	0.320435
0.354333	0.162456	0.170771	0.32891	0.253918	0.389351	0.377838	0.31324	0.319795	0.18724
0.354013	0.304925	0.29629	0.31292	0.26575	0.400863	0.383914	0.305884	0.315638	0.317077
0.320275	0.300128	0.295811	0.331788	0.304125	0.342501	0.3433	0.308443	0.321874	0.333706
0.223057	0.259514	0.260953	0.324113	0.255516	0.2819	0.259514	0.257915	0.31196	0.293892
0.210745	0.356252	0.367765	0.361369	0.365046	0.145827	0.238887	0.283818	0.375919	0.323153
0.37448	0.307323	0.293732	0.318036	0.295651	0.415894	0.389511	0.308922	0.303646	0.331788
0.306684	0.302047	0.268788	0.324432	0.303006	0.349376	0.327151	0.291653	0.29709	0.308123
0.291174	0.335465	0.350815	0.34394	0.333387	0.318836	0.298529	0.330508	0.327311	0.350815
0.2811	0.309882	0.326511	0.347298	0.321874	0.290374	0.286856	0.288455	0.333387	0.335785
0.319476	0.35961	0.355453	0.351455	0.356252	0.334666	0.298049	0.313879	0.348897	0.366006
0.309722	0.308283	0.316437	0.32747	0.306844	0.330668	0.331948	0.299808	0.326351	0.329229
0.291973	0.315798	0.32747	0.317237	0.342181	0.314839	0.286856	0.318836	0.31292	0.348737
0.378318	0.306044	0.282379	0.323153	0.300927	0.412376	0.388551	0.317397	0.309402	0.320435
0.379917	0.302846	0.295811	0.313879	0.230892	0.423249	0.392229	0.318836	0.320275	0.31292
0.339942	0.303646	0.307004	0.341701	0.295811	0.3748	0.331468	0.311641	0.309722	0.319156
0.356732	0.255996	0.258395	0.300128	0.170611	0.389031	0.361848	0.304605	0.290854	0.284458
0.338503	0.298049	0.298529	0.322514	0.287816	0.367125	0.330189	0.316597	0.292453	0.331468
0.35913	0.268628	0.258874	0.305085	0.227854	0.401023	0.365366	0.298369	0.295491	0.264471
0	0.309402	0.310361	0.345219	0.326831	0.224496	0.239207	0.28078	0.347138	0.321714
0.309402	0	0.193636	0.301087	0.247362	0.352894	0.344899	0.291014	0.287656	0.243364
0.310361	0.193636	0	0.317877	0.246402	0.355932	0.339623	0.301407	0.298849	0.231532
0.345219	0.301087	0.317877	0	0.301247	0.368724	0.362648	0.308283	0.170931	0.3118
0.326831	0.247362	0.246402	0.301247	0	0.364886	0.336265	0.280301	0.29629	0.274065
0.224496	0.352894	0.355932	0.368724	0.364886	0	0.225136	0.285417	0.384234	0.321714
0.239207	0.344899	0.339623	0.362648	0.336265	0.225136	0	0.292933	0.362968	0.335785

For Review Only

0.28078	0.291014	0.301407	0.308283	0.280301	0.285417	0.292933	0	0.314679	0.299648
0.347138	0.287656	0.298849	0.170931	0.29629	0.384234	0.362968	0.314679	0	0.321714
0.321714	0.243364	0.231532	0.3118	0.274065	0.321714	0.335785	0.299648	0.321714	0
0.317717	0.308443	0.308762	0.309722	0.294532	0.321394	0.330508	0.300448	0.329229	0.29757
0.315798	0.191238	0.227534	0.308602	0.239687	0.35945	0.346498	0.290694	0.307483	0.238567
0.10889	0.300128	0.304925	0.341701	0.330508	0.232651	0.253598	0.283658	0.340742	0.310361
0.352894	0.236808	0.244803	0.310201	0.188839	0.391909	0.362168	0.288136	0.307963	0.273905
0.325392	0.208986	0.226415	0.309722	0.247042	0.364407	0.354653	0.288775	0.310521	0.240006
0.345859	0.282219	0.26623	0.309562	0.23473	0.391589	0.366485	0.291813	0.310841	0.304445
0.272945	0.252958	0.237128	0.289095	0.239847	0.304925	0.302526	0.277263	0.293092	0.276943
0.28158	0.261113	0.182443	0.339942	0.28126	0.295651	0.304445	0.306044	0.332587	0.276303
0.270227	0.252159	0.267189	0.319476	0.284778	0.290534	0.284618	0.273105	0.310841	0.288136
0.292933	0.306204	0.290374	0.315958	0.29757	0.336105	0.318196	0.295971	0.283179	0.316278
0.295491	0.254397	0.264631	0.294212	0.261113	0.31244	0.309402	0.277422	0.286057	0.262232
0.320595	0.289575	0.289415	0.325552	0.284138	0.355133	0.316757	0.31228	0.285098	0.314519
0.345379	0.251999	0.268788	0.291653	0.216981	0.376399	0.347937	0.290534	0.276623	0.277263
0.314679	0.288295	0.282699	0.31196	0.260313	0.346818	0.319156	0.293412	0.307483	0.304285
0.300288	0.226415	0.222098	0.31196	0.258075	0.319795	0.323633	0.279501	0.305245	0.225935
0.367125	0.295331	0.298689	0.300288	0.310042	0.404061	0.362168	0.306204	0.2496	0.31308
0.302047	0.241126	0.233291	0.294851	0.240646	0.326831	0.324272	0.268628	0.284618	0.236009
0.098017	0.301087	0.307803	0.346019	0.32779	0.23505	0.248481	0.285737	0.345059	0.317077
0.342341	0.336745	0.335785	0.322194	0.289894	0.361529	0.344899	0.303965	0.300927	0.309722
0.287176	0.240166	0.248801	0.287016	0.246722	0.305405	0.302846	0.260793	0.282539	0.249121
0.353054	0.301727	0.299968	0.304605	0.271027	0.397026	0.376239	0.290054	0.307643	0.313719
0.333547	0.261912	0.26495	0.314998	0.225616	0.371282	0.352894	0.292933	0.294691	0.285737
0.209786	0.349536	0.36009	0.369843	0.364407	0.142309	0.238088	0.2819	0.381036	0.321394
0.319316	0.237928	0.261593	0.278542	0.284458	0.319156	0.32779	0.291334	0.330988	0.21842
0.293092	0.297889	0.311161	0.275823	0.310361	0.273905	0.314839	0.288295	0.322674	0.279661
0.347777	0.306044	0.282699	0.305724	0.277422	0.383914	0.364567	0.287176	0.298529	0.324113
0.276943	0.269747	0.278862	0.299169	0.256156	0.341222	0.314998	0.255357	0.297889	0.310042
0.378957	0.262072	0.260633	0.320595	0.243364	0.415414	0.388711	0.303806	0.302367	0.297729
0.360889	0.304125	0.299009	0.294052	0.286377	0.395427	0.372562	0.298529	0.29677	0.304765
0.172849	0.342981	0.348897	0.362168	0.355772	0.173649	0.238567	0.274544	0.369044	0.32763
0.275184	0.25008	0.26591	0.291653	0.252478	0.294212	0.286217	0.263671	0.290374	0.271346
0.382155	0.306524	0.283499	0.322194	0.303006	0.414934	0.393348	0.317397	0.310681	0.322034
0.303006	0.231052	0.208027	0.302367	0.240486	0.339623	0.330029	0.274704	0.290054	0.21874
0.145187	0.300288	0.309882	0.346978	0.309722	0.243204	0.256956	0.274225	0.34314	0.326671
0.266869	0.298209	0.293092	0.321874	0.303006	0.288935	0.307163	0.295331	0.319316	0.314359
0.090342	0.309402	0.320915	0.346019	0.339623	0.230253	0.241445	0.283179	0.347458	0.324432
0.350176	0.256156	0.26575	0.289095	0.213943	0.382315	0.354813	0.302207	0.273265	0.278542
0.306364	0.2811	0.280141	0.331468	0.307643	0.286537	0.320115	0.292933	0.323313	0.253278
0.25008	0.364247	0.369364	0.371122	0.367445	0.171091	0.260313	0.298529	0.378957	0.318836
0.31292	0.308602	0.305085	0.303166	0.307004	0.290534	0.323793	0.302047	0.351455	0.266869
0.102494	0.31308	0.320915	0.349376	0.336585	0.227854	0.232811	0.279661	0.352894	0.325232
0.35945	0.262072	0.278222	0.266709	0.251359	0.395587	0.364567	0.309562	0.198433	0.295651
0.24992	0.329549	0.334026	0.358171	0.346019	0.269747	0.256796	0.304125	0.35993	0.340422
0.314679	0.240966	0.266709	0.325712	0.283019	0.334346	0.325232	0.299968	0.327311	0.278222
0.319156	0.274225	0.287496	0.305724	0.266869	0.368884	0.333706	0.279181	0.275664	0.29757
0.356572	0.170291	0.17205	0.323473	0.246722	0.397825	0.376399	0.31292	0.313879	0.160217
0.357371	0.186281	0.207547	0.301727	0.220659	0.388391	0.379117	0.309722	0.293732	0.21842
0.21938	0.290054	0.298369	0.307163	0.307963	0.253438	0.247362	0.26559	0.307803	0.309722
0.357691	0.192517	0.193476	0.333387	0.23505	0.389831	0.376399	0.306524	0.323793	0.207387
0.335465	0.177966	0.168692	0.316597	0.251359	0.380876	0.364886	0.302686	0.310201	0.183563
0.25008	0.316278	0.316118	0.339463	0.352255	0.226575	0.235369	0.307483	0.332587	0.303965
0.290214	0.271346	0.273905	0.305085	0.276143	0.329549	0.333387	0.28206	0.309082	0.293892

For Review Only

0.356732	0.211864	0.236808	0.303806	0.215702	0.387112	0.363767	0.315798	0.303326	0.282859
0.249121	0.287496	0.290854	0.320595	0.290534	0.245763	0.241445	0.263032	0.319635	0.293732
0.366645	0.241126	0.257755	0.305564	0.268788	0.398945	0.388551	0.305884	0.274704	0.272306
0.294851	0.307963	0.325072	0.322674	0.32827	0.278542	0.306044	0.326991	0.329709	0.283978
0.326991	0.31292	0.294372	0.365526	0.335625	0.347298	0.31308	0.318196	0.34362	0.32843
0.299968	0.321554	0.318836	0.304765	0.313559	0.280461	0.319955	0.290694	0.32859	0.308922
0.348737	0.268148	0.257755	0.310201	0.231052	0.396386	0.365846	0.305405	0.299169	0.289255
0.286377	0.31212	0.302047	0.331788	0.308922	0.29757	0.275983	0.290854	0.339623	0.287656
0.269108	0.333387	0.331628	0.333547	0.333067	0.210106	0.272306	0.297889	0.326031	0.301727
0.273585	0.235209	0.233291	0.290854	0.242565	0.307643	0.302846	0.264471	0.285257	0.2504
0.261273	0.342821	0.326831	0.356732	0.32875	0.238567	0.301407	0.305405	0.362328	0.317397
0.373361	0.305564	0.29757	0.318996	0.266709	0.410777	0.381516	0.303646	0.322034	0.326191
0.331948	0.200352	0.184362	0.305884	0.235369	0.360569	0.346658	0.282539	0.304285	0.200831
0.237768	0.287816	0.28206	0.306524	0.292613	0.237768	0.275344	0.279341	0.297729	0.278702
0.338663	0.232491	0.226895	0.301087	0.229933	0.381196	0.345699	0.293092	0.29709	0.254557
0.2819	0.286217	0.303326	0.302526	0.291174	0.3441	0.318036	0.290054	0.274544	0.295651
0.098977	0.308922	0.319156	0.345059	0.334026	0.225136	0.23489	0.273745	0.340262	0.318356
0.306204	0.362808	0.34426	0.354173	0.350176	0.212344	0.29757	0.322993	0.357691	0.310042
0.272785	0.267189	0.274864	0.298689	0.273105	0.308762	0.313559	0.275024	0.304445	0.287016
0.363927	0.264791	0.289735	0.320915	0.224976	0.398785	0.360409	0.292133	0.31276	0.291813
0.303965	0.210745	0.198913	0.310201	0.247362	0.347777	0.32859	0.291493	0.283978	0.23409
0.338503	0.278222	0.260953	0.303646	0.231852	0.385034	0.35961	0.286057	0.304765	0.299648
0.276303	0.253758	0.247362	0.285098	0.246083	0.304925	0.300448	0.259354	0.277582	0.259034
0.204189	0.3118	0.329709	0.349217	0.324592	0.184842	0.232651	0.273105	0.364087	0.317077
0.284618	0.321874	0.339783	0.267509	0.339623	0.246242	0.28078	0.294851	0.348737	0.293412
0.350336	0.257115	0.258075	0.291973	0.18804	0.388551	0.364567	0.307963	0.298209	0.292293
0.316118	0.320915	0.332587	0.345379	0.305884	0.34346	0.318836	0.300608	0.336105	0.34314
0.141829	0.305245	0.31276	0.346019	0.314998	0.244484	0.254557	0.283019	0.346178	0.319635
0.329549	0.225776	0.242245	0.271186	0.214743	0.378798	0.346498	0.287016	0.2496	0.286537
0.293572	0.284458	0.288455	0.307004	0.298849	0.342181	0.320595	0.292933	0.269907	0.311001
0.322833	0.268628	0.261593	0.306844	0.272785	0.353854	0.330029	0.293732	0.288455	0.288295
0.354333	0.302526	0.279661	0.285737	0.289575	0.391589	0.371762	0.303006	0.282699	0.308762
0.32859	0.261753	0.28206	0.305564	0.269747	0.365046	0.324272	0.290694	0.272466	0.299968
0.370643	0.283978	0.262552	0.303486	0.282379	0.423409	0.395267	0.31308	0.268308	0.305564
0.326511	0.304125	0.273105	0.311321	0.273265	0.379917	0.336265	0.289255	0.298209	0.301247
0.104093	0.309722	0.318516	0.342501	0.332267	0.224656	0.236329	0.273265	0.341382	0.313879
0.336265	0.295331	0.275823	0.313719	0.298049	0.385034	0.35913	0.29613	0.303646	0.315958
0.350016	0.255996	0.258874	0.291653	0.18692	0.388391	0.364407	0.307323	0.297729	0.292453
0.342981	0.269747	0.256796	0.309402	0.256796	0.35881	0.332267	0.307483	0.324432	0.240646
0.320915	0.29757	0.279021	0.316278	0.284618	0.365526	0.320115	0.286856	0.295971	0.323793
0.35961	0.193476	0.208666	0.303326	0.212024	0.386792	0.380397	0.309082	0.292933	0.227374
0.345219	0.253278	0.267349	0.299648	0.226255	0.380077	0.358491	0.287336	0.289735	0.31196
0.364886	0.255516	0.247202	0.320755	0.236169	0.404541	0.381196	0.29693	0.300128	0.288295
0.35913	0.153502	0.167733	0.324912	0.24992	0.395587	0.379277	0.31196	0.31324	0.165334
0.206108	0.355133	0.363288	0.361049	0.35913	0.139431	0.231372	0.283339	0.39063	0.322034
0.329869	0.268948	0.251999	0.310361	0.273745	0.339942	0.348097	0.294372	0.321714	0.271027
0.28174	0.271506	0.277902	0.317877	0.277902	0.337544	0.326991	0.284938	0.323793	0.314039
0.314679	0.325712	0.321394	0.327311	0.2811	0.345859	0.305884	0.28174	0.320435	0.329549
0.306684	0.362648	0.342821	0.353694	0.350176	0.210585	0.299009	0.322514	0.357531	0.308922
0.24944	0.335305	0.346498	0.358331	0.35993	0.181484	0.248961	0.294212	0.356892	0.332267
0.157659	0.288295	0.29677	0.338663	0.306844	0.222098	0.217621	0.271027	0.338343	0.31308
0.300768	0.288295	0.295171	0.321714	0.282859	0.335625	0.321394	0.294691	0.326191	0.31292
0.369204	0.267349	0.274065	0.315318	0.270387	0.407579	0.377678	0.298529	0.29757	0.31308
0.210585	0.337864	0.336265	0.34314	0.342661	0.263511	0.190438	0.259994	0.338823	0.32795
0.267189	0.32891	0.350656	0.273425	0.355612	0.215062	0.274704	0.282219	0.350016	0.299648

For Review Only

0.356412	0.255676	0.257275	0.299648	0.169492	0.387912	0.361049	0.303006	0.290374	0.283818
0.21842	0.35913	0.36009	0.365526	0.358331	0.100096	0.235529	0.285417	0.387752	0.330668
0.275344	0.304765	0.294532	0.324432	0.301087	0.313879	0.323473	0.297889	0.315478	0.309722
0.298049	0.316757	0.285257	0.337864	0.315478	0.326831	0.323153	0.29661	0.339783	0.315638
0.379597	0.283339	0.275344	0.313559	0.28158	0.424528	0.393348	0.302367	0.299808	0.305405
0.320275	0.325712	0.340422	0.2811	0.333227	0.310042	0.322674	0.315318	0.333706	0.304445
0.295011	0.325072	0.287816	0.340262	0.307163	0.32875	0.313719	0.291334	0.322354	0.309242
0.356892	0.236968	0.221618	0.306844	0.263351	0.4055	0.37432	0.29725	0.293732	0.240966
0.2819	0.317877	0.324592	0.346019	0.341861	0.260313	0.305405	0.304605	0.34362	0.305405
0.261113	0.347458	0.360889	0.382955	0.372562	0.259354	0.262712	0.31324	0.383275	0.368564
0.214743	0.275344	0.237608	0.329229	0.301087	0.258555	0.261113	0.283339	0.321554	0.288615
0.213463	0.353694	0.369843	0.370643	0.358331	0.127279	0.23377	0.2811	0.380556	0.32875
0.275024	0.247202	0.245123	0.286217	0.235369	0.29709	0.295971	0.255516	0.279021	0.260153
0.325392	0.287656	0.313879	0.326191	0.302526	0.333227	0.324592	0.291174	0.304125	0.314519
0.334026	0.294212	0.294532	0.324272	0.303006	0.340902	0.34394	0.300768	0.327151	0.314839
0.309082	0.20243	0.224656	0.310521	0.254397	0.347777	0.346019	0.283978	0.295971	0.249121
0.367125	0.262872	0.25056	0.321394	0.235369	0.393668	0.380237	0.310521	0.302047	0.287976
0.32811	0.321554	0.307483	0.31228	0.332267	0.340742	0.336585	0.315638	0.324272	0.300768
0.242245	0.314839	0.313559	0.348097	0.32795	0.158459	0.24928	0.289575	0.357851	0.295651
0.268628	0.348737	0.34378	0.324592	0.32891	0.28206	0.290374	0.289095	0.336904	0.321714
0.263671	0.322833	0.320595	0.354813	0.333866	0.231532	0.291653	0.303166	0.351295	0.302526
0.31276	0.316597	0.313719	0.308602	0.320275	0.324272	0.317557	0.317717	0.291334	0.313399
0.314998	0.287656	0.329709	0.34314	0.292773	0.292933	0.326671	0.29629	0.344579	0.316597
0.342981	0.175088	0.180365	0.318836	0.251199	0.384874	0.366326	0.307483	0.306524	0.184042
0.345859	0.300927	0.294532	0.3118	0.268148	0.392389	0.360409	0.289575	0.313719	0.321874
0.303166	0.316437	0.301567	0.289255	0.299169	0.267189	0.314839	0.301407	0.26543	0.28078
0.217461	0.334026	0.3433	0.357052	0.352574	0.192677	0.143268	0.277422	0.364886	0.338503
0.335785	0.300768	0.300768	0.344899	0.261113	0.36009	0.34346	0.302846	0.331308	0.318516
0.214263	0.356572	0.366485	0.369683	0.368084	0.14039	0.238248	0.279661	0.387272	0.333067
0.364407	0.222258	0.238567	0.318836	0.208986	0.402462	0.368084	0.293092	0.314199	0.272945
0.326671	0.309402	0.289735	0.32747	0.293732	0.360889	0.349057	0.290374	0.313559	0.313879
0.366006	0.274384	0.273745	0.311001	0.259514	0.410777	0.385673	0.301087	0.293732	0.310361
0.230413	0.339463	0.348417	0.348577	0.355772	0.139111	0.261113	0.294851	0.362488	0.320755
0.3126	0.216501	0.236649	0.339623	0.286057	0.310681	0.330189	0.298529	0.331788	0.25072
0.229613	0.302047	0.314839	0.29629	0.31324	0.309882	0.293892	0.298209	0.271186	0.291334
0.334666	0.190918	0.214423	0.311001	0.259674	0.355293	0.339783	0.291813	0.304125	0.215862
0.291334	0.318676	0.337704	0.247042	0.34394	0.25072	0.294851	0.293732	0.342021	0.275024
0.213144	0.35961	0.376879	0.369524	0.368724	0.142949	0.245443	0.289415	0.393028	0.330349
0.362648	0.28142	0.284778	0.295171	0.260793	0.396067	0.37528	0.303646	0.292933	0.31308
0.351135	0.257435	0.259674	0.293252	0.18772	0.38999	0.366006	0.309402	0.298689	0.293252
0.256796	0.228174	0.225616	0.29613	0.246722	0.304285	0.293252	0.283818	0.302526	0.285417
0.216501	0.358331	0.362168	0.372082	0.362648	0.081708	0.232971	0.284298	0.389191	0.316917
0.356732	0.291653	0.275024	0.294052	0.272626	0.409338	0.379437	0.298529	0.267029	0.300608
0.274544	0.256636	0.26495	0.298209	0.267029	0.330189	0.306844	0.28078	0.263032	0.302207
0.298689	0.323953	0.305724	0.292613	0.300768	0.267349	0.306524	0.300927	0.290054	0.275983
0.380716	0.309722	0.299488	0.309402	0.290214	0.416374	0.386952	0.31276	0.309722	0.333067
0.336105	0.261433	0.276783	0.329389	0.239367	0.347138	0.335305	0.278702	0.322674	0.302047
0.310042	0.305724	0.300448	0.323633	0.287816	0.332747	0.337224	0.314839	0.334186	0.308602
0.316437	0.237608	0.23505	0.321714	0.279021	0.275024	0.32827	0.308922	0.326351	0.232012
0.324912	0.266869	0.294851	0.294212	0.269747	0.366965	0.347777	0.305564	0.235689	0.307963
0.338983	0.29757	0.29757	0.333866	0.272785	0.368564	0.347937	0.319955	0.308443	0.301727
0.213943	0.352894	0.365686	0.365366	0.366805	0.143748	0.235689	0.28206	0.381676	0.332427
0.377199	0.309082	0.294052	0.288775	0.277582	0.424209	0.392709	0.309082	0.29741	0.319156
0.209306	0.354493	0.363767	0.369044	0.368244	0.084906	0.232171	0.287016	0.385993	0.326351
0.366326	0.256316	0.248801	0.319795	0.236808	0.4055	0.382955	0.298529	0.299968	0.288615

For Review Only

0.387752	0.236169	0.260313	0.308283	0.228014	0.42213	0.387592	0.303166	0.301567	0.288136
0.286217	0.32875	0.332907	0.338024	0.333866	0.21954	0.308123	0.300608	0.354333	0.285098
0.365046	0.305245	0.288136	0.314199	0.294532	0.415094	0.380077	0.31308	0.315318	0.321234
0.362328	0.244963	0.269428	0.305245	0.208826	0.401343	0.379437	0.279501	0.29613	0.295651

For Review Only

0.330029	0.305884	0.330349	0.294212	0.299009	0.282859	0.286217	0.314679	0.317557	0.212824
0.300288	0.299328	0.299328	0.247202	0.3126	0.302367	0.309242	0.304765	0.304445	0.329389
0.32779	0.254077	0.360889	0.205309	0.229613	0.20275	0.266709	0.291334	0.29757	0.322833
0.29693	0.259994	0.330508	0.240006	0.261273	0.227694	0.268148	0.29709	0.288136	0.310521
0.336105	0.322354	0.324272	0.351295	0.325392	0.327151	0.306044	0.324912	0.326671	0.292293
0.310841	0.275664	0.330508	0.255996	0.271826	0.255516	0.244484	0.307323	0.316278	0.251039
0.317717	0.300288	0.349856	0.308123	0.29757	0.291334	0.301567	0.326031	0.321394	0.246242
0.287016	0.300768	0.291014	0.337384	0.3118	0.31196	0.29757	0.299808	0.277263	0.330349
0.327311	0.292133	0.337064	0.310841	0.302686	0.283658	0.298689	0.331468	0.318196	0.251039
0.320435	0.334666	0.294532	0.363927	0.341222	0.345379	0.323473	0.304285	0.295171	0.357211
0.315958	0.335305	0.3126	0.355293	0.345539	0.32811	0.295491	0.32875	0.308602	0.318356
0.317717	0.245923	0.356732	0.195555	0.227534	0.190278	0.26543	0.292453	0.291014	0.320435
0.309882	0.288136	0.295811	0.316757	0.305085	0.310841	0.294372	0.298849	0.299968	0.261113
0.303486	0.291653	0.198913	0.317877	0.307004	0.306364	0.262552	0.279981	0.279341	0.259994
0.318196	0.267189	0.32811	0.278062	0.267669	0.26543	0.26543	0.307483	0.305245	0.266549
0.32779	0.272466	0.299648	0.291813	0.276783	0.135433	0.26575	0.295171	0.280301	0.331628
0.349057	0.336425	0.340102	0.335625	0.335465	0.291813	0.320435	0.351295	0.329069	0.309402
0.315158	0.298049	0.316437	0.304765	0.303326	0.304765	0.288136	0.305884	0.299968	0.244803
0.337704	0.287656	0.371122	0.235209	0.270867	0.241605	0.264631	0.326991	0.307483	0.325552
0.323633	0.336105	0.3126	0.365846	0.341861	0.355932	0.318196	0.329709	0.303006	0.340102
0.323793	0.329869	0.304285	0.346338	0.32763	0.34362	0.306044	0.326671	0.307323	0.300288
0.351135	0.326671	0.331788	0.322034	0.313399	0.298849	0.326831	0.336265	0.321234	0.298689
0.319156	0.361689	0.280461	0.39063	0.35977	0.385353	0.327311	0.346178	0.329549	0.339303
0.290694	0.28174	0.209946	0.32795	0.292933	0.327151	0.236968	0.285577	0.252159	0.29741
0.332427	0.268468	0.354013	0.275344	0.274384	0.242565	0.263192	0.32875	0.307803	0.274225
0.322993	0.167253	0.349057	0.2189	0.149344	0.262072	0.279181	0.258395	0.270387	0.32891
0.28094	0.213303	0.335945	0.174288	0.239847	0.232811	0.236649	0.267988	0.288455	0.308762
0.290694	0.244324	0.274864	0.251519	0.242725	0.246083	0.244004	0.273745	0.257435	0.271506
0.320115	0.290054	0.289735	0.3126	0.309402	0.292933	0.302047	0.316437	0.276143	0.286377
0.310201	0.377518	0.264791	0.413495	0.368564	0.408698	0.339143	0.318196	0.304125	0.369044
0.323313	0.24976	0.356252	0.230253	0.2496	0.227055	0.244324	0.31244	0.301247	0.310201
0.330349	0.302367	0.377518	0.261753	0.293892	0.262392	0.28174	0.346178	0.319156	0.298369
0.342181	0.304925	0.350975	0.293572	0.294372	0.287976	0.307323	0.337064	0.32795	0.254877
0.324912	0.292293	0.177646	0.338343	0.301407	0.32859	0.26511	0.287816	0.260633	0.292773
0.319156	0.303646	0.308283	0.31324	0.293732	0.322514	0.309242	0.321554	0.304605	0.318836
0.289255	0.153822	0.275983	0.264151	0.23473	0.270547	0.21938	0.267829	0.191557	0.298209
0.289894	0.285257	0.283818	0.300448	0.301247	0.307004	0.273745	0.307963	0.295331	0.26495
0.259514	0.287176	0.304605	0.29741	0.292133	0.29757	0.286537	0.307803	0.302047	0.284298
0.309242	0.357211	0.224017	0.386633	0.355293	0.380876	0.298209	0.285897	0.282859	0.329389
0.287176	0.342341	0.295491	0.34378	0.325232	0.334186	0.313719	0.326991	0.316278	0.322833
0.306204	0.367285	0.257275	0.40662	0.355453	0.395107	0.325072	0.305405	0.295811	0.357851
0.29677	0.318036	0.291174	0.340422	0.330988	0.329229	0.297729	0.315158	0.291493	0.323633
0.31244	0.317077	0.278542	0.350176	0.332587	0.336105	0.306044	0.298849	0.300448	0.331948
0.323953	0.338823	0.331148	0.355293	0.340262	0.339143	0.304765	0.333706	0.331948	0.293572
0.319476	0.32875	0.29645	0.370323	0.346178	0.342821	0.306844	0.308922	0.321234	0.344579
0.316917	0.199232	0.351775	0.204669	0.197793	0.216022	0.267988	0.288455	0.279981	0.318516
0.326191	0.230093	0.338024	0.209626	0.228014	0.252319	0.270227	0.279021	0.271826	0.314359
0.308123	0.250879	0.287976	0.293892	0.261753	0.301567	0.257275	0.24944	0.267509	0.307323
0.330508	0.326991	0.322833	0.353054	0.341382	0.320755	0.325552	0.318036	0.319476	0.301407
0.32795	0.286856	0.344739	0.284618	0.278382	0.257435	0.287176	0.322833	0.302686	0.288136

For Review Only

0.337064	0.193156	0.355932	0.201791	0.202111	0.253758	0.280461	0.282859	0.264311	0.307163
0.321394	0.291174	0.330828	0.270067	0.29629	0.277902	0.298689	0.338024	0.316917	0.313879
0.347458	0.292293	0.364407	0.299328	0.299488	0.26543	0.300288	0.338343	0.329389	0.244324
0.342021	0.304925	0.349217	0.293732	0.294052	0.287336	0.306844	0.336585	0.327311	0.253438
0.319795	0.256796	0.352734	0.222578	0.259034	0.243044	0.259514	0.323473	0.301567	0.308922
0.334186	0.325552	0.338343	0.332107	0.322034	0.286697	0.308283	0.338184	0.32795	0.300128
0.337224	0.287336	0.370323	0.236009	0.270547	0.242565	0.264471	0.326031	0.306844	0.324432
0.316597	0.217141	0.35929	0.198113	0.224336	0.260793	0.262552	0.295491	0.290054	0.321714
0.308283	0.181324	0.345059	0.20355	0.185801	0.2504	0.257115	0.26575	0.275823	0.300448
0.303486	0.350656	0.225616	0.382955	0.34426	0.380077	0.29661	0.290374	0.264791	0.325552
0.315958	0.292773	0.266869	0.326351	0.304765	0.32891	0.287176	0.300608	0.268308	0.264631
0.294212	0.240646	0.287496	0.24928	0.245443	0.204669	0.239687	0.283019	0.264631	0.277582
0.307483	0.243204	0.353694	0.175568	0.247042	0.231852	0.255676	0.301087	0.290854	0.324272
0.321394	0.248641	0.353054	0.205149	0.253118	0.244803	0.261912	0.307323	0.290214	0.333387
0.346818	0.291653	0.364247	0.298849	0.299808	0.26527	0.300448	0.337544	0.32875	0.243684
0.320275	0.289095	0.289095	0.31196	0.308762	0.292133	0.300927	0.315158	0.275823	0.286537
0.292773	0.268788	0.303166	0.263032	0.256156	0.270227	0.240006	0.284138	0.283658	0.262552
0.31196	0.302846	0.283499	0.339143	0.3126	0.329069	0.295491	0.294052	0.291493	0.26559
0.318516	0.291174	0.325871	0.288455	0.289095	0.290374	0.295331	0.316278	0.305085	0.320115
0.352095	0.270227	0.369683	0.284618	0.267029	0.25008	0.284778	0.336265	0.322993	0.291014
0.329709	0.286537	0.372402	0.276303	0.292933	0.244484	0.303806	0.352734	0.324432	0.299808
0.339942	0.304605	0.354333	0.298689	0.29645	0.268948	0.291973	0.341382	0.339783	0.257275
0.301887	0.236489	0.320115	0.207067	0.245603	0.238248	0.245603	0.293252	0.271186	0.309082
0.293572	0.321714	0.286377	0.345219	0.322034	0.348257	0.293732	0.31276	0.287656	0.32875
0.322993	0.295651	0.307323	0.3118	0.306844	0.294532	0.299169	0.313559	0.287656	0.291973
0.335625	0.287816	0.333227	0.270707	0.273265	0.307483	0.279501	0.333866	0.289894	0.308762
0.318836	0.32843	0.295491	0.369843	0.346498	0.342661	0.306524	0.308922	0.320275	0.3441
0.315318	0.226575	0.356092	0.196994	0.238407	0.228174	0.256956	0.309562	0.287496	0.323473
0.338024	0.35993	0.329869	0.352095	0.334346	0.347618	0.340422	0.355453	0.330668	0.324432
0.323153	0.357211	0.225616	0.39079	0.352414	0.386633	0.305405	0.313879	0.287496	0.334506
0.331148	0.18772	0.342821	0.224976	0.17221	0.276623	0.282859	0.247042	0.262872	0.323153
0.333067	0.285417	0.355612	0.279661	0.283499	0.215702	0.269268	0.337224	0.315318	0.316597
0.316118	0.294052	0.316917	0.322674	0.306204	0.291174	0.293092	0.331948	0.321874	0.302047
0.297889	0.252958	0.245443	0.295331	0.233131	0.295651	0.229613	0.289894	0.237928	0.280301
0.325232	0.365846	0.212344	0.403422	0.35881	0.397825	0.314359	0.317717	0.285257	0.337384
0.346338	0.291493	0.363128	0.298369	0.298849	0.264631	0.300288	0.338024	0.32811	0.243044
0.323153	0.292293	0.303166	0.297729	0.290854	0.298369	0.283658	0.314359	0.294851	0.216981
0.318676	0.32875	0.295811	0.370163	0.346658	0.34314	0.307483	0.309242	0.320435	0.345539
0.302526	0.314039	0.29677	0.338663	0.320435	0.331468	0.302367	0.309722	0.29661	0.315318
0.342341	0.335625	0.313719	0.369044	0.342021	0.342021	0.334026	0.339143	0.316757	0.310681
0.322674	0.292293	0.332747	0.307963	0.288775	0.307643	0.292133	0.337064	0.298849	0.338503
0.309562	0.332907	0.286377	0.365526	0.338983	0.346978	0.298529	0.301407	0.308602	0.321714
0.344899	0.280301	0.35961	0.29693	0.277422	0.255357	0.300448	0.342501	0.334026	0.285737
0.327151	0.258714	0.388232	0.252319	0.272146	0.237288	0.273905	0.345219	0.318356	0.347777
0.336585	0.302846	0.34394	0.300608	0.302526	0.292133	0.315478	0.302686	0.330988	0.303326
0.308443	0.244324	0.354493	0.176047	0.248321	0.233131	0.256316	0.302367	0.292133	0.325232
0.345219	0.269268	0.342821	0.305085	0.287976	0.293572	0.285257	0.309722	0.294851	0.285098
0.310042	0.26511	0.346658	0.191398	0.258874	0.252638	0.274544	0.318516	0.306364	0.295491
0.317717	0.315798	0.10889	0.352894	0.325392	0.345859	0.272945	0.28158	0.270227	0.292933
0.308443	0.191238	0.300128	0.236808	0.208986	0.282219	0.252958	0.261113	0.252159	0.306204
0.308762	0.227534	0.304925	0.244803	0.226415	0.26623	0.237128	0.182443	0.267189	0.290374
0.309722	0.308602	0.341701	0.310201	0.309722	0.309562	0.289095	0.339942	0.319476	0.315958
0.294532	0.239687	0.330508	0.188839	0.247042	0.23473	0.239847	0.28126	0.284778	0.29757
0.321394	0.35945	0.232651	0.391909	0.364407	0.391589	0.304925	0.295651	0.290534	0.336105
0.330508	0.346498	0.253598	0.362168	0.354653	0.366485	0.302526	0.304445	0.284618	0.318196

For Review Only

0.300448	0.290694	0.283658	0.288136	0.288775	0.291813	0.277263	0.306044	0.273105	0.295971
0.329229	0.307483	0.340742	0.307963	0.310521	0.310841	0.293092	0.332587	0.310841	0.283179
0.29757	0.238567	0.310361	0.273905	0.240006	0.304445	0.276943	0.276303	0.288136	0.316278
0	0.305405	0.31308	0.313719	0.302047	0.32779	0.284138	0.320275	0.313879	0.321075
0.305405	0	0.325392	0.236968	0.169492	0.253438	0.252159	0.277902	0.240806	0.308922
0.31308	0.325392	0	0.352734	0.325392	0.345699	0.275504	0.276783	0.270867	0.287656
0.313719	0.236968	0.352734	0	0.236169	0.236489	0.26623	0.292133	0.2811	0.297889
0.302047	0.169492	0.325392	0.236169	0	0.260953	0.260633	0.292933	0.255197	0.300128
0.32779	0.253438	0.345699	0.236489	0.260953	0	0.269268	0.315478	0.318516	0.32763
0.284138	0.252159	0.275504	0.26623	0.260633	0.269268	0	0.273745	0.262392	0.277103
0.320275	0.277902	0.276783	0.292133	0.292933	0.315478	0.273745	0	0.269907	0.315158
0.313879	0.240806	0.270867	0.2811	0.255197	0.318516	0.262392	0.269907	0	0.305884
0.321075	0.308922	0.287656	0.297889	0.300128	0.32763	0.277103	0.315158	0.305884	0
0.285737	0.257275	0.284938	0.263192	0.260953	0.268468	0.258874	0.282379	0.275024	0.274065
0.336105	0.291653	0.322833	0.299808	0.303965	0.295171	0.307963	0.284938	0.31324	0.319156
0.308762	0.236489	0.346818	0.204509	0.243364	0.238567	0.254717	0.3126	0.280461	0.304925
0.309242	0.26559	0.313399	0.268468	0.289415	0.283658	0.264791	0.298529	0.299488	0.318996
0.298849	0.224496	0.294691	0.244324	0.220979	0.270547	0.263032	0.266869	0.25024	0.290214
0.342981	0.305405	0.348417	0.316597	0.292293	0.306044	0.318356	0.335945	0.317397	0.317077
0.294691	0.23393	0.290534	0.239687	0.219859	0.244643	0.245443	0.277742	0.254397	0.284938
0.314039	0.329229	0.067157	0.355453	0.326351	0.34426	0.274384	0.284778	0.277902	0.283658
0.32859	0.299488	0.337704	0.31196	0.315958	0.275024	0.310042	0.352414	0.326511	0.31196
0.290694	0.240966	0.28094	0.246882	0.240166	0.255197	0.246562	0.273585	0.256636	0.284138
0.314998	0.276623	0.347937	0.269907	0.279181	0.195075	0.272146	0.341382	0.313719	0.300448
0.310361	0.252798	0.32795	0.225456	0.243364	0.225776	0.267029	0.310042	0.282699	0.302686
0.327311	0.357211	0.214902	0.391749	0.361049	0.385513	0.310201	0.316118	0.286217	0.348417
0.307803	0.242885	0.308443	0.275983	0.226575	0.304925	0.278862	0.290214	0.262712	0.324912
0.261113	0.297729	0.299488	0.324912	0.300128	0.323153	0.280301	0.321075	0.291653	0.310361
0.337544	0.282699	0.349536	0.294851	0.292613	0.261433	0.291334	0.339623	0.31308	0.295331
0.293892	0.261113	0.299808	0.280461	0.28174	0.264631	0.242245	0.306044	0.276143	0.279501
0.326191	0.271666	0.372721	0.211385	0.242085	0.20371	0.28158	0.325232	0.306364	0.313559
0.335785	0.303166	0.351935	0.307643	0.305724	0.277422	0.295171	0.34426	0.32827	0.303006
0.320275	0.344739	0.189958	0.388391	0.346019	0.376559	0.299009	0.307483	0.289415	0.329389
0.286856	0.259994	0.276143	0.263831	0.261593	0.271986	0.258075	0.284618	0.262072	0.273905
0.345379	0.28062	0.361689	0.298209	0.277582	0.254557	0.302207	0.344739	0.335305	0.286697
0.301567	0.223377	0.298529	0.240806	0.220659	0.254237	0.238887	0.267029	0.257755	0.285897
0.323473	0.290694	0.181004	0.335305	0.299328	0.32779	0.264311	0.287016	0.260153	0.292293
0.324592	0.29645	0.263671	0.322034	0.289735	0.314679	0.275024	0.31196	0.291334	0.29693
0.318196	0.32827	0.087144	0.366326	0.336585	0.362648	0.284938	0.290374	0.276783	0.301887
0.292933	0.24992	0.35977	0.214583	0.252159	0.237128	0.248961	0.303806	0.292293	0.295651
0.32795	0.257915	0.290214	0.306044	0.270547	0.319476	0.290214	0.298849	0.286377	0.314359
0.325552	0.35913	0.254717	0.398145	0.364247	0.383754	0.318836	0.331308	0.31292	0.349696
0.286217	0.319635	0.316118	0.331148	0.303646	0.324432	0.306684	0.31276	0.316597	0.32891
0.315318	0.319955	0.124081	0.363288	0.323153	0.352894	0.271826	0.288295	0.267988	0.303806
0.336105	0.259834	0.361049	0.276303	0.279181	0.272466	0.292773	0.307163	0.291334	0.288615
0.311161	0.336425	0.270387	0.37432	0.345539	0.366326	0.309242	0.289415	0.28062	0.333706
0.310681	0.230572	0.307004	0.292613	0.267029	0.292133	0.28158	0.29709	0.291334	0.307004
0.321075	0.256636	0.306364	0.270867	0.266709	0.272945	0.28078	0.300128	0.306844	0.292613
0.317397	0.17253	0.342661	0.207067	0.167253	0.263511	0.266869	0.26639	0.258235	0.315478
0.29629	0.212184	0.34426	0.212824	0.210106	0.245923	0.255197	0.279341	0.284938	0.318196
0.295491	0.291653	0.23473	0.327151	0.299169	0.323313	0.26639	0.290854	0.257275	0.271666
0.325232	0.161976	0.349057	0.207227	0.160058	0.251039	0.267189	0.259834	0.269428	0.321714
0.314359	0.162776	0.32795	0.228814	0.184522	0.275664	0.262072	0.260953	0.250879	0.304925
0.317557	0.332267	0.247681	0.369364	0.310681	0.362968	0.298849	0.301247	0.289415	0.319795
0.31292	0.261273	0.286377	0.28174	0.261273	0.26495	0.26575	0.288136	0.283818	0.288136

For Review Only

0.335465	0.220179	0.349057	0.209146	0.224656	0.26607	0.279661	0.293252	0.266549	0.325072
0.282219	0.292773	0.258235	0.317717	0.299169	0.310521	0.269587	0.285098	0.269108	0.29629
0.330988	0.259834	0.349376	0.243524	0.242565	0.252478	0.289735	0.311161	0.294372	0.299808
0.293892	0.330988	0.293412	0.35865	0.340742	0.362488	0.300448	0.311321	0.316917	0.32747
0.335146	0.317237	0.321234	0.341541	0.337064	0.347937	0.31308	0.298849	0.3126	0.325871
0.284778	0.313559	0.303646	0.340102	0.313719	0.318676	0.298849	0.311321	0.300608	0.314199
0.3118	0.272785	0.338503	0.206428	0.257755	0.253278	0.272466	0.309882	0.29757	0.294052
0.314839	0.322674	0.275344	0.318516	0.306044	0.326671	0.279981	0.315318	0.299648	0.279341
0.311641	0.335305	0.263351	0.35897	0.332427	0.358171	0.307163	0.307163	0.292933	0.324752
0.287496	0.225776	0.273265	0.241126	0.233451	0.256956	0.236329	0.26591	0.246083	0.26639
0.317557	0.32875	0.276463	0.34362	0.335625	0.332747	0.289575	0.294851	0.316118	0.338184
0.341062	0.283339	0.375919	0.279821	0.279501	0.23457	0.278862	0.34378	0.323153	0.313879
0.302846	0.18788	0.322833	0.208027	0.183083	0.245923	0.241286	0.264471	0.253918	0.302526
0.285897	0.286057	0.253598	0.317237	0.287176	0.321874	0.253438	0.282859	0.263192	0.284298
0.307803	0.245603	0.330189	0.228494	0.231692	0.242405	0.247362	0.273745	0.280301	0.2811
0.316118	0.269428	0.287656	0.304125	0.284138	0.302367	0.276463	0.313399	0.29757	0.299808
0.311481	0.315638	0.133195	0.354813	0.320595	0.346338	0.273905	0.295651	0.271027	0.299328
0.322993	0.353214	0.306524	0.368564	0.362488	0.350336	0.31292	0.336904	0.325232	0.325712
0.305405	0.256636	0.270067	0.278702	0.255996	0.268948	0.262712	0.28142	0.270707	0.278222
0.326031	0.256956	0.370003	0.179405	0.260153	0.255516	0.286217	0.324272	0.282219	0.325072
0.302047	0.189799	0.301087	0.240806	0.191717	0.254557	0.252478	0.259994	0.251679	0.293892
0.318196	0.248481	0.338983	0.235529	0.255996	0.025104	0.26575	0.309882	0.310841	0.323153
0.285257	0.244004	0.274704	0.251839	0.240966	0.253918	0.244963	0.277263	0.259514	0.264631
0.301087	0.303646	0.220819	0.364727	0.31308	0.35961	0.28062	0.310841	0.26623	0.31324
0.301727	0.324432	0.286856	0.357371	0.329229	0.357691	0.295171	0.324592	0.294212	0.330508
0.322194	0.24992	0.353854	0.205948	0.254397	0.245443	0.262392	0.306524	0.290854	0.333547
0.293092	0.306044	0.319476	0.32827	0.317237	0.309722	0.303006	0.333706	0.305564	0.290534
0.324592	0.29693	0.179725	0.347937	0.31228	0.321394	0.26575	0.291334	0.261433	0.299169
0.292773	0.250879	0.319955	0.21922	0.242085	0.2189	0.263671	0.295491	0.284938	0.307643
0.321874	0.291973	0.285737	0.308762	0.28158	0.303486	0.290534	0.301087	0.291334	0.282219
0.322833	0.263032	0.334506	0.274225	0.271186	0.29677	0.277103	0.306044	0.314519	0.282539
0.32827	0.286697	0.339463	0.290854	0.271826	0.256796	0.28078	0.317717	0.316597	0.273745
0.327151	0.261753	0.317237	0.255516	0.254237	0.291014	0.275024	0.293252	0.2819	0.269268
0.3433	0.291653	0.35945	0.299968	0.287496	0.246722	0.28078	0.341062	0.329069	0.322833
0.321234	0.289575	0.340102	0.286217	0.29629	0.268948	0.29741	0.322993	0.294052	0.279981
0.311001	0.318996	0.133515	0.356732	0.326831	0.348417	0.271666	0.292613	0.279021	0.302367
0.34394	0.291813	0.334506	0.289894	0.293892	0.291813	0.29645	0.322993	0.302846	0.28142
0.321554	0.2496	0.353054	0.204989	0.253598	0.244324	0.262552	0.306684	0.289575	0.332587
0.278382	0.233131	0.348577	0.255197	0.264471	0.277742	0.267509	0.303965	0.289415	0.313719
0.320275	0.289255	0.32795	0.290534	0.304605	0.277103	0.279501	0.303006	0.303326	0.2504
0.297729	0.220979	0.347298	0.213623	0.208986	0.244164	0.253758	0.279181	0.28142	0.319476
0.324592	0.2496	0.344899	0.229133	0.24992	0.268628	0.2811	0.305884	0.289255	0.305085
0.32811	0.251999	0.360249	0.204989	0.228494	0.201791	0.26559	0.290694	0.297729	0.324432
0.317557	0.178446	0.342661	0.213303	0.176367	0.260793	0.275344	0.255676	0.26639	0.310042
0.320595	0.362808	0.208666	0.393828	0.363128	0.384874	0.301727	0.307163	0.287816	0.344739
0.318836	0.268148	0.330029	0.278542	0.252958	0.284938	0.278062	0.300927	0.288455	0.309722
0.317237	0.253278	0.28174	0.274704	0.271027	0.285897	0.276143	0.29741	0.282859	0.29677
0.338024	0.317237	0.32891	0.295171	0.317877	0.303006	0.305245	0.318196	0.3126	0.316118
0.322674	0.351935	0.306204	0.368244	0.360889	0.349217	0.311321	0.334186	0.325552	0.324912
0.32827	0.345219	0.23473	0.381676	0.3433	0.37496	0.31308	0.305245	0.293092	0.331148
0.289095	0.299009	0.175088	0.341382	0.303166	0.324912	0.257275	0.2819	0.256476	0.290534
0.303006	0.297729	0.310521	0.282539	0.29725	0.244803	0.287816	0.297729	0.302047	0.318836
0.330828	0.28078	0.371282	0.245923	0.244324	0.252159	0.285098	0.335465	0.314839	0.305405
0.311001	0.327151	0.212344	0.360569	0.349856	0.365206	0.286217	0.291813	0.284458	0.291334
0.31244	0.333227	0.269907	0.376079	0.333547	0.377359	0.303646	0.318676	0.287016	0.335465

For Review Only

0.307803	0.244164	0.354173	0.174768	0.247362	0.230892	0.255357	0.301247	0.290694	0.324113
0.318196	0.350815	0.227055	0.385673	0.365846	0.385353	0.307163	0.302367	0.288775	0.340902
0.318516	0.289255	0.270227	0.304925	0.285257	0.272626	0.262072	0.29613	0.295331	0.277422
0.274225	0.331308	0.29613	0.329709	0.329549	0.330828	0.282219	0.285577	0.299808	0.302526
0.345059	0.284458	0.370963	0.251359	0.261753	0.217941	0.291493	0.325712	0.315318	0.316597
0.317237	0.332107	0.315478	0.345699	0.329709	0.337864	0.321234	0.335146	0.318836	0.334186
0.285098	0.322993	0.291334	0.330508	0.305564	0.318516	0.291493	0.302526	0.304925	0.310841
0.327311	0.2189	0.350016	0.25024	0.220819	0.261753	0.28094	0.291014	0.291813	0.300128
0.323953	0.327311	0.268948	0.333706	0.307803	0.354973	0.306844	0.324912	0.29725	0.319156
0.302686	0.363447	0.28174	0.387432	0.354173	0.391749	0.331308	0.299808	0.310361	0.35945
0.294691	0.277103	0.21922	0.321714	0.28142	0.315958	0.260473	0.255676	0.258714	0.308602
0.322194	0.361049	0.214583	0.400064	0.365526	0.396386	0.304925	0.307643	0.293252	0.337384
0.289415	0.244484	0.270707	0.252319	0.244484	0.24944	0.239367	0.271666	0.251679	0.26623
0.314039	0.292133	0.306524	0.299648	0.287496	0.322194	0.293732	0.322194	0.278702	0.291973
0.332427	0.290374	0.345539	0.293092	0.290854	0.304765	0.293892	0.340582	0.299808	0.320115
0.304285	0.194276	0.29741	0.239527	0.190278	0.253118	0.2504	0.267829	0.245603	0.292773
0.335465	0.258395	0.357691	0.212504	0.230093	0.220179	0.28206	0.291493	0.303965	0.31212
0.326831	0.306044	0.332907	0.346178	0.313719	0.319635	0.301567	0.335146	0.329069	0.321234
0.298049	0.31196	0.246083	0.351455	0.316597	0.358331	0.283658	0.2811	0.286057	0.314199
0.320755	0.334346	0.262712	0.351775	0.3441	0.330189	0.295491	0.326031	0.273745	0.304445
0.319955	0.340742	0.237288	0.362808	0.330029	0.363447	0.314359	0.29645	0.28174	0.322034
0.257755	0.298049	0.303166	0.313399	0.303486	0.31212	0.293572	0.316278	0.306044	0.299808
0.294691	0.318516	0.308762	0.305245	0.29725	0.341861	0.300927	0.325871	0.307803	0.330988
0.316597	0.179725	0.32891	0.21954	0.167733	0.269587	0.267189	0.259674	0.260473	0.315158
0.333067	0.268948	0.349217	0.275024	0.299648	0.237928	0.287336	0.32843	0.297889	0.282859
0.298529	0.309882	0.310521	0.321714	0.316278	0.32795	0.293412	0.306684	0.300927	0.280301
0.303166	0.350656	0.224336	0.383275	0.34442	0.379277	0.29645	0.289735	0.26495	0.325552
0.325232	0.272146	0.341062	0.277263	0.287176	0.276943	0.286217	0.313399	0.308922	0.288935
0.324272	0.367925	0.217461	0.403902	0.365846	0.397346	0.314679	0.319316	0.292773	0.34426
0.304285	0.240166	0.358011	0.18724	0.238567	0.178286	0.269907	0.299488	0.288775	0.325072
0.315478	0.295171	0.325552	0.310681	0.29677	0.285098	0.278542	0.32811	0.31244	0.269268
0.336904	0.285257	0.366326	0.238248	0.244963	0.224017	0.273425	0.334346	0.316597	0.299328
0.320595	0.351615	0.225296	0.383115	0.348097	0.385353	0.304925	0.308922	0.286217	0.322674
0.324432	0.219699	0.300128	0.254877	0.216821	0.290054	0.278542	0.272146	0.252798	0.316437
0.301887	0.283818	0.253278	0.335945	0.301887	0.330668	0.270067	0.320115	0.291014	0.306524
0.305724	0.198113	0.316437	0.237928	0.204509	0.28206	0.261113	0.260313	0.260313	0.302367
0.302846	0.332267	0.286856	0.357691	0.321394	0.35897	0.289415	0.332107	0.291174	0.320755
0.322514	0.362648	0.208347	0.402462	0.368724	0.397186	0.311161	0.324113	0.29629	0.34378
0.339783	0.254877	0.357691	0.269907	0.263671	0.224017	0.280461	0.310042	0.314519	0.277902
0.322354	0.250879	0.354493	0.206428	0.254877	0.245603	0.264151	0.308443	0.291334	0.334346
0.287336	0.152382	0.275344	0.262872	0.23473	0.269268	0.217621	0.266549	0.189799	0.297729
0.319635	0.367445	0.232012	0.392229	0.369843	0.391749	0.31212	0.300927	0.293732	0.345859
0.321554	0.288136	0.369524	0.280141	0.278702	0.26639	0.292773	0.324752	0.314519	0.297889
0.302526	0.268788	0.272466	0.270547	0.261593	0.284138	0.252638	0.303166	0.273745	0.276783
0.29645	0.306204	0.306044	0.324432	0.309722	0.325232	0.29629	0.310521	0.301887	0.301407
0.346178	0.283499	0.366965	0.29693	0.287176	0.243044	0.300927	0.349376	0.337384	0.291653
0.321075	0.255676	0.333387	0.23409	0.257915	0.271186	0.275024	0.293092	0.285897	0.315158
0.317237	0.294532	0.324752	0.306044	0.314998	0.306204	0.284938	0.314519	0.306684	0.322674
0.307963	0.26607	0.299488	0.283818	0.273265	0.304125	0.251039	0.285577	0.297729	0.319316
0.311001	0.276143	0.324752	0.276303	0.284138	0.294212	0.304765	0.277263	0.300448	0.294851
0.31324	0.279981	0.330189	0.293252	0.29741	0.285577	0.305884	0.299648	0.308283	0.330828
0.321714	0.365206	0.217141	0.403742	0.355293	0.399904	0.307963	0.317557	0.290854	0.337224
0.336105	0.290054	0.386633	0.28206	0.291973	0.231052	0.278542	0.342661	0.320435	0.298689
0.323313	0.367445	0.227055	0.393668	0.367605	0.387272	0.311161	0.304605	0.294691	0.34442
0.327311	0.253758	0.360889	0.205788	0.228814	0.20243	0.266869	0.291174	0.29693	0.322993

For Review Only

0.320115	0.240166	0.380716	0.225136	0.250879	0.235849	0.297889	0.317397	0.300608	0.334826
0.273425	0.329549	0.276943	0.354333	0.320115	0.338343	0.308283	0.317557	0.309082	0.319635
0.326991	0.282699	0.372402	0.295331	0.298529	0.256636	0.293412	0.34426	0.32875	0.29741
0.314039	0.229293	0.368404	0.195875	0.238248	0.240166	0.264151	0.318676	0.298049	0.323473

For Review Only

0.272945	0.301887	0.288775	0.3126	0.2819	0.301087	0.274225	0.334986	0.299968	0.273265
0.286377	0.324272	0.273585	0.28062	0.291334	0.339303	0.290214	0.305564	0.309082	0.285098
0.26559	0.306364	0.225136	0.287816	0.259834	0.293252	0.228174	0.368244	0.3118	0.255996
0.264311	0.31276	0.255037	0.283339	0.270387	0.319316	0.255676	0.32859	0.321075	0.258874
0.303486	0.335146	0.331628	0.324592	0.314359	0.369044	0.307004	0.319156	0.332107	0.301087
0.267189	0.299009	0.241286	0.290694	0.283179	0.291334	0.261273	0.337864	0.295491	0.260473
0.276463	0.307483	0.283978	0.305724	0.301567	0.300288	0.276303	0.361529	0.298209	0.279661
0.299169	0.291973	0.31244	0.306524	0.29757	0.349536	0.294372	0.288295	0.310361	0.285737
0.274225	0.31292	0.288615	0.323473	0.299169	0.299169	0.276303	0.347458	0.285257	0.271506
0.320915	0.29645	0.347138	0.324752	0.332587	0.381036	0.327151	0.294691	0.339303	0.308123
0.297729	0.365366	0.333387	0.339942	0.310042	0.335465	0.293892	0.314679	0.311321	0.294691
0.259354	0.290534	0.217141	0.284138	0.253438	0.290854	0.222258	0.360249	0.303166	0.248481
0.272306	0.310201	0.293732	0.304925	0.289255	0.323633	0.279981	0.299488	0.318356	0.278062
0.276623	0.305245	0.319795	0.29645	0.277582	0.335146	0.272785	0.179725	0.318996	0.269907
0.255676	0.271186	0.257915	0.298209	0.261912	0.254877	0.240966	0.336585	0.26639	0.251519
0.279501	0.336105	0.284298	0.295971	0.277103	0.345859	0.259674	0.300448	0.331308	0.270387
0.292133	0.334506	0.322993	0.325712	0.314519	0.317717	0.298369	0.346978	0.284138	0.292133
0.276463	0.313559	0.295331	0.302047	0.285897	0.310521	0.28078	0.318676	0.320435	0.280301
0.284618	0.294052	0.239847	0.290214	0.280141	0.298849	0.254397	0.373841	0.295331	0.270387
0.314199	0.354493	0.361848	0.340742	0.316757	0.355453	0.318516	0.309402	0.347618	0.306044
0.294851	0.32747	0.332427	0.317397	0.304605	0.333547	0.29725	0.310681	0.308283	0.287496
0.290214	0.330189	0.314519	0.335146	0.314039	0.325072	0.289735	0.3433	0.287656	0.288136
0.3126	0.341701	0.379117	0.34346	0.336745	0.369204	0.332907	0.283818	0.329869	0.319316
0.275823	0.307483	0.314519	0.29613	0.28142	0.342981	0.285897	0.200032	0.334346	0.271666
0.272146	0.31292	0.255996	0.274544	0.279981	0.302207	0.259354	0.35977	0.28078	0.256956
0.254877	0.299328	0.237768	0.293732	0.196514	0.294691	0.226575	0.354493	0.324752	0.238407
0.263671	0.276463	0.211705	0.270067	0.247362	0.318356	0.236169	0.336904	0.310841	0.243364
0.233291	0.28142	0.246722	0.273105	0.235849	0.280141	0.221458	0.277742	0.269268	0.217781
0.271986	0.320435	0.299328	0.3118	0.295491	0.307323	0.284778	0.299009	0.300288	0.268628
0.335785	0.346178	0.398465	0.353214	0.354013	0.394468	0.353214	0.257915	0.382795	0.331468
0.271027	0.295811	0.247042	0.269268	0.269268	0.310201	0.25072	0.356412	0.299328	0.258235
0.289415	0.333866	0.274225	0.31324	0.290374	0.29677	0.271826	0.373681	0.301087	0.287496
0.28078	0.331948	0.299169	0.324592	0.292293	0.304925	0.273585	0.361848	0.314039	0.283179
0.293732	0.321394	0.331468	0.307004	0.291014	0.362168	0.294691	0.161976	0.336425	0.275983
0.294212	0.32875	0.310361	0.332907	0.274065	0.327151	0.28078	0.309402	0.334506	0.28078
0.268468	0.29741	0.259034	0.261273	0.260313	0.342661	0.248961	0.277422	0.32763	0.24928
0.271186	0.304445	0.311321	0.300927	0.28126	0.327311	0.28094	0.271506	0.326031	0.274704
0.246242	0.317077	0.316917	0.311321	0.289415	0.316597	0.276623	0.3118	0.314679	0.28126
0.307483	0.34378	0.365046	0.332907	0.311481	0.397346	0.319156	0.229453	0.349217	0.29693
0.277742	0.331948	0.342021	0.336265	0.29693	0.330029	0.307004	0.308922	0.315478	0.297729
0.331628	0.347138	0.387752	0.345539	0.336105	0.389671	0.340742	0.253918	0.381516	0.323313
0.298209	0.335945	0.333706	0.326351	0.305884	0.342661	0.308762	0.293092	0.347937	0.295331
0.303006	0.302367	0.32779	0.310521	0.319476	0.366326	0.306524	0.277582	0.32843	0.298209
0.294691	0.350815	0.345539	0.340102	0.321554	0.342821	0.303965	0.32875	0.318836	0.300608
0.316278	0.289415	0.342181	0.321394	0.333706	0.382315	0.323953	0.292773	0.318676	0.308283
0.252478	0.29709	0.224656	0.286217	0.232491	0.291014	0.220819	0.35977	0.309882	0.242085
0.268468	0.304445	0.235209	0.293892	0.208347	0.290694	0.225456	0.342501	0.318676	0.240966
0.262232	0.298849	0.295491	0.298049	0.257915	0.311481	0.264791	0.294052	0.311161	0.257595
0.295971	0.347777	0.331468	0.329069	0.329869	0.324432	0.309562	0.329229	0.313719	0.29709
0.26607	0.275823	0.283339	0.302207	0.279021	0.273105	0.263831	0.350975	0.28158	0.26543

For Review Only

0.260473	0.285417	0.231052	0.294532	0.199073	0.301727	0.223217	0.362328	0.290854	0.233451
0.28078	0.291813	0.196994	0.298209	0.295811	0.331788	0.274704	0.327151	0.3126	0.279181
0.268788	0.319476	0.2811	0.304445	0.292933	0.306364	0.272785	0.372402	0.26575	0.274384
0.280301	0.330668	0.299808	0.325072	0.291813	0.304285	0.272945	0.360569	0.31308	0.282859
0.274384	0.292293	0.23377	0.28142	0.26639	0.288935	0.2504	0.354973	0.282699	0.24928
0.289575	0.314839	0.314359	0.316278	0.31244	0.325072	0.292133	0.348257	0.285897	0.290054
0.284138	0.294691	0.240166	0.289735	0.280301	0.298849	0.252958	0.373521	0.29645	0.270067
0.271826	0.313559	0.216661	0.286377	0.216022	0.295811	0.221458	0.357211	0.326511	0.239847
0.250879	0.31292	0.224496	0.285577	0.18772	0.29645	0.221778	0.342181	0.319156	0.238727
0.305724	0.324113	0.367285	0.319156	0.321554	0.339143	0.325392	0.216342	0.346178	0.299009
0.289415	0.31196	0.320915	0.314998	0.290534	0.313399	0.285098	0.258714	0.336585	0.2819
0.233131	0.269747	0.246242	0.274065	0.242405	0.278542	0.226895	0.288775	0.271826	0.224017
0.259834	0.307963	0.196994	0.266709	0.252478	0.31244	0.216821	0.352095	0.31324	0.248001
0.276623	0.308922	0.21874	0.279181	0.262712	0.327311	0.232171	0.348257	0.330349	0.256316
0.267829	0.318996	0.28094	0.302686	0.292293	0.305724	0.272306	0.372082	0.26527	0.273425
0.271666	0.319476	0.298529	0.311001	0.294851	0.306684	0.283818	0.298209	0.299968	0.267988
0.243524	0.28078	0.247681	0.278542	0.253118	0.28142	0.243204	0.307323	0.290054	0.240966
0.28142	0.323153	0.323153	0.310042	0.294372	0.322194	0.29757	0.279661	0.325232	0.287176
0.279021	0.304925	0.277582	0.308602	0.26591	0.300128	0.262552	0.326991	0.290694	0.270707
0.269587	0.29677	0.279661	0.322354	0.269428	0.188679	0.266709	0.37464	0.295011	0.272466
0.28174	0.295491	0.264471	0.303646	0.300448	0.292133	0.273745	0.380556	0.258235	0.269108
0.276303	0.320435	0.287336	0.306684	0.292453	0.301087	0.276303	0.364567	0.283978	0.274544
0.257115	0.295491	0.210425	0.262712	0.248321	0.309882	0.222418	0.317237	0.306684	0.241765
0.290854	0.363128	0.337224	0.330988	0.304925	0.338503	0.307803	0.289894	0.340262	0.284778
0.263671	0.319476	0.299488	0.316437	0.294691	0.295171	0.278382	0.319476	0.284618	0.269108
0.29725	0.331148	0.264311	0.303326	0.288295	0.317397	0.274384	0.326351	0.336265	0.278062
0.316118	0.289575	0.342181	0.321394	0.332907	0.381676	0.323473	0.291493	0.319476	0.307963
0.275024	0.307963	0.134474	0.274544	0.272306	0.337384	0.246882	0.353694	0.307643	0.258075
0.31292	0.330668	0.34442	0.351135	0.319635	0.340422	0.314199	0.335305	0.329389	0.307004
0.318516	0.346818	0.366166	0.329709	0.319635	0.388232	0.324432	0.226735	0.355133	0.302526
0.259194	0.316597	0.251199	0.300288	0.18676	0.292773	0.219859	0.350496	0.336265	0.239367
0.28078	0.329229	0.268628	0.310841	0.29741	0.325552	0.271666	0.360409	0.279821	0.276783
0.282379	0.335146	0.307803	0.3126	0.29613	0.356252	0.288615	0.315958	0.301407	0.288136
0.274225	0.302047	0.283499	0.274225	0.283179	0.32827	0.263511	0.238727	0.306524	0.257435
0.324432	0.351935	0.373041	0.3433	0.32827	0.389351	0.334506	0.214263	0.351935	0.3118
0.267669	0.318996	0.279981	0.302846	0.292933	0.304925	0.272146	0.370963	0.26495	0.273585
0.278222	0.339303	0.302526	0.307323	0.282699	0.292933	0.26527	0.301407	0.345539	0.275823
0.315958	0.289894	0.342021	0.320915	0.333866	0.381996	0.323953	0.291813	0.319955	0.308283
0.302686	0.295491	0.329869	0.315158	0.306844	0.348417	0.307004	0.285897	0.334666	0.298689
0.31212	0.314839	0.348417	0.320435	0.333706	0.352414	0.317717	0.316278	0.318196	0.305245
0.292773	0.313719	0.295971	0.309402	0.295331	0.368884	0.282219	0.329069	0.31212	0.285257
0.305085	0.291653	0.3441	0.317077	0.321554	0.353054	0.31228	0.283179	0.324113	0.298689
0.275344	0.32891	0.286217	0.319476	0.286697	0.305245	0.263831	0.370963	0.264791	0.276303
0.299648	0.326511	0.257435	0.29757	0.304925	0.32763	0.270227	0.389511	0.304605	0.279181
0.284298	0.145507	0.299328	0.310201	0.285098	0.294851	0.278542	0.341861	0.268468	0.282219
0.261113	0.308443	0.198433	0.267349	0.253598	0.31308	0.21874	0.352894	0.313719	0.248801
0.288455	0.334506	0.287976	0.318356	0.293572	0.321394	0.28078	0.348097	0.325232	0.286377
0.261433	0.305245	0.240326	0.279341	0.256316	0.272466	0.247681	0.35881	0.293412	0.255836
0.295491	0.320595	0.345379	0.314679	0.300288	0.367125	0.302047	0.098017	0.342341	0.287176
0.254397	0.289575	0.251999	0.288295	0.226415	0.295331	0.241126	0.301087	0.336745	0.240166
0.264631	0.289415	0.268788	0.282699	0.222098	0.298689	0.233291	0.307803	0.335785	0.248801
0.294212	0.325552	0.291653	0.31196	0.31196	0.300288	0.294851	0.346019	0.322194	0.287016
0.261113	0.284138	0.216981	0.260313	0.258075	0.310042	0.240646	0.32779	0.289894	0.246722
0.31244	0.355133	0.376399	0.346818	0.319795	0.404061	0.326831	0.23505	0.361529	0.305405
0.309402	0.316757	0.347937	0.319156	0.323633	0.362168	0.324272	0.248481	0.344899	0.302846

For Review Only

0.277422	0.31228	0.290534	0.293412	0.279501	0.306204	0.268628	0.285737	0.303965	0.260793
0.286057	0.285098	0.276623	0.307483	0.305245	0.2496	0.284618	0.345059	0.300927	0.282539
0.262232	0.314519	0.277263	0.304285	0.225935	0.31308	0.236009	0.317077	0.309722	0.249121
0.285737	0.336105	0.308762	0.309242	0.298849	0.342981	0.294691	0.314039	0.32859	0.290694
0.257275	0.291653	0.236489	0.26559	0.224496	0.305405	0.23393	0.329229	0.299488	0.240966
0.284938	0.322833	0.346818	0.313399	0.294691	0.348417	0.290534	0.067157	0.337704	0.28094
0.263192	0.299808	0.204509	0.268468	0.244324	0.316597	0.239687	0.355453	0.31196	0.246882
0.260953	0.303965	0.243364	0.289415	0.220979	0.292293	0.219859	0.326351	0.315958	0.240166
0.268468	0.295171	0.238567	0.283658	0.270547	0.306044	0.244643	0.34426	0.275024	0.255197
0.258874	0.307963	0.254717	0.264791	0.263032	0.318356	0.245443	0.274384	0.310042	0.246562
0.282379	0.284938	0.3126	0.298529	0.266869	0.335945	0.277742	0.284778	0.352414	0.273585
0.275024	0.31324	0.280461	0.299488	0.25024	0.317397	0.254397	0.277902	0.326511	0.256636
0.274065	0.319156	0.304925	0.318996	0.290214	0.317077	0.284938	0.283658	0.31196	0.284138
0	0.291334	0.263671	0.277263	0.243524	0.278062	0.23425	0.290694	0.276783	0.232971
0.291334	0	0.29709	0.307643	0.295491	0.276783	0.279021	0.326351	0.262552	0.28142
0.263671	0.29709	0	0.26495	0.255516	0.310361	0.23457	0.349856	0.284138	0.248321
0.277263	0.307643	0.26495	0	0.289255	0.323313	0.270707	0.3126	0.325552	0.268148
0.243524	0.295491	0.255516	0.289255	0	0.291973	0.217781	0.29757	0.295331	0.227854
0.278062	0.276783	0.310361	0.323313	0.291973	0	0.291334	0.360729	0.298689	0.288775
0.23425	0.279021	0.23457	0.270707	0.217781	0.291334	0	0.29709	0.284778	0.220339
0.290694	0.326351	0.349856	0.3126	0.29757	0.360729	0.29709	0	0.349856	0.283658
0.276783	0.262552	0.284138	0.325552	0.295331	0.298689	0.284778	0.349856	0	0.280141
0.232971	0.28142	0.248321	0.268148	0.227854	0.288775	0.220339	0.283658	0.280141	0
0.271346	0.311641	0.271346	0.294851	0.282219	0.291493	0.268468	0.352414	0.277902	0.271506
0.270067	0.285577	0.229613	0.278862	0.26591	0.299648	0.227854	0.330508	0.295011	0.255676
0.316917	0.355932	0.376239	0.336265	0.322514	0.391429	0.330668	0.216022	0.365206	0.303486
0.264151	0.333067	0.288295	0.306684	0.242245	0.317077	0.252319	0.313559	0.331148	0.252638
0.267988	0.332747	0.313879	0.316917	0.289095	0.310361	0.288615	0.301247	0.29757	0.278702
0.28062	0.307323	0.283339	0.310201	0.274065	0.298689	0.268468	0.35929	0.286057	0.277582
0.26543	0.288935	0.26543	0.283978	0.267988	0.314199	0.263511	0.29709	0.288615	0.257595
0.28158	0.276463	0.23425	0.29677	0.26559	0.295971	0.244324	0.37464	0.303166	0.259514
0.284618	0.320595	0.307803	0.31212	0.299488	0.272146	0.276783	0.352734	0.307323	0.28126
0.313559	0.347937	0.365686	0.330349	0.322034	0.379117	0.326831	0.188359	0.355612	0.302686
0.243364	0.280141	0.248001	0.282539	0.252638	0.295971	0.242885	0.276303	0.283978	0.228494
0.275664	0.330508	0.287496	0.319635	0.286856	0.305724	0.261433	0.373041	0.264631	0.277902
0.238407	0.298369	0.25008	0.284618	0.207547	0.289735	0.220179	0.305405	0.291973	0.230892
0.292613	0.322833	0.329709	0.306524	0.289575	0.361369	0.294532	0.164535	0.337064	0.274544
0.285098	0.306844	0.31308	0.319156	0.285417	0.32795	0.288295	0.258555	0.324272	0.275664
0.29709	0.322514	0.355453	0.317397	0.306364	0.366326	0.309242	0.088903	0.348417	0.294532
0.255676	0.290054	0.210585	0.28174	0.251679	0.285897	0.235689	0.35865	0.271346	0.254077
0.273105	0.341541	0.295331	0.3126	0.261113	0.303806	0.272945	0.294851	0.297729	0.275344
0.320915	0.362328	0.3756	0.358011	0.32795	0.380397	0.32859	0.261912	0.336745	0.308922
0.28158	0.333387	0.319795	0.325712	0.302207	0.301567	0.292773	0.322034	0.315958	0.286057
0.297889	0.324113	0.351615	0.310681	0.307163	0.365686	0.303646	0.114167	0.345219	0.293572
0.285737	0.292933	0.259354	0.31196	0.276463	0.272626	0.260633	0.35945	0.317557	0.269907
0.309722	0.335785	0.358011	0.330189	0.318996	0.358011	0.32763	0.261433	0.350496	0.311481
0.263991	0.273745	0.283339	0.306524	0.255516	0.292933	0.256796	0.31228	0.306364	0.257275
0.270707	0.298369	0.267988	0.293572	0.271186	0.305884	0.256476	0.31292	0.302846	0.261912
0.252159	0.308602	0.23377	0.29693	0.167893	0.295011	0.201151	0.348897	0.318996	0.226095
0.263991	0.313719	0.23377	0.289575	0.210265	0.29613	0.233131	0.351455	0.319635	0.23393
0.272466	0.316917	0.309562	0.289735	0.286537	0.330189	0.284778	0.227854	0.340742	0.271027
0.256956	0.307803	0.229133	0.292293	0.197634	0.306044	0.215222	0.357371	0.310201	0.236169
0.256636	0.315318	0.252319	0.290054	0.190118	0.291014	0.225616	0.334346	0.338184	0.236489
0.292293	0.348897	0.355453	0.337224	0.294532	0.324752	0.304285	0.252638	0.332587	0.288136
0.261273	0.314039	0.279501	0.287336	0.26511	0.294052	0.257915	0.285737	0.308443	0.254557

For Review Only

0.272945	0.306844	0.231692	0.293572	0.241605	0.320435	0.230892	0.352894	0.332907	0.252478
0.269587	0.301887	0.309402	0.294212	0.284138	0.310201	0.279661	0.257115	0.294851	0.267029
0.267829	0.294851	0.264471	0.311641	0.228174	0.261113	0.246083	0.356092	0.307163	0.24992
0.292293	0.23473	0.341861	0.329709	0.304125	0.339463	0.314998	0.299968	0.275823	0.29741
0.317397	0.32779	0.344739	0.32763	0.319316	0.365526	0.303166	0.325871	0.350496	0.301727
0.286537	0.335625	0.32875	0.318836	0.299648	0.310681	0.300288	0.304765	0.329869	0.290054
0.270867	0.316757	0.238407	0.253278	0.264471	0.31292	0.246402	0.344739	0.316118	0.258714
0.274225	0.332267	0.325392	0.31324	0.283818	0.309402	0.284778	0.267988	0.311321	0.274704
0.291493	0.32875	0.341222	0.329709	0.304445	0.348097	0.303326	0.272785	0.332427	0.292773
0.237928	0.283339	0.245123	0.272626	0.221778	0.288935	0.21874	0.274225	0.288455	0.216661
0.306044	0.339623	0.34426	0.331468	0.308123	0.366645	0.308762	0.276463	0.322833	0.299488
0.28062	0.332427	0.271826	0.301727	0.291493	0.309242	0.276943	0.383435	0.300448	0.273425
0.246882	0.301407	0.226575	0.283978	0.190438	0.300448	0.211225	0.333067	0.305085	0.226095
0.262712	0.322674	0.305884	0.29709	0.274544	0.318196	0.279341	0.245443	0.326351	0.263032
0.254877	0.285098	0.243044	0.285577	0.249121	0.301727	0.23393	0.334666	0.309402	0.242725
0.282699	0.304925	0.294851	0.301887	0.284298	0.308123	0.270867	0.287976	0.305564	0.276783
0.297729	0.32827	0.342661	0.310841	0.301247	0.364567	0.301247	0.118324	0.349057	0.285577
0.303486	0.369683	0.350016	0.347298	0.321714	0.349856	0.306684	0.31228	0.321554	0.304125
0.258555	0.299968	0.283658	0.288295	0.260313	0.29629	0.25072	0.271666	0.303965	0.241445
0.285897	0.318676	0.196514	0.274864	0.271346	0.31276	0.257115	0.371922	0.314359	0.261593
0.254077	0.286217	0.237288	0.276303	0.222578	0.285897	0.228973	0.309562	0.289894	0.240646
0.262552	0.287816	0.236329	0.280141	0.26639	0.300768	0.241765	0.338823	0.270707	0.251679
0.229933	0.274704	0.252798	0.269108	0.233611	0.2819	0.224976	0.28062	0.274544	0.221938
0.294212	0.32763	0.340742	0.309882	0.299648	0.366805	0.307163	0.220179	0.339623	0.283818
0.289415	0.362968	0.342501	0.335146	0.307483	0.339623	0.315158	0.291334	0.34362	0.290534
0.276303	0.308443	0.21906	0.279981	0.262872	0.32779	0.232811	0.349376	0.329549	0.256796
0.290054	0.326511	0.3126	0.310521	0.306204	0.331948	0.293092	0.320595	0.307163	0.291334
0.290534	0.325552	0.32891	0.306524	0.294052	0.364727	0.294851	0.161337	0.325232	0.277422
0.261753	0.266869	0.232331	0.287496	0.263192	0.293412	0.246402	0.322993	0.301567	0.247202
0.273585	0.288455	0.304445	0.302686	0.283658	0.17205	0.279501	0.288295	0.31212	0.276783
0.267988	0.270707	0.279821	0.306044	0.275504	0.246242	0.270227	0.333227	0.303486	0.26607
0.271186	0.292293	0.278382	0.316437	0.282539	0.278062	0.26639	0.351775	0.283179	0.263192
0.286697	0.306524	0.258395	0.300608	0.273745	0.31196	0.258714	0.32811	0.322833	0.271186
0.282539	0.304445	0.289095	0.325232	0.290374	0.232811	0.266709	0.369364	0.298369	0.28158
0.276943	0.306684	0.284778	0.318836	0.274384	0.305245	0.270227	0.337704	0.307163	0.273265
0.299648	0.327151	0.341541	0.3126	0.302526	0.365686	0.303965	0.12456	0.347458	0.285897
0.282219	0.336585	0.303965	0.310841	0.283499	0.295331	0.278382	0.334026	0.32811	0.277742
0.275823	0.308123	0.21858	0.279341	0.262232	0.326191	0.232171	0.347937	0.329549	0.255996
0.26623	0.330668	0.25008	0.285257	0.261273	0.333067	0.24976	0.348257	0.309722	0.260473
0.271506	0.316597	0.292453	0.286217	0.2819	0.3118	0.280141	0.325712	0.303646	0.273745
0.267829	0.308922	0.233131	0.289255	0.217461	0.298529	0.239687	0.353854	0.319156	0.235209
0.276303	0.275504	0.244643	0.275983	0.268468	0.305405	0.253118	0.346978	0.302207	0.254397
0.26495	0.303646	0.225296	0.287816	0.259994	0.292293	0.227694	0.367125	0.31228	0.255037
0.255676	0.316597	0.235849	0.301567	0.157979	0.295971	0.207067	0.350016	0.326991	0.228814
0.315798	0.358491	0.372562	0.337704	0.320595	0.385034	0.323313	0.212504	0.357371	0.306844
0.26639	0.330189	0.276623	0.314039	0.251359	0.299009	0.255197	0.335305	0.29741	0.258555
0.286856	0.338024	0.288455	0.305884	0.287976	0.34362	0.279021	0.279821	0.332907	0.280301
0.292133	0.299328	0.295811	0.304445	0.298209	0.303965	0.280301	0.321714	0.317397	0.275983
0.301887	0.369843	0.348577	0.346498	0.320595	0.349057	0.305564	0.3126	0.321075	0.303486
0.311001	0.342181	0.365206	0.32747	0.319476	0.377838	0.316757	0.244803	0.358331	0.304285
0.28174	0.304925	0.327151	0.303166	0.287176	0.353694	0.295331	0.160857	0.330828	0.274384
0.276303	0.277742	0.291334	0.292293	0.290214	0.299328	0.271346	0.299169	0.295491	0.266869
0.28206	0.305564	0.256476	0.309402	0.271346	0.307963	0.260953	0.377199	0.300288	0.26559
0.295171	0.319156	0.356892	0.314998	0.314839	0.324432	0.317237	0.210265	0.32779	0.295651
0.295171	0.365686	0.351135	0.333866	0.310361	0.355612	0.316757	0.271826	0.335945	0.289415

For Review Only

0.260153	0.307643	0.197474	0.26623	0.252319	0.31244	0.217141	0.352574	0.31244	0.247522
0.310841	0.350975	0.372562	0.341382	0.322034	0.401343	0.324912	0.232491	0.354173	0.308762
0.275504	0.32891	0.31324	0.305405	0.280461	0.31196	0.277742	0.264311	0.332267	0.272945
0.297889	0.329229	0.326351	0.322514	0.289575	0.310361	0.301087	0.301887	0.340902	0.293252
0.28206	0.301247	0.258555	0.31196	0.269108	0.291653	0.247522	0.377518	0.299169	0.268628
0.294212	0.303806	0.331948	0.335146	0.309242	0.325871	0.310521	0.321874	0.242725	0.295491
0.285098	0.302686	0.327151	0.321394	0.28158	0.290694	0.286537	0.286856	0.300128	0.283978
0.263192	0.272466	0.264471	0.288935	0.236329	0.259514	0.239047	0.35881	0.311001	0.244803
0.29693	0.354813	0.348417	0.347777	0.300288	0.333227	0.309242	0.28174	0.331788	0.290854
0.324432	0.318516	0.379117	0.3433	0.339623	0.386153	0.348897	0.272945	0.362808	0.323153
0.284778	0.304125	0.310841	0.300128	0.275983	0.350336	0.279981	0.214902	0.337544	0.271186
0.324113	0.351615	0.376399	0.337704	0.329709	0.388391	0.333706	0.222578	0.365526	0.308602
0.23489	0.270707	0.25024	0.270547	0.238248	0.28126	0.225935	0.273745	0.278382	0.217781
0.289894	0.301727	0.306524	0.324752	0.28142	0.2811	0.284298	0.314839	0.314199	0.277902
0.290694	0.323953	0.295651	0.308922	0.284618	0.350496	0.274065	0.340262	0.315958	0.277742
0.254397	0.310521	0.229933	0.264791	0.217141	0.298849	0.223537	0.304605	0.302526	0.23425
0.274544	0.293572	0.237288	0.295971	0.257595	0.294372	0.241126	0.364567	0.316757	0.257915
0.293092	0.366006	0.322674	0.31244	0.302367	0.350496	0.285577	0.331148	0.342981	0.290374
0.29741	0.339463	0.342021	0.326511	0.300128	0.386153	0.295331	0.255357	0.35865	0.285098
0.297729	0.344739	0.320115	0.319156	0.309722	0.330349	0.308443	0.269268	0.31212	0.295171
0.291334	0.339783	0.348577	0.331788	0.29645	0.345219	0.308283	0.253278	0.332747	0.295651
0.241286	0.322194	0.32827	0.324752	0.298369	0.304925	0.286057	0.31244	0.306844	0.288775
0.303806	0.336585	0.295171	0.320915	0.289894	0.311481	0.298369	0.314519	0.346338	0.287496
0.251519	0.293732	0.241445	0.290534	0.183403	0.279341	0.209306	0.337224	0.316278	0.232171
0.274384	0.303326	0.274544	0.288935	0.279341	0.308922	0.268948	0.353534	0.268948	0.266709
0.275184	0.314039	0.310681	0.329229	0.284938	0.291174	0.286377	0.315798	0.298049	0.277263
0.305724	0.324752	0.367445	0.318196	0.321554	0.339463	0.325072	0.215062	0.346658	0.299169
0.289415	0.310521	0.266709	0.29693	0.300288	0.323473	0.279181	0.342661	0.308283	0.275823
0.324272	0.35977	0.377199	0.341382	0.333706	0.392869	0.340102	0.21842	0.364886	0.317557
0.261593	0.276943	0.211225	0.278222	0.256156	0.309082	0.23425	0.360729	0.29661	0.246562
0.269268	0.319795	0.299009	0.310042	0.282539	0.3126	0.274704	0.324912	0.277582	0.273425
0.285098	0.291014	0.240006	0.303326	0.277422	0.29677	0.255197	0.371122	0.303166	0.261593
0.302367	0.3433	0.370163	0.335305	0.314519	0.364247	0.317877	0.238727	0.346818	0.301567
0.261273	0.309722	0.271986	0.308443	0.225136	0.329389	0.237288	0.309082	0.326671	0.245603
0.286217	0.304765	0.316278	0.306044	0.29725	0.308602	0.284298	0.244324	0.303326	0.286057
0.256636	0.290854	0.251039	0.273425	0.209946	0.290854	0.226095	0.326991	0.322833	0.232811
0.283019	0.356092	0.339942	0.333547	0.306684	0.332427	0.304125	0.289095	0.333067	0.28206
0.325232	0.356572	0.376719	0.342501	0.32875	0.405341	0.337064	0.213943	0.360569	0.315478
0.272785	0.320275	0.259514	0.298689	0.269907	0.290534	0.259354	0.360409	0.298849	0.26511
0.277103	0.309242	0.219699	0.279821	0.263351	0.32795	0.232971	0.349696	0.330508	0.257275
0.267509	0.29677	0.257595	0.259514	0.259674	0.341701	0.248161	0.276623	0.327151	0.248161
0.310841	0.351775	0.3748	0.347777	0.318516	0.404861	0.329869	0.23409	0.35881	0.306844
0.280141	0.302526	0.285577	0.316437	0.283019	0.267669	0.275823	0.369204	0.292133	0.276783
0.269268	0.320435	0.274065	0.266869	0.26511	0.321234	0.261433	0.270067	0.321874	0.252638
0.272466	0.314039	0.314998	0.325392	0.288136	0.318196	0.285257	0.315958	0.319316	0.279021
0.268628	0.322194	0.277582	0.311481	0.290054	0.306524	0.263671	0.378318	0.243684	0.269587
0.278862	0.323473	0.249121	0.279981	0.262072	0.325232	0.243844	0.337704	0.319316	0.261113
0.282859	0.297889	0.303326	0.297729	0.304925	0.337704	0.28174	0.323793	0.318356	0.285098
0.275344	0.32747	0.303326	0.309562	0.255676	0.319316	0.270867	0.307803	0.337864	0.26575
0.291653	0.232651	0.278222	0.305724	0.291334	0.303646	0.28142	0.332107	0.313719	0.274225
0.303006	0.313399	0.279181	0.295331	0.284938	0.352255	0.278542	0.334666	0.314359	0.2811
0.322034	0.355772	0.373521	0.342341	0.32875	0.391429	0.335146	0.215862	0.363288	0.314519
0.282699	0.316118	0.271027	0.303806	0.295811	0.294372	0.275184	0.388551	0.288455	0.275664
0.314359	0.345059	0.374001	0.34362	0.323793	0.403102	0.330668	0.229773	0.356572	0.307963
0.26543	0.306364	0.226415	0.287816	0.259674	0.293412	0.228654	0.368244	0.311641	0.255676

0.280461	0.267029	0.233131	0.294052	0.274065	0.29629	0.247202	0.39047	0.299968	0.257915
0.28142	0.345859	0.341701	0.341382	0.294851	0.340582	0.307963	0.287816	0.329069	0.293092
0.276943	0.340422	0.277103	0.302367	0.29629	0.317717	0.270387	0.372562	0.293732	0.276943
0.270547	0.308283	0.200672	0.240166	0.260793	0.313719	0.24928	0.368724	0.315798	0.256956

For Review Only

0.279981	0.284938	0.387912	0.32779	0.331788	0.276943	0.253758	0.292293	0.29709	0.364087
0.330508	0.280141	0.316118	0.314039	0.333067	0.345379	0.302686	0.319795	0.333227	0.314039
0.276463	0.170131	0.405181	0.288935	0.342981	0.293732	0.295651	0.223697	0.304605	0.395587
0.269587	0.252958	0.367605	0.289894	0.321075	0.307004	0.283978	0.222897	0.293892	0.35945
0.295331	0.347298	0.344579	0.346178	0.333547	0.31292	0.300768	0.347298	0.348097	0.330988
0.269747	0.2504	0.386633	0.31212	0.330988	0.285737	0.275823	0.251039	0.276463	0.37432
0.267988	0.303166	0.389351	0.32779	0.316118	0.280461	0.278702	0.303166	0.306524	0.389351
0.329229	0.319476	0.282699	0.331948	0.318356	0.326351	0.286377	0.346178	0.34442	0.291653
0.26591	0.299808	0.370643	0.322514	0.29645	0.267988	0.246562	0.303486	0.293732	0.361848
0.372562	0.348417	0.294372	0.350975	0.32827	0.353694	0.302047	0.374161	0.354653	0.287816
0.315318	0.353694	0.28174	0.315158	0.269907	0.336105	0.337224	0.363447	0.336904	0.298049
0.264151	0.192996	0.401343	0.286697	0.339942	0.287816	0.292933	0.195395	0.290854	0.388232
0.293892	0.295971	0.327311	0.332907	0.321714	0.293572	0.287336	0.318516	0.31292	0.320115
0.320755	0.319476	0.267829	0.31276	0.303806	0.333227	0.277103	0.335785	0.329549	0.245283
0.25008	0.26591	0.363927	0.302846	0.290534	0.254237	0.260953	0.26607	0.274065	0.352894
0.259674	0.276463	0.325552	0.299488	0.316917	0.291813	0.29613	0.299808	0.318356	0.31228
0.273265	0.310841	0.379437	0.349376	0.337704	0.294851	0.277263	0.314519	0.307323	0.378318
0.298689	0.293092	0.356892	0.329869	0.310841	0.308922	0.29741	0.301567	0.298209	0.347937
0.255516	0.244164	0.414455	0.317877	0.348097	0.285737	0.275504	0.240326	0.26607	0.402143
0.369044	0.358331	0.313399	0.32875	0.330189	0.355932	0.333067	0.371122	0.333547	0.316917
0.322034	0.316597	0.318196	0.324752	0.313879	0.326831	0.305245	0.338184	0.335465	0.321075
0.28126	0.299488	0.367925	0.348097	0.3433	0.294212	0.298049	0.290214	0.3126	0.365046
0.370803	0.365366	0.210106	0.314519	0.228174	0.364567	0.347937	0.407579	0.378478	0.235529
0.321394	0.31212	0.220659	0.307323	0.273105	0.320915	0.26623	0.354013	0.34394	0.223217
0.232811	0.260953	0.397346	0.324113	0.316118	0.262232	0.260793	0.263991	0.275664	0.386952
0.29677	0.267829	0.384714	0.209786	0.330029	0.29645	0.304125	0.267988	0.310201	0.382315
0.273265	0.222897	0.367445	0.275024	0.305245	0.287496	0.255357	0.222897	0.299488	0.362968
0.264791	0.252638	0.301887	0.26591	0.279181	0.275823	0.242565	0.258714	0.275664	0.300128
0.291014	0.29693	0.309562	0.305245	0.299488	0.284618	0.261593	0.310681	0.294691	0.309882
0.407419	0.394148	0.231372	0.354173	0.330508	0.399264	0.358491	0.428686	0.4063	0.243204
0.229773	0.227215	0.394787	0.29661	0.330508	0.275504	0.262552	0.232171	0.279981	0.389351
0.259994	0.262232	0.403902	0.323953	0.333547	0.276303	0.274225	0.258395	0.288615	0.403262
0.248001	0.300768	0.401023	0.332747	0.329549	0.279181	0.267189	0.295811	0.29757	0.394308
0.336585	0.31308	0.227534	0.315638	0.284938	0.330189	0.268948	0.354653	0.347777	0.211705
0.329869	0.3126	0.317877	0.287656	0.285577	0.334346	0.319795	0.337384	0.335146	0.310521
0.290694	0.253758	0.309882	0.287016	0.298689	0.285897	0.246562	0.285257	0.308283	0.291653
0.303326	0.291813	0.334986	0.300927	0.276623	0.286057	0.199712	0.327151	0.313559	0.309242
0.293412	0.303646	0.334506	0.304925	0.286856	0.302367	0.289735	0.318036	0.309242	0.332267
0.391429	0.364727	0.144228	0.307004	0.258555	0.375919	0.32827	0.413655	0.389351	0.166294
0.331468	0.351775	0.2811	0.28206	0.225616	0.335785	0.325871	0.371602	0.344899	0.28142
0.405341	0.378318	0.236329	0.341382	0.324113	0.393828	0.355453	0.411417	0.39047	0.243364
0.345859	0.330988	0.293892	0.319635	0.302207	0.329389	0.286377	0.353694	0.336904	0.290054
0.355932	0.323793	0.297889	0.342181	0.317877	0.353694	0.29693	0.366645	0.342341	0.293892
0.320755	0.334986	0.358491	0.344899	0.332427	0.331148	0.318516	0.350656	0.325712	0.346338
0.376239	0.344739	0.315158	0.352574	0.32827	0.371442	0.322194	0.380237	0.353374	0.307963
0.270707	0.211545	0.397346	0.26527	0.329069	0.28174	0.292293	0.228494	0.293892	0.391429
0.295811	0.235369	0.400544	0.259834	0.32779	0.291174	0.303486	0.240166	0.304285	0.386792
0.301887	0.302207	0.302047	0.276783	0.29677	0.325871	0.311481	0.300128	0.323633	0.311001
0.320435	0.344579	0.34442	0.346498	0.350496	0.318516	0.31308	0.340102	0.316437	0.333706
0.248001	0.26639	0.394627	0.311641	0.304605	0.262392	0.247042	0.260633	0.259834	0.383754

For Review Only

0.279021	0.25008	0.391589	0.248481	0.332747	0.303326	0.294532	0.239527	0.305085	0.384874
0.289415	0.276303	0.347937	0.314039	0.330189	0.292453	0.277103	0.275823	0.323793	0.335625
0.225456	0.289575	0.418452	0.340582	0.330189	0.247202	0.262552	0.271666	0.269268	0.411737
0.247681	0.300608	0.400384	0.332747	0.329389	0.278062	0.267349	0.295971	0.29757	0.393668
0.26591	0.247522	0.392069	0.301567	0.32843	0.283019	0.284138	0.247362	0.28126	0.380556
0.269108	0.31212	0.370003	0.341861	0.32763	0.28126	0.280141	0.304285	0.297729	0.362648
0.255996	0.244164	0.414295	0.318196	0.347138	0.285417	0.275504	0.240166	0.26575	0.401503
0.287176	0.247042	0.396386	0.257275	0.330508	0.29661	0.301727	0.230572	0.294851	0.389671
0.287976	0.263831	0.387112	0.215222	0.314359	0.288935	0.294052	0.23393	0.292293	0.381676
0.383754	0.363927	0.191877	0.325072	0.299648	0.374001	0.324752	0.402942	0.373521	0.199712
0.307323	0.299808	0.294052	0.306684	0.287016	0.309562	0.259674	0.324272	0.331788	0.287496
0.245443	0.245443	0.31196	0.268948	0.277422	0.267669	0.251839	0.248001	0.277422	0.309242
0.270387	0.21826	0.396226	0.285257	0.336105	0.285098	0.292453	0.215382	0.294212	0.388551
0.292293	0.228334	0.391589	0.291973	0.331308	0.29677	0.294212	0.235209	0.302047	0.381356
0.224177	0.289415	0.417333	0.339463	0.329869	0.247202	0.262872	0.271186	0.268308	0.411257
0.291174	0.295971	0.309562	0.304765	0.298849	0.284618	0.260793	0.309882	0.294052	0.309402
0.270867	0.267988	0.338663	0.278222	0.294851	0.277103	0.263192	0.272785	0.28126	0.32827
0.332907	0.311641	0.308922	0.315638	0.285737	0.327151	0.277103	0.357052	0.342181	0.293412
0.302686	0.286537	0.32811	0.290054	0.286856	0.323313	0.309562	0.305245	0.335465	0.333706
0.254557	0.2504	0.42181	0.301407	0.326511	0.26639	0.271506	0.252958	0.247681	0.42165
0.220019	0.279341	0.407739	0.324912	0.323313	0.260633	0.269268	0.238887	0.267029	0.404061
0.251199	0.292613	0.412216	0.326351	0.331948	0.268308	0.258075	0.285098	0.29613	0.398625
0.271346	0.223057	0.356412	0.28078	0.307643	0.279181	0.270707	0.231852	0.290534	0.350496
0.340102	0.340422	0.253438	0.204349	0.193476	0.344579	0.310521	0.374161	0.346818	0.271666
0.277422	0.308602	0.32875	0.295971	0.290694	0.279661	0.263991	0.315318	0.292773	0.331788
0.311481	0.272306	0.35977	0.302367	0.321394	0.283179	0.295331	0.286856	0.309882	0.360889
0.376079	0.34426	0.315158	0.352734	0.32763	0.371122	0.321554	0.380876	0.352574	0.307323
0.284298	0.230413	0.379917	0.29757	0.322514	0.298049	0.2811	0.246562	0.310361	0.373361
0.352095	0.347138	0.351455	0.362968	0.374001	0.337704	0.329549	0.354173	0.333706	0.358171
0.39127	0.367285	0.10953	0.319316	0.291014	0.380876	0.339783	0.412216	0.391749	0.141829
0.311001	0.270067	0.383275	0.216501	0.336105	0.307963	0.300768	0.271027	0.306364	0.39015
0.131116	0.262392	0.398145	0.315158	0.335146	0.271666	0.252478	0.252798	0.284938	0.384234
0.267189	0.307483	0.351295	0.334986	0.310521	0.287336	0.277103	0.318196	0.32827	0.34378
0.292453	0.28206	0.272945	0.298849	0.2819	0.302846	0.247681	0.305564	0.314998	0.256796
0.399105	0.378957	0.128238	0.323473	0.287976	0.39047	0.341062	0.419891	0.395587	0.150464
0.224177	0.289415	0.416693	0.339783	0.329229	0.247042	0.261912	0.270387	0.266709	0.410617
0.287016	0.286537	0.349536	0.311641	0.3126	0.288455	0.260313	0.311481	0.283019	0.342981
0.376399	0.34426	0.314839	0.352095	0.32747	0.371762	0.321075	0.381036	0.352574	0.307963
0.326031	0.327151	0.289575	0.336265	0.323793	0.331308	0.29757	0.341062	0.354493	0.303006
0.323313	0.347458	0.326831	0.353694	0.349217	0.330508	0.331788	0.352894	0.336265	0.321714
0.301407	0.31292	0.330189	0.307323	0.287816	0.331788	0.292453	0.31292	0.353374	0.322034
0.367125	0.339783	0.314199	0.336265	0.317557	0.350975	0.322514	0.371922	0.350336	0.300608
0.223857	0.286537	0.413655	0.325072	0.322194	0.239847	0.261912	0.276143	0.288136	0.412696
0.21826	0.209786	0.42117	0.317717	0.338823	0.291653	0.284298	0.25008	0.302367	0.4055
0.31244	0.288615	0.369843	0.34426	0.34362	0.307163	0.2811	0.286377	0.303806	0.353534
0.271506	0.220339	0.396226	0.285897	0.337704	0.285897	0.293412	0.216501	0.29645	0.387912
0.267509	0.284618	0.369843	0.326031	0.32747	0.289735	0.275024	0.298369	0.315318	0.368084
0.261113	0.240006	0.396067	0.285417	0.308762	0.258075	0.272306	0.236489	0.272626	0.39063
0.353054	0.333547	0.209786	0.319316	0.293092	0.347777	0.276943	0.378957	0.360889	0.172849
0.301727	0.261912	0.349536	0.237928	0.297889	0.306044	0.269747	0.262072	0.304125	0.342981
0.299968	0.26495	0.36009	0.261593	0.311161	0.282699	0.278862	0.260633	0.299009	0.348897
0.304605	0.314998	0.369843	0.278542	0.275823	0.305724	0.299169	0.320595	0.294052	0.362168
0.271027	0.225616	0.364407	0.284458	0.310361	0.277422	0.256156	0.243364	0.286377	0.355772
0.397026	0.371282	0.142309	0.319156	0.273905	0.383914	0.341222	0.415414	0.395427	0.173649
0.376239	0.352894	0.238088	0.32779	0.314839	0.364567	0.314998	0.388711	0.372562	0.238567

For Review Only

0.290054	0.292933	0.2819	0.291334	0.288295	0.287176	0.255357	0.303806	0.298529	0.274544
0.307643	0.294691	0.381036	0.330988	0.322674	0.298529	0.297889	0.302367	0.29677	0.369044
0.313719	0.285737	0.321394	0.21842	0.279661	0.324113	0.310042	0.297729	0.304765	0.32763
0.314998	0.310361	0.327311	0.307803	0.261113	0.337544	0.293892	0.326191	0.335785	0.320275
0.276623	0.252798	0.357211	0.242885	0.297729	0.282699	0.261113	0.271666	0.303166	0.344739
0.347937	0.32795	0.214902	0.308443	0.299488	0.349536	0.299808	0.372721	0.351935	0.189958
0.269907	0.225456	0.391749	0.275983	0.324912	0.294851	0.280461	0.211385	0.307643	0.388391
0.279181	0.243364	0.361049	0.226575	0.300128	0.292613	0.28174	0.242085	0.305724	0.346019
0.195075	0.225776	0.385513	0.304925	0.323153	0.261433	0.264631	0.20371	0.277422	0.376559
0.272146	0.267029	0.310201	0.278862	0.280301	0.291334	0.242245	0.28158	0.295171	0.299009
0.341382	0.310042	0.316118	0.290214	0.321075	0.339623	0.306044	0.325232	0.34426	0.307483
0.313719	0.282699	0.286217	0.262712	0.291653	0.31308	0.276143	0.306364	0.32827	0.289415
0.300448	0.302686	0.348417	0.324912	0.310361	0.295331	0.279501	0.313559	0.303006	0.329389
0.271346	0.270067	0.316917	0.264151	0.267988	0.28062	0.26543	0.28158	0.284618	0.313559
0.311641	0.285577	0.355932	0.333067	0.332747	0.307323	0.288935	0.276463	0.320595	0.347937
0.271346	0.229613	0.376239	0.288295	0.313879	0.283339	0.26543	0.23425	0.307803	0.365686
0.294851	0.278862	0.336265	0.306684	0.316917	0.310201	0.283978	0.29677	0.31212	0.330349
0.282219	0.26591	0.322514	0.242245	0.289095	0.274065	0.267988	0.26559	0.299488	0.322034
0.291493	0.299648	0.391429	0.317077	0.310361	0.298689	0.314199	0.295971	0.272146	0.379117
0.268468	0.227854	0.330668	0.252319	0.288615	0.268468	0.263511	0.244324	0.276783	0.326831
0.352414	0.330508	0.216022	0.313559	0.301247	0.35929	0.29709	0.37464	0.352734	0.188359
0.277902	0.295011	0.365206	0.331148	0.29757	0.286057	0.288615	0.303166	0.307323	0.355612
0.271506	0.255676	0.303486	0.252638	0.278702	0.277582	0.257595	0.259514	0.28126	0.302686
0	0.271346	0.392709	0.317397	0.316278	0.274225	0.240486	0.238248	0.261593	0.381836
0.271346	0	0.370323	0.297889	0.311481	0.297729	0.258235	0.226735	0.293252	0.360729
0.392709	0.370323	0	0.318356	0.284458	0.39015	0.333227	0.416693	0.392709	0.161337
0.317397	0.297889	0.318356	0	0.248321	0.320275	0.301887	0.308602	0.322514	0.331308
0.316278	0.311481	0.284458	0.248321	0	0.301247	0.278222	0.349376	0.32875	0.288136
0.274225	0.297729	0.39015	0.320275	0.301247	0	0.25008	0.287496	0.290054	0.380876
0.240486	0.258235	0.333227	0.301887	0.278222	0.25008	0	0.286697	0.269108	0.320755
0.238248	0.226735	0.416693	0.308602	0.349376	0.287496	0.286697	0	0.301727	0.402622
0.261593	0.293252	0.392709	0.322514	0.32875	0.290054	0.269108	0.301727	0	0.389351
0.381836	0.360729	0.161337	0.331308	0.288136	0.380876	0.320755	0.402622	0.389351	0
0.283019	0.263991	0.300288	0.272466	0.271027	0.284138	0.256476	0.275024	0.287176	0.292453
0.221938	0.288455	0.417973	0.326831	0.321234	0.240326	0.263991	0.276943	0.287016	0.416054
0.26543	0.257115	0.335945	0.241126	0.276303	0.269428	0.255836	0.268308	0.278862	0.337864
0.336265	0.313559	0.228174	0.314359	0.284778	0.32891	0.269268	0.354173	0.348097	0.214743
0.304125	0.309562	0.277263	0.313399	0.303486	0.307323	0.289255	0.319635	0.318196	0.288935
0.360409	0.347298	0.208507	0.316278	0.301087	0.362968	0.29725	0.389511	0.369524	0.181324
0.251359	0.240166	0.379597	0.302526	0.31324	0.273905	0.276623	0.243524	0.290854	0.377998
0.32763	0.315318	0.290854	0.277422	0.279661	0.331788	0.324113	0.335465	0.334826	0.292773
0.372402	0.384074	0.177327	0.326831	0.287976	0.380556	0.347777	0.415734	0.386313	0.181324
0.315638	0.32779	0.308922	0.258075	0.199712	0.310361	0.294851	0.351135	0.323953	0.314199
0.357052	0.335785	0.201791	0.319635	0.293572	0.35897	0.283499	0.383754	0.369683	0.165174
0.280141	0.255516	0.381196	0.303006	0.32827	0.284458	0.260953	0.270067	0.272945	0.385034
0.376079	0.354813	0.260953	0.337064	0.308602	0.362488	0.323793	0.383435	0.365846	0.253438
0.31292	0.272466	0.32875	0.2819	0.29645	0.313559	0.278542	0.314039	0.325552	0.333547
0.26559	0.260793	0.349536	0.302846	0.314519	0.26639	0.238088	0.289575	0.300768	0.340902
0.299968	0.254557	0.391909	0.207227	0.321075	0.298369	0.303965	0.244484	0.29757	0.39127
0.284618	0.244004	0.385513	0.240806	0.325232	0.293572	0.298529	0.244963	0.304925	0.386473
0.316757	0.308922	0.245443	0.295011	0.284938	0.322354	0.263511	0.340742	0.331308	0.240006
0.302367	0.252159	0.387432	0.226735	0.322034	0.294532	0.301567	0.250879	0.309402	0.383914
0.29757	0.272306	0.374161	0.221298	0.316757	0.294691	0.293892	0.267349	0.290854	0.37464
0.35865	0.354013	0.254717	0.303486	0.26511	0.349217	0.331948	0.384074	0.34394	0.259354
0.271666	0.264311	0.322674	0.278542	0.279181	0.285577	0.254557	0.288136	0.288775	0.319316

For Review Only

0.309562	0.239207	0.395107	0.279181	0.341382	0.301727	0.295171	0.24944	0.316278	0.381036
0.314199	0.306844	0.253598	0.293732	0.259674	0.313719	0.282219	0.32763	0.318356	0.246882
0.239527	0.263991	0.402782	0.28078	0.333067	0.290854	0.274544	0.224017	0.269268	0.393828
0.362648	0.349856	0.290054	0.293732	0.21842	0.353854	0.323633	0.380237	0.35945	0.295491
0.363447	0.337224	0.348737	0.332107	0.332907	0.334186	0.323473	0.361369	0.338663	0.337064
0.309882	0.315478	0.290534	0.284938	0.229293	0.31244	0.298049	0.338823	0.321714	0.300128
0.267349	0.248961	0.401183	0.300927	0.326511	0.291334	0.279661	0.254077	0.286537	0.39127
0.311321	0.322354	0.29613	0.300128	0.303326	0.331308	0.298209	0.340422	0.299648	0.293732
0.353534	0.334346	0.215382	0.307643	0.251199	0.355133	0.325072	0.386153	0.362328	0.23505
0.271186	0.253598	0.305245	0.252638	0.276783	0.279181	0.255996	0.262072	0.280141	0.304925
0.337224	0.340422	0.258075	0.322674	0.279981	0.357531	0.324912	0.363447	0.364886	0.271186
0.18772	0.282219	0.413495	0.324752	0.333866	0.255357	0.253118	0.247202	0.266549	0.401823
0.288295	0.245283	0.363927	0.217461	0.298689	0.286217	0.280141	0.247362	0.299328	0.357691
0.322034	0.315798	0.240646	0.289255	0.242245	0.322993	0.291653	0.344899	0.330508	0.239207
0.251039	0.239207	0.375919	0.262392	0.298849	0.285257	0.279821	0.227215	0.301247	0.366166
0.289255	0.283658	0.32779	0.304285	0.308602	0.294052	0.256156	0.319955	0.310681	0.320755
0.355453	0.330189	0.205149	0.318676	0.295651	0.357851	0.283019	0.371442	0.370483	0.14023
0.335945	0.370803	0.263671	0.324113	0.287016	0.35897	0.35961	0.382315	0.348417	0.276943
0.276303	0.26591	0.300768	0.283179	0.277103	0.288615	0.260473	0.279821	0.295491	0.298049
0.276943	0.253278	0.395587	0.294691	0.330189	0.272785	0.274544	0.251679	0.304445	0.386313
0.271666	0.2181	0.341701	0.273585	0.291174	0.289575	0.258235	0.2496	0.307323	0.335945
0.192837	0.227374	0.377838	0.300608	0.314998	0.259994	0.259194	0.204509	0.275024	0.369204
0.261433	0.2504	0.302367	0.267669	0.273745	0.268628	0.249121	0.258714	0.275823	0.292933
0.356732	0.339942	0.173649	0.311481	0.287496	0.354173	0.295651	0.388551	0.371442	0.189958
0.347937	0.348897	0.258235	0.204509	0.176687	0.342981	0.305724	0.388391	0.344739	0.275823
0.292133	0.229133	0.391749	0.292453	0.331628	0.29677	0.293732	0.236009	0.301567	0.381356
0.298529	0.311641	0.337544	0.338823	0.299009	0.303646	0.286697	0.326351	0.322194	0.334506
0.342021	0.315158	0.228334	0.313879	0.284458	0.329869	0.273905	0.369204	0.339463	0.213783
0.262072	0.243044	0.368404	0.284778	0.303166	0.289255	0.275664	0.208666	0.287336	0.357531
0.29741	0.287176	0.333227	0.314199	0.289255	0.305245	0.288615	0.302686	0.292613	0.318836
0.301727	0.286217	0.354653	0.294532	0.285897	0.289735	0.248481	0.28206	0.291813	0.347138
0.23473	0.273105	0.388391	0.314519	0.304925	0.251679	0.246402	0.262232	0.26639	0.382315
0.301407	0.263351	0.35865	0.295651	0.303965	0.289894	0.276943	0.287176	0.315158	0.346978
0.211864	0.266869	0.420371	0.311001	0.333067	0.277263	0.283818	0.263671	0.276943	0.407579
0.277582	0.288295	0.376719	0.306684	0.297889	0.164055	0.237128	0.286057	0.29709	0.363927
0.355453	0.339623	0.209466	0.314998	0.29725	0.355772	0.279501	0.377359	0.370003	0.154461
0.283019	0.284458	0.380237	0.306684	0.313399	0.263671	0.248961	0.286057	0.276303	0.377518
0.292133	0.228494	0.39095	0.292133	0.331308	0.295811	0.292453	0.23505	0.300927	0.381196
0.305564	0.258874	0.363128	0.264311	0.253758	0.307323	0.287656	0.287016	0.313719	0.35945
0.26607	0.289575	0.370483	0.321714	0.320755	0.268468	0.202111	0.29741	0.300927	0.357531
0.286377	0.241126	0.382955	0.244963	0.323313	0.293892	0.294691	0.240966	0.307803	0.386313
0.280301	0.246882	0.378478	0.300927	0.322833	0.290054	0.283818	0.257435	0.302047	0.370483
0.276623	0.17173	0.404061	0.288775	0.34346	0.293572	0.29629	0.223537	0.303965	0.394627
0.295971	0.260633	0.39047	0.205469	0.324912	0.29677	0.304925	0.251839	0.297729	0.39079
0.38999	0.370003	0.135114	0.317077	0.280301	0.39031	0.334346	0.421011	0.387432	0.161017
0.289255	0.277103	0.351455	0.271826	0.28126	0.28094	0.290534	0.286856	0.301407	0.34442
0.317557	0.286537	0.322034	0.31276	0.331788	0.302686	0.29629	0.3118	0.322993	0.309242
0.295491	0.268308	0.348897	0.334346	0.342181	0.295811	0.262552	0.301247	0.284938	0.338503
0.335146	0.369843	0.264791	0.322833	0.284938	0.358331	0.358491	0.381196	0.347618	0.276303
0.369683	0.342661	0.182443	0.326511	0.303486	0.369044	0.32827	0.404061	0.368244	0.200032
0.330349	0.320435	0.215062	0.311641	0.28174	0.326671	0.25072	0.358491	0.34426	0.185961
0.266869	0.293732	0.336105	0.315158	0.303166	0.288775	0.286697	0.26591	0.307004	0.332427
0.252478	0.26559	0.400704	0.310681	0.334986	0.295491	0.272466	0.147106	0.308283	0.389031
0.355453	0.351935	0.245283	0.319156	0.299488	0.349217	0.295491	0.387112	0.351135	0.238407
0.357371	0.350016	0.227055	0.221458	0.208507	0.35897	0.310361	0.398625	0.357371	0.239527

For Review Only

0.270227	0.21874	0.394627	0.286057	0.335305	0.284938	0.292133	0.215542	0.294052	0.387432
0.392869	0.370643	0.149984	0.321394	0.276783	0.378798	0.331948	0.417973	0.402622	0.165174
0.275664	0.283818	0.303006	0.306684	0.291334	0.300608	0.282699	0.298529	0.311481	0.297889
0.331308	0.316917	0.321714	0.330508	0.306844	0.318676	0.291014	0.341222	0.303486	0.315958
0.236808	0.21938	0.417813	0.304605	0.344739	0.275823	0.267669	0.173009	0.300288	0.409978
0.330349	0.330508	0.322833	0.262872	0.229773	0.326351	0.304605	0.35897	0.334026	0.324592
0.320275	0.319156	0.314039	0.318996	0.295651	0.307004	0.288615	0.334346	0.326191	0.3118
0.274544	0.268468	0.400224	0.26559	0.321234	0.268628	0.256636	0.253438	0.276463	0.389831
0.361529	0.344899	0.264151	0.298049	0.266709	0.338663	0.331468	0.358011	0.354493	0.266869
0.403902	0.369044	0.255676	0.351775	0.322993	0.384554	0.330988	0.403102	0.384074	0.253438
0.336425	0.303006	0.243044	0.301087	0.299488	0.329869	0.276303	0.336265	0.334346	0.235209
0.402782	0.380397	0.117045	0.32859	0.291014	0.387272	0.339143	0.419572	0.39095	0.126959
0.259834	0.252958	0.29613	0.261273	0.275344	0.272466	0.251359	0.257755	0.275344	0.29677
0.322194	0.304925	0.324432	0.294212	0.26511	0.317237	0.299648	0.322034	0.336745	0.320595
0.293252	0.308602	0.347618	0.299169	0.279501	0.313879	0.305085	0.286537	0.34394	0.345219
0.240966	0.25056	0.346178	0.2504	0.300288	0.285897	0.261593	0.246402	0.302207	0.337544
0.282699	0.206908	0.400704	0.290694	0.34442	0.293412	0.317877	0.235369	0.3126	0.39047
0.286217	0.337384	0.32779	0.314359	0.302367	0.317557	0.316278	0.342501	0.339783	0.326511
0.372402	0.340102	0.20291	0.303326	0.272785	0.358011	0.307963	0.381996	0.354813	0.220659
0.321874	0.337864	0.26575	0.325871	0.291334	0.318516	0.300288	0.353694	0.332587	0.276623
0.35865	0.348417	0.235849	0.305085	0.287496	0.373681	0.32827	0.378798	0.3748	0.235369
0.308602	0.313399	0.333067	0.304925	0.268628	0.323313	0.305564	0.324432	0.320435	0.330988
0.34362	0.317237	0.303965	0.3126	0.307643	0.338503	0.338983	0.329389	0.348577	0.303646
0.301087	0.256476	0.379437	0.209466	0.320755	0.306364	0.293732	0.255516	0.29693	0.381036
0.193476	0.279021	0.39015	0.323953	0.307483	0.255037	0.231532	0.255197	0.274544	0.386952
0.316118	0.330668	0.286537	0.293572	0.236329	0.324432	0.302207	0.356252	0.322993	0.300448
0.383435	0.362968	0.191717	0.324912	0.299968	0.373841	0.323633	0.403102	0.374001	0.199073
0.303965	0.275983	0.362328	0.322514	0.336585	0.302207	0.289095	0.287656	0.313399	0.358491
0.402143	0.377359	0.11001	0.331948	0.290534	0.391749	0.340422	0.42261	0.396067	0.142309
0.258714	0.240646	0.400064	0.285098	0.342501	0.29661	0.29661	0.152382	0.287336	0.386792
0.270387	0.293892	0.355772	0.321394	0.29629	0.270387	0.263831	0.302367	0.294691	0.353214
0.225136	0.227854	0.4055	0.307323	0.341222	0.274225	0.257755	0.098337	0.308123	0.389511
0.385034	0.353694	0.15622	0.317077	0.261433	0.384554	0.338343	0.401503	0.384554	0.190598
0.316118	0.273905	0.319156	0.258395	0.31276	0.322833	0.302207	0.286697	0.32859	0.326191
0.318516	0.317237	0.293572	0.31244	0.292613	0.326991	0.277742	0.354493	0.316597	0.284458
0.288615	0.271027	0.353854	0.231692	0.308123	0.283658	0.284778	0.26527	0.302846	0.35865
0.338823	0.340902	0.258395	0.192197	0.17125	0.335146	0.306684	0.378638	0.339623	0.275344
0.401503	0.3756	0.119923	0.329549	0.290694	0.387912	0.34346	0.425328	0.399424	0.154621
0.239527	0.240006	0.394627	0.307323	0.318676	0.243844	0.251359	0.240646	0.259354	0.397825
0.292613	0.229453	0.392869	0.293412	0.331468	0.29693	0.294372	0.236808	0.302207	0.382475
0.289735	0.253598	0.308762	0.285577	0.29677	0.284938	0.244324	0.285098	0.307483	0.291653
0.397666	0.377039	0.146306	0.320915	0.264471	0.385833	0.334666	0.42149	0.394787	0.169012
0.251679	0.283019	0.409978	0.309082	0.315478	0.257435	0.233611	0.255836	0.26527	0.396067
0.283339	0.238248	0.319476	0.29677	0.290214	0.270867	0.231532	0.29645	0.278382	0.320755
0.322993	0.330828	0.275344	0.280461	0.199712	0.320115	0.306204	0.360409	0.338024	0.289415
0.211225	0.282699	0.418452	0.331308	0.3118	0.228973	0.260473	0.26511	0.272146	0.414135
0.29693	0.244963	0.351135	0.302686	0.337064	0.319316	0.298849	0.270227	0.310201	0.344579
0.295491	0.310681	0.345219	0.308762	0.299009	0.317077	0.277902	0.304125	0.321554	0.3433
0.327311	0.306524	0.301727	0.259354	0.301887	0.338343	0.338823	0.308443	0.347298	0.308762
0.305564	0.28126	0.360729	0.324592	0.319955	0.318996	0.295971	0.295011	0.331148	0.35945
0.322514	0.229773	0.362328	0.326991	0.351615	0.333227	0.321394	0.306524	0.332427	0.352095
0.399584	0.37576	0.1244	0.330828	0.291493	0.393668	0.335465	0.418132	0.394787	0.143108
0.226095	0.282859	0.423089	0.325232	0.321394	0.245763	0.245603	0.253438	0.236009	0.408539
0.398305	0.380237	0.14135	0.321234	0.271986	0.379757	0.333067	0.419412	0.398625	0.165814
0.276783	0.171091	0.404541	0.289095	0.34362	0.294691	0.295971	0.223857	0.305245	0.395107

0.287176	0.258555	0.418132	0.294372	0.336904	0.292293	0.311001	0.191717	0.287336	0.40662
0.342341	0.34426	0.248801	0.288935	0.209946	0.344579	0.311161	0.383275	0.354653	0.25072
0.204509	0.284778	0.416693	0.325871	0.307803	0.259994	0.252478	0.276303	0.280461	0.401343
0.28142	0.239047	0.399744	0.293572	0.340582	0.286377	0.277263	0.233451	0.310521	0.387432

For Review Only

0.277422	0.253438	0.271186	0.321075	0.295651	0.349376	0.269428	0.318836	0.37576	0.330828
0.289575	0.360889	0.306204	0.307323	0.319476	0.301087	0.28142	0.321874	0.329069	0.34394
0.272466	0.285257	0.257595	0.361848	0.333547	0.378478	0.233451	0.31196	0.402462	0.335945
0.272466	0.306524	0.262552	0.319955	0.309722	0.346338	0.260633	0.314199	0.365046	0.318516
0.291493	0.245923	0.315478	0.308283	0.295491	0.317557	0.334186	0.342661	0.338503	0.360729
0.278222	0.280461	0.270707	0.320915	0.304765	0.356412	0.240326	0.318676	0.388232	0.320435
0.286697	0.192517	0.285577	0.347618	0.304285	0.365206	0.28142	0.336904	0.389511	0.327151
0.286057	0.348577	0.310521	0.26623	0.303486	0.279661	0.305405	0.329869	0.307963	0.330508
0.275504	0.18724	0.277742	0.313879	0.287336	0.348097	0.289415	0.326831	0.363128	0.308283
0.298849	0.392869	0.337704	0.273265	0.32779	0.285897	0.350975	0.362168	0.331468	0.352894
0.29693	0.333387	0.314998	0.304925	0.333706	0.314998	0.330349	0.26495	0.186281	0.290854
0.26623	0.288615	0.254877	0.352414	0.325712	0.373361	0.221938	0.308443	0.396226	0.32747
0.280461	0.258235	0.278862	0.294851	0.283339	0.303646	0.300768	0.317717	0.342821	0.327311
0.258075	0.321874	0.282219	0.209306	0.283978	0.200192	0.292293	0.292933	0.292773	0.311161
0.26511	0.209786	0.246882	0.319476	0.272306	0.339303	0.241765	0.294691	0.35865	0.306684
0.280141	0.313719	0.267509	0.291493	0.311321	0.310681	0.287976	0.307803	0.311001	0.32763
0.294532	0.179405	0.299009	0.334346	0.3126	0.360249	0.319635	0.348417	0.369364	0.34362
0.283339	0.301887	0.274864	0.307803	0.303965	0.342181	0.29693	0.315318	0.367605	0.3433
0.282379	0.279021	0.28142	0.362008	0.339143	0.383914	0.23441	0.339783	0.403422	0.339463
0.315958	0.362008	0.319795	0.31324	0.332427	0.310681	0.346658	0.34394	0.331948	0.350656
0.289095	0.263032	0.307643	0.304765	0.291334	0.309562	0.326831	0.333227	0.325552	0.334346
0.289255	0.169012	0.300927	0.319955	0.285417	0.346818	0.315158	0.339783	0.370323	0.350656
0.303166	0.394148	0.336585	0.278542	0.311001	0.279181	0.384234	0.29661	0.231532	0.266709
0.263032	0.352095	0.287496	0.196674	0.258395	0.200192	0.323793	0.289575	0.264311	0.298209
0.268628	0.216501	0.255357	0.337544	0.308762	0.373361	0.279821	0.308922	0.388871	0.334666
0.268148	0.295651	0.195715	0.337864	0.323313	0.365046	0.248481	0.258075	0.391909	0.298049
0.256316	0.289415	0.233611	0.313559	0.302367	0.342341	0.21858	0.309402	0.372242	0.311641
0.23457	0.271666	0.233291	0.269108	0.273425	0.288136	0.255676	0.26543	0.311481	0.286057
0.272306	0.267029	0.279181	0.267029	0.28078	0.29613	0.303806	0.315158	0.336425	0.29709
0.324912	0.432683	0.366805	0.261593	0.322034	0.251199	0.40662	0.350656	0.277742	0.357691
0.280461	0.269747	0.251199	0.332267	0.314519	0.363927	0.236329	0.320595	0.396546	0.331628
0.286856	0.288935	0.291334	0.352894	0.329229	0.389671	0.257755	0.337224	0.396866	0.334186
0.29677	0.185801	0.283818	0.338983	0.298849	0.374001	0.295331	0.339463	0.382315	0.337384
0.276623	0.351935	0.298049	0.011832	0.237448	0.164695	0.331308	0.299808	0.268948	0.324272
0.292773	0.341861	0.28206	0.318996	0.308762	0.31212	0.316597	0.290694	0.309722	0.305085
0.250879	0.327311	0.256316	0.248961	0.285098	0.277582	0.273585	0.302846	0.317557	0.314519
0.2811	0.303646	0.277742	0.288775	0.300448	0.279661	0.309882	0.326191	0.34378	0.303486
0.273585	0.307963	0.274065	0.322514	0.321394	0.318836	0.291493	0.306044	0.323153	0.307323
0.290854	0.407419	0.329229	0.232171	0.290054	0.224177	0.377199	0.288455	0.160857	0.279981
0.29757	0.362968	0.295971	0.329229	0.32747	0.303326	0.334346	0.285257	0.271666	0.240806
0.314679	0.426607	0.354493	0.260473	0.316597	0.252159	0.39015	0.337224	0.28126	0.346019
0.299169	0.354333	0.309722	0.286377	0.311161	0.287976	0.336425	0.334826	0.322354	0.322674
0.289415	0.373201	0.321394	0.269268	0.311481	0.285098	0.336265	0.334506	0.325072	0.34362
0.306684	0.246083	0.3126	0.341701	0.315958	0.336745	0.346498	0.344899	0.353694	0.364886
0.301407	0.388391	0.337384	0.289255	0.320595	0.295651	0.350975	0.338503	0.335465	0.357691
0.266869	0.282379	0.23377	0.351775	0.321234	0.368724	0.227694	0.290374	0.394308	0.32811
0.270547	0.298529	0.211385	0.342021	0.317717	0.360889	0.251199	0.29741	0.391589	0.336425
0.270227	0.319635	0.252478	0.293732	0.291493	0.300128	0.293732	0.235849	0.305564	0.300768
0.303806	0.247362	0.319795	0.333706	0.322354	0.337704	0.333706	0.35993	0.344899	0.351455
0.278542	0.23457	0.263671	0.337384	0.298049	0.35897	0.274544	0.315638	0.379437	0.310681

For Review Only

0.264471	0.288935	0.20259	0.345859	0.325552	0.370003	0.244803	0.278062	0.393828	0.324113
0.273425	0.301887	0.288295	0.325392	0.316118	0.337864	0.26575	0.306684	0.34314	0.318516
0.278062	0.127279	0.262392	0.348417	0.290854	0.386952	0.282539	0.326031	0.407099	0.346818
0.295811	0.18692	0.283339	0.338024	0.297889	0.372562	0.295651	0.338823	0.381996	0.336904
0.26591	0.291973	0.275184	0.346978	0.320275	0.369843	0.228174	0.314839	0.384394	0.334026
0.288455	0.170611	0.295011	0.34346	0.301887	0.35865	0.315798	0.355453	0.373521	0.340102
0.28126	0.279021	0.28142	0.361369	0.339303	0.383754	0.23457	0.339463	0.404061	0.338823
0.267829	0.308762	0.211225	0.350176	0.317717	0.368724	0.226735	0.300927	0.391589	0.323793
0.268308	0.288935	0.20243	0.330988	0.308283	0.35961	0.231052	0.26559	0.387432	0.306684
0.2811	0.410138	0.337704	0.226895	0.295971	0.210905	0.372881	0.308283	0.233291	0.326671
0.280461	0.311481	0.294532	0.24976	0.279501	0.248321	0.329549	0.319316	0.319476	0.307323
0.237608	0.26623	0.237928	0.274065	0.275344	0.294851	0.25024	0.274065	0.319476	0.289735
0.262072	0.309242	0.255516	0.339942	0.323473	0.362968	0.192677	0.318836	0.389671	0.330029
0.270867	0.32747	0.26495	0.337704	0.330508	0.354813	0.228014	0.330828	0.391589	0.333706
0.277582	0.125999	0.261912	0.347138	0.289735	0.386633	0.28158	0.324752	0.40614	0.346338
0.271506	0.267029	0.278702	0.267189	0.280301	0.295491	0.303166	0.314998	0.336265	0.29709
0.254717	0.284938	0.242725	0.292773	0.286217	0.315478	0.254877	0.292133	0.337704	0.288775
0.285577	0.323793	0.298529	0.275504	0.294052	0.278382	0.319795	0.293572	0.322833	0.315958
0.284938	0.313559	0.271506	0.321075	0.321874	0.331628	0.295331	0.267988	0.318836	0.297889
0.289255	0.259834	0.24928	0.361369	0.298209	0.386473	0.249121	0.316757	0.419252	0.32859
0.275983	0.192197	0.280461	0.351775	0.293252	0.382315	0.260793	0.337384	0.391909	0.333387
0.286377	0.165174	0.272785	0.336425	0.282859	0.370323	0.279181	0.327151	0.397666	0.330349
0.254237	0.307323	0.247681	0.307004	0.304925	0.325712	0.220179	0.310361	0.35929	0.308762
0.286697	0.372082	0.302846	0.291653	0.322674	0.290534	0.352574	0.2819	0.260313	0.217141
0.274384	0.241126	0.274704	0.290694	0.28062	0.316118	0.293732	0.319156	0.330189	0.299808
0.286856	0.319316	0.290854	0.307483	0.333067	0.340422	0.279501	0.32795	0.369044	0.302047
0.301087	0.387752	0.337064	0.288615	0.320115	0.294851	0.350496	0.338024	0.334986	0.357691
0.256476	0.303965	0.272785	0.331148	0.325712	0.357052	0.207547	0.315638	0.39063	0.347618
0.315638	0.302686	0.341861	0.339463	0.32747	0.351615	0.337544	0.349057	0.357851	0.377518
0.290854	0.418772	0.335785	0.236968	0.2819	0.221938	0.380237	0.290054	0.165494	0.302047
0.270707	0.304125	0.180844	0.344899	0.32763	0.366166	0.247522	0.251199	0.39063	0.301087
0.286697	0.240806	0.273425	0.337384	0.306364	0.365686	0.246722	0.333067	0.37576	0.309562
0.293412	0.240646	0.285417	0.303006	0.288615	0.326991	0.300448	0.325072	0.353694	0.34314
0.257595	0.326191	0.269428	0.21906	0.267349	0.243364	0.291653	0.299488	0.295331	0.308443
0.299808	0.429325	0.346818	0.229293	0.294851	0.213783	0.39127	0.29693	0.170291	0.313879
0.276783	0.125839	0.261113	0.346178	0.289255	0.385513	0.282539	0.324912	0.404221	0.345379
0.2819	0.267829	0.263032	0.284298	0.289415	0.320435	0.304765	0.298849	0.352894	0.329549
0.301567	0.388072	0.337704	0.289255	0.320595	0.295011	0.351295	0.338663	0.335625	0.356892
0.29693	0.351615	0.31196	0.278382	0.304125	0.284778	0.317557	0.324432	0.319635	0.346178
0.301407	0.290214	0.337864	0.303806	0.302207	0.313719	0.358011	0.347618	0.333706	0.377039
0.292933	0.318196	0.305564	0.306844	0.326191	0.325232	0.321554	0.316917	0.32891	0.302846
0.295011	0.367605	0.325392	0.299328	0.325712	0.288455	0.351775	0.324752	0.329709	0.354173
0.283339	0.01583	0.264471	0.348577	0.285897	0.384714	0.278862	0.326351	0.397026	0.332907
0.29661	0.273905	0.286377	0.362488	0.337704	0.393508	0.25072	0.344739	0.399424	0.331148
0.285577	0.307483	0.291014	0.350016	0.319955	0.340422	0.29645	0.326031	0.373201	0.331628
0.262872	0.309562	0.257275	0.339623	0.324912	0.363607	0.193476	0.319795	0.39015	0.332427
0.293252	0.248321	0.287176	0.31324	0.304125	0.342021	0.287816	0.316437	0.35881	0.332907
0.267349	0.26623	0.251039	0.333866	0.320435	0.360569	0.232971	0.318836	0.386952	0.319795
0.275184	0.382155	0.303006	0.145187	0.266869	0.090342	0.350176	0.306364	0.25008	0.31292
0.25008	0.306524	0.231052	0.300288	0.298209	0.309402	0.256156	0.2811	0.364247	0.308602
0.26591	0.283499	0.208027	0.309882	0.293092	0.320915	0.26575	0.280141	0.369364	0.305085
0.291653	0.322194	0.302367	0.346978	0.321874	0.346019	0.289095	0.331468	0.371122	0.303166
0.252478	0.303006	0.240486	0.309722	0.303006	0.339623	0.213943	0.307643	0.367445	0.307004
0.294212	0.414934	0.339623	0.243204	0.288935	0.230253	0.382315	0.286537	0.171091	0.290534
0.286217	0.393348	0.330029	0.256956	0.307163	0.241445	0.354813	0.320115	0.260313	0.323793

For Review Only

0.263671	0.317397	0.274704	0.274225	0.295331	0.283179	0.302207	0.292933	0.298529	0.302047
0.290374	0.310681	0.290054	0.34314	0.319316	0.347458	0.273265	0.323313	0.378957	0.351455
0.271346	0.322034	0.21874	0.326671	0.314359	0.324432	0.278542	0.253278	0.318836	0.266869
0.286856	0.345379	0.301567	0.323473	0.324592	0.318196	0.292933	0.32795	0.325552	0.286217
0.259994	0.28062	0.223377	0.290694	0.29645	0.32827	0.24992	0.257915	0.35913	0.319635
0.276143	0.361689	0.298529	0.181004	0.263671	0.087144	0.35977	0.290214	0.254717	0.316118
0.263831	0.298209	0.240806	0.335305	0.322034	0.366326	0.214583	0.306044	0.398145	0.331148
0.261593	0.277582	0.220659	0.299328	0.289735	0.336585	0.252159	0.270547	0.364247	0.303646
0.271986	0.254557	0.254237	0.32779	0.314679	0.362648	0.237128	0.319476	0.383754	0.324432
0.258075	0.302207	0.238887	0.264311	0.275024	0.284938	0.248961	0.290214	0.318836	0.306684
0.284618	0.344739	0.267029	0.287016	0.31196	0.290374	0.303806	0.298849	0.331308	0.31276
0.262072	0.335305	0.257755	0.260153	0.291334	0.276783	0.292293	0.286377	0.31292	0.316597
0.273905	0.286697	0.285897	0.292293	0.29693	0.301887	0.295651	0.314359	0.349696	0.32891
0.243364	0.275664	0.238407	0.292613	0.285098	0.29709	0.255676	0.273105	0.320915	0.28158
0.280141	0.330508	0.298369	0.322833	0.306844	0.322514	0.290054	0.341541	0.362328	0.333387
0.248001	0.287496	0.25008	0.329709	0.31308	0.355453	0.210585	0.295331	0.3756	0.319795
0.282539	0.319635	0.284618	0.306524	0.319156	0.317397	0.28174	0.3126	0.358011	0.325712
0.252638	0.286856	0.207547	0.289575	0.285417	0.306364	0.251679	0.261113	0.32795	0.302207
0.295971	0.305724	0.289735	0.361369	0.32795	0.366326	0.285897	0.303806	0.380397	0.301567
0.242885	0.261433	0.220179	0.294532	0.288295	0.309242	0.235689	0.272945	0.32859	0.292773
0.276303	0.373041	0.305405	0.164535	0.258555	0.088903	0.35865	0.294851	0.261912	0.322034
0.283978	0.264631	0.291973	0.337064	0.324272	0.348417	0.271346	0.297729	0.336745	0.315958
0.228494	0.277902	0.230892	0.274544	0.275664	0.294532	0.254077	0.275344	0.308922	0.286057
0.283019	0.221938	0.26543	0.336265	0.304125	0.360409	0.251359	0.32763	0.372402	0.315638
0.263991	0.288455	0.257115	0.313559	0.309562	0.347298	0.240166	0.315318	0.384074	0.32779
0.300288	0.417973	0.335945	0.228174	0.277263	0.208507	0.379597	0.290854	0.177327	0.308922
0.272466	0.326831	0.241126	0.314359	0.313399	0.316278	0.302526	0.277422	0.326831	0.258075
0.271027	0.321234	0.276303	0.284778	0.303486	0.301087	0.31324	0.279661	0.287976	0.199712
0.284138	0.240326	0.269428	0.32891	0.307323	0.362968	0.273905	0.331788	0.380556	0.310361
0.256476	0.263991	0.255836	0.269268	0.289255	0.29725	0.276623	0.324113	0.347777	0.294851
0.275024	0.276943	0.268308	0.354173	0.319635	0.389511	0.243524	0.335465	0.415734	0.351135
0.287176	0.287016	0.278862	0.348097	0.318196	0.369524	0.290854	0.334826	0.386313	0.323953
0.292453	0.416054	0.337864	0.214743	0.288935	0.181324	0.377998	0.292773	0.181324	0.314199
0	0.284938	0.251519	0.276303	0.279821	0.285737	0.261433	0.282699	0.309882	0.284458
0.284938	0	0.264151	0.351775	0.285897	0.388072	0.279661	0.326191	0.399584	0.332747
0.251519	0.264151	0	0.295171	0.289575	0.318836	0.246242	0.263671	0.342181	0.291334
0.276303	0.351775	0.295171	0	0.237128	0.169012	0.329389	0.298209	0.269428	0.324272
0.279821	0.285897	0.289575	0.237128	0	0.273585	0.306844	0.300927	0.31228	0.332747
0.285737	0.388072	0.318836	0.169012	0.273585	0	0.371282	0.305405	0.257435	0.325712
0.261433	0.279661	0.246242	0.329389	0.306844	0.371282	0	0.300128	0.380237	0.313719
0.282699	0.326191	0.263671	0.298209	0.300927	0.305405	0.300128	0	0.292613	0.301567
0.309882	0.399584	0.342181	0.269428	0.31228	0.257435	0.380237	0.292613	0	0.297889
0.284458	0.332747	0.291334	0.324272	0.332747	0.325712	0.313719	0.301567	0.297889	0
0.282539	0.383595	0.315158	0.157819	0.268788	0.104573	0.354973	0.300768	0.248321	0.317077
0.276943	0.295651	0.257435	0.346818	0.315958	0.369524	0.235209	0.313719	0.388072	0.32891
0.29645	0.394308	0.333866	0.258235	0.309562	0.259514	0.362488	0.324592	0.295811	0.341382
0.273265	0.299328	0.252478	0.299648	0.303806	0.316118	0.277103	0.278542	0.34362	0.307004
0.273425	0.249121	0.264631	0.313399	0.298209	0.322034	0.275344	0.316278	0.368564	0.323313
0.26559	0.285417	0.15686	0.346658	0.318356	0.361848	0.240806	0.260313	0.392229	0.320275
0.263032	0.306364	0.216022	0.345699	0.320755	0.361689	0.231532	0.285417	0.384074	0.315798
0.262712	0.342181	0.277582	0.247362	0.270707	0.228814	0.320595	0.299488	0.280141	0.301247
0.26543	0.275823	0.18756	0.345059	0.316437	0.370003	0.235369	0.261912	0.394947	0.314519
0.268948	0.302207	0.188519	0.320275	0.298529	0.345059	0.254717	0.268308	0.376879	0.314039
0.282539	0.361848	0.310201	0.263511	0.305405	0.248801	0.357851	0.290534	0.244164	0.287816
0.261273	0.286057	0.268148	0.271666	0.270227	0.29709	0.279821	0.286057	0.319156	0.292293

For Review Only

0.267669	0.322354	0.260473	0.347138	0.335945	0.35961	0.240486	0.308123	0.396226	0.339783
0.254717	0.330508	0.28126	0.260953	0.277902	0.256156	0.314679	0.28158	0.267189	0.289255
0.274544	0.26527	0.243044	0.350496	0.319955	0.367925	0.242085	0.298689	0.394627	0.336265
0.288775	0.379917	0.305724	0.320435	0.323313	0.299808	0.350656	0.314199	0.282699	0.263511
0.304765	0.346818	0.317557	0.330349	0.319316	0.325552	0.352734	0.351935	0.341062	0.354173
0.283818	0.334026	0.29741	0.290534	0.306844	0.299169	0.32843	0.311641	0.291334	0.259834
0.269428	0.267029	0.248001	0.324912	0.319955	0.35977	0.240646	0.321075	0.396866	0.342341
0.274704	0.314679	0.285897	0.288136	0.305405	0.289735	0.322993	0.307323	0.301887	0.29613
0.289575	0.362808	0.310521	0.278542	0.29661	0.269747	0.3433	0.283818	0.228174	0.286537
0.236169	0.274065	0.227694	0.269747	0.272785	0.278702	0.251039	0.26623	0.317237	0.290854
0.299968	0.356412	0.318836	0.273745	0.293412	0.277582	0.342661	0.290854	0.258874	0.315958
0.287976	0.228174	0.279661	0.34362	0.315798	0.383754	0.255676	0.324113	0.39047	0.322034
0.248961	0.275504	0.184202	0.319156	0.29613	0.336265	0.238887	0.259194	0.361689	0.299488
0.267029	0.339623	0.283499	0.242885	0.272785	0.247042	0.31212	0.273905	0.264791	0.262072
0.26639	0.280301	0.231852	0.317877	0.292613	0.346978	0.243844	0.295011	0.374161	0.305884
0.290214	0.290694	0.277742	0.288295	0.287656	0.290694	0.301567	0.31244	0.341062	0.322194
0.269907	0.387592	0.31212	0.159738	0.26575	0.112568	0.350815	0.300128	0.248481	0.314039
0.305085	0.361209	0.330189	0.317237	0.333227	0.314839	0.348257	0.266549	0.1559	0.289255
0.256796	0.293732	0.267029	0.256636	0.259834	0.2811	0.273105	0.284938	0.307004	0.291014
0.26639	0.303326	0.272945	0.342021	0.337864	0.37496	0.245123	0.32811	0.387272	0.331468
0.256796	0.283499	0.205788	0.300288	0.294532	0.31276	0.253438	0.266869	0.360409	0.311321
0.26639	0.251999	0.2496	0.321874	0.306684	0.353854	0.237128	0.314039	0.376239	0.318196
0.235369	0.267189	0.239367	0.269907	0.26639	0.2811	0.249121	0.271826	0.305405	0.286697
0.272785	0.378158	0.314679	0.224656	0.277902	0.226255	0.347618	0.300128	0.223057	0.305405
0.290374	0.370483	0.308762	0.287816	0.323953	0.290694	0.354813	0.292613	0.260473	0.216661
0.270867	0.326991	0.264151	0.338184	0.330988	0.355612	0.228014	0.330189	0.39127	0.333866
0.300448	0.257595	0.297729	0.295331	0.304285	0.318196	0.316437	0.340262	0.35977	0.330349
0.277103	0.352095	0.29613	0.050048	0.251359	0.168852	0.32747	0.287976	0.257595	0.316278
0.258714	0.292133	0.2496	0.316917	0.300128	0.335465	0.23377	0.304605	0.374001	0.301247
0.279501	0.308283	0.285737	0.257435	0.289575	0.299009	0.284778	0.291174	0.34442	0.301087
0.264151	0.293572	0.268948	0.313719	0.289415	0.335305	0.278382	0.316278	0.362168	0.285098
0.270547	0.17253	0.26495	0.330828	0.260633	0.366965	0.268948	0.32747	0.391749	0.318196
0.275504	0.290694	0.277582	0.310521	0.300288	0.32763	0.264311	0.31324	0.364727	0.321714
0.291174	0.254557	0.263032	0.362008	0.3118	0.376879	0.258874	0.316118	0.399904	0.304925
0.274704	0.259354	0.275823	0.318676	0.298529	0.344739	0.283019	0.3433	0.367605	0.311161
0.271346	0.384394	0.307803	0.162936	0.26575	0.115126	0.354333	0.298209	0.246562	0.31308
0.295331	0.24928	0.275024	0.319955	0.293732	0.342501	0.306364	0.32779	0.377518	0.335465
0.270387	0.326511	0.264311	0.337544	0.330668	0.354813	0.227854	0.330668	0.39127	0.332427
0.277103	0.309242	0.245603	0.336904	0.329069	0.351295	0.268948	0.309722	0.357531	0.295171
0.278222	0.264791	0.278382	0.31292	0.303646	0.325232	0.277902	0.307803	0.37448	0.319955
0.262712	0.307803	0.216661	0.345859	0.321394	0.365046	0.232811	0.290054	0.386473	0.317237
0.263991	0.295651	0.276623	0.315798	0.316757	0.351775	0.23377	0.311161	0.380876	0.329389
0.271666	0.285257	0.258555	0.36009	0.332267	0.377678	0.232651	0.311481	0.401023	0.336265
0.26559	0.283818	0.164215	0.346019	0.315478	0.364247	0.242565	0.261433	0.39127	0.319156
0.300448	0.419252	0.338024	0.222737	0.285257	0.207547	0.384074	0.288295	0.173009	0.306524
0.271346	0.274704	0.247522	0.322354	0.306844	0.336425	0.274704	0.28142	0.340742	0.29645
0.275823	0.319476	0.284778	0.286217	0.307323	0.279821	0.299328	0.318196	0.336904	0.345059
0.290694	0.31244	0.303326	0.313719	0.313719	0.333387	0.308922	0.356732	0.36009	0.336745
0.304445	0.35961	0.329069	0.316437	0.334026	0.314679	0.346978	0.26495	0.154141	0.287336
0.295331	0.369044	0.332427	0.259034	0.287016	0.238407	0.378798	0.293412	0.209146	0.335146
0.251199	0.355612	0.300288	0.173969	0.247681	0.164375	0.337384	0.297729	0.252958	0.305405
0.271986	0.270547	0.285577	0.300608	0.29757	0.311481	0.282539	0.317237	0.336265	0.299009
0.284778	0.285897	0.273745	0.345059	0.325232	0.379597	0.269907	0.326831	0.403742	0.351295
0.293892	0.383275	0.316278	0.238727	0.294212	0.200512	0.356092	0.322514	0.277902	0.330349
0.291493	0.382155	0.315478	0.277103	0.308283	0.267509	0.369364	0.286057	0.248961	0.235209

For Review Only

0.261433	0.309242	0.255516	0.339463	0.323953	0.363288	0.193156	0.318036	0.389031	0.330189
0.298209	0.415734	0.341062	0.236329	0.294052	0.230253	0.383275	0.298369	0.170451	0.29709
0.276143	0.306204	0.284618	0.252638	0.255836	0.272146	0.292453	0.29661	0.314519	0.319156
0.300768	0.346338	0.287496	0.299648	0.322514	0.298849	0.31292	0.318196	0.339942	0.323793
0.284458	0.268148	0.26639	0.368724	0.331468	0.387112	0.26591	0.334986	0.413655	0.342501
0.301567	0.334986	0.311481	0.32779	0.332267	0.323313	0.338184	0.320115	0.315958	0.252958
0.286217	0.323473	0.288136	0.291813	0.29741	0.295331	0.307323	0.305405	0.320595	0.319955
0.276463	0.271666	0.21938	0.338503	0.308762	0.362808	0.271666	0.29725	0.400384	0.309562
0.290534	0.364087	0.299169	0.288775	0.315158	0.282539	0.344579	0.292773	0.26511	0.29741
0.299328	0.431884	0.356092	0.267029	0.325871	0.263671	0.381036	0.350336	0.294851	0.346178
0.26559	0.341222	0.28206	0.226255	0.274704	0.216342	0.316757	0.298529	0.283499	0.31292
0.302047	0.420371	0.345859	0.23457	0.291813	0.216342	0.389671	0.295331	0.154141	0.310201
0.227694	0.267349	0.23425	0.268628	0.270067	0.282219	0.255676	0.276303	0.306524	0.284458
0.288295	0.338184	0.287176	0.304925	0.298049	0.316278	0.302526	0.303326	0.330189	0.320435
0.285577	0.297729	0.290694	0.323153	0.326671	0.350016	0.317717	0.299968	0.32827	0.282859
0.253598	0.272306	0.215862	0.29645	0.300768	0.315798	0.256476	0.270707	0.353374	0.309882
0.273905	0.284778	0.263032	0.348737	0.317077	0.376559	0.244164	0.313399	0.394148	0.347618
0.29677	0.294851	0.299169	0.326831	0.322674	0.336425	0.324272	0.287496	0.284298	0.295171
0.272306	0.370643	0.306364	0.256316	0.294532	0.256956	0.342981	0.298689	0.226735	0.288455
0.299009	0.348897	0.3126	0.277103	0.302047	0.275664	0.331628	0.291653	0.290214	0.324912
0.293892	0.372242	0.320755	0.270547	0.299169	0.257435	0.348737	0.278542	0.241126	0.318516
0.285897	0.321234	0.285897	0.324113	0.319795	0.314998	0.29693	0.298369	0.311161	0.304285
0.286537	0.371282	0.315478	0.316917	0.327311	0.311001	0.286697	0.322034	0.315478	0.29741
0.26495	0.301087	0.176847	0.335625	0.320115	0.351135	0.244484	0.247202	0.383435	0.300128
0.282219	0.221458	0.273905	0.322034	0.301247	0.357211	0.264151	0.321714	0.377518	0.324432
0.272306	0.322833	0.285098	0.322354	0.319795	0.314519	0.29661	0.2811	0.286697	0.232651
0.28126	0.409818	0.338024	0.226255	0.294851	0.210265	0.372402	0.308283	0.23393	0.326191
0.295011	0.308602	0.29677	0.321394	0.314199	0.345859	0.288295	0.342501	0.371282	0.344899
0.305564	0.431724	0.351135	0.232012	0.295491	0.213144	0.386952	0.297889	0.166613	0.314998
0.269268	0.302367	0.256316	0.349696	0.318676	0.375919	0.221298	0.308762	0.400544	0.323153
0.278702	0.211545	0.26575	0.305245	0.28206	0.334026	0.287656	0.31308	0.348417	0.326191
0.273905	0.268628	0.268788	0.342021	0.317877	0.372721	0.259674	0.329229	0.405021	0.349696
0.295811	0.40694	0.334506	0.256316	0.290694	0.236009	0.379277	0.278062	0.190598	0.289255
0.266869	0.324272	0.245443	0.304125	0.297889	0.314679	0.28142	0.259994	0.327151	0.319635
0.299648	0.336425	0.286856	0.2504	0.278542	0.242885	0.318996	0.298689	0.303166	0.313879
0.266709	0.299968	0.216981	0.318836	0.304605	0.337704	0.247681	0.273425	0.366326	0.303486
0.28174	0.361369	0.306204	0.299009	0.318996	0.287656	0.355133	0.289575	0.254397	0.194915
0.302047	0.429805	0.346978	0.224336	0.285577	0.212984	0.39063	0.298689	0.181484	0.315478
0.278382	0.200672	0.258874	0.326191	0.278382	0.369843	0.25024	0.319156	0.39063	0.323793
0.271826	0.327311	0.26543	0.338823	0.332107	0.356252	0.228654	0.332267	0.392709	0.333387
0.25008	0.326511	0.255197	0.247522	0.285737	0.276623	0.272785	0.302367	0.316278	0.31324
0.29709	0.420051	0.341222	0.237608	0.289415	0.227055	0.387112	0.291813	0.164855	0.287816
0.289575	0.266549	0.260953	0.334346	0.310361	0.378478	0.252638	0.346019	0.39015	0.315158
0.260793	0.268788	0.262552	0.258235	0.270867	0.287176	0.280141	0.307803	0.337384	0.318836
0.28126	0.335945	0.277902	0.303965	0.307483	0.31324	0.290694	0.292293	0.278382	0.236649
0.278382	0.066677	0.263351	0.347618	0.276143	0.389191	0.272626	0.320755	0.395107	0.337064
0.262872	0.320755	0.270227	0.321234	0.314359	0.341861	0.255357	0.294851	0.367605	0.344579
0.291493	0.289575	0.289095	0.315158	0.320115	0.334826	0.308123	0.329229	0.34442	0.278222
0.275344	0.348097	0.259194	0.323153	0.307163	0.317877	0.294372	0.264791	0.304925	0.295811
0.285737	0.304445	0.269907	0.317237	0.305085	0.335785	0.257755	0.326191	0.37512	0.340422
0.292773	0.331308	0.286057	0.335305	0.334186	0.3433	0.288295	0.324912	0.377678	0.352255
0.302846	0.432363	0.348897	0.228973	0.291334	0.215062	0.385833	0.298529	0.174288	0.315158
0.287176	0.225296	0.278062	0.351455	0.31228	0.395907	0.273105	0.329869	0.396386	0.318036
0.300608	0.418292	0.340262	0.231212	0.293252	0.228814	0.381836	0.293572	0.163575	0.295011
0.272466	0.285577	0.258714	0.362168	0.333547	0.378957	0.23377	0.31324	0.401823	0.335785

For Review Only

0.280461	0.294532	0.271666	0.363288	0.322514	0.394148	0.245123	0.315798	0.412856	0.329389
0.29709	0.361209	0.302686	0.310201	0.315798	0.2819	0.340102	0.279821	0.244484	0.242565
0.283818	0.20371	0.275983	0.354973	0.329869	0.381996	0.271986	0.315318	0.346818	0.305245
0.273745	0.308922	0.266549	0.342661	0.32811	0.37512	0.222258	0.314519	0.400544	0.332587

For Review Only

0.351935	0.287496	0.35977	0.303965	0.266709	0.291493	0.299328	0.302686	0.307643	0.291014
0.302207	0.331148	0.301567	0.329069	0.309562	0.315798	0.31324	0.294372	0.314039	0.315958
0.376079	0.263671	0.382635	0.288615	0.287496	0.223057	0.219859	0.331148	0.213463	0.252798
0.339942	0.305245	0.346338	0.289095	0.286697	0.251039	0.261273	0.279341	0.259034	0.263351
0.318676	0.339303	0.301887	0.331308	0.287176	0.3433	0.352255	0.303006	0.355293	0.329549
0.350496	0.285257	0.363927	0.293252	0.258075	0.276943	0.275184	0.308602	0.267988	0.28078
0.364087	0.309882	0.358491	0.290214	0.261753	0.307643	0.323633	0.308123	0.303486	0.311001
0.275024	0.339143	0.247522	0.310681	0.300927	0.341222	0.322034	0.291813	0.339783	0.331308
0.34378	0.285737	0.351935	0.303486	0.248801	0.322514	0.322194	0.300128	0.31196	0.314839
0.277902	0.373681	0.236009	0.334186	0.338823	0.369683	0.355293	0.299328	0.369524	0.35993
0.31324	0.347777	0.320275	0.324912	0.346498	0.349536	0.353054	0.299488	0.353054	0.345859
0.370803	0.26559	0.37464	0.289894	0.28062	0.217781	0.21826	0.326671	0.211545	0.244643
0.305405	0.31276	0.298849	0.302207	0.276623	0.315478	0.324432	0.278862	0.303965	0.317077
0.191717	0.29677	0.286856	0.272306	0.29677	0.309402	0.318516	0.255037	0.31228	0.290534
0.333547	0.238088	0.348897	0.28142	0.247522	0.273585	0.279181	0.306524	0.271666	0.280461
0.304445	0.318516	0.326991	0.301727	0.307323	0.28174	0.278382	0.29661	0.283658	0.278542
0.349376	0.341861	0.346498	0.333067	0.284298	0.340582	0.345539	0.331948	0.319955	0.355612
0.342021	0.308443	0.316118	0.317557	0.320275	0.298849	0.305245	0.307963	0.31292	0.298529
0.380397	0.271186	0.384234	0.3118	0.28094	0.270387	0.276463	0.334026	0.269907	0.283019
0.305724	0.368404	0.277902	0.350176	0.346019	0.349057	0.338343	0.316437	0.35945	0.34378
0.309882	0.342021	0.300448	0.306684	0.3126	0.332427	0.345699	0.301407	0.331468	0.346658
0.344739	0.334026	0.331148	0.324752	0.278382	0.326511	0.340902	0.314359	0.321394	0.335785
0.276943	0.387912	0.304605	0.325871	0.362968	0.394308	0.396706	0.285577	0.392229	0.376559
0.188519	0.332907	0.241925	0.288775	0.308123	0.340742	0.325871	0.233451	0.336105	0.310361
0.365366	0.286377	0.365846	0.300448	0.271986	0.284938	0.284138	0.31308	0.272306	0.287176
0.360409	0.276463	0.3748	0.239527	0.279661	0.092261	0.152542	0.329869	0.098337	0.121202
0.338663	0.263991	0.353054	0.277902	0.263671	0.214423	0.20275	0.295811	0.212824	0.221458
0.28158	0.264311	0.305724	0.262392	0.259034	0.246083	0.251679	0.26591	0.25056	0.253438
0.295491	0.303326	0.298369	0.305884	0.284458	0.325871	0.322194	0.278062	0.31276	0.316597
0.244004	0.416374	0.195875	0.350496	0.366485	0.411577	0.408858	0.279341	0.409018	0.394627
0.362808	0.252638	0.368244	0.315158	0.271986	0.253758	0.244484	0.319156	0.245923	0.259034
0.380237	0.292933	0.378957	0.331948	0.300448	0.291174	0.288136	0.331468	0.298209	0.299009
0.373361	0.300288	0.383754	0.300927	0.261113	0.302686	0.319795	0.310681	0.301407	0.304925
0.153822	0.348097	0.256316	0.300128	0.314839	0.349856	0.349376	0.246722	0.348897	0.323153
0.313879	0.317237	0.338663	0.307963	0.315798	0.295491	0.297889	0.29725	0.304285	0.298209
0.262072	0.284138	0.295171	0.280141	0.285417	0.263671	0.257595	0.261912	0.267509	0.221138
0.276463	0.303326	0.320275	0.272306	0.260153	0.321874	0.323313	0.226095	0.323633	0.310841
0.32811	0.32779	0.321554	0.288615	0.291653	0.301567	0.305884	0.280141	0.306524	0.302846
0.21906	0.380077	0.261753	0.329069	0.35929	0.385833	0.382315	0.256316	0.382475	0.371922
0.300768	0.367285	0.323473	0.330349	0.321554	0.342661	0.345379	0.31244	0.342501	0.339623
0.24976	0.411737	0.179565	0.347298	0.370643	0.394308	0.391589	0.279501	0.392069	0.381836
0.284778	0.339623	0.266869	0.311001	0.334186	0.34426	0.335945	0.285737	0.336745	0.336745
0.277422	0.356412	0.254397	0.332747	0.327311	0.366006	0.341062	0.284778	0.35865	0.349856
0.335785	0.34426	0.318836	0.320275	0.310521	0.348577	0.350496	0.309562	0.34426	0.355133
0.29629	0.370803	0.253758	0.340102	0.335945	0.370643	0.354493	0.31292	0.370483	0.355932
0.366485	0.259834	0.374001	0.261113	0.277582	0.176047	0.205628	0.323313	0.17221	0.208027
0.365046	0.268628	0.37512	0.293892	0.288615	0.162296	0.177167	0.323953	0.18708	0.1882
0.298049	0.300288	0.315478	0.276783	0.300448	0.243684	0.273745	0.294851	0.255357	0.242405
0.32843	0.34442	0.313719	0.344579	0.299009	0.35897	0.361848	0.305564	0.350336	0.358491
0.35945	0.255357	0.371922	0.288615	0.270067	0.286856	0.298849	0.31308	0.300927	0.290854

For Review Only

0.370163	0.262552	0.370483	0.253118	0.273585	0.119923	0.175887	0.32763	0.151743	0.162136
0.335305	0.298529	0.365686	0.308443	0.310681	0.294691	0.290214	0.315638	0.291334	0.297889
0.379117	0.304445	0.382155	0.31228	0.25056	0.293252	0.310201	0.329869	0.282699	0.301407
0.372402	0.299488	0.382795	0.300448	0.260633	0.302686	0.320275	0.310681	0.301247	0.305245
0.367285	0.252319	0.379597	0.294532	0.273585	0.258395	0.252478	0.322993	0.260313	0.272146
0.349217	0.334666	0.32875	0.334346	0.29693	0.32795	0.335785	0.318836	0.319795	0.341541
0.380077	0.271986	0.383435	0.310521	0.28078	0.269747	0.276143	0.333387	0.269907	0.282539
0.370803	0.267829	0.371602	0.292933	0.300608	0.144068	0.162296	0.330668	0.17221	0.168212
0.36009	0.271186	0.363447	0.255197	0.280301	0.103454	0.147905	0.316437	0.137512	0.12536
0.200032	0.37528	0.217941	0.323953	0.326511	0.389031	0.384874	0.226895	0.384394	0.366006
0.244803	0.307963	0.300128	0.293092	0.278542	0.341062	0.345059	0.232331	0.340102	0.320755
0.287976	0.263192	0.310042	0.26591	0.257755	0.256316	0.251999	0.268948	0.251839	0.258395
0.361848	0.253918	0.377518	0.29629	0.271027	0.232331	0.204829	0.333067	0.227694	0.251999
0.356412	0.267669	0.371602	0.307163	0.291653	0.245283	0.21874	0.322354	0.242565	0.260473
0.379117	0.304605	0.381676	0.31276	0.250879	0.293092	0.309242	0.329549	0.28174	0.300608
0.295011	0.302686	0.298689	0.304765	0.283818	0.325072	0.321874	0.277742	0.311481	0.316278
0.314839	0.28126	0.329389	0.269268	0.26511	0.259354	0.269108	0.276783	0.260793	0.263991
0.283179	0.315798	0.288455	0.289575	0.289575	0.338343	0.344579	0.252478	0.332107	0.322674
0.336904	0.297889	0.34442	0.282379	0.324912	0.28062	0.271346	0.299169	0.293412	0.29677
0.389671	0.17237	0.395907	0.283179	0.26511	0.25072	0.259354	0.332107	0.275983	0.256316
0.3756	0.28158	0.384234	0.323953	0.297889	0.303166	0.299328	0.331628	0.301887	0.303965
0.368404	0.302686	0.385993	0.314359	0.245443	0.294052	0.305564	0.322194	0.290694	0.303166
0.326831	0.258075	0.34378	0.290374	0.271346	0.245123	0.21906	0.294691	0.243204	0.251999
0.290214	0.342821	0.315158	0.320115	0.345699	0.350656	0.335146	0.269108	0.34314	0.337704
0.317717	0.310042	0.324272	0.293412	0.284618	0.325552	0.325552	0.285257	0.314839	0.320915
0.339942	0.310042	0.352414	0.294372	0.314839	0.29661	0.294372	0.320915	0.282539	0.300288
0.295171	0.370163	0.253278	0.340102	0.336904	0.370163	0.353694	0.31276	0.370483	0.355133
0.350176	0.263192	0.358171	0.29645	0.28078	0.252958	0.241286	0.319795	0.232811	0.26543
0.34346	0.341382	0.309242	0.349217	0.315638	0.361689	0.350176	0.326991	0.361848	0.35977
0.208027	0.381036	0.261912	0.336904	0.351935	0.388551	0.384394	0.251359	0.382795	0.371282
0.360409	0.270867	0.376079	0.23393	0.283978	0.074832	0.144867	0.32843	0.10905	0.111129
0.35913	0.275184	0.377998	0.318676	0.28174	0.29725	0.276623	0.32747	0.294691	0.303006
0.320275	0.330668	0.313559	0.31324	0.294052	0.319316	0.317877	0.297889	0.324592	0.31196
0.227055	0.294372	0.28126	0.284618	0.274065	0.300448	0.291973	0.261593	0.301567	0.278862
0.199872	0.387912	0.253118	0.342821	0.364567	0.403422	0.397026	0.255676	0.396546	0.382155
0.377838	0.303166	0.380397	0.31244	0.25024	0.293412	0.309562	0.32859	0.28206	0.300608
0.321234	0.286377	0.336425	0.284298	0.284138	0.294691	0.309882	0.274225	0.304925	0.290694
0.295811	0.370643	0.253918	0.340422	0.336425	0.370643	0.354333	0.3126	0.370483	0.355772
0.283179	0.352574	0.254237	0.304765	0.317557	0.354973	0.339623	0.282219	0.355772	0.336265
0.31212	0.369524	0.283978	0.344899	0.313879	0.368564	0.372082	0.295491	0.370163	0.371442
0.319955	0.31276	0.333866	0.314998	0.300608	0.314998	0.31276	0.285897	0.307004	0.314199
0.283818	0.351455	0.253278	0.322674	0.317717	0.357691	0.356092	0.298049	0.364087	0.347458
0.379597	0.295171	0.392709	0.299488	0.248321	0.284618	0.307323	0.339303	0.276303	0.301567
0.382315	0.287336	0.394947	0.317877	0.283818	0.290694	0.272945	0.345379	0.2819	0.295971
0.336904	0.294052	0.346818	0.282859	0.290534	0.301567	0.31308	0.319795	0.301887	0.31196
0.362168	0.255836	0.378158	0.29645	0.272306	0.233611	0.204989	0.333547	0.228973	0.253278
0.340582	0.259674	0.355293	0.292613	0.257275	0.298849	0.326831	0.302846	0.298849	0.298209
0.362808	0.28094	0.3756	0.295651	0.259994	0.244803	0.238887	0.324113	0.263511	0.260953
0.102494	0.35945	0.24992	0.314679	0.319156	0.356572	0.357371	0.21938	0.357691	0.335465
0.31308	0.262072	0.329549	0.240966	0.274225	0.170291	0.186281	0.290054	0.192517	0.177966
0.320915	0.278222	0.334026	0.266709	0.287496	0.17205	0.207547	0.298369	0.193476	0.168692
0.349376	0.266709	0.358171	0.325712	0.305724	0.323473	0.301727	0.307163	0.333387	0.316597
0.336585	0.251359	0.346019	0.283019	0.266869	0.246722	0.220659	0.307963	0.23505	0.251359
0.227854	0.395587	0.269747	0.334346	0.368884	0.397825	0.388391	0.253438	0.389831	0.380876
0.232811	0.364567	0.256796	0.325232	0.333706	0.376399	0.379117	0.247362	0.376399	0.364886

For Review Only

0.279661	0.309562	0.304125	0.299968	0.279181	0.31292	0.309722	0.26559	0.306524	0.302686
0.352894	0.198433	0.35993	0.327311	0.275664	0.313879	0.293732	0.307803	0.323793	0.310201
0.325232	0.295651	0.340422	0.278222	0.29757	0.160217	0.21842	0.309722	0.207387	0.183563
0.315318	0.336105	0.311161	0.310681	0.321075	0.317397	0.29629	0.295491	0.325232	0.314359
0.319955	0.259834	0.336425	0.230572	0.256636	0.17253	0.212184	0.291653	0.161976	0.162776
0.124081	0.361049	0.270387	0.307004	0.306364	0.342661	0.34426	0.23473	0.349057	0.32795
0.363288	0.276303	0.37432	0.292613	0.270867	0.207067	0.212824	0.327151	0.207227	0.228814
0.323153	0.279181	0.345539	0.267029	0.266709	0.167253	0.210106	0.299169	0.160058	0.184522
0.352894	0.272466	0.366326	0.292133	0.272945	0.263511	0.245923	0.323313	0.251039	0.275664
0.271826	0.292773	0.309242	0.28158	0.28078	0.266869	0.255197	0.26639	0.267189	0.262072
0.288295	0.307163	0.289415	0.29709	0.300128	0.26639	0.279341	0.290854	0.259834	0.260953
0.267988	0.291334	0.28062	0.291334	0.306844	0.258235	0.284938	0.257275	0.269428	0.250879
0.303806	0.288615	0.333706	0.307004	0.292613	0.315478	0.318196	0.271666	0.321714	0.304925
0.297889	0.285737	0.309722	0.263991	0.270707	0.252159	0.263991	0.272466	0.256956	0.256636
0.324113	0.292933	0.335785	0.273745	0.298369	0.308602	0.313719	0.316917	0.307803	0.315318
0.351615	0.259354	0.358011	0.283339	0.267988	0.23377	0.23377	0.309562	0.229133	0.252319
0.310681	0.31196	0.330189	0.306524	0.293572	0.29693	0.289575	0.289735	0.292293	0.290054
0.307163	0.276463	0.318996	0.255516	0.271186	0.167893	0.210265	0.286537	0.197634	0.190118
0.365686	0.272626	0.358011	0.292933	0.305884	0.295011	0.29613	0.330189	0.306044	0.291014
0.303646	0.260633	0.32763	0.256796	0.256476	0.201151	0.233131	0.284778	0.215222	0.225616
0.114167	0.35945	0.261433	0.31228	0.31292	0.348897	0.351455	0.227854	0.357371	0.334346
0.345219	0.317557	0.350496	0.306364	0.302846	0.318996	0.319635	0.340742	0.310201	0.338184
0.293572	0.269907	0.311481	0.257275	0.261912	0.226095	0.23393	0.271027	0.236169	0.236489
0.357052	0.280141	0.376079	0.31292	0.26559	0.299968	0.284618	0.316757	0.302367	0.29757
0.335785	0.255516	0.354813	0.272466	0.260793	0.254557	0.244004	0.308922	0.252159	0.272306
0.201791	0.381196	0.260953	0.32875	0.349536	0.391909	0.385513	0.245443	0.387432	0.374161
0.319635	0.303006	0.337064	0.2819	0.302846	0.207227	0.240806	0.295011	0.226735	0.221298
0.293572	0.32827	0.308602	0.29645	0.314519	0.321075	0.325232	0.284938	0.322034	0.316757
0.35897	0.284458	0.362488	0.313559	0.26639	0.298369	0.293572	0.322354	0.294532	0.294691
0.283499	0.260953	0.323793	0.278542	0.238088	0.303965	0.298529	0.263511	0.301567	0.293892
0.383754	0.270067	0.383435	0.314039	0.289575	0.244484	0.244963	0.340742	0.250879	0.267349
0.369683	0.272945	0.365846	0.325552	0.300768	0.29757	0.304925	0.331308	0.309402	0.290854
0.165174	0.385034	0.253438	0.333547	0.340902	0.39127	0.386473	0.240006	0.383914	0.37464
0.282539	0.276943	0.29645	0.273265	0.273425	0.26559	0.263032	0.262712	0.26543	0.268948
0.383595	0.295651	0.394308	0.299328	0.249121	0.285417	0.306364	0.342181	0.275823	0.302207
0.315158	0.257435	0.333866	0.252478	0.264631	0.15686	0.216022	0.277582	0.18756	0.188519
0.157819	0.346818	0.258235	0.299648	0.313399	0.346658	0.345699	0.247362	0.345059	0.320275
0.268788	0.315958	0.309562	0.303806	0.298209	0.318356	0.320755	0.270707	0.316437	0.298529
0.104573	0.369524	0.259514	0.316118	0.322034	0.361848	0.361689	0.228814	0.370003	0.345059
0.354973	0.235209	0.362488	0.277103	0.275344	0.240806	0.231532	0.320595	0.235369	0.254717
0.300768	0.313719	0.324592	0.278542	0.316278	0.260313	0.285417	0.299488	0.261912	0.268308
0.248321	0.388072	0.295811	0.34362	0.368564	0.392229	0.384074	0.280141	0.394947	0.376879
0.317077	0.32891	0.341382	0.307004	0.323313	0.320275	0.315798	0.301247	0.314519	0.314039
0	0.369683	0.246402	0.319795	0.314679	0.367285	0.368564	0.228494	0.364567	0.34346
0.369683	0	0.377039	0.295491	0.260153	0.26559	0.251999	0.318836	0.273105	0.271666
0.246402	0.377039	0	0.336585	0.349376	0.37432	0.369364	0.269428	0.372402	0.357052
0.319795	0.295491	0.336585	0	0.284138	0.232971	0.278542	0.283499	0.236649	0.254557
0.314679	0.260153	0.349376	0.284138	0	0.274384	0.297889	0.282219	0.278222	0.285098
0.367285	0.26559	0.37432	0.232971	0.274384	0	0.145987	0.326991	0.098337	0.086345
0.368564	0.251999	0.369364	0.278542	0.297889	0.145987	0	0.325392	0.170451	0.168852
0.228494	0.318836	0.269428	0.283499	0.282219	0.326991	0.325392	0	0.330828	0.306044
0.364567	0.273105	0.372402	0.236649	0.278222	0.098337	0.170451	0.330828	0	0.142629
0.34346	0.271666	0.357052	0.254557	0.285098	0.086345	0.168852	0.306044	0.142629	0
0.264151	0.372082	0.28158	0.336745	0.338184	0.337384	0.337544	0.259194	0.344579	0.32795
0.295971	0.226735	0.32859	0.266869	0.274704	0.294851	0.285737	0.280141	0.287496	0.282859

For Review Only

0.360889	0.272146	0.371122	0.28142	0.276303	0.204989	0.204989	0.322354	0.20387	0.209786
0.255996	0.326671	0.257115	0.304285	0.294691	0.320755	0.311321	0.239687	0.316917	0.307803
0.371442	0.253758	0.384234	0.283019	0.268148	0.198113	0.232331	0.321075	0.241445	0.231372
0.304285	0.346338	0.305564	0.298849	0.338503	0.334026	0.346658	0.29645	0.345859	0.337384
0.334346	0.342181	0.305724	0.32811	0.317557	0.331308	0.349376	0.31308	0.339783	0.325072
0.29677	0.31212	0.324113	0.317077	0.314519	0.336904	0.336585	0.28142	0.338024	0.325871
0.355133	0.279181	0.369204	0.314039	0.274384	0.241286	0.246242	0.323473	0.252638	0.259514
0.292453	0.339303	0.317557	0.311001	0.318836	0.317397	0.314998	0.267189	0.323793	0.31228
0.258874	0.337704	0.298049	0.318516	0.326031	0.357211	0.351455	0.28174	0.351135	0.351295
0.283339	0.259034	0.305245	0.255996	0.263991	0.221618	0.223537	0.264631	0.230253	0.223377
0.267829	0.360569	0.315318	0.323793	0.333547	0.358011	0.354653	0.299488	0.347298	0.342661
0.383914	0.275184	0.383595	0.325072	0.2819	0.29661	0.289095	0.330508	0.293412	0.295331
0.336904	0.272466	0.350815	0.242405	0.274864	0.105692	0.18772	0.29709	0.130157	0.153662
0.242245	0.300927	0.270387	0.294212	0.307643	0.324592	0.308443	0.258395	0.322674	0.300288
0.339463	0.2496	0.35865	0.287336	0.268468	0.204989	0.229133	0.300128	0.222737	0.228174
0.287016	0.282699	0.332907	0.298049	0.153662	0.299808	0.311001	0.273585	0.302686	0.29709
0.102015	0.353854	0.254717	0.315958	0.31212	0.364247	0.362328	0.221138	0.362648	0.346978
0.309882	0.365846	0.326031	0.335465	0.362488	0.368884	0.369843	0.304285	0.369204	0.365686
0.269268	0.246882	0.310201	0.259674	0.273905	0.291174	0.289255	0.267349	0.289575	0.279981
0.368084	0.285737	0.361529	0.304605	0.287816	0.260633	0.252159	0.330349	0.251039	0.260313
0.301887	0.267669	0.332587	0.23473	0.270547	0.182443	0.217941	0.282859	0.184042	0.210585
0.346178	0.268148	0.35913	0.288775	0.269587	0.261433	0.239687	0.314839	0.250879	0.271186
0.277422	0.26511	0.299328	0.267988	0.257755	0.24992	0.257435	0.260473	0.255197	0.254077
0.208507	0.358331	0.254557	0.303806	0.323473	0.362008	0.351775	0.24928	0.354013	0.338024
0.287496	0.347458	0.310681	0.319635	0.353534	0.358011	0.354173	0.272146	0.349696	0.346178
0.356732	0.266869	0.371442	0.306844	0.290694	0.245603	0.220339	0.323153	0.243044	0.261433
0.306044	0.317397	0.301887	0.301567	0.306684	0.346978	0.341062	0.298049	0.325871	0.347458
0.158139	0.351295	0.263991	0.299169	0.318356	0.348577	0.345379	0.239207	0.347618	0.32811
0.338024	0.246083	0.353694	0.28094	0.275504	0.241126	0.229613	0.307004	0.239527	0.253758
0.293252	0.288455	0.315478	0.289255	0.29629	0.306684	0.302686	0.289095	0.310361	0.294851
0.330508	0.286057	0.339463	0.275823	0.2811	0.274704	0.291014	0.300768	0.28094	0.275344
0.354493	0.271027	0.375919	0.292133	0.264471	0.294372	0.29645	0.306684	0.286537	0.29741
0.325552	0.269587	0.3441	0.260793	0.251999	0.269907	0.266709	0.278382	0.269108	0.279821
0.379757	0.25056	0.385993	0.314998	0.289255	0.26495	0.256636	0.335465	0.287016	0.26607
0.342181	0.2811	0.361209	0.31292	0.276783	0.293412	0.307163	0.307483	0.302047	0.284298
0.108251	0.352414	0.258874	0.315478	0.313719	0.361848	0.35961	0.222578	0.360249	0.34378
0.336265	0.283818	0.364247	0.317877	0.251999	0.301887	0.31212	0.292613	0.303646	0.289735
0.356572	0.266709	0.370483	0.306844	0.290694	0.244963	0.21906	0.321874	0.242245	0.260793
0.339623	0.305405	0.348097	0.291973	0.301407	0.217941	0.256316	0.311161	0.229933	0.229293
0.324432	0.28206	0.345859	0.291493	0.254237	0.313559	0.315638	0.261113	0.305564	0.309562
0.369683	0.254397	0.370323	0.285737	0.29709	0.1567	0.026063	0.326991	0.178446	0.181004
0.352095	0.26607	0.360249	0.286217	0.28126	0.272785	0.232651	0.314679	0.260953	0.272466
0.37576	0.263831	0.381676	0.286697	0.287336	0.222098	0.21874	0.329229	0.212824	0.251519
0.369524	0.264151	0.373361	0.232971	0.276463	0.033579	0.134794	0.322993	0.10921	0.085225
0.197634	0.389351	0.255836	0.331628	0.356412	0.39015	0.381676	0.251359	0.38999	0.376239
0.339143	0.29709	0.350975	0.298209	0.3118	0.253438	0.240966	0.309722	0.264631	0.257595
0.276463	0.306684	0.333227	0.31324	0.297889	0.288455	0.292293	0.289095	0.293732	0.28142
0.326991	0.303006	0.339303	0.322514	0.286377	0.32875	0.325072	0.29757	0.325392	0.332107
0.310201	0.365206	0.326351	0.333227	0.361209	0.368084	0.369364	0.303965	0.368404	0.364727
0.23377	0.37496	0.277263	0.32827	0.322034	0.377998	0.3748	0.268948	0.376239	0.368244
0.145507	0.34378	0.236009	0.290374	0.307803	0.349696	0.341062	0.224976	0.341382	0.323473
0.308443	0.326351	0.31212	0.303965	0.299648	0.3126	0.301407	0.282859	0.309882	0.319316
0.37448	0.287336	0.382635	0.309402	0.307004	0.262552	0.26495	0.330029	0.257115	0.28126
0.202111	0.35977	0.26575	0.305564	0.292933	0.376559	0.374161	0.228174	0.372402	0.356092
0.26575	0.348417	0.298849	0.322833	0.340742	0.371922	0.366326	0.25024	0.365686	0.354653

For Review Only

0.361848	0.254237	0.377199	0.29661	0.271027	0.232971	0.204669	0.333067	0.228014	0.252798
0.220019	0.392709	0.263991	0.334506	0.363447	0.397825	0.393348	0.253918	0.387752	0.377678
0.269108	0.245443	0.318996	0.294532	0.283658	0.316917	0.310042	0.285257	0.316118	0.306684
0.300927	0.335465	0.275983	0.310681	0.330189	0.329549	0.321874	0.288295	0.332107	0.316437
0.387912	0.267509	0.388711	0.308443	0.287336	0.246242	0.246562	0.335305	0.263671	0.271506
0.322194	0.339783	0.335625	0.326511	0.332267	0.346658	0.347458	0.310681	0.345219	0.352734
0.289415	0.341861	0.285098	0.317397	0.3118	0.321554	0.315798	0.298689	0.338823	0.317397
0.362968	0.263192	0.3756	0.262552	0.279821	0.176847	0.21922	0.322034	0.199073	0.189319
0.282219	0.347298	0.318996	0.31276	0.337544	0.313879	0.330029	0.28126	0.325712	0.320915
0.261273	0.400224	0.21874	0.339623	0.35929	0.399584	0.380077	0.275184	0.387272	0.385673
0.199392	0.317557	0.261912	0.294691	0.293092	0.302367	0.31244	0.249121	0.301247	0.285897
0.212664	0.39095	0.26511	0.342821	0.362008	0.403262	0.39015	0.260153	0.397346	0.382155
0.28062	0.264311	0.300608	0.269428	0.259674	0.248321	0.254077	0.262872	0.24976	0.251999
0.316118	0.301407	0.329709	0.214902	0.304925	0.29709	0.300128	0.289095	0.309882	0.308922
0.344739	0.31308	0.348897	0.318356	0.303006	0.290854	0.303326	0.29677	0.300927	0.293412
0.3126	0.263032	0.334506	0.262392	0.261593	0.193476	0.204029	0.272466	0.193796	0.208826
0.376399	0.263831	0.3748	0.288455	0.29757	0.225776	0.209306	0.332747	0.222897	0.244164
0.326831	0.319316	0.351615	0.325552	0.313719	0.32795	0.335945	0.305245	0.330189	0.324113
0.251519	0.358331	0.2819	0.318516	0.308443	0.345059	0.346818	0.251519	0.34314	0.332267
0.270867	0.333547	0.300448	0.330189	0.316917	0.357531	0.349856	0.274704	0.35913	0.345379
0.254557	0.369843	0.294052	0.326511	0.333387	0.351775	0.358011	0.294052	0.355453	0.342341
0.322993	0.333706	0.318676	0.288775	0.319316	0.317397	0.315958	0.288136	0.315958	0.316917
0.303006	0.333547	0.333227	0.317077	0.316757	0.314998	0.31244	0.31244	0.316917	0.317237
0.347777	0.275664	0.365686	0.226255	0.276943	0.073233	0.161976	0.320755	0.115286	0.114647
0.356732	0.287496	0.365046	0.299169	0.269907	0.299808	0.31276	0.302207	0.299968	0.301247
0.305724	0.272626	0.327311	0.302686	0.32875	0.322674	0.31292	0.302047	0.316437	0.319955
0.199712	0.3756	0.217301	0.323793	0.326191	0.39031	0.385513	0.225136	0.385193	0.367125
0.342341	0.303326	0.347298	0.321075	0.299169	0.307803	0.292613	0.320275	0.289894	0.302367
0.20355	0.393508	0.254877	0.345699	0.367605	0.408698	0.400064	0.264631	0.402303	0.384074
0.371282	0.278382	0.368884	0.300608	0.283499	0.210106	0.216821	0.32811	0.211705	0.236649
0.32843	0.320115	0.334666	0.302047	0.289255	0.306044	0.299968	0.303965	0.305085	0.305245
0.370803	0.26623	0.377199	0.318676	0.286057	0.272785	0.256316	0.324912	0.268468	0.290374
0.232012	0.369524	0.277902	0.320115	0.334666	0.381356	0.380237	0.261433	0.37576	0.362968
0.319795	0.304925	0.323953	0.267669	0.306044	0.183403	0.238407	0.300768	0.211705	0.201791
0.245603	0.317397	0.307004	0.301727	0.298049	0.32763	0.322674	0.259034	0.32763	0.314199
0.339623	0.259194	0.346178	0.246882	0.277902	0.157179	0.127918	0.302047	0.169332	0.168852
0.283499	0.351135	0.314839	0.315158	0.346019	0.352894	0.341062	0.260793	0.352894	0.342981
0.204669	0.401663	0.262552	0.333706	0.365526	0.4055	0.394148	0.257275	0.394627	0.386313
0.365366	0.200672	0.37448	0.277582	0.24992	0.271666	0.282379	0.320115	0.270547	0.287656
0.358011	0.267669	0.372082	0.308123	0.291334	0.245603	0.220659	0.323473	0.243204	0.261593
0.260633	0.282379	0.294691	0.279021	0.283978	0.262232	0.256156	0.260633	0.267189	0.21938
0.226575	0.389031	0.261593	0.342181	0.367285	0.399264	0.395587	0.259194	0.393348	0.383435
0.366645	0.237448	0.381516	0.319955	0.275983	0.279981	0.295651	0.319795	0.289415	0.275344
0.283658	0.269428	0.330668	0.288455	0.228654	0.276143	0.273105	0.261912	0.278702	0.28094
0.303006	0.275184	0.327151	0.304125	0.309882	0.322034	0.317717	0.299328	0.315798	0.315318
0.382635	0.298529	0.386633	0.294691	0.240166	0.293572	0.308602	0.336904	0.283658	0.311641
0.342181	0.289415	0.349696	0.299488	0.295011	0.251679	0.235689	0.29645	0.251679	0.262872
0.323953	0.305085	0.353054	0.306844	0.288615	0.315638	0.322194	0.292293	0.304125	0.308762
0.318996	0.304925	0.340102	0.292133	0.327151	0.228814	0.154301	0.302686	0.243204	0.228014
0.32859	0.261433	0.339303	0.2811	0.2819	0.287176	0.279981	0.311161	0.284938	0.288455
0.341701	0.299808	0.301567	0.318836	0.309082	0.278222	0.264151	0.326031	0.28078	0.285577
0.200352	0.391429	0.256796	0.342341	0.361369	0.407419	0.395587	0.26543	0.399904	0.383115
0.387752	0.274544	0.381836	0.313719	0.291813	0.29661	0.293252	0.32763	0.293572	0.301727
0.21858	0.391589	0.262392	0.339783	0.364567	0.399424	0.392229	0.257755	0.398465	0.383435
0.376239	0.263511	0.382955	0.288136	0.287976	0.222258	0.21874	0.330828	0.213623	0.252159

For Review Only

0.392229	0.267189	0.38999	0.29709	0.299488	0.233291	0.232331	0.34346	0.228494	0.252319
0.284938	0.363288	0.310521	0.31196	0.338823	0.347458	0.338663	0.279341	0.34394	0.337704
0.376239	0.279021	0.388551	0.307323	0.279981	0.301087	0.309402	0.32859	0.29645	0.309242
0.371282	0.26559	0.377678	0.301727	0.277902	0.243204	0.221138	0.324912	0.237928	0.254397

For Review Only

0.324912	0.270387	0.313879	0.307163	0.290214	0.342661	0.334826	0.325232	0.264791	0.300128
0.325232	0.314359	0.29693	0.286697	0.32763	0.334666	0.333387	0.3433	0.244803	0.319635
0.37576	0.270867	0.227374	0.331788	0.236808	0.370323	0.338983	0.340742	0.252798	0.333866
0.337384	0.271986	0.269907	0.294212	0.276303	0.349856	0.32891	0.324752	0.263991	0.272146
0.307643	0.311641	0.362648	0.290534	0.330189	0.350656	0.303646	0.316757	0.337064	0.304445
0.357052	0.263511	0.277103	0.314359	0.271506	0.358331	0.336265	0.332907	0.257915	0.306364
0.338024	0.310681	0.315798	0.31292	0.289894	0.356892	0.327151	0.333706	0.276303	0.317237
0.295331	0.300288	0.339303	0.282699	0.341861	0.309242	0.310841	0.339463	0.321714	0.314998
0.330828	0.291973	0.323633	0.308443	0.29613	0.345699	0.337384	0.3118	0.299169	0.307803
0.298689	0.334666	0.3748	0.289735	0.363128	0.316118	0.294691	0.339783	0.358171	0.330508
0.245283	0.278542	0.35913	0.278222	0.335625	0.287656	0.352414	0.288136	0.349696	0.320595
0.369843	0.275983	0.223377	0.319795	0.23377	0.364247	0.337704	0.340102	0.242565	0.332107
0.317557	0.292933	0.331148	0.282859	0.306364	0.336265	0.325712	0.311641	0.295811	0.316278
0.280461	0.270227	0.310841	0.26639	0.323473	0.290214	0.323313	0.317397	0.310201	0.286217
0.337544	0.266869	0.283019	0.299968	0.244164	0.324592	0.32795	0.307803	0.274225	0.303806
0.309402	0.272785	0.299169	0.292293	0.304925	0.351935	0.32827	0.304765	0.292773	0.295651
0.336425	0.313719	0.349696	0.315958	0.307803	0.353374	0.341222	0.333547	0.300608	0.295651
0.300288	0.287976	0.320275	0.305245	0.310042	0.332587	0.305085	0.325072	0.26511	0.305884
0.373201	0.282539	0.270867	0.334186	0.255197	0.372562	0.357691	0.336904	0.258395	0.325072
0.307163	0.341062	0.370803	0.300128	0.367605	0.349376	0.299328	0.348577	0.339143	0.3126
0.286377	0.325232	0.361529	0.283179	0.324272	0.322354	0.324432	0.325072	0.334666	0.325232
0.322354	0.31276	0.339783	0.305405	0.29661	0.3756	0.330189	0.327311	0.306524	0.325232
0.274065	0.331628	0.394787	0.264151	0.396226	0.261593	0.362488	0.275184	0.394308	0.336904
0.253438	0.268788	0.319156	0.244963	0.34378	0.283339	0.307004	0.276303	0.334826	0.283978
0.363927	0.275983	0.282219	0.31276	0.272146	0.36009	0.330029	0.3126	0.257275	0.318996
0.345699	0.284778	0.215702	0.319635	0.240326	0.346818	0.348097	0.335146	0.262072	0.32795
0.357851	0.285897	0.231212	0.302207	0.270387	0.352255	0.325072	0.322674	0.254877	0.333706
0.293572	0.25072	0.26607	0.264151	0.262072	0.299169	0.303326	0.289575	0.263032	0.271666
0.309402	0.294372	0.329389	0.285897	0.307483	0.345379	0.333387	0.288935	0.304125	0.301407
0.283818	0.353054	0.40678	0.26559	0.417493	0.303486	0.308762	0.337864	0.408698	0.326351
0.369204	0.271186	0.245123	0.31228	0.254237	0.362168	0.354013	0.32859	0.267988	0.323793
0.369204	0.280301	0.295651	0.332907	0.272785	0.382955	0.352255	0.330029	0.291973	0.313399
0.341861	0.290054	0.314679	0.329869	0.268788	0.37576	0.330668	0.329069	0.287816	0.311481
0.263032	0.270227	0.348897	0.259834	0.353854	0.319955	0.331308	0.291014	0.326031	0.287816
0.291813	0.294691	0.299808	0.299009	0.306364	0.31212	0.353854	0.294372	0.326511	0.308443
0.304445	0.26591	0.232491	0.270387	0.29613	0.321714	0.298689	0.314359	0.268948	0.314039
0.291653	0.260473	0.323953	0.278382	0.302846	0.301087	0.308123	0.304765	0.31324	0.276943
0.290694	0.290534	0.322354	0.278382	0.306844	0.297889	0.317557	0.307323	0.304925	0.279341
0.221938	0.319156	0.385833	0.244643	0.393028	0.26511	0.338823	0.26543	0.382795	0.291813
0.275823	0.315798	0.371122	0.282699	0.351935	0.259194	0.337864	0.284298	0.342021	0.262712
0.269907	0.347618	0.396067	0.26559	0.403902	0.299648	0.301727	0.32747	0.401183	0.323633
0.291973	0.315158	0.351935	0.286377	0.348257	0.310681	0.322993	0.310681	0.334026	0.303166
0.285737	0.326511	0.351615	0.28174	0.356412	0.316437	0.269747	0.326831	0.337224	0.306524
0.317077	0.32763	0.358171	0.299968	0.341382	0.329229	0.307803	0.347138	0.340262	0.321075
0.301247	0.336904	0.362488	0.290054	0.367285	0.29629	0.232171	0.351455	0.355293	0.32795
0.357851	0.263671	0.221298	0.321394	0.243364	0.357851	0.333547	0.332587	0.243204	0.326671
0.335305	0.291813	0.178286	0.321554	0.223857	0.351775	0.342501	0.334666	0.235689	0.315158
0.304285	0.269268	0.287976	0.28206	0.286377	0.290534	0.334506	0.310042	0.309402	0.294851
0.31196	0.34346	0.366006	0.299328	0.330189	0.355453	0.311641	0.34442	0.338663	0.302367
0.342501	0.242885	0.306044	0.308443	0.257115	0.330668	0.333706	0.300927	0.278542	0.298689

For Review Only

0.361209	0.276943	0.20339	0.321394	0.204029	0.331148	0.347138	0.342661	0.255996	0.309242
0.354333	0.311641	0.300768	0.308762	0.3126	0.351615	0.363927	0.329229	0.277422	0.301887
0.367125	0.295651	0.322993	0.325072	0.264151	0.378798	0.341541	0.330508	0.253438	0.318676
0.340422	0.290214	0.313719	0.32875	0.268308	0.37448	0.329709	0.329069	0.288136	0.3118
0.366965	0.274225	0.245123	0.325392	0.255197	0.358331	0.342981	0.337384	0.254717	0.32747
0.332747	0.333866	0.340102	0.307323	0.313879	0.340582	0.333866	0.349376	0.294212	0.332747
0.371922	0.28158	0.271186	0.333866	0.255676	0.372562	0.354973	0.336265	0.259034	0.323313
0.344899	0.309562	0.17173	0.319476	0.244324	0.361848	0.360729	0.341382	0.240166	0.319156
0.333387	0.283179	0.219699	0.31228	0.227055	0.350016	0.330668	0.332907	0.251839	0.307323
0.216022	0.319635	0.37432	0.189639	0.395107	0.287336	0.308123	0.299968	0.389671	0.268468
0.275344	0.2811	0.331948	0.26495	0.325392	0.317557	0.326511	0.29645	0.341222	0.295331
0.294052	0.244324	0.26591	0.263831	0.261273	0.299009	0.31276	0.285257	0.259194	0.282539
0.369843	0.278702	0.15702	0.319635	0.247681	0.362968	0.344579	0.34314	0.211864	0.336585
0.368564	0.299009	0.116246	0.321714	0.273265	0.364407	0.354173	0.346498	0.243204	0.3433
0.367125	0.294851	0.323473	0.325232	0.264311	0.378318	0.341541	0.329709	0.251999	0.318516
0.308762	0.293892	0.32843	0.285417	0.307004	0.345539	0.332267	0.288935	0.303965	0.300608
0.310042	0.258395	0.280301	0.283658	0.271826	0.308443	0.317237	0.298849	0.26511	0.284618
0.295171	0.261273	0.333866	0.276943	0.329229	0.306044	0.299648	0.309402	0.32859	0.298049
0.300288	0.287816	0.304285	0.291334	0.275184	0.263032	0.357531	0.3118	0.301407	0.295171
0.362008	0.217141	0.292453	0.337224	0.207547	0.361209	0.35929	0.307004	0.275664	0.304125
0.365686	0.278862	0.295651	0.318196	0.26607	0.372562	0.355293	0.322034	0.286377	0.323473
0.336745	0.288295	0.326991	0.324432	0.279501	0.373681	0.347937	0.335305	0.267669	0.301727
0.340422	0.280301	0.148065	0.294372	0.262392	0.341382	0.333547	0.320115	0.237928	0.315798
0.256956	0.295011	0.356252	0.272146	0.349217	0.262072	0.336105	0.252319	0.358171	0.274225
0.307323	0.291174	0.336904	0.295491	0.305724	0.334026	0.336745	0.293092	0.319316	0.308922
0.347298	0.292933	0.279021	0.326191	0.310361	0.365206	0.331628	0.316917	0.314039	0.320275
0.300927	0.336265	0.363128	0.290374	0.366965	0.29629	0.231852	0.350656	0.354813	0.32747
0.36009	0.287816	0.232491	0.31292	0.271346	0.357851	0.354333	0.340902	0.231372	0.335785
0.320435	0.339783	0.353054	0.310361	0.316437	0.349856	0.334026	0.358331	0.332587	0.335146
0.251359	0.316917	0.386153	0.259194	0.397346	0.285897	0.339463	0.291973	0.392229	0.288455
0.336904	0.289575	0.212184	0.324432	0.223377	0.346978	0.34442	0.34426	0.259194	0.331148
0.372562	0.282379	0.307643	0.316917	0.26639	0.366965	0.366326	0.332587	0.287176	0.334986
0.32795	0.287016	0.34426	0.300608	0.313879	0.339143	0.320275	0.329869	0.305245	0.309402
0.294372	0.256796	0.284618	0.259194	0.314519	0.309722	0.299968	0.291813	0.298369	0.283658
0.243844	0.329709	0.395907	0.254557	0.409658	0.284138	0.347777	0.294052	0.402462	0.29693
0.365846	0.293412	0.324432	0.324113	0.264151	0.377518	0.340742	0.32827	0.251839	0.317877
0.311001	0.247362	0.322034	0.303326	0.305085	0.349696	0.305724	0.297889	0.279181	0.257115
0.301727	0.336425	0.362808	0.290694	0.367605	0.29741	0.232651	0.350975	0.354813	0.32811
0.314519	0.298689	0.355932	0.28142	0.357052	0.309882	0.317877	0.323633	0.346658	0.322514
0.31212	0.350496	0.373361	0.297729	0.349856	0.34346	0.324592	0.329709	0.347777	0.344899
0.321234	0.290054	0.335945	0.298049	0.308123	0.289894	0.34394	0.320115	0.317717	0.323953
0.295171	0.317077	0.346338	0.285257	0.347138	0.28078	0.278222	0.3433	0.346658	0.332907
0.360889	0.285417	0.321554	0.32811	0.26543	0.377678	0.346338	0.333067	0.268468	0.313719
0.393828	0.293572	0.267988	0.341541	0.287176	0.385673	0.377518	0.339143	0.284458	0.348737
0.361689	0.322674	0.317077	0.307643	0.292773	0.252958	0.331468	0.351295	0.321554	0.320915
0.371762	0.279821	0.158778	0.319476	0.247042	0.363927	0.345219	0.344739	0.212984	0.337064
0.349376	0.279181	0.302207	0.305724	0.283179	0.35865	0.333706	0.320115	0.292933	0.349536
0.344899	0.275823	0.258395	0.316757	0.251839	0.358171	0.337704	0.322993	0.189958	0.306844
0.25008	0.290214	0.356732	0.249121	0.366645	0.294851	0.326991	0.299968	0.348737	0.286377
0.316278	0.271346	0.211864	0.287496	0.241126	0.307963	0.31292	0.321554	0.268148	0.31212
0.316118	0.273905	0.236808	0.290854	0.257755	0.325072	0.294372	0.318836	0.257755	0.302047
0.339463	0.305085	0.303806	0.320595	0.305564	0.322674	0.365526	0.304765	0.310201	0.331788
0.352255	0.276143	0.215702	0.290534	0.268788	0.32827	0.335625	0.313559	0.231052	0.308922
0.226575	0.329549	0.387112	0.245763	0.398945	0.278542	0.347298	0.280461	0.396386	0.29757
0.235369	0.333387	0.363767	0.241445	0.388551	0.306044	0.31308	0.319955	0.365846	0.275983

For Review Only

0.307483	0.28206	0.315798	0.263032	0.305884	0.326991	0.318196	0.290694	0.305405	0.290854
0.332587	0.309082	0.303326	0.319635	0.274704	0.329709	0.34362	0.32859	0.299169	0.339623
0.303965	0.293892	0.282859	0.293732	0.272306	0.283978	0.32843	0.308922	0.289255	0.287656
0.317557	0.31292	0.335465	0.282219	0.330988	0.293892	0.335146	0.284778	0.3118	0.314839
0.332267	0.261273	0.220179	0.292773	0.259834	0.330988	0.317237	0.313559	0.272785	0.322674
0.247681	0.286377	0.349057	0.258235	0.349376	0.293412	0.321234	0.303646	0.338503	0.275344
0.369364	0.28174	0.209146	0.317717	0.243524	0.35865	0.341541	0.340102	0.206428	0.318516
0.310681	0.261273	0.224656	0.299169	0.242565	0.340742	0.337064	0.313719	0.257755	0.306044
0.362968	0.26495	0.26607	0.310521	0.252478	0.362488	0.347937	0.318676	0.253278	0.326671
0.298849	0.26575	0.279661	0.269587	0.289735	0.300448	0.31308	0.298849	0.272466	0.279981
0.301247	0.288136	0.293252	0.285098	0.311161	0.311321	0.298849	0.311321	0.309882	0.315318
0.289415	0.283818	0.266549	0.269108	0.294372	0.316917	0.3126	0.300608	0.29757	0.299648
0.319795	0.288136	0.325072	0.29629	0.299808	0.32747	0.325871	0.314199	0.294052	0.279341
0.292293	0.261273	0.272945	0.269587	0.267829	0.292293	0.317397	0.286537	0.270867	0.274225
0.348897	0.314039	0.306844	0.301887	0.294851	0.23473	0.32779	0.335625	0.316757	0.332267
0.355453	0.279501	0.231692	0.309402	0.264471	0.341861	0.344739	0.32875	0.238407	0.325392
0.337224	0.287336	0.293572	0.294212	0.311641	0.329709	0.32763	0.318836	0.253278	0.31324
0.294532	0.26511	0.241605	0.284138	0.228174	0.304125	0.319316	0.299648	0.264471	0.283818
0.324752	0.294052	0.320435	0.310201	0.261113	0.339463	0.365526	0.310681	0.31292	0.309402
0.304285	0.257915	0.230892	0.279661	0.246083	0.314998	0.303166	0.300288	0.246402	0.284778
0.252638	0.285737	0.352894	0.257115	0.356092	0.299968	0.325871	0.304765	0.344739	0.267988
0.332587	0.308443	0.332907	0.294851	0.307163	0.275823	0.350496	0.329869	0.316118	0.311321
0.288136	0.254557	0.252478	0.267029	0.24992	0.29741	0.301727	0.290054	0.258714	0.274704
0.35865	0.271666	0.309562	0.314199	0.239527	0.362648	0.363447	0.309882	0.267349	0.311321
0.354013	0.264311	0.239207	0.306844	0.263991	0.349856	0.337224	0.315478	0.248961	0.322354
0.254717	0.322674	0.395107	0.253598	0.402782	0.290054	0.348737	0.290534	0.401183	0.29613
0.303486	0.278542	0.279181	0.293732	0.28078	0.293732	0.332107	0.284938	0.300927	0.300128
0.26511	0.279181	0.341382	0.259674	0.333067	0.21842	0.332907	0.229293	0.326511	0.303326
0.349217	0.285577	0.301727	0.313719	0.290854	0.353854	0.334186	0.31244	0.291334	0.331308
0.331948	0.254557	0.295171	0.282219	0.274544	0.323633	0.323473	0.298049	0.279661	0.298209
0.384074	0.288136	0.24944	0.32763	0.224017	0.380237	0.361369	0.338823	0.254077	0.340422
0.34394	0.288775	0.316278	0.318356	0.269268	0.35945	0.338663	0.321714	0.286537	0.299648
0.259354	0.319316	0.381036	0.246882	0.393828	0.295491	0.337064	0.300128	0.39127	0.293732
0.282539	0.261273	0.267669	0.254717	0.274544	0.288775	0.304765	0.283818	0.269428	0.274704
0.361848	0.286057	0.322354	0.330508	0.26527	0.379917	0.346818	0.334026	0.267029	0.314679
0.310201	0.268148	0.260473	0.28126	0.243044	0.305724	0.317557	0.29741	0.248001	0.285897
0.263511	0.271666	0.347138	0.260953	0.350496	0.320435	0.330349	0.290534	0.324912	0.288136
0.305405	0.270227	0.335945	0.277902	0.319955	0.323313	0.319316	0.306844	0.319955	0.305405
0.248801	0.29709	0.35961	0.256156	0.367925	0.299808	0.325552	0.299169	0.35977	0.289735
0.357851	0.279821	0.240486	0.314679	0.242085	0.350656	0.352734	0.32843	0.240646	0.322993
0.290534	0.286057	0.308123	0.28158	0.298689	0.314199	0.351935	0.311641	0.321075	0.307323
0.244164	0.319156	0.396226	0.267189	0.394627	0.282699	0.341062	0.291334	0.396866	0.301887
0.287816	0.292293	0.339783	0.289255	0.336265	0.263511	0.354173	0.259834	0.342341	0.29613
0.264151	0.295971	0.360889	0.255996	0.371442	0.304285	0.334346	0.29677	0.355133	0.292453
0.372082	0.226735	0.272146	0.326671	0.253758	0.346338	0.342181	0.31212	0.279181	0.339303
0.28158	0.32859	0.371122	0.257115	0.384234	0.305564	0.305724	0.324113	0.369204	0.317557
0.336745	0.266869	0.28142	0.304285	0.283019	0.298849	0.32811	0.317077	0.314039	0.311001
0.338184	0.274704	0.276303	0.294691	0.268148	0.338503	0.317557	0.314519	0.274384	0.318836
0.337384	0.294851	0.204989	0.320755	0.198113	0.334026	0.331308	0.336904	0.241286	0.317397
0.337544	0.285737	0.204989	0.311321	0.232331	0.346658	0.349376	0.336585	0.246242	0.314998
0.259194	0.280141	0.322354	0.239687	0.321075	0.29645	0.31308	0.28142	0.323473	0.267189
0.344579	0.287496	0.20387	0.316917	0.241445	0.345859	0.339783	0.338024	0.252638	0.323793
0.32795	0.282859	0.209786	0.307803	0.231372	0.337384	0.325072	0.325871	0.259514	0.31228
0	0.317877	0.361209	0.245763	0.348577	0.279501	0.314679	0.289255	0.351775	0.266549
0.317877	0	0.295811	0.292293	0.283339	0.306044	0.325871	0.262872	0.278862	0.283179

For Review Only

0.361209	0.295811	0	0.325392	0.255836	0.361369	0.347937	0.349217	0.250879	0.360889
0.245763	0.292293	0.325392	0	0.323793	0.273105	0.306044	0.275184	0.316757	0.275664
0.348577	0.283339	0.255836	0.323793	0	0.353054	0.346498	0.335945	0.272466	0.317397
0.279501	0.306044	0.361369	0.273105	0.353054	0	0.337064	0.28174	0.35897	0.319955
0.314679	0.325871	0.347937	0.306044	0.346498	0.337064	0	0.337864	0.332587	0.308123
0.289255	0.262872	0.349217	0.275184	0.335945	0.28174	0.337864	0	0.338823	0.299648
0.351775	0.278862	0.250879	0.316757	0.272466	0.35897	0.332587	0.338823	0	0.308602
0.266549	0.283179	0.360889	0.275664	0.317397	0.319955	0.308123	0.299648	0.308602	0
0.252159	0.310521	0.358171	0.272626	0.364727	0.267189	0.334826	0.28158	0.360569	0.29709
0.286856	0.251359	0.251359	0.26639	0.257435	0.304765	0.305245	0.289894	0.255836	0.274704
0.285737	0.294212	0.361209	0.283339	0.360729	0.311481	0.346818	0.307963	0.34394	0.319955
0.369524	0.273105	0.299808	0.331948	0.245603	0.39095	0.355772	0.324432	0.279981	0.322034
0.326351	0.276943	0.223057	0.290694	0.227854	0.3118	0.318836	0.318196	0.244643	0.306204
0.257115	0.280301	0.320755	0.253758	0.336105	0.277422	0.313399	0.270067	0.332267	0.26591
0.339303	0.269587	0.24928	0.300608	0.241445	0.32843	0.319795	0.322194	0.251839	0.31324
0.321554	0.284298	0.313399	0.291653	0.299808	0.321554	0.331308	0.320595	0.300288	0.314359
0.268948	0.292613	0.350656	0.254397	0.369524	0.300288	0.333387	0.304445	0.35961	0.284778
0.254237	0.29661	0.37496	0.287496	0.352095	0.28078	0.358331	0.29741	0.364886	0.324272
0.305405	0.143748	0.289095	0.279341	0.282219	0.303486	0.314039	0.271826	0.285257	0.284298
0.366805	0.291334	0.243204	0.323793	0.277263	0.361529	0.345859	0.339623	0.213783	0.32811
0.334666	0.28078	0.226095	0.283978	0.262232	0.324592	0.330349	0.310361	0.255516	0.309722
0.354173	0.261273	0.267669	0.302367	0.24992	0.353694	0.342661	0.31292	0.251679	0.318036
0.286697	0.244484	0.263032	0.262872	0.259034	0.29661	0.301247	0.287816	0.261273	0.274225
0.254077	0.307963	0.349217	0.257755	0.370323	0.279821	0.326351	0.293892	0.365686	0.299009
0.258235	0.301727	0.365846	0.273425	0.364247	0.255037	0.339783	0.25008	0.357691	0.286217
0.369044	0.299328	0.116565	0.322674	0.273745	0.364727	0.353694	0.346658	0.244004	0.34442
0.322833	0.319156	0.340742	0.301407	0.338343	0.32811	0.331308	0.323473	0.323953	0.330349
0.242405	0.274704	0.350975	0.257435	0.352894	0.305724	0.32795	0.29613	0.32811	0.287016
0.347138	0.274864	0.228334	0.297889	0.259994	0.324592	0.340742	0.323793	0.261753	0.324752
0.298209	0.273745	0.316437	0.286377	0.285897	0.319955	0.352095	0.294532	0.29757	0.295811
0.310361	0.277422	0.304125	0.295491	0.292613	0.301407	0.325072	0.314519	0.316757	0.287816
0.348097	0.256796	0.301407	0.314199	0.258075	0.351775	0.337384	0.305405	0.272785	0.297889
0.324752	0.28158	0.285737	0.31212	0.276143	0.338663	0.32859	0.32891	0.283658	0.330508
0.352574	0.286377	0.304285	0.323313	0.235529	0.362968	0.357211	0.339303	0.284618	0.326351
0.339783	0.295011	0.300128	0.306044	0.299648	0.353534	0.276463	0.304765	0.29693	0.307803
0.266549	0.290374	0.352574	0.253438	0.368244	0.293092	0.337384	0.304445	0.357211	0.284138
0.332427	0.270867	0.309242	0.31308	0.279021	0.369204	0.309082	0.313879	0.297889	0.290694
0.368244	0.298689	0.115766	0.321394	0.271986	0.364087	0.354013	0.346338	0.243524	0.342341
0.334666	0.311481	0.258395	0.302207	0.288775	0.316118	0.329869	0.313399	0.279341	0.325552
0.332107	0.25072	0.323153	0.294691	0.275504	0.34346	0.330988	0.316278	0.286057	0.301087
0.340262	0.287816	0.215542	0.3118	0.228654	0.3441	0.347777	0.336745	0.25072	0.314998
0.347777	0.263032	0.229773	0.31292	0.268468	0.348577	0.358011	0.336745	0.267988	0.338184
0.3748	0.271186	0.226415	0.330189	0.236808	0.369843	0.335945	0.340902	0.253278	0.334506
0.334666	0.290054	0.204029	0.318836	0.188839	0.335945	0.333547	0.335465	0.248481	0.316118
0.251679	0.321874	0.398145	0.255516	0.397346	0.291334	0.346019	0.280461	0.394148	0.294851
0.306044	0.28126	0.287016	0.29725	0.280141	0.338343	0.327311	0.307163	0.292133	0.287496
0.316118	0.298529	0.286377	0.29645	0.31244	0.350815	0.32763	0.321075	0.294532	0.290374
0.346658	0.292133	0.301087	0.307004	0.303326	0.35945	0.338184	0.31212	0.303486	0.244643
0.253598	0.294532	0.373681	0.287336	0.351455	0.279981	0.357531	0.295491	0.364087	0.322833
0.239367	0.315958	0.378957	0.26527	0.380237	0.298529	0.323953	0.29645	0.377199	0.302367
0.255197	0.269747	0.336745	0.240006	0.350336	0.282699	0.303486	0.283818	0.346178	0.283339
0.307323	0.297729	0.299488	0.287336	0.289415	0.305085	0.332907	0.316597	0.302846	0.302367
0.369843	0.29645	0.270547	0.324272	0.238567	0.381356	0.365046	0.335945	0.260793	0.319156
0.268628	0.297889	0.373841	0.246562	0.376559	0.298369	0.29661	0.292773	0.368244	0.256956
0.255197	0.29693	0.371442	0.269428	0.368404	0.249121	0.341062	0.26575	0.378158	0.275664

For Review Only

0.369843	0.278222	0.157179	0.318036	0.247522	0.362328	0.344739	0.34314	0.211545	0.335945
0.242405	0.329389	0.39047	0.251999	0.400064	0.284618	0.350016	0.28062	0.397506	0.291493
0.322993	0.153182	0.32811	0.294052	0.290214	0.315958	0.347298	0.259834	0.303006	0.306684
0.300927	0.316437	0.338503	0.284138	0.334026	0.326831	0.31292	0.308123	0.332427	0.294052
0.389511	0.28174	0.276143	0.336745	0.217941	0.378478	0.353214	0.345219	0.263192	0.337384
0.307963	0.31276	0.352894	0.298049	0.3433	0.255197	0.348257	0.295331	0.340582	0.307643
0.273905	0.300768	0.348577	0.28094	0.323793	0.304765	0.31196	0.318196	0.318996	0.283978
0.349536	0.272785	0.268308	0.317877	0.236009	0.34362	0.338343	0.329389	0.272945	0.321394
0.270707	0.307803	0.353694	0.289575	0.340102	0.292133	0.340422	0.287016	0.356572	0.294532
0.278222	0.332427	0.389031	0.267189	0.402622	0.294052	0.297729	0.331948	0.387432	0.324272
0.2811	0.272146	0.313559	0.261113	0.333866	0.303806	0.309082	0.302207	0.314679	0.301407
0.251679	0.331468	0.396706	0.24928	0.402143	0.286377	0.341541	0.291174	0.399744	0.298849
0.280301	0.254877	0.262232	0.255676	0.256476	0.294691	0.301087	0.282699	0.26511	0.268468
0.325871	0.271826	0.317557	0.309402	0.302207	0.302047	0.335305	0.308283	0.32843	0.304605
0.318676	0.284138	0.324432	0.299488	0.287816	0.302526	0.332107	0.311321	0.303486	0.324113
0.319635	0.246242	0.236649	0.289735	0.223057	0.317877	0.337704	0.309402	0.244963	0.304125
0.365526	0.276463	0.231052	0.333387	0.25072	0.362488	0.345539	0.349376	0.251199	0.340582
0.302846	0.285098	0.355932	0.302207	0.31308	0.329709	0.337064	0.309402	0.335785	0.302367
0.236489	0.3126	0.354493	0.25072	0.356732	0.279821	0.322833	0.274864	0.350016	0.286856
0.293572	0.300608	0.363288	0.276943	0.332907	0.322354	0.364247	0.318036	0.348097	0.316278
0.258235	0.278702	0.352255	0.283658	0.34346	0.28078	0.330029	0.295651	0.349696	0.294372
0.293732	0.284138	0.334506	0.28126	0.316597	0.284298	0.336265	0.301567	0.325392	0.285897
0.314359	0.3118	0.318836	0.29661	0.336425	0.32811	0.353054	0.292133	0.327311	0.325392
0.327151	0.283818	0.213303	0.314998	0.207387	0.331468	0.338663	0.334826	0.253118	0.315158
0.354653	0.270227	0.31324	0.31308	0.247681	0.333547	0.350656	0.329229	0.28158	0.317717
0.291653	0.292613	0.326351	0.2819	0.318836	0.262232	0.339623	0.276623	0.336425	0.272306
0.216661	0.319795	0.373681	0.189159	0.395427	0.288455	0.308283	0.300288	0.39015	0.267029
0.351455	0.295651	0.303486	0.311161	0.305405	0.361049	0.335625	0.331308	0.291653	0.340902
0.252958	0.331628	0.396067	0.253278	0.408219	0.293572	0.352414	0.292293	0.404381	0.302846
0.376399	0.292773	0.225296	0.314679	0.24976	0.356252	0.349856	0.349376	0.233131	0.340422
0.311321	0.285417	0.339303	0.292933	0.299328	0.333067	0.320755	0.315958	0.287496	0.284458
0.3748	0.277422	0.26591	0.321714	0.225776	0.382315	0.364567	0.333866	0.261593	0.331948
0.240326	0.300927	0.370803	0.256156	0.373201	0.259194	0.342021	0.279821	0.377199	0.300608
0.282379	0.271346	0.237448	0.289735	0.256476	0.31196	0.332267	0.325392	0.300608	0.29741
0.29613	0.288455	0.347618	0.284458	0.335785	0.29725	0.334346	0.323473	0.32811	0.298369
0.337384	0.269907	0.217941	0.302846	0.251679	0.322514	0.332907	0.313719	0.266709	0.317717
0.254397	0.289415	0.360729	0.272945	0.351455	0.244484	0.333547	0.24976	0.361848	0.272626
0.258555	0.325072	0.395267	0.26511	0.404381	0.286537	0.347618	0.302846	0.404381	0.290534
0.370803	0.17157	0.294691	0.324752	0.239207	0.364886	0.346658	0.285417	0.264791	0.326671
0.369204	0.300448	0.117045	0.322993	0.273905	0.365366	0.354493	0.347298	0.244324	0.34394
0.303326	0.264791	0.232331	0.269428	0.294212	0.321234	0.29741	0.31292	0.267509	0.31228
0.213943	0.322833	0.395107	0.252159	0.400863	0.279981	0.342021	0.276943	0.395747	0.292453
0.363927	0.286057	0.305085	0.326671	0.2504	0.368404	0.337704	0.322514	0.285098	0.327151
0.299968	0.261912	0.28078	0.28126	0.276943	0.332267	0.287496	0.303166	0.236968	0.29661
0.275664	0.31324	0.322674	0.284618	0.335465	0.258555	0.331308	0.263991	0.335785	0.29693
0.364567	0.283179	0.316118	0.323633	0.261753	0.368084	0.349536	0.32779	0.263991	0.327151
0.337224	0.279661	0.224656	0.299169	0.274384	0.355932	0.335785	0.340902	0.258075	0.284298
0.358331	0.288615	0.335945	0.307163	0.29677	0.291493	0.348897	0.315958	0.29629	0.322993
0.29645	0.290854	0.28062	0.290694	0.29709	0.316118	0.335305	0.308123	0.307643	0.302526
0.341222	0.295651	0.284618	0.309402	0.295171	0.29709	0.345219	0.330508	0.288935	0.327311
0.356092	0.310521	0.297729	0.318516	0.306844	0.336585	0.3126	0.345699	0.29629	0.347458
0.251199	0.325392	0.392869	0.255197	0.404861	0.29645	0.352255	0.293732	0.404061	0.29613
0.368084	0.283818	0.301727	0.319635	0.277582	0.373681	0.355772	0.322993	0.269428	0.309722
0.228014	0.326031	0.397026	0.245603	0.399264	0.282699	0.352894	0.2819	0.396546	0.288935
0.375919	0.271186	0.227215	0.331468	0.236009	0.369364	0.338503	0.340582	0.252638	0.333706

For Review Only

0.377838	0.301407	0.241445	0.331628	0.255676	0.348097	0.358331	0.358171	0.255996	0.350496
0.259354	0.311001	0.366485	0.273745	0.356892	0.26527	0.332907	0.262712	0.362808	0.232491
0.357531	0.279181	0.315318	0.325552	0.261912	0.358011	0.34442	0.324752	0.284458	0.32795
0.369204	0.285737	0.225456	0.330189	0.274864	0.362328	0.349057	0.341382	0.243524	0.347937

For Review Only

0.346178	0.26607	0.346019	0.28142	0.285737	0.320915	0.279181	0.287976	0.345699	0.354653
0.32779	0.279341	0.318676	0.332587	0.301407	0.309242	0.299968	0.306524	0.29629	0.32747
0.362808	0.253598	0.349536	0.28062	0.213623	0.340422	0.229933	0.314199	0.365366	0.366006
0.341861	0.262392	0.336585	0.288136	0.240646	0.319316	0.220019	0.29693	0.330988	0.354973
0.329389	0.293732	0.324272	0.308762	0.335305	0.308922	0.32875	0.302526	0.322354	0.354973
0.353694	0.26575	0.337224	0.273905	0.263671	0.317877	0.243044	0.286856	0.350656	0.360569
0.344899	0.275344	0.365206	0.276943	0.298529	0.320435	0.289255	0.298529	0.362008	0.362968
0.302367	0.280461	0.31276	0.356412	0.319476	0.283978	0.338503	0.288615	0.276303	0.335785
0.337864	0.272306	0.339303	0.273105	0.298369	0.294691	0.29677	0.279981	0.34394	0.345699
0.325392	0.305405	0.336105	0.383115	0.357211	0.306524	0.37432	0.330508	0.284618	0.37544
0.261593	0.301087	0.276463	0.325871	0.331948	0.288455	0.334826	0.329549	0.321394	0.061561
0.361209	0.246562	0.347618	0.275184	0.210106	0.332107	0.216661	0.307323	0.357691	0.362648
0.31196	0.269587	0.330189	0.305245	0.292133	0.305564	0.294052	0.298049	0.299488	0.347618
0.290214	0.257755	0.291174	0.346338	0.302367	0.237768	0.311161	0.277582	0.196514	0.295651
0.320915	0.2504	0.32779	0.257915	0.262072	0.29709	0.259674	0.277742	0.338823	0.332587
0.315958	0.273265	0.309082	0.290214	0.271346	0.282219	0.285897	0.314039	0.307483	0.305085
0.356572	0.29693	0.35993	0.273265	0.318676	0.349057	0.32763	0.316437	0.358491	0.355772
0.331788	0.263511	0.335465	0.31196	0.301087	0.301567	0.290374	0.314359	0.338663	0.341062
0.373201	0.279021	0.35977	0.246882	0.259514	0.349217	0.259354	0.311641	0.3756	0.361529
0.342981	0.298369	0.340742	0.362968	0.340422	0.314679	0.346818	0.332747	0.305405	0.361209
0.322993	0.286057	0.329389	0.32827	0.31292	0.310521	0.316757	0.319476	0.310841	0.333067
0.348897	0.286217	0.345699	0.270547	0.307323	0.333227	0.306044	0.315798	0.349376	0.363767
0.247362	0.322514	0.262072	0.394148	0.361689	0.267349	0.368084	0.334506	0.26623	0.254877
0.267189	0.255197	0.272466	0.338663	0.307323	0.176207	0.316437	0.286537	0.200512	0.308123
0.339783	0.267509	0.354653	0.248321	0.269268	0.313879	0.254557	0.295811	0.365686	0.361209
0.356892	0.230253	0.353694	0.300288	0.139431	0.326831	0.236489	0.303646	0.36009	0.372881
0.339143	0.239687	0.315958	0.276623	0.208666	0.297729	0.199872	0.286697	0.331308	0.371282
0.290694	0.221938	0.29661	0.26527	0.240646	0.262392	0.245123	0.271666	0.277103	0.302526
0.323793	0.269428	0.32763	0.308123	0.306684	0.290214	0.314199	0.292453	0.293092	0.350176
0.304125	0.333706	0.324912	0.423089	0.381196	0.284938	0.397346	0.349856	0.24944	0.339783
0.357851	0.262232	0.358331	0.241925	0.242245	0.321554	0.229613	0.29725	0.362328	0.363927
0.377039	0.283818	0.363607	0.259514	0.279501	0.337864	0.278702	0.318836	0.372721	0.370483
0.346019	0.278382	0.366805	0.256316	0.284778	0.326511	0.293732	0.295011	0.365846	0.350815
0.279341	0.270547	0.274704	0.346818	0.322514	0.243044	0.319795	0.288295	0.155261	0.317557
0.292773	0.28206	0.304925	0.34442	0.288455	0.275024	0.300608	0.31244	0.307163	0.287656
0.311001	0.240486	0.306844	0.29709	0.253758	0.245283	0.263671	0.28078	0.258235	0.318036
0.321394	0.278542	0.321234	0.304925	0.301087	0.288935	0.279341	0.261912	0.274704	0.337544
0.308283	0.280301	0.329229	0.335785	0.291973	0.28078	0.291174	0.302047	0.324752	0.301407
0.204509	0.29757	0.238088	0.404061	0.354013	0.224017	0.366326	0.335305	0.220019	0.223697
0.238567	0.303486	0.289894	0.360729	0.315478	0.251999	0.330668	0.323793	0.302846	0.258395
0.295011	0.314998	0.317077	0.417173	0.370483	0.279181	0.388391	0.349057	0.25056	0.330828
0.31212	0.284938	0.318996	0.356732	0.322354	0.287016	0.342661	0.316917	0.287336	0.340422
0.309882	0.295011	0.330668	0.371122	0.34442	0.288935	0.354493	0.316437	0.279661	0.352255
0.335945	0.303006	0.336265	0.317397	0.326991	0.310841	0.308762	0.314998	0.342501	0.333227
0.329229	0.309722	0.34394	0.381516	0.351295	0.298049	0.365846	0.323633	0.292293	0.355293
0.35897	0.239047	0.345219	0.271346	0.182123	0.329869	0.206588	0.29709	0.363288	0.362168
0.356252	0.235209	0.354973	0.294691	0.190438	0.317237	0.244643	0.31308	0.354173	0.366326
0.288295	0.251679	0.283019	0.321874	0.235689	0.271027	0.28094	0.295651	0.29661	0.276943
0.350176	0.300927	0.364727	0.326831	0.338343	0.323473	0.337704	0.321394	0.32891	0.342501
0.346978	0.26623	0.34314	0.240006	0.275024	0.323793	0.26511	0.292293	0.358171	0.350336

For Review Only

0.361529	0.238567	0.356412	0.282699	0.165494	0.324113	0.228494	0.304125	0.364407	0.366166
0.32843	0.277263	0.339303	0.301407	0.294052	0.31324	0.292773	0.319156	0.320915	0.346498
0.366326	0.270547	0.363128	0.228973	0.283179	0.348577	0.28142	0.295651	0.387272	0.366965
0.346658	0.278542	0.367125	0.256476	0.284298	0.326671	0.292613	0.294691	0.364567	0.350815
0.356412	0.253278	0.349696	0.269907	0.252638	0.313559	0.245443	0.300927	0.360889	0.352414
0.340902	0.29725	0.369683	0.272785	0.311641	0.340422	0.311321	0.322354	0.354493	0.353214
0.372721	0.278702	0.35993	0.247202	0.259034	0.349057	0.259514	0.311161	0.37496	0.362328
0.370323	0.238248	0.35945	0.284298	0.185801	0.307643	0.230413	0.321234	0.355772	0.37528
0.361209	0.221938	0.348417	0.282379	0.150943	0.310841	0.204189	0.299968	0.355453	0.361369
0.261753	0.299488	0.286377	0.399584	0.35993	0.243204	0.372082	0.319316	0.20355	0.290534
0.293892	0.277742	0.308283	0.323313	0.319316	0.262552	0.293092	0.278062	0.251999	0.331468
0.297729	0.222737	0.29677	0.260953	0.241765	0.26623	0.240486	0.270707	0.284458	0.305724
0.35945	0.244004	0.355293	0.271027	0.227854	0.320435	0.225935	0.299808	0.353374	0.356252
0.364727	0.254397	0.357531	0.283339	0.239047	0.31228	0.240806	0.317397	0.34314	0.371762
0.365366	0.269907	0.362808	0.228654	0.282219	0.347937	0.279821	0.295971	0.387752	0.366645
0.323793	0.268628	0.327311	0.307323	0.305724	0.290214	0.314199	0.291973	0.292773	0.349376
0.308602	0.244963	0.310361	0.273425	0.248641	0.283499	0.242725	0.279501	0.314839	0.319316
0.305405	0.278862	0.319316	0.346978	0.314839	0.272945	0.307323	0.28206	0.282219	0.326831
0.306684	0.276143	0.292133	0.319156	0.283818	0.288295	0.272146	0.302846	0.327311	0.288775
0.375919	0.268628	0.370483	0.258075	0.261273	0.34314	0.263192	0.288775	0.387592	0.384394
0.371122	0.276303	0.356252	0.220659	0.283978	0.336585	0.267029	0.311321	0.380077	0.355932
0.360729	0.269907	0.35961	0.258395	0.280461	0.329869	0.282539	0.287016	0.363927	0.367125
0.338343	0.239047	0.329709	0.267829	0.235689	0.284298	0.230892	0.293572	0.314998	0.347777
0.246882	0.290854	0.289415	0.352574	0.315638	0.258714	0.314839	0.32827	0.288615	0.274225
0.322514	0.270387	0.325392	0.292453	0.302047	0.29645	0.295971	0.289894	0.31196	0.324432
0.357052	0.275344	0.35993	0.301887	0.277263	0.301087	0.288615	0.321394	0.333547	0.36009
0.32843	0.308922	0.342981	0.380876	0.350815	0.29757	0.365846	0.324113	0.291334	0.354173
0.353214	0.252638	0.34314	0.279181	0.237128	0.311001	0.240806	0.302686	0.341222	0.370643
0.345859	0.313399	0.355133	0.333866	0.347777	0.339942	0.357851	0.332587	0.352894	0.35865
0.20387	0.305085	0.256156	0.413176	0.356572	0.236968	0.3748	0.332267	0.209946	0.254397
0.35945	0.228174	0.355293	0.31308	0.139271	0.331148	0.23489	0.307323	0.363288	0.362488
0.356892	0.274704	0.334666	0.161657	0.279661	0.336585	0.226575	0.299328	0.355772	0.339143
0.329069	0.285257	0.345379	0.304925	0.302047	0.29757	0.292133	0.304285	0.322674	0.340582
0.289894	0.247522	0.293412	0.303486	0.286217	0.221298	0.286537	0.274065	0.224816	0.329389
0.201791	0.3126	0.256796	0.420691	0.370643	0.248161	0.381036	0.339783	0.205788	0.264151
0.364727	0.269747	0.362488	0.228174	0.282539	0.346498	0.280141	0.295171	0.386792	0.365366
0.329549	0.262872	0.326671	0.29629	0.284458	0.295491	0.285417	0.292613	0.315638	0.324752
0.329549	0.309722	0.34426	0.380876	0.351615	0.298049	0.366645	0.324432	0.292133	0.355293
0.314839	0.289575	0.316437	0.356732	0.335785	0.274065	0.332107	0.306524	0.284618	0.334666
0.329709	0.305724	0.351935	0.338184	0.351775	0.324912	0.339942	0.336265	0.318196	0.356572
0.329389	0.28126	0.307643	0.301567	0.29677	0.307004	0.290214	0.298689	0.314998	0.311161
0.320755	0.305724	0.340102	0.378957	0.341222	0.293572	0.346019	0.316757	0.287016	0.342501
0.361529	0.274384	0.355133	0.229453	0.275184	0.338823	0.279341	0.288935	0.383754	0.35993
0.379277	0.286537	0.345859	0.208826	0.282379	0.34378	0.23505	0.309242	0.369683	0.364567
0.340582	0.282859	0.342021	0.319635	0.301727	0.326991	0.282699	0.307963	0.338983	0.368564
0.360409	0.244324	0.355612	0.271506	0.229453	0.320915	0.227534	0.300608	0.354013	0.356892
0.334826	0.278862	0.338823	0.244963	0.288295	0.309402	0.269587	0.288295	0.347618	0.323313
0.361049	0.259354	0.354333	0.261593	0.247681	0.329389	0.245923	0.292293	0.355133	0.357851
0.269108	0.273585	0.261273	0.373361	0.331948	0.237768	0.338663	0.2819	0.098977	0.306204
0.333387	0.235209	0.342821	0.305564	0.200352	0.287816	0.232491	0.286217	0.308922	0.362808
0.331628	0.233291	0.326831	0.29757	0.184362	0.28206	0.226895	0.303326	0.319156	0.34426
0.333547	0.290854	0.356732	0.318996	0.305884	0.306524	0.301087	0.302526	0.345059	0.354173
0.333067	0.242565	0.32875	0.266709	0.235369	0.292613	0.229933	0.291174	0.334026	0.350176
0.210106	0.307643	0.238567	0.410777	0.360569	0.237768	0.381196	0.3441	0.225136	0.212344
0.272306	0.302846	0.301407	0.381516	0.346658	0.275344	0.345699	0.318036	0.23489	0.29757

For Review Only

0.297889	0.264471	0.305405	0.303646	0.282539	0.279341	0.293092	0.290054	0.273745	0.322993
0.326031	0.285257	0.362328	0.322034	0.304285	0.297729	0.29709	0.274544	0.340262	0.357691
0.301727	0.2504	0.317397	0.326191	0.200831	0.278702	0.254557	0.295651	0.318356	0.310042
0.311641	0.287496	0.317557	0.341062	0.302846	0.285897	0.307803	0.316118	0.311481	0.322993
0.335305	0.225776	0.32875	0.283339	0.18788	0.286057	0.245603	0.269428	0.315638	0.353214
0.263351	0.273265	0.276463	0.375919	0.322833	0.253598	0.330189	0.287656	0.133195	0.306524
0.35897	0.241126	0.34362	0.279821	0.208027	0.317237	0.228494	0.304125	0.354813	0.368564
0.332427	0.233451	0.335625	0.279501	0.183083	0.287176	0.231692	0.284138	0.320595	0.362488
0.358171	0.256956	0.332747	0.23457	0.245923	0.321874	0.242405	0.302367	0.346338	0.350336
0.307163	0.236329	0.289575	0.278862	0.241286	0.253438	0.247362	0.276463	0.273905	0.31292
0.307163	0.26591	0.294851	0.34378	0.264471	0.282859	0.273745	0.313399	0.295651	0.336904
0.292933	0.246083	0.316118	0.323153	0.253918	0.263192	0.280301	0.29757	0.271027	0.325232
0.324752	0.26639	0.338184	0.313879	0.302526	0.284298	0.2811	0.299808	0.299328	0.325712
0.291493	0.237928	0.306044	0.28062	0.246882	0.262712	0.254877	0.282699	0.297729	0.303486
0.32875	0.283339	0.339623	0.332427	0.301407	0.322674	0.285098	0.304925	0.32827	0.369683
0.341222	0.245123	0.34426	0.271826	0.226575	0.305884	0.243044	0.294851	0.342661	0.350016
0.329709	0.272626	0.331468	0.301727	0.283978	0.29709	0.285577	0.301887	0.310841	0.347298
0.304445	0.221778	0.308123	0.291493	0.190438	0.274544	0.249121	0.284298	0.301247	0.321714
0.348097	0.288935	0.366645	0.309242	0.300448	0.318196	0.301727	0.308123	0.364567	0.349856
0.303326	0.21874	0.308762	0.276943	0.211225	0.279341	0.23393	0.270867	0.301247	0.306684
0.272785	0.274225	0.276463	0.383435	0.333067	0.245443	0.334666	0.287976	0.118324	0.31228
0.332427	0.288455	0.322833	0.300448	0.305085	0.326351	0.309402	0.305564	0.349057	0.321554
0.292773	0.216661	0.299488	0.273425	0.226095	0.263032	0.242725	0.276783	0.285577	0.304125
0.353534	0.271186	0.337224	0.18772	0.288295	0.322034	0.251039	0.289255	0.355453	0.335945
0.334346	0.253598	0.340422	0.282219	0.245283	0.315798	0.239207	0.283658	0.330189	0.370803
0.215382	0.305245	0.258075	0.413495	0.363927	0.240646	0.375919	0.32779	0.205149	0.263671
0.307643	0.252638	0.322674	0.324752	0.217461	0.289255	0.262392	0.304285	0.318676	0.324113
0.251199	0.276783	0.279981	0.333866	0.298689	0.242245	0.298849	0.308602	0.295651	0.287016
0.355133	0.279181	0.357531	0.255357	0.286217	0.322993	0.285257	0.294052	0.357851	0.35897
0.325072	0.255996	0.324912	0.253118	0.280141	0.291653	0.279821	0.256156	0.283019	0.35961
0.386153	0.262072	0.363447	0.247202	0.247362	0.344899	0.227215	0.319955	0.371442	0.382315
0.362328	0.280141	0.364886	0.266549	0.299328	0.330508	0.301247	0.310681	0.370483	0.348417
0.23505	0.304925	0.271186	0.401823	0.357691	0.239207	0.366166	0.320755	0.14023	0.276943
0.289575	0.236169	0.299968	0.287976	0.248961	0.267029	0.26639	0.290214	0.269907	0.305085
0.362808	0.274065	0.356412	0.228174	0.275504	0.339623	0.280301	0.290694	0.387592	0.361209
0.310521	0.227694	0.318836	0.279661	0.184202	0.283499	0.231852	0.277742	0.31212	0.330189
0.278542	0.269747	0.273745	0.34362	0.319156	0.242885	0.317877	0.288295	0.159738	0.317237
0.29661	0.272785	0.293412	0.315798	0.29613	0.272785	0.292613	0.287656	0.26575	0.333227
0.269747	0.278702	0.277582	0.383754	0.336265	0.247042	0.346978	0.290694	0.112568	0.314839
0.3433	0.251039	0.342661	0.255676	0.238887	0.31212	0.243844	0.301567	0.350815	0.348257
0.283818	0.26623	0.290854	0.324113	0.259194	0.273905	0.295011	0.31244	0.300128	0.266549
0.228174	0.317237	0.258874	0.39047	0.361689	0.264791	0.374161	0.341062	0.248481	0.1559
0.286537	0.290854	0.315958	0.322034	0.299488	0.262072	0.305884	0.322194	0.314039	0.289255
0.258874	0.283339	0.267829	0.383914	0.336904	0.242245	0.339463	0.287016	0.102015	0.309882
0.337704	0.259034	0.360569	0.275184	0.272466	0.300927	0.2496	0.282699	0.353854	0.365846
0.298049	0.305245	0.315318	0.383595	0.350815	0.270387	0.35865	0.332907	0.254717	0.326031
0.318516	0.255996	0.323793	0.325072	0.242405	0.294212	0.287336	0.298049	0.315958	0.335465
0.326031	0.263991	0.333547	0.2819	0.274864	0.307643	0.268468	0.153662	0.31212	0.362488
0.357211	0.221618	0.358011	0.29661	0.105692	0.324592	0.204989	0.299808	0.364247	0.368884
0.351455	0.223537	0.354653	0.289095	0.18772	0.308443	0.229133	0.311001	0.362328	0.369843
0.28174	0.264631	0.299488	0.330508	0.29709	0.258395	0.300128	0.273585	0.221138	0.304285
0.351135	0.230253	0.347298	0.293412	0.130157	0.322674	0.222737	0.302686	0.362648	0.369204
0.351295	0.223377	0.342661	0.295331	0.153662	0.300288	0.228174	0.29709	0.346978	0.365686
0.252159	0.286856	0.285737	0.369524	0.326351	0.257115	0.339303	0.321554	0.268948	0.254237
0.310521	0.251359	0.294212	0.273105	0.276943	0.280301	0.269587	0.284298	0.292613	0.29661

For Review Only

0.358171	0.251359	0.361209	0.299808	0.223057	0.320755	0.24928	0.313399	0.350656	0.37496
0.272626	0.26639	0.283339	0.331948	0.290694	0.253758	0.300608	0.291653	0.254397	0.287496
0.364727	0.257435	0.360729	0.245603	0.227854	0.336105	0.241445	0.299808	0.369524	0.352095
0.267189	0.304765	0.311481	0.39095	0.3118	0.277422	0.32843	0.321554	0.300288	0.28078
0.334826	0.305245	0.346818	0.355772	0.318836	0.313399	0.319795	0.331308	0.333387	0.358331
0.28158	0.289894	0.307963	0.324432	0.318196	0.270067	0.322194	0.320595	0.304445	0.29741
0.360569	0.255836	0.34394	0.279981	0.244643	0.332267	0.251839	0.300288	0.35961	0.364886
0.29709	0.274704	0.319955	0.322034	0.306204	0.26591	0.31324	0.314359	0.284778	0.324272
0	0.297729	0.268628	0.380716	0.333387	0.233131	0.337064	0.316757	0.267829	0.247522
0.297729	0	0.295171	0.278222	0.219699	0.252638	0.245283	0.274864	0.276143	0.314519
0.268628	0.295171	0	0.341701	0.334666	0.276463	0.329069	0.316118	0.277263	0.271826
0.380716	0.278222	0.341701	0	0.288775	0.34426	0.263032	0.308123	0.376079	0.349057
0.333387	0.219699	0.334666	0.288775	0	0.305564	0.207227	0.293892	0.335625	0.348417
0.233131	0.252638	0.276463	0.34426	0.305564	0	0.300927	0.298849	0.232651	0.28158
0.337064	0.245283	0.329069	0.263032	0.207227	0.300927	0	0.28094	0.339143	0.355932
0.316757	0.274864	0.316118	0.308123	0.293892	0.298849	0.28094	0	0.28094	0.339303
0.267829	0.276143	0.277263	0.376079	0.335625	0.232651	0.339143	0.28094	0	0.308762
0.247522	0.314519	0.271826	0.349057	0.348417	0.28158	0.355932	0.339303	0.308762	0
0.302846	0.240806	0.290054	0.2811	0.277263	0.269907	0.26623	0.280141	0.272146	0.293412
0.360569	0.269268	0.356732	0.274384	0.251359	0.323953	0.262712	0.310841	0.360569	0.362648
0.318516	0.239687	0.319316	0.282699	0.192037	0.276943	0.239687	0.276943	0.303646	0.346818
0.351135	0.251839	0.32875	0.232651	0.243684	0.315798	0.241765	0.29725	0.339942	0.345379
0.289095	0.223057	0.297889	0.267669	0.240646	0.257595	0.244484	0.26591	0.274225	0.295971
0.21954	0.285737	0.275344	0.378638	0.332427	0.217301	0.348737	0.307803	0.20259	0.273425
0.255357	0.29661	0.292933	0.361848	0.322833	0.259834	0.324912	0.329389	0.286537	0.274384
0.364407	0.255516	0.358491	0.283499	0.239367	0.31276	0.240646	0.317397	0.34362	0.371602
0.338823	0.283179	0.352255	0.307323	0.309722	0.29677	0.321554	0.302846	0.304125	0.357531
0.273265	0.273425	0.278382	0.357371	0.319795	0.24928	0.318516	0.283339	0.159578	0.292613
0.336745	0.247522	0.349057	0.277902	0.221938	0.298689	0.202271	0.294691	0.324752	0.363288
0.317077	0.271186	0.325712	0.31308	0.302207	0.282699	0.300608	0.288935	0.293252	0.326671
0.331788	0.267829	0.336105	0.295171	0.269907	0.29661	0.277902	0.295011	0.326831	0.368244
0.354013	0.262232	0.342021	0.244963	0.288935	0.320755	0.245283	0.290854	0.355133	0.366485
0.320595	0.263192	0.337064	0.321234	0.262392	0.287656	0.252958	0.275823	0.318676	0.350496
0.364886	0.279021	0.347777	0.189958	0.270227	0.346178	0.243204	0.305085	0.376239	0.363447
0.34394	0.274704	0.345699	0.267509	0.285897	0.31292	0.28158	0.288136	0.332747	0.36009
0.267829	0.275983	0.275664	0.377199	0.333547	0.237928	0.338663	0.282379	0.043652	0.307963
0.358011	0.274384	0.35913	0.267829	0.293572	0.308922	0.267669	0.2811	0.34314	0.362808
0.364727	0.254557	0.35881	0.283339	0.238727	0.31196	0.240966	0.316757	0.34346	0.371282
0.317717	0.254877	0.333547	0.308922	0.226575	0.295331	0.242245	0.311481	0.339783	0.345379
0.34378	0.267829	0.326991	0.251839	0.285098	0.320275	0.267988	0.272785	0.326031	0.347937
0.351615	0.227374	0.355453	0.286217	0.191078	0.307803	0.224816	0.31292	0.363288	0.370003
0.354173	0.262872	0.340262	0.278062	0.263831	0.31276	0.240646	0.305564	0.347138	0.352734
0.362488	0.252798	0.347777	0.280301	0.212664	0.340262	0.229293	0.313879	0.365046	0.365046
0.355932	0.223377	0.358331	0.300927	0.117685	0.322194	0.214743	0.302526	0.364247	0.367605
0.217301	0.305564	0.257755	0.416693	0.364567	0.239367	0.374001	0.331948	0.207387	0.248001
0.314359	0.257115	0.314998	0.287656	0.2504	0.292933	0.276143	0.311321	0.337384	0.315638
0.338663	0.268308	0.333227	0.303326	0.28158	0.293572	0.303806	0.308922	0.2811	0.345699
0.344579	0.287176	0.350496	0.285098	0.317877	0.322034	0.287336	0.305724	0.324752	0.37544
0.246882	0.313879	0.271666	0.347458	0.347298	0.28094	0.355612	0.338503	0.309402	0.010233
0.137512	0.307163	0.263351	0.393348	0.357052	0.267988	0.364886	0.322354	0.247042	0.24944
0.263991	0.262392	0.273745	0.354013	0.317557	0.229133	0.313719	0.279341	0.145987	0.310042
0.331788	0.274065	0.323473	0.290054	0.298849	0.29661	0.276783	0.305085	0.303965	0.32763
0.369843	0.268628	0.366166	0.258235	0.257595	0.342821	0.278062	0.333706	0.362168	0.379437
0.287176	0.292933	0.299328	0.376879	0.347777	0.266709	0.34442	0.278382	0.211065	0.325712
0.246882	0.299009	0.283818	0.373041	0.336425	0.25008	0.331628	0.320755	0.26495	0.2811

For Review Only

0.35897	0.243684	0.354653	0.270707	0.227854	0.320435	0.225935	0.299169	0.353534	0.355293
0.220339	0.306844	0.237768	0.411896	0.365046	0.236329	0.377359	0.336425	0.220659	0.231212
0.309082	0.262072	0.287176	0.298529	0.303326	0.275664	0.284458	0.284938	0.264791	0.292773
0.315798	0.288136	0.318516	0.32891	0.31196	0.286856	0.315638	0.319635	0.301727	0.330349
0.376879	0.266549	0.35977	0.236808	0.24992	0.357211	0.260953	0.318196	0.380237	0.382155
0.297889	0.305405	0.317397	0.345699	0.32763	0.301727	0.318196	0.323313	0.324752	0.314679
0.313559	0.276463	0.317717	0.334986	0.302686	0.2811	0.322354	0.307643	0.289735	0.325552
0.362648	0.246402	0.362808	0.280301	0.212344	0.32779	0.251999	0.303486	0.362808	0.377678
0.271666	0.29693	0.290694	0.360569	0.310042	0.262232	0.334186	0.336425	0.278062	0.289255
0.307323	0.320115	0.321394	0.408379	0.373201	0.286217	0.39111	0.348097	0.262232	0.336425
0.280301	0.261273	0.289894	0.353374	0.282219	0.24944	0.295651	0.284458	0.209946	0.314998
0.221618	0.310521	0.259354	0.415414	0.370323	0.242085	0.381356	0.339783	0.206108	0.258395
0.287336	0.216821	0.29677	0.270067	0.238567	0.256796	0.239367	0.271027	0.275823	0.300288
0.302526	0.278382	0.329069	0.337864	0.292133	0.289415	0.29613	0.305724	0.306684	0.317717
0.334186	0.274065	0.307643	0.282699	0.283978	0.309562	0.278542	0.309882	0.337864	0.290054
0.322674	0.227694	0.330349	0.269747	0.196354	0.288775	0.23505	0.278542	0.308443	0.333387
0.360409	0.255676	0.342661	0.275823	0.220339	0.333387	0.228494	0.325392	0.366485	0.364886
0.311161	0.292453	0.324113	0.283019	0.306044	0.287176	0.306044	0.304605	0.322194	0.231212
0.240006	0.282859	0.264631	0.382955	0.319795	0.244324	0.335305	0.31324	0.251839	0.253598
0.292133	0.292613	0.303646	0.330828	0.342821	0.280461	0.334506	0.308762	0.270547	0.290374
0.225456	0.298369	0.273745	0.373041	0.341062	0.262072	0.333547	0.326991	0.261753	0.273905
0.307643	0.287976	0.321554	0.340102	0.302686	0.277422	0.29693	0.310201	0.322833	0.283339
0.304445	0.285737	0.310361	0.358011	0.300448	0.272146	0.306204	0.329549	0.303965	0.339143
0.351295	0.223217	0.349057	0.306844	0.135433	0.322514	0.233611	0.295651	0.349536	0.356412
0.356572	0.272945	0.337544	0.193796	0.286697	0.32811	0.270547	0.290854	0.355453	0.335305
0.216981	0.278222	0.292133	0.346498	0.305085	0.204669	0.306204	0.317877	0.307963	0.251519
0.261912	0.299488	0.286697	0.399264	0.36009	0.243364	0.372402	0.319156	0.20259	0.291493
0.346818	0.273425	0.339623	0.29629	0.290214	0.315478	0.287496	0.31244	0.3433	0.35897
0.209946	0.318836	0.260793	0.42229	0.37448	0.247522	0.385513	0.339942	0.204349	0.26495
0.368404	0.2496	0.352095	0.270227	0.208347	0.332747	0.191238	0.305245	0.35977	0.369683
0.332107	0.264631	0.32747	0.271986	0.277902	0.300608	0.294052	0.302367	0.332587	0.340262
0.378158	0.268788	0.360569	0.221298	0.26495	0.341062	0.251839	0.319476	0.357531	0.380077
0.205788	0.307803	0.239687	0.410457	0.357531	0.233131	0.352255	0.32875	0.235209	0.223857
0.295491	0.247522	0.313399	0.323153	0.212824	0.291973	0.271186	0.316597	0.315478	0.299169
0.297729	0.283499	0.294052	0.334986	0.315318	0.276783	0.291973	0.164215	0.233611	0.304285
0.322034	0.225456	0.342181	0.29725	0.192996	0.290214	0.238088	0.294212	0.334986	0.350336
0.253758	0.289575	0.290534	0.351935	0.316917	0.259034	0.315798	0.321554	0.28142	0.26511
0.208027	0.315158	0.259674	0.423249	0.372721	0.254557	0.385353	0.34442	0.210905	0.264791
0.362808	0.263032	0.340262	0.223537	0.26495	0.336904	0.259034	0.288295	0.366805	0.351135
0.365846	0.256156	0.35945	0.283658	0.239367	0.313399	0.241286	0.317557	0.344899	0.372562
0.310042	0.239527	0.306204	0.29645	0.252638	0.244164	0.262392	0.279181	0.257275	0.317397
0.199392	0.306524	0.238567	0.415734	0.365206	0.225935	0.381996	0.3433	0.224017	0.223057
0.361369	0.28206	0.35865	0.241605	0.279021	0.333387	0.257275	0.295171	0.367605	0.371602
0.31276	0.246402	0.323473	0.287976	0.269587	0.290374	0.254557	0.259514	0.283499	0.350016
0.181484	0.279501	0.301727	0.352894	0.303486	0.1882	0.29757	0.31276	0.299648	0.268788
0.356572	0.274864	0.347138	0.213144	0.280461	0.34362	0.268948	0.284938	0.389511	0.351615
0.333387	0.259354	0.331948	0.298529	0.247681	0.302526	0.263032	0.31196	0.333067	0.35929
0.34346	0.282539	0.309882	0.295171	0.293092	0.305245	0.271346	0.290694	0.323953	0.314679
0.302367	0.257275	0.302846	0.337224	0.239847	0.273265	0.275024	0.332587	0.315478	0.301087
0.331628	0.274704	0.335146	0.334826	0.292613	0.301567	0.261273	0.284298	0.331788	0.369204
0.352255	0.286057	0.365526	0.32843	0.273905	0.314039	0.299808	0.322674	0.338024	0.367285
0.201311	0.315958	0.259354	0.42181	0.373041	0.248161	0.380716	0.338663	0.206268	0.266709
0.368724	0.277902	0.361369	0.215382	0.2811	0.326991	0.276943	0.309242	0.384394	0.346978
0.210425	0.307963	0.23489	0.417173	0.368884	0.240006	0.379277	0.337704	0.212344	0.217941
0.362328	0.253438	0.349536	0.28126	0.213303	0.340422	0.231052	0.314679	0.365846	0.365846

For Review Only

0.384714	0.266549	0.374161	0.274225	0.229293	0.345699	0.208507	0.318516	0.381676	0.3748
0.212024	0.293092	0.283019	0.381356	0.32763	0.213623	0.332747	0.32859	0.276623	0.248321
0.363128	0.28078	0.347138	0.189639	0.28094	0.338983	0.273905	0.301887	0.37528	0.262072
0.372402	0.259514	0.354813	0.278702	0.241925	0.322194	0.237928	0.306204	0.364727	0.384074

For Review Only

0.280301	0.298849	0.302047	0.28078	0.259834	0.353854	0.357052	0.322194	0.318996	0.32811
0.299808	0.246562	0.285098	0.297889	0.277902	0.298529	0.330828	0.279981	0.31196	0.307963
0.2811	0.243844	0.244004	0.204029	0.254077	0.377518	0.374161	0.215862	0.347618	0.363128
0.272306	0.260793	0.251359	0.228814	0.252319	0.349057	0.340742	0.263351	0.329389	0.31196
0.300288	0.356092	0.32827	0.321554	0.295331	0.331148	0.355453	0.35945	0.323793	0.304765
0.272146	0.268468	0.271027	0.257115	0.254557	0.35993	0.351295	0.275664	0.305085	0.323953
0.300608	0.310521	0.29677	0.288775	0.269268	0.360249	0.358331	0.345539	0.259834	0.352414
0.290854	0.326831	0.31196	0.304125	0.28094	0.274704	0.336585	0.339783	0.299328	0.264311
0.288935	0.310841	0.29645	0.28078	0.260953	0.340102	0.327151	0.327151	0.230413	0.316917
0.321234	0.369044	0.345059	0.335945	0.311161	0.283658	0.338663	0.376399	0.315318	0.280461
0.28078	0.354013	0.341062	0.325871	0.289095	0.291493	0.277902	0.356572	0.342821	0.275983
0.28158	0.239687	0.241286	0.190918	0.248801	0.373521	0.373841	0.206748	0.339942	0.355453
0.290054	0.322674	0.283019	0.305405	0.271826	0.305245	0.335146	0.338024	0.251519	0.303326
0.25056	0.335945	0.298049	0.302047	0.261433	0.255197	0.309722	0.314039	0.311641	0.209146
0.26575	0.283179	0.264791	0.258555	0.243044	0.335945	0.324592	0.2819	0.28206	0.326991
0.275823	0.299169	0.274384	0.144068	0.270867	0.310841	0.325232	0.290534	0.34346	0.283339
0.308762	0.337544	0.313399	0.289894	0.284138	0.358491	0.348737	0.358171	0.269587	0.337064
0.283339	0.316597	0.284938	0.298689	0.274544	0.332587	0.350496	0.319795	0.291493	0.32843
0.288775	0.245283	0.274384	0.244803	0.267669	0.377359	0.367925	0.25056	0.347937	0.355612
0.323313	0.363288	0.341701	0.350656	0.307323	0.300288	0.308443	0.35881	0.301407	0.314039
0.310042	0.34362	0.311161	0.339303	0.282699	0.31292	0.322194	0.365526	0.230253	0.305245
0.298049	0.336105	0.293732	0.29693	0.279341	0.355293	0.356572	0.351135	0.269587	0.318196
0.319635	0.389511	0.341222	0.378478	0.308123	0.241445	0.249121	0.388871	0.346818	0.279821
0.259994	0.333387	0.271346	0.321554	0.261753	0.185162	0.283339	0.317557	0.284618	0.200192
0.283339	0.26511	0.270547	0.241765	0.256476	0.35993	0.352734	0.282699	0.290374	0.338983
0.280461	0.258714	0.190278	0.260153	0.256156	0.354813	0.357052	0.256476	0.345699	0.339942
0.279181	0.244164	0.226255	0.230253	0.24928	0.335305	0.342501	0.2181	0.31324	0.320595
0.241765	0.270547	0.237608	0.242405	0.21858	0.28062	0.293892	0.268468	0.28206	0.268148
0.286697	0.31196	0.29757	0.288295	0.26543	0.299968	0.305245	0.324113	0.217301	0.276783
0.32859	0.413495	0.368884	0.400863	0.329549	0.240646	0.31244	0.401183	0.300768	0.268148
0.271346	0.24928	0.255676	0.225935	0.253278	0.361369	0.361049	0.23457	0.321714	0.340262
0.291813	0.286537	0.280301	0.264791	0.276943	0.376399	0.356732	0.291653	0.341062	0.365526
0.292933	0.300448	0.298369	0.287176	0.273905	0.373841	0.366166	0.331148	0.28174	0.347298
0.255676	0.342341	0.300927	0.323473	0.271027	0.223057	0.287976	0.338823	0.295331	0.04765
0.294851	0.323313	0.294851	0.316917	0.28174	0.306524	0.290374	0.282219	0.338503	0.31324
0.262072	0.267988	0.231532	0.264631	0.242405	0.255676	0.315318	0.236489	0.315318	0.241605
0.263511	0.307323	0.294212	0.300288	0.267349	0.301247	0.284298	0.325712	0.29757	0.279821
0.288136	0.320915	0.301407	0.288615	0.273265	0.325072	0.314359	0.322674	0.32843	0.321874
0.298849	0.384714	0.338983	0.37496	0.294052	0.180844	0.233451	0.380237	0.340262	0.232651
0.308443	0.354813	0.31292	0.32747	0.294532	0.28078	0.239847	0.366965	0.348737	0.323473
0.321075	0.404861	0.362488	0.388232	0.317397	0.243364	0.303326	0.394947	0.298209	0.261433
0.298689	0.339942	0.325871	0.321714	0.292133	0.291973	0.297889	0.341382	0.231052	0.287656
0.307963	0.352095	0.32747	0.329069	0.294851	0.28142	0.32891	0.354653	0.306364	0.272626
0.319476	0.352414	0.325712	0.335146	0.294691	0.331948	0.34362	0.358331	0.261273	0.338343
0.325552	0.368084	0.345219	0.335305	0.302846	0.29661	0.34442	0.362008	0.318356	0.290854
0.267509	0.245923	0.220179	0.216342	0.245603	0.368404	0.356572	0.226255	0.332587	0.356732
0.292133	0.254397	0.224177	0.251039	0.252319	0.367605	0.360889	0.133515	0.340582	0.342821
0.260953	0.307963	0.255836	0.294212	0.259514	0.295811	0.31212	0.31244	0.336425	0.303326
0.326191	0.349217	0.338184	0.317237	0.289735	0.330189	0.342341	0.356412	0.262872	0.329229
0.248641	0.292933	0.274384	0.254077	0.257595	0.362648	0.339303	0.307163	0.303965	0.332267

For Review Only

0.278542	0.241286	0.205948	0.251519	0.256316	0.352414	0.365046	0.233451	0.339303	0.349217
0.308283	0.276623	0.292453	0.276303	0.277582	0.324592	0.331788	0.295331	0.316118	0.326991
0.301567	0.306524	0.284458	0.263351	0.26527	0.378318	0.370803	0.329229	0.2504	0.349536
0.292453	0.300927	0.297729	0.286057	0.273425	0.373041	0.366006	0.330508	0.28094	0.346338
0.277902	0.248481	0.269907	0.241925	0.254557	0.352414	0.357371	0.245123	0.330349	0.350336
0.318676	0.340102	0.313399	0.284938	0.282859	0.345379	0.350016	0.344579	0.252478	0.341382
0.287816	0.245923	0.274065	0.245603	0.267349	0.376879	0.368564	0.250879	0.346818	0.354813
0.302686	0.231692	0.223537	0.260153	0.261753	0.364407	0.364727	0.112088	0.326671	0.357052
0.28206	0.254717	0.20323	0.24928	0.251679	0.354493	0.351455	0.242885	0.32827	0.338503
0.301727	0.384074	0.333547	0.372562	0.29741	0.208986	0.279981	0.382155	0.308762	0.229613
0.266869	0.321714	0.285897	0.322354	0.267509	0.278062	0.285417	0.326671	0.277422	0.260633
0.241605	0.262552	0.244963	0.201471	0.21954	0.292293	0.298849	0.260473	0.2811	0.273745
0.2811	0.201311	0.24928	0.232491	0.245763	0.361689	0.362968	0.082187	0.331788	0.348737
0.292613	0.226415	0.254237	0.246562	0.260153	0.362488	0.361369	0.005916	0.330828	0.344899
0.301567	0.305405	0.285257	0.263351	0.264471	0.378318	0.369683	0.32811	0.25024	0.348897
0.286537	0.31196	0.29645	0.287336	0.264631	0.299968	0.304765	0.323313	0.216501	0.276943
0.257755	0.273105	0.259514	0.26639	0.238727	0.311161	0.307963	0.279341	0.305724	0.295971
0.267509	0.330668	0.313559	0.324592	0.272785	0.298369	0.305884	0.340902	0.283658	0.275024
0.286057	0.304285	0.285098	0.285098	0.279981	0.32843	0.29725	0.293412	0.325712	0.311321
0.242885	0.300128	0.272146	0.246882	0.264631	0.39127	0.358331	0.290694	0.326671	0.372082
0.274864	0.283019	0.275823	0.240806	0.26575	0.39127	0.355932	0.28078	0.274384	0.34426
0.291334	0.301407	0.295171	0.26591	0.26527	0.372721	0.369204	0.332107	0.275344	0.340422
0.273905	0.228814	0.247202	0.236968	0.245123	0.329389	0.335465	0.059002	0.309242	0.314359
0.287496	0.347458	0.332747	0.339623	0.287336	0.267669	0.084426	0.345379	0.336265	0.284458
0.282859	0.297889	0.305085	0.288775	0.26639	0.318036	0.298209	0.329229	0.235849	0.285417
0.286377	0.256316	0.301087	0.304605	0.285737	0.337064	0.330029	0.270387	0.307643	0.311001
0.325552	0.367925	0.345219	0.335305	0.302686	0.29613	0.34394	0.362008	0.318996	0.290374
0.295651	0.197793	0.251999	0.228654	0.264631	0.346818	0.349057	0.207867	0.310681	0.329389
0.332267	0.363767	0.355612	0.342661	0.311481	0.320915	0.360729	0.37448	0.319955	0.340742
0.301247	0.38999	0.339463	0.379117	0.299009	0.165174	0.258714	0.388871	0.34426	0.239207
0.284298	0.274225	0.183722	0.273905	0.262552	0.357211	0.357851	0.267189	0.355932	0.340902
0.279661	0.292933	0.277103	0.214263	0.271826	0.35977	0.357531	0.29645	0.311321	0.336425
0.291813	0.32795	0.299488	0.285737	0.28174	0.313399	0.341861	0.339942	0.292293	0.29725
0.254557	0.295651	0.230253	0.290054	0.257915	0.229773	0.308443	0.290054	0.293412	0.228814
0.307004	0.398145	0.341861	0.39095	0.308762	0.154781	0.24928	0.401183	0.346338	0.226415
0.300927	0.304285	0.284778	0.262552	0.264311	0.377678	0.368884	0.32843	0.249121	0.347937
0.254717	0.306044	0.275983	0.29725	0.266709	0.322674	0.322674	0.324272	0.29725	0.287976
0.325712	0.367765	0.345699	0.335625	0.302686	0.29661	0.342821	0.362488	0.318996	0.291174
0.294691	0.335625	0.321554	0.325392	0.288455	0.263192	0.32875	0.348577	0.272306	0.287816
0.325392	0.372562	0.349856	0.337704	0.300608	0.321075	0.351775	0.378318	0.291174	0.304285
0.288775	0.32779	0.305564	0.299009	0.283019	0.318516	0.305884	0.333067	0.325871	0.313879
0.306204	0.365206	0.339623	0.339143	0.295651	0.303326	0.330988	0.364247	0.307803	0.295011
0.292613	0.302526	0.282219	0.253118	0.26607	0.37528	0.369524	0.325232	0.257915	0.349696
0.29613	0.255357	0.271506	0.236649	0.279821	0.378318	0.367285	0.248641	0.332267	0.360889
0.307323	0.309882	0.280141	0.284778	0.273425	0.333706	0.370803	0.321394	0.324752	0.347298
0.28126	0.200831	0.251519	0.23393	0.246722	0.362808	0.364087	0.084426	0.333547	0.348417
0.2819	0.313399	0.283339	0.291174	0.270227	0.339783	0.342021	0.31244	0.29709	0.315478
0.272785	0.195075	0.254397	0.248321	0.242245	0.368884	0.344899	0.244643	0.320755	0.334986
0.272785	0.363927	0.303965	0.338503	0.276303	0.204189	0.284618	0.350336	0.316118	0.141829
0.267189	0.264791	0.210745	0.278222	0.253758	0.3118	0.321874	0.257115	0.320915	0.305245
0.274864	0.289735	0.198913	0.260953	0.247362	0.329709	0.339783	0.258075	0.332587	0.31276
0.298689	0.320915	0.310201	0.303646	0.285098	0.349217	0.267509	0.291973	0.345379	0.346019
0.273105	0.224976	0.247362	0.231852	0.246083	0.324592	0.339623	0.18804	0.305884	0.314998
0.308762	0.398785	0.347777	0.385034	0.304925	0.184842	0.246242	0.388551	0.34346	0.244484
0.313559	0.360409	0.32859	0.35961	0.300448	0.232651	0.28078	0.364567	0.318836	0.254557

For Review Only

0.275024	0.292133	0.291493	0.286057	0.259354	0.273105	0.294851	0.307963	0.300608	0.283019
0.304445	0.31276	0.283978	0.304765	0.277582	0.364087	0.348737	0.298209	0.336105	0.346178
0.287016	0.291813	0.23409	0.299648	0.259034	0.317077	0.293412	0.292293	0.34314	0.319635
0.305405	0.326031	0.302047	0.318196	0.285257	0.301087	0.301727	0.322194	0.293092	0.324592
0.256636	0.256956	0.189799	0.248481	0.244004	0.303646	0.324432	0.24992	0.306044	0.29693
0.270067	0.370003	0.301087	0.338983	0.274704	0.220819	0.286856	0.353854	0.319476	0.179725
0.278702	0.179405	0.240806	0.235529	0.251839	0.364727	0.357371	0.205948	0.32827	0.347937
0.255996	0.260153	0.191717	0.255996	0.240966	0.31308	0.329229	0.254397	0.317237	0.31228
0.268948	0.255516	0.254557	0.025104	0.253918	0.35961	0.357691	0.245443	0.309722	0.321394
0.262712	0.286217	0.252478	0.26575	0.244963	0.28062	0.295171	0.262392	0.303006	0.26575
0.28142	0.324272	0.259994	0.309882	0.277263	0.310841	0.324592	0.306524	0.333706	0.291334
0.270707	0.282219	0.251679	0.310841	0.259514	0.26623	0.294212	0.290854	0.305564	0.261433
0.278222	0.325072	0.293892	0.323153	0.264631	0.31324	0.330508	0.333547	0.290534	0.299169
0.258555	0.285897	0.254077	0.262552	0.229933	0.294212	0.289415	0.276303	0.290054	0.290534
0.299968	0.318676	0.286217	0.287816	0.274704	0.32763	0.362968	0.308443	0.326511	0.325552
0.283658	0.196514	0.237288	0.236329	0.252798	0.340742	0.342501	0.21906	0.3126	0.32891
0.288295	0.274864	0.276303	0.280141	0.269108	0.309882	0.335146	0.279981	0.310521	0.306524
0.260313	0.271346	0.222578	0.26639	0.233611	0.299648	0.307483	0.262872	0.306204	0.294052
0.29629	0.31276	0.285897	0.300768	0.2819	0.366805	0.339623	0.32779	0.331948	0.364727
0.25072	0.257115	0.228973	0.241765	0.224976	0.307163	0.315158	0.232811	0.293092	0.294851
0.271666	0.371922	0.309562	0.338823	0.28062	0.220179	0.291334	0.349376	0.320595	0.161337
0.303965	0.314359	0.289894	0.270707	0.274544	0.339623	0.34362	0.329549	0.307163	0.325232
0.241445	0.261593	0.240646	0.251679	0.221938	0.283818	0.290534	0.256796	0.291334	0.277422
0.276303	0.276943	0.271666	0.192837	0.261433	0.356732	0.347937	0.292133	0.298529	0.342021
0.26591	0.253278	0.2181	0.227374	0.2504	0.339942	0.348897	0.229133	0.311641	0.315158
0.300768	0.395587	0.341701	0.377838	0.302367	0.173649	0.258235	0.391749	0.337544	0.228334
0.283179	0.294691	0.273585	0.300608	0.267669	0.311481	0.204509	0.292453	0.338823	0.313879
0.277103	0.330189	0.291174	0.314998	0.273745	0.287496	0.176687	0.331628	0.299009	0.284458
0.288615	0.272785	0.289575	0.259994	0.268628	0.354173	0.342981	0.29677	0.303646	0.329869
0.260473	0.274544	0.258235	0.259194	0.249121	0.295651	0.305724	0.293732	0.286697	0.273905
0.279821	0.251679	0.2496	0.204509	0.258714	0.388551	0.388391	0.236009	0.326351	0.369204
0.295491	0.304445	0.307323	0.275024	0.275823	0.371442	0.344739	0.301567	0.322194	0.339463
0.298049	0.386313	0.335945	0.369204	0.292933	0.189958	0.275823	0.381356	0.334506	0.213783
0.256796	0.26639	0.256796	0.26639	0.235369	0.272785	0.290374	0.270867	0.300448	0.277103
0.293732	0.303326	0.283499	0.251999	0.267189	0.378158	0.370483	0.326991	0.257595	0.352095
0.267029	0.272945	0.205788	0.2496	0.239367	0.314679	0.308762	0.264151	0.297729	0.29613
0.256636	0.342021	0.300288	0.321874	0.269907	0.224656	0.287816	0.338184	0.295331	0.050048
0.259834	0.337864	0.294532	0.306684	0.26639	0.277902	0.323953	0.330988	0.304285	0.251359
0.2811	0.37496	0.31276	0.353854	0.2811	0.226255	0.290694	0.355612	0.318196	0.168852
0.273105	0.245123	0.253438	0.237128	0.249121	0.347618	0.354813	0.228014	0.316437	0.32747
0.284938	0.32811	0.266869	0.314039	0.271826	0.300128	0.292613	0.330189	0.340262	0.287976
0.307004	0.387272	0.360409	0.376239	0.305405	0.223057	0.260473	0.39127	0.35977	0.257595
0.291014	0.331468	0.311321	0.318196	0.286697	0.305405	0.216661	0.333866	0.330349	0.316278
0.269268	0.368084	0.301887	0.346178	0.277422	0.208507	0.287496	0.356732	0.306044	0.158139
0.246882	0.285737	0.267669	0.268148	0.26511	0.358331	0.347458	0.266869	0.317397	0.351295
0.310201	0.361529	0.332587	0.35913	0.299328	0.254557	0.310681	0.371442	0.301887	0.263991
0.259674	0.304605	0.23473	0.288775	0.267988	0.303806	0.319635	0.306844	0.301567	0.299169
0.273905	0.287816	0.270547	0.269587	0.257755	0.323473	0.353534	0.290694	0.306684	0.318356
0.291174	0.260633	0.182443	0.261433	0.24992	0.362008	0.358011	0.245603	0.346978	0.348577
0.289255	0.252159	0.217941	0.239687	0.257435	0.351775	0.354173	0.220339	0.341062	0.345379
0.267349	0.330349	0.282859	0.314839	0.260473	0.24928	0.272146	0.323153	0.298049	0.239207
0.289575	0.251039	0.184042	0.250879	0.255197	0.354013	0.349696	0.243044	0.325871	0.347618
0.279981	0.260313	0.210585	0.271186	0.254077	0.338024	0.346178	0.261433	0.347458	0.32811
0.305405	0.366805	0.334666	0.354173	0.286697	0.254077	0.258235	0.369044	0.322833	0.242405
0.143748	0.291334	0.28078	0.261273	0.244484	0.307963	0.301727	0.299328	0.319156	0.274704

For Review Only

0.289095	0.243204	0.226095	0.267669	0.263032	0.349217	0.365846	0.116565	0.340742	0.350975
0.279341	0.323793	0.283978	0.302367	0.262872	0.257755	0.273425	0.322674	0.301407	0.257435
0.282219	0.277263	0.262232	0.24992	0.259034	0.370323	0.364247	0.273745	0.338343	0.352894
0.303486	0.361529	0.324592	0.353694	0.29661	0.279821	0.255037	0.364727	0.32811	0.305724
0.314039	0.345859	0.330349	0.342661	0.301247	0.326351	0.339783	0.353694	0.331308	0.32795
0.271826	0.339623	0.310361	0.31292	0.287816	0.293892	0.25008	0.346658	0.323473	0.29613
0.285257	0.213783	0.255516	0.251679	0.261273	0.365686	0.357691	0.244004	0.323953	0.32811
0.284298	0.32811	0.309722	0.318036	0.274225	0.299009	0.286217	0.34442	0.330349	0.287016
0.302846	0.360569	0.318516	0.351135	0.289095	0.21954	0.255357	0.364407	0.338823	0.273265
0.240806	0.269268	0.239687	0.251839	0.223057	0.285737	0.29661	0.255516	0.283179	0.273425
0.290054	0.356732	0.319316	0.32875	0.297889	0.275344	0.292933	0.358491	0.352255	0.278382
0.2811	0.274384	0.282699	0.232651	0.267669	0.378638	0.361848	0.283499	0.307323	0.357371
0.277263	0.251359	0.192037	0.243684	0.240646	0.332427	0.322833	0.239367	0.309722	0.319795
0.269907	0.323953	0.276943	0.315798	0.257595	0.217301	0.259834	0.31276	0.29677	0.24928
0.26623	0.262712	0.239687	0.241765	0.244484	0.348737	0.324912	0.240646	0.321554	0.318516
0.280141	0.310841	0.276943	0.29725	0.26591	0.307803	0.329389	0.317397	0.302846	0.283339
0.272146	0.360569	0.303646	0.339942	0.274225	0.20259	0.286537	0.34362	0.304125	0.159578
0.293412	0.362648	0.346818	0.345379	0.295971	0.273425	0.274384	0.371602	0.357531	0.292613
0	0.286856	0.262232	0.26511	0.238248	0.288935	0.294851	0.293412	0.307163	0.26511
0.286856	0	0.258874	0.253118	0.263831	0.355293	0.350496	0.227534	0.318996	0.347618
0.262232	0.258874	0	0.256156	0.238088	0.305405	0.336105	0.254397	0.307323	0.304125
0.26511	0.253118	0.256156	0	0.248961	0.351935	0.350176	0.247202	0.305245	0.316118
0.238248	0.263831	0.238088	0.248961	0	0.279981	0.291493	0.260633	0.280141	0.273425
0.288935	0.355293	0.305405	0.351935	0.279981	0	0.267189	0.362648	0.3118	0.223057
0.294851	0.350496	0.336105	0.350176	0.291493	0.267189	0	0.361049	0.329069	0.275184
0.293412	0.227534	0.254397	0.247202	0.260633	0.362648	0.361049	0	0.329389	0.344899
0.307163	0.318996	0.307323	0.305245	0.280141	0.3118	0.329069	0.329389	0	0.300288
0.26511	0.347618	0.304125	0.316118	0.273425	0.223057	0.275184	0.344899	0.300288	0
0.272466	0.259674	0.256636	0.216182	0.248641	0.348897	0.348417	0.209946	0.309882	0.322993
0.268628	0.308762	0.276623	0.29757	0.264311	0.310521	0.323633	0.315318	0.302207	0.271186
0.279181	0.29709	0.259354	0.292293	0.261753	0.334506	0.311641	0.291813	0.316437	0.322833
0.257435	0.299169	0.284938	0.251199	0.259034	0.35929	0.347298	0.29757	0.280461	0.340902
0.273905	0.252958	0.262392	0.286697	0.26527	0.324272	0.339303	0.28174	0.307483	0.309562
0.288935	0.302526	0.285577	0.244164	0.275664	0.389511	0.360729	0.301727	0.340902	0.362648
0.290054	0.254077	0.290374	0.262872	0.26495	0.346019	0.331308	0.29629	0.298529	0.316437
0.275504	0.362008	0.305724	0.342021	0.275983	0.210585	0.283499	0.3441	0.31244	0.163096
0.278062	0.283978	0.295171	0.285417	0.260633	0.36009	0.338983	0.307004	0.300768	0.321075
0.292453	0.227055	0.253438	0.246083	0.259834	0.362008	0.360889	0.006716	0.330189	0.34442
0.302846	0.258075	0.230572	0.274864	0.262392	0.340262	0.295971	0.262392	0.314519	0.335785
0.264471	0.278862	0.274544	0.270707	0.256476	0.340902	0.327151	0.329229	0.303166	0.307963
0.290054	0.254877	0.221618	0.238407	0.258075	0.350016	0.353694	0.21922	0.345219	0.345859
0.271826	0.229293	0.269747	0.263192	0.260793	0.336265	0.352574	0.231052	0.331628	0.321714
0.280461	0.242565	0.242885	0.20387	0.253438	0.376399	0.37448	0.215542	0.348737	0.362488
0.285897	0.262072	0.180524	0.258714	0.253118	0.364567	0.353534	0.254877	0.342981	0.349217
0.295651	0.393988	0.338823	0.376559	0.300448	0.177966	0.254237	0.387912	0.339623	0.222737
0.279821	0.289575	0.26607	0.277902	0.256476	0.324432	0.288935	0.289894	0.323313	0.321075
0.28206	0.303166	0.278862	0.28094	0.267349	0.310361	0.334826	0.290534	0.337064	0.289735
0.298049	0.288615	0.301727	0.298849	0.277742	0.334986	0.340582	0.277103	0.318836	0.315478
0.292293	0.361689	0.345699	0.3441	0.294212	0.273745	0.273425	0.370163	0.357052	0.292613
0.303326	0.379597	0.334506	0.367125	0.301087	0.175408	0.28078	0.393508	0.349057	0.254717
0.248001	0.34314	0.287656	0.316757	0.264471	0.196354	0.285897	0.337864	0.289255	0.17253
0.283499	0.306204	0.290854	0.243044	0.258555	0.313399	0.325552	0.29661	0.271986	0.293572
0.286537	0.260953	0.25072	0.247042	0.26543	0.374001	0.368084	0.273745	0.31324	0.367605
0.293412	0.366965	0.329069	0.35929	0.293092	0.243524	0.285737	0.367285	0.320755	0.235209
0.285098	0.367765	0.337544	0.369364	0.293732	0.247841	0.092901	0.367605	0.326671	0.26623

For Review Only

0.28062	0.201151	0.2496	0.231372	0.245443	0.361369	0.362808	0.082667	0.331468	0.348097
0.31292	0.389511	0.345699	0.377678	0.306844	0.192197	0.255357	0.388072	0.348897	0.238887
0.169651	0.315318	0.291813	0.269747	0.261113	0.295011	0.32859	0.320915	0.320595	0.266549
0.299808	0.325232	0.301247	0.325392	0.277582	0.306044	0.310361	0.329549	0.303806	0.299328
0.282379	0.274704	0.268948	0.215702	0.267509	0.388391	0.372562	0.277103	0.344739	0.372562
0.306044	0.346498	0.329709	0.330988	0.294691	0.309402	0.180365	0.354173	0.315958	0.319955
0.295651	0.3126	0.314679	0.31308	0.269587	0.29645	0.320435	0.340262	0.288775	0.284938
0.272306	0.269587	0.229773	0.254557	0.255197	0.37432	0.363447	0.294052	0.334186	0.338343
0.287176	0.349856	0.283818	0.348897	0.293732	0.279181	0.285577	0.365206	0.347618	0.294851
0.322194	0.392549	0.352095	0.384074	0.323633	0.259674	0.322833	0.388871	0.31324	0.269108
0.264311	0.332747	0.270547	0.309722	0.261113	0.244164	0.301727	0.322514	0.311481	0.227215
0.306844	0.397026	0.353694	0.388232	0.306524	0.173649	0.26559	0.394947	0.346498	0.238727
0.246882	0.269428	0.245123	0.244963	0.214583	0.2811	0.285897	0.257755	0.280301	0.269587
0.262552	0.298689	0.290214	0.317557	0.280461	0.307323	0.310521	0.320435	0.323793	0.309242
0.28206	0.320275	0.307483	0.298529	0.274864	0.338024	0.303965	0.333866	0.348737	0.332747
0.255357	0.248641	0.186441	0.247841	0.240966	0.31308	0.319955	0.248961	0.3118	0.298049
0.278862	0.243364	0.257115	0.217781	0.263192	0.365846	0.37576	0.21954	0.34426	0.360409
0.2819	0.34346	0.299968	0.31212	0.279181	0.330508	0.292933	0.342181	0.342661	0.308123
0.294372	0.357211	0.320755	0.352414	0.291334	0.2189	0.256956	0.357371	0.334346	0.260313
0.294212	0.345859	0.314519	0.324752	0.285737	0.274384	0.290694	0.362488	0.319156	0.26591
0.268468	0.364087	0.311001	0.35993	0.283499	0.227534	0.262872	0.37464	0.357531	0.263831
0.282699	0.338823	0.306204	0.303166	0.274384	0.319316	0.298529	0.330828	0.323793	0.318356
0.302367	0.316278	0.303806	0.339942	0.299169	0.286057	0.324432	0.309882	0.354013	0.316757
0.282699	0.267349	0.185961	0.26543	0.251839	0.350656	0.349856	0.261433	0.347298	0.330029
0.271986	0.272466	0.278702	0.236808	0.255197	0.355293	0.32859	0.302367	0.291813	0.316278
0.285257	0.333866	0.286697	0.324432	0.271186	0.284778	0.255357	0.316917	0.323473	0.321874
0.301407	0.383914	0.333227	0.371762	0.29741	0.208666	0.279661	0.381516	0.306684	0.228654
0.292453	0.295971	0.290534	0.275504	0.273905	0.335945	0.346818	0.291174	0.292293	0.324113
0.311001	0.401503	0.348897	0.388711	0.311161	0.157819	0.256476	0.399424	0.3433	0.232331
0.287336	0.237128	0.244324	0.181964	0.251999	0.374001	0.382635	0.210265	0.331148	0.353214
0.294372	0.299968	0.293092	0.28078	0.260473	0.327151	0.329229	0.32811	0.254237	0.302047
0.273265	0.253598	0.253758	0.21954	0.264151	0.377838	0.372082	0.259994	0.319635	0.355612
0.289575	0.388711	0.32795	0.377838	0.293252	0.18772	0.260953	0.385513	0.349217	0.256956
0.273745	0.277742	0.253438	0.284138	0.260953	0.309562	0.330029	0.278702	0.332747	0.298849
0.278862	0.336904	0.284138	0.322194	0.271826	0.283179	0.301087	0.336105	0.291014	0.233291
0.273425	0.254237	0.220659	0.277902	0.257275	0.323793	0.330508	0.255197	0.321554	0.329389
0.283339	0.355133	0.332747	0.350176	0.283658	0.277902	0.07755	0.354973	0.337864	0.286217
0.307163	0.397666	0.347618	0.38999	0.31196	0.167893	0.255996	0.395747	0.345539	0.228014
0.204029	0.279341	0.263351	0.225136	0.252798	0.366805	0.350815	0.289735	0.291014	0.337704
0.293732	0.227694	0.254717	0.247362	0.261113	0.363927	0.362488	0.006876	0.329709	0.345699
0.260633	0.266709	0.230732	0.263032	0.241286	0.254397	0.31308	0.236489	0.314039	0.240166
0.304925	0.396546	0.348577	0.385993	0.306524	0.184842	0.247042	0.393028	0.347298	0.237928
0.283339	0.275664	0.277902	0.260153	0.26607	0.379117	0.346658	0.294052	0.317397	0.346658
0.256956	0.285098	0.254077	0.278862	0.251679	0.290214	0.310521	0.272626	0.310681	0.259834
0.295811	0.336265	0.300288	0.320275	0.273425	0.278542	0.238727	0.316278	0.317237	0.305884
0.290534	0.301727	0.28174	0.242085	0.261753	0.383275	0.361529	0.322993	0.254877	0.339942
0.288455	0.255996	0.256636	0.267669	0.262072	0.334186	0.35977	0.20291	0.350496	0.32747
0.290054	0.317557	0.307163	0.301407	0.283019	0.326991	0.314359	0.329549	0.311321	0.318516
0.302047	0.314679	0.268468	0.29741	0.275344	0.291813	0.311001	0.292933	0.363128	0.325072
0.279021	0.29677	0.269587	0.289415	0.277103	0.341701	0.36009	0.286217	0.31308	0.330349
0.305405	0.306204	0.269428	0.284618	0.289255	0.335146	0.3748	0.288935	0.318516	0.338343
0.309242	0.396706	0.34378	0.391909	0.307643	0.159898	0.254237	0.397186	0.345059	0.233131
0.286377	0.284458	0.277103	0.228174	0.269587	0.392229	0.3433	0.288615	0.307643	0.350656
0.306524	0.397825	0.349856	0.381036	0.307963	0.183083	0.25008	0.392229	0.348897	0.232491
0.28094	0.244004	0.244324	0.20339	0.254077	0.376879	0.37496	0.216342	0.348417	0.363927

FOR REVIEW ONLY

0.294052	0.247202	0.259834	0.23473	0.262392	0.385993	0.372721	0.231372	0.330349	0.362968
0.302526	0.358331	0.317877	0.331148	0.287336	0.258555	0.239367	0.363767	0.339783	0.308762
0.283978	0.301087	0.283499	0.253758	0.264471	0.37464	0.337384	0.305724	0.307963	0.32747
0.292133	0.220819	0.257115	0.236489	0.263511	0.362328	0.35977	0.200032	0.332907	0.346498

For Review Only

0.284938	0.287016	0.272306	0.256316	0.271027	0.284778	0.257755	0.340422	0.271986	0.320915
0.304445	0.314679	0.319955	0.342501	0.323313	0.361848	0.336105	0.299648	0.349536	0.279341
0.226575	0.308922	0.29661	0.267669	0.257915	0.271986	0.292613	0.367605	0.304445	0.215542
0.213303	0.300927	0.292613	0.291174	0.272466	0.287816	0.290214	0.332107	0.283339	0.263192
0.333227	0.338983	0.321874	0.28126	0.322354	0.323793	0.311321	0.323633	0.315318	0.35881
0.245923	0.284458	0.287176	0.251839	0.268628	0.28094	0.29677	0.351775	0.293092	0.274384
0.299968	0.301087	0.290054	0.220019	0.268788	0.288775	0.263032	0.365366	0.266549	0.34442
0.315478	0.31244	0.314039	0.337544	0.307323	0.354493	0.321394	0.279341	0.341701	0.339303
0.292453	0.289415	0.274704	0.216981	0.279661	0.294851	0.269587	0.344579	0.260313	0.326671
0.342181	0.338503	0.332107	0.364567	0.332107	0.39127	0.351135	0.290854	0.37496	0.3756
0.345379	0.313399	0.362648	0.352574	0.341062	0.341541	0.336585	0.318836	0.34394	0.355932
0.190758	0.301887	0.290534	0.272945	0.257915	0.270227	0.284458	0.35913	0.299808	0.206108
0.307803	0.304605	0.29613	0.274864	0.299009	0.309402	0.299968	0.303486	0.304445	0.337864
0.293572	0.279181	0.302367	0.308283	0.304925	0.337544	0.314359	0.196514	0.314199	0.31324
0.241126	0.269907	0.259194	0.21938	0.272626	0.253598	0.273425	0.338343	0.274384	0.28206
0.285417	0.324912	0.308283	0.306844	0.315958	0.300768	0.302367	0.303006	0.321234	0.289415
0.320915	0.326031	0.316757	0.232651	0.342981	0.304925	0.309082	0.354653	0.290374	0.357691
0.301087	0.280141	0.299328	0.277902	0.291014	0.302207	0.302686	0.345539	0.310841	0.319476
0.251839	0.302367	0.310841	0.288295	0.277103	0.25024	0.300768	0.376079	0.294532	0.25024
0.358331	0.335465	0.332427	0.353374	0.357691	0.369683	0.350176	0.306844	0.342181	0.357851
0.329389	0.319156	0.334826	0.290214	0.307643	0.334346	0.317397	0.314519	0.318996	0.365206
0.315478	0.325392	0.305724	0.233611	0.316917	0.291334	0.289415	0.352894	0.286856	0.350656
0.364407	0.321874	0.335625	0.380077	0.361689	0.388391	0.347618	0.26591	0.377678	0.389031
0.303806	0.283978	0.31276	0.327151	0.303326	0.352574	0.313879	0.205149	0.319156	0.316597
0.258075	0.290374	0.28174	0.237768	0.274065	0.273105	0.274704	0.361529	0.274384	0.282859
0.247202	0.305245	0.268148	0.298369	0.268148	0.283339	0.295811	0.357531	0.311001	0.255516
0.20387	0.302207	0.253278	0.285098	0.263192	0.284618	0.268628	0.329549	0.292933	0.217301
0.24992	0.267988	0.261273	0.260633	0.266549	0.274704	0.268948	0.277422	0.271986	0.267829
0.292453	0.283499	0.294212	0.259674	0.290534	0.322674	0.282699	0.291174	0.282219	0.322833
0.382155	0.340582	0.365526	0.410457	0.385513	0.425807	0.392709	0.250879	0.393028	0.400863
0.230253	0.304765	0.294691	0.271826	0.270707	0.248481	0.263991	0.362648	0.282699	0.23377
0.278702	0.309082	0.301247	0.272626	0.309562	0.270867	0.270867	0.376879	0.29693	0.290374
0.299328	0.299648	0.291174	0.241765	0.283499	0.279181	0.264311	0.368564	0.259354	0.330349
0.317237	0.259354	0.313719	0.331148	0.310201	0.363767	0.319795	0.159098	0.319476	0.338343
0.310521	0.308762	0.324592	0.322354	0.309402	0.338983	0.32891	0.307004	0.330189	0.28206
0.259674	0.295011	0.29709	0.311321	0.270387	0.311321	0.285897	0.264791	0.291174	0.235849
0.29725	0.294052	0.268308	0.287496	0.261753	0.320915	0.267829	0.276143	0.263351	0.325232
0.287816	0.304125	0.292933	0.314039	0.284618	0.331148	0.305085	0.325072	0.29725	0.321714
0.369524	0.331948	0.340742	0.384714	0.356412	0.414934	0.369843	0.216342	0.379277	0.380077
0.335945	0.326511	0.318036	0.342501	0.342501	0.352095	0.34346	0.299328	0.348737	0.366326
0.369843	0.340742	0.361049	0.399744	0.363607	0.425328	0.391749	0.252798	0.388072	0.393988
0.326671	0.318676	0.322354	0.324912	0.31244	0.360249	0.317237	0.289255	0.332427	0.340262
0.326671	0.316437	0.329069	0.350016	0.323473	0.383595	0.348737	0.285417	0.349856	0.353694
0.339623	0.334986	0.341222	0.289095	0.32779	0.326191	0.318836	0.341541	0.298049	0.358171
0.332587	0.336904	0.334826	0.364886	0.348577	0.399584	0.360729	0.300768	0.363607	0.361049
0.209306	0.302846	0.271506	0.272306	0.264151	0.275504	0.293732	0.360729	0.29693	0.225776
0.238248	0.293092	0.289575	0.28126	0.277902	0.269428	0.289575	0.352894	0.295491	0.132715
0.267509	0.29661	0.304765	0.31212	0.303806	0.311481	0.32747	0.29645	0.309082	0.31276
0.334506	0.334026	0.334026	0.267189	0.333387	0.347618	0.307963	0.331468	0.279181	0.355133
0.263351	0.290694	0.240006	0.215862	0.289894	0.247362	0.257915	0.35961	0.263831	0.306204

For Review Only

0.240966	0.305884	0.288455	0.286217	0.267988	0.2819	0.303326	0.365206	0.307643	0.232331
0.287976	0.322354	0.305884	0.279021	0.318356	0.306844	0.289894	0.317717	0.32795	0.295011
0.297889	0.300608	0.290214	0.188359	0.306364	0.267988	0.268148	0.383115	0.260953	0.32891
0.298529	0.298849	0.290534	0.242085	0.283339	0.278542	0.263192	0.367605	0.258555	0.329709
0.238407	0.301727	0.291174	0.278542	0.264791	0.271506	0.292453	0.363767	0.293572	0.244484
0.308602	0.325712	0.313559	0.216022	0.326671	0.303326	0.291813	0.350975	0.284938	0.344579
0.251519	0.302047	0.310201	0.288136	0.276783	0.25072	0.299808	0.3756	0.295011	0.2504
0.2189	0.300927	0.283019	0.29693	0.278222	0.272785	0.302846	0.354813	0.303965	0.110649
0.216182	0.298369	0.258874	0.286377	0.271826	0.270867	0.289255	0.352734	0.292453	0.242245
0.360409	0.301727	0.32827	0.381996	0.349057	0.403102	0.365526	0.208666	0.35865	0.381516
0.303486	0.275983	0.271986	0.292133	0.267189	0.342821	0.283658	0.260473	0.28142	0.325552
0.246083	0.264311	0.274065	0.259354	0.26623	0.264631	0.268628	0.285257	0.272785	0.259354
0.212024	0.309562	0.286537	0.286377	0.273585	0.274864	0.286697	0.352574	0.298529	0.080748
0.208666	0.314839	0.292453	0.297729	0.28142	0.300128	0.29645	0.34362	0.306524	0.005437
0.29725	0.299968	0.288455	0.18804	0.306364	0.267669	0.267669	0.383115	0.260473	0.32795
0.291653	0.283179	0.293572	0.259194	0.290214	0.322194	0.28206	0.290854	0.282219	0.321874
0.257115	0.277103	0.263032	0.256636	0.268308	0.275983	0.272146	0.311321	0.279981	0.278542
0.31324	0.284458	0.300927	0.307643	0.26527	0.339942	0.311481	0.288136	0.291973	0.340102
0.298529	0.299488	0.305564	0.322993	0.29613	0.301727	0.332267	0.325232	0.324752	0.292773
0.26559	0.247681	0.246402	0.224656	0.285737	0.212024	0.266869	0.388232	0.26511	0.290374
0.259674	0.306364	0.284938	0.206908	0.303486	0.248001	0.256316	0.376239	0.267829	0.28126
0.287976	0.302846	0.271986	0.211385	0.278702	0.273745	0.267988	0.362168	0.241765	0.331148
0.211864	0.29725	0.279501	0.284138	0.270227	0.275983	0.28126	0.314679	0.290214	0.058203
0.329869	0.322034	0.316437	0.338184	0.337384	0.347777	0.337224	0.285098	0.335785	0.344739
0.29725	0.291653	0.298209	0.242565	0.28142	0.311161	0.277263	0.31292	0.26575	0.32811
0.282539	0.307643	0.291174	0.298369	0.261912	0.316597	0.284298	0.336425	0.289095	0.270067
0.332107	0.336904	0.334986	0.364567	0.348417	0.399105	0.35945	0.299648	0.363128	0.361209
0.226575	0.323153	0.295491	0.295491	0.257595	0.299488	0.303166	0.342181	0.303326	0.207707
0.344739	0.337064	0.349057	0.321714	0.339623	0.338983	0.351135	0.348737	0.330988	0.373361
0.368244	0.334986	0.345859	0.388072	0.357531	0.414135	0.37448	0.213144	0.380077	0.388391
0.256636	0.304285	0.278862	0.301887	0.269268	0.274864	0.3126	0.36009	0.311481	0.26639
0.263032	0.323953	0.306844	0.255197	0.301407	0.130157	0.270227	0.353854	0.278382	0.29613
0.301727	0.319955	0.323633	0.267669	0.287016	0.316757	0.29629	0.324912	0.29629	0.340742
0.278222	0.2811	0.295811	0.301567	0.28206	0.320595	0.293092	0.230093	0.293252	0.289095
0.376399	0.334666	0.354333	0.395747	0.364087	0.419731	0.388551	0.210425	0.39111	0.400384
0.29709	0.299169	0.287656	0.186441	0.304925	0.267349	0.266869	0.382315	0.259034	0.32795
0.303646	0.271346	0.284618	0.277902	0.261593	0.29693	0.263032	0.318516	0.259194	0.322674
0.332587	0.336904	0.334986	0.364247	0.348897	0.399264	0.360249	0.300448	0.362968	0.361529
0.320435	0.309722	0.319795	0.331148	0.302686	0.347298	0.322354	0.287816	0.330349	0.348417
0.340902	0.333227	0.348897	0.304765	0.334506	0.355133	0.32891	0.318356	0.311161	0.377678
0.294691	0.336585	0.325232	0.322034	0.314998	0.318996	0.322993	0.316757	0.332107	0.330668
0.326831	0.318356	0.349536	0.342661	0.325712	0.384394	0.354333	0.291174	0.34346	0.363288
0.292293	0.307163	0.292773	0.175887	0.291014	0.256156	0.257915	0.380716	0.248481	0.325072
0.264631	0.335146	0.301887	0.269907	0.294691	0.222098	0.294052	0.370963	0.301247	0.248161
0.290054	0.306524	0.267988	0.283499	0.31228	0.315478	0.310681	0.338024	0.322194	0.320275
0.213783	0.310201	0.288935	0.286697	0.274544	0.275024	0.287336	0.352734	0.299488	0.082987
0.29757	0.298049	0.29757	0.292133	0.288935	0.273745	0.29613	0.347458	0.277582	0.31228
0.247841	0.284458	0.263511	0.26495	0.266869	0.259674	0.254557	0.352894	0.254877	0.244004
0.329549	0.293572	0.322833	0.354333	0.32859	0.370643	0.326511	0.104093	0.336265	0.350016
0.225776	0.284458	0.268628	0.302526	0.261753	0.283978	0.304125	0.309722	0.295331	0.255996
0.242245	0.288455	0.261593	0.279661	0.28206	0.262552	0.273105	0.318516	0.275823	0.258874
0.271186	0.307004	0.306844	0.285737	0.305564	0.303486	0.311321	0.342501	0.313719	0.291653
0.214743	0.298849	0.272785	0.289575	0.269747	0.282379	0.273265	0.332267	0.298049	0.18692
0.378798	0.342181	0.353854	0.391589	0.365046	0.423409	0.379917	0.224656	0.385034	0.388391
0.346498	0.320595	0.330029	0.371762	0.324272	0.395267	0.336265	0.236329	0.35913	0.364407

For Review Only

0.287016	0.292933	0.293732	0.303006	0.290694	0.31308	0.289255	0.273265	0.29613	0.307323
0.2496	0.269907	0.288455	0.282699	0.272466	0.268308	0.298209	0.341382	0.303646	0.297729
0.286537	0.311001	0.288295	0.308762	0.299968	0.305564	0.301247	0.313879	0.315958	0.292453
0.292773	0.321874	0.322833	0.32827	0.327151	0.3433	0.321234	0.311001	0.34394	0.321554
0.250879	0.291973	0.263032	0.286697	0.261753	0.291653	0.289575	0.318996	0.291813	0.2496
0.319955	0.285737	0.334506	0.339463	0.317237	0.35945	0.340102	0.133515	0.334506	0.353054
0.21922	0.308762	0.274225	0.290854	0.255516	0.299968	0.286217	0.356732	0.289894	0.204989
0.242085	0.28158	0.271186	0.271826	0.254237	0.287496	0.29629	0.326831	0.293892	0.253598
0.2189	0.303486	0.29677	0.256796	0.291014	0.246722	0.268948	0.348417	0.291813	0.244324
0.263671	0.290534	0.277103	0.28078	0.275024	0.28078	0.29741	0.271666	0.29645	0.262552
0.295491	0.301087	0.306044	0.317717	0.293252	0.341062	0.322993	0.292613	0.322993	0.306684
0.284938	0.291334	0.314519	0.316597	0.2819	0.329069	0.294052	0.279021	0.302846	0.289575
0.307643	0.282219	0.282539	0.273745	0.269268	0.322833	0.279981	0.302367	0.28142	0.332587
0.261753	0.273585	0.267988	0.271186	0.286697	0.282539	0.276943	0.299648	0.282219	0.275823
0.266869	0.288455	0.270707	0.292293	0.306524	0.304445	0.306684	0.327151	0.336585	0.308123
0.232331	0.304445	0.279821	0.278382	0.258395	0.289095	0.284778	0.341541	0.303965	0.21858
0.287496	0.302686	0.306044	0.316437	0.300608	0.325232	0.318836	0.3126	0.310841	0.279341
0.263192	0.283658	0.275504	0.282539	0.273745	0.290374	0.274384	0.302526	0.283499	0.262232
0.293412	0.17205	0.246242	0.278062	0.31196	0.232811	0.305245	0.365686	0.295331	0.326191
0.246402	0.279501	0.270227	0.26639	0.258714	0.266709	0.270227	0.303965	0.278382	0.232171
0.322993	0.288295	0.333227	0.351775	0.32811	0.369364	0.337704	0.12456	0.334026	0.347937
0.301567	0.31212	0.303486	0.283179	0.322833	0.298369	0.307163	0.347458	0.32811	0.329549
0.247202	0.276783	0.26607	0.263192	0.271186	0.28158	0.273265	0.285897	0.277742	0.255996
0.262072	0.29741	0.301727	0.23473	0.301407	0.211864	0.277582	0.355453	0.283019	0.292133
0.243044	0.287176	0.286217	0.273105	0.263351	0.266869	0.288295	0.339623	0.284458	0.228494
0.368404	0.333227	0.354653	0.388391	0.35865	0.420371	0.376719	0.209466	0.380237	0.39095
0.284778	0.314199	0.294532	0.314519	0.295651	0.311001	0.306684	0.314998	0.306684	0.292133
0.303166	0.289255	0.285897	0.304925	0.303965	0.333067	0.297889	0.29725	0.313399	0.331308
0.289255	0.305245	0.289735	0.251679	0.289894	0.277263	0.164055	0.355772	0.263671	0.295811
0.275664	0.288615	0.248481	0.246402	0.276943	0.283818	0.237128	0.279501	0.248961	0.292453
0.208666	0.302686	0.28206	0.262232	0.287176	0.263671	0.286057	0.377359	0.286057	0.23505
0.287336	0.292613	0.291813	0.26639	0.315158	0.276943	0.29709	0.370003	0.276303	0.300927
0.357531	0.318836	0.347138	0.382315	0.346978	0.407579	0.363927	0.154461	0.377518	0.381196
0.258714	0.279501	0.264151	0.270547	0.275504	0.291174	0.274704	0.271346	0.295331	0.270387
0.292133	0.308283	0.293572	0.17253	0.290694	0.254557	0.259354	0.384394	0.24928	0.326511
0.2496	0.285737	0.268948	0.26495	0.277582	0.263032	0.275823	0.307803	0.275024	0.264311
0.316917	0.257435	0.313719	0.330828	0.310521	0.362008	0.318676	0.162936	0.319955	0.337544
0.300128	0.289575	0.289415	0.260633	0.300288	0.3118	0.298529	0.26575	0.293732	0.330668
0.335465	0.299009	0.335305	0.366965	0.32763	0.376879	0.344739	0.115126	0.342501	0.354813
0.23377	0.284778	0.278382	0.268948	0.264311	0.258874	0.283019	0.354333	0.306364	0.227854
0.304605	0.291174	0.316278	0.32747	0.31324	0.316118	0.3433	0.298209	0.32779	0.330668
0.374001	0.34442	0.362168	0.391749	0.364727	0.399904	0.367605	0.246562	0.377518	0.39127
0.301247	0.301087	0.285098	0.318196	0.321714	0.304925	0.311161	0.31308	0.335465	0.332427
0.338024	0.293252	0.330508	0.354493	0.325552	0.379757	0.342181	0.108251	0.336265	0.356572
0.246083	0.288455	0.286057	0.271027	0.269587	0.25056	0.2811	0.352414	0.283818	0.266709
0.353694	0.315478	0.339463	0.375919	0.3441	0.385993	0.361209	0.258874	0.364247	0.370483
0.28094	0.289255	0.275823	0.292133	0.260793	0.314998	0.31292	0.315478	0.317877	0.306844
0.275504	0.29629	0.2811	0.264471	0.251999	0.289255	0.276783	0.313719	0.251999	0.290694
0.241126	0.306684	0.274704	0.294372	0.269907	0.26495	0.293412	0.361848	0.301887	0.244963
0.229613	0.302686	0.291014	0.29645	0.266709	0.256636	0.307163	0.35961	0.31212	0.21906
0.307004	0.289095	0.300768	0.306684	0.278382	0.335465	0.307483	0.222578	0.292613	0.321874
0.239527	0.310361	0.28094	0.286537	0.269108	0.287016	0.302047	0.360249	0.303646	0.242245
0.253758	0.294851	0.275344	0.29741	0.279821	0.26607	0.284298	0.34378	0.289735	0.260793
0.347138	0.298209	0.310361	0.348097	0.324752	0.352574	0.339783	0.266549	0.332427	0.368244
0.274864	0.273745	0.277422	0.256796	0.28158	0.286377	0.295011	0.290374	0.270867	0.298689

For Review Only

0.228334	0.316437	0.304125	0.301407	0.285737	0.304285	0.300128	0.352574	0.309242	0.115766
0.297889	0.286377	0.295491	0.314199	0.31212	0.323313	0.306044	0.253438	0.31308	0.321394
0.259994	0.285897	0.292613	0.258075	0.276143	0.235529	0.299648	0.368244	0.279021	0.271986
0.324592	0.319955	0.301407	0.351775	0.338663	0.362968	0.353534	0.293092	0.369204	0.364087
0.340742	0.352095	0.325072	0.337384	0.32859	0.357211	0.276463	0.337384	0.309082	0.354013
0.323793	0.294532	0.314519	0.305405	0.32891	0.339303	0.304765	0.304445	0.313879	0.346338
0.261753	0.29757	0.316757	0.272785	0.283658	0.284618	0.29693	0.357211	0.297889	0.243524
0.324752	0.295811	0.287816	0.297889	0.330508	0.326351	0.307803	0.284138	0.290694	0.342341
0.336745	0.317077	0.331788	0.354013	0.320595	0.364886	0.34394	0.267829	0.358011	0.364727
0.247522	0.271186	0.267829	0.262232	0.263192	0.279021	0.274704	0.275983	0.274384	0.254557
0.349057	0.325712	0.336105	0.342021	0.337064	0.347777	0.345699	0.275664	0.35913	0.35881
0.277902	0.31308	0.295171	0.244963	0.321234	0.189958	0.267509	0.377199	0.267829	0.283339
0.221938	0.302207	0.269907	0.288935	0.262392	0.270227	0.285897	0.333547	0.293572	0.238727
0.298689	0.282699	0.29661	0.320755	0.287656	0.346178	0.31292	0.237928	0.308922	0.31196
0.202271	0.300608	0.277902	0.245283	0.252958	0.243204	0.28158	0.338663	0.267669	0.240966
0.294691	0.288935	0.295011	0.290854	0.275823	0.305085	0.288136	0.282379	0.2811	0.316757
0.324752	0.293252	0.326831	0.355133	0.318676	0.376239	0.332747	0.043652	0.34314	0.34346
0.363288	0.326671	0.368244	0.366485	0.350496	0.363447	0.36009	0.307963	0.362808	0.371282
0.272466	0.268628	0.279181	0.257435	0.273905	0.288935	0.290054	0.275504	0.278062	0.292453
0.259674	0.308762	0.29709	0.299169	0.252958	0.302526	0.254077	0.362008	0.283978	0.227055
0.256636	0.276623	0.259354	0.284938	0.262392	0.285577	0.290374	0.305724	0.295171	0.253438
0.216182	0.29757	0.292293	0.251199	0.286697	0.244164	0.262872	0.342021	0.285417	0.246083
0.248641	0.264311	0.261753	0.259034	0.26527	0.275664	0.26495	0.275983	0.260633	0.259834
0.348897	0.310521	0.334506	0.35929	0.324272	0.389511	0.346019	0.210585	0.36009	0.362008
0.348417	0.323633	0.311641	0.347298	0.339303	0.360729	0.331308	0.283499	0.338983	0.360889
0.209946	0.315318	0.291813	0.29757	0.28174	0.301727	0.29629	0.3441	0.307004	0.006716
0.309882	0.302207	0.316437	0.280461	0.307483	0.340902	0.298529	0.31244	0.300768	0.330189
0.322993	0.271186	0.322833	0.340902	0.309562	0.362648	0.316437	0.163096	0.321075	0.34442
0	0.289255	0.272945	0.273105	0.260953	0.268628	0.280141	0.32747	0.290054	0.208347
0.289255	0	0.272626	0.292133	0.290694	0.2819	0.305884	0.297729	0.300128	0.314199
0.272945	0.272626	0	0.263351	0.299648	0.274225	0.274065	0.324272	0.287336	0.291014
0.273105	0.292133	0.263351	0	0.284778	0.255676	0.268308	0.351775	0.246882	0.29677
0.260953	0.290694	0.299648	0.284778	0	0.302207	0.274225	0.326511	0.267829	0.282219
0.268628	0.2819	0.274225	0.255676	0.302207	0	0.2811	0.373361	0.278542	0.300608
0.280141	0.305884	0.274065	0.268308	0.274225	0.2811	0	0.336265	0.246882	0.295491
0.32747	0.297729	0.324272	0.351775	0.326511	0.373361	0.336265	0	0.340742	0.34362
0.290054	0.300128	0.287336	0.246882	0.267829	0.278542	0.246882	0.340742	0	0.305884
0.208347	0.314199	0.291014	0.29677	0.282219	0.300608	0.295491	0.34362	0.305884	0
0.269108	0.322034	0.288295	0.300768	0.287496	0.309082	0.285897	0.342341	0.309082	0.262072
0.294212	0.294691	0.273425	0.268628	0.279341	0.292613	0.251519	0.325072	0.25024	0.32795
0.230413	0.304605	0.288775	0.299648	0.267988	0.262232	0.302047	0.360729	0.313399	0.217941
0.241605	0.287496	0.29629	0.288935	0.251359	0.285897	0.306364	0.345539	0.302526	0.230732
0.226575	0.307963	0.293412	0.267829	0.256796	0.271186	0.291174	0.367125	0.303806	0.215062
0.244164	0.305405	0.277742	0.295971	0.268628	0.262712	0.295331	0.362808	0.302846	0.254077
0.377518	0.329869	0.35961	0.389031	0.35929	0.416533	0.377039	0.207547	0.377678	0.386633
0.290214	0.303166	0.273745	0.277263	0.299009	0.283339	0.277742	0.335945	0.278062	0.288455
0.291813	0.315478	0.304445	0.322354	0.317237	0.326191	0.280141	0.282859	0.29709	0.289575
0.287816	0.302047	0.292773	0.290054	0.31308	0.29613	0.283179	0.326351	0.290214	0.276143
0.362648	0.326191	0.366805	0.366326	0.350176	0.361848	0.35945	0.308283	0.361848	0.370163
0.364247	0.330988	0.352095	0.376559	0.322034	0.385353	0.35865	0.24944	0.363927	0.393668
0.315318	0.279661	0.302526	0.341062	0.3126	0.358331	0.311001	0.151263	0.322034	0.336904
0.253758	0.286856	0.302686	0.268308	0.311321	0.306364	0.287496	0.307803	0.302047	0.295971
0.26607	0.308443	0.289575	0.267509	0.293732	0.280301	0.295331	0.369843	0.29725	0.272945
0.344579	0.303166	0.321234	0.362648	0.323793	0.381676	0.321554	0.209306	0.322354	0.366006
0.352894	0.32875	0.330189	0.363288	0.345059	0.372562	0.349696	0.262232	0.336745	0.367285

For Review Only

0.212504	0.309242	0.286856	0.286377	0.274065	0.275664	0.286697	0.352414	0.298689	0.081228
0.37512	0.340262	0.350656	0.392229	0.363607	0.420531	0.367125	0.223217	0.382475	0.387592
0.308443	0.270387	0.314199	0.251199	0.289255	0.305405	0.308922	0.26607	0.291493	0.320115
0.325712	0.29693	0.305085	0.310201	0.337544	0.333547	0.316278	0.297729	0.307643	0.32875
0.262072	0.31292	0.300128	0.26607	0.279181	0.224496	0.28078	0.384394	0.289415	0.276623
0.331948	0.323953	0.320115	0.318996	0.338983	0.337384	0.326511	0.321714	0.340902	0.354013
0.317877	0.275983	0.292933	0.316118	0.315798	0.320115	0.294212	0.289255	0.305245	0.339303
0.254397	0.276943	0.263511	0.25024	0.277263	0.261753	0.260313	0.361689	0.270227	0.293732
0.336745	0.311641	0.320595	0.345059	0.318836	0.362008	0.332107	0.285417	0.335625	0.364407
0.363128	0.333387	0.346498	0.393188	0.367125	0.419412	0.385673	0.267669	0.393028	0.387912
0.29629	0.29709	0.310361	0.319156	0.302846	0.349376	0.314199	0.212824	0.313719	0.322194
0.371762	0.331628	0.352414	0.394627	0.367125	0.42165	0.373681	0.20291	0.385193	0.394308
0.242885	0.267829	0.264791	0.255676	0.267029	0.276783	0.26591	0.275184	0.267829	0.256956
0.287976	0.284778	0.29693	0.317877	0.176847	0.336105	0.307803	0.31308	0.307163	0.319316
0.291014	0.326831	0.314839	0.313559	0.3118	0.295011	0.315318	0.339143	0.31324	0.332267
0.253278	0.285257	0.282379	0.268308	0.259674	0.256156	0.305405	0.307803	0.29709	0.247522
0.229933	0.307803	0.29613	0.267029	0.242245	0.264791	0.308922	0.368084	0.307963	0.21874
0.324912	0.332107	0.323313	0.322194	0.334826	0.3118	0.314199	0.320275	0.304765	0.341062
0.341861	0.334826	0.310841	0.356892	0.316278	0.384394	0.346978	0.252638	0.346178	0.356892
0.338024	0.302526	0.355772	0.331788	0.333866	0.338343	0.332427	0.277263	0.321874	0.362008
0.357211	0.314359	0.334506	0.356572	0.341382	0.358331	0.366965	0.26591	0.356732	0.37432
0.289735	0.293572	0.311001	0.321234	0.304445	0.338983	0.32891	0.324432	0.311641	0.330668
0.311481	0.29725	0.297729	0.341222	0.279981	0.364886	0.338663	0.309402	0.347937	0.309082
0.251519	0.289415	0.26591	0.299009	0.267349	0.275344	0.305724	0.346338	0.305564	0.260793
0.276303	0.293252	0.292773	0.26511	0.285257	0.257755	0.252478	0.356252	0.264471	0.302367
0.299968	0.295331	0.289415	0.297889	0.311001	0.32859	0.318676	0.305564	0.339143	0.315798
0.360569	0.301727	0.32779	0.381996	0.348577	0.402782	0.366166	0.208027	0.358171	0.380716
0.294052	0.309882	0.318676	0.301087	0.2819	0.318676	0.290214	0.34362	0.31212	0.290374
0.379117	0.336904	0.361209	0.402622	0.367285	0.42117	0.385673	0.20355	0.392389	0.398625
0.135114	0.309722	0.292133	0.286217	0.273265	0.273265	0.288775	0.360249	0.302047	0.209306
0.301407	0.290854	0.273105	0.251999	0.304765	0.288775	0.270547	0.32827	0.274864	0.32763
0.256796	0.304925	0.279501	0.256796	0.286537	0.243204	0.283499	0.364407	0.283019	0.259034
0.361529	0.318836	0.348897	0.376079	0.342661	0.396706	0.381036	0.240006	0.364727	0.385034
0.266549	0.303006	0.308602	0.316757	0.279981	0.31212	0.316118	0.314679	0.314679	0.277263
0.31196	0.276623	0.303486	0.317717	0.306044	0.326831	0.308602	0.237608	0.31212	0.335146
0.247681	0.290054	0.291973	0.291813	0.267669	0.28062	0.300288	0.333866	0.299808	0.254717
0.342181	0.321874	0.31324	0.32795	0.336425	0.345699	0.326991	0.279021	0.321714	0.354493
0.384874	0.340742	0.367445	0.394627	0.357691	0.425648	0.378798	0.215382	0.384554	0.395427
0.270067	0.286537	0.28158	0.198753	0.273585	0.26559	0.271506	0.365846	0.263351	0.288775
0.210265	0.315638	0.292933	0.298049	0.282539	0.300608	0.29677	0.345219	0.307323	0.006396
0.258714	0.293412	0.295651	0.310521	0.268628	0.309882	0.284618	0.263351	0.289575	0.235689
0.381356	0.340102	0.352414	0.393348	0.368084	0.427566	0.374001	0.225136	0.383115	0.392869
0.262072	0.287016	0.276623	0.246402	0.283978	0.240806	0.222258	0.365206	0.184842	0.293732
0.256796	0.288615	0.272466	0.279821	0.271027	0.288455	0.252958	0.284138	0.254877	0.271826
0.293892	0.308123	0.295171	0.320435	0.307483	0.32763	0.316597	0.297729	0.334506	0.316278
0.284138	0.31212	0.294372	0.158938	0.288295	0.250879	0.260153	0.384394	0.24944	0.322833
0.257275	0.308283	0.285577	0.304765	0.283658	0.314199	0.319156	0.32891	0.31212	0.202271
0.288455	0.316597	0.333547	0.297889	0.324272	0.319476	0.306684	0.322194	0.313879	0.32795
0.287816	0.313399	0.302846	0.335625	0.31212	0.308922	0.334186	0.31196	0.332267	0.292613
0.245443	0.283019	0.287496	0.286057	0.292453	0.31212	0.311161	0.331468	0.306204	0.285897
0.284778	0.332907	0.32891	0.32891	0.310841	0.326351	0.334986	0.339303	0.335465	0.287656
0.3748	0.334826	0.35993	0.399744	0.360729	0.42149	0.387592	0.208507	0.39063	0.396067
0.272785	0.301247	0.273905	0.221458	0.291653	0.252159	0.244484	0.380397	0.252319	0.288455
0.377039	0.338184	0.351295	0.39047	0.366006	0.423569	0.374161	0.209786	0.383754	0.392069
0.227215	0.308762	0.29725	0.268308	0.258075	0.272146	0.293572	0.368244	0.305085	0.215542

For Review Only

0.167093	0.31324	0.299488	0.282219	0.277263	0.278542	0.304925	0.381676	0.307483	0.230093
0.336105	0.321394	0.336745	0.333547	0.340262	0.378158	0.344579	0.273585	0.347777	0.362328
0.283339	0.314839	0.308283	0.261113	0.305245	0.239207	0.253278	0.374001	0.275184	0.305405
0.231212	0.317717	0.273905	0.298049	0.259994	0.293732	0.29757	0.363767	0.307323	0.200991

For Review Only

0.307483	0.18804	0.299648	0.290214	0.28062	0.293732	0.379437	0.293252	0.307323	0.298849
0.305564	0.299328	0.315318	0.308123	0.31228	0.320275	0.309402	0.32795	0.326671	0.310681
0.257755	0.285737	0.213463	0.248161	0.008634	0.228174	0.402942	0.270547	0.284618	0.290854
0.268948	0.271027	0.262392	0.267029	0.238567	0.255197	0.376079	0.290054	0.285737	0.266549
0.34426	0.311161	0.356252	0.342821	0.356252	0.3441	0.345859	0.32875	0.32859	0.330668
0.268788	0.248641	0.278222	0.276783	0.261113	0.280461	0.39111	0.293892	0.326031	0.294532
0.292773	0.267669	0.326511	0.302367	0.314839	0.305564	0.395107	0.310201	0.32859	0.317877
0.336904	0.319955	0.321874	0.307004	0.339942	0.337864	0.285417	0.337704	0.326031	0.320435
0.305085	0.262072	0.324592	0.303965	0.320595	0.321714	0.37512	0.287336	0.315638	0.302686
0.358171	0.337384	0.354813	0.344739	0.372082	0.369364	0.294372	0.357371	0.334506	0.333706
0.335785	0.332107	0.354333	0.342181	0.350336	0.346658	0.269428	0.302207	0.334346	0.372721
0.255676	0.288775	0.213463	0.243044	0.070675	0.224656	0.398305	0.273425	0.286856	0.290214
0.299808	0.267829	0.326191	0.315798	0.316278	0.314998	0.32843	0.313399	0.315958	0.295331
0.330508	0.29741	0.316757	0.295491	0.326671	0.310042	0.270067	0.307643	0.256636	0.32859
0.292133	0.258075	0.28142	0.255357	0.269268	0.276143	0.367605	0.272306	0.307004	0.288295
0.292933	0.307483	0.280301	0.31292	0.26591	0.286697	0.31228	0.290694	0.283818	0.32875
0.334666	0.282859	0.349057	0.32811	0.32875	0.340902	0.382475	0.317077	0.335305	0.310201
0.304925	0.276623	0.307004	0.315478	0.306524	0.298689	0.361689	0.309402	0.306524	0.333547
0.295331	0.282699	0.274225	0.252798	0.245443	0.276943	0.409658	0.290054	0.314679	0.283339
0.338503	0.338343	0.341222	0.342501	0.366326	0.348257	0.322354	0.329549	0.315958	0.361689
0.318036	0.299328	0.351935	0.330668	0.34442	0.332107	0.317557	0.329389	0.352255	0.316118
0.333387	0.285897	0.3433	0.325552	0.327311	0.324912	0.379757	0.313879	0.32875	0.310361
0.347458	0.364886	0.398145	0.363927	0.397346	0.394308	0.209626	0.332907	0.362968	0.356572
0.318516	0.314998	0.325232	0.31324	0.353374	0.341541	0.229613	0.316278	0.286377	0.307963
0.275184	0.259354	0.287176	0.251519	0.269747	0.287976	0.391589	0.288935	0.321234	0.28174
0.238887	0.31308	0.163895	0.266549	0.229613	0.09402	0.382155	0.269747	0.291493	0.337704
0.220179	0.288775	0.201311	0.246882	0.216182	0.216661	0.367125	0.278222	0.273265	0.284778
0.268948	0.261273	0.251679	0.262392	0.257435	0.247202	0.304605	0.257595	0.272626	0.277742
0.306364	0.293892	0.326191	0.309882	0.330189	0.325712	0.318036	0.29661	0.324272	0.294851
0.384554	0.380237	0.408379	0.39095	0.429485	0.409018	0.240966	0.384554	0.355293	0.363767
0.251199	0.283179	0.242405	0.261593	0.23441	0.256476	0.387752	0.292453	0.300288	0.28126
0.29741	0.267669	0.290854	0.294532	0.251359	0.29661	0.398785	0.293412	0.318356	0.302526
0.311641	0.24944	0.321874	0.298369	0.289575	0.299169	0.396546	0.29677	0.333387	0.306524
0.337064	0.313399	0.349696	0.317717	0.361848	0.349217	0.223217	0.324752	0.287176	0.31196
0.306684	0.325072	0.29741	0.322194	0.321234	0.290854	0.309082	0.291813	0.322514	0.32891
0.238088	0.290214	0.260313	0.260313	0.274065	0.278062	0.31292	0.276303	0.242885	0.3118
0.294691	0.141989	0.322514	0.309402	0.31324	0.316437	0.334026	0.295651	0.295011	0.284298
0.29725	0.292293	0.306364	0.29709	0.284938	0.300288	0.339303	0.285098	0.300448	0.325871
0.348737	0.357371	0.379437	0.368244	0.396546	0.384234	0.127119	0.333866	0.329869	0.340582
0.295651	0.346498	0.345059	0.364886	0.347937	0.340262	0.275983	0.292773	0.345379	0.368884
0.374001	0.379437	0.391589	0.384554	0.407899	0.395747	0.240486	0.371602	0.352255	0.361049
0.333227	0.322833	0.339143	0.3433	0.34394	0.338983	0.300288	0.320595	0.323313	0.324592
0.346498	0.327151	0.339463	0.325552	0.360409	0.362488	0.304445	0.345859	0.321874	0.335945
0.341382	0.284778	0.352574	0.339143	0.353054	0.349536	0.363128	0.319795	0.335945	0.323153
0.356412	0.345859	0.353214	0.350815	0.373201	0.370483	0.320115	0.356092	0.342181	0.362808
0.251359	0.276143	0.205628	0.247042	0.10889	0.191078	0.398145	0.266709	0.28094	0.307004
0.251199	0.316917	0.177486	0.261113	0.225935	0.165014	0.39079	0.26591	0.292773	0.29693
0.297889	0.315638	0.276143	0.291973	0.291174	0.247042	0.311321	0.284618	0.307643	0.346178
0.334186	0.306524	0.361369	0.354333	0.348417	0.362648	0.360729	0.326671	0.317717	0.31308
0.303166	0.257435	0.300608	0.288455	0.272945	0.287976	0.395267	0.273425	0.310521	0.285737

For Review Only

0.256796	0.298689	0.178446	0.206108	0.216501	0.128078	0.385034	0.269428	0.298209	0.305085
0.302846	0.326511	0.294052	0.305405	0.286217	0.299328	0.341701	0.301887	0.315478	0.305884
0.302686	0.237928	0.315158	0.286856	0.29709	0.29741	0.42197	0.291014	0.330189	0.305405
0.3118	0.2496	0.322194	0.298049	0.289735	0.299328	0.396226	0.29629	0.332587	0.306524
0.280301	0.290214	0.252958	0.257755	0.240486	0.263671	0.386473	0.276143	0.291813	0.304285
0.334666	0.300448	0.337544	0.330349	0.329389	0.32843	0.378318	0.319635	0.324113	0.326991
0.295011	0.28206	0.274065	0.251999	0.245923	0.276303	0.409818	0.288615	0.314359	0.283658
0.235369	0.330029	0.170611	0.251039	0.23393	0.148865	0.394468	0.261113	0.29629	0.289575
0.237448	0.300288	0.159578	0.264471	0.232012	0.087944	0.388391	0.255676	0.279981	0.332907
0.356252	0.342661	0.386313	0.367925	0.402143	0.388711	0.186121	0.353694	0.322194	0.325712
0.303646	0.232012	0.3441	0.319635	0.336105	0.337704	0.294212	0.316437	0.304125	0.302367
0.267189	0.257115	0.251039	0.257595	0.242885	0.257755	0.311321	0.263831	0.272785	0.279661
0.267988	0.307483	0.204989	0.213463	0.204989	0.239207	0.389671	0.287176	0.295011	0.278542
0.262712	0.329229	0.217941	0.229933	0.214583	0.254237	0.387592	0.288615	0.289415	0.276783
0.302207	0.237928	0.314199	0.284618	0.29757	0.29709	0.420211	0.290694	0.330029	0.304285
0.305724	0.292773	0.325712	0.310042	0.32875	0.324752	0.317717	0.29613	0.323473	0.293892
0.267029	0.257115	0.270547	0.271826	0.26575	0.26607	0.335785	0.267829	0.300128	0.287656
0.330189	0.253118	0.345219	0.3118	0.338184	0.332427	0.309082	0.314519	0.325712	0.327311
0.313559	0.299648	0.270867	0.275184	0.259994	0.277103	0.325072	0.279981	0.313719	0.333387
0.31228	0.263511	0.262872	0.29645	0.240166	0.24976	0.42165	0.276143	0.31292	0.301887
0.301407	0.278862	0.299009	0.285257	0.290054	0.301407	0.412536	0.280141	0.319476	0.303166
0.304925	0.225456	0.308443	0.292453	0.292933	0.29677	0.409338	0.293252	0.326991	0.307163
0.25072	0.302047	0.216342	0.223217	0.21858	0.251039	0.356732	0.278222	0.275024	0.26559
0.300288	0.331308	0.335305	0.346818	0.360569	0.345059	0.254557	0.282699	0.330349	0.338343
0.298849	0.28142	0.327311	0.295491	0.326351	0.324912	0.332107	0.2819	0.323793	0.292773
0.300608	0.306844	0.293412	0.268148	0.284138	0.304925	0.365366	0.298369	0.32795	0.303486
0.356092	0.344899	0.352574	0.351135	0.372562	0.370163	0.319795	0.355293	0.341222	0.362008
0.264631	0.300927	0.241925	0.227694	0.213783	0.253758	0.382475	0.291813	0.280461	0.301727
0.373361	0.323793	0.350815	0.334506	0.339143	0.364247	0.349057	0.341062	0.341541	0.340742
0.356572	0.369364	0.383595	0.37544	0.398945	0.387592	0.121362	0.3433	0.315958	0.340582
0.249121	0.314199	0.15574	0.271826	0.236329	0.064918	0.383914	0.26495	0.294851	0.344739
0.300768	0.270547	0.277902	0.28158	0.282539	0.29693	0.394468	0.283818	0.318516	0.302526
0.320595	0.285417	0.317557	0.310201	0.32827	0.315158	0.353854	0.301727	0.31244	0.336265
0.29677	0.298369	0.293252	0.276783	0.316437	0.3118	0.279981	0.292133	0.283818	0.29693
0.366645	0.382315	0.396706	0.385513	0.419572	0.402782	0.127598	0.35865	0.32779	0.355133
0.302846	0.237928	0.314679	0.285257	0.29725	0.29741	0.419412	0.290214	0.329549	0.303965
0.304285	0.236169	0.31244	0.302207	0.29693	0.295011	0.339463	0.290854	0.302846	0.284778
0.355932	0.345539	0.353054	0.351455	0.373521	0.370803	0.320115	0.355932	0.341861	0.362648
0.339143	0.317397	0.338663	0.311321	0.350016	0.350336	0.293892	0.324752	0.320115	0.339143
0.362968	0.326511	0.373841	0.3441	0.365686	0.368884	0.322674	0.349696	0.341861	0.310521
0.324113	0.291973	0.310841	0.290694	0.301087	0.317877	0.323953	0.324752	0.311641	0.335305
0.351455	0.321075	0.358171	0.338343	0.355293	0.358011	0.305245	0.353214	0.333387	0.357211
0.307803	0.26527	0.308123	0.293572	0.285417	0.284138	0.417653	0.275983	0.317237	0.310361
0.255197	0.287496	0.263671	0.255357	0.216821	0.300128	0.422929	0.294212	0.31276	0.292453
0.32747	0.280461	0.305405	0.260473	0.301567	0.31276	0.365846	0.323153	0.331948	0.294212
0.269747	0.308123	0.205469	0.213623	0.205628	0.240326	0.388551	0.287816	0.295811	0.280141
0.316278	0.276463	0.326351	0.302207	0.294532	0.303166	0.371282	0.298049	0.306844	0.321234
0.275504	0.287016	0.241605	0.263192	0.258874	0.248961	0.394148	0.260473	0.286217	0.290534
0.342981	0.320915	0.35961	0.345219	0.364886	0.35913	0.206108	0.329869	0.28174	0.314679
0.269747	0.29757	0.193476	0.253278	0.255516	0.153502	0.355133	0.268948	0.271506	0.325712
0.256796	0.279021	0.208666	0.267349	0.247202	0.167733	0.363288	0.251999	0.277902	0.321394
0.309402	0.316278	0.303326	0.299648	0.320755	0.324912	0.361049	0.310361	0.317877	0.327311
0.256796	0.284618	0.212024	0.226255	0.236169	0.24992	0.35913	0.273745	0.277902	0.2811
0.35881	0.365526	0.386792	0.380077	0.404541	0.395587	0.139431	0.339942	0.337544	0.345859
0.332267	0.320115	0.380397	0.358491	0.381196	0.379277	0.231372	0.348097	0.326991	0.305884

For Review Only

0.307483	0.286856	0.309082	0.287336	0.29693	0.31196	0.283339	0.294372	0.284938	0.28174
0.324432	0.295971	0.292933	0.289735	0.300128	0.31324	0.39063	0.321714	0.323793	0.320435
0.240646	0.323793	0.227374	0.31196	0.288295	0.165334	0.322034	0.271027	0.314039	0.329549
0.278382	0.320275	0.297729	0.324592	0.32811	0.317557	0.320595	0.318836	0.317237	0.338024
0.233131	0.289255	0.220979	0.2496	0.251999	0.178446	0.362808	0.268148	0.253278	0.317237
0.348577	0.32795	0.347298	0.344899	0.360249	0.342661	0.208666	0.330029	0.28174	0.32891
0.255197	0.290534	0.213623	0.229133	0.204989	0.213303	0.393828	0.278542	0.274704	0.295171
0.264471	0.304605	0.208986	0.24992	0.228494	0.176367	0.363128	0.252958	0.271027	0.317877
0.277742	0.277103	0.244164	0.268628	0.201791	0.260793	0.384874	0.284938	0.285897	0.303006
0.267509	0.279501	0.253758	0.2811	0.26559	0.275344	0.301727	0.278062	0.276143	0.305245
0.303965	0.303006	0.279181	0.305884	0.290694	0.255676	0.307163	0.300927	0.29741	0.318196
0.289415	0.303326	0.28142	0.289255	0.297729	0.26639	0.287816	0.288455	0.282859	0.3126
0.313719	0.2504	0.319476	0.305085	0.324432	0.310042	0.344739	0.309722	0.29677	0.316118
0.26623	0.271506	0.267829	0.276303	0.26495	0.255676	0.315798	0.26639	0.286856	0.292133
0.330668	0.316597	0.308922	0.275504	0.303646	0.316597	0.358491	0.330189	0.338024	0.299328
0.25008	0.292453	0.233131	0.244643	0.225296	0.235849	0.372562	0.276623	0.288455	0.295811
0.285257	0.286217	0.289255	0.275983	0.287816	0.301567	0.337704	0.314039	0.305884	0.304445
0.261273	0.2819	0.217461	0.268468	0.259994	0.157979	0.320595	0.251359	0.287976	0.298209
0.333067	0.3118	0.298529	0.305405	0.292293	0.295971	0.385034	0.299009	0.34362	0.303965
0.24976	0.280141	0.239687	0.253118	0.227694	0.207067	0.323313	0.255197	0.279021	0.280301
0.348257	0.325712	0.353854	0.346978	0.367125	0.350016	0.212504	0.335305	0.279821	0.321714
0.309722	0.303646	0.319156	0.302207	0.31228	0.326991	0.357371	0.29741	0.332907	0.317397
0.260473	0.273745	0.235209	0.254397	0.255037	0.228814	0.306844	0.258555	0.280301	0.275983
0.305564	0.26607	0.286377	0.280301	0.276623	0.295971	0.38999	0.289255	0.317557	0.295491
0.258874	0.289575	0.241126	0.246882	0.17173	0.260633	0.370003	0.277103	0.286537	0.268308
0.363128	0.370483	0.382955	0.378478	0.404061	0.39047	0.135114	0.351455	0.322034	0.348897
0.264311	0.321714	0.244963	0.300927	0.288775	0.205469	0.317077	0.271826	0.31276	0.334346
0.253758	0.320755	0.323313	0.322833	0.34346	0.324912	0.280301	0.28126	0.331788	0.342181
0.307323	0.268468	0.293892	0.290054	0.293572	0.29677	0.39031	0.28094	0.302686	0.295811
0.287656	0.202111	0.294691	0.283818	0.29629	0.304925	0.334346	0.290534	0.29629	0.262552
0.287016	0.29741	0.240966	0.257435	0.223537	0.251839	0.421011	0.286856	0.3118	0.301247
0.313719	0.300927	0.307803	0.302047	0.303965	0.297729	0.387432	0.301407	0.322993	0.284938
0.35945	0.357531	0.386313	0.370483	0.394627	0.39079	0.161017	0.34442	0.309242	0.338503
0.277103	0.278222	0.262712	0.263991	0.271666	0.26559	0.300448	0.271346	0.275823	0.290694
0.309242	0.264791	0.307803	0.295651	0.285257	0.283818	0.419252	0.274704	0.319476	0.31244
0.245603	0.278382	0.216661	0.276623	0.258555	0.164215	0.338024	0.247522	0.284778	0.303326
0.336904	0.31292	0.345859	0.315798	0.36009	0.346019	0.222737	0.322354	0.286217	0.313719
0.329069	0.303646	0.321394	0.316757	0.332267	0.315478	0.285257	0.306844	0.307323	0.313719
0.351295	0.325232	0.365046	0.351775	0.377678	0.364247	0.207547	0.336425	0.279821	0.333387
0.268948	0.277902	0.232811	0.23377	0.232651	0.242565	0.384074	0.274704	0.299328	0.308922
0.309722	0.307803	0.290054	0.311161	0.311481	0.261433	0.288295	0.28142	0.318196	0.356732
0.357531	0.37448	0.386473	0.380876	0.401023	0.39127	0.173009	0.340742	0.336904	0.36009
0.295171	0.319955	0.317237	0.329389	0.336265	0.319156	0.306524	0.29645	0.345059	0.336745
0.339623	0.324432	0.369683	0.352095	0.37576	0.369524	0.197634	0.339143	0.276463	0.326991
0.305405	0.28206	0.254397	0.26607	0.263831	0.264151	0.389351	0.29709	0.306684	0.303006
0.348097	0.345859	0.370323	0.360249	0.381676	0.373361	0.255836	0.350975	0.333227	0.339303
0.291973	0.291493	0.285737	0.286217	0.286697	0.232971	0.331628	0.298209	0.31324	0.322514
0.301407	0.254237	0.29709	0.28126	0.287336	0.276463	0.356412	0.3118	0.297889	0.286377
0.217941	0.313559	0.1567	0.272785	0.222098	0.033579	0.39015	0.253438	0.288455	0.32875
0.256316	0.315638	0.026063	0.232651	0.21874	0.134794	0.381676	0.240966	0.292293	0.325072
0.311161	0.261113	0.326991	0.314679	0.329229	0.322993	0.251359	0.309722	0.289095	0.29757
0.229933	0.305564	0.178446	0.260953	0.212824	0.10921	0.38999	0.264631	0.293732	0.325392
0.229293	0.309562	0.181004	0.272466	0.251519	0.085225	0.376239	0.257595	0.28142	0.332107
0.334666	0.332107	0.340262	0.347777	0.3748	0.334666	0.251679	0.306044	0.316118	0.346658
0.311481	0.25072	0.287816	0.263032	0.271186	0.290054	0.321874	0.28126	0.298529	0.292133

For Review Only

0.258395	0.323153	0.215542	0.229773	0.226415	0.204029	0.398145	0.287016	0.286377	0.301087
0.302207	0.294691	0.3118	0.31292	0.330189	0.318836	0.255516	0.29725	0.29645	0.307004
0.288775	0.275504	0.228654	0.268468	0.236808	0.188839	0.397346	0.280141	0.31244	0.303326
0.316118	0.34346	0.3441	0.348577	0.369843	0.335945	0.291334	0.338343	0.350815	0.35945
0.329869	0.330988	0.347777	0.358011	0.335945	0.333547	0.346019	0.327311	0.32763	0.338184
0.313399	0.316278	0.336745	0.336745	0.340902	0.335465	0.280461	0.307163	0.321075	0.31212
0.279341	0.286057	0.25072	0.267988	0.253278	0.248481	0.394148	0.292133	0.294532	0.303486
0.325552	0.301087	0.314998	0.338184	0.334506	0.316118	0.294851	0.287496	0.290374	0.244643
0.317717	0.34378	0.351615	0.354173	0.362488	0.355932	0.217301	0.314359	0.338663	0.344579
0.254877	0.267829	0.227374	0.262872	0.252798	0.223377	0.305564	0.257115	0.268308	0.287176
0.333547	0.326991	0.355453	0.340262	0.347777	0.358331	0.257755	0.314998	0.333227	0.350496
0.308922	0.251839	0.286217	0.278062	0.280301	0.300927	0.416693	0.287656	0.303326	0.285098
0.226575	0.285098	0.191078	0.263831	0.212664	0.117685	0.364567	0.2504	0.28158	0.317877
0.295331	0.320275	0.307803	0.31276	0.340262	0.322194	0.239367	0.292933	0.293572	0.322034
0.242245	0.267988	0.224816	0.240646	0.229293	0.214743	0.374001	0.276143	0.303806	0.287336
0.311481	0.272785	0.31292	0.305564	0.313879	0.302526	0.331948	0.311321	0.308922	0.305724
0.339783	0.326031	0.363288	0.347138	0.365046	0.364247	0.207387	0.337384	0.2811	0.324752
0.345379	0.347937	0.370003	0.352734	0.365046	0.367605	0.248001	0.315638	0.345699	0.37544
0.302846	0.264471	0.290054	0.271826	0.280461	0.285897	0.295651	0.279821	0.28206	0.298049
0.258075	0.278862	0.254877	0.229293	0.242565	0.262072	0.393988	0.289575	0.303166	0.288615
0.230572	0.274544	0.221618	0.269747	0.242885	0.180524	0.338823	0.26607	0.278862	0.301727
0.274864	0.270707	0.238407	0.263192	0.20387	0.258714	0.376559	0.277902	0.28094	0.298849
0.262392	0.256476	0.258075	0.260793	0.253438	0.253118	0.300448	0.256476	0.267349	0.277742
0.340262	0.340902	0.350016	0.336265	0.376399	0.364567	0.177966	0.324432	0.310361	0.334986
0.295971	0.327151	0.353694	0.352574	0.37448	0.353534	0.254237	0.288935	0.334826	0.340582
0.262392	0.329229	0.21922	0.231052	0.215542	0.254877	0.387912	0.289894	0.290534	0.277103
0.314519	0.303166	0.345219	0.331628	0.348737	0.342981	0.339623	0.323313	0.337064	0.318836
0.335785	0.307963	0.345859	0.321714	0.362488	0.349217	0.222737	0.321075	0.289735	0.315478
0.269108	0.294212	0.230413	0.241605	0.226575	0.244164	0.377518	0.290214	0.291813	0.287816
0.322034	0.294691	0.304605	0.287496	0.307963	0.305405	0.329869	0.303166	0.315478	0.302047
0.288295	0.273425	0.288775	0.29629	0.293412	0.277742	0.35961	0.273745	0.304445	0.292773
0.300768	0.268628	0.299648	0.288935	0.267829	0.295971	0.389031	0.277263	0.322354	0.290054
0.287496	0.279341	0.267988	0.251359	0.256796	0.268628	0.35929	0.299009	0.317237	0.31308
0.309082	0.292613	0.262232	0.285897	0.271186	0.262712	0.416533	0.283339	0.326191	0.29613
0.285897	0.251519	0.302047	0.306364	0.291174	0.295331	0.377039	0.277742	0.280141	0.283179
0.342341	0.325072	0.360729	0.345539	0.367125	0.362808	0.207547	0.335945	0.282859	0.326351
0.309082	0.25024	0.313399	0.302526	0.303806	0.302846	0.377678	0.278062	0.29709	0.290214
0.262072	0.32795	0.217941	0.230732	0.215062	0.254077	0.386633	0.288455	0.289575	0.276143
0	0.290534	0.26575	0.301727	0.257275	0.225456	0.358171	0.279021	0.318516	0.31292
0.290534	0	0.316437	0.284618	0.284618	0.311001	0.364567	0.29709	0.308762	0.283818
0.26575	0.316437	0	0.229453	0.212344	0.147106	0.380077	0.240966	0.287656	0.322993
0.301727	0.284618	0.229453	0	0.247202	0.271986	0.380556	0.291334	0.306204	0.291813
0.257275	0.284618	0.212344	0.247202	0	0.227055	0.402782	0.270067	0.284298	0.290694
0.225456	0.311001	0.147106	0.271986	0.227055	0	0.388711	0.250879	0.288136	0.32891
0.358171	0.364567	0.380077	0.380556	0.402782	0.388711	0	0.348257	0.330029	0.34442
0.279021	0.29709	0.240966	0.291334	0.270067	0.250879	0.348257	0	0.291813	0.323313
0.318516	0.308762	0.287656	0.306204	0.284298	0.288136	0.330029	0.291813	0	0.326671
0.31292	0.283818	0.322993	0.291813	0.290694	0.32891	0.34442	0.323313	0.326671	0
0.342821	0.347138	0.369524	0.351615	0.363607	0.366485	0.247841	0.315158	0.345059	0.373681
0.354333	0.351135	0.372562	0.356732	0.372242	0.376719	0.179245	0.339463	0.335785	0.332107
0.326351	0.301087	0.342021	0.329069	0.366805	0.347777	0.223697	0.323313	0.270547	0.308443
0.317077	0.291174	0.298689	0.278542	0.294052	0.307963	0.34394	0.315958	0.29613	0.293732
0.29661	0.280301	0.266549	0.264151	0.256636	0.262712	0.401503	0.294532	0.309562	0.306044
0.336745	0.314839	0.3756	0.356092	0.379117	0.376879	0.239687	0.333067	0.309722	0.305405
0.314679	0.330189	0.364886	0.353214	0.387592	0.367445	0.232811	0.305405	0.336425	0.331468

For Review Only

0.268948	0.306844	0.204509	0.212824	0.204509	0.239687	0.388391	0.286697	0.294851	0.278222
0.354973	0.361848	0.393668	0.378478	0.404861	0.396706	0.14071	0.342661	0.330029	0.34378
0.333547	0.270867	0.310841	0.287336	0.288295	0.311001	0.299968	0.311001	0.308762	0.321554
0.332267	0.301087	0.320595	0.337064	0.339143	0.32827	0.310521	0.278382	0.306044	0.331308
0.302367	0.28094	0.244803	0.278702	0.17205	0.248321	0.420211	0.279661	0.307643	0.298209
0.314679	0.325392	0.345699	0.335625	0.354653	0.348417	0.313719	0.31276	0.353534	0.329869
0.325072	0.291813	0.316437	0.331308	0.338343	0.325712	0.315478	0.295491	0.307004	0.321394
0.26511	0.263831	0.229773	0.270067	0.256956	0.182603	0.400544	0.2811	0.305405	0.300927
0.317077	0.332427	0.332587	0.35913	0.354493	0.308283	0.251839	0.329869	0.308762	0.350656
0.37432	0.361689	0.378798	0.370003	0.398945	0.394787	0.266549	0.369204	0.345539	0.363607
0.306364	0.303326	0.31228	0.311481	0.325871	0.304925	0.257435	0.315158	0.269108	0.314998
0.372881	0.375919	0.388391	0.381996	0.413655	0.402303	0.129997	0.350016	0.319156	0.347618
0.26607	0.266869	0.253438	0.261912	0.260793	0.251999	0.299968	0.261753	0.271506	0.279341
0.321075	0.302846	0.304285	0.292773	0.31212	0.299328	0.323953	0.306044	0.337704	0.323313
0.313559	0.287976	0.301887	0.278702	0.302526	0.293892	0.350336	0.307643	0.31276	0.34378
0.258075	0.264791	0.212984	0.232651	0.240966	0.186441	0.345539	0.268468	0.295171	0.298849
0.272785	0.306044	0.204029	0.211545	0.098337	0.228494	0.395747	0.279981	0.303326	0.310361
0.311641	0.29645	0.336265	0.321394	0.337544	0.332427	0.329869	0.291174	0.304125	0.346658
0.315158	0.315798	0.34362	0.345059	0.357851	0.342821	0.196674	0.316118	0.305884	0.318996
0.326831	0.3118	0.353534	0.342021	0.360409	0.35913	0.26527	0.329549	0.326991	0.339303
0.342341	0.340422	0.356892	0.351135	0.377199	0.345859	0.215222	0.322034	0.332907	0.346818
0.304285	0.29725	0.316917	0.308123	0.293092	0.315158	0.334186	0.289095	0.320755	0.337864
0.318036	0.347618	0.307483	0.304445	0.317077	0.318516	0.295491	0.326511	0.329709	0.329069
0.241605	0.303646	0.174928	0.262872	0.23409	0.081548	0.381836	0.269907	0.297729	0.335146
0.295331	0.221938	0.307163	0.264151	0.284618	0.295491	0.393508	0.299968	0.308123	0.284618
0.289575	0.310521	0.31292	0.317077	0.330988	0.322993	0.287656	0.26559	0.317717	0.345379
0.356412	0.341861	0.386792	0.367125	0.401823	0.388871	0.185641	0.354013	0.321874	0.324592
0.292613	0.288136	0.289735	0.284618	0.271506	0.310361	0.362168	0.307323	0.310841	0.293252
0.372562	0.380876	0.399584	0.386792	0.417173	0.407419	0.123121	0.360569	0.329389	0.358171
0.257755	0.299328	0.215702	0.23441	0.194116	0.21954	0.404221	0.289415	0.301087	0.300128
0.298529	0.254237	0.303646	0.303006	0.315158	0.307803	0.360729	0.278222	0.307803	0.310521
0.302047	0.270067	0.256956	0.262712	0.246242	0.273265	0.409658	0.2819	0.31292	0.300128
0.35865	0.361049	0.379757	0.362168	0.388072	0.381036	0.152223	0.335785	0.339303	0.354653
0.279341	0.322674	0.245763	0.26559	0.257755	0.18788	0.324752	0.278222	0.304285	0.327151
0.318036	0.292453	0.325712	0.333387	0.341062	0.332747	0.293892	0.307803	0.315158	0.323153
0.24992	0.301247	0.137672	0.239207	0.251999	0.151423	0.351775	0.275664	0.311641	0.319156
0.291493	0.322674	0.340582	0.350176	0.370483	0.348417	0.258555	0.285577	0.329709	0.336904
0.369843	0.373841	0.394308	0.387912	0.416374	0.404221	0.1252	0.354813	0.332107	0.350496
0.29741	0.228174	0.284938	0.260153	0.237928	0.269747	0.396386	0.28158	0.299648	0.294851
0.263032	0.329869	0.21954	0.230732	0.216342	0.255037	0.388391	0.289735	0.291493	0.277582
0.236649	0.288935	0.259194	0.260153	0.273905	0.276463	0.31212	0.274704	0.242405	0.310681
0.354173	0.366965	0.391589	0.381836	0.404221	0.397346	0.128398	0.338503	0.336745	0.346978
0.291973	0.251199	0.295171	0.292933	0.280301	0.277103	0.40646	0.29693	0.314839	0.28158
0.288295	0.261433	0.272146	0.275024	0.284618	0.283499	0.319635	0.293572	0.272785	0.284938
0.272945	0.340422	0.318676	0.327311	0.342981	0.319316	0.278062	0.285417	0.324272	0.340422
0.302367	0.260953	0.313399	0.28142	0.287336	0.292933	0.42197	0.269587	0.322514	0.304445
0.277103	0.283339	0.23489	0.228494	0.209146	0.254877	0.353534	0.28142	0.289255	0.271186
0.305085	0.284618	0.315478	0.301887	0.295811	0.315638	0.341222	0.327311	0.332747	0.309562
0.287336	0.34426	0.15686	0.300927	0.288136	0.221458	0.297889	0.26495	0.301887	0.362968
0.314519	0.310841	0.274384	0.26607	0.288935	0.283818	0.361209	0.319635	0.304445	0.317077
0.313559	0.333706	0.263991	0.301087	0.261273	0.2819	0.357851	0.324752	0.300768	0.322674
0.3748	0.376399	0.395587	0.380397	0.415574	0.40646	0.130636	0.355133	0.32763	0.354813
0.306364	0.263671	0.295971	0.306204	0.278382	0.299488	0.419731	0.278062	0.313559	0.308123
0.35993	0.368724	0.388391	0.379437	0.407099	0.396386	0.132875	0.339143	0.337224	0.348897
0.257915	0.285098	0.212664	0.248641	0.009114	0.227374	0.402303	0.271346	0.285417	0.290694

For Review Only

0.26575	0.305564	0.229453	0.204829	0.221618	0.238887	0.423729	0.291334	0.314839	0.309562
0.292293	0.333227	0.337544	0.351135	0.348097	0.342181	0.241286	0.263831	0.323633	0.355932
0.300448	0.267988	0.311001	0.290854	0.290534	0.305085	0.413335	0.284778	0.307963	0.31244
0.248801	0.291653	0.223857	0.216661	0.21874	0.246562	0.394627	0.289255	0.301087	0.293572

For Review Only

0.353694	0.352574	0.322034	0.307004	0.305724	0.32811	0.365046	0.29741	0.378638	0.293732
0.327151	0.331148	0.292933	0.307163	0.323953	0.307323	0.320275	0.251199	0.322833	0.310681
0.364567	0.372562	0.367605	0.295171	0.255836	0.381036	0.387112	0.204509	0.40614	0.288615
0.354493	0.353374	0.309242	0.248481	0.267988	0.330828	0.352095	0.244803	0.37464	0.300288
0.354013	0.308283	0.293572	0.321714	0.349217	0.307163	0.346019	0.353694	0.353534	0.307163
0.35881	0.375919	0.329389	0.308443	0.270227	0.349217	0.362008	0.251359	0.396226	0.295811
0.361689	0.354333	0.336105	0.272945	0.295171	0.35993	0.360889	0.321714	0.386792	0.315478
0.335785	0.286537	0.262712	0.298689	0.339623	0.288455	0.325232	0.320435	0.293572	0.302207
0.34426	0.361049	0.327311	0.287816	0.303326	0.341222	0.326511	0.32747	0.372402	0.320595
0.3748	0.303326	0.26591	0.29661	0.358171	0.286377	0.321234	0.37464	0.295011	0.337544
0.060281	0.256636	0.300608	0.311641	0.37432	0.321075	0.277263	0.34362	0.248481	0.289095
0.361209	0.37528	0.354653	0.267509	0.247522	0.373841	0.387912	0.190118	0.400704	0.29757
0.346978	0.29629	0.29645	0.289255	0.319476	0.319476	0.335146	0.320755	0.330508	0.305884
0.294532	0.304285	0.213783	0.280141	0.341062	0.270227	0.308602	0.31276	0.280301	0.262712
0.331148	0.346818	0.320435	0.282859	0.272626	0.336585	0.333067	0.268148	0.363128	0.278862
0.303166	0.321554	0.284778	0.310361	0.319476	0.317877	0.334026	0.285257	0.305405	0.269268
0.354333	0.357052	0.329389	0.261113	0.310681	0.350815	0.354973	0.338823	0.386473	0.337384
0.340422	0.338823	0.323313	0.303326	0.304765	0.346338	0.35977	0.302207	0.361529	0.282219
0.36009	0.389351	0.356252	0.304925	0.277582	0.369843	0.381676	0.240166	0.413975	0.324432
0.361049	0.340102	0.298689	0.324272	0.361049	0.286537	0.321714	0.357052	0.336585	0.344899
0.330988	0.307163	0.291653	0.287336	0.332107	0.322993	0.318516	0.342021	0.316757	0.315958
0.362168	0.340422	0.326031	0.270707	0.299808	0.35929	0.352095	0.332107	0.370643	0.32827
0.252958	0.245123	0.270227	0.322674	0.396226	0.292453	0.228654	0.392229	0.185002	0.318036
0.308602	0.260953	0.160217	0.2819	0.351455	0.233451	0.258874	0.327311	0.227854	0.257115
0.35961	0.355133	0.340262	0.289735	0.270387	0.364247	0.357211	0.275983	0.395107	0.29677
0.371282	0.382475	0.341861	0.319795	0.258714	0.377838	0.370003	0.245603	0.388232	0.315958
0.370803	0.363288	0.308922	0.271506	0.262552	0.338503	0.356412	0.214902	0.35929	0.305724
0.302047	0.299488	0.26639	0.261433	0.263831	0.289894	0.290534	0.255996	0.307323	0.266549
0.349057	0.325552	0.276943	0.289894	0.319795	0.301727	0.297729	0.323633	0.316917	0.302047
0.341222	0.275024	0.236808	0.31324	0.412856	0.246083	0.283499	0.411097	0.261912	0.336745
0.362808	0.383275	0.339463	0.283339	0.268628	0.367445	0.37448	0.235689	0.389671	0.286377
0.368884	0.393028	0.35993	0.31308	0.273905	0.373521	0.379757	0.278382	0.4055	0.314519
0.348737	0.350016	0.348097	0.308443	0.280461	0.358331	0.369524	0.310681	0.404541	0.322034
0.316917	0.258235	0.171091	0.300927	0.346658	0.238727	0.276303	0.340422	0.235369	0.252159
0.286856	0.314359	0.310361	0.330189	0.3441	0.317397	0.288455	0.295171	0.308283	0.302367
0.316597	0.315478	0.235689	0.298049	0.311641	0.286057	0.314998	0.248961	0.298369	0.291973
0.337064	0.319795	0.266869	0.288295	0.32859	0.245443	0.283978	0.324113	0.324592	0.28174
0.300448	0.31244	0.31324	0.301247	0.309082	0.295811	0.32843	0.310841	0.329069	0.285577
0.221618	0.185321	0.21858	0.332427	0.398465	0.257595	0.20243	0.383275	0.107931	0.309722
0.256956	0.282379	0.295491	0.338983	0.364247	0.29629	0.255676	0.358011	0.258395	0.310042
0.331308	0.267189	0.239207	0.318676	0.403742	0.261912	0.279341	0.397506	0.260153	0.335945
0.339942	0.310361	0.269108	0.299968	0.349696	0.298369	0.299648	0.3441	0.293412	0.320115
0.351295	0.301247	0.268628	0.295811	0.356252	0.285417	0.320435	0.350016	0.307643	0.341222
0.333227	0.32891	0.316757	0.294372	0.356732	0.306364	0.338024	0.345699	0.365206	0.322034
0.354973	0.323793	0.279981	0.3118	0.371122	0.300128	0.334666	0.35897	0.32747	0.347298
0.361369	0.372562	0.352255	0.299169	0.256636	0.372562	0.371922	0.204669	0.394947	0.285417
0.365366	0.384394	0.348257	0.304285	0.275024	0.376879	0.372562	0.165654	0.392229	0.317557
0.275344	0.29629	0.293092	0.307803	0.303006	0.310361	0.306684	0.300448	0.317077	0.271506
0.342021	0.340742	0.322034	0.283499	0.348737	0.300288	0.338823	0.349376	0.356572	0.35897
0.349696	0.361209	0.334826	0.288775	0.267829	0.345859	0.348737	0.286537	0.398625	0.271666

For Review Only

0.365846	0.378957	0.349376	0.305405	0.259514	0.367445	0.353214	0.228014	0.387272	0.297889
0.346178	0.335785	0.330668	0.308443	0.292613	0.348417	0.345219	0.267988	0.340422	0.325232
0.365526	0.373201	0.361848	0.289255	0.278702	0.374161	0.378478	0.316278	0.420531	0.31244
0.348737	0.349856	0.347138	0.308602	0.28078	0.357531	0.369044	0.310521	0.404381	0.321874
0.352734	0.376879	0.349057	0.29645	0.26591	0.364727	0.367765	0.227694	0.39031	0.300288
0.352414	0.336904	0.337544	0.220979	0.303166	0.368724	0.363128	0.333227	0.376879	0.34362
0.361049	0.389671	0.355453	0.304125	0.277902	0.368724	0.381996	0.239687	0.413975	0.324113
0.37448	0.397985	0.347777	0.305085	0.271506	0.370803	0.373521	0.161657	0.395267	0.329549
0.360729	0.385034	0.333387	0.285257	0.255996	0.366645	0.365046	0.225616	0.391589	0.307803
0.291334	0.221938	0.186441	0.306844	0.383115	0.167893	0.2504	0.381836	0.201951	0.298529
0.330508	0.295171	0.251519	0.302047	0.324592	0.252638	0.269108	0.329069	0.29613	0.284458
0.304125	0.307323	0.270067	0.260953	0.266709	0.302367	0.300128	0.244963	0.316118	0.262072
0.355133	0.386633	0.338823	0.287656	0.263192	0.368884	0.37496	0.007035	0.393188	0.298209
0.370643	0.393988	0.337864	0.29613	0.273265	0.366645	0.367925	0.081068	0.388072	0.319955
0.365366	0.372082	0.362488	0.289415	0.278062	0.373521	0.377039	0.315478	0.419252	0.3118
0.347937	0.325552	0.276623	0.288775	0.319476	0.300608	0.29757	0.322833	0.315798	0.301727
0.316757	0.323473	0.29661	0.294532	0.28142	0.307643	0.310521	0.262392	0.337864	0.283658
0.326031	0.313559	0.270547	0.291493	0.350496	0.261593	0.280141	0.331788	0.310361	0.277263
0.287976	0.335785	0.321394	0.299328	0.323473	0.336105	0.285737	0.287336	0.329229	0.309082
0.383435	0.404861	0.368724	0.314998	0.26623	0.373361	0.366485	0.272146	0.427886	0.247841
0.354813	0.388391	0.349696	0.278862	0.263511	0.377998	0.364407	0.277422	0.415574	0.31308
0.365206	0.37576	0.346338	0.292293	0.287816	0.364407	0.372721	0.310201	0.40678	0.318036
0.346178	0.362808	0.307004	0.283978	0.26495	0.334826	0.339143	0.10953	0.355612	0.301407
0.272785	0.274704	0.288935	0.321874	0.362968	0.287656	0.113687	0.349217	0.250879	0.321714
0.323153	0.337384	0.298049	0.283339	0.320915	0.306684	0.295971	0.316437	0.336904	0.311001
0.35913	0.380397	0.313559	0.299488	0.29741	0.342021	0.334346	0.276463	0.368084	0.321554
0.353854	0.322993	0.279821	0.3118	0.371602	0.300288	0.334826	0.358491	0.326191	0.346818
0.370323	0.371922	0.329549	0.29693	0.279021	0.354493	0.361848	0.185321	0.377518	0.3118
0.358011	0.320915	0.325712	0.337064	0.351295	0.322833	0.354813	0.355932	0.35929	0.353374
0.252958	0.158139	0.215542	0.335465	0.393188	0.254077	0.224017	0.387272	0.135593	0.305085
0.361848	0.376239	0.344739	0.333547	0.268628	0.382475	0.367925	0.248641	0.38999	0.313879
0.338503	0.366485	0.330189	0.287976	0.280141	0.361689	0.35945	0.272626	0.394308	0.292933
0.340582	0.324912	0.295491	0.284778	0.321075	0.321874	0.34346	0.326991	0.347618	0.287176
0.329229	0.291014	0.206108	0.302047	0.302686	0.246083	0.290534	0.289894	0.285257	0.269108
0.264151	0.152702	0.213463	0.344739	0.403102	0.251519	0.221778	0.400064	0.145987	0.315958
0.363927	0.371442	0.361689	0.288455	0.277263	0.372562	0.376239	0.315158	0.418452	0.311001
0.323953	0.326671	0.303646	0.31228	0.304605	0.295171	0.327151	0.318516	0.352894	0.259354
0.354973	0.324272	0.279821	0.31228	0.371602	0.300448	0.333706	0.35929	0.326831	0.347458
0.333387	0.304285	0.255836	0.285577	0.342661	0.2819	0.319156	0.336265	0.293252	0.294212
0.357052	0.284298	0.300768	0.291653	0.362968	0.31196	0.337224	0.364407	0.336585	0.340742
0.309082	0.341382	0.304445	0.280141	0.308283	0.340582	0.297889	0.311641	0.323793	0.303965
0.342341	0.308602	0.278702	0.29757	0.36009	0.299968	0.327311	0.350336	0.322993	0.319635
0.358171	0.366485	0.353214	0.271186	0.286057	0.380397	0.381516	0.307963	0.413975	0.304125
0.362488	0.396067	0.362808	0.318036	0.287816	0.378957	0.380237	0.235209	0.419092	0.31212
0.368724	0.347458	0.318036	0.282859	0.29693	0.34314	0.35993	0.315158	0.368244	0.332747
0.355932	0.387112	0.339623	0.287816	0.263511	0.368084	0.376239	0.008954	0.393028	0.298049
0.321714	0.334186	0.308123	0.324113	0.307643	0.334986	0.342501	0.299648	0.368084	0.300768
0.356892	0.376879	0.346658	0.276143	0.254877	0.353694	0.364567	0.215862	0.401663	0.303806
0.306684	0.24944	0.157659	0.300768	0.369204	0.210585	0.267189	0.356412	0.21842	0.275344
0.362648	0.335305	0.288295	0.288295	0.267349	0.337864	0.32891	0.255676	0.35913	0.304765
0.342821	0.346498	0.29677	0.295171	0.274065	0.336265	0.350656	0.257275	0.36009	0.294532
0.353694	0.358331	0.338663	0.321714	0.315318	0.34314	0.273425	0.299648	0.365526	0.324432
0.350176	0.35993	0.306844	0.282859	0.270387	0.342661	0.355612	0.169492	0.358331	0.301087
0.210585	0.181484	0.222098	0.335625	0.407579	0.263511	0.215062	0.387912	0.100096	0.313879
0.299009	0.248961	0.217621	0.321394	0.377678	0.190438	0.274704	0.361049	0.235529	0.323473

For Review Only

0.322514	0.294212	0.271027	0.294691	0.298529	0.259994	0.282219	0.303006	0.285417	0.297889
0.357531	0.356892	0.338343	0.326191	0.29757	0.338823	0.350016	0.290374	0.387752	0.315478
0.308922	0.332267	0.31308	0.31292	0.31308	0.32795	0.299648	0.283818	0.330668	0.309722
0.322674	0.32827	0.289095	0.303006	0.330828	0.311001	0.31244	0.307803	0.318196	0.318516
0.351935	0.345219	0.299009	0.297729	0.28078	0.327151	0.333227	0.244164	0.350815	0.289255
0.306204	0.23473	0.175088	0.310521	0.371282	0.212344	0.269907	0.354173	0.227055	0.270227
0.368244	0.381676	0.341382	0.282539	0.245923	0.360569	0.376079	0.174768	0.385673	0.304925
0.360889	0.3433	0.303166	0.29725	0.244324	0.349856	0.333547	0.247362	0.365846	0.285257
0.349217	0.37496	0.324912	0.244803	0.252159	0.365206	0.377359	0.230892	0.385353	0.272626
0.311321	0.31308	0.257275	0.287816	0.285098	0.286217	0.303646	0.255357	0.307163	0.262072
0.334186	0.305245	0.2819	0.297729	0.335465	0.291813	0.318676	0.301247	0.302367	0.29613
0.325552	0.293092	0.256476	0.302047	0.314839	0.284458	0.287016	0.290694	0.288775	0.295331
0.324912	0.331148	0.290534	0.318836	0.305405	0.291334	0.335465	0.324113	0.340902	0.277422
0.301887	0.311001	0.28174	0.276303	0.28206	0.295171	0.295171	0.260153	0.310841	0.275504
0.369843	0.342181	0.304925	0.277742	0.305564	0.319156	0.365686	0.307643	0.350975	0.32891
0.348577	0.365206	0.327151	0.291334	0.256476	0.356892	0.351135	0.197474	0.372562	0.31324
0.346498	0.32747	0.303166	0.292293	0.309402	0.314998	0.333866	0.26623	0.341382	0.305405
0.320595	0.319476	0.287176	0.290214	0.271346	0.314839	0.310361	0.252319	0.322034	0.280461
0.349057	0.377838	0.353694	0.299328	0.307963	0.324432	0.355612	0.31244	0.401343	0.31196
0.305564	0.316757	0.295331	0.271346	0.260953	0.317237	0.316757	0.217141	0.324912	0.277742
0.3126	0.244803	0.160857	0.299169	0.377199	0.210265	0.271826	0.352574	0.232491	0.264311
0.321075	0.358331	0.330828	0.295491	0.300288	0.32779	0.335945	0.31244	0.354173	0.332267
0.303486	0.304285	0.274384	0.266869	0.26559	0.295651	0.289415	0.247522	0.308762	0.272945
0.335146	0.369683	0.330349	0.266869	0.252478	0.355453	0.357371	0.270227	0.392869	0.275664
0.369843	0.342661	0.320435	0.293732	0.26559	0.351935	0.350016	0.21874	0.370643	0.283818
0.264791	0.182443	0.215062	0.336105	0.400704	0.245283	0.227055	0.394627	0.149984	0.303006
0.322833	0.326511	0.311641	0.315158	0.310681	0.319156	0.221458	0.286057	0.321394	0.306684
0.284938	0.303486	0.28174	0.303166	0.334986	0.299488	0.208507	0.335305	0.276783	0.291334
0.358331	0.369044	0.326671	0.288775	0.295491	0.349217	0.35897	0.284938	0.378798	0.300608
0.358491	0.32827	0.25072	0.286697	0.272466	0.295491	0.310361	0.292133	0.331948	0.282699
0.381196	0.404061	0.358491	0.26591	0.147106	0.387112	0.398625	0.215542	0.417973	0.298529
0.347618	0.368244	0.34426	0.307004	0.308283	0.351135	0.357371	0.294052	0.402622	0.311481
0.276303	0.200032	0.185961	0.332427	0.389031	0.238407	0.239527	0.387432	0.165174	0.297889
0.304445	0.295331	0.251199	0.271986	0.284778	0.293892	0.291493	0.261433	0.298209	0.276143
0.35961	0.369044	0.355612	0.270547	0.285897	0.383275	0.382155	0.309242	0.415734	0.306204
0.329069	0.332427	0.300288	0.285577	0.273745	0.316278	0.315478	0.255516	0.341062	0.284618
0.316437	0.259034	0.173969	0.300608	0.345059	0.238727	0.277103	0.339463	0.236329	0.252638
0.334026	0.287016	0.247681	0.29757	0.325232	0.294212	0.308283	0.323953	0.294052	0.255836
0.314679	0.238407	0.164375	0.311481	0.379597	0.200512	0.267509	0.363288	0.230253	0.272146
0.346978	0.378798	0.337384	0.282539	0.269907	0.356092	0.369364	0.193156	0.383275	0.292453
0.26495	0.293412	0.297729	0.317237	0.326831	0.322514	0.286057	0.318036	0.298369	0.29661
0.154141	0.209146	0.252958	0.336265	0.403742	0.277902	0.248961	0.389031	0.170451	0.314519
0.287336	0.335146	0.305405	0.299009	0.351295	0.330349	0.235209	0.330189	0.29709	0.319156
0.310201	0.23377	0.145507	0.308443	0.37448	0.202111	0.26575	0.361848	0.220019	0.269108
0.365206	0.37496	0.34378	0.326351	0.287336	0.35977	0.348417	0.254237	0.392709	0.245443
0.326351	0.277263	0.236009	0.31212	0.382635	0.26575	0.298849	0.377199	0.263991	0.318996
0.333227	0.32827	0.290374	0.303965	0.309402	0.305564	0.322833	0.29661	0.334506	0.294532
0.361209	0.322034	0.307803	0.299648	0.307004	0.292933	0.340742	0.271027	0.363447	0.283658
0.368084	0.377998	0.349696	0.3126	0.262552	0.376559	0.371922	0.232971	0.397825	0.316917
0.369364	0.3748	0.341062	0.301407	0.26495	0.374161	0.366326	0.204669	0.393348	0.310042
0.303965	0.268948	0.224976	0.282859	0.330029	0.228174	0.25024	0.333067	0.253918	0.285257
0.368404	0.376239	0.341382	0.309882	0.257115	0.372402	0.365686	0.228014	0.387752	0.316118
0.364727	0.368244	0.323473	0.319316	0.28126	0.356092	0.354653	0.252798	0.377678	0.306684
0.253598	0.239367	0.255197	0.307323	0.369843	0.268628	0.255197	0.369843	0.242405	0.322993
0.294532	0.315958	0.269747	0.297729	0.29645	0.297889	0.29693	0.278222	0.329389	0.153182

For Review Only

0.373681	0.378957	0.336745	0.299488	0.270547	0.373841	0.371442	0.157179	0.39047	0.32811
0.287336	0.26527	0.240006	0.287336	0.324272	0.246562	0.269428	0.318036	0.251999	0.294052
0.351455	0.380237	0.350336	0.289415	0.238567	0.376559	0.368404	0.247522	0.400064	0.290214
0.279981	0.298529	0.282699	0.305085	0.381356	0.298369	0.249121	0.362328	0.284618	0.315958
0.357531	0.323953	0.303486	0.332907	0.365046	0.29661	0.341062	0.344739	0.350016	0.347298
0.295491	0.29645	0.283818	0.316597	0.335945	0.292773	0.26575	0.34314	0.28062	0.259834
0.364087	0.377199	0.346178	0.302846	0.260793	0.368244	0.378158	0.211545	0.397506	0.303006
0.322833	0.302367	0.283339	0.302367	0.319156	0.256956	0.275664	0.335945	0.291493	0.306684
0.246882	0.137512	0.263991	0.331788	0.369843	0.287176	0.246882	0.35897	0.220339	0.309082
0.313879	0.307163	0.262392	0.274065	0.268628	0.292933	0.299009	0.243684	0.306844	0.262072
0.271666	0.263351	0.273745	0.323473	0.366166	0.299328	0.283818	0.354653	0.237768	0.287176
0.347458	0.393348	0.354013	0.290054	0.258235	0.376879	0.373041	0.270707	0.411896	0.298529
0.347298	0.357052	0.317557	0.298849	0.257595	0.347777	0.336425	0.227854	0.365046	0.303326
0.28094	0.267988	0.229133	0.29661	0.342821	0.266709	0.25008	0.320435	0.236329	0.275664
0.355612	0.364886	0.313719	0.276783	0.278062	0.34442	0.331628	0.225935	0.377359	0.284458
0.338503	0.322354	0.279341	0.305085	0.333706	0.278382	0.320755	0.299169	0.336425	0.284938
0.309402	0.247042	0.145987	0.303965	0.362168	0.211065	0.26495	0.353534	0.220659	0.264791
0.010233	0.24944	0.310042	0.32763	0.379437	0.325712	0.2811	0.355293	0.231212	0.292773
0.292293	0.303326	0.248001	0.283499	0.286537	0.293412	0.285098	0.28062	0.31292	0.169651
0.361689	0.379597	0.34314	0.306204	0.260953	0.366965	0.367765	0.201151	0.389511	0.315318
0.345699	0.334506	0.287656	0.290854	0.25072	0.329069	0.337544	0.2496	0.345699	0.291813
0.3441	0.367125	0.316757	0.243044	0.247042	0.35929	0.369364	0.231372	0.377678	0.269747
0.294212	0.301087	0.264471	0.258555	0.26543	0.293092	0.293732	0.245443	0.306844	0.261113
0.273745	0.175408	0.196354	0.313399	0.374001	0.243524	0.247841	0.361369	0.192197	0.295011
0.273425	0.28078	0.285897	0.325552	0.368084	0.285737	0.092901	0.362808	0.255357	0.32859
0.370163	0.393508	0.337864	0.29661	0.273745	0.367285	0.367605	0.082667	0.388072	0.320915
0.357052	0.349057	0.289255	0.271986	0.31324	0.320755	0.326671	0.331468	0.348897	0.320595
0.292613	0.254717	0.17253	0.293572	0.367605	0.235209	0.26623	0.348097	0.238887	0.266549
0.362648	0.364247	0.315318	0.253758	0.26607	0.344579	0.352894	0.212504	0.37512	0.308443
0.326191	0.330988	0.279661	0.286856	0.308443	0.303166	0.32875	0.309242	0.340262	0.270387
0.366805	0.352095	0.302526	0.302686	0.289575	0.321234	0.330189	0.286856	0.350656	0.314199
0.366326	0.376559	0.341062	0.268308	0.267509	0.362648	0.363288	0.286377	0.392229	0.251199
0.350176	0.322034	0.3126	0.311321	0.293732	0.323793	0.345059	0.274065	0.363607	0.289255
0.361848	0.385353	0.358331	0.306364	0.280301	0.381676	0.372562	0.275664	0.420531	0.305405
0.35945	0.35865	0.311001	0.287496	0.295331	0.321554	0.349696	0.286697	0.367125	0.308922
0.308283	0.24944	0.151263	0.307803	0.369843	0.209306	0.262232	0.352414	0.223217	0.26607
0.361848	0.363927	0.322034	0.302047	0.29725	0.322354	0.336745	0.298689	0.382475	0.291493
0.370163	0.393668	0.336904	0.295971	0.272945	0.366006	0.367285	0.081228	0.387592	0.320115
0.342821	0.354333	0.326351	0.317077	0.29661	0.336745	0.314679	0.268948	0.354973	0.333547
0.347138	0.351135	0.301087	0.291174	0.280301	0.314839	0.330189	0.306844	0.361848	0.270867
0.369524	0.372562	0.342021	0.298689	0.266549	0.3756	0.364886	0.204509	0.393668	0.310841
0.351615	0.356732	0.329069	0.278542	0.264151	0.356092	0.353214	0.212824	0.378478	0.287336
0.363607	0.372242	0.366805	0.294052	0.256636	0.379117	0.387592	0.204509	0.404861	0.288295
0.366485	0.376719	0.347777	0.307963	0.262712	0.376879	0.367445	0.239687	0.396706	0.311001
0.247841	0.179245	0.223697	0.34394	0.401503	0.239687	0.232811	0.388391	0.14071	0.299968
0.315158	0.339463	0.323313	0.315958	0.294532	0.333067	0.305405	0.286697	0.342661	0.311001
0.345059	0.335785	0.270547	0.29613	0.309562	0.309722	0.336425	0.294851	0.330029	0.308762
0.373681	0.332107	0.308443	0.293732	0.306044	0.305405	0.331468	0.278222	0.34378	0.321554
0	0.247681	0.311001	0.326671	0.377678	0.324592	0.28078	0.354173	0.230093	0.292453
0.247681	0	0.246083	0.345859	0.384554	0.268308	0.25056	0.386313	0.196514	0.314839
0.311001	0.246083	0	0.289095	0.350656	0.215702	0.267189	0.338983	0.217781	0.240966
0.326671	0.345859	0.289095	0	0.315958	0.323953	0.32811	0.287816	0.341541	0.292613
0.377678	0.384554	0.350656	0.315958	0	0.391749	0.387112	0.262552	0.407099	0.303646
0.324592	0.268308	0.215702	0.323953	0.391749	0	0.259194	0.367925	0.253598	0.300288
0.28078	0.25056	0.267189	0.32811	0.387112	0.259194	0	0.37496	0.227055	0.324113

For Review Only

0.354173	0.386313	0.338983	0.287816	0.262552	0.367925	0.37496	0	0.392549	0.297729
0.230093	0.196514	0.217781	0.341541	0.407099	0.253598	0.227055	0.392549	0	0.313719
0.292453	0.314839	0.240966	0.292613	0.303646	0.300288	0.324113	0.297729	0.313719	0
0.330189	0.332747	0.293892	0.308123	0.336265	0.302686	0.313559	0.338983	0.325712	0.307163
0.380716	0.387112	0.365526	0.302367	0.180844	0.382955	0.379437	0.256956	0.426767	0.298369
0.313559	0.324272	0.316917	0.306684	0.349536	0.319316	0.205948	0.355293	0.311321	0.334186
0.325871	0.325072	0.28158	0.274704	0.336904	0.269428	0.318516	0.332747	0.332747	0.303646
0.376239	0.381196	0.349696	0.305724	0.251359	0.377199	0.368884	0.277902	0.4063	0.304605
0.288775	0.272306	0.28062	0.330349	0.336585	0.320115	0.276463	0.360409	0.235689	0.293412
0.337704	0.284298	0.247042	0.318516	0.388711	0.274065	0.291973	0.393348	0.263032	0.336265
0.314519	0.269747	0.195555	0.300128	0.338663	0.261433	0.291973	0.319795	0.262072	0.263192
0.258235	0.179245	0.204509	0.346019	0.40598	0.247042	0.229293	0.397985	0.137512	0.316917
0.299169	0.29645	0.262712	0.258395	0.270067	0.288615	0.285737	0.251359	0.302686	0.261273
0.317077	0.317877	0.305085	0.323313	0.323793	0.276623	0.307163	0.314039	0.332267	0.274384
0.287656	0.352734	0.327311	0.256796	0.298849	0.362328	0.310681	0.310042	0.342661	0.300608
0.331308	0.32795	0.283499	0.286057	0.248481	0.333387	0.317877	0.236169	0.349696	0.253278
0.363607	0.366485	0.360249	0.294212	0.26495	0.388232	0.392069	0.205149	0.399264	0.294691
0.229293	0.319156	0.31244	0.303806	0.342661	0.329549	0.278062	0.32827	0.331308	0.297729
0.253118	0.217781	0.246083	0.334186	0.371442	0.272466	0.23441	0.357371	0.168052	0.317557
0.289735	0.290374	0.26527	0.337224	0.350975	0.286217	0.276463	0.353854	0.279181	0.29613
0.271027	0.193636	0.245763	0.318516	0.376399	0.279821	0.242885	0.356572	0.23377	0.288136
0.28174	0.324752	0.308283	0.300288	0.318676	0.292293	0.308283	0.319476	0.325712	0.279981
0.338343	0.314519	0.28126	0.322674	0.34362	0.316757	0.320595	0.298689	0.290214	0.300927
0.356252	0.371442	0.337864	0.322354	0.262552	0.367285	0.358011	0.243204	0.387752	0.308443
0.333547	0.360889	0.32747	0.272626	0.270867	0.350336	0.338663	0.28206	0.389511	0.290054
0.2504	0.307323	0.304125	0.322993	0.34394	0.305564	0.260473	0.317557	0.275184	0.309882
0.292293	0.221778	0.185641	0.306524	0.382955	0.166773	0.24928	0.381036	0.201951	0.298369
0.35913	0.349856	0.319476	0.298849	0.303486	0.336265	0.353694	0.279021	0.362968	0.316757
0.26559	0.157659	0.217621	0.348097	0.4055	0.260313	0.228494	0.398785	0.137832	0.315318
0.368884	0.39031	0.341222	0.23505	0.240486	0.373521	0.392229	0.18708	0.401183	0.319156
0.339783	0.346019	0.313399	0.280141	0.300768	0.314519	0.333067	0.325712	0.364886	0.303806
0.378478	0.392229	0.345219	0.29741	0.095299	0.379277	0.380397	0.25056	0.408219	0.283179
0.222418	0.176047	0.233451	0.341382	0.39079	0.271986	0.227534	0.381356	0.138951	0.288295
0.297729	0.295011	0.305085	0.325392	0.284138	0.332107	0.313719	0.28062	0.321554	0.29741
0.303965	0.311321	0.240486	0.305405	0.360729	0.24992	0.294372	0.322194	0.299648	0.292933
0.349217	0.336585	0.319795	0.307483	0.267189	0.338823	0.336425	0.236329	0.357691	0.286856
0.263991	0.279021	0.285577	0.305245	0.367765	0.28142	0.096898	0.356572	0.259034	0.316917
0.264631	0.158139	0.220819	0.349217	0.408698	0.253438	0.228174	0.398945	0.139591	0.318836
0.350176	0.368084	0.339303	0.286537	0.253758	0.365206	0.35913	0.26607	0.396546	0.189639
0.371442	0.394947	0.338823	0.29693	0.273745	0.367765	0.368884	0.082028	0.389511	0.321714
0.316118	0.314679	0.235529	0.29709	0.310841	0.286697	0.314039	0.248001	0.297729	0.292133
0.222258	0.181164	0.216501	0.344899	0.413176	0.259834	0.213783	0.394627	0.104733	0.309242
0.370483	0.393828	0.3433	0.301247	0.262872	0.371442	0.362808	0.271826	0.404861	0.302047
0.349536	0.29725	0.251359	0.299808	0.295811	0.301247	0.309882	0.26511	0.331308	0.284458
0.267349	0.298049	0.294052	0.32875	0.345219	0.311481	0.255676	0.316917	0.268628	0.316917
0.350336	0.364567	0.35961	0.260793	0.279981	0.382155	0.372402	0.301247	0.417813	0.306844
0.357052	0.345539	0.32843	0.295491	0.279821	0.331788	0.348417	0.217141	0.347618	0.303965
0.31292	0.362968	0.316278	0.271027	0.322993	0.346178	0.311001	0.310521	0.339303	0.299648
0.300448	0.318516	0.304285	0.315798	0.322993	0.330029	0.322034	0.279341	0.293412	0.304285
0.368244	0.344579	0.316757	0.294532	0.301087	0.339303	0.352255	0.287336	0.361049	0.295491
0.366326	0.351935	0.331948	0.321234	0.319955	0.352894	0.368564	0.270067	0.376399	0.326351
0.267189	0.153022	0.216182	0.347777	0.401983	0.253598	0.225616	0.395107	0.146306	0.306044
0.346019	0.397186	0.357531	0.303326	0.261273	0.379117	0.369843	0.284458	0.420371	0.299968
0.216661	0.186441	0.223697	0.338823	0.409978	0.261593	0.216342	0.391749	0.106172	0.310681
0.364407	0.371922	0.367445	0.29613	0.255197	0.380716	0.387272	0.204989	0.405341	0.287176

For Review Only

0.373681	0.407419	0.367925	0.289255	0.255516	0.398305	0.385034	0.219699	0.42133	0.334346
0.246562	0.252478	0.271826	0.339942	0.369524	0.279341	0.244803	0.361209	0.221458	0.305564
0.259994	0.37464	0.347138	0.277742	0.28174	0.377678	0.354333	0.284138	0.408219	0.307483
0.381996	0.388871	0.351455	0.29613	0.26543	0.367445	0.368564	0.195075	0.396386	0.31212

For Review Only

0.318836	0.292453	0.340102	0.278382	0.26495	0.347458	0.387752	0.319156	0.379917	0.267509
0.305245	0.337064	0.322833	0.3118	0.334506	0.347298	0.297729	0.309242	0.321874	0.279661
0.339463	0.17141	0.354173	0.340262	0.257275	0.355453	0.400224	0.32763	0.414295	0.261113
0.315638	0.26623	0.339303	0.313719	0.256636	0.334986	0.364087	0.298049	0.379277	0.256796
0.321234	0.345059	0.364087	0.310841	0.333227	0.350336	0.317397	0.317237	0.338823	0.288136
0.325392	0.269268	0.341701	0.31308	0.252319	0.336745	0.383115	0.317237	0.393668	0.256476
0.329709	0.301407	0.339143	0.301567	0.278062	0.338503	0.385513	0.326351	0.394627	0.272146
0.310201	0.348257	0.336585	0.240166	0.330828	0.323633	0.222098	0.274065	0.299328	0.283818
0.314359	0.303806	0.316118	0.29709	0.283339	0.3441	0.37464	0.325072	0.370163	0.262872
0.294212	0.3748	0.3441	0.28078	0.352894	0.324912	0.178286	0.287816	0.299488	0.303326
0.317877	0.361529	0.316757	0.31276	0.353054	0.302367	0.337384	0.309562	0.274225	0.287176
0.335305	0.191078	0.350336	0.335625	0.247681	0.351295	0.392069	0.321874	0.407579	0.251359
0.316278	0.316917	0.335625	0.304765	0.298849	0.331148	0.319795	0.298209	0.325072	0.270227
0.285737	0.340582	0.32779	0.279181	0.315638	0.302846	0.301887	0.229293	0.272945	0.260473
0.311481	0.263032	0.303326	0.301887	0.261113	0.32779	0.380397	0.313559	0.365046	0.248801
0.306204	0.29741	0.348737	0.316437	0.301247	0.310681	0.355453	0.277902	0.319795	0.26575
0.349376	0.303326	0.330029	0.321714	0.318196	0.353214	0.381516	0.353054	0.384554	0.282859
0.304605	0.3126	0.345539	0.259194	0.279181	0.315798	0.319635	0.316597	0.362488	0.271986
0.345219	0.257435	0.341222	0.338983	0.28126	0.376399	0.40678	0.34346	0.417493	0.270067
0.271986	0.361049	0.336904	0.254557	0.368084	0.331788	0.26511	0.311161	0.32891	0.305564
0.332587	0.344739	0.324912	0.298369	0.331148	0.302047	0.309082	0.317077	0.322674	0.276783
0.332587	0.307323	0.3433	0.336105	0.300128	0.323473	0.35897	0.324432	0.374161	0.274384
0.330668	0.403582	0.286217	0.330828	0.404221	0.291174	0.311321	0.301247	0.208507	0.308443
0.283658	0.367285	0.317397	0.271346	0.3441	0.295171	0.25072	0.213943	0.228174	0.256956
0.324113	0.268308	0.336745	0.309562	0.26591	0.353854	0.396866	0.326671	0.400704	0.256316
0.341382	0.267189	0.352894	0.341861	0.164375	0.326671	0.393508	0.299488	0.393348	0.254397
0.317557	0.26575	0.335945	0.315318	0.241925	0.32875	0.372721	0.295171	0.371282	0.245923
0.286697	0.261753	0.294212	0.280461	0.248961	0.29757	0.321874	0.267509	0.307004	0.216182
0.298209	0.330029	0.317237	0.286856	0.29725	0.309722	0.307963	0.290854	0.320115	0.260633
0.294212	0.434282	0.34314	0.294372	0.415254	0.32811	0.178286	0.274065	0.232811	0.324912
0.323953	0.269907	0.338503	0.340262	0.255836	0.358331	0.397026	0.316278	0.396386	0.257595
0.316597	0.262072	0.348737	0.32795	0.26511	0.349536	0.397026	0.338983	0.411737	0.28062
0.335945	0.284298	0.352414	0.311641	0.279021	0.344739	0.414135	0.341541	0.407099	0.280461
0.299808	0.369204	0.32827	0.290534	0.340102	0.287816	0.267189	0.226255	0.23393	0.269268
0.322674	0.34426	0.319955	0.306364	0.319955	0.284458	0.341222	0.315158	0.323793	0.285257
0.303646	0.300128	0.336105	0.304125	0.28094	0.318836	0.316118	0.242405	0.308443	0.241765
0.295651	0.311641	0.297889	0.278222	0.29693	0.324752	0.330189	0.280461	0.340422	0.273105
0.319635	0.313399	0.325232	0.295811	0.304925	0.319635	0.331148	0.302526	0.339783	0.275024
0.315958	0.417173	0.29677	0.315478	0.398305	0.253758	0.261593	0.252638	0.12424	0.287656
0.315158	0.356252	0.274544	0.314839	0.362488	0.274704	0.329869	0.311481	0.277263	0.29613
0.279501	0.420851	0.340102	0.284938	0.40614	0.325392	0.175568	0.269268	0.239527	0.310841
0.290694	0.355612	0.326511	0.269587	0.337544	0.305724	0.263351	0.291493	0.298369	0.287176
0.280461	0.372721	0.336265	0.247841	0.348897	0.322354	0.196514	0.287976	0.310521	0.291174
0.311641	0.337864	0.335625	0.304125	0.352574	0.356572	0.336745	0.311321	0.35929	0.295491
0.301887	0.389031	0.339783	0.245443	0.357851	0.346658	0.216981	0.298849	0.320595	0.303166
0.332107	0.215862	0.34426	0.330828	0.233291	0.336105	0.392709	0.311001	0.404861	0.25072
0.325871	0.251839	0.346978	0.311161	0.221298	0.340902	0.388871	0.313719	0.40566	0.2504
0.293892	0.305724	0.331788	0.300288	0.262392	0.311001	0.342341	0.268468	0.320115	0.255357
0.325072	0.34442	0.339143	0.295651	0.337544	0.365686	0.336904	0.31212	0.348417	0.286856
0.324592	0.249121	0.317717	0.31276	0.2496	0.346338	0.391909	0.322674	0.391909	0.263032

For Review Only

0.339783	0.248961	0.330988	0.342181	0.208507	0.316917	0.385513	0.313879	0.396546	0.250879
0.334666	0.306044	0.32827	0.32891	0.295331	0.352095	0.379757	0.321075	0.345539	0.273425
0.338024	0.273265	0.330668	0.324272	0.271666	0.371442	0.42181	0.345859	0.423729	0.26559
0.334826	0.283978	0.351935	0.310841	0.279021	0.344739	0.413176	0.340902	0.40614	0.279821
0.332427	0.261273	0.326511	0.318036	0.262392	0.337064	0.388711	0.32763	0.397186	0.259514
0.324912	0.29709	0.319476	0.327151	0.305884	0.35881	0.351615	0.331148	0.371442	0.28078
0.3441	0.257755	0.342181	0.337544	0.28158	0.376239	0.404861	0.342661	0.417013	0.270387
0.326191	0.275664	0.356732	0.320915	0.224976	0.342021	0.400064	0.319476	0.398625	0.254557
0.322194	0.260953	0.34426	0.320755	0.196994	0.325392	0.380716	0.307004	0.397985	0.253758
0.291334	0.411737	0.321874	0.279821	0.389671	0.284938	0.200192	0.240806	0.193956	0.289735
0.303646	0.337224	0.317877	0.293252	0.311001	0.300448	0.303166	0.274704	0.301087	0.268628
0.290854	0.257755	0.301247	0.284938	0.25072	0.305085	0.32843	0.276943	0.315798	0.21826
0.338503	0.257755	0.355772	0.333866	0.277422	0.360569	0.394627	0.320115	0.399105	0.251839
0.329549	0.276463	0.354813	0.339143	0.294372	0.364886	0.388711	0.322833	0.394947	0.257115
0.337224	0.272785	0.330029	0.323793	0.271666	0.371442	0.42229	0.345379	0.42245	0.264791
0.29757	0.329549	0.316597	0.287176	0.29709	0.309402	0.307803	0.290374	0.320115	0.259994
0.302686	0.282699	0.309882	0.294372	0.257435	0.314039	0.347298	0.293412	0.339463	0.235689
0.285737	0.34394	0.316597	0.269907	0.314998	0.324592	0.275664	0.285737	0.31244	0.279981
0.321075	0.29645	0.304445	0.314679	0.309562	0.324752	0.35945	0.325712	0.333067	0.271346
0.319955	0.232331	0.336105	0.31196	0.225616	0.346658	0.417813	0.345219	0.432044	0.267829
0.337384	0.257115	0.329869	0.331788	0.260793	0.35913	0.404861	0.3441	0.413335	0.258235
0.338184	0.2819	0.339942	0.29677	0.26639	0.351295	0.412696	0.339942	0.413335	0.271506
0.31308	0.266709	0.333547	0.3126	0.284458	0.340102	0.364567	0.300448	0.35993	0.242405
0.307163	0.35993	0.206108	0.314679	0.356092	0.283179	0.314998	0.302367	0.260153	0.28206
0.304605	0.320755	0.307803	0.278702	0.286057	0.314839	0.334986	0.306364	0.339783	0.263991
0.301247	0.306684	0.338983	0.313399	0.290854	0.341861	0.346019	0.317557	0.367605	0.2819
0.301727	0.388391	0.339783	0.245603	0.357851	0.345699	0.216981	0.298209	0.320115	0.302846
0.329389	0.276303	0.350496	0.32811	0.288775	0.367605	0.3748	0.311481	0.386952	0.254877
0.330668	0.337544	0.358171	0.316437	0.362488	0.370803	0.323153	0.325712	0.360729	0.307483
0.325232	0.414295	0.321075	0.320115	0.399105	0.251839	0.259354	0.252638	0.096418	0.295171
0.332107	0.261912	0.353214	0.338663	0.158459	0.315638	0.389351	0.300288	0.399264	0.258395
0.346818	0.23489	0.332587	0.329069	0.286537	0.371602	0.402942	0.337864	0.401183	0.268308
0.324912	0.316118	0.339463	0.280141	0.310201	0.348417	0.32891	0.303166	0.356252	0.283339
0.304605	0.323633	0.322354	0.280301	0.301407	0.303326	0.290534	0.241286	0.273585	0.2496
0.323313	0.426607	0.313399	0.318196	0.413495	0.26495	0.26543	0.253598	0.116565	0.305884
0.336105	0.272466	0.329389	0.323633	0.271027	0.370323	0.420531	0.345059	0.42133	0.264311
0.292933	0.300768	0.342821	0.279501	0.279661	0.310201	0.364727	0.302526	0.351135	0.26591
0.302047	0.389191	0.339303	0.246722	0.357531	0.346658	0.217621	0.298369	0.320275	0.303166
0.299328	0.355453	0.34378	0.240006	0.34442	0.329389	0.232651	0.284138	0.29757	0.291493
0.354493	0.354653	0.347777	0.315478	0.34362	0.347138	0.29741	0.317877	0.32763	0.299648
0.342021	0.303806	0.315318	0.333866	0.322034	0.310521	0.332107	0.305884	0.333866	0.284458
0.329869	0.366805	0.326511	0.272785	0.340262	0.326671	0.251519	0.286057	0.3118	0.29645
0.34426	0.268948	0.334986	0.321554	0.271666	0.362328	0.431244	0.339143	0.417653	0.26607
0.351135	0.248641	0.34394	0.361049	0.288935	0.385513	0.425008	0.344899	0.427247	0.278382
0.331628	0.302207	0.306204	0.310201	0.279821	0.347777	0.335146	0.323313	0.367605	0.276303
0.340582	0.258395	0.356732	0.333387	0.278062	0.361848	0.393828	0.320435	0.398145	0.252798
0.342821	0.288295	0.355453	0.34394	0.299968	0.376239	0.384234	0.306524	0.370483	0.272146
0.285577	0.262392	0.330508	0.290854	0.258395	0.339143	0.391749	0.331628	0.400384	0.258714
0.298049	0.379597	0.320275	0.295011	0.356892	0.2819	0.261113	0.214743	0.213463	0.275024
0.316757	0.283339	0.325712	0.325072	0.236968	0.317877	0.347458	0.275344	0.353694	0.247202
0.285257	0.275344	0.340422	0.287816	0.221618	0.324592	0.360889	0.237608	0.369843	0.245123
0.337864	0.313559	0.2811	0.340262	0.306844	0.346019	0.382955	0.329229	0.370643	0.286217
0.315478	0.28158	0.333227	0.307163	0.263351	0.341861	0.372562	0.301087	0.358331	0.235369
0.326831	0.424528	0.310042	0.32875	0.4055	0.260313	0.259354	0.258555	0.127279	0.29709
0.323153	0.393348	0.322674	0.313719	0.37432	0.305405	0.262712	0.261113	0.23377	0.295971

For Review Only

0.29661	0.302367	0.315318	0.291334	0.29725	0.304605	0.31324	0.283339	0.2811	0.255516
0.339783	0.299808	0.333706	0.322354	0.293732	0.34362	0.383275	0.321554	0.380556	0.279021
0.315638	0.305405	0.304445	0.309242	0.240966	0.305405	0.368564	0.288615	0.32875	0.260153
0.274225	0.345059	0.317237	0.285098	0.327311	0.323953	0.302686	0.294691	0.322194	0.289415
0.331308	0.284458	0.332107	0.322993	0.2189	0.327311	0.363447	0.277103	0.361049	0.244484
0.29613	0.370963	0.315478	0.291334	0.350016	0.268948	0.28174	0.21922	0.214583	0.270707
0.329709	0.251359	0.345699	0.330508	0.25024	0.333706	0.387432	0.321714	0.400064	0.252319
0.329549	0.261753	0.329709	0.305564	0.220819	0.307803	0.354173	0.28142	0.365526	0.244484
0.330828	0.217941	0.337864	0.318516	0.261753	0.354973	0.391749	0.315958	0.396386	0.24944
0.282219	0.291493	0.321234	0.291493	0.28094	0.306844	0.331308	0.260473	0.304925	0.239367
0.285577	0.325712	0.335146	0.302526	0.291014	0.324912	0.299808	0.255676	0.307643	0.271666
0.299808	0.315318	0.318836	0.304925	0.291813	0.29725	0.310361	0.258714	0.293252	0.251679
0.302526	0.316597	0.334186	0.310841	0.300128	0.319156	0.35945	0.308602	0.337384	0.26623
0.297889	0.28206	0.294212	0.285098	0.263192	0.29693	0.324432	0.284778	0.324113	0.23489
0.329229	0.301247	0.303806	0.302686	0.272466	0.354813	0.318516	0.304125	0.351615	0.270707
0.326351	0.258555	0.331948	0.327151	0.264471	0.348417	0.379117	0.310841	0.376399	0.25024
0.322514	0.31196	0.335146	0.321394	0.288935	0.347777	0.3433	0.300128	0.337704	0.270547
0.289575	0.269108	0.309242	0.28158	0.236329	0.300288	0.339623	0.275983	0.329709	0.238248
0.310361	0.291653	0.325871	0.290694	0.259514	0.333227	0.386153	0.350336	0.388391	0.28126
0.301087	0.247522	0.310521	0.286537	0.239047	0.309242	0.348897	0.279981	0.333706	0.225935
0.301887	0.377518	0.321874	0.286856	0.35881	0.28174	0.272945	0.214902	0.222578	0.273745
0.340902	0.299169	0.242725	0.300128	0.311001	0.331788	0.362808	0.337544	0.365526	0.278382
0.293252	0.268628	0.295491	0.283978	0.244803	0.290854	0.323153	0.271186	0.308602	0.217781
0.331308	0.236808	0.330349	0.320275	0.274544	0.361529	0.403902	0.336425	0.402782	0.259834
0.316917	0.21938	0.330508	0.319156	0.268468	0.344899	0.369044	0.303006	0.380397	0.252958
0.321714	0.417813	0.322833	0.314039	0.400224	0.264151	0.255676	0.243044	0.117045	0.29613
0.330508	0.304605	0.262872	0.318996	0.26559	0.298049	0.351775	0.301087	0.32859	0.261273
0.306844	0.344739	0.229773	0.295651	0.321234	0.266709	0.322993	0.299488	0.291014	0.275344
0.318676	0.275823	0.326351	0.307004	0.268628	0.338663	0.384554	0.329869	0.387272	0.272466
0.291014	0.267669	0.304605	0.288615	0.256636	0.331468	0.330988	0.276303	0.339143	0.251359
0.341222	0.173009	0.35897	0.334346	0.253438	0.358011	0.403102	0.336265	0.419572	0.257755
0.303486	0.300288	0.334026	0.326191	0.276463	0.354493	0.384074	0.334346	0.39095	0.275344
0.315958	0.409978	0.324592	0.3118	0.389831	0.266869	0.253438	0.235209	0.126959	0.29677
0.300768	0.284458	0.301567	0.286217	0.276463	0.290534	0.299328	0.26559	0.302047	0.227694
0.346338	0.268148	0.334986	0.323473	0.271666	0.364087	0.431884	0.341222	0.420371	0.267349
0.287496	0.26639	0.311481	0.288136	0.21938	0.299169	0.356092	0.28206	0.345859	0.23425
0.299648	0.368724	0.32779	0.291813	0.338503	0.288775	0.267029	0.226255	0.23457	0.268628
0.322514	0.331468	0.332267	0.29741	0.308762	0.315158	0.325871	0.274704	0.291813	0.270067
0.298849	0.387112	0.323313	0.295331	0.362808	0.282539	0.263671	0.216342	0.216342	0.282219
0.31292	0.26591	0.338184	0.307323	0.271666	0.344579	0.381036	0.316757	0.389671	0.255676
0.318196	0.334986	0.320115	0.305405	0.29725	0.292773	0.350336	0.298529	0.295331	0.276303
0.339942	0.413655	0.315958	0.320595	0.400384	0.26511	0.294851	0.283499	0.154141	0.306524
0.323793	0.342501	0.252958	0.319955	0.309562	0.29741	0.346178	0.31292	0.310201	0.284458
0.300927	0.387912	0.322194	0.289415	0.362968	0.282219	0.261273	0.199392	0.212664	0.28062
0.335465	0.267509	0.339783	0.341861	0.263192	0.347298	0.400224	0.317557	0.39095	0.264311
0.275983	0.388711	0.335625	0.285098	0.3756	0.318996	0.21874	0.261912	0.26511	0.300608
0.310681	0.308443	0.326511	0.317397	0.262552	0.31276	0.339623	0.294691	0.342821	0.269428
0.330189	0.287336	0.332267	0.3118	0.279821	0.337544	0.35929	0.293092	0.362008	0.259674
0.329549	0.246242	0.346658	0.321554	0.176847	0.313879	0.399584	0.302367	0.403262	0.248321
0.321874	0.246562	0.347458	0.315798	0.21922	0.330029	0.380077	0.31244	0.39015	0.254077
0.288295	0.335305	0.310681	0.298689	0.322034	0.28126	0.275184	0.249121	0.260153	0.262872
0.332107	0.263671	0.345219	0.338823	0.199073	0.325712	0.387272	0.301247	0.397346	0.24976
0.316437	0.271506	0.352734	0.317397	0.189319	0.320915	0.385673	0.285897	0.382155	0.251999
0.300927	0.389511	0.307963	0.273905	0.349536	0.270707	0.278222	0.2811	0.251679	0.280301
0.316437	0.28174	0.31276	0.300768	0.272785	0.307803	0.332427	0.272146	0.331468	0.254877

For Review Only

0.338503	0.276143	0.352894	0.348577	0.268308	0.353694	0.389031	0.313559	0.396706	0.262232
0.284138	0.336745	0.298049	0.28094	0.317877	0.289575	0.267189	0.261113	0.24928	0.255676
0.334026	0.217941	0.3433	0.323793	0.236009	0.340102	0.402622	0.333866	0.402143	0.256476
0.326831	0.378478	0.255197	0.304765	0.34362	0.292133	0.294052	0.303806	0.286377	0.294691
0.31292	0.353214	0.348257	0.31196	0.338343	0.340422	0.297729	0.309082	0.341541	0.301087
0.308123	0.345219	0.295331	0.318196	0.329389	0.287016	0.331948	0.302207	0.291174	0.282699
0.332427	0.263192	0.340582	0.318996	0.272945	0.356572	0.387432	0.314679	0.399744	0.26511
0.294052	0.337384	0.307643	0.283978	0.321394	0.294532	0.324272	0.301407	0.298849	0.268468
0.315798	0.376879	0.297889	0.313559	0.362648	0.271666	0.307323	0.280301	0.221618	0.287336
0.288136	0.266549	0.305405	0.276463	0.246402	0.29693	0.320115	0.261273	0.310521	0.216821
0.318516	0.35977	0.317397	0.317717	0.362808	0.290694	0.321394	0.289894	0.259354	0.29677
0.32891	0.236808	0.345699	0.334986	0.280301	0.360569	0.408379	0.353374	0.415414	0.270067
0.31196	0.24992	0.32763	0.302686	0.212344	0.310042	0.373201	0.282219	0.370323	0.238567
0.286856	0.357211	0.301727	0.2811	0.32779	0.262232	0.286217	0.24944	0.242085	0.256796
0.315638	0.260953	0.318196	0.322354	0.251999	0.334186	0.39111	0.295651	0.381356	0.239367
0.319635	0.318196	0.323313	0.307643	0.303486	0.336425	0.348097	0.284458	0.339783	0.271027
0.301727	0.380237	0.324752	0.289735	0.362808	0.278062	0.262232	0.209946	0.206108	0.275823
0.330349	0.382155	0.314679	0.325552	0.377678	0.289255	0.336425	0.314998	0.258395	0.300288
0.299808	0.282379	0.306044	0.295651	0.272306	0.287176	0.322194	0.264311	0.306844	0.246882
0.325232	0.274704	0.346498	0.3126	0.269587	0.349856	0.392549	0.332747	0.397026	0.269428
0.301247	0.268948	0.329709	0.314679	0.229773	0.283818	0.352095	0.270547	0.353694	0.245123
0.325392	0.215702	0.330988	0.31308	0.254557	0.348897	0.384074	0.309722	0.388232	0.244963
0.277582	0.267509	0.294691	0.269587	0.255197	0.293732	0.323633	0.261113	0.306524	0.214583
0.306044	0.388391	0.309402	0.29645	0.37432	0.279181	0.259674	0.244164	0.173649	0.2811
0.310361	0.372562	0.180365	0.320435	0.363447	0.285577	0.322833	0.301727	0.26559	0.285897
0.329549	0.277103	0.354173	0.340262	0.294052	0.365206	0.388871	0.322514	0.394947	0.257755
0.303806	0.344739	0.315958	0.288775	0.334186	0.347618	0.31324	0.311481	0.346498	0.280301
0.299328	0.372562	0.319955	0.284938	0.338343	0.294851	0.269108	0.227215	0.238727	0.269587
0.325712	0.262072	0.331948	0.317877	0.254397	0.336745	0.363128	0.29629	0.371762	0.242885
0.29693	0.31292	0.323953	0.275983	0.276943	0.311641	0.333387	0.29709	0.331628	0.267829
0.305085	0.300128	0.320115	0.292933	0.263511	0.320595	0.346498	0.310361	0.352414	0.264791
0.310201	0.26607	0.318996	0.316118	0.25024	0.345059	0.393188	0.319156	0.394627	0.255676
0.337544	0.279181	0.338983	0.315798	0.277263	0.318836	0.367125	0.302846	0.367125	0.267029
0.333547	0.224496	0.337384	0.320115	0.261753	0.362008	0.419412	0.349376	0.42165	0.276783
0.316278	0.28078	0.326511	0.294212	0.260313	0.332107	0.385673	0.314199	0.373681	0.26591
0.297729	0.384394	0.321714	0.289255	0.361689	0.285417	0.267669	0.212824	0.20291	0.275184
0.307643	0.289415	0.340902	0.305245	0.270227	0.335625	0.393028	0.313719	0.385193	0.267829
0.32875	0.276623	0.354013	0.339303	0.293732	0.364407	0.387912	0.322194	0.394308	0.256956
0.332267	0.302367	0.314679	0.325072	0.26511	0.317077	0.37432	0.306364	0.372881	0.26607
0.301087	0.28094	0.325392	0.291813	0.263831	0.332427	0.361689	0.303326	0.375919	0.266869
0.320595	0.244803	0.345699	0.316437	0.229773	0.332587	0.378798	0.31228	0.388391	0.253438
0.337064	0.278702	0.335625	0.331308	0.270067	0.35913	0.370003	0.311481	0.381996	0.261912
0.339143	0.17205	0.354653	0.338343	0.256956	0.354493	0.398945	0.325871	0.413655	0.260793
0.32827	0.248321	0.348417	0.325712	0.182603	0.308283	0.394787	0.304925	0.402303	0.251999
0.310521	0.420211	0.313719	0.315478	0.400544	0.251839	0.266549	0.257435	0.129997	0.299968
0.278382	0.279661	0.31276	0.295491	0.2811	0.329869	0.369204	0.315158	0.350016	0.261753
0.306044	0.307643	0.353534	0.307004	0.305405	0.308762	0.345539	0.269108	0.319156	0.271506
0.331308	0.298209	0.329869	0.321394	0.300927	0.350656	0.363607	0.314998	0.347618	0.279341
0.330189	0.380716	0.313559	0.325871	0.376239	0.288775	0.337704	0.314519	0.258235	0.299169
0.332747	0.387112	0.324272	0.325072	0.381196	0.272306	0.284298	0.269747	0.179245	0.29645
0.293892	0.365526	0.316917	0.28158	0.349696	0.28062	0.247042	0.195555	0.204509	0.262712
0.308123	0.302367	0.306684	0.274704	0.305724	0.330349	0.318516	0.300128	0.346019	0.258395
0.336265	0.180844	0.349536	0.336904	0.251359	0.336585	0.388711	0.338663	0.40598	0.270067
0.302686	0.382955	0.319316	0.269428	0.377199	0.320115	0.274065	0.261433	0.247042	0.288615
0.313559	0.379437	0.205948	0.318516	0.368884	0.276463	0.291973	0.291973	0.229293	0.285737

0.338983	0.256956	0.355293	0.332747	0.277902	0.360409	0.393348	0.319795	0.397985	0.251359
0.325712	0.426767	0.311321	0.332747	0.4063	0.235689	0.263032	0.262072	0.137512	0.302686
0.307163	0.298369	0.334186	0.303646	0.304605	0.293412	0.336265	0.263192	0.316917	0.261273
0	0.342021	0.331628	0.259034	0.319955	0.319476	0.287336	0.291493	0.329389	0.286537
0.342021	0	0.346818	0.338663	0.255357	0.363288	0.408858	0.346178	0.424368	0.274065
0.331628	0.346818	0	0.324592	0.342821	0.317717	0.341861	0.325392	0.326031	0.294851
0.259034	0.338663	0.324592	0	0.316597	0.321554	0.277263	0.300448	0.323633	0.28062
0.319955	0.255357	0.342821	0.316597	0	0.335465	0.399264	0.314039	0.40694	0.255037
0.319476	0.363288	0.317717	0.321554	0.335465	0	0.303486	0.311321	0.263192	0.291813
0.287336	0.408858	0.341861	0.277263	0.399264	0.303486	0	0.28158	0.262392	0.317557
0.291493	0.346178	0.325392	0.300448	0.314039	0.311321	0.28158	0	0.255197	0.263991
0.329389	0.424368	0.326031	0.323633	0.40694	0.263192	0.262392	0.255197	0	0.300128
0.286537	0.274065	0.294851	0.28062	0.255037	0.291813	0.317557	0.263991	0.300128	0
0.316278	0.319635	0.319955	0.292613	0.31228	0.286537	0.338823	0.31276	0.332747	0.28206
0.341701	0.28094	0.31308	0.335465	0.303965	0.307004	0.351935	0.321874	0.353694	0.278862
0.318036	0.252638	0.321394	0.323153	0.243204	0.321874	0.358171	0.274384	0.346818	0.242405
0.348257	0.210265	0.355772	0.34362	0.272146	0.358491	0.394627	0.322833	0.409338	0.26575
0.316757	0.334826	0.335465	0.310201	0.334506	0.338983	0.373201	0.313399	0.331948	0.284778
0.329709	0.377518	0.313719	0.311001	0.362008	0.271826	0.277422	0.258235	0.18724	0.279181
0.330349	0.344899	0.320275	0.320595	0.337384	0.29645	0.324752	0.28094	0.260953	0.283658
0.318036	0.379437	0.307963	0.29725	0.358491	0.273905	0.303326	0.287336	0.229773	0.290534
0.318676	0.323153	0.31244	0.302846	0.315318	0.317717	0.325232	0.311001	0.339942	0.28206
0.315638	0.349057	0.339143	0.315958	0.332107	0.301087	0.338343	0.307803	0.305245	0.291334
0.323313	0.255676	0.338663	0.325552	0.129357	0.318196	0.384074	0.29693	0.393188	0.24992
0.333067	0.25072	0.317237	0.317077	0.260633	0.354813	0.39015	0.326831	0.394787	0.261113
0.291174	0.345379	0.282859	0.297729	0.330349	0.288136	0.347618	0.305245	0.288136	0.273265
0.291014	0.411257	0.321714	0.280301	0.39095	0.285417	0.200352	0.240006	0.194116	0.289415
0.338823	0.303486	0.347458	0.317877	0.286537	0.358011	0.360409	0.322833	0.361689	0.272466
0.32779	0.426767	0.321234	0.32843	0.414775	0.264471	0.269587	0.253598	0.101215	0.310521
0.34442	0.239047	0.350496	0.340902	0.241765	0.35961	0.39047	0.314839	0.4055	0.248001
0.292613	0.304445	0.322993	0.177007	0.293092	0.336585	0.351135	0.315958	0.361848	0.266549
0.335146	0.144068	0.351935	0.32763	0.260633	0.349217	0.394787	0.336585	0.40678	0.263991
0.335146	0.399744	0.305724	0.324113	0.393508	0.252798	0.284778	0.258395	0.139271	0.294532
0.332587	0.294372	0.338343	0.31324	0.253118	0.309082	0.335465	0.285257	0.334346	0.253278
0.306044	0.356252	0.3126	0.298529	0.330189	0.326831	0.326831	0.267029	0.302047	0.276143
0.319316	0.274544	0.330988	0.32827	0.216022	0.322034	0.352414	0.286057	0.363288	0.255037
0.310521	0.35929	0.186441	0.313559	0.352095	0.269268	0.321874	0.301567	0.268308	0.2811
0.331468	0.427247	0.317717	0.326511	0.413335	0.269268	0.268468	0.259194	0.115606	0.306524
0.339783	0.232811	0.337064	0.323633	0.245923	0.345539	0.399105	0.323473	0.403742	0.259514
0.330029	0.277742	0.354813	0.340582	0.295171	0.366166	0.389831	0.323633	0.396546	0.258395
0.303166	0.299009	0.334826	0.303166	0.279501	0.317717	0.316437	0.241605	0.307483	0.240486
0.326671	0.431884	0.308283	0.319955	0.408219	0.25072	0.261912	0.258075	0.132875	0.298689
0.324912	0.255676	0.325232	0.332267	0.242885	0.333067	0.401023	0.32859	0.411417	0.270067
0.304445	0.289095	0.323953	0.299009	0.283179	0.300448	0.327151	0.266869	0.329069	0.25072
0.305405	0.360729	0.283339	0.310201	0.338503	0.283818	0.342181	0.300927	0.285577	0.275664
0.346818	0.259354	0.325392	0.332587	0.270547	0.362968	0.425168	0.348417	0.422769	0.263032
0.334026	0.276143	0.358011	0.318516	0.288295	0.34314	0.355612	0.305564	0.353214	0.261433
0.334666	0.304445	0.305245	0.347138	0.307004	0.331788	0.358491	0.307004	0.347138	0.278222
0.314359	0.315478	0.335305	0.309082	0.282699	0.288935	0.354333	0.294052	0.294052	0.272945
0.303326	0.310201	0.339623	0.314998	0.28206	0.322194	0.334986	0.294372	0.369524	0.273265
0.339463	0.306044	0.357052	0.319795	0.291493	0.345859	0.325871	0.309242	0.35881	0.283818
0.325552	0.423729	0.322194	0.318996	0.413176	0.272466	0.273265	0.252958	0.10873	0.306204
0.315638	0.244004	0.326191	0.337704	0.260473	0.362648	0.419092	0.345699	0.42117	0.270547
0.324432	0.426447	0.309242	0.325552	0.408858	0.254237	0.259194	0.259994	0.128558	0.301727
0.340422	0.17157	0.354333	0.339303	0.256956	0.354973	0.399105	0.32811	0.414775	0.261113

For Review Only

0.356412	0.262232	0.346498	0.362168	0.244324	0.35881	0.401023	0.334026	0.424049	0.262392
0.286856	0.377039	0.284458	0.307483	0.367605	0.261912	0.310201	0.29709	0.2496	0.292293
0.341062	0.26543	0.32875	0.344899	0.275184	0.379597	0.42245	0.340582	0.412376	0.269268
0.354653	0.267189	0.347458	0.352734	0.266549	0.374001	0.398945	0.325072	0.400704	0.260313

For Review Only

0.306364	0.306684	0.293732	0.289095	0.314519	0.335785	0.323313	0.32875	0.31228	0.32779
0.321874	0.345539	0.292773	0.314359	0.347618	0.321075	0.308602	0.305085	0.333067	0.326351
0.31292	0.302846	0.241765	0.098337	0.337224	0.35929	0.360729	0.377838	0.294052	0.317877
0.303166	0.300608	0.259994	0.268308	0.320595	0.341062	0.345859	0.340102	0.276783	0.315478
0.334346	0.330029	0.323473	0.35929	0.309722	0.34362	0.350656	0.314199	0.320915	0.347298
0.301247	0.305405	0.268468	0.271666	0.338983	0.356572	0.3118	0.341701	0.313399	0.308762
0.297729	0.308283	0.287656	0.315158	0.325232	0.355133	0.32795	0.347937	0.31324	0.337384
0.318196	0.331308	0.310042	0.336105	0.339783	0.294052	0.304925	0.311321	0.318516	0.322674
0.311641	0.323793	0.310681	0.326191	0.307803	0.342661	0.315478	0.350016	0.317557	0.345219
0.337544	0.334986	0.34362	0.37448	0.365686	0.29693	0.339783	0.318676	0.329869	0.339942
0.315158	0.279021	0.31196	0.349696	0.239527	0.272945	0.289255	0.279021	0.283339	0.32859
0.309882	0.292613	0.244324	0.136872	0.332267	0.356732	0.349536	0.370963	0.298689	0.308762
0.325712	0.316437	0.280141	0.320275	0.325392	0.294052	0.323153	0.323953	0.322354	0.316917
0.302686	0.298209	0.304445	0.329229	0.293092	0.273905	0.294851	0.286377	0.299009	0.319476
0.292773	0.308602	0.262072	0.269907	0.298369	0.337064	0.318996	0.331468	0.300927	0.31196
0.323473	0.327311	0.266869	0.278542	0.293572	0.29661	0.327311	0.314998	0.303006	0.326511
0.35945	0.320755	0.315958	0.341701	0.348097	0.364407	0.331788	0.334666	0.324272	0.379757
0.310042	0.308602	0.302526	0.29629	0.329869	0.323313	0.316917	0.322354	0.306364	0.323633
0.326511	0.304125	0.26527	0.258714	0.337224	0.379757	0.347618	0.366006	0.339143	0.338823
0.34346	0.350656	0.342821	0.366645	0.341062	0.331308	0.329389	0.337544	0.349856	0.332587
0.305564	0.320435	0.307803	0.338823	0.355453	0.315798	0.304445	0.322354	0.318036	0.323793
0.345059	0.306204	0.298529	0.333227	0.330349	0.338343	0.32875	0.338983	0.334506	0.354333
0.308123	0.31244	0.352574	0.401023	0.293572	0.232651	0.29677	0.274384	0.327311	0.304445
0.302846	0.316917	0.275184	0.345379	0.307483	0.252159	0.277582	0.26559	0.314679	0.291653
0.311321	0.307483	0.258395	0.275504	0.305564	0.361209	0.333227	0.342661	0.303166	0.329869
0.311481	0.300448	0.191877	0.231692	0.334666	0.349057	0.361049	0.358331	0.32827	0.307483
0.307803	0.287336	0.247841	0.233291	0.330029	0.326351	0.346978	0.354813	0.310681	0.292933
0.275823	0.278862	0.248801	0.268628	0.292933	0.282859	0.292133	0.292293	0.279501	0.293412
0.299169	0.332587	0.29709	0.332107	0.346658	0.314679	0.300448	0.326831	0.319795	0.313399
0.348737	0.363767	0.35881	0.418292	0.366006	0.277263	0.318676	0.300448	0.337224	0.341701
0.331148	0.303326	0.245603	0.244164	0.319955	0.364727	0.347777	0.368084	0.332267	0.317237
0.340262	0.307004	0.286537	0.262712	0.32843	0.37544	0.351135	0.371762	0.335465	0.339303
0.310042	0.305884	0.291334	0.286377	0.31276	0.354813	0.350815	0.350176	0.316597	0.366805
0.303326	0.323953	0.299009	0.349856	0.32763	0.255996	0.276623	0.270547	0.325232	0.317077
0.295971	0.313399	0.291014	0.32795	0.294851	0.307483	0.320115	0.306044	0.31292	0.315798
0.299808	0.29693	0.243524	0.273105	0.298529	0.286697	0.304765	0.317877	0.297889	0.316597
0.263192	0.302367	0.278222	0.32875	0.31292	0.295011	0.323153	0.335625	0.261433	0.335465
0.290694	0.316917	0.288295	0.291014	0.316757	0.285897	0.330029	0.32811	0.067317	0.334186
0.325552	0.337864	0.338823	0.39079	0.335146	0.145667	0.273105	0.221618	0.320115	0.28158
0.31308	0.342501	0.323633	0.344739	0.308762	0.28174	0.289095	0.262072	0.262872	0.284938
0.333706	0.354173	0.356892	0.40614	0.354813	0.263671	0.324113	0.289575	0.323473	0.333547
0.288935	0.339623	0.322034	0.348257	0.353374	0.29709	0.32747	0.321394	0.326831	0.308443
0.32827	0.327151	0.334186	0.368244	0.346818	0.295811	0.327151	0.326671	0.324912	0.347777
0.333866	0.333067	0.310361	0.354493	0.31276	0.332107	0.320755	0.347937	0.318356	0.362008
0.341382	0.329549	0.350815	0.369524	0.348897	0.319156	0.336745	0.334026	0.322354	0.355293
0.29613	0.287656	0.215062	0.160217	0.321075	0.345539	0.361369	0.370163	0.291493	0.311161
0.309402	0.314359	0.206108	0.227215	0.345859	0.356092	0.351775	0.345539	0.332907	0.325552
0.290854	0.304125	0.262712	0.297729	0.29677	0.306844	0.300927	0.282219	0.280141	0.315638
0.355293	0.340582	0.329549	0.354813	0.320755	0.324432	0.320595	0.361848	0.330189	0.372402
0.302367	0.303006	0.282379	0.290374	0.309402	0.364087	0.329229	0.339303	0.301087	0.34378

For Review Only

0.29661	0.294691	0.194436	0.221938	0.333227	0.351135	0.349057	0.354973	0.313879	0.317877
0.342981	0.32859	0.295811	0.302686	0.338983	0.334826	0.323473	0.339463	0.330508	0.334986
0.350815	0.31324	0.275983	0.309082	0.307963	0.383275	0.323953	0.355772	0.321075	0.372562
0.309402	0.306044	0.290854	0.286856	0.31244	0.354813	0.350496	0.350656	0.316437	0.367125
0.3118	0.303646	0.260793	0.251839	0.321714	0.355772	0.342821	0.347458	0.325552	0.298849
0.362008	0.301407	0.299009	0.331148	0.333866	0.345379	0.326671	0.356412	0.336904	0.384874
0.325072	0.304125	0.26527	0.259834	0.336745	0.379277	0.348097	0.365846	0.338823	0.338184
0.317717	0.325871	0.21938	0.221298	0.340102	0.354813	0.353214	0.367445	0.341861	0.313719
0.31324	0.280141	0.209466	0.238727	0.325392	0.345059	0.357851	0.356732	0.311481	0.305564
0.314679	0.353694	0.341062	0.395267	0.330349	0.23441	0.272945	0.253118	0.305724	0.299968
0.278862	0.299328	0.293252	0.335785	0.297729	0.274225	0.287496	0.323473	0.318676	0.322674
0.282699	0.277902	0.241286	0.255037	0.290214	0.29645	0.288455	0.301407	0.279021	0.298049
0.314839	0.311161	0.235369	0.205309	0.32827	0.357211	0.354013	0.357211	0.320435	0.299648
0.319635	0.332107	0.248481	0.21826	0.341382	0.357851	0.362488	0.37432	0.330508	0.308922
0.350496	0.31276	0.276303	0.308443	0.307483	0.381676	0.323313	0.354333	0.320755	0.372721
0.298529	0.332587	0.29629	0.331468	0.345379	0.314359	0.301087	0.326831	0.319955	0.313719
0.283179	0.298209	0.260793	0.268788	0.303646	0.299009	0.302367	0.307483	0.295811	0.295331
0.273105	0.323633	0.293572	0.337704	0.331308	0.29757	0.303965	0.290694	0.295491	0.331308
0.294212	0.269108	0.26575	0.262552	0.299808	0.300288	0.313879	0.306044	0.300128	0.321234
0.302526	0.316597	0.264471	0.253598	0.329869	0.383435	0.352734	0.377039	0.319635	0.348417
0.326511	0.316757	0.299488	0.301887	0.303006	0.389671	0.323473	0.372082	0.332587	0.350336
0.326831	0.314199	0.299169	0.298049	0.314359	0.363447	0.335785	0.354973	0.330668	0.35929
0.302686	0.3126	0.233131	0.220979	0.323153	0.327311	0.334666	0.348737	0.313719	0.295651
0.306844	0.305564	0.310201	0.35913	0.295651	0.254717	0.286697	0.253758	0.301727	0.317237
0.285897	0.334826	0.303166	0.32891	0.318356	0.324912	0.295811	0.325712	0.302686	0.318036
0.302047	0.313719	0.289894	0.284618	0.341222	0.34362	0.365366	0.377199	0.331948	0.259034
0.340742	0.329229	0.350656	0.368724	0.348897	0.317397	0.336904	0.333866	0.321874	0.354813
0.310042	0.304925	0.23393	0.224336	0.333227	0.347298	0.349217	0.365526	0.330828	0.304445
0.350815	0.324113	0.326671	0.331948	0.342501	0.32891	0.322034	0.341382	0.352095	0.348577
0.329389	0.346818	0.336585	0.39111	0.325072	0.194755	0.257115	0.217141	0.334666	0.295651
0.311001	0.301407	0.196834	0.245443	0.335146	0.340742	0.354653	0.351935	0.329069	0.31196
0.331948	0.298689	0.256156	0.285897	0.283818	0.369843	0.331788	0.360889	0.323793	0.352574
0.304445	0.315638	0.283339	0.331628	0.305884	0.32891	0.335785	0.32779	0.289575	0.337864
0.294212	0.316917	0.256476	0.320275	0.313879	0.273905	0.289255	0.289255	0.303646	0.289575
0.325232	0.362008	0.346178	0.409818	0.338024	0.208666	0.266869	0.204669	0.346338	0.29645
0.350016	0.311481	0.275504	0.308443	0.307163	0.381036	0.322514	0.354493	0.319316	0.370483
0.275024	0.308283	0.284458	0.305724	0.294212	0.305085	0.321075	0.330668	0.301247	0.347458
0.341541	0.329549	0.350656	0.369683	0.349536	0.318516	0.336904	0.334506	0.322194	0.354973
0.302047	0.338983	0.322034	0.34442	0.335945	0.294212	0.319476	0.315318	0.311641	0.303965
0.34378	0.338343	0.330828	0.364567	0.353534	0.319476	0.313879	0.317237	0.330349	0.352095
0.310042	0.082187	0.282699	0.309722	0.257915	0.302047	0.314679	0.347138	0.314199	0.342661
0.319795	0.318996	0.326351	0.349856	0.3433	0.318836	0.314839	0.329709	0.308123	0.3441
0.336585	0.299009	0.272466	0.284938	0.294212	0.369204	0.347618	0.369044	0.322354	0.367125
0.338024	0.320275	0.278542	0.229773	0.319795	0.392389	0.363128	0.387272	0.334506	0.341861
0.32827	0.31196	0.29677	0.29725	0.334186	0.337384	0.346178	0.344899	0.329869	0.333067
0.315958	0.31212	0.236808	0.205628	0.32875	0.358491	0.355293	0.357211	0.320915	0.300448
0.336265	0.293572	0.279981	0.300128	0.269747	0.339783	0.308922	0.348417	0.319795	0.352255
0.298849	0.315958	0.254237	0.261593	0.318996	0.35961	0.355293	0.348897	0.310681	0.326991
0.325392	0.334026	0.309082	0.367125	0.32811	0.242245	0.268628	0.263671	0.31276	0.314998
0.287656	0.294212	0.20243	0.262872	0.321554	0.314839	0.348737	0.322833	0.316597	0.287656
0.313879	0.294532	0.224656	0.25056	0.307483	0.313559	0.34378	0.320595	0.313719	0.329709
0.326191	0.324272	0.310521	0.321394	0.31228	0.348097	0.324592	0.354813	0.308602	0.34314
0.302526	0.303006	0.254397	0.235369	0.332267	0.32795	0.32891	0.333866	0.320275	0.292773
0.333227	0.340902	0.347777	0.393668	0.340742	0.158459	0.28206	0.231532	0.324272	0.292933
0.324592	0.34394	0.346019	0.380237	0.336585	0.24928	0.290374	0.291653	0.317557	0.326671

For Review Only

0.291174	0.300768	0.283978	0.310521	0.315638	0.289575	0.289095	0.303166	0.317717	0.29629
0.304125	0.327151	0.295971	0.302047	0.324272	0.357851	0.336904	0.351295	0.291334	0.344579
0.314519	0.314839	0.249121	0.287976	0.300768	0.295651	0.321714	0.302526	0.313399	0.316597
0.314039	0.332427	0.304285	0.335465	0.326831	0.298049	0.320755	0.319955	0.257755	0.294691
0.292133	0.290374	0.194276	0.258395	0.306044	0.31196	0.334346	0.340742	0.298049	0.318516
0.306524	0.345539	0.29741	0.357691	0.332907	0.246083	0.262712	0.237288	0.303166	0.308762
0.299648	0.293092	0.239527	0.212504	0.346178	0.351455	0.351775	0.362808	0.313399	0.305245
0.287496	0.290854	0.190278	0.230093	0.313719	0.316597	0.3441	0.330029	0.303486	0.29725
0.322194	0.304765	0.253118	0.220179	0.319635	0.358331	0.330189	0.363447	0.31212	0.341861
0.293732	0.293892	0.2504	0.28206	0.301567	0.283658	0.295491	0.314359	0.293572	0.300927
0.322194	0.340582	0.267829	0.291493	0.335146	0.2811	0.326031	0.29645	0.316278	0.325871
0.278702	0.299808	0.245603	0.303965	0.329069	0.286057	0.273745	0.28174	0.306044	0.307803
0.291973	0.320115	0.292773	0.31212	0.321234	0.314199	0.304445	0.322034	0.299808	0.330988
0.289894	0.290694	0.254397	0.274544	0.293092	0.29741	0.297729	0.291334	0.241286	0.303806
0.301727	0.323953	0.310521	0.293572	0.366006	0.339463	0.344739	0.339783	0.322194	0.336585
0.306524	0.295651	0.229933	0.237288	0.322674	0.342021	0.320115	0.348577	0.32827	0.295171
0.324752	0.308922	0.264791	0.295971	0.31244	0.326511	0.319156	0.331788	0.324752	0.320915
0.28142	0.284618	0.217141	0.257595	0.302367	0.300128	0.309722	0.29645	0.298369	0.289894
0.2811	0.350496	0.298849	0.294372	0.350496	0.386153	0.330349	0.345219	0.304925	0.311481
0.284298	0.274065	0.223537	0.241126	0.285577	0.295331	0.308443	0.308283	0.286057	0.298369
0.314839	0.340262	0.304605	0.364567	0.331148	0.255357	0.269268	0.253278	0.31244	0.314519
0.314199	0.315958	0.302526	0.316757	0.342981	0.35865	0.31212	0.332747	0.306844	0.346338
0.277902	0.277742	0.23425	0.257915	0.290374	0.285098	0.295171	0.295651	0.288775	0.287496
0.322194	0.293252	0.240966	0.282699	0.286217	0.372402	0.321874	0.35865	0.308602	0.34362
0.304925	0.308602	0.25056	0.206908	0.337384	0.340102	0.337864	0.348417	0.313399	0.317237
0.324432	0.347618	0.346178	0.400704	0.32779	0.20291	0.26575	0.235849	0.333067	0.303965
0.294212	0.299169	0.2504	0.290694	0.314359	0.303326	0.325871	0.305085	0.304925	0.3126
0.26511	0.279501	0.300288	0.34442	0.302367	0.272785	0.291334	0.287496	0.268628	0.307643
0.317237	0.313879	0.285897	0.293412	0.317557	0.358011	0.318516	0.373681	0.323313	0.338503
0.299648	0.305085	0.261593	0.317877	0.316278	0.307963	0.300288	0.32827	0.305564	0.338983
0.322034	0.286537	0.246402	0.235369	0.342501	0.381996	0.353694	0.378798	0.324432	0.329389
0.336745	0.34394	0.302207	0.3126	0.339783	0.354813	0.332587	0.3748	0.320435	0.348577
0.320595	0.345219	0.337544	0.39047	0.326511	0.220659	0.276623	0.235369	0.330988	0.303646
0.288295	0.285577	0.253598	0.273905	0.29677	0.272306	0.299009	0.293892	0.285897	0.286537
0.338184	0.297729	0.272306	0.284778	0.294851	0.370643	0.348897	0.372242	0.321234	0.371282
0.287176	0.290694	0.215862	0.263032	0.299169	0.306364	0.3126	0.320755	0.285897	0.315478
0.304925	0.323153	0.29645	0.348737	0.326831	0.256316	0.277103	0.270547	0.324113	0.316917
0.298049	0.326671	0.300768	0.317077	0.322674	0.294532	0.302047	0.299169	0.319795	0.327311
0.316278	0.350016	0.315798	0.376559	0.336425	0.256956	0.275664	0.257435	0.314998	0.311001
0.302526	0.317717	0.256476	0.244164	0.324272	0.342981	0.331628	0.348737	0.29693	0.286697
0.303326	0.299968	0.270707	0.313399	0.287496	0.298689	0.291653	0.278542	0.298369	0.322034
0.330189	0.32827	0.353374	0.394148	0.284298	0.226735	0.290214	0.241126	0.311161	0.315478
0.320435	0.282859	0.309882	0.347618	0.295171	0.288455	0.324912	0.318516	0.304285	0.29741
0.316118	0.344739	0.3126	0.376399	0.326831	0.251519	0.270867	0.254557	0.322993	0.303006
0.301407	0.31308	0.263032	0.263831	0.319316	0.358331	0.333547	0.369843	0.333706	0.333547
0.329709	0.348897	0.334506	0.3748	0.351615	0.2819	0.300448	0.294052	0.318676	0.333227
0.214902	0.318356	0.262392	0.288455	0.325552	0.318516	0.330189	0.326511	0.288775	0.317077
0.304925	0.303006	0.261593	0.29757	0.313719	0.308443	0.316917	0.333387	0.319316	0.316757
0.29709	0.290854	0.193476	0.225776	0.32795	0.345059	0.357531	0.351775	0.317397	0.314998
0.300128	0.303326	0.204029	0.209306	0.335945	0.346818	0.349856	0.358011	0.315958	0.31244
0.289095	0.29677	0.272466	0.332747	0.305245	0.251519	0.274704	0.294052	0.288136	0.31244
0.309882	0.300927	0.193796	0.222897	0.330189	0.34314	0.35913	0.355453	0.315958	0.316917
0.308922	0.293412	0.208826	0.244164	0.324113	0.332267	0.345379	0.342341	0.316917	0.317237
0.325871	0.318676	0.319635	0.365526	0.302846	0.236489	0.293572	0.258235	0.293732	0.314359
0.271826	0.284138	0.246242	0.276463	0.285098	0.3126	0.300608	0.278702	0.284138	0.3118

For Review Only

0.317557	0.324432	0.236649	0.231052	0.355932	0.354493	0.363288	0.352255	0.334506	0.318836
0.309402	0.299488	0.289735	0.333387	0.302207	0.25072	0.276943	0.283658	0.28126	0.29661
0.302207	0.287816	0.223057	0.25072	0.31308	0.356732	0.332907	0.34346	0.316597	0.336425
0.302047	0.302526	0.317877	0.362488	0.329709	0.279821	0.322354	0.28078	0.284298	0.32811
0.335305	0.332107	0.337704	0.345539	0.337064	0.322833	0.364247	0.330029	0.336265	0.353054
0.308283	0.311321	0.309402	0.349376	0.309402	0.274864	0.318036	0.295651	0.301567	0.292133
0.32843	0.303486	0.244963	0.251199	0.335785	0.350016	0.348097	0.349696	0.325392	0.327311
0.304605	0.324113	0.304125	0.340582	0.302367	0.286856	0.316278	0.294372	0.285897	0.325392
0.302526	0.334186	0.322674	0.360409	0.311161	0.240006	0.292133	0.225456	0.307643	0.304445
0.278382	0.274065	0.227694	0.255676	0.292453	0.282859	0.292613	0.298369	0.287976	0.285737
0.329069	0.307643	0.330349	0.342661	0.324113	0.264631	0.303646	0.273745	0.321554	0.310361
0.337864	0.282699	0.269747	0.275823	0.283019	0.382955	0.330828	0.373041	0.340102	0.358011
0.292133	0.283978	0.196354	0.220339	0.306044	0.319795	0.342821	0.341062	0.302686	0.300448
0.289415	0.309562	0.288775	0.333387	0.287176	0.244324	0.280461	0.262072	0.277422	0.272146
0.29613	0.278542	0.23505	0.228494	0.306044	0.335305	0.334506	0.333547	0.29693	0.306204
0.305724	0.309882	0.278542	0.325392	0.304605	0.31324	0.308762	0.326991	0.310201	0.329549
0.306684	0.337864	0.308443	0.366485	0.322194	0.251839	0.270547	0.261753	0.322833	0.303965
0.317717	0.290054	0.333387	0.364886	0.231212	0.253598	0.290374	0.273905	0.283339	0.339143
0.262552	0.28206	0.255357	0.278862	0.2819	0.294372	0.294212	0.268468	0.282699	0.302367
0.298689	0.320275	0.248641	0.243364	0.34346	0.357211	0.345859	0.364087	0.338823	0.316278
0.290214	0.307483	0.186441	0.257115	0.299968	0.320755	0.314519	0.311001	0.306204	0.303806
0.317557	0.298529	0.247841	0.217781	0.31212	0.352414	0.324752	0.35993	0.303166	0.339942
0.280461	0.274864	0.240966	0.263192	0.279181	0.291334	0.285737	0.283499	0.274384	0.299169
0.307323	0.338024	0.31308	0.365846	0.330508	0.2189	0.274384	0.227534	0.319316	0.286057
0.310521	0.303965	0.319955	0.37576	0.292933	0.256956	0.290694	0.262872	0.298529	0.324432
0.320435	0.333866	0.248961	0.21954	0.342181	0.357371	0.362488	0.37464	0.330828	0.309882
0.323793	0.348737	0.3118	0.34426	0.342661	0.334346	0.319156	0.357531	0.323793	0.354013
0.309242	0.332747	0.298049	0.360409	0.308123	0.260313	0.26591	0.263831	0.318356	0.316757
0.287976	0.291014	0.253278	0.229933	0.324912	0.341861	0.338024	0.357211	0.289735	0.311481
0.284778	0.326831	0.285257	0.307803	0.332107	0.334826	0.302526	0.314359	0.293572	0.29725
0.29693	0.314839	0.282379	0.29613	0.323313	0.310841	0.355772	0.334506	0.311001	0.297729
0.317877	0.313559	0.268308	0.267029	0.322194	0.356892	0.331788	0.356572	0.321234	0.341222
0.176847	0.3118	0.259674	0.242245	0.334826	0.316278	0.333866	0.341382	0.304445	0.279981
0.336105	0.295011	0.256156	0.264791	0.3118	0.384394	0.338343	0.358331	0.338983	0.364886
0.307803	0.315318	0.305405	0.308922	0.314199	0.346978	0.332427	0.366965	0.32891	0.338663
0.31308	0.339143	0.307803	0.368084	0.320275	0.252638	0.277263	0.26591	0.324432	0.309402
0.307163	0.31324	0.29709	0.307963	0.304765	0.346178	0.321874	0.356732	0.311641	0.347937
0.319316	0.332267	0.247522	0.21874	0.341062	0.356892	0.362008	0.37432	0.330668	0.309082
0.321075	0.313559	0.258075	0.272785	0.311641	0.315158	0.326831	0.342341	0.304285	0.318036
0.302846	0.287976	0.264791	0.306044	0.29645	0.315798	0.3118	0.340422	0.29725	0.347618
0.304285	0.301887	0.212984	0.204029	0.336265	0.34362	0.353534	0.356892	0.316917	0.307483
0.292773	0.278702	0.232651	0.211545	0.321394	0.345059	0.342021	0.351135	0.308123	0.304445
0.31212	0.302526	0.240966	0.098337	0.337544	0.357851	0.360409	0.377199	0.293092	0.317077
0.299328	0.293892	0.186441	0.228494	0.332427	0.342821	0.35913	0.345859	0.315158	0.318516
0.323953	0.350336	0.345539	0.395747	0.329869	0.196674	0.26527	0.215222	0.334186	0.295491
0.306044	0.307643	0.268468	0.279981	0.291174	0.316118	0.329549	0.322034	0.289095	0.326511
0.337704	0.31276	0.295171	0.303326	0.304125	0.305884	0.326991	0.332907	0.320755	0.329709
0.323313	0.34378	0.298849	0.310361	0.346658	0.318996	0.339303	0.346818	0.337864	0.329069
0.317077	0.287656	0.331308	0.363607	0.229293	0.253118	0.289735	0.271027	0.28174	0.338343
0.317877	0.352734	0.32795	0.366485	0.319156	0.217781	0.290374	0.193636	0.324752	0.314519
0.305085	0.327311	0.283499	0.360249	0.31244	0.246083	0.26527	0.245763	0.308283	0.28126
0.323313	0.256796	0.286057	0.294212	0.303806	0.334186	0.337224	0.318516	0.300288	0.322674
0.323793	0.298849	0.248481	0.26495	0.342661	0.371442	0.350975	0.376399	0.318676	0.34362
0.276623	0.362328	0.333387	0.388232	0.329549	0.272466	0.286217	0.279821	0.292293	0.316757
0.307163	0.310681	0.317877	0.392069	0.278062	0.23441	0.276463	0.242885	0.308283	0.320595

For Review Only

0.314039	0.310042	0.236169	0.205149	0.32827	0.357371	0.353854	0.356572	0.319476	0.298689
0.332267	0.342661	0.349696	0.399264	0.331308	0.168052	0.279181	0.23377	0.325712	0.290214
0.274384	0.300608	0.253278	0.294691	0.297729	0.317557	0.29613	0.288136	0.279981	0.300927
0.316278	0.341701	0.318036	0.348257	0.316757	0.329709	0.330349	0.318036	0.318676	0.315638
0.319635	0.28094	0.252638	0.210265	0.334826	0.377518	0.344899	0.379437	0.323153	0.349057
0.319955	0.31308	0.321394	0.355772	0.335465	0.313719	0.320275	0.307963	0.31244	0.339143
0.292613	0.335465	0.323153	0.34362	0.310201	0.311001	0.320595	0.29725	0.302846	0.315958
0.31228	0.303965	0.243204	0.272146	0.334506	0.362008	0.337384	0.358491	0.315318	0.332107
0.286537	0.307004	0.321874	0.358491	0.338983	0.271826	0.29645	0.273905	0.317717	0.301087
0.338823	0.351935	0.358171	0.394627	0.373201	0.277422	0.324752	0.303326	0.325232	0.338343
0.31276	0.321874	0.274384	0.322833	0.313399	0.258235	0.28094	0.287336	0.311001	0.307803
0.332747	0.353694	0.346818	0.409338	0.331948	0.18724	0.260953	0.229773	0.339942	0.305245
0.28206	0.278862	0.242405	0.26575	0.284778	0.279181	0.283658	0.290534	0.28206	0.291334
0	0.314519	0.283339	0.303486	0.346978	0.315798	0.321554	0.3118	0.288935	0.305405
0.314519	0	0.277263	0.308123	0.240486	0.31324	0.320915	0.339463	0.31292	0.350016
0.283339	0.277263	0	0.240326	0.306844	0.313879	0.316757	0.318196	0.302686	0.307004
0.303486	0.308123	0.240326	0	0.341062	0.35881	0.361049	0.369843	0.303646	0.315798
0.346978	0.240486	0.306844	0.341062	0	0.309882	0.310681	0.326511	0.315478	0.335465
0.315798	0.31324	0.313879	0.35881	0.309882	0	0.302207	0.254237	0.305724	0.302686
0.321554	0.320915	0.316757	0.361049	0.310681	0.302207	0	0.293732	0.319635	0.340902
0.3118	0.339463	0.318196	0.369843	0.326511	0.254237	0.293732	0	0.316278	0.286217
0.288935	0.31292	0.302686	0.303646	0.315478	0.305724	0.319635	0.316278	0	0.333227
0.305405	0.350016	0.307004	0.315798	0.335465	0.302686	0.340902	0.286217	0.333227	0
0.299808	0.298209	0.193316	0.245283	0.336904	0.347138	0.350656	0.339463	0.316757	0.310361
0.322354	0.273425	0.253278	0.293092	0.280301	0.363128	0.26543	0.354493	0.302207	0.351135
0.293572	0.319795	0.305245	0.336265	0.284458	0.255836	0.313879	0.286057	0.269108	0.299009
0.313559	0.354013	0.339783	0.395427	0.330828	0.23425	0.272626	0.253118	0.307004	0.300128
0.326671	0.324272	0.283179	0.280141	0.348737	0.340102	0.337224	0.341382	0.34378	0.338184
0.335625	0.366645	0.354813	0.410937	0.337224	0.205948	0.263032	0.213783	0.351775	0.300608
0.316118	0.2819	0.246882	0.2181	0.336265	0.362968	0.346818	0.364087	0.310361	0.306044
0.314998	0.314359	0.293572	0.323313	0.298209	0.330668	0.317237	0.321554	0.301407	0.342981
0.318356	0.295491	0.229933	0.246242	0.32891	0.373681	0.342661	0.370003	0.331628	0.337864
0.31244	0.32811	0.322514	0.382155	0.330988	0.188839	0.254077	0.212504	0.310201	0.305724
0.285098	0.292453	0.248001	0.272466	0.310681	0.289255	0.322034	0.299009	0.310201	0.319795
0.295971	0.321554	0.298689	0.348097	0.286537	0.305724	0.289415	0.309402	0.287496	0.339303
0.282379	0.304125	0.185641	0.228334	0.339143	0.320595	0.332587	0.342981	0.307004	0.313559
0.298529	0.279501	0.318036	0.370323	0.276783	0.260633	0.288136	0.259194	0.293572	0.31308
0.323473	0.367445	0.350815	0.411257	0.346658	0.193636	0.262712	0.208826	0.346818	0.305724
0.307963	0.289095	0.244324	0.252319	0.308922	0.347937	0.331148	0.364407	0.321075	0.350975
0.320275	0.334186	0.248801	0.219699	0.342341	0.358491	0.363927	0.376079	0.330828	0.310681
0.299009	0.295811	0.242085	0.272785	0.299009	0.286217	0.303326	0.316757	0.29693	0.316118
0.339783	0.354333	0.355293	0.399424	0.332427	0.15638	0.279821	0.221298	0.32779	0.279341
0.317557	0.292933	0.293412	0.280301	0.326671	0.369524	0.331308	0.369364	0.318196	0.351775
0.301887	0.288295	0.256636	0.298049	0.310841	0.300288	0.314359	0.302526	0.316917	0.308762
0.300128	0.321874	0.307963	0.340262	0.285577	0.263831	0.314519	0.284458	0.286217	0.299169
0.333547	0.297889	0.272626	0.295011	0.292133	0.378478	0.330189	0.360889	0.311641	0.369524
0.316118	0.295971	0.244324	0.232651	0.320115	0.313559	0.333227	0.345859	0.324912	0.315798
0.353374	0.15686	0.288935	0.308123	0.2819	0.324272	0.321234	0.346658	0.293572	0.347618
0.317397	0.307643	0.277263	0.275664	0.316757	0.28094	0.338663	0.3126	0.298209	0.298689
0.307803	0.317717	0.295011	0.2811	0.326831	0.338823	0.340262	0.341701	0.317397	0.321234
0.333387	0.315638	0.270387	0.276143	0.342021	0.340102	0.337224	0.361529	0.329549	0.34314
0.326031	0.361209	0.347777	0.407579	0.331468	0.196354	0.263351	0.205628	0.350815	0.297729
0.333387	0.313879	0.294691	0.290534	0.29693	0.380237	0.32779	0.388871	0.31244	0.364727
0.333866	0.346338	0.351775	0.399904	0.334346	0.15686	0.284298	0.233611	0.324592	0.292133
0.31308	0.303006	0.242085	0.097697	0.336904	0.35897	0.360729	0.377678	0.293892	0.318516

For Review Only

0.311481	0.283818	0.243364	0.211385	0.348257	0.377518	0.349696	0.39095	0.309562	0.325392
0.303965	0.354493	0.319955	0.354653	0.302207	0.245443	0.303486	0.258395	0.253918	0.28158
0.349696	0.260793	0.275983	0.299648	0.163735	0.383754	0.326031	0.365206	0.303326	0.358171
0.305884	0.308283	0.23473	0.225616	0.350496	0.356252	0.35929	0.376399	0.340582	0.290534

For Review Only

0.29741	0.241765	0.310841	0.35897	0.270387	0.388871	0.290534	0.242725	0.298369	0.369044
0.31212	0.322674	0.336105	0.28158	0.307803	0.318036	0.287816	0.336745	0.326191	0.321554
0.23505	0.283019	0.331948	0.403422	0.271186	0.417973	0.195235	0.315318	0.246083	0.388391
0.258395	0.276783	0.332587	0.34426	0.282859	0.382795	0.15558	0.293252	0.269907	0.371442
0.346019	0.29677	0.334826	0.303965	0.333866	0.341861	0.355772	0.26511	0.333866	0.357531
0.268148	0.260793	0.322514	0.3756	0.269907	0.395907	0.240966	0.278382	0.255516	0.376559
0.306844	0.254397	0.317877	0.361848	0.303326	0.404061	0.308123	0.259354	0.288935	0.362648
0.323953	0.329389	0.339623	0.262392	0.316597	0.294212	0.32811	0.310201	0.345859	0.308123
0.317877	0.248481	0.275823	0.360729	0.287496	0.384554	0.315638	0.2181	0.294851	0.363128
0.358491	0.366326	0.356252	0.252798	0.333866	0.298689	0.357531	0.333067	0.368244	0.326031
0.337864	0.307004	0.273105	0.29645	0.337224	0.285737	0.351615	0.323473	0.362008	0.247841
0.231372	0.279021	0.333227	0.394148	0.268948	0.411896	0.137832	0.313879	0.240006	0.385993
0.316118	0.275823	0.332907	0.313399	0.286377	0.326671	0.313879	0.267029	0.311641	0.330349
0.305564	0.319795	0.241605	0.268948	0.332107	0.277902	0.325392	0.29725	0.350016	0.263831
0.278382	0.258714	0.268788	0.36009	0.295171	0.371122	0.271506	0.244643	0.260473	0.350336
0.286697	0.291813	0.308443	0.321554	0.319955	0.32859	0.274544	0.309882	0.294851	0.327151
0.34378	0.261912	0.32843	0.347618	0.318036	0.392709	0.318196	0.237128	0.308283	0.373361
0.295811	0.295811	0.310361	0.333067	0.29677	0.363447	0.298209	0.269268	0.306844	0.346178
0.276463	0.254397	0.338823	0.402143	0.284298	0.416374	0.226575	0.309402	0.260153	0.40582
0.350496	0.352894	0.351615	0.283339	0.341701	0.32843	0.363128	0.303006	0.365046	0.351455
0.339942	0.292773	0.341062	0.292773	0.316118	0.329389	0.340422	0.285257	0.333067	0.325871
0.325871	0.252958	0.350336	0.350656	0.31324	0.381356	0.313879	0.254077	0.287016	0.365846
0.392709	0.367125	0.275024	0.255996	0.37528	0.206748	0.394468	0.357371	0.396067	0.189319
0.336105	0.324432	0.295651	0.204669	0.318676	0.232012	0.338823	0.298689	0.345059	0.23473
0.294532	0.247522	0.303166	0.381836	0.271506	0.404541	0.263991	0.222098	0.260793	0.381676
0.071954	0.314199	0.327151	0.386473	0.29629	0.397186	0.221778	0.320115	0.278062	0.381036
0.229933	0.278862	0.324272	0.35945	0.267988	0.37512	0.183722	0.291334	0.248481	0.372402
0.244484	0.261593	0.271027	0.297729	0.275823	0.314679	0.25056	0.269907	0.256956	0.295971
0.321714	0.299808	0.304925	0.295331	0.273105	0.325072	0.305405	0.25072	0.311481	0.329869
0.400704	0.401983	0.357371	0.175408	0.368724	0.237608	0.410457	0.358011	0.418292	0.270227
0.257595	0.258235	0.323633	0.385673	0.267349	0.396226	0.21842	0.29661	0.238567	0.389511
0.29677	0.268948	0.319635	0.397825	0.291334	0.411257	0.258874	0.299488	0.259994	0.397186
0.311481	0.25024	0.322993	0.389191	0.305245	0.411896	0.311001	0.237128	0.280141	0.371442
0.337864	0.322514	0.323633	0.224816	0.322514	0.230413	0.351615	0.305085	0.342661	0.255836
0.291174	0.336105	0.261273	0.330988	0.339143	0.321075	0.32891	0.319635	0.339303	0.310841
0.263671	0.279021	0.297729	0.291813	0.285737	0.313719	0.258555	0.305405	0.299648	0.303166
0.311321	0.271027	0.305245	0.303486	0.319476	0.346978	0.330349	0.275344	0.306044	0.325392
0.304125	0.302047	0.277742	0.310201	0.330029	0.353214	0.303166	0.294691	0.322993	0.316278
0.377359	0.383914	0.255197	0.195715	0.356572	0.134474	0.399744	0.349376	0.403262	0.133195
0.339463	0.347937	0.212184	0.300608	0.363288	0.277422	0.344899	0.330349	0.365686	0.244963
0.384714	0.399744	0.341861	0.193796	0.367285	0.237448	0.398945	0.354333	0.407259	0.269428
0.340262	0.348737	0.321234	0.280461	0.304925	0.301407	0.341701	0.304285	0.349536	0.316757
0.350815	0.349057	0.335465	0.252319	0.326351	0.309402	0.352095	0.305564	0.366645	0.316757
0.352574	0.305085	0.317557	0.309402	0.334506	0.367605	0.349856	0.271506	0.342181	0.348257
0.35945	0.361369	0.342181	0.271186	0.333387	0.32875	0.35945	0.310521	0.379597	0.330828
0.189319	0.278702	0.32763	0.392869	0.28174	0.407899	0.179885	0.310521	0.256316	0.379437
0.168052	0.293892	0.314998	0.395107	0.290374	0.40662	0.228973	0.300768	0.266549	0.377838
0.23409	0.302367	0.278222	0.307163	0.324272	0.315798	0.282699	0.301567	0.305884	0.288455
0.363767	0.304285	0.321714	0.307483	0.325232	0.361848	0.346338	0.283179	0.341222	0.366805
0.286217	0.23457	0.318036	0.370323	0.303166	0.399424	0.271346	0.259514	0.24976	0.370963

For Review Only

0.150144	0.277263	0.307163	0.378798	0.294851	0.400704	0.21938	0.313879	0.261273	0.371442
0.300927	0.294212	0.329069	0.355133	0.298049	0.34378	0.266549	0.304285	0.275024	0.362968
0.300128	0.211385	0.315318	0.400224	0.271027	0.432044	0.286537	0.155421	0.267988	0.400224
0.311481	0.25024	0.323153	0.388072	0.305724	0.411257	0.310681	0.237288	0.279661	0.370803
0.26511	0.273425	0.317877	0.386952	0.284458	0.39111	0.224177	0.299169	0.254397	0.382635
0.337384	0.255357	0.333067	0.346338	0.319156	0.382155	0.317717	0.263831	0.29709	0.35865
0.276623	0.253918	0.338184	0.401823	0.283499	0.416693	0.226415	0.308602	0.259834	0.40582
0.175887	0.298849	0.318676	0.391429	0.285737	0.405181	0.202271	0.310681	0.262712	0.395267
0.113687	0.301247	0.324752	0.380397	0.294372	0.400544	0.165814	0.29661	0.26559	0.386633
0.377838	0.379597	0.316437	0.007035	0.350975	0.190278	0.387912	0.335945	0.389191	0.214263
0.334346	0.283339	0.300448	0.246562	0.301407	0.307483	0.338503	0.289735	0.309402	0.287336
0.253598	0.253278	0.282859	0.305884	0.270707	0.322674	0.240326	0.268148	0.253758	0.303166
0.242885	0.282219	0.318516	0.382795	0.279501	0.399264	0.18676	0.326991	0.25072	0.382475
0.261113	0.303166	0.317237	0.381996	0.290534	0.399264	0.209466	0.327311	0.259354	0.385833
0.299808	0.210585	0.314679	0.400064	0.270387	0.431724	0.285737	0.153822	0.267669	0.399264
0.320915	0.299009	0.305085	0.294532	0.272306	0.324272	0.304445	0.2504	0.311001	0.329389
0.257435	0.270547	0.284938	0.329709	0.284938	0.341222	0.255037	0.274704	0.273905	0.324912
0.326031	0.307323	0.291813	0.285577	0.330029	0.31196	0.336265	0.291493	0.346338	0.304125
0.28094	0.295011	0.261912	0.340102	0.314199	0.342181	0.293732	0.311481	0.311001	0.317077
0.256636	0.264311	0.317717	0.411896	0.309882	0.429325	0.280141	0.287816	0.244324	0.403422
0.300608	0.221938	0.319156	0.400224	0.290214	0.425488	0.252319	0.248641	0.236808	0.410297
0.29693	0.23425	0.323153	0.389511	0.29709	0.42261	0.29661	0.212664	0.278062	0.385673
0.257755	0.279021	0.295331	0.350496	0.272306	0.364727	0.214423	0.298369	0.248001	0.355772
0.342341	0.339463	0.244484	0.284458	0.34346	0.261433	0.364567	0.32811	0.356092	0.255037
0.319635	0.277902	0.299328	0.329069	0.292133	0.349696	0.3126	0.246402	0.31292	0.345219
0.294691	0.31228	0.31308	0.349057	0.3126	0.370963	0.297889	0.307483	0.286537	0.37576
0.35961	0.361049	0.341541	0.271027	0.332747	0.32795	0.35945	0.310361	0.380556	0.329869
0.264471	0.283019	0.331148	0.364727	0.253438	0.387272	0.212024	0.303326	0.260313	0.377678
0.357531	0.329549	0.357851	0.300128	0.35993	0.348417	0.355612	0.322993	0.348417	0.337544
0.382315	0.385833	0.274225	0.194116	0.357531	0.101535	0.394627	0.362328	0.398945	0.147586
0.052446	0.314039	0.329709	0.386792	0.314679	0.401503	0.231532	0.326351	0.285257	0.37576
0.307803	0.21906	0.326351	0.384714	0.302367	0.400704	0.252638	0.277103	0.233131	0.395427
0.322034	0.258874	0.324592	0.332587	0.309882	0.353534	0.317717	0.244324	0.307483	0.34426
0.300448	0.310361	0.290694	0.245603	0.306684	0.277582	0.298529	0.289255	0.303166	0.279501
0.391909	0.399584	0.292773	0.185641	0.372881	0.074033	0.407739	0.367445	0.404381	0.146946
0.299808	0.209306	0.313399	0.398625	0.269587	0.430445	0.285737	0.153502	0.266709	0.398305
0.292933	0.280461	0.305564	0.332907	0.295491	0.35961	0.324592	0.259194	0.305884	0.336745
0.35977	0.361689	0.341701	0.271346	0.333067	0.32811	0.35977	0.310841	0.380556	0.330029
0.344739	0.323793	0.321394	0.259994	0.310042	0.291493	0.332747	0.295971	0.336585	0.307004
0.370483	0.308123	0.382635	0.287496	0.330988	0.338983	0.350016	0.305245	0.349856	0.344899
0.319635	0.285417	0.320275	0.330349	0.310201	0.345539	0.299009	0.324752	0.31212	0.318836
0.348257	0.346498	0.336425	0.256476	0.332907	0.306684	0.351295	0.335945	0.366166	0.301887
0.300927	0.221618	0.322514	0.4063	0.307323	0.429005	0.301887	0.212184	0.268148	0.404861
0.289894	0.248641	0.340902	0.4063	0.301407	0.422929	0.243364	0.310361	0.251999	0.415734
0.292613	0.295011	0.309402	0.338343	0.322514	0.371602	0.287016	0.309562	0.300608	0.35881
0.243364	0.28206	0.319476	0.381836	0.279821	0.399264	0.1882	0.327311	0.251519	0.381996
0.301887	0.223217	0.332267	0.348737	0.303806	0.37544	0.31292	0.298209	0.287016	0.351775
0.259514	0.277103	0.324912	0.389191	0.286217	0.40598	0.239527	0.274544	0.244963	0.39079
0.342981	0.345859	0.303166	0.217461	0.335785	0.214263	0.364407	0.326671	0.366006	0.230413
0.175088	0.300927	0.316437	0.334026	0.300768	0.356572	0.222258	0.309402	0.274384	0.339463
0.180365	0.294532	0.301567	0.3433	0.300768	0.366485	0.238567	0.289735	0.273745	0.348417
0.318836	0.3118	0.289255	0.357052	0.344899	0.369683	0.318836	0.32747	0.311001	0.348577
0.251199	0.268148	0.299169	0.352574	0.261113	0.368084	0.208986	0.293732	0.259514	0.355772
0.384874	0.392389	0.267189	0.192677	0.36009	0.14039	0.402462	0.360889	0.410777	0.139111
0.366326	0.360409	0.314839	0.143268	0.34346	0.238248	0.368084	0.349057	0.385673	0.261113

For Review Only

0.307483	0.289575	0.301407	0.277422	0.302846	0.279661	0.293092	0.290374	0.301087	0.294851
0.306524	0.313719	0.26543	0.364886	0.331308	0.387272	0.314199	0.313559	0.293732	0.362488
0.184042	0.321874	0.28078	0.338503	0.318516	0.333067	0.272945	0.313879	0.310361	0.320755
0.316597	0.333067	0.298529	0.303166	0.325232	0.324272	0.304285	0.315478	0.336904	0.320595
0.179725	0.268948	0.309882	0.350656	0.272146	0.367925	0.240166	0.295171	0.285257	0.351615
0.32891	0.349217	0.310521	0.224336	0.341062	0.217461	0.358011	0.325552	0.366326	0.225296
0.21954	0.275024	0.321714	0.383275	0.277263	0.403902	0.18724	0.310681	0.238248	0.383115
0.167733	0.299648	0.316278	0.34442	0.287176	0.365846	0.238567	0.29677	0.244963	0.348097
0.269587	0.237928	0.32795	0.379277	0.276943	0.397346	0.178286	0.285098	0.224017	0.385353
0.267189	0.287336	0.293412	0.29645	0.286217	0.314679	0.269907	0.278542	0.273425	0.304925
0.259674	0.32843	0.306684	0.289735	0.313399	0.319316	0.299488	0.32811	0.334346	0.308922
0.260473	0.297889	0.300927	0.26495	0.308922	0.292773	0.288775	0.31244	0.316597	0.286217
0.315158	0.282859	0.280301	0.325552	0.288935	0.34426	0.325072	0.269268	0.299328	0.322674
0.251519	0.274384	0.275184	0.305724	0.289415	0.324272	0.261593	0.269268	0.285098	0.302367
0.293732	0.303326	0.314039	0.324752	0.310521	0.35977	0.276943	0.319795	0.291014	0.3433
0.241445	0.274544	0.310681	0.367445	0.266709	0.377199	0.211225	0.299009	0.240006	0.370163
0.290534	0.288935	0.329229	0.318196	0.29693	0.341382	0.278222	0.310042	0.303326	0.335305
0.183403	0.279341	0.284938	0.321554	0.300288	0.333706	0.256156	0.282539	0.277422	0.314519
0.279341	0.308922	0.291174	0.339463	0.323473	0.392869	0.309082	0.3126	0.29677	0.364247
0.209306	0.268948	0.286377	0.325072	0.279181	0.340102	0.23425	0.274704	0.255197	0.317877
0.337224	0.353534	0.315798	0.215062	0.342661	0.21842	0.360729	0.324912	0.371122	0.238727
0.316278	0.268948	0.298049	0.346658	0.308283	0.364886	0.29661	0.277582	0.303166	0.346818
0.232171	0.266709	0.277263	0.299169	0.275823	0.317557	0.246562	0.273425	0.261593	0.301567
0.301087	0.193476	0.316118	0.383435	0.303965	0.402143	0.258714	0.270387	0.225136	0.385034
0.256476	0.279021	0.330668	0.362968	0.275983	0.377359	0.240646	0.293892	0.227854	0.353694
0.379437	0.39015	0.286537	0.191717	0.362328	0.11001	0.400064	0.355772	0.4055	0.15622
0.209466	0.323953	0.293572	0.324912	0.322514	0.331948	0.285098	0.321394	0.307323	0.317077
0.320755	0.307483	0.236329	0.299968	0.336585	0.290534	0.342501	0.29629	0.341222	0.261433
0.306364	0.255037	0.324432	0.373841	0.302207	0.391749	0.29661	0.270387	0.274225	0.384554
0.293732	0.231532	0.302207	0.323633	0.289095	0.340422	0.29661	0.263831	0.257755	0.338343
0.255516	0.255197	0.356252	0.403102	0.287656	0.42261	0.152382	0.302367	0.098337	0.401503
0.29693	0.274544	0.322993	0.374001	0.313399	0.396067	0.287336	0.294691	0.308123	0.384554
0.381036	0.386952	0.300448	0.199073	0.358491	0.142309	0.386792	0.353214	0.389511	0.190598
0.26495	0.282219	0.272306	0.28126	0.295011	0.305564	0.269268	0.278702	0.273905	0.295811
0.301087	0.221458	0.322833	0.409818	0.308602	0.431724	0.302367	0.211545	0.268628	0.40694
0.176847	0.273905	0.285098	0.338024	0.29677	0.351135	0.256316	0.26575	0.268788	0.334506
0.335625	0.322034	0.322354	0.226255	0.321394	0.232012	0.349696	0.305245	0.342021	0.256316
0.320115	0.301247	0.319795	0.294851	0.314199	0.295491	0.318676	0.28206	0.317877	0.290694
0.351135	0.357211	0.314519	0.210265	0.345859	0.213144	0.375919	0.334026	0.372721	0.236009
0.244484	0.264151	0.29661	0.372402	0.288295	0.386952	0.221298	0.287656	0.259674	0.379277
0.247202	0.321714	0.2811	0.308283	0.342501	0.297889	0.308762	0.31308	0.329229	0.278062
0.383435	0.377518	0.286697	0.23393	0.371282	0.166613	0.400544	0.348417	0.405021	0.190598
0.300128	0.324432	0.232651	0.326191	0.344899	0.314998	0.323153	0.326191	0.349696	0.289255
0.347777	0.356732	0.305724	0.199712	0.342341	0.20355	0.371282	0.32843	0.370803	0.232012
0.275664	0.287496	0.272626	0.3756	0.303326	0.393508	0.278382	0.320115	0.26623	0.369524
0.365686	0.365046	0.327311	0.217301	0.347298	0.254877	0.368884	0.334666	0.377199	0.277902
0.226255	0.299169	0.302686	0.323793	0.321075	0.345699	0.300608	0.302047	0.318676	0.320115
0.276943	0.269907	0.32875	0.326191	0.299169	0.367605	0.283499	0.289255	0.286057	0.334666
0.073233	0.299808	0.322674	0.39031	0.307803	0.408698	0.210106	0.306044	0.272785	0.381356
0.161976	0.31276	0.31292	0.385513	0.292613	0.400064	0.216821	0.299968	0.256316	0.380237
0.320755	0.302207	0.302047	0.225136	0.320275	0.264631	0.32811	0.303965	0.324912	0.261433
0.115286	0.299968	0.316437	0.385193	0.289894	0.402303	0.211705	0.305085	0.268468	0.37576
0.114647	0.301247	0.319955	0.367125	0.302367	0.384074	0.236649	0.305245	0.290374	0.362968
0.327151	0.354653	0.291653	0.216661	0.351455	0.252958	0.376399	0.311321	0.3748	0.240326
0.283818	0.270227	0.292613	0.319795	0.295651	0.331628	0.292773	0.285417	0.277422	0.300927

For Review Only

0.213303	0.31324	0.326351	0.373681	0.303486	0.396067	0.225296	0.339303	0.26591	0.370803
0.314998	0.31308	0.2819	0.189159	0.311161	0.253278	0.314679	0.292933	0.321714	0.256156
0.207387	0.247681	0.318836	0.395427	0.305405	0.408219	0.24976	0.299328	0.225776	0.373201
0.331468	0.333547	0.262232	0.288455	0.361049	0.293572	0.356252	0.333067	0.382315	0.259194
0.338663	0.350656	0.339623	0.308283	0.335625	0.352414	0.349856	0.320755	0.364567	0.342021
0.334826	0.329229	0.276623	0.300288	0.331308	0.292293	0.349376	0.315958	0.333866	0.279821
0.253118	0.28158	0.336425	0.39015	0.291653	0.404381	0.233131	0.287496	0.261593	0.377199
0.315158	0.317717	0.272306	0.267029	0.340902	0.302846	0.340422	0.284458	0.331948	0.300608
0.351295	0.356572	0.216981	0.261912	0.346818	0.209946	0.368404	0.332107	0.378158	0.205788
0.223217	0.272945	0.278222	0.299488	0.273425	0.318836	0.2496	0.264631	0.268788	0.307803
0.349057	0.337544	0.292133	0.286697	0.339623	0.260793	0.352095	0.32747	0.360569	0.239687
0.306844	0.193796	0.346498	0.399264	0.29629	0.42229	0.270227	0.271986	0.221298	0.410457
0.135433	0.286697	0.305085	0.36009	0.290214	0.37448	0.208347	0.277902	0.26495	0.357531
0.322514	0.32811	0.204669	0.243364	0.315478	0.247522	0.332747	0.300608	0.341062	0.233131
0.233611	0.270547	0.306204	0.372402	0.287496	0.385513	0.191238	0.294052	0.251839	0.352255
0.295651	0.290854	0.317877	0.319156	0.31244	0.339942	0.305245	0.302367	0.319476	0.32875
0.349536	0.355453	0.307963	0.20259	0.3433	0.204349	0.35977	0.332587	0.357531	0.235209
0.356412	0.335305	0.251519	0.291493	0.35897	0.26495	0.369683	0.340262	0.380077	0.223857
0.282699	0.271986	0.285257	0.301407	0.292453	0.311001	0.287336	0.294372	0.273265	0.289575
0.267349	0.272466	0.333866	0.383914	0.295971	0.401503	0.237128	0.299968	0.253598	0.388711
0.185961	0.278702	0.286697	0.333227	0.290534	0.348897	0.244324	0.293092	0.253758	0.32795
0.26543	0.236808	0.324432	0.371762	0.275504	0.388711	0.181964	0.28078	0.21954	0.377838
0.251839	0.255197	0.271186	0.29741	0.273905	0.311161	0.251999	0.260473	0.264151	0.293252
0.350656	0.355293	0.284778	0.208666	0.335945	0.157819	0.374001	0.327151	0.377838	0.18772
0.349856	0.32859	0.255357	0.279661	0.346818	0.256476	0.382635	0.329229	0.372082	0.260953
0.261433	0.302367	0.316917	0.381516	0.291174	0.399424	0.210265	0.32811	0.259994	0.385513
0.347298	0.291813	0.323473	0.306684	0.292293	0.3433	0.331148	0.254237	0.319635	0.349217
0.330029	0.316278	0.321874	0.228654	0.324113	0.232331	0.353214	0.302047	0.355612	0.256956
0.251519	0.276303	0.299968	0.360569	0.294052	0.379117	0.135114	0.301407	0.256796	0.361529
0.289415	0.293252	0.295331	0.301727	0.309882	0.336904	0.309722	0.290854	0.304925	0.318836
0.26591	0.292773	0.289415	0.32779	0.318676	0.361209	0.292133	0.273105	0.279501	0.348897
0.299009	0.26511	0.297889	0.381996	0.301087	0.402622	0.286217	0.251999	0.256796	0.376079
0.267349	0.285257	0.311001	0.348577	0.2819	0.367285	0.273265	0.304765	0.286537	0.342661
0.275344	0.257755	0.32859	0.402782	0.318676	0.42117	0.273265	0.288775	0.243204	0.396706
0.305724	0.252478	0.318676	0.366166	0.290214	0.385673	0.288775	0.270547	0.283499	0.381036
0.346338	0.356252	0.305564	0.208027	0.34362	0.20355	0.360249	0.32827	0.364407	0.240006
0.305564	0.264471	0.339143	0.358171	0.31212	0.392389	0.302047	0.274864	0.283019	0.364727
0.260793	0.302367	0.315798	0.380716	0.290374	0.398625	0.209306	0.32763	0.259034	0.385034
0.241605	0.295331	0.289575	0.356412	0.292613	0.372562	0.257755	0.298529	0.302047	0.35865
0.303646	0.221938	0.310521	0.341861	0.288136	0.380876	0.299328	0.254237	0.270067	0.361049
0.174928	0.307163	0.31292	0.386792	0.289735	0.399584	0.215702	0.303646	0.256956	0.379757
0.262872	0.264151	0.317077	0.367125	0.284618	0.386792	0.23441	0.303006	0.262712	0.362168
0.23409	0.284618	0.330988	0.401823	0.271506	0.417173	0.194116	0.315158	0.246242	0.388072
0.081548	0.295491	0.322993	0.388871	0.310361	0.407419	0.21954	0.307803	0.273265	0.381036
0.381836	0.393508	0.287656	0.185641	0.362168	0.123121	0.404221	0.360729	0.409658	0.152223
0.269907	0.299968	0.26559	0.354013	0.307323	0.360569	0.289415	0.278222	0.2819	0.335785
0.297729	0.308123	0.317717	0.321874	0.310841	0.329389	0.301087	0.307803	0.31292	0.339303
0.335146	0.284618	0.345379	0.324592	0.293252	0.358171	0.300128	0.310521	0.300128	0.354653
0.356252	0.333547	0.2504	0.292293	0.35913	0.26559	0.368884	0.339783	0.378478	0.222418
0.371442	0.360889	0.307323	0.221778	0.349856	0.157659	0.39031	0.346019	0.392229	0.176047
0.337864	0.32747	0.304125	0.185641	0.319476	0.217621	0.341222	0.313399	0.345219	0.233451
0.322354	0.272626	0.322993	0.306524	0.298849	0.348097	0.23505	0.280141	0.29741	0.341382
0.262552	0.270867	0.34394	0.382955	0.303486	0.4055	0.240486	0.300768	0.095299	0.39079
0.367285	0.350336	0.305564	0.166773	0.336265	0.260313	0.373521	0.314519	0.379277	0.271986
0.358011	0.338663	0.260473	0.24928	0.353694	0.228494	0.392229	0.333067	0.380397	0.227534

For Review Only

0.243204	0.28206	0.317557	0.381036	0.279021	0.398785	0.18708	0.325712	0.25056	0.381356
0.387752	0.389511	0.275184	0.201951	0.362968	0.137832	0.401183	0.364886	0.408219	0.138951
0.308443	0.290054	0.309882	0.298369	0.316757	0.315318	0.319156	0.303806	0.283179	0.288295
0.323313	0.333067	0.291174	0.291014	0.338823	0.32779	0.34442	0.292613	0.335146	0.335146
0.255676	0.25072	0.345379	0.411257	0.303486	0.426767	0.239047	0.304445	0.144068	0.399744
0.338663	0.317237	0.282859	0.321714	0.347458	0.321234	0.350496	0.322993	0.351935	0.305724
0.325552	0.317077	0.297729	0.280301	0.317877	0.32843	0.340902	0.177007	0.32763	0.324113
0.129357	0.260633	0.330349	0.39095	0.286537	0.414775	0.241765	0.293092	0.260633	0.393508
0.318196	0.354813	0.288136	0.285417	0.358011	0.264471	0.35961	0.336585	0.349217	0.252798
0.384074	0.39015	0.347618	0.200352	0.360409	0.269587	0.39047	0.351135	0.394787	0.284778
0.29693	0.326831	0.305245	0.240006	0.322833	0.253598	0.314839	0.315958	0.336585	0.258395
0.393188	0.394787	0.288136	0.194116	0.361689	0.101215	0.4055	0.361848	0.40678	0.139271
0.24992	0.261113	0.273265	0.289415	0.272466	0.310521	0.248001	0.266549	0.263991	0.294532
0.299808	0.322354	0.293572	0.313559	0.326671	0.335625	0.316118	0.314998	0.318356	0.31244
0.298209	0.273425	0.319795	0.354013	0.324272	0.366645	0.2819	0.314359	0.295491	0.32811
0.193316	0.253278	0.305245	0.339783	0.283179	0.354813	0.246882	0.293572	0.229933	0.322514
0.245283	0.293092	0.336265	0.395427	0.280141	0.410937	0.2181	0.323313	0.246242	0.382155
0.336904	0.280301	0.284458	0.330828	0.348737	0.337224	0.336265	0.298209	0.32891	0.330988
0.347138	0.363128	0.255836	0.23425	0.340102	0.205948	0.362968	0.330668	0.373681	0.188839
0.350656	0.26543	0.313879	0.272626	0.337224	0.263032	0.346818	0.317237	0.342661	0.254077
0.339463	0.354493	0.286057	0.253118	0.341382	0.213783	0.364087	0.321554	0.370003	0.212504
0.316757	0.302207	0.269108	0.307004	0.34378	0.351775	0.310361	0.301407	0.331628	0.310201
0.310361	0.351135	0.299009	0.300128	0.338184	0.300608	0.306044	0.342981	0.337864	0.305724
0	0.301887	0.319795	0.378798	0.305724	0.395747	0.225935	0.31324	0.274065	0.372562
0.301887	0	0.340582	0.378957	0.286537	0.401343	0.269907	0.25072	0.240966	0.377678
0.319795	0.340582	0	0.316278	0.335465	0.300448	0.346978	0.301567	0.352255	0.264311
0.378798	0.378957	0.316278	0	0.350815	0.191078	0.387912	0.335465	0.389031	0.213943
0.305724	0.286537	0.335465	0.350815	0	0.367445	0.272306	0.280461	0.29677	0.362808
0.395747	0.401343	0.300448	0.191078	0.367445	0	0.409978	0.371762	0.409018	0.14103
0.225935	0.269907	0.346978	0.387912	0.272306	0.409978	0	0.313559	0.233451	0.38999
0.31324	0.25072	0.301567	0.335465	0.280461	0.371762	0.313559	0	0.287336	0.356412
0.274065	0.240966	0.352255	0.389031	0.29677	0.409018	0.233451	0.287336	0	0.391749
0.372562	0.377678	0.264311	0.213943	0.362808	0.14103	0.38999	0.356412	0.391749	0
0.192677	0.308443	0.307803	0.318036	0.314039	0.334666	0.257595	0.306524	0.290694	0.305564
0.318196	0.317717	0.294212	0.29629	0.324912	0.301407	0.329709	0.311321	0.356092	0.311001
0.161017	0.29677	0.31324	0.353374	0.302367	0.368244	0.243844	0.319635	0.274704	0.347138
0.342181	0.333547	0.243684	0.276623	0.356412	0.269747	0.3756	0.324752	0.360729	0.257595
0.392069	0.397985	0.300608	0.209466	0.368564	0.10889	0.410617	0.366166	0.411577	0.131276
0.2811	0.222098	0.331308	0.395587	0.280141	0.40582	0.271027	0.257115	0.228654	0.379597
0.261912	0.303006	0.317397	0.382635	0.291653	0.400384	0.210745	0.32827	0.260473	0.386633
0.262232	0.278062	0.29629	0.292293	0.285098	0.31228	0.258235	0.304765	0.298849	0.303006
0.39079	0.395107	0.25072	0.199392	0.367125	0.137992	0.407259	0.35897	0.416853	0.136553
0.284138	0.244803	0.332907	0.396866	0.299808	0.412536	0.269268	0.295331	0.256636	0.397825
0.278062	0.279981	0.326511	0.313559	0.299648	0.331628	0.290054	0.274065	0.292773	0.321234
0.326031	0.347777	0.128718	0.316278	0.334986	0.292133	0.341541	0.313399	0.353214	0.259194
0.305724	0.196194	0.319795	0.414934	0.299328	0.432523	0.284938	0.198593	0.255996	0.401823
0.262392	0.291653	0.314998	0.336745	0.292773	0.362808	0.23473	0.302207	0.282699	0.342501
0.318036	0.26575	0.321714	0.352574	0.3126	0.361049	0.287016	0.322993	0.322034	0.32827
0.246882	0.355453	0.268788	0.316437	0.330668	0.298529	0.284298	0.319316	0.320115	0.274544
0.278862	0.315798	0.310681	0.344899	0.329549	0.369524	0.286697	0.323953	0.305405	0.358491
0.28062	0.316278	0.336425	0.338343	0.300288	0.358331	0.282699	0.326351	0.320595	0.361529
0.392389	0.399264	0.295171	0.191557	0.369524	0.052446	0.40662	0.368084	0.40678	0.142629
0.297889	0.223537	0.306044	0.413176	0.297729	0.432203	0.26559	0.271666	0.243844	0.417333
0.391429	0.389671	0.263032	0.195875	0.366166	0.135433	0.402782	0.35897	0.411417	0.139111
0.233451	0.284618	0.332107	0.402942	0.271346	0.417333	0.195715	0.315318	0.246083	0.387432

For Review Only

0.239207	0.267029	0.347458	0.409018	0.28206	0.431244	0.129037	0.32763	0.246882	0.401663
0.3441	0.368244	0.180684	0.278542	0.358491	0.255197	0.355612	0.332427	0.377838	0.220499
0.304605	0.177486	0.326031	0.403902	0.303965	0.416853	0.279981	0.270547	0.260633	0.401183
0.244484	0.295171	0.34378	0.39047	0.271027	0.405341	0.201311	0.315958	0.257915	0.39047

For Review Only

0.319795	0.311481	0.29725	0.347937	0.386313	0.248321	0.322194	0.301727	0.377518	0.261593
0.309242	0.292133	0.316437	0.337704	0.323313	0.32843	0.280301	0.301727	0.317877	0.337864
0.257755	0.341222	0.253758	0.369843	0.416853	0.236968	0.216821	0.274225	0.404861	0.28094
0.279661	0.310201	0.275664	0.334506	0.379597	0.272785	0.264471	0.252159	0.37496	0.288935
0.32779	0.320275	0.34426	0.341861	0.346978	0.310201	0.35945	0.316118	0.349057	0.329229
0.291493	0.306204	0.2819	0.338663	0.39095	0.248001	0.275823	0.291653	0.400224	0.274384
0.332267	0.334826	0.313719	0.34362	0.395267	0.258075	0.345539	0.322034	0.39111	0.275504
0.31244	0.271506	0.308283	0.339303	0.301407	0.334506	0.341541	0.278222	0.287976	0.345859
0.329709	0.309882	0.330828	0.314998	0.381996	0.250879	0.32795	0.303166	0.3756	0.270707
0.331948	0.316597	0.351615	0.339623	0.294851	0.362328	0.377199	0.299648	0.298529	0.369524
0.283019	0.306524	0.335945	0.267669	0.283339	0.323633	0.357211	0.303806	0.242245	0.358491
0.255357	0.334826	0.248481	0.369843	0.410297	0.247522	0.207547	0.26591	0.401343	0.270227
0.314519	0.317077	0.300128	0.338024	0.326671	0.261753	0.338823	0.307643	0.32891	0.313879
0.291174	0.247841	0.305884	0.303646	0.278382	0.329229	0.315158	0.246242	0.283019	0.320115
0.302207	0.308602	0.269428	0.319156	0.37576	0.225456	0.282699	0.300608	0.366326	0.261273
0.28206	0.313879	0.282699	0.32827	0.325232	0.286217	0.291014	0.230732	0.308922	0.324912
0.366805	0.341541	0.349376	0.338184	0.395747	0.263671	0.358171	0.352255	0.385034	0.31276
0.309722	0.303326	0.302526	0.345219	0.358011	0.289575	0.320435	0.299009	0.353214	0.301407
0.287656	0.344579	0.28142	0.364247	0.417813	0.264631	0.25056	0.276783	0.418932	0.272146
0.345859	0.31292	0.337224	0.318196	0.32843	0.348097	0.35897	0.314839	0.337064	0.37448
0.347618	0.320595	0.321874	0.324432	0.330828	0.299968	0.365526	0.34346	0.315158	0.333706
0.340422	0.352414	0.326191	0.347138	0.383754	0.253278	0.350975	0.348897	0.370803	0.304765
0.338184	0.294691	0.369044	0.241286	0.217781	0.393828	0.389671	0.334986	0.204029	0.385034
0.286697	0.260153	0.300768	0.278862	0.232171	0.32843	0.318196	0.213623	0.223217	0.34314
0.293572	0.323633	0.284298	0.350815	0.398625	0.251199	0.282859	0.295491	0.397985	0.285257
0.214423	0.325712	0.157339	0.353534	0.393508	0.284458	0.256156	0.26495	0.395107	0.286377
0.270867	0.309882	0.232491	0.350975	0.382635	0.270867	0.21874	0.246722	0.370483	0.268308
0.2504	0.274544	0.251519	0.287176	0.31212	0.258874	0.268628	0.248801	0.309402	0.275184
0.31228	0.303965	0.309882	0.307004	0.318996	0.274384	0.324752	0.284458	0.324272	0.280301
0.346338	0.321714	0.372242	0.31196	0.248961	0.413815	0.401823	0.329229	0.260633	0.423409
0.283019	0.331628	0.258555	0.364886	0.397026	0.248161	0.23473	0.26591	0.393188	0.251359
0.318836	0.334506	0.29709	0.34426	0.405341	0.24976	0.292453	0.300927	0.409338	0.261273
0.316278	0.322674	0.310841	0.356412	0.410617	0.253598	0.330668	0.330189	0.400064	0.263991
0.304925	0.248641	0.322674	0.298689	0.223537	0.32763	0.339623	0.246402	0.236808	0.334986
0.282219	0.299488	0.29613	0.278862	0.325072	0.332107	0.283179	0.311001	0.308443	0.339783
0.229133	0.270387	0.255357	0.320595	0.314519	0.295011	0.237768	0.008315	0.311481	0.295971
0.307643	0.253278	0.308283	0.272626	0.336425	0.28158	0.325712	0.276143	0.333866	0.276303
0.304765	0.299169	0.295651	0.31212	0.34362	0.311321	0.322674	0.282379	0.331628	0.314519
0.314359	0.299968	0.347618	0.244164	0.134794	0.39079	0.381516	0.303486	0.059322	0.398465
0.31324	0.314039	0.329389	0.23505	0.269428	0.374001	0.367285	0.315798	0.232651	0.351615
0.332587	0.314998	0.367925	0.303646	0.251679	0.410617	0.395427	0.319795	0.246083	0.418772
0.320595	0.29709	0.332587	0.31196	0.300927	0.338983	0.341701	0.305724	0.301887	0.342341
0.323473	0.291973	0.334986	0.32827	0.307004	0.353374	0.355293	0.288455	0.301247	0.353534
0.354013	0.309882	0.341062	0.329069	0.361848	0.31228	0.358331	0.335625	0.358331	0.34426
0.331628	0.294851	0.350975	0.341062	0.321075	0.366326	0.362808	0.29757	0.321234	0.369524
0.241445	0.319476	0.205788	0.357052	0.407419	0.244803	0.227055	0.255516	0.404701	0.278222
0.252958	0.337544	0.211864	0.353374	0.404861	0.282699	0.133515	0.266869	0.396226	0.292933
0.222897	0.288615	0.255516	0.304765	0.315958	0.318196	0.314199	0.267988	0.303965	0.308922
0.367125	0.333227	0.361209	0.334186	0.364087	0.297889	0.356092	0.320275	0.353854	0.320755
0.295171	0.316597	0.295811	0.326351	0.401023	0.200352	0.307963	0.301407	0.398465	0.248321

For Review Only

0.211545	0.335625	0.188359	0.356412	0.399424	0.266549	0.233611	0.267349	0.39031	0.29693
0.318676	0.317557	0.299009	0.330668	0.340582	0.299328	0.295651	0.303965	0.340902	0.298689
0.322993	0.345539	0.317237	0.368084	0.425488	0.217781	0.329069	0.324113	0.42245	0.274544
0.315798	0.322354	0.311001	0.356252	0.410138	0.252958	0.330029	0.329549	0.400064	0.263671
0.283019	0.329069	0.274225	0.348097	0.388551	0.262392	0.246083	0.279821	0.397825	0.272945
0.351935	0.342981	0.329549	0.339783	0.385193	0.256796	0.344899	0.334026	0.381356	0.29693
0.287816	0.34426	0.28094	0.364407	0.418132	0.264311	0.25072	0.276463	0.418292	0.271826
0.246242	0.337544	0.195875	0.362008	0.40566	0.290534	0.111129	0.251679	0.396546	0.283339
0.221458	0.320275	0.163096	0.341701	0.398625	0.273585	0.243524	0.269747	0.397506	0.272146
0.318356	0.29693	0.353214	0.277103	0.210425	0.395907	0.383435	0.292613	0.199232	0.396386
0.29757	0.275344	0.316917	0.285417	0.304605	0.293412	0.327311	0.276623	0.291174	0.292133
0.258075	0.279021	0.261113	0.291493	0.325232	0.246882	0.260953	0.244324	0.318196	0.274864
0.28142	0.323633	0.235369	0.357052	0.400224	0.26607	0.081548	0.247841	0.395267	0.271346
0.277902	0.335785	0.255037	0.354813	0.395747	0.289575	0.005756	0.235529	0.393668	0.294691
0.323633	0.345059	0.315318	0.367445	0.424209	0.217141	0.32827	0.324912	0.42213	0.273745
0.311321	0.303166	0.309882	0.306524	0.318676	0.273745	0.323793	0.284138	0.324272	0.280141
0.272466	0.282699	0.262392	0.298209	0.335785	0.264311	0.279821	0.266869	0.332107	0.269747
0.31292	0.26575	0.315798	0.298529	0.311161	0.309242	0.341382	0.307163	0.306684	0.326031
0.28062	0.271027	0.294372	0.288295	0.34378	0.308762	0.293892	0.315478	0.320275	0.335146
0.31196	0.329069	0.26607	0.350815	0.433003	0.172689	0.291653	0.318356	0.426127	0.235209
0.319156	0.322194	0.313879	0.345859	0.416214	0.237768	0.28158	0.29725	0.416693	0.252958
0.331948	0.330828	0.310361	0.353534	0.415894	0.233291	0.331948	0.326511	0.407099	0.253598
0.269587	0.308123	0.243844	0.330029	0.362328	0.274864	0.059002	0.226095	0.35977	0.275823
0.314519	0.304285	0.325871	0.09338	0.258075	0.349696	0.346498	0.314199	0.237768	0.34346
0.322674	0.291653	0.307803	0.290854	0.337864	0.269428	0.329549	0.303326	0.341222	0.269428
0.308283	0.321234	0.28094	0.318516	0.368564	0.303166	0.270707	0.292613	0.367765	0.31228
0.331468	0.294532	0.351135	0.340742	0.320595	0.366166	0.362808	0.29661	0.320275	0.368884
0.267988	0.324113	0.269428	0.351615	0.383754	0.276943	0.208507	0.244803	0.386633	0.293252
0.34346	0.338983	0.356892	0.360569	0.35993	0.32795	0.37464	0.34442	0.352574	0.347618
0.324752	0.29725	0.356732	0.255676	0.105053	0.399105	0.39063	0.302846	0.133994	0.403742
0.204829	0.333547	0.157819	0.351775	0.397186	0.283339	0.267349	0.274065	0.393188	0.29725
0.314519	0.318996	0.305085	0.341382	0.401023	0.248161	0.29645	0.285417	0.402942	0.25072
0.303326	0.31196	0.304605	0.345059	0.357211	0.270067	0.340742	0.292133	0.348257	0.311161
0.286537	0.26527	0.272785	0.310681	0.28078	0.299648	0.290854	0.199712	0.276943	0.309082
0.331148	0.303326	0.362648	0.262392	0.110169	0.407899	0.402143	0.308443	0.135114	0.414934
0.323313	0.34426	0.316437	0.366805	0.423729	0.217141	0.32827	0.324592	0.420851	0.272945
0.291493	0.302526	0.291653	0.329549	0.346658	0.246242	0.324912	0.289095	0.348737	0.292133
0.331148	0.295491	0.351135	0.339783	0.320915	0.365686	0.363288	0.29661	0.320915	0.369044
0.319476	0.292453	0.316597	0.332587	0.306524	0.330349	0.350496	0.291813	0.29677	0.346019
0.352414	0.349856	0.350975	0.34346	0.331468	0.330349	0.379117	0.342021	0.336745	0.356252
0.302047	0.293892	0.315958	0.297889	0.341861	0.306204	0.333067	0.301567	0.333067	0.308762
0.325712	0.303806	0.337064	0.326351	0.310201	0.346658	0.364886	0.29709	0.324912	0.361049
0.322354	0.333866	0.299169	0.360889	0.426447	0.201311	0.325552	0.323953	0.417813	0.26527
0.293412	0.336904	0.305245	0.360249	0.425807	0.267988	0.248641	0.252638	0.428526	0.269747
0.313399	0.322194	0.303806	0.361689	0.373201	0.303965	0.322194	0.316917	0.370643	0.306044
0.2811	0.324113	0.235849	0.357691	0.399584	0.267829	0.083786	0.248801	0.395427	0.272945
0.304765	0.320915	0.303486	0.354653	0.383754	0.229933	0.31276	0.278062	0.368884	0.280301
0.293252	0.324912	0.261433	0.336105	0.402462	0.256316	0.244963	0.279181	0.405021	0.259834
0.3126	0.229613	0.334666	0.291334	0.213144	0.362648	0.351135	0.256796	0.216501	0.356732
0.216501	0.302047	0.190918	0.318676	0.35961	0.28142	0.257435	0.228174	0.358331	0.291653
0.236649	0.314839	0.214423	0.337704	0.376879	0.284778	0.259674	0.225616	0.362168	0.275024
0.339623	0.29629	0.311001	0.247042	0.369524	0.295171	0.293252	0.29613	0.372082	0.294052
0.286057	0.31324	0.259674	0.34394	0.368724	0.260793	0.18772	0.246722	0.362648	0.272626
0.310681	0.309882	0.355293	0.25072	0.142949	0.396067	0.38999	0.304285	0.081708	0.409338
0.330189	0.293892	0.339783	0.294851	0.245443	0.37528	0.366006	0.293252	0.232971	0.379437

For Review Only

0.298529	0.298209	0.291813	0.293732	0.289415	0.303646	0.309402	0.283818	0.284298	0.298529
0.331788	0.271186	0.304125	0.342021	0.393028	0.292933	0.298689	0.302526	0.389191	0.267029
0.25072	0.291334	0.215862	0.275024	0.330349	0.31308	0.293252	0.285417	0.316917	0.300608
0.324432	0.301887	0.305724	0.302846	0.322514	0.339783	0.322354	0.287336	0.319635	0.321554
0.219699	0.283818	0.198113	0.332267	0.362648	0.254877	0.250879	0.152382	0.367445	0.288136
0.300128	0.253278	0.316437	0.286856	0.208347	0.357691	0.354493	0.275344	0.232012	0.369524
0.254877	0.335945	0.237928	0.357691	0.402462	0.269907	0.206428	0.262872	0.392229	0.280141
0.216821	0.301887	0.204509	0.321394	0.368724	0.263671	0.254877	0.23473	0.369843	0.278702
0.290054	0.330668	0.28206	0.35897	0.397186	0.224017	0.245603	0.269268	0.391749	0.26639
0.278542	0.270067	0.261113	0.289415	0.311161	0.280461	0.264151	0.217621	0.31212	0.292773
0.272146	0.320115	0.260313	0.332107	0.324113	0.310042	0.308443	0.266549	0.300927	0.324752
0.252798	0.291014	0.260313	0.291174	0.29629	0.314519	0.291334	0.189799	0.293732	0.314519
0.316437	0.306524	0.302367	0.320755	0.34378	0.277902	0.334346	0.297729	0.345859	0.297889
0.261273	0.286217	0.256636	0.283019	0.325232	0.272785	0.277103	0.267509	0.310841	0.280141
0.309722	0.304765	0.290854	0.356092	0.356572	0.320275	0.309242	0.29677	0.351775	0.302526
0.271986	0.316278	0.251039	0.339942	0.376719	0.259514	0.219699	0.257595	0.3748	0.285577
0.308443	0.306044	0.273425	0.333547	0.342501	0.298689	0.279821	0.259514	0.347777	0.316437
0.225136	0.29725	0.209946	0.306684	0.32875	0.269907	0.263351	0.259674	0.318516	0.283019
0.329389	0.308602	0.290854	0.332427	0.405341	0.290534	0.32795	0.341701	0.404861	0.267669
0.237288	0.284298	0.226095	0.304125	0.337064	0.259354	0.232971	0.248161	0.329869	0.275823
0.309082	0.244324	0.326991	0.289095	0.213943	0.360409	0.349696	0.276623	0.23409	0.369204
0.326671	0.303326	0.322833	0.333067	0.360569	0.298849	0.330508	0.327151	0.35881	0.292133
0.245603	0.286057	0.232811	0.28206	0.315478	0.26511	0.257275	0.248161	0.306844	0.276783
0.316118	0.318516	0.288615	0.338823	0.401503	0.239527	0.292613	0.289735	0.397666	0.251679
0.273905	0.317237	0.271027	0.340902	0.3756	0.240006	0.229453	0.253598	0.377039	0.283019
0.319156	0.293572	0.353854	0.258395	0.119923	0.394627	0.392869	0.308762	0.146306	0.409978
0.258395	0.31244	0.231692	0.192197	0.329549	0.307323	0.293412	0.285577	0.320915	0.309082
0.31276	0.292613	0.308123	0.17125	0.290694	0.318676	0.331468	0.29677	0.264471	0.315478
0.322833	0.326991	0.283658	0.335146	0.387912	0.243844	0.29693	0.284938	0.385833	0.257435
0.302207	0.277742	0.284778	0.306684	0.34346	0.251359	0.294372	0.244324	0.334666	0.233611
0.286697	0.354493	0.26527	0.378638	0.425328	0.240646	0.236808	0.285098	0.42149	0.255836
0.32859	0.316597	0.302846	0.339623	0.399424	0.259354	0.302207	0.307483	0.394787	0.26527
0.326191	0.284458	0.35865	0.275344	0.154621	0.397825	0.382475	0.291653	0.169012	0.396067
0.266869	0.299648	0.266709	0.28174	0.302047	0.278382	0.271826	0.25008	0.29709	0.289575
0.324272	0.336425	0.299968	0.361369	0.429805	0.200672	0.327311	0.326511	0.420051	0.266549
0.245443	0.286856	0.216981	0.306204	0.346978	0.258874	0.26543	0.255197	0.341222	0.260953
0.304125	0.2504	0.318836	0.299009	0.224336	0.326191	0.338823	0.247522	0.237608	0.334346
0.297889	0.278542	0.304605	0.318996	0.285577	0.278382	0.332107	0.285737	0.289415	0.310361
0.314679	0.242885	0.337704	0.287656	0.212984	0.369843	0.356252	0.276623	0.227055	0.378478
0.28142	0.318996	0.247681	0.355133	0.39063	0.25024	0.228654	0.272785	0.387112	0.252638
0.259994	0.298689	0.273425	0.289575	0.298689	0.319156	0.332267	0.302367	0.291813	0.346019
0.327151	0.303166	0.366326	0.254397	0.181484	0.39063	0.392709	0.316278	0.164855	0.39015
0.319635	0.313879	0.303486	0.194915	0.315478	0.323793	0.333387	0.31324	0.287816	0.315158
0.319795	0.245603	0.339623	0.283499	0.204669	0.365366	0.358011	0.260633	0.226575	0.366645
0.304925	0.317397	0.259194	0.351135	0.401663	0.200672	0.267669	0.282379	0.389031	0.237448
0.323953	0.307004	0.346178	0.314839	0.262552	0.37448	0.372082	0.294691	0.261593	0.381516
0.267669	0.301727	0.246882	0.315158	0.333706	0.277582	0.308123	0.279021	0.342181	0.319955
0.306044	0.298049	0.277902	0.346019	0.365526	0.24992	0.291334	0.283978	0.367285	0.275983
0.183403	0.32763	0.157179	0.352894	0.4055	0.271666	0.245603	0.262232	0.399264	0.279981
0.238407	0.322674	0.127918	0.341062	0.394148	0.282379	0.220659	0.256156	0.395587	0.295651
0.300768	0.259034	0.302047	0.260793	0.257275	0.320115	0.323473	0.260633	0.259194	0.319795
0.211705	0.32763	0.169332	0.352894	0.394627	0.270547	0.243204	0.267189	0.393348	0.289415
0.201791	0.314199	0.168852	0.342981	0.386313	0.287656	0.261593	0.21938	0.383435	0.275344
0.282379	0.29613	0.337384	0.254397	0.258555	0.370803	0.369204	0.303326	0.213943	0.363927
0.271346	0.288455	0.269907	0.289415	0.325072	0.17157	0.300448	0.264791	0.322833	0.286057

For Review Only

0.237448	0.347618	0.217941	0.360729	0.395267	0.294691	0.117045	0.232331	0.395107	0.305085
0.289735	0.284458	0.302846	0.272945	0.26511	0.324752	0.322993	0.269428	0.252159	0.326671
0.256476	0.335785	0.251679	0.351455	0.404381	0.239207	0.273905	0.294212	0.400863	0.2504
0.31196	0.29725	0.322514	0.244484	0.286537	0.364886	0.365366	0.321234	0.279981	0.368404
0.332267	0.334346	0.332907	0.333547	0.347618	0.346658	0.354493	0.29741	0.342021	0.337704
0.325392	0.323473	0.313719	0.24976	0.302846	0.285417	0.347298	0.31292	0.276943	0.322514
0.300608	0.32811	0.266709	0.361848	0.404381	0.264791	0.244324	0.267509	0.395747	0.285098
0.29741	0.298369	0.317717	0.272626	0.290534	0.326671	0.34394	0.31228	0.292453	0.327151
0.295491	0.297729	0.322034	0.253758	0.208027	0.362808	0.365846	0.310042	0.199392	0.361369
0.247522	0.283499	0.225456	0.289575	0.315158	0.263032	0.256156	0.239527	0.306524	0.28206
0.313399	0.294052	0.342181	0.290534	0.259674	0.340262	0.35945	0.306204	0.238567	0.35865
0.323153	0.334986	0.29725	0.351935	0.423249	0.223537	0.283658	0.29645	0.415734	0.241605
0.212824	0.315318	0.192996	0.316917	0.372721	0.26495	0.239367	0.252638	0.365206	0.279021
0.291973	0.276783	0.290214	0.259034	0.254557	0.336904	0.313399	0.244164	0.225935	0.333387
0.271186	0.291973	0.238088	0.315798	0.385353	0.259034	0.241286	0.262392	0.381996	0.257275
0.316597	0.164215	0.294212	0.321554	0.34442	0.288295	0.317557	0.279181	0.3433	0.295171
0.315478	0.233611	0.334986	0.28142	0.210905	0.366805	0.344899	0.257275	0.224017	0.367605
0.299169	0.304285	0.350336	0.26511	0.264791	0.351135	0.372562	0.317397	0.223057	0.371602
0.273745	0.278862	0.273425	0.283339	0.307163	0.204029	0.293732	0.260633	0.304925	0.283339
0.277742	0.336904	0.254237	0.355133	0.397666	0.279341	0.227694	0.266709	0.396546	0.275664
0.253438	0.284138	0.220659	0.332747	0.347618	0.263351	0.254717	0.230732	0.348577	0.277902
0.284138	0.322194	0.277902	0.350176	0.38999	0.225136	0.247362	0.263032	0.385993	0.260153
0.260953	0.271826	0.257275	0.283658	0.31196	0.252798	0.261113	0.241286	0.306524	0.26607
0.309562	0.283179	0.323793	0.277902	0.167893	0.366805	0.363927	0.254397	0.184842	0.379117
0.330029	0.301087	0.330508	0.07755	0.255996	0.350815	0.362488	0.31308	0.247042	0.346658
0.278702	0.336105	0.255197	0.354973	0.395747	0.289735	0.006876	0.236489	0.393028	0.294052
0.332747	0.291014	0.321554	0.337864	0.345539	0.291014	0.329709	0.314039	0.347298	0.317397
0.298849	0.233291	0.329389	0.286217	0.228014	0.337704	0.345699	0.240166	0.237928	0.346658
0.266549	0.31196	0.247681	0.342181	0.384874	0.270067	0.210265	0.258714	0.381356	0.262072
0.303006	0.276623	0.290054	0.321874	0.340742	0.286537	0.315638	0.293412	0.340102	0.287016
0.308602	0.303486	0.291973	0.31324	0.367445	0.28158	0.292933	0.295651	0.352414	0.276623
0.316757	0.317717	0.291813	0.32795	0.394627	0.198753	0.298049	0.310521	0.393348	0.246402
0.279981	0.306044	0.267669	0.336425	0.357691	0.273585	0.282539	0.268628	0.368084	0.283978
0.31212	0.326831	0.28062	0.345699	0.425648	0.26559	0.300608	0.309882	0.427566	0.240806
0.316118	0.308602	0.300288	0.326991	0.378798	0.271506	0.29677	0.284618	0.374001	0.222258
0.314679	0.237608	0.333866	0.279021	0.215382	0.365846	0.345219	0.263351	0.225136	0.365206
0.314679	0.31212	0.299808	0.321714	0.384554	0.263351	0.307323	0.289575	0.383115	0.184842
0.277263	0.335146	0.254717	0.354493	0.395427	0.288775	0.006396	0.235689	0.392869	0.293732
0.279341	0.318036	0.24992	0.291493	0.369843	0.29741	0.263032	0.236649	0.354173	0.291973
0.322674	0.292453	0.301247	0.322674	0.373841	0.228174	0.329869	0.288935	0.366965	0.251199
0.245763	0.325712	0.137672	0.340582	0.394308	0.284938	0.21954	0.259194	0.391589	0.295171
0.26559	0.333387	0.239207	0.350176	0.387912	0.260153	0.230732	0.260153	0.381836	0.292933
0.257755	0.341062	0.251999	0.370483	0.416374	0.237928	0.216342	0.273905	0.404221	0.280301
0.18788	0.332747	0.151423	0.348417	0.404221	0.269747	0.255037	0.276463	0.397346	0.277103
0.324752	0.293892	0.351775	0.258555	0.1252	0.396386	0.388391	0.31212	0.128398	0.40646
0.278222	0.307803	0.275664	0.285577	0.354813	0.28158	0.289735	0.274704	0.338503	0.29693
0.304285	0.315158	0.311641	0.329709	0.332107	0.299648	0.291493	0.242405	0.336745	0.314839
0.327151	0.323153	0.319156	0.336904	0.350496	0.294851	0.277582	0.310681	0.346978	0.28158
0.297729	0.303965	0.349217	0.263991	0.264631	0.350176	0.371442	0.316118	0.222258	0.370483
0.295011	0.311321	0.336585	0.279021	0.158139	0.368084	0.394947	0.314679	0.181164	0.393828
0.305085	0.240486	0.319795	0.285577	0.220819	0.339303	0.338823	0.235529	0.216501	0.3433
0.325392	0.305405	0.307483	0.305245	0.349217	0.286537	0.29693	0.29709	0.344899	0.301247
0.284138	0.360729	0.267189	0.367765	0.408698	0.253758	0.273745	0.310841	0.413176	0.262872
0.332107	0.24992	0.338823	0.28142	0.253438	0.365206	0.367765	0.286697	0.259834	0.371442
0.313719	0.294372	0.336425	0.096898	0.228174	0.35913	0.368884	0.314039	0.213783	0.362808

For Review Only

0.28062	0.322194	0.236329	0.356572	0.398945	0.26607	0.082028	0.248001	0.394627	0.271826
0.321554	0.299648	0.357691	0.259034	0.139591	0.396546	0.389511	0.297729	0.104733	0.404861
0.29741	0.292933	0.286856	0.316917	0.318836	0.189639	0.321714	0.292133	0.309242	0.302047
0.332587	0.306044	0.319316	0.310521	0.331468	0.339783	0.330029	0.303166	0.326671	0.324912
0.294372	0.356252	0.274544	0.35929	0.427247	0.232811	0.277742	0.299009	0.431884	0.255676
0.338343	0.3126	0.330988	0.186441	0.317717	0.337064	0.354813	0.334826	0.308283	0.325232
0.31324	0.298529	0.32827	0.313559	0.326511	0.323633	0.340582	0.303166	0.319955	0.332267
0.253118	0.330189	0.216022	0.352095	0.413335	0.245923	0.295171	0.279501	0.408219	0.242885
0.309082	0.326831	0.322034	0.269268	0.269268	0.345539	0.366166	0.317717	0.25072	0.333067
0.335465	0.326831	0.352414	0.321874	0.268468	0.399105	0.389831	0.316437	0.261912	0.401023
0.285257	0.267029	0.286057	0.301567	0.259194	0.323473	0.323633	0.241605	0.258075	0.32859
0.334346	0.302047	0.363288	0.268308	0.115606	0.403742	0.396546	0.307483	0.132875	0.411417
0.253278	0.276143	0.255037	0.2811	0.306524	0.259514	0.258395	0.240486	0.298689	0.270067
0.285098	0.295971	0.282379	0.298529	0.323473	0.307963	0.320275	0.299009	0.339783	0.317557
0.292453	0.321554	0.304125	0.279501	0.367445	0.289095	0.334186	0.295811	0.354333	0.292933
0.248001	0.298689	0.185641	0.318036	0.350815	0.244324	0.248801	0.242085	0.355293	0.293412
0.272466	0.348097	0.228334	0.370323	0.411257	0.252319	0.219699	0.272785	0.399424	0.280301
0.310681	0.286537	0.339143	0.276783	0.346658	0.308922	0.342341	0.299009	0.332427	0.326671
0.289255	0.305724	0.320595	0.260633	0.193636	0.347937	0.358491	0.286217	0.15638	0.369524
0.322034	0.289415	0.332587	0.288136	0.262712	0.331148	0.363927	0.303326	0.279821	0.331308
0.299009	0.309402	0.342981	0.259194	0.208826	0.364407	0.376079	0.316757	0.221298	0.369364
0.310201	0.287496	0.307004	0.293572	0.346818	0.321075	0.330828	0.29693	0.32779	0.318196
0.319795	0.339303	0.313559	0.31308	0.305724	0.350975	0.310681	0.316118	0.279341	0.351775
0.192677	0.318196	0.161017	0.342181	0.392069	0.2811	0.261912	0.262232	0.39079	0.284138
0.308443	0.317717	0.29677	0.333547	0.397985	0.222098	0.303006	0.278062	0.395107	0.244803
0.307803	0.294212	0.31324	0.243684	0.300608	0.331308	0.317397	0.29629	0.25072	0.332907
0.318036	0.29629	0.353374	0.276623	0.209466	0.395587	0.382635	0.292293	0.199392	0.396866
0.314039	0.324912	0.302367	0.356412	0.368564	0.280141	0.291653	0.285098	0.367125	0.299808
0.334666	0.301407	0.368244	0.269747	0.10889	0.40582	0.400384	0.31228	0.137992	0.412536
0.257595	0.329709	0.243844	0.3756	0.410617	0.271027	0.210745	0.258235	0.407259	0.269268
0.306524	0.311321	0.319635	0.324752	0.366166	0.257115	0.32827	0.304765	0.35897	0.295331
0.290694	0.356092	0.274704	0.360729	0.411577	0.228654	0.260473	0.298849	0.416853	0.256636
0.305564	0.311001	0.347138	0.257595	0.131276	0.379597	0.386633	0.303006	0.136553	0.397825
0	0.324912	0.238248	0.325392	0.326191	0.305724	0.279181	0.228014	0.313879	0.317077
0.324912	0	0.308443	0.290854	0.307483	0.337704	0.336425	0.269108	0.307963	0.320115
0.238248	0.308443	0	0.32891	0.361529	0.267988	0.255836	0.254877	0.360249	0.291493
0.325392	0.290854	0.32891	0	0.263671	0.351295	0.356092	0.318516	0.251679	0.341062
0.326191	0.307483	0.361529	0.263671	0	0.410937	0.397346	0.313719	0.137992	0.410937
0.305724	0.337704	0.267988	0.351295	0.410937	0	0.290374	0.293572	0.396866	0.259194
0.279181	0.336425	0.255836	0.356092	0.397346	0.290374	0	0.237608	0.394468	0.294691
0.228014	0.269108	0.254877	0.318516	0.313719	0.293572	0.237608	0	0.310681	0.294532
0.313879	0.307963	0.360249	0.251679	0.137992	0.396866	0.394468	0.310681	0	0.4055
0.317077	0.320115	0.291493	0.341062	0.410937	0.259194	0.294691	0.294532	0.4055	0
0.294691	0.286537	0.275983	0.309402	0.330508	0.263351	0.272945	0.254077	0.331628	0.267349
0.29725	0.304445	0.295651	0.241445	0.291973	0.3441	0.317237	0.295171	0.243524	0.31228
0.324752	0.333387	0.306204	0.353694	0.430125	0.183243	0.323153	0.322034	0.42149	0.262872
0.263831	0.326351	0.259994	0.360409	0.361209	0.273105	0.20387	0.264631	0.352255	0.320755
0.32843	0.294851	0.301567	0.311481	0.360889	0.289575	0.329869	0.299488	0.350496	0.287336
0.273265	0.329549	0.201791	0.298369	0.314998	0.329869	0.294052	0.277902	0.285737	0.34378
0.303166	0.287336	0.274384	0.355612	0.37576	0.304445	0.287496	0.300128	0.360729	0.29645
0.301407	0.32747	0.292293	0.3748	0.365686	0.305884	0.288455	0.288935	0.372881	0.332267
0.326671	0.304125	0.364727	0.267349	0.102175	0.403902	0.397985	0.308443	0.136073	0.409818
0.324432	0.330349	0.309242	0.340102	0.424049	0.229133	0.289415	0.292933	0.423729	0.228494
0.316597	0.298849	0.35897	0.250879	0.14039	0.395907	0.393508	0.310361	0.066517	0.40566
0.257275	0.341701	0.252478	0.370323	0.417493	0.236968	0.216821	0.274864	0.404701	0.28206

For Review Only

0.289575	0.340742	0.233291	0.367925	0.428206	0.26511	0.231692	0.278382	0.426607	0.284458
0.297889	0.304765	0.320275	0.231212	0.244484	0.363447	0.363607	0.305724	0.196674	0.362968
0.315478	0.327151	0.310201	0.32891	0.425168	0.238088	0.305884	0.292933	0.417173	0.261433
0.269428	0.334186	0.259674	0.362008	0.404061	0.279821	0.201151	0.259034	0.396866	0.290214

For Review Only

0.274225	0.337224	0.262712	0.297889	0.313399	0.320115	0.29693	0.319955	0.379917	0.26623
0.300288	0.335625	0.364087	0.301887	0.314519	0.350016	0.317557	0.29693	0.322514	0.357531
0.284138	0.34346	0.286537	0.208986	0.295651	0.290694	0.289735	0.260633	0.416214	0.278062
0.273745	0.336105	0.294052	0.254717	0.31228	0.294372	0.306684	0.306684	0.379597	0.276783
0.302207	0.340742	0.226255	0.345859	0.353374	0.353374	0.326351	0.326991	0.342661	0.324912
0.270707	0.337704	0.269747	0.28126	0.304125	0.321394	0.29741	0.304125	0.386952	0.268308
0.288295	0.322354	0.184682	0.333067	0.294691	0.353694	0.29757	0.335785	0.399424	0.26607
0.289735	0.326511	0.357691	0.320435	0.321075	0.338343	0.293092	0.294372	0.295011	0.351775
0.292773	0.298369	0.170611	0.32859	0.3126	0.346338	0.311481	0.332587	0.383914	0.253918
0.31324	0.342021	0.389031	0.338823	0.353374	0.364567	0.316278	0.304285	0.300448	0.382635
0.330668	0.288775	0.322993	0.352894	0.29725	0.306844	0.355932	0.358011	0.286537	0.32875
0.284138	0.336904	0.284138	0.216661	0.291973	0.287016	0.287816	0.26607	0.408539	0.270227
0.275823	0.331148	0.24944	0.316757	0.303965	0.332747	0.304605	0.314679	0.325871	0.305724
0.270387	0.270547	0.326991	0.297889	0.298849	0.284938	0.274864	0.315638	0.28094	0.331948
0.26639	0.287336	0.193316	0.294691	0.299968	0.305564	0.272626	0.309722	0.368724	0.257595
0.288935	0.299328	0.308602	0.299328	0.339143	0.285737	0.317877	0.321075	0.325712	0.283499
0.286537	0.355293	0.154301	0.330828	0.304125	0.369044	0.351615	0.341541	0.396546	0.277742
0.283978	0.323793	0.311321	0.299009	0.331308	0.32747	0.285577	0.297729	0.356252	0.306364
0.29645	0.34314	0.28142	0.274384	0.317557	0.340422	0.316917	0.313559	0.414934	0.24976
0.310201	0.340902	0.362008	0.341062	0.361369	0.340102	0.3441	0.339783	0.326191	0.3748
0.28206	0.347618	0.253118	0.351135	0.314839	0.344739	0.320115	0.337864	0.332267	0.332427
0.28206	0.361209	0.128238	0.338823	0.313879	0.353214	0.334026	0.331148	0.385993	0.284298
0.334026	0.257115	0.392229	0.357211	0.320755	0.320435	0.353854	0.387592	0.21826	0.399264
0.270387	0.278062	0.354013	0.306044	0.31228	0.292453	0.315318	0.321714	0.227374	0.355612
0.261273	0.309242	0.188999	0.273105	0.305724	0.331148	0.279981	0.304925	0.400544	0.245283
0.288935	0.320115	0.307643	0.267509	0.31308	0.240006	0.288775	0.277902	0.396866	0.303965
0.244164	0.29725	0.291653	0.248481	0.272306	0.267349	0.267189	0.279821	0.371602	0.280301
0.253438	0.28062	0.26511	0.264151	0.28206	0.277103	0.275024	0.286377	0.311321	0.26639
0.279501	0.318836	0.262712	0.32827	0.317877	0.337544	0.322034	0.330988	0.331628	0.298689
0.339783	0.346978	0.431404	0.372721	0.366645	0.356572	0.363927	0.331628	0.242725	0.43732
0.288615	0.313879	0.264791	0.268148	0.292613	0.300448	0.285257	0.289735	0.394947	0.251679
0.292133	0.350016	0.287656	0.302207	0.318996	0.32875	0.323953	0.317877	0.409018	0.235529
0.272306	0.32811	0.195715	0.319476	0.300448	0.354013	0.305884	0.338503	0.408858	0.263671
0.259514	0.306044	0.349376	0.322354	0.313879	0.325552	0.318836	0.335305	0.227374	0.351935
0.305884	0.263192	0.336425	0.305724	0.339783	0.301567	0.314679	0.330508	0.316757	0.346178
0.255516	0.29629	0.323153	0.264631	0.300608	0.278702	0.301407	0.289575	0.310042	0.293892
0.256156	0.323473	0.303326	0.315798	0.298369	0.34314	0.320275	0.341701	0.339623	0.298689
0.290534	0.287176	0.307803	0.307163	0.29677	0.299169	0.311481	0.313559	0.348737	0.302846
0.319795	0.247042	0.408858	0.347458	0.333547	0.286377	0.355612	0.362968	0.133834	0.412536
0.339143	0.211545	0.357851	0.357052	0.32795	0.295491	0.352734	0.349856	0.270387	0.339463
0.333067	0.336425	0.42197	0.370003	0.363607	0.347138	0.353374	0.3118	0.240966	0.424209
0.299968	0.325232	0.35977	0.342501	0.333547	0.341382	0.320115	0.323313	0.310201	0.347777
0.298209	0.319635	0.380876	0.318356	0.339303	0.340422	0.294372	0.284458	0.303646	0.37576
0.302686	0.341861	0.23425	0.340422	0.333866	0.347777	0.337224	0.339623	0.368564	0.316437
0.315478	0.335625	0.386153	0.339143	0.338663	0.349376	0.319635	0.26527	0.32843	0.376079
0.271826	0.333227	0.282699	0.226095	0.284138	0.275823	0.273905	0.271027	0.405341	0.272306
0.264791	0.324272	0.299009	0.231052	0.323633	0.257275	0.291174	0.271186	0.403902	0.292133
0.301887	0.293252	0.307163	0.303806	0.306364	0.266869	0.29741	0.304765	0.316917	0.319156
0.315638	0.351775	0.252159	0.346338	0.322993	0.367605	0.32843	0.331788	0.364567	0.314199
0.271986	0.320275	0.211225	0.300768	0.298049	0.322354	0.321714	0.321075	0.398465	0.21874

For Review Only

0.293412	0.324432	0.287336	0.230253	0.300608	0.278062	0.251199	0.277263	0.400704	0.290694
0.308602	0.319635	0.295331	0.309242	0.310521	0.325392	0.314359	0.322833	0.340902	0.255676
0.274704	0.342181	0.088264	0.306204	0.300608	0.357691	0.315478	0.333387	0.430604	0.224177
0.271186	0.32843	0.196194	0.318836	0.301087	0.353534	0.305405	0.338343	0.408858	0.263671
0.271506	0.318676	0.285417	0.256636	0.322034	0.309242	0.289575	0.288295	0.39063	0.262392
0.284778	0.340422	0.134314	0.332107	0.278222	0.355772	0.324752	0.334826	0.388711	0.274225
0.29645	0.342501	0.28094	0.273745	0.318036	0.338983	0.317397	0.313719	0.414775	0.2504
0.288775	0.320435	0.316118	0.225616	0.32779	0.248321	0.278062	0.275983	0.4055	0.289735
0.28126	0.321394	0.300448	0.253598	0.302686	0.221458	0.292453	0.293252	0.398625	0.284938
0.314039	0.315958	0.415254	0.337064	0.352734	0.315638	0.345219	0.338024	0.190918	0.412696
0.288295	0.289255	0.309562	0.320115	0.298529	0.347618	0.293732	0.324432	0.303806	0.314519
0.258235	0.284618	0.258714	0.263351	0.271826	0.28142	0.276303	0.285257	0.319635	0.267349
0.26511	0.317077	0.301727	0.217621	0.311161	0.279501	0.286377	0.270387	0.396067	0.284458
0.271506	0.316917	0.323473	0.202111	0.329549	0.292773	0.285737	0.288295	0.396386	0.289735
0.273105	0.341861	0.087304	0.305884	0.299968	0.355932	0.315158	0.333706	0.429965	0.224177
0.279341	0.318516	0.262712	0.327311	0.317397	0.337224	0.322514	0.330508	0.331308	0.29757
0.26527	0.295811	0.272945	0.275184	0.294532	0.292933	0.293252	0.305405	0.335465	0.275184
0.286856	0.307963	0.334666	0.310841	0.314359	0.345379	0.305564	0.313879	0.309882	0.342981
0.314679	0.289575	0.301887	0.270227	0.28062	0.302526	0.293092	0.31212	0.346818	0.314998
0.274225	0.330828	0.263192	0.293252	0.31212	0.308922	0.288935	0.323473	0.428686	0.243364
0.301727	0.337224	0.157179	0.307483	0.321234	0.353694	0.29645	0.322674	0.424848	0.21938
0.278862	0.338663	0.158778	0.315798	0.29741	0.340262	0.313879	0.324592	0.417013	0.243364
0.251359	0.295171	0.302686	0.205309	0.308762	0.276303	0.275344	0.279821	0.361848	0.28142
0.310201	0.23393	0.364727	0.347618	0.315478	0.297729	0.35913	0.373681	0.256796	0.341382
0.285577	0.298689	0.21922	0.32875	0.311641	0.339623	0.311161	0.34362	0.349696	0.283499
0.290694	0.325712	0.320595	0.293092	0.31228	0.323153	0.323793	0.323793	0.371442	0.299808
0.314359	0.335146	0.385993	0.338823	0.338663	0.348097	0.319316	0.264151	0.326991	0.376079
0.272945	0.319635	0.294212	0.240326	0.308602	0.315958	0.287816	0.278862	0.382795	0.290054
0.318676	0.370963	0.301727	0.337224	0.336585	0.372082	0.323153	0.321554	0.349057	0.348097
0.321554	0.279181	0.418452	0.34426	0.346178	0.289255	0.361049	0.346978	0.101695	0.420851
0.287016	0.335305	0.317237	0.267349	0.321394	0.237288	0.286697	0.287496	0.400224	0.307643
0.283499	0.336105	0.224976	0.31228	0.301567	0.331468	0.317877	0.317077	0.400704	0.231052
0.287816	0.322993	0.205948	0.313879	0.324752	0.332427	0.335305	0.308283	0.353214	0.2819
0.24976	0.286217	0.320755	0.290534	0.302686	0.293412	0.29661	0.309242	0.272306	0.315958
0.330508	0.282379	0.432683	0.365686	0.360889	0.306844	0.364247	0.356572	0.071954	0.430764
0.273425	0.341222	0.087144	0.305564	0.299169	0.356092	0.314359	0.333227	0.429005	0.223697
0.258235	0.313879	0.293092	0.286377	0.314519	0.322674	0.295011	0.330508	0.357052	0.282539
0.315318	0.335146	0.385833	0.339623	0.337864	0.349856	0.320435	0.264631	0.32779	0.375919
0.316118	0.325072	0.344899	0.327311	0.345699	0.32827	0.308762	0.301567	0.294372	0.34346
0.300608	0.367765	0.251519	0.353854	0.339143	0.385193	0.32795	0.336425	0.339623	0.351775
0.289735	0.321874	0.321394	0.294212	0.15606	0.320915	0.308283	0.303806	0.338184	0.32875
0.321714	0.342021	0.369044	0.329869	0.327311	0.332107	0.311001	0.247362	0.308443	0.367925
0.269268	0.335945	0.072434	0.320915	0.290214	0.347777	0.304765	0.329229	0.428526	0.227215
0.304925	0.342021	0.257595	0.288136	0.306204	0.331948	0.3118	0.298689	0.426767	0.257115
0.302526	0.321234	0.308762	0.303806	0.295171	0.330508	0.245603	0.330029	0.369364	0.300128
0.26639	0.318356	0.301567	0.217781	0.3118	0.28062	0.287496	0.272146	0.395907	0.285897
0.269108	0.316118	0.242085	0.309402	0.288775	0.350975	0.302526	0.330029	0.373681	0.274065
0.26559	0.334026	0.262232	0.2811	0.325072	0.300288	0.291334	0.307803	0.40614	0.252478
0.274544	0.298689	0.380716	0.336105	0.310042	0.316437	0.324912	0.338983	0.213943	0.377199
0.256636	0.323953	0.309722	0.261433	0.305724	0.237608	0.266869	0.29757	0.352894	0.309082
0.26495	0.305724	0.299488	0.276783	0.300448	0.23505	0.294851	0.29757	0.365686	0.294052
0.298209	0.292613	0.309402	0.329389	0.323633	0.321714	0.294212	0.333866	0.365366	0.288775
0.267029	0.300768	0.290214	0.239367	0.287816	0.279021	0.269747	0.272785	0.366805	0.277582
0.330189	0.267349	0.416374	0.347138	0.332747	0.275024	0.366965	0.368564	0.143748	0.424209
0.306844	0.306524	0.386952	0.335305	0.337224	0.32827	0.347777	0.347937	0.235689	0.392709

For Review Only

0.28078	0.300927	0.31276	0.278702	0.314839	0.308922	0.305564	0.319955	0.28206	0.309082
0.263032	0.290054	0.309722	0.322674	0.334186	0.326351	0.235689	0.308443	0.381676	0.29741
0.302207	0.275983	0.333067	0.302047	0.308602	0.232012	0.307963	0.301727	0.332427	0.319156
0.302526	0.29645	0.346178	0.321075	0.317237	0.307963	0.311001	0.31324	0.321714	0.336105
0.268788	0.306204	0.283499	0.255676	0.294532	0.26607	0.276143	0.279981	0.365206	0.290054
0.272466	0.306044	0.366965	0.333387	0.324752	0.299488	0.324752	0.330189	0.217141	0.386633
0.270547	0.324432	0.29693	0.23409	0.306044	0.283818	0.276303	0.293252	0.403742	0.28206
0.261593	0.309722	0.287176	0.257915	0.314998	0.273265	0.284138	0.29741	0.355293	0.291973
0.284138	0.325232	0.243044	0.271186	0.306204	0.304125	0.294212	0.285577	0.399904	0.231052
0.252638	0.29629	0.300927	0.275024	0.284938	0.251039	0.304765	0.305884	0.307963	0.278542
0.303166	0.310521	0.349376	0.293092	0.314519	0.285577	0.277263	0.299648	0.317557	0.342661
0.273745	0.301887	0.337384	0.285897	0.306684	0.297729	0.300448	0.308283	0.290854	0.320435
0.276783	0.301407	0.291653	0.315158	0.322674	0.319316	0.294851	0.330828	0.337224	0.298689
0.269268	0.272466	0.268628	0.278862	0.282859	0.275344	0.291653	0.303006	0.322034	0.282699
0.320435	0.314039	0.322194	0.323473	0.297889	0.32747	0.232651	0.313399	0.355772	0.316118
0.274065	0.314998	0.277582	0.249121	0.303326	0.303326	0.278222	0.279181	0.373521	0.271027
0.266869	0.325392	0.311481	0.279981	0.297729	0.309562	0.305724	0.295331	0.342341	0.303806
0.26511	0.288136	0.290054	0.262072	0.304925	0.255676	0.291334	0.284938	0.32875	0.295811
0.321234	0.318196	0.306524	0.325232	0.337704	0.319316	0.303646	0.352255	0.391429	0.294372
0.261433	0.285257	0.263671	0.243844	0.28174	0.270867	0.28142	0.278542	0.335146	0.275184
0.270067	0.315958	0.378318	0.337704	0.323793	0.307803	0.332107	0.334666	0.215862	0.388551
0.321874	0.319316	0.243684	0.319316	0.318356	0.337864	0.313719	0.314359	0.363288	0.288455
0.252638	0.279021	0.269587	0.261113	0.285098	0.26575	0.274225	0.2811	0.314519	0.275664
0.283339	0.322993	0.211225	0.29693	0.295491	0.327311	0.305564	0.322514	0.399584	0.226095
0.238248	0.330828	0.282699	0.244963	0.310681	0.306524	0.28126	0.229773	0.37576	0.282859
0.319476	0.275344	0.418452	0.351135	0.345219	0.301727	0.360729	0.362328	0.1244	0.423089
0.29677	0.280461	0.331308	0.302686	0.308762	0.259354	0.324592	0.326991	0.330828	0.325232
0.290214	0.199712	0.3118	0.337064	0.299009	0.301887	0.319955	0.351615	0.291493	0.321394
0.270867	0.320115	0.228973	0.319316	0.317077	0.338343	0.318996	0.333227	0.393668	0.245763
0.231532	0.306204	0.260473	0.298849	0.277902	0.338823	0.295971	0.321394	0.335465	0.245603
0.29645	0.360409	0.26511	0.270227	0.304125	0.308443	0.295011	0.306524	0.418132	0.253438
0.278382	0.338024	0.272146	0.310201	0.321554	0.347298	0.331148	0.332427	0.394787	0.236009
0.320755	0.289415	0.414135	0.344579	0.3433	0.308762	0.35945	0.352095	0.143108	0.408539
0.260793	0.28126	0.278382	0.262872	0.291493	0.275344	0.285737	0.292773	0.302846	0.287176
0.268788	0.335945	0.066677	0.320755	0.289575	0.348097	0.304445	0.331308	0.432363	0.225296
0.262552	0.277902	0.263351	0.270227	0.289095	0.259194	0.269907	0.286057	0.348897	0.278062
0.258235	0.303965	0.347618	0.321234	0.315158	0.323153	0.317237	0.335305	0.228973	0.351455
0.270867	0.307483	0.276143	0.314359	0.320115	0.307163	0.305085	0.334186	0.291334	0.31228
0.287176	0.31324	0.389191	0.341861	0.334826	0.317877	0.335785	0.3433	0.215062	0.395907
0.280141	0.290694	0.272626	0.255357	0.308123	0.294372	0.257755	0.288295	0.385833	0.273105
0.307803	0.292293	0.320755	0.294851	0.329229	0.264791	0.326191	0.324912	0.298529	0.329869
0.337384	0.278382	0.395107	0.367605	0.34442	0.304925	0.37512	0.377678	0.174288	0.396386
0.318836	0.236649	0.337064	0.344579	0.278222	0.295811	0.340422	0.352255	0.315158	0.318036
0.283658	0.303006	0.382635	0.342181	0.323953	0.318996	0.32859	0.341701	0.200352	0.387752
0.269428	0.275184	0.298529	0.289415	0.305085	0.304925	0.261433	0.299808	0.391429	0.274544
0.330668	0.327151	0.386633	0.349696	0.353054	0.340102	0.339303	0.301567	0.256796	0.381836
0.288455	0.304125	0.294691	0.299488	0.306844	0.292133	0.2811	0.318836	0.342341	0.313719
0.228654	0.309882	0.240166	0.295011	0.288615	0.327151	0.2819	0.309082	0.361369	0.291813
0.276143	0.322034	0.293572	0.251679	0.315638	0.228814	0.287176	0.278222	0.407419	0.29661
0.273105	0.317717	0.308602	0.235689	0.322194	0.154301	0.279981	0.264151	0.395587	0.293252
0.261912	0.299328	0.336904	0.29645	0.292293	0.302686	0.311161	0.326031	0.26543	0.32763
0.278702	0.315798	0.283658	0.251679	0.304125	0.243204	0.284938	0.28078	0.399904	0.293572
0.28094	0.315318	0.311641	0.262872	0.308762	0.228014	0.288455	0.285577	0.383115	0.301727
0.299968	0.275664	0.364567	0.337224	0.358331	0.29645	0.341222	0.356092	0.251199	0.368084
0.261912	0.31324	0.283179	0.279661	0.288615	0.290854	0.295651	0.310521	0.325392	0.283818

For Review Only

0.28078	0.322674	0.316118	0.224656	0.335945	0.28062	0.284618	0.297729	0.392869	0.301727
0.28126	0.284618	0.323633	0.299169	0.307163	0.290694	0.309402	0.318516	0.255197	0.319635
0.276943	0.335465	0.261753	0.274384	0.29677	0.29709	0.295171	0.306844	0.404861	0.277582
0.332267	0.258555	0.368084	0.355932	0.291493	0.316118	0.29709	0.336585	0.29645	0.373681
0.287496	0.331308	0.349536	0.335785	0.348897	0.335305	0.345219	0.3126	0.352255	0.355772
0.303166	0.263991	0.32779	0.340902	0.315958	0.308123	0.330508	0.345699	0.293732	0.322993
0.236968	0.335785	0.263991	0.258075	0.29629	0.307643	0.288935	0.29629	0.404061	0.269428
0.29661	0.29693	0.327151	0.284298	0.322993	0.302526	0.327311	0.347458	0.29613	0.309722
0.31276	0.181484	0.356572	0.333387	0.34346	0.302367	0.331628	0.352255	0.201311	0.368724
0.246402	0.279501	0.274864	0.259354	0.282539	0.257275	0.274704	0.286057	0.315958	0.277902
0.323473	0.301727	0.347138	0.331948	0.309882	0.302846	0.335146	0.365526	0.259354	0.361369
0.287976	0.352894	0.213144	0.298529	0.295171	0.337224	0.334826	0.32843	0.42181	0.215382
0.269587	0.303486	0.280461	0.247681	0.293092	0.239847	0.292613	0.273905	0.373041	0.2811
0.290374	0.1882	0.34362	0.302526	0.305245	0.273265	0.301567	0.314039	0.248161	0.326991
0.254557	0.29757	0.268948	0.263032	0.271346	0.275024	0.261273	0.299808	0.380716	0.276943
0.259514	0.31276	0.284938	0.31196	0.290694	0.332587	0.284298	0.322674	0.338663	0.309242
0.283499	0.299648	0.389511	0.333067	0.323953	0.315478	0.331788	0.338024	0.206268	0.384394
0.350016	0.268788	0.351615	0.35929	0.314679	0.301087	0.369204	0.367285	0.266709	0.346978
0.256956	0.295811	0.290534	0.288455	0.290054	0.302047	0.279021	0.305405	0.309242	0.286377
0.285098	0.336265	0.301727	0.255996	0.317557	0.314679	0.29677	0.306204	0.396706	0.284458
0.254077	0.300288	0.28174	0.256636	0.307163	0.268468	0.269587	0.269428	0.34378	0.277103
0.278862	0.320275	0.242085	0.267669	0.301407	0.29741	0.289415	0.284618	0.391909	0.228174
0.251679	0.273425	0.261753	0.262072	0.283019	0.275344	0.277103	0.289255	0.307643	0.269587
0.290214	0.278542	0.383275	0.334186	0.326991	0.291813	0.341701	0.335146	0.159898	0.392229
0.310521	0.238727	0.361529	0.35977	0.314359	0.311001	0.36009	0.3748	0.254237	0.3433
0.272626	0.316278	0.322993	0.20291	0.329549	0.292933	0.286217	0.288935	0.397186	0.288615
0.310681	0.317237	0.254877	0.350496	0.311321	0.363128	0.31308	0.318516	0.345059	0.307643
0.259834	0.305884	0.339942	0.32747	0.318516	0.325072	0.330349	0.338343	0.233131	0.350656
0.256796	0.293892	0.284138	0.257275	0.288455	0.287816	0.245443	0.284778	0.3748	0.272785
0.288615	0.308123	0.31212	0.308283	0.316597	0.313399	0.283019	0.332907	0.334826	0.301247
0.272466	0.295171	0.294372	0.285577	0.333547	0.302846	0.287496	0.32891	0.35993	0.273905
0.279821	0.320435	0.158938	0.304765	0.297889	0.335625	0.286057	0.32891	0.399744	0.221458
0.271027	0.307483	0.288295	0.283658	0.324272	0.31212	0.292453	0.310841	0.360729	0.291653
0.288455	0.32763	0.250879	0.314199	0.319476	0.308922	0.31212	0.326351	0.42149	0.252159
0.252958	0.316597	0.260153	0.319156	0.306684	0.334186	0.311161	0.334986	0.387592	0.244484
0.284138	0.297729	0.384394	0.32891	0.322194	0.31196	0.331468	0.339303	0.208507	0.380397
0.254877	0.334506	0.24944	0.31212	0.313879	0.332267	0.306204	0.335465	0.39063	0.252319
0.271826	0.316278	0.322833	0.202271	0.32795	0.292613	0.285897	0.287656	0.396067	0.288455
0.288295	0.272945	0.302367	0.277103	0.305085	0.287336	0.314519	0.313559	0.3748	0.306364
0.261433	0.340422	0.260953	0.283339	0.284618	0.34426	0.310841	0.333706	0.376399	0.263671
0.272146	0.318676	0.313399	0.23489	0.315478	0.15686	0.274384	0.263991	0.395587	0.295971
0.275024	0.327311	0.28142	0.228494	0.301887	0.300927	0.26607	0.301087	0.380397	0.306204
0.284618	0.342981	0.287336	0.209146	0.295811	0.288136	0.288935	0.261273	0.415574	0.278382
0.283499	0.319316	0.292933	0.254877	0.315638	0.221458	0.283818	0.2819	0.40646	0.299488
0.319635	0.278062	0.42197	0.353534	0.341222	0.297889	0.361209	0.357851	0.130636	0.419731
0.293572	0.285417	0.269587	0.28142	0.327311	0.26495	0.319635	0.324752	0.355133	0.278062
0.272785	0.324272	0.322514	0.289255	0.332747	0.301887	0.304445	0.300768	0.32763	0.313559
0.284938	0.340422	0.304445	0.271186	0.309562	0.362968	0.317077	0.322674	0.354813	0.308123
0.349536	0.267349	0.350336	0.357052	0.31292	0.300448	0.368244	0.366326	0.267189	0.346019
0.29725	0.298049	0.364567	0.345539	0.362968	0.318516	0.344579	0.351935	0.153022	0.397186
0.251359	0.294052	0.35961	0.32843	0.316278	0.304285	0.316757	0.331948	0.216182	0.357531
0.299808	0.32875	0.260793	0.295491	0.271027	0.315798	0.294532	0.321234	0.347777	0.303326
0.295811	0.345219	0.279981	0.279821	0.322993	0.322993	0.301087	0.319955	0.401983	0.261273
0.301247	0.311481	0.382155	0.331788	0.346178	0.330029	0.339303	0.352894	0.253598	0.379117
0.309882	0.255676	0.372402	0.348417	0.311001	0.322034	0.352255	0.368564	0.225616	0.369843

For Review Only

0.26511	0.316917	0.301247	0.217141	0.310521	0.279341	0.287336	0.270067	0.395107	0.284458
0.331308	0.268628	0.417813	0.347618	0.339303	0.293412	0.361049	0.376399	0.146306	0.420371
0.284458	0.316917	0.306844	0.303965	0.299648	0.304285	0.295491	0.326351	0.306044	0.299968
0.304445	0.305405	0.346818	0.334026	0.334666	0.314359	0.303326	0.339463	0.325552	0.315638
0.289095	0.360729	0.259354	0.276143	0.304445	0.315478	0.310201	0.306044	0.423729	0.244004
0.323953	0.283339	0.325392	0.358011	0.305245	0.335305	0.339623	0.357052	0.322194	0.326191
0.299009	0.310201	0.332587	0.318516	0.347138	0.309082	0.314998	0.319795	0.318996	0.337704
0.283179	0.338503	0.270547	0.288295	0.307004	0.282699	0.28206	0.291493	0.413176	0.260473
0.300448	0.283818	0.362968	0.34314	0.331788	0.288935	0.322194	0.345859	0.272466	0.362648
0.327151	0.342181	0.425168	0.355612	0.358491	0.354333	0.334986	0.325871	0.273265	0.419092
0.266869	0.300927	0.348417	0.305564	0.307004	0.294052	0.294372	0.309242	0.252958	0.345699
0.329069	0.285577	0.422769	0.353214	0.347138	0.294052	0.369524	0.35881	0.10873	0.42117
0.25072	0.275664	0.263032	0.261433	0.278222	0.272945	0.273265	0.283818	0.306204	0.270547
0.301887	0.300128	0.333547	0.316118	0.353374	0.317397	0.307803	0.333387	0.326031	0.333387
0.288295	0.321874	0.297889	0.295971	0.15686	0.307643	0.317717	0.315638	0.361209	0.313879
0.256636	0.307963	0.272626	0.244324	0.288935	0.277263	0.295011	0.270387	0.347777	0.294691
0.298049	0.340262	0.295011	0.232651	0.308123	0.275664	0.2811	0.276143	0.407579	0.290534
0.310841	0.285577	0.292133	0.320115	0.2819	0.316757	0.326831	0.342021	0.331468	0.29693
0.300288	0.263831	0.378478	0.313559	0.324272	0.28094	0.338823	0.340102	0.196354	0.380237
0.314359	0.314519	0.330189	0.333227	0.321234	0.338663	0.340262	0.337224	0.263351	0.32779
0.302526	0.284458	0.360889	0.345859	0.346658	0.3126	0.341701	0.361529	0.205628	0.388871
0.316917	0.286217	0.311641	0.324912	0.293572	0.298209	0.317397	0.329549	0.350815	0.31244
0.308762	0.299169	0.369524	0.315798	0.347618	0.298689	0.321234	0.34314	0.297729	0.364727
0.278062	0.326031	0.305724	0.262392	0.318036	0.246882	0.278862	0.28062	0.392389	0.297889
0.279981	0.347777	0.196194	0.291653	0.26575	0.355453	0.315798	0.316278	0.399264	0.223537
0.326511	0.128718	0.319795	0.314998	0.321714	0.268788	0.310681	0.336425	0.295171	0.306044
0.313559	0.316278	0.414934	0.336745	0.352574	0.316437	0.344899	0.338343	0.191557	0.413176
0.299648	0.334986	0.299328	0.292773	0.3126	0.330668	0.329549	0.300288	0.369524	0.297729
0.331628	0.292133	0.432523	0.362808	0.361049	0.298529	0.369524	0.358331	0.052446	0.432203
0.290054	0.341541	0.284938	0.23473	0.287016	0.284298	0.286697	0.282699	0.40662	0.26559
0.274065	0.313399	0.198593	0.302207	0.322993	0.319316	0.323953	0.326351	0.368084	0.271666
0.292773	0.353214	0.255996	0.282699	0.322034	0.320115	0.305405	0.320595	0.40678	0.243844
0.321234	0.259194	0.401823	0.342501	0.32827	0.274544	0.358491	0.361529	0.142629	0.417333
0.294691	0.29725	0.324752	0.263831	0.32843	0.273265	0.303166	0.301407	0.326671	0.324432
0.286537	0.304445	0.333387	0.326351	0.294851	0.329549	0.287336	0.32747	0.304125	0.330349
0.275983	0.295651	0.306204	0.259994	0.301567	0.201791	0.274384	0.292293	0.364727	0.309242
0.309402	0.241445	0.353694	0.360409	0.311481	0.298369	0.355612	0.3748	0.267349	0.340102
0.330508	0.291973	0.430125	0.361209	0.360889	0.314998	0.37576	0.365686	0.102175	0.424049
0.263351	0.3441	0.183243	0.273105	0.289575	0.329869	0.304445	0.305884	0.403902	0.229133
0.272945	0.317237	0.323153	0.20387	0.329869	0.294052	0.287496	0.288455	0.397985	0.289415
0.254077	0.295171	0.322034	0.264631	0.299488	0.277902	0.300128	0.288935	0.308443	0.292933
0.331628	0.243524	0.42149	0.352255	0.350496	0.285737	0.360729	0.372881	0.136073	0.423729
0.267349	0.31228	0.262872	0.320755	0.287336	0.34378	0.29645	0.332267	0.409818	0.228494
0	0.313719	0.269587	0.28142	0.302207	0.305724	0.282219	0.305085	0.330988	0.290694
0.313719	0	0.334026	0.31308	0.315798	0.288935	0.298049	0.351455	0.287976	0.327151
0.269587	0.334026	0	0.313399	0.290534	0.353374	0.318836	0.334666	0.434762	0.207227
0.28142	0.31308	0.313399	0	0.319955	0.275184	0.284938	0.279981	0.361848	0.310841
0.302207	0.315798	0.290534	0.319955	0	0.321394	0.301247	0.320275	0.35945	0.306684
0.305724	0.288935	0.353374	0.275184	0.321394	0	0.330828	0.315638	0.301087	0.341062
0.282219	0.298049	0.318836	0.284938	0.301247	0.330828	0	0.298209	0.369044	0.31308
0.305085	0.351455	0.334666	0.279981	0.320275	0.315638	0.298209	0	0.357531	0.334826
0.330988	0.287976	0.434762	0.361848	0.35945	0.301087	0.369044	0.357531	0	0.431884
0.290694	0.327151	0.207227	0.310841	0.306684	0.341062	0.31308	0.334826	0.431884	0
0.331468	0.257755	0.417493	0.347618	0.339463	0.279341	0.363767	0.376719	0.138152	0.42245
0.284298	0.342341	0.287336	0.208507	0.29661	0.290214	0.289575	0.259834	0.414934	0.279181

For Review Only

0.299169	0.349536	0.277263	0.244643	0.28062	0.302047	0.273905	0.298849	0.427726	0.284138
0.326831	0.172849	0.370163	0.338663	0.345539	0.286856	0.339143	0.357371	0.247202	0.361529
0.282219	0.32811	0.191717	0.320435	0.259834	0.347458	0.331148	0.334346	0.419412	0.207387
0.279021	0.34362	0.300288	0.238088	0.31212	0.302686	0.290214	0.287656	0.400224	0.283019

For Review Only

0.376079	0.28078	0.300448	0.357531	0.277422	0.308602
0.319316	0.31212	0.316757	0.311161	0.357851	0.282379
0.408698	0.006876	0.222737	0.348417	0.289735	0.21874
0.371602	0.240486	0.219699	0.330828	0.291973	0.258555
0.342661	0.355612	0.373041	0.354813	0.290214	0.354973
0.395907	0.261113	0.25024	0.354013	0.291653	0.258714
0.388551	0.314839	0.303326	0.355612	0.270547	0.316597
0.292453	0.340742	0.342661	0.315638	0.352734	0.334986
0.373361	0.319795	0.31292	0.336745	0.258235	0.313399
0.29741	0.371602	0.379277	0.335465	0.384554	0.373201
0.240166	0.351615	0.356092	0.26511	0.233611	0.364407
0.401823	0.070515	0.189159	0.344739	0.28126	0.211065
0.324752	0.315638	0.316757	0.339463	0.304605	0.3126
0.279181	0.32875	0.339783	0.299328	0.332747	0.32875
0.368564	0.269268	0.270867	0.323153	0.259994	0.270067
0.307163	0.267349	0.31228	0.302846	0.29661	0.298689
0.380556	0.32875	0.325392	0.365526	0.268308	0.34314
0.356732	0.306844	0.314199	0.333227	0.322034	0.313559
0.417173	0.245603	0.247362	0.377678	0.253758	0.236489
0.329869	0.366965	0.366485	0.32795	0.381356	0.373201
0.315798	0.345859	0.321554	0.345379	0.32763	0.349376
0.368884	0.32747	0.315158	0.392549	0.26623	0.333706
0.204029	0.397186	0.404701	0.255836	0.37512	0.393508
0.232651	0.354173	0.35993	0.272466	0.342181	0.341382
0.397825	0.267988	0.272785	0.350336	0.246402	0.255197
0.396866	0.230253	0.240326	0.355293	0.309882	0.236808
0.373521	0.21842	0.224816	0.341062	0.288455	0.205788
0.306844	0.257755	0.26575	0.287336	0.269428	0.254237
0.319955	0.330988	0.31212	0.313399	0.31276	0.313399
0.25024	0.429965	0.427087	0.326511	0.430285	0.416054
0.398625	0.235849	0.253118	0.365846	0.257115	0.21842
0.409338	0.251839	0.293252	0.369044	0.268628	0.29629
0.402143	0.289415	0.325232	0.367445	0.245763	0.318836
0.230892	0.363767	0.364247	0.31212	0.356092	0.34346
0.310042	0.321714	0.336745	0.274225	0.337704	0.324272
0.31212	0.275024	0.278702	0.306524	0.293412	0.260793
0.330508	0.314359	0.340422	0.282539	0.308123	0.304605
0.32859	0.284938	0.311161	0.252958	0.303006	0.325712
0.082028	0.397186	0.416054	0.212344	0.409658	0.385833
0.248641	0.346818	0.365846	0.130796	0.342821	0.366645
0.246083	0.408858	0.42165	0.313559	0.42245	0.400544
0.298529	0.345539	0.345379	0.301567	0.366006	0.340582
0.299648	0.361049	0.366006	0.310681	0.378798	0.35865
0.355293	0.354653	0.344739	0.334826	0.309242	0.352414
0.323953	0.37448	0.367445	0.32859	0.383435	0.373521
0.404701	0.10889	0.196194	0.346178	0.279821	0.221938
0.397346	0.226095	0.244963	0.354813	0.307163	0.229613
0.299169	0.291334	0.292293	0.289095	0.316118	0.306364
0.348097	0.349856	0.344899	0.345059	0.316917	0.350815
0.393828	0.273425	0.283019	0.349217	0.235689	0.302526

For Review Only

0.397186	0.216022	0.228494	0.351135	0.298209	0.244324
0.334186	0.287816	0.283499	0.324912	0.290534	0.275504
0.420691	0.295971	0.285257	0.376719	0.21874	0.295971
0.401023	0.289735	0.324432	0.367605	0.246242	0.319635
0.393828	0.241445	0.251359	0.356092	0.271027	0.21842
0.370803	0.330189	0.317877	0.367925	0.263351	0.333866
0.416214	0.246562	0.247362	0.377039	0.254077	0.236329
0.400384	0.23457	0.224017	0.352095	0.309882	0.217301
0.39047	0.232971	0.210425	0.341222	0.295651	0.229933
0.195875	0.403422	0.409018	0.278702	0.403262	0.391589
0.295491	0.336904	0.339942	0.317237	0.321234	0.325392
0.316757	0.243044	0.257755	0.292133	0.267509	0.257435
0.392389	0.205469	0.21938	0.361529	0.282859	0.194755
0.392549	0.215062	0.230732	0.363128	0.306044	0.199552
0.420371	0.29661	0.283499	0.377039	0.21858	0.295331
0.319955	0.329549	0.311481	0.31308	0.31212	0.313399
0.334346	0.264631	0.270387	0.307163	0.283019	0.262872
0.308922	0.337544	0.346658	0.287656	0.335785	0.337064
0.321714	0.258714	0.280141	0.294691	0.310681	0.291014
0.428846	0.241126	0.277263	0.377518	0.266549	0.290054
0.415574	0.290534	0.26527	0.376719	0.227215	0.276783
0.40694	0.292133	0.295651	0.369204	0.262872	0.306364
0.358331	0.2181	0.232491	0.332907	0.292453	0.199392
0.241765	0.360889	0.364886	0.221618	0.335945	0.353854
0.341541	0.32747	0.307163	0.316278	0.286377	0.318196
0.370483	0.285417	0.305405	0.361209	0.309082	0.26623
0.323313	0.373681	0.367445	0.327311	0.382795	0.373201
0.383754	0.215542	0.237608	0.355133	0.299648	0.185321
0.348737	0.340102	0.356412	0.364407	0.353374	0.362488
0.131436	0.399424	0.414615	0.239847	0.411417	0.393188
0.393348	0.235849	0.24992	0.356412	0.309242	0.253598
0.399264	0.283179	0.283339	0.357851	0.208507	0.28142
0.345539	0.32747	0.329869	0.323793	0.284618	0.325072
0.283658	0.317397	0.316917	0.301247	0.324912	0.303006
0.134474	0.420051	0.428846	0.246242	0.417973	0.399584
0.418132	0.29613	0.283818	0.376399	0.217621	0.294372
0.350975	0.298209	0.321874	0.323633	0.279341	0.318356
0.323633	0.37464	0.367765	0.32811	0.383115	0.373041
0.299808	0.351455	0.354013	0.308283	0.35881	0.336585
0.331628	0.366326	0.346338	0.373681	0.338024	0.356732
0.325552	0.300768	0.287816	0.348737	0.287336	0.305405
0.317877	0.355133	0.342661	0.333067	0.364407	0.363607
0.416214	0.286217	0.295011	0.362008	0.205948	0.307004
0.426767	0.216342	0.248641	0.364567	0.245763	0.241925
0.366645	0.303326	0.274065	0.339143	0.316278	0.316278
0.392709	0.205948	0.220339	0.362008	0.285098	0.196035
0.37512	0.295491	0.308283	0.365846	0.211705	0.319955
0.4063	0.259674	0.254877	0.353854	0.282699	0.248321
0.209306	0.366326	0.387752	0.286217	0.365046	0.362328
0.354493	0.256316	0.236169	0.32875	0.305245	0.244963
0.363767	0.248801	0.260313	0.332907	0.288136	0.269428
0.369044	0.319795	0.308283	0.338024	0.314199	0.305245
0.368244	0.236808	0.228014	0.333866	0.294532	0.208826
0.084906	0.4055	0.42213	0.21954	0.415094	0.401343
0.232171	0.382955	0.387592	0.308123	0.380077	0.379437

For Review Only

0.287016	0.298529	0.303166	0.300608	0.31308	0.279501
0.385993	0.299968	0.301567	0.354333	0.315318	0.29613
0.326351	0.288615	0.288136	0.285098	0.321234	0.295651
0.323313	0.327311	0.320115	0.273425	0.326991	0.314039
0.367445	0.253758	0.240166	0.329549	0.282699	0.229293
0.227055	0.360889	0.380716	0.276943	0.372402	0.368404
0.393668	0.205788	0.225136	0.354333	0.295331	0.195875
0.367605	0.228814	0.250879	0.320115	0.298529	0.238248
0.387272	0.20243	0.235849	0.338343	0.256636	0.240166
0.311161	0.266869	0.297889	0.308283	0.293412	0.264151
0.304605	0.291174	0.317397	0.317557	0.34426	0.318676
0.294691	0.29693	0.300608	0.309082	0.32875	0.298049
0.34442	0.322993	0.334826	0.319635	0.29741	0.323473
0.314359	0.26543	0.280461	0.28142	0.276943	0.270547
0.345059	0.306364	0.267029	0.345859	0.340422	0.308283
0.374001	0.226415	0.233131	0.341701	0.277103	0.200672
0.34362	0.287816	0.294052	0.341382	0.302367	0.240166
0.323793	0.259674	0.274065	0.294851	0.29629	0.260793
0.403102	0.293412	0.29629	0.340582	0.317717	0.313719
0.330668	0.228654	0.247202	0.307963	0.270387	0.24928
0.229773	0.368244	0.39047	0.287816	0.372562	0.368724
0.356572	0.311641	0.299968	0.329069	0.293732	0.315798
0.307963	0.255676	0.257915	0.293092	0.276943	0.256956
0.398305	0.276783	0.287176	0.342341	0.204509	0.28142
0.380237	0.171091	0.258555	0.34426	0.284778	0.239047
0.14135	0.404541	0.418132	0.248801	0.416693	0.399744
0.321234	0.289095	0.294372	0.288935	0.325871	0.293572
0.271986	0.34362	0.336904	0.209946	0.307803	0.340582
0.379757	0.294691	0.292293	0.344579	0.259994	0.286377
0.333067	0.295971	0.311001	0.311161	0.252478	0.277263
0.419412	0.223857	0.191717	0.383275	0.276303	0.233451
0.398625	0.305245	0.287336	0.354653	0.280461	0.310521
0.165814	0.395107	0.40662	0.25072	0.401343	0.387432
0.300608	0.272466	0.280461	0.29709	0.283818	0.273745
0.418292	0.285577	0.294532	0.361209	0.20371	0.308922
0.340262	0.258714	0.271666	0.302686	0.275983	0.266549
0.231212	0.362168	0.363288	0.310201	0.354973	0.342661
0.293252	0.333547	0.322514	0.315798	0.329869	0.32811
0.228814	0.378957	0.394148	0.2819	0.381996	0.37512
0.381836	0.23377	0.245123	0.340102	0.271986	0.222258
0.293572	0.31324	0.315798	0.279821	0.315318	0.314519
0.163575	0.401823	0.412856	0.244484	0.346818	0.400544
0.295011	0.335785	0.329389	0.242565	0.305245	0.332587
0.21858	0.376239	0.392229	0.284938	0.376239	0.371282
0.391589	0.263511	0.267189	0.363288	0.279021	0.26559
0.262392	0.382955	0.38999	0.310521	0.388551	0.377678
0.339783	0.288136	0.29709	0.31196	0.307323	0.301727
0.364567	0.287976	0.299488	0.338823	0.279981	0.277902
0.399424	0.222258	0.233291	0.347458	0.301087	0.243204
0.392229	0.21874	0.232331	0.338663	0.309402	0.221138
0.257755	0.330828	0.34346	0.279341	0.32859	0.324912
0.398465	0.213623	0.228494	0.34394	0.29645	0.237928
0.383435	0.252159	0.252319	0.337704	0.309242	0.254397
0.228014	0.375919	0.377838	0.259354	0.357531	0.369204
0.326031	0.271186	0.301407	0.311001	0.279181	0.285737

For Review Only

0.397026	0.227215	0.241445	0.366485	0.315318	0.225456
0.245603	0.331468	0.331628	0.273745	0.325552	0.330189
0.399264	0.236009	0.255676	0.356892	0.261912	0.274864
0.282699	0.369364	0.348097	0.26527	0.358011	0.362328
0.352894	0.338503	0.358331	0.332907	0.34442	0.349057
0.2819	0.340582	0.358171	0.262712	0.324752	0.341382
0.396546	0.252638	0.255996	0.362808	0.284458	0.243524
0.288935	0.333706	0.350496	0.232491	0.32795	0.347937
0.210425	0.362328	0.384714	0.212024	0.363128	0.372402
0.307963	0.253438	0.266549	0.293092	0.28078	0.259514
0.23489	0.349536	0.374161	0.283019	0.347138	0.354813
0.417173	0.28126	0.274225	0.381356	0.189639	0.278702
0.368884	0.213303	0.229293	0.32763	0.28094	0.241925
0.240006	0.340422	0.345699	0.213623	0.338983	0.322194
0.379277	0.231052	0.208507	0.332747	0.273905	0.237928
0.337704	0.314679	0.318516	0.32859	0.301887	0.306204
0.212344	0.365846	0.381676	0.276623	0.37528	0.364727
0.217941	0.365846	0.3748	0.248321	0.262072	0.384074
0.306524	0.28094	0.294052	0.302526	0.283978	0.292133
0.397825	0.244004	0.247202	0.358331	0.301087	0.220819
0.349856	0.244324	0.259834	0.317877	0.283499	0.257115
0.381036	0.20339	0.23473	0.331148	0.253758	0.236489
0.307963	0.254077	0.262392	0.287336	0.264471	0.263511
0.183083	0.376879	0.385993	0.258555	0.37464	0.362328
0.25008	0.37496	0.372721	0.239367	0.337384	0.35977
0.392229	0.216342	0.231372	0.363767	0.305724	0.200032
0.348897	0.348417	0.330349	0.339783	0.307963	0.332907
0.232491	0.363927	0.362968	0.308762	0.32747	0.346498
0.377039	0.227215	0.167093	0.336105	0.283339	0.231212
0.338184	0.308762	0.31324	0.321394	0.314839	0.317717
0.351295	0.29725	0.299488	0.336745	0.308283	0.273905
0.39047	0.268308	0.282219	0.333547	0.261113	0.298049
0.366006	0.258075	0.277263	0.340262	0.305245	0.259994
0.423569	0.272146	0.278542	0.378158	0.239207	0.293732
0.374161	0.293572	0.304925	0.344579	0.253278	0.29757
0.209786	0.368244	0.381676	0.273585	0.374001	0.363767
0.383754	0.305085	0.307483	0.347777	0.275184	0.307323
0.392069	0.215542	0.230093	0.362328	0.305405	0.200991
0.35993	0.257915	0.26575	0.292293	0.300448	0.248801
0.368724	0.285098	0.305564	0.333227	0.267988	0.291653
0.388391	0.212664	0.229453	0.337544	0.311001	0.223857
0.379437	0.248641	0.204829	0.351135	0.290854	0.216661
0.407099	0.009114	0.221618	0.348097	0.290534	0.21874
0.396386	0.227374	0.238887	0.342181	0.305085	0.246562
0.132875	0.402303	0.423729	0.241286	0.413335	0.394627
0.339143	0.271346	0.291334	0.263831	0.284778	0.289255
0.337224	0.285417	0.314839	0.323633	0.307963	0.301087
0.348897	0.290694	0.309562	0.355932	0.31244	0.293572
0.216661	0.364407	0.373681	0.246562	0.259994	0.381996
0.186441	0.371922	0.407419	0.252478	0.37464	0.388871
0.223697	0.367445	0.367925	0.271826	0.347138	0.351455
0.338823	0.29613	0.289255	0.339942	0.277742	0.29613
0.409978	0.255197	0.255516	0.369524	0.28174	0.26543
0.261593	0.380716	0.398305	0.279341	0.377678	0.367445
0.216342	0.387272	0.385034	0.244803	0.354333	0.368564

For Review Only

0.391749	0.204989	0.219699	0.361209	0.284138	0.195075
0.106172	0.405341	0.42133	0.221458	0.408219	0.396386
0.310681	0.287176	0.334346	0.305564	0.307483	0.31212
0.324432	0.340422	0.356412	0.286856	0.341062	0.354653
0.426447	0.17157	0.262232	0.377039	0.26543	0.267189
0.309242	0.354333	0.346498	0.284458	0.32875	0.347458
0.325552	0.339303	0.362168	0.307483	0.344899	0.352734
0.408858	0.256956	0.244324	0.367605	0.275184	0.266549
0.254237	0.354973	0.35881	0.261912	0.379597	0.374001
0.259194	0.399105	0.401023	0.310201	0.42245	0.398945
0.259994	0.32811	0.334026	0.29709	0.340582	0.325072
0.128558	0.414775	0.424049	0.2496	0.412376	0.400704
0.301727	0.261113	0.262392	0.292293	0.269268	0.260313
0.333866	0.31308	0.311481	0.303965	0.349696	0.305884
0.346338	0.303006	0.283818	0.354493	0.260793	0.308283
0.351775	0.242085	0.243364	0.319955	0.275983	0.23473
0.399904	0.097697	0.211385	0.354653	0.299648	0.225616
0.334346	0.336904	0.348257	0.302207	0.163735	0.350496
0.15686	0.35897	0.377518	0.245443	0.383754	0.356252
0.284298	0.360729	0.349696	0.303486	0.326031	0.35929
0.233611	0.377678	0.39095	0.258395	0.365206	0.376399
0.324592	0.293892	0.309562	0.253918	0.303326	0.340582
0.292133	0.318516	0.325392	0.28158	0.358171	0.290534
0.391429	0.233451	0.239207	0.3441	0.304605	0.244484
0.389671	0.284618	0.267029	0.368244	0.177486	0.295171
0.263032	0.332107	0.347458	0.180684	0.326031	0.34378
0.195875	0.402942	0.409018	0.278542	0.403902	0.39047
0.366166	0.271346	0.28206	0.358491	0.303965	0.271027
0.135433	0.417333	0.431244	0.255197	0.416853	0.405341
0.402782	0.195715	0.129037	0.355612	0.279981	0.201311
0.35897	0.315318	0.32763	0.332427	0.270547	0.315958
0.411417	0.246083	0.246882	0.377838	0.260633	0.257915
0.139111	0.387432	0.401663	0.220499	0.401183	0.39047
0.316597	0.257275	0.289575	0.297889	0.315478	0.269428
0.298849	0.341701	0.340742	0.304765	0.327151	0.334186
0.35897	0.252478	0.233291	0.320275	0.310201	0.259674
0.250879	0.370323	0.367925	0.231212	0.32891	0.362008
0.14039	0.417493	0.428206	0.244484	0.425168	0.404061
0.395907	0.236968	0.26511	0.363447	0.238088	0.279821
0.393508	0.216821	0.231692	0.363607	0.305884	0.201151
0.310361	0.274864	0.278382	0.305724	0.292933	0.259034
0.066517	0.404701	0.426607	0.196674	0.417173	0.396866
0.40566	0.28206	0.284458	0.362968	0.261433	0.290214
0.331468	0.284298	0.299169	0.326831	0.282219	0.279021
0.257755	0.342341	0.349536	0.172849	0.32811	0.34362
0.417493	0.287336	0.277263	0.370163	0.191717	0.300288
0.347618	0.208507	0.244643	0.338663	0.320435	0.238088
0.339463	0.29661	0.28062	0.345539	0.259834	0.31212
0.279341	0.290214	0.302047	0.286856	0.347458	0.302686
0.363767	0.289575	0.273905	0.339143	0.331148	0.290214
0.376719	0.259834	0.298849	0.357371	0.334346	0.287656
0.138152	0.414934	0.427726	0.247202	0.419412	0.400224
0.42245	0.279181	0.284138	0.361529	0.207387	0.283019
0	0.408539	0.420051	0.213623	0.417493	0.397985
0.408539	0	0.223377	0.347618	0.290854	0.21954

For Review Only

0.420051	0.223377	0	0.368884	0.291813	0.224017
0.213623	0.347618	0.368884	0	0.360889	0.372562
0.417493	0.290854	0.291813	0.360889	0	0.301087
0.397985	0.21954	0.224017	0.372562	0.301087	0

For Review Only

Table S4 :genotype data

ID	SNP1	SNP1	SNP2	SNP2	SNP3	SNP3	SNP4	SNP4
1	4	4	3	3	1	1	1	1
2	4	4	4	4	4	4	1	1
3	4	4	3	3	1	1	1	1
4	4	4	3	3	4	4	3	3
5	4	4	3	3	4	4	3	3
6	4	4	4	4	4	4	1	1
7	4	4	3	3	4	4	1	1
8	4	4	3	3	4	4	1	1
9	4	4	3	3	4	4	1	1
10	4	4	3	3	4	4	3	3
11	1	1	3	3	4	4	1	1
12	4	4	3	3	4	4	1	1
13	4	4	3	3	4	4	3	3
14	4	4	3	3	4	4	1	1
15	4	4	3	3	4	4	1	1
16	4	4	3	3	1	1	3	3
17	4	4	3	3	1	1	3	3
18	1	1	3	3	4	4	1	1
19	4	4	3	3	4	4	1	1
20	1	1	3	3	4	4	3	3
21	4	4	3	3	4	4	3	3
22	4	4	3	3	4	4	3	3
23	1	1	3	3	4	4	1	1
24	1	1	3	3	4	4	3	3
25	4	4	3	3	4	4	1	1
26	4	4	3	3	4	4	1	1
27	4	4	3	3	4	4	1	1
28	4	4	3	3	4	4	1	1
29	4	4	3	3	4	4	3	3
30	1	1	3	3	4	4	3	3
31	4	4	3	3	4	4	1	1
32	4	4	4	4	1	1	1	1
33	4	4	3	3	4	4	1	1
34	4	4	3	3	4	4	3	3
35	4	4	3	3	4	4	1	1
36	4	4	3	3	4	4	1	1
37	4	4	3	3	4	4	1	1
38	4	4	3	3	4	4	1	1
39	1	1	3	3	4	4	3	3
40	4	4	3	3	4	4	1	1
41	1	1	3	3	4	4	3	3
42	1	1	3	3	4	4	3	3
43	4	4	3	3	4	4	3	3
44	1	1	3	3	4	4	1	1
45	1	1	3	3	4	4	1	1
46	4	4	3	3	1	1	1	1
47	4	4	3	3	4	4	1	1
48	1	1	3	3	4	4	1	1
49	4	4	3	3	4	4	3	3
50	4	4	3	3	1	1	1	1
51	4	4	3	3	4	4	1	1
52	4	4	3	3	4	4	1	1

For Review Only

53	4	4	3	3	4	4	1	1
54	4	4	3	3	4	4	1	1
55	4	4	3	3	4	4	1	1
56	4	4	3	3	4	4	1	1
57	4	4	3	3	4	4	1	1
58	4	4	3	3	4	4	1	1
59	4	4	3	3	4	4	1	1
60	1	1	3	3	4	4	3	3
61	4	4	3	3	4	4	1	1
62	4	4	3	3	4	4	1	1
63	4	4	4	4	4	4	1	1
64	4	4	3	3	4	4	1	1
65	4	4	3	3	4	4	1	1
66	4	4	3	3	4	4	3	3
67	4	4	3	3	4	4	1	1
68	4	4	3	3	4	4	1	1
69	1	1	3	3	4	4	1	1
70	4	4	3	3	1	1	1	1
71	4	4	3	3	4	4	1	1
72	4	4	3	3	1	1	1	1
73	4	4	3	3	4	4	1	1
74	1	1	3	3	4	4	3	3
75	4	4	3	3	4	4	3	3
76	4	4	4	4	4	4	3	3
77	1	1	3	3	4	4	1	1
78	4	4	4	4	4	4	1	1
79	4	4	3	3	4	4	1	1
80	1	1	3	3	4	4	3	3
81	4	4	3	3	4	4	1	1
82	4	4	3	3	4	4	1	1
83	1	1	3	3	4	4	1	1
84	4	4	3	3	4	4	3	3
85	1	1	3	3	4	4	3	3
86	4	4	3	3	4	4	1	1
87	1	1	3	3	4	4	1	1
88	1	1	3	3	4	4	1	1
89	1	1	3	3	4	4	1	1
90	4	4	3	3	4	4	3	3
91	1	1	3	3	4	4	1	1
92	1	1	3	3	4	4	1	1
93	4	4	3	3	4	4	1	1
94	4	4	3	3	1	1	1	1
95	4	4	3	3	4	4	1	1
96	4	4	4	4	4	4	1	1
97	4	4	3	3	4	4	1	1
98	4	4	4	4	4	4	1	1
99	4	4	3	3	4	4	3	3
100	4	4	3	3	4	4	1	1
101	4	4	3	3	4	4	1	1
102	4	4	3	3	1	1	1	1
103	4	4	4	4	4	4	1	1
104	1	1	3	3	4	4	3	3
105	4	4	3	3	4	4	1	1
106	4	4	3	3	4	4	1	1
107	4	4	3	3	4	4	1	1

For Review Only

108	4	4	3	3	4	4	1	1
109	4	4	3	3	4	4	1	1
110	4	4	3	3	4	4	1	1
111	4	4	3	3	4	4	3	3
112	4	4	3	3	4	4	1	1
113	4	4	3	3	4	4	1	1
114	4	4	3	3	1	1	1	1
115	1	1	4	4	1	1	1	1
116	4	4	3	3	4	4	3	3
117	4	4	3	3	4	4	1	1
118	4	4	3	3	4	4	1	1
119	4	4	3	3	4	4	1	1
120	4	4	3	3	4	4	1	1
121	4	4	4	4	4	4	1	1
122	4	4	3	3	4	4	1	1
123	4	4	3	3	4	4	1	1
124	4	4	3	3	4	4	1	1
125	4	4	3	3	4	4	1	1
126	4	4	3	3	4	4	3	3
127	4	4	3	3	1	1	1	1
128	4	4	3	3	4	4	1	1
129	4	4	3	3	4	4	1	1
130	4	4	3	3	1	1	1	1
131	1	1	3	3	4	4	3	3
132	4	4	3	3	4	4	1	1
133	1	1	3	3	4	4	1	1
134	4	4	3	3	4	4	1	1
135	4	4	3	3	4	4	1	1
136	4	4	3	3	4	4	1	1
137	4	4	3	3	4	4	1	1
138	1	1	3	3	4	4	3	3
139	4	4	3	3	4	4	1	1
140	4	4	3	3	4	4	1	1
141	4	4	3	3	4	4	1	1
142	4	4	3	3	4	4	3	3
143	4	4	3	3	1	1	1	1
144	4	4	3	3	4	4	3	3
145	4	4	4	4	4	4	1	1
146	4	4	3	3	4	4	1	1
147	1	1	3	3	4	4	3	3
148	4	4	3	3	4	4	1	1
149	4	4	3	3	4	4	3	3
150	4	4	3	3	4	4	1	1
151	4	4	3	3	4	4	1	1
152	1	1	3	3	4	4	1	1
153	4	4	4	4	4	4	1	1
154	1	1	3	3	4	4	1	1
155	4	4	3	3	4	4	1	1
156	4	4	3	3	4	4	3	3
157	4	4	3	3	4	4	1	1
158	4	4	3	3	4	4	1	1
159	1	1	3	3	4	4	3	3
160	4	4	3	3	4	4	1	1
161	4	4	3	3	4	4	1	1
162	4	4	3	3	4	4	1	1

For Review Only

163	4	4	3	3	4	4	1	1
164	1	1	3	3	4	4	1	1
165	4	4	3	3	4	4	3	3
166	4	4	3	3	1	1	3	3
167	4	4	3	3	4	4	1	1
168	1	1	3	3	4	4	3	3
169	1	1	3	3	4	4	1	1
170	4	4	3	3	4	4	1	1
171	4	4	3	3	4	4	3	3
172	4	4	3	3	4	4	1	1
173	1	1	3	3	4	4	1	1
174	1	1	3	3	4	4	1	1
175	4	4	3	3	4	4	1	1
176	4	4	3	3	4	4	1	1
177	4	4	3	3	4	4	3	3
178	1	1	3	3	4	4	1	1
179	4	4	3	3	1	1	1	1
180	4	4	4	4	4	4	1	1
181	4	4	3	3	4	4	1	1
182	4	4	3	3	1	1	1	1
183	4	4	3	3	4	4	1	1
184	1	1	3	3	4	4	3	3
185	1	1	3	3	4	4	3	3
186	4	4	3	3	4	4	1	1
187	4	4	3	3	4	4	1	1
188	4	4	3	3	4	4	3	3
189	4	4	3	3	4	4	1	1
190	4	4	3	3	4	4	1	1
191	4	4	3	3	4	4	1	1
192	4	4	3	3	1	1	1	1
193	4	4	4	4	4	4	1	1
194	4	4	3	3	4	4	1	1
195	4	4	3	3	4	4	1	1
196	4	4	3	3	4	4	3	3
197	4	4	3	3	4	4	1	1
198	4	4	3	3	4	4	1	1
199	1	1	3	3	4	4	1	1
200	4	4	3	3	4	4	1	1
201	4	4	3	3	4	4	1	1
202	4	4	3	3	4	4	1	1
203	4	4	3	3	1	1	1	1
204	1	1	3	3	4	4	1	1
205	1	1	3	3	4	4	3	3
206	4	4	3	3	4	4	1	1
207	4	4	3	3	4	4	3	3
208	4	4	3	3	4	4	3	3
209	1	1	3	3	4	4	1	1
210	1	1	3	3	4	4	3	3
211	4	4	3	3	4	4	3	3
212	1	1	3	3	4	4	1	1
213	4	4	3	3	4	4	1	1
214	4	4	3	3	4	4	3	3
215	1	1	3	3	4	4	3	3
216	4	4	4	4	4	4	1	1
217	1	1	3	3	4	4	3	3

For Review Only

218	4	4	3	3	1	1	1	1
219	1	1	3	3	4	4	1	1
220	4	4	3	3	1	1	1	1
221	4	4	3	3	1	1	1	1
222	1	1	3	3	4	4	1	1
223	4	4	3	3	4	4	1	1
224	1	1	3	3	4	4	3	3
225	1	1	3	3	4	4	3	3
226	4	4	3	3	4	4	1	1
227	1	1	3	3	4	4	3	3
228	4	4	3	3	4	4	1	1
229	1	1	3	3	4	4	1	1
230	1	1	3	3	4	4	1	1
231	4	4	3	3	4	4	1	1
232	4	4	3	3	1	1	1	1
233	1	1	3	3	4	4	1	1
234	1	1	3	3	4	4	3	3
235	4	4	3	3	4	4	1	1
236	1	1	3	3	4	4	3	3
237	4	4	3	3	4	4	1	1
238	4	4	4	4	4	4	3	3
239	4	4	3	3	4	4	1	1
240	4	4	3	3	4	4	1	1
241	4	4	3	3	4	4	1	1
242	1	1	3	3	4	4	3	3
243	4	4	3	3	4	4	1	1
244	1	1	3	3	4	4	3	3
245	4	4	3	3	4	4	1	1
246	1	1	3	3	4	4	1	1
247	4	4	3	3	4	4	1	1
248	1	1	3	3	4	4	1	1
249	1	1	3	3	4	4	1	1
250	4	4	3	3	4	4	1	1
251	4	4	3	3	4	4	1	1
252	1	1	3	3	4	4	3	3
253	1	1	3	3	4	4	3	3
254	4	4	3	3	1	1	1	1
255	4	4	3	3	4	4	1	1
256	4	4	3	3	4	4	1	1
257	1	1	3	3	4	4	3	3
258	4	4	3	3	4	4	1	1
259	4	4	3	3	4	4	3	3
260	1	1	3	3	4	4	1	1
261	4	4	3	3	4	4	1	1
262	4	4	3	3	4	4	1	1
263	4	4	3	3	4	4	1	1
264	1	1	3	3	4	4	1	1
265	4	4	3	3	4	4	1	1
266	4	4	3	3	4	4	1	1
267	1	1	3	3	4	4	3	3
268	4	4	3	3	1	1	1	1
269	1	1	3	3	4	4	3	3
270	4	4	3	3	1	1	1	1
271	4	4	3	3	4	4	1	1
272	1	1	3	3	4	4	1	1

For Review Only

273	4	4	3	3	4	4	1	1
274	4	4	3	3	4	4	1	1

For Review Only

SNP5	SNP5	SNP6	SNP6	SNP7	SNP7	SNP8	SNP8	SNP9	SNP9
1	1	1	1	4	4	2	2	3	3
1	1	1	1	4	4	2	2	3	3
1	1	2	2	4	4	2	2	1	1
1	1	1	1	4	4	2	2	3	3
1	1	1	1	4	4	2	2	3	3
4	4	1	1	4	4	2	2	3	3
4	4	1	1	4	4	2	2	3	3
1	1	1	1	4	4	2	2	3	3
4	4	1	1	4	4	2	2	3	3
1	1	1	1	4	4	2	2	3	3
1	1	1	1	4	4	2	2	3	3
1	1	2	2	4	4	2	2	1	1
1	1	1	1	4	4	2	2	1	1
1	1	1	1	4	4	2	2	3	3
4	4	1	1	4	4	2	2	3	3
1	1	1	1	4	4	2	2	3	3
4	4	1	1	4	4	2	2	3	3
1	1	1	1	4	4	2	2	3	3
1	1	1	1	4	4	2	2	1	1
1	1	1	1	4	4	2	2	3	3
1	1	1	1	4	4	2	2	3	3
4	4	1	1	4	4	2	2	3	3
1	1	1	1	4	4	2	2	1	1
1	1	1	1	4	4	2	2	3	3
1	1	1	1	4	4	2	2	1	1
1	1	2	2	4	4	2	2	1	1
1	1	2	2	4	4	2	2	1	1
1	1	1	1	4	4	2	2	1	1
1	1	1	1	4	4	2	2	3	3
1	1	1	1	4	4	2	2	3	3
1	1	2	2	4	4	2	2	3	3
1	1	1	1	4	4	2	2	3	3
4	4	1	1	4	4	2	2	3	3
1	1	1	1	4	4	2	2	3	3
4	4	2	2	4	4	2	2	3	3
1	1	1	1	4	4	2	2	3	3
4	4	1	1	4	4	2	2	3	3
1	1	1	1	4	4	2	2	1	1
1	1	1	1	4	4	2	2	3	3
1	1	1	1	4	4	2	2	3	3
1	1	1	1	4	4	2	2	3	3
1	1	1	1	4	4	2	2	3	3
1	1	1	1	4	4	2	2	3	3
1	1	1	1	4	4	2	2	3	3
1	1	1	1	4	4	2	2	3	3
1	1	1	1	4	4	2	2	1	1
1	1	1	1	4	4	2	2	3	3
1	1	1	1	4	4	2	2	1	1
4	4	1	1	4	4	2	2	3	3
1	1	1	1	4	4	2	2	1	1
1	1	2	2	4	4	2	2	3	3
1	1	1	1	4	4	2	2	1	1

For Review Only

1	1	1	1	4	4	2	2	3	3
4	4	1	1	4	4	2	2	3	3
4	4	1	1	4	4	2	2	3	3
1	1	1	1	4	4	2	2	3	3
1	1	1	1	4	4	2	2	1	1
1	1	2	2	4	4	2	2	3	3
1	1	1	1	4	4	2	2	3	3
1	1	1	1	4	4	2	2	3	3
1	1	1	1	4	4	2	2	3	3
1	1	1	1	4	4	2	2	1	1
1	1	1	1	4	4	2	2	1	1
1	1	2	2	4	4	2	2	3	3
1	1	1	1	4	4	2	2	3	3
1	1	1	1	4	4	2	2	3	3
4	4	1	1	4	4	2	2	3	3
4	4	1	1	4	4	2	2	3	3
1	1	1	1	4	4	2	2	3	3
4	4	1	1	3	3	2	2	3	3
4	4	1	1	4	4	2	2	3	3
4	4	1	1	4	4	2	2	3	3
1	1	2	2	4	4	2	2	3	3
4	4	1	1	4	4	2	2	3	3
1	1	1	1	4	4	2	2	3	3
1	1	1	1	4	4	2	2	1	1
1	1	1	1	4	4	2	2	3	3
1	1	2	2	4	4	2	2	3	3
1	1	1	1	4	4	2	2	3	3
1	1	1	1	4	4	2	2	3	3
1	1	2	2	4	4	2	2	1	1
1	1	1	1	4	4	2	2	1	1
4	4	1	1	4	4	2	2	3	3
1	1	2	2	3	3	2	2	3	3
1	1	1	1	4	4	2	2	3	3
1	1	1	1	4	4	2	2	3	3
1	1	1	1	3	3	2	2	3	3
1	1	1	1	4	4	2	2	3	3
1	1	1	1	4	4	2	2	3	3
4	4	1	1	4	4	2	2	3	3
1	1	1	1	4	4	2	2	1	1
1	1	1	1	4	4	2	2	3	3
4	4	1	1	4	4	2	2	3	3
1	1	1	1	4	4	2	2	1	1
4	4	1	1	4	4	2	2	3	3
1	1	1	1	4	4	2	2	1	1
4	4	1	1	4	4	2	2	3	3
1	1	1	1	4	4	2	2	1	1
1	1	1	1	4	4	2	2	3	3
1	1	1	1	4	4	2	2	3	3
1	1	1	1	4	4	2	2	1	1
4	4	1	1	4	4	2	2	3	3
1	1	2	2	4	4	2	2	3	3
1	1	1	1	4	4	2	2	3	3
1	1	1	1	4	4	2	2	3	3
1	1	1	1	4	4	2	2	1	1
4	4	1	1	4	4	2	2	3	3

For Review Only

1	1	1	1	4	4	2	2	3	3
1	1	1	1	4	4	2	2	1	1
1	1	1	1	4	4	2	2	1	1
1	1	1	1	4	4	2	2	3	3
1	1	1	1	4	4	2	2	1	1
1	1	1	1	4	4	2	2	3	3
1	1	1	1	4	4	2	2	3	3
1	1	1	1	4	4	2	2	3	3
1	1	1	1	4	4	2	2	3	3
1	1	1	1	4	4	2	2	1	1
1	1	1	1	4	4	2	2	3	3
1	1	1	1	4	4	2	2	3	3
4	4	1	1	4	4	2	2	3	3
1	1	1	1	4	4	2	2	1	1
1	1	1	1	4	4	2	2	3	3
1	1	1	1	4	4	2	2	3	3
4	4	1	1	4	4	2	2	3	3
4	4	1	1	4	4	2	2	1	1
1	1	1	1	4	4	2	2	3	3
1	1	1	1	4	4	2	2	1	1
1	1	1	1	4	4	2	2	1	1
1	1	1	1	4	4	2	2	3	3
1	1	1	1	4	4	2	2	1	1
1	1	1	1	4	4	2	2	3	3
4	4	1	1	4	4	2	2	3	3
1	1	1	1	4	4	2	2	1	1
4	4	1	1	4	4	2	2	3	3
1	1	1	1	3	3	2	2	3	3
1	1	1	1	4	4	2	2	1	1
1	1	1	1	4	4	2	2	3	3
1	1	1	1	4	4	2	2	3	3
1	1	1	1	4	4	2	2	1	1
4	4	1	1	4	4	2	2	3	3
1	1	1	1	4	4	2	2	1	1
1	1	1	1	4	4	2	2	3	3
1	1	1	1	4	4	2	2	3	3
1	1	1	1	4	4	2	2	3	3
1	1	1	1	4	4	2	2	1	1
1	1	2	2	4	4	2	2	3	3
1	1	1	1	4	4	2	2	3	3
1	1	1	1	4	4	2	2	1	1
1	1	1	1	4	4	2	2	3	3
4	4	2	2	3	3	2	2	3	3
1	1	1	1	4	4	3	3	3	3
1	1	2	2	4	4	2	2	1	1
4	4	1	1	3	3	2	2	3	3
1	1	1	1	4	4	2	2	3	3
1	1	2	2	4	4	2	2	3	3
1	1	1	1	4	4	2	2	3	3
1	1	1	1	4	4	2	2	1	1
1	1	1	1	4	4	2	2	3	3
1	1	1	1	4	4	2	2	3	3
1	1	1	1	3	3	2	2	1	1
1	1	1	1	4	4	2	2	3	3
1	1	1	1	4	4	2	2	3	3

For Review Only

4	4	1	1	4	4	2	2	3	3
1	1	1	1	4	4	2	2	1	1
4	4	1	1	4	4	2	2	3	3
1	1	1	1	4	4	2	2	1	1
1	1	1	1	4	4	2	2	1	1
1	1	1	1	4	4	2	2	3	3
4	4	1	1	4	4	2	2	3	3
1	1	1	1	4	4	2	2	1	1
1	1	1	1	4	4	2	2	1	1
4	4	1	1	4	4	2	2	3	3
1	1	1	1	4	4	2	2	1	1
1	1	2	2	4	4	2	2	3	3
4	4	1	1	4	4	2	2	3	3
4	4	1	1	4	4	2	2	3	3
1	1	1	1	4	4	2	2	3	3
1	1	1	1	4	4	2	2	3	3
1	1	1	1	3	3	2	2	1	1
1	1	1	1	4	4	2	2	1	1
1	1	2	2	4	4	2	2	1	1
1	1	1	1	4	4	2	2	3	3
4	4	1	1	4	4	2	2	1	1
1	1	1	1	4	4	2	2	3	3
4	4	1	1	4	4	2	2	3	3
1	1	2	2	4	4	2	2	3	3
4	4	1	1	4	4	2	2	3	3
1	1	1	1	4	4	2	2	3	3
4	4	1	1	4	4	2	2	3	3
4	4	1	1	4	4	2	2	3	3
1	1	1	1	4	4	2	2	3	3
1	1	1	1	4	4	2	2	3	3
4	4	2	2	4	4	2	2	3	3
4	4	1	1	4	4	2	2	3	3
4	4	1	1	4	4	2	2	3	3
1	1	1	1	4	4	2	2	3	3
4	4	1	1	3	3	2	2	3	3
1	1	2	2	4	4	2	2	3	3
1	1	1	1	4	4	2	2	1	1
1	1	1	1	3	3	2	2	3	3
1	1	2	2	4	4	2	2	3	3
1	1	1	1	4	4	2	2	3	3
1	1	2	2	4	4	2	2	1	1
1	1	2	2	4	4	2	2	3	3
1	1	1	1	4	4	2	2	3	3
4	4	1	1	4	4	2	2	3	3
1	1	1	1	4	4	2	2	3	3
1	1	2	2	4	4	2	2	3	3
1	1	1	1	4	4	2	2	3	3
1	1	1	1	4	4	2	2	3	3
1	1	1	1	4	4	2	2	3	3
1	1	1	1	4	4	2	2	3	3
1	1	1	1	4	4	2	2	1	1
1	1	1	1	4	4	2	2	3	3
4	4	1	1	4	4	2	2	3	3
1	1	1	1	4	4	2	2	1	1
1	1	1	1	4	4	2	2	3	3

For Review Only

1	1	1	1	3	3	2	2	1	1
1	1	1	1	4	4	2	2	3	3
4	4	1	1	4	4	2	2	3	3
4	4	1	1	4	4	2	2	1	1
1	1	1	1	4	4	2	2	3	3
1	1	1	1	4	4	2	2	1	1
1	1	2	2	4	4	2	2	3	3
1	1	1	1	4	4	2	2	3	3
1	1	1	1	4	4	2	2	1	1
1	1	1	1	4	4	2	2	3	3
1	1	1	1	4	4	2	2	3	3
4	4	2	2	4	4	2	2	3	3
1	1	1	1	4	4	2	2	1	1
1	1	1	1	4	4	2	2	1	1
1	1	2	2	4	4	2	2	3	3
1	1	1	1	4	4	2	2	1	1
1	1	1	1	4	4	2	2	3	3
1	1	1	1	4	4	2	2	3	3
1	1	1	1	4	4	2	2	3	3
1	1	1	1	3	3	2	2	1	1
1	1	1	1	4	4	2	2	3	3
1	1	1	1	4	4	2	2	1	1
1	1	1	1	4	4	2	2	3	3
4	4	2	2	4	4	2	2	3	3
1	1	1	1	4	4	2	2	3	3
1	1	1	1	4	4	2	2	1	1
1	1	1	1	4	4	2	2	3	3
1	1	1	1	4	4	2	2	3	3
1	1	1	1	4	4	2	2	3	3
1	1	1	1	3	3	2	2	1	1
1	1	1	1	4	4	2	2	3	3
1	1	1	1	4	4	2	2	1	1
1	1	1	1	4	4	2	2	3	3
1	1	1	1	4	4	2	2	3	3
4	4	1	1	4	4	2	2	3	3
1	1	1	1	4	4	2	2	3	3
1	1	1	1	4	4	2	2	1	1
1	1	2	2	4	4	2	2	3	3
1	1	1	1	4	4	2	2	3	3
1	1	1	1	4	4	2	2	3	3
4	4	1	1	4	4	2	2	3	3
1	1	1	1	4	4	2	2	3	3
4	4	2	2	4	4	2	2	3	3
4	4	1	1	4	4	2	2	3	3
1	1	1	1	4	4	2	2	3	3
1	1	1	1	4	4	2	2	1	1
1	1	1	1	4	4	2	2	3	3
1	1	2	2	4	4	2	2	3	3
1	1	1	1	4	4	2	2	1	1
1	1	1	1	4	4	2	2	3	3
4	4	1	1	4	4	2	2	3	3
1	1	1	1	4	4	2	2	3	3
1	1	2	2	4	4	2	2	1	1
1	1	1	1	4	4	2	2	3	3
1	1	1	1	4	4	2	2	3	3

For Review Only

1	1	1	1	4	4	2	2	1	1
1	1	1	1	4	4	2	2	1	1

For Review Only

SNP010	SNP010	SNP011	SNP011	SNP012	SNP012	SNP013	SNP013	SNP014	SNP014
1	1	2	2	1	1	1	1	1	1
1	1	2	2	1	1	4	4	1	1
1	1	2	2	1	1	1	1	4	4
1	1	2	2	1	1	1	1	1	1
1	1	2	2	1	1	1	1	1	1
1	1	3	3	1	1	4	4	1	1
1	1	2	2	1	1	1	1	1	1
4	4	2	2	3	3	4	4	1	1
1	1	2	2	1	1	1	1	1	1
1	1	3	3	1	1	4	4	1	1
1	1	2	2	1	1	1	1	1	1
1	1	2	2	1	1	1	1	1	1
1	1	2	2	3	3	1	1	1	1
1	1	2	2	1	1	1	1	1	1
1	1	2	2	1	1	4	4	1	1
1	1	2	2	1	1	1	1	1	1
1	1	2	2	1	1	1	1	1	1
1	1	2	2	1	1	1	1	1	1
1	1	2	2	1	1	1	1	1	1
1	1	2	2	1	1	1	1	1	1
1	1	2	2	1	1	1	1	1	1
1	1	2	2	1	1	1	1	1	1
1	1	2	2	1	1	1	1	1	1
1	1	2	2	1	1	1	1	1	1
1	1	2	2	1	1	1	1	1	1
1	1	2	2	3	3	4	4	4	4
1	1	2	2	1	1	1	1	4	4
1	1	2	2	1	1	1	1	1	1
1	1	2	2	1	1	1	1	1	1
1	1	3	3	1	1	4	4	1	1
1	1	2	2	1	1	1	1	4	4
1	1	2	2	1	1	1	1	1	1
1	1	2	2	1	1	1	1	1	1
1	1	3	3	1	1	4	4	1	1
1	1	2	2	1	1	1	1	1	1
1	1	2	2	1	1	1	1	1	1
1	1	2	2	1	1	1	1	1	1
1	1	2	2	1	1	1	1	1	1
4	4	2	2	1	1	1	1	1	1
1	1	2	2	1	1	1	1	1	1
4	4	2	2	3	3	4	4	1	1
1	1	2	2	1	1	1	1	1	1
1	1	3	3	1	1	1	1	1	1
1	1	3	3	1	1	4	4	1	1
1	1	3	3	1	1	4	4	1	1
1	1	2	2	1	1	1	1	1	1
1	1	2	2	1	1	1	1	1	1
1	1	2	2	3	3	1	1	1	1
1	1	2	2	1	1	4	4	1	1
1	1	2	2	1	1	1	1	1	1
1	1	2	2	3	3	4	4	4	4
1	1	2	2	1	1	1	1	1	1

1	1	2	2	1	1	1	1	1	1
1	1	2	2	1	1	1	1	1	1
1	1	2	2	1	1	1	1	1	1
1	1	2	2	1	1	1	1	1	1
1	1	2	2	1	1	1	1	1	1
1	1	2	2	1	1	1	1	4	4
1	1	2	2	3	3	4	4	1	1
1	1	3	3	1	1	4	4	1	1
1	1	2	2	1	1	1	1	1	1
1	1	2	2	1	1	1	1	1	1
1	1	2	2	3	3	4	4	1	1
1	1	2	2	1	1	1	1	4	4
1	1	2	2	1	1	1	1	1	1
1	1	2	2	1	1	1	1	1	1
1	1	2	2	1	1	1	1	1	1
1	1	2	2	1	1	1	1	1	1
1	1	2	2	3	3	4	4	1	1
1	1	2	2	3	3	1	1	1	1
1	1	2	2	1	1	1	1	1	1
1	1	2	2	1	1	1	1	1	1
1	1	2	2	1	1	1	1	1	1
1	1	2	2	1	1	1	1	1	1
1	1	2	2	1	1	1	1	1	1
1	1	2	2	1	1	4	4	1	1
1	1	3	3	1	1	4	4	1	1
1	1	2	2	1	1	1	1	4	4
1	1	3	3	1	1	4	4	1	1
4	4	2	2	1	1	1	1	1	1
1	1	2	2	3	3	4	4	4	4
1	1	2	2	1	1	1	1	1	1
4	4	2	2	1	1	1	1	1	1
1	1	2	2	3	3	4	4	4	4
4	4	2	2	1	1	1	1	1	1
1	1	2	2	1	1	1	1	1	1
1	1	2	2	1	1	1	1	1	1
1	1	3	3	1	1	4	4	1	1
1	1	3	3	1	1	4	4	1	1
1	1	2	2	1	1	1	1	1	1
1	1	3	3	1	1	4	4	1	1
1	1	3	3	1	1	1	1	1	1
1	1	2	2	1	1	1	1	1	1
1	1	2	2	1	1	1	1	1	1
1	1	2	2	1	1	1	1	1	1
1	1	2	2	3	3	4	4	1	1
1	1	2	2	1	1	1	1	1	1
1	1	2	2	3	3	4	4	1	1
1	1	2	2	3	3	4	4	1	1
1	1	2	2	3	3	4	4	1	1
1	1	2	2	3	3	4	4	1	1
1	1	3	3	1	1	4	4	1	1
1	1	2	2	1	1	1	1	4	4
4	4	2	2	1	1	1	1	1	1
1	1	2	2	1	1	1	1	1	1
1	1	3	3	1	1	1	1	1	1
1	1	3	3	1	1	4	4	1	1

For Review Only

1	1	2	2	1	1	4	4	1	1
1	1	2	2	1	1	1	1	1	1
1	1	2	2	3	3	1	1	1	1
1	1	2	2	3	3	1	1	1	1
1	1	2	2	1	1	4	4	1	1
1	1	2	2	1	1	4	4	1	1
1	1	2	2	1	1	1	1	1	1
1	1	2	2	1	1	4	4	1	1
1	1	3	3	1	1	4	4	1	1
1	1	2	2	1	1	1	1	1	1
1	1	2	2	1	1	4	4	1	1
1	1	2	2	1	1	1	1	1	1
1	1	2	2	1	1	1	1	1	1
1	1	2	2	1	1	1	1	1	1
1	1	2	2	1	1	1	1	1	1
1	1	2	2	1	1	1	1	1	1
1	1	2	2	3	3	4	4	1	1
1	1	2	2	1	1	1	1	1	1
1	1	2	2	3	3	4	4	1	1
1	1	2	2	1	1	1	1	1	1
1	1	2	2	1	1	1	1	1	1
1	1	2	2	1	1	1	1	1	1
1	1	2	2	1	1	1	1	1	1
4	4	2	2	1	1	1	1	1	1
1	1	2	2	1	1	1	1	1	1
1	1	2	2	1	1	1	1	1	1
1	1	2	2	1	1	1	1	1	1
1	1	2	2	1	1	1	1	1	1
1	1	2	2	1	1	1	1	1	1
1	1	3	3	1	1	4	4	1	1
4	4	2	2	1	1	1	1	1	1
1	1	2	2	1	1	1	1	1	1
1	1	2	2	1	1	1	1	1	1
1	1	2	2	1	1	1	1	1	1
1	1	3	3	1	1	4	4	1	1
1	1	2	2	1	1	1	1	1	1
1	1	2	2	3	3	1	1	1	1
1	1	2	2	1	1	4	4	1	1
1	1	2	2	1	1	1	1	1	1
4	4	2	2	1	1	1	1	1	1
1	1	2	2	1	1	1	1	1	1
1	1	2	2	1	1	1	1	1	1
1	1	2	2	1	1	1	1	4	4
1	1	2	2	3	3	1	1	1	1
1	1	2	2	3	3	1	1	4	4
1	1	3	3	1	1	4	4	1	1
1	1	2	2	3	3	4	4	4	4
1	1	2	2	3	3	4	4	4	4
1	1	2	2	1	1	1	1	1	1
1	1	2	2	3	3	4	4	1	1
1	1	2	2	3	3	4	4	1	1
1	1	2	2	1	1	1	1	1	1
1	1	2	2	1	1	1	1	1	1
1	1	2	2	3	3	4	4	1	1
1	1	2	2	1	1	1	1	1	1

For Review Only

1	1	2	2	1	1	4	4	1	1
1	1	2	2	3	3	4	4	1	1
1	1	2	2	1	1	1	1	1	1
1	1	2	2	1	1	1	1	1	1
1	1	2	2	1	1	1	1	1	1
1	1	3	3	1	1	4	4	1	1
1	1	2	2	1	1	1	1	1	1
1	1	2	2	1	1	1	1	1	1
1	1	2	2	1	1	1	1	1	1
1	1	2	2	1	1	1	1	1	1
1	1	2	2	1	1	1	1	1	1
1	1	2	2	1	1	4	4	1	1
1	1	2	2	1	1	1	1	4	4
1	1	2	2	1	1	1	1	1	1
1	1	2	2	3	3	4	4	1	1
1	1	2	2	1	1	1	1	1	1
1	1	2	2	1	1	1	1	1	1
1	1	2	2	3	3	1	1	1	1
1	1	2	2	3	3	4	4	4	4
1	1	2	2	1	1	1	1	1	1
1	1	2	2	1	1	1	1	1	1
1	1	2	2	1	1	1	1	1	1
1	1	2	2	1	1	1	1	1	1
1	1	2	2	1	1	1	1	4	4
1	1	2	2	1	1	1	1	1	1
1	1	3	3	1	1	4	4	1	1
1	1	2	2	1	1	1	1	1	1
1	1	3	3	1	1	4	4	1	1
1	1	2	2	3	3	4	4	1	1
1	1	2	2	1	1	1	1	1	1
1	1	2	2	1	1	4	4	4	4
1	1	2	2	3	3	4	4	1	1
1	1	2	2	1	1	1	1	1	1
1	1	2	2	3	3	4	4	1	1
1	1	2	2	1	1	1	1	1	1
1	1	2	2	1	1	1	1	4	4
1	1	2	2	3	3	4	4	1	1
1	1	2	2	1	1	1	1	1	1
1	1	2	2	1	1	1	1	4	4
1	1	2	2	1	1	1	1	1	1
1	1	2	2	1	1	1	1	4	4
1	1	2	2	3	3	4	4	4	4
4	4	2	2	1	1	1	1	1	1
1	1	2	2	1	1	1	1	1	1
1	1	2	2	1	1	1	1	4	4
1	1	2	2	1	1	4	4	4	4
1	1	2	2	1	1	1	1	1	1
1	1	2	2	1	1	1	1	1	1
1	1	2	2	1	1	1	1	1	1
1	1	2	2	1	1	1	1	1	1
1	1	2	2	1	1	1	1	1	1
1	1	3	3	1	1	4	4	1	1
1	1	2	2	1	1	1	1	1	1
1	1	2	2	1	1	4	4	1	1
1	1	2	2	1	1	1	1	1	1

For Review Only

1	1	2	2	1	1	1	1	1	1
1	1	2	2	3	3	4	4	1	1
1	1	2	2	1	1	1	1	1	1
1	1	2	2	1	1	1	1	1	1
4	4	3	3	1	1	4	4	1	1
1	1	2	2	3	3	1	1	1	1
1	1	2	2	1	1	1	1	4	4
1	1	3	3	1	1	4	4	1	1
1	1	2	2	1	1	1	1	1	1
4	4	2	2	1	1	1	1	1	1
1	1	2	2	1	1	1	1	1	1
1	1	2	2	1	1	1	1	4	4
1	1	3	3	1	1	4	4	1	1
1	1	2	2	3	3	4	4	1	1
1	1	2	2	1	1	1	1	4	4
1	1	2	2	1	1	1	1	1	1
1	1	2	2	1	1	1	1	1	1
1	1	2	2	1	1	1	1	1	1
1	1	2	2	1	1	1	1	1	1
1	1	2	2	1	1	1	1	1	1
1	1	2	2	1	1	4	4	1	1
1	1	2	2	3	3	4	4	1	1
1	1	2	2	1	1	1	1	1	1
1	1	2	2	1	1	1	1	4	4
1	1	3	3	1	1	4	4	1	1
1	1	2	2	1	1	1	1	1	1
4	4	2	2	1	1	1	1	1	1
1	1	2	2	1	1	1	1	1	1
1	1	3	3	1	1	4	4	1	1
1	1	2	2	1	1	1	1	1	1
1	1	2	2	1	1	1	1	1	1
1	1	2	2	1	1	1	1	1	1
1	1	3	3	1	1	4	4	1	1
1	1	2	2	3	3	4	4	1	1
1	1	2	2	1	1	1	1	1	1
4	4	2	2	1	1	1	1	1	1
1	1	2	2	1	1	1	1	1	1
1	1	2	2	1	1	1	1	4	4
1	1	2	2	1	1	1	1	1	1
1	1	2	2	1	1	1	1	1	1
1	1	2	2	1	1	1	1	1	1
1	1	2	2	1	1	1	1	1	1
1	1	2	2	1	1	1	1	4	4
1	1	2	2	1	1	1	1	1	1
1	1	2	2	1	1	1	1	1	1
1	1	3	3	1	1	4	4	1	1
1	1	2	2	1	1	1	1	1	1
1	1	2	2	1	1	1	1	4	4
1	1	2	2	1	1	1	1	1	1
4	4	2	2	1	1	1	1	1	1
1	1	2	2	1	1	1	1	1	1
1	1	2	2	1	1	1	1	1	1
1	1	2	2	1	1	1	1	4	4
1	1	2	2	1	1	1	1	4	4
1	1	2	2	1	1	1	1	1	1

1	1	2	2	1	1	1	1	1	1
1	1	2	2	3	3	4	4	1	1

For Review Only

SNP015	SNP015	SNP016	SNP016	SNP017	SNP017	SNP018	SNP018	SNP019	SNP019
3	3	4	4	4	4	1	1	2	2
3	3	4	4	3	3	1	1	2	2
2	2	4	4	4	4	1	1	2	2
3	3	4	4	4	4	1	1	2	2
3	3	4	4	4	4	1	1	2	2
3	3	4	4	3	3	1	1	2	2
3	3	4	4	4	4	1	1	2	2
3	3	4	4	4	4	1	1	2	2
3	3	4	4	4	4	1	1	2	2
3	3	1	1	3	3	3	3	2	2
3	3	4	4	4	4	1	1	4	4
3	3	4	4	4	4	1	1	2	2
3	3	4	4	4	4	1	1	2	2
3	3	4	4	4	4	1	1	2	2
3	3	4	4	4	4	1	1	2	2
3	3	4	4	4	4	1	1	2	2
3	3	4	4	4	4	1	1	2	2
3	3	4	4	4	4	1	1	2	2
3	3	4	4	4	4	1	1	2	2
3	3	4	4	4	4	1	1	2	2
3	3	4	4	4	4	1	1	2	2
3	3	4	4	4	4	1	1	2	2
3	3	4	4	4	4	1	1	2	2
3	3	4	4	4	4	1	1	2	2
3	3	4	4	4	4	1	1	2	2
2	2	4	4	4	4	1	1	2	2
2	2	4	4	4	4	1	1	2	2
3	3	4	4	4	4	1	1	2	2
3	3	4	4	4	4	1	1	2	2
3	3	4	4	3	3	1	1	2	2
2	2	4	4	4	4	1	1	2	2
3	3	4	4	4	4	1	1	2	2
3	3	4	4	4	4	1	1	2	2
3	3	4	4	3	3	1	1	2	2
3	3	4	4	4	4	1	1	2	2
3	3	4	4	4	4	1	1	2	2
3	3	1	1	4	4	3	3	2	2
3	3	1	1	4	4	3	3	2	2
3	3	4	4	4	4	1	1	4	4
3	3	4	4	4	4	1	1	2	2
3	3	4	4	4	4	1	1	2	2
3	3	4	4	4	4	1	1	4	4
3	3	4	4	3	3	1	1	2	2
3	3	4	4	3	3	1	1	2	2
3	3	1	1	3	3	3	3	2	2
3	3	4	4	4	4	1	1	2	2
3	3	4	4	4	4	1	1	2	2
3	3	4	4	4	4	1	1	2	2
3	3	4	4	3	3	1	1	2	2
3	3	1	1	4	4	1	1	2	2
2	2	4	4	4	4	1	1	2	2
3	3	4	4	4	4	1	1	2	2

For Review Only

3	3	4	4	4	4	1	1	2	2
3	3	4	4	4	4	1	1	2	2
3	3	4	4	4	4	1	1	2	2
3	3	4	4	4	4	1	1	2	2
3	3	4	4	4	4	1	1	2	2
2	2	4	4	4	4	1	1	2	2
3	3	4	4	4	4	1	1	2	2
3	3	4	4	3	3	1	1	2	2
3	3	4	4	4	4	1	1	2	2
3	3	4	4	4	4	1	1	2	2
3	3	4	4	4	4	1	1	2	2
2	2	4	4	4	4	1	1	2	2
3	3	4	4	4	4	1	1	2	2
3	3	4	4	4	4	1	1	2	2
3	3	4	4	3	3	1	1	2	2
3	3	1	1	3	3	3	3	2	2
3	3	4	4	4	4	1	1	2	2
3	3	1	1	4	4	3	3	2	2
3	3	4	4	4	4	1	1	2	2
3	3	4	4	4	4	1	1	2	2
3	3	4	4	4	4	1	1	2	2
3	3	4	4	4	4	1	1	2	2
3	3	4	4	4	4	1	1	2	2
3	3	4	4	4	4	1	1	2	2
3	3	1	1	3	3	3	3	2	2
2	2	4	4	4	4	1	1	2	2
3	3	4	4	3	3	1	1	2	2
3	3	4	4	4	4	1	1	2	2
2	2	4	4	4	4	1	1	2	2
3	3	4	4	4	4	1	1	2	2
3	3	4	4	3	3	1	1	2	2
2	2	1	1	4	4	3	3	2	2
3	3	4	4	4	4	1	1	2	2
3	3	4	4	4	4	1	1	2	2
3	3	1	1	4	4	3	3	2	2
3	3	1	1	3	3	3	3	2	2
3	3	4	4	3	3	1	1	2	2
3	3	4	4	4	4	1	1	2	2
3	3	4	4	3	3	1	1	2	2
3	3	1	1	3	3	1	1	2	2
3	3	4	4	4	4	1	1	2	2
3	3	4	4	4	4	1	1	2	2
3	3	4	4	4	4	1	1	2	2
3	3	4	4	4	4	1	1	2	2
3	3	4	4	4	4	1	1	2	2
3	3	1	1	4	4	3	3	2	2
3	3	4	4	4	4	1	1	2	2
3	3	4	4	4	4	1	1	2	2
3	3	4	4	4	4	1	1	2	2
3	3	4	4	3	3	1	1	2	2
3	3	4	4	4	4	1	1	2	2
3	3	4	4	4	4	1	1	4	4
3	3	4	4	4	4	1	1	2	2
3	3	4	4	4	4	1	1	2	2
3	3	4	4	3	3	1	1	2	2

For Review Only

3	3	4	4	4	4	1	1	2	2
3	3	4	4	4	4	1	1	2	2
3	3	4	4	4	4	1	1	2	2
3	3	4	4	4	4	1	1	4	4
3	3	4	4	4	4	1	1	2	2
3	3	4	4	4	4	1	1	2	2
3	3	4	4	4	4	1	1	2	2
3	3	4	4	4	4	1	1	2	2
3	3	4	4	3	3	1	1	2	2
3	3	4	4	4	4	1	1	2	2
3	3	4	4	4	4	1	1	2	2
3	3	4	4	4	4	1	1	2	2
3	3	1	1	4	4	1	1	2	2
3	3	4	4	4	4	1	1	2	2
3	3	4	4	4	4	1	1	2	2
3	3	4	4	4	4	1	1	2	2
3	3	4	4	4	4	1	1	2	2
3	3	4	4	4	4	1	1	2	2
3	3	4	4	4	4	1	1	4	4
3	3	1	1	4	4	1	1	2	2
3	3	4	4	4	4	1	1	2	2
3	3	4	4	4	4	1	1	2	2
3	3	4	4	4	4	1	1	2	2
3	3	4	4	4	4	1	1	2	2
3	3	4	4	4	4	1	1	2	2
3	3	4	4	4	4	1	1	2	2
3	3	4	4	4	4	1	1	2	2
3	3	1	1	4	4	3	3	2	2
3	3	1	1	4	4	1	1	2	2
3	3	4	4	3	3	1	1	2	2
3	3	4	4	4	4	1	1	4	4
3	3	4	4	4	4	1	1	2	2
3	3	4	4	4	4	1	1	2	2
3	3	4	4	4	4	1	1	2	2
3	3	4	4	3	3	1	1	2	2
3	3	4	4	4	4	1	1	2	2
3	3	4	4	4	4	1	1	4	4
3	3	4	4	4	4	1	1	2	2
3	3	4	4	4	4	1	1	4	4
3	3	4	4	4	4	1	1	4	4
3	3	4	4	4	4	1	1	2	2
3	3	4	4	4	4	1	1	2	2
2	2	1	1	4	4	3	3	2	2
3	3	4	4	3	3	1	1	2	2
2	2	4	4	4	4	1	1	2	2
3	3	1	1	3	3	3	3	2	2
2	2	4	4	4	4	1	1	2	2
2	2	4	4	4	4	1	1	2	2
3	3	4	4	4	4	1	1	2	2
3	3	4	4	4	4	1	1	2	2
3	3	4	4	4	4	1	1	2	2
3	3	4	4	4	4	1	1	2	2
3	3	1	1	4	4	3	3	2	2
3	3	4	4	4	4	1	1	2	2
3	3	4	4	4	4	1	1	2	2

For Review Only

3	3	4	4	4	4	1	1	2	2
3	3	4	4	4	4	1	1	2	2
3	3	1	1	3	3	3	3	2	2
3	3	4	4	4	4	1	1	2	2
3	3	4	4	4	4	1	1	2	2
3	3	4	4	3	3	1	1	2	2
3	3	4	4	4	4	1	1	2	2
3	3	4	4	4	4	1	1	2	2
3	3	4	4	4	4	1	1	2	2
3	3	4	4	4	4	1	1	2	2
3	3	4	4	4	4	1	1	2	2
2	2	4	4	3	3	1	1	2	2
2	2	4	4	4	4	1	1	2	2
3	3	1	1	3	3	1	1	2	2
3	3	4	4	4	4	1	1	2	2
3	3	4	4	4	4	1	1	4	4
3	3	4	4	4	4	1	1	2	2
3	3	4	4	4	4	1	1	2	2
2	2	4	4	4	4	1	1	2	2
3	3	4	4	4	4	1	1	2	2
3	3	4	4	4	4	1	1	2	2
3	3	4	4	4	4	1	1	2	2
3	3	4	4	4	4	1	1	2	2
2	2	4	4	4	4	1	1	2	2
3	3	4	4	4	4	1	1	2	2
3	3	4	4	3	3	1	1	2	2
3	3	4	4	4	4	1	1	2	2
3	3	4	4	3	3	1	1	2	2
3	3	4	4	4	4	1	1	2	2
3	3	4	4	4	4	1	1	2	2
2	2	4	4	4	4	1	1	2	2
3	3	4	4	4	4	1	1	2	2
3	3	4	4	4	4	1	1	2	2
3	3	4	4	4	4	1	1	2	2
3	3	1	1	4	4	3	3	2	2
2	2	4	4	4	4	1	1	2	2
3	3	4	4	4	4	1	1	2	2
3	3	1	1	4	4	1	1	2	2
2	2	4	4	4	4	1	1	2	2
3	3	4	4	4	4	1	1	2	2
2	2	4	4	4	4	1	1	2	2
2	2	4	4	4	4	1	1	2	2
3	3	4	4	4	4	1	1	4	4
3	3	1	1	4	4	1	1	2	2
3	3	4	4	4	4	1	1	2	2
2	2	4	4	3	3	1	1	2	2
3	3	4	4	4	4	1	1	4	4
3	3	4	4	4	4	1	1	4	4
3	3	4	4	4	4	1	1	2	2
3	3	4	4	4	4	1	1	2	2
3	3	1	1	4	4	1	1	2	2
3	3	4	4	3	3	1	1	2	2
3	3	4	4	4	4	1	1	2	2
3	3	4	4	4	4	1	1	2	2
3	3	4	4	4	4	1	1	4	4

For Review Only

3	3	1	1	4	4	3	3	2	2
3	3	4	4	4	4	1	1	4	4
3	3	4	4	4	4	1	1	2	2
3	3	1	1	4	4	1	1	2	2
3	3	1	1	3	3	3	3	2	2
3	3	4	4	4	4	3	3	2	2
2	2	4	4	4	4	1	1	4	4
3	3	1	1	4	4	3	3	2	2
3	3	4	4	4	4	1	1	2	2
3	3	4	4	4	4	1	1	4	4
3	3	4	4	4	4	1	1	2	2
2	2	4	4	4	4	1	1	2	2
3	3	4	4	3	3	1	1	2	2
3	3	4	4	4	4	1	1	2	2
2	2	4	4	4	4	1	1	2	2
3	3	4	4	4	4	1	1	2	2
3	3	4	4	4	4	1	1	4	4
3	3	4	4	4	4	1	1	2	2
3	3	4	4	4	4	1	1	2	2
3	3	1	1	4	4	3	3	2	2
3	3	4	4	4	4	1	1	2	2
3	3	4	4	4	4	1	1	2	2
3	3	4	4	4	4	1	1	2	2
2	2	4	4	4	4	1	1	2	2
3	3	4	4	3	3	1	1	2	2
3	3	4	4	4	4	1	1	2	2
3	3	4	4	4	4	1	1	4	4
3	3	4	4	4	4	1	1	2	2
3	3	1	1	3	3	1	1	2	2
3	3	1	1	4	4	3	3	2	2
3	3	4	4	4	4	1	1	4	4
3	3	4	4	4	4	1	1	2	2
3	3	4	4	3	3	1	1	2	2
3	3	4	4	4	4	1	1	2	2
3	3	4	4	4	4	1	1	2	2
3	3	4	4	4	4	1	1	2	2
3	3	4	4	4	4	1	1	2	2
2	2	4	4	4	4	1	1	2	2
3	3	4	4	4	4	1	1	2	2
3	3	4	4	4	4	1	1	4	4
3	3	1	1	4	4	3	3	2	2
3	3	4	4	4	4	1	1	2	2
2	2	4	4	4	4	1	1	2	2
3	3	4	4	4	4	1	1	2	2
3	3	4	4	3	3	1	1	2	2
3	3	4	4	3	3	1	1	2	2
3	3	4	4	4	4	1	1	2	2
2	2	4	4	4	4	1	1	2	2
3	3	4	4	3	3	1	1	2	2
3	3	4	4	4	4	1	1	4	4
3	3	4	4	4	4	1	1	2	2
3	3	4	4	4	4	1	1	4	4
2	2	4	4	4	4	1	1	2	2
2	2	4	4	4	4	1	1	2	2
3	3	4	4	4	4	1	1	2	2

For Review Only

3	3	4	4	4	4	1	1	2	2
3	3	4	4	4	4	1	1	2	2

For Review Only

SNP020	SNP020	SNP021	SNP021	SNP022	SNP022	SNP023	SNP023	SNP024	SNP024
2	2	4	4	4	4	4	4	1	1
2	2	4	4	1	1	1	1	1	1
2	2	4	4	4	4	4	4	1	1
2	2	4	4	4	4	4	4	1	1
2	2	4	4	4	4	4	4	1	1
2	2	4	4	4	4	4	4	1	1
3	3	4	4	4	4	4	4	1	1
2	2	4	4	4	4	4	4	1	1
3	3	4	4	4	4	4	4	1	1
2	2	4	4	4	4	1	1	1	1
2	2	4	4	4	4	4	4	1	1
2	2	4	4	4	4	4	4	1	1
2	2	4	4	4	4	4	4	1	1
2	2	4	4	4	4	4	4	1	1
2	2	2	2	4	4	4	4	1	1
2	2	4	4	4	4	4	4	1	1
2	2	4	4	4	4	4	4	1	1
2	2	4	4	4	4	4	4	1	1
2	2	4	4	4	4	4	4	1	1
2	2	4	4	4	4	4	4	1	1
3	3	4	4	4	4	4	4	1	1
3	3	4	4	4	4	4	4	1	1
2	2	4	4	4	4	4	4	1	1
2	2	4	4	4	4	4	4	1	1
3	3	4	4	4	4	4	4	1	1
2	2	4	4	4	4	4	4	1	1
2	2	4	4	4	4	4	4	1	1
2	2	4	4	4	4	4	4	1	1
3	3	4	4	4	4	4	4	1	1
2	2	4	4	4	4	4	4	1	1
2	2	2	2	4	4	4	4	1	1
2	2	4	4	4	4	4	4	1	1
3	3	4	4	4	4	4	4	1	1
2	2	4	4	4	4	4	4	1	1
2	2	2	2	1	1	4	4	1	1
2	2	4	4	4	4	4	4	1	1
2	2	4	4	1	1	1	1	3	3
2	2	4	4	4	4	4	4	1	1
2	2	4	4	4	4	4	4	1	1
2	2	4	4	4	4	4	4	1	1
2	2	4	4	4	4	4	4	1	1
2	2	4	4	4	4	4	4	1	1
2	2	4	4	4	4	4	4	1	1
2	2	4	4	4	4	4	4	1	1
2	2	4	4	4	4	1	1	1	1
2	2	4	4	4	4	4	4	1	1
2	2	4	4	4	4	4	4	1	1
2	2	4	4	4	4	4	4	1	1
2	2	4	4	4	4	4	4	1	1
2	2	4	4	4	4	1	1	1	1
2	2	4	4	4	4	4	4	1	1
2	2	4	4	4	4	4	4	1	1

3	3	4	4	4	4	4	4	1	1
3	3	4	4	4	4	4	4	1	1
3	3	2	2	4	4	4	4	1	1
2	2	4	4	4	4	4	4	1	1
2	2	4	4	4	4	4	4	1	1
2	2	4	4	4	4	4	4	1	1
2	2	4	4	4	4	4	4	1	1
2	2	4	4	4	4	4	4	1	1
2	2	4	4	1	1	1	1	1	1
2	2	4	4	4	4	4	4	1	1
2	2	4	4	4	4	4	4	1	1
2	2	4	4	4	4	4	4	1	1
3	3	4	4	4	4	4	4	1	1
3	3	4	4	4	4	4	4	1	1
2	2	4	4	4	4	4	4	1	1
2	2	4	4	1	1	1	1	1	1
2	2	4	4	4	4	4	4	1	1
2	2	2	2	4	4	1	1	3	3
3	3	4	4	4	4	4	4	1	1
3	3	4	4	4	4	4	4	1	1
2	2	4	4	4	4	4	4	1	1
2	2	4	4	4	4	4	4	1	1
3	3	4	4	4	4	4	4	1	1
2	2	4	4	4	4	4	4	1	1
2	2	4	4	4	4	1	1	1	1
2	2	4	4	4	4	4	4	1	1
2	2	4	4	4	4	4	4	1	1
2	2	4	4	4	4	4	4	1	1
2	2	4	4	4	4	4	4	1	1
2	2	4	4	4	4	4	4	1	1
2	2	4	4	4	4	4	4	1	1
2	2	4	4	4	4	1	1	3	3
2	2	4	4	4	4	4	4	1	1
3	3	4	4	4	4	4	4	1	1
2	2	4	4	4	4	1	1	3	3
2	2	4	4	4	4	1	1	1	1
2	2	4	4	4	4	4	4	1	1
3	3	4	4	4	4	4	4	1	1
2	2	4	4	4	4	4	4	1	1
2	2	4	4	4	4	1	1	1	1
3	3	4	4	4	4	4	4	1	1
2	2	4	4	4	4	4	4	1	1
2	2	4	4	4	4	1	1	1	1
2	2	4	4	4	4	4	4	1	1
3	3	4	4	4	4	4	4	1	1
2	2	4	4	4	4	4	4	1	1
2	2	4	4	4	4	4	4	1	1
2	2	4	4	4	4	4	4	1	1
2	2	4	4	4	4	4	4	1	1
2	2	2	2	1	1	4	4	1	1
2	2	4	4	4	4	4	4	1	1
2	2	4	4	4	4	4	4	1	1
2	2	4	4	1	1	4	4	1	1
2	2	4	4	4	4	4	4	1	1
2	2	2	2	1	1	4	4	1	1

For Review Only

3	3	4	4	4	4	4	4	1	1
2	2	4	4	4	4	4	4	1	1
2	2	4	4	4	4	4	4	1	1
2	2	4	4	4	4	4	4	1	1
2	2	4	4	4	4	4	4	1	1
2	2	4	4	4	4	4	4	1	1
2	2	4	4	4	4	4	4	1	1
2	2	4	4	4	4	4	4	1	1
2	2	4	4	4	4	4	4	1	1
2	2	4	4	4	4	4	4	1	1
2	2	4	4	4	4	4	4	1	1
2	2	4	4	4	4	4	4	1	1
2	2	2	2	4	4	4	4	1	1
2	2	4	4	4	4	4	4	1	1
2	2	4	4	4	4	4	4	1	1
2	2	4	4	4	4	4	4	1	1
2	2	2	2	4	4	4	4	1	1
2	2	4	4	4	4	4	4	1	1
2	2	4	4	4	4	4	4	1	1
2	2	2	2	4	4	4	4	1	1
2	2	4	4	4	4	4	4	1	1
3	3	4	4	4	4	4	4	1	1
2	2	4	4	4	4	4	4	1	1
2	2	4	4	4	4	4	4	1	1
2	2	4	4	4	4	4	4	1	1
2	2	4	4	4	4	4	4	1	1
2	2	4	4	4	4	4	4	1	1
2	2	4	4	4	4	1	1	3	3
2	2	4	4	4	4	1	1	1	1
2	2	2	2	4	4	4	4	1	1
2	2	4	4	4	4	4	4	1	1
2	2	4	4	4	4	4	4	1	1
3	3	4	4	4	4	4	4	1	1
2	2	4	4	4	4	1	1	3	3
2	2	4	4	4	4	4	4	1	1
2	2	4	4	1	1	4	4	1	1
2	2	4	4	4	4	4	4	1	1
2	2	4	4	4	4	4	4	1	1
3	3	4	4	4	4	4	4	1	1
2	2	4	4	4	4	4	4	1	1
2	2	4	4	4	4	4	4	1	1
2	2	4	4	4	4	4	4	1	1
2	2	2	2	4	4	1	1	3	3
2	2	4	4	4	4	4	4	1	1
2	2	4	4	4	4	4	4	1	1
2	2	4	4	4	4	4	4	1	1
2	2	4	4	4	4	4	4	1	1
2	2	4	4	4	4	4	4	1	1
2	2	4	4	1	1	4	4	1	1
2	2	4	4	4	4	4	4	1	1
2	2	4	4	4	4	4	4	1	1
2	2	4	4	1	1	4	4	1	1
2	2	4	4	4	4	4	4	1	1
2	2	4	4	4	4	4	4	1	1
2	2	4	4	4	4	4	4	1	1

For Review Only

3	3	2	2	4	4	4	4	1	1
2	2	4	4	4	4	4	4	1	1
3	3	4	4	4	4	1	1	1	1
2	2	4	4	4	4	4	4	1	1
2	2	4	4	4	4	4	4	1	1
2	2	4	4	4	4	4	4	1	1
2	2	2	2	4	4	4	4	1	1
2	2	4	4	4	4	4	4	1	1
2	2	4	4	4	4	4	4	1	1
3	3	4	4	4	4	4	4	1	1
2	2	4	4	4	4	4	4	1	1
2	2	4	4	4	4	4	4	1	1
2	2	4	4	4	4	4	4	1	1
2	2	4	4	1	1	4	4	1	1
2	2	4	4	4	4	4	4	1	1
2	2	4	4	4	4	4	4	1	1
2	2	4	4	4	4	4	4	1	1
2	2	4	4	4	4	4	4	1	1
2	2	4	4	4	4	4	4	1	1
2	2	4	4	4	4	4	4	1	1
2	2	4	4	4	4	4	4	1	1
2	2	4	4	4	4	4	4	1	1
2	2	4	4	4	4	4	4	1	1
2	2	4	4	4	4	4	4	1	1
2	2	4	4	4	4	4	4	1	1
2	2	4	4	4	4	4	4	1	1
2	2	4	4	4	4	4	4	1	1
2	2	2	2	4	4	4	4	1	1
2	2	4	4	4	4	4	4	1	1
2	2	4	4	4	4	4	4	1	1
2	2	4	4	4	4	4	4	1	1
2	2	2	2	4	4	4	4	1	1
2	2	4	4	4	4	4	4	1	1
2	2	4	4	1	1	4	4	1	1
2	2	4	4	4	4	1	1	3	3
2	2	4	4	4	4	4	4	1	1
2	2	4	4	4	4	4	4	1	1
2	2	4	4	4	4	1	1	3	3
2	2	4	4	4	4	4	4	1	1
2	2	4	4	4	4	4	4	1	1
2	2	4	4	4	4	4	4	1	1
2	2	4	4	4	4	4	4	1	1
2	2	4	4	4	4	4	4	1	1
2	2	4	4	4	4	1	1	1	1
2	2	4	4	1	1	4	4	1	1
2	2	4	4	4	4	4	4	1	1
2	2	4	4	4	4	4	4	1	1
2	2	4	4	4	4	4	4	1	1
2	2	4	4	1	1	4	4	1	1
2	2	4	4	4	4	4	4	1	1
2	2	4	4	4	4	1	1	1	1
2	2	4	4	1	1	4	4	1	1
2	2	4	4	4	4	1	1	1	1
2	2	4	4	4	4	4	4	1	1
2	2	4	4	4	4	4	4	1	1

For Review Only

2	2	4	4	4	4	1	1	3	3
2	2	4	4	4	4	4	4	1	1
2	2	4	4	4	4	4	4	1	1
2	2	2	2	4	4	1	1	1	1
2	2	4	4	4	4	1	1	1	1
2	2	4	4	4	4	1	1	3	3
2	2	4	4	4	4	4	4	1	1
2	2	4	4	4	4	1	1	1	1
2	2	4	4	1	1	4	4	1	1
2	2	4	4	4	4	4	4	1	1
2	2	4	4	4	4	4	4	1	1
2	2	4	4	4	4	4	4	1	1
2	2	4	4	4	4	4	4	1	1
2	2	4	4	4	4	4	4	1	1
2	2	4	4	4	4	4	4	1	1
3	3	4	4	4	4	4	4	1	1
2	2	4	4	4	4	4	4	1	1
2	2	4	4	4	4	4	4	1	1
2	2	4	4	4	4	4	4	1	1
2	2	4	4	4	4	4	4	1	1
2	2	4	4	4	4	4	4	1	1
2	2	4	4	4	4	4	4	1	1
3	3	4	4	4	4	4	4	1	1
2	2	2	2	4	4	4	4	1	1
2	2	4	4	4	4	4	4	1	1
2	2	4	4	4	4	4	4	1	1
2	2	4	4	4	4	4	4	1	1
2	2	4	4	4	4	4	4	1	1
2	2	4	4	4	4	1	1	1	1
2	2	4	4	4	4	1	1	3	3
2	2	4	4	4	4	4	4	1	1
2	2	4	4	4	4	4	4	1	1
2	2	4	4	1	1	4	4	1	1
2	2	4	4	4	4	4	4	1	1
2	2	4	4	4	4	4	4	1	1
2	2	4	4	4	4	4	4	1	1
2	2	4	4	4	4	4	4	1	1
2	2	4	4	4	4	4	4	1	1
2	2	4	4	4	4	4	4	1	1
2	2	4	4	4	4	4	4	1	1
2	2	4	4	4	4	1	1	3	3
2	2	4	4	4	4	4	4	1	1
2	2	2	2	4	4	4	4	1	1
3	3	4	4	4	4	4	4	1	1
2	2	4	4	4	4	4	4	1	1
2	2	4	4	4	4	4	4	1	1
2	2	4	4	4	4	4	4	1	1
2	2	4	4	4	4	4	4	1	1
2	2	4	4	4	4	4	4	1	1
2	2	4	4	4	4	4	4	1	1
2	2	4	4	4	4	4	4	1	1
2	2	4	4	4	4	4	4	1	1
2	2	4	4	4	4	4	4	1	1
2	2	4	4	4	4	4	4	1	1
2	2	4	4	4	4	4	4	1	1

3	3	4	4	4	4	4	4	1	1
2	2	4	4	4	4	4	4	1	1

For Review Only

SNP025	SNP025	SNP026	SNP026	SNP027	SNP027	SNP028	SNP028	SNP029	SNP029
3	3	4	4	3	3	1	1	3	3
2	2	1	1	3	3	1	1	3	3
3	3	4	4	3	3	1	1	3	3
3	3	4	4	3	3	1	1	3	3
3	3	4	4	3	3	1	1	3	3
3	3	4	4	3	3	1	1	3	3
3	3	1	1	3	3	1	1	3	3
3	3	4	4	2	2	3	3	3	3
3	3	4	4	3	3	1	1	3	3
3	3	4	4	2	2	1	1	3	3
3	3	4	4	3	3	1	1	2	2
3	3	4	4	3	3	1	1	3	3
3	3	4	4	3	3	1	1	3	3
3	3	4	4	3	3	3	3	3	3
3	3	4	4	3	3	3	3	3	3
3	3	1	1	3	3	1	1	3	3
3	3	1	1	2	2	1	1	3	3
3	3	4	4	3	3	3	3	3	3
3	3	4	4	3	3	1	1	3	3
3	3	1	1	3	3	1	1	3	3
3	3	1	1	2	2	1	1	3	3
3	3	1	1	3	3	1	1	3	3
3	3	4	4	3	3	3	3	3	3
3	3	4	4	3	3	3	3	3	3
3	3	1	1	3	3	3	3	3	3
3	3	1	1	3	3	1	1	3	3
3	3	4	4	3	3	1	1	3	3
3	3	4	4	3	3	1	1	3	3
3	3	1	1	3	3	1	1	3	3
3	3	1	1	2	2	1	1	3	3
3	3	4	4	3	3	3	3	3	3
3	3	4	4	3	3	1	1	3	3
3	3	1	1	3	3	1	1	3	3
3	3	4	4	3	3	1	1	3	3
3	3	4	4	3	3	3	3	3	3
3	3	4	4	3	3	1	1	3	3
3	3	4	4	3	3	1	1	3	3
3	3	4	4	3	3	1	1	3	3
3	3	1	1	3	3	3	3	3	3
3	3	4	4	3	3	1	1	3	3
3	3	4	4	2	2	3	3	3	3
3	3	4	4	3	3	1	1	3	3
3	3	4	4	3	3	1	1	3	3
3	3	1	1	3	3	1	1	3	3
3	3	4	4	3	3	1	1	3	3
3	3	1	1	3	3	1	1	3	3
3	3	1	1	3	3	3	3	3	3
3	3	4	4	3	3	1	1	3	3
3	3	1	1	2	2	1	1	3	3
3	3	4	4	3	3	1	1	3	3
3	3	1	1	3	3	1	1	3	3
3	3	4	4	3	3	1	1	3	3

For Review Only

3	3	1	1	3	3	1	1	3	3
3	3	1	1	3	3	1	1	3	3
3	3	4	4	3	3	3	3	3	3
3	3	1	1	3	3	1	1	3	3
3	3	4	4	3	3	1	1	3	3
3	3	4	4	3	3	1	1	3	3
3	3	4	4	3	3	1	1	3	3
3	3	1	1	3	3	1	1	3	3
2	2	1	1	3	3	1	1	3	3
3	3	4	4	3	3	1	1	3	3
3	3	4	4	3	3	1	1	3	3
3	3	4	4	3	3	1	1	3	3
3	3	1	1	3	3	1	1	3	3
3	3	1	1	3	3	1	1	3	3
3	3	4	4	3	3	3	3	3	3
3	3	4	4	3	3	1	1	3	3
3	3	4	4	3	3	1	1	3	3
3	3	4	4	3	3	1	1	3	3
3	3	4	4	2	2	1	1	3	3
3	3	4	4	3	3	1	1	3	3
3	3	4	4	3	3	1	1	3	3
3	3	4	4	3	3	1	1	3	3
3	3	4	4	3	3	1	1	3	3
3	3	4	4	3	3	1	1	3	3
3	3	4	4	3	3	1	1	3	3
3	3	4	4	3	3	1	1	3	3
3	3	1	1	3	3	3	3	3	3
3	3	1	1	3	3	3	3	2	2
3	3	1	1	3	3	1	1	3	3
3	3	4	4	3	3	1	1	3	3
3	3	4	4	3	3	1	1	3	3
3	3	4	4	3	3	1	1	3	3
3	3	1	1	3	3	3	3	2	2
3	3	1	1	3	3	1	1	3	3
3	3	4	4	3	3	1	1	3	3
3	3	4	4	3	3	1	1	3	3
3	3	4	4	3	3	1	1	3	3
3	3	1	1	2	2	1	1	3	3
3	3	4	4	2	2	1	1	3	3
3	3	4	4	2	2	1	1	3	3
3	3	4	4	3	3	1	1	3	3
3	3	4	4	3	3	1	1	3	3
3	3	4	4	3	3	1	1	3	3
3	3	4	4	3	3	1	1	3	3
3	3	1	1	3	3	1	1	3	3
3	3	4	4	3	3	1	1	3	3
2	2	4	4	3	3	1	1	3	3
3	3	4	4	3	3	1	1	3	3
3	3	1	1	3	3	3	3	3	3
3	3	4	4	2	2	1	1	3	3
3	3	4	4	3	3	1	1	3	3
3	3	1	1	3	3	3	3	2	2
2	2	4	4	3	3	3	3	3	3
3	3	4	4	3	3	1	1	3	3
2	2	4	4	2	2	1	1	3	3

For Review Only

3	3	4	4	3	3	3	3	3	3
3	3	4	4	3	3	1	1	3	3
3	3	1	1	3	3	1	1	3	3
2	2	4	4	3	3	1	1	3	3
3	3	4	4	3	3	1	1	3	3
3	3	1	1	3	3	3	3	3	3
3	3	4	4	3	3	1	1	3	3
3	3	4	4	3	3	3	3	3	3
3	3	1	1	3	3	3	3	3	3
3	3	4	4	3	3	1	1	3	3
3	3	4	4	3	3	1	1	3	3
3	3	4	4	3	3	1	1	3	3
3	3	4	4	3	3	1	1	3	3
3	3	4	4	3	3	1	1	3	3
3	3	4	4	3	3	1	1	3	3
3	3	4	4	3	3	3	3	3	3
3	3	4	4	3	3	1	1	3	3
3	3	4	4	3	3	1	1	3	3
2	2	4	4	3	3	3	3	3	3
3	3	4	4	3	3	1	1	3	3
3	3	4	4	3	3	1	1	3	3
3	3	4	4	3	3	1	1	3	3
3	3	4	4	3	3	1	1	3	3
3	3	1	1	3	3	3	3	3	3
3	3	4	4	3	3	1	1	3	3
3	3	4	4	3	3	3	3	3	3
3	3	4	4	3	3	1	1	3	3
3	3	4	4	3	3	1	1	3	3
3	3	4	4	3	3	1	1	3	3
3	3	4	4	3	3	3	3	3	3
3	3	4	4	3	3	3	3	3	3
3	3	4	4	3	3	1	1	3	3
3	3	4	4	3	3	1	1	3	3
3	3	4	4	3	3	1	1	3	3
3	3	4	4	3	3	1	1	3	3
2	2	4	4	3	3	1	1	3	3
2	2	4	4	3	3	1	1	3	3
3	3	4	4	3	3	3	3	3	3
3	3	4	4	3	3	3	3	3	3
3	3	1	1	3	3	1	1	3	3
3	3	4	4	3	3	1	1	3	3
2	2	4	4	3	3	1	1	3	3
3	3	4	4	3	3	1	1	3	3
3	3	4	4	2	2	3	3	3	3
3	3	1	1	3	3	1	1	3	3
3	3	1	1	3	3	1	1	3	3
3	3	1	1	3	3	1	1	3	3
3	3	1	1	3	3	1	1	3	3
3	3	1	1	3	3	3	3	3	3
3	3	1	1	3	3	1	1	3	3
3	3	4	4	3	3	1	1	3	3
2	2	4	4	2	2	3	3	3	3
3	3	4	4	3	3	1	1	3	3
3	3	4	4	3	3	1	1	3	3
3	3	4	4	3	3	1	1	3	3

For Review Only

3	3	1	1	3	3	3	3	3	3
3	3	4	4	3	3	1	1	3	3
3	3	1	1	2	2	1	1	3	3
3	3	4	4	3	3	1	1	3	3
3	3	4	4	3	3	1	1	3	3
3	3	4	4	3	3	3	3	3	3
3	3	4	4	3	3	1	1	2	2
3	3	4	4	3	3	1	1	3	3
3	3	4	4	3	3	1	1	3	3
3	3	4	4	3	3	1	1	3	3
3	3	4	4	3	3	1	1	3	3
3	3	4	4	3	3	3	3	3	3
3	3	4	4	3	3	1	1	3	3
3	3	4	4	3	3	1	1	3	3
2	2	4	4	3	3	3	3	3	3
3	3	4	4	3	3	1	1	3	3
3	3	4	4	3	3	1	1	3	3
3	3	4	4	3	3	1	1	3	3
3	3	1	1	3	3	3	3	3	3
3	3	4	4	3	3	1	1	3	3
3	3	4	4	3	3	1	1	3	3
3	3	4	4	3	3	3	3	2	2
3	3	4	4	3	3	1	1	3	3
3	3	4	4	3	3	1	1	3	3
3	3	4	4	2	2	1	1	3	3
3	3	4	4	3	3	1	1	3	3
3	3	4	4	3	3	1	1	3	3
3	3	4	4	3	3	1	1	3	3
3	3	1	1	3	3	1	1	3	3
3	3	4	4	3	3	1	1	3	3
3	3	4	4	3	3	1	1	3	3
3	3	4	4	3	3	1	1	3	3
3	3	4	4	3	3	1	1	3	3
2	2	1	1	3	3	3	3	3	3
3	3	4	4	3	3	1	1	3	3
3	3	4	4	3	3	1	1	3	3
3	3	4	4	3	3	1	1	3	3
3	3	4	4	3	3	1	1	3	3
3	3	1	1	3	3	1	1	3	3
3	3	4	4	3	3	1	1	3	3
3	3	4	4	3	3	1	1	3	3
3	3	1	1	3	3	1	1	3	3
3	3	1	1	3	3	3	3	2	2
3	3	4	4	3	3	1	1	3	3
3	3	4	4	3	3	1	1	3	3
3	3	4	4	3	3	3	3	3	3
3	3	4	4	3	3	1	1	3	3
3	3	1	1	3	3	3	3	2	2
2	2	4	4	3	3	3	3	3	3
3	3	4	4	3	3	1	1	3	3
3	3	4	4	3	3	1	1	3	3
2	2	4	4	3	3	1	1	3	3
3	3	4	4	3	3	1	1	3	3
3	3	4	4	3	3	1	1	3	3
3	3	1	1	3	3	3	3	2	2

For Review Only

3	3	4	4	3	3	1	1	3	3
3	3	4	4	3	3	3	3	3	3
3	3	4	4	3	3	1	1	3	3
3	3	4	4	3	3	1	1	3	3
3	3	4	4	3	3	1	1	3	3
3	3	4	4	3	3	1	1	3	3
3	3	4	4	3	3	3	3	2	2
3	3	1	1	3	3	3	3	3	3
3	3	1	1	3	3	1	1	3	3
3	3	1	1	3	3	3	3	2	2
3	3	4	4	3	3	1	1	3	3
3	3	4	4	3	3	1	1	3	3
3	3	4	4	3	3	1	1	3	3
3	3	1	1	3	3	1	1	3	3
3	3	4	4	3	3	1	1	3	3
3	3	4	4	3	3	3	3	3	3
3	3	4	4	3	3	3	3	3	3
3	3	4	4	3	3	1	1	3	3
3	3	1	1	3	3	3	3	3	3
3	3	4	4	3	3	1	1	3	3
3	3	4	4	3	3	1	1	3	3
3	3	1	1	3	3	1	1	3	3
3	3	4	4	3	3	1	1	3	3
3	3	4	4	3	3	1	1	3	3
3	3	1	1	3	3	1	1	3	3
3	3	4	4	3	3	1	1	3	3
3	3	1	1	3	3	3	3	2	2
3	3	4	4	2	2	1	1	3	3
3	3	4	4	3	3	1	1	3	3
3	3	4	4	3	3	1	1	3	3
3	3	1	1	3	3	3	3	3	3
3	3	1	1	3	3	1	1	3	3
2	2	4	4	3	3	1	1	3	3
3	3	1	1	3	3	1	1	3	3
3	3	4	4	3	3	1	1	3	3
3	3	1	1	3	3	3	3	3	3
3	3	4	4	3	3	1	1	3	3
3	3	4	4	3	3	1	1	3	3
3	3	4	4	3	3	1	1	3	3
3	3	1	1	3	3	3	3	3	3
3	3	4	4	3	3	1	1	3	3
3	3	1	1	3	3	3	3	3	3
3	3	4	4	3	3	1	1	3	3
3	3	1	1	3	3	1	1	3	3
3	3	1	1	3	3	1	1	3	3
3	3	4	4	3	3	1	1	3	3
3	3	1	1	3	3	3	3	2	2
3	3	1	1	3	3	1	1	3	3
3	3	4	4	2	2	1	1	3	3
3	3	1	1	3	3	3	3	3	3
3	3	4	4	3	3	1	1	3	3
3	3	1	1	3	3	3	3	3	3
3	3	4	4	3	3	1	1	3	3
3	3	4	4	3	3	1	1	3	3
3	3	4	4	3	3	3	3	3	3

For Review Only

3	3	4	4	3	3	1	1	3	3
3	3	4	4	3	3	3	3	3	3

For Review Only

SNP030	SNP030	SNP031	SNP031	SNP032	SNP032	SNP033	SNP033	SNP034	SNP034
1	1	4	4	4	4	1	1	2	2
1	1	4	4	4	4	1	1	2	2
1	1	4	4	4	4	1	1	2	2
1	1	4	4	4	4	1	1	2	2
1	1	4	4	4	4	1	1	2	2
1	1	4	4	3	3	1	1	2	2
1	1	4	4	4	4	1	1	2	2
1	1	4	4	4	4	1	1	2	2
1	1	1	1	4	4	1	1	2	2
3	3	4	4	4	4	1	1	2	2
1	1	1	1	4	4	1	1	2	2
1	1	4	4	4	4	1	1	2	2
1	1	4	4	4	4	1	1	2	2
1	1	4	4	4	4	1	1	2	2
1	1	4	4	4	4	1	1	2	2
1	1	4	4	4	4	1	1	2	2
1	1	4	4	4	4	1	1	2	2
1	1	4	4	4	4	1	1	2	2
1	1	4	4	4	4	4	4	3	3
1	1	4	4	4	4	1	1	2	2
1	1	1	1	4	4	1	1	2	2
1	1	1	1	4	4	1	1	2	2
1	1	1	1	4	4	1	1	2	2
1	1	4	4	4	4	1	1	2	2
1	1	4	4	4	4	1	1	2	2
1	1	4	4	3	3	1	1	2	2
1	1	4	4	4	4	1	1	2	2
1	1	4	4	4	4	1	1	2	2
1	1	4	4	4	4	1	1	2	2
1	1	4	4	4	4	1	1	2	2
1	1	4	4	4	4	4	4	3	3
1	1	4	4	3	3	1	1	2	2
1	1	4	4	4	4	1	1	2	2
1	1	1	1	4	4	1	1	2	2
1	1	4	4	4	4	1	1	3	3
1	1	4	4	4	4	1	1	2	2
3	3	4	4	3	3	1	1	2	2
1	1	4	4	3	3	1	1	2	2
1	1	4	4	4	4	1	1	2	2
1	1	4	4	3	3	1	1	2	2
1	1	4	4	4	4	4	4	2	2
1	1	4	4	4	4	1	1	2	2
1	1	4	4	4	4	1	1	2	2
1	1	1	1	4	4	1	1	2	2
3	3	4	4	4	4	1	1	2	2
1	1	4	4	4	4	1	1	2	2
1	1	4	4	4	4	1	1	2	2
1	1	4	4	4	4	1	1	2	2
1	1	4	4	4	4	1	1	2	2
1	1	4	4	4	4	1	1	2	2
1	1	4	4	3	3	1	1	2	2
1	1	4	4	4	4	1	1	2	2

For Review Only

1	1	4	4	4	4	1	1	2	2
1	1	4	4	4	4	1	1	2	2
1	1	4	4	4	4	4	4	3	3
1	1	4	4	4	4	4	4	2	2
1	1	4	4	4	4	4	4	3	3
1	1	4	4	4	4	1	1	2	2
1	1	4	4	3	3	1	1	2	2
1	1	4	4	3	3	1	1	2	2
1	1	4	4	4	4	1	1	2	2
1	1	4	4	4	4	1	1	2	2
1	1	4	4	4	4	1	1	2	2
1	1	4	4	4	4	1	1	2	2
1	1	4	4	4	4	1	1	2	2
1	1	4	4	4	4	1	1	2	2
1	1	4	4	4	4	1	1	2	2
3	3	4	4	4	4	1	1	2	2
1	1	1	1	4	4	1	1	2	2
1	1	4	4	4	4	4	4	3	3
1	1	4	4	4	4	1	1	2	2
1	1	4	4	4	4	1	1	2	2
1	1	4	4	4	4	1	1	2	2
1	1	1	1	4	4	1	1	2	2
1	1	1	1	4	4	1	1	2	2
1	1	4	4	4	4	1	1	2	2
3	3	4	4	4	4	1	1	2	2
1	1	4	4	4	4	1	1	2	2
1	1	4	4	4	4	1	1	2	2
1	1	4	4	4	4	1	1	2	2
1	1	4	4	3	3	1	1	2	2
1	1	4	4	4	4	1	1	2	2
1	1	1	1	4	4	1	1	2	2
1	1	4	4	4	4	1	1	2	2
1	1	4	4	4	4	1	1	2	2
1	1	4	4	4	4	1	1	2	2
3	3	4	4	4	4	1	1	2	2
3	3	4	4	4	4	1	1	2	2
1	1	4	4	4	4	1	1	2	2
1	1	1	1	4	4	1	1	2	2
1	1	1	1	4	4	1	1	2	2
1	1	4	4	4	4	1	1	2	2
1	1	4	4	4	4	1	1	2	2
1	1	4	4	4	4	1	1	2	2
1	1	4	4	4	4	1	1	3	3
1	1	4	4	4	4	1	1	2	2
1	1	4	4	3	3	1	1	2	2
1	1	4	4	4	4	1	1	2	2
1	1	4	4	4	4	1	1	2	2
1	1	4	4	3	3	1	1	2	2
1	1	4	4	4	4	1	1	2	2
1	1	1	1	4	4	1	1	3	3
1	1	4	4	4	4	1	1	2	2
1	1	4	4	4	4	1	1	2	2
1	1	4	4	3	3	1	1	2	2
1	1	4	4	4	4	1	1	2	2
1	1	4	4	4	4	4	4	3	3

For Review Only

1	1	4	4	4	4	1	1	2	2
1	1	4	4	4	4	1	1	2	2
1	1	4	4	4	4	1	1	2	2
1	1	4	4	4	4	1	1	2	2
1	1	4	4	4	4	1	1	2	2
1	1	4	4	3	3	1	1	2	2
1	1	4	4	4	4	1	1	2	2
1	1	4	4	4	4	1	1	2	2
1	1	4	4	4	4	1	1	2	2
1	1	4	4	4	4	1	1	2	2
1	1	4	4	4	4	1	1	2	2
1	1	4	4	4	4	1	1	2	2
1	1	4	4	4	4	4	4	3	3
1	1	4	4	4	4	1	1	2	2
1	1	4	4	4	4	1	1	2	2
1	1	4	4	4	4	1	1	2	2
1	1	4	4	3	3	4	4	3	3
1	1	4	4	4	4	1	1	2	2
1	1	4	4	4	4	1	1	2	2
1	1	1	1	4	4	1	1	2	2
1	1	4	4	4	4	1	1	2	2
1	1	4	4	4	4	1	1	2	2
1	1	4	4	4	4	1	1	2	2
1	1	4	4	4	4	1	1	2	2
1	1	4	4	4	4	1	1	2	2
1	1	1	1	4	4	1	1	2	2
1	1	4	4	4	4	1	1	2	2
3	3	4	4	4	4	1	1	2	2
1	1	4	4	4	4	1	1	2	2
1	1	4	4	4	4	4	4	3	3
1	1	4	4	4	4	1	1	2	2
1	1	4	4	4	4	1	1	2	2
1	1	4	4	4	4	1	1	2	2
1	1	4	4	4	4	1	1	2	2
1	1	1	1	4	4	1	1	2	2
1	1	4	4	4	4	1	1	2	2
1	1	4	4	4	4	1	1	2	2
1	1	4	4	4	4	4	4	3	3
1	1	4	4	4	4	1	1	2	2
1	1	4	4	4	4	1	1	2	2
1	1	4	4	4	4	1	1	2	2
1	1	4	4	4	4	1	1	2	2
1	1	4	4	4	4	4	4	3	3
1	1	4	4	4	4	1	1	2	2
1	1	4	4	4	4	1	1	2	2
1	1	4	4	4	4	1	1	2	2
1	1	4	4	3	3	1	1	2	2
1	1	4	4	4	4	1	1	2	2
1	1	4	4	4	4	1	1	2	2
1	1	4	4	3	3	1	1	2	2
1	1	4	4	3	3	1	1	2	2
1	1	1	1	3	3	1	1	2	2
1	1	4	4	4	4	1	1	2	2
1	1	4	4	4	4	1	1	2	2
1	1	4	4	4	4	1	1	2	2

For Review Only

1	1	4	4	4	4	4	4	3	3
1	1	1	1	4	4	1	1	2	2
3	3	4	4	4	4	1	1	2	2
1	1	4	4	4	4	1	1	2	2
1	1	4	4	4	4	1	1	2	2
1	1	4	4	4	4	1	1	2	2
1	1	4	4	4	4	1	1	3	3
1	1	4	4	4	4	1	1	2	2
1	1	4	4	4	4	1	1	2	2
1	1	4	4	4	4	1	1	2	2
1	1	4	4	3	3	1	1	2	2
1	1	4	4	4	4	1	1	2	2
1	1	4	4	4	4	1	1	2	2
1	1	4	4	4	4	1	1	2	2
1	1	4	4	4	4	1	1	2	2
1	1	1	1	4	4	1	1	2	2
1	1	4	4	4	4	1	1	2	2
1	1	4	4	4	4	1	1	2	2
1	1	4	4	4	4	1	1	2	2
1	1	4	4	4	4	1	1	2	2
1	1	4	4	4	4	1	1	2	2
1	1	4	4	4	4	1	1	2	2
1	1	1	1	4	4	1	1	2	2
1	1	4	4	4	4	1	1	2	2
1	1	4	4	4	4	1	1	2	2
1	1	1	1	4	4	1	1	2	2
1	1	4	4	4	4	1	1	2	2
1	1	4	4	4	4	1	1	3	3
1	1	4	4	4	4	1	1	2	2
1	1	1	1	4	4	1	1	2	2
1	1	1	1	4	4	1	1	3	3
1	1	4	4	4	4	4	4	3	3
1	1	4	4	4	4	1	1	2	2
1	1	4	4	4	4	1	1	2	2
3	3	4	4	4	4	1	1	2	2
1	1	4	4	4	4	1	1	2	2
1	1	4	4	4	4	1	1	2	2
3	3	4	4	4	4	1	1	2	2
1	1	4	4	4	4	1	1	2	2
1	1	4	4	4	4	1	1	2	2
1	1	4	4	4	4	1	1	2	2
1	1	4	4	3	3	1	1	2	2
1	1	4	4	4	4	1	1	2	2
1	1	4	4	4	4	1	1	2	2
1	1	1	1	4	4	1	1	2	2
1	1	4	4	4	4	1	1	2	2
1	1	1	1	4	4	1	1	2	2
1	1	4	4	4	4	1	1	2	2
1	1	4	4	4	4	1	1	2	2
1	1	4	4	4	4	1	1	2	2
1	1	4	4	4	4	1	1	2	2
1	1	4	4	4	4	1	1	2	2
1	1	1	1	4	4	1	1	2	2
1	1	4	4	4	4	1	1	2	2
1	1	4	4	4	4	1	1	2	2

For Review Only

1	1	4	4	4	4	1	1	2	2
1	1	4	4	4	4	1	1	2	2
1	1	4	4	4	4	1	1	2	2
1	1	4	4	4	4	1	1	3	3
3	3	4	4	4	4	1	1	2	2
1	1	4	4	4	4	1	1	2	2
1	1	4	4	4	4	1	1	2	2
3	3	4	4	4	4	1	1	2	2
1	1	4	4	4	4	1	1	2	2
1	1	4	4	4	4	1	1	2	2
1	1	4	4	4	4	1	1	2	2
1	1	1	1	4	4	1	1	2	2
1	1	4	4	4	4	1	1	2	2
1	1	4	4	4	4	1	1	2	2
1	1	4	4	4	4	1	1	2	2
1	1	4	4	4	4	1	1	2	2
1	1	4	4	4	4	1	1	2	2
1	1	1	1	4	4	1	1	2	2
1	1	4	4	4	4	1	1	2	2
1	1	4	4	3	3	1	1	2	2
1	1	4	4	3	3	1	1	2	2
1	1	4	4	3	3	1	1	2	2
1	1	1	1	4	4	1	1	2	2
1	1	4	4	4	4	4	4	3	3
1	1	4	4	3	3	1	1	2	2
1	1	4	4	4	4	1	1	2	2
1	1	4	4	4	4	1	1	2	2
1	1	4	4	4	4	1	1	2	2
1	1	4	4	4	4	1	1	2	2
3	3	4	4	4	4	1	1	2	2
1	1	4	4	4	4	1	1	2	2
1	1	4	4	3	3	1	1	2	2
1	1	4	4	4	4	1	1	2	2
1	1	4	4	4	4	1	1	2	2
1	1	1	1	4	4	1	1	2	2
1	1	4	4	4	4	1	1	2	2
1	1	4	4	4	4	1	1	2	2
1	1	4	4	4	4	1	1	2	2
1	1	4	4	4	4	1	1	2	2
1	1	4	4	4	4	1	1	2	2
3	3	4	4	4	4	4	4	3	3
1	1	4	4	4	4	1	1	2	2
1	1	4	4	4	4	4	4	3	3
1	1	1	1	4	4	1	1	2	2
1	1	4	4	4	4	1	1	2	2
1	1	4	4	4	4	1	1	2	2
1	1	4	4	4	4	1	1	2	2
1	1	4	4	4	4	1	1	2	2
1	1	4	4	4	4	1	1	2	2
1	1	4	4	4	4	1	1	2	2
1	1	4	4	4	4	1	1	2	2
1	1	4	4	4	4	1	1	2	2
1	1	4	4	4	4	1	1	2	2
1	1	1	1	4	4	1	1	2	2
1	1	1	1	3	3	1	1	2	2

For Review Only

1	1	1	1	4	4	1	1	2	2
1	1	4	4	4	4	1	1	2	2

For Review Only

SNP035	SNP035	SNP036	SNP036	SNP037	SNP037	SNP038	SNP038	SNP039	SNP039
3	3	3	3	2	2	1	1	2	2
3	3	3	3	2	2	4	4	3	3
3	3	3	3	3	3	1	1	2	2
3	3	3	3	2	2	1	1	2	2
3	3	3	3	2	2	1	1	2	2
3	3	3	3	2	2	1	1	2	2
3	3	3	3	2	2	1	1	2	2
3	3	3	3	2	2	1	1	2	2
3	3	3	3	2	2	1	1	2	2
3	3	3	3	2	2	1	1	2	2
3	3	3	3	3	3	1	1	2	2
3	3	3	3	2	2	1	1	2	2
3	3	3	3	2	2	1	1	2	2
3	3	3	3	2	2	1	1	2	2
3	3	1	1	2	2	1	1	2	2
3	3	3	3	2	2	1	1	3	3
3	3	3	3	2	2	1	1	2	2
3	3	3	3	2	2	1	1	2	2
2	2	1	1	2	2	1	1	2	2
3	3	3	3	2	2	1	1	2	2
3	3	3	3	2	2	1	1	2	2
3	3	3	3	2	2	1	1	2	2
3	3	3	3	3	3	1	1	2	2
3	3	3	3	2	2	1	1	2	2
3	3	3	3	2	2	1	1	2	2
3	3	3	3	2	2	4	4	3	3
3	3	3	3	3	3	4	4	3	3
3	3	3	3	2	2	1	1	2	2
3	3	3	3	2	2	1	1	2	2
3	3	3	3	2	2	1	1	2	2
2	2	1	1	2	2	1	1	2	2
3	3	3	3	2	2	1	1	2	2
3	3	3	3	2	2	1	1	2	2
3	3	3	3	2	2	1	1	2	2
3	3	1	1	2	2	1	1	2	2
3	3	3	3	3	3	1	1	2	2
3	3	3	3	2	2	1	1	2	2
3	3	3	3	2	2	1	1	2	2
3	3	3	3	3	3	1	1	2	2
3	3	3	3	2	2	1	1	2	2
3	3	3	3	2	2	1	1	2	2
3	3	3	3	2	2	1	1	2	2
3	3	3	3	2	2	1	1	2	2
3	3	3	3	2	2	1	1	2	2
3	3	3	3	2	2	1	1	2	2
3	3	3	3	2	2	1	1	2	2
3	3	3	3	2	2	1	1	2	2
3	3	3	3	2	2	1	1	2	2
3	3	3	3	2	2	1	1	2	2
3	3	3	3	2	2	1	1	2	2
3	3	3	3	2	2	4	4	3	3
3	3	3	3	3	3	1	1	2	2

For Review Only

3	3	3	3	2	2	1	1	2	2
3	3	3	3	2	2	1	1	2	2
2	2	1	1	2	2	1	1	2	2
3	3	3	3	2	2	1	1	2	2
2	2	1	1	2	2	1	1	2	2
3	3	3	3	2	2	1	1	2	2
3	3	3	3	2	2	4	4	3	3
3	3	3	3	2	2	1	1	2	2
3	3	3	3	2	2	1	1	2	2
3	3	3	3	2	2	1	1	2	2
3	3	3	3	3	3	1	1	2	2
3	3	3	3	2	2	1	1	2	2
3	3	3	3	2	2	1	1	2	2
3	3	3	3	2	2	1	1	2	2
3	3	3	3	2	2	1	1	2	2
3	3	3	3	2	2	1	1	2	2
3	3	3	3	3	3	1	1	2	2
2	2	1	1	2	2	1	1	2	2
3	3	3	3	2	2	1	1	2	2
3	3	3	3	2	2	1	1	2	2
3	3	3	3	2	2	1	1	2	2
3	3	3	3	2	2	1	1	2	2
3	3	3	3	2	2	1	1	2	2
3	3	3	3	2	2	1	1	2	2
3	3	3	3	2	2	1	1	2	2
3	3	3	3	2	2	1	1	2	2
3	3	3	3	2	2	1	1	2	2
3	3	3	3	3	3	1	1	2	2
3	3	3	3	2	2	4	4	3	3
3	3	3	3	2	2	1	1	2	2
3	3	3	3	2	2	1	1	2	2
3	3	3	3	2	2	1	1	2	2
3	3	3	3	3	3	1	1	2	2
3	3	3	3	2	2	1	1	2	2
3	3	3	3	2	2	1	1	2	2
3	3	3	3	2	2	1	1	2	2
3	3	3	3	2	2	1	1	2	2
3	3	3	3	2	2	1	1	2	2
3	3	3	3	2	2	1	1	2	2
3	3	3	3	2	2	1	1	2	2
3	3	3	3	2	2	1	1	2	2
3	3	3	3	3	3	1	1	2	2
3	3	3	3	2	2	1	1	2	2
3	3	3	3	3	3	1	1	2	2
3	3	3	3	2	2	1	1	2	2
3	3	3	3	2	2	1	1	2	2
3	3	3	3	2	2	1	1	2	2
3	3	3	3	2	2	1	1	2	2
3	3	3	3	2	2	1	1	2	2
3	3	1	1	3	3	1	1	2	2
3	3	3	3	2	2	1	1	2	2
3	3	3	3	3	3	1	1	2	2
3	3	3	3	2	2	1	1	2	2
3	3	3	3	2	2	1	1	2	2
2	2	1	1	3	3	1	1	2	2

For Review Only

3	3	3	3	2	2	4	4	3	3
3	3	3	3	3	3	1	1	2	2
3	3	3	3	2	2	4	4	3	3
3	3	3	3	2	2	1	1	2	2
3	3	3	3	2	2	4	4	3	3
3	3	3	3	2	2	1	1	2	2
3	3	3	3	2	2	4	4	3	3
3	3	3	3	2	2	1	1	2	2
3	3	3	3	2	2	1	1	2	2
3	3	3	3	2	2	1	1	2	2
3	3	3	3	2	2	1	1	2	2
3	3	3	3	2	2	1	1	2	2
2	2	1	1	2	2	1	1	2	2
3	3	3	3	2	2	1	1	2	2
3	3	3	3	2	2	1	1	2	2
3	3	3	3	2	2	1	1	2	2
2	2	1	1	3	3	1	1	2	2
3	3	3	3	2	2	1	1	2	2
3	3	3	3	2	2	1	1	2	2
3	3	1	1	2	2	4	4	3	3
3	3	3	3	2	2	1	1	2	2
3	3	3	3	2	2	1	1	2	2
3	3	3	3	2	2	1	1	2	2
3	3	3	3	3	3	1	1	2	2
3	3	3	3	2	2	1	1	3	3
3	3	3	3	2	2	1	1	2	2
3	3	3	3	2	2	1	1	2	2
3	3	3	3	2	2	1	1	2	2
3	3	3	3	2	2	1	1	2	2
2	2	1	1	2	2	1	1	2	2
3	3	3	3	2	2	1	1	2	2
3	3	3	3	2	2	1	1	2	2
3	3	3	3	2	2	1	1	2	2
3	3	3	3	2	2	1	1	2	2
3	3	3	3	2	2	1	1	2	2
3	3	3	3	2	2	1	1	2	2
3	3	3	3	2	2	1	1	2	2
2	2	1	1	2	2	1	1	3	3
3	3	3	3	2	2	1	1	3	3
3	3	3	3	3	3	1	1	2	2
3	3	3	3	2	2	1	1	2	2
3	3	3	3	2	2	1	1	2	2
2	2	1	1	3	3	1	1	2	2
3	3	3	3	2	2	1	1	2	2
3	3	3	3	3	3	1	1	2	2
3	3	3	3	2	2	1	1	2	2
3	3	3	3	2	2	4	4	3	3
3	3	3	3	2	2	4	4	3	3
3	3	3	3	2	2	1	1	2	2
3	3	3	3	2	2	4	4	3	3
3	3	3	3	2	2	4	4	3	3
3	3	3	3	2	2	1	1	2	2
3	3	3	3	2	2	1	1	2	2
3	3	3	3	2	2	4	4	3	3
3	3	3	3	2	2	1	1	2	2

For Review Only

2	2	1	1	2	2	1	1	2	2
3	3	3	3	2	2	1	1	2	2
3	3	3	3	2	2	1	1	2	2
3	3	3	3	2	2	1	1	2	2
3	3	3	3	2	2	4	4	3	3
3	3	3	3	2	2	1	1	2	2
3	3	1	1	3	3	1	1	2	2
3	3	3	3	2	2	1	1	2	2
3	3	3	3	2	2	1	1	2	2
3	3	3	3	2	2	1	1	2	2
3	3	3	3	2	2	1	1	3	3
3	3	3	3	2	2	1	1	2	2
3	3	3	3	2	2	4	4	2	2
3	3	3	3	2	2	1	1	2	2
3	3	3	3	2	2	1	1	2	2
3	3	3	3	3	3	1	1	2	2
3	3	3	3	2	2	1	1	2	2
3	3	3	3	2	2	4	4	3	3
3	3	3	3	3	3	4	4	3	3
3	3	3	3	2	2	4	4	2	2
3	3	3	3	2	2	1	1	2	2
3	3	3	3	3	3	1	1	2	2
3	3	3	3	3	3	1	1	2	2
3	3	3	3	2	2	1	1	2	2
3	3	3	3	2	2	1	1	2	2
3	3	3	3	2	2	1	1	2	2
3	3	3	3	2	2	1	1	2	2
3	3	1	1	3	3	1	1	2	2
3	3	3	3	2	2	1	1	2	2
3	3	3	3	2	2	1	1	2	2
3	3	3	3	2	2	1	1	2	2
2	2	1	1	2	2	1	1	2	2
3	3	3	3	3	3	1	1	2	2
3	3	3	3	2	2	1	1	2	2
3	3	3	3	2	2	1	1	2	2
3	3	3	3	2	2	1	1	2	2
3	3	3	3	2	2	4	4	3	3
3	3	3	3	2	2	1	1	2	2
3	3	3	3	2	2	4	4	3	3
3	3	3	3	3	3	1	1	2	2
3	3	3	3	3	3	1	1	2	2
3	3	3	3	2	2	4	4	3	3
3	3	3	3	3	3	1	1	2	2
3	3	3	3	3	3	1	1	2	2
3	3	3	3	2	2	1	1	3	3
3	3	3	3	2	2	1	1	2	2
3	3	3	3	3	3	1	1	2	2
3	3	3	3	3	3	1	1	2	2
3	3	3	3	2	2	1	1	2	2
3	3	3	3	2	2	1	1	2	2
3	3	3	3	2	2	1	1	2	2
3	3	3	3	2	2	1	1	2	2
3	3	3	3	3	3	1	1	2	2
3	3	3	3	3	3	1	1	2	2
3	3	3	3	3	3	1	1	2	2

For Review Only

3	3	3	3	2	2	1	1	2	2
3	3	3	3	2	2	1	1	2	2
3	3	3	3	2	2	1	1	2	2
3	3	1	1	2	2	1	1	2	2
3	3	3	3	2	2	1	1	2	2
3	3	3	3	2	2	1	1	2	2
3	3	3	3	2	2	4	4	2	2
3	3	3	3	2	2	1	1	2	2
3	3	3	3	2	2	1	1	2	2
3	3	3	3	3	3	1	1	2	2
3	3	3	3	2	2	1	1	2	2
3	3	3	3	2	2	1	1	2	2
3	3	3	3	2	2	1	1	2	2
3	3	3	3	2	2	1	1	2	2
3	3	3	3	2	2	1	1	2	2
3	3	3	3	2	2	1	1	2	2
3	3	3	3	2	2	1	1	2	2
3	3	3	3	2	2	1	1	2	2
3	3	3	3	2	2	1	1	2	2
3	3	3	3	2	2	1	1	2	2
3	3	3	3	2	2	1	1	2	2
3	3	3	3	2	2	1	1	2	2
3	3	3	3	2	2	1	1	2	2
2	2	1	1	3	3	1	1	2	2
3	3	3	3	2	2	1	1	2	2
3	3	3	3	2	2	1	1	2	2
3	3	3	3	3	3	1	1	2	2
3	3	3	3	2	2	1	1	2	2
3	3	3	3	2	2	1	1	2	2
3	3	3	3	2	2	1	1	2	2
3	3	3	3	3	3	1	1	2	2
3	3	3	3	2	2	1	1	2	2
3	3	3	3	2	2	1	1	2	2
3	3	3	3	2	2	1	1	2	2
3	3	3	3	3	3	1	1	2	2
3	3	3	3	2	2	1	1	2	2
3	3	3	3	2	2	1	1	2	2
3	3	3	3	2	2	1	1	2	2
3	3	3	3	3	3	1	1	2	2
3	3	3	3	3	3	1	1	2	2
2	2	1	1	2	2	1	1	2	2
3	3	3	3	2	2	1	1	2	2
2	2	1	1	3	3	1	1	2	2
3	3	3	3	2	2	1	1	2	2
3	3	3	3	2	2	1	1	2	2
3	3	3	3	2	2	1	1	2	2
3	3	3	3	2	2	4	4	3	3
3	3	3	3	2	2	1	1	2	2
3	3	3	3	2	2	1	1	2	2
3	3	3	3	3	3	1	1	2	2
3	3	3	3	2	2	1	1	2	2
3	3	3	3	3	3	1	1	2	2
3	3	3	3	3	3	1	1	2	2
3	3	3	3	2	2	1	1	2	2
3	3	3	3	3	3	1	1	2	2

For Review Only

3	3	3	3	2	2	1	1	2	2
3	3	3	3	2	2	1	1	2	2

For Review Only

SNP040	SNP040	SNP041	SNP041	SNP042	SNP042	SNP043	SNP043	SNP044	SNP044
3	3	1	1	2	2	3	3	1	1
3	3	1	1	2	2	3	3	1	1
3	3	1	1	2	2	3	3	1	1
3	3	1	1	2	2	3	3	1	1
2	2	1	1	2	2	3	3	1	1
3	3	3	3	3	3	3	3	1	1
3	3	3	3	3	3	3	3	1	1
2	2	1	1	2	2	3	3	1	1
3	3	1	1	2	2	3	3	1	1
3	3	1	1	2	2	3	3	1	1
3	3	1	1	2	2	2	2	1	1
3	3	1	1	2	2	3	3	1	1
3	3	1	1	2	2	3	3	1	1
3	3	1	1	2	2	3	3	1	1
3	3	1	1	2	2	3	3	1	1
2	2	1	1	2	2	2	2	1	1
3	3	1	1	2	2	3	3	1	1
2	2	1	1	2	2	3	3	1	1
3	3	1	1	2	2	3	3	2	2
2	2	1	1	2	2	3	3	2	2
2	2	1	1	2	2	2	2	2	2
2	2	1	1	2	2	2	2	2	2
2	2	1	1	2	2	2	2	1	1
2	2	1	1	2	2	3	3	1	1
3	3	1	1	2	2	3	3	1	1
3	3	3	3	3	3	3	3	1	1
3	3	1	1	2	2	3	3	1	1
3	3	1	1	2	2	3	3	1	1
3	3	1	1	2	2	3	3	1	1
2	2	1	1	2	2	3	3	2	2
3	3	1	1	2	2	3	3	2	2
2	2	1	1	2	2	3	3	1	1
3	3	1	1	2	2	3	3	1	1
2	2	1	1	2	2	3	3	1	1
3	3	1	1	2	2	2	2	1	1
2	2	1	1	2	2	3	3	1	1
3	3	3	3	3	3	3	3	1	1
3	3	3	3	3	3	3	3	1	1
2	2	1	1	2	2	3	3	1	1
3	3	3	3	3	3	3	3	1	1
2	2	1	1	2	2	3	3	2	2
3	3	1	1	2	2	3	3	1	1
3	3	1	1	2	2	3	3	1	1
3	3	1	1	2	2	2	2	1	1
3	3	1	1	2	2	3	3	1	1
3	3	1	1	2	2	3	3	1	1
3	3	1	1	2	2	3	3	1	1
3	3	1	1	2	2	3	3	1	1
3	3	3	3	3	3	3	3	1	1
3	3	1	1	2	2	3	3	1	1
2	2	3	3	3	3	3	3	1	1
2	2	1	1	2	2	3	3	1	1

For Review Only

3	3	1	1	2	2	3	3	1	1
3	3	1	1	2	2	3	3	1	1
3	3	1	1	2	2	3	3	2	2
3	3	1	1	2	2	3	3	2	2
3	3	1	1	2	2	3	3	2	2
2	2	1	1	2	2	3	3	1	1
3	3	3	3	3	3	3	3	1	1
2	2	3	3	3	3	3	3	1	1
3	3	1	1	2	2	3	3	1	1
3	3	1	1	2	2	3	3	1	1
2	2	1	1	2	2	3	3	1	1
2	2	1	1	2	2	3	3	1	1
3	3	1	1	2	2	3	3	1	1
3	3	1	1	2	2	3	3	1	1
3	3	1	1	2	2	3	3	1	1
3	3	1	1	2	2	3	3	1	1
3	3	1	1	2	2	3	3	1	1
3	3	1	1	2	2	3	3	2	2
3	3	1	1	2	2	3	3	1	1
3	3	1	1	2	2	3	3	1	1
2	2	1	1	2	2	3	3	1	1
2	2	1	1	2	2	2	2	1	1
3	3	1	1	2	2	2	2	1	1
3	3	1	1	2	2	3	3	1	1
3	3	1	1	2	2	3	3	1	1
3	3	1	1	2	2	3	3	1	1
2	2	1	1	2	2	3	3	1	1
2	2	1	1	2	2	3	3	1	1
3	3	3	3	3	3	3	3	1	1
3	3	1	1	2	2	3	3	1	1
2	2	1	1	2	2	3	3	1	1
3	3	1	1	2	2	3	3	1	1
2	2	1	1	2	2	3	3	1	1
3	3	1	1	2	2	3	3	1	1
3	3	1	1	2	2	3	3	1	1
3	3	1	1	2	2	3	3	1	1
2	2	1	1	2	2	3	3	1	1
3	3	1	1	2	2	2	2	1	1
3	3	1	1	2	2	2	2	1	1
3	3	1	1	2	2	3	3	1	1
3	3	1	1	2	2	3	3	1	1
2	2	1	1	2	2	3	3	1	1
3	3	1	1	2	2	3	3	1	1
2	2	1	1	2	2	3	3	1	1
3	3	3	3	2	2	3	3	1	1
3	3	1	1	2	2	3	3	1	1
3	3	1	1	2	2	3	3	1	1
3	3	3	3	3	3	3	3	1	1
3	3	1	1	2	2	3	3	1	1
3	3	1	1	2	2	2	2	1	1
3	3	1	1	2	2	3	3	1	1
2	2	1	1	2	2	3	3	1	1
3	3	3	3	3	3	3	3	1	1
3	3	1	1	2	2	3	3	1	1
3	3	1	1	2	2	3	3	2	2

3	3	1	1	2	2	3	3	1	1
3	3	1	1	2	2	3	3	1	1
3	3	3	3	3	3	3	3	1	1
3	3	1	1	2	2	3	3	1	1
3	3	1	1	2	2	3	3	1	1
3	3	3	3	3	3	3	3	1	1
3	3	1	1	2	2	2	2	1	1
3	3	1	1	2	2	3	3	1	1
3	3	1	1	2	2	3	3	1	1
3	3	1	1	3	3	3	3	1	1
3	3	1	1	2	2	3	3	1	1
3	3	1	1	2	2	3	3	1	1
3	3	1	1	2	2	3	3	2	2
3	3	1	1	2	2	3	3	1	1
3	3	1	1	2	2	3	3	1	1
3	3	1	1	2	2	3	3	1	1
3	3	3	3	2	2	3	3	2	2
3	3	1	1	2	2	3	3	1	1
3	3	1	1	2	2	3	3	1	1
3	3	1	1	2	2	3	3	2	2
3	3	1	1	2	2	3	3	1	1
3	3	1	1	2	2	3	3	1	1
2	2	1	1	2	2	3	3	1	1
2	2	1	1	2	2	3	3	1	1
3	3	1	1	3	3	3	3	1	1
3	3	1	1	2	2	2	2	1	1
3	3	1	1	2	2	3	3	1	1
3	3	1	1	2	2	3	3	1	1
3	3	1	1	2	2	3	3	1	1
3	3	1	1	2	2	3	3	2	2
3	3	1	1	2	2	3	3	1	1
3	3	1	1	2	2	3	3	1	1
3	3	1	1	2	2	3	3	1	1
3	3	1	1	2	2	3	3	1	1
2	2	1	1	2	2	3	3	1	1
3	3	1	1	2	2	3	3	1	1
3	3	1	1	2	2	3	3	1	1
3	3	1	1	2	2	3	3	2	2
3	3	1	1	2	2	3	3	1	1
2	2	1	1	2	2	3	3	1	1
3	3	1	1	2	2	3	3	1	1
3	3	1	1	2	2	3	3	1	1
3	3	1	1	2	2	3	3	2	2
2	2	1	1	2	2	3	3	1	1
2	2	1	1	2	2	3	3	1	1
3	3	1	1	2	2	3	3	1	1
3	3	3	3	3	3	3	3	1	1
3	3	1	1	2	2	3	3	1	1
3	3	1	1	2	2	2	2	1	1
3	3	3	3	3	3	3	3	1	1
2	2	3	3	3	3	3	3	1	1
3	3	3	3	3	3	2	2	1	1
3	3	1	1	2	2	3	3	1	1
2	2	1	1	2	2	3	3	1	1
3	3	1	1	2	2	3	3	1	1

For Review Only

3	3	1	1	2	2	2	2	2	2
3	3	1	1	2	2	2	2	1	1
3	3	1	1	2	2	3	3	1	1
3	3	1	1	2	2	3	3	1	1
3	3	1	1	2	2	3	3	1	1
3	3	1	1	2	2	3	3	1	1
2	2	1	1	2	2	3	3	1	1
3	3	1	1	2	2	3	3	1	1
2	2	1	1	2	2	3	3	1	1
3	3	1	1	2	2	3	3	1	1
3	3	3	3	2	2	3	3	1	1
2	2	1	1	2	2	3	3	1	1
3	3	1	1	2	2	3	3	1	1
3	3	1	1	2	2	3	3	1	1
3	3	1	1	2	2	3	3	1	1
3	3	1	1	2	2	2	2	1	1
3	3	1	1	2	2	3	3	1	1
3	3	1	1	2	2	3	3	1	1
3	3	1	1	2	2	3	3	1	1
3	3	1	1	2	2	3	3	1	1
3	3	1	1	2	2	3	3	1	1
2	2	1	1	2	2	3	3	1	1
2	2	1	1	2	2	2	2	1	1
2	2	1	1	2	2	3	3	1	1
3	3	1	1	2	2	3	3	1	1
2	2	1	1	2	2	2	2	1	1
3	3	1	1	2	2	3	3	1	1
3	3	1	1	2	2	3	3	1	1
3	3	1	1	2	2	3	3	1	1
3	3	1	1	2	2	2	2	1	1
3	3	1	1	2	2	3	3	1	1
3	3	1	1	2	2	3	3	2	2
3	3	1	1	2	2	3	3	1	1
3	3	1	1	2	2	3	3	1	1
3	3	1	1	2	2	3	3	1	1
2	2	1	1	2	2	3	3	1	1
3	3	1	1	3	3	3	3	1	1
3	3	1	1	2	2	3	3	1	1
3	3	1	1	2	2	3	3	1	1
2	2	1	1	2	2	3	3	1	1
3	3	1	1	2	2	3	3	1	1
3	3	3	3	3	3	3	3	1	1
2	2	1	1	2	2	3	3	1	1
3	3	3	3	3	3	3	3	1	1
3	3	1	1	2	2	3	3	1	1
3	3	1	1	2	2	3	3	1	1
3	3	1	1	2	2	2	2	1	1
2	2	1	1	2	2	3	3	1	1
3	3	1	1	2	2	3	3	1	1
3	3	1	1	2	2	3	3	1	1
3	3	1	1	2	2	3	3	1	1
3	3	1	1	2	2	3	3	1	1
2	2	1	1	2	2	2	2	1	1
2	2	1	1	2	2	3	3	1	1
2	2	1	1	2	2	3	3	1	1

For Review Only

3	3	1	1	2	2	3	3	1	1
2	2	1	1	2	2	3	3	2	2
3	3	1	1	2	2	3	3	1	1
3	3	1	1	2	2	3	3	1	1
3	3	1	1	2	2	3	3	1	1
3	3	1	1	2	2	3	3	1	1
2	2	1	1	2	2	3	3	1	1
3	3	1	1	2	2	3	3	1	1
3	3	1	1	2	2	3	3	1	1
2	2	1	1	2	2	3	3	1	1
3	3	1	1	2	2	3	3	1	1
3	3	1	1	2	2	2	2	1	1
3	3	1	1	2	2	3	3	1	1
3	3	1	1	2	2	3	3	1	1
2	2	1	1	2	2	3	3	1	1
3	3	1	1	2	2	2	2	1	1
3	3	1	1	2	2	3	3	1	1
3	3	1	1	2	2	2	2	1	1
2	2	1	1	2	2	3	3	1	1
3	3	3	3	3	3	3	3	1	1
3	3	1	1	3	3	3	3	1	1
3	3	3	3	3	3	3	3	1	1
2	2	1	1	2	2	2	2	1	1
3	3	1	1	2	2	3	3	2	2
2	2	3	3	3	3	3	3	1	1
3	3	1	1	2	2	3	3	1	1
2	2	1	1	2	2	3	3	1	1
3	3	1	1	2	2	3	3	1	1
3	3	1	1	2	2	3	3	1	1
3	3	1	1	2	2	3	3	1	1
2	2	1	1	2	2	3	3	1	1
2	2	3	3	3	3	3	3	1	1
3	3	1	1	2	2	3	3	1	1
3	3	1	1	2	2	3	3	1	1
2	2	1	1	2	2	2	2	1	1
2	2	1	1	2	2	3	3	1	1
3	3	1	1	2	2	3	3	1	1
2	2	1	1	2	2	3	3	1	1
2	2	1	1	2	2	3	3	1	1
2	2	1	1	2	2	3	3	1	1
3	3	1	1	2	2	3	3	2	2
2	2	1	1	2	2	3	3	1	1
2	2	1	1	2	2	3	3	2	2
3	3	1	1	2	2	2	2	1	1
3	3	1	1	2	2	3	3	1	1
3	3	1	1	2	2	3	3	1	1
3	3	1	1	2	2	3	3	1	1
3	3	1	1	2	2	3	3	1	1
2	2	1	1	2	2	3	3	1	1
2	2	1	1	2	2	3	3	1	1
3	3	1	1	2	2	3	3	1	1
2	2	1	1	2	2	3	3	1	1
3	3	1	1	2	2	3	3	1	1
2	2	1	1	2	2	2	2	1	1
2	2	3	3	3	3	3	3	1	1

For Review Only

3	3	1	1	2	2	2	2	1	1
3	3	1	1	2	2	3	3	1	1

For Review Only

SNP045	SNP045	SNP046	SNP046	SNP047	SNP047	SNP048	SNP048	SNP049	SNP049
4	4	1	1	1	1	4	4	2	2
4	4	1	1	1	1	1	1	2	2
4	4	1	1	4	4	4	4	2	2
4	4	1	1	1	1	1	1	2	2
4	4	1	1	4	4	4	4	3	3
4	4	1	1	1	1	4	4	2	2
4	4	1	1	1	1	4	4	3	3
4	4	1	1	4	4	1	1	2	2
4	4	3	3	1	1	4	4	3	3
4	4	1	1	1	1	1	1	2	2
4	4	3	3	1	1	1	1	2	2
4	4	1	1	4	4	4	4	2	2
4	4	1	1	1	1	4	4	3	3
4	4	1	1	4	4	4	4	3	3
2	2	1	1	1	1	4	4	3	3
4	4	3	3	4	4	4	4	2	2
4	4	1	1	1	1	1	1	2	2
4	4	1	1	4	4	4	4	2	2
2	2	1	1	1	1	4	4	2	2
4	4	1	1	4	4	4	4	3	3
4	4	3	3	4	4	4	4	3	3
4	4	3	3	1	1	4	4	3	3
4	4	3	3	1	1	4	4	2	2
4	4	1	1	1	1	1	1	2	2
4	4	1	1	1	1	4	4	3	3
4	4	1	1	4	4	4	4	3	3
4	4	1	1	4	4	4	4	3	3
4	4	1	1	1	1	4	4	3	3
4	4	1	1	1	1	4	4	3	3
4	4	1	1	4	4	1	1	2	2
2	2	1	1	1	1	4	4	3	3
2	2	1	1	4	4	4	4	2	2
4	4	1	1	1	1	4	4	3	3
4	4	1	1	1	1	4	4	2	2
4	4	3	3	1	1	4	4	3	3
4	4	1	1	4	4	1	1	2	2
4	4	1	1	1	1	1	1	2	2
4	4	1	1	1	1	4	4	3	3
4	4	1	1	1	1	4	4	3	3
4	4	1	1	1	1	4	4	3	3
4	4	1	1	4	4	4	4	3	3
4	4	1	1	1	1	4	4	3	3
4	4	1	1	1	1	1	1	2	2
4	4	3	3	1	1	1	1	2	2
4	4	1	1	1	1	1	1	2	2
4	4	1	1	1	1	4	4	2	2
4	4	1	1	4	4	4	4	3	3
4	4	1	1	4	4	4	4	2	2
4	4	1	1	1	1	1	1	2	2
2	2	1	1	4	4	4	4	2	2
4	4	1	1	4	4	4	4	3	3
4	4	1	1	4	4	4	4	2	2

For Review Only

4	4	1	1	1	1	4	4	3	3
4	4	1	1	1	1	4	4	3	3
2	2	1	1	1	1	4	4	2	2
4	4	1	1	1	1	1	1	2	2
2	2	1	1	1	1	4	4	2	2
4	4	1	1	4	4	4	4	3	3
4	4	1	1	1	1	4	4	2	2
4	4	1	1	4	4	1	1	2	2
4	4	1	1	1	1	4	4	3	3
4	4	1	1	1	1	4	4	3	3
4	4	1	1	4	4	4	4	2	2
4	4	1	1	4	4	4	4	3	3
4	4	1	1	1	1	4	4	3	3
4	4	1	1	1	1	4	4	3	3
4	4	1	1	1	1	4	4	3	3
4	4	1	1	1	1	1	1	2	2
4	4	3	3	1	1	4	4	3	3
4	4	1	1	4	4	4	4	2	2
2	2	1	1	1	1	4	4	2	2
4	4	1	1	1	1	4	4	3	3
4	4	1	1	1	1	4	4	3	3
4	4	3	3	1	1	4	4	3	3
4	4	3	3	1	1	4	4	3	3
4	4	1	1	4	4	1	1	2	2
4	4	1	1	1	1	1	1	2	2
4	4	1	1	4	4	4	4	2	2
4	4	1	1	4	4	1	1	2	2
4	4	1	1	4	4	4	4	2	2
4	4	1	1	4	4	4	4	3	3
2	2	1	1	1	1	4	4	2	2
4	4	3	3	4	4	4	4	2	2
4	4	1	1	1	1	4	4	3	3
4	4	1	1	1	1	4	4	3	3
4	4	1	1	1	1	4	4	3	3
4	4	1	1	1	1	4	4	3	3
4	4	1	1	1	1	1	1	2	2
4	4	1	1	4	4	4	4	2	2
4	4	3	3	1	1	4	4	3	3
4	4	3	3	1	1	1	1	2	2
4	4	1	1	1	1	1	1	2	2
4	4	1	1	1	1	4	4	3	3
2	2	1	1	4	4	4	4	2	2
2	2	1	1	1	1	4	4	3	3
4	4	1	1	4	4	4	4	2	2
4	4	3	3	1	1	4	4	2	2
4	4	1	1	1	1	4	4	2	2
4	4	1	1	1	1	4	4	3	3
4	4	1	1	4	4	4	4	2	2
4	4	1	1	4	4	1	1	2	2
2	2	3	3	1	1	1	1	2	2
4	4	1	1	1	1	4	4	3	3
4	4	1	1	1	1	1	1	2	2
4	4	1	1	1	1	1	1	2	2
4	4	1	1	4	4	4	4	3	3
2	2	1	1	1	1	1	1	2	2

For Review Only

4	4	1	1	1	1	4	4	3	3
4	4	1	1	1	1	4	4	3	3
4	4	1	1	1	1	4	4	2	2
4	4	1	1	1	1	4	4	3	3
4	4	1	1	4	4	4	4	2	2
4	4	1	1	1	1	4	4	2	2
4	4	3	3	1	1	4	4	2	2
4	4	1	1	1	1	4	4	2	2
4	4	1	1	4	4	4	4	3	3
4	4	1	1	4	4	4	4	2	2
4	4	1	1	1	1	4	4	3	3
4	4	1	1	1	1	4	4	2	2
2	2	1	1	1	1	4	4	3	3
4	4	1	1	1	1	4	4	2	2
4	4	1	1	1	1	4	4	3	3
4	4	1	1	1	1	4	4	3	3
2	2	1	1	4	4	4	4	2	2
4	4	1	1	1	1	4	4	3	3
4	4	1	1	1	1	4	4	3	3
2	2	1	1	1	1	4	4	3	3
4	4	1	1	1	1	4	4	3	3
4	4	1	1	1	1	4	4	2	2
4	4	1	1	4	4	4	4	2	2
4	4	1	1	1	1	4	4	2	2
4	4	3	3	1	1	4	4	3	3
4	4	3	3	1	1	4	4	2	2
2	2	1	1	1	1	4	4	2	2
4	4	1	1	1	1	1	1	2	2
4	4	1	1	1	1	4	4	2	2
2	2	1	1	4	4	4	4	2	2
4	4	1	1	1	1	4	4	3	3
4	4	1	1	1	1	4	4	3	3
4	4	1	1	1	1	4	4	3	3
4	4	1	1	1	1	4	4	3	3
4	4	1	1	1	1	4	4	2	2
4	4	1	1	1	1	4	4	2	2
4	4	1	1	1	1	4	4	3	3
2	2	1	1	4	4	4	4	2	2
4	4	3	3	1	1	4	4	2	2
4	4	3	3	4	4	4	4	3	3
4	4	1	1	4	4	4	4	3	3
4	4	1	1	1	1	4	4	3	3
2	2	1	1	4	4	4	4	3	3
4	4	1	1	4	4	1	1	3	3
4	4	3	3	1	1	4	4	3	3
4	4	1	1	1	1	4	4	3	3
4	4	1	1	1	1	4	4	3	3
4	4	1	1	4	4	4	4	3	3
4	4	3	3	1	1	4	4	2	2
4	4	1	1	1	1	4	4	3	3
4	4	1	1	4	4	4	4	2	2
4	4	3	3	1	1	4	4	3	3
4	4	1	1	4	4	4	4	2	2
4	4	1	1	4	4	4	4	2	2
4	4	1	1	4	4	4	4	3	3

For Review Only

4	4	3	3	1	1	4	4	2	2
4	4	3	3	1	1	4	4	3	3
4	4	1	1	1	1	4	4	3	3
4	4	1	1	1	1	4	4	2	2
4	4	1	1	1	1	4	4	3	3
4	4	1	1	1	1	1	1	2	2
4	4	1	1	1	1	4	4	3	3
4	4	1	1	1	1	4	4	3	3
4	4	3	3	1	1	4	4	3	3
2	2	1	1	1	1	4	4	2	2
4	4	1	1	1	1	4	4	2	2
4	4	1	1	1	1	4	4	3	3
4	4	3	3	1	1	4	4	3	3
4	4	1	1	1	1	4	4	3	3
4	4	1	1	1	1	4	4	3	3
4	4	3	3	1	1	1	1	2	2
4	4	1	1	1	1	4	4	3	3
4	4	1	1	4	4	4	4	2	2
4	4	1	1	4	4	4	4	3	3
4	4	3	3	1	1	4	4	2	2
4	4	1	1	1	1	4	4	3	3
4	4	1	1	1	1	4	4	2	2
4	4	3	3	4	4	4	4	3	3
4	4	1	1	4	4	4	4	3	3
4	4	1	1	1	1	4	4	3	3
4	4	3	3	1	1	4	4	3	3
2	2	1	1	1	1	4	4	3	3
2	2	1	1	1	1	4	4	2	2
4	4	1	1	1	1	4	4	3	3
4	4	1	1	1	1	4	4	2	2
4	4	3	3	4	4	4	4	3	3
2	2	1	1	4	4	4	4	2	2
4	4	1	1	1	1	4	4	3	3
4	4	1	1	1	1	4	4	3	3
2	2	1	1	1	1	4	4	2	2
4	4	1	1	4	4	4	4	3	3
4	4	1	1	1	1	4	4	3	3
4	4	1	1	1	1	1	1	2	2
4	4	1	1	4	4	4	4	3	3
2	2	1	1	4	4	4	4	2	2
4	4	1	1	4	4	4	4	2	2
4	4	1	1	1	1	4	4	3	3
4	4	1	1	1	1	4	4	3	3
4	4	1	1	1	1	4	4	3	3
4	4	3	3	1	1	4	4	2	2
2	2	3	3	1	1	4	4	3	3
4	4	3	3	1	1	1	1	2	2
4	4	1	1	1	1	4	4	2	2
4	4	1	1	1	1	4	4	2	2
4	4	1	1	1	1	1	1	2	2
4	4	1	1	1	1	4	4	2	2
4	4	1	1	1	1	4	4	3	3
4	4	3	3	1	1	4	4	3	3
4	4	1	1	4	4	4	4	2	2
4	4	1	1	1	1	4	4	3	3

For Review Only

4	4	1	1	4	4	4	4	2	2
4	4	1	1	4	4	4	4	3	3
4	4	1	1	1	1	4	4	2	2
2	2	3	3	1	1	4	4	3	3
4	4	1	1	1	1	4	4	3	3
4	4	1	1	4	4	4	4	3	3
2	2	1	1	4	4	4	4	3	3
4	4	1	1	1	1	1	1	2	2
4	4	1	1	4	4	4	4	2	2
4	4	1	1	1	1	4	4	3	3
4	4	1	1	1	1	4	4	3	3
4	4	3	3	1	1	4	4	3	3
4	4	1	1	1	1	1	1	2	2
4	4	1	1	1	1	1	1	2	2
2	2	1	1	4	4	4	4	2	2
4	4	3	3	1	1	4	4	3	3
4	4	1	1	1	1	4	4	2	2
4	4	3	3	1	1	4	4	2	2
4	4	3	3	1	1	4	4	2	2
4	4	1	1	1	1	4	4	3	3
4	4	1	1	4	4	4	4	3	3
4	4	1	1	4	4	4	4	3	3
4	4	3	3	4	4	4	4	2	2
4	4	1	1	4	4	4	4	3	3
4	4	1	1	4	4	1	1	2	2
4	4	1	1	1	1	4	4	3	3
4	4	1	1	4	4	4	4	2	2
4	4	1	1	1	1	4	4	2	2
4	4	1	1	1	1	4	4	3	3
4	4	1	1	1	1	4	4	2	2
4	4	1	1	4	4	4	4	3	3
4	4	1	1	4	4	4	4	2	2
4	4	1	1	1	1	4	4	2	2
4	4	1	1	4	4	4	4	3	3
4	4	3	3	4	4	4	4	2	2
4	4	1	1	1	1	4	4	2	2
4	4	1	1	1	1	4	4	2	2
4	4	1	1	4	4	4	4	3	3
4	4	1	1	4	4	1	1	2	2
4	4	1	1	1	1	4	4	3	3
2	2	1	1	1	1	4	4	2	2
4	4	1	1	1	1	4	4	2	2
4	4	1	1	1	1	4	4	3	3
4	4	3	3	1	1	4	4	3	3
4	4	1	1	1	1	4	4	2	2
4	4	1	1	1	1	1	1	2	2
4	4	1	1	4	4	4	4	2	2
4	4	1	1	1	1	4	4	3	3
4	4	1	1	4	4	4	4	2	2
4	4	1	1	4	4	4	4	2	2
4	4	1	1	4	4	4	4	2	2
4	4	1	1	1	1	1	1	2	2
4	4	1	1	4	4	4	4	2	2
2	2	3	3	4	4	4	4	2	2
4	4	1	1	1	1	1	1	2	2

For Review Only

4	4	3	3	1	1	4	4	3	3
4	4	1	1	1	1	4	4	3	3

For Review Only

SNP050	SNP050	SNP051	SNP051	SNP052	SNP052	SNP053	SNP053	SNP054	SNP054
1	1	4	4	3	3	4	4	3	3
1	1	4	4	3	3	4	4	3	3
1	1	4	4	3	3	4	4	3	3
1	1	4	4	3	3	4	4	3	3
1	1	4	4	3	3	4	4	3	3
1	1	4	4	3	3	4	4	3	3
4	4	4	4	3	3	4	4	3	3
1	1	4	4	3	3	4	4	3	3
4	4	1	1	3	3	4	4	3	3
1	1	4	4	3	3	4	4	3	3
1	1	4	4	3	3	4	4	3	3
1	1	4	4	3	3	4	4	3	3
1	1	4	4	3	3	4	4	3	3
4	4	1	1	3	3	4	4	3	3
4	4	1	1	3	3	4	4	3	3
1	1	4	4	3	3	4	4	3	3
1	1	4	4	3	3	4	4	3	3
1	1	4	4	3	3	4	4	3	3
1	1	4	4	3	3	4	4	3	3
4	4	1	1	3	3	1	1	2	2
4	4	4	4	3	3	4	4	3	3
4	4	4	4	3	3	4	4	3	3
1	1	4	4	3	3	4	4	3	3
1	1	4	4	3	3	4	4	3	3
4	4	4	4	3	3	4	4	3	3
4	4	4	4	3	3	4	4	3	3
4	4	4	4	3	3	4	4	3	3
4	4	4	4	3	3	4	4	3	3
4	4	4	4	3	3	4	4	3	3
1	1	4	4	3	3	4	4	3	3
4	4	4	4	3	3	4	4	3	3
1	1	4	4	3	3	4	4	3	3
4	4	4	4	3	3	4	4	3	3
1	1	1	1	2	2	1	1	2	2
4	4	4	4	3	3	4	4	3	3
1	1	4	4	3	3	4	4	3	3
1	1	4	4	3	3	4	4	3	3
4	4	4	4	3	3	4	4	3	3
4	4	4	4	3	3	4	4	3	3
4	4	4	4	3	3	1	1	2	2
4	4	4	4	3	3	4	4	3	3
4	4	4	4	3	3	4	4	3	3
1	1	1	1	3	3	1	1	2	2
1	1	4	4	3	3	4	4	3	3
1	1	1	1	3	3	1	1	2	2
1	1	4	4	3	3	4	4	3	3
4	4	4	4	3	3	4	4	3	3
1	1	4	4	3	3	4	4	3	3
1	1	4	4	3	3	4	4	3	3
1	1	4	4	3	3	1	1	3	3
4	4	4	4	3	3	4	4	3	3
1	1	4	4	3	3	4	4	3	3

For Review Only

4	4	4	4	3	3	4	4	3	3
4	4	4	4	3	3	4	4	3	3
1	1	4	4	3	3	4	4	3	3
1	1	4	4	3	3	4	4	3	3
1	1	4	4	3	3	4	4	3	3
4	4	4	4	3	3	4	4	3	3
1	1	4	4	3	3	4	4	3	3
1	1	4	4	3	3	4	4	3	3
4	4	1	1	3	3	4	4	3	3
1	1	4	4	3	3	4	4	3	3
1	1	4	4	3	3	4	4	3	3
4	4	4	4	3	3	4	4	3	3
4	4	4	4	3	3	4	4	3	3
4	4	4	4	3	3	4	4	3	3
1	1	4	4	3	3	4	4	3	3
1	1	1	1	3	3	4	4	3	3
4	4	4	4	3	3	1	1	2	2
1	1	4	4	3	3	4	4	3	3
1	1	4	4	3	3	4	4	3	3
4	4	4	4	3	3	4	4	3	3
1	1	4	4	3	3	4	4	3	3
4	4	1	1	3	3	1	1	2	2
4	4	1	1	3	3	4	4	3	3
1	1	4	4	3	3	4	4	3	3
1	1	1	1	3	3	1	1	2	2
1	1	4	4	3	3	4	4	3	3
1	1	4	4	3	3	4	4	3	3
1	1	4	4	3	3	4	4	3	3
4	4	4	4	3	3	4	4	3	3
1	1	4	4	3	3	4	4	3	3
1	1	4	4	3	3	4	4	3	3
4	4	4	4	3	3	4	4	3	3
4	4	4	4	3	3	4	4	3	3
4	4	4	4	3	3	4	4	3	3
4	4	4	4	3	3	4	4	3	3
1	1	1	1	3	3	1	1	2	2
1	1	4	4	3	3	4	4	3	3
4	4	4	4	3	3	4	4	3	3
1	1	4	4	3	3	4	4	3	3
1	1	1	1	3	3	1	1	2	2
4	4	4	4	3	3	4	4	3	3
1	1	4	4	3	3	4	4	3	3
4	4	1	1	3	3	1	1	2	2
1	1	4	4	3	3	4	4	3	3
1	1	4	4	3	3	4	4	3	3
1	1	1	1	2	2	1	1	3	3
4	4	1	1	2	2	4	4	2	2
1	1	4	4	3	3	4	4	3	3
1	1	4	4	3	3	4	4	3	3
1	1	4	4	3	3	4	4	3	3
4	4	4	4	3	3	4	4	3	3
1	1	4	4	3	3	4	4	3	3
1	1	4	4	3	3	4	4	3	3
1	1	4	4	3	3	4	4	3	3
1	1	1	1	3	3	4	4	3	3

For Review Only

4	4	4	4	3	3	4	4	3	3
4	4	4	4	3	3	4	4	3	3
1	1	4	4	3	3	4	4	3	3
4	4	1	1	2	2	4	4	3	3
1	1	4	4	3	3	4	4	3	3
1	1	4	4	3	3	4	4	3	3
1	1	4	4	3	3	4	4	3	3
1	1	4	4	3	3	4	4	3	3
4	4	1	1	3	3	1	1	2	2
1	1	4	4	3	3	4	4	3	3
4	4	4	4	3	3	4	4	3	3
1	1	4	4	3	3	4	4	3	3
4	4	1	1	3	3	1	1	2	2
1	1	4	4	3	3	4	4	3	3
1	1	4	4	3	3	4	4	3	3
1	1	4	4	3	3	4	4	3	3
1	1	4	4	3	3	4	4	3	3
4	4	4	4	3	3	4	4	3	3
4	4	1	1	3	3	4	4	3	3
4	4	4	4	3	3	4	4	3	3
1	1	4	4	3	3	4	4	3	3
1	1	4	4	3	3	4	4	3	3
1	1	4	4	3	3	4	4	3	3
1	1	4	4	3	3	4	4	3	3
1	1	4	4	3	3	1	1	2	2
1	1	1	1	3	3	1	1	2	2
1	1	1	1	2	2	4	4	3	3
1	1	4	4	3	3	4	4	3	3
1	1	4	4	3	3	4	4	3	3
1	1	4	4	3	3	4	4	3	3
1	1	4	4	3	3	4	4	3	3
1	1	4	4	3	3	4	4	3	3
4	4	4	4	3	3	4	4	3	3
4	4	4	4	3	3	4	4	3	3
1	1	1	1	2	2	1	1	2	2
1	1	4	4	2	2	1	1	2	2
4	4	1	1	2	2	1	1	2	2
1	1	4	4	3	3	4	4	3	3
4	4	4	4	3	3	4	4	3	3
4	4	4	4	3	3	4	4	3	3
1	1	4	4	3	3	4	4	3	3
4	4	4	4	3	3	4	4	3	3
4	4	1	1	3	3	4	4	3	3
1	1	4	4	3	3	4	4	3	3
4	4	4	4	3	3	4	4	3	3
4	4	1	1	3	3	4	4	3	3
4	4	4	4	3	3	4	4	3	3
1	1	4	4	3	3	4	4	3	3
1	1	4	4	3	3	4	4	3	3
1	1	4	4	3	3	4	4	3	3
1	1	4	4	3	3	4	4	3	3
4	4	4	4	3	3	4	4	3	3
1	1	4	4	3	3	4	4	3	3
1	1	4	4	3	3	4	4	3	3
1	1	4	4	3	3	4	4	3	3

For Review Only

1	1	4	4	3	3	4	4	3	3
4	4	1	1	3	3	1	1	2	2
4	4	4	4	3	3	4	4	3	3
1	1	1	1	3	3	1	1	2	2
4	4	4	4	3	3	4	4	3	3
1	1	4	4	3	3	4	4	3	3
1	1	4	4	3	3	4	4	3	3
1	1	4	4	3	3	4	4	3	3
4	4	4	4	3	3	4	4	3	3
1	1	4	4	3	3	4	4	3	3
1	1	4	4	3	3	4	4	3	3
4	4	4	4	3	3	4	4	3	3
4	4	4	4	3	3	4	4	3	3
1	1	1	1	2	2	4	4	3	3
4	4	1	1	2	2	1	1	2	2
1	1	4	4	3	3	4	4	3	3
1	1	4	4	3	3	4	4	3	3
1	1	4	4	3	3	4	4	3	3
4	4	4	4	3	3	4	4	3	3
1	1	4	4	2	2	4	4	3	3
1	1	4	4	3	3	4	4	3	3
1	1	4	4	3	3	4	4	3	3
4	4	1	1	3	3	1	1	2	2
4	4	4	4	3	3	4	4	3	3
4	4	4	4	3	3	4	4	3	3
4	4	1	1	2	2	1	1	2	2
1	1	4	4	3	3	4	4	3	3
1	1	4	4	3	3	4	4	3	3
4	4	1	1	3	3	1	1	2	2
1	1	4	4	3	3	4	4	3	3
4	4	1	1	3	3	4	4	3	3
1	1	4	4	3	3	4	4	3	3
4	4	1	1	2	2	1	1	3	3
1	1	1	1	2	2	1	1	2	2
1	1	4	4	3	3	4	4	3	3
4	4	4	4	3	3	4	4	3	3
4	4	4	4	3	3	4	4	3	3
1	1	4	4	3	3	4	4	3	3
1	1	4	4	3	3	4	4	3	3
1	1	4	4	3	3	4	4	3	3
1	1	4	4	3	3	4	4	3	3
4	4	4	4	3	3	4	4	3	3
4	4	4	4	3	3	4	4	3	3
1	1	4	4	3	3	4	4	3	3
1	1	4	4	2	2	4	4	3	3
4	4	4	4	3	3	4	4	3	3
1	1	4	4	3	3	4	4	3	3
1	1	4	4	3	3	4	4	3	3
1	1	4	4	3	3	4	4	3	3
1	1	1	1	3	3	1	1	2	2
1	1	4	4	3	3	4	4	3	3
4	4	4	4	3	3	4	4	3	3
4	4	4	4	3	3	4	4	3	3
1	1	4	4	3	3	4	4	3	3
4	4	4	4	3	3	4	4	3	3

For Review Only

1	1	4	4	3	3	4	4	3	3
4	4	4	4	3	3	4	4	3	3
1	1	4	4	3	3	4	4	3	3
4	4	4	4	3	3	4	4	3	3
4	4	1	1	3	3	1	1	2	2
4	4	4	4	3	3	4	4	3	3
4	4	4	4	3	3	4	4	3	3
1	1	4	4	3	3	4	4	3	3
1	1	4	4	3	3	4	4	3	3
4	4	4	4	3	3	4	4	3	3
1	1	4	4	3	3	4	4	3	3
4	4	4	4	3	3	4	4	3	3
1	1	4	4	3	3	4	4	3	3
1	1	4	4	3	3	4	4	3	3
1	1	4	4	3	3	4	4	3	3
4	4	4	4	3	3	4	4	3	3
1	1	4	4	3	3	4	4	3	3
1	1	4	4	3	3	4	4	3	3
1	1	4	4	3	3	4	4	3	3
4	4	4	4	3	3	4	4	3	3
4	4	4	4	3	3	4	4	3	3
4	4	4	4	3	3	4	4	3	3
1	1	4	4	3	3	4	4	3	3
4	4	1	1	3	3	4	4	3	3
1	1	4	4	3	3	4	4	3	3
1	1	4	4	3	3	4	4	3	3
1	1	4	4	3	3	4	4	3	3
1	1	4	4	3	3	4	4	3	3
4	4	4	4	3	3	4	4	3	3
1	1	4	4	3	3	4	4	3	3
4	4	4	4	3	3	4	4	3	3
1	1	4	4	3	3	4	4	3	3
1	1	1	1	2	2	1	1	2	2
1	1	4	4	3	3	4	4	3	3
1	1	1	1	3	3	1	1	2	2
1	1	4	4	3	3	4	4	3	3
1	1	4	4	3	3	4	4	3	3
4	4	4	4	3	3	4	4	3	3
1	1	4	4	3	3	4	4	3	3
4	4	4	4	3	3	4	4	3	3
1	1	4	4	3	3	4	4	3	3
1	1	4	4	3	3	4	4	3	3
4	4	1	1	3	3	1	1	2	2
4	4	4	4	3	3	4	4	3	3
1	1	4	4	3	3	4	4	3	3
1	1	4	4	3	3	4	4	3	3
1	1	4	4	3	3	4	4	3	3
4	4	1	1	3	3	1	1	2	2
1	1	1	1	3	3	1	1	2	2
1	1	4	4	3	3	4	4	3	3
1	1	4	4	3	3	4	4	3	3
1	1	4	4	3	3	4	4	3	3
1	1	4	4	3	3	4	4	3	3
1	1	4	4	3	3	4	4	3	3
1	1	1	1	3	3	1	1	2	2

For Review Only

4	4	4	4	3	3	4	4	3	3
4	4	4	4	3	3	4	4	3	3

For Review Only

SNP055	SNP055	SNP056	SNP056	SNP057	SNP057	SNP058	SNP058	SNP059	SNP059
1	1	4	4	2	2	1	1	3	3
1	1	4	4	2	2	1	1	2	2
4	4	4	4	2	2	1	1	3	3
1	1	4	4	2	2	1	1	3	3
1	1	4	4	2	2	1	1	3	3
1	1	4	4	2	2	1	1	3	3
1	1	4	4	2	2	1	1	3	3
1	1	4	4	2	2	1	1	3	3
1	1	4	4	2	2	1	1	3	3
1	1	4	4	2	2	1	1	3	3
1	1	1	1	2	2	4	4	3	3
4	4	4	4	2	2	1	1	3	3
1	1	4	4	2	2	1	1	3	3
1	1	4	4	2	2	1	1	2	2
1	1	4	4	2	2	1	1	3	3
1	1	1	1	2	2	4	4	2	2
1	1	4	4	2	2	1	1	3	3
1	1	4	4	2	2	1	1	3	3
4	4	1	1	3	3	4	4	3	3
1	1	4	4	2	2	1	1	2	2
1	1	4	4	2	2	1	1	3	3
1	1	4	4	2	2	1	1	3	3
1	1	4	4	2	2	1	1	3	3
1	1	4	4	2	2	1	1	3	3
1	1	4	4	2	2	1	1	3	3
4	4	4	4	2	2	1	1	3	3
4	4	4	4	2	2	1	1	2	2
1	1	4	4	2	2	1	1	3	3
1	1	4	4	2	2	1	1	3	3
1	1	4	4	2	2	1	1	3	3
1	1	4	4	3	3	1	1	3	3
1	1	4	4	2	2	1	1	3	3
1	1	4	4	2	2	1	1	3	3
1	1	4	4	2	2	1	1	3	3
1	1	4	4	3	3	1	1	3	3
1	1	4	4	2	2	1	1	2	2
1	1	4	4	2	2	1	1	2	2
1	1	4	4	2	2	1	1	3	3
1	1	4	4	2	2	1	1	3	3
1	1	4	4	2	2	1	1	3	3
1	1	4	4	2	2	1	1	3	3
1	1	4	4	2	2	1	1	3	3
1	1	4	4	2	2	1	1	2	2
1	1	4	4	2	2	1	1	3	3
1	1	4	4	2	2	1	1	3	3
1	1	4	4	2	2	1	1	3	3
4	4	4	4	2	2	1	1	3	3
1	1	4	4	2	2	1	1	3	3
1	1	4	4	2	2	1	1	3	3
1	1	4	4	2	2	1	1	3	3
1	1	4	4	2	2	1	1	3	3
1	1	4	4	2	2	1	1	3	3

For Review Only

1	1	4	4	2	2	1	1	3	3
1	1	4	4	2	2	1	1	3	3
4	4	4	4	3	3	1	1	3	3
1	1	4	4	2	2	1	1	3	3
4	4	1	1	3	3	4	4	3	3
1	1	4	4	2	2	1	1	2	2
1	1	4	4	2	2	1	1	3	3
1	1	4	4	2	2	1	1	2	2
1	1	4	4	2	2	1	1	2	2
1	1	4	4	2	2	1	1	3	3
1	1	4	4	2	2	1	1	2	2
1	1	4	4	2	2	1	1	2	2
1	1	4	4	2	2	1	1	3	3
1	1	4	4	2	2	1	1	3	3
1	1	4	4	2	2	1	1	3	3
1	1	4	4	2	2	1	1	3	3
1	1	4	4	2	2	1	1	3	3
1	1	4	4	3	3	1	1	3	3
1	1	1	1	2	2	4	4	3	3
1	1	4	4	2	2	1	1	3	3
1	1	4	4	2	2	1	1	2	2
1	1	4	4	2	2	1	1	3	3
1	1	4	4	2	2	1	1	3	3
1	1	4	4	2	2	4	4	2	2
1	1	4	4	2	2	1	1	3	3
1	1	4	4	2	2	1	1	2	2
1	1	4	4	2	2	1	1	3	3
1	1	1	1	2	2	1	1	2	2
4	4	4	4	2	2	1	1	3	3
1	1	1	1	2	2	4	4	3	3
1	1	4	4	2	2	1	1	3	3
1	1	4	4	2	2	1	1	2	2
1	1	1	1	2	2	1	1	2	2
1	1	4	4	2	2	1	1	3	3
1	1	4	4	2	2	1	1	3	3
1	1	4	4	2	2	1	1	3	3
1	1	4	4	2	2	1	1	3	3
1	1	4	4	2	2	1	1	3	3
4	4	4	4	2	2	1	1	3	3
1	1	4	4	2	2	1	1	3	3
1	1	4	4	2	2	1	1	3	3
1	1	4	4	2	2	1	1	3	3
1	1	4	4	2	2	1	1	3	3
1	1	4	4	2	2	1	1	2	2
1	1	4	4	2	2	1	1	3	3
1	1	4	4	2	2	1	1	2	2
1	1	4	4	2	2	1	1	2	2
1	1	4	4	2	2	1	1	3	3
1	1	4	4	2	2	1	1	3	3
1	1	4	4	3	3	1	1	3	3
1	1	4	4	2	2	1	1	3	3
1	1	4	4	2	2	1	1	3	3
1	1	4	4	2	2	1	1	2	2
1	1	4	4	2	2	1	1	2	2
1	1	4	4	3	3	1	1	3	3

1	1	4	4	2	2	1	1	3	3
1	1	4	4	2	2	1	1	3	3
1	1	4	4	2	2	1	1	3	3
1	1	4	4	2	2	1	1	3	3
1	1	4	4	2	2	1	1	2	2
4	4	4	4	2	2	1	1	3	3
1	1	1	1	2	2	4	4	3	3
1	1	4	4	2	2	1	1	3	3
1	1	4	4	2	2	1	1	3	3
1	1	4	4	2	2	1	1	3	3
1	1	4	4	2	2	1	1	3	3
1	1	4	4	2	2	1	1	3	3
1	1	4	4	3	3	1	1	3	3
1	1	4	4	2	2	1	1	2	2
1	1	4	4	2	2	1	1	3	3
1	1	4	4	2	2	1	1	3	3
1	1	4	4	3	3	1	1	3	3
1	1	4	4	2	2	1	1	3	3
1	1	4	4	2	2	1	1	2	2
1	1	4	4	3	3	1	1	3	3
1	1	4	4	2	2	1	1	3	3
1	1	1	1	3	3	4	4	3	3
1	1	1	1	2	2	4	4	3	3
1	1	1	1	2	2	1	1	2	2
1	1	4	4	2	2	1	1	3	3
1	1	4	4	2	2	1	1	3	3
1	1	4	4	2	2	1	1	3	3
1	1	4	4	2	2	1	1	2	2
1	1	4	4	2	2	4	4	3	3
1	1	4	4	3	3	1	1	3	3
1	1	4	4	2	2	1	1	2	2
1	1	4	4	2	2	1	1	3	3
1	1	4	4	2	2	1	1	3	3
1	1	4	4	2	2	1	1	3	3
1	1	4	4	2	2	1	1	3	3
1	1	4	4	2	2	1	1	3	3
1	1	4	4	2	2	1	1	3	3
4	4	4	4	3	3	1	1	3	3
1	1	4	4	2	2	1	1	3	3
1	1	1	1	2	2	4	4	2	2
1	1	4	4	2	2	1	1	3	3
1	1	4	4	2	2	1	1	2	2
1	1	1	1	3	3	4	4	3	3
1	1	4	4	2	2	1	1	3	3
1	1	4	4	2	2	1	1	3	3
1	1	4	4	2	2	1	1	3	3
1	1	4	4	2	2	1	1	3	3
4	4	4	4	2	2	1	1	3	3
1	1	4	4	2	2	1	1	3	3
4	4	4	4	2	2	1	1	3	3
1	1	4	4	2	2	1	1	3	3
1	1	4	4	2	2	1	1	3	3
1	1	1	1	2	2	4	4	3	3
1	1	4	4	2	2	1	1	2	2
1	1	4	4	2	2	1	1	3	3

For Review Only

4	4	1	1	3	3	4	4	3	3
1	1	4	4	2	2	1	1	3	3
1	1	4	4	2	2	1	1	3	3
1	1	1	1	2	2	1	1	3	3
1	1	4	4	2	2	1	1	2	2
1	1	4	4	2	2	1	1	3	3
1	1	1	1	3	3	1	1	3	3
1	1	4	4	2	2	1	1	3	3
1	1	4	4	3	3	1	1	3	3
1	1	1	1	3	3	4	4	3	3
4	4	4	4	2	2	1	1	3	3
1	1	4	4	2	2	1	1	3	3
1	1	4	4	2	2	1	1	3	3
1	1	4	4	2	2	1	1	3	3
1	1	4	4	2	2	1	1	2	2
1	1	1	1	2	2	4	4	3	3
1	1	1	1	2	2	4	4	3	3
1	1	4	4	2	2	1	1	2	2
1	1	4	4	2	2	1	1	3	3
1	1	1	1	2	2	4	4	3	3
1	1	4	4	2	2	1	1	3	3
1	1	4	4	2	2	1	1	3	3
1	1	4	4	2	2	1	1	3	3
1	1	4	4	2	2	1	1	2	2
1	1	4	4	2	2	1	1	3	3
1	1	1	1	2	2	4	4	3	3
1	1	4	4	2	2	1	1	3	3
1	1	4	4	3	3	1	1	3	3
1	1	4	4	2	2	1	1	2	2
1	1	4	4	2	2	1	1	3	3
1	1	4	4	2	2	1	1	3	3
1	1	1	1	3	3	4	4	3	3
1	1	4	4	2	2	1	1	2	2
1	1	4	4	2	2	1	1	2	2
1	1	4	4	2	2	1	1	3	3
1	1	4	4	2	2	1	1	2	2
1	1	4	4	2	2	1	1	2	2
1	1	4	4	2	2	1	1	3	3
4	4	4	4	2	2	1	1	3	3
4	4	4	4	2	2	1	1	3	3
4	4	4	4	2	2	1	1	3	3
1	1	4	4	2	2	1	1	3	3
1	1	1	1	2	2	1	1	3	3
1	1	4	4	2	2	1	1	3	3
1	1	4	4	2	2	1	1	3	3
1	1	4	4	2	2	1	1	3	3
1	1	1	1	2	2	4	4	3	3
1	1	1	1	2	2	1	1	3	3
1	1	4	4	2	2	1	1	2	2
1	1	4	4	2	2	1	1	3	3
4	4	4	4	2	2	1	1	3	3
1	1	4	4	2	2	1	1	2	2
1	1	4	4	2	2	1	1	3	3
1	1	4	4	2	2	1	1	2	2
1	1	1	1	2	2	1	1	2	2

For Review Only

1	1	1	1	2	2	4	4	3	3
1	1	4	4	2	2	1	1	2	2
4	4	4	4	2	2	1	1	3	3
1	1	4	4	3	3	1	1	3	3
1	1	1	1	2	2	1	1	3	3
1	1	4	4	2	2	1	1	3	3
1	1	1	1	2	2	1	1	3	3
1	1	4	4	2	2	1	1	2	2
1	1	4	4	2	2	1	1	3	3
1	1	1	1	2	2	1	1	2	2
1	1	4	4	2	2	1	1	3	3
1	1	4	4	2	2	1	1	3	3
4	4	4	4	2	2	1	1	3	3
4	4	4	4	2	2	1	1	3	3
4	4	4	4	2	2	1	1	3	3
1	1	1	1	2	2	4	4	3	3
1	1	1	1	2	2	1	1	3	3
1	1	1	1	2	2	4	4	3	3
1	1	4	4	2	2	1	1	3	3
1	1	4	4	2	2	1	1	3	3
1	1	4	4	2	2	1	1	3	3
4	4	4	4	2	2	1	1	3	3
1	1	1	1	2	2	4	4	3	3
1	1	4	4	3	3	1	1	3	3
1	1	4	4	2	2	1	1	2	2
1	1	4	4	2	2	1	1	3	3
1	1	1	1	2	2	1	1	2	2
4	4	4	4	2	2	1	1	3	3
1	1	4	4	2	2	1	1	3	3
1	1	1	1	2	2	4	4	3	3
1	1	1	1	2	2	1	1	3	3
4	4	4	4	2	2	1	1	3	3
1	1	4	4	2	2	1	1	2	2
1	1	4	4	2	2	1	1	3	3
1	1	4	4	2	2	1	1	3	3
1	1	1	1	2	2	1	1	3	3
1	1	1	1	2	2	4	4	3	3
1	1	4	4	2	2	1	1	2	2
1	1	4	4	2	2	1	1	2	2
1	1	1	1	2	2	1	1	2	2
1	1	4	4	3	3	1	1	3	3
1	1	4	4	2	2	1	1	3	3
1	1	1	1	3	3	1	1	3	3
1	1	4	4	2	2	1	1	3	3
4	4	1	1	2	2	1	1	3	3
1	1	4	4	2	2	1	1	3	3
1	1	1	1	2	2	1	1	3	3
1	1	4	4	2	2	1	1	2	2
1	1	4	4	2	2	1	1	3	3
1	1	1	1	2	2	1	1	2	2
1	1	4	4	2	2	1	1	3	3
1	1	1	1	2	2	1	1	2	2
4	4	4	4	2	2	1	1	3	3
4	4	4	4	2	2	1	1	3	3
1	1	4	4	2	2	1	1	3	3

For Review Only

1	1	1	1	2	2	4	4	3	3
1	1	4	4	2	2	1	1	3	3

For Review Only

SNP060	SNP060	SNP061	SNP061	SNP062	SNP062	SNP063	SNP063	SNP064	SNP064
4	4	1	1	3	3	3	3	2	2
4	4	1	1	3	3	3	3	3	3
4	4	1	1	3	3	3	3	2	2
4	4	1	1	3	3	3	3	2	2
4	4	1	1	3	3	3	3	2	2
4	4	1	1	3	3	3	3	2	2
4	4	1	1	3	3	3	3	3	3
4	4	1	1	3	3	3	3	3	3
4	4	1	1	3	3	3	3	2	2
4	4	1	1	3	3	3	3	2	2
4	4	1	1	3	3	3	3	3	3
4	4	1	1	3	3	3	3	2	2
4	4	1	1	3	3	3	3	3	3
4	4	1	1	3	3	3	3	2	2
4	4	1	1	3	3	3	3	3	3
4	4	1	1	3	3	3	3	2	2
4	4	1	1	3	3	3	3	3	3
4	4	1	1	3	3	3	3	2	2
3	3	4	4	1	1	3	3	2	2
4	4	1	1	3	3	3	3	3	3
4	4	1	1	3	3	3	3	3	3
4	4	1	1	3	3	3	3	3	3
4	4	1	1	3	3	3	3	3	3
4	4	1	1	3	3	3	3	2	2
4	4	1	1	3	3	3	3	3	3
4	4	1	1	3	3	3	3	2	2
4	4	1	1	3	3	3	3	2	2
4	4	1	1	3	3	3	3	2	2
4	4	1	1	3	3	3	3	3	3
4	4	1	1	3	3	3	3	3	3
4	4	1	1	3	3	3	3	2	2
4	4	1	1	3	3	3	3	2	2
4	4	1	1	3	3	3	3	3	3
4	4	1	1	3	3	3	3	3	3
4	4	1	1	3	3	3	3	2	2
4	4	1	1	3	3	3	3	3	3
4	4	1	1	3	3	3	3	3	3
4	4	1	1	3	3	3	3	2	2
4	4	1	1	3	3	3	3	3	3
4	4	1	1	3	3	3	3	3	3
4	4	1	1	3	3	3	3	3	3
4	4	1	1	3	3	3	3	3	3
4	4	1	1	3	3	3	3	3	3
4	4	1	1	3	3	3	3	3	3
4	4	1	1	3	3	3	3	3	3
4	4	1	1	3	3	3	3	2	2
4	4	1	1	3	3	3	3	2	2
4	4	1	1	3	3	3	3	3	3
4	4	1	1	3	3	3	3	3	3
4	4	1	1	3	3	3	3	3	3
4	4	1	1	3	3	3	3	2	2
4	4	1	1	3	3	3	3	3	3

For Review Only

4	4	1	1	3	3	3	3	3	3
4	4	1	1	3	3	3	3	3	3
3	3	4	4	1	1	3	3	2	2
4	4	1	1	3	3	3	3	3	3
3	3	4	4	1	1	3	3	2	2
4	4	1	1	3	3	3	3	2	2
4	4	1	1	3	3	3	3	2	2
4	4	1	1	3	3	3	3	3	3
4	4	1	1	3	3	3	3	3	3
4	4	1	1	3	3	3	3	2	2
4	4	1	1	3	3	3	3	2	2
4	4	1	1	3	3	3	3	2	2
4	4	1	1	3	3	3	3	3	3
4	4	1	1	3	3	3	3	3	3
4	4	1	1	3	3	3	3	2	2
4	4	1	1	3	3	3	3	3	3
4	4	1	1	3	3	3	3	3	3
3	3	4	4	1	1	3	3	3	3
4	4	1	1	3	3	3	3	3	3
4	4	1	1	3	3	3	3	3	3
4	4	1	1	3	3	3	3	2	2
4	4	1	1	3	3	3	3	3	3
4	4	1	1	3	3	3	3	3	3
4	4	1	1	3	3	3	3	3	3
4	4	1	1	3	3	3	3	3	3
4	4	1	1	3	3	3	3	2	2
4	4	1	1	3	3	3	3	3	3
4	4	1	1	3	3	3	3	2	2
4	4	1	1	3	3	3	3	2	2
4	4	1	1	3	3	3	3	2	2
4	4	1	1	3	3	3	3	2	2
4	4	1	1	3	3	3	3	2	2
4	4	1	1	3	3	3	3	3	3
4	4	1	1	3	3	3	3	3	3
4	4	1	1	3	3	3	3	2	2
4	4	1	1	3	3	3	3	3	3
4	4	1	1	3	3	3	3	2	2
4	4	1	1	3	3	3	3	3	3
4	4	1	1	3	3	3	3	3	3
4	4	1	1	3	3	3	3	3	3
4	4	1	1	3	3	3	3	3	3
4	4	1	1	3	3	3	3	2	2
4	4	1	1	3	3	3	3	3	3
4	4	1	1	3	3	3	3	2	2
4	4	1	1	3	3	3	3	3	3
4	4	1	1	3	3	3	3	2	2
4	4	1	1	3	3	1	1	3	3
4	4	1	1	3	3	3	3	2	2
4	4	1	1	3	3	3	3	3	3
4	4	1	1	3	3	3	3	3	3
4	4	1	1	3	3	3	3	2	2
4	4	1	1	3	3	3	3	3	3
4	4	1	1	3	3	3	3	3	3
4	4	1	1	3	3	3	3	2	2
3	3	4	4	1	1	1	1	3	3

For Review Only

4	4	1	1	3	3	3	3	3	3
4	4	1	1	3	3	3	3	3	3
4	4	1	1	3	3	3	3	3	3
4	4	1	1	3	3	1	1	3	3
4	4	1	1	3	3	3	3	2	2
4	4	1	1	3	3	3	3	3	3
4	4	1	1	3	3	3	3	2	2
4	4	1	1	3	3	3	3	3	3
4	4	1	1	3	3	3	3	3	3
4	4	1	1	3	3	3	3	3	3
4	4	1	1	3	3	3	3	2	2
4	4	1	1	3	3	3	3	2	2
3	3	4	4	1	1	3	3	3	3
4	4	1	1	3	3	3	3	2	2
4	4	1	1	3	3	3	3	2	2
4	4	1	1	3	3	3	3	2	2
3	3	4	4	1	1	3	3	3	3
4	4	1	1	3	3	3	3	2	2
4	4	1	1	3	3	1	1	3	3
4	4	1	1	3	3	3	3	3	3
4	4	1	1	3	3	3	3	2	2
4	4	1	1	3	3	3	3	2	2
4	4	1	1	3	3	3	3	2	2
4	4	1	1	3	3	3	3	2	2
4	4	1	1	3	3	3	3	3	3
4	4	1	1	3	3	3	3	3	3
4	4	1	1	3	3	3	3	3	3
4	4	1	1	3	3	3	3	2	2
4	4	1	1	3	3	3	3	3	3
4	4	1	1	3	3	3	3	3	3
4	4	1	1	3	3	3	3	3	3
4	4	1	1	3	3	3	3	2	2
4	4	1	1	3	3	3	3	3	3
4	4	1	1	3	3	3	3	2	2
4	4	1	1	3	3	3	3	3	3
4	4	1	1	3	3	3	3	3	3
4	4	1	1	3	3	1	1	3	3
4	4	4	4	1	1	3	3	2	2
4	4	1	1	3	3	3	3	3	3
4	4	1	1	3	3	3	3	3	3
4	4	1	1	3	3	3	3	2	2
4	4	1	1	3	3	3	3	3	3
3	3	4	4	1	1	3	3	2	2
4	4	1	1	3	3	3	3	3	3
4	4	1	1	3	3	3	3	2	2
4	4	1	1	3	3	3	3	2	2
4	4	1	1	3	3	3	3	2	2
4	4	1	1	3	3	3	3	2	2
4	4	1	1	3	3	3	3	3	3
4	4	1	1	3	3	3	3	2	2
4	4	1	1	3	3	3	3	2	2
4	4	1	1	3	3	3	3	3	3
4	4	1	1	3	3	3	3	3	3
4	4	1	1	3	3	3	3	2	2
4	4	1	1	3	3	3	3	3	3

For Review Only

3	3	4	4	1	1	3	3	2	2
4	4	1	1	3	3	3	3	3	3
4	4	1	1	3	3	3	3	3	3
4	4	1	1	3	3	3	3	3	3
4	4	1	1	3	3	3	3	3	3
4	4	1	1	3	3	3	3	3	3
4	4	1	1	3	3	3	3	2	2
4	4	1	1	3	3	3	3	2	2
4	4	4	4	1	1	3	3	3	3
3	3	4	4	1	1	3	3	3	3
4	4	1	1	3	3	3	3	2	2
4	4	1	1	3	3	3	3	2	2
4	4	1	1	3	3	3	3	2	2
4	4	1	1	3	3	3	3	2	2
4	4	1	1	3	3	1	1	3	3
4	4	1	1	3	3	1	1	3	3
4	4	1	1	3	3	3	3	3	3
4	4	1	1	3	3	3	3	2	2
4	4	1	1	3	3	3	3	2	2
4	4	1	1	3	3	3	3	2	2
4	4	1	1	3	3	3	3	2	2
4	4	1	1	3	3	3	3	2	2
4	4	1	1	3	3	3	3	3	3
4	4	1	1	3	3	3	3	2	2
4	4	1	1	3	3	3	3	2	2
4	4	1	1	3	3	3	3	3	3
4	4	1	1	3	3	3	3	2	2
4	4	1	1	3	3	3	3	3	3
4	4	1	1	3	3	3	3	3	3
4	4	1	1	3	3	1	1	3	3
4	4	1	1	3	3	3	3	2	2
3	3	4	4	1	1	3	3	2	2
4	4	1	1	3	3	3	3	3	3
4	4	1	1	3	3	1	1	3	3
4	4	1	1	3	3	3	3	2	2
4	4	1	1	3	3	3	3	2	2
4	4	1	1	3	3	3	3	2	2
4	4	1	1	3	3	3	3	3	3
4	4	1	1	3	3	3	3	2	2
4	4	1	1	3	3	3	3	3	3
4	4	1	1	3	3	3	3	2	2
4	4	1	1	3	3	3	3	2	2
4	4	1	1	3	3	3	3	3	3
4	4	1	1	3	3	3	3	3	3
4	4	1	1	3	3	3	3	2	2
4	4	1	1	3	3	3	3	2	2
4	4	1	1	3	3	1	1	3	3
4	4	1	1	3	3	3	3	2	2
4	4	1	1	3	3	3	3	3	3
4	4	1	1	3	3	3	3	3	3
4	4	1	1	3	3	3	3	2	2
4	4	1	1	3	3	3	3	3	3
4	4	1	1	3	3	3	3	3	3
4	4	1	1	3	3	3	3	2	2
4	4	1	1	3	3	3	3	3	3

For Review Only

4	4	1	1	3	3	3	3	3	3
4	4	1	1	3	3	3	3	3	3
4	4	1	1	3	3	3	3	2	2
4	4	1	1	3	3	3	3	3	3
4	4	1	1	3	3	3	3	3	3
4	4	1	1	3	3	3	3	3	3
4	4	1	1	3	3	3	3	2	2
4	4	1	1	3	3	1	1	3	3
4	4	1	1	3	3	1	1	3	3
4	4	1	1	3	3	3	3	3	3
4	4	1	1	3	3	3	3	2	2
4	4	1	1	3	3	3	3	2	2
4	4	1	1	3	3	3	3	3	3
4	4	1	1	3	3	3	3	3	3
4	4	1	1	3	3	3	3	3	3
4	4	1	1	3	3	3	3	2	2
4	4	1	1	3	3	1	1	3	3
4	4	1	1	3	3	3	3	2	2
4	4	1	1	3	3	3	3	2	2
4	4	1	1	3	3	3	3	2	2
4	4	1	1	3	3	3	3	2	2
4	4	1	1	3	3	3	3	3	3
4	4	1	1	3	3	3	3	2	2
3	3	4	4	1	1	3	3	2	2
4	4	1	1	3	3	3	3	3	3
4	4	1	1	3	3	3	3	2	2
4	4	1	1	3	3	3	3	2	2
4	4	1	1	3	3	3	3	2	2
4	4	1	1	3	3	3	3	3	3
4	4	1	1	3	3	3	3	3	3
4	4	1	1	3	3	3	3	3	3
4	4	1	1	3	3	3	3	2	2
4	4	1	1	3	3	1	1	3	3
4	4	1	1	3	3	3	3	3	3
4	4	1	1	3	3	3	3	3	3
4	4	1	1	3	3	3	3	2	2
4	4	1	1	3	3	3	3	3	3
4	4	1	1	3	3	3	3	2	2
4	4	1	1	3	3	3	3	3	3
4	4	1	1	3	3	3	3	3	3
3	3	4	4	1	1	3	3	3	3
4	4	1	1	3	3	3	3	3	3
3	3	4	4	1	1	3	3	2	2
4	4	1	1	3	3	3	3	3	3
4	4	1	1	3	3	3	3	2	2
4	4	1	1	3	3	3	3	3	3
4	4	1	1	3	3	3	3	3	3
4	4	1	1	3	3	3	3	2	2
4	4	1	1	3	3	3	3	2	2
4	4	1	1	3	3	3	3	2	2
4	4	1	1	3	3	3	3	3	3
4	4	1	1	3	3	3	3	3	3
4	4	1	1	3	3	3	3	2	2
4	4	1	1	3	3	3	3	3	3
4	4	1	1	3	3	3	3	3	3

For Review Only

4	4	1	1	3	3	3	3	2	2
4	4	1	1	3	3	3	3	2	2

For Review Only

SNP065	SNP065	SNP066	SNP066	SNP067	SNP067	SNP068	SNP068	SNP069	SNP069
1	1	4	4	4	4	3	3	2	2
1	1	4	4	4	4	3	3	2	2
1	1	4	4	4	4	3	3	2	2
1	1	4	4	4	4	3	3	2	2
1	1	4	4	4	4	3	3	3	3
1	1	4	4	4	4	3	3	2	2
1	1	4	4	4	4	3	3	3	3
1	1	3	3	4	4	2	2	2	2
1	1	4	4	4	4	3	3	2	2
1	1	3	3	4	4	2	2	3	3
1	1	4	4	4	4	3	3	2	2
1	1	4	4	4	4	3	3	2	2
1	1	4	4	4	4	3	3	2	2
1	1	4	4	4	4	3	3	2	2
1	1	4	4	4	4	3	3	2	2
1	1	4	4	4	4	3	3	2	2
1	1	4	4	4	4	3	3	2	2
1	1	3	3	4	4	2	2	2	2
3	3	4	4	1	1	3	3	3	3
1	1	3	3	4	4	2	2	3	3
1	1	3	3	4	4	2	2	3	3
1	1	3	3	4	4	2	2	2	2
3	3	4	4	1	1	3	3	2	2
1	1	4	4	4	4	3	3	2	2
1	1	4	4	4	4	3	3	2	2
1	1	4	4	4	4	3	3	2	2
1	1	4	4	4	4	3	3	2	2
1	1	4	4	4	4	3	3	2	2
1	1	4	4	4	4	3	3	2	2
1	1	3	3	4	4	2	2	3	3
1	1	4	4	4	4	3	3	2	2
1	1	4	4	4	4	3	3	2	2
1	1	4	4	4	4	3	3	2	2
1	1	4	4	4	4	3	3	2	2
1	1	4	4	4	4	3	3	2	2
1	1	4	4	4	4	3	3	2	2
1	1	4	4	4	4	3	3	2	2
3	3	4	4	4	4	3	3	2	2
1	1	4	4	4	4	3	3	2	2
1	1	4	4	4	4	3	3	3	3
1	1	3	3	4	4	2	2	2	2
1	1	4	4	4	4	3	3	3	3
1	1	3	3	4	4	2	2	2	2
1	1	4	4	4	4	3	3	2	2
1	1	3	3	4	4	2	2	2	2
1	1	4	4	4	4	3	3	2	2
1	1	4	4	4	4	3	3	2	2
1	1	4	4	4	4	3	3	2	2
1	1	4	4	4	4	3	3	2	2
1	1	4	4	4	4	3	3	2	2
1	1	4	4	4	4	3	3	2	2
1	1	4	4	4	4	3	3	2	2

For Review Only

1	1	4	4	4	4	3	3	2	2
1	1	4	4	4	4	3	3	2	2
3	3	4	4	1	1	3	3	2	2
1	1	3	3	4	4	2	2	2	2
3	3	4	4	1	1	3	3	3	3
1	1	4	4	4	4	3	3	2	2
1	1	4	4	4	4	3	3	2	2
1	1	4	4	4	4	3	3	3	3
1	1	4	4	4	4	3	3	3	3
1	1	4	4	4	4	3	3	2	2
1	1	4	4	4	4	3	3	2	2
1	1	4	4	4	4	3	3	2	2
1	1	4	4	4	4	3	3	2	2
1	1	4	4	4	4	3	3	2	2
1	1	4	4	4	4	3	3	2	2
1	1	4	4	4	4	3	3	2	2
1	1	4	4	4	4	3	3	2	2
3	3	4	4	1	1	3	3	2	2
1	1	4	4	4	4	3	3	2	2
1	1	4	4	4	4	3	3	2	2
1	1	4	4	4	4	3	3	2	2
1	1	4	4	4	4	3	3	2	2
1	1	4	4	4	4	3	3	2	2
1	1	4	4	4	4	3	3	2	2
1	1	3	3	4	4	2	2	2	2
1	1	4	4	4	4	3	3	2	2
1	1	4	4	4	4	3	3	2	2
1	1	4	4	4	4	3	3	2	2
1	1	4	4	4	4	3	3	2	2
1	1	4	4	4	4	3	3	2	2
1	1	4	4	4	4	3	3	2	2
1	1	4	4	4	4	3	3	2	2
1	1	4	4	4	4	3	3	2	2
1	1	4	4	4	4	3	3	2	2
1	1	4	4	4	4	3	3	2	2
1	1	3	3	4	4	2	2	2	2
1	1	4	4	4	4	3	3	2	2
1	1	4	4	4	4	3	3	3	3
1	1	4	4	4	4	3	3	2	2
1	1	4	4	4	4	3	3	3	3
1	1	4	4	4	4	3	3	2	2
1	1	4	4	4	4	3	3	2	2
1	1	3	3	4	4	2	2	3	3
1	1	4	4	4	4	3	3	2	2
3	3	4	4	1	1	3	3	2	2
1	1	4	4	4	4	3	3	2	2
1	1	4	4	4	4	3	3	2	2
1	1	4	4	4	4	3	3	2	2
1	1	4	4	4	4	3	3	2	2
1	1	4	4	4	4	3	3	2	2
1	1	4	4	4	4	3	3	2	2
1	1	4	4	4	4	3	3	2	2
1	1	4	4	4	4	3	3	2	2
1	1	4	4	4	4	3	3	2	2
3	3	4	4	1	1	3	3	2	2

For Review Only

1	1	4	4	4	4	3	3	2	2
3	3	4	4	1	1	3	3	2	2
1	1	4	4	4	4	3	3	2	2
1	1	4	4	4	4	3	3	2	2
1	1	4	4	4	4	3	3	2	2
1	1	4	4	4	4	3	3	2	2
1	1	4	4	4	4	3	3	2	2
1	1	4	4	4	4	3	3	2	2
1	1	3	3	4	4	2	2	2	2
1	1	4	4	4	4	3	3	2	2
1	1	4	4	4	4	3	3	2	2
1	1	4	4	4	4	3	3	2	2
3	3	4	4	1	1	2	2	3	3
1	1	4	4	4	4	3	3	2	2
1	1	4	4	4	4	3	3	2	2
1	1	4	4	4	4	3	3	2	2
3	3	4	4	1	1	3	3	2	2
1	1	4	4	4	4	3	3	2	2
1	1	4	4	4	4	3	3	2	2
3	3	4	4	1	1	3	3	2	2
1	1	4	4	4	4	3	3	2	2
3	3	4	4	4	4	3	3	2	2
1	1	4	4	4	4	3	3	2	2
1	1	4	4	4	4	3	3	2	2
1	1	4	4	4	4	3	3	2	2
3	3	4	4	1	1	3	3	2	2
1	1	4	4	4	4	3	3	3	3
1	1	4	4	4	4	3	3	2	2
1	1	4	4	4	4	3	3	2	2
1	1	4	4	4	4	3	3	2	2
1	1	4	4	4	4	3	3	2	2
1	1	4	4	4	4	3	3	2	2
1	1	4	4	4	4	3	3	2	2
1	1	4	4	4	4	3	3	2	2
1	1	4	4	4	4	3	3	2	2
1	1	4	4	4	4	3	3	2	2
1	1	4	4	4	4	3	3	2	2
3	3	4	4	1	1	3	3	2	2
1	1	4	4	4	4	3	3	2	2
1	1	4	4	4	4	3	3	2	2
3	3	4	4	1	1	3	3	2	2
1	1	4	4	4	4	3	3	2	2
3	3	4	4	1	1	3	3	2	2
1	1	4	4	4	4	3	3	3	3
1	1	4	4	4	4	3	3	3	3
1	1	4	4	4	4	3	3	2	2
1	1	4	4	4	4	3	3	2	2
1	1	4	4	4	4	3	3	2	2
1	1	4	4	4	4	3	3	2	2
1	1	4	4	4	4	3	3	2	2
1	1	4	4	4	4	3	3	2	2
1	1	4	4	4	4	3	3	2	2
1	1	4	4	4	4	3	3	2	2
1	1	4	4	4	4	3	3	2	2
1	1	4	4	4	4	3	3	2	2

For Review Only

3	3	4	4	1	1	3	3	2	2
1	1	3	3	4	4	2	2	3	3
1	1	4	4	4	4	3	3	2	2
1	1	4	4	4	4	3	3	2	2
1	1	4	4	4	4	3	3	2	2
1	1	4	4	4	4	3	3	2	2
1	1	4	4	4	4	3	3	2	2
1	1	4	4	4	4	3	3	2	2
1	1	4	4	4	4	3	3	2	2
1	1	4	4	4	4	3	3	2	2
1	1	4	4	4	4	3	3	2	2
1	1	4	4	4	4	3	3	2	2
1	1	4	4	4	4	3	3	2	2
1	1	4	4	4	4	3	3	2	2
1	1	4	4	4	4	3	3	2	2
1	1	4	4	4	4	3	3	2	2
1	1	4	4	4	4	3	3	2	2
1	1	4	4	4	4	3	3	2	2
1	1	4	4	4	4	3	3	2	2
1	1	4	4	4	4	3	3	2	2
1	1	4	4	4	4	3	3	2	2
1	1	4	4	4	4	3	3	2	2
1	1	4	4	4	4	3	3	2	2
1	1	4	4	4	4	3	3	2	2
1	1	4	4	4	4	3	3	2	2
1	1	4	4	4	4	3	3	2	2
1	1	4	4	4	4	3	3	2	2
1	1	4	4	4	4	3	3	2	2
1	1	4	4	4	4	3	3	2	2
1	1	4	4	4	4	3	3	2	2
1	1	4	4	4	4	3	3	2	2
3	3	4	4	1	1	3	3	2	2
1	1	4	4	4	4	3	3	2	2
1	1	4	4	4	4	3	3	2	2
1	1	4	4	4	4	3	3	2	2
1	1	4	4	4	4	3	3	2	2
1	1	4	4	4	4	3	3	2	2
1	1	4	4	4	4	3	3	2	2
1	1	4	4	4	4	3	3	2	2
1	1	4	4	4	4	3	3	2	2
1	1	4	4	4	4	3	3	2	2
1	1	4	4	4	4	3	3	2	2
1	1	4	4	4	4	3	3	2	2
1	1	4	4	4	4	3	3	2	2
1	1	4	4	4	4	3	3	2	2
1	1	4	4	4	4	3	3	2	2
1	1	4	4	4	4	3	3	2	2
1	1	4	4	4	4	3	3	2	2
1	1	4	4	4	4	3	3	2	2
1	1	4	4	4	4	3	3	2	2
1	1	4	4	4	4	3	3	2	2
1	1	4	4	4	4	3	3	2	2
1	1	4	4	4	4	3	3	2	2
1	1	4	4	4	4	3	3	2	2
1	1	4	4	4	4	3	3	3	3

For Review Only

1	1	4	4	4	4	3	3	2	2
1	1	3	3	4	4	2	2	2	2
1	1	4	4	4	4	3	3	2	2
1	1	4	4	4	4	3	3	2	2
1	1	4	4	4	4	3	3	2	2
1	1	4	4	4	4	3	3	2	2
1	1	4	4	4	4	3	3	2	2
1	1	3	3	4	4	2	2	3	3
1	1	4	4	4	4	3	3	2	2
1	1	4	4	4	4	3	3	2	2
1	1	4	4	4	4	3	3	2	2
1	1	4	4	4	4	3	3	3	3
1	1	4	4	4	4	3	3	2	2
1	1	4	4	4	4	3	3	2	2
1	1	4	4	4	4	3	3	2	2
3	3	4	4	1	1	3	3	2	2
1	1	4	4	4	4	3	3	2	2
1	1	4	4	4	4	3	3	2	2
1	1	4	4	4	4	3	3	2	2
3	3	4	4	4	4	3	3	3	3
1	1	4	4	4	4	3	3	3	3
1	1	4	4	4	4	3	3	2	2
1	1	4	4	4	4	3	3	2	2
3	3	4	4	1	1	3	3	2	2
1	1	4	4	4	4	3	3	3	3
1	1	4	4	4	4	3	3	2	2
1	1	4	4	4	4	3	3	2	2
1	1	4	4	4	4	3	3	2	2
1	1	4	4	4	4	3	3	2	2
1	1	4	4	4	4	3	3	2	2
1	1	4	4	4	4	3	3	2	2
1	1	4	4	4	4	3	3	2	2
1	1	4	4	4	4	3	3	2	2
1	1	4	4	4	4	3	3	2	2
1	1	4	4	4	4	3	3	2	2
1	1	4	4	4	4	3	3	3	3
1	1	4	4	4	4	3	3	2	2
1	1	4	4	4	4	3	3	2	2
1	1	4	4	4	4	3	3	2	2
1	1	4	4	4	4	3	3	2	2
3	3	4	4	1	1	3	3	2	2
1	1	4	4	4	4	3	3	2	2
3	3	4	4	1	1	3	3	2	2
1	1	4	4	4	4	3	3	2	2
1	1	4	4	4	4	3	3	2	2
1	1	4	4	4	4	3	3	2	2
1	1	4	4	4	4	3	3	2	2
1	1	3	3	4	4	2	2	2	2
1	1	4	4	4	4	3	3	2	2
1	1	4	4	4	4	3	3	2	2
1	1	4	4	4	4	3	3	2	2
1	1	4	4	4	4	3	3	3	3
1	1	4	4	4	4	3	3	2	2
1	1	4	4	4	4	3	3	3	3
1	1	4	4	4	4	3	3	3	3

For Review Only

3	3	4	4	1	1	3	3	2	2
1	1	4	4	4	4	3	3	2	2

For Review Only

SNP070	SNP070	SNP071	SNP071	SNP072	SNP072	SNP073	SNP073	SNP074	SNP074
2	2	2	2	1	1	4	4	4	4
2	2	2	2	1	1	2	2	1	1
2	2	2	2	1	1	4	4	4	4
2	2	2	2	1	1	4	4	4	4
3	3	3	3	2	2	2	2	1	1
2	2	2	2	1	1	4	4	4	4
3	3	3	3	2	2	2	2	1	1
2	2	2	2	1	1	4	4	4	4
2	2	2	2	1	1	2	2	1	1
3	3	3	3	2	2	2	2	1	1
2	2	2	2	2	2	4	4	4	4
2	2	2	2	2	2	2	2	4	4
2	2	2	2	2	2	2	2	4	4
2	2	2	2	1	1	2	2	1	1
2	2	2	2	2	2	2	2	4	4
2	2	2	2	1	1	4	4	4	4
2	2	2	2	1	1	2	2	1	1
2	2	2	2	1	1	4	4	4	4
2	2	3	3	2	2	4	4	4	4
3	3	3	3	2	2	2	2	1	1
3	3	3	3	2	2	4	4	4	4
2	2	2	2	1	1	4	4	4	4
2	2	2	2	2	2	4	4	4	4
2	2	2	2	1	1	2	2	1	1
2	2	2	2	1	1	4	4	4	4
2	2	2	2	1	1	4	4	4	4
2	2	2	2	2	2	2	2	1	1
2	2	2	2	1	1	4	4	4	4
2	2	2	2	1	1	2	2	1	1
3	3	3	3	2	2	2	2	1	1
2	2	2	2	2	2	4	4	4	4
2	2	2	2	1	1	2	2	1	1
2	2	2	2	1	1	2	2	1	1
2	2	2	2	1	1	2	2	1	1
2	2	2	2	2	2	4	4	4	4
2	2	2	2	1	1	2	2	1	1
2	2	2	2	1	1	2	2	1	1
3	3	3	3	2	2	2	2	1	1
2	2	2	2	1	1	2	2	1	1
3	3	3	3	2	2	2	2	1	1
2	2	2	2	1	1	2	2	1	1
3	3	3	3	2	2	2	2	1	1
2	2	2	2	1	1	2	2	1	1
2	2	2	2	1	1	4	4	4	4
2	2	2	2	1	1	2	2	1	1
2	2	2	2	1	1	2	2	4	4
2	2	2	2	1	1	4	4	4	4
2	2	2	2	1	1	4	4	4	4
2	2	2	2	1	1	2	2	1	1
2	2	2	2	1	1	4	4	4	4
2	2	2	2	2	2	4	4	4	4
2	2	2	2	1	1	2	2	4	4

For Review Only

2	2	2	2	1	1	4	4	4	4
2	2	2	2	1	1	2	2	1	1
2	2	2	2	2	2	4	4	4	4
2	2	2	2	1	1	2	2	1	1
2	2	3	3	2	2	4	4	4	4
2	2	2	2	1	1	2	2	4	4
2	2	2	2	1	1	2	2	1	1
3	3	3	3	2	2	2	2	1	1
3	3	3	3	2	2	2	2	1	1
2	2	2	2	1	1	2	2	4	4
2	2	2	2	1	1	2	2	1	1
2	2	2	2	1	1	2	2	1	1
2	2	2	2	1	1	4	4	4	4
2	2	2	2	1	1	2	2	1	1
2	2	2	2	1	1	2	2	4	4
2	2	2	2	1	1	2	2	1	1
2	2	3	3	1	1	4	4	4	4
2	2	2	2	1	1	4	4	4	4
2	2	2	2	1	1	4	4	4	4
2	2	2	2	1	1	4	4	4	4
2	2	2	2	1	1	2	2	1	1
2	2	2	2	1	1	2	2	1	1
2	2	2	2	1	1	2	2	1	1
2	2	2	2	2	2	4	4	4	4
2	2	2	2	1	1	2	2	1	1
2	2	2	2	1	1	4	4	4	4
2	2	3	3	1	1	2	2	1	1
2	2	2	2	2	2	2	2	1	1
2	2	2	2	1	1	4	4	4	4
2	2	2	2	1	1	4	4	4	4
2	2	2	2	1	1	4	4	4	4
2	2	2	2	1	1	2	2	1	1
2	2	2	2	2	2	2	2	1	1
2	2	2	2	1	1	4	4	4	4
2	2	2	2	1	1	4	4	4	4
2	2	2	2	1	1	2	2	1	1
2	2	2	2	1	1	4	4	4	4
3	3	3	3	2	2	2	2	1	1
2	2	2	2	1	1	2	2	1	1
3	3	3	3	2	2	2	2	1	1
2	2	2	2	1	1	4	4	4	4
2	2	2	2	1	1	2	2	1	1
3	3	3	3	2	2	4	4	4	4
2	2	2	2	1	1	2	2	1	1
2	2	2	2	2	2	2	2	4	4
2	2	2	2	1	1	2	2	1	1
2	2	2	2	1	1	2	2	1	1
2	2	2	2	1	1	2	2	1	1
2	2	2	2	1	1	4	4	4	4
2	2	2	2	1	1	2	2	1	1
2	2	2	2	1	1	2	2	1	1
2	2	2	2	2	2	2	2	1	1
2	2	2	2	1	1	2	2	1	1
2	2	2	2	1	1	2	2	4	4
2	2	2	2	1	1	2	2	4	4

For Review Only

2	2	2	2	2	2	4	4	4	4
2	2	2	2	1	1	2	2	1	1
2	2	2	2	1	1	2	2	1	1
2	2	2	2	1	1	2	2	1	1
2	2	2	2	1	1	2	2	1	1
2	2	2	2	1	1	4	4	4	4
2	2	2	2	1	1	4	4	4	4
2	2	2	2	1	1	2	2	1	1
2	2	2	2	1	1	4	4	4	4
2	2	2	2	2	2	2	2	1	1
2	2	2	2	1	1	2	2	4	4
2	2	2	2	1	1	2	2	4	4
3	3	3	3	2	2	4	4	4	4
2	2	2	2	1	1	2	2	4	4
2	2	2	2	1	1	2	2	4	4
2	2	2	2	2	2	2	2	4	4
2	2	2	2	1	1	4	4	4	4
2	2	2	2	2	2	4	4	1	1
2	2	2	2	1	1	2	2	1	1
2	2	2	2	1	1	2	2	1	1
2	2	2	2	1	1	2	2	4	4
2	2	2	2	1	1	4	4	4	4
2	2	2	2	1	1	4	4	4	4
2	2	2	2	2	2	2	2	1	1
2	2	2	2	1	1	2	2	1	1
2	2	2	2	1	1	2	2	1	1
3	3	3	3	2	2	2	2	1	1
2	2	2	2	1	1	2	2	1	1
2	2	2	2	1	1	4	4	4	4
2	2	2	2	1	1	4	4	4	4
2	2	2	2	1	1	2	2	1	1
2	2	2	2	1	1	2	2	4	4
2	2	2	2	1	1	4	4	4	4
2	2	2	2	1	1	2	2	4	4
2	2	2	2	1	1	2	2	1	1
2	2	2	2	1	1	2	2	4	4
2	2	2	2	1	1	2	2	1	1
2	2	2	2	2	2	2	2	1	1
2	2	2	2	1	1	2	2	4	4
2	2	2	2	2	2	2	2	4	4
2	2	2	2	1	1	4	4	4	4
2	2	2	2	1	1	2	2	1	1
2	2	2	2	2	2	4	4	4	4
2	2	2	2	1	1	2	2	1	1
3	3	3	3	2	2	4	4	4	4
2	2	2	2	2	2	2	2	1	1
2	2	2	2	2	2	4	4	4	4
2	2	2	2	1	1	4	4	4	4
2	2	2	2	1	1	2	2	4	4
2	2	2	2	1	1	2	2	4	4
2	2	2	2	2	2	4	4	4	4
2	2	2	2	1	1	2	2	1	1
2	2	2	2	1	1	4	4	4	4
2	2	2	2	2	2	2	2	1	1
2	2	2	2	2	2	2	2	4	4

For Review Only

2	2	2	2	1	1	4	4	4	4
3	3	3	3	2	2	2	2	1	1
2	2	2	2	1	1	2	2	1	1
2	2	2	2	1	1	2	2	4	4
2	2	2	2	1	1	2	2	1	1
2	2	2	2	1	1	2	2	1	1
2	2	2	2	2	2	2	2	1	1
2	2	2	2	1	1	2	2	4	4
2	2	2	2	1	1	2	2	1	1
2	2	2	2	1	1	4	4	4	4
2	2	2	2	2	2	4	4	4	4
2	2	2	2	1	1	2	2	1	1
2	2	2	2	1	1	4	4	4	4
2	2	2	2	1	1	2	2	4	4
2	2	2	2	1	1	2	2	1	1
2	2	2	2	2	2	4	4	4	4
2	2	2	2	1	1	2	2	4	4
2	2	2	2	1	1	2	2	1	1
2	2	2	2	2	2	4	4	4	4
2	2	2	2	1	1	4	4	4	4
2	2	2	2	1	1	2	2	4	4
2	2	2	2	2	2	2	2	1	1
2	2	2	2	1	1	2	2	1	1
2	2	2	2	1	1	2	2	1	1
2	2	2	2	1	1	2	2	1	1
2	2	2	2	1	1	4	4	4	4
2	2	2	2	2	2	2	2	1	1
2	2	2	2	1	1	2	2	4	4
2	2	2	2	1	1	2	2	1	1
2	2	2	2	1	1	4	4	4	4
2	2	2	2	1	1	4	4	4	4
2	2	2	2	1	1	4	4	4	4
2	2	2	2	1	1	2	2	1	1
2	2	2	2	1	1	2	2	1	1
2	2	2	2	1	1	4	4	4	4
2	2	2	2	1	1	2	2	1	1
2	2	2	2	2	2	2	2	1	1
2	2	2	2	1	1	4	4	4	4
2	2	2	2	1	1	4	4	4	4
2	2	2	2	1	1	2	2	1	1
2	2	2	2	1	1	4	4	4	4
2	2	2	2	2	2	4	4	4	4
2	2	2	2	2	2	2	2	1	1
2	2	2	2	1	1	2	2	4	4
2	2	2	2	1	1	2	2	1	1
2	2	2	2	1	1	2	2	4	4
2	2	2	2	2	2	4	4	4	4
2	2	2	2	2	2	2	2	1	1
2	2	2	2	2	2	2	2	1	1
2	2	2	2	1	1	2	2	1	1
2	2	2	2	1	1	2	2	1	1
2	2	2	2	1	1	2	2	1	1
2	2	2	2	1	1	2	2	1	1
2	2	2	2	1	1	2	2	1	1
3	3	3	3	2	2	2	2	1	1

For Review Only

2	2	2	2	1	1	4	4	4	4
2	2	2	2	1	1	2	2	1	1
2	2	2	2	1	1	4	4	4	4
2	2	2	2	1	1	2	2	1	1
2	2	2	2	1	1	2	2	4	4
2	2	2	2	1	1	4	4	4	4
3	3	2	2	2	2	2	2	1	1
3	3	3	3	2	2	2	2	1	1
2	2	2	2	1	1	2	2	4	4
2	2	2	2	2	2	2	2	1	1
2	2	2	2	1	1	2	2	4	4
3	3	3	3	2	2	4	4	4	4
2	2	2	2	1	1	2	2	1	1
2	2	2	2	1	1	4	4	4	4
2	2	2	2	1	1	4	4	4	4
2	2	2	2	1	1	4	4	4	4
2	2	2	2	1	1	2	2	1	1
2	2	2	2	2	2	4	4	4	4
2	2	2	2	1	1	2	2	1	1
3	3	3	3	2	2	2	2	1	1
3	3	3	3	2	2	4	4	4	4
2	2	2	2	2	2	4	4	4	4
2	2	2	2	1	1	4	4	4	4
2	2	2	2	2	2	2	2	1	1
3	3	3	3	2	2	2	2	1	1
2	2	2	2	1	1	4	4	4	4
2	2	2	2	2	2	2	2	1	1
2	2	2	2	1	1	2	2	1	1
2	2	2	2	1	1	2	2	4	4
2	2	2	2	1	1	4	4	4	4
2	2	2	2	1	1	2	2	1	1
2	2	2	2	1	1	2	2	1	1
2	2	2	2	1	1	4	4	4	4
2	2	2	2	1	1	4	4	4	4
2	2	2	2	1	1	2	2	1	1
3	3	3	3	2	2	2	2	1	1
2	2	2	2	1	1	4	4	4	4
2	2	2	2	1	1	2	2	1	1
2	2	2	2	1	1	2	2	1	1
2	2	2	2	2	2	2	2	1	1
2	2	2	2	1	1	2	2	1	1
2	2	2	2	1	1	4	4	4	4
2	2	2	2	2	2	2	2	1	1
2	2	2	2	1	1	4	4	4	4
2	2	2	2	1	1	2	2	1	1
2	2	2	2	1	1	2	2	1	1
2	2	2	2	1	1	2	2	1	1
2	2	2	2	2	2	2	2	1	1
2	2	2	2	1	1	4	4	4	4
2	2	2	2	2	2	2	2	1	1
2	2	2	2	1	1	4	4	4	4
3	3	3	3	2	2	2	2	1	1
2	2	2	2	1	1	4	4	4	4
3	3	3	3	2	2	4	4	4	4
3	3	3	3	2	2	2	2	1	1

For Review Only

2	2	2	2	1	1	4	4	4	4
2	2	2	2	1	1	4	4	4	4

For Review Only

SNP075	SNP075	SNP076	SNP076	SNP077	SNP077	SNP078	SNP078	SNP079	SNP079
1	1	3	3	3	3	1	1	1	1
2	2	3	3	3	3	1	1	1	1
1	1	2	2	3	3	3	3	1	1
1	1	3	3	3	3	1	1	1	1
1	1	3	3	3	3	1	1	1	1
1	1	2	2	3	3	3	3	1	1
1	1	3	3	3	3	1	1	1	1
1	1	3	3	3	3	1	1	1	1
1	1	3	3	3	3	1	1	1	1
1	1	3	3	3	3	1	1	1	1
1	1	3	3	3	3	1	1	1	1
1	1	2	2	3	3	3	3	1	1
1	1	2	2	3	3	3	3	1	1
2	2	3	3	4	4	1	1	1	1
1	1	3	3	3	3	3	3	1	1
2	2	3	3	3	3	1	1	4	4
1	1	3	3	3	3	1	1	1	1
1	1	3	3	3	3	1	1	4	4
1	1	2	2	4	4	3	3	4	4
1	1	3	3	3	3	1	1	4	4
1	1	3	3	3	3	1	1	1	1
1	1	3	3	3	3	1	1	1	1
2	2	3	3	3	3	3	3	1	1
2	2	3	3	3	3	3	3	1	1
1	1	3	3	3	3	1	1	1	1
1	1	3	3	3	3	1	1	1	1
2	2	3	3	3	3	1	1	1	1
1	1	3	3	3	3	1	1	1	1
1	1	3	3	3	3	1	1	1	1
2	2	3	3	3	3	3	3	1	1
1	1	2	2	3	3	1	1	4	4
1	1	3	3	4	4	1	1	1	1
1	1	3	3	3	3	1	1	1	1
1	1	3	3	3	3	3	3	1	1
1	1	3	3	3	3	3	3	1	1
2	2	3	3	4	4	1	1	1	1
2	2	3	3	3	3	1	1	1	1
1	1	3	3	4	4	1	1	1	1
1	1	3	3	3	3	3	3	1	1
1	1	3	3	3	3	3	3	1	1
1	1	2	2	3	3	3	3	1	1
1	1	3	3	3	3	1	1	1	1
2	2	2	2	3	3	3	3	1	1
1	1	3	3	3	3	1	1	1	1
1	1	3	3	3	3	3	3	1	1
1	1	3	3	3	3	3	3	1	1
1	1	3	3	3	3	3	3	1	1
1	1	3	3	3	3	1	1	1	1
1	1	3	3	3	3	1	1	1	1
1	1	3	3	3	3	1	1	1	1
1	1	3	3	4	4	1	1	1	1
1	1	3	3	3	3	3	3	1	1

For Review Only

1	1	3	3	3	3	1	1	1	1
1	1	3	3	3	3	1	1	1	1
1	1	2	2	3	3	3	3	1	1
1	1	3	3	3	3	1	1	1	1
1	1	2	2	4	4	3	3	4	4
2	2	3	3	4	4	3	3	1	1
1	1	3	3	3	3	1	1	1	1
2	2	3	3	3	3	1	1	1	1
2	2	3	3	3	3	1	1	1	1
1	1	3	3	3	3	1	1	1	1
2	2	3	3	4	4	3	3	1	1
2	2	3	3	4	4	3	3	1	1
1	1	3	3	3	3	1	1	1	1
1	1	3	3	3	3	1	1	1	1
1	1	3	3	3	3	3	3	1	1
1	1	3	3	3	3	1	1	1	1
1	1	3	3	3	3	1	1	1	1
1	1	3	3	3	3	1	1	1	1
1	1	3	3	3	3	1	1	1	1
1	1	3	3	3	3	1	1	1	1
2	2	3	3	3	3	3	3	1	1
2	2	3	3	3	3	3	3	1	1
1	1	3	3	3	3	1	1	1	1
2	2	3	3	4	4	3	3	1	1
1	1	3	3	3	3	3	3	1	1
2	2	3	3	3	3	3	3	1	1
1	1	3	3	3	3	1	1	1	1
2	2	3	3	3	3	3	3	1	1
1	1	3	3	3	3	1	1	1	1
1	1	3	3	3	3	1	1	1	1
1	1	3	3	3	3	1	1	1	1
2	2	3	3	3	3	1	1	1	1
2	2	3	3	3	3	3	3	1	1
1	1	3	3	3	3	1	1	1	1
1	1	3	3	3	3	1	1	1	1
1	1	3	3	3	3	3	3	1	1
1	1	3	3	3	3	1	1	1	1
1	1	3	3	3	3	1	1	1	1
1	1	3	3	3	3	1	1	1	1
1	1	3	3	3	3	1	1	1	1
1	1	3	3	3	3	1	1	4	4
1	1	2	2	4	4	3	3	1	1
1	1	3	3	3	3	3	3	1	1
2	2	3	3	4	4	3	3	1	1
1	1	3	3	3	3	1	1	1	1
2	2	3	3	4	4	3	3	1	1
2	2	3	3	3	3	3	3	1	1
1	1	3	3	3	3	1	1	1	1
1	1	3	3	3	3	3	3	1	1
1	1	3	3	3	3	3	3	1	1
1	1	2	2	3	3	3	3	1	1
1	1	3	3	3	3	3	3	1	1
2	2	3	3	3	3	1	1	1	1
1	1	3	3	3	3	1	1	1	1
1	1	3	3	3	3	3	3	1	1

For Review Only

2	2	3	3	3	3	3	3	1	1
2	2	3	3	3	3	1	1	1	1
1	1	3	3	3	3	1	1	1	1
1	1	3	3	3	3	3	3	4	4
1	1	3	3	3	3	3	3	1	1
1	1	3	3	3	3	3	3	1	1
1	1	3	3	3	3	1	1	4	4
1	1	2	2	3	3	3	3	1	1
1	1	3	3	3	3	1	1	1	1
1	1	3	3	3	3	1	1	1	1
1	1	3	3	3	3	1	1	1	1
1	1	3	3	3	3	1	1	1	1
1	1	3	3	3	3	3	3	1	1
2	2	3	3	3	3	3	3	1	1
1	1	3	3	3	3	1	1	1	1
1	1	3	3	3	3	1	1	1	1
1	1	3	3	4	4	1	1	1	1
1	1	3	3	3	3	1	1	1	1
2	2	3	3	3	3	3	3	4	4
1	1	3	3	3	3	1	1	1	1
1	1	3	3	3	3	1	1	1	1
1	1	3	3	3	3	1	1	1	1
1	1	2	2	4	4	3	3	1	1
2	2	3	3	3	3	3	3	1	1
1	1	3	3	3	3	1	1	1	1
2	2	3	3	3	3	3	3	1	1
1	1	3	3	3	3	1	1	1	1
2	2	3	3	3	3	1	1	1	1
1	1	3	3	3	3	1	1	1	1
1	1	3	3	4	4	1	1	1	1
2	2	3	3	3	3	3	3	1	1
2	2	3	3	3	3	1	1	1	1
1	1	3	3	3	3	1	1	4	4
1	1	3	3	3	3	1	1	1	1
1	1	3	3	3	3	3	3	1	1
1	1	3	3	3	3	3	3	1	1
1	1	3	3	3	3	3	3	1	1
1	1	3	3	3	3	3	3	1	1
1	1	3	3	3	3	3	3	1	1
2	2	3	3	3	3	1	1	1	1
1	1	3	3	4	4	1	1	1	1
2	2	3	3	3	3	1	1	1	1
1	1	3	3	3	3	1	1	1	1
1	1	3	3	3	3	1	1	1	1
1	1	3	3	3	3	1	1	1	1
1	1	3	3	3	3	1	1	1	1
1	1	3	3	3	3	1	1	1	1
1	1	3	3	3	3	1	1	1	1
1	1	3	3	3	3	1	1	1	1
1	1	3	3	3	3	1	1	1	1
1	1	3	3	4	4	1	1	1	1
1	1	3	3	3	3	3	3	1	1
1	1	3	3	3	3	1	1	1	1
2	2	3	3	3	3	1	1	1	1
1	1	3	3	3	3	1	1	1	1

For Review Only

1	1	3	3	3	3	3	3	1	1
2	2	3	3	3	3	1	1	1	1
1	1	3	3	3	3	1	1	1	1
1	1	3	3	3	3	1	1	1	1
2	2	3	3	3	3	3	3	1	1
1	1	3	3	3	3	1	1	1	1
1	1	3	3	3	3	3	3	1	1
1	1	3	3	3	3	1	1	1	1
1	1	3	3	3	3	1	1	1	1
1	1	3	3	4	4	1	1	1	1
1	1	3	3	3	3	1	1	1	1
1	1	3	3	3	3	3	3	1	1
1	1	3	3	3	3	3	3	1	1
1	1	3	3	3	3	1	1	1	1
2	2	3	3	3	3	3	3	1	1
1	1	2	2	3	3	3	3	1	1
1	1	3	3	3	3	1	1	1	1
2	2	3	3	3	3	3	3	1	1
1	1	2	2	3	3	3	3	1	1
1	1	3	3	3	3	1	1	4	4
1	1	3	3	3	3	1	1	1	1
2	2	3	3	3	3	3	3	1	1
1	1	3	3	3	3	3	3	1	1
2	2	3	3	4	4	3	3	1	1
1	1	3	3	3	3	1	1	1	1
1	1	3	3	3	3	3	3	1	1
1	1	3	3	3	3	3	3	1	1
1	1	3	3	4	4	3	3	1	1
2	2	3	3	3	3	3	3	1	1
1	1	3	3	3	3	3	3	1	1
1	1	3	3	4	4	3	3	1	1
1	1	3	3	4	4	1	1	1	1
2	2	3	3	3	3	1	1	1	1
2	2	3	3	3	3	3	3	1	1
1	1	3	3	3	3	1	1	1	1
2	2	3	3	4	4	3	3	1	1
2	2	2	2	3	3	3	3	1	1
1	1	3	3	3	3	1	1	1	1
1	1	2	2	3	3	3	3	1	1
1	1	3	3	4	4	3	3	1	1
1	1	2	2	3	3	3	3	1	1
1	1	3	3	3	3	1	1	1	1
1	1	3	3	3	3	3	3	1	1
1	1	3	3	3	3	1	1	1	1
1	1	3	3	3	3	1	1	1	1
1	1	3	3	3	3	1	1	1	1
1	1	2	2	3	3	3	3	1	1
1	1	3	3	3	3	3	3	1	1
2	2	3	3	3	3	1	1	1	1
1	1	3	3	3	3	1	1	1	1
1	1	3	3	3	3	1	1	1	1
2	2	3	3	3	3	1	1	1	1
1	1	3	3	3	3	3	3	1	1
2	2	3	3	4	4	3	3	1	1
2	2	3	3	3	3	3	3	1	1

For Review Only

1	1	3	3	3	3	1	1	1	1
1	1	2	2	3	3	3	3	1	1
1	1	3	3	3	3	1	1	1	1
1	1	3	3	3	3	1	1	1	1
1	1	3	3	3	3	1	1	4	4
1	1	3	3	3	3	1	1	1	1
1	1	3	3	3	3	3	3	1	1
2	2	3	3	3	3	3	3	1	1
2	2	3	3	3	3	1	1	1	1
2	2	3	3	3	3	3	3	1	1
1	1	3	3	3	3	1	1	1	1
1	1	3	3	3	3	3	3	1	1
1	1	3	3	3	3	1	1	1	1
1	1	3	3	3	3	1	1	1	1
1	1	3	3	4	4	3	3	1	1
1	1	3	3	3	3	3	3	1	1
2	2	3	3	3	3	3	3	1	1
1	1	3	3	3	3	3	3	4	4
1	1	3	3	3	3	3	3	1	1
1	1	3	3	4	4	1	1	1	1
1	1	3	3	4	4	3	3	1	1
1	1	3	3	3	3	1	1	1	1
1	1	3	3	3	3	1	1	1	1
1	1	3	3	4	4	3	3	1	1
2	2	3	3	3	3	1	1	1	1
1	1	3	3	3	3	1	1	1	1
2	2	3	3	3	3	3	3	1	1
1	1	3	3	3	3	3	3	1	1
1	1	3	3	3	3	1	1	1	1
1	1	3	3	3	3	1	1	1	1
1	1	3	3	3	3	3	3	1	1
1	1	3	3	3	3	3	3	1	1
2	2	3	3	3	3	3	3	1	1
1	1	3	3	3	3	1	1	1	1
1	1	3	3	3	3	3	3	1	1
1	1	3	3	3	3	3	3	1	1
1	1	3	3	3	3	1	1	1	1
2	2	3	3	4	4	3	3	1	1
2	2	3	3	4	4	1	1	1	1
2	2	3	3	3	3	3	3	1	1
1	1	3	3	3	3	1	1	1	1
1	1	2	2	3	3	3	3	1	1
1	1	3	3	3	3	3	3	1	1
1	1	3	3	3	3	1	1	1	1
1	1	3	3	3	3	1	1	1	1
1	1	3	3	3	3	1	1	1	1
1	1	3	3	3	3	3	3	1	1
2	2	3	3	3	3	3	3	1	1
1	1	3	3	4	4	1	1	1	1
2	2	3	3	3	3	3	3	1	1
1	1	3	3	4	4	1	1	1	1
2	2	3	3	3	3	3	3	1	1
1	1	2	2	3	3	3	3	1	1
1	1	3	3	4	4	3	3	1	1
1	1	3	3	3	3	3	3	1	1

For Review Only

1	1	3	3	3	3	1	1	1	1
1	1	2	2	3	3	3	3	1	1

For Review Only

SNP080	SNP080	SNP081	SNP081	SNP082	SNP082	SNP083	SNP083	SNP084	SNP084
4	4	3	3	3	3	1	1	4	4
4	4	3	3	3	3	3	3	4	4
4	4	3	3	3	3	1	1	4	4
4	4	3	3	3	3	1	1	4	4
4	4	3	3	3	3	1	1	4	4
4	4	3	3	3	3	1	1	4	4
4	4	3	3	3	3	1	1	4	4
4	4	3	3	4	4	1	1	4	4
4	4	3	3	3	3	1	1	4	4
4	4	3	3	4	4	1	1	4	4
4	4	3	3	3	3	1	1	4	4
4	4	3	3	3	3	1	1	4	4
4	4	3	3	3	3	1	1	4	4
1	1	1	1	3	3	1	1	4	4
4	4	3	3	3	3	1	1	4	4
4	4	3	3	4	4	3	3	4	4
4	4	3	3	3	3	1	1	4	4
4	4	3	3	3	3	1	1	4	4
1	1	1	1	3	3	1	1	4	4
4	4	3	3	3	3	1	1	4	4
4	4	3	3	3	3	1	1	4	4
4	4	3	3	3	3	1	1	4	4
4	4	3	3	3	3	1	1	1	1
4	4	3	3	3	3	3	3	1	1
4	4	3	3	3	3	1	1	4	4
4	4	3	3	3	3	1	1	4	4
4	4	3	3	3	3	1	1	4	4
4	4	3	3	3	3	1	1	4	4
4	4	3	3	3	3	1	1	4	4
4	4	3	3	3	3	3	3	4	4
4	4	3	3	3	3	1	1	4	4
4	4	1	1	3	3	1	1	4	4
4	4	3	3	3	3	1	1	4	4
4	4	3	3	3	3	1	1	1	1
4	4	3	3	3	3	1	1	1	1
1	1	1	1	3	3	1	1	4	4
4	4	3	3	3	3	1	1	4	4
4	4	3	3	3	3	1	1	4	4
4	4	3	3	4	4	3	3	1	1
4	4	3	3	3	3	1	1	1	1
4	4	3	3	4	4	1	1	4	4
4	4	3	3	3	3	1	1	4	4
4	4	3	3	3	3	3	3	4	4
4	4	3	3	3	3	1	1	4	4
4	4	3	3	4	4	1	1	1	1
4	4	3	3	3	3	1	1	4	4
4	4	3	3	3	3	1	1	4	4
4	4	3	3	3	3	1	1	4	4
4	4	3	3	3	3	1	1	4	4
4	4	3	3	3	3	1	1	4	4
1	1	1	1	3	3	1	1	4	4
4	4	3	3	3	3	1	1	1	1

For Review Only

4	4	3	3	3	3	1	1	4	4
4	4	3	3	3	3	1	1	4	4
4	4	3	3	3	3	1	1	4	4
4	4	3	3	3	3	1	1	4	4
1	1	1	1	3	3	1	1	4	4
1	1	1	1	3	3	1	1	1	1
4	4	3	3	3	3	1	1	4	4
4	4	3	3	3	3	3	3	4	4
4	4	3	3	3	3	3	3	4	4
4	4	3	3	3	3	1	1	4	4
1	1	1	1	3	3	1	1	1	1
1	1	1	1	3	3	1	1	1	1
4	4	3	3	3	3	1	1	4	4
4	4	3	3	3	3	1	1	4	4
4	4	3	3	3	3	1	1	4	4
4	4	3	3	3	3	1	1	4	4
4	4	3	3	3	3	1	1	4	4
4	4	3	3	3	3	1	1	4	4
4	4	3	3	3	3	1	1	4	4
4	4	3	3	3	3	1	1	4	4
1	1	3	3	3	3	1	1	4	4
4	4	3	3	3	3	1	1	1	1
4	4	3	3	3	3	1	1	1	1
1	1	1	1	3	3	3	3	4	4
4	4	3	3	4	4	1	1	1	1
4	4	3	3	3	3	1	1	1	1
4	4	3	3	3	3	1	1	4	4
4	4	3	3	3	3	3	3	1	1
4	4	3	3	3	3	1	1	4	4
4	4	3	3	3	3	1	1	4	4
4	4	3	3	3	3	1	1	4	4
4	4	3	3	3	3	1	1	4	4
4	4	3	3	3	3	3	3	1	1
4	4	3	3	3	3	1	1	4	4
4	4	3	3	3	3	1	1	4	4
4	4	3	3	4	4	1	1	1	1
4	4	3	3	4	4	1	1	4	4
4	4	3	3	3	3	1	1	4	4
4	4	3	3	3	3	1	1	4	4
4	4	3	3	3	3	1	1	4	4
4	4	3	3	3	3	1	1	4	4
1	1	1	1	3	3	1	1	4	4
4	4	3	3	3	3	1	1	4	4
1	1	1	1	3	3	1	1	1	1
4	4	3	3	3	3	1	1	4	4
1	1	3	3	3	3	1	1	1	1
4	4	3	3	3	3	1	1	1	1
4	4	3	3	3	3	1	1	4	4
4	4	3	3	3	3	1	1	1	1
4	4	3	3	3	3	1	1	1	1
4	4	3	3	3	3	1	1	4	4
4	4	3	3	4	4	3	3	1	1
4	4	3	3	3	3	1	1	1	1
4	4	3	3	3	3	1	1	4	4
4	4	3	3	3	3	1	1	1	1

For Review Only

4	4	3	3	3	3	1	1	4	4
4	4	3	3	3	3	1	1	4	4
4	4	3	3	3	3	1	1	4	4
4	4	3	3	3	3	1	1	1	1
4	4	3	3	3	3	1	1	1	1
4	4	3	3	3	3	1	1	4	4
4	4	3	3	3	3	1	1	4	4
4	4	3	3	3	3	1	1	4	4
4	4	3	3	3	3	1	1	1	1
4	4	3	3	3	3	1	1	4	4
4	4	3	3	3	3	1	1	4	4
4	4	3	3	3	3	1	1	4	4
4	4	3	3	3	3	1	1	1	1
4	4	3	3	3	3	1	1	1	1
4	4	3	3	3	3	1	1	1	1
4	4	3	3	3	3	1	1	4	4
4	4	1	1	3	3	1	1	1	1
4	4	3	3	3	3	1	1	4	4
4	4	3	3	3	3	1	1	1	1
4	4	3	3	3	3	1	1	4	4
4	4	3	3	3	3	1	1	4	4
4	4	3	3	3	3	1	1	4	4
4	4	1	1	3	3	1	1	4	4
4	4	3	3	3	3	3	3	4	4
4	4	3	3	3	3	1	1	4	4
4	4	3	3	3	3	1	1	1	1
4	4	3	3	3	3	1	1	4	4
4	4	3	3	3	3	1	1	4	4
4	4	3	3	3	3	1	1	4	4
1	1	1	1	3	3	1	1	4	4
4	4	3	3	3	3	3	3	1	1
4	4	3	3	3	3	1	1	4	4
4	4	3	3	3	3	1	1	4	4
4	4	3	3	3	3	1	1	4	4
4	4	3	3	3	3	1	1	1	1
4	4	3	3	3	3	1	1	1	1
4	4	3	3	3	3	1	1	1	1
1	1	1	1	3	3	1	1	4	4
4	4	3	3	3	3	1	1	1	1
4	4	3	3	3	3	3	3	4	4
1	1	1	1	3	3	1	1	4	4
4	4	3	3	3	3	1	1	4	4
4	4	3	3	3	3	1	1	4	4
4	4	3	3	3	3	1	1	4	4
4	4	3	3	3	3	1	1	4	4
4	4	3	3	3	3	1	1	4	4
4	4	3	3	3	3	1	1	4	4
4	4	3	3	3	3	1	1	4	4
4	4	3	3	3	3	3	3	4	4
4	4	3	3	3	3	1	1	4	4
1	1	1	1	3	3	1	1	4	4
4	4	3	3	3	3	1	1	1	1
4	4	3	3	3	3	1	1	4	4
4	4	3	3	3	3	1	1	4	4
4	4	3	3	3	3	1	1	4	4

For Review Only

4	4	3	3	3	3	1	1	4	4
4	4	3	3	3	3	1	1	4	4
4	4	3	3	3	3	1	1	4	4
4	4	3	3	3	3	1	1	4	4
4	4	3	3	3	3	1	1	4	4
4	4	3	3	3	3	1	1	4	4
4	4	3	3	3	3	1	1	1	1
4	4	3	3	3	3	1	1	4	4
4	4	3	3	3	3	1	1	1	1
1	1	1	1	3	3	1	1	4	4
4	4	3	3	3	3	1	1	4	4
4	4	1	1	3	3	1	1	1	1
4	4	3	3	3	3	1	1	1	1
4	4	3	3	3	3	1	1	4	4
4	4	3	3	3	3	1	1	1	1
4	4	3	3	3	3	1	1	4	4
4	4	3	3	3	3	1	1	4	4
4	4	3	3	3	3	1	1	1	1
4	4	3	3	3	3	1	1	4	4
4	4	3	3	3	3	1	1	4	4
4	4	3	3	3	3	1	1	4	4
4	4	3	3	4	4	1	1	1	1
4	4	3	3	3	3	1	1	1	1
1	1	1	1	3	3	1	1	1	1
4	4	3	3	3	3	1	1	4	4
4	4	3	3	3	3	1	1	1	1
4	4	3	3	3	3	1	1	1	1
1	1	1	1	3	3	1	1	1	1
4	4	3	3	3	3	3	3	4	4
4	4	3	3	3	3	1	1	1	1
1	1	1	1	3	3	1	1	4	4
1	1	1	1	3	3	1	1	4	4
4	4	3	3	3	3	1	1	4	4
4	4	3	3	3	3	1	1	1	1
4	4	3	3	3	3	1	1	4	4
1	1	1	1	3	3	1	1	1	1
4	4	3	3	3	3	1	1	4	4
4	4	3	3	3	3	1	1	4	4
4	4	3	3	3	3	1	1	4	4
1	1	1	1	3	3	1	1	4	4
4	4	3	3	3	3	1	1	4	4
4	4	3	3	3	3	1	1	4	4
4	4	3	3	4	4	3	3	1	1
4	4	3	3	3	3	1	1	4	4
4	4	3	3	3	3	1	1	4	4
4	4	3	3	3	3	1	1	4	4
4	4	3	3	3	3	1	1	4	4
4	4	3	3	3	3	1	1	1	1
4	4	3	3	3	3	1	1	4	4
4	4	3	3	3	3	1	1	4	4
4	4	3	3	3	3	1	1	4	4
4	4	3	3	3	3	1	1	4	4
4	4	3	3	3	3	1	1	1	1
1	1	1	1	3	3	1	1	1	1
4	4	3	3	3	3	3	3	1	1

For Review Only

4	4	3	3	3	3	1	1	4	4
4	4	3	3	3	3	1	1	4	4
4	4	3	3	3	3	1	1	4	4
4	4	3	3	3	3	1	1	4	4
4	4	3	3	4	4	1	1	4	4
4	4	3	3	3	3	1	1	4	4
4	4	3	3	3	3	1	1	1	1
4	4	3	3	3	3	3	3	1	1
4	4	3	3	3	3	1	1	4	4
4	4	3	3	3	3	3	3	1	1
4	4	3	3	3	3	1	1	4	4
4	4	3	3	3	3	1	1	1	1
4	4	3	3	3	3	1	1	4	4
4	4	3	3	3	3	1	1	4	4
1	1	1	1	3	3	1	1	4	4
4	4	3	3	3	3	1	1	1	1
4	4	3	3	3	3	1	1	4	4
4	4	3	3	3	3	1	1	1	1
4	4	3	3	3	3	1	1	1	1
1	1	1	1	3	3	1	1	4	4
1	1	1	1	3	3	1	1	4	4
4	4	3	3	3	3	1	1	4	4
4	4	3	3	3	3	1	1	4	4
1	1	1	1	3	3	1	1	1	1
4	4	3	3	3	3	3	3	4	4
4	4	3	3	3	3	1	1	4	4
4	4	3	3	3	3	3	3	1	1
4	4	3	3	3	3	1	1	1	1
4	4	3	3	4	4	1	1	4	4
4	4	3	3	3	3	1	1	4	4
4	4	3	3	3	3	1	1	1	1
4	4	3	3	3	3	1	1	1	1
4	4	3	3	3	3	1	1	1	1
4	4	3	3	3	3	1	1	4	4
4	4	3	3	3	3	1	1	1	1
4	4	3	3	3	3	3	3	1	1
4	4	3	3	3	3	1	1	4	4
1	1	1	1	3	3	1	1	1	1
1	1	1	1	3	3	1	1	4	4
4	4	3	3	3	3	3	3	1	1
4	4	3	3	3	3	1	1	4	4
4	4	3	3	3	3	1	1	4	4
4	4	3	3	3	3	1	1	1	1
4	4	3	3	3	3	1	1	4	4
4	4	3	3	3	3	1	1	4	4
4	4	3	3	3	3	1	1	4	4
4	4	3	3	3	3	1	1	4	4
4	4	3	3	3	3	1	1	1	1
1	1	1	1	3	3	1	1	4	4
4	4	3	3	3	3	3	3	1	1
1	1	1	1	3	3	1	1	4	4
4	4	3	3	3	3	3	3	1	1
4	4	3	3	3	3	1	1	4	4
1	1	1	1	3	3	1	1	1	1
4	4	3	3	3	3	1	1	1	1

For Review Only

4	4	3	3	3	3	1	1	4	4
4	4	3	3	3	3	1	1	4	4

For Review Only

SNP085	SNP085	SNP086	SNP086	SNP087	SNP087	SNP088	SNP088	SNP089	SNP089
4	4	3	3	3	3	4	4	1	1
4	4	3	3	4	4	2	2	4	4
4	4	3	3	3	3	4	4	1	1
4	4	2	2	3	3	2	2	1	1
4	4	3	3	4	4	2	2	4	4
4	4	2	2	3	3	2	2	1	1
4	4	3	3	4	4	4	4	1	1
4	4	3	3	3	3	2	2	4	4
4	4	3	3	4	4	4	4	1	1
4	4	3	3	4	4	2	2	4	4
4	4	3	3	3	3	2	2	4	4
4	4	3	3	4	4	2	2	1	1
4	4	3	3	4	4	4	4	1	1
4	4	3	3	4	4	4	4	1	1
4	4	3	3	3	3	2	2	4	4
4	4	3	3	3	3	2	2	4	4
4	4	3	3	4	4	2	2	4	4
1	1	3	3	3	3	4	4	1	1
4	4	3	3	3	3	2	2	1	1
4	4	3	3	4	4	2	2	4	4
4	4	3	3	4	4	2	2	4	4
4	4	3	3	4	4	2	2	4	4
4	4	3	3	4	4	2	2	4	4
1	1	3	3	4	4	2	2	4	4
4	4	3	3	3	3	4	4	1	1
1	1	3	3	3	3	4	4	1	1
4	4	3	3	3	3	4	4	1	1
4	4	3	3	4	4	2	2	1	1
4	4	2	2	4	4	2	2	1	1
4	4	3	3	4	4	2	2	4	4
4	4	3	3	3	3	2	2	1	1
4	4	3	3	4	4	2	2	1	1
4	4	3	3	4	4	4	4	1	1
4	4	3	3	3	3	2	2	4	4
4	4	3	3	4	4	2	2	4	4
4	4	3	3	4	4	2	2	1	1
4	4	3	3	4	4	2	2	1	1
4	4	3	3	4	4	4	4	1	1
4	4	3	3	4	4	2	2	4	4
4	4	3	3	3	3	4	4	1	1
4	4	3	3	4	4	2	2	4	4
4	4	2	2	4	4	2	2	1	1
4	4	3	3	4	4	2	2	4	4
4	4	3	3	3	3	4	4	1	1
4	4	3	3	4	4	2	2	4	4
1	1	3	3	4	4	4	4	1	1
4	4	3	3	3	3	4	4	1	1
1	1	3	3	3	3	2	2	1	1
4	4	3	3	4	4	4	4	1	1
4	4	3	3	3	3	2	2	1	1
1	1	3	3	3	3	2	2	1	1
1	1	3	3	3	3	2	2	4	4

4	4	3	3	3	3	4	4	1	1
4	4	3	3	4	4	4	4	1	1
4	4	3	3	3	3	2	2	1	1
4	4	3	3	4	4	2	2	4	4
4	4	3	3	3	3	2	2	1	1
1	1	3	3	4	4	2	2	1	1
1	1	2	2	4	4	2	2	1	1
4	4	3	3	4	4	2	2	4	4
4	4	3	3	4	4	2	2	4	4
4	4	3	3	4	4	4	4	1	1
1	1	3	3	4	4	2	2	1	1
1	1	3	3	4	4	2	2	1	1
4	4	3	3	3	3	4	4	1	1
4	4	2	2	4	4	2	2	1	1
4	4	3	3	3	3	2	2	1	1
4	4	3	3	4	4	2	2	4	4
1	1	3	3	4	4	2	2	4	4
1	1	3	3	3	3	4	4	1	1
4	4	2	2	4	4	2	2	1	1
4	4	3	3	3	3	4	4	1	1
4	4	3	3	4	4	2	2	1	1
1	1	3	3	4	4	2	2	4	4
4	4	3	3	4	4	2	2	1	1
4	4	3	3	3	3	4	4	1	1
4	4	3	3	4	4	2	2	4	4
1	1	3	3	3	3	4	4	1	1
4	4	2	2	4	4	2	2	1	1
4	4	3	3	4	4	2	2	4	4
1	1	3	3	3	3	4	4	1	1
4	4	3	3	3	3	4	4	1	1
4	4	3	3	4	4	2	2	4	4
4	4	3	3	4	4	4	4	1	1
4	4	3	3	4	4	2	2	4	4
4	4	3	3	3	3	4	4	1	1
4	4	3	3	3	3	2	2	1	1
4	4	3	3	4	4	2	2	4	4
4	4	3	3	3	3	2	2	4	4
4	4	3	3	4	4	4	4	1	1
4	4	3	3	4	4	2	2	4	4
4	4	3	3	4	4	2	2	1	1
4	4	3	3	3	3	2	2	4	4
4	4	3	3	4	4	2	2	1	1
4	4	2	2	3	3	2	2	1	1
1	1	3	3	4	4	2	2	1	1
4	4	3	3	4	4	2	2	1	1
1	1	3	3	4	4	2	2	1	1
4	4	3	3	4	4	2	2	4	4
1	1	3	3	4	4	4	4	1	1
1	1	3	3	3	3	4	4	1	1
4	4	3	3	4	4	2	2	1	1
4	4	3	3	4	4	2	2	1	1
4	4	3	3	4	4	2	2	4	4
4	4	3	3	4	4	2	2	4	4
4	4	3	3	4	4	2	2	1	1
4	4	3	3	3	3	2	2	4	4

For Review Only

4	4	3	3	3	3	2	2	4	4
4	4	2	2	4	4	2	2	1	1
1	1	3	3	4	4	2	2	1	1
4	4	3	3	4	4	2	2	4	4
4	4	3	3	4	4	4	4	1	1
4	4	3	3	3	3	2	2	4	4
4	4	3	3	3	3	4	4	1	1
4	4	3	3	4	4	2	2	1	1
4	4	3	3	3	3	4	4	1	1
4	4	3	3	4	4	2	2	4	4
4	4	3	3	4	4	2	2	4	4
4	4	3	3	4	4	4	4	1	1
4	4	2	2	3	3	2	2	1	1
1	1	3	3	3	3	2	2	1	1
4	4	3	3	4	4	2	2	1	1
4	4	3	3	3	3	2	2	4	4
4	4	3	3	3	3	4	4	1	1
4	4	3	3	3	3	2	2	1	1
4	4	3	3	4	4	2	2	4	4
4	4	2	2	4	4	2	2	1	1
4	4	3	3	4	4	2	2	1	1
4	4	3	3	4	4	4	4	1	1
4	4	3	3	3	3	2	2	1	1
4	4	3	3	4	4	2	2	4	4
1	1	3	3	4	4	2	2	4	4
4	4	2	2	4	4	2	2	1	1
4	4	3	3	4	4	2	2	1	1
4	4	3	3	4	4	2	2	4	4
1	1	3	3	3	3	2	2	1	1
4	4	3	3	3	3	2	2	1	1
4	4	3	3	4	4	2	2	4	4
4	4	3	3	4	4	2	2	1	1
4	4	3	3	3	3	2	2	4	4
4	4	3	3	3	3	4	4	1	1
4	4	3	3	3	3	2	2	4	4
4	4	3	3	4	4	2	2	4	4
4	4	3	3	4	4	2	2	4	4
4	4	3	3	3	3	4	4	1	1
4	4	3	3	3	3	2	2	4	4
4	4	3	3	3	3	2	2	4	4
4	4	3	3	3	3	4	4	1	1
4	4	3	3	4	4	2	2	4	4
4	4	3	3	4	4	4	4	1	1
4	4	3	3	4	4	2	2	4	4
1	1	2	2	3	3	2	2	1	1
4	4	2	2	3	3	2	2	1	1
1	1	3	3	3	3	2	2	4	4
1	1	3	3	3	3	4	4	1	1
4	4	3	3	4	4	2	2	4	4
4	4	3	3	3	3	2	2	1	1
1	1	3	3	3	3	2	2	1	1
4	4	3	3	4	4	2	2	4	4
4	4	3	3	4	4	4	4	1	1
1	1	3	3	4	4	2	2	4	4
4	4	3	3	4	4	2	2	4	4

For Review Only

4	4	3	3	3	3	2	2	4	4
1	1	3	3	4	4	2	2	4	4
4	4	3	3	4	4	2	2	4	4
4	4	2	2	4	4	2	2	1	1
4	4	3	3	4	4	4	4	1	1
4	4	3	3	4	4	2	2	4	4
4	4	3	3	4	4	2	2	4	4
4	4	3	3	4	4	4	4	1	1
4	4	3	3	4	4	2	2	1	1
4	4	3	3	3	3	2	2	4	4
4	4	3	3	3	3	2	2	4	4
4	4	3	3	4	4	4	4	1	1
4	4	3	3	3	3	2	2	4	4
4	4	3	3	3	3	2	2	1	1
4	4	3	3	4	4	2	2	4	4
4	4	3	3	3	3	2	2	4	4
4	4	3	3	4	4	2	2	1	1
1	1	3	3	4	4	4	4	1	1
4	4	3	3	3	3	2	2	4	4
4	4	3	3	3	3	4	4	1	1
4	4	3	3	4	4	2	2	1	1
4	4	3	3	4	4	2	2	4	4
1	1	3	3	4	4	2	2	4	4
1	1	3	3	4	4	2	2	1	1
4	4	2	2	4	4	2	2	1	1
4	4	3	3	3	3	2	2	4	4
1	1	3	3	3	3	2	2	1	1
4	4	3	3	4	4	4	4	1	1
4	4	2	2	4	4	2	2	1	1
4	4	3	3	3	3	2	2	1	1
4	4	3	3	3	3	4	4	1	1
1	1	3	3	3	3	4	4	1	1
4	4	3	3	4	4	2	2	4	4
4	4	3	3	4	4	2	2	4	4
4	4	2	2	4	4	2	2	1	1
1	1	3	3	4	4	2	2	1	1
4	4	3	3	4	4	2	2	1	1
4	4	3	3	3	3	2	2	1	1
4	4	3	3	3	3	4	4	1	1
4	4	3	3	3	3	2	2	1	1
4	4	3	3	3	3	4	4	1	1
1	1	3	3	3	3	2	2	4	4
4	4	3	3	4	4	2	2	4	4
4	4	3	3	4	4	2	2	1	1
4	4	3	3	4	4	2	2	4	4
4	4	3	3	3	3	2	2	1	1
4	4	3	3	3	3	2	2	4	4
4	4	3	3	4	4	2	2	4	4
4	4	3	3	4	4	2	2	4	4
4	4	3	3	4	4	2	2	4	4
4	4	3	3	4	4	2	2	4	4
4	4	3	3	4	4	2	2	4	4
1	1	3	3	4	4	2	2	4	4
1	1	3	3	4	4	2	2	1	1
4	4	3	3	4	4	2	2	4	4

For Review Only

4	4	3	3	4	4	4	4	1	1
4	4	3	3	4	4	2	2	4	4
4	4	3	3	3	3	4	4	1	1
4	4	3	3	4	4	2	2	4	4
4	4	3	3	3	3	2	2	4	4
1	1	3	3	3	3	4	4	1	1
4	4	3	3	4	4	2	2	4	4
4	4	3	3	4	4	2	2	4	4
4	4	3	3	3	3	2	2	4	4
4	4	3	3	4	4	2	2	4	4
4	4	3	3	4	4	2	2	1	1
1	1	2	2	3	3	2	2	1	1
4	4	3	3	4	4	2	2	4	4
1	1	3	3	3	3	4	4	1	1
4	4	3	3	3	3	4	4	1	1
4	4	3	3	3	3	2	2	4	4
4	4	2	2	4	4	2	2	1	1
4	4	3	3	3	3	2	2	4	4
4	4	3	3	4	4	2	2	4	4
4	4	3	3	4	4	4	4	1	1
4	4	3	3	3	3	4	4	1	1
1	1	3	3	3	3	4	4	1	1
4	4	3	3	3	3	2	2	4	4
4	4	3	3	3	3	4	4	1	1
4	4	3	3	4	4	2	2	4	4
4	4	3	3	3	3	2	2	1	1
4	4	3	3	4	4	2	2	4	4
1	1	2	2	4	4	2	2	1	1
4	4	3	3	3	3	2	2	1	1
1	1	3	3	3	3	2	2	4	4
4	4	3	3	4	4	2	2	4	4
4	4	3	3	4	4	2	2	4	4
4	4	3	3	3	3	2	2	4	4
1	1	3	3	4	4	4	4	1	1
1	1	3	3	4	4	2	2	4	4
4	4	3	3	4	4	2	2	4	4
4	4	3	3	4	4	2	2	4	4
1	1	3	3	4	4	2	2	1	1
4	4	3	3	4	4	2	2	1	1
4	4	3	3	4	4	2	2	4	4
4	4	2	2	3	3	2	2	1	1
4	4	3	3	3	3	2	2	4	4
4	4	3	3	4	4	2	2	4	4
4	4	3	3	4	4	2	2	4	4
4	4	3	3	4	4	2	2	4	4
4	4	2	2	4	4	2	2	1	1
4	4	3	3	4	4	4	4	1	1
1	1	2	2	4	4	2	2	1	1
4	4	3	3	3	3	2	2	1	1
4	4	3	3	4	4	2	2	4	4
4	4	3	3	3	3	2	2	1	1
4	4	3	3	4	4	2	2	4	4
4	4	3	3	3	3	4	4	1	1
1	1	3	3	4	4	2	2	1	1
4	4	3	3	4	4	2	2	4	4

For Review Only

4	4	3	3	3	3	2	2	4	4
4	4	3	3	3	3	2	2	1	1

For Review Only

SNP090	SNP090	SNP091	SNP091	SNP092	SNP092	SNP093	SNP093	SNP094	SNP094
3	3	4	4	3	3	4	4	4	4
3	3	1	1	2	2	4	4	1	1
4	4	1	1	2	2	4	4	1	1
3	3	1	1	2	2	4	4	1	1
4	4	1	1	2	2	4	4	1	1
3	3	1	1	2	2	4	4	1	1
4	4	1	1	2	2	4	4	1	1
3	3	1	1	2	2	4	4	1	1
4	4	1	1	2	2	4	4	1	1
3	3	1	1	2	2	4	4	1	1
3	3	1	1	2	2	4	4	1	1
4	4	1	1	2	2	4	4	1	1
4	4	1	1	2	2	4	4	1	1
3	3	1	1	2	2	4	4	1	1
4	4	1	1	2	2	4	4	1	1
3	3	4	4	3	3	1	1	4	4
3	3	1	1	2	2	4	4	1	1
4	4	4	4	3	3	1	1	4	4
3	3	4	4	3	3	1	1	4	4
3	3	4	4	3	3	1	1	4	4
4	4	1	1	2	2	4	4	1	1
4	4	1	1	2	2	4	4	1	1
3	3	1	1	2	2	4	4	1	1
4	4	1	1	2	2	4	4	1	1
4	4	1	1	2	2	4	4	1	1
3	3	4	4	3	3	1	1	4	4
3	3	1	1	2	2	4	4	1	1
4	4	1	1	2	2	4	4	1	1
4	4	1	1	2	2	4	4	1	1
3	3	1	1	2	2	4	4	4	4
3	3	1	1	2	2	1	1	4	4
3	3	1	1	2	2	4	4	1	1
4	4	1	1	2	2	4	4	1	1
3	3	1	1	2	2	4	4	1	1
3	3	1	1	2	2	4	4	1	1
3	3	1	1	2	2	4	4	1	1
3	3	1	1	2	2	4	4	1	1
3	3	1	1	2	2	4	4	1	1
3	3	1	1	2	2	4	4	1	1
4	4	1	1	2	2	4	4	1	1
3	3	1	1	2	2	4	4	1	1
3	3	1	1	2	2	4	4	1	1
3	3	1	1	2	2	4	4	1	1
3	3	1	1	2	2	4	4	1	1
3	3	1	1	2	2	4	4	1	1
4	4	4	4	2	2	4	4	1	1
3	3	4	4	2	2	4	4	4	4
4	4	1	1	2	2	4	4	1	1
3	3	1	1	2	2	4	4	1	1
4	4	1	1	2	2	4	4	1	1
3	3	1	1	2	2	4	4	1	1
4	4	1	1	2	2	4	4	1	1

For Review Only

4	4	1	1	2	2	4	4	1	1
4	4	1	1	2	2	4	4	1	1
3	3	1	1	2	2	4	4	1	1
3	3	1	1	2	2	4	4	1	1
3	3	4	4	3	3	1	1	4	4
3	3	4	4	3	3	1	1	4	4
4	4	4	4	3	3	1	1	4	4
3	3	1	1	2	2	4	4	1	1
3	3	1	1	2	2	4	4	1	1
4	4	1	1	2	2	4	4	4	4
4	4	1	1	2	2	4	4	1	1
3	3	1	1	2	2	4	4	1	1
4	4	1	1	2	2	4	4	1	1
4	4	1	1	2	2	4	4	1	1
4	4	1	1	2	2	4	4	1	1
3	3	1	1	2	2	4	4	1	1
3	3	1	1	2	2	4	4	1	1
4	4	4	4	3	3	1	1	4	4
3	3	1	1	2	2	4	4	1	1
4	4	4	4	3	3	1	1	4	4
4	4	1	1	2	2	4	4	1	1
3	3	1	1	2	2	4	4	1	1
4	4	1	1	2	2	4	4	1	1
3	3	1	1	2	2	4	4	1	1
3	3	1	1	2	2	4	4	1	1
4	4	1	1	2	2	4	4	1	1
3	3	1	1	2	2	4	4	1	1
3	3	1	1	2	2	4	4	1	1
3	3	4	4	3	3	1	1	4	4
3	3	1	1	2	2	4	4	1	1
4	4	1	1	2	2	4	4	1	1
4	4	1	1	2	2	4	4	1	1
3	3	1	1	2	2	4	4	1	1
4	4	1	1	2	2	4	4	1	1
3	3	4	4	3	3	1	1	4	4
3	3	1	1	2	2	4	4	1	1
4	4	1	1	2	2	4	4	1	1
4	4	1	1	2	2	4	4	1	1
3	3	1	1	2	2	4	4	1	1
3	3	1	1	2	2	4	4	1	1
4	4	4	4	3	3	1	1	4	4
4	4	1	1	2	2	4	4	1	1
3	3	1	1	2	2	4	4	1	1
4	4	1	1	2	2	4	4	1	1
4	4	1	1	2	2	4	4	1	1
4	4	1	1	2	2	4	4	1	1
3	3	1	1	2	2	4	4	4	4
4	4	1	1	2	2	4	4	1	1
3	3	4	4	3	3	1	1	4	4
4	4	1	1	2	2	4	4	1	1
3	3	1	1	2	2	4	4	1	1
3	3	1	1	2	2	4	4	1	1
3	3	1	1	2	2	4	4	1	1
4	4	1	1	2	2	4	4	1	1
4	4	1	1	2	2	4	4	1	1

For Review Only

4	4	1	1	3	3	4	4	1	1
3	3	1	1	2	2	4	4	1	1
4	4	1	1	2	2	4	4	1	1
3	3	1	1	2	2	4	4	4	4
4	4	1	1	2	2	4	4	1	1
3	3	4	4	3	3	1	1	4	4
3	3	4	4	3	3	1	1	4	4
4	4	1	1	2	2	4	4	1	1
3	3	1	1	2	2	4	4	1	1
3	3	1	1	2	2	4	4	1	1
4	4	1	1	2	2	4	4	1	1
4	4	1	1	2	2	4	4	1	1
3	3	1	1	2	2	4	4	1	1
4	4	1	1	2	2	4	4	1	1
4	4	1	1	2	2	4	4	1	1
3	3	4	4	2	2	4	4	1	1
4	4	4	4	3	3	1	1	4	4
4	4	1	1	2	2	4	4	1	1
3	3	1	1	2	2	4	4	4	4
3	3	1	1	2	2	4	4	1	1
4	4	1	1	2	2	4	4	1	1
3	3	1	1	2	2	4	4	1	1
3	3	1	1	2	2	4	4	1	1
3	3	1	1	2	2	4	4	1	1
4	4	1	1	2	2	4	4	1	1
3	3	1	1	2	2	4	4	1	1
3	3	1	1	2	2	4	4	4	4
3	3	1	1	2	2	4	4	1	1
3	3	1	1	2	2	4	4	1	1
3	3	1	1	2	2	4	4	1	1
3	3	1	1	2	2	4	4	1	1
4	4	1	1	2	2	4	4	1	1
4	4	4	4	3	3	1	1	4	4
3	3	4	4	2	2	4	4	1	1
3	3	1	1	2	2	4	4	1	1
4	4	1	1	3	3	1	1	4	4
3	3	1	1	2	2	4	4	4	4
4	4	1	1	2	2	4	4	1	1
3	3	4	4	3	3	4	4	1	1
3	3	1	1	2	2	4	4	1	1
4	4	1	1	2	2	4	4	1	1
3	3	1	1	2	2	4	4	4	4
3	3	1	1	2	2	4	4	1	1
4	4	1	1	2	2	4	4	1	1
3	3	1	1	2	2	4	4	1	1
3	3	1	1	2	2	4	4	1	1
3	3	4	4	3	3	1	1	4	4
3	3	4	4	3	3	1	1	4	4
3	3	1	1	2	2	4	4	1	1
4	4	4	4	3	3	1	1	4	4
4	4	1	1	3	3	1	1	4	4
3	3	4	4	3	3	1	1	4	4
3	3	1	1	2	2	4	4	1	1
3	3	1	1	2	2	4	4	1	1
3	3	1	1	2	2	4	4	1	1

For Review Only

3	3	1	1	2	2	4	4	1	1
3	3	1	1	2	2	4	4	1	1
4	4	1	1	2	2	4	4	1	1
3	3	1	1	2	2	4	4	1	1
3	3	4	4	3	3	1	1	4	4
3	3	4	4	3	3	1	1	4	4
3	3	1	1	2	2	4	4	1	1
4	4	1	1	3	3	4	4	1	1
3	3	1	1	2	2	4	4	1	1
4	4	1	1	2	2	4	4	4	4
4	4	4	4	2	2	1	1	4	4
3	3	1	1	2	2	4	4	1	1
3	3	1	1	2	2	4	4	1	1
3	3	1	1	2	2	4	4	1	1
3	3	1	1	2	2	4	4	4	4
3	3	1	1	2	2	4	4	1	1
3	3	1	1	2	2	4	4	1	1
4	4	1	1	2	2	4	4	1	1
3	3	1	1	2	2	4	4	1	1
3	3	4	4	3	3	1	1	4	4
4	4	1	1	2	2	4	4	1	1
3	3	1	1	2	2	4	4	1	1
3	3	1	1	2	2	4	4	1	1
3	3	1	1	2	2	4	4	1	1
3	3	1	1	2	2	4	4	1	1
3	3	1	1	2	2	4	4	1	1
4	4	1	1	2	2	4	4	1	1
4	4	4	4	3	3	1	1	4	4
4	4	1	1	2	2	4	4	1	1
3	3	1	1	2	2	4	4	1	1
3	3	1	1	2	2	4	4	1	1
3	3	4	4	3	3	1	1	4	4
3	3	1	1	2	2	4	4	1	1
3	3	1	1	2	2	4	4	4	4
3	3	1	1	2	2	4	4	1	1
3	3	1	1	2	2	4	4	1	1
4	4	1	1	2	2	4	4	1	1
3	3	1	1	2	2	4	4	1	1
3	3	4	4	3	3	1	1	4	4
4	4	1	1	2	2	4	4	1	1
4	4	1	1	2	2	4	4	1	1
3	3	4	4	3	3	1	1	4	4
3	3	1	1	2	2	4	4	1	1
3	3	1	1	2	2	4	4	1	1
4	4	1	1	2	2	4	4	4	4
3	3	1	1	2	2	4	4	1	1
3	3	1	1	2	2	4	4	1	1
3	3	1	1	2	2	4	4	1	1
3	3	1	1	2	2	4	4	1	1
3	3	1	1	2	2	4	4	1	1
3	3	1	1	2	2	4	4	1	1
3	3	1	1	2	2	4	4	1	1
3	3	1	1	2	2	4	4	1	1
4	4	1	1	2	2	4	4	1	1
3	3	1	1	2	2	4	4	1	1

For Review Only

3	3	1	1	2	2	4	4	1	1
3	3	1	1	2	2	4	4	1	1
3	3	1	1	2	2	4	4	1	1
3	3	1	1	2	2	4	4	1	1
3	3	4	4	3	3	1	1	4	4
3	3	1	1	3	3	1	1	4	4
3	3	1	1	2	2	4	4	1	1
3	3	1	1	2	2	4	4	1	1
3	3	1	1	2	2	4	4	1	1
3	3	1	1	2	2	4	4	1	1
4	4	1	1	2	2	4	4	1	1
3	3	1	1	2	2	4	4	1	1
3	3	1	1	2	2	4	4	1	1
3	3	1	1	2	2	4	4	1	1
4	4	4	4	3	3	1	1	4	4
4	4	1	1	2	2	4	4	1	1
3	3	1	1	2	2	4	4	1	1
3	3	1	1	2	2	4	4	1	1
3	3	1	1	2	2	4	4	1	1
3	3	1	1	2	2	4	4	1	1
3	3	1	1	2	2	4	4	1	1
3	3	4	4	3	3	1	1	4	4
3	3	1	1	2	2	4	4	1	1
3	3	1	1	2	2	4	4	1	1
3	3	1	1	2	2	4	4	1	1
3	3	1	1	2	2	4	4	1	1
3	3	1	1	2	2	4	4	1	1
4	4	1	1	2	2	4	4	1	1
4	4	1	1	2	2	4	4	1	1
3	3	1	1	2	2	4	4	1	1
3	3	1	1	2	2	4	4	1	1
4	4	1	1	2	2	4	4	4	4
3	3	1	1	2	2	4	4	4	4
4	4	4	4	3	3	4	4	4	4
3	3	1	1	2	2	4	4	1	1
3	3	1	1	2	2	4	4	1	1
3	3	1	1	2	2	4	4	1	1
3	3	1	1	2	2	4	4	1	1
3	3	1	1	2	2	4	4	1	1
3	3	1	1	2	2	4	4	1	1
4	4	1	1	2	2	4	4	1	1
3	3	1	1	2	2	4	4	1	1
3	3	1	1	2	2	4	4	1	1
4	4	1	1	2	2	4	4	1	1
4	4	1	1	2	2	4	4	1	1
3	3	1	1	2	2	4	4	1	1
4	4	4	4	3	3	1	1	4	4
3	3	1	1	2	2	4	4	1	1
3	3	1	1	2	2	4	4	1	1
3	3	1	1	2	2	4	4	1	1
3	3	1	1	2	2	4	4	1	1
3	3	1	1	2	2	4	4	1	1
4	4	1	1	2	2	4	4	1	1
4	4	1	1	2	2	4	4	1	1
3	3	1	1	2	2	4	4	1	1

For Review Only

4	4	1	1	2	2	4	4	1	1
4	4	1	1	2	2	4	4	1	1

For Review Only

SNP095	SNP095	SNP096	SNP096	SNP097	SNP097	SNP098	SNP098	SNP099	SNP099
2	2	1	1	2	2	3	3	1	1
2	2	4	4	4	4	2	2	3	3
3	3	1	1	2	2	3	3	1	1
3	3	4	4	4	4	2	2	1	1
2	2	1	1	2	2	3	3	3	3
2	2	1	1	2	2	3	3	1	1
2	2	1	1	2	2	3	3	1	1
3	3	4	4	4	4	2	2	3	3
2	2	1	1	2	2	3	3	1	1
2	2	4	4	4	4	2	2	1	1
3	3	4	4	4	4	2	2	3	3
3	3	1	1	2	2	3	3	1	1
2	2	1	1	2	2	2	2	1	1
2	2	4	4	4	4	2	2	3	3
2	2	1	1	2	2	3	3	1	1
3	3	4	4	4	4	3	3	1	1
2	2	4	4	4	4	2	2	1	1
2	2	1	1	2	2	3	3	1	1
3	3	1	1	4	4	3	3	3	3
3	3	4	4	4	4	2	2	1	1
3	3	1	1	2	2	3	3	1	1
3	3	1	1	2	2	3	3	1	1
2	2	1	1	2	2	3	3	3	3
3	3	1	1	2	2	3	3	1	1
2	2	1	1	2	2	3	3	1	1
3	3	1	1	4	4	3	3	3	3
3	3	1	1	2	2	3	3	1	1
2	2	1	1	4	4	2	2	1	1
2	2	1	1	2	2	3	3	1	1
2	2	4	4	4	4	2	2	3	3
2	2	1	1	2	2	3	3	1	1
2	2	1	1	4	4	3	3	1	1
2	2	1	1	2	2	3	3	3	3
2	2	4	4	4	4	2	2	3	3
2	2	1	1	4	4	2	2	1	1
2	2	4	4	2	2	2	2	1	1
2	2	4	4	4	4	2	2	3	3
2	2	1	1	2	2	3	3	3	3
3	3	4	4	4	4	2	2	3	3
3	3	4	4	4	4	2	2	3	3
3	3	4	4	4	4	2	2	3	3
2	2	4	4	4	4	2	2	1	1
2	2	4	4	4	4	2	2	1	1
2	2	4	4	4	4	2	2	1	1
2	2	4	4	4	4	2	2	1	1
3	3	1	1	2	2	3	3	1	1
2	2	4	4	4	4	2	2	1	1
3	3	4	4	2	2	2	2	1	1
2	2	4	4	4	4	2	2	3	3
2	2	1	1	2	2	3	3	1	1
2	2	1	1	4	4	3	3	3	3
2	2	1	1	2	2	3	3	3	3

For Review Only

2	2	1	1	2	2	3	3	1	1
2	2	1	1	2	2	3	3	3	3
2	2	4	4	2	2	2	2	1	1
3	3	4	4	4	4	2	2	1	1
3	3	1	1	4	4	3	3	3	3
2	2	1	1	2	2	3	3	1	1
3	3	1	1	2	2	3	3	3	3
2	2	4	4	4	4	2	2	3	3
2	2	1	1	2	2	3	3	1	1
2	2	1	1	4	4	2	2	1	1
2	2	1	1	2	2	3	3	1	1
2	2	1	1	2	2	3	3	1	1
2	2	1	1	2	2	3	3	1	1
2	2	1	1	2	2	3	3	1	1
2	2	1	1	4	4	2	2	1	1
2	2	4	4	4	4	2	2	1	1
2	2	4	4	4	4	2	2	3	3
2	2	1	1	2	2	3	3	1	1
2	2	1	1	2	2	3	3	1	1
2	2	1	1	2	2	3	3	1	1
2	2	1	1	2	2	3	3	1	1
3	3	4	4	4	4	2	2	3	3
2	2	1	1	2	2	3	3	1	1
2	2	1	1	2	2	3	3	1	1
2	2	4	4	4	4	2	2	1	1
2	2	1	1	2	2	3	3	1	1
2	2	4	4	4	4	2	2	1	1
2	2	4	4	4	4	2	2	3	3
3	3	1	1	4	4	3	3	3	3
2	2	1	1	2	2	2	2	1	1
2	2	1	1	4	4	2	2	1	1
2	2	1	1	2	2	3	3	1	1
3	3	4	4	4	4	2	2	3	3
2	2	1	1	2	2	3	3	1	1
3	3	1	1	2	2	3	3	3	3
2	2	4	4	4	4	2	2	1	1
2	2	1	1	2	2	3	3	1	1
2	2	1	1	2	2	3	3	1	1
3	3	4	4	4	4	2	2	1	1
2	2	4	4	4	4	2	2	1	1
2	2	1	1	2	2	3	3	1	1
2	2	1	1	2	2	3	3	1	1
3	3	4	4	4	4	2	2	1	1
2	2	1	1	2	2	3	3	1	1
2	2	1	1	2	2	3	3	1	1
2	2	1	1	2	2	3	3	1	1
2	2	4	4	4	4	2	2	3	3
3	3	1	1	2	2	3	3	3	3
3	3	4	4	4	4	2	2	1	1
2	2	1	1	2	2	3	3	1	1
2	2	1	1	4	4	3	3	3	3
3	3	4	4	4	4	2	2	3	3
2	2	4	4	4	4	2	2	3	3
2	2	1	1	2	2	2	2	1	1
2	2	1	1	2	2	3	3	1	1

For Review Only

3	3	1	1	2	2	3	3	3	3
2	2	4	4	4	4	2	2	3	3
3	3	1	1	2	2	3	3	3	3
2	2	4	4	4	4	2	2	3	3
3	3	1	1	4	4	3	3	1	1
2	2	1	1	2	2	3	3	3	3
3	3	1	1	4	4	3	3	1	1
3	3	4	4	4	4	2	2	1	1
3	3	4	4	4	4	2	2	1	1
2	2	4	4	4	4	2	2	1	1
2	2	1	1	2	2	3	3	1	1
2	2	1	1	4	4	3	3	1	1
2	2	4	4	4	4	2	2	1	1
2	2	1	1	2	2	3	3	1	1
2	2	1	1	2	2	2	2	1	1
2	2	4	4	4	4	2	2	1	1
2	2	1	1	2	2	3	3	3	3
2	2	1	1	4	4	3	3	1	1
2	2	4	4	4	4	2	2	3	3
2	2	4	4	4	4	2	2	3	3
2	2	1	1	4	4	2	2	1	1
2	2	4	4	2	2	2	2	1	1
2	2	1	1	4	4	3	3	1	1
2	2	4	4	4	4	2	2	3	3
3	3	1	1	4	4	3	3	3	3
2	2	1	1	2	2	3	3	3	3
2	2	1	1	2	2	3	3	1	1
2	2	4	4	4	4	2	2	1	1
2	2	1	1	2	2	3	3	1	1
3	3	1	1	2	2	3	3	1	1
2	2	4	4	4	4	2	2	3	3
2	2	1	1	4	4	2	2	1	1
2	2	1	1	2	2	3	3	1	1
3	3	1	1	4	4	3	3	1	1
2	2	4	4	4	4	2	2	3	3
2	2	4	4	2	2	3	3	1	1
2	2	4	4	4	4	2	2	3	3
2	2	1	1	2	2	3	3	1	1
2	2	1	1	4	4	3	3	3	3
2	2	4	4	4	4	2	2	3	3
2	2	1	1	4	4	2	2	1	1
2	2	4	4	4	4	2	2	3	3
2	2	1	1	2	2	3	3	1	1
2	2	4	4	4	4	2	2	1	1
3	3	1	1	4	4	3	3	3	3
2	2	1	1	2	2	3	3	1	1
3	3	1	1	4	4	3	3	3	3
2	2	1	1	4	4	3	3	3	3
2	2	4	4	4	4	2	2	3	3
3	3	1	1	4	4	3	3	3	3
3	3	1	1	2	2	2	2	3	3
2	2	4	4	4	4	2	2	3	3
2	2	1	1	2	2	3	3	1	1
2	2	1	1	4	4	3	3	1	1
2	2	4	4	4	4	2	2	3	3

For Review Only

2	2	4	4	4	4	2	2	1	1
3	3	4	4	4	4	2	2	3	3
2	2	1	1	2	2	3	3	1	1
2	2	1	1	2	2	3	3	3	3
2	2	4	4	2	2	2	2	1	1
2	2	4	4	4	4	2	2	1	1
2	2	4	4	4	4	2	2	1	1
2	2	1	1	4	4	2	2	1	1
2	2	4	4	4	4	2	2	1	1
2	2	1	1	2	2	3	3	1	1
3	3	1	1	2	2	3	3	3	3
2	2	1	1	2	2	3	3	1	1
2	2	1	1	4	4	3	3	1	1
2	2	1	1	2	2	2	2	1	1
2	2	4	4	4	4	2	2	3	3
3	3	4	4	4	4	2	2	3	3
2	2	1	1	2	2	3	3	1	1
2	2	1	1	2	2	3	3	1	1
3	3	1	1	4	4	3	3	1	1
2	2	1	1	4	4	3	3	1	1
2	2	1	1	4	4	2	2	1	1
3	3	4	4	4	4	2	2	3	3
3	3	4	4	4	4	2	2	3	3
2	2	1	1	2	2	3	3	1	1
2	2	1	1	2	2	3	3	1	1
2	2	4	4	4	4	2	2	3	3
2	2	1	1	2	2	3	3	1	1
2	2	1	1	2	2	2	2	3	3
2	2	1	1	2	2	3	3	1	1
2	2	1	1	2	2	3	3	1	1
2	2	1	1	2	2	3	3	1	1
2	2	1	1	2	2	3	3	1	1
2	2	1	1	2	2	3	3	1	1
2	2	4	4	4	4	2	2	3	3
2	2	1	1	2	2	3	3	1	1
2	2	1	1	2	2	3	3	1	1
2	2	1	1	2	2	3	3	1	1
2	2	4	4	4	4	2	2	1	1
2	2	1	1	4	4	3	3	3	3
2	2	1	1	2	2	3	3	1	1
3	3	1	1	2	2	3	3	1	1
3	3	1	1	4	4	3	3	3	3
3	3	4	4	4	4	2	2	3	3
2	2	1	1	4	4	3	3	1	1
2	2	4	4	2	2	2	2	1	1
2	2	1	1	2	2	3	3	1	1
3	3	4	4	4	4	2	2	3	3
3	3	4	4	4	4	2	2	3	3
2	2	4	4	4	4	2	2	3	3
3	3	4	4	4	4	2	2	1	1
3	3	1	1	2	2	3	3	1	1
2	2	4	4	4	4	2	2	1	1
3	3	4	4	4	4	2	2	3	3
2	2	1	1	2	2	3	3	1	1
3	3	4	4	4	4	2	2	3	3

For Review Only

2	2	1	1	2	2	3	3	1	1
3	3	4	4	4	4	2	2	1	1
2	2	1	1	4	4	2	2	1	1
2	2	4	4	4	4	2	2	3	3
2	2	4	4	4	4	2	2	1	1
3	3	1	1	4	4	2	2	1	1
2	2	1	1	4	4	3	3	1	1
2	2	4	4	4	4	2	2	1	1
2	2	4	4	4	4	2	2	1	1
2	2	4	4	4	4	2	2	3	3
2	2	1	1	4	4	2	2	1	1
2	2	1	1	2	2	3	3	1	1
3	3	4	4	4	4	2	2	1	1
2	2	4	4	4	4	2	2	1	1
2	2	1	1	2	2	3	3	1	1
2	2	1	1	2	2	3	3	1	1
3	3	4	4	4	4	2	2	3	3
3	3	4	4	4	4	2	2	1	1
3	3	4	4	4	4	2	2	1	1
2	2	1	1	2	2	3	3	3	3
3	3	1	1	2	2	3	3	3	3
3	3	4	4	4	4	2	2	3	3
2	2	4	4	2	2	2	2	1	1
2	2	4	4	4	4	2	2	1	1
2	2	4	4	4	4	2	2	3	3
2	2	1	1	2	2	3	3	1	1
2	2	4	4	4	4	2	2	3	3
3	3	1	1	2	2	3	3	1	1
2	2	1	1	2	2	2	2	1	1
2	2	1	1	2	2	3	3	1	1
2	2	4	4	4	4	2	2	3	3
3	3	1	1	2	2	3	3	3	3
2	2	4	4	2	2	2	2	3	3
2	2	1	1	2	2	3	3	3	3
3	3	4	4	4	4	2	2	3	3
3	3	4	4	4	4	2	2	3	3
2	2	1	1	2	2	3	3	1	1
2	2	1	1	2	2	3	3	1	1
2	2	4	4	2	2	2	2	1	1
3	3	4	4	4	4	2	2	3	3
2	2	1	1	2	2	3	3	1	1
2	2	4	4	2	2	2	2	1	1
2	2	1	1	2	2	3	3	1	1
2	2	1	1	2	2	3	3	1	1
2	2	1	1	4	4	2	2	1	1
3	3	4	4	4	4	2	2	3	3
3	3	1	1	2	2	3	3	3	3
3	3	1	1	2	2	3	3	1	1
2	2	4	4	4	4	3	3	1	1
2	2	4	4	4	4	2	2	3	3
2	2	1	1	2	2	3	3	1	1
2	2	4	4	4	4	2	2	3	3
3	3	1	1	2	2	3	3	1	1
3	3	1	1	2	2	3	3	1	1
2	2	4	4	4	4	2	2	3	3

For Review Only

2	2	1	1	2	2	3	3	1	1
2	2	1	1	2	2	3	3	1	1

For Review Only

SNP100	SNP100	SNP1000	SNP1000	SNP1001	SNP1001	SNP1002	SNP1002	SNP1003	SNP1003
1	1	4	4	3	3	4	4	3	3
3	3	4	4	3	3	4	4	3	3
3	3	4	4	1	1	4	4	3	3
3	3	4	4	1	1	4	4	3	3
3	3	4	4	3	3	4	4	3	3
3	3	3	3	3	3	4	4	3	3
3	3	4	4	3	3	4	4	3	3
3	3	4	4	3	3	4	4	3	3
3	3	4	4	3	3	4	4	3	3
3	3	4	4	3	3	4	4	2	2
3	3	4	4	3	3	4	4	3	3
3	3	4	4	1	1	4	4	3	3
3	3	4	4	3	3	4	4	3	3
3	3	4	4	3	3	4	4	3	3
3	3	4	4	3	3	4	4	3	3
1	1	4	4	1	1	4	4	3	3
3	3	4	4	3	3	4	4	3	3
1	1	4	4	3	3	4	4	3	3
1	1	3	3	3	3	4	4	3	3
3	3	4	4	1	1	4	4	3	3
3	3	4	4	3	3	4	4	3	3
3	3	4	4	3	3	4	4	3	3
3	3	4	4	3	3	4	4	3	3
3	3	3	3	3	3	4	4	3	3
3	3	4	4	1	1	4	4	3	3
1	1	4	4	3	3	4	4	3	3
3	3	4	4	3	3	1	1	3	3
3	3	4	4	3	3	4	4	3	3
3	3	4	4	3	3	4	4	3	3
3	3	4	4	3	3	4	4	3	3
1	1	3	3	3	3	4	4	3	3
3	3	4	4	3	3	4	4	3	3
3	3	4	4	3	3	4	4	3	3
3	3	4	4	3	3	4	4	3	3
3	3	4	4	1	1	4	4	3	3
3	3	4	4	3	3	4	4	3	3
3	3	4	4	3	3	4	4	3	3
3	3	4	4	1	1	4	4	3	3
3	3	4	4	3	3	4	4	3	3
3	3	3	3	1	1	4	4	3	3
3	3	4	4	3	3	4	4	3	3
3	3	4	4	3	3	4	4	3	3
3	3	4	4	3	3	4	4	3	3
3	3	4	4	3	3	4	4	3	3
3	3	4	4	3	3	4	4	2	2
3	3	4	4	1	1	4	4	3	3
1	1	4	4	1	1	4	4	3	3
3	3	4	4	3	3	4	4	3	3
3	3	4	4	3	3	4	4	3	3
3	3	4	4	1	1	4	4	3	3
3	3	4	4	3	3	4	4	3	3
3	3	4	4	1	1	4	4	3	3

For Review Only

3	3	4	4	3	3	4	4	3	3
3	3	4	4	3	3	4	4	3	3
3	3	4	4	1	1	4	4	3	3
3	3	4	4	3	3	4	4	3	3
1	1	3	3	3	3	4	4	3	3
1	1	4	4	3	3	4	4	3	3
1	1	4	4	1	1	4	4	3	3
3	3	4	4	3	3	4	4	3	3
3	3	4	4	3	3	4	4	3	3
3	3	4	4	1	1	4	4	3	3
3	3	4	4	3	3	4	4	3	3
3	3	4	4	3	3	4	4	3	3
3	3	4	4	3	3	4	4	3	3
3	3	4	4	3	3	4	4	3	3
3	3	4	4	3	3	4	4	3	3
3	3	4	4	3	3	4	4	3	3
3	3	3	3	3	3	4	4	3	3
3	3	4	4	1	1	4	4	3	3
3	3	4	4	3	3	4	4	3	3
1	1	4	4	3	3	4	4	3	3
3	3	4	4	3	3	4	4	3	3
3	3	3	3	1	1	4	4	3	3
3	3	4	4	3	3	4	4	3	3
3	3	4	4	3	3	4	4	3	3
3	3	4	4	3	3	4	4	2	2
3	3	3	3	3	3	4	4	3	3
3	3	4	4	3	3	4	4	3	3
3	3	4	4	3	3	4	4	3	3
1	1	4	4	3	3	4	4	3	3
3	3	4	4	3	3	4	4	3	3
3	3	4	4	3	3	4	4	3	3
3	3	3	3	3	3	4	4	3	3
3	3	3	3	3	3	4	4	3	3
3	3	4	4	3	3	4	4	3	3
1	1	4	4	3	3	4	4	3	3
3	3	4	4	3	3	4	4	2	2
3	3	4	4	3	3	4	4	3	3
3	3	4	4	3	3	4	4	3	3
3	3	4	4	3	3	4	4	2	2
3	3	4	4	3	3	4	4	2	2
1	1	4	4	3	3	4	4	3	3
3	3	4	4	1	1	4	4	3	3
3	3	4	4	3	3	4	4	3	3
3	3	4	4	3	3	4	4	3	3
3	3	3	3	3	3	1	1	3	3
3	3	4	4	1	1	4	4	3	3
3	3	4	4	3	3	4	4	3	3
3	3	4	4	1	1	4	4	3	3
1	1	4	4	1	1	4	4	3	3
3	3	4	4	3	3	4	4	3	3
3	3	3	3	3	3	4	4	3	3
3	3	4	4	3	3	4	4	3	3
3	3	4	4	3	3	4	4	3	3
3	3	4	4	3	3	4	4	3	3
3	3	4	4	3	3	4	4	3	3

For Review Only

1	1	4	4	3	3	4	4	3	3
3	3	4	4	3	3	4	4	3	3
3	3	4	4	3	3	4	4	3	3
1	1	4	4	3	3	4	4	3	3
3	3	4	4	3	3	4	4	3	3
1	1	4	4	3	3	4	4	3	3
1	1	4	4	1	1	4	4	3	3
3	3	3	3	3	3	1	1	3	3
3	3	4	4	3	3	4	4	3	3
3	3	4	4	3	3	1	1	3	3
3	3	4	4	3	3	4	4	3	3
3	3	4	4	1	1	4	4	3	3
3	3	4	4	1	1	4	4	2	2
3	3	4	4	3	3	1	1	3	3
3	3	4	4	3	3	4	4	3	3
3	3	4	4	1	1	4	4	3	3
1	1	4	4	1	1	4	4	3	3
3	3	4	4	3	3	4	4	3	3
1	1	4	4	3	3	4	4	3	3
3	3	4	4	3	3	4	4	2	2
3	3	4	4	1	1	4	4	3	3
3	3	4	4	3	3	4	4	3	3
3	3	4	4	1	1	4	4	3	3
3	3	3	3	3	3	4	4	3	3
3	3	3	3	1	1	4	4	3	3
3	3	3	3	3	3	4	4	3	3
3	3	4	4	3	3	4	4	3	3
3	3	3	3	3	3	1	1	3	3
3	3	4	4	1	1	4	4	3	3
3	3	4	4	3	3	4	4	3	3
3	3	4	4	3	3	4	4	3	3
3	3	4	4	1	1	4	4	3	3
1	1	4	4	3	3	4	4	3	3
3	3	4	4	3	3	4	4	3	3
3	3	4	4	3	3	4	4	3	3
3	3	4	4	3	3	4	4	3	3
3	3	4	4	3	3	4	4	3	3
3	3	4	4	3	3	4	4	3	3
1	1	4	4	3	3	4	4	3	3
3	3	4	4	3	3	4	4	3	3
3	3	4	4	3	3	4	4	3	3
3	3	4	4	3	3	4	4	3	3
3	3	4	4	3	3	4	4	3	3
3	3	4	4	3	3	4	4	3	3
3	3	4	4	3	3	4	4	3	3
3	3	4	4	3	3	4	4	3	3
1	1	4	4	3	3	4	4	3	3
1	1	4	4	1	1	4	4	3	3
3	3	4	4	3	3	4	4	3	3
1	1	4	4	3	3	4	4	3	3
1	1	4	4	3	3	4	4	3	3
1	1	4	4	1	1	4	4	3	3
3	3	4	4	3	3	4	4	3	3
3	3	4	4	3	3	4	4	3	3
3	3	4	4	3	3	4	4	3	3

For Review Only

3	3	4	4	1	1	4	4	3	3
3	3	4	4	3	3	4	4	2	2
3	3	4	4	3	3	4	4	3	3
3	3	4	4	3	3	4	4	3	3
1	1	4	4	3	3	4	4	3	3
1	1	3	3	1	1	4	4	3	3
3	3	3	3	3	3	4	4	3	3
3	3	4	4	1	1	4	4	3	3
1	1	4	4	3	3	4	4	3	3
3	3	4	4	1	1	4	4	3	3
1	1	4	4	1	1	4	4	3	3
3	3	3	3	3	3	4	4	3	3
3	3	4	4	1	1	4	4	3	3
3	3	4	4	3	3	4	4	3	3
3	3	4	4	3	3	4	4	3	3
3	3	4	4	3	3	4	4	3	3
3	3	4	4	3	3	4	4	3	3
3	3	3	3	3	3	1	1	3	3
3	3	4	4	3	3	4	4	3	3
1	1	4	4	1	1	4	4	3	3
3	3	4	4	1	1	4	4	3	3
3	3	3	3	3	3	4	4	3	3
3	3	3	3	3	3	4	4	3	3
3	3	4	4	3	3	4	4	3	3
3	3	4	4	3	3	4	4	3	3
3	3	4	4	3	3	4	4	3	3
3	3	4	4	1	1	4	4	3	3
1	1	4	4	1	1	4	4	3	3
3	3	4	4	1	1	4	4	3	3
3	3	4	4	3	3	4	4	3	3
3	3	4	4	3	3	4	4	3	3
1	1	4	4	1	1	4	4	3	3
3	3	4	4	3	3	1	1	3	3
3	3	4	4	3	3	4	4	3	3
3	3	3	3	1	1	4	4	3	3
3	3	4	4	3	3	4	4	3	3
3	3	4	4	3	3	4	4	3	3
3	3	4	4	3	3	4	4	3	3
1	1	3	3	1	1	1	1	3	3
3	3	4	4	3	3	4	4	3	3
3	3	4	4	1	1	4	4	3	3
1	1	4	4	3	3	4	4	3	3
3	3	3	3	3	3	4	4	3	3
3	3	4	4	1	1	4	4	3	3
3	3	4	4	3	3	4	4	3	3
3	3	4	4	3	3	4	4	3	3
3	3	4	4	3	3	4	4	3	3
3	3	3	3	3	3	4	4	3	3
3	3	4	4	3	3	4	4	3	3
3	3	4	4	3	3	4	4	3	3
3	3	4	4	1	1	4	4	3	3
3	3	4	4	3	3	4	4	3	3
3	3	3	3	3	3	4	4	3	3
3	3	4	4	3	3	4	4	3	3
3	3	4	4	3	3	4	4	3	3

For Review Only

3	3	4	4	3	3	4	4	3	3
3	3	4	4	1	1	4	4	3	3
3	3	4	4	1	1	4	4	3	3
3	3	4	4	3	3	4	4	3	3
1	1	4	4	1	1	4	4	3	3
3	3	4	4	3	3	4	4	3	3
3	3	4	4	3	3	4	4	2	2
3	3	4	4	3	3	4	4	3	3
3	3	4	4	3	3	4	4	3	3
3	3	4	4	3	3	4	4	3	3
3	3	4	4	3	3	4	4	3	3
3	3	4	4	1	1	4	4	2	2
3	3	4	4	3	3	4	4	3	3
3	3	4	4	3	3	4	4	3	3
1	1	4	4	1	1	4	4	3	3
3	3	4	4	3	3	4	4	3	3
3	3	4	4	3	3	4	4	3	3
3	3	4	4	3	3	4	4	3	3
3	3	3	3	3	3	4	4	3	3
3	3	4	4	1	1	4	4	3	3
3	3	3	3	3	3	4	4	3	3
1	1	4	4	3	3	4	4	3	3
3	3	4	4	3	3	4	4	3	3
3	3	3	3	3	3	4	4	3	3
3	3	4	4	3	3	4	4	3	3
3	3	4	4	3	3	4	4	3	3
3	3	3	3	3	3	4	4	3	3
3	3	4	4	1	1	4	4	3	3
3	3	4	4	1	1	4	4	3	3
3	3	4	4	1	1	4	4	3	3
3	3	4	4	3	3	4	4	3	3
3	3	4	4	3	3	4	4	3	3
3	3	4	4	3	3	4	4	3	3
3	3	3	3	3	3	1	1	3	3
3	3	3	3	3	3	4	4	3	3
3	3	4	4	3	3	4	4	3	3
3	3	4	4	3	3	4	4	3	3
3	3	4	4	3	3	4	4	3	3
3	3	4	4	3	3	4	4	3	3
3	3	3	3	3	3	4	4	3	3
3	3	4	4	3	3	4	4	3	3
3	3	3	3	3	3	1	1	3	3
3	3	3	3	3	3	4	4	3	3
3	3	4	4	3	3	4	4	3	3
3	3	4	4	3	3	4	4	3	3
3	3	4	4	3	3	4	4	3	3
1	1	3	3	1	1	1	1	3	3
3	3	4	4	3	3	4	4	3	3
3	3	4	4	3	3	4	4	3	3
3	3	3	3	3	3	4	4	3	3
3	3	4	4	1	1	4	4	3	3
3	3	3	3	3	3	4	4	3	3
3	3	4	4	1	1	4	4	3	3
3	3	4	4	1	1	4	4	3	3
3	3	3	3	3	3	4	4	3	3

For Review Only

3	3	4	4	3	3	4	4	3	3
3	3	4	4	3	3	4	4	3	3

For Review Only

SNP1004	SNP1004	SNP1005	SNP1005	SNP1006	SNP1006	SNP1007	SNP1007	SNP1008	SNP1008
2	2	1	1	1	1	2	2	2	2
2	2	1	1	1	1	2	2	2	2
2	2	1	1	1	1	2	2	2	2
2	2	1	1	1	1	2	2	2	2
2	2	1	1	1	1	2	2	2	2
2	2	1	1	1	1	2	2	2	2
2	2	1	1	1	1	2	2	2	2
2	2	1	1	1	1	2	2	2	2
2	2	1	1	1	1	2	2	2	2
2	2	1	1	1	1	1	1	2	2
2	2	1	1	1	1	2	2	4	4
2	2	1	1	1	1	2	2	2	2
2	2	1	1	1	1	2	2	2	2
2	2	1	1	1	1	1	1	2	2
2	2	1	1	1	1	2	2	2	2
2	2	1	1	1	1	1	1	2	2
2	2	1	1	1	1	2	2	2	2
2	2	1	1	1	1	2	2	2	2
2	2	1	1	1	1	1	1	2	2
2	2	1	1	1	1	2	2	2	2
2	2	1	1	1	1	2	2	2	2
2	2	1	1	1	1	2	2	2	2
2	2	1	1	1	1	2	2	4	4
2	2	1	1	4	4	2	2	2	2
2	2	1	1	1	1	2	2	2	2
4	4	3	3	4	4	2	2	4	4
2	2	1	1	1	1	2	2	2	2
2	2	1	1	1	1	2	2	2	2
2	2	1	1	1	1	1	1	2	2
2	2	1	1	1	1	1	1	2	2
2	2	1	1	1	1	2	2	2	2
2	2	1	1	1	1	2	2	2	2
2	2	1	1	1	1	2	2	2	2
2	2	1	1	1	1	1	1	2	2
4	4	3	3	4	4	2	2	4	4
2	2	1	1	1	1	1	1	2	2
2	2	1	1	1	1	1	1	2	2
2	2	1	1	1	1	2	2	2	2
2	2	1	1	1	1	2	2	4	4
2	2	1	1	1	1	2	2	4	4
2	2	1	1	1	1	1	1	2	2
2	2	1	1	1	1	1	1	2	2
2	2	1	1	1	1	1	1	2	2
2	2	1	1	1	1	2	2	2	2
2	2	1	1	1	1	1	1	2	2
2	2	1	1	1	1	2	2	2	2
4	4	3	3	4	4	2	2	4	4
2	2	1	1	1	1	2	2	4	4
2	2	1	1	1	1	2	2	2	2
2	2	1	1	1	1	2	2	2	2
2	2	1	1	1	1	2	2	2	2
2	2	1	1	1	1	2	2	2	2

For Review Only

2	2	1	1	1	1	2	2	2	2
2	2	1	1	1	1	2	2	2	2
2	2	1	1	1	1	2	2	2	2
2	2	1	1	1	1	1	1	2	2
2	2	1	1	1	1	1	1	2	2
2	2	1	1	1	1	2	2	2	2
4	4	3	3	4	4	2	2	4	4
2	2	1	1	1	1	1	1	2	2
2	2	1	1	1	1	1	1	2	2
2	2	1	1	1	1	2	2	2	2
2	2	1	1	1	1	2	2	2	2
2	2	1	1	1	1	1	1	2	2
2	2	1	1	1	1	2	2	2	2
2	2	1	1	1	1	1	1	2	2
2	2	1	1	1	1	2	2	2	2
2	2	1	1	1	1	2	2	2	2
2	2	1	1	1	1	2	2	4	4
2	2	1	1	1	1	2	2	2	2
2	2	1	1	1	1	2	2	2	2
2	2	1	1	1	1	2	2	2	2
2	2	1	1	1	1	1	1	2	2
2	2	1	1	1	1	1	1	4	4
2	2	1	1	1	1	2	2	2	2
2	2	1	1	1	1	1	1	2	2
2	2	1	1	1	1	1	1	2	2
2	2	1	1	1	1	2	2	2	2
2	2	1	1	1	1	2	2	2	2
2	2	1	1	1	1	2	2	4	4
4	4	3	3	4	4	2	2	4	4
2	2	1	1	1	1	2	2	2	2
2	2	1	1	1	1	2	2	2	2
2	2	1	1	1	1	1	1	4	4
2	2	1	1	1	1	2	2	4	4
2	2	1	1	1	1	2	2	2	2
2	2	1	1	1	1	2	2	2	2
2	2	1	1	1	1	1	1	2	2
2	2	1	1	1	1	2	2	2	2
2	2	1	1	1	1	2	2	2	2
4	4	3	3	4	4	2	2	4	4
2	2	1	1	1	1	1	1	2	2
2	2	1	1	1	1	2	2	2	2
2	2	1	1	1	1	2	2	2	2
2	2	1	1	1	1	2	2	2	2
2	2	1	1	1	1	2	2	2	2
2	2	1	1	1	1	2	2	2	2
2	2	1	1	1	1	2	2	2	2
2	2	1	1	1	1	2	2	2	2
2	2	1	1	1	1	1	1	2	2
4	4	3	3	4	4	2	2	4	4
2	2	1	1	1	1	1	1	2	2
2	2	1	1	1	1	2	2	2	2
2	2	1	1	1	1	2	2	4	4
4	4	1	1	1	1	2	2	4	4
2	2	1	1	1	1	2	2	2	2
2	2	1	1	1	1	2	2	2	2

For Review Only

2	2	1	1	4	4	2	2	4	4
2	2	1	1	1	1	2	2	2	2
2	2	1	1	1	1	2	2	2	2
2	2	1	1	1	1	2	2	2	2
2	2	1	1	1	1	2	2	2	2
2	2	1	1	1	1	2	2	2	2
2	2	1	1	1	1	1	1	2	2
2	2	1	1	1	1	2	2	2	2
2	2	1	1	1	1	2	2	4	4
2	2	1	1	1	1	2	2	2	2
2	2	1	1	1	1	2	2	2	2
2	2	1	1	1	1	2	2	2	2
2	2	1	1	1	1	2	2	2	2
2	2	1	1	1	1	2	2	2	2
2	2	1	1	1	1	2	2	2	2
4	4	3	3	1	1	2	2	2	2
2	2	1	1	1	1	2	2	2	2
2	2	1	1	1	1	2	2	2	2
2	2	1	1	1	1	2	2	2	2
2	2	1	1	1	1	2	2	4	4
2	2	1	1	1	1	2	2	2	2
2	2	1	1	1	1	2	2	2	2
2	2	1	1	1	1	2	2	2	2
2	2	1	1	1	1	2	2	4	4
2	2	1	1	1	1	2	2	4	4
2	2	1	1	1	1	2	2	4	4
2	2	1	1	1	1	2	2	2	2
2	2	1	1	1	1	2	2	2	2
2	2	1	1	1	1	2	2	2	2
2	2	1	1	1	1	2	2	2	2
2	2	1	1	1	1	1	1	4	4
2	2	1	1	1	1	2	2	2	2
2	2	1	1	1	1	2	2	2	2
4	4	1	1	1	1	2	2	4	4
2	2	1	1	1	1	1	1	2	2
2	2	1	1	1	1	2	2	2	2
2	2	1	1	1	1	2	2	2	2
2	2	1	1	1	1	2	2	2	2
2	2	1	1	1	1	2	2	4	4
2	2	1	1	1	1	2	2	4	4
2	2	1	1	1	1	1	1	4	4
2	2	1	1	1	1	1	1	2	2
4	4	3	3	4	4	2	2	4	4
2	2	1	1	1	1	2	2	2	2
2	2	1	1	1	1	2	2	2	2
2	2	1	1	1	1	2	2	2	2
4	4	3	3	4	4	2	2	4	4
2	2	1	1	1	1	2	2	2	2
2	2	1	1	1	1	2	2	2	2
2	2	1	1	1	1	2	2	2	2
4	4	3	3	4	4	2	2	4	4
2	2	1	1	1	1	1	1	2	2
2	2	1	1	1	1	2	2	4	4
2	2	1	1	1	1	1	1	2	2
2	2	1	1	1	1	2	2	2	2

2	2	1	1	1	1	2	2	2	2
2	2	1	1	1	1	2	2	4	4
2	2	1	1	1	1	2	2	2	2
2	2	1	1	1	1	2	2	4	4
2	2	1	1	1	1	2	2	2	2
2	2	1	1	1	1	2	2	2	2
2	2	1	1	1	1	2	2	4	4
4	4	1	1	1	1	2	2	2	2
2	2	1	1	1	1	2	2	4	4
2	2	1	1	1	1	2	2	2	2
2	2	1	1	1	1	2	2	2	2
2	2	1	1	1	1	2	2	4	4
4	4	3	3	4	4	2	2	4	4
2	2	1	1	1	1	2	2	2	2
2	2	1	1	1	1	1	1	4	4
2	2	1	1	1	1	2	2	4	4
2	2	1	1	1	1	2	2	2	2
2	2	1	1	1	1	2	2	2	2
2	2	1	1	1	1	2	2	2	2
2	2	1	1	1	1	1	1	2	2
2	2	1	1	1	1	2	2	2	2
4	4	1	1	4	4	2	2	2	2
2	2	1	1	1	1	1	1	4	4
2	2	1	1	1	1	1	1	2	2
2	2	1	1	1	1	1	1	2	2
2	2	1	1	1	1	1	1	2	2
2	2	1	1	1	1	2	2	2	2
2	2	1	1	1	1	2	2	2	2
2	2	1	1	1	1	2	2	2	2
4	4	3	3	4	4	2	2	4	4
2	2	1	1	1	1	2	2	2	2
2	2	1	1	1	1	2	2	2	2
4	4	3	3	4	4	2	2	4	4
2	2	1	1	1	1	1	1	4	4
2	2	1	1	1	1	2	2	2	2
2	2	1	1	1	1	1	1	2	2
4	4	3	3	1	1	2	2	4	4
2	2	1	1	1	1	2	2	2	2
2	2	1	1	1	1	2	2	2	2
2	2	1	1	1	1	2	2	2	2
2	2	1	1	1	1	2	2	2	2
4	4	3	3	4	4	2	2	4	4
2	2	1	1	1	1	2	2	4	4
2	2	1	1	1	1	2	2	2	2
2	2	1	1	1	1	2	2	2	2
2	2	1	1	1	1	2	2	2	2
2	2	1	1	1	1	2	2	4	4
2	2	1	1	1	1	2	2	2	2
2	2	1	1	1	1	1	1	4	4
2	2	1	1	1	1	1	1	2	2
2	2	1	1	1	1	2	2	2	2
2	2	1	1	1	1	2	2	2	2
2	2	1	1	1	1	1	1	4	4
2	2	1	1	1	1	2	2	2	2
2	2	1	1	1	1	2	2	4	4

For Review Only

2	2	1	1	1	1	2	2	4	4
2	2	1	1	1	1	2	2	2	2
2	2	1	1	1	1	2	2	2	2
2	2	1	1	1	1	1	1	4	4
2	2	1	1	1	1	2	2	2	2
4	4	1	1	1	1	2	2	2	2
2	2	1	1	1	1	2	2	4	4
2	2	1	1	1	1	1	1	2	2
2	2	1	1	1	1	2	2	2	2
2	2	1	1	1	1	2	2	2	2
2	2	1	1	1	1	2	2	2	2
2	2	1	1	1	1	2	2	2	2
4	4	3	3	4	4	2	2	4	4
2	2	1	1	1	1	2	2	2	2
2	2	1	1	1	1	2	2	2	2
2	2	1	1	1	1	2	2	2	2
2	2	1	1	1	1	2	2	2	2
2	2	1	1	1	1	2	2	2	2
2	2	1	1	1	1	2	2	4	4
2	2	1	1	1	1	2	2	2	2
2	2	1	1	1	1	2	2	4	4
4	4	3	3	4	4	2	2	4	4
2	2	1	1	1	1	2	2	2	2
2	2	1	1	1	1	2	2	4	4
2	2	1	1	1	1	1	1	2	2
2	2	1	1	1	1	2	2	2	2
2	2	1	1	1	1	2	2	4	4
2	2	1	1	1	1	2	2	2	2
2	2	1	1	1	1	2	2	2	2
2	2	1	1	1	1	2	2	2	2
2	2	1	1	1	1	2	2	4	4
2	2	1	1	1	1	1	1	2	2
2	2	1	1	1	1	2	2	2	2
2	2	1	1	1	1	2	2	2	2
2	2	1	1	1	1	1	1	4	4
2	2	1	1	1	1	2	2	2	2
2	2	1	1	1	1	2	2	2	2
2	2	1	1	1	1	1	1	2	2
2	2	1	1	1	1	1	1	2	2
2	2	1	1	1	1	2	2	4	4
4	4	3	3	4	4	2	2	4	4
4	4	3	3	4	4	2	2	2	2
2	2	1	1	1	1	2	2	2	2
2	2	1	1	1	1	2	2	2	2
2	2	1	1	1	1	2	2	2	2
2	2	1	1	1	1	2	2	2	2
2	2	1	1	1	1	2	2	4	4
2	2	1	1	1	1	2	2	2	2
2	2	1	1	1	1	2	2	2	2
2	2	1	1	1	1	2	2	4	4
2	2	1	1	1	1	1	1	2	2
2	2	1	1	1	1	2	2	4	4
2	2	1	1	1	1	2	2	2	2
2	2	1	1	1	1	2	2	2	2
2	2	1	1	1	1	2	2	4	4

For Review Only

2	2	1	1	1	1	2	2	2	2
2	2	1	1	1	1	2	2	2	2

For Review Only

SNP1009	SNP1009	SNP101	SNP101	SNP1010	SNP1010	SNP1011	SNP1011	SNP1012	SNP1012
3	3	4	4	4	4	2	2	1	1
3	3	4	4	4	4	3	3	1	1
3	3	4	4	4	4	2	2	1	1
3	3	4	4	4	4	2	2	1	1
3	3	4	4	4	4	2	2	1	1
3	3	4	4	4	4	2	2	1	1
3	3	4	4	4	4	3	3	1	1
3	3	4	4	4	4	3	3	1	1
3	3	4	4	4	4	3	3	1	1
3	3	4	4	4	4	2	2	1	1
3	3	4	4	1	1	2	2	4	4
3	3	4	4	4	4	2	2	1	1
3	3	4	4	4	4	3	3	1	1
3	3	4	4	4	4	2	2	1	1
3	3	4	4	1	1	2	2	1	1
3	3	1	1	4	4	3	3	1	1
3	3	4	4	4	4	2	2	1	1
3	3	1	1	4	4	3	3	1	1
3	3	1	1	4	4	3	3	1	1
3	3	1	1	4	4	2	2	1	1
3	3	4	4	4	4	3	3	1	1
3	3	4	4	4	4	2	2	1	1
3	3	4	4	1	1	2	2	4	4
3	3	4	4	1	1	3	3	1	1
3	3	4	4	4	4	3	3	1	1
3	3	1	1	4	4	2	2	1	1
3	3	4	4	4	4	2	2	1	1
3	3	4	4	1	1	3	3	1	1
3	3	4	4	4	4	3	3	1	1
3	3	4	4	4	4	3	3	1	1
3	3	1	1	4	4	3	3	1	1
3	3	4	4	4	4	3	3	1	1
3	3	4	4	4	4	3	3	1	1
2	2	4	4	4	4	3	3	1	1
3	3	4	4	4	4	2	2	1	1
3	3	4	4	4	4	3	3	1	1
2	2	4	4	4	4	2	2	1	1
3	3	4	4	4	4	2	2	1	1
3	3	4	4	1	1	2	2	4	4
3	3	4	4	1	1	2	2	4	4
3	3	4	4	4	4	2	2	1	1
3	3	4	4	4	4	3	3	1	1
3	3	4	4	4	4	3	3	1	1
3	3	4	4	4	4	2	2	1	1
3	3	4	4	4	4	3	3	1	1
3	3	4	4	4	4	2	2	1	1
3	3	4	4	4	4	2	2	1	1
3	3	4	4	1	1	3	3	4	4
3	3	4	4	4	4	2	2	1	1
3	3	4	4	4	4	3	3	1	1
3	3	4	4	4	4	3	3	1	1
3	3	4	4	4	4	3	3	1	1

For Review Only

3	3	4	4	4	4	2	2	1	1
3	3	4	4	4	4	3	3	1	1
3	3	4	4	4	4	3	3	1	1
3	3	4	4	4	4	2	2	1	1
3	3	1	1	4	4	3	3	1	1
3	3	1	1	4	4	2	2	1	1
3	3	1	1	4	4	2	2	1	1
3	3	4	4	4	4	3	3	1	1
2	2	4	4	4	4	3	3	1	1
3	3	4	4	1	1	3	3	1	1
3	3	4	4	4	4	3	3	1	1
3	3	4	4	4	4	2	2	1	1
3	3	4	4	4	4	2	2	1	1
3	3	4	4	4	4	3	3	1	1
3	3	4	4	1	1	2	2	1	1
3	3	4	4	4	4	3	3	1	1
3	3	4	4	1	1	3	3	4	4
3	3	4	4	4	4	2	2	1	1
3	3	4	4	4	4	2	2	1	1
3	3	1	1	4	4	2	2	1	1
3	3	4	4	4	4	3	3	1	1
3	3	4	4	1	1	2	2	4	4
3	3	4	4	4	4	3	3	1	1
3	3	4	4	4	4	2	2	1	1
3	3	4	4	4	4	3	3	1	1
3	3	4	4	4	4	3	3	1	1
3	3	4	4	4	4	3	3	1	1
3	3	4	4	1	1	3	3	4	4
3	3	1	1	4	4	2	2	1	1
3	3	4	4	4	4	3	3	1	1
3	3	4	4	4	4	3	3	1	1
3	3	4	4	1	1	2	2	4	4
2	2	4	4	1	1	2	2	4	4
3	3	4	4	4	4	2	2	1	1
3	3	1	1	4	4	2	2	1	1
3	3	4	4	4	4	3	3	1	1
3	3	4	4	4	4	3	3	1	1
3	3	4	4	4	4	3	3	1	1
3	3	4	4	4	4	3	3	1	1
3	3	4	4	4	4	3	3	1	1
3	3	1	1	4	4	2	2	1	1
3	3	4	4	4	4	2	2	1	1
3	3	4	4	4	4	2	2	1	1
3	3	4	4	4	4	2	2	1	1
3	3	4	4	4	4	3	3	1	1
3	3	4	4	4	4	2	2	1	1
2	2	4	4	4	4	2	2	1	1
3	3	4	4	4	4	3	3	1	1
3	3	1	1	4	4	2	2	1	1
3	3	4	4	4	4	3	3	1	1
3	3	4	4	4	4	2	2	1	1
3	3	4	4	1	1	2	2	4	4
3	3	4	4	4	4	3	3	1	1
3	3	4	4	1	1	2	2	1	1
3	3	4	4	4	4	2	2	1	1

For Review Only

3	3	4	4	1	1	2	2	1	1
3	3	4	4	4	4	2	2	1	1
3	3	4	4	4	4	2	2	1	1
2	2	4	4	4	4	3	3	1	1
3	3	4	4	4	4	2	2	1	1
3	3	1	1	4	4	2	2	1	1
3	3	4	4	4	4	3	3	1	1
3	3	4	4	1	1	3	3	1	1
3	3	4	4	4	4	3	3	1	1
3	3	4	4	1	1	3	3	4	4
3	3	4	4	4	4	2	2	1	1
3	3	4	4	1	1	3	3	1	1
3	3	4	4	4	4	3	3	1	1
3	3	4	4	1	1	3	3	1	1
3	3	4	4	4	4	3	3	1	1
3	3	4	4	1	1	3	3	1	1
3	3	1	1	1	1	2	2	1	1
3	3	4	4	1	1	3	3	1	1
2	2	1	1	4	4	3	3	1	1
3	3	4	4	1	1	3	3	4	4
3	3	4	4	1	1	2	2	1	1
3	3	4	4	1	1	3	3	1	1
3	3	4	4	4	4	2	2	1	1
3	3	4	4	1	1	3	3	4	4
3	3	1	1	1	1	2	2	4	4
3	3	4	4	1	1	2	2	4	4
3	3	4	4	4	4	2	2	1	1
3	3	4	4	4	4	3	3	1	1
3	3	4	4	4	4	2	2	1	1
3	3	4	4	4	4	2	2	1	1
3	3	4	4	4	4	2	2	1	1
3	3	4	4	4	4	2	2	1	1
3	3	1	1	4	4	2	2	1	1
3	3	4	4	1	1	3	3	1	1
3	3	4	4	4	4	3	3	1	1
3	3	4	4	4	4	3	3	1	1
2	2	4	4	4	4	3	3	1	1
3	3	4	4	4	4	3	3	1	1
3	3	1	1	1	1	3	3	1	1
3	3	4	4	1	1	2	2	4	4
3	3	4	4	1	1	2	2	4	4
2	2	4	4	4	4	3	3	1	1
3	3	4	4	4	4	3	3	1	1
3	3	4	4	4	4	3	3	1	1
3	3	4	4	4	4	2	2	1	1
3	3	4	4	4	4	2	2	1	1
3	3	1	1	4	4	2	2	1	1
3	3	1	1	4	4	2	2	1	1
3	3	4	4	4	4	3	3	1	1
3	3	1	1	4	4	2	2	1	1
3	3	1	1	4	4	2	2	1	1
3	3	1	1	4	4	2	2	1	1
3	3	4	4	1	1	3	3	4	4
3	3	4	4	4	4	3	3	1	1
3	3	4	4	4	4	2	2	1	1

For Review Only

3	3	4	4	4	4	2	2	1	1
3	3	4	4	1	1	3	3	4	4
3	3	4	4	4	4	2	2	1	1
3	3	4	4	1	1	2	2	4	4
3	3	1	1	4	4	2	2	1	1
3	3	1	1	4	4	2	2	1	1
3	3	4	4	1	1	3	3	1	1
3	3	4	4	4	4	2	2	1	1
3	3	4	4	1	1	2	2	4	4
3	3	4	4	4	4	2	2	1	1
3	3	4	4	4	4	2	2	1	1
3	3	4	4	1	1	3	3	1	1
3	3	4	4	4	4	3	3	1	1
3	3	4	4	4	4	2	2	1	1
2	2	4	4	4	4	2	2	1	1
3	3	4	4	1	1	2	2	4	4
3	3	4	4	1	1	3	3	1	1
3	3	4	4	4	4	2	2	1	1
3	3	4	4	4	4	3	3	1	1
3	3	4	4	4	4	3	3	1	1
3	3	4	4	4	4	3	3	1	1
2	2	4	4	1	1	3	3	1	1
3	3	4	4	1	1	2	2	4	4
3	3	4	4	4	4	3	3	1	1
3	3	4	4	4	4	3	3	1	1
3	3	4	4	4	4	3	3	1	1
3	3	4	4	4	4	2	2	1	1
3	3	1	1	4	4	2	2	1	1
3	3	4	4	1	1	2	2	1	1
3	3	4	4	4	4	2	2	1	1
3	3	4	4	4	4	2	2	1	1
3	3	1	1	4	4	2	2	1	1
3	3	4	4	4	4	2	2	1	1
2	2	4	4	4	4	2	2	1	1
3	3	4	4	4	4	3	3	1	1
3	3	4	4	4	4	3	3	1	1
3	3	4	4	4	4	2	2	1	1
3	3	4	4	4	4	2	2	1	1
3	3	1	1	4	4	2	2	1	1
3	3	4	4	4	4	2	2	1	1
3	3	4	4	4	4	2	2	1	1
3	3	1	1	4	4	2	2	1	1
3	3	4	4	1	1	3	3	4	4
3	3	4	4	1	1	2	2	1	1
3	3	4	4	4	4	2	2	1	1
3	3	4	4	4	4	2	2	1	1
3	3	4	4	1	1	2	2	4	4
2	2	4	4	1	1	3	3	1	1
3	3	4	4	4	4	3	3	1	1
3	3	4	4	1	1	2	2	1	1
3	3	4	4	4	4	2	2	1	1
3	3	4	4	4	4	3	3	1	1
3	3	4	4	1	1	3	3	4	4
3	3	4	4	4	4	3	3	1	1
3	3	4	4	1	1	2	2	4	4

For Review Only

3	3	4	4	1	1	3	3	4	4
3	3	4	4	4	4	3	3	1	1
3	3	4	4	4	4	2	2	1	1
3	3	4	4	1	1	3	3	4	4
3	3	1	1	4	4	2	2	1	1
3	3	4	4	4	4	2	2	1	1
3	3	4	4	1	1	2	2	1	1
3	3	4	4	4	4	3	3	1	1
3	3	4	4	4	4	3	3	1	1
3	3	4	4	4	4	3	3	1	1
3	3	4	4	4	4	3	3	1	1
3	3	4	4	4	4	3	3	1	1
3	3	4	4	4	4	2	2	1	1
3	3	4	4	4	4	3	3	1	1
3	3	1	1	4	4	2	2	1	1
3	3	4	4	4	4	2	2	1	1
3	3	4	4	4	4	2	2	1	1
3	3	4	4	1	1	3	3	1	1
3	3	4	4	1	1	2	2	4	4
3	3	4	4	4	4	2	2	1	1
3	3	4	4	1	1	2	2	4	4
3	3	1	1	4	4	2	2	1	1
3	3	4	4	4	4	3	3	1	1
3	3	4	4	1	1	2	2	4	4
3	3	4	4	4	4	3	3	1	1
3	3	4	4	4	4	3	3	1	1
2	2	4	4	1	1	2	2	4	4
3	3	4	4	4	4	2	2	1	1
3	3	4	4	4	4	2	2	1	1
3	3	4	4	4	4	2	2	1	1
3	3	4	4	1	1	3	3	4	4
3	3	4	4	4	4	3	3	1	1
3	3	4	4	4	4	3	3	1	1
3	3	4	4	4	4	3	3	1	1
3	3	4	4	1	1	2	2	4	4
3	3	4	4	4	4	3	3	1	1
3	3	4	4	4	4	3	3	1	1
3	3	4	4	4	4	3	3	1	1
3	3	4	4	4	4	3	3	1	1
3	3	4	4	1	1	2	2	4	4
3	3	4	4	4	4	3	3	1	1
3	3	4	4	4	4	2	2	1	1
3	3	4	4	4	4	2	2	1	1
3	3	4	4	4	4	2	2	1	1
3	3	4	4	4	4	3	3	1	1
3	3	4	4	1	1	3	3	1	1
3	3	1	1	1	1	2	2	4	4
3	3	4	4	4	4	3	3	1	1
3	3	4	4	4	4	3	3	1	1
2	2	4	4	1	1	2	2	4	4
3	3	4	4	4	4	3	3	1	1
3	3	4	4	1	1	3	3	4	4
3	3	4	4	4	4	2	2	1	1
3	3	4	4	4	4	2	2	1	1
3	3	4	4	1	1	2	2	4	4

For Review Only

3	3	4	4	4	4	2	2	1	1
3	3	4	4	4	4	3	3	1	1

For Review Only

SNP1013	SNP1013	SNP1014	SNP1014	SNP1015	SNP1015	SNP1016	SNP1016	SNP1017	SNP1017
4	4	1	1	4	4	4	4	2	2
4	4	1	1	4	4	4	4	2	2
4	4	1	1	3	3	2	2	3	3
4	4	1	1	4	4	2	2	2	2
4	4	1	1	4	4	4	4	2	2
4	4	1	1	3	3	4	4	2	2
4	4	1	1	4	4	2	2	2	2
4	4	1	1	4	4	2	2	2	2
4	4	3	3	4	4	4	4	2	2
1	1	3	3	4	4	2	2	2	2
4	4	1	1	4	4	4	4	2	2
4	4	1	1	3	3	2	2	3	3
4	4	1	1	4	4	2	2	2	2
4	4	1	1	4	4	4	4	2	2
4	4	1	1	4	4	2	2	2	2
4	4	3	3	4	4	2	2	2	2
4	4	1	1	4	4	2	2	2	2
4	4	1	1	4	4	4	4	2	2
4	4	1	1	3	3	2	2	2	2
4	4	1	1	4	4	2	2	2	2
4	4	1	1	4	4	2	2	2	2
4	4	1	1	4	4	4	4	2	2
4	4	1	1	4	4	2	2	3	3
4	4	1	1	4	4	2	2	2	2
4	4	1	1	4	4	4	4	2	2
4	4	1	1	4	4	4	4	2	2
4	4	1	1	4	4	4	4	2	2
4	4	1	1	4	4	2	2	2	2
4	4	3	3	4	4	2	2	2	2
4	4	3	3	4	4	2	2	2	2
4	4	1	1	4	4	2	2	2	2
4	4	1	1	4	4	2	2	2	2
4	4	1	1	4	4	2	2	2	2
4	4	1	1	4	4	2	2	2	2
4	4	3	3	4	4	4	4	2	2
4	4	1	1	4	4	2	2	2	2
4	4	1	1	4	4	4	4	2	2
4	4	1	1	4	4	2	2	2	2
4	4	1	1	4	4	2	2	2	2
4	4	1	1	4	4	2	2	2	2
4	4	1	1	4	4	2	2	2	2
4	4	1	1	4	4	2	2	2	2
4	4	1	1	4	4	2	2	2	2
4	4	1	1	4	4	4	4	2	2
1	1	1	1	4	4	2	2	2	2
4	4	1	1	3	3	2	2	3	3
4	4	1	1	4	4	2	2	2	2
4	4	1	1	4	4	2	2	2	2
4	4	1	1	4	4	4	4	2	2
4	4	1	1	4	4	2	2	2	2
4	4	1	1	4	4	2	2	2	2
4	4	1	1	4	4	2	2	2	2

For Review Only

4	4	1	1	4	4	4	4	2	2
4	4	1	1	4	4	2	2	2	2
4	4	1	1	3	3	2	2	2	2
4	4	1	1	4	4	4	4	2	2
4	4	1	1	3	3	2	2	2	2
4	4	1	1	4	4	4	4	2	2
4	4	1	1	3	3	2	2	2	2
4	4	1	1	4	4	2	2	2	2
4	4	1	1	4	4	2	2	2	2
4	4	1	1	4	4	2	2	2	2
4	4	1	1	4	4	2	2	2	2
4	4	1	1	4	4	2	2	2	2
4	4	1	1	4	4	4	4	2	2
4	4	3	3	4	4	2	2	2	2
4	4	1	1	4	4	4	4	2	2
4	4	1	1	4	4	2	2	2	2
4	4	1	1	3	3	2	2	3	3
4	4	1	1	4	4	2	2	2	2
4	4	1	1	4	4	4	4	2	2
4	4	1	1	4	4	4	4	3	3
4	4	1	1	4	4	2	2	2	2
4	4	1	1	4	4	2	2	3	3
4	4	3	3	4	4	2	2	2	2
4	4	1	1	3	3	4	4	2	2
1	1	1	1	4	4	2	2	2	2
4	4	3	3	3	3	2	2	2	2
4	4	1	1	3	3	2	2	2	2
4	4	1	1	4	4	2	2	2	2
4	4	1	1	4	4	4	4	2	2
4	4	1	1	4	4	2	2	2	2
4	4	1	1	4	4	2	2	2	2
4	4	1	1	4	4	2	2	2	2
4	4	1	1	4	4	2	2	2	2
4	4	1	1	4	4	4	4	2	2
4	4	1	1	4	4	4	4	2	2
1	1	1	1	4	4	2	2	2	2
4	4	1	1	4	4	2	2	2	2
4	4	1	1	4	4	2	2	2	2
1	1	1	1	3	3	2	2	2	2
1	1	1	1	4	4	2	2	2	2
4	4	1	1	4	4	4	4	2	2
4	4	1	1	4	4	4	4	2	2
4	4	1	1	4	4	2	2	2	2
4	4	1	1	4	4	2	2	2	2
4	4	1	1	4	4	2	2	2	2
4	4	1	1	4	4	2	2	2	2
4	4	1	1	4	4	2	2	2	2
4	4	1	1	3	3	2	2	2	2
4	4	1	1	4	4	2	2	2	2
4	4	1	1	4	4	2	2	2	2
4	4	1	1	3	3	4	4	2	2
4	4	1	1	4	4	2	2	2	2
4	4	1	1	4	4	2	2	2	2
4	4	1	1	4	4	2	2	2	2
4	4	1	1	4	4	4	4	2	2

For Review Only

4	4	1	1	4	4	4	4	2	2
4	4	1	1	4	4	2	2	2	2
4	4	1	1	4	4	2	2	2	2
4	4	1	1	4	4	2	2	2	2
4	4	1	1	3	3	4	4	2	2
4	4	1	1	4	4	4	4	2	2
4	4	1	1	4	4	2	2	2	2
4	4	1	1	4	4	2	2	2	2
4	4	1	1	4	4	2	2	2	2
4	4	1	1	4	4	2	2	2	2
4	4	1	1	4	4	4	4	2	2
4	4	1	1	4	4	2	2	2	2
1	1	1	1	4	4	2	2	2	2
4	4	1	1	3	3	2	2	2	2
4	4	1	1	4	4	2	2	2	2
4	4	1	1	4	4	2	2	2	2
4	4	1	1	4	4	2	2	2	2
4	4	1	1	4	4	2	2	2	2
4	4	1	1	4	4	2	2	2	2
1	1	1	1	4	4	2	2	2	2
4	4	1	1	4	4	4	4	2	2
4	4	1	1	4	4	2	2	2	2
4	4	1	1	3	3	2	2	3	3
4	4	1	1	4	4	2	2	2	2
4	4	1	1	4	4	2	2	2	2
4	4	1	1	4	4	4	4	2	2
4	4	1	1	3	3	4	4	3	3
4	4	1	1	4	4	2	2	2	2
4	4	1	1	4	4	2	2	2	2
4	4	1	1	4	4	4	4	2	2
4	4	1	1	4	4	2	2	2	2
4	4	1	1	4	4	4	4	2	2
4	4	1	1	4	4	4	4	2	2
4	4	1	1	4	4	4	4	2	2
4	4	1	1	4	4	2	2	2	2
4	4	1	1	4	4	2	2	2	2
4	4	1	1	4	4	2	2	2	2
4	4	1	1	4	4	2	2	2	2
4	4	1	1	4	4	2	2	2	2
4	4	1	1	4	4	4	4	2	2
4	4	1	1	4	4	4	4	2	2
4	4	1	1	4	4	2	2	2	2
4	4	1	1	4	4	2	2	2	2
4	4	1	1	4	4	2	2	2	2
4	4	1	1	4	4	2	2	2	2
4	4	1	1	4	4	2	2	2	2
4	4	1	1	4	4	4	4	2	2
4	4	1	1	3	3	2	2	2	2
4	4	1	1	4	4	2	2	2	2
4	4	1	1	4	4	4	4	2	2
4	4	1	1	4	4	4	4	2	2
4	4	1	1	4	4	2	2	2	2
4	4	1	1	4	4	2	2	2	2
4	4	1	1	4	4	2	2	2	2
4	4	1	1	4	4	2	2	2	2

For Review Only

4	4	1	1	4	4	2	2	2	2
1	1	1	1	4	4	2	2	2	2
4	4	1	1	4	4	4	4	2	2
4	4	1	1	4	4	4	4	2	2
4	4	1	1	4	4	4	4	2	2
4	4	1	1	4	4	2	2	2	2
4	4	1	1	4	4	2	2	2	2
4	4	1	1	4	4	2	2	2	2
4	4	1	1	4	4	4	4	2	2
4	4	1	1	4	4	2	2	2	2
4	4	1	1	4	4	2	2	2	2
4	4	1	1	4	4	4	4	2	2
4	4	1	1	4	4	2	2	2	2
4	4	1	1	4	4	2	2	2	2
4	4	1	1	4	4	2	2	2	2
4	4	1	1	4	4	4	4	2	2
4	4	1	1	4	4	2	2	2	2
4	4	1	1	3	3	4	4	2	2
4	4	1	1	4	4	2	2	2	2
4	4	1	1	4	4	2	2	2	2
4	4	1	1	4	4	2	2	2	2
4	4	1	1	4	4	2	2	2	2
4	4	1	1	4	4	4	4	3	3
4	4	1	1	4	4	2	2	2	2
4	4	3	3	4	4	2	2	2	2
4	4	1	1	4	4	2	2	2	2
4	4	1	1	4	4	2	2	2	2
4	4	1	1	4	4	2	2	2	2
4	4	1	1	4	4	2	2	3	3
4	4	1	1	4	4	4	4	2	2
4	4	1	1	3	3	2	2	2	2
4	4	1	1	4	4	2	2	2	2
4	4	1	1	4	4	4	4	3	3
4	4	1	1	4	4	2	2	2	2
4	4	1	1	4	4	2	2	2	2
4	4	1	1	4	4	2	2	2	2
4	4	1	1	4	4	4	4	2	2
4	4	1	1	4	4	4	4	2	2
4	4	1	1	4	4	2	2	2	2
4	4	1	1	3	3	4	4	2	2
4	4	1	1	3	3	2	2	3	3
4	4	1	1	4	4	4	4	2	2
4	4	1	1	4	4	2	2	2	2
4	4	3	3	4	4	2	2	2	2
4	4	1	1	4	4	4	4	2	2
4	4	1	1	4	4	2	2	2	2
4	4	1	1	4	4	4	4	2	2
4	4	1	1	4	4	2	2	2	2
4	4	1	1	4	4	2	2	2	2
4	4	1	1	4	4	4	4	2	2
4	4	1	1	4	4	2	2	2	2
4	4	1	1	4	4	2	2	2	2
4	4	1	1	4	4	2	2	3	3
4	4	1	1	4	4	2	2	2	2
4	4	3	3	4	4	2	2	2	2

For Review Only

4	4	1	1	4	4	2	2	2	2
4	4	3	3	4	4	2	2	2	2
4	4	1	1	3	3	2	2	2	2
4	4	1	1	4	4	2	2	2	2
4	4	1	1	4	4	2	2	2	2
4	4	1	1	4	4	4	4	2	2
1	1	1	1	4	4	4	4	2	2
4	4	1	1	4	4	2	2	2	2
4	4	1	1	4	4	2	2	2	2
4	4	1	1	4	4	2	2	2	2
4	4	1	1	4	4	2	2	2	2
1	1	1	1	4	4	2	2	2	2
4	4	1	1	3	3	4	4	2	2
4	4	1	1	4	4	2	2	2	2
4	4	1	1	3	3	2	2	3	3
4	4	1	1	4	4	4	4	2	2
4	4	1	1	4	4	2	2	2	2
4	4	1	1	4	4	2	2	2	2
4	4	1	1	4	4	2	2	2	2
4	4	1	1	4	4	2	2	2	2
4	4	1	1	3	3	4	4	2	2
4	4	1	1	4	4	4	4	2	2
4	4	1	1	4	4	2	2	2	2
4	4	3	3	4	4	4	4	2	2
4	4	1	1	4	4	2	2	2	2
4	4	3	3	4	4	2	2	2	2
4	4	1	1	4	4	2	2	2	2
4	4	1	1	4	4	2	2	2	2
4	4	1	1	4	4	2	2	2	2
4	4	1	1	4	4	2	2	2	2
4	4	1	1	4	4	2	2	2	2
4	4	1	1	4	4	2	2	2	2
4	4	1	1	4	4	2	2	2	2
4	4	1	1	4	4	2	2	2	2
4	4	1	1	4	4	4	4	3	3
4	4	1	1	4	4	2	2	2	2
4	4	1	1	4	4	2	2	2	2
4	4	1	1	4	4	2	2	2	2
4	4	1	1	4	4	2	2	2	2
4	4	1	1	4	4	2	2	2	2
4	4	1	1	4	4	2	2	2	2
4	4	1	1	4	4	4	4	2	2
4	4	3	3	4	4	4	4	2	2
4	4	1	1	4	4	4	4	2	2
4	4	1	1	3	3	2	2	2	2
4	4	1	1	4	4	2	2	2	2
4	4	1	1	4	4	2	2	2	2
4	4	1	1	4	4	2	2	2	2
1	1	1	1	4	4	2	2	2	2
4	4	1	1	4	4	2	2	2	2
4	4	1	1	4	4	2	2	3	3
4	4	1	1	4	4	2	2	2	2
4	4	1	1	3	3	2	2	3	3
4	4	1	1	3	3	2	2	2	2
4	4	3	3	4	4	4	4	2	2

For Review Only

4	4	1	1	4	4	2	2	2	2
4	4	1	1	3	3	2	2	2	2

For Review Only

SNP1018	SNP1018	SNP1019	SNP1019	SNP102	SNP102	SNP1020	SNP1020	SNP1021	SNP1021
4	4	4	4	2	2	3	3	1	1
4	4	4	4	1	1	3	3	1	1
4	4	1	1	2	2	3	3	1	1
4	4	1	1	2	2	3	3	1	1
4	4	4	4	2	2	3	3	1	1
4	4	4	4	1	1	3	3	1	1
4	4	4	4	2	2	3	3	1	1
4	4	4	4	2	2	4	4	1	1
4	4	4	4	2	2	3	3	1	1
3	3	4	4	2	2	4	4	1	1
4	4	4	4	2	2	3	3	2	2
4	4	1	1	2	2	3	3	1	1
4	4	4	4	2	2	3	3	1	1
4	4	4	4	2	2	3	3	1	1
4	4	4	4	2	2	3	3	1	1
4	4	1	1	1	1	3	3	1	1
4	4	4	4	2	2	3	3	1	1
4	4	4	4	2	2	3	3	1	1
4	4	4	4	1	1	3	3	1	1
4	4	4	4	1	1	3	3	1	1
4	4	4	4	2	2	4	4	1	1
4	4	4	4	2	2	3	3	1	1
4	4	4	4	2	2	3	3	2	2
4	4	4	4	1	1	3	3	1	1
4	4	4	4	2	2	3	3	1	1
4	4	4	4	2	2	3	3	1	1
4	4	4	4	2	2	3	3	1	1
4	4	4	4	2	2	3	3	1	1
4	4	4	4	2	2	3	3	1	1
4	4	4	4	2	2	4	4	1	1
4	4	4	4	2	2	3	3	1	1
4	4	4	4	1	1	3	3	1	1
4	4	4	4	2	2	3	3	1	1
4	4	4	4	2	2	3	3	1	1
4	4	4	4	2	2	3	3	1	1
4	4	4	4	1	1	3	3	1	1
4	4	4	4	2	2	3	3	1	1
4	4	4	4	2	2	3	3	1	1
4	4	4	4	1	1	3	3	2	2
4	4	1	1	1	1	3	3	2	2
4	4	4	4	2	2	4	4	1	1
4	4	4	4	2	2	4	4	1	1
4	4	4	4	2	2	4	4	1	1
4	4	4	4	2	2	3	3	1	1
3	3	4	4	2	2	3	3	1	1
4	4	1	1	2	2	3	3	1	1
4	4	4	4	2	2	3	3	1	1
4	4	4	4	2	2	3	3	1	1
4	4	4	4	2	2	3	3	1	1
4	4	4	4	1	1	3	3	1	1
4	4	4	4	2	2	3	3	1	1
4	4	4	4	1	1	3	3	1	1

For Review Only

4	4	4	4	2	2	3	3	1	1
4	4	4	4	2	2	3	3	1	1
4	4	1	1	2	2	3	3	1	1
4	4	4	4	2	2	4	4	1	1
4	4	4	4	1	1	3	3	1	1
4	4	4	4	2	2	3	3	1	1
4	4	4	4	2	2	3	3	1	1
4	4	4	4	2	2	3	3	1	1
4	4	4	4	2	2	3	3	1	1
4	4	4	4	2	2	3	3	1	1
4	4	4	4	2	2	3	3	1	1
4	4	4	4	2	2	3	3	1	1
4	4	4	4	2	2	3	3	1	1
4	4	4	4	2	2	3	3	1	1
4	4	4	4	1	1	3	3	1	1
4	4	4	4	2	2	3	3	1	1
4	4	4	4	2	2	3	3	2	2
4	4	4	4	2	2	3	3	1	1
4	4	4	4	2	2	3	3	1	1
4	4	4	4	2	2	3	3	1	1
4	4	4	4	2	2	3	3	1	1
4	4	4	4	1	1	3	3	2	2
4	4	4	4	2	2	3	3	1	1
4	4	4	4	2	2	3	3	1	1
3	3	4	4	2	2	3	3	1	1
4	4	4	4	2	2	3	3	1	1
4	4	4	4	1	1	3	3	1	1
4	4	4	4	1	1	3	3	2	2
4	4	4	4	2	2	3	3	1	1
4	4	4	4	2	2	3	3	1	1
4	4	4	4	2	2	4	4	1	1
4	4	4	4	2	2	3	3	2	2
4	4	4	4	1	1	3	3	2	2
4	4	4	4	2	2	3	3	1	1
4	4	4	4	1	1	3	3	1	1
3	3	4	4	2	2	3	3	1	1
4	4	4	4	2	2	4	4	1	1
4	4	4	4	2	2	3	3	1	1
3	3	4	4	2	2	3	3	1	1
3	3	4	4	2	2	4	4	1	1
4	4	4	4	2	2	3	3	1	1
4	4	4	4	2	2	3	3	1	1
4	4	4	4	2	2	3	3	1	1
4	4	4	4	2	2	3	3	1	1
4	4	4	4	2	2	3	3	2	2
4	4	4	4	1	1	3	3	1	1
4	4	4	4	2	2	3	3	1	1
4	4	4	4	2	2	3	3	1	1
4	4	4	4	2	2	3	3	1	1
4	4	4	4	1	1	3	3	1	1
4	4	4	4	1	1	3	3	1	1
4	4	4	4	2	2	3	3	2	2
4	4	4	4	2	2	3	3	1	1
4	4	4	4	2	2	3	3	1	1
4	4	4	4	2	2	3	3	1	1

For Review Only

4	4	4	4	2	2	3	3	2	2
4	4	4	4	2	2	3	3	1	1
4	4	4	4	2	2	3	3	1	1
4	4	4	4	2	2	3	3	1	1
4	4	4	4	1	1	3	3	1	1
4	4	4	4	2	2	3	3	1	1
4	4	1	1	2	2	3	3	1	1
4	4	4	4	1	1	3	3	1	1
4	4	4	4	2	2	3	3	1	1
4	4	4	4	2	2	3	3	1	1
4	4	4	4	2	2	3	3	1	1
4	4	4	4	2	2	3	3	1	1
3	3	4	4	2	2	3	3	1	1
4	4	4	4	1	1	3	3	1	1
4	4	4	4	1	1	3	3	1	1
4	4	4	4	1	1	3	3	1	1
4	4	4	4	2	2	4	4	1	1
4	4	4	4	2	2	3	3	1	1
4	4	4	4	2	2	3	3	1	1
3	3	4	4	2	2	3	3	2	2
4	4	4	4	2	2	3	3	1	1
4	4	4	4	2	2	3	3	1	1
4	4	4	4	2	2	3	3	1	1
4	4	4	4	2	2	3	3	2	2
4	4	4	4	1	1	3	3	2	2
4	4	4	4	2	2	3	3	2	2
4	4	4	4	1	1	3	3	1	1
4	4	4	4	2	2	3	3	1	1
4	4	4	4	2	2	3	3	1	1
4	4	4	4	1	1	3	3	1	1
4	4	4	4	2	2	3	3	1	1
4	4	4	4	1	1	3	3	1	1
4	4	4	4	2	2	3	3	1	1
4	4	4	4	2	2	3	3	1	1
4	4	4	4	2	2	3	3	1	1
4	4	4	4	1	1	3	3	1	1
4	4	4	4	2	2	3	3	1	1
4	4	4	4	2	2	3	3	1	1
4	4	4	4	2	2	3	3	1	1
4	4	4	4	1	1	3	3	2	2
4	4	4	4	2	2	3	3	2	2
4	4	4	4	2	2	3	3	1	1
4	4	4	4	2	2	3	3	1	1
4	4	4	4	2	2	4	4	1	1
4	4	4	4	2	2	3	3	1	1
4	4	4	4	2	2	3	3	1	1
4	4	4	4	2	2	3	3	1	1
4	4	4	4	2	2	3	3	1	1
4	4	4	4	2	2	3	3	1	1
4	4	4	4	2	2	3	3	1	1
4	4	4	4	2	2	3	3	1	1
4	4	4	4	2	2	4	4	1	1
4	4	4	4	1	1	3	3	1	1
4	4	4	4	2	2	3	3	1	1
4	4	4	4	2	2	3	3	1	1

For Review Only

4	4	4	4	2	2	3	3	1	1
3	3	4	4	2	2	3	3	2	2
4	4	4	4	2	2	3	3	1	1
4	4	4	4	1	1	3	3	2	2
4	4	4	4	1	1	3	3	1	1
4	4	4	4	2	2	3	3	1	1
4	4	4	4	2	2	3	3	1	1
4	4	4	4	2	2	3	3	1	1
4	4	4	4	1	1	3	3	2	2
4	4	1	1	2	2	3	3	1	1
4	4	1	1	1	1	3	3	1	1
4	4	4	4	2	2	3	3	1	1
4	4	1	1	2	2	3	3	1	1
4	4	4	4	2	2	3	3	1	1
4	4	4	4	2	2	3	3	1	1
4	4	4	4	1	1	3	3	2	2
4	4	4	4	1	1	3	3	1	1
4	4	4	4	1	1	3	3	1	1
4	4	4	4	2	2	3	3	1	1
4	4	1	1	2	2	3	3	1	1
4	4	4	4	2	2	3	3	1	1
4	4	4	4	1	1	3	3	1	1
4	4	4	4	1	1	3	3	2	2
4	4	4	4	2	2	3	3	1	1
4	4	4	4	2	2	4	4	1	1
4	4	4	4	2	2	3	3	1	1
4	4	1	1	2	2	3	3	1	1
4	4	4	4	2	2	3	3	1	1
4	4	4	4	2	2	3	3	1	1
4	4	4	4	1	1	3	3	1	1
4	4	4	4	2	2	3	3	1	1
4	4	4	4	2	2	3	3	1	1
4	4	4	4	2	2	3	3	1	1
4	4	4	4	2	2	3	3	1	1
4	4	4	4	2	2	3	3	1	1
4	4	4	4	2	2	3	3	1	1
4	4	4	4	2	2	3	3	1	1
4	4	4	4	2	2	3	3	1	1
4	4	4	4	2	2	3	3	1	1
4	4	4	4	2	2	3	3	1	1
4	4	1	1	2	2	3	3	1	1
4	4	4	4	2	2	3	3	1	1
4	4	4	4	1	1	3	3	2	2
4	4	4	4	2	2	3	3	1	1
4	4	4	4	1	1	3	3	1	1
4	4	4	4	2	2	3	3	1	1
4	4	4	4	1	1	3	3	2	2
4	4	4	4	2	2	3	3	1	1
4	4	4	4	2	2	3	3	1	1
4	4	4	4	2	2	3	3	1	1
4	4	4	4	2	2	3	3	1	1
4	4	4	4	2	2	3	3	1	1
4	4	4	4	1	1	3	3	2	2
4	4	4	4	2	2	3	3	1	1
4	4	4	4	2	2	3	3	2	2

For Review Only

4	4	4	4	1	1	3	3	2	2
4	4	1	1	2	2	3	3	1	1
4	4	4	4	2	2	3	3	1	1
4	4	4	4	1	1	3	3	2	2
4	4	4	4	2	2	3	3	1	1
4	4	4	4	2	2	3	3	1	1
4	4	4	4	2	2	3	3	1	1
4	4	4	4	2	2	3	3	1	1
4	4	4	4	2	2	3	3	1	1
4	4	4	4	1	1	3	3	2	2
4	4	4	4	2	2	3	3	1	1
3	3	4	4	2	2	3	3	1	1
4	4	4	4	2	2	3	3	1	1
4	4	4	4	2	2	3	3	1	1
4	4	1	1	2	2	3	3	1	1
4	4	4	4	2	2	3	3	1	1
4	4	4	4	1	1	3	3	1	1
4	4	4	4	1	1	3	3	1	1
4	4	4	4	1	1	3	3	2	2
4	4	4	4	2	2	3	3	1	1
4	4	4	4	1	1	3	3	2	2
4	4	4	4	2	2	3	3	1	1
4	4	4	4	2	2	3	3	1	1
4	4	4	4	2	2	3	3	2	2
4	4	4	4	2	2	3	3	1	1
4	4	4	4	2	2	3	3	1	1
4	4	4	4	2	2	3	3	2	2
4	4	1	1	2	2	3	3	1	1
4	4	4	4	2	2	3	3	1	1
4	4	4	4	2	2	3	3	1	1
4	4	4	4	2	2	3	3	2	2
4	4	4	4	1	1	3	3	1	1
4	4	4	4	2	2	3	3	1	1
4	4	4	4	2	2	3	3	1	1
4	4	4	4	1	1	3	3	2	2
4	4	4	4	1	1	3	3	1	1
4	4	4	4	1	1	3	3	1	1
4	4	4	4	2	2	3	3	1	1
4	4	4	4	1	1	3	3	1	1
4	4	4	4	1	1	3	3	2	2
4	4	4	4	2	2	3	3	1	1
4	4	4	4	2	2	3	3	1	1
4	4	4	4	2	2	3	3	1	1
4	4	4	4	2	2	3	3	1	1
4	4	4	4	2	2	3	3	1	1
4	4	4	4	2	2	3	3	1	1
4	4	4	4	2	2	3	3	2	2
4	4	4	4	2	2	3	3	1	1
4	4	4	4	2	2	3	3	1	1
4	4	4	4	1	1	3	3	2	2
4	4	1	1	2	2	3	3	1	1
4	4	4	4	2	2	3	3	2	2
4	4	1	1	2	2	3	3	1	1
4	4	1	1	2	2	3	3	1	1
4	4	4	4	2	2	3	3	2	2

For Review Only

4	4	4	4	2	2	3	3	1	1
4	4	4	4	2	2	3	3	1	1

For Review Only

SNP1022	SNP1022	SNP1023	SNP1023	SNP1024	SNP1024	SNP1025	SNP1025	SNP1026	SNP1026
3	3	3	3	3	3	4	4	2	2
3	3	3	3	3	3	1	1	2	2
3	3	2	2	3	3	1	1	2	2
3	3	3	3	3	3	1	1	2	2
3	3	2	2	3	3	1	1	2	2
2	2	3	3	3	3	4	4	2	2
3	3	3	3	3	3	4	4	2	2
2	2	3	3	3	3	1	1	2	2
3	3	2	2	3	3	4	4	2	2
2	2	2	2	3	3	1	1	2	2
3	3	3	3	3	3	1	1	2	2
3	3	2	2	3	3	1	1	2	2
3	3	3	3	3	3	1	1	2	2
3	3	2	2	3	3	1	1	2	2
3	3	3	3	3	3	1	1	2	2
3	3	2	2	3	3	1	1	2	2
3	3	3	3	3	3	1	1	2	2
3	3	3	3	3	3	1	1	2	2
2	2	2	2	3	3	1	1	2	2
3	3	3	3	3	3	1	1	2	2
2	2	3	3	3	3	1	1	2	2
3	3	2	2	3	3	1	1	2	2
3	3	2	2	3	3	4	4	2	2
3	3	2	2	3	3	1	1	2	2
3	3	3	3	3	3	1	1	2	2
3	3	3	3	3	3	1	1	2	2
3	3	3	3	3	3	1	1	2	2
3	3	3	3	3	3	1	1	2	2
3	3	2	2	3	3	1	1	2	2
2	2	2	2	3	3	1	1	2	2
3	3	3	3	3	3	1	1	2	2
3	3	2	2	3	3	4	4	2	2
3	3	2	2	3	3	4	4	2	2
3	3	2	2	3	3	1	1	2	2
3	3	3	3	3	3	1	1	2	2
3	3	2	2	3	3	1	1	2	2
3	3	2	2	3	3	4	4	3	3
3	3	2	2	3	3	1	1	2	2
3	3	2	2	3	3	1	1	2	2
3	3	2	2	3	3	1	1	2	2
2	2	2	2	3	3	1	1	2	2
3	3	2	2	3	3	1	1	2	2
3	3	2	2	3	3	1	1	2	2
3	3	2	2	3	3	1	1	2	2
3	3	2	2	3	3	1	1	2	2
2	2	3	3	3	3	1	1	2	2
3	3	3	3	3	3	1	1	2	2
3	3	3	3	3	3	1	1	2	2
3	3	2	2	3	3	1	1	2	2
3	3	3	3	3	3	1	1	2	2
3	3	3	3	3	3	1	1	2	2
3	3	3	3	3	3	1	1	2	2

For Review Only

3	3	3	3	3	3	1	1	2	2
3	3	2	2	3	3	4	4	2	2
2	2	3	3	3	3	1	1	2	2
2	2	2	2	3	3	1	1	2	2
2	2	2	2	3	3	1	1	2	2
3	3	3	3	3	3	1	1	2	2
2	2	3	3	3	3	1	1	2	2
3	3	2	2	3	3	1	1	2	2
3	3	2	2	3	3	4	4	3	3
3	3	3	3	3	3	1	1	2	2
3	3	3	3	3	3	1	1	2	2
3	3	2	2	3	3	1	1	2	2
3	3	3	3	3	3	1	1	2	2
3	3	2	2	3	3	1	1	2	2
3	3	3	3	3	3	1	1	2	2
3	3	3	3	3	3	4	4	2	2
3	3	2	2	3	3	1	1	2	2
3	3	3	3	3	3	4	4	2	2
3	3	2	2	3	3	1	1	2	2
3	3	3	3	3	3	4	4	2	2
3	3	2	2	3	3	1	1	2	2
3	3	2	2	3	3	1	1	2	2
3	3	3	3	3	3	1	1	2	2
3	3	2	2	3	3	1	1	2	2
3	3	2	2	3	3	1	1	2	2
2	2	3	3	3	3	1	1	2	2
3	3	2	2	3	3	4	4	2	2
3	3	2	2	3	3	1	1	2	2
3	3	3	3	3	3	1	1	2	2
3	3	3	3	3	3	1	1	2	2
2	2	3	3	3	3	1	1	2	2
3	3	3	3	3	3	1	1	2	2
3	3	2	2	3	3	1	1	3	3
3	3	3	3	3	3	1	1	2	2
3	3	2	2	3	3	1	1	2	2
3	3	2	2	3	3	1	1	2	2
2	2	3	3	3	3	1	1	2	2
3	3	3	3	3	3	1	1	2	2
3	3	3	3	3	3	1	1	2	2
2	2	2	2	3	3	1	1	2	2
3	3	2	2	3	3	1	1	2	2
3	3	3	3	3	3	1	1	2	2
3	3	2	2	3	3	1	1	2	2
3	3	3	3	3	3	1	1	2	2
3	3	2	2	3	3	4	4	2	2
3	3	2	2	3	3	1	1	2	2
3	3	2	2	2	2	1	1	2	2
2	2	2	2	3	3	1	1	2	2
3	3	3	3	3	3	1	1	2	2
3	3	2	2	2	2	1	1	2	2
3	3	3	3	3	3	1	1	2	2
3	3	2	2	3	3	1	1	2	2
3	3	2	2	3	3	1	1	2	2
3	3	3	3	3	3	1	1	2	2
3	3	2	2	2	2	4	4	2	2

For Review Only

3	3	3	3	3	3	1	1	2	2
3	3	3	3	3	3	1	1	2	2
3	3	3	3	3	3	1	1	2	2
3	3	2	2	2	2	1	1	2	2
2	2	2	2	3	3	1	1	2	2
3	3	2	2	3	3	1	1	2	2
3	3	2	2	3	3	1	1	2	2
3	3	3	3	3	3	1	1	2	2
3	3	3	3	3	3	1	1	2	2
3	3	3	3	3	3	1	1	2	2
3	3	2	2	3	3	4	4	2	2
3	3	3	3	3	3	1	1	2	2
3	3	3	3	3	3	1	1	2	2
3	3	3	3	3	3	1	1	2	2
3	3	3	3	3	3	1	1	2	2
3	3	3	3	3	3	1	1	2	2
3	3	3	3	3	3	4	4	2	2
3	3	3	3	3	3	1	1	2	2
3	3	2	2	2	2	1	1	3	3
3	3	3	3	3	3	4	4	2	2
3	3	3	3	3	3	1	1	2	2
3	3	3	3	3	3	1	1	2	2
3	3	3	3	3	3	1	1	2	2
3	3	2	2	3	3	1	1	2	2
3	3	3	3	3	3	1	1	2	2
3	3	2	2	3	3	1	1	2	2
3	3	2	2	3	3	4	4	2	2
3	3	3	3	3	3	1	1	2	2
3	3	2	2	3	3	1	1	2	2
3	3	2	2	3	3	1	1	2	2
3	3	2	2	3	3	1	1	2	2
3	3	3	3	3	3	1	1	2	2
3	3	2	2	3	3	1	1	2	2
3	3	3	3	3	3	1	1	2	2
3	3	2	2	3	3	1	1	2	2
3	3	2	2	3	3	4	4	2	2
3	3	2	2	2	2	1	1	3	3
3	3	3	3	3	3	4	4	2	2
3	3	3	3	3	3	1	1	2	2
3	3	3	3	3	3	1	1	2	2
3	3	2	2	3	3	1	1	2	2
3	3	2	2	3	3	1	1	3	3
3	3	3	3	3	3	1	1	2	2
2	2	2	2	3	3	1	1	2	2
3	3	3	3	3	3	1	1	2	2
3	3	2	2	3	3	1	1	2	2
3	3	3	3	3	3	1	1	2	2
3	3	3	3	3	3	1	1	2	2
3	3	2	2	3	3	1	1	2	2
3	3	3	3	3	3	1	1	2	2
3	3	3	3	3	3	1	1	2	2
2	2	2	2	3	3	1	1	2	2
3	3	3	3	3	3	1	1	2	2
3	3	2	2	3	3	1	1	2	2
3	3	2	2	3	3	1	1	2	2

For Review Only

3	3	3	3	3	3	1	1	2	2
3	3	2	2	3	3	1	1	2	2
3	3	2	2	3	3	1	1	2	2
3	3	3	3	3	3	1	1	2	2
3	3	3	3	3	3	1	1	2	2
3	3	2	2	3	3	1	1	2	2
3	3	2	2	3	3	1	1	2	2
3	3	3	3	3	3	1	1	2	2
3	3	2	2	3	3	1	1	2	2
3	3	3	3	3	3	1	1	2	2
3	3	3	3	3	3	1	1	2	2
3	3	2	2	3	3	1	1	2	2
3	3	3	3	3	3	1	1	2	2
3	3	2	2	3	3	1	1	2	2
3	3	2	2	3	3	1	1	3	3
3	3	3	3	3	3	1	1	2	2
3	3	3	3	3	3	1	1	2	2
2	2	3	3	3	3	1	1	2	2
3	3	3	3	3	3	1	1	2	2
3	3	2	2	3	3	1	1	2	2
3	3	3	3	3	3	1	1	2	2
3	3	2	2	3	3	1	1	2	2
3	3	2	2	3	3	1	1	2	2
3	3	2	2	3	3	1	1	2	2
2	2	2	2	3	3	1	1	2	2
3	3	2	2	3	3	1	1	2	2
3	3	2	2	3	3	1	1	2	2
3	3	2	2	3	3	4	4	2	2
3	3	3	3	3	3	4	4	2	2
3	3	2	2	3	3	1	1	2	2
3	3	2	2	3	3	4	4	2	2
3	3	3	3	3	3	1	1	2	2
3	3	2	2	3	3	4	4	2	2
3	3	2	2	3	3	1	1	3	3
3	3	2	2	3	3	1	1	2	2
3	3	2	2	3	3	1	1	2	2
3	3	3	3	3	3	1	1	2	2
3	3	3	3	3	3	4	4	2	2
3	3	3	3	3	3	1	1	2	2
3	3	3	3	3	3	1	1	2	2
3	3	2	2	3	3	1	1	2	2
3	3	3	3	3	3	1	1	2	2
3	3	2	2	3	3	1	1	3	3
3	3	3	3	3	3	1	1	2	2
3	3	2	2	3	3	1	1	3	3
3	3	3	3	3	3	1	1	2	2
3	3	3	3	3	3	1	1	2	2
3	3	2	2	3	3	1	1	3	3
3	3	2	2	3	3	1	1	2	2
3	3	2	2	3	3	1	1	2	2
3	3	2	2	3	3	1	1	2	2
3	3	2	2	2	2	1	1	2	2
3	3	2	2	3	3	1	1	2	2
3	3	3	3	3	3	1	1	2	2
3	3	2	2	3	3	1	1	2	2

For Review Only

3	3	3	3	3	3	1	1	2	2
3	3	2	2	3	3	1	1	2	2
3	3	2	2	3	3	1	1	2	2
3	3	2	2	3	3	1	1	2	2
3	3	3	3	3	3	1	1	2	2
3	3	3	3	3	3	1	1	2	2
3	3	2	2	3	3	1	1	2	2
2	2	2	2	3	3	1	1	2	2
3	3	3	3	3	3	1	1	2	2
3	3	2	2	3	3	1	1	2	2
3	3	3	3	3	3	1	1	2	2
3	3	2	2	3	3	4	4	2	2
3	3	3	3	3	3	1	1	2	2
3	3	3	3	3	3	1	1	2	2
3	3	2	2	3	3	1	1	2	2
3	3	3	3	3	3	1	1	2	2
3	3	2	2	3	3	1	1	2	2
3	3	3	3	3	3	1	1	2	2
3	3	3	3	3	3	1	1	2	2
3	3	2	2	2	2	1	1	2	2
3	3	3	3	3	3	4	4	2	2
3	3	3	3	3	3	1	1	2	2
3	3	3	3	3	3	4	4	2	2
3	3	2	2	3	3	1	1	2	2
3	3	2	2	3	3	1	1	2	2
3	3	3	3	3	3	1	1	2	2
3	3	2	2	3	3	1	1	3	3
3	3	3	3	3	3	1	1	2	2
3	3	3	3	3	3	1	1	2	2
3	3	2	2	3	3	1	1	2	2
3	3	2	2	3	3	1	1	2	2
3	3	2	2	3	3	1	1	2	2
3	3	2	2	2	2	4	4	2	2
3	3	3	3	3	3	1	1	2	2
3	3	2	2	3	3	4	4	2	2
3	3	2	2	3	3	1	1	2	2
3	3	3	3	3	3	1	1	2	2
3	3	2	2	3	3	1	1	2	2
3	3	2	2	3	3	1	1	2	2
3	3	2	2	3	3	1	1	2	2
3	3	2	2	3	3	1	1	2	2
3	3	2	2	3	3	1	1	2	2
3	3	2	2	3	3	1	1	2	2
3	3	2	2	3	3	1	1	2	2
3	3	3	3	3	3	1	1	2	2
3	3	3	3	3	3	1	1	2	2
3	3	3	3	3	3	1	1	2	2
3	3	2	2	3	3	1	1	2	2
3	3	3	3	3	3	1	1	2	2
3	3	2	2	3	3	1	1	2	2
3	3	2	2	3	3	4	4	2	2
3	3	2	2	3	3	1	1	2	2
3	3	2	2	3	3	1	1	2	2
3	3	3	3	3	3	1	1	2	2
3	3	2	2	3	3	1	1	2	2

3	3	3	3	3	3	1	1	2	2
3	3	3	3	3	3	1	1	2	2

For Review Only

SNP1027	SNP1027	SNP1028	SNP1028	SNP1029	SNP1029	SNP103	SNP103	SNP1030	SNP1030
3	3	3	3	2	2	2	2	2	2
3	3	2	2	3	3	2	2	2	2
1	1	2	2	2	2	2	2	2	2
3	3	2	2	2	2	2	2	4	4
1	1	2	2	2	2	2	2	2	2
3	3	3	3	2	2	3	3	2	2
3	3	3	3	2	2	2	2	2	2
3	3	2	2	2	2	2	2	2	2
1	1	2	2	2	2	2	2	2	2
1	1	2	2	2	2	2	2	2	2
1	1	2	2	2	2	2	2	2	2
1	1	2	2	2	2	2	2	2	2
3	3	2	2	2	2	2	2	2	2
1	1	2	2	2	2	2	2	2	2
3	3	2	2	2	2	2	2	2	2
1	1	2	2	2	2	3	3	2	2
3	3	2	2	2	2	2	2	2	2
3	3	2	2	2	2	2	2	2	2
1	1	2	2	2	2	3	3	2	2
3	3	2	2	2	2	2	2	2	2
3	3	2	2	2	2	2	2	2	2
1	1	2	2	2	2	2	2	2	2
1	1	3	3	3	3	2	2	2	2
1	1	2	2	2	2	2	2	2	2
3	3	2	2	2	2	2	2	4	4
3	3	2	2	2	2	3	3	2	2
3	3	2	2	2	2	2	2	2	2
3	3	2	2	2	2	2	2	2	2
1	1	2	2	2	2	2	2	2	2
1	1	2	2	2	2	2	2	2	2
3	3	2	2	2	2	2	2	2	2
3	3	3	3	2	2	3	3	2	2
3	3	3	3	2	2	2	2	2	2
1	1	2	2	2	2	2	2	2	2
3	3	2	2	3	3	2	2	2	2
1	1	2	2	2	2	2	2	4	4
1	1	3	3	2	2	2	2	2	2
1	1	2	2	3	3	2	2	2	2
1	1	2	2	3	3	2	2	2	2
1	1	2	2	3	3	2	2	2	2
1	1	2	2	2	2	2	2	2	2
1	1	2	2	2	2	2	2	2	2
1	1	2	2	2	2	2	2	2	2
1	1	2	2	2	2	2	2	2	2
1	1	2	2	2	2	2	2	2	2
3	3	2	2	2	2	2	2	2	2
3	3	2	2	2	2	2	2	2	2
3	3	2	2	3	3	2	2	2	2
1	1	2	2	2	2	2	2	2	2
3	3	2	2	2	2	2	2	2	2
3	3	2	2	2	2	3	3	4	4
3	3	2	2	3	3	2	2	2	2

For Review Only

3	3	2	2	2	2	2	2	2	2
3	3	3	3	2	2	2	2	2	2
3	3	2	2	2	2	2	2	2	2
1	1	2	2	2	2	2	2	2	2
1	1	2	2	2	2	3	3	2	2
3	3	2	2	2	2	2	2	2	2
3	3	2	2	2	2	3	3	2	2
1	1	2	2	3	3	2	2	2	2
1	1	3	3	3	3	2	2	2	2
3	3	2	2	2	2	2	2	2	2
3	3	2	2	2	2	3	3	2	2
1	1	2	2	2	2	2	2	2	2
3	3	2	2	2	2	2	2	2	2
1	1	2	2	2	2	2	2	2	2
3	3	2	2	2	2	2	2	2	2
3	3	2	2	2	2	2	2	2	2
1	1	2	2	2	2	2	2	2	2
3	3	3	3	2	2	2	2	4	4
1	1	2	2	2	2	2	2	4	4
3	3	3	3	2	2	2	2	2	2
1	1	2	2	2	2	2	2	2	2
1	1	2	2	3	3	2	2	2	2
1	1	2	2	2	2	2	2	2	2
1	1	2	2	2	2	2	2	2	2
1	1	2	2	2	2	2	2	2	2
3	3	2	2	2	2	2	2	2	2
1	1	3	3	2	2	2	2	2	2
1	1	2	2	3	3	2	2	2	2
3	3	2	2	2	2	3	3	2	2
3	3	2	2	2	2	2	2	4	4
3	3	2	2	2	2	2	2	2	2
3	3	2	2	2	2	2	2	2	2
1	1	2	2	3	3	2	2	2	2
3	3	2	2	2	2	2	2	2	2
1	1	2	2	3	3	2	2	2	2
1	1	2	2	2	2	2	2	2	2
3	3	2	2	2	2	2	2	2	2
3	3	2	2	2	2	2	2	2	2
1	1	2	2	2	2	2	2	2	2
1	1	2	2	2	2	2	2	2	2
1	1	2	2	2	2	2	2	2	2
3	3	2	2	2	2	2	2	4	4
1	1	2	2	2	2	2	2	2	2
3	3	2	2	2	2	3	3	2	2
1	1	3	3	2	2	2	2	2	2
1	1	2	2	2	2	2	2	2	2
1	1	2	2	3	3	2	2	2	2
1	1	2	2	2	2	3	3	2	2
3	3	2	2	2	2	2	2	2	2
1	1	2	2	2	2	2	2	2	2
3	3	2	2	2	2	2	2	2	2
1	1	2	2	3	3	2	2	2	2
1	1	2	2	2	2	2	2	2	2
3	3	2	2	2	2	2	2	4	4
1	1	3	3	2	2	2	2	2	2

For Review Only

3	3	2	2	3	3	2	2	2	2
3	3	2	2	2	2	2	2	2	2
3	3	2	2	2	2	2	2	2	2
1	1	2	2	3	3	2	2	2	2
3	3	2	2	2	2	2	2	2	2
1	1	2	2	2	2	2	2	2	2
1	1	2	2	2	2	3	3	2	2
3	3	2	2	2	2	2	2	4	4
3	3	2	2	2	2	2	2	2	2
3	3	2	2	2	2	2	2	2	2
3	3	3	3	2	2	2	2	2	2
3	3	2	2	2	2	2	2	2	2
3	3	2	2	2	2	2	2	4	4
3	3	2	2	2	2	2	2	2	2
3	3	2	2	2	2	2	2	2	2
3	3	2	2	2	2	2	2	2	2
3	3	3	3	2	2	2	2	2	2
3	3	2	2	2	2	2	2	2	2
1	1	2	2	3	3	2	2	2	2
3	3	3	3	2	2	2	2	2	2
3	3	2	2	2	2	2	2	2	2
3	3	2	2	2	2	2	2	4	4
3	3	2	2	2	2	3	3	4	4
1	1	2	2	3	3	2	2	2	2
3	3	2	2	3	3	2	2	2	2
1	1	2	2	2	2	2	2	2	2
1	1	3	3	2	2	2	2	2	2
3	3	2	2	2	2	2	2	2	2
1	1	2	2	2	2	2	2	2	2
1	1	2	2	2	2	2	2	2	2
1	1	2	2	3	3	2	2	2	2
3	3	2	2	2	2	2	2	2	2
1	1	2	2	2	2	2	2	2	2
3	3	2	2	2	2	2	2	2	2
1	1	2	2	2	2	2	2	2	2
1	1	2	2	2	2	2	2	2	2
1	1	2	2	3	3	2	2	2	2
3	3	3	3	2	2	3	3	2	2
3	3	2	2	2	2	2	2	2	2
1	1	2	2	2	2	2	2	2	2
1	1	2	2	2	2	2	2	2	2
1	1	2	2	2	2	2	2	2	2
3	3	2	2	3	3	2	2	2	2
3	3	2	2	2	2	2	2	2	2
3	3	2	2	2	2	2	2	2	2
1	1	2	2	3	3	2	2	2	2
3	3	2	2	2	2	3	3	2	2
3	3	2	2	2	2	3	3	2	2
1	1	2	2	2	2	2	2	2	2
3	3	2	2	2	2	3	3	2	2
3	3	2	2	2	2	2	2	2	2
1	1	2	2	2	2	2	2	2	2
3	3	2	2	2	2	2	2	2	2
1	1	2	2	2	2	3	3	2	2
1	1	2	2	2	2	2	2	2	2

For Review Only

1	1	2	2	2	2	2	2	2	2
3	3	2	2	2	2	2	2	2	2
1	1	2	2	2	2	2	2	2	2
3	3	2	2	2	2	2	2	2	2
3	3	2	2	2	2	2	2	2	2
1	1	2	2	3	3	2	2	4	4
1	1	2	2	2	2	2	2	2	2
3	3	2	2	2	2	2	2	2	2
1	1	2	2	2	2	2	2	2	2
3	3	2	2	2	2	2	2	4	4
3	3	2	2	2	2	2	2	2	2
1	1	2	2	3	3	2	2	2	2
3	3	2	2	2	2	3	3	2	2
1	1	2	2	2	2	2	2	2	2
1	1	2	2	3	3	2	2	2	2
1	1	2	2	2	2	2	2	2	2
3	3	2	2	2	2	2	2	2	2
3	3	2	2	2	2	2	2	2	2
3	3	2	2	3	3	3	3	2	2
1	1	2	2	2	2	2	2	2	2
3	3	2	2	2	2	2	2	2	2
1	1	2	2	3	3	2	2	2	2
1	1	2	2	3	3	2	2	2	2
1	1	2	2	2	2	2	2	2	2
1	1	2	2	2	2	2	2	2	2
1	1	2	2	2	2	2	2	2	2
1	1	2	2	2	2	2	2	2	2
1	1	3	3	2	2	2	2	2	2
3	3	3	3	2	2	2	2	4	4
1	1	2	2	2	2	2	2	2	2
1	1	3	3	2	2	2	2	2	2
3	3	2	2	2	2	2	2	4	4
1	1	3	3	2	2	2	2	2	2
1	1	2	2	3	3	2	2	2	2
1	1	2	2	2	2	2	2	2	2
1	1	2	2	2	2	2	2	2	2
3	3	2	2	2	2	2	2	2	2
3	3	3	3	2	2	2	2	2	2
3	3	2	2	2	2	3	3	2	2
3	3	2	2	2	2	2	2	2	2
1	1	2	2	2	2	2	2	2	2
3	3	2	2	2	2	3	3	2	2
1	1	2	2	3	3	2	2	2	2
3	3	2	2	2	2	2	2	2	2
1	1	2	2	2	2	2	2	2	2
3	3	2	2	2	2	2	2	4	4
1	1	2	2	2	2	2	2	2	2
1	1	2	2	2	2	2	2	2	2
1	1	2	2	2	2	2	2	2	2
1	1	2	2	2	2	2	2	2	2
1	1	2	2	2	2	2	2	4	4
1	1	2	2	2	2	2	2	2	2
1	1	2	2	3	3	2	2	2	2
3	3	2	2	2	2	3	3	2	2
1	1	2	2	3	3	2	2	2	2

For Review Only

3	3	2	2	2	2	2	2	2	2
3	3	2	2	2	2	2	2	2	2
1	1	2	2	2	2	2	2	4	4
1	1	2	2	2	2	2	2	2	2
3	3	2	2	2	2	2	2	2	2
3	3	2	2	2	2	2	2	2	2
1	1	2	2	3	3	3	3	2	2
1	1	2	2	2	2	2	2	2	2
3	3	2	2	2	2	2	2	2	2
1	1	2	2	3	3	2	2	2	2
3	3	2	2	2	2	2	2	4	4
3	3	3	3	2	2	2	2	2	2
1	1	2	2	2	2	2	2	2	2
3	3	2	2	2	2	2	2	2	2
1	1	2	2	2	2	2	2	2	2
1	1	2	2	3	3	2	2	2	2
1	1	2	2	3	3	2	2	2	2
3	3	2	2	3	3	2	2	2	2
3	3	2	2	2	2	2	2	2	2
1	1	2	2	3	3	2	2	2	2
3	3	3	3	2	2	2	2	2	2
3	3	2	2	2	2	2	2	2	2
3	3	3	3	2	2	2	2	2	2
1	1	2	2	3	3	2	2	2	2
1	1	2	2	3	3	2	2	2	2
3	3	2	2	2	2	2	2	2	2
1	1	2	2	3	3	2	2	2	2
3	3	2	2	2	2	3	3	2	2
3	3	2	2	2	2	2	2	2	2
1	1	2	2	2	2	2	2	4	4
1	1	2	2	3	3	2	2	2	2
1	1	2	2	2	2	3	3	2	2
1	1	3	3	2	2	2	2	2	2
3	3	2	2	2	2	2	2	2	2
1	1	3	3	2	2	2	2	2	2
1	1	2	2	3	3	2	2	2	2
3	3	2	2	2	2	2	2	2	2
1	1	2	2	2	2	2	2	2	2
1	1	2	2	2	2	2	2	4	4
1	1	2	2	3	3	2	2	2	2
1	1	2	2	2	2	2	2	2	2
1	1	2	2	2	2	2	2	2	2
1	1	2	2	3	3	2	2	4	4
1	1	2	2	2	2	2	2	2	2
3	3	2	2	2	2	2	2	2	2
1	1	2	2	3	3	2	2	2	2
3	3	2	2	3	3	2	2	2	2
1	1	2	2	2	2	2	2	2	2
3	3	2	2	2	2	3	3	4	4
1	1	2	2	3	3	2	2	2	2
1	1	3	3	2	2	2	2	2	2
1	1	2	2	3	3	2	2	2	2
1	1	2	2	2	2	2	2	2	2
3	3	2	2	2	2	2	2	2	2
1	1	2	2	3	3	2	2	2	2

For Review Only

CSSA 5585 Guilford Rd., Madison, WI 53711

1	1	2	2	2	2	2	2	2	2
3	3	2	2	2	2	2	2	4	4

For Review Only

SNP1031	SNP1031	SNP1032	SNP1032	SNP1033	SNP1033	SNP1034	SNP1034	SNP1035	SNP1035
2	2	4	4	3	3	3	3	2	2
4	4	4	4	4	4	1	1	2	2
2	2	4	4	4	4	1	1	2	2
2	2	4	4	4	4	1	1	2	2
2	2	4	4	4	4	1	1	2	2
4	4	4	4	3	3	3	3	2	2
2	2	4	4	3	3	3	3	2	2
2	2	4	4	4	4	1	1	2	2
2	2	4	4	3	3	3	3	2	2
4	4	4	4	4	4	1	1	3	3
2	2	4	4	4	4	1	1	2	2
2	2	4	4	4	4	1	1	2	2
2	2	4	4	4	4	1	1	2	2
2	2	4	4	4	4	1	1	2	2
2	2	4	4	4	4	1	1	3	3
2	2	4	4	4	4	1	1	2	2
2	2	4	4	4	4	1	1	2	2
2	2	1	1	4	4	1	1	2	2
2	2	4	4	4	4	1	1	2	2
2	2	4	4	4	4	1	1	2	2
2	2	4	4	4	4	1	1	2	2
2	2	4	4	4	4	1	1	2	2
2	2	4	4	3	3	3	3	2	2
2	2	4	4	4	4	1	1	2	2
2	2	4	4	4	4	1	1	3	3
4	4	4	4	4	4	1	1	2	2
2	2	4	4	4	4	1	1	2	2
2	2	4	4	4	4	1	1	2	2
2	2	4	4	4	4	1	1	2	2
2	2	4	4	4	4	1	1	2	2
2	2	4	4	4	4	1	1	2	2
2	2	4	4	3	3	3	3	2	2
2	2	4	4	3	3	3	3	2	2
2	2	4	4	4	4	1	1	2	2
2	2	4	4	4	4	1	1	2	2
2	2	4	4	4	4	1	1	2	2
2	2	4	4	3	3	3	3	2	2
2	2	4	4	4	4	1	1	2	2
2	2	4	4	4	4	1	1	2	2
2	2	4	4	4	4	1	1	2	2
4	4	4	4	4	4	1	1	2	2
2	2	4	4	4	4	1	1	2	2
2	2	1	1	4	4	1	1	2	2
2	2	4	4	4	4	1	1	2	2
2	2	1	1	4	4	1	1	2	2
2	2	4	4	4	4	1	1	2	2
2	2	4	4	4	4	1	1	2	2
2	2	4	4	4	4	1	1	2	2
2	2	4	4	4	4	1	1	2	2
2	2	4	4	4	4	1	1	2	2
2	2	4	4	4	4	1	1	3	3
4	4	4	4	4	4	1	1	2	2

For Review Only

2	2	4	4	4	4	1	1	2	2
2	2	4	4	3	3	3	3	2	2
2	2	4	4	4	4	1	1	3	3
2	2	4	4	4	4	1	1	2	2
2	2	4	4	4	4	1	1	2	2
4	4	4	4	4	4	1	1	2	2
2	2	4	4	4	4	1	1	2	2
2	2	4	4	4	4	1	1	2	2
2	2	4	4	3	3	3	3	2	2
2	2	4	4	4	4	1	1	2	2
2	2	4	4	4	4	1	1	2	2
2	2	4	4	4	4	1	1	2	2
2	2	4	4	4	4	1	1	2	2
2	2	4	4	4	4	1	1	2	2
2	2	4	4	3	3	1	1	2	2
4	4	4	4	3	3	3	3	2	2
4	4	4	4	4	4	1	1	2	2
2	2	4	4	4	4	3	3	3	3
2	2	1	1	4	4	1	1	3	3
2	2	4	4	3	3	3	3	2	2
2	2	4	4	4	4	1	1	2	2
2	2	4	4	4	4	1	1	2	2
2	2	4	4	4	4	1	1	2	2
2	2	4	4	4	4	1	1	2	2
2	2	1	1	4	4	1	1	2	2
2	2	4	4	4	4	1	1	2	2
2	2	4	4	3	3	3	3	2	2
2	2	4	4	4	4	1	1	2	2
4	4	4	4	4	4	1	1	2	2
2	2	4	4	4	4	1	1	3	3
2	2	4	4	4	4	1	1	2	2
2	2	4	4	4	4	1	1	2	2
2	2	4	4	4	4	1	1	2	2
2	2	4	4	4	4	1	1	2	2
2	2	4	4	4	4	1	1	2	2
2	2	1	1	4	4	1	1	2	2
2	2	4	4	4	4	1	1	2	2
2	2	4	4	4	4	1	1	2	2
2	2	4	4	4	4	1	1	2	2
2	2	4	4	4	4	1	1	2	2
2	2	4	4	4	4	1	1	2	2
4	4	4	4	4	4	1	1	2	2
2	2	1	1	4	4	1	1	3	3
2	2	4	4	4	4	1	1	2	2
2	2	4	4	3	3	3	3	2	2
2	2	4	4	4	4	1	1	2	2
2	2	4	4	4	4	1	1	2	2
2	2	4	4	4	4	1	1	2	2
2	2	4	4	4	4	1	1	2	2
2	2	4	4	4	4	1	1	2	2
4	4	4	4	4	4	1	1	2	2
2	2	4	4	4	4	1	1	2	2
2	2	4	4	4	4	1	1	2	2
2	2	4	4	4	4	1	1	2	2
2	2	1	1	3	3	3	3	2	2

For Review Only

2	2	4	4	4	4	1	1	2	2
2	2	4	4	4	4	1	1	2	2
2	2	4	4	4	4	1	1	2	2
2	2	4	4	4	4	1	1	2	2
2	2	4	4	4	4	1	1	2	2
4	4	4	4	4	4	1	1	2	2
2	2	4	4	4	4	1	1	2	2
2	2	4	4	4	4	1	1	3	3
2	2	4	4	4	4	1	1	2	2
2	2	4	4	4	4	1	1	2	2
2	2	4	4	4	4	1	1	2	2
2	2	4	4	4	4	1	1	2	2
2	2	1	1	4	4	1	1	3	3
4	4	4	4	4	4	1	1	2	2
2	2	4	4	4	4	1	1	2	2
2	2	4	4	4	4	1	1	2	2
2	2	1	1	3	3	3	3	2	2
2	2	4	4	4	4	1	1	2	2
2	2	4	4	4	4	1	1	2	2
2	2	1	1	3	3	3	3	2	2
2	2	4	4	4	4	1	1	2	2
2	2	4	4	4	4	1	1	3	3
2	2	4	4	4	4	1	1	2	2
2	2	4	4	4	4	1	1	2	2
2	2	4	4	4	4	1	1	2	2
2	2	4	4	4	4	1	1	2	2
2	2	4	4	3	3	3	3	2	2
2	2	4	4	4	4	1	1	2	2
2	2	4	4	4	4	1	1	3	3
4	4	4	4	4	4	1	1	2	2
2	2	4	4	4	4	1	1	2	2
2	2	4	4	4	4	1	1	2	2
2	2	4	4	4	4	1	1	2	2
4	4	4	4	4	4	1	1	3	3
2	2	4	4	4	4	1	1	2	2
2	2	4	4	4	4	3	3	2	2
2	2	4	4	4	4	1	1	2	2
2	2	4	4	4	4	1	1	2	2
2	2	4	4	4	4	1	1	2	2
2	2	4	4	4	4	1	1	2	2
2	2	4	4	4	4	1	1	2	2
2	2	4	4	4	4	1	1	2	2
2	2	4	4	4	4	1	1	2	2
2	2	4	4	4	4	1	1	2	2
2	2	4	4	4	4	3	3	2	2
2	2	4	4	4	4	1	1	2	2
4	4	4	4	4	4	1	1	2	2
2	2	4	4	4	4	1	1	2	2
2	2	4	4	4	4	1	1	2	2
4	4	4	4	4	4	1	1	2	2
4	4	4	4	4	4	1	1	2	2
2	2	4	4	4	4	1	1	2	2
2	2	4	4	4	4	1	1	2	2
2	2	4	4	4	4	1	1	2	2
2	2	4	4	4	4	1	1	2	2

For Review Only

2	2	4	4	4	4	1	1	2	2
2	2	4	4	4	4	1	1	2	2
2	2	4	4	4	4	1	1	2	2
2	2	4	4	4	4	1	1	2	2
2	2	4	4	4	4	1	1	2	2
2	2	4	4	4	4	1	1	3	3
2	2	4	4	4	4	1	1	2	2
2	2	4	4	4	4	1	1	2	2
2	2	4	4	4	4	1	1	2	2
2	2	4	4	4	4	1	1	3	3
2	2	4	4	4	4	1	1	2	2
2	2	4	4	4	4	1	1	2	2
2	2	4	4	4	4	1	1	2	2
2	2	4	4	4	4	1	1	2	2
2	2	4	4	4	4	1	1	2	2
2	2	4	4	4	4	1	1	2	2
2	2	4	4	4	4	1	1	2	2
4	4	4	4	4	4	1	1	3	3
2	2	4	4	4	4	1	1	2	2
2	2	4	4	4	4	1	1	2	2
2	2	4	4	4	4	1	1	2	2
2	2	4	4	4	4	1	1	2	2
2	2	4	4	4	4	1	1	2	2
2	2	4	4	4	4	1	1	2	2
2	2	4	4	4	4	1	1	2	2
2	2	4	4	4	4	1	1	2	2
2	2	4	4	4	4	1	1	3	3
2	2	4	4	4	4	1	1	3	3
2	2	4	4	3	3	3	3	3	3
2	2	1	1	4	4	1	1	2	2
4	4	4	4	3	3	3	3	2	2
2	2	4	4	4	4	1	1	3	3
2	2	4	4	3	3	3	3	2	2
2	2	4	4	4	4	1	1	2	2
2	2	4	4	4	4	1	1	2	2
2	2	4	4	4	4	1	1	2	2
4	4	4	4	4	4	1	1	2	2
2	2	4	4	3	3	3	3	2	2
2	2	4	4	4	4	1	1	2	2
2	2	4	4	4	4	1	1	2	2
2	2	4	4	4	4	1	1	2	2
4	4	4	4	4	4	1	1	2	2
2	2	4	4	4	4	1	1	2	2
2	2	4	4	4	4	1	1	2	2
2	2	4	4	4	4	1	1	2	2
2	2	4	4	4	4	1	1	3	3
2	2	4	4	4	4	1	1	2	2
2	2	4	4	4	4	1	1	2	2
2	2	4	4	4	4	1	1	2	2
2	2	4	4	4	4	1	1	2	2
2	2	4	4	4	4	1	1	3	3
2	2	4	4	3	3	3	3	2	2
2	2	4	4	4	4	1	1	2	2
2	2	4	4	4	4	1	1	2	2
2	2	4	4	4	4	1	1	2	2

For Review Only

2	2	4	4	4	4	1	1	2	2
2	2	4	4	4	4	1	1	2	2
2	2	4	4	4	4	1	1	3	3
2	2	4	4	4	4	1	1	2	2
2	2	1	1	4	4	1	1	2	2
4	4	4	4	4	4	1	1	2	2
2	2	4	4	4	4	1	1	2	2
2	2	4	4	4	4	1	1	2	2
2	2	4	4	4	4	1	1	2	2
2	2	4	4	4	4	1	1	2	2
2	2	4	4	4	4	1	1	2	2
2	2	4	4	3	3	3	3	2	2
2	2	4	4	4	4	1	1	2	2
2	2	4	4	4	4	1	1	2	2
2	2	4	4	4	4	1	1	2	2
2	2	4	4	4	4	1	1	2	2
2	2	4	4	4	4	1	1	2	2
2	2	4	4	4	4	1	1	2	2
4	4	4	4	4	4	1	1	2	2
2	2	4	4	4	4	1	1	2	2
2	2	4	4	3	3	3	3	2	2
4	4	4	4	4	4	1	1	2	2
2	2	4	4	3	3	3	3	2	2
2	2	4	4	4	4	1	1	2	2
2	2	4	4	4	4	1	1	2	2
2	2	4	4	4	4	1	1	2	2
2	2	4	4	4	4	1	1	2	2
2	2	4	4	4	4	1	1	2	2
2	2	4	4	4	4	1	1	2	2
2	2	4	4	4	4	1	1	3	3
2	2	4	4	4	4	1	1	2	2
2	2	4	4	4	4	1	1	2	2
2	2	4	4	3	3	3	3	2	2
2	2	4	4	4	4	1	1	2	2
2	2	4	4	4	4	1	1	2	2
2	2	4	4	4	4	1	1	2	2
2	2	4	4	4	4	1	1	2	2
2	2	4	4	4	4	1	1	2	2
2	2	4	4	4	4	1	1	2	2
2	2	4	4	4	4	1	1	2	2
2	2	4	4	4	4	1	1	2	2
2	2	4	4	4	4	1	1	2	2
2	2	4	4	4	4	1	1	3	3
2	2	4	4	4	4	1	1	2	2
2	2	4	4	4	4	1	1	2	2
2	2	4	4	4	4	1	1	2	2
2	2	4	4	4	4	1	1	2	2
2	2	1	1	4	4	1	1	3	3
2	2	4	4	4	4	1	1	2	2
2	2	4	4	4	4	1	1	2	2
2	2	4	4	4	4	1	1	2	2
2	2	4	4	4	4	1	1	2	2
2	2	4	4	4	4	1	1	2	2
2	2	4	4	4	4	1	1	2	2
2	2	4	4	4	4	1	1	2	2

For Review Only

2	2	4	4	4	4	1	1	2	2
2	2	4	4	4	4	1	1	2	2

For Review Only

SNP1036	SNP1036	SNP1037	SNP1037	SNP1038	SNP1038	SNP1039	SNP1039	SNP104	SNP104
1	1	1	1	2	2	2	2	1	1
4	4	1	1	2	2	2	2	1	1
4	4	4	4	2	2	2	2	1	1
4	4	4	4	2	2	2	2	1	1
4	4	1	1	2	2	2	2	1	1
1	1	1	1	2	2	2	2	3	3
4	4	1	1	2	2	2	2	1	1
1	1	1	1	3	3	4	4	1	1
4	4	4	4	2	2	2	2	1	1
4	4	4	4	3	3	4	4	1	1
4	4	4	4	2	2	2	2	1	1
4	4	4	4	2	2	2	2	1	1
4	4	1	1	3	3	4	4	1	1
4	4	4	4	2	2	2	2	1	1
4	4	4	4	2	2	2	2	1	1
4	4	4	4	2	2	2	2	3	3
4	4	1	1	2	2	2	2	1	1
4	4	1	1	2	2	2	2	1	1
4	4	1	1	3	3	4	4	3	3
4	4	1	1	2	2	2	2	1	1
4	4	1	1	3	3	4	4	1	1
4	4	1	1	2	2	2	2	1	1
1	1	1	1	3	3	4	4	1	1
4	4	4	4	3	3	4	4	1	1
4	4	1	1	3	3	4	4	1	1
4	4	1	1	3	3	4	4	3	3
4	4	1	1	2	2	2	2	1	1
4	4	4	4	3	3	4	4	1	1
4	4	4	4	3	3	4	4	1	1
4	4	4	4	3	3	4	4	1	1
4	4	1	1	3	3	4	4	1	1
4	4	4	4	2	2	2	2	3	3
1	1	4	4	2	2	2	2	1	1
4	4	4	4	3	3	4	4	1	1
4	4	4	4	2	2	2	2	1	1
4	4	4	4	3	3	4	4	1	1
1	1	4	4	2	2	2	2	1	1
4	4	4	4	2	2	2	2	1	1
4	4	1	1	3	3	4	4	1	1
4	4	4	4	2	2	2	2	1	1
4	4	4	4	3	3	4	4	1	1
4	4	4	4	3	3	4	4	1	1
4	4	4	4	3	3	4	4	1	1
4	4	1	1	2	2	2	2	1	1
4	4	4	4	3	3	4	4	1	1
4	4	4	4	2	2	2	2	1	1
4	4	1	1	2	2	2	2	1	1
4	4	1	1	3	3	4	4	1	1
4	4	4	4	2	2	2	2	1	1
4	4	4	4	2	2	2	2	1	1
4	4	1	1	3	3	4	4	3	3
4	4	1	1	2	2	4	4	1	1

For Review Only

4	4	1	1	2	2	2	2	1	1
1	1	4	4	2	2	2	2	1	1
4	4	1	1	2	2	2	2	1	1
4	4	4	4	2	2	2	2	1	1
4	4	1	1	3	3	4	4	3	3
4	4	1	1	2	2	4	4	1	1
4	4	1	1	2	2	2	2	3	3
4	4	4	4	3	3	4	4	1	1
1	1	4	4	3	3	4	4	1	1
4	4	4	4	3	3	4	4	1	1
4	4	1	1	2	2	2	2	1	1
4	4	4	4	2	2	2	2	1	1
4	4	1	1	2	2	2	2	1	1
4	4	4	4	3	3	4	4	1	1
1	1	4	4	3	3	4	4	1	1
4	4	1	1	2	2	2	2	1	1
4	4	1	1	2	2	2	2	1	1
4	4	4	4	2	2	2	2	1	1
4	4	1	1	2	2	2	2	1	1
4	4	1	1	2	2	2	2	1	1
4	4	4	4	3	3	2	2	1	1
4	4	4	4	2	2	2	2	1	1
4	4	1	1	3	3	4	4	1	1
1	1	4	4	3	3	4	4	1	1
4	4	4	4	3	3	4	4	1	1
4	4	1	1	3	3	4	4	1	1
4	4	4	4	2	2	2	2	1	1
4	4	1	1	3	3	4	4	1	1
4	4	1	1	3	3	4	4	3	3
4	4	1	1	3	3	4	4	3	3
4	4	1	1	3	3	4	4	1	1
4	4	4	4	3	3	4	4	1	1
4	4	1	1	3	3	4	4	1	1
4	4	1	1	2	2	2	2	1	1
1	1	4	4	2	2	2	2	1	1
4	4	4	4	3	3	4	4	1	1
4	4	1	1	3	3	4	4	1	1
4	4	1	1	3	3	2	2	1	1
4	4	4	4	3	3	4	4	1	1
4	4	4	4	3	3	4	4	1	1
4	4	1	1	2	2	2	2	1	1
4	4	1	1	2	2	2	2	1	1
4	4	4	4	3	3	4	4	1	1
4	4	1	1	2	2	2	2	1	1
1	1	4	4	3	3	4	4	1	1
4	4	4	4	2	2	2	2	1	1
4	4	4	4	3	3	4	4	1	1
4	4	4	4	3	3	4	4	3	3
4	4	1	1	2	2	2	2	1	1
4	4	4	4	2	2	2	2	1	1
4	4	1	1	2	2	2	2	1	1
4	4	1	1	3	3	4	4	1	1
4	4	4	4	3	3	4	4	1	1
4	4	4	4	3	3	4	4	1	1
1	1	4	4	2	2	2	2	1	1

For Review Only

4	4	1	1	2	2	4	4	1	1
4	4	4	4	2	2	2	2	1	1
4	4	1	1	3	3	4	4	1	1
4	4	4	4	3	3	4	4	1	1
4	4	4	4	2	2	2	2	1	1
4	4	4	4	3	3	4	4	1	1
4	4	4	4	2	2	2	2	3	3
4	4	1	1	3	3	4	4	1	1
4	4	1	1	3	3	4	4	1	1
4	4	4	4	3	3	4	4	1	1
4	4	4	4	2	2	2	2	1	1
4	4	4	4	3	3	2	2	1	1
4	4	4	4	3	3	4	4	1	1
4	4	1	1	3	3	2	2	1	1
4	4	1	1	3	3	4	4	1	1
4	4	1	1	3	3	4	4	1	1
4	4	4	4	2	2	2	2	1	1
4	4	1	1	3	3	4	4	1	1
4	4	4	4	3	3	4	4	1	1
1	1	1	1	3	3	4	4	1	1
4	4	4	4	3	3	4	4	1	1
4	4	1	1	3	3	4	4	1	1
4	4	1	1	3	3	4	4	3	3
4	4	1	1	3	3	4	4	1	1
4	4	4	4	3	3	4	4	1	1
4	4	4	4	2	2	2	2	1	1
1	1	4	4	2	2	2	2	1	1
4	4	1	1	3	3	4	4	1	1
4	4	4	4	2	2	2	2	1	1
4	4	4	4	3	3	4	4	1	1
4	4	4	4	3	3	4	4	1	1
4	4	4	4	3	3	4	4	1	1
4	4	1	1	2	2	2	2	1	1
4	4	1	1	3	3	4	4	1	1
4	4	4	4	3	3	4	4	1	1
4	4	4	4	3	3	4	4	1	1
4	4	4	4	3	3	4	4	1	1
4	4	1	1	2	2	4	4	3	3
4	4	1	1	2	2	2	2	1	1
4	4	1	1	3	3	2	2	1	1
4	4	4	4	2	2	2	2	1	1
4	4	4	4	3	3	4	4	1	1
4	4	1	1	3	3	4	4	1	1
4	4	4	4	3	3	4	4	1	1
1	1	4	4	3	3	4	4	1	1
4	4	4	4	3	3	4	4	1	1
4	4	1	1	3	3	4	4	3	3
4	4	1	1	2	2	2	2	3	3
4	4	4	4	3	3	4	4	1	1
4	4	1	1	3	3	4	4	3	3
4	4	1	1	3	3	4	4	1	1
4	4	4	4	2	2	2	2	1	1
4	4	1	1	3	3	4	4	1	1
4	4	4	4	2	2	2	2	3	3
4	4	4	4	3	3	4	4	1	1

For Review Only

4	4	4	4	2	2	2	2	1	1
4	4	4	4	3	3	4	4	1	1
4	4	4	4	2	2	2	2	1	1
4	4	4	4	2	2	2	2	1	1
4	4	1	1	2	2	2	2	1	1
4	4	4	4	2	2	2	2	1	1
4	4	4	4	2	2	4	4	1	1
4	4	1	1	3	3	4	4	1	1
4	4	1	1	3	3	4	4	1	1
4	4	1	1	2	2	4	4	1	1
4	4	1	1	3	3	2	2	1	1
4	4	4	4	3	3	2	2	1	1
4	4	1	1	2	2	2	2	1	1
4	4	1	1	3	3	4	4	1	1
4	4	4	4	3	3	4	4	1	1
4	4	4	4	2	2	2	2	1	1
4	4	4	4	3	3	4	4	1	1
4	4	1	1	3	3	2	2	1	1
4	4	1	1	3	3	4	4	3	3
4	4	4	4	2	2	2	2	1	1
4	4	4	4	2	2	4	4	1	1
4	4	1	1	3	3	4	4	1	1
4	4	4	4	2	2	2	2	1	1
4	4	4	4	2	2	2	2	1	1
4	4	4	4	3	3	4	4	1	1
4	4	4	4	3	3	4	4	1	1
4	4	4	4	2	2	2	2	1	1
4	4	4	4	2	2	4	4	1	1
4	4	1	1	2	2	2	2	1	1
4	4	1	1	2	2	2	2	1	1
1	1	4	4	2	2	2	2	1	1
4	4	1	1	2	2	2	2	1	1
1	1	4	4	2	2	2	2	1	1
4	4	4	4	3	3	4	4	1	1
4	4	4	4	3	3	4	4	1	1
4	4	4	4	2	2	2	2	1	1
4	4	1	1	2	2	2	2	1	1
1	1	1	1	2	2	2	2	1	1
4	4	1	1	2	2	2	2	3	3
4	4	1	1	3	3	4	4	1	1
4	4	4	4	2	2	2	2	1	1
4	4	1	1	3	3	4	4	3	3
4	4	1	1	3	3	4	4	1	1
4	4	1	1	2	2	4	4	1	1
4	4	4	4	2	2	2	2	1	1
4	4	4	4	3	3	4	4	1	1
4	4	4	4	2	2	2	2	1	1
4	4	4	4	3	3	4	4	1	1
4	4	4	4	3	3	4	4	1	1
4	4	4	4	2	2	2	2	1	1
4	4	4	4	2	2	2	2	1	1
1	1	4	4	3	3	4	4	1	1
4	4	4	4	3	3	4	4	1	1
4	4	1	1	2	2	2	2	1	1
4	4	1	1	3	3	4	4	1	1

For Review Only

4	4	1	1	3	3	4	4	1	1
4	4	4	4	2	2	4	4	1	1
4	4	4	4	2	2	2	2	3	3
4	4	4	4	3	3	4	4	1	1
1	1	1	1	2	2	2	2	1	1
4	4	1	1	3	3	4	4	1	1
1	1	4	4	2	2	2	2	3	3
4	4	4	4	3	3	4	4	1	1
4	4	4	4	3	3	4	4	1	1
4	4	1	1	3	3	4	4	1	1
4	4	4	4	3	3	4	4	1	1
1	1	4	4	3	3	4	4	1	1
4	4	4	4	2	2	2	2	1	1
4	4	1	1	3	3	4	4	1	1
4	4	4	4	2	2	2	2	1	1
4	4	4	4	2	2	2	2	1	1
1	1	1	1	3	3	4	4	1	1
4	4	1	1	3	3	4	4	1	1
4	4	1	1	2	2	2	2	1	1
4	4	4	4	2	2	2	2	1	1
1	1	1	1	3	3	4	4	1	1
4	4	1	1	3	3	4	4	1	1
1	1	1	1	3	3	4	4	1	1
4	4	4	4	2	2	2	2	1	1
4	4	4	4	3	3	4	4	1	1
4	4	4	4	3	3	4	4	1	1
4	4	1	1	3	3	4	4	1	1
4	4	4	4	2	2	2	2	3	3
4	4	1	1	2	2	2	2	1	1
4	4	4	4	2	2	2	2	1	1
4	4	1	1	3	3	4	4	1	1
4	4	4	4	3	3	4	4	3	3
1	1	1	1	3	3	4	4	1	1
4	4	1	1	3	3	4	4	1	1
4	4	4	4	2	2	2	2	1	1
4	4	1	1	3	3	4	4	1	1
4	4	1	1	3	3	4	4	1	1
4	4	4	4	2	2	2	2	1	1
4	4	4	4	3	3	4	4	1	1
1	1	1	1	3	3	4	4	1	1
1	1	4	4	3	3	4	4	1	1
4	4	4	4	2	2	2	2	1	1
4	4	4	4	2	2	2	2	1	1
4	4	1	1	2	2	2	2	1	1
4	4	1	1	3	3	4	4	1	1
4	4	4	4	3	3	4	4	1	1
4	4	1	1	2	2	2	2	1	1
4	4	4	4	3	3	4	4	1	1
4	4	1	1	3	3	4	4	3	3
4	4	1	1	3	3	4	4	1	1
4	4	4	4	2	2	4	4	1	1
1	1	1	1	3	3	4	4	1	1
4	4	4	4	2	2	2	2	1	1
4	4	1	1	2	2	2	2	1	1
4	4	4	4	2	2	2	2	1	1

For Review Only

4	4	4	4	2	2	2	2	1	1
4	4	1	1	3	3	4	4	1	1

For Review Only

SNP1040	SNP1040	SNP1041	SNP1041	SNP1042	SNP1042	SNP1043	SNP1043	SNP1044	SNP1044
2	2	1	1	3	3	4	4	3	3
2	2	1	1	2	2	1	1	1	1
2	2	3	3	3	3	1	1	1	1
2	2	1	1	3	3	1	1	3	3
2	2	3	3	3	3	4	4	3	3
2	2	3	3	3	3	1	1	1	1
2	2	3	3	3	3	4	4	3	3
2	2	1	1	3	3	1	1	1	1
2	2	1	1	2	2	4	4	3	3
2	2	1	1	3	3	4	4	1	1
2	2	3	3	3	3	4	4	3	3
2	2	3	3	3	3	1	1	1	1
2	2	3	3	3	3	4	4	3	3
1	1	1	1	2	2	1	1	1	1
2	2	3	3	3	3	4	4	3	3
2	2	1	1	3	3	4	4	3	3
2	2	3	3	3	3	4	4	3	3
2	2	3	3	3	3	1	1	1	1
2	2	3	3	3	3	1	1	1	1
2	2	1	1	3	3	4	4	3	3
2	2	3	3	3	3	4	4	3	3
2	2	3	3	3	3	4	4	3	3
2	2	1	1	2	2	1	1	1	1
2	2	3	3	3	3	4	4	3	3
2	2	3	3	3	3	1	1	1	1
2	2	3	3	3	3	1	1	1	1
2	2	3	3	3	3	1	1	1	1
2	2	3	3	2	2	1	1	1	1
2	2	3	3	3	3	4	4	3	3
2	2	1	1	3	3	1	1	1	1
2	2	1	1	3	3	4	4	3	3
2	2	3	3	3	3	1	1	1	1
2	2	3	3	3	3	1	1	1	1
2	2	3	3	3	3	4	4	3	3
2	2	1	1	2	2	1	1	1	1
2	2	3	3	3	3	4	4	3	3
2	2	3	3	3	3	4	4	3	3
2	2	1	1	2	2	1	1	1	1
2	2	1	1	2	2	1	1	1	1
2	2	1	1	2	2	1	1	1	1
2	2	1	1	3	3	1	1	1	1
2	2	3	3	3	3	1	1	1	1
2	2	3	3	3	3	1	1	1	1
2	2	3	3	3	3	1	1	1	1
2	2	1	1	3	3	1	1	1	1
2	2	1	1	3	3	4	4	3	3
2	2	3	3	3	3	1	1	1	1
2	2	1	1	2	2	1	1	1	1
2	2	3	3	3	3	4	4	3	3
2	2	3	3	3	3	4	4	3	3
2	2	3	3	3	3	1	1	1	1
1	1	1	1	2	2	1	1	1	1

For Review Only

2	2	3	3	3	3	4	4	3	3
2	2	3	3	3	3	1	1	1	1
2	2	3	3	3	3	1	1	1	1
2	2	1	1	3	3	1	1	1	1
2	2	3	3	3	3	1	1	1	1
2	2	3	3	3	3	1	1	1	1
2	2	1	1	3	3	4	4	3	3
2	2	1	1	2	2	1	1	1	1
2	2	3	3	3	3	4	4	3	3
2	2	3	3	3	3	1	1	1	1
2	2	3	3	3	3	1	1	1	1
2	2	3	3	3	3	1	1	1	1
2	2	3	3	3	3	4	4	3	3
2	2	3	3	3	3	4	4	3	3
2	2	3	3	3	3	1	1	1	1
2	2	3	3	3	3	4	4	3	3
2	2	3	3	3	3	1	1	1	1
2	2	1	1	3	3	4	4	3	3
2	2	3	3	3	3	4	4	3	3
2	2	1	1	2	2	4	4	3	3
2	2	3	3	3	3	1	1	1	1
2	2	1	1	2	2	1	1	1	1
2	2	1	1	3	3	4	4	3	3
2	2	3	3	3	3	1	1	1	1
2	2	1	1	3	3	1	1	1	1
2	2	1	1	3	3	4	4	3	3
2	2	3	3	3	3	1	1	1	1
2	2	1	1	2	2	1	1	1	1
2	2	3	3	3	3	1	1	1	1
2	2	3	3	3	3	1	1	1	1
2	2	1	1	3	3	1	1	1	1
2	2	3	3	2	2	1	1	1	1
2	2	1	1	2	2	1	1	1	1
2	2	3	3	3	3	4	4	3	3
2	2	1	1	2	2	1	1	1	1
2	2	1	1	3	3	1	1	1	1
2	2	3	3	3	3	4	4	3	3
2	2	1	1	3	3	4	4	1	1
2	2	3	3	3	3	1	1	1	1
2	2	1	1	3	3	1	1	1	1
2	2	3	3	3	3	4	4	1	1
2	2	3	3	3	3	1	1	1	1
2	2	1	1	3	3	4	4	3	3
2	2	3	3	3	3	1	1	1	1
2	2	3	3	3	3	4	4	3	3
2	2	3	3	3	3	1	1	1	1
1	1	1	1	2	2	1	1	1	1
2	2	1	1	3	3	4	4	3	3
2	2	3	3	3	3	4	4	3	3
2	2	3	3	2	2	1	1	1	1
2	2	3	3	3	3	1	1	1	1
2	2	1	1	2	2	1	1	1	1
2	2	1	1	3	3	4	4	3	3
2	2	1	1	3	3	4	4	3	3
2	2	3	3	3	3	1	1	1	1

For Review Only

2	2	1	1	2	2	1	1	1	1
2	2	3	3	3	3	4	4	3	3
2	2	3	3	3	3	4	4	3	3
1	1	1	1	2	2	1	1	1	1
2	2	3	3	3	3	1	1	1	1
2	2	3	3	3	3	1	1	1	1
2	2	1	1	3	3	1	1	1	1
2	2	3	3	3	3	1	1	1	1
2	2	3	3	3	3	4	4	3	3
2	2	3	3	3	3	1	1	1	1
2	2	1	1	3	3	4	4	3	3
2	2	3	3	2	2	1	1	1	1
2	2	1	1	3	3	4	4	3	3
2	2	3	3	3	3	1	1	1	1
2	2	3	3	2	2	1	1	1	1
2	2	3	3	3	3	1	1	1	1
2	2	3	3	3	3	1	1	1	1
2	2	3	3	2	2	1	1	1	1
1	1	1	1	2	2	1	1	1	1
2	2	1	1	3	3	4	4	3	3
2	2	3	3	3	3	1	1	1	1
2	2	3	3	3	3	1	1	1	1
2	2	3	3	3	3	1	1	1	1
2	2	1	1	2	2	1	1	1	1
2	2	3	3	2	2	1	1	1	1
2	2	3	3	3	3	1	1	1	1
2	2	1	1	3	3	4	4	3	3
2	2	3	3	3	3	4	4	3	3
2	2	3	3	3	3	1	1	1	1
2	2	3	3	3	3	4	4	3	3
1	1	1	1	2	2	1	1	1	1
2	2	3	3	2	2	1	1	1	1
2	2	3	3	3	3	4	4	3	3
2	2	3	3	2	2	1	1	1	1
2	2	3	3	3	3	4	4	3	3
2	2	3	3	3	3	1	1	1	1
1	1	1	1	3	3	4	4	3	3
2	2	3	3	3	3	1	1	1	1
2	2	3	3	2	2	1	1	1	1
1	1	1	1	2	2	1	1	1	1
1	1	1	1	2	2	1	1	1	1
2	2	3	3	3	3	4	4	3	3
2	2	1	1	2	2	1	1	1	1
2	2	3	3	3	3	4	4	1	1
2	2	3	3	3	3	4	4	3	3
2	2	1	1	2	2	1	1	1	1
2	2	3	3	3	3	1	1	1	1
2	2	3	3	3	3	1	1	1	1
2	2	1	1	2	2	1	1	1	1
2	2	3	3	3	3	1	1	1	1
2	2	3	3	3	3	1	1	1	1
2	2	3	3	3	3	1	1	1	1
2	2	3	3	3	3	4	4	3	3
2	2	3	3	3	3	1	1	1	1
2	2	3	3	2	2	1	1	1	1

For Review Only

2	2	3	3	3	3	1	1	1	1
1	1	1	1	2	2	1	1	1	1
2	2	3	3	3	3	4	4	3	3
2	2	3	3	2	2	1	1	1	1
2	2	3	3	3	3	1	1	1	1
2	2	1	1	2	2	1	1	1	1
2	2	3	3	2	2	1	1	1	1
2	2	3	3	3	3	1	1	1	1
1	1	3	3	2	2	1	1	1	1
2	2	3	3	3	3	1	1	1	1
2	2	3	3	3	3	1	1	1	1
2	2	1	1	2	2	1	1	1	1
2	2	3	3	3	3	1	1	1	1
1	1	1	1	2	2	1	1	1	1
1	1	1	1	2	2	1	1	1	1
1	1	1	1	2	2	1	1	1	1
2	2	1	1	3	3	1	1	1	1
2	2	3	3	3	3	1	1	1	1
2	2	1	1	2	2	1	1	1	1
2	2	1	1	3	3	1	1	1	1
2	2	3	3	3	3	1	1	3	3
2	2	1	1	2	2	1	1	1	1
2	2	1	1	2	2	1	1	1	1
2	2	3	3	3	3	1	1	1	1
2	2	3	3	3	3	4	4	3	3
2	2	3	3	3	3	4	4	3	3
2	2	3	3	3	3	1	1	1	1
2	2	3	3	2	2	1	1	1	1
2	2	1	1	3	3	4	4	3	3
2	2	3	3	3	3	1	1	1	1
2	2	3	3	3	3	1	1	1	1
2	2	3	3	3	3	1	1	1	1
2	2	3	3	3	3	4	4	3	3
1	1	1	1	2	2	1	1	1	1
2	2	3	3	3	3	4	4	3	3
2	2	3	3	3	3	1	1	1	1
2	2	3	3	3	3	4	4	3	3
2	2	3	3	3	3	4	4	3	3
2	2	3	3	3	3	1	1	1	1
2	2	3	3	3	3	1	1	1	1
2	2	3	3	3	3	1	1	1	1
2	2	3	3	3	3	1	1	1	1
2	2	1	1	2	2	1	1	1	1
2	2	3	3	3	3	1	1	1	1
2	2	1	1	3	3	4	4	3	3
2	2	1	1	3	3	1	1	3	3
1	1	1	1	2	2	1	1	1	1
2	2	3	3	3	3	4	4	3	3
2	2	3	3	3	3	4	4	3	3
1	1	1	1	2	2	1	1	1	1
2	2	3	3	3	3	1	1	1	1
2	2	1	1	3	3	4	4	3	3
2	2	1	1	2	2	1	1	1	1
2	2	3	3	3	3	1	1	1	1
2	2	1	1	2	2	1	1	1	1

For Review Only

2	2	1	1	2	2	1	1	1	1
2	2	3	3	3	3	4	4	3	3
2	2	3	3	3	3	1	1	1	1
2	2	1	1	2	2	1	1	1	1
2	2	3	3	3	3	1	1	1	1
2	2	3	3	3	3	4	4	3	3
2	2	1	1	2	2	1	1	1	1
2	2	1	1	3	3	1	1	1	1
2	2	3	3	3	3	4	4	3	3
2	2	1	1	2	2	1	1	1	1
2	2	3	3	2	2	1	1	1	1
2	2	3	3	3	3	1	1	1	1
2	2	3	3	3	3	1	1	1	1
2	2	3	3	3	3	1	1	1	1
2	2	3	3	3	3	1	1	1	1
2	2	1	1	2	2	1	1	1	1
2	2	1	1	2	2	1	1	1	1
2	2	3	3	2	2	1	1	1	1
2	2	1	1	2	2	1	1	1	1
2	2	1	1	2	2	1	1	1	1
2	2	3	3	3	3	1	1	1	1
2	2	3	3	3	3	1	1	1	1
2	2	3	3	3	3	4	4	3	3
2	2	1	1	2	2	1	1	1	1
2	2	1	1	2	2	1	1	1	1
2	2	3	3	3	3	4	4	3	3
2	2	1	1	2	2	1	1	1	1
2	2	3	3	3	3	1	1	1	1
2	2	3	3	3	3	4	4	3	3
2	2	3	3	3	3	1	1	1	1
2	2	1	1	2	2	1	1	1	1
2	2	3	3	3	3	1	1	1	1
1	1	1	1	2	2	1	1	1	1
2	2	3	3	3	3	1	1	1	1
1	1	1	1	2	2	1	1	1	1
2	2	1	1	2	2	1	1	1	1
2	2	3	3	3	3	4	4	3	3
2	2	3	3	3	3	1	1	1	1
2	2	3	3	3	3	4	4	3	3
2	2	1	1	2	2	1	1	1	1
2	2	3	3	3	3	1	1	1	1
2	2	3	3	3	3	4	4	3	3
2	2	1	1	2	2	1	1	1	1
2	2	3	3	3	3	4	4	3	3
2	2	3	3	3	3	1	1	1	1
2	2	1	1	2	2	1	1	1	1
2	2	1	1	2	2	1	1	1	1
2	2	3	3	3	3	4	4	3	3
2	2	1	1	3	3	1	1	1	1
2	2	1	1	2	2	1	1	1	1
2	2	3	3	3	3	4	4	3	3
1	1	1	1	2	2	1	1	1	1
2	2	3	3	3	3	1	1	1	1
2	2	3	3	3	3	1	1	1	1
2	2	1	1	2	2	1	1	1	1

For Review Only

CSSA 5585 Guilford Rd., Madison, WI 53711

2	2	3	3	3	3	4	4	3	3
2	2	3	3	3	3	1	1	1	1

For Review Only

SNP1045	SNP1045	SNP1046	SNP1046	SNP1047	SNP1047	SNP1048	SNP1048	SNP1049	SNP1049
4	4	3	3	1	1	2	2	4	4
4	4	1	1	1	1	2	2	4	4
4	4	1	1	1	1	2	2	4	4
4	4	1	1	1	1	2	2	4	4
4	4	1	1	1	1	2	2	4	4
4	4	1	1	4	4	2	2	4	4
4	4	1	1	4	4	2	2	4	4
4	4	1	1	1	1	2	2	4	4
4	4	3	3	1	1	2	2	4	4
4	4	1	1	1	1	2	2	4	4
4	4	1	1	1	1	2	2	4	4
4	4	1	1	1	1	2	2	4	4
4	4	1	1	1	1	2	2	4	4
4	4	1	1	1	1	2	2	4	4
4	4	1	1	1	1	2	2	4	4
4	4	1	1	1	1	2	2	4	4
4	4	1	1	1	1	2	2	4	4
4	4	1	1	1	1	2	2	4	4
4	4	1	1	1	1	2	2	4	4
4	4	1	1	1	1	2	2	4	4
4	4	1	1	1	1	2	2	4	4
4	4	1	1	1	1	2	2	4	4
4	4	1	1	4	4	2	2	4	4
4	4	1	1	1	1	2	2	4	4
4	4	1	1	1	1	2	2	4	4
4	4	1	1	1	1	2	2	4	4
4	4	1	1	1	1	2	2	4	4
4	4	1	1	1	1	2	2	4	4
4	4	1	1	1	1	2	2	4	4
4	4	1	1	1	1	2	2	4	4
4	4	1	1	1	1	2	2	4	4
4	4	1	1	1	1	2	2	4	4
4	4	1	1	4	4	2	2	4	4
4	4	1	1	1	1	2	2	4	4
4	4	1	1	1	1	2	2	4	4
4	4	1	1	1	1	2	2	4	4
4	4	1	1	4	4	2	2	4	4
1	1	1	1	1	1	2	2	4	4
4	4	1	1	1	1	2	2	4	4
4	4	1	1	1	1	2	2	4	4
4	4	1	1	1	1	2	2	4	4
4	4	1	1	1	1	2	2	4	4
4	4	1	1	1	1	2	2	4	4
4	4	1	1	1	1	2	2	4	4
4	4	1	1	1	1	2	2	4	4
4	4	1	1	1	1	2	2	4	4
4	4	1	1	1	1	2	2	4	4
4	4	1	1	1	1	2	2	4	4
4	4	1	1	1	1	2	2	4	4
4	4	1	1	1	1	2	2	4	4
4	4	1	1	1	1	2	2	4	4
4	4	1	1	1	1	2	2	4	4

For Review Only

4	4	1	1	1	1	2	2	4	4
4	4	1	1	4	4	2	2	4	4
4	4	1	1	1	1	2	2	4	4
4	4	1	1	1	1	2	2	4	4
4	4	1	1	1	1	2	2	4	4
4	4	1	1	1	1	2	2	4	4
4	4	1	1	1	1	2	2	4	4
4	4	1	1	1	1	2	2	4	4
4	4	1	1	4	4	2	2	4	4
4	4	1	1	1	1	2	2	4	4
4	4	1	1	1	1	2	2	4	4
4	4	1	1	1	1	2	2	4	4
4	4	1	1	1	1	2	2	4	4
4	4	1	1	1	1	2	2	4	4
4	4	1	1	1	1	2	2	4	4
4	4	1	1	4	4	2	2	4	4
4	4	1	1	1	1	2	2	4	4
4	4	1	1	4	4	2	2	4	4
4	4	1	1	1	1	2	2	4	4
4	4	1	1	1	1	2	2	4	4
4	4	1	1	1	1	2	2	4	4
4	4	1	1	1	1	2	2	4	4
4	4	1	1	1	1	2	2	4	4
4	4	1	1	4	4	2	2	4	4
4	4	1	1	1	1	2	2	4	4
4	4	1	1	1	1	2	2	4	4
4	4	1	1	1	1	2	2	4	4
4	4	1	1	1	1	2	2	2	2
4	4	3	3	1	1	2	2	4	4
4	4	1	1	1	1	2	2	4	4
4	4	1	1	1	1	2	2	4	4
4	4	1	1	1	1	2	2	4	4
4	4	1	1	1	1	2	2	2	2
4	4	1	1	1	1	2	2	4	4
4	4	1	1	1	1	2	2	4	4
4	4	1	1	1	1	2	2	4	4
4	4	1	1	1	1	2	2	4	4
4	4	1	1	1	1	2	2	4	4
4	4	1	1	1	1	2	2	4	4
4	4	1	1	1	1	2	2	4	4
4	4	1	1	1	1	2	2	4	4
4	4	1	1	1	1	2	2	4	4
4	4	1	1	1	1	2	2	4	4
4	4	1	1	1	1	2	2	4	4
4	4	1	1	4	4	2	2	4	4
4	4	1	1	1	1	2	2	4	4
1	1	1	1	1	1	2	2	4	4
4	4	1	1	1	1	2	2	4	4
4	4	1	1	1	1	2	2	4	4
1	1	1	1	1	1	2	2	4	4
4	4	1	1	1	1	2	2	4	4
4	4	1	1	1	1	2	2	4	4
4	4	1	1	1	1	2	2	4	4
4	4	1	1	1	1	2	2	4	4
1	1	1	1	4	4	2	2	4	4

For Review Only

4	4	1	1	1	1	2	2	2	2
4	4	1	1	1	1	2	2	4	4
4	4	1	1	1	1	2	2	4	4
1	1	1	1	1	1	3	3	4	4
4	4	1	1	1	1	2	2	4	4
4	4	3	3	1	1	2	2	4	4
4	4	1	1	1	1	2	2	4	4
4	4	1	1	1	1	2	2	4	4
4	4	1	1	1	1	2	2	4	4
4	4	1	1	1	1	2	2	4	4
4	4	1	1	4	4	2	2	4	4
4	4	1	1	1	1	2	2	4	4
4	4	1	1	1	1	2	2	4	4
4	4	1	1	1	1	2	2	4	4
4	4	1	1	1	1	2	2	4	4
4	4	1	1	1	1	2	2	4	4
4	4	1	1	4	4	2	2	4	4
4	4	1	1	1	1	2	2	4	4
1	1	1	1	1	1	2	2	4	4
4	4	1	1	4	4	2	2	4	4
4	4	1	1	1	1	2	2	4	4
4	4	1	1	1	1	2	2	4	4
4	4	1	1	1	1	2	2	4	4
4	4	1	1	1	1	2	2	4	4
4	4	1	1	1	1	2	2	4	4
4	4	1	1	1	1	2	2	2	2
4	4	1	1	4	4	2	2	4	4
4	4	1	1	1	1	2	2	4	4
4	4	1	1	1	1	2	2	4	4
4	4	1	1	1	1	2	2	4	4
4	4	1	1	1	1	2	2	4	4
4	4	1	1	1	1	2	2	4	4
4	4	3	3	1	1	2	2	4	4
4	4	1	1	1	1	3	3	4	4
4	4	1	1	1	1	2	2	4	4
4	4	1	1	1	1	2	2	4	4
1	1	1	1	1	1	3	3	4	4
4	4	1	1	1	1	2	2	4	4
4	4	1	1	1	1	2	2	2	2
4	4	1	1	1	1	2	2	4	4
4	4	1	1	1	1	2	2	4	4
4	4	1	1	1	1	2	2	4	4
4	4	1	1	1	1	2	2	4	4
4	4	1	1	1	1	2	2	4	4
4	4	1	1	4	4	2	2	4	4
4	4	1	1	1	1	2	2	4	4
4	4	1	1	1	1	2	2	4	4
4	4	1	1	1	1	2	2	4	4
4	4	1	1	1	1	2	2	4	4
4	4	1	1	1	1	2	2	4	4
4	4	1	1	1	1	2	2	4	4
4	4	1	1	1	1	2	2	4	4
4	4	1	1	1	1	2	2	4	4
4	4	1	1	1	1	2	2	4	4
4	4	1	1	1	1	2	2	4	4

For Review Only

4	4	1	1	1	1	2	2	4	4
4	4	1	1	1	1	2	2	2	2
4	4	1	1	1	1	2	2	4	4
4	4	1	1	1	1	2	2	2	2
4	4	1	1	1	1	2	2	4	4
4	4	1	1	1	1	2	2	4	4
4	4	1	1	1	1	2	2	4	4
4	4	1	1	1	1	2	2	4	4
4	4	1	1	1	1	2	2	2	2
4	4	1	1	1	1	2	2	4	4
4	4	1	1	1	1	2	2	4	4
4	4	1	1	1	1	2	2	4	4
4	4	1	1	1	1	2	2	4	4
4	4	1	1	1	1	2	2	4	4
4	4	1	1	1	1	2	2	4	4
4	4	1	1	1	1	2	2	4	4
4	4	1	1	1	1	2	2	4	4
4	4	1	1	1	1	2	2	4	4
4	4	1	1	1	1	2	2	4	4
4	4	1	1	1	1	2	2	4	4
4	4	1	1	1	1	2	2	4	4
4	4	1	1	1	1	2	2	4	4
4	4	1	1	1	1	2	2	4	4
4	4	1	1	1	1	2	2	4	4
4	4	1	1	1	1	2	2	4	4
4	4	1	1	1	1	2	2	4	4
4	4	1	1	1	1	2	2	4	4
4	4	1	1	1	1	2	2	4	4
4	4	1	1	1	1	2	2	4	4
4	4	1	1	1	1	2	2	4	4
4	4	1	1	4	4	2	2	4	4
4	4	1	1	1	1	2	2	4	4
4	4	1	1	4	4	2	2	4	4
4	4	1	1	1	1	2	2	4	4
4	4	1	1	1	1	2	2	4	4
4	4	1	1	1	1	2	2	4	4
4	4	1	1	1	1	2	2	4	4
4	4	1	1	4	4	2	2	4	4
4	4	1	1	1	1	2	2	4	4
4	4	1	1	1	1	2	2	4	4
4	4	1	1	1	1	2	2	4	4
4	4	1	1	1	1	2	2	4	4
4	4	1	1	1	1	2	2	4	4
4	4	1	1	1	1	2	2	4	4
4	4	1	1	1	1	2	2	4	4
4	4	1	1	1	1	2	2	4	4
4	4	1	1	1	1	2	2	4	4
4	4	1	1	1	1	2	2	2	2
4	4	1	1	1	1	2	2	4	4
4	4	1	1	1	1	2	2	4	4
4	4	1	1	1	1	2	2	4	4
1	1	1	1	4	4	2	2	4	4
4	4	1	1	1	1	2	2	4	4
4	4	1	1	1	1	2	2	4	4
4	4	1	1	1	1	2	2	4	4

For Review Only

4	4	1	1	1	1	2	2	4	4
4	4	1	1	1	1	2	2	4	4
4	4	1	1	1	1	2	2	4	4
4	4	1	1	1	1	2	2	4	4
4	4	1	1	1	1	2	2	4	4
4	4	1	1	1	1	2	2	4	4
4	4	1	1	1	1	2	2	4	4
4	4	1	1	1	1	2	2	4	4
4	4	1	1	1	1	2	2	4	4
4	4	1	1	1	1	2	2	4	4
4	4	1	1	1	1	2	2	4	4
4	4	1	1	4	4	2	2	4	4
4	4	1	1	1	1	2	2	4	4
4	4	1	1	1	1	2	2	4	4
4	4	1	1	1	1	2	2	4	4
4	4	1	1	1	1	2	2	4	4
4	4	1	1	1	1	2	2	4	4
4	4	1	1	1	1	2	2	4	4
4	4	1	1	1	1	2	2	4	4
1	1	1	1	1	1	2	2	4	4
4	4	1	1	4	4	2	2	4	4
4	4	1	1	1	1	2	2	4	4
4	4	1	1	4	4	2	2	4	4
4	4	1	1	1	1	2	2	4	4
4	4	1	1	1	1	2	2	4	4
4	4	1	1	1	1	2	2	4	4
4	4	1	1	1	1	2	2	2	2
4	4	1	1	1	1	2	2	4	4
4	4	1	1	1	1	2	2	4	4
4	4	1	1	1	1	2	2	4	4
4	4	1	1	1	1	2	2	4	4
4	4	1	1	1	1	2	2	4	4
1	1	1	1	4	4	2	2	4	4
4	4	1	1	1	1	2	2	4	4
4	4	1	1	1	1	2	2	4	4
4	4	1	1	1	1	2	2	4	4
4	4	1	1	1	1	2	2	4	4
4	4	1	1	1	1	2	2	4	4
4	4	1	1	1	1	2	2	4	4
4	4	1	1	1	1	2	2	4	4
4	4	1	1	4	4	2	2	4	4
4	4	1	1	1	1	2	2	4	4
4	4	1	1	1	1	2	2	4	4
4	4	1	1	1	1	2	2	4	4
4	4	1	1	1	1	2	2	4	4
4	4	1	1	1	1	2	2	4	4
4	4	1	1	1	1	2	2	4	4
4	4	1	1	1	1	2	2	4	4
4	4	1	1	1	1	2	2	4	4
4	4	1	1	1	1	2	2	4	4
4	4	1	1	1	1	2	2	4	4
4	4	1	1	1	1	2	2	4	4
4	4	1	1	1	1	2	2	4	4
4	4	1	1	1	1	2	2	4	4
4	4	1	1	1	1	2	2	4	4

For Review Only

4	4	1	1	1	1	2	2	4	4
4	4	1	1	1	1	2	2	4	4

For Review Only

SNP105	SNP105	SNP1050	SNP1050	SNP1051	SNP1051	SNP1052	SNP1052	SNP1053	SNP1053
2	2	1	1	2	2	2	2	2	2
2	2	1	1	3	3	2	2	3	3
2	2	1	1	3	3	2	2	2	2
2	2	1	1	3	3	2	2	2	2
2	2	1	1	3	3	2	2	2	2
2	2	1	1	2	2	2	2	2	2
2	2	1	1	2	2	2	2	2	2
2	2	1	1	3	3	2	2	2	2
2	2	4	4	2	2	2	2	2	2
2	2	4	4	2	2	2	2	2	2
2	2	1	1	3	3	3	3	2	2
2	2	1	1	3	3	2	2	2	2
2	2	1	1	3	3	2	2	2	2
3	3	1	1	3	3	2	2	2	2
2	2	1	1	3	3	2	2	2	2
2	2	4	4	3	3	2	2	3	3
2	2	1	1	3	3	2	2	2	2
2	2	1	1	2	2	2	2	2	2
2	2	1	1	3	3	2	2	2	2
2	2	1	1	3	3	2	2	2	2
2	2	1	1	3	3	2	2	3	3
2	2	1	1	3	3	2	2	2	2
2	2	1	1	2	2	3	3	2	2
2	2	1	1	3	3	2	2	3	3
2	2	1	1	3	3	2	2	2	2
2	2	1	1	3	3	2	2	2	2
2	2	1	1	3	3	2	2	2	2
2	2	1	1	3	3	2	2	2	2
2	2	4	4	3	3	2	2	2	2
2	2	4	4	3	3	2	2	2	2
2	2	1	1	3	3	2	2	2	2
2	2	1	1	2	2	2	2	2	2
2	2	1	1	2	2	2	2	2	2
3	3	1	1	3	3	2	2	3	3
2	2	1	1	3	3	2	2	2	2
2	2	1	1	3	3	2	2	3	3
2	2	1	1	2	2	2	2	3	3
2	2	1	1	3	3	2	2	2	2
3	3	1	1	3	3	3	3	2	2
2	2	1	1	3	3	2	2	2	2
2	2	1	1	3	3	2	2	2	2
2	2	1	1	3	3	2	2	2	2
2	2	1	1	3	3	2	2	2	2
2	2	1	1	3	3	2	2	3	3
2	2	1	1	3	3	2	2	2	2
2	2	1	1	3	3	2	2	2	2
3	3	1	1	3	3	2	2	2	2
2	2	1	1	3	3	2	2	2	2
2	2	1	1	3	3	2	2	2	2
2	2	1	1	3	3	2	2	2	2
2	2	1	1	3	3	2	2	2	2
2	2	1	1	3	3	2	2	2	2

For Review Only

2	2	1	1	3	3	2	2	2	2
2	2	1	1	2	2	2	2	2	2
2	2	1	1	3	3	2	2	2	2
2	2	1	1	3	3	2	2	2	2
2	2	1	1	3	3	2	2	2	2
2	2	1	1	3	3	2	2	2	2
2	2	1	1	3	3	2	2	2	2
2	2	1	1	3	3	2	2	3	3
2	2	1	1	2	2	2	2	3	3
2	2	1	1	3	3	2	2	2	2
2	2	1	1	3	3	2	2	2	2
2	2	1	1	3	3	2	2	2	2
2	2	1	1	3	3	2	2	2	2
2	2	4	4	3	3	2	2	2	2
2	2	1	1	2	2	2	2	2	2
2	2	1	1	2	2	2	2	3	3
3	3	1	1	3	3	2	2	3	3
2	2	1	1	2	2	2	2	3	3
2	2	1	1	3	3	2	2	2	2
2	2	1	1	2	2	2	2	2	2
2	2	1	1	3	3	2	2	2	2
2	2	1	1	3	3	2	2	2	2
2	2	4	4	3	3	2	2	2	2
2	2	1	1	2	2	2	2	2	2
2	2	1	1	3	3	2	2	2	2
2	2	4	4	3	3	2	2	3	3
2	2	1	1	2	2	2	2	2	2
3	3	1	1	3	3	2	2	2	2
2	2	1	1	3	3	2	2	2	2
2	2	1	1	3	3	2	2	2	2
2	2	1	1	2	2	2	2	3	3
2	2	1	1	3	3	2	2	2	2
2	2	1	1	3	3	2	2	2	2
2	2	1	1	3	3	2	2	2	2
2	2	1	1	3	3	2	2	3	3
2	2	1	1	3	3	2	2	2	2
2	2	1	1	2	2	2	2	3	3
2	2	1	1	3	3	2	2	2	2
2	2	1	1	3	3	2	2	2	2
2	2	1	1	3	3	2	2	2	2
2	2	1	1	3	3	2	2	2	2
2	2	1	1	3	3	2	2	3	3
2	2	1	1	3	3	2	2	2	2
2	2	1	1	3	3	2	2	2	2
2	2	1	1	2	2	2	2	2	2
2	2	1	1	3	3	2	2	2	2
3	3	1	1	3	3	2	2	2	2
2	2	1	1	3	3	2	2	2	2
2	2	1	1	3	3	2	2	3	3
2	2	1	1	3	3	2	2	2	2
2	2	1	1	3	3	2	2	2	2
2	2	1	1	3	3	3	3	2	2
2	2	1	1	3	3	2	2	2	2
2	2	1	1	3	3	2	2	2	2
2	2	1	1	2	2	2	2	3	3

For Review Only

2	2	1	1	3	3	2	2	2	2
2	2	1	1	3	3	2	2	3	3
2	2	1	1	3	3	2	2	2	2
3	3	1	1	3	3	2	2	2	2
2	2	1	1	3	3	2	2	2	2
2	2	1	1	3	3	2	2	2	2
2	2	1	1	3	3	2	2	2	2
2	2	1	1	3	3	2	2	2	2
2	2	1	1	3	3	2	2	2	2
2	2	1	1	3	3	2	2	2	2
2	2	1	1	2	2	2	2	2	2
2	2	1	1	3	3	2	2	2	2
2	2	1	1	3	3	2	2	2	2
2	2	1	1	3	3	2	2	3	3
2	2	1	1	3	3	2	2	3	3
2	2	1	1	3	3	2	2	2	2
2	2	1	1	2	2	2	2	2	2
2	2	1	1	3	3	2	2	2	2
3	3	1	1	3	3	2	2	2	2
2	2	1	1	2	2	2	2	2	2
2	2	1	1	3	3	2	2	2	2
2	2	1	1	3	3	2	2	2	2
2	2	1	1	3	3	2	2	2	2
3	3	1	1	3	3	3	3	2	2
2	2	1	1	3	3	2	2	2	2
2	2	1	1	3	3	2	2	2	2
2	2	1	1	3	3	2	2	2	2
2	2	1	1	3	3	2	2	2	2
2	2	1	1	3	3	2	2	2	2
2	2	1	1	3	3	2	2	2	2
3	3	1	1	3	3	2	2	3	3
2	2	1	1	3	3	2	2	2	2
2	2	1	1	3	3	2	2	2	2
2	2	1	1	3	3	2	2	2	2
3	3	1	1	3	3	2	2	2	2
2	2	1	1	3	3	2	2	2	2
3	3	1	1	3	3	2	2	3	3
2	2	1	1	3	3	2	2	2	2
2	2	1	1	3	3	2	2	2	2
3	3	1	1	3	3	2	2	2	2
2	2	1	1	3	3	2	2	2	2
3	3	1	1	3	3	2	2	2	2
2	2	1	1	3	3	2	2	2	2
2	2	4	4	3	3	2	2	2	2
2	2	1	1	3	3	2	2	3	3
2	2	1	1	3	3	2	2	3	3
2	2	1	1	3	3	2	2	2	2
2	2	1	1	3	3	2	2	2	2
2	2	1	1	3	3	2	2	2	2
2	2	1	1	3	3	2	2	2	2
2	2	1	1	3	3	2	2	2	2
3	3	1	1	3	3	2	2	2	2
2	2	1	1	3	3	2	2	2	2
2	2	1	1	3	3	2	2	2	2
2	2	1	1	3	3	2	2	2	2

For Review Only

2	2	1	1	3	3	2	2	2	2
2	2	1	1	3	3	2	2	2	2
2	2	1	1	3	3	2	2	2	2
2	2	1	1	3	3	2	2	2	2
2	2	1	1	3	3	2	2	2	2
2	2	1	1	3	3	2	2	2	2
3	3	1	1	3	3	2	2	2	2
2	2	1	1	3	3	2	2	2	2
2	2	1	1	3	3	2	2	2	2
2	2	1	1	3	3	2	2	2	2
2	2	1	1	3	3	2	2	2	2
2	2	1	1	3	3	2	2	2	2
2	2	1	1	3	3	2	2	2	2
2	2	1	1	3	3	2	2	2	2
3	3	1	1	3	3	2	2	3	3
2	2	1	1	3	3	3	3	2	2
2	2	1	1	3	3	2	2	2	2
2	2	1	1	3	3	2	2	3	3
2	2	1	1	3	3	2	2	2	2
2	2	1	1	3	3	2	2	2	2
2	2	1	1	3	3	2	2	2	2
3	3	1	1	3	3	2	2	2	2
2	2	1	1	3	3	2	2	2	2
2	2	1	1	3	3	2	2	2	2
2	2	4	4	3	3	2	2	3	3
3	3	1	1	3	3	2	2	3	3
2	2	1	1	3	3	2	2	2	2
2	2	1	1	3	3	2	2	2	2
2	2	1	1	2	2	2	2	3	3
2	2	1	1	3	3	2	2	3	3
2	2	1	1	2	2	2	2	2	2
2	2	1	1	3	3	2	2	2	2
2	2	1	1	2	2	2	2	2	2
3	3	1	1	3	3	2	2	2	2
2	2	1	1	3	3	2	2	3	3
2	2	1	1	3	3	2	2	2	2
2	2	1	1	3	3	2	2	3	3
2	2	1	1	2	2	2	2	3	3
2	2	1	1	3	3	2	2	2	2
2	2	1	1	3	3	2	2	2	2
2	2	1	1	3	3	2	2	2	2
2	2	1	1	3	3	2	2	2	2
3	3	1	1	3	3	2	2	2	2
2	2	1	1	3	3	2	2	2	2
2	2	1	1	3	3	2	2	2	2
2	2	1	1	3	3	2	2	2	2
2	2	1	1	3	3	3	3	2	2
3	3	1	1	3	3	2	2	2	2
3	3	1	1	3	3	2	2	3	3
2	2	1	1	3	3	2	2	2	2
2	2	1	1	3	3	2	2	2	2
3	3	1	1	2	2	2	2	2	2
2	2	1	1	3	3	2	2	2	2
2	2	1	1	3	3	2	2	2	2
2	2	1	1	3	3	3	3	2	2

For Review Only

2	2	1	1	3	3	2	2	2	2
2	2	4	4	3	3	2	2	2	2
2	2	1	1	3	3	2	2	3	3
2	2	1	1	3	3	3	3	2	2
2	2	4	4	3	3	2	2	2	2
2	2	1	1	3	3	2	2	3	3
2	2	1	1	3	3	2	2	2	2
2	2	1	1	3	3	2	2	2	2
2	2	1	1	3	3	2	2	3	3
3	3	1	1	3	3	2	2	2	2
2	2	1	1	3	3	2	2	2	2
2	2	1	1	2	2	2	2	2	2
2	2	1	1	3	3	2	2	2	2
2	2	1	1	3	3	2	2	3	3
2	2	1	1	3	3	2	2	2	2
2	2	1	1	3	3	2	2	2	2
3	3	1	1	3	3	3	3	2	2
2	2	1	1	3	3	2	2	2	2
3	3	1	1	3	3	2	2	2	2
2	2	1	1	3	3	2	2	2	2
2	2	1	1	2	2	3	3	2	2
3	3	1	1	3	3	2	2	2	2
2	2	1	1	2	2	2	2	2	2
3	3	1	1	3	3	3	3	2	2
2	2	1	1	3	3	2	2	3	3
2	2	1	1	3	3	2	2	2	2
3	3	1	1	3	3	2	2	2	2
2	2	1	1	3	3	2	2	2	2
2	2	4	4	3	3	2	2	2	2
2	2	1	1	3	3	2	2	2	2
2	2	1	1	3	3	3	3	2	2
2	2	1	1	3	3	2	2	2	2
3	3	1	1	2	2	2	2	2	2
2	2	1	1	3	3	2	2	2	2
2	2	1	1	2	2	2	2	2	2
3	3	1	1	3	3	3	3	2	2
2	2	1	1	3	3	2	2	2	2
2	2	1	1	3	3	2	2	2	2
2	2	1	1	3	3	2	2	3	3
3	3	1	1	3	3	3	3	2	2
2	2	1	1	2	2	2	2	2	2
2	2	1	1	3	3	2	2	2	2
2	2	1	1	3	3	3	3	2	2
2	2	1	1	3	3	2	2	2	2
2	2	1	1	3	3	2	2	2	2
2	2	1	1	3	3	2	2	2	2
2	2	1	1	3	3	2	2	2	2
2	2	1	1	3	3	2	2	2	2
2	2	1	1	3	3	2	2	2	2
2	2	1	1	3	3	2	2	2	2
3	3	1	1	3	3	3	3	2	2
2	2	1	1	3	3	2	2	2	2
2	2	1	1	3	3	3	3	2	2
2	2	1	1	3	3	2	2	2	2
2	2	1	1	3	3	2	2	3	3
2	2	1	1	3	3	3	3	2	2

For Review Only

2	2	1	1	3	3	2	2	2	2
2	2	1	1	3	3	2	2	3	3

For Review Only

SNP1054	SNP1054	SNP1055	SNP1055	SNP1056	SNP1056	SNP1057	SNP1057	SNP1058	SNP1058
4	4	1	1	1	1	3	3	3	3
1	1	1	1	3	3	3	3	2	2
1	1	1	1	1	1	3	3	2	2
1	1	1	1	1	1	3	3	2	2
1	1	1	1	3	3	3	3	2	2
4	4	1	1	1	1	3	3	2	2
4	4	1	1	1	1	3	3	3	3
1	1	4	4	1	1	2	2	2	2
1	1	1	1	1	1	3	3	3	3
1	1	1	1	1	1	2	2	2	2
1	1	1	1	3	3	3	3	3	3
1	1	1	1	1	1	3	3	2	2
1	1	1	1	1	1	3	3	2	2
1	1	1	1	1	1	3	3	2	2
1	1	1	1	1	1	3	3	2	2
1	1	1	1	1	1	3	3	2	2
1	1	4	4	1	1	3	3	3	3
4	4	1	1	3	3	3	3	2	2
1	1	1	1	1	1	3	3	2	2
1	1	1	1	3	3	3	3	2	2
1	1	1	1	3	3	3	3	2	2
1	1	1	1	1	1	3	3	3	3
4	4	1	1	3	3	2	2	2	2
1	1	1	1	1	1	3	3	2	2
1	1	1	1	1	1	3	3	2	2
1	1	1	1	1	1	3	3	2	2
1	1	1	1	1	1	3	3	2	2
1	1	1	1	1	1	3	3	2	2
1	1	4	4	3	3	3	3	3	3
1	1	1	1	3	3	3	3	2	2
1	1	1	1	1	1	3	3	2	2
1	1	1	1	1	1	3	3	3	3
4	4	1	1	1	1	3	3	3	3
1	1	1	1	3	3	3	3	2	2
1	1	1	1	3	3	3	3	2	2
1	1	1	1	1	1	3	3	2	2
4	4	1	1	1	1	3	3	2	2
1	1	1	1	1	1	3	3	2	2
1	1	1	1	3	3	3	3	2	2
1	1	1	1	3	3	3	3	2	2
1	1	4	4	3	3	2	2	2	2
1	1	4	4	3	3	3	3	2	2
1	1	4	4	1	1	3	3	2	2
1	1	4	4	3	3	3	3	2	2
1	1	4	4	1	1	3	3	2	2
1	1	1	1	1	1	3	3	2	2
1	1	1	1	1	1	3	3	2	2
1	1	1	1	1	1	3	3	2	2
1	1	1	1	3	3	3	3	2	2
1	1	1	1	3	3	3	3	2	2
1	1	1	1	1	1	3	3	2	2
1	1	1	1	1	1	3	3	2	2

1	1	1	1	1	1	3	3	3	3
4	4	1	1	1	1	3	3	3	3
1	1	1	1	1	1	3	3	2	2
1	1	1	1	1	1	3	3	2	2
1	1	1	1	1	1	3	3	2	2
1	1	1	1	1	1	3	3	2	2
1	1	1	1	1	1	3	3	2	2
1	1	1	1	3	3	3	3	2	2
4	4	1	1	3	3	3	3	2	2
1	1	1	1	1	1	3	3	2	2
1	1	1	1	1	1	3	3	2	2
1	1	1	1	1	1	3	3	2	2
1	1	1	1	1	1	3	3	3	3
1	1	4	4	3	3	3	3	3	3
1	1	1	1	1	1	3	3	2	2
4	4	1	1	1	1	3	3	2	2
1	1	1	1	1	1	3	3	2	2
4	4	1	1	1	1	3	3	2	2
1	1	1	1	1	1	3	3	3	3
4	4	1	1	1	1	3	3	3	3
1	1	1	1	1	1	3	3	2	2
1	1	1	1	1	1	3	3	2	2
1	1	4	4	3	3	3	3	3	3
4	4	1	1	1	1	3	3	2	2
1	1	4	4	1	1	3	3	2	2
1	1	1	1	1	1	3	3	2	2
1	1	1	1	1	1	3	3	2	2
1	1	1	1	3	3	3	3	2	2
1	1	1	1	1	1	3	3	2	2
1	1	1	1	1	1	3	3	2	2
4	4	1	1	1	1	3	3	2	2
1	1	1	1	3	3	3	3	2	2
1	1	1	1	3	3	3	3	2	2
1	1	1	1	1	1	3	3	3	3
1	1	1	1	1	1	3	3	2	2
1	1	4	4	1	1	3	3	2	2
4	4	1	1	1	1	3	3	2	2
1	1	1	1	1	1	3	3	2	2
1	1	1	1	1	1	3	3	2	2
1	1	1	1	1	1	3	3	2	2
1	1	1	1	1	1	3	3	3	3
1	1	1	1	1	1	3	3	2	2
1	1	1	1	1	1	3	3	2	2
1	1	1	1	1	1	3	3	2	2
4	4	1	1	1	1	3	3	3	3
4	4	1	1	1	1	3	3	2	2
1	1	1	1	3	3	3	3	2	2
1	1	1	1	1	1	3	3	2	2
1	1	1	1	1	1	3	3	2	2
1	1	1	1	1	1	3	3	2	2
1	1	1	1	1	1	3	3	2	2
1	1	1	1	3	3	3	3	2	2
1	1	1	1	3	3	3	3	2	2
1	1	1	1	1	1	3	3	2	2
4	4	1	1	1	1	3	3	2	2

For Review Only

1	1	1	1	3	3	3	3	2	2
1	1	1	1	3	3	3	3	2	2
1	1	1	1	1	1	3	3	2	2
1	1	1	1	3	3	3	3	2	2
1	1	1	1	1	1	3	3	2	2
1	1	1	1	1	1	3	3	2	2
1	1	1	1	1	1	3	3	2	2
1	1	1	1	1	1	3	3	2	2
1	1	1	1	1	1	3	3	2	2
1	1	1	1	1	1	3	3	2	2
4	4	4	4	1	1	3	3	3	3
1	1	1	1	1	1	3	3	2	2
1	1	1	1	1	1	3	3	2	2
1	1	1	1	1	1	3	3	2	2
1	1	1	1	1	1	3	3	2	2
1	1	1	1	1	1	3	3	2	2
4	4	1	1	3	3	3	3	2	2
1	1	1	1	1	1	3	3	2	2
1	1	1	1	3	3	3	3	2	2
4	4	1	1	1	1	3	3	2	2
1	1	1	1	1	1	3	3	2	2
1	1	1	1	1	1	3	3	2	2
1	1	1	1	1	1	3	3	2	2
1	1	1	1	3	3	3	3	2	2
1	1	1	1	1	1	3	3	2	2
1	1	1	1	3	3	3	3	2	2
1	1	1	1	1	1	3	3	2	2
1	1	1	1	3	3	3	3	2	2
1	1	1	1	1	1	3	3	2	2
1	1	1	1	3	3	3	3	2	2
1	1	1	1	3	3	3	3	2	2
1	1	1	1	1	1	3	3	2	2
1	1	1	1	1	1	3	3	3	3
1	1	1	1	1	1	3	3	2	2
1	1	1	1	3	3	3	3	2	2
1	1	1	1	3	3	3	3	2	2
1	1	1	1	3	3	3	3	2	2
1	1	1	1	1	1	3	3	2	2
1	1	1	1	3	3	3	3	2	2
1	1	1	1	3	3	3	3	2	2
1	1	1	1	1	1	3	3	2	2
1	1	1	1	3	3	3	3	2	2
1	1	1	1	1	1	3	3	2	2
1	1	1	1	3	3	3	3	2	2
4	4	1	1	3	3	3	3	2	2
1	1	1	1	1	1	3	3	2	2
1	1	1	1	1	1	3	3	2	2
1	1	1	1	3	3	3	3	2	2
1	1	1	1	1	1	3	3	2	2
1	1	1	1	1	1	3	3	2	2
1	1	1	1	1	1	3	3	2	2
1	1	1	1	3	3	3	3	2	2
1	1	1	1	1	1	3	3	2	2
1	1	1	1	1	1	3	3	2	2
1	1	1	1	3	3	3	3	2	2

For Review Only

1	1	1	1	1	1	3	3	2	2
1	1	1	1	3	3	3	3	2	2
1	1	1	1	1	1	3	3	2	2
1	1	1	1	3	3	3	3	2	2
1	1	1	1	1	1	3	3	2	2
1	1	1	1	1	1	3	3	2	2
1	1	1	1	3	3	3	3	2	2
1	1	1	1	1	1	3	3	2	2
1	1	1	1	3	3	3	3	2	2
1	1	1	1	1	1	3	3	2	2
1	1	1	1	1	1	3	3	2	2
1	1	1	1	3	3	3	3	2	2
1	1	1	1	1	1	3	3	2	2
1	1	1	1	1	1	3	3	2	2
1	1	1	1	3	3	3	3	2	2
1	1	1	1	3	3	3	3	2	2
1	1	1	1	1	1	3	3	2	2
1	1	1	1	1	1	3	3	2	2
1	1	1	1	1	1	3	3	2	2
1	1	1	1	1	1	3	3	2	2
1	1	1	1	1	1	3	3	2	2
1	1	1	1	3	3	3	3	2	2
1	1	1	1	1	1	3	3	2	2
1	1	1	1	1	1	3	3	2	2
1	1	4	4	3	3	3	3	2	2
1	1	1	1	3	3	3	3	2	2
1	1	1	1	1	1	3	3	2	2
1	1	1	1	3	3	3	3	2	2
4	4	1	1	1	1	3	3	2	2
1	1	1	1	1	1	3	3	2	2
4	4	1	1	1	1	3	3	2	2
1	1	1	1	1	1	3	3	2	2
4	4	1	1	1	1	3	3	3	3
1	1	1	1	3	3	3	3	2	2
1	1	1	1	1	1	3	3	2	2
1	1	1	1	1	1	3	3	2	2
1	1	1	1	3	3	3	3	2	2
4	4	1	1	1	1	3	3	2	2
1	1	1	1	3	3	3	3	2	2
1	1	1	1	1	1	3	3	2	2
1	1	1	1	1	1	3	3	2	2
1	1	1	1	1	1	3	3	2	2
1	1	1	1	3	3	3	3	2	2
1	1	1	1	1	1	3	3	2	2
1	1	1	1	1	1	3	3	2	2
1	1	1	1	1	1	3	3	2	2
1	1	1	1	3	3	3	3	2	2
1	1	1	1	3	3	3	3	3	3
1	1	1	1	3	3	3	3	2	2
1	1	1	1	1	1	3	3	2	2
1	1	1	1	1	1	3	3	2	2
4	4	1	1	3	3	3	3	2	2
1	1	1	1	1	1	3	3	2	2
1	1	1	1	1	1	3	3	2	2
1	1	1	1	1	1	2	2	2	2

For Review Only

1	1	1	1	3	3	3	3	2	2
1	1	1	1	3	3	3	3	2	2
1	1	1	1	1	1	3	3	2	2
1	1	1	1	1	1	3	3	2	2
1	1	1	1	1	1	3	3	2	2
1	1	1	1	1	1	3	3	2	2
1	1	1	1	3	3	2	2	2	2
1	1	1	1	3	3	2	2	2	2
1	1	1	1	1	1	3	3	2	2
1	1	1	1	3	3	3	3	2	2
1	1	1	1	1	1	3	3	2	2
4	4	1	1	1	1	3	3	2	2
1	1	1	1	1	1	3	3	2	2
1	1	1	1	1	1	3	3	2	2
1	1	1	1	1	1	3	3	2	2
1	1	1	1	1	1	2	2	2	2
1	1	1	1	3	3	3	3	2	2
1	1	1	1	3	3	3	3	2	2
1	1	1	1	1	1	3	3	2	2
1	1	1	1	3	3	3	3	2	2
4	4	1	1	3	3	3	3	2	2
1	1	1	1	1	1	3	3	2	2
4	4	1	1	1	1	3	3	3	3
1	1	1	1	3	3	3	3	2	2
1	1	1	1	3	3	3	3	2	2
1	1	1	1	1	1	3	3	3	3
1	1	1	1	3	3	3	3	2	2
1	1	1	1	1	1	3	3	2	2
1	1	1	1	1	1	3	3	3	3
1	1	1	1	1	1	3	3	2	2
1	1	1	1	3	3	2	2	2	2
1	1	1	1	1	1	3	3	2	2
4	4	1	1	3	3	3	3	2	2
1	1	1	1	3	3	3	3	2	2
4	4	1	1	1	1	3	3	2	2
1	1	1	1	3	3	3	3	2	2
1	1	1	1	1	1	3	3	2	2
1	1	1	1	1	1	3	3	2	2
1	1	1	1	1	1	3	3	2	2
1	1	1	1	3	3	3	3	2	2
4	4	1	1	1	1	3	3	2	2
1	1	1	1	1	1	3	3	2	2
1	1	1	1	3	3	3	3	2	2
1	1	1	1	1	1	3	3	3	3
1	1	1	1	1	1	3	3	2	2
1	1	1	1	1	1	2	2	2	2
1	1	1	1	3	3	3	3	2	2
1	1	1	1	1	1	3	3	3	3
1	1	1	1	1	1	3	3	2	2
1	1	1	1	3	3	3	3	2	2
1	1	1	1	1	1	3	3	2	2
1	1	1	1	3	3	3	3	2	2
1	1	1	1	1	1	3	3	2	2
1	1	1	1	1	1	3	3	2	2
1	1	1	1	3	3	3	3	2	2

For Review Only

1	1	1	1	1	1	3	3	3	3
1	1	1	1	1	1	3	3	2	2

For Review Only

SNP1059	SNP1059	SNP106	SNP106	SNP1060	SNP1060	SNP1061	SNP1061	SNP1062	SNP1062
1	1	1	1	2	2	1	1	3	3
1	1	1	1	2	2	1	1	3	3
1	1	1	1	2	2	1	1	3	3
1	1	1	1	2	2	1	1	3	3
1	1	1	1	2	2	1	1	3	3
1	1	3	3	2	2	1	1	3	3
1	1	1	1	2	2	1	1	3	3
1	1	1	1	2	2	1	1	3	3
1	1	1	1	2	2	1	1	3	3
4	4	1	1	2	2	1	1	3	3
1	1	1	1	2	2	4	4	4	4
1	1	1	1	2	2	1	1	3	3
1	1	1	1	2	2	1	1	3	3
1	1	1	1	2	2	1	1	4	4
1	1	1	1	2	2	1	1	3	3
4	4	3	3	2	2	1	1	3	3
1	1	1	1	2	2	1	1	3	3
1	1	1	1	2	2	1	1	3	3
1	1	3	3	2	2	1	1	3	3
1	1	1	1	2	2	1	1	4	4
1	1	1	1	2	2	1	1	3	3
1	1	1	1	2	2	1	1	3	3
1	1	1	1	2	2	1	1	3	3
1	1	1	1	2	2	1	1	4	4
1	1	1	1	2	2	1	1	3	3
1	1	1	1	3	3	1	1	3	3
1	1	1	1	3	3	1	1	3	3
1	1	1	1	2	2	1	1	3	3
4	4	1	1	2	2	1	1	3	3
4	4	1	1	2	2	1	1	4	4
1	1	1	1	2	2	1	1	3	3
1	1	3	3	2	2	1	1	3	3
1	1	1	1	2	2	1	1	3	3
1	1	1	1	2	2	1	1	4	4
1	1	1	1	2	2	1	1	4	4
1	1	1	1	2	2	1	1	3	3
1	1	1	1	2	2	1	1	3	3
1	1	1	1	2	2	1	1	3	3
1	1	1	1	2	2	1	1	4	4
1	1	1	1	2	2	1	1	4	4
1	1	1	1	2	2	1	1	4	4
4	4	1	1	2	2	1	1	4	4
1	1	1	1	2	2	1	1	3	3
1	1	1	1	2	2	1	1	4	4
1	1	1	1	2	2	1	1	3	3
1	1	1	1	2	2	1	1	3	3
1	1	1	1	3	3	1	1	4	4
1	1	1	1	3	3	1	1	4	4
1	1	1	1	2	2	1	1	3	3
1	1	1	1	2	2	1	1	3	3
1	1	1	1	2	2	1	1	3	3
1	1	1	1	3	3	1	1	3	3

For Review Only

1	1	1	1	2	2	1	1	3	3
1	1	1	1	2	2	1	1	3	3
1	1	1	1	2	2	1	1	3	3
1	1	1	1	2	2	1	1	3	3
1	1	3	3	2	2	1	1	3	3
1	1	1	1	3	3	1	1	4	4
1	1	1	1	3	3	1	1	3	3
1	1	1	1	2	2	1	1	3	3
1	1	1	1	2	2	1	1	3	3
1	1	1	1	2	2	1	1	3	3
1	1	1	1	3	3	1	1	3	3
1	1	1	1	3	3	1	1	4	4
1	1	1	1	2	2	1	1	3	3
4	4	1	1	2	2	1	1	3	3
1	1	1	1	2	2	1	1	3	3
1	1	1	1	2	2	1	1	3	3
1	1	1	1	2	2	1	1	4	4
1	1	1	1	2	2	1	1	3	3
1	1	1	1	2	2	1	1	3	3
1	1	1	1	2	2	1	1	3	3
1	1	1	1	2	2	1	1	3	3
1	1	1	1	2	2	1	1	4	4
1	1	1	1	2	2	1	1	3	3
1	1	1	1	2	2	1	1	3	3
1	1	1	1	2	2	1	1	3	3
1	1	1	1	3	3	1	1	3	3
1	1	1	1	2	2	1	1	3	3
1	1	1	1	2	2	1	1	3	3
1	1	1	1	3	3	1	1	3	3
1	1	3	3	2	2	1	1	3	3
1	1	1	1	2	2	1	1	3	3
1	1	1	1	2	2	1	1	3	3
1	1	1	1	2	2	1	1	4	4
1	1	1	1	2	2	1	1	3	3
1	1	1	1	2	2	1	1	4	4
1	1	1	1	2	2	1	1	3	3
1	1	1	1	2	2	1	1	3	3
1	1	1	1	2	2	1	1	3	3
1	1	1	1	3	3	4	4	3	3
1	1	1	1	2	2	1	1	3	3
1	1	1	1	2	2	1	1	3	3
1	1	1	1	2	2	1	1	3	3
1	1	1	1	2	2	1	1	3	3
1	1	1	1	3	3	1	1	3	3
1	1	1	1	2	2	4	4	3	3
1	1	1	1	2	2	1	1	3	3
1	1	1	1	2	2	1	1	3	3
1	1	1	1	3	3	1	1	3	3
1	1	1	1	3	3	1	1	3	3
1	1	1	1	2	2	1	1	3	3
1	1	1	1	3	3	1	1	3	3
1	1	1	1	2	2	1	1	4	4
1	1	1	1	2	2	1	1	3	3
1	1	1	1	2	2	1	1	3	3
1	1	1	1	2	2	1	1	3	3

For Review Only

1	1	1	1	3	3	1	1	3	3
1	1	1	1	2	2	1	1	3	3
1	1	1	1	2	2	1	1	3	3
1	1	1	1	2	2	1	1	3	3
1	1	1	1	3	3	1	1	3	3
1	1	1	1	3	3	1	1	3	3
1	1	3	3	3	3	1	1	3	3
1	1	1	1	2	2	1	1	4	4
1	1	1	1	2	2	1	1	3	3
1	1	1	1	2	2	1	1	3	3
1	1	1	1	2	2	1	1	3	3
1	1	1	1	2	2	1	1	3	3
1	1	1	1	3	3	1	1	3	3
1	1	1	1	2	2	1	1	3	3
1	1	1	1	2	2	1	1	3	3
1	1	1	1	2	2	1	1	3	3
1	1	1	1	2	2	1	1	3	3
1	1	1	1	2	2	1	1	3	3
1	1	1	1	2	2	1	1	3	3
1	1	1	1	2	2	1	1	3	3
1	1	1	1	2	2	1	1	3	3
1	1	1	1	2	2	1	1	3	3
1	1	3	3	2	2	1	1	3	3
1	1	1	1	2	2	1	1	4	4
1	1	1	1	2	2	1	1	3	3
1	1	1	1	2	2	1	1	4	4
1	1	1	1	2	2	1	1	3	3
1	1	1	1	2	2	1	1	3	3
1	1	1	1	2	2	4	4	3	3
1	1	1	1	2	2	1	1	3	3
1	1	1	1	2	2	1	1	3	3
1	1	1	1	2	2	1	1	3	3
1	1	1	1	2	2	1	1	3	3
1	1	1	1	3	3	1	1	3	3
1	1	1	1	2	2	1	1	4	4
1	1	1	1	2	2	1	1	3	3
1	1	1	1	2	2	1	1	3	3
1	1	1	1	2	2	1	1	3	3
1	1	1	1	2	2	1	1	3	3
1	1	1	1	2	2	1	1	4	4
1	1	1	1	2	2	1	1	3	3
1	1	1	1	2	2	1	1	4	4
1	1	1	1	2	2	1	1	3	3
1	1	1	1	2	2	1	1	3	3
1	1	1	1	2	2	1	1	3	3
1	1	1	1	2	2	1	1	3	3
1	1	1	1	3	3	1	1	3	3
1	1	3	3	2	2	1	1	3	3
1	1	1	1	2	2	1	1	3	3
1	1	1	1	3	3	1	1	3	3
1	1	1	1	2	2	1	1	3	3
1	1	1	1	2	2	1	1	4	4
1	1	1	1	2	2	4	4	3	3
1	1	1	1	2	2	1	1	4	4
1	1	1	1	2	2	1	1	3	3

For Review Only

1	1	1	1	2	2	1	1	3	3
1	1	1	1	3	3	1	1	4	4
1	1	1	1	2	2	1	1	3	3
1	1	1	1	2	2	1	1	3	3
1	1	1	1	2	2	1	1	3	3
1	1	1	1	2	2	1	1	3	3
1	1	1	1	2	2	1	1	4	4
1	1	1	1	2	2	1	1	3	3
1	1	1	1	2	2	1	1	4	4
1	1	1	1	2	2	1	1	3	3
1	1	1	1	3	3	1	1	3	3
1	1	1	1	2	2	1	1	3	3
1	1	1	1	2	2	1	1	3	3
1	1	1	1	2	2	1	1	3	3
1	1	1	1	2	2	1	1	4	4
1	1	1	1	2	2	4	4	4	4
1	1	1	1	2	2	4	4	3	3
1	1	1	1	2	2	1	1	3	3
1	1	1	1	2	2	1	1	3	3
1	1	3	3	3	3	1	1	3	3
1	1	1	1	2	2	1	1	3	3
1	1	1	1	2	2	1	1	4	4
1	1	1	1	2	2	1	1	4	4
1	1	1	1	3	3	1	1	4	4
4	4	1	1	2	2	1	1	3	3
1	1	1	1	2	2	1	1	4	4
1	1	1	1	3	3	1	1	3	3
1	1	1	1	2	2	1	1	3	3
1	1	1	1	2	2	1	1	3	3
1	1	1	1	2	2	1	1	3	3
1	1	1	1	2	2	1	1	3	3
1	1	1	1	2	2	1	1	3	3
1	1	1	1	2	2	1	1	3	3
1	1	1	1	2	2	1	1	4	4
1	1	1	1	2	2	1	1	3	3
1	1	1	1	3	3	1	1	4	4
1	1	1	1	2	2	1	1	3	3
1	1	1	1	2	2	1	1	3	3
1	1	3	3	2	2	1	1	3	3
1	1	1	1	2	2	1	1	3	3
1	1	1	1	2	2	1	1	3	3
1	1	1	1	3	3	1	1	3	3
1	1	1	1	2	2	1	1	3	3
1	1	1	1	2	2	1	1	3	3
1	1	1	1	2	2	1	1	3	3
1	1	1	1	2	2	1	1	3	3
1	1	1	1	2	2	4	4	4	4
1	1	1	1	2	2	1	1	4	4
1	1	1	1	2	2	1	1	3	3
1	1	1	1	2	2	1	1	3	3
1	1	1	1	2	2	4	4	3	3
1	1	1	1	2	2	1	1	3	3
1	1	1	1	2	2	1	1	4	4
1	1	1	1	3	3	1	1	3	3
1	1	1	1	2	2	1	1	3	3

For Review Only

1	1	1	1	2	2	4	4	3	3
4	4	1	1	2	2	1	1	3	3
1	1	3	3	2	2	1	1	3	3
1	1	1	1	2	2	1	1	3	3
4	4	1	1	2	2	1	1	4	4
1	1	1	1	2	2	1	1	3	3
1	1	1	1	2	2	1	1	3	3
1	1	1	1	2	2	1	1	3	3
1	1	1	1	2	2	1	1	3	3
1	1	1	1	2	2	1	1	4	4
1	1	1	1	2	2	1	1	3	3
1	1	1	1	2	2	1	1	3	3
1	1	1	1	3	3	4	4	3	3
1	1	1	1	3	3	1	1	3	3
1	1	1	1	3	3	1	1	3	3
1	1	1	1	2	2	4	4	4	4
1	1	1	1	2	2	1	1	4	4
1	1	1	1	2	2	1	1	3	3
1	1	1	1	2	2	1	1	4	4
1	1	1	1	2	2	1	1	3	3
1	1	1	1	2	2	1	1	3	3
1	1	1	1	3	3	1	1	3	3
1	1	1	1	2	2	1	1	3	3
1	1	1	1	2	2	1	1	3	3
1	1	1	1	2	2	1	1	3	3
4	4	1	1	3	3	1	1	3	3
1	1	1	1	2	2	1	1	4	4
1	1	1	1	3	3	1	1	3	3
4	4	1	1	2	2	1	1	4	4
1	1	1	1	2	2	4	4	3	3
1	1	1	1	2	2	1	1	4	4
1	1	1	1	2	2	1	1	4	4
1	1	1	1	2	2	1	1	3	3
1	1	1	1	3	3	1	1	3	3
1	1	1	1	2	2	1	1	4	4
1	1	1	1	2	2	1	1	4	4
1	1	1	1	2	2	4	4	3	3
1	1	1	1	3	3	1	1	4	4
1	1	1	1	2	2	1	1	3	3
1	1	1	1	2	2	1	1	4	4
1	1	1	1	2	2	1	1	3	3
1	1	1	1	2	2	1	1	3	3
1	1	1	1	2	2	1	1	4	4
1	1	1	1	2	2	1	1	3	3
1	1	1	1	2	2	1	1	3	3
1	1	1	1	3	3	4	4	3	3
1	1	1	1	3	3	1	1	3	3
1	1	1	1	3	3	1	1	3	3
1	1	3	3	2	2	1	1	3	3
1	1	1	1	2	2	1	1	4	4
1	1	1	1	2	2	1	1	3	3
1	1	1	1	2	2	1	1	4	4
1	1	1	1	2	2	1	1	3	3
1	1	1	1	3	3	1	1	3	3
1	1	1	1	2	2	1	1	4	4

For Review Only

1	1	1	1	2	2	4	4	3	3
1	1	1	1	2	2	1	1	3	3

For Review Only

SNP1063	SNP1063	SNP1064	SNP1064	SNP1065	SNP1065	SNP1066	SNP1066	SNP1067	SNP1067
1	1	2	2	3	3	2	2	3	3
1	1	1	1	3	3	2	2	3	3
1	1	2	2	3	3	2	2	3	3
1	1	2	2	3	3	2	2	3	3
1	1	2	2	2	2	2	2	3	3
1	1	2	2	3	3	2	2	3	3
1	1	2	2	3	3	2	2	3	3
1	1	2	2	3	3	2	2	3	3
1	1	2	2	3	3	2	2	3	3
1	1	2	2	3	3	2	2	3	3
1	1	2	2	2	2	3	3	3	3
1	1	2	2	3	3	2	2	3	3
1	1	2	2	3	3	2	2	3	3
1	1	2	2	2	2	3	3	3	3
1	1	2	2	2	2	2	2	3	3
1	1	2	2	3	3	2	2	3	3
1	1	2	2	3	3	2	2	3	3
1	1	2	2	3	3	2	2	3	3
1	1	2	2	3	3	2	2	3	3
1	1	2	2	2	2	3	3	3	3
1	1	2	2	3	3	2	2	2	2
1	1	2	2	3	3	2	2	3	3
1	1	1	1	3	3	2	2	3	3
1	1	2	2	2	2	3	3	3	3
1	1	2	2	3	3	2	2	3	3
1	1	2	2	3	3	2	2	3	3
1	1	2	2	3	3	2	2	3	3
1	1	2	2	2	2	2	2	3	3
1	1	2	2	3	3	2	2	2	2
1	1	1	1	2	2	3	3	3	3
1	1	2	2	3	3	2	2	3	3
1	1	2	2	3	3	2	2	3	3
1	1	2	2	3	3	2	2	3	3
1	1	2	2	2	2	3	3	3	3
1	1	2	2	2	2	3	3	3	3
1	1	2	2	3	3	2	2	3	3
3	3	2	2	3	3	3	3	3	3
3	3	2	2	3	3	3	3	3	3
1	1	1	1	2	2	3	3	3	3
1	1	1	1	2	2	3	3	3	3
1	1	2	2	2	2	3	3	3	3
1	1	2	2	2	2	3	3	2	2
1	1	1	1	3	3	2	2	2	2
1	1	2	2	2	2	3	3	3	3
1	1	2	2	3	3	2	2	3	3
1	1	2	2	3	3	2	2	3	3
1	1	2	2	2	2	3	3	3	3
1	1	1	1	2	2	3	3	3	3
1	1	2	2	3	3	2	2	3	3
1	1	2	2	3	3	2	2	3	3
1	1	2	2	3	3	2	2	2	2
1	1	1	1	3	3	2	2	3	3

For Review Only

1	1	2	2	3	3	2	2	3	3
1	1	2	2	3	3	2	2	3	3
1	1	2	2	3	3	2	2	3	3
1	1	1	1	3	3	2	2	3	3
1	1	2	2	3	3	2	2	3	3
1	1	2	2	2	2	3	3	3	3
1	1	2	2	3	3	2	2	3	3
1	1	1	1	3	3	2	2	3	3
1	1	1	1	3	3	2	2	3	3
1	1	2	2	2	2	2	2	3	3
1	1	2	2	3	3	2	2	3	3
1	1	2	2	2	2	3	3	3	3
1	1	2	2	3	3	2	2	3	3
1	1	2	2	3	3	2	2	2	2
1	1	2	2	2	2	2	2	3	3
1	1	2	2	3	3	2	2	3	3
1	1	1	1	2	2	2	2	3	3
1	1	2	2	3	3	2	2	3	3
1	1	2	2	3	3	2	2	3	3
1	1	2	2	3	3	2	2	3	3
1	1	2	2	2	2	3	3	3	3
1	1	1	1	2	2	3	3	3	3
1	1	2	2	3	3	2	2	2	2
1	1	2	2	3	3	2	2	3	3
1	1	2	2	3	3	2	2	3	3
1	1	2	2	3	3	2	2	3	3
1	1	2	2	3	3	2	2	3	3
3	3	1	1	3	3	3	3	3	3
1	1	2	2	3	3	2	2	3	3
1	1	2	2	3	3	2	2	3	3
1	1	2	2	3	3	2	2	3	3
1	1	2	2	3	3	2	2	3	3
1	1	1	1	2	2	3	3	3	3
1	1	2	2	3	3	2	2	3	3
1	1	2	2	2	2	3	3	3	3
1	1	2	2	3	3	2	2	3	3
1	1	2	2	3	3	2	2	3	3
1	1	2	2	3	3	2	2	3	3
1	1	2	2	3	3	2	2	3	3
1	1	2	2	3	3	2	2	3	3
1	1	2	2	3	3	2	2	3	3
1	1	2	2	3	3	2	2	3	3
1	1	1	1	3	3	2	2	2	2
1	1	2	2	3	3	2	2	3	3
1	1	2	2	3	3	2	2	3	3
1	1	2	2	3	3	2	2	3	3
3	3	2	2	3	3	3	3	3	3
1	1	2	2	2	2	2	2	3	3
1	1	2	2	3	3	2	2	3	3
3	3	1	1	3	3	3	3	3	3
1	1	2	2	3	3	2	2	3	3
1	1	1	1	2	2	3	3	3	3
1	1	1	1	3	3	2	2	3	3
1	1	2	2	3	3	2	2	3	3
3	3	2	2	3	3	3	3	3	3

For Review Only

1	1	1	1	2	2	2	2	3	3
1	1	2	2	3	3	2	2	3	3
1	1	2	2	3	3	2	2	3	3
3	3	1	1	3	3	3	3	3	3
1	1	2	2	3	3	2	2	3	3
1	1	2	2	3	3	2	2	3	3
1	1	2	2	3	3	2	2	3	3
1	1	2	2	2	2	2	2	3	3
1	1	2	2	3	3	2	2	3	3
1	1	2	2	3	3	2	2	3	3
1	1	2	2	3	3	2	2	3	3
1	1	2	2	2	2	2	2	3	3
1	1	1	1	3	3	2	2	2	2
1	1	2	2	3	3	2	2	3	3
1	1	2	2	3	3	2	2	3	3
1	1	2	2	2	2	2	2	3	3
1	1	1	1	3	3	2	2	3	3
1	1	2	2	2	2	2	2	3	3
3	3	1	1	3	3	3	3	3	3
1	1	2	2	3	3	2	2	3	3
1	1	2	2	2	2	2	2	3	3
1	1	2	2	3	3	2	2	3	3
1	1	2	2	3	3	2	2	3	3
1	1	1	1	2	2	3	3	3	3
1	1	2	2	2	2	2	2	3	3
1	1	2	2	2	2	3	3	3	3
1	1	2	2	3	3	2	2	3	3
1	1	2	2	3	3	2	2	3	3
1	1	2	2	3	3	2	2	3	3
1	1	2	2	3	3	2	2	3	3
1	1	2	2	3	3	3	3	3	3
1	1	2	2	3	3	2	2	3	3
1	1	2	2	3	3	2	2	3	3
1	1	2	2	2	2	2	2	3	3
1	1	2	2	2	2	3	3	3	3
1	1	1	1	2	2	3	3	3	3
3	3	2	2	3	3	3	3	3	3
1	1	2	2	3	3	2	2	3	3
1	1	2	2	2	2	3	3	3	3
1	1	1	1	2	2	3	3	3	3
1	1	1	1	3	3	2	2	3	3
1	1	2	2	2	2	3	3	3	3
1	1	2	2	3	3	2	2	3	3
1	1	2	2	2	2	2	2	3	3
1	1	2	2	2	2	3	3	3	3
1	1	2	2	3	3	2	2	3	3
1	1	2	2	3	3	2	2	3	3
1	1	2	2	3	3	2	2	3	3
1	1	2	2	3	3	2	2	3	3
1	1	2	2	3	3	2	2	3	3
1	1	2	2	3	3	2	2	3	3
1	1	2	2	2	2	3	3	3	3
1	1	2	2	3	3	2	2	3	3
1	1	2	2	2	2	3	3	3	3
1	1	2	2	3	3	2	2	3	3

For Review Only

1	1	2	2	3	3	2	2	3	3
1	1	1	1	2	2	3	3	2	2
1	1	2	2	3	3	2	2	3	3
1	1	2	2	2	2	2	2	3	3
1	1	2	2	3	3	2	2	3	3
1	1	1	1	3	3	2	2	3	3
1	1	1	1	2	2	3	3	3	3
1	1	2	2	2	2	2	2	3	3
1	1	2	2	2	2	3	3	3	3
1	1	2	2	3	3	2	2	3	3
1	1	2	2	2	2	2	2	3	3
1	1	1	1	2	2	3	3	3	3
1	1	2	2	3	3	2	2	3	3
1	1	2	2	3	3	2	2	3	3
1	1	2	2	2	2	3	3	3	3
1	1	1	1	2	2	3	3	3	3
1	1	2	2	3	3	2	2	3	3
1	1	2	2	3	3	2	2	3	3
1	1	1	1	3	3	2	2	3	3
1	1	2	2	3	3	2	2	3	3
1	1	2	2	3	3	2	2	3	3
1	1	1	1	2	2	3	3	3	3
1	1	1	1	2	2	3	3	3	3
1	1	2	2	2	2	3	3	3	3
1	1	2	2	2	2	3	3	2	2
1	1	2	2	2	2	3	3	3	3
1	1	2	2	3	3	2	2	3	3
1	1	2	2	2	2	2	2	3	3
1	1	2	2	3	3	2	2	3	3
1	1	2	2	3	3	2	2	3	3
1	1	2	2	3	3	2	2	3	3
1	1	2	2	3	3	2	2	3	3
1	1	2	2	3	3	2	2	3	3
1	1	1	1	2	2	3	3	3	3
1	1	2	2	3	3	2	2	3	3
1	1	2	2	2	2	3	3	3	3
1	1	2	2	2	2	2	2	3	3
1	1	2	2	3	3	2	2	3	3
1	1	2	2	3	3	2	2	3	3
1	1	2	2	3	3	2	2	3	3
1	1	2	2	3	3	2	2	3	3
1	1	2	2	3	3	2	2	3	3
3	3	1	1	3	3	3	3	3	3
1	1	2	2	2	2	2	2	3	3
1	1	2	2	3	3	2	2	3	3
1	1	2	2	3	3	2	2	3	3
1	1	1	1	2	2	3	3	3	3
1	1	1	1	2	2	3	3	3	3
1	1	2	2	3	3	2	2	3	3
1	1	1	1	3	3	2	2	3	3
1	1	2	2	3	3	2	2	3	3
3	3	1	1	3	3	3	3	3	3
1	1	1	1	2	2	3	3	3	3
1	1	2	2	3	3	2	2	3	3
3	3	1	1	3	3	3	3	3	3

For Review Only

1	1	2	2	3	3	2	2	3	3
1	1	2	2	2	2	3	3	3	3
1	1	2	2	3	3	2	2	3	3
1	1	1	1	2	2	2	2	3	3
1	1	2	2	2	2	3	3	3	3
1	1	2	2	3	3	2	2	3	3
3	3	1	1	3	3	3	3	3	3
1	1	1	1	3	3	2	2	3	3
1	1	2	2	3	3	2	2	3	3
1	1	1	1	2	2	3	3	3	3
1	1	2	2	2	2	2	2	3	3
1	1	2	2	2	2	3	3	3	3
1	1	2	2	3	3	2	2	3	3
1	1	2	2	3	3	2	2	3	3
1	1	2	2	3	3	2	2	3	3
1	1	1	1	2	2	3	3	3	3
1	1	1	1	2	2	3	3	3	3
1	1	2	2	3	3	2	2	3	3
1	1	2	2	2	2	3	3	3	3
3	3	2	2	3	3	3	3	3	3
1	1	2	2	2	2	2	2	3	3
1	1	2	2	3	3	2	2	3	3
1	1	2	2	3	3	2	2	3	3
1	1	1	1	3	3	2	2	3	3
1	1	1	1	3	3	2	2	3	3
1	1	2	2	3	3	2	2	3	3
1	1	1	1	2	2	3	3	3	3
1	1	2	2	3	3	2	2	3	3
1	1	2	2	2	2	3	3	3	3
1	1	2	2	3	3	2	2	3	3
1	1	1	1	2	2	3	3	3	3
1	1	2	2	2	2	3	3	3	3
3	3	2	2	3	3	3	3	3	3
1	1	2	2	3	3	2	2	3	3
1	1	1	1	2	2	3	3	3	3
1	1	1	1	2	2	3	3	3	3
1	1	2	2	3	3	2	2	3	3
1	1	2	2	2	2	3	3	3	3
1	1	2	2	3	3	2	2	3	3
1	1	1	1	2	2	3	3	3	3
1	1	2	2	3	3	2	2	3	3
1	1	2	2	3	3	2	2	3	3
1	1	1	1	2	2	3	3	3	3
1	1	2	2	3	3	2	2	3	3
1	1	2	2	3	3	2	2	3	3
1	1	1	1	3	3	2	2	3	3
1	1	1	1	2	2	3	3	3	3
1	1	2	2	3	3	2	2	3	3
1	1	2	2	3	3	2	2	3	3
1	1	1	1	2	2	3	3	3	3
1	1	2	2	3	3	2	2	3	3
1	1	1	1	2	2	3	3	3	3
1	1	2	2	3	3	2	2	3	3
1	1	2	2	3	3	2	2	3	3
1	1	1	1	2	2	3	3	3	3

For Review Only

1	1	2	2	3	3	2	2	3	3
1	1	2	2	3	3	2	2	3	3

For Review Only

SNP1068	SNP1068	SNP1069	SNP1069	SNP107	SNP107	SNP1070	SNP1070	SNP1071	SNP1071
1	1	3	3	3	3	1	1	3	3
1	1	2	2	3	3	1	1	1	1
1	1	3	3	3	3	1	1	3	3
1	1	2	2	3	3	4	4	3	3
1	1	2	2	3	3	1	1	3	3
1	1	3	3	2	2	1	1	3	3
1	1	3	3	3	3	1	1	3	3
1	1	2	2	3	3	1	1	1	1
1	1	3	3	3	3	1	1	3	3
1	1	2	2	3	3	1	1	3	3
4	4	2	2	3	3	1	1	3	3
1	1	2	2	3	3	1	1	3	3
1	1	2	2	3	3	1	1	3	3
1	1	2	2	3	3	1	1	3	3
1	1	2	2	3	3	1	1	3	3
1	1	2	2	2	2	4	4	3	3
1	1	2	2	3	3	1	1	3	3
1	1	2	2	3	3	1	1	3	3
1	1	2	2	2	2	1	1	3	3
1	1	3	3	3	3	1	1	1	1
1	1	2	2	3	3	1	1	3	3
1	1	2	2	3	3	1	1	3	3
1	1	2	2	3	3	1	1	1	1
1	1	2	2	3	3	1	1	1	1
1	1	2	2	3	3	4	4	3	3
1	1	2	2	3	3	1	1	3	3
1	1	2	2	3	3	1	1	3	3
1	1	2	2	3	3	1	1	1	1
1	1	2	2	3	3	1	1	3	3
1	1	2	2	3	3	1	1	1	1
1	1	2	2	3	3	1	1	3	3
1	1	3	3	2	2	1	1	3	3
1	1	3	3	3	3	1	1	3	3
1	1	2	2	3	3	1	1	3	3
1	1	2	2	3	3	1	1	3	3
1	1	2	2	3	3	1	1	3	3
1	1	3	3	3	3	1	1	3	3
1	1	2	2	3	3	1	1	1	1
1	1	2	2	3	3	1	1	1	1
1	1	2	2	3	3	1	1	1	1
1	1	2	2	3	3	1	1	1	1
1	1	2	2	3	3	1	1	3	3
1	1	2	2	3	3	1	1	3	3
1	1	2	2	3	3	1	1	3	3
1	1	2	2	3	3	1	1	1	1
1	1	3	3	3	3	1	1	3	3
1	1	2	2	3	3	1	1	3	3
1	1	2	2	3	3	1	1	3	3
1	1	2	2	3	3	1	1	1	1
1	1	3	3	3	3	1	1	3	3
1	1	2	2	3	3	1	1	3	3
1	1	2	2	3	3	1	1	1	1

For Review Only

1	1	2	2	3	3	1	1	3	3
1	1	3	3	3	3	1	1	3	3
1	1	2	2	2	2	1	1	3	3
1	1	2	2	3	3	1	1	1	1
1	1	2	2	2	2	1	1	3	3
1	1	2	2	3	3	1	1	3	3
1	1	2	2	3	3	1	1	3	3
1	1	2	2	3	3	1	1	1	1
1	1	3	3	3	3	1	1	1	1
1	1	2	2	3	3	1	1	1	1
1	1	2	2	3	3	1	1	3	3
1	1	2	2	3	3	1	1	3	3
1	1	2	2	3	3	1	1	3	3
1	1	2	2	3	3	1	1	3	3
1	1	2	2	3	3	1	1	1	1
1	1	3	3	3	3	1	1	3	3
1	1	3	3	3	3	1	1	1	1
1	1	3	3	3	3	1	1	3	3
1	1	2	2	3	3	1	1	3	3
1	1	2	2	3	3	1	1	3	3
1	1	2	2	3	3	1	1	3	3
1	1	2	2	3	3	1	1	1	1
1	1	2	2	3	3	1	1	3	3
1	1	3	3	3	3	1	1	3	3
1	1	2	2	3	3	1	1	1	1
1	1	2	2	3	3	1	1	3	3
1	1	3	3	3	3	1	1	3	3
1	1	2	2	3	3	1	1	1	1
1	1	2	2	3	3	1	1	3	3
1	1	3	3	2	2	1	1	3	3
1	1	2	2	3	3	1	1	3	3
1	1	2	2	2	2	1	1	1	1
1	1	2	2	3	3	1	1	1	1
1	1	2	2	3	3	1	1	3	3
1	1	2	2	3	3	1	1	3	3
1	1	2	2	3	3	1	1	1	1
1	1	2	2	3	3	1	1	3	3
1	1	2	2	3	3	1	1	1	1
4	4	2	2	3	3	1	1	3	3
1	1	2	2	3	3	1	1	1	1
1	1	2	2	3	3	1	1	3	3
1	1	3	3	3	3	1	1	3	3
1	1	2	2	3	3	4	4	1	1
1	1	2	2	3	3	1	1	3	3
4	4	3	3	3	3	1	1	3	3
1	1	2	2	3	3	1	1	1	1
1	1	2	2	3	3	1	1	1	1
1	1	2	2	3	3	1	1	3	3
1	1	2	2	3	3	1	1	3	3
1	1	2	2	3	3	1	1	1	1
1	1	2	2	3	3	1	1	3	3
1	1	2	2	3	3	1	1	1	1
1	1	2	2	3	3	1	1	1	1
1	1	2	2	3	3	1	1	1	1
1	1	3	3	3	3	1	1	3	3

For Review Only

1	1	2	2	3	3	1	1	1	1
1	1	2	2	3	3	1	1	3	3
1	1	2	2	3	3	1	1	3	3
1	1	2	2	3	3	1	1	1	1
1	1	2	2	3	3	1	1	3	3
1	1	2	2	3	3	1	1	3	3
1	1	3	3	2	2	4	4	3	3
1	1	2	2	3	3	4	4	3	3
1	1	2	2	3	3	1	1	3	3
1	1	2	2	3	3	1	1	3	3
1	1	3	3	3	3	1	1	3	3
1	1	2	2	3	3	1	1	1	1
1	1	2	2	3	3	1	1	1	1
1	1	2	2	3	3	1	1	3	3
1	1	2	2	3	3	1	1	1	1
1	1	2	2	3	3	1	1	3	3
1	1	3	3	3	3	1	1	1	1
1	1	2	2	3	3	1	1	3	3
1	1	2	2	3	3	1	1	1	1
1	1	3	3	3	3	1	1	1	1
1	1	2	2	3	3	1	1	1	1
1	1	2	2	2	2	1	1	1	1
1	1	3	3	2	2	1	1	3	3
1	1	2	2	3	3	1	1	1	1
1	1	2	2	3	3	1	1	1	1
1	1	2	2	3	3	1	1	3	3
1	1	2	2	3	3	1	1	3	3
1	1	2	2	3	3	1	1	3	3
4	4	2	2	3	3	1	1	3	3
1	1	3	3	3	3	1	1	1	1
1	1	2	2	3	3	1	1	1	1
1	1	2	2	3	3	1	1	1	1
1	1	2	2	3	3	1	1	3	3
1	1	2	2	3	3	1	1	1	1
1	1	2	2	3	3	1	1	3	3
1	1	2	2	3	3	1	1	3	3
1	1	2	2	3	3	1	1	1	1
1	1	2	2	3	3	1	1	3	3
1	1	2	2	3	3	1	1	3	3
1	1	2	2	3	3	1	1	1	1
1	1	2	2	3	3	1	1	1	1
1	1	2	2	3	3	1	1	3	3
4	4	2	2	3	3	1	1	3	3
1	1	2	2	3	3	1	1	1	1
1	1	2	2	3	3	1	1	3	3
1	1	2	2	3	3	1	1	1	1
1	1	2	2	3	3	1	1	3	3
1	1	2	2	2	2	1	1	3	3
1	1	2	2	3	3	1	1	1	1
1	1	2	2	3	3	1	1	3	3
1	1	2	2	3	3	1	1	3	3
1	1	2	2	3	3	1	1	3	3
4	4	2	2	3	3	1	1	3	3
1	1	2	2	3	3	1	1	3	3
1	1	2	2	3	3	1	1	1	1

For Review Only

1	1	2	2	3	3	1	1	3	3
1	1	2	2	3	3	1	1	1	1
1	1	2	2	3	3	1	1	1	1
1	1	2	2	3	3	1	1	1	1
1	1	2	2	3	3	1	1	3	3
1	1	2	2	3	3	1	1	1	1
1	1	2	2	3	3	1	1	1	1
1	1	2	2	3	3	1	1	3	3
1	1	3	3	3	3	1	1	1	1
1	1	2	2	3	3	1	1	3	3
1	1	2	2	3	3	1	1	3	3
1	1	2	2	3	3	1	1	1	1
1	1	3	3	3	3	1	1	3	3
1	1	2	2	3	3	1	1	1	1
1	1	2	2	3	3	1	1	3	3
4	4	2	2	3	3	1	1	1	1
4	4	2	2	3	3	1	1	3	3
1	1	2	2	3	3	1	1	3	3
1	1	2	2	3	3	1	1	1	1
1	1	2	2	3	3	4	4	3	3
1	1	2	2	3	3	1	1	1	1
1	1	2	2	3	3	1	1	1	1
1	1	2	2	3	3	1	1	1	1
1	1	2	2	3	3	1	1	3	3
1	1	2	2	3	3	1	1	3	3
1	1	2	2	3	3	1	1	3	3
1	1	2	2	3	3	1	1	3	3
1	1	2	2	3	3	1	1	1	1
1	1	2	2	3	3	1	1	3	3
1	1	3	3	3	3	1	1	3	3
1	1	3	3	3	3	1	1	3	3
1	1	3	3	3	3	1	1	3	3
1	1	2	2	3	3	1	1	3	3
1	1	2	2	3	3	1	1	1	1
1	1	3	3	3	3	1	1	1	1
1	1	2	2	3	3	1	1	3	3
1	1	2	2	3	3	1	1	3	3
1	1	3	3	3	3	1	1	3	3
1	1	2	2	2	2	1	1	3	3
1	1	2	2	3	3	1	1	3	3
1	1	3	3	3	3	1	1	3	3
1	1	2	2	3	3	1	1	3	3
1	1	2	2	3	3	1	1	1	1
1	1	2	2	3	3	1	1	3	3
1	1	2	2	3	3	1	1	3	3
1	1	2	2	3	3	1	1	3	3
4	4	2	2	3	3	1	1	1	1
1	1	2	2	3	3	1	1	1	1
1	1	2	2	3	3	1	1	3	3
1	1	2	2	3	3	1	1	1	1
4	4	2	2	3	3	1	1	3	3
1	1	3	3	3	3	1	1	1	1
1	1	2	2	3	3	1	1	1	1
1	1	2	2	3	3	1	1	3	3
1	1	2	2	3	3	1	1	1	1

For Review Only

4	4	2	2	3	3	1	1	3	3
1	1	3	3	3	3	1	1	1	1
1	1	2	2	2	2	1	1	3	3
1	1	2	2	3	3	1	1	1	1
1	1	2	2	3	3	1	1	1	1
1	1	2	2	3	3	1	1	3	3
1	1	2	2	3	3	1	1	3	3
1	1	2	2	3	3	1	1	3	3
1	1	2	2	3	3	1	1	3	3
1	1	2	2	3	3	1	1	1	1
1	1	2	2	3	3	1	1	1	1
1	1	3	3	3	3	1	1	3	3
4	4	2	2	3	3	1	1	3	3
1	1	2	2	3	3	1	1	3	3
1	1	2	2	3	3	1	1	3	3
4	4	2	2	3	3	1	1	1	1
1	1	2	2	3	3	1	1	1	1
1	1	2	2	3	3	1	1	3	3
1	1	2	2	3	3	1	1	3	3
1	1	2	2	3	3	1	1	1	1
1	1	3	3	3	3	1	1	3	3
1	1	2	2	3	3	1	1	3	3
1	1	3	3	2	2	1	1	3	3
1	1	2	2	3	3	1	1	1	1
1	1	2	2	3	3	1	1	1	1
1	1	2	2	3	3	1	1	3	3
1	1	2	2	3	3	1	1	1	1
1	1	2	2	3	3	1	1	3	3
1	1	2	2	3	3	1	1	3	3
4	4	2	2	3	3	1	1	3	3
1	1	2	2	3	3	1	1	1	1
1	1	2	2	3	3	1	1	3	3
1	1	3	3	3	3	1	1	1	1
1	1	2	2	3	3	1	1	1	1
1	1	3	3	3	3	1	1	1	1
1	1	2	2	3	3	1	1	1	1
4	4	2	2	3	3	1	1	3	3
1	1	2	2	3	3	1	1	3	3
1	1	2	2	3	3	1	1	3	3
1	1	2	2	3	3	1	1	1	1
1	1	3	3	3	3	1	1	3	3
1	1	2	2	3	3	1	1	3	3
1	1	2	2	3	3	1	1	1	1
1	1	2	2	3	3	1	1	3	3
1	1	2	2	3	3	1	1	3	3
4	4	2	2	3	3	1	1	1	1
1	1	2	2	3	3	1	1	1	1
1	1	2	2	3	3	4	4	3	3
1	1	2	2	2	2	1	1	1	1
1	1	2	2	3	3	1	1	1	1
1	1	2	2	3	3	1	1	1	1
1	1	2	2	3	3	1	1	1	1
1	1	3	3	3	3	1	1	3	3
1	1	2	2	3	3	1	1	3	3
1	1	2	2	3	3	1	1	1	1

For Review Only

4	4	2	2	3	3	1	1	3	3
1	1	2	2	3	3	1	1	3	3

For Review Only

SNP1072	SNP1072	SNP1073	SNP1073	SNP1074	SNP1074	SNP1075	SNP1075	SNP1076	SNP1076
2	2	3	3	2	2	4	4	3	3
2	2	3	3	2	2	1	1	4	4
2	2	3	3	2	2	4	4	3	3
2	2	3	3	2	2	4	4	3	3
2	2	3	3	2	2	4	4	3	3
2	2	3	3	4	4	4	4	3	3
2	2	3	3	2	2	4	4	3	3
2	2	3	3	2	2	4	4	3	3
2	2	3	3	2	2	4	4	3	3
2	2	2	2	2	2	4	4	3	3
2	2	2	2	2	2	4	4	3	3
2	2	3	3	2	2	4	4	3	3
2	2	3	3	2	2	4	4	3	3
2	2	3	3	2	2	4	4	3	3
2	2	3	3	2	2	4	4	3	3
2	2	3	3	2	2	4	4	3	3
2	2	3	3	2	2	4	4	3	3
2	2	3	3	2	2	4	4	3	3
2	2	3	3	4	4	4	4	3	3
1	1	3	3	4	4	4	4	3	3
2	2	3	3	2	2	4	4	3	3
2	2	3	3	2	2	4	4	3	3
2	2	2	2	2	2	1	1	4	4
2	2	3	3	2	2	4	4	3	3
2	2	3	3	2	2	4	4	3	3
2	2	3	3	2	2	4	4	3	3
2	2	3	3	2	2	4	4	3	3
2	2	3	3	2	2	4	4	3	3
2	2	3	3	2	2	4	4	3	3
2	2	3	3	2	2	4	4	3	3
2	2	3	3	4	4	4	4	3	3
1	1	3	3	4	4	4	4	3	3
1	1	3	3	4	4	4	4	3	3
2	2	3	3	2	2	4	4	3	3
2	2	3	3	2	2	4	4	4	4
2	2	3	3	2	2	4	4	3	3
2	2	3	3	2	2	4	4	3	3
2	2	3	3	2	2	4	4	3	3
2	2	2	2	2	2	1	1	4	4
2	2	2	2	2	2	1	1	4	4
1	1	2	2	2	2	4	4	3	3
2	2	3	3	2	2	4	4	3	3
2	2	3	3	2	2	4	4	3	3
2	2	2	2	2	2	4	4	3	3
2	2	3	3	2	2	4	4	3	3
2	2	3	3	2	2	4	4	3	3
2	2	3	3	2	2	4	4	3	3
2	2	3	3	2	2	1	1	4	4
2	2	3	3	2	2	4	4	3	3
2	2	3	3	2	2	4	4	3	3
2	2	3	3	2	2	4	4	3	3
1	1	3	3	2	2	1	1	4	4

For Review Only

2	2	3	3	2	2	4	4	3	3
1	1	3	3	4	4	4	4	3	3
2	2	3	3	4	4	4	4	3	3
2	2	3	3	2	2	4	4	3	3
2	2	3	3	4	4	4	4	3	3
2	2	3	3	2	2	4	4	3	3
2	2	3	3	2	2	4	4	3	3
2	2	3	3	2	2	4	4	3	3
2	2	3	3	2	2	4	4	3	3
2	2	3	3	2	2	4	4	3	3
2	2	3	3	2	2	4	4	3	3
2	2	3	3	2	2	4	4	3	3
2	2	3	3	2	2	4	4	3	3
2	2	3	3	2	2	4	4	3	3
2	2	3	3	2	2	4	4	3	3
2	2	3	3	2	2	4	4	3	3
2	2	3	3	2	2	4	4	4	4
2	2	3	3	2	2	4	4	3	3
2	2	3	3	2	2	4	4	3	3
2	2	3	3	2	2	4	4	3	3
2	2	3	3	2	2	4	4	3	3
2	2	3	3	2	2	1	1	4	4
2	2	3	3	2	2	4	4	3	3
2	2	3	3	2	2	4	4	3	3
2	2	3	3	2	2	4	4	3	3
2	2	3	3	2	2	4	4	3	3
2	2	3	3	2	2	4	4	3	3
2	2	3	3	2	2	1	1	4	4
2	2	3	3	2	2	4	4	3	3
2	2	3	3	4	4	4	4	3	3
2	2	3	3	2	2	4	4	3	3
2	2	3	3	2	2	4	4	3	3
2	2	3	3	2	2	1	1	4	4
2	2	3	3	2	2	4	4	3	3
2	2	3	3	2	2	4	4	3	3
2	2	3	3	2	2	4	4	3	3
2	2	3	3	2	2	4	4	3	3
2	2	3	3	2	2	4	4	3	3
2	2	3	3	2	2	4	4	3	3
2	2	3	3	2	2	4	4	3	3
2	2	3	3	2	2	4	4	3	3
2	2	3	3	2	2	4	4	3	3
2	2	3	3	2	2	1	1	4	4
2	2	3	3	2	2	4	4	3	3
2	2	3	3	2	2	4	4	3	3
2	2	3	3	2	2	4	4	3	3
2	2	3	3	2	2	1	1	4	4
2	2	3	3	2	2	4	4	3	3
2	2	3	3	2	2	4	4	3	3
2	2	3	3	2	2	4	4	3	3
2	2	3	3	2	2	4	4	3	3
2	2	2	2	2	2	1	1	4	4
2	2	2	2	2	2	1	1	4	4
2	2	3	3	2	2	4	4	3	3
2	2	3	3	2	2	4	4	3	3

For Review Only

2	2	3	3	2	2	1	1	4	4
2	2	3	3	2	2	4	4	3	3
2	2	3	3	2	2	4	4	3	3
2	2	3	3	2	2	1	1	4	4
2	2	3	3	2	2	4	4	3	3
2	2	3	3	2	2	4	4	3	3
2	2	3	3	2	2	4	4	3	3
2	2	3	3	2	2	4	4	3	3
2	2	3	3	2	2	4	4	3	3
2	2	3	3	2	2	4	4	3	3
2	2	3	3	2	2	4	4	3	3
2	2	3	3	2	2	4	4	3	3
2	2	3	3	2	2	1	1	4	4
2	2	3	3	2	2	4	4	3	3
2	2	3	3	2	2	1	1	3	3
2	2	3	3	2	2	4	4	3	3
2	2	3	3	2	2	4	4	3	3
2	2	3	3	2	2	4	4	3	3
2	2	3	3	2	2	1	1	4	4
2	2	3	3	2	2	4	4	3	3
2	2	3	3	2	2	4	4	3	3
2	2	3	3	4	4	4	4	3	3
2	2	3	3	2	2	4	4	3	3
2	2	2	2	2	2	1	1	4	4
2	2	3	3	2	2	4	4	3	3
2	2	3	3	2	2	4	4	3	3
2	2	3	3	2	2	4	4	3	3
2	2	3	3	2	2	4	4	3	3
2	2	3	3	2	2	4	4	3	3
2	2	3	3	4	4	4	4	3	3
2	2	3	3	2	2	1	1	4	4
2	2	3	3	2	2	4	4	3	3
2	2	3	3	2	2	4	4	3	3
2	2	3	3	2	2	4	4	3	3
2	2	3	3	2	2	4	4	3	3
2	2	3	3	2	2	4	4	3	3
2	2	3	3	2	2	1	1	4	4
2	2	3	3	2	2	4	4	3	3
2	2	3	3	2	2	4	4	3	3
2	2	3	3	2	2	1	1	4	4
2	2	3	3	2	2	1	1	4	4
2	2	3	3	2	2	1	1	4	4
2	2	3	3	2	2	4	4	3	3
2	2	3	3	2	2	4	4	3	3
2	2	3	3	2	2	4	4	3	3
2	2	3	3	2	2	4	4	3	3
2	2	3	3	2	2	4	4	3	3
2	2	3	3	2	2	4	4	3	3
2	2	3	3	2	2	1	1	3	3
2	2	3	3	2	2	4	4	3	3
2	2	3	3	2	2	4	4	3	3
2	2	2	2	2	2	4	4	3	3
2	2	3	3	2	2	4	4	3	3
2	2	3	3	2	2	4	4	3	3
2	2	3	3	2	2	4	4	3	3

For Review Only

2	2	3	3	2	2	4	4	3	3
2	2	3	3	2	2	1	1	4	4
2	2	3	3	2	2	4	4	3	3
2	2	3	3	2	2	4	4	3	3
2	2	3	3	2	2	4	4	3	3
2	2	3	3	2	2	1	1	4	4
2	2	3	3	2	2	1	1	4	4
2	2	3	3	2	2	4	4	3	3
2	2	3	3	2	2	4	4	3	3
2	2	3	3	4	4	4	4	3	3
2	2	3	3	2	2	4	4	3	3
2	2	3	3	2	2	1	1	4	4
2	2	3	3	2	2	4	4	3	3
2	2	3	3	2	2	4	4	3	3
2	2	3	3	2	2	4	4	4	4
2	2	2	2	2	2	1	1	4	4
2	2	3	3	2	2	4	4	3	3
1	1	3	3	2	2	4	4	3	3
2	2	3	3	2	2	4	4	3	3
2	2	3	3	2	2	4	4	3	3
2	2	3	3	2	2	4	4	3	3
2	2	3	3	2	2	1	1	4	4
2	2	3	3	2	2	1	1	4	4
2	2	3	3	2	2	4	4	3	3
2	2	3	3	2	2	4	4	3	3
2	2	3	3	2	2	4	4	3	3
2	2	3	3	2	2	4	4	3	3
2	2	3	3	2	2	4	4	3	3
2	2	3	3	2	2	4	4	3	3
2	2	3	3	2	2	4	4	3	3
2	2	3	3	2	2	4	4	3	3
2	2	3	3	4	4	4	4	3	3
1	1	3	3	2	2	4	4	3	3
2	2	3	3	2	2	4	4	3	3
2	2	3	3	2	2	4	4	3	3
2	2	3	3	2	2	4	4	3	3
2	2	3	3	2	2	4	4	3	3
2	2	3	3	2	2	4	4	3	3
2	2	3	3	2	2	4	4	3	3
2	2	3	3	2	2	4	4	3	3
2	2	3	3	2	2	4	4	3	3
2	2	3	3	2	2	4	4	3	3
2	2	3	3	2	2	4	4	4	4
2	2	3	3	2	2	4	4	3	3
1	1	3	3	2	2	4	4	3	3
2	2	3	3	2	2	4	4	3	3
2	2	2	2	2	2	1	1	4	4
2	2	3	3	2	2	1	1	4	4
2	2	3	3	2	2	4	4	3	3
2	2	3	3	2	2	4	4	3	3
2	2	3	3	2	2	4	4	3	3
2	2	2	2	2	2	1	1	4	4
2	2	3	3	2	2	1	1	4	4
2	2	3	3	2	2	4	4	3	3
2	2	2	2	2	2	1	1	4	4

For Review Only

2	2	3	3	2	2	4	4	3	3
2	2	3	3	4	4	4	4	3	3
2	2	3	3	2	2	4	4	3	3
2	2	3	3	2	2	1	1	4	4
2	2	3	3	4	4	4	4	3	3
2	2	3	3	2	2	4	4	3	3
2	2	3	3	2	2	4	4	3	3
2	2	3	3	2	2	4	4	3	3
2	2	3	3	2	2	4	4	3	3
2	2	3	3	2	2	4	4	4	4
2	2	3	3	2	2	4	4	3	3
2	2	3	3	2	2	4	4	3	3
2	2	3	3	2	2	4	4	3	3
2	2	3	3	2	2	4	4	3	3
2	2	3	3	2	2	4	4	3	3
2	2	3	3	2	2	4	4	4	4
2	2	2	2	2	2	1	1	4	4
2	2	3	3	2	2	4	4	3	3
2	2	3	3	2	2	1	1	4	4
2	2	3	3	2	2	1	1	4	4
2	2	2	2	2	2	4	4	3	3
2	2	3	3	2	2	4	4	3	3
2	2	3	3	2	2	4	4	3	3
2	2	2	2	2	2	1	1	4	4
2	2	3	3	2	2	4	4	3	3
2	2	3	3	2	2	4	4	3	3
2	2	3	3	2	2	1	1	4	4
2	2	3	3	2	2	4	4	3	3
2	2	3	3	2	2	4	4	3	3
2	2	3	3	2	2	4	4	3	3
2	2	2	2	2	2	1	1	4	4
2	2	3	3	2	2	4	4	3	3
2	2	3	3	2	2	1	1	4	4
2	2	3	3	2	2	1	1	4	4
2	2	3	3	2	2	1	1	4	4
2	2	2	2	2	2	1	1	4	4
2	2	3	3	2	2	4	4	3	3
2	2	3	3	2	2	4	4	3	3
2	2	3	3	2	2	4	4	3	3
2	2	2	2	2	2	1	1	4	4
2	2	3	3	2	2	4	4	3	3
2	2	3	3	2	2	4	4	3	3
2	2	2	2	2	2	1	1	4	4
2	2	3	3	2	2	4	4	3	3
2	2	3	3	2	2	4	4	3	3
2	2	3	3	2	2	1	1	4	4
2	2	3	3	2	2	1	1	4	4
2	2	3	3	2	2	4	4	3	3
2	2	3	3	2	2	4	4	3	3
2	2	3	3	2	2	1	1	4	4
1	1	3	3	4	4	4	4	3	3
2	2	2	2	2	2	1	1	4	4
2	2	3	3	2	2	4	4	3	3
2	2	3	3	2	2	4	4	3	3
2	2	2	2	2	2	1	1	4	4

For Review Only

2	2	3	3	2	2	4	4	3	3
2	2	3	3	2	2	4	4	3	3

For Review Only

SNP1077	SNP1077	SNP1078	SNP1078	SNP1079	SNP1079	SNP108	SNP108	SNP1080	SNP1080
3	3	4	4	3	3	3	3	4	4
1	1	4	4	1	1	3	3	1	1
3	3	4	4	3	3	3	3	4	4
3	3	4	4	3	3	3	3	4	4
3	3	4	4	3	3	2	2	4	4
3	3	4	4	3	3	3	3	1	1
3	3	4	4	3	3	3	3	4	4
1	1	1	1	3	3	2	2	1	1
3	3	4	4	3	3	3	3	4	4
3	3	1	1	3	3	2	2	4	4
3	3	4	4	3	3	3	3	4	4
3	3	4	4	3	3	3	3	4	4
3	3	4	4	3	3	3	3	1	1
3	3	4	4	3	3	3	3	4	4
3	3	4	4	3	3	3	3	1	1
3	3	4	4	3	3	3	3	4	4
3	3	1	1	3	3	2	2	4	4
3	3	1	1	3	3	3	3	4	4
3	3	4	4	3	3	3	3	1	1
3	3	4	4	3	3	3	3	1	1
3	3	1	1	3	3	3	3	4	4
3	3	4	4	3	3	3	3	4	4
1	1	1	1	1	1	3	3	1	1
1	1	4	4	1	1	3	3	1	1
3	3	4	4	3	3	3	3	1	1
3	3	4	4	3	3	3	3	4	4
3	3	1	1	3	3	3	3	4	4
3	3	4	4	3	3	3	3	1	1
3	3	4	4	3	3	2	2	4	4
1	1	4	4	3	3	2	2	1	1
3	3	1	1	3	3	3	3	1	1
1	1	1	1	3	3	2	2	1	1
1	1	4	4	3	3	2	2	1	1
3	3	4	4	3	3	3	3	4	4
1	1	4	4	3	3	3	3	1	1
3	3	4	4	3	3	3	3	4	4
3	3	4	4	3	3	3	3	4	4
1	1	4	4	3	3	2	2	1	1
1	1	4	4	1	1	2	2	1	1
1	1	1	1	1	1	3	3	1	1
1	1	4	4	1	1	2	2	1	1
3	3	4	4	3	3	2	2	4	4
3	3	1	1	3	3	2	2	4	4
3	3	4	4	3	3	3	3	4	4
1	1	4	4	3	3	2	2	1	1
3	3	4	4	3	3	3	3	4	4
3	3	1	1	3	3	2	2	4	4
1	1	4	4	1	1	3	3	1	1
1	1	4	4	3	3	2	2	1	1
3	3	4	4	3	3	3	3	4	4
3	3	4	4	3	3	3	3	4	4
1	1	4	4	1	1	3	3	1	1

For Review Only

3	3	4	4	3	3	3	3	4	4
1	1	4	4	3	3	2	2	1	1
3	3	1	1	3	3	3	3	1	1
1	1	4	4	1	1	2	2	1	1
3	3	4	4	3	3	3	3	1	1
3	3	4	4	3	3	3	3	4	4
3	3	4	4	3	3	3	3	4	4
1	1	4	4	1	1	2	2	1	1
1	1	4	4	1	1	3	3	1	1
3	3	4	4	3	3	3	3	1	1
3	3	4	4	3	3	3	3	4	4
3	3	4	4	3	3	3	3	4	4
3	3	4	4	3	3	3	3	4	4
3	3	4	4	3	3	2	2	4	4
3	3	4	4	3	3	3	3	1	1
3	3	4	4	3	3	3	3	4	4
1	1	4	4	3	3	3	3	1	1
3	3	4	4	3	3	2	2	4	4
3	3	4	4	3	3	2	2	4	4
3	3	4	4	3	3	3	3	4	4
3	3	4	4	3	3	3	3	4	4
1	1	4	4	1	1	3	3	1	1
3	3	4	4	3	3	3	3	4	4
3	3	4	4	3	3	3	3	4	4
1	1	4	4	3	3	2	2	1	1
3	3	4	4	3	3	3	3	4	4
3	3	1	1	3	3	2	2	4	4
1	1	1	1	1	1	2	2	1	1
3	3	1	1	3	3	3	3	4	4
1	1	4	4	3	3	3	3	1	1
3	3	1	1	3	3	3	3	1	1
3	3	4	4	3	3	2	2	1	1
1	1	1	1	1	1	2	2	1	1
3	3	4	4	3	3	3	3	4	4
3	3	4	4	3	3	3	3	4	4
1	1	4	4	3	3	2	2	1	1
1	1	1	1	3	3	3	3	1	1
1	1	1	1	3	3	3	3	1	1
3	3	1	1	3	3	2	2	4	4
3	3	1	1	3	3	3	3	1	1
3	3	4	4	3	3	3	3	4	4
3	3	4	4	3	3	3	3	4	4
1	1	1	1	3	3	3	3	1	1
3	3	4	4	3	3	3	3	4	4
3	3	4	4	3	3	3	3	4	4
3	3	1	1	3	3	3	3	4	4
1	1	4	4	1	1	3	3	1	1
3	3	4	4	3	3	3	3	4	4
3	3	4	4	3	3	3	3	4	4
3	3	4	4	3	3	3	3	1	1
3	3	1	1	3	3	3	3	4	4
1	1	1	1	1	1	2	2	1	1
1	1	4	4	1	1	3	3	1	1
3	3	1	1	3	3	3	3	1	1
3	3	4	4	3	3	3	3	4	4

For Review Only

1	1	4	4	1	1	3	3	1	1
3	3	4	4	3	3	3	3	4	4
3	3	4	4	3	3	3	3	4	4
1	1	4	4	1	1	3	3	1	1
3	3	1	1	3	3	3	3	4	4
3	3	4	4	3	3	3	3	4	4
3	3	4	4	3	3	2	2	4	4
3	3	4	4	3	3	3	3	4	4
1	1	4	4	3	3	3	3	1	1
3	3	4	4	3	3	3	3	1	1
3	3	4	4	3	3	3	3	1	1
3	3	4	4	3	3	3	3	1	1
1	1	1	1	1	1	2	2	1	1
3	3	4	4	3	3	3	3	4	4
1	1	4	4	1	1	3	3	1	1
3	3	4	4	3	3	3	3	1	1
1	1	4	4	1	1	3	3	1	1
3	3	4	4	3	3	3	3	1	1
1	1	4	4	1	1	3	3	1	1
3	3	4	4	3	3	3	3	1	1
3	3	4	4	3	3	3	3	1	1
1	1	4	4	3	3	3	3	1	1
3	3	4	4	3	3	3	3	4	4
1	1	1	1	1	1	2	2	1	1
3	3	4	4	1	1	3	3	4	4
3	3	1	1	3	3	3	3	4	4
3	3	1	1	3	3	3	3	4	4
3	3	4	4	3	3	3	3	4	4
3	3	4	4	3	3	3	3	4	4
1	1	4	4	3	3	2	2	1	1
1	1	1	1	1	1	3	3	1	1
3	3	4	4	3	3	3	3	1	1
3	3	4	4	3	3	3	3	4	4
3	3	4	4	3	3	3	3	4	4
3	3	4	4	3	3	3	3	4	4
3	3	4	4	1	1	3	3	1	1
1	1	4	4	1	1	3	3	1	1
3	3	4	4	3	3	3	3	1	1
1	1	4	4	3	3	3	3	1	1
1	1	1	1	1	1	3	3	1	1
1	1	4	4	1	1	2	2	1	1
1	1	4	4	1	1	3	3	1	1
3	3	4	4	3	3	2	2	4	4
1	1	4	4	3	3	3	3	1	1
3	3	4	4	3	3	3	3	4	4
3	3	1	1	3	3	3	3	4	4
3	3	4	4	3	3	3	3	4	4
3	3	4	4	3	3	3	3	4	4
1	1	4	4	1	1	3	3	1	1
3	3	4	4	3	3	3	3	4	4
3	3	4	4	3	3	3	3	4	4
1	1	4	4	1	1	3	3	1	1
3	3	4	4	3	3	2	2	4	4
3	3	1	1	3	3	3	3	4	4
3	3	4	4	1	1	3	3	1	1

For Review Only

3	3	1	1	3	3	2	2	4	4
1	1	1	1	1	1	3	3	1	1
3	3	4	4	3	3	3	3	1	1
3	3	4	4	3	3	2	2	1	1
3	3	4	4	3	3	3	3	4	4
1	1	4	4	1	1	2	2	1	1
1	1	4	4	1	1	3	3	1	1
3	3	4	4	3	3	3	3	1	1
3	3	4	4	3	3	3	3	1	1
1	1	4	4	3	3	3	3	1	1
3	3	4	4	3	3	3	3	4	4
1	1	4	4	1	1	3	3	1	1
3	3	4	4	3	3	3	3	4	4
3	3	4	4	3	3	3	3	1	1
1	1	4	4	3	3	3	3	1	1
1	1	4	4	1	1	3	3	1	1
3	3	4	4	3	3	3	3	1	1
3	3	4	4	3	3	3	3	4	4
1	1	4	4	1	1	3	3	1	1
3	3	4	4	3	3	2	2	4	4
3	3	4	4	3	3	3	3	1	1
1	1	4	4	1	1	3	3	1	1
1	1	4	4	1	1	3	3	1	1
3	3	4	4	3	3	3	3	4	4
3	3	4	4	3	3	3	3	1	1
3	3	4	4	3	3	3	3	4	4
3	3	4	4	3	3	3	3	4	4
3	3	4	4	3	3	3	3	1	1
3	3	4	4	3	3	3	3	4	4
3	3	4	4	3	3	3	3	4	4
3	3	4	4	3	3	3	3	4	4
1	1	4	4	3	3	3	3	1	1
3	3	1	1	3	3	3	3	4	4
1	1	4	4	3	3	3	3	1	1
3	3	4	4	3	3	3	3	1	1
3	3	4	4	3	3	3	3	4	4
3	3	4	4	3	3	3	3	4	4
3	3	4	4	3	3	3	3	1	1
3	3	1	1	3	3	3	3	4	4
3	3	4	4	3	3	3	3	4	4
3	3	4	4	3	3	3	3	4	4
3	3	4	4	3	3	3	3	4	4
1	1	1	1	3	3	2	2	1	1
3	3	1	1	3	3	3	3	1	1
3	3	1	1	3	3	3	3	4	4
3	3	4	4	3	3	3	3	4	4
1	1	4	4	1	1	3	3	1	1
1	1	1	1	1	1	2	2	1	1
3	3	4	4	3	3	3	3	4	4
1	1	4	4	1	1	3	3	1	1
3	3	4	4	3	3	3	3	4	4
1	1	4	4	1	1	3	3	1	1
1	1	4	4	1	1	3	3	1	1
3	3	4	4	3	3	3	3	4	4
1	1	1	1	1	1	2	2	1	1

For Review Only

3	3	4	4	3	3	2	2	4	4
3	3	1	1	3	3	3	3	1	1
3	3	4	4	3	3	3	3	4	4
1	1	4	4	1	1	3	3	1	1
3	3	4	4	3	3	3	3	1	1
3	3	4	4	3	3	2	2	4	4
3	3	1	1	3	3	3	3	4	4
1	1	1	1	1	1	2	2	1	1
3	3	4	4	3	3	3	3	4	4
1	1	1	1	1	1	2	2	1	1
3	3	4	4	3	3	3	3	1	1
3	3	4	4	3	3	3	3	4	4
3	3	1	1	3	3	3	3	4	4
3	3	4	4	3	3	3	3	4	4
3	3	1	1	3	3	3	3	4	4
1	1	4	4	1	1	3	3	1	1
1	1	4	4	1	1	2	2	1	1
3	3	4	4	3	3	3	3	1	1
1	1	1	1	1	1	3	3	1	1
1	1	4	4	1	1	3	3	1	1
3	3	4	4	3	3	3	3	4	4
3	3	4	4	3	3	2	2	4	4
3	3	4	4	3	3	3	3	4	4
1	1	4	4	1	1	3	3	1	1
1	1	4	4	1	1	2	2	1	1
3	3	1	1	3	3	3	3	4	4
1	1	1	1	1	1	2	2	1	1
3	3	4	4	3	3	3	3	4	4
3	3	4	4	3	3	3	3	4	4
3	3	4	4	3	3	3	3	4	4
1	1	1	1	1	1	2	2	1	1
3	3	4	4	3	3	3	3	4	4
1	1	4	4	1	1	3	3	1	1
1	1	4	4	1	1	3	3	1	1
1	1	4	4	1	1	3	3	1	1
1	1	1	1	1	1	2	2	1	1
3	3	4	4	3	3	2	2	4	4
3	3	4	4	3	3	3	3	4	4
3	3	4	4	3	3	3	3	4	4
1	1	1	1	1	1	2	2	1	1
3	3	4	4	3	3	3	3	4	4
3	3	4	4	3	3	3	3	4	4
1	1	4	4	1	1	3	3	1	1
3	3	4	4	3	3	3	3	4	4
3	3	1	1	3	3	3	3	4	4
1	1	1	1	1	1	2	2	1	1
1	1	4	4	3	3	2	2	4	4
3	3	1	1	3	3	3	3	1	1
3	3	4	4	3	3	3	3	4	4
1	1	1	1	1	1	2	2	1	1
3	3	4	4	3	3	3	3	1	1
1	1	1	1	1	1	2	2	1	1
3	3	4	4	3	3	3	3	4	4
3	3	1	1	3	3	3	3	4	4
1	1	1	1	1	1	2	2	1	1

For Review Only

3	3	4	4	3	3	3	3	4	4
3	3	4	4	3	3	3	3	4	4

For Review Only

SNP1081	SNP1081	SNP1082	SNP1082	SNP1083	SNP1083	SNP1084	SNP1084	SNP1085	SNP1085
1	1	1	1	4	4	1	1	2	2
4	4	2	2	4	4	1	1	2	2
1	1	1	1	4	4	1	1	2	2
1	1	1	1	4	4	1	1	2	2
1	1	1	1	4	4	1	1	2	2
1	1	1	1	1	1	1	1	2	2
1	1	1	1	4	4	1	1	2	2
4	4	2	2	4	4	1	1	3	3
1	1	1	1	4	4	1	1	2	2
4	4	2	2	4	4	1	1	2	2
1	1	1	1	4	4	1	1	2	2
1	1	1	1	4	4	1	1	2	2
1	1	1	1	4	4	1	1	2	2
1	1	1	1	4	4	3	3	2	2
1	1	1	1	4	4	1	1	2	2
4	4	2	2	4	4	1	1	2	2
1	1	1	1	4	4	1	1	2	2
1	1	1	1	4	4	1	1	2	2
1	1	1	1	1	1	1	1	2	2
1	1	1	1	4	4	1	1	2	2
4	4	2	2	4	4	1	1	2	2
1	1	1	1	4	4	1	1	2	2
1	1	1	1	4	4	1	1	2	2
1	1	1	1	4	4	1	1	2	2
1	1	1	1	4	4	1	1	2	2
1	1	1	1	4	4	1	1	2	2
1	1	1	1	4	4	1	1	2	2
1	1	1	1	4	4	1	1	2	2
1	1	1	1	4	4	1	1	2	2
1	1	1	1	4	4	1	1	2	2
1	1	1	1	4	4	1	1	2	2
1	1	1	1	1	1	1	1	2	2
1	1	1	1	1	1	1	1	2	2
1	1	1	1	4	4	3	3	2	2
1	1	1	1	4	4	1	1	2	2
1	1	1	1	4	4	1	1	2	2
1	1	1	1	4	4	1	1	2	2
1	1	1	1	4	4	1	1	2	2
4	4	2	2	4	4	1	1	3	3
4	4	2	2	4	4	1	1	3	3
1	1	1	1	4	4	1	1	2	2
1	1	1	1	4	4	1	1	2	2
1	1	1	1	4	4	1	1	2	2
1	1	1	1	4	4	1	1	2	2
1	1	1	1	4	4	1	1	3	3
1	1	1	1	4	4	1	1	2	2
1	1	1	1	4	4	1	1	2	2
1	1	1	1	4	4	1	1	3	3
1	1	1	1	4	4	1	1	3	3
1	1	1	1	4	4	1	1	2	2
1	1	1	1	4	4	1	1	2	2
1	1	1	1	4	4	1	1	3	3

For Review Only

1	1	1	1	4	4	1	1	2	2
1	1	1	1	1	1	1	1	2	2
1	1	1	1	4	4	1	1	2	2
1	1	1	1	4	4	1	1	2	2
1	1	1	1	1	1	1	1	2	2
1	1	1	1	4	4	1	1	2	2
1	1	1	1	4	4	1	1	2	2
1	1	1	1	4	4	1	1	2	2
1	1	1	1	4	4	3	3	2	2
1	1	1	1	4	4	1	1	2	2
1	1	1	1	4	4	1	1	2	2
1	1	1	1	4	4	1	1	2	2
1	1	1	1	4	4	1	1	2	2
1	1	1	1	4	4	1	1	2	2
1	1	1	1	4	4	1	1	2	2
1	1	1	1	4	4	3	3	2	2
1	1	1	1	4	4	1	1	2	2
1	1	1	1	4	4	1	1	2	2
1	1	1	1	4	4	1	1	2	2
1	1	1	1	4	4	1	1	2	2
1	1	1	1	4	4	1	1	2	2
4	4	2	2	4	4	1	1	2	2
1	1	1	1	4	4	1	1	2	2
1	1	1	1	4	4	1	1	2	2
1	1	1	1	4	4	1	1	3	3
1	1	1	1	4	4	1	1	2	2
1	1	1	1	4	4	1	1	2	2
4	4	2	2	4	4	1	1	2	2
1	1	1	1	4	4	1	1	2	2
1	1	1	1	1	1	1	1	2	2
1	1	1	1	4	4	1	1	2	2
1	1	1	1	4	4	1	1	2	2
4	4	2	2	4	4	3	3	3	3
1	1	1	1	4	4	1	1	2	2
1	1	1	1	4	4	1	1	2	2
1	1	1	1	4	4	1	1	3	3
1	1	1	1	4	4	1	1	3	3
4	4	2	2	4	4	1	1	3	3
1	1	1	1	4	4	1	1	2	2
4	4	2	2	4	4	1	1	3	3
1	1	1	1	4	4	1	1	2	2
1	1	1	1	4	4	1	1	2	2
1	1	1	1	4	4	1	1	3	3
1	1	1	1	4	4	1	1	2	2
1	1	1	1	4	4	1	1	2	2
4	4	2	2	4	4	1	1	2	2
1	1	1	1	4	4	1	1	3	3
1	1	1	1	4	4	1	1	2	2
1	1	1	1	4	4	1	1	2	2
1	1	1	1	4	4	1	1	2	2
4	4	1	1	4	4	1	1	2	2
4	4	2	2	4	4	1	1	2	2
1	1	1	1	4	4	1	1	3	3
4	4	1	1	4	4	1	1	2	2
1	1	1	1	4	4	1	1	2	2

For Review Only

1	1	1	1	4	4	1	1	2	2
1	1	1	1	4	4	1	1	2	2
1	1	1	1	4	4	1	1	2	2
1	1	1	1	4	4	1	1	3	3
1	1	1	1	4	4	1	1	2	2
1	1	1	1	4	4	1	1	2	2
1	1	1	1	4	4	1	1	2	2
1	1	1	1	4	4	1	1	2	2
4	4	2	2	4	4	1	1	2	2
1	1	1	1	4	4	1	1	2	2
1	1	1	1	4	4	1	1	2	2
1	1	1	1	4	4	1	1	2	2
1	1	1	1	4	4	1	1	3	3
1	1	1	1	4	4	1	1	2	2
1	1	1	1	4	4	1	1	2	2
4	4	1	1	4	4	1	1	2	2
1	1	1	1	4	4	1	1	2	2
1	1	1	1	4	4	1	1	2	2
1	1	1	1	4	4	1	1	3	3
1	1	1	1	4	4	1	1	2	2
1	1	1	1	4	4	1	1	2	2
1	1	1	1	1	1	1	1	2	2
1	1	1	1	4	4	1	1	2	2
4	4	2	2	4	4	1	1	3	3
1	1	1	1	4	4	1	1	2	2
4	4	1	1	4	4	1	1	2	2
1	1	1	1	4	4	1	1	2	2
1	1	1	1	4	4	1	1	2	2
1	1	1	1	4	4	1	1	2	2
1	1	1	1	1	1	1	1	2	2
4	4	2	2	4	4	1	1	2	2
1	1	1	1	4	4	1	1	2	2
1	1	1	1	4	4	1	1	2	2
1	1	1	1	4	4	1	1	2	2
1	1	1	1	4	4	3	3	2	2
1	1	1	1	4	4	1	1	2	2
1	1	1	1	4	4	3	3	3	3
1	1	1	1	4	4	1	1	2	2
4	4	1	1	4	4	1	1	2	2
4	4	2	2	4	4	1	1	2	2
1	1	1	1	4	4	1	1	2	2
1	1	1	1	4	4	3	3	3	3
1	1	1	1	4	4	1	1	2	2
4	4	1	1	4	4	1	1	2	2
1	1	1	1	4	4	1	1	2	2
4	4	1	1	4	4	1	1	2	2
1	1	1	1	4	4	1	1	2	2
1	1	1	1	4	4	1	1	2	2
1	1	1	1	4	4	1	1	2	2
1	1	1	1	4	4	1	1	2	2
1	1	1	1	4	4	1	1	2	2
1	1	1	1	4	4	1	1	2	2
1	1	1	1	4	4	1	1	2	2
1	1	1	1	4	4	1	1	2	2
1	1	1	1	4	4	1	1	2	2

For Review Only

1	1	1	1	4	4	1	1	2	2
1	1	1	1	4	4	1	1	2	2
1	1	1	1	4	4	1	1	2	2
1	1	1	1	4	4	1	1	2	2
1	1	1	1	4	4	1	1	2	2
1	1	1	1	4	4	1	1	2	2
4	4	1	1	4	4	1	1	2	2
1	1	1	1	4	4	1	1	2	2
4	4	2	2	4	4	1	1	2	2
1	1	1	1	1	1	1	1	2	2
1	1	1	1	4	4	1	1	2	2
1	1	1	1	4	4	1	1	2	2
1	1	1	1	4	4	1	1	2	2
4	4	1	1	4	4	1	1	2	2
1	1	1	1	4	4	1	1	3	3
1	1	1	1	4	4	1	1	3	3
1	1	1	1	4	4	1	1	2	2
1	1	1	1	4	4	1	1	2	2
1	1	1	1	4	4	1	1	2	2
1	1	1	1	4	4	1	1	2	2
1	1	1	1	4	4	1	1	2	2
1	1	1	1	4	4	1	1	3	3
4	4	2	2	4	4	1	1	2	2
1	1	1	1	4	4	1	1	2	2
1	1	1	1	4	4	1	1	2	2
1	1	1	1	4	4	3	3	2	2
1	1	1	1	4	4	1	1	2	2
1	1	1	1	4	4	1	1	2	2
1	1	1	1	4	4	1	1	2	2
1	1	1	1	4	4	1	1	2	2
1	1	1	1	4	4	1	1	2	2
1	1	1	1	1	1	1	1	2	2
1	1	1	1	4	4	1	1	2	2
1	1	1	1	4	4	1	1	2	2
1	1	1	1	1	1	1	1	2	2
1	1	1	1	4	4	1	1	2	2
1	1	1	1	4	4	1	1	2	2
1	1	1	1	4	4	1	1	2	2
1	1	1	1	4	4	1	1	2	2
1	1	1	1	4	4	1	1	2	2
1	1	1	1	4	4	1	1	2	2
1	1	1	1	4	4	1	1	2	2
4	4	2	2	4	4	1	1	3	3
4	4	2	2	4	4	1	1	2	2
4	4	1	1	4	4	1	1	2	2
1	1	1	1	4	4	1	1	2	2
1	1	1	1	4	4	1	1	3	3
4	4	2	2	4	4	1	1	3	3
1	1	1	1	4	4	1	1	2	2
1	1	1	1	4	4	1	1	2	2
1	1	1	1	4	4	1	1	2	2
1	1	1	1	4	4	1	1	3	3
4	4	2	2	4	4	1	1	2	2
1	1	1	1	4	4	1	1	2	2
4	4	2	2	4	4	3	3	3	3

For Review Only

1	1	1	1	4	4	3	3	2	2
4	4	2	2	4	4	1	1	2	2
1	1	1	1	4	4	1	1	2	2
4	4	1	1	4	4	1	1	2	2
1	1	1	1	4	4	1	1	2	2
1	1	1	1	4	4	1	1	2	2
4	4	2	2	4	4	1	1	2	2
4	4	2	2	4	4	1	1	2	2
4	4	1	1	4	4	1	1	2	2
4	4	2	2	4	4	1	1	2	2
1	1	1	1	4	4	1	1	2	2
1	1	1	1	4	4	3	3	2	2
1	1	1	1	4	4	1	1	2	2
1	1	1	1	4	4	1	1	2	2
1	1	1	1	4	4	1	1	2	2
1	1	1	1	4	4	1	1	2	2
1	1	1	1	4	4	1	1	2	2
1	1	1	1	4	4	1	1	2	2
4	4	2	2	4	4	1	1	3	3
1	1	1	1	4	4	1	1	2	2
1	1	1	1	4	4	1	1	2	2
1	1	1	1	4	4	1	1	2	2
1	1	1	1	4	4	1	1	2	2
1	1	1	1	4	4	1	1	2	2
1	1	1	1	4	4	1	1	2	2
4	4	1	1	4	4	1	1	2	2
4	4	2	2	4	4	1	1	3	3
1	1	1	1	4	4	1	1	2	2
1	1	1	1	4	4	1	1	2	2
1	1	1	1	4	4	1	1	2	2
4	4	2	2	4	4	1	1	2	2
1	1	1	1	4	4	1	1	2	2
1	1	1	1	4	4	3	3	3	3
1	1	1	1	4	4	1	1	3	3
1	1	1	1	4	4	1	1	2	2
4	4	2	2	4	4	3	3	3	3
1	1	1	1	4	4	1	1	2	2
1	1	1	1	4	4	1	1	2	2
1	1	1	1	4	4	1	1	2	2
4	4	2	2	4	4	1	1	3	3
1	1	1	1	4	4	1	1	2	2
1	1	1	1	4	4	1	1	2	2
1	1	1	1	4	4	1	1	2	2
1	1	1	1	4	4	1	1	2	2
1	1	1	1	4	4	1	1	2	2
1	1	1	1	4	4	1	1	2	2
1	1	1	1	4	4	1	1	2	2
1	1	1	1	4	4	1	1	2	2
1	1	1	1	4	4	1	1	2	2
4	4	2	2	4	4	3	3	3	3
1	1	1	1	1	1	1	1	2	2
4	4	2	2	4	4	3	3	3	3
1	1	1	1	4	4	1	1	2	2
1	1	1	1	4	4	1	1	2	2
4	4	2	2	4	4	1	1	2	2

For Review Only

1	1	1	1	4	4	1	1	2	2
1	1	1	1	4	4	1	1	2	2

For Review Only

SNP1086	SNP1086	SNP1087	SNP1087	SNP1088	SNP1088	SNP1089	SNP1089	SNP109	SNP109
2	2	2	2	1	1	4	4	4	4
2	2	2	2	1	1	4	4	4	4
2	2	2	2	1	1	4	4	4	4
2	2	2	2	1	1	4	4	4	4
2	2	2	2	1	1	4	4	4	4
2	2	2	2	1	1	1	1	4	4
2	2	2	2	1	1	4	4	2	2
2	2	3	3	1	1	1	1	4	4
2	2	2	2	4	4	4	4	4	4
2	2	2	2	4	4	4	4	4	4
2	2	2	2	1	1	4	4	2	2
2	2	2	2	1	1	4	4	4	4
2	2	2	2	1	1	4	4	4	4
2	2	2	2	1	1	4	4	4	4
2	2	2	2	1	1	4	4	4	4
2	2	2	2	4	4	4	4	4	4
2	2	2	2	1	1	4	4	4	4
2	2	2	2	1	1	4	4	4	4
2	2	2	2	1	1	4	4	4	4
2	2	3	3	1	1	1	1	4	4
2	2	2	2	1	1	4	4	4	4
2	2	2	2	1	1	4	4	2	2
2	2	2	2	1	1	4	4	4	4
2	2	2	2	1	1	4	4	4	4
2	2	2	2	1	1	4	4	4	4
2	2	2	2	1	1	4	4	4	4
2	2	2	2	1	1	4	4	4	4
2	2	2	2	1	1	4	4	4	4
2	2	2	2	4	4	4	4	4	4
2	2	2	2	4	4	4	4	4	4
2	2	2	2	1	1	4	4	2	2
2	2	2	2	1	1	4	4	2	2
2	2	2	2	1	1	4	4	2	2
2	2	2	2	1	1	4	4	4	4
1	1	3	3	1	1	1	1	2	2
2	2	2	2	1	1	4	4	4	4
2	2	2	2	1	1	4	4	4	4
2	2	2	2	1	1	4	4	4	4
1	1	3	3	1	1	1	1	4	4
1	1	3	3	1	1	1	1	4	4
2	2	2	2	4	4	4	4	4	4
2	2	2	2	4	4	4	4	4	4
2	2	2	2	1	1	4	4	4	4
2	2	2	2	1	1	4	4	4	4
2	2	3	3	1	1	1	1	4	4
2	2	2	2	1	1	4	4	4	4
2	2	2	2	1	1	4	4	4	4
1	1	3	3	4	4	1	1	4	4
2	2	3	3	1	1	1	1	4	4
2	2	2	2	1	1	4	4	4	4
2	2	2	2	1	1	4	4	4	4
1	1	3	3	1	1	1	1	2	2

For Review Only

2	2	2	2	1	1	4	4	2	2
2	2	2	2	1	1	4	4	2	2
2	2	2	2	1	1	4	4	4	4
2	2	2	2	1	1	4	4	2	2
2	2	2	2	1	1	4	4	4	4
2	2	2	2	1	1	4	4	4	4
2	2	2	2	1	1	4	4	4	4
2	2	2	2	1	1	4	4	4	4
2	2	2	2	1	1	4	4	4	4
2	2	2	2	1	1	4	4	4	4
2	2	2	2	1	1	4	4	4	4
2	2	2	2	1	1	4	4	4	4
2	2	2	2	1	1	4	4	2	2
2	2	2	2	4	4	4	4	4	4
2	2	2	2	1	1	4	4	4	4
2	2	2	2	1	1	4	4	4	4
2	2	2	2	1	1	4	4	4	4
2	2	2	2	1	1	4	4	4	4
2	2	2	2	1	1	4	4	2	2
2	2	2	2	1	1	4	4	4	4
2	2	2	2	1	1	4	4	4	4
2	2	2	2	1	1	4	4	4	4
2	2	2	2	4	4	4	4	4	4
2	2	2	2	1	1	4	4	4	4
2	2	3	3	1	1	1	1	4	4
2	2	2	2	4	4	4	4	4	4
2	2	2	2	1	1	4	4	4	4
2	2	2	2	1	1	4	4	4	4
2	2	2	2	1	1	4	4	4	4
2	2	2	2	1	1	4	4	2	2
2	2	2	2	1	1	4	4	2	2
2	2	2	2	1	1	4	4	4	4
1	1	3	3	1	1	1	1	4	4
2	2	2	2	1	1	4	4	2	2
2	2	2	2	1	1	4	4	4	4
2	2	3	3	1	1	1	1	4	4
2	2	3	3	1	1	1	1	4	4
2	2	3	3	1	1	1	1	4	4
2	2	2	2	1	1	4	4	4	4
2	2	3	3	1	1	1	1	4	4
2	2	2	2	1	1	4	4	4	4
2	2	2	2	1	1	4	4	4	4
1	1	2	2	1	1	1	1	4	4
2	2	2	2	1	1	4	4	4	4
2	2	2	2	1	1	4	4	4	4
2	2	2	2	1	1	4	4	2	2
1	1	3	3	1	1	1	1	4	4
2	2	2	2	1	1	4	4	4	4
2	2	2	2	1	1	4	4	4	4
2	2	2	2	1	1	4	4	4	4
2	2	2	2	1	1	4	4	4	4
2	2	2	2	1	1	4	4	4	4
1	1	3	3	1	1	1	1	4	4
1	1	2	2	1	1	4	4	4	4
2	2	2	2	1	1	4	4	4	4

For Review Only

2	2	2	2	1	1	4	4	4	4
2	2	2	2	1	1	4	4	4	4
2	2	2	2	1	1	4	4	4	4
1	1	3	3	1	1	1	1	4	4
2	2	2	2	1	1	4	4	4	4
2	2	2	2	1	1	4	4	4	4
2	2	2	2	1	1	4	4	4	4
2	2	2	2	1	1	4	4	4	4
2	2	2	2	1	1	4	4	4	4
2	2	2	2	1	1	4	4	4	4
2	2	2	2	1	1	4	4	2	2
2	2	2	2	1	1	4	4	4	4
1	1	3	3	1	1	1	1	4	4
2	2	2	2	1	1	4	4	4	4
2	2	2	2	1	1	4	4	4	4
2	2	2	2	1	1	4	4	4	4
2	2	2	2	1	1	4	4	4	4
2	2	2	2	1	1	4	4	4	4
1	1	3	3	1	1	1	1	4	4
2	2	2	2	1	1	4	4	2	2
2	2	2	2	1	1	4	4	4	4
2	2	2	2	1	1	4	4	2	2
2	2	2	2	1	1	4	4	4	4
1	1	3	3	1	1	4	4	4	4
2	2	2	2	1	1	4	4	4	4
2	2	2	2	1	1	4	4	4	4
2	2	2	2	1	1	4	4	4	4
2	2	2	2	1	1	4	4	4	4
2	2	2	2	1	1	4	4	4	4
2	2	2	2	1	1	4	4	4	4
1	1	3	3	1	1	1	1	4	4
2	2	2	2	1	1	4	4	4	4
2	2	2	2	1	1	4	4	4	4
2	2	2	2	1	1	4	4	4	4
2	2	2	2	1	1	4	4	4	4
2	2	2	2	1	1	4	4	2	2
1	1	3	3	1	1	1	1	4	4
2	2	2	2	1	1	4	4	4	4
2	2	2	2	1	1	4	4	2	2
2	2	3	3	1	1	1	1	4	4
2	2	2	2	1	1	4	4	4	4
1	1	3	3	1	1	1	1	2	2
2	2	2	2	1	1	4	4	4	4
2	2	2	2	4	4	1	1	4	4
2	2	2	2	1	1	4	4	4	4
2	2	2	2	1	1	4	4	4	4
2	2	2	2	1	1	4	4	4	4
2	2	2	2	1	1	4	4	4	4
2	2	2	2	1	1	4	4	4	4
2	2	2	2	1	1	4	4	4	4
2	2	2	2	1	1	4	4	4	4
2	2	2	2	1	1	4	4	4	4
2	2	2	2	1	1	4	4	4	4
2	2	2	2	1	1	4	4	4	4
2	2	2	2	1	1	4	4	4	4

2	2	2	2	1	1	4	4	4	4
2	2	2	2	1	1	4	4	4	4
2	2	2	2	1	1	4	4	4	4
2	2	2	2	1	1	4	4	4	4
2	2	2	2	1	1	4	4	4	4
2	2	2	2	1	1	4	4	4	4
2	2	2	2	1	1	4	4	4	4
2	2	2	2	1	1	4	4	4	4
2	2	2	2	1	1	4	4	4	4
2	2	2	2	1	1	4	4	4	4
2	2	2	2	1	1	4	4	4	4
2	2	2	2	1	1	4	4	4	4
2	2	2	2	1	1	4	4	2	2
2	2	2	2	1	1	4	4	2	2
1	1	2	2	1	1	1	1	4	4
1	1	3	3	1	1	1	1	2	2
2	2	2	2	1	1	4	4	4	4
2	2	2	2	1	1	4	4	4	4
2	2	2	2	1	1	4	4	4	4
2	2	2	2	1	1	4	4	4	4
2	2	2	2	1	1	4	4	4	4
1	1	3	3	1	1	1	1	4	4
2	2	2	2	1	1	4	4	4	4
2	2	2	2	1	1	4	4	4	4
2	2	2	2	4	4	4	4	4	4
2	2	2	2	1	1	4	4	4	4
2	2	2	2	1	1	4	4	4	4
2	2	2	2	1	1	4	4	4	4
2	2	2	2	1	1	4	4	4	4
2	2	2	2	1	1	4	4	2	2
2	2	2	2	1	1	4	4	4	4
2	2	2	2	1	1	4	4	4	4
2	2	2	2	1	1	4	4	4	4
2	2	2	2	1	1	4	4	2	2
2	2	2	2	1	1	1	1	4	4
2	2	2	2	1	1	4	4	4	4
2	2	2	2	1	1	4	4	4	4
2	2	2	2	1	1	4	4	4	4
2	2	2	2	1	1	4	4	4	4
2	2	2	2	1	1	4	4	4	4
2	2	2	2	1	1	4	4	4	4
2	2	2	2	1	1	4	4	4	4
1	1	3	3	1	1	1	1	4	4
2	2	2	2	1	1	4	4	4	4
2	2	2	2	1	1	4	4	4	4
2	2	2	2	1	1	4	4	4	4
1	1	3	3	1	1	1	1	2	2
1	1	3	3	1	1	1	1	4	4
2	2	2	2	1	1	4	4	4	4
2	2	2	2	1	1	4	4	4	4
2	2	2	2	1	1	4	4	4	4
1	1	3	3	1	1	1	1	4	4
2	2	2	2	1	1	1	1	4	4
2	2	2	2	1	1	4	4	4	4
1	1	3	3	1	1	1	1	4	4

For Review Only

2	2	2	2	1	1	4	4	4	4
2	2	2	2	4	4	4	4	2	2
2	2	2	2	1	1	4	4	4	4
2	2	2	2	1	1	4	4	4	4
2	2	2	2	4	4	4	4	4	4
2	2	2	2	1	1	4	4	4	4
2	2	2	2	1	1	4	4	4	4
2	2	2	2	1	1	4	4	4	4
2	2	2	2	1	1	4	4	4	4
2	2	2	2	1	1	4	4	4	4
2	2	2	2	1	1	4	4	4	4
2	2	2	2	1	1	4	4	4	4
2	2	2	2	1	1	4	4	2	2
2	2	2	2	1	1	4	4	4	4
2	2	2	2	1	1	4	4	4	4
2	2	2	2	1	1	4	4	2	2
2	2	2	2	1	1	4	4	4	4
2	2	2	2	1	1	4	4	4	4
1	1	3	3	1	1	1	1	4	4
2	2	2	2	1	1	4	4	4	4
2	2	2	2	1	1	4	4	4	4
2	2	2	2	1	1	4	4	4	4
2	2	2	2	1	1	4	4	4	4
2	2	2	2	1	1	4	4	4	4
2	2	2	2	1	1	4	4	4	4
2	2	2	2	1	1	4	4	2	2
1	1	3	3	1	1	1	1	4	4
2	2	2	2	1	1	4	4	4	4
2	2	2	2	4	4	4	4	4	4
2	2	2	2	1	1	4	4	4	4
2	2	2	2	1	1	4	4	4	4
2	2	2	2	4	4	4	4	4	4
1	1	3	3	1	1	4	4	2	2
1	1	3	3	1	1	1	1	4	4
2	2	2	2	1	1	4	4	4	4
1	1	3	3	1	1	1	1	4	4
2	2	2	2	1	1	4	4	4	4
2	2	2	2	1	1	4	4	4	4
2	2	2	2	1	1	4	4	4	4
1	1	3	3	1	1	1	1	4	4
2	2	2	2	1	1	4	4	4	4
2	2	2	2	1	1	4	4	4	4
2	2	2	2	1	1	4	4	4	4
2	2	2	2	1	1	4	4	2	2
2	2	2	2	1	1	4	4	4	4
2	2	2	2	1	1	4	4	4	4
2	2	2	2	1	1	4	4	4	4
2	2	2	2	1	1	4	4	4	4
2	2	2	2	1	1	4	4	4	4
1	1	3	3	1	1	1	1	4	4
2	2	2	2	1	1	1	1	2	2
1	1	3	3	1	1	1	1	4	4
2	2	2	2	1	1	4	4	4	4
2	2	2	2	1	1	4	4	4	4
2	2	2	2	1	1	4	4	4	4

For Review Only

2	2	2	2	1	1	4	4	2	2
2	2	2	2	1	1	4	4	4	4

For Review Only

SNP1090	SNP1090	SNP1091	SNP1091	SNP1092	SNP1092	SNP1093	SNP1093	SNP1094	SNP1094
1	1	1	1	3	3	2	2	1	1
1	1	1	1	2	2	2	2	3	3
1	1	1	1	3	3	2	2	3	3
1	1	2	2	3	3	2	2	3	3
1	1	1	1	2	2	2	2	3	3
1	1	1	1	3	3	3	3	3	3
1	1	1	1	2	2	2	2	3	3
1	1	2	2	3	3	2	2	3	3
1	1	1	1	2	2	2	2	1	1
1	1	2	2	2	2	2	2	3	3
1	1	1	1	3	3	2	2	3	3
1	1	2	2	3	3	2	2	3	3
1	1	2	2	2	2	2	2	3	3
1	1	1	1	2	2	2	2	3	3
1	1	2	2	2	2	2	2	3	3
1	1	2	2	3	3	2	2	3	3
1	1	1	1	3	3	2	2	3	3
1	1	2	2	2	2	2	2	3	3
1	1	1	1	3	3	3	3	3	3
1	1	1	1	3	3	2	2	3	3
1	1	2	2	3	3	2	2	3	3
1	1	1	1	2	2	2	2	3	3
1	1	1	1	3	3	2	2	3	3
1	1	1	1	3	3	2	2	3	3
1	1	1	1	2	2	2	2	3	3
1	1	1	1	2	2	2	2	1	1
1	1	1	1	3	3	2	2	3	3
1	1	1	1	3	3	2	2	3	3
1	1	1	1	3	3	2	2	1	1
4	4	1	1	3	3	2	2	3	3
1	1	1	1	3	3	3	3	1	1
4	4	1	1	2	2	3	3	3	3
4	4	1	1	2	2	3	3	3	3
1	1	1	1	2	2	2	2	3	3
1	1	1	1	2	2	2	2	3	3
1	1	1	1	2	2	2	2	3	3
1	1	1	1	2	2	2	2	3	3
1	1	1	1	3	3	2	2	3	3
1	1	2	2	3	3	2	2	3	3
1	1	2	2	3	3	2	2	3	3
1	1	2	2	3	3	2	2	3	3
1	1	1	1	3	3	2	2	3	3
4	4	1	1	3	3	2	2	3	3
1	1	1	1	3	3	2	2	1	1
1	1	1	1	3	3	2	2	3	3
1	1	1	1	3	3	2	2	3	3
1	1	1	1	2	2	2	2	1	1
1	1	1	1	2	2	2	2	3	3
1	1	1	1	3	3	2	2	3	3
1	1	1	1	2	2	2	2	3	3
1	1	1	1	3	3	2	2	1	1
1	1	1	1	2	2	2	2	3	3

1	1	1	1	2	2	2	2	1	1
4	4	1	1	2	2	3	3	3	3
1	1	1	1	2	2	2	2	3	3
4	4	1	1	2	2	2	2	3	3
1	1	1	1	3	3	3	3	3	3
1	1	1	1	2	2	2	2	3	3
1	1	2	2	2	2	2	2	3	3
1	1	1	1	3	3	2	2	3	3
1	1	1	1	3	3	2	2	3	3
1	1	1	1	3	3	2	2	3	3
1	1	1	1	2	2	2	2	3	3
1	1	1	1	2	2	2	2	3	3
1	1	1	1	2	2	2	2	1	1
1	1	1	1	3	3	2	2	1	1
1	1	1	1	3	3	3	3	3	3
1	1	1	1	3	3	2	2	3	3
1	1	1	1	3	3	2	2	3	3
1	1	1	1	3	3	2	2	3	3
1	1	1	1	3	3	2	2	3	3
1	1	1	1	2	2	2	2	1	1
1	1	1	1	2	2	2	2	3	3
1	1	2	2	2	2	2	2	3	3
1	1	1	1	3	3	2	2	1	1
1	1	1	1	3	3	2	2	3	3
1	1	1	1	3	3	2	2	3	3
1	1	1	1	2	2	2	2	3	3
1	1	1	1	2	2	2	2	3	3
1	1	2	2	2	2	2	2	3	3
1	1	2	2	2	2	2	2	1	1
4	4	1	1	2	2	3	3	3	3
1	1	1	1	2	2	2	2	3	3
1	1	1	1	3	3	2	2	3	3
1	1	2	2	2	2	2	2	3	3
1	1	1	1	2	2	2	2	1	1
1	1	1	1	2	2	2	2	3	3
1	1	1	1	3	3	2	2	3	3
1	1	1	1	3	3	2	2	3	3
1	1	2	2	2	2	2	2	3	3
1	1	1	1	2	2	2	2	3	3
1	1	2	2	3	3	2	2	3	3
1	1	1	1	2	2	2	2	3	3
1	1	1	1	2	2	2	2	3	3
1	1	1	1	3	3	2	2	3	3
1	1	1	1	2	2	2	2	3	3
1	1	1	1	2	2	2	2	3	3
1	1	2	2	2	2	2	2	3	3
1	1	1	1	2	2	2	2	3	3
1	1	2	2	2	2	2	2	3	3
1	1	1	1	2	2	2	2	3	3
1	1	1	1	2	2	2	2	3	3
1	1	1	1	2	2	2	2	3	3
1	1	2	2	2	2	2	2	3	3
1	1	1	1	3	3	2	2	3	3
1	1	2	2	3	3	2	2	3	3
1	1	1	1	3	3	2	2	3	3

For Review Only

1	1	1	1	2	2	2	2	3	3
1	1	1	1	3	3	2	2	3	3
1	1	1	1	2	2	2	2	3	3
1	1	1	1	3	3	2	2	3	3
1	1	1	1	2	2	2	2	3	3
1	1	1	1	2	2	2	2	1	1
1	1	1	1	3	3	2	2	3	3
1	1	1	1	3	3	2	2	3	3
1	1	2	2	2	2	2	2	3	3
1	1	2	2	2	2	2	2	1	1
1	1	1	1	2	2	2	2	1	1
1	1	2	2	3	3	2	2	3	3
1	1	1	1	3	3	2	2	3	3
1	1	1	1	2	2	2	2	3	3
1	1	1	1	3	3	2	2	3	3
1	1	1	1	3	3	2	2	3	3
1	1	1	1	3	3	2	2	3	3
1	1	1	1	3	3	2	2	3	3
1	1	1	1	2	2	2	2	3	3
1	1	1	1	2	2	2	2	1	1
1	1	1	1	3	3	2	2	3	3
4	4	1	1	3	3	3	3	3	3
1	1	1	1	2	2	2	2	3	3
1	1	2	2	3	3	2	2	3	3
1	1	2	2	2	2	2	2	3	3
1	1	1	1	2	2	2	2	3	3
1	1	1	1	2	2	2	2	3	3
1	1	1	1	2	2	2	2	3	3
1	1	1	1	3	3	2	2	3	3
4	4	1	1	3	3	3	3	3	3
1	1	2	2	3	3	2	2	3	3
1	1	1	1	3	3	2	2	3	3
1	1	1	1	2	2	2	2	3	3
1	1	1	1	2	2	2	2	3	3
1	1	1	1	2	2	2	2	3	3
1	1	1	1	2	2	2	2	3	3
1	1	1	1	3	3	2	2	3	3
1	1	1	1	3	3	2	2	3	3
1	1	2	2	3	3	2	2	1	1
1	1	2	2	3	3	2	2	3	3
1	1	2	2	2	2	2	2	3	3
1	1	1	1	2	2	2	2	3	3
1	1	1	1	2	2	2	2	3	3
1	1	2	2	2	2	2	2	3	3
1	1	1	1	3	3	2	2	3	3
1	1	1	1	2	2	2	2	3	3
1	1	1	1	2	2	2	2	1	1
1	1	1	1	3	3	2	2	3	3
1	1	1	1	2	2	2	2	3	3
1	1	1	1	2	2	2	2	1	1
1	1	1	1	2	2	2	2	3	3
4	4	1	1	3	3	2	2	3	3
1	1	1	1	3	3	2	2	3	3
1	1	1	1	2	2	2	2	3	3
1	1	1	1	3	3	2	2	3	3

For Review Only

1	1	1	1	2	2	2	2	1	1
1	1	1	1	2	2	2	2	3	3
1	1	1	1	3	3	2	2	3	3
1	1	1	1	3	3	2	2	3	3
1	1	1	1	2	2	2	2	3	3
1	1	1	1	2	2	2	2	3	3
1	1	2	2	2	2	2	2	3	3
1	1	1	1	3	3	2	2	3	3
1	1	2	2	3	3	2	2	3	3
4	4	1	1	3	3	2	2	3	3
1	1	1	1	2	2	2	2	1	1
1	1	2	2	3	3	2	2	3	3
1	1	2	2	2	2	2	2	3	3
1	1	1	1	2	2	2	2	3	3
1	1	1	1	3	3	2	2	3	3
1	1	1	1	3	3	2	2	3	3
1	1	1	1	3	3	2	2	3	3
1	1	1	1	2	2	2	2	3	3
1	1	1	1	2	2	2	2	3	3
1	1	1	1	3	3	2	2	3	3
1	1	1	1	3	3	2	2	3	3
1	1	1	1	2	2	2	2	3	3
1	1	2	2	2	2	2	2	3	3
1	1	1	1	2	2	2	2	3	3
1	1	1	1	3	3	2	2	3	3
1	1	1	1	2	2	2	2	3	3
1	1	2	2	3	3	2	2	3	3
1	1	1	1	3	3	2	2	3	3
1	1	1	1	2	2	2	2	3	3
1	1	1	1	2	2	2	2	3	3
1	1	1	1	3	3	2	2	3	3
4	4	1	1	3	3	3	3	3	3
1	1	1	1	3	3	2	2	3	3
1	1	1	1	3	3	2	2	3	3
1	1	1	1	2	2	3	3	3	3
1	1	1	1	2	2	2	2	3	3
1	1	1	1	2	2	2	2	3	3
1	1	1	1	2	2	2	2	3	3
1	1	1	1	3	3	2	2	3	3
1	1	1	1	3	3	2	2	3	3
1	1	1	1	3	3	2	2	3	3
1	1	1	1	2	2	2	2	1	1
1	1	2	2	2	2	2	2	3	3
1	1	2	2	3	3	2	2	3	3
1	1	2	2	2	2	2	2	3	3
1	1	1	1	2	2	2	2	3	3
1	1	1	1	3	3	2	2	3	3
1	1	2	2	2	2	2	2	3	3
1	1	1	1	3	3	2	2	3	3
4	4	1	1	2	2	2	2	3	3
1	1	1	1	3	3	2	2	1	1
1	1	1	1	3	3	2	2	3	3
1	1	2	2	2	2	2	2	3	3
1	1	1	1	2	2	2	2	3	3
1	1	2	2	2	2	2	2	3	3

For Review Only

1	1	1	1	2	2	2	2	3	3
1	1	1	1	2	2	3	3	3	3
1	1	1	1	3	3	2	2	3	3
1	1	2	2	2	2	2	2	3	3
1	1	1	1	3	3	2	2	3	3
1	1	1	1	2	2	2	2	3	3
1	1	2	2	2	2	2	2	1	1
4	4	2	2	3	3	2	2	3	3
1	1	2	2	2	2	2	2	3	3
1	1	2	2	3	3	2	2	3	3
1	1	1	1	3	3	2	2	3	3
1	1	1	1	2	2	2	2	3	3
1	1	1	1	2	2	2	2	3	3
1	1	1	1	3	3	2	2	3	3
1	1	1	1	3	3	2	2	3	3
1	1	1	1	2	2	2	2	3	3
1	1	1	1	2	2	2	2	3	3
1	1	1	1	3	3	2	2	3	3
1	1	2	2	3	3	2	2	3	3
1	1	1	1	3	3	2	2	3	3
1	1	1	1	3	3	2	2	3	3
1	1	1	1	2	2	2	2	1	1
1	1	1	1	2	2	2	2	3	3
1	1	1	1	2	2	2	2	3	3
1	1	1	1	3	3	2	2	3	3
1	1	1	1	3	3	2	2	3	3
1	1	2	2	3	3	2	2	3	3
1	1	2	2	3	3	2	2	3	3
1	1	1	1	3	3	2	2	1	1
1	1	1	1	3	3	2	2	3	3
1	1	2	2	3	3	2	2	3	3
1	1	1	1	2	2	2	2	1	1
1	1	1	1	2	2	2	2	3	3
1	1	1	1	3	3	2	2	3	3
1	1	1	1	2	2	2	2	3	3
1	1	2	2	2	2	2	2	3	3
1	1	1	1	2	2	2	2	3	3
1	1	1	1	2	2	2	2	3	3
1	1	1	1	2	2	2	2	3	3
1	1	2	2	3	3	2	2	3	3
1	1	1	1	2	2	2	2	3	3
1	1	1	1	2	2	2	2	3	3
1	1	1	1	2	2	2	2	3	3
1	1	1	1	2	2	2	2	3	3
1	1	1	1	3	3	2	2	3	3
1	1	1	1	2	2	2	2	3	3
1	1	1	1	3	3	2	2	3	3
1	1	1	1	2	2	2	2	3	3
1	1	1	1	2	2	2	2	3	3
1	1	2	2	2	2	2	2	3	3
1	1	1	1	3	3	3	3	3	3
1	1	2	2	2	2	2	2	3	3
1	1	1	1	3	3	2	2	3	3
1	1	1	1	3	3	2	2	3	3
1	1	2	2	3	3	2	2	3	3

For Review Only

1	1	1	1	3	3	2	2	3	3
1	1	1	1	3	3	2	2	3	3

For Review Only

SNP1095	SNP1095	SNP1096	SNP1096	SNP1097	SNP1097	SNP1098	SNP1098	SNP1099	SNP1099
1	1	4	4	2	2	1	1	2	2
1	1	4	4	2	2	1	1	3	3
4	4	4	4	2	2	1	1	2	2
4	4	4	4	2	2	1	1	3	3
1	1	4	4	2	2	1	1	3	3
1	1	4	4	2	2	1	1	2	2
1	1	4	4	2	2	1	1	2	2
1	1	4	4	2	2	1	1	3	3
4	4	4	4	2	2	3	3	2	2
4	4	3	3	2	2	1	1	2	2
1	1	4	4	2	2	1	1	2	2
4	4	4	4	2	2	1	1	2	2
4	4	4	4	2	2	1	1	2	2
4	4	4	4	2	2	1	1	3	3
4	4	4	4	2	2	1	1	2	2
4	4	3	3	2	2	1	1	2	2
1	1	4	4	2	2	1	1	2	2
1	1	4	4	2	2	1	1	2	2
4	4	4	4	2	2	1	1	2	2
1	1	4	4	2	2	1	1	3	3
1	1	4	4	2	2	1	1	2	2
1	1	4	4	2	2	1	1	2	2
4	4	4	4	2	2	1	1	2	2
4	4	4	4	2	2	1	1	2	2
1	1	4	4	2	2	1	1	2	2
4	4	4	4	2	2	1	1	2	2
4	4	4	4	2	2	1	1	2	2
4	4	4	4	2	2	1	1	2	2
4	4	3	3	2	2	3	3	2	2
4	4	3	3	2	2	3	3	2	2
4	4	4	4	2	2	1	1	2	2
1	1	4	4	2	2	1	1	2	2
1	1	4	4	2	2	1	1	2	2
4	4	4	4	2	2	1	1	2	2
4	4	4	4	2	2	1	1	2	2
4	4	4	4	2	2	1	1	2	2
4	4	4	4	2	2	1	1	3	3
4	4	4	4	2	2	1	1	2	2
4	4	4	4	2	2	1	1	2	2
4	4	4	4	2	2	1	1	2	2
4	4	3	3	2	2	3	3	2	2
4	4	4	4	2	2	3	3	2	2
4	4	4	4	2	2	1	1	2	2
1	1	4	4	2	2	1	1	2	2
4	4	4	4	1	1	1	1	3	3
4	4	4	4	2	2	1	1	2	2
4	4	4	4	2	2	1	1	2	2
1	1	3	3	2	2	1	1	2	2
4	4	4	4	2	2	1	1	3	3
4	4	4	4	2	2	1	1	2	2
4	4	4	4	2	2	1	1	2	2
1	1	4	4	2	2	1	1	2	2

For Review Only

1	1	4	4	2	2	1	1	2	2
1	1	4	4	2	2	1	1	2	2
1	1	4	4	2	2	1	1	2	2
4	4	4	4	2	2	1	1	2	2
4	4	4	4	2	2	1	1	2	2
4	4	4	4	2	2	1	1	2	2
4	4	4	4	2	2	1	1	2	2
4	4	4	4	2	2	1	1	2	2
4	4	4	4	2	2	1	1	2	2
4	4	4	4	2	2	1	1	2	2
1	1	4	4	2	2	1	1	2	2
4	4	4	4	2	2	1	1	2	2
1	1	4	4	2	2	1	1	2	2
4	4	3	3	2	2	3	3	2	2
4	4	4	4	2	2	1	1	2	2
4	4	4	4	2	2	1	1	3	3
4	4	4	4	2	2	1	1	2	2
4	4	4	4	2	2	1	1	2	2
1	1	4	4	2	2	1	1	2	2
1	1	4	4	2	2	1	1	2	2
4	4	4	4	2	2	1	1	2	2
4	4	4	4	2	2	1	1	2	2
4	4	4	4	2	2	3	3	2	2
4	4	4	4	2	2	1	1	2	2
4	4	4	4	1	1	1	1	3	3
1	1	3	3	2	2	3	3	2	2
1	1	4	4	2	2	1	1	2	2
4	4	4	4	2	2	1	1	2	2
4	4	4	4	2	2	1	1	2	2
1	1	4	4	2	2	1	1	2	2
1	1	4	4	2	2	3	3	3	3
4	4	4	4	2	2	1	1	2	2
4	4	4	4	2	2	1	1	2	2
1	1	4	4	2	2	1	1	2	2
4	4	4	4	2	2	1	1	2	2
4	4	4	4	2	2	1	1	3	3
1	1	3	3	2	2	3	3	2	2
1	1	4	4	2	2	1	1	2	2
1	1	4	4	2	2	1	1	2	2
4	4	4	4	2	2	1	1	3	3
4	4	4	4	2	2	1	1	2	2
1	1	4	4	2	2	1	1	2	2
4	4	4	4	2	2	1	1	2	2
1	1	4	4	2	2	1	1	2	2
1	1	4	4	2	2	1	1	2	2
4	4	4	4	2	2	1	1	2	2
4	4	4	4	2	2	1	1	3	3
4	4	4	4	2	2	1	1	2	2
4	4	4	4	2	2	1	1	2	2
4	4	4	4	2	2	1	1	2	2
1	1	4	4	2	2	1	1	2	2
4	4	4	4	2	2	1	1	2	2
4	4	4	4	2	2	1	1	2	2
4	4	4	4	2	2	1	1	2	2
4	4	4	4	2	2	1	1	3	3

For Review Only

4	4	4	4	2	2	1	1	2	2
1	1	4	4	2	2	1	1	2	2
4	4	4	4	2	2	1	1	2	2
4	4	4	4	2	2	1	1	3	3
4	4	4	4	2	2	1	1	2	2
4	4	4	4	2	2	1	1	2	2
4	4	4	4	2	2	1	1	2	2
4	4	4	4	2	2	1	1	2	2
4	4	4	4	2	2	1	1	2	2
4	4	4	4	2	2	1	1	2	2
4	4	4	4	2	2	1	1	2	2
4	4	4	4	2	2	1	1	2	2
4	4	4	4	2	2	1	1	2	2
1	1	4	4	2	2	1	1	2	2
1	1	4	4	2	2	1	1	3	3
4	4	4	4	2	2	1	1	2	2
4	4	4	4	2	2	1	1	2	2
4	4	4	4	2	2	1	1	2	2
4	4	4	4	2	2	1	1	3	3
1	1	4	4	2	2	1	1	2	2
4	4	4	4	2	2	1	1	2	2
1	1	4	4	2	2	1	1	2	2
4	4	4	4	2	2	1	1	2	2
4	4	4	4	2	2	1	1	2	2
4	4	4	4	2	2	1	1	2	2
4	4	4	4	2	2	1	1	2	2
4	4	4	4	2	2	1	1	2	2
4	4	4	4	2	2	1	1	2	2
1	1	4	4	2	2	1	1	2	2
4	4	4	4	2	2	1	1	2	2
4	4	4	4	2	2	1	1	2	2
4	4	4	4	2	2	1	1	2	2
4	4	4	4	2	2	1	1	2	2
4	4	4	4	2	2	1	1	2	2
4	4	4	4	2	2	1	1	2	2
4	4	4	4	2	2	1	1	2	2
4	4	4	4	2	2	1	1	3	3
1	1	4	4	2	2	1	1	2	2
4	4	4	4	2	2	1	1	2	2
4	4	4	4	2	2	1	1	2	2
4	4	4	4	2	2	1	1	2	2
4	4	4	4	2	2	1	1	3	3
1	1	4	4	2	2	1	1	2	2
4	4	4	4	2	2	1	1	2	2
4	4	4	4	2	2	1	1	2	2
4	4	4	4	2	2	1	1	3	3
4	4	4	4	2	2	1	1	2	2
4	4	4	4	2	2	1	1	2	2
4	4	4	4	2	2	1	1	2	2
4	4	4	4	2	2	1	1	2	2
4	4	4	4	2	2	1	1	2	2
4	4	4	4	2	2	1	1	2	2
1	1	4	4	2	2	1	1	2	2
4	4	4	4	2	2	1	1	2	2
4	4	4	4	2	2	1	1	2	2

For Review Only

4	4	4	4	2	2	1	1	2	2
4	4	4	4	2	2	1	1	2	2
4	4	4	4	2	2	1	1	2	2
4	4	4	4	2	2	1	1	2	2
1	1	4	4	2	2	1	1	2	2
4	4	4	4	2	2	1	1	2	2
4	4	4	4	2	2	1	1	2	2
4	4	4	4	2	2	1	1	2	2
1	1	4	4	2	2	1	1	2	2
1	1	4	4	2	2	1	1	2	2
4	4	4	4	2	2	1	1	2	2
4	4	4	4	2	2	1	1	2	2
4	4	4	4	2	2	1	1	2	2
4	4	4	4	2	2	1	1	3	3
4	4	4	4	2	2	1	1	3	3
1	1	4	4	2	2	1	1	2	2
4	4	4	4	2	2	1	1	2	2
1	1	4	4	2	2	1	1	2	2
4	4	4	4	2	2	1	1	2	2
4	4	4	4	2	2	1	1	2	2
4	4	4	4	2	2	1	1	2	2
4	4	4	4	2	2	1	1	2	2
4	4	4	4	2	2	1	1	2	2
4	4	4	4	2	2	1	1	2	2
4	4	3	3	2	2	3	3	2	2
4	4	4	4	2	2	1	1	2	2
4	4	4	4	2	2	1	1	2	2
4	4	4	4	2	2	1	1	2	2
4	4	4	4	2	2	1	1	2	2
4	4	4	4	2	2	1	1	2	2
4	4	4	4	2	2	1	1	2	2
1	1	4	4	2	2	1	1	2	2
4	4	4	4	2	2	1	1	2	2
4	4	4	4	2	2	1	1	3	3
4	4	4	4	2	2	1	1	2	2
4	4	4	4	2	2	1	1	2	2
1	1	4	4	2	2	1	1	2	2
1	1	4	4	2	2	1	1	2	2
4	4	4	4	2	2	1	1	2	2
1	1	4	4	2	2	1	1	2	2
4	4	4	4	2	2	1	1	2	2
4	4	4	4	2	2	1	1	2	2
4	4	4	4	2	2	1	1	2	2
1	1	4	4	2	2	1	1	2	2
4	4	4	4	2	2	1	1	2	2
4	4	4	4	2	2	1	1	3	3
1	1	4	4	2	2	1	1	2	2
4	4	4	4	2	2	1	1	2	2
4	4	4	4	2	2	1	1	2	2
4	4	4	4	2	2	1	1	2	2
1	1	4	4	2	2	1	1	2	2
4	4	4	4	2	2	1	1	2	2
4	4	4	4	2	2	1	1	2	2
1	1	4	4	2	2	1	1	2	2
4	4	4	4	2	2	1	1	2	2

For Review Only

1	1	4	4	2	2	1	1	3	3
4	4	3	3	2	2	3	3	2	2
1	1	4	4	2	2	1	1	2	2
4	4	4	4	2	2	1	1	2	2
1	1	3	3	2	2	1	1	2	2
4	4	4	4	2	2	1	1	2	2
4	4	4	4	2	2	1	1	2	2
4	4	4	4	2	2	1	1	2	2
4	4	4	4	2	2	1	1	2	2
4	4	4	4	2	2	1	1	2	2
4	4	4	4	2	2	1	1	2	2
4	4	4	4	2	2	1	1	2	2
4	4	4	4	2	2	1	1	2	2
4	4	4	4	2	2	1	1	2	2
4	4	4	4	2	2	1	1	2	2
1	1	4	4	2	2	1	1	2	2
4	4	4	4	2	2	1	1	2	2
1	1	4	4	2	2	1	1	2	2
1	1	4	4	2	2	1	1	3	3
4	4	4	4	2	2	1	1	2	2
1	1	4	4	2	2	1	1	2	2
4	4	4	4	2	2	1	1	2	2
1	1	4	4	2	2	1	1	2	2
4	4	4	4	2	2	1	1	2	2
4	4	4	4	2	2	1	1	2	2
4	4	4	4	2	2	3	3	2	2
4	4	4	4	2	2	1	1	2	2
1	1	4	4	2	2	1	1	2	2
1	1	3	3	2	2	1	1	2	2
1	1	4	4	2	2	1	1	2	2
4	4	4	4	2	2	1	1	2	2
4	4	3	3	2	2	1	1	2	2
1	1	4	4	2	2	1	1	3	3
4	4	4	4	2	2	1	1	2	2
4	4	4	4	2	2	1	1	2	2
4	4	4	4	2	2	1	1	2	2
1	1	4	4	2	2	1	1	2	2
4	4	4	4	2	2	1	1	2	2
4	4	4	4	2	2	1	1	2	2
4	4	4	4	2	2	1	1	2	2
4	4	4	4	2	2	1	1	2	2
4	4	4	4	2	2	1	1	2	2
4	4	4	4	2	2	1	1	2	2
1	1	4	4	2	2	1	1	2	2
1	1	4	4	2	2	1	1	2	2
1	1	4	4	2	2	1	1	2	2
4	4	4	4	2	2	1	1	2	2
4	4	4	4	2	2	1	1	2	2
4	4	4	4	2	2	1	1	2	2
4	4	4	4	2	2	1	1	2	2
4	4	4	4	2	2	1	1	2	2
4	4	4	4	2	2	1	1	2	2
4	4	4	4	2	2	1	1	2	2
1	1	4	4	2	2	1	1	2	2
4	4	4	4	2	2	1	1	2	2

For Review Only

1	1	4	4	2	2	1	1	2	2
1	1	4	4	2	2	1	1	2	2

For Review Only

SNP110	SNP110	SNP1100	SNP1100	SNP1101	SNP1101	SNP1102	SNP1102	SNP1103	SNP1103
2	2	3	3	1	1	3	3	4	4
3	3	3	3	1	1	3	3	4	4
3	3	3	3	1	1	3	3	1	1
3	3	3	3	1	1	3	3	4	4
2	2	3	3	1	1	3	3	4	4
3	3	3	3	1	1	3	3	1	1
2	2	3	3	1	1	3	3	4	4
3	3	1	1	4	4	3	3	1	1
2	2	3	3	1	1	3	3	4	4
3	3	1	1	4	4	2	2	1	1
2	2	3	3	1	1	3	3	4	4
3	3	3	3	1	1	3	3	1	1
2	2	3	3	1	1	3	3	4	4
3	3	1	1	4	4	2	2	1	1
2	2	3	3	1	1	3	3	4	4
2	2	3	3	1	1	2	2	4	4
3	3	3	3	1	1	3	3	4	4
2	2	3	3	1	1	3	3	1	1
2	2	3	3	1	1	2	2	4	4
2	2	1	1	4	4	3	3	1	1
3	3	3	3	1	1	3	3	1	1
2	2	3	3	1	1	3	3	4	4
2	2	3	3	1	1	2	2	4	4
2	2	3	3	1	1	2	2	4	4
2	2	3	3	1	1	3	3	1	1
2	2	3	3	1	1	2	2	1	1
3	3	3	3	1	1	2	2	4	4
2	2	3	3	1	1	3	3	4	4
2	2	3	3	1	1	2	2	4	4
2	2	3	3	1	1	2	2	4	4
2	2	3	3	1	1	2	2	4	4
2	2	3	3	1	1	3	3	4	4
2	2	3	3	1	1	3	3	4	4
2	2	1	1	4	4	2	2	1	1
2	2	1	1	4	4	3	3	1	1
3	3	1	1	4	4	2	2	1	1
2	2	3	3	1	1	2	2	4	4
3	3	1	1	1	1	3	3	4	4
3	3	3	3	1	1	2	2	4	4
3	3	3	3	1	1	3	3	1	1
3	3	3	3	1	1	2	2	1	1
2	2	3	3	1	1	2	2	1	1
3	3	3	3	1	1	2	2	1	1
2	2	3	3	1	1	3	3	4	4
3	3	1	1	4	4	2	2	1	1
2	2	3	3	1	1	3	3	4	4
2	2	3	3	1	1	2	2	1	1
3	3	1	1	4	4	3	3	1	1
2	2	1	1	4	4	3	3	1	1
2	2	3	3	1	1	3	3	4	4
3	3	3	3	1	1	2	2	1	1
2	2	3	3	1	1	3	3	4	4

For Review Only

2	2	3	3	1	1	3	3	4	4
2	2	3	3	1	1	3	3	4	4
3	3	3	3	1	1	3	3	4	4
2	2	3	3	1	1	2	2	4	4
2	2	3	3	1	1	2	2	4	4
2	2	3	3	1	1	2	2	4	4
2	2	3	3	1	1	2	2	4	4
3	3	3	3	1	1	2	2	4	4
3	3	3	3	1	1	2	2	4	4
2	2	3	3	1	1	3	3	4	4
3	3	3	3	1	1	3	3	4	4
3	3	3	3	1	1	2	2	4	4
2	2	3	3	1	1	3	3	4	4
2	2	3	3	1	1	2	2	4	4
2	2	3	3	1	1	3	3	4	4
3	3	3	3	1	1	3	3	4	4
3	3	3	3	1	1	3	3	4	4
2	2	3	3	1	1	3	3	4	4
2	2	3	3	1	1	3	3	4	4
2	2	3	3	1	1	3	3	4	4
3	3	3	3	1	1	3	3	4	4
3	3	3	3	1	1	2	2	4	4
2	2	3	3	1	1	3	3	4	4
3	3	3	3	1	1	2	2	1	1
3	3	1	1	4	4	2	2	1	1
3	3	3	3	1	1	3	3	1	1
2	2	3	3	1	1	3	3	1	1
2	2	3	3	1	1	2	2	1	1
2	2	3	3	1	1	2	2	1	1
2	2	3	3	1	1	3	3	4	4
2	2	3	3	1	1	3	3	4	4
3	3	3	3	1	1	3	3	4	4
2	2	3	3	1	1	2	2	1	1
2	2	3	3	1	1	3	3	4	4
2	2	3	3	1	1	3	3	4	4
3	3	1	1	4	4	2	2	1	1
2	2	3	3	1	1	3	3	1	1
2	2	3	3	1	1	3	3	4	4
3	3	3	3	1	1	3	3	4	4
3	3	1	1	4	4	2	2	1	1
2	2	3	3	1	1	2	2	4	4
3	3	3	3	1	1	3	3	4	4
3	3	3	3	1	1	2	2	4	4
3	3	3	3	1	1	3	3	4	4
3	3	3	3	1	1	3	3	4	4
2	2	3	3	1	1	3	3	4	4
3	3	1	1	4	4	2	2	1	1
3	3	3	3	1	1	2	2	4	4
2	2	3	3	1	1	2	2	4	4
2	2	3	3	1	1	2	2	4	4
3	3	3	3	1	1	3	3	4	4
2	2	3	3	1	1	2	2	4	4
3	3	3	3	1	1	2	2	4	4
2	2	3	3	1	1	3	3	4	4
3	3	3	3	1	1	3	3	4	4

For Review Only

2	2	3	3	1	1	2	2	1	1
3	3	3	3	1	1	3	3	1	1
3	3	1	1	4	4	2	2	1	1
2	2	3	3	4	4	2	2	1	1
3	3	3	3	1	1	3	3	4	4
2	2	3	3	4	4	2	2	1	1
2	2	3	3	1	1	2	2	1	1
3	3	3	3	1	1	2	2	4	4
3	3	3	3	1	1	2	2	4	4
3	3	3	3	4	4	2	2	4	4
2	2	3	3	1	1	3	3	4	4
2	2	3	3	1	1	3	3	4	4
3	3	3	3	1	1	2	2	4	4
3	3	3	3	1	1	3	3	1	1
3	3	3	3	1	1	3	3	4	4
2	2	3	3	1	1	3	3	1	1
2	2	3	3	1	1	3	3	4	4
2	2	3	3	1	1	2	2	4	4
2	2	1	1	4	4	2	2	1	1
2	2	1	1	1	1	2	2	1	1
2	2	3	3	1	1	3	3	4	4
2	2	3	3	1	1	3	3	4	4
3	3	3	3	1	1	2	2	1	1
2	2	3	3	1	1	2	2	1	1
3	3	3	3	1	1	2	2	4	4
3	3	3	3	1	1	2	2	4	4
2	2	3	3	1	1	3	3	4	4
3	3	3	3	1	1	2	2	4	4
3	3	3	3	1	1	3	3	1	1
2	2	3	3	1	1	2	2	4	4
2	2	1	1	4	4	2	2	4	4
2	2	3	3	1	1	3	3	4	4
2	2	3	3	1	1	2	2	4	4
2	2	3	3	1	1	3	3	1	1
2	2	1	1	4	4	2	2	1	1
2	2	3	3	1	1	3	3	4	4
3	3	3	3	1	1	2	2	4	4
3	3	3	3	1	1	3	3	1	1
2	2	3	3	4	4	3	3	1	1
2	2	1	1	4	4	3	3	4	4
3	3	3	3	1	1	2	2	4	4
3	3	1	1	4	4	2	2	1	1
3	3	3	3	1	1	3	3	4	4
2	2	1	1	4	4	2	2	4	4
3	3	3	3	1	1	2	2	4	4
3	3	3	3	1	1	3	3	4	4
2	2	3	3	1	1	2	2	1	1
2	2	3	3	1	1	2	2	1	1
2	2	3	3	1	1	3	3	4	4
2	2	3	3	1	1	2	2	1	1
2	2	1	1	4	4	2	2	1	1
2	2	3	3	1	1	2	2	4	4
2	2	3	3	1	1	3	3	4	4
3	3	3	3	1	1	2	2	4	4
2	2	3	3	1	1	3	3	4	4

For Review Only

3	3	3	3	1	1	3	3	1	1
3	3	3	3	1	1	2	2	4	4
2	2	3	3	1	1	3	3	4	4
3	3	1	1	4	4	3	3	4	4
2	2	3	3	1	1	3	3	4	4
2	2	3	3	1	1	2	2	4	4
2	2	3	3	1	1	2	2	4	4
2	2	3	3	1	1	3	3	4	4
2	2	3	3	1	1	3	3	4	4
2	2	3	3	1	1	3	3	4	4
2	2	3	3	1	1	2	2	1	1
3	3	3	3	1	1	2	2	4	4
2	2	3	3	1	1	3	3	4	4
3	3	1	1	4	4	3	3	4	4
3	3	1	1	4	4	2	2	1	1
2	2	3	3	1	1	3	3	4	4
2	2	3	3	1	1	3	3	4	4
3	3	3	3	1	1	3	3	4	4
3	3	3	3	1	1	2	2	1	1
2	2	3	3	1	1	2	2	1	1
2	2	3	3	1	1	3	3	4	4
2	2	3	3	1	1	2	2	1	1
3	3	3	3	1	1	2	2	4	4
3	3	3	3	1	1	2	2	4	4
2	2	3	3	1	1	2	2	4	4
2	2	1	1	4	4	2	2	1	1
3	3	3	3	1	1	2	2	4	4
2	2	3	3	1	1	3	3	4	4
3	3	3	3	1	1	2	2	4	4
2	2	3	3	1	1	2	2	4	4
3	3	3	3	1	1	3	3	1	1
2	2	3	3	1	1	3	3	4	4
2	2	3	3	1	1	3	3	4	4
3	3	1	1	4	4	2	2	4	4
2	2	3	3	1	1	3	3	4	4
3	3	3	3	1	1	2	2	4	4
3	3	1	1	4	4	3	3	4	4
2	2	3	3	1	1	3	3	4	4
2	2	3	3	1	1	2	2	1	1
3	3	3	3	1	1	3	3	4	4
3	3	3	3	1	1	3	3	1	1
2	2	3	3	4	4	2	2	1	1
2	2	3	3	1	1	2	2	1	1
2	2	3	3	1	1	3	3	4	4
2	2	1	1	4	4	2	2	4	4
2	2	3	3	1	1	3	3	4	4
2	2	3	3	1	1	3	3	4	4
2	2	3	3	1	1	2	2	1	1
2	2	3	3	1	1	2	2	4	4
3	3	3	3	1	1	2	2	4	4
3	3	3	3	1	1	3	3	1	1
3	3	3	3	1	1	3	3	4	4
2	2	3	3	1	1	2	2	4	4
3	3	3	3	1	1	3	3	4	4
2	2	1	1	4	4	2	2	4	4

For Review Only

3	3	3	3	1	1	3	3	4	4
2	2	3	3	1	1	2	2	4	4
3	3	3	3	1	1	3	3	1	1
2	2	3	3	1	1	2	2	4	4
2	2	3	3	1	1	3	3	1	1
2	2	3	3	1	1	2	2	4	4
3	3	3	3	1	1	3	3	1	1
2	2	3	3	1	1	2	2	1	1
2	2	1	1	4	4	2	2	4	4
2	2	3	3	1	1	2	2	4	4
2	2	3	3	1	1	3	3	4	4
3	3	3	3	1	1	3	3	1	1
2	2	3	3	1	1	3	3	4	4
2	2	3	3	1	1	2	2	4	4
2	2	3	3	1	1	3	3	1	1
2	2	3	3	1	1	3	3	4	4
2	2	3	3	1	1	2	2	4	4
2	2	3	3	1	1	3	3	4	4
2	2	1	1	4	4	3	3	1	1
3	3	3	3	1	1	2	2	4	4
3	3	3	3	1	1	3	3	1	1
2	2	3	3	1	1	2	2	1	1
2	2	3	3	1	1	3	3	4	4
3	3	3	3	1	1	2	2	4	4
3	3	3	3	1	1	2	2	4	4
2	2	3	3	1	1	3	3	4	4
2	2	3	3	1	1	2	2	1	1
3	3	3	3	1	1	3	3	4	4
2	2	3	3	1	1	3	3	4	4
3	3	3	3	1	1	3	3	1	1
2	2	3	3	1	1	2	2	4	4
2	2	3	3	1	1	2	2	1	1
3	3	1	1	4	4	3	3	4	4
2	2	3	3	1	1	2	2	4	4
3	3	3	3	1	1	2	2	4	4
2	2	3	3	1	1	2	2	1	1
3	3	3	3	1	1	3	3	4	4
3	3	3	3	1	1	2	2	4	4
3	3	1	1	4	4	2	2	1	1
2	2	3	3	1	1	2	2	4	4
2	2	3	3	1	1	3	3	4	4
3	3	3	3	1	1	3	3	4	4
3	3	3	3	1	1	2	2	4	4
2	2	3	3	1	1	3	3	4	4
2	2	3	3	1	1	3	3	4	4
3	3	3	3	1	1	3	3	4	4
2	2	3	3	1	1	2	2	1	1
3	3	3	3	4	4	3	3	4	4
3	3	1	1	4	4	2	2	1	1
2	2	3	3	1	1	2	2	1	1
2	2	3	3	1	1	2	2	4	4
2	2	3	3	1	1	2	2	4	4
3	3	3	3	1	1	3	3	1	1
3	3	3	3	1	1	3	3	4	4
3	3	3	3	1	1	2	2	4	4

For Review Only

2	2	3	3	1	1	3	3	4	4
3	3	3	3	1	1	3	3	4	4

For Review Only

SNP1104	SNP1104	SNP1105	SNP1105	SNP1106	SNP1106	SNP1107	SNP1107	SNP1108	SNP1108
3	3	2	2	4	4	1	1	2	2
3	3	2	2	4	4	1	1	3	3
4	4	1	1	4	4	4	4	3	3
3	3	2	2	4	4	4	4	3	3
3	3	2	2	4	4	1	1	3	3
4	4	1	1	4	4	1	1	3	3
3	3	2	2	4	4	1	1	3	3
4	4	1	1	4	4	1	1	3	3
3	3	2	2	4	4	1	1	3	3
4	4	1	1	4	4	1	1	3	3
3	3	2	2	4	4	1	1	2	2
3	3	2	2	4	4	1	1	3	3
4	4	1	1	4	4	1	1	3	3
3	3	1	1	4	4	1	1	3	3
3	3	2	2	4	4	1	1	2	2
3	3	2	2	4	4	4	4	2	2
3	3	2	2	4	4	1	1	3	3
4	4	1	1	4	4	1	1	3	3
3	3	2	2	4	4	1	1	3	3
4	4	1	1	4	4	1	1	2	2
4	4	1	1	4	4	1	1	3	3
3	3	2	2	4	4	1	1	3	3
3	3	2	2	4	4	1	1	3	3
3	3	2	2	4	4	1	1	2	2
4	4	1	1	4	4	1	1	3	3
4	4	1	1	4	4	1	1	2	2
3	3	2	2	4	4	4	4	3	3
3	3	1	1	4	4	1	1	3	3
3	3	2	2	4	4	1	1	3	3
3	3	2	2	4	4	1	1	2	2
3	3	2	2	4	4	1	1	3	3
3	3	2	2	4	4	1	1	3	3
3	3	2	2	4	4	1	1	3	3
4	4	1	1	4	4	1	1	2	2
3	3	1	1	4	4	1	1	2	2
4	4	1	1	4	4	1	1	2	2
3	3	2	2	4	4	1	1	2	2
3	3	2	2	4	4	4	4	2	2
3	3	2	2	4	4	1	1	2	2
4	4	1	1	4	4	1	1	2	2
4	4	1	1	4	4	1	1	2	2
4	4	1	1	4	4	1	1	2	2
4	4	1	1	1	1	1	1	3	3
3	3	2	2	4	4	1	1	2	2
4	4	1	1	4	4	1	1	3	3
3	3	2	2	4	4	4	4	3	3
4	4	1	1	1	1	1	1	2	2
4	4	1	1	4	4	1	1	3	3
4	4	1	1	4	4	1	1	3	3
3	3	2	2	4	4	1	1	3	3
4	4	1	1	4	4	1	1	2	2
3	3	2	2	4	4	1	1	3	3

For Review Only

3	3	2	2	4	4	1	1	3	3
3	3	2	2	4	4	1	1	3	3
3	3	2	2	4	4	1	1	3	3
3	3	2	2	4	4	1	1	3	3
3	3	2	2	4	4	1	1	3	3
3	3	2	2	4	4	1	1	2	2
3	3	2	2	4	4	1	1	2	2
3	3	2	2	4	4	1	1	3	3
3	3	2	2	4	4	1	1	3	3
3	3	2	2	4	4	1	1	3	3
3	3	2	2	4	4	1	1	3	3
3	3	2	2	4	4	1	1	2	2
3	3	2	2	4	4	1	1	3	3
3	3	2	2	4	4	1	1	3	3
3	3	2	2	4	4	1	1	3	3
3	3	2	2	4	4	1	1	3	3
3	3	2	2	4	4	1	1	3	3
3	3	2	2	4	4	1	1	2	2
3	3	2	2	4	4	1	1	3	3
3	3	2	2	4	4	1	1	3	3
3	3	2	2	4	4	1	1	2	2
3	3	2	2	4	4	1	1	2	2
3	3	2	2	4	4	1	1	3	3
4	4	1	1	4	4	1	1	2	2
4	4	1	1	4	4	1	1	3	3
4	4	1	1	4	4	1	1	3	3
4	4	1	1	4	4	1	1	3	3
3	3	1	1	4	4	1	1	2	2
4	4	1	1	4	4	1	1	2	2
3	3	2	2	4	4	1	1	3	3
3	3	2	2	1	1	1	1	3	3
3	3	2	2	4	4	1	1	3	3
4	4	1	1	4	4	1	1	2	2
3	3	2	2	4	4	1	1	3	3
3	3	2	2	4	4	1	1	2	2
4	4	1	1	4	4	1	1	3	3
3	3	1	1	1	1	1	1	3	3
3	3	2	2	4	4	1	1	3	3
3	3	2	2	4	4	1	1	3	3
4	4	1	1	4	4	1	1	3	3
3	3	2	2	4	4	1	1	3	3
3	3	2	2	4	4	1	1	2	2
3	3	2	2	4	4	1	1	2	2
3	3	2	2	4	4	1	1	3	3
3	3	2	2	4	4	1	1	2	2
3	3	2	2	4	4	1	1	3	3
3	3	1	1	4	4	1	1	2	2
3	3	2	2	4	4	1	1	2	2
3	3	2	2	4	4	1	1	2	2
3	3	2	2	4	4	1	1	2	2
3	3	2	2	4	4	1	1	3	3
3	3	2	2	4	4	1	1	2	2
3	3	2	2	4	4	1	1	3	3
3	3	2	2	4	4	1	1	3	3
3	3	2	2	4	4	1	1	2	2

For Review Only

3	3	2	2	1	1	1	1	2	2
4	4	1	1	4	4	1	1	3	3
4	4	1	1	4	4	1	1	2	2
4	4	1	1	4	4	1	1	2	2
3	3	2	2	4	4	1	1	3	3
4	4	1	1	4	4	1	1	3	3
4	4	1	1	4	4	4	4	3	3
3	3	2	2	4	4	1	1	2	2
3	3	2	2	4	4	1	1	2	2
4	4	1	1	4	4	1	1	3	3
3	3	2	2	4	4	1	1	3	3
3	3	2	2	4	4	1	1	3	3
3	3	2	2	4	4	1	1	2	2
3	3	2	2	1	1	1	1	3	3
3	3	2	2	4	4	1	1	3	3
4	4	1	1	4	4	1	1	2	2
3	3	2	2	4	4	1	1	2	2
3	3	2	2	4	4	1	1	3	3
3	3	1	1	4	4	1	1	2	2
4	4	1	1	4	4	1	1	3	3
3	3	2	2	4	4	1	1	3	3
3	3	2	2	4	4	1	1	3	3
4	4	1	1	4	4	1	1	3	3
4	4	2	2	4	4	1	1	2	2
3	3	2	2	1	1	1	1	2	2
3	3	2	2	4	4	1	1	2	2
3	3	2	2	4	4	1	1	3	3
3	3	2	2	4	4	1	1	3	3
4	4	1	1	4	4	1	1	3	3
3	3	2	2	4	4	4	4	3	3
4	4	1	1	4	4	1	1	2	2
3	3	2	2	4	4	1	1	3	3
3	3	2	2	4	4	1	1	3	3
3	3	1	1	4	4	1	1	2	2
4	4	1	1	4	4	1	1	2	2
3	3	2	2	4	4	1	1	2	2
3	3	2	2	4	4	1	1	2	2
4	4	1	1	4	4	1	1	3	3
4	4	1	1	4	4	1	1	2	2
3	3	2	2	4	4	1	1	2	2
3	3	2	2	4	4	1	1	3	3
4	4	1	1	4	4	1	1	2	2
3	3	2	2	4	4	1	1	2	2
4	4	1	1	4	4	1	1	2	2
3	3	2	2	4	4	4	4	2	2
3	3	2	2	4	4	1	1	3	3
4	4	1	1	4	4	1	1	2	2
4	4	1	1	4	4	1	1	2	2
3	3	2	2	4	4	1	1	2	2
4	4	1	1	4	4	1	1	2	2
4	4	2	2	4	4	1	1	2	2
3	3	2	2	4	4	1	1	2	2
3	3	2	2	4	4	1	1	2	2
3	3	2	2	4	4	1	1	2	2
3	3	2	2	4	4	1	1	3	3

For Review Only

4	4	1	1	4	4	1	1	3	3
3	3	2	2	4	4	1	1	2	2
3	3	2	2	4	4	1	1	3	3
3	3	2	2	4	4	1	1	3	3
3	3	2	2	4	4	1	1	3	3
3	3	2	2	4	4	1	1	3	3
3	3	2	2	4	4	1	1	2	2
3	3	2	2	4	4	1	1	3	3
3	3	2	2	4	4	1	1	2	2
3	3	2	2	4	4	1	1	3	3
4	4	1	1	1	1	1	1	2	2
3	3	2	2	4	4	1	1	3	3
3	3	2	2	4	4	1	1	2	2
3	3	2	2	4	4	1	1	3	3
4	4	1	1	4	4	1	1	3	3
3	3	2	2	4	4	1	1	2	2
3	3	2	2	4	4	1	1	2	2
4	4	2	2	4	4	1	1	3	3
4	4	1	1	4	4	1	1	2	2
4	4	1	1	4	4	4	4	3	3
3	3	2	2	4	4	1	1	3	3
4	4	1	1	4	4	1	1	3	3
3	3	2	2	4	4	1	1	2	2
3	3	2	2	4	4	1	1	2	2
4	4	1	1	4	4	1	1	2	2
4	4	1	1	4	4	1	1	2	2
3	3	2	2	4	4	1	1	3	3
3	3	2	2	4	4	1	1	3	3
3	3	2	2	4	4	1	1	2	2
3	3	2	2	4	4	1	1	3	3
4	4	1	1	4	4	1	1	3	3
3	3	2	2	4	4	1	1	3	3
3	3	2	2	4	4	1	1	3	3
3	3	2	2	4	4	1	1	3	3
3	3	2	2	4	4	1	1	3	3
3	3	2	2	4	4	1	1	2	2
4	4	1	1	4	4	1	1	2	2
3	3	2	2	4	4	1	1	2	2
4	4	1	1	4	4	1	1	2	2
3	3	2	2	4	4	1	1	2	2
4	4	1	1	4	4	4	4	3	3
4	4	1	1	4	4	1	1	2	2
4	4	1	1	4	4	1	1	2	2
3	3	2	2	4	4	1	1	2	2
4	4	2	2	4	4	1	1	3	3
3	3	2	2	4	4	1	1	3	3
3	3	2	2	4	4	1	1	2	2
4	4	1	1	4	4	1	1	2	2
3	3	2	2	4	4	1	1	2	2
3	3	2	2	4	4	1	1	3	3
4	4	1	1	4	4	1	1	3	3
3	3	2	2	4	4	1	1	2	2
3	3	2	2	4	4	1	1	2	2
3	3	2	2	4	4	1	1	3	3
3	3	2	2	4	4	1	1	2	2

For Review Only

3	3	2	2	4	4	1	1	2	2
3	3	2	2	4	4	1	1	2	2
4	4	1	1	4	4	1	1	3	3
3	3	2	2	4	4	1	1	3	3
4	4	1	1	4	4	1	1	2	2
3	3	2	2	4	4	1	1	3	3
4	4	1	1	4	4	1	1	2	2
3	3	1	1	4	4	1	1	3	3
3	3	2	2	4	4	1	1	3	3
3	3	2	2	4	4	1	1	2	2
3	3	2	2	4	4	1	1	3	3
4	4	1	1	4	4	1	1	2	2
3	3	2	2	4	4	1	1	3	3
3	3	2	2	4	4	1	1	2	2
4	4	1	1	4	4	1	1	3	3
3	3	2	2	4	4	1	1	2	2
3	3	2	2	4	4	1	1	2	2
3	3	2	2	4	4	1	1	3	3
4	4	1	1	4	4	1	1	2	2
3	3	2	2	4	4	4	4	2	2
4	4	1	1	4	4	1	1	3	3
4	4	1	1	4	4	1	1	2	2
3	3	2	2	4	4	1	1	3	3
3	3	2	2	4	4	1	1	2	2
3	3	2	2	4	4	1	1	3	3
3	3	2	2	4	4	1	1	2	2
4	4	1	1	4	4	1	1	2	2
3	3	2	2	4	4	1	1	3	3
3	3	2	2	4	4	1	1	2	2
4	4	1	1	4	4	1	1	3	3
3	3	2	2	4	4	1	1	2	2
4	4	1	1	4	4	1	1	2	2
3	3	2	2	4	4	1	1	2	2
3	3	2	2	4	4	1	1	2	2
3	3	2	2	4	4	1	1	2	2
4	4	1	1	4	4	1	1	2	2
3	3	2	2	4	4	1	1	3	3
3	3	2	2	4	4	1	1	2	2
4	4	1	1	4	4	1	1	2	2
3	3	2	2	4	4	1	1	2	2
3	3	2	2	4	4	1	1	3	3
3	3	2	2	4	4	1	1	2	2
3	3	2	2	4	4	1	1	2	2
3	3	2	2	4	4	1	1	3	3
3	3	2	2	4	4	4	4	3	3
3	3	2	2	4	4	1	1	3	3
4	4	1	1	4	4	1	1	2	2
4	4	1	1	4	4	1	1	3	3
4	4	1	1	4	4	1	1	3	3
4	4	1	1	4	4	1	1	2	2
3	3	2	2	4	4	1	1	3	3
3	3	2	2	4	4	1	1	2	2
4	4	1	1	4	4	4	4	3	3
3	3	2	2	4	4	1	1	3	3
3	3	2	2	4	4	4	4	2	2

For Review Only

3	3	2	2	4	4	1	1	3	3
4	4	1	1	4	4	1	1	3	3

For Review Only

SNP1109	SNP1109	SNP111	SNP111	SNP1110	SNP1110	SNP1111	SNP1111	SNP1112	SNP1112
3	3	1	1	3	3	2	2	4	4
2	2	1	1	3	3	2	2	1	1
3	3	1	1	3	3	2	2	4	4
3	3	1	1	3	3	2	2	4	4
3	3	1	1	3	3	2	2	1	1
3	3	1	1	3	3	2	2	1	1
3	3	1	1	3	3	2	2	1	1
3	3	3	3	3	3	2	2	1	1
3	3	1	1	3	3	2	2	4	4
3	3	1	1	3	3	2	2	1	1
3	3	3	3	3	3	3	3	1	1
3	3	1	1	3	3	2	2	1	1
3	3	1	1	3	3	2	2	4	4
3	3	1	1	3	3	2	2	1	1
3	3	1	1	3	3	2	2	1	1
2	2	1	1	3	3	2	2	1	1
3	3	1	1	3	3	2	2	1	1
3	3	3	3	3	3	2	2	1	1
3	3	1	1	3	3	2	2	1	1
3	3	3	3	3	3	2	2	1	1
3	3	3	3	3	3	2	2	1	1
3	3	1	1	3	3	2	2	1	1
2	2	3	3	3	3	2	2	1	1
3	3	1	1	3	3	2	2	1	1
3	3	1	1	3	3	2	2	1	1
3	3	1	1	3	3	2	2	1	1
3	3	1	1	3	3	2	2	1	1
3	3	1	1	3	3	2	2	1	1
3	3	1	1	3	3	2	2	4	4
3	3	1	1	3	3	2	2	1	1
3	3	1	1	3	3	2	2	1	1
3	3	1	1	3	3	2	2	1	1
3	3	1	1	3	3	2	2	1	1
3	3	3	3	3	3	2	2	1	1
3	3	1	1	3	3	2	2	4	4
3	3	1	1	3	3	2	2	1	1
3	3	1	1	3	3	2	2	1	1
3	3	1	1	2	2	3	3	4	4
2	2	3	3	3	3	2	2	1	1
2	2	1	1	2	2	3	3	1	1
3	3	1	1	3	3	2	2	4	4
3	3	3	3	3	3	2	2	1	1
3	3	1	1	3	3	2	2	4	4
2	2	1	1	3	3	2	2	1	1
3	3	1	1	3	3	2	2	1	1
3	3	1	1	3	3	2	2	1	1
3	3	1	1	3	3	2	2	1	1
3	3	3	3	3	3	2	2	4	4
3	3	1	1	2	2	3	3	4	4
3	3	1	1	3	3	2	2	1	1
3	3	1	1	3	3	2	2	1	1
3	3	1	1	3	3	2	2	4	4

For Review Only

3	3	1	1	3	3	2	2	4	4
3	3	1	1	3	3	2	2	1	1
3	3	1	1	3	3	2	2	1	1
3	3	1	1	3	3	2	2	1	1
3	3	1	1	3	3	2	2	1	1
3	3	1	1	3	3	2	2	1	1
3	3	1	1	3	3	2	2	1	1
2	2	3	3	3	3	2	2	1	1
2	2	3	3	3	3	2	2	1	1
3	3	1	1	3	3	2	2	1	1
3	3	1	1	3	3	2	2	1	1
3	3	1	1	3	3	2	2	1	1
3	3	1	1	3	3	2	2	4	4
3	3	1	1	3	3	2	2	4	4
3	3	1	1	2	2	2	2	1	1
3	3	1	1	3	3	2	2	1	1
3	3	1	1	3	3	2	2	1	1
3	3	1	1	2	2	3	3	1	1
3	3	1	1	3	3	2	2	1	1
3	3	1	1	3	3	2	2	4	4
3	3	1	1	3	3	2	2	1	1
3	3	1	1	3	3	2	2	4	4
3	3	1	1	3	3	2	2	4	4
3	3	1	1	3	3	2	2	1	1
3	3	1	1	3	3	2	2	1	1
3	3	1	1	3	3	2	2	1	1
3	3	1	1	3	3	2	2	1	1
2	2	3	3	3	3	2	2	4	4
3	3	1	1	3	3	2	2	1	1
3	3	1	1	3	3	2	2	1	1
3	3	1	1	3	3	2	2	1	1
3	3	1	1	3	3	2	2	1	1
3	3	3	3	3	3	2	2	1	1
3	3	1	1	3	3	2	2	4	4
3	3	1	1	2	2	3	3	4	4
3	3	1	1	3	3	2	2	1	1
3	3	1	1	3	3	2	2	1	1
3	3	3	3	3	3	2	2	1	1
2	2	1	1	3	3	3	3	1	1
3	3	1	1	3	3	2	2	1	1
3	3	1	1	3	3	2	2	1	1
3	3	1	1	3	3	2	2	1	1
3	3	1	1	3	3	2	2	1	1
3	3	1	1	3	3	2	2	1	1
3	3	1	1	2	2	3	3	1	1
3	3	1	1	2	2	3	3	1	1
3	3	1	1	3	3	2	2	1	1
3	3	1	1	3	3	2	2	1	1
3	3	1	1	3	3	2	2	1	1
3	3	1	1	3	3	2	2	1	1
3	3	1	1	3	3	2	2	1	1
2	2	3	3	3	3	2	2	4	4
3	3	1	1	3	3	2	2	1	1
3	3	1	1	2	2	2	2	1	1
3	3	1	1	2	2	3	3	1	1

For Review Only

3	3	1	1	3	3	2	2	1	1
3	3	1	1	3	3	2	2	1	1
3	3	1	1	3	3	2	2	1	1
3	3	1	1	3	3	2	2	1	1
3	3	1	1	3	3	3	3	1	1
3	3	1	1	3	3	2	2	1	1
3	3	1	1	3	3	2	2	1	1
3	3	1	1	3	3	2	2	1	1
2	2	1	1	3	3	2	2	1	1
3	3	1	1	3	3	2	2	1	1
3	3	1	1	2	2	3	3	4	4
3	3	1	1	3	3	2	2	1	1
3	3	1	1	3	3	2	2	1	1
3	3	1	1	3	3	2	2	1	1
3	3	1	1	3	3	2	2	1	1
3	3	1	1	3	3	2	2	1	1
3	3	1	1	2	2	3	3	1	1
3	3	1	1	3	3	2	2	1	1
3	3	1	1	3	3	2	2	1	1
3	3	1	1	3	3	2	2	1	1
3	3	1	1	3	3	2	2	1	1
3	3	1	1	3	3	2	2	1	1
3	3	1	1	3	3	2	2	4	4
2	2	3	3	3	3	2	2	4	4
3	3	1	1	3	3	2	2	1	1
2	2	1	1	3	3	2	2	1	1
3	3	1	1	2	2	3	3	1	1
3	3	1	1	3	3	2	2	1	1
3	3	1	1	3	3	3	3	1	1
3	3	1	1	3	3	2	2	1	1
2	2	1	1	3	3	2	2	1	1
3	3	1	1	3	3	2	2	1	1
3	3	1	1	3	3	2	2	1	1
3	3	1	1	3	3	2	2	1	1
3	3	3	3	3	3	2	2	1	1
3	3	1	1	3	3	2	2	1	1
3	3	1	1	3	3	2	2	1	1
3	3	1	1	3	3	2	2	1	1
3	3	1	1	3	3	2	2	1	1
2	2	3	3	3	3	2	2	4	4
3	3	1	1	3	3	2	2	4	4
2	2	1	1	3	3	2	2	1	1
3	3	1	1	2	2	3	3	1	1
3	3	1	1	3	3	2	2	1	1
3	3	3	3	3	3	2	2	1	1
2	2	1	1	2	2	3	3	1	1
3	3	1	1	3	3	2	2	1	1
3	3	1	1	3	3	2	2	4	4
3	3	1	1	3	3	2	2	1	1
3	3	1	1	3	3	2	2	1	1
3	3	1	1	3	3	2	2	1	1
2	2	1	1	3	3	2	2	1	1
3	3	1	1	3	3	2	2	1	1
3	3	1	1	3	3	2	2	1	1
3	3	1	1	3	3	2	2	1	1

For Review Only

3	3	1	1	2	2	3	3	1	1
3	3	1	1	3	3	2	2	4	4
3	3	1	1	2	2	3	3	1	1
3	3	1	1	3	3	2	2	1	1
3	3	1	1	3	3	2	2	1	1
3	3	1	1	3	3	2	2	1	1
3	3	1	1	3	3	2	2	4	4
3	3	1	1	3	3	2	2	1	1
3	3	1	1	3	3	2	2	1	1
3	3	1	1	3	3	2	2	1	1
3	3	1	1	3	3	2	2	1	1
3	3	1	1	3	3	2	2	1	1
3	3	1	1	3	3	2	2	1	1
3	3	1	1	2	2	2	2	1	1
3	3	1	1	3	3	2	2	1	1
2	2	3	3	3	3	3	3	4	4
3	3	1	1	3	3	2	2	1	1
3	3	1	1	3	3	2	2	1	1
3	3	1	1	3	3	2	2	1	1
3	3	1	1	3	3	2	2	1	1
3	3	1	1	3	3	2	2	1	1
3	3	3	3	3	3	2	2	4	4
3	3	1	1	3	3	2	2	1	1
3	3	1	1	3	3	2	2	1	1
3	3	1	1	3	3	2	2	1	1
3	3	3	3	3	3	2	2	1	1
3	3	1	1	3	3	2	2	1	1
3	3	1	1	2	2	2	2	1	1
2	2	1	1	3	3	2	2	1	1
3	3	1	1	3	3	2	2	1	1
3	3	1	1	2	2	3	3	1	1
3	3	1	1	3	3	2	2	1	1
3	3	1	1	2	2	3	3	1	1
3	3	1	1	3	3	2	2	1	1
3	3	1	1	2	2	3	3	1	1
3	3	1	1	3	3	2	2	1	1
3	3	1	1	3	3	2	2	1	1
3	3	1	1	3	3	2	2	1	1
3	3	1	1	3	3	2	2	4	4
3	3	1	1	3	3	2	2	1	1
3	3	1	1	3	3	2	2	4	4
3	3	1	1	3	3	2	2	1	1
3	3	3	3	3	3	2	2	1	1
3	3	1	1	3	3	2	2	4	4
3	3	1	1	2	2	2	2	1	1
3	3	1	1	3	3	2	2	4	4
2	2	3	3	3	3	3	3	4	4
3	3	3	3	3	3	2	2	4	4
2	2	1	1	3	3	2	2	1	1
3	3	1	1	3	3	2	2	1	1
3	3	1	1	3	3	3	3	1	1
3	3	1	1	2	2	3	3	1	1
3	3	1	1	3	3	2	2	1	1
3	3	1	1	3	3	2	2	1	1
2	2	3	3	3	3	2	2	1	1

For Review Only

3	3	1	1	2	2	3	3	1	1
3	3	1	1	3	3	2	2	1	1
3	3	1	1	3	3	3	3	1	1
3	3	1	1	3	3	2	2	1	1
3	3	1	1	3	3	2	2	1	1
3	3	1	1	3	3	2	2	1	1
2	2	1	1	2	2	2	2	1	1
3	3	1	1	3	3	2	2	4	4
2	2	1	1	3	3	2	2	1	1
2	2	3	3	3	3	2	2	1	1
3	3	1	1	2	2	2	2	1	1
3	3	3	3	2	2	3	3	1	1
2	2	1	1	3	3	3	3	1	1
3	3	1	1	3	3	2	2	1	1
3	3	1	1	3	3	2	2	1	1
2	2	1	1	3	3	3	3	1	1
2	2	3	3	3	3	2	2	4	4
3	3	3	3	3	3	2	2	1	1
3	3	1	1	3	3	2	2	1	1
3	3	1	1	3	3	2	2	4	4
3	3	3	3	3	3	2	2	1	1
3	3	1	1	3	3	2	2	1	1
3	3	1	1	3	3	2	2	1	1
2	2	1	1	3	3	2	2	4	4
2	2	3	3	3	3	2	2	1	1
3	3	1	1	2	2	2	2	1	1
2	2	3	3	3	3	2	2	1	1
3	3	1	1	3	3	2	2	1	1
3	3	1	1	3	3	2	2	4	4
2	2	1	1	3	3	3	3	1	1
2	2	1	1	3	3	2	2	4	4
3	3	1	1	3	3	2	2	1	1
3	3	1	1	3	3	2	2	1	1
3	3	1	1	3	3	2	2	1	1
3	3	1	1	3	3	2	2	4	4
3	3	3	3	3	3	2	2	4	4
3	3	1	1	3	3	3	3	1	1
3	3	1	1	3	3	2	2	1	1
3	3	1	1	3	3	2	2	1	1
2	2	3	3	3	3	2	2	1	1
3	3	1	1	2	2	3	3	1	1
3	3	1	1	2	2	3	3	1	1
2	2	1	1	3	3	2	2	1	1
3	3	1	1	3	3	2	2	1	1
3	3	1	1	3	3	2	2	1	1
3	3	1	1	3	3	3	3	1	1
2	2	1	1	3	3	2	2	4	4
3	3	1	1	2	2	3	3	1	1
3	3	1	1	3	3	2	2	4	4
2	2	3	3	3	3	2	2	1	1
3	3	1	1	3	3	2	2	1	1
2	2	3	3	3	3	2	2	4	4
3	3	1	1	3	3	2	2	4	4
3	3	1	1	3	3	2	2	1	1
2	2	1	1	3	3	2	2	4	4

For Review Only

3	3	1	1	3	3	3	3	1	1
3	3	1	1	3	3	2	2	1	1

For Review Only

SNP1113	SNP1113	SNP1114	SNP1114	SNP1115	SNP1115	SNP1116	SNP1116	SNP1117	SNP1117
3	3	3	3	1	1	1	1	2	2
4	4	2	2	1	1	1	1	2	2
3	3	3	3	2	2	1	1	2	2
3	3	3	3	1	1	1	1	3	3
4	4	2	2	1	1	1	1	2	2
4	4	2	2	2	2	1	1	3	3
4	4	2	2	2	2	1	1	2	2
4	4	2	2	2	2	1	1	3	3
3	3	3	3	2	2	1	1	2	2
4	4	2	2	2	2	1	1	3	3
4	4	2	2	2	2	1	1	2	2
4	4	2	2	2	2	1	1	2	2
4	4	2	2	1	1	1	1	2	2
3	3	3	3	1	1	1	1	3	3
4	4	2	2	2	2	1	1	2	2
4	4	2	2	2	2	1	1	3	3
3	3	3	3	2	2	1	1	2	2
4	4	2	2	2	2	1	1	2	2
4	4	2	2	2	2	3	3	2	2
4	4	2	2	2	2	1	1	2	2
4	4	2	2	2	2	1	1	2	2
4	4	2	2	2	2	1	1	2	2
3	3	3	3	2	2	1	1	3	3
4	4	2	2	2	2	1	1	3	3
3	3	3	3	1	1	1	1	2	2
4	4	2	2	2	2	1	1	2	2
4	4	2	2	2	2	1	1	2	2
4	4	2	2	2	2	1	1	2	2
3	3	3	3	1	1	1	1	2	2
4	4	2	2	2	2	1	1	2	2
4	4	2	2	2	2	1	1	2	2
4	4	2	2	2	2	1	1	2	2
4	4	2	2	2	2	1	1	2	2
4	4	2	2	2	2	1	1	3	3
4	4	2	2	2	2	1	1	2	2
4	4	2	2	2	2	1	1	3	3
4	4	2	2	1	1	1	1	2	2
3	3	3	3	2	2	1	1	3	3
3	3	3	3	2	2	1	1	3	3
4	4	2	2	2	2	1	1	3	3
3	3	3	3	2	2	1	1	3	3
4	4	2	2	2	2	1	1	2	2
3	3	3	3	1	1	1	1	2	2
4	4	2	2	2	2	1	1	2	2
3	3	3	3	2	2	1	1	3	3
4	4	2	2	2	2	1	1	2	2
4	4	2	2	2	2	1	1	2	2
3	3	3	3	1	1	1	1	2	2
3	3	3	3	1	1	1	1	2	2
4	4	2	2	2	2	1	1	2	2
4	4	2	2	2	2	1	1	2	2
3	3	3	3	1	1	1	1	2	2

3	3	3	3	1	1	1	1	2	2
4	4	2	2	2	2	1	1	2	2
4	4	2	2	1	1	1	1	2	2
4	4	2	2	2	2	1	1	2	2
4	4	2	2	2	2	3	3	2	2
4	4	2	2	2	2	1	1	2	2
4	4	2	2	2	2	1	1	2	2
4	4	2	2	2	2	1	1	3	3
4	4	2	2	2	2	1	1	2	2
4	4	2	2	2	2	1	1	2	2
4	4	2	2	2	2	1	1	2	2
4	4	2	2	2	2	1	1	2	2
3	3	3	3	1	1	1	1	2	2
3	3	3	3	1	1	1	1	2	2
4	4	2	2	2	2	1	1	2	2
4	4	2	2	2	2	1	1	2	2
3	3	3	3	2	2	1	1	2	2
4	4	2	2	2	2	3	3	2	2
4	4	2	2	2	2	1	1	2	2
3	3	3	3	1	1	1	1	2	2
4	4	2	2	2	2	1	1	2	2
3	3	3	3	2	2	1	1	3	3
3	3	2	2	1	1	1	1	2	2
4	4	2	2	2	2	1	1	2	2
3	3	3	3	2	2	1	1	3	3
4	4	2	2	2	2	1	1	2	2
4	4	2	2	2	2	1	1	2	2
3	3	3	3	2	2	1	1	2	2
4	4	2	2	2	2	1	1	2	2
4	4	2	2	2	2	1	1	2	2
4	4	2	2	1	1	1	1	2	2
4	4	2	2	2	2	1	1	2	2
4	4	2	2	1	1	1	1	2	2
3	3	3	3	1	1	1	1	2	2
3	3	3	3	2	2	1	1	2	2
3	3	3	3	2	2	1	1	3	3
4	4	2	2	2	2	1	1	3	3
4	4	2	2	2	2	1	1	2	2
3	3	3	3	2	2	1	1	2	2
4	4	2	2	2	2	1	1	3	3
4	4	2	2	2	2	1	1	2	2
4	4	2	2	2	2	3	3	2	2
4	4	2	2	2	2	3	3	3	3
4	4	2	2	2	2	1	1	2	2
4	4	2	2	2	2	1	1	2	2
4	4	2	2	2	2	1	1	2	2
3	3	3	3	2	2	1	1	3	3
4	4	2	2	2	2	1	1	2	2
4	4	2	2	2	2	1	1	2	2
4	4	2	2	1	1	1	1	2	2
4	4	2	2	2	2	1	1	2	2
3	3	3	3	1	1	1	1	3	3
4	4	2	2	2	2	1	1	3	3
4	4	2	2	2	2	1	1	2	2
4	4	2	2	1	1	1	1	2	2

For Review Only

4	4	2	2	2	2	1	1	3	3
4	4	2	2	2	2	1	1	2	2
4	4	2	2	2	2	1	1	3	3
3	3	2	2	1	1	1	1	2	2
4	4	2	2	2	2	1	1	2	2
4	4	2	2	2	2	1	1	2	2
4	4	2	2	2	2	3	3	2	2
4	4	2	2	2	2	1	1	3	3
4	4	2	2	2	2	1	1	2	2
4	4	2	2	2	2	1	1	2	2
3	3	3	3	1	1	1	1	2	2
4	4	2	2	2	2	1	1	2	2
4	4	2	2	2	2	3	3	3	3
4	4	2	2	2	2	1	1	2	2
4	4	2	2	2	2	1	1	3	3
4	4	2	2	2	2	1	1	2	2
4	4	2	2	2	2	1	1	2	2
4	4	2	2	2	2	1	1	2	2
3	3	3	3	1	1	1	1	2	2
4	4	2	2	2	2	1	1	2	2
4	4	2	2	2	2	1	1	2	2
4	4	2	2	2	2	1	1	2	2
3	3	3	3	2	2	1	1	2	2
3	3	3	3	2	2	1	1	2	2
4	4	3	3	2	2	1	1	2	2
4	4	2	2	2	2	1	1	2	2
4	4	2	2	2	2	1	1	2	2
4	4	2	2	2	2	1	1	2	2
4	4	2	2	2	2	3	3	2	2
4	4	2	2	2	2	3	3	2	2
3	3	3	3	2	2	1	1	2	2
4	4	2	2	2	2	1	1	2	2
4	4	2	2	2	2	1	1	2	2
4	4	2	2	2	2	1	1	2	2
4	4	2	2	2	2	1	1	3	3
4	4	2	2	2	2	1	1	2	2
4	4	2	2	2	2	1	1	2	2
4	4	2	2	2	2	1	1	2	2
4	4	2	2	2	2	1	1	2	2
3	3	3	3	2	2	1	1	3	3
3	3	3	3	1	1	3	3	3	3
4	4	2	2	1	1	1	1	2	2
4	4	2	2	2	2	1	1	2	2
4	4	2	2	2	2	1	1	3	3
4	4	2	2	2	2	1	1	2	2
4	4	2	2	2	2	1	1	2	2
4	4	2	2	2	2	1	1	2	2
3	3	3	3	2	2	1	1	2	2
4	4	2	2	2	2	1	1	2	2
4	4	2	2	2	2	1	1	2	2
4	4	2	2	2	2	1	1	2	2
4	4	2	2	2	2	1	1	3	3
4	4	2	2	2	2	1	1	2	2
4	4	2	2	2	2	1	1	2	2
4	4	2	2	2	2	1	1	2	2

For Review Only

4	4	2	2	2	2	3	3	2	2
3	3	3	3	2	2	1	1	3	3
4	4	2	2	2	2	1	1	2	2
4	4	3	3	2	2	1	1	3	3
4	4	2	2	2	2	1	1	3	3
3	3	3	3	2	2	1	1	3	3
3	3	3	3	2	2	1	1	2	2
4	4	2	2	2	2	1	1	2	2
4	4	2	2	2	2	1	1	2	2
4	4	2	2	2	2	1	1	2	2
4	4	2	2	2	2	1	1	2	2
3	3	3	3	2	2	1	1	3	3
4	4	2	2	2	2	1	1	2	2
4	4	2	2	2	2	1	1	2	2
3	3	2	2	1	1	1	1	2	2
3	3	3	3	1	1	1	1	2	2
4	4	2	2	2	2	1	1	2	2
4	4	2	2	2	2	1	1	2	2
4	4	2	2	2	2	1	1	2	2
4	4	2	2	2	2	3	3	2	2
4	4	2	2	2	2	1	1	2	2
3	3	3	3	2	2	1	1	2	2
4	4	2	2	1	1	1	1	2	2
4	4	2	2	2	2	1	1	2	2
4	4	2	2	2	2	1	1	2	2
4	4	2	2	2	2	1	1	3	3
4	4	2	2	2	2	1	1	2	2
4	4	2	2	2	2	1	1	2	2
4	4	2	2	1	1	1	1	2	2
4	4	2	2	2	2	1	1	2	2
4	4	2	2	2	2	1	1	2	2
4	4	2	2	2	2	1	1	2	2
4	4	2	2	2	2	1	1	2	2
4	4	2	2	1	1	1	1	2	2
4	4	2	2	1	1	1	1	3	3
4	4	2	2	2	2	1	1	2	2
4	4	2	2	2	2	1	1	3	3
4	4	2	2	2	2	1	1	2	2
3	3	3	3	2	2	1	1	2	2
4	4	2	2	2	2	1	1	2	2
3	3	3	3	2	2	1	1	2	2
4	4	2	2	2	2	1	1	2	2
4	4	3	3	2	2	1	1	3	3
4	4	2	2	2	2	1	1	2	2
4	4	2	2	2	2	1	1	2	2
3	3	3	3	2	2	1	1	3	3
3	3	3	3	1	1	1	1	3	3
3	3	3	3	2	2	1	1	2	2
4	4	2	2	2	2	1	1	2	2
4	4	2	2	2	2	1	1	2	2
4	4	2	2	2	2	1	1	2	2
4	4	2	2	2	2	1	1	3	3
3	3	3	3	2	2	1	1	2	2
4	4	2	2	2	2	1	1	2	2
3	3	3	3	2	2	1	1	3	3

For Review Only

4	4	2	2	2	2	1	1	3	3
4	4	2	2	2	2	1	1	3	3
4	4	2	2	2	2	1	1	2	2
4	4	2	2	2	2	1	1	3	3
4	4	2	2	2	2	1	1	2	2
4	4	2	2	2	2	1	1	2	2
4	4	2	2	2	2	1	1	2	2
4	4	3	3	2	2	1	1	3	3
4	4	2	2	2	2	1	1	2	2
3	3	3	3	1	1	1	1	3	3
4	4	2	2	2	2	1	1	2	2
4	4	2	2	2	2	1	1	2	2
3	3	3	3	2	2	1	1	3	3
4	4	2	2	2	2	1	1	3	3
4	4	2	2	2	2	1	1	2	2
3	3	3	3	2	2	1	1	3	3
3	3	3	3	1	1	1	1	3	3
4	4	2	2	2	2	1	1	2	2
4	4	2	2	2	2	1	1	3	3
3	3	3	3	2	2	1	1	3	3
4	4	2	2	2	2	1	1	2	2
4	4	2	2	2	2	1	1	2	2
4	4	2	2	2	2	3	3	2	2
3	3	3	3	2	2	1	1	3	3
4	4	2	2	2	2	1	1	3	3
4	4	2	2	1	1	1	1	2	2
4	4	2	2	1	1	1	1	2	2
4	4	2	2	2	2	1	1	2	2
3	3	3	3	1	1	1	1	2	2
4	4	2	2	2	2	1	1	2	2
3	3	3	3	1	1	1	1	3	3
4	4	2	2	2	2	1	1	2	2
4	4	2	2	2	2	1	1	2	2
4	4	2	2	2	2	1	1	2	2
3	3	3	3	1	1	1	1	3	3
3	3	3	3	2	2	1	1	2	2
4	4	2	2	2	2	1	1	2	2
4	4	2	2	2	2	1	1	2	2
4	4	2	2	2	2	1	1	3	3
3	3	3	3	2	2	1	1	3	3
4	4	2	2	1	1	1	1	3	3
4	4	2	2	2	2	1	1	2	2
3	3	3	3	2	2	1	1	3	3
4	4	2	2	2	2	1	1	2	2
4	4	2	2	2	2	1	1	2	2
3	3	3	3	2	2	1	1	3	3
3	3	3	3	2	2	1	1	2	2
4	4	2	2	2	2	1	1	2	2
4	4	2	2	2	2	1	1	2	2
4	4	2	2	1	1	1	1	2	2
4	4	2	2	2	2	3	3	2	2
3	3	3	3	1	1	1	1	3	3
3	3	3	3	2	2	1	1	2	2
4	4	2	2	2	2	3	3	2	2
3	3	3	3	2	2	1	1	3	3

For Review Only

4	4	2	2	2	2	1	1	2	2
4	4	2	2	2	2	1	1	2	2

For Review Only

SNP1118	SNP1118	SNP1119	SNP1119	SNP112	SNP112	SNP1120	SNP1120	SNP1121	SNP1121
3	3	3	3	1	1	1	1	3	3
2	2	3	3	3	3	1	1	3	3
3	3	3	3	1	1	1	1	3	3
3	3	3	3	1	1	1	1	3	3
2	2	3	3	1	1	1	1	3	3
3	3	3	3	1	1	1	1	3	3
3	3	3	3	1	1	1	1	3	3
3	3	3	3	3	3	1	1	3	3
3	3	3	3	1	1	1	1	3	3
3	3	3	3	3	3	1	1	1	1
3	3	3	3	3	3	1	1	1	1
3	3	3	3	1	1	1	1	3	3
3	3	3	3	1	1	1	1	3	3
2	2	3	3	3	3	1	1	1	1
3	3	3	3	1	1	1	1	3	3
3	3	3	3	1	1	3	3	1	1
3	3	3	3	3	3	1	1	3	3
3	3	3	3	1	1	1	1	3	3
3	3	2	2	1	1	1	1	1	1
3	3	3	3	3	3	1	1	3	3
3	3	3	3	1	1	1	1	3	3
3	3	3	3	1	1	1	1	3	3
3	3	3	3	1	1	1	1	1	1
3	3	3	3	1	1	1	1	1	1
3	3	3	3	1	1	1	1	3	3
3	3	3	3	1	1	1	1	1	1
3	3	3	3	1	1	1	1	1	1
3	3	3	3	1	1	1	1	3	3
3	3	3	3	1	1	1	1	1	1
3	3	3	3	3	3	1	1	1	1
3	3	3	3	1	1	1	1	1	1
3	3	2	2	1	1	1	1	3	3
3	3	2	2	1	1	1	1	3	3
2	2	3	3	3	3	3	3	1	1
3	3	3	3	1	1	1	1	3	3
3	3	3	3	3	3	1	1	1	1
2	2	3	3	3	3	1	1	1	1
3	3	3	3	1	1	1	1	1	1
3	3	3	3	3	3	3	3	1	1
3	3	3	3	3	3	1	1	3	3
3	3	3	3	3	3	1	1	1	1
3	3	3	3	1	1	1	1	1	1
3	3	3	3	3	3	1	1	1	1
3	3	3	3	3	3	1	1	3	3
3	3	3	3	3	3	1	1	1	1
3	3	3	3	1	1	1	1	3	3
3	3	3	3	1	1	1	1	1	1
3	3	3	3	1	1	1	1	3	3
3	3	3	3	3	3	1	1	3	3
3	3	3	3	1	1	1	1	3	3
3	3	3	3	1	1	1	1	1	1
2	2	3	3	1	1	1	1	3	3

For Review Only

3	3	3	3	1	1	1	1	3	3
3	3	2	2	1	1	1	1	3	3
3	3	3	3	1	1	1	1	3	3
3	3	3	3	3	3	1	1	1	1
3	3	2	2	1	1	1	1	1	1
3	3	3	3	1	1	1	1	1	1
3	3	3	3	1	1	1	1	1	1
3	3	3	3	3	3	1	1	1	1
3	3	3	3	1	1	1	1	1	1
3	3	3	3	1	1	1	1	3	3
3	3	3	3	1	1	1	1	3	3
3	3	3	3	1	1	1	1	1	1
3	3	3	3	1	1	1	1	3	3
3	3	3	3	1	1	1	1	1	1
3	3	3	3	1	1	1	1	3	3
2	2	3	3	3	3	1	1	3	3
3	3	3	3	3	3	1	1	3	3
3	3	3	3	1	1	1	1	1	1
3	3	3	3	1	1	1	1	3	3
3	3	3	3	1	1	1	1	3	3
2	2	3	3	1	1	1	1	3	3
3	3	3	3	3	3	3	3	1	1
3	3	3	3	1	1	1	1	3	3
3	3	3	3	1	1	1	1	1	1
3	3	3	3	3	3	1	1	1	1
3	3	3	3	1	1	1	1	3	3
3	3	3	3	3	3	1	1	3	3
3	3	3	3	3	3	1	1	1	1
3	3	3	3	1	1	1	1	1	1
3	3	3	3	3	3	1	1	3	3
2	2	3	3	3	3	1	1	3	3
3	3	3	3	1	1	1	1	3	3
2	2	3	3	3	3	1	1	1	1
3	3	3	3	1	1	1	1	3	3
3	3	3	3	1	1	1	1	3	3
3	3	3	3	3	3	1	1	1	1
3	3	3	3	1	1	1	1	3	3
3	3	3	3	1	1	1	1	3	3
3	3	3	3	3	3	1	1	1	1
3	3	3	3	3	3	1	1	1	1
3	3	3	3	1	1	1	1	1	1
3	3	2	2	1	1	1	1	3	3
3	3	3	3	3	3	1	1	1	1
3	3	3	3	1	1	1	1	3	3
3	3	3	3	1	1	1	1	1	1
3	3	3	3	1	1	1	1	3	3
3	3	3	3	3	3	1	1	1	1
3	3	3	3	1	1	1	1	1	1
3	3	3	3	3	3	1	1	1	1
2	2	3	3	1	1	1	1	1	1
3	3	3	3	1	1	1	1	3	3
3	3	3	3	3	3	3	3	1	1
3	3	3	3	3	3	1	1	1	1
3	3	3	3	1	1	1	1	3	3
2	2	3	3	1	1	1	1	3	3

For Review Only

3	3	3	3	1	1	1	1	1	1
3	3	3	3	3	3	3	3	3	3
3	3	3	3	1	1	1	1	1	1
2	2	3	3	3	3	3	3	1	1
3	3	3	3	1	1	1	1	3	3
3	3	3	3	1	1	1	1	1	1
3	3	2	2	1	1	1	1	1	1
3	3	3	3	3	3	1	1	1	1
3	3	3	3	3	3	3	3	1	1
3	3	3	3	3	3	1	1	1	1
3	3	3	3	1	1	1	1	3	3
3	3	3	3	1	1	1	1	3	3
3	3	3	3	3	3	1	1	1	1
3	3	3	3	1	1	1	1	3	3
3	3	3	3	1	1	1	1	3	3
3	3	3	3	1	1	1	1	1	1
3	3	3	3	1	1	1	1	3	3
3	3	3	3	1	1	1	1	3	3
2	2	3	3	3	3	3	3	1	1
3	3	3	3	3	3	1	1	3	3
3	3	3	3	1	1	1	1	3	3
3	3	3	3	1	1	1	1	3	3
3	3	3	3	1	1	1	1	1	1
3	3	3	3	3	3	1	1	1	1
3	3	3	3	3	3	1	1	1	1
3	3	3	3	1	1	1	1	1	1
3	3	3	3	1	1	1	1	3	3
3	3	3	3	3	3	1	1	1	1
3	3	2	2	1	1	1	1	1	1
3	3	3	3	1	1	1	1	1	1
2	2	3	3	3	3	1	1	1	1
3	3	3	3	1	1	1	1	3	3
3	3	3	3	1	1	1	1	1	1
3	3	3	3	1	1	1	1	1	1
2	2	3	3	3	3	3	3	1	1
3	3	3	3	3	3	1	1	3	3
2	2	3	3	3	3	3	3	1	1
3	3	3	3	1	1	1	1	3	3
3	3	3	3	1	1	1	1	3	3
3	3	3	3	3	3	1	1	1	1
3	3	2	2	1	1	1	1	1	1
2	2	3	3	3	3	3	3	1	1
3	3	3	3	1	1	1	1	1	1
3	3	3	3	3	3	1	1	1	1
3	3	3	3	1	1	1	1	1	1
3	3	3	3	1	1	1	1	3	3
3	3	3	3	1	1	1	1	1	1
3	3	3	3	1	1	3	3	1	1
3	3	3	3	3	3	1	1	1	1
3	3	3	3	1	1	1	1	1	1
3	3	3	3	1	1	1	1	1	1
3	3	3	3	3	3	1	1	1	1
3	3	3	3	1	1	1	1	1	1
3	3	3	3	1	1	1	1	1	1
3	3	3	3	3	3	1	1	3	3

For Review Only

3	3	3	3	1	1	1	1	3	3
3	3	3	3	3	3	1	1	1	1
3	3	3	3	1	1	1	1	3	3
3	3	3	3	1	1	1	1	1	1
3	3	3	3	3	3	1	1	3	3
3	3	3	3	3	3	1	1	1	1
3	3	3	3	3	3	1	1	1	1
3	3	3	3	1	1	1	1	3	3
3	3	3	3	3	3	1	1	3	3
3	3	3	3	1	1	1	1	3	3
3	3	3	3	1	1	1	1	1	1
3	3	3	3	1	1	1	1	1	1
3	3	3	3	1	1	1	1	3	3
3	3	3	3	1	1	1	1	3	3
2	2	3	3	3	3	1	1	1	1
3	3	3	3	3	3	1	1	1	1
3	3	3	3	1	1	1	1	1	1
3	3	3	3	1	1	1	1	3	3
3	3	3	3	1	1	1	1	1	1
3	3	3	3	1	1	1	1	1	1
3	3	3	3	1	1	1	1	3	3
2	2	3	3	3	3	1	1	1	1
3	3	3	3	3	3	3	3	1	1
3	3	3	3	1	1	1	1	1	1
3	3	3	3	1	1	1	1	1	1
2	2	3	3	3	3	3	3	1	1
3	3	3	3	1	1	1	1	1	1
3	3	3	3	1	1	1	1	3	3
3	3	3	3	1	1	1	1	1	1
3	3	3	3	1	1	1	1	1	1
2	2	3	3	1	1	1	1	3	3
3	3	3	3	1	1	1	1	3	3
3	3	3	3	1	1	1	1	3	3
3	3	3	3	3	3	1	1	1	1
3	3	3	3	1	1	1	1	3	3
3	3	3	3	1	1	1	1	1	1
3	3	3	3	3	3	1	1	3	3
3	3	3	3	3	3	1	1	3	3
3	3	3	3	1	1	3	3	1	1
3	3	3	3	1	1	1	1	3	3
3	3	3	3	1	1	1	1	3	3
3	3	3	3	1	1	1	1	1	1
3	3	3	3	3	3	1	1	1	1
3	3	3	3	1	1	1	1	3	3
3	3	3	3	1	1	1	1	1	1
3	3	3	3	1	1	1	1	3	3
3	3	3	3	3	3	1	1	1	1
2	2	3	3	3	3	1	1	1	1
3	3	3	3	3	3	1	1	1	1
3	3	3	3	3	3	1	1	1	1
3	3	3	3	1	1	1	1	1	1
3	3	3	3	3	3	1	1	3	3
2	2	3	3	3	3	3	3	1	1
3	3	3	3	1	1	1	1	3	3
3	3	3	3	3	3	3	3	1	1

For Review Only

3	3	3	3	1	1	1	1	1	1
3	3	3	3	3	3	1	1	1	1
3	3	3	3	3	3	1	1	3	3
3	3	3	3	3	3	1	1	1	1
3	3	3	3	3	3	1	1	3	3
3	3	3	3	1	1	1	1	1	1
3	3	3	3	1	1	1	1	3	3
3	3	3	3	3	3	1	1	1	1
3	3	3	3	3	3	1	1	1	1
2	2	3	3	3	3	1	1	1	1
3	3	3	3	1	1	1	1	3	3
2	2	3	3	1	1	1	1	3	3
3	3	3	3	3	3	1	1	1	1
3	3	3	3	3	3	1	1	1	1
3	3	3	3	1	1	1	1	3	3
3	3	3	3	1	1	1	1	1	1
3	3	3	3	3	3	1	1	1	1
3	3	3	3	3	3	1	1	3	3
3	3	3	3	3	3	1	1	3	3
3	3	3	3	1	1	1	1	1	1
3	3	3	3	1	1	1	1	3	3
3	3	3	3	3	3	1	1	1	1
3	3	3	3	1	1	1	1	3	3
3	3	3	3	3	3	1	1	1	1
3	3	3	3	3	3	1	1	1	1
3	3	3	3	1	1	1	1	3	3
2	2	3	3	3	3	1	1	1	1
3	3	2	2	1	1	1	1	3	3
3	3	3	3	1	1	1	1	3	3
3	3	3	3	1	1	1	1	1	1
3	3	3	3	3	3	1	1	1	1
3	3	3	3	1	1	1	1	1	1
3	3	3	3	3	3	1	1	3	3
3	3	3	3	1	1	1	1	1	1
3	3	3	3	3	3	1	1	1	1
2	2	3	3	3	3	1	1	1	1
3	3	2	2	1	1	1	1	1	1
3	3	3	3	1	1	1	1	1	1
3	3	3	3	3	3	1	1	1	1
3	3	3	3	3	3	1	1	1	1
3	3	3	3	1	1	1	1	3	3
3	3	3	3	3	3	1	1	3	3
3	3	3	3	1	1	1	1	1	1
3	3	3	3	1	1	1	1	3	3
3	3	3	3	1	1	3	3	3	3
3	3	3	3	3	3	1	1	1	1
3	3	3	3	1	1	3	3	1	1
3	3	3	3	1	1	1	1	3	3
3	3	3	3	1	1	1	1	1	1
2	2	3	3	3	3	1	1	1	1
3	3	3	3	1	1	1	1	1	1
3	3	3	3	3	3	1	1	1	1
3	3	3	3	1	1	1	1	3	3
3	3	2	2	1	1	1	1	3	3
3	3	3	3	3	3	1	1	1	1

For Review Only

3	3	3	3	1	1	1	1	1	1
3	3	3	3	1	1	1	1	3	3

For Review Only

SNP1122	SNP1122	SNP1123	SNP1123	SNP1124	SNP1124	SNP1125	SNP1125	SNP1126	SNP1126
4	4	3	3	2	2	4	4	1	1
4	4	4	4	2	2	4	4	1	1
1	1	3	3	2	2	3	3	1	1
4	4	4	4	2	2	3	3	1	1
4	4	4	4	2	2	4	4	1	1
4	4	3	3	2	2	4	4	1	1
4	4	3	3	2	2	4	4	1	1
4	4	4	4	2	2	4	4	1	1
1	1	3	3	2	2	3	3	1	1
1	1	4	4	2	2	3	3	1	1
1	1	3	3	2	2	3	3	4	4
1	1	3	3	2	2	3	3	1	1
4	4	3	3	2	2	3	3	1	1
1	1	3	3	2	2	3	3	1	1
4	4	3	3	2	2	4	4	1	1
1	1	4	4	2	2	3	3	1	1
4	4	4	4	2	2	4	4	1	1
4	4	3	3	2	2	4	4	1	1
1	1	3	3	2	2	3	3	1	1
4	4	3	3	2	2	4	4	1	1
4	4	3	3	2	2	4	4	1	1
4	4	3	3	2	2	4	4	1	1
1	1	4	4	2	2	3	3	1	1
1	1	4	4	4	4	3	3	1	1
4	4	4	4	2	2	4	4	1	1
1	1	3	3	2	2	3	3	1	1
1	1	3	3	2	2	3	3	1	1
4	4	4	4	2	2	3	3	1	1
1	1	3	3	2	2	3	3	1	1
1	1	4	4	2	2	3	3	1	1
1	1	3	3	2	2	3	3	1	1
1	1	3	3	2	2	3	3	1	1
1	1	3	3	2	2	3	3	1	1
1	1	3	3	2	2	3	3	1	1
4	4	3	3	2	2	3	3	1	1
1	1	3	3	2	2	3	3	1	1
1	1	4	4	2	2	3	3	1	1
1	1	4	4	2	2	3	3	1	1
1	1	4	4	2	2	3	3	1	1
1	1	4	4	2	2	3	3	1	1
1	1	3	3	2	2	3	3	1	1
1	1	4	4	2	2	3	3	1	1
1	1	3	3	2	2	3	3	1	1
4	4	3	3	2	2	4	4	1	1
1	1	3	3	2	2	3	3	1	1
1	1	3	3	2	2	4	4	1	1
1	1	3	3	2	2	3	3	1	1
4	4	4	4	2	2	4	4	1	1
1	1	3	3	2	2	3	3	1	1
4	4	3	3	2	2	3	3	1	1
1	1	3	3	2	2	3	3	1	1
4	4	3	3	2	2	4	4	1	1

For Review Only

4	4	3	3	2	2	4	4	1	1
1	1	3	3	2	2	3	3	1	1
4	4	3	3	2	2	4	4	1	1
1	1	4	4	2	2	3	3	1	1
1	1	3	3	2	2	3	3	1	1
1	1	3	3	2	2	3	3	1	1
1	1	3	3	2	2	3	3	1	1
1	1	4	4	2	2	3	3	1	1
1	1	3	3	4	4	3	3	1	1
1	1	4	4	2	2	3	3	1	1
4	4	3	3	2	2	4	4	1	1
1	1	3	3	2	2	3	3	1	1
4	4	3	3	2	2	4	4	1	1
1	1	3	3	2	2	3	3	1	1
4	4	4	4	2	2	3	3	1	1
4	4	4	4	2	2	4	4	1	1
4	4	4	4	2	2	4	4	1	1
1	1	3	3	2	2	3	3	1	1
4	4	3	3	2	2	4	4	1	1
4	4	3	3	2	2	4	4	1	1
1	1	3	3	2	2	3	3	1	1
1	1	4	4	2	2	3	3	1	1
4	4	3	3	2	2	4	4	1	1
1	1	3	3	2	2	3	3	1	1
1	1	3	3	2	2	3	3	1	1
4	4	3	3	2	2	4	4	1	1
1	1	3	3	2	2	3	3	1	1
1	1	4	4	2	2	3	3	1	1
1	1	3	3	2	2	3	3	1	1
4	4	3	3	2	2	4	4	1	1
4	4	4	4	2	2	4	4	1	1
4	4	4	4	4	4	4	4	1	1
1	1	3	3	2	2	3	3	1	1
4	4	3	3	2	2	4	4	1	1
1	1	4	4	2	2	3	3	1	1
1	1	3	3	2	2	3	3	1	1
4	4	4	4	2	2	4	4	1	1
4	4	4	4	2	2	4	4	1	1
1	1	4	4	2	2	3	3	4	4
1	1	4	4	2	2	3	3	1	1
1	1	3	3	2	2	3	3	1	1
4	4	3	3	2	2	4	4	1	1
1	1	4	4	2	2	3	3	1	1
4	4	3	3	2	2	4	4	1	1
1	1	3	3	2	2	3	3	4	4
1	1	3	3	2	2	3	3	1	1
1	1	4	4	2	2	3	3	1	1
1	1	3	3	4	4	3	3	1	1
1	1	3	3	4	4	3	3	1	1
1	1	4	4	2	2	3	3	1	1
4	4	3	3	2	2	4	4	1	1
1	1	4	4	2	2	3	3	1	1
1	1	4	4	2	2	3	3	1	1
1	1	4	4	2	2	3	3	1	1
1	1	4	4	4	4	3	3	1	1

For Review Only

1	1	3	3	2	2	3	3	1	1
4	4	4	4	2	2	4	4	1	1
1	1	3	3	2	2	3	3	1	1
1	1	3	3	2	2	3	3	1	1
1	1	4	4	2	2	4	4	1	1
1	1	3	3	2	2	3	3	1	1
1	1	3	3	2	2	3	3	1	1
1	1	4	4	2	2	3	3	1	1
1	1	4	4	4	4	3	3	1	1
1	1	4	4	2	2	3	3	1	1
1	1	4	4	2	2	3	3	1	1
4	4	4	4	2	2	3	3	1	1
1	1	4	4	2	2	3	3	1	1
4	4	3	3	2	2	4	4	1	1
4	4	4	4	2	2	4	4	1	1
1	1	3	3	2	2	3	3	1	1
1	1	3	3	2	2	3	3	1	1
1	1	4	4	2	2	3	3	1	1
1	1	3	3	2	2	3	3	1	1
4	4	3	3	2	2	4	4	1	1
1	1	4	4	2	2	3	3	1	1
4	4	3	3	2	2	4	4	1	1
1	1	3	3	2	2	3	3	1	1
1	1	4	4	2	2	3	3	1	1
1	1	3	3	2	2	3	3	1	1
1	1	3	3	2	2	3	3	1	1
1	1	3	3	2	2	3	3	1	1
1	1	3	3	2	2	3	3	1	1
1	1	3	3	2	2	3	3	4	4
1	1	3	3	2	2	3	3	1	1
1	1	4	4	2	2	3	3	1	1
4	4	4	4	2	2	3	3	1	1
1	1	3	3	2	2	3	3	1	1
1	1	4	4	2	2	3	3	1	1
1	1	3	3	2	2	3	3	1	1
4	4	3	3	2	2	4	4	1	1
1	1	3	3	2	2	3	3	1	1
4	4	3	3	2	2	4	4	1	1
4	4	4	4	2	2	4	4	4	4
1	1	4	4	2	2	3	3	4	4
1	1	4	4	2	2	3	3	1	1
1	1	4	4	2	2	3	3	1	1
1	1	3	3	2	2	3	3	4	4
1	1	4	4	2	2	3	3	1	1
1	1	3	3	2	2	3	3	1	1
1	1	3	3	2	2	3	3	4	4
1	1	3	3	2	2	3	3	1	1
1	1	3	3	2	2	3	3	1	1
1	1	4	4	2	2	3	3	1	1
1	1	3	3	2	2	3	3	1	1
1	1	3	3	2	2	3	3	1	1
1	1	4	4	2	2	3	3	1	1
1	1	4	4	2	2	3	3	4	4
1	1	3	3	2	2	3	3	1	1
4	4	4	4	2	2	3	3	1	1

For Review Only

1	1	3	3	2	2	3	3	1	1
1	1	4	4	2	2	3	3	1	1
1	1	3	3	2	2	3	3	1	1
1	1	4	4	2	2	3	3	4	4
4	4	4	4	2	2	4	4	1	1
1	1	4	4	2	2	3	3	1	1
1	1	4	4	2	2	3	3	1	1
1	1	3	3	2	2	3	3	1	1
4	4	3	3	2	2	4	4	1	1
4	4	3	3	2	2	4	4	1	1
1	1	3	3	2	2	3	3	1	1
1	1	4	4	2	2	3	3	1	1
4	4	4	4	2	2	4	4	1	1
4	4	4	4	4	4	3	3	1	1
1	1	3	3	2	2	3	3	1	1
1	1	4	4	2	2	3	3	4	4
1	1	4	4	2	2	3	3	4	4
4	4	3	3	2	2	4	4	1	1
1	1	3	3	4	4	3	3	1	1
1	1	3	3	2	2	3	3	1	1
4	4	4	4	2	2	3	3	1	1
1	1	4	4	2	2	3	3	1	1
1	1	3	3	2	2	3	3	1	1
1	1	3	3	2	2	3	3	1	1
1	1	4	4	2	2	3	3	1	1
1	1	3	3	2	2	3	3	1	1
1	1	3	3	2	2	3	3	1	1
1	1	4	4	2	2	3	3	1	1
1	1	3	3	2	2	3	3	1	1
1	1	3	3	2	2	3	3	1	1
1	1	4	4	2	2	3	3	1	1
4	4	3	3	2	2	4	4	1	1
1	1	3	3	2	2	3	3	1	1
1	1	4	4	2	2	3	3	1	1
1	1	4	4	2	2	3	3	1	1
1	1	3	3	2	2	3	3	1	1
4	4	3	3	2	2	4	4	1	1
4	4	3	3	2	2	4	4	1	1
1	1	3	3	2	2	3	3	1	1
4	4	3	3	2	2	4	4	1	1
1	1	3	3	2	2	3	3	1	1
1	1	3	3	2	2	3	3	1	1
1	1	4	4	2	2	3	3	1	1
4	4	3	3	2	2	3	3	1	1
1	1	3	3	2	2	3	3	1	1
1	1	4	4	2	2	3	3	1	1
1	1	4	4	2	2	3	3	4	4
1	1	4	4	2	2	3	3	1	1
1	1	3	3	2	2	3	3	1	1
1	1	4	4	2	2	3	3	1	1
1	1	3	3	2	2	3	3	4	4
1	1	4	4	2	2	3	3	1	1
1	1	4	4	2	2	3	3	1	1
4	4	3	3	2	2	4	4	1	1
1	1	4	4	2	2	3	3	1	1

For Review Only

1	1	4	4	2	2	3	3	4	4
1	1	4	4	2	2	3	3	1	1
1	1	3	3	2	2	3	3	4	4
1	1	4	4	2	2	3	3	1	1
4	4	3	3	2	2	4	4	1	1
1	1	3	3	2	2	3	3	1	1
1	1	3	3	2	2	3	3	1	1
1	1	4	4	2	2	3	3	1	1
1	1	3	3	2	2	3	3	1	1
1	1	4	4	2	2	3	3	1	1
1	1	4	4	2	2	3	3	1	1
1	1	4	4	2	2	3	3	1	1
1	1	4	4	2	2	3	3	4	4
1	1	4	4	4	4	3	3	1	1
1	1	3	3	2	2	3	3	1	1
1	1	4	4	2	2	3	3	4	4
1	1	4	4	2	2	3	3	1	1
4	4	3	3	2	2	4	4	1	1
4	4	4	4	2	2	4	4	1	1
1	1	4	4	2	2	3	3	1	1
4	4	3	3	2	2	4	4	1	1
1	1	3	3	2	2	3	3	1	1
4	4	3	3	2	2	4	4	1	1
1	1	4	4	2	2	3	3	1	1
1	1	4	4	2	2	3	3	1	1
4	4	3	3	2	2	4	4	1	1
1	1	3	3	2	2	3	3	1	1
4	4	4	4	2	2	4	4	1	1
4	4	3	3	2	2	4	4	1	1
1	1	3	3	2	2	3	3	4	4
1	1	4	4	2	2	3	3	1	1
1	1	3	3	2	2	3	3	1	1
4	4	4	4	2	2	4	4	1	1
1	1	4	4	2	2	3	3	1	1
1	1	4	4	2	2	3	3	1	1
1	1	4	4	2	2	3	3	1	1
1	1	3	3	2	2	3	3	4	4
1	1	3	3	2	2	3	3	1	1
1	1	3	3	2	2	3	3	1	1
1	1	4	4	2	2	3	3	1	1
1	1	4	4	2	2	3	3	1	1
1	1	3	3	2	2	3	3	1	1
1	1	4	4	2	2	3	3	1	1
4	4	3	3	2	2	4	4	1	1
4	4	3	3	2	2	4	4	1	1
1	1	4	4	2	2	3	3	4	4
1	1	3	3	2	2	3	3	1	1
1	1	3	3	4	4	3	3	1	1
1	1	3	3	2	2	3	3	1	1
1	1	3	3	2	2	3	3	1	1
1	1	3	3	2	2	3	3	1	1
1	1	4	4	2	2	3	3	1	1
1	1	3	3	2	2	3	3	1	1
4	4	3	3	2	2	4	4	1	1
1	1	4	4	2	2	3	3	1	1

For Review Only

1	1	3	3	2	2	3	3	4	4
4	4	3	3	2	2	4	4	1	1

For Review Only

SNP1127	SNP1127	SNP1128	SNP1128	SNP1129	SNP1129	SNP113	SNP113	SNP1130	SNP1130
2	2	2	2	2	2	2	2	4	4
3	3	2	2	2	2	2	2	4	4
2	2	2	2	3	3	2	2	4	4
2	2	2	2	2	2	2	2	4	4
2	2	2	2	2	2	2	2	4	4
2	2	2	2	2	2	2	2	4	4
2	2	2	2	2	2	2	2	4	4
3	3	2	2	2	2	3	3	4	4
2	2	3	3	2	2	2	2	4	4
3	3	2	2	2	2	2	2	4	4
2	2	2	2	2	2	3	3	4	4
2	2	2	2	2	2	2	2	4	4
2	2	2	2	2	2	2	2	4	4
2	2	2	2	3	3	2	2	4	4
2	2	2	2	2	2	2	2	4	4
2	2	2	2	3	3	2	2	4	4
2	2	2	2	2	2	2	2	4	4
2	2	2	2	2	2	3	3	4	4
2	2	2	2	2	2	2	2	1	1
2	2	2	2	2	2	3	3	4	4
3	3	2	2	2	2	3	3	4	4
2	2	2	2	2	2	2	2	4	4
3	3	2	2	3	3	3	3	4	4
3	3	2	2	2	2	2	2	4	4
2	2	2	2	2	2	2	2	4	4
2	2	2	2	3	3	2	2	4	4
2	2	2	2	3	3	2	2	4	4
2	2	2	2	2	2	2	2	4	4
3	3	3	3	2	2	2	2	4	4
3	3	2	2	2	2	2	2	4	4
3	3	2	2	2	2	2	2	4	4
2	2	2	2	2	2	2	2	1	1
2	2	2	2	2	2	2	2	1	1
3	3	2	2	2	2	3	3	4	4
2	2	2	2	2	2	2	2	4	4
3	3	2	2	3	3	2	2	4	4
2	2	2	2	3	3	2	2	4	4
2	2	2	2	3	3	2	2	4	4
3	3	2	2	2	2	3	3	4	4
2	2	2	2	2	2	2	2	4	4
2	2	2	2	2	2	2	2	4	4
3	3	2	2	2	2	3	3	4	4
3	3	2	2	2	2	2	2	4	4
2	2	2	2	2	2	2	2	4	4
3	3	2	2	2	2	2	2	4	4
2	2	2	2	3	3	2	2	4	4
2	2	2	2	3	3	2	2	4	4
3	3	2	2	3	3	2	2	4	4
2	2	2	2	2	2	2	2	4	4
2	2	2	2	2	2	2	2	4	4
3	3	2	2	3	3	2	2	4	4
2	2	2	2	2	2	3	3	4	4

For Review Only

2	2	3	3	2	2	2	2	4	4
2	2	2	2	2	2	2	2	1	1
2	2	2	2	2	2	2	2	4	4
2	2	2	2	2	2	2	2	4	4
2	2	2	2	2	2	2	2	1	1
2	2	2	2	3	3	2	2	4	4
2	2	2	2	3	3	2	2	4	4
3	3	2	2	2	2	3	3	4	4
3	3	3	3	2	2	3	3	4	4
2	2	2	2	2	2	2	2	4	4
2	2	2	2	2	2	2	2	4	4
2	2	2	2	3	3	2	2	4	4
2	2	3	3	2	2	2	2	4	4
3	3	3	3	2	2	2	2	4	4
2	2	2	2	2	2	2	2	4	4
2	2	2	2	3	3	2	2	4	4
2	2	2	2	2	2	2	2	4	4
2	2	2	2	2	2	2	2	4	4
2	2	2	2	2	2	2	2	4	4
2	2	3	3	2	2	2	2	4	4
2	2	2	2	2	2	2	2	4	4
2	2	2	2	2	2	2	2	4	4
3	3	3	3	2	2	2	2	4	4
3	3	2	2	2	2	2	2	4	4
3	3	2	2	2	2	2	2	4	4
3	3	2	2	2	2	2	2	4	4
2	2	2	2	2	2	2	2	4	4
3	3	2	2	3	3	3	3	4	4
2	2	2	2	3	3	2	2	4	4
3	3	2	2	2	2	2	2	4	4
2	2	2	2	2	2	2	2	4	4
3	3	2	2	3	3	2	2	4	4
3	3	2	2	3	3	3	3	4	4
2	2	3	3	2	2	2	2	4	4
2	2	2	2	3	3	2	2	4	4
3	3	2	2	2	2	2	2	4	4
3	3	2	2	2	2	2	2	4	4
2	2	2	2	2	2	3	3	4	4
3	3	2	2	2	2	2	2	4	4
3	3	2	2	2	2	2	2	4	4
2	2	2	2	2	2	2	2	4	4
3	3	2	2	2	2	2	2	4	4
3	3	2	2	2	2	2	2	4	4
2	2	2	2	2	2	2	2	4	4
2	2	2	2	3	3	2	2	4	4
2	2	2	2	2	2	2	2	4	4
3	3	2	2	3	3	2	2	4	4
2	2	2	2	2	2	2	2	4	4
2	2	2	2	3	3	2	2	4	4
2	2	2	2	3	3	2	2	4	4
3	3	2	2	2	2	2	2	4	4
3	3	2	2	2	2	3	3	4	4
3	3	2	2	2	2	2	2	4	4
2	2	2	2	2	2	2	2	4	4
2	2	2	2	3	3	2	2	4	4

For Review Only

2	2	2	2	2	2	2	2	4	4
2	2	2	2	2	2	2	2	4	4
3	3	2	2	3	3	2	2	4	4
3	3	2	2	3	3	2	2	4	4
3	3	2	2	2	2	2	2	4	4
2	2	2	2	3	3	2	2	4	4
2	2	2	2	2	2	2	2	1	1
3	3	2	2	3	3	2	2	4	4
3	3	2	2	3	3	2	2	4	4
3	3	2	2	3	3	2	2	4	4
2	2	2	2	3	3	2	2	4	4
2	2	2	2	2	2	2	2	4	4
3	3	2	2	2	2	2	2	4	4
2	2	2	2	2	2	2	2	4	4
3	3	2	2	2	2	2	2	4	4
2	2	2	2	2	2	2	2	4	4
2	2	3	3	2	2	2	2	4	4
2	2	2	2	2	2	2	2	4	4
3	3	2	2	3	3	2	2	4	4
3	3	2	2	2	2	2	2	4	4
2	2	2	2	2	2	2	2	4	4
3	3	2	2	2	2	2	2	4	4
3	3	2	2	3	3	2	2	4	4
3	3	2	2	2	2	3	3	4	4
2	2	2	2	2	2	2	2	4	4
2	2	2	2	2	2	2	2	4	4
2	2	2	2	2	2	2	2	4	4
3	3	2	2	2	2	2	2	4	4
2	2	2	2	2	2	2	2	1	1
2	2	2	2	2	2	2	2	1	1
3	3	2	2	3	3	2	2	4	4
2	2	2	2	2	2	2	2	4	4
2	2	2	2	2	2	2	2	4	4
2	2	2	2	3	3	2	2	4	4
3	3	2	2	2	2	3	3	4	4
2	2	2	2	2	2	2	2	4	4
3	3	2	2	3	3	2	2	4	4
2	2	2	2	2	2	2	2	4	4
2	2	2	2	2	2	3	3	4	4
2	2	2	2	2	2	3	3	4	4
2	2	2	2	2	2	2	2	1	1
3	3	2	2	3	3	2	2	4	4
2	2	2	2	3	3	2	2	4	4
2	2	2	2	2	2	2	2	4	4
3	3	2	2	2	2	3	3	4	4
3	3	2	2	2	2	2	2	4	4
2	2	2	2	3	3	2	2	4	4
2	2	2	2	3	3	2	2	4	4
2	2	2	2	3	3	2	2	4	4
2	2	2	2	3	3	2	2	4	4
2	2	2	2	3	3	2	2	4	4
2	2	2	2	2	2	2	2	4	4
2	2	2	2	2	2	2	2	4	4
2	2	2	2	3	3	2	2	4	4
2	2	2	2	2	2	2	2	4	4

For Review Only

2	2	2	2	2	2	2	2	4	4
3	3	2	2	2	2	2	2	4	4
2	2	2	2	2	2	2	2	4	4
2	2	2	2	2	2	2	2	4	4
2	2	2	2	2	2	2	2	4	4
2	2	2	2	2	2	2	2	4	4
2	2	2	2	2	2	3	3	4	4
2	2	2	2	2	2	2	2	4	4
2	2	2	2	2	2	2	2	4	4
2	2	2	2	2	2	2	2	4	4
2	2	2	2	3	3	2	2	4	4
2	2	2	2	2	2	2	2	4	4
2	2	2	2	3	3	2	2	4	4
3	3	2	2	2	2	2	2	4	4
3	3	2	2	3	3	2	2	4	4
2	2	2	2	2	2	3	3	4	4
2	2	2	2	2	2	2	2	4	4
3	3	2	2	2	2	2	2	4	4
3	3	2	2	3	3	2	2	4	4
2	2	2	2	2	2	2	2	4	4
2	2	2	2	2	2	2	2	4	4
3	3	2	2	3	3	3	3	4	4
2	2	2	2	2	2	2	2	4	4
2	2	2	2	3	3	2	2	4	4
3	3	2	2	2	2	2	2	4	4
3	3	2	2	2	2	3	3	4	4
2	2	2	2	2	2	2	2	4	4
2	2	2	2	2	2	2	2	4	4
2	2	2	2	3	3	2	2	4	4
2	2	2	2	2	2	2	2	4	4
2	2	2	2	2	2	2	2	4	4
2	2	2	2	2	2	2	2	4	4
2	2	2	2	2	2	2	2	4	4
3	3	2	2	2	2	2	2	4	4
2	2	2	2	2	2	2	2	4	4
2	2	2	2	3	3	2	2	4	4
3	3	2	2	3	3	2	2	4	4
2	2	2	2	2	2	2	2	4	4
2	2	2	2	3	3	2	2	4	4
3	3	2	2	3	3	2	2	4	4
2	2	2	2	3	3	2	2	4	4
2	2	2	2	3	3	2	2	4	4
3	3	2	2	2	2	3	3	4	4
2	2	2	2	2	2	2	2	4	4
2	2	2	2	3	3	2	2	4	4
3	3	2	2	2	2	2	2	4	4
2	2	2	2	2	2	3	3	4	4
3	3	2	2	2	2	3	3	4	4
3	3	2	2	3	3	2	2	4	4
2	2	2	2	2	2	2	2	4	4
2	2	2	2	2	2	2	2	4	4
3	3	2	2	3	3	2	2	4	4
3	3	2	2	2	2	2	2	4	4
2	2	2	2	2	2	2	2	4	4
3	3	2	2	2	2	3	3	4	4

For Review Only

2	2	2	2	2	2	2	2	4	4
2	2	2	2	2	2	2	2	4	4
2	2	2	2	2	2	2	2	4	4
2	2	2	2	2	2	2	2	4	4
2	2	2	2	2	2	2	2	4	4
2	2	2	2	2	2	2	2	4	4
2	2	2	2	2	2	2	2	4	4
3	3	2	2	2	2	2	2	4	4
3	3	2	2	3	3	2	2	4	4
3	3	2	2	2	2	3	3	4	4
2	2	2	2	2	2	2	2	4	4
3	3	2	2	2	2	3	3	4	4
2	2	2	2	2	2	2	2	4	4
3	3	2	2	3	3	2	2	4	4
2	2	2	2	3	3	2	2	4	4
2	2	2	2	2	2	2	2	4	4
3	3	2	2	2	2	3	3	4	4
3	3	2	2	2	2	3	3	4	4
2	2	2	2	2	2	2	2	4	4
2	2	2	2	3	3	2	2	4	4
3	3	2	2	2	2	3	3	4	4
2	2	2	2	3	3	2	2	4	4
3	3	2	2	2	2	2	2	4	4
2	2	2	2	3	3	2	2	4	4
3	3	2	2	2	2	3	3	4	4
2	2	3	3	2	2	2	2	4	4
3	3	2	2	2	2	3	3	4	4
2	2	2	2	2	2	2	2	4	4
2	2	3	3	2	2	2	2	4	4
2	2	2	2	2	2	2	2	4	4
3	3	2	2	2	2	2	2	4	4
2	2	2	2	2	2	2	2	4	4
3	3	2	2	3	3	2	2	4	4
3	3	2	2	3	3	2	2	4	4
2	2	2	2	2	2	2	2	4	4
3	3	2	2	2	2	3	3	4	4
2	2	2	2	2	2	2	2	1	1
2	2	2	2	3	3	2	2	4	4
3	3	2	2	3	3	2	2	4	4
3	3	2	2	2	2	3	3	4	4
2	2	2	2	2	2	2	2	4	4
2	2	2	2	3	3	2	2	4	4
2	2	2	2	2	2	2	2	4	4
2	2	3	3	2	2	2	2	4	4
2	2	2	2	2	2	2	2	4	4
3	3	2	2	2	2	2	2	4	4
2	2	2	2	2	2	2	2	4	4
3	3	2	2	3	3	2	2	4	4
3	3	2	2	3	3	2	2	4	4
3	3	2	2	2	2	3	3	4	4
2	2	2	2	2	2	2	2	4	4
3	3	2	2	2	2	3	3	4	4
2	2	2	2	3	3	2	2	4	4
2	2	2	2	2	2	2	2	1	1
2	2	2	2	2	2	2	2	4	4

For Review Only

2	2	2	2	2	2	2	2	4	4
3	3	2	2	2	2	2	2	4	4

For Review Only

SNP1131	SNP1131	SNP1132	SNP1132	SNP1133	SNP1133	SNP1134	SNP1134	SNP1135	SNP1135
4	4	3	3	1	1	4	4	2	2
1	1	3	3	4	4	1	1	2	2
4	4	3	3	1	1	4	4	2	2
4	4	3	3	1	1	1	1	2	2
4	4	3	3	4	4	4	4	2	2
4	4	3	3	1	1	4	4	2	2
4	4	3	3	1	1	4	4	2	2
4	4	3	3	1	1	4	4	3	3
4	4	3	3	1	1	4	4	2	2
4	4	3	3	1	1	4	4	3	3
4	4	3	3	4	4	4	4	2	2
4	4	3	3	1	1	4	4	2	2
4	4	3	3	1	1	4	4	2	2
4	4	3	3	4	4	4	4	2	2
4	4	3	3	1	1	4	4	2	2
4	4	3	3	1	1	4	4	2	2
4	4	3	3	4	4	4	4	2	2
4	4	3	3	1	1	1	1	2	2
1	1	3	3	1	1	4	4	2	2
4	4	3	3	1	1	4	4	2	2
4	4	3	3	4	4	4	4	2	2
4	4	3	3	1	1	4	4	2	2
4	4	3	3	4	4	4	4	2	2
4	4	2	2	1	1	4	4	2	2
4	4	3	3	1	1	4	4	2	2
4	4	3	3	4	4	4	4	2	2
4	4	3	3	1	1	4	4	2	2
4	4	3	3	1	1	4	4	2	2
4	4	3	3	1	1	4	4	3	3
4	4	3	3	4	4	4	4	2	2
4	4	3	3	1	1	4	4	2	2
1	1	3	3	1	1	4	4	2	2
1	1	3	3	4	4	4	4	2	2
4	4	3	3	1	1	4	4	2	2
4	4	3	3	4	4	4	4	2	2
4	4	3	3	1	1	4	4	2	2
4	4	3	3	4	4	4	4	2	2
4	4	2	2	1	1	4	4	2	2
4	4	3	3	4	4	4	4	2	2
4	4	2	2	4	4	4	4	2	2
4	4	3	3	1	1	4	4	2	2
4	4	3	3	4	4	4	4	3	3
4	4	3	3	1	1	4	4	2	2
4	4	3	3	4	4	4	4	3	3
4	4	3	3	1	1	4	4	2	2
4	4	3	3	1	1	4	4	2	2
4	4	3	3	4	4	4	4	2	2
4	4	3	3	4	4	4	4	2	2
4	4	3	3	1	1	4	4	2	2
1	1	3	3	1	1	4	4	2	2
4	4	3	3	1	1	4	4	2	2
4	4	3	3	1	1	4	4	2	2

For Review Only

4	4	3	3	1	1	4	4	2	2
1	1	3	3	4	4	4	4	2	2
4	4	3	3	1	1	1	1	2	2
4	4	3	3	1	1	4	4	3	3
1	1	3	3	1	1	4	4	2	2
4	4	3	3	4	4	4	4	2	2
4	4	3	3	4	4	4	4	2	2
4	4	3	3	4	4	1	1	2	2
4	4	3	3	4	4	1	1	2	2
1	1	3	3	1	1	4	4	2	2
1	1	3	3	4	4	1	1	2	2
4	4	3	3	4	4	4	4	2	2
4	4	3	3	1	1	4	4	2	2
4	4	3	3	1	1	4	4	3	3
4	4	3	3	1	1	1	1	2	2
4	4	3	3	1	1	4	4	2	2
4	4	3	3	4	4	4	4	2	2
4	4	3	3	1	1	4	4	2	2
4	4	3	3	1	1	4	4	2	2
4	4	2	2	4	4	4	4	2	2
4	4	3	3	1	1	4	4	2	2
4	4	2	2	4	4	4	4	2	2
4	4	3	3	1	1	4	4	3	3
4	4	3	3	4	4	4	4	2	2
4	4	3	3	1	1	4	4	2	2
1	1	3	3	1	1	1	1	2	2
4	4	3	3	1	1	4	4	2	2
4	4	3	3	1	1	4	4	3	3
4	4	3	3	4	4	4	4	2	2
4	4	3	3	1	1	4	4	2	2
4	4	2	2	1	1	1	1	2	2
4	4	3	3	1	1	4	4	2	2
4	4	2	2	1	1	4	4	2	2
4	4	3	3	1	1	4	4	2	2
4	4	3	3	4	4	4	4	2	2
4	4	3	3	1	1	4	4	2	2
4	4	2	2	4	4	1	1	2	2
4	4	3	3	1	1	4	4	3	3
4	4	3	3	4	4	4	4	2	2
4	4	3	3	4	4	4	4	3	3
4	4	3	3	1	1	4	4	2	2
4	4	3	3	1	1	4	4	2	2
4	4	3	3	1	1	4	4	2	2
1	1	3	3	1	1	1	1	2	2
4	4	3	3	1	1	4	4	2	2
1	1	3	3	4	4	1	1	2	2
4	4	3	3	4	4	4	4	2	2
4	4	3	3	4	4	4	4	2	2
4	4	3	3	4	4	4	4	2	2
4	4	2	2	1	1	4	4	2	2
1	1	3	3	1	1	1	1	2	2
4	4	2	2	4	4	4	4	2	2
4	4	3	3	4	4	1	1	3	3
4	4	2	2	4	4	4	4	2	2
4	4	3	3	1	1	4	4	2	2

For Review Only

4	4	3	3	4	4	4	4	2	2
4	4	3	3	1	1	4	4	2	2
4	4	3	3	4	4	4	4	2	2
4	4	2	2	1	1	4	4	2	2
1	1	3	3	4	4	1	1	2	2
4	4	3	3	1	1	4	4	2	2
1	1	3	3	1	1	4	4	2	2
4	4	2	2	1	1	4	4	2	2
4	4	2	2	1	1	4	4	2	2
4	4	3	3	1	1	4	4	3	3
4	4	3	3	1	1	4	4	2	2
4	4	3	3	1	1	4	4	2	2
4	4	3	3	1	1	4	4	2	2
4	4	3	3	1	1	4	4	2	2
4	4	3	3	1	1	4	4	2	2
4	4	3	3	1	1	4	4	2	2
4	4	3	3	4	4	4	4	3	3
4	4	3	3	1	1	4	4	2	2
4	4	2	2	4	4	4	4	2	2
4	4	3	3	1	1	4	4	2	2
4	4	3	3	1	1	4	4	2	2
4	4	3	3	1	1	4	4	2	2
4	4	3	3	1	1	4	4	2	2
4	4	2	2	4	4	4	4	2	2
4	4	2	2	4	4	4	4	2	2
4	4	3	3	4	4	4	4	2	2
4	4	2	2	1	1	4	4	2	2
4	4	3	3	4	4	4	4	2	2
1	1	3	3	1	1	4	4	2	2
1	1	3	3	4	4	4	4	2	2
4	4	3	3	1	1	4	4	3	3
4	4	3	3	1	1	4	4	2	2
4	4	3	3	1	1	4	4	2	2
4	4	3	3	1	1	4	4	2	2
4	4	3	3	1	1	4	4	2	2
4	4	3	3	1	1	4	4	2	2
4	4	2	2	4	4	4	4	2	2
1	1	3	3	1	1	1	1	2	2
4	4	3	3	4	4	4	4	2	2
4	4	3	3	4	4	4	4	2	2
1	1	3	3	4	4	4	4	2	2
4	4	2	2	4	4	4	4	2	2
4	4	3	3	1	1	4	4	2	2
4	4	2	2	1	1	4	4	2	2
4	4	3	3	1	1	4	4	2	2
4	4	3	3	4	4	4	4	2	2
4	4	3	3	4	4	4	4	2	2
4	4	3	3	1	1	4	4	2	2
4	4	2	2	1	1	4	4	2	2
4	4	3	3	4	4	4	4	2	2
4	4	3	3	4	4	4	4	2	2
4	4	2	2	4	4	4	4	2	2
4	4	3	3	1	1	4	4	2	2
4	4	3	3	4	4	4	4	2	2
4	4	3	3	4	4	4	4	2	2

For Review Only

4	4	3	3	1	1	4	4	2	2
4	4	3	3	4	4	4	4	2	2
4	4	3	3	1	1	1	1	2	2
4	4	3	3	4	4	4	4	2	2
4	4	3	3	4	4	4	4	2	2
4	4	3	3	1	1	1	1	3	3
4	4	3	3	4	4	4	4	2	2
4	4	3	3	1	1	4	4	2	2
4	4	2	2	1	1	4	4	2	2
4	4	3	3	1	1	4	4	2	2
4	4	3	3	4	4	4	4	2	2
4	4	3	3	1	1	4	4	2	2
4	4	3	3	1	1	4	4	2	2
4	4	3	3	4	4	4	4	2	2
4	4	3	3	4	4	4	4	2	2
4	4	3	3	4	4	4	4	2	2
4	4	3	3	1	1	4	4	2	2
4	4	3	3	1	1	4	4	2	2
4	4	3	3	4	4	4	4	2	2
1	1	3	3	1	1	4	4	2	2
4	4	3	3	1	1	4	4	2	2
4	4	2	2	4	4	4	4	2	2
4	4	2	2	4	4	4	4	2	2
4	4	3	3	4	4	4	4	2	2
4	4	3	3	1	1	4	4	3	3
4	4	3	3	1	1	4	4	2	2
4	4	3	3	1	1	4	4	2	2
4	4	3	3	4	4	4	4	2	2
4	4	3	3	4	4	4	4	2	2
4	4	3	3	1	1	4	4	2	2
4	4	2	2	1	1	1	1	2	2
4	4	3	3	1	1	4	4	2	2
4	4	3	3	1	1	4	4	2	2
4	4	2	2	4	4	4	4	2	2
4	4	3	3	4	4	4	4	2	2
4	4	3	3	4	4	4	4	2	2
4	4	3	3	4	4	4	4	2	2
4	4	3	3	4	4	4	4	2	2
4	4	3	3	1	1	4	4	2	2
4	4	3	3	1	1	4	4	2	2
4	4	3	3	1	1	4	4	2	2
4	4	3	3	4	4	4	4	2	2
4	4	3	3	4	4	4	4	3	3
4	4	2	2	1	1	4	4	2	2
4	4	3	3	1	1	4	4	2	2
4	4	3	3	1	1	4	4	3	3
4	4	3	3	4	4	4	4	2	2
4	4	2	2	4	4	4	4	2	2
4	4	3	3	4	4	4	4	2	2
4	4	3	3	4	4	4	4	3	3
4	4	3	3	1	1	4	4	2	2
4	4	3	3	4	4	4	4	2	2
4	4	2	2	4	4	4	4	2	2
1	1	3	3	4	4	1	1	2	2
4	4	2	2	1	1	4	4	2	2

For Review Only

4	4	3	3	1	1	4	4	2	2
4	4	3	3	1	1	4	4	2	2
4	4	3	3	1	1	4	4	2	2
4	4	2	2	4	4	4	4	2	2
4	4	3	3	4	4	4	4	2	2
4	4	3	3	1	1	4	4	2	2
4	4	2	2	4	4	4	4	2	2
4	4	3	3	4	4	1	1	3	3
4	4	2	2	1	1	4	4	2	2
4	4	3	3	4	4	4	4	3	3
4	4	3	3	1	1	4	4	2	2
4	4	3	3	1	1	4	4	2	2
4	4	3	3	4	4	4	4	2	2
4	4	3	3	1	1	4	4	2	2
4	4	3	3	1	1	4	4	2	2
4	4	3	3	1	1	4	4	2	2
4	4	3	3	4	4	4	4	2	2
4	4	3	3	1	1	4	4	2	2
4	4	2	2	4	4	4	4	2	2
4	4	3	3	1	1	4	4	2	2
4	4	3	3	4	4	4	4	2	2
4	4	3	3	4	4	4	4	2	2
4	4	3	3	1	1	4	4	2	2
4	4	3	3	4	4	4	4	2	2
4	4	3	3	4	4	1	1	2	2
4	4	3	3	1	1	4	4	2	2
4	4	2	2	4	4	4	4	2	2
1	1	3	3	1	1	1	1	2	2
4	4	3	3	1	1	4	4	2	2
4	4	3	3	1	1	4	4	2	2
4	4	2	2	4	4	4	4	2	2
4	4	3	3	1	1	4	4	2	2
4	4	3	3	4	4	4	4	2	2
4	4	3	3	1	1	4	4	2	2
4	4	3	3	4	4	4	4	2	2
4	4	2	2	1	1	4	4	2	2
1	1	3	3	1	1	4	4	2	2
4	4	3	3	4	4	4	4	2	2
4	4	3	3	1	1	4	4	2	2
4	4	2	2	4	4	4	4	2	2
4	4	3	3	4	4	4	4	2	2
4	4	3	3	4	4	4	4	2	2
4	4	3	3	4	4	4	4	2	2
4	4	3	3	1	1	4	4	2	2
4	4	3	3	1	1	4	4	2	2
4	4	3	3	4	4	4	4	2	2
4	4	2	2	4	4	4	4	2	2
4	4	3	3	1	1	4	4	2	2
4	4	3	3	1	1	4	4	2	2
4	4	2	2	1	1	4	4	2	2
4	4	3	3	1	1	4	4	2	2
4	4	3	3	4	4	4	4	3	3
4	4	3	3	1	1	4	4	2	2
1	1	3	3	1	1	4	4	2	2
4	4	2	2	1	1	4	4	2	2

For Review Only

4	4	3	3	1	1	4	4	2	2
4	4	3	3	1	1	4	4	2	2

For Review Only

SNP1136	SNP1136	SNP1137	SNP1137	SNP1138	SNP1138	SNP1139	SNP1139	SNP114	SNP114
1	1	1	1	3	3	3	3	1	1
4	4	2	2	1	1	1	1	1	1
1	1	1	1	3	3	1	1	1	1
4	4	1	1	3	3	1	1	1	1
4	4	2	2	1	1	1	1	1	1
1	1	1	1	3	3	1	1	1	1
1	1	1	1	3	3	3	3	3	3
4	4	1	1	3	3	3	3	1	1
4	4	1	1	3	3	3	3	3	3
4	4	1	1	3	3	3	3	1	1
1	1	1	1	3	3	3	3	3	3
1	1	1	1	3	3	1	1	1	1
1	1	1	1	3	3	3	3	1	1
1	1	1	1	3	3	3	3	1	1
1	1	1	1	3	3	3	3	1	1
1	1	1	1	3	3	1	1	1	1
1	1	1	1	3	3	1	1	3	3
1	1	1	1	3	3	1	1	1	1
1	1	1	1	3	3	1	1	1	1
1	1	1	1	3	3	3	3	1	1
1	1	1	1	3	3	3	3	1	1
1	1	1	1	3	3	3	3	3	3
1	1	2	2	1	1	1	1	1	1
1	1	1	1	3	3	3	3	1	1
1	1	1	1	3	3	1	1	1	1
1	1	1	1	3	3	1	1	1	1
1	1	1	1	3	3	1	1	1	1
1	1	1	1	3	3	3	3	1	1
4	4	1	1	3	3	3	3	1	1
1	1	1	1	3	3	3	3	1	1
1	1	1	1	3	3	1	1	1	1
1	1	1	1	3	3	1	1	3	3
1	1	1	1	3	3	1	1	3	3
1	1	1	1	3	3	1	1	1	1
1	1	2	2	3	3	1	1	1	1
1	1	1	1	3	3	3	3	1	1
1	1	2	2	1	1	1	1	1	1
1	1	1	1	3	3	1	1	1	1
1	1	1	1	3	3	1	1	1	1
1	1	1	1	3	3	1	1	1	1
1	1	1	1	3	3	3	3	1	1
4	4	1	1	3	3	3	3	1	1
1	1	1	1	3	3	3	3	1	1
4	4	1	1	3	3	1	1	1	1
1	1	1	1	3	3	3	3	1	1
1	1	1	1	3	3	1	1	1	1
1	1	1	1	3	3	1	1	1	1
1	1	1	1	3	3	1	1	1	1
1	1	1	1	3	3	3	3	1	1
1	1	1	1	3	3	3	3	3	3
1	1	1	1	3	3	1	1	1	1
1	1	2	2	1	1	1	1	3	3

For Review Only

1	1	1	1	3	3	3	3	3	3
1	1	1	1	3	3	1	1	3	3
1	1	1	1	3	3	1	1	1	1
4	4	1	1	3	3	1	1	3	3
1	1	1	1	3	3	1	1	1	1
1	1	1	1	3	3	1	1	1	1
1	1	1	1	3	3	1	1	1	1
1	1	1	1	3	3	1	1	1	1
1	1	1	1	3	3	1	1	1	1
1	1	1	1	3	3	3	3	1	1
1	1	1	1	3	3	1	1	1	1
1	1	1	1	3	3	1	1	1	1
1	1	1	1	3	3	3	3	3	3
4	4	1	1	3	3	3	3	1	1
1	1	1	1	3	3	1	1	1	1
1	1	2	2	3	3	1	1	1	1
1	1	1	1	3	3	1	1	1	1
1	1	1	1	3	3	3	3	1	1
1	1	1	1	3	3	3	3	3	3
1	1	1	1	3	3	3	3	1	1
1	1	1	1	3	3	1	1	1	1
1	1	1	1	3	3	1	1	1	1
4	4	1	1	3	3	3	3	3	3
1	1	1	1	3	3	1	1	1	1
1	1	1	1	3	3	3	3	1	1
1	1	2	2	3	3	1	1	1	1
1	1	1	1	3	3	1	1	3	3
4	4	1	1	3	3	1	1	1	1
1	1	1	1	3	3	1	1	1	1
1	1	1	1	3	3	3	3	3	3
1	1	2	2	1	1	1	1	3	3
1	1	1	1	3	3	3	3	1	1
1	1	2	2	1	1	1	1	1	1
1	1	1	1	3	3	3	3	3	3
1	1	1	1	3	3	3	3	1	1
1	1	1	1	3	3	3	3	1	1
1	1	1	1	3	3	3	3	1	1
4	4	1	1	3	3	3	3	3	3
1	1	2	2	1	1	1	1	1	1
4	4	1	1	3	3	1	1	1	1
1	1	1	1	3	3	3	3	1	1
1	1	1	1	3	3	3	3	1	1
4	4	1	1	3	3	1	1	1	1
1	1	1	1	3	3	1	1	1	1
1	1	1	1	3	3	3	3	1	1
1	1	1	1	3	3	1	1	3	3
1	1	1	1	3	3	3	3	1	1
1	1	1	1	3	3	1	1	1	1
1	1	1	1	3	3	3	3	1	1
1	1	2	2	3	3	1	1	1	1
1	1	1	1	3	3	1	1	1	1
1	1	1	1	3	3	1	1	1	1
4	4	1	1	3	3	1	1	1	1
1	1	1	1	3	3	3	3	1	1
1	1	2	2	1	1	1	1	1	1

For Review Only

1	1	2	2	3	3	1	1	3	3
1	1	1	1	3	3	3	3	1	1
1	1	1	1	3	3	3	3	1	1
1	1	1	1	3	3	3	3	1	1
1	1	1	1	3	3	1	1	1	1
1	1	1	1	3	3	1	1	1	1
1	1	1	1	3	3	3	3	1	1
1	1	1	1	3	3	1	1	1	1
1	1	1	1	3	3	3	3	1	1
1	1	1	1	3	3	1	1	1	1
1	1	1	1	3	3	3	3	3	3
1	1	1	1	3	3	3	3	1	1
4	4	1	1	3	3	1	1	1	1
1	1	2	2	3	3	1	1	1	1
4	4	2	2	3	3	3	3	1	1
1	1	1	1	3	3	1	1	1	1
4	4	1	1	3	3	1	1	1	1
1	1	1	1	3	3	1	1	1	1
1	1	1	1	3	3	3	3	1	1
1	1	1	1	3	3	3	3	3	3
1	1	1	1	3	3	3	3	1	1
1	1	1	1	3	3	3	3	3	3
1	1	1	1	3	3	1	1	1	1
1	1	1	1	3	3	1	1	1	1
1	1	1	1	3	3	1	1	1	1
1	1	1	1	3	3	3	3	1	1
1	1	1	1	3	3	3	3	3	3
1	1	1	1	3	3	1	1	1	1
1	1	1	1	3	3	3	3	1	1
1	1	1	1	3	3	3	3	1	1
4	4	2	2	3	3	1	1	1	1
1	1	1	1	3	3	3	3	1	1
1	1	1	1	3	3	3	3	1	1
1	1	1	1	3	3	1	1	1	1
1	1	1	1	3	3	1	1	1	1
1	1	2	2	3	3	3	3	1	1
1	1	1	1	3	3	3	3	1	1
1	1	1	1	3	3	3	3	1	1
1	1	2	2	3	3	1	1	1	1
1	1	1	1	3	3	1	1	3	3
1	1	1	1	3	3	1	1	1	1
1	1	1	1	3	3	3	3	1	1
1	1	1	1	3	3	1	1	1	1
4	4	1	1	3	3	3	3	1	1
1	1	1	1	3	3	1	1	1	1
1	1	1	1	3	3	3	3	1	1
1	1	1	1	3	3	1	1	1	1
1	1	1	1	3	3	3	3	1	1
1	1	2	2	3	3	1	1	1	1
1	1	1	1	3	3	1	1	1	1
1	1	1	1	3	3	3	3	1	1
1	1	1	1	3	3	1	1	1	1
1	1	1	1	3	3	3	3	1	1
1	1	1	1	3	3	1	1	1	1
1	1	1	1	3	3	3	3	1	1

For Review Only

1	1	1	1	3	3	1	1	1	1
1	1	1	1	3	3	1	1	1	1
1	1	1	1	3	3	3	3	1	1
1	1	1	1	3	3	1	1	1	1
1	1	1	1	3	3	3	3	1	1
4	4	1	1	3	3	1	1	1	1
1	1	1	1	3	3	1	1	1	1
1	1	1	1	3	3	3	3	1	1
1	1	1	1	3	3	3	3	1	1
1	1	1	1	3	3	3	3	1	1
1	1	1	1	3	3	1	1	1	1
1	1	1	1	3	3	1	1	1	1
1	1	1	1	3	3	1	1	3	3
1	1	1	1	3	3	3	3	1	1
1	1	2	2	1	1	1	1	1	1
1	1	1	1	3	3	1	1	3	3
1	1	1	1	3	3	3	3	1	1
1	1	1	1	3	3	1	1	1	1
1	1	1	1	3	3	3	3	1	1
1	1	1	1	3	3	3	3	1	1
1	1	1	1	3	3	3	3	1	1
1	1	1	1	3	3	1	1	1	1
1	1	2	2	3	3	1	1	1	1
1	1	1	1	3	3	1	1	1	1
4	4	1	1	3	3	3	3	1	1
1	1	1	1	3	3	1	1	1	1
1	1	1	1	3	3	1	1	1	1
4	4	1	1	3	3	1	1	1	1
1	1	1	1	3	3	3	3	1	1
1	1	1	1	3	3	3	3	3	3
1	1	2	2	1	1	1	1	1	1
1	1	1	1	3	3	3	3	1	1
1	1	1	1	3	3	3	3	1	1
1	1	2	2	1	1	1	1	1	1
1	1	1	1	3	3	1	1	1	1
1	1	1	1	3	3	1	1	1	1
1	1	1	1	3	3	3	3	1	1
1	1	1	1	3	3	3	3	1	1
1	1	1	1	3	3	3	3	1	1
1	1	1	1	3	3	1	1	1	1
1	1	1	1	3	3	1	1	1	1
1	1	1	1	3	3	1	1	1	1
4	4	1	1	3	3	1	1	1	1
1	1	1	1	3	3	3	3	3	3
1	1	1	1	3	3	3	3	1	1
4	4	1	1	3	3	1	1	1	1
1	1	1	1	3	3	1	1	3	3
1	1	1	1	3	3	1	1	1	1
4	4	1	1	3	3	3	3	1	1
4	4	1	1	3	3	1	1	1	1
1	1	1	1	3	3	1	1	1	1
1	1	1	1	3	3	3	3	1	1
1	1	2	2	1	1	1	1	1	1
1	1	1	1	3	3	1	1	1	1
1	1	1	1	3	3	1	1	1	1

For Review Only

1	1	1	1	3	3	3	3	1	1
1	1	1	1	3	3	3	3	3	3
1	1	1	1	3	3	1	1	1	1
1	1	1	1	3	3	1	1	3	3
1	1	1	1	3	3	1	1	1	1
1	1	1	1	3	3	1	1	1	1
1	1	1	1	3	3	1	1	1	1
4	4	1	1	3	3	3	3	1	1
1	1	1	1	3	3	3	3	1	1
4	4	1	1	3	3	1	1	1	1
1	1	1	1	3	3	3	3	1	1
1	1	2	2	1	1	1	1	1	1
1	1	2	2	1	1	1	1	3	3
1	1	1	1	3	3	1	1	1	1
1	1	1	1	3	3	1	1	1	1
1	1	2	2	1	1	1	1	3	3
1	1	1	1	3	3	1	1	1	1
1	1	1	1	3	3	3	3	1	1
1	1	1	1	3	3	3	3	1	1
1	1	1	1	3	3	1	1	1	1
1	1	1	1	3	3	1	1	1	1
1	1	1	1	3	3	1	1	1	1
1	1	1	1	3	3	3	3	1	1
1	1	1	1	3	3	1	1	1	1
1	1	1	1	3	3	1	1	1	1
4	4	1	1	3	3	3	3	3	3
1	1	2	2	1	1	1	1	1	1
1	1	1	1	3	3	1	1	1	1
1	1	1	1	3	3	3	3	3	3
1	1	1	1	3	3	3	3	1	1
1	1	1	1	3	3	1	1	1	1
1	1	1	1	3	3	1	1	1	1
1	1	1	1	3	3	1	1	1	1
1	1	1	1	3	3	1	1	1	1
1	1	1	1	3	3	1	1	1	1
1	1	2	2	1	1	1	1	1	1
1	1	1	1	3	3	3	3	1	1
1	1	1	1	3	3	1	1	1	1
1	1	1	1	3	3	3	3	1	1
1	1	1	1	3	3	1	1	1	1
1	1	1	1	3	3	1	1	1	1
1	1	1	1	3	3	1	1	1	1
1	1	1	1	3	3	1	1	1	1
1	1	1	1	3	3	3	3	3	3
1	1	1	1	3	3	1	1	1	1
1	1	2	2	1	1	1	1	1	1
1	1	1	1	3	3	3	3	1	1
1	1	1	1	3	3	1	1	1	1
1	1	1	1	3	3	1	1	1	1
1	1	2	2	1	1	1	1	1	1
1	1	1	1	3	3	3	3	3	3
4	4	1	1	3	3	1	1	1	1
1	1	1	1	3	3	1	1	1	1
1	1	1	1	3	3	1	1	1	1
1	1	1	1	3	3	1	1	1	1

For Review Only

1	1	1	1	3	3	3	3	3	3
1	1	1	1	3	3	1	1	1	1

For Review Only

SNP1140	SNP1140	SNP1141	SNP1141	SNP1142	SNP1142	SNP1143	SNP1143	SNP1144	SNP1144
4	4	2	2	2	2	1	1	1	1
4	4	2	2	2	2	4	4	1	1
4	4	2	2	2	2	1	1	1	1
4	4	2	2	2	2	1	1	1	1
4	4	2	2	2	2	4	4	1	1
4	4	2	2	2	2	1	1	1	1
4	4	2	2	2	2	1	1	1	1
4	4	2	2	2	2	1	1	1	1
1	1	1	1	2	2	1	1	1	1
1	1	1	1	2	2	1	1	1	1
1	1	2	2	3	3	1	1	1	1
4	4	2	2	2	2	1	1	1	1
4	4	2	2	2	2	1	1	1	1
1	1	1	1	2	2	1	1	1	1
4	4	2	2	2	2	1	1	1	1
1	1	1	1	2	2	1	1	1	1
4	4	2	2	2	2	1	1	1	1
4	4	2	2	2	2	1	1	1	1
1	1	1	1	2	2	1	1	1	1
4	4	2	2	2	2	1	1	2	2
4	4	2	2	2	2	1	1	2	2
4	4	2	2	2	2	1	1	1	1
1	1	1	1	3	3	4	4	1	1
1	1	1	1	2	2	1	1	1	1
4	4	2	2	2	2	1	1	1	1
1	1	1	1	2	2	1	1	1	1
1	1	1	1	2	2	1	1	1	1
4	4	2	2	2	2	1	1	1	1
1	1	1	1	2	2	1	1	1	1
1	1	1	1	2	2	1	1	1	1
1	1	1	1	2	2	1	1	1	1
4	4	1	1	2	2	1	1	1	1
4	4	2	2	2	2	1	1	1	1
1	1	1	1	2	2	1	1	1	1
4	4	2	2	3	3	4	4	1	1
1	1	1	1	2	2	1	1	1	1
1	1	1	1	2	2	1	1	1	1
1	1	2	2	2	2	1	1	1	1
1	1	1	1	3	3	1	1	1	1
1	1	2	2	2	2	1	1	1	1
1	1	1	1	2	2	1	1	2	2
1	1	1	1	2	2	1	1	2	2
1	1	1	1	2	2	1	1	2	2
4	4	2	2	2	2	1	1	1	1
1	1	1	1	2	2	1	1	2	2
4	4	2	2	2	2	1	1	1	1
1	1	1	1	2	2	1	1	1	1
4	4	2	2	3	3	1	1	1	1
4	4	2	2	2	2	1	1	2	2
4	4	2	2	2	2	1	1	1	1
1	1	1	1	2	2	1	1	1	1
4	4	2	2	2	2	4	4	1	1

For Review Only

4	4	2	2	2	2	1	1	1	1
4	4	2	2	2	2	1	1	1	1
4	4	2	2	2	2	1	1	1	1
1	1	2	2	2	2	1	1	1	1
1	1	1	1	2	2	1	1	1	1
1	1	1	1	2	2	1	1	1	1
1	1	1	1	2	2	1	1	1	1
1	1	1	1	2	2	1	1	1	1
1	1	1	1	2	2	1	1	1	1
4	4	2	2	2	2	1	1	1	1
4	4	2	2	2	2	1	1	1	1
4	4	1	1	2	2	1	1	1	1
4	4	2	2	2	2	1	1	1	1
1	1	1	1	2	2	1	1	1	1
4	4	2	2	2	2	1	1	1	1
4	4	2	2	2	2	1	1	1	1
4	4	2	2	3	3	1	1	1	1
1	1	1	1	2	2	1	1	1	1
4	4	2	2	2	2	1	1	1	1
4	4	2	2	2	2	1	1	1	1
4	4	2	2	2	2	1	1	1	1
1	1	1	1	3	3	1	1	1	1
4	4	2	2	2	2	1	1	1	1
1	1	1	1	2	2	1	1	1	1
1	1	1	1	2	2	1	1	2	2
4	4	2	2	2	2	1	1	1	1
4	4	2	2	2	2	1	1	1	1
1	1	1	1	3	3	1	1	1	1
1	1	1	1	2	2	1	1	1	1
4	4	2	2	2	2	1	1	1	1
4	4	2	2	2	2	4	4	1	1
1	1	2	2	2	2	1	1	1	1
1	1	1	1	2	2	4	4	1	1
4	4	2	2	2	2	1	1	1	1
1	1	2	2	2	2	1	1	1	1
1	1	1	1	2	2	1	1	2	2
4	4	2	2	2	2	1	1	2	2
4	4	2	2	2	2	1	1	2	2
1	1	2	2	3	3	4	4	1	1
1	1	1	1	2	2	1	1	1	1
1	1	1	1	2	2	1	1	1	1
4	4	2	2	2	2	1	1	1	1
1	1	1	1	2	2	1	1	1	1
4	4	2	2	2	2	1	1	1	1
1	1	2	2	2	2	1	1	1	1
4	4	2	2	2	2	1	1	1	1
1	1	1	1	2	2	1	1	1	1
1	1	1	1	2	2	1	1	1	1
1	1	1	1	2	2	1	1	1	1
1	1	1	1	2	2	1	1	1	1
4	4	2	2	2	2	1	1	1	1
1	1	1	1	3	3	1	1	1	1
1	1	1	1	2	2	1	1	1	1
4	4	1	1	2	2	1	1	1	1
1	1	1	1	2	2	4	4	1	1

For Review Only

1	1	1	1	2	2	1	1	1	1
4	4	1	1	2	2	1	1	1	1
1	1	1	1	2	2	1	1	1	1
1	1	1	1	2	2	1	1	1	1
4	4	2	2	2	2	1	1	1	1
1	1	1	1	2	2	1	1	1	1
1	1	1	1	2	2	1	1	1	1
4	4	1	1	2	2	1	1	1	1
1	1	1	1	2	2	1	1	1	1
4	4	1	1	2	2	1	1	1	1
1	1	2	2	2	2	1	1	1	1
1	1	2	2	2	2	1	1	1	1
1	1	1	1	2	2	1	1	1	1
4	4	2	2	2	2	1	1	1	1
4	4	2	2	2	2	1	1	1	1
4	4	2	2	2	2	1	1	1	1
1	1	1	1	2	2	1	1	1	1
4	4	2	2	2	2	1	1	1	1
1	1	1	1	2	2	1	1	1	1
4	4	2	2	2	2	1	1	1	1
4	4	2	2	2	2	1	1	1	1
4	4	2	2	2	2	1	1	1	1
1	1	1	1	2	2	1	1	1	1
1	1	1	1	3	3	1	1	1	1
1	1	1	1	2	2	1	1	1	1
1	1	1	1	3	3	1	1	1	1
1	1	2	2	2	2	1	1	1	1
1	1	1	1	2	2	1	1	1	1
1	1	2	2	2	2	1	1	1	1
1	1	1	1	2	2	1	1	1	1
1	1	1	1	2	2	4	4	1	1
1	1	2	2	2	2	1	1	1	1
1	1	1	1	2	2	1	1	1	1
1	1	1	1	2	2	1	1	1	1
1	1	1	1	2	2	1	1	1	1
4	4	2	2	2	2	1	1	1	1
1	1	1	1	2	2	1	1	1	1
4	4	2	2	2	2	1	1	1	1
4	4	1	1	3	3	1	1	1	1
1	1	2	2	3	3	1	1	1	1
1	1	1	1	3	3	1	1	1	1
1	1	1	1	2	2	1	1	1	1
1	1	2	2	2	2	1	1	1	1
1	1	1	1	2	2	1	1	1	1
1	1	1	1	2	2	1	1	1	1
1	1	2	2	2	2	1	1	1	1
1	1	1	1	2	2	1	1	1	1
1	1	1	1	2	2	1	1	1	1
1	1	1	1	2	2	1	1	1	1
1	1	1	1	2	2	1	1	1	1
1	1	1	1	2	2	1	1	1	1
1	1	1	1	3	3	1	1	1	1
1	1	2	2	2	2	1	1	1	1
1	1	1	1	2	2	1	1	1	1
4	4	1	1	2	2	1	1	1	1

For Review Only

1	1	1	1	2	2	1	1	1	1
4	4	1	1	3	3	1	1	1	1
1	1	2	2	2	2	1	1	1	1
1	1	2	2	3	3	1	1	1	1
4	4	2	2	2	2	1	1	1	1
1	1	2	2	2	2	1	1	1	1
4	4	1	1	2	2	1	1	1	1
4	4	2	2	2	2	1	1	1	1
4	4	2	2	2	2	1	1	1	1
4	4	2	2	2	2	1	1	1	1
1	1	1	1	2	2	1	1	1	1
1	1	1	1	2	2	1	1	1	1
4	4	2	2	2	2	1	1	1	1
4	4	2	2	2	2	1	1	1	1
1	1	1	1	2	2	4	4	1	1
1	1	2	2	3	3	1	1	1	1
1	1	2	2	2	2	1	1	1	1
4	4	2	2	2	2	1	1	1	1
1	1	1	1	2	2	1	1	1	1
1	1	1	1	2	2	1	1	1	1
4	4	2	2	2	2	1	1	1	1
1	1	1	1	2	2	1	1	1	1
1	1	1	1	3	3	1	1	1	1
1	1	1	1	2	2	1	1	1	1
1	1	1	1	2	2	1	1	2	2
1	1	1	1	2	2	1	1	1	1
1	1	1	1	2	2	1	1	1	1
1	1	1	1	2	2	1	1	1	1
1	1	1	1	2	2	1	1	1	1
1	1	1	1	2	2	1	1	1	1
4	4	2	2	2	2	4	4	1	1
4	4	2	2	2	2	1	1	1	1
1	1	2	2	2	2	1	1	1	1
1	1	1	1	2	2	4	4	1	1
1	1	2	2	2	2	1	1	1	1
1	1	1	1	2	2	1	1	1	1
4	4	2	2	2	2	1	1	1	1
4	4	2	2	2	2	1	1	1	1
1	1	1	1	2	2	1	1	1	1
4	4	2	2	2	2	1	1	1	1
4	4	2	2	2	2	1	1	1	1
1	1	1	1	2	2	1	1	1	1
1	1	1	1	3	3	4	4	1	1
4	4	2	2	2	2	1	1	1	1
1	1	2	2	2	2	1	1	1	1
4	4	2	2	2	2	1	1	1	1
1	1	2	2	3	3	1	1	1	1
1	1	1	1	2	2	1	1	1	1
1	1	1	1	2	2	1	1	1	1
1	1	1	1	2	2	1	1	1	1
1	1	2	2	2	2	1	1	1	1
1	1	2	2	2	2	1	1	1	1
1	1	1	1	3	3	4	4	1	1
4	4	2	2	2	2	1	1	1	1
1	1	1	1	2	2	1	1	1	1

For Review Only

1	1	2	2	2	2	1	1	1	1
1	1	1	1	2	2	1	1	1	1
1	1	2	2	2	2	1	1	1	1
1	1	1	1	3	3	1	1	1	1
4	4	2	2	2	2	1	1	1	1
1	1	1	1	2	2	1	1	1	1
1	1	1	1	3	3	1	1	1	1
1	1	1	1	2	2	1	1	1	1
1	1	1	1	2	2	1	1	1	1
1	1	1	1	3	3	1	1	1	1
4	4	2	2	2	2	1	1	1	1
1	1	2	2	2	2	4	4	1	1
1	1	2	2	3	3	4	4	1	1
1	1	1	1	2	2	1	1	1	1
4	4	2	2	2	2	1	1	1	1
1	1	2	2	2	2	4	4	1	1
1	1	1	1	2	2	1	1	1	1
4	4	2	2	2	2	1	1	1	1
4	4	2	2	3	3	1	1	1	1
1	1	1	1	2	2	1	1	1	1
4	4	2	2	3	3	1	1	1	1
1	1	1	1	2	2	1	1	1	1
4	4	2	2	2	2	1	1	1	1
1	1	1	1	3	3	1	1	1	1
1	1	1	1	2	2	1	1	1	1
4	4	2	2	2	2	1	1	1	1
1	1	1	1	3	3	4	4	1	1
4	4	2	2	2	2	1	1	1	1
4	4	2	2	2	2	1	1	1	1
1	1	2	2	2	2	1	1	1	1
1	1	1	1	3	3	1	1	1	1
1	1	1	1	2	2	1	1	1	1
4	4	2	2	2	2	4	4	1	1
1	1	1	1	2	2	1	1	1	1
1	1	1	1	3	3	1	1	1	1
1	1	1	1	2	2	1	1	1	1
1	1	2	2	2	2	1	1	1	1
1	1	1	1	2	2	1	1	1	1
1	1	1	1	2	2	1	1	1	1
1	1	1	1	3	3	1	1	1	1
1	1	1	1	2	2	1	1	1	1
1	1	2	2	2	2	1	1	1	1
1	1	1	1	3	3	1	1	1	1
4	4	2	2	2	2	1	1	1	1
4	4	2	2	2	2	1	1	1	1
1	1	2	2	3	3	4	4	1	1
1	1	1	1	3	3	1	1	1	1
1	1	1	1	2	2	1	1	1	1
1	1	1	1	2	2	1	1	1	1
1	1	1	1	2	2	4	4	1	1
1	1	1	1	2	2	1	1	1	1
1	1	1	1	3	3	1	1	1	1
4	4	2	2	2	2	1	1	1	1
4	4	2	2	2	2	1	1	1	1
1	1	2	2	2	2	1	1	1	1

For Review Only

1	1	2	2	2	2	1	1	1	1
4	4	2	2	2	2	1	1	1	1

For Review Only

SNP1145	SNP1145	SNP1146	SNP1146	SNP1147	SNP1147	SNP1148	SNP1148	SNP1149	SNP1149
1	1	4	4	1	1	2	2	2	2
1	1	2	2	2	2	3	3	4	4
4	4	4	4	1	1	2	2	2	2
1	1	4	4	1	1	2	2	2	2
1	1	2	2	1	1	2	2	4	4
1	1	4	4	2	2	3	3	2	2
1	1	4	4	1	1	2	2	2	2
1	1	4	4	1	1	2	2	2	2
4	4	4	4	1	1	2	2	2	2
4	4	4	4	1	1	2	2	2	2
4	4	4	4	1	1	2	2	2	2
4	4	4	4	1	1	2	2	2	2
1	1	2	2	1	1	2	2	4	4
4	4	4	4	1	1	2	2	2	2
1	1	4	4	1	1	2	2	2	2
4	4	4	4	1	1	2	2	2	2
1	1	4	4	1	1	2	2	2	2
1	1	4	4	1	1	2	2	2	2
1	1	4	4	1	1	2	2	2	2
1	1	4	4	1	1	2	2	4	4
1	1	2	2	1	1	2	2	4	4
1	1	4	4	1	1	2	2	2	2
1	1	2	2	1	1	2	2	4	4
4	4	4	4	1	1	2	2	2	2
1	1	4	4	1	1	2	2	2	2
1	1	4	4	1	1	2	2	2	2
1	1	4	4	2	2	2	2	2	2
1	1	4	4	1	1	2	2	2	2
4	4	4	4	1	1	2	2	2	2
4	4	4	4	1	1	2	2	2	2
1	1	4	4	1	1	2	2	2	2
4	4	4	4	1	1	2	2	2	2
4	4	4	4	1	1	2	2	2	2
4	4	4	4	1	1	2	2	2	2
1	1	2	2	1	1	2	2	2	2
4	4	4	4	1	1	2	2	2	2
4	4	2	2	1	1	2	2	4	4
4	4	4	4	1	1	2	2	4	4
1	1	4	4	1	1	2	2	4	4
4	4	4	4	1	1	2	2	4	4
4	4	2	2	1	1	2	2	4	4
4	4	2	2	1	1	2	2	4	4
4	4	2	2	1	1	2	2	4	4
1	1	4	4	1	1	2	2	2	2
4	4	2	2	1	1	2	2	4	4
1	1	4	4	1	1	2	2	2	2
1	1	4	4	1	1	2	2	2	2
1	1	4	4	1	1	2	2	4	4
4	4	2	2	1	1	2	2	4	4
4	4	4	4	1	1	2	2	2	2
1	1	4	4	1	1	2	2	2	2
1	1	2	2	1	1	2	2	4	4

For Review Only

1	1	4	4	1	1	2	2	2	2
4	4	4	4	1	1	2	2	2	2
1	1	4	4	2	2	2	2	2	2
4	4	4	4	1	1	2	2	2	2
1	1	4	4	1	1	2	2	2	2
1	1	4	4	1	1	2	2	2	2
1	1	4	4	1	1	2	2	2	2
4	4	4	4	1	1	2	2	2	2
4	4	4	4	1	1	2	2	2	2
1	1	4	4	1	1	2	2	2	2
1	1	4	4	2	2	3	3	2	2
4	4	4	4	1	1	2	2	2	2
1	1	4	4	1	1	2	2	2	2
4	4	4	4	1	1	2	2	2	2
1	1	4	4	1	1	2	2	2	2
1	1	2	2	1	1	2	2	4	4
1	1	4	4	1	1	2	2	4	4
4	4	4	4	1	1	2	2	2	2
1	1	4	4	1	1	2	2	2	2
1	1	4	4	1	1	2	2	2	2
1	1	4	4	1	1	2	2	2	2
4	4	4	4	1	1	2	2	4	4
1	1	4	4	1	1	2	2	2	2
4	4	4	4	1	1	2	2	2	2
4	4	2	2	1	1	2	2	4	4
1	1	4	4	2	2	3	3	2	2
4	4	4	4	1	1	2	2	2	2
1	1	2	2	1	1	2	2	4	4
1	1	4	4	1	1	2	2	2	2
1	1	4	4	1	1	2	2	2	2
1	1	2	2	1	1	2	2	2	2
1	1	4	4	1	1	2	2	2	2
1	1	2	2	1	1	2	2	4	4
1	1	4	4	1	1	2	2	2	2
4	4	4	4	1	1	2	2	2	2
4	4	2	2	1	1	2	2	4	4
1	1	4	4	1	1	2	2	2	2
1	1	4	4	1	1	2	2	2	2
4	4	2	2	1	1	2	2	4	4
4	4	4	4	1	1	2	2	2	2
1	1	4	4	1	1	2	2	2	2
1	1	4	4	2	2	2	2	2	2
4	4	4	4	1	1	2	2	2	2
1	1	4	4	2	2	3	3	2	2
4	4	4	4	1	1	2	2	2	2
4	4	4	4	2	2	2	2	2	2
4	4	4	4	1	1	2	2	2	2
4	4	4	4	1	1	2	2	2	2
4	4	4	4	1	1	2	2	2	2
4	4	4	4	1	1	2	2	4	4
1	1	4	4	2	2	3	3	2	2
1	1	2	2	1	1	2	2	4	4
4	4	4	4	1	1	2	2	2	2
1	1	4	4	1	1	2	2	2	2
4	4	2	2	1	1	2	2	4	4

For Review Only

1	1	4	4	1	1	2	2	4	4
1	1	4	4	2	2	2	2	2	2
1	1	4	4	1	1	2	2	2	2
4	4	4	4	1	1	2	2	2	2
1	1	4	4	1	1	3	3	2	2
4	4	4	4	1	1	2	2	2	2
4	4	4	4	1	1	2	2	2	2
1	1	4	4	1	1	2	2	2	2
1	1	4	4	1	1	2	2	2	2
1	1	4	4	1	1	2	2	2	2
4	4	4	4	1	1	2	2	2	2
1	1	4	4	1	1	2	2	2	2
4	4	4	4	1	1	2	2	2	2
1	1	4	4	1	1	2	2	2	2
1	1	2	2	1	1	2	2	2	2
1	1	4	4	1	1	2	2	2	2
4	4	4	4	1	1	2	2	2	2
1	1	4	4	1	1	2	2	2	2
4	4	4	4	1	1	2	2	2	2
1	1	4	4	1	1	2	2	2	2
1	1	4	4	1	1	2	2	2	2
1	1	4	4	1	1	2	2	2	2
4	4	4	4	1	1	2	2	2	2
1	1	2	2	1	1	2	2	4	4
4	4	4	4	1	1	2	2	2	2
4	4	4	4	1	1	2	2	2	2
4	4	4	4	1	1	2	2	2	2
4	4	4	4	1	1	2	2	2	2
4	4	4	4	1	1	2	2	2	2
1	1	4	4	1	1	2	2	2	2
4	4	2	2	1	1	2	2	4	4
1	1	4	4	1	1	2	2	2	2
1	1	4	4	1	1	2	2	2	2
1	1	4	4	1	1	2	2	2	2
4	4	4	4	1	1	2	2	2	2
1	1	2	2	1	1	2	2	2	2
4	4	4	4	1	1	2	2	2	2
1	1	4	4	2	2	2	2	2	2
1	1	4	4	1	1	2	2	4	4
1	1	2	2	1	1	2	2	4	4
4	4	4	4	1	1	2	2	4	4
4	4	4	4	1	1	2	2	2	2
4	4	4	4	1	1	2	2	2	2
1	1	4	4	1	1	2	2	2	2
4	4	4	4	1	1	2	2	2	2
4	4	4	4	1	1	2	2	2	2
1	1	4	4	1	1	2	2	2	2
1	1	4	4	1	1	2	2	2	2
4	4	2	2	1	1	2	2	2	2
1	1	4	4	1	1	2	2	2	2
1	1	4	4	1	1	2	2	2	2
4	4	4	4	1	1	2	2	2	2
4	4	4	4	1	1	2	2	2	2
4	4	4	4	1	1	2	2	2	2
1	1	4	4	1	1	2	2	2	2

For Review Only

4	4	4	4	1	1	2	2	2	2
4	4	4	4	1	1	2	2	2	2
4	4	4	4	1	1	2	2	2	2
4	4	4	4	1	1	2	2	2	2
1	1	4	4	1	1	2	2	2	2
4	4	4	4	1	1	2	2	4	4
4	4	4	4	1	1	2	2	4	4
1	1	4	4	1	1	2	2	2	2
1	1	4	4	1	1	2	2	2	2
1	1	4	4	1	1	2	2	2	2
1	1	4	4	1	1	2	2	2	2
1	1	4	4	1	1	2	2	4	4
1	1	4	4	1	1	2	2	2	2
1	1	4	4	1	1	2	2	2	2
4	4	2	2	1	1	2	2	4	4
4	4	2	2	1	1	2	2	4	4
4	4	4	4	1	1	2	2	2	2
1	1	4	4	1	1	2	2	2	2
1	1	4	4	1	1	2	2	2	2
4	4	4	4	1	1	2	2	2	2
1	1	4	4	1	1	2	2	2	2
1	1	4	4	1	1	2	2	4	4
4	4	4	4	1	1	2	2	4	4
4	4	4	4	1	1	2	2	2	2
4	4	4	4	1	1	2	2	4	4
4	4	4	4	1	1	2	2	2	2
4	4	4	4	1	1	2	2	2	2
4	4	4	4	1	1	2	2	2	2
4	4	4	4	1	1	2	2	2	2
1	1	4	4	1	1	2	2	2	2
4	4	2	2	1	1	2	2	4	4
1	1	4	4	1	1	2	2	2	2
4	4	4	4	1	1	2	2	2	2
4	4	2	2	1	1	2	2	4	4
4	4	4	4	1	1	2	2	2	2
4	4	4	4	1	1	2	2	2	2
1	1	4	4	1	1	2	2	2	2
1	1	4	4	1	1	2	2	2	2
1	1	4	4	1	1	2	2	2	2
1	1	4	4	1	1	2	2	2	2
4	4	4	4	1	1	2	2	2	2
1	1	4	4	1	1	2	2	2	2
1	1	2	2	1	1	2	2	4	4
1	1	4	4	1	1	2	2	2	2
4	4	4	4	1	1	2	2	2	2
1	1	4	4	1	1	2	2	2	2
4	4	2	2	1	1	2	2	4	4
4	4	4	4	1	1	2	2	4	4
4	4	4	4	1	1	2	2	2	2
4	4	4	4	1	1	2	2	2	2
4	4	4	4	1	1	2	2	2	2
4	4	4	4	1	1	2	2	2	2
4	4	2	2	1	1	2	2	4	4
1	1	4	4	2	2	3	3	2	2
1	1	2	2	1	1	2	2	4	4

For Review Only

4	4	4	4	1	1	2	2	2	2
4	4	4	4	1	1	2	2	2	2
4	4	4	4	1	1	2	2	2	2
4	4	4	4	1	1	2	2	2	2
1	1	4	4	1	1	2	2	2	2
1	1	4	4	1	1	2	2	2	2
1	1	4	4	1	1	2	2	2	2
4	4	4	4	1	1	2	2	2	2
4	4	4	4	1	1	2	2	2	2
1	1	4	4	1	1	2	2	4	4
1	1	4	4	1	1	2	2	2	2
4	4	2	2	1	1	2	2	4	4
4	4	2	2	1	1	2	2	4	4
1	1	4	4	1	1	2	2	2	2
4	4	4	4	2	2	3	3	2	2
4	4	2	2	1	1	2	2	4	4
1	1	4	4	1	1	2	2	4	4
1	1	4	4	1	1	2	2	2	2
1	1	4	4	1	1	2	2	2	2
4	4	4	4	1	1	2	2	4	4
1	1	4	4	1	1	2	2	2	2
1	1	4	4	1	1	2	2	2	2
1	1	4	4	1	1	2	2	2	2
4	4	4	4	1	1	2	2	4	4
4	4	4	4	1	1	2	2	2	2
1	1	4	4	1	1	2	2	2	2
1	1	2	2	1	1	2	2	4	4
1	1	4	4	1	1	2	2	2	2
1	1	4	4	1	1	2	2	2	2
4	4	4	4	1	1	2	2	2	2
1	1	4	4	1	1	2	2	4	4
4	4	4	4	1	1	2	2	2	2
1	1	2	2	1	1	2	2	4	4
1	1	4	4	1	1	2	2	2	2
4	4	4	4	1	1	2	2	4	4
1	1	4	4	1	1	2	2	4	4
4	4	4	4	1	1	2	2	2	2
4	4	4	4	1	1	2	2	2	2
4	4	4	4	1	1	2	2	2	2
1	1	4	4	1	1	2	2	4	4
4	4	4	4	1	1	2	2	2	2
4	4	4	4	1	1	2	2	2	2
4	4	4	4	1	1	2	2	4	4
1	1	4	4	1	1	2	2	2	2
1	1	4	4	1	1	2	2	2	2
4	4	4	4	1	1	2	2	4	4
1	1	4	4	1	1	2	2	2	2
4	4	4	4	2	2	2	2	2	2
1	1	4	4	1	1	2	2	2	2
1	1	2	2	1	1	2	2	4	4
4	4	4	4	1	1	2	2	2	2
1	1	2	2	1	1	2	2	4	4
4	4	4	4	1	1	2	2	2	2
1	1	4	4	1	1	2	2	2	2
4	4	4	4	1	1	2	2	4	4

For Review Only

4	4	4	4	1	1	2	2	2	2
1	1	4	4	2	2	3	3	2	2

For Review Only

SNP115	SNP115	SNP1150	SNP1150	SNP1151	SNP1151	SNP1152	SNP1152	SNP1153	SNP1153
2	2	4	4	4	4	2	2	2	2
2	2	2	2	3	3	3	3	2	2
3	3	4	4	4	4	2	2	2	2
3	3	4	4	4	4	2	2	2	2
3	3	2	2	4	4	2	2	2	2
3	3	4	4	3	3	2	2	2	2
3	3	4	4	4	4	2	2	2	2
2	2	4	4	3	3	3	3	2	2
3	3	4	4	4	4	2	2	2	2
2	2	4	4	3	3	2	2	3	3
2	2	4	4	4	4	3	3	2	2
3	3	4	4	4	4	2	2	2	2
2	2	2	2	4	4	2	2	2	2
3	3	4	4	4	4	3	3	2	2
2	2	4	4	4	4	2	2	2	2
2	2	4	4	4	4	2	2	2	2
3	3	4	4	4	4	3	3	2	2
2	2	4	4	4	4	2	2	2	2
2	2	4	4	4	4	2	2	2	2
2	2	2	2	4	4	3	3	3	3
2	2	2	2	3	3	3	3	2	2
3	3	4	4	4	4	2	2	2	2
3	3	2	2	3	3	3	3	2	2
2	2	4	4	3	3	2	2	2	2
2	2	4	4	3	3	2	2	2	2
3	3	4	4	3	3	3	3	2	2
3	3	4	4	4	4	2	2	2	2
2	2	2	2	4	4	2	2	2	2
3	3	4	4	3	3	2	2	2	2
2	2	4	4	3	3	3	3	3	3
2	2	4	4	3	3	2	2	2	2
2	2	4	4	4	4	3	3	2	2
3	3	4	4	4	4	2	2	2	2
3	3	4	4	3	3	2	2	2	2
2	2	2	2	4	4	2	2	2	2
2	2	4	4	3	3	2	2	2	2
3	3	2	2	4	4	2	2	2	2
3	3	2	2	4	4	2	2	2	2
2	2	2	2	3	3	3	3	2	2
2	2	2	2	4	4	3	3	2	2
2	2	2	2	3	3	3	3	3	3
2	2	2	2	3	3	3	3	2	2
2	2	2	2	3	3	2	2	3	3
2	2	4	4	4	4	3	3	2	2
2	2	2	2	3	3	3	3	3	3
3	3	4	4	4	4	2	2	2	2
2	2	4	4	4	4	3	3	2	2
3	3	2	2	3	3	3	3	2	2
3	3	2	2	4	4	2	2	2	2
2	2	4	4	4	4	3	3	2	2
2	2	4	4	3	3	2	2	2	2
2	2	2	2	3	3	2	2	2	2

For Review Only

3	3	4	4	4	4	2	2	2	2
3	3	4	4	4	4	2	2	2	2
2	2	4	4	4	4	2	2	2	2
3	3	4	4	4	4	2	2	2	2
2	2	4	4	4	4	2	2	2	2
2	2	4	4	3	3	3	3	2	2
3	3	4	4	4	4	3	3	2	2
2	2	4	4	3	3	3	3	2	2
2	2	4	4	3	3	3	3	2	2
2	2	2	2	4	4	2	2	2	2
2	2	4	4	4	4	3	3	2	2
2	2	4	4	4	4	3	3	2	2
3	3	4	4	4	4	2	2	2	2
3	3	4	4	3	3	2	2	2	2
2	2	2	2	3	3	2	2	2	2
2	2	2	2	4	4	3	3	3	3
2	2	2	2	3	3	3	3	2	2
2	2	4	4	4	4	2	2	2	2
3	3	4	4	4	4	2	2	2	2
3	3	4	4	4	4	2	2	2	2
2	2	4	4	4	4	2	2	2	2
2	2	2	2	4	4	3	3	2	2
3	3	4	4	3	3	2	2	2	2
2	2	4	4	3	3	3	3	2	2
2	2	2	2	3	3	3	3	3	3
2	2	4	4	3	3	2	2	2	2
2	2	4	4	4	4	2	2	2	2
2	2	2	2	3	3	3	3	2	2
3	3	4	4	3	3	3	3	2	2
2	2	4	4	3	3	2	2	2	2
3	3	2	2	4	4	2	2	2	2
2	2	4	4	3	3	2	2	2	2
2	2	2	2	3	3	2	2	2	2
3	3	4	4	4	4	2	2	2	2
2	2	4	4	4	4	3	3	2	2
2	2	2	2	3	3	3	3	3	3
2	2	4	4	3	3	3	3	2	2
3	3	2	2	4	4	2	2	2	2
3	3	2	2	3	3	3	3	2	2
2	2	4	4	3	3	3	3	2	2
3	3	4	4	4	4	2	2	2	2
2	2	4	4	3	3	2	2	2	2
2	2	4	4	3	3	3	3	2	2
2	2	4	4	4	4	3	3	2	2
3	3	4	4	3	3	2	2	2	2
2	2	4	4	4	4	3	3	2	2
3	3	4	4	3	3	3	3	2	2
3	3	4	4	4	4	3	3	2	2
3	3	4	4	4	4	3	3	2	2
2	2	2	2	4	4	2	2	2	2
2	2	4	4	3	3	2	2	2	2
2	2	2	2	3	3	3	3	2	2
2	2	4	4	3	3	3	3	2	2
2	2	4	4	4	4	2	2	2	2
2	2	2	2	4	4	2	2	2	2

For Review Only

3	3	2	2	4	4	3	3	2	2
3	3	4	4	4	4	2	2	2	2
3	3	4	4	3	3	3	3	2	2
3	3	4	4	3	3	2	2	2	2
3	3	4	4	4	4	3	3	2	2
2	2	4	4	3	3	2	2	2	2
3	3	4	4	4	4	2	2	2	2
2	2	4	4	3	3	2	2	2	2
3	3	4	4	3	3	3	3	2	2
2	2	4	4	3	3	2	2	2	2
2	2	4	4	4	4	3	3	2	2
3	3	2	2	4	4	3	3	2	2
2	2	4	4	3	3	2	2	3	3
2	2	4	4	3	3	2	2	2	2
2	2	2	2	3	3	2	2	2	2
2	2	4	4	4	4	2	2	2	2
2	2	4	4	4	4	3	3	2	2
2	2	2	2	4	4	2	2	2	2
3	3	4	4	3	3	3	3	2	2
2	2	4	4	4	4	2	2	2	2
2	2	2	2	4	4	2	2	2	2
3	3	4	4	3	3	2	2	2	2
2	2	4	4	3	3	2	2	2	2
3	3	2	2	3	3	3	3	2	2
2	2	2	2	4	4	3	3	2	2
3	3	4	4	4	4	3	3	2	2
2	2	4	4	4	4	2	2	2	2
3	3	4	4	3	3	2	2	2	2
2	2	4	4	4	4	2	2	2	2
2	2	4	4	3	3	2	2	2	2
2	2	2	2	3	3	2	2	2	2
2	2	2	2	4	4	2	2	2	2
3	3	4	4	4	4	2	2	2	2
3	3	4	4	3	3	2	2	2	2
3	3	4	4	3	3	2	2	2	2
2	2	2	2	3	3	2	2	2	2
3	3	4	4	3	3	3	3	2	2
2	2	4	4	4	4	2	2	2	2
2	2	2	2	4	4	3	3	2	2
2	2	2	2	4	4	3	3	2	2
3	3	2	2	4	4	3	3	2	2
3	3	4	4	3	3	3	3	2	2
2	2	4	4	4	4	2	2	2	2
2	2	2	2	3	3	2	2	2	2
3	3	2	2	3	3	2	2	2	2
3	3	4	4	3	3	2	2	2	2
3	3	4	4	3	3	3	3	2	2
2	2	4	4	4	4	2	2	2	2
2	2	2	2	3	3	2	2	2	2
3	3	4	4	3	3	3	3	2	2
3	3	4	4	3	3	3	3	2	2
2	2	4	4	4	4	3	3	2	2
2	2	4	4	4	4	2	2	2	2
2	2	4	4	4	4	3	3	2	2
2	2	2	2	4	4	2	2	2	2

For Review Only

2	2	4	4	4	4	2	2	2	2
2	2	2	2	3	3	3	3	2	2
3	3	2	2	4	4	2	2	2	2
2	2	2	2	4	4	3	3	2	2
2	2	4	4	4	4	3	3	2	2
2	2	2	2	4	4	2	2	2	2
2	2	2	2	4	4	3	3	2	2
2	2	4	4	4	4	2	2	2	2
2	2	4	4	3	3	3	3	2	2
2	2	4	4	4	4	2	2	2	2
3	3	4	4	4	4	3	3	2	2
2	2	2	2	4	4	3	3	2	2
2	2	4	4	4	4	3	3	2	2
3	3	2	2	3	3	2	2	2	2
3	3	2	2	3	3	3	3	2	2
2	2	2	2	4	4	3	3	2	2
2	2	4	4	4	4	3	3	2	2
2	2	4	4	3	3	3	3	2	2
3	3	4	4	3	3	3	3	2	2
3	3	4	4	4	4	2	2	2	2
3	3	2	2	4	4	2	2	2	2
2	2	2	2	3	3	3	3	2	2
2	2	2	2	4	4	3	3	2	2
2	2	4	4	4	4	3	3	2	2
3	3	2	2	3	3	3	3	2	2
3	3	4	4	3	3	2	2	2	2
3	3	4	4	4	4	2	2	2	2
2	2	4	4	4	4	3	3	2	2
3	3	4	4	4	4	3	3	2	2
2	2	4	4	4	4	2	2	2	2
2	2	2	2	3	3	3	3	2	2
2	2	4	4	4	4	2	2	2	2
3	3	4	4	4	4	2	2	2	2
3	3	2	2	3	3	3	3	2	2
3	3	4	4	4	4	2	2	2	2
2	2	4	4	4	4	3	3	2	2
3	3	4	4	3	3	3	3	2	2
3	3	4	4	4	4	2	2	2	2
2	2	4	4	4	4	2	2	2	2
2	2	4	4	3	3	2	2	2	2
3	3	4	4	4	4	2	2	2	2
3	3	4	4	3	3	3	3	2	2
2	2	2	2	3	3	3	3	2	2
2	2	4	4	4	4	2	2	2	2
2	2	4	4	4	4	2	2	2	2
2	2	4	4	3	3	2	2	2	2
2	2	2	2	4	4	3	3	2	2
2	2	2	2	3	3	3	3	2	2
3	3	4	4	3	3	3	3	2	2
3	3	4	4	4	4	3	3	2	2
3	3	4	4	4	4	2	2	2	2
2	2	4	4	3	3	2	2	2	2
2	2	2	2	3	3	3	3	2	2
2	2	4	4	4	4	3	3	2	2
2	2	2	2	3	3	2	2	2	2

For Review Only

2	2	4	4	4	4	3	3	2	2
2	2	4	4	4	4	2	2	3	3
3	3	4	4	4	4	2	2	2	2
2	2	2	2	4	4	3	3	2	2
2	2	4	4	4	4	3	3	2	2
3	3	4	4	3	3	2	2	2	2
2	2	4	4	4	4	3	3	2	2
2	2	4	4	3	3	3	3	3	3
3	3	4	4	3	3	2	2	2	2
2	2	2	2	3	3	3	3	2	2
2	2	4	4	4	4	2	2	2	2
2	2	2	2	3	3	2	2	2	2
3	3	2	2	4	4	3	3	2	2
2	2	4	4	3	3	2	2	2	2
2	2	4	4	3	3	2	2	2	2
3	3	2	2	4	4	2	2	2	2
2	2	2	2	3	3	2	2	2	2
2	2	4	4	3	3	2	2	2	2
2	2	4	4	4	4	3	3	2	2
3	3	2	2	4	4	2	2	2	2
2	2	4	4	3	3	2	2	2	2
3	3	4	4	3	3	3	3	2	2
3	3	4	4	3	3	2	2	2	2
2	2	2	2	4	4	3	3	2	2
2	2	4	4	3	3	3	3	2	2
2	2	4	4	3	3	3	3	2	2
2	2	2	2	3	3	3	3	2	2
3	3	4	4	4	4	2	2	2	2
2	2	4	4	4	4	2	2	2	2
2	2	4	4	4	4	2	2	2	2
2	2	2	2	3	3	3	3	2	2
2	2	4	4	3	3	2	2	2	2
3	3	2	2	3	3	3	3	2	2
2	2	4	4	3	3	2	2	2	2
2	2	2	2	4	4	3	3	2	2
2	2	2	2	3	3	2	2	2	2
2	2	4	4	4	4	2	2	2	2
2	2	4	4	4	4	3	3	2	2
2	2	4	4	3	3	2	2	2	2
2	2	2	2	3	3	3	3	2	2
3	3	4	4	4	4	2	2	2	2
3	3	4	4	4	4	3	3	2	2
2	2	2	2	4	4	3	3	2	2
3	3	4	4	4	4	2	2	2	2
2	2	4	4	4	4	2	2	2	2
3	3	2	2	3	3	3	3	2	2
2	2	4	4	4	4	3	3	2	2
3	3	4	4	3	3	2	2	2	2
2	2	4	4	3	3	2	2	2	2
2	2	2	2	3	3	2	2	2	2
2	2	4	4	4	4	2	2	2	2
2	2	2	2	3	3	3	3	2	2
3	3	4	4	4	4	2	2	2	2
3	3	4	4	3	3	2	2	2	2
2	2	2	2	4	4	2	2	2	2

For Review Only

3	3	4	4	4	4	2	2	2	2
2	2	4	4	3	3	2	2	2	2

For Review Only

SNP1154	SNP1154	SNP1155	SNP1155	SNP1156	SNP1156	SNP1157	SNP1157	SNP1158	SNP1158
4	4	4	4	3	3	2	2	3	3
2	2	4	4	2	2	3	3	1	1
2	2	4	4	3	3	2	2	3	3
4	4	4	4	3	3	2	2	3	3
4	4	4	4	3	3	3	3	1	1
2	2	4	4	2	2	2	2	3	3
4	4	4	4	3	3	2	2	3	3
4	4	4	4	3	3	2	2	3	3
4	4	4	4	3	3	2	2	3	3
4	4	4	4	3	3	2	2	3	3
2	2	4	4	3	3	2	2	3	3
2	2	4	4	3	3	2	2	3	3
4	4	4	4	3	3	2	2	3	3
2	2	1	1	3	3	3	3	1	1
4	4	4	4	3	3	2	2	3	3
2	2	1	1	3	3	2	2	3	3
4	4	4	4	3	3	2	2	3	3
2	2	4	4	3	3	2	2	3	3
4	4	4	4	3	3	2	2	3	3
2	2	4	4	3	3	2	2	3	3
4	4	4	4	3	3	2	2	3	3
4	4	4	4	3	3	2	2	3	3
2	2	4	4	3	3	2	2	3	3
2	2	4	4	3	3	3	3	3	3
4	4	4	4	3	3	2	2	3	3
2	2	1	1	3	3	2	2	3	3
2	2	4	4	2	2	2	2	3	3
4	4	4	4	3	3	2	2	3	3
4	4	4	4	3	3	2	2	3	3
2	2	4	4	3	3	2	2	3	3
4	4	4	4	3	3	2	2	3	3
4	4	4	4	3	3	2	2	3	3
4	4	4	4	3	3	2	2	3	3
2	2	4	4	3	3	2	2	3	3
2	2	4	4	3	3	3	3	1	1
2	2	4	4	3	3	2	2	3	3
2	2	4	4	3	3	3	3	1	1
2	2	4	4	3	3	2	2	3	3
2	2	4	4	3	3	2	2	3	3
2	2	4	4	3	3	2	2	3	3
2	2	4	4	3	3	2	2	3	3
2	2	4	4	3	3	2	2	3	3
4	4	4	4	3	3	2	2	3	3
2	2	4	4	3	3	2	2	3	3
4	4	4	4	3	3	2	2	3	3
2	2	4	4	3	3	2	2	3	3
2	2	1	1	3	3	2	2	3	3
2	2	1	1	3	3	2	2	3	3
4	4	4	4	3	3	2	2	3	3
4	4	4	4	3	3	2	2	3	3
2	2	1	1	3	3	2	2	3	3
4	4	4	4	3	3	2	2	3	3

For Review Only

4	4	4	4	3	3	2	2	3	3
4	4	4	4	3	3	2	2	3	3
2	2	4	4	2	2	2	2	3	3
4	4	4	4	3	3	2	2	3	3
4	4	4	4	3	3	2	2	3	3
2	2	4	4	3	3	2	2	3	3
2	2	1	1	3	3	2	2	3	3
2	2	4	4	3	3	2	2	3	3
2	2	4	4	3	3	2	2	3	3
2	2	4	4	3	3	2	2	3	3
2	2	4	4	2	2	2	2	3	3
2	2	4	4	3	3	2	2	3	3
4	4	4	4	3	3	2	2	3	3
4	4	4	4	3	3	2	2	3	3
4	4	4	4	3	3	2	2	3	3
2	2	4	4	3	3	3	3	3	3
2	2	1	1	3	3	2	2	3	3
4	4	1	1	3	3	2	2	3	3
4	4	4	4	3	3	2	2	3	3
4	4	4	4	3	3	2	2	3	3
2	2	4	4	3	3	2	2	3	3
2	2	4	4	3	3	2	2	3	3
4	4	4	4	3	3	2	2	3	3
2	2	1	1	3	3	2	2	3	3
4	4	4	4	3	3	2	2	3	3
2	2	4	4	2	2	2	2	3	3
4	4	4	4	3	3	2	2	3	3
2	2	4	4	3	3	2	2	3	3
2	2	1	1	3	3	2	2	3	3
4	4	4	4	3	3	2	2	3	3
2	2	4	4	3	3	3	3	1	1
2	2	4	4	3	3	2	2	3	3
2	2	4	4	3	3	2	2	3	3
4	4	4	4	3	3	2	2	3	3
2	2	1	1	3	3	2	2	1	1
4	4	4	4	3	3	2	2	3	3
2	2	4	4	3	3	2	2	3	3
4	4	4	4	3	3	2	2	3	3
4	4	4	4	3	3	2	2	3	3
4	4	4	4	3	3	2	2	3	3
4	4	4	4	3	3	2	2	3	3
2	2	4	4	3	3	2	2	3	3
4	4	1	1	3	3	2	2	3	3
2	2	4	4	2	2	2	2	3	3
2	2	4	4	3	3	2	2	3	3
2	2	4	4	3	3	2	2	3	3
2	2	1	1	3	3	2	2	3	3
2	2	4	4	3	3	2	2	3	3
2	2	4	4	3	3	2	2	3	3
4	4	4	4	3	3	3	3	3	3
2	2	4	4	3	3	2	2	3	3
2	2	4	4	3	3	2	2	3	3
2	2	1	1	3	3	2	2	3	3
4	4	4	4	3	3	2	2	3	3
4	4	4	4	3	3	3	3	1	1

For Review Only

2	2	1	1	3	3	2	2	3	3
2	2	4	4	3	3	2	2	3	3
2	2	4	4	3	3	2	2	3	3
2	2	1	1	3	3	3	3	1	1
2	2	4	4	3	3	2	2	3	3
2	2	4	4	3	3	2	2	3	3
2	2	1	1	3	3	2	2	3	3
2	2	4	4	3	3	2	2	3	3
2	2	4	4	3	3	2	2	3	3
4	4	4	4	3	3	2	2	3	3
2	2	1	1	3	3	2	2	3	3
4	4	4	4	3	3	2	2	3	3
4	4	4	4	3	3	2	2	3	3
4	4	4	4	3	3	2	2	3	3
4	4	4	4	3	3	3	3	1	1
2	2	4	4	3	3	2	2	3	3
4	4	4	4	3	3	2	2	3	3
4	4	4	4	3	3	2	2	3	3
2	2	1	1	3	3	3	3	1	1
4	4	4	4	3	3	2	2	3	3
4	4	4	4	3	3	2	2	3	3
4	4	4	4	3	3	2	2	3	3
2	2	4	4	3	3	2	2	3	3
2	2	4	4	3	3	2	2	3	3
2	2	4	4	3	3	2	2	3	3
2	2	4	4	3	3	2	2	3	3
4	4	4	4	3	3	2	2	3	3
4	4	4	4	3	3	2	2	3	3
2	2	4	4	3	3	2	2	3	3
4	4	4	4	3	3	2	2	3	3
2	2	4	4	3	3	3	3	3	3
4	4	4	4	3	3	2	2	3	3
4	4	4	4	3	3	2	2	3	3
2	2	4	4	3	3	2	2	3	3
2	2	4	4	3	3	2	2	3	3
4	4	4	4	3	3	2	2	3	3
2	2	1	1	3	3	3	3	1	1
2	2	4	4	2	2	2	2	3	3
2	2	1	1	3	3	2	2	3	3
2	2	4	4	3	3	2	2	3	3
2	2	4	4	3	3	2	2	3	3
2	2	4	4	3	3	3	3	1	1
2	2	1	1	3	3	2	2	3	3
4	4	4	4	3	3	2	2	3	3
2	2	4	4	3	3	2	2	3	3
4	4	4	4	3	3	2	2	3	3
2	2	1	1	3	3	2	2	3	3
2	2	1	1	3	3	2	2	3	3
4	4	4	4	3	3	2	2	1	1
2	2	4	4	3	3	2	2	3	3
2	2	1	1	3	3	2	2	3	3
2	2	4	4	3	3	2	2	3	3
2	2	4	4	3	3	2	2	3	3
2	2	4	4	3	3	2	2	3	3
2	2	4	4	3	3	2	2	3	3

For Review Only

4	4	1	1	3	3	2	2	3	3
2	2	1	1	3	3	2	2	3	3
2	2	4	4	3	3	2	2	3	3
2	2	4	4	3	3	3	3	3	3
4	4	4	4	3	3	2	2	1	1
2	2	1	1	3	3	2	2	3	3
2	2	4	4	3	3	2	2	3	3
4	4	4	4	3	3	2	2	3	3
2	2	4	4	3	3	2	2	3	3
4	4	4	4	3	3	2	2	3	3
2	2	4	4	3	3	2	2	3	3
2	2	4	4	3	3	2	2	3	3
2	2	4	4	3	3	2	2	3	3
4	4	4	4	3	3	3	3	3	3
2	2	4	4	3	3	3	3	1	1
2	2	4	4	3	3	2	2	3	3
2	2	4	4	3	3	3	3	3	3
4	4	4	4	3	3	2	2	3	3
2	2	4	4	3	3	2	2	3	3
2	2	1	1	3	3	2	2	3	3
4	4	4	4	3	3	2	2	3	3
2	2	4	4	3	3	2	2	3	3
2	2	4	4	3	3	2	2	3	3
2	2	4	4	3	3	2	2	3	3
2	2	4	4	3	3	2	2	3	3
2	2	4	4	3	3	2	2	3	3
2	2	4	4	3	3	2	2	3	3
4	4	4	4	3	3	2	2	3	3
2	2	4	4	3	3	2	2	3	3
4	4	4	4	3	3	2	2	3	3
4	4	4	4	3	3	3	3	1	1
4	4	1	1	3	3	2	2	3	3
4	4	4	4	3	3	2	2	3	3
2	2	4	4	3	3	2	2	3	3
4	4	4	4	3	3	2	2	3	3
2	2	4	4	3	3	2	2	3	3
2	2	4	4	3	3	2	2	3	3
4	4	1	1	3	3	2	2	3	3
2	2	1	1	3	3	2	2	3	3
2	2	4	4	3	3	2	2	3	3
2	2	4	4	3	3	2	2	3	3
2	2	1	1	3	3	2	2	3	3
2	2	4	4	3	3	2	2	3	3
2	2	4	4	3	3	2	2	3	3
2	2	4	4	3	3	2	2	3	3
4	4	4	4	3	3	2	2	3	3
2	2	4	4	3	3	2	2	3	3
2	2	4	4	3	3	2	2	3	3
2	2	4	4	3	3	3	3	1	1
4	4	4	4	3	3	2	2	3	3
4	4	4	4	3	3	2	2	3	3
2	2	4	4	3	3	2	2	3	3
2	2	4	4	3	3	2	2	3	3
2	2	4	4	2	2	2	2	3	3
2	2	4	4	3	3	2	2	3	3

For Review Only

2	2	4	4	3	3	3	3	1	1
2	2	4	4	3	3	2	2	3	3
4	4	4	4	3	3	2	2	3	3
4	4	4	4	3	3	2	2	3	3
4	4	4	4	3	3	2	2	3	3
4	4	1	1	3	3	2	2	3	3
2	2	4	4	3	3	2	2	3	3
2	2	4	4	3	3	2	2	3	3
2	2	4	4	3	3	3	3	3	3
2	2	4	4	3	3	2	2	3	3
4	4	4	4	3	3	2	2	3	3
2	2	4	4	3	3	3	3	1	1
4	4	4	4	3	3	2	2	3	3
2	2	4	4	3	3	2	2	3	3
2	2	4	4	2	2	2	2	3	3
2	2	4	4	3	3	2	2	3	3
2	2	4	4	3	3	2	2	3	3
4	4	1	1	3	3	2	2	3	3
2	2	4	4	3	3	3	3	1	1
2	2	4	4	3	3	2	2	3	3
4	4	4	4	3	3	2	2	3	3
2	2	1	1	3	3	2	2	3	3
4	4	1	1	3	3	2	2	3	3
2	2	4	4	3	3	2	2	3	3
2	2	4	4	3	3	2	2	3	3
4	4	4	4	3	3	2	2	3	3
2	2	4	4	3	3	2	2	3	3
4	4	4	4	3	3	2	2	3	3
4	4	4	4	3	3	2	2	3	3
4	4	4	4	3	3	2	2	3	3
2	2	4	4	3	3	2	2	3	3
2	2	1	1	3	3	2	2	3	3
2	2	4	4	3	3	3	3	1	1
2	2	1	1	3	3	2	2	3	3
2	2	4	4	3	3	2	2	3	3
2	2	4	4	3	3	2	2	3	3
2	2	1	1	3	3	2	2	3	3
2	2	4	4	3	3	2	2	3	3
2	2	4	4	3	3	2	2	3	3
2	2	4	4	3	3	2	2	3	3
4	4	4	4	3	3	2	2	3	3
2	2	4	4	3	3	2	2	3	3
2	2	4	4	3	3	2	2	3	3
4	4	4	4	3	3	2	2	3	3
2	2	4	4	3	3	2	2	3	3
4	4	1	1	3	3	2	2	3	3
2	2	4	4	3	3	2	2	3	3
2	2	4	4	2	2	2	2	3	3
2	2	4	4	3	3	2	2	3	3
2	2	4	4	3	3	2	2	3	3
4	4	4	4	3	3	2	2	3	3
2	2	4	4	3	3	2	2	3	3
2	2	4	4	3	3	2	2	3	3
2	2	4	4	3	3	2	2	3	3
2	2	4	4	3	3	2	2	3	3

For Review Only

4	4	4	4	3	3	2	2	3	3
2	2	4	4	2	2	2	2	3	3

For Review Only

SNP1159	SNP1159	SNP116	SNP116	SNP1160	SNP1160	SNP1161	SNP1161	SNP1162	SNP1162
4	4	3	3	4	4	3	3	3	3
4	4	2	2	4	4	3	3	3	3
4	4	3	3	4	4	3	3	4	4
4	4	3	3	4	4	3	3	4	4
4	4	3	3	4	4	3	3	3	3
4	4	3	3	4	4	3	3	3	3
4	4	3	3	4	4	3	3	3	3
4	4	2	2	4	4	3	3	3	3
4	4	3	3	4	4	3	3	3	3
1	1	2	2	4	4	3	3	3	3
4	4	2	2	4	4	2	2	3	3
4	4	3	3	4	4	3	3	4	4
4	4	3	3	4	4	3	3	3	3
4	4	3	3	4	4	3	3	3	3
4	4	2	2	4	4	3	3	3	3
4	4	2	2	4	4	3	3	4	4
4	4	3	3	4	4	2	2	3	3
4	4	2	2	4	4	3	3	3	3
4	4	2	2	4	4	3	3	3	3
4	4	2	2	4	4	2	2	4	4
4	4	3	3	4	4	2	2	3	3
4	4	3	3	4	4	3	3	3	3
4	4	3	3	4	4	2	2	3	3
4	4	2	2	4	4	3	3	3	3
4	4	2	2	4	4	3	3	3	3
4	4	3	3	4	4	3	3	4	4
4	4	3	3	4	4	3	3	3	3
4	4	2	2	4	4	3	3	3	3
4	4	3	3	4	4	3	3	3	3
4	4	2	2	4	4	3	3	3	3
4	4	2	2	4	4	3	3	3	3
4	4	2	2	4	4	3	3	3	3
4	4	3	3	4	4	3	3	3	3
4	4	3	3	4	4	3	3	3	3
4	4	2	2	4	4	2	2	4	4
4	4	2	2	4	4	3	3	3	3
4	4	3	3	4	4	3	3	3	3
4	4	3	3	4	4	3	3	4	4
4	4	2	2	4	4	2	2	3	3
4	4	2	2	4	4	2	2	4	4
4	4	2	2	4	4	3	3	3	3
4	4	2	2	4	4	2	2	3	3
4	4	2	2	4	4	3	3	3	3
1	1	2	2	4	4	3	3	3	3
4	4	2	2	1	1	3	3	3	3
4	4	3	3	4	4	3	3	4	4
4	4	2	2	4	4	3	3	4	4
4	4	3	3	4	4	2	2	4	4
4	4	3	3	4	4	3	3	3	3
4	4	2	2	4	4	2	2	3	3
4	4	2	2	4	4	3	3	3	3
4	4	2	2	4	4	3	3	4	4

For Review Only

4	4	3	3	4	4	3	3	3	3
4	4	3	3	4	4	3	3	3	3
4	4	2	2	4	4	3	3	4	4
1	1	3	3	4	4	3	3	3	3
4	4	2	2	4	4	3	3	3	3
4	4	2	2	4	4	3	3	4	4
4	4	3	3	4	4	3	3	4	4
4	4	2	2	4	4	3	3	3	3
4	4	2	2	4	4	3	3	3	3
4	4	2	2	4	4	3	3	3	3
4	4	2	2	4	4	3	3	3	3
4	4	2	2	4	4	3	3	3	3
4	4	3	3	4	4	3	3	3	3
4	4	3	3	4	4	3	3	3	3
4	4	2	2	4	4	2	2	3	3
4	4	2	2	4	4	2	2	3	3
4	4	2	2	4	4	2	2	4	4
4	4	2	2	4	4	3	3	4	4
4	4	3	3	4	4	3	3	3	3
4	4	3	3	4	4	3	3	3	3
4	4	2	2	4	4	3	3	3	3
4	4	2	2	4	4	2	2	4	4
1	1	3	3	4	4	3	3	3	3
4	4	2	2	4	4	2	2	3	3
4	4	2	2	4	4	3	3	3	3
4	4	2	2	4	4	3	3	3	3
4	4	2	2	4	4	3	3	3	3
4	4	2	2	4	4	2	2	3	3
4	4	3	3	4	4	3	3	4	4
4	4	2	2	4	4	3	3	3	3
4	4	3	3	4	4	3	3	3	3
4	4	2	2	4	4	3	3	3	3
4	4	2	2	4	4	2	2	3	3
4	4	3	3	4	4	3	3	3	3
4	4	2	2	4	4	3	3	3	3
4	4	2	2	4	4	3	3	3	3
4	4	2	2	4	4	2	2	3	3
1	1	3	3	4	4	3	3	3	3
4	4	3	3	4	4	2	2	3	3
4	4	2	2	4	4	2	2	3	3
4	4	3	3	4	4	3	3	3	3
4	4	2	2	4	4	3	3	3	3
4	4	2	2	4	4	3	3	3	3
4	4	2	2	4	4	3	3	3	3
4	4	3	3	4	4	3	3	3	3
4	4	2	2	4	4	3	3	4	4
4	4	3	3	4	4	2	2	3	3
4	4	3	3	4	4	3	3	4	4
4	4	3	3	4	4	3	3	4	4
4	4	2	2	4	4	3	3	3	3
4	4	2	2	4	4	3	3	3	3
4	4	2	2	4	4	2	2	3	3
1	1	2	2	4	4	3	3	3	3
4	4	2	2	4	4	2	2	3	3
4	4	2	2	4	4	3	3	3	3

For Review Only

4	4	3	3	4	4	2	2	3	3
4	4	3	3	4	4	2	2	4	4
4	4	3	3	4	4	3	3	3	3
4	4	3	3	4	4	3	3	3	3
4	4	2	2	4	4	3	3	3	3
4	4	2	2	4	4	3	3	4	4
4	4	3	3	4	4	3	3	4	4
4	4	2	2	4	4	3	3	3	3
4	4	3	3	4	4	3	3	3	3
4	4	2	2	4	4	3	3	3	3
4	4	2	2	4	4	3	3	3	3
4	4	2	2	4	4	3	3	4	4
4	4	2	2	1	1	3	3	3	3
4	4	2	2	4	4	3	3	3	3
4	4	3	3	4	4	3	3	3	3
4	4	2	2	4	4	2	2	4	4
1	1	2	2	4	4	3	3	4	4
4	4	2	2	4	4	3	3	3	3
4	4	3	3	4	4	2	2	3	3
4	4	3	3	4	4	3	3	3	3
4	4	2	2	4	4	2	2	3	3
4	4	3	3	4	4	3	3	3	3
4	4	2	2	4	4	3	3	3	3
4	4	3	3	4	4	2	2	3	3
4	4	2	2	4	4	2	2	4	4
4	4	3	3	4	4	2	2	3	3
4	4	2	2	4	4	3	3	3	3
4	4	3	3	4	4	3	3	3	3
4	4	2	2	4	4	3	3	4	4
4	4	2	2	4	4	3	3	3	3
4	4	2	2	4	4	2	2	3	3
4	4	2	2	4	4	2	2	3	3
4	4	3	3	4	4	3	3	3	3
4	4	2	2	4	4	3	3	3	3
4	4	3	3	4	4	3	3	3	3
4	4	2	2	4	4	3	3	3	3
4	4	3	3	4	4	2	2	3	3
4	4	2	2	4	4	3	3	3	3
4	4	2	2	4	4	2	2	3	3
4	4	2	2	4	4	2	2	3	3
4	4	3	3	4	4	2	2	3	3
4	4	3	3	4	4	3	3	3	3
4	4	2	2	4	4	3	3	3	3
4	4	2	2	4	4	3	3	3	3
4	4	3	3	4	4	2	2	3	3
4	4	3	3	4	4	3	3	3	3
4	4	3	3	4	4	3	3	4	4
4	4	2	2	4	4	3	3	4	4
4	4	2	2	4	4	3	3	3	3
4	4	3	3	4	4	3	3	4	4
4	4	3	3	4	4	3	3	4	4
4	4	2	2	4	4	2	2	4	4
4	4	2	2	4	4	2	2	3	3
4	4	2	2	4	4	3	3	3	3
4	4	2	2	4	4	3	3	3	3

For Review Only

4	4	2	2	4	4	3	3	4	4
4	4	2	2	4	4	2	2	3	3
4	4	3	3	4	4	3	3	3	3
4	4	2	2	4	4	2	2	3	3
4	4	2	2	4	4	3	3	3	3
1	1	2	2	4	4	3	3	3	3
4	4	2	2	4	4	2	2	3	3
4	4	2	2	4	4	3	3	3	3
4	4	2	2	4	4	2	2	3	3
4	4	2	2	4	4	3	3	3	3
4	4	3	3	4	4	3	3	4	4
4	4	2	2	4	4	2	2	3	3
4	4	2	2	4	4	3	3	4	4
4	4	3	3	4	4	3	3	3	3
4	4	3	3	4	4	2	2	3	3
4	4	2	2	4	4	2	2	3	3
4	4	2	2	4	4	2	2	3	3
4	4	2	2	4	4	3	3	3	3
4	4	3	3	4	4	3	3	3	3
4	4	3	3	4	4	3	3	4	4
4	4	2	2	4	4	3	3	4	4
4	4	2	2	4	4	3	3	3	3
4	4	2	2	4	4	2	2	3	3
4	4	2	2	4	4	3	3	3	3
1	1	3	3	4	4	2	2	3	3
4	4	3	3	4	4	3	3	3	3
4	4	3	3	4	4	3	3	4	4
4	4	2	2	4	4	3	3	4	4
4	4	3	3	4	4	2	2	4	4
4	4	2	2	4	4	3	3	3	3
4	4	2	2	4	4	3	3	3	3
4	4	2	2	4	4	3	3	4	4
4	4	3	3	4	4	3	3	3	3
4	4	3	3	4	4	2	2	3	3
4	4	3	3	4	4	3	3	4	4
4	4	2	2	4	4	3	3	3	3
4	4	3	3	4	4	3	3	3	3
4	4	3	3	4	4	3	3	3	3
4	4	2	2	4	4	3	3	4	4
4	4	2	2	4	4	3	3	3	3
4	4	3	3	4	4	3	3	4	4
4	4	3	3	4	4	3	3	4	4
4	4	2	2	4	4	2	2	3	3
4	4	2	2	4	4	3	3	4	4
4	4	3	3	4	4	3	3	3	3
4	4	2	2	4	4	3	3	3	3
4	4	2	2	4	4	2	2	3	3
4	4	2	2	4	4	3	3	3	3
4	4	3	3	4	4	3	3	3	3
1	1	3	3	4	4	2	2	3	3
4	4	3	3	4	4	3	3	4	4
4	4	2	2	4	4	3	3	3	3
4	4	2	2	4	4	2	2	3	3
4	4	2	2	4	4	3	3	3	3
4	4	2	2	4	4	3	3	3	3

For Review Only

4	4	2	2	4	4	3	3	3	3
4	4	2	2	4	4	3	3	3	3
4	4	3	3	4	4	3	3	4	4
4	4	2	2	4	4	2	2	3	3
4	4	2	2	4	4	2	2	4	4
4	4	3	3	4	4	3	3	4	4
4	4	2	2	4	4	2	2	3	3
1	1	2	2	4	4	2	2	3	3
4	4	3	3	4	4	3	3	3	3
4	4	2	2	4	4	2	2	3	3
4	4	2	2	4	4	3	3	3	3
4	4	2	2	4	4	2	2	3	3
4	4	3	3	4	4	2	2	3	3
4	4	2	2	4	4	3	3	3	3
4	4	2	2	4	4	3	3	4	4
4	4	3	3	4	4	3	3	3	3
4	4	2	2	4	4	3	3	3	3
1	1	2	2	4	4	3	3	3	3
4	4	2	2	4	4	2	2	4	4
4	4	3	3	4	4	3	3	4	4
4	4	2	2	4	4	2	2	3	3
4	4	3	3	4	4	3	3	4	4
4	4	3	3	4	4	3	3	3	3
4	4	2	2	4	4	2	2	3	3
1	1	2	2	4	4	3	3	3	3
4	4	2	2	4	4	2	2	3	3
4	4	2	2	4	4	2	2	3	3
4	4	3	3	4	4	3	3	4	4
4	4	2	2	4	4	2	2	4	4
4	4	2	2	4	4	3	3	4	4
4	4	2	2	4	4	2	2	3	3
4	4	2	2	4	4	3	3	4	4
4	4	3	3	4	4	3	3	3	3
4	4	2	2	4	4	3	3	3	3
4	4	2	2	4	4	2	2	3	3
4	4	2	2	4	4	2	2	3	3
4	4	2	2	4	4	3	3	3	3
4	4	2	2	4	4	3	3	3	3
4	4	2	2	4	4	3	3	3	3
4	4	2	2	4	4	2	2	3	3
4	4	3	3	4	4	3	3	3	3
4	4	3	3	4	4	3	3	3	3
4	4	2	2	4	4	2	2	3	3
4	4	3	3	4	4	3	3	3	3
4	4	2	2	4	4	3	3	3	3
4	4	3	3	4	4	2	2	3	3
4	4	2	2	4	4	2	2	4	4
4	4	3	3	4	4	3	3	3	3
4	4	2	2	4	4	3	3	3	3
4	4	2	2	4	4	2	2	3	3
4	4	2	2	4	4	3	3	4	4
4	4	2	2	4	4	2	2	3	3
4	4	3	3	4	4	3	3	4	4
4	4	3	3	4	4	3	3	4	4
4	4	2	2	4	4	3	3	3	3

For Review Only

4	4	3	3	4	4	3	3	3	3
4	4	2	2	4	4	3	3	3	3

For Review Only

SNP1163	SNP1163	SNP1164	SNP1164	SNP1165	SNP1165	SNP1166	SNP1166	SNP1167	SNP1167
4	4	1	1	2	2	2	2	2	2
3	3	4	4	3	3	3	3	2	2
3	3	4	4	2	2	3	3	2	2
4	4	1	1	2	2	2	2	2	2
4	4	1	1	2	2	2	2	4	4
4	4	1	1	2	2	2	2	2	2
4	4	1	1	2	2	3	3	2	2
4	4	1	1	2	2	3	3	2	2
4	4	1	1	2	2	2	2	2	2
4	4	1	1	3	3	2	2	4	4
3	3	4	4	2	2	3	3	2	2
4	4	1	1	2	2	3	3	2	2
4	4	1	1	2	2	2	2	2	2
3	3	4	4	2	2	2	2	2	2
4	4	1	1	2	2	2	2	2	2
3	3	4	4	2	2	2	2	2	2
4	4	1	1	2	2	3	3	2	2
3	3	4	4	3	3	3	3	2	2
4	4	1	1	2	2	2	2	2	2
3	3	4	4	3	3	3	3	4	4
4	4	1	1	2	2	3	3	2	2
4	4	1	1	2	2	2	2	2	2
3	3	4	4	2	2	3	3	2	2
3	3	4	4	2	2	2	2	2	2
4	4	1	1	2	2	2	2	2	2
3	3	4	4	2	2	3	3	2	2
3	3	4	4	2	2	3	3	2	2
4	4	1	1	2	2	2	2	2	2
4	4	1	1	3	3	2	2	2	2
3	3	4	4	2	2	2	2	4	4
4	4	1	1	2	2	3	3	2	2
4	4	1	1	2	2	3	3	2	2
4	4	1	1	2	2	3	3	2	2
3	3	4	4	2	2	2	2	2	2
3	3	4	4	2	2	2	2	2	2
3	3	4	4	2	2	2	2	2	2
3	3	4	4	2	2	2	2	2	2
3	3	4	4	2	2	3	3	2	2
3	3	4	4	2	2	2	2	2	2
3	3	1	1	2	2	3	3	2	2
3	3	4	4	2	2	2	2	4	4
3	3	1	1	3	3	2	2	2	2
4	4	1	1	2	2	2	2	4	4
3	3	4	4	3	3	2	2	2	2
4	4	1	1	2	2	2	2	4	4
3	3	4	4	2	2	3	3	2	2
3	3	4	4	2	2	3	3	2	2
3	3	4	4	2	2	2	2	2	2
4	4	1	1	2	2	3	3	2	2
4	4	1	1	2	2	2	2	2	2
3	3	4	4	2	2	3	3	2	2
4	4	1	1	2	2	3	3	2	2

For Review Only

4	4	1	1	2	2	2	2	2	2
4	4	1	1	2	2	3	3	2	2
3	3	4	4	2	2	3	3	2	2
4	4	1	1	2	2	2	2	2	2
4	4	1	1	2	2	2	2	2	2
3	3	4	4	2	2	3	3	2	2
3	3	4	4	2	2	3	3	2	2
3	3	4	4	3	3	2	2	2	2
3	3	4	4	3	3	2	2	2	2
4	4	1	1	2	2	2	2	2	2
3	3	4	4	2	2	3	3	2	2
3	3	4	4	2	2	2	2	2	2
4	4	1	1	2	2	2	2	2	2
4	4	1	1	3	3	2	2	2	2
4	4	1	1	2	2	2	2	2	2
3	3	1	1	2	2	3	3	2	2
3	3	4	4	2	2	2	2	2	2
4	4	1	1	2	2	3	3	2	2
4	4	1	1	2	2	2	2	2	2
4	4	1	1	2	2	2	2	2	2
3	3	4	4	2	2	2	2	2	2
3	3	4	4	2	2	2	2	2	2
4	4	1	1	2	2	2	2	2	2
3	3	4	4	2	2	2	2	2	2
4	4	1	1	2	2	2	2	4	4
3	3	4	4	2	2	3	3	2	2
4	4	1	1	2	2	3	3	2	2
3	3	4	4	2	2	3	3	2	2
3	3	4	4	2	2	3	3	2	2
4	4	1	1	2	2	2	2	2	2
4	4	4	4	2	2	3	3	2	2
3	3	4	4	2	2	2	2	2	2
3	3	4	4	2	2	3	3	2	2
4	4	1	1	2	2	2	2	2	2
3	3	4	4	2	2	3	3	2	2
4	4	1	1	2	2	2	2	4	4
3	3	4	4	2	2	3	3	4	4
4	4	1	1	2	2	2	2	2	2
4	4	1	1	2	2	3	3	2	2
4	4	1	1	2	2	2	2	2	2
4	4	1	1	2	2	3	3	2	2
3	3	1	1	2	2	2	2	2	2
4	4	1	1	2	2	2	2	2	2
3	3	4	4	2	2	3	3	2	2
3	3	1	1	2	2	3	3	2	2
4	4	4	4	2	2	3	3	2	2
3	3	4	4	2	2	2	2	2	2
3	3	4	4	2	2	2	2	2	2
3	3	4	4	2	2	2	2	2	2
4	4	1	1	2	2	2	2	2	2
3	3	4	4	2	2	3	3	2	2
3	3	4	4	2	2	3	3	2	2
3	3	4	4	3	3	2	2	2	2
4	4	1	1	2	2	3	3	2	2
4	4	1	1	2	2	3	3	2	2

For Review Only

3	3	4	4	2	2	3	3	2	2
3	3	4	4	2	2	2	2	2	2
3	3	4	4	2	2	3	3	2	2
3	3	4	4	2	2	2	2	2	2
3	3	4	4	2	2	3	3	2	2
3	3	4	4	2	2	2	2	2	2
4	4	1	1	2	2	2	2	2	2
3	3	4	4	2	2	2	2	2	2
3	3	4	4	2	2	2	2	2	2
3	3	4	4	2	2	3	3	2	2
3	3	4	4	2	2	3	3	2	2
4	4	1	1	2	2	2	2	2	2
4	4	1	1	2	2	2	2	4	4
3	3	4	4	2	2	3	3	2	2
3	3	4	4	2	2	2	2	2	2
4	4	1	1	2	2	3	3	2	2
4	4	1	1	2	2	3	3	2	2
3	3	1	1	2	2	2	2	2	2
3	3	4	4	2	2	2	2	2	2
4	4	1	1	2	2	3	3	2	2
4	4	1	1	2	2	2	2	2	2
4	4	1	1	2	2	2	2	2	2
3	3	4	4	2	2	2	2	2	2
3	3	4	4	2	2	3	3	2	2
3	3	4	4	2	2	2	2	2	2
3	3	4	4	2	2	2	2	2	2
4	4	1	1	2	2	3	3	2	2
4	4	1	1	2	2	2	2	2	2
4	4	1	1	2	2	3	3	2	2
4	4	1	1	3	3	2	2	2	2
3	3	4	4	2	2	2	2	2	2
4	4	1	1	2	2	2	2	2	2
4	4	1	1	2	2	3	3	2	2
3	3	4	4	2	2	3	3	2	2
3	3	4	4	2	2	2	2	2	2
4	4	1	1	2	2	2	2	2	2
3	3	4	4	2	2	2	2	2	2
3	3	4	4	2	2	3	3	2	2
3	3	4	4	2	2	2	2	2	2
3	3	4	4	2	2	3	3	2	2
4	4	1	1	2	2	2	2	2	2
3	3	4	4	2	2	2	2	2	2
3	3	4	4	2	2	3	3	2	2
4	4	1	1	3	3	2	2	2	2
3	3	4	4	2	2	2	2	2	2
4	4	1	1	2	2	3	3	2	2
3	3	4	4	2	2	3	3	2	2
3	3	4	4	2	2	3	3	2	2
3	3	1	1	2	2	2	2	2	2
3	3	4	4	2	2	3	3	2	2
3	3	4	4	2	2	3	3	2	2
3	3	4	4	3	3	2	2	2	2
3	3	4	4	2	2	2	2	2	2
3	3	4	4	2	2	2	2	2	2
4	4	1	1	2	2	2	2	2	2

For Review Only

4	4	1	1	2	2	3	3	2	2
3	3	4	4	2	2	2	2	2	2
3	3	4	4	2	2	3	3	2	2
4	4	1	1	2	2	2	2	2	2
4	4	4	4	2	2	3	3	2	2
3	3	4	4	2	2	3	3	2	2
3	3	4	4	2	2	2	2	2	2
4	4	1	1	2	2	2	2	2	2
3	3	4	4	2	2	2	2	2	2
4	4	1	1	2	2	2	2	2	2
3	3	4	4	2	2	3	3	2	2
3	3	4	4	2	2	2	2	2	2
3	3	4	4	2	2	3	3	2	2
4	4	1	1	2	2	2	2	2	2
3	3	4	4	2	2	2	2	2	2
3	3	4	4	2	2	3	3	2	2
3	3	1	1	2	2	2	2	2	2
3	3	4	4	2	2	3	3	2	2
3	3	4	4	2	2	3	3	2	2
4	4	1	1	2	2	2	2	2	2
4	4	1	1	2	2	2	2	2	2
3	3	4	4	2	2	3	3	2	2
3	3	4	4	2	2	2	2	2	2
3	3	4	4	2	2	2	2	2	2
3	3	4	4	3	3	2	2	2	2
3	3	4	4	2	2	2	2	2	2
4	4	1	1	2	2	2	2	2	2
4	4	1	1	2	2	2	2	2	2
3	3	4	4	2	2	2	2	2	2
4	4	1	1	2	2	3	3	2	2
4	4	1	1	2	2	3	3	2	2
4	4	1	1	2	2	2	2	2	2
4	4	1	1	2	2	3	3	2	2
3	3	4	4	2	2	2	2	2	2
4	4	1	1	2	2	3	3	2	2
3	3	4	4	2	2	2	2	2	2
3	3	4	4	2	2	3	3	2	2
4	4	1	1	2	2	2	2	2	2
3	3	4	4	2	2	3	3	2	2
3	3	4	4	2	2	2	2	2	2
3	3	4	4	2	2	3	3	2	2
3	3	4	4	2	2	3	3	2	2
3	3	4	4	2	2	3	3	2	2
3	3	4	4	2	2	2	2	2	2
3	3	4	4	2	2	2	2	2	2
4	4	1	1	2	2	2	2	2	2
3	3	4	4	2	2	3	3	2	2
3	3	4	4	2	2	2	2	2	2
3	3	4	4	2	2	2	2	2	2
4	4	1	1	2	2	2	2	2	2
4	4	1	1	2	2	3	3	2	2
3	3	4	4	2	2	3	3	2	2
3	3	4	4	2	2	2	2	2	2
3	3	4	4	2	2	3	3	2	2
3	3	4	4	2	2	3	3	2	2

For Review Only

3	3	4	4	2	2	3	3	2	2
3	3	4	4	2	2	2	2	4	4
4	4	1	1	2	2	3	3	2	2
4	4	1	1	2	2	2	2	2	2
4	4	1	1	2	2	3	3	2	2
4	4	1	1	2	2	3	3	2	2
3	3	4	4	2	2	3	3	2	2
3	3	4	4	3	3	2	2	4	4
3	3	4	4	2	2	2	2	2	2
3	3	4	4	2	2	3	3	2	2
4	4	1	1	2	2	2	2	2	2
4	4	1	1	2	2	3	3	2	2
4	4	1	1	2	2	3	3	2	2
3	3	4	4	2	2	3	3	2	2
3	3	4	4	2	2	3	3	2	2
3	3	4	4	2	2	3	3	2	2
3	3	4	4	2	2	3	3	2	2
4	4	1	1	2	2	3	3	2	2
3	3	4	4	2	2	2	2	2	2
3	3	4	4	2	2	2	2	2	2
4	4	1	1	2	2	3	3	2	2
3	3	4	4	2	2	3	3	2	2
4	4	1	1	2	2	3	3	2	2
3	3	4	4	2	2	2	2	2	2
3	3	4	4	3	3	2	2	2	2
4	4	1	1	2	2	2	2	2	2
3	3	4	4	2	2	3	3	2	2
4	4	1	1	2	2	3	3	2	2
4	4	1	1	2	2	2	2	2	2
4	4	1	1	2	2	3	3	2	2
3	3	4	4	2	2	3	3	2	2
3	3	4	4	2	2	2	2	2	2
3	3	4	4	2	2	2	2	2	2
3	3	4	4	2	2	3	3	2	2
3	3	4	4	2	2	2	2	2	2
3	3	4	4	2	2	3	3	2	2
4	4	1	1	2	2	3	3	2	2
3	3	4	4	2	2	2	2	2	2
3	3	4	4	2	2	2	2	2	2
3	3	4	4	2	2	3	3	2	2
4	4	1	1	2	2	3	3	2	2
3	3	4	4	2	2	3	3	2	2
3	3	4	4	2	2	2	2	2	2
4	4	1	1	2	2	2	2	2	2
3	3	4	4	2	2	3	3	2	2
4	4	1	1	2	2	3	3	2	2
3	3	4	4	2	2	3	3	2	2
3	3	4	4	2	2	3	3	2	2
3	3	4	4	2	2	3	3	2	2
3	3	4	4	2	2	3	3	2	2
4	4	1	1	2	2	2	2	2	2
3	3	4	4	2	2	3	3	2	2
3	3	4	4	2	2	3	3	2	2
4	4	1	1	2	2	3	3	2	2
3	3	4	4	2	2	3	3	2	2

For Review Only

4	4	1	1	2	2	3	3	2	2
3	3	4	4	2	2	3	3	2	2

For Review Only

SNP1168	SNP1168	SNP1169	SNP1169	SNP117	SNP117	SNP1170	SNP1170	SNP1171	SNP1171
1	1	3	3	2	2	4	4	2	2
4	4	3	3	4	4	3	3	2	2
4	4	3	3	2	2	3	3	2	2
4	4	3	3	2	2	4	4	2	2
4	4	3	3	2	2	4	4	2	2
1	1	3	3	2	2	4	4	2	2
1	1	3	3	2	2	3	3	2	2
1	1	2	2	2	2	3	3	2	2
4	4	3	3	2	2	4	4	2	2
1	1	2	2	2	2	4	4	2	2
4	4	3	3	4	4	3	3	1	1
4	4	3	3	2	2	3	3	2	2
4	4	3	3	4	4	4	4	2	2
4	4	3	3	2	2	4	4	2	2
4	4	3	3	2	2	4	4	2	2
4	4	3	3	4	4	4	4	1	1
4	4	3	3	2	2	3	3	2	2
4	4	3	3	4	4	4	4	2	2
1	1	3	3	4	4	4	4	2	2
4	4	2	2	2	2	4	4	2	2
1	1	3	3	2	2	3	3	2	2
1	1	3	3	2	2	4	4	2	2
4	4	3	3	2	2	3	3	2	2
4	4	3	3	4	4	4	4	2	2
1	1	3	3	4	4	4	4	2	2
4	4	3	3	2	2	3	3	2	2
4	4	3	3	2	2	3	3	2	2
4	4	3	3	2	2	4	4	2	2
1	1	3	3	2	2	4	4	2	2
4	4	3	3	2	2	4	4	2	2
1	1	3	3	4	4	4	4	2	2
1	1	3	3	4	4	3	3	2	2
1	1	3	3	2	2	3	3	2	2
4	4	3	3	2	2	4	4	2	2
4	4	3	3	4	4	4	4	2	2
4	4	3	3	4	4	4	4	2	2
4	4	3	3	2	2	4	4	2	2
4	4	3	3	2	2	4	4	2	2
4	4	3	3	4	4	4	4	1	1
4	4	3	3	4	4	4	4	2	2
4	4	2	2	2	2	4	4	2	2
4	4	3	3	2	2	4	4	2	2
1	1	2	2	2	2	4	4	2	2
4	4	3	3	4	4	4	4	2	2
1	1	2	2	2	2	4	4	2	2
4	4	3	3	2	2	3	3	2	2
4	4	3	3	4	4	4	4	2	2
4	4	3	3	2	2	4	4	2	2
1	1	3	3	2	2	4	4	2	2
1	1	3	3	4	4	4	4	2	2
4	4	3	3	4	4	3	3	2	2
4	4	3	3	4	4	3	3	2	2

For Review Only

1	1	3	3	2	2	4	4	2	2
1	1	3	3	2	2	3	3	2	2
4	4	3	3	4	4	3	3	2	2
1	1	3	3	2	2	4	4	2	2
1	1	3	3	4	4	4	4	2	2
4	4	3	3	4	4	3	3	2	2
4	4	3	3	2	2	3	3	2	2
4	4	3	3	4	4	4	4	2	2
4	4	3	3	4	4	4	4	2	2
4	4	3	3	2	2	4	4	2	2
4	4	3	3	4	4	3	3	2	2
4	4	3	3	4	4	4	4	2	2
1	1	3	3	2	2	4	4	2	2
1	1	3	3	2	2	4	4	2	2
4	4	3	3	4	4	4	4	2	2
4	4	3	3	4	4	4	4	2	2
4	4	3	3	4	4	4	4	2	2
1	1	3	3	4	4	3	3	2	2
1	1	3	3	2	2	4	4	2	2
1	1	3	3	2	2	4	4	2	2
4	4	3	3	4	4	4	4	2	2
4	4	3	3	4	4	4	4	2	2
1	1	3	3	2	2	4	4	2	2
4	4	3	3	4	4	4	4	2	2
4	4	2	2	2	2	4	4	2	2
4	4	3	3	4	4	3	3	2	2
1	1	3	3	4	4	3	3	2	2
4	4	3	3	4	4	3	3	1	1
4	4	3	3	2	2	3	3	2	2
1	1	3	3	4	4	4	4	2	2
4	4	3	3	2	2	3	3	2	2
4	4	3	3	4	4	4	4	2	2
4	4	3	3	4	4	3	3	1	1
1	1	3	3	2	2	4	4	2	2
4	4	3	3	4	4	3	3	2	2
1	1	2	2	2	2	4	4	2	2
4	4	3	3	4	4	3	3	2	2
1	1	3	3	2	2	4	4	2	2
1	1	3	3	2	2	3	3	2	2
1	1	3	3	4	4	4	4	2	2
1	1	3	3	2	2	3	3	2	2
4	4	3	3	4	4	4	4	2	2
1	1	3	3	2	2	4	4	2	2
4	4	3	3	4	4	3	3	2	2
4	4	3	3	2	2	3	3	2	2
4	4	3	3	4	4	3	3	2	2
4	4	3	3	2	2	4	4	2	2
4	4	3	3	2	2	4	4	2	2
4	4	3	3	2	2	4	4	2	2
1	1	3	3	4	4	4	4	2	2
4	4	3	3	4	4	3	3	2	2
4	4	3	3	4	4	3	3	1	1
4	4	3	3	4	4	4	4	2	2
4	4	3	3	4	4	3	3	2	2
1	1	3	3	4	4	3	3	2	2

For Review Only

4	4	3	3	2	2	3	3	2	2
4	4	2	2	2	2	4	4	2	2
4	4	3	3	2	2	3	3	2	2
4	4	3	3	2	2	4	4	2	2
4	4	3	3	4	4	3	3	2	2
4	4	3	3	4	4	4	4	2	2
1	1	3	3	2	2	4	4	2	2
4	4	3	3	4	4	4	4	2	2
4	4	3	3	2	2	4	4	2	2
4	4	3	3	4	4	4	4	2	2
4	4	3	3	4	4	3	3	2	2
4	4	3	3	4	4	4	4	2	2
1	1	3	3	4	4	4	4	2	2
4	4	3	3	4	4	3	3	2	2
1	1	3	3	4	4	4	4	2	2
4	4	3	3	4	4	4	4	2	2
1	1	3	3	4	4	3	3	2	2
4	4	3	3	4	4	3	3	2	2
4	4	3	3	2	2	4	4	2	2
1	1	3	3	4	4	3	3	2	2
4	4	3	3	4	4	4	4	2	2
1	1	3	3	2	2	4	4	2	2
4	4	3	3	4	4	4	4	2	2
4	4	3	3	2	2	3	3	1	1
4	4	3	3	4	4	4	4	2	2
4	4	3	3	2	2	4	4	2	2
4	4	3	3	4	4	4	4	2	2
1	1	3	3	2	2	4	4	2	2
1	1	3	3	4	4	3	3	2	2
1	1	3	3	4	4	4	4	2	2
4	4	3	3	4	4	4	4	1	1
4	4	3	3	4	4	4	4	2	2
1	1	3	3	2	2	3	3	2	2
4	4	3	3	4	4	4	4	2	2
4	4	3	3	2	2	4	4	2	2
4	4	3	3	4	4	4	4	2	2
4	4	3	3	2	2	4	4	2	2
4	4	3	3	4	4	3	3	2	2
4	4	3	3	2	2	4	4	2	2
4	4	3	3	4	4	3	3	1	1
1	1	3	3	2	2	4	4	2	2
4	4	3	3	2	2	4	4	2	2
4	4	3	3	4	4	3	3	2	2
4	4	3	3	4	4	4	4	2	2
4	4	3	3	2	2	4	4	2	2
1	1	3	3	2	2	4	4	2	2
4	4	3	3	4	4	3	3	2	2
4	4	3	3	4	4	3	3	2	2
4	4	3	3	4	4	4	4	2	2
4	4	3	3	2	2	3	3	2	2
4	4	3	3	2	2	3	3	2	2
4	4	3	3	4	4	4	4	1	1
4	4	3	3	4	4	4	4	2	2
4	4	3	3	4	4	4	4	2	2
4	4	3	3	4	4	4	4	2	2

For Review Only

1	1	3	3	4	4	3	3	2	2
4	4	3	3	2	2	4	4	2	2
4	4	3	3	2	2	3	3	2	2
4	4	3	3	4	4	4	4	2	2
4	4	3	3	4	4	3	3	2	2
4	4	3	3	4	4	3	3	2	2
4	4	3	3	4	4	4	4	2	2
4	4	3	3	4	4	4	4	2	2
4	4	3	3	4	4	4	4	1	1
1	1	3	3	4	4	4	4	2	2
4	4	3	3	2	2	4	4	2	2
4	4	3	3	4	4	4	4	2	2
4	4	3	3	4	4	4	4	2	2
4	4	3	3	2	2	4	4	2	2
4	4	3	3	2	2	4	4	2	2
4	4	3	3	4	4	3	3	1	1
4	4	3	3	4	4	4	4	2	2
4	4	3	3	4	4	3	3	2	2
4	4	3	3	2	2	3	3	2	2
1	1	3	3	2	2	4	4	2	2
4	4	3	3	4	4	4	4	2	2
4	4	3	3	4	4	3	3	2	2
4	4	3	3	4	4	4	4	2	2
4	4	3	3	4	4	4	4	2	2
4	4	3	3	2	2	4	4	2	2
4	4	3	3	2	2	4	4	2	2
4	4	3	3	2	2	4	4	2	2
4	4	3	3	4	4	4	4	2	2
4	4	3	3	2	2	4	4	2	2
1	1	3	3	4	4	3	3	2	2
4	4	3	3	4	4	3	3	2	2
1	1	3	3	4	4	4	4	2	2
4	4	3	3	2	2	3	3	2	2
4	4	3	3	2	2	4	4	2	2
4	4	3	3	2	2	3	3	2	2
4	4	3	3	4	4	4	4	2	2
4	4	3	3	4	4	3	3	2	2
1	1	3	3	2	2	4	4	2	2
4	4	3	3	4	4	3	3	2	2
4	4	3	3	4	4	3	3	2	2
4	4	3	3	2	2	3	3	2	2
4	4	3	3	2	2	3	3	2	2
4	4	3	3	4	4	3	3	1	1
4	4	3	3	4	4	4	4	2	2
4	4	3	3	2	2	3	3	2	2
1	1	3	3	4	4	4	4	2	2
4	4	3	3	4	4	3	3	1	1
4	4	3	3	4	4	4	4	1	1
4	4	3	3	2	2	4	4	2	2
1	1	3	3	2	2	4	4	2	2
1	1	3	3	2	2	3	3	2	2
4	4	3	3	4	4	3	3	2	2
4	4	3	3	4	4	4	4	2	2
4	4	3	3	4	4	3	3	2	2
4	4	3	3	4	4	3	3	1	1

For Review Only

4	4	3	3	4	4	3	3	2	2
4	4	2	2	2	2	4	4	2	2
4	4	3	3	2	2	3	3	2	2
4	4	3	3	4	4	4	4	2	2
1	1	3	3	4	4	3	3	2	2
1	1	3	3	2	2	4	4	2	2
4	4	3	3	4	4	3	3	1	1
4	4	2	2	4	4	4	4	2	2
4	4	3	3	2	2	4	4	2	2
4	4	3	3	4	4	3	3	1	1
4	4	3	3	4	4	4	4	2	2
4	4	3	3	4	4	3	3	2	2
1	1	3	3	2	2	3	3	2	2
4	4	3	3	4	4	3	3	2	2
4	4	3	3	4	4	3	3	2	2
4	4	3	3	2	2	3	3	2	2
4	4	3	3	4	4	3	3	2	2
1	1	3	3	4	4	3	3	2	2
4	4	3	3	4	4	4	4	1	1
4	4	3	3	2	2	4	4	2	2
4	4	3	3	4	4	3	3	2	2
4	4	3	3	2	2	3	3	2	2
1	1	3	3	2	2	3	3	2	2
4	4	3	3	4	4	4	4	2	2
4	4	3	3	4	4	4	4	2	2
1	1	3	3	4	4	4	4	2	2
4	4	3	3	4	4	3	3	1	1
1	1	3	3	2	2	3	3	2	2
1	1	3	3	4	4	4	4	2	2
1	1	3	3	4	4	3	3	2	2
4	4	3	3	4	4	3	3	1	1
4	4	3	3	4	4	4	4	2	2
4	4	3	3	2	2	4	4	2	2
4	4	3	3	4	4	3	3	2	2
4	4	3	3	4	4	4	4	2	2
4	4	3	3	4	4	3	3	1	1
1	1	3	3	4	4	3	3	2	2
4	4	3	3	4	4	4	4	2	2
4	4	3	3	4	4	4	4	2	2
4	4	3	3	4	4	3	3	1	1
1	1	3	3	2	2	3	3	2	2
4	4	3	3	2	2	3	3	2	2
4	4	3	3	4	4	4	4	2	2
1	1	3	3	2	2	4	4	2	2
4	4	3	3	4	4	3	3	2	2
1	1	3	3	2	2	3	3	2	2
4	4	3	3	4	4	3	3	2	2
4	4	3	3	2	2	3	3	2	2
4	4	3	3	4	4	3	3	2	2
4	4	3	3	4	4	3	3	1	1
1	1	3	3	4	4	4	4	2	2
4	4	3	3	4	4	3	3	1	1
4	4	3	3	2	2	3	3	2	2
1	1	3	3	2	2	3	3	2	2
4	4	3	3	4	4	3	3	1	1

For Review Only

1	1	3	3	2	2	4	4	2	2
4	4	3	3	4	4	3	3	2	2

For Review Only

SNP1172	SNP1172	SNP1173	SNP1173	SNP1174	SNP1174	SNP1175	SNP1175	SNP1176	SNP1176
3	3	2	2	4	4	4	4	2	2
3	3	2	2	1	1	1	1	3	3
3	3	2	2	1	1	1	1	3	3
3	3	2	2	1	1	4	4	3	3
3	3	1	1	4	4	4	4	3	3
3	3	2	2	4	4	4	4	3	3
3	3	2	2	4	4	4	4	3	3
3	3	2	2	4	4	4	4	3	3
3	3	2	2	4	4	4	4	2	2
3	3	1	1	4	4	4	4	2	2
3	3	2	2	1	1	1	1	3	3
3	3	2	2	1	1	4	4	3	3
3	3	2	2	4	4	4	4	2	2
3	3	2	2	1	1	1	1	3	3
3	3	2	2	1	1	4	4	2	2
3	3	2	2	1	1	1	1	3	3
3	3	2	2	4	4	4	4	2	2
3	3	2	2	1	1	1	1	3	3
3	3	2	2	4	4	4	4	3	3
3	3	1	1	1	1	1	1	2	2
3	3	2	2	1	1	1	1	3	3
3	3	2	2	4	4	4	4	3	3
3	3	2	2	1	1	1	1	3	3
3	3	2	2	1	1	1	1	3	3
3	3	2	2	1	1	1	1	3	3
3	3	2	2	1	1	1	1	3	3
3	3	2	2	1	1	1	1	3	3
3	3	2	2	1	1	4	4	3	3
3	3	2	2	4	4	4	4	2	2
3	3	1	1	1	1	1	1	2	2
3	3	2	2	4	4	4	4	3	3
3	3	2	2	4	4	4	4	3	3
3	3	2	2	4	4	4	4	3	3
3	3	2	2	1	1	1	1	3	3
3	3	2	2	1	1	1	1	3	3
3	3	2	2	1	1	1	1	3	3
3	3	2	2	1	1	1	1	3	3
2	2	2	2	1	1	1	1	3	3
3	3	2	2	1	1	1	1	3	3
2	2	2	2	1	1	1	1	3	3
3	3	1	1	1	1	4	4	3	3
3	3	2	2	1	1	1	1	3	3
3	3	1	1	4	4	4	4	3	3
3	3	2	2	1	1	1	1	3	3
3	3	1	1	4	4	4	4	3	3
3	3	2	2	1	1	1	1	3	3
3	3	2	2	1	1	1	1	3	3
3	3	2	2	1	1	1	1	3	3
2	2	2	2	4	4	4	4	3	3
3	3	2	2	4	4	4	4	3	3
3	3	2	2	1	1	1	1	3	3
3	3	2	2	4	4	4	4	3	3

For Review Only

3	3	2	2	4	4	4	4	2	2
3	3	2	2	4	4	4	4	3	3
3	3	2	2	1	1	1	1	3	3
3	3	2	2	4	4	4	4	2	2
3	3	2	2	4	4	4	4	3	3
3	3	2	2	1	1	1	1	3	3
3	3	2	2	1	1	1	1	2	2
3	3	2	2	1	1	1	1	2	2
3	3	2	2	1	1	1	1	3	3
3	3	2	2	1	1	4	4	3	3
3	3	2	2	1	1	1	1	3	3
3	3	2	2	1	1	1	1	3	3
3	3	2	2	4	4	4	4	2	2
3	3	2	2	4	4	4	4	2	2
3	3	2	2	1	1	4	4	3	3
3	3	1	1	1	1	1	1	3	3
3	3	2	2	1	1	1	1	3	3
3	3	2	2	4	4	4	4	3	3
3	3	2	2	4	4	4	4	3	3
3	3	2	2	4	4	4	4	2	2
3	3	2	2	1	1	4	4	3	3
3	3	2	2	1	1	1	1	3	3
3	3	2	2	4	4	4	4	2	2
3	3	2	2	1	1	1	1	3	3
3	3	1	1	4	4	4	4	3	3
3	3	2	2	1	1	1	1	3	3
3	3	2	2	4	4	4	4	3	3
3	3	2	2	1	1	1	1	3	3
3	3	2	2	1	1	1	1	3	3
3	3	2	2	4	4	1	1	3	3
3	3	2	2	1	1	1	1	3	3
3	3	2	2	1	1	1	1	3	3
3	3	2	2	1	1	1	1	3	3
3	3	2	2	4	4	4	4	2	2
2	2	2	2	1	1	1	1	3	3
3	3	1	1	4	4	4	4	3	3
3	3	2	2	1	1	1	1	3	3
3	3	2	2	4	4	4	4	3	3
3	3	2	2	4	4	4	4	3	3
3	3	2	2	4	4	4	4	2	2
3	3	2	2	4	4	4	4	3	3
3	3	2	2	4	4	4	4	3	3
3	3	2	2	4	4	4	4	2	2
3	3	2	2	1	1	1	1	3	3
3	3	2	2	1	1	1	1	3	3
2	2	2	2	1	1	1	1	3	3
3	3	2	2	1	1	1	1	3	3
3	3	2	2	1	1	1	1	3	3
3	3	2	2	1	1	1	1	3	3
3	3	2	2	4	4	4	4	3	3
3	3	2	2	1	1	1	1	3	3
3	3	2	2	1	1	1	1	3	3
3	3	2	2	1	1	1	1	2	2
3	3	2	2	1	1	4	4	3	3
3	3	2	2	4	4	4	4	3	3

For Review Only

3	3	2	2	1	1	1	1	3	3
3	3	2	2	1	1	1	1	3	3
3	3	2	2	1	1	1	1	3	3
3	3	2	2	1	1	1	1	3	3
3	3	2	2	1	1	1	1	3	3
3	3	2	2	1	1	1	1	3	3
3	3	2	2	4	4	4	4	3	3
3	3	2	2	1	1	1	1	3	3
3	3	2	2	1	1	1	1	3	3
3	3	2	2	1	1	1	1	3	3
2	2	2	2	1	1	1	1	2	2
3	3	2	2	1	1	4	4	3	3
3	3	2	2	4	4	4	4	2	2
3	3	2	2	1	1	4	4	3	3
3	3	2	2	4	4	1	1	3	3
3	3	2	2	1	1	1	1	3	3
3	3	2	2	4	4	4	4	2	2
3	3	2	2	1	1	4	4	3	3
3	3	2	2	1	1	1	1	3	3
3	3	2	2	4	4	4	4	3	3
3	3	2	2	1	1	4	4	3	3
3	3	2	2	4	4	4	4	3	3
3	3	2	2	1	1	1	1	3	3
3	3	2	2	1	1	1	1	3	3
3	3	2	2	1	1	1	1	3	3
3	3	2	2	1	1	1	1	3	3
2	2	2	2	4	4	4	4	3	3
3	3	2	2	4	4	4	4	3	3
3	3	2	2	4	4	4	4	3	3
3	3	2	2	4	4	4	4	3	3
3	3	2	2	1	1	1	1	3	3
3	3	2	2	1	1	1	1	3	3
3	3	2	2	4	4	4	4	3	3
3	3	2	2	1	1	4	4	3	3
3	3	2	2	1	1	1	1	3	3
3	3	2	2	1	1	4	4	3	3
3	3	2	2	1	1	1	1	3	3
3	3	2	2	1	1	1	1	3	3
3	3	2	2	1	1	1	1	3	3
3	3	2	2	1	1	1	1	3	3
3	3	2	2	4	4	4	4	3	3
3	3	2	2	1	1	1	1	3	3
3	3	2	2	1	1	1	1	2	2
3	3	2	2	1	1	4	4	3	3
3	3	2	2	1	1	1	1	3	3
2	2	2	2	4	4	4	4	3	3
3	3	2	2	1	1	1	1	3	3
3	3	2	2	1	1	1	1	3	3
3	3	2	2	1	1	4	4	3	3
3	3	2	2	1	1	1	1	3	3
3	3	2	2	1	1	1	1	3	3
3	3	2	2	1	1	1	1	3	3
3	3	2	2	1	1	4	4	3	3
3	3	2	2	1	1	1	1	3	3
3	3	2	2	1	1	4	4	3	3

For Review Only

3	3	2	2	4	4	4	4	3	3
3	3	2	2	1	1	1	1	3	3
2	2	2	2	1	1	1	1	3	3
3	3	2	2	1	1	4	4	3	3
3	3	2	2	1	1	4	4	2	2
2	2	2	2	1	1	1	1	3	3
3	3	2	2	1	1	1	1	3	3
3	3	2	2	1	1	4	4	3	3
3	3	2	2	1	1	1	1	3	3
3	3	2	2	4	4	4	4	3	3
3	3	2	2	1	1	1	1	3	3
3	3	2	2	1	1	1	1	3	3
3	3	2	2	1	1	1	1	3	3
2	2	2	2	1	1	4	4	3	3
3	3	2	2	1	1	1	1	3	3
3	3	2	2	1	1	1	1	2	2
3	3	2	2	1	1	4	4	3	3
3	3	2	2	1	1	1	1	3	3
3	3	2	2	1	1	1	1	3	3
3	3	2	2	4	4	4	4	3	3
3	3	2	2	1	1	4	4	3	3
3	3	2	2	1	1	1	1	3	3
3	3	2	2	1	1	1	1	3	3
3	3	2	2	1	1	1	1	3	3
3	3	2	2	1	1	1	1	3	3
3	3	2	2	1	1	1	1	3	3
3	3	2	2	1	1	4	4	3	3
3	3	2	2	1	1	4	4	3	3
3	3	2	2	1	1	1	1	3	3
3	3	2	2	4	4	4	4	3	3
2	2	2	2	1	1	1	1	3	3
3	3	2	2	4	4	1	1	3	3
2	2	2	2	4	4	4	4	3	3
3	3	2	2	1	1	1	1	3	3
2	2	2	2	1	1	4	4	3	3
3	3	2	2	1	1	4	4	3	3
3	3	2	2	1	1	1	1	3	3
3	3	2	2	4	4	4	4	3	3
3	3	2	2	1	1	1	1	3	3
3	3	2	2	1	1	1	1	3	3
3	3	2	2	1	1	1	1	3	3
3	3	2	2	1	1	1	1	3	3
3	3	2	2	1	1	1	1	3	3
3	3	2	2	1	1	1	1	3	3
3	3	2	2	1	1	1	1	3	3
3	3	2	2	4	4	4	4	3	3
3	3	2	2	1	1	1	1	2	2
3	3	2	2	1	1	1	1	3	3
3	3	2	2	1	1	1	1	3	3
3	3	2	2	4	4	4	4	2	2
3	3	2	2	4	4	4	4	3	3
2	2	2	2	1	1	1	1	3	3
3	3	2	2	1	1	1	1	3	3
3	3	2	2	1	1	1	1	3	3
3	3	2	2	1	1	1	1	3	3

For Review Only

3	3	2	2	1	1	1	1	3	3
3	3	1	1	1	1	1	1	3	3
3	3	2	2	4	4	4	4	3	3
3	3	2	2	1	1	4	4	3	3
3	3	2	2	1	1	1	1	3	3
3	3	2	2	4	4	4	4	3	3
3	3	2	2	1	1	1	1	3	3
3	3	1	1	1	1	1	1	3	3
3	3	2	2	1	1	1	1	3	3
3	3	2	2	1	1	1	1	3	3
3	3	2	2	1	1	4	4	3	3
2	2	2	2	1	1	1	1	3	3
3	3	2	2	4	4	4	4	3	3
3	3	2	2	1	1	1	1	3	3
3	3	2	2	1	1	1	1	3	3
3	3	2	2	1	1	1	1	3	3
3	3	2	2	1	1	1	1	3	3
3	3	2	2	1	1	1	1	3	3
3	3	2	2	1	1	1	1	2	2
3	3	2	2	1	1	1	1	3	3
3	3	2	2	1	1	1	1	3	3
3	3	2	2	1	1	1	1	3	3
3	3	2	2	4	4	4	4	3	3
3	3	2	2	1	1	1	1	3	3
3	3	2	2	1	1	1	1	2	2
3	3	2	2	4	4	4	4	3	3
3	3	2	2	1	1	1	1	3	3
3	3	2	2	1	1	4	4	2	2
3	3	2	2	1	1	1	1	2	2
3	3	2	2	4	4	4	4	3	3
3	3	2	2	1	1	1	1	3	3
3	3	2	2	1	1	1	1	3	3
3	3	2	2	1	1	1	1	3	3
3	3	2	2	1	1	1	1	3	3
3	3	2	2	1	1	1	1	3	3
3	3	2	2	1	1	1	1	3	3
3	3	2	2	4	4	4	4	3	3
3	3	2	2	1	1	1	1	3	3
3	3	2	2	1	1	1	1	3	3
3	3	2	2	1	1	1	1	3	3
3	3	2	2	4	4	4	4	3	3
2	2	2	2	1	1	1	1	3	3
3	3	2	2	1	1	1	1	3	3
3	3	2	2	4	4	4	4	3	3
3	3	2	2	1	1	1	1	3	3
3	3	2	2	4	4	4	4	3	3
3	3	2	2	1	1	1	1	3	3
3	3	2	2	1	1	1	1	3	3
3	3	2	2	1	1	1	1	3	3
3	3	2	2	1	1	1	1	3	3
3	3	2	2	4	4	4	4	3	3
3	3	2	2	1	1	1	1	3	3
3	3	2	2	1	1	1	1	3	3
3	3	2	2	4	4	4	4	3	3
2	2	2	2	1	1	1	1	3	3

For Review Only

3	3	2	2	4	4	4	4	3	3
3	3	2	2	1	1	1	1	3	3

For Review Only

SNP1177	SNP1177	SNP1178	SNP1178	SNP1179	SNP1179	SNP118	SNP118	SNP1180	SNP1180
3	3	3	3	3	3	1	1	4	4
2	2	3	3	3	3	1	1	2	2
3	3	3	3	3	3	4	4	4	4
3	3	3	3	3	3	4	4	4	4
3	3	3	3	2	2	4	4	4	4
3	3	3	3	3	3	4	4	4	4
2	2	3	3	3	3	4	4	4	4
2	2	3	3	3	3	4	4	4	4
3	3	3	3	3	3	4	4	4	4
3	3	3	3	2	2	4	4	4	4
2	2	3	3	2	2	1	1	2	2
3	3	3	3	3	3	4	4	4	4
3	3	3	3	3	3	1	1	4	4
3	3	3	3	3	3	4	4	2	2
3	3	3	3	3	3	1	1	4	4
3	3	2	2	2	2	1	1	4	4
2	2	3	3	2	2	4	4	4	4
2	2	3	3	3	3	1	1	4	4
3	3	3	3	3	3	1	1	2	2
2	2	3	3	3	3	4	4	2	2
2	2	3	3	2	2	4	4	2	2
3	3	3	3	3	3	4	4	4	4
3	3	2	2	3	3	4	4	4	4
3	3	3	3	3	3	1	1	2	2
3	3	3	3	3	3	1	1	4	4
2	2	3	3	3	3	4	4	4	4
2	2	3	3	3	3	4	4	4	4
3	3	3	3	3	3	1	1	4	4
3	3	3	3	3	3	4	4	4	4
3	3	3	3	2	2	4	4	2	2
3	3	3	3	3	3	1	1	4	4
2	2	3	3	3	3	1	1	4	4
2	2	3	3	3	3	4	4	2	2
3	3	3	3	2	2	4	4	2	2
3	3	3	3	3	3	1	1	2	2
3	3	3	3	3	3	1	1	4	4
3	3	3	3	3	3	4	4	2	2
2	2	3	3	2	2	4	4	4	4
3	3	2	2	2	2	1	1	2	2
2	2	2	2	3	3	1	1	4	4
3	3	3	3	2	2	4	4	2	2
3	3	3	3	3	3	4	4	2	2
3	3	3	3	2	2	4	4	2	2
3	3	3	3	3	3	1	1	2	2
3	3	3	3	3	3	4	4	2	2
3	3	3	3	3	3	4	4	4	4
2	2	3	3	3	3	1	1	2	2
3	3	3	3	3	3	4	4	4	4
2	2	3	3	2	2	4	4	2	2
3	3	3	3	3	3	1	1	4	4
2	2	3	3	3	3	1	1	4	4
2	2	3	3	3	3	1	1	4	4

For Review Only

3	3	3	3	3	3	4	4	4	4
2	2	3	3	3	3	4	4	2	2
2	2	3	3	3	3	1	1	4	4
3	3	3	3	2	2	4	4	2	2
3	3	3	3	3	3	1	1	2	2
2	2	3	3	3	3	1	1	4	4
2	2	3	3	3	3	4	4	4	4
3	3	3	3	2	2	1	1	2	2
3	3	3	3	3	3	1	1	2	2
3	3	3	3	3	3	1	1	4	4
2	2	3	3	3	3	1	1	2	2
3	3	3	3	3	3	1	1	2	2
3	3	3	3	3	3	4	4	4	4
3	3	3	3	3	3	4	4	4	4
3	3	3	3	3	3	1	1	4	4
2	2	3	3	3	3	1	1	2	2
3	3	3	3	3	3	1	1	4	4
3	3	3	3	3	3	1	1	4	4
3	3	3	3	3	3	4	4	2	2
3	3	3	3	3	3	4	4	4	4
3	3	3	3	3	3	1	1	4	4
3	3	3	3	3	3	1	1	2	2
3	3	3	3	3	3	4	4	2	2
3	3	3	3	3	3	1	1	4	4
3	3	3	3	3	3	4	4	2	2
2	2	3	3	3	3	1	1	2	2
2	2	3	3	3	3	1	1	2	2
3	3	2	2	2	2	1	1	4	4
2	2	3	3	3	3	4	4	4	4
3	3	3	3	3	3	1	1	4	4
2	2	3	3	2	2	4	4	4	4
3	3	3	3	3	3	1	1	2	2
3	3	2	2	2	2	1	1	2	2
3	3	3	3	3	3	4	4	4	4
2	2	3	3	3	3	1	1	2	2
3	3	3	3	3	3	4	4	2	2
2	2	3	3	2	2	1	1	4	4
3	3	3	3	2	2	4	4	2	2
2	2	3	3	3	3	4	4	2	2
3	3	3	3	2	2	1	1	2	2
3	3	3	3	3	3	4	4	4	4
3	3	3	3	3	3	1	1	2	2
3	3	3	3	3	3	1	1	4	4
2	2	3	3	3	3	1	1	2	2
2	2	3	3	3	3	4	4	2	2
2	2	3	3	3	3	1	1	2	2
3	3	3	3	2	2	4	4	2	2
3	3	3	3	3	3	4	4	2	2
2	2	3	3	3	3	4	4	2	2
3	3	3	3	3	3	1	1	2	2
2	2	3	3	3	3	1	1	2	2
3	3	2	2	2	2	1	1	4	4
3	3	3	3	2	2	1	1	2	2
2	2	3	3	3	3	1	1	4	4
3	3	3	3	3	3	1	1	2	2

For Review Only

2	2	3	3	3	3	4	4	2	2
3	3	3	3	3	3	4	4	2	2
2	2	3	3	3	3	4	4	4	4
3	3	3	3	2	2	4	4	2	2
2	2	3	3	3	3	4	4	4	4
3	3	3	3	3	3	1	1	2	2
3	3	3	3	3	3	4	4	4	4
3	3	3	3	3	3	1	1	4	4
3	3	3	3	2	2	4	4	2	2
3	3	3	3	3	3	1	1	2	2
2	2	3	3	3	3	1	1	2	2
3	3	3	3	2	2	1	1	4	4
3	3	3	3	3	3	1	1	2	2
2	2	3	3	3	3	1	1	4	4
3	3	3	3	3	3	4	4	4	4
2	2	3	3	3	3	1	1	4	4
3	3	3	3	2	2	1	1	2	2
2	2	3	3	3	3	1	1	4	4
3	3	3	3	2	2	4	4	2	2
2	2	3	3	3	3	1	1	2	2
3	3	3	3	3	3	1	1	4	4
3	3	3	3	3	3	1	1	4	4
3	3	3	3	3	3	1	1	2	2
3	3	2	2	2	2	4	4	4	4
2	2	3	3	3	3	1	1	2	2
3	3	3	3	3	3	4	4	4	4
2	2	3	3	3	3	1	1	4	4
2	2	3	3	3	3	4	4	2	2
2	2	3	3	3	3	1	1	2	2
3	3	3	3	3	3	1	1	4	4
3	3	2	2	2	2	1	1	4	4
3	3	3	3	3	3	1	1	4	4
3	3	3	3	3	3	4	4	4	4
2	2	3	3	3	3	1	1	4	4
3	3	3	3	2	2	4	4	2	2
3	3	3	3	3	3	4	4	4	4
3	3	3	3	2	2	4	4	2	2
2	2	3	3	3	3	1	1	4	4
2	2	3	3	3	3	1	1	4	4
3	3	2	2	2	2	1	1	2	2
3	3	3	3	3	3	4	4	4	4
3	3	2	2	2	2	4	4	2	2
2	2	3	3	3	3	1	1	4	4
3	3	3	3	2	2	1	1	4	4
2	2	3	3	3	3	4	4	4	4
2	2	3	3	3	3	4	4	4	4
2	2	3	3	3	3	4	4	4	4
2	2	3	3	3	3	1	1	4	4
3	3	3	3	3	3	1	1	4	4
2	2	3	3	3	3	4	4	4	4
2	2	3	3	3	3	4	4	4	4
3	3	3	3	2	2	1	1	2	2
2	2	3	3	3	3	1	1	2	2
3	3	3	3	3	3	1	1	2	2
3	3	3	3	3	3	1	1	4	4

For Review Only

3	3	3	3	3	3	1	1	4	4
3	3	3	3	3	3	1	1	2	2
2	2	3	3	3	3	4	4	2	2
3	3	3	3	3	3	1	1	4	4
2	2	3	3	3	3	1	1	2	2
2	2	3	3	2	2	1	1	4	4
3	3	2	2	3	3	1	1	4	4
2	2	3	3	3	3	1	1	4	4
3	3	2	2	3	3	1	1	4	4
3	3	3	3	3	3	1	1	2	2
2	2	3	3	3	3	4	4	4	4
3	3	3	3	3	3	1	1	4	4
3	3	3	3	3	3	1	1	4	4
3	3	3	3	3	3	4	4	4	4
3	3	3	3	2	2	4	4	2	2
2	2	2	2	2	2	1	1	2	2
3	3	3	3	3	3	1	1	4	4
2	2	3	3	3	3	1	1	2	2
2	2	3	3	3	3	4	4	2	2
3	3	3	3	3	3	4	4	4	4
3	3	3	3	3	3	1	1	4	4
3	3	2	2	3	3	1	1	4	4
3	3	3	3	3	3	1	1	2	2
3	3	3	3	3	3	1	1	2	2
3	3	3	3	3	3	4	4	2	2
3	3	3	3	2	2	4	4	2	2
3	3	3	3	3	3	4	4	2	2
3	3	3	3	2	2	1	1	2	2
2	2	3	3	3	3	4	4	4	4
3	3	3	3	3	3	1	1	4	4
2	2	3	3	3	3	1	1	4	4
3	3	3	3	3	3	1	1	4	4
2	2	3	3	3	3	4	4	2	2
3	3	3	3	2	2	4	4	2	2
2	2	3	3	3	3	4	4	4	4
3	3	3	3	3	3	1	1	2	2
2	2	3	3	3	3	1	1	4	4
3	3	3	3	3	3	4	4	4	4
2	2	3	3	3	3	1	1	4	4
2	2	3	3	3	3	1	1	2	2
3	3	3	3	3	3	4	4	4	4
2	2	3	3	3	3	4	4	4	4
3	3	2	2	2	2	1	1	4	4
3	3	3	3	3	3	1	1	4	4
3	3	3	3	2	2	4	4	4	4
3	3	3	3	2	2	1	1	2	2
2	2	2	2	2	2	1	1	2	2
3	3	2	2	2	2	1	1	4	4
3	3	2	2	2	2	4	4	2	2
3	3	3	3	3	3	4	4	2	2
2	2	3	3	3	3	4	4	2	2
2	2	3	3	3	3	1	1	2	2
3	3	3	3	3	3	1	1	2	2
2	2	3	3	3	3	1	1	2	2
3	3	2	2	2	2	1	1	4	4

For Review Only

2	2	3	3	3	3	1	1	4	4
3	3	3	3	3	3	4	4	4	4
3	3	3	3	3	3	4	4	4	4
3	3	3	3	3	3	1	1	2	2
2	2	3	3	3	3	1	1	2	2
2	2	3	3	3	3	4	4	4	4
3	3	2	2	2	2	1	1	2	2
3	3	3	3	2	2	1	1	2	2
3	3	3	3	3	3	4	4	4	4
3	3	2	2	2	2	1	1	4	4
3	3	3	3	3	3	1	1	4	4
2	2	3	3	3	3	1	1	4	4
2	2	3	3	3	3	4	4	4	4
2	2	3	3	3	3	1	1	2	2
3	3	3	3	3	3	1	1	4	4
2	2	2	2	3	3	4	4	2	2
3	3	2	2	3	3	1	1	4	4
2	2	3	3	2	2	1	1	4	4
3	3	2	2	2	2	1	1	2	2
3	3	3	3	2	2	4	4	2	2
2	2	2	2	3	3	1	1	4	4
2	2	3	3	3	3	4	4	4	4
2	2	3	3	3	3	4	4	2	2
3	3	2	2	3	3	1	1	4	4
3	3	3	3	2	2	1	1	2	2
3	3	3	3	3	3	1	1	4	4
3	3	2	2	2	2	1	1	4	4
2	2	3	3	3	3	4	4	4	4
3	3	3	3	3	3	1	1	4	4
2	2	3	3	3	3	1	1	2	2
3	3	2	2	2	2	1	1	4	4
3	3	3	3	3	3	1	1	4	4
3	3	3	3	3	3	4	4	2	2
2	2	3	3	3	3	1	1	4	4
3	3	3	3	3	3	1	1	2	2
3	3	2	2	2	2	1	1	4	4
2	2	3	3	3	3	1	1	4	4
3	3	3	3	3	3	1	1	2	2
3	3	3	3	3	3	1	1	4	4
3	3	2	2	2	2	1	1	4	4
3	3	3	3	3	3	4	4	4	4
2	2	3	3	3	3	4	4	4	4
3	3	2	2	3	3	1	1	4	4
3	3	3	3	3	3	4	4	4	4
2	2	3	3	3	3	1	1	4	4
2	2	3	3	3	3	4	4	2	2
2	2	2	2	3	3	1	1	4	4
3	3	3	3	3	3	4	4	2	2
2	2	3	3	3	3	1	1	2	2
3	3	2	2	2	2	1	1	4	4
3	3	3	3	3	3	1	1	4	4
3	3	2	2	2	2	1	1	4	4
3	3	3	3	3	3	4	4	4	4
2	2	3	3	3	3	4	4	2	2
2	2	2	2	2	2	1	1	4	4

For Review Only

2	2	3	3	3	3	4	4	2	2
2	2	3	3	3	3	1	1	4	4

For Review Only

SNP1181	SNP1181	SNP1182	SNP1182	SNP1183	SNP1183	SNP1184	SNP1184	SNP1185	SNP1185
3	3	3	3	1	1	2	2	1	1
3	3	3	3	1	1	2	2	4	4
3	3	3	3	1	1	2	2	4	4
3	3	1	1	1	1	2	2	4	4
3	3	1	1	1	1	2	2	1	1
3	3	3	3	1	1	2	2	1	1
3	3	3	3	1	1	2	2	1	1
3	3	3	3	1	1	2	2	1	1
3	3	3	3	1	1	2	2	1	1
3	3	3	3	1	1	2	2	1	1
3	3	1	1	1	1	2	2	4	4
3	3	3	3	1	1	2	2	1	1
2	2	3	3	1	1	2	2	1	1
2	2	1	1	1	1	2	2	4	4
3	3	3	3	1	1	2	2	1	1
3	3	1	1	1	1	2	2	4	4
3	3	3	3	1	1	2	2	1	1
3	3	1	1	1	1	2	2	4	4
3	3	3	3	1	1	2	2	1	1
3	3	1	1	1	1	2	2	4	4
3	3	1	1	1	1	2	2	1	1
3	3	3	3	1	1	2	2	1	1
3	3	1	1	1	1	2	2	4	4
2	2	1	1	1	1	2	2	4	4
3	3	3	3	1	1	2	2	1	1
3	3	1	1	2	2	3	3	4	4
3	3	3	3	1	1	2	2	4	4
3	3	1	1	1	1	2	2	1	1
3	3	3	3	1	1	2	2	1	1
3	3	1	1	1	1	2	2	4	4
3	3	3	3	1	1	2	2	1	1
3	3	3	3	1	1	2	2	1	1
3	3	3	3	1	1	2	2	1	1
3	3	1	1	1	1	2	2	4	4
3	3	1	1	1	1	2	2	4	4
3	3	1	1	1	1	2	2	4	4
3	3	1	1	1	1	2	2	4	4
3	3	1	1	1	1	2	2	4	4
3	3	1	1	1	1	2	2	4	4
3	3	1	1	1	1	2	2	4	4
2	2	1	1	1	1	2	2	4	4
3	3	1	1	1	1	2	2	4	4
3	3	3	3	1	1	2	2	1	1
3	3	1	1	1	1	2	2	4	4
3	3	3	3	1	1	2	2	1	1
3	3	1	1	1	1	2	2	4	4
3	3	1	1	1	1	2	2	4	4
3	3	1	1	1	1	2	2	4	4
2	2	3	3	1	1	2	2	1	1
3	3	3	3	1	1	2	2	1	1
3	3	3	3	1	1	2	2	4	4
3	3	3	3	1	1	2	2	1	1

For Review Only

3	3	3	3	1	1	2	2	1	1
3	3	3	3	1	1	2	2	1	1
3	3	3	3	1	1	2	2	1	1
3	3	3	3	1	1	2	2	1	1
3	3	3	3	1	1	2	2	1	1
3	3	1	1	2	2	3	3	4	4
3	3	1	1	1	1	2	2	4	4
3	3	1	1	1	1	2	2	4	4
3	3	1	1	1	1	2	2	4	4
3	3	1	1	1	1	2	2	1	1
3	3	3	3	1	1	2	2	1	1
3	3	1	1	1	1	2	2	4	4
3	3	3	3	1	1	2	2	1	1
3	3	3	3	1	1	2	2	1	1
3	3	1	1	1	1	2	2	1	1
3	3	1	1	1	1	2	2	4	4
2	2	1	1	1	1	2	2	4	4
3	3	3	3	1	1	2	2	1	1
3	3	3	3	1	1	2	2	1	1
3	3	3	3	1	1	2	2	1	1
3	3	1	1	1	1	2	2	4	4
3	3	1	1	1	1	2	2	4	4
3	3	3	3	1	1	2	2	1	1
3	3	1	1	1	1	2	2	4	4
3	3	3	3	1	1	2	2	1	1
3	3	3	3	1	1	2	2	1	1
3	3	3	3	1	1	2	2	1	1
3	3	1	1	1	1	2	2	4	4
3	3	1	1	2	2	3	3	4	4
3	3	3	3	1	1	2	2	1	1
3	3	1	1	1	1	2	2	4	4
3	3	1	1	1	1	2	2	4	4
3	3	1	1	1	1	2	2	4	4
3	3	3	3	1	1	2	2	1	1
3	3	1	1	1	1	2	2	4	4
3	3	3	3	1	1	3	3	1	1
3	3	1	1	1	1	2	2	4	4
3	3	3	3	1	1	2	2	1	1
3	3	3	3	1	1	2	2	1	1
3	3	3	3	1	1	2	2	1	1
3	3	3	3	1	1	2	2	1	1
3	3	3	3	1	1	2	2	1	1
3	3	3	3	1	1	2	2	1	1
3	3	3	3	1	1	2	2	1	1
3	3	3	3	1	1	2	2	4	4
3	3	3	3	1	1	2	2	4	4
2	2	1	1	1	1	2	2	4	4
3	3	1	1	1	1	2	2	4	4
3	3	1	1	1	1	2	2	4	4
3	3	3	3	1	1	2	2	1	1
3	3	3	3	1	1	2	2	4	4
3	3	1	1	1	1	2	2	4	4
3	3	1	1	1	1	2	2	4	4
3	3	1	1	1	1	2	2	4	4
3	3	3	3	1	1	2	2	1	1

For Review Only

3	3	1	1	1	1	3	3	4	4
3	3	1	1	1	1	2	2	4	4
3	3	1	1	1	1	2	2	4	4
2	2	1	1	1	1	2	2	4	4
3	3	3	3	1	1	2	2	4	4
3	3	1	1	2	2	3	3	4	4
3	3	3	3	1	1	2	2	1	1
3	3	1	1	1	1	2	2	4	4
3	3	1	1	1	1	2	2	4	4
3	3	1	1	1	1	2	2	4	4
3	3	1	1	1	1	2	2	4	4
3	3	1	1	1	1	2	2	1	1
3	3	3	3	1	1	2	2	1	1
3	3	3	3	1	1	2	2	1	1
3	3	1	1	1	1	2	2	1	1
3	3	1	1	1	1	2	2	4	4
3	3	3	3	1	1	2	2	1	1
3	3	1	1	2	2	3	3	4	4
2	2	1	1	1	1	2	2	4	4
3	3	3	3	1	1	2	2	1	1
3	3	1	1	1	1	2	2	1	1
3	3	3	3	1	1	2	2	1	1
3	3	3	3	1	1	2	2	4	4
3	3	1	1	1	1	2	2	4	4
3	3	1	1	1	1	3	3	4	4
3	3	1	1	1	1	2	2	4	4
3	3	3	3	1	1	2	2	1	1
3	3	3	3	1	1	2	2	1	1
3	3	3	3	1	1	2	2	1	1
3	3	3	3	2	2	3	3	1	1
3	3	1	1	1	1	2	2	4	4
3	3	1	1	1	1	2	2	1	1
3	3	3	3	1	1	2	2	1	1
3	3	1	1	2	2	3	3	4	4
3	3	1	1	1	1	2	2	4	4
3	3	1	1	1	1	2	2	1	1
2	2	1	1	1	1	2	2	4	4
3	3	3	3	1	1	2	2	1	1
3	3	1	1	1	1	2	2	4	4
3	3	1	1	1	1	2	2	4	4
3	3	3	3	1	1	2	2	1	1
3	3	1	1	1	1	2	2	4	4
3	3	3	3	1	1	2	2	4	4
3	3	1	1	1	1	2	2	4	4
3	3	1	1	1	1	2	2	4	4
3	3	3	3	1	1	2	2	1	1
3	3	1	1	2	2	3	3	4	4
3	3	3	3	1	1	2	2	4	4
2	2	1	1	1	1	2	2	4	4
3	3	1	1	2	2	3	3	4	4
3	3	1	1	2	2	3	3	4	4
3	3	1	1	1	1	2	2	4	4
3	3	1	1	1	1	2	2	4	4
3	3	1	1	1	1	2	2	4	4
3	3	1	1	1	1	2	2	4	4

For Review Only

3	3	3	3	1	1	2	2	1	1
3	3	1	1	1	1	2	2	4	4
3	3	1	1	1	1	2	2	4	4
3	3	1	1	1	1	2	2	1	1
3	3	3	3	1	1	2	2	1	1
2	2	1	1	1	1	2	2	4	4
3	3	1	1	1	1	2	2	4	4
3	3	1	1	1	1	2	2	4	4
3	3	1	1	1	1	3	3	4	4
3	3	3	3	1	1	2	2	1	1
3	3	1	1	1	1	2	2	4	4
3	3	1	1	1	1	2	2	4	4
3	3	1	1	1	1	2	2	4	4
3	3	1	1	1	1	2	2	1	1
3	3	1	1	1	1	2	2	1	1
3	3	1	1	1	1	2	2	4	4
3	3	1	1	1	1	2	2	4	4
3	3	3	3	1	1	2	2	1	1
3	3	1	1	1	1	2	2	4	4
3	3	3	3	1	1	2	2	1	1
3	3	1	1	1	1	2	2	1	1
3	3	1	1	1	1	2	2	4	4
3	3	1	1	1	1	2	2	4	4
3	3	1	1	1	1	2	2	4	4
3	3	1	1	1	1	2	2	4	4
3	3	1	1	1	1	2	2	4	4
3	3	3	3	1	1	2	2	1	1
3	3	1	1	1	1	2	2	1	1
3	3	1	1	1	1	2	2	4	4
3	3	3	3	1	1	2	2	1	1
2	2	1	1	1	1	2	2	4	4
3	3	3	3	1	1	2	2	1	1
3	3	3	3	1	1	2	2	1	1
3	3	1	1	1	1	2	2	4	4
2	2	1	1	1	1	2	2	4	4
3	3	1	1	1	1	2	2	4	4
3	3	1	1	1	1	2	2	4	4
3	3	3	3	1	1	2	2	1	1
3	3	3	3	1	1	2	2	4	4
3	3	3	3	1	1	2	2	1	1
3	3	3	3	1	1	2	2	4	4
3	3	1	1	2	2	3	3	4	4
3	3	1	1	1	1	2	2	4	4
3	3	1	1	1	1	2	2	4	4
3	3	1	1	1	1	2	2	4	4
3	3	3	3	1	1	2	2	1	1
3	3	1	1	1	1	2	2	4	4
3	3	1	1	1	1	2	2	4	4
3	3	1	1	1	1	2	2	4	4
3	3	3	3	1	1	2	2	1	1
3	3	3	3	1	1	2	2	1	1
3	3	1	1	1	1	2	2	4	4
3	3	1	1	1	1	2	2	4	4
3	3	3	3	1	1	2	2	1	1
3	3	1	1	1	1	2	2	4	4

For Review Only

3	3	1	1	1	1	2	2	4	4
3	3	1	1	1	1	2	2	4	4
3	3	3	3	1	1	2	2	1	1
3	3	1	1	1	1	2	2	1	1
3	3	1	1	1	1	2	2	4	4
3	3	3	3	2	2	3	3	1	1
3	3	1	1	1	1	2	2	4	4
3	3	1	1	1	1	2	2	4	4
3	3	1	1	1	1	2	2	4	4
3	3	1	1	1	1	2	2	4	4
3	3	1	1	1	1	2	2	1	1
3	3	1	1	1	1	2	2	4	4
3	3	3	3	1	1	2	2	1	1
3	3	1	1	1	1	2	2	4	4
3	3	3	3	1	1	2	2	4	4
3	3	1	1	1	1	2	2	4	4
3	3	1	1	1	1	2	2	4	4
3	3	1	1	1	1	2	2	4	4
3	3	1	1	1	1	2	2	4	4
3	3	1	1	1	1	2	2	4	4
3	3	1	1	1	1	2	2	4	4
3	3	1	1	2	2	3	3	4	4
3	3	3	3	1	1	2	2	1	1
3	3	1	1	1	1	2	2	4	4
3	3	1	1	1	1	2	2	4	4
3	3	3	3	1	1	2	2	1	1
3	3	1	1	1	1	2	2	4	4
3	3	3	3	1	1	2	2	1	1
3	3	1	1	1	1	2	2	4	4
3	3	3	3	1	1	2	2	1	1
3	3	1	1	1	1	2	2	4	4
3	3	1	1	1	1	3	3	4	4
3	3	1	1	1	1	2	2	4	4
3	3	1	1	1	1	2	2	4	4
3	3	1	1	1	1	2	2	4	4
3	3	1	1	1	1	2	2	4	4
3	3	3	3	1	1	2	2	1	1
3	3	1	1	1	1	2	2	4	4
3	3	1	1	1	1	2	2	4	4
3	3	1	1	1	1	2	2	4	4
3	3	3	3	1	1	2	2	1	1
3	3	3	3	1	1	2	2	4	4
3	3	1	1	1	1	2	2	4	4
3	3	3	3	1	1	2	2	1	1
3	3	1	1	1	1	2	2	4	4
3	3	3	3	1	1	2	2	1	1
3	3	1	1	1	1	2	2	4	4
3	3	3	3	1	1	2	2	4	4
3	3	3	3	1	1	2	2	1	1
3	3	1	1	1	1	2	2	4	4
3	3	3	3	1	1	2	2	1	1
3	3	1	1	1	1	2	2	4	4
3	3	3	3	1	1	2	2	4	4
3	3	3	3	1	1	2	2	1	1
3	3	1	1	1	1	2	2	4	4

For Review Only

3	3	3	3	1	1	2	2	1	1
3	3	3	3	1	1	2	2	1	1

For Review Only

SNP1186	SNP1186	SNP1187	SNP1187	SNP1188	SNP1188	SNP1189	SNP1189	SNP119	SNP119
4	4	1	1	4	4	1	1	4	4
1	1	2	2	3	3	1	1	4	4
1	1	2	2	4	4	1	1	4	4
1	1	2	2	4	4	1	1	4	4
4	4	1	1	4	4	1	1	4	4
4	4	1	1	4	4	1	1	4	4
4	4	1	1	4	4	1	1	4	4
4	4	1	1	4	4	1	1	4	4
4	4	1	1	3	3	1	1	4	4
4	4	1	1	4	4	1	1	4	4
1	1	2	2	4	4	1	1	4	4
4	4	1	1	4	4	1	1	4	4
4	4	1	1	3	3	1	1	4	4
1	1	2	2	3	3	1	1	1	1
4	4	1	1	4	4	1	1	4	4
1	1	2	2	3	3	1	1	4	4
4	4	1	1	4	4	1	1	4	4
1	1	2	2	3	3	1	1	4	4
4	4	1	1	4	4	1	1	4	4
1	1	2	2	4	4	1	1	4	4
1	1	2	2	4	4	1	1	4	4
4	4	1	1	4	4	1	1	4	4
1	1	2	2	3	3	1	1	4	4
1	1	2	2	3	3	4	4	4	4
1	1	1	1	4	4	1	1	4	4
1	1	2	2	4	4	1	1	4	4
1	1	2	2	4	4	1	1	4	4
4	4	1	1	4	4	1	1	4	4
4	4	1	1	4	4	1	1	4	4
1	1	2	2	4	4	4	4	4	4
4	4	1	1	4	4	1	1	4	4
1	1	1	1	4	4	1	1	4	4
1	1	1	1	4	4	1	1	4	4
1	1	2	2	3	3	1	1	1	1
1	1	2	2	3	3	1	1	4	4
1	1	2	2	4	4	4	4	4	4
1	1	2	2	4	4	1	1	1	1
1	1	2	2	4	4	1	1	4	4
1	1	2	2	3	3	1	1	4	4
1	1	2	2	3	3	1	1	4	4
1	1	2	2	4	4	1	1	4	4
1	1	2	2	4	4	1	1	4	4
4	4	1	1	4	4	1	1	4	4
1	1	2	2	4	4	4	4	4	4
4	4	1	1	4	4	1	1	4	4
1	1	2	2	4	4	1	1	4	4
1	1	2	2	4	4	1	1	4	4
1	1	2	2	3	3	1	1	4	4
4	4	1	1	4	4	1	1	4	4
4	4	1	1	3	3	1	1	4	4
1	1	2	2	4	4	1	1	4	4
4	4	1	1	4	4	1	1	4	4

For Review Only

4	4	1	1	4	4	1	1	4	4
1	1	1	1	4	4	1	1	4	4
1	1	1	1	4	4	1	1	4	4
4	4	1	1	4	4	4	4	4	4
4	4	1	1	4	4	1	1	4	4
1	1	2	2	4	4	1	1	4	4
1	1	2	2	4	4	1	1	4	4
1	1	2	2	3	3	1	1	4	4
1	1	2	2	3	3	1	1	1	1
4	4	1	1	4	4	1	1	4	4
4	4	1	1	4	4	1	1	4	4
1	1	2	2	4	4	1	1	4	4
4	4	1	1	4	4	1	1	4	4
4	4	1	1	4	4	1	1	4	4
4	4	1	1	4	4	1	1	4	4
1	1	2	2	3	3	1	1	4	4
1	1	2	2	4	4	1	1	4	4
4	4	1	1	4	4	1	1	4	4
4	4	1	1	4	4	1	1	4	4
4	4	1	1	4	4	1	1	4	4
1	1	2	2	4	4	1	1	4	4
1	1	2	2	4	4	1	1	4	4
4	4	1	1	4	4	1	1	4	4
1	1	2	2	4	4	1	1	4	4
4	4	1	1	4	4	1	1	4	4
4	4	1	1	4	4	1	1	4	4
4	4	1	1	3	3	1	1	4	4
4	4	2	2	3	3	1	1	4	4
1	1	2	2	4	4	1	1	4	4
1	1	1	1	4	4	1	1	4	4
1	1	2	2	4	4	1	1	4	4
1	1	2	2	4	4	1	1	4	4
1	1	2	2	3	3	1	1	4	4
4	4	1	1	4	4	1	1	4	4
1	1	2	2	4	4	1	1	4	4
4	4	1	1	4	4	1	1	4	4
1	1	2	2	4	4	1	1	4	4
4	4	1	1	4	4	1	1	4	4
4	4	1	1	3	3	1	1	4	4
4	4	1	1	4	4	1	1	4	4
4	4	1	1	4	4	1	1	4	4
4	4	1	1	4	4	1	1	4	4
4	4	1	1	4	4	1	1	4	4
4	4	1	1	4	4	1	1	4	4
1	1	1	1	4	4	1	1	4	4
1	1	2	2	4	4	1	1	4	4
1	1	2	2	3	3	1	1	1	1
1	1	2	2	4	4	1	1	4	4
1	1	2	2	4	4	1	1	4	4
4	4	1	1	4	4	1	1	4	4
1	1	2	2	4	4	1	1	4	4
1	1	2	2	3	3	1	1	4	4
1	1	2	2	4	4	1	1	1	1
4	4	1	1	3	3	1	1	4	4
4	4	1	1	4	4	1	1	4	4

For Review Only

1	1	2	2	4	4	1	1	4	4
1	1	2	2	4	4	1	1	4	4
1	1	2	2	4	4	4	4	4	4
1	1	2	2	3	3	1	1	1	1
1	1	2	2	4	4	1	1	4	4
1	1	2	2	4	4	1	1	4	4
4	4	1	1	4	4	1	1	4	4
1	1	2	2	4	4	1	1	4	4
1	1	2	2	3	3	1	1	4	4
1	1	2	2	3	3	1	1	4	4
1	1	2	2	3	3	1	1	4	4
4	4	1	1	3	3	1	1	4	4
4	4	1	1	4	4	1	1	4	4
4	4	1	1	4	4	1	1	4	4
4	4	2	2	4	4	1	1	4	4
4	4	1	1	3	3	1	1	4	4
4	4	1	1	4	4	1	1	4	4
4	4	1	1	3	3	1	1	4	4
1	1	2	2	3	3	1	1	1	1
4	4	1	1	4	4	1	1	4	4
4	4	1	1	3	3	1	1	4	4
4	4	1	1	4	4	1	1	4	4
1	1	2	2	4	4	1	1	4	4
1	1	2	2	3	3	1	1	4	4
1	1	2	2	4	4	1	1	4	4
1	1	2	2	3	3	1	1	4	4
4	4	1	1	4	4	1	1	4	4
4	4	1	1	4	4	1	1	4	4
4	4	1	1	4	4	1	1	4	4
4	4	1	1	4	4	1	1	4	4
1	1	2	2	3	3	1	1	1	1
4	4	1	1	3	3	1	1	4	4
4	4	1	1	4	4	1	1	4	4
1	1	2	2	4	4	1	1	4	4
1	1	2	2	3	3	1	1	1	1
1	1	2	2	4	4	1	1	4	4
1	1	2	2	3	3	1	1	1	1
4	4	2	2	4	4	1	1	4	4
1	1	2	2	4	4	1	1	4	4
1	1	2	2	3	3	1	1	4	4
1	1	2	2	3	3	1	1	4	4
1	1	2	2	3	3	4	4	1	1
1	1	2	2	4	4	1	1	4	4
1	1	2	2	3	3	1	1	4	4
1	1	2	2	4	4	1	1	4	4
4	4	1	1	3	3	1	1	4	4
1	1	2	2	4	4	1	1	4	4
1	1	2	2	4	4	1	1	4	4
1	1	2	2	3	3	1	1	4	4
1	1	2	2	4	4	1	1	4	4
1	1	2	2	4	4	1	1	4	4
1	1	2	2	3	3	1	1	4	4
1	1	2	2	3	3	1	1	4	4
1	1	2	2	4	4	1	1	4	4
1	1	1	1	3	3	1	1	4	4

For Review Only

4	4	1	1	4	4	1	1	4	4
1	1	2	2	4	4	1	1	4	4
1	1	2	2	3	3	1	1	4	4
4	4	1	1	3	3	1	1	4	4
4	4	1	1	4	4	1	1	4	4
1	1	2	2	4	4	1	1	4	4
1	1	2	2	3	3	1	1	4	4
1	1	1	1	3	3	1	1	4	4
1	1	2	2	3	3	1	1	4	4
4	4	1	1	4	4	1	1	4	4
1	1	2	2	4	4	1	1	4	4
1	1	2	2	3	3	1	1	4	4
1	1	2	2	4	4	1	1	4	4
4	4	1	1	3	3	1	1	4	4
1	1	2	2	3	3	1	1	1	1
1	1	2	2	3	3	1	1	4	4
1	1	2	2	3	3	1	1	4	4
4	4	1	1	4	4	1	1	4	4
1	1	2	2	4	4	1	1	4	4
4	4	1	1	4	4	1	1	4	4
4	4	1	1	3	3	1	1	4	4
1	1	2	2	3	3	1	1	4	4
1	1	2	2	4	4	1	1	4	4
1	1	2	2	4	4	1	1	4	4
1	1	2	2	4	4	1	1	4	4
1	1	2	2	3	3	1	1	4	4
4	4	1	1	4	4	1	1	4	4
4	4	1	1	3	3	1	1	4	4
1	1	2	2	3	3	1	1	4	4
4	4	1	1	4	4	1	1	4	4
1	1	2	2	4	4	1	1	4	4
1	1	1	1	4	4	1	1	4	4
4	4	1	1	4	4	1	1	4	4
1	1	2	2	3	3	1	1	1	1
4	4	1	1	4	4	1	1	4	4
1	1	2	2	4	4	1	1	4	4
1	1	2	2	4	4	4	4	4	4
4	4	1	1	4	4	1	1	4	4
1	1	2	2	4	4	1	1	4	4
1	1	1	1	4	4	1	1	4	4
1	1	2	2	4	4	1	1	4	4
1	1	2	2	4	4	1	1	4	4
1	1	2	2	3	3	1	1	4	4
1	1	2	2	4	4	1	1	4	4
1	1	2	2	3	3	1	1	4	4
4	4	1	1	4	4	1	1	4	4
1	1	2	2	3	3	1	1	4	4
1	1	2	2	3	3	1	1	4	4
1	1	2	2	3	3	1	1	4	4
4	4	1	1	4	4	4	4	4	4
4	4	1	1	4	4	1	1	4	4
1	1	2	2	4	4	1	1	1	1
1	1	2	2	4	4	1	1	4	4
4	4	1	1	4	4	1	1	4	4
1	1	2	2	3	3	1	1	4	4

For Review Only

1	1	2	2	3	3	1	1	4	4
1	1	2	2	4	4	1	1	4	4
4	4	1	1	4	4	1	1	4	4
4	4	1	1	4	4	1	1	4	4
1	1	2	2	4	4	1	1	1	1
4	4	1	1	4	4	1	1	4	4
1	1	2	2	3	3	1	1	4	4
1	1	2	2	4	4	4	4	4	4
1	1	2	2	4	4	1	1	4	4
1	1	2	2	3	3	1	1	4	4
4	4	1	1	3	3	1	1	4	4
1	1	2	2	3	3	1	1	4	4
4	4	1	1	3	3	1	1	4	4
1	1	1	1	4	4	1	1	4	4
1	1	1	1	4	4	1	1	4	4
1	1	2	2	3	3	1	1	4	4
1	1	2	2	3	3	1	1	1	1
1	1	1	1	3	3	1	1	4	4
1	1	2	2	3	3	1	1	4	4
1	1	2	2	4	4	1	1	4	4
4	4	2	2	3	3	1	1	4	4
1	1	2	2	4	4	1	1	4	4
4	4	1	1	4	4	1	1	4	4
1	1	2	2	3	3	1	1	4	4
1	1	2	2	3	3	1	1	4	4
4	4	1	1	4	4	1	1	4	4
1	1	2	2	3	3	1	1	4	4
4	4	1	1	4	4	1	1	4	4
1	1	2	2	4	4	1	1	4	4
4	4	1	1	4	4	1	1	4	4
4	4	2	2	3	3	1	1	4	4
1	1	2	2	4	4	1	1	4	4
1	1	2	2	4	4	1	1	4	4
1	1	2	2	4	4	1	1	4	4
1	1	2	2	4	4	1	1	4	4
1	1	2	2	3	3	1	1	4	4
4	4	1	1	4	4	1	1	4	4
1	1	2	2	4	4	1	1	4	4
1	1	2	2	4	4	4	4	4	4
1	1	2	2	3	3	1	1	4	4
1	1	1	1	4	4	1	1	4	4
1	1	2	2	4	4	1	1	4	4
1	1	2	2	3	3	1	1	4	4
4	4	1	1	4	4	1	1	4	4
4	4	2	2	4	4	1	1	4	4
4	4	1	1	4	4	1	1	4	4
1	1	2	2	3	3	1	1	4	4
1	1	2	2	4	4	1	1	4	4
1	1	2	2	4	4	1	1	4	4
1	1	2	2	3	3	1	1	4	4
4	4	1	1	4	4	1	1	4	4
1	1	2	2	3	3	1	1	4	4
1	1	2	2	4	4	1	1	4	4
4	4	1	1	4	4	1	1	4	4
1	1	2	2	3	3	1	1	4	4

For Review Only

4	4	1	1	4	4	1	1	4	4
4	4	1	1	4	4	1	1	4	4

For Review Only

SNP1190	SNP1190	SNP1191	SNP1191	SNP1192	SNP1192	SNP1193	SNP1193	SNP1194	SNP1194
3	3	1	1	3	3	3	3	4	4
2	2	2	2	2	2	2	2	4	4
2	2	2	2	2	2	3	3	4	4
2	2	2	2	2	2	3	3	4	4
3	3	1	1	3	3	3	3	4	4
2	2	2	2	2	2	3	3	4	4
2	2	2	2	3	3	3	3	4	4
2	2	2	2	3	3	3	3	4	4
3	3	1	1	3	3	2	2	4	4
2	2	2	2	3	3	3	3	4	4
3	3	1	1	2	2	2	2	4	4
2	2	2	2	3	3	3	3	4	4
2	2	2	2	3	3	3	3	4	4
3	3	1	1	2	2	2	2	4	4
3	3	1	1	3	3	2	2	4	4
2	2	2	2	2	2	3	3	2	2
3	3	1	1	3	3	2	2	4	4
3	3	1	1	2	2	3	3	4	4
2	2	1	1	3	3	2	2	4	4
3	3	1	1	2	2	2	2	4	4
2	2	2	2	2	2	2	2	4	4
3	3	1	1	3	3	3	3	4	4
2	2	2	2	2	2	3	3	4	4
2	2	2	2	2	2	2	2	4	4
2	2	2	2	2	2	3	3	4	4
3	3	1	1	2	2	3	3	4	4
2	2	2	2	2	2	3	3	4	4
3	3	1	1	3	3	2	2	4	4
2	2	2	2	3	3	3	3	4	4
2	2	2	2	2	2	2	2	4	4
2	2	2	2	3	3	3	3	4	4
2	2	2	2	3	3	3	3	4	4
2	2	2	2	3	3	2	2	4	4
2	2	2	2	2	2	2	2	4	4
3	3	1	1	2	2	2	2	4	4
2	2	2	2	2	2	3	3	4	4
2	2	2	2	2	2	2	2	4	4
2	2	2	2	2	2	2	2	4	4
2	2	2	2	2	2	2	2	4	4
2	2	2	2	2	2	3	3	4	4
2	2	2	2	2	2	2	2	4	4
2	2	2	2	3	3	2	2	4	4
2	2	2	2	3	3	2	2	4	4
3	3	1	1	2	2	2	2	4	4
2	2	2	2	3	3	2	2	4	4
2	2	2	2	2	2	3	3	4	4
3	3	1	1	2	2	2	2	4	4
2	2	2	2	2	2	3	3	4	4
3	3	1	1	3	3	2	2	4	4
3	3	1	1	3	3	2	2	4	4
2	2	2	2	2	2	3	3	4	4
3	3	1	1	3	3	3	3	4	4

For Review Only

3	3	1	1	3	3	3	3	4	4
2	2	2	2	3	3	2	2	4	4
2	2	2	2	3	3	3	3	4	4
3	3	1	1	3	3	2	2	4	4
2	2	1	1	3	3	2	2	4	4
3	3	1	1	2	2	2	2	4	4
3	3	1	1	2	2	3	3	4	4
2	2	2	2	2	2	2	2	4	4
2	2	2	2	2	2	2	2	4	4
2	2	2	2	3	3	2	2	4	4
3	3	1	1	2	2	2	2	4	4
3	3	1	1	2	2	2	2	4	4
3	3	1	1	3	3	3	3	4	4
2	2	2	2	3	3	3	3	4	4
2	2	2	2	2	2	2	2	4	4
2	2	2	2	2	2	2	2	4	4
2	2	2	2	2	2	2	2	4	4
3	3	1	1	3	3	3	3	4	4
3	3	1	1	3	3	2	2	4	4
3	3	1	1	3	3	3	3	4	4
3	3	1	1	2	2	2	2	4	4
3	3	1	1	2	2	2	2	4	4
2	2	2	2	3	3	2	2	4	4
2	2	2	2	2	2	3	3	4	4
2	2	2	2	3	3	2	2	4	4
2	2	2	2	2	2	2	2	4	4
2	2	2	2	3	3	2	2	4	4
2	2	2	2	2	2	3	3	4	4
3	3	1	1	2	2	3	3	4	4
2	2	2	2	2	2	3	3	4	4
2	2	1	1	2	2	3	3	4	4
2	2	2	2	2	2	2	2	4	4
2	2	2	2	2	2	2	2	2	2
3	3	1	1	3	3	3	3	4	4
3	3	1	1	2	2	2	2	4	4
2	2	2	2	3	3	2	2	4	4
2	2	2	2	2	2	3	3	4	4
3	3	1	1	3	3	2	2	4	4
2	2	2	2	3	3	2	2	4	4
2	2	2	2	3	3	2	2	4	4
3	3	1	1	3	3	3	3	4	4
2	2	2	2	2	2	2	2	4	4
2	2	2	2	3	3	3	3	4	4
3	3	1	1	2	2	2	2	4	4
2	2	2	2	2	2	2	2	4	4
3	3	1	1	2	2	2	2	4	4
2	2	2	2	2	2	2	2	4	4
3	3	1	1	2	2	2	2	4	4
3	3	1	1	2	2	2	2	4	4
3	3	1	1	3	3	2	2	4	4
2	2	2	2	2	2	2	2	4	4
2	2	2	2	2	2	3	3	4	4
2	2	2	2	2	2	2	2	4	4
2	2	2	2	3	3	2	2	4	4
3	3	1	1	3	3	2	2	4	4

For Review Only

3	3	1	1	2	2	2	2	4	4
2	2	2	2	2	2	2	2	4	4
2	2	2	2	2	2	3	3	4	4
2	2	2	2	2	2	2	2	4	4
3	3	1	1	2	2	3	3	4	4
2	2	1	1	2	2	2	2	4	4
2	2	2	2	3	3	3	3	4	4
2	2	2	2	2	2	2	2	4	4
2	2	2	2	2	2	2	2	2	2
2	2	2	2	2	2	3	3	4	4
3	3	1	1	2	2	2	2	4	4
3	3	1	1	3	3	2	2	4	4
2	2	2	2	3	3	2	2	4	4
2	2	2	2	2	2	2	2	4	4
2	2	2	2	3	3	3	3	4	4
3	3	1	1	2	2	2	2	4	4
3	3	1	1	3	3	2	2	4	4
3	3	1	1	3	3	2	2	4	4
2	2	2	2	2	2	2	2	4	4
3	3	1	1	3	3	2	2	4	4
3	3	1	1	3	3	2	2	4	4
2	2	2	2	3	3	3	3	4	4
2	2	2	2	2	2	2	2	4	4
2	2	2	2	2	2	3	3	4	4
3	3	1	1	2	2	2	2	4	4
2	2	2	2	2	2	3	3	2	2
3	3	1	1	3	3	3	3	4	4
2	2	2	2	3	3	2	2	4	4
3	3	1	1	3	3	2	2	4	4
3	3	1	1	3	3	3	3	4	4
2	2	2	2	2	2	2	2	4	4
3	3	1	1	2	2	2	2	4	4
3	3	1	1	3	3	3	3	4	4
2	2	1	1	2	2	3	3	4	4
2	2	2	2	2	2	2	2	4	4
2	2	2	2	2	2	3	3	4	4
2	2	2	2	2	2	2	2	4	4
2	2	1	1	2	2	3	3	4	4
3	3	1	1	2	2	3	3	4	4
3	3	1	1	2	2	2	2	4	4
3	3	1	1	2	2	2	2	4	4
2	2	2	2	2	2	2	2	4	4
3	3	1	1	3	3	3	3	4	4
2	2	2	2	2	2	2	2	4	4
2	2	2	2	2	2	3	3	4	4
2	2	2	2	3	3	3	3	4	4
3	3	1	1	2	2	3	3	4	4
3	3	1	1	2	2	3	3	4	4
2	2	2	2	2	2	3	3	4	4
2	2	1	1	2	2	3	3	4	4
3	3	1	1	2	2	3	3	4	4
3	3	1	1	2	2	2	2	2	2
3	3	1	1	2	2	2	2	4	4
3	3	1	1	2	2	2	2	4	4
2	2	2	2	2	2	2	2	4	4

For Review Only

3	3	1	1	3	3	3	3	4	4
2	2	2	2	2	2	2	2	4	4
3	3	1	1	2	2	2	2	4	4
2	2	2	2	3	3	2	2	4	4
3	3	1	1	3	3	2	2	4	4
3	3	1	1	2	2	3	3	4	4
2	2	2	2	2	2	3	3	4	4
3	3	1	1	3	3	2	2	4	4
2	2	2	2	2	2	2	2	4	4
3	3	1	1	3	3	2	2	4	4
2	2	2	2	2	2	2	2	4	4
3	3	1	1	2	2	3	3	4	4
2	2	2	2	2	2	2	2	4	4
2	2	2	2	3	3	2	2	4	4
2	2	2	2	2	2	2	2	4	4
3	3	1	1	2	2	2	2	4	4
3	3	1	1	2	2	2	2	4	4
2	2	2	2	2	2	2	2	4	4
2	2	2	2	2	2	2	2	4	4
2	2	2	2	3	3	3	3	4	4
3	3	1	1	3	3	2	2	4	4
2	2	2	2	2	2	2	2	4	4
2	2	2	2	2	2	2	2	4	4
3	3	1	1	2	2	2	2	4	4
2	2	2	2	2	2	2	2	4	4
2	2	2	2	2	2	2	2	4	4
2	2	2	2	3	3	2	2	4	4
3	3	1	1	3	3	2	2	4	4
3	3	1	1	2	2	3	3	4	4
3	3	1	1	3	3	3	3	4	4
2	2	2	2	2	2	3	3	4	4
3	3	1	1	2	2	3	3	4	4
3	3	1	1	3	3	2	2	4	4
2	2	2	2	2	2	2	2	4	4
3	3	1	1	3	3	3	3	4	4
3	3	1	1	2	2	2	2	4	4
2	2	2	2	2	2	2	2	4	4
2	2	2	2	3	3	3	3	4	4
3	3	1	1	2	2	3	3	4	4
2	2	2	2	2	2	2	2	4	4
2	2	2	2	2	2	3	3	4	4
3	3	1	1	2	2	2	2	4	4
2	2	2	2	2	2	3	3	2	2
3	3	1	1	2	2	3	3	4	4
3	3	1	1	2	2	2	2	4	4
2	2	2	2	3	3	2	2	4	4
3	3	1	1	2	2	2	2	4	4
2	2	2	2	2	2	3	3	2	2
2	2	2	2	2	2	2	2	4	4
3	3	1	1	3	3	2	2	4	4
3	3	1	1	3	3	2	2	4	4
2	2	2	2	2	2	2	2	4	4
2	2	2	2	2	2	2	2	4	4
3	3	1	1	2	2	2	2	4	4
2	2	2	2	2	2	3	3	4	4

For Review Only

3	3	1	1	2	2	3	3	4	4
2	2	2	2	2	2	3	3	4	4
3	3	1	1	3	3	3	3	4	4
2	2	2	2	3	3	2	2	4	4
3	3	1	1	2	2	2	2	4	4
3	3	1	1	3	3	3	3	4	4
3	3	1	1	2	2	2	2	4	4
2	2	2	2	2	2	2	2	4	4
2	2	2	2	2	2	2	2	4	4
2	2	2	2	2	2	3	3	4	4
3	3	1	1	3	3	2	2	4	4
2	2	2	2	2	2	3	3	4	4
3	3	1	1	3	3	3	3	4	4
2	2	2	2	2	2	2	2	4	4
2	2	2	2	2	2	3	3	4	4
3	3	1	1	2	2	2	2	4	4
2	2	2	2	2	2	3	3	4	4
2	2	2	2	2	2	2	2	4	4
3	3	1	1	2	2	2	2	2	2
2	2	2	2	2	2	2	2	4	4
2	2	2	2	2	2	3	3	4	4
3	3	1	1	2	2	3	3	4	4
2	2	2	2	3	3	2	2	4	4
3	3	1	1	2	2	3	3	4	4
2	2	2	2	2	2	2	2	4	4
2	2	2	2	3	3	2	2	4	4
2	2	2	2	2	2	3	3	2	2
2	2	2	2	3	3	3	3	4	4
3	3	1	1	2	2	2	2	4	4
3	3	1	1	3	3	2	2	4	4
2	2	2	2	2	2	3	3	2	2
2	2	1	1	2	2	3	3	4	4
2	2	2	2	2	2	2	2	4	4
2	2	1	1	2	2	3	3	4	4
3	3	1	1	2	2	2	2	2	2
2	2	2	2	2	2	3	3	2	2
3	3	1	1	3	3	3	3	4	4
3	3	1	1	2	2	2	2	4	4
2	2	2	2	2	2	3	3	4	4
2	2	2	2	2	2	3	3	2	2
3	3	1	1	3	3	3	3	4	4
3	3	1	1	2	2	3	3	4	4
2	2	2	2	2	2	3	3	4	4
3	3	1	1	3	3	3	3	4	4
3	3	1	1	2	2	3	3	4	4
2	2	2	2	3	3	2	2	4	4
3	3	1	1	2	2	3	3	4	4
2	2	2	2	2	2	2	2	4	4
2	2	2	2	2	2	2	2	4	4
2	2	2	2	2	2	3	3	2	2
3	3	1	1	3	3	3	3	4	4
2	2	2	2	2	2	3	3	2	2
2	2	2	2	2	2	3	3	4	4
2	2	2	2	3	3	2	2	4	4
2	2	2	2	2	2	3	3	2	2

For Review Only

3	3	1	1	3	3	2	2	4	4
2	2	2	2	2	2	3	3	4	4

For Review Only

SNP1195	SNP1195	SNP1196	SNP1196	SNP1197	SNP1197	SNP1198	SNP1198	SNP1199	SNP1199
4	4	2	2	3	3	2	2	1	1
4	4	2	2	3	3	2	2	1	1
3	3	2	2	3	3	3	3	1	1
4	4	3	3	3	3	3	3	1	1
3	3	3	3	3	3	2	2	1	1
3	3	2	2	3	3	3	3	1	1
4	4	2	2	3	3	2	2	1	1
3	3	2	2	3	3	2	2	1	1
4	4	2	2	3	3	2	2	1	1
3	3	2	2	3	3	2	2	1	1
3	3	3	3	2	2	3	3	1	1
4	4	2	2	3	3	3	3	1	1
3	3	2	2	3	3	3	3	1	1
4	4	3	3	2	2	2	2	1	1
4	4	2	2	3	3	3	3	1	1
3	3	3	3	2	2	3	3	1	1
4	4	2	2	3	3	3	3	1	1
4	4	3	3	2	2	2	2	1	1
3	3	2	2	3	3	3	3	1	1
4	4	3	3	2	2	2	2	1	1
3	3	3	3	2	2	2	2	1	1
3	3	2	2	3	3	3	3	1	1
3	3	3	3	2	2	2	2	1	1
3	3	3	3	2	2	2	2	1	1
4	4	2	2	3	3	3	3	1	1
4	4	3	3	2	2	3	3	1	1
4	4	3	3	3	3	3	3	1	1
4	4	3	3	3	3	3	3	1	1
3	3	2	2	3	3	2	2	1	1
4	4	3	3	2	2	2	2	1	1
4	4	2	2	3	3	3	3	1	1
4	4	2	2	3	3	3	3	1	1
4	4	2	2	3	3	2	2	1	1
3	3	3	3	2	2	2	2	1	1
4	4	3	3	2	2	3	3	1	1
4	4	3	3	2	2	3	3	1	1
4	4	3	3	2	2	3	3	1	1
3	3	3	3	2	2	2	2	1	1
3	3	3	3	2	2	2	2	1	1
4	4	3	3	2	2	2	2	1	1
3	3	3	3	2	2	2	2	1	1
4	4	3	3	2	2	2	2	1	1
3	3	2	2	3	3	2	2	2	2
3	3	3	3	2	2	2	2	1	1
4	4	3	3	3	3	2	2	2	2
4	4	3	3	3	3	3	3	1	1
4	4	3	3	2	2	3	3	1	1
4	4	3	3	2	2	3	3	1	1
4	4	2	2	3	3	2	2	1	1
4	4	2	2	3	3	3	3	1	1
4	4	2	2	3	3	3	3	1	1
4	4	3	3	3	3	3	3	1	1

For Review Only

4	4	2	2	3	3	2	2	1	1
4	4	2	2	3	3	2	2	1	1
3	3	3	3	3	3	3	3	1	1
3	3	2	2	3	3	3	3	1	1
3	3	3	3	3	3	3	3	1	1
4	4	3	3	2	2	3	3	1	1
4	4	3	3	2	2	3	3	1	1
3	3	3	3	2	2	2	2	1	1
4	4	3	3	2	2	2	2	1	1
4	4	3	3	3	3	3	3	1	1
4	4	2	2	3	3	3	3	1	1
4	4	3	3	2	2	3	3	1	1
4	4	2	2	3	3	2	2	1	1
3	3	2	2	3	3	2	2	1	1
4	4	3	3	2	2	3	3	1	1
4	4	3	3	2	2	2	2	1	1
4	4	3	3	3	3	3	3	1	1
4	4	2	2	3	3	3	3	1	1
4	4	3	3	3	3	3	3	1	1
4	4	2	2	3	3	2	2	1	1
4	4	3	3	2	2	3	3	1	1
3	3	3	3	2	2	3	3	1	1
3	3	3	3	3	3	3	3	1	1
3	3	3	3	2	2	3	3	1	1
4	4	2	2	3	3	2	2	2	2
4	4	2	2	3	3	3	3	1	1
4	4	3	3	3	3	2	2	1	1
3	3	3	3	2	2	2	2	1	1
4	4	3	3	2	2	3	3	1	1
4	4	2	2	3	3	3	3	1	1
4	4	3	3	2	2	2	2	1	1
3	3	3	3	2	2	3	3	1	1
3	3	3	3	2	2	2	2	1	1
4	4	2	2	3	3	2	2	1	1
3	3	3	3	2	2	3	3	1	1
4	4	2	2	3	3	2	2	2	2
4	4	3	3	2	2	2	2	1	1
3	3	3	3	3	3	3	3	1	1
4	4	2	2	3	3	2	2	2	2
3	3	2	2	3	3	2	2	2	2
4	4	3	3	3	3	3	3	1	1
4	4	2	2	3	3	3	3	1	1
4	4	2	2	3	3	2	2	1	1
4	4	2	2	3	3	3	3	1	1
4	4	3	3	3	3	3	3	1	1
3	3	2	2	3	3	3	3	1	1
3	3	3	3	2	2	2	2	1	1
4	4	3	3	2	2	3	3	1	1
4	4	3	3	2	2	3	3	1	1
4	4	2	2	3	3	3	3	1	1
4	4	2	2	3	3	3	3	1	1
3	3	3	3	2	2	2	2	1	1
3	3	3	3	2	2	2	2	1	1
3	3	3	3	3	3	3	3	1	1
4	4	2	2	3	3	2	2	1	1

For Review Only

4	4	3	3	2	2	3	3	1	1
3	3	3	3	3	3	2	2	1	1
4	4	3	3	2	2	3	3	1	1
3	3	3	3	2	2	2	2	1	1
4	4	2	2	3	3	3	3	1	1
3	3	3	3	2	2	3	3	1	1
4	4	3	3	3	3	3	3	1	1
4	4	3	3	2	2	3	3	1	1
3	3	3	3	2	2	2	2	1	1
3	3	3	3	2	2	3	3	1	1
4	4	3	3	2	2	2	2	1	1
4	4	3	3	3	3	3	3	1	1
4	4	2	2	3	3	2	2	2	2
4	4	2	2	3	3	3	3	1	1
4	4	3	3	3	3	3	3	1	1
4	4	3	3	2	2	3	3	1	1
3	3	2	2	3	3	3	3	1	1
4	4	3	3	3	3	3	3	1	1
3	3	3	3	2	2	2	2	1	1
4	4	3	3	3	3	2	2	2	2
4	4	3	3	3	3	3	3	1	1
4	4	2	2	3	3	3	3	1	1
3	3	2	2	3	3	3	3	1	1
3	3	3	3	2	2	2	2	1	1
4	4	3	3	2	2	3	3	1	1
3	3	3	3	2	2	2	2	1	1
3	3	3	3	3	3	3	3	1	1
3	3	2	2	3	3	3	3	1	1
4	4	2	2	3	3	3	3	1	1
4	4	2	2	3	3	3	3	1	1
3	3	3	3	2	2	2	2	1	1
4	4	3	3	3	3	3	3	1	1
4	4	2	2	3	3	3	3	1	1
4	4	3	3	2	2	3	3	1	1
3	3	3	3	2	2	2	2	1	1
4	4	2	2	3	3	2	2	1	1
3	3	3	3	2	2	2	2	1	1
4	4	2	2	3	3	3	3	1	1
4	4	3	3	2	2	3	3	1	1
3	3	3	3	2	2	3	3	1	1
4	4	3	3	2	2	3	3	1	1
3	3	3	3	2	2	2	2	1	1
4	4	2	2	3	3	3	3	1	1
3	3	3	3	2	2	2	2	1	1
4	4	3	3	2	2	3	3	1	1
4	4	2	2	3	3	2	2	1	1
4	4	3	3	2	2	3	3	1	1
4	4	2	2	3	3	3	3	1	1
3	3	3	3	2	2	3	3	1	1
4	4	3	3	2	2	3	3	1	1
4	4	3	3	2	2	3	3	1	1
3	3	3	3	2	2	2	2	1	1
4	4	3	3	2	2	3	3	1	1
4	4	3	3	2	2	3	3	1	1
4	4	3	3	2	2	3	3	1	1

For Review Only

4	4	3	3	3	3	3	3	1	1
4	4	3	3	2	2	2	2	2	2
4	4	3	3	2	2	3	3	1	1
3	3	3	3	3	3	2	2	1	1
4	4	2	2	3	3	3	3	1	1
3	3	3	3	2	2	2	2	1	1
4	4	3	3	2	2	2	2	1	1
4	4	3	3	3	3	3	3	1	1
3	3	3	3	2	2	2	2	1	1
4	4	2	2	3	3	3	3	1	1
4	4	3	3	2	2	3	3	1	1
3	3	3	3	2	2	2	2	1	1
4	4	3	3	2	2	3	3	1	1
4	4	3	3	3	3	2	2	1	1
3	3	3	3	2	2	2	2	1	1
3	3	3	3	2	2	3	3	1	1
4	4	3	3	3	3	3	3	1	1
4	4	3	3	3	3	3	3	1	1
3	3	3	3	2	2	2	2	1	1
4	4	2	2	3	3	3	3	1	1
4	4	3	3	3	3	3	3	1	1
3	3	3	3	2	2	2	2	1	1
3	3	3	3	2	2	3	3	1	1
4	4	3	3	2	2	3	3	1	1
3	3	3	3	2	2	2	2	1	1
3	3	3	3	2	2	3	3	1	1
4	4	2	2	3	3	3	3	1	1
3	3	3	3	3	3	2	2	1	1
4	4	3	3	2	2	2	2	1	1
4	4	2	2	3	3	3	3	1	1
3	3	3	3	2	2	2	2	1	1
4	4	3	3	3	3	3	3	1	1
3	3	2	2	3	3	3	3	1	1
3	3	3	3	2	2	2	2	1	1
4	4	3	3	3	3	2	2	1	1
4	4	3	3	2	2	3	3	1	1
4	4	3	3	2	2	3	3	1	1
4	4	2	2	3	3	3	3	1	1
4	4	2	2	3	3	3	3	1	1
4	4	2	2	3	3	3	3	1	1
3	3	3	3	3	3	3	3	1	1
4	4	3	3	2	2	3	3	1	1
3	3	3	3	2	2	2	2	1	1
3	3	3	3	3	3	3	3	1	1
3	3	3	3	3	3	3	3	1	1
3	3	2	2	3	3	2	2	1	1
3	3	3	3	2	2	3	3	1	1
3	3	3	3	2	2	2	2	1	1
3	3	3	3	2	2	2	2	1	1
3	3	2	2	3	3	3	3	1	1
4	4	2	2	3	3	3	3	1	1
4	4	3	3	2	2	2	2	1	1
3	3	3	3	2	2	3	3	1	1
4	4	2	2	3	3	3	3	1	1
3	3	3	3	2	2	2	2	1	1

For Review Only

4	4	3	3	2	2	2	2	1	1
3	3	3	3	2	2	2	2	1	1
4	4	2	2	3	3	3	3	1	1
4	4	3	3	3	3	3	3	1	1
4	4	3	3	2	2	2	2	2	2
4	4	2	2	3	3	3	3	1	1
3	3	3	3	2	2	2	2	2	2
3	3	3	3	2	2	2	2	2	2
4	4	3	3	2	2	3	3	1	1
3	3	3	3	2	2	2	2	1	1
4	4	3	3	3	3	3	3	1	1
4	4	3	3	2	2	2	2	1	1
4	4	2	2	3	3	2	2	1	1
3	3	3	3	2	2	3	3	1	1
4	4	2	2	3	3	3	3	1	1
3	3	3	3	3	3	3	3	1	1
3	3	3	3	2	2	3	3	1	1
3	3	3	3	2	2	2	2	1	1
3	3	3	3	2	2	2	2	1	1
3	3	3	3	2	2	2	2	1	1
4	4	3	3	2	2	2	2	1	1
4	4	3	3	2	2	3	3	1	1
4	4	2	2	3	3	3	3	1	1
3	3	3	3	2	2	2	2	1	1
3	3	3	3	2	2	2	2	1	1
3	3	2	2	3	3	3	3	1	1
3	3	3	3	2	2	2	2	1	1
4	4	2	2	3	3	3	3	1	1
4	4	3	3	2	2	2	2	1	1
4	4	3	3	3	3	3	3	1	1
3	3	3	3	2	2	2	2	1	1
4	4	3	3	2	2	3	3	1	1
4	4	3	3	2	2	2	2	1	1
4	4	3	3	2	2	3	3	1	1
3	3	3	3	2	2	3	3	1	1
3	3	3	3	2	2	2	2	1	1
4	4	2	2	3	3	3	3	1	1
4	4	3	3	2	2	3	3	1	1
4	4	3	3	2	2	3	3	1	1
3	3	3	3	2	2	2	2	1	1
4	4	2	2	3	3	3	3	1	1
3	3	2	2	3	3	3	3	1	1
4	4	3	3	2	2	2	2	1	1
4	4	2	2	3	3	3	3	1	1
3	3	3	3	3	3	3	3	1	1
4	4	2	2	3	3	3	3	2	2
4	4	3	3	2	2	2	2	1	1
4	4	2	2	3	3	2	2	1	1
4	4	2	2	3	3	2	2	1	1
3	3	3	3	2	2	2	2	1	1
4	4	2	2	3	3	3	3	1	1
3	3	3	3	2	2	2	2	1	1
3	3	2	2	3	3	3	3	1	1
4	4	2	2	3	3	3	3	1	1
3	3	3	3	2	2	2	2	1	1

For Review Only

3	3	2	2	3	3	3	3	1	1
4	4	2	2	3	3	3	3	1	1

For Review Only

SNP120	SNP120	SNP1200	SNP1200	SNP1201	SNP1201	SNP1202	SNP1202	SNP1203	SNP1203
4	4	1	1	1	1	2	2	2	2
4	4	4	4	4	4	2	2	2	2
4	4	4	4	4	4	2	2	2	2
4	4	4	4	4	4	2	2	2	2
4	4	4	4	4	4	1	1	2	2
4	4	4	4	4	4	1	1	2	2
4	4	1	1	1	1	1	1	2	2
4	4	1	1	1	1	1	1	2	2
4	4	1	1	1	1	1	1	2	2
4	4	1	1	1	1	1	1	2	2
4	4	4	4	4	4	2	2	3	3
4	4	4	4	4	4	1	1	2	2
4	4	4	4	4	4	2	2	2	2
1	1	4	4	4	4	2	2	2	2
4	4	4	4	4	4	1	1	2	2
4	4	4	4	4	4	2	2	2	2
4	4	1	1	1	1	1	1	2	2
4	4	4	4	4	4	2	2	2	2
4	4	1	1	1	1	1	1	2	2
4	4	4	4	4	4	2	2	2	2
4	4	4	4	4	4	2	2	2	2
4	4	1	1	1	1	1	1	2	2
4	4	4	4	4	4	2	2	3	3
4	4	4	4	4	4	2	2	2	2
4	4	1	1	1	1	1	1	2	2
4	4	4	4	4	4	2	2	2	2
4	4	4	4	4	4	2	2	2	2
4	4	4	4	4	4	1	1	2	2
4	4	1	1	1	1	1	1	2	2
4	4	4	4	4	4	1	1	2	2
4	4	4	4	4	4	2	2	2	2
4	4	1	1	1	1	1	1	2	2
4	4	1	1	1	1	1	1	2	2
1	1	4	4	4	4	2	2	2	2
4	4	4	4	4	4	2	2	2	2
4	4	4	4	4	4	2	2	2	2
1	1	4	4	4	4	2	2	2	2
4	4	4	4	4	4	2	2	2	2
4	4	4	4	4	4	2	2	2	2
4	4	4	4	4	4	2	2	2	2
4	4	4	4	4	4	2	2	2	2
4	4	4	4	4	4	2	2	2	2
4	4	1	1	1	1	1	1	2	2
4	4	4	4	4	4	2	2	2	2
4	4	1	1	1	1	1	1	2	2
4	4	4	4	4	4	2	2	2	2
4	4	4	4	4	4	2	2	2	2
4	4	4	4	4	4	2	2	2	2
4	4	1	1	1	1	1	1	2	2
4	4	1	1	1	1	1	1	2	2
4	4	1	1	1	1	2	2	2	2
4	4	4	4	4	4	2	2	3	3

For Review Only

4	4	1	1	1	1	1	1	2	2
4	4	1	1	1	1	1	1	2	2
4	4	1	1	1	1	1	1	2	2
4	4	1	1	1	1	1	1	2	2
4	4	1	1	1	1	1	1	2	2
4	4	4	4	4	4	2	2	2	2
4	4	4	4	4	4	2	2	2	2
4	4	4	4	4	4	2	2	2	2
1	1	4	4	4	4	2	2	2	2
4	4	4	4	4	4	1	1	2	2
4	4	4	4	4	4	2	2	2	2
4	4	4	4	4	4	2	2	2	2
4	4	1	1	1	1	1	1	2	2
4	4	1	1	1	1	1	1	2	2
4	4	4	4	4	4	2	2	2	2
4	4	4	4	4	4	2	2	2	2
4	4	4	4	4	4	2	2	2	2
4	4	1	1	1	1	1	1	2	2
4	4	1	1	1	1	1	1	2	2
4	4	1	1	1	1	1	1	2	2
4	4	4	4	4	4	2	2	2	2
4	4	4	4	4	4	2	2	2	2
4	4	1	1	4	4	1	1	2	2
4	4	4	4	4	4	2	2	2	2
4	4	1	1	1	1	1	1	2	2
4	4	4	4	4	4	2	2	2	2
4	4	1	1	1	1	1	1	2	2
4	4	4	4	4	4	2	2	2	2
4	4	4	4	4	4	2	2	2	2
4	4	4	4	4	4	2	2	2	2
4	4	4	4	4	4	2	2	2	2
4	4	4	4	4	4	2	2	2	2
4	4	4	4	4	4	2	2	2	2
4	4	1	1	1	1	1	1	2	2
4	4	4	4	4	4	2	2	2	2
4	4	1	1	1	1	1	1	2	2
4	4	4	4	4	4	2	2	2	2
4	4	1	1	1	1	1	1	2	2
4	4	1	1	1	1	1	1	2	2
4	4	1	1	1	1	1	1	2	2
4	4	1	1	1	1	1	1	2	2
4	4	1	1	4	4	2	2	2	2
4	4	1	1	1	1	1	1	2	2
4	4	4	4	4	4	2	2	2	2
4	4	4	4	4	4	2	2	2	2
4	4	4	4	4	4	2	2	2	2
1	1	4	4	4	4	2	2	2	2
4	4	4	4	4	4	2	2	2	2
4	4	4	4	4	4	2	2	2	2
4	4	1	1	1	1	1	1	2	2
4	4	4	4	4	4	2	2	2	2
4	4	4	4	4	4	2	2	2	2
1	1	4	4	4	4	2	2	2	2
4	4	1	1	4	4	2	2	2	2
4	4	1	1	1	1	1	1	2	2

For Review Only

4	4	4	4	4	4	2	2	2	2
4	4	4	4	4	4	2	2	3	3
4	4	4	4	4	4	2	2	2	2
1	1	4	4	4	4	2	2	2	2
4	4	4	4	4	4	2	2	2	2
4	4	4	4	4	4	2	2	2	2
4	4	1	1	1	1	1	1	2	2
4	4	4	4	4	4	2	2	2	2
4	4	4	4	4	4	2	2	2	2
4	4	4	4	4	4	2	2	2	2
1	1	4	4	4	4	2	2	2	2
4	4	4	4	4	4	1	1	2	2
4	4	1	1	1	1	1	1	2	2
4	4	4	4	4	4	2	2	2	2
4	4	4	4	4	4	1	1	2	2
4	4	4	4	4	4	2	2	2	2
4	4	1	1	1	1	1	1	2	2
4	4	4	4	4	4	1	1	2	2
1	1	4	4	4	4	2	2	2	2
4	4	1	1	1	1	1	1	2	2
4	4	4	4	4	4	1	1	2	2
4	4	4	4	4	4	1	1	2	2
4	4	4	4	4	4	2	2	2	2
4	4	4	4	4	4	2	2	2	2
4	4	4	4	4	4	2	2	2	2
4	4	4	4	4	4	2	2	3	3
4	4	1	1	1	1	2	2	2	2
4	4	1	1	1	1	1	1	2	2
4	4	1	1	1	1	1	1	2	2
4	4	1	1	1	1	1	1	2	2
1	1	4	4	4	4	2	2	2	2
4	4	4	4	4	4	1	1	2	2
4	4	1	1	1	1	1	1	2	2
4	4	4	4	4	4	2	2	2	2
1	1	4	4	4	4	2	2	2	2
4	4	4	4	1	1	1	1	2	2
1	1	4	4	4	4	2	2	2	2
4	4	4	4	4	4	2	2	2	2
4	4	4	4	4	4	2	2	2	2
4	4	4	4	4	4	2	2	3	3
4	4	4	4	4	4	2	2	3	3
1	1	4	4	4	4	2	2	2	2
4	4	1	1	1	1	1	1	2	2
4	4	4	4	4	4	2	2	2	2
4	4	4	4	4	4	2	2	2	2
4	4	4	4	1	1	1	1	2	2
4	4	4	4	4	4	2	2	2	2
4	4	4	4	4	4	2	2	2	2
4	4	4	4	4	4	2	2	2	2
4	4	4	4	4	4	2	2	2	2
4	4	4	4	4	4	2	2	2	2
4	4	4	4	4	4	2	2	2	2
4	4	4	4	4	4	2	2	2	2
4	4	4	4	4	4	2	2	2	2
4	4	4	4	4	4	2	2	2	2

For Review Only

4	4	1	1	1	1	1	1	2	2
4	4	4	4	4	4	2	2	3	3
4	4	4	4	4	4	2	2	2	2
4	4	4	4	4	4	2	2	3	3
4	4	4	4	4	4	1	1	2	2
4	4	4	4	4	4	2	2	2	2
4	4	4	4	4	4	2	2	2	2
4	4	4	4	4	4	1	1	2	2
4	4	4	4	4	4	2	2	3	3
4	4	1	1	1	1	1	1	2	2
4	4	4	4	4	4	2	2	2	2
4	4	4	4	4	4	2	2	2	2
4	4	4	4	4	4	2	2	2	2
4	4	4	4	4	4	1	1	2	2
1	1	4	4	4	4	2	2	2	2
4	4	4	4	4	4	2	2	3	3
4	4	4	4	4	4	2	2	2	2
4	4	4	4	4	4	2	2	2	2
4	4	4	4	4	4	2	2	2	2
4	4	1	1	1	1	1	1	2	2
4	4	4	4	4	4	1	1	2	2
4	4	4	4	4	4	2	2	2	2
4	4	4	4	4	4	2	2	3	3
4	4	4	4	4	4	2	2	2	2
4	4	4	4	4	4	2	2	2	2
4	4	4	4	4	4	2	2	2	2
4	4	4	4	4	4	1	1	2	2
4	4	4	4	4	4	1	1	2	2
4	4	4	4	4	4	2	2	2	2
4	4	1	1	1	1	1	1	2	2
4	4	4	4	4	4	2	2	2	2
4	4	4	4	4	4	2	2	2	2
4	4	1	1	1	1	2	2	2	2
1	1	4	4	4	4	2	2	2	2
4	4	4	4	4	4	1	1	2	2
4	4	4	4	4	4	2	2	2	2
4	4	4	4	4	4	2	2	3	3
4	4	1	1	1	1	2	2	2	2
4	4	4	4	4	4	2	2	2	2
4	4	1	1	1	1	2	2	2	2
4	4	4	4	4	4	2	2	2	2
4	4	4	4	4	4	2	2	2	2
4	4	4	4	4	4	2	2	3	3
4	4	4	4	4	4	2	2	2	2
1	1	4	4	4	4	2	2	2	2
4	4	1	1	1	1	1	1	2	2
4	4	4	4	4	4	2	2	3	3
4	4	4	4	4	4	2	2	2	2
4	4	4	4	4	4	2	2	2	2
4	4	1	1	1	1	1	1	2	2
4	4	1	1	1	1	1	1	2	2
1	1	4	4	4	4	2	2	2	2
4	4	4	4	4	4	2	2	2	2
4	4	4	4	4	4	2	2	2	2
4	4	4	4	4	4	2	2	2	2

4	4	4	4	4	4	2	2	2	2
4	4	4	4	4	4	2	2	2	2
4	4	1	1	1	1	1	1	2	2
4	4	4	4	4	4	1	1	3	3
4	4	4	4	4	4	2	2	2	2
4	4	1	1	1	1	1	1	2	2
4	4	4	4	4	4	2	2	2	2
4	4	4	4	4	4	2	2	2	2
1	1	4	4	4	4	2	2	2	2
4	4	4	4	4	4	2	2	2	2
4	4	4	4	4	4	1	1	2	2
4	4	4	4	4	4	2	2	2	2
4	4	1	1	1	1	1	1	2	2
4	4	4	4	4	4	2	2	2	2
4	4	4	4	4	4	2	2	2	2
4	4	4	4	4	4	2	2	2	2
4	4	4	4	4	4	2	2	3	3
4	4	4	4	4	4	2	2	2	2
4	4	4	4	4	4	2	2	3	3
4	4	4	4	4	4	2	2	2	2
4	4	4	4	4	4	2	2	2	2
4	4	4	4	4	4	2	2	2	2
4	4	1	1	1	1	1	1	2	2
4	4	4	4	4	4	2	2	2	2
4	4	4	4	4	4	2	2	2	2
4	4	1	1	1	1	2	2	2	2
4	4	4	4	4	4	2	2	2	2
4	4	1	1	1	1	1	1	2	2
4	4	4	4	4	4	2	2	2	2
4	4	1	1	1	1	1	1	2	2
4	4	4	4	4	4	2	2	2	2
4	4	4	4	4	4	2	2	2	2
4	4	4	4	4	4	2	2	2	2
4	4	4	4	4	4	2	2	2	2
4	4	4	4	4	4	2	2	2	2
4	4	4	4	4	4	2	2	2	2
4	4	1	1	1	1	2	2	2	2
4	4	4	4	4	4	2	2	2	2
4	4	4	4	4	4	2	2	2	2
4	4	4	4	4	4	2	2	2	2
4	4	1	1	1	1	1	1	2	2
4	4	4	4	4	4	2	2	2	2
4	4	4	4	4	4	2	2	2	2
4	4	1	1	1	1	1	1	2	2
4	4	4	4	4	4	2	2	2	2
4	4	1	1	1	1	1	1	2	2
4	4	4	4	4	4	2	2	2	2
4	4	4	4	4	4	2	2	2	2
4	4	4	4	4	4	2	2	2	2
4	4	4	4	4	4	2	2	2	2
4	4	1	1	1	1	1	1	2	2
4	4	4	4	4	4	2	2	2	2
4	4	4	4	4	4	2	2	2	2
4	4	1	1	1	1	1	1	2	2
4	4	4	4	4	4	2	2	2	2

For Review Only

4	4	1	1	1	1	1	1	2	2
4	4	4	4	4	4	2	2	2	2

For Review Only

SNP1204	SNP1204	SNP1205	SNP1205	SNP1206	SNP1206	SNP1207	SNP1207	SNP1208	SNP1208
2	2	2	2	4	4	3	3	2	2
1	1	3	3	1	1	4	4	2	2
2	2	2	2	1	1	4	4	2	2
2	2	2	2	1	1	4	4	2	2
1	1	3	3	4	4	3	3	2	2
2	2	2	2	1	1	4	4	2	2
2	2	2	2	4	4	4	4	2	2
2	2	2	2	1	1	4	4	2	2
2	2	2	2	4	4	3	3	2	2
1	1	3	3	1	1	4	4	2	2
2	2	2	2	4	4	4	4	2	2
2	2	2	2	1	1	4	4	2	2
2	2	2	2	4	4	4	4	2	2
2	2	2	2	4	4	4	4	2	2
2	2	2	2	1	1	4	4	2	2
2	2	2	2	1	1	4	4	2	2
2	2	2	2	4	4	4	4	2	2
2	2	2	2	4	4	4	4	2	2
2	2	2	2	1	1	4	4	2	2
1	1	3	3	4	4	4	4	2	2
2	2	2	2	4	4	4	4	2	2
2	2	2	2	4	4	4	4	2	2
2	2	2	2	4	4	3	3	2	2
2	2	2	2	4	4	4	4	2	2
2	2	2	2	4	4	3	3	2	2
2	2	2	2	4	4	4	4	2	2
2	2	2	2	1	1	4	4	2	2
2	2	2	2	1	1	4	4	2	2
2	2	2	2	4	4	3	3	2	2
1	1	3	3	4	4	4	4	2	2
2	2	2	2	4	4	4	4	2	2
2	2	2	2	1	1	4	4	2	2
2	2	2	2	1	1	3	3	2	2
2	2	2	2	4	4	4	4	2	2
2	2	2	2	4	4	4	4	2	2
2	2	2	2	4	4	4	4	2	2
2	2	2	2	4	4	3	3	2	2
2	2	2	2	4	4	3	3	3	3
2	2	2	2	4	4	3	3	2	2
2	2	2	2	4	4	3	3	3	3
1	1	3	3	4	4	4	4	2	2
2	2	2	2	4	4	4	4	2	2
1	1	3	3	1	1	4	4	2	2
2	2	2	2	4	4	4	4	2	2
1	1	3	3	1	1	4	4	2	2
2	2	2	2	1	1	4	4	2	2
2	2	2	2	4	4	4	4	2	2
2	2	2	2	4	4	3	3	2	2
2	2	2	2	4	4	4	4	2	2
2	2	2	2	4	4	4	4	2	2
2	2	2	2	4	4	4	4	2	2
2	2	2	2	4	4	3	3	2	2

For Review Only

2	2	2	2	4	4	3	3	2	2
2	2	2	2	1	1	3	3	2	2
2	2	2	2	1	1	4	4	2	2
2	2	2	2	4	4	4	4	2	2
2	2	2	2	1	1	4	4	2	2
2	2	2	2	4	4	4	4	2	2
2	2	2	2	4	4	4	4	2	2
2	2	2	2	4	4	3	3	2	2
2	2	2	2	4	4	3	3	2	2
2	2	2	2	1	1	4	4	2	2
2	2	2	2	1	1	4	4	2	2
2	2	2	2	4	4	4	4	2	2
2	2	2	2	4	4	3	3	2	2
2	2	2	2	4	4	3	3	2	2
2	2	2	2	1	1	4	4	2	2
1	1	3	3	4	4	3	3	2	2
2	2	2	2	1	1	3	3	2	2
2	2	2	2	1	1	4	4	3	3
2	2	2	2	4	4	4	4	2	2
2	2	2	2	4	4	3	3	2	2
2	2	2	2	4	4	4	4	2	2
2	2	2	2	4	4	3	3	2	2
2	2	2	2	4	4	3	3	2	2
2	2	2	2	4	4	4	4	2	2
1	1	3	3	1	1	4	4	2	2
2	2	2	2	1	1	4	4	2	2
2	2	2	2	4	4	4	4	2	2
2	2	2	2	4	4	4	4	2	2
2	2	2	2	4	4	4	4	2	2
2	2	2	2	1	1	4	4	2	2
2	2	2	2	4	4	3	3	2	2
2	2	2	2	4	4	4	4	2	2
2	2	2	2	4	4	3	3	2	2
2	2	2	2	4	4	3	3	2	2
2	2	2	2	4	4	4	4	3	3
1	1	3	3	1	1	4	4	2	2
2	2	2	2	4	4	4	4	2	2
2	2	2	2	4	4	4	4	2	2
2	2	2	2	1	1	3	3	2	2
2	2	2	2	1	1	4	4	2	2
2	2	2	2	4	4	3	3	2	2
2	2	2	2	4	4	4	4	2	2
2	2	2	2	1	1	4	4	3	3
2	2	2	2	1	1	4	4	2	2
2	2	2	2	1	1	4	4	2	2
2	2	2	2	1	1	4	4	3	3
2	2	2	2	4	4	4	4	2	2
2	2	2	2	4	4	4	4	2	2
2	2	2	2	4	4	4	4	2	2
2	2	2	2	1	1	3	3	2	2
2	2	2	2	1	1	4	4	2	2
2	2	2	2	4	4	3	3	2	2
2	2	2	2	4	4	4	4	2	2
2	2	2	2	1	1	4	4	2	2
2	2	2	2	1	1	3	3	3	3

For Review Only

2	2	2	2	4	4	4	4	2	2
2	2	2	2	1	1	3	3	2	2
2	2	2	2	4	4	4	4	2	2
2	2	2	2	4	4	4	4	2	2
2	2	2	2	1	1	4	4	2	2
2	2	2	2	4	4	4	4	2	2
2	2	2	2	1	1	4	4	2	2
2	2	2	2	4	4	4	4	2	2
1	1	3	3	4	4	4	4	2	2
2	2	2	2	4	4	4	4	2	2
2	2	2	2	4	4	3	3	3	3
2	2	2	2	1	1	4	4	2	2
2	2	2	2	1	1	4	4	2	2
2	2	2	2	1	1	4	4	2	2
2	2	2	2	4	4	4	4	2	2
2	2	2	2	4	4	4	4	2	2
2	2	2	2	1	1	4	4	3	3
2	2	2	2	4	4	4	4	2	2
2	2	2	2	4	4	4	4	2	2
2	2	2	2	1	1	4	4	2	2
2	2	2	2	1	1	4	4	2	2
2	2	2	2	1	1	4	4	2	2
2	2	2	2	1	1	4	4	2	2
2	2	2	2	4	4	3	3	2	2
2	2	2	2	4	4	4	4	2	2
2	2	2	2	4	4	4	4	2	2
2	2	2	2	4	4	4	4	3	3
2	2	2	2	1	1	4	4	2	2
2	2	2	2	1	1	4	4	2	2
2	2	2	2	1	1	4	4	2	2
2	2	2	2	4	4	4	4	2	2
2	2	2	2	1	1	4	4	2	2
2	2	2	2	4	4	3	3	2	2
2	2	2	2	4	4	4	4	2	2
2	2	2	2	4	4	4	4	2	2
2	2	2	2	4	4	4	4	2	2
2	2	2	2	4	4	4	4	2	2
2	2	2	2	1	1	4	4	2	2
2	2	2	2	4	4	4	4	2	2
2	2	2	2	4	4	3	3	2	2
2	2	2	2	4	4	3	3	2	2
2	2	2	2	4	4	4	4	2	2
2	2	2	2	1	1	3	3	2	2
1	1	2	2	4	4	4	4	2	2
2	2	2	2	4	4	4	4	2	2
2	2	2	2	4	4	4	4	3	3
2	2	2	2	4	4	4	4	2	2
2	2	2	2	1	1	4	4	2	2
2	2	2	2	4	4	4	4	2	2
2	2	2	2	4	4	4	4	2	2
2	2	2	2	4	4	4	4	2	2
2	2	2	2	4	4	3	3	2	2
2	2	2	2	4	4	4	4	2	2
2	2	2	2	4	4	4	4	2	2
2	2	2	2	4	4	4	4	2	2

For Review Only

2	2	2	2	1	1	4	4	3	3
2	2	2	2	4	4	3	3	2	2
1	1	3	3	4	4	4	4	3	3
2	2	2	2	4	4	4	4	2	2
2	2	2	2	4	4	4	4	2	2
2	2	2	2	4	4	3	3	2	2
2	2	2	2	4	4	3	3	2	2
2	2	2	2	1	1	4	4	2	2
2	2	2	2	4	4	4	4	2	2
2	2	2	2	1	1	4	4	2	2
2	2	2	2	4	4	4	4	2	2
2	2	2	2	4	4	3	3	2	2
2	2	2	2	1	1	4	4	2	2
2	2	2	2	4	4	4	4	2	2
2	2	2	2	4	4	4	4	2	2
2	2	2	2	4	4	3	3	2	2
2	2	2	2	4	4	4	4	2	2
2	2	2	2	1	1	4	4	2	2
2	2	2	2	4	4	4	4	2	2
2	2	2	2	1	1	4	4	2	2
2	2	2	2	1	1	4	4	2	2
2	2	2	2	4	4	3	3	2	2
2	2	2	2	4	4	3	3	2	2
2	2	2	2	4	4	4	4	2	2
2	2	2	2	4	4	4	4	2	2
2	2	2	2	4	4	4	4	2	2
2	2	2	2	1	1	4	4	2	2
2	2	2	2	1	1	4	4	2	2
2	2	2	2	4	4	4	4	2	2
2	2	2	2	4	4	4	4	2	2
2	2	2	2	4	4	4	4	3	3
2	2	2	2	1	1	4	4	2	2
2	2	2	2	4	4	4	4	2	2
2	2	2	2	4	4	4	4	2	2
2	2	2	2	1	1	4	4	3	3
2	2	2	2	4	4	4	4	2	2
2	2	2	2	4	4	4	4	2	2
2	2	2	2	4	4	3	3	2	2
2	2	2	2	1	1	4	4	2	2
2	2	2	2	4	4	4	4	2	2
2	2	2	2	1	1	4	4	2	2
2	2	2	2	4	4	4	4	2	2
2	2	2	2	4	4	3	3	2	2
2	2	2	2	1	1	4	4	2	2
2	2	2	2	1	1	4	4	2	2
2	2	2	2	1	1	4	4	2	2
2	2	2	2	4	4	3	3	2	2
2	2	2	2	4	4	3	3	2	2
2	2	2	2	4	4	4	4	2	2
2	2	2	2	4	4	4	4	2	2
2	2	2	2	1	1	4	4	2	2
2	2	2	2	4	4	4	4	3	3
2	2	2	2	4	4	3	3	2	2
2	2	2	2	1	1	4	4	2	2
2	2	2	2	4	4	3	3	2	2

For Review Only

2	2	2	2	4	4	3	3	2	2
1	1	3	3	4	4	3	3	2	2
2	2	2	2	1	1	4	4	2	2
2	2	2	2	4	4	4	4	2	2
2	2	2	2	4	4	4	4	2	2
2	2	2	2	1	1	4	4	2	2
2	2	2	2	4	4	4	4	2	2
1	1	3	3	4	4	4	4	2	2
2	2	2	2	4	4	4	4	2	2
2	2	2	2	4	4	4	4	2	2
2	2	2	2	1	1	4	4	2	2
2	2	2	2	4	4	4	4	3	3
2	2	2	2	1	1	3	3	2	2
2	2	2	2	4	4	4	4	2	2
2	2	2	2	1	1	4	4	2	2
2	2	2	2	4	4	3	3	2	2
2	2	2	2	4	4	3	3	2	2
2	2	2	2	4	4	4	4	2	2
2	2	2	2	4	4	3	3	2	2
2	2	2	2	4	4	3	3	3	3
2	2	2	2	4	4	4	4	2	2
2	2	2	2	4	4	4	4	2	2
2	2	2	2	1	1	4	4	2	2
2	2	2	2	4	4	3	3	2	2
2	2	2	2	4	4	3	3	2	2
2	2	2	2	4	4	4	4	3	3
2	2	2	2	4	4	3	3	2	2
2	2	2	2	1	1	4	4	2	2
2	2	2	2	4	4	3	3	2	2
2	2	2	2	1	1	4	4	2	2
2	2	2	2	4	4	3	3	2	2
2	2	2	2	4	4	4	4	2	2
2	2	2	2	4	4	3	3	2	2
2	2	2	2	4	4	4	4	2	2
2	2	2	2	4	4	3	3	2	2
2	2	2	2	4	4	4	4	2	2
2	2	2	2	4	4	4	4	2	2
2	2	2	2	4	4	4	4	2	2
2	2	2	2	4	4	4	4	2	2
2	2	2	2	4	4	3	3	2	2
2	2	2	2	1	1	4	4	3	3
2	2	2	2	1	1	4	4	3	3
2	2	2	2	4	4	3	3	2	2
2	2	2	2	4	4	4	4	2	2
2	2	2	2	1	1	4	4	2	2
2	2	2	2	4	4	3	3	2	2
2	2	2	2	4	4	4	4	2	2
2	2	2	2	1	1	4	4	3	3
2	2	2	2	1	1	4	4	2	2
2	2	2	2	4	4	3	3	2	2
2	2	2	2	4	4	4	4	3	3
2	2	2	2	4	4	3	3	2	2
2	2	2	2	1	1	3	3	2	2
2	2	2	2	1	1	4	4	2	2
2	2	2	2	4	4	3	3	2	2

For Review Only

2	2	2	2	1	1	4	4	2	2
2	2	2	2	1	1	4	4	2	2

For Review Only

SNP1209	SNP1209	SNP121	SNP121	SNP1210	SNP1210	SNP1211	SNP1211	SNP1212	SNP1212
2	2	3	3	2	2	2	2	2	2
2	2	3	3	2	2	2	2	2	2
1	1	3	3	2	2	2	2	2	2
2	2	3	3	2	2	2	2	2	2
2	2	3	3	2	2	2	2	2	2
2	2	3	3	2	2	2	2	2	2
2	2	3	3	2	2	2	2	2	2
1	1	3	3	2	2	1	1	2	2
2	2	3	3	2	2	2	2	2	2
1	1	3	3	2	2	2	2	2	2
1	1	3	3	2	2	2	2	2	2
1	1	3	3	2	2	2	2	2	2
2	2	3	3	2	2	2	2	2	2
2	2	2	2	2	2	2	2	2	2
2	2	3	3	2	2	2	2	2	2
1	1	3	3	1	1	2	2	2	2
1	1	3	3	2	2	1	1	2	2
2	2	3	3	2	2	2	2	2	2
2	2	3	3	2	2	2	2	2	2
1	1	3	3	2	2	2	2	2	2
1	1	3	3	2	2	2	2	2	2
1	1	3	3	2	2	2	2	2	2
1	1	3	3	2	2	2	2	2	2
1	1	3	3	2	2	2	2	2	2
2	2	3	3	2	2	2	2	3	3
2	2	3	3	1	1	2	2	2	2
2	2	3	3	2	2	2	2	2	2
2	2	3	3	2	2	2	2	2	2
2	2	3	3	2	2	2	2	2	2
1	1	3	3	2	2	1	1	2	2
1	1	3	3	2	2	2	2	2	2
2	2	3	3	2	2	2	2	3	3
2	2	3	3	2	2	2	2	3	3
2	2	2	2	2	2	2	2	2	2
2	2	3	3	2	2	2	2	2	2
2	2	3	3	2	2	2	2	2	2
2	2	2	2	2	2	2	2	2	2
1	1	3	3	2	2	2	2	2	2
1	1	3	3	2	2	2	2	2	2
1	1	3	3	2	2	2	2	2	2
1	1	3	3	2	2	1	1	2	2
1	1	3	3	2	2	1	1	2	2
2	2	3	3	2	2	1	1	2	2
1	1	3	3	2	2	2	2	2	2
1	1	3	3	2	2	1	1	2	2
1	1	3	3	2	2	2	2	2	2
2	2	3	3	2	2	2	2	2	2
1	1	3	3	2	2	2	2	2	2
1	1	3	3	2	2	2	2	2	2
1	1	3	3	2	2	2	2	2	2
2	2	3	3	2	2	2	2	3	3
1	1	3	3	2	2	2	2	2	2

For Review Only

2	2	3	3	2	2	2	2	2	2
2	2	3	3	2	2	2	2	3	3
1	1	3	3	2	2	2	2	2	2
1	1	3	3	2	2	2	2	2	2
2	2	3	3	2	2	2	2	2	2
2	2	3	3	2	2	2	2	2	2
2	2	3	3	1	1	2	2	2	2
1	1	3	3	2	2	2	2	2	2
1	1	2	2	2	2	2	2	2	2
2	2	3	3	2	2	2	2	2	2
2	2	3	3	2	2	2	2	2	2
2	2	3	3	2	2	2	2	2	2
2	2	3	3	2	2	2	2	2	2
2	2	3	3	2	2	2	2	2	2
2	2	3	3	2	2	2	2	2	2
1	1	3	3	2	2	2	2	2	2
1	1	3	3	2	2	2	2	2	2
1	1	3	3	2	2	2	2	2	2
1	1	3	3	2	2	2	2	2	2
2	2	3	3	2	2	2	2	2	2
2	2	3	3	2	2	2	2	2	2
1	1	3	3	2	2	2	2	2	2
2	2	3	3	2	2	2	2	2	2
1	1	3	3	2	2	2	2	2	2
1	1	3	3	2	2	1	1	2	2
1	1	3	3	2	2	2	2	2	2
2	2	3	3	2	2	2	2	2	2
1	1	3	3	2	2	2	2	2	2
2	2	3	3	1	1	2	2	2	2
2	2	3	3	2	2	2	2	2	2
1	1	3	3	2	2	2	2	2	2
2	2	3	3	2	2	2	2	2	2
2	2	3	3	2	2	2	2	2	2
2	2	3	3	2	2	2	2	2	2
2	2	3	3	2	2	2	2	2	2
1	1	3	3	2	2	1	1	2	2
1	1	3	3	2	2	1	1	2	2
1	1	3	3	2	2	2	2	2	2
1	1	3	3	2	2	2	2	2	2
1	1	3	3	2	2	1	1	2	2
2	2	3	3	2	2	2	2	2	2
2	2	3	3	2	2	2	2	3	3
2	2	3	3	2	2	1	1	2	2
2	2	3	3	2	2	2	2	2	2
1	1	3	3	2	2	2	2	2	2
2	2	3	3	2	2	2	2	2	2
2	2	2	2	2	2	2	2	2	2
2	2	3	3	2	2	2	2	2	2
2	2	3	3	1	1	2	2	2	2
2	2	3	3	2	2	2	2	2	2
1	1	3	3	2	2	2	2	2	2
1	1	3	3	2	2	2	2	2	2
1	1	2	2	2	2	2	2	2	2
2	2	3	3	2	2	2	2	2	2
1	1	3	3	2	2	2	2	2	2

For Review Only

1	1	3	3	1	1	2	2	2	2
2	2	3	3	2	2	2	2	2	2
2	2	3	3	2	2	2	2	2	2
2	2	2	2	2	2	2	2	2	2
2	2	3	3	2	2	2	2	2	2
1	1	3	3	2	2	2	2	2	2
1	1	3	3	2	2	2	2	2	2
2	2	3	3	2	2	2	2	2	2
2	2	3	3	1	1	1	1	2	2
2	2	3	3	1	1	2	2	2	2
2	2	2	2	2	2	2	2	2	2
2	2	3	3	2	2	2	2	2	2
1	1	3	3	2	2	2	2	2	2
2	2	3	3	2	2	2	2	2	2
2	2	3	3	2	2	2	2	2	2
2	2	3	3	2	2	2	2	2	2
1	1	3	3	2	2	2	2	2	2
2	2	3	3	2	2	2	2	2	2
2	2	2	2	2	2	2	2	2	2
2	2	3	3	2	2	2	2	2	2
2	2	3	3	2	2	2	2	3	3
2	2	3	3	2	2	2	2	2	2
2	2	3	3	2	2	2	2	2	2
1	1	3	3	2	2	2	2	2	2
2	2	3	3	1	1	2	2	2	2
1	1	3	3	2	2	2	2	2	2
1	1	3	3	2	2	2	2	2	2
2	2	3	3	2	2	2	2	3	3
2	2	3	3	2	2	2	2	2	2
2	2	3	3	2	2	2	2	2	2
2	2	2	2	2	2	2	2	2	2
2	2	3	3	2	2	2	2	2	2
2	2	3	3	2	2	2	2	2	2
2	2	3	3	2	2	2	2	2	2
2	2	2	2	2	2	2	2	2	2
2	2	3	3	2	2	2	2	2	2
1	1	2	2	2	2	2	2	2	2
2	2	3	3	2	2	2	2	2	2
1	1	3	3	2	2	2	2	2	2
1	1	3	3	2	2	2	2	2	2
1	1	3	3	2	2	2	2	2	2
2	2	2	2	2	2	2	2	2	2
1	1	3	3	2	2	2	2	2	2
2	2	3	3	2	2	1	1	2	2
1	1	3	3	2	2	2	2	2	2
2	2	3	3	2	2	2	2	2	2
1	1	3	3	1	1	2	2	2	2
2	2	3	3	1	1	2	2	2	2
2	2	3	3	2	2	2	2	2	2
2	2	3	3	1	1	2	2	2	2
2	2	3	3	2	2	2	2	2	2
1	1	3	3	2	2	2	2	2	2
2	2	3	3	2	2	2	2	2	2
2	2	3	3	2	2	2	2	2	2
2	2	3	3	2	2	2	2	2	2

For Review Only

1	1	3	3	2	2	2	2	3	3
1	1	3	3	2	2	2	2	2	2
2	2	3	3	2	2	1	1	2	2
1	1	3	3	2	2	2	2	2	2
2	2	3	3	2	2	2	2	2	2
2	2	3	3	2	2	2	2	2	2
2	2	3	3	2	2	2	2	2	2
2	2	3	3	2	2	2	2	2	2
2	2	3	3	2	2	2	2	2	2
1	1	3	3	2	2	2	2	3	3
2	2	3	3	2	2	2	2	2	2
1	1	3	3	2	2	2	2	2	2
2	2	3	3	1	1	2	2	2	2
2	2	3	3	2	2	2	2	2	2
2	2	2	2	2	2	2	2	2	2
1	1	3	3	2	2	2	2	2	2
2	2	3	3	2	2	2	2	2	2
2	2	3	3	2	2	2	2	2	2
2	2	3	3	2	2	2	2	2	2
1	1	3	3	2	2	2	2	2	2
2	2	3	3	2	2	2	2	2	2
2	2	3	3	2	2	2	2	2	2
1	1	3	3	2	2	2	2	2	2
2	2	3	3	2	2	2	2	2	2
1	1	3	3	2	2	1	1	2	2
2	2	3	3	2	2	2	2	2	2
2	2	3	3	2	2	2	2	2	2
2	2	3	3	2	2	2	2	2	2
1	1	3	3	2	2	2	2	2	2
1	1	3	3	2	2	2	2	2	2
2	2	3	3	2	2	2	2	2	2
1	1	3	3	2	2	2	2	2	2
2	2	3	3	2	2	2	2	2	2
2	2	2	2	2	2	2	2	2	2
2	2	3	3	2	2	2	2	2	2
2	2	3	3	2	2	2	2	2	2
1	1	3	3	2	2	2	2	2	2
2	2	3	3	2	2	2	2	2	2
2	2	3	3	1	1	2	2	2	2
2	2	3	3	2	2	2	2	3	3
1	1	3	3	2	2	2	2	2	2
1	1	3	3	1	1	2	2	2	2
1	1	3	3	2	2	2	2	2	2
1	1	3	3	2	2	2	2	2	2
2	2	2	2	2	2	2	2	2	2
2	2	3	3	2	2	2	2	3	3
1	1	3	3	2	2	2	2	2	2
2	2	3	3	2	2	2	2	2	2
2	2	3	3	2	2	2	2	2	2
1	1	3	3	2	2	1	1	2	2
1	1	3	3	2	2	2	2	3	3
2	2	2	2	2	2	2	2	2	2
1	1	3	3	2	2	2	2	2	2
2	2	3	3	2	2	2	2	2	2
2	2	3	3	2	2	2	2	2	2

2	2	3	3	2	2	2	2	2	2
1	1	3	3	2	2	2	2	2	2
1	1	3	3	2	2	2	2	3	3
1	1	3	3	2	2	2	2	2	2
1	1	3	3	2	2	1	1	2	2
1	1	3	3	2	2	2	2	3	3
1	1	3	3	2	2	2	2	2	2
1	1	3	3	2	2	1	1	2	2
2	2	3	3	2	2	2	2	2	2
1	1	3	3	2	2	2	2	2	2
2	2	3	3	2	2	2	2	2	2
2	2	3	3	2	2	2	2	2	2
1	1	3	3	2	2	2	2	2	2
2	2	3	3	1	1	2	2	2	2
1	1	3	3	2	2	2	2	2	2
2	2	3	3	2	2	2	2	2	2
2	2	3	3	2	2	2	2	2	2
2	2	3	3	2	2	2	2	2	2
1	1	3	3	2	2	2	2	2	2
1	1	3	3	2	2	2	2	2	2
1	1	3	3	2	2	2	2	2	2
2	2	3	3	1	1	2	2	2	2
2	2	3	3	2	2	2	2	3	3
1	1	3	3	2	2	2	2	2	2
1	1	3	3	2	2	2	2	2	2
1	1	3	3	2	2	1	1	2	2
1	1	3	3	2	2	2	2	2	2
2	2	3	3	2	2	2	2	2	2
2	2	3	3	2	2	1	1	2	2
1	1	3	3	2	2	2	2	3	3
1	1	3	3	2	2	2	2	2	2
2	2	3	3	2	2	2	2	2	2
2	2	3	3	2	2	2	2	2	2
2	2	3	3	1	1	2	2	2	2
1	1	3	3	2	2	2	2	2	2
2	2	3	3	2	2	2	2	2	2
1	1	3	3	2	2	2	2	2	2
2	2	3	3	2	2	2	2	2	2
2	2	3	3	2	2	2	2	2	2
1	1	3	3	2	2	2	2	2	2
2	2	3	3	2	2	2	2	3	3
2	2	3	3	2	2	2	2	2	2
1	1	3	3	2	2	2	2	2	2
1	1	3	3	2	2	2	2	2	2
1	1	3	3	2	2	2	2	2	2
1	1	3	3	2	2	2	2	2	2
1	1	3	3	2	2	2	2	2	2
2	2	3	3	2	2	2	2	2	2
2	2	3	3	2	2	1	1	2	2
2	2	3	3	2	2	2	2	2	2
1	1	3	3	2	2	2	2	2	2
1	1	3	3	2	2	2	2	2	2
1	1	3	3	2	2	2	2	2	2
2	2	3	3	2	2	2	2	2	2
1	1	3	3	2	2	2	2	2	2

For Review Only

2	2	3	3	2	2	2	2	3	3
2	2	3	3	2	2	2	2	2	2

For Review Only

SNP1213	SNP1213	SNP1214	SNP1214	SNP1215	SNP1215	SNP1216	SNP1216	SNP1217	SNP1217
2	2	1	1	4	4	2	2	3	3
2	2	1	1	4	4	4	4	3	3
2	2	1	1	4	4	2	2	3	3
2	2	1	1	4	4	2	2	3	3
2	2	1	1	4	4	4	4	3	3
2	2	1	1	4	4	2	2	3	3
2	2	1	1	4	4	2	2	3	3
4	4	3	3	4	4	2	2	3	3
2	2	1	1	4	4	2	2	3	3
4	4	3	3	4	4	4	4	2	2
2	2	1	1	4	4	2	2	3	3
2	2	1	1	4	4	2	2	3	3
2	2	1	1	4	4	2	2	3	3
2	2	1	1	4	4	2	2	3	3
2	2	1	1	4	4	2	2	3	3
2	2	1	1	4	4	2	2	3	3
4	4	3	3	4	4	2	2	2	2
2	2	1	1	4	4	2	2	3	3
2	2	1	1	4	4	2	2	3	3
4	4	3	3	4	4	2	2	3	3
2	2	1	1	4	4	2	2	3	3
2	2	1	1	4	4	2	2	2	2
2	2	1	1	4	4	2	2	3	3
2	2	1	1	4	4	2	2	3	3
2	2	1	1	1	1	2	2	3	3
2	2	1	1	4	4	2	2	3	3
2	2	1	1	1	1	2	2	3	3
2	2	1	1	1	1	2	2	3	3
2	2	1	1	4	4	2	2	3	3
4	4	3	3	4	4	2	2	2	2
2	2	1	1	4	4	2	2	3	3
2	2	1	1	1	1	2	2	3	3
2	2	1	1	1	1	2	2	3	3
2	2	1	1	4	4	2	2	3	3
2	2	1	1	4	4	2	2	3	3
2	2	1	1	4	4	2	2	3	3
2	2	1	1	4	4	2	2	3	3
2	2	1	1	4	4	2	2	3	3
2	2	1	1	4	4	2	2	3	3
2	2	1	1	4	4	2	2	3	3
4	4	3	3	4	4	4	4	3	3
4	4	3	3	4	4	2	2	2	2
4	4	3	3	4	4	4	4	3	3
4	4	3	3	4	4	2	2	2	2
4	4	3	3	4	4	4	4	3	3
2	2	1	1	4	4	2	2	3	3
2	2	1	1	4	4	2	2	3	3
2	2	1	1	4	4	2	2	3	3
2	2	1	1	4	4	2	2	2	2
2	2	1	1	4	4	2	2	3	3
2	2	1	1	1	1	2	2	3	3
2	2	1	1	4	4	2	2	3	3

For Review Only

2	2	1	1	4	4	2	2	3	3
2	2	1	1	1	1	2	2	3	3
2	2	1	1	4	4	2	2	3	3
2	2	1	1	4	4	2	2	2	2
2	2	1	1	4	4	2	2	3	3
2	2	1	1	4	4	2	2	3	3
2	2	1	1	4	4	2	2	3	3
4	4	3	3	4	4	2	2	3	3
4	4	3	3	4	4	2	2	3	3
2	2	1	1	4	4	2	2	3	3
2	2	1	1	4	4	2	2	3	3
2	2	1	1	4	4	2	2	3	3
2	2	1	1	4	4	2	2	3	3
2	2	1	1	4	4	2	2	2	2
2	2	1	1	4	4	2	2	3	3
2	2	1	1	4	4	4	4	3	3
2	2	1	1	4	4	2	2	3	3
2	2	1	1	4	4	2	2	3	3
2	2	1	1	4	4	2	2	3	3
2	2	1	1	4	4	2	2	3	3
2	2	1	1	4	4	2	2	3	3
2	2	1	1	4	4	2	2	3	3
2	2	1	1	4	4	2	2	2	2
4	4	3	3	4	4	2	2	2	2
4	4	3	3	4	4	4	4	3	3
2	2	1	1	4	4	2	2	3	3
2	2	1	1	4	4	2	2	3	3
2	2	1	1	4	4	2	2	3	3
2	2	1	1	4	4	2	2	3	3
2	2	1	1	4	4	2	2	3	3
2	2	1	1	4	4	2	2	3	3
2	2	1	1	4	4	2	2	3	3
2	2	1	1	4	4	2	2	3	3
2	2	1	1	4	4	2	2	3	3
2	2	1	1	4	4	2	2	3	3
4	4	3	3	4	4	4	4	3	3
4	4	3	3	4	4	2	2	3	3
2	2	1	1	4	4	2	2	2	2
2	2	1	1	4	4	2	2	3	3
4	4	3	3	4	4	2	2	2	2
2	2	1	1	4	4	2	2	3	3
2	2	1	1	1	1	2	2	3	3
4	4	3	3	4	4	2	2	3	3
2	2	1	1	4	4	2	2	3	3
2	2	1	1	4	4	2	2	3	3
2	2	1	1	4	4	2	2	3	3
2	2	1	1	4	4	2	2	3	3
2	2	1	1	4	4	2	2	3	3
2	2	1	1	4	4	2	2	3	3
2	2	1	1	4	4	2	2	3	3
2	2	1	1	4	4	2	2	3	3
2	2	1	1	4	4	2	2	3	3
4	4	1	1	4	4	2	2	3	3
2	2	3	3	4	4	2	2	3	3
2	2	1	1	4	4	2	2	3	3

For Review Only

2	2	1	1	4	4	2	2	3	3
2	2	1	1	4	4	2	2	3	3
2	2	1	1	4	4	2	2	3	3
2	2	1	1	4	4	2	2	3	3
2	2	1	1	4	4	2	2	3	3
2	2	1	1	4	4	2	2	3	3
2	2	1	1	4	4	2	2	2	2
2	2	1	1	4	4	2	2	3	3
2	2	1	1	4	4	4	4	3	3
2	2	3	3	4	4	2	2	3	3
2	2	1	1	4	4	2	2	3	3
2	2	1	1	4	4	2	2	3	3
2	2	1	1	4	4	2	2	3	3
2	2	1	1	4	4	2	2	3	3
2	2	1	1	4	4	2	2	3	3
2	2	1	1	4	4	2	2	3	3
4	4	1	1	4	4	2	2	3	3
2	2	1	1	4	4	2	2	3	3
2	2	1	1	4	4	2	2	3	3
2	2	1	1	4	4	2	2	3	3
2	2	1	1	4	4	2	2	3	3
2	2	1	1	4	4	2	2	3	3
2	2	1	1	4	4	2	2	3	3
2	2	1	1	4	4	2	2	3	3
2	2	1	1	4	4	2	2	3	3
2	2	1	1	4	4	2	2	3	3
2	2	1	1	4	4	2	2	3	3
2	2	1	1	1	1	2	2	3	3
2	2	1	1	4	4	2	2	3	3
2	2	1	1	1	1	2	2	2	2
2	2	1	1	4	4	2	2	3	3
2	2	1	1	4	4	2	2	3	3
2	2	1	1	4	4	2	2	3	3
2	2	1	1	4	4	2	2	3	3
2	2	1	1	4	4	2	2	3	3
2	2	1	1	4	4	2	2	3	3
2	2	1	1	4	4	2	2	3	3
2	2	1	1	4	4	2	2	3	3
2	2	1	1	4	4	2	2	3	3
2	2	1	1	4	4	2	2	3	3
2	2	1	1	4	4	2	2	3	3
4	4	3	3	4	4	2	2	3	3
2	2	1	1	4	4	2	2	3	3
4	4	1	1	4	4	2	2	3	3
2	2	1	1	4	4	2	2	3	3
2	2	1	1	4	4	2	2	3	3
2	2	1	1	4	4	2	2	3	3
2	2	1	1	4	4	2	2	3	3
2	2	1	1	4	4	2	2	3	3
2	2	1	1	4	4	2	2	3	3
2	2	1	1	4	4	2	2	3	3
2	2	1	1	4	4	2	2	3	3
2	2	1	1	4	4	2	2	3	3
2	2	1	1	4	4	2	2	3	3
2	2	1	1	4	4	2	2	3	3

For Review Only

2	2	1	1	1	1	2	2	3	3
2	2	1	1	4	4	2	2	3	3
2	2	1	1	4	4	4	4	3	3
2	2	1	1	4	4	2	2	3	3
2	2	1	1	4	4	2	2	3	3
2	2	1	1	4	4	2	2	3	3
2	2	1	1	4	4	2	2	3	3
2	2	1	1	4	4	2	2	3	3
2	2	1	1	4	4	2	2	3	3
2	2	1	1	1	1	2	2	3	3
2	2	1	1	4	4	2	2	3	3
2	2	1	1	4	4	2	2	3	3
2	2	1	1	4	4	2	2	3	3
2	2	1	1	4	4	2	2	3	3
2	2	1	1	4	4	2	2	3	3
2	2	1	1	4	4	2	2	2	2
2	2	1	1	4	4	2	2	3	3
2	2	1	1	4	4	2	2	3	3
2	2	1	1	4	4	2	2	3	3
2	2	1	1	4	4	2	2	2	2
2	2	1	1	4	4	2	2	3	3
2	2	1	1	4	4	2	2	3	3
2	2	1	1	4	4	2	2	3	3
2	2	1	1	4	4	2	2	3	3
2	2	3	3	4	4	2	2	2	2
2	2	1	1	4	4	2	2	3	3
2	2	1	1	4	4	2	2	3	3
2	2	1	1	4	4	2	2	3	3
2	2	1	1	4	4	2	2	3	3
2	2	1	1	4	4	2	2	3	3
2	2	1	1	4	4	2	2	3	3
2	2	1	1	4	4	2	2	3	3
2	2	1	1	4	4	2	2	3	3
2	2	1	1	4	4	2	2	3	3
2	2	1	1	4	4	2	2	3	3
2	2	1	1	4	4	2	2	3	3
2	2	1	1	4	4	2	2	3	3
2	2	1	1	4	4	2	2	3	3
2	2	1	1	4	4	2	2	3	3
2	2	1	1	1	1	2	2	3	3
2	2	1	1	4	4	2	2	3	3
2	2	1	1	4	4	2	2	3	3
2	2	1	1	4	4	2	2	3	3
2	2	1	1	4	4	2	2	3	3
2	2	3	3	4	4	2	2	3	3
2	2	1	1	1	1	2	2	3	3
2	2	1	1	4	4	2	2	2	2
2	2	1	1	4	4	2	2	3	3
2	2	1	1	4	4	2	2	3	3
4	4	3	3	4	4	2	2	2	2
2	2	1	1	1	1	2	2	3	3
2	2	1	1	4	4	2	2	3	3
2	2	1	1	4	4	2	2	3	3
2	2	1	1	4	4	2	2	3	3
2	2	1	1	4	4	2	2	3	3

For Review Only

2	2	1	1	4	4	2	2	3	3
2	2	1	1	4	4	4	4	3	3
2	2	1	1	1	1	2	2	3	3
2	2	1	1	4	4	2	2	3	3
4	4	3	3	4	4	2	2	3	3
2	2	1	1	1	1	2	2	3	3
2	2	1	1	4	4	2	2	3	3
4	4	3	3	4	4	4	4	3	3
2	2	1	1	4	4	2	2	3	3
2	2	1	1	4	4	2	2	3	3
2	2	1	1	4	4	2	2	3	3
2	2	1	1	4	4	2	2	3	3
2	2	1	1	4	4	2	2	3	3
2	2	1	1	4	4	2	2	3	3
2	2	1	1	1	1	2	2	3	3
2	2	1	1	4	4	2	2	2	2
2	2	1	1	4	4	2	2	3	3
2	2	1	1	4	4	2	2	3	3
2	2	1	1	4	4	2	2	3	3
2	2	1	1	4	4	2	2	3	3
2	2	1	1	4	4	2	2	3	3
2	2	1	1	4	4	2	2	3	3
2	2	1	1	1	1	2	2	3	3
2	2	1	1	4	4	2	2	3	3
2	2	3	3	4	4	2	2	3	3
2	2	3	3	4	4	2	2	3	3
2	2	1	1	4	4	2	2	3	3
2	2	1	1	4	4	2	2	3	3
2	2	1	1	4	4	2	2	3	3
2	2	1	1	1	1	2	2	3	3
2	2	1	1	4	4	2	2	3	3
2	2	1	1	4	4	2	2	3	3
2	2	1	1	4	4	2	2	3	3
2	2	1	1	4	4	2	2	3	3
2	2	1	1	4	4	2	2	3	3
2	2	1	1	4	4	2	2	3	3
2	2	1	1	4	4	2	2	3	3
2	2	1	1	4	4	2	2	3	3
2	2	1	1	4	4	2	2	3	3
2	2	1	1	4	4	2	2	3	3
2	2	1	1	4	4	2	2	3	3
2	2	1	1	4	4	2	2	3	3
2	2	1	1	4	4	2	2	3	3
2	2	1	1	4	4	2	2	3	3
2	2	1	1	4	4	2	2	3	3
2	2	1	1	4	4	2	2	3	3
2	2	1	1	4	4	2	2	3	3
2	2	1	1	1	1	2	2	3	3
2	2	1	1	4	4	2	2	3	3
2	2	1	1	4	4	2	2	3	3
2	2	1	1	4	4	2	2	3	3
2	2	1	1	4	4	2	2	3	3
2	2	1	1	4	4	2	2	3	3
2	2	1	1	4	4	2	2	3	3
2	2	1	1	4	4	2	2	3	3

For Review Only

2	2	1	1	1	1	2	2	2	2
2	2	1	1	4	4	2	2	3	3

For Review Only

SNP1218	SNP1218	SNP1219	SNP1219	SNP122	SNP122	SNP1220	SNP1220	SNP1221	SNP1221
4	4	4	4	1	1	1	1	2	2
4	4	1	1	1	1	4	4	1	1
4	4	1	1	3	3	1	1	2	2
4	4	1	1	3	3	1	1	2	2
4	4	4	4	3	3	4	4	1	1
4	4	4	4	3	3	1	1	2	2
1	1	4	4	3	3	1	1	2	2
4	4	1	1	3	3	1	1	2	2
4	4	4	4	3	3	1	1	2	2
4	4	1	1	3	3	4	4	1	1
4	4	4	4	1	1	1	1	2	2
4	4	4	4	3	3	1	1	2	2
4	4	1	1	1	1	1	1	2	2
4	4	4	4	3	3	1	1	2	2
4	4	4	4	1	1	1	1	2	2
4	4	4	4	1	1	1	1	2	2
4	4	4	4	3	3	1	1	2	2
4	4	4	4	1	1	1	1	2	2
4	4	1	1	1	1	1	1	2	2
1	1	4	4	3	3	1	1	2	2
1	1	1	1	3	3	1	1	2	2
4	4	4	4	3	3	1	1	2	2
4	4	1	1	3	3	1	1	2	2
4	4	1	1	1	1	1	1	2	2
4	4	4	4	1	1	1	1	2	2
4	4	1	1	3	3	1	1	2	2
4	4	1	1	3	3	1	1	2	2
4	4	4	4	1	1	1	1	2	2
4	4	1	1	3	3	1	1	2	2
1	1	1	1	3	3	1	1	2	2
4	4	1	1	1	1	1	1	2	2
4	4	4	4	1	1	1	1	2	2
4	4	4	4	3	3	1	1	2	2
4	4	1	1	3	3	1	1	2	2
4	4	4	4	1	1	1	1	2	2
4	4	1	1	1	1	1	1	2	2
4	4	1	1	3	3	1	1	2	2
4	4	1	1	3	3	1	1	2	2
4	4	1	1	1	1	1	1	2	2
4	4	1	1	1	1	1	1	2	2
1	1	1	1	1	1	4	4	1	1
4	4	1	1	3	3	1	1	2	2
1	1	1	1	3	3	4	4	1	1
1	1	4	4	1	1	1	1	2	2
4	4	4	4	3	3	4	4	1	1
4	4	1	1	3	3	1	1	2	2
4	4	4	4	1	1	1	1	2	2
4	4	1	1	3	3	1	1	2	2
1	1	4	4	3	3	1	1	2	2
4	4	4	4	1	1	1	1	2	2
4	4	4	4	1	1	1	1	2	2
4	4	4	4	1	1	1	1	2	2

For Review Only

4	4	4	4	3	3	1	1	2	2
4	4	4	4	3	3	1	1	2	2
4	4	4	4	1	1	1	1	2	2
4	4	4	4	3	3	1	1	2	2
4	4	1	1	1	1	1	1	2	2
4	4	1	1	1	1	1	1	2	2
4	4	4	4	3	3	1	1	2	2
4	4	1	1	1	1	1	1	2	2
4	4	1	1	1	1	1	1	2	2
4	4	4	4	1	1	1	1	2	2
4	4	4	4	1	1	1	1	2	2
4	4	4	4	1	1	1	1	2	2
4	4	4	4	3	3	1	1	2	2
4	4	1	1	3	3	1	1	2	2
4	4	4	4	1	1	1	1	2	2
4	4	1	1	1	1	4	4	1	1
4	4	4	4	1	1	1	1	2	2
4	4	4	4	1	1	1	1	2	2
4	4	4	4	3	3	1	1	2	2
4	4	4	4	3	3	1	1	2	2
4	4	4	4	1	1	1	1	2	2
4	4	4	4	1	1	1	1	2	2
4	4	1	1	3	3	1	1	2	2
4	4	4	4	1	1	1	1	2	2
4	4	4	4	3	3	4	4	1	1
4	4	1	1	1	1	1	1	2	2
4	4	4	4	1	1	1	1	2	2
4	4	1	1	1	1	1	1	2	2
4	4	1	1	3	3	1	1	2	2
4	4	1	1	1	1	1	1	2	2
4	4	1	1	3	3	1	1	2	2
4	4	1	1	1	1	1	1	2	2
4	4	1	1	1	1	1	1	2	2
4	4	4	4	3	3	1	1	2	2
4	4	1	1	1	1	1	1	2	2
4	4	4	4	3	3	4	4	1	1
4	4	1	1	1	1	1	1	2	2
4	4	1	1	3	3	1	1	2	2
4	4	1	1	3	3	1	1	2	2
4	4	4	4	1	1	1	1	2	2
4	4	4	4	3	3	1	1	2	2
4	4	1	1	1	1	1	1	2	2
4	4	1	1	3	3	1	1	2	2
4	4	4	4	1	1	1	1	2	2
4	4	1	1	3	3	1	1	2	2
4	4	4	4	1	1	1	1	2	2
4	4	1	1	3	3	1	1	2	2
4	4	4	4	3	3	1	1	2	2
4	4	4	4	3	3	1	1	2	2
4	4	4	4	1	1	1	1	2	2
4	4	1	1	1	1	1	1	2	2
4	4	1	1	1	1	1	1	2	2
4	4	1	1	1	1	1	1	2	2
4	4	4	4	1	1	1	1	2	2
4	4	4	4	1	1	1	1	2	2

For Review Only

4	4	4	4	1	1	1	1	2	2
4	4	1	1	3	3	1	1	2	2
4	4	1	1	3	3	1	1	2	2
1	1	1	1	3	3	1	1	2	2
4	4	1	1	3	3	1	1	2	2
4	4	1	1	1	1	1	1	2	2
4	4	4	4	3	3	1	1	2	2
4	4	4	4	1	1	1	1	2	2
4	4	1	1	3	3	4	4	1	1
4	4	1	1	1	1	1	1	2	2
4	4	4	4	1	1	1	1	2	2
4	4	4	4	1	1	1	1	2	2
4	4	1	1	1	1	1	1	2	2
4	4	4	4	1	1	1	1	2	2
4	4	1	1	1	1	1	1	2	2
4	4	4	4	1	1	1	1	2	2
4	4	4	4	1	1	1	1	2	2
4	4	4	4	1	1	1	1	2	2
1	1	1	1	3	3	1	1	2	2
4	4	4	4	1	1	1	1	2	2
4	4	4	4	1	1	1	1	2	2
4	4	1	1	3	3	1	1	2	2
4	4	4	4	1	1	1	1	2	2
4	4	1	1	3	3	1	1	2	2
4	4	4	4	1	1	1	1	2	2
4	4	1	1	3	3	1	1	2	2
4	4	4	4	1	1	1	1	2	2
4	4	1	1	3	3	1	1	2	2
4	4	4	4	1	1	1	1	2	2
4	4	4	4	1	1	1	1	2	2
4	4	1	1	1	1	1	1	2	2
4	4	4	4	1	1	1	1	2	2
4	4	4	4	3	3	1	1	2	2
4	4	1	1	3	3	1	1	2	2
4	4	1	1	3	3	1	1	2	2
4	4	1	1	1	1	1	1	2	2
1	1	1	1	3	3	1	1	2	2
4	4	1	1	1	1	1	1	2	2
4	4	1	1	1	1	1	1	2	2
4	4	4	4	1	1	1	1	2	2
4	4	4	4	3	3	1	1	2	2
1	1	1	1	3	3	1	1	2	2
4	4	1	1	1	1	1	1	2	2
4	4	4	4	1	1	1	1	2	2
4	4	1	1	3	3	1	1	2	2
4	4	1	1	3	3	1	1	2	2
4	4	1	1	3	3	1	1	2	2
4	4	4	4	1	1	1	1	2	2
4	4	1	1	1	1	1	1	2	2
4	4	1	1	3	3	1	1	2	2
4	4	1	1	3	3	1	1	2	2
4	4	4	4	1	1	1	1	2	2
4	4	4	4	1	1	1	1	2	2
4	4	4	4	1	1	1	1	2	2
4	4	4	4	1	1	1	1	2	2

For Review Only

4	4	4	4	1	1	1	1	2	2
4	4	1	1	3	3	1	1	2	2
4	4	4	4	3	3	4	4	1	1
4	4	1	1	1	1	1	1	2	2
4	4	4	4	1	1	1	1	2	2
4	4	4	4	1	1	1	1	2	2
4	4	1	1	1	1	1	1	2	2
4	4	4	4	1	1	1	1	2	2
4	4	4	4	1	1	1	1	2	2
4	4	1	1	1	1	1	1	2	2
4	4	1	1	3	3	1	1	2	2
4	4	4	4	1	1	1	1	2	2
4	4	1	1	1	1	1	1	2	2
4	4	1	1	3	3	1	1	2	2
4	4	1	1	3	3	1	1	2	2
4	4	4	4	1	1	1	1	2	2
4	4	4	4	1	1	1	1	2	2
4	4	1	1	1	1	1	1	2	2
4	4	1	1	3	3	1	1	2	2
4	4	4	4	3	3	1	1	2	2
4	4	4	4	1	1	1	1	2	2
4	4	1	1	1	1	1	1	2	2
4	4	1	1	1	1	1	1	2	2
4	4	4	4	1	1	1	1	2	2
1	1	1	1	3	3	1	1	2	2
4	4	1	1	3	3	1	1	2	2
4	4	1	1	3	3	1	1	2	2
4	4	4	4	1	1	1	1	2	2
4	4	4	4	3	3	1	1	2	2
4	4	4	4	1	1	1	1	2	2
4	4	1	1	1	1	1	1	2	2
4	4	4	4	1	1	1	1	2	2
4	4	4	4	3	3	1	1	2	2
1	1	1	1	3	3	1	1	2	2
4	4	4	4	3	3	1	1	2	2
4	4	4	4	1	1	1	1	2	2
4	4	1	1	1	1	1	1	2	2
4	4	1	1	3	3	1	1	2	2
4	4	4	4	1	1	1	1	2	2
4	4	1	1	1	1	1	1	2	2
4	4	1	1	3	3	1	1	2	2
4	4	1	1	3	3	1	1	2	2
4	4	1	1	1	1	1	1	2	2
4	4	4	4	1	1	1	1	2	2
4	4	4	4	3	3	1	1	2	2
4	4	1	1	1	1	1	1	2	2
4	4	4	4	1	1	1	1	2	2
4	4	1	1	1	1	1	1	2	2
1	1	1	1	3	3	1	1	2	2
4	4	4	4	3	3	1	1	2	2
4	4	4	4	3	3	1	1	2	2
4	4	1	1	1	1	1	1	2	2
4	4	1	1	1	1	1	1	2	2
4	4	4	4	1	1	1	1	2	2
4	4	1	1	1	1	1	1	2	2

For Review Only

1	1	1	1	1	1	1	1	2	2
4	4	4	4	3	3	4	4	1	1
4	4	4	4	3	3	1	1	2	2
4	4	4	4	1	1	1	1	2	2
1	1	4	4	1	1	1	1	2	2
4	4	1	1	3	3	1	1	2	2
4	4	4	4	1	1	1	1	2	2
4	4	1	1	1	1	4	4	1	1
4	4	1	1	3	3	1	1	2	2
4	4	1	1	1	1	1	1	2	2
4	4	4	4	1	1	1	1	2	2
4	4	1	1	1	1	1	1	2	2
4	4	4	4	3	3	1	1	2	2
4	4	1	1	1	1	1	1	2	2
4	4	1	1	1	1	1	1	2	2
4	4	4	4	3	3	1	1	2	2
4	4	1	1	1	1	1	1	2	2
4	4	1	1	1	1	1	1	2	2
4	4	4	4	1	1	1	1	2	2
4	4	1	1	3	3	1	1	2	2
4	4	4	4	1	1	1	1	2	2
4	4	1	1	3	3	1	1	2	2
4	4	1	1	3	3	1	1	2	2
4	4	4	4	1	1	1	1	2	2
4	4	1	1	1	1	1	1	2	2
4	4	1	1	1	1	1	1	2	2
4	4	1	1	1	1	1	1	2	2
4	4	1	1	3	3	1	1	2	2
4	4	4	4	1	1	1	1	2	2
4	4	4	4	1	1	1	1	2	2
4	4	1	1	1	1	1	1	2	2
4	4	1	1	1	1	1	1	2	2
4	4	1	1	3	3	1	1	2	2
4	4	1	1	1	1	1	1	2	2
4	4	4	4	1	1	1	1	2	2
4	4	1	1	1	1	1	1	2	2
4	4	1	1	1	1	1	1	2	2
4	4	4	4	1	1	1	1	2	2
4	4	1	1	1	1	1	1	2	2
4	4	1	1	1	1	1	1	2	2
4	4	1	1	3	3	1	1	2	2
4	4	4	4	3	3	1	1	2	2
4	4	1	1	1	1	1	1	2	2
4	4	4	4	3	3	1	1	2	2
4	4	4	4	1	1	1	1	2	2
4	4	1	1	3	3	1	1	2	2
4	4	4	4	1	1	1	1	2	2
4	4	1	1	3	3	1	1	2	2
4	4	4	4	1	1	4	4	1	1
4	4	1	1	1	1	1	1	2	2
4	4	4	4	1	1	1	1	2	2
4	4	1	1	1	1	1	1	2	2
4	4	1	1	3	3	1	1	2	2
4	4	1	1	3	3	1	1	2	2
4	4	1	1	1	1	1	1	2	2

For Review Only

4	4	4	4	3	3	1	1	2	2
4	4	1	1	1	1	1	1	2	2

For Review Only

SNP1222	SNP1222	SNP1223	SNP1223	SNP1224	SNP1224	SNP1225	SNP1225	SNP1226	SNP1226
2	2	3	3	4	4	4	4	3	3
2	2	3	3	4	4	4	4	1	1
2	2	3	3	4	4	4	4	3	3
2	2	3	3	4	4	4	4	3	3
2	2	3	3	1	1	4	4	1	1
2	2	3	3	1	1	4	4	3	3
2	2	3	3	1	1	4	4	3	3
2	2	3	3	4	4	4	4	3	3
2	2	3	3	1	1	4	4	3	3
2	2	3	3	4	4	4	4	1	1
2	2	3	3	1	1	4	4	3	3
2	2	3	3	4	4	4	4	3	3
2	2	3	3	4	4	4	4	3	3
2	2	3	3	4	4	4	4	3	3
2	2	3	3	4	4	4	4	3	3
2	2	3	3	4	4	4	4	3	3
2	2	3	3	1	1	4	4	3	3
2	2	3	3	1	1	4	4	3	3
2	2	3	3	4	4	4	4	3	3
2	2	3	3	1	1	4	4	3	3
2	2	3	3	4	4	4	4	3	3
2	2	3	3	1	1	4	4	3	3
2	2	3	3	4	4	4	4	3	3
2	2	3	3	4	4	4	4	3	3
2	2	3	3	1	1	4	4	3	3
2	2	3	3	4	4	4	4	3	3
2	2	3	3	4	4	4	4	3	3
2	2	3	3	4	4	4	4	3	3
2	2	3	3	4	4	4	4	3	3
2	2	3	3	4	4	4	4	1	1
1	1	3	3	4	4	4	4	3	3
2	2	3	3	1	1	4	4	3	3
2	2	3	3	1	1	4	4	3	3
2	2	3	3	4	4	4	4	3	3
2	2	3	3	1	1	4	4	3	3
2	2	3	3	4	4	4	4	3	3
2	2	3	3	4	4	4	4	3	3
2	2	3	3	4	4	4	4	3	3
2	2	3	3	4	4	4	4	3	3
2	2	3	3	4	4	4	4	3	3
2	2	3	3	4	4	4	4	1	1
2	2	3	3	4	4	4	4	3	3
2	2	3	3	4	4	4	4	1	1
2	2	3	3	1	1	4	4	3	3
2	2	3	3	1	1	4	4	1	1
2	2	3	3	4	4	4	4	3	3
2	2	3	3	1	1	4	4	3	3
2	2	3	3	4	4	4	4	3	3
2	2	3	3	1	1	4	4	3	3
2	2	3	3	4	4	1	1	3	3
2	2	3	3	4	4	4	4	3	3
1	1	3	3	4	4	4	4	3	3

For Review Only

2	2	3	3	1	1	4	4	3	3
2	2	3	3	1	1	4	4	3	3
2	2	3	3	1	1	4	4	3	3
2	2	3	3	1	1	4	4	3	3
2	2	3	3	4	4	4	4	3	3
2	2	3	3	4	4	4	4	3	3
2	2	3	3	1	1	4	4	3	3
2	2	3	3	4	4	4	4	3	3
2	2	3	3	4	4	4	4	3	3
2	2	3	3	4	4	1	1	3	3
2	2	3	3	1	1	4	4	3	3
2	2	3	3	1	1	4	4	3	3
2	2	3	3	1	1	4	4	3	3
2	2	3	3	4	4	4	4	3	3
2	2	3	3	4	4	4	4	3	3
2	2	3	3	4	4	4	4	1	1
2	2	3	3	4	4	4	4	3	3
2	2	2	2	4	4	1	1	3	3
2	2	3	3	1	1	4	4	3	3
2	2	3	3	1	1	4	4	3	3
2	2	3	3	1	1	4	4	3	3
2	2	3	3	4	4	4	4	3	3
2	2	3	3	4	4	4	4	3	3
2	2	3	3	1	1	4	4	3	3
2	2	3	3	1	1	4	4	1	1
1	1	3	3	4	4	4	4	3	3
2	2	3	3	1	1	4	4	3	3
2	2	3	3	4	4	4	4	3	3
2	2	3	3	4	4	4	4	3	3
2	2	2	2	4	4	1	1	3	3
2	2	3	3	4	4	4	4	3	3
2	2	3	3	4	4	4	4	3	3
2	2	3	3	4	4	4	4	3	3
2	2	3	3	1	1	4	4	3	3
2	2	3	3	4	4	4	4	3	3
2	2	3	3	1	1	4	4	1	1
2	2	3	3	4	4	4	4	3	3
2	2	3	3	4	4	4	4	3	3
2	2	3	3	4	4	4	4	3	3
2	2	3	3	1	1	4	4	3	3
2	2	3	3	1	1	4	4	3	3
2	2	3	3	4	4	4	4	3	3
2	2	2	2	4	4	1	1	3	3
2	2	3	3	1	1	4	4	3	3
2	2	3	3	4	4	4	4	3	3
2	2	3	3	1	1	4	4	3	3
2	2	3	3	4	4	4	4	3	3
2	2	3	3	4	4	4	4	3	3
2	2	3	3	1	1	4	4	3	3
2	2	2	2	1	1	1	1	3	3
1	1	3	3	4	4	4	4	3	3
2	2	3	3	4	4	4	4	3	3
2	2	3	3	4	4	4	4	3	3
2	2	3	3	4	4	4	4	3	3
2	2	2	2	1	1	1	1	3	3

For Review Only

2	2	3	3	4	4	4	4	3	3
2	2	3	3	4	4	4	4	3	3
2	2	3	3	4	4	4	4	3	3
2	2	3	3	4	4	4	4	3	3
2	2	3	3	4	4	4	4	3	3
2	2	3	3	4	4	4	4	3	3
2	2	2	2	4	4	1	1	3	3
2	2	3	3	1	1	4	4	3	3
2	2	3	3	4	4	4	4	1	1
2	2	3	3	4	4	4	4	3	3
2	2	3	3	1	1	4	4	3	3
2	2	3	3	4	4	4	4	3	3
2	2	2	2	4	4	1	1	3	3
2	2	3	3	4	4	4	4	3	3
2	2	3	3	4	4	4	4	3	3
2	2	3	3	4	4	4	4	3	3
2	2	2	2	1	1	1	1	3	3
2	2	3	3	4	4	4	4	3	3
2	2	3	3	4	4	4	4	3	3
2	2	3	3	4	4	4	4	3	3
2	2	3	3	4	4	4	4	3	3
2	2	3	3	4	4	1	1	3	3
2	2	3	3	4	4	4	4	3	3
2	2	3	3	4	4	4	4	3	3
2	2	3	3	4	4	4	4	3	3
2	2	3	3	4	4	4	4	3	3
2	2	3	3	1	1	4	4	3	3
2	2	3	3	4	4	4	4	3	3
2	2	2	2	1	1	1	1	3	3
2	2	2	2	1	1	1	1	3	3
2	2	3	3	4	4	4	4	3	3
2	2	3	3	4	4	4	4	3	3
2	2	3	3	1	1	4	4	3	3
2	2	3	3	4	4	4	4	3	3
2	2	3	3	4	4	4	4	3	3
2	2	3	3	4	4	4	4	3	3
2	2	3	3	4	4	4	4	3	3
1	1	3	3	4	4	4	4	3	3
2	2	3	3	1	1	4	4	3	3
2	2	3	3	4	4	4	4	3	3
2	2	3	3	4	4	4	4	3	3
2	2	3	3	4	4	4	4	3	3
1	1	2	2	4	4	1	1	3	3
1	1	3	3	4	4	4	4	1	1
2	2	3	3	4	4	4	4	3	3
2	2	3	3	4	4	4	4	3	3
2	2	3	3	4	4	4	4	3	3
2	2	3	3	1	1	4	4	3	3
2	2	3	3	4	4	4	4	3	3
2	2	3	3	4	4	4	4	3	3
2	2	3	3	4	4	4	4	3	3
2	2	3	3	1	1	4	4	3	3
2	2	3	3	4	4	4	4	3	3
2	2	3	3	1	1	4	4	3	3
2	2	3	3	4	4	4	4	3	3

For Review Only

2	2	2	2	1	1	1	1	3	3
2	2	3	3	4	4	4	4	3	3
2	2	3	3	1	1	4	4	1	1
2	2	3	3	4	4	4	4	3	3
2	2	3	3	4	4	4	4	3	3
2	2	3	3	1	1	4	4	3	3
2	2	3	3	4	4	4	4	3	3
2	2	3	3	4	4	4	4	3	3
2	2	3	3	1	1	4	4	3	3
2	2	3	3	1	1	4	4	3	3
2	2	3	3	4	4	4	4	3	3
2	2	3	3	1	1	4	4	3	3
2	2	3	3	4	4	4	4	3	3
2	2	3	3	4	4	4	4	3	3
2	2	3	3	4	4	4	4	3	3
2	2	3	3	1	1	4	4	3	3
2	2	3	3	4	4	4	4	3	3
1	1	3	3	4	4	4	4	3	3
2	2	3	3	4	4	4	4	3	3
2	2	2	2	4	4	1	1	3	3
2	2	3	3	4	4	4	4	3	3
2	2	3	3	4	4	4	4	3	3
2	2	3	3	4	4	4	4	3	3
2	2	3	3	1	1	4	4	3	3
2	2	3	3	4	4	4	4	3	3
2	2	3	3	4	4	4	4	3	3
2	2	3	3	4	4	4	4	3	3
2	2	2	2	4	4	4	4	3	3
2	2	3	3	1	1	4	4	3	3
2	2	3	3	1	1	4	4	3	3
1	1	3	3	4	4	4	4	3	3
2	2	2	2	1	1	1	1	3	3
2	2	3	3	1	1	4	4	3	3
2	2	3	3	4	4	4	4	3	3
2	2	3	3	4	4	4	4	3	3
2	2	3	3	1	1	4	4	3	3
2	2	3	3	4	4	4	4	3	3
2	2	3	3	4	4	4	4	3	3
2	2	3	3	1	1	4	4	3	3
2	2	3	3	4	4	4	4	3	3
2	2	3	3	4	4	4	4	3	3
2	2	3	3	4	4	4	4	3	3
2	2	3	3	4	4	4	4	3	3
2	2	3	3	1	1	4	4	3	3
2	2	3	3	1	1	4	4	3	3
2	2	3	3	4	4	4	4	3	3
2	2	3	3	1	1	4	4	3	3
2	2	3	3	4	4	4	4	3	3
2	2	3	3	4	4	4	4	3	3
2	2	3	3	1	1	4	4	3	3
2	2	2	2	1	1	1	1	3	3
2	2	3	3	4	4	4	4	3	3
2	2	3	3	4	4	4	4	3	3
2	2	3	3	1	1	4	4	3	3
1	1	3	3	4	4	4	4	3	3

For Review Only

2	2	3	3	4	4	4	4	3	3
2	2	3	3	1	1	4	4	1	1
2	2	2	2	1	1	1	1	3	3
2	2	3	3	4	4	4	4	3	3
2	2	3	3	1	1	4	4	3	3
2	2	2	2	4	4	1	1	3	3
2	2	3	3	1	1	4	4	3	3
2	2	3	3	4	4	4	4	1	1
2	2	3	3	4	4	4	4	3	3
2	2	3	3	4	4	4	4	3	3
2	2	3	3	4	4	4	4	3	3
2	2	3	3	4	4	4	4	3	3
2	2	3	3	1	1	4	4	3	3
2	2	3	3	4	4	4	4	3	3
2	2	3	3	4	4	4	4	3	3
2	2	3	3	1	1	4	4	3	3
2	2	3	3	4	4	4	4	3	3
2	2	3	3	4	4	4	4	3	3
2	2	3	3	1	1	4	4	3	3
2	2	3	3	4	4	4	4	3	3
1	1	3	3	1	1	4	4	3	3
2	2	3	3	4	4	4	4	3	3
2	2	3	3	4	4	4	4	3	3
2	2	3	3	1	1	4	4	3	3
2	2	3	3	4	4	4	4	3	3
1	1	3	3	4	4	4	4	3	3
2	2	3	3	4	4	4	4	3	3
2	2	3	3	4	4	4	4	3	3
2	2	3	3	1	1	4	4	3	3
2	2	2	2	1	1	1	1	3	3
2	2	3	3	4	4	4	4	3	3
2	2	3	3	4	4	4	4	3	3
2	2	3	3	4	4	4	4	3	3
2	2	3	3	4	4	4	4	3	3
2	2	3	3	4	4	4	4	3	3
2	2	3	3	4	4	4	4	3	3
2	2	2	2	4	4	1	1	3	3
2	2	3	3	1	1	4	4	3	3
2	2	3	3	4	4	4	4	3	3
2	2	3	3	4	4	4	4	3	3
1	1	3	3	4	4	4	4	3	3
2	2	3	3	1	1	4	4	3	3
2	2	3	3	4	4	4	4	3	3
2	2	3	3	1	1	4	4	3	3
2	2	3	3	4	4	4	4	3	3
2	2	3	3	4	4	4	4	3	3
2	2	3	3	1	1	4	4	3	3
2	2	3	3	4	4	4	4	3	3
2	2	3	3	1	1	4	4	3	3
2	2	3	3	4	4	4	4	3	3
2	2	3	3	1	1	4	4	3	3
2	2	3	3	4	4	4	4	3	3
2	2	3	3	4	4	4	4	3	3
2	2	2	2	4	4	1	1	3	3
2	2	3	3	4	4	4	4	3	3

2	2	3	3	1	1	4	4	3	3
2	2	3	3	4	4	4	4	3	3

For Review Only

SNP1227	SNP1227	SNP1228	SNP1228	SNP1229	SNP1229	SNP123	SNP123	SNP1230	SNP1230
1	1	1	1	2	2	1	1	1	1
1	1	3	3	3	3	4	4	3	3
1	1	1	1	2	2	1	1	3	3
1	1	1	1	2	2	4	4	3	3
1	1	3	3	3	3	1	1	1	1
1	1	1	1	2	2	4	4	1	1
1	1	1	1	2	2	1	1	1	1
1	1	1	1	2	2	4	4	3	3
1	1	1	1	2	2	1	1	1	1
1	1	3	3	3	3	4	4	3	3
1	1	1	1	2	2	4	4	1	1
1	1	1	1	2	2	1	1	3	3
1	1	1	1	2	2	4	4	3	3
1	1	1	1	2	2	4	4	1	1
1	1	1	1	2	2	4	4	1	1
1	1	1	1	2	2	4	4	3	3
1	1	1	1	2	2	4	4	1	1
1	1	1	1	2	2	1	1	1	1
1	1	1	1	2	2	4	4	3	3
1	1	1	1	2	2	4	4	1	1
1	1	1	1	2	2	1	1	3	3
1	1	1	1	2	2	1	1	1	1
1	1	1	1	2	2	1	1	3	3
1	1	1	1	2	2	1	1	3	3
1	1	1	1	2	2	1	1	1	1
1	1	1	1	2	2	1	1	3	3
1	1	1	1	2	2	4	4	3	3
1	1	1	1	2	2	4	4	1	1
1	1	1	1	2	2	1	1	3	3
1	1	3	3	3	3	4	4	3	3
1	1	1	1	2	2	4	4	3	3
1	1	1	1	2	2	4	4	1	1
1	1	1	1	2	2	1	1	1	1
1	1	1	1	2	2	4	4	3	3
1	1	1	1	2	2	4	4	3	3
1	1	1	1	2	2	4	4	3	3
1	1	1	1	2	2	4	4	3	3
1	1	1	1	2	2	4	4	3	3
1	1	1	1	2	2	4	4	3	3
1	1	1	1	2	2	4	4	3	3
1	1	3	3	3	3	4	4	1	1
1	1	1	1	2	2	4	4	3	3
1	1	3	3	3	3	4	4	1	1
1	1	1	1	2	2	4	4	1	1
1	1	3	3	3	3	4	4	1	1
1	1	1	1	2	2	1	1	3	3
1	1	1	1	2	2	4	4	1	1
1	1	1	1	2	2	4	4	3	3
1	1	1	1	2	2	4	4	1	1
4	4	1	1	2	2	1	1	1	1
1	1	1	1	2	2	1	1	3	3
1	1	1	1	2	2	1	1	1	1

For Review Only

1	1	1	1	2	2	1	1	1	1
1	1	1	1	2	2	1	1	1	1
1	1	1	1	2	2	1	1	1	1
1	1	1	1	2	2	4	4	1	1
1	1	1	1	2	2	4	4	3	3
1	1	1	1	2	2	4	4	1	1
1	1	1	1	2	2	1	1	1	1
1	1	1	1	2	2	4	4	3	3
1	1	1	1	2	2	1	1	3	3
1	1	1	1	2	2	4	4	1	1
1	1	1	1	2	2	1	1	1	1
1	1	1	1	2	2	4	4	1	1
1	1	1	1	2	2	1	1	1	1
1	1	1	1	2	2	1	1	3	3
1	1	1	1	2	2	4	4	1	1
1	1	1	1	2	2	4	4	1	1
1	1	1	1	2	2	4	4	3	3
4	4	1	1	2	2	1	1	1	1
1	1	1	1	2	2	1	1	1	1
1	1	1	1	2	2	1	1	1	1
1	1	1	1	2	2	4	4	1	1
1	1	1	1	2	2	4	4	1	1
1	1	1	1	2	2	1	1	1	1
1	1	1	1	2	2	1	1	1	1
1	1	3	3	3	3	4	4	1	1
1	1	1	1	2	2	1	1	3	3
1	1	1	1	2	2	4	4	1	1
1	1	1	1	2	2	4	4	3	3
1	1	1	1	2	2	1	1	1	1
4	4	1	1	2	2	4	4	3	3
1	1	1	1	2	2	1	1	1	1
1	1	1	1	2	2	4	4	3	3
1	1	1	1	2	2	4	4	3	3
1	1	1	1	2	2	1	1	1	1
1	1	1	1	2	2	1	1	3	3
1	1	3	3	3	3	4	4	1	1
1	1	1	1	2	2	1	1	1	1
1	1	1	1	2	2	1	1	1	1
1	1	1	1	2	2	4	4	3	3
1	1	1	1	2	2	4	4	1	1
1	1	1	1	2	2	1	1	1	1
1	1	1	1	2	2	4	4	3	3
4	4	1	1	2	2	4	4	3	3
1	1	1	1	2	2	1	1	1	1
1	1	1	1	2	2	1	1	3	3
1	1	1	1	2	2	1	1	1	1
1	1	1	1	2	2	4	4	3	3
1	1	1	1	2	2	1	1	1	1
1	1	1	1	2	2	4	4	1	1
4	4	1	1	2	2	1	1	1	1
1	1	1	1	2	2	4	4	3	3
1	1	1	1	2	2	4	4	3	3
1	1	1	1	2	2	4	4	3	3
1	1	1	1	2	2	1	1	1	1
4	4	1	1	2	2	1	1	1	1

For Review Only

1	1	1	1	2	2	1	1	1	1
1	1	1	1	2	2	4	4	3	3
1	1	1	1	2	2	1	1	3	3
1	1	1	1	2	2	4	4	1	1
1	1	1	1	2	2	1	1	1	1
1	1	1	1	2	2	1	1	3	3
1	1	1	1	2	2	4	4	3	3
1	1	1	1	2	2	4	4	1	1
1	1	3	3	3	3	4	4	3	3
1	1	1	1	2	2	4	4	3	3
1	1	1	1	2	2	1	1	1	1
1	1	1	1	2	2	1	1	1	1
1	1	1	1	2	2	4	4	3	3
1	1	1	1	2	2	4	4	1	1
1	1	1	1	2	2	4	4	1	1
1	1	1	1	2	2	4	4	1	1
4	4	1	1	2	2	1	1	1	1
1	1	1	1	2	2	4	4	1	1
1	1	1	1	2	2	4	4	3	3
1	1	1	1	2	2	4	4	1	1
1	1	1	1	2	2	4	4	1	1
4	4	1	1	2	2	1	1	3	3
1	1	1	1	2	2	4	4	3	3
1	1	1	1	2	2	4	4	3	3
1	1	1	1	2	2	4	4	3	3
1	1	1	1	2	2	1	1	3	3
1	1	1	1	2	2	1	1	1	1
1	1	1	1	2	2	4	4	3	3
4	4	1	1	2	2	1	1	1	1
4	4	1	1	2	2	1	1	1	1
1	1	1	1	2	2	4	4	3	3
1	1	1	1	2	2	4	4	1	1
1	1	1	1	2	2	1	1	1	1
1	1	1	1	2	2	1	1	1	1
1	1	1	1	2	2	4	4	3	3
1	1	1	1	2	2	4	4	3	3
1	1	1	1	2	2	4	4	3	3
1	1	1	1	2	2	1	1	3	3
1	1	1	1	2	2	4	4	3	3
1	1	1	1	2	2	4	4	1	1
1	1	1	1	2	2	4	4	1	1
1	1	1	1	2	2	4	4	3	3
4	4	1	1	2	2	1	1	3	3
1	1	3	3	3	3	4	4	1	1
1	1	1	1	2	2	1	1	3	3
1	1	1	1	2	2	4	4	3	3
1	1	1	1	2	2	1	1	1	1
1	1	1	1	2	2	4	4	1	1
1	1	1	1	2	2	4	4	3	3
1	1	1	1	2	2	1	1	3	3
1	1	1	1	2	2	4	4	1	1
1	1	1	1	2	2	4	4	1	1
1	1	1	1	2	2	4	4	1	1
1	1	1	1	2	2	1	1	1	1
1	1	1	1	2	2	4	4	3	3

For Review Only

4	4	1	1	2	2	1	1	1	1
1	1	1	1	2	2	4	4	3	3
1	1	3	3	3	3	1	1	1	1
1	1	1	1	2	2	1	1	3	3
1	1	1	1	2	2	4	4	1	1
1	1	1	1	2	2	4	4	1	1
1	1	1	1	2	2	4	4	3	3
1	1	1	1	2	2	4	4	1	1
1	1	1	1	2	2	4	4	1	1
1	1	1	1	2	2	1	1	1	1
1	1	1	1	2	2	4	4	1	1
1	1	1	1	2	2	4	4	1	1
1	1	1	1	2	2	1	1	3	3
1	1	1	1	2	2	4	4	3	3
1	1	1	1	2	2	4	4	3	3
1	1	1	1	2	2	4	4	1	1
1	1	1	1	2	2	4	4	1	1
1	1	1	1	2	2	1	1	3	3
1	1	1	1	2	2	1	1	3	3
1	1	1	1	2	2	4	4	3	3
1	1	1	1	2	2	4	4	1	1
1	1	1	1	2	2	4	4	3	3
1	1	1	1	2	2	4	4	1	1
1	1	1	1	2	2	4	4	1	1
1	1	1	1	2	2	1	1	3	3
1	1	1	1	2	2	4	4	3	3
1	1	1	1	2	2	1	1	3	3
1	1	1	1	2	2	4	4	1	1
1	1	1	1	2	2	1	1	1	1
1	1	1	1	2	2	1	1	1	1
1	1	1	1	2	2	1	1	3	3
4	4	1	1	2	2	1	1	1	1
1	1	1	1	2	2	1	1	1	1
1	1	1	1	2	2	4	4	3	3
1	1	1	1	2	2	1	1	1	1
1	1	1	1	2	2	4	4	1	1
1	1	1	1	2	2	4	4	3	3
4	4	1	1	2	2	4	4	3	3
1	1	1	1	2	2	4	4	1	1
1	1	1	1	2	2	1	1	3	3
1	1	1	1	2	2	1	1	3	3
1	1	1	1	2	2	1	1	1	1
1	1	1	1	2	2	4	4	3	3
1	1	1	1	2	2	4	4	1	1
1	1	1	1	2	2	4	4	1	1
1	1	1	1	2	2	4	4	3	3
1	1	1	1	2	2	4	4	1	1
1	1	1	1	2	2	4	4	3	3
1	1	1	1	2	2	4	4	3	3
1	1	1	1	2	2	4	4	1	1
4	4	1	1	2	2	1	1	1	1
1	1	1	1	2	2	4	4	3	3
1	1	1	1	2	2	4	4	3	3
1	1	1	1	2	2	1	1	1	1
1	1	1	1	2	2	4	4	3	3

For Review Only

1	1	1	1	2	2	1	1	3	3
1	1	1	1	3	3	4	4	1	1
4	4	1	1	2	2	4	4	1	1
1	1	1	1	2	2	4	4	1	1
1	1	1	1	2	2	4	4	1	1
1	1	1	1	2	2	4	4	1	1
1	1	1	1	2	2	1	1	1	1
1	1	3	3	3	3	4	4	3	3
1	1	1	1	2	2	4	4	1	1
1	1	1	1	2	2	4	4	3	3
1	1	1	1	2	2	4	4	1	1
1	1	1	1	2	2	4	4	3	3
1	1	1	1	2	2	4	4	1	1
1	1	1	1	2	2	4	4	3	3
1	1	1	1	2	2	1	1	3	3
1	1	1	1	2	2	1	1	1	1
1	1	1	1	2	2	4	4	3	3
4	4	1	1	2	2	4	4	3	3
1	1	1	1	2	2	4	4	1	1
1	1	1	1	2	2	4	4	3	3
1	1	1	1	2	2	4	4	1	1
1	1	1	1	2	2	4	4	1	1
1	1	1	1	2	2	1	1	3	3
1	1	1	1	2	2	4	4	1	1
1	1	1	1	2	2	4	4	3	3
1	1	1	1	2	2	1	1	3	3
1	1	1	1	2	2	4	4	3	3
4	4	1	1	2	2	1	1	3	3
1	1	1	1	2	2	4	4	1	1
4	4	1	1	2	2	1	1	1	1
1	1	1	1	2	2	4	4	3	3
1	1	1	1	2	2	1	1	1	1
1	1	1	1	2	2	4	4	3	3
1	1	1	1	2	2	1	1	3	3
1	1	1	1	2	2	4	4	1	1
1	1	1	1	2	2	4	4	3	3
4	4	1	1	2	2	1	1	3	3
1	1	1	1	2	2	4	4	1	1
1	1	1	1	2	2	4	4	3	3
1	1	1	1	2	2	4	4	3	3
4	4	1	1	2	2	1	1	3	3
1	1	1	1	2	2	4	4	1	1
1	1	1	1	2	2	4	4	3	3
1	1	1	1	2	2	1	1	1	1
1	1	1	1	2	2	4	4	1	1
1	1	1	1	2	2	4	4	3	3
1	1	1	1	2	2	1	1	1	1
1	1	1	1	2	2	4	4	3	3
1	1	1	1	2	2	4	4	1	1
1	1	1	1	2	2	4	4	3	3
1	1	1	1	2	2	1	1	1	1
1	1	1	1	2	2	4	4	3	3
1	1	1	1	2	2	1	1	3	3
4	4	1	1	2	2	1	1	3	3
1	1	1	1	2	2	4	4	3	3

For Review Only

1	1	1	1	2	2	1	1	1	1
1	1	1	1	2	2	1	1	1	1

For Review Only

SNP1231	SNP1231	SNP1232	SNP1232	SNP1233	SNP1233	SNP1234	SNP1234	SNP1235	SNP1235
2	2	4	4	2	2	3	3	2	2
2	2	3	3	3	3	3	3	2	2
2	2	3	3	2	2	3	3	2	2
2	2	3	3	2	2	3	3	2	2
2	2	4	4	3	3	3	3	2	2
2	2	4	4	2	2	3	3	2	2
2	2	4	4	2	2	3	3	2	2
3	3	3	3	2	2	3	3	4	4
2	2	4	4	2	2	3	3	2	2
3	3	3	3	3	3	3	3	2	2
2	2	4	4	2	2	3	3	2	2
2	2	3	3	2	2	3	3	2	2
2	2	3	3	2	2	3	3	4	4
3	3	4	4	2	2	3	3	2	2
2	2	4	4	2	2	4	4	2	2
2	2	3	3	2	2	3	3	2	2
3	3	4	4	2	2	3	3	4	4
3	3	4	4	2	2	3	3	2	2
2	2	3	3	2	2	3	3	2	2
2	2	4	4	2	2	3	3	2	2
2	2	3	3	2	2	3	3	2	2
2	2	4	4	2	2	3	3	2	2
2	2	3	3	2	2	3	3	2	2
3	3	3	3	2	2	3	3	4	4
2	2	4	4	2	2	3	3	2	2
2	2	3	3	2	2	3	3	2	2
2	2	3	3	2	2	3	3	2	2
2	2	4	4	2	2	3	3	2	2
3	3	3	3	2	2	3	3	2	2
3	3	3	3	3	3	3	3	4	4
2	2	3	3	2	2	3	3	2	2
2	2	4	4	2	2	3	3	2	2
2	2	4	4	2	2	3	3	2	2
2	2	3	3	2	2	3	3	2	2
2	2	4	4	2	2	3	3	2	2
2	2	3	3	2	2	3	3	2	2
2	2	3	3	2	2	3	3	2	2
2	2	3	3	2	2	3	3	2	2
2	2	3	3	2	2	3	3	2	2
2	2	3	3	2	2	3	3	2	2
2	2	3	3	3	3	3	3	2	2
3	3	3	3	2	2	3	3	4	4
3	3	3	3	3	3	3	3	4	4
3	3	4	4	2	2	3	3	2	2
3	3	4	4	3	3	3	3	4	4
2	2	3	3	2	2	3	3	2	2
2	2	4	4	2	2	3	3	2	2
2	2	4	4	2	2	3	3	2	2
2	2	4	4	2	2	3	3	2	2
2	2	4	4	2	2	4	4	2	2
2	2	3	3	2	2	3	3	2	2
2	2	4	4	2	2	3	3	2	2

For Review Only

3	3	4	4	2	2	3	3	2	2
2	2	4	4	2	2	3	3	2	2
2	2	4	4	2	2	3	3	2	2
2	2	4	4	2	2	3	3	2	2
2	2	3	3	2	2	3	3	2	2
2	2	4	4	2	2	3	3	2	2
2	2	4	4	2	2	3	3	2	2
3	3	3	3	2	2	3	3	2	2
3	3	3	3	2	2	3	3	2	2
2	2	4	4	2	2	3	3	2	2
2	2	4	4	2	2	3	3	2	2
2	2	4	4	2	2	3	3	2	2
3	3	4	4	2	2	3	3	2	2
3	3	3	3	2	2	3	3	2	2
2	2	4	4	2	2	3	3	2	2
2	2	4	4	3	3	3	3	2	2
2	2	4	4	2	2	3	3	2	2
2	2	4	4	2	2	4	4	2	2
2	2	4	4	2	2	3	3	2	2
2	2	4	4	2	2	3	3	2	2
2	2	4	4	2	2	3	3	2	2
2	2	4	4	2	2	3	3	2	2
3	3	4	4	2	2	3	3	2	2
3	3	4	4	2	2	3	3	2	2
3	3	4	4	3	3	3	3	4	4
2	2	3	3	2	2	3	3	2	2
3	3	4	4	2	2	3	3	2	2
2	2	3	3	2	2	3	3	2	2
2	2	4	4	2	2	3	3	2	2
2	2	3	3	2	2	4	4	2	2
2	2	4	4	2	2	3	3	4	4
2	2	3	3	2	2	3	3	2	2
2	2	3	3	2	2	3	3	2	2
3	3	4	4	2	2	3	3	2	2
2	2	3	3	2	2	3	3	2	2
3	3	4	4	3	3	3	3	4	4
3	3	3	3	2	2	3	3	4	4
2	2	4	4	2	2	3	3	2	2
2	2	3	3	2	2	3	3	2	2
3	3	4	4	2	2	3	3	4	4
2	2	4	4	2	2	3	3	2	2
2	2	3	3	2	2	3	3	2	2
3	3	3	3	2	2	4	4	4	4
2	2	4	4	2	2	3	3	2	2
2	2	3	3	2	2	3	3	2	2
2	2	4	4	2	2	3	3	2	2
3	3	3	3	2	2	3	3	2	2
2	2	4	4	2	2	3	3	2	2
2	2	4	4	2	2	3	3	2	2
2	2	4	4	2	2	4	4	2	2
2	2	3	3	2	2	3	3	2	2
2	2	3	3	2	2	3	3	2	2
3	3	3	3	2	2	3	3	2	2
2	2	4	4	2	2	3	3	2	2
2	2	4	4	2	2	4	4	2	2

For Review Only

2	2	4	4	2	2	3	3	2	2
2	2	3	3	2	2	3	3	2	2
2	2	3	3	2	2	3	3	2	2
2	2	4	4	2	2	3	3	2	2
2	2	4	4	2	2	3	3	2	2
2	2	3	3	2	2	3	3	2	2
2	2	3	3	2	2	4	4	2	2
2	2	4	4	2	2	3	3	2	2
2	2	3	3	3	3	3	3	4	4
2	2	3	3	2	2	3	3	2	2
3	3	4	4	2	2	3	3	2	2
2	2	4	4	2	2	3	3	2	2
2	2	3	3	2	2	4	4	2	2
2	2	3	3	2	2	3	3	2	2
2	2	3	3	2	2	3	3	2	2
2	2	4	4	2	2	3	3	2	2
3	3	4	4	2	2	4	4	2	2
2	2	4	4	2	2	3	3	2	2
3	3	3	3	2	2	3	3	2	2
2	2	4	4	2	2	3	3	2	2
2	2	4	4	2	2	3	3	2	2
2	2	3	3	2	2	4	4	2	2
2	2	3	3	2	2	3	3	2	2
2	2	3	3	2	2	3	3	2	2
2	2	4	4	2	2	3	3	2	2
2	2	3	3	2	2	3	3	2	2
2	2	4	4	2	2	3	3	2	2
2	2	3	3	2	2	3	3	2	2
2	2	4	4	2	2	4	4	2	2
2	2	4	4	2	2	4	4	2	2
2	2	3	3	2	2	3	3	2	2
2	2	4	4	2	2	3	3	2	2
2	2	4	4	2	2	3	3	2	2
2	2	4	4	2	2	3	3	2	2
2	2	3	3	2	2	3	3	2	2
2	2	3	3	2	2	3	3	2	2
3	3	3	3	2	2	3	3	2	2
3	3	3	3	2	2	3	3	2	2
2	2	4	4	2	2	3	3	2	2
2	2	4	4	2	2	3	3	2	2
2	2	4	4	2	2	3	3	2	2
3	3	3	3	2	2	3	3	2	2
2	2	3	3	2	2	4	4	2	2
3	3	3	3	3	3	3	3	4	4
2	2	3	3	2	2	3	3	2	2
2	2	3	3	2	2	3	3	2	2
2	2	4	4	2	2	3	3	2	2
2	2	4	4	2	2	3	3	2	2
2	2	3	3	2	2	3	3	2	2
2	2	3	3	2	2	3	3	2	2
2	2	4	4	2	2	3	3	2	2
2	2	4	4	2	2	3	3	2	2
2	2	3	3	2	2	3	3	2	2
2	2	4	4	2	2	3	3	2	2
2	2	3	3	2	2	3	3	2	2

For Review Only

2	2	4	4	2	2	4	4	2	2
2	2	3	3	2	2	3	3	2	2
2	2	4	4	3	3	3	3	4	4
2	2	3	3	2	2	3	3	2	2
2	2	4	4	2	2	3	3	2	2
2	2	4	4	2	2	3	3	2	2
2	2	3	3	2	2	3	3	2	2
2	2	4	4	2	2	3	3	2	2
2	2	4	4	2	2	3	3	2	2
2	2	4	4	2	2	3	3	2	2
2	2	4	4	2	2	3	3	2	2
2	2	4	4	2	2	3	3	4	4
2	2	3	3	2	2	3	3	2	2
2	2	3	3	2	2	3	3	2	2
2	2	3	3	2	2	3	3	2	2
2	2	4	4	2	2	3	3	2	2
2	2	3	3	2	2	3	3	2	2
2	2	3	3	2	2	3	3	2	2
2	2	3	3	2	2	3	3	2	2
2	2	3	3	2	2	4	4	2	2
2	2	4	4	2	2	3	3	2	2
2	2	3	3	2	2	3	3	2	2
2	2	4	4	2	2	3	3	2	2
2	2	4	4	2	2	3	3	2	2
3	3	3	3	2	2	3	3	4	4
2	2	3	3	2	2	3	3	2	2
2	2	3	3	2	2	4	4	2	2
3	3	4	4	2	2	3	3	2	2
3	3	4	4	2	2	3	3	2	2
2	2	4	4	2	2	3	3	2	2
2	2	3	3	2	2	3	3	2	2
2	2	4	4	2	2	4	4	2	2
2	2	4	4	2	2	3	3	2	2
2	2	3	3	2	2	3	3	2	2
2	2	4	4	2	2	3	3	2	2
2	2	4	4	2	2	3	3	2	2
2	2	3	3	2	2	3	3	2	2
2	2	3	3	2	2	3	3	2	2
2	2	4	4	2	2	3	3	2	2
2	2	3	3	2	2	3	3	2	2
2	2	3	3	2	2	3	3	2	2
2	2	4	4	2	2	3	3	2	2
2	2	3	3	2	2	3	3	2	2
2	2	4	4	2	2	3	3	2	2
2	2	4	4	2	2	3	3	2	2
2	2	3	3	2	2	3	3	2	2
2	2	4	4	2	2	3	3	2	2
2	2	3	3	2	2	3	3	2	2
2	2	3	3	2	2	3	3	2	2
2	2	4	4	2	2	3	3	4	4
2	2	4	4	2	2	4	4	2	2
2	2	3	3	2	2	3	3	2	2
2	2	3	3	2	2	3	3	2	2
2	2	4	4	2	2	3	3	2	2
2	2	3	3	2	2	3	3	2	2

For Review Only

3	3	3	3	2	2	3	3	2	2
2	2	4	4	3	3	3	3	2	2
2	2	4	4	2	2	4	4	2	2
2	2	4	4	2	2	3	3	2	2
3	3	4	4	2	2	3	3	4	4
2	2	4	4	2	2	4	4	2	2
2	2	4	4	2	2	3	3	2	2
3	3	3	3	3	3	3	3	4	4
2	2	3	3	2	2	3	3	2	2
2	2	3	3	2	2	3	3	2	2
2	2	4	4	2	2	3	3	2	2
2	2	3	3	2	2	3	3	2	2
2	2	4	4	2	2	3	3	2	2
2	2	3	3	2	2	3	3	2	2
2	2	3	3	2	2	3	3	2	2
2	2	4	4	2	2	3	3	2	2
2	2	3	3	2	2	3	3	2	2
2	2	3	3	2	2	3	3	2	2
2	2	4	4	2	2	3	3	2	2
2	2	3	3	2	2	3	3	2	2
2	2	4	4	2	2	3	3	2	2
2	2	4	4	2	2	3	3	2	2
2	2	3	3	2	2	3	3	2	2
2	2	4	4	2	2	3	3	2	2
3	3	3	3	2	2	3	3	2	2
3	3	3	3	2	2	3	3	4	4
2	2	4	4	2	2	3	3	2	2
2	2	3	3	2	2	3	3	2	2
3	3	4	4	2	2	3	3	4	4
2	2	4	4	2	2	3	3	2	2
2	2	3	3	2	2	3	3	2	2
2	2	4	4	2	2	3	3	2	2
3	3	3	3	2	2	3	3	2	2
2	2	3	3	2	2	3	3	2	2
2	2	4	4	2	2	3	3	2	2
2	2	3	3	2	2	3	3	2	2
2	2	3	3	2	2	4	4	2	2
2	2	4	4	2	2	3	3	2	2
2	2	3	3	2	2	3	3	2	2
2	2	3	3	2	2	3	3	2	2
2	2	3	3	2	2	3	3	2	2
2	2	4	4	2	2	3	3	2	2
2	2	3	3	2	2	3	3	2	2
2	2	4	4	2	2	3	3	2	2
2	2	4	4	2	2	3	3	2	2
2	2	3	3	2	2	3	3	2	2
2	2	4	4	2	2	3	3	2	2
3	3	3	3	2	2	3	3	2	2
2	2	4	4	2	2	3	3	4	4
2	2	3	3	2	2	3	3	2	2
3	3	4	4	2	2	3	3	2	2
2	2	3	3	2	2	3	3	2	2
2	2	3	3	2	2	3	3	2	2
2	2	3	3	2	2	4	4	2	2
2	2	3	3	2	2	3	3	2	2

For Review Only

2	2	4	4	2	2	3	3	2	2
2	2	3	3	2	2	3	3	2	2

For Review Only

SNP1236	SNP1236	SNP1237	SNP1237	SNP1238	SNP1238	SNP1239	SNP1239	SNP124	SNP124
1	1	2	2	1	1	4	4	1	1
1	1	2	2	1	1	4	4	1	1
2	2	2	2	1	1	4	4	1	1
2	2	2	2	1	1	4	4	1	1
2	2	4	4	1	1	4	4	1	1
2	2	2	2	1	1	4	4	1	1
1	1	2	2	1	1	4	4	1	1
2	2	2	2	1	1	4	4	1	1
2	2	2	2	1	1	4	4	1	1
2	2	2	2	1	1	4	4	1	1
2	2	4	4	1	1	4	4	4	4
2	2	2	2	3	3	4	4	1	1
2	2	2	2	1	1	4	4	1	1
2	2	2	2	1	1	4	4	1	1
2	2	2	2	3	3	4	4	1	1
2	2	4	4	1	1	4	4	1	1
2	2	2	2	1	1	4	4	1	1
2	2	2	2	1	1	4	4	4	4
2	2	2	2	1	1	4	4	1	1
2	2	2	2	1	1	4	4	1	1
2	2	2	2	1	1	4	4	4	4
2	2	2	2	1	1	4	4	1	1
2	2	2	2	1	1	4	4	1	1
2	2	2	2	1	1	4	4	1	1
2	2	2	2	1	1	2	2	1	1
1	1	2	2	1	1	4	4	1	1
1	1	2	2	1	1	4	4	1	1
2	2	2	2	1	1	4	4	1	1
2	2	2	2	1	1	4	4	1	1
2	2	2	2	1	1	4	4	1	1
2	2	2	2	1	1	4	4	1	1
2	2	2	2	1	1	4	4	1	1
1	1	2	2	1	1	4	4	1	1
2	2	2	2	1	1	4	4	1	1
2	2	2	2	1	1	4	4	1	1
2	2	2	2	1	1	4	4	1	1
2	2	2	2	1	1	4	4	1	1
2	2	4	4	1	1	4	4	1	1
2	2	4	4	1	1	4	4	1	1
2	2	2	2	1	1	4	4	1	1
2	2	2	2	1	1	4	4	1	1
2	2	2	2	1	1	4	4	4	4
2	2	2	2	1	1	4	4	1	1
1	1	2	2	1	1	4	4	1	1
2	2	2	2	1	1	4	4	1	1
2	2	2	2	1	1	4	4	1	1
2	2	2	2	1	1	4	4	1	1
2	2	2	2	1	1	4	4	1	1
1	1	2	2	1	1	4	4	1	1
2	2	2	2	3	3	4	4	1	1
2	2	2	2	1	1	4	4	1	1
2	2	2	2	1	1	4	4	1	1

For Review Only

2	2	2	2	1	1	4	4	1	1
1	1	2	2	1	1	4	4	1	1
2	2	2	2	1	1	2	2	1	1
2	2	2	2	1	1	4	4	1	1
2	2	2	2	1	1	4	4	1	1
2	2	2	2	1	1	4	4	1	1
1	1	2	2	1	1	4	4	1	1
2	2	4	4	1	1	4	4	1	1
2	2	2	2	1	1	4	4	4	4
2	2	2	2	3	3	4	4	1	1
2	2	2	2	1	1	4	4	1	1
2	2	2	2	1	1	4	4	1	1
2	2	2	2	1	1	4	4	1	1
2	2	2	2	1	1	4	4	1	1
2	2	2	2	1	1	4	4	1	1
2	2	2	2	1	1	4	4	1	1
2	2	2	2	1	1	4	4	1	1
2	2	2	2	3	3	4	4	1	1
2	2	2	2	1	1	4	4	1	1
1	1	2	2	1	1	4	4	1	1
2	2	2	2	1	1	4	4	1	1
2	2	2	2	1	1	4	4	1	1
2	2	2	2	1	1	4	4	1	1
2	2	2	2	1	1	4	4	1	1
2	2	2	2	1	1	4	4	1	1
2	2	2	2	1	1	4	4	1	1
1	1	2	2	1	1	4	4	1	1
2	2	4	4	1	1	4	4	1	1
1	1	2	2	1	1	4	4	1	1
2	2	2	2	3	3	4	4	1	1
2	2	2	2	1	1	4	4	1	1
2	2	2	2	1	1	4	4	1	1
2	2	4	4	1	1	4	4	4	4
2	2	2	2	1	1	4	4	1	1
2	2	2	2	1	1	4	4	1	1
2	2	2	2	1	1	4	4	1	1
2	2	2	2	1	1	4	4	1	1
2	2	2	2	1	1	4	4	1	1
2	2	2	2	1	1	4	4	1	1
1	1	2	2	1	1	4	4	1	1
1	1	2	2	1	1	4	4	1	1
2	2	2	2	1	1	4	4	1	1
2	2	2	2	3	3	4	4	1	1
2	2	2	2	1	1	4	4	1	1
1	1	2	2	1	1	4	4	1	1
1	1	2	2	1	1	4	4	1	1
2	2	2	2	1	1	4	4	1	1
1	1	2	2	1	1	4	4	1	1
1	1	2	2	1	1	4	4	1	1
1	1	2	2	3	3	4	4	1	1
1	1	2	2	1	1	2	2	1	1
2	2	4	4	1	1	4	4	4	4
2	2	4	4	1	1	4	4	1	1
2	2	2	2	1	1	4	4	1	1
2	2	2	2	3	3	4	4	1	1

For Review Only

1	1	2	2	1	1	4	4	1	1
2	2	2	2	1	1	4	4	1	1
2	2	2	2	1	1	4	4	1	1
2	2	2	2	1	1	4	4	1	1
1	1	2	2	1	1	4	4	1	1
1	1	2	2	1	1	4	4	1	1
2	2	2	2	3	3	4	4	1	1
2	2	2	2	1	1	4	4	1	1
1	1	2	2	1	1	4	4	1	1
2	2	2	2	1	1	4	4	1	1
2	2	2	2	1	1	4	4	1	1
2	2	2	2	1	1	4	4	1	1
2	2	2	2	3	3	4	4	1	1
2	2	2	2	1	1	4	4	1	1
1	1	2	2	1	1	4	4	1	1
2	2	2	2	1	1	4	4	1	1
2	2	4	4	3	3	4	4	1	1
2	2	2	2	1	1	4	4	1	1
2	2	2	2	1	1	4	4	1	1
2	2	2	2	1	1	4	4	1	1
2	2	2	2	1	1	4	4	1	1
2	2	2	2	3	3	4	4	1	1
2	2	2	2	1	1	4	4	1	1
2	2	4	4	1	1	4	4	1	1
1	1	2	2	1	1	4	4	1	1
2	2	4	4	1	1	4	4	1	1
2	2	2	2	1	1	2	2	1	1
2	2	2	2	1	1	4	4	1	1
2	2	2	2	3	3	4	4	1	1
2	2	2	2	3	3	4	4	1	1
2	2	4	4	1	1	4	4	1	1
2	2	2	2	1	1	4	4	1	1
1	1	2	2	1	1	4	4	1	1
2	2	2	2	1	1	4	4	1	1
2	2	2	2	1	1	4	4	1	1
2	2	2	2	1	1	4	4	1	1
2	2	2	2	1	1	4	4	1	1
2	2	2	2	1	1	4	4	1	1
2	2	2	2	1	1	4	4	1	1
2	2	2	2	1	1	4	4	4	4
2	2	2	2	1	1	4	4	1	1
2	2	2	2	1	1	4	4	1	1
2	2	2	2	3	3	4	4	1	1
2	2	2	2	1	1	4	4	1	1
1	1	2	2	1	1	4	4	1	1
2	2	2	2	1	1	4	4	1	1
1	1	2	2	1	1	4	4	1	1
2	2	2	2	1	1	4	4	1	1
2	2	2	2	1	1	4	4	1	1
1	1	2	2	1	1	4	4	1	1
1	1	2	2	1	1	4	4	1	1
2	2	4	4	1	1	4	4	1	1
2	2	2	2	1	1	4	4	1	1
2	2	2	2	1	1	4	4	1	1
2	2	2	2	1	1	4	4	1	1

For Review Only

2	2	2	2	3	3	4	4	1	1
2	2	2	2	1	1	4	4	1	1
2	2	2	2	1	1	4	4	1	1
2	2	2	2	1	1	4	4	1	1
1	1	2	2	1	1	4	4	1	1
2	2	4	4	1	1	4	4	1	1
2	2	2	2	1	1	4	4	1	1
2	2	2	2	1	1	4	4	1	1
2	2	2	2	1	1	4	4	1	1
2	2	2	2	1	1	2	2	1	1
2	2	2	2	1	1	4	4	1	1
2	2	2	2	1	1	4	4	1	1
1	1	2	2	1	1	4	4	1	1
2	2	2	2	1	1	4	4	1	1
2	2	2	2	1	1	4	4	1	1
2	2	2	2	1	1	4	4	4	4
2	2	2	2	1	1	4	4	1	1
1	1	2	2	1	1	4	4	1	1
2	2	2	2	1	1	4	4	1	1
2	2	2	2	3	3	4	4	1	1
2	2	2	2	1	1	4	4	1	1
2	2	2	2	1	1	4	4	1	1
2	2	2	2	1	1	4	4	1	1
2	2	2	2	1	1	4	4	1	1
2	2	2	2	1	1	4	4	1	1
2	2	2	2	1	1	4	4	1	1
2	2	2	2	1	1	4	4	1	1
2	2	2	2	1	1	4	4	1	1
2	2	2	2	1	1	4	4	1	1
2	2	2	2	1	1	4	4	1	1
2	2	2	2	1	1	4	4	1	1
2	2	2	2	3	3	4	4	1	1
2	2	2	2	1	1	2	2	1	1
2	2	2	2	1	1	4	4	1	1
2	2	2	2	1	1	4	4	1	1
2	2	2	2	1	1	4	4	1	1
2	2	2	2	1	1	4	4	1	1
2	2	2	2	1	1	4	4	1	1
2	2	2	2	1	1	4	4	1	1
2	2	2	2	1	1	2	2	1	1
2	2	2	2	1	1	4	4	1	1
1	1	2	2	1	1	4	4	1	1
2	2	2	2	1	1	4	4	1	1
2	2	2	2	1	1	4	4	1	1
1	1	2	2	1	1	4	4	1	1
2	2	4	4	1	1	4	4	1	1
2	2	2	2	1	1	4	4	4	4
2	2	4	4	1	1	4	4	1	1
1	1	4	4	1	1	4	4	1	1
2	2	2	2	1	1	4	4	1	1
2	2	2	2	3	3	4	4	1	1
2	2	2	2	1	1	4	4	1	1
2	2	2	2	1	1	4	4	1	1
2	2	2	2	1	1	4	4	1	1
2	2	4	4	1	1	4	4	1	1

For Review Only

2	2	2	2	1	1	4	4	1	1
2	2	2	2	1	1	4	4	1	1
2	2	2	2	3	3	4	4	1	1
2	2	2	2	1	1	4	4	1	1
2	2	2	2	1	1	4	4	1	1
2	2	2	2	3	3	4	4	1	1
2	2	4	4	1	1	4	4	1	1
2	2	2	2	1	1	2	2	1	1
1	1	2	2	1	1	4	4	1	1
2	2	4	4	1	1	4	4	1	1
2	2	2	2	1	1	4	4	1	1
2	2	2	2	1	1	4	4	1	1
2	2	2	2	1	1	4	4	1	1
2	2	2	2	1	1	2	2	1	1
2	2	2	2	1	1	2	2	1	1
2	2	2	2	1	1	4	4	1	1
2	2	2	2	1	1	4	4	1	1
2	2	2	2	1	1	4	4	1	1
2	2	4	4	1	1	4	4	1	1
2	2	4	4	1	1	4	4	1	1
2	2	2	2	1	1	4	4	1	1
2	2	2	2	1	1	4	4	1	1
2	2	2	2	1	1	4	4	1	1
2	2	2	2	1	1	4	4	1	1
2	2	4	4	1	1	4	4	1	1
2	2	2	2	1	1	4	4	1	1
2	2	4	4	1	1	4	4	1	1
2	2	2	2	1	1	4	4	1	1
2	2	2	2	1	1	4	4	1	1
2	2	2	2	3	3	4	4	1	1
2	2	4	4	1	1	4	4	1	1
2	2	2	2	1	1	4	4	1	1
2	2	2	2	1	1	4	4	1	1
2	2	2	2	1	1	2	2	1	1
2	2	4	4	1	1	4	4	1	1
2	2	4	4	1	1	4	4	1	1
2	2	2	2	3	3	4	4	1	1
2	2	2	2	1	1	4	4	1	1
2	2	2	2	1	1	4	4	1	1
2	2	4	4	1	1	4	4	4	4
2	2	2	2	1	1	4	4	1	1
2	2	2	2	1	1	4	4	1	1
2	2	2	2	1	1	4	4	1	1
2	2	2	2	1	1	4	4	1	1
2	2	2	2	1	1	2	2	1	1
2	2	2	2	1	1	4	4	1	1
2	2	2	2	1	1	4	4	1	1
2	2	2	2	1	1	4	4	1	1
2	2	2	2	1	1	4	4	1	1
2	2	4	4	1	1	4	4	1	1
2	2	2	2	1	1	4	4	1	1
2	2	4	4	1	1	4	4	1	1
2	2	2	2	1	1	4	4	1	1
2	2	2	2	3	3	2	2	1	1
2	2	4	4	1	1	4	4	1	1

For Review Only

2	2	2	2	1	1	4	4	1	1
2	2	2	2	1	1	2	2	1	1

For Review Only

SNP1240	SNP1240	SNP1241	SNP1241	SNP1242	SNP1242	SNP1243	SNP1243	SNP1244	SNP1244
4	4	2	2	4	4	4	4	3	3
4	4	2	2	2	2	4	4	1	1
4	4	2	2	4	4	4	4	3	3
4	4	2	2	4	4	4	4	3	3
4	4	1	1	4	4	4	4	3	3
4	4	2	2	4	4	4	4	3	3
4	4	1	1	4	4	4	4	1	1
4	4	2	2	2	2	4	4	3	3
4	4	2	2	4	4	4	4	3	3
4	4	1	1	2	2	4	4	3	3
1	1	1	1	2	2	1	1	3	3
4	4	2	2	4	4	4	4	3	3
4	4	1	1	2	2	4	4	3	3
4	4	1	1	4	4	4	4	3	3
4	4	2	2	2	2	4	4	3	3
1	1	2	2	4	4	4	4	3	3
4	4	2	2	4	4	4	4	3	3
4	4	1	1	2	2	4	4	3	3
4	4	2	2	2	2	4	4	3	3
4	4	1	1	2	2	4	4	1	1
1	1	1	1	2	2	1	1	1	1
1	1	2	2	4	4	1	1	3	3
4	4	1	1	2	2	4	4	3	3
4	4	1	1	2	2	1	1	3	3
4	4	1	1	4	4	4	4	3	3
4	4	2	2	4	4	4	4	3	3
4	4	2	2	4	4	4	4	3	3
4	4	2	2	2	2	4	4	3	3
4	4	1	1	2	2	4	4	3	3
1	1	1	1	2	2	1	1	1	1
4	4	2	2	4	4	4	4	3	3
4	4	2	2	4	4	4	4	3	3
4	4	2	2	4	4	4	4	3	3
4	4	2	2	4	4	4	4	3	3
4	4	2	2	2	2	4	4	3	3
4	4	1	1	4	4	4	4	3	3
4	4	1	1	4	4	4	4	1	1
1	1	2	2	4	4	4	4	3	3
1	1	1	1	2	2	1	1	3	3
4	4	1	1	2	2	4	4	3	3
1	1	1	1	2	2	1	1	1	1
4	4	1	1	2	2	4	4	3	3
4	4	1	1	4	4	4	4	1	1
4	4	1	1	2	2	4	4	1	1
4	4	2	2	4	4	4	4	3	3
4	4	2	2	4	4	4	4	3	3
4	4	2	2	4	4	4	4	3	3
4	4	2	2	2	2	4	4	3	3
4	4	1	1	4	4	4	4	3	3
4	4	2	2	4	4	4	4	3	3
4	4	2	2	4	4	4	4	3	3
4	4	1	1	4	4	4	4	3	3

For Review Only

4	4	1	1	4	4	4	4	3	3
4	4	2	2	4	4	4	4	3	3
4	4	2	2	2	2	4	4	3	3
1	1	1	1	4	4	1	1	1	1
4	4	2	2	2	2	4	4	3	3
4	4	2	2	4	4	4	4	3	3
4	4	1	1	2	2	4	4	3	3
1	1	1	1	2	2	4	4	3	3
4	4	1	1	2	2	4	4	1	1
4	4	2	2	2	2	4	4	3	3
4	4	2	2	2	2	4	4	3	3
4	4	2	2	4	4	4	4	3	3
4	4	1	1	4	4	4	4	3	3
4	4	1	1	2	2	4	4	3	3
4	4	2	2	2	2	4	4	3	3
4	4	1	1	4	4	4	4	1	1
4	4	1	1	4	4	4	4	3	3
4	4	2	2	4	4	4	4	3	3
4	4	2	2	4	4	4	4	3	3
4	4	2	2	4	4	4	4	3	3
4	4	2	2	4	4	4	4	3	3
4	4	2	2	2	2	4	4	3	3
4	4	2	2	4	4	4	4	3	3
4	4	2	2	4	4	4	4	3	3
4	4	2	2	4	4	4	4	3	3
4	4	2	2	4	4	4	4	3	3
4	4	2	2	2	2	4	4	3	3
4	4	1	1	2	2	4	4	3	3
4	4	2	2	2	2	4	4	3	3
4	4	2	2	2	2	4	4	3	3
4	4	2	2	4	4	4	4	3	3
4	4	2	2	2	2	4	4	3	3
1	1	1	1	2	2	4	4	3	3
4	4	1	1	4	4	4	4	3	3
4	4	2	2	4	4	4	4	3	3
4	4	2	2	4	4	4	4	3	3
4	4	2	2	4	4	4	4	3	3
1	1	1	1	4	4	1	1	1	1
4	4	1	1	4	4	4	4	3	3
4	4	2	2	4	4	4	4	1	1
4	4	1	1	4	4	4	4	3	3
4	4	2	2	2	2	4	4	3	3
4	4	1	1	4	4	4	4	1	1
4	4	2	2	2	2	4	4	3	3
4	4	2	2	2	2	4	4	3	3
4	4	2	2	2	2	4	4	3	3
4	4	1	1	2	2	4	4	3	3
4	4	1	1	2	2	4	4	3	3
4	4	2	2	2	2	4	4	3	3
4	4	2	2	2	2	4	4	3	3
4	4	2	2	2	2	4	4	3	3
1	1	1	1	2	2	1	1	3	3
1	1	1	1	2	2	4	4	3	3
4	4	2	2	2	2	4	4	3	3
4	4	1	1	4	4	4	4	3	3

For Review Only

4	4	2	2	2	2	4	4	3	3
4	4	2	2	2	2	4	4	3	3
4	4	1	1	4	4	4	4	3	3
4	4	2	2	2	2	4	4	3	3
4	4	2	2	2	2	4	4	3	3
4	4	2	2	2	2	4	4	3	3
4	4	2	2	4	4	4	4	3	3
4	4	2	2	2	2	4	4	3	3
4	4	2	2	2	2	4	4	3	3
1	1	1	1	2	2	4	4	3	3
4	4	1	1	4	4	4	4	3	3
4	4	2	2	4	4	4	4	3	3
4	4	1	1	4	4	4	4	1	1
4	4	2	2	4	4	4	4	3	3
4	4	2	2	2	2	4	4	3	3
4	4	2	2	2	2	4	4	3	3
1	1	2	2	2	2	4	4	3	3
4	4	2	2	2	2	4	4	3	3
4	4	1	1	2	2	4	4	3	3
4	4	2	2	4	4	4	4	3	3
4	4	2	2	2	2	4	4	3	3
4	4	2	2	4	4	4	4	3	3
4	4	2	2	4	4	4	4	3	3
4	4	1	1	2	2	4	4	3	3
4	4	2	2	2	2	4	4	3	3
1	1	2	2	2	2	1	1	3	3
4	4	2	2	2	2	4	4	3	3
4	4	2	2	4	4	4	4	3	3
4	4	2	2	4	4	4	4	3	3
4	4	2	2	2	2	4	4	3	3
1	1	1	1	2	2	4	4	3	3
4	4	2	2	2	2	4	4	3	3
4	4	1	1	4	4	4	4	3	3
4	4	2	2	2	2	4	4	3	3
4	4	2	2	4	4	4	4	3	3
4	4	2	2	4	4	4	4	3	3
4	4	1	1	2	2	4	4	1	1
4	4	2	2	4	4	4	4	3	3
4	4	1	1	4	4	4	4	3	3
4	4	2	2	2	2	4	4	3	3
4	4	2	2	2	2	4	4	3	3
1	1	1	1	2	2	4	4	1	1
4	4	1	1	2	2	4	4	3	3
1	1	2	2	2	2	4	4	3	3
4	4	2	2	4	4	4	4	3	3
4	4	2	2	4	4	4	4	3	3
4	4	2	2	2	2	4	4	3	3
4	4	2	2	2	2	4	4	3	3
4	4	1	1	2	2	4	4	3	3
4	4	2	2	4	4	4	4	3	3
4	4	2	2	2	2	4	4	3	3
1	1	1	1	2	2	4	4	3	3
4	4	2	2	4	4	4	4	3	3
4	4	2	2	2	2	4	4	3	3
4	4	2	2	2	2	4	4	3	3

For Review Only

4	4	2	2	2	2	4	4	3	3
4	4	2	2	2	2	4	4	3	3
4	4	2	2	2	2	4	4	3	3
4	4	2	2	2	2	4	4	3	3
4	4	1	1	2	2	4	4	1	1
1	1	1	1	4	4	4	4	3	3
1	1	2	2	2	2	4	4	3	3
4	4	1	1	2	2	4	4	3	3
4	4	2	2	2	2	4	4	3	3
4	4	2	2	2	2	4	4	1	1
4	4	2	2	4	4	4	4	3	3
4	4	1	1	2	2	1	1	3	3
4	4	2	2	2	2	4	4	3	3
4	4	2	2	4	4	4	4	3	3
4	4	1	1	2	2	4	4	3	3
4	4	2	2	2	2	4	4	3	3
4	4	1	1	4	4	4	4	3	3
4	4	2	2	2	2	4	4	3	3
4	4	2	2	4	4	4	4	3	3
4	4	2	2	4	4	4	4	3	3
4	4	2	2	2	2	4	4	3	3
4	4	2	2	2	2	1	1	3	3
4	4	2	2	2	2	4	4	3	3
4	4	2	2	4	4	4	4	3	3
4	4	1	1	2	2	4	4	1	1
4	4	2	2	4	4	4	4	3	3
4	4	2	2	4	4	4	4	3	3
4	4	1	1	2	2	4	4	3	3
4	4	2	2	4	4	4	4	3	3
4	4	2	2	4	4	4	4	3	3
4	4	2	2	4	4	4	4	3	3
4	4	2	2	2	2	4	4	3	3
4	4	2	2	4	4	4	4	1	1
4	4	1	1	2	2	4	4	3	3
4	4	2	2	4	4	4	4	3	3
4	4	2	2	4	4	4	4	3	3
4	4	1	1	2	2	4	4	3	3
4	4	1	1	4	4	4	4	1	1
4	4	2	2	2	2	4	4	3	3
4	4	2	2	4	4	4	4	3	3
4	4	2	2	4	4	4	4	3	3
4	4	2	2	2	2	4	4	3	3
4	4	2	2	2	2	1	1	3	3
4	4	2	2	2	2	4	4	3	3
4	4	1	1	4	4	4	4	1	1
1	1	1	1	4	4	4	4	3	3
4	4	2	2	2	2	4	4	3	3
1	1	2	2	2	2	4	4	3	3
1	1	1	1	2	2	4	4	3	3
4	4	1	1	4	4	4	4	1	1
4	4	2	2	4	4	4	4	3	3
4	4	1	1	2	2	4	4	3	3
4	4	1	1	2	2	4	4	3	3
4	4	2	2	2	2	4	4	3	3
1	1	1	1	2	2	4	4	3	3

For Review Only

4	4	1	1	2	2	4	4	3	3
4	4	2	2	2	2	4	4	3	3
4	4	2	2	2	2	4	4	3	3
4	4	2	2	2	2	4	4	3	3
4	4	1	1	2	2	4	4	1	1
4	4	2	2	4	4	4	4	3	3
1	1	2	2	2	2	1	1	3	3
4	4	1	1	2	2	4	4	1	1
4	4	2	2	2	2	4	4	3	3
1	1	1	1	2	2	4	4	3	3
4	4	2	2	2	2	4	4	3	3
4	4	2	2	4	4	4	4	3	3
4	4	1	1	4	4	4	4	3	3
4	4	2	2	4	4	4	4	3	3
4	4	2	2	4	4	4	4	3	3
4	4	1	1	4	4	4	4	3	3
4	4	1	1	2	2	1	1	1	1
1	1	2	2	2	2	4	4	3	3
1	1	1	1	2	2	4	4	3	3
1	1	2	2	4	4	4	4	3	3
4	4	1	1	2	2	4	4	1	1
4	4	2	2	2	2	4	4	3	3
4	4	2	2	4	4	4	4	3	3
4	4	1	1	2	2	4	4	3	3
1	1	1	1	2	2	4	4	3	3
4	4	2	2	4	4	4	4	3	3
1	1	2	2	2	2	4	4	3	3
4	4	1	1	4	4	4	4	3	3
4	4	1	1	4	4	4	4	3	3
4	4	2	2	2	2	4	4	3	3
1	1	2	2	2	2	4	4	3	3
4	4	2	2	2	2	4	4	3	3
4	4	1	1	4	4	4	4	1	1
4	4	2	2	2	2	4	4	3	3
4	4	1	1	2	2	1	1	3	3
1	1	2	2	2	2	4	4	3	3
4	4	2	2	4	4	4	4	3	3
4	4	2	2	4	4	4	4	3	3
4	4	1	1	4	4	4	4	3	3
1	1	1	1	2	2	1	1	3	3
4	4	2	2	4	4	4	4	3	3
4	4	1	1	2	2	4	4	3	3
4	4	1	1	2	2	4	4	3	3
4	4	2	2	4	4	4	4	3	3
4	4	2	2	4	4	4	4	3	3
4	4	1	1	4	4	4	4	3	3
4	4	2	2	2	2	4	4	3	3
4	4	1	1	4	4	4	4	1	1
4	4	2	2	4	4	4	4	1	1
1	1	2	2	2	2	4	4	3	3
4	4	2	2	4	4	4	4	3	3
1	1	1	1	2	2	1	1	3	3
4	4	2	2	4	4	4	4	3	3
4	4	2	2	4	4	4	4	3	3
1	1	1	1	2	2	1	1	3	3

For Review Only

4	4	2	2	4	4	4	4	3	3
4	4	2	2	4	4	4	4	3	3

For Review Only

SNP1245	SNP1245	SNP1246	SNP1246	SNP1247	SNP1247	SNP1248	SNP1248	SNP1249	SNP1249
2	2	2	2	2	2	3	3	4	4
1	1	2	2	2	2	2	2	4	4
2	2	2	2	2	2	3	3	4	4
1	1	1	1	3	3	3	3	4	4
2	2	2	2	2	2	2	2	4	4
2	2	2	2	3	3	3	3	4	4
2	2	2	2	2	2	2	2	4	4
2	2	2	2	3	3	3	3	4	4
2	2	2	2	2	2	3	3	4	4
2	2	1	1	2	2	3	3	2	2
2	2	1	1	2	2	3	3	4	4
2	2	2	2	2	2	3	3	4	4
2	2	2	2	2	2	3	3	2	2
2	2	2	2	2	2	3	3	4	4
2	2	2	2	2	2	3	3	4	4
2	2	2	2	2	2	3	3	4	4
2	2	2	2	2	2	3	3	4	4
1	1	2	2	2	2	2	2	2	2
2	2	2	2	3	3	3	3	4	4
1	1	1	1	2	2	2	2	4	4
1	1	2	2	2	2	2	2	4	4
2	2	2	2	2	2	3	3	4	4
2	2	2	2	2	2	2	2	4	4
2	2	2	2	2	2	3	3	4	4
2	2	2	2	2	2	2	2	4	4
2	2	2	2	2	2	3	3	4	4
2	2	2	2	2	2	3	3	4	4
2	2	2	2	2	2	3	3	4	4
2	2	2	2	2	2	2	2	2	2
1	1	2	2	2	2	2	2	4	4
2	2	2	2	3	3	3	3	4	4
2	2	2	2	2	2	3	3	4	4
2	2	2	2	2	2	3	3	4	4
1	1	2	2	2	2	3	3	4	4
2	2	2	2	2	2	3	3	4	4
2	2	2	2	2	2	3	3	4	4
1	1	1	1	2	2	3	3	2	2
2	2	1	1	2	2	3	3	4	4
2	2	1	1	2	2	3	3	4	4
2	2	2	2	2	2	3	3	4	4
2	2	2	2	2	2	2	2	4	4
1	1	2	2	2	2	2	2	2	2
1	1	1	1	2	2	3	3	4	4
1	1	2	2	2	2	2	2	4	4
2	2	1	1	2	2	2	2	2	2
2	2	2	2	2	2	3	3	4	4
2	2	2	2	2	2	3	3	4	4
2	2	2	2	2	2	3	3	4	4
1	1	2	2	2	2	2	2	2	2
2	2	2	2	2	2	3	3	4	4
2	2	2	2	3	3	3	3	4	4
2	2	2	2	2	2	3	3	4	4

For Review Only

2	2	2	2	2	2	2	2	4	4
2	2	2	2	2	2	3	3	4	4
2	2	2	2	3	3	3	3	4	4
1	1	2	2	2	2	2	2	4	4
2	2	2	2	3	3	3	3	4	4
2	2	2	2	2	2	3	3	4	4
2	2	2	2	2	2	3	3	4	4
2	2	1	1	2	2	2	2	4	4
1	1	1	1	2	2	3	3	2	2
2	2	2	2	2	2	3	3	4	4
2	2	2	2	2	2	3	3	4	4
2	2	2	2	2	2	3	3	4	4
2	2	2	2	2	2	2	2	4	4
2	2	2	2	2	2	2	2	2	2
2	2	2	2	3	3	3	3	4	4
1	1	1	1	2	2	3	3	4	4
2	2	2	2	2	2	2	2	4	4
2	2	2	2	2	2	3	3	4	4
2	2	2	2	2	2	3	3	4	4
2	2	2	2	2	2	3	3	4	4
2	2	2	2	2	2	3	3	4	4
2	2	2	2	2	2	3	3	4	4
2	2	2	2	2	2	3	3	4	4
2	2	2	2	2	2	3	3	4	4
2	2	1	1	2	2	2	2	4	4
2	2	2	2	2	2	3	3	4	4
2	2	2	2	2	2	3	3	4	4
2	2	1	1	2	2	3	3	4	4
2	2	2	2	2	2	3	3	4	4
2	2	2	2	2	2	3	3	4	4
1	1	1	1	2	2	3	3	2	2
1	1	2	2	2	2	3	3	4	4
2	2	2	2	2	2	3	3	4	4
2	2	2	2	2	2	2	2	4	4
2	2	2	2	2	2	2	2	2	2
2	2	1	1	2	2	2	2	4	4
2	2	1	1	2	2	2	2	2	2
1	1	2	2	2	2	2	2	4	4
2	2	1	1	2	2	2	2	4	4
1	1	1	1	2	2	2	2	4	4
2	2	2	2	2	2	2	2	4	4
2	2	2	2	2	2	3	3	4	4
1	1	2	2	2	2	2	2	4	4
2	2	2	2	2	2	3	3	4	4
2	2	1	1	2	2	3	3	4	4
2	2	2	2	2	2	3	3	4	4
2	2	2	2	2	2	3	3	4	4
2	2	2	2	2	2	3	3	4	4
1	1	2	2	2	2	3	3	4	4
2	2	2	2	2	2	3	3	4	4
2	2	2	2	2	2	3	3	4	4
2	2	1	1	2	2	3	3	4	4
2	2	1	1	2	2	3	3	4	4
2	2	2	2	2	2	3	3	4	4
1	1	2	2	2	2	3	3	4	4

For Review Only

2	2	2	2	2	2	3	3	4	4
2	2	2	2	2	2	3	3	4	4
2	2	2	2	2	2	3	3	4	4
2	2	1	1	2	2	3	3	4	4
2	2	2	2	2	2	3	3	4	4
2	2	2	2	2	2	3	3	4	4
2	2	2	2	2	2	3	3	4	4
2	2	2	2	3	3	3	3	4	4
1	1	2	2	2	2	3	3	4	4
2	2	2	2	2	2	3	3	4	4
2	2	2	2	2	2	2	2	2	2
2	2	2	2	2	2	3	3	4	4
2	2	2	2	2	2	2	2	4	4
2	2	2	2	2	2	3	3	4	4
1	1	2	2	2	2	3	3	4	4
2	2	2	2	2	2	3	3	4	4
2	2	2	2	2	2	3	3	4	4
2	2	2	2	2	2	3	3	4	4
1	1	1	1	2	2	3	3	4	4
2	2	2	2	2	2	3	3	4	4
2	2	2	2	2	2	3	3	4	4
2	2	2	2	2	2	3	3	4	4
2	2	2	2	2	2	3	3	4	4
2	2	1	1	2	2	3	3	4	4
2	2	2	2	2	2	3	3	4	4
2	2	2	2	2	2	3	3	4	4
2	2	2	2	2	2	3	3	4	4
2	2	2	2	2	2	3	3	4	4
2	2	2	2	2	2	3	3	4	4
1	1	2	2	2	2	3	3	4	4
2	2	1	1	2	2	3	3	4	4
2	2	2	2	2	2	3	3	4	4
2	2	2	2	2	2	2	2	4	4
2	2	2	2	2	2	3	3	4	4
1	1	2	2	2	2	3	3	4	4
2	2	2	2	2	2	3	3	4	4
1	1	1	1	2	2	3	3	4	4
2	2	2	2	2	2	3	3	4	4
2	2	2	2	2	2	3	3	4	4
2	2	1	1	2	2	3	3	4	4
2	2	1	1	2	2	3	3	4	4
1	1	1	1	2	2	3	3	4	4
1	1	2	2	2	2	3	3	4	4
2	2	2	2	2	2	3	3	4	4
2	2	2	2	2	2	3	3	4	4
2	2	1	1	3	3	3	3	4	4
2	2	2	2	2	2	3	3	4	4
2	2	2	2	2	2	3	3	4	4
2	2	2	2	3	3	3	3	2	2
2	2	2	2	2	2	3	3	4	4
2	2	2	2	2	2	3	3	4	4
2	2	1	1	2	2	3	3	4	4
2	2	2	2	2	2	3	3	4	4
2	2	2	2	2	2	3	3	4	4
2	2	2	2	2	2	3	3	4	4

For Review Only

2	2	2	2	2	2	3	3	4	4
2	2	2	2	2	2	3	3	4	4
2	2	1	1	2	2	3	3	4	4
1	1	2	2	2	2	3	3	4	4
1	1	2	2	2	2	3	3	4	4
2	2	1	1	2	2	3	3	4	4
2	2	1	1	2	2	3	3	4	4
2	2	2	2	2	2	3	3	4	4
2	2	1	1	2	2	3	3	4	4
2	2	2	2	2	2	3	3	4	4
2	2	2	2	2	2	3	3	4	4
2	2	2	2	2	2	3	3	4	4
2	2	2	2	2	2	3	3	4	4
2	2	1	1	3	3	3	3	4	4
2	2	1	1	2	2	3	3	4	4
2	2	1	1	2	2	3	3	4	4
2	2	2	2	2	2	3	3	4	4
2	2	2	2	2	2	3	3	4	4
1	1	2	2	2	2	3	3	4	4
2	2	2	2	2	2	3	3	4	4
2	2	2	2	2	2	3	3	4	4
2	2	2	2	2	2	3	3	4	4
2	2	2	2	2	2	3	3	4	4
2	2	2	2	2	2	3	3	4	4
1	1	2	2	2	2	2	2	2	2
1	1	2	2	2	2	3	3	4	4
2	2	2	2	2	2	3	3	4	4
1	1	2	2	2	2	2	2	4	4
2	2	2	2	2	2	3	3	2	2
2	2	2	2	2	2	3	3	4	4
2	2	1	1	3	3	3	3	4	4
2	2	2	2	2	2	3	3	4	4
2	2	2	2	2	2	3	3	4	4
2	2	2	2	2	2	3	3	4	4
2	2	2	2	2	2	3	3	4	4
2	2	2	2	2	2	3	3	4	4
2	2	2	2	2	2	3	3	4	4
1	1	2	2	2	2	3	3	2	2
2	2	2	2	2	2	3	3	4	4
2	2	2	2	3	3	3	3	4	4
2	2	2	2	2	2	3	3	4	4
2	2	2	2	2	2	3	3	4	4
2	2	1	1	2	2	3	3	4	4
2	2	2	2	2	2	3	3	4	4
1	1	2	2	2	2	3	3	4	4
2	2	2	2	2	2	3	3	4	4
2	2	1	1	2	2	3	3	4	4
2	2	1	1	2	2	3	3	4	4
2	2	2	2	2	2	3	3	4	4
1	1	2	2	2	2	2	2	2	2
2	2	2	2	2	2	3	3	4	4
2	2	1	1	2	2	3	3	2	2
2	2	2	2	2	2	3	3	4	4
2	2	2	2	2	2	3	3	4	4
2	2	1	1	2	2	3	3	4	4

For Review Only

2	2	2	2	2	2	3	3	4	4
2	2	1	1	2	2	3	3	4	4
2	2	2	2	2	2	3	3	4	4
2	2	2	2	2	2	3	3	4	4
1	1	1	1	2	2	2	2	4	4
2	2	2	2	2	2	3	3	4	4
2	2	1	1	2	2	3	3	4	4
1	1	1	1	2	2	2	2	2	2
2	2	2	2	2	2	3	3	4	4
2	2	2	2	2	2	3	3	4	4
2	2	2	2	2	2	3	3	4	4
2	2	1	1	2	2	3	3	4	4
2	2	1	1	2	2	2	2	4	4
2	2	2	2	2	2	3	3	4	4
2	2	2	2	2	2	3	3	4	4
2	2	2	2	2	2	2	2	4	4
2	2	1	1	2	2	3	3	4	4
2	2	2	2	2	2	3	3	4	4
2	2	2	2	2	2	2	2	4	4
2	2	1	1	2	2	3	3	4	4
1	1	1	1	3	3	2	2	4	4
2	2	2	2	2	2	3	3	4	4
2	2	2	2	2	2	3	3	4	4
2	2	1	1	2	2	3	3	4	4
2	2	1	1	2	2	2	2	4	4
2	2	2	2	2	2	3	3	4	4
2	2	2	2	2	2	3	3	4	4
2	2	2	2	3	3	3	3	4	4
2	2	1	1	2	2	2	2	4	4
2	2	2	2	2	2	3	3	4	4
2	2	1	1	2	2	3	3	4	4
2	2	1	1	2	2	3	3	4	4
1	1	1	1	2	2	3	3	4	4
2	2	2	2	2	2	3	3	4	4
2	2	1	1	2	2	3	3	4	4
2	2	1	1	2	2	3	3	4	4
2	2	2	2	2	2	3	3	4	4
2	2	2	2	2	2	3	3	4	4
2	2	2	2	2	2	3	3	4	4
2	2	1	1	2	2	3	3	4	4
2	2	2	2	2	2	3	3	4	4
2	2	2	2	2	2	3	3	4	4
2	2	1	1	2	2	3	3	4	4
2	2	2	2	2	2	3	3	4	4
2	2	2	2	2	2	3	3	4	4
2	2	2	2	2	2	2	2	4	4
2	2	2	2	2	2	3	3	4	4
1	1	2	2	2	2	2	2	4	4
2	2	2	2	2	2	2	2	4	4
2	2	1	1	2	2	3	3	4	4
2	2	2	2	2	2	3	3	4	4
2	2	1	1	2	2	3	3	4	4
2	2	2	2	2	2	3	3	4	4
2	2	2	2	2	2	3	3	4	4
2	2	1	1	2	2	3	3	4	4

For Review Only

2	2	2	2	2	2	3	3	4	4
2	2	2	2	2	2	3	3	4	4

For Review Only

SNP125	SNP125	SNP1250	SNP1250	SNP1251	SNP1251	SNP1252	SNP1252	SNP1253	SNP1253
1	1	4	4	3	3	3	3	1	1
1	1	4	4	3	3	3	3	1	1
1	1	4	4	3	3	1	1	1	1
3	3	3	3	3	3	3	3	1	1
3	3	4	4	3	3	3	3	1	1
1	1	4	4	3	3	1	1	1	1
1	1	3	3	3	3	3	3	1	1
1	1	4	4	3	3	1	1	1	1
3	3	4	4	3	3	1	1	1	1
1	1	4	4	3	3	1	1	3	3
1	1	4	4	3	3	1	1	1	1
1	1	3	3	3	3	1	1	1	1
1	1	3	3	3	3	1	1	1	1
1	1	3	3	3	3	1	1	1	1
3	3	4	4	3	3	1	1	1	1
3	3	4	4	3	3	1	1	1	1
1	1	4	4	3	3	1	1	1	1
3	3	3	3	3	3	3	3	3	3
1	1	4	4	3	3	1	1	1	1
1	1	3	3	3	3	3	3	1	1
3	3	3	3	2	2	3	3	1	1
3	3	4	4	2	2	1	1	1	1
3	3	3	3	3	3	3	3	1	1
1	1	4	4	3	3	1	1	1	1
1	1	4	4	3	3	3	3	1	1
1	1	4	4	3	3	1	1	1	1
1	1	3	3	3	3	3	3	1	1
1	1	4	4	3	3	1	1	1	1
1	1	4	4	3	3	3	3	3	3
1	1	3	3	2	2	3	3	1	1
3	3	4	4	3	3	1	1	1	1
1	1	4	4	3	3	1	1	1	1
1	1	4	4	3	3	1	1	1	1
1	1	4	4	3	3	1	1	1	1
3	3	4	4	3	3	1	1	1	1
1	1	4	4	3	3	1	1	1	1
1	1	3	3	3	3	3	3	3	3
1	1	4	4	3	3	1	1	1	1
1	1	4	4	3	3	1	1	1	1
1	1	4	4	3	3	1	1	3	3
1	1	3	3	2	2	1	1	1	1
1	1	3	3	3	3	1	1	3	3
1	1	4	4	2	2	1	1	1	1
3	3	3	3	3	3	3	3	3	3
1	1	3	3	2	2	3	3	1	1
1	1	4	4	3	3	1	1	1	1
3	3	4	4	3	3	1	1	1	1
1	1	4	4	3	3	1	1	1	1
3	3	3	3	3	3	3	3	3	3
1	1	4	4	3	3	1	1	1	1
1	1	4	4	3	3	1	1	1	1
1	1	4	4	3	3	1	1	1	1

For Review Only

1	1	4	4	3	3	3	3	1	1
1	1	4	4	3	3	1	1	1	1
1	1	4	4	3	3	1	1	1	1
1	1	3	3	2	2	3	3	1	1
1	1	4	4	3	3	1	1	1	1
3	3	4	4	3	3	1	1	1	1
1	1	3	3	3	3	3	3	1	1
1	1	4	4	3	3	3	3	1	1
1	1	3	3	3	3	3	3	3	3
3	3	4	4	3	3	1	1	1	1
3	3	4	4	3	3	1	1	1	1
3	3	4	4	3	3	1	1	1	1
1	1	4	4	3	3	3	3	1	1
1	1	4	4	3	3	3	3	3	3
1	1	4	4	3	3	1	1	1	1
1	1	3	3	3	3	3	3	1	1
3	3	3	3	3	3	3	3	1	1
1	1	4	4	3	3	1	1	3	3
3	3	4	4	3	3	1	1	1	1
1	1	4	4	3	3	1	1	1	1
3	3	4	4	3	3	1	1	1	1
3	3	4	4	3	3	1	1	1	1
3	3	4	4	3	3	1	1	3	3
1	1	4	4	3	3	1	1	1	1
1	1	3	3	2	2	3	3	1	1
1	1	4	4	3	3	1	1	1	1
1	1	4	4	3	3	1	1	1	1
1	1	4	4	3	3	1	1	1	1
1	1	4	4	3	3	1	1	1	1
1	1	4	4	3	3	1	1	1	1
3	3	3	3	3	3	1	1	3	3
1	1	3	3	3	3	1	1	1	1
1	1	4	4	3	3	1	1	1	1
1	1	4	4	3	3	3	3	1	1
1	1	4	4	3	3	3	3	3	3
1	1	3	3	2	2	3	3	1	1
1	1	3	3	3	3	3	3	3	3
3	3	3	3	2	2	3	3	1	1
1	1	3	3	3	3	3	3	1	1
3	3	3	3	2	2	3	3	1	1
3	3	4	4	3	3	3	3	1	1
1	1	4	4	3	3	1	1	1	1
1	1	3	3	3	3	1	1	1	1
3	3	4	4	3	3	1	1	1	1
1	1	4	4	3	3	1	1	1	1
1	1	4	4	3	3	1	1	3	3
1	1	4	4	3	3	1	1	1	1
1	1	3	3	3	3	3	3	1	1
1	1	3	3	3	3	3	3	1	1
3	3	4	4	3	3	1	1	1	1
1	1	4	4	3	3	1	1	1	1
1	1	4	4	3	3	1	1	1	1
1	1	4	4	3	3	3	3	1	1
1	1	4	4	3	3	3	3	1	1
1	1	3	3	3	3	3	3	1	1

For Review Only

1	1	4	4	3	3	1	1	1	1
3	3	4	4	3	3	1	1	1	1
1	1	4	4	3	3	1	1	1	1
1	1	4	4	3	3	1	1	1	1
1	1	4	4	3	3	1	1	1	1
1	1	4	4	3	3	1	1	1	1
3	3	4	4	3	3	1	1	1	1
1	1	4	4	3	3	1	1	1	1
1	1	3	3	3	3	3	3	1	1
1	1	4	4	3	3	1	1	1	1
1	1	4	4	3	3	3	3	3	3
3	3	4	4	3	3	1	1	1	1
1	1	3	3	3	3	1	1	1	1
1	1	4	4	3	3	1	1	1	1
1	1	4	4	3	3	3	3	1	1
1	1	4	4	3	3	1	1	1	1
1	1	4	4	3	3	1	1	1	1
3	3	4	4	3	3	1	1	1	1
1	1	3	3	3	3	3	3	1	1
3	3	4	4	3	3	1	1	1	1
1	1	4	4	3	3	1	1	1	1
3	3	4	4	3	3	1	1	1	1
3	3	4	4	3	3	1	1	1	1
1	1	4	4	3	3	1	1	1	1
3	3	4	4	3	3	1	1	1	1
3	3	4	4	3	3	1	1	1	1
1	1	4	4	3	3	1	1	1	1
1	1	4	4	3	3	1	1	1	1
3	3	4	4	3	3	1	1	1	1
3	3	4	4	3	3	1	1	1	1
1	1	3	3	3	3	3	3	1	1
3	3	4	4	3	3	1	1	1	1
3	3	4	4	3	3	3	3	1	1
1	1	4	4	3	3	1	1	1	1
1	1	4	4	3	3	1	1	1	1
3	3	4	4	3	3	1	1	1	1
1	1	3	3	3	3	3	3	1	1
1	1	4	4	3	3	1	1	1	1
1	1	4	4	3	3	1	1	1	1
1	1	4	4	3	3	1	1	1	1
1	1	4	4	3	3	1	1	1	1
1	1	4	4	3	3	1	1	1	1
3	3	3	3	3	3	3	3	1	1
1	1	4	4	2	2	1	1	1	1
1	1	4	4	3	3	1	1	1	1
1	1	4	4	3	3	1	1	3	3
1	1	4	4	3	3	1	1	1	1
1	1	4	4	3	3	1	1	1	1
1	1	3	3	3	3	1	1	1	1
1	1	4	4	3	3	1	1	1	1
1	1	4	4	3	3	1	1	1	1
3	3	4	4	3	3	1	1	1	1
3	3	4	4	3	3	1	1	1	1
3	3	4	4	3	3	1	1	1	1
1	1	3	3	3	3	1	1	1	1

For Review Only

3	3	4	4	3	3	1	1	1	1
3	3	4	4	3	3	1	1	1	1
1	1	3	3	2	2	3	3	1	1
3	3	4	4	3	3	1	1	1	1
1	1	4	4	3	3	1	1	1	1
1	1	4	4	3	3	1	1	1	1
3	3	4	4	3	3	1	1	1	1
1	1	4	4	3	3	1	1	1	1
1	1	4	4	3	3	1	1	1	1
1	1	4	4	3	3	1	1	1	1
1	1	4	4	3	3	1	1	1	1
3	3	4	4	3	3	1	1	1	1
3	3	4	4	3	3	1	1	1	1
1	1	4	4	3	3	1	1	3	3
1	1	3	3	3	3	3	3	1	1
1	1	4	4	3	3	1	1	1	1
3	3	4	4	3	3	1	1	1	1
1	1	4	4	3	3	1	1	1	1
1	1	3	3	3	3	3	3	1	1
3	3	4	4	3	3	1	1	1	1
3	3	4	4	3	3	1	1	1	1
1	1	4	4	3	3	1	1	1	1
3	3	4	4	3	3	1	1	1	1
3	3	4	4	3	3	1	1	1	1
3	3	3	3	3	3	3	3	3	3
1	1	4	4	3	3	1	1	1	1
1	1	4	4	3	3	3	3	1	1
3	3	3	3	3	3	1	1	1	1
1	1	4	4	3	3	1	1	3	3
3	3	4	4	3	3	1	1	1	1
1	1	4	4	3	3	1	1	1	1
1	1	4	4	3	3	1	1	1	1
1	1	3	3	3	3	3	3	1	1
1	1	4	4	3	3	1	1	1	1
1	1	4	4	3	3	1	1	1	1
3	3	4	4	3	3	1	1	1	1
3	3	4	4	3	3	1	1	1	1
1	1	3	3	3	3	3	3	3	3
1	1	4	4	3	3	1	1	1	1
1	1	4	4	3	3	1	1	1	1
1	1	4	4	3	3	1	1	1	1
1	1	4	4	3	3	1	1	1	1
1	1	4	4	3	3	1	1	1	1
3	3	4	4	3	3	1	1	1	1
3	3	4	4	3	3	1	1	1	1
1	1	4	4	3	3	1	1	1	1
1	1	4	4	3	3	1	1	1	1
1	1	4	4	3	3	1	1	1	1
1	1	3	3	3	3	3	3	1	1
1	1	3	3	3	3	3	3	3	3
1	1	4	4	3	3	1	1	1	1
1	1	4	4	3	3	3	3	3	3
3	3	4	4	3	3	1	1	1	1
3	3	4	4	3	3	1	1	1	1
1	1	4	4	3	3	1	1	1	1

For Review Only

3	3	3	3	3	3	3	3	1	1
1	1	4	4	3	3	1	1	1	1
1	1	4	4	3	3	1	1	1	1
3	3	4	4	3	3	1	1	1	1
1	1	3	3	3	3	3	3	3	3
1	1	4	4	3	3	1	1	1	1
1	1	3	3	3	3	1	1	1	1
1	1	3	3	3	3	3	3	3	3
1	1	4	4	3	3	1	1	1	1
1	1	4	4	3	3	1	1	1	1
3	3	4	4	3	3	1	1	1	1
1	1	4	4	3	3	1	1	1	1
1	1	3	3	3	3	3	3	1	1
1	1	4	4	3	3	1	1	1	1
1	1	4	4	3	3	1	1	1	1
1	1	3	3	3	3	3	3	1	1
1	1	4	4	3	3	1	1	1	1
1	1	4	4	3	3	1	1	1	1
3	3	4	4	3	3	3	3	1	1
3	3	4	4	3	3	1	1	1	1
1	1	3	3	3	3	3	3	1	1
1	1	4	4	3	3	1	1	1	1
1	1	4	4	3	3	1	1	1	1
1	1	4	4	3	3	1	1	1	1
1	1	4	4	3	3	3	3	1	1
1	1	4	4	3	3	3	3	1	1
1	1	4	4	3	3	1	1	1	1
1	1	3	3	3	3	3	3	1	1
1	1	4	4	3	3	3	3	1	1
3	3	4	4	3	3	1	1	1	1
1	1	4	4	3	3	1	1	1	1
1	1	4	4	3	3	1	1	1	1
1	1	3	3	3	3	3	3	1	1
1	1	4	4	3	3	1	1	1	1
3	3	4	4	3	3	1	1	1	1
1	1	4	4	3	3	1	1	1	1
3	3	4	4	3	3	1	1	1	1
3	3	4	4	3	3	1	1	1	1
1	1	4	4	3	3	1	1	1	1
1	1	4	4	3	3	1	1	1	1
1	1	4	4	3	3	1	1	1	1
1	1	4	4	3	3	1	1	1	1
3	3	4	4	3	3	1	1	1	1
3	3	4	4	3	3	1	1	1	1
1	1	4	4	3	3	1	1	1	1
1	1	3	3	3	3	3	3	1	1
1	1	4	4	3	3	1	1	1	1
1	1	3	3	3	3	3	3	1	1
1	1	4	4	3	3	1	1	1	1
1	1	4	4	3	3	1	1	1	1
1	1	4	4	3	3	1	1	1	1
1	1	4	4	3	3	1	1	1	1
1	1	4	4	3	3	1	1	1	1
3	3	4	4	3	3	1	1	1	1
1	1	4	4	3	3	1	1	1	1

For Review Only

1	1	4	4	3	3	1	1	1	1
1	1	4	4	3	3	1	1	1	1

For Review Only

SNP1254	SNP1254	SNP1255	SNP1255	SNP1256	SNP1256	SNP1257	SNP1257	SNP1258	SNP1258
4	4	2	2	4	4	3	3	1	1
4	4	2	2	4	4	3	3	2	2
4	4	2	2	2	2	3	3	2	2
4	4	2	2	2	2	3	3	2	2
4	4	2	2	4	4	3	3	1	1
4	4	2	2	4	4	3	3	2	2
4	4	2	2	4	4	3	3	2	2
4	4	2	2	4	4	3	3	1	1
4	4	2	2	4	4	3	3	2	2
4	4	2	2	4	4	3	3	2	2
4	4	2	2	4	4	3	3	2	2
4	4	2	2	2	2	3	3	2	2
4	4	2	2	2	2	3	3	2	2
4	4	2	2	4	4	3	3	2	2
4	4	2	2	2	2	2	2	2	2
4	4	2	2	2	2	3	3	2	2
4	4	2	2	4	4	3	3	2	2
4	4	2	2	4	4	3	3	2	2
4	4	2	2	4	4	3	3	1	1
2	2	2	2	2	2	3	3	2	2
4	4	2	2	4	4	3	3	2	2
4	4	2	2	4	4	3	3	2	2
4	4	2	2	4	4	3	3	2	2
4	4	2	2	4	4	3	3	2	2
4	4	2	2	4	4	3	3	1	1
4	4	2	2	4	4	3	3	2	2
4	4	2	2	4	4	3	3	2	2
4	4	2	2	2	2	3	3	2	2
4	4	2	2	4	4	3	3	1	1
4	4	2	2	4	4	3	3	2	2
4	4	2	2	4	4	3	3	2	2
2	2	2	2	2	2	3	3	2	2
4	4	2	2	4	4	3	3	2	2
4	4	2	2	4	4	3	3	2	2
4	4	2	2	4	4	3	3	2	2
4	4	2	2	2	2	3	3	2	2
4	4	2	2	4	4	3	3	2	2
4	4	2	2	2	2	2	2	2	2
4	4	2	2	4	4	3	3	2	2
4	4	2	2	4	4	3	3	2	2
4	4	2	2	4	4	3	3	2	2
4	4	2	2	4	4	3	3	2	2
4	4	2	2	2	2	3	3	1	1
4	4	2	2	4	4	3	3	2	2
4	4	2	2	4	4	3	3	2	2
4	4	2	2	2	2	3	3	2	2
4	4	2	2	4	4	3	3	2	2
4	4	2	2	4	4	3	3	2	2
2	2	2	2	2	2	3	3	2	2
4	4	2	2	4	4	3	3	2	2
2	2	2	2	2	2	3	3	2	2
4	4	2	2	4	4	3	3	2	2

For Review Only

4	4	2	2	4	4	3	3	1	1
4	4	2	2	4	4	3	3	2	2
4	4	2	2	4	4	3	3	2	2
2	2	2	2	2	2	3	3	2	2
4	4	2	2	4	4	3	3	1	1
4	4	2	2	4	4	3	3	2	2
4	4	2	2	4	4	3	3	2	2
4	4	2	2	4	4	3	3	1	1
4	4	2	2	4	4	3	3	2	2
4	4	2	2	2	2	3	3	2	2
4	4	2	2	4	4	3	3	2	2
4	4	2	2	4	4	3	3	2	2
4	4	2	2	4	4	3	3	1	1
4	4	2	2	4	4	3	3	1	1
4	4	2	2	4	4	3	3	2	2
4	4	2	2	4	4	3	3	2	2
4	4	2	2	4	4	3	3	2	2
4	4	2	2	4	4	2	2	2	2
4	4	2	2	4	4	3	3	2	2
4	4	2	2	4	4	3	3	2	2
4	4	2	2	4	4	3	3	2	2
4	4	2	2	4	4	3	3	2	2
4	4	2	2	4	4	3	3	2	2
2	2	2	2	2	2	3	3	2	2
4	4	2	2	4	4	3	3	2	2
2	2	2	2	2	2	2	2	2	2
4	4	2	2	4	4	3	3	1	1
4	4	2	2	4	4	3	3	2	2
4	4	2	2	4	4	3	3	2	2
2	2	2	2	2	2	3	3	2	2
2	2	2	2	2	2	3	3	2	2
4	4	2	2	4	4	3	3	2	2
4	4	2	2	4	4	3	3	2	2
4	4	2	2	4	4	3	3	1	1
4	4	2	2	4	4	3	3	1	1
4	4	2	2	4	4	3	3	2	2
4	4	2	2	4	4	3	3	2	2
2	2	2	2	2	2	3	3	2	2
4	4	2	2	4	4	3	3	2	2
4	4	2	2	4	4	3	3	2	2
4	4	2	2	4	4	3	3	1	1
4	4	2	2	2	2	3	3	2	2
4	4	2	2	4	4	3	3	2	2
4	4	2	2	4	4	3	3	2	2
2	2	2	2	2	2	3	3	2	2
4	4	2	2	4	4	3	3	2	2
4	4	2	2	4	4	3	3	2	2
4	4	2	2	4	4	3	3	2	2
4	4	2	2	4	4	3	3	2	2
4	4	2	2	4	4	2	2	2	2
4	4	2	2	4	4	3	3	2	2
4	4	2	2	4	4	3	3	2	2
4	4	2	2	4	4	3	3	2	2
4	4	2	2	4	4	3	3	2	2
4	4	2	2	4	4	3	3	2	2

For Review Only

4	4	2	2	2	2	3	3	2	2
4	4	2	2	2	2	3	3	1	1
4	4	2	2	2	2	3	3	2	2
4	4	2	2	4	4	3	3	2	2
4	4	2	2	4	4	3	3	2	2
4	4	2	2	4	4	3	3	2	2
4	4	2	2	2	2	3	3	2	2
4	4	2	2	4	4	3	3	2	2
4	4	2	2	4	4	3	3	2	2
4	4	2	2	4	4	3	3	2	2
4	4	2	2	4	4	3	3	1	1
4	4	2	2	2	2	3	3	2	2
4	4	2	2	4	4	3	3	2	2
2	2	3	3	2	2	3	3	2	2
4	4	2	2	4	4	3	3	2	2
4	4	2	2	4	4	3	3	2	2
4	4	2	2	4	4	2	2	2	2
4	4	2	2	2	2	3	3	2	2
4	4	2	2	4	4	3	3	2	2
2	2	2	2	2	2	3	3	2	2
4	4	2	2	2	2	3	3	2	2
2	2	3	3	2	2	3	3	2	2
4	4	2	2	2	2	3	3	2	2
4	4	2	2	4	4	3	3	2	2
4	4	2	2	4	4	3	3	2	2
4	4	2	2	4	4	3	3	2	2
4	4	2	2	2	2	3	3	2	2
4	4	2	2	4	4	3	3	2	2
4	4	2	2	2	2	3	3	2	2
4	4	2	2	4	4	3	3	2	2
4	4	2	2	4	4	3	3	2	2
4	4	2	2	2	2	3	3	2	2
4	4	2	2	4	4	3	3	1	1
4	4	2	2	2	2	3	3	2	2
4	4	2	2	4	4	3	3	2	2
4	4	2	2	4	4	3	3	2	2
4	4	2	2	4	4	3	3	2	2
2	2	2	2	2	2	3	3	2	2
4	4	2	2	4	4	3	3	2	2
4	4	2	2	4	4	3	3	1	1
4	4	2	2	4	4	3	3	2	2
4	4	2	2	4	4	3	3	2	2
4	4	2	2	4	4	3	3	2	2
4	4	2	2	4	4	3	3	2	2
4	4	2	2	2	2	2	2	2	2
4	4	2	2	4	4	3	3	2	2
4	4	2	2	2	2	3	3	2	2
2	2	2	2	2	2	3	3	2	2
4	4	2	2	4	4	3	3	2	2
4	4	2	2	2	2	3	3	2	2
4	4	2	2	4	4	3	3	2	2
4	4	2	2	4	4	3	3	2	2
4	4	2	2	4	4	3	3	2	2
4	4	2	2	4	4	3	3	2	2
4	4	2	2	4	4	3	3	2	2

For Review Only

4	4	2	2	4	4	3	3	2	2
4	4	2	2	4	4	2	2	2	2
4	4	2	2	4	4	3	3	2	2
4	4	2	2	4	4	3	3	2	2
4	4	2	2	4	4	3	3	1	1
4	4	2	2	4	4	3	3	2	2
4	4	2	2	2	2	3	3	1	1
4	4	2	2	4	4	3	3	2	2
4	4	2	2	4	4	3	3	2	2
4	4	2	2	4	4	3	3	2	2
4	4	2	2	2	2	3	3	2	2
4	4	2	2	4	4	3	3	2	2
2	2	3	3	2	2	3	3	2	2
4	4	2	2	4	4	3	3	2	2
4	4	2	2	4	4	3	3	2	2
4	4	2	2	4	4	3	3	1	1
4	4	2	2	4	4	3	3	2	2
4	4	2	2	4	4	2	2	2	2
4	4	2	2	2	2	3	3	2	2
4	4	2	2	2	2	3	3	2	2
4	4	2	2	4	4	3	3	2	2
4	4	2	2	4	4	3	3	1	1
4	4	2	2	4	4	2	2	2	2
4	4	2	2	4	4	3	3	2	2
4	4	2	2	4	4	3	3	2	2
4	4	2	2	4	4	3	3	2	2
4	4	2	2	2	2	3	3	2	2
4	4	2	2	4	4	3	3	2	2
4	4	2	2	4	4	2	2	2	2
4	4	2	2	4	4	3	3	2	2
4	4	2	2	4	4	3	3	1	1
4	4	2	2	4	4	3	3	2	2
4	4	2	2	4	4	3	3	2	2
4	4	2	2	4	4	3	3	2	2
4	4	2	2	4	4	3	3	2	2
4	4	2	2	4	4	3	3	2	2
4	4	2	2	2	2	3	3	2	2
4	4	2	2	4	4	3	3	2	2
2	2	2	2	2	2	3	3	2	2
2	2	2	2	2	2	3	3	2	2
4	4	2	2	2	2	3	3	2	2
4	4	2	2	2	2	3	3	2	2
4	4	2	2	4	4	3	3	2	2
4	4	2	2	2	2	3	3	2	2
4	4	2	2	2	2	3	3	2	2
4	4	2	2	4	4	3	3	2	2
4	4	2	2	4	4	3	3	1	1
2	2	2	2	2	2	3	3	1	1
4	4	2	2	4	4	3	3	2	2
4	4	2	2	4	4	3	3	2	2
2	2	2	2	2	2	3	3	2	2
4	4	2	2	4	4	3	3	1	1
4	4	2	2	4	4	2	2	2	2
4	4	2	2	4	4	3	3	2	2
4	4	2	2	4	4	3	3	2	2

For Review Only

4	4	2	2	4	4	3	3	2	2
4	4	2	2	4	4	3	3	2	2
2	2	2	2	2	2	3	3	2	2
4	4	2	2	4	4	3	3	2	2
4	4	2	2	4	4	3	3	2	2
4	4	2	2	4	4	3	3	2	2
4	4	2	2	4	4	3	3	2	2
2	2	2	2	2	2	3	3	2	2
4	4	2	2	4	4	3	3	2	2
4	4	2	2	4	4	3	3	2	2
4	4	2	2	4	4	3	3	2	2
4	4	2	2	4	4	3	3	2	2
4	4	2	2	4	4	3	3	2	2
2	2	2	2	2	2	3	3	2	2
4	4	2	2	2	2	3	3	2	2
4	4	2	2	4	4	3	3	2	2
4	4	2	2	4	4	3	3	2	2
4	4	2	2	4	4	3	3	2	2
4	4	2	2	4	4	3	3	1	1
4	4	2	2	2	2	2	2	2	2
4	4	2	2	4	4	3	3	2	2
4	4	2	2	4	4	3	3	2	2
2	2	2	2	2	2	3	3	2	2
4	4	2	2	4	4	3	3	2	2
4	4	2	2	4	4	3	3	1	1
4	4	2	2	4	4	3	3	1	1
4	4	2	2	4	4	3	3	1	1
4	4	2	2	2	2	3	3	2	2
4	4	2	2	4	4	3	3	1	1
2	2	2	2	2	2	3	3	2	2
4	4	2	2	4	4	2	2	2	2
4	4	2	2	2	2	3	3	2	2
4	4	2	2	4	4	3	3	2	2
2	2	2	2	2	2	3	3	2	2
4	4	2	2	4	4	2	2	2	2
4	4	2	2	4	4	3	3	2	2
2	2	2	2	2	2	3	3	2	2
4	4	2	2	4	4	3	3	2	2
4	4	2	2	2	2	3	3	2	2
4	4	2	2	4	4	3	3	2	2
4	4	2	2	4	4	3	3	2	2
4	4	2	2	4	4	3	3	2	2
4	4	2	2	4	4	3	3	2	2
2	2	2	2	2	2	3	3	2	2
4	4	2	2	2	2	3	3	2	2
4	4	2	2	4	4	3	3	2	2
4	4	2	2	4	4	3	3	2	2
2	2	2	2	2	2	3	3	2	2
4	4	2	2	2	2	3	3	2	2
4	4	2	2	4	4	3	3	1	1
4	4	2	2	4	4	3	3	2	2
4	4	2	2	4	4	3	3	1	1
4	4	2	2	2	2	3	3	2	2
4	4	2	2	4	4	3	3	2	2
4	4	2	2	2	2	3	3	2	2

For Review Only

4	4	2	2	4	4	3	3	1	1
4	4	2	2	4	4	2	2	2	2

For Review Only

SNP1259	SNP1259	SNP126	SNP126	SNP1260	SNP1260	SNP1261	SNP1261	SNP1262	SNP1262
2	2	4	4	3	3	4	4	3	3
1	1	4	4	2	2	4	4	3	3
2	2	4	4	3	3	4	4	3	3
2	2	4	4	3	3	4	4	3	3
1	1	4	4	2	2	4	4	3	3
2	2	4	4	3	3	4	4	3	3
2	2	4	4	3	3	4	4	3	3
2	2	2	2	3	3	4	4	3	3
2	2	4	4	3	3	4	4	3	3
1	1	2	2	2	2	3	3	3	3
2	2	4	4	3	3	4	4	3	3
2	2	4	4	3	3	4	4	3	3
2	2	4	4	3	3	4	4	3	3
2	2	4	4	3	3	4	4	3	3
2	2	4	4	3	3	4	4	3	3
2	2	4	4	3	3	4	4	3	3
2	2	4	4	3	3	3	3	3	3
2	2	4	4	3	3	3	3	3	3
2	2	4	4	3	3	4	4	3	3
2	2	2	2	3	3	4	4	3	3
2	2	2	2	3	3	4	4	3	3
2	2	2	2	3	3	4	4	3	3
2	2	4	4	3	3	4	4	3	3
2	2	4	4	3	3	4	4	3	3
2	2	4	4	3	3	4	4	3	3
2	2	4	4	3	3	4	4	3	3
2	2	4	4	3	3	4	4	3	3
2	2	4	4	3	3	4	4	3	3
2	2	4	4	3	3	3	3	3	3
1	1	2	2	2	2	4	4	3	3
2	2	4	4	3	3	4	4	3	3
2	2	4	4	3	3	4	4	3	3
2	2	4	4	3	3	4	4	3	3
2	2	4	4	3	3	4	4	3	3
2	2	4	4	3	3	4	4	3	3
2	2	4	4	3	3	4	4	3	3
2	2	4	4	3	3	4	4	3	3
2	2	4	4	3	3	4	4	3	3
2	2	4	4	3	3	4	4	3	3
2	2	4	4	3	3	4	4	3	3
1	1	2	2	2	2	4	4	3	3
2	2	2	2	3	3	3	3	3	3
1	1	2	2	2	2	4	4	3	3
2	2	4	4	3	3	3	3	3	3
1	1	2	2	2	2	4	4	3	3
2	2	4	4	3	3	4	4	3	3
2	2	4	4	3	3	4	4	3	3
2	2	4	4	3	3	4	4	3	3
2	2	4	4	3	3	3	3	3	3
2	2	4	4	3	3	4	4	3	3
2	2	4	4	3	3	4	4	3	3
2	2	4	4	3	3	4	4	3	3

For Review Only

2	2	4	4	3	3	4	4	3	3
2	2	4	4	3	3	4	4	3	3
2	2	4	4	3	3	4	4	3	3
2	2	4	4	3	3	4	4	3	3
2	2	4	4	3	3	4	4	3	3
2	2	4	4	3	3	4	4	3	3
2	2	4	4	3	3	4	4	3	3
2	2	4	4	3	3	4	4	3	3
2	2	4	4	3	3	4	4	3	3
2	2	4	4	3	3	4	4	3	3
2	2	4	4	3	3	4	4	2	2
2	2	4	4	3	3	4	4	2	2
2	2	4	4	3	3	4	4	3	3
2	2	4	4	3	3	3	3	3	3
2	2	4	4	3	3	4	4	3	3
1	1	4	4	2	2	4	4	3	3
2	2	4	4	3	3	4	4	3	3
2	2	4	4	3	3	4	4	3	3
2	2	4	4	3	3	4	4	3	3
2	2	4	4	3	3	4	4	3	3
2	2	4	4	3	3	4	4	2	2
2	2	4	4	3	3	4	4	3	3
2	2	4	4	3	3	3	3	3	3
2	2	4	4	3	3	4	4	3	3
1	1	2	2	2	2	4	4	3	3
2	2	4	4	3	3	4	4	2	2
2	2	4	4	3	3	4	4	3	3
2	2	4	4	3	3	3	3	3	3
2	2	4	4	3	3	4	4	3	3
2	2	4	4	3	3	4	4	3	3
2	2	4	4	3	3	3	3	3	3
2	2	4	4	3	3	4	4	3	3
2	2	4	4	3	3	4	4	3	3
2	2	4	4	3	3	4	4	3	3
2	2	4	4	3	3	4	4	3	3
1	1	2	2	2	2	4	4	3	3
2	2	4	4	3	3	3	3	3	3
2	2	4	4	3	3	4	4	3	3
2	2	4	4	3	3	4	4	3	3
2	2	4	4	3	3	4	4	3	3
2	2	4	4	3	3	4	4	3	3
2	2	4	4	3	3	4	4	3	3
2	2	4	4	3	3	4	4	3	3
2	2	4	4	3	3	4	4	2	2
2	2	4	4	3	3	4	4	3	3
2	2	4	4	3	3	4	4	2	2
2	2	4	4	3	3	4	4	3	3
2	2	4	4	3	3	4	4	3	3
2	2	4	4	3	3	4	4	3	3
2	2	4	4	3	3	4	4	2	2
2	2	4	4	3	3	4	4	3	3
2	2	4	4	3	3	3	3	3	3
2	2	4	4	3	3	4	4	3	3
2	2	4	4	3	3	4	4	3	3
2	2	4	4	3	3	4	4	3	3

For Review Only

2	2	4	4	3	3	4	4	3	3
2	2	4	4	3	3	4	4	3	3
2	2	4	4	3	3	4	4	3	3
2	2	4	4	3	3	4	4	3	3
2	2	4	4	3	3	4	4	2	2
2	2	4	4	3	3	4	4	3	3
2	2	4	4	3	3	4	4	3	3
2	2	4	4	3	3	4	4	3	3
1	1	4	4	2	2	4	4	3	3
2	2	4	4	3	3	4	4	3	3
2	2	4	4	3	3	4	4	3	3
2	2	4	4	3	3	4	4	3	3
2	2	4	4	3	3	4	4	3	3
2	2	4	4	3	3	4	4	2	2
2	2	4	4	3	3	4	4	3	3
2	2	4	4	3	3	4	4	3	3
2	2	4	4	3	3	4	4	2	2
2	2	4	4	3	3	4	4	3	3
2	2	4	4	3	3	4	4	3	3
2	2	4	4	3	3	4	4	3	3
2	2	4	4	3	3	4	4	3	3
2	2	4	4	3	3	4	4	3	3
2	2	4	4	3	3	4	4	3	3
2	2	4	4	3	3	4	4	3	3
2	2	4	4	3	3	4	4	3	3
2	2	4	4	3	3	4	4	3	3
2	2	4	4	3	3	4	4	3	3
2	2	4	4	3	3	4	4	3	3
2	2	4	4	3	3	4	4	3	3
2	2	4	4	3	3	4	4	3	3
2	2	4	4	3	3	3	3	3	3
2	2	4	4	3	3	4	4	3	3
2	2	4	4	3	3	4	4	3	3
2	2	4	4	3	3	4	4	3	3
2	2	4	4	3	3	4	4	3	3
2	2	4	4	3	3	4	4	3	3
2	2	4	4	3	3	4	4	3	3
2	2	4	4	3	3	4	4	3	3
2	2	4	4	3	3	4	4	3	3
2	2	4	4	3	3	4	4	3	3
2	2	4	4	3	3	4	4	3	3
2	2	4	4	3	3	3	3	3	3
2	2	4	4	3	3	4	4	3	3
2	2	2	2	3	3	4	4	3	3
2	2	4	4	3	3	4	4	2	2
2	2	4	4	3	3	4	4	3	3
2	2	4	4	3	3	4	4	3	3
2	2	4	4	3	3	4	4	3	3
2	2	4	4	3	3	4	4	3	3
2	2	4	4	3	3	4	4	3	3
2	2	4	4	3	3	4	4	3	3
2	2	4	4	3	3	4	4	3	3
2	2	4	4	3	3	4	4	3	3
2	2	4	4	3	3	4	4	3	3
2	2	4	4	3	3	4	4	3	3

For Review Only

2	2	4	4	3	3	4	4	3	3
2	2	2	2	3	3	4	4	2	2
1	1	4	4	2	2	4	4	3	3
2	2	4	4	3	3	4	4	3	3
2	2	4	4	3	3	4	4	3	3
2	2	4	4	3	3	4	4	3	3
2	2	4	4	3	3	4	4	3	3
2	2	4	4	3	3	4	4	3	3
2	2	4	4	3	3	4	4	3	3
2	2	4	4	3	3	4	4	3	3
2	2	4	4	3	3	4	4	3	3
2	2	4	4	3	3	4	4	3	3
2	2	4	4	3	3	4	4	3	3
2	2	4	4	3	3	4	4	3	3
2	2	4	4	3	3	3	3	3	3
2	2	4	4	3	3	4	4	3	3
2	2	4	4	3	3	4	4	3	3
2	2	4	4	3	3	4	4	3	3
2	2	4	4	3	3	4	4	3	3
2	2	4	4	3	3	4	4	3	3
2	2	4	4	3	3	4	4	3	3
2	2	4	4	3	3	4	4	3	3
2	2	4	4	3	3	4	4	2	2
2	2	4	4	3	3	4	4	2	2
2	2	4	4	3	3	4	4	3	3
2	2	4	4	3	3	4	4	3	3
2	2	4	4	3	3	4	4	3	3
2	2	4	4	3	3	4	4	3	3
2	2	4	4	3	3	4	4	2	2
2	2	4	4	3	3	4	4	3	3
2	2	4	4	3	3	4	4	3	3
2	2	4	4	3	3	4	4	3	3
2	2	4	4	3	3	4	4	3	3
2	2	4	4	3	3	3	3	3	3
2	2	4	4	3	3	4	4	3	3
2	2	4	4	3	3	4	4	2	2
2	2	4	4	3	3	4	4	3	3
2	2	4	4	3	3	4	4	3	3
2	2	4	4	3	3	4	4	3	3
2	2	4	4	3	3	4	4	3	3
2	2	4	4	3	3	4	4	3	3
2	2	4	4	3	3	4	4	3	3
2	2	4	4	3	3	4	4	3	3
2	2	4	4	3	3	4	4	3	3
2	2	4	4	3	3	4	4	3	3
2	2	4	4	3	3	4	4	3	3
2	2	4	4	3	3	4	4	3	3
2	2	4	4	3	3	4	4	3	3
2	2	4	4	3	3	4	4	3	3
2	2	4	4	3	3	3	3	3	3
2	2	4	4	3	3	4	4	3	3
2	2	4	4	3	3	4	4	3	3
2	2	4	4	3	3	4	4	2	2
2	2	4	4	3	3	4	4	2	2
2	2	4	4	3	3	4	4	3	3

For Review Only

2	2	4	4	3	3	4	4	3	3
1	1	2	2	2	2	4	4	3	3
2	2	4	4	3	3	4	4	3	3
2	2	4	4	3	3	4	4	3	3
2	2	4	4	3	3	3	3	3	3
2	2	4	4	3	3	4	4	3	3
2	2	4	4	3	3	4	4	3	3
1	1	4	4	2	2	3	3	3	3
2	2	4	4	3	3	4	4	3	3
2	2	4	4	3	3	4	4	3	3
2	2	4	4	3	3	4	4	3	3
2	2	4	4	3	3	4	4	3	3
2	2	4	4	3	3	4	4	3	3
2	2	4	4	3	3	4	4	3	3
2	2	4	4	3	3	4	4	3	3
2	2	4	4	3	3	4	4	3	3
2	2	4	4	3	3	4	4	3	3
2	2	4	4	3	3	4	4	3	3
2	2	4	4	3	3	4	4	3	3
2	2	4	4	3	3	4	4	2	2
2	2	4	4	3	3	4	4	3	3
2	2	4	4	3	3	4	4	3	3
2	2	4	4	3	3	4	4	3	3
2	2	4	4	3	3	4	4	3	3
2	2	4	4	3	3	4	4	3	3
2	2	4	4	3	3	4	4	3	3
2	2	4	4	3	3	4	4	3	3
2	2	4	4	3	3	4	4	3	3
2	2	4	4	3	3	4	4	3	3
2	2	4	4	3	3	4	4	3	3
2	2	4	4	3	3	4	4	2	2
2	2	4	4	3	3	4	4	3	3
2	2	4	4	3	3	4	4	3	3
2	2	4	4	3	3	4	4	3	3
2	2	4	4	3	3	4	4	2	2
2	2	4	4	3	3	4	4	3	3
2	2	4	4	3	3	4	4	3	3
2	2	4	4	3	3	4	4	2	2
2	2	4	4	3	3	4	4	3	3
2	2	4	4	3	3	4	4	3	3
2	2	4	4	3	3	4	4	3	3
2	2	4	4	3	3	4	4	3	3
2	2	4	4	3	3	4	4	3	3
2	2	4	4	3	3	4	4	3	3
2	2	4	4	3	3	4	4	3	3
2	2	4	4	3	3	4	4	3	3
2	2	4	4	3	3	4	4	3	3
2	2	4	4	3	3	4	4	3	3
2	2	4	4	2	2	4	4	3	3
2	2	4	4	3	3	4	4	3	3
2	2	4	4	3	3	4	4	3	3
2	2	4	4	3	3	4	4	3	3
2	2	4	4	3	3	4	4	3	3
2	2	4	4	3	3	4	4	3	3
2	2	4	4	3	3	4	4	3	3

For Review Only

2	2	4	4	3	3	4	4	3	3
2	2	4	4	3	3	4	4	2	2

For Review Only

SNP1263	SNP1263	SNP1264	SNP1264	SNP1265	SNP1265	SNP1266	SNP1266	SNP1267	SNP1267
1	1	1	1	1	1	4	4	2	2
1	1	1	1	2	2	1	1	2	2
1	1	1	1	1	1	1	1	2	2
1	1	1	1	1	1	1	1	2	2
1	1	4	4	1	1	4	4	2	2
1	1	1	1	1	1	1	1	2	2
1	1	1	1	2	2	1	1	2	2
1	1	1	1	1	1	1	1	2	2
1	1	1	1	1	1	1	1	2	2
1	1	4	4	2	2	1	1	2	2
1	1	4	4	1	1	4	4	2	2
1	1	1	1	1	1	1	1	2	2
1	1	1	1	1	1	1	1	2	2
1	1	4	4	1	1	1	1	2	2
3	3	1	1	1	1	1	1	2	2
1	1	1	1	1	1	1	1	2	2
1	1	1	1	1	1	1	1	2	2
1	1	1	1	2	2	1	1	2	2
1	1	1	1	1	1	4	4	2	2
1	1	4	4	2	2	1	1	2	2
1	1	4	4	2	2	1	1	2	2
1	1	1	1	1	1	1	1	2	2
1	1	1	1	2	2	1	1	2	2
1	1	4	4	1	1	1	1	2	2
1	1	1	1	1	1	4	4	2	2
1	1	1	1	1	1	1	1	2	2
1	1	1	1	1	1	1	1	2	2
1	1	1	1	1	1	1	1	2	2
1	1	1	1	1	1	4	4	2	2
1	1	4	4	2	2	1	1	2	2
1	1	1	1	1	1	1	1	2	2
1	1	1	1	1	1	1	1	2	2
1	1	1	1	1	1	1	1	2	2
1	1	4	4	1	1	1	1	2	2
1	1	1	1	1	1	4	4	2	2
1	1	4	4	1	1	1	1	2	2
1	1	4	4	1	1	1	1	2	2
3	3	4	4	1	1	1	1	2	2
1	1	4	4	1	1	4	4	2	2
1	1	1	1	1	1	4	4	2	2
1	1	4	4	2	2	1	1	2	2
1	1	1	1	2	2	1	1	2	2
1	1	4	4	1	1	4	4	2	2
1	1	4	4	2	2	1	1	2	2
1	1	4	4	2	2	1	1	2	2
1	1	1	1	1	1	1	1	2	2
1	1	1	1	1	1	1	1	2	2
1	1	1	1	1	1	4	4	2	2
1	1	1	1	2	2	1	1	3	3
1	1	1	1	1	1	1	1	2	2
1	1	1	1	1	1	1	1	2	2
1	1	1	1	1	1	4	4	2	2

For Review Only

1	1	1	1	1	1	4	4	2	2
1	1	1	1	1	1	1	1	2	2
1	1	1	1	1	1	1	1	2	2
1	1	1	1	2	2	1	1	2	2
1	1	1	1	1	1	4	4	2	2
1	1	1	1	1	1	1	1	2	2
1	1	1	1	1	1	1	1	2	2
1	1	4	4	1	1	4	4	3	3
1	1	4	4	1	1	1	1	3	3
1	1	1	1	1	1	1	1	2	2
3	3	1	1	1	1	1	1	2	2
3	3	1	1	1	1	1	1	2	2
1	1	1	1	1	1	4	4	2	2
1	1	1	1	1	1	4	4	2	2
1	1	1	1	1	1	1	1	2	2
1	1	1	1	1	1	1	1	3	3
1	1	1	1	2	2	1	1	2	2
3	3	1	1	1	1	1	1	2	2
1	1	1	1	1	1	1	1	2	2
1	1	1	1	1	1	1	1	2	2
3	3	1	1	1	1	1	1	2	2
1	1	4	4	1	1	1	1	2	2
3	3	1	1	1	1	1	1	2	2
1	1	1	1	1	1	1	1	2	2
1	1	4	4	2	2	1	1	2	2
3	3	1	1	1	1	4	4	2	2
1	1	1	1	1	1	4	4	2	2
1	1	4	4	1	1	4	4	2	2
1	1	1	1	1	1	1	1	2	2
1	1	1	1	1	1	1	1	2	2
1	1	4	4	1	1	1	1	2	2
1	1	1	1	1	1	1	1	2	2
1	1	1	1	1	1	4	4	2	2
1	1	1	1	1	1	4	4	2	2
1	1	1	1	1	1	4	4	2	2
1	1	4	4	2	2	1	1	2	2
1	1	4	4	2	2	1	1	2	2
1	1	1	1	2	2	1	1	2	2
1	1	4	4	2	2	1	1	3	3
1	1	4	4	2	2	1	1	2	2
1	1	1	1	1	1	4	4	2	2
1	1	1	1	1	1	1	1	2	2
1	1	1	1	2	2	1	1	2	2
3	3	1	1	1	1	1	1	2	2
1	1	4	4	1	1	1	1	2	2
3	3	1	1	1	1	1	1	2	2
1	1	4	4	1	1	4	4	2	2
1	1	1	1	1	1	1	1	2	2
1	1	1	1	1	1	1	1	2	2
3	3	1	1	1	1	1	1	2	2
1	1	1	1	1	1	1	1	2	2
1	1	4	4	1	1	4	4	2	2
1	1	4	4	1	1	4	4	2	2
1	1	1	1	1	1	4	4	2	2
1	1	1	1	1	1	4	4	2	2

For Review Only

1	1	1	1	1	1	1	1	2	2
1	1	1	1	1	1	4	4	2	2
1	1	4	4	1	1	1	1	2	2
1	1	4	4	1	1	4	4	2	2
3	3	1	1	1	1	1	1	2	2
1	1	1	1	1	1	1	1	2	2
1	1	1	1	1	1	1	1	2	2
1	1	1	1	1	1	1	1	2	2
1	1	1	1	2	2	1	1	3	3
1	1	1	1	1	1	1	1	2	2
1	1	1	1	1	1	4	4	2	2
1	1	1	1	1	1	1	1	2	2
1	1	1	1	2	2	1	1	2	2
3	3	1	1	1	1	4	4	2	2
1	1	1	1	2	2	1	1	2	2
1	1	1	1	1	1	1	1	2	2
3	3	1	1	1	1	1	1	3	3
1	1	1	1	1	1	1	1	2	2
1	1	4	4	1	1	4	4	2	2
1	1	1	1	1	1	1	1	2	2
1	1	1	1	1	1	1	1	2	2
1	1	1	1	1	1	1	1	2	2
1	1	1	1	1	1	1	1	2	2
1	1	1	1	1	1	4	4	2	2
1	1	1	1	1	1	1	1	2	2
1	1	4	4	1	1	1	1	3	3
1	1	1	1	1	1	1	1	2	2
1	1	1	1	1	1	1	1	2	2
1	1	1	1	1	1	1	1	2	2
1	1	1	1	1	1	1	1	2	2
1	1	1	1	1	1	1	1	2	2
1	1	1	1	1	1	1	1	2	2
1	1	1	1	1	1	4	4	2	2
1	1	1	1	1	1	1	1	2	2
1	1	4	4	1	1	1	1	2	2
1	1	1	1	1	1	1	1	2	2
1	1	4	4	1	1	4	4	2	2
1	1	1	1	1	1	1	1	2	2
1	1	1	1	1	1	1	1	2	2
1	1	4	4	1	1	4	4	2	2
1	1	4	4	1	1	1	1	2	2
1	1	4	4	1	1	1	1	2	2
1	1	1	1	1	1	4	4	2	2
1	1	4	4	1	1	1	1	2	2
3	3	1	1	1	1	1	1	2	2
1	1	1	1	1	1	1	1	3	3
1	1	1	1	1	1	1	1	2	2
1	1	1	1	1	1	1	1	2	2
1	1	4	4	1	1	1	1	2	2
1	1	1	1	1	1	1	1	2	2
1	1	4	4	1	1	1	1	2	2
1	1	4	4	1	1	4	4	3	3
1	1	1	1	1	1	1	1	2	2
1	1	1	1	1	1	1	1	2	2
1	1	4	4	1	1	1	1	2	2

For Review Only

1	1	1	1	1	1	1	1	2	2
3	3	4	4	1	1	1	1	2	2
1	1	4	4	2	2	1	1	2	2
1	1	1	1	1	1	1	1	2	2
1	1	1	1	1	1	4	4	2	2
1	1	4	4	1	1	4	4	2	2
1	1	4	4	1	1	4	4	2	2
1	1	1	1	1	1	1	1	2	2
1	1	4	4	2	2	1	1	2	2
1	1	1	1	1	1	1	1	2	2
1	1	1	1	1	1	1	1	2	2
1	1	1	1	1	1	1	1	2	2
1	1	1	1	1	1	1	1	2	2
1	1	1	1	1	1	1	1	2	2
1	1	4	4	1	1	1	1	2	2
1	1	4	4	1	1	4	4	2	2
1	1	1	1	1	1	1	1	2	2
3	3	1	1	1	1	4	4	2	2
1	1	1	1	1	1	1	1	2	2
1	1	1	1	1	1	1	1	2	2
1	1	1	1	1	1	1	1	2	2
1	1	1	1	1	1	4	4	2	2
3	3	1	1	1	1	1	1	2	2
3	3	1	1	1	1	1	1	2	2
1	1	1	1	1	1	1	1	2	2
1	1	4	4	1	1	1	1	2	2
1	1	1	1	1	1	1	1	2	2
1	1	1	1	1	1	1	1	2	2
3	3	1	1	1	1	1	1	3	3
1	1	1	1	1	1	1	1	2	2
1	1	1	1	1	1	4	4	2	2
1	1	1	1	1	1	1	1	2	2
1	1	1	1	1	1	1	1	2	2
1	1	4	4	1	1	4	4	2	2
1	1	1	1	1	1	1	1	2	2
3	3	1	1	1	1	1	1	2	2
1	1	4	4	1	1	1	1	2	2
1	1	1	1	1	1	1	1	2	2
1	1	1	1	1	1	1	1	2	2
1	1	1	1	1	1	1	1	2	2
1	1	1	1	1	1	1	1	2	2
1	1	1	1	1	1	1	1	2	2
1	1	1	1	1	1	4	4	2	2
1	1	4	4	1	1	1	1	2	2
1	1	1	1	1	1	4	4	2	2
1	1	1	1	1	1	1	1	2	2
1	1	4	4	1	1	4	4	2	2
1	1	4	4	1	1	4	4	2	2
1	1	1	1	1	1	1	1	2	2
1	1	1	1	2	2	1	1	2	2
1	1	1	1	1	1	1	1	2	2
1	1	4	4	1	1	4	4	2	2
3	3	1	1	1	1	1	1	2	2
3	3	1	1	1	1	1	1	2	2
1	1	4	4	1	1	4	4	2	2

For Review Only

1	1	1	1	1	1	1	1	2	2
1	1	4	4	1	1	4	4	2	2
1	1	1	1	1	1	1	1	2	2
1	1	1	1	1	1	1	1	2	2
1	1	1	1	2	2	1	1	2	2
1	1	1	1	1	1	1	1	2	2
1	1	4	4	1	1	4	4	2	2
1	1	4	4	2	2	1	1	2	2
1	1	4	4	1	1	1	1	2	2
1	1	1	1	1	1	4	4	2	2
1	1	1	1	1	1	1	1	2	2
1	1	1	1	1	1	4	4	2	2
1	1	4	4	2	2	1	1	3	3
1	1	1	1	1	1	1	1	2	2
1	1	1	1	1	1	1	1	2	2
1	1	4	4	2	2	1	1	2	2
1	1	4	4	1	1	1	1	2	2
1	1	1	1	1	1	1	1	2	2
1	1	1	1	1	1	4	4	2	2
3	3	4	4	1	1	1	1	2	2
1	1	1	1	2	2	1	1	2	2
1	1	1	1	1	1	1	1	2	2
1	1	1	1	1	1	1	1	2	2
1	1	4	4	1	1	4	4	2	2
1	1	4	4	1	1	4	4	3	3
1	1	1	1	1	1	4	4	2	2
1	1	1	1	1	1	4	4	2	2
1	1	1	1	1	1	1	1	2	2
1	1	1	1	1	1	4	4	2	2
1	1	1	1	1	1	1	1	2	2
3	3	1	1	1	1	1	1	2	2
1	1	4	4	1	1	1	1	2	2
1	1	4	4	1	1	1	1	2	2
1	1	1	1	1	1	1	1	2	2
3	3	4	4	1	1	1	1	2	2
1	1	1	1	1	1	4	4	2	2
1	1	1	1	1	1	1	1	2	2
3	3	1	1	1	1	1	1	2	2
1	1	4	4	1	1	1	1	2	2
1	1	4	4	1	1	4	4	2	2
1	1	1	1	1	1	1	1	2	2
1	1	1	1	1	1	1	1	3	3
1	1	4	4	1	1	4	4	2	2
1	1	1	1	1	1	1	1	2	2
1	1	1	1	1	1	1	1	2	2
1	1	1	1	2	2	1	1	2	2
1	1	4	4	1	1	1	1	2	2
1	1	1	1	2	2	1	1	2	2
1	1	1	1	1	1	1	1	2	2
1	1	1	1	1	1	4	4	2	2
1	1	1	1	1	1	1	1	2	2
1	1	4	4	1	1	4	4	2	2
1	1	1	1	1	1	1	1	2	2
1	1	1	1	1	1	1	1	2	2
1	1	4	4	1	1	4	4	2	2

For Review Only

1	1	1	1	1	1	4	4	2	2
3	3	1	1	1	1	4	4	2	2

For Review Only

SNP1268	SNP1268	SNP1269	SNP1269	SNP127	SNP127	SNP1270	SNP1270	SNP1271	SNP1271
3	3	1	1	1	1	4	4	4	4
2	2	1	1	3	3	1	1	4	4
2	2	4	4	1	1	4	4	4	4
2	2	1	1	1	1	4	4	4	4
3	3	1	1	1	1	4	4	4	4
3	3	1	1	1	1	4	4	4	4
3	3	1	1	1	1	1	1	4	4
2	2	1	1	1	1	1	1	1	1
3	3	1	1	1	1	4	4	4	4
2	2	1	1	1	1	1	1	4	4
3	3	4	4	1	1	4	4	4	4
2	2	1	1	1	1	4	4	4	4
2	2	1	1	1	1	4	4	4	4
3	3	1	1	1	1	4	4	4	4
2	2	1	1	1	1	4	4	4	4
2	2	4	4	1	1	4	4	4	4
3	3	1	1	1	1	4	4	1	1
3	3	1	1	1	1	1	1	4	4
3	3	1	1	1	1	4	4	4	4
2	2	1	1	1	1	1	1	4	4
2	2	1	1	1	1	1	1	4	4
3	3	1	1	1	1	4	4	4	4
2	2	1	1	3	3	1	1	4	4
2	2	1	1	1	1	4	4	4	4
2	2	4	4	1	1	4	4	4	4
2	2	1	1	1	1	4	4	4	4
2	2	1	1	3	3	4	4	4	4
2	2	1	1	1	1	4	4	4	4
3	3	1	1	1	1	4	4	4	4
2	2	1	1	1	1	1	1	1	1
3	3	1	1	1	1	4	4	4	4
3	3	4	4	1	1	4	4	4	4
3	3	1	1	1	1	4	4	4	4
2	2	4	4	1	1	4	4	4	4
2	2	1	1	1	1	4	4	4	4
2	2	1	1	1	1	4	4	4	4
2	2	1	1	1	1	4	4	4	4
2	2	1	1	1	1	4	4	4	4
3	3	4	4	1	1	4	4	4	4
2	2	1	1	1	1	4	4	4	4
2	2	1	1	1	1	1	1	4	4
3	3	1	1	1	1	1	1	1	1
2	2	1	1	1	1	1	1	4	4
3	3	1	1	1	1	1	1	4	4
3	3	1	1	1	1	1	1	4	4
2	2	1	1	1	1	4	4	4	4
2	2	1	1	1	1	4	4	4	4
2	2	1	1	1	1	4	4	4	4
3	3	1	1	1	1	1	1	4	4
2	2	4	4	1	1	4	4	4	4
2	2	4	4	1	1	4	4	4	4
2	2	1	1	1	1	4	4	4	4

For Review Only

3	3	4	4	1	1	4	4	4	4
3	3	1	1	1	1	4	4	4	4
3	3	4	4	1	1	4	4	4	4
3	3	1	1	1	1	1	1	4	4
3	3	1	1	1	1	4	4	4	4
2	2	1	1	1	1	4	4	4	4
2	2	1	1	1	1	4	4	4	4
2	2	4	4	1	1	4	4	4	4
2	2	1	1	1	1	4	4	4	4
2	2	1	1	1	1	4	4	4	4
3	3	1	1	1	1	4	4	4	4
3	3	1	1	1	1	4	4	4	4
3	3	4	4	1	1	4	4	4	4
3	3	1	1	1	1	4	4	4	4
2	2	1	1	1	1	4	4	4	4
2	2	1	1	1	1	4	4	4	4
2	2	1	1	1	1	1	1	4	4
2	2	4	4	1	1	4	4	4	4
3	3	4	4	1	1	4	4	4	4
3	3	4	4	1	1	4	4	4	4
2	2	1	1	1	1	4	4	4	4
2	2	4	4	1	1	4	4	4	4
2	2	1	1	1	1	4	4	4	4
2	2	4	4	1	1	4	4	4	4
3	3	1	1	1	1	1	1	4	4
3	3	1	1	3	3	4	4	4	4
3	3	1	1	1	1	4	4	4	4
3	3	4	4	1	1	4	4	4	4
2	2	1	1	1	1	4	4	4	4
2	2	4	4	1	1	4	4	4	4
2	2	1	1	1	1	4	4	4	4
2	2	1	1	1	1	4	4	4	4
3	3	4	4	1	1	4	4	4	4
3	3	4	4	1	1	4	4	4	4
2	2	4	4	1	1	4	4	4	4
3	3	1	1	1	1	1	1	4	4
2	2	1	1	1	1	1	1	1	1
2	2	1	1	1	1	1	1	4	4
2	2	1	1	1	1	1	1	4	4
3	3	1	1	1	1	1	1	1	1
3	3	4	4	1	1	4	4	4	4
2	2	4	4	1	1	4	4	4	4
2	2	4	4	1	1	1	1	1	1
3	3	1	1	1	1	4	4	4	4
2	2	1	1	1	1	4	4	4	4
2	2	1	1	3	3	4	4	4	4
2	2	4	4	1	1	4	4	4	4
2	2	1	1	1	1	4	4	4	4
2	2	1	1	1	1	4	4	4	4
3	3	1	1	1	1	4	4	4	4
2	2	1	1	1	1	4	4	4	4
3	3	4	4	1	1	4	4	4	4
2	2	1	1	1	1	4	4	4	4
2	2	1	1	1	1	4	4	4	4
3	3	1	1	1	1	4	4	4	4

For Review Only

2	2	1	1	3	3	4	4	4	4
2	2	1	1	3	3	4	4	4	4
2	2	1	1	1	1	4	4	4	4
2	2	4	4	1	1	4	4	4	4
2	2	1	1	3	3	4	4	4	4
2	2	1	1	1	1	4	4	4	4
2	2	4	4	1	1	4	4	4	4
2	2	1	1	3	3	4	4	4	4
2	2	1	1	1	1	1	1	1	1
2	2	1	1	1	1	4	4	4	4
3	3	1	1	1	1	4	4	4	4
2	2	1	1	1	1	4	4	4	4
2	2	1	1	1	1	1	1	4	4
2	2	1	1	1	1	4	4	4	4
3	3	1	1	3	3	1	1	4	4
2	2	1	1	1	1	4	4	4	4
2	2	4	4	1	1	4	4	4	4
2	2	1	1	1	1	4	4	4	4
2	2	4	4	1	1	4	4	4	4
2	2	4	4	1	1	4	4	4	4
2	2	1	1	1	1	4	4	4	4
2	2	1	1	1	1	4	4	4	4
2	2	1	1	1	1	4	4	4	4
3	3	1	1	1	1	4	4	4	4
2	2	4	4	1	1	4	4	4	4
2	2	4	4	3	3	4	4	4	4
3	3	4	4	1	1	4	4	4	4
2	2	1	1	1	1	4	4	4	4
2	2	1	1	1	1	4	4	4	4
3	3	4	4	1	1	4	4	4	4
3	3	1	1	1	1	4	4	4	4
2	2	1	1	1	1	4	4	4	4
3	3	4	4	1	1	4	4	4	4
2	2	1	1	1	1	4	4	4	4
2	2	4	4	1	1	4	4	4	4
3	3	1	1	1	1	4	4	4	4
2	2	4	4	1	1	4	4	4	4
3	3	1	1	3	3	4	4	4	4
2	2	1	1	1	1	4	4	4	4
3	3	1	1	1	1	4	4	4	4
2	2	1	1	1	1	4	4	4	4
2	2	1	1	1	1	4	4	4	4
3	3	1	1	1	1	4	4	4	4
2	2	1	1	1	1	1	1	1	1
2	2	1	1	1	1	4	4	4	4
2	2	1	1	1	1	4	4	4	4
2	2	1	1	1	1	4	4	4	4
2	2	1	1	1	1	4	4	4	4
2	2	1	1	1	1	4	4	4	4
2	2	1	1	1	1	4	4	4	4
2	2	1	1	1	1	4	4	4	4
2	2	4	4	1	1	4	4	4	4
3	3	4	4	1	1	4	4	4	4
3	3	1	1	1	1	4	4	4	4
2	2	1	1	1	1	4	4	4	4

For Review Only

2	2	1	1	1	1	4	4	4	4
2	2	1	1	3	3	1	1	4	4
3	3	1	1	1	1	1	1	4	4
2	2	1	1	1	1	4	4	4	4
2	2	1	1	3	3	1	1	4	4
3	3	4	4	1	1	4	4	4	4
3	3	1	1	1	1	4	4	4	4
2	2	1	1	1	1	4	4	4	4
2	2	4	4	3	3	1	1	4	4
3	3	4	4	1	1	4	4	4	4
2	2	1	1	1	1	4	4	4	4
2	2	1	1	1	1	4	4	4	4
2	2	1	1	1	1	4	4	4	4
2	2	1	1	1	1	4	4	4	4
2	2	4	4	1	1	4	4	4	4
3	3	4	4	1	1	4	4	4	4
3	3	1	1	1	1	4	4	4	4
2	2	1	1	3	3	4	4	4	4
2	2	1	1	1	1	4	4	4	4
2	2	1	1	1	1	4	4	4	4
2	2	1	1	1	1	4	4	4	4
2	2	4	4	1	1	4	4	4	4
2	2	4	4	1	1	4	4	4	4
3	3	1	1	1	1	4	4	4	4
2	2	1	1	1	1	4	4	1	1
2	2	4	4	1	1	4	4	4	4
2	2	1	1	1	1	4	4	4	4
2	2	1	1	1	1	4	4	4	4
2	2	4	4	1	1	4	4	4	4
3	3	4	4	1	1	4	4	4	4
2	2	1	1	1	1	4	4	4	4
2	2	4	4	1	1	4	4	4	4
3	3	1	1	1	1	4	4	4	4
2	2	1	1	1	1	4	4	4	4
2	2	4	4	1	1	4	4	4	4
3	3	1	1	1	1	4	4	4	4
2	2	1	1	1	1	4	4	4	4
2	2	1	1	1	1	4	4	4	4
2	2	1	1	1	1	4	4	4	4
2	2	4	4	1	1	4	4	4	4
2	2	4	4	1	1	4	4	4	4
2	2	1	1	1	1	4	4	4	4
2	2	4	4	1	1	4	4	4	4
2	2	4	4	1	1	4	4	4	4
3	3	1	1	1	1	4	4	4	4
2	2	4	4	1	1	4	4	4	4
3	3	4	4	1	1	4	4	4	4
3	3	4	4	1	1	4	4	4	4
2	2	1	1	1	1	4	4	4	4
3	3	1	1	1	1	1	1	1	1
2	2	1	1	1	1	4	4	4	4
2	2	1	1	1	1	4	4	4	4
3	3	4	4	1	1	4	4	4	4
3	3	1	1	1	1	4	4	4	4
2	2	4	4	1	1	4	4	4	4

For Review Only

3	3	4	4	1	1	4	4	4	4
2	2	1	1	1	1	4	4	4	4
2	2	1	1	1	1	4	4	4	4
2	2	4	4	1	1	4	4	4	4
2	2	1	1	1	1	1	1	1	1
2	2	1	1	1	1	4	4	4	4
3	3	4	4	1	1	4	4	4	4
2	2	1	1	1	1	1	1	4	4
3	3	1	1	1	1	4	4	4	4
3	3	4	4	1	1	4	4	4	4
2	2	1	1	1	1	4	4	4	4
2	2	1	1	1	1	4	4	4	4
3	3	1	1	1	1	1	1	4	4
2	2	1	1	1	1	4	4	4	4
2	2	4	4	1	1	4	4	4	4
3	3	1	1	1	1	1	1	4	4
2	2	4	4	3	3	4	4	4	4
2	2	4	4	1	1	4	4	4	4
2	2	4	4	1	1	4	4	4	4
2	2	1	1	1	1	4	4	4	4
2	2	4	4	3	3	1	1	4	4
2	2	1	1	1	1	4	4	4	4
3	3	4	4	1	1	4	4	4	4
3	3	4	4	1	1	4	4	4	4
2	2	4	4	1	1	4	4	4	4
2	2	1	1	1	1	4	4	1	1
3	3	4	4	1	1	4	4	4	4
2	2	1	1	1	1	4	4	4	4
2	2	1	1	1	1	4	4	1	1
2	2	1	1	1	1	4	4	4	4
3	3	4	4	1	1	4	4	4	4
2	2	1	1	1	1	4	4	4	4
2	2	1	1	1	1	4	4	4	4
2	2	1	1	1	1	4	4	4	4
3	3	4	4	1	1	4	4	4	4
3	3	4	4	1	1	4	4	4	4
3	3	4	4	1	1	4	4	4	4
3	3	1	1	1	1	4	4	4	4
2	2	1	1	1	1	4	4	4	4
2	2	4	4	1	1	4	4	4	4
3	3	1	1	1	1	4	4	4	4
2	2	1	1	1	1	4	4	4	4
2	2	1	1	1	1	4	4	4	4
3	3	4	4	1	1	4	4	4	4
2	2	1	1	1	1	4	4	4	4
2	2	1	1	1	1	1	1	4	4
2	2	1	1	1	1	4	4	4	4
2	2	1	1	1	1	1	1	4	4
3	3	1	1	1	1	4	4	1	1
3	3	4	4	1	1	4	4	4	4
3	3	4	4	1	1	4	4	4	4
3	3	4	4	1	1	4	4	4	4
2	2	4	4	1	1	4	4	4	4
2	2	1	1	1	1	4	4	4	4
2	2	4	4	1	1	4	4	4	4

For Review Only

3	3	1	1	1	1	4	4	4	4
3	3	1	1	1	1	4	4	4	4

For Review Only

SNP1272	SNP1272	SNP1273	SNP1273	SNP1274	SNP1274	SNP1275	SNP1275	SNP1276	SNP1276
4	4	1	1	1	1	3	3	4	4
4	4	4	4	4	4	3	3	4	4
4	4	4	4	4	4	3	3	1	1
4	4	1	1	1	1	3	3	1	1
1	1	1	1	1	1	3	3	1	1
4	4	1	1	4	4	4	4	1	1
4	4	1	1	4	4	3	3	4	4
4	4	1	1	4	4	3	3	1	1
4	4	4	4	4	4	3	3	1	1
1	1	1	1	1	1	3	3	1	1
4	4	4	4	1	1	3	3	1	1
4	4	4	4	4	4	3	3	1	1
4	4	4	4	1	1	3	3	1	1
4	4	4	4	1	1	4	4	1	1
4	4	4	4	4	4	3	3	1	1
4	4	4	4	1	1	3	3	1	1
4	4	1	1	4	4	3	3	1	1
4	4	1	1	1	1	3	3	1	1
4	4	1	1	4	4	3	3	4	4
4	4	1	1	1	1	3	3	4	4
4	4	1	1	1	1	3	3	1	1
4	4	1	1	4	4	3	3	1	1
4	4	1	1	1	1	3	3	4	4
4	4	1	1	1	1	3	3	4	4
4	4	4	4	4	4	3	3	1	1
4	4	1	1	1	1	4	4	1	1
4	4	1	1	4	4	4	4	1	1
4	4	4	4	1	1	3	3	1	1
4	4	1	1	4	4	3	3	1	1
4	4	1	1	1	1	3	3	1	1
4	4	4	4	1	1	4	4	1	1
4	4	4	4	1	1	3	3	1	1
4	4	1	1	1	1	3	3	4	4
4	4	4	4	1	1	3	3	4	4
4	4	4	4	1	1	3	3	1	1
4	4	1	1	1	1	4	4	1	1
4	4	1	1	1	1	3	3	1	1
4	4	1	1	1	1	3	3	1	1
4	4	4	4	1	1	3	3	4	4
4	4	1	1	1	1	3	3	1	1
1	1	1	1	1	1	3	3	1	1
4	4	1	1	1	1	3	3	1	1
1	1	1	1	1	1	3	3	1	1
4	4	1	1	1	1	3	3	1	1
1	1	1	1	1	1	3	3	1	1
4	4	4	4	1	1	4	4	1	1
4	4	1	1	4	4	3	3	1	1
4	4	1	1	1	1	4	4	1	1
4	4	1	1	4	4	3	3	1	1
4	4	4	4	1	1	3	3	4	4
4	4	4	4	1	1	3	3	4	4
4	4	1	1	4	4	3	3	4	4

For Review Only

4	4	4	4	4	4	3	3	1	1
4	4	1	1	1	1	3	3	4	4
4	4	4	4	4	4	3	3	1	1
4	4	1	1	4	4	3	3	1	1
4	4	1	1	1	1	3	3	4	4
4	4	1	1	4	4	4	4	1	1
4	4	1	1	1	1	4	4	1	1
4	4	4	4	1	1	3	3	4	4
4	4	1	1	1	1	3	3	1	1
4	4	4	4	4	4	3	3	1	1
4	4	1	1	4	4	3	3	4	4
4	4	1	1	4	4	3	3	4	4
4	4	4	4	4	4	3	3	1	1
4	4	1	1	4	4	3	3	1	1
4	4	4	4	4	4	4	4	1	1
1	1	1	1	1	1	3	3	1	1
4	4	4	4	1	1	3	3	1	1
4	4	4	4	1	1	3	3	1	1
4	4	4	4	4	4	3	3	4	4
4	4	4	4	4	4	3	3	4	4
4	4	1	1	1	1	3	3	4	4
4	4	4	4	1	1	3	3	4	4
4	4	1	1	1	1	3	3	1	1
4	4	4	4	1	1	3	3	1	1
1	1	1	1	1	1	3	3	1	1
4	4	1	1	4	4	3	3	4	4
4	4	4	4	4	4	3	3	1	1
4	4	1	1	1	1	3	3	4	4
4	4	1	1	1	1	4	4	1	1
4	4	4	4	1	1	3	3	1	1
4	4	1	1	1	1	3	3	1	1
4	4	1	1	1	1	3	3	4	4
4	4	4	4	1	1	3	3	4	4
4	4	4	4	4	4	3	3	1	1
4	4	4	4	1	1	3	3	1	1
1	1	1	1	1	1	3	3	1	1
4	4	1	1	1	1	3	3	1	1
4	4	1	1	4	4	3	3	1	1
4	4	1	1	1	1	4	4	1	1
4	4	1	1	1	1	3	3	1	1
4	4	4	4	4	4	3	3	1	1
4	4	4	4	4	4	3	3	1	1
4	4	4	4	4	4	3	3	4	4
4	4	1	1	4	4	3	3	4	4
4	4	1	1	1	1	3	3	1	1
4	4	4	4	4	4	3	3	1	1
4	4	4	4	1	1	3	3	4	4
4	4	1	1	1	1	4	4	1	1
4	4	1	1	1	1	4	4	1	1
4	4	4	4	4	4	3	3	1	1
4	4	1	1	4	4	4	4	1	1
4	4	4	4	1	1	3	3	1	1
4	4	1	1	1	1	3	3	1	1
4	4	4	4	1	1	3	3	1	1
4	4	1	1	4	4	3	3	4	4

For Review Only

4	4	1	1	1	1	4	4	1	1
4	4	1	1	1	1	4	4	1	1
4	4	1	1	1	1	4	4	1	1
4	4	4	4	1	1	3	3	1	1
4	4	1	1	4	4	3	3	1	1
4	4	1	1	1	1	3	3	1	1
4	4	4	4	4	4	3	3	1	1
4	4	1	1	1	1	3	3	1	1
1	1	1	1	1	1	4	4	1	1
4	4	4	4	1	1	3	3	1	1
4	4	4	4	1	1	3	3	4	4
4	4	4	4	1	1	3	3	1	1
4	4	1	1	4	4	4	4	1	1
4	4	1	1	4	4	3	3	4	4
4	4	1	1	1	1	3	3	4	4
4	4	4	4	1	1	3	3	1	1
4	4	4	4	4	4	3	3	1	1
4	4	4	4	1	1	3	3	1	1
4	4	4	4	1	1	3	3	1	1
4	4	4	4	4	4	3	3	1	1
4	4	4	4	1	1	3	3	1	1
4	4	4	4	4	4	3	3	1	1
4	4	1	1	4	4	3	3	4	4
4	4	1	1	1	1	3	3	4	4
4	4	1	1	1	1	3	3	1	1
4	4	4	4	1	1	3	3	4	4
4	4	4	4	4	4	3	3	1	1
4	4	1	1	4	4	3	3	4	4
4	4	1	1	4	4	3	3	1	1
4	4	4	4	4	4	3	3	1	1
4	4	1	1	1	1	3	3	4	4
4	4	4	4	1	1	3	3	1	1
4	4	4	4	4	4	3	3	1	1
4	4	1	1	1	1	4	4	1	1
4	4	4	4	1	1	3	3	4	4
4	4	4	4	1	1	3	3	1	1
4	4	4	4	1	1	3	3	1	1
4	4	1	1	4	4	4	4	1	1
4	4	4	4	1	1	4	4	1	1
4	4	1	1	1	1	3	3	4	4
4	4	4	4	1	1	3	3	1	1
4	4	1	1	1	1	3	3	1	1
4	4	1	1	4	4	4	4	1	1
4	4	1	1	1	1	3	3	1	1
4	4	1	1	1	1	4	4	1	1
4	4	1	1	4	4	3	3	1	1
4	4	1	1	1	1	4	4	1	1
4	4	1	1	1	1	4	4	1	1
4	4	1	1	1	1	3	3	1	1
4	4	1	1	1	1	4	4	1	1
4	4	1	1	1	1	4	4	1	1
4	4	4	4	1	1	3	3	4	4
4	4	4	4	1	1	3	3	1	1
4	4	1	1	4	4	4	4	1	1
4	4	4	4	1	1	3	3	4	4

For Review Only

4	4	4	4	4	4	3	3	1	1
4	4	1	1	1	1	4	4	1	1
1	1	1	1	1	1	3	3	1	1
4	4	4	4	1	1	3	3	1	1
4	4	1	1	4	4	3	3	4	4
4	4	4	4	1	1	3	3	1	1
4	4	4	4	1	1	3	3	4	4
4	4	4	4	1	1	3	3	1	1
4	4	4	4	1	1	3	3	1	1
4	4	4	4	4	4	3	3	1	1
4	4	1	1	1	1	3	3	1	1
4	4	4	4	1	1	3	3	4	4
4	4	4	4	1	1	4	4	1	1
4	4	1	1	1	1	3	3	1	1
4	4	1	1	1	1	3	3	4	4
4	4	1	1	1	1	3	3	4	4
4	4	4	4	1	1	3	3	1	1
4	4	1	1	4	4	3	3	4	4
4	4	1	1	1	1	3	3	4	4
4	4	4	4	4	4	3	3	1	1
4	4	4	4	4	4	3	3	1	1
4	4	4	4	1	1	3	3	1	1
4	4	4	4	1	1	3	3	4	4
4	4	1	1	4	4	3	3	4	4
4	4	1	1	4	4	3	3	1	1
4	4	4	4	1	1	3	3	4	4
4	4	1	1	4	4	4	4	1	1
4	4	4	4	1	1	3	3	1	1
4	4	4	4	1	1	3	3	1	1
4	4	4	4	4	4	3	3	1	1
4	4	1	1	1	1	3	3	4	4
4	4	4	4	1	1	3	3	1	1
4	4	1	1	4	4	3	3	4	4
4	4	1	1	1	1	3	3	4	4
4	4	4	4	1	1	3	3	4	4
4	4	1	1	4	4	3	3	4	4
4	4	1	1	1	1	4	4	1	1
4	4	1	1	1	1	3	3	1	1
4	4	1	1	1	1	4	4	1	1
4	4	4	4	1	1	3	3	4	4
4	4	4	4	4	4	3	3	1	1
4	4	1	1	1	1	4	4	1	1
4	4	4	4	1	1	3	3	1	1
4	4	4	4	1	1	3	3	1	1
4	4	1	1	1	1	3	3	4	4
4	4	4	4	4	4	3	3	1	1
4	4	1	1	1	1	3	3	4	4
4	4	4	4	1	1	3	3	4	4
4	4	1	1	1	1	3	3	4	4
4	4	1	1	4	4	3	3	1	1
4	4	1	1	4	4	3	3	4	4
4	4	1	1	1	1	3	3	1	1
4	4	4	4	1	1	3	3	4	4
4	4	1	1	4	4	3	3	4	4
4	4	4	4	1	1	3	3	1	1

For Review Only

4	4	4	4	1	1	3	3	1	1
1	1	4	4	1	1	3	3	1	1
4	4	4	4	4	4	3	3	1	1
4	4	4	4	1	1	3	3	1	1
4	4	1	1	1	1	3	3	4	4
4	4	1	1	1	1	3	3	1	1
4	4	4	4	1	1	3	3	4	4
1	1	1	1	1	1	3	3	1	1
4	4	1	1	1	1	3	3	1	1
4	4	1	1	1	1	3	3	4	4
4	4	4	4	4	4	3	3	1	1
4	4	1	1	1	1	4	4	1	1
4	4	1	1	1	1	4	4	1	1
4	4	1	1	1	1	3	3	4	4
4	4	4	4	4	4	3	3	1	1
4	4	1	1	1	1	3	3	4	4
4	4	4	4	1	1	3	3	1	1
4	4	4	4	4	4	3	3	4	4
4	4	4	4	1	1	3	3	1	1
4	4	1	1	1	1	3	3	1	1
4	4	4	4	1	1	3	3	1	1
4	4	1	1	1	1	4	4	1	1
4	4	4	4	4	4	3	3	1	1
4	4	4	4	1	1	3	3	4	4
4	4	4	4	1	1	3	3	4	4
4	4	4	4	1	1	3	3	4	4
4	4	4	4	1	1	3	3	4	4
4	4	1	1	1	1	4	4	1	1
4	4	4	4	1	1	3	3	1	1
4	4	1	1	4	4	3	3	4	4
4	4	4	4	1	1	3	3	4	4
4	4	4	4	1	1	3	3	1	1
4	4	1	1	1	1	3	3	4	4
4	4	1	1	1	1	4	4	1	1
4	4	4	4	1	1	3	3	4	4
4	4	4	4	1	1	3	3	4	4
4	4	4	4	1	1	3	3	1	1
4	4	1	1	4	4	3	3	4	4
4	4	1	1	1	1	4	4	1	1
4	4	4	4	1	1	3	3	4	4
4	4	1	1	4	4	4	4	1	1
4	4	1	1	1	1	3	3	4	4
4	4	1	1	1	1	3	3	4	4
4	4	4	4	4	4	3	3	1	1
4	4	1	1	1	1	3	3	1	1
4	4	1	1	4	4	4	4	1	1
4	4	1	1	1	1	3	3	1	1
4	4	1	1	4	4	4	4	1	1
4	4	1	1	4	4	4	4	1	1
4	4	4	4	1	1	3	3	4	4
4	4	4	4	4	4	3	3	4	4
4	4	1	1	1	1	3	3	4	4
4	4	4	4	4	4	3	3	1	1
4	4	1	1	4	4	4	4	1	1
4	4	4	4	1	1	3	3	4	4

For Review Only

4	4	1	1	4	4	3	3	4	4
4	4	1	1	4	4	4	4	1	1

For Review Only

SNP1277	SNP1277	SNP1278	SNP1278	SNP1279	SNP1279	SNP128	SNP128	SNP1280	SNP1280
1	1	1	1	3	3	3	3	3	3
1	1	4	4	3	3	3	3	3	3
1	1	4	4	3	3	3	3	2	2
1	1	1	1	3	3	3	3	3	3
1	1	4	4	3	3	3	3	3	3
1	1	4	4	2	2	2	2	3	3
1	1	4	4	3	3	3	3	3	3
1	1	4	4	3	3	2	2	3	3
4	4	4	4	3	3	3	3	3	3
4	4	4	4	3	3	3	3	3	3
1	1	4	4	3	3	3	3	3	3
1	1	4	4	3	3	3	3	3	3
1	1	1	1	3	3	3	3	3	3
1	1	4	4	2	2	2	2	3	3
1	1	4	4	3	3	3	3	3	3
1	1	4	4	3	3	3	3	3	3
4	4	4	4	3	3	2	2	3	3
4	4	1	1	3	3	3	3	3	3
1	1	4	4	3	3	3	3	3	3
1	1	4	4	3	3	3	3	3	3
1	1	4	4	3	3	2	2	3	3
4	4	4	4	3	3	3	3	3	3
1	1	4	4	3	3	2	2	3	3
1	1	4	4	3	3	2	2	3	3
1	1	4	4	3	3	3	3	2	2
1	1	4	4	2	2	3	3	3	3
1	1	4	4	2	2	3	3	3	3
1	1	4	4	3	3	3	3	3	3
4	4	4	4	3	3	2	2	3	3
4	4	4	4	3	3	3	3	3	3
1	1	4	4	2	2	3	3	3	3
1	1	1	1	3	3	3	3	2	2
1	1	1	1	3	3	3	3	3	3
1	1	4	4	3	3	2	2	3	3
1	1	4	4	3	3	3	3	3	3
1	1	4	4	3	3	2	2	3	3
1	1	4	4	3	3	2	2	2	2
1	1	4	4	3	3	3	3	3	3
1	1	4	4	3	3	2	2	3	3
1	1	4	4	3	3	3	3	2	2
4	4	4	4	3	3	2	2	3	3
1	1	4	4	3	3	3	3	3	3
4	4	4	4	3	3	2	2	3	3
4	4	4	4	3	3	2	2	3	3
4	4	4	4	3	3	2	2	3	3
1	1	4	4	3	3	3	3	3	3
1	1	4	4	3	3	3	3	3	3
1	1	4	4	2	2	3	3	3	3
4	4	4	4	3	3	2	2	3	3
1	1	4	4	3	3	2	2	2	2
1	1	1	1	3	3	2	2	2	2
1	1	4	4	3	3	3	3	3	3

For Review Only

1	1	4	4	3	3	3	3	2	2
1	1	1	1	3	3	3	3	3	3
1	1	4	4	3	3	3	3	3	3
4	4	4	4	3	3	3	3	3	3
1	1	4	4	3	3	3	3	3	3
1	1	4	4	2	2	3	3	3	3
1	1	4	4	2	2	3	3	3	3
1	1	4	4	3	3	2	2	3	3
1	1	4	4	3	3	2	2	2	2
1	1	4	4	3	3	3	3	3	3
1	1	4	4	3	3	3	3	3	3
1	1	4	4	3	3	3	3	3	3
1	1	4	4	3	3	3	3	2	2
4	4	4	4	3	3	2	2	3	3
1	1	4	4	3	3	3	3	3	3
1	1	4	4	3	3	2	2	3	3
1	1	4	4	3	3	2	2	2	2
1	1	4	4	3	3	3	3	2	2
1	1	4	4	3	3	3	3	3	3
1	1	4	4	3	3	3	3	3	3
1	1	4	4	3	3	3	3	3	3
1	1	4	4	3	3	3	3	3	3
1	1	4	4	3	3	2	2	3	3
1	1	1	1	3	3	3	3	3	3
4	4	4	4	3	3	2	2	3	3
1	1	4	4	3	3	3	3	3	3
1	1	4	4	3	3	3	3	3	3
1	1	4	4	3	3	2	2	3	3
1	1	4	4	2	2	3	3	3	3
1	1	1	1	3	3	3	3	3	3
4	4	4	4	3	3	3	3	3	3
1	1	4	4	3	3	2	2	3	3
1	1	4	4	3	3	2	2	3	3
1	1	4	4	3	3	3	3	2	2
1	1	4	4	3	3	3	3	2	2
4	4	4	4	3	3	2	2	3	3
4	4	4	4	3	3	3	3	3	3
4	4	4	4	3	3	3	3	3	3
1	1	4	4	2	2	2	2	3	3
4	4	4	4	3	3	2	2	3	3
1	1	4	4	3	3	3	3	2	2
1	1	4	4	3	3	3	3	2	2
1	1	4	4	3	3	2	2	2	2
1	1	4	4	3	3	3	3	3	3
1	1	4	4	3	3	2	2	3	3
1	1	4	4	3	3	3	3	2	2
1	1	4	4	3	3	2	2	3	3
1	1	4	4	2	2	2	2	3	3
1	1	4	4	2	2	3	3	3	3
1	1	4	4	3	3	3	3	3	3
1	1	4	4	2	2	3	3	3	3
1	1	4	4	3	3	2	2	3	3
1	1	4	4	3	3	2	2	3	3
1	1	4	4	3	3	3	3	3	3
1	1	4	4	3	3	2	2	3	3

For Review Only

1	1	4	4	2	2	3	3	3	3
1	1	4	4	2	2	3	3	3	3
1	1	4	4	3	3	2	2	3	3
1	1	4	4	3	3	2	2	3	3
1	1	4	4	3	3	3	3	3	3
1	1	4	4	3	3	3	3	2	2
1	1	4	4	3	3	3	3	3	3
1	1	4	4	3	3	3	3	3	3
1	1	4	4	2	2	3	3	3	3
1	1	4	4	3	3	2	2	3	3
1	1	1	1	3	3	3	3	3	3
1	1	4	4	3	3	3	3	3	3
1	1	4	4	2	2	2	2	3	3
1	1	4	4	3	3	3	3	3	3
1	1	4	4	3	3	3	3	3	3
1	1	4	4	3	3	3	3	3	3
1	1	4	4	3	3	3	3	2	2
1	1	4	4	3	3	3	3	3	3
1	1	4	4	3	3	2	2	3	3
1	1	4	4	3	3	3	3	3	3
1	1	4	4	3	3	3	3	3	3
1	1	4	4	3	3	3	3	3	3
1	1	4	4	3	3	3	3	3	3
1	1	4	4	3	3	2	2	3	3
1	1	4	4	3	3	3	3	3	3
1	1	4	4	3	3	3	3	3	3
1	1	4	4	3	3	3	3	2	2
1	1	4	4	3	3	2	2	3	3
1	1	4	4	3	3	3	3	3	3
1	1	4	4	3	3	3	3	3	3
1	1	4	4	3	3	2	2	3	3
1	1	4	4	3	3	3	3	3	3
1	1	4	4	3	3	3	3	2	2
1	1	4	4	3	3	3	3	3	3
1	1	4	4	3	3	2	2	3	3
1	1	4	4	3	3	3	3	3	3
1	1	4	4	3	3	2	2	3	3
1	1	4	4	2	2	3	3	3	3
1	1	4	4	3	3	3	3	3	3
1	1	4	4	3	3	3	3	3	3
1	1	4	4	2	2	3	3	3	3
1	1	4	4	3	3	2	2	3	3
1	1	4	4	2	2	2	2	3	3
1	1	4	4	3	3	2	2	3	3
1	1	4	4	2	2	2	2	3	3
1	1	4	4	3	3	2	2	2	2
1	1	4	4	2	2	3	3	3	3
1	1	4	4	2	2	3	3	3	3
1	1	4	4	3	3	3	3	3	3
1	1	4	4	2	2	3	3	3	3
1	1	4	4	2	2	3	3	3	3
1	1	4	4	3	3	3	3	3	3
1	1	4	4	3	3	3	3	3	3
1	1	4	4	2	2	3	3	3	3
1	1	4	4	3	3	2	2	3	3

For Review Only

1	1	4	4	2	2	2	2	3	3
1	1	4	4	2	2	2	2	3	3
4	4	4	4	3	3	3	3	3	3
1	1	4	4	3	3	3	3	3	3
1	1	4	4	3	3	3	3	3	3
1	1	4	4	3	3	3	3	2	2
1	1	4	4	3	3	3	3	3	3
1	1	4	4	3	3	3	3	3	3
1	1	4	4	3	3	3	3	3	3
1	1	4	4	3	3	3	3	2	2
1	1	4	4	3	3	3	3	3	3
1	1	4	4	3	3	2	2	3	3
1	1	4	4	3	3	3	3	3	3
1	1	4	4	3	3	2	2	3	3
1	1	4	4	3	3	2	2	3	3
1	1	4	4	3	3	3	3	3	3
1	1	4	4	3	3	3	3	3	3
1	1	4	4	3	3	3	3	3	3
1	1	4	4	3	3	2	2	3	3
1	1	4	4	3	3	3	3	3	3
1	1	4	4	3	3	3	3	3	3
1	1	4	4	3	3	2	2	2	2
1	1	4	4	3	3	3	3	3	3
1	1	4	4	3	3	3	3	3	3
4	4	4	4	3	3	2	2	3	3
1	1	4	4	3	3	2	2	3	3
1	1	4	4	2	2	3	3	3	3
1	1	4	4	3	3	3	3	3	3
1	1	4	4	3	3	2	2	2	2
1	1	4	4	3	3	3	3	2	2
1	1	4	4	3	3	2	2	3	3
1	1	1	1	3	3	3	3	3	3
1	1	4	4	3	3	3	3	3	3
1	1	4	4	3	3	2	2	3	3
1	1	4	4	3	3	3	3	3	3
1	1	4	4	3	3	3	3	3	3
1	1	4	4	3	3	3	3	3	3
1	1	1	1	3	3	2	2	2	2
1	1	4	4	2	2	3	3	3	3
1	1	1	1	3	3	2	2	2	2
1	1	4	4	3	3	3	3	2	2
1	1	4	4	2	2	3	3	3	3
1	1	4	4	3	3	2	2	3	3
1	1	4	4	3	3	3	3	3	3
1	1	4	4	3	3	3	3	3	3
1	1	4	4	3	3	2	2	2	2
1	1	4	4	3	3	3	3	3	3
1	1	4	4	3	3	2	2	3	3
1	1	4	4	3	3	3	3	3	3
4	4	4	4	3	3	3	3	3	3
1	1	4	4	3	3	3	3	3	3
1	1	4	4	3	3	2	2	2	2
1	1	4	4	3	3	2	2	3	3
1	1	4	4	3	3	3	3	3	3
1	1	4	4	3	3	2	2	3	3

1	1	4	4	3	3	3	3	2	2
1	1	4	4	3	3	3	3	2	2
1	1	4	4	3	3	3	3	3	3
1	1	4	4	3	3	3	3	3	3
1	1	4	4	3	3	3	3	3	3
1	1	1	1	3	3	3	3	3	3
1	1	4	4	3	3	3	3	3	3
4	4	4	4	3	3	3	3	3	3
1	1	4	4	3	3	2	2	3	3
1	1	4	4	3	3	2	2	3	3
1	1	4	4	3	3	3	3	3	3
1	1	4	4	3	3	2	2	3	3
1	1	4	4	2	2	3	3	3	3
1	1	4	4	3	3	3	3	3	3
1	1	4	4	3	3	3	3	2	2
1	1	4	4	3	3	3	3	3	3
1	1	4	4	3	3	2	2	3	3
1	1	4	4	3	3	3	3	3	3
1	1	4	4	3	3	2	2	3	3
1	1	4	4	3	3	3	3	3	3
1	1	4	4	3	3	3	3	2	2
1	1	4	4	2	2	3	3	3	3
1	1	4	4	3	3	3	3	2	2
1	1	4	4	3	3	2	2	2	2
1	1	4	4	3	3	2	2	3	3
1	1	1	1	3	3	3	3	3	3
1	1	4	4	3	3	2	2	3	3
1	1	4	4	2	2	3	3	3	3
1	1	4	4	3	3	3	3	3	3
1	1	4	4	3	3	3	3	3	3
1	1	4	4	3	3	2	2	3	3
1	1	4	4	3	3	3	3	3	3
1	1	4	4	3	3	2	2	3	3
1	1	4	4	2	2	3	3	3	3
1	1	4	4	3	3	3	3	3	3
1	1	4	4	3	3	2	2	3	3
1	1	4	4	3	3	3	3	2	2
1	1	4	4	3	3	3	3	3	3
1	1	4	4	3	3	2	2	3	3
1	1	4	4	3	3	2	2	3	3
1	1	4	4	2	2	3	3	3	3
1	1	1	1	3	3	2	2	3	3
1	1	4	4	3	3	3	3	3	3
1	1	4	4	3	3	3	3	2	2
1	1	4	4	3	3	3	3	3	3
1	1	4	4	2	2	2	2	3	3
1	1	4	4	2	2	3	3	3	3
1	1	4	4	2	2	2	2	3	3
1	1	4	4	2	2	2	2	3	3
1	1	4	4	3	3	2	2	3	3
1	1	4	4	3	3	3	3	3	3
1	1	4	4	3	3	2	2	3	3
1	1	4	4	3	3	3	3	2	2
1	1	4	4	2	2	3	3	3	3
1	1	4	4	3	3	3	3	3	3

For Review Only

1	1	4	4	3	3	3	3	3	3
1	1	4	4	2	2	2	2	3	3

For Review Only

SNP1281	SNP1281	SNP1282	SNP1282	SNP1283	SNP1283	SNP1284	SNP1284	SNP1285	SNP1285
4	4	2	2	3	3	2	2	3	3
1	1	2	2	3	3	2	2	3	3
4	4	2	2	3	3	2	2	3	3
4	4	2	2	3	3	2	2	3	3
4	4	4	4	3	3	3	3	3	3
1	1	2	2	3	3	2	2	3	3
1	1	2	2	1	1	3	3	4	4
1	1	2	2	3	3	2	2	3	3
1	1	2	2	3	3	3	3	3	3
4	4	4	4	3	3	3	3	3	3
4	4	2	2	3	3	2	2	3	3
4	4	2	2	3	3	2	2	3	3
4	4	2	2	3	3	3	3	3	3
4	4	2	2	3	3	2	2	3	3
4	4	2	2	3	3	2	2	3	3
4	4	2	2	1	1	2	2	3	3
1	1	2	2	1	1	3	3	4	4
4	4	2	2	3	3	2	2	3	3
1	1	2	2	1	1	3	3	4	4
4	4	2	2	1	1	2	2	3	3
4	4	2	2	1	1	3	3	4	4
1	1	2	2	1	1	3	3	4	4
4	4	2	2	3	3	2	2	3	3
4	4	2	2	3	3	2	2	3	3
4	4	2	2	1	1	3	3	4	4
4	4	2	2	3	3	2	2	3	3
4	4	2	2	3	3	2	2	3	3
4	4	2	2	3	3	2	2	3	3
1	1	2	2	3	3	2	2	3	3
4	4	2	2	1	1	2	2	3	3
4	4	2	2	3	3	2	2	3	3
4	4	2	2	3	3	2	2	3	3
4	4	2	2	1	1	3	3	4	4
4	4	2	2	3	3	2	2	3	3
4	4	2	2	3	3	2	2	3	3
4	4	2	2	3	3	2	2	3	3
4	4	2	2	1	1	3	3	4	4
4	4	2	2	3	3	2	2	3	3
4	4	2	2	3	3	2	2	3	3
4	4	2	2	3	3	2	2	3	3
4	4	4	4	3	3	3	3	3	3
4	4	2	2	3	3	2	2	3	3
4	4	4	4	3	3	2	2	3	3
4	4	2	2	3	3	3	3	3	3
4	4	4	4	3	3	2	2	3	3
4	4	2	2	3	3	2	2	3	3
1	1	2	2	3	3	2	2	3	3
4	4	2	2	3	3	2	2	3	3
1	1	2	2	3	3	2	2	3	3
4	4	2	2	3	3	2	2	3	3
4	4	2	2	3	3	2	2	3	3
1	1	2	2	3	3	2	2	3	3

For Review Only

1	1	2	2	1	1	3	3	4	4
4	4	2	2	1	1	3	3	4	4
1	1	2	2	3	3	2	2	3	3
1	1	2	2	1	1	3	3	4	4
1	1	2	2	1	1	3	3	4	4
1	1	2	2	3	3	2	2	3	3
4	4	2	2	3	3	2	2	3	3
4	4	2	2	3	3	2	2	3	3
4	4	2	2	1	1	3	3	4	4
4	4	2	2	3	3	2	2	3	3
1	1	2	2	3	3	2	2	3	3
1	1	2	2	3	3	2	2	3	3
1	1	2	2	1	1	3	3	4	4
1	1	2	2	3	3	2	2	3	3
4	4	2	2	3	3	2	2	3	3
4	4	4	4	3	3	2	2	3	3
4	4	2	2	3	3	2	2	3	3
4	4	2	2	3	3	2	2	3	3
1	1	2	2	3	3	2	2	3	3
1	1	2	2	1	1	3	3	4	4
4	4	2	2	3	3	2	2	3	3
4	4	2	2	3	3	2	2	3	3
1	1	2	2	3	3	2	2	3	3
4	4	2	2	3	3	2	2	3	3
4	4	4	4	3	3	2	2	3	3
1	1	2	2	3	3	2	2	3	3
1	1	2	2	1	1	3	3	4	4
4	4	2	2	3	3	2	2	3	3
4	4	2	2	3	3	2	2	3	3
4	4	2	2	1	1	3	3	4	4
4	4	2	2	3	3	3	3	4	4
4	4	2	2	3	3	2	2	3	3
4	4	2	2	3	3	2	2	3	3
1	1	2	2	1	1	3	3	4	4
4	4	2	2	3	3	2	2	3	3
4	4	4	4	3	3	2	2	3	3
4	4	2	2	3	3	2	2	3	3
1	1	2	2	1	1	3	3	4	4
4	4	2	2	3	3	2	2	3	3
4	4	2	2	3	3	2	2	3	3
1	1	2	2	3	3	2	2	3	3
4	4	2	2	1	1	3	3	4	4
1	1	2	2	3	3	2	2	3	3
1	1	2	2	3	3	2	2	3	3
4	4	2	2	3	3	2	2	3	3
1	1	2	2	3	3	2	2	3	3
4	4	2	2	3	3	2	2	3	3
4	4	2	2	3	3	2	2	3	3
4	4	2	2	3	3	2	2	3	3
1	1	2	2	3	3	2	2	3	3
1	1	2	2	3	3	2	2	3	3
4	4	2	2	3	3	2	2	3	3
4	4	2	2	3	3	2	2	3	3
4	4	2	2	3	3	3	3	3	3
1	1	2	2	3	3	2	2	3	3

For Review Only

4	4	2	2	3	3	2	2	3	3
4	4	2	2	3	3	2	2	3	3
4	4	2	2	3	3	2	2	3	3
4	4	2	2	3	3	2	2	3	3
1	1	2	2	3	3	2	2	3	3
4	4	2	2	3	3	2	2	3	3
1	1	2	2	3	3	2	2	3	3
4	4	2	2	3	3	3	3	3	3
4	4	4	4	3	3	2	2	3	3
4	4	2	2	3	3	2	2	3	3
4	4	2	2	3	3	2	2	3	3
4	4	2	2	3	3	2	2	3	3
1	1	2	2	3	3	2	2	3	3
1	1	2	2	3	3	2	2	3	3
4	4	2	2	3	3	2	2	3	3
4	4	2	2	3	3	2	2	3	3
1	1	2	2	3	3	2	2	3	3
4	4	2	2	3	3	2	2	3	3
4	4	2	2	3	3	2	2	3	3
1	1	2	2	3	3	2	2	3	3
4	4	2	2	3	3	2	2	3	3
1	1	2	2	3	3	3	3	3	3
4	4	2	2	3	3	2	2	3	3
4	4	2	2	3	3	2	2	3	3
4	4	2	2	3	3	2	2	3	3
4	4	2	2	3	3	2	2	3	3
1	1	2	2	3	3	2	2	3	3
1	1	2	2	3	3	2	2	3	3
1	1	2	2	3	3	2	2	3	3
1	1	2	2	3	3	3	3	4	4
4	4	2	2	3	3	2	2	3	3
4	4	2	2	3	3	2	2	3	3
1	1	2	2	3	3	2	2	3	3
4	4	2	2	3	3	2	2	3	3
4	4	2	2	3	3	2	2	3	3
4	4	2	2	1	1	3	3	3	3
4	4	2	2	3	3	2	2	3	3
1	1	2	2	3	3	2	2	3	3
4	4	2	2	3	3	2	2	3	3
4	4	2	2	3	3	2	2	3	3
4	4	2	2	3	3	2	2	3	3
4	4	2	2	3	3	2	2	3	3
1	1	2	2	3	3	2	2	3	3
4	4	4	4	3	3	3	3	3	3
4	4	2	2	3	3	2	2	3	3
1	1	2	2	1	1	3	3	4	4
4	4	2	2	3	3	2	2	3	3
4	4	2	2	3	3	2	2	3	3
4	4	2	2	3	3	3	3	3	3
4	4	2	2	3	3	3	3	3	3
4	4	2	2	3	3	2	2	3	3
4	4	2	2	1	1	2	2	3	3
4	4	2	2	3	3	2	2	3	3
1	1	2	2	3	3	2	2	3	3
4	4	2	2	3	3	3	3	3	3

For Review Only

1	1	2	2	3	3	2	2	3	3
4	4	2	2	3	3	2	2	3	3
4	4	4	4	3	3	3	3	3	3
4	4	2	2	3	3	3	3	4	4
1	1	2	2	3	3	2	2	3	3
4	4	2	2	3	3	2	2	3	3
4	4	2	2	1	1	2	2	3	3
4	4	2	2	3	3	2	2	3	3
4	4	2	2	3	3	2	2	3	3
1	1	2	2	1	1	3	3	4	4
4	4	2	2	3	3	2	2	3	3
4	4	2	2	3	3	2	2	3	3
4	4	2	2	3	3	2	2	3	3
4	4	2	2	3	3	3	3	3	3
4	4	2	2	3	3	2	2	3	3
4	4	2	2	3	3	2	2	3	3
4	4	2	2	3	3	2	2	3	3
1	1	2	2	3	3	2	2	3	3
4	4	2	2	3	3	2	2	3	3
1	1	2	2	3	3	2	2	3	3
4	4	2	2	3	3	2	2	3	3
4	4	2	2	3	3	2	2	3	3
4	4	2	2	3	3	2	2	3	3
1	1	2	2	3	3	2	2	3	3
4	4	2	2	3	3	3	3	3	3
4	4	2	2	3	3	2	2	3	3
4	4	2	2	3	3	2	2	3	3
4	4	2	2	3	3	2	2	3	3
4	4	2	2	3	3	2	2	3	3
1	1	2	2	3	3	2	2	3	3
4	4	2	2	1	1	3	3	4	4
4	4	2	2	3	3	2	2	3	3
1	1	2	2	3	3	2	2	3	3
4	4	2	2	3	3	2	2	3	3
1	1	2	2	3	3	3	3	3	3
1	1	2	2	3	3	2	2	3	3
4	4	2	2	3	3	2	2	3	3
4	4	2	2	1	1	3	3	4	4
4	4	2	2	3	3	2	2	3	3
4	4	2	2	3	3	2	2	3	3
4	4	2	2	3	3	2	2	3	3
4	4	2	2	3	3	2	2	3	3
4	4	2	2	3	3	2	2	3	3
4	4	2	2	3	3	2	2	3	3
4	4	2	2	3	3	2	2	3	3
1	1	2	2	1	1	3	3	4	4
4	4	2	2	3	3	2	2	3	3
4	4	2	2	1	1	3	3	4	4
4	4	2	2	3	3	2	2	3	3
1	1	2	2	3	3	2	2	3	3
1	1	2	2	3	3	2	2	3	3
4	4	2	2	1	1	2	2	3	3
4	4	2	2	3	3	2	2	3	3
1	1	2	2	3	3	2	2	3	3
4	4	2	2	3	3	2	2	3	3

For Review Only

4	4	2	2	3	3	2	2	3	3
4	4	4	4	3	3	3	3	3	3
1	1	2	2	3	3	2	2	3	3
4	4	2	2	3	3	2	2	3	3
4	4	2	2	3	3	2	2	3	3
4	4	2	2	3	3	2	2	3	3
4	4	2	2	3	3	3	3	3	3
4	4	4	4	3	3	3	3	3	3
4	4	2	2	3	3	2	2	3	3
4	4	2	2	3	3	2	2	3	3
4	4	2	2	3	3	2	2	3	3
4	4	2	2	3	3	3	3	4	4
4	4	2	2	3	3	2	2	3	3
4	4	2	2	3	3	2	2	3	3
4	4	2	2	3	3	2	2	3	3
4	4	2	2	3	3	2	2	3	3
4	4	2	2	3	3	2	2	3	3
1	1	2	2	3	3	2	2	3	3
4	4	2	2	3	3	2	2	3	3
4	4	2	2	3	3	2	2	3	3
4	4	2	2	3	3	2	2	3	3
4	4	2	2	3	3	2	2	3	3
1	1	2	2	1	1	3	3	4	4
4	4	2	2	3	3	2	2	3	3
4	4	2	2	3	3	2	2	3	3
4	4	2	2	3	3	2	2	3	3
4	4	2	2	3	3	2	2	3	3
4	4	2	2	3	3	2	2	3	3
4	4	2	2	3	3	2	2	3	3
1	1	2	2	3	3	2	2	3	3
4	4	2	2	3	3	2	2	3	3
4	4	2	2	3	3	2	2	3	3
4	4	2	2	3	3	2	2	3	3
4	4	2	2	3	3	2	2	3	3
4	4	2	2	3	3	2	2	3	3
4	4	2	2	3	3	2	2	3	3
4	4	2	2	3	3	2	2	3	3
1	1	2	2	3	3	2	2	3	3
4	4	2	2	3	3	2	2	3	3
4	4	2	2	3	3	2	2	3	3
1	1	2	2	3	3	2	2	3	3
4	4	2	2	1	1	3	3	4	4
4	4	2	2	3	3	2	2	3	3
1	1	2	2	1	1	3	3	4	4
4	4	2	2	3	3	2	2	3	3
1	1	2	2	3	3	2	2	3	3
4	4	2	2	3	3	2	2	3	3
4	4	2	2	3	3	2	2	3	3
4	4	2	2	3	3	3	3	3	3
4	4	2	2	3	3	2	2	3	3
1	1	2	2	3	3	3	3	3	3
4	4	2	2	3	3	2	2	3	3
4	4	2	2	3	3	2	2	3	3
1	1	2	2	3	3	2	2	3	3
4	4	2	2	3	3	2	2	3	3

For Review Only

1	1	2	2	3	3	2	2	3	3
1	1	2	2	3	3	2	2	3	3

For Review Only

SNP1286	SNP1286	SNP1287	SNP1287	SNP1288	SNP1288	SNP1289	SNP1289	SNP129	SNP129
3	3	4	4	4	4	4	4	2	2
3	3	1	1	3	3	3	3	2	2
3	3	4	4	4	4	4	4	4	4
3	3	4	4	4	4	4	4	4	4
3	3	4	4	4	4	4	4	2	2
1	1	1	1	3	3	4	4	4	4
3	3	1	1	3	3	3	3	2	2
3	3	1	1	3	3	3	3	2	2
3	3	1	1	3	3	4	4	2	2
3	3	4	4	4	4	3	3	2	2
3	3	4	4	4	4	4	4	2	2
3	3	4	4	4	4	4	4	4	4
3	3	4	4	4	4	3	3	2	2
1	1	4	4	4	4	4	4	2	2
3	3	1	1	4	4	4	4	2	2
3	3	4	4	4	4	4	4	4	4
3	3	1	1	3	3	4	4	2	2
3	3	4	4	4	4	3	3	2	2
3	3	1	1	3	3	4	4	4	4
3	3	4	4	4	4	3	3	4	4
3	3	4	4	4	4	3	3	2	2
3	3	1	1	3	3	4	4	2	2
3	3	4	4	4	4	3	3	2	2
3	3	4	4	4	4	4	4	2	2
3	3	4	4	4	4	4	4	2	2
1	1	4	4	4	4	4	4	4	4
1	1	4	4	4	4	4	4	4	4
3	3	4	4	4	4	4	4	2	2
3	3	1	1	3	3	4	4	2	2
3	3	4	4	4	4	3	3	2	2
1	1	4	4	4	4	4	4	2	2
3	3	4	4	4	4	4	4	2	2
3	3	1	1	4	4	4	4	2	2
3	3	4	4	4	4	4	4	2	2
3	3	4	4	4	4	3	3	2	2
3	3	4	4	4	4	4	4	2	2
3	3	4	4	4	4	4	4	2	2
3	3	4	4	4	4	4	4	2	2
3	3	4	4	4	4	4	4	2	2
3	3	4	4	4	4	4	4	4	4
3	3	4	4	4	4	3	3	2	2
3	3	4	4	4	4	3	3	4	4
3	3	4	4	4	4	3	3	4	4
3	3	4	4	4	4	3	3	4	4
3	3	4	4	4	4	3	3	4	4
3	3	4	4	4	4	4	4	4	4
3	3	1	1	3	3	4	4	4	4
1	1	4	4	4	4	4	4	4	4
3	3	1	1	3	3	3	3	2	2
3	3	4	4	4	4	4	4	2	2
3	3	4	4	4	4	4	4	2	2
3	3	1	1	3	3	4	4	2	2

For Review Only

3	3	1	1	3	3	4	4	2	2
3	3	1	1	4	4	4	4	2	2
3	3	1	1	3	3	4	4	2	2
3	3	1	1	3	3	3	3	2	2
3	3	1	1	3	3	4	4	4	4
1	1	1	1	3	3	4	4	4	4
1	1	4	4	4	4	4	4	4	4
3	3	4	4	4	4	4	4	4	4
3	3	4	4	4	4	4	4	4	4
3	3	4	4	4	4	4	4	2	2
3	3	1	1	3	3	4	4	2	2
3	3	1	1	3	3	4	4	4	4
3	3	1	1	3	3	4	4	2	2
3	3	1	1	3	3	4	4	2	2
3	3	4	4	4	4	4	4	2	2
3	3	4	4	4	4	4	4	4	4
3	3	4	4	4	4	3	3	2	2
3	3	1	1	3	3	4	4	4	4
3	3	1	1	3	3	4	4	2	2
3	3	1	1	3	3	4	4	2	2
3	3	1	1	3	3	4	4	2	2
3	3	4	4	4	4	4	4	2	2
3	3	4	4	3	3	4	4	2	2
3	3	4	4	4	4	4	4	2	2
3	3	4	4	4	4	3	3	4	4
3	3	1	1	3	3	4	4	2	2
3	3	1	1	3	3	3	3	2	2
3	3	4	4	4	4	4	4	2	2
1	1	4	4	4	4	4	4	4	4
3	3	4	4	4	4	4	4	2	2
1	1	4	4	4	4	4	4	2	2
3	3	4	4	4	4	4	4	2	2
3	3	4	4	4	4	4	4	2	2
3	3	1	1	3	3	4	4	2	2
3	3	4	4	4	4	4	4	4	4
3	3	4	4	4	4	3	3	4	4
1	1	4	4	4	4	4	4	4	4
3	3	1	1	3	3	3	3	2	2
1	1	4	4	4	4	3	3	4	4
3	3	4	4	4	4	3	3	4	4
3	3	1	1	3	3	4	4	2	2
3	3	4	4	4	4	4	4	2	2
3	3	1	1	3	3	3	3	2	2
3	3	1	1	3	3	4	4	2	2
3	3	4	4	4	4	4	4	2	2
3	3	1	1	3	3	4	4	2	2
3	3	4	4	4	4	4	4	2	2
1	1	4	4	4	4	4	4	4	4
1	1	4	4	4	4	4	4	4	4
3	3	1	1	3	3	4	4	2	2
1	1	4	4	4	4	4	4	4	4
3	3	4	4	4	4	4	4	2	2
3	3	4	4	4	4	4	4	4	4
3	3	4	4	4	4	4	4	4	4
3	3	1	1	3	3	4	4	4	4

For Review Only

1	1	4	4	4	4	4	4	4	4
1	1	1	1	3	3	3	3	2	2
3	3	4	4	4	4	4	4	4	4
3	3	4	4	4	4	4	4	2	2
3	3	4	4	4	4	4	4	2	2
3	3	4	4	4	4	4	4	2	2
3	3	1	1	3	3	4	4	4	4
3	3	4	4	4	4	4	4	4	4
1	1	4	4	4	4	3	3	4	4
3	3	4	4	4	4	4	4	2	2
3	3	4	4	4	4	4	4	2	2
3	3	4	4	4	4	4	4	2	2
3	3	1	1	3	3	3	3	4	4
3	3	1	1	3	3	4	4	2	2
3	3	4	4	4	4	3	3	2	2
3	3	4	4	4	4	4	4	2	2
3	3	1	1	3	3	4	4	4	4
3	3	4	4	4	4	4	4	2	2
3	3	4	4	4	4	4	4	2	2
3	3	1	1	3	3	4	4	2	2
3	3	4	4	4	4	4	4	2	2
3	3	1	1	3	3	4	4	2	2
3	3	4	4	4	4	4	4	2	2
3	3	4	4	4	4	4	4	2	2
3	3	4	4	4	4	4	4	4	4
3	3	4	4	4	4	4	4	2	2
3	3	1	1	3	3	4	4	2	2
3	3	1	1	3	3	4	4	2	2
3	3	1	1	3	3	4	4	2	2
3	3	1	1	3	3	4	4	4	4
3	3	4	4	4	4	4	4	2	2
3	3	4	4	4	4	4	4	2	2
3	3	1	1	3	3	4	4	2	2
3	3	4	4	4	4	4	4	2	2
3	3	4	4	4	4	4	4	2	2
3	3	1	1	4	4	4	4	2	2
3	3	4	4	4	4	4	4	2	2
3	3	1	1	3	3	4	4	4	4
3	3	4	4	4	4	4	4	2	2
3	3	4	4	4	4	4	4	2	2
3	3	4	4	4	4	4	4	2	2
3	3	4	4	4	4	4	4	2	2
1	1	1	1	3	3	4	4	4	4
3	3	4	4	4	4	4	4	2	2
1	1	4	4	4	4	4	4	4	4
3	3	1	1	3	3	4	4	2	2
1	1	4	4	4	4	4	4	4	4
1	1	4	4	4	4	4	4	2	2
3	3	4	4	4	4	4	4	2	2
1	1	4	4	4	4	4	4	4	4
1	1	4	4	4	4	4	4	4	4
3	3	4	4	4	4	3	3	4	4
3	3	4	4	4	4	4	4	2	2
3	3	1	1	3	3	4	4	4	4
3	3	4	4	4	4	4	4	2	2

For Review Only

3	3	1	1	3	3	4	4	2	2
1	1	4	4	4	4	3	3	2	2
3	3	4	4	4	4	3	3	2	2
3	3	4	4	4	4	4	4	2	2
3	3	1	1	3	3	3	3	2	2
3	3	4	4	4	4	4	4	4	4
3	3	4	4	4	4	4	4	2	2
3	3	4	4	4	4	4	4	2	2
3	3	4	4	4	4	3	3	2	2
3	3	1	1	3	3	4	4	2	2
3	3	4	4	4	4	4	4	2	2
3	3	4	4	4	4	4	4	2	2
1	1	4	4	4	4	4	4	2	2
3	3	4	4	4	4	4	4	2	2
3	3	4	4	4	4	4	4	2	2
3	3	4	4	4	4	4	4	2	2
3	3	4	4	4	4	4	4	2	2
3	3	4	4	3	3	4	4	2	2
3	3	4	4	4	4	4	4	4	4
3	3	1	1	3	3	4	4	2	2
3	3	4	4	4	4	4	4	2	2
3	3	4	4	4	4	4	4	2	2
3	3	4	4	4	4	4	4	2	2
3	3	1	1	3	3	4	4	4	4
3	3	1	1	3	3	3	3	2	2
3	3	4	4	4	4	4	4	2	2
1	1	4	4	4	4	4	4	2	2
3	3	4	4	4	4	4	4	2	2
3	3	4	4	4	4	4	4	2	2
3	3	1	1	3	3	4	4	2	2
3	3	4	4	4	4	4	4	4	4
3	3	1	1	4	4	4	4	4	4
3	3	1	1	3	3	4	4	2	2
3	3	4	4	4	4	4	4	2	2
3	3	4	4	4	4	4	4	2	2
3	3	1	1	3	3	4	4	4	4
3	3	4	4	4	4	4	4	2	2
3	3	4	4	4	4	4	4	2	2
1	1	4	4	4	4	4	4	2	2
3	3	4	4	4	4	4	4	4	4
3	3	4	4	4	4	4	4	4	4
1	1	4	4	4	4	4	4	4	4
3	3	4	4	4	4	4	4	2	2
3	3	4	4	4	4	4	4	2	2
3	3	4	4	4	4	4	4	2	2
3	3	1	1	3	3	4	4	4	4
3	3	4	4	4	4	4	4	2	2
3	3	4	4	4	4	4	4	2	2
3	3	4	4	4	4	4	4	2	2
3	3	1	1	3	3	3	3	4	4
3	3	1	1	3	3	4	4	4	4
3	3	4	4	4	4	4	4	4	4
3	3	4	4	4	4	4	4	2	2
3	3	1	1	3	3	4	4	2	2
3	3	4	4	4	4	4	4	2	2

For Review Only

3	3	4	4	4	4	4	4	2	2
3	3	4	4	4	4	3	3	4	4
3	3	1	1	3	3	4	4	2	2
3	3	4	4	4	4	4	4	2	2
3	3	4	4	4	4	3	3	2	2
1	1	4	4	4	4	4	4	4	4
3	3	4	4	4	4	4	4	2	2
3	3	4	4	4	4	3	3	2	2
3	3	4	4	4	4	4	4	4	4
3	3	4	4	4	4	4	4	2	2
3	3	4	4	4	4	4	4	2	2
1	1	4	4	4	4	4	4	4	4
1	1	4	4	4	4	3	3	2	2
3	3	4	4	4	4	4	4	2	2
3	3	4	4	4	4	4	4	2	2
3	3	4	4	4	4	3	3	2	2
3	3	4	4	4	4	4	4	2	2
3	3	1	1	3	3	4	4	2	2
3	3	4	4	4	4	4	4	4	4
3	3	4	4	4	4	4	4	2	2
3	3	4	4	4	4	3	3	4	4
1	1	4	4	4	4	4	4	4	4
3	3	1	1	3	3	4	4	2	2
3	3	4	4	4	4	4	4	4	4
3	3	4	4	4	4	4	4	4	4
3	3	1	1	4	4	4	4	4	4
3	3	4	4	4	4	4	4	2	2
1	1	1	1	4	4	4	4	4	4
3	3	4	4	4	4	4	4	2	2
3	3	1	1	3	3	4	4	2	2
3	3	4	4	4	4	4	4	2	2
3	3	4	4	4	4	4	4	4	4
3	3	4	4	4	4	4	4	2	2
1	1	4	4	4	4	4	4	2	2
3	3	4	4	4	4	4	4	2	2
3	3	4	4	4	4	4	4	2	2
3	3	4	4	4	4	4	4	2	2
3	3	1	1	3	3	4	4	4	4
3	3	4	4	4	4	4	4	2	2
3	3	4	4	4	4	4	4	2	2
1	1	1	1	3	3	4	4	2	2
3	3	4	4	4	4	4	4	2	2
3	3	4	4	4	4	4	4	4	4
3	3	1	1	3	3	4	4	2	2
3	3	4	4	4	4	4	4	4	4
1	1	1	1	3	3	3	3	4	4
1	1	4	4	4	4	4	4	2	2
1	1	4	4	4	4	3	3	4	4
1	1	4	4	4	4	4	4	2	2
3	3	4	4	4	4	4	4	2	2
3	3	1	1	3	3	4	4	2	2
3	3	4	4	4	4	4	4	2	2
3	3	4	4	4	4	4	4	4	4
1	1	1	1	3	3	4	4	4	4
3	3	4	4	4	4	4	4	4	4

For Review Only

3	3	1	1	3	3	4	4	2	2
1	1	1	1	3	3	4	4	2	2

For Review Only

SNP1290	SNP1290	SNP1291	SNP1291	SNP1292	SNP1292	SNP1293	SNP1293	SNP1294	SNP1294
1	1	2	2	4	4	2	2	4	4
3	3	2	2	4	4	4	4	2	2
1	1	2	2	4	4	2	2	4	4
1	1	2	2	1	1	2	2	4	4
1	1	2	2	1	1	2	2	2	2
1	1	2	2	4	4	2	2	2	2
3	3	2	2	4	4	4	4	2	2
3	3	2	2	4	4	4	4	2	2
1	1	2	2	4	4	2	2	4	4
3	3	2	2	1	1	4	4	2	2
1	1	2	2	1	1	2	2	4	4
1	1	2	2	4	4	2	2	4	4
3	3	2	2	4	4	4	4	2	2
1	1	3	3	1	1	2	2	4	4
1	1	2	2	4	4	2	2	2	2
1	1	3	3	1	1	2	2	2	2
1	1	2	2	4	4	2	2	4	4
3	3	2	2	4	4	4	4	2	2
1	1	2	2	4	4	2	2	4	4
3	3	2	2	1	1	4	4	4	4
3	3	2	2	1	1	4	4	2	2
1	1	2	2	4	4	2	2	2	2
3	3	3	3	1	1	4	4	2	2
1	1	2	2	1	1	2	2	2	2
1	1	2	2	1	1	2	2	4	4
1	1	2	2	1	1	2	2	4	4
1	1	2	2	4	4	2	2	4	4
1	1	2	2	4	4	2	2	2	2
1	1	2	2	4	4	2	2	4	4
1	1	2	2	1	1	4	4	2	2
1	1	2	2	1	1	2	2	4	4
1	1	2	2	4	4	2	2	2	2
1	1	2	2	4	4	2	2	2	2
1	1	2	2	1	1	2	2	2	2
3	3	3	3	4	4	4	4	2	2
1	1	2	2	1	1	2	2	2	2
1	1	2	2	1	1	2	2	2	2
1	1	2	2	1	1	2	2	2	2
1	1	2	2	1	1	2	2	2	2
3	3	3	3	1	1	2	2	2	2
3	3	2	2	1	1	4	4	2	2
3	3	2	2	4	4	4	4	4	4
3	3	2	2	1	1	4	4	2	2
3	3	2	2	1	1	2	2	2	2
3	3	2	2	1	1	4	4	2	2
1	1	2	2	4	4	2	2	2	2
1	1	2	2	4	4	2	2	4	4
1	1	3	3	1	1	2	2	4	4
3	3	3	3	4	4	4	4	4	4
1	1	2	2	4	4	2	2	2	2
1	1	2	2	4	4	2	2	4	4
1	1	2	2	4	4	2	2	4	4

For Review Only

1	1	2	2	4	4	2	2	4	4
1	1	2	2	4	4	2	2	2	2
1	1	2	2	4	4	2	2	2	2
3	3	2	2	4	4	4	4	2	2
1	1	2	2	4	4	2	2	4	4
1	1	2	2	4	4	2	2	4	4
1	1	2	2	1	1	2	2	4	4
3	3	2	2	1	1	2	2	2	2
1	1	2	2	1	1	2	2	2	2
1	1	2	2	4	4	2	2	2	2
1	1	2	2	4	4	2	2	4	4
1	1	2	2	4	4	2	2	4	4
1	1	2	2	4	4	2	2	4	4
1	1	2	2	4	4	2	2	4	4
1	1	2	2	4	4	2	2	2	2
1	1	2	2	1	1	2	2	2	2
3	3	2	2	1	1	4	4	4	4
1	1	2	2	4	4	2	2	4	4
1	1	2	2	4	4	2	2	4	4
1	1	2	2	4	4	2	2	4	4
1	1	2	2	4	4	2	2	4	4
1	1	2	2	1	1	2	2	2	2
1	1	2	2	4	4	2	2	4	4
1	1	2	2	4	4	2	2	2	2
3	3	2	2	1	1	4	4	2	2
1	1	2	2	4	4	2	2	4	4
3	3	2	2	4	4	4	4	2	2
1	1	3	3	1	1	2	2	2	2
1	1	2	2	1	1	2	2	4	4
1	1	2	2	4	4	2	2	2	2
1	1	2	2	1	1	2	2	4	4
1	1	2	2	1	1	2	2	4	4
1	1	3	3	1	1	2	2	2	2
1	1	2	2	4	4	2	2	4	4
1	1	2	2	1	1	2	2	4	4
3	3	2	2	1	1	4	4	2	2
1	1	2	2	1	1	4	4	4	4
3	3	2	2	4	4	4	4	2	2
3	3	2	2	1	1	4	4	4	4
3	3	2	2	1	1	4	4	2	2
1	1	2	2	1	1	2	2	4	4
1	1	2	2	4	4	2	2	4	4
3	3	3	3	4	4	4	4	4	4
1	1	2	2	4	4	2	2	4	4
1	1	2	2	1	1	2	2	2	2
1	1	2	2	4	4	2	2	4	4
1	1	3	3	1	1	2	2	2	2
1	1	2	2	1	1	2	2	4	4
1	1	2	2	1	1	2	2	4	4
1	1	2	2	4	4	2	2	2	2
1	1	2	2	4	4	2	2	4	4
1	1	3	3	1	1	2	2	2	2
1	1	3	3	1	1	2	2	2	2
1	1	2	2	4	4	2	2	2	2
1	1	2	2	4	4	2	2	2	2

For Review Only

1	1	3	3	1	1	2	2	4	4
3	3	2	2	4	4	4	4	4	4
1	1	2	2	1	1	2	2	2	2
1	1	3	3	1	1	2	2	2	2
1	1	2	2	4	4	2	2	2	2
1	1	2	2	1	1	2	2	4	4
1	1	2	2	4	4	2	2	2	2
1	1	2	2	1	1	2	2	2	2
3	3	2	2	1	1	4	4	4	4
1	1	3	3	1	1	2	2	2	2
1	1	3	3	4	4	2	2	2	2
1	1	2	2	4	4	2	2	2	2
3	3	2	2	4	4	4	4	4	4
1	1	2	2	4	4	2	2	2	2
3	3	2	2	4	4	4	4	2	2
1	1	2	2	1	1	2	2	2	2
3	3	2	2	4	4	4	4	4	4
1	1	2	2	1	1	2	2	2	2
1	1	3	3	1	1	2	2	2	2
1	1	2	2	4	4	2	2	4	4
1	1	2	2	4	4	2	2	2	2
1	1	2	2	4	4	2	2	2	2
1	1	2	2	4	4	2	2	2	2
1	1	3	3	1	1	2	2	2	2
1	1	2	2	1	1	2	2	2	2
1	1	2	2	1	1	2	2	2	2
1	1	2	2	4	4	2	2	4	4
1	1	2	2	4	4	2	2	2	2
1	1	2	2	4	4	2	2	2	2
1	1	2	2	4	4	2	2	2	2
1	1	3	3	1	1	2	2	2	2
1	1	2	2	4	4	2	2	2	2
1	1	2	2	1	1	2	2	4	4
1	1	2	2	4	4	2	2	4	4
1	1	2	2	1	1	2	2	2	2
1	1	3	3	4	4	2	2	2	2
1	1	3	3	1	1	2	2	2	2
1	1	2	2	4	4	2	2	4	4
1	1	3	3	1	1	2	2	2	2
1	1	3	3	1	1	2	2	4	4
3	3	2	2	1	1	4	4	4	4
1	1	3	3	1	1	2	2	2	2
1	1	2	2	4	4	2	2	4	4
3	3	2	2	1	1	4	4	2	2
1	1	2	2	1	1	2	2	4	4
1	1	2	2	4	4	2	2	2	2
1	1	2	2	1	1	2	2	4	4
1	1	2	2	1	1	2	2	4	4
1	1	2	2	4	4	2	2	2	2
1	1	2	2	1	1	2	2	4	4
1	1	2	2	1	1	2	2	4	4
3	3	2	2	1	1	4	4	2	2
1	1	2	2	1	1	2	2	4	4
1	1	2	2	4	4	2	2	4	4
1	1	2	2	1	1	4	4	2	2

1	1	2	2	4	4	2	2	2	2
3	3	3	3	1	1	4	4	4	4
3	3	2	2	1	1	4	4	2	2
1	1	2	2	1	1	2	2	4	4
3	3	2	2	4	4	4	4	2	2
1	1	2	2	1	1	2	2	4	4
1	1	3	3	1	1	2	2	2	2
1	1	2	2	4	4	2	2	2	2
3	3	3	3	1	1	4	4	2	2
1	1	2	2	4	4	2	2	4	4
1	1	2	2	1	1	2	2	4	4
1	1	3	3	1	1	2	2	2	2
1	1	2	2	4	4	2	2	2	2
1	1	2	2	4	4	2	2	2	2
1	1	3	3	1	1	2	2	2	2
1	1	3	3	1	1	2	2	4	4
1	1	2	2	1	1	2	2	2	2
1	1	2	2	4	4	2	2	2	2
1	1	3	3	1	1	2	2	2	2
1	1	2	2	4	4	2	2	2	2
1	1	2	2	4	4	2	2	2	2
1	1	3	3	1	1	2	2	4	4
1	1	2	2	1	1	2	2	2	2
1	1	2	2	4	4	2	2	4	4
3	3	2	2	4	4	4	4	2	2
1	1	2	2	1	1	2	2	2	2
1	1	2	2	4	4	2	2	4	4
3	3	2	2	4	4	4	4	2	2
1	1	2	2	1	1	2	2	4	4
1	1	2	2	4	4	2	2	4	4
1	1	2	2	1	1	2	2	4	4
1	1	2	2	4	4	2	2	4	4
1	1	2	2	4	4	2	2	4	4
1	1	3	3	1	1	2	2	2	2
1	1	2	2	4	4	2	2	4	4
1	1	2	2	4	4	2	2	4	4
1	1	2	2	1	1	2	2	2	2
1	1	3	3	4	4	2	2	2	2
1	1	2	2	1	1	2	2	4	4
1	1	2	2	4	4	2	2	4	4
1	1	2	2	4	4	2	2	4	4
1	1	2	2	1	1	2	2	4	4
1	1	3	3	1	1	2	2	2	2
1	1	2	2	1	1	2	2	2	2
1	1	3	3	4	4	2	2	2	2
1	1	2	2	4	4	2	2	4	4
1	1	3	3	1	1	2	2	4	4
1	1	3	3	1	1	2	2	2	2
1	1	2	2	1	1	2	2	2	2
3	3	2	2	4	4	4	4	4	4
1	1	2	2	4	4	2	2	2	2
1	1	2	2	1	1	2	2	2	2
1	1	2	2	1	1	2	2	2	2
1	1	2	2	4	4	2	2	4	4
1	1	3	3	1	1	2	2	2	2

For Review Only

1	1	3	3	1	1	2	2	4	4
3	3	2	2	1	1	4	4	2	2
1	1	2	2	4	4	2	2	2	2
3	3	2	2	1	1	4	4	2	2
3	3	2	2	1	1	4	4	4	4
1	1	2	2	4	4	2	2	4	4
1	1	3	3	1	1	2	2	2	2
3	3	2	2	1	1	4	4	2	2
1	1	2	2	1	1	2	2	2	2
1	1	3	3	1	1	2	2	2	2
1	1	2	2	4	4	2	2	2	2
1	1	2	2	1	1	2	2	4	4
3	3	2	2	1	1	4	4	4	4
1	1	2	2	1	1	2	2	2	2
1	1	2	2	4	4	2	2	4	4
3	3	3	3	1	1	4	4	4	4
1	1	3	3	4	4	2	2	2	2
1	1	2	2	4	4	2	2	2	2
1	1	3	3	1	1	2	2	2	2
1	1	2	2	1	1	2	2	2	2
3	3	3	3	1	1	4	4	4	4
1	1	2	2	1	1	2	2	4	4
1	1	2	2	4	4	2	2	4	4
1	1	3	3	1	1	2	2	4	4
3	3	2	2	1	1	2	2	2	2
1	1	2	2	4	4	2	2	4	4
1	1	3	3	1	1	2	2	2	2
1	1	2	2	4	4	2	2	4	4
1	1	2	2	1	1	2	2	4	4
1	1	2	2	4	4	2	2	2	2
1	1	3	3	1	1	2	2	2	2
1	1	2	2	1	1	2	2	4	4
1	1	2	2	1	1	2	2	2	2
1	1	2	2	1	1	2	2	2	2
1	1	2	2	1	1	2	2	2	2
1	1	3	3	1	1	2	2	2	2
1	1	2	2	1	1	2	2	4	4
1	1	2	2	4	4	2	2	4	4
1	1	2	2	1	1	2	2	2	2
1	1	3	3	1	1	2	2	2	2
1	1	2	2	4	4	2	2	4	4
1	1	3	3	4	4	2	2	2	2
1	1	3	3	1	1	2	2	2	2
1	1	2	2	4	4	2	2	4	4
1	1	2	2	1	1	2	2	2	2
3	3	2	2	4	4	4	4	4	4
1	1	3	3	1	1	2	2	4	4
3	3	2	2	4	4	4	4	4	4
3	3	2	2	1	1	4	4	4	4
1	1	3	3	1	1	2	2	2	2
1	1	2	2	4	4	2	2	4	4
1	1	3	3	1	1	2	2	2	2
1	1	2	2	4	4	2	2	4	4
1	1	2	2	4	4	2	2	4	4
1	1	3	3	1	1	2	2	2	2

For Review Only

1	1	2	2	4	4	2	2	4	4
1	1	2	2	4	4	2	2	4	4

For Review Only

SNP1295	SNP1295	SNP1296	SNP1296	SNP1297	SNP1297	SNP1298	SNP1298	SNP1299	SNP1299
4	4	4	4	1	1	2	2	2	2
1	1	1	1	4	4	2	2	2	2
4	4	4	4	1	1	2	2	2	2
4	4	4	4	1	1	2	2	2	2
4	4	4	4	1	1	2	2	2	2
4	4	4	4	4	4	2	2	2	2
1	1	1	1	4	4	2	2	2	2
1	1	1	1	4	4	2	2	2	2
4	4	4	4	4	4	2	2	2	2
1	1	1	1	1	1	2	2	2	2
4	4	4	4	1	1	2	2	2	2
4	4	4	4	1	1	2	2	2	2
1	1	1	1	1	1	2	2	2	2
4	4	4	4	1	1	3	3	2	2
4	4	4	4	1	1	2	2	2	2
4	4	4	4	1	1	3	3	2	2
4	4	4	4	4	4	2	2	2	2
1	1	1	1	1	1	2	2	2	2
4	4	4	4	1	1	2	2	2	2
1	1	1	1	1	1	2	2	1	1
1	1	1	1	1	1	2	2	2	2
4	4	4	4	4	4	2	2	2	2
1	1	1	1	1	1	3	3	2	2
4	4	4	4	1	1	2	2	2	2
4	4	4	4	1	1	2	2	2	2
4	4	4	4	1	1	2	2	2	2
4	4	4	4	1	1	2	2	2	2
4	4	4	4	1	1	2	2	2	2
4	4	4	4	4	4	2	2	2	2
1	1	1	1	1	1	2	2	2	2
4	4	4	4	1	1	2	2	2	2
4	4	4	4	1	1	2	2	1	1
4	4	4	4	1	1	2	2	2	2
4	4	4	4	1	1	2	2	2	2
4	4	4	4	1	1	2	2	2	2
4	4	4	4	1	1	2	2	2	2
4	4	4	4	1	1	2	2	2	2
4	4	4	4	1	1	2	2	2	2
4	4	4	4	1	1	3	3	2	2
4	4	4	4	1	1	3	3	2	2
1	1	1	1	1	1	2	2	2	2
4	4	4	4	1	1	2	2	2	2
1	1	1	1	1	1	2	2	1	1
4	4	4	4	1	1	2	2	2	2
1	1	1	1	1	1	2	2	2	2
4	4	4	4	1	1	2	2	2	2
4	4	4	4	4	4	2	2	2	2
4	4	4	4	1	1	2	2	2	2
4	4	4	4	4	4	2	2	1	1
4	4	4	4	4	4	2	2	2	2
4	4	4	4	1	1	2	2	1	1
4	4	4	4	4	4	2	2	2	2

For Review Only

4	4	4	4	4	4	2	2	2	2
4	4	4	4	1	1	2	2	2	2
4	4	4	4	4	4	2	2	2	2
1	1	1	1	4	4	2	2	1	1
4	4	4	4	1	1	2	2	2	2
4	4	4	4	4	4	2	2	2	2
4	4	4	4	1	1	2	2	2	2
4	4	4	4	1	1	2	2	2	2
4	4	4	4	1	1	2	2	2	2
4	4	4	4	1	1	2	2	2	2
4	4	4	4	4	4	2	2	2	2
4	4	4	4	4	4	2	2	2	2
4	4	4	4	4	4	2	2	2	2
4	4	4	4	4	4	2	2	2	2
4	4	4	4	1	1	2	2	2	2
4	4	4	4	1	1	2	2	2	2
1	1	1	1	1	1	2	2	2	2
4	4	4	4	4	4	2	2	2	2
4	4	4	4	4	4	2	2	2	2
4	4	4	4	4	4	2	2	2	2
4	4	4	4	4	4	2	2	2	2
4	4	4	4	1	1	2	2	2	2
4	4	4	4	4	4	2	2	2	2
4	4	4	4	1	1	2	2	1	1
1	1	4	4	1	1	2	2	2	2
4	4	4	4	4	4	2	2	2	2
1	1	1	1	4	4	2	2	2	2
4	4	4	4	1	1	3	3	2	2
4	4	4	4	1	1	2	2	2	2
4	4	4	4	1	1	2	2	1	1
4	4	4	4	1	1	2	2	1	1
4	4	4	4	1	1	2	2	2	2
4	4	4	4	1	1	3	3	2	2
4	4	4	4	4	4	2	2	2	2
4	4	4	4	1	1	2	2	2	2
1	1	1	1	1	1	2	2	2	2
1	1	1	1	1	1	2	2	2	2
1	1	1	1	4	4	2	2	1	1
1	1	1	1	1	1	2	2	2	2
1	1	1	1	1	1	2	2	2	2
4	4	4	4	1	1	2	2	2	2
4	4	4	4	1	1	2	2	2	2
1	1	1	1	4	4	3	3	2	2
4	4	4	4	4	4	2	2	2	2
4	4	4	4	1	1	2	2	1	1
4	4	4	4	4	4	2	2	2	2
4	4	4	4	1	1	3	3	2	2
4	4	4	4	1	1	2	2	2	2
4	4	4	4	1	1	2	2	2	2
4	4	4	4	4	4	2	2	2	2
4	4	4	4	1	1	2	2	2	2
4	4	4	4	1	1	3	3	2	2
4	4	4	4	1	1	3	3	2	2
4	4	4	4	1	1	2	2	2	2
4	4	4	4	4	4	2	2	2	2

For Review Only

4	4	4	4	1	1	2	2	2	2
1	1	1	1	4	4	2	2	2	2
4	4	4	4	1	1	2	2	2	2
4	4	4	4	1	1	3	3	2	2
4	4	4	4	1	1	2	2	2	2
4	4	4	4	1	1	2	2	2	2
4	4	4	4	4	4	2	2	2	2
4	4	4	4	1	1	2	2	2	2
1	1	1	1	1	1	2	2	2	2
4	4	4	4	1	1	2	2	2	2
4	4	4	4	1	1	3	3	2	2
4	4	4	4	1	1	2	2	2	2
1	1	1	1	4	4	2	2	2	2
4	4	4	4	4	4	2	2	2	2
1	1	1	1	4	4	2	2	2	2
4	4	4	4	1	1	2	2	2	2
4	4	4	4	4	4	2	2	2	2
4	4	4	4	1	1	2	2	2	2
4	4	4	4	1	1	3	3	2	2
4	4	4	4	4	4	2	2	1	1
4	4	4	4	1	1	2	2	2	2
4	4	4	4	4	4	2	2	1	1
4	4	4	4	1	1	2	2	2	2
4	4	4	4	1	1	3	3	2	2
4	4	4	4	1	1	2	2	2	2
4	4	4	4	1	1	2	2	2	2
4	4	4	4	4	4	2	2	2	2
4	4	4	4	4	4	2	2	2	2
4	4	4	4	4	4	2	2	2	2
4	4	4	4	4	4	2	2	2	2
4	4	4	4	1	1	3	3	2	2
4	4	4	4	1	1	2	2	2	2
4	4	4	4	1	1	2	2	2	2
4	4	4	4	1	1	2	2	2	2
4	4	4	4	1	1	2	2	2	2
4	4	4	4	1	1	3	3	2	2
4	4	4	4	1	1	3	3	2	2
4	4	4	4	4	4	2	2	2	2
4	4	4	4	1	1	3	3	2	2
4	4	4	4	1	1	3	3	2	2
4	4	4	4	1	1	2	2	2	2
4	4	4	4	1	1	3	3	2	2
4	4	4	4	4	4	2	2	2	2
1	1	4	4	1	1	2	2	2	2
4	4	4	4	1	1	2	2	2	2
4	4	4	4	4	4	2	2	2	2
4	4	4	4	1	1	2	2	2	2
4	4	4	4	1	1	2	2	1	1
4	4	4	4	1	1	2	2	2	2
4	4	4	4	1	1	2	2	2	2
4	4	4	4	1	1	2	2	2	2
1	1	1	1	1	1	2	2	2	2
4	4	4	4	1	1	2	2	2	2
4	4	4	4	4	4	2	2	2	2
4	4	4	4	1	1	2	2	2	2

For Review Only

4	4	4	4	4	4	2	2	2	2
1	1	1	1	1	1	3	3	2	2
1	1	1	1	1	1	2	2	2	2
4	4	4	4	1	1	2	2	2	2
1	1	1	1	4	4	2	2	2	2
4	4	4	4	1	1	2	2	2	2
4	4	4	4	1	1	3	3	2	2
4	4	4	4	1	1	2	2	2	2
1	1	1	1	1	1	3	3	2	2
4	4	4	4	1	1	2	2	2	2
4	4	4	4	1	1	2	2	2	2
4	4	4	4	1	1	3	3	2	2
4	4	4	4	1	1	2	2	2	2
4	4	4	4	1	1	2	2	2	2
4	4	4	4	1	1	3	3	2	2
4	4	4	4	1	1	3	3	2	2
4	4	4	4	1	1	2	2	2	2
4	4	4	4	4	4	2	2	2	2
4	4	4	4	1	1	3	3	2	2
4	4	4	4	4	4	2	2	2	2
4	4	4	4	1	1	2	2	2	2
4	4	4	4	1	1	3	3	2	2
4	4	4	4	1	1	2	2	2	2
4	4	4	4	4	4	2	2	2	2
1	1	1	1	4	4	2	2	2	2
4	4	4	4	1	1	2	2	2	2
4	4	4	4	1	1	2	2	2	2
4	4	4	4	1	1	2	2	2	2
4	4	4	4	1	1	2	2	2	2
4	4	4	4	4	4	2	2	2	2
4	4	4	4	1	1	2	2	2	2
4	4	4	4	1	1	2	2	2	2
4	4	4	4	4	4	2	2	2	2
4	4	4	4	1	1	3	3	2	2
4	4	4	4	1	1	2	2	2	2
4	4	4	4	4	4	2	2	2	2
4	4	4	4	1	1	2	2	2	2
4	4	4	4	1	1	3	3	2	2
4	4	4	4	1	1	2	2	1	1
4	4	4	4	1	1	2	2	1	1
4	4	4	4	1	1	2	2	2	2
4	4	4	4	1	1	2	2	2	2
4	4	4	4	1	1	3	3	2	2
4	4	4	4	1	1	2	2	2	2
4	4	4	4	1	1	2	2	2	2
4	4	4	4	4	4	2	2	2	2
4	4	4	4	1	1	3	3	2	2
4	4	4	4	1	1	3	3	1	1
4	4	4	4	1	1	3	3	2	2
1	1	1	1	4	4	2	2	2	2
4	4	4	4	4	4	2	2	1	1
4	4	4	4	1	1	2	2	2	2
4	4	4	4	1	1	2	2	2	2
4	4	4	4	4	4	2	2	2	2
4	4	4	4	1	1	3	3	2	2

For Review Only

4	4	4	4	1	1	3	3	2	2
1	1	1	1	1	1	2	2	2	2
4	4	4	4	4	4	2	2	1	1
4	4	4	4	1	1	2	2	2	2
1	1	1	1	1	1	2	2	2	2
4	4	4	4	1	1	2	2	2	2
4	4	4	4	1	1	3	3	2	2
1	1	1	1	1	1	2	2	1	1
4	4	4	4	1	1	2	2	2	2
4	4	4	4	1	1	3	3	2	2
4	4	4	4	1	1	2	2	2	2
4	4	4	4	1	1	2	2	2	2
1	1	1	1	1	1	2	2	2	2
4	4	4	4	1	1	2	2	1	1
4	4	4	4	1	1	2	2	2	2
1	1	1	1	1	1	3	3	2	2
4	4	4	4	1	1	3	3	2	2
4	4	4	4	4	4	2	2	2	2
4	4	4	4	1	1	3	3	2	2
4	4	4	4	1	1	2	2	2	2
1	1	1	1	1	1	3	3	2	2
4	4	4	4	1	1	2	2	2	2
4	4	4	4	4	4	2	2	1	1
4	4	4	4	1	1	3	3	2	2
4	4	4	4	1	1	2	2	2	2
4	4	4	4	1	1	2	2	2	2
4	4	4	4	1	1	3	3	2	2
4	4	4	4	1	1	2	2	2	2
4	4	4	4	1	1	2	2	2	2
4	4	4	4	4	4	2	2	1	1
4	4	4	4	1	1	3	3	2	2
4	4	4	4	1	1	2	2	2	2
4	4	4	4	1	1	2	2	2	2
4	4	4	4	1	1	2	2	1	1
4	4	4	4	1	1	2	2	2	2
4	4	4	4	1	1	3	3	2	2
4	4	4	4	1	1	2	2	1	1
4	4	4	4	4	4	2	2	2	2
4	4	4	4	1	1	2	2	2	2
4	4	4	4	1	1	3	3	2	2
4	4	4	4	4	4	2	2	2	2
4	4	4	4	1	1	2	2	2	2
4	4	4	4	1	1	3	3	2	2
4	4	4	4	4	4	2	2	1	1
4	4	4	4	1	1	2	2	2	2
1	1	1	1	4	4	2	2	2	2
4	4	4	4	1	1	3	3	2	2
1	1	4	4	1	1	2	2	1	1
4	4	4	4	1	1	2	2	2	2
4	4	4	4	1	1	3	3	2	2
4	4	4	4	4	4	2	2	2	2
4	4	4	4	1	1	3	3	2	2
4	4	4	4	1	1	2	2	2	2
4	4	4	4	4	4	2	2	2	2
4	4	4	4	1	1	3	3	2	2

For Review Only

4	4	4	4	1	1	2	2	2	2
4	4	4	4	4	4	2	2	2	2

For Review Only

SNP130	SNP130	SNP1300	SNP1300	SNP1301	SNP1301	SNP1302	SNP1302	SNP1303	SNP1303
3	3	2	2	2	2	2	2	3	3
1	1	2	2	3	3	2	2	3	3
3	3	2	2	2	2	3	3	3	3
3	3	2	2	2	2	3	3	3	3
1	1	2	2	2	2	2	2	3	3
3	3	2	2	2	2	3	3	3	3
1	1	4	4	3	3	2	2	1	1
3	3	2	2	3	3	2	2	3	3
3	3	2	2	2	2	3	3	3	3
3	3	2	2	3	3	2	2	3	3
1	1	2	2	2	2	3	3	3	3
3	3	2	2	2	2	3	3	3	3
3	3	2	2	3	3	3	3	1	1
3	3	2	2	2	2	3	3	3	3
3	3	2	2	2	2	3	3	3	3
3	3	2	2	2	2	3	3	3	3
3	3	4	4	2	2	3	3	1	1
3	3	2	2	3	3	2	2	3	3
3	3	2	2	2	2	3	3	1	1
3	3	2	2	3	3	2	2	3	3
3	3	2	2	3	3	2	2	1	1
3	3	4	4	2	2	3	3	1	1
3	3	2	2	2	2	3	3	3	3
3	3	2	2	2	2	3	3	3	3
3	3	2	2	2	2	3	3	1	1
3	3	2	2	2	2	3	3	3	3
3	3	2	2	2	2	3	3	3	3
3	3	2	2	2	2	3	3	3	3
3	3	4	4	2	2	3	3	3	3
1	1	2	2	3	3	2	2	3	3
3	3	2	2	2	2	3	3	3	3
3	3	2	2	2	2	3	3	3	3
3	3	2	2	2	2	3	3	1	1
3	3	2	2	2	2	3	3	3	3
3	3	2	2	2	2	3	3	3	3
3	3	2	2	2	2	3	3	3	3
1	1	2	2	2	2	3	3	1	1
1	1	2	2	2	2	3	3	3	3
1	1	2	2	2	2	2	2	3	3
1	1	2	2	2	2	2	2	3	3
1	1	2	2	3	3	2	2	3	3
3	3	2	2	3	3	2	2	3	3
1	1	2	2	3	3	2	2	3	3
3	3	2	2	2	2	3	3	3	3
1	1	2	2	3	3	2	2	3	3
3	3	2	2	2	2	3	3	1	1
3	3	2	2	2	2	3	3	3	3
3	3	2	2	2	2	3	3	3	3
1	1	4	4	3	3	2	2	3	3
3	3	2	2	2	2	3	3	3	3
3	3	2	2	2	2	3	3	3	3
1	1	2	2	2	2	2	2	3	3

For Review Only

3	3	4	4	2	2	3	3	1	1
3	3	2	2	2	2	3	3	1	1
3	3	2	2	2	2	3	3	3	3
3	3	4	4	3	3	2	2	1	1
3	3	2	2	2	2	3	3	1	1
3	3	2	2	2	2	3	3	3	3
3	3	2	2	2	2	3	3	3	3
1	1	2	2	2	2	2	2	3	3
3	3	2	2	2	2	3	3	1	1
3	3	2	2	2	2	3	3	3	3
3	3	2	2	2	2	3	3	3	3
3	3	2	2	2	2	3	3	3	3
3	3	4	4	2	2	3	3	1	1
3	3	4	4	2	2	3	3	3	3
3	3	2	2	2	2	3	3	3	3
3	3	2	2	2	2	3	3	3	3
1	1	2	2	2	2	3	3	3	3
3	3	2	2	2	2	3	3	3	3
3	3	4	4	2	2	3	3	3	3
3	3	4	4	2	2	3	3	1	1
3	3	2	2	2	2	3	3	3	3
1	1	2	2	2	2	3	3	3	3
3	3	4	4	2	2	3	3	3	3
3	3	2	2	2	2	3	3	3	3
1	1	2	2	3	3	2	2	3	3
3	3	2	2	2	2	3	3	3	3
3	3	4	4	3	3	2	2	1	1
1	1	2	2	2	2	3	3	3	3
3	3	2	2	2	2	3	3	3	3
3	3	2	2	2	2	3	3	1	1
3	3	2	2	2	2	3	3	1	1
3	3	2	2	2	2	3	3	3	3
1	1	2	2	2	2	3	3	3	3
3	3	4	4	2	2	3	3	1	1
3	3	2	2	2	2	2	2	3	3
1	1	2	2	3	3	2	2	3	3
3	3	2	2	3	3	2	2	3	3
3	3	4	4	3	3	2	2	1	1
3	3	2	2	2	2	3	3	3	3
3	3	2	2	3	3	2	2	3	3
3	3	2	2	2	2	3	3	1	1
3	3	2	2	2	2	3	3	1	1
3	3	2	2	3	3	2	2	3	3
3	3	2	2	2	2	3	3	3	3
3	3	2	2	2	2	3	3	3	3
3	3	2	2	2	2	3	3	3	3
3	3	2	2	2	2	3	3	3	3
3	3	2	2	2	2	3	3	3	3
3	3	2	2	2	2	3	3	3	3
3	3	2	2	2	2	3	3	3	3
1	1	2	2	2	2	3	3	3	3
1	1	2	2	2	2	2	2	3	3
1	1	2	2	2	2	3	3	3	3
3	3	2	2	2	2	3	3	3	3
3	3	2	2	2	2	3	3	3	3

For Review Only

1	1	2	2	2	2	3	3	3	3
3	3	2	2	3	3	2	2	3	3
3	3	2	2	2	2	3	3	3	3
3	3	2	2	2	2	3	3	3	3
3	3	2	2	2	2	3	3	3	3
3	3	2	2	2	2	3	3	3	3
3	3	2	2	2	2	3	3	3	3
3	3	2	2	2	2	3	3	3	3
3	3	2	2	3	3	2	2	3	3
3	3	2	2	2	2	3	3	3	3
3	3	2	2	2	2	3	3	3	3
3	3	2	2	2	2	3	3	3	3
3	3	2	2	3	3	2	2	3	3
3	3	2	2	2	2	3	3	3	3
3	3	2	2	3	3	3	3	3	3
3	3	2	2	2	2	3	3	3	3
3	3	2	2	2	2	3	3	3	3
3	3	2	2	2	2	3	3	3	3
1	1	2	2	2	2	3	3	3	3
1	1	4	4	2	2	3	3	3	3
3	3	2	2	2	2	3	3	3	3
3	3	4	4	2	2	3	3	1	1
3	3	2	2	2	2	3	3	3	3
1	1	2	2	2	2	3	3	3	3
3	3	2	2	2	2	3	3	3	3
1	1	2	2	2	2	3	3	3	3
3	3	2	2	2	2	3	3	3	3
3	3	2	2	2	2	3	3	3	3
3	3	2	2	2	2	3	3	3	3
3	3	4	4	2	2	3	3	1	1
1	1	2	2	2	2	3	3	3	3
3	3	2	2	2	2	3	3	3	3
3	3	2	2	2	2	3	3	1	1
3	3	2	2	2	2	3	3	3	3
3	3	2	2	2	2	3	3	3	3
3	3	2	2	2	2	3	3	3	3
3	3	2	2	2	2	3	3	3	3
3	3	2	2	2	2	3	3	3	3
3	3	2	2	2	2	3	3	3	3
1	1	2	2	2	2	3	3	3	3
3	3	2	2	2	2	3	3	3	3
3	3	2	2	2	2	3	3	3	3
3	3	2	2	2	2	3	3	3	3
3	3	2	2	3	3	3	3	3	3
1	1	2	2	2	2	3	3	3	3
3	3	2	2	2	2	3	3	1	1
3	3	2	2	2	2	3	3	3	3
1	1	2	2	2	2	3	3	3	3
1	1	2	2	2	2	3	3	1	1
3	3	2	2	2	2	3	3	1	1
3	3	2	2	2	2	3	3	3	3
3	3	2	2	2	2	3	3	3	3
3	3	2	2	2	2	3	3	3	3
3	3	2	2	2	2	3	3	3	3
3	3	2	2	2	2	3	3	3	3

For Review Only

3	3	4	4	2	2	3	3	3	3
1	1	2	2	3	3	2	2	3	3
3	3	2	2	3	3	2	2	3	3
1	1	2	2	2	2	3	3	3	3
3	3	2	2	3	3	2	2	3	3
3	3	2	2	2	2	2	2	3	3
3	3	2	2	2	2	2	2	1	1
3	3	2	2	2	2	3	3	3	3
3	3	2	2	2	2	3	3	3	3
3	3	2	2	2	2	3	3	1	1
3	3	2	2	2	2	3	3	3	3
3	3	2	2	2	2	3	3	3	3
3	3	2	2	2	2	3	3	3	3
3	3	2	2	2	2	3	3	1	1
3	3	2	2	2	2	3	3	3	3
1	1	2	2	2	2	3	3	3	3
3	3	2	2	2	2	3	3	3	3
3	3	2	2	2	2	3	3	3	3
1	1	2	2	2	2	3	3	3	3
3	3	2	2	2	2	3	3	3	3
3	3	2	2	2	2	3	3	3	3
1	1	2	2	2	2	3	3	3	3
1	1	2	2	2	2	3	3	3	3
3	3	2	2	2	2	3	3	3	3
3	3	4	4	3	3	2	2	3	3
1	1	2	2	2	2	3	3	3	3
3	3	2	2	2	2	3	3	3	3
3	3	2	2	2	2	3	3	3	3
3	3	2	2	2	2	3	3	3	3
3	3	4	4	2	2	3	3	3	3
3	3	2	2	2	2	3	3	1	1
3	3	2	2	2	2	3	3	3	3
3	3	2	2	2	2	3	3	3	3
3	3	2	2	2	2	3	3	3	3
3	3	2	2	2	2	3	3	1	1
3	3	2	2	2	2	3	3	3	3
3	3	2	2	2	2	3	3	3	3
3	3	2	2	2	2	3	3	1	1
1	1	2	2	2	2	3	3	3	3
3	3	2	2	2	2	3	3	3	3
3	3	2	2	2	2	3	3	3	3
3	3	2	2	2	2	3	3	3	3
1	1	2	2	2	2	3	3	3	3
3	3	2	2	2	2	3	3	3	3
3	3	2	2	2	2	3	3	3	3
3	3	2	2	2	2	3	3	1	1
1	1	2	2	2	2	3	3	3	3
1	1	2	2	2	2	3	3	1	1
1	1	2	2	2	2	3	3	3	3
3	3	4	4	3	3	2	2	3	3
3	3	2	2	2	2	3	3	3	3
3	3	2	2	2	2	3	3	3	3
1	1	2	2	2	2	3	3	3	3
3	3	2	2	2	2	3	3	3	3
3	3	2	2	2	2	3	3	3	3

3	3	2	2	2	2	3	3	3	3
3	3	2	2	3	3	2	2	3	3
3	3	2	2	2	2	3	3	3	3
1	1	2	2	2	2	3	3	3	3
3	3	2	2	3	3	2	2	3	3
3	3	2	2	2	2	3	3	3	3
3	3	2	2	2	2	3	3	3	3
1	1	2	2	3	3	2	2	3	3
3	3	2	2	2	2	3	3	3	3
1	1	2	2	2	2	3	3	3	3
3	3	2	2	2	2	3	3	3	3
3	3	2	2	2	2	3	3	1	1
3	3	2	2	2	2	3	3	3	3
3	3	2	2	2	2	3	3	3	3
3	3	2	2	2	2	3	3	3	3
3	3	2	2	2	2	3	3	3	3
3	3	2	2	2	2	2	2	3	3
3	3	2	2	2	2	3	3	3	3
3	3	2	2	2	2	2	2	3	3
1	1	2	2	2	2	3	3	3	3
3	3	2	2	2	2	3	3	3	3
3	3	2	2	2	2	3	3	3	3
3	3	2	2	2	2	3	3	1	1
3	3	2	2	2	2	2	2	3	3
1	1	2	2	2	2	2	2	3	3
3	3	2	2	2	2	2	2	3	3
1	1	2	2	2	2	2	2	3	3
3	3	2	2	2	2	3	3	3	3
3	3	2	2	2	2	3	3	3	3
3	3	2	2	2	2	3	3	3	3
1	1	2	2	2	2	3	3	3	3
3	3	2	2	2	2	3	3	3	3
1	1	2	2	2	2	3	3	3	3
1	1	2	2	2	2	3	3	3	3
1	1	2	2	2	2	3	3	3	3
1	1	2	2	2	2	3	3	3	3
3	3	2	2	2	2	3	3	3	3
3	3	2	2	2	2	3	3	3	3
3	3	2	2	2	2	3	3	3	3
1	1	2	2	2	2	2	2	3	3
3	3	2	2	2	2	3	3	3	3
3	3	2	2	2	2	3	3	1	1
3	3	2	2	2	2	2	2	3	3
3	3	4	4	2	2	3	3	1	1
3	3	2	2	2	2	3	3	3	3
1	1	4	4	2	2	3	3	3	3
1	1	2	2	2	2	3	3	3	3
3	3	2	2	3	3	2	2	3	3
3	3	2	2	2	2	3	3	3	3
1	1	2	2	2	2	2	2	3	3
3	3	2	2	2	2	3	3	1	1
1	1	2	2	2	2	2	2	3	3
3	3	2	2	2	2	3	3	3	3
3	3	2	2	2	2	3	3	3	3
1	1	2	2	2	2	2	2	3	3

For Review Only

3	3	2	2	2	2	3	3	3	3
3	3	2	2	2	2	3	3	3	3

For Review Only

SNP1304	SNP1304	SNP1305	SNP1305	SNP1306	SNP1306	SNP1307	SNP1307	SNP1308	SNP1308
1	1	2	2	3	3	1	1	1	1
1	1	2	2	3	3	1	1	1	1
1	1	2	2	3	3	1	1	4	4
1	1	2	2	3	3	1	1	1	1
1	1	2	2	3	3	1	1	1	1
1	1	2	2	3	3	1	1	1	1
4	4	4	4	1	1	4	4	4	4
1	1	4	4	1	1	1	1	4	4
1	1	2	2	3	3	1	1	1	1
1	1	4	4	1	1	1	1	4	4
1	1	2	2	3	3	1	1	1	1
1	1	2	2	3	3	1	1	4	4
4	4	2	2	3	3	1	1	4	4
1	1	2	2	3	3	1	1	1	1
4	4	2	2	3	3	1	1	1	1
1	1	2	2	3	3	1	1	1	1
4	4	2	2	3	3	4	4	1	1
1	1	4	4	1	1	1	1	4	4
4	4	2	2	3	3	4	4	1	1
1	1	4	4	1	1	1	1	4	4
4	4	4	4	1	1	4	4	4	4
1	1	2	2	3	3	4	4	1	1
1	1	2	2	3	3	1	1	1	1
1	1	2	2	3	3	1	1	1	1
4	4	2	2	3	3	4	4	1	1
1	1	2	2	3	3	1	1	1	1
1	1	2	2	3	3	1	1	1	1
1	1	2	2	3	3	1	1	1	1
1	1	2	2	3	3	1	1	1	1
1	1	4	4	1	1	1	1	4	4
1	1	2	2	3	3	1	1	4	4
1	1	2	2	3	3	1	1	4	4
4	4	2	2	3	3	4	4	4	4
1	1	2	2	3	3	1	1	1	1
1	1	2	2	3	3	1	1	1	1
1	1	2	2	3	3	1	1	1	1
4	4	2	2	3	3	4	4	1	1
1	1	2	2	3	3	1	1	1	1
1	1	2	2	3	3	1	1	1	1
1	1	2	2	3	3	1	1	1	1
1	1	4	4	1	1	1	1	4	4
1	1	4	4	1	1	1	1	4	4
1	1	4	4	1	1	1	1	4	4
1	1	2	2	3	3	4	4	1	1
1	1	4	4	1	1	1	1	4	4
4	4	2	2	3	3	1	1	4	4
1	1	2	2	3	3	1	1	1	1
1	1	2	2	3	3	1	1	1	1
1	1	4	4	1	1	1	1	4	4
1	1	2	2	3	3	1	1	1	1
1	1	2	2	3	3	1	1	1	1
1	1	2	2	3	3	1	1	1	1

For Review Only

4	4	2	2	3	3	4	4	1	1
4	4	2	2	3	3	4	4	4	4
1	1	2	2	3	3	1	1	1	1
4	4	4	4	1	1	4	4	4	4
4	4	2	2	3	3	4	4	1	1
1	1	2	2	3	3	1	1	1	1
1	1	2	2	3	3	1	1	1	1
1	1	2	2	3	3	1	1	1	1
4	4	2	2	3	3	4	4	1	1
1	1	2	2	3	3	1	1	1	1
1	1	2	2	3	3	1	1	1	1
1	1	2	2	3	3	1	1	1	1
4	4	2	2	3	3	4	4	1	1
1	1	2	2	3	3	1	1	1	1
1	1	2	2	3	3	1	1	1	1
1	1	2	2	3	3	1	1	1	1
1	1	2	2	3	3	1	1	1	1
1	1	2	2	3	3	1	1	4	4
1	1	2	2	3	3	1	1	1	1
4	4	2	2	3	3	4	4	1	1
1	1	2	2	3	3	1	1	1	1
1	1	2	2	3	3	1	1	1	1
1	1	2	2	3	3	1	1	1	1
1	1	2	2	3	3	1	1	4	4
1	1	4	4	1	1	1	1	4	4
1	1	2	2	3	3	1	1	1	1
4	4	4	4	1	1	4	4	4	4
1	1	2	2	3	3	1	1	1	1
1	1	2	2	3	3	1	1	1	1
4	4	2	2	3	3	4	4	4	4
4	4	2	2	3	3	4	4	1	1
1	1	2	2	3	3	1	1	1	1
1	1	2	2	3	3	1	1	1	1
4	4	2	2	3	3	4	4	1	1
1	1	2	2	3	3	1	1	1	1
1	1	4	4	1	1	1	1	4	4
1	1	4	4	1	1	1	1	4	4
4	4	4	4	1	1	4	4	4	4
1	1	2	2	3	3	1	1	1	1
1	1	4	4	1	1	1	1	4	4
4	4	2	2	3	3	4	4	1	1
4	4	2	2	3	3	4	4	4	4
1	1	4	4	1	1	1	1	4	4
1	1	2	2	3	3	1	1	1	1
1	1	2	2	3	3	1	1	1	1
1	1	2	2	3	3	1	1	4	4
1	1	2	2	3	3	1	1	1	1
1	1	2	2	3	3	1	1	1	1
1	1	2	2	3	3	1	1	1	1
1	1	2	2	3	3	1	1	1	1
1	1	2	2	3	3	1	1	1	1
1	1	2	2	3	3	1	1	1	1
1	1	2	2	3	3	1	1	1	1
1	1	2	2	3	3	1	1	1	1
1	1	2	2	3	3	1	1	1	1

For Review Only

1	1	2	2	3	3	1	1	1	1
1	1	4	4	1	1	1	1	4	4
1	1	2	2	3	3	1	1	1	1
1	1	2	2	3	3	1	1	1	1
1	1	2	2	3	3	1	1	1	1
1	1	2	2	3	3	1	1	1	1
1	1	2	2	3	3	1	1	1	1
1	1	2	2	3	3	1	1	1	1
1	1	4	4	1	1	1	1	4	4
1	1	2	2	3	3	1	1	1	1
1	1	2	2	3	3	1	1	1	1
1	1	2	2	3	3	1	1	1	1
1	1	4	4	1	1	1	1	4	4
1	1	2	2	3	3	1	1	1	1
1	1	2	2	3	3	1	1	4	4
1	1	2	2	3	3	1	1	1	1
1	1	2	2	3	3	1	1	1	1
1	1	2	2	3	3	1	1	1	1
1	1	2	2	3	3	1	1	1	1
1	1	2	2	3	3	1	1	1	1
1	1	2	2	3	3	1	1	1	1
4	4	2	2	3	3	4	4	4	4
1	1	2	2	3	3	1	1	4	4
1	1	2	2	3	3	1	1	1	1
1	1	2	2	3	3	1	1	1	1
1	1	2	2	3	3	1	1	1	1
1	1	2	2	3	3	1	1	1	1
1	1	2	2	3	3	1	1	1	1
1	1	2	2	3	3	1	1	1	1
4	4	2	2	3	3	1	1	4	4
1	1	2	2	3	3	1	1	1	1
1	1	2	2	3	3	1	1	1	1
4	4	2	2	3	3	4	4	1	1
1	1	2	2	3	3	1	1	1	1
1	1	2	2	3	3	1	1	1	1
1	1	2	2	3	3	1	1	1	1
1	1	2	2	3	3	1	1	1	1
1	1	2	2	3	3	1	1	4	4
1	1	2	2	3	3	1	1	1	1
1	1	2	2	3	3	1	1	1	1
1	1	2	2	3	3	1	1	1	1
1	1	2	2	3	3	1	1	1	1
1	1	2	2	3	3	1	1	1	1
1	1	4	4	3	3	1	1	4	4
1	1	2	2	3	3	1	1	1	1
4	4	2	2	3	3	4	4	1	1
1	1	2	2	3	3	1	1	1	1
1	1	2	2	3	3	1	1	1	1
1	1	2	2	3	3	1	1	1	1
1	1	2	2	3	3	1	1	1	1
1	1	2	2	3	3	1	1	1	1
1	1	2	2	3	3	1	1	1	1
1	1	2	2	3	3	1	1	1	1
1	1	2	2	3	3	1	1	1	1
1	1	2	2	3	3	1	1	1	1

1	1	2	2	3	3	1	1	1	1
1	1	4	4	1	1	1	1	4	4
1	1	4	4	1	1	1	1	4	4
1	1	2	2	3	3	1	1	1	1
1	1	4	4	3	3	1	1	1	1
1	1	2	2	3	3	1	1	1	1
4	4	2	2	3	3	1	1	1	1
1	1	2	2	3	3	1	1	1	1
1	1	2	2	3	3	1	1	1	1
4	4	2	2	3	3	4	4	1	1
1	1	2	2	3	3	1	1	1	1
1	1	2	2	3	3	1	1	1	1
1	1	2	2	3	3	1	1	1	1
1	1	2	2	3	3	1	1	1	1
1	1	2	2	3	3	1	1	1	1
1	1	2	2	3	3	1	1	1	1
1	1	2	2	3	3	1	1	4	4
1	1	2	2	3	3	1	1	1	1
1	1	2	2	3	3	1	1	1	1
1	1	2	2	3	3	1	1	1	1
1	1	2	2	3	3	1	1	4	4
1	1	2	2	3	3	1	1	1	1
1	1	2	2	3	3	1	1	1	1
1	1	2	2	3	3	1	1	1	1
1	1	4	4	1	1	1	1	4	4
1	1	2	2	3	3	1	1	1	1
1	1	2	2	3	3	1	1	1	1
1	1	2	2	3	3	1	1	1	1
1	1	2	2	3	3	1	1	1	1
1	1	2	2	3	3	1	1	1	1
4	4	2	2	3	3	4	4	1	1
1	1	2	2	3	3	1	1	4	4
1	1	2	2	3	3	1	1	1	1
1	1	2	2	3	3	1	1	1	1
1	1	2	2	3	3	1	1	1	1
1	1	2	2	3	3	1	1	1	1
1	1	2	2	3	3	1	1	1	1
4	4	2	2	3	3	4	4	1	1
1	1	2	2	3	3	1	1	1	1
1	1	2	2	3	3	1	1	1	1
1	1	2	2	3	3	1	1	4	4
1	1	2	2	3	3	1	1	1	1
1	1	2	2	3	3	1	1	1	1
1	1	2	2	3	3	1	1	1	1
1	1	2	2	3	3	1	1	4	4
1	1	2	2	3	3	4	4	1	1
1	1	2	2	3	3	1	1	1	1
4	4	2	2	3	3	4	4	1	1
1	1	2	2	3	3	1	1	1	1
1	1	4	4	1	1	1	1	4	4
1	1	2	2	3	3	1	1	1	1
1	1	2	2	3	3	1	1	1	1
1	1	2	2	3	3	1	1	1	1
1	1	2	2	3	3	1	1	1	1
1	1	2	2	3	3	1	1	1	1

For Review Only

1	1	2	2	3	3	1	1	1	1
1	1	4	4	1	1	1	1	4	4
1	1	2	2	3	3	1	1	1	1
1	1	2	2	3	3	1	1	4	4
1	1	4	4	1	1	1	1	4	4
1	1	2	2	3	3	1	1	1	1
4	4	2	2	3	3	1	1	4	4
1	1	4	4	1	1	1	1	4	4
1	1	2	2	3	3	1	1	1	1
1	1	2	2	3	3	1	1	1	1
1	1	2	2	3	3	1	1	1	1
4	4	2	2	3	3	4	4	1	1
1	1	2	2	3	3	1	1	1	1
1	1	2	2	3	3	1	1	1	1
1	1	2	2	3	3	1	1	4	4
1	1	2	2	3	3	1	1	1	1
1	1	2	2	3	3	1	1	1	1
1	1	2	2	3	3	1	1	1	1
1	1	2	2	3	3	1	1	1	1
1	1	2	2	3	3	1	1	1	1
1	1	2	2	3	3	1	1	1	1
1	1	2	2	3	3	1	1	1	1
4	4	2	2	3	3	4	4	1	1
1	1	2	2	3	3	1	1	1	1
1	1	2	2	3	3	1	1	1	1
1	1	2	2	3	3	1	1	1	1
1	1	2	2	3	3	1	1	1	1
1	1	2	2	3	3	1	1	1	1
1	1	2	2	3	3	1	1	1	1
1	1	2	2	3	3	1	1	1	1
1	1	2	2	3	3	1	1	1	1
1	1	2	2	3	3	1	1	1	1
1	1	2	2	3	3	1	1	1	1
1	1	2	2	3	3	1	1	1	1
1	1	2	2	3	3	1	1	1	1
1	1	2	2	3	3	1	1	1	1
1	1	2	2	3	3	1	1	4	4
1	1	2	2	3	3	1	1	1	1
1	1	2	2	3	3	1	1	1	1
1	1	2	2	3	3	1	1	1	1
1	1	2	2	3	3	1	1	1	1
4	4	2	2	3	3	4	4	4	4
1	1	2	2	3	3	1	1	1	1
4	4	2	2	3	3	4	4	1	1
1	1	2	2	3	3	1	1	1	1
1	1	2	2	3	3	1	1	1	1
1	1	2	2	3	3	1	1	1	1
1	1	4	4	1	1	1	1	4	4
1	1	2	2	3	3	1	1	4	4
1	1	2	2	3	3	1	1	1	1
4	4	2	2	3	3	4	4	4	4
1	1	2	2	3	3	1	1	1	1
1	1	2	2	3	3	1	1	4	4
1	1	2	2	3	3	1	1	1	1
1	1	2	2	3	3	1	1	1	1

For Review Only

1	1	2	2	3	3	1	1	1	1
1	1	2	2	3	3	1	1	1	1

For Review Only

SNP1309	SNP1309	SNP131	SNP131	SNP1310	SNP1310	SNP1311	SNP1311	SNP1312	SNP1312
1	1	3	3	1	1	1	1	4	4
1	1	3	3	3	3	1	1	1	1
1	1	3	3	3	3	4	4	1	1
1	1	3	3	3	3	4	4	1	1
4	4	3	3	3	3	4	4	1	1
1	1	3	3	3	3	1	1	1	1
4	4	3	3	3	3	1	1	1	1
1	1	3	3	3	3	1	1	1	1
4	4	3	3	3	3	1	1	1	1
1	1	3	3	3	3	4	4	1	1
1	1	3	3	3	3	4	4	1	1
1	1	3	3	3	3	4	4	1	1
4	4	3	3	3	3	4	4	4	4
1	1	3	3	3	3	4	4	1	1
4	4	3	3	3	3	4	4	1	1
1	1	3	3	3	3	4	4	1	1
4	4	3	3	3	3	4	4	1	1
1	1	3	3	1	1	1	1	4	4
4	4	3	3	3	3	4	4	1	1
1	1	3	3	3	3	4	4	1	1
4	4	3	3	3	3	4	4	1	1
4	4	3	3	3	3	1	1	1	1
1	1	1	1	3	3	4	4	1	1
1	1	3	3	3	3	4	4	1	1
4	4	3	3	3	3	4	4	1	1
1	1	3	3	3	3	4	4	1	1
1	1	3	3	3	3	4	4	1	1
1	1	3	3	3	3	4	4	1	1
1	1	3	3	3	3	1	1	1	1
1	1	3	3	3	3	4	4	1	1
1	1	3	3	3	3	4	4	1	1
1	1	3	3	1	1	1	1	4	4
4	4	3	3	1	1	1	1	4	4
1	1	3	3	3	3	4	4	1	1
1	1	3	3	3	3	4	4	1	1
1	1	3	3	3	3	4	4	1	1
4	4	3	3	3	3	4	4	1	1
1	1	3	3	3	3	4	4	1	1
1	1	3	3	3	3	4	4	1	1
1	1	3	3	3	3	4	4	1	1
4	4	3	3	3	3	4	4	1	1
4	4	3	3	3	3	4	4	1	1
1	1	3	3	3	3	4	4	1	1
4	4	3	3	3	3	4	4	1	1
1	1	3	3	3	3	4	4	1	1
4	4	3	3	3	3	4	4	1	1
1	1	3	3	3	3	1	1	1	1
1	1	1	1	3	3	4	4	1	1
4	4	3	3	3	3	1	1	1	1
1	1	3	3	3	3	4	4	1	1
1	1	3	3	1	1	1	1	4	4
1	1	1	1	3	3	1	1	1	1

For Review Only

4	4	3	3	3	3	1	1	1	1
4	4	3	3	1	1	1	1	4	4
1	1	3	3	3	3	4	4	1	1
4	4	3	3	3	3	1	1	1	1
4	4	3	3	3	3	4	4	1	1
1	1	3	3	3	3	1	1	1	1
1	1	3	3	3	3	4	4	1	1
1	1	3	3	3	3	4	4	1	1
4	4	3	3	3	3	4	4	1	1
1	1	3	3	3	3	4	4	1	1
1	1	1	1	3	3	1	1	1	1
1	1	3	3	3	3	1	1	1	1
4	4	3	3	3	3	1	1	1	1
1	1	3	3	3	3	1	1	1	1
1	1	3	3	1	1	4	4	1	1
1	1	3	3	3	3	4	4	1	1
1	1	3	3	3	3	4	4	1	1
1	1	3	3	3	3	4	4	1	1
1	1	3	3	3	3	1	1	1	1
4	4	3	3	3	3	1	1	1	1
1	1	3	3	3	3	1	1	1	1
1	1	3	3	3	3	4	4	1	1
1	1	3	3	3	3	1	1	1	1
1	1	3	3	1	1	1	1	4	4
1	1	3	3	3	3	4	4	1	1
1	1	3	3	3	3	1	1	1	1
4	4	3	3	3	3	1	1	1	1
1	1	3	3	3	3	4	4	1	1
1	1	3	3	3	3	4	4	1	1
4	4	3	3	1	1	1	1	4	4
4	4	3	3	3	3	4	4	1	1
1	1	3	3	3	3	4	4	1	1
1	1	3	3	3	3	4	4	1	1
4	4	3	3	3	3	1	1	1	1
1	1	3	3	3	3	4	4	1	1
1	1	3	3	3	3	4	4	1	1
1	1	3	3	3	3	4	4	1	1
4	4	3	3	3	3	1	1	1	1
1	1	3	3	3	3	4	4	1	1
1	1	3	3	3	3	4	4	1	1
4	4	3	3	3	3	4	4	1	1
4	4	3	3	3	3	4	4	1	1
1	1	3	3	3	3	4	4	1	1
1	1	1	1	3	3	1	1	1	1
1	1	3	3	3	3	4	4	1	1
1	1	3	3	3	3	1	1	1	1
1	1	3	3	3	3	4	4	1	1
1	1	3	3	3	3	4	4	1	1
1	1	3	3	3	3	4	4	1	1
1	1	3	3	3	3	4	4	1	1
1	1	3	3	3	3	1	1	1	1
1	1	3	3	3	3	4	4	1	1
1	1	3	3	3	3	4	4	1	1
4	4	3	3	3	3	4	4	1	1
1	1	3	3	3	3	4	4	1	1

For Review Only

1	1	3	3	3	3	4	4	1	1
1	1	3	3	3	3	4	4	1	1
1	1	3	3	3	3	4	4	1	1
1	1	3	3	3	3	4	4	1	1
1	1	3	3	3	3	1	1	1	1
1	1	3	3	3	3	4	4	1	1
1	1	3	3	3	3	1	1	1	1
1	1	3	3	3	3	4	4	1	1
1	1	3	3	3	3	4	4	1	1
1	1	3	3	3	3	4	4	1	1
1	1	3	3	1	1	4	4	4	4
1	1	3	3	3	3	4	4	1	1
1	1	3	3	3	3	4	4	1	1
1	1	1	1	3	3	1	1	1	1
1	1	3	3	3	3	4	4	1	1
4	4	3	3	3	3	4	4	1	1
1	1	3	3	3	3	4	4	1	1
1	1	3	3	3	3	4	4	1	1
1	1	3	3	3	3	4	4	1	1
1	1	3	3	3	3	1	1	1	1
1	1	3	3	3	3	4	4	1	1
4	4	3	3	1	1	1	1	4	4
1	1	3	3	3	3	4	4	1	1
1	1	3	3	3	3	4	4	1	1
1	1	3	3	3	3	4	4	1	1
1	1	3	3	3	3	4	4	1	1
1	1	3	3	3	3	1	1	1	1
1	1	3	3	3	3	1	1	1	1
1	1	3	3	3	3	1	1	1	1
4	4	3	3	3	3	1	1	1	1
1	1	3	3	3	3	4	4	1	1
1	1	3	3	3	3	4	4	1	1
4	4	3	3	3	3	4	4	1	1
1	1	3	3	3	3	4	4	1	1
1	1	3	3	3	3	4	4	1	1
4	4	3	3	3	3	1	1	1	1
1	1	3	3	3	3	4	4	1	1
1	1	3	3	3	3	1	1	1	1
1	1	3	3	3	3	4	4	1	1
1	1	3	3	3	3	4	4	1	1
1	1	3	3	3	3	4	4	1	1
1	1	3	3	3	3	4	4	1	1
1	1	3	3	3	3	4	4	1	1
4	4	3	3	3	3	4	4	1	1
1	1	3	3	3	3	4	4	1	1
4	4	3	3	3	3	1	1	1	1
1	1	3	3	3	3	4	4	1	1
1	1	3	3	3	3	4	4	1	1
4	4	3	3	3	3	4	4	1	1
4	4	3	3	3	3	4	4	1	1
1	1	3	3	3	3	4	4	1	1
1	1	3	3	3	3	4	4	1	1
1	1	3	3	3	3	4	4	1	1
1	1	3	3	3	3	1	1	1	1
1	1	3	3	3	3	4	4	1	1

For Review Only

1	1	3	3	3	3	1	1	1	1
1	1	3	3	3	3	4	4	1	1
4	4	3	3	3	3	4	4	1	1
4	4	3	3	3	3	4	4	1	1
1	1	3	3	3	3	1	1	1	1
1	1	3	3	3	3	4	4	1	1
4	4	3	3	3	3	4	4	1	1
1	1	3	3	3	3	4	4	1	1
1	1	3	3	3	3	4	4	1	1
4	4	3	3	3	3	4	4	1	1
1	1	3	3	3	3	4	4	1	1
1	1	3	3	3	3	4	4	1	1
1	1	3	3	3	3	4	4	1	1
4	4	3	3	3	3	4	4	1	1
1	1	3	3	3	3	4	4	1	1
1	1	3	3	3	3	4	4	1	1
1	1	3	3	3	3	4	4	1	1
1	1	3	3	3	3	1	1	1	1
1	1	3	3	3	3	4	4	1	1
1	1	3	3	3	3	1	1	1	1
1	1	3	3	3	3	4	4	1	1
1	1	3	3	3	3	4	4	1	1
1	1	1	1	3	3	4	4	1	1
1	1	3	3	3	3	1	1	1	1
1	1	3	3	3	3	1	1	1	1
1	1	3	3	3	3	4	4	1	1
1	1	3	3	3	3	4	4	1	1
1	1	3	3	3	3	4	4	1	1
1	1	3	3	3	3	4	4	1	1
1	1	3	3	3	3	1	1	1	1
4	4	3	3	3	3	4	4	1	1
1	1	3	3	1	1	1	1	4	4
1	1	3	3	3	3	1	1	1	1
1	1	3	3	3	3	4	4	1	1
4	4	3	3	3	3	4	4	1	1
1	1	3	3	3	3	1	1	1	1
1	1	3	3	3	3	4	4	1	1
4	4	3	3	1	1	1	1	4	4
1	1	3	3	3	3	4	4	1	1
1	1	3	3	1	1	1	1	4	4
1	1	3	3	3	3	4	4	1	1
1	1	3	3	3	3	4	4	1	1
1	1	3	3	3	3	4	4	1	1
1	1	3	3	3	3	4	4	1	1
1	1	3	3	3	3	4	4	1	1
4	4	3	3	3	3	1	1	1	1
1	1	3	3	3	3	4	4	1	1
4	4	3	3	3	3	4	4	1	1
1	1	3	3	3	3	4	4	1	1
1	1	3	3	3	3	1	1	1	1
1	1	3	3	3	3	4	4	1	1
1	1	3	3	3	3	4	4	1	1
1	1	1	1	3	3	4	4	1	1
1	1	1	1	3	3	1	1	1	1
1	1	3	3	3	3	4	4	1	1

For Review Only

1	1	3	3	3	3	4	4	1	1
4	4	3	3	3	3	4	4	1	1
1	1	3	3	3	3	4	4	1	1
1	1	3	3	3	3	4	4	1	1
1	1	3	3	3	3	4	4	1	1
1	1	3	3	1	1	1	1	4	4
4	4	3	3	3	3	4	4	1	1
4	4	3	3	3	3	4	4	1	1
1	1	1	1	3	3	4	4	1	1
1	1	3	3	3	3	4	4	1	1
1	1	3	3	3	3	4	4	1	1
4	4	3	3	3	3	4	4	1	1
1	1	3	3	3	3	4	4	1	1
1	1	3	3	3	3	4	4	1	1
1	1	3	3	3	3	4	4	1	1
1	1	3	3	3	3	4	4	1	1
1	1	1	1	3	3	4	4	1	1
1	1	3	3	3	3	1	1	1	1
1	1	3	3	3	3	4	4	1	1
1	1	3	3	3	3	4	4	1	1
1	1	1	1	3	3	4	4	1	1
1	1	3	3	3	3	4	4	1	1
4	4	3	3	3	3	1	1	1	1
1	1	3	3	3	3	4	4	1	1
1	1	3	3	3	3	4	4	1	1
1	1	3	3	3	3	1	1	4	4
1	1	3	3	3	3	4	4	1	1
1	1	1	1	3	3	1	1	1	1
1	1	3	3	3	3	4	4	1	1
1	1	3	3	3	3	4	4	1	1
1	1	3	3	3	3	4	4	1	1
1	1	3	3	3	3	4	4	1	1
1	1	3	3	3	3	4	4	1	1
1	1	3	3	3	3	4	4	1	1
1	1	3	3	3	3	4	4	1	1
1	1	3	3	3	3	4	4	1	1
1	1	3	3	3	3	4	4	1	1
1	1	3	3	3	3	1	1	1	1
1	1	3	3	3	3	4	4	1	1
1	1	3	3	3	3	4	4	1	1
1	1	3	3	3	3	1	1	1	1
4	4	3	3	1	1	1	1	4	4
1	1	3	3	3	3	4	4	1	1
4	4	3	3	3	3	1	1	1	1
1	1	3	3	3	3	4	4	1	1
1	1	3	3	3	3	1	1	1	1
1	1	3	3	3	3	4	4	1	1
1	1	3	3	3	3	4	4	1	1
4	4	3	3	3	3	4	4	1	1
1	1	3	3	3	3	4	4	1	1
4	4	3	3	3	3	1	1	1	1
1	1	1	1	3	3	4	4	1	1
1	1	3	3	3	3	4	4	1	1
1	1	1	1	3	3	1	1	1	1
1	1	3	3	3	3	4	4	1	1

For Review Only

1	1	3	3	3	3	4	4	1	1
1	1	1	1	3	3	1	1	1	1

For Review Only

SNP1313	SNP1313	SNP1314	SNP1314	SNP1315	SNP1315	SNP1316	SNP1316	SNP1317	SNP1317
4	4	4	4	1	1	1	1	4	4
1	1	1	1	4	4	1	1	4	4
4	4	1	1	4	4	1	1	4	4
4	4	1	1	4	4	1	1	4	4
4	4	4	4	1	1	1	1	3	3
1	1	1	1	4	4	1	1	4	4
4	4	4	4	4	4	1	1	4	4
1	1	4	4	4	4	1	1	3	3
4	4	1	1	4	4	1	1	3	3
4	4	4	4	4	4	1	1	3	3
4	4	1	1	4	4	1	1	4	4
4	4	1	1	4	4	1	1	4	4
4	4	4	4	1	1	1	1	3	3
4	4	1	1	1	1	1	1	4	4
4	4	1	1	4	4	1	1	4	4
4	4	1	1	4	4	1	1	4	4
4	4	1	1	4	4	1	1	4	4
4	4	4	4	4	4	1	1	3	3
4	4	1	1	4	4	1	1	4	4
4	4	4	4	1	1	4	4	4	4
4	4	4	4	4	4	1	1	4	4
4	4	1	1	4	4	1	1	3	3
4	4	4	4	1	1	1	1	4	4
4	4	1	1	4	4	1	1	4	4
4	4	1	1	4	4	1	1	4	4
4	4	1	1	4	4	1	1	4	4
4	4	1	1	4	4	1	1	4	4
4	4	1	1	4	4	1	1	4	4
4	4	1	1	4	4	1	1	3	3
4	4	1	1	4	4	1	1	3	3
4	4	1	1	4	4	1	1	4	4
4	4	1	1	4	4	4	4	4	4
4	4	1	1	4	4	1	1	4	4
4	4	1	1	4	4	1	1	4	4
4	4	1	1	1	1	1	1	3	3
4	4	1	1	4	4	1	1	4	4
4	4	4	4	1	1	1	1	4	4
4	4	1	1	4	4	1	1	4	4
4	4	4	4	1	1	1	1	4	4
4	4	1	1	4	4	1	1	4	4
4	4	4	4	4	4	1	1	3	3
4	4	1	1	4	4	1	1	3	3
4	4	4	4	4	4	4	4	3	3
4	4	4	4	1	1	1	1	4	4
4	4	4	4	4	4	1	1	4	4
4	4	1	1	4	4	1	1	4	4
1	1	1	1	4	4	1	1	4	4
4	4	1	1	4	4	1	1	4	4
4	4	4	4	4	4	1	1	4	4
4	4	1	1	4	4	1	1	4	4
4	4	1	1	4	4	4	4	4	4
1	1	4	4	1	1	1	1	4	4

For Review Only

4	4	1	1	4	4	1	1	4	4
4	4	1	1	4	4	1	1	4	4
4	4	1	1	4	4	1	1	4	4
4	4	4	4	4	4	1	1	3	3
4	4	1	1	4	4	1	1	4	4
1	1	1	1	4	4	1	1	4	4
4	4	1	1	4	4	1	1	4	4
4	4	4	4	1	1	1	1	4	4
4	4	1	1	1	1	1	1	4	4
4	4	1	1	4	4	1	1	4	4
1	1	1	1	4	4	1	1	4	4
1	1	1	1	4	4	1	1	4	4
4	4	1	1	4	4	1	1	4	4
4	4	1	1	4	4	1	1	3	3
4	4	1	1	4	4	1	1	4	4
4	4	4	4	1	1	1	1	4	4
4	4	1	1	1	1	1	1	4	4
4	4	1	1	4	4	1	1	4	4
4	4	1	1	4	4	1	1	4	4
4	4	1	1	4	4	1	1	4	4
1	1	1	1	4	4	1	1	4	4
4	4	1	1	4	4	1	1	4	4
4	4	1	1	4	4	1	1	3	3
4	4	1	1	4	4	4	4	4	4
4	4	4	4	4	4	1	1	4	4
1	1	1	1	4	4	1	1	4	4
4	4	4	4	1	1	1	1	4	4
4	4	4	4	1	1	1	1	4	4
4	4	1	1	4	4	1	1	4	4
4	4	1	1	4	4	1	1	4	4
4	4	1	1	4	4	1	1	3	3
4	4	1	1	4	4	1	1	4	4
4	4	4	4	1	1	1	1	4	4
4	4	1	1	4	4	1	1	4	4
4	4	4	4	1	1	1	1	4	4
4	4	4	4	4	4	1	1	4	4
4	4	4	4	4	4	1	1	3	3
4	4	4	4	4	4	1	1	3	3
4	4	4	4	1	1	1	1	3	3
4	4	4	4	4	4	1	1	4	4
4	4	1	1	4	4	1	1	4	4
4	4	1	1	4	4	1	1	4	4
4	4	4	4	4	4	1	1	4	4
1	1	1	1	4	4	1	1	4	4
4	4	1	1	4	4	1	1	3	3
1	1	1	1	4	4	1	1	4	4
4	4	4	4	1	1	1	1	4	4
4	4	1	1	4	4	1	1	4	4
4	4	1	1	4	4	1	1	4	4
4	4	1	1	4	4	1	1	4	4
1	1	1	1	4	4	1	1	4	4
4	4	4	4	1	1	1	1	4	4
4	4	4	4	1	1	1	1	4	4
4	4	4	4	1	1	4	4	4	4
4	4	1	1	4	4	1	1	4	4

For Review Only

4	4	1	1	4	4	1	1	4	4
4	4	4	4	1	1	1	1	4	4
4	4	1	1	4	4	1	1	4	4
4	4	4	4	1	1	1	1	4	4
4	4	1	1	4	4	1	1	4	4
4	4	1	1	4	4	1	1	4	4
4	4	1	1	4	4	1	1	4	4
4	4	1	1	4	4	1	1	4	4
4	4	4	4	4	4	1	1	4	4
4	4	1	1	4	4	1	1	3	3
4	4	4	4	1	1	1	1	4	4
4	4	1	1	4	4	1	1	4	4
4	4	4	4	4	4	1	1	4	4
4	4	1	1	4	4	1	1	4	4
4	4	1	1	4	4	1	1	4	4
4	4	1	1	1	1	1	1	4	4
4	4	1	1	4	4	1	1	4	4
4	4	1	1	1	1	1	1	4	4
4	4	4	4	1	1	1	1	4	4
4	4	1	1	4	4	1	1	4	4
4	4	1	1	4	4	1	1	4	4
4	4	1	1	4	4	1	1	4	4
4	4	1	1	4	4	1	1	4	4
4	4	4	4	1	1	1	1	4	4
4	4	1	1	4	4	1	1	4	4
4	4	1	1	4	4	1	1	4	4
4	4	1	1	4	4	1	1	4	4
1	1	1	1	4	4	1	1	4	4
4	4	1	1	4	4	1	1	4	4
4	4	1	1	4	4	1	1	4	4
4	4	4	4	1	1	1	1	4	4
4	4	1	1	4	4	1	1	4	4
4	4	1	1	4	4	1	1	4	4
4	4	1	1	4	4	4	4	4	4
4	4	1	1	4	4	1	1	4	4
4	4	1	1	1	1	1	1	4	4
4	4	4	4	1	1	1	1	4	4
1	1	1	1	4	4	1	1	4	4
4	4	1	1	1	1	1	1	4	4
4	4	1	1	1	1	1	1	4	4
4	4	1	1	1	1	1	1	4	4
4	4	4	4	1	1	1	1	4	4
4	4	1	1	4	4	1	1	4	4
4	4	4	4	4	4	1	1	3	3
4	4	1	1	4	4	1	1	4	4
4	4	4	4	1	1	1	1	4	4
4	4	1	1	4	4	1	1	4	4
4	4	1	1	4	4	4	4	4	4
4	4	4	4	1	1	1	1	4	4
4	4	1	1	4	4	1	1	4	4
4	4	1	1	4	4	1	1	4	4
4	4	4	4	1	1	1	1	4	4
4	4	1	1	4	4	1	1	4	4
1	1	1	1	4	4	1	1	4	4
4	4	4	4	1	1	1	1	4	4

For Review Only

4	4	1	1	4	4	1	1	4	4
4	4	4	4	4	4	1	1	4	4
4	4	4	4	4	4	1	1	4	4
4	4	1	1	1	1	1	1	4	4
4	4	1	1	4	4	1	1	4	4
4	4	1	1	4	4	1	1	4	4
4	4	1	1	1	1	1	1	4	4
4	4	1	1	1	1	1	1	4	4
4	4	4	4	1	1	1	1	4	4
4	4	1	1	4	4	1	1	4	4
4	4	1	1	4	4	1	1	4	4
4	4	1	1	4	4	1	1	4	4
4	4	1	1	4	4	4	4	4	4
4	4	4	4	1	1	1	1	4	4
4	4	4	4	1	1	1	1	4	4
4	4	1	1	4	4	1	1	4	4
4	4	4	4	1	1	1	1	4	4
4	4	1	1	4	4	1	1	4	4
4	4	1	1	4	4	1	1	4	4
4	4	1	1	4	4	1	1	4	4
4	4	1	1	4	4	1	1	4	4
4	4	4	4	1	1	1	1	3	3
4	4	1	1	4	4	1	1	4	4
1	1	1	1	4	4	1	1	4	4
4	4	4	4	4	4	1	1	4	4
4	4	1	1	4	4	1	1	4	4
4	4	1	1	4	4	1	1	4	4
4	4	1	1	1	1	1	1	4	4
4	4	1	1	4	4	1	1	4	4
4	4	4	4	1	1	1	1	4	4
4	4	1	1	4	4	1	1	4	4
4	4	1	1	4	4	1	1	4	4
1	1	1	1	4	4	1	1	4	4
4	4	4	4	1	1	1	1	4	4
4	4	1	1	4	4	1	1	4	4
1	1	1	1	4	4	1	1	4	4
4	4	1	1	4	4	1	1	4	4
4	4	4	4	1	1	1	1	4	4
4	4	1	1	4	4	4	4	4	4
4	4	1	1	4	4	4	4	4	4
4	4	1	1	4	4	1	1	4	4
4	4	1	1	4	4	1	1	4	4
4	4	4	4	1	1	1	1	4	4
4	4	1	1	4	4	1	1	4	4
4	4	1	1	4	4	1	1	4	4
1	1	1	1	4	4	1	1	4	4
4	4	1	1	4	4	1	1	4	4
4	4	4	4	1	1	1	1	4	4
4	4	4	4	1	1	1	1	4	4
4	4	4	4	4	4	1	1	4	4
4	4	1	1	4	4	4	4	4	4
4	4	4	4	1	1	1	1	4	4
4	4	1	1	4	4	1	1	4	4
1	1	1	1	4	4	1	1	4	4
4	4	4	4	1	1	1	1	4	4

For Review Only

4	4	4	4	1	1	1	1	4	4
4	4	4	4	1	1	1	1	4	4
4	4	1	1	4	4	4	4	4	4
4	4	1	1	4	4	1	1	4	4
4	4	4	4	1	1	1	1	4	4
4	4	1	1	4	4	1	1	4	4
4	4	1	1	4	4	1	1	4	4
4	4	4	4	4	4	4	4	3	3
4	4	1	1	4	4	1	1	4	4
4	4	4	4	1	1	1	1	4	4
4	4	1	1	4	4	1	1	4	4
4	4	1	1	4	4	1	1	4	4
4	4	1	1	4	4	1	1	4	4
4	4	1	1	4	4	4	4	4	4
4	4	1	1	4	4	1	1	4	4
4	4	1	1	1	1	1	1	4	4
4	4	4	4	1	1	1	1	4	4
1	1	1	1	4	4	1	1	4	4
4	4	4	4	1	1	1	1	4	4
4	4	1	1	4	4	1	1	4	4
4	4	4	4	1	1	1	1	4	4
4	4	1	1	4	4	1	1	4	4
1	1	1	1	4	4	1	1	4	4
4	4	1	1	4	4	1	1	4	4
4	4	4	4	1	1	1	1	4	4
4	4	1	1	1	1	1	1	4	4
4	4	4	4	1	1	1	1	4	4
4	4	1	1	4	4	1	1	4	4
4	4	1	1	4	4	1	1	4	4
4	4	1	1	4	4	4	4	4	4
4	4	1	1	4	4	1	1	4	4
4	4	1	1	4	4	1	1	4	4
4	4	4	4	1	1	1	1	4	4
4	4	1	1	4	4	1	1	4	4
4	4	1	1	4	4	1	1	4	4
4	4	4	4	1	1	1	1	4	4
4	4	1	1	4	4	1	1	4	4
1	1	1	1	4	4	1	1	4	4
4	4	1	1	4	4	1	1	4	4
4	4	4	4	1	1	1	1	4	4
1	1	1	1	4	4	1	1	4	4
4	4	1	1	4	4	1	1	4	4
4	4	1	1	4	4	1	1	4	4
4	4	1	1	4	4	1	1	4	4
4	4	1	1	4	4	1	1	4	4
4	4	4	4	1	1	1	1	4	4
4	4	1	1	4	4	1	1	4	4
4	4	4	4	4	4	4	4	4	4
4	4	1	1	4	4	1	1	4	4
4	4	4	4	1	1	1	1	4	4
1	1	1	1	4	4	1	1	4	4
4	4	4	4	1	1	1	1	4	4
4	4	1	1	4	4	1	1	4	4
4	4	1	1	4	4	1	1	4	4
4	4	4	4	1	1	1	1	4	4

For Review Only

4	4	1	1	4	4	1	1	4	4
1	1	1	1	4	4	1	1	4	4

For Review Only

SNP1318	SNP1318	SNP1319	SNP1319	SNP132	SNP132	SNP1320	SNP1320	SNP1321	SNP1321
1	1	2	2	4	4	2	2	3	3
1	1	2	2	4	4	2	2	3	3
4	4	1	1	4	4	2	2	2	2
4	4	1	1	4	4	2	2	2	2
1	1	2	2	1	1	2	2	3	3
4	4	1	1	4	4	3	3	2	2
1	1	1	1	1	1	2	2	3	3
1	1	1	1	4	4	2	2	3	3
4	4	1	1	1	1	2	2	2	2
1	1	1	1	4	4	2	2	3	3
4	4	1	1	4	4	2	2	2	2
4	4	1	1	4	4	2	2	2	2
1	1	1	1	1	1	2	2	3	3
1	1	2	2	4	4	2	2	3	3
4	4	1	1	1	1	2	2	2	2
4	4	1	1	4	4	3	3	2	2
4	4	1	1	4	4	3	3	2	2
1	1	1	1	1	1	2	2	3	3
4	4	1	1	4	4	3	3	2	2
1	1	1	1	4	4	3	3	3	3
1	1	1	1	1	1	2	2	3	3
4	4	1	1	1	1	2	2	2	2
1	1	2	2	1	1	2	2	3	3
1	1	2	2	4	4	2	2	3	3
4	4	1	1	1	1	3	3	2	2
4	4	1	1	4	4	2	2	2	2
4	4	1	1	4	4	2	2	2	2
4	4	1	1	4	4	2	2	2	2
4	4	1	1	1	1	2	2	2	2
1	1	1	1	4	4	2	2	3	3
4	4	1	1	4	4	2	2	2	2
4	4	1	1	4	4	2	2	2	2
4	4	1	1	1	1	2	2	2	2
1	1	2	2	4	4	2	2	3	3
4	4	2	2	4	4	2	2	3	3
4	4	1	1	4	4	2	2	2	2
1	1	2	2	4	4	2	2	3	3
4	4	1	1	4	4	2	2	2	2
1	1	1	1	4	4	2	2	3	3
4	4	1	1	4	4	2	2	2	2
1	1	1	1	4	4	2	2	3	3
1	1	1	1	1	1	2	2	3	3
1	1	1	1	4	4	2	2	3	3
1	1	2	2	4	4	2	2	3	3
1	1	1	1	4	4	2	2	2	2
4	4	1	1	1	1	2	2	2	2
4	4	1	1	4	4	2	2	2	2
4	4	1	1	1	1	2	2	2	2
1	1	1	1	4	4	2	2	3	3
4	4	1	1	1	1	2	2	2	2
4	4	1	1	4	4	2	2	2	2
1	1	1	1	1	1	2	2	3	3

For Review Only

4	4	1	1	1	1	3	3	2	2
4	4	1	1	1	1	2	2	2	2
4	4	1	1	1	1	2	2	2	2
1	1	1	1	4	4	2	2	3	3
4	4	1	1	4	4	3	3	2	2
4	4	1	1	4	4	2	2	2	2
4	4	1	1	4	4	2	2	2	2
1	1	2	2	1	1	2	2	3	3
1	1	2	2	4	4	2	2	3	3
4	4	1	1	4	4	2	2	2	2
4	4	1	1	1	1	2	2	2	2
4	4	1	1	4	4	2	2	2	2
4	4	1	1	1	1	3	3	2	2
4	4	1	1	1	1	2	2	2	2
4	4	1	1	4	4	2	2	2	2
1	1	2	2	4	4	2	2	3	3
1	1	1	1	4	4	2	2	3	3
4	4	1	1	1	1	2	2	2	2
4	4	1	1	4	4	3	3	2	2
4	4	1	1	1	1	2	2	2	2
4	4	1	1	4	4	2	2	2	2
4	4	1	1	4	4	2	2	2	2
4	4	1	1	1	1	2	2	2	2
4	4	1	1	1	1	3	3	2	2
1	1	1	1	4	4	2	2	2	2
4	4	1	1	4	4	2	2	2	2
1	1	1	1	4	4	2	2	3	3
1	1	1	1	4	4	2	2	2	2
4	4	1	1	4	4	2	2	2	2
4	4	1	1	4	4	3	3	2	2
1	1	2	2	1	1	2	2	3	3
4	4	1	1	4	4	3	3	2	2
1	1	1	1	4	4	2	2	3	3
4	4	1	1	1	1	3	3	2	2
1	1	2	2	4	4	2	2	3	3
1	1	1	1	4	4	2	2	2	2
1	1	1	1	1	1	2	2	3	3
1	1	1	1	1	1	2	2	3	3
1	1	2	2	4	4	2	2	3	3
1	1	1	1	1	1	2	2	2	2
1	1	2	2	1	1	3	3	3	3
4	4	1	1	4	4	3	3	2	2
1	1	1	1	4	4	2	2	3	3
4	4	1	1	1	1	2	2	2	2
4	4	1	1	1	1	2	2	2	2
4	4	1	1	4	4	2	2	2	2
1	1	2	2	4	4	2	2	3	3
4	4	1	1	4	4	2	2	2	2
1	1	2	2	4	4	2	2	3	3
4	4	1	1	1	1	2	2	2	2
4	4	1	1	4	4	2	2	2	2
1	1	1	1	4	4	2	2	3	3
1	1	2	2	4	4	2	2	3	3
4	4	1	1	1	1	2	2	2	2
4	4	1	1	1	1	2	2	2	2

For Review Only

4	4	1	1	1	1	2	2	2	2
1	1	1	1	4	4	2	2	3	3
4	4	1	1	1	1	2	2	2	2
1	1	2	2	4	4	2	2	3	3
4	4	1	1	4	4	2	2	2	2
4	4	1	1	1	1	3	3	2	2
4	4	1	1	4	4	2	2	2	2
4	4	1	1	4	4	2	2	2	2
1	1	1	1	4	4	2	2	3	3
4	4	1	1	4	4	2	2	2	2
4	4	2	2	4	4	2	2	3	3
4	4	1	1	1	1	2	2	2	2
1	1	1	1	4	4	2	2	3	3
4	4	1	1	4	4	2	2	2	2
4	4	1	1	4	4	2	2	2	2
4	4	1	1	4	4	2	2	2	2
4	4	1	1	1	1	2	2	2	2
4	4	1	1	4	4	2	2	2	2
1	1	2	2	4	4	2	2	3	3
4	4	1	1	4	4	2	2	2	2
4	4	1	1	1	1	2	2	2	2
4	4	1	1	1	1	3	3	2	2
4	4	1	1	4	4	2	2	2	2
1	1	1	1	4	4	2	2	2	2
4	4	1	1	4	4	2	2	2	2
4	4	1	1	1	1	2	2	2	2
1	1	2	2	1	1	2	2	3	3
4	4	2	2	4	4	2	2	3	3
4	4	1	1	1	1	2	2	2	2
4	4	1	1	1	1	2	2	2	2
1	1	2	2	4	4	2	2	3	3
4	4	1	1	1	1	2	2	2	2
1	1	2	2	1	1	3	3	3	3
4	4	1	1	4	4	2	2	2	2
1	1	2	2	4	4	2	2	3	3
4	4	1	1	1	1	2	2	3	3
1	1	2	2	4	4	2	2	3	3
4	4	1	1	4	4	2	2	2	2
4	4	1	1	1	1	2	2	2	2
4	4	1	1	4	4	2	2	2	2
4	4	1	1	4	4	2	2	2	2
1	1	2	2	4	4	2	2	3	3
4	4	1	1	1	1	2	2	2	2
4	4	1	1	4	4	2	2	3	3
4	4	1	1	4	4	2	2	2	2
4	4	1	1	4	4	2	2	2	2
4	4	1	1	4	4	2	2	2	2
4	4	1	1	4	4	2	2	2	2
4	4	1	1	4	4	2	2	2	2
4	4	1	1	4	4	2	2	2	2
4	4	1	1	1	1	2	2	2	2
1	1	2	2	4	4	2	2	3	3
4	4	1	1	1	1	2	2	2	2
4	4	1	1	4	4	2	2	2	2
4	4	1	1	4	4	2	2	2	2

For Review Only

4	4	1	1	1	1	2	2	2	2
1	1	1	1	4	4	2	2	3	3
1	1	1	1	4	4	2	2	2	2
1	1	2	2	1	1	2	2	3	3
4	4	1	1	4	4	2	2	2	2
4	4	1	1	4	4	2	2	2	2
4	4	1	1	4	4	2	2	2	2
4	4	1	1	1	1	2	2	2	2
1	1	1	1	4	4	2	2	3	3
4	4	1	1	1	1	2	2	2	2
4	4	1	1	4	4	2	2	2	2
4	4	1	1	4	4	2	2	2	2
4	4	1	1	4	4	2	2	2	2
4	4	1	1	4	4	2	2	2	2
1	1	2	2	4	4	2	2	3	3
4	4	1	1	4	4	3	3	2	2
4	4	1	1	1	1	2	2	2	2
4	4	1	1	4	4	2	2	2	2
4	4	1	1	4	4	3	3	2	2
4	4	1	1	4	4	2	2	2	2
4	4	1	1	1	1	2	2	2	2
1	1	1	1	4	4	2	2	3	3
4	4	1	1	4	4	2	2	2	2
4	4	1	1	4	4	2	2	2	2
1	1	1	1	4	4	2	2	3	3
1	1	2	2	4	4	2	2	3	3
4	4	1	1	4	4	2	2	2	2
4	4	1	1	1	1	2	2	2	2
4	4	1	1	1	1	2	2	2	2
1	1	1	1	1	1	3	3	3	3
4	4	1	1	4	4	2	2	2	2
4	4	1	1	1	1	2	2	2	2
1	1	2	2	1	1	2	2	3	3
1	1	2	2	4	4	2	2	3	3
4	4	1	1	4	4	2	2	2	2
4	4	1	1	4	4	2	2	2	2
4	4	1	1	4	4	2	2	2	2
1	1	2	2	4	4	2	2	3	3
4	4	1	1	4	4	2	2	2	2
4	4	1	1	1	1	2	2	2	2
4	4	1	1	4	4	2	2	2	2
4	4	1	1	4	4	2	2	2	2
1	1	1	1	4	4	2	2	3	3
4	4	1	1	1	1	2	2	2	2
1	1	1	1	4	4	2	2	2	2
1	1	2	2	4	4	2	2	3	3
4	4	1	1	4	4	3	3	2	2
1	1	1	1	4	4	2	2	3	3
1	1	2	2	4	4	2	2	3	3
1	1	1	1	4	4	2	2	3	3
4	4	1	1	1	1	2	2	2	2
1	1	2	2	4	4	2	2	3	3
4	4	1	1	4	4	2	2	2	2
4	4	1	1	1	1	2	2	2	2
1	1	1	1	4	4	2	2	3	3

For Review Only

1	1	2	2	1	1	2	2	3	3
1	1	1	1	4	4	2	2	3	3
4	4	1	1	4	4	2	2	2	2
4	4	1	1	4	4	2	2	2	2
1	1	1	1	4	4	2	2	3	3
4	4	1	1	1	1	2	2	2	2
4	4	1	1	4	4	2	2	2	2
1	1	1	1	4	4	2	2	3	3
1	1	2	2	4	4	2	2	3	3
1	1	1	1	4	4	2	2	3	3
4	4	1	1	4	4	2	2	2	2
4	4	1	1	4	4	2	2	2	2
4	4	1	1	4	4	2	2	2	2
4	4	1	1	4	4	2	2	2	2
4	4	1	1	1	1	2	2	2	2
1	1	2	2	1	1	3	3	3	3
1	1	1	1	4	4	2	2	3	3
4	4	1	1	4	4	2	2	2	2
1	1	2	2	4	4	2	2	3	3
4	4	1	1	4	4	2	2	2	2
1	1	2	2	4	4	2	2	3	3
4	4	1	1	4	4	2	2	2	2
4	4	1	1	1	1	2	2	2	2
4	4	1	1	4	4	2	2	2	2
1	1	2	2	4	4	2	2	3	3
4	4	1	1	4	4	2	2	2	2
1	1	1	1	4	4	2	2	3	3
4	4	1	1	4	4	2	2	2	2
4	4	1	1	1	1	2	2	2	2
4	4	1	1	1	1	2	2	2	2
4	4	1	1	4	4	2	2	2	2
4	4	1	1	4	4	2	2	2	2
1	1	2	2	4	4	2	2	3	3
4	4	2	2	1	1	2	2	2	2
4	4	1	1	4	4	2	2	2	2
1	1	1	1	4	4	2	2	3	3
4	4	1	1	1	1	2	2	2	2
4	4	1	1	4	4	2	2	2	2
4	4	1	1	4	4	2	2	2	2
1	1	1	1	4	4	2	2	3	3
4	4	1	1	1	1	2	2	2	2
4	4	1	1	4	4	2	2	2	2
4	4	1	1	4	4	2	2	2	2
4	4	1	1	1	1	3	3	2	2
4	4	1	1	4	4	2	2	2	2
1	1	2	2	4	4	2	2	3	3
4	4	1	1	1	1	2	2	2	2
1	1	1	1	4	4	2	2	3	3
4	4	1	1	4	4	2	2	2	2
1	1	1	1	4	4	2	2	3	3
4	4	1	1	4	4	3	3	2	2
1	1	1	1	4	4	2	2	3	3
4	4	1	1	4	4	2	2	2	2
4	4	1	1	1	1	2	2	2	2
1	1	1	1	4	4	2	2	3	3

For Review Only

4	4	1	1	1	1	3	3	2	2
4	4	1	1	4	4	2	2	2	2

For Review Only

SNP1322	SNP1322	SNP1323	SNP1323	SNP1324	SNP1324	SNP1325	SNP1325	SNP1326	SNP1326
2	2	1	1	3	3	4	4	1	1
2	2	1	1	3	3	4	4	4	4
2	2	1	1	3	3	3	3	1	1
2	2	1	1	3	3	4	4	4	4
2	2	1	1	4	4	4	4	1	1
2	2	1	1	3	3	3	3	4	4
2	2	1	1	4	4	4	4	4	4
2	2	1	1	3	3	3	3	4	4
2	2	1	1	4	4	4	4	4	4
2	2	1	1	4	4	4	4	1	1
2	2	1	1	3	3	3	3	1	1
2	2	1	1	3	3	3	3	4	4
2	2	1	1	4	4	4	4	4	4
2	2	1	1	4	4	4	4	1	1
3	3	4	4	3	3	3	3	4	4
3	3	4	4	3	3	3	3	1	1
3	3	4	4	3	3	3	3	4	4
2	2	1	1	4	4	4	4	1	1
3	3	4	4	3	3	3	3	4	4
3	3	4	4	3	3	3	3	1	1
2	2	1	1	4	4	4	4	1	1
2	2	1	1	4	4	4	4	4	4
3	3	4	4	3	3	3	3	1	1
2	2	1	1	3	3	3	3	1	1
3	3	4	4	3	3	3	3	1	1
2	2	1	1	3	3	3	3	1	1
2	2	1	1	3	3	3	3	1	1
2	2	1	1	3	3	3	3	4	4
2	2	1	1	4	4	4	4	4	4
2	2	1	1	4	4	4	4	1	1
2	2	1	1	3	3	3	3	4	4
2	2	1	1	4	4	4	4	1	1
2	2	1	1	4	4	4	4	1	1
2	2	1	1	3	3	3	3	1	1
3	3	1	1	3	3	4	4	1	1
2	2	1	1	3	3	3	3	1	1
3	3	4	4	3	3	3	3	1	1
2	2	1	1	4	4	4	4	1	1
2	2	1	1	4	4	4	4	1	1
3	3	4	4	3	3	3	3	1	1
2	2	1	1	4	4	4	4	1	1
2	2	1	1	4	4	4	4	1	1
2	2	1	1	3	3	4	4	1	1
2	2	1	1	4	4	4	4	1	1
2	2	1	1	3	3	3	3	1	1
2	2	1	1	3	3	3	3	1	1
2	2	1	1	3	3	3	3	4	4
2	2	1	1	3	3	3	3	1	1
2	2	1	1	4	4	4	4	4	4
3	3	1	1	3	3	3	3	1	1
3	3	4	4	3	3	3	3	1	1
2	2	1	1	3	3	3	3	4	4

For Review Only

3	3	4	4	3	3	3	3	4	4
2	2	1	1	4	4	4	4	1	1
2	2	1	1	3	3	3	3	4	4
2	2	1	1	4	4	4	4	4	4
3	3	4	4	3	3	3	3	4	4
2	2	1	1	3	3	3	3	4	4
2	2	1	1	3	3	3	3	1	1
2	2	1	1	4	4	4	4	1	1
2	2	1	1	3	3	3	3	1	1
3	3	1	1	3	3	3	3	4	4
2	2	1	1	3	3	3	3	4	4
2	2	1	1	3	3	3	3	4	4
3	3	4	4	3	3	3	3	4	4
2	2	1	1	4	4	4	4	4	4
2	2	1	1	3	3	3	3	4	4
2	2	1	1	4	4	4	4	1	1
2	2	1	1	3	3	3	3	1	1
2	2	1	1	4	4	4	4	1	1
3	3	4	4	3	3	3	3	4	4
2	2	1	1	3	3	3	3	4	4
2	2	1	1	3	3	3	3	4	4
2	2	1	1	4	4	4	4	1	1
2	2	1	1	4	4	4	4	4	4
3	3	4	4	3	3	3	3	1	1
2	2	1	1	3	3	3	3	1	1
2	2	1	1	3	3	3	3	4	4
2	2	1	1	4	4	4	4	4	4
2	2	1	1	3	3	3	3	1	1
2	2	1	1	3	3	3	3	1	1
3	3	4	4	3	3	3	3	1	1
3	3	4	4	3	3	3	3	1	1
3	3	4	4	3	3	3	3	1	1
2	2	1	1	4	4	4	4	1	1
3	3	4	4	3	3	3	3	4	4
2	2	1	1	3	3	3	3	1	1
2	2	1	1	3	3	3	3	1	1
3	3	4	4	3	3	3	3	1	1
2	2	1	1	4	4	4	4	4	4
2	2	1	1	4	4	4	4	1	1
2	2	1	1	3	3	3	3	1	1
3	3	4	4	3	3	3	3	1	1
3	3	4	4	3	3	3	3	1	1
3	3	4	4	3	3	3	3	1	1
2	2	1	1	3	3	3	3	4	4
2	2	1	1	4	4	4	4	1	1
2	2	1	1	3	3	3	3	4	4
2	2	1	1	3	3	3	3	1	1
2	2	1	1	3	3	3	3	1	1
2	2	1	1	3	3	3	3	1	1
2	2	1	1	4	4	4	4	1	1
2	2	1	1	3	3	3	3	4	4
2	2	1	1	3	3	3	3	1	1
2	2	1	1	4	4	4	4	1	1
3	3	1	1	3	3	3	3	4	4
2	2	1	1	4	4	4	4	1	1

For Review Only

2	2	1	1	3	3	3	3	1	1
2	2	1	1	4	4	4	4	1	1
2	2	1	1	3	3	3	3	1	1
2	2	1	1	3	3	3	3	1	1
2	2	1	1	3	3	3	3	4	4
3	3	4	4	3	3	3	3	1	1
3	3	4	4	3	3	3	3	4	4
2	2	1	1	3	3	3	3	1	1
2	2	1	1	3	3	3	3	1	1
2	2	1	1	3	3	4	4	1	1
2	2	1	1	3	3	4	4	1	1
2	2	1	1	3	3	4	4	1	1
2	2	1	1	3	3	3	3	1	1
2	2	1	1	3	3	3	3	4	4
2	2	1	1	3	3	3	3	1	1
2	2	1	1	3	3	3	3	1	1
2	2	1	1	4	4	4	4	1	1
2	2	1	1	3	3	3	3	1	1
2	2	1	1	3	3	3	3	1	1
3	3	4	4	3	3	3	3	4	4
3	3	1	1	3	3	3	3	4	4
3	3	4	4	3	3	3	3	4	4
2	2	1	1	3	3	3	3	1	1
2	2	1	1	3	3	3	3	1	1
2	2	1	1	3	3	3	3	1	1
2	2	1	1	3	3	3	3	1	1
3	3	1	1	3	3	3	3	4	4
2	2	1	1	3	3	3	3	4	4
2	2	1	1	3	3	3	3	4	4
2	2	1	1	3	3	3	3	4	4
2	2	1	1	3	3	4	4	1	1
2	2	1	1	3	3	4	4	4	4
3	3	4	4	3	3	3	3	1	1
2	2	1	1	3	3	3	3	1	1
2	2	1	1	3	3	3	3	1	1
2	2	1	1	3	3	3	3	4	4
2	2	1	1	3	3	3	3	1	1
2	2	1	1	4	4	4	4	4	4
2	2	1	1	3	3	3	3	1	1
3	3	1	1	3	3	4	4	1	1
3	3	1	1	3	3	3	3	1	1
2	2	1	1	3	3	3	3	1	1
2	2	1	1	4	4	4	4	1	1
2	2	1	1	4	4	4	4	1	1
3	3	4	4	3	3	3	3	1	1
3	3	4	4	3	3	3	3	4	4
2	2	1	1	3	3	3	3	1	1
2	2	1	1	3	3	3	3	1	1
2	2	1	1	3	3	3	3	1	1
2	2	1	1	3	3	3	3	1	1
2	2	1	1	3	3	3	3	1	1
2	2	1	1	4	4	4	4	1	1
2	2	1	1	3	3	3	3	1	1
2	2	1	1	3	3	3	3	4	4
2	2	1	1	3	3	4	4	1	1

For Review Only

3	3	1	1	3	3	3	3	4	4
2	2	1	1	3	3	3	3	1	1
3	3	4	4	3	3	3	3	1	1
3	3	1	1	3	3	3	3	1	1
2	2	1	1	3	3	4	4	4	4
2	2	1	1	4	4	4	4	1	1
2	2	1	1	4	4	4	4	1	1
2	2	1	1	3	3	3	3	1	1
3	3	1	1	3	3	3	3	1	1
2	2	1	1	3	3	3	3	4	4
2	2	1	1	3	3	3	3	1	1
2	2	1	1	3	3	4	4	1	1
2	2	1	1	3	3	3	3	1	1
3	3	1	1	3	3	4	4	4	4
2	2	1	1	3	3	3	3	1	1
3	3	4	4	3	3	3	3	1	1
2	2	1	1	3	3	3	3	1	1
3	3	4	4	3	3	3	3	4	4
3	3	4	4	3	3	3	3	1	1
3	3	4	4	3	3	3	3	4	4
2	2	1	1	3	3	3	3	4	4
3	3	4	4	3	3	3	3	1	1
2	2	1	1	4	4	4	4	1	1
2	2	1	1	3	3	3	3	4	4
2	2	1	1	4	4	4	4	4	4
2	2	1	1	3	3	3	3	1	1
2	2	1	1	3	3	3	3	4	4
2	2	1	1	4	4	4	4	1	1
2	2	1	1	4	4	4	4	1	1
3	3	4	4	3	3	3	3	4	4
2	2	1	1	3	3	3	3	4	4
2	2	1	1	4	4	4	4	1	1
3	3	4	4	3	3	3	3	4	4
2	2	1	1	3	3	3	3	1	1
2	2	1	1	3	3	3	3	4	4
2	2	1	1	3	3	3	3	4	4
2	2	1	1	3	3	3	3	1	1
3	3	4	4	3	3	3	3	1	1
2	2	1	1	3	3	3	3	1	1
3	3	4	4	3	3	3	3	1	1
2	2	1	1	3	3	3	3	1	1
2	2	1	1	3	3	3	3	1	1
2	2	1	1	3	3	3	3	1	1
3	3	1	1	3	3	3	3	1	1
3	3	1	1	3	3	3	3	1	1
2	2	1	1	4	4	4	4	4	4
3	3	4	4	3	3	3	3	1	1
2	2	1	1	4	4	4	4	1	1
2	2	1	1	3	3	3	3	1	1
2	2	1	1	4	4	4	4	4	4
2	2	1	1	3	3	3	3	1	1
3	3	4	4	3	3	3	3	1	1
2	2	1	1	4	4	4	4	1	1
2	2	1	1	3	3	3	3	4	4
2	2	1	1	3	3	3	3	1	1

For Review Only

2	2	1	1	3	3	3	3	1	1
2	2	1	1	3	3	3	3	1	1
2	2	1	1	3	3	3	3	1	1
3	3	1	1	3	3	4	4	4	4
2	2	1	1	4	4	4	4	1	1
2	2	1	1	3	3	3	3	1	1
2	2	1	1	3	3	3	3	1	1
2	2	1	1	4	4	4	4	1	1
2	2	1	1	3	3	4	4	1	1
2	2	1	1	3	3	3	3	1	1
2	2	1	1	3	3	4	4	4	4
3	3	1	1	3	3	3	3	1	1
2	2	1	1	3	3	3	3	1	1
2	2	1	1	3	3	3	3	1	1
2	2	1	1	3	3	3	3	1	1
3	3	4	4	3	3	3	3	1	1
2	2	1	1	4	4	4	4	1	1
2	2	1	1	4	4	4	4	4	4
2	2	1	1	4	4	4	4	1	1
2	2	1	1	4	4	4	4	1	1
2	2	1	1	4	4	4	4	4	4
2	2	1	1	3	3	3	3	1	1
2	2	1	1	3	3	3	3	4	4
3	3	4	4	3	3	3	3	1	1
2	2	1	1	4	4	4	4	1	1
2	2	1	1	3	3	3	3	4	4
2	2	1	1	4	4	4	4	1	1
2	2	1	1	3	3	3	3	4	4
3	3	1	1	3	3	4	4	1	1
2	2	1	1	3	3	3	3	1	1
2	2	1	1	4	4	4	4	1	1
3	3	1	1	3	3	3	3	1	1
2	2	1	1	4	4	4	4	1	1
2	2	1	1	3	3	3	3	1	1
2	2	1	1	3	3	3	3	1	1
2	2	1	1	4	4	4	4	1	1
2	2	1	1	3	3	3	3	1	1
2	2	1	1	3	3	3	3	4	4
2	2	1	1	3	3	3	3	1	1
2	2	1	1	3	3	3	3	1	1
2	2	1	1	4	4	4	4	4	4
2	2	1	1	4	4	4	4	1	1
2	2	1	1	4	4	4	4	1	1
3	3	4	4	3	3	3	3	4	4
2	2	1	1	4	4	4	4	1	1
2	2	1	1	3	3	3	3	4	4
2	2	1	1	3	3	3	3	1	1
2	2	1	1	3	3	3	3	1	1
2	2	1	1	3	3	3	3	1	1
2	2	1	1	4	4	4	4	1	1
3	3	4	4	3	3	3	3	4	4
2	2	1	1	3	3	3	3	1	1
2	2	1	1	3	3	3	3	1	1
2	2	1	1	3	3	3	3	4	4
3	3	4	4	3	3	3	3	1	1

For Review Only

3	3	4	4	3	3	3	3	4	4
2	2	1	1	3	3	3	3	4	4

For Review Only

SNP1327	SNP1327	SNP1328	SNP1328	SNP1329	SNP1329	SNP133	SNP133	SNP1330	SNP1330
1	1	2	2	3	3	2	2	2	2
4	4	2	2	3	3	1	1	2	2
1	1	1	1	2	2	1	1	2	2
1	1	1	1	2	2	1	1	2	2
1	1	2	2	3	3	1	1	2	2
1	1	1	1	2	2	1	1	2	2
1	1	1	1	2	2	2	2	2	2
4	4	2	2	3	3	1	1	2	2
1	1	1	1	2	2	2	2	2	2
4	4	2	2	3	3	1	1	2	2
1	1	1	1	2	2	2	2	1	1
1	1	1	1	2	2	1	1	2	2
1	1	2	2	3	3	2	2	2	2
1	1	2	2	3	3	1	1	2	2
1	1	1	1	2	2	2	2	2	2
1	1	1	1	2	2	2	2	1	1
1	1	1	1	2	2	1	1	2	2
1	1	2	2	3	3	2	2	2	2
4	4	1	1	2	2	2	2	2	2
4	4	2	2	3	3	2	2	2	2
4	4	2	2	3	3	1	1	2	2
1	1	1	1	2	2	2	2	2	2
4	4	1	1	2	2	1	1	2	2
4	4	2	2	3	3	1	1	2	2
1	1	1	1	2	2	1	1	2	2
1	1	1	1	2	2	2	2	2	2
4	4	1	1	2	2	1	1	2	2
1	1	1	1	2	2	1	1	2	2
4	4	1	1	2	2	1	1	2	2
4	4	2	2	3	3	1	1	2	2
4	4	1	1	2	2	2	2	2	2
1	1	1	1	2	2	2	2	2	2
1	1	1	1	2	2	2	2	2	2
4	4	1	1	2	2	1	1	2	2
4	4	1	1	2	2	2	2	2	2
4	4	1	1	2	2	1	1	2	2
4	4	2	2	3	3	1	1	2	2
1	1	1	1	2	2	1	1	2	2
4	4	2	2	3	3	1	1	1	1
1	1	1	1	2	2	2	2	2	2
4	4	2	2	3	3	1	1	2	2
4	4	2	2	2	2	2	2	2	2
4	4	2	2	2	2	1	1	2	2
1	1	2	2	3	3	1	1	2	2
1	1	1	1	2	2	1	1	2	2
1	1	1	1	2	2	2	2	2	2
4	4	1	1	2	2	2	2	2	2
1	1	1	1	2	2	1	1	2	2
1	1	2	2	3	3	1	1	2	2
1	1	1	1	2	2	2	2	2	2
1	1	1	1	2	2	1	1	2	2
1	1	2	2	3	3	2	2	2	2

For Review Only

1	1	1	1	2	2	2	2	2	2
1	1	1	1	2	2	2	2	2	2
1	1	1	1	2	2	2	2	2	2
1	1	2	2	3	3	2	2	2	2
4	4	1	1	2	2	2	2	2	2
4	4	1	1	2	2	2	2	2	2
4	4	1	1	2	2	2	2	2	2
4	4	2	2	3	3	1	1	2	2
4	4	1	1	2	2	2	2	2	2
1	1	1	1	2	2	2	2	2	2
1	1	1	1	2	2	1	1	2	2
1	1	1	1	2	2	1	1	2	2
1	1	1	1	2	2	2	2	2	2
4	4	1	1	2	2	1	1	2	2
1	1	1	1	2	2	1	1	2	2
1	1	2	2	3	3	1	1	2	2
1	1	1	1	2	2	1	1	1	1
4	4	1	1	2	2	2	2	2	2
1	1	1	1	2	2	2	2	2	2
1	1	1	1	2	2	2	2	2	2
1	1	1	1	2	2	1	1	2	2
4	4	1	1	2	2	2	2	1	1
4	4	1	1	2	2	1	1	2	2
1	1	1	1	2	2	1	1	2	2
1	1	1	1	2	2	1	1	2	2
1	1	1	1	2	2	1	1	2	2
1	1	2	2	3	3	2	2	2	2
1	1	2	2	3	3	1	1	1	1
4	4	1	1	2	2	2	2	2	2
1	1	1	1	2	2	2	2	2	2
4	4	2	2	3	3	2	2	2	2
4	4	1	1	2	2	1	1	1	1
4	4	2	2	3	3	1	1	2	2
1	1	1	1	2	2	2	2	2	2
4	4	2	2	3	3	2	2	2	2
1	1	1	1	2	2	1	1	2	2
4	4	2	2	3	3	1	1	2	2
1	1	2	2	3	3	2	2	2	2
1	1	1	1	2	2	1	1	2	2
1	1	2	2	3	3	1	1	2	2
1	1	2	2	3	3	2	2	2	2
4	4	1	1	2	2	1	1	2	2
1	1	2	2	3	3	1	1	2	2
1	1	1	1	2	2	1	1	2	2
1	1	1	1	2	2	1	1	2	2
4	4	1	1	2	2	2	2	2	2
1	1	2	2	3	3	1	1	2	2
4	4	1	1	2	2	1	1	2	2
4	4	2	2	3	3	2	2	2	2
1	1	1	1	2	2	2	2	2	2
4	4	1	1	2	2	1	1	2	2
4	4	2	2	3	3	1	1	1	1
4	4	1	1	2	2	1	1	1	1
4	4	1	1	2	2	2	2	2	2
1	1	1	1	2	2	1	1	2	2

For Review Only

4	4	2	2	2	2	2	2	2	2
1	1	2	2	3	3	2	2	2	2
4	4	1	1	2	2	1	1	2	2
4	4	2	2	3	3	2	2	2	2
1	1	1	1	2	2	2	2	2	2
1	1	1	1	2	2	2	2	2	2
1	1	1	1	2	2	2	2	2	2
1	1	1	1	2	2	1	1	2	2
4	4	2	2	3	3	2	2	2	2
1	1	1	1	2	2	1	1	2	2
1	1	2	2	3	3	2	2	2	2
1	1	1	1	2	2	2	2	2	2
1	1	2	2	3	3	1	1	2	2
1	1	1	1	2	2	1	1	2	2
4	4	1	1	2	2	1	1	2	2
4	4	1	1	2	2	2	2	2	2
4	4	1	1	2	2	2	2	2	2
1	1	1	1	2	2	2	2	2	2
4	4	2	2	3	3	2	2	2	2
1	1	1	1	2	2	2	2	2	2
1	1	1	1	2	2	2	2	2	2
1	1	1	1	2	2	2	2	2	2
4	4	1	1	2	2	2	2	2	2
4	4	2	2	3	3	1	1	1	1
1	1	1	1	2	2	2	2	2	2
4	4	1	1	2	2	2	2	2	2
1	1	2	2	3	3	2	2	2	2
4	4	2	2	3	3	1	1	2	2
4	4	1	1	2	2	1	1	2	2
1	1	1	1	2	2	2	2	2	2
1	1	2	2	3	3	1	1	1	1
1	1	1	1	2	2	2	2	2	2
1	1	2	2	3	3	2	2	2	2
4	4	1	1	2	2	2	2	2	2
4	4	1	1	2	2	1	1	2	2
4	4	1	1	2	2	1	1	1	1
4	4	2	2	3	3	1	1	2	2
1	1	1	1	2	2	2	2	2	2
1	1	1	1	2	2	2	2	1	1
1	1	1	1	2	2	2	2	1	1
1	1	1	1	2	2	2	2	2	2
4	4	2	2	3	3	1	1	2	2
4	4	1	1	2	2	1	1	2	2
4	4	2	2	3	3	1	1	2	2
4	4	1	1	2	2	1	1	2	2
4	4	1	1	2	2	1	1	2	2
1	1	1	1	2	2	2	2	2	2
4	4	1	1	2	2	2	2	2	2
4	4	1	1	3	3	1	1	2	2
1	1	1	1	2	2	2	2	2	2
4	4	1	1	2	2	2	2	2	2
4	4	1	1	2	2	2	2	2	2
4	4	1	1	2	2	2	2	2	2
1	1	1	1	2	2	1	1	2	2
1	1	1	1	2	2	2	2	2	2

For Review Only

4	4	1	1	2	2	1	1	2	2
1	1	2	2	3	3	1	1	2	2
1	1	1	1	2	2	1	1	2	2
4	4	1	1	2	2	2	2	2	2
1	1	1	1	2	2	2	2	2	2
1	1	1	1	2	2	2	2	2	2
4	4	2	2	3	3	2	2	2	2
1	1	1	1	2	2	2	2	2	2
4	4	1	1	2	2	2	2	2	2
1	1	1	1	2	2	2	2	2	2
1	1	1	1	2	2	2	2	2	2
4	4	1	1	2	2	1	1	2	2
1	1	1	1	2	2	2	2	2	2
4	4	1	1	2	2	1	1	2	2
4	4	2	2	3	3	1	1	2	2
1	1	1	1	2	2	2	2	1	1
1	1	1	1	2	2	2	2	2	2
4	4	1	1	2	2	1	1	2	2
4	4	1	1	2	2	1	1	2	2
1	1	1	1	2	2	2	2	2	2
1	1	1	1	2	2	2	2	2	2
4	4	2	2	3	3	1	1	2	2
4	4	1	1	2	2	2	2	1	1
1	1	1	1	2	2	1	1	2	2
1	1	2	2	3	3	1	1	2	2
4	4	1	1	2	2	1	1	2	2
1	1	1	1	2	2	1	1	2	2
4	4	1	1	2	2	2	2	2	2
4	4	1	1	2	2	1	1	2	2
1	1	2	2	3	3	2	2	2	2
4	4	1	1	2	2	1	1	2	2
4	4	1	1	2	2	2	2	2	2
1	1	2	2	3	3	2	2	2	2
4	4	2	2	3	3	1	1	2	2
4	4	1	1	2	2	2	2	2	2
1	1	1	1	2	2	1	1	2	2
4	4	1	1	2	2	1	1	2	2
4	4	2	2	3	3	1	1	2	2
4	4	1	1	2	2	2	2	2	2
1	1	1	1	2	2	1	1	2	2
1	1	1	1	2	2	1	1	2	2
4	4	1	1	2	2	2	2	2	2
4	4	2	2	3	3	1	1	1	1
4	4	1	1	2	2	2	2	2	2
1	1	1	1	2	2	2	2	2	2
4	4	2	2	3	3	1	1	2	2
1	1	1	1	2	2	2	2	1	1
4	4	2	2	3	3	1	1	2	2
4	4	2	2	3	3	1	1	1	1
1	1	2	2	3	3	2	2	2	2
4	4	1	1	2	2	1	1	2	2
4	4	2	2	3	3	1	1	2	2
4	4	1	1	2	2	1	1	1	1
1	1	1	1	2	2	1	1	2	2
4	4	2	2	3	3	1	1	2	2

For Review Only

4	4	2	2	3	3	2	2	2	2
4	4	2	2	3	3	2	2	2	2
4	4	1	1	2	2	1	1	2	2
1	1	1	1	2	2	2	2	2	2
4	4	2	2	3	3	2	2	2	2
4	4	1	1	2	2	2	2	2	2
4	4	1	1	2	2	2	2	2	2
4	4	2	2	3	3	1	1	2	2
1	1	2	2	3	3	1	1	2	2
1	1	2	2	3	3	1	1	1	1
1	1	1	1	2	2	2	2	2	2
4	4	1	1	2	2	1	1	2	2
1	1	1	1	2	2	2	2	2	2
4	4	1	1	2	2	2	2	2	2
1	1	1	1	2	2	2	2	2	2
1	1	1	1	2	2	2	2	2	2
4	4	2	2	3	3	1	1	1	1
1	1	1	1	2	2	2	2	2	2
4	4	2	2	3	3	1	1	1	1
1	1	1	1	2	2	1	1	2	2
1	1	1	1	2	2	1	1	2	2
4	4	1	1	2	2	2	2	2	2
1	1	1	1	2	2	2	2	2	2
1	1	1	1	2	2	1	1	2	2
4	4	2	2	3	3	1	1	2	2
4	4	1	1	2	2	2	2	2	2
4	4	2	2	3	3	1	1	1	1
1	1	1	1	2	2	1	1	2	2
1	1	1	1	2	2	2	2	2	2
4	4	1	1	2	2	1	1	2	2
4	4	1	1	2	2	1	1	1	1
4	4	1	1	2	2	2	2	2	2
4	4	2	2	3	3	1	1	2	2
4	4	2	2	2	2	2	2	2	2
1	1	1	1	2	2	2	2	2	2
4	4	2	2	3	3	1	1	1	1
4	4	1	1	2	2	2	2	2	2
1	1	1	1	2	2	1	1	2	2
4	4	1	1	2	2	1	1	2	2
4	4	2	2	3	3	1	1	1	1
4	4	1	1	2	2	2	2	2	2
1	1	1	1	2	2	1	1	2	2
4	4	1	1	2	2	2	2	2	2
1	1	1	1	2	2	2	2	2	2
1	1	1	1	2	2	1	1	2	2
1	1	1	1	2	2	1	1	2	2
4	4	1	1	2	2	2	2	2	2
1	1	2	2	3	3	1	1	2	2
1	1	2	2	2	2	1	1	2	2
4	4	2	2	3	3	1	1	1	1
1	1	1	1	2	2	2	2	2	2
4	4	2	2	3	3	1	1	1	1
1	1	1	1	2	2	1	1	2	2
1	1	1	1	2	2	1	1	2	2
4	4	2	2	3	3	2	2	2	2

For Review Only

1	1	1	1	2	2	2	2	2	2
4	4	1	1	2	2	1	1	2	2

For Review Only

SNP1331	SNP1331	SNP1332	SNP1332	SNP1333	SNP1333	SNP1334	SNP1334	SNP1335	SNP1335
1	1	3	3	4	4	3	3	1	1
4	4	2	2	4	4	1	1	4	4
4	4	3	3	4	4	3	3	1	1
4	4	3	3	4	4	1	1	4	4
4	4	3	3	4	4	3	3	1	1
1	1	3	3	4	4	3	3	1	1
4	4	3	3	4	4	3	3	1	1
4	4	2	2	4	4	3	3	4	4
4	4	3	3	4	4	3	3	1	1
4	4	2	2	4	4	3	3	4	4
4	4	2	2	1	1	3	3	1	1
4	4	3	3	4	4	1	1	1	1
1	1	3	3	4	4	3	3	4	4
4	4	2	2	4	4	3	3	1	1
4	4	3	3	4	4	3	3	1	1
4	4	2	2	4	4	1	1	1	1
4	4	3	3	4	4	3	3	1	1
4	4	3	3	1	1	3	3	1	1
4	4	3	3	4	4	3	3	4	4
4	4	2	2	4	4	3	3	4	4
4	4	3	3	4	4	3	3	4	4
4	4	3	3	4	4	3	3	1	1
4	4	3	3	4	4	3	3	4	4
4	4	2	2	4	4	3	3	4	4
4	4	3	3	4	4	3	3	1	1
4	4	3	3	4	4	1	1	4	4
4	4	3	3	4	4	1	1	4	4
4	4	3	3	4	4	1	1	4	4
4	4	3	3	4	4	3	3	4	4
4	4	2	2	4	4	3	3	4	4
4	4	3	3	4	4	3	3	4	4
1	1	3	3	1	1	3	3	1	1
1	1	3	3	4	4	3	3	1	1
4	4	2	2	4	4	3	3	4	4
4	4	3	3	4	4	1	1	4	4
4	4	3	3	4	4	1	1	4	4
4	4	3	3	1	1	3	3	4	4
4	4	3	3	1	1	3	3	4	4
4	4	2	2	4	4	3	3	4	4
4	4	3	3	1	1	3	3	1	1
4	4	3	3	4	4	3	3	1	1
4	4	3	3	4	4	3	3	4	4
4	4	3	3	4	4	1	1	4	4
4	4	3	3	1	1	3	3	1	1
4	4	3	3	4	4	1	1	1	1
4	4	3	3	4	4	1	1	1	1
4	4	3	3	4	4	1	1	4	4
4	4	2	2	4	4	1	1	1	1
4	4	3	3	4	4	3	3	1	1
4	4	3	3	1	1	1	1	4	4
1	1	3	3	4	4	1	1	1	1
4	4	3	3	4	4	1	1	1	1

For Review Only

4	4	3	3	1	1	3	3	1	1
1	1	3	3	4	4	3	3	1	1
4	4	3	3	1	1	3	3	1	1
4	4	3	3	1	1	3	3	1	1
4	4	3	3	4	4	3	3	4	4
4	4	3	3	4	4	1	1	4	4
4	4	3	3	4	4	1	1	4	4
4	4	2	2	4	4	3	3	4	4
4	4	3	3	1	1	3	3	4	4
4	4	3	3	4	4	1	1	4	4
4	4	3	3	4	4	1	1	1	1
4	4	3	3	4	4	1	1	1	1
4	4	3	3	1	1	3	3	1	1
4	4	3	3	4	4	3	3	4	4
4	4	3	3	4	4	3	3	1	1
4	4	3	3	1	1	3	3	4	4
4	4	3	3	4	4	3	3	1	1
4	4	3	3	4	4	3	3	4	4
4	4	3	3	1	1	1	1	1	1
4	4	3	3	4	4	3	3	1	1
4	4	3	3	4	4	1	1	1	1
4	4	3	3	4	4	3	3	4	4
4	4	3	3	4	4	3	3	4	4
1	1	3	3	4	4	1	1	1	1
4	4	3	3	4	4	1	1	1	1
4	4	3	3	4	4	1	1	4	4
4	4	3	3	1	1	3	3	4	4
4	4	2	2	4	4	3	3	4	4
4	4	3	3	4	4	1	1	4	4
4	4	3	3	4	4	1	1	1	1
4	4	3	3	4	4	3	3	1	1
4	4	2	2	4	4	3	3	4	4
4	4	2	2	4	4	3	3	4	4
4	4	3	3	1	1	3	3	1	1
4	4	3	3	4	4	3	3	4	4
4	4	3	3	4	4	1	1	1	1
4	4	3	3	4	4	3	3	4	4
4	4	3	3	4	4	3	3	4	4
4	4	2	2	4	4	3	3	1	1
4	4	2	2	4	4	1	1	1	1
4	4	3	3	1	1	3	3	1	1
4	4	3	3	4	4	1	1	4	4
4	4	3	3	1	1	1	1	1	1
4	4	3	3	4	4	1	1	1	1
4	4	2	2	1	1	3	3	4	4
4	4	3	3	4	4	3	3	4	4
4	4	2	2	4	4	3	3	4	4
4	4	3	3	4	4	1	1	4	4
4	4	3	3	4	4	1	1	4	4
4	4	3	3	4	4	3	3	1	1
4	4	3	3	4	4	1	1	4	4
4	4	2	2	4	4	3	3	4	4
4	4	2	2	4	4	3	3	4	4
4	4	3	3	4	4	3	3	4	4
4	4	2	2	4	4	3	3	1	1

For Review Only

4	4	3	3	4	4	1	1	4	4
4	4	3	3	4	4	3	3	4	4
4	4	3	3	4	4	1	1	4	4
4	4	2	2	4	4	3	3	4	4
4	4	3	3	4	4	1	1	4	4
4	4	3	3	4	4	3	3	4	4
4	4	3	3	4	4	1	1	1	1
4	4	3	3	4	4	3	3	4	4
4	4	3	3	4	4	1	1	4	4
4	4	3	3	4	4	3	3	4	4
1	1	3	3	1	1	3	3	1	1
4	4	3	3	4	4	1	1	4	4
4	4	3	3	4	4	1	1	1	1
4	4	3	3	4	4	1	1	1	1
4	4	3	3	4	4	1	1	1	1
4	4	3	3	4	4	1	1	4	4
4	4	3	3	1	1	3	3	4	4
4	4	3	3	4	4	1	1	4	4
4	4	2	2	4	4	3	3	4	4
4	4	3	3	4	4	3	3	1	1
4	4	3	3	4	4	1	1	4	4
4	4	3	3	4	4	1	1	1	1
4	4	3	3	4	4	1	1	4	4
4	4	2	2	4	4	3	3	4	4
4	4	3	3	4	4	1	1	4	4
4	4	3	3	4	4	3	3	4	4
4	4	3	3	1	1	3	3	1	1
4	4	2	2	4	4	3	3	4	4
4	4	3	3	1	1	1	1	4	4
4	4	3	3	4	4	3	3	1	1
4	4	2	2	4	4	3	3	4	4
4	4	3	3	4	4	1	1	4	4
4	4	3	3	1	1	3	3	1	1
4	4	3	3	4	4	1	1	4	4
4	4	2	2	4	4	3	3	4	4
4	4	2	2	4	4	1	1	4	4
4	4	2	2	4	4	3	3	4	4
4	4	3	3	4	4	3	3	4	4
4	4	2	2	4	4	1	1	4	4
4	4	2	2	4	4	1	1	1	1
4	4	3	3	4	4	1	1	4	4
4	4	2	2	4	4	3	3	4	4
4	4	2	2	1	1	3	3	4	4
4	4	3	3	4	4	3	3	4	4
4	4	3	3	4	4	3	3	4	4
4	4	3	3	4	4	3	3	4	4
4	4	3	3	4	4	1	1	4	4
4	4	3	3	4	4	1	1	4	4
4	4	3	3	4	4	3	3	4	4
4	4	3	3	4	4	1	1	4	4
4	4	3	3	4	4	1	1	4	4
4	4	2	2	4	4	3	3	4	4
4	4	3	3	1	1	3	3	4	4
4	4	3	3	4	4	1	1	1	1
4	4	3	3	4	4	1	1	4	4

For Review Only

4	4	3	3	4	4	1	1	4	4
4	4	3	3	4	4	1	1	1	1
4	4	3	3	4	4	3	3	1	1
4	4	3	3	4	4	3	3	4	4
4	4	3	3	4	4	1	1	1	1
4	4	3	3	1	1	3	3	1	1
4	4	3	3	4	4	3	3	4	4
4	4	3	3	4	4	1	1	4	4
4	4	3	3	4	4	1	1	4	4
4	4	3	3	1	1	1	1	1	1
4	4	3	3	4	4	1	1	1	1
4	4	2	2	4	4	3	3	4	4
4	4	3	3	4	4	1	1	1	1
4	4	3	3	4	4	3	3	4	4
4	4	2	2	4	4	3	3	4	4
4	4	2	2	1	1	1	1	1	1
4	4	3	3	1	1	3	3	4	4
4	4	3	3	4	4	3	3	4	4
4	4	3	3	4	4	3	3	4	4
4	4	3	3	4	4	1	1	4	4
4	4	3	3	4	4	3	3	4	4
4	4	2	2	4	4	3	3	4	4
4	4	3	3	4	4	3	3	4	4
4	4	3	3	4	4	1	1	1	1
4	4	3	3	1	1	3	3	4	4
4	4	2	2	4	4	3	3	4	4
4	4	2	2	4	4	1	1	1	1
4	4	3	3	1	1	3	3	4	4
4	4	3	3	4	4	3	3	4	4
4	4	3	3	4	4	3	3	1	1
4	4	3	3	4	4	3	3	4	4
4	4	3	3	4	4	3	3	4	4
4	4	2	2	4	4	3	3	1	1
4	4	2	2	4	4	3	3	4	4
4	4	3	3	1	1	3	3	4	4
4	4	3	3	4	4	1	1	1	1
4	4	3	3	4	4	1	1	4	4
1	1	3	3	1	1	3	3	4	4
4	4	3	3	4	4	1	1	4	4
1	1	3	3	4	4	1	1	4	4
4	4	3	3	4	4	3	3	1	1
4	4	3	3	4	4	1	1	4	4
4	4	2	2	4	4	3	3	4	4
4	4	3	3	1	1	1	1	4	4
4	4	3	3	1	1	3	3	1	1
4	4	3	3	4	4	3	3	4	4
4	4	2	2	1	1	1	1	1	1
4	4	3	3	4	4	3	3	4	4
4	4	2	2	4	4	3	3	4	4
4	4	3	3	4	4	3	3	1	1
4	4	3	3	1	1	1	1	4	4
4	4	2	2	4	4	3	3	4	4
4	4	2	2	4	4	3	3	4	4
4	4	3	3	4	4	1	1	1	1
4	4	2	2	4	4	3	3	4	4

For Review Only

4	4	2	2	1	1	3	3	4	4
4	4	3	3	4	4	3	3	4	4
4	4	3	3	1	1	1	1	4	4
4	4	3	3	4	4	3	3	4	4
4	4	3	3	4	4	3	3	4	4
1	1	3	3	4	4	1	1	4	4
4	4	3	3	4	4	3	3	4	4
4	4	3	3	4	4	3	3	4	4
4	4	3	3	4	4	1	1	1	1
4	4	2	2	4	4	3	3	4	4
4	4	3	3	4	4	1	1	4	4
4	4	3	3	4	4	3	3	4	4
4	4	3	3	4	4	1	1	1	1
4	4	3	3	4	4	3	3	4	4
4	4	3	3	4	4	3	3	1	1
4	4	2	2	1	1	1	1	1	1
4	4	2	2	4	4	3	3	4	4
4	4	2	2	1	1	3	3	1	1
4	4	2	2	4	4	3	3	4	4
4	4	3	3	1	1	3	3	4	4
4	4	3	3	4	4	3	3	1	1
4	4	3	3	4	4	1	1	4	4
4	4	3	3	1	1	3	3	1	1
4	4	2	2	4	4	1	1	1	1
4	4	2	2	4	4	3	3	4	4
4	4	3	3	4	4	3	3	4	4
4	4	2	2	4	4	3	3	4	4
4	4	3	3	4	4	1	1	1	1
4	4	3	3	1	1	3	3	4	4
4	4	3	3	1	1	1	1	4	4
4	4	2	2	4	4	3	3	4	4
4	4	2	2	1	1	1	1	4	4
4	4	2	2	4	4	3	3	4	4
4	4	3	3	4	4	1	1	4	4
4	4	3	3	4	4	3	3	1	1
4	4	2	2	4	4	3	3	4	4
4	4	3	3	1	1	3	3	4	4
4	4	3	3	4	4	1	1	1	1
4	4	3	3	4	4	1	1	4	4
4	4	2	2	4	4	3	3	4	4
4	4	2	2	1	1	3	3	4	4
4	4	3	3	4	4	3	3	4	4
4	4	2	2	4	4	3	3	4	4
4	4	3	3	1	1	3	3	1	1
4	4	3	3	4	4	3	3	4	4
4	4	3	3	4	4	1	1	1	1
4	4	3	3	4	4	1	1	4	4
4	4	2	2	4	4	1	1	1	1
4	4	3	3	1	1	1	1	1	1
4	4	2	2	4	4	3	3	4	4
4	4	3	3	1	1	1	1	1	1
4	4	2	2	4	4	3	3	4	4
4	4	3	3	4	4	3	3	1	1
4	4	3	3	4	4	1	1	1	1
4	4	3	3	1	1	1	1	4	4

For Review Only

4	4	3	3	1	1	1	1	1	1
4	4	3	3	4	4	3	3	1	1

For Review Only

SNP1336	SNP1336	SNP1337	SNP1337	SNP1338	SNP1338	SNP1339	SNP1339	SNP134	SNP134
3	3	3	3	2	2	1	1	3	3
3	3	3	3	2	2	1	1	1	1
3	3	1	1	1	1	4	4	1	1
4	4	1	1	1	1	4	4	1	1
3	3	3	3	2	2	1	1	1	1
3	3	1	1	1	1	4	4	1	1
3	3	1	1	1	1	4	4	3	3
3	3	3	3	2	2	1	1	1	1
4	4	1	1	1	1	4	4	3	3
4	4	3	3	2	2	1	1	1	1
4	4	1	1	1	1	4	4	3	3
3	3	1	1	1	1	4	4	1	1
4	4	1	1	2	2	1	1	3	3
4	4	3	3	2	2	1	1	1	1
4	4	1	1	1	1	4	4	3	3
3	3	1	1	1	1	1	1	3	3
3	3	1	1	1	1	4	4	1	1
3	3	3	3	2	2	1	1	3	3
3	3	1	1	1	1	4	4	3	3
3	3	3	3	2	2	1	1	3	3
3	3	3	3	2	2	1	1	1	1
3	3	1	1	1	1	4	4	3	3
3	3	1	1	1	1	4	4	1	1
4	4	3	3	2	2	1	1	1	1
3	3	1	1	1	1	4	4	1	1
3	3	1	1	1	1	4	4	3	3
3	3	1	1	1	1	4	4	1	1
4	4	1	1	1	1	4	4	3	3
4	4	1	1	1	1	4	4	1	1
4	4	3	3	2	2	1	1	1	1
3	3	1	1	1	1	4	4	3	3
4	4	1	1	1	1	4	4	3	3
4	4	1	1	1	1	4	4	3	3
3	3	1	1	1	1	4	4	1	1
4	4	1	1	1	1	4	4	3	3
4	4	1	1	1	1	4	4	1	1
4	4	3	3	2	2	1	1	1	1
4	4	1	1	1	1	4	4	1	1
3	3	3	3	2	2	1	1	1	1
4	4	1	1	1	1	4	4	3	3
4	4	3	3	1	1	1	1	1	1
4	4	3	3	2	2	1	1	1	1
4	4	1	1	2	2	4	4	1	1
3	3	3	3	2	2	1	1	1	1
4	4	1	1	1	1	4	4	1	1
3	3	1	1	1	1	4	4	3	3
3	3	1	1	1	1	4	4	3	3
3	3	1	1	1	1	4	4	1	1
4	4	3	3	2	2	1	1	3	3
4	4	1	1	1	1	4	4	3	3
3	3	1	1	1	1	4	4	1	1
3	3	3	3	2	2	1	1	3	3

For Review Only

3	3	1	1	1	1	4	4	3	3
4	4	1	1	1	1	4	4	3	3
3	3	1	1	1	1	4	4	3	3
4	4	3	3	2	2	1	1	3	3
3	3	1	1	1	1	4	4	3	3
3	3	1	1	1	1	4	4	3	3
3	3	1	1	1	1	4	4	3	3
4	4	3	3	2	2	1	1	1	1
4	4	1	1	1	1	4	4	1	1
4	4	1	1	1	1	4	4	3	3
3	3	1	1	1	1	4	4	1	1
4	4	1	1	1	1	4	4	1	1
3	3	1	1	1	1	4	4	3	3
4	4	1	1	1	1	4	4	1	1
3	3	1	1	1	1	4	4	3	3
4	4	1	1	2	2	1	1	1	1
3	3	1	1	1	1	4	4	1	1
4	4	1	1	1	1	4	4	3	3
3	3	1	1	1	1	4	4	3	3
3	3	1	1	1	1	4	4	3	3
4	4	1	1	1	1	4	4	1	1
4	4	1	1	1	1	4	4	3	3
4	4	1	1	1	1	4	4	3	3
4	4	1	1	1	1	4	4	1	1
4	4	1	1	1	1	4	4	1	1
3	3	1	1	1	1	4	4	1	1
4	4	1	1	2	2	4	4	3	3
3	3	3	3	2	2	1	1	1	1
3	3	1	1	1	1	4	4	3	3
3	3	1	1	1	1	4	4	3	3
3	3	3	3	2	2	1	1	3	3
4	4	1	1	1	1	4	4	1	1
3	3	3	3	2	2	1	1	1	1
3	3	1	1	1	1	4	4	3	3
4	4	3	3	2	2	1	1	3	3
4	4	1	1	1	1	4	4	1	1
3	3	3	3	2	2	1	1	1	1
3	3	3	3	1	1	1	1	3	3
4	4	1	1	1	1	4	4	1	1
4	4	3	3	2	2	1	1	1	1
3	3	3	3	2	2	1	1	3	3
3	3	1	1	1	1	4	4	1	1
4	4	3	3	2	2	1	1	1	1
3	3	1	1	1	1	4	4	1	1
4	4	1	1	1	1	4	4	1	1
4	4	1	1	1	1	4	4	3	3
4	4	3	3	2	2	1	1	1	1
4	4	1	1	1	1	4	4	1	1
4	4	3	3	2	2	1	1	3	3
4	4	1	1	1	1	4	4	3	3
3	3	1	1	1	1	4	4	3	3
3	3	3	3	2	2	1	1	1	1
4	4	1	1	1	1	4	4	1	1
4	4	1	1	1	1	4	4	3	3
4	4	1	1	1	1	4	4	1	1

For Review Only

3	3	1	1	1	1	4	4	3	3
4	4	3	3	2	2	1	1	3	3
3	3	1	1	1	1	4	4	1	1
4	4	3	3	2	2	1	1	3	3
4	4	1	1	1	1	4	4	3	3
4	4	1	1	1	1	4	4	3	3
3	3	1	1	1	1	4	4	3	3
3	3	1	1	1	1	4	4	3	3
4	4	3	3	2	2	1	1	3	3
3	3	1	1	1	1	4	4	1	1
4	4	3	3	2	2	1	1	3	3
4	4	1	1	1	1	4	4	3	3
4	4	3	3	2	2	1	1	1	1
3	3	1	1	1	1	4	4	3	3
3	3	1	1	1	1	4	4	1	1
4	4	1	1	1	1	4	4	3	3
4	4	1	1	1	1	4	4	3	3
4	4	1	1	1	1	4	4	3	3
4	4	3	3	2	2	1	1	3	3
3	3	1	1	1	1	4	4	3	3
3	3	1	1	1	1	4	4	3	3
3	3	1	1	1	1	4	4	3	3
4	4	1	1	1	1	4	4	3	3
3	3	3	3	2	2	1	1	1	1
4	4	1	1	1	1	4	4	3	3
4	4	1	1	1	1	4	4	3	3
4	4	3	3	2	2	1	1	3	3
4	4	3	3	2	2	1	1	1	1
4	4	1	1	1	1	4	4	1	1
3	3	1	1	1	1	4	4	3	3
4	4	3	3	2	2	1	1	1	1
4	4	1	1	1	1	4	4	3	3
3	3	3	3	2	2	1	1	3	3
4	4	1	1	1	1	4	4	3	3
3	3	1	1	1	1	4	4	1	1
3	3	1	1	1	1	4	4	3	3
4	4	3	3	2	2	1	1	1	1
3	3	1	1	1	1	4	4	3	3
3	3	1	1	1	1	4	4	3	3
4	4	1	1	1	1	4	4	3	3
4	4	1	1	1	1	4	4	3	3
4	4	3	3	2	2	1	1	1	1
4	4	1	1	1	1	4	4	1	1
4	4	1	1	2	2	1	1	1	1
4	4	1	1	1	1	4	4	1	1
4	4	1	1	1	1	4	4	1	1
3	3	1	1	1	1	4	4	3	3
3	3	1	1	1	1	4	4	3	3
4	4	1	1	1	1	4	4	1	1
3	3	1	1	1	1	4	4	3	3
3	3	1	1	1	1	4	4	3	3
4	4	1	1	1	1	4	4	3	3
4	4	1	1	1	1	4	4	3	3
4	4	1	1	1	1	4	4	1	1
4	4	1	1	1	1	4	4	3	3

For Review Only

4	4	1	1	1	1	4	4	1	1
4	4	3	3	1	1	1	1	1	1
4	4	1	1	1	1	4	4	1	1
4	4	1	1	1	1	4	4	3	3
3	3	1	1	1	1	4	4	3	3
4	4	1	1	1	1	4	4	3	3
4	4	1	1	1	1	4	4	3	3
4	4	1	1	1	1	4	4	3	3
3	3	1	1	1	1	4	4	3	3
3	3	1	1	1	1	4	4	3	3
3	3	1	1	1	1	4	4	3	3
4	4	1	1	1	1	1	1	1	1
3	3	1	1	1	1	4	4	3	3
4	4	1	1	1	1	4	4	1	1
4	4	3	3	2	2	1	1	1	1
4	4	1	1	1	1	4	4	3	3
4	4	1	1	1	1	4	4	3	3
3	3	1	1	1	1	4	4	1	1
3	3	1	1	1	1	4	4	1	1
3	3	1	1	1	1	4	4	3	3
4	4	1	1	1	1	4	4	3	3
3	3	3	3	2	2	1	1	1	1
4	4	1	1	1	1	4	4	3	3
4	4	1	1	1	1	4	4	1	1
4	4	3	3	2	2	1	1	1	1
3	3	1	1	1	1	4	4	1	1
4	4	1	1	1	1	4	4	1	1
4	4	1	1	1	1	4	4	3	3
4	4	1	1	1	1	4	4	1	1
3	3	3	3	2	2	1	1	3	3
4	4	1	1	1	1	4	4	1	1
3	3	1	1	1	1	4	4	3	3
4	4	3	3	2	2	1	1	3	3
4	4	3	3	2	2	1	1	1	1
4	4	1	1	1	1	4	4	3	3
4	4	1	1	1	1	4	4	1	1
3	3	1	1	1	1	4	4	3	3
3	3	3	3	2	2	1	1	1	1
3	3	1	1	1	1	4	4	3	3
3	3	1	1	1	1	4	4	1	1
3	3	1	1	1	1	4	4	1	1
3	3	1	1	1	1	4	4	3	3
3	3	3	3	2	2	1	1	1	1
4	4	1	1	1	1	4	4	3	3
4	4	1	1	1	1	1	1	3	3
4	4	3	3	2	2	1	1	1	1
4	4	1	1	1	1	4	4	3	3
4	4	3	3	2	2	1	1	1	1
4	4	3	3	2	2	1	1	1	1
4	4	3	3	2	2	1	1	3	3
4	4	1	1	1	1	4	4	1	1
4	4	3	3	2	2	1	1	1	1
4	4	1	1	1	1	4	4	1	1
3	3	1	1	1	1	4	4	1	1
3	3	3	3	2	2	1	1	1	1

For Review Only

4	4	3	3	2	2	1	1	3	3
4	4	3	3	2	2	1	1	3	3
4	4	1	1	1	1	4	4	1	1
4	4	1	1	1	1	4	4	3	3
3	3	3	3	2	2	4	4	3	3
3	3	1	1	1	1	4	4	3	3
4	4	1	1	1	1	4	4	3	3
4	4	3	3	2	2	1	1	1	1
4	4	3	3	2	2	1	1	1	1
3	3	3	3	2	2	1	1	1	1
4	4	1	1	1	1	4	4	3	3
4	4	1	1	1	1	4	4	1	1
4	4	1	1	1	1	4	4	3	3
3	3	1	1	1	1	4	4	3	3
3	3	1	1	1	1	4	4	3	3
4	4	1	1	1	1	4	4	3	3
3	3	3	3	2	2	1	1	1	1
3	3	1	1	1	1	4	4	3	3
3	3	3	3	2	2	1	1	1	1
4	4	1	1	1	1	4	4	1	1
3	3	1	1	1	1	4	4	1	1
3	3	1	1	1	1	4	4	3	3
3	3	1	1	1	1	4	4	3	3
4	4	1	1	1	1	4	4	1	1
4	4	3	3	2	2	1	1	1	1
3	3	1	1	1	1	1	1	3	3
3	3	3	3	2	2	1	1	1	1
3	3	1	1	1	1	4	4	1	1
3	3	1	1	1	1	4	4	3	3
4	4	1	1	1	1	4	4	1	1
3	3	1	1	1	1	4	4	1	1
4	4	1	1	1	1	4	4	3	3
3	3	3	3	2	2	1	1	1	1
3	3	3	3	2	2	1	1	3	3
4	4	1	1	1	1	4	4	3	3
3	3	3	3	2	2	1	1	1	1
4	4	1	1	1	1	4	4	3	3
4	4	1	1	1	1	4	4	1	1
4	4	1	1	1	1	4	4	1	1
3	3	3	3	2	2	1	1	1	1
4	4	1	1	1	1	4	4	3	3
4	4	1	1	1	1	4	4	1	1
4	4	1	1	1	1	4	4	3	3
3	3	1	1	1	1	4	4	3	3
3	3	1	1	1	1	4	4	1	1
4	4	1	1	1	1	4	4	1	1
3	3	1	1	1	1	4	4	3	3
4	4	3	3	2	2	1	1	1	1
3	3	1	1	1	1	4	4	1	1
3	3	3	3	2	2	1	1	1	1
4	4	1	1	1	1	4	4	3	3
3	3	3	3	2	2	1	1	1	1
3	3	1	1	1	1	4	4	1	1
3	3	1	1	1	1	4	4	1	1
4	4	3	3	2	2	1	1	3	3

For Review Only

4	4	1	1	1	1	4	4	3	3
3	3	1	1	1	1	4	4	1	1

For Review Only

SNP1340	SNP1340	SNP1341	SNP1341	SNP1342	SNP1342	SNP1343	SNP1343	SNP1344	SNP1344
3	3	4	4	2	2	3	3	2	2
1	1	3	3	2	2	3	3	2	2
3	3	4	4	2	2	3	3	2	2
3	3	4	4	2	2	3	3	2	2
1	1	3	3	1	1	3	3	2	2
1	1	4	4	2	2	1	1	3	3
1	1	3	3	1	1	3	3	3	3
1	1	3	3	2	2	3	3	2	2
3	3	3	3	1	1	3	3	3	3
1	1	3	3	1	1	3	3	2	2
1	1	3	3	2	2	3	3	2	2
3	3	4	4	2	2	3	3	2	2
1	1	3	3	2	2	3	3	2	2
3	3	4	4	2	2	3	3	2	2
1	1	3	3	2	2	3	3	2	2
1	1	4	4	2	2	3	3	3	3
1	1	3	3	2	2	1	1	3	3
1	1	3	3	1	1	3	3	2	2
3	3	4	4	2	2	3	3	2	2
1	1	3	3	2	2	3	3	2	2
1	1	3	3	2	2	3	3	2	2
1	1	3	3	2	2	3	3	2	2
3	3	4	4	2	2	3	3	2	2
1	1	4	4	2	2	3	3	3	3
1	1	3	3	2	2	1	1	3	3
1	1	4	4	2	2	3	3	2	2
1	1	4	4	2	2	3	3	2	2
1	1	3	3	2	2	3	3	2	2
1	1	3	3	2	2	1	1	3	3
1	1	3	3	2	2	3	3	2	2
3	3	4	4	2	2	3	3	2	2
3	3	4	4	2	2	3	3	2	2
1	1	3	3	1	1	3	3	3	3
1	1	3	3	2	2	3	3	2	2
1	1	4	4	2	2	3	3	2	2
1	1	4	4	2	2	3	3	3	3
3	3	4	4	2	2	3	3	2	2
3	3	3	3	1	1	3	3	2	2
1	1	3	3	2	2	3	3	2	2
3	3	4	4	2	2	3	3	2	2
1	1	3	3	2	2	3	3	2	2
1	1	3	3	1	1	3	3	2	2
1	1	4	4	2	2	1	1	2	2
3	3	3	3	2	2	3	3	2	2
1	1	4	4	2	2	1	1	3	3
3	3	4	4	2	2	3	3	2	2
1	1	4	4	2	2	3	3	2	2
1	1	4	4	2	2	3	3	2	2
3	3	4	4	2	2	3	3	2	2
1	1	4	4	2	2	3	3	3	3
1	1	4	4	2	2	1	1	3	3
1	1	3	3	1	1	3	3	3	3

For Review Only

1	1	3	3	2	2	1	1	3	3
1	1	3	3	1	1	3	3	3	3
3	3	4	4	2	2	3	3	2	2
1	1	3	3	2	2	3	3	2	2
3	3	4	4	2	2	3	3	2	2
1	1	4	4	2	2	3	3	2	2
1	1	4	4	2	2	3	3	2	2
1	1	3	3	2	2	3	3	2	2
1	1	3	3	2	2	3	3	2	2
3	3	3	3	2	2	3	3	2	2
1	1	4	4	2	2	3	3	2	2
1	1	4	4	2	2	3	3	2	2
1	1	3	3	2	2	1	1	3	3
1	1	3	3	2	2	1	1	3	3
1	1	4	4	2	2	3	3	2	2
3	3	3	3	2	2	3	3	3	3
1	1	3	3	2	2	3	3	2	2
3	3	4	4	2	2	3	3	2	2
1	1	4	4	2	2	3	3	3	3
3	3	4	4	2	2	3	3	2	2
1	1	4	4	2	2	3	3	2	2
1	1	3	3	2	2	3	3	2	2
1	1	3	3	2	2	3	3	2	2
1	1	4	4	2	2	1	1	3	3
1	1	4	4	2	2	1	1	3	3
1	1	3	3	1	1	1	1	3	3
3	3	3	3	2	2	3	3	2	2
1	1	3	3	2	2	3	3	2	2
1	1	4	4	2	2	3	3	2	2
1	1	4	4	2	2	1	1	3	3
1	1	4	4	2	2	1	1	3	3
1	1	3	3	1	1	3	3	2	2
1	1	3	3	2	2	3	3	2	2
1	1	3	3	2	2	3	3	3	3
3	3	4	4	2	2	3	3	2	2
1	1	4	4	2	2	1	1	3	3
1	1	3	3	2	2	1	1	2	2
1	1	3	3	2	2	3	3	2	2
1	1	3	3	2	2	3	3	2	2
1	1	4	4	2	2	1	1	3	3
1	1	3	3	2	2	1	1	3	3
1	1	4	4	2	2	1	1	3	3
1	1	4	4	2	2	1	1	3	3
1	1	4	4	2	2	3	3	2	2
1	1	3	3	1	1	3	3	2	2
3	3	4	4	2	2	3	3	2	2
1	1	3	3	2	2	3	3	2	2
1	1	4	4	2	2	3	3	2	2
1	1	4	4	2	2	3	3	2	2
1	1	3	3	1	1	3	3	3	3
1	1	4	4	2	2	3	3	2	2
1	1	3	3	2	2	3	3	2	2
1	1	3	3	2	2	3	3	2	2
3	3	3	3	2	2	3	3	2	2
1	1	3	3	1	1	3	3	3	3

For Review Only

1	1	4	4	2	2	3	3	2	2
3	3	4	4	2	2	3	3	2	2
1	1	4	4	2	2	1	1	3	3
1	1	3	3	2	2	3	3	2	2
1	1	4	4	2	2	3	3	2	2
1	1	3	3	1	1	3	3	2	2
1	1	4	4	2	2	3	3	3	3
1	1	3	3	1	1	3	3	2	2
1	1	4	4	2	2	3	3	2	2
1	1	3	3	2	2	3	3	2	2
3	3	3	3	2	2	3	3	2	2
3	3	3	3	2	2	3	3	2	2
1	1	4	4	2	2	3	3	2	2
1	1	3	3	1	1	3	3	3	3
1	1	3	3	2	2	3	3	2	2
1	1	3	3	2	2	3	3	2	2
3	3	4	4	2	2	3	3	2	2
3	3	3	3	2	2	3	3	2	2
1	1	3	3	2	2	3	3	2	2
1	1	3	3	2	2	1	1	3	3
1	1	3	3	2	2	3	3	2	2
1	1	4	4	2	2	1	1	2	2
3	3	4	4	2	2	3	3	2	2
1	1	3	3	2	2	3	3	2	2
1	1	3	3	2	2	3	3	2	2
1	1	3	3	2	2	3	3	2	2
3	3	3	3	2	2	1	1	2	2
1	1	3	3	1	1	3	3	2	2
1	1	3	3	2	2	3	3	2	2
3	3	4	4	2	2	3	3	2	2
1	1	3	3	2	2	3	3	2	2
3	3	3	3	2	2	3	3	2	2
1	1	3	3	2	2	1	1	3	3
3	3	4	4	2	2	3	3	2	2
3	3	3	3	2	2	3	3	2	2
1	1	3	3	2	2	3	3	2	2
1	1	3	3	2	2	3	3	2	2
3	3	4	4	2	2	3	3	2	2
1	1	3	3	2	2	3	3	2	2
1	1	3	3	2	2	3	3	2	2
1	1	4	4	2	2	3	3	2	2
1	1	3	3	2	2	3	3	2	2
3	3	4	4	2	2	3	3	2	2
1	1	3	3	2	2	3	3	2	2
3	3	4	4	2	2	3	3	2	2
3	3	4	4	2	2	3	3	2	2
1	1	4	4	2	2	3	3	2	2
1	1	4	4	2	2	3	3	2	2
3	3	3	3	1	1	3	3	2	2
1	1	4	4	2	2	3	3	2	2
1	1	4	4	2	2	3	3	2	2
1	1	3	3	1	1	3	3	3	3
1	1	4	4	2	2	3	3	2	2
1	1	4	4	2	2	1	1	3	3
1	1	3	3	2	2	3	3	2	2

For Review Only

1	1	4	4	2	2	3	3	2	2
1	1	4	4	2	2	3	3	2	2
3	3	4	4	2	2	3	3	2	2
1	1	4	4	2	2	3	3	2	2
1	1	3	3	2	2	3	3	2	2
3	3	4	4	2	2	3	3	2	2
3	3	3	3	2	2	3	3	2	2
1	1	4	4	2	2	3	3	2	2
1	1	3	3	2	2	3	3	2	2
1	1	4	4	2	2	1	1	3	3
1	1	4	4	2	2	3	3	2	2
1	1	3	3	1	1	3	3	3	3
1	1	4	4	2	2	3	3	2	2
3	3	3	3	2	2	3	3	2	2
3	3	3	3	2	2	3	3	2	2
1	1	4	4	2	2	1	1	3	3
1	1	4	4	2	2	3	3	2	2
3	3	4	4	2	2	3	3	2	2
1	1	3	3	2	2	3	3	2	2
1	1	4	4	2	2	1	1	3	3
3	3	3	3	2	2	3	3	2	2
1	1	4	4	2	2	3	3	3	3
1	1	3	3	2	2	3	3	2	2
1	1	4	4	2	2	3	3	2	2
1	1	3	3	1	1	3	3	2	2
1	1	3	3	2	2	3	3	2	2
1	1	4	4	2	2	3	3	2	2
3	3	3	3	2	2	3	3	2	2
1	1	3	3	2	2	3	3	2	2
1	1	3	3	2	2	1	1	3	3
3	3	3	3	1	1	3	3	3	3
3	3	4	4	2	2	3	3	2	2
3	3	4	4	2	2	3	3	2	2
3	3	3	3	2	2	3	3	2	2
1	1	3	3	2	2	3	3	2	2
1	1	4	4	2	2	3	3	2	2
1	1	4	4	2	2	1	1	3	3
3	3	4	4	2	2	3	3	2	2
1	1	4	4	2	2	3	3	2	2
1	1	4	4	2	2	1	1	3	3
3	3	4	4	2	2	3	3	2	2
1	1	4	4	2	2	3	3	2	2
1	1	3	3	2	2	3	3	2	2
1	1	4	4	2	2	3	3	3	3
3	3	4	4	2	2	3	3	2	2
3	3	4	4	2	2	3	3	2	2
1	1	4	4	2	2	1	1	3	3
1	1	3	3	2	2	3	3	2	2
1	1	3	3	1	1	3	3	3	3
3	3	4	4	2	2	3	3	2	2
1	1	3	3	2	2	3	3	2	2
3	3	4	4	2	2	3	3	2	2
1	1	3	3	2	2	3	3	2	2
1	1	4	4	2	2	3	3	2	2
1	1	3	3	2	2	3	3	2	2

For Review Only

1	1	4	4	2	2	3	3	3	3
3	3	4	4	2	2	3	3	2	2
3	3	3	3	2	2	3	3	2	2
1	1	3	3	2	2	3	3	2	2
3	3	4	4	2	2	3	3	2	2
3	3	4	4	2	2	3	3	2	2
1	1	3	3	2	2	3	3	2	2
1	1	3	3	1	1	3	3	2	2
1	1	3	3	2	2	3	3	2	2
1	1	3	3	2	2	3	3	2	2
1	1	3	3	2	2	3	3	2	2
3	3	4	4	2	2	3	3	2	2
1	1	4	4	2	2	3	3	2	2
1	1	3	3	2	2	3	3	2	2
1	1	3	3	2	2	3	3	3	3
1	1	4	4	2	2	1	1	3	3
1	1	3	3	2	2	3	3	2	2
1	1	3	3	2	2	3	3	2	2
1	1	3	3	2	2	3	3	2	2
1	1	3	3	1	1	3	3	3	3
3	3	4	4	2	2	3	3	2	2
1	1	4	4	2	2	3	3	2	2
1	1	3	3	2	2	3	3	2	2
1	1	4	4	2	2	1	1	3	3
1	1	3	3	2	2	3	3	2	2
1	1	3	3	1	1	3	3	2	2
1	1	3	3	2	2	3	3	2	2
1	1	4	4	2	2	3	3	2	2
3	3	3	3	2	2	3	3	2	2
1	1	3	3	2	2	3	3	2	2
1	1	3	3	2	2	3	3	2	2
1	1	3	3	2	2	3	3	2	2
1	1	3	3	1	1	3	3	3	3
1	1	4	4	2	2	3	3	2	2
1	1	3	3	2	2	3	3	2	2
1	1	3	3	2	2	3	3	2	2
1	1	4	4	2	2	3	3	3	3
1	1	4	4	2	2	3	3	2	2
1	1	4	4	2	2	3	3	3	3
1	1	3	3	2	2	3	3	2	2
3	3	4	4	2	2	3	3	2	2
1	1	3	3	2	2	3	3	2	2
3	3	4	4	2	2	3	3	2	2
1	1	3	3	2	2	1	1	3	3
3	3	4	4	2	2	3	3	2	2
1	1	4	4	2	2	3	3	2	2
1	1	4	4	2	2	3	3	2	2
1	1	4	4	2	2	3	3	2	2
1	1	4	4	2	2	3	3	2	2
1	1	3	3	2	2	3	3	2	2
1	1	4	4	2	2	1	1	3	3
1	1	3	3	2	2	3	3	2	2
3	3	4	4	2	2	3	3	2	2
1	1	4	4	2	2	3	3	2	2
1	1	4	4	2	2	3	3	3	3

For Review Only

1	1	4	4	2	2	1	1	3	3
1	1	3	3	2	2	3	3	3	3

For Review Only

SNP1345	SNP1345	SNP1346	SNP1346	SNP1347	SNP1347	SNP1348	SNP1348	SNP1349	SNP1349
2	2	3	3	1	1	3	3	3	3
3	3	1	1	4	4	3	3	2	2
2	2	1	1	4	4	3	3	2	2
3	3	3	3	4	4	3	3	2	2
3	3	3	3	4	4	3	3	3	3
2	2	3	3	1	1	2	2	2	2
2	2	3	3	4	4	3	3	2	2
3	3	1	1	4	4	3	3	2	2
2	2	3	3	4	4	3	3	2	2
3	3	3	3	4	4	3	3	3	3
3	3	1	1	4	4	3	3	3	3
2	2	3	3	4	4	3	3	2	2
3	3	3	3	4	4	3	3	2	2
2	2	1	1	4	4	3	3	2	2
3	3	3	3	4	4	3	3	2	2
2	2	1	1	4	4	3	3	2	2
2	2	3	3	4	4	3	3	2	2
3	3	3	3	1	1	2	2	2	2
3	3	3	3	1	1	3	3	2	2
2	2	3	3	4	4	3	3	3	3
3	3	3	3	4	4	3	3	3	3
3	3	3	3	4	4	3	3	2	2
2	2	1	1	4	4	3	3	2	2
3	3	1	1	4	4	3	3	3	3
2	2	1	1	4	4	3	3	2	2
3	3	1	1	4	4	3	3	2	2
3	3	1	1	4	4	3	3	2	2
3	3	3	3	4	4	3	3	2	2
2	2	3	3	4	4	3	3	2	2
3	3	1	1	4	4	3	3	2	2
2	2	1	1	4	4	3	3	2	2
2	2	3	3	1	1	2	2	2	2
2	2	3	3	1	1	2	2	2	2
3	3	1	1	4	4	3	3	2	2
2	2	1	1	4	4	3	3	2	2
2	2	3	3	4	4	3	3	2	2
3	3	1	1	4	4	3	3	3	3
2	2	1	1	4	4	3	3	3	3
3	3	1	1	4	4	3	3	3	3
2	2	1	1	4	4	3	3	2	2
3	3	3	3	4	4	3	3	3	3
3	3	3	3	4	4	3	3	3	3
2	2	3	3	4	4	3	3	2	2
2	2	1	1	4	4	3	3	3	3
2	2	3	3	4	4	3	3	2	2
2	2	1	1	4	4	3	3	2	2
3	3	1	1	4	4	3	3	2	2
3	3	1	1	4	4	3	3	2	2
2	2	3	3	4	4	3	3	2	2
2	2	1	1	4	4	3	3	2	2
2	2	3	3	1	1	2	2	2	2
2	2	3	3	4	4	3	3	2	2

For Review Only

2	2	3	3	4	4	3	3	2	2
2	2	3	3	1	1	2	2	2	2
3	3	1	1	4	4	3	3	2	2
3	3	3	3	4	4	3	3	2	2
3	3	3	3	1	1	3	3	2	2
3	3	1	1	4	4	3	3	2	2
3	3	1	1	4	4	3	3	2	2
3	3	3	3	4	4	3	3	3	3
3	3	3	3	4	4	3	3	3	3
3	3	3	3	4	4	3	3	2	2
3	3	1	1	4	4	3	3	2	2
3	3	1	1	4	4	3	3	2	2
2	2	3	3	4	4	3	3	2	2
2	2	3	3	4	4	3	3	2	2
3	3	3	3	4	4	3	3	2	2
2	2	1	1	4	4	3	3	2	2
2	2	1	1	4	4	3	3	2	2
3	3	3	3	4	4	3	3	2	2
2	2	3	3	4	4	3	3	2	2
2	2	3	3	4	4	3	3	2	2
3	3	1	1	4	4	3	3	2	2
3	3	1	1	4	4	3	3	2	2
2	2	1	1	4	4	3	3	2	2
2	2	3	3	1	1	2	2	2	2
2	2	3	3	4	4	3	3	2	2
2	2	1	1	4	4	3	3	2	2
2	2	3	3	4	4	3	3	2	2
3	3	1	1	4	4	3	3	3	3
3	3	1	1	4	4	3	3	2	2
2	2	1	1	4	4	3	3	2	2
2	2	3	3	4	4	3	3	3	3
3	3	1	1	4	4	3	3	2	2
3	3	1	1	4	4	3	3	2	2
2	2	3	3	4	4	3	3	2	2
2	2	3	3	4	4	3	3	3	3
2	2	3	3	4	4	3	3	2	2
2	2	3	3	4	4	3	3	2	2
3	3	3	3	4	4	3	3	2	2
3	3	1	1	4	4	3	3	3	3
2	2	3	3	4	4	3	3	2	2
2	2	1	1	4	4	3	3	2	2
2	2	1	1	4	4	3	3	2	2
2	2	3	3	4	4	3	3	2	2
3	3	1	1	4	4	3	3	2	2
3	3	3	3	4	4	3	3	3	3
2	2	1	1	4	4	3	3	2	2
3	3	1	1	4	4	3	3	2	2
3	3	1	1	4	4	3	3	2	2
3	3	1	1	4	4	3	3	2	2
2	2	3	3	4	4	3	3	2	2
3	3	3	3	4	4	3	3	2	2
3	3	1	1	4	4	3	3	3	3
3	3	3	3	4	4	3	3	3	3
3	3	3	3	4	4	3	3	2	2
2	2	3	3	4	4	3	3	2	2

For Review Only

3	3	1	1	4	4	3	3	2	2
2	2	3	3	4	4	3	3	2	2
2	2	3	3	4	4	3	3	2	2
3	3	1	1	4	4	3	3	2	2
3	3	1	1	4	4	3	3	2	2
3	3	1	1	4	4	3	3	2	2
2	2	3	3	4	4	3	3	2	2
3	3	1	1	4	4	3	3	2	2
3	3	1	1	4	4	3	3	2	2
3	3	1	1	4	4	3	3	2	2
2	2	3	3	4	4	3	3	2	2
3	3	1	1	4	4	3	3	2	2
3	3	3	3	4	4	3	3	2	2
3	3	1	1	4	4	3	3	2	2
3	3	3	3	4	4	3	3	2	2
3	3	1	1	4	4	3	3	2	2
3	3	3	3	4	4	3	3	2	2
3	3	1	1	4	4	3	3	2	2
3	3	1	1	4	4	3	3	2	2
2	2	3	3	4	4	3	3	2	2
3	3	3	3	4	4	3	3	2	2
2	2	3	3	4	4	3	3	2	2
3	3	1	1	4	4	3	3	2	2
3	3	1	1	4	4	3	3	3	3
3	3	1	1	4	4	3	3	2	2
3	3	1	1	4	4	3	3	3	3
2	2	1	1	4	4	3	3	2	2
3	3	3	3	4	4	3	3	3	3
3	3	3	3	4	4	3	3	2	2
2	2	3	3	4	4	3	3	2	2
3	3	1	1	4	4	3	3	2	2
3	3	3	3	4	4	3	3	2	2
2	2	1	1	4	4	3	3	2	2
3	3	1	1	4	4	3	3	2	2
3	3	1	1	4	4	3	3	2	2
3	3	3	3	4	4	3	3	2	2
3	3	1	1	4	4	3	3	2	2
3	3	1	1	4	4	3	3	2	2
2	2	1	1	4	4	3	3	2	2
2	2	1	1	4	4	3	3	3	3
3	3	1	1	4	4	3	3	3	3
3	3	1	1	4	4	3	3	2	2
2	2	3	3	4	4	3	3	2	2
3	3	3	3	4	4	3	3	2	2
2	2	1	1	4	4	3	3	2	2
2	2	1	1	4	4	3	3	2	2
3	3	1	1	4	4	3	3	2	2
3	3	3	3	4	4	3	3	3	3
2	2	1	1	4	4	3	3	2	2
3	3	1	1	4	4	3	3	2	2
3	3	3	3	4	4	3	3	2	2
2	2	1	1	4	4	3	3	3	3
2	2	1	1	4	4	3	3	3	3
2	2	1	1	4	4	3	3	2	2
3	3	3	3	4	4	3	3	2	2

For Review Only

3	3	3	3	4	4	3	3	2	2
3	3	1	1	4	4	3	3	3	3
2	2	3	3	4	4	3	3	3	3
2	2	1	1	4	4	3	3	2	2
3	3	3	3	4	4	3	3	2	2
3	3	3	3	4	4	3	3	3	3
3	3	1	1	4	4	3	3	3	3
3	3	3	3	4	4	3	3	2	2
3	3	1	1	4	4	3	3	2	2
2	2	3	3	1	1	2	2	2	2
3	3	1	1	4	4	3	3	2	2
3	3	1	1	4	4	3	3	2	2
3	3	1	1	4	4	3	3	2	2
2	2	1	1	4	4	3	3	2	2
3	3	1	1	4	4	3	3	2	2
2	2	1	1	4	4	3	3	3	3
2	2	1	1	4	4	3	3	2	2
3	3	1	1	4	4	3	3	2	2
3	3	1	1	4	4	3	3	2	2
2	2	3	3	4	4	3	3	2	2
3	3	3	3	4	4	3	3	2	2
2	2	1	1	4	4	3	3	3	3
3	3	1	1	4	4	3	3	2	2
3	3	1	1	4	4	3	3	2	2
2	2	3	3	4	4	3	3	2	2
3	3	1	1	4	4	3	3	2	2
3	3	3	3	4	4	3	3	2	2
3	3	3	3	4	4	3	3	2	2
3	3	1	1	4	4	3	3	2	2
2	2	3	3	4	4	3	3	2	2
2	2	1	1	4	4	3	3	2	2
3	3	1	1	4	4	3	3	2	2
2	2	1	1	4	4	3	3	2	2
3	3	1	1	4	4	3	3	2	2
3	3	3	3	4	4	3	3	2	2
3	3	1	1	4	4	3	3	2	2
2	2	3	3	4	4	3	3	2	2
3	3	3	3	1	1	2	2	3	3
3	3	3	3	4	4	3	3	3	3
2	2	3	3	1	1	2	2	2	2
2	2	1	1	4	4	3	3	2	2
3	3	1	1	4	4	3	3	2	2
3	3	1	1	4	4	3	3	3	3
2	2	3	3	4	4	3	3	3	3
3	3	1	1	4	4	3	3	2	2
3	3	3	3	4	4	3	3	3	3
2	2	1	1	4	4	3	3	3	3
3	3	1	1	4	4	3	3	3	3
2	2	1	1	4	4	3	3	2	2
2	2	3	3	4	4	3	3	2	2
3	3	1	1	4	4	3	3	2	2
3	3	1	1	4	4	3	3	3	3
3	3	1	1	4	4	3	3	2	2
3	3	1	1	4	4	3	3	2	2
3	3	1	1	4	4	3	3	3	3

For Review Only

2	2	1	1	4	4	3	3	3	3
3	3	3	3	4	4	3	3	3	3
3	3	1	1	4	4	3	3	2	2
3	3	3	3	4	4	3	3	2	2
3	3	3	3	4	4	3	3	3	3
3	3	3	3	4	4	2	2	2	2
3	3	1	1	4	4	3	3	2	2
3	3	3	3	4	4	3	3	3	3
3	3	1	1	4	4	3	3	2	2
3	3	1	1	4	4	3	3	3	3
3	3	3	3	4	4	3	3	2	2
2	2	1	1	4	4	3	3	3	3
3	3	1	1	4	4	3	3	3	3
3	3	1	1	4	4	3	3	2	2
2	2	1	1	4	4	3	3	2	2
2	2	1	1	4	4	3	3	3	3
3	3	1	1	4	4	3	3	3	3
3	3	1	1	4	4	3	3	2	2
3	3	1	1	4	4	3	3	2	2
2	2	1	1	4	4	3	3	3	3
2	2	1	1	4	4	3	3	2	2
3	3	1	1	4	4	3	3	2	2
3	3	3	3	1	1	2	2	2	2
2	2	1	1	4	4	3	3	3	3
3	3	3	3	4	4	3	3	3	3
2	2	3	3	4	4	3	3	3	3
3	3	1	1	4	4	3	3	2	2
3	3	3	3	4	4	3	3	2	2
3	3	3	3	4	4	3	3	3	3
3	3	1	1	4	4	3	3	2	2
3	3	1	1	4	4	3	3	3	3
3	3	1	1	4	4	3	3	3	3
2	2	1	1	4	4	3	3	2	2
3	3	1	1	4	4	3	3	2	2
3	3	1	1	4	4	3	3	3	3
3	3	1	1	4	4	3	3	3	3
2	2	1	1	4	4	3	3	2	2
3	3	1	1	4	4	3	3	2	2
2	2	3	3	4	4	3	3	2	2
3	3	1	1	4	4	3	3	3	3
2	2	3	3	4	4	3	3	2	2
3	3	1	1	1	1	3	3	2	2
2	2	1	1	4	4	3	3	3	3
2	2	3	3	4	4	3	3	2	2
3	3	3	3	4	4	3	3	2	2
3	3	3	3	4	4	3	3	2	2
3	3	1	1	4	4	3	3	3	3
3	3	1	1	4	4	3	3	2	2
3	3	1	1	4	4	3	3	2	2
3	3	1	1	4	4	3	3	3	3
2	2	1	1	4	4	3	3	2	2
3	3	1	1	4	4	3	3	3	3
2	2	1	1	4	4	3	3	2	2
3	3	3	3	4	4	3	3	2	2
2	2	1	1	4	4	3	3	3	3

For Review Only

2	2	3	3	1	1	2	2	2	2
2	2	1	1	4	4	3	3	2	2

For Review Only

SNP135	SNP135	SNP1350	SNP1350	SNP1351	SNP1351	SNP1352	SNP1352	SNP1353	SNP1353
4	4	3	3	4	4	3	3	4	4
4	4	3	3	4	4	3	3	4	4
4	4	3	3	4	4	3	3	4	4
2	2	3	3	4	4	3	3	4	4
2	2	3	3	4	4	3	3	4	4
4	4	3	3	4	4	3	3	4	4
4	4	3	3	4	4	3	3	4	4
4	4	3	3	4	4	3	3	4	4
4	4	3	3	4	4	3	3	4	4
2	2	3	3	4	4	3	3	4	4
4	4	3	3	4	4	3	3	4	4
4	4	3	3	4	4	3	3	4	4
2	2	3	3	4	4	3	3	4	4
4	4	3	3	4	4	3	3	4	4
4	4	3	3	4	4	3	3	4	4
2	2	3	3	4	4	3	3	4	4
2	2	3	3	4	4	3	3	4	4
4	4	3	3	4	4	3	3	4	4
4	4	3	3	4	4	3	3	4	4
2	2	2	2	1	1	2	2	2	2
2	2	3	3	4	4	3	3	4	4
2	2	3	3	4	4	3	3	4	4
4	4	3	3	4	4	3	3	4	4
2	2	3	3	4	4	3	3	4	4
4	4	3	3	4	4	3	3	4	4
4	4	3	3	4	4	3	3	4	4
4	4	3	3	4	4	3	3	4	4
2	2	3	3	4	4	3	3	4	4
2	2	3	3	4	4	3	3	4	4
2	2	2	2	1	1	2	2	2	2
4	4	3	3	4	4	3	3	4	4
4	4	3	3	4	4	3	3	4	4
4	4	3	3	4	4	3	3	4	4
2	2	3	3	4	4	3	3	4	4
4	4	3	3	4	4	3	3	4	4
4	4	3	3	4	4	3	3	4	4
4	4	3	3	4	4	3	3	4	4
4	4	3	3	4	4	3	3	4	4
2	2	3	3	4	4	3	3	4	4
4	4	3	3	4	4	3	3	4	4
2	2	2	2	1	1	2	2	2	2
2	2	3	3	4	4	3	3	4	4
2	2	3	3	4	4	3	3	4	4
4	4	3	3	4	4	3	3	4	4
4	4	2	2	1	1	2	2	2	2
4	4	3	3	4	4	3	3	4	4
4	4	3	3	4	4	3	3	4	4
4	4	3	3	4	4	3	3	4	4
2	2	3	3	4	4	3	3	4	4
4	4	3	3	4	4	3	3	4	4
4	4	3	3	4	4	3	3	4	4
2	2	3	3	4	4	3	3	4	4

For Review Only

4	4	3	3	4	4	3	3	4	4
4	4	3	3	4	4	3	3	4	4
4	4	3	3	4	4	3	3	4	4
4	4	3	3	4	4	3	3	4	4
4	4	3	3	4	4	3	3	4	4
4	4	3	3	4	4	3	3	4	4
4	4	3	3	4	4	3	3	4	4
2	2	3	3	4	4	3	3	4	4
4	4	3	3	4	4	3	3	4	4
4	4	3	3	4	4	3	3	4	4
4	4	3	3	4	4	3	3	4	4
4	4	3	3	4	4	3	3	4	4
4	4	3	3	4	4	3	3	4	4
2	2	3	3	4	4	3	3	4	4
4	4	3	3	4	4	3	3	4	4
4	4	3	3	4	4	3	3	4	4
4	4	3	3	4	4	3	3	4	4
4	4	3	3	4	4	3	3	4	4
4	4	3	3	4	4	3	3	4	4
4	4	3	3	4	4	3	3	4	4
4	4	3	3	4	4	3	3	4	4
2	2	3	3	4	4	3	3	4	4
2	2	3	3	4	4	3	3	4	4
2	2	3	3	4	4	3	3	4	4
4	4	2	2	1	1	2	2	2	2
4	4	3	3	4	4	3	3	4	4
4	4	2	2	1	1	2	2	2	2
2	2	2	2	1	1	2	2	4	4
4	4	3	3	4	4	3	3	4	4
4	4	3	3	4	4	3	3	4	4
4	4	3	3	4	4	3	3	4	4
2	2	3	3	4	4	3	3	4	4
2	2	3	3	4	4	3	3	4	4
4	4	3	3	4	4	3	3	4	4
4	4	2	2	1	1	2	2	2	2
4	4	2	2	1	1	2	2	2	2
4	4	3	3	4	4	3	3	4	4
2	2	2	2	1	1	2	2	2	2
4	4	3	3	4	4	3	3	4	4
4	4	2	2	1	1	2	2	2	2
4	4	3	3	4	4	3	3	4	4
4	4	3	3	4	4	3	3	4	4
4	4	3	3	4	4	3	3	4	4
4	4	3	3	4	4	3	3	4	4
4	4	3	3	4	4	3	3	4	4
4	4	3	3	4	4	3	3	4	4
2	2	3	3	4	4	3	3	4	4
4	4	3	3	4	4	3	3	4	4
4	4	3	3	4	4	3	3	4	4
4	4	3	3	4	4	3	3	4	4
4	4	3	3	4	4	3	3	4	4
2	2	3	3	4	4	3	3	4	4
2	2	3	3	4	4	3	3	4	4
2	2	3	3	4	4	3	3	4	4
4	4	3	3	4	4	3	3	4	4

For Review Only

4	4	2	2	1	1	3	3	4	4
4	4	2	2	1	1	2	2	4	4
4	4	3	3	4	4	3	3	4	4
2	2	3	3	4	4	3	3	4	4
4	4	3	3	4	4	3	3	4	4
4	4	3	3	4	4	3	3	4	4
4	4	3	3	4	4	3	3	4	4
4	4	3	3	4	4	3	3	4	4
2	2	3	3	4	4	3	3	4	4
2	2	3	3	4	4	3	3	4	4
4	4	3	3	4	4	3	3	4	4
2	2	3	3	4	4	3	3	4	4
4	4	3	3	4	4	3	3	4	4
4	4	3	3	4	4	3	3	4	4
2	2	3	3	4	4	3	3	4	4
4	4	3	3	4	4	3	3	4	4
4	4	3	3	4	4	3	3	4	4
2	2	3	3	4	4	3	3	4	4
2	2	3	3	4	4	3	3	4	4
4	4	3	3	4	4	3	3	4	4
2	2	3	3	4	4	3	3	4	4
4	4	3	3	4	4	3	3	4	4
4	4	3	3	4	4	3	3	4	4
2	2	3	3	4	4	3	3	4	4
2	2	3	3	4	4	3	3	4	4
4	4	3	3	4	4	3	3	4	4
4	4	3	3	4	4	3	3	4	4
4	4	3	3	4	4	3	3	4	4
4	4	3	3	4	4	3	3	4	4
4	4	3	3	4	4	3	3	4	4
2	2	3	3	4	4	3	3	4	4
2	2	3	3	4	4	3	3	4	4
4	4	3	3	4	4	3	3	4	4
4	4	3	3	4	4	3	3	4	4
2	2	3	3	4	4	3	3	4	4
2	2	2	2	1	1	2	2	4	4
2	2	3	3	4	4	3	3	4	4
4	4	3	3	4	4	3	3	4	4
4	4	2	2	1	1	3	3	4	4
2	2	3	3	4	4	3	3	4	4
2	2	3	3	4	4	3	3	4	4
2	2	3	3	4	4	3	3	4	4
4	4	3	3	4	4	3	3	4	4
2	2	2	2	1	1	3	3	4	4
4	4	3	3	4	4	3	3	4	4
2	2	3	3	4	4	3	3	4	4
4	4	3	3	4	4	3	3	4	4
4	4	3	3	4	4	3	3	4	4
2	2	3	3	4	4	3	3	4	4
4	4	3	3	4	4	3	3	4	4
4	4	3	3	4	4	3	3	4	4
2	2	3	3	4	4	3	3	4	4
4	4	3	3	4	4	3	3	4	4
4	4	3	3	4	4	3	3	4	4
2	2	3	3	4	4	3	3	4	4

For Review Only

4	4	3	3	4	4	3	3	4	4
4	4	3	3	4	4	3	3	4	4
2	2	2	2	1	1	2	2	2	2
2	2	3	3	4	4	3	3	4	4
4	4	3	3	4	4	3	3	4	4
2	2	3	3	4	4	3	3	4	4
2	2	3	3	4	4	3	3	4	4
2	2	3	3	4	4	3	3	4	4
2	2	2	2	1	1	3	3	4	4
4	4	3	3	4	4	3	3	4	4
4	4	3	3	4	4	3	3	4	4
2	2	3	3	4	4	3	3	4	4
4	4	3	3	4	4	3	3	4	4
4	4	3	3	4	4	3	3	4	4
2	2	3	3	4	4	3	3	4	4
4	4	3	3	4	4	3	3	4	4
2	2	3	3	4	4	3	3	4	4
4	4	3	3	4	4	3	3	4	4
4	4	3	3	4	4	3	3	4	4
4	4	3	3	4	4	3	3	4	4
4	4	3	3	4	4	3	3	4	4
2	2	3	3	4	4	3	3	4	4
2	2	3	3	4	4	3	3	4	4
4	4	3	3	4	4	3	3	4	4
4	4	3	3	4	4	3	3	4	4
2	2	3	3	4	4	3	3	4	4
4	4	3	3	4	4	3	3	4	4
4	4	3	3	4	4	3	3	4	4
4	4	3	3	4	4	3	3	4	4
4	4	3	3	4	4	3	3	4	4
4	4	3	3	4	4	3	3	4	4
4	4	3	3	4	4	3	3	4	4
4	4	3	3	4	4	3	3	4	4
2	2	3	3	4	4	3	3	4	4
4	4	3	3	4	4	3	3	4	4
4	4	3	3	4	4	3	3	4	4
4	4	3	3	4	4	3	3	4	4
4	4	3	3	4	4	3	3	4	4
4	4	3	3	4	4	3	3	4	4
4	4	3	3	4	4	3	3	4	4
4	4	3	3	4	4	3	3	4	4
4	4	3	3	4	4	3	3	4	4
2	2	2	2	1	1	2	2	2	2
4	4	3	3	4	4	3	3	4	4
2	2	3	3	4	4	3	3	4	4
2	2	3	3	4	4	3	3	4	4
4	4	3	3	4	4	3	3	4	4
2	2	3	3	4	4	3	3	4	4
2	2	3	3	4	4	3	3	4	4
2	2	3	3	4	4	3	3	4	4
4	4	3	3	4	4	3	3	4	4
2	2	3	3	4	4	3	3	4	4
2	2	3	3	4	4	3	3	4	4
4	4	3	3	4	4	3	3	4	4
2	2	3	3	4	4	3	3	4	4

For Review Only

4	4	3	3	4	4	3	3	4	4
4	4	2	2	1	1	2	2	2	2
4	4	3	3	4	4	3	3	4	4
2	2	3	3	4	4	3	3	4	4
4	4	2	2	1	1	2	2	2	2
4	4	3	3	4	4	3	3	4	4
2	2	3	3	4	4	3	3	4	4
2	2	3	3	4	4	3	3	4	4
2	2	3	3	4	4	3	3	4	4
2	2	3	3	4	4	3	3	4	4
2	2	3	3	4	4	3	3	4	4
4	4	3	3	4	4	3	3	4	4
4	4	3	3	4	4	3	3	4	4
4	4	3	3	4	4	3	3	4	4
4	4	3	3	4	4	3	3	4	4
4	4	3	3	4	4	3	3	4	4
2	2	3	3	4	4	3	3	4	4
4	4	3	3	4	4	3	3	4	4
2	2	2	2	1	1	2	2	2	2
4	4	3	3	4	4	3	3	4	4
2	2	3	3	4	4	3	3	4	4
4	4	3	3	4	4	3	3	4	4
4	4	3	3	4	4	3	3	4	4
4	4	3	3	4	4	3	3	4	4
2	2	3	3	4	4	3	3	4	4
2	2	2	2	1	1	3	3	4	4
2	2	3	3	4	4	3	3	4	4
4	4	3	3	4	4	3	3	4	4
4	4	3	3	4	4	3	3	4	4
4	4	3	3	4	4	3	3	4	4
4	4	3	3	4	4	3	3	4	4
4	4	3	3	4	4	3	3	4	4
4	4	3	3	4	4	3	3	4	4
4	4	3	3	4	4	3	3	4	4
2	2	3	3	4	4	3	3	4	4
2	2	2	2	1	1	2	2	2	2
4	4	3	3	4	4	3	3	4	4
4	4	3	3	4	4	3	3	4	4
4	4	3	3	4	4	3	3	4	4
2	2	3	3	4	4	3	3	4	4
4	4	3	3	4	4	3	3	4	4
2	2	3	3	4	4	3	3	4	4
4	4	3	3	4	4	3	3	4	4
4	4	3	3	4	4	3	3	4	4
4	4	3	3	4	4	3	3	4	4
4	4	3	3	4	4	3	3	4	4
4	4	3	3	4	4	3	3	4	4
4	4	3	3	4	4	3	3	4	4
4	4	2	2	1	1	3	3	4	4
2	2	3	3	4	4	3	3	4	4
4	4	3	3	4	4	3	3	4	4
2	2	3	3	4	4	3	3	4	4
4	4	3	3	4	4	3	3	4	4
4	4	3	3	4	4	3	3	4	4
4	4	3	3	4	4	3	3	4	4

For Review Only

4	4	3	3	4	4	3	3	4	4
4	4	3	3	4	4	3	3	4	4

For Review Only

SNP1354	SNP1354	SNP1355	SNP1355	SNP1356	SNP1356	SNP1357	SNP1357	SNP1358	SNP1358
2	2	3	3	1	1	3	3	2	2
4	4	4	4	4	4	1	1	4	4
4	4	4	4	1	1	1	1	2	2
4	4	3	3	1	1	3	3	2	2
2	2	3	3	1	1	3	3	2	2
4	4	4	4	1	1	1	1	2	2
4	4	4	4	1	1	1	1	2	2
4	4	4	4	1	1	1	1	2	2
4	4	4	4	1	1	1	1	2	2
2	2	3	3	1	1	3	3	2	2
2	2	3	3	1	1	3	3	2	2
4	4	4	4	1	1	1	1	2	2
2	2	3	3	1	1	1	1	2	2
4	4	4	4	1	1	1	1	2	2
4	4	3	3	1	1	1	1	2	2
4	4	4	4	1	1	3	3	2	2
4	4	4	4	1	1	1	1	2	2
4	4	4	4	1	1	1	1	2	2
4	4	4	4	1	1	1	1	2	2
2	2	3	3	1	1	3	3	2	2
2	2	3	3	1	1	3	3	2	2
4	4	4	4	1	1	1	1	2	2
2	2	3	3	1	1	3	3	2	2
2	2	3	3	1	1	3	3	2	2
4	4	4	4	1	1	1	1	2	2
4	4	4	4	1	1	1	1	2	2
4	4	4	4	1	1	1	1	2	2
4	4	4	4	1	1	3	3	2	2
4	4	4	4	1	1	1	1	2	2
2	2	3	3	1	1	3	3	2	2
4	4	4	4	1	1	1	1	2	2
4	4	4	4	1	1	3	3	2	2
4	4	4	4	1	1	3	3	2	2
4	4	4	4	1	1	1	1	2	2
4	4	3	3	1	1	3	3	4	4
4	4	4	4	1	1	1	1	2	2
2	2	3	3	1	1	3	3	2	2
2	2	3	3	1	1	3	3	2	2
2	2	3	3	1	1	3	3	2	2
2	2	3	3	1	1	3	3	2	2
2	2	3	3	1	1	3	3	2	2
2	2	3	3	1	1	3	3	2	2
2	2	3	3	1	1	3	3	2	2
2	2	3	3	1	1	3	3	2	2
2	2	3	3	1	1	3	3	2	2
4	4	4	4	1	1	1	1	2	2
4	4	4	4	4	4	1	1	4	4
4	4	4	4	1	1	1	1	2	2
4	4	4	4	1	1	1	1	2	2
4	4	3	3	1	1	3	3	2	2
4	4	4	4	1	1	1	1	2	2
4	4	3	3	1	1	3	3	2	2

For Review Only

4	4	4	4	1	1	1	1	2	2
4	4	4	4	1	1	3	3	2	2
4	4	4	4	1	1	1	1	2	2
4	4	4	4	1	1	1	1	2	2
4	4	4	4	1	1	1	1	2	2
4	4	4	4	4	4	1	1	4	4
4	4	4	4	1	1	1	1	2	2
2	2	3	3	1	1	3	3	2	2
2	2	3	3	1	1	3	3	2	2
4	4	3	3	1	1	3	3	2	2
4	4	4	4	4	4	1	1	4	4
4	4	4	4	4	4	1	1	4	4
4	4	4	4	1	1	1	1	2	2
4	4	4	4	1	1	1	1	2	2
4	4	3	3	1	1	3	3	2	2
2	2	3	3	1	1	3	3	2	2
4	4	3	3	1	1	3	3	2	2
4	4	4	4	1	1	1	1	2	2
4	4	4	4	1	1	1	1	2	2
4	4	4	4	1	1	1	1	2	2
4	4	4	4	1	1	1	1	2	2
2	2	3	3	1	1	3	3	2	2
4	4	4	4	1	1	1	1	2	2
4	4	4	4	1	1	3	3	2	2
2	2	3	3	1	1	3	3	2	2
4	4	4	4	4	4	1	1	4	4
4	4	4	4	1	1	1	1	2	2
2	2	3	3	1	1	3	3	2	2
4	4	4	4	1	1	1	1	2	2
4	4	4	4	1	1	3	3	2	2
2	2	3	3	1	1	3	3	2	2
4	4	4	4	1	1	1	1	2	2
2	2	3	3	1	1	3	3	2	2
4	4	4	4	1	1	1	1	2	2
2	2	3	3	1	1	3	3	2	2
2	2	3	3	1	1	3	3	2	2
2	2	3	3	1	1	3	3	2	2
4	4	4	4	1	1	1	1	2	2
2	2	3	3	1	1	3	3	2	2
2	2	3	3	1	1	3	3	2	2
4	4	4	4	1	1	1	1	2	2
4	4	4	4	1	1	1	1	2	2
4	4	3	3	1	1	1	1	2	2
4	4	4	4	4	4	1	1	4	4
2	2	3	3	1	1	3	3	2	2
4	4	4	4	4	4	1	1	4	4
4	4	4	4	1	1	1	1	2	2
4	4	3	3	1	1	3	3	2	2
4	4	4	4	1	1	1	1	2	2
4	4	3	3	1	1	1	1	2	2
2	2	3	3	1	1	3	3	2	2
2	2	3	3	1	1	3	3	2	2
2	2	3	3	1	1	3	3	2	2
4	4	3	3	1	1	3	3	2	2
4	4	4	4	1	1	1	1	2	2

For Review Only

4	4	3	3	1	1	3	3	2	2
4	4	4	4	1	1	3	3	2	2
4	4	4	4	1	1	1	1	2	2
4	4	4	4	1	1	1	1	2	2
4	4	4	4	1	1	1	1	2	2
4	4	4	4	1	1	1	1	2	2
4	4	4	4	1	1	1	1	2	2
4	4	4	4	1	1	3	3	2	2
4	4	4	4	1	1	1	1	2	2
4	4	3	3	1	1	1	1	2	2
4	4	4	4	1	1	1	1	2	2
4	4	3	3	1	1	3	3	2	2
4	4	4	4	1	1	1	1	2	2
4	4	4	4	1	1	1	1	2	2
4	4	4	4	1	1	1	1	2	2
4	4	3	3	1	1	3	3	2	2
4	4	4	4	1	1	1	1	2	2
4	4	3	3	1	1	3	3	2	2
4	4	4	4	1	1	1	1	2	2
4	4	4	4	1	1	1	1	2	2
4	4	3	3	1	1	3	3	2	2
4	4	4	4	1	1	3	3	2	2
4	4	4	4	1	1	1	1	2	2
2	2	3	3	1	1	3	3	2	2
4	4	3	3	1	1	3	3	2	2
2	2	3	3	1	1	3	3	2	2
2	2	3	3	1	1	3	3	2	2
2	2	3	3	1	1	3	3	2	2
4	4	4	4	1	1	1	1	2	2
4	4	4	4	1	1	1	1	2	2
4	4	3	3	1	1	3	3	2	2
4	4	3	3	1	1	3	3	2	2
4	4	4	4	1	1	1	1	2	2
4	4	3	3	1	1	3	3	2	2
4	4	4	4	1	1	1	1	2	2
4	4	3	3	1	1	3	3	2	2
4	4	3	3	1	1	1	1	2	2
4	4	4	4	4	4	1	1	4	4
4	4	3	3	1	1	3	3	2	2
2	2	3	3	1	1	3	3	2	2
2	2	3	3	1	1	3	3	2	2
4	4	4	4	1	1	1	1	2	2
4	4	4	4	1	1	1	1	2	2
4	4	3	3	1	1	3	3	2	2
4	4	3	3	1	1	3	3	2	2
4	4	4	4	1	1	1	1	2	2
4	4	4	4	1	1	1	1	2	2
2	2	3	3	1	1	3	3	2	2
4	4	3	3	1	1	3	3	2	2
4	4	3	3	1	1	3	3	2	2
4	4	4	4	1	1	1	1	2	2
2	2	3	3	1	1	3	3	2	2
2	2	3	3	1	1	3	3	2	2
4	4	4	4	4	4	1	1	4	4
2	2	3	3	1	1	3	3	2	2

For Review Only

4	4	4	4	1	1	1	1	2	2
2	2	3	3	1	1	3	3	2	2
2	2	3	3	1	1	3	3	2	2
2	2	3	3	1	1	3	3	2	2
4	4	4	4	4	4	1	1	4	4
2	2	3	3	1	1	3	3	2	2
2	2	3	3	1	1	3	3	2	2
4	4	3	3	1	1	3	3	2	2
4	4	3	3	1	1	3	3	2	2
2	2	3	3	1	1	3	3	2	2
4	4	3	3	1	1	1	1	2	2
4	4	3	3	1	1	3	3	2	2
4	4	4	4	1	1	1	1	2	2
4	4	4	4	1	1	1	1	2	2
4	4	4	4	1	1	1	1	2	2
2	2	3	3	1	1	3	3	2	2
2	2	3	3	1	1	3	3	2	2
4	4	4	4	1	1	1	1	2	2
4	4	4	4	1	1	1	1	2	2
4	4	4	4	1	1	1	1	2	2
4	4	3	3	1	1	3	3	2	2
2	2	3	3	1	1	3	3	2	2
2	2	3	3	1	1	3	3	2	2
4	4	4	4	4	4	1	1	4	4
4	4	4	4	1	1	1	1	2	2
4	4	4	4	1	1	1	1	2	2
4	4	4	4	1	1	1	1	2	2
4	4	4	4	1	1	1	1	2	2
2	2	3	3	1	1	3	3	2	2
4	4	4	4	1	1	1	1	2	2
4	4	4	4	1	1	1	1	2	2
4	4	4	4	1	1	1	1	2	2
2	2	3	3	1	1	3	3	2	2
4	4	4	4	1	1	1	1	2	2
4	4	4	4	1	1	1	1	2	2
4	4	4	4	4	4	1	1	4	4
4	4	4	4	1	1	1	1	2	2
2	2	3	3	1	1	3	3	2	2
2	2	3	3	1	1	3	3	2	2
4	4	4	4	1	1	1	1	2	2
4	4	4	4	1	1	1	1	2	2
4	4	4	4	1	1	1	1	2	2
2	2	3	3	1	1	3	3	2	2
2	2	3	3	1	1	3	3	2	2
4	4	4	4	1	1	1	1	2	2
2	2	3	3	1	1	3	3	2	2
2	2	3	3	1	1	3	3	2	2
2	2	3	3	1	1	3	3	2	2
2	2	3	3	1	1	3	3	2	2
4	4	4	4	1	1	1	1	2	2
2	2	3	3	1	1	3	3	2	2
2	2	3	3	1	1	3	3	2	2
2	2	3	3	1	1	3	3	2	2
4	4	4	4	4	4	1	1	4	4
2	2	3	3	1	1	3	3	2	2

For Review Only

2	2	3	3	1	1	3	3	2	2
2	2	3	3	1	1	3	3	2	2
2	2	3	3	1	1	3	3	2	2
4	4	3	3	1	1	3	3	2	2
2	2	3	3	1	1	3	3	2	2
4	4	3	3	1	1	1	1	2	2
2	2	3	3	1	1	3	3	2	2
2	2	3	3	1	1	3	3	2	2
4	4	4	4	1	1	1	1	2	2
2	2	3	3	1	1	3	3	2	2
4	4	3	3	1	1	3	3	2	2
2	2	3	3	1	1	3	3	2	2
2	2	3	3	1	1	3	3	2	2
4	4	4	4	1	1	1	1	2	2
4	4	4	4	1	1	1	1	2	2
2	2	3	3	1	1	3	3	2	2
2	2	3	3	1	1	3	3	2	2
2	2	3	3	1	1	3	3	2	2
2	2	3	3	1	1	3	3	2	2
2	2	3	3	1	1	3	3	2	2
4	4	3	3	1	1	3	3	2	2
4	4	4	4	1	1	1	1	2	2
2	2	3	3	1	1	3	3	2	2
2	2	3	3	1	1	3	3	2	2
2	2	3	3	1	1	3	3	2	2
2	2	3	3	1	1	3	3	2	2
2	2	3	3	1	1	3	3	2	2
4	4	4	4	1	1	1	1	2	2
2	2	3	3	1	1	3	3	2	2
2	2	3	3	1	1	3	3	2	2
2	2	3	3	1	1	3	3	2	2
2	2	3	3	1	1	3	3	2	2
4	4	4	4	1	1	1	1	2	2
4	4	4	4	1	1	1	1	2	2
2	2	3	3	1	1	3	3	2	2
2	2	3	3	1	1	3	3	2	2
2	2	3	3	1	1	3	3	2	2
4	4	4	4	4	4	1	1	4	4
4	4	4	4	1	1	1	1	2	2
2	2	3	3	1	1	3	3	2	2
4	4	4	4	1	1	1	1	2	2
4	4	4	4	4	4	1	1	4	4
2	2	3	3	1	1	3	3	2	2
4	4	4	4	1	1	1	1	2	2
4	4	3	3	1	1	3	3	2	2
4	4	4	4	1	1	1	1	2	2
2	2	3	3	1	1	3	3	2	2
4	4	4	4	1	1	1	1	2	2
4	4	4	4	1	1	1	1	2	2
2	2	3	3	1	1	3	3	2	2
2	2	3	3	1	1	3	3	2	2
2	2	3	3	1	1	3	3	2	2
4	4	4	4	1	1	1	1	2	2
4	4	4	4	1	1	1	1	2	2
2	2	3	3	1	1	3	3	2	2

For Review Only

2	2	3	3	1	1	3	3	2	2
4	4	4	4	1	1	1	1	2	2

For Review Only

SNP1359	SNP1359	SNP136	SNP136	SNP1360	SNP1360	SNP1361	SNP1361	SNP1362	SNP1362
1	1	3	3	1	1	4	4	1	1
1	1	2	2	1	1	4	4	1	1
1	1	3	3	3	3	1	1	1	1
1	1	3	3	1	1	4	4	1	1
1	1	3	3	1	1	4	4	4	4
1	1	2	2	1	1	4	4	1	1
1	1	3	3	1	1	4	4	1	1
1	1	3	3	1	1	4	4	1	1
1	1	3	3	1	1	4	4	4	4
1	1	3	3	1	1	4	4	1	1
4	4	2	2	3	3	1	1	1	1
1	1	3	3	1	1	4	4	1	1
1	1	3	3	1	1	4	4	4	4
1	1	2	2	1	1	1	1	1	1
1	1	3	3	3	3	4	4	4	4
1	1	2	2	3	3	1	1	1	1
1	1	2	2	3	3	1	1	4	4
1	1	3	3	1	1	4	4	1	1
1	1	3	3	3	3	4	4	1	1
1	1	2	2	3	3	1	1	1	1
1	1	3	3	1	1	4	4	1	1
1	1	3	3	1	1	4	4	4	4
1	1	3	3	1	1	4	4	1	1
1	1	3	3	1	1	4	4	1	1
1	1	3	3	3	3	1	1	1	1
1	1	3	3	1	1	4	4	1	1
1	1	3	3	1	1	4	4	1	1
1	1	3	3	1	1	4	4	1	1
1	1	3	3	3	3	1	1	1	1
4	4	2	2	1	1	4	4	1	1
1	1	3	3	1	1	4	4	1	1
1	1	2	2	3	3	4	4	1	1
1	1	3	3	1	1	4	4	1	1
1	1	3	3	1	1	4	4	1	1
1	1	3	3	1	1	4	4	1	1
1	1	3	3	1	1	4	4	1	1
4	4	2	2	1	1	1	1	1	1
1	1	2	2	1	1	4	4	1	1
4	4	3	3	1	1	4	4	1	1
1	1	2	2	3	3	1	1	1	1
1	1	3	3	1	1	4	4	1	1
1	1	3	3	1	1	4	4	1	1
1	1	2	2	1	1	4	4	1	1
4	4	2	2	3	3	1	1	1	1
1	1	2	2	1	1	4	4	1	1
1	1	3	3	1	1	4	4	1	1
1	1	3	3	1	1	4	4	1	1
1	1	3	3	1	1	4	4	1	1
1	1	3	3	3	3	1	1	1	1
1	1	3	3	1	1	1	1	1	1
1	1	3	3	3	3	4	4	1	1
1	1	3	3	1	1	4	4	1	1

For Review Only

1	1	3	3	3	3	1	1	4	4
1	1	3	3	1	1	4	4	1	1
1	1	3	3	1	1	4	4	1	1
1	1	2	2	1	1	4	4	1	1
1	1	3	3	3	3	4	4	1	1
1	1	3	3	1	1	4	4	1	1
1	1	3	3	1	1	4	4	1	1
1	1	2	2	1	1	4	4	1	1
1	1	3	3	1	1	4	4	1	1
1	1	3	3	1	1	4	4	1	1
1	1	3	3	1	1	4	4	1	1
1	1	3	3	1	1	4	4	1	1
1	1	3	3	3	3	1	1	4	4
1	1	3	3	3	3	1	1	1	1
1	1	3	3	1	1	4	4	1	1
1	1	2	2	1	1	4	4	1	1
1	1	3	3	1	1	4	4	1	1
1	1	3	3	3	3	4	4	1	1
1	1	3	3	1	1	4	4	1	1
1	1	3	3	1	1	4	4	4	4
1	1	3	3	1	1	4	4	1	1
1	1	2	2	1	1	4	4	1	1
1	1	3	3	3	3	4	4	4	4
1	1	3	3	1	1	4	4	1	1
1	1	2	2	1	1	4	4	1	1
1	1	3	3	1	1	4	4	1	1
1	1	2	2	1	1	4	4	1	1
4	4	2	2	1	1	4	4	1	1
1	1	3	3	1	1	4	4	1	1
1	1	2	2	1	1	4	4	4	4
1	1	2	2	3	3	4	4	4	4
1	1	3	3	1	1	4	4	1	1
1	1	2	2	1	1	4	4	1	1
1	1	3	3	3	3	1	1	4	4
1	1	3	3	1	1	4	4	1	1
1	1	2	2	1	1	4	4	1	1
1	1	3	3	3	3	4	4	1	1
1	1	3	3	1	1	4	4	1	1
4	4	2	2	1	1	4	4	1	1
1	1	2	2	1	1	4	4	1	1
1	1	3	3	3	3	1	1	4	4
1	1	3	3	1	1	4	4	4	4
1	1	2	2	3	3	4	4	1	1
1	1	3	3	1	1	4	4	1	1
1	1	3	3	1	1	4	4	1	1
1	1	3	3	1	1	4	4	1	1
1	1	2	2	1	1	4	4	1	1
1	1	3	3	1	1	4	4	1	1
1	1	3	3	1	1	4	4	1	1
1	1	3	3	1	1	4	4	1	1
1	1	3	3	1	1	4	4	1	1
4	4	2	2	1	1	4	4	1	1
1	1	2	2	1	1	4	4	1	1
1	1	3	3	1	1	4	4	1	1
1	1	3	3	1	1	4	4	1	1

For Review Only

1	1	3	3	1	1	4	4	1	1
1	1	2	2	1	1	1	1	1	1
1	1	3	3	1	1	4	4	1	1
1	1	2	2	1	1	4	4	1	1
1	1	3	3	1	1	4	4	1	1
1	1	3	3	1	1	4	4	1	1
1	1	2	2	3	3	4	4	1	1
1	1	3	3	1	1	4	4	1	1
1	1	2	2	1	1	4	4	1	1
1	1	3	3	1	1	4	4	1	1
1	1	3	3	1	1	4	4	1	1
1	1	3	3	1	1	4	4	1	1
1	1	2	2	1	1	4	4	1	1
1	1	3	3	1	1	4	4	1	1
1	1	3	3	1	1	4	4	1	1
1	1	3	3	1	1	4	4	1	1
1	1	3	3	3	3	4	4	1	1
1	1	3	3	1	1	4	4	1	1
1	1	2	2	1	1	4	4	1	1
1	1	2	2	3	3	4	4	1	1
1	1	3	3	1	1	4	4	1	1
1	1	3	3	3	3	1	1	4	4
1	1	3	3	1	1	4	4	1	1
4	4	2	2	1	1	4	4	1	1
1	1	3	3	1	1	4	4	1	1
4	4	3	3	1	1	4	4	1	1
1	1	3	3	3	3	4	4	4	4
1	1	3	3	3	3	4	4	1	1
1	1	3	3	1	1	4	4	1	1
1	1	3	3	3	3	1	1	4	4
4	4	2	2	1	1	4	4	1	1
1	1	3	3	1	1	4	4	1	1
1	1	3	3	3	3	1	1	4	4
1	1	3	3	1	1	4	4	1	1
1	1	3	3	1	1	4	4	1	1
1	1	3	3	1	1	4	4	1	1
1	1	2	2	1	1	4	4	1	1
1	1	2	2	1	1	4	4	1	1
4	4	3	3	1	1	4	4	1	1
4	4	2	2	1	1	4	4	1	1
4	4	3	3	1	1	4	4	1	1
1	1	2	2	1	1	4	4	1	1
1	1	3	3	1	1	1	1	1	1
1	1	3	3	1	1	4	4	1	1
1	1	3	3	1	1	4	4	1	1
1	1	3	3	3	3	1	1	4	4
1	1	3	3	1	1	4	4	1	1
1	1	3	3	1	1	4	4	1	1
1	1	2	2	1	1	4	4	1	1
1	1	3	3	1	1	4	4	1	1
1	1	3	3	1	1	4	4	1	1
4	4	3	3	1	1	4	4	1	1
4	4	3	3	1	1	1	1	1	1
1	1	3	3	1	1	4	4	1	1
1	1	2	2	1	1	4	4	1	1

For Review Only

1	1	3	3	1	1	4	4	1	1
4	4	2	2	1	1	4	4	1	1
1	1	2	2	1	1	4	4	1	1
1	1	3	3	1	1	4	4	1	1
1	1	3	3	1	1	4	4	1	1
1	1	2	2	3	3	4	4	1	1
4	4	3	3	1	1	4	4	1	1
1	1	3	3	1	1	4	4	1	1
1	1	3	3	1	1	4	4	1	1
1	1	3	3	1	1	4	4	1	1
1	1	3	3	1	1	4	4	1	1
1	1	3	3	1	1	4	4	1	1
1	1	3	3	1	1	4	4	1	1
1	1	3	3	1	1	4	4	4	4
1	1	2	2	1	1	4	4	1	1
4	4	3	3	3	3	4	4	1	1
1	1	3	3	1	1	4	4	1	1
1	1	3	3	1	1	4	4	1	1
1	1	3	3	1	1	4	4	1	1
1	1	2	2	3	3	4	4	1	1
1	1	3	3	1	1	4	4	1	1
1	1	2	2	3	3	4	4	1	1
1	1	2	2	1	1	4	4	1	1
1	1	3	3	1	1	4	4	1	1
1	1	3	3	1	1	4	4	1	1
1	1	2	2	1	1	4	4	1	1
1	1	3	3	1	1	4	4	1	1
1	1	3	3	1	1	4	4	1	1
1	1	3	3	1	1	4	4	1	1
1	1	3	3	3	3	4	4	1	1
1	1	3	3	1	1	4	4	1	1
1	1	3	3	1	1	4	4	4	4
1	1	3	3	1	1	4	4	1	1
1	1	2	2	1	1	4	4	1	1
1	1	3	3	1	1	4	4	1	1
1	1	3	3	1	1	4	4	1	1
1	1	3	3	1	1	4	4	1	1
1	1	2	2	1	1	4	4	1	1
1	1	3	3	1	1	4	4	1	1
1	1	3	3	3	3	1	1	1	1
1	1	3	3	3	3	1	1	1	1
1	1	3	3	1	1	4	4	1	1
4	4	2	2	1	1	4	4	1	1
1	1	3	3	3	3	1	1	1	1
1	1	2	2	1	1	4	4	1	1
1	1	3	3	3	3	4	4	1	1
4	4	3	3	3	3	4	4	1	1
4	4	2	2	1	1	4	4	1	1
1	1	2	2	1	1	4	4	1	1
1	1	2	2	3	3	1	1	1	1
1	1	3	3	1	1	4	4	1	1
4	4	2	2	1	1	1	1	1	1
1	1	3	3	1	1	4	4	1	1
1	1	3	3	1	1	4	4	1	1
4	4	3	3	1	1	4	4	1	1

For Review Only

4	4	3	3	3	3	1	1	1	1
1	1	2	2	1	1	1	1	1	1
1	1	2	2	1	1	4	4	1	1
1	1	2	2	1	1	4	4	4	4
1	1	3	3	3	3	4	4	1	1
1	1	3	3	1	1	4	4	1	1
1	1	3	3	1	1	4	4	1	1
1	1	2	2	1	1	4	4	1	1
1	1	2	2	1	1	4	4	1	1
4	4	2	2	1	1	4	4	1	1
1	1	3	3	1	1	4	4	1	1
4	4	3	3	1	1	4	4	1	1
4	4	2	2	1	1	4	4	1	1
1	1	3	3	1	1	4	4	1	1
1	1	3	3	1	1	4	4	1	1
4	4	3	3	3	3	1	1	1	1
4	4	3	3	1	1	4	4	1	1
1	1	2	2	1	1	4	4	1	1
1	1	2	2	1	1	4	4	1	1
4	4	2	2	1	1	4	4	1	1
1	1	3	3	1	1	1	1	1	1
1	1	3	3	1	1	4	4	1	1
1	1	3	3	1	1	4	4	1	1
4	4	3	3	3	3	1	1	1	1
1	1	2	2	1	1	4	4	1	1
1	1	3	3	1	1	4	4	1	1
1	1	2	2	1	1	4	4	1	1
1	1	3	3	1	1	4	4	1	1
1	1	3	3	3	3	4	4	4	4
1	1	3	3	1	1	4	4	1	1
4	4	3	3	1	1	4	4	1	1
4	4	3	3	1	1	4	4	1	1
1	1	2	2	1	1	4	4	1	1
1	1	3	3	1	1	4	4	1	1
4	4	2	2	1	1	4	4	1	1
4	4	2	2	1	1	4	4	1	1
1	1	3	3	3	3	1	1	1	1
1	1	3	3	1	1	4	4	1	1
1	1	3	3	1	1	4	4	1	1
4	4	3	3	1	1	4	4	1	1
1	1	3	3	1	1	1	1	1	1
1	1	3	3	1	1	4	4	1	1
4	4	3	3	1	1	4	4	1	1
1	1	3	3	3	3	1	1	4	4
1	1	3	3	3	3	4	4	1	1
1	1	2	2	1	1	4	4	1	1
4	4	3	3	1	1	4	4	1	1
1	1	3	3	1	1	4	4	1	1
1	1	3	3	1	1	4	4	1	1
4	4	2	2	1	1	4	4	1	1
1	1	3	3	3	3	4	4	1	1
4	4	2	2	1	1	4	4	1	1
1	1	3	3	3	3	1	1	1	1
1	1	3	3	1	1	4	4	1	1
4	4	2	2	3	3	1	1	1	1

For Review Only

1	1	3	3	3	3	4	4	1	1
1	1	3	3	1	1	4	4	1	1

For Review Only

SNP1363	SNP1363	SNP1364	SNP1364	SNP1365	SNP1365	SNP1366	SNP1366	SNP1367	SNP1367
3	3	2	2	2	2	2	2	4	4
3	3	2	2	2	2	3	3	4	4
3	3	2	2	1	1	3	3	4	4
3	3	2	2	1	1	3	3	4	4
3	3	2	2	2	2	2	2	4	4
3	3	2	2	2	2	3	3	4	4
3	3	2	2	2	2	3	3	4	4
3	3	2	2	1	1	2	2	4	4
3	3	2	2	2	2	3	3	4	4
3	3	2	2	2	2	2	2	4	4
3	3	3	3	2	2	3	3	4	4
3	3	2	2	1	1	3	3	4	4
3	3	2	2	2	2	3	3	4	4
3	3	2	2	2	2	3	3	4	4
3	3	2	2	2	2	2	2	4	4
3	3	2	2	2	2	3	3	4	4
3	3	2	2	2	2	3	3	4	4
3	3	2	2	2	2	2	2	4	4
3	3	2	2	1	1	3	3	4	4
3	3	2	2	2	2	2	2	4	4
3	3	2	2	2	2	3	3	4	4
3	3	2	2	2	2	3	3	4	4
3	3	2	2	2	2	3	3	4	4
3	3	2	2	2	2	3	3	4	4
3	3	2	2	2	2	3	3	4	4
3	3	2	2	2	2	3	3	4	4
3	3	2	2	2	2	3	3	4	4
3	3	2	2	2	2	3	3	4	4
3	3	2	2	2	2	3	3	4	4
3	3	2	2	2	2	2	2	4	4
3	3	2	2	2	2	3	3	4	4
1	1	3	3	1	1	3	3	4	4
1	1	3	3	2	2	2	2	4	4
3	3	2	2	2	2	3	3	4	4
3	3	2	2	2	2	3	3	4	4
3	3	2	2	2	2	3	3	4	4
3	3	2	2	1	1	3	3	4	4
1	1	3	3	2	2	2	2	4	4
3	3	2	2	2	2	3	3	4	4
3	3	2	2	2	2	3	3	4	4
3	3	2	2	2	2	2	2	4	4
3	3	2	2	2	2	3	3	4	4
3	3	2	2	2	2	2	2	4	4
3	3	2	2	2	2	2	2	4	4
3	3	2	2	2	2	2	2	4	4
3	3	2	2	1	1	3	3	4	4
3	3	2	2	2	2	3	3	4	4
3	3	2	2	2	2	3	3	4	4
1	1	3	3	2	2	2	2	4	4
3	3	2	2	2	2	3	3	4	4
3	3	2	2	2	2	3	3	4	4
3	3	2	2	2	2	3	3	4	4

For Review Only

3	3	2	2	2	2	2	2	4	4
1	1	3	3	2	2	2	2	4	4
3	3	2	2	1	1	3	3	4	4
3	3	2	2	2	2	3	3	4	4
3	3	2	2	1	1	3	3	4	4
3	3	2	2	2	2	3	3	4	4
3	3	2	2	2	2	3	3	4	4
3	3	2	2	2	2	2	2	4	4
3	3	2	2	2	2	3	3	4	4
3	3	2	2	2	2	3	3	4	4
3	3	2	2	2	2	2	2	4	4
3	3	2	2	2	2	3	3	4	4
3	3	2	2	2	2	2	2	4	4
3	3	2	2	2	2	3	3	4	4
3	3	2	2	2	2	3	3	4	4
3	3	2	2	2	2	3	3	4	4
3	3	2	2	2	2	3	3	4	4
3	3	3	3	1	1	3	3	4	4
3	3	2	2	2	2	3	3	4	4
3	3	2	2	2	2	2	2	4	4
3	3	2	2	2	2	3	3	4	4
3	3	2	2	2	2	3	3	4	4
3	3	2	2	2	2	3	3	4	4
3	3	2	2	2	2	3	3	4	4
3	3	2	2	2	2	2	2	4	4
3	3	2	2	2	2	3	3	4	4
1	1	3	3	1	1	2	2	4	4
3	3	2	2	2	2	3	3	4	4
3	3	2	2	2	2	3	3	4	4
3	3	2	2	2	2	3	3	4	4
3	3	2	2	2	2	3	3	4	4
3	3	2	2	2	2	3	3	4	4
3	3	2	2	2	2	3	3	4	4
3	3	2	2	2	2	2	2	4	4
1	1	3	3	2	2	3	3	4	4
3	3	2	2	2	2	2	2	4	4
3	3	2	2	2	2	3	3	4	4
3	3	2	2	2	2	3	3	4	4
3	3	3	3	2	2	3	3	4	4
3	3	2	2	2	2	2	2	4	4
3	3	2	2	2	2	3	3	4	4
3	3	2	2	2	2	3	3	4	4
3	3	2	2	2	2	3	3	4	4
3	3	2	2	2	2	2	2	4	4
3	3	3	3	2	2	3	3	4	4
1	1	3	3	2	2	3	3	4	4
3	3	2	2	2	2	3	3	4	4
3	3	2	2	2	2	3	3	4	4
3	3	2	2	2	2	2	2	4	4
3	3	2	2	2	2	3	3	4	4
3	3	2	2	2	2	3	3	4	4
3	3	2	2	2	2	3	3	4	4
3	3	2	2	2	2	3	3	4	4
3	3	2	2	1	1	3	3	2	2
1	1	3	3	2	2	2	2	4	4

For Review Only

3	3	2	2	2	2	3	3	4	4
3	3	2	2	2	2	2	2	4	4
3	3	2	2	2	2	3	3	4	4
3	3	2	2	2	2	3	3	4	4
1	1	3	3	2	2	3	3	4	4
3	3	2	2	2	2	3	3	4	4
3	3	2	2	2	2	3	3	4	4
3	3	2	2	2	2	3	3	4	4
3	3	2	2	2	2	2	2	4	4
3	3	2	2	2	2	3	3	4	4
1	1	3	3	2	2	3	3	4	4
3	3	2	2	2	2	3	3	4	4
3	3	2	2	2	2	2	2	4	4
3	3	2	2	2	2	3	3	4	4
3	3	2	2	2	2	3	3	4	4
3	3	2	2	2	2	3	3	4	4
1	1	3	3	1	1	3	3	4	4
3	3	2	2	2	2	3	3	4	4
3	3	2	2	2	2	3	3	4	4
3	3	2	2	2	2	2	2	4	4
3	3	2	2	2	2	3	3	4	4
3	3	2	2	2	2	3	3	4	4
3	3	2	2	1	1	3	3	4	4
3	3	2	2	2	2	3	3	4	4
3	3	2	2	2	2	3	3	4	4
3	3	2	2	2	2	3	3	4	4
3	3	2	2	1	1	3	3	4	4
3	3	2	2	2	2	3	3	4	4
3	3	3	3	2	2	3	3	4	4
3	3	2	2	2	2	3	3	4	4
3	3	2	2	2	2	3	3	4	4
3	3	2	2	2	2	3	3	4	4
3	3	2	2	2	2	3	3	4	4
3	3	2	2	2	2	3	3	2	2
3	3	2	2	2	2	3	3	4	4
3	3	2	2	2	2	3	3	4	4
3	3	2	2	2	2	3	3	4	4
3	3	2	2	2	2	2	2	4	4
3	3	2	2	2	2	3	3	4	4
3	3	3	3	2	2	3	3	4	4
3	3	2	2	2	2	3	3	4	4
3	3	2	2	2	2	3	3	4	4
3	3	2	2	2	2	3	3	4	4
3	3	2	2	2	2	3	3	4	4
3	3	2	2	2	2	3	3	4	4
3	3	2	2	2	2	3	3	4	4
3	3	2	2	2	2	3	3	4	4
3	3	2	2	2	2	3	3	4	4
3	3	2	2	2	2	3	3	4	4
3	3	2	2	2	2	3	3	4	4
3	3	2	2	2	2	3	3	4	4
3	3	2	2	2	2	3	3	4	4
3	3	2	2	1	1	3	3	4	4
3	3	2	2	2	2	3	3	4	4
3	3	2	2	2	2	2	2	4	4

For Review Only

1	1	3	3	2	2	3	3	4	4
3	3	2	2	2	2	3	3	4	4
3	3	2	2	2	2	3	3	4	4
3	3	2	2	1	1	3	3	4	4
3	3	2	2	2	2	2	2	4	4
3	3	2	2	1	1	3	3	4	4
3	3	2	2	2	2	3	3	4	4
3	3	2	2	2	2	3	3	4	4
3	3	2	2	2	2	3	3	4	4
3	3	2	2	2	2	3	3	4	4
3	3	2	2	2	2	3	3	4	4
3	3	2	2	2	2	3	3	4	4
3	3	2	2	2	2	3	3	4	4
3	3	2	2	2	2	3	3	4	4
3	3	2	2	2	2	3	3	4	4
3	3	3	3	2	2	3	3	4	4
3	3	2	2	1	1	3	3	4	4
3	3	2	2	1	1	2	2	4	4
3	3	2	2	2	2	3	3	4	4
3	3	2	2	2	2	3	3	4	4
3	3	2	2	2	2	3	3	4	4
3	3	2	2	2	2	3	3	4	4
3	3	2	2	2	2	3	3	4	4
3	3	2	2	2	2	3	3	4	4
3	3	2	2	2	2	2	2	4	4
3	3	2	2	2	2	3	3	4	4
3	3	2	2	2	2	3	3	4	4
3	3	2	2	1	1	3	3	4	4
3	3	2	2	2	2	3	3	4	4
3	3	2	2	2	2	3	3	4	4
1	1	3	3	2	2	3	3	4	4
3	3	2	2	2	2	3	3	4	4
3	3	2	2	2	2	3	3	4	4
3	3	2	2	2	2	3	3	4	4
1	1	3	3	1	1	3	3	4	4
3	3	2	2	2	2	3	3	4	4
3	3	2	2	2	2	3	3	4	4
3	3	2	2	1	1	3	3	4	4
3	3	2	2	2	2	3	3	4	4
3	3	2	2	2	2	2	2	4	4
3	3	2	2	1	1	3	3	4	4
3	3	2	2	2	2	3	3	4	4
3	3	2	2	2	2	3	3	4	4
3	3	2	2	2	2	3	3	4	4
3	3	2	2	1	1	3	3	4	4
3	3	2	2	1	1	3	3	4	4
3	3	3	3	2	2	3	3	4	4
3	3	2	2	2	2	3	3	4	4
3	3	2	2	2	2	3	3	4	4
3	3	2	2	2	2	3	3	4	4
3	3	3	3	2	2	3	3	4	4
1	1	3	3	1	1	2	2	4	4
3	3	2	2	2	2	3	3	4	4
3	3	2	2	2	2	2	2	4	4
3	3	2	2	1	1	3	3	4	4

For Review Only

3	3	3	3	1	1	3	3	4	4
3	3	2	2	2	2	2	2	4	4
3	3	3	3	1	1	3	3	4	4
3	3	2	2	2	2	3	3	4	4
3	3	2	2	1	1	2	2	4	4
3	3	2	2	1	1	3	3	4	4
3	3	2	2	2	2	3	3	4	4
3	3	2	2	2	2	2	2	4	4
3	3	2	2	2	2	3	3	4	4
3	3	2	2	2	2	3	3	4	4
3	3	2	2	2	2	3	3	4	4
3	3	3	3	2	2	3	3	4	4
3	3	3	3	2	2	3	3	4	4
3	3	2	2	2	2	3	3	4	4
3	3	2	2	2	2	3	3	4	4
3	3	3	3	2	2	3	3	4	4
3	3	2	2	2	2	3	3	4	4
3	3	2	2	2	2	3	3	4	4
3	3	2	2	2	2	3	3	4	4
3	3	3	3	2	2	2	2	4	4
3	3	2	2	2	2	3	3	4	4
3	3	2	2	2	2	3	3	4	4
3	3	2	2	2	2	3	3	4	4
3	3	2	2	1	1	3	3	4	4
3	3	2	2	2	2	2	2	4	4
3	3	2	2	2	2	3	3	4	4
3	3	2	2	2	2	3	3	4	4
3	3	2	2	2	2	3	3	4	4
3	3	2	2	1	1	2	2	4	4
3	3	3	3	2	2	3	3	4	4
3	3	2	2	2	2	3	3	4	4
3	3	2	2	2	2	3	3	4	4
3	3	2	2	2	2	2	2	4	4
3	3	2	2	2	2	3	3	4	4
3	3	2	2	2	2	3	3	4	4
3	3	2	2	2	2	3	3	4	4
3	3	3	3	1	1	3	3	4	4
3	3	2	2	2	2	3	3	4	4
3	3	2	2	2	2	3	3	4	4
3	3	2	2	2	2	3	3	4	4
1	1	3	3	2	2	3	3	4	4
3	3	2	2	2	2	3	3	4	4
3	3	2	2	2	2	3	3	4	4
3	3	2	2	2	2	3	3	4	4
3	3	2	2	1	1	3	3	4	4
3	3	3	3	2	2	3	3	4	4
3	3	2	2	2	2	3	3	4	4
1	1	3	3	2	2	2	2	4	4
3	3	2	2	2	2	2	2	4	4
3	3	2	2	2	2	3	3	4	4
3	3	2	2	2	2	3	3	4	4
3	3	2	2	2	2	3	3	4	4
3	3	2	2	1	1	3	3	4	4
3	3	2	2	2	2	3	3	4	4
3	3	2	2	2	2	3	3	4	4

For Review Only

3	3	3	3	2	2	3	3	4	4
3	3	2	2	2	2	3	3	4	4

For Review Only

SNP1368	SNP1368	SNP1369	SNP1369	SNP137	SNP137	SNP1370	SNP1370	SNP1371	SNP1371
1	1	2	2	1	1	2	2	1	1
4	4	3	3	1	1	3	3	1	1
4	4	3	3	1	1	2	2	1	1
4	4	3	3	1	1	2	2	2	2
4	4	2	2	1	1	3	3	1	1
4	4	2	2	1	1	3	3	1	1
4	4	2	2	1	1	3	3	1	1
4	4	2	2	1	1	3	3	1	1
1	1	2	2	1	1	2	2	1	1
4	4	2	2	1	1	3	3	1	1
4	4	3	3	1	1	2	2	1	1
4	4	3	3	1	1	2	2	2	2
4	4	3	3	1	1	3	3	1	1
4	4	2	2	1	1	3	3	1	1
4	4	2	2	1	1	2	2	2	2
4	4	3	3	1	1	3	3	1	1
1	1	2	2	1	1	2	2	1	1
4	4	2	2	1	1	3	3	1	1
1	1	3	3	1	1	2	2	2	2
1	1	2	2	1	1	2	2	1	1
4	4	2	2	1	1	2	2	1	1
4	4	2	2	1	1	3	3	1	1
4	4	2	2	4	4	3	3	2	2
4	4	2	2	1	1	3	3	1	1
1	1	3	3	1	1	2	2	1	1
4	4	2	2	1	1	3	3	1	1
4	4	2	2	4	4	3	3	1	1
4	4	3	3	1	1	2	2	2	2
1	1	2	2	1	1	2	2	1	1
4	4	2	2	1	1	3	3	1	1
4	4	3	3	1	1	3	3	2	2
4	4	2	2	1	1	2	2	2	2
4	4	2	2	1	1	3	3	1	1
4	4	2	2	1	1	3	3	1	1
1	1	2	2	1	1	2	2	1	1
4	4	3	3	1	1	3	3	1	1
1	1	3	3	1	1	2	2	1	1
1	1	3	3	1	1	2	2	1	1
4	4	2	2	1	1	3	3	1	1
1	1	2	2	1	1	2	2	2	2
4	4	2	2	1	1	3	3	1	1
4	4	2	2	1	1	3	3	1	1
4	4	2	2	1	1	3	3	1	1
4	4	2	2	1	1	2	2	1	1
4	4	2	2	1	1	3	3	1	1
4	4	3	3	1	1	2	2	1	1
4	4	2	2	1	1	3	3	2	2
4	4	3	3	1	1	3	3	1	1
1	1	3	3	1	1	2	2	1	1
1	1	2	2	1	1	2	2	2	2
1	1	2	2	1	1	2	2	1	1
4	4	3	3	4	4	3	3	2	2

For Review Only

1	1	2	2	1	1	2	2	1	1
4	4	2	2	1	1	3	3	1	1
1	1	3	3	1	1	2	2	2	2
4	4	3	3	1	1	3	3	1	1
1	1	3	3	1	1	2	2	2	2
4	4	3	3	1	1	3	3	2	2
4	4	2	2	1	1	3	3	1	1
4	4	2	2	1	1	3	3	1	1
4	4	2	2	1	1	3	3	1	1
4	4	3	3	1	1	2	2	2	2
4	4	3	3	1	1	3	3	2	2
4	4	3	3	1	1	3	3	2	2
1	1	2	2	1	1	2	2	1	1
1	1	2	2	1	1	2	2	1	1
4	4	2	2	1	1	3	3	2	2
4	4	2	2	1	1	3	3	1	1
4	4	3	3	4	4	3	3	2	2
1	1	2	2	1	1	2	2	1	1
4	4	3	3	1	1	3	3	2	2
1	1	2	2	1	1	2	2	1	1
4	4	3	3	1	1	3	3	2	2
4	4	2	2	4	4	3	3	2	2
1	1	2	2	1	1	2	2	1	1
4	4	2	2	1	1	3	3	2	2
4	4	2	2	1	1	3	3	1	1
4	4	3	3	4	4	3	3	1	1
4	4	2	2	1	1	2	2	1	1
4	4	2	2	1	1	3	3	1	1
4	4	2	2	1	1	3	3	1	1
4	4	3	3	1	1	2	2	2	2
1	1	3	3	1	1	2	2	1	1
4	4	2	2	1	1	3	3	1	1
4	4	2	2	1	1	3	3	1	1
1	1	2	2	1	1	2	2	1	1
1	1	2	2	1	1	2	2	1	1
4	4	2	2	1	1	3	3	1	1
1	1	2	2	1	1	2	2	1	1
4	4	3	3	1	1	3	3	1	1
4	4	2	2	1	1	3	3	1	1
4	4	2	2	1	1	3	3	1	1
1	1	3	3	1	1	2	2	1	1
4	4	3	3	1	1	2	2	2	2
1	1	2	2	1	1	2	2	2	2
4	4	3	3	1	1	3	3	2	2
4	4	3	3	1	1	3	3	1	1
1	1	3	3	4	4	2	2	2	2
4	4	2	2	1	1	3	3	1	1
4	4	2	2	4	4	3	3	2	2
4	4	2	2	1	1	3	3	1	1
4	4	2	2	1	1	3	3	2	2
4	4	2	2	1	1	3	3	2	2
4	4	2	2	1	1	3	3	1	1
4	4	2	2	1	1	3	3	1	1
4	4	3	3	1	1	2	2	2	2
4	4	2	2	1	1	3	3	1	1

For Review Only

4	4	3	3	4	4	3	3	1	1
4	4	3	3	4	4	3	3	1	1
4	4	3	3	1	1	3	3	1	1
4	4	2	2	1	1	3	3	1	1
4	4	2	2	4	4	3	3	2	2
4	4	2	2	1	1	3	3	1	1
4	4	3	3	1	1	3	3	1	1
4	4	2	2	4	4	3	3	2	2
4	4	2	2	1	1	3	3	1	1
4	4	2	2	1	1	3	3	1	1
4	4	2	2	1	1	3	3	1	1
4	4	2	2	1	1	2	2	2	2
4	4	2	2	1	1	3	3	2	2
4	4	3	3	4	4	3	3	2	2
4	4	3	3	4	4	3	3	2	2
4	4	3	3	1	1	2	2	1	1
1	1	2	2	1	1	2	2	2	2
4	4	3	3	1	1	2	2	2	2
4	4	2	2	1	1	3	3	1	1
1	1	3	3	1	1	2	2	1	1
4	4	3	3	1	1	2	2	2	2
1	1	3	3	1	1	2	2	2	2
4	4	3	3	4	4	3	3	1	1
4	4	2	2	1	1	3	3	1	1
4	4	2	2	1	1	3	3	2	2
4	4	2	2	4	4	3	3	2	2
1	1	3	3	1	1	2	2	2	2
1	1	2	2	1	1	2	2	1	1
4	4	3	3	1	1	3	3	2	2
1	1	2	2	1	1	2	2	1	1
4	4	2	2	1	1	3	3	1	1
4	4	3	3	4	4	2	2	2	2
1	1	3	3	1	1	2	2	1	1
1	1	3	3	1	1	2	2	2	2
4	4	2	2	1	1	3	3	1	1
4	4	2	2	1	1	3	3	1	1
4	4	2	2	1	1	3	3	1	1
4	4	3	3	4	4	3	3	2	2
4	4	3	3	1	1	3	3	1	1
4	4	3	3	1	1	2	2	1	1
4	4	2	2	1	1	2	2	2	2
4	4	2	2	1	1	3	3	1	1
4	4	2	2	1	1	3	3	1	1
4	4	2	2	1	1	3	3	1	1
4	4	3	3	1	1	3	3	1	1
1	1	2	2	1	1	2	2	1	1
4	4	3	3	1	1	3	3	1	1
4	4	2	2	1	1	3	3	1	1
4	4	2	2	1	1	3	3	2	2
4	4	3	3	1	1	3	3	1	1
4	4	2	2	1	1	3	3	1	1
4	4	2	2	1	1	3	3	1	1
4	4	2	2	1	1	2	2	1	1
4	4	2	2	1	1	3	3	1	1
4	4	3	3	1	1	3	3	2	2

For Review Only

4	4	2	2	1	1	2	2	1	1
4	4	2	2	4	4	3	3	2	2
4	4	2	2	1	1	2	2	1	1
4	4	2	2	1	1	2	2	2	2
4	4	3	3	4	4	3	3	2	2
1	1	2	2	1	1	2	2	2	2
4	4	3	3	1	1	3	3	1	1
4	4	2	2	1	1	3	3	1	1
4	4	2	2	4	4	3	3	1	1
4	4	2	2	1	1	3	3	2	2
4	4	3	3	1	1	3	3	1	1
4	4	2	2	1	1	3	3	1	1
4	4	2	2	1	1	3	3	1	1
4	4	2	2	1	1	2	2	1	1
4	4	2	2	1	1	3	3	1	1
4	4	3	3	1	1	2	2	1	1
4	4	2	2	1	1	2	2	1	1
1	1	3	3	4	4	2	2	2	2
4	4	3	3	1	1	3	3	1	1
4	4	3	3	1	1	3	3	1	1
4	4	3	3	1	1	2	2	2	2
1	1	2	2	1	1	2	2	1	1
4	4	2	2	4	4	3	3	2	2
4	4	3	3	1	1	3	3	2	2
4	4	2	2	1	1	3	3	1	1
4	4	2	2	1	1	3	3	1	1
4	4	3	3	1	1	3	3	2	2
4	4	2	2	1	1	2	2	2	2
4	4	2	2	1	1	3	3	2	2
1	1	2	2	1	1	2	2	1	1
4	4	3	3	4	4	3	3	1	1
4	4	2	2	1	1	2	2	2	2
4	4	2	2	1	1	3	3	1	1
4	4	2	2	1	1	2	2	1	1
1	1	2	2	1	1	2	2	1	1
4	4	3	3	1	1	3	3	2	2
4	4	3	3	1	1	3	3	1	1
1	1	2	2	1	1	2	2	1	1
4	4	2	2	1	1	3	3	1	1
1	1	2	2	1	1	2	2	2	2
4	4	3	3	1	1	2	2	1	1
4	4	3	3	1	1	3	3	1	1
4	4	2	2	1	1	3	3	1	1
1	1	3	3	1	1	2	2	1	1
4	4	3	3	4	4	2	2	1	1
1	1	2	2	1	1	2	2	2	2
4	4	3	3	1	1	2	2	1	1
4	4	3	3	1	1	3	3	1	1
4	4	2	2	1	1	3	3	1	1
1	1	2	2	1	1	2	2	2	2
4	4	3	3	4	4	3	3	2	2
1	1	2	2	1	1	2	2	1	1
4	4	2	2	1	1	3	3	1	1
4	4	3	3	1	1	3	3	2	2
4	4	2	2	1	1	3	3	1	1

For Review Only

4	4	2	2	1	1	2	2	1	1
1	1	2	2	1	1	2	2	1	1
4	4	3	3	4	4	2	2	2	2
4	4	2	2	1	1	2	2	2	2
1	1	2	2	1	1	2	2	1	1
4	4	2	2	1	1	3	3	2	2
4	4	2	2	1	1	3	3	1	1
4	4	2	2	1	1	3	3	1	1
4	4	2	2	1	1	3	3	1	1
4	4	2	2	1	1	3	3	1	1
4	4	3	3	1	1	2	2	2	2
4	4	2	2	1	1	3	3	1	1
4	4	2	2	1	1	3	3	2	2
4	4	2	2	1	1	3	3	2	2
4	4	3	3	1	1	3	3	1	1
4	4	3	3	1	1	2	2	1	1
4	4	2	2	4	4	3	3	1	1
4	4	2	2	1	1	3	3	1	1
4	4	2	2	1	1	3	3	1	1
1	1	3	3	1	1	2	2	1	1
4	4	2	2	4	4	3	3	2	2
4	4	2	2	1	1	3	3	1	1
4	4	3	3	1	1	2	2	2	2
1	1	2	2	1	1	2	2	1	1
4	4	2	2	1	1	3	3	1	1
4	4	2	2	1	1	2	2	1	1
4	4	2	2	1	1	3	3	1	1
4	4	3	3	1	1	3	3	2	2
1	1	2	2	1	1	2	2	1	1
4	4	3	3	4	4	3	3	2	2
4	4	2	2	1	1	3	3	1	1
4	4	3	3	1	1	3	3	1	1
4	4	2	2	1	1	3	3	1	1
4	4	2	2	1	1	3	3	1	1
4	4	2	2	1	1	3	3	2	2
4	4	2	2	1	1	3	3	1	1
4	4	3	3	1	1	2	2	1	1
4	4	3	3	1	1	3	3	2	2
4	4	3	3	1	1	3	3	1	1
4	4	2	2	1	1	3	3	1	1
4	4	2	2	1	1	3	3	1	1
4	4	2	2	1	1	3	3	1	1
4	4	2	2	1	1	3	3	1	1
1	1	3	3	1	1	2	2	1	1
1	1	3	3	1	1	2	2	2	2
4	4	2	2	1	1	3	3	2	2
4	4	2	2	1	1	3	3	1	1
4	4	2	2	1	1	3	3	1	1
4	4	3	3	4	4	3	3	1	1
4	4	2	2	1	1	3	3	1	1
1	1	3	3	1	1	2	2	2	2
4	4	2	2	1	1	2	2	1	1
4	4	3	3	1	1	2	2	1	1
4	4	3	3	1	1	3	3	2	2
1	1	3	3	1	1	2	2	1	1

For Review Only

4	4	3	3	1	1	2	2	2	2
4	4	3	3	1	1	3	3	2	2

For Review Only

SNP1372	SNP1372	SNP1373	SNP1373	SNP1374	SNP1374	SNP1375	SNP1375	SNP1376	SNP1376
2	2	3	3	4	4	2	2	2	2
4	4	3	3	4	4	2	2	3	3
2	2	2	2	1	1	3	3	2	2
4	4	2	2	1	1	3	3	3	3
2	2	3	3	4	4	2	2	3	3
2	2	2	2	1	1	3	3	3	3
2	2	2	2	1	1	3	3	3	3
4	4	2	2	1	1	3	3	3	3
2	2	2	2	1	1	3	3	2	2
4	4	3	3	4	4	2	2	3	3
2	2	2	2	1	1	3	3	3	3
2	2	2	2	1	1	3	3	2	2
4	4	3	3	4	4	2	2	3	3
2	2	2	2	1	1	3	3	3	3
2	2	2	2	1	1	3	3	2	2
2	2	2	2	4	4	3	3	2	2
2	2	2	2	1	1	3	3	2	2
2	2	2	2	1	1	3	3	3	3
4	4	2	2	1	1	3	3	2	2
4	4	2	2	1	1	3	3	2	2
4	4	3	3	4	4	2	2	3	3
4	4	2	2	1	1	3	3	3	3
4	4	2	2	1	1	3	3	3	3
4	4	2	2	1	1	3	3	3	3
2	2	2	2	1	1	3	3	2	2
4	4	2	2	1	1	3	3	3	3
4	4	2	2	1	1	3	3	3	3
4	4	2	2	1	1	3	3	3	3
4	4	2	2	1	1	3	3	2	2
4	4	2	2	1	1	3	3	3	3
4	4	2	2	1	1	3	3	3	3
2	2	2	2	1	1	3	3	2	2
2	2	2	2	1	1	3	3	3	3
4	4	2	2	1	1	3	3	3	3
4	4	3	3	1	1	3	3	3	3
4	4	2	2	1	1	3	3	3	3
4	4	3	3	4	4	2	2	2	2
2	2	2	2	1	1	3	3	2	2
4	4	3	3	4	4	2	2	3	3
4	4	2	2	1	1	3	3	2	2
4	4	3	3	4	4	2	2	3	3
4	4	3	3	4	4	2	2	3	3
4	4	2	2	1	1	3	3	3	3
2	2	3	3	4	4	2	2	2	2
2	2	2	2	1	1	3	3	3	3
2	2	2	2	1	1	3	3	3	3
4	4	2	2	1	1	3	3	3	3
4	4	2	2	1	1	3	3	3	3
2	2	2	2	1	1	3	3	2	2
4	4	2	2	1	1	3	3	3	3
2	2	2	2	1	1	3	3	2	2
2	2	3	3	4	4	2	2	3	3

For Review Only

2	2	2	2	1	1	3	3	2	2
2	2	2	2	1	1	3	3	3	3
2	2	2	2	1	1	3	3	2	2
2	2	2	2	1	1	3	3	3	3
4	4	2	2	1	1	3	3	2	2
4	4	2	2	1	1	3	3	3	3
4	4	2	2	1	1	3	3	3	3
4	4	2	2	1	1	3	3	3	3
4	4	2	2	1	1	3	3	3	3
4	4	2	2	1	1	3	3	3	3
2	2	2	2	1	1	3	3	3	3
2	2	2	2	1	1	3	3	3	3
2	2	2	2	1	1	3	3	2	2
4	4	2	2	1	1	3	3	2	2
4	4	2	2	1	1	3	3	3	3
4	4	3	3	4	4	2	2	3	3
2	2	2	2	1	1	3	3	3	3
4	4	2	2	1	1	3	3	2	2
2	2	2	2	1	1	3	3	3	3
2	2	2	2	1	1	3	3	2	2
4	4	2	2	1	1	3	3	3	3
4	4	2	2	1	1	3	3	3	3
4	4	2	2	1	1	3	3	2	2
2	2	2	2	1	1	3	3	3	3
2	2	2	2	1	1	3	3	3	3
4	4	2	2	1	1	3	3	3	3
2	2	3	3	4	4	2	2	2	2
4	4	2	2	1	1	3	3	3	3
4	4	2	2	1	1	3	3	3	3
2	2	2	2	1	1	3	3	3	3
4	4	2	2	1	1	3	3	2	2
4	4	2	2	1	1	3	3	3	3
4	4	3	3	4	4	2	2	3	3
2	2	2	2	1	1	3	3	2	2
4	4	2	2	1	1	3	3	2	2
2	2	2	2	1	1	3	3	3	3
4	4	3	3	4	4	2	2	2	2
4	4	3	3	4	4	2	2	3	3
2	2	2	2	1	1	3	3	3	3
2	2	2	2	1	1	3	3	3	3
2	2	2	2	1	1	3	3	2	2
4	4	2	2	1	1	3	3	3	3
2	2	3	3	4	4	2	2	2	2
2	2	2	2	1	1	3	3	3	3
4	4	2	2	1	1	3	3	3	3
4	4	2	2	1	1	3	3	2	2
4	4	2	2	1	1	3	3	3	3
4	4	2	2	1	1	3	3	3	3
4	4	2	2	1	1	3	3	3	3
2	2	2	2	1	1	3	3	3	3
4	4	2	2	1	1	3	3	3	3
4	4	3	3	4	4	2	2	3	3
4	4	2	2	1	1	3	3	3	3
4	4	3	3	1	1	2	2	3	3
2	2	2	2	1	1	3	3	3	3

For Review Only

4	4	2	2	1	1	3	3	3	3
4	4	3	3	4	4	2	2	3	3
4	4	2	2	1	1	3	3	3	3
4	4	3	3	4	4	2	2	3	3
4	4	2	2	1	1	3	3	3	3
4	4	2	2	1	1	3	3	3	3
2	2	2	2	1	1	3	3	2	2
4	4	2	2	1	1	3	3	3	3
4	4	2	2	1	1	3	3	3	3
4	4	2	2	1	1	3	3	3	3
2	2	3	3	4	4	2	2	3	3
4	4	2	2	1	1	3	3	3	3
2	2	3	3	4	4	2	2	3	3
2	2	2	2	1	1	3	3	3	3
4	4	3	3	1	1	3	3	3	3
4	4	2	2	1	1	3	3	3	3
4	4	2	2	1	1	3	3	2	2
2	2	2	2	1	1	3	3	3	3
4	4	3	3	4	4	2	2	3	3
2	2	2	2	1	1	3	3	2	2
4	4	2	2	1	1	3	3	3	3
2	2	2	2	1	1	3	3	2	2
4	4	2	2	1	1	3	3	3	3
4	4	2	2	1	1	3	3	3	3
4	4	2	2	1	1	3	3	3	3
4	4	2	2	1	1	3	3	3	3
2	2	2	2	1	1	3	3	2	2
4	4	3	3	4	4	2	2	2	2
4	4	2	2	1	1	3	3	3	3
2	2	2	2	1	1	3	3	2	2
4	4	2	2	1	1	3	3	3	3
2	2	2	2	1	1	3	3	3	3
2	2	2	2	1	1	3	3	2	2
4	4	2	2	1	1	3	3	3	3
4	4	2	2	1	1	3	3	3	3
4	4	2	2	1	1	3	3	3	3
4	4	3	3	4	4	2	2	3	3
4	4	2	2	1	1	3	3	3	3
2	2	2	2	1	1	3	3	3	3
4	4	2	2	1	1	3	3	3	3
4	4	2	2	1	1	3	3	3	3
4	4	2	2	1	1	3	3	3	3
4	4	2	2	1	1	3	3	3	3
4	4	2	2	1	1	3	3	3	3
4	4	3	3	4	4	2	2	3	3
4	4	2	2	1	1	3	3	2	2
4	4	2	2	1	1	3	3	3	3
4	4	2	2	1	1	3	3	3	3
4	4	2	2	1	1	2	2	3	3
4	4	2	2	1	1	3	3	3	3
4	4	2	2	1	1	3	3	3	3
4	4	2	2	1	1	3	3	3	3
4	4	2	2	1	1	3	3	2	2
2	2	2	2	1	1	3	3	3	3
4	4	2	2	1	1	3	3	3	3

For Review Only

4	4	2	2	1	1	3	3	3	3
2	2	3	3	4	4	2	2	3	3
2	2	2	2	1	1	3	3	3	3
4	4	2	2	1	1	3	3	3	3
4	4	2	2	1	1	3	3	3	3
2	2	2	2	1	1	3	3	2	2
4	4	2	2	1	1	3	3	3	3
4	4	2	2	1	1	3	3	3	3
4	4	3	3	1	1	3	3	3	3
2	2	2	2	1	1	3	3	3	3
2	2	2	2	1	1	3	3	3	3
4	4	2	2	1	1	3	3	3	3
2	2	2	2	1	1	3	3	3	3
4	4	2	2	1	1	3	3	3	3
4	4	2	2	1	1	3	3	3	3
2	2	2	2	1	1	3	3	3	3
4	4	2	2	1	1	3	3	3	3
4	4	2	2	1	1	3	3	2	2
4	4	2	2	1	1	3	3	3	3
2	2	2	2	1	1	3	3	2	2
4	4	2	2	1	1	3	3	3	3
4	4	3	3	4	4	2	2	2	2
4	4	2	2	1	1	3	3	3	3
2	2	2	2	1	1	3	3	3	3
4	4	3	3	4	4	2	2	3	3
4	4	2	2	1	1	3	3	3	3
2	2	2	2	1	1	3	3	3	3
4	4	2	2	1	1	3	3	3	3
4	4	2	2	1	1	3	3	3	3
2	2	3	3	4	4	2	2	2	2
4	4	2	2	1	1	3	3	3	3
4	4	2	2	1	1	3	3	3	3
2	2	2	2	1	1	3	3	3	3
4	4	2	2	4	4	3	3	3	3
4	4	2	2	1	1	3	3	2	2
2	2	2	2	1	1	3	3	3	3
4	4	2	2	1	1	3	3	3	3
4	4	3	3	4	4	2	2	2	2
4	4	2	2	1	1	3	3	3	3
4	4	2	2	1	1	3	3	2	2
2	2	2	2	1	1	3	3	2	2
4	4	2	2	1	1	3	3	3	3
4	4	3	3	4	4	2	2	3	3
4	4	2	2	1	1	3	3	2	2
4	4	2	2	1	1	3	3	3	3
4	4	2	2	1	1	3	3	2	2
2	2	2	2	1	1	3	3	3	3
4	4	3	3	4	4	2	2	3	3
4	4	2	2	1	1	3	3	3	3
2	2	2	2	1	1	3	3	2	2
4	4	2	2	1	1	3	3	3	3
4	4	3	3	4	4	2	2	2	2
4	4	2	2	1	1	3	3	3	3
2	2	2	2	1	1	3	3	3	3
4	4	3	3	4	4	2	2	3	3

For Review Only

4	4	2	2	1	1	3	3	2	2
4	4	3	3	4	4	2	2	2	2
4	4	2	2	1	1	3	3	3	3
4	4	2	2	1	1	3	3	3	3
4	4	2	2	1	1	3	3	2	2
4	4	2	2	1	1	3	3	3	3
4	4	2	2	1	1	3	3	3	3
4	4	3	3	1	1	2	2	3	3
4	4	2	2	1	1	3	3	3	3
4	4	3	3	4	4	2	2	3	3
4	4	2	2	1	1	3	3	3	3
4	4	2	2	1	1	3	3	3	3
2	2	2	2	1	1	3	3	3	3
4	4	2	2	1	1	3	3	3	3
2	2	2	2	1	1	3	3	3	3
2	2	2	2	1	1	3	3	3	3
4	4	3	3	4	4	2	2	3	3
4	4	2	2	1	1	3	3	3	3
4	4	2	2	1	1	3	3	3	3
2	2	2	2	1	1	3	3	2	2
2	2	2	2	1	1	3	3	3	3
4	4	2	2	1	1	3	3	3	3
2	2	2	2	1	1	3	3	3	3
2	2	2	2	1	1	3	3	2	2
4	4	2	2	1	1	3	3	3	3
4	4	2	2	1	1	3	3	3	3
4	4	3	3	4	4	2	2	3	3
2	2	2	2	1	1	3	3	3	3
4	4	2	2	1	1	3	3	2	2
4	4	2	2	1	1	3	3	3	3
4	4	2	2	1	1	3	3	3	3
4	4	2	2	1	1	3	3	3	3
4	4	3	3	4	4	2	2	3	3
4	4	3	3	4	4	2	2	3	3
2	2	2	2	1	1	3	3	3	3
4	4	3	3	4	4	2	2	3	3
4	4	2	2	1	1	3	3	2	2
2	2	2	2	1	1	3	3	3	3
4	4	2	2	1	1	3	3	3	3
4	4	3	3	4	4	2	2	3	3
4	4	2	2	1	1	3	3	3	3
4	4	2	2	1	1	3	3	3	3
4	4	2	2	1	1	3	3	3	3
2	2	2	2	1	1	3	3	2	2
4	4	2	2	1	1	3	3	2	2
2	2	2	2	1	1	3	3	3	3
4	4	2	2	1	1	3	3	3	3
2	2	3	3	4	4	2	2	3	3
2	2	2	2	1	1	3	3	3	3
4	4	3	3	4	4	2	2	3	3
2	2	2	2	1	1	3	3	2	2
4	4	3	3	4	4	2	2	3	3
2	2	2	2	1	1	3	3	2	2
2	2	2	2	1	1	3	3	3	3
4	4	3	3	4	4	2	2	2	2

For Review Only

2	2	2	2	1	1	3	3	3	3
4	4	2	2	1	1	3	3	3	3

For Review Only

SNP1377	SNP1377	SNP1378	SNP1378	SNP1379	SNP1379	SNP138	SNP138	SNP1380	SNP1380
1	1	4	4	3	3	2	2	1	1
1	1	1	1	2	2	3	3	1	1
1	1	1	1	2	2	3	3	1	1
4	4	1	1	2	2	3	3	2	2
1	1	4	4	3	3	3	3	1	1
1	1	4	4	3	3	3	3	1	1
4	4	4	4	3	3	2	2	1	1
1	1	4	4	3	3	2	2	1	1
1	1	4	4	3	3	3	3	1	1
1	1	4	4	3	3	2	2	1	1
1	1	1	1	2	2	3	3	1	1
1	1	1	1	2	2	3	3	1	1
1	1	4	4	3	3	3	3	1	1
1	1	4	4	3	3	3	3	1	1
4	4	4	4	3	3	3	3	1	1
1	1	1	1	2	2	3	3	1	1
1	1	4	4	3	3	2	2	1	1
1	1	4	4	3	3	3	3	1	1
4	4	1	1	2	2	3	3	2	2
1	1	4	4	3	3	2	2	1	1
1	1	4	4	3	3	3	3	1	1
1	1	4	4	3	3	3	3	1	1
4	4	4	4	3	3	3	3	2	2
1	1	4	4	3	3	3	3	1	1
1	1	1	1	3	3	3	3	1	1
4	4	4	4	3	3	3	3	2	2
4	4	4	4	3	3	3	3	2	2
4	4	4	4	3	3	3	3	2	2
1	1	4	4	3	3	2	2	1	1
1	1	4	4	3	3	3	3	1	1
1	1	4	4	3	3	3	3	1	1
4	4	4	4	3	3	2	2	2	2
1	1	4	4	3	3	2	2	1	1
1	1	4	4	3	3	3	3	1	1
4	4	4	4	3	3	3	3	2	2
1	1	1	1	2	2	3	3	1	1
4	4	4	4	3	3	3	3	1	1
4	4	1	1	2	2	3	3	2	2
1	1	4	4	3	3	3	3	1	1
4	4	4	4	3	3	3	3	2	2
1	1	4	4	3	3	2	2	1	1
1	1	4	4	3	3	2	2	1	1
1	1	4	4	3	3	2	2	1	1
1	1	4	4	3	3	3	3	1	1
1	1	4	4	3	3	2	2	1	1
1	1	1	1	2	2	3	3	1	1
4	4	4	4	3	3	3	3	2	2
1	1	1	1	2	2	3	3	1	1
1	1	1	1	3	3	3	3	1	1
4	4	4	4	3	3	3	3	2	2
4	4	4	4	3	3	3	3	2	2
4	4	1	1	2	2	3	3	2	2

For Review Only

1	1	4	4	3	3	2	2	1	1
1	1	4	4	3	3	2	2	1	1
4	4	1	1	2	2	3	3	2	2
1	1	1	1	2	2	3	3	1	1
4	4	1	1	2	2	3	3	2	2
4	4	1	1	2	2	3	3	2	2
4	4	4	4	3	3	3	3	2	2
1	1	4	4	3	3	2	2	1	1
1	1	4	4	3	3	3	3	1	1
4	4	1	1	3	3	3	3	1	1
4	4	1	1	2	2	3	3	1	1
4	4	1	1	2	2	3	3	2	2
1	1	4	4	3	3	2	2	1	1
1	1	4	4	3	3	2	2	1	1
4	4	4	4	3	3	3	3	2	2
1	1	4	4	3	3	2	2	1	1
4	4	4	4	3	3	3	3	2	2
1	1	4	4	3	3	3	3	1	1
1	1	4	4	3	3	3	3	1	1
1	1	4	4	3	3	2	2	1	1
4	4	4	4	3	3	3	3	2	2
4	4	4	4	3	3	3	3	2	2
1	1	4	4	3	3	3	3	2	2
4	4	4	4	3	3	2	2	2	2
1	1	4	4	3	3	2	2	1	1
1	1	1	1	2	2	3	3	1	1
1	1	4	4	3	3	2	2	1	1
1	1	4	4	3	3	3	3	1	1
4	4	4	4	3	3	3	3	2	2
4	4	1	1	2	2	3	3	2	2
1	1	4	4	2	2	3	3	1	1
1	1	4	4	3	3	2	2	1	1
1	1	4	4	3	3	3	3	1	1
1	1	4	4	3	3	2	2	1	1
1	1	4	4	3	3	2	2	1	1
1	1	4	4	3	3	2	2	1	1
1	1	4	4	3	3	2	2	1	1
1	1	1	1	2	2	3	3	1	1
1	1	4	4	3	3	2	2	1	1
1	1	4	4	3	3	3	3	1	1
1	1	1	1	2	2	3	3	1	1
4	4	1	1	2	2	3	3	2	2
4	4	4	4	3	3	2	2	2	2
4	4	1	1	2	2	3	3	1	1
1	1	1	1	2	2	3	3	1	1
4	4	1	1	2	2	3	3	2	2
1	1	4	4	3	3	3	3	1	1
4	4	4	4	3	3	3	3	2	2
4	4	4	4	3	3	3	3	2	2
4	4	4	4	3	3	3	3	2	2
4	4	4	4	3	3	3	3	2	2
1	1	4	4	3	3	3	3	1	1
1	1	4	4	3	3	3	3	1	1
4	4	4	4	3	3	3	3	1	1
1	1	4	4	3	3	3	3	1	1

For Review Only

4	4	4	4	3	3	3	3	2	2
4	4	4	4	3	3	3	3	2	2
4	4	1	1	2	2	3	3	2	2
1	1	4	4	3	3	3	3	1	1
4	4	4	4	3	3	3	3	2	2
4	4	4	4	3	3	3	3	2	2
1	1	1	1	2	2	3	3	1	1
4	4	1	1	3	3	3	3	1	1
1	1	4	4	3	3	3	3	1	1
1	1	4	4	3	3	3	3	1	1
1	1	4	4	3	3	2	2	1	1
4	4	4	4	3	3	3	3	2	2
4	4	4	4	3	3	3	3	2	2
4	4	1	1	2	2	3	3	2	2
4	4	4	4	3	3	3	3	2	2
4	4	4	4	3	3	3	3	2	2
4	4	4	4	3	3	3	3	2	2
4	4	4	4	2	2	3	3	2	2
1	1	4	4	3	3	3	3	1	1
1	1	1	1	3	3	3	3	1	1
4	4	4	4	3	3	3	3	2	2
4	4	1	1	2	2	3	3	2	2
1	1	1	1	2	2	3	3	1	1
4	4	4	4	3	3	3	3	1	1
4	4	4	4	3	3	3	3	2	2
4	4	4	4	3	3	3	3	2	2
1	1	4	4	2	2	3	3	2	2
1	1	4	4	3	3	2	2	1	1
4	4	4	4	2	2	3	3	2	2
4	4	4	4	3	3	3	3	2	2
1	1	4	4	3	3	3	3	1	1
4	4	4	4	3	3	3	3	2	2
4	4	1	1	2	2	3	3	1	1
4	4	4	4	3	3	3	3	2	2
1	1	4	4	3	3	3	3	1	1
1	1	4	4	3	3	3	3	1	1
1	1	4	4	3	3	3	3	1	1
4	4	1	1	2	2	3	3	1	1
4	4	1	1	3	3	2	2	2	2
1	1	4	4	2	2	3	3	1	1
4	4	4	4	3	3	3	3	2	2
1	1	4	4	3	3	3	3	1	1
1	1	4	4	3	3	3	3	1	1
1	1	4	4	3	3	3	3	1	1
4	4	1	1	2	2	3	3	2	2
1	1	4	4	3	3	3	3	1	1
4	4	1	1	2	2	3	3	2	2
4	4	4	4	3	3	3	3	2	2
4	4	4	4	3	3	3	3	1	1
4	4	4	4	3	3	3	3	2	2
1	1	4	4	3	3	3	3	1	1
1	1	4	4	3	3	3	3	1	1
1	1	4	4	3	3	3	3	1	1
1	1	4	4	3	3	3	3	1	1
4	4	4	4	3	3	3	3	2	2

For Review Only

4	4	4	4	3	3	2	2	2	2
4	4	4	4	3	3	3	3	2	2
1	1	4	4	3	3	3	3	1	1
4	4	4	4	3	3	3	3	2	2
4	4	1	1	2	2	3	3	2	2
4	4	4	4	3	3	2	2	2	2
1	1	4	4	2	2	3	3	1	1
4	4	4	4	3	3	3	3	2	2
4	4	4	4	3	3	3	3	2	2
4	4	4	4	3	3	3	3	2	2
4	4	1	1	3	3	3	3	2	2
1	1	4	4	3	3	3	3	1	1
4	4	4	4	3	3	3	3	2	2
1	1	4	4	3	3	3	3	1	1
1	1	4	4	3	3	3	3	1	1
1	1	1	1	2	2	3	3	1	1
1	1	4	4	3	3	3	3	1	1
4	4	1	1	2	2	3	3	2	2
4	4	1	1	2	2	3	3	2	2
1	1	1	1	2	2	3	3	1	1
4	4	4	4	3	3	3	3	2	2
1	1	4	4	3	3	3	3	1	1
4	4	4	4	3	3	3	3	2	2
4	4	1	1	2	2	3	3	2	2
1	1	4	4	3	3	3	3	1	1
1	1	4	4	3	3	3	3	1	1
4	4	4	4	3	3	3	3	2	2
4	4	4	4	3	3	3	3	2	2
4	4	4	4	3	3	2	2	2	2
1	1	4	4	3	3	2	2	1	1
4	4	1	1	2	2	3	3	1	1
4	4	4	4	3	3	3	3	2	2
1	1	4	4	3	3	3	3	1	1
1	1	4	4	3	3	3	3	1	1
1	1	4	4	3	3	2	2	1	1
4	4	1	1	2	2	3	3	2	2
4	4	1	1	2	2	3	3	2	2
1	1	4	4	3	3	2	2	1	1
4	4	4	4	3	3	3	3	2	2
4	4	4	4	3	3	3	3	2	2
1	1	1	1	2	2	3	3	1	1
4	4	1	1	2	2	3	3	2	2
1	1	4	4	3	3	3	3	1	1
4	4	1	1	2	2	3	3	2	2
1	1	4	4	3	3	3	3	2	2
4	4	4	4	3	3	3	3	2	2
1	1	1	1	2	2	3	3	1	1
1	1	4	4	2	2	3	3	1	1
1	1	4	4	3	3	2	2	1	1
4	4	4	4	3	3	3	3	2	2
4	4	1	1	2	2	3	3	2	2
1	1	4	4	3	3	3	3	1	1
1	1	4	4	3	3	3	3	1	1
4	4	1	1	2	2	3	3	1	1
1	1	4	4	3	3	3	3	1	1

For Review Only

1	1	4	4	3	3	3	3	1	1
1	1	4	4	3	3	2	2	1	1
1	1	1	1	2	2	3	3	2	2
4	4	4	4	3	3	3	3	2	2
1	1	4	4	3	3	2	2	1	1
4	4	4	4	3	3	3	3	1	1
4	4	4	4	3	3	3	3	2	2
1	1	4	4	3	3	2	2	1	1
4	4	4	4	3	3	3	3	2	2
1	1	4	4	3	3	3	3	1	1
4	4	4	4	3	3	3	3	2	2
4	4	4	4	3	3	3	3	1	1
4	4	4	4	3	3	3	3	2	2
4	4	4	4	3	3	3	3	2	2
1	1	1	1	2	2	3	3	1	1
1	1	1	1	2	2	3	3	1	1
4	4	4	4	3	3	2	2	1	1
1	1	4	4	3	3	3	3	1	1
1	1	4	4	3	3	3	3	1	1
4	4	1	1	2	2	3	3	2	2
4	4	4	4	3	3	3	3	2	2
4	4	4	4	3	3	3	3	2	2
4	4	1	1	2	2	3	3	2	2
1	1	4	4	3	3	2	2	1	1
1	1	4	4	3	3	2	2	1	1
1	1	4	4	3	3	3	3	1	1
1	1	4	4	3	3	3	3	1	1
4	4	1	1	2	2	3	3	2	2
1	1	4	4	3	3	2	2	1	1
4	4	1	1	2	2	3	3	2	2
1	1	4	4	3	3	3	3	1	1
1	1	1	1	2	2	3	3	1	1
1	1	4	4	3	3	3	3	1	1
4	4	4	4	3	3	3	3	2	2
4	4	4	4	3	3	3	3	2	2
1	1	4	4	3	3	3	3	1	1
1	1	1	1	2	2	3	3	1	1
4	4	1	1	2	2	3	3	2	2
1	1	1	1	2	2	3	3	1	1
1	1	4	4	3	3	3	3	1	1
1	1	4	4	3	3	3	3	1	1
1	1	4	4	3	3	3	3	1	1
1	1	4	4	3	3	3	3	1	1
1	1	1	1	2	2	3	3	1	1
4	4	1	1	2	2	3	3	2	2
4	4	4	4	3	3	2	2	2	2
4	4	4	4	3	3	3	3	2	2
1	1	4	4	3	3	3	3	1	1
1	1	4	4	3	3	3	3	1	1
1	1	4	4	3	3	3	3	1	1
4	4	1	1	2	2	2	2	2	2
1	1	4	4	3	3	3	3	1	1
1	1	1	1	2	2	3	3	1	1
4	4	1	1	2	2	3	3	2	2
4	4	1	1	2	2	2	2	2	2

For Review Only

4	4	1	1	2	2	3	3	2	2
4	4	1	1	2	2	3	3	2	2

For Review Only

SNP1381	SNP1381	SNP1382	SNP1382	SNP1383	SNP1383	SNP1384	SNP1384	SNP1385	SNP1385
3	3	4	4	2	2	1	1	1	1
3	3	4	4	3	3	1	1	1	1
3	3	4	4	2	2	1	1	1	1
3	3	4	4	2	2	1	1	1	1
2	2	4	4	3	3	1	1	1	1
3	3	4	4	2	2	1	1	1	1
3	3	4	4	3	3	1	1	1	1
3	3	4	4	2	2	1	1	1	1
3	3	3	3	2	2	1	1	1	1
3	3	4	4	2	2	1	1	1	1
3	3	4	4	2	2	1	1	1	1
3	3	4	4	2	2	1	1	1	1
2	2	4	4	2	2	1	1	1	1
3	3	4	4	3	3	1	1	1	1
3	3	4	4	2	2	4	4	1	1
3	3	3	3	2	2	4	4	1	1
2	2	3	3	2	2	4	4	1	1
3	3	4	4	3	3	1	1	3	3
3	3	3	3	2	2	4	4	1	1
3	3	3	3	2	2	4	4	3	3
3	3	4	4	2	2	1	1	3	3
3	3	4	4	2	2	1	1	1	1
3	3	3	3	2	2	4	4	1	1
3	3	4	4	2	2	1	1	1	1
3	3	3	3	2	2	4	4	1	1
3	3	4	4	2	2	1	1	1	1
3	3	4	4	2	2	1	1	1	1
3	3	4	4	2	2	1	1	1	1
3	3	3	3	2	2	4	4	3	3
3	3	4	4	2	2	1	1	3	3
3	3	4	4	2	2	1	1	1	1
3	3	4	4	2	2	1	1	1	1
3	3	4	4	3	3	1	1	1	1
3	3	4	4	3	3	1	1	1	1
3	3	3	3	2	2	1	1	1	1
3	3	4	4	2	2	1	1	1	1
3	3	3	3	2	2	4	4	1	1
3	3	3	3	2	2	4	4	1	1
3	3	4	4	3	3	1	1	1	1
3	3	3	3	2	2	4	4	1	1
3	3	4	4	2	2	1	1	1	1
3	3	4	4	3	3	1	1	3	3
3	3	4	4	2	2	1	1	1	1
3	3	3	3	2	2	4	4	3	3
3	3	4	4	2	2	1	1	1	1
3	3	4	4	2	2	1	1	1	1
3	3	4	4	2	2	1	1	1	1
3	3	4	4	2	2	1	1	1	1
3	3	3	3	2	2	4	4	1	1
3	3	4	4	2	2	1	1	1	1
3	3	3	3	2	2	4	4	1	1
3	3	4	4	2	2	1	1	1	1

For Review Only

2	2	3	3	2	2	4	4	1	1
3	3	4	4	3	3	1	1	1	1
3	3	3	3	2	2	1	1	1	1
3	3	4	4	3	3	1	1	1	1
3	3	3	3	2	2	4	4	1	1
3	3	4	4	2	2	1	1	1	1
3	3	4	4	2	2	1	1	1	1
3	3	4	4	3	3	1	1	3	3
3	3	4	4	3	3	1	1	1	1
3	3	4	4	3	3	1	1	1	1
3	3	4	4	2	2	1	1	1	1
3	3	4	4	2	2	1	1	1	1
2	2	3	3	2	2	4	4	1	1
3	3	3	3	2	2	4	4	3	3
3	3	4	4	2	2	1	1	1	1
3	3	4	4	3	3	1	1	1	1
3	3	4	4	2	2	1	1	1	1
3	3	3	3	2	2	1	1	1	1
3	3	4	4	2	2	1	1	1	1
2	2	3	3	2	2	4	4	1	1
3	3	4	4	2	2	1	1	1	1
3	3	4	4	3	3	1	1	1	1
3	3	3	3	2	2	4	4	1	1
3	3	4	4	2	2	1	1	1	1
3	3	4	4	2	2	1	1	1	1
3	3	4	4	2	2	1	1	1	1
3	3	4	4	2	2	1	1	3	3
3	3	4	4	2	2	1	1	1	1
3	3	4	4	2	2	1	1	1	1
2	2	4	4	2	2	1	1	1	1
3	3	3	3	2	2	4	4	1	1
3	3	4	4	3	3	1	1	1	1
3	3	4	4	3	3	1	1	3	3
2	2	3	3	2	2	4	4	1	1
3	3	3	3	2	2	4	4	1	1
3	3	4	4	2	2	1	1	1	1
3	3	3	3	2	2	4	4	1	1
3	3	4	4	2	2	1	1	1	1
3	3	4	4	2	2	1	1	1	1
3	3	4	4	2	2	1	1	1	1
2	2	3	3	2	2	4	4	1	1
2	2	4	4	2	2	1	1	1	1
3	3	3	3	2	2	4	4	1	1
3	3	4	4	2	2	1	1	1	1
3	3	4	4	3	3	1	1	1	1
3	3	3	3	2	2	4	4	1	1
3	3	4	4	2	2	1	1	1	1
3	3	4	4	2	2	1	1	1	1
3	3	4	4	2	2	1	1	1	1
3	3	4	4	3	3	1	1	1	1
3	3	4	4	2	2	1	1	1	1
3	3	4	4	2	2	1	1	1	1
3	3	4	4	2	2	1	1	3	3
3	3	3	3	3	3	1	1	1	1
3	3	4	4	3	3	1	1	1	1

For Review Only

3	3	4	4	2	2	1	1	1	1
3	3	4	4	2	2	1	1	1	1
3	3	4	4	2	2	1	1	1	1
3	3	4	4	3	3	1	1	1	1
3	3	4	4	2	2	1	1	1	1
3	3	4	4	3	3	1	1	1	1
3	3	3	3	2	2	4	4	1	1
3	3	4	4	3	3	1	1	1	1
3	3	4	4	2	2	1	1	1	1
3	3	4	4	3	3	1	1	1	1
3	3	4	4	3	3	1	1	1	1
3	3	4	4	2	2	1	1	1	1
3	3	4	4	2	2	1	1	1	1
3	3	4	4	2	2	1	1	1	1
3	3	4	4	3	3	1	1	1	1
3	3	4	4	2	2	1	1	1	1
3	3	3	3	2	2	1	1	1	1
3	3	4	4	3	3	1	1	1	1
3	3	4	4	3	3	1	1	1	1
3	3	3	3	2	2	4	4	1	1
3	3	4	4	2	2	1	1	1	1
3	3	4	4	2	2	4	4	1	1
3	3	4	4	3	3	1	1	1	1
3	3	4	4	2	2	1	1	1	1
3	3	4	4	3	3	1	1	1	1
3	3	4	4	3	3	1	1	1	1
3	3	3	3	2	2	4	4	1	1
3	3	3	3	2	2	4	4	1	1
3	3	4	4	2	2	1	1	1	1
2	2	3	3	2	2	4	4	3	3
3	3	4	4	3	3	1	1	1	1
3	3	4	4	2	2	1	1	1	1
2	2	3	3	2	2	4	4	1	1
3	3	4	4	2	2	1	1	1	1
3	3	4	4	3	3	1	1	1	1
3	3	4	4	2	2	1	1	1	1
3	3	4	4	3	3	1	1	1	1
3	3	4	4	2	2	1	1	1	1
3	3	3	3	3	3	1	1	1	1
3	3	4	4	2	2	1	1	1	1
3	3	4	4	2	2	1	1	1	1
3	3	4	4	3	3	1	1	1	1
3	3	4	4	3	3	1	1	1	1
3	3	4	4	3	3	1	1	3	3
3	3	3	3	2	2	1	1	1	1
2	2	3	3	2	2	4	4	1	1
3	3	4	4	2	2	1	1	1	1
3	3	4	4	2	2	1	1	1	1
3	3	4	4	3	3	1	1	1	1
3	3	4	4	2	2	1	1	1	1
3	3	4	4	2	2	1	1	1	1
3	3	4	4	3	3	1	1	3	3
3	3	3	3	2	2	1	1	1	1
3	3	4	4	2	2	1	1	1	1
3	3	4	4	3	3	1	1	3	3

For Review Only

3	3	4	4	2	2	1	1	1	1
3	3	4	4	2	2	1	1	1	1
3	3	4	4	2	2	1	1	1	1
3	3	3	3	2	2	4	4	1	1
3	3	4	4	2	2	1	1	1	1
3	3	3	3	2	2	1	1	1	1
3	3	4	4	3	3	1	1	3	3
3	3	4	4	2	2	1	1	1	1
3	3	4	4	2	2	1	1	1	1
3	3	3	3	2	2	4	4	1	1
3	3	4	4	2	2	1	1	1	1
3	3	4	4	2	2	1	1	1	1
3	3	4	4	2	2	1	1	1	1
3	3	4	4	3	3	1	1	1	1
3	3	4	4	3	3	1	1	1	1
3	3	4	4	2	2	1	1	1	1
3	3	4	4	3	3	1	1	1	1
3	3	3	3	2	2	4	4	1	1
3	3	4	4	3	3	1	1	1	1
3	3	3	3	2	2	4	4	1	1
3	3	3	3	2	2	1	1	1	1
3	3	3	3	2	2	4	4	3	3
3	3	4	4	3	3	1	1	1	1
3	3	4	4	2	2	1	1	1	1
3	3	4	4	3	3	1	1	3	3
3	3	4	4	3	3	1	1	1	1
3	3	4	4	2	2	1	1	1	1
3	3	4	4	2	2	1	1	3	3
3	3	4	4	2	2	1	1	1	1
3	3	4	4	2	2	4	4	1	1
3	3	4	4	2	2	1	1	1	1
2	2	4	4	2	2	1	1	1	1
3	3	3	3	2	2	4	4	1	1
3	3	4	4	3	3	1	1	1	1
3	3	3	3	2	2	4	4	1	1
3	3	4	4	2	2	1	1	1	1
3	3	4	4	2	2	1	1	1	1
3	3	3	3	2	2	4	4	1	1
3	3	4	4	2	2	1	1	1	1
3	3	3	3	2	2	4	4	1	1
3	3	4	4	2	2	1	1	1	1
3	3	4	4	2	2	1	1	1	1
3	3	4	4	3	3	1	1	1	1
3	3	3	3	2	2	4	4	1	1
3	3	4	4	2	2	4	4	1	1
3	3	3	3	2	2	1	1	1	1
3	3	4	4	2	2	1	1	1	1
3	3	4	4	3	3	1	1	3	3
3	3	4	4	2	2	1	1	1	1
3	3	3	3	2	2	4	4	1	1
3	3	4	4	2	2	1	1	1	1
3	3	3	3	3	3	4	4	1	1
3	3	4	4	3	3	1	1	1	1
3	3	4	4	2	2	1	1	1	1
3	3	4	4	2	2	1	1	1	1

For Review Only

3	3	3	3	2	2	4	4	1	1
3	3	3	3	2	2	4	4	3	3
3	3	4	4	2	2	1	1	1	1
3	3	4	4	3	3	1	1	1	1
3	3	3	3	2	2	4	4	1	1
3	3	4	4	2	2	1	1	1	1
3	3	4	4	3	3	1	1	1	1
3	3	4	4	2	2	1	1	3	3
3	3	4	4	2	2	1	1	1	1
3	3	4	4	2	2	1	1	1	1
3	3	4	4	2	2	1	1	1	1
3	3	3	3	2	2	4	4	1	1
3	3	4	4	2	2	1	1	1	1
3	3	4	4	2	2	1	1	1	1
3	3	4	4	2	2	1	1	1	1
3	3	4	4	2	2	1	1	1	1
3	3	4	4	2	2	1	1	1	1
3	3	4	4	3	3	1	1	1	1
3	3	4	4	3	3	1	1	3	3
3	3	3	3	2	2	4	4	1	1
3	3	4	4	2	2	1	1	1	1
3	3	4	4	2	2	1	1	1	1
3	3	4	4	2	2	1	1	1	1
3	3	3	3	2	2	4	4	1	1
3	3	4	4	3	3	1	1	3	3
3	3	4	4	2	2	1	1	3	3
3	3	4	4	3	3	1	1	3	3
3	3	4	4	2	2	1	1	1	1
3	3	3	3	2	2	4	4	1	1
3	3	4	4	2	2	1	1	1	1
3	3	4	4	3	3	1	1	1	1
3	3	4	4	2	2	1	1	1	1
3	3	4	4	3	3	1	1	1	1
3	3	4	4	2	2	1	1	1	1
3	3	4	4	3	3	1	1	1	1
3	3	4	4	3	3	1	1	1	1
3	3	3	3	2	2	4	4	1	1
3	3	4	4	2	2	1	1	1	1
3	3	4	4	2	2	1	1	1	1
3	3	4	4	3	3	1	1	1	1
3	3	4	4	2	2	1	1	1	1
3	3	4	4	2	2	1	1	1	1
3	3	4	4	2	2	1	1	1	1
2	2	3	3	2	2	4	4	1	1
3	3	3	3	2	2	1	1	1	1
3	3	4	4	2	2	1	1	1	1
3	3	4	4	2	2	1	1	1	1
3	3	4	4	2	2	1	1	1	1
3	3	4	4	2	2	1	1	1	1
3	3	4	4	3	3	1	1	1	1
3	3	3	3	2	2	4	4	1	1
3	3	4	4	3	3	1	1	1	1
3	3	4	4	2	2	1	1	1	1
3	3	4	4	2	2	1	1	1	1
3	3	3	3	2	2	4	4	1	1

For Review Only

3	3	4	4	2	2	1	1	1	1
3	3	3	3	2	2	4	4	1	1

For Review Only

SNP1386	SNP1386	SNP1387	SNP1387	SNP1388	SNP1388	SNP1389	SNP1389	SNP139	SNP139
1	1	2	2	2	2	2	2	1	1
3	3	2	2	3	3	2	2	1	1
1	1	2	2	2	2	2	2	1	1
1	1	1	1	2	2	2	2	1	1
1	1	2	2	2	2	2	2	1	1
1	1	2	2	3	3	2	2	1	1
1	1	2	2	3	3	2	2	4	4
3	3	2	2	2	2	1	1	4	4
1	1	2	2	2	2	2	2	1	1
3	3	2	2	2	2	1	1	4	4
1	1	2	2	2	2	2	2	1	1
1	1	2	2	2	2	2	2	1	1
1	1	2	2	2	2	1	1	1	1
1	1	2	2	2	2	2	2	1	1
1	1	2	2	2	2	2	2	1	1
1	1	2	2	2	2	2	2	1	1
1	1	2	2	2	2	2	2	4	4
1	1	2	2	2	2	1	1	1	1
1	1	2	2	2	2	2	2	1	1
3	3	2	2	2	2	2	2	4	4
3	3	2	2	2	2	1	1	1	1
1	1	2	2	2	2	1	1	1	1
3	3	2	2	2	2	2	2	1	1
3	3	2	2	3	3	2	2	1	1
1	1	2	2	2	2	2	2	1	1
1	1	1	1	2	2	2	2	1	1
3	3	1	1	2	2	2	2	1	1
1	1	2	2	2	2	2	2	1	1
3	3	2	2	2	2	2	2	4	4
3	3	2	2	2	2	1	1	1	1
1	1	2	2	2	2	2	2	1	1
1	1	2	2	2	2	2	2	4	4
1	1	2	2	3	3	2	2	4	4
3	3	2	2	3	3	2	2	1	1
1	1	2	2	2	2	2	2	1	1
3	3	2	2	3	3	2	2	1	1
1	1	2	2	2	2	2	2	1	1
1	1	2	2	2	2	2	2	4	4
3	3	2	2	3	3	2	2	1	1
1	1	2	2	2	2	2	2	1	1
3	3	2	2	2	2	1	1	4	4
3	3	2	2	2	2	1	1	4	4
3	3	2	2	3	3	2	2	4	4
1	1	2	2	2	2	2	2	1	1
1	1	2	2	3	3	2	2	4	4
1	1	2	2	2	2	2	2	1	1
3	3	2	2	3	3	2	2	1	1
1	1	1	1	2	2	2	2	1	1
1	1	2	2	2	2	2	2	1	1
1	1	2	2	2	2	2	2	1	1
1	1	2	2	2	2	2	2	1	1
1	1	2	2	3	3	2	2	1	1

For Review Only

1	1	2	2	2	2	2	2	4	4
1	1	2	2	3	3	2	2	4	4
1	1	2	2	2	2	2	2	1	1
1	1	2	2	2	2	1	1	1	1
1	1	2	2	2	2	2	2	1	1
3	3	1	1	2	2	2	2	1	1
3	3	1	1	2	2	2	2	1	1
3	3	2	2	3	3	2	2	4	4
3	3	2	2	2	2	1	1	1	1
1	1	2	2	2	2	2	2	1	1
3	3	2	2	3	3	2	2	1	1
3	3	2	2	3	3	2	2	1	1
1	1	2	2	2	2	2	2	4	4
3	3	2	2	2	2	2	2	4	4
1	1	2	2	2	2	2	2	1	1
3	3	2	2	2	2	2	2	4	4
1	1	1	1	2	2	2	2	1	1
3	3	2	2	2	2	2	2	1	1
1	1	2	2	3	3	2	2	1	1
1	1	2	2	3	3	2	2	4	4
3	3	2	2	3	3	2	2	1	1
3	3	2	2	3	3	2	2	1	1
3	3	2	2	2	2	2	2	1	1
1	1	2	2	2	2	1	1	4	4
1	1	2	2	3	3	2	2	4	4
1	1	2	2	3	3	2	2	1	1
1	1	2	2	2	2	2	2	4	4
3	3	2	2	3	3	2	2	1	1
3	3	1	1	2	2	2	2	1	1
1	1	2	2	2	2	2	2	1	1
1	1	2	2	2	2	2	2	1	1
3	3	2	2	3	3	2	2	4	4
3	3	2	2	3	3	2	2	1	1
1	1	2	2	2	2	2	2	4	4
3	3	2	2	2	2	2	2	4	4
1	1	2	2	3	3	2	2	4	4
3	3	2	2	2	2	2	2	4	4
1	1	2	2	2	2	1	1	1	1
1	1	2	2	2	2	1	1	4	4
1	1	2	2	3	3	2	2	1	1
1	1	2	2	2	2	2	2	1	1
3	3	2	2	2	2	2	2	1	1
1	1	2	2	2	2	2	2	4	4
3	3	2	2	3	3	2	2	1	1
3	3	2	2	2	2	1	1	1	1
3	3	2	2	2	2	2	2	1	1
3	3	2	2	3	3	2	2	1	1
3	3	1	1	2	2	2	2	1	1
3	3	1	1	2	2	2	2	1	1
1	1	2	2	3	3	2	2	1	1
3	3	2	2	2	2	2	2	1	1
3	3	2	2	3	3	2	2	1	1
3	3	2	2	2	2	1	1	1	1
1	1	2	2	2	2	2	2	1	1
1	1	2	2	3	3	2	2	1	1

For Review Only

1	1	1	1	2	2	2	2	1	1
3	3	2	2	2	2	2	2	1	1
3	3	2	2	3	3	2	2	1	1
3	3	2	2	3	3	2	2	1	1
1	1	2	2	3	3	2	2	4	4
1	1	2	2	3	3	2	2	1	1
1	1	2	2	2	2	2	2	1	1
3	3	2	2	3	3	2	2	1	1
3	3	1	1	2	2	2	2	1	1
3	3	2	2	2	2	2	2	1	1
1	1	2	2	2	2	2	2	1	1
1	1	2	2	2	2	2	2	1	1
1	1	1	1	2	2	2	2	1	1
1	1	2	2	3	3	2	2	1	1
1	1	2	2	3	3	2	2	1	1
1	1	2	2	2	2	2	2	1	1
3	3	2	2	2	2	2	2	1	1
1	1	2	2	2	2	2	2	1	1
3	3	2	2	3	3	2	2	1	1
1	1	2	2	2	2	2	2	1	1
1	1	1	1	2	2	2	2	1	1
1	1	2	2	2	2	2	2	1	1
3	3	2	2	3	3	2	2	1	1
3	3	2	2	2	2	1	1	1	1
1	1	2	2	2	2	2	2	1	1
3	3	2	2	3	3	2	2	1	1
1	1	2	2	2	2	2	2	1	1
3	3	2	2	2	2	2	2	4	4
3	3	2	2	2	2	2	2	1	1
1	1	2	2	2	2	2	2	1	1
3	3	2	2	2	2	1	1	1	1
1	1	2	2	2	2	2	2	1	1
1	1	2	2	2	2	2	2	1	1
3	3	2	2	2	2	2	2	1	1
3	3	2	2	3	3	2	2	1	1
1	1	2	2	2	2	2	2	1	1
3	3	2	2	3	3	2	2	1	1
1	1	2	2	2	2	2	2	1	1
1	1	2	2	3	3	2	2	1	1
1	1	2	2	2	2	2	2	1	1
1	1	2	2	2	2	2	2	1	1
3	3	2	2	3	3	2	2	1	1
1	1	2	2	2	2	2	2	1	1
1	1	2	2	2	2	2	2	1	1
3	3	2	2	2	2	2	2	1	1
3	3	2	2	2	2	2	2	1	1
3	3	1	1	2	2	2	2	1	1
3	3	1	1	2	2	2	2	1	1
3	3	2	2	3	3	2	2	1	1
1	1	1	1	2	2	2	2	1	1
3	3	1	1	2	2	2	2	1	1
3	3	2	2	3	3	2	2	1	1
1	1	2	2	2	2	2	2	1	1
3	3	2	2	3	3	2	2	1	1
1	1	2	2	2	2	2	2	1	1

For Review Only

3	3	2	2	2	2	2	2	4	4
1	1	1	1	2	2	2	2	1	1
1	1	2	2	2	2	2	2	1	1
3	3	2	2	2	2	2	2	1	1
1	1	2	2	3	3	2	2	1	1
1	1	2	2	2	2	2	2	4	4
3	3	2	2	2	2	2	2	1	1
1	1	2	2	2	2	2	2	1	1
1	1	2	2	2	2	2	2	1	1
1	1	2	2	2	2	2	2	1	1
1	1	1	1	2	2	2	2	1	1
1	1	2	2	2	2	2	2	1	1
1	1	1	1	2	2	2	2	1	1
3	3	2	2	2	2	2	2	1	1
3	3	2	2	3	3	2	2	1	1
1	1	2	2	2	2	2	2	1	1
1	1	2	2	2	2	2	2	1	1
3	3	2	2	2	2	2	2	1	1
3	3	2	2	3	3	2	2	1	1
1	1	2	2	2	2	2	2	1	1
1	1	2	2	2	2	2	2	1	1
3	3	2	2	2	2	2	2	1	1
3	3	2	2	3	3	2	2	1	1
3	3	2	2	3	3	2	2	1	1
3	3	2	2	3	3	2	2	1	1
3	3	2	2	3	3	2	2	1	1
1	1	1	1	2	2	2	2	1	1
3	3	2	2	2	2	2	2	1	1
3	3	2	2	2	2	1	1	4	4
1	1	2	2	2	2	2	2	4	4
3	3	1	1	2	2	2	2	1	1
3	3	2	2	2	2	2	2	1	1
1	1	2	2	2	2	2	2	1	1
3	3	2	2	3	3	2	2	1	1
1	1	2	2	2	2	2	2	4	4
3	3	2	2	3	3	2	2	1	1
3	3	2	2	3	3	2	2	1	1
1	1	2	2	2	2	2	2	4	4
3	3	1	1	2	2	2	2	1	1
3	3	2	2	2	2	2	2	1	1
1	1	2	2	2	2	2	2	1	1
3	3	1	1	2	2	2	2	1	1
3	3	2	2	3	3	2	2	1	1
3	3	2	2	2	2	2	2	1	1
1	1	2	2	2	2	1	1	1	1
3	3	2	2	2	2	2	2	1	1
1	1	2	2	2	2	2	2	1	1
3	3	2	2	3	3	2	2	1	1
3	3	2	2	3	3	2	2	4	4
1	1	2	2	2	2	2	2	1	1
3	3	2	2	3	3	2	2	1	1
3	3	2	2	2	2	2	2	1	1
3	3	2	2	3	3	2	2	1	1
3	3	2	2	3	3	2	2	1	1
3	3	2	2	3	3	2	2	1	1

For Review Only

1	1	2	2	2	2	2	2	1	1
3	3	2	2	2	2	2	2	4	4
3	3	2	2	2	2	2	2	1	1
1	1	2	2	2	2	2	2	1	1
3	3	2	2	2	2	2	2	1	1
1	1	1	1	2	2	2	2	1	1
1	1	2	2	3	3	2	2	1	1
3	3	2	2	2	2	1	1	4	4
1	1	2	2	2	2	1	1	1	1
3	3	2	2	2	2	1	1	1	1
1	1	2	2	2	2	2	2	1	1
3	3	2	2	2	2	2	2	1	1
1	1	2	2	2	2	1	1	1	1
3	3	2	2	3	3	2	2	1	1
1	1	1	1	2	2	2	2	1	1
1	1	2	2	2	2	2	2	1	1
3	3	2	2	2	2	1	1	4	4
1	1	2	2	3	3	2	2	1	1
3	3	2	2	3	3	2	2	1	1
1	1	2	2	2	2	2	2	1	1
1	1	2	2	2	2	2	2	1	1
3	3	1	1	2	2	2	2	1	1
1	1	2	2	2	2	2	2	1	1
1	1	2	2	2	2	2	2	4	4
3	3	2	2	3	3	2	2	4	4
3	3	2	2	2	2	2	2	1	1
3	3	2	2	3	3	2	2	1	1
1	1	1	1	2	2	2	2	1	1
1	1	2	2	2	2	2	2	1	1
3	3	2	2	3	3	2	2	1	1
3	3	2	2	3	3	2	2	1	1
3	3	1	1	2	2	2	2	1	1
3	3	2	2	3	3	2	2	4	4
3	3	2	2	2	2	2	2	1	1
1	1	2	2	3	3	2	2	1	1
3	3	2	2	3	3	2	2	1	1
1	1	2	2	2	2	2	2	1	1
3	3	2	2	3	3	2	2	1	1
3	3	2	2	3	3	2	2	1	1
1	1	2	2	3	3	2	2	1	1
1	1	2	2	2	2	2	2	1	1
1	1	2	2	3	3	2	2	1	1
3	3	2	2	2	2	2	2	1	1
1	1	2	2	2	2	2	2	1	1
3	3	2	2	2	2	2	2	1	1
1	1	1	1	2	2	2	2	4	4
3	3	1	1	2	2	2	2	1	1
1	1	1	1	2	2	2	2	1	1
1	1	1	1	2	2	2	2	1	1
3	3	2	2	3	3	2	2	1	1
1	1	2	2	2	2	2	2	4	4
3	3	2	2	3	3	2	2	1	1
1	1	2	2	2	2	2	2	1	1
1	1	1	1	2	2	2	2	1	1
3	3	2	2	2	2	2	2	4	4

For Review Only

1	1	2	2	2	2	2	2	1	1
1	1	2	2	2	2	2	2	1	1

For Review Only

SNP1390	SNP1390	SNP1391	SNP1391	SNP1392	SNP1392	SNP1393	SNP1393	SNP1394	SNP1394
4	4	1	1	4	4	4	4	3	3
2	2	1	1	4	4	4	4	3	3
4	4	1	1	4	4	4	4	3	3
4	4	1	1	4	4	4	4	3	3
4	4	1	1	4	4	4	4	3	3
2	2	1	1	4	4	4	4	3	3
2	2	1	1	4	4	4	4	3	3
2	2	1	1	4	4	4	4	3	3
4	4	1	1	4	4	4	4	3	3
4	4	4	4	1	1	4	4	3	3
4	4	1	1	4	4	4	4	2	2
4	4	1	1	4	4	4	4	3	3
4	4	4	4	4	4	4	4	3	3
4	4	1	1	4	4	4	4	3	3
4	4	1	1	4	4	4	4	3	3
4	4	1	1	4	4	4	4	3	3
4	4	1	1	4	4	4	4	3	3
4	4	4	4	1	1	4	4	3	3
4	4	1	1	4	4	4	4	3	3
4	4	1	1	4	4	4	4	3	3
2	2	1	1	4	4	1	1	3	3
4	4	4	4	1	1	4	4	3	3
4	4	1	1	4	4	4	4	3	3
2	2	1	1	4	4	4	4	2	2
4	4	1	1	4	4	4	4	3	3
4	4	1	1	4	4	4	4	3	3
4	4	1	1	4	4	4	4	3	3
4	4	1	1	4	4	4	4	3	3
4	4	1	1	4	4	4	4	3	3
4	4	4	4	1	1	4	4	3	3
4	4	1	1	4	4	4	4	3	3
4	4	1	1	4	4	1	1	3	3
2	2	1	1	4	4	1	1	3	3
2	2	1	1	4	4	4	4	3	3
4	4	1	1	4	4	4	4	3	3
2	2	1	1	4	4	4	4	3	3
4	4	1	1	4	4	4	4	3	3
4	4	1	1	4	4	4	4	3	3
2	2	1	1	4	4	4	4	2	2
4	4	1	1	4	4	4	4	3	3
4	4	4	4	1	1	4	4	3	3
4	4	1	1	1	1	1	1	3	3
2	2	1	1	4	4	4	4	3	3
4	4	1	1	4	4	4	4	3	3
2	2	1	1	4	4	4	4	3	3
4	4	1	1	4	4	4	4	3	3
2	2	1	1	4	4	4	4	3	3
4	4	1	1	4	4	4	4	3	3
4	4	1	1	4	4	4	4	3	3
4	4	1	1	4	4	4	4	3	3
4	4	1	1	4	4	4	4	3	3
2	2	1	1	4	4	4	4	3	3

For Review Only

4	4	1	1	4	4	4	4	3	3
2	2	1	1	4	4	1	1	3	3
4	4	1	1	4	4	4	4	3	3
4	4	4	4	1	1	4	4	3	3
4	4	1	1	4	4	4	4	3	3
4	4	1	1	4	4	4	4	3	3
4	4	1	1	4	4	4	4	3	3
2	2	1	1	4	4	4	4	3	3
4	4	1	1	1	1	4	4	3	3
4	4	1	1	4	4	4	4	3	3
2	2	1	1	4	4	4	4	3	3
2	2	1	1	4	4	4	4	3	3
4	4	1	1	4	4	4	4	3	3
4	4	1	1	4	4	4	4	3	3
4	4	1	1	4	4	4	4	3	3
2	2	1	1	4	4	4	4	3	3
4	4	1	1	4	4	4	4	3	3
4	4	1	1	4	4	4	4	3	3
2	2	1	1	4	4	4	4	3	3
2	2	1	1	4	4	4	4	3	3
2	2	1	1	4	4	4	4	3	3
2	2	1	1	4	4	4	4	3	3
4	4	1	1	4	4	4	4	3	3
4	4	4	4	1	1	1	1	3	3
2	2	1	1	4	4	4	4	3	3
2	2	1	1	4	4	4	4	3	3
4	4	1	1	4	4	4	4	3	3
2	2	1	1	4	4	4	4	2	2
4	4	1	1	4	4	4	4	3	3
4	4	1	1	4	4	4	4	3	3
4	4	1	1	4	4	4	4	3	3
2	2	1	1	4	4	4	4	3	3
2	2	1	1	4	4	4	4	3	3
4	4	1	1	4	4	4	4	3	3
4	4	1	1	4	4	4	4	3	3
2	2	1	1	4	4	4	4	3	3
4	4	1	1	4	4	4	4	3	3
2	2	1	1	4	4	4	4	3	3
4	4	4	4	1	1	4	4	2	2
2	2	1	1	4	4	4	4	3	3
4	4	1	1	4	4	4	4	3	3
4	4	1	1	4	4	4	4	3	3
4	4	1	1	4	4	4	4	3	3
2	2	1	1	4	4	4	4	3	3
4	4	4	4	1	1	4	4	3	3
4	4	1	1	4	4	4	4	3	3
2	2	1	1	4	4	4	4	3	3
4	4	1	1	4	4	4	4	3	3
4	4	1	1	4	4	4	4	3	3
2	2	1	1	4	4	4	4	3	3
2	2	1	1	4	4	1	1	3	3
2	2	1	1	4	4	4	4	2	2
2	2	1	1	4	4	4	4	3	3
4	4	1	1	4	4	1	1	3	3
2	2	1	1	4	4	4	4	3	3

For Review Only

4	4	1	1	4	4	4	4	3	3
4	4	1	1	4	4	4	4	3	3
2	2	1	1	4	4	4	4	3	3
2	2	1	1	4	4	4	4	3	3
2	2	1	1	4	4	4	4	3	3
2	2	1	1	4	4	4	4	3	3
4	4	1	1	4	4	4	4	3	3
2	2	1	1	4	4	4	4	3	3
4	4	1	1	4	4	4	4	3	3
4	4	1	1	4	4	4	4	3	3
2	2	1	1	4	4	4	4	3	3
4	4	1	1	4	4	4	4	3	3
4	4	1	1	4	4	4	4	3	3
2	2	1	1	4	4	4	4	3	3
2	2	1	1	4	4	4	4	3	3
4	4	1	1	4	4	4	4	3	3
4	4	1	1	4	4	4	4	3	3
4	4	1	1	4	4	4	4	3	3
2	2	1	1	4	4	4	4	3	3
4	4	1	1	4	4	4	4	3	3
4	4	1	1	4	4	4	4	3	3
4	4	1	1	4	4	4	4	3	3
2	2	1	1	4	4	4	4	3	3
4	4	1	1	1	1	4	4	2	2
4	4	1	1	4	4	4	4	3	3
2	2	1	1	4	4	4	4	2	2
4	4	1	1	4	4	1	1	3	3
4	4	1	1	4	4	1	1	3	3
4	4	1	1	4	4	4	4	3	3
4	4	1	1	4	4	4	4	3	3
4	4	1	1	4	4	4	4	2	2
4	4	1	1	4	4	4	4	3	3
4	4	1	1	4	4	4	4	3	3
4	4	1	1	4	4	4	4	3	3
2	2	1	1	4	4	4	4	3	3
4	4	1	1	4	4	4	4	2	2
2	2	1	1	4	4	4	4	3	3
4	4	1	1	4	4	4	4	3	3
2	2	1	1	4	4	4	4	2	2
4	4	1	1	4	4	4	4	2	2
4	4	1	1	4	4	4	4	2	2
2	2	1	1	4	4	4	4	3	3
4	4	1	1	4	4	4	4	3	3
4	4	1	1	4	4	4	4	3	3
4	4	1	1	4	4	4	4	3	3
4	4	1	1	4	4	4	4	3	3
4	4	1	1	4	4	4	4	3	3
4	4	1	1	4	4	4	4	3	3
2	2	1	1	4	4	4	4	3	3
4	4	1	1	4	4	4	4	3	3
4	4	1	1	4	4	4	4	3	3
2	2	1	1	4	4	4	4	2	2
4	4	1	1	4	4	4	4	3	3
2	2	1	1	4	4	4	4	3	3
4	4	1	1	4	4	4	4	3	3

For Review Only

4	4	1	1	4	4	4	4	3	3
4	4	1	1	4	4	4	4	2	2
4	4	1	1	4	4	4	4	3	3
4	4	1	1	4	4	4	4	3	3
2	2	1	1	4	4	4	4	3	3
4	4	1	1	4	4	4	4	3	3
4	4	1	1	4	4	4	4	2	2
2	2	1	1	4	4	4	4	3	3
4	4	1	1	4	4	4	4	3	3
4	4	1	1	4	4	4	4	3	3
4	4	1	1	4	4	4	4	3	3
2	2	1	1	4	4	4	4	2	2
4	4	1	1	4	4	4	4	3	3
4	4	1	1	4	4	4	4	3	3
2	2	1	1	4	4	4	4	3	3
4	4	1	1	4	4	4	4	2	2
2	2	1	1	4	4	4	4	3	3
4	4	1	1	4	4	4	4	3	3
2	2	1	1	4	4	4	4	3	3
4	4	1	1	4	4	4	4	3	3
4	4	1	1	4	4	4	4	3	3
4	4	1	1	4	4	4	4	2	2
2	2	1	1	4	4	4	4	3	3
2	2	1	1	4	4	4	4	3	3
2	2	1	1	4	4	4	4	3	3
2	2	1	1	4	4	4	4	3	3
4	4	1	1	4	4	4	4	3	3
4	4	1	1	4	4	4	4	3	3
4	4	4	4	1	1	4	4	3	3
4	4	1	1	4	4	4	4	3	3
4	4	1	1	4	4	4	4	3	3
4	4	1	1	4	4	4	4	3	3
4	4	1	1	4	4	1	1	3	3
2	2	1	1	4	4	4	4	3	3
4	4	1	1	4	4	4	4	3	3
2	2	1	1	4	4	4	4	3	3
2	2	1	1	4	4	4	4	3	3
4	4	1	1	4	4	4	4	3	3
4	4	1	1	4	4	4	4	3	3
4	4	1	1	4	4	4	4	3	3
4	4	1	1	4	4	4	4	3	3
4	4	1	1	4	4	4	4	3	3
2	2	1	1	4	4	4	4	3	3
4	4	1	1	4	4	4	4	3	3
4	4	1	1	4	4	4	4	3	3
4	4	1	1	4	4	1	1	3	3
4	4	1	1	4	4	4	4	2	2
2	2	1	1	4	4	4	4	2	2
2	2	1	1	4	4	4	4	3	3
4	4	1	1	4	4	4	4	3	3
2	2	1	1	4	4	1	1	3	3
4	4	1	1	4	4	4	4	3	3
2	2	1	1	4	4	4	4	3	3
2	2	1	1	4	4	4	4	3	3
2	2	1	1	4	4	4	4	2	2

For Review Only

4	4	1	1	4	4	4	4	3	3
4	4	1	1	4	4	4	4	3	3
4	4	1	1	4	4	1	1	3	3
4	4	1	1	4	4	4	4	3	3
4	4	1	1	4	4	4	4	3	3
4	4	1	1	4	4	1	1	3	3
2	2	1	1	4	4	4	4	3	3
4	4	4	4	1	1	4	4	3	3
4	4	1	1	4	4	4	4	3	3
2	2	1	1	1	1	4	4	2	2
4	4	1	1	4	4	4	4	3	3
4	4	1	1	4	4	4	4	2	2
4	4	4	4	1	1	4	4	2	2
2	2	1	1	4	4	4	4	3	3
4	4	1	1	4	4	4	4	3	3
4	4	1	1	4	4	4	4	2	2
2	2	1	1	1	1	4	4	2	2
2	2	1	1	4	4	4	4	3	3
2	2	1	1	4	4	4	4	3	3
4	4	1	1	4	4	4	4	3	3
4	4	1	1	4	4	4	4	3	3
4	4	1	1	4	4	4	4	3	3
4	4	1	1	4	4	4	4	3	3
4	4	1	1	4	4	4	4	2	2
2	2	1	1	4	4	4	4	3	3
4	4	1	1	4	4	4	4	3	3
2	2	1	1	4	4	4	4	3	3
4	4	1	1	4	4	4	4	3	3
4	4	1	1	4	4	4	4	3	3
2	2	1	1	4	4	1	1	3	3
2	2	1	1	4	4	4	4	2	2
4	4	1	1	4	4	4	4	2	2
2	2	1	1	4	4	4	4	3	3
4	4	1	1	4	4	4	4	3	3
2	2	1	1	4	4	4	4	2	2
2	2	1	1	4	4	4	4	2	2
4	4	1	1	4	4	4	4	3	3
2	2	1	1	4	4	4	4	3	3
2	2	1	1	4	4	4	4	3	3
2	2	1	1	4	4	4	4	2	2
4	4	1	1	4	4	4	4	3	3
2	2	1	1	4	4	4	4	3	3
4	4	1	1	4	4	4	4	2	2
4	4	1	1	4	4	4	4	3	3
4	4	1	1	4	4	4	4	3	3
4	4	1	1	4	4	4	4	3	3
4	4	1	1	4	4	4	4	2	2
4	4	1	1	4	4	4	4	3	3
4	4	1	1	4	4	4	4	3	3
2	2	1	1	4	4	4	4	2	2
4	4	1	1	4	4	1	1	3	3
2	2	1	1	4	4	4	4	2	2
4	4	1	1	4	4	4	4	3	3
4	4	1	1	4	4	4	4	3	3
4	4	1	1	4	4	4	4	2	2

For Review Only

4	4	1	1	4	4	4	4	3	3
4	4	1	1	4	4	4	4	3	3

For Review Only

SNP1395	SNP1395	SNP1396	SNP1396	SNP1397	SNP1397	SNP1398	SNP1398	SNP1399	SNP1399
4	4	4	4	1	1	3	3	2	2
4	4	4	4	4	4	3	3	3	3
4	4	4	4	1	1	3	3	3	3
4	4	4	4	4	4	3	3	3	3
4	4	3	3	1	1	3	3	2	2
4	4	4	4	4	4	3	3	2	2
4	4	4	4	1	1	3	3	2	2
4	4	4	4	1	1	3	3	3	3
4	4	3	3	1	1	3	3	2	2
4	4	4	4	1	1	3	3	3	3
4	4	4	4	1	1	3	3	3	3
4	4	4	4	1	1	3	3	3	3
4	4	3	3	1	1	3	3	3	3
4	4	4	4	1	1	3	3	3	3
4	4	4	4	1	1	3	3	2	2
4	4	4	4	1	1	3	3	3	3
4	4	4	4	1	1	3	3	3	3
4	4	4	4	1	1	3	3	2	2
4	4	4	4	1	1	3	3	3	3
4	4	4	4	1	1	3	3	2	2
1	1	4	4	1	1	1	1	3	3
4	4	4	4	1	1	3	3	2	2
4	4	4	4	1	1	3	3	2	2
4	4	4	4	1	1	3	3	2	2
4	4	4	4	1	1	3	3	3	3
4	4	4	4	1	1	3	3	2	2
4	4	4	4	1	1	3	3	2	2
4	4	4	4	1	1	3	3	2	2
4	4	4	4	1	1	3	3	2	2
4	4	4	4	1	1	3	3	3	3
4	4	4	4	4	4	3	3	2	2
1	1	4	4	1	1	1	1	2	2
1	1	4	4	1	1	1	1	2	2
4	4	4	4	1	1	3	3	2	2
4	4	4	4	1	1	3	3	2	2
4	4	4	4	1	1	3	3	3	3
4	4	4	4	1	1	3	3	2	2
4	4	4	4	1	1	3	3	3	3
4	4	4	4	1	1	3	3	2	2
4	4	4	4	1	1	3	3	2	2
4	4	4	4	1	1	3	3	3	3
1	1	4	4	1	1	1	1	2	2
4	4	4	4	1	1	3	3	2	2
4	4	4	4	1	1	3	3	2	2
4	4	4	4	1	1	3	3	2	2
4	4	4	4	1	1	3	3	3	3
4	4	4	4	1	1	3	3	2	2
4	4	4	4	1	1	3	3	3	3
4	4	4	4	1	1	3	3	3	3
4	4	4	4	4	4	3	3	2	2
4	4	4	4	1	1	3	3	2	2
1	1	4	4	1	1	3	3	3	3

For Review Only

For Review Only

4	4	3	3	1	1	3	3	2	2
1	1	4	4	1	1	1	1	2	2
4	4	4	4	1	1	3	3	3	3
4	4	4	4	1	1	3	3	3	3
4	4	4	4	1	1	3	3	3	3
4	4	4	4	1	1	3	3	3	3
4	4	4	4	1	1	3	3	2	2
4	4	4	4	1	1	3	3	2	2
4	4	4	4	1	1	3	3	2	2
4	4	4	4	1	1	3	3	2	2
4	4	4	4	1	1	3	3	3	3
4	4	4	4	1	1	3	3	3	3
4	4	3	3	1	1	3	3	2	2
4	4	4	4	1	1	3	3	2	2
4	4	4	4	1	1	3	3	2	2
4	4	4	4	1	1	3	3	3	3
4	4	4	4	1	1	3	3	2	2
4	4	4	4	1	1	3	3	2	2
4	4	4	4	4	4	3	3	2	2
4	4	3	3	1	1	3	3	2	2
4	4	4	4	1	1	3	3	3	3
4	4	4	4	1	1	3	3	2	2
4	4	4	4	1	1	3	3	2	2
1	1	4	4	1	1	1	1	2	2
4	4	4	4	1	1	3	3	2	2
4	4	4	4	1	1	3	3	3	3
4	4	4	4	1	1	3	3	2	2
4	4	4	4	1	1	3	3	2	2
4	4	4	4	1	1	3	3	2	2
4	4	3	3	1	1	3	3	3	3
4	4	4	4	1	1	3	3	3	3
4	4	4	4	1	1	3	3	2	2
4	4	4	4	1	1	3	3	2	2
4	4	3	3	1	1	3	3	2	2
4	4	4	4	1	1	3	3	2	2
4	4	4	4	1	1	3	3	2	2
4	4	4	4	1	1	3	3	3	3
4	4	4	4	1	1	3	3	3	3
4	4	4	4	1	1	3	3	2	2
4	4	4	4	1	1	3	3	3	3
4	4	3	3	1	1	3	3	3	3
4	4	3	3	1	1	3	3	3	3
4	4	4	4	1	1	3	3	2	2
4	4	4	4	1	1	3	3	3	3
4	4	4	4	1	1	3	3	3	3
4	4	4	4	4	4	3	3	3	3
4	4	4	4	1	1	3	3	2	2
4	4	4	4	1	1	3	3	2	2
4	4	4	4	1	1	3	3	2	2
4	4	4	4	1	1	3	3	2	2
1	1	4	4	1	1	3	3	2	2
4	4	4	4	1	1	3	3	2	2
4	4	4	4	1	1	3	3	2	2
1	1	4	4	1	1	3	3	2	2
4	4	4	4	1	1	3	3	2	2

4	4	4	4	1	1	3	3	2	2
4	4	4	4	1	1	3	3	2	2
4	4	4	4	1	1	3	3	3	3
4	4	4	4	1	1	3	3	2	2
4	4	4	4	1	1	3	3	2	2
4	4	4	4	1	1	3	3	2	2
4	4	4	4	1	1	3	3	3	3
4	4	4	4	1	1	3	3	3	3
4	4	4	4	1	1	3	3	2	2
4	4	4	4	1	1	3	3	2	2
4	4	4	4	1	1	3	3	2	2
4	4	4	4	1	1	3	3	2	2
4	4	4	4	1	1	3	3	2	2
4	4	4	4	1	1	3	3	3	3
4	4	4	4	1	1	3	3	2	2
4	4	4	4	1	1	3	3	2	2
4	4	4	4	1	1	3	3	2	2
4	4	4	4	1	1	3	3	3	3
4	4	4	4	1	1	3	3	2	2
4	4	4	4	1	1	3	3	3	3
4	4	4	4	1	1	3	3	2	2
4	4	3	3	1	1	3	3	3	3
4	4	4	4	1	1	3	3	3	3
4	4	4	4	1	1	3	3	2	2
4	4	4	4	1	1	3	3	2	2
4	4	4	4	1	1	3	3	2	2
1	1	4	4	1	1	1	1	3	3
1	1	4	4	1	1	1	1	2	2
4	4	4	4	1	1	3	3	3	3
4	4	3	3	1	1	3	3	2	2
4	4	4	4	1	1	3	3	3	3
4	4	4	4	1	1	3	3	2	2
4	4	3	3	1	1	3	3	3	3
4	4	4	4	1	1	3	3	2	2
4	4	4	4	1	1	3	3	2	2
4	4	4	4	1	1	3	3	2	2
4	4	4	4	1	1	3	3	3	3
4	4	4	4	1	1	3	3	3	3
4	4	4	4	1	1	3	3	3	3
4	4	4	4	1	1	3	3	3	3
4	4	4	4	1	1	3	3	2	2
4	4	4	4	1	1	3	3	3	3
4	4	4	4	1	1	3	3	2	2
4	4	4	4	1	1	3	3	3	3
4	4	4	4	1	1	3	3	3	3
4	4	3	3	1	1	3	3	2	2
4	4	4	4	1	1	3	3	3	3
4	4	4	4	1	1	3	3	2	2
4	4	4	4	1	1	3	3	2	2
4	4	4	4	1	1	3	3	2	2
4	4	4	4	1	1	3	3	2	2
4	4	4	4	1	1	3	3	2	2
4	4	4	4	4	4	3	3	2	2
4	4	4	4	1	1	3	3	2	2
4	4	4	4	1	1	3	3	2	2

For Review Only

4	4	4	4	4	4	3	3	2	2
4	4	4	4	1	1	3	3	2	2
4	4	4	4	1	1	3	3	2	2
4	4	4	4	1	1	3	3	2	2
4	4	4	4	1	1	3	3	3	3
4	4	4	4	1	1	3	3	2	2
4	4	4	4	1	1	3	3	2	2
4	4	4	4	1	1	3	3	2	2
4	4	4	4	1	1	3	3	2	2
4	4	4	4	1	1	3	3	2	2
4	4	4	4	1	1	3	3	3	3
4	4	4	4	1	1	3	3	2	2
4	4	4	4	1	1	3	3	2	2
4	4	4	4	1	1	3	3	2	2
4	4	4	4	1	1	3	3	3	3
4	4	4	4	1	1	3	3	3	3
4	4	4	4	4	4	3	3	2	2
4	4	4	4	4	4	3	3	3	3
4	4	4	4	1	1	3	3	3	3
4	4	4	4	1	1	3	3	3	3
4	4	4	4	1	1	3	3	3	3
4	4	4	4	1	1	3	3	2	2
4	4	4	4	1	1	3	3	2	2
4	4	4	4	1	1	3	3	3	3
4	4	4	4	1	1	3	3	3	3
4	4	4	4	1	1	3	3	2	2
4	4	4	4	1	1	3	3	2	2
4	4	4	4	1	1	3	3	2	2
4	4	4	4	1	1	3	3	2	2
4	4	4	4	4	4	3	3	2	2
4	4	4	4	1	1	3	3	3	3
4	4	3	3	1	1	3	3	2	2
1	1	4	4	1	1	1	1	2	2
4	4	4	4	1	1	3	3	3	3
4	4	4	4	1	1	3	3	2	2
4	4	4	4	1	1	3	3	3	3
4	4	4	4	1	1	3	3	3	3
4	4	4	4	1	1	3	3	2	2
4	4	4	4	1	1	3	3	2	2
4	4	4	4	1	1	3	3	2	2
4	4	4	4	1	1	3	3	3	3
4	4	4	4	1	1	3	3	3	3
4	4	4	4	1	1	3	3	3	3
4	4	4	4	1	1	3	3	3	3
4	4	4	4	1	1	3	3	2	2
1	1	4	4	1	1	1	1	2	2
4	4	4	4	1	1	3	3	3	3
4	4	4	4	1	1	3	3	3	3
4	4	4	4	1	1	3	3	2	2
4	4	4	4	4	4	3	3	3	3
1	1	4	4	1	1	1	1	3	3
4	4	4	4	1	1	3	3	3	3
4	4	4	4	1	1	3	3	2	2
4	4	4	4	1	1	3	3	3	3
4	4	4	4	1	1	3	3	2	2

4	4	4	4	4	4	3	3	2	2
4	4	4	4	1	1	3	3	2	2
1	1	4	4	1	1	1	1	3	3
4	4	4	4	1	1	3	3	2	2
4	4	4	4	1	1	3	3	2	2
1	1	4	4	1	1	3	3	2	2
4	4	4	4	1	1	3	3	2	2
4	4	4	4	1	1	3	3	2	2
4	4	4	4	1	1	3	3	2	2
4	4	4	4	1	1	3	3	2	2
4	4	4	4	1	1	3	3	2	2
4	4	4	4	1	1	3	3	2	2
4	4	4	4	1	1	3	3	2	2
4	4	4	4	1	1	3	3	2	2
4	4	4	4	1	1	3	3	3	3
4	4	4	4	1	1	3	3	3	3
4	4	4	4	1	1	3	3	2	2
4	4	4	4	1	1	3	3	2	2
4	4	4	4	4	4	3	3	2	2
4	4	4	4	1	1	3	3	3	3
4	4	4	4	1	1	3	3	2	2
4	4	4	4	1	1	3	3	2	2
4	4	4	4	1	1	3	3	3	3
4	4	4	4	1	1	3	3	2	2
4	4	4	4	1	1	3	3	2	2
1	1	4	4	1	1	3	3	2	2
4	4	4	4	1	1	3	3	2	2
4	4	4	4	1	1	3	3	3	3
4	4	4	4	1	1	3	3	2	2
1	1	4	4	1	1	1	1	3	3
4	4	4	4	1	1	3	3	2	2
4	4	4	4	1	1	3	3	3	3
4	4	4	4	1	1	3	3	3	3
4	4	4	4	1	1	3	3	2	2
4	4	4	4	1	1	3	3	2	2
4	4	4	4	1	1	3	3	2	2
4	4	4	4	4	4	3	3	3	3
4	4	4	4	1	1	3	3	3	3
4	4	4	4	1	1	3	3	3	3
4	4	4	4	1	1	3	3	2	2
4	4	4	4	1	1	3	3	2	2
4	4	4	4	1	1	3	3	2	2
4	4	4	4	1	1	3	3	2	2
4	4	3	3	1	1	3	3	3	3
4	4	4	4	1	1	3	3	3	3
4	4	4	4	1	1	3	3	2	2
4	4	4	4	1	1	3	3	2	2
4	4	4	4	1	1	3	3	2	2
4	4	4	4	1	1	3	3	3	3
4	4	4	4	1	1	3	3	2	2
1	1	4	4	1	1	3	3	3	3
4	4	4	4	1	1	3	3	2	2
4	4	4	4	1	1	3	3	3	3
4	4	4	4	1	1	3	3	3	3
4	4	4	4	1	1	3	3	3	3

4	4	4	4	1	1	3	3	3	3
4	4	4	4	1	1	3	3	3	3

For Review Only

SNP140	SNP140	SNP1400	SNP1400	SNP1401	SNP1401	SNP1402	SNP1402	SNP1403	SNP1403
2	2	3	3	2	2	4	4	1	1
4	4	2	2	3	3	1	1	4	4
2	2	2	2	2	2	4	4	4	4
2	2	2	2	2	2	1	1	4	4
2	2	2	2	2	2	1	1	4	4
2	2	3	3	3	3	1	1	1	1
2	2	2	2	2	2	4	4	4	4
4	4	2	2	3	3	4	4	4	4
2	2	2	2	2	2	1	1	1	1
4	4	2	2	3	3	4	4	4	4
4	4	2	2	2	2	4	4	1	1
2	2	2	2	2	2	4	4	1	1
4	4	2	2	3	3	4	4	1	1
2	2	2	2	2	2	4	4	1	1
4	4	2	2	2	2	1	1	1	1
4	4	2	2	2	2	4	4	4	4
2	2	2	2	2	2	1	1	4	4
2	2	2	2	2	2	4	4	4	4
2	2	2	2	2	2	4	4	4	4
4	4	2	2	3	3	4	4	4	4
4	4	2	2	3	3	4	4	4	4
4	4	2	2	2	2	4	4	4	4
2	2	2	2	3	3	4	4	4	4
4	4	2	2	3	3	4	4	4	4
2	2	2	2	3	3	4	4	4	4
2	2	3	3	2	2	4	4	1	1
2	2	3	3	3	3	4	4	1	1
4	4	2	2	2	2	4	4	1	1
2	2	2	2	3	3	4	4	4	4
4	4	2	2	3	3	4	4	4	4
4	4	2	2	2	2	1	1	1	1
4	4	2	2	2	2	4	4	4	4
2	2	2	2	2	2	4	4	4	4
2	2	2	2	3	3	4	4	4	4
4	4	2	2	2	2	4	4	4	4
2	2	3	3	3	3	4	4	4	4
2	2	2	2	2	2	4	4	4	4
2	2	2	2	2	2	4	4	4	4
4	4	2	2	3	3	4	4	4	4
4	4	2	2	2	2	4	4	4	4
4	4	2	2	3	3	4	4	4	4
4	4	2	2	2	2	4	4	4	4
4	4	2	2	2	2	4	4	4	4
4	4	2	2	2	2	4	4	4	4
4	4	2	2	2	2	4	4	4	4
2	2	2	2	2	2	4	4	1	1
2	2	3	3	3	3	4	4	4	4
2	2	3	3	2	2	4	4	1	1
2	2	2	2	2	2	4	4	4	4
4	4	2	2	2	2	4	4	4	4
2	2	2	2	2	2	4	4	4	4
2	2	2	2	2	2	4	4	4	4

For Review Only

2	2	2	2	2	2	1	1	4	4
2	2	2	2	2	2	4	4	4	4
2	2	2	2	2	2	4	4	4	4
4	4	2	2	2	2	4	4	4	4
2	2	2	2	2	2	4	4	4	4
2	2	3	3	3	3	4	4	1	1
2	2	3	3	3	3	4	4	1	1
4	4	2	2	3	3	4	4	4	4
2	2	2	2	3	3	4	4	4	4
4	4	2	2	2	2	4	4	1	1
2	2	3	3	3	3	4	4	4	4
2	2	3	3	3	3	4	4	4	4
2	2	2	2	2	2	1	1	4	4
2	2	2	2	3	3	4	4	4	4
4	4	2	2	2	2	4	4	1	1
4	4	2	2	3	3	4	4	4	4
2	2	3	3	2	2	4	4	4	4
2	2	2	2	3	3	4	4	4	4
2	2	3	3	2	2	1	1	1	1
2	2	3	3	2	2	1	1	1	1
2	2	3	3	3	3	4	4	4	4
4	4	2	2	3	3	4	4	4	4
2	2	2	2	3	3	4	4	1	1
2	2	2	2	3	3	4	4	1	1
4	4	2	2	2	2	4	4	4	4
2	2	2	2	2	2	4	4	4	4
4	4	2	2	2	2	4	4	1	1
4	4	2	2	3	3	4	4	4	4
2	2	3	3	3	3	4	4	1	1
2	2	3	3	2	2	1	1	1	1
2	2	2	2	2	2	4	4	4	4
2	2	2	2	3	3	4	4	4	4
4	4	2	2	2	2	4	4	4	4
2	2	2	2	2	2	1	1	4	4
2	2	2	2	3	3	4	4	4	4
4	4	2	2	2	2	4	4	4	4
4	4	2	2	3	3	4	4	4	4
2	2	2	2	2	2	4	4	4	4
2	2	2	2	2	2	4	4	4	4
4	4	2	2	2	2	4	4	4	4
2	2	2	2	2	2	1	1	4	4
2	2	3	3	3	3	1	1	1	1
4	4	2	2	2	2	4	4	4	4
2	2	3	3	3	3	4	4	4	4
2	2	2	2	3	3	4	4	1	1
2	2	2	2	3	3	1	1	4	4
2	2	2	2	3	3	4	4	4	4
2	2	3	3	3	3	4	4	1	1
2	2	3	3	3	3	4	4	1	1
2	2	2	2	2	2	4	4	1	1
4	4	2	2	2	2	4	4	1	1
4	4	2	2	3	3	4	4	4	4
4	4	2	2	3	3	4	4	4	4
4	4	2	2	3	3	4	4	4	4
2	2	2	2	2	2	4	4	1	1

For Review Only

2	2	3	3	2	2	4	4	1	1
2	2	2	2	3	3	4	4	1	1
2	2	3	3	3	3	4	4	4	4
2	2	2	2	3	3	4	4	4	4
2	2	2	2	2	2	4	4	4	4
2	2	2	2	2	2	4	4	4	4
2	2	2	2	2	2	4	4	4	4
4	4	2	2	2	2	4	4	1	1
2	2	3	3	3	3	4	4	1	1
2	2	2	2	3	3	4	4	4	4
4	4	2	2	2	2	4	4	1	1
4	4	2	2	2	2	4	4	4	4
2	2	2	2	2	2	4	4	1	1
2	2	2	2	2	2	4	4	4	4
4	4	3	3	2	2	4	4	4	4
4	4	2	2	3	3	4	4	1	1
2	2	2	2	3	3	4	4	4	4
2	2	2	2	2	2	4	4	1	1
2	2	2	2	2	2	4	4	4	4
2	2	2	2	2	2	4	4	4	4
4	4	2	2	2	2	4	4	1	1
2	2	2	2	2	2	1	1	4	4
2	2	3	3	3	3	4	4	4	4
4	4	2	2	3	3	4	4	4	4
2	2	3	3	3	3	4	4	1	1
2	2	2	2	3	3	4	4	4	4
4	4	2	2	3	3	4	4	4	4
2	2	2	2	2	2	4	4	4	4
2	2	2	2	3	3	4	4	1	1
4	4	2	2	2	2	1	1	1	1
4	4	2	2	2	2	4	4	4	4
4	4	2	2	2	2	4	4	1	1
2	2	2	2	2	2	1	1	4	4
2	2	3	3	3	3	4	4	1	1
2	2	2	2	3	3	4	4	4	4
4	4	2	2	2	2	4	4	1	1
2	2	2	2	3	3	4	4	4	4
2	2	2	2	2	2	4	4	1	1
4	4	2	2	3	3	4	4	1	1
4	4	2	2	2	2	4	4	1	1
2	2	2	2	2	2	4	4	1	1
2	2	2	2	3	3	4	4	4	4
2	2	2	2	2	2	4	4	1	1
4	4	2	2	2	2	4	4	4	4
2	2	2	2	3	3	4	4	1	1
4	4	2	2	3	3	1	1	4	4
2	2	3	3	3	3	4	4	1	1
2	2	3	3	3	3	4	4	1	1
4	4	2	2	2	2	4	4	4	4
2	2	3	3	2	2	4	4	1	1
2	2	3	3	3	3	4	4	1	1
4	4	2	2	3	3	4	4	4	4
4	4	2	2	2	2	1	1	4	4
2	2	2	2	3	3	4	4	4	4
4	4	2	2	2	2	4	4	4	4

For Review Only

2	2	2	2	3	3	1	1	1	1
4	4	3	3	2	2	4	4	1	1
4	4	2	2	3	3	4	4	4	4
4	4	2	2	3	3	4	4	4	4
2	2	2	2	2	2	4	4	4	4
4	4	2	2	2	2	4	4	4	4
4	4	2	2	3	3	4	4	1	1
4	4	2	2	2	2	4	4	1	1
2	2	2	2	2	2	4	4	1	1
4	4	2	2	2	2	4	4	4	4
4	4	3	3	2	2	4	4	1	1
4	4	2	2	3	3	4	4	4	4
2	2	3	3	2	2	4	4	1	1
4	4	2	2	3	3	4	4	4	4
2	2	2	2	3	3	4	4	4	4
4	4	2	2	2	2	4	4	1	1
4	4	2	2	2	2	1	1	4	4
2	2	2	2	2	2	1	1	4	4
2	2	2	2	3	3	4	4	4	4
2	2	2	2	2	2	4	4	4	4
4	4	2	2	2	2	4	4	4	4
4	4	2	2	3	3	4	4	4	4
4	4	2	2	3	3	4	4	4	4
2	2	3	3	3	3	4	4	4	4
4	4	2	2	3	3	4	4	4	4
2	2	2	2	3	3	4	4	4	4
2	2	3	3	2	2	4	4	1	1
2	2	2	2	3	3	4	4	4	4
2	2	2	2	3	3	4	4	4	4
4	4	2	2	2	2	1	1	4	4
4	4	3	3	3	3	4	4	4	4
2	2	3	3	3	3	1	1	1	1
2	2	2	2	3	3	4	4	4	4
2	2	2	2	3	3	4	4	1	1
2	2	2	2	2	2	4	4	4	4
2	2	3	3	3	3	4	4	4	4
2	2	2	2	3	3	4	4	4	4
4	4	2	2	2	2	4	4	4	4
2	2	3	3	3	3	4	4	1	1
2	2	2	2	3	3	1	1	4	4
2	2	2	2	2	2	4	4	4	4
2	2	3	3	3	3	4	4	1	1
4	4	2	2	2	2	4	4	4	4
2	2	2	2	3	3	4	4	4	4
2	2	2	2	2	2	4	4	4	4
4	4	2	2	3	3	4	4	4	4
4	4	2	2	2	2	4	4	1	1
4	4	2	2	3	3	4	4	4	4
2	2	2	2	3	3	4	4	4	4
2	2	2	2	2	2	1	1	4	4
2	2	2	2	3	3	4	4	4	4
4	4	2	2	3	3	4	4	4	4
2	2	2	2	3	3	4	4	4	4
2	2	3	3	3	3	4	4	4	4
4	4	2	2	3	3	4	4	4	4

For Review Only

4	4	2	2	2	2	1	1	4	4
4	4	2	2	3	3	4	4	1	1
2	2	2	2	3	3	4	4	4	4
2	2	2	2	2	2	4	4	4	4
2	2	2	2	3	3	4	4	4	4
2	2	3	3	3	3	4	4	1	1
4	4	2	2	2	2	4	4	4	4
4	4	2	2	3	3	4	4	4	4
2	2	3	3	2	2	4	4	1	1
4	4	2	2	3	3	4	4	4	4
4	4	2	2	2	2	4	4	1	1
4	4	2	2	3	3	4	4	4	4
2	2	2	2	2	2	4	4	4	4
2	2	2	2	3	3	4	4	4	4
2	2	3	3	2	2	1	1	4	4
2	2	2	2	2	2	4	4	1	1
4	4	2	2	3	3	4	4	4	4
2	2	2	2	2	2	4	4	4	4
4	4	2	2	3	3	1	1	4	4
2	2	2	2	2	2	4	4	4	4
4	4	2	2	3	3	4	4	1	1
2	2	3	3	3	3	4	4	1	1
2	2	2	2	2	2	4	4	4	4
2	2	2	2	2	2	4	4	4	4
4	4	2	2	3	3	4	4	4	4
2	2	3	3	3	3	4	4	4	4
4	4	2	2	3	3	4	4	4	4
2	2	3	3	2	2	4	4	1	1
2	2	2	2	2	2	4	4	4	4
2	2	2	2	3	3	4	4	4	4
4	4	2	2	3	3	4	4	4	4
4	4	3	3	3	3	4	4	1	1
2	2	2	2	3	3	4	4	4	4
4	4	3	3	3	3	4	4	1	1
2	2	2	2	2	2	4	4	4	4
4	4	2	2	3	3	4	4	4	4
4	4	2	2	2	2	1	1	4	4
2	2	3	3	3	3	4	4	4	4
2	2	3	3	3	3	4	4	4	4
4	4	2	2	3	3	4	4	4	4
2	2	2	2	2	2	4	4	1	1
2	2	2	2	2	2	4	4	4	4
2	2	2	2	3	3	4	4	1	1
2	2	2	2	2	2	1	1	4	4
2	2	2	2	3	3	4	4	4	4
2	2	3	3	2	2	4	4	1	1
4	4	3	3	3	3	4	4	1	1
2	2	3	3	2	2	4	4	1	1
2	2	3	3	2	2	4	4	1	1
4	4	2	2	3	3	4	4	4	4
2	2	2	2	2	2	4	4	4	4
4	4	2	2	3	3	4	4	4	4
2	2	2	2	2	2	4	4	4	4
2	2	3	3	2	2	4	4	1	1
4	4	2	2	3	3	4	4	4	4

2	2	2	2	2	2	4	4	1	1
2	2	2	2	2	2	4	4	4	4

For Review Only

SNP1404	SNP1404	SNP1405	SNP1405	SNP1406	SNP1406	SNP1407	SNP1407	SNP1408	SNP1408
2	2	1	1	3	3	3	3	4	4
2	2	4	4	2	2	3	3	4	4
2	2	1	1	2	2	2	2	4	4
2	2	4	4	2	2	3	3	4	4
2	2	4	4	2	2	3	3	4	4
2	2	4	4	3	3	3	3	4	4
2	2	1	1	2	2	3	3	4	4
2	2	1	1	2	2	3	3	4	4
2	2	4	4	2	2	2	2	4	4
2	2	1	1	2	2	3	3	4	4
2	2	1	1	3	3	2	2	4	4
2	2	1	1	2	2	2	2	4	4
2	2	4	4	2	2	3	3	4	4
2	2	1	1	3	3	2	2	4	4
2	2	4	4	2	2	3	3	4	4
2	2	1	1	2	2	2	2	4	4
2	2	4	4	2	2	2	2	4	4
2	2	1	1	2	2	3	3	4	4
2	2	1	1	2	2	2	2	4	4
2	2	1	1	2	2	3	3	4	4
2	2	1	1	2	2	3	3	4	4
2	2	4	4	2	2	2	2	4	4
4	4	1	1	2	2	3	3	3	3
2	2	1	1	2	2	3	3	4	4
2	2	1	1	2	2	2	2	4	4
2	2	1	1	3	3	2	2	4	4
2	2	1	1	3	3	2	2	3	3
2	2	1	1	2	2	2	2	4	4
2	2	1	1	2	2	3	3	4	4
2	2	1	1	2	2	3	3	4	4
2	2	4	4	3	3	2	2	4	4
2	2	1	1	2	2	3	3	4	4
2	2	1	1	2	2	3	3	4	4
2	2	1	1	2	2	2	2	4	4
2	2	1	1	2	2	2	2	3	3
2	2	1	1	2	2	3	3	4	4
2	2	1	1	2	2	2	2	3	3
2	2	1	1	2	2	3	3	4	4
4	4	1	1	2	2	2	2	4	4
4	4	1	1	2	2	2	2	4	4
2	2	1	1	2	2	3	3	4	4
2	2	1	1	2	2	3	3	4	4
2	2	1	1	2	2	3	3	4	4
2	2	1	1	2	2	3	3	4	4
2	2	1	1	2	2	3	3	4	4
2	2	1	1	2	2	2	2	4	4
2	2	1	1	2	2	2	2	4	4
2	2	1	1	3	3	2	2	4	4
2	2	1	1	2	2	3	3	4	4
2	2	4	4	2	2	2	2	4	4
2	2	1	1	2	2	2	2	4	4
2	2	1	1	2	2	3	3	4	4

For Review Only

2	2	4	4	2	2	3	3	4	4
2	2	1	1	2	2	3	3	4	4
2	2	1	1	2	2	2	2	4	4
2	2	1	1	2	2	2	2	4	4
2	2	1	1	2	2	2	2	4	4
2	2	1	1	3	3	3	3	4	4
2	2	1	1	3	3	2	2	4	4
2	2	1	1	2	2	3	3	4	4
2	2	4	4	2	2	2	2	3	3
2	2	1	1	2	2	2	2	4	4
2	2	1	1	2	2	3	3	4	4
2	2	1	1	2	2	3	3	4	4
2	2	4	4	2	2	3	3	4	4
2	2	1	1	2	2	3	3	4	4
2	2	4	4	3	3	2	2	4	4
2	2	1	1	2	2	2	2	3	3
4	4	1	1	2	2	3	3	4	4
2	2	1	1	2	2	2	2	4	4
2	2	4	4	3	3	3	3	4	4
2	2	4	4	3	3	3	3	4	4
2	2	1	1	2	2	3	3	4	4
4	4	1	1	2	2	2	2	4	4
2	2	4	4	2	2	2	2	4	4
2	2	1	1	3	3	2	2	4	4
2	2	1	1	2	2	3	3	4	4
2	2	1	1	2	2	3	3	4	4
2	2	1	1	2	2	3	3	4	4
2	2	1	1	2	2	2	2	4	4
2	2	1	1	3	3	2	2	4	4
2	2	4	4	3	3	3	3	4	4
4	4	1	1	2	2	3	3	4	4
2	2	1	1	2	2	2	2	4	4
2	2	1	1	2	2	3	3	4	4
2	2	4	4	2	2	3	3	4	4
2	2	1	1	2	2	2	2	4	4
2	2	1	1	2	2	3	3	4	4
2	2	1	1	2	2	3	3	4	4
2	2	1	1	2	2	2	2	4	4
4	4	1	1	2	2	2	2	3	3
2	2	1	1	2	2	3	3	4	4
2	2	4	4	2	2	2	2	4	4
2	2	4	4	3	3	3	3	4	4
2	2	1	1	2	2	2	2	4	4
2	2	1	1	2	2	3	3	4	4
2	2	1	1	2	2	2	2	4	4
2	2	4	4	2	2	2	2	4	4
2	2	1	1	2	2	2	2	4	4
2	2	1	1	3	3	3	3	4	4
2	2	1	1	3	3	3	3	4	4
2	2	1	1	3	3	2	2	3	3
2	2	1	1	2	2	3	3	4	4
2	2	1	1	2	2	2	2	4	4
2	2	1	1	2	2	2	2	3	3
2	2	1	1	2	2	2	2	4	4
2	2	1	1	3	3	3	3	3	3

For Review Only

2	2	1	1	3	3	2	2	4	4
2	2	1	1	3	3	3	3	4	4
2	2	1	1	2	2	2	2	4	4
2	2	1	1	2	2	2	2	4	4
2	2	1	1	2	2	3	3	4	4
2	2	1	1	2	2	2	2	4	4
2	2	1	1	2	2	2	2	4	4
2	2	1	1	2	2	3	3	4	4
2	2	1	1	3	3	2	2	4	4
2	2	1	1	2	2	3	3	4	4
2	2	1	1	2	2	2	2	4	4
2	2	1	1	2	2	2	2	4	4
2	2	1	1	3	3	3	3	4	4
2	2	1	1	2	2	2	2	4	4
2	2	1	1	2	2	2	2	4	4
2	2	1	1	2	2	2	2	4	4
2	2	1	1	2	2	2	2	4	4
2	2	1	1	2	2	2	2	4	4
2	2	1	1	2	2	2	2	4	4
2	2	1	1	2	2	3	3	4	4
2	2	1	1	2	2	2	2	4	4
2	2	4	4	2	2	3	3	4	4
2	2	1	1	2	2	2	2	4	4
2	2	1	1	2	2	3	3	4	4
4	4	1	1	3	3	2	2	4	4
4	4	1	1	2	2	2	2	4	4
2	2	4	4	2	2	2	2	4	4
2	2	1	1	2	2	2	2	4	4
2	2	1	1	2	2	2	2	4	4
2	2	4	4	3	3	2	2	4	4
2	2	1	1	2	2	2	2	4	4
2	2	1	1	2	2	2	2	4	4
2	2	4	4	2	2	2	2	4	4
2	2	1	1	2	2	2	2	4	4
2	2	1	1	2	2	2	2	4	4
2	2	4	4	2	2	2	2	4	4
2	2	1	1	2	2	2	2	4	4
2	2	1	1	2	2	3	3	4	4
2	2	1	1	2	2	2	2	4	4
2	2	1	1	3	3	2	2	4	4
4	4	1	1	2	2	2	2	4	4
2	2	1	1	2	2	2	2	4	4
2	2	1	1	3	3	2	2	3	3
2	2	1	1	2	2	2	2	4	4
2	2	1	1	2	2	2	2	4	4
2	2	4	4	2	2	3	3	4	4
2	2	1	1	3	3	2	2	4	4
2	2	1	1	3	3	2	2	4	4
2	2	1	1	2	2	2	2	4	4
2	2	1	1	3	3	2	2	4	4
2	2	1	1	3	3	2	2	4	4
2	2	1	1	2	2	3	3	4	4
4	4	4	4	2	2	2	2	4	4
2	2	1	1	2	2	3	3	4	4
2	2	1	1	2	2	2	2	4	4

For Review Only

2	2	4	4	2	2	2	2	4	4
4	4	1	1	3	3	2	2	4	4
2	2	1	1	2	2	2	2	4	4
2	2	1	1	2	2	2	2	4	4
2	2	1	1	2	2	3	3	4	4
2	2	1	1	2	2	3	3	4	4
2	2	1	1	2	2	3	3	4	4
2	2	1	1	2	2	2	2	4	4
4	4	4	4	2	2	2	2	4	4
2	2	1	1	2	2	2	2	4	4
2	2	1	1	3	3	2	2	4	4
2	2	1	1	2	2	2	2	4	4
2	2	4	4	3	3	3	3	4	4
2	2	4	4	2	2	3	3	4	4
2	2	1	1	2	2	2	2	4	4
2	2	1	1	3	3	2	2	4	4
2	2	4	4	2	2	2	2	4	4
2	2	4	4	2	2	3	3	4	4
2	2	1	1	2	2	2	2	4	4
2	2	1	1	2	2	2	2	4	4
2	2	1	1	2	2	2	2	4	4
2	2	1	1	2	2	3	3	4	4
4	4	1	1	2	2	2	2	4	4
2	2	1	1	2	2	3	3	4	4
2	2	1	1	2	2	3	3	4	4
2	2	1	1	2	2	2	2	4	4
2	2	1	1	3	3	3	3	4	4
2	2	1	1	2	2	2	2	4	4
2	2	1	1	2	2	2	2	4	4
2	2	4	4	2	2	3	3	4	4
2	2	1	1	2	2	2	2	4	4
2	2	4	4	3	3	2	2	4	4
2	2	1	1	2	2	2	2	4	4
2	2	1	1	2	2	2	2	4	4
2	2	1	1	2	2	2	2	4	4
2	2	1	1	2	2	3	3	4	4
2	2	1	1	2	2	2	2	4	4
2	2	1	1	2	2	2	2	4	4
2	2	1	1	3	3	2	2	4	4
2	2	4	4	2	2	3	3	4	4
2	2	1	1	2	2	2	2	4	4
2	2	1	1	3	3	2	2	4	4
2	2	1	1	2	2	2	2	4	4
2	2	1	1	2	2	2	2	4	4
2	2	1	1	2	2	2	2	4	4
2	2	1	1	2	2	2	2	4	4
2	2	1	1	3	3	2	2	4	4
2	2	1	1	2	2	3	3	4	4
2	2	1	1	2	2	2	2	3	3
2	2	4	4	2	2	2	2	4	4
2	2	1	1	2	2	3	3	4	4
2	2	1	1	2	2	3	3	4	4
4	4	1	1	2	2	2	2	4	4
2	2	1	1	2	2	3	3	4	4
2	2	1	1	2	2	2	2	4	4

For Review Only

2	2	4	4	2	2	2	2	4	4
2	2	1	1	2	2	3	3	4	4
2	2	1	1	2	2	2	2	4	4
4	4	4	4	2	2	2	2	4	4
2	2	1	1	2	2	3	3	4	4
2	2	1	1	3	3	2	2	4	4
4	4	1	1	2	2	3	3	4	4
2	2	1	1	2	2	3	3	4	4
2	2	1	1	2	2	2	2	4	4
2	2	1	1	2	2	2	2	4	4
2	2	1	1	2	2	2	2	4	4
2	2	1	1	2	2	2	2	4	4
4	4	1	1	2	2	2	2	4	4
2	2	1	1	2	2	3	3	4	4
2	2	4	4	2	2	2	2	4	4
2	2	1	1	3	3	2	2	4	4
4	4	1	1	2	2	2	2	4	4
2	2	1	1	2	2	2	2	4	4
4	4	4	4	2	2	3	3	4	4
2	2	1	1	2	2	3	3	4	4
4	4	1	1	3	3	3	3	4	4
2	2	1	1	3	3	2	2	4	4
2	2	1	1	2	2	3	3	4	4
4	4	1	1	2	2	3	3	4	4
2	2	1	1	2	2	3	3	4	4
2	2	1	1	2	2	3	3	4	4
2	2	1	1	2	2	2	2	4	4
2	2	4	4	3	3	3	3	4	4
2	2	4	4	2	2	3	3	4	4
2	2	1	1	2	2	3	3	4	4
4	4	1	1	2	2	3	3	4	4
2	2	1	1	3	3	2	2	4	4
2	2	1	1	2	2	3	3	3	3
2	2	1	1	3	3	2	2	4	4
4	4	1	1	2	2	2	2	4	4
2	2	1	1	2	2	2	2	4	4
2	2	4	4	2	2	2	2	4	4
2	2	1	1	2	2	3	3	4	4
2	2	1	1	2	2	3	3	4	4
4	4	1	1	2	2	2	2	4	4
2	2	1	1	3	3	2	2	4	4
2	2	1	1	2	2	3	3	4	4
4	4	1	1	3	3	3	3	4	4
2	2	4	4	2	2	2	2	4	4
2	2	1	1	2	2	2	2	4	4
4	4	1	1	3	3	2	2	3	3
2	2	1	1	3	3	2	2	4	4
2	2	1	1	3	3	3	3	4	4
2	2	1	1	3	3	3	3	4	4
2	2	1	1	2	2	2	2	4	4
2	2	1	1	2	2	2	2	4	4
2	2	1	1	2	2	2	2	4	4
2	2	1	1	2	2	2	2	4	4
2	2	1	1	3	3	3	3	4	4
2	2	1	1	2	2	3	3	4	4

For Review Only

2	2	1	1	3	3	2	2	4	4
2	2	1	1	2	2	2	2	4	4

For Review Only

SNP1409	SNP1409	SNP141	SNP141	SNP1410	SNP1410	SNP1411	SNP1411	SNP1412	SNP1412
3	3	1	1	3	3	4	4	3	3
3	3	2	2	2	2	4	4	2	2
3	3	2	2	2	2	1	1	2	2
3	3	2	2	2	2	1	1	2	2
3	3	2	2	2	2	4	4	2	2
3	3	2	2	3	3	4	4	3	3
3	3	2	2	2	2	4	4	2	2
3	3	2	2	2	2	4	4	2	2
3	3	2	2	2	2	4	4	2	2
1	1	2	2	3	3	4	4	2	2
3	3	1	1	3	3	4	4	2	2
3	3	2	2	2	2	1	1	2	2
3	3	2	2	2	2	4	4	2	2
3	3	2	2	3	3	4	4	2	2
3	3	1	1	2	2	4	4	2	2
3	3	1	1	2	2	1	1	2	2
3	3	2	2	2	2	4	4	2	2
3	3	1	1	2	2	4	4	3	3
3	3	1	1	2	2	1	1	2	2
3	3	1	1	2	2	4	4	2	2
3	3	2	2	2	2	4	4	2	2
1	1	2	2	3	3	4	4	2	2
3	3	2	2	2	2	1	1	2	2
3	3	2	2	2	2	4	4	2	2
3	3	2	2	2	2	4	4	3	3
3	3	1	1	3	3	1	1	2	2
3	3	2	2	3	3	1	1	3	3
3	3	1	1	2	2	1	1	2	2
3	3	2	2	2	2	4	4	2	2
1	1	2	2	3	3	4	4	2	2
3	3	1	1	3	3	4	4	3	3
3	3	1	1	2	2	1	1	3	3
3	3	1	1	2	2	4	4	3	3
3	3	2	2	2	2	4	4	2	2
3	3	1	1	2	2	1	1	2	2
3	3	2	2	2	2	1	1	2	2
3	3	2	2	2	2	1	1	2	2
3	3	2	2	2	2	1	1	2	2
3	3	2	2	2	2	4	4	2	2
3	3	1	1	2	2	1	1	2	2
1	1	2	2	3	3	4	4	2	2
1	1	2	2	3	3	4	4	2	2
1	1	2	2	3	3	4	4	2	2
3	3	2	2	2	2	4	4	2	2
1	1	2	2	3	3	4	4	2	2
3	3	1	1	2	2	1	1	2	2
3	3	1	1	3	3	1	1	2	2
3	3	2	2	3	3	4	4	2	2
3	3	2	2	2	2	1	1	2	2
3	3	2	2	2	2	1	1	2	2
3	3	2	2	2	2	1	1	2	2
3	3	1	1	2	2	1	1	2	2

For Review Only

3	3	1	1	2	2	4	4	2	2
3	3	1	1	2	2	4	4	3	3
3	3	1	1	2	2	1	1	3	3
1	1	1	1	3	3	4	4	2	2
3	3	1	1	2	2	1	1	2	2
3	3	1	1	3	3	1	1	2	2
3	3	1	1	3	3	1	1	2	2
3	3	2	2	2	2	4	4	2	2
3	3	2	2	2	2	4	4	2	2
3	3	1	1	2	2	1	1	2	2
3	3	2	2	3	3	4	4	2	2
3	3	2	2	3	3	1	1	2	2
3	3	1	1	2	2	4	4	2	2
3	3	2	2	2	2	4	4	2	2
3	3	2	2	3	3	4	4	2	2
3	3	2	2	2	2	4	4	2	2
3	3	2	2	2	2	1	1	2	2
3	3	1	1	2	2	1	1	2	2
3	3	1	1	3	3	4	4	2	2
3	3	1	1	3	3	4	4	2	2
3	3	2	2	2	2	1	1	2	2
3	3	1	1	2	2	1	1	2	2
3	3	1	1	2	2	4	4	2	2
3	3	2	2	3	3	1	1	3	3
1	1	2	2	3	3	4	4	2	2
3	3	2	2	2	2	4	4	2	2
3	3	2	2	2	2	4	4	2	2
3	3	2	2	2	2	4	4	2	2
3	3	1	1	3	3	1	1	2	2
3	3	1	1	3	3	1	1	2	2
3	3	1	1	2	2	4	4	2	2
3	3	2	2	2	2	4	4	2	2
3	3	2	2	2	2	4	4	2	2
3	3	1	1	2	2	4	4	2	2
3	3	1	1	2	2	4	4	2	2
1	1	2	2	3	3	4	4	2	2
3	3	2	2	2	2	4	4	2	2
3	3	2	2	2	2	4	4	2	2
1	1	2	2	3	3	4	4	2	2
1	1	2	2	3	3	4	4	2	2
3	3	1	1	2	2	4	4	3	3
3	3	2	2	3	3	1	1	2	2
3	3	2	2	2	2	1	1	2	2
3	3	2	2	3	3	4	4	2	2
1	1	2	2	3	3	4	4	2	2
3	3	1	1	2	2	1	1	2	2
3	3	2	2	2	2	4	4	2	2
3	3	2	2	3	3	1	1	2	2
3	3	1	1	3	3	1	1	2	2
3	3	1	1	3	3	1	1	2	2
3	3	2	2	3	3	1	1	2	2
3	3	2	2	2	2	4	4	2	2
3	3	2	2	2	2	4	4	2	2
3	3	1	1	2	2	1	1	2	2
3	3	2	2	3	3	4	4	2	2

For Review Only

3	3	1	1	3	3	1	1	2	2
3	3	1	1	3	3	1	1	2	2
3	3	2	2	2	2	1	1	2	2
3	3	2	2	2	2	4	4	2	2
3	3	1	1	2	2	1	1	3	3
3	3	1	1	2	2	1	1	2	2
3	3	1	1	2	2	1	1	2	2
3	3	2	2	2	2	4	4	2	2
3	3	2	2	3	3	4	4	2	2
3	3	2	2	2	2	1	1	2	2
3	3	1	1	2	2	4	4	2	2
3	3	1	1	2	2	1	1	2	2
3	3	2	2	3	3	1	1	2	2
3	3	2	2	2	2	1	1	2	2
3	3	1	1	2	2	1	1	2	2
3	3	1	1	2	2	1	1	2	2
3	3	1	1	2	2	1	1	2	2
3	3	1	1	2	2	1	1	2	2
3	3	2	2	2	2	4	4	2	2
3	3	1	1	2	2	4	4	2	2
3	3	1	1	2	2	1	1	2	2
3	3	1	1	2	2	1	1	3	3
3	3	2	2	2	2	1	1	2	2
3	3	2	2	2	2	4	4	2	2
3	3	1	1	2	2	1	1	2	2
3	3	1	1	2	2	1	1	2	2
3	3	1	1	2	2	1	1	2	2
3	3	2	2	2	2	4	4	2	2
3	3	2	2	2	2	1	1	2	2
3	3	1	1	2	2	1	1	2	2
3	3	2	2	2	2	4	4	2	2
3	3	1	1	2	2	1	1	2	2
3	3	1	1	2	2	4	4	3	3
3	3	1	1	2	2	1	1	2	2
3	3	2	2	2	2	4	4	2	2
3	3	1	1	3	3	4	4	2	2
3	3	2	2	2	2	4	4	2	2
3	3	1	1	3	3	4	4	2	2
3	3	1	1	2	2	1	1	2	2
3	3	1	1	3	3	4	4	2	2
3	3	1	1	2	2	1	1	2	2
3	3	2	2	2	2	4	4	2	2
3	3	2	2	3	3	4	4	2	2
3	3	2	2	2	2	4	4	2	2
3	3	2	2	2	2	1	1	2	2
3	3	2	2	2	2	4	4	3	3
3	3	1	1	3	3	1	1	2	2
3	3	1	1	3	3	1	1	3	3
3	3	2	2	2	2	1	1	2	2
3	3	1	1	3	3	1	1	2	2
3	3	1	1	3	3	1	1	2	2
3	3	1	1	2	2	4	4	2	2
3	3	1	1	2	2	1	1	2	2
3	3	2	2	2	2	1	1	2	2
3	3	1	1	2	2	1	1	2	2

For Review Only

3	3	2	2	2	2	1	1	2	2
3	3	2	2	2	2	1	1	2	2
3	3	2	2	2	2	4	4	2	2
3	3	1	1	2	2	1	1	2	2
3	3	1	1	2	2	1	1	2	2
3	3	1	1	2	2	4	4	2	2
3	3	1	1	2	2	4	4	2	2
3	3	1	1	2	2	1	1	2	2
3	3	1	1	2	2	1	1	2	2
3	3	1	1	2	2	1	1	2	2
3	3	1	1	3	3	1	1	2	2
3	3	2	2	2	2	4	4	2	2
3	3	2	2	2	2	1	1	2	2
3	3	2	2	2	2	4	4	2	2
3	3	2	2	2	2	4	4	2	2
3	3	1	1	3	3	4	4	2	2
3	3	2	2	2	2	1	1	2	2
3	3	2	2	2	2	1	1	2	2
3	3	2	2	2	2	1	1	2	2
3	3	1	1	2	2	1	1	2	2
3	3	1	1	2	2	1	1	2	2
3	3	2	2	2	2	4	4	2	2
3	3	1	1	2	2	1	1	2	2
3	3	2	2	3	3	1	1	2	2
3	3	2	2	2	2	4	4	2	2
3	3	2	2	2	2	4	4	2	2
3	3	2	2	3	3	1	1	2	2
3	3	1	1	2	2	1	1	2	2
1	1	2	2	3	3	4	4	2	2
3	3	1	1	2	2	1	1	2	2
3	3	2	2	2	2	1	1	3	3
3	3	1	1	3	3	1	1	2	2
3	3	1	1	2	2	4	4	2	2
3	3	2	2	2	2	4	4	2	2
3	3	1	1	2	2	4	4	2	2
3	3	2	2	3	3	1	1	2	2
3	3	2	2	2	2	1	1	2	2
3	3	2	2	2	2	4	4	3	3
3	3	1	1	3	3	1	1	3	3
3	3	2	2	2	2	1	1	2	2
3	3	2	2	2	2	1	1	2	2
3	3	1	1	3	3	1	1	2	2
3	3	2	2	2	2	4	4	2	2
3	3	1	1	2	2	1	1	2	2
3	3	1	1	2	2	1	1	2	2
3	3	2	2	2	2	1	1	2	2
3	3	1	1	3	3	4	4	2	2
3	3	2	2	2	2	4	4	2	2
3	3	2	2	2	2	4	4	2	2
3	3	2	2	2	2	1	1	2	2
3	3	2	2	2	2	1	1	2	2
3	3	2	2	2	2	4	4	2	2
3	3	2	2	2	2	4	4	2	2
3	3	2	2	3	3	4	4	2	2
3	3	2	2	2	2	4	4	2	2

For Review Only

3	3	1	1	2	2	4	4	2	2
3	3	1	1	2	2	4	4	2	2
3	3	2	2	2	2	1	1	2	2
3	3	1	1	2	2	1	1	2	2
3	3	1	1	2	2	4	4	2	2
3	3	1	1	2	2	1	1	2	2
3	3	1	1	2	2	1	1	2	2
1	1	2	2	3	3	4	4	2	2
3	3	2	2	2	2	4	4	2	2
3	3	2	2	2	2	4	4	2	2
3	3	1	1	2	2	1	1	2	2
3	3	2	2	2	2	1	1	2	2
1	1	1	1	3	3	1	1	2	2
3	3	2	2	2	2	1	1	2	2
3	3	1	1	2	2	1	1	3	3
3	3	1	1	3	3	4	4	2	2
3	3	2	2	2	2	4	4	2	2
3	3	1	1	2	2	4	4	2	2
3	3	2	2	2	2	4	4	2	2
3	3	2	2	2	2	1	1	2	2
3	3	2	2	3	3	1	1	2	2
3	3	1	1	3	3	1	1	2	2
3	3	1	1	2	2	1	1	3	3
3	3	2	2	2	2	4	4	2	2
3	3	2	2	2	2	4	4	2	2
3	3	2	2	2	2	4	4	2	2
3	3	2	2	2	2	4	4	2	2
3	3	2	2	3	3	1	1	2	2
3	3	1	1	2	2	4	4	2	2
3	3	2	2	2	2	1	1	2	2
3	3	2	2	2	2	4	4	2	2
3	3	1	1	3	3	4	4	2	2
3	3	2	2	2	2	4	4	2	2
3	3	1	1	3	3	1	1	2	2
3	3	1	1	2	2	1	1	2	2
3	3	2	2	2	2	4	4	2	2
3	3	1	1	2	2	1	1	2	2
3	3	2	2	3	3	1	1	2	2
3	3	2	2	2	2	1	1	2	2
3	3	2	2	2	2	4	4	2	2
3	3	1	1	3	3	4	4	2	2
3	3	2	2	2	2	4	4	3	3
3	3	1	1	3	3	4	4	2	2
3	3	1	1	2	2	4	4	2	2
3	3	2	2	2	2	1	1	2	2
3	3	2	2	3	3	1	1	2	2
3	3	1	1	3	3	1	1	2	2
3	3	2	2	3	3	4	4	3	3
3	3	2	2	3	3	1	1	2	2
3	3	2	2	2	2	4	4	2	2
3	3	1	1	2	2	1	1	2	2
3	3	2	2	2	2	4	4	2	2
3	3	2	2	2	2	1	1	2	2
3	3	2	2	3	3	1	1	2	2
3	3	1	1	2	2	1	1	2	2

For Review Only

3	3	1	1	3	3	1	1	3	3
3	3	2	2	2	2	1	1	2	2

For Review Only

SNP1413	SNP1413	SNP1414	SNP1414	SNP1415	SNP1415	SNP1416	SNP1416	SNP1417	SNP1417
3	3	2	2	2	2	4	4	4	4
3	3	2	2	2	2	1	1	1	1
4	4	2	2	2	2	1	1	1	1
3	3	2	2	1	1	1	1	1	1
4	4	2	2	1	1	1	1	1	1
3	3	2	2	2	2	4	4	4	4
4	4	2	2	2	2	1	1	1	1
4	4	2	2	1	1	1	1	1	1
4	4	2	2	2	2	1	1	1	1
4	4	2	2	2	2	4	4	1	1
4	4	2	2	1	1	1	1	1	1
4	4	2	2	2	2	1	1	1	1
4	4	2	2	1	1	4	4	1	1
4	4	2	2	1	1	4	4	4	4
3	3	2	2	2	2	1	1	1	1
3	3	2	2	1	1	1	1	1	1
4	4	2	2	2	2	1	1	1	1
4	4	2	2	2	2	1	1	1	1
3	3	4	4	2	2	1	1	1	1
4	4	2	2	1	1	1	1	1	1
4	4	2	2	2	2	1	1	1	1
4	4	2	2	2	2	4	4	1	1
4	4	2	2	1	1	1	1	1	1
3	3	2	2	1	1	1	1	1	1
4	4	2	2	2	2	1	1	1	1
4	4	2	2	1	1	4	4	4	4
4	4	2	2	2	2	4	4	4	4
4	4	2	2	1	1	1	1	1	1
4	4	2	2	2	2	1	1	1	1
4	4	2	2	1	1	4	4	1	1
4	4	4	4	2	2	4	4	4	4
3	3	2	2	2	2	1	1	1	1
4	4	2	2	2	2	1	1	1	1
3	3	2	2	1	1	1	1	1	1
4	4	2	2	1	1	1	1	1	1
3	3	2	2	1	1	1	1	1	1
3	3	4	4	1	1	1	1	1	1
3	3	2	2	1	1	1	1	1	1
4	4	2	2	1	1	1	1	1	1
4	4	2	2	1	1	1	1	1	1
4	4	2	2	1	1	4	4	1	1
4	4	2	2	2	2	1	1	1	1
4	4	2	2	2	2	4	4	1	1
4	4	2	2	1	1	1	1	1	1
4	4	2	2	2	2	4	4	1	1
4	4	2	2	1	1	1	1	1	1
4	4	2	2	2	2	1	1	1	1
3	3	2	2	1	1	4	4	4	4
4	4	2	2	2	2	1	1	1	1
4	4	2	2	2	2	1	1	1	1
4	4	2	2	2	2	1	1	1	1
3	3	2	2	2	2	4	4	4	4

For Review Only

4	4	2	2	2	2	1	1	1	1
4	4	2	2	2	2	1	1	1	1
3	3	2	2	2	2	1	1	1	1
4	4	2	2	2	2	4	4	1	1
3	3	4	4	2	2	1	1	1	1
3	3	2	2	2	2	4	4	4	4
4	4	2	2	1	1	4	4	4	4
4	4	2	2	1	1	1	1	1	1
4	4	2	2	1	1	1	1	1	1
4	4	2	2	1	1	1	1	1	1
3	3	2	2	2	2	1	1	1	1
3	3	2	2	2	2	1	1	1	1
4	4	2	2	2	2	1	1	1	1
4	4	2	2	2	2	1	1	1	1
3	3	2	2	1	1	1	1	1	1
4	4	2	2	1	1	1	1	1	1
3	3	2	2	1	1	1	1	1	1
4	4	2	2	2	2	1	1	1	1
4	4	2	2	2	2	4	4	4	4
4	4	2	2	2	2	4	4	4	4
3	3	2	2	2	2	1	1	1	1
4	4	2	2	1	1	1	1	1	1
4	4	2	2	2	2	1	1	1	1
4	4	2	2	2	2	1	1	1	1
4	4	2	2	2	2	4	4	1	1
3	3	2	2	2	2	4	4	4	4
4	4	2	2	2	2	1	1	1	1
3	3	2	2	1	1	1	1	1	1
4	4	2	2	1	1	4	4	4	4
3	3	2	2	2	2	1	1	1	1
4	4	2	2	2	2	1	1	1	1
3	3	2	2	2	2	1	1	1	1
4	4	2	2	1	1	1	1	1	1
4	4	2	2	2	2	1	1	1	1
4	4	2	2	1	1	1	1	1	1
4	4	2	2	2	2	4	4	1	1
4	4	2	2	2	2	1	1	1	1
4	4	2	2	2	2	1	1	1	1
4	4	2	2	1	1	4	4	1	1
4	4	2	2	2	2	4	4	4	4
4	4	2	2	2	2	1	1	1	1
3	3	2	2	2	2	1	1	1	1
3	3	2	2	2	2	1	1	1	1
3	3	2	2	2	2	1	1	1	1
4	4	2	2	1	1	4	4	1	1
4	4	2	2	2	2	1	1	1	1
4	4	2	2	1	1	1	1	1	1
4	4	2	2	1	1	4	4	4	4
3	3	2	2	1	1	4	4	4	4
4	4	2	2	2	2	4	4	4	4
3	3	2	2	2	2	1	1	1	1
4	4	2	2	1	1	1	1	1	1
3	3	2	2	1	1	1	1	1	1
4	4	2	2	1	1	1	1	1	1
4	4	2	2	2	2	4	4	4	4

For Review Only

3	3	2	2	1	1	4	4	4	4
4	4	2	2	1	1	4	4	4	4
4	4	2	2	1	1	1	1	1	1
4	4	2	2	1	1	1	1	1	1
3	3	4	4	2	2	1	1	1	1
3	3	2	2	1	1	1	1	1	1
4	4	2	2	2	2	1	1	1	1
3	3	2	2	1	1	1	1	1	1
4	4	2	2	1	1	4	4	4	4
4	4	4	4	1	1	1	1	1	1
3	3	2	2	1	1	1	1	1	1
4	4	2	2	1	1	1	1	1	1
4	4	2	2	2	2	4	4	4	4
3	3	2	2	2	2	4	4	1	1
3	3	2	2	2	2	1	1	1	1
4	4	2	2	1	1	1	1	1	1
4	4	2	2	2	2	1	1	1	1
3	3	2	2	1	1	1	1	1	1
4	4	2	2	1	1	1	1	1	1
4	4	2	2	2	2	1	1	1	1
4	4	2	2	1	1	1	1	1	1
4	4	2	2	2	2	1	1	1	1
4	4	2	2	2	2	1	1	1	1
4	4	2	2	1	1	1	1	1	1
4	4	2	2	1	1	1	1	1	1
4	4	2	2	1	1	1	1	1	1
4	4	4	4	2	2	1	1	1	1
4	4	4	4	2	2	1	1	1	1
3	3	2	2	2	2	1	1	1	1
4	4	2	2	2	2	1	1	1	1
4	4	2	2	1	1	1	1	1	1
3	3	2	2	1	1	1	1	1	1
4	4	2	2	2	2	1	1	1	1
4	4	2	2	1	1	1	1	1	1
3	3	2	2	1	1	1	1	1	1
3	3	4	4	1	1	1	1	1	1
4	4	4	4	1	1	1	1	1	1
3	3	4	4	2	2	4	4	4	4
3	3	2	2	1	1	1	1	1	1
3	3	2	2	1	1	1	1	1	1
4	4	2	2	1	1	1	1	1	1
4	4	2	2	1	1	1	1	1	1
4	4	4	4	2	2	4	4	4	4
4	4	2	2	1	1	4	4	1	1
4	4	2	2	1	1	1	1	1	1
4	4	2	2	2	2	1	1	1	1
4	4	2	2	1	1	4	4	4	4
4	4	2	2	2	2	4	4	4	4
4	4	2	2	1	1	1	1	1	1
4	4	2	2	1	1	4	4	4	4
4	4	2	2	1	1	4	4	4	4
4	4	2	2	1	1	1	1	1	1
4	4	4	4	1	1	1	1	1	1
3	3	2	2	2	2	1	1	1	1
4	4	2	2	1	1	1	1	1	1

For Review Only

4	4	2	2	2	2	1	1	1	1
3	3	2	2	1	1	4	4	4	4
4	4	4	4	1	1	1	1	1	1
4	4	2	2	1	1	1	1	1	1
3	3	2	2	2	2	1	1	1	1
3	3	2	2	1	1	1	1	1	1
4	4	2	2	1	1	1	1	1	1
4	4	2	2	1	1	1	1	1	1
4	4	2	2	1	1	1	1	1	1
4	4	2	2	2	2	1	1	1	1
4	4	2	2	1	1	1	1	4	4
3	3	2	2	1	1	1	1	1	1
4	4	2	2	1	1	4	4	1	1
4	4	2	2	1	1	1	1	1	1
4	4	2	2	1	1	1	1	1	1
3	3	2	2	1	1	1	1	1	1
4	4	4	4	1	1	1	1	1	1
3	3	2	2	2	2	1	1	1	1
3	3	2	2	1	1	1	1	1	1
4	4	2	2	2	2	1	1	1	1
4	4	2	2	1	1	1	1	1	1
4	4	2	2	1	1	1	1	1	1
4	4	2	2	1	1	1	1	1	1
3	3	2	2	2	2	1	1	1	1
4	4	2	2	2	2	1	1	1	1
3	3	2	2	1	1	1	1	1	1
4	4	2	2	2	2	4	4	4	4
3	3	2	2	1	1	1	1	1	1
3	3	2	2	1	1	4	4	4	4
4	4	2	2	2	2	1	1	1	1
4	4	2	2	2	2	1	1	1	1
4	4	2	2	2	2	1	1	1	1
4	4	4	4	2	2	1	1	1	1
4	4	2	2	1	1	1	1	1	1
4	4	4	4	1	1	1	1	1	1
3	3	2	2	2	2	1	1	1	1
4	4	2	2	1	1	4	4	4	4
3	3	4	4	2	2	1	1	1	1
4	4	4	4	2	2	4	4	4	4
3	3	2	2	2	2	1	1	1	1
4	4	2	2	2	2	1	1	1	1
4	4	2	2	1	1	4	4	4	4
3	3	2	2	1	1	1	1	1	1
4	4	2	2	1	1	1	1	1	1
3	3	4	4	2	2	1	1	1	1
3	3	2	2	2	2	1	1	1	1
3	3	2	2	1	1	1	1	1	1
4	4	2	2	1	1	1	1	1	1
4	4	2	2	1	1	1	1	1	1
4	4	2	2	2	2	1	1	1	1
3	3	2	2	2	2	1	1	1	1
4	4	2	2	1	1	1	1	1	1
4	4	2	2	1	1	1	1	1	1
3	3	2	2	2	2	1	1	1	1
4	4	2	2	1	1	1	1	1	1

For Review Only

3	3	4	4	1	1	1	1	1	1
4	4	2	2	1	1	1	1	1	1
4	4	2	2	2	2	1	1	1	1
4	4	2	2	1	1	1	1	1	1
4	4	2	2	2	2	1	1	1	1
4	4	2	2	2	2	4	4	4	4
4	4	4	4	1	1	1	1	1	1
4	4	2	2	1	1	4	4	1	1
3	3	2	2	1	1	1	1	1	1
4	4	2	2	1	1	1	1	1	1
3	3	2	2	1	1	1	1	1	1
4	4	2	2	1	1	1	1	1	1
4	4	2	2	1	1	4	4	1	1
3	3	2	2	1	1	1	1	1	1
4	4	2	2	2	2	1	1	1	1
4	4	2	2	1	1	1	1	1	1
3	3	2	2	1	1	1	1	1	1
3	3	2	2	2	2	1	1	1	1
4	4	2	2	1	1	1	1	1	1
4	4	2	2	1	1	1	1	1	1
4	4	2	2	1	1	4	4	4	4
4	4	2	2	1	1	4	4	4	4
3	3	2	2	2	2	1	1	1	1
4	4	2	2	1	1	1	1	1	1
4	4	2	2	1	1	1	1	1	1
4	4	2	2	2	2	1	1	1	1
4	4	2	2	1	1	1	1	1	1
3	3	2	2	2	2	1	1	4	4
4	4	2	2	2	2	1	1	1	1
3	3	2	2	2	2	1	1	1	1
4	4	2	2	1	1	1	1	1	1
3	3	2	2	1	1	4	4	4	4
4	4	2	2	1	1	1	1	1	1
4	4	2	2	1	1	4	4	4	4
4	4	2	2	1	1	1	1	1	1
3	3	2	2	1	1	1	1	1	1
4	4	4	4	2	2	1	1	1	1
3	3	2	2	2	2	1	1	1	1
3	3	2	2	1	1	1	1	1	1
4	4	4	4	1	1	1	1	1	1
4	4	4	4	2	2	4	4	4	4
3	3	4	4	2	2	1	1	1	1
4	4	2	2	1	1	4	4	4	4
4	4	2	2	2	2	1	1	1	1
4	4	2	2	1	1	1	1	1	1
4	4	2	2	1	1	4	4	4	4
4	4	2	2	1	1	4	4	4	4
4	4	2	2	2	2	4	4	4	4
4	4	2	2	2	2	4	4	4	4
4	4	2	2	1	1	1	1	1	1
4	4	2	2	2	2	1	1	1	1
4	4	2	2	1	1	1	1	1	1
4	4	2	2	2	2	1	1	1	1
3	3	2	2	2	2	4	4	4	4
4	4	2	2	1	1	1	1	1	1

For Review Only

4	4	2	2	2	2	1	1	1	1
4	4	2	2	2	2	4	4	4	4

For Review Only

SNP1418	SNP1418	SNP1419	SNP1419	SNP142	SNP142	SNP1420	SNP1420	SNP1421	SNP1421
4	4	1	1	3	3	3	3	4	4
4	4	4	4	3	3	2	2	1	1
4	4	4	4	3	3	2	2	1	1
4	4	4	4	3	3	2	2	1	1
4	4	4	4	4	4	2	2	1	1
4	4	1	1	3	3	3	3	4	4
4	4	1	1	3	3	3	3	4	4
4	4	1	1	3	3	3	3	4	4
4	4	1	1	3	3	3	3	4	4
4	4	4	4	4	4	2	2	1	1
4	4	1	1	3	3	3	3	4	4
4	4	4	4	3	3	2	2	1	1
4	4	1	1	4	4	3	3	4	4
4	4	1	1	3	3	3	3	4	4
4	4	1	1	3	3	2	2	4	4
4	4	1	1	4	4	3	3	4	4
4	4	1	1	4	4	3	3	4	4
4	4	1	1	3	3	3	3	4	4
4	4	4	4	3	3	2	2	1	1
4	4	4	4	4	4	2	2	1	1
4	4	1	1	4	4	3	3	4	4
4	4	4	4	4	4	2	2	4	4
4	4	4	4	3	3	2	2	1	1
4	4	1	1	4	4	2	2	1	1
4	4	1	1	3	3	3	3	4	4
3	3	4	4	3	3	2	2	1	1
3	3	4	4	3	3	2	2	1	1
4	4	4	4	3	3	3	3	4	4
4	4	1	1	4	4	3	3	4	4
4	4	4	4	4	4	2	2	1	1
4	4	4	4	3	3	2	2	1	1
4	4	1	1	3	3	3	3	4	4
4	4	1	1	3	3	3	3	4	4
4	4	1	1	4	4	3	3	4	4
4	4	4	4	3	3	2	2	4	4
4	4	4	4	3	3	2	2	1	1
4	4	4	4	3	3	2	2	1	1
4	4	4	4	3	3	2	2	4	4
4	4	1	1	4	4	3	3	4	4
4	4	4	4	3	3	2	2	1	1
4	4	1	1	4	4	3	3	4	4
4	4	1	1	4	4	3	3	4	4
4	4	1	1	4	4	3	3	4	4
4	4	4	4	3	3	2	2	4	4
4	4	1	1	3	3	3	3	4	4
4	4	4	4	3	3	2	2	1	1
4	4	4	4	3	3	2	2	1	1
4	4	1	1	3	3	3	3	4	4
4	4	1	1	3	3	3	3	4	4
4	4	4	4	3	3	2	2	4	4
4	4	4	4	3	3	2	2	1	1
4	4	4	4	3	3	2	2	1	1

For Review Only

4	4	1	1	3	3	3	3	4	4
4	4	1	1	3	3	3	3	4	4
4	4	4	4	3	3	2	2	1	1
4	4	1	1	3	3	3	3	4	4
4	4	4	4	3	3	2	2	1	1
3	3	4	4	3	3	2	2	1	1
3	3	4	4	3	3	2	2	1	1
4	4	1	1	4	4	3	3	4	4
4	4	1	1	3	3	3	3	4	4
4	4	4	4	3	3	2	2	4	4
4	4	1	1	3	3	3	3	4	4
4	4	4	4	3	3	2	2	1	1
4	4	1	1	3	3	3	3	4	4
4	4	1	1	4	4	3	3	4	4
4	4	4	4	3	3	2	2	4	4
4	4	4	4	3	3	2	2	1	1
4	4	4	4	3	3	2	2	1	1
4	4	1	1	3	3	3	3	4	4
4	4	4	4	3	3	2	2	1	1
3	3	1	1	3	3	3	3	4	4
4	4	4	4	3	3	2	2	4	4
4	4	4	4	4	4	2	2	4	4
4	4	1	1	4	4	3	3	4	4
4	4	4	4	4	4	2	2	1	1
4	4	1	1	3	3	3	3	4	4
4	4	1	1	3	3	3	3	4	4
4	4	1	1	3	3	3	3	4	4
4	4	1	1	4	4	3	3	4	4
3	3	4	4	3	3	2	2	1	1
4	4	1	1	3	3	3	3	4	4
4	4	1	1	3	3	3	3	4	4
4	4	1	1	4	4	3	3	4	4
4	4	1	1	4	4	3	3	4	4
4	4	1	1	3	3	3	3	4	4
4	4	1	1	3	3	3	3	4	4
4	4	1	1	3	3	3	3	4	4
4	4	1	1	3	3	3	3	4	4
4	4	1	1	4	4	3	3	4	4
4	4	1	1	3	3	3	3	4	4
4	4	1	1	3	3	3	3	4	4
4	4	1	1	3	3	3	3	4	4
4	4	1	1	3	3	3	3	4	4
4	4	4	4	3	3	2	2	1	1
4	4	1	1	3	3	3	3	4	4
4	4	1	1	3	3	3	3	4	4
4	4	4	4	3	3	2	2	1	1
4	4	1	1	4	4	2	2	1	1
3	3	4	4	3	3	2	2	1	1
3	3	4	4	3	3	2	2	1	1
4	4	4	4	3	3	2	2	1	1
4	4	1	1	3	3	3	3	4	4
4	4	1	1	4	4	3	3	4	4
4	4	1	1	4	4	3	3	4	4
4	4	4	4	3	3	2	2	1	1
4	4	1	1	3	3	3	3	4	4

For Review Only

3	3	4	4	3	3	2	2	1	1
4	4	4	4	3	3	2	2	1	1
4	4	4	4	3	3	2	2	1	1
4	4	1	1	4	4	3	3	4	4
4	4	4	4	3	3	2	2	1	1
4	4	4	4	3	3	2	2	1	1
4	4	1	1	3	3	3	3	4	4
4	4	1	1	3	3	3	3	4	4
3	3	1	1	4	4	3	3	4	4
4	4	4	4	3	3	2	2	1	1
4	4	1	1	3	3	3	3	4	4
4	4	4	4	3	3	2	2	4	4
3	3	4	4	3	3	2	2	1	1
4	4	4	4	3	3	2	2	4	4
4	4	4	4	3	3	2	2	1	1
4	4	4	4	3	3	2	2	4	4
4	4	4	4	3	3	2	2	1	1
4	4	4	4	3	3	2	2	4	4
4	4	1	1	4	4	2	2	4	4
4	4	1	1	3	3	3	3	4	4
4	4	4	4	3	3	2	2	4	4
4	4	1	1	3	3	2	2	4	4
4	4	4	4	3	3	2	2	1	1
4	4	1	1	4	4	3	3	4	4
4	4	4	4	4	4	2	2	1	1
4	4	4	4	3	3	2	2	1	1
4	4	1	1	3	3	2	2	4	4
4	4	1	1	3	3	3	3	4	4
4	4	4	4	3	3	2	2	1	1
4	4	1	1	3	3	3	3	4	4
4	4	4	4	4	4	2	2	1	1
4	4	4	4	3	3	2	2	4	4
4	4	1	1	3	3	3	3	4	4
4	4	4	4	3	3	2	2	4	4
4	4	1	1	4	4	3	3	4	4
4	4	1	1	3	3	3	3	4	4
4	4	4	4	4	4	2	2	1	1
4	4	1	1	3	3	2	2	4	4
4	4	4	4	3	3	2	2	4	4
4	4	1	1	4	4	3	3	4	4
4	4	4	4	3	3	2	2	1	1
4	4	1	1	4	4	2	2	1	1
4	4	1	1	3	3	3	3	4	4
4	4	4	4	4	4	2	2	4	4
4	4	4	4	3	3	2	2	1	1
4	4	1	1	3	3	3	3	4	4
3	3	4	4	3	3	2	2	1	1
3	3	4	4	3	3	2	2	1	1
4	4	4	4	4	4	2	2	1	1
3	3	4	4	3	3	2	2	4	4
3	3	4	4	3	3	2	2	1	1
4	4	1	1	4	4	3	3	4	4
4	4	1	1	3	3	3	3	4	4
4	4	4	4	3	3	2	2	1	1
4	4	4	4	3	3	2	2	4	4

For Review Only

4	4	4	4	3	3	2	2	1	1
3	3	1	1	3	3	3	3	4	4
4	4	4	4	4	4	2	2	1	1
4	4	4	4	4	4	2	2	4	4
4	4	4	4	3	3	2	2	1	1
4	4	1	1	4	4	3	3	4	4
4	4	1	1	3	3	3	3	4	4
4	4	4	4	3	3	2	2	4	4
4	4	4	4	3	3	2	2	4	4
4	4	4	4	3	3	2	2	1	1
4	4	4	4	3	3	2	2	1	1
4	4	1	1	3	3	2	2	4	4
4	4	4	4	3	3	2	2	1	1
4	4	1	1	3	3	3	3	4	4
4	4	1	1	4	4	2	2	1	1
4	4	1	1	3	3	3	3	4	4
4	4	1	1	3	3	2	2	4	4
4	4	4	4	3	3	2	2	1	1
4	4	4	4	3	3	2	2	1	1
4	4	1	1	3	3	3	3	4	4
4	4	4	4	3	3	2	2	4	4
4	4	1	1	4	4	2	2	4	4
4	4	4	4	4	4	2	2	4	4
4	4	4	4	3	3	2	2	1	1
4	4	1	1	3	3	3	3	4	4
4	4	1	1	4	4	3	3	4	4
3	3	4	4	3	3	2	2	4	4
4	4	4	4	3	3	2	2	1	1
4	4	1	1	3	3	3	3	4	4
4	4	1	1	3	3	3	3	4	4
4	4	4	4	3	3	2	2	1	1
4	4	4	4	3	3	2	2	1	1
4	4	1	1	3	3	3	3	4	4
4	4	1	1	4	4	2	2	4	4
4	4	1	1	3	3	3	3	4	4
4	4	4	4	3	3	2	2	4	4
3	3	4	4	3	3	2	2	1	1
4	4	1	1	3	3	3	3	4	4
4	4	4	4	3	3	2	2	1	1
4	4	4	4	3	3	2	2	1	1
4	4	4	4	3	3	2	2	1	1
3	3	4	4	3	3	2	2	1	1
4	4	1	1	4	4	3	3	4	4
4	4	4	4	3	3	2	2	1	1
4	4	4	4	3	3	2	2	4	4
4	4	4	4	4	4	2	2	1	1
4	4	1	1	3	3	3	3	4	4
4	4	1	1	4	4	3	3	4	4
4	4	1	1	4	4	3	3	4	4
4	4	4	4	3	3	2	2	1	1
4	4	4	4	3	3	2	2	1	1
4	4	1	1	4	4	3	3	4	4
4	4	4	4	4	4	2	2	1	1
4	4	1	1	3	3	3	3	4	4
4	4	1	1	4	4	3	3	4	4

For Review Only

4	4	1	1	3	3	3	3	4	4
4	4	4	4	3	3	2	2	1	1
4	4	4	4	3	3	2	2	1	1
4	4	4	4	3	3	2	2	1	1
4	4	1	1	3	3	3	3	4	4
4	4	4	4	3	3	2	2	1	1
4	4	4	4	4	4	2	2	1	1
4	4	4	4	4	4	2	2	1	1
4	4	1	1	3	3	2	2	4	4
4	4	1	1	4	4	3	3	4	4
4	4	4	4	3	3	2	2	4	4
4	4	4	4	3	3	2	2	1	1
4	4	4	4	3	3	2	2	1	1
4	4	4	4	3	3	2	2	1	1
4	4	4	4	3	3	2	2	1	1
4	4	1	1	3	3	3	3	4	4
4	4	1	1	4	4	3	3	4	4
4	4	1	1	3	3	3	3	4	4
4	4	1	1	4	4	3	3	4	4
4	4	4	4	3	3	2	2	1	1
4	4	4	4	4	4	2	2	1	1
3	3	4	4	3	3	2	2	1	1
4	4	4	4	3	3	2	2	1	1
4	4	1	1	3	3	3	3	4	4
4	4	1	1	4	4	3	3	4	4
4	4	1	1	3	3	3	3	4	4
4	4	1	1	4	4	3	3	4	4
3	3	4	4	3	3	2	2	1	1
4	4	1	1	3	3	3	3	4	4
4	4	4	4	3	3	2	2	1	1
4	4	1	1	3	3	3	3	4	4
3	3	1	1	3	3	3	3	4	4
4	4	1	1	3	3	3	3	4	4
4	4	4	4	3	3	2	2	1	1
4	4	4	4	4	4	2	2	1	1
4	4	1	1	4	4	3	3	4	4
4	4	1	1	3	3	3	3	4	4
4	4	4	4	3	3	2	2	1	1
4	4	4	4	3	3	2	2	1	1
4	4	1	1	4	4	3	3	4	4
4	4	1	1	3	3	3	3	4	4
4	4	1	1	4	4	3	3	4	4
4	4	1	1	3	3	3	3	4	4
4	4	1	1	3	3	3	3	4	4
4	4	4	4	3	3	2	2	4	4
3	3	4	4	3	3	2	2	1	1
3	3	4	4	3	3	2	2	1	1
4	4	1	1	3	3	3	3	4	4
3	3	4	4	3	3	3	3	4	4
4	4	1	1	4	4	3	3	4	4
4	4	1	1	3	3	3	3	4	4
4	4	1	1	4	4	3	3	4	4
4	4	4	4	3	3	2	2	1	1
3	3	4	4	3	3	2	2	1	1
4	4	4	4	3	3	2	2	1	1

For Review Only

4	4	4	4	3	3	2	2	1	1
3	3	4	4	3	3	2	2	1	1

For Review Only

SNP1422	SNP1422	SNP1423	SNP1423	SNP1424	SNP1424	SNP1425	SNP1425	SNP1426	SNP1426
2	2	3	3	2	2	1	1	4	4
2	2	2	2	2	2	1	1	4	4
3	3	2	2	2	2	3	3	1	1
3	3	2	2	2	2	1	1	1	1
2	2	3	3	2	2	1	1	4	4
2	2	3	3	2	2	1	1	4	4
2	2	3	3	2	2	3	3	1	1
2	2	2	2	2	2	1	1	4	4
2	2	3	3	2	2	3	3	1	1
2	2	2	2	2	2	3	3	1	1
2	2	2	2	2	2	1	1	4	4
3	3	2	2	2	2	3	3	1	1
2	2	2	2	2	2	3	3	4	4
2	2	2	2	2	2	1	1	4	4
2	2	3	3	2	2	3	3	1	1
2	2	2	2	2	2	3	3	4	4
2	2	2	2	2	2	3	3	1	1
2	2	3	3	2	2	1	1	4	4
3	3	2	2	2	2	1	1	4	4
2	2	3	3	2	2	1	1	4	4
2	2	2	2	2	2	3	3	1	1
2	2	3	3	2	2	3	3	1	1
3	3	3	3	2	2	1	1	4	4
2	2	3	3	2	2	1	1	4	4
2	2	2	2	2	2	1	1	4	4
3	3	3	3	3	3	1	1	4	4
3	3	3	3	3	3	1	1	4	4
3	3	2	2	2	2	3	3	1	1
2	2	3	3	2	2	3	3	1	1
2	2	2	2	2	2	3	3	4	4
3	3	3	3	2	2	1	1	4	4
2	2	3	3	2	2	1	1	4	4
2	2	3	3	2	2	1	1	4	4
2	2	3	3	2	2	1	1	4	4
2	2	3	3	2	2	1	1	4	4
3	3	2	2	2	2	1	1	4	4
3	3	3	3	2	2	1	1	4	4
3	3	2	2	2	2	1	1	4	4
2	2	3	3	2	2	1	1	4	4
3	3	3	3	2	2	1	1	4	4
2	2	2	2	2	2	1	1	4	4
2	2	2	2	2	2	3	3	1	1
2	2	3	3	2	2	3	3	1	1
2	2	3	3	2	2	1	1	4	4
2	2	3	3	2	2	3	3	1	1
3	3	2	2	2	2	3	3	1	1
3	3	3	3	2	2	1	1	4	4
2	2	2	2	3	3	3	3	1	1
2	2	2	2	2	2	3	3	1	1
2	2	3	3	2	2	3	3	1	1
3	3	3	3	2	2	1	1	4	4
3	3	2	2	2	2	3	3	1	1

For Review Only

2	2	3	3	2	2	3	3	1	1
2	2	3	3	2	2	1	1	4	4
3	3	2	2	2	2	1	1	4	4
2	2	2	2	2	2	3	3	1	1
3	3	2	2	2	2	1	1	4	4
3	3	2	2	3	3	1	1	4	4
3	3	3	3	3	3	1	1	4	4
2	2	3	3	2	2	1	1	4	4
2	2	3	3	2	2	1	1	4	4
3	3	2	2	2	2	3	3	1	1
2	2	2	2	2	2	1	1	4	4
3	3	2	2	2	2	1	1	4	4
2	2	3	3	2	2	3	3	1	1
2	2	3	3	2	2	3	3	1	1
3	3	3	3	2	2	1	1	1	1
2	2	2	2	2	2	1	1	4	4
3	3	3	3	2	2	3	3	1	1
2	2	3	3	2	2	3	3	1	1
3	3	3	3	2	2	3	3	1	1
2	2	3	3	2	2	3	3	1	1
3	3	2	2	2	2	1	1	4	4
3	3	3	3	2	2	1	1	4	4
2	2	3	3	2	2	3	3	1	1
3	3	3	3	2	2	1	1	4	4
2	2	3	3	2	2	3	3	1	1
3	3	2	2	2	2	1	1	4	4
2	2	3	3	2	2	3	3	1	1
2	2	2	2	2	2	1	1	4	4
3	3	3	3	3	3	1	1	4	4
2	2	2	2	2	2	3	3	1	1
2	2	2	2	2	2	3	3	1	1
2	2	3	3	2	2	3	3	1	1
2	2	3	3	2	2	1	1	4	4
2	2	3	3	2	2	3	3	1	1
2	2	3	3	2	2	1	1	4	4
2	2	3	3	2	2	3	3	1	1
2	2	2	2	2	2	3	3	1	1
2	2	2	2	2	2	3	3	1	1
2	2	3	3	2	2	1	1	4	4
2	2	2	2	2	2	3	3	1	1
2	2	2	2	2	2	1	1	4	4
2	2	2	2	2	2	1	1	4	4
3	3	3	3	2	2	3	3	1	1
2	2	2	2	2	2	1	1	4	4
2	2	2	2	2	2	1	1	4	4
3	3	2	2	2	2	1	1	4	4
2	2	3	3	2	2	1	1	1	1
3	3	3	3	3	3	1	1	4	4
2	2	3	3	3	3	1	1	4	4
3	3	2	2	2	2	3	3	1	1
2	2	3	3	2	2	3	3	1	1
2	2	3	3	2	2	1	1	4	4
2	2	3	3	2	2	1	1	4	4
3	3	2	2	2	2	3	3	1	1
2	2	3	3	2	2	3	3	1	1

For Review Only

3	3	2	2	3	3	1	1	4	4
3	3	2	2	2	2	1	1	4	4
3	3	2	2	2	2	1	1	4	4
2	2	3	3	2	2	1	1	4	4
3	3	3	3	2	2	1	1	4	4
3	3	3	3	2	2	1	1	4	4
2	2	2	2	2	2	3	3	1	1
2	2	2	2	2	2	1	1	4	4
2	2	3	3	3	3	1	1	4	4
3	3	2	2	2	2	3	3	1	1
2	2	3	3	2	2	1	1	4	4
3	3	2	2	2	2	3	3	1	1
3	3	3	3	3	3	3	3	1	1
3	3	2	2	2	2	3	3	1	1
3	3	2	2	2	2	1	1	4	4
3	3	2	2	2	2	1	1	4	4
3	3	3	3	2	2	3	3	1	1
3	3	2	2	3	3	3	3	1	1
2	2	3	3	2	2	1	1	4	4
2	2	2	2	2	2	3	3	1	1
3	3	2	2	2	2	3	3	1	1
2	2	2	2	2	2	1	1	1	1
3	3	2	2	2	2	3	3	1	1
2	2	3	3	2	2	1	1	4	4
3	3	3	3	2	2	1	1	4	4
3	3	3	3	2	2	1	1	4	4
2	2	2	2	2	2	3	3	1	1
2	2	3	3	2	2	3	3	1	1
3	3	2	2	2	2	3	3	1	1
2	2	2	2	2	2	3	3	1	1
2	2	2	2	2	2	1	1	4	4
3	3	2	2	2	2	3	3	1	1
2	2	2	2	2	2	1	1	4	4
3	3	2	2	2	2	3	3	1	1
2	2	3	3	2	2	1	1	4	4
2	2	3	3	2	2	3	3	4	4
2	2	2	2	2	2	1	1	4	4
2	2	2	2	2	2	1	1	4	4
3	3	2	2	2	2	3	3	4	4
2	2	2	2	2	2	1	1	4	4
3	3	3	3	3	3	1	1	4	4
2	2	3	3	2	2	1	1	4	4
2	2	3	3	2	2	3	3	1	1
2	2	2	2	2	2	3	3	1	1
3	3	2	2	2	2	1	1	4	4
2	2	3	3	2	2	1	1	4	4
3	3	2	2	3	3	1	1	4	4
3	3	3	3	3	3	3	3	1	1
3	3	3	3	2	2	1	1	4	4
3	3	2	2	3	3	1	1	4	4
3	3	3	3	3	3	1	1	4	4
2	2	3	3	2	2	1	1	4	4
2	2	3	3	2	2	1	1	4	4
3	3	3	3	2	2	1	1	4	4
3	3	3	3	2	2	3	3	1	1

For Review Only

3	3	3	3	2	2	3	3	1	1
2	2	3	3	2	2	1	1	4	4
2	2	3	3	2	2	1	1	4	4
3	3	3	3	2	2	3	3	1	1
3	3	2	2	2	2	1	1	4	4
2	2	3	3	2	2	1	1	4	4
2	2	2	2	2	2	1	1	4	4
3	3	2	2	2	2	3	3	1	1
3	3	3	3	2	2	1	1	4	4
3	3	3	3	2	2	1	1	4	4
3	3	2	2	3	3	1	1	4	4
2	2	3	3	2	2	1	1	4	4
3	3	3	3	2	2	1	1	1	1
2	2	2	2	2	2	1	1	4	4
2	2	2	2	2	2	1	1	4	4
2	2	2	2	2	2	1	1	4	4
3	3	3	3	2	2	3	3	4	4
3	3	2	2	2	2	1	1	4	4
3	3	2	2	2	2	1	1	4	4
2	2	2	2	2	2	3	3	1	1
3	3	2	2	2	2	3	3	1	1
2	2	3	3	2	2	1	1	4	4
3	3	3	3	2	2	1	1	4	4
3	3	2	2	2	2	1	1	4	4
2	2	2	2	2	2	3	3	1	1
2	2	3	3	2	2	1	1	4	4
3	3	2	2	3	3	3	3	1	1
3	3	3	3	2	2	3	3	1	1
2	2	3	3	2	2	1	1	1	1
2	2	3	3	2	2	3	3	1	1
3	3	2	2	2	2	1	1	4	4
3	3	3	3	2	2	3	3	1	1
2	2	3	3	2	2	3	3	1	1
2	2	2	2	2	2	1	1	4	4
2	2	3	3	2	2	3	3	1	1
3	3	2	2	2	2	1	1	4	4
3	3	2	2	2	2	1	1	4	4
2	2	3	3	2	2	1	1	4	4
3	3	3	3	3	3	3	3	1	1
3	3	3	3	2	2	1	1	4	4
3	3	2	2	2	2	3	3	1	1
3	3	2	2	3	3	1	1	4	4
2	2	2	2	2	2	1	1	4	4
3	3	2	2	2	2	3	3	1	1
3	3	2	2	2	2	3	3	4	4
3	3	3	3	2	2	3	3	1	1
2	2	2	2	2	2	1	1	4	4
2	2	2	2	2	2	1	1	4	4
2	2	3	3	2	2	1	1	4	4
3	3	2	2	2	2	3	3	1	1
3	3	2	2	2	2	3	3	1	1
2	2	2	2	2	2	1	1	4	4
2	2	3	3	2	2	1	1	4	4
2	2	2	2	2	2	1	1	4	4
2	2	3	3	2	2	1	1	4	4

For Review Only

2	2	3	3	2	2	1	1	4	4
2	2	3	3	2	2	1	1	4	4
3	3	2	2	2	2	3	3	1	1
3	3	3	3	2	2	3	3	1	1
2	2	3	3	2	2	3	3	1	1
3	3	3	3	3	3	3	3	1	1
3	3	3	3	2	2	1	1	4	4
2	2	3	3	2	2	1	1	4	4
2	2	3	3	2	2	1	1	4	4
2	2	3	3	2	2	1	1	4	4
3	3	2	2	2	2	3	3	1	1
3	3	2	2	2	2	1	1	4	4
3	3	3	3	2	2	1	1	4	4
3	3	2	2	2	2	1	1	4	4
3	3	2	2	2	2	1	1	4	4
2	2	2	2	2	2	1	1	4	4
2	2	3	3	2	2	1	1	4	4
2	2	3	3	2	2	3	3	1	1
2	2	3	3	2	2	1	1	4	4
3	3	2	2	2	2	1	1	4	4
3	3	3	3	2	2	1	1	4	4
3	3	3	3	3	3	1	1	4	4
3	3	2	2	2	2	1	1	4	4
2	2	3	3	2	2	1	1	4	4
2	2	3	3	2	2	1	1	4	4
2	2	2	2	2	2	3	3	1	1
2	2	2	2	2	2	1	1	4	4
3	3	2	2	3	3	3	3	1	1
2	2	3	3	2	2	3	3	1	1
3	3	2	2	2	2	3	3	1	1
2	2	3	3	2	2	1	1	4	4
2	2	2	2	3	3	1	1	4	4
2	2	2	2	2	2	1	1	4	4
3	3	3	3	3	3	1	1	4	4
3	3	3	3	2	2	1	1	4	4
2	2	3	3	2	2	1	1	4	4
2	2	2	2	2	2	3	3	1	1
3	3	2	2	2	2	1	1	4	4
3	3	2	2	2	2	1	1	4	4
2	2	3	3	2	2	1	1	4	4
2	2	3	3	2	2	3	3	1	1
2	2	3	3	2	2	1	1	4	4
2	2	3	3	2	2	1	1	4	4
2	2	2	2	2	2	3	3	1	1
3	3	2	2	2	2	1	1	4	4
3	3	3	3	3	3	3	3	1	1
3	3	3	3	3	3	1	1	4	4
2	2	3	3	3	3	1	1	4	4
2	2	2	2	3	3	3	3	1	1
2	2	3	3	2	2	1	1	4	4
2	2	2	2	2	2	3	3	1	1
2	2	3	3	2	2	1	1	4	4
3	3	2	2	2	2	3	3	1	1
3	3	2	2	3	3	3	3	1	1
3	3	2	2	2	2	1	1	4	4

For Review Only

3	3	2	2	2	2	1	1	4	4
3	3	2	2	3	3	3	3	4	4

For Review Only

SNP1427	SNP1427	SNP1428	SNP1428	SNP1429	SNP1429	SNP143	SNP143	SNP1430	SNP1430
1	1	2	2	1	1	2	2	2	2
1	1	3	3	1	1	2	2	1	1
1	1	2	2	1	1	2	2	2	2
1	1	2	2	4	4	1	1	2	2
1	1	3	3	4	4	1	1	1	1
1	1	2	2	4	4	2	2	2	2
1	1	2	2	4	4	2	2	2	2
1	1	2	2	4	4	2	2	2	2
1	1	2	2	4	4	2	2	2	2
1	1	3	3	4	4	1	1	1	1
1	1	2	2	4	4	2	2	2	2
1	1	2	2	1	1	2	2	2	2
1	1	2	2	1	1	1	1	2	2
4	4	2	2	1	1	1	1	2	2
1	1	2	2	1	1	1	1	2	2
1	1	2	2	4	4	1	1	2	2
1	1	2	2	4	4	1	1	2	2
1	1	2	2	4	4	1	1	2	2
1	1	2	2	1	1	2	2	2	2
1	1	3	3	4	4	1	1	1	1
1	1	2	2	1	1	1	1	2	2
1	1	2	2	4	4	1	1	1	1
1	1	2	2	1	1	2	2	2	2
1	1	3	3	1	1	1	1	1	1
1	1	2	2	4	4	2	2	2	2
1	1	2	2	1	1	2	2	2	2
1	1	2	2	4	4	2	2	2	2
1	1	2	2	1	1	1	1	2	2
1	1	2	2	4	4	1	1	2	2
1	1	3	3	1	1	1	1	2	2
4	4	2	2	4	4	2	2	2	2
1	1	2	2	4	4	2	2	2	2
1	1	2	2	4	4	2	2	2	2
1	1	2	2	4	4	1	1	2	2
1	1	2	2	1	1	2	2	2	2
1	1	2	2	4	4	2	2	2	2
1	1	2	2	1	1	2	2	1	1
1	1	2	2	1	1	2	2	2	2
1	1	2	2	1	1	1	1	2	2
1	1	2	2	1	1	2	2	2	2
1	1	2	2	1	1	1	1	2	2
1	1	2	2	1	1	1	1	2	2
1	1	2	2	4	4	1	1	2	2
1	1	2	2	1	1	2	2	2	2
1	1	2	2	4	4	2	2	2	2
1	1	2	2	1	1	2	2	2	2
1	1	2	2	1	1	2	2	2	2
1	1	2	2	1	1	1	1	2	2
1	1	2	2	1	1	1	1	2	2
1	1	2	2	1	1	2	2	2	2
1	1	2	2	1	1	2	2	2	2
4	4	2	2	1	1	2	2	2	2

For Review Only

1	1	2	2	4	4	2	2	2	2
1	1	2	2	4	4	2	2	2	2
1	1	2	2	4	4	2	2	2	2
1	1	2	2	1	1	2	2	2	2
1	1	2	2	1	1	2	2	2	2
1	1	2	2	1	1	2	2	2	2
1	1	2	2	1	1	2	2	2	2
1	1	2	2	1	1	1	1	2	2
1	1	2	2	1	1	2	2	2	2
1	1	2	2	1	1	2	2	2	2
1	1	2	2	1	1	2	2	2	2
1	1	2	2	1	1	2	2	2	2
1	1	2	2	4	4	2	2	2	2
1	1	2	2	4	4	1	1	2	2
1	1	2	2	1	1	1	1	2	2
1	1	3	3	1	1	1	1	1	1
1	1	2	2	1	1	2	2	2	2
1	1	2	2	1	1	2	2	2	2
1	1	2	2	4	4	2	2	2	2
1	1	2	2	4	4	2	2	2	2
1	1	2	2	1	1	1	1	2	2
1	1	2	2	1	1	1	1	2	2
1	1	2	2	4	4	1	1	2	2
1	1	2	2	4	4	1	1	2	2
1	1	2	2	4	4	2	2	2	2
4	4	2	2	1	1	2	2	2	2
1	1	2	2	1	1	2	2	2	2
1	1	2	2	1	1	1	1	2	2
1	1	2	2	1	1	2	2	2	2
1	1	2	2	4	4	2	2	2	2
1	1	2	2	4	4	2	2	2	2
1	1	2	2	4	4	1	1	2	2
1	1	2	2	1	1	1	1	2	2
1	1	2	2	4	4	2	2	2	2
1	1	2	2	4	4	2	2	2	2
1	1	2	2	4	4	2	2	2	2
1	1	2	2	1	1	2	2	2	2
1	1	2	2	1	1	1	1	2	2
1	1	2	2	1	1	2	2	2	2
1	1	2	2	1	1	2	2	2	2
1	1	2	2	4	4	2	2	2	2
1	1	2	2	4	4	2	2	2	2
1	1	2	2	1	1	2	2	2	2
1	1	2	2	1	1	2	2	2	2
1	1	2	2	1	1	2	2	2	2
1	1	2	2	4	4	2	2	2	2
1	1	3	3	1	1	1	1	1	1
1	1	2	2	1	1	2	2	2	2
1	1	3	3	1	1	2	2	1	1
1	1	2	2	1	1	2	2	2	2
1	1	2	2	1	1	1	1	2	2
1	1	2	2	1	1	1	1	2	2
1	1	2	2	1	1	1	1	2	2
1	1	2	2	1	1	1	1	2	2
4	4	2	2	1	1	2	2	2	2

For Review Only

1	1	2	2	1	1	2	2	2	2
4	4	2	2	1	1	2	2	2	2
1	1	2	2	1	1	2	2	2	2
1	1	2	2	1	1	1	1	2	2
1	1	2	2	1	1	2	2	2	2
1	1	2	2	1	1	2	2	2	2
1	1	2	2	4	4	2	2	2	2
1	1	2	2	1	1	1	1	2	2
1	1	2	2	1	1	1	1	2	2
1	1	2	2	1	1	1	1	2	2
1	1	2	2	1	1	2	2	2	2
1	1	2	2	1	1	1	1	2	2
1	1	2	2	1	1	2	2	2	2
1	1	2	2	1	1	2	2	2	2
1	1	2	2	4	4	1	1	2	2
1	1	2	2	1	1	2	2	2	2
1	1	2	2	1	1	2	2	2	2
1	1	2	2	1	1	1	1	2	2
1	1	2	2	1	1	1	1	2	2
1	1	2	2	4	4	2	2	2	2
1	1	2	2	1	1	1	1	2	2
1	1	2	2	4	4	2	2	2	2
1	1	2	2	4	4	2	2	2	2
1	1	2	2	1	1	1	1	2	2
1	1	2	2	1	1	2	2	2	2
1	1	2	2	4	4	2	2	2	2
1	1	2	2	4	4	2	2	2	2
1	1	2	2	4	4	2	2	2	2
1	1	2	2	4	4	2	2	2	2
1	1	2	2	4	4	2	2	2	2
1	1	3	3	1	1	1	1	1	1
1	1	2	2	1	1	1	1	2	2
1	1	2	2	4	4	2	2	2	2
1	1	2	2	1	1	2	2	2	2
1	1	2	2	4	4	1	1	2	2
1	1	2	2	1	1	1	1	2	2
1	1	3	3	1	1	1	1	1	1
4	4	2	2	4	4	2	2	2	2
1	1	2	2	1	1	2	2	2	2
1	1	2	2	1	1	1	1	2	2
1	1	2	2	1	1	1	1	2	2
1	1	2	2	1	1	1	1	2	2
4	4	2	2	1	1	2	2	2	2
1	1	3	3	1	1	1	1	1	1
1	1	2	2	1	1	2	2	2	2
1	1	2	2	4	4	2	2	2	2
1	1	2	2	1	1	2	2	2	2
1	1	2	2	1	1	2	2	2	2
1	1	2	2	1	1	1	1	2	2
1	1	2	2	1	1	2	2	2	2
1	1	2	2	1	1	2	2	2	2
1	1	2	2	1	1	1	1	2	2
1	1	2	2	4	4	2	2	2	2
1	1	2	2	1	1	2	2	2	2
1	1	2	2	1	1	1	1	2	2

For Review Only

1	1	2	2	1	1	2	2	2	2
1	1	2	2	1	1	2	2	2	2
1	1	3	3	4	4	1	1	1	1
1	1	2	2	1	1	1	1	2	2
1	1	2	2	4	4	2	2	2	2
1	1	2	2	1	1	1	1	2	2
1	1	2	2	1	1	1	1	2	2
1	1	2	2	1	1	1	1	2	2
1	1	2	2	1	1	2	2	2	2
1	1	2	2	4	4	2	2	2	2
1	1	2	2	1	1	2	2	2	2
1	1	2	2	1	1	1	1	1	1
1	1	2	2	4	4	2	2	2	2
1	1	2	2	1	1	2	2	2	2
1	1	3	3	1	1	1	1	1	1
1	1	2	2	4	4	2	2	2	2
1	1	2	2	4	4	1	1	2	2
1	1	2	2	1	1	2	2	2	2
1	1	2	2	4	4	2	2	2	2
1	1	2	2	1	1	2	2	2	2
1	1	2	2	4	4	2	2	2	2
1	1	2	2	1	1	1	1	2	2
1	1	2	2	1	1	1	1	2	2
1	1	2	2	1	1	2	2	2	2
1	1	2	2	1	1	2	2	2	2
1	1	2	2	4	4	1	1	2	2
1	1	2	2	1	1	2	2	2	2
1	1	2	2	1	1	2	2	2	2
1	1	2	2	1	1	2	2	2	2
1	1	2	2	1	1	2	2	2	2
1	1	2	2	4	4	2	2	2	2
1	1	2	2	4	4	2	2	2	2
1	1	2	2	1	1	2	2	2	2
1	1	2	2	1	1	1	1	2	2
1	1	2	2	4	4	2	2	2	2
1	1	2	2	1	1	2	2	2	2
1	1	2	2	4	4	2	2	2	2
1	1	2	2	4	4	2	2	2	2
1	1	2	2	1	1	2	2	2	2
1	1	2	2	1	1	2	2	2	2
1	1	2	2	1	1	2	2	2	2
1	1	2	2	1	1	2	2	2	2
1	1	2	2	1	1	1	1	2	2
1	1	2	2	1	1	2	2	2	2
1	1	2	2	1	1	1	1	2	2
1	1	2	2	1	1	1	1	2	2
1	1	2	2	4	4	2	2	2	2
1	1	2	2	1	1	1	1	2	2
1	1	2	2	1	1	1	1	2	2
1	1	2	2	1	1	1	1	2	2
1	1	2	2	4	4	2	2	2	2
1	1	2	2	1	1	1	1	2	2
1	1	3	3	1	1	1	1	1	1
1	1	2	2	1	1	2	2	2	2
1	1	2	2	1	1	1	1	2	2

For Review Only

1	1	2	2	1	1	2	2	2	2
1	1	3	3	1	1	2	2	1	1
1	1	2	2	4	4	2	2	2	2
1	1	2	2	1	1	1	1	2	2
1	1	2	2	4	4	2	2	2	2
1	1	2	2	1	1	2	2	1	1
1	1	2	2	4	4	1	1	2	2
1	1	3	3	4	4	1	1	1	1
1	1	2	2	1	1	1	1	2	2
1	1	2	2	1	1	1	1	2	2
1	1	2	2	1	1	1	1	2	2
1	1	2	2	1	1	2	2	2	2
1	1	2	2	4	4	2	2	2	2
1	1	2	2	1	1	2	2	2	2
1	1	2	2	1	1	2	2	2	2
1	1	2	2	4	4	2	2	2	2
1	1	2	2	1	1	1	1	2	2
1	1	2	2	4	4	2	2	2	2
1	1	2	2	4	4	1	1	2	2
1	1	2	2	1	1	2	2	2	2
4	4	2	2	1	1	1	1	2	2
1	1	2	2	1	1	2	2	2	2
1	1	2	2	1	1	2	2	2	2
1	1	2	2	1	1	2	2	2	2
1	1	2	2	1	1	1	1	2	2
1	1	2	2	1	1	2	2	2	2
1	1	2	2	1	1	1	1	2	2
1	1	2	2	1	1	2	2	2	2
1	1	2	2	4	4	2	2	2	2
1	1	2	2	4	4	2	2	2	2
1	1	2	2	1	1	2	2	2	2
1	1	2	2	1	1	2	2	2	2
1	1	2	2	1	1	2	2	2	2
1	1	2	2	4	4	2	2	2	2
1	1	2	2	1	1	1	1	2	2
1	1	2	2	1	1	1	1	2	2
1	1	2	2	4	4	2	2	2	2
1	1	2	2	1	1	2	2	2	2
1	1	2	2	4	4	2	2	2	2
1	1	2	2	1	1	1	1	2	2
4	4	2	2	1	1	2	2	2	2
1	1	2	2	4	4	1	1	2	2
4	4	2	2	1	1	2	2	2	2
1	1	2	2	4	4	2	2	2	2
1	1	2	2	1	1	2	2	2	2
1	1	2	2	1	1	2	2	2	2
1	1	2	2	1	1	2	2	2	2
1	1	2	2	1	1	2	2	2	2
1	1	2	2	1	1	2	2	2	2
1	1	2	2	1	1	1	1	2	2
1	1	2	2	1	1	2	2	2	2
1	1	2	2	1	1	1	1	2	2
1	1	2	2	1	1	2	2	2	2
1	1	2	2	1	1	2	2	2	2
1	1	2	2	1	1	2	2	2	2

For Review Only

1	1	2	2	4	4	2	2	2	2
1	1	2	2	1	1	2	2	2	2

For Review Only

SNP1431	SNP1431	SNP1432	SNP1432	SNP1433	SNP1433	SNP1434	SNP1434	SNP1435	SNP1435
3	3	1	1	3	3	1	1	3	3
1	1	1	1	3	3	1	1	3	3
3	3	3	3	1	1	4	4	3	3
3	3	3	3	3	3	1	1	3	3
1	1	3	3	3	3	1	1	3	3
3	3	1	1	3	3	1	1	3	3
3	3	3	3	3	3	4	4	3	3
3	3	1	1	3	3	1	1	3	3
3	3	3	3	3	3	4	4	3	3
1	1	3	3	1	1	4	4	3	3
3	3	3	3	3	3	1	1	4	4
3	3	3	3	3	3	4	4	3	3
3	3	3	3	3	3	4	4	3	3
3	3	1	1	3	3	1	1	3	3
3	3	3	3	3	3	4	4	3	3
3	3	3	3	3	3	1	1	3	3
3	3	3	3	1	1	4	4	3	3
3	3	1	1	3	3	1	1	3	3
1	1	1	1	3	3	1	1	3	3
1	1	3	3	3	3	1	1	4	4
3	3	3	3	1	1	4	4	3	3
1	1	3	3	1	1	4	4	3	3
3	3	3	3	3	3	1	1	4	4
1	1	3	3	3	3	1	1	3	3
3	3	1	1	3	3	1	1	3	3
3	3	1	1	3	3	1	1	3	3
3	3	1	1	3	3	1	1	3	3
3	3	3	3	3	3	4	4	3	3
3	3	3	3	3	3	4	4	3	3
1	1	3	3	3	3	1	1	3	3
3	3	1	1	3	3	1	1	3	3
3	3	1	1	3	3	1	1	3	3
3	3	1	1	3	3	1	1	3	3
3	3	1	1	3	3	1	1	3	3
3	3	3	3	3	3	1	1	3	3
3	3	1	1	3	3	1	1	3	3
1	1	3	3	3	3	1	1	3	3
3	3	3	3	3	3	1	1	3	3
3	3	3	3	3	3	1	1	3	3
3	3	3	3	3	3	1	1	3	3
3	3	3	3	3	3	1	1	4	4
3	3	3	3	1	1	4	4	3	3
3	3	3	3	1	1	4	4	3	3
3	3	3	3	3	3	1	1	3	3
3	3	3	3	1	1	4	4	3	3
3	3	1	1	3	3	1	1	3	3
1	1	1	1	3	3	1	1	3	3
3	3	3	3	3	3	4	4	3	3
3	3	3	3	1	1	4	4	3	3
3	3	3	3	1	1	4	4	3	3
3	3	1	1	3	3	1	1	3	3
3	3	3	3	1	1	4	4	3	3

3	3	3	3	1	1	4	4	3	3
3	3	1	1	3	3	1	1	3	3
3	3	1	1	3	3	1	1	3	3
3	3	3	3	1	1	4	4	3	3
1	1	1	1	3	3	1	1	3	3
1	1	1	1	3	3	1	1	3	3
3	3	1	1	3	3	1	1	3	3
3	3	3	3	3	3	1	1	3	3
3	3	3	3	3	3	1	1	3	3
3	3	3	3	3	3	4	4	3	3
3	3	1	1	3	3	1	1	3	3
1	1	1	1	3	3	1	1	3	3
3	3	3	3	1	1	4	4	3	3
3	3	3	3	3	3	4	4	3	3
3	3	3	3	3	3	1	1	4	4
1	1	1	1	3	3	1	1	3	3
3	3	3	3	3	3	4	4	3	3
3	3	3	3	1	1	4	4	3	3
3	3	3	3	1	1	4	4	3	3
3	3	3	3	1	1	4	4	3	3
3	3	3	3	3	3	1	1	3	3
3	3	3	3	3	3	1	1	3	3
3	3	3	3	3	3	4	4	3	3
3	3	1	1	3	3	1	1	3	3
3	3	3	3	1	1	4	4	3	3
3	3	1	1	3	3	1	1	3	3
3	3	3	3	1	1	4	4	3	3
3	3	3	3	3	3	1	1	3	3
3	3	1	1	3	3	1	1	3	3
3	3	3	3	1	1	4	4	3	3
3	3	3	3	1	1	4	4	3	3
3	3	3	3	1	1	4	4	3	3
3	3	3	3	3	3	1	1	3	3
3	3	3	3	1	1	4	4	3	3
3	3	1	1	3	3	1	1	3	3
3	3	3	3	1	1	4	4	3	3
3	3	3	3	1	1	4	4	3	3
3	3	3	3	1	1	4	4	3	3
3	3	3	3	3	3	1	1	3	3
3	3	3	3	1	1	4	4	3	3
3	3	1	1	3	3	1	1	3	3
3	3	3	3	3	3	1	1	3	3
3	3	3	3	1	1	4	4	3	3
3	3	1	1	3	3	1	1	3	3
3	3	3	3	3	3	1	1	3	3
3	3	1	1	3	3	1	1	3	3
1	1	3	3	3	3	1	1	3	3
3	3	3	3	3	3	1	1	3	3
1	1	1	1	3	3	1	1	3	3
3	3	3	3	1	1	4	4	3	3
3	3	3	3	1	1	4	4	3	3
3	3	3	3	3	3	1	1	4	4
3	3	3	3	3	3	1	1	3	3
3	3	3	3	3	3	4	4	3	3
3	3	3	3	1	1	4	4	3	3

For Review Only

3	3	3	3	3	3	1	1	3	3
3	3	1	1	3	3	1	1	3	3
3	3	1	1	3	3	1	1	3	3
3	3	1	1	3	3	1	1	3	3
3	3	1	1	3	3	1	1	3	3
3	3	3	3	3	3	1	1	3	3
3	3	3	3	1	1	4	4	3	3
3	3	3	3	3	3	1	1	4	4
3	3	1	1	3	3	1	1	3	3
3	3	3	3	3	3	1	1	3	3
3	3	1	1	3	3	1	1	3	3
3	3	1	1	3	3	4	4	3	3
3	3	3	3	1	1	4	4	3	3
3	3	3	3	3	3	1	1	3	3
3	3	1	1	3	3	1	1	3	3
3	3	1	1	3	3	1	1	3	3
3	3	3	3	1	1	4	4	3	3
3	3	3	3	3	3	4	4	3	3
3	3	1	1	3	3	1	1	3	3
3	3	3	3	1	1	4	4	3	3
3	3	3	3	3	3	4	4	3	3
3	3	1	1	3	3	4	4	3	3
3	3	3	3	3	3	4	4	3	3
3	3	3	3	3	3	1	1	4	4
3	3	3	3	3	3	1	1	3	3
3	3	3	3	3	3	1	1	3	3
3	3	3	3	1	1	4	4	3	3
3	3	3	3	1	1	4	4	3	3
3	3	3	3	1	1	4	4	3	3
3	3	3	3	1	1	4	4	3	3
1	1	3	3	3	3	1	1	3	3
3	3	3	3	3	3	4	4	3	3
3	3	1	1	3	3	1	1	3	3
3	3	3	3	3	3	4	4	3	3
3	3	1	1	3	3	1	1	3	3
3	3	3	3	3	3	1	1	3	3
1	1	1	1	3	3	1	1	3	3
3	3	1	1	3	3	1	1	3	3
3	3	3	3	3	3	1	1	3	3
3	3	3	3	3	3	1	1	3	3
3	3	3	3	3	3	1	1	3	3
3	3	1	1	3	3	1	1	3	3
3	3	3	3	1	1	4	4	3	3
3	3	3	3	3	3	4	4	3	3
3	3	3	3	3	3	1	1	3	3
3	3	1	1	3	3	1	1	3	3
3	3	1	1	3	3	1	1	3	3
3	3	3	3	1	1	4	4	3	3
1	1	3	3	3	3	1	1	3	3
3	3	3	3	3	3	1	1	4	4
3	3	1	1	3	3	1	1	3	3
3	3	3	3	3	3	1	1	4	4
3	3	1	1	3	3	1	1	3	3
3	3	1	1	3	3	1	1	3	3
3	3	3	3	3	3	4	4	3	3

For Review Only

3	3	3	3	1	1	4	4	3	3
3	3	3	3	3	3	1	1	3	3
1	1	1	1	3	3	4	4	3	3
3	3	3	3	3	3	4	4	3	3
3	3	1	1	3	3	1	1	3	3
3	3	3	3	3	3	1	1	3	3
3	3	3	3	3	3	1	1	4	4
3	3	3	3	3	3	4	4	3	3
3	3	3	3	3	3	1	1	4	4
1	1	1	1	3	3	1	1	3	3
3	3	1	1	3	3	1	1	3	3
3	3	3	3	3	3	1	1	3	3
3	3	3	3	3	3	1	1	3	3
3	3	1	1	3	3	1	1	3	3
1	1	1	1	3	3	1	1	3	3
3	3	3	3	3	3	1	1	4	4
3	3	3	3	3	3	1	1	3	3
3	3	3	3	3	3	1	1	3	3
3	3	3	3	3	3	1	1	3	3
3	3	3	3	1	1	4	4	3	3
3	3	3	3	3	3	4	4	3	3
3	3	3	3	3	3	1	1	3	3
3	3	3	3	3	3	1	1	4	4
1	1	1	1	3	3	1	1	3	3
3	3	3	3	3	3	4	4	3	3
3	3	1	1	3	3	1	1	3	3
3	3	3	3	3	3	4	4	3	3
3	3	3	3	3	3	4	4	3	3
3	3	1	1	3	3	1	1	3	3
3	3	3	3	1	1	4	4	3	3
3	3	1	1	3	3	1	1	3	3
3	3	3	3	1	1	4	4	3	3
3	3	3	3	1	1	4	4	3	3
3	3	1	1	3	3	1	1	3	3
3	3	3	3	3	3	4	4	3	3
1	1	1	1	3	3	1	1	3	3
3	3	3	3	3	3	1	1	3	3
3	3	1	1	3	3	1	1	3	3
3	3	3	3	1	1	4	4	3	3
3	3	1	1	3	3	1	1	3	3
3	3	3	3	1	1	4	4	3	3
3	3	1	1	3	3	1	1	3	3
3	3	3	3	3	3	1	1	4	4
3	3	3	3	3	3	4	4	3	3
3	3	3	3	3	3	1	1	3	3
1	1	3	3	1	1	4	4	3	3
3	3	3	3	3	3	1	1	4	4
3	3	3	3	3	3	1	1	4	4
3	3	1	1	3	3	1	1	3	3
3	3	3	3	1	1	4	4	3	3
3	3	3	3	1	1	4	4	3	3
3	3	3	3	3	3	1	1	3	3
1	1	3	3	3	3	1	1	3	3
3	3	1	1	3	3	1	1	3	3
3	3	3	3	3	3	1	1	3	3

For Review Only

3	3	1	1	3	3	1	1	3	3
1	1	1	1	3	3	4	4	3	3
3	3	3	3	1	1	4	4	3	3
3	3	3	3	1	1	4	4	3	3
3	3	3	3	1	1	4	4	3	3
3	3	3	3	1	1	4	4	3	3
3	3	3	3	3	3	1	1	4	4
1	1	3	3	3	3	1	1	3	3
3	3	1	1	3	3	1	1	3	3
3	3	3	3	3	3	1	1	4	4
3	3	3	3	3	3	4	4	4	4
3	3	3	3	3	3	1	1	3	3
3	3	3	3	3	3	1	1	3	3
3	3	1	1	3	3	1	1	3	3
3	3	1	1	3	3	1	1	3	3
3	3	3	3	3	3	1	1	3	3
3	3	3	3	3	3	1	1	4	4
3	3	3	3	1	1	4	4	3	3
3	3	3	3	3	3	1	1	4	4
3	3	3	3	3	3	1	1	3	3
3	3	3	3	3	3	1	1	3	3
3	3	1	1	3	3	1	1	3	3
1	1	1	1	3	3	1	1	3	3
3	3	3	3	3	3	1	1	3	3
3	3	3	3	3	3	1	1	3	3
3	3	3	3	3	3	1	1	3	3
3	3	3	3	3	3	1	1	3	3
3	3	3	3	3	3	4	4	3	3
3	3	3	3	1	1	4	4	3	3
3	3	3	3	1	1	4	4	3	3
3	3	3	3	3	3	1	1	4	4
3	3	3	3	3	3	1	1	4	4
3	3	1	1	3	3	1	1	3	3
3	3	3	3	3	3	1	1	3	3
3	3	3	3	3	3	1	1	3	3
3	3	3	3	3	3	1	1	4	4
3	3	3	3	1	1	4	4	3	3
1	1	1	1	3	3	1	1	3	3
3	3	1	1	3	3	1	1	3	3
3	3	3	3	3	3	1	1	4	4
3	3	3	3	1	1	4	4	3	3
3	3	1	1	3	3	1	1	3	3
3	3	3	3	3	3	1	1	3	3
3	3	3	3	1	1	4	4	3	3
3	3	3	3	3	3	1	1	3	3
3	3	3	3	1	1	4	4	3	3
3	3	3	3	3	3	1	1	3	3
3	3	1	1	3	3	1	1	3	3
1	1	3	3	1	1	4	4	3	3
3	3	3	3	3	3	1	1	4	4
3	3	3	3	1	1	4	4	3	3
3	3	3	3	3	3	1	1	3	3
3	3	3	3	1	1	4	4	3	3
3	3	3	3	1	1	4	4	3	3
3	3	3	3	3	3	1	1	3	3

For Review Only

1	1	1	1	3	3	1	1	3	3
3	3	1	1	3	3	1	1	3	3

For Review Only

SNP1436	SNP1436	SNP1437	SNP1437	SNP1438	SNP1438	SNP1439	SNP1439	SNP144	SNP144
2	2	3	3	1	1	1	1	3	3
2	2	3	3	4	4	4	4	3	3
2	2	3	3	1	1	4	4	3	3
3	3	3	3	1	1	1	1	3	3
3	3	4	4	4	4	4	4	3	3
2	2	4	4	4	4	1	1	3	3
2	2	4	4	4	4	1	1	3	3
2	2	4	4	4	4	1	1	2	2
2	2	3	3	1	1	1	1	2	2
2	2	4	4	4	4	1	1	3	3
2	2	3	3	1	1	1	1	2	2
2	2	3	3	1	1	4	4	3	3
2	2	3	3	4	4	4	4	2	2
2	2	3	3	1	1	4	4	3	3
2	2	3	3	1	1	4	4	2	2
2	2	3	3	1	1	1	1	2	2
2	2	3	3	1	1	1	1	2	2
2	2	4	4	4	4	1	1	2	2
2	2	3	3	1	1	1	1	3	3
2	2	3	3	1	1	1	1	3	3
2	2	4	4	4	4	4	4	3	3
2	2	4	4	4	4	1	1	2	2
2	2	4	4	4	4	4	4	3	3
2	2	4	4	4	4	4	4	3	3
2	2	3	3	1	1	1	1	2	2
2	2	3	3	1	1	4	4	2	2
2	2	3	3	1	1	1	1	3	3
2	2	3	3	1	1	4	4	2	2
2	2	3	3	1	1	1	1	3	3
3	3	4	4	4	4	4	4	2	2
2	2	3	3	1	1	4	4	3	3
2	2	4	4	4	4	1	1	2	2
2	2	4	4	4	4	1	1	2	2
2	2	4	4	4	4	1	1	3	3
2	2	3	3	1	1	4	4	3	3
2	2	3	3	1	1	1	1	3	3
3	3	3	3	1	1	4	4	3	3
3	3	3	3	1	1	4	4	3	3
2	2	4	4	4	4	4	4	3	3
2	2	3	3	1	1	4	4	3	3
2	2	4	4	4	4	4	4	3	3
2	2	3	3	1	1	4	4	2	2
2	2	4	4	4	4	1	1	3	3
3	3	3	3	1	1	4	4	2	2
2	2	3	3	1	1	1	1	2	2
2	2	3	3	1	1	4	4	2	2
2	2	3	3	1	1	4	4	3	3
2	2	3	3	1	1	4	4	2	2
2	2	3	3	1	1	4	4	2	2
2	2	3	3	1	1	4	4	3	3
3	3	3	3	1	1	4	4	2	2
2	2	4	4	4	4	4	4	3	3

For Review Only

2	2	3	3	1	1	1	1	2	2
2	2	4	4	4	4	1	1	2	2
2	2	3	3	1	1	1	1	3	3
2	2	4	4	4	4	4	4	2	2
2	2	3	3	1	1	1	1	3	3
2	2	3	3	1	1	4	4	2	2
2	2	3	3	1	1	4	4	2	2
3	3	4	4	4	4	4	4	3	3
3	3	3	3	1	1	4	4	3	3
2	2	3	3	1	1	4	4	2	2
2	2	3	3	1	1	4	4	3	3
2	2	3	3	1	1	4	4	2	2
2	2	3	3	1	1	1	1	2	2
2	2	3	3	1	1	1	1	3	3
2	2	3	3	1	1	4	4	3	3
2	2	3	3	1	1	4	4	3	3
2	2	3	3	1	1	4	4	2	2
2	2	3	3	1	1	4	4	2	2
2	2	3	3	1	1	1	1	2	2
2	2	3	3	1	1	1	1	2	2
2	2	3	3	1	1	4	4	2	2
2	2	3	3	1	1	4	4	3	3
2	2	3	3	1	1	1	1	3	3
2	2	3	3	1	1	1	1	3	3
2	2	3	3	1	1	1	1	2	2
2	2	4	4	4	4	4	4	3	3
2	2	4	4	4	4	4	4	3	3
2	2	4	4	4	4	4	4	3	3
2	2	3	3	1	1	4	4	2	2
2	2	3	3	1	1	4	4	2	2
2	2	3	3	1	1	1	1	2	2
2	2	4	4	4	4	4	4	3	3
2	2	4	4	4	4	4	4	3	3
2	2	3	3	1	1	1	1	2	2
2	2	4	4	4	4	1	1	3	3
2	2	3	3	1	1	1	1	2	2
2	2	3	3	1	1	4	4	2	2
2	2	4	4	4	4	4	4	2	2
2	2	4	4	4	4	4	4	2	2
2	2	3	3	1	1	4	4	3	3
2	2	3	3	1	1	1	1	2	2
2	2	3	3	1	1	4	4	3	3
2	2	3	3	1	1	4	4	3	3
2	2	3	3	1	1	4	4	3	3
3	3	3	3	1	1	4	4	2	2
2	2	3	3	1	1	1	1	3	3
2	2	4	4	4	4	4	4	3	3
2	2	3	3	1	1	4	4	2	2
2	2	3	3	1	1	4	4	3	3
2	2	3	3	1	1	4	4	3	3
2	2	3	3	1	1	4	4	3	3
2	2	4	4	4	4	4	4	3	3
3	3	3	3	1	1	4	4	3	3
2	2	3	3	1	1	4	4	2	2
2	2	3	3	1	1	4	4	3	3

For Review Only

2	2	3	3	1	1	4	4	3	3
2	2	3	3	1	1	4	4	3	3
2	2	3	3	1	1	4	4	2	2
2	2	4	4	4	4	4	4	3	3
2	2	3	3	1	1	4	4	3	3
2	2	4	4	4	4	4	4	3	3
2	2	3	3	1	1	1	1	2	2
2	2	4	4	4	4	4	4	3	3
2	2	3	3	1	1	4	4	3	3
2	2	3	3	1	1	4	4	2	2
2	2	4	4	4	4	4	4	3	3
2	2	3	3	1	1	4	4	2	2
2	2	3	3	1	1	4	4	2	2
2	2	4	4	1	1	4	4	3	3
2	2	3	3	1	1	4	4	3	3
2	2	3	3	1	1	4	4	2	2
2	2	3	3	1	1	4	4	2	2
2	2	3	3	1	1	4	4	2	2
2	2	4	4	4	4	4	4	3	3
2	2	3	3	1	1	1	1	2	2
2	2	3	3	1	1	4	4	2	2
2	2	3	3	1	1	4	4	2	2
2	2	4	4	4	4	1	1	3	3
2	2	4	4	4	4	4	4	3	3
2	2	3	3	1	1	4	4	2	2
2	2	4	4	4	4	4	4	3	3
2	2	3	3	1	1	1	1	2	2
2	2	3	3	1	1	1	1	2	2
2	2	3	3	1	1	1	1	2	2
2	2	3	3	1	1	4	4	3	3
2	2	4	4	4	4	4	4	3	3
2	2	3	3	1	1	4	4	3	3
2	2	3	3	1	1	1	1	2	2
2	2	3	3	1	1	4	4	2	2
2	2	4	4	4	4	1	1	3	3
2	2	3	3	1	1	4	4	3	3
2	2	4	4	4	4	4	4	3	3
2	2	3	3	1	1	4	4	3	3
2	2	3	3	1	1	4	4	3	3
2	2	3	3	1	1	1	1	2	2
2	2	3	3	1	1	4	4	3	3
2	2	4	4	4	4	4	4	3	3
2	2	3	3	1	1	4	4	3	3
2	2	4	4	4	4	4	4	3	3
2	2	4	4	4	4	4	4	2	2
2	2	3	3	1	1	4	4	2	2
2	2	3	3	1	1	4	4	2	2
2	2	3	3	1	1	4	4	3	3
3	3	3	3	1	1	4	4	3	3
2	2	3	3	1	1	4	4	2	2
2	2	3	3	1	1	4	4	2	2
2	2	4	4	4	4	4	4	3	3
2	2	3	3	1	1	1	1	3	3
2	2	4	4	4	4	4	4	2	2
3	3	3	3	1	1	4	4	3	3

For Review Only

2	2	3	3	1	1	4	4	3	3
2	2	3	3	1	1	4	4	2	2
2	2	3	3	1	1	1	1	3	3
2	2	3	3	1	1	4	4	2	2
2	2	3	3	1	1	1	1	3	3
3	3	3	3	1	1	4	4	3	3
2	2	3	3	4	4	4	4	3	3
2	2	3	3	1	1	4	4	2	2
2	2	3	3	1	1	4	4	2	2
2	2	4	4	4	4	4	4	2	2
2	2	3	3	1	1	4	4	2	2
2	2	4	4	4	4	4	4	3	3
2	2	3	3	1	1	4	4	2	2
2	2	3	3	1	1	4	4	2	2
2	2	4	4	4	4	4	4	3	3
2	2	3	3	1	1	1	1	3	3
2	2	3	3	1	1	4	4	3	3
2	2	3	3	1	1	4	4	3	3
2	2	4	4	4	4	1	1	3	3
2	2	3	3	1	1	4	4	2	2
2	2	3	3	1	1	4	4	3	3
2	2	4	4	4	4	4	4	3	3
2	2	3	3	1	1	4	4	3	3
2	2	3	3	1	1	4	4	2	2
2	2	4	4	4	4	4	4	2	2
2	2	4	4	4	4	1	1	3	3
2	2	3	3	1	1	4	4	2	2
2	2	3	3	1	1	4	4	3	3
2	2	4	4	4	4	4	4	3	3
2	2	3	3	1	1	4	4	3	3
2	2	3	3	1	1	4	4	3	3
2	2	3	3	1	1	4	4	2	2
2	2	3	3	1	1	4	4	3	3
2	2	4	4	4	4	4	4	3	3
2	2	3	3	1	1	1	1	2	2
2	2	3	3	1	1	4	4	2	2
2	2	3	3	1	1	1	1	3	3
2	2	3	3	1	1	1	1	3	3
2	2	3	3	1	1	4	4	3	3
2	2	3	3	1	1	4	4	3	3
2	2	3	3	1	1	4	4	3	3
2	2	3	3	1	1	4	4	2	2
2	2	4	4	4	4	4	4	3	3
2	2	3	3	1	1	4	4	2	2
2	2	3	3	1	1	4	4	3	3
2	2	3	3	1	1	4	4	3	3
2	2	3	3	1	1	1	1	3	3
2	2	4	4	4	4	4	4	3	3
2	2	4	4	4	4	4	4	3	3
2	2	3	3	1	1	4	4	2	2
2	2	3	3	1	1	1	1	3	3
3	3	3	3	1	1	4	4	2	2
2	2	3	3	1	1	4	4	3	3
2	2	3	3	1	1	4	4	3	3
2	2	4	4	4	4	4	4	3	3

For Review Only

2	2	3	3	1	1	4	4	3	3
2	2	3	3	1	1	4	4	3	3
2	2	3	3	1	1	1	1	2	2
2	2	3	3	1	1	4	4	3	3
2	2	3	3	1	1	1	1	2	2
2	2	3	3	1	1	4	4	2	2
2	2	4	4	4	4	1	1	3	3
3	3	4	4	4	4	1	1	3	3
3	3	3	3	1	1	4	4	3	3
2	2	4	4	4	4	4	4	3	3
2	2	3	3	1	1	4	4	2	2
2	2	4	4	4	4	4	4	2	2
2	2	4	4	4	4	1	1	2	2
2	2	3	3	1	1	4	4	3	3
2	2	3	3	1	1	4	4	2	2
2	2	3	3	1	1	1	1	3	3
2	2	4	4	4	4	4	4	3	3
2	2	4	4	4	4	4	4	3	3
2	2	4	4	4	4	1	1	3	3
3	3	3	3	1	1	4	4	3	3
2	2	3	3	1	1	4	4	3	3
2	2	3	3	1	1	4	4	2	2
2	2	3	3	1	1	4	4	2	2
2	2	3	3	1	1	4	4	3	3
3	3	4	4	4	4	4	4	3	3
2	2	3	3	1	1	4	4	2	2
2	2	4	4	4	4	4	4	3	3
2	2	3	3	1	1	4	4	2	2
2	2	3	3	1	1	1	1	2	2
2	2	3	3	1	1	1	1	3	3
2	2	4	4	4	4	4	4	3	3
2	2	3	3	1	1	4	4	3	3
2	2	4	4	4	4	4	4	3	3
2	2	3	3	1	1	1	1	2	2
2	2	4	4	4	4	4	4	3	3
2	2	4	4	4	4	4	4	3	3
2	2	3	3	1	1	1	1	3	3
2	2	3	3	1	1	4	4	2	2
2	2	3	3	1	1	1	1	3	3
2	2	4	4	4	4	4	4	3	3
2	2	3	3	1	1	4	4	3	3
2	2	3	3	1	1	1	1	3	3
2	2	3	3	1	1	4	4	3	3
2	2	3	3	1	1	1	1	2	2
3	3	3	3	1	1	4	4	3	3
2	2	3	3	1	1	4	4	2	2
2	2	3	3	1	1	4	4	3	3
2	2	3	3	1	1	4	4	2	2
2	2	3	3	1	1	4	4	3	3
2	2	4	4	4	4	4	4	3	3
2	2	3	3	1	1	4	4	3	3
2	2	4	4	4	4	4	4	3	3
2	2	3	3	1	1	4	4	3	3
2	2	3	3	1	1	4	4	2	2
2	2	3	3	1	1	4	4	3	3

For Review Only

2	2	3	3	1	1	1	1	2	2
2	2	3	3	1	1	4	4	2	2

For Review Only

SNP1440	SNP1440	SNP1441	SNP1441	SNP1442	SNP1442	SNP1443	SNP1443	SNP1444	SNP1444
2	2	3	3	1	1	1	1	1	1
1	1	3	3	4	4	4	4	1	1
1	1	3	3	1	1	4	4	1	1
1	1	3	3	4	4	1	1	1	1
2	2	3	3	4	4	4	4	1	1
1	1	3	3	1	1	1	1	1	1
2	2	3	3	1	1	1	1	1	1
1	1	4	4	4	4	1	1	1	1
2	2	3	3	1	1	1	1	1	1
1	1	4	4	1	1	1	1	1	1
1	1	3	3	4	4	4	4	1	1
1	1	3	3	1	1	4	4	1	1
1	1	3	3	1	1	1	1	1	1
2	2	4	4	4	4	1	1	1	1
2	2	3	3	1	1	4	4	1	1
1	1	3	3	4	4	4	4	1	1
2	2	4	4	1	1	1	1	1	1
2	2	3	3	1	1	1	1	1	1
1	1	3	3	4	4	4	4	1	1
1	1	3	3	4	4	4	4	1	1
1	1	3	3	1	1	1	1	1	1
2	2	3	3	1	1	4	4	1	1
1	1	3	3	4	4	4	4	1	1
1	1	3	3	4	4	4	4	1	1
1	1	3	3	4	4	1	1	1	1
1	1	3	3	4	4	4	4	3	3
1	1	3	3	1	1	4	4	3	3
1	1	3	3	1	1	4	4	1	1
1	1	3	3	1	1	1	1	1	1
1	1	4	4	4	4	4	4	1	1
1	1	3	3	1	1	4	4	3	3
2	2	3	3	1	1	1	1	1	1
2	2	3	3	1	1	1	1	1	1
1	1	3	3	4	4	1	1	1	1
1	1	3	3	1	1	4	4	3	3
1	1	3	3	4	4	4	4	1	1
1	1	3	3	4	4	4	4	1	1
1	1	3	3	4	4	4	4	1	1
2	2	3	3	4	4	4	4	1	1
1	1	3	3	4	4	4	4	1	1
1	1	4	4	4	4	4	4	1	1
1	1	4	4	1	1	1	1	1	1
1	1	3	3	4	4	1	1	1	1
2	2	3	3	4	4	4	4	1	1
1	1	4	4	4	4	1	1	1	1
1	1	3	3	4	4	4	4	1	1
1	1	3	3	1	1	4	4	3	3
2	2	3	3	4	4	4	4	1	1
2	2	3	3	1	1	1	1	1	1
1	1	3	3	1	1	4	4	1	1
1	1	3	3	4	4	4	4	3	3
1	1	3	3	1	1	1	1	1	1

For Review Only

2	2	3	3	1	1	1	1	1	1
2	2	3	3	1	1	1	1	1	1
1	1	3	3	1	1	4	4	1	1
2	2	3	3	1	1	1	1	1	1
1	1	3	3	4	4	4	4	1	1
1	1	3	3	1	1	4	4	3	3
1	1	3	3	4	4	1	1	3	3
1	1	3	3	4	4	4	4	1	1
1	1	3	3	4	4	1	1	1	1
1	1	3	3	1	1	4	4	1	1
1	1	3	3	4	4	1	1	1	1
1	1	3	3	1	1	4	4	1	1
2	2	3	3	1	1	1	1	1	1
1	1	3	3	1	1	1	1	1	1
1	1	3	3	1	1	4	4	1	1
1	1	4	4	4	4	4	4	1	1
1	1	3	3	1	1	4	4	1	1
1	1	3	3	1	1	4	4	1	1
2	2	3	3	1	1	4	4	1	1
2	2	3	3	1	1	1	1	1	1
1	1	3	3	1	1	4	4	1	1
1	1	3	3	4	4	4	4	1	1
1	1	3	3	1	1	4	4	1	1
1	1	4	4	1	1	1	1	1	1
1	1	4	4	4	4	1	1	1	1
1	1	3	3	4	4	1	1	1	1
2	2	3	3	1	1	4	4	1	1
1	1	4	4	4	4	4	4	1	1
1	1	3	3	4	4	4	4	3	3
2	2	3	3	1	1	1	1	1	1
1	1	3	3	4	4	1	1	1	1
1	1	3	3	4	4	1	1	1	1
1	1	3	3	4	4	4	4	1	1
2	2	3	3	1	1	1	1	1	1
1	1	3	3	4	4	1	1	1	1
1	1	4	4	4	4	1	1	1	1
1	1	4	4	4	4	1	1	1	1
2	2	3	3	1	1	1	1	1	1
1	1	3	3	4	4	1	1	1	1
1	1	4	4	4	4	1	1	1	1
2	2	3	3	1	1	1	1	1	1
2	2	3	3	1	1	1	1	1	1
1	1	3	3	1	1	4	4	1	1
1	1	3	3	4	4	1	1	1	1
1	1	3	3	4	4	1	1	1	1
1	1	3	3	4	4	1	1	1	1
1	1	3	3	4	4	4	4	1	1
1	1	3	3	4	4	4	4	1	1
1	1	3	3	4	4	4	4	1	1
2	2	3	3	1	1	4	4	1	1
1	1	3	3	1	1	1	1	1	1
1	1	3	3	4	4	4	4	1	1
1	1	3	3	4	4	1	1	1	1
1	1	3	3	1	1	4	4	1	1
2	2	3	3	1	1	1	1	1	1

For Review Only

1	1	3	3	4	4	4	4	3	3
1	1	3	3	1	1	4	4	1	1
1	1	3	3	4	4	4	4	3	3
1	1	3	3	4	4	1	1	1	1
1	1	3	3	4	4	1	1	1	1
1	1	3	3	4	4	4	4	1	1
1	1	3	3	1	1	1	1	1	1
1	1	3	3	1	1	4	4	3	3
1	1	3	3	4	4	4	4	1	1
1	1	3	3	1	1	4	4	1	1
2	2	3	3	4	4	1	1	1	1
1	1	3	3	1	1	4	4	1	1
1	1	3	3	1	1	4	4	1	1
1	1	3	3	1	1	1	1	1	1
1	1	3	3	1	1	1	1	1	1
1	1	3	3	1	1	4	4	3	3
1	1	3	3	1	1	4	4	1	1
1	1	3	3	1	1	4	4	1	1
1	1	3	3	4	4	1	1	1	1
2	2	3	3	1	1	1	1	1	1
1	1	3	3	1	1	4	4	1	1
2	2	3	3	4	4	1	1	1	1
1	1	3	3	1	1	4	4	1	1
1	1	4	4	4	4	4	4	1	1
1	1	3	3	4	4	4	4	1	1
1	1	3	3	1	1	4	4	1	1
1	1	3	3	1	1	1	1	1	1
1	1	3	3	1	1	1	1	1	1
1	1	3	3	1	1	1	1	1	1
2	2	3	3	1	1	1	1	1	1
1	1	3	3	4	4	4	4	1	1
2	2	3	3	1	1	4	4	1	1
2	2	3	3	1	1	1	1	1	1
1	1	3	3	1	1	4	4	3	3
1	1	3	3	4	4	1	1	1	1
1	1	3	3	1	1	4	4	1	1
1	1	4	4	4	4	4	4	1	1
1	1	3	3	4	4	1	1	1	1
1	1	3	3	4	4	4	4	1	1
1	1	3	3	4	4	4	4	1	1
1	1	3	3	4	4	4	4	1	1
1	1	3	3	4	4	1	1	1	1
1	1	3	3	1	1	1	1	1	1
1	1	3	3	1	1	4	4	1	1
1	1	3	3	4	4	4	4	1	1
1	1	3	3	4	4	1	1	1	1
1	1	3	3	4	4	4	4	3	3
1	1	3	3	1	1	4	4	3	3
1	1	3	3	4	4	4	4	1	1
1	1	3	3	4	4	4	4	3	3
1	1	3	3	4	4	4	4	3	3
1	1	3	3	4	4	4	4	1	1
1	1	3	3	4	4	1	1	1	1
1	1	3	3	1	1	4	4	1	1
1	1	3	3	1	1	4	4	1	1

For Review Only

1	1	3	3	1	1	4	4	1	1
1	1	3	3	4	4	4	4	1	1
1	1	3	3	4	4	4	4	1	1
1	1	3	3	1	1	4	4	1	1
2	2	3	3	4	4	1	1	1	1
2	2	3	3	4	4	4	4	1	1
1	1	3	3	4	4	1	1	1	1
2	2	3	3	1	1	4	4	1	1
2	2	3	3	4	4	4	4	1	1
1	1	3	3	1	1	4	4	1	1
1	1	3	3	4	4	4	4	3	3
1	1	3	3	4	4	4	4	1	1
1	1	3	3	1	1	4	4	1	1
1	1	3	3	4	4	1	1	1	1
1	1	3	3	4	4	4	4	1	1
1	1	3	3	4	4	4	4	1	1
1	1	3	3	4	4	4	4	1	1
1	1	3	3	4	4	1	1	1	1
1	1	3	3	4	4	4	4	3	3
1	1	3	3	1	1	1	1	1	1
1	1	3	3	1	1	4	4	1	1
1	1	3	3	4	4	4	4	1	1
1	1	3	3	4	4	4	4	1	1
1	1	3	3	1	1	4	4	1	1
1	1	4	4	1	1	1	1	1	1
1	1	3	3	4	4	1	1	1	1
1	1	3	3	1	1	1	1	1	1
1	1	3	3	1	1	4	4	1	1
1	1	3	3	4	4	4	4	1	1
2	2	3	3	1	1	1	1	1	1
1	1	3	3	1	1	1	1	1	1
1	1	3	3	1	1	4	4	1	1
1	1	3	3	1	1	1	1	1	1
1	1	3	3	4	4	4	4	1	1
1	1	3	3	1	1	1	1	1	1
1	1	3	3	1	1	4	4	1	1
1	1	3	3	1	1	4	4	1	1
1	1	3	3	4	4	1	1	1	1
1	1	3	3	1	1	4	4	3	3
1	1	3	3	4	4	4	4	1	1
1	1	3	3	1	1	4	4	1	1
1	1	3	3	4	4	4	4	3	3
1	1	4	4	4	4	4	4	1	1
1	1	3	3	1	1	4	4	1	1
2	2	3	3	4	4	4	4	1	1
1	1	3	3	1	1	4	4	1	1
1	1	3	3	4	4	4	4	1	1
1	1	3	3	4	4	1	1	1	1
1	1	3	3	4	4	1	1	1	1
1	1	4	4	1	1	1	1	1	1
1	1	3	3	1	1	4	4	1	1
1	1	4	4	4	4	4	4	1	1
1	1	3	3	4	4	4	4	1	1
1	1	3	3	4	4	1	1	1	1
1	1	3	3	4	4	4	4	1	1

For Review Only

1	1	3	3	4	4	1	1	1	1
1	1	3	3	4	4	4	4	1	1
1	1	3	3	1	1	4	4	1	1
1	1	3	3	1	1	4	4	1	1
1	1	3	3	1	1	1	1	1	1
1	1	3	3	1	1	4	4	3	3
1	1	3	3	4	4	1	1	1	1
1	1	3	3	4	4	4	4	1	1
1	1	3	3	4	4	4	4	1	1
1	1	4	4	4	4	4	4	1	1
1	1	3	3	1	1	4	4	1	1
1	1	4	4	4	4	4	4	1	1
1	1	3	3	4	4	4	4	1	1
1	1	3	3	4	4	4	4	3	3
1	1	3	3	1	1	4	4	1	1
1	1	3	3	4	4	4	4	1	1
1	1	3	3	4	4	4	4	1	1
2	2	3	3	1	1	1	1	1	1
1	1	3	3	4	4	4	4	1	1
1	1	3	3	4	4	4	4	1	1
1	1	3	3	1	1	1	1	1	1
1	1	3	3	4	4	4	4	3	3
2	2	3	3	4	4	4	4	1	1
2	2	3	3	4	4	4	4	1	1
1	1	3	3	4	4	4	4	1	1
1	1	4	4	1	1	1	1	1	1
1	1	4	4	4	4	4	4	1	1
1	1	3	3	1	1	1	1	1	1
2	2	3	3	1	1	1	1	1	1
1	1	3	3	1	1	4	4	1	1
1	1	3	3	4	4	4	4	1	1
1	1	3	3	4	4	4	4	1	1
1	1	4	4	4	4	1	1	1	1
1	1	3	3	4	4	4	4	3	3
1	1	3	3	4	4	4	4	1	1
1	1	3	3	4	4	4	4	1	1
1	1	3	3	1	1	1	1	1	1
1	1	3	3	1	1	4	4	1	1
1	1	3	3	4	4	4	4	1	1
2	2	3	3	4	4	4	4	1	1
1	1	3	3	1	1	1	1	1	1
1	1	3	3	4	4	1	1	1	1
1	1	3	3	4	4	4	4	1	1
2	2	3	3	1	1	1	1	1	1
1	1	3	3	1	1	4	4	1	1
1	1	3	3	4	4	4	4	1	1
1	1	3	3	4	4	4	4	3	3
1	1	3	3	1	1	1	1	1	1
1	1	4	4	1	1	4	4	3	3
1	1	3	3	4	4	4	4	1	1
1	1	3	3	1	1	1	1	1	1
1	1	3	3	4	4	4	4	1	1
1	1	3	3	1	1	4	4	1	1
1	1	3	3	1	1	1	1	1	1
1	1	3	3	4	4	4	4	1	1

For Review Only

1	1	3	3	4	4	4	4	1	1
1	1	3	3	4	4	1	1	1	1

For Review Only

SNP1445	SNP1445	SNP1446	SNP1446	SNP1447	SNP1447	SNP1448	SNP1448	SNP1449	SNP1449
3	3	3	3	1	1	2	2	1	1
4	4	3	3	3	3	3	3	4	4
3	3	3	3	1	1	2	2	1	1
4	4	3	3	1	1	2	2	1	1
4	4	3	3	3	3	3	3	4	4
3	3	3	3	1	1	2	2	1	1
3	3	3	3	1	1	2	2	1	1
4	4	3	3	1	1	2	2	1	1
3	3	3	3	1	1	2	2	1	1
3	3	3	3	1	1	2	2	1	1
4	4	3	3	1	1	3	3	4	4
3	3	3	3	1	1	2	2	1	1
3	3	3	3	1	1	2	2	1	1
4	4	3	3	1	1	2	2	1	1
3	3	3	3	1	1	2	2	1	1
4	4	3	3	1	1	3	3	4	4
3	3	3	3	1	1	2	2	1	1
3	3	3	3	1	1	2	2	1	1
4	4	3	3	1	1	2	2	1	1
3	3	3	3	3	3	3	3	4	4
3	3	3	3	1	1	2	2	1	1
3	3	3	3	1	1	2	2	4	4
4	4	3	3	1	1	2	2	1	1
4	4	3	3	1	1	2	2	1	1
4	4	3	3	1	1	2	2	1	1
4	4	2	2	1	1	2	2	1	1
4	4	3	3	1	1	2	2	1	1
3	3	3	3	1	1	2	2	1	1
3	3	3	3	1	1	2	2	1	1
4	4	3	3	3	3	3	3	4	4
3	3	3	3	1	1	2	2	1	1
4	4	3	3	1	1	2	2	1	1
4	4	3	3	1	1	2	2	1	1
4	4	3	3	1	1	2	2	1	1
4	4	3	3	1	1	2	2	4	4
4	4	3	3	1	1	2	2	1	1
4	4	3	3	1	1	2	2	1	1
4	4	2	2	1	1	2	2	1	1
4	4	3	3	1	1	3	3	4	4
4	4	3	3	1	1	2	2	1	1
4	4	3	3	1	1	2	2	1	1
3	3	3	3	1	1	2	2	1	1
4	4	3	3	1	1	2	2	1	1
4	4	3	3	1	1	2	2	4	4
4	4	3	3	1	1	2	2	1	1
4	4	2	2	1	1	2	2	1	1
3	3	3	3	1	1	2	2	1	1
4	4	2	2	1	1	2	2	1	1
3	3	3	3	1	1	2	2	1	1
3	3	3	3	1	1	2	2	1	1
4	4	3	3	1	1	2	2	1	1
3	3	3	3	1	1	2	2	1	1

For Review Only

CSSA 5585 Guilford Rd., Madison, WI 53711

3	3	3	3	1	1	2	2	1	1
4	4	3	3	1	1	2	2	1	1
3	3	3	3	1	1	2	2	1	1
3	3	3	3	1	1	2	2	1	1
4	4	3	3	1	1	2	2	1	1
3	3	3	3	1	1	2	2	1	1
4	4	2	2	1	1	2	2	1	1
4	4	3	3	1	1	2	2	1	1
4	4	3	3	1	1	2	2	1	1
3	3	3	3	1	1	2	2	1	1
4	4	3	3	1	1	2	2	1	1
3	3	3	3	1	1	2	2	1	1
3	3	3	3	1	1	2	2	1	1
3	3	3	3	1	1	2	2	1	1
4	4	3	3	1	1	2	2	1	1
4	4	3	3	3	3	3	3	4	4
3	3	3	3	1	1	2	2	1	1
3	3	2	2	1	1	2	2	1	1
3	3	3	3	1	1	2	2	1	1
3	3	3	3	1	1	2	2	1	1
3	3	3	3	1	1	2	2	1	1
4	4	3	3	1	1	2	2	4	4
3	3	3	3	1	1	2	2	1	1
4	4	3	3	1	1	2	2	1	1
4	4	3	3	1	1	2	2	1	1
4	4	3	3	1	1	2	2	1	1
3	3	3	3	1	1	2	2	1	1
4	4	3	3	1	1	3	3	4	4
4	4	2	2	1	1	2	2	1	1
3	3	3	3	1	1	2	2	1	1
4	4	3	3	1	1	2	2	4	4
4	4	2	2	1	1	2	2	1	1
4	4	3	3	1	1	3	3	4	4
3	3	3	3	1	1	2	2	1	1
4	4	3	3	1	1	2	2	1	1
4	4	3	3	1	1	2	2	1	1
4	4	3	3	1	1	2	2	1	1
3	3	3	3	1	1	2	2	1	1
4	4	3	3	1	1	2	2	1	1
4	4	3	3	1	1	2	2	1	1
3	3	3	3	1	1	2	2	1	1
4	4	3	3	1	1	2	2	1	1
3	3	3	3	1	1	2	2	1	1
4	4	3	3	1	1	2	2	1	1
4	4	3	3	1	1	2	2	1	1
4	4	3	3	1	1	2	2	1	1
4	4	3	3	1	1	2	2	1	1
4	4	2	2	1	1	2	2	1	1
4	4	3	3	1	1	2	2	1	1
3	3	3	3	1	1	2	2	1	1
3	3	3	3	1	1	2	2	1	1
4	4	3	3	1	1	3	3	4	4
4	4	3	3	1	1	2	2	1	1
4	4	3	3	1	1	2	2	1	1
3	3	3	3	1	1	2	2	1	1

For Review Only

4	4	3	3	1	1	2	2	1	1
4	4	3	3	1	1	2	2	1	1
4	4	3	3	1	1	2	2	1	1
4	4	3	3	1	1	2	2	1	1
4	4	3	3	1	1	2	2	1	1
4	4	2	2	1	1	2	2	1	1
3	3	3	3	1	1	2	2	1	1
4	4	3	3	1	1	2	2	1	1
4	4	3	3	1	1	2	2	1	1
4	4	3	3	1	1	2	2	1	1
4	4	3	3	1	1	2	2	1	1
3	3	2	2	1	1	2	2	1	1
3	3	3	3	1	1	2	2	1	1
4	4	3	3	1	1	2	2	1	1
3	3	3	3	1	1	2	2	1	1
3	3	3	3	1	1	2	2	1	1
3	3	2	2	1	1	2	2	1	1
3	3	3	3	1	1	2	2	1	1
4	4	3	3	1	1	2	2	1	1
3	3	3	3	1	1	2	2	1	1
3	3	3	3	1	1	2	2	1	1
4	4	3	3	1	1	2	2	1	1
3	3	2	2	1	1	2	2	1	1
4	4	3	3	1	1	3	3	4	4
4	4	3	3	1	1	2	2	1	1
4	4	2	2	1	1	2	2	1	1
3	3	3	3	1	1	2	2	1	1
3	3	2	2	1	1	2	2	1	1
3	3	3	3	1	1	2	2	1	1
3	3	3	3	1	1	2	2	1	1
4	4	3	3	1	1	2	2	1	1
3	3	3	3	1	1	2	2	1	1
3	3	3	3	1	1	2	2	1	1
4	4	2	2	1	1	2	2	1	1
4	4	3	3	1	1	2	2	1	1
4	4	3	3	1	1	3	3	1	1
4	4	3	3	1	1	2	2	1	1
4	4	2	2	1	1	2	2	1	1
4	4	3	3	1	1	2	2	1	1
4	4	3	3	1	1	3	3	4	4
4	4	2	2	1	1	2	2	1	1
4	4	3	3	1	1	2	2	1	1
3	3	3	3	1	1	2	2	1	1
4	4	3	3	3	3	2	2	4	4
4	4	2	2	1	1	2	2	1	1
4	4	3	3	1	1	2	2	1	1
4	4	2	2	1	1	2	2	1	1
3	3	3	3	1	1	2	2	1	1
4	4	3	3	1	1	2	2	1	1
4	4	2	2	1	1	2	2	1	1
4	4	3	3	1	1	2	2	1	1
4	4	3	3	1	1	2	2	1	1
4	4	3	3	1	1	2	2	1	1
3	3	3	3	1	1	2	2	1	1
3	3	3	3	1	1	2	2	1	1

For Review Only

3	3	3	3	1	1	2	2	1	1
4	4	3	3	1	1	2	2	1	1
4	4	3	3	3	3	3	3	4	4
3	3	2	2	1	1	2	2	1	1
4	4	3	3	1	1	2	2	1	1
4	4	3	3	1	1	2	2	1	1
4	4	3	3	1	1	2	2	1	1
3	3	3	3	1	1	2	2	1	1
4	4	3	3	1	1	2	2	1	1
3	3	2	2	1	1	2	2	1	1
4	4	2	2	1	1	2	2	1	1
4	4	3	3	1	1	3	3	4	4
4	4	3	3	1	1	2	2	1	1
4	4	3	3	1	1	2	2	1	1
4	4	3	3	1	1	2	2	1	1
4	4	3	3	1	1	3	3	4	4
4	4	3	3	1	1	2	2	4	4
4	4	3	3	1	1	2	2	1	1
4	4	2	2	1	1	2	2	1	1
3	3	3	3	1	1	2	2	1	1
4	4	3	3	1	1	2	2	1	1
4	4	3	3	1	1	2	2	4	4
4	4	3	3	1	1	2	2	4	4
3	3	3	3	1	1	2	2	1	1
3	3	3	3	1	1	2	2	1	1
4	4	3	3	1	1	2	2	1	1
3	3	2	2	1	1	2	2	1	1
3	3	2	2	1	1	2	2	1	1
4	4	2	2	1	1	2	2	1	1
3	3	3	3	1	1	2	2	1	1
3	3	3	3	1	1	2	2	1	1
3	3	3	3	1	1	2	2	1	1
3	3	3	3	1	1	2	2	1	1
4	4	3	3	1	1	2	2	1	1
3	3	3	3	1	1	2	2	1	1
3	3	3	3	1	1	2	2	1	1
4	4	3	3	1	1	2	2	1	1
4	4	3	3	1	1	2	2	1	1
3	3	3	3	1	1	2	2	1	1
4	4	3	3	1	1	2	2	1	1
3	3	3	3	1	1	2	2	1	1
4	4	3	3	1	1	2	2	1	1
4	4	3	3	1	1	3	3	4	4
3	3	3	3	1	1	2	2	1	1
4	4	3	3	1	1	2	2	4	4
3	3	3	3	1	1	2	2	1	1
4	4	3	3	1	1	3	3	4	4
4	4	3	3	1	1	2	2	1	1
4	4	3	3	1	1	2	2	1	1
3	3	3	3	1	1	2	2	1	1
3	3	2	2	1	1	2	2	1	1
4	4	3	3	1	1	2	2	1	1
4	4	3	3	3	3	3	3	4	4
4	4	3	3	1	1	2	2	1	1
4	4	3	3	1	1	2	2	1	1

4	4	3	3	1	1	2	2	1	1
4	4	3	3	3	3	3	3	4	4
3	3	3	3	1	1	2	2	1	1
3	3	3	3	1	1	2	2	1	1
3	3	3	3	1	1	2	2	1	1
3	3	2	2	1	1	2	2	1	1
4	4	3	3	1	1	2	2	1	1
4	4	3	3	3	3	3	3	4	4
4	4	3	3	1	1	2	2	1	1
4	4	3	3	1	1	3	3	4	4
3	3	3	3	1	1	2	2	1	1
4	4	2	2	1	1	2	2	1	1
4	4	3	3	1	1	2	2	1	1
4	4	3	3	1	1	2	2	1	1
4	4	3	3	1	1	2	2	1	1
4	4	3	3	1	1	3	3	4	4
4	4	3	3	1	1	3	3	4	4
3	3	3	3	1	1	2	2	1	1
4	4	3	3	1	1	3	3	4	4
4	4	2	2	1	1	2	2	1	1
3	3	2	2	1	1	2	2	1	1
4	4	2	2	1	1	2	2	1	1
4	4	3	3	1	1	2	2	1	1
4	4	2	2	1	1	3	3	4	4
4	4	3	3	1	1	2	2	1	1
3	3	3	3	1	1	2	2	4	4
4	4	3	3	1	1	3	3	4	4
3	3	3	3	1	1	2	2	1	1
3	3	3	3	1	1	2	2	1	1
3	3	3	3	1	1	2	2	1	1
4	4	3	3	1	1	3	3	4	4
4	4	3	3	1	1	2	2	1	1
4	4	3	3	1	1	2	2	1	1
4	4	2	2	1	1	2	2	1	1
4	4	3	3	1	1	2	2	1	1
4	4	3	3	1	1	3	3	4	4
3	3	3	3	1	1	2	2	1	1
3	3	3	3	1	1	2	2	1	1
4	4	3	3	1	1	2	2	1	1
4	4	3	3	3	3	3	3	4	4
3	3	3	3	1	1	2	2	1	1
4	4	3	3	1	1	2	2	1	1
4	4	3	3	1	1	3	3	4	4
3	3	3	3	1	1	2	2	1	1
4	4	3	3	1	1	2	2	1	1
4	4	3	3	1	1	2	2	1	1
4	4	3	3	1	1	2	2	1	1
4	4	2	2	1	1	2	2	1	1
3	3	3	3	3	3	3	3	4	4
4	4	3	3	1	1	3	3	4	4
3	3	3	3	1	1	2	2	1	1
4	4	3	3	1	1	3	3	4	4
3	3	3	3	1	1	2	2	1	1
3	3	3	3	1	1	2	2	1	1
4	4	3	3	1	1	2	2	1	1

For Review Only

4	4	2	2	1	1	2	2	1	1
4	4	3	3	1	1	2	2	1	1

For Review Only

SNP145	SNP145	SNP1450	SNP1450	SNP1451	SNP1451	SNP1452	SNP1452	SNP1453	SNP1453
1	1	2	2	4	4	3	3	4	4
1	1	4	4	4	4	3	3	4	4
1	1	2	2	1	1	2	2	4	4
1	1	2	2	4	4	3	3	3	3
1	1	2	2	4	4	3	3	4	4
1	1	4	4	4	4	3	3	4	4
4	4	2	2	4	4	3	3	4	4
1	1	2	2	4	4	3	3	4	4
4	4	4	4	4	4	3	3	4	4
1	1	4	4	4	4	3	3	4	4
4	4	2	2	1	1	2	2	4	4
1	1	2	2	1	1	2	2	4	4
4	4	4	4	4	4	3	3	4	4
1	1	4	4	4	4	3	3	4	4
4	4	2	2	1	1	2	2	4	4
1	1	2	2	4	4	3	3	4	4
1	1	4	4	4	4	3	3	4	4
1	1	4	4	4	4	3	3	4	4
1	1	4	4	4	4	3	3	4	4
1	1	4	4	4	4	3	3	4	4
1	1	4	4	4	4	3	3	4	4
4	4	4	4	4	4	3	3	4	4
1	1	4	4	4	4	3	3	4	4
1	1	4	4	4	4	3	3	4	4
1	1	4	4	4	4	3	3	4	4
1	1	2	2	1	1	2	2	3	3
1	1	2	2	4	4	3	3	4	4
1	1	2	2	1	1	2	2	4	4
1	1	4	4	4	4	3	3	4	4
1	1	4	4	4	4	3	3	4	4
4	4	2	2	1	1	2	2	4	4
4	4	2	2	4	4	3	3	4	4
4	4	2	2	4	4	3	3	4	4
4	4	4	4	4	4	3	3	4	4
1	1	2	2	4	4	3	3	4	4
1	1	4	4	4	4	3	3	4	4
1	1	4	4	4	4	3	3	4	4
1	1	2	2	1	1	2	2	4	4
1	1	4	4	4	4	3	3	4	4
1	1	2	2	1	1	2	2	4	4
1	1	4	4	4	4	3	3	4	4
1	1	2	2	4	4	3	3	4	4
1	1	4	4	4	4	3	3	4	4
1	1	2	2	4	4	3	3	4	4
1	1	2	2	4	4	3	3	4	4
1	1	2	2	1	1	2	2	4	4
1	1	2	2	1	1	2	2	3	3
1	1	2	2	1	1	2	2	4	4
4	4	2	2	4	4	3	3	4	4
4	4	2	2	1	1	2	2	4	4
1	1	2	2	1	1	2	2	4	4
4	4	2	2	4	4	3	3	3	3

For Review Only

4	4	4	4	4	4	3	3	4	4
4	4	2	2	4	4	3	3	4	4
4	4	2	2	1	1	2	2	4	4
4	4	4	4	4	4	3	3	4	4
1	1	4	4	4	4	3	3	4	4
1	1	2	2	1	1	2	2	3	3
1	1	2	2	1	1	2	2	3	3
1	1	4	4	4	4	3	3	4	4
1	1	4	4	4	4	3	3	4	4
1	1	2	2	1	1	3	3	4	4
1	1	2	2	1	1	2	2	3	3
1	1	2	2	1	1	2	2	3	3
4	4	4	4	4	4	3	3	4	4
1	1	4	4	4	4	3	3	4	4
4	4	2	2	4	4	3	3	4	4
1	1	2	2	1	1	3	3	4	4
1	1	2	2	4	4	3	3	4	4
1	1	2	2	1	1	2	2	4	4
4	4	4	4	4	4	3	3	4	4
1	1	2	2	4	4	3	3	4	4
1	1	2	2	1	1	2	2	4	4
1	1	2	2	1	1	2	2	4	4
1	1	2	2	4	4	3	3	4	4
1	1	2	2	4	4	3	3	4	4
1	1	2	2	4	4	3	3	4	4
1	1	2	2	1	1	2	2	3	3
4	4	2	2	4	4	3	3	4	4
1	1	4	4	4	4	3	3	4	4
1	1	2	2	1	1	2	2	3	3
4	4	2	2	1	1	2	2	4	4
4	4	2	2	4	4	3	3	4	4
1	1	4	4	4	4	3	3	3	3
1	1	4	4	4	4	3	3	4	4
4	4	4	4	4	4	3	3	4	4
1	1	2	2	4	4	3	3	3	3
1	1	2	2	4	4	3	3	4	4
1	1	2	2	4	4	3	3	4	4
4	4	4	4	4	4	3	3	4	4
1	1	4	4	4	4	3	3	4	4
1	1	2	2	4	4	3	3	4	4
1	1	4	4	4	4	3	3	4	4
1	1	2	2	1	1	2	2	4	4
1	1	2	2	4	4	3	3	4	4
1	1	2	2	1	1	2	2	3	3
1	1	4	4	4	4	3	3	4	4
4	4	2	2	4	4	3	3	4	4
4	4	4	4	4	4	3	3	4	4
1	1	2	2	1	1	2	2	3	3
1	1	2	2	1	1	2	2	3	3
1	1	2	2	1	1	2	2	4	4
1	1	2	2	1	1	2	2	3	3
1	1	4	4	4	4	3	3	4	4
1	1	4	4	4	4	3	3	4	4
1	1	2	2	4	4	3	3	4	4
1	1	4	4	1	1	2	2	4	4

For Review Only

1	1	2	2	1	1	2	2	3	3
1	1	2	2	4	4	3	3	4	4
1	1	4	4	1	1	2	2	4	4
1	1	4	4	4	4	3	3	4	4
1	1	4	4	1	1	2	2	4	4
1	1	4	4	1	1	2	2	4	4
1	1	2	2	4	4	3	3	4	4
1	1	2	2	1	1	2	2	4	4
1	1	2	2	1	1	2	2	3	3
1	1	2	2	1	1	2	2	4	4
4	4	4	4	4	4	3	3	4	4
1	1	2	2	1	1	2	2	4	4
1	1	2	2	4	4	3	3	3	3
1	1	2	2	1	1	2	2	4	4
1	1	2	2	4	4	3	3	4	4
1	1	2	2	1	1	2	2	4	4
1	1	2	2	1	1	2	2	3	3
1	1	2	2	4	4	2	2	4	4
1	1	4	4	4	4	3	3	4	4
4	4	4	4	1	1	3	3	4	4
1	1	2	2	1	1	2	2	4	4
4	4	2	2	1	1	2	2	4	4
1	1	2	2	4	4	3	3	4	4
1	1	4	4	4	4	3	3	4	4
1	1	2	2	1	1	2	2	4	4
1	1	2	2	4	4	3	3	4	4
4	4	4	4	4	4	3	3	4	4
1	1	2	2	4	4	3	3	4	4
1	1	2	2	4	4	3	3	4	4
1	1	2	2	4	4	3	3	4	4
1	1	4	4	4	4	3	3	4	4
1	1	2	2	1	1	2	2	4	4
1	1	4	4	4	4	3	3	4	4
1	1	2	2	1	1	2	2	4	4
4	4	4	4	4	4	2	2	4	4
4	4	2	2	4	4	3	3	4	4
4	4	4	4	4	4	3	3	4	4
4	4	4	4	1	1	2	2	4	4
1	1	2	2	1	1	2	2	4	4
1	1	2	2	1	1	2	2	4	4
1	1	2	2	1	1	2	2	4	4
1	1	4	4	4	4	3	3	4	4
1	1	4	4	1	1	2	2	4	4
1	1	2	2	4	4	3	3	4	4
1	1	4	4	4	4	3	3	4	4
1	1	2	2	4	4	3	3	4	4
1	1	2	2	1	1	2	2	3	3
1	1	2	2	1	1	2	2	3	3
1	1	2	2	4	4	3	3	4	4
1	1	2	2	1	1	2	2	4	4
1	1	2	2	1	1	2	2	3	3
1	1	4	4	4	4	3	3	4	4
4	4	2	2	4	4	3	3	4	4
1	1	4	4	1	1	2	2	4	4
1	1	2	2	4	4	3	3	4	4

For Review Only

1	1	2	2	1	1	2	2	4	4
1	1	2	2	1	1	2	2	3	3
1	1	2	2	4	4	3	3	4	4
1	1	2	2	4	4	3	3	4	4
1	1	2	2	4	4	3	3	3	3
1	1	2	2	4	4	3	3	3	3
1	1	4	4	1	1	2	2	4	4
1	1	2	2	1	1	2	2	4	4
1	1	4	4	4	4	3	3	4	4
4	4	2	2	4	4	3	3	4	4
1	1	2	2	1	1	2	2	4	4
1	1	4	4	1	1	3	3	4	4
4	4	2	2	1	1	2	2	4	4
4	4	2	2	4	4	3	3	3	3
4	4	4	4	4	4	3	3	4	4
4	4	2	2	1	1	2	2	4	4
4	4	2	2	4	4	3	3	4	4
1	1	2	2	4	4	3	3	4	4
1	1	4	4	4	4	3	3	4	4
1	1	2	2	4	4	3	3	4	4
4	4	2	2	1	1	2	2	4	4
1	1	4	4	4	4	3	3	4	4
1	1	2	2	1	1	2	2	4	4
1	1	2	2	1	1	2	2	3	3
1	1	2	2	4	4	3	3	3	3
4	4	4	4	4	4	3	3	4	4
1	1	2	2	1	1	2	2	3	3
1	1	2	2	4	4	3	3	4	4
1	1	4	4	1	1	2	2	4	4
4	4	4	4	4	4	3	3	4	4
1	1	4	4	1	1	2	2	4	4
1	1	2	2	1	1	2	2	4	4
4	4	2	2	4	4	3	3	3	3
4	4	4	4	4	4	3	3	4	4
1	1	2	2	4	4	3	3	4	4
1	1	2	2	1	1	2	2	3	3
1	1	2	2	4	4	3	3	3	3
1	1	4	4	4	4	3	3	4	4
1	1	2	2	1	1	2	2	3	3
1	1	2	2	4	4	3	3	4	4
1	1	2	2	1	1	2	2	4	4
1	1	2	2	1	1	2	2	3	3
1	1	4	4	4	4	3	3	4	4
4	4	2	2	1	1	2	2	4	4
4	4	2	2	4	4	3	3	4	4
1	1	2	2	4	4	3	3	3	3
1	1	2	2	1	1	2	2	4	4
1	1	4	4	4	4	3	3	4	4
1	1	4	4	4	4	3	3	4	4
1	1	2	2	4	4	3	3	4	4
1	1	2	2	4	4	3	3	4	4
1	1	4	4	4	4	3	3	4	4
1	1	2	2	1	1	2	2	4	4
1	1	2	2	1	1	2	2	3	3
1	1	4	4	4	4	3	3	4	4

For Review Only

4	4	2	2	4	4	3	3	4	4
4	4	2	2	4	4	3	3	4	4
1	1	2	2	4	4	3	3	4	4
4	4	2	2	1	1	2	2	4	4
1	1	2	2	4	4	3	3	4	4
1	1	2	2	1	1	2	2	3	3
1	1	4	4	4	4	3	3	4	4
1	1	4	4	4	4	3	3	4	4
1	1	2	2	4	4	2	2	4	4
1	1	4	4	4	4	3	3	4	4
1	1	2	2	1	1	2	2	4	4
1	1	4	4	1	1	2	2	4	4
1	1	4	4	4	4	3	3	4	4
1	1	2	2	1	1	2	2	4	4
1	1	2	2	1	1	2	2	4	4
4	4	2	2	1	1	2	2	4	4
1	1	4	4	1	1	2	2	4	4
1	1	4	4	4	4	3	3	4	4
1	1	4	4	4	4	3	3	4	4
1	1	4	4	1	1	2	2	4	4
1	1	2	2	4	4	3	3	4	4
1	1	2	2	1	1	2	2	3	3
1	1	2	2	4	4	3	3	4	4
1	1	2	2	1	1	2	2	4	4
1	1	4	4	4	4	3	3	4	4
4	4	4	4	4	4	3	3	4	4
1	1	4	4	4	4	3	3	4	4
1	1	2	2	1	1	2	2	3	3
4	4	2	2	4	4	3	3	4	4
1	1	2	2	4	4	3	3	4	4
1	1	4	4	4	4	3	3	4	4
1	1	2	2	1	1	2	2	4	4
4	4	2	2	4	4	3	3	3	3
1	1	2	2	1	1	2	2	4	4
1	1	2	2	1	1	2	2	4	4
1	1	4	4	1	1	3	3	4	4
4	4	2	2	4	4	3	3	4	4
1	1	2	2	1	1	2	2	3	3
1	1	4	4	4	4	3	3	4	4
1	1	4	4	4	4	3	3	4	4
1	1	4	4	1	1	2	2	4	4
1	1	2	2	4	4	3	3	4	4
1	1	4	4	1	1	2	2	4	4
4	4	4	4	4	4	3	3	4	4
1	1	2	2	4	4	3	3	3	3
1	1	2	2	4	4	3	3	3	3
1	1	2	2	1	1	2	2	3	3
1	1	2	2	4	4	3	3	4	4
1	1	2	2	1	1	2	2	4	4
1	1	4	4	4	4	3	3	4	4
4	4	2	2	4	4	3	3	4	4
1	1	4	4	4	4	3	3	4	4
1	1	2	2	1	1	2	2	4	4
1	1	2	2	4	4	3	3	3	3
1	1	2	2	1	1	2	2	4	4

For Review Only

4	4	2	2	1	1	2	2	4	4
1	1	4	4	1	1	2	2	3	3

For Review Only

SNP1454	SNP1454	SNP1455	SNP1455	SNP1456	SNP1456	SNP1457	SNP1457	SNP1458	SNP1458
4	4	4	4	2	2	4	4	4	4
1	1	4	4	4	4	3	3	2	2
1	1	4	4	4	4	3	3	2	2
4	4	4	4	4	4	4	4	4	4
1	1	4	4	4	4	3	3	2	2
4	4	4	4	4	4	4	4	4	4
4	4	4	4	4	4	4	4	4	4
1	1	1	1	4	4	3	3	2	2
4	4	4	4	4	4	4	4	2	2
4	4	4	4	4	4	4	4	4	4
1	1	4	4	4	4	3	3	2	2
1	1	4	4	4	4	4	4	2	2
1	1	4	4	4	4	4	4	2	2
4	4	4	4	4	4	3	3	2	2
1	1	4	4	4	4	4	4	2	2
4	4	4	4	4	4	4	4	4	4
4	4	4	4	4	4	4	4	4	4
4	4	4	4	4	4	4	4	4	4
4	4	4	4	4	4	4	4	4	4
4	4	4	4	4	4	4	4	4	4
1	1	4	4	4	4	3	3	2	2
4	4	4	4	4	4	4	4	4	4
1	1	4	4	2	2	3	3	2	2
4	4	1	1	4	4	3	3	2	2
4	4	4	4	4	4	4	4	4	4
1	1	4	4	4	4	4	4	4	4
4	4	4	4	4	4	4	4	4	4
1	1	4	4	4	4	4	4	2	2
4	4	4	4	4	4	4	4	4	4
1	1	4	4	4	4	3	3	2	2
1	1	4	4	4	4	3	3	2	2
4	4	4	4	4	4	4	4	4	4
4	4	4	4	4	4	4	4	4	4
4	4	4	4	4	4	4	4	4	4
1	1	4	4	2	2	4	4	2	2
4	4	4	4	4	4	4	4	4	4
1	1	4	4	2	2	3	3	2	2
1	1	4	4	4	4	3	3	2	2
1	1	4	4	4	4	3	3	2	2
1	1	4	4	2	2	3	3	2	2
1	1	4	4	4	4	3	3	2	2
1	1	4	4	4	4	3	3	2	2
4	4	1	1	4	4	4	4	4	4
1	1	4	4	4	4	3	3	2	2
4	4	1	1	4	4	4	4	4	4
1	1	4	4	4	4	3	3	2	2
1	1	4	4	4	4	4	4	4	4
1	1	4	4	4	4	4	4	2	2
1	1	4	4	4	4	3	3	2	2
1	1	4	4	4	4	4	4	2	2
1	1	4	4	4	4	4	4	4	4
1	1	4	4	2	2	3	3	2	2

For Review Only

4	4	4	4	4	4	4	4	4	4
4	4	4	4	4	4	4	4	4	4
1	1	4	4	4	4	3	3	2	2
1	1	4	4	4	4	3	3	2	2
4	4	4	4	4	4	4	4	4	4
1	1	4	4	4	4	3	3	2	2
1	1	4	4	4	4	4	4	4	4
1	1	4	4	4	4	3	3	2	2
1	1	4	4	2	2	3	3	2	2
1	1	4	4	4	4	4	4	2	2
1	1	4	4	4	4	3	3	2	2
1	1	4	4	4	4	3	3	2	2
4	4	4	4	4	4	4	4	4	4
4	4	4	4	4	4	4	4	4	4
1	1	4	4	4	4	4	4	2	2
1	1	4	4	2	2	3	3	2	2
1	1	4	4	4	4	3	3	2	2
1	1	4	4	4	4	3	3	2	2
4	4	4	4	4	4	4	4	4	4
4	4	4	4	4	4	4	4	4	4
1	1	4	4	4	4	3	3	2	2
1	1	4	4	2	2	3	3	2	2
4	4	4	4	4	4	4	4	4	4
4	4	4	4	4	4	4	4	4	4
4	4	1	1	4	4	4	4	4	4
1	1	4	4	4	4	3	3	2	2
1	1	4	4	4	4	3	3	2	2
1	1	4	4	4	4	3	3	2	2
1	1	4	4	4	4	4	4	4	4
1	1	4	4	4	4	3	3	2	2
4	4	4	4	4	4	4	4	4	4
4	4	1	1	4	4	4	4	4	4
1	1	4	4	4	4	3	3	2	2
4	4	4	4	4	4	4	4	4	4
4	4	4	4	4	4	4	4	4	4
4	4	1	1	4	4	4	4	4	4
1	1	4	4	4	4	3	3	2	2
1	1	4	4	4	4	3	3	2	2
1	1	4	4	4	4	3	3	2	2
1	1	1	1	4	4	3	3	2	2
4	4	4	4	4	4	4	4	4	4
1	1	4	4	4	4	3	3	2	2
4	4	4	4	4	4	3	3	4	4
1	1	4	4	4	4	3	3	2	2
1	1	4	4	4	4	3	3	2	2
4	4	4	4	4	4	4	4	4	4
4	4	1	1	4	4	3	3	2	2
1	1	4	4	4	4	4	4	4	4
1	1	4	4	4	4	4	4	4	4
1	1	4	4	2	2	3	3	2	2
4	4	4	4	4	4	4	4	4	4
1	1	4	4	4	4	3	3	4	4
1	1	4	4	4	4	3	3	2	2
1	1	4	4	2	2	4	4	2	2
1	1	4	4	4	4	3	3	2	2

For Review Only

1	1	4	4	2	2	3	3	2	2
1	1	4	4	4	4	3	3	2	2
1	1	4	4	4	4	4	4	4	4
1	1	4	4	4	4	3	3	2	2
4	4	4	4	4	4	4	4	4	4
1	1	1	1	4	4	4	4	4	4
4	4	4	4	4	4	4	4	4	4
1	1	4	4	4	4	4	4	2	2
1	1	4	4	4	4	3	3	2	2
1	1	4	4	4	4	4	4	4	4
1	1	4	4	2	2	4	4	4	4
1	1	4	4	4	4	4	4	2	2
4	4	4	4	4	4	3	3	4	4
1	1	4	4	4	4	4	4	4	4
1	1	4	4	4	4	4	4	2	2
1	1	4	4	4	4	4	4	2	2
1	1	4	4	4	4	3	3	2	2
1	1	4	4	4	4	4	4	2	2
1	1	4	4	4	4	3	3	2	2
4	4	4	4	4	4	4	4	4	4
1	1	4	4	4	4	4	4	2	2
1	1	4	4	4	4	3	3	2	2
4	4	4	4	4	4	4	4	4	4
1	1	4	4	4	4	3	3	2	2
1	1	4	4	2	2	3	3	2	2
1	1	4	4	4	4	3	3	2	2
1	1	4	4	4	4	4	4	2	2
4	4	4	4	4	4	4	4	4	4
4	4	4	4	4	4	4	4	4	4
4	4	4	4	4	4	4	4	4	4
1	1	4	4	4	4	3	3	2	2
1	1	4	4	4	4	4	4	2	2
4	4	4	4	4	4	4	4	4	4
1	1	4	4	4	4	4	4	2	2
4	4	4	4	4	4	4	4	4	4
1	1	4	4	4	4	4	4	2	2
1	1	4	4	4	4	3	3	2	2
1	1	4	4	4	4	3	3	2	2
1	1	4	4	4	4	4	4	4	4
1	1	4	4	4	4	3	3	2	2
1	1	4	4	4	4	3	3	2	2
1	1	4	4	4	4	3	3	2	2
1	1	4	4	4	4	3	3	2	2
1	1	4	4	4	4	4	4	2	2
4	4	4	4	4	4	3	3	4	4
1	1	4	4	4	4	3	3	2	2
1	1	4	4	4	4	4	4	4	4
1	1	4	4	4	4	4	4	4	4
1	1	4	4	2	2	4	4	2	2
1	1	4	4	4	4	4	4	4	4
1	1	4	4	4	4	4	4	4	4
1	1	4	4	4	4	3	3	2	2
4	4	4	4	4	4	4	4	4	4
1	1	4	4	4	4	4	4	4	4
1	1	4	4	4	4	4	4	2	2

For Review Only

1	1	4	4	4	4	3	3	2	2
1	1	4	4	4	4	3	3	2	2
4	4	1	1	4	4	4	4	4	4
1	1	4	4	4	4	4	4	2	2
4	4	4	4	4	4	4	4	4	4
1	1	4	4	2	2	3	3	2	2
1	1	4	4	4	4	3	3	2	2
1	1	4	4	4	4	4	4	2	2
1	1	4	4	4	4	3	3	2	2
4	4	4	4	4	4	4	4	4	4
1	1	4	4	4	4	4	4	2	2
1	1	4	4	4	4	3	3	2	2
1	1	1	1	4	4	3	3	2	2
1	1	4	4	4	4	3	3	2	2
1	1	4	4	4	4	3	3	2	2
1	1	4	4	4	4	3	3	2	2
4	4	4	4	4	4	4	4	2	2
4	4	4	4	4	4	4	4	4	4
4	4	1	1	4	4	4	4	4	4
4	4	4	4	4	4	4	4	4	4
1	1	4	4	4	4	4	4	2	2
1	1	1	1	4	4	3	3	2	2
1	1	4	4	2	2	3	3	2	2
1	1	4	4	4	4	3	3	2	2
1	1	4	4	4	4	3	3	2	2
4	4	4	4	4	4	4	4	4	4
1	1	4	4	4	4	4	4	2	2
1	1	4	4	4	4	4	4	2	2
1	1	4	4	4	4	3	3	2	2
1	1	4	4	4	4	3	3	2	2
1	1	4	4	4	4	3	3	2	2
1	1	4	4	4	4	3	3	2	2
1	1	4	4	4	4	3	3	2	2
1	1	4	4	4	4	3	3	2	2
4	4	4	4	2	2	4	4	4	4
1	1	4	4	4	4	3	3	2	2
4	4	4	4	2	2	4	4	2	2
4	4	4	4	2	2	4	4	4	4
1	1	4	4	4	4	4	4	4	4
4	4	4	4	4	4	4	4	4	4
1	1	4	4	4	4	3	3	2	2
1	1	4	4	4	4	4	4	4	4
1	1	4	4	4	4	3	3	2	2
1	1	4	4	2	2	4	4	2	2
1	1	4	4	4	4	4	4	4	4
4	4	4	4	2	2	3	3	2	2
1	1	4	4	4	4	3	3	2	2
1	1	4	4	4	4	3	3	2	2
4	4	4	4	4	4	4	4	4	4
1	1	4	4	4	4	3	3	2	2
4	4	4	4	4	4	4	4	4	4
1	1	4	4	2	2	3	3	2	2
1	1	4	4	2	2	3	3	2	2
1	1	4	4	4	4	4	4	2	2
1	1	4	4	4	4	3	3	2	2

For Review Only

4	4	4	4	4	4	3	3	4	4
1	1	1	1	4	4	3	3	2	2
4	4	4	4	4	4	4	4	4	4
1	1	4	4	2	2	3	3	2	2
1	1	4	4	4	4	3	3	2	2
1	1	4	4	4	4	4	4	4	4
4	4	4	4	4	4	4	4	4	4
4	4	4	4	4	4	4	4	4	4
1	1	4	4	4	4	4	4	2	2
1	1	4	4	4	4	3	3	2	2
1	1	4	4	4	4	4	4	2	2
1	1	4	4	4	4	3	3	2	2
4	4	4	4	4	4	4	4	4	4
1	1	4	4	4	4	3	3	2	2
1	1	4	4	4	4	3	3	2	2
1	1	4	4	4	4	3	3	2	2
1	1	4	4	4	4	3	3	2	2
4	4	4	4	4	4	4	4	4	4
1	1	4	4	4	4	4	4	4	4
1	1	4	4	4	4	3	3	2	2
1	1	4	4	4	4	3	3	2	2
1	1	4	4	4	4	4	4	4	4
4	4	4	4	4	4	4	4	4	4
1	1	4	4	4	4	3	3	2	2
1	1	4	4	4	4	3	3	2	2
4	4	4	4	2	2	4	4	4	4
1	1	4	4	4	4	3	3	2	2
1	1	4	4	4	4	4	4	4	4
4	4	4	4	4	4	4	4	4	4
4	4	4	4	4	4	4	4	4	4
1	1	4	4	4	4	3	3	2	2
1	1	4	4	4	4	4	4	4	4
1	1	4	4	4	4	3	3	2	2
1	1	4	4	4	4	4	4	4	4
1	1	4	4	2	2	3	3	2	2
1	1	4	4	4	4	3	3	2	2
4	4	4	4	4	4	4	4	4	4
1	1	4	4	4	4	3	3	2	2
4	4	4	4	4	4	4	4	4	4
1	1	4	4	4	4	3	3	2	2
4	4	4	4	2	2	4	4	4	4
4	4	4	4	4	4	4	4	4	4
1	1	4	4	4	4	3	3	2	2
4	4	4	4	4	4	4	4	4	4
4	4	4	4	4	4	4	4	4	4
1	1	4	4	4	4	3	3	2	2
1	1	4	4	4	4	4	4	4	4
4	4	4	4	4	4	3	3	4	4
1	1	4	4	4	4	4	4	2	2
1	1	4	4	4	4	3	3	2	2
4	4	4	4	2	2	4	4	4	4
1	1	4	4	4	4	3	3	2	2
1	1	4	4	4	4	3	3	2	2
4	4	4	4	4	4	4	4	4	4
1	1	4	4	2	2	3	3	2	2

For Review Only

1	1	4	4	4	4	3	3	2	2
1	1	4	4	4	4	3	3	2	2

For Review Only

SNP1459	SNP1459	SNP146	SNP146	SNP1460	SNP1460	SNP1461	SNP1461	SNP1462	SNP1462
2	2	1	1	3	3	1	1	3	3
3	3	1	1	3	3	3	3	3	3
2	2	1	1	3	3	1	1	3	3
2	2	1	1	2	2	1	1	3	3
3	3	1	1	3	3	3	3	3	3
2	2	1	1	3	3	1	1	2	2
2	2	1	1	3	3	1	1	3	3
2	2	1	1	3	3	1	1	3	3
2	2	1	1	3	3	1	1	3	3
2	2	1	1	3	3	1	1	3	3
2	2	1	1	3	3	1	1	3	3
2	2	1	1	3	3	1	1	3	3
2	2	1	1	3	3	1	1	3	3
2	2	1	1	3	3	1	1	3	3
2	2	1	1	3	3	1	1	3	3
2	2	1	1	3	3	1	1	3	3
2	2	1	1	2	2	1	1	3	3
2	2	1	1	3	3	1	1	3	3
2	2	1	1	2	2	1	1	2	2
3	3	1	1	2	2	3	3	3	3
2	2	1	1	3	3	1	1	3	3
3	3	1	1	3	3	1	1	3	3
2	2	1	1	2	2	1	1	3	3
2	2	1	1	3	3	1	1	3	3
2	2	1	1	2	2	1	1	3	3
2	2	1	1	3	3	1	1	3	3
2	2	1	1	3	3	1	1	3	3
2	2	1	1	3	3	1	1	3	3
2	2	1	1	2	2	1	1	3	3
3	3	1	1	3	3	3	3	3	3
2	2	1	1	3	3	1	1	3	3
2	2	1	1	3	3	1	1	3	3
2	2	1	1	3	3	1	1	3	3
2	2	4	4	3	3	1	1	3	3
2	2	1	1	3	3	1	1	3	3
2	2	1	1	2	2	1	1	3	3
2	2	1	1	3	3	3	3	3	3
2	2	1	1	3	3	3	3	3	3
2	2	1	1	3	3	1	1	3	3
3	3	1	1	2	2	3	3	3	3
3	3	1	1	3	3	3	3	3	3
2	2	1	1	3	3	1	1	3	3
2	2	1	1	3	3	1	1	3	3
3	3	1	1	3	3	1	1	3	3
2	2	1	1	3	3	1	1	3	3
2	2	1	1	3	3	1	1	3	3
2	2	1	1	2	2	1	1	3	3
3	3	1	1	3	3	3	3	3	3
2	2	1	1	3	3	1	1	3	3
2	2	1	1	3	3	1	1	3	3
2	2	1	1	3	3	1	1	3	3
2	2	1	1	2	2	1	1	3	3

For Review Only

2	2	1	1	2	2	1	1	3	3
2	2	1	1	3	3	1	1	3	3
2	2	1	1	3	3	1	1	3	3
2	2	1	1	3	3	1	1	3	3
2	2	1	1	2	2	1	1	2	2
2	2	1	1	3	3	1	1	3	3
2	2	1	1	3	3	1	1	3	3
3	3	1	1	3	3	3	3	3	3
2	2	1	1	3	3	1	1	3	3
2	2	1	1	3	3	1	1	3	3
2	2	1	1	2	2	1	1	2	2
2	2	1	1	2	2	1	1	3	3
2	2	1	1	2	2	1	1	3	3
2	2	1	1	2	2	1	1	3	3
2	2	1	1	3	3	1	1	3	3
2	2	1	1	3	3	3	3	3	3
3	3	1	1	3	3	1	1	3	3
2	2	1	1	3	3	1	1	3	3
2	2	1	1	2	2	1	1	3	3
2	2	1	1	3	3	1	1	3	3
2	2	1	1	2	2	1	1	3	3
2	2	1	1	3	3	1	1	3	3
2	2	1	1	3	3	1	1	3	3
2	2	1	1	3	3	1	1	3	3
2	2	1	1	3	3	1	1	3	3
2	2	1	1	2	2	1	1	3	3
2	2	1	1	3	3	1	1	3	3
2	2	1	1	3	3	1	1	3	3
2	2	1	1	3	3	1	1	3	3
2	2	1	1	3	3	1	1	3	3
2	2	1	1	3	3	1	1	3	3
2	2	1	1	3	3	1	1	3	3
2	2	1	1	3	3	1	1	3	3
2	2	1	1	2	2	1	1	3	3
2	2	1	1	3	3	1	1	3	3
2	2	1	1	3	3	1	1	3	3
2	2	1	1	3	3	1	1	3	3
2	2	1	1	3	3	1	1	3	3
2	2	1	1	3	3	1	1	3	3
2	2	1	1	3	3	1	1	3	3
2	2	1	1	2	2	1	1	3	3
2	2	1	1	3	3	1	1	3	3
2	2	1	1	3	3	1	1	3	3
2	2	1	1	2	2	1	1	2	2
2	2	1	1	3	3	1	1	3	3
2	2	4	4	3	3	1	1	3	3
2	2	4	4	3	3	1	1	3	3
2	2	1	1	3	3	1	1	3	3
2	2	1	1	3	3	1	1	3	3
2	2	1	1	3	3	1	1	3	3
2	2	1	1	2	2	1	1	3	3
2	2	1	1	3	3	1	1	3	3
2	2	1	1	3	3	1	1	3	3
2	2	1	1	3	3	1	1	3	3
2	2	1	1	3	3	1	1	3	3

For Review Only

2	2	1	1	3	3	1	1	3	3
2	2	1	1	3	3	3	3	3	3
2	2	1	1	2	2	1	1	3	3
2	2	1	1	3	3	1	1	3	3
2	2	1	1	3	3	1	1	3	3
2	2	1	1	3	3	1	1	3	3
2	2	1	1	3	3	1	1	3	3
2	2	1	1	3	3	1	1	2	2
3	3	1	1	3	3	3	3	3	3
2	2	1	1	3	3	1	1	3	3
2	2	1	1	3	3	1	1	3	3
2	2	1	1	3	3	1	1	3	3
2	2	1	1	3	3	1	1	3	3
2	2	1	1	3	3	1	1	3	3
2	2	1	1	3	3	1	1	3	3
2	2	1	1	3	3	1	1	3	3
2	2	1	1	2	2	1	1	3	3
2	2	1	1	3	3	1	1	3	3
2	2	1	1	3	3	1	1	3	3
2	2	1	1	2	2	1	1	3	3
2	2	1	1	3	3	1	1	3	3
2	2	1	1	3	3	1	1	3	3
2	2	1	1	3	3	1	1	3	3
2	2	1	1	3	3	1	1	3	3
2	2	1	1	3	3	1	1	3	3
2	2	1	1	3	3	1	1	3	3
2	2	1	1	2	2	1	1	3	3
2	2	1	1	3	3	1	1	3	3
2	2	1	1	3	3	1	1	3	3
2	2	1	1	3	3	1	1	3	3
2	2	1	1	3	3	1	1	3	3
2	2	1	1	3	3	1	1	3	3
2	2	1	1	2	2	1	1	3	3
2	2	1	1	3	3	1	1	3	3
2	2	1	1	3	3	1	1	3	3
3	3	1	1	3	3	3	3	3	3
2	2	4	4	3	3	1	1	3	3
2	2	1	1	3	3	1	1	3	3
2	2	1	1	3	3	1	1	3	3
2	2	1	1	3	3	1	1	3	3
2	2	1	1	3	3	1	1	3	3
2	2	1	1	3	3	1	1	2	2
2	2	1	1	3	3	1	1	3	3
3	3	1	1	3	3	1	1	3	3
2	2	1	1	2	2	1	1	3	3
2	2	1	1	3	3	1	1	3	3
2	2	1	1	3	3	1	1	3	3
2	2	1	1	3	3	1	1	3	3
2	2	1	1	3	3	1	1	3	3
2	2	1	1	3	3	1	1	3	3
2	2	1	1	3	3	1	1	3	3
3	3	1	1	3	3	3	3	3	3
2	2	1	1	3	3	1	1	3	3
2	2	1	1	3	3	1	1	3	3
3	3	1	1	2	2	1	1	3	3

For Review Only

2	2	1	1	3	3	1	1	3	3
3	3	1	1	3	3	3	3	3	3
3	3	1	1	3	3	3	3	3	3
3	3	1	1	3	3	1	1	3	3
2	2	1	1	3	3	1	1	2	2
2	2	1	1	2	2	1	1	3	3
2	2	1	1	3	3	1	1	3	3
2	2	1	1	3	3	1	1	3	3
3	3	1	1	3	3	1	1	3	3
2	2	1	1	3	3	1	1	3	3
2	2	1	1	3	3	1	1	3	3
2	2	1	1	3	3	1	1	3	3
2	2	1	1	3	3	1	1	3	3
2	2	1	1	3	3	1	1	3	3
2	2	4	4	3	3	1	1	3	3
2	2	1	1	3	3	1	1	3	3
2	2	1	1	3	3	1	1	3	3
2	2	1	1	3	3	1	1	2	2
2	2	1	1	3	3	1	1	3	3
2	2	1	1	3	3	1	1	3	3
2	2	1	1	3	3	1	1	3	3
2	2	1	1	3	3	1	1	3	3
2	2	1	1	3	3	1	1	3	3
2	2	1	1	2	2	1	1	3	3
2	2	1	1	3	3	1	1	3	3
2	2	4	4	3	3	1	1	3	3
2	2	1	1	3	3	1	1	3	3
2	2	1	1	3	3	1	1	3	3
3	3	1	1	3	3	3	3	3	3
2	2	1	1	2	2	1	1	3	3
2	2	1	1	3	3	1	1	3	3
2	2	1	1	3	3	1	1	3	3
2	2	4	4	3	3	1	1	3	3
2	2	4	4	3	3	1	1	3	3
2	2	1	1	3	3	1	1	3	3
2	2	1	1	2	2	1	1	3	3
2	2	1	1	3	3	1	1	3	3
2	2	1	1	3	3	1	1	3	3
2	2	1	1	3	3	1	1	3	3
2	2	1	1	3	3	1	1	3	3
2	2	1	1	3	3	1	1	3	3
2	2	1	1	3	3	1	1	3	3
2	2	1	1	3	3	1	1	3	3
2	2	1	1	3	3	1	1	3	3
2	2	1	1	3	3	1	1	3	3
2	2	1	1	3	3	1	1	3	3
2	2	1	1	3	3	1	1	3	3
2	2	1	1	3	3	1	1	3	3
2	2	1	1	3	3	1	1	3	3
2	2	1	1	3	3	1	1	3	3
2	2	1	1	3	3	1	1	3	3
3	3	1	1	3	3	3	3	3	3
2	2	1	1	3	3	1	1	3	3
2	2	1	1	2	2	1	1	2	2
2	2	1	1	3	3	1	1	3	3

For Review Only

2	2	1	1	3	3	1	1	3	3
3	3	4	4	3	3	3	3	3	3
2	2	1	1	3	3	1	1	3	3
2	2	1	1	3	3	1	1	3	3
2	2	1	1	3	3	1	1	3	3
2	2	1	1	3	3	1	1	3	3
2	2	1	1	3	3	1	1	3	3
2	2	1	1	3	3	1	1	3	3
2	2	1	1	3	3	1	1	3	3
2	2	1	1	3	3	1	1	3	3
2	2	1	1	3	3	1	1	3	3
2	2	1	1	2	2	1	1	3	3
2	2	1	1	3	3	1	1	3	3
2	2	1	1	3	3	1	1	3	3
2	2	1	1	3	3	1	1	3	3
2	2	1	1	3	3	1	1	2	2
2	2	1	1	3	3	1	1	3	3
2	2	1	1	3	3	1	1	3	3
2	2	1	1	3	3	1	1	3	3
2	2	1	1	2	2	3	3	3	3
2	2	1	1	3	3	1	1	3	3
2	2	1	1	3	3	1	1	3	3
2	2	1	1	3	3	1	1	3	3
2	2	1	1	3	3	1	1	3	3
3	3	1	1	3	3	3	3	3	3
2	2	1	1	3	3	1	1	3	3
2	2	1	1	3	3	1	1	3	3
2	2	1	1	3	3	1	1	3	3
2	2	1	1	3	3	1	1	3	3
2	2	1	1	3	3	1	1	3	3
2	2	1	1	3	3	1	1	3	3
3	3	1	1	3	3	3	3	3	3
2	2	4	4	3	3	1	1	3	3
2	2	1	1	3	3	1	1	3	3
2	2	1	1	3	3	1	1	3	3
2	2	1	1	3	3	1	1	3	3
2	2	1	1	3	3	1	1	3	3
2	2	1	1	2	2	1	1	3	3
2	2	1	1	2	2	1	1	3	3
2	2	1	1	3	3	1	1	3	3
2	2	1	1	3	3	1	1	3	3
2	2	1	1	3	3	1	1	3	3
2	2	1	1	3	3	1	1	3	3
2	2	1	1	2	2	1	1	3	3
3	3	1	1	2	2	1	1	3	3
2	2	1	1	3	3	1	1	3	3
2	2	1	1	3	3	1	1	3	3
2	2	1	1	3	3	1	1	3	3
3	3	1	1	3	3	1	1	3	3
2	2	1	1	3	3	1	1	3	3
2	2	1	1	3	3	1	1	3	3
2	2	1	1	3	3	1	1	3	3
2	2	1	1	3	3	1	1	3	3
2	2	1	1	3	3	1	1	3	3
2	2	1	1	3	3	3	3	3	3

For Review Only

2	2	1	1	3	3	1	1	3	3
2	2	1	1	2	2	1	1	3	3

For Review Only

SNP1463	SNP1463	SNP1464	SNP1464	SNP1465	SNP1465	SNP1466	SNP1466	SNP1467	SNP1467
1	1	2	2	1	1	3	3	3	3
4	4	3	3	3	3	2	2	3	3
1	1	3	3	3	3	2	2	3	3
4	4	3	3	1	1	2	2	3	3
1	1	2	2	3	3	3	3	4	4
4	4	3	3	1	1	2	2	3	3
1	1	2	2	1	1	3	3	4	4
1	1	3	3	3	3	3	3	4	4
4	4	2	2	1	1	2	2	3	3
1	1	3	3	1	1	3	3	4	4
1	1	3	3	3	3	2	2	3	3
1	1	3	3	1	1	2	2	3	3
1	1	3	3	1	1	3	3	3	3
1	1	2	2	3	3	2	2	3	3
4	4	3	3	1	1	2	2	3	3
1	1	3	3	1	1	2	2	3	3
4	4	2	2	1	1	2	2	3	3
1	1	2	2	1	1	3	3	4	4
4	4	3	3	3	3	2	2	3	3
4	4	3	3	3	3	3	3	4	4
1	1	3	3	3	3	2	2	3	3
1	1	2	2	1	1	2	2	3	3
4	4	3	3	3	3	2	2	3	3
4	4	3	3	3	3	2	2	3	3
4	4	3	3	1	1	2	2	3	3
1	1	3	3	1	1	2	2	3	3
1	1	3	3	1	1	2	2	3	3
4	4	3	3	1	1	2	2	3	3
4	4	3	3	1	1	2	2	3	3
1	1	3	3	3	3	3	3	4	4
1	1	3	3	3	3	2	2	3	3
1	1	2	2	1	1	2	2	3	3
1	1	2	2	1	1	3	3	4	4
1	1	3	3	1	1	2	2	3	3
4	4	3	3	1	1	2	2	3	3
4	4	3	3	1	1	2	2	3	3
1	1	3	3	3	3	3	3	4	4
4	4	3	3	3	3	3	3	4	4
1	1	3	3	3	3	2	2	3	3
4	4	3	3	3	3	2	2	3	3
1	1	3	3	3	3	3	3	4	4
1	1	3	3	3	3	2	2	3	3
1	1	3	3	1	1	3	3	4	4
1	1	2	2	3	3	3	3	4	4
1	1	3	3	1	1	3	3	4	4
4	4	3	3	3	3	2	2	3	3
4	4	3	3	1	1	2	2	3	3
1	1	3	3	1	1	2	2	3	3
1	1	2	2	3	3	3	3	4	4
1	1	3	3	1	1	2	2	3	3
1	1	3	3	1	1	2	2	3	3
4	4	3	3	3	3	2	2	3	3

For Review Only

4	4	2	2	1	1	3	3	4	4
1	1	2	2	1	1	3	3	4	4
1	1	3	3	1	1	2	2	3	3
1	1	2	2	1	1	2	2	3	3
4	4	3	3	3	3	2	2	3	3
1	1	3	3	3	3	2	2	3	3
1	1	3	3	1	1	2	2	3	3
1	1	3	3	3	3	3	3	4	4
1	1	3	3	3	3	2	2	3	3
4	4	3	3	1	1	2	2	3	3
4	4	3	3	3	3	2	2	3	3
4	4	3	3	3	3	2	2	3	3
4	4	2	2	1	1	3	3	4	4
4	4	3	3	1	1	2	2	3	3
4	4	3	3	1	1	2	2	3	3
1	1	3	3	3	3	2	2	3	3
1	1	3	3	3	3	2	2	3	3
1	1	3	3	3	3	2	2	3	3
4	4	2	2	1	1	2	2	3	3
1	1	2	2	1	1	3	3	4	4
4	4	3	3	3	3	2	2	3	3
1	1	3	3	3	3	2	2	3	3
4	4	3	3	1	1	2	2	3	3
1	1	3	3	1	1	2	2	3	3
1	1	3	3	1	1	3	3	4	4
4	4	3	3	3	3	2	2	3	3
1	1	2	2	3	3	3	3	4	4
1	1	3	3	3	3	2	2	3	3
1	1	3	3	1	1	2	2	3	3
1	1	3	3	3	3	2	2	3	3
1	1	3	3	1	1	2	2	3	3
1	1	3	3	1	1	2	2	3	3
1	1	3	3	3	3	2	2	3	3
4	4	2	2	1	1	3	3	4	4
1	1	3	3	1	1	2	2	3	3
1	1	3	3	1	1	3	3	4	4
1	1	3	3	3	3	2	2	3	3
1	1	3	3	3	3	2	2	3	3
1	1	3	3	3	3	2	2	3	3
1	1	3	3	3	3	3	3	4	4
4	4	2	2	1	1	2	2	3	3
1	1	2	2	3	3	2	2	3	3
1	1	3	3	1	1	2	2	3	3
4	4	3	3	3	3	2	2	3	3
1	1	3	3	1	1	2	2	3	3
1	1	3	3	1	1	2	2	3	3
1	1	3	3	3	3	2	2	3	3
1	1	3	3	1	1	2	2	3	3
1	1	3	3	1	1	2	2	3	3
1	1	3	3	3	3	2	2	3	3
4	4	3	3	1	1	2	2	3	3
1	1	3	3	3	3	2	2	3	3
4	4	3	3	3	3	2	2	3	3
4	4	3	3	3	3	2	2	3	3
1	1	2	2	3	3	3	3	4	4

For Review Only

1	1	3	3	3	3	2	2	3	3
1	1	3	3	3	3	3	3	4	4
4	4	3	3	1	1	2	2	3	3
1	1	3	3	3	3	2	2	3	3
4	4	3	3	1	1	2	2	3	3
1	1	3	3	1	1	2	2	3	3
1	1	3	3	1	1	2	2	3	3
1	1	3	3	1	1	2	2	3	3
1	1	3	3	3	3	2	2	3	3
4	4	3	3	1	1	2	2	3	3
1	1	3	3	1	1	2	2	3	3
4	4	3	3	1	1	3	3	3	3
1	1	3	3	3	3	2	2	3	3
4	4	3	3	1	1	2	2	3	3
4	4	3	3	3	3	3	3	3	3
4	4	3	3	1	1	2	2	3	3
4	4	3	3	3	3	2	2	3	3
4	4	3	3	1	1	2	2	3	3
1	1	3	3	3	3	2	2	3	3
4	4	2	2	1	1	3	3	4	4
1	1	3	3	1	1	2	2	3	3
1	1	3	3	3	3	2	2	3	3
1	1	3	3	1	1	2	2	3	3
1	1	3	3	3	3	2	2	3	3
1	1	3	3	1	1	2	2	3	3
1	1	3	3	3	3	2	2	3	3
4	4	3	3	3	3	3	3	4	4
1	1	3	3	1	1	2	2	3	3
1	1	3	3	1	1	2	2	3	3
1	1	2	2	1	1	2	2	3	3
1	1	3	3	3	3	2	2	3	3
4	4	3	3	1	1	2	2	3	3
4	4	2	2	1	1	2	2	3	3
4	4	3	3	1	1	2	2	3	3
1	1	3	3	1	1	2	2	3	3
1	1	3	3	1	1	2	2	3	3
1	1	3	3	3	3	2	2	3	3
1	1	3	3	1	1	2	2	3	3
4	4	3	3	1	1	2	2	3	3
1	1	3	3	3	3	2	2	3	3
1	1	3	3	3	3	2	2	3	3
1	1	3	3	3	3	2	2	3	3
1	1	3	3	3	3	2	2	3	3
1	1	3	3	3	3	3	3	3	3
4	4	3	3	3	3	2	2	3	3
4	4	3	3	3	3	3	3	4	4
1	1	3	3	1	1	2	2	3	3
1	1	3	3	1	1	2	2	3	3
1	1	3	3	1	1	2	2	3	3
4	4	3	3	1	1	2	2	3	3
1	1	3	3	1	1	2	2	3	3
1	1	3	3	3	3	3	3	4	4
1	1	3	3	1	1	2	2	3	3
1	1	3	3	1	1	2	2	3	3
4	4	3	3	1	1	2	2	3	3

For Review Only

1	1	3	3	3	3	2	2	3	3
1	1	3	3	3	3	2	2	3	3
4	4	3	3	1	1	2	2	3	3
4	4	3	3	1	1	2	2	3	3
4	4	3	3	1	1	2	2	3	3
4	4	2	2	3	3	2	2	3	3
1	1	3	3	3	3	2	2	3	3
4	4	3	3	1	1	2	2	3	3
4	4	3	3	3	3	2	2	3	3
1	1	3	3	1	1	2	2	3	3
1	1	3	3	1	1	2	2	3	3
1	1	3	3	3	3	2	2	3	3
1	1	3	3	3	3	2	2	3	3
4	4	3	3	3	3	3	3	4	4
1	1	3	3	3	3	2	2	3	3
1	1	3	3	3	3	2	2	3	3
1	1	3	3	1	1	2	2	3	3
1	1	3	3	1	1	2	2	3	3
1	1	3	3	1	1	2	2	3	3
1	1	3	3	1	1	2	2	3	3
1	1	3	3	1	1	2	2	3	3
1	1	3	3	3	3	2	2	3	3
1	1	3	3	3	3	2	2	3	3
4	4	3	3	3	3	2	2	3	3
1	1	3	3	3	3	3	3	4	4
1	1	3	3	1	1	2	2	3	3
1	1	3	3	1	1	2	2	3	3
4	4	3	3	1	1	2	2	3	3
1	1	3	3	3	3	2	2	3	3
4	4	2	2	3	3	2	2	3	3
4	4	3	3	1	1	2	2	3	3
1	1	3	3	3	3	2	2	3	3
1	1	3	3	3	3	2	2	3	3
4	4	3	3	3	3	2	2	3	3
1	1	3	3	1	1	2	2	3	3
4	4	3	3	3	3	2	2	3	3
1	1	3	3	1	1	2	2	3	3
1	1	3	3	1	1	2	2	3	3
1	1	3	3	1	1	2	2	3	3
1	1	3	3	1	1	2	2	3	3
1	1	3	3	3	3	2	2	3	3
1	1	3	3	1	1	2	2	3	3
1	1	3	3	3	3	2	2	3	3
1	1	3	3	1	1	2	2	3	3
1	1	3	3	1	1	2	2	3	3
4	4	3	3	3	3	2	2	3	3
1	1	3	3	3	3	2	2	3	3
1	1	3	3	3	3	2	2	3	3
1	1	3	3	3	3	2	2	3	3
1	1	3	3	1	1	2	2	3	3
1	1	3	3	1	1	3	3	3	3
1	1	3	3	3	3	3	3	4	4
1	1	3	3	3	3	2	2	3	3
4	4	3	3	3	3	2	2	3	3
1	1	3	3	3	3	2	2	3	3

For Review Only

1	1	3	3	3	3	2	2	3	3
1	1	3	3	3	3	3	3	4	4
1	1	3	3	1	1	2	2	3	3
4	4	3	3	3	3	2	2	3	3
1	1	3	3	3	3	3	3	4	4
1	1	3	3	1	1	2	2	3	3
1	1	3	3	1	1	2	2	3	3
1	1	3	3	1	1	3	3	4	4
1	1	3	3	1	1	2	2	3	3
1	1	3	3	3	3	2	2	3	3
4	4	3	3	1	1	2	2	3	3
4	4	3	3	1	1	2	2	3	3
1	1	3	3	1	1	2	2	3	3
1	1	3	3	3	3	2	2	3	3
1	1	3	3	3	3	2	2	3	3
1	1	3	3	3	3	2	2	3	3
1	1	3	3	3	3	2	2	3	3
1	1	3	3	1	1	2	2	3	3
1	1	3	3	1	1	2	2	3	3
4	4	3	3	3	3	3	3	4	4
1	1	3	3	3	3	2	2	3	3
1	1	3	3	1	1	2	2	3	3
1	1	3	3	1	1	2	2	3	3
1	1	2	2	3	3	2	2	3	3
1	1	3	3	3	3	3	3	4	4
4	4	3	3	1	1	2	2	3	3
1	1	3	3	3	3	2	2	3	3
1	1	3	3	1	1	2	2	3	3
4	4	3	3	1	1	3	3	4	4
1	1	3	3	1	1	2	2	3	3
1	1	3	3	3	3	2	2	3	3
1	1	3	3	1	1	2	2	3	3
1	1	3	3	3	3	3	3	4	4
1	1	3	3	1	1	2	2	3	3
1	1	3	3	3	3	2	2	3	3
1	1	3	3	3	3	2	2	3	3
1	1	3	3	1	1	2	2	3	3
4	4	3	3	3	3	2	2	3	3
4	4	3	3	1	1	2	2	3	3
1	1	3	3	3	3	2	2	3	3
1	1	3	3	1	1	3	3	4	4
1	1	3	3	1	1	2	2	3	3
1	1	3	3	3	3	2	2	3	3
4	4	2	2	1	1	2	2	3	3
4	4	3	3	1	1	2	2	3	3
1	1	3	3	3	3	2	2	3	3
1	1	3	3	1	1	2	2	3	3
1	1	3	3	3	3	2	2	3	3
1	1	3	3	3	3	3	3	4	4
1	1	3	3	3	3	2	2	3	3
1	1	3	3	1	1	2	2	3	3
1	1	3	3	3	3	2	2	3	3
1	1	3	3	3	3	2	2	3	3
1	1	3	3	1	1	2	2	3	3
1	1	3	3	3	3	2	2	3	3

For Review Only

1	1	3	3	3	3	2	2	3	3
4	4	3	3	3	3	2	2	3	3

For Review Only

SNP1468	SNP1468	SNP1469	SNP1469	SNP147	SNP147	SNP1470	SNP1470	SNP1471	SNP1471
3	3	4	4	3	3	4	4	4	4
3	3	3	3	3	3	4	4	1	1
3	3	3	3	3	3	4	4	1	1
3	3	4	4	3	3	4	4	4	4
4	4	3	3	3	3	4	4	1	1
3	3	4	4	3	3	4	4	1	1
4	4	4	4	1	1	4	4	1	1
3	3	3	3	3	3	4	4	1	1
4	4	3	3	3	3	3	3	4	4
3	3	4	4	3	3	3	3	4	4
3	3	3	3	3	3	4	4	1	1
3	3	3	3	3	3	4	4	1	1
3	3	3	3	3	3	4	4	1	1
4	4	3	3	3	3	3	3	1	1
3	3	3	3	3	3	4	4	1	1
3	3	4	4	3	3	4	4	1	1
4	4	4	4	3	3	4	4	1	1
4	4	4	4	3	3	4	4	4	4
3	3	3	3	3	3	4	4	1	1
3	3	3	3	3	3	4	4	1	1
3	3	3	3	3	3	4	4	1	1
4	4	4	4	1	1	4	4	1	1
3	3	3	3	3	3	3	3	1	1
3	3	3	3	3	3	3	3	1	1
3	3	4	4	3	3	4	4	1	1
3	3	4	4	3	3	4	4	1	1
3	3	3	3	3	3	4	4	1	1
3	3	3	3	3	3	4	4	1	1
3	3	4	4	3	3	3	3	4	4
3	3	3	3	3	3	3	3	1	1
3	3	4	4	1	1	4	4	1	1
4	4	4	4	1	1	4	4	4	4
4	4	4	4	1	1	4	4	4	4
3	3	4	4	3	3	3	3	4	4
3	3	3	3	3	3	4	4	1	1
3	3	4	4	3	3	3	3	4	4
3	3	3	3	3	3	3	3	1	1
3	3	3	3	3	3	3	3	1	1
3	3	3	3	3	3	3	3	1	1
3	3	3	3	3	3	4	4	1	1
3	3	3	3	3	3	3	3	1	1
3	3	3	3	3	3	3	3	1	1
3	3	4	4	3	3	3	3	4	4
4	4	3	3	3	3	4	4	1	1
3	3	3	3	3	3	3	3	1	1
3	3	3	3	3	3	4	4	1	1
3	3	4	4	3	3	4	4	1	1
3	3	3	3	3	3	4	4	1	1
4	4	3	3	3	3	3	3	1	1
3	3	4	4	3	3	4	4	1	1
3	3	4	4	3	3	4	4	1	1
3	3	3	3	3	3	4	4	1	1

For Review Only

4	4	4	4	1	1	4	4	4	4
4	4	4	4	1	1	4	4	4	4
3	3	4	4	1	1	4	4	4	4
4	4	4	4	1	1	3	3	1	1
3	3	3	3	3	3	4	4	1	1
3	3	3	3	3	3	4	4	1	1
3	3	4	4	3	3	4	4	1	1
3	3	3	3	3	3	3	3	1	1
3	3	3	3	3	3	3	3	1	1
3	3	3	3	3	3	4	4	1	1
3	3	3	3	3	3	4	4	1	1
3	3	3	3	3	3	3	3	1	1
4	4	4	4	1	1	4	4	4	4
3	3	4	4	3	3	3	3	4	4
3	3	3	3	3	3	4	4	1	1
3	3	3	3	3	3	4	4	1	1
3	3	3	3	3	3	4	4	1	1
3	3	3	3	3	3	3	3	1	1
4	4	4	4	1	1	4	4	1	1
4	4	4	4	3	3	4	4	4	4
3	3	3	3	3	3	4	4	1	1
3	3	3	3	3	3	3	3	1	1
3	3	4	4	3	3	4	4	1	1
3	3	4	4	3	3	3	3	4	4
3	3	3	3	3	3	3	3	1	1
3	3	3	3	3	3	4	4	1	1
4	4	3	3	3	3	4	4	1	1
3	3	3	3	3	3	3	3	1	1
3	3	4	4	3	3	4	4	1	1
3	3	4	4	1	1	4	4	1	1
3	3	4	4	1	1	4	4	1	1
3	3	4	4	3	3	4	4	4	4
3	3	3	3	3	3	3	3	1	1
4	4	4	4	1	1	4	4	4	4
3	3	4	4	3	3	3	3	4	4
3	3	3	3	3	3	3	3	1	1
3	3	3	3	3	3	4	4	1	1
3	3	3	3	3	3	4	4	1	1
3	3	3	3	3	3	4	4	1	1
3	3	3	3	3	3	3	3	1	1
4	4	4	4	3	3	3	3	1	1
4	4	4	4	3	3	4	4	1	1
3	3	3	3	3	3	4	4	1	1
3	3	3	3	3	3	4	4	1	1
3	3	3	3	3	3	4	4	1	1
3	3	4	4	3	3	3	3	4	4
3	3	3	3	3	3	3	3	1	1
3	3	4	4	3	3	3	3	1	1
3	3	4	4	3	3	3	3	1	1
3	3	3	3	3	3	3	3	1	1
3	3	4	4	3	3	4	4	1	1
3	3	3	3	3	3	3	3	1	1
3	3	3	3	3	3	3	3	1	1
3	3	3	3	3	3	4	4	1	1
4	4	3	3	3	3	3	3	1	1

For Review Only

CSSA 5585 Guilford Rd., Madison, WI 53711

3	3	3	3	3	3	4	4	1	1
3	3	3	3	3	3	4	4	1	1
3	3	4	4	3	3	4	4	1	1
3	3	3	3	3	3	3	3	1	1
3	3	4	4	3	3	4	4	1	1
3	3	4	4	3	3	3	3	1	1
3	3	4	4	3	3	4	4	1	1
3	3	3	3	3	3	4	4	1	1
3	3	3	3	3	3	3	3	1	1
3	3	4	4	3	3	3	3	1	1
4	4	3	3	1	1	3	3	1	1
3	3	3	3	3	3	4	4	1	1
3	3	3	3	3	3	3	3	1	1
3	3	3	3	3	3	4	4	1	1
3	3	3	3	3	3	4	4	1	1
3	3	3	3	3	3	4	4	1	1
3	3	3	3	3	3	3	3	1	1
3	3	3	3	3	3	4	4	1	1
3	3	3	3	3	3	3	3	1	1
3	3	4	4	1	1	4	4	1	1
3	3	3	3	3	3	4	4	1	1
3	3	3	3	3	3	4	4	1	1
3	3	4	4	1	1	3	3	1	1
3	3	3	3	3	3	3	3	1	1
3	3	4	4	3	3	4	4	1	1
3	3	3	3	3	3	3	3	1	1
3	3	4	4	1	1	3	3	4	4
3	3	4	4	3	3	3	3	4	4
3	3	4	4	3	3	4	4	1	1
4	4	4	4	3	3	3	3	4	4
3	3	3	3	3	3	3	3	1	1
4	4	3	3	3	3	4	4	1	1
4	4	4	4	3	3	3	3	1	1
3	3	3	3	3	3	4	4	1	1
3	3	4	4	3	3	3	3	4	4
3	3	4	4	3	3	4	4	1	1
3	3	3	3	3	3	3	3	1	1
3	3	4	4	1	1	4	4	1	1
3	3	3	3	3	3	4	4	1	1
3	3	3	3	3	3	4	4	1	1
3	3	3	3	3	3	3	3	1	1
3	3	3	3	3	3	3	3	1	1
3	3	3	3	3	3	3	3	1	1
3	3	3	3	3	3	3	3	1	1
3	3	3	3	3	3	3	3	1	1
3	3	3	3	3	3	4	4	1	1
3	3	4	4	3	3	4	4	1	1
3	3	4	4	3	3	4	4	1	1
3	3	3	3	3	3	3	3	1	1
3	3	4	4	3	3	4	4	1	1
3	3	3	3	3	3	4	4	1	1
3	3	3	3	3	3	3	3	1	1
3	3	3	3	1	1	4	4	4	4
3	3	4	4	3	3	3	3	1	1
3	3	3	3	3	3	4	4	1	1

For Review Only

3	3	3	3	3	3	3	3	1	1
3	3	3	3	3	3	3	3	1	1
3	3	3	3	3	3	3	3	1	1
3	3	3	3	3	3	4	4	1	1
3	3	4	4	3	3	4	4	4	4
4	4	3	3	3	3	3	3	1	1
3	3	3	3	3	3	3	3	1	1
3	3	3	3	3	3	4	4	1	1
3	3	3	3	3	3	4	4	1	1
3	3	4	4	1	1	4	4	4	4
3	3	4	4	3	3	4	4	1	1
3	3	3	3	3	3	3	3	1	1
3	3	3	3	1	1	4	4	1	1
3	3	3	3	3	3	4	4	1	1
3	3	3	3	3	3	3	3	1	1
3	3	3	3	3	3	4	4	1	1
3	3	3	3	3	3	4	4	1	1
3	3	4	4	3	3	4	4	1	1
3	3	4	4	3	3	4	4	4	4
3	3	4	4	3	3	4	4	1	1
3	3	3	3	3	3	4	4	1	1
3	3	3	3	3	3	3	3	1	1
3	3	3	3	3	3	3	3	1	1
3	3	3	3	3	3	3	3	1	1
3	3	3	3	3	3	3	3	1	1
3	3	4	4	3	3	3	3	4	4
3	3	3	3	3	3	3	3	1	1
3	3	3	3	3	3	3	3	1	1
3	3	3	3	3	3	3	3	1	1
4	4	3	3	1	1	3	3	1	1
3	3	4	4	3	3	3	3	1	1
3	3	4	4	3	3	4	4	1	1
3	3	4	4	3	3	3	3	4	4
3	3	3	3	3	3	3	3	1	1
3	3	4	4	3	3	3	3	1	1
3	3	3	3	3	3	3	3	1	1
3	3	4	4	3	3	4	4	4	4
3	3	4	4	3	3	4	4	1	1
3	3	4	4	3	3	4	4	1	1
3	3	4	4	3	3	4	4	1	1
3	3	3	3	3	3	4	4	1	1
3	3	4	4	3	3	4	4	1	1
3	3	3	3	3	3	3	3	1	1
3	3	3	3	3	3	4	4	1	1
3	3	3	3	3	3	3	3	1	1
3	3	3	3	3	3	4	4	1	1
3	3	3	3	3	3	4	4	1	1
3	3	3	3	3	3	3	3	1	1
3	3	3	3	3	3	3	3	1	1
3	3	4	4	3	3	3	3	1	1
3	3	4	4	3	3	4	4	4	4
3	3	3	3	3	3	3	3	1	1
3	3	3	3	3	3	3	3	1	1
3	3	3	3	3	3	4	4	1	1
3	3	3	3	3	3	3	3	1	1

For Review Only

3	3	3	3	1	1	4	4	1	1
3	3	3	3	3	3	3	3	1	1
3	3	4	4	3	3	4	4	1	1
3	3	3	3	3	3	3	3	1	1
3	3	3	3	3	3	4	4	1	1
3	3	4	4	3	3	4	4	1	1
3	3	4	4	3	3	3	3	4	4
3	3	4	4	3	3	3	3	4	4
3	3	3	3	3	3	3	3	1	1
3	3	3	3	3	3	3	3	1	1
3	3	3	3	3	3	4	4	1	1
3	3	4	4	3	3	3	3	1	1
3	3	4	4	3	3	4	4	4	4
3	3	3	3	3	3	4	4	1	1
3	3	3	3	3	3	4	4	1	1
3	3	3	3	3	3	4	4	1	1
3	3	3	3	3	3	3	3	1	1
3	3	4	4	3	3	4	4	4	4
3	3	4	4	3	3	4	4	4	4
3	3	3	3	3	3	3	3	1	1
3	3	3	3	3	3	4	4	1	1
3	3	4	4	3	3	4	4	1	1
3	3	4	4	3	3	4	4	4	4
4	4	3	3	3	3	3	3	1	1
3	3	3	3	3	3	3	3	1	1
3	3	3	3	3	3	4	4	1	1
3	3	3	3	3	3	3	3	1	1
3	3	4	4	3	3	4	4	1	1
3	3	4	4	3	3	4	4	4	4
3	3	4	4	3	3	4	4	1	1
3	3	3	3	3	3	3	3	1	1
3	3	4	4	3	3	3	3	1	1
3	3	3	3	3	3	4	4	1	1
3	3	4	4	3	3	4	4	1	1
3	3	3	3	3	3	3	3	1	1
3	3	3	3	3	3	3	3	1	1
3	3	4	4	1	1	4	4	4	4
3	3	3	3	3	3	3	3	1	1
3	3	4	4	3	3	3	3	4	4
3	3	3	3	3	3	3	3	1	1
3	3	4	4	3	3	3	3	4	4
3	3	4	4	3	3	4	4	4	4
3	3	3	3	3	3	3	3	1	1
4	4	4	4	1	1	4	4	1	1
3	3	4	4	3	3	4	4	1	1
3	3	3	3	3	3	4	4	1	1
3	3	4	4	3	3	4	4	1	1
3	3	3	3	3	3	3	3	1	1
3	3	3	3	3	3	4	4	1	1
3	3	3	3	3	3	3	3	1	1
3	3	4	4	3	3	4	4	1	1
3	3	3	3	3	3	3	3	1	1
3	3	3	3	3	3	4	4	1	1
3	3	4	4	3	3	4	4	1	1
3	3	3	3	3	3	3	3	1	1

3	3	3	3	3	3	4	4	1	1
3	3	3	3	3	3	4	4	1	1

For Review Only

SNP1472	SNP1472	SNP1473	SNP1473	SNP1474	SNP1474	SNP1475	SNP1475	SNP1476	SNP1476
2	2	2	2	4	4	2	2	4	4
2	2	2	2	4	4	3	3	4	4
2	2	2	2	4	4	2	2	4	4
2	2	2	2	4	4	2	2	2	2
2	2	2	2	4	4	2	2	2	2
2	2	2	2	4	4	2	2	4	4
2	2	2	2	4	4	2	2	4	4
2	2	2	2	4	4	2	2	2	2
2	2	2	2	4	4	2	2	4	4
2	2	2	2	4	4	2	2	4	4
2	2	3	3	4	4	2	2	2	2
2	2	2	2	4	4	2	2	4	4
2	2	2	2	4	4	2	2	4	4
2	2	2	2	4	4	2	2	2	2
2	2	2	2	4	4	2	2	4	4
2	2	2	2	4	4	2	2	2	2
2	2	2	2	4	4	2	2	4	4
2	2	2	2	4	4	2	2	4	4
2	2	3	3	4	4	2	2	4	4
2	2	2	2	4	4	2	2	4	4
2	2	2	2	4	4	2	2	4	4
2	2	2	2	4	4	2	2	4	4
2	2	2	2	2	2	2	2	2	2
2	2	2	2	4	4	2	2	2	2
2	2	2	2	4	4	2	2	4	4
4	4	2	2	4	4	2	2	2	2
2	2	2	2	4	4	2	2	4	4
2	2	2	2	4	4	2	2	4	4
2	2	2	2	4	4	2	2	4	4
2	2	2	2	4	4	2	2	2	2
2	2	3	3	4	4	3	3	4	4
2	2	2	2	4	4	2	2	4	4
2	2	2	2	4	4	2	2	4	4
2	2	2	2	2	2	2	2	2	2
2	2	2	2	4	4	2	2	2	2
2	2	2	2	4	4	2	2	2	2
2	2	3	3	4	4	2	2	2	2
2	2	2	2	4	4	2	2	2	2
2	2	2	2	2	2	2	2	2	2
2	2	2	2	4	4	3	3	2	2
2	2	2	2	4	4	2	2	2	2
2	2	2	2	4	4	2	2	4	4
2	2	2	2	4	4	2	2	2	2
2	2	2	2	4	4	2	2	4	4
2	2	2	2	4	4	2	2	2	2
4	4	2	2	4	4	2	2	2	2
2	2	2	2	4	4	2	2	4	4
4	4	3	3	4	4	2	2	2	2
2	2	2	2	4	4	2	2	4	4
2	2	2	2	4	4	2	2	4	4
2	2	2	2	4	4	2	2	4	4
2	2	2	2	4	4	3	3	4	4

For Review Only

2	2	2	2	4	4	2	2	4	4
2	2	2	2	4	4	2	2	4	4
2	2	2	2	4	4	2	2	4	4
2	2	2	2	4	4	2	2	4	4
2	2	3	3	4	4	2	2	4	4
2	2	2	2	4	4	2	2	4	4
4	4	2	2	4	4	2	2	2	2
2	2	2	2	4	4	2	2	2	2
2	2	2	2	4	4	2	2	2	2
2	2	2	2	4	4	2	2	4	4
2	2	2	2	4	4	2	2	4	4
2	2	2	2	4	4	2	2	4	4
2	2	2	2	4	4	2	2	4	4
2	2	2	2	4	4	2	2	4	4
2	2	2	2	4	4	2	2	4	4
2	2	2	2	4	4	2	2	2	2
2	2	2	2	4	4	2	2	4	4
2	2	2	2	4	4	2	2	4	4
2	2	2	2	4	4	2	2	4	4
2	2	2	2	4	4	2	2	4	4
2	2	2	2	4	4	2	2	4	4
2	2	2	2	4	4	2	2	2	2
2	2	2	2	4	4	2	2	4	4
2	2	2	2	4	4	2	2	4	4
2	2	2	2	4	4	2	2	2	2
2	2	2	2	4	4	3	3	4	4
2	2	2	2	4	4	2	2	4	4
2	2	2	2	4	4	2	2	2	2
4	4	2	2	4	4	2	2	2	2
2	2	3	3	4	4	2	2	4	4
2	2	2	2	4	4	2	2	4	4
2	2	2	2	4	4	2	2	2	2
2	2	2	2	4	4	2	2	2	2
2	2	2	2	4	4	2	2	4	4
2	2	2	2	4	4	2	2	4	4
2	2	2	2	4	4	2	2	2	2
2	2	2	2	4	4	2	2	4	4
2	2	2	2	4	4	2	2	4	4
2	2	2	2	4	4	2	2	2	2
2	2	2	2	4	4	2	2	2	2
2	2	2	2	4	4	2	2	4	4
2	2	2	2	4	4	2	2	4	4
2	2	2	2	4	4	2	2	4	4
2	2	2	2	4	4	2	2	4	4
2	2	2	2	4	4	2	2	2	2
2	2	2	2	4	4	2	2	4	4
2	2	2	2	2	2	2	2	2	2
4	4	2	2	4	4	2	2	2	2
2	2	2	2	4	4	2	2	2	2
2	2	2	2	4	4	2	2	4	4
2	2	3	3	4	4	2	2	4	4
2	2	2	2	2	2	2	2	2	2
2	2	2	2	4	4	2	2	4	4
2	2	2	2	4	4	2	2	4	4
2	2	2	2	4	4	2	2	4	4

2	2	2	2	4	4	2	2	2	2
2	2	2	2	4	4	3	3	4	4
4	4	2	2	4	4	3	3	2	2
2	2	2	2	4	4	2	2	2	2
2	2	3	3	4	4	3	3	4	4
4	4	2	2	4	4	2	2	2	2
2	2	2	2	4	4	2	2	4	4
4	4	2	2	4	4	2	2	2	2
2	2	2	2	4	4	2	2	2	2
2	2	2	2	4	4	2	2	4	4
2	2	2	2	4	4	2	2	2	2
2	2	2	2	4	4	2	2	4	4
2	2	2	2	4	4	2	2	4	4
2	2	2	2	4	4	2	2	4	4
2	2	2	2	4	4	2	2	4	4
2	2	2	2	4	4	2	2	4	4
2	2	2	2	4	4	2	2	4	4
2	2	2	2	4	4	2	2	4	4
2	2	2	2	4	4	2	2	2	2
2	2	2	2	4	4	2	2	4	4
2	2	2	2	4	4	2	2	4	4
2	2	2	2	4	4	2	2	4	4
2	2	2	2	4	4	2	2	4	4
2	2	2	2	4	4	2	2	2	2
2	2	2	2	4	4	2	2	2	2
2	2	2	2	4	4	2	2	4	4
2	2	3	3	4	4	2	2	4	4
4	4	3	3	4	4	2	2	4	4
2	2	2	2	4	4	2	2	4	4
2	2	2	2	4	4	2	2	4	4
2	2	2	2	4	4	2	2	2	2
2	2	2	2	4	4	2	2	4	4
2	2	2	2	4	4	2	2	4	4
4	4	2	2	4	4	2	2	4	4
2	2	2	2	2	2	2	2	2	2
2	2	2	2	2	2	2	2	2	2
2	2	2	2	4	4	2	2	2	2
2	2	2	2	4	4	2	2	4	4
2	2	2	2	4	4	2	2	2	2
2	2	2	2	4	4	2	2	2	2
2	2	2	2	4	4	2	2	2	2
2	2	2	2	2	2	2	2	2	2
2	2	3	3	4	4	2	2	4	4
2	2	2	2	4	4	2	2	4	4
4	4	2	2	4	4	2	2	2	2
2	2	2	2	4	4	3	3	4	4
4	4	2	2	4	4	2	2	2	2
2	2	2	2	4	4	2	2	4	4
2	2	2	2	4	4	2	2	2	2
4	4	2	2	4	4	2	2	2	2
2	2	2	2	4	4	2	2	2	2
2	2	2	2	4	4	2	2	2	2
2	2	3	3	4	4	2	2	2	2
2	2	2	2	4	4	2	2	4	4
2	2	2	2	4	4	2	2	4	4

For Review Only

2	2	2	2	4	4	2	2	4	4
2	2	2	2	4	4	2	2	2	2
2	2	3	3	4	4	2	2	2	2
2	2	2	2	4	4	2	2	4	4
2	2	2	2	4	4	3	3	4	4
2	2	2	2	4	4	2	2	2	2
2	2	2	2	2	2	2	2	2	2
2	2	2	2	4	4	2	2	4	4
2	2	2	2	2	2	2	2	2	2
2	2	3	3	4	4	2	2	4	4
4	4	2	2	4	4	2	2	2	2
2	2	2	2	4	4	2	2	2	2
2	2	2	2	4	4	2	2	4	4
2	2	2	2	4	4	2	2	4	4
2	2	2	2	2	2	2	2	2	2
2	2	3	3	4	4	2	2	2	2
2	2	3	3	4	4	2	2	2	2
2	2	2	2	4	4	2	2	4	4
4	4	2	2	4	4	2	2	2	2
2	2	2	2	4	4	2	2	4	4
2	2	2	2	4	4	2	2	4	4
2	2	2	2	2	2	2	2	2	2
2	2	2	2	2	2	2	2	2	2
2	2	2	2	4	4	2	2	4	4
2	2	2	2	4	4	2	2	4	4
2	2	2	2	2	2	2	2	2	2
2	2	2	2	4	4	2	2	4	4
2	2	2	2	4	4	2	2	4	4
2	2	2	2	4	4	2	2	2	2
2	2	2	2	4	4	2	2	4	4
2	2	2	2	4	4	3	3	4	4
2	2	2	2	4	4	2	2	4	4
2	2	3	3	4	4	2	2	4	4
2	2	2	2	2	2	2	2	2	2
2	2	2	2	4	4	2	2	4	4
2	2	2	2	4	4	2	2	4	4
2	2	2	2	4	4	2	2	4	4
2	2	3	3	4	4	2	2	2	2
2	2	2	2	4	4	2	2	4	4
2	2	3	3	4	4	2	2	4	4
2	2	2	2	4	4	2	2	4	4
2	2	2	2	4	4	2	2	2	2
2	2	2	2	4	4	2	2	2	2
2	2	2	2	4	4	2	2	4	4
2	2	2	2	4	4	2	2	4	4
2	2	2	2	4	4	2	2	4	4
2	2	3	3	4	4	2	2	2	2
2	2	2	2	2	2	2	2	2	2
2	2	2	2	4	4	2	2	2	2
2	2	3	3	4	4	2	2	4	4
4	4	2	2	4	4	2	2	4	4
2	2	2	2	4	4	2	2	2	2
2	2	2	2	2	2	2	2	2	2
2	2	2	2	4	4	2	2	4	4
2	2	2	2	2	2	2	2	2	2

For Review Only

2	2	3	3	4	4	2	2	2	2
2	2	2	2	4	4	2	2	4	4
2	2	2	2	4	4	2	2	4	4
2	2	2	2	2	2	2	2	4	4
2	2	2	2	4	4	2	2	4	4
4	4	2	2	4	4	2	2	4	4
2	2	3	3	4	4	2	2	2	2
2	2	2	2	4	4	2	2	2	2
2	2	2	2	4	4	2	2	2	2
2	2	2	2	4	4	2	2	2	2
2	2	2	2	4	4	2	2	4	4
2	2	2	2	4	4	2	2	2	2
2	2	3	3	4	4	2	2	2	2
2	2	2	2	4	4	2	2	2	2
2	2	2	2	4	4	2	2	4	4
2	2	3	3	4	4	2	2	2	2
2	2	2	2	4	4	2	2	2	2
2	2	2	2	4	4	2	2	4	4
2	2	2	2	4	4	2	2	2	2
2	2	2	2	4	4	2	2	2	2
2	2	2	2	4	4	3	3	4	4
4	4	2	2	4	4	2	2	2	2
2	2	2	2	4	4	2	2	4	4
2	2	2	2	4	4	2	2	2	2
2	2	2	2	4	4	2	2	2	2
2	2	2	2	4	4	3	3	4	4
2	2	2	2	2	2	2	2	2	2
2	2	2	2	4	4	2	2	4	4
2	2	2	2	4	4	2	2	4	4
2	2	2	2	4	4	2	2	4	4
2	2	2	2	2	2	2	2	2	2
2	2	2	2	4	4	2	2	2	2
2	2	2	2	4	4	2	2	2	2
4	4	2	2	4	4	2	2	2	2
2	2	3	3	4	4	2	2	2	2
2	2	2	2	2	2	2	2	2	2
2	2	3	3	4	4	2	2	4	4
2	2	2	2	4	4	2	2	4	4
2	2	2	2	4	4	3	3	2	2
2	2	2	2	4	4	2	2	2	2
2	2	3	3	4	4	2	2	4	4
2	2	2	2	4	4	2	2	2	2
2	2	2	2	4	4	2	2	2	2
2	2	2	2	4	4	2	2	4	4
2	2	2	2	4	4	2	2	4	4
2	2	2	2	4	4	2	2	2	2
2	2	2	2	4	4	2	2	2	2
2	2	2	2	4	4	2	2	4	4
4	4	2	2	4	4	2	2	4	4
2	2	2	2	2	2	2	2	2	2
2	2	2	2	4	4	2	2	4	4
2	2	2	2	2	2	2	2	2	2
2	2	2	2	4	4	2	2	4	4
2	2	2	2	4	4	2	2	4	4
2	2	2	2	4	4	2	2	2	2

For Review Only

4	4	3	3	4	4	2	2	4	4
2	2	3	3	4	4	3	3	4	4

For Review Only

SNP1477	SNP1477	SNP1478	SNP1478	SNP1479	SNP1479	SNP148	SNP148	SNP1480	SNP1480
3	3	3	3	4	4	3	3	4	4
2	2	2	2	4	4	1	1	4	4
3	3	3	3	4	4	1	1	4	4
3	3	3	3	4	4	3	3	4	4
3	3	3	3	4	4	3	3	4	4
3	3	3	3	4	4	1	1	4	4
3	3	3	3	4	4	3	3	4	4
3	3	3	3	4	4	3	3	4	4
3	3	3	3	4	4	3	3	4	4
3	3	3	3	4	4	1	1	4	4
3	3	2	2	4	4	3	3	1	1
3	3	3	3	4	4	1	1	4	4
3	3	3	3	4	4	1	1	4	4
3	3	2	2	4	4	1	1	4	4
3	3	3	3	4	4	1	1	4	4
3	3	3	3	4	4	3	3	4	4
3	3	3	3	4	4	3	3	4	4
3	3	3	3	1	1	3	3	4	4
3	3	3	3	4	4	1	1	4	4
3	3	2	2	4	4	3	3	1	1
3	3	3	3	4	4	3	3	4	4
3	3	3	3	1	1	3	3	4	4
3	3	2	2	4	4	1	1	4	4
3	3	2	2	4	4	3	3	4	4
3	3	3	3	4	4	3	3	4	4
2	2	3	3	4	4	3	3	4	4
2	2	3	3	4	4	1	1	4	4
3	3	3	3	4	4	3	3	4	4
3	3	3	3	4	4	3	3	4	4
3	3	3	3	4	4	3	3	1	1
2	2	3	3	1	1	1	1	4	4
3	3	3	3	4	4	3	3	4	4
3	3	3	3	4	4	3	3	4	4
3	3	2	2	4	4	1	1	4	4
3	3	3	3	4	4	1	1	4	4
3	3	2	2	1	1	1	1	4	4
3	3	2	2	4	4	1	1	4	4
3	3	2	2	4	4	1	1	4	4
3	3	2	2	4	4	1	1	1	1
3	3	2	2	1	1	1	1	4	4
3	3	2	2	4	4	1	1	1	1
3	3	3	3	4	4	3	3	4	4
3	3	3	3	1	1	1	1	4	4
3	3	3	3	4	4	3	3	4	4
3	3	3	3	4	4	3	3	4	4
3	3	3	3	4	4	3	3	4	4
2	2	2	2	4	4	1	1	4	4
3	3	3	3	4	4	3	3	4	4
3	3	3	3	4	4	3	3	1	1
3	3	3	3	4	4	1	1	4	4
2	2	2	2	4	4	3	3	4	4
2	2	3	3	4	4	1	1	4	4

For Review Only

3	3	3	3	4	4	3	3	4	4
3	3	3	3	4	4	3	3	4	4
3	3	3	3	4	4	1	1	4	4
3	3	3	3	4	4	3	3	1	1
3	3	3	3	4	4	1	1	4	4
2	2	2	2	4	4	3	3	4	4
2	2	3	3	4	4	3	3	4	4
3	3	2	2	4	4	1	1	1	1
3	3	2	2	4	4	3	3	4	4
3	3	3	3	4	4	3	3	4	4
2	2	2	2	4	4	1	1	4	4
3	3	2	2	4	4	3	3	4	4
3	3	3	3	4	4	3	3	4	4
3	3	3	3	4	4	3	3	4	4
3	3	3	3	4	4	3	3	4	4
3	3	2	2	4	4	3	3	4	4
3	3	3	3	4	4	3	3	4	4
3	3	3	3	4	4	3	3	4	4
3	3	3	3	1	1	3	3	4	4
3	3	3	3	4	4	3	3	4	4
3	3	2	2	4	4	3	3	4	4
3	3	3	3	4	4	3	3	4	4
3	3	3	3	4	4	3	3	4	4
3	3	3	3	4	4	1	1	4	4
3	3	3	3	4	4	3	3	4	4
3	3	2	2	4	4	1	1	4	4
3	3	3	3	4	4	1	1	1	1
3	3	2	2	4	4	1	1	4	4
2	2	3	3	4	4	3	3	4	4
3	3	3	3	4	4	3	3	4	4
3	3	3	3	4	4	3	3	4	4
3	3	3	3	4	4	1	1	4	4
3	3	2	2	1	1	1	1	1	1
3	3	3	3	4	4	3	3	4	4
3	3	3	3	4	4	1	1	4	4
3	3	3	3	4	4	3	3	4	4
3	3	3	3	4	4	3	3	4	4
3	3	3	3	4	4	3	3	1	1
3	3	2	2	4	4	3	3	1	1
3	3	3	3	4	4	3	3	1	1
3	3	3	3	4	4	3	3	4	4
3	3	3	3	4	4	1	1	4	4
3	3	3	3	4	4	1	1	4	4
2	2	2	2	4	4	1	1	4	4
3	3	2	2	4	4	3	3	4	4
3	3	2	2	4	4	1	1	4	4
3	3	3	3	4	4	1	1	4	4
3	3	3	3	4	4	3	3	4	4
3	3	3	3	4	4	1	1	4	4
3	3	3	3	4	4	1	1	4	4
2	2	2	2	4	4	1	1	4	4
3	3	2	2	4	4	1	1	1	1
3	3	2	2	4	4	3	3	1	1
3	3	3	3	4	4	3	3	4	4
3	3	3	3	4	4	1	1	4	4

For Review Only

2	2	3	3	4	4	1	1	4	4
3	3	3	3	1	1	1	1	4	4
2	2	2	2	4	4	3	3	4	4
3	3	2	2	4	4	1	1	4	4
3	3	3	3	1	1	1	1	4	4
3	3	3	3	1	1	1	1	4	4
3	3	3	3	4	4	3	3	4	4
3	3	2	2	4	4	3	3	4	4
3	3	3	3	4	4	1	1	4	4
3	3	2	2	4	4	3	3	4	4
3	3	2	2	4	4	1	1	4	4
3	3	3	3	4	4	3	3	4	4
3	3	3	3	4	4	3	3	4	4
3	3	3	3	4	4	1	1	4	4
3	3	3	3	1	1	3	3	4	4
2	2	3	3	4	4	1	1	4	4
3	3	3	3	4	4	3	3	4	4
3	3	3	3	4	4	1	1	4	4
3	3	2	2	4	4	1	1	4	4
3	3	3	3	4	4	3	3	4	4
3	3	2	2	4	4	3	3	4	4
3	3	3	3	4	4	3	3	4	4
3	3	3	3	4	4	1	1	4	4
3	3	2	2	4	4	1	1	1	1
3	3	3	3	4	4	3	3	4	4
3	3	3	3	4	4	3	3	4	4
3	3	3	3	4	4	3	3	4	4
3	3	3	3	4	4	1	1	4	4
3	3	3	3	4	4	3	3	4	4
3	3	3	3	4	4	1	1	4	4
3	3	2	2	4	4	1	1	1	1
3	3	2	2	4	4	3	3	4	4
3	3	3	3	4	4	3	3	4	4
3	3	3	3	4	4	3	3	4	4
3	3	2	2	4	4	1	1	4	4
3	3	3	3	4	4	1	1	4	4
3	3	2	2	4	4	1	1	4	4
3	3	2	2	4	4	1	1	4	4
3	3	3	3	4	4	3	3	4	4
3	3	2	2	4	4	3	3	1	1
3	3	3	3	4	4	3	3	4	4
3	3	2	2	4	4	1	1	4	4
3	3	3	3	4	4	1	1	4	4
3	3	3	3	4	4	3	3	1	1
3	3	3	3	4	4	3	3	4	4
3	3	3	3	1	1	3	3	4	4
2	2	3	3	4	4	3	3	4	4
2	2	3	3	4	4	1	1	4	4
3	3	2	2	4	4	3	3	1	1
2	2	3	3	4	4	3	3	4	4
2	2	2	2	4	4	3	3	4	4
3	3	2	2	1	1	1	1	4	4
3	3	2	2	4	4	1	1	4	4
3	3	2	2	1	1	3	3	4	4
3	3	3	3	4	4	3	3	1	1

For Review Only

3	3	3	3	4	4	1	1	4	4
3	3	2	2	4	4	3	3	4	4
3	3	2	2	4	4	1	1	1	1
3	3	3	3	4	4	3	3	4	4
2	2	2	2	4	4	3	3	4	4
3	3	2	2	4	4	1	1	4	4
3	3	3	3	4	4	1	1	4	4
3	3	3	3	4	4	3	3	4	4
3	3	3	3	4	4	3	3	4	4
3	3	3	3	4	4	3	3	4	4
3	3	3	3	4	4	3	3	4	4
3	3	3	3	4	4	1	1	4	4
3	3	2	2	4	4	3	3	4	4
3	3	3	3	4	4	3	3	4	4
3	3	2	2	4	4	1	1	4	4
3	3	2	2	4	4	1	1	1	1
3	3	2	2	4	4	1	1	4	4
3	3	3	3	4	4	1	1	4	4
2	2	3	3	1	1	1	1	4	4
3	3	3	3	4	4	3	3	4	4
3	3	3	3	4	4	3	3	4	4
3	3	2	2	4	4	1	1	1	1
3	3	2	2	4	4	3	3	4	4
3	3	2	2	4	4	3	3	4	4
3	3	3	3	4	4	3	3	4	4
3	3	2	2	4	4	1	1	4	4
3	3	3	3	4	4	3	3	4	4
3	3	3	3	4	4	1	1	4	4
3	3	2	2	4	4	1	1	4	4
3	3	3	3	4	4	1	1	4	4
3	3	3	3	1	1	1	1	4	4
3	3	3	3	4	4	3	3	4	4
3	3	3	3	4	4	1	1	4	4
3	3	2	2	4	4	1	1	4	4
3	3	3	3	4	4	3	3	4	4
3	3	2	2	4	4	3	3	4	4
3	3	3	3	4	4	1	1	4	4
3	3	2	2	4	4	1	1	4	4
2	2	3	3	4	4	1	1	4	4
2	2	2	2	4	4	1	1	4	4
3	3	3	3	4	4	1	1	4	4
2	2	3	3	4	4	3	3	4	4
3	3	2	2	4	4	1	1	4	4
3	3	3	3	4	4	3	3	4	4
3	3	3	3	4	4	3	3	4	4
3	3	3	3	4	4	1	1	4	4
3	3	2	2	4	4	1	1	1	1
3	3	2	2	1	1	1	1	1	1
3	3	2	2	4	4	1	1	4	4
3	3	3	3	4	4	3	3	4	4
3	3	3	3	4	4	1	1	4	4
3	3	2	2	4	4	3	3	4	4
3	3	2	2	4	4	3	3	4	4
2	2	2	2	4	4	1	1	4	4
3	3	2	2	4	4	1	1	1	1

For Review Only

3	3	2	2	4	4	1	1	4	4
3	3	3	3	4	4	1	1	4	4
3	3	3	3	4	4	3	3	4	4
3	3	3	3	4	4	3	3	4	4
3	3	3	3	4	4	3	3	4	4
3	3	3	3	4	4	3	3	4	4
3	3	3	3	4	4	1	1	1	1
3	3	3	3	4	4	1	1	1	1
3	3	2	2	4	4	1	1	4	4
3	3	2	2	4	4	1	1	4	4
3	3	3	3	4	4	3	3	4	4
3	3	2	2	4	4	3	3	4	4
3	3	2	2	4	4	3	3	1	1
2	2	3	3	4	4	3	3	4	4
3	3	3	3	4	4	3	3	4	4
3	3	2	2	4	4	1	1	4	4
3	3	2	2	4	4	1	1	1	1
2	2	3	3	4	4	3	3	4	4
3	3	2	2	4	4	1	1	4	4
3	3	2	2	4	4	1	1	4	4
3	3	3	3	1	1	1	1	4	4
2	2	3	3	4	4	3	3	4	4
2	2	3	3	4	4	3	3	4	4
3	3	3	3	4	4	1	1	4	4
3	3	2	2	4	4	1	1	1	1
3	3	3	3	1	1	3	3	4	4
3	3	2	2	1	1	1	1	1	1
2	2	3	3	4	4	3	3	4	4
3	3	3	3	4	4	3	3	4	4
3	3	3	3	4	4	1	1	4	4
3	3	2	2	1	1	1	1	1	1
3	3	3	3	4	4	1	1	4	4
3	3	2	2	4	4	1	1	4	4
2	2	2	2	4	4	3	3	4	4
3	3	2	2	4	4	3	3	4	4
3	3	2	2	1	1	1	1	1	1
3	3	3	3	4	4	1	1	4	4
3	3	2	2	4	4	3	3	4	4
3	3	2	2	1	1	1	1	4	4
3	3	2	2	4	4	1	1	1	1
3	3	3	3	4	4	1	1	4	4
3	3	2	2	4	4	1	1	4	4
3	3	3	3	4	4	1	1	4	4
3	3	3	3	4	4	3	3	4	4
2	2	3	3	4	4	1	1	4	4
3	3	3	3	4	4	3	3	1	1
2	2	2	2	4	4	1	1	1	1
3	3	3	3	4	4	3	3	4	4
2	2	3	3	4	4	1	1	4	4
3	3	2	2	1	1	1	1	1	1
3	3	3	3	4	4	1	1	4	4
3	3	3	3	4	4	1	1	1	1
3	3	3	3	4	4	1	1	4	4
2	2	3	3	4	4	3	3	4	4
3	3	3	3	4	4	1	1	4	4

3	3	3	3	4	4	3	3	4	4
3	3	3	3	1	1	3	3	4	4

For Review Only

SNP1481	SNP1481	SNP1482	SNP1482	SNP1483	SNP1483	SNP1484	SNP1484	SNP1485	SNP1485
2	2	2	2	1	1	1	1	1	1
1	1	2	2	1	1	4	4	1	1
2	2	4	4	1	1	4	4	4	4
1	1	4	4	1	1	1	1	4	4
1	1	2	2	1	1	4	4	4	4
2	2	2	2	1	1	1	1	1	1
2	2	4	4	4	4	4	4	4	4
1	1	4	4	1	1	4	4	4	4
1	1	4	4	1	1	1	1	4	4
1	1	2	2	1	1	1	1	1	1
1	1	4	4	1	1	1	1	4	4
2	2	4	4	1	1	4	4	4	4
2	2	2	2	1	1	4	4	4	4
1	1	4	4	1	1	4	4	4	4
1	1	4	4	1	1	4	4	4	4
2	2	4	4	1	1	4	4	4	4
2	2	4	4	4	4	4	4	4	4
1	1	2	2	1	1	1	1	1	1
2	2	4	4	1	1	1	1	1	1
1	1	4	4	1	1	1	1	4	4
2	2	2	2	1	1	4	4	1	1
1	1	2	2	4	4	4	4	4	4
1	1	2	2	1	1	4	4	4	4
1	1	2	2	1	1	4	4	1	1
2	2	4	4	1	1	1	1	4	4
1	1	2	2	4	4	4	4	1	1
1	1	2	2	1	1	4	4	1	1
1	1	4	4	1	1	4	4	4	4
1	1	4	4	1	1	1	1	4	4
1	1	2	2	1	1	4	4	4	4
1	1	2	2	1	1	4	4	1	1
2	2	4	4	1	1	1	1	4	4
2	2	4	4	1	1	1	1	4	4
1	1	2	2	1	1	1	1	1	1
1	1	4	4	1	1	4	4	4	4
1	1	2	2	1	1	1	1	1	1
1	1	4	4	1	1	4	4	4	4
1	1	4	4	1	1	1	1	4	4
1	1	2	2	1	1	4	4	1	1
2	2	2	2	1	1	1	1	1	1
1	1	2	2	1	1	4	4	1	1
1	1	4	4	1	1	4	4	4	4
1	1	2	2	1	1	4	4	1	1
1	1	4	4	1	1	4	4	4	4
1	1	4	4	1	1	4	4	1	1
2	2	4	4	1	1	4	4	1	1
1	1	4	4	4	4	4	4	1	1
1	1	2	2	1	1	1	1	1	1
1	1	4	4	1	1	4	4	4	4
2	2	4	4	1	1	1	1	4	4
1	1	4	4	4	4	4	4	4	4
1	1	4	4	1	1	4	4	1	1

For Review Only

2	2	4	4	1	1	4	4	4	4
2	2	4	4	1	1	1	1	4	4
1	1	4	4	1	1	1	1	4	4
1	1	2	2	4	4	4	4	1	1
2	2	4	4	1	1	1	1	1	1
1	1	2	2	1	1	4	4	1	1
1	1	2	2	4	4	4	4	1	1
1	1	2	2	1	1	4	4	1	1
1	1	2	2	1	1	4	4	1	1
2	2	4	4	1	1	4	4	4	4
1	1	4	4	1	1	4	4	1	1
1	1	4	4	1	1	4	4	1	1
2	2	4	4	1	1	1	1	4	4
1	1	4	4	1	1	1	1	4	4
2	2	4	4	1	1	4	4	4	4
1	1	4	4	1	1	4	4	4	4
1	1	4	4	1	1	4	4	4	4
1	1	4	4	1	1	1	1	4	4
1	1	2	2	4	4	4	4	1	1
2	2	2	2	1	1	1	1	1	1
1	1	4	4	1	1	4	4	1	1
1	1	4	4	1	1	1	1	4	4
1	1	4	4	1	1	1	1	4	4
1	1	4	4	1	1	1	1	4	4
1	1	4	4	1	1	4	4	1	1
1	1	4	4	4	4	4	4	1	1
2	2	4	4	1	1	4	4	1	1
1	1	2	2	1	1	4	4	1	1
1	1	2	2	4	4	4	4	1	1
2	2	4	4	1	1	4	4	4	4
1	1	2	2	4	4	4	4	1	1
1	1	4	4	1	1	1	1	4	4
1	1	2	2	1	1	4	4	1	1
2	2	4	4	1	1	1	1	4	4
1	1	4	4	1	1	1	1	4	4
1	1	4	4	1	1	4	4	1	1
1	1	4	4	1	1	4	4	4	4
2	2	2	2	1	1	4	4	1	1
1	1	2	2	1	1	4	4	1	1
1	1	4	4	1	1	4	4	1	1
1	1	4	4	4	4	4	4	4	4
2	2	4	4	1	1	4	4	4	4
2	2	4	4	1	1	4	4	4	4
1	1	4	4	1	1	4	4	1	1
1	1	2	2	1	1	1	1	1	1
1	1	4	4	1	1	1	1	4	4
1	1	2	2	1	1	4	4	1	1
1	1	2	2	4	4	4	4	1	1
1	1	2	2	4	4	4	4	1	1
1	1	4	4	1	1	1	1	4	4
1	1	4	4	4	4	4	4	1	1
1	1	2	2	1	1	4	4	1	1
1	1	2	2	1	1	4	4	1	1
1	1	2	2	1	1	1	1	4	4
1	1	4	4	1	1	1	1	4	4

For Review Only

1	1	2	2	1	1	1	1	1	1
1	1	2	2	1	1	4	4	1	1
1	1	2	2	4	4	4	4	4	4
1	1	2	2	1	1	4	4	1	1
1	1	2	2	4	4	4	4	1	1
1	1	2	2	4	4	4	4	1	1
2	2	4	4	4	4	4	4	4	4
1	1	4	4	1	1	4	4	1	1
1	1	2	2	1	1	4	4	1	1
1	1	2	2	4	4	1	1	4	4
1	1	4	4	1	1	1	1	4	4
2	2	4	4	1	1	4	4	4	4
1	1	2	2	1	1	4	4	1	1
1	1	4	4	1	1	4	4	1	1
2	2	2	2	1	1	1	1	4	4
1	1	4	4	1	1	4	4	4	4
1	1	4	4	1	1	4	4	4	4
1	1	4	4	1	1	4	4	4	4
1	1	2	2	1	1	4	4	1	1
1	1	4	4	4	4	1	1	4	4
1	1	4	4	1	1	4	4	4	4
1	1	4	4	1	1	4	4	4	4
1	1	4	4	4	4	4	4	4	4
1	1	2	2	1	1	4	4	4	4
1	1	4	4	1	1	4	4	4	4
1	1	4	4	1	1	1	1	4	4
1	1	4	4	1	1	1	1	4	4
1	1	4	4	1	1	1	1	4	4
1	1	4	4	4	4	4	4	4	4
1	1	4	4	1	1	1	1	4	4
1	1	2	2	1	1	4	4	1	1
1	1	4	4	1	1	4	4	4	4
1	1	4	4	4	4	4	4	4	4
1	1	4	4	4	4	4	4	4	4
1	1	2	2	1	1	1	1	1	1
1	1	2	2	4	4	4	4	1	1
1	1	2	2	1	1	4	4	1	1
1	1	4	4	1	1	1	1	1	1
1	1	4	4	1	1	1	1	4	4
1	1	4	4	1	1	1	1	4	4
1	1	4	4	1	1	1	1	4	4
1	1	2	2	1	1	4	4	1	1
1	1	4	4	1	1	1	1	4	4
1	1	4	4	1	1	4	4	4	4
1	1	4	4	1	1	4	4	4	4
2	2	2	2	1	1	1	1	1	1
1	1	2	2	4	4	4	4	1	1
1	1	2	2	4	4	4	4	1	1
1	1	4	4	1	1	4	4	4	4
1	1	4	4	4	4	4	4	4	4
1	1	2	2	4	4	4	4	1	1
1	1	2	2	1	1	1	1	1	1
1	1	4	4	1	1	1	1	4	4
1	1	2	2	4	4	4	4	1	1
1	1	2	2	1	1	4	4	4	4

For Review Only

For Review Only

1	1	4	4	1	1	1	1	4	4
1	1	2	2	1	1	1	1	1	1
1	1	4	4	1	1	4	4	1	1
1	1	4	4	1	1	4	4	4	4
1	1	2	2	1	1	1	1	4	4
1	1	4	4	1	1	4	4	4	4
1	1	4	4	1	1	1	1	4	4
1	1	4	4	1	1	4	4	4	4
1	1	2	2	1	1	4	4	1	1
2	2	4	4	1	1	1	1	4	4
1	1	2	2	4	4	4	4	1	1
1	1	2	2	1	1	1	1	1	1
1	1	2	2	1	1	4	4	1	1
1	1	2	2	1	1	4	4	4	4
1	1	2	2	1	1	4	4	1	1
1	1	4	4	1	1	1	1	4	4
1	1	4	4	1	1	4	4	4	4
1	1	4	4	1	1	1	1	4	4
1	1	2	2	4	4	4	4	1	1
2	2	4	4	4	4	4	4	4	4
1	1	4	4	1	1	4	4	4	4
1	1	2	2	1	1	4	4	1	1
1	1	4	4	1	1	1	1	4	4
1	1	4	4	1	1	4	4	1	1
1	1	4	4	1	1	4	4	4	4
1	1	2	2	1	1	1	1	1	1
1	1	2	2	1	1	4	4	1	1
1	1	4	4	1	1	4	4	1	1
1	1	4	4	1	1	1	1	4	4
1	1	4	4	1	1	4	4	4	4
1	1	2	2	1	1	1	1	1	1
2	2	4	4	1	1	4	4	4	4
1	1	2	2	1	1	4	4	4	4
1	1	4	4	1	1	4	4	1	1
1	1	4	4	4	4	4	4	4	4
1	1	4	4	1	1	4	4	1	1
2	2	2	2	1	1	1	1	1	1
2	2	4	4	1	1	1	1	4	4
1	1	2	2	4	4	4	4	1	1
1	1	4	4	1	1	1	1	4	4
2	2	4	4	1	1	4	4	4	4
1	1	2	2	4	4	4	4	1	1
1	1	2	2	1	1	4	4	1	1
1	1	4	4	1	1	4	4	4	4
1	1	4	4	1	1	4	4	4	4
2	2	4	4	1	1	4	4	4	4
1	1	4	4	1	1	1	1	4	4
1	1	2	2	1	1	4	4	4	4
1	1	2	2	1	1	4	4	1	1
1	1	2	2	4	4	4	4	1	1
1	1	4	4	1	1	1	1	4	4
1	1	2	2	1	1	4	4	4	4
1	1	4	4	1	1	1	1	4	4
1	1	4	4	1	1	4	4	1	1
1	1	2	2	1	1	4	4	1	1

1	1	4	4	1	1	4	4	4	4
1	1	4	4	1	1	1	1	4	4
2	2	4	4	4	4	4	4	4	4
1	1	4	4	1	1	1	1	4	4
1	1	4	4	1	1	4	4	4	4
1	1	2	2	1	1	4	4	1	1
1	1	2	2	1	1	1	1	1	1
1	1	2	2	1	1	1	1	1	1
1	1	2	2	1	1	4	4	1	1
1	1	2	2	1	1	4	4	4	4
1	1	4	4	1	1	4	4	4	4
1	1	2	2	1	1	1	1	1	1
1	1	2	2	1	1	1	1	1	1
1	1	4	4	1	1	4	4	4	4
2	2	4	4	1	1	4	4	4	4
1	1	4	4	1	1	1	1	4	4
1	1	2	2	1	1	1	1	1	1
1	1	2	2	1	1	1	1	1	1
2	2	2	2	1	1	1	1	1	1
1	1	4	4	1	1	1	1	4	4
1	1	2	2	1	1	4	4	1	1
1	1	2	2	4	4	4	4	1	1
1	1	4	4	1	1	1	1	4	4
1	1	4	4	1	1	1	1	4	4
1	1	2	2	1	1	4	4	1	1
2	2	2	2	1	1	4	4	1	1
1	1	2	2	1	1	4	4	1	1
1	1	2	2	4	4	4	4	1	1
2	2	4	4	1	1	1	1	4	4
1	1	4	4	4	4	4	4	4	4
1	1	2	2	1	1	4	4	1	1
1	1	2	2	4	4	4	4	1	1
1	1	2	2	1	1	4	4	4	4
1	1	2	2	1	1	1	1	1	1
1	1	4	4	1	1	1	1	4	4
1	1	2	2	1	1	4	4	1	1
1	1	4	4	1	1	1	1	4	4
1	1	4	4	1	1	4	4	1	1
1	1	2	2	1	1	1	1	1	1
1	1	2	2	1	1	4	4	1	1
1	1	4	4	1	1	1	1	4	4
2	2	2	2	1	1	1	1	4	4
1	1	4	4	1	1	1	1	4	4
1	1	4	4	4	4	4	4	4	4
1	1	4	4	1	1	1	1	4	4
1	1	2	2	1	1	4	4	1	1
1	1	2	2	4	4	4	4	1	1
1	1	2	2	1	1	4	4	1	1
1	1	2	2	1	1	4	4	1	1
1	1	2	2	1	1	4	4	1	1
2	2	4	4	1	1	1	1	4	4
1	1	2	2	1	1	4	4	1	1
2	2	4	4	1	1	4	4	4	4
1	1	2	2	1	1	1	1	1	1
1	1	4	4	1	1	1	1	4	4

For Review Only

1	1	4	4	1	1	1	1	4	4
1	1	2	2	1	1	4	4	1	1

For Review Only

SNP1486	SNP1486	SNP1487	SNP1487	SNP1488	SNP1488	SNP1489	SNP1489	SNP149	SNP149
1	1	2	2	3	3	3	3	1	1
1	1	1	1	3	3	2	2	3	3
1	1	2	2	3	3	3	3	3	3
1	1	2	2	3	3	2	2	3	3
2	2	2	2	1	1	3	3	3	3
1	1	2	2	3	3	3	3	3	3
1	1	2	2	3	3	3	3	3	3
2	2	2	2	3	3	3	3	3	3
2	2	2	2	3	3	2	2	3	3
2	2	1	1	3	3	3	3	3	3
2	2	2	2	3	3	2	2	3	3
1	1	2	2	3	3	2	2	3	3
2	2	2	2	3	3	3	3	3	3
2	2	2	2	3	3	2	2	3	3
2	2	2	2	3	3	3	3	3	3
2	2	2	2	3	3	2	2	1	1
2	2	2	2	1	1	2	2	3	3
2	2	1	1	3	3	3	3	1	1
1	1	2	2	3	3	3	3	1	1
2	2	2	2	1	1	3	3	1	1
2	2	2	2	3	3	2	2	3	3
2	2	2	2	3	3	2	2	3	3
1	1	2	2	3	3	2	2	3	3
2	2	1	1	3	3	2	2	3	3
1	1	2	2	3	3	2	2	3	3
2	2	2	2	3	3	3	3	1	1
1	1	2	2	3	3	3	3	3	3
2	2	2	2	3	3	2	2	3	3
2	2	1	1	3	3	2	2	3	3
2	2	2	2	1	1	3	3	3	3
1	1	2	2	3	3	3	3	3	3
1	1	2	2	3	3	2	2	3	3
1	1	2	2	3	3	3	3	3	3
2	2	1	1	3	3	2	2	3	3
1	1	2	2	3	3	2	2	3	3
2	2	2	2	3	3	2	2	3	3
1	1	1	1	3	3	3	3	3	3
1	1	1	1	3	3	3	3	3	3
2	2	2	2	3	3	2	2	3	3
1	1	1	1	3	3	2	2	3	3
2	2	2	2	3	3	3	3	3	3
2	2	1	1	3	3	2	2	3	3
2	2	2	2	3	3	3	3	3	3
2	2	2	2	1	1	3	3	3	3
2	2	1	1	3	3	3	3	3	3
2	2	2	2	3	3	3	3	3	3
1	1	2	2	3	3	3	3	1	1
2	2	2	2	3	3	2	2	1	1
2	2	1	1	3	3	3	3	3	3
1	1	2	2	3	3	3	3	3	3
1	1	2	2	3	3	3	3	3	3
1	1	2	2	3	3	3	3	3	3

For Review Only

2	2	2	2	3	3	3	3	3	3
1	1	2	2	3	3	3	3	3	3
1	1	2	2	3	3	2	2	3	3
2	2	2	2	1	1	2	2	3	3
1	1	2	2	3	3	3	3	1	1
1	1	2	2	3	3	3	3	1	1
2	2	2	2	3	3	3	3	1	1
2	2	1	1	3	3	2	2	3	3
1	1	2	2	3	3	3	3	3	3
2	2	2	2	3	3	3	3	3	3
1	1	2	2	3	3	3	3	3	3
1	1	2	2	3	3	3	3	3	3
2	2	2	2	3	3	3	3	3	3
2	2	1	1	3	3	2	2	3	3
1	1	2	2	3	3	3	3	3	3
1	1	2	2	1	1	3	3	3	3
1	1	2	2	3	3	2	2	3	3
1	1	2	2	3	3	2	2	1	1
1	1	2	2	3	3	2	2	3	3
2	2	2	2	3	3	3	3	1	1
1	1	2	2	3	3	3	3	3	3
1	1	2	2	3	3	3	3	3	3
2	2	2	2	3	3	2	2	3	3
1	1	2	2	3	3	2	2	3	3
2	2	1	1	3	3	3	3	3	3
1	1	1	1	3	3	3	3	3	3
2	2	2	2	1	1	3	3	3	3
2	2	2	2	3	3	2	2	3	3
2	2	2	2	3	3	3	3	1	1
1	1	2	2	3	3	2	2	3	3
2	2	2	2	1	1	2	2	3	3
2	2	2	2	3	3	2	2	3	3
2	2	2	2	3	3	3	3	3	3
2	2	2	2	3	3	3	3	3	3
2	2	2	2	3	3	3	3	1	1
2	2	1	1	3	3	3	3	3	3
2	2	1	1	3	3	2	2	3	3
2	2	2	2	1	1	2	2	3	3
2	2	2	2	3	3	3	3	3	3
2	2	2	2	3	3	3	3	3	3
2	2	2	2	3	3	2	2	3	3
1	1	2	2	3	3	2	2	3	3
2	2	2	2	3	3	3	3	3	3
1	1	2	2	3	3	3	3	3	3
1	1	2	2	3	3	2	2	3	3
1	1	2	2	3	3	2	2	3	3
2	2	1	1	3	3	3	3	3	3
2	2	2	2	3	3	2	2	3	3
2	2	2	2	3	3	3	3	1	1
1	1	2	2	3	3	3	3	3	3
1	1	2	2	3	3	3	3	3	3
2	2	2	2	3	3	2	2	3	3
2	2	2	2	3	3	2	2	3	3
2	2	2	2	3	3	2	2	1	1
1	1	2	2	3	3	3	3	3	3

For Review Only

1	1	2	2	3	3	2	2	1	1
1	1	1	1	3	3	3	3	3	3
2	2	2	2	3	3	3	3	3	3
2	2	2	2	3	3	2	2	3	3
1	1	2	2	3	3	3	3	3	3
2	2	2	2	3	3	2	2	1	1
1	1	2	2	3	3	2	2	1	1
1	1	2	2	3	3	2	2	3	3
1	1	1	1	3	3	2	2	3	3
2	2	2	2	3	3	3	3	3	3
1	1	1	1	3	3	3	3	3	3
2	2	2	2	3	3	3	3	3	3
2	2	2	2	3	3	3	3	3	3
1	1	2	2	3	3	3	3	3	3
2	2	2	2	3	3	2	2	3	3
1	1	2	2	3	3	3	3	3	3
1	1	2	2	3	3	3	3	1	1
2	2	2	2	3	3	2	2	3	3
2	2	2	2	3	3	2	2	3	3
2	2	2	2	3	3	3	3	3	3
2	2	2	2	3	3	2	2	3	3
1	1	2	2	3	3	2	2	3	3
1	1	2	2	3	3	2	2	3	3
2	2	2	2	3	3	2	2	3	3
1	1	2	2	3	3	3	3	3	3
1	1	2	2	3	3	3	3	3	3
2	2	2	2	3	3	3	3	3	3
1	1	2	2	3	3	3	3	3	3
1	1	2	2	3	3	2	2	1	1
2	2	2	2	3	3	3	3	3	3
2	2	1	1	3	3	2	2	3	3
2	2	2	2	3	3	2	2	3	3
2	2	2	2	3	3	2	2	1	1
2	2	2	2	3	3	3	3	1	1
2	2	1	1	3	3	2	2	3	3
2	2	1	1	3	3	2	2	3	3
2	2	1	1	3	3	3	3	3	3
1	1	2	2	3	3	3	3	3	3
2	2	2	2	3	3	2	2	3	3
2	2	2	2	3	3	2	2	3	3
1	1	2	2	3	3	3	3	3	3
2	2	2	2	3	3	2	2	3	3
1	1	2	2	3	3	2	2	3	3
2	2	2	2	3	3	3	3	3	3
2	2	2	2	3	3	3	3	3	3
2	2	2	2	3	3	2	2	3	3
2	2	2	2	3	3	3	3	1	1
1	1	2	2	3	3	3	3	1	1
2	2	1	1	3	3	3	3	3	3
2	2	2	2	3	3	3	3	1	1
2	2	2	2	3	3	3	3	1	1
2	2	1	1	3	3	3	3	1	1
1	1	2	2	3	3	2	2	3	3
1	1	2	2	3	3	3	3	3	3
2	2	1	1	3	3	2	2	3	3

For Review Only

1	1	2	2	3	3	3	3	3	3
2	2	1	1	3	3	3	3	3	3
2	2	2	2	1	1	3	3	3	3
2	2	1	1	3	3	2	2	3	3
2	2	2	2	3	3	3	3	1	1
1	1	2	2	3	3	2	2	1	1
2	2	2	2	3	3	2	2	3	3
2	2	2	2	3	3	3	3	3	3
2	2	2	2	3	3	2	2	3	3
2	2	2	2	3	3	2	2	3	3
2	2	2	2	3	3	3	3	1	1
1	1	1	1	3	3	2	2	3	3
2	2	2	2	3	3	2	2	3	3
2	2	2	2	3	3	2	2	3	3
2	2	1	1	3	3	2	2	3	3
2	2	2	2	3	3	2	2	3	3
1	1	2	2	3	3	2	2	3	3
1	1	2	2	3	3	3	3	3	3
2	2	2	2	3	3	2	2	3	3
1	1	2	2	3	3	2	2	1	1
2	2	2	2	3	3	2	2	3	3
2	2	2	2	3	3	3	3	3	3
1	1	2	2	3	3	3	3	3	3
1	1	2	2	3	3	3	3	3	3
2	2	1	1	3	3	3	3	3	3
2	2	1	1	3	3	2	2	3	3
1	1	2	2	3	3	2	2	3	3
1	1	2	2	3	3	2	2	1	1
1	1	1	1	3	3	2	2	3	3
2	2	2	2	3	3	2	2	3	3
1	1	2	2	3	3	3	3	3	3
1	1	2	2	3	3	3	3	1	1
1	1	2	2	3	3	3	3	3	3
2	2	2	2	3	3	2	2	3	3
2	2	2	2	3	3	3	3	3	3
1	1	2	2	3	3	3	3	3	3
1	1	2	2	3	3	3	3	3	3
1	1	2	2	3	3	2	2	3	3
1	1	2	2	3	3	3	3	1	1
1	1	2	2	3	3	3	3	3	3
1	1	2	2	3	3	3	3	3	3
1	1	2	2	3	3	3	3	1	1
2	2	2	2	3	3	2	2	3	3
1	1	2	2	3	3	3	3	3	3
2	2	1	1	3	3	2	2	3	3
1	1	2	2	3	3	2	2	3	3
2	2	2	2	3	3	2	2	3	3
2	2	2	2	3	3	3	3	3	3
2	2	1	1	3	3	2	2	3	3
1	1	2	2	3	3	2	2	3	3
2	2	2	2	3	3	3	3	1	1
1	1	1	1	3	3	3	3	3	3
1	1	1	1	3	3	3	3	3	3
1	1	2	2	3	3	3	3	3	3
2	2	1	1	3	3	2	2	3	3

For Review Only

1	1	1	1	3	3	2	2	3	3
2	2	1	1	1	1	3	3	3	3
1	1	2	2	3	3	3	3	1	1
2	2	2	2	3	3	3	3	3	3
2	2	1	1	3	3	3	3	1	1
2	2	2	2	3	3	3	3	1	1
2	2	2	2	3	3	2	2	1	1
2	2	1	1	3	3	3	3	3	3
2	2	2	2	3	3	2	2	3	3
2	2	2	2	3	3	2	2	3	3
2	2	2	2	3	3	2	2	3	3
1	1	2	2	3	3	2	2	3	3
2	2	2	2	3	3	3	3	3	3
1	1	2	2	3	3	3	3	3	3
1	1	2	2	3	3	3	3	1	1
2	2	2	2	3	3	2	2	3	3
2	2	1	1	3	3	2	2	3	3
2	2	2	2	3	3	2	2	3	3
1	1	2	2	3	3	2	2	3	3
1	1	1	1	3	3	3	3	3	3
1	1	2	2	3	3	2	2	3	3
2	2	2	2	3	3	3	3	1	1
1	1	2	2	3	3	3	3	1	1
1	1	1	1	3	3	2	2	3	3
2	2	1	1	3	3	2	2	3	3
1	1	2	2	3	3	3	3	3	3
2	2	2	2	3	3	2	2	3	3
1	1	2	2	3	3	2	2	3	3
2	2	1	1	3	3	3	3	3	3
1	1	2	2	3	3	3	3	1	1
2	2	2	2	3	3	2	2	3	3
1	1	2	2	3	3	2	2	3	3
2	2	1	1	3	3	3	3	3	3
2	2	2	2	3	3	3	3	1	1
1	1	2	2	3	3	3	3	3	3
2	2	2	2	3	3	2	2	3	3
1	1	2	2	3	3	2	2	3	3
1	1	2	2	3	3	3	3	3	3
2	2	2	2	3	3	2	2	3	3
2	2	2	2	3	3	2	2	3	3
1	1	2	2	3	3	3	3	1	1
2	2	2	2	3	3	3	3	3	3
1	1	1	1	3	3	2	2	3	3
2	2	2	2	3	3	2	2	3	3
1	1	2	2	3	3	2	2	3	3
2	2	2	2	3	3	3	3	3	3
2	2	2	2	3	3	2	2	1	1
1	1	2	2	3	3	2	2	3	3
2	2	2	2	3	3	3	3	3	3
2	2	2	2	3	3	2	2	3	3
1	1	1	1	3	3	3	3	3	3
2	2	2	2	3	3	2	2	3	3
1	1	2	2	3	3	3	3	3	3
2	2	2	2	3	3	2	2	3	3
1	1	1	1	3	3	2	2	3	3

For Review Only

1	1	2	2	3	3	2	2	3	3
1	1	2	2	3	3	3	3	3	3

For Review Only

SNP1490	SNP1490	SNP1491	SNP1491	SNP1492	SNP1492	SNP1493	SNP1493	SNP1494	SNP1494
1	1	4	4	1	1	1	1	4	4
1	1	4	4	1	1	1	1	4	4
1	1	4	4	1	1	4	4	4	4
1	1	4	4	1	1	1	1	4	4
3	3	1	1	4	4	4	4	4	4
1	1	4	4	1	1	1	1	4	4
1	1	4	4	1	1	1	1	4	4
1	1	4	4	1	1	1	1	1	1
1	1	4	4	1	1	1	1	4	4
1	1	4	4	1	1	1	1	1	1
1	1	4	4	1	1	4	4	4	4
1	1	4	4	1	1	1	1	4	4
1	1	4	4	1	1	1	1	4	4
1	1	4	4	1	1	4	4	4	4
1	1	4	4	1	1	1	1	4	4
1	1	4	4	1	1	4	4	4	4
3	3	1	1	4	4	1	1	4	4
1	1	4	4	1	1	1	1	4	4
1	1	4	4	1	1	1	1	4	4
3	3	1	1	4	4	4	4	1	1
1	1	4	4	1	1	1	1	1	1
1	1	4	4	1	1	1	1	4	4
1	1	4	4	1	1	4	4	1	1
1	1	4	4	1	1	4	4	4	4
1	1	4	4	1	1	1	1	1	1
1	1	4	4	1	1	4	4	4	4
1	1	4	4	1	1	1	1	1	1
1	1	4	4	1	1	1	1	4	4
1	1	4	4	1	1	1	1	1	1
3	3	1	1	4	4	4	4	1	1
1	1	4	4	1	1	1	1	4	4
1	1	4	4	1	1	1	1	4	4
1	1	4	4	1	1	1	1	4	4
1	1	4	4	1	1	4	4	4	4
1	1	4	4	1	1	4	4	4	4
1	1	4	4	1	1	4	4	4	4
1	1	4	4	1	1	4	4	4	4
1	1	4	4	1	1	4	4	4	4
1	1	4	4	1	1	4	4	1	1
1	1	4	4	1	1	4	4	4	4
1	1	4	4	1	1	4	4	1	1
1	1	4	4	1	1	1	1	1	1
1	1	4	4	1	1	1	1	1	1
3	3	4	4	4	4	4	4	4	4
1	1	4	4	1	1	1	1	1	1
1	1	4	4	1	1	4	4	4	4
1	1	4	4	1	1	1	1	4	4
1	1	4	4	1	1	4	4	4	4
1	1	4	4	1	1	1	1	4	4
1	1	4	4	1	1	1	1	4	4
1	1	4	4	1	1	1	1	4	4
1	1	4	4	1	1	1	1	4	4

For Review Only

1	1	4	4	1	1	1	1	4	4
1	1	4	4	1	1	1	1	4	4
1	1	4	4	1	1	1	1	4	4
3	3	1	1	4	4	1	1	4	4
1	1	4	4	1	1	1	1	4	4
1	1	4	4	1	1	1	1	4	4
1	1	4	4	1	1	4	4	4	4
1	1	4	4	1	1	4	4	1	1
1	1	4	4	1	1	4	4	4	4
1	1	4	4	1	1	1	1	4	4
1	1	4	4	1	1	1	1	4	4
1	1	4	4	1	1	1	1	4	4
1	1	4	4	1	1	1	1	4	4
1	1	4	4	1	1	1	1	1	1
1	1	4	4	1	1	1	1	4	4
3	3	4	4	4	4	4	4	1	1
1	1	4	4	1	1	4	4	4	4
1	1	4	4	1	1	1	1	4	4
1	1	4	4	1	1	1	1	4	4
1	1	4	4	1	1	1	1	4	4
1	1	4	4	1	1	1	1	4	4
1	1	4	4	1	1	4	4	1	1
1	1	4	4	1	1	1	1	1	1
1	1	4	4	1	1	1	1	1	1
1	1	4	4	1	1	1	1	1	1
1	1	4	4	1	1	1	1	4	4
3	3	1	1	4	4	1	1	4	4
1	1	4	4	1	1	4	4	1	1
1	1	4	4	1	1	4	4	4	4
1	1	4	4	1	1	1	1	4	4
3	3	1	1	4	4	4	4	4	4
1	1	4	4	1	1	4	4	1	1
1	1	4	4	1	1	4	4	1	1
1	1	4	4	1	1	1	1	4	4
1	1	4	4	1	1	1	1	4	4
1	1	4	4	1	1	1	1	1	1
1	1	4	4	1	1	4	4	1	1
3	3	1	1	4	4	1	1	4	4
1	1	4	4	1	1	4	4	4	4
1	1	4	4	1	1	1	1	1	1
1	1	4	4	1	1	1	1	4	4
1	1	4	4	1	1	1	1	4	4
1	1	4	4	1	1	1	1	4	4
1	1	4	4	1	1	1	1	4	4
1	1	4	4	1	1	1	1	1	1
1	1	4	4	1	1	1	1	4	4
1	1	4	4	1	1	4	4	4	4
1	1	4	4	1	1	4	4	4	4
1	1	4	4	1	1	4	4	4	4
1	1	4	4	1	1	1	1	4	4
1	1	4	4	1	1	1	1	4	4
1	1	4	4	1	1	4	4	1	1
1	1	4	4	1	1	4	4	1	1
1	1	4	4	1	1	1	1	4	4
1	1	4	4	1	1	1	1	4	4

For Review Only

1	1	4	4	1	1	4	4	4	4
1	1	4	4	1	1	1	1	4	4
1	1	4	4	1	1	4	4	4	4
1	1	4	4	1	1	4	4	4	4
1	1	4	4	1	1	1	1	4	4
1	1	4	4	1	1	4	4	4	4
1	1	4	4	1	1	1	1	4	4
1	1	4	4	1	1	4	4	4	4
1	1	4	4	1	1	4	4	1	1
1	1	4	4	1	1	4	4	1	1
1	1	4	4	1	1	4	4	4	4
1	1	4	4	1	1	1	1	4	4
1	1	4	4	1	1	1	1	4	4
1	1	4	4	1	1	1	1	4	4
1	1	4	4	1	1	1	1	4	4
1	1	4	4	1	1	1	1	4	4
1	1	4	4	1	1	1	1	4	4
1	1	4	4	1	1	1	1	4	4
1	1	4	4	1	1	4	4	4	4
1	1	4	4	1	1	1	1	4	4
1	1	4	4	1	1	1	1	4	4
1	1	4	4	1	1	1	1	4	4
1	1	4	4	1	1	4	4	4	4
1	1	4	4	1	1	4	4	1	1
1	1	4	4	1	1	4	4	4	4
1	1	4	4	1	1	1	1	1	1
1	1	4	4	1	1	1	1	4	4
1	1	4	4	1	1	1	1	4	4
1	1	4	4	1	1	1	1	4	4
1	1	4	4	1	1	1	1	4	4
1	1	4	4	1	1	4	4	4	4
1	1	4	4	1	1	1	1	4	4
1	1	4	4	1	1	1	1	4	4
1	1	4	4	1	1	1	1	4	4
1	1	4	4	1	1	4	4	4	4
1	1	4	4	1	1	1	1	4	4
1	1	4	4	1	1	4	4	4	4
1	1	4	4	1	1	1	1	4	4
1	1	4	4	1	1	4	4	4	4
1	1	4	4	1	1	4	4	4	4
1	1	4	4	1	1	4	4	4	4
1	1	4	4	1	1	4	4	4	4
1	1	4	4	1	1	1	1	4	4
1	1	1	1	1	1	4	4	4	4
1	1	4	4	1	1	4	4	4	4
1	1	4	4	1	1	1	1	1	1
1	1	4	4	1	1	4	4	4	4
1	1	4	4	1	1	1	1	4	4
1	1	4	4	1	1	4	4	4	4
1	1	4	4	1	1	4	4	4	4
1	1	4	4	1	1	4	4	4	4
1	1	4	4	1	1	4	4	4	4
1	1	4	4	1	1	4	4	4	4
1	1	4	4	1	1	1	1	4	4
1	1	4	4	1	1	4	4	4	4

For Review Only

1	1	4	4	1	1	1	1	4	4
1	1	4	4	1	1	4	4	4	4
3	3	1	1	4	4	4	4	4	4
1	1	4	4	1	1	1	1	1	1
1	1	4	4	1	1	1	1	4	4
1	1	4	4	1	1	4	4	4	4
1	1	4	4	1	1	4	4	4	4
1	1	4	4	1	1	1	1	4	4
1	1	4	4	1	1	4	4	4	4
1	1	4	4	1	1	1	1	4	4
1	1	4	4	1	1	4	4	4	4
1	1	4	4	1	1	4	4	1	1
1	1	4	4	1	1	1	1	4	4
1	1	4	4	1	1	1	1	1	1
1	1	4	4	1	1	4	4	4	4
1	1	4	4	1	1	4	4	4	4
1	1	4	4	1	1	1	1	4	4
1	1	4	4	1	1	1	1	4	4
1	1	4	4	1	1	4	4	4	4
1	1	4	4	1	1	1	1	4	4
1	1	4	4	1	1	1	1	4	4
1	1	4	4	1	1	4	4	1	1
1	1	4	4	1	1	4	4	1	1
1	1	4	4	1	1	1	1	4	4
1	1	4	4	1	1	1	1	4	4
1	1	4	4	1	1	4	4	4	4
1	1	4	4	1	1	1	1	4	4
1	1	4	4	1	1	1	1	1	1
1	1	4	4	1	1	4	4	4	4
1	1	4	4	1	1	1	1	4	4
1	1	4	4	1	1	1	1	4	4
1	1	4	4	1	1	1	1	4	4
1	1	4	4	1	1	1	1	4	4
1	1	4	4	1	1	4	4	4	4
1	1	4	4	1	1	1	1	4	4
1	1	4	4	1	1	1	1	4	4
1	1	4	4	1	1	1	1	4	4
1	1	4	4	1	1	4	4	4	4
1	1	4	4	1	1	1	1	4	4
1	1	4	4	1	1	1	1	4	4
1	1	4	4	1	1	4	4	4	4
1	1	4	4	1	1	4	4	4	4
1	1	4	4	1	1	4	4	1	1
1	1	4	4	1	1	1	1	4	4
1	1	4	4	1	1	1	1	4	4
1	1	4	4	1	1	1	1	1	1
1	1	4	4	1	1	4	4	4	4
1	1	4	4	1	1	4	4	4	4
1	1	4	4	1	1	4	4	1	1
1	1	4	4	1	1	1	1	4	4
1	1	4	4	1	1	1	1	4	4
1	1	4	4	1	1	4	4	1	1
1	1	4	4	1	1	4	4	1	1
1	1	4	4	1	1	1	1	4	4
1	1	4	4	1	1	4	4	1	1

For Review Only

For Review Only

1	1	4	4	1	1	4	4	4	4
3	3	1	1	4	4	4	4	4	4
1	1	4	4	1	1	1	1	4	4
1	1	4	4	1	1	1	1	4	4
1	1	4	4	1	1	1	1	4	4
1	1	4	4	1	1	1	1	4	4
1	1	4	4	1	1	4	4	4	4
1	1	4	4	1	1	4	4	1	1
1	1	4	4	1	1	4	4	1	1
1	1	4	4	1	1	4	4	1	1
1	1	4	4	1	1	1	1	4	4
1	1	4	4	1	1	4	4	1	1
1	1	4	4	1	1	4	4	4	4
1	1	4	4	1	1	4	4	1	1
1	1	4	4	1	1	1	1	4	4
1	1	4	4	1	1	4	4	4	4
1	1	4	4	1	1	4	4	1	1
1	1	4	4	1	1	1	1	4	4
1	1	4	4	1	1	4	4	4	4
1	1	4	4	1	1	4	4	4	4
1	1	4	4	1	1	1	1	1	1
1	1	4	4	1	1	4	4	4	4
1	1	4	4	1	1	1	1	4	4
1	1	4	4	1	1	4	4	4	4
1	1	4	4	1	1	4	4	1	1
1	1	4	4	1	1	1	1	4	4
1	1	4	4	1	1	4	4	4	4
1	1	4	4	1	1	1	1	4	4
1	1	4	4	1	1	1	1	4	4
1	1	4	4	1	1	1	1	4	4
1	1	4	4	1	1	4	4	4	4
1	1	4	4	1	1	4	4	4	4
1	1	4	4	1	1	4	4	1	1
1	1	4	4	1	1	4	4	4	4
1	1	4	4	1	1	4	4	4	4
1	1	4	4	1	1	4	4	4	4
1	1	4	4	1	1	1	1	4	4
1	1	4	4	1	1	1	1	4	4
1	1	4	4	1	1	4	4	4	4
1	1	4	4	1	1	4	4	4	4
1	1	4	4	1	1	1	1	4	4
1	1	4	4	1	1	4	4	1	1
1	1	4	4	1	1	4	4	1	1
1	1	4	4	1	1	1	1	4	4
1	1	4	4	1	1	1	1	4	4
1	1	4	4	1	1	4	4	4	4
1	1	4	4	1	1	4	4	4	4
1	1	4	4	1	1	1	1	4	4
3	3	1	1	1	1	4	4	1	1
1	1	4	4	1	1	4	4	4	4
1	1	4	4	1	1	1	1	4	4
1	1	4	4	1	1	4	4	4	4
1	1	4	4	1	1	4	4	4	4
1	1	4	4	1	1	1	1	4	4
1	1	4	4	1	1	4	4	1	1

1	1	4	4	1	1	1	1	4	4
1	1	4	4	1	1	1	1	4	4

For Review Only

SNP1495	SNP1495	SNP1496	SNP1496	SNP1497	SNP1497	SNP1498	SNP1498	SNP1499	SNP1499
3	3	1	1	2	2	3	3	3	3
3	3	3	3	2	2	3	3	3	3
3	3	1	1	4	4	3	3	3	3
3	3	1	1	4	4	3	3	3	3
3	3	1	1	2	2	1	1	3	3
3	3	3	3	4	4	3	3	2	2
4	4	3	3	4	4	3	3	3	3
4	4	3	3	2	2	3	3	3	3
3	3	1	1	4	4	3	3	3	3
3	3	1	1	2	2	3	3	3	3
3	3	1	1	2	2	3	3	3	3
3	3	1	1	4	4	3	3	3	3
3	3	3	3	2	2	3	3	3	3
3	3	1	1	2	2	3	3	3	3
3	3	1	1	4	4	3	3	3	3
3	3	1	1	4	4	1	1	3	3
3	3	1	1	4	4	3	3	3	3
3	3	1	1	2	2	3	3	3	3
4	4	3	3	2	2	3	3	3	3
3	3	1	1	2	2	3	3	3	3
3	3	3	3	2	2	3	3	2	2
3	3	3	3	4	4	3	3	2	2
3	3	1	1	2	2	3	3	3	3
4	4	3	3	2	2	3	3	3	3
3	3	1	1	2	2	3	3	3	3
4	4	3	3	4	4	3	3	3	3
4	4	3	3	2	2	3	3	3	3
3	3	1	1	4	4	3	3	3	3
3	3	1	1	2	2	3	3	3	3
3	3	3	3	2	2	3	3	2	2
4	4	3	3	2	2	3	3	3	3
4	4	3	3	2	2	3	3	3	3
3	3	1	1	2	2	1	1	3	3
3	3	3	3	2	2	3	3	2	2
3	3	1	1	4	4	3	3	3	3
4	4	3	3	2	2	3	3	3	3
3	3	1	1	2	2	1	1	3	3
3	3	1	1	4	4	3	3	3	3
3	3	3	3	2	2	3	3	2	2
4	4	3	3	4	4	3	3	3	3
3	3	3	3	2	2	3	3	2	2
3	3	1	1	2	2	3	3	3	3
3	3	1	1	2	2	3	3	3	3
4	4	3	3	2	2	3	3	3	3
4	4	3	3	2	2	3	3	3	3
3	3	1	1	4	4	3	3	3	3
3	3	3	3	4	4	3	3	2	2
4	4	3	3	4	4	3	3	3	3
4	4	3	3	2	2	3	3	3	3
3	3	1	1	4	4	3	3	3	3
3	3	1	1	4	4	3	3	3	3
3	3	3	3	2	2	3	3	3	3

For Review Only

For Review Only

3	3	1	1	2	2	3	3	3	3
3	3	1	1	2	2	1	1	3	3
3	3	1	1	4	4	3	3	3	3
3	3	3	3	4	4	3	3	2	2
4	4	3	3	2	2	3	3	3	3
4	4	3	3	4	4	3	3	3	3
4	4	3	3	4	4	3	3	3	3
4	4	3	3	2	2	3	3	2	2
4	4	3	3	2	2	3	3	2	2
3	3	1	1	4	4	3	3	3	3
3	3	3	3	4	4	3	3	2	2
3	3	3	3	4	4	3	3	2	2
3	3	1	1	2	2	3	3	3	3
3	3	1	1	2	2	3	3	3	3
3	3	3	3	4	4	3	3	3	3
3	3	1	1	2	2	1	1	3	3
3	3	1	1	2	2	3	3	3	3
3	3	1	1	4	4	3	3	3	3
4	4	3	3	4	4	3	3	3	3
3	3	1	1	4	4	3	3	3	3
3	3	3	3	4	4	3	3	2	2
3	3	1	1	4	4	3	3	3	3
3	3	1	1	4	4	3	3	3	3
3	3	1	1	2	2	3	3	3	3
4	4	3	3	2	2	3	3	3	3
3	3	3	3	4	4	3	3	3	3
4	4	3	3	2	2	3	3	3	3
3	3	1	1	2	2	3	3	3	3
4	4	3	3	4	4	3	3	3	3
3	3	1	1	2	2	3	3	3	3
3	3	1	1	4	4	3	3	3	3
3	3	1	1	2	2	3	3	3	3
3	3	1	1	2	2	3	3	3	3
3	3	1	1	2	2	3	3	3	3
3	3	1	1	2	2	3	3	3	3
4	4	3	3	2	2	3	3	3	3
4	4	3	3	2	2	3	3	2	2
3	3	3	3	2	2	3	3	2	2
3	3	1	1	2	2	3	3	3	3
4	4	3	3	2	2	3	3	3	3
3	3	1	1	4	4	3	3	3	3
3	3	1	1	2	2	3	3	3	3
3	3	1	1	4	4	3	3	3	3
3	3	3	3	4	4	3	3	2	2
4	4	3	3	2	2	3	3	2	2
3	3	1	1	2	2	3	3	3	3
4	4	3	3	2	2	3	3	2	2
4	4	3	3	4	4	3	3	3	3
4	4	3	3	4	4	3	3	3	3
3	3	1	1	4	4	3	3	3	3
3	3	3	3	4	4	3	3	2	2
4	4	3	3	2	2	3	3	2	2
4	4	3	3	2	2	3	3	2	2
3	3	1	1	2	2	3	3	3	3
3	3	1	1	4	4	3	3	3	3

4	4	3	3	4	4	3	3	3	3
4	4	3	3	4	4	3	3	3	3
3	3	1	1	4	4	3	3	3	3
4	4	3	3	2	2	3	3	2	2
4	4	3	3	4	4	3	3	3	3
4	4	1	1	4	4	3	3	3	3
3	3	1	1	4	4	1	1	3	3
4	4	3	3	2	2	3	3	2	2
4	4	3	3	4	4	3	3	3	3
3	3	3	3	2	2	3	3	2	2
3	3	3	3	2	2	3	3	2	2
3	3	1	1	4	4	3	3	3	3
4	4	3	3	2	2	3	3	3	3
3	3	3	3	4	4	3	3	3	3
4	4	1	1	2	2	3	3	3	3
3	3	1	1	4	4	3	3	3	3
3	3	1	1	4	4	3	3	3	3
3	3	1	1	4	4	3	3	3	3
4	4	3	3	2	2	3	3	2	2
3	3	1	1	4	4	3	3	3	3
4	4	1	1	4	4	3	3	3	3
3	3	1	1	2	2	1	1	3	3
3	3	1	1	4	4	3	3	3	3
4	4	3	3	2	2	3	3	2	2
3	3	1	1	4	4	3	3	3	3
3	3	1	1	4	4	3	3	3	3
3	3	1	1	2	2	3	3	3	3
3	3	1	1	2	2	3	3	3	3
3	3	1	1	4	4	1	1	3	3
3	3	1	1	4	4	3	3	3	3
3	3	1	1	2	2	3	3	3	3
3	3	1	1	4	4	3	3	3	3
3	3	1	1	4	4	3	3	3	3
3	3	1	1	4	4	3	3	3	3
3	3	3	3	2	2	3	3	2	2
4	4	3	3	4	4	3	3	3	3
4	4	3	3	2	2	3	3	2	2
4	4	3	3	4	4	3	3	3	3
3	3	1	1	4	4	3	3	3	3
3	3	1	1	2	2	3	3	3	3
3	3	1	1	4	4	3	3	3	3
4	4	3	3	2	2	3	3	2	2
3	3	1	1	4	4	3	3	3	3
3	3	1	1	2	2	3	3	3	3
3	3	1	1	2	2	3	3	3	3
4	4	3	3	2	2	3	3	3	3
4	4	3	3	4	4	3	3	3	3
4	4	3	3	4	4	3	3	3	3
3	3	1	1	2	2	1	1	3	3
3	3	1	1	4	4	3	3	3	3
4	4	3	3	4	4	3	3	3	3
3	3	1	1	4	4	3	3	3	3
3	3	1	1	4	4	3	3	3	3
4	4	3	3	4	4	3	3	3	3
3	3	1	1	2	2	3	3	3	3

For Review Only

3	3	1	1	4	4	1	1	3	3
4	4	3	3	4	4	3	3	3	3
3	3	1	1	2	2	3	3	3	3
3	3	1	1	2	2	3	3	3	3
3	3	1	1	2	2	3	3	3	3
3	3	1	1	2	2	3	3	3	3
3	3	1	1	4	4	3	3	3	3
3	3	1	1	4	4	3	3	3	3
3	3	1	1	2	2	3	3	3	3
3	3	1	1	2	2	1	1	3	3
4	4	3	3	4	4	3	3	3	3
3	3	3	3	2	2	3	3	2	2
4	4	3	3	2	2	3	3	3	3
3	3	1	1	2	2	1	1	3	3
3	3	3	3	2	2	3	3	2	2
3	3	1	1	2	2	3	3	3	3
4	4	1	1	4	4	3	3	3	3
3	3	1	1	4	4	3	3	3	3
4	4	3	3	4	4	3	3	3	3
3	3	1	1	4	4	1	1	3	3
3	3	1	1	4	4	3	3	3	3
4	4	3	3	2	2	3	3	2	2
3	3	1	1	4	4	3	3	3	3
3	3	3	3	4	4	3	3	2	2
3	3	1	1	2	2	1	1	3	3
3	3	3	3	2	2	3	3	2	2
4	4	3	3	4	4	3	3	3	3
3	3	1	1	4	4	3	3	3	3
3	3	1	1	4	4	3	3	3	3
3	3	1	1	2	2	3	3	3	3
4	4	3	3	4	4	3	3	3	3
3	3	1	1	2	2	3	3	3	3
3	3	1	1	2	2	3	3	3	3
3	3	3	3	2	2	3	3	2	2
3	3	1	1	4	4	3	3	3	3
3	3	3	3	4	4	3	3	2	2
4	4	3	3	2	2	3	3	3	3
3	3	1	1	2	2	1	1	3	3
4	4	3	3	4	4	3	3	3	3
3	3	1	1	4	4	3	3	3	3
3	3	1	1	4	4	3	3	3	3
4	4	3	3	4	4	3	3	3	3
3	3	1	1	2	2	3	3	3	3
3	3	1	1	4	4	3	3	3	3
3	3	1	1	2	2	3	3	2	2
3	3	1	1	2	2	3	3	3	3
3	3	1	1	2	2	3	3	3	3
3	3	1	1	2	2	3	3	3	3
4	4	3	3	2	2	3	3	2	2
4	4	3	3	4	4	3	3	2	2
3	3	1	1	2	2	1	1	3	3
3	3	1	1	2	2	1	1	3	3
3	3	1	1	4	4	3	3	3	3
3	3	3	3	4	4	3	3	2	2
3	3	1	1	2	2	3	3	3	3

For Review Only

3	3	1	1	2	2	3	3	3	3
3	3	1	1	2	2	3	3	3	3
3	3	1	1	4	4	1	1	3	3
3	3	1	1	4	4	3	3	3	3
3	3	1	1	2	2	3	3	3	3
4	4	3	3	4	4	3	3	3	3
4	4	3	3	2	2	3	3	2	2
3	3	3	3	2	2	3	3	2	2
4	4	3	3	4	4	3	3	3	3
4	4	3	3	2	2	3	3	2	2
3	3	3	3	4	4	3	3	3	3
3	3	1	1	4	4	3	3	3	3
3	3	1	1	2	2	3	3	3	3
3	3	1	1	4	4	3	3	3	3
4	4	3	3	4	4	3	3	3	3
3	3	1	1	2	2	3	3	3	3
3	3	3	3	4	4	3	3	2	2
4	4	3	3	2	2	3	3	2	2
3	3	1	1	2	2	3	3	3	3
3	3	1	1	4	4	3	3	3	3
4	4	3	3	2	2	3	3	3	3
4	4	3	3	4	4	3	3	3	3
4	4	3	3	2	2	3	3	3	3
3	3	1	1	4	4	3	3	3	3
4	4	3	3	2	2	3	3	2	2
4	4	1	1	2	2	3	3	3	3
3	3	1	1	2	2	3	3	3	3
4	4	3	3	4	4	3	3	3	3
3	3	1	1	2	2	3	3	3	3
3	3	1	1	4	4	1	1	3	3
3	3	1	1	2	2	3	3	3	3
4	4	3	3	4	4	3	3	3	3
3	3	1	1	2	2	1	1	3	3
4	4	3	3	4	4	3	3	3	3
3	3	1	1	4	4	1	1	3	3
3	3	1	1	2	2	3	3	3	3
3	3	1	1	4	4	3	3	3	3
3	3	3	3	4	4	3	3	2	2
4	4	3	3	2	2	3	3	3	3
3	3	1	1	2	2	3	3	3	3
3	3	1	1	2	2	3	3	3	3
3	3	1	1	2	2	3	3	3	3
3	3	1	1	4	4	3	3	3	3
3	3	1	1	4	4	3	3	3	3
3	3	1	1	4	4	3	3	3	3
4	4	3	3	2	2	3	3	3	3
4	4	3	3	4	4	3	3	2	2
4	4	3	3	2	2	3	3	3	3
4	4	3	3	4	4	3	3	3	3
3	3	1	1	4	4	3	3	3	3
3	3	1	1	2	2	3	3	3	3
3	3	1	1	2	2	3	3	3	3
3	3	1	1	4	4	3	3	3	3
4	4	3	3	4	4	3	3	3	3
3	3	1	1	4	4	3	3	3	3

For Review Only

3	3	1	1	2	2	3	3	3	3
4	4	3	3	4	4	3	3	3	3

For Review Only

SNP150	SNP150	SNP1500	SNP1500	SNP1501	SNP1501	SNP1502	SNP1502	SNP1503	SNP1503
4	4	3	3	2	2	2	2	4	4
2	2	3	3	2	2	2	2	4	4
2	2	3	3	2	2	2	2	4	4
4	4	3	3	2	2	2	2	4	4
4	4	3	3	2	2	2	2	2	2
2	2	3	3	2	2	2	2	4	4
4	4	3	3	2	2	2	2	4	4
4	4	3	3	2	2	2	2	4	4
4	4	3	3	2	2	2	2	2	2
4	4	3	3	2	2	2	2	4	4
4	4	2	2	2	2	3	3	4	4
4	4	3	3	2	2	2	2	4	4
4	4	3	3	2	2	2	2	4	4
4	4	3	3	2	2	2	2	4	4
4	4	3	3	2	2	2	2	2	2
2	2	3	3	2	2	2	2	2	2
4	4	3	3	2	2	2	2	4	4
4	4	2	2	2	2	2	2	4	4
2	2	3	3	2	2	2	2	4	4
4	4	3	3	2	2	2	2	2	2
4	4	3	3	2	2	2	2	4	4
4	4	3	3	2	2	2	2	4	4
4	4	3	3	1	1	2	2	4	4
4	4	2	2	2	2	2	2	4	4
4	4	3	3	2	2	2	2	2	2
2	2	3	3	2	2	2	2	4	4
4	4	3	3	2	2	2	2	4	4
4	4	3	3	2	2	2	2	4	4
4	4	3	3	2	2	2	2	2	2
4	4	3	3	2	2	2	2	4	4
2	2	3	3	2	2	2	2	4	4
4	4	3	3	2	2	2	2	4	4
4	4	3	3	2	2	2	2	2	2
4	4	2	2	2	2	2	2	4	4
4	4	3	3	2	2	3	3	2	2
2	2	3	3	2	2	2	2	4	4
4	4	2	2	2	2	2	2	2	2
4	4	2	2	2	2	2	2	2	2
4	4	2	2	2	2	2	2	2	2
4	4	2	2	2	2	3	3	4	4
4	4	2	2	2	2	2	2	4	4
4	4	3	3	2	2	2	2	4	4
4	4	3	3	2	2	2	2	4	4
4	4	3	3	2	2	2	2	2	2
4	4	3	3	2	2	2	2	4	4
4	4	3	3	2	2	2	2	4	4
2	2	3	3	2	2	2	2	4	4
2	2	3	3	2	2	2	2	4	4
4	4	3	3	2	2	2	2	4	4
4	4	3	3	2	2	2	2	2	2
2	2	3	3	2	2	2	2	2	2
4	4	3	3	2	2	2	2	4	4

For Review Only

4	4	3	3	2	2	2	2	2	2
4	4	3	3	2	2	2	2	2	2
2	2	3	3	2	2	2	2	4	4
4	4	3	3	2	2	2	2	4	4
2	2	3	3	2	2	2	2	4	4
4	4	3	3	2	2	2	2	4	4
4	4	3	3	2	2	2	2	4	4
4	4	2	2	2	2	2	2	4	4
4	4	3	3	2	2	2	2	4	4
4	4	3	3	2	2	2	2	2	2
4	4	3	3	2	2	2	2	4	4
4	4	3	3	2	2	2	2	4	4
4	4	3	3	2	2	2	2	2	2
4	4	3	3	2	2	2	2	2	2
2	2	3	3	2	2	2	2	4	4
4	4	3	3	2	2	2	2	2	2
4	4	3	3	1	1	2	2	2	2
2	2	3	3	2	2	2	2	4	4
2	2	3	3	2	2	2	2	2	2
4	4	3	3	2	2	2	2	4	4
4	4	3	3	2	2	2	2	4	4
4	4	3	3	2	2	3	3	2	2
4	4	3	3	2	2	2	2	2	2
4	4	3	3	2	2	2	2	2	2
4	4	3	3	2	2	2	2	4	4
4	4	3	3	2	2	2	2	4	4
4	4	3	3	2	2	2	2	4	4
4	4	3	3	2	2	2	2	4	4
2	2	3	3	2	2	2	2	4	4
4	4	3	3	2	2	2	2	2	2
4	4	3	3	2	2	2	2	4	4
4	4	3	3	2	2	2	2	4	4
4	4	2	2	2	2	2	2	4	4
4	4	3	3	2	2	2	2	2	2
4	4	3	3	2	2	2	2	4	4
4	4	3	3	2	2	2	2	4	4
4	4	3	3	2	2	2	2	4	4
4	4	3	3	2	2	2	2	4	4
4	4	3	3	2	2	2	2	2	2
4	4	3	3	2	2	2	2	4	4
4	4	3	3	2	2	2	2	2	2
2	2	3	3	2	2	2	2	2	2
4	4	3	3	2	2	2	2	2	2
4	4	3	3	2	2	2	2	4	4
4	4	3	3	2	2	2	2	4	4
2	2	3	3	2	2	2	2	4	4
4	4	2	2	2	2	2	2	4	4
4	4	3	3	2	2	2	2	4	4
2	2	3	3	2	2	2	2	4	4
4	4	3	3	1	1	2	2	2	2
4	4	3	3	2	2	2	2	4	4
4	4	2	2	2	2	3	3	4	4
4	4	2	2	2	2	2	2	4	4
4	4	3	3	2	2	2	2	2	2
4	4	3	3	2	2	2	2	4	4

For Review Only

2	2	3	3	2	2	2	2	4	4
4	4	2	2	2	2	2	2	4	4
2	2	3	3	2	2	2	2	2	2
4	4	3	3	2	2	2	2	4	4
4	4	3	3	2	2	2	2	4	4
2	2	3	3	2	2	2	2	2	2
2	2	3	3	2	2	2	2	2	2
4	4	3	3	2	2	2	2	4	4
2	2	2	2	2	2	2	2	4	4
4	4	3	3	2	2	2	2	4	4
4	4	3	3	2	2	2	2	4	4
4	4	3	3	2	2	2	2	2	2
2	2	3	3	2	2	2	2	4	4
4	4	3	3	2	2	2	2	4	4
2	2	3	3	2	2	2	2	4	4
2	2	3	3	2	2	2	2	4	4
2	2	3	3	2	2	2	2	4	4
4	4	3	3	2	2	2	2	4	4
4	4	3	3	2	2	2	2	4	4
4	4	3	3	2	2	2	2	2	2
4	4	3	3	2	2	2	2	4	4
2	2	3	3	2	2	2	2	2	2
2	2	3	3	2	2	2	2	4	4
4	4	3	3	2	2	2	2	4	4
4	4	3	3	2	2	2	2	2	2
4	4	3	3	1	1	2	2	2	2
4	4	3	3	2	2	2	2	2	2
4	4	3	3	2	2	2	2	4	4
2	2	3	3	2	2	2	2	2	2
4	4	3	3	2	2	2	2	4	4
4	4	2	2	2	2	2	2	2	2
4	4	3	3	2	2	2	2	4	4
4	4	3	3	2	2	2	2	2	2
2	2	3	3	2	2	2	2	2	2
4	4	2	2	2	2	2	2	4	4
4	4	3	3	2	2	2	2	2	2
4	4	2	2	2	2	2	2	4	4
4	4	3	3	2	2	2	2	4	4
4	4	3	3	2	2	2	2	2	2
4	4	3	3	2	2	2	2	4	4
2	2	3	3	1	1	3	3	2	2
4	4	3	3	2	2	2	2	4	4
2	2	3	3	2	2	2	2	4	4
4	4	3	3	2	2	2	2	4	4
2	2	3	3	2	2	2	2	2	2
4	4	3	3	2	2	2	2	4	4
2	2	3	3	2	2	2	2	4	4
2	2	3	3	2	2	2	2	4	4
4	4	3	3	2	2	2	2	2	2
2	2	3	3	2	2	3	3	2	2
2	2	3	3	2	2	2	2	4	4
4	4	2	2	2	2	3	3	4	4
2	2	3	3	2	2	2	2	4	4
4	4	3	3	2	2	2	2	4	4
4	4	3	3	1	1	2	2	2	2

4	4	3	3	2	2	2	2	2	2
4	4	2	2	2	2	2	2	4	4
4	4	3	3	2	2	2	2	4	4
4	4	3	3	2	2	2	2	4	4
2	2	3	3	2	2	2	2	4	4
4	4	2	2	2	2	2	2	4	4
4	4	3	3	2	2	3	3	4	4
4	4	3	3	2	2	2	2	4	4
4	4	3	3	2	2	3	3	2	2
4	4	3	3	2	2	2	2	2	2
4	4	3	3	2	2	2	2	4	4
4	4	3	3	2	2	3	3	4	4
4	4	3	3	2	2	2	2	4	4
4	4	3	3	2	2	2	2	2	2
4	4	2	2	2	2	2	2	4	4
4	4	2	2	2	2	3	3	4	4
2	2	3	3	2	2	2	2	4	4
2	2	3	3	2	2	2	2	4	4
2	2	3	3	2	2	2	2	2	2
2	2	3	3	2	2	2	2	2	2
4	4	3	3	2	2	2	2	4	4
4	4	3	3	2	2	2	2	4	4
4	4	3	3	2	2	3	3	2	2
4	4	3	3	2	2	2	2	4	4
4	4	3	3	2	2	2	2	2	2
4	4	2	2	2	2	2	2	4	4
4	4	3	3	2	2	2	2	4	4
4	4	3	3	2	2	2	2	4	4
4	4	2	2	2	2	2	2	2	2
2	2	3	3	2	2	2	2	2	2
2	2	3	3	2	2	2	2	4	4
4	4	3	3	2	2	2	2	2	2
4	4	3	3	2	2	2	2	4	4
4	4	2	2	2	2	2	2	4	4
4	4	3	3	2	2	2	2	4	4
4	4	3	3	2	2	2	2	4	4
4	4	3	3	2	2	2	2	4	4
4	4	3	3	2	2	2	2	2	2
2	2	3	3	2	2	2	2	4	4
4	4	3	3	1	1	2	2	2	2
4	4	3	3	2	2	2	2	4	4
2	2	3	3	2	2	2	2	4	4
4	4	3	3	2	2	2	2	4	4
4	4	3	3	2	2	3	3	2	2
4	4	3	3	2	2	2	2	4	4
4	4	3	3	2	2	2	2	4	4
4	4	2	2	2	2	3	3	4	4
4	4	3	3	2	2	3	3	4	4
4	4	2	2	2	2	2	2	4	4
4	4	3	3	2	2	2	2	2	2
4	4	3	3	2	2	2	2	2	2
4	4	2	2	2	2	2	2	2	2
4	4	2	2	2	2	2	2	2	2
4	4	3	3	2	2	2	2	4	4
4	4	2	2	2	2	2	2	4	4

2	2	2	2	2	2	2	2	4	4
4	4	3	3	2	2	2	2	4	4
2	2	3	3	2	2	2	2	2	2
4	4	3	3	2	2	2	2	2	2
4	4	3	3	2	2	2	2	4	4
4	4	3	3	2	2	2	2	4	4
4	4	3	3	2	2	2	2	4	4
4	4	2	2	2	2	2	2	4	4
4	4	3	3	2	2	2	2	4	4
4	4	2	2	2	2	2	2	4	4
4	4	3	3	2	2	2	2	4	4
2	2	3	3	2	2	2	2	4	4
4	4	3	3	1	1	2	2	2	2
2	2	3	3	2	2	2	2	2	2
2	2	3	3	2	2	2	2	4	4
4	4	2	2	2	2	2	2	4	4
4	4	2	2	2	2	2	2	4	4
4	4	3	3	2	2	2	2	4	4
4	4	3	3	2	2	2	2	4	4
4	4	2	2	2	2	2	2	2	2
2	2	3	3	2	2	3	3	4	4
2	2	3	3	2	2	2	2	4	4
4	4	3	3	2	2	2	2	4	4
4	4	2	2	2	2	3	3	2	2
4	4	2	2	2	2	2	2	4	4
4	4	3	3	2	2	2	2	4	4
4	4	3	3	2	2	2	2	4	4
4	4	3	3	2	2	2	2	4	4
4	4	3	3	2	2	2	2	2	2
2	2	3	3	2	2	2	2	2	2
4	4	3	3	2	2	2	2	4	4
2	2	3	3	2	2	2	2	4	4
2	2	2	2	2	2	2	2	2	2
2	2	3	3	2	2	2	2	4	4
4	4	3	3	1	1	2	2	2	2
4	4	3	3	2	2	2	2	4	4
2	2	3	3	2	2	2	2	4	4
4	4	3	3	2	2	2	2	4	4
2	2	3	3	2	2	2	2	4	4
4	4	2	2	2	2	3	3	4	4
4	4	3	3	2	2	2	2	4	4
4	4	3	3	2	2	2	2	4	4
4	4	2	2	2	2	3	3	2	2
4	4	3	3	2	2	2	2	2	2
4	4	3	3	2	2	2	2	4	4
4	4	3	3	1	1	2	2	4	4
4	4	3	3	2	2	3	3	4	4
4	4	3	3	2	2	2	2	4	4
2	2	3	3	2	2	2	2	4	4
4	4	2	2	2	2	2	2	4	4
4	4	3	3	2	2	2	2	4	4
4	4	2	2	2	2	3	3	4	4
2	2	3	3	2	2	2	2	4	4
4	4	3	3	2	2	2	2	4	4
4	4	2	2	2	2	3	3	2	2

For Review Only

4	4	3	3	2	2	2	2	4	4
2	2	3	3	2	2	2	2	4	4

For Review Only

SNP1504	SNP1504	SNP1505	SNP1505	SNP1506	SNP1506	SNP1507	SNP1507	SNP1508	SNP1508
1	1	3	3	4	4	4	4	1	1
1	1	3	3	4	4	4	4	4	4
1	1	3	3	3	3	4	4	1	1
1	1	3	3	4	4	3	3	4	4
3	3	3	3	4	4	4	4	4	4
1	1	3	3	4	4	3	3	4	4
1	1	3	3	4	4	3	3	1	1
3	3	2	2	4	4	3	3	4	4
1	1	3	3	4	4	3	3	4	4
3	3	2	2	4	4	3	3	4	4
3	3	3	3	4	4	4	4	1	1
1	1	3	3	4	4	3	3	1	1
3	3	2	2	4	4	4	4	1	1
3	3	2	2	4	4	4	4	4	4
1	1	3	3	4	4	3	3	1	1
3	3	3	3	4	4	3	3	1	1
3	3	2	2	3	3	3	3	1	1
3	3	3	3	4	4	4	4	4	4
1	1	3	3	4	4	3	3	1	1
3	3	2	2	4	4	3	3	4	4
3	3	3	3	4	4	4	4	1	1
3	3	3	3	4	4	3	3	4	4
1	1	3	3	4	4	4	4	4	4
3	3	3	3	4	4	4	4	4	4
1	1	3	3	4	4	4	4	1	1
3	3	3	3	4	4	4	4	4	4
1	1	3	3	4	4	4	4	4	4
1	1	3	3	4	4	3	3	4	4
3	3	3	3	4	4	4	4	4	4
3	3	2	2	4	4	4	4	4	4
1	1	3	3	4	4	4	4	4	4
1	1	3	3	4	4	4	4	1	1
1	1	3	3	4	4	4	4	1	1
3	3	2	2	4	4	4	4	4	4
1	1	3	3	4	4	4	4	4	4
3	3	3	3	4	4	4	4	4	4
1	1	3	3	4	4	3	3	4	4
1	1	3	3	4	4	3	3	4	4
3	3	3	3	3	3	4	4	4	4
1	1	3	3	4	4	3	3	4	4
3	3	2	2	4	4	4	4	4	4
3	3	2	2	3	3	4	4	4	4
3	3	3	3	4	4	4	4	4	4
3	3	3	3	4	4	4	4	4	4
3	3	2	2	4	4	3	3	4	4
3	3	3	3	4	4	4	4	1	1
1	1	3	3	3	3	4	4	4	4
3	3	3	3	4	4	3	3	4	4
3	3	3	3	4	4	4	4	4	4
1	1	3	3	3	3	3	3	4	4
1	1	3	3	4	4	4	4	4	4
1	1	3	3	4	4	4	4	4	4

For Review Only

3	3	3	3	4	4	4	4	1	1
1	1	3	3	4	4	4	4	1	1
1	1	3	3	4	4	3	3	4	4
3	3	3	3	4	4	3	3	4	4
1	1	3	3	4	4	3	3	1	1
1	1	3	3	4	4	4	4	4	4
3	3	3	3	4	4	3	3	4	4
3	3	3	3	4	4	4	4	4	4
1	1	3	3	4	4	4	4	4	4
1	1	3	3	4	4	3	3	1	1
1	1	3	3	3	3	4	4	4	4
1	1	3	3	3	3	4	4	4	4
3	3	3	3	4	4	4	4	1	1
3	3	3	3	4	4	4	4	4	4
1	1	3	3	4	4	3	3	4	4
1	1	2	2	4	4	3	3	4	4
1	1	3	3	4	4	3	3	4	4
1	1	3	3	3	3	3	3	1	1
1	1	3	3	4	4	3	3	4	4
3	3	3	3	4	4	3	3	1	1
1	1	3	3	3	3	4	4	4	4
1	1	3	3	4	4	3	3	4	4
3	3	3	3	4	4	3	3	4	4
3	3	2	2	4	4	4	4	4	4
3	3	2	2	4	4	3	3	4	4
1	1	3	3	4	4	4	4	4	4
3	3	3	3	4	4	4	4	1	1
3	3	2	2	4	4	4	4	4	4
3	3	3	3	4	4	4	4	4	4
1	1	3	3	4	4	4	4	1	1
3	3	3	3	4	4	3	3	4	4
3	3	3	3	4	4	4	4	4	4
3	3	2	2	4	4	4	4	4	4
3	3	3	3	4	4	4	4	1	1
3	3	3	3	4	4	4	4	4	4
3	3	2	2	4	4	3	3	4	4
3	3	2	2	4	4	4	4	4	4
3	3	3	3	4	4	4	4	1	1
3	3	3	3	4	4	4	4	1	1
3	3	2	2	4	4	4	4	4	4
3	3	3	3	4	4	3	3	4	4
1	1	3	3	4	4	4	4	1	1
3	3	3	3	4	4	4	4	1	1
1	1	3	3	3	3	4	4	4	4
1	1	3	3	4	4	4	4	1	1
1	1	3	3	4	4	3	3	4	4
3	3	3	3	4	4	4	4	4	4
3	3	3	3	4	4	4	4	4	4
3	3	3	3	4	4	4	4	4	4
1	1	3	3	4	4	3	3	4	4
1	1	3	3	3	3	4	4	4	4
3	3	3	3	4	4	4	4	4	4
3	3	3	3	4	4	4	4	4	4
1	1	2	2	4	4	3	3	4	4
1	1	3	3	4	4	3	3	1	1

For Review Only

1	1	3	3	4	4	3	3	4	4
1	1	2	2	4	4	3	3	4	4
3	3	3	3	4	4	4	4	4	4
3	3	3	3	4	4	4	4	4	4
1	1	3	3	4	4	4	4	4	4
3	3	3	3	4	4	4	4	4	4
1	1	3	3	4	4	3	3	1	1
3	3	3	3	4	4	3	3	4	4
1	1	3	3	4	4	3	3	4	4
3	3	2	2	4	4	3	3	4	4
1	1	3	3	4	4	4	4	4	4
1	1	3	3	4	4	3	3	1	1
3	3	3	3	4	4	4	4	4	4
1	1	3	3	4	4	3	3	4	4
3	3	2	2	4	4	4	4	1	1
1	1	3	3	4	4	3	3	4	4
1	1	3	3	3	3	3	3	1	1
1	1	3	3	4	4	3	3	4	4
3	3	3	3	4	4	4	4	4	4
3	3	3	3	4	4	3	3	4	4
1	1	3	3	4	4	3	3	4	4
1	1	3	3	4	4	4	4	4	4
1	1	3	3	3	3	3	3	4	4
3	3	2	2	4	4	4	4	4	4
1	1	3	3	4	4	3	3	4	4
1	1	3	3	4	4	3	3	4	4
3	3	3	3	4	4	3	3	4	4
1	1	3	3	3	3	4	4	4	4
1	1	3	3	4	4	3	3	1	1
3	3	3	3	3	3	3	3	4	4
3	3	3	3	4	4	4	4	4	4
1	1	3	3	4	4	3	3	4	4
3	3	3	3	4	4	3	3	4	4
3	3	3	3	4	4	3	3	4	4
3	3	2	2	4	4	4	4	4	4
3	3	2	2	4	4	3	3	4	4
3	3	2	2	4	4	4	4	4	4
1	1	3	3	4	4	3	3	4	4
3	3	3	3	4	4	3	3	4	4
3	3	2	2	4	4	4	4	1	1
1	1	3	3	4	4	3	3	4	4
3	3	2	2	4	4	4	4	4	4
1	1	3	3	4	4	3	3	4	4
3	3	2	2	4	4	4	4	4	4
1	1	3	3	4	4	4	4	4	4
3	3	3	3	4	4	3	3	4	4
3	3	3	3	4	4	4	4	4	4
1	1	3	3	4	4	4	4	4	4
1	1	3	3	4	4	4	4	4	4
3	3	3	3	4	4	4	4	4	4
1	1	3	3	4	4	4	4	4	4
3	3	3	3	4	4	3	3	4	4
1	1	3	3	3	3	3	3	1	1
1	1	3	3	4	4	4	4	4	4
3	3	3	3	4	4	3	3	4	4

For Review Only

1	1	3	3	4	4	3	3	1	1
3	3	3	3	4	4	3	3	4	4
3	3	2	2	4	4	3	3	4	4
1	1	3	3	4	4	4	4	1	1
3	3	3	3	4	4	4	4	4	4
1	1	3	3	3	3	4	4	4	4
1	1	3	3	4	4	3	3	4	4
1	1	3	3	4	4	3	3	4	4
1	1	3	3	4	4	3	3	4	4
3	3	3	3	4	4	3	3	1	1
3	3	3	3	4	4	4	4	4	4
1	1	3	3	4	4	3	3	4	4
1	1	3	3	4	4	4	4	4	4
3	3	2	2	4	4	3	3	4	4
3	3	3	3	4	4	4	4	4	4
3	3	3	3	4	4	4	4	1	1
1	1	2	2	4	4	3	3	1	1
1	1	3	3	4	4	3	3	4	4
3	3	3	3	4	4	4	4	4	4
1	1	3	3	4	4	3	3	1	1
1	1	3	3	4	4	3	3	4	4
3	3	3	3	4	4	4	4	4	4
1	1	3	3	4	4	3	3	4	4
1	1	3	3	3	3	4	4	4	4
3	3	2	2	4	4	4	4	4	4
3	3	2	2	4	4	4	4	4	4
1	1	3	3	4	4	3	3	4	4
1	1	3	3	4	4	3	3	1	1
1	1	3	3	4	4	3	3	4	4
3	3	3	3	4	4	4	4	4	4
1	1	3	3	4	4	3	3	4	4
1	1	3	3	4	4	4	4	1	1
1	1	3	3	4	4	3	3	4	4
3	3	3	3	4	4	4	4	4	4
1	1	3	3	3	3	3	3	4	4
1	1	3	3	3	3	4	4	4	4
1	1	3	3	4	4	4	4	4	4
1	1	3	3	4	4	4	4	1	1
1	1	3	3	4	4	4	4	4	4
1	1	3	3	4	4	4	4	4	4
1	1	3	3	3	3	4	4	1	1
1	1	3	3	4	4	4	4	4	4
3	3	2	2	4	4	4	4	4	4
1	1	3	3	4	4	3	3	4	4
3	3	3	3	4	4	4	4	4	4
1	1	3	3	3	3	4	4	1	1
3	3	3	3	4	4	4	4	1	1
3	3	3	3	4	4	4	4	4	4
3	3	3	3	4	4	4	4	4	4
1	1	2	2	4	4	3	3	4	4
1	1	3	3	4	4	4	4	1	1
1	1	2	2	4	4	3	3	4	4
1	1	3	3	4	4	3	3	4	4
1	1	3	3	3	3	4	4	4	4
3	3	3	3	4	4	4	4	4	4

For Review Only

1	1	3	3	3	3	4	4	1	1
3	3	3	3	4	4	4	4	4	4
1	1	3	3	4	4	3	3	1	1
1	1	3	3	4	4	3	3	4	4
3	3	3	3	3	3	3	3	4	4
3	3	3	3	4	4	3	3	4	4
3	3	3	3	4	4	4	4	4	4
3	3	3	3	4	4	3	3	4	4
3	3	2	2	4	4	4	4	4	4
3	3	2	2	4	4	3	3	4	4
1	1	3	3	4	4	3	3	4	4
1	1	2	2	4	4	3	3	4	4
3	3	3	3	4	4	3	3	1	1
1	1	3	3	4	4	4	4	4	4
1	1	3	3	4	4	4	4	1	1
3	3	3	3	4	4	4	4	1	1
3	3	3	3	4	4	3	3	4	4
1	1	3	3	4	4	3	3	4	4
1	1	3	3	4	4	4	4	1	1
1	1	3	3	4	4	3	3	1	1
1	1	3	3	4	4	4	4	4	4
3	3	3	3	4	4	4	4	4	4
1	1	3	3	4	4	3	3	4	4
1	1	3	3	4	4	3	3	4	4
3	3	3	3	4	4	4	4	4	4
1	1	2	2	4	4	4	4	1	1
3	3	2	2	4	4	4	4	4	4
1	1	3	3	4	4	3	3	4	4
3	3	3	3	4	4	4	4	4	4
1	1	3	3	4	4	3	3	1	1
3	3	3	3	4	4	4	4	4	4
1	1	3	3	4	4	4	4	4	4
3	3	2	2	4	4	4	4	4	4
3	3	3	3	4	4	4	4	4	4
1	1	3	3	4	4	3	3	4	4
3	3	2	2	4	4	4	4	4	4
1	1	3	3	3	3	3	3	1	1
1	1	3	3	3	3	4	4	4	4
3	3	3	3	4	4	4	4	4	4
3	3	3	3	4	4	4	4	4	4
1	1	3	3	4	4	3	3	1	1
3	3	3	3	3	3	3	3	4	4
1	1	3	3	4	4	3	3	4	4
3	3	3	3	4	4	3	3	4	4
1	1	3	3	3	3	4	4	4	4
3	3	3	3	4	4	4	4	1	1
3	3	3	3	4	4	4	4	4	4
1	1	3	3	4	4	4	4	4	4
1	1	2	2	4	4	4	4	4	4
3	3	2	2	4	4	4	4	4	4
1	1	3	3	4	4	3	3	1	1
3	3	3	3	4	4	4	4	4	4
1	1	3	3	3	3	4	4	1	1
3	3	3	3	4	4	4	4	1	1
1	1	3	3	4	4	3	3	4	4

For Review Only

1	1	3	3	4	4	4	4	1	1
1	1	3	3	4	4	4	4	4	4

For Review Only

SNP1509	SNP1509	SNP151	SNP151	SNP1510	SNP1510	SNP1511	SNP1511	SNP1512	SNP1512
3	3	2	2	4	4	1	1	4	4
3	3	2	2	4	4	1	1	4	4
2	2	2	2	4	4	2	2	1	1
3	3	2	2	4	4	1	1	4	4
3	3	2	2	1	1	1	1	4	4
3	3	2	2	4	4	1	1	4	4
2	2	2	2	4	4	1	1	4	4
3	3	2	2	4	4	1	1	4	4
3	3	2	2	4	4	1	1	4	4
3	3	2	2	4	4	1	1	4	4
2	2	2	2	4	4	1	1	4	4
3	3	2	2	4	4	1	1	1	1
3	3	2	2	4	4	1	1	4	4
3	3	2	2	4	4	1	1	4	4
3	3	1	1	4	4	1	1	4	4
3	3	2	2	4	4	2	2	4	4
2	2	2	2	4	4	2	2	4	4
3	3	2	2	4	4	1	1	4	4
2	2	1	1	4	4	1	1	4	4
3	3	2	2	4	4	1	1	4	4
2	2	2	2	4	4	1	1	4	4
3	3	2	2	4	4	1	1	4	4
3	3	2	2	4	4	2	2	1	1
3	3	2	2	4	4	1	1	4	4
3	3	2	2	4	4	1	1	4	4
3	3	2	2	4	4	1	1	4	4
3	3	2	2	4	4	1	1	4	4
3	3	2	2	4	4	1	1	4	4
3	3	2	2	4	4	1	1	4	4
3	3	2	2	1	1	1	1	4	4
3	3	1	1	4	4	1	1	4	4
2	2	1	1	4	4	2	2	4	4
2	2	2	2	4	4	2	2	4	4
3	3	2	2	4	4	1	1	4	4
3	3	2	2	4	4	1	1	4	4
3	3	2	2	4	4	1	1	4	4
3	3	2	2	4	4	2	2	4	4
3	3	2	2	4	4	1	1	4	4
3	3	2	2	4	4	1	1	4	4
3	3	2	2	1	1	1	1	4	4
3	3	2	2	4	4	1	1	4	4
3	3	2	2	4	4	2	2	4	4
3	3	2	2	4	4	1	1	4	4
3	3	2	2	1	1	1	1	4	4
3	3	2	2	4	4	1	1	4	4
3	3	2	2	4	4	1	1	4	4
3	3	2	2	4	4	1	1	4	4
3	3	2	2	4	4	1	1	4	4
3	3	2	2	4	4	1	1	4	4
3	3	2	2	4	4	2	2	4	4
3	3	2	2	4	4	1	1	4	4
3	3	2	2	4	4	1	1	4	4

For Review Only

2	2	2	2	4	4	1	1	4	4
2	2	2	2	4	4	2	2	4	4
3	3	2	2	4	4	1	1	4	4
3	3	2	2	4	4	1	1	4	4
2	2	1	1	4	4	1	1	4	4
3	3	2	2	4	4	1	1	4	4
3	3	2	2	4	4	1	1	4	4
3	3	2	2	4	4	1	1	4	4
3	3	2	2	4	4	1	1	4	4
3	3	2	2	4	4	1	1	4	4
3	3	2	2	4	4	1	1	4	4
3	3	2	2	4	4	1	1	4	4
2	2	2	2	4	4	1	1	4	4
3	3	2	2	4	4	1	1	4	4
3	3	2	2	4	4	1	1	4	4
3	3	2	2	4	4	2	2	4	4
3	3	2	2	4	4	1	1	4	4
2	2	2	2	4	4	2	2	1	1
3	3	1	1	4	4	1	1	4	4
2	2	2	2	4	4	1	1	4	4
3	3	2	2	4	4	1	1	4	4
3	3	2	2	1	1	1	1	4	4
3	3	2	2	4	4	1	1	4	4
3	3	2	2	4	4	1	1	4	4
3	3	2	2	4	4	1	1	4	4
3	3	2	2	4	4	1	1	4	4
2	2	2	2	4	4	1	1	4	4
3	3	2	2	1	1	1	1	4	4
3	3	2	2	4	4	1	1	4	4
3	3	1	1	4	4	1	1	4	4
3	3	2	2	1	1	1	1	4	4
3	3	2	2	4	4	1	1	4	4
3	3	2	2	1	1	1	1	4	4
2	2	2	2	4	4	1	1	4	4
3	3	2	2	4	4	2	2	1	1
3	3	2	2	4	4	1	1	4	4
3	3	2	2	1	1	1	1	4	4
2	2	2	2	4	4	1	1	4	4
2	2	2	2	4	4	1	1	4	4
3	3	2	2	4	4	1	1	4	4
3	3	2	2	4	4	1	1	4	4
2	2	1	1	4	4	1	1	4	4
2	2	1	1	4	4	1	1	4	4
3	3	2	2	4	4	1	1	4	4
2	2	2	2	4	4	1	1	4	4
3	3	2	2	4	4	2	2	4	4
3	3	2	2	4	4	1	1	4	4
3	3	2	2	4	4	1	1	4	4
3	3	2	2	4	4	1	1	4	4
3	3	2	2	4	4	1	1	4	4
3	3	1	1	4	4	1	1	4	4
3	3	2	2	4	4	1	1	4	4
3	3	2	2	4	4	1	1	4	4
3	3	2	2	4	4	1	1	4	4
2	2	1	1	4	4	1	1	4	4

For Review Only

3	3	2	2	4	4	1	1	4	4
3	3	2	2	4	4	1	1	4	4
3	3	2	2	4	4	1	1	4	4
3	3	2	2	4	4	1	1	4	4
3	3	2	2	4	4	1	1	4	4
3	3	2	2	4	4	1	1	4	4
2	2	2	2	4	4	2	2	4	4
3	3	2	2	4	4	1	1	4	4
3	3	2	2	4	4	1	1	4	4
3	3	2	2	4	4	1	1	4	4
3	3	2	2	4	4	1	1	4	4
3	3	2	2	4	4	1	1	4	4
3	3	1	1	4	4	1	1	4	4
3	3	2	2	4	4	1	1	4	4
3	3	2	2	4	4	1	1	4	4
3	3	2	2	4	4	1	1	4	4
3	3	1	1	4	4	2	2	1	1
3	3	2	2	4	4	1	1	4	4
3	3	2	2	4	4	1	1	4	4
3	3	1	1	4	4	1	1	4	4
3	3	2	2	4	4	1	1	4	4
3	3	1	1	4	4	2	2	4	4
3	3	2	2	4	4	2	2	1	1
3	3	2	2	4	4	1	1	4	4
3	3	2	2	4	4	1	1	4	4
3	3	2	2	4	4	1	1	4	4
3	3	1	1	4	4	1	1	4	4
3	3	1	1	4	4	2	2	4	4
2	2	2	2	4	4	1	1	4	4
3	3	1	1	4	4	2	2	4	4
3	3	2	2	1	1	1	1	4	4
3	3	2	2	4	4	1	1	4	4
3	3	2	2	4	4	1	1	4	4
3	3	2	2	4	4	1	1	4	4
3	3	2	2	4	4	1	1	4	4
3	3	2	2	4	4	1	1	4	4
3	3	2	2	4	4	1	1	4	4
3	3	2	2	4	4	1	1	4	4
3	3	2	2	4	4	1	1	4	4
3	3	2	2	4	4	1	1	4	4
3	3	2	2	4	4	1	1	4	4
3	3	2	2	4	4	1	1	4	4
3	3	1	1	4	4	1	1	4	4
3	3	2	2	4	4	1	1	4	4
3	3	2	2	4	4	1	1	1	1
3	3	2	2	4	4	1	1	4	4
3	3	2	2	4	4	1	1	4	4
3	3	2	2	4	4	1	1	4	4
3	3	2	2	4	4	1	1	4	4
3	3	2	2	4	4	1	1	4	4
3	3	2	2	4	4	1	1	4	4
3	3	2	2	4	4	1	1	4	4
3	3	2	2	4	4	2	2	1	1
3	3	2	2	4	4	1	1	4	4
3	3	2	2	4	4	1	1	4	4

For Review Only

2	2	2	2	4	4	2	2	4	4
3	3	2	2	4	4	1	1	4	4
3	3	2	2	4	4	1	1	4	4
3	3	2	2	4	4	2	2	1	1
3	3	2	2	4	4	1	1	4	4
3	3	2	2	4	4	2	2	1	1
3	3	2	2	1	1	1	1	4	4
3	3	2	2	4	4	1	1	4	4
3	3	2	2	4	4	1	1	4	4
2	2	1	1	4	4	2	2	4	4
3	3	2	2	4	4	1	1	4	4
3	3	2	2	4	4	1	1	4	4
3	3	2	2	4	4	1	1	4	4
3	3	2	2	4	4	1	1	4	4
3	3	2	2	4	4	1	1	4	4
2	2	2	2	4	4	1	1	4	4
3	3	2	2	4	4	1	1	4	4
3	3	2	2	4	4	2	2	4	4
3	3	2	2	4	4	1	1	4	4
3	3	2	2	4	4	2	2	4	4
3	3	2	2	4	4	1	1	4	4
3	3	2	2	4	4	1	1	4	4
3	3	2	2	4	4	1	1	4	4
3	3	2	2	4	4	1	1	4	4
3	3	2	2	4	4	1	1	4	4
3	3	2	2	4	4	1	1	4	4
3	3	1	1	4	4	1	1	4	4
3	3	1	1	4	4	1	1	4	4
3	3	2	2	4	4	1	1	4	4
3	3	2	2	4	4	1	1	4	4
3	3	1	1	4	4	1	1	4	4
2	2	1	1	4	4	1	1	4	4
3	3	2	2	4	4	2	2	1	1
3	3	2	2	4	4	1	1	4	4
3	3	2	2	4	4	1	1	4	4
3	3	2	2	4	4	1	1	4	4
3	3	2	2	4	4	1	1	4	4
2	2	2	2	4	4	2	2	4	4
3	3	2	2	4	4	1	1	4	4
3	3	1	1	4	4	1	1	4	4
2	2	2	2	4	4	2	2	1	1
3	3	2	2	4	4	1	1	4	4
3	3	2	2	4	4	1	1	4	4
3	3	2	2	4	4	1	1	4	4
3	3	2	2	4	4	1	1	4	4
3	3	1	1	4	4	2	2	1	1
2	2	2	2	4	4	1	1	4	4
3	3	2	2	4	4	1	1	4	4
3	3	2	2	4	4	1	1	4	4
3	3	2	2	4	4	1	1	4	4
2	2	2	2	4	4	1	1	4	4
3	3	2	2	4	4	2	2	4	4
3	3	2	2	1	1	1	1	4	4
3	3	2	2	4	4	1	1	4	4
3	3	2	2	4	4	1	1	4	4

For Review Only

2	2	2	2	4	4	2	2	1	1
3	3	2	2	1	1	2	2	4	4
2	2	2	2	4	4	1	1	4	4
3	3	1	1	4	4	1	1	4	4
3	3	2	2	4	4	2	2	4	4
3	3	2	2	4	4	1	1	4	4
3	3	1	1	4	4	1	1	4	4
3	3	2	2	1	1	1	1	4	4
3	3	2	2	4	4	1	1	4	4
3	3	2	2	4	4	1	1	4	4
3	3	2	2	4	4	1	1	4	4
3	3	1	1	1	1	2	2	1	1
2	2	2	2	4	4	1	1	4	4
3	3	2	2	4	4	1	1	4	4
2	2	1	1	4	4	1	1	4	4
2	2	2	2	4	4	1	1	4	4
3	3	2	2	4	4	1	1	4	4
3	3	2	2	4	4	1	1	4	4
2	2	1	1	1	1	1	1	4	4
2	2	2	2	4	4	1	1	4	4
3	3	2	2	4	4	1	1	4	4
3	3	2	2	4	4	1	1	4	4
3	3	2	2	4	4	1	1	4	4
3	3	2	2	4	4	1	1	4	4
3	3	2	2	4	4	1	1	4	4
3	3	2	2	4	4	1	1	4	4
3	3	2	2	1	1	1	1	4	4
3	3	2	2	4	4	1	1	4	4
3	3	2	2	4	4	1	1	4	4
2	2	2	2	4	4	1	1	4	4
3	3	2	2	1	1	1	1	4	4
3	3	2	2	4	4	1	1	4	4
3	3	2	2	4	4	2	2	4	4
3	3	2	2	4	4	1	1	4	4
3	3	2	2	4	4	1	1	4	4
3	3	2	2	1	1	1	1	4	4
2	2	2	2	4	4	2	2	1	1
3	3	2	2	4	4	1	1	4	4
3	3	2	2	4	4	1	1	4	4
3	3	2	2	4	4	1	1	4	4
2	2	2	2	4	4	1	1	4	4
3	3	2	2	4	4	2	2	1	1
3	3	2	2	4	4	1	1	4	4
3	3	2	2	4	4	1	1	4	4
3	3	2	2	4	4	1	1	4	4
2	2	2	2	4	4	1	1	4	4
3	3	2	2	4	4	1	1	4	4
3	3	2	2	4	4	1	1	4	4
3	3	2	2	4	4	1	1	4	4
3	3	2	2	1	1	1	1	4	4
2	2	2	2	4	4	1	1	4	4
3	3	2	2	4	4	1	1	4	4
2	2	2	2	4	4	2	2	1	1
3	3	1	1	4	4	1	1	4	4
3	3	2	2	4	4	1	1	4	4

For Review Only

2	2	2	2	4	4	1	1	4	4
3	3	2	2	4	4	1	1	4	4

For Review Only

SNP1513	SNP1513	SNP1514	SNP1514	SNP1515	SNP1515	SNP1516	SNP1516	SNP1517	SNP1517
3	3	1	1	2	2	4	4	1	1
2	2	1	1	2	2	4	4	1	1
2	2	4	4	2	2	4	4	1	1
2	2	1	1	2	2	4	4	1	1
2	2	1	1	2	2	4	4	1	1
3	3	1	1	2	2	4	4	1	1
2	2	1	1	2	2	4	4	1	1
2	2	1	1	2	2	4	4	1	1
2	2	1	1	2	2	4	4	1	1
2	2	1	1	2	2	4	4	4	4
3	3	1	1	2	2	4	4	1	1
2	2	1	1	2	2	4	4	1	1
2	2	1	1	2	2	4	4	1	1
2	2	1	1	2	2	4	4	1	1
2	2	1	1	2	2	4	4	1	1
2	2	4	4	2	2	4	4	1	1
2	2	1	1	2	2	4	4	4	4
3	3	1	1	2	2	4	4	4	4
2	2	1	1	2	2	4	4	1	1
3	3	1	1	2	2	4	4	1	1
2	2	1	1	2	2	1	1	1	1
2	2	1	1	2	2	1	1	1	1
2	2	4	4	2	2	1	1	1	1
2	2	1	1	2	2	4	4	1	1
3	3	1	1	2	2	4	4	1	1
2	2	1	1	2	2	4	4	1	1
2	2	1	1	2	2	4	4	1	1
2	2	1	1	2	2	4	4	1	1
3	3	1	1	2	2	4	4	1	1
2	2	1	1	2	2	4	4	1	1
2	2	1	1	2	2	4	4	1	1
3	3	4	4	2	2	4	4	1	1
3	3	4	4	2	2	4	4	1	1
3	3	1	1	2	2	1	1	1	1
2	2	1	1	2	2	4	4	1	1
2	2	1	1	2	2	4	4	1	1
2	2	4	4	3	3	4	4	1	1
2	2	1	1	2	2	4	4	1	1
2	2	1	1	2	2	1	1	1	1
2	2	1	1	2	2	1	1	1	1
2	2	1	1	2	2	4	4	1	1
2	2	1	1	2	2	4	4	4	4
2	2	1	1	2	2	4	4	4	4
2	2	1	1	2	2	4	4	1	1
3	3	1	1	2	2	4	4	1	1
2	2	1	1	2	2	4	4	1	1
2	2	1	1	2	2	4	4	1	1
2	2	1	1	2	2	4	4	1	1
2	2	1	1	2	2	4	4	1	1
2	2	1	1	2	2	4	4	1	1
2	2	1	1	2	2	4	4	1	1
2	2	4	4	2	2	4	4	1	1

For Review Only

3	3	1	1	2	2	4	4	1	1
3	3	4	4	2	2	4	4	1	1
2	2	1	1	2	2	4	4	1	1
2	2	1	1	2	2	4	4	4	4
2	2	1	1	2	2	4	4	1	1
2	2	1	1	2	2	4	4	1	1
3	3	1	1	2	2	4	4	1	1
2	2	1	1	2	2	1	1	1	1
2	2	1	1	2	2	4	4	1	1
2	2	1	1	2	2	4	4	1	1
2	2	1	1	3	3	4	4	1	1
2	2	1	1	2	2	4	4	1	1
3	3	1	1	2	2	4	4	1	1
3	3	1	1	2	2	4	4	1	1
2	2	1	1	2	2	4	4	1	1
3	3	4	4	2	2	4	4	1	1
2	2	1	1	2	2	1	1	1	1
2	2	4	4	2	2	4	4	1	1
2	2	1	1	2	2	4	4	4	4
2	2	1	1	2	2	4	4	1	1
2	2	1	1	2	2	4	4	1	1
2	2	1	1	2	2	1	1	1	1
2	2	1	1	2	2	4	4	1	1
3	3	1	1	2	2	4	4	1	1
3	3	1	1	2	2	4	4	1	1
2	2	4	4	2	2	4	4	1	1
2	2	1	1	2	2	4	4	1	1
2	2	1	1	2	2	1	1	1	1
2	2	1	1	2	2	4	4	1	1
2	2	1	1	2	2	4	4	1	1
2	2	1	1	2	2	4	4	1	1
2	2	1	1	2	2	4	4	1	1
2	2	1	1	2	2	1	1	1	1
3	3	1	1	2	2	4	4	1	1
2	2	4	4	2	2	4	4	1	1
3	3	1	1	2	2	4	4	1	1
2	2	1	1	2	2	4	4	1	1
2	2	1	1	2	2	4	4	1	1
2	2	1	1	2	2	1	1	1	1
2	2	1	1	2	2	4	4	1	1
2	2	1	1	2	2	4	4	1	1
2	2	1	1	3	3	4	4	1	1
2	2	1	1	2	2	4	4	1	1
2	2	1	1	3	3	4	4	1	1
3	3	1	1	2	2	4	4	1	1
2	2	1	1	2	2	4	4	4	4
2	2	1	1	2	2	4	4	1	1
2	2	1	1	2	2	4	4	1	1
2	2	1	1	2	2	4	4	1	1
2	2	1	1	2	2	4	4	1	1
2	2	1	1	3	3	4	4	1	1
2	2	1	1	2	2	1	1	1	1
2	2	1	1	2	2	4	4	1	1
3	3	1	1	2	2	4	4	1	1
2	2	1	1	2	2	4	4	1	1

For Review Only

2	2	1	1	2	2	4	4	1	1
2	2	1	1	2	2	4	4	1	1
2	2	1	1	2	2	4	4	1	1
3	3	1	1	2	2	4	4	1	1
2	2	1	1	3	3	4	4	1	1
2	2	1	1	2	2	4	4	1	1
2	2	4	4	2	2	4	4	1	1
2	2	1	1	2	2	4	4	1	1
2	2	1	1	2	2	4	4	1	1
2	2	1	1	2	2	4	4	1	1
3	3	1	1	2	2	4	4	1	1
2	2	1	1	2	2	4	4	1	1
2	2	1	1	2	2	4	4	1	1
3	3	4	4	2	2	4	4	1	1
2	2	1	1	2	2	4	4	1	1
2	2	1	1	2	2	4	4	1	1
2	2	4	4	2	2	4	4	1	1
2	2	1	1	2	2	4	4	1	1
2	2	1	1	2	2	4	4	1	1
2	2	1	1	2	2	4	4	1	1
2	2	1	1	2	2	4	4	1	1
2	2	4	4	2	2	4	4	1	1
2	2	1	1	2	2	4	4	1	1
2	2	1	1	2	2	1	1	1	1
2	2	1	1	2	2	4	4	1	1
2	2	1	1	2	2	1	1	1	1
3	3	1	1	3	3	4	4	1	1
2	2	1	1	2	2	4	4	4	4
2	2	1	1	2	2	4	4	1	1
3	3	1	1	2	2	4	4	4	4
2	2	1	1	2	2	4	4	1	1
2	2	1	1	2	2	4	4	1	1
2	2	1	1	2	2	4	4	1	1
2	2	1	1	2	2	4	4	1	1
3	3	1	1	2	2	1	1	1	1
2	2	1	1	2	2	1	1	1	1
2	2	1	1	2	2	4	4	1	1
2	2	1	1	2	2	4	4	1	1
2	2	1	1	2	2	4	4	1	1
2	2	1	1	2	2	4	4	1	1
2	2	1	1	2	2	1	1	1	1
2	2	1	1	2	2	1	1	1	1
2	2	1	1	2	2	4	4	1	1
2	2	1	1	2	2	4	4	1	1
2	2	1	1	2	2	4	4	1	1
3	3	1	1	2	2	4	4	1	1
2	2	1	1	2	2	4	4	1	1
2	2	1	1	2	2	4	4	1	1
2	2	1	1	2	2	4	4	1	1
2	2	1	1	2	2	4	4	1	1
2	2	1	1	2	2	4	4	1	1
2	2	1	1	2	2	1	1	1	1
2	2	4	4	2	2	4	4	1	1
2	2	1	1	2	2	4	4	1	1
2	2	1	1	2	2	4	4	1	1

For Review Only

2	2	4	4	2	2	4	4	1	1
2	2	1	1	2	2	1	1	1	1
3	3	1	1	2	2	4	4	1	1
2	2	4	4	2	2	1	1	1	1
3	3	1	1	2	2	4	4	4	4
2	2	4	4	2	2	4	4	1	1
2	2	1	1	2	2	4	4	1	1
2	2	1	1	2	2	4	4	1	1
2	2	1	1	2	2	1	1	1	1
3	3	1	1	2	2	4	4	4	4
2	2	1	1	2	2	4	4	1	1
2	2	1	1	2	2	4	4	1	1
2	2	1	1	2	2	4	4	1	1
2	2	1	1	2	2	4	4	1	1
2	2	1	1	2	2	4	4	1	1
3	3	1	1	2	2	4	4	1	1
2	2	1	1	2	2	4	4	1	1
3	3	1	1	3	3	4	4	4	4
2	2	1	1	2	2	4	4	1	1
2	2	1	1	2	2	4	4	1	1
2	2	1	1	2	2	4	4	1	1
2	2	1	1	2	2	4	4	1	1
2	2	1	1	2	2	1	1	1	1
2	2	1	1	2	2	4	4	1	1
2	2	1	1	2	2	4	4	1	1
2	2	1	1	2	2	1	1	1	1
2	2	1	1	2	2	4	4	1	1
2	2	1	1	2	2	4	4	1	1
2	2	1	1	2	2	4	4	1	1
2	2	1	1	2	2	4	4	1	1
2	2	1	1	2	2	4	4	1	1
2	2	1	1	2	2	4	4	1	1
3	3	4	4	2	2	4	4	1	1
2	2	1	1	2	2	4	4	1	1
2	2	1	1	2	2	4	4	4	4
2	2	1	1	2	2	4	4	1	1
2	2	1	1	2	2	4	4	1	1
2	2	4	4	2	2	4	4	1	1
2	2	1	1	2	2	4	4	1	1
2	2	1	1	2	2	4	4	1	1
2	2	4	4	2	2	4	4	1	1
2	2	1	1	2	2	4	4	1	1
2	2	1	1	2	2	1	1	1	1
2	2	1	1	2	2	4	4	1	1
2	2	1	1	2	2	4	4	4	4
2	2	4	4	2	2	4	4	1	1
3	3	1	1	2	2	4	4	1	1
2	2	1	1	2	2	4	4	1	1
2	2	1	1	2	2	4	4	1	1
2	2	1	1	2	2	4	4	1	1
2	2	1	1	2	2	4	4	1	1
3	3	4	4	2	2	4	4	1	1
2	2	1	1	2	2	1	1	1	1
2	2	1	1	3	3	4	4	1	1
2	2	1	1	2	2	1	1	1	1

For Review Only

2	2	4	4	2	2	4	4	1	1
2	2	1	1	2	2	4	4	4	4
2	2	1	1	2	2	4	4	1	1
2	2	1	1	2	2	1	1	1	1
2	2	1	1	2	2	4	4	4	4
2	2	1	1	2	2	4	4	1	1
2	2	1	1	2	2	1	1	1	1
3	3	1	1	2	2	4	4	1	1
2	2	1	1	2	2	4	4	1	1
2	2	1	1	2	2	1	1	1	1
2	2	1	1	2	2	4	4	1	1
2	2	4	4	2	2	1	1	1	1
3	3	1	1	2	2	1	1	1	1
2	2	1	1	3	3	4	4	1	1
2	2	1	1	2	2	4	4	1	1
3	3	1	1	2	2	4	4	1	1
2	2	1	1	2	2	1	1	1	1
3	3	1	1	2	2	4	4	1	1
3	3	1	1	2	2	1	1	1	1
2	2	1	1	2	2	4	4	1	1
2	2	1	1	2	2	1	1	1	1
2	2	1	1	2	2	4	4	1	1
3	3	1	1	2	2	4	4	1	1
2	2	1	1	2	2	1	1	1	1
2	2	1	1	2	2	1	1	1	1
2	2	1	1	2	2	4	4	1	1
2	2	1	1	2	2	1	1	1	1
3	3	1	1	2	2	4	4	1	1
3	3	1	1	2	2	4	4	4	4
2	2	1	1	3	3	4	4	1	1
2	2	1	1	2	2	1	1	1	1
2	2	1	1	2	2	4	4	1	1
2	2	4	4	2	2	4	4	1	1
2	2	1	1	2	2	4	4	1	1
2	2	1	1	2	2	1	1	1	1
2	2	1	1	2	2	1	1	1	1
3	3	4	4	2	2	4	4	1	1
2	2	1	1	2	2	4	4	1	1
2	2	1	1	2	2	4	4	1	1
2	2	1	1	2	2	1	1	1	1
3	3	1	1	3	3	4	4	1	1
3	3	1	1	2	2	4	4	4	4
2	2	1	1	2	2	1	1	1	1
2	2	1	1	2	2	4	4	1	1
2	2	1	1	2	2	4	4	1	1
2	2	1	1	2	2	1	1	1	1
2	2	1	1	2	2	1	1	1	1
2	2	1	1	2	2	4	4	1	1
2	2	1	1	2	2	4	4	1	1
2	2	1	1	2	2	1	1	1	1
2	2	1	1	2	2	4	4	1	1
2	2	1	1	2	2	1	1	1	1
2	2	4	4	2	2	4	4	1	1
2	2	1	1	2	2	4	4	1	1
2	2	1	1	2	2	4	4	1	1

For Review Only

3	3	1	1	2	2	1	1	1	1
2	2	1	1	2	2	4	4	1	1

For Review Only

SNP1518	SNP1518	SNP1519	SNP1519	SNP152	SNP152	SNP1520	SNP1520	SNP1521	SNP1521
4	4	1	1	2	2	3	3	4	4
4	4	1	1	3	3	2	2	4	4
3	3	4	4	2	2	3	3	4	4
3	3	1	1	2	2	2	2	4	4
3	3	1	1	2	2	3	3	4	4
4	4	1	1	2	2	3	3	4	4
4	4	1	1	2	2	3	3	4	4
4	4	1	1	2	2	2	2	4	4
3	3	1	1	2	2	2	2	4	4
3	3	1	1	3	3	2	2	4	4
3	3	1	1	2	2	3	3	1	1
3	3	1	1	2	2	3	3	4	4
4	4	1	1	2	2	3	3	4	4
4	4	1	1	2	2	2	2	4	4
3	3	1	1	2	2	3	3	4	4
3	3	1	1	2	2	3	3	4	4
3	3	4	4	2	2	3	3	4	4
3	3	1	1	2	2	2	2	4	4
4	4	1	1	2	2	3	3	4	4
3	3	1	1	2	2	3	3	4	4
4	4	1	1	3	3	2	2	4	4
3	3	1	1	2	2	3	3	4	4
3	3	4	4	2	2	3	3	4	4
4	4	1	1	2	2	2	2	4	4
3	3	1	1	2	2	3	3	4	4
4	4	1	1	2	2	3	3	4	4
4	4	1	1	2	2	3	3	4	4
3	3	1	1	2	2	3	3	4	4
3	3	1	1	3	3	2	2	4	4
4	4	1	1	3	3	2	2	4	4
4	4	1	1	2	2	3	3	4	4
3	3	1	1	2	2	2	2	4	4
3	3	1	1	2	2	3	3	4	4
4	4	1	1	2	2	3	3	4	4
3	3	1	1	2	2	3	3	4	4
4	4	1	1	2	2	2	2	4	4
3	3	1	1	2	2	3	3	4	4
3	3	1	1	2	2	2	2	4	4
4	4	1	1	2	2	3	3	4	4
4	4	1	1	2	2	2	2	4	4
4	4	1	1	2	2	2	2	4	4
3	3	4	4	3	3	2	2	4	4
3	3	1	1	3	3	2	2	4	4
3	3	1	1	2	2	3	3	4	4
4	4	1	1	3	3	2	2	4	4
3	3	1	1	2	2	3	3	4	4
4	4	1	1	2	2	3	3	4	4
4	4	1	1	2	2	3	3	4	4
4	4	1	1	2	2	3	3	4	4
3	3	1	1	2	2	3	3	4	4
3	3	1	1	2	2	3	3	4	4
3	3	4	4	3	3	3	3	4	4

For Review Only

3	3	1	1	2	2	3	3	4	4
3	3	1	1	2	2	3	3	4	4
4	4	1	1	3	3	2	2	4	4
4	4	1	1	2	2	3	3	4	4
4	4	1	1	2	2	3	3	4	4
4	4	1	1	2	2	3	3	4	4
4	4	1	1	2	2	3	3	4	4
4	4	1	1	3	3	2	2	4	4
4	4	1	1	3	3	3	3	4	4
3	3	1	1	2	2	3	3	4	4
4	4	1	1	2	2	3	3	4	4
4	4	1	1	2	2	3	3	4	4
3	3	1	1	2	2	3	3	4	4
3	3	1	1	3	3	2	2	4	4
3	3	1	1	2	2	3	3	4	4
3	3	1	1	3	3	3	3	4	4
3	3	1	1	2	2	3	3	4	4
3	3	4	4	2	2	3	3	4	4
3	3	4	4	2	2	3	3	4	4
4	4	1	1	2	2	3	3	4	4
4	4	1	1	2	2	3	3	4	4
3	3	1	1	2	2	3	3	4	4
3	3	1	1	3	3	3	3	4	4
3	3	1	1	2	2	3	3	4	4
4	4	1	1	3	3	2	2	4	4
3	3	4	4	2	2	3	3	4	4
4	4	1	1	2	2	2	2	4	4
4	4	1	1	2	2	2	2	1	1
4	4	1	1	2	2	3	3	4	4
3	3	1	1	2	2	3	3	4	4
4	4	1	1	2	2	2	2	4	4
4	4	1	1	2	2	2	2	4	4
4	4	1	1	3	3	3	3	1	1
3	3	1	1	2	2	3	3	4	4
3	3	4	4	2	2	3	3	4	4
4	4	1	1	3	3	2	2	4	4
4	4	1	1	2	2	2	2	1	1
4	4	1	1	2	2	3	3	4	4
4	4	1	1	2	2	3	3	4	4
4	4	1	1	2	2	2	2	4	4
3	3	1	1	2	2	3	3	4	4
3	3	1	1	2	2	3	3	4	4
3	3	1	1	2	2	3	3	4	4
4	4	1	1	2	2	3	3	4	4
4	4	1	1	2	2	3	3	1	1
3	3	1	1	2	2	3	3	4	4
4	4	1	1	3	3	3	3	4	4
4	4	1	1	2	2	2	2	4	4
4	4	1	1	2	2	2	2	4	4
3	3	1	1	3	3	3	3	4	4
4	4	1	1	2	2	3	3	4	4
4	4	1	1	3	3	2	2	4	4
4	4	1	1	3	3	3	3	4	4
3	3	1	1	2	2	3	3	4	4
4	4	1	1	3	3	2	2	4	4

For Review Only

4	4	1	1	2	2	3	3	4	4
4	4	1	1	2	2	2	2	4	4
3	3	1	1	2	2	3	3	4	4
4	4	1	1	3	3	2	2	4	4
3	3	1	1	2	2	3	3	4	4
4	4	1	1	2	2	2	2	4	4
3	3	1	1	2	2	3	3	4	4
4	4	1	1	2	2	3	3	4	4
4	4	1	1	2	2	3	3	4	4
4	4	1	1	3	3	3	3	4	4
3	3	1	1	2	2	3	3	4	4
3	3	1	1	2	2	3	3	4	4
4	4	1	1	2	2	2	2	4	4
3	3	1	1	3	3	3	3	4	4
3	3	1	1	2	2	3	3	4	4
3	3	1	1	2	2	3	3	4	4
3	3	4	4	2	2	3	3	4	4
3	3	1	1	2	2	3	3	4	4
4	4	1	1	3	3	2	2	1	1
3	3	1	1	2	2	2	2	4	4
3	3	1	1	2	2	3	3	4	4
3	3	1	1	2	2	3	3	4	4
3	3	4	4	2	2	3	3	4	4
4	4	1	1	3	3	2	2	1	1
3	3	1	1	2	2	3	3	4	4
3	3	1	1	2	2	3	3	4	4
3	3	1	1	2	2	3	3	4	4
3	3	4	4	2	2	3	3	4	4
4	4	1	1	2	2	3	3	4	4
3	3	4	4	2	2	3	3	4	4
4	4	1	1	3	3	3	3	4	4
3	3	1	1	2	2	3	3	4	4
3	3	1	1	2	2	3	3	4	4
3	3	1	1	2	2	3	3	4	4
4	4	1	1	2	2	3	3	4	4
4	4	1	1	3	3	2	2	4	4
4	4	1	1	3	3	2	2	1	1
4	4	1	1	2	2	2	2	4	4
3	3	1	1	3	3	3	3	4	4
3	3	1	1	2	2	3	3	1	1
3	3	1	1	2	2	3	3	4	4
4	4	1	1	3	3	2	2	4	4
4	4	1	1	2	2	3	3	4	4
4	4	1	1	2	2	2	2	4	4
3	3	1	1	2	2	3	3	4	4
4	4	1	1	2	2	2	2	4	4
4	4	1	1	2	2	3	3	4	4
4	4	1	1	2	2	3	3	4	4
3	3	1	1	3	3	3	3	4	4
3	3	1	1	2	2	3	3	4	4
4	4	1	1	2	2	3	3	4	4
4	4	1	1	3	3	2	2	4	4
3	3	4	4	2	2	3	3	4	4
4	4	1	1	2	2	3	3	4	4
3	3	4	4	3	3	3	3	4	4

For Review Only

3	3	1	1	2	2	3	3	4	4
4	4	1	1	2	2	3	3	4	4
3	3	1	1	2	2	3	3	4	4
3	3	4	4	2	2	3	3	4	4
3	3	1	1	2	2	3	3	4	4
3	3	4	4	2	2	2	2	4	4
4	4	1	1	2	2	3	3	4	4
3	3	1	1	2	2	3	3	4	4
3	3	1	1	2	2	3	3	4	4
3	3	4	4	2	2	3	3	4	4
3	3	1	1	2	2	3	3	4	4
4	4	1	1	2	2	2	2	4	4
4	4	1	1	2	2	3	3	4	4
3	3	1	1	2	2	2	2	4	4
4	4	1	1	3	3	2	2	4	4
3	3	1	1	2	2	3	3	1	1
3	3	1	1	2	2	3	3	4	4
3	3	4	4	3	3	3	3	4	4
4	4	1	1	2	2	2	2	4	4
3	3	1	1	2	2	3	3	4	4
3	3	1	1	2	2	3	3	4	4
4	4	1	1	3	3	3	3	4	4
3	3	1	1	2	2	3	3	4	4
4	4	1	1	2	2	3	3	4	4
3	3	1	1	2	2	3	3	4	4
4	4	1	1	2	2	3	3	4	4
4	4	1	1	2	2	2	2	4	4
3	3	1	1	2	2	3	3	4	4
3	3	1	1	2	2	3	3	4	4
3	3	1	1	2	2	3	3	4	4
4	4	1	1	2	2	3	3	4	4
3	3	1	1	2	2	3	3	4	4
3	3	4	4	2	2	3	3	4	4
4	4	1	1	3	3	3	3	4	4
3	3	4	4	2	2	3	3	4	4
4	4	1	1	2	2	3	3	4	4
4	4	1	1	2	2	3	3	4	4
3	3	1	1	2	2	3	3	4	4
4	4	1	1	2	2	3	3	4	4
3	3	1	1	2	2	2	2	4	4
3	3	4	4	2	2	3	3	4	4
4	4	1	1	2	2	3	3	4	4
4	4	1	1	3	3	3	3	4	4
3	3	1	1	2	2	3	3	4	4
3	3	1	1	2	2	3	3	4	4
3	3	4	4	2	2	2	2	4	4
3	3	1	1	2	2	3	3	1	1
4	4	1	1	3	3	3	3	1	1
4	4	1	1	2	2	2	2	4	4
4	4	1	1	2	2	3	3	4	4
4	4	1	1	2	2	3	3	4	4
3	3	1	1	3	3	3	3	4	4
3	3	1	1	2	2	3	3	4	4
4	4	1	1	2	2	3	3	4	4
4	4	1	1	2	2	2	2	1	1

For Review Only

3	3	4	4	2	2	2	2	4	4
3	3	1	1	2	2	2	2	4	4
3	3	1	1	2	2	3	3	4	4
3	3	1	1	2	2	3	3	4	4
3	3	4	4	2	2	2	2	4	4
4	4	1	1	2	2	3	3	4	4
4	4	1	1	2	2	2	2	1	1
4	4	1	1	2	2	2	2	1	1
4	4	1	1	3	3	2	2	4	4
4	4	1	1	2	2	3	3	4	4
3	3	1	1	2	2	3	3	4	4
3	3	4	4	2	2	3	3	4	4
4	4	1	1	2	2	3	3	4	4
3	3	1	1	2	2	3	3	4	4
4	4	1	1	2	2	3	3	4	4
3	3	1	1	2	2	2	2	4	4
4	4	1	1	2	2	3	3	4	4
4	4	1	1	3	3	3	3	4	4
4	4	1	1	3	3	3	3	1	1
3	3	1	1	2	2	2	2	4	4
4	4	1	1	2	2	2	2	4	4
4	4	1	1	2	2	3	3	4	4
4	4	1	1	2	2	3	3	4	4
3	3	1	1	2	2	2	2	4	4
4	4	1	1	3	3	2	2	4	4
4	4	1	1	2	2	3	3	4	4
4	4	1	1	3	3	2	2	1	1
4	4	1	1	2	2	2	2	4	4
3	3	1	1	2	2	3	3	4	4
4	4	1	1	2	2	3	3	4	4
4	4	1	1	2	2	3	3	1	1
4	4	1	1	3	3	3	3	4	4
3	3	1	1	3	3	2	2	4	4
4	4	1	1	2	2	3	3	4	4
3	3	1	1	2	2	3	3	4	4
4	4	1	1	3	3	3	3	1	1
3	3	4	4	2	2	3	3	4	4
4	4	1	1	2	2	3	3	4	4
4	4	1	1	2	2	2	2	4	4
4	4	1	1	2	2	2	2	1	1
4	4	1	1	2	2	3	3	4	4
3	3	4	4	3	3	3	3	4	4
3	3	1	1	2	2	2	2	4	4
3	3	1	1	2	2	3	3	4	4
3	3	1	1	3	3	3	3	4	4
4	4	1	1	2	2	3	3	4	4
4	4	1	1	2	2	3	3	4	4
4	4	1	1	2	2	2	2	4	4
4	4	1	1	2	2	2	2	4	4
4	4	1	1	3	3	2	2	1	1
4	4	1	1	2	2	3	3	4	4
4	4	1	1	2	2	2	2	1	1
3	3	4	4	2	2	3	3	4	4
4	4	1	1	2	2	2	2	4	4
3	3	1	1	2	2	2	2	4	4

For Review Only

3	3	1	1	2	2	3	3	4	4
4	4	1	1	2	2	3	3	4	4

For Review Only

SNP1522	SNP1522	SNP1523	SNP1523	SNP1524	SNP1524	SNP1525	SNP1525	SNP1526	SNP1526
1	1	3	3	1	1	2	2	2	2
2	2	3	3	1	1	4	4	4	4
1	1	3	3	1	1	4	4	4	4
1	1	3	3	1	1	4	4	4	4
2	2	3	3	1	1	2	2	2	2
1	1	3	3	1	1	4	4	4	4
1	1	3	3	1	1	2	2	2	2
1	1	3	3	1	1	4	4	4	4
1	1	3	3	3	3	2	2	2	2
2	2	3	3	3	3	4	4	4	4
1	1	3	3	1	1	2	2	2	2
1	1	3	3	1	1	4	4	4	4
1	1	3	3	1	1	4	4	4	4
1	1	3	3	1	1	2	2	2	2
1	1	3	3	3	3	4	4	2	2
1	1	3	3	1	1	4	4	4	4
1	1	3	3	1	1	2	2	2	2
1	1	3	3	1	1	2	2	2	2
1	1	3	3	1	1	2	2	2	2
1	1	2	2	3	3	4	4	4	4
1	1	3	3	1	1	4	4	4	4
1	1	3	3	1	1	2	2	2	2
1	1	3	3	1	1	4	4	4	4
1	1	3	3	1	1	4	4	4	4
1	1	2	2	3	3	2	2	2	2
1	1	3	3	1	1	4	4	2	2
1	1	3	3	1	1	4	4	4	4
1	1	3	3	3	3	4	4	2	2
1	1	2	2	3	3	4	4	4	4
1	1	3	3	1	1	4	4	4	4
1	1	3	3	1	1	4	4	4	4
1	1	3	3	1	1	2	2	2	2
1	1	3	3	1	1	2	2	2	2
1	1	3	3	1	1	4	4	4	4
2	2	2	2	3	3	4	4	2	2
1	1	3	3	1	1	4	4	4	4
1	1	3	3	1	1	4	4	4	4
1	1	2	2	3	3	4	4	4	4
1	1	3	3	1	1	4	4	4	4
1	1	3	3	1	1	4	4	4	4
1	1	3	3	1	1	4	4	4	4
1	1	3	3	1	1	4	4	4	4
1	1	3	3	3	3	4	4	4	4
1	1	3	3	1	1	2	2	2	2
1	1	3	3	1	1	4	4	4	4
1	1	3	3	1	1	4	4	4	4
1	1	3	3	1	1	2	2	2	2
1	1	3	3	1	1	2	2	2	2
1	1	3	3	1	1	2	2	2	2
1	1	3	3	3	3	4	4	4	4
1	1	2	2	3	3	4	4	4	4
1	1	3	3	3	3	4	4	2	2

For Review Only

1	1	2	2	3	3	2	2	2	2
1	1	3	3	1	1	2	2	2	2
1	1	3	3	1	1	4	4	4	4
1	1	3	3	1	1	2	2	2	2
1	1	3	3	1	1	2	2	2	2
1	1	3	3	1	1	2	2	2	2
1	1	3	3	1	1	2	2	2	2
2	2	3	3	1	1	4	4	4	4
1	1	3	3	1	1	4	4	4	4
1	1	3	3	1	1	4	4	2	2
1	1	3	3	1	1	4	4	4	4
1	1	3	3	1	1	4	4	4	4
1	1	2	2	3	3	2	2	2	2
1	1	2	2	3	3	4	4	4	4
1	1	3	3	1	1	4	4	2	2
1	1	3	3	1	1	4	4	4	4
1	1	2	2	3	3	2	2	2	2
1	1	3	3	1	1	2	2	2	2
1	1	3	3	1	1	2	2	2	2
1	1	3	3	1	1	2	2	2	2
1	1	3	3	1	1	4	4	4	4
1	1	2	2	3	3	4	4	4	4
1	1	2	2	3	3	4	4	4	4
1	1	2	2	3	3	2	2	2	2
1	1	3	3	1	1	4	4	4	4
1	1	3	3	3	3	4	4	2	2
2	2	3	3	1	1	2	2	2	2
1	1	3	3	1	1	4	4	4	4
1	1	3	3	1	1	2	2	2	2
1	1	2	2	3	3	2	2	2	2
1	1	3	3	1	1	4	4	2	2
1	1	3	3	1	1	4	4	4	4
1	1	3	3	3	3	4	4	4	4
1	1	2	2	3	3	2	2	2	2
2	2	3	3	1	1	2	2	2	2
1	1	3	3	1	1	4	4	4	4
1	1	3	3	1	1	4	4	4	4
1	1	3	3	1	1	4	4	2	2
1	1	3	3	1	1	4	4	4	4
2	2	3	3	1	1	4	4	4	4
1	1	2	2	3	3	2	2	2	2
1	1	2	2	3	3	2	2	2	2
1	1	2	2	3	3	4	4	4	4
1	1	3	3	1	1	4	4	4	4
1	1	3	3	1	1	4	4	2	2
1	1	3	3	3	3	2	2	2	2
1	1	3	3	1	1	4	4	4	4
1	1	3	3	1	1	4	4	2	2
1	1	3	3	1	1	2	2	2	2
1	1	3	3	3	3	4	4	2	2
1	1	3	3	1	1	4	4	4	4
2	2	3	3	1	1	4	4	4	4
2	2	3	3	1	1	4	4	4	4
1	1	3	3	3	3	4	4	4	4
1	1	3	3	1	1	2	2	2	2

For Review Only

1	1	3	3	1	1	2	2	2	2
1	1	3	3	1	1	4	4	4	4
1	1	3	3	1	1	4	4	4	4
1	1	3	3	1	1	4	4	4	4
1	1	3	3	1	1	4	4	4	4
1	1	3	3	1	1	4	4	2	2
1	1	3	3	1	1	4	4	4	4
1	1	3	3	1	1	2	2	2	2
1	1	3	3	1	1	4	4	4	4
1	1	3	3	1	1	4	4	4	4
1	1	3	3	1	1	2	2	2	2
1	1	3	3	3	3	4	4	2	2
1	1	3	3	1	1	4	4	4	4
1	1	3	3	3	3	2	2	2	2
1	1	3	3	1	1	4	4	4	4
1	1	3	3	1	1	4	4	2	2
1	1	3	3	1	1	2	2	2	2
1	1	3	3	3	3	4	4	2	2
1	1	3	3	1	1	4	4	4	4
1	1	2	2	3	3	4	4	2	2
1	1	3	3	3	3	4	4	2	2
1	1	3	3	1	1	2	2	2	2
1	1	3	3	1	1	4	4	4	4
1	1	3	3	1	1	4	4	4	4
1	1	3	3	3	3	4	4	4	4
1	1	2	2	3	3	4	4	4	4
1	1	3	3	3	3	4	4	4	4
1	1	3	3	3	3	4	4	4	4
1	1	3	3	1	1	2	2	2	2
1	1	3	3	3	3	2	2	2	2
1	1	3	3	1	1	4	4	4	4
1	1	3	3	3	3	4	4	2	2
1	1	2	2	3	3	2	2	2	2
1	1	3	3	3	3	4	4	2	2
1	1	3	3	1	1	4	4	4	4
1	1	3	3	1	1	4	4	2	2
1	1	3	3	1	1	4	4	4	4
1	1	3	3	1	1	2	2	2	2
1	1	3	3	3	3	4	4	4	4
1	1	3	3	1	1	4	4	2	2
1	1	3	3	3	3	4	4	2	2
1	1	3	3	1	1	4	4	4	4
1	1	3	3	1	1	4	4	4	4
1	1	2	2	1	1	4	4	4	4
1	1	2	2	3	3	4	4	2	2
1	1	3	3	1	1	4	4	4	4
1	1	3	3	1	1	2	2	2	2
1	1	3	3	1	1	2	2	2	2
1	1	3	3	1	1	4	4	4	4
1	1	3	3	3	3	4	4	2	2
1	1	3	3	1	1	2	2	2	2
1	1	3	3	1	1	2	2	2	2
1	1	3	3	1	1	4	4	4	4
1	1	3	3	1	1	4	4	4	4
1	1	3	3	1	1	4	4	4	4

For Review Only

1	1	3	3	1	1	2	2	2	2
1	1	3	3	1	1	4	4	4	4
1	1	3	3	1	1	4	4	4	4
1	1	3	3	1	1	4	4	4	4
1	1	3	3	1	1	2	2	2	2
1	1	3	3	1	1	2	2	2	2
1	1	3	3	1	1	4	4	4	4
1	1	3	3	3	3	4	4	2	2
1	1	3	3	3	3	4	4	2	2
1	1	3	3	3	3	2	2	2	2
1	1	3	3	1	1	4	4	4	4
1	1	3	3	1	1	4	4	2	2
1	1	3	3	3	3	4	4	4	4
1	1	3	3	1	1	4	4	4	4
1	1	3	3	1	1	4	4	4	4
1	1	3	3	1	1	2	2	2	2
1	1	3	3	1	1	4	4	4	4
1	1	3	3	1	1	4	4	4	4
1	1	3	3	1	1	2	2	2	2
1	1	3	3	1	1	4	4	4	4
1	1	3	3	1	1	4	4	2	2
1	1	3	3	1	1	4	4	4	4
1	1	2	2	3	3	4	4	4	4
1	1	3	3	1	1	4	4	4	4
1	1	3	3	1	1	4	4	4	4
1	1	3	3	1	1	4	4	4	4
1	1	3	3	1	1	4	4	4	4
1	1	3	3	1	1	4	4	2	2
1	1	2	2	3	3	2	2	2	2
1	1	2	2	3	3	2	2	2	2
1	1	3	3	1	1	4	4	4	4
1	1	2	2	3	3	2	2	2	2
1	1	3	3	1	1	4	4	4	4
1	1	3	3	1	1	4	4	4	4
1	1	3	3	3	3	4	4	2	2
1	1	3	3	1	1	4	4	4	4
1	1	3	3	1	1	4	4	4	4
2	2	3	3	1	1	4	4	4	4
1	1	3	3	1	1	2	2	2	2
1	1	2	2	3	3	4	4	4	4
1	1	3	3	1	1	4	4	4	4
1	1	3	3	1	1	2	2	2	2
1	1	3	3	1	1	4	4	4	4
1	1	2	2	3	3	2	2	2	2
1	1	3	3	1	1	4	4	2	2
1	1	3	3	1	1	4	4	4	4
1	1	3	3	1	1	2	2	2	2
1	1	3	3	1	1	4	4	4	4
1	1	3	3	1	1	4	4	4	4
1	1	3	3	1	1	2	2	2	2
2	2	3	3	1	1	4	4	2	2
1	1	3	3	1	1	4	4	4	4
1	1	2	2	3	3	4	4	4	4
1	1	3	3	1	1	4	4	4	4
1	1	3	3	1	1	4	4	4	4

For Review Only

1	1	3	3	1	1	4	4	4	4
2	2	3	3	3	3	2	2	2	2
1	1	3	3	1	1	2	2	2	2
1	1	2	2	3	3	4	4	4	4
1	1	3	3	1	1	2	2	2	2
1	1	3	3	1	1	4	4	2	2
1	1	3	3	1	1	4	4	4	4
2	2	3	3	1	1	4	4	4	4
1	1	3	3	1	1	4	4	4	4
1	1	3	3	1	1	4	4	4	4
1	1	3	3	1	1	4	4	2	2
1	1	3	3	1	1	4	4	4	4
1	1	3	3	1	1	2	2	2	2
1	1	2	2	3	3	4	4	4	4
1	1	3	3	1	1	4	4	4	4
1	1	2	2	3	3	2	2	2	2
1	1	3	3	1	1	4	4	4	4
1	1	3	3	1	1	4	4	4	4
1	1	3	3	1	1	2	2	2	2
1	1	2	2	3	3	4	4	4	4
1	1	3	3	1	1	2	2	2	2
1	1	3	3	1	1	2	2	2	2
1	1	3	3	3	3	4	4	4	4
1	1	2	2	3	3	2	2	2	2
2	2	3	3	1	1	4	4	4	4
1	1	3	3	1	1	4	4	4	4
1	1	3	3	1	1	4	4	4	4
1	1	3	3	1	1	4	4	4	4
1	1	3	3	3	3	2	2	2	2
1	1	3	3	1	1	2	2	2	2
1	1	3	3	1	1	4	4	4	4
1	1	3	3	1	1	4	4	2	2
1	1	3	3	1	1	4	4	4	4
1	1	3	3	1	1	4	4	4	4
1	1	2	2	3	3	2	2	2	2
1	1	3	3	1	1	4	4	4	4
1	1	3	3	1	1	4	4	4	4
1	1	3	3	1	1	4	4	4	4
1	1	3	3	1	1	4	4	4	4
1	1	3	3	1	1	4	4	4	4
1	1	3	3	1	1	4	4	4	4
1	1	3	3	1	1	4	4	4	4
1	1	2	2	3	3	4	4	4	4
1	1	2	2	3	3	2	2	2	2
1	1	3	3	1	1	4	4	4	4
1	1	3	3	1	1	4	4	4	4
1	1	3	3	1	1	2	2	2	2
1	1	3	3	1	1	4	4	4	4
1	1	3	3	1	1	4	4	4	4
1	1	3	3	1	1	4	4	4	4
1	1	3	3	1	1	2	2	2	2
1	1	3	3	1	1	4	4	4	4
1	1	3	3	1	1	4	4	4	4
1	1	3	3	1	1	4	4	4	4
1	1	2	2	3	3	4	4	4	4

For Review Only

1	1	2	2	3	3	2	2	2	2
1	1	3	3	1	1	4	4	4	4

For Review Only

SNP1527	SNP1527	SNP1528	SNP1528	SNP1529	SNP1529	SNP153	SNP153	SNP1530	SNP1530
1	1	2	2	4	4	2	2	2	2
1	1	2	2	4	4	2	2	2	2
1	1	2	2	4	4	2	2	2	2
1	1	2	2	3	3	2	2	3	3
1	1	1	1	4	4	2	2	2	2
3	3	2	2	4	4	2	2	2	2
1	1	2	2	4	4	2	2	2	2
1	1	1	1	4	4	3	3	3	3
1	1	2	2	4	4	2	2	2	2
1	1	1	1	4	4	2	2	2	2
1	1	1	1	4	4	2	2	2	2
1	1	2	2	3	3	2	2	3	3
1	1	1	1	4	4	2	2	2	2
1	1	1	1	4	4	2	2	2	2
1	1	2	2	4	4	2	2	2	2
1	1	1	1	4	4	2	2	2	2
1	1	1	1	4	4	2	2	2	2
1	1	1	1	4	4	2	2	2	2
3	3	2	2	4	4	2	2	2	2
3	3	1	1	4	4	2	2	2	2
1	1	1	1	4	4	2	2	2	2
1	1	1	1	4	4	2	2	2	2
1	1	2	2	3	3	3	3	3	3
1	1	1	1	4	4	2	2	2	2
1	1	2	2	4	4	2	2	2	2
1	1	1	1	4	4	2	2	2	2
1	1	2	2	4	4	2	2	2	2
1	1	2	2	4	4	2	2	2	2
1	1	1	1	4	4	2	2	2	2
3	3	1	1	4	4	2	2	2	2
1	1	2	2	3	3	2	2	3	3
1	1	2	2	4	4	2	2	2	2
1	1	2	2	4	4	2	2	2	2
1	1	1	1	4	4	2	2	2	2
1	1	2	2	4	4	2	2	2	2
1	1	1	1	4	4	2	2	2	2
1	1	2	2	4	4	2	2	2	2
1	1	2	2	4	4	2	2	2	2
1	1	1	1	4	4	3	3	2	2
1	1	1	1	4	4	3	3	2	2
1	1	1	1	4	4	2	2	2	2
1	1	1	1	4	4	2	2	2	2
1	1	1	1	4	4	2	2	2	2
1	1	1	1	4	4	2	2	2	2
3	3	1	1	4	4	2	2	2	2
1	1	1	1	4	4	2	2	2	2
1	1	2	2	4	4	2	2	2	2
1	1	1	1	4	4	2	2	2	2
3	3	1	1	4	4	3	3	2	2
1	1	2	2	4	4	2	2	2	2
1	1	2	2	4	4	2	2	2	2
1	1	1	1	4	4	2	2	2	2

For Review Only

1	1	1	1	4	4	2	2	2	2
1	1	2	2	4	4	2	2	2	2
1	1	2	2	4	4	2	2	2	2
1	1	1	1	4	4	2	2	2	2
3	3	2	2	4	4	2	2	2	2
3	3	2	2	4	4	2	2	2	2
1	1	1	1	3	3	2	2	3	3
1	1	1	1	4	4	2	2	2	2
1	1	2	2	4	4	2	2	2	2
1	1	2	2	4	4	2	2	2	2
3	3	2	2	4	4	2	2	2	2
3	3	2	2	4	4	2	2	2	2
1	1	1	1	4	4	2	2	2	2
1	1	1	1	4	4	2	2	2	2
3	3	2	2	4	4	2	2	2	2
3	3	2	2	4	4	2	2	2	2
1	1	2	2	3	3	2	2	3	3
1	1	2	2	4	4	2	2	2	2
1	1	2	2	3	3	2	2	3	3
1	1	1	1	4	4	2	2	2	2
3	3	2	2	4	4	2	2	2	2
1	1	2	2	3	3	3	3	3	3
1	1	2	2	4	4	2	2	2	2
1	1	1	1	4	4	2	2	2	2
3	3	1	1	4	4	2	2	2	2
1	1	2	2	4	4	2	2	2	2
1	1	1	1	4	4	2	2	2	2
1	1	1	1	4	4	3	3	2	2
1	1	1	1	4	4	2	2	2	2
1	1	2	2	4	4	2	2	2	2
1	1	1	1	4	4	2	2	2	2
1	1	1	1	4	4	2	2	2	2
1	1	1	1	4	4	2	2	2	2
1	1	1	1	4	4	2	2	2	2
1	1	1	1	4	4	2	2	2	2
3	3	1	1	4	4	2	2	2	2
1	1	1	1	4	4	2	2	2	2
1	1	1	1	4	4	2	2	2	2
1	1	1	1	4	4	2	2	2	2
1	1	1	1	4	4	2	2	2	2
1	1	1	1	4	4	2	2	2	2
1	1	2	2	4	4	2	2	2	2
1	1	1	1	4	4	2	2	2	2
3	3	2	2	4	4	2	2	2	2
1	1	2	2	4	4	2	2	2	2
1	1	2	2	4	4	2	2	2	2
1	1	1	1	4	4	2	2	2	2
1	1	1	1	4	4	2	2	2	2
1	1	1	1	4	4	2	2	2	2
1	1	2	2	3	3	2	2	3	3
1	1	2	2	4	4	2	2	2	2
1	1	1	1	4	4	2	2	2	2
1	1	1	1	4	4	2	2	2	2
1	1	2	2	4	4	2	2	2	2
1	1	2	2	4	4	2	2	2	2

For Review Only

1	1	2	2	4	4	2	2	2	2
1	1	2	2	4	4	2	2	3	3
1	1	1	1	4	4	2	2	2	2
1	1	1	1	4	4	2	2	2	2
1	1	2	2	4	4	2	2	2	2
1	1	1	1	4	4	2	2	2	2
1	1	2	2	4	4	2	2	2	2
1	1	2	2	4	4	2	2	2	2
1	1	2	2	4	4	2	2	2	2
1	1	2	2	4	4	2	2	2	2
1	1	2	2	4	4	2	2	2	2
1	1	2	2	4	4	2	2	2	2
1	1	1	1	4	4	2	2	2	2
1	1	2	2	4	4	2	2	2	2
1	1	2	2	4	4	2	2	2	2
1	1	2	2	4	4	2	2	2	2
1	1	2	2	4	4	2	2	2	2
1	1	2	2	4	4	2	2	2	2
1	1	1	1	4	4	2	2	2	2
1	1	1	1	4	4	2	2	2	2
1	1	2	2	4	4	2	2	2	2
1	1	2	2	4	4	2	2	2	2
1	1	2	2	4	4	2	2	2	2
1	1	1	1	4	4	2	2	2	2
1	1	2	2	4	4	2	2	3	3
3	3	2	2	3	3	2	2	3	3
1	1	1	1	4	4	2	2	2	2
1	1	1	1	4	4	2	2	2	2
1	1	2	2	3	3	2	2	3	3
1	1	1	1	4	4	2	2	2	2
1	1	1	1	4	4	2	2	2	2
1	1	2	2	4	4	2	2	2	2
1	1	1	1	4	4	2	2	2	2
1	1	2	2	4	4	2	2	2	2
1	1	1	1	4	4	2	2	2	2
1	1	1	1	4	4	2	2	2	2
1	1	1	1	4	4	2	2	2	2
1	1	2	2	4	4	2	2	2	2
1	1	2	2	4	4	2	2	2	2
1	1	1	1	4	4	3	3	2	2
1	1	2	2	3	3	2	2	3	3
1	1	1	1	4	4	2	2	2	2
1	1	2	2	4	4	2	2	2	2
1	1	1	1	4	4	2	2	2	2
1	1	1	1	4	4	2	2	2	2
1	1	1	1	4	4	2	2	2	2
1	1	1	1	4	4	2	2	2	2
1	1	2	2	4	4	2	2	2	2
1	1	2	2	4	4	2	2	2	2
1	1	1	1	4	4	2	2	2	2
1	1	2	2	4	4	2	2	2	2
1	1	1	1	4	4	2	2	2	2
1	1	2	2	4	4	2	2	2	2
1	1	2	2	4	4	2	2	2	2
1	1	2	2	4	4	2	2	2	2

For Review Only

1	1	2	2	4	4	2	2	2	2
1	1	1	1	4	4	2	2	2	2
3	3	1	1	4	4	2	2	2	2
1	1	2	2	4	4	2	2	2	2
1	1	1	1	4	4	2	2	2	2
1	1	1	1	4	4	3	3	2	2
1	1	2	2	4	4	3	3	2	2
1	1	2	2	4	4	2	2	2	2
1	1	2	2	4	4	3	3	2	2
1	1	1	1	4	4	2	2	2	2
1	1	2	2	4	4	2	2	2	2
1	1	2	2	4	4	3	3	2	2
1	1	2	2	4	4	2	2	3	3
1	1	1	1	4	4	2	2	2	2
1	1	1	1	4	4	2	2	2	2
1	1	1	1	4	4	2	2	2	2
1	1	2	2	4	4	2	2	2	2
1	1	2	2	4	4	2	2	2	2
1	1	1	1	4	4	2	2	2	2
1	1	2	2	4	4	2	2	2	2
1	1	2	2	4	4	2	2	2	2
1	1	1	1	4	4	2	2	2	2
1	1	2	2	3	3	2	2	3	3
3	3	2	2	4	4	2	2	2	2
1	1	1	1	4	4	2	2	2	2
1	1	1	1	4	4	2	2	2	2
1	1	2	2	3	3	2	2	3	3
1	1	2	2	4	4	2	2	2	2
1	1	2	2	4	4	2	2	2	2
1	1	1	1	4	4	2	2	2	2
1	1	2	2	4	4	2	2	2	2
1	1	2	2	4	4	2	2	2	2
3	3	2	2	4	4	2	2	2	2
1	1	1	1	4	4	2	2	2	2
1	1	2	2	4	4	2	2	2	2
3	3	2	2	4	4	2	2	2	2
1	1	2	2	4	4	2	2	2	2
1	1	2	2	4	4	2	2	2	2
1	1	2	2	4	4	2	2	2	2
1	1	1	1	3	3	2	2	3	3
1	1	2	2	4	4	2	2	2	2
1	1	2	2	4	4	2	2	2	2
1	1	1	1	4	4	2	2	2	2
1	1	2	2	4	4	2	2	2	2
1	1	1	1	4	4	2	2	2	2
1	1	2	2	4	4	2	2	2	2
1	1	1	1	4	4	2	2	2	2
1	1	1	1	4	4	2	2	2	2
1	1	1	1	4	4	3	3	2	2
1	1	2	2	3	3	2	2	3	3
1	1	2	2	4	4	2	2	2	2
3	3	1	1	4	4	2	2	2	2
1	1	2	2	4	4	3	3	2	2
3	3	2	2	4	4	2	2	2	2
1	1	1	1	4	4	3	3	2	2

For Review Only

1	1	2	2	4	4	2	2	2	2
1	1	2	2	4	4	2	2	2	2
1	1	2	2	4	4	2	2	2	2
1	1	2	2	3	3	2	2	3	3
1	1	1	1	4	4	2	2	2	2
1	1	1	1	4	4	2	2	2	2
1	1	1	1	4	4	2	2	2	2
1	1	1	1	4	4	2	2	2	2
1	1	1	1	4	4	2	2	2	2
1	1	1	1	4	4	2	2	2	2
1	1	2	2	4	4	2	2	2	2
1	1	2	2	4	4	2	2	2	2
1	1	1	1	3	3	2	2	3	3
1	1	2	2	4	4	2	2	2	2
1	1	1	1	4	4	2	2	2	2
1	1	1	1	4	4	2	2	2	2
1	1	1	1	4	4	2	2	2	2
1	1	2	2	4	4	2	2	2	2
1	1	2	2	4	4	2	2	2	2
1	1	2	2	4	4	2	2	2	2
1	1	2	2	4	4	2	2	2	2
1	1	1	1	4	4	2	2	2	2
1	1	2	2	4	4	2	2	2	2
1	1	2	2	4	4	2	2	2	2
1	1	1	1	4	4	2	2	2	2
3	3	2	2	4	4	2	2	2	2
1	1	1	1	4	4	2	2	2	2
1	1	2	2	3	3	2	2	3	3
1	1	1	1	4	4	2	2	2	2
1	1	2	2	4	4	2	2	2	2
1	1	1	1	4	4	3	3	2	2
1	1	2	2	4	4	2	2	2	2
1	1	1	1	4	4	2	2	2	2
1	1	1	1	4	4	2	2	2	2
1	1	2	2	3	3	2	2	3	3
1	1	1	1	4	4	2	2	2	2
1	1	2	2	4	4	2	2	2	2
3	3	2	2	4	4	2	2	2	2
1	1	1	1	4	4	2	2	2	2
1	1	1	1	4	4	3	3	2	2
1	1	2	2	4	4	2	2	2	2
3	3	1	1	4	4	2	2	2	2
1	1	2	2	4	4	3	3	2	2
1	1	1	1	4	4	2	2	2	2
1	1	2	2	4	4	2	2	2	2
1	1	1	1	3	3	2	2	3	3
1	1	2	2	4	4	2	2	2	2
1	1	2	2	4	4	2	2	2	2
1	1	1	1	4	4	2	2	2	2
1	1	1	1	4	4	2	2	2	2
1	1	2	2	4	4	2	2	2	2
1	1	1	1	4	4	3	3	2	2
1	1	2	2	4	4	2	2	2	2
1	1	1	1	3	3	2	2	3	3
1	1	1	1	4	4	3	3	2	2

For Review Only

1	1	1	1	4	4	2	2	2	2
1	1	2	2	4	4	2	2	2	2

For Review Only

SNP1531	SNP1531	SNP1532	SNP1532	SNP1533	SNP1533	SNP1534	SNP1534	SNP1535	SNP1535
4	4	2	2	3	3	2	2	3	3
4	4	3	3	3	3	2	2	3	3
4	4	2	2	2	2	2	2	3	3
4	4	2	2	3	3	2	2	3	3
4	4	3	3	3	3	2	2	3	3
4	4	2	2	3	3	2	2	3	3
4	4	2	2	3	3	2	2	3	3
4	4	2	2	3	3	2	2	3	3
4	4	2	2	3	3	3	3	3	3
4	4	3	3	3	3	3	3	3	3
4	4	2	2	3	3	2	2	3	3
4	4	2	2	2	2	2	2	3	3
4	4	2	2	3	3	3	3	3	3
4	4	2	2	3	3	2	2	3	3
4	4	2	2	3	3	3	3	3	3
4	4	2	2	3	3	2	2	3	3
4	4	2	2	2	2	2	2	3	3
4	4	2	2	3	3	3	3	3	3
4	4	2	2	3	3	2	2	3	3
1	1	2	2	3	3	3	3	4	4
4	4	2	2	3	3	2	2	3	3
4	4	2	2	3	3	2	2	3	3
4	4	2	2	2	2	2	2	3	3
4	4	2	2	3	3	2	2	3	3
4	4	2	2	3	3	3	3	3	3
4	4	2	2	3	3	2	2	3	3
4	4	2	2	3	3	2	2	3	3
4	4	2	2	3	3	2	2	3	3
4	4	2	2	3	3	3	3	3	3
4	4	2	2	3	3	2	2	3	3
4	4	2	2	3	3	2	2	3	3
4	4	2	2	3	3	2	2	3	3
4	4	2	2	3	3	2	2	3	3
4	4	2	2	3	3	2	2	3	3
4	4	2	2	3	3	3	3	3	3
4	4	2	2	3	3	2	2	3	3
4	4	2	2	3	3	2	2	3	3
4	4	2	2	3	3	3	3	3	3
4	4	2	2	3	3	2	2	3	3
4	4	2	2	3	3	2	2	3	3
4	4	2	2	3	3	2	2	3	3
4	4	2	2	2	2	2	2	3	3
4	4	2	2	3	3	3	3	3	3
4	4	2	2	3	3	3	3	3	3
1	1	2	2	3	3	2	2	4	4
4	4	2	2	3	3	2	2	3	3
4	4	2	2	3	3	2	2	3	3
4	4	2	2	3	3	2	2	3	3
4	4	2	2	3	3	3	3	3	3
4	4	2	2	3	3	3	3	3	3
4	4	2	2	3	3	3	3	3	3
4	4	2	2	3	3	2	2	3	3

For Review Only

4	4	2	2	3	3	3	3	3	3
4	4	2	2	3	3	2	2	3	3
4	4	2	2	2	2	2	2	3	3
4	4	2	2	3	3	2	2	3	3
4	4	2	2	3	3	2	2	3	3
1	1	2	2	3	3	2	2	4	4
4	4	2	2	3	3	2	2	3	3
4	4	3	3	3	3	2	2	3	3
4	4	2	2	3	3	2	2	3	3
4	4	2	2	3	3	2	2	3	3
1	1	2	2	3	3	2	2	4	4
1	1	2	2	3	3	2	2	4	4
4	4	2	2	3	3	3	3	3	3
4	4	2	2	3	3	3	3	3	3
4	4	2	2	3	3	3	3	3	3
4	4	2	2	3	3	3	3	4	4
4	4	2	2	3	3	3	3	3	3
4	4	2	2	2	2	2	2	3	3
4	4	2	2	2	2	3	3	3	3
4	4	2	2	3	3	2	2	3	3
4	4	2	2	3	3	2	2	4	4
4	4	2	2	3	3	3	3	3	3
4	4	2	2	3	3	3	3	3	3
4	4	2	2	3	3	3	3	3	3
1	1	2	2	3	3	2	2	4	4
4	4	2	2	3	3	2	2	3	3
4	4	3	3	3	3	2	2	3	3
4	4	2	2	3	3	2	2	3	3
4	4	2	2	3	3	2	2	3	3
4	4	2	2	3	3	3	3	3	3
4	4	2	2	3	3	2	2	3	3
4	4	2	2	3	3	2	2	3	3
4	4	2	2	3	3	2	2	3	3
4	4	2	2	3	3	3	3	3	3
4	4	3	3	2	2	2	2	3	3
1	1	2	2	3	3	2	2	4	4
4	4	2	2	3	3	2	2	3	3
4	4	2	2	3	3	2	2	3	3
4	4	2	2	3	3	2	2	3	3
4	4	3	3	3	3	2	2	3	3
4	4	2	2	3	3	3	3	3	3
4	4	2	2	3	3	3	3	3	3
4	4	2	2	3	3	3	3	3	3
1	1	2	2	3	3	2	2	4	4
4	4	2	2	3	3	2	2	3	3
4	4	2	2	3	3	2	2	3	3
4	4	2	2	3	3	2	2	3	3
4	4	2	2	3	3	2	2	3	3
4	4	2	2	3	3	2	2	3	3
4	4	2	2	3	3	2	2	3	3
4	4	2	2	3	3	2	2	3	3
4	4	2	2	3	3	2	2	3	3
4	4	3	3	3	3	2	2	3	3
4	4	2	2	3	3	2	2	3	3
4	4	2	2	3	3	2	2	3	3

For Review Only

4	4	2	2	3	3	2	2	3	3
4	4	3	3	3	3	2	2	3	3
4	4	2	2	3	3	3	3	3	3
4	4	2	2	3	3	2	2	3	3
4	4	2	2	3	3	2	2	3	3
4	4	2	2	3	3	2	2	3	3
4	4	2	2	3	3	2	2	3	3
4	4	2	2	3	3	2	2	3	3
4	4	2	2	3	3	2	2	3	3
4	4	2	2	3	3	2	2	3	3
4	4	2	2	3	3	2	2	3	3
4	4	2	2	3	3	3	3	3	3
4	4	2	2	3	3	2	2	3	3
4	4	2	2	3	3	2	2	3	3
4	4	2	2	2	2	3	3	3	3
4	4	2	2	3	3	2	2	3	3
4	4	2	2	2	2	2	2	3	3
4	4	2	2	3	3	2	2	3	3
4	4	2	2	3	3	2	2	3	3
4	4	2	2	3	3	3	3	3	3
4	4	2	2	3	3	2	2	3	3
4	4	2	2	3	3	2	2	3	3
4	4	2	2	2	2	2	2	3	3
4	4	2	2	3	3	2	2	3	3
4	4	2	2	3	3	2	2	3	3
4	4	2	2	3	3	3	3	3	3
4	4	2	2	3	3	3	3	3	3
4	4	2	2	3	3	2	2	3	3
4	4	2	2	3	3	2	2	3	3
4	4	2	2	3	3	2	2	3	3
4	4	2	2	3	3	2	2	3	3
4	4	2	2	3	3	2	2	3	3
4	4	2	2	3	3	3	3	3	3
4	4	2	2	3	3	3	3	3	3
4	4	2	2	3	3	2	2	3	3
4	4	2	2	3	3	2	2	3	3
4	4	2	2	3	3	2	2	3	3
4	4	2	2	3	3	2	2	3	3
4	4	2	2	3	3	3	3	3	3
4	4	2	2	3	3	2	2	3	3
4	4	2	2	3	3	3	3	3	3
4	4	2	2	3	3	2	2	3	3
4	4	2	2	3	3	2	2	3	3
4	4	2	2	3	3	2	2	3	3
4	4	2	2	3	3	3	3	3	3
4	4	2	2	3	3	2	2	3	3
4	4	2	2	3	3	2	2	3	3
4	4	2	2	3	3	2	2	3	3
4	4	2	2	3	3	2	2	3	3
4	4	2	2	3	3	3	3	3	3
4	4	2	2	3	3	2	2	3	3
4	4	2	2	3	3	2	2	3	3
4	4	2	2	3	3	2	2	3	3
4	4	2	2	3	3	2	2	3	3
4	4	3	3	3	3	2	2	3	3

For Review Only

4	4	2	2	3	3	2	2	3	3
4	4	2	2	3	3	2	2	3	3
1	1	2	2	3	3	2	2	4	4
4	4	2	2	2	2	2	2	3	3
4	4	2	2	3	3	2	2	3	3
4	4	2	2	2	2	2	2	3	3
4	4	2	2	3	3	2	2	3	3
4	4	2	2	3	3	2	2	3	3
4	4	2	2	3	3	3	3	3	3
4	4	2	2	3	3	2	2	3	3
4	4	2	2	3	3	2	2	3	3
4	4	2	2	3	3	2	2	3	3
4	4	2	2	3	3	2	2	3	3
4	4	2	2	3	3	2	2	3	3
4	4	2	2	3	3	2	2	3	3
4	4	2	2	3	3	2	2	3	3
4	4	2	2	3	3	2	2	3	3
4	4	2	2	2	2	2	2	4	4
4	4	2	2	3	3	3	3	3	3
4	4	2	2	3	3	2	2	3	3
4	4	2	2	3	3	2	2	3	3
4	4	2	2	3	3	2	2	3	3
4	4	2	2	3	3	3	3	3	3
4	4	2	2	3	3	2	2	4	4
4	4	2	2	3	3	3	3	3	3
4	4	2	2	3	3	2	2	3	3
4	4	2	2	3	3	2	2	3	3
4	4	2	2	3	3	2	2	3	3
4	4	2	2	3	3	3	3	3	3
4	4	2	2	3	3	3	3	3	3
4	4	2	2	3	3	2	2	3	3
4	4	2	2	3	3	3	3	3	3
4	4	2	2	2	2	2	2	3	3
4	4	2	2	3	3	2	2	3	3
4	4	2	2	3	3	2	2	4	4
1	1	2	2	3	3	2	2	4	4
4	4	2	2	3	3	2	2	3	3
4	4	3	3	3	3	2	2	3	3
4	4	2	2	3	3	2	2	3	3
4	4	2	2	3	3	3	3	3	3
4	4	2	2	2	2	2	2	3	3
4	4	2	2	3	3	2	2	3	3
4	4	2	2	3	3	2	2	3	3
4	4	2	2	3	3	3	3	3	3
4	4	2	2	3	3	2	2	3	3
4	4	2	2	2	2	2	2	3	3
4	4	2	2	3	3	2	2	3	3
4	4	2	2	3	3	2	2	3	3
4	4	2	2	3	3	2	2	3	3
4	4	2	2	3	3	2	2	3	3
4	4	3	3	3	3	2	2	3	3
1	1	2	2	3	3	2	2	4	4
4	4	2	2	3	3	3	3	3	3
1	1	2	2	3	3	2	2	4	4
4	4	2	2	3	3	2	2	3	3

For Review Only

4	4	2	2	2	2	2	2	3	3
4	4	3	3	3	3	2	2	3	3
4	4	2	2	3	3	2	2	3	3
4	4	2	2	3	3	3	3	3	3
4	4	2	2	2	2	2	2	3	3
4	4	2	2	3	3	2	2	3	3
4	4	2	2	3	3	2	2	3	3
4	4	3	3	3	3	2	2	3	3
4	4	2	2	3	3	2	2	3	3
4	4	2	2	3	3	2	2	3	3
4	4	2	2	3	3	2	2	3	3
4	4	2	2	2	2	2	2	3	3
4	4	2	2	3	3	2	2	3	3
4	4	2	2	3	3	3	3	3	3
4	4	2	2	3	3	2	2	3	3
4	4	2	2	3	3	3	3	3	3
4	4	2	2	3	3	2	2	3	3
4	4	2	2	3	3	2	2	3	3
4	4	2	2	3	3	2	2	3	3
4	4	2	2	3	3	3	3	3	3
4	4	2	2	3	3	2	2	3	3
4	4	2	2	3	3	2	2	3	3
4	4	2	2	3	3	3	3	3	3
4	4	2	2	3	3	3	3	3	3
4	4	3	3	3	3	2	2	3	3
4	4	2	2	3	3	2	2	3	3
4	4	2	2	3	3	2	2	3	3
4	4	2	2	3	3	2	2	3	3
4	4	2	2	3	3	3	3	3	3
4	4	2	2	3	3	2	2	3	3
4	4	2	2	3	3	2	2	3	3
4	4	2	2	3	3	2	2	3	3
4	4	2	2	3	3	2	2	3	3
4	4	2	2	3	3	2	2	3	3
4	4	2	2	3	3	3	3	3	3
4	4	2	2	3	3	2	2	3	3
4	4	2	2	2	2	2	2	3	3
1	1	2	2	3	3	2	2	4	4
4	4	2	2	3	3	2	2	3	3
4	4	2	2	3	3	2	2	3	3
4	4	2	2	3	3	2	2	4	4
4	4	2	2	2	2	2	2	3	3
4	4	2	2	3	3	3	3	3	3
4	4	2	2	3	3	3	3	3	3
4	4	2	2	3	3	2	2	3	3
4	4	2	2	3	3	2	2	3	3
4	4	2	2	3	3	2	2	3	3
4	4	2	2	3	3	2	2	3	3
4	4	2	2	3	3	2	2	3	3
4	4	2	2	3	3	2	2	3	3
4	4	2	2	3	3	2	2	3	3
4	4	2	2	3	3	2	2	3	3
4	4	2	2	2	2	2	2	3	3
4	4	2	2	3	3	2	2	3	3
4	4	2	2	3	3	3	3	3	3

For Review Only

4	4	2	2	3	3	3	3	3	3
4	4	2	2	3	3	2	2	3	3

For Review Only

SNP1536	SNP1536	SNP1537	SNP1537	SNP1538	SNP1538	SNP1539	SNP1539	SNP154	SNP154
4	4	3	3	3	3	1	1	1	1
1	1	1	1	2	2	1	1	1	1
1	1	3	3	2	2	1	1	1	1
1	1	1	1	2	2	1	1	1	1
1	1	3	3	3	3	4	4	1	1
4	4	3	3	2	2	1	1	1	1
4	4	3	3	3	3	1	1	1	1
4	4	3	3	2	2	4	4	1	1
1	1	3	3	3	3	4	4	1	1
1	1	3	3	2	2	4	4	1	1
4	4	3	3	3	3	4	4	1	1
1	1	3	3	2	2	1	1	1	1
4	4	3	3	3	3	4	4	1	1
4	4	3	3	3	3	4	4	1	1
1	1	3	3	3	3	4	4	4	4
4	4	3	3	2	2	4	4	1	1
1	1	3	3	3	3	4	4	1	1
1	1	3	3	3	3	4	4	1	1
1	1	3	3	3	3	1	1	4	4
1	1	3	3	2	2	4	4	1	1
4	4	3	3	2	2	4	4	1	1
4	4	3	3	3	3	4	4	1	1
1	1	1	1	2	2	1	1	1	1
4	4	3	3	2	2	4	4	1	1
1	1	3	3	3	3	1	1	1	1
4	4	3	3	3	3	4	4	1	1
4	4	3	3	2	2	1	1	1	1
1	1	3	3	3	3	1	1	1	1
1	1	3	3	2	2	4	4	1	1
4	4	3	3	2	2	4	4	1	1
1	1	3	3	2	2	1	1	4	4
4	4	3	3	2	2	1	1	4	4
4	4	3	3	2	2	1	1	1	1
4	4	3	3	2	2	4	4	1	1
1	1	3	3	3	3	1	1	1	1
4	4	3	3	2	2	4	4	1	1
4	4	3	3	2	2	1	1	1	1
1	1	3	3	2	2	1	1	1	1
1	1	3	3	2	2	4	4	1	1
4	4	3	3	3	3	4	4	1	1
4	4	3	3	2	2	4	4	1	1
1	1	3	3	2	2	4	4	1	1
1	1	3	3	2	2	4	4	1	1
1	1	3	3	3	3	4	4	1	1
4	4	3	3	2	2	4	4	1	1
1	1	3	3	2	2	4	4	1	1
1	1	3	3	3	3	1	1	1	1
4	4	3	3	3	3	1	1	1	1
1	1	3	3	3	3	4	4	1	1
1	1	3	3	3	3	1	1	4	4
1	1	3	3	3	3	1	1	1	1
4	4	3	3	3	3	1	1	1	1

For Review Only

1	1	3	3	3	3	4	4	1	1
4	4	3	3	2	2	1	1	1	1
4	4	3	3	2	2	1	1	1	1
4	4	3	3	3	3	4	4	1	1
1	1	3	3	3	3	1	1	4	4
4	4	3	3	3	3	1	1	1	1
4	4	1	1	3	3	4	4	1	1
4	4	3	3	2	2	4	4	1	1
4	4	3	3	2	2	1	1	1	1
1	1	3	3	3	3	1	1	1	1
1	1	3	3	2	2	1	1	1	1
1	1	3	3	2	2	1	1	1	1
1	1	3	3	3	3	4	4	1	1
1	1	3	3	2	2	4	4	1	1
1	1	3	3	3	3	1	1	1	1
4	4	3	3	2	2	1	1	1	1
1	1	1	1	3	3	1	1	1	1
1	1	3	3	3	3	1	1	1	1
1	1	1	1	3	3	1	1	4	4
4	4	3	3	3	3	4	4	1	1
1	1	3	3	2	2	1	1	1	1
1	1	3	3	2	2	1	1	1	1
1	1	3	3	2	2	4	4	1	1
1	1	3	3	3	3	4	4	1	1
4	4	3	3	2	2	4	4	1	1
4	4	3	3	3	3	1	1	1	1
4	4	3	3	3	3	4	4	1	1
4	4	3	3	2	2	4	4	1	1
4	4	3	3	3	3	4	4	1	1
1	1	3	3	3	3	1	1	4	4
4	4	3	3	3	3	4	4	1	1
4	4	3	3	2	2	4	4	1	1
4	4	3	3	2	2	4	4	1	1
1	1	3	3	3	3	4	4	1	1
1	1	3	3	3	3	4	4	1	1
4	4	3	3	2	2	4	4	1	1
1	1	3	3	2	2	4	4	1	1
1	1	3	3	3	3	4	4	1	1
4	4	3	3	2	2	4	4	1	1
4	4	3	3	2	2	4	4	1	1
1	1	3	3	3	3	4	4	1	1
1	1	3	3	3	3	1	1	4	4
1	1	3	3	3	3	4	4	4	4
1	1	3	3	2	2	1	1	1	1
1	1	3	3	2	2	1	1	1	1
1	1	3	3	3	3	1	1	1	1
4	4	3	3	2	2	4	4	1	1
4	4	3	3	3	3	4	4	1	1
4	4	3	3	3	3	4	4	1	1
1	1	1	1	3	3	1	1	1	1
1	1	3	3	2	2	1	1	1	1
4	4	3	3	2	2	4	4	1	1
4	4	3	3	2	2	4	4	1	1
1	1	3	3	2	2	1	1	1	1
4	4	3	3	3	3	1	1	4	4

For Review Only

4	4	3	3	3	3	1	1	1	1
1	1	3	3	2	2	1	1	1	1
1	1	3	3	2	2	4	4	1	1
4	4	3	3	2	2	4	4	1	1
1	1	3	3	2	2	1	1	1	1
4	4	3	3	3	3	4	4	1	1
4	4	3	3	2	2	1	1	1	1
1	1	3	3	3	3	4	4	1	1
4	4	3	3	2	2	1	1	1	1
1	1	3	3	2	2	1	1	1	1
4	4	3	3	3	3	1	1	1	1
1	1	3	3	3	3	1	1	1	1
4	4	3	3	3	3	4	4	4	4
4	4	3	3	3	3	1	1	1	1
1	1	3	3	2	2	1	1	1	1
1	1	3	3	3	3	1	1	1	1
1	1	3	3	3	3	1	1	4	4
1	1	3	3	3	3	1	1	1	1
4	4	3	3	2	2	4	4	1	1
1	1	3	3	3	3	4	4	4	4
1	1	3	3	3	3	1	1	1	1
4	4	3	3	3	3	1	1	4	4
1	1	3	3	3	3	1	1	1	1
4	4	3	3	2	2	4	4	1	1
1	1	3	3	2	2	1	1	1	1
1	1	1	1	2	2	1	1	1	1
1	1	3	3	2	2	4	4	4	4
1	1	3	3	2	2	4	4	1	1
1	1	1	1	3	3	1	1	1	1
1	1	3	3	3	3	4	4	4	4
4	4	3	3	2	2	4	4	1	1
1	1	3	3	3	3	1	1	1	1
1	1	3	3	3	3	4	4	1	1
1	1	3	3	3	3	1	1	1	1
4	4	3	3	2	2	4	4	1	1
4	4	3	3	2	2	4	4	1	1
4	4	3	3	2	2	4	4	1	1
4	4	3	3	3	3	1	1	1	1
1	1	3	3	3	3	1	1	1	1
4	4	3	3	3	3	4	4	1	1
1	1	1	1	3	3	1	1	1	1
4	4	3	3	2	2	4	4	1	1
4	4	3	3	2	2	1	1	4	4
4	4	3	3	2	2	4	4	1	1
1	1	3	3	3	3	1	1	1	1
4	4	3	3	2	2	4	4	1	1
4	4	3	3	3	3	4	4	1	1
4	4	3	3	3	3	1	1	1	1
4	4	3	3	2	2	1	1	1	1
1	1	3	3	3	3	4	4	1	1
4	4	3	3	3	3	1	1	1	1
4	4	3	3	3	3	4	4	1	1
1	1	3	3	2	2	1	1	1	1
4	4	3	3	2	2	1	1	1	1
1	1	3	3	2	2	1	1	1	1

For Review Only

4	4	3	3	3	3	1	1	1	1
4	4	3	3	3	3	4	4	1	1
1	1	3	3	2	2	4	4	1	1
1	1	3	3	2	2	1	1	1	1
1	1	3	3	3	3	4	4	1	1
1	1	3	3	3	3	4	4	1	1
4	4	3	3	2	2	1	1	1	1
1	1	3	3	3	3	1	1	1	1
1	1	3	3	3	3	1	1	1	1
1	1	3	3	3	3	4	4	4	4
4	4	3	3	2	2	4	4	1	1
4	4	3	3	2	2	1	1	1	1
4	4	3	3	2	2	1	1	1	1
4	4	3	3	2	2	4	4	1	1
4	4	3	3	2	2	4	4	1	1
4	4	3	3	3	3	4	4	1	1
1	1	3	3	3	3	1	1	1	1
1	1	3	3	3	3	1	1	1	1
1	1	3	3	3	3	4	4	1	1
4	4	3	3	2	2	1	1	1	1
1	1	3	3	3	3	1	1	1	1
4	4	3	3	2	2	4	4	1	1
1	1	3	3	2	2	1	1	1	1
1	1	3	3	2	2	1	1	1	1
4	4	3	3	2	2	4	4	1	1
4	4	3	3	2	2	4	4	1	1
1	1	3	3	2	2	1	1	4	4
1	1	3	3	3	3	1	1	4	4
1	1	3	3	3	3	1	1	1	1
1	1	3	3	3	3	4	4	1	1
1	1	3	3	2	2	1	1	4	4
1	1	3	3	3	3	1	1	4	4
1	1	3	3	2	2	1	1	1	1
4	4	3	3	2	2	4	4	1	1
1	1	3	3	3	3	1	1	1	1
1	1	3	3	2	2	1	1	1	1
4	4	3	3	2	2	1	1	1	1
4	4	3	3	2	2	1	1	1	1
4	4	3	3	3	3	1	1	1	1
1	1	1	1	2	2	4	4	4	4
1	1	3	3	2	2	1	1	1	1
4	4	3	3	3	3	1	1	1	1
4	4	3	3	2	2	4	4	1	1
1	1	3	3	3	3	1	1	1	1
1	1	3	3	3	3	4	4	1	1
1	1	3	3	2	2	1	1	4	4
4	4	3	3	3	3	4	4	1	1
4	4	3	3	2	2	4	4	1	1
4	4	3	3	2	2	4	4	1	1
4	4	1	1	3	3	1	1	1	1
1	1	3	3	3	3	1	1	1	1
4	4	3	3	2	2	4	4	1	1
1	1	3	3	2	2	1	1	1	1
1	1	3	3	2	2	1	1	1	1
4	4	3	3	2	2	4	4	1	1

For Review Only

For Review Only

1	1	3	3	2	2	1	1	1	1
1	1	3	3	3	3	4	4	1	1
1	1	3	3	3	3	1	1	1	1
1	1	3	3	3	3	1	1	4	4
4	4	3	3	3	3	4	4	1	1
4	4	3	3	3	3	1	1	1	1
4	4	3	3	2	2	4	4	4	4
1	1	3	3	2	2	4	4	1	1
4	4	3	3	2	2	4	4	1	1
1	1	3	3	2	2	4	4	1	1
1	1	3	3	3	3	1	1	1	1
1	1	3	3	2	2	1	1	4	4
4	4	1	1	3	3	4	4	1	1
1	1	3	3	2	2	1	1	1	1
4	4	3	3	2	2	4	4	4	4
1	1	3	3	3	3	4	4	1	1
1	1	3	3	2	2	4	4	1	1
4	4	3	3	2	2	1	1	1	1
4	4	3	3	3	3	1	1	1	1
1	1	3	3	2	2	1	1	1	1
1	1	3	3	3	3	1	1	1	1
4	4	3	3	3	3	4	4	1	1
1	1	3	3	2	2	1	1	1	1
1	1	3	3	3	3	1	1	1	1
4	4	3	3	2	2	4	4	1	1
1	1	3	3	2	2	1	1	1	1
4	4	3	3	2	2	4	4	1	1
1	1	1	1	2	2	1	1	1	1
1	1	3	3	3	3	4	4	1	1
1	1	3	3	3	3	1	1	1	1
4	4	3	3	2	2	4	4	1	1
4	4	3	3	3	3	1	1	1	1
4	4	3	3	2	2	4	4	1	1
4	4	3	3	2	2	4	4	1	1
1	1	1	1	3	3	1	1	1	1
4	4	3	3	2	2	4	4	1	1
1	1	3	3	2	2	1	1	1	1
1	1	3	3	2	2	1	1	1	1
4	4	3	3	2	2	4	4	1	1
4	4	3	3	2	2	4	4	1	1
1	1	3	3	2	2	1	1	1	1
1	1	3	3	2	2	4	4	1	1
1	1	3	3	2	2	1	1	1	1
1	1	3	3	3	3	4	4	1	1
1	1	3	3	2	2	1	1	1	1
4	4	1	1	2	2	4	4	1	1
4	4	3	3	3	3	4	4	1	1
4	4	3	3	3	3	1	1	1	1
4	4	3	3	2	2	4	4	1	1
4	4	3	3	2	2	4	4	1	1
4	4	3	3	3	3	1	1	1	1
4	4	3	3	2	2	4	4	1	1
1	1	3	3	2	2	1	1	1	1
1	1	1	1	2	2	4	4	4	4
1	1	3	3	2	2	4	4	1	1

1	1	3	3	3	3	4	4	1	1
4	4	3	3	2	2	1	1	1	1

For Review Only

SNP1540	SNP1540	SNP1541	SNP1541	SNP1542	SNP1542	SNP1543	SNP1543	SNP1544	SNP1544
3	3	2	2	2	2	4	4	1	1
2	2	2	2	2	2	4	4	4	4
3	3	2	2	2	2	4	4	1	1
3	3	2	2	2	2	4	4	4	4
3	3	2	2	2	2	4	4	4	4
3	3	2	2	2	2	4	4	4	4
3	3	2	2	2	2	4	4	4	4
2	2	2	2	2	2	4	4	4	4
2	2	2	2	2	2	4	4	4	4
2	2	3	3	3	3	4	4	4	4
3	3	3	3	3	3	4	4	1	1
3	3	2	2	2	2	4	4	1	1
2	2	2	2	2	2	4	4	4	4
2	2	2	2	2	2	4	4	4	4
3	3	2	2	2	2	4	4	4	4
2	2	2	2	2	2	4	4	4	4
3	3	2	2	2	2	4	4	4	4
3	3	2	2	2	2	1	1	1	1
3	3	2	2	2	2	4	4	4	4
2	2	2	2	2	2	4	4	4	4
2	2	2	2	2	2	4	4	4	4
3	3	2	2	2	2	4	4	4	4
3	3	3	3	3	3	4	4	4	4
3	3	2	2	2	2	4	4	4	4
3	3	2	2	2	2	4	4	1	1
3	3	2	2	2	2	4	4	1	1
3	3	2	2	2	2	4	4	4	4
2	2	2	2	2	2	4	4	4	4
2	2	2	2	2	2	4	4	1	1
2	2	2	2	2	2	4	4	4	4
3	3	2	2	2	2	4	4	4	4
3	3	2	2	2	2	4	4	4	4
3	3	2	2	2	2	4	4	4	4
2	2	2	2	2	2	4	4	4	4
3	3	2	2	3	3	4	4	4	4
3	3	2	2	2	2	4	4	1	1
3	3	2	2	2	2	1	1	4	4
2	2	2	2	2	2	4	4	1	1
3	3	3	3	3	3	4	4	4	4
2	2	3	3	3	3	4	4	1	1
2	2	2	2	2	2	4	4	4	4
2	2	2	2	2	2	4	4	4	4
2	2	2	2	2	2	4	4	4	4
3	3	2	2	2	2	4	4	4	4
2	2	2	2	2	2	4	4	4	4
3	3	2	2	2	2	4	4	1	1
3	3	2	2	2	2	4	4	1	1
3	3	2	2	2	2	4	4	4	4
3	3	2	2	2	2	4	4	4	4
3	3	2	2	2	2	4	4	1	1
3	3	2	2	2	2	4	4	1	1
3	3	2	2	2	2	4	4	1	1

For Review Only

3	3	2	2	2	2	4	4	1	1
3	3	2	2	2	2	4	4	4	4
3	3	2	2	2	2	1	1	4	4
3	3	2	2	2	2	4	4	4	4
3	3	2	2	2	2	4	4	4	4
3	3	2	2	2	2	4	4	1	1
3	3	2	2	2	2	4	4	1	1
3	3	2	2	2	2	4	4	4	4
3	3	2	2	2	2	4	4	4	4
2	2	2	2	2	2	4	4	4	4
3	3	2	2	2	2	1	1	1	1
3	3	2	2	2	2	4	4	1	1
3	3	2	2	2	2	4	4	1	1
2	2	2	2	2	2	4	4	1	1
3	3	2	2	2	2	4	4	4	4
3	3	2	2	2	2	4	4	4	4
3	3	2	2	2	2	4	4	1	1
3	3	2	2	2	2	4	4	1	1
3	3	2	2	2	2	4	4	4	4
3	3	2	2	2	2	4	4	4	4
3	3	2	2	2	2	4	4	1	1
3	3	3	3	3	3	4	4	1	1
2	2	2	2	3	3	4	4	4	4
3	3	2	2	2	2	4	4	4	4
2	2	2	2	2	2	4	4	4	4
3	3	2	2	2	2	4	4	1	1
2	2	2	2	2	2	4	4	4	4
2	2	3	3	3	3	4	4	1	1
3	3	2	2	2	2	4	4	1	1
3	3	2	2	2	2	4	4	4	4
3	3	2	2	2	2	4	4	4	4
3	3	2	2	2	2	4	4	4	4
2	2	3	3	2	2	4	4	1	1
3	3	2	2	2	2	4	4	1	1
3	3	2	2	2	2	4	4	4	4
2	2	2	2	2	2	4	4	4	4
2	2	2	2	2	2	4	4	4	4
3	3	2	2	2	2	4	4	4	4
3	3	2	2	2	2	4	4	4	4
2	2	2	2	2	2	4	4	4	4
3	3	2	2	2	2	4	4	4	4
3	3	2	2	2	2	4	4	4	4
3	3	2	2	2	2	4	4	4	4
3	3	2	2	2	2	1	1	1	1
3	3	2	2	2	2	4	4	1	1
3	3	2	2	2	2	4	4	1	1
2	2	2	2	2	2	4	4	4	4
3	3	2	2	2	2	4	4	1	1
3	3	2	2	2	2	4	4	1	1
2	2	2	2	3	3	4	4	1	1
3	3	2	2	2	2	1	1	1	1
3	3	3	3	3	3	4	4	1	1
3	3	2	2	2	2	4	4	4	4
2	2	2	2	2	2	4	4	4	4
2	2	2	2	2	2	4	4	1	1

For Review Only

3	3	2	2	2	2	4	4	1	1
2	2	3	3	3	3	4	4	4	4
3	3	2	2	2	2	4	4	1	1
3	3	2	2	2	2	4	4	4	4
3	3	2	2	2	2	4	4	1	1
2	2	2	2	2	2	4	4	1	1
3	3	2	2	2	2	4	4	4	4
3	3	2	2	2	2	4	4	4	4
3	3	2	2	2	2	4	4	1	1
2	2	2	2	2	2	4	4	1	1
2	2	2	2	2	2	4	4	1	1
2	2	2	2	2	2	4	4	4	4
3	3	2	2	2	2	4	4	4	4
3	3	2	2	2	2	4	4	1	1
3	3	2	2	2	2	4	4	4	4
2	2	2	2	2	2	4	4	4	4
2	2	2	2	2	2	4	4	1	1
3	3	2	2	2	2	4	4	4	4
3	3	2	2	2	2	4	4	4	4
2	2	2	2	2	2	4	4	4	4
3	3	2	2	2	2	4	4	4	4
3	3	2	2	2	2	4	4	4	4
3	3	2	2	2	2	4	4	4	4
3	3	2	2	2	2	4	4	4	4
3	3	2	2	3	3	4	4	1	1
3	3	3	3	3	3	4	4	4	4
2	2	2	2	2	2	4	4	4	4
3	3	2	2	2	2	4	4	4	4
3	3	2	2	2	2	4	4	4	4
3	3	2	2	2	2	4	4	1	1
3	3	2	2	3	3	4	4	4	4
3	3	2	2	2	2	4	4	4	4
3	3	2	2	2	2	4	4	4	4
3	3	2	2	2	2	4	4	4	4
2	2	2	2	2	2	4	4	4	4
3	3	2	2	2	2	4	4	4	4
3	3	2	2	2	2	4	4	4	4
2	2	2	2	2	2	4	4	1	1
2	2	2	2	2	2	4	4	4	4
3	3	3	3	3	3	4	4	1	1
3	3	2	2	3	3	4	4	1	1
2	2	2	2	2	2	4	4	4	4
3	3	2	2	2	2	4	4	1	1
2	2	2	2	2	2	4	4	4	4
3	3	2	2	2	2	4	4	4	4
3	3	2	2	2	2	1	1	4	4
3	3	2	2	2	2	4	4	1	1
3	3	2	2	2	2	4	4	1	1
2	2	2	2	2	2	4	4	4	4
3	3	2	2	2	2	4	4	1	1
3	3	2	2	2	2	4	4	1	1
3	3	2	2	2	2	1	1	1	1
3	3	2	2	2	2	4	4	1	1
3	3	2	2	2	2	4	4	1	1
3	3	2	2	3	3	4	4	4	4

For Review Only

3	3	2	2	2	2	4	4	1	1
3	3	3	3	3	3	4	4	1	1
3	3	2	2	2	2	4	4	4	4
2	2	3	3	3	3	4	4	1	1
3	3	2	2	2	2	4	4	1	1
3	3	2	2	2	2	4	4	4	4
2	2	3	3	3	3	4	4	1	1
3	3	2	2	2	2	4	4	4	4
2	2	2	2	3	3	4	4	4	4
3	3	2	2	2	2	4	4	4	4
3	3	2	2	2	2	4	4	1	1
2	2	2	2	2	2	4	4	1	1
3	3	2	2	2	2	4	4	4	4
2	2	2	2	2	2	4	4	4	4
2	2	2	2	2	2	4	4	4	4
3	3	3	3	3	3	4	4	1	1
2	2	2	2	2	2	4	4	4	4
3	3	2	2	2	2	1	1	1	1
3	3	2	2	2	2	4	4	1	1
3	3	2	2	2	2	4	4	4	4
3	3	2	2	2	2	4	4	4	4
2	2	2	2	2	2	4	4	1	1
3	3	3	3	3	3	4	4	1	1
3	3	2	2	2	2	4	4	1	1
3	3	2	2	2	2	4	4	4	4
2	2	2	2	2	2	4	4	4	4
3	3	2	2	2	2	4	4	1	1
2	2	2	2	2	2	4	4	4	4
3	3	2	2	2	2	4	4	1	1
3	3	2	2	2	2	4	4	4	4
3	3	2	2	2	2	4	4	1	1
3	3	2	2	2	2	4	4	4	4
3	3	2	2	2	2	4	4	4	4
2	2	2	2	2	2	4	4	4	4
3	3	2	2	2	2	4	4	4	4
3	3	2	2	2	2	4	4	1	1
2	2	3	3	3	3	4	4	1	1
3	3	2	2	2	2	4	4	4	4
3	3	2	2	2	2	4	4	1	1
2	2	2	2	2	2	4	4	1	1
3	3	2	2	2	2	4	4	1	1
3	3	2	2	2	2	4	4	1	1
3	3	3	3	3	3	4	4	4	4
3	3	2	2	2	2	4	4	4	4
3	3	2	2	2	2	4	4	4	4
3	3	2	2	2	2	4	4	4	4
3	3	3	3	3	3	4	4	1	1
2	2	3	3	2	2	4	4	1	1
3	3	2	2	2	2	4	4	4	4
3	3	2	2	2	2	4	4	4	4
2	2	2	2	2	2	4	4	4	4
3	3	2	2	2	2	4	4	4	4
3	3	3	3	3	3	4	4	1	1
3	3	2	2	2	2	4	4	1	1
3	3	3	3	3	3	4	4	4	4

For Review Only

3	3	2	2	2	2	4	4	4	4
2	2	2	2	2	2	4	4	4	4
3	3	2	2	2	2	4	4	4	4
3	3	3	3	3	3	4	4	1	1
2	2	2	2	2	2	4	4	4	4
3	3	2	2	2	2	4	4	1	1
2	2	3	3	3	3	4	4	4	4
2	2	2	2	2	2	4	4	4	4
3	3	2	2	2	2	4	4	4	4
3	3	3	3	3	3	4	4	1	1
3	3	2	2	2	2	4	4	4	4
2	2	2	2	2	2	4	4	1	1
3	3	2	2	2	2	4	4	4	4
3	3	2	2	2	2	4	4	1	1
3	3	2	2	2	2	4	4	1	1
3	3	2	2	2	2	4	4	1	1
3	3	3	3	3	3	4	4	1	1
2	2	3	3	3	3	4	4	1	1
2	2	3	3	3	3	4	4	1	1
2	2	2	2	2	2	4	4	1	1
2	2	3	3	3	3	4	4	4	4
3	3	2	2	2	2	4	4	1	1
3	3	2	2	2	2	4	4	4	4
2	2	3	3	3	3	4	4	1	1
3	3	2	2	2	2	4	4	4	4
3	3	2	2	3	3	4	4	1	1
3	3	3	3	2	2	4	4	1	1
3	3	2	2	2	2	4	4	1	1
3	3	2	2	2	2	4	4	1	1
3	3	2	2	2	2	4	4	4	4
3	3	3	3	3	3	4	4	4	4
3	3	2	2	2	2	4	4	1	1
2	2	2	2	2	2	4	4	1	1
3	3	2	2	2	2	4	4	1	1
3	3	3	3	3	3	4	4	1	1
3	3	3	3	3	3	4	4	1	1
3	3	2	2	2	2	4	4	1	1
3	3	2	2	2	2	4	4	1	1
3	3	2	2	2	2	4	4	1	1
3	3	3	3	3	3	4	4	4	4
3	3	2	2	2	2	1	1	4	4
3	3	2	2	2	2	4	4	4	4
2	2	3	3	3	3	4	4	1	1
3	3	2	2	2	2	4	4	4	4
3	3	2	2	2	2	4	4	1	1
3	3	2	2	2	2	4	4	4	4
3	3	2	2	2	2	4	4	1	1
2	2	2	2	2	2	4	4	4	4
2	2	2	2	2	2	4	4	1	1
3	3	3	3	3	3	4	4	1	1
3	3	2	2	2	2	4	4	1	1
3	3	3	3	3	3	4	4	4	4
3	3	2	2	2	2	4	4	1	1
3	3	2	2	2	2	4	4	1	1
2	2	3	3	3	3	4	4	4	4

3	3	2	2	2	2	4	4	1	1
3	3	2	2	2	2	4	4	1	1

For Review Only

SNP1545	SNP1545	SNP1546	SNP1546	SNP1547	SNP1547	SNP1548	SNP1548	SNP1549	SNP1549
1	1	1	1	4	4	4	4	1	1
1	1	1	1	2	2	4	4	1	1
1	1	1	1	2	2	4	4	1	1
1	1	1	1	4	4	1	1	4	4
1	1	1	1	4	4	4	4	1	1
1	1	1	1	2	2	4	4	1	1
1	1	1	1	2	2	4	4	1	1
1	1	1	1	2	2	4	4	1	1
1	1	1	1	4	4	4	4	1	1
2	2	1	1	4	4	4	4	1	1
1	1	1	1	2	2	4	4	1	1
1	1	1	1	2	2	4	4	1	1
1	1	1	1	4	4	4	4	1	1
2	2	1	1	4	4	4	4	1	1
1	1	1	1	4	4	4	4	1	1
1	1	1	1	2	2	4	4	1	1
1	1	1	1	4	4	4	4	1	1
1	1	1	1	4	4	4	4	1	1
2	2	1	1	4	4	4	4	1	1
1	1	1	1	2	2	4	4	1	1
2	2	1	1	4	4	4	4	1	1
1	1	1	1	4	4	4	4	1	1
1	1	1	1	2	2	4	4	1	1
2	2	1	1	2	2	4	4	1	1
1	1	1	1	2	2	4	4	1	1
1	1	1	1	4	4	4	4	1	1
2	2	1	1	2	2	4	4	1	1
1	1	1	1	4	4	4	4	1	1
1	1	1	1	2	2	4	4	1	1
2	2	1	1	2	2	4	4	1	1
2	2	1	1	2	2	4	4	1	1
1	1	1	1	2	2	4	4	1	1
1	1	1	1	2	2	4	4	1	1
2	2	1	1	4	4	4	4	1	1
1	1	1	1	4	4	4	4	1	1
1	1	1	1	2	2	4	4	1	1
2	2	2	2	2	2	1	1	4	4
1	1	2	2	2	2	1	1	4	4
2	2	1	1	2	2	4	4	1	1
1	1	1	1	2	2	4	4	1	1
1	1	1	1	2	2	4	4	1	1
2	2	1	1	2	2	4	4	1	1
2	2	1	1	2	2	4	4	1	1
1	1	1	1	4	4	4	4	4	4
1	1	1	1	4	4	4	4	1	1
1	1	1	1	2	2	4	4	1	1
1	1	1	1	4	4	4	4	1	1
1	1	1	1	4	4	4	4	1	1
1	1	1	1	4	4	4	4	1	1
1	1	1	1	4	4	4	4	1	1
1	1	1	1	4	4	4	4	1	1
1	1	1	1	4	4	4	4	1	1

For Review Only

1	1	1	1	4	4	4	4	1	1
1	1	1	1	2	2	4	4	1	1
1	1	1	1	4	4	4	4	1	1
1	1	1	1	4	4	4	4	1	1
2	2	1	1	4	4	4	4	1	1
1	1	1	1	4	4	4	4	1	1
1	1	1	1	2	2	4	4	1	1
1	1	2	2	2	2	1	1	4	4
2	2	2	2	4	4	1	1	4	4
1	1	1	1	4	4	4	4	1	1
1	1	1	1	2	2	4	4	1	1
1	1	1	1	2	2	4	4	1	1
1	1	1	1	4	4	4	4	1	1
1	1	1	1	2	2	4	4	1	1
1	1	1	1	2	2	4	4	1	1
1	1	1	1	4	4	4	4	1	1
1	1	1	1	4	4	4	4	1	1
1	1	1	1	4	4	4	4	1	1
1	1	1	1	4	4	4	4	1	1
1	1	1	1	4	4	4	4	1	1
1	1	1	1	2	2	4	4	1	1
1	1	1	1	2	2	4	4	1	1
1	1	1	1	2	2	4	4	1	1
1	1	1	1	2	2	4	4	1	1
1	1	1	1	4	4	4	4	1	1
1	1	1	1	2	2	4	4	1	1
1	1	1	1	2	2	4	4	1	1
1	1	1	1	2	2	4	4	1	1
1	1	1	1	4	4	4	4	1	1
2	2	1	1	4	4	4	4	1	1
1	1	1	1	4	4	4	4	1	1
2	2	1	1	4	4	4	4	1	1
1	1	1	1	2	2	4	4	1	1
1	1	1	1	4	4	4	4	1	1
1	1	1	1	4	4	4	4	1	1
1	1	1	1	4	4	4	4	1	1
2	2	1	1	4	4	4	4	1	1
1	1	1	1	4	4	4	4	1	1
2	2	1	1	2	2	4	4	1	1
1	1	1	1	4	4	4	4	1	1
1	1	1	1	4	4	4	4	1	1
2	2	1	1	2	2	4	4	1	1
2	2	1	1	4	4	4	4	1	1
1	1	1	1	2	2	4	4	1	1
1	1	1	1	2	2	4	4	1	1
1	1	1	1	4	4	4	4	1	1
2	2	2	2	4	4	1	1	4	4
1	1	1	1	4	4	4	4	1	1
1	1	1	1	4	4	4	4	1	1
1	1	1	1	4	4	4	4	1	1
1	1	1	1	2	2	4	4	1	1
1	1	1	1	2	2	4	4	1	1
1	1	2	2	2	2	1	1	4	4
1	1	1	1	2	2	4	4	1	1
1	1	1	1	4	4	4	4	1	1

For Review Only

1	1	1	1	4	4	4	4	1	1
1	1	1	1	2	2	4	4	1	1
1	1	1	1	2	2	4	4	1	1
2	2	2	2	4	4	1	1	4	4
1	1	1	1	4	4	4	4	1	1
1	1	1	1	2	2	4	4	1	1
1	1	1	1	2	2	4	4	1	1
1	1	1	1	4	4	4	4	1	1
1	1	1	1	4	4	4	4	1	1
1	1	2	2	4	4	4	4	1	1
1	1	1	1	4	4	4	4	1	1
1	1	1	1	4	4	4	4	1	1
2	2	1	1	4	4	4	4	1	1
1	1	1	1	4	4	4	4	1	1
1	1	1	1	2	2	4	4	1	1
1	1	1	1	4	4	4	4	1	1
1	1	1	1	4	4	4	4	1	1
1	1	1	1	4	4	4	4	1	1
2	2	2	2	4	4	1	1	4	4
1	1	1	1	4	4	4	4	1	1
1	1	1	1	4	4	4	4	1	1
2	2	1	1	4	4	4	4	1	1
1	1	1	1	4	4	4	4	1	1
2	2	1	1	2	2	4	4	1	1
1	1	1	1	2	2	4	4	1	1
1	1	1	1	2	2	4	4	1	1
1	1	1	1	2	2	4	4	1	1
2	2	1	1	2	2	4	4	1	1
1	1	1	1	4	4	4	4	1	1
1	1	1	1	2	2	4	4	1	1
2	2	1	1	2	2	4	4	1	1
1	1	1	1	4	4	1	1	4	4
1	1	1	1	4	4	4	4	1	1
1	1	1	1	4	4	4	4	1	1
2	2	1	1	4	4	4	4	1	1
2	2	1	1	2	2	1	1	4	4
2	2	2	2	4	4	4	4	1	1
1	1	1	1	4	4	4	4	1	1
1	1	1	1	2	2	4	4	1	1
1	1	1	1	2	2	4	4	1	1
1	1	1	1	2	2	4	4	1	1
2	2	2	2	4	4	1	1	4	4
1	1	1	1	2	2	4	4	1	1
1	1	1	1	4	4	1	1	4	4
2	2	1	1	4	4	4	4	1	1
1	1	1	1	2	2	4	4	1	1
1	1	1	1	4	4	4	4	1	1
1	1	1	1	4	4	4	4	1	1
1	1	2	2	2	2	1	1	4	4
1	1	1	1	2	2	4	4	1	1
1	1	1	1	4	4	4	4	1	1
1	1	1	1	4	4	4	4	1	1
1	1	1	1	2	2	1	1	4	4
1	1	1	1	2	2	4	4	1	1
1	1	1	1	4	4	4	4	1	1

For Review Only

1	1	1	1	4	4	4	4	1	1
1	1	1	1	4	4	4	4	1	1
1	1	1	1	2	2	4	4	1	1
1	1	1	1	2	2	4	4	1	1
1	1	1	1	4	4	4	4	1	1
2	2	2	2	4	4	1	1	4	4
1	1	1	1	2	2	4	4	1	1
1	1	1	1	4	4	4	4	1	1
2	2	1	1	2	2	4	4	1	1
1	1	1	1	2	2	4	4	1	1
1	1	1	1	2	2	4	4	1	1
1	1	1	1	4	4	4	4	1	1
2	2	1	1	2	2	4	4	1	1
1	1	1	1	2	2	4	4	1	1
2	2	2	2	4	4	1	1	4	4
1	1	1	1	2	2	4	4	1	1
1	1	1	1	4	4	1	1	4	4
1	1	1	1	2	2	4	4	1	1
1	1	1	1	4	4	4	4	1	1
1	1	1	1	2	2	4	4	1	1
1	1	1	1	4	4	4	4	1	1
1	1	1	1	2	2	4	4	1	1
1	1	1	1	2	2	4	4	1	1
1	1	1	1	2	2	4	4	1	1
1	1	1	1	4	4	4	4	1	1
2	2	1	1	4	4	4	4	1	1
1	1	1	1	2	2	4	4	1	1
1	1	1	1	4	4	4	4	1	1
1	1	2	2	4	4	1	1	4	4
2	2	1	1	4	4	4	4	1	1
1	1	1	1	2	2	4	4	1	1
2	2	1	1	4	4	4	4	1	1
2	2	1	1	2	2	4	4	1	1
2	2	2	2	4	4	1	1	4	4
1	1	1	1	4	4	4	4	1	1
1	1	1	1	2	2	4	4	1	1
1	1	1	1	2	2	4	4	1	1
1	1	2	2	2	2	1	1	4	4
1	1	1	1	4	4	4	4	1	1
1	1	1	1	2	2	4	4	1	1
1	1	1	1	2	2	4	4	1	1
1	1	1	1	4	4	4	4	1	1
2	2	1	1	2	2	4	4	1	1
1	1	1	1	4	4	4	4	1	1
1	1	1	1	4	4	4	4	1	1
2	2	1	1	4	4	4	4	1	1
1	1	1	1	2	2	4	4	1	1
1	1	1	1	2	2	4	4	1	1
2	2	2	2	2	2	1	1	4	4
1	1	1	1	2	2	4	4	1	1
1	1	1	1	4	4	4	4	1	1
1	1	2	2	2	2	1	1	4	4
1	1	1	1	2	2	4	4	1	1
1	1	1	1	2	2	4	4	1	1
1	1	1	1	2	2	4	4	1	1

2	2	2	2	2	2	1	1	4	4
1	1	1	1	4	4	4	4	1	1
1	1	1	1	4	4	4	4	1	1
1	1	1	1	4	4	4	4	1	1
1	1	1	1	4	4	4	4	1	1
1	1	1	1	4	4	4	4	1	1
2	2	2	2	2	2	1	1	4	4
1	1	1	1	4	4	4	4	1	1
1	1	2	2	2	2	1	1	4	4
1	1	1	1	2	2	4	4	1	1
1	1	1	1	4	4	4	4	1	1
1	1	1	1	2	2	4	4	1	1
1	1	1	1	2	2	4	4	1	1
1	1	1	1	2	2	4	4	1	1
1	1	1	1	2	2	4	4	1	1
1	1	1	1	2	2	4	4	1	1
1	1	1	1	4	4	4	4	1	1
1	1	1	1	4	4	4	4	1	1
1	1	1	1	4	4	4	4	1	1
1	1	2	2	2	2	1	1	4	4
2	2	1	1	2	2	4	4	1	1
1	1	1	1	4	4	4	4	1	1
1	1	1	1	4	4	4	4	1	1
1	1	1	1	4	4	4	4	1	1
1	1	2	2	2	2	1	1	4	4
1	1	1	1	4	4	4	4	1	1
1	1	1	1	2	2	4	4	1	1
1	1	1	1	2	2	4	4	1	1
1	1	1	1	4	4	4	4	1	1
1	1	1	1	4	4	4	4	1	1
2	2	1	1	2	2	4	4	1	1
1	1	1	1	4	4	4	4	1	1
1	1	1	1	2	2	4	4	1	1
1	1	1	1	2	2	4	4	1	1
1	1	1	1	2	2	4	4	1	1
1	1	1	1	2	2	4	4	1	1
1	1	1	1	2	2	4	4	1	1
1	1	1	1	2	2	4	4	1	1
1	1	1	1	2	2	4	4	1	1
2	2	1	1	2	2	4	4	1	1
1	1	1	1	2	2	4	4	1	1
2	2	1	1	2	2	4	4	1	1
1	1	1	1	2	2	4	4	1	1
1	1	1	1	4	4	4	4	1	1
1	1	1	1	4	4	1	1	4	4
2	2	1	1	2	2	4	4	1	1
1	1	1	1	4	4	4	4	1	1
2	2	1	1	4	4	4	4	1	1
1	1	1	1	4	4	4	4	1	1
1	1	1	1	2	2	4	4	1	1
1	1	1	1	4	4	4	4	1	1
2	2	1	1	2	2	4	4	1	1
1	1	1	1	2	2	4	4	1	1
1	1	1	1	2	2	4	4	1	1
2	2	1	1	2	2	4	4	1	1

For Review Only

1	1	1	1	4	4	4	4	1	1
1	1	1	1	2	2	4	4	1	1

For Review Only

SNP155	SNP155	SNP1550	SNP1550	SNP1551	SNP1551	SNP1552	SNP1552	SNP1553	SNP1553
2	2	4	4	1	1	3	3	3	3
2	2	4	4	3	3	4	4	2	2
3	3	4	4	3	3	3	3	3	3
2	2	2	2	3	3	3	3	3	3
2	2	4	4	1	1	3	3	2	2
3	3	4	4	3	3	4	4	3	3
2	2	4	4	3	3	3	3	3	3
2	2	4	4	3	3	3	3	2	2
2	2	4	4	3	3	3	3	3	3
2	2	4	4	3	3	4	4	2	2
2	2	4	4	3	3	3	3	3	3
3	3	4	4	3	3	3	3	3	3
2	2	4	4	3	3	3	3	3	3
2	2	4	4	1	1	3	3	2	2
2	2	4	4	3	3	3	3	3	3
2	2	4	4	3	3	3	3	3	3
2	2	4	4	1	1	3	3	3	3
2	2	4	4	1	1	3	3	3	3
3	3	4	4	1	1	3	3	3	3
2	2	4	4	3	3	3	3	2	2
2	2	4	4	1	1	3	3	2	2
2	2	4	4	1	1	3	3	3	3
2	2	2	2	3	3	3	3	2	2
2	2	4	4	3	3	3	3	2	2
2	2	4	4	3	3	3	3	3	3
3	3	4	4	1	1	4	4	3	3
3	3	4	4	3	3	4	4	2	2
2	2	4	4	3	3	3	3	3	3
2	2	4	4	3	3	3	3	3	3
2	2	4	4	3	3	3	3	2	2
3	3	2	2	3	3	4	4	3	3
2	2	4	4	3	3	3	3	3	3
2	2	4	4	3	3	3	3	3	3
2	2	4	4	3	3	3	3	2	2
2	2	4	4	3	3	3	3	2	2
2	2	4	4	3	3	3	3	3	3
2	2	4	4	3	3	4	4	2	2
2	2	4	4	3	3	3	3	3	3
2	2	4	4	3	3	3	3	2	2
2	2	4	4	3	3	3	3	3	3
2	2	4	4	3	3	3	3	2	2
2	2	4	4	3	3	3	3	2	2
2	2	4	4	3	3	4	4	2	2
2	2	4	4	1	1	3	3	2	2
2	2	4	4	3	3	4	4	2	2
3	3	4	4	3	3	3	3	3	3
3	3	4	4	1	1	3	3	3	3
2	2	4	4	1	1	3	3	3	3
2	2	4	4	1	1	3	3	3	3
2	2	4	4	1	1	3	3	3	3
2	2	4	4	1	1	3	3	3	3
2	2	4	4	3	3	3	3	2	2

For Review Only

2	2	4	4	1	1	3	3	3	3
2	2	4	4	3	3	3	3	3	3
2	2	4	4	3	3	4	4	3	3
2	2	4	4	1	1	3	3	3	3
3	3	4	4	1	1	3	3	3	3
2	2	4	4	1	1	3	3	3	3
3	3	2	2	3	3	3	3	3	3
2	2	4	4	3	3	3	3	2	2
2	2	4	4	3	3	3	3	2	2
2	2	4	4	3	3	3	3	3	3
2	2	4	4	3	3	3	3	3	3
2	2	4	4	3	3	3	3	3	3
2	2	4	4	1	1	3	3	3	3
2	2	4	4	3	3	3	3	3	3
2	2	4	4	3	3	3	3	3	3
2	2	4	4	3	3	4	4	3	3
2	2	2	2	1	1	3	3	3	3
2	2	4	4	1	1	3	3	3	3
2	2	2	2	1	1	3	3	3	3
2	2	4	4	1	1	3	3	3	3
2	2	4	4	3	3	3	3	3	3
2	2	4	4	3	3	3	3	3	3
2	2	4	4	3	3	3	3	3	3
2	2	4	4	3	3	3	3	3	3
2	2	4	4	3	3	4	4	2	2
2	2	4	4	3	3	4	4	3	3
2	2	4	4	3	3	3	3	2	2
2	2	4	4	3	3	3	3	2	2
3	3	4	4	1	1	3	3	3	3
2	2	4	4	1	1	3	3	3	3
2	2	4	4	3	3	3	3	3	3
2	2	4	4	3	3	3	3	2	2
2	2	4	4	3	3	3	3	2	2
2	2	4	4	1	1	3	3	3	3
2	2	4	4	1	1	3	3	2	2
2	2	4	4	3	3	4	4	2	2
2	2	4	4	3	3	3	3	2	2
2	2	4	4	1	1	3	3	2	2
2	2	4	4	3	3	3	3	2	2
2	2	4	4	3	3	4	4	2	2
2	2	4	4	1	1	3	3	3	3
2	2	4	4	3	3	4	4	3	3
2	2	4	4	1	1	3	3	2	2
2	2	4	4	3	3	3	3	3	3
3	3	4	4	3	3	3	3	3	3
3	3	4	4	1	1	3	3	3	3
2	2	4	4	3	3	3	3	2	2
3	3	4	4	3	3	3	3	3	3
3	3	4	4	1	1	3	3	3	3
2	2	2	2	3	3	3	3	3	3
2	2	4	4	3	3	4	4	3	3
2	2	4	4	3	3	3	3	2	2
2	2	4	4	3	3	3	3	2	2
2	2	4	4	3	3	4	4	3	3
2	2	4	4	1	1	3	3	3	3

For Review Only

2	2	4	4	3	3	3	3	3	3
2	2	4	4	3	3	3	3	3	3
2	2	4	4	3	3	3	3	3	3
2	2	4	4	3	3	3	3	3	3
2	2	4	4	3	3	4	4	3	3
2	2	4	4	3	3	4	4	3	3
2	2	4	4	3	3	3	3	3	3
3	3	4	4	3	3	3	3	3	3
3	3	4	4	3	3	3	3	3	3
2	2	4	4	3	3	3	3	3	3
2	2	4	4	1	1	3	3	3	3
2	2	4	4	3	3	3	3	3	3
2	2	4	4	1	1	3	3	2	2
2	2	4	4	3	3	3	3	3	3
2	2	4	4	3	3	3	3	3	3
2	2	4	4	1	1	3	3	3	3
2	2	4	4	1	1	3	3	3	3
2	2	4	4	3	3	3	3	3	3
2	2	4	4	3	3	3	3	2	2
2	2	4	4	1	1	3	3	3	3
2	2	4	4	3	3	3	3	3	3
2	2	4	4	1	1	3	3	3	3
2	2	4	4	3	3	4	4	3	3
2	2	4	4	3	3	3	3	2	2
2	2	4	4	3	3	3	3	3	3
2	2	2	2	3	3	3	3	3	3
2	2	4	4	3	3	4	4	3	3
2	2	4	4	3	3	4	4	2	2
2	2	2	2	1	1	3	3	3	3
2	2	4	4	3	3	4	4	3	3
2	2	4	4	3	3	3	3	2	2
2	2	4	4	3	3	3	3	3	3
2	2	4	4	1	1	3	3	3	3
2	2	4	4	3	3	3	3	3	3
2	2	4	4	3	3	3	3	2	2
2	2	4	4	3	3	3	3	2	2
2	2	4	4	3	3	3	3	2	2
2	2	4	4	1	1	3	3	3	3
2	2	4	4	3	3	3	3	3	3
2	2	4	4	3	3	3	3	3	3
2	2	2	2	3	3	3	3	3	3
2	2	4	4	3	3	3	3	2	2
2	2	4	4	3	3	4	4	3	3
2	2	4	4	3	3	3	3	2	2
2	2	4	4	1	1	3	3	2	2
3	3	4	4	3	3	3	3	3	3
2	2	4	4	1	1	3	3	3	3
3	3	4	4	1	1	3	3	3	3
2	2	4	4	3	3	3	3	2	2
2	2	4	4	3	3	4	4	3	3
2	2	4	4	3	3	3	3	3	3
2	2	4	4	1	1	3	3	3	3
2	2	4	4	3	3	4	4	3	3
2	2	4	4	3	3	3	3	3	3
2	2	4	4	3	3	3	3	3	3

For Review Only

2	2	4	4	1	1	3	3	3	3
2	2	4	4	1	1	3	3	3	3
2	2	4	4	3	3	4	4	2	2
2	2	4	4	3	3	3	3	3	3
2	2	4	4	3	3	3	3	3	3
2	2	4	4	1	1	3	3	2	2
2	2	4	4	3	3	3	3	3	3
2	2	4	4	3	3	3	3	3	3
2	2	4	4	3	3	3	3	2	2
2	2	4	4	3	3	3	3	3	3
2	2	4	4	3	3	3	3	3	3
2	2	4	4	3	3	3	3	2	2
2	2	4	4	3	3	3	3	3	3
2	2	4	4	3	3	3	3	3	3
2	2	4	4	3	3	3	3	2	2
2	2	4	4	3	3	3	3	3	3
2	2	4	4	3	3	4	4	3	3
2	2	4	4	3	3	4	4	3	3
2	2	4	4	1	1	3	3	3	3
2	2	4	4	3	3	3	3	3	3
2	2	4	4	3	3	3	3	3	3
2	2	4	4	3	3	3	3	2	2
2	2	4	4	3	3	3	3	3	3
2	2	4	4	3	3	3	3	3	3
2	2	4	4	3	3	3	3	2	2
2	2	4	4	3	3	3	3	2	2
2	2	4	4	3	3	3	3	3	3
2	2	4	4	3	3	3	3	3	3
2	2	4	4	1	1	3	3	3	3
2	2	4	4	1	1	3	3	2	2
3	3	4	4	3	3	4	4	3	3
2	2	4	4	1	1	3	3	3	3
2	2	4	4	3	3	4	4	2	2
2	2	4	4	3	3	3	3	2	2
2	2	4	4	3	3	3	3	3	3
2	2	4	4	3	3	3	3	3	3
3	3	4	4	3	3	3	3	3	3
2	2	4	4	3	3	4	4	2	2
3	3	4	4	1	1	3	3	3	3
2	2	2	2	3	3	4	4	3	3
3	3	4	4	3	3	3	3	3	3
2	2	4	4	1	1	3	3	3	3
2	2	4	4	3	3	3	3	2	2
2	2	4	4	1	1	3	3	3	3
2	2	4	4	3	3	3	3	3	3
2	2	4	4	3	3	3	3	2	2
2	2	4	4	3	3	3	3	3	3
2	2	4	4	3	3	3	3	2	2
2	2	4	4	3	3	3	3	2	2
2	2	2	2	3	3	3	3	3	3
2	2	4	4	1	1	3	3	2	2
2	2	4	4	3	3	3	3	2	2
2	2	4	4	3	3	3	3	3	3
2	2	4	4	3	3	3	3	3	3
2	2	4	4	3	3	3	3	2	2

For Review Only

2	2	4	4	3	3	4	4	2	2
2	2	4	4	1	1	3	3	2	2
2	2	4	4	1	1	3	3	3	3
2	2	4	4	3	3	3	3	3	3
3	3	4	4	1	1	3	3	3	3
2	2	4	4	1	1	3	3	3	3
2	2	4	4	3	3	4	4	3	3
2	2	4	4	3	3	4	4	2	2
2	2	4	4	3	3	3	3	2	2
2	2	4	4	3	3	3	3	2	2
2	2	4	4	3	3	3	3	3	3
2	2	4	4	3	3	3	3	3	3
2	2	2	2	3	3	3	3	3	3
2	2	4	4	3	3	3	3	3	3
2	2	4	4	3	3	3	3	3	3
2	2	4	4	3	3	3	3	3	3
2	2	4	4	3	3	3	3	3	3
2	2	4	4	3	3	3	3	3	3
2	2	4	4	1	1	3	3	3	3
2	2	4	4	3	3	3	3	3	3
2	2	4	4	3	3	3	3	2	2
3	3	4	4	1	1	3	3	3	3
2	2	4	4	1	1	3	3	3	3
2	2	4	4	1	1	3	3	3	3
2	2	4	4	3	3	3	3	2	2
3	3	4	4	3	3	4	4	3	3
2	2	4	4	3	3	3	3	2	2
3	3	2	2	3	3	3	3	3	3
2	2	4	4	1	1	3	3	3	3
2	2	4	4	1	1	3	3	3	3
2	2	4	4	3	3	3	3	2	2
2	2	4	4	3	3	3	3	3	3
2	2	4	4	3	3	3	3	3	3
2	2	4	4	3	3	3	3	3	3
2	2	2	2	3	3	3	3	3	3
2	2	4	4	3	3	3	3	2	2
2	2	4	4	3	3	4	4	3	3
2	2	4	4	3	3	3	3	3	3
2	2	4	4	3	3	3	3	3	3
2	2	4	4	3	3	4	4	2	2
2	2	4	4	3	3	4	4	3	3
2	2	4	4	3	3	4	4	3	3
2	2	4	4	3	3	3	3	3	3
2	2	4	4	1	1	3	3	3	3
2	2	4	4	3	3	3	3	3	3
2	2	2	2	3	3	3	3	2	2
3	3	4	4	1	1	3	3	3	3
2	2	4	4	1	1	3	3	2	2
2	2	4	4	3	3	4	4	3	3
2	2	4	4	3	3	3	3	2	2
3	3	4	4	1	1	3	3	3	3
2	2	4	4	3	3	3	3	2	2
3	3	4	4	3	3	3	3	3	3
2	2	2	2	3	3	3	3	3	3
2	2	4	4	3	3	3	3	2	2

For Review Only

2	2	4	4	3	3	3	3	3	3
3	3	2	2	3	3	4	4	3	3

For Review Only

SNP1554	SNP1554	SNP1555	SNP1555	SNP1556	SNP1556	SNP1557	SNP1557	SNP1558	SNP1558
4	4	4	4	3	3	3	3	4	4
1	1	3	3	1	1	2	2	4	4
1	1	3	3	3	3	3	3	4	4
1	1	3	3	3	3	3	3	4	4
1	1	4	4	3	3	2	2	4	4
1	1	3	3	3	3	3	3	4	4
1	1	3	3	3	3	3	3	4	4
1	1	3	3	3	3	3	3	2	2
1	1	4	4	3	3	3	3	4	4
1	1	3	3	1	1	2	2	2	2
1	1	3	3	1	1	3	3	4	4
1	1	3	3	3	3	3	3	4	4
1	1	3	3	1	1	3	3	4	4
1	1	4	4	3	3	3	3	4	4
1	1	3	3	3	3	3	3	4	4
1	1	3	3	3	3	3	3	4	4
1	1	4	4	3	3	3	3	4	4
1	1	4	4	1	1	3	3	4	4
1	1	4	4	3	3	3	3	4	4
1	1	3	3	3	3	2	2	4	4
1	1	4	4	3	3	3	3	4	4
1	1	4	4	3	3	3	3	4	4
4	4	3	3	1	1	3	3	2	2
1	1	3	3	3	3	3	3	4	4
1	1	3	3	3	3	3	3	4	4
1	1	3	3	3	3	3	3	4	4
1	1	3	3	3	3	3	3	4	4
1	1	3	3	3	3	3	3	4	4
1	1	3	3	3	3	3	3	4	4
1	1	3	3	3	3	3	3	4	4
1	1	3	3	3	3	3	3	4	4
1	1	3	3	3	3	3	3	4	4
1	1	3	3	3	3	3	3	4	4
1	1	3	3	3	3	3	3	2	2
4	4	3	3	3	3	3	3	4	4
1	1	3	3	3	3	3	3	4	4
4	4	3	3	3	3	3	3	4	4
1	1	3	3	3	3	3	3	4	4
1	1	3	3	1	1	3	3	2	2
1	1	3	3	1	1	3	3	4	4
1	1	3	3	1	1	2	2	2	2
1	1	3	3	3	3	3	3	4	4
1	1	3	3	3	3	2	2	2	2
1	1	4	4	3	3	3	3	4	4
1	1	3	3	3	3	2	2	2	2
1	1	3	3	3	3	3	3	4	4
1	1	4	4	3	3	3	3	4	4
1	1	4	4	3	3	3	3	4	4
1	1	4	4	3	3	3	3	4	4
4	4	3	3	3	3	3	3	4	4
1	1	4	4	3	3	3	3	4	4
4	4	3	3	3	3	3	3	4	4

1	1	4	4	3	3	3	3	4	4
1	1	3	3	3	3	3	3	4	4
1	1	3	3	3	3	3	3	4	4
1	1	4	4	3	3	3	3	4	4
1	1	4	4	3	3	3	3	4	4
1	1	4	4	3	3	3	3	4	4
1	1	3	3	3	3	3	3	4	4
1	1	3	3	3	3	2	2	4	4
4	4	3	3	1	1	3	3	4	4
1	1	3	3	3	3	3	3	4	4
1	1	3	3	3	3	3	3	4	4
1	1	3	3	3	3	3	3	4	4
1	1	4	4	3	3	3	3	4	4
1	1	3	3	3	3	3	3	4	4
1	1	3	3	3	3	3	3	4	4
4	4	3	3	3	3	3	3	4	4
1	1	4	4	3	3	3	3	4	4
1	1	4	4	3	3	3	3	4	4
1	1	4	4	3	3	3	3	4	4
1	1	4	4	3	3	3	3	4	4
1	1	3	3	3	3	3	3	4	4
4	4	3	3	1	1	3	3	4	4
1	1	3	3	1	1	3	3	4	4
1	1	3	3	3	3	3	3	4	4
1	1	3	3	3	3	2	2	2	2
1	1	3	3	3	3	3	3	4	4
1	1	3	3	3	3	2	2	4	4
1	1	3	3	1	1	3	3	2	2
1	1	4	4	3	3	3	3	4	4
1	1	4	4	3	3	3	3	4	4
1	1	3	3	3	3	3	3	2	2
1	1	3	3	3	3	3	3	4	4
1	1	3	3	1	1	3	3	2	2
1	1	4	4	3	3	3	3	4	4
1	1	4	4	1	1	2	2	4	4
1	1	3	3	3	3	2	2	2	2
1	1	3	3	3	3	3	3	4	4
1	1	3	3	3	3	2	2	4	4
1	1	3	3	3	3	3	3	4	4
1	1	3	3	3	3	2	2	2	2
1	1	4	4	3	3	3	3	4	4
1	1	3	3	3	3	3	3	4	4
1	1	4	4	3	3	3	3	4	4
1	1	3	3	3	3	3	3	4	4
1	1	3	3	3	3	3	3	4	4
1	1	4	4	3	3	3	3	4	4
1	1	3	3	3	3	3	3	2	2
1	1	3	3	3	3	3	3	4	4
1	1	4	4	3	3	3	3	4	4
4	4	3	3	1	1	3	3	4	4
1	1	3	3	3	3	3	3	4	4
1	1	3	3	1	1	3	3	2	2
1	1	3	3	3	3	2	2	4	4
1	1	3	3	3	3	3	3	4	4
1	1	4	4	3	3	3	3	4	4

For Review Only

4	4	4	4	3	3	3	3	4	4
1	1	3	3	1	1	3	3	4	4
1	1	3	3	3	3	3	3	4	4
1	1	3	3	3	3	3	3	2	2
1	1	3	3	3	3	3	3	4	4
1	1	3	3	3	3	3	3	4	4
1	1	3	3	3	3	3	3	4	4
1	1	3	3	3	3	3	3	4	4
1	1	3	3	3	3	3	3	4	4
1	1	3	3	1	1	3	3	4	4
4	4	4	4	3	3	3	3	4	4
1	1	3	3	3	3	3	3	4	4
1	1	4	4	3	3	3	3	4	4
1	1	3	3	3	3	3	3	4	4
1	1	3	3	1	1	2	2	4	4
1	1	3	3	3	3	3	3	4	4
1	1	4	4	3	3	3	3	4	4
1	1	3	3	3	3	3	3	4	4
1	1	3	3	3	3	3	3	2	2
1	1	4	4	3	3	3	3	4	4
1	1	3	3	3	3	3	3	4	4
1	1	3	3	3	3	3	3	4	4
1	1	3	3	3	3	3	3	4	4
1	1	3	3	1	1	3	3	4	4
4	4	3	3	1	1	3	3	4	4
1	1	3	3	1	1	3	3	4	4
1	1	3	3	3	3	3	3	4	4
1	1	3	3	3	3	3	3	4	4
1	1	4	4	3	3	3	3	4	4
1	1	3	3	3	3	3	3	4	4
1	1	3	3	3	3	3	3	2	2
1	1	3	3	3	3	3	3	4	4
1	1	4	4	3	3	3	3	4	4
1	1	3	3	3	3	3	3	4	4
1	1	3	3	3	3	3	3	2	2
1	1	3	3	3	3	3	3	4	4
1	1	3	3	3	3	3	3	2	2
1	1	4	4	3	3	3	3	4	4
4	4	3	3	3	3	3	3	4	4
1	1	3	3	1	1	3	3	4	4
1	1	3	3	1	1	3	3	4	4
1	1	3	3	3	3	3	3	2	2
1	1	3	3	3	3	3	3	4	4
1	1	3	3	1	1	3	3	4	4
1	1	4	4	3	3	3	3	4	4
1	1	3	3	3	3	3	3	2	2
1	1	4	4	3	3	3	3	4	4
1	1	4	4	3	3	3	3	4	4
1	1	3	3	3	3	3	3	4	4
1	1	3	3	3	3	3	3	4	4
1	1	3	3	3	3	3	3	4	4
1	1	4	4	3	3	3	3	2	2
1	1	3	3	3	3	3	3	4	4
1	1	3	3	3	3	3	3	4	4
1	1	3	3	3	3	3	3	4	4

For Review Only

1	1	4	4	3	3	3	3	4	4
1	1	4	4	1	1	3	3	4	4
1	1	3	3	3	3	2	2	4	4
1	1	3	3	1	1	3	3	4	4
1	1	3	3	3	3	3	3	4	4
1	1	4	4	3	3	3	3	4	4
1	1	3	3	1	1	3	3	2	2
1	1	3	3	3	3	3	3	4	4
1	1	3	3	1	1	3	3	2	2
1	1	3	3	3	3	3	3	4	4
1	1	3	3	3	3	3	3	4	4
1	1	3	3	3	3	3	3	4	4
1	1	3	3	3	3	3	3	4	4
1	1	3	3	3	3	3	3	2	2
1	1	3	3	3	3	3	3	2	2
1	1	3	3	1	1	3	3	4	4
1	1	3	3	3	3	3	3	4	4
1	1	3	3	3	3	3	3	4	4
1	1	4	4	3	3	3	3	4	4
1	1	3	3	3	3	3	3	4	4
1	1	3	3	3	3	3	3	4	4
1	1	3	3	1	1	3	3	4	4
4	4	3	3	1	1	3	3	4	4
1	1	3	3	3	3	3	3	4	4
1	1	3	3	3	3	3	3	4	4
1	1	3	3	3	3	3	3	2	2
1	1	3	3	3	3	3	3	4	4
1	1	3	3	3	3	3	3	4	4
1	1	4	4	3	3	3	3	4	4
1	1	4	4	3	3	3	3	4	4
1	1	3	3	3	3	3	3	4	4
1	1	4	4	3	3	3	3	4	4
4	4	3	3	3	3	3	3	4	4
1	1	3	3	3	3	3	3	2	2
4	4	3	3	3	3	3	3	4	4
1	1	3	3	3	3	3	3	4	4
1	1	3	3	1	1	3	3	4	4
4	4	3	3	3	3	2	2	4	4
1	1	4	4	3	3	3	3	4	4
1	1	3	3	3	3	3	3	4	4
1	1	3	3	3	3	3	3	4	4
1	1	4	4	3	3	3	3	4	4
1	1	3	3	1	1	3	3	2	2
4	4	4	4	3	3	3	3	4	4
1	1	3	3	3	3	3	3	4	4
1	1	3	3	3	3	3	3	4	4
1	1	3	3	1	1	3	3	4	4
1	1	3	3	1	1	3	3	2	2
1	1	3	3	3	3	3	3	4	4
1	1	3	3	3	3	3	3	4	4
1	1	4	4	3	3	2	2	4	4
1	1	3	3	3	3	3	3	4	4
4	4	3	3	1	1	3	3	2	2
1	1	3	3	3	3	3	3	4	4
1	1	3	3	1	1	3	3	2	2

For Review Only

1	1	3	3	3	3	3	3	4	4
1	1	4	4	3	3	2	2	4	4
1	1	4	4	3	3	3	3	4	4
4	4	3	3	1	1	3	3	4	4
1	1	4	4	3	3	3	3	4	4
1	1	4	4	3	3	3	3	4	4
1	1	3	3	1	1	3	3	4	4
1	1	3	3	3	3	2	2	4	4
1	1	3	3	3	3	3	3	2	2
1	1	3	3	3	3	3	3	2	2
1	1	3	3	3	3	3	3	4	4
1	1	3	3	1	1	3	3	4	4
1	1	3	3	3	3	3	3	4	4
1	1	3	3	3	3	3	3	4	4
1	1	3	3	3	3	3	3	4	4
1	1	3	3	3	3	3	3	4	4
1	1	3	3	1	1	3	3	4	4
1	1	3	3	3	3	3	3	4	4
1	1	4	4	1	1	3	3	4	4
1	1	3	3	3	3	3	3	4	4
1	1	3	3	1	1	3	3	4	4
1	1	4	4	3	3	3	3	4	4
1	1	4	4	3	3	3	3	4	4
1	1	4	4	1	1	3	3	4	4
1	1	3	3	3	3	2	2	4	4
4	4	3	3	1	1	3	3	4	4
1	1	3	3	1	1	3	3	2	2
1	1	3	3	3	3	3	3	4	4
1	1	4	4	3	3	3	3	4	4
1	1	4	4	3	3	3	3	4	4
1	1	3	3	1	1	3	3	2	2
1	1	3	3	3	3	3	3	4	4
1	1	3	3	3	3	3	3	2	2
1	1	3	3	3	3	3	3	4	4
4	4	3	3	1	1	3	3	4	4
1	1	3	3	1	1	3	3	2	2
1	1	3	3	3	3	3	3	4	4
1	1	3	3	3	3	3	3	4	4
1	1	3	3	3	3	3	3	4	4
1	1	3	3	1	1	3	3	2	2
4	4	3	3	3	3	3	3	4	4
1	1	3	3	3	3	3	3	4	4
1	1	3	3	1	1	3	3	4	4
1	1	4	4	3	3	3	3	4	4
1	1	3	3	3	3	3	3	4	4
1	1	3	3	3	3	3	3	4	4
1	1	4	4	3	3	3	3	4	4
1	1	4	4	3	3	3	3	4	4
1	1	3	3	3	3	3	3	2	2
1	1	3	3	3	3	3	3	2	2
4	4	4	4	3	3	3	3	4	4
1	1	3	3	1	1	3	3	2	2
1	1	3	3	3	3	3	3	4	4
1	1	3	3	3	3	3	3	4	4
1	1	3	3	1	1	3	3	4	4

For Review Only

1	1	3	3	3	3	3	3	4	4
1	1	3	3	3	3	3	3	4	4

For Review Only

SNP1559	SNP1559	SNP156	SNP156	SNP1560	SNP1560	SNP1561	SNP1561	SNP1562	SNP1562
4	4	1	1	2	2	3	3	2	2
1	1	1	1	4	4	2	2	2	2
4	4	1	1	2	2	3	3	2	2
1	1	1	1	2	2	3	3	2	2
1	1	1	1	2	2	3	3	2	2
1	1	1	1	2	2	3	3	2	2
4	4	1	1	2	2	3	3	2	2
4	4	1	1	2	2	3	3	4	4
4	4	1	1	2	2	3	3	2	2
1	1	1	1	4	4	2	2	4	4
4	4	1	1	4	4	2	2	2	2
4	4	1	1	2	2	3	3	2	2
4	4	1	1	2	2	3	3	2	2
1	1	4	4	2	2	3	3	4	4
4	4	1	1	2	2	3	3	2	2
1	1	1	1	2	2	3	3	2	2
4	4	1	1	2	2	3	3	4	4
1	1	1	1	4	4	2	2	2	2
4	4	1	1	2	2	3	3	2	2
1	1	1	1	2	2	3	3	4	4
1	1	1	1	2	2	3	3	2	2
4	4	1	1	2	2	3	3	2	2
1	1	1	1	4	4	2	2	2	2
4	4	1	1	2	2	3	3	4	4
1	1	1	1	2	2	3	3	2	2
4	4	1	1	2	2	3	3	2	2
1	1	1	1	2	2	3	3	2	2
4	4	1	1	2	2	3	3	2	2
1	1	1	1	2	2	3	3	2	2
1	1	4	4	2	2	3	3	4	4
4	4	1	1	2	2	3	3	2	2
4	4	1	1	2	2	3	3	2	2
4	4	1	1	2	2	3	3	2	2
1	1	4	4	2	2	3	3	4	4
4	4	1	1	2	2	3	3	2	2
4	4	1	1	2	2	3	3	2	2
1	1	1	1	2	2	3	3	2	2
4	4	1	1	2	2	3	3	2	2
1	1	4	4	4	4	2	2	2	2
4	4	1	1	4	4	2	2	2	2
1	1	1	1	4	4	2	2	4	4
1	1	1	1	2	2	3	3	4	4
1	1	1	1	2	2	3	3	2	2
1	1	1	1	2	2	3	3	2	2
1	1	1	1	2	2	3	3	4	4
4	4	1	1	2	2	3	3	2	2
4	4	1	1	2	2	3	3	2	2
4	4	1	1	2	2	3	3	2	2
4	4	1	1	2	2	3	3	2	2
4	4	1	1	2	2	3	3	2	2
4	4	1	1	2	2	3	3	2	2
1	1	1	1	2	2	3	3	2	2

For Review Only

1	1	4	4	2	2	3	3	2	2
4	4	1	1	2	2	3	3	2	2
4	4	1	1	2	2	3	3	2	2
4	4	4	4	2	2	3	3	2	2
4	4	1	1	2	2	3	3	2	2
4	4	1	1	2	2	3	3	2	2
1	1	1	1	2	2	3	3	2	2
1	1	4	4	2	2	3	3	2	2
1	1	1	1	4	4	2	2	4	4
4	4	1	1	2	2	3	3	2	2
4	4	1	1	2	2	3	3	2	2
4	4	1	1	2	2	3	3	2	2
1	1	4	4	2	2	3	3	2	2
1	1	1	1	2	2	3	3	2	2
4	4	1	1	2	2	3	3	2	2
4	4	1	1	2	2	3	3	4	4
4	4	1	1	2	2	3	3	2	2
4	4	1	1	2	2	3	3	2	2
4	4	1	1	2	2	3	3	2	2
4	4	1	1	2	2	3	3	2	2
4	4	1	1	2	2	3	3	2	2
4	4	1	1	4	4	2	2	2	2
4	4	1	1	2	2	3	3	2	2
4	4	1	1	2	2	3	3	4	4
1	1	1	1	2	2	3	3	4	4
1	1	1	1	2	2	3	3	2	2
1	1	1	1	2	2	3	3	2	2
1	1	4	4	4	4	2	2	4	4
4	4	1	1	2	2	3	3	2	2
4	4	1	1	2	2	3	3	2	2
4	4	1	1	2	2	3	3	2	2
4	4	1	1	2	2	3	3	4	4
1	1	4	4	4	4	2	2	4	4
1	1	4	4	2	2	3	3	2	2
1	1	4	4	4	4	2	2	4	4
1	1	1	1	2	2	3	3	4	4
1	1	1	1	2	2	3	3	4	4
1	1	1	1	2	2	3	3	2	2
1	1	1	1	2	2	3	3	2	2
1	1	1	1	2	2	3	3	4	4
4	4	1	1	2	2	3	3	2	2
4	4	1	1	2	2	3	3	2	2
1	1	1	1	2	2	3	3	2	2
4	4	1	1	2	2	3	3	2	2
4	4	1	1	2	2	3	3	2	2
1	1	1	1	2	2	3	3	2	2
1	1	1	1	2	2	3	3	4	4
4	4	1	1	2	2	3	3	2	2
4	4	1	1	2	2	3	3	2	2
4	4	1	1	2	2	3	3	2	2
4	4	1	1	2	2	3	3	2	2
1	1	1	1	4	4	3	3	2	2
1	1	1	1	2	2	3	3	4	4
1	1	1	1	2	2	3	3	2	2
4	4	1	1	2	2	3	3	2	2

For Review Only

4	4	1	1	2	2	3	3	2	2
4	4	1	1	4	4	2	2	2	2
4	4	1	1	2	2	3	3	2	2
1	1	1	1	2	2	3	3	2	2
4	4	1	1	2	2	3	3	2	2
4	4	1	1	2	2	3	3	2	2
1	1	1	1	2	2	3	3	2	2
4	4	1	1	2	2	3	3	2	2
4	4	1	1	2	2	3	3	2	2
4	4	4	4	2	2	3	3	4	4
1	1	1	1	2	2	3	3	2	2
4	4	1	1	2	2	3	3	2	2
1	1	1	1	2	2	3	3	2	2
1	1	1	1	2	2	3	3	2	2
1	1	1	1	4	4	3	3	2	2
4	4	1	1	2	2	3	3	2	2
4	4	1	1	2	2	3	3	4	4
1	1	1	1	2	2	3	3	2	2
1	1	1	1	2	2	3	3	4	4
4	4	1	1	2	2	3	3	2	2
4	4	1	1	2	2	3	3	2	2
4	4	1	1	2	2	3	3	2	2
1	1	1	1	2	2	3	3	2	2
1	1	4	4	4	4	2	2	4	4
4	4	1	1	2	2	2	2	2	2
4	4	1	1	4	4	2	2	2	2
1	1	1	1	2	2	3	3	2	2
1	1	1	1	2	2	3	3	2	2
1	1	1	1	2	2	3	3	2	2
4	4	1	1	2	2	3	3	2	2
1	1	1	1	2	2	3	3	4	4
4	4	1	1	2	2	3	3	2	2
4	4	1	1	2	2	3	3	2	2
4	4	1	1	2	2	3	3	2	2
1	1	4	4	2	2	3	3	4	4
1	1	1	1	2	2	3	3	2	2
1	1	1	1	2	2	3	3	4	4
4	4	1	1	2	2	3	3	2	2
4	4	1	1	2	2	3	3	2	2
4	4	1	1	2	2	3	3	2	2
4	4	1	1	2	2	3	3	2	2
1	1	4	4	2	2	3	3	4	4
4	4	1	1	2	2	3	3	2	2
1	1	1	1	2	2	3	3	4	4
1	1	1	1	2	2	3	3	2	2
1	1	1	1	2	2	3	3	2	2
4	4	1	1	2	2	3	3	2	2
4	4	1	1	2	2	3	3	2	2
1	1	1	1	2	2	3	3	2	2
4	4	1	1	2	2	3	3	2	2
4	4	1	1	2	2	3	3	2	2
4	4	1	1	2	2	3	3	2	2
4	4	1	1	2	2	3	3	2	2
4	4	1	1	2	2	3	3	4	4
1	1	1	1	2	2	3	3	2	2

4	4	1	1	2	2	3	3	2	2
4	4	1	1	4	4	2	2	2	2
1	1	1	1	2	2	3	3	2	2
1	1	1	1	4	4	2	2	2	2
1	1	1	1	2	2	3	3	2	2
1	1	1	1	2	2	3	3	2	2
4	4	1	1	4	4	2	2	2	2
4	4	1	1	2	2	3	3	2	2
1	1	1	1	2	2	3	3	2	2
4	4	1	1	2	2	3	3	2	2
4	4	1	1	2	2	3	3	2	2
4	4	1	1	2	2	3	3	2	2
4	4	1	1	2	2	3	3	2	2
4	4	1	1	2	2	3	3	2	2
1	1	4	4	2	2	3	3	4	4
4	4	1	1	4	4	2	2	2	2
4	4	1	1	2	2	3	3	2	2
4	4	1	1	2	2	3	3	2	2
4	4	1	1	2	2	3	3	2	2
1	1	1	1	2	2	3	3	2	2
4	4	1	1	2	2	3	3	2	2
1	1	4	4	4	4	2	2	4	4
4	4	1	1	4	4	2	2	2	2
4	4	1	1	2	2	3	3	2	2
1	1	1	1	2	2	3	3	4	4
1	1	1	1	2	2	3	3	4	4
4	4	1	1	2	2	3	3	2	2
4	4	1	1	2	2	3	3	4	4
4	4	1	1	2	2	3	3	2	2
1	1	1	1	2	2	3	3	2	2
4	4	1	1	2	2	3	3	2	2
4	4	1	1	2	2	3	3	2	2
1	1	1	1	2	2	3	3	2	2
1	1	1	1	2	2	3	3	4	4
4	4	1	1	2	2	3	3	2	2
4	4	1	1	2	2	3	3	2	2
4	4	1	1	2	2	3	3	2	2
1	1	4	4	2	2	3	3	2	2
4	4	1	1	2	2	3	3	2	2
4	4	1	1	2	2	3	3	2	2
4	4	1	1	2	2	3	3	2	2
4	4	1	1	2	2	3	3	2	2
1	1	4	4	4	4	2	2	2	2
4	4	1	1	2	2	3	3	2	2
1	1	1	1	2	2	3	3	2	2
1	1	1	1	2	2	3	3	2	2
4	4	1	1	4	4	2	2	2	2
1	1	1	1	4	4	2	2	2	2
1	1	1	1	2	2	3	3	4	4
4	4	1	1	2	2	3	3	4	4
1	1	4	4	2	2	3	3	2	2
1	1	1	1	2	2	3	3	4	4
4	4	1	1	4	4	2	2	2	2
4	4	1	1	2	2	3	3	2	2
1	1	4	4	4	4	2	2	2	2

For Review Only

1	1	1	1	2	2	3	3	4	4
1	1	1	1	2	2	3	3	2	2
1	1	1	1	2	2	3	3	2	2
4	4	1	1	4	4	2	2	2	2
4	4	1	1	2	2	3	3	4	4
4	4	1	1	2	2	3	3	2	2
1	1	1	1	4	4	2	2	2	2
1	1	1	1	2	2	3	3	2	2
4	4	1	1	2	2	3	3	2	2
1	1	4	4	2	2	3	3	4	4
4	4	1	1	2	2	3	3	2	2
4	4	1	1	4	4	3	3	2	2
1	1	1	1	2	2	3	3	2	2
4	4	1	1	2	2	3	3	2	2
4	4	1	1	2	2	3	3	2	2
4	4	1	1	2	2	3	3	2	2
4	4	4	4	4	4	2	2	2	2
1	1	1	1	2	2	3	3	2	2
4	4	1	1	4	4	2	2	2	2
4	4	1	1	2	2	3	3	2	2
4	4	1	1	4	4	2	2	2	2
4	4	1	1	2	2	3	3	2	2
4	4	1	1	2	2	3	3	2	2
4	4	4	4	4	4	2	2	2	2
1	1	4	4	2	2	3	3	2	2
1	1	1	1	2	2	3	3	4	4
1	1	4	4	4	4	2	2	4	4
1	1	1	1	2	2	3	3	2	2
1	1	1	1	2	2	3	3	2	2
1	1	1	1	2	2	3	3	2	2
1	1	4	4	4	4	2	2	2	2
4	4	1	1	2	2	3	3	2	2
4	4	1	1	2	2	3	3	4	4
4	4	1	1	2	2	3	3	2	2
4	4	1	1	4	4	2	2	2	2
1	1	4	4	4	4	2	2	4	4
4	4	1	1	2	2	3	3	2	2
4	4	1	1	2	2	3	3	2	2
4	4	1	1	2	2	3	3	2	2
1	1	4	4	4	4	2	2	2	2
1	1	1	1	2	2	3	3	2	2
1	1	1	1	2	2	3	3	2	2
4	4	1	1	4	4	2	2	2	2
4	4	1	1	2	2	3	3	2	2
4	4	1	1	2	2	3	3	2	2
1	1	1	1	2	2	3	3	2	2
4	4	4	4	2	2	3	3	2	2
1	1	1	1	2	2	3	3	2	2
4	4	1	1	2	2	3	3	2	2
1	1	4	4	2	2	3	3	4	4
4	4	1	1	2	2	3	3	2	2
1	1	4	4	4	4	2	2	2	2
4	4	1	1	2	2	3	3	2	2
4	4	1	1	2	2	3	3	2	2
1	1	1	1	4	4	2	2	2	2

For Review Only

4	4	1	1	2	2	3	3	2	2
1	1	1	1	2	2	3	3	2	2

For Review Only

SNP1563	SNP1563	SNP1564	SNP1564	SNP1565	SNP1565	SNP1566	SNP1566	SNP1567	SNP1567
1	1	1	1	1	1	2	2	4	4
1	1	1	1	1	1	2	2	4	4
1	1	1	1	1	1	2	2	4	4
1	1	1	1	1	1	2	2	4	4
1	1	1	1	1	1	2	2	4	4
1	1	1	1	1	1	2	2	4	4
1	1	4	4	4	4	2	2	4	4
1	1	4	4	1	1	2	2	4	4
1	1	1	1	1	1	2	2	4	4
1	1	4	4	1	1	2	2	4	4
4	4	1	1	4	4	2	2	3	3
1	1	1	1	1	1	2	2	4	4
1	1	1	1	1	1	2	2	4	4
4	4	1	1	1	1	2	2	4	4
1	1	1	1	1	1	2	2	4	4
4	4	1	1	1	1	2	2	4	4
1	1	4	4	1	1	2	2	4	4
1	1	4	4	1	1	2	2	4	4
1	1	1	1	1	1	2	2	4	4
1	1	1	1	1	1	2	2	4	4
1	1	4	4	1	1	2	2	4	4
1	1	4	4	1	1	2	2	4	4
4	4	1	1	4	4	1	1	4	4
1	1	1	1	1	1	2	2	4	4
1	1	1	1	1	1	2	2	4	4
4	4	1	1	1	1	2	2	4	4
1	1	1	1	1	1	2	2	4	4
1	1	1	1	1	1	2	2	4	4
1	1	1	1	4	4	2	2	4	4
1	1	4	4	1	1	2	2	4	4
1	1	1	1	1	1	2	2	4	4
1	1	1	1	1	1	2	2	4	4
1	1	1	1	1	1	2	2	4	4
4	4	1	1	1	1	2	2	4	4
1	1	1	1	1	1	1	1	4	4
1	1	1	1	1	1	2	2	4	4
4	4	1	1	1	1	1	1	4	4
4	4	1	1	1	1	2	2	4	4
1	1	1	1	4	4	1	1	4	4
4	4	1	1	4	4	2	2	4	4
1	1	4	4	1	1	2	2	4	4
1	1	4	4	4	4	2	2	4	4
1	1	4	4	1	1	2	2	4	4
1	1	4	4	1	1	2	2	4	4
1	1	4	4	1	1	2	2	4	4
4	4	1	1	1	1	2	2	4	4
1	1	1	1	1	1	2	2	4	4
1	1	1	1	1	1	2	2	4	4
1	1	4	4	4	4	2	2	4	4
1	1	1	1	1	1	2	2	4	4
1	1	1	1	1	1	2	2	4	4
1	1	1	1	1	1	1	1	4	4

For Review Only

1	1	1	1	1	1	2	2	4	4
1	1	1	1	1	1	2	2	4	4
1	1	1	1	1	1	2	2	4	4
1	1	4	4	1	1	2	2	4	4
1	1	1	1	1	1	2	2	4	4
1	1	1	1	1	1	2	2	4	4
4	4	1	1	1	1	2	2	4	4
4	4	1	1	1	1	2	2	4	4
4	4	1	1	1	1	1	1	4	4
1	1	1	1	1	1	2	2	4	4
1	1	1	1	1	1	2	2	4	4
1	1	1	1	1	1	2	2	4	4
1	1	1	1	1	1	2	2	4	4
1	1	1	1	4	4	2	2	4	4
1	1	1	1	1	1	2	2	4	4
1	1	1	1	1	1	1	1	4	4
1	1	1	1	1	1	2	2	4	4
1	1	1	1	1	1	2	2	4	4
1	1	4	4	1	1	2	2	4	4
1	1	1	1	1	1	2	2	4	4
1	1	1	1	1	1	2	2	4	4
1	1	1	1	4	4	2	2	4	4
1	1	1	1	1	1	2	2	4	4
1	1	4	4	1	1	2	2	4	4
1	1	4	4	1	1	2	2	4	4
1	1	1	1	1	1	2	2	4	4
1	1	4	4	1	1	2	2	4	4
4	4	1	1	4	4	1	1	4	4
4	4	1	1	1	1	2	2	4	4
1	1	4	4	1	1	2	2	4	4
1	1	1	1	1	1	2	2	4	4
4	4	1	1	1	1	2	2	4	4
4	4	1	1	1	1	1	1	4	4
1	1	1	1	1	1	2	2	4	4
1	1	1	1	1	1	2	2	4	4
1	1	4	4	1	1	2	2	4	4
1	1	4	4	1	1	2	2	4	4
1	1	4	4	1	1	2	2	4	4
1	1	1	1	1	1	2	2	3	3
1	1	4	4	1	1	2	2	4	4
1	1	1	1	1	1	2	2	4	4
1	1	4	4	1	1	2	2	4	4
1	1	1	1	1	1	2	2	4	4
1	1	1	1	1	1	2	2	4	4
1	1	1	1	1	1	2	2	3	3
1	1	1	1	1	1	2	2	4	4
4	4	1	1	1	1	2	2	4	4
4	4	1	1	1	1	2	2	4	4
4	4	1	1	1	1	2	2	4	4
1	1	1	1	1	1	2	2	4	4
1	1	1	1	1	1	2	2	4	4
4	4	1	1	4	4	2	2	4	4
4	4	1	1	1	1	2	2	4	4
1	1	1	1	1	1	2	2	4	4
1	1	1	1	1	1	2	2	4	4

For Review Only

1	1	1	1	1	1	2	2	4	4
1	1	1	1	1	1	2	2	4	4
1	1	1	1	1	1	2	2	4	4
4	4	1	1	1	1	2	2	4	4
1	1	1	1	1	1	2	2	4	4
4	4	1	1	1	1	2	2	4	4
1	1	1	1	1	1	2	2	4	4
1	1	1	1	1	1	2	2	4	4
4	4	1	1	1	1	2	2	4	4
1	1	1	1	1	1	2	2	4	4
1	1	1	1	1	1	2	2	4	4
1	1	1	1	1	1	2	2	4	4
1	1	1	1	1	1	2	2	4	4
1	1	1	1	1	1	2	2	4	4
1	1	1	1	1	1	2	2	4	4
1	1	1	1	1	1	2	2	4	4
1	1	1	1	1	1	1	1	4	4
1	1	1	1	1	1	2	2	4	4
4	4	1	1	1	1	2	2	4	4
1	1	1	1	1	1	2	2	4	4
1	1	1	1	1	1	2	2	4	4
1	1	1	1	1	1	2	2	4	4
1	1	1	1	1	1	2	2	4	4
4	4	1	1	1	1	1	1	4	4
4	4	1	1	4	4	2	2	4	4
1	1	1	1	4	4	2	2	4	4
1	1	1	1	1	1	2	2	4	4
1	1	1	1	1	1	2	2	4	4
1	1	1	1	1	1	2	2	3	3
1	1	1	1	4	4	2	2	4	4
1	1	1	1	1	1	1	1	4	4
4	4	1	1	1	1	2	2	4	4
1	1	1	1	1	1	2	2	4	4
1	1	1	1	1	1	2	2	4	4
4	4	1	1	1	1	2	2	4	4
1	1	1	1	1	1	1	1	4	4
4	4	1	1	1	1	2	2	4	4
1	1	1	1	1	1	2	2	4	4
1	1	1	1	1	1	2	2	4	4
1	1	1	1	1	1	1	1	3	3
1	1	1	1	1	1	2	2	4	4
4	4	1	1	1	1	2	2	4	4
1	1	1	1	1	1	2	2	4	4
4	4	1	1	1	1	2	2	4	4
4	4	1	1	1	1	2	2	4	4
1	1	1	1	1	1	2	2	4	4
4	4	1	1	1	1	2	2	4	4
1	1	1	1	1	1	2	2	4	4
1	1	1	1	1	1	2	2	4	4
1	1	1	1	1	1	2	2	4	4
1	1	1	1	1	1	2	2	4	4
4	4	1	1	1	1	2	2	4	4
1	1	1	1	1	1	2	2	3	3
1	1	1	1	1	1	2	2	4	4
1	1	1	1	1	1	2	2	4	4

For Review Only

1	1	1	1	1	1	2	2	4	4
1	1	1	1	4	4	2	2	4	4
1	1	1	1	1	1	2	2	4	4
1	1	1	1	4	4	1	1	3	3
1	1	1	1	1	1	2	2	4	4
4	4	1	1	1	1	1	1	4	4
1	1	1	1	4	4	1	1	4	4
1	1	1	1	1	1	2	2	4	4
1	1	1	1	1	1	1	1	4	4
1	1	1	1	1	1	2	2	4	4
4	4	1	1	1	1	2	2	4	4
1	1	1	1	1	1	2	2	4	4
1	1	1	1	1	1	2	2	4	4
1	1	1	1	1	1	2	2	4	4
4	4	1	1	1	1	2	2	4	4
4	4	1	1	4	4	2	2	3	3
1	1	1	1	1	1	2	2	3	3
1	1	1	1	1	1	2	2	4	4
4	4	1	1	1	1	2	2	4	4
1	1	1	1	1	1	2	2	4	4
1	1	1	1	1	1	2	2	4	4
4	4	1	1	1	1	1	1	4	4
4	4	1	1	4	4	2	2	4	4
1	1	1	1	1	1	2	2	4	4
1	1	1	1	1	1	2	2	4	4
4	4	1	1	1	1	2	2	4	4
1	1	1	1	1	1	2	2	4	4
1	1	1	1	1	1	1	1	4	4
4	4	1	1	1	1	2	2	4	4
1	1	1	1	1	1	1	1	4	4
1	1	1	1	1	1	2	2	4	4
1	1	4	4	1	1	2	2	4	4
1	1	1	1	1	1	2	2	4	4
4	4	1	1	1	1	2	2	4	4
1	1	1	1	1	1	2	2	4	4
1	1	1	1	1	1	2	2	4	4
1	1	1	1	4	4	2	2	4	4
4	4	1	1	1	1	2	2	4	4
1	1	1	1	1	1	2	2	4	4
1	1	1	1	1	1	2	2	4	4
1	1	1	1	1	1	2	2	4	4
4	4	1	1	1	1	2	2	4	4
1	1	1	1	4	4	1	1	4	4
1	1	1	1	1	1	2	2	4	4
1	1	1	1	1	1	2	2	4	4
1	1	1	1	1	1	2	2	4	4
4	4	1	1	4	4	2	2	3	3
4	4	1	1	4	4	1	1	4	4
4	4	1	1	1	1	2	2	4	4
1	1	1	1	1	1	2	2	4	4
1	1	1	1	1	1	2	2	3	3
1	1	1	1	1	1	2	2	4	4
4	4	1	1	4	4	2	2	4	4
1	1	1	1	1	1	2	2	4	4
1	1	1	1	4	4	2	2	4	4

For Review Only

1	1	1	1	1	1	2	2	3	3
1	1	1	1	1	1	2	2	4	4
1	1	1	1	1	1	2	2	3	3
1	1	1	1	4	4	2	2	4	4
1	1	4	4	1	1	2	2	4	4
1	1	1	1	1	1	2	2	4	4
1	1	1	1	4	4	2	2	4	4
4	4	4	4	4	4	2	2	4	4
1	1	1	1	1	1	2	2	4	4
1	1	1	1	1	1	1	1	4	4
1	1	1	1	1	1	2	2	4	4
4	4	1	1	4	4	2	2	4	4
1	1	1	1	1	1	2	2	3	3
4	4	1	1	1	1	2	2	4	4
1	1	1	1	1	1	2	2	4	4
1	1	1	1	1	1	2	2	3	3
4	4	1	1	4	4	2	2	4	4
4	4	1	1	1	1	2	2	4	4
4	4	1	1	4	4	2	2	4	4
4	4	1	1	1	1	2	2	4	4
1	1	1	1	1	1	1	1	4	4
4	4	1	1	1	1	2	2	4	4
1	1	1	1	1	1	2	2	4	4
1	1	1	1	4	4	2	2	4	4
4	4	1	1	1	1	2	2	4	4
1	1	1	1	1	1	2	2	4	4
4	4	1	1	1	1	1	1	4	4
1	1	1	1	1	1	2	2	4	4
1	1	1	1	1	1	2	2	4	4
1	1	1	1	1	1	2	2	3	3
4	4	1	1	4	4	2	2	4	4
4	4	1	1	1	1	2	2	4	4
1	1	1	1	1	1	2	2	4	4
4	4	1	1	1	1	2	2	4	4
1	1	1	1	4	4	2	2	4	4
4	4	1	1	4	4	1	1	4	4
1	1	1	1	1	1	2	2	3	3
1	1	1	1	1	1	2	2	4	4
1	1	1	1	1	1	2	2	4	4
1	1	1	1	4	4	1	1	4	4
1	1	1	1	1	1	2	2	4	4
1	1	1	1	1	1	2	2	4	4
1	1	1	1	4	4	2	2	4	4
1	1	1	1	1	1	2	2	4	4
1	1	1	1	1	1	2	2	4	4
1	1	1	1	1	1	2	2	3	3
4	4	1	1	1	1	2	2	4	4
1	1	1	1	1	1	2	2	4	4
1	1	1	1	1	1	2	2	4	4
4	4	1	1	1	1	1	1	4	4
1	1	1	1	1	1	2	2	4	4
1	1	1	1	4	4	1	1	4	4
1	1	1	1	1	1	2	2	4	4
1	1	1	1	1	1	2	2	4	4
1	1	1	1	4	4	1	1	4	4

For Review Only

1	1	1	1	1	1	2	2	3	3
1	1	1	1	1	1	2	2	4	4

For Review Only

SNP1568	SNP1568	SNP1569	SNP1569	SNP157	SNP157	SNP1570	SNP1570	SNP1571	SNP1571
3	3	3	3	2	2	2	2	1	1
3	3	3	3	2	2	3	3	4	4
3	3	3	3	2	2	2	2	1	1
3	3	3	3	2	2	2	2	4	4
3	3	3	3	4	4	3	3	4	4
3	3	3	3	2	2	2	2	1	1
3	3	3	3	2	2	2	2	4	4
3	3	2	2	2	2	3	3	4	4
3	3	3	3	2	2	3	3	4	4
3	3	3	3	4	4	3	3	4	4
3	3	3	3	2	2	3	3	4	4
3	3	3	3	2	2	2	2	1	1
3	3	3	3	2	2	3	3	4	4
3	3	3	3	2	2	3	3	4	4
3	3	3	3	2	2	3	3	4	4
3	3	3	3	2	2	3	3	4	4
3	3	3	3	4	4	2	2	4	4
3	3	3	3	2	2	2	2	1	1
3	3	3	3	2	2	2	2	1	1
3	3	3	3	4	4	3	3	4	4
3	3	3	3	4	4	3	3	4	4
3	3	3	3	4	4	2	2	4	4
3	3	3	3	2	2	2	2	4	4
3	3	2	2	4	4	3	3	4	4
3	3	3	3	2	2	2	2	1	1
3	3	3	3	2	2	2	2	1	1
3	3	3	3	2	2	2	2	4	4
3	3	3	3	2	2	3	3	4	4
3	3	3	3	4	4	2	2	1	1
2	2	3	3	4	4	2	2	4	4
3	3	3	3	2	2	2	2	1	1
3	3	3	3	2	2	2	2	4	4
3	3	3	3	2	2	2	2	4	4
3	3	2	2	4	4	3	3	4	4
3	3	3	3	2	2	3	3	4	4
3	3	3	3	2	2	2	2	1	1
3	3	2	2	2	2	3	3	4	4
3	3	3	3	2	2	2	2	1	1
3	3	3	3	4	4	3	3	4	4
3	3	3	3	2	2	2	2	1	1
3	3	3	3	4	4	3	3	4	4
3	3	3	3	2	2	3	3	4	4
3	3	3	3	4	4	3	3	4	4
3	3	3	3	2	2	2	2	4	4
3	3	3	3	2	2	3	3	4	4
3	3	3	3	2	2	2	2	1	1
3	3	3	3	2	2	2	2	1	1
3	3	3	3	2	2	3	3	4	4
3	3	3	3	4	4	2	2	4	4
3	3	3	3	2	2	2	2	4	4
3	3	3	3	2	2	2	2	1	1
3	3	3	3	2	2	3	3	4	4

For Review Only

3	3	3	3	2	2	2	2	1	1
3	3	3	3	2	2	2	2	4	4
3	3	3	3	2	2	2	2	1	1
3	3	3	3	2	2	2	2	4	4
3	3	3	3	2	2	2	2	1	1
3	3	3	3	2	2	2	2	1	1
3	3	3	3	2	2	2	2	1	1
3	3	3	3	4	4	3	3	4	4
3	3	2	2	2	2	3	3	4	4
3	3	3	3	2	2	3	3	4	4
3	3	3	3	2	2	2	2	1	1
3	3	3	3	2	2	2	2	1	1
3	3	3	3	2	2	2	2	1	1
3	3	3	3	4	4	2	2	1	1
3	3	3	3	2	2	2	2	1	1
3	3	3	3	2	2	3	3	4	4
3	3	3	3	2	2	2	2	4	4
3	3	3	3	2	2	2	2	1	1
3	3	3	3	2	2	2	2	4	4
3	3	3	3	2	2	2	2	4	4
3	3	3	3	2	2	2	2	1	1
3	3	3	3	4	4	3	3	4	4
3	3	3	3	4	4	2	2	4	4
3	3	3	3	4	4	2	2	4	4
3	3	3	3	2	2	3	3	4	4
3	3	3	3	2	2	2	2	1	1
3	3	3	3	2	2	2	2	4	4
3	3	3	3	4	4	2	2	4	4
3	3	3	3	2	2	2	2	1	1
3	3	3	3	2	2	2	2	1	1
3	3	3	3	2	2	2	2	4	4
3	3	2	2	4	4	3	3	4	4
3	3	3	3	4	4	3	3	4	4
3	3	3	3	2	2	2	2	1	1
2	2	3	3	2	2	2	2	4	4
3	3	3	3	2	2	3	3	4	4
3	3	2	2	2	2	3	3	4	4
3	3	3	3	4	4	3	3	4	4
3	3	2	2	2	2	3	3	4	4
2	2	3	3	2	2	2	2	4	4
3	3	3	3	2	2	2	2	4	4
3	3	3	3	2	2	2	2	1	1
2	2	3	3	2	2	3	3	4	4
3	3	3	3	2	2	2	2	1	1
3	3	3	3	2	2	2	2	4	4
3	3	3	3	2	2	2	2	1	1
3	3	2	2	4	4	3	3	4	4
3	3	3	3	2	2	2	2	1	1
3	3	3	3	2	2	2	2	1	1
3	3	3	3	2	2	2	2	1	1
3	3	3	3	2	2	2	2	1	1
3	3	2	2	4	4	3	3	4	4
3	3	3	3	4	4	3	3	4	4
3	3	3	3	2	2	3	3	4	4
3	3	3	3	2	2	2	2	1	1

For Review Only

3	3	3	3	2	2	2	2	1	1
3	3	3	3	2	2	3	3	4	4
3	3	3	3	2	2	2	2	1	1
3	3	2	2	4	4	3	3	4	4
3	3	3	3	2	2	2	2	1	1
3	3	3	3	2	2	2	2	1	1
3	3	2	2	2	2	3	3	4	4
3	3	3	3	2	2	3	3	4	4
3	3	3	3	4	4	2	2	1	1
3	3	3	3	2	2	3	3	4	4
3	3	3	3	2	2	3	3	1	1
3	3	3	3	2	2	2	2	4	4
2	2	3	3	2	2	3	3	4	4
3	3	3	3	2	2	3	3	4	4
3	3	3	3	2	2	3	3	4	4
3	3	3	3	2	2	3	3	4	4
3	3	3	3	2	2	2	2	1	1
3	3	3	3	2	2	3	3	4	4
3	3	2	2	4	4	3	3	4	4
3	3	3	3	2	2	2	2	1	1
3	3	3	3	2	2	3	3	4	4
3	3	3	3	2	2	2	2	4	4
3	3	2	2	2	2	3	3	4	4
3	3	3	3	4	4	3	3	4	4
3	3	3	3	2	2	3	3	4	4
3	3	3	3	2	2	3	3	4	4
3	3	2	2	2	2	3	3	4	4
3	3	2	2	2	2	3	3	4	4
3	3	2	2	2	2	3	3	4	4
3	3	3	3	2	2	2	2	1	1
3	3	2	2	4	4	3	3	4	4
3	3	3	3	2	2	3	3	4	4
3	3	3	3	2	2	2	2	4	4
3	3	3	3	2	2	2	2	1	1
3	3	3	3	4	4	3	3	4	4
3	3	3	3	2	2	3	3	4	4
3	3	2	2	4	4	3	3	4	4
3	3	3	3	2	2	2	2	1	1
3	3	3	3	2	2	2	2	4	4
3	3	3	3	4	4	3	3	4	4
3	3	3	3	2	2	2	2	1	1
3	3	2	2	4	4	3	3	4	4
3	3	3	3	2	2	2	2	1	1
3	3	3	3	2	2	3	3	4	4
2	2	3	3	2	2	2	2	4	4
3	3	3	3	2	2	3	3	4	4
3	3	3	3	2	2	2	2	1	1
3	3	3	3	2	2	2	2	1	1
3	3	3	3	4	4	3	3	4	4
3	3	3	3	2	2	2	2	1	1
3	3	3	3	2	2	2	2	1	1
3	3	3	3	4	4	2	2	1	1
3	3	3	3	2	2	3	3	4	4
3	3	3	3	2	2	2	2	1	1
3	3	3	3	2	2	3	3	4	4

For Review Only

3	3	3	3	2	2	2	2	1	1
3	3	3	3	2	2	2	2	1	1
3	3	3	3	4	4	3	3	4	4
3	3	3	3	4	4	3	3	4	4
3	3	3	3	2	2	2	2	1	1
3	3	3	3	4	4	3	3	4	4
3	3	3	3	2	2	3	3	4	4
3	3	3	3	2	2	3	3	4	4
3	3	2	2	2	2	3	3	4	4
3	3	3	3	2	2	2	2	4	4
3	3	3	3	2	2	2	2	1	1
3	3	3	3	2	2	3	3	4	4
3	3	3	3	2	2	2	2	1	1
3	3	3	3	2	2	3	3	4	4
3	3	2	2	4	4	3	3	4	4
3	3	3	3	2	2	3	3	4	4
3	3	3	3	2	2	3	3	4	4
3	3	3	3	2	2	2	2	1	1
3	3	3	3	2	2	2	2	4	4
3	3	3	3	2	2	3	3	4	4
3	3	3	3	2	2	3	3	4	4
3	3	3	3	4	4	3	3	4	4
3	3	3	3	4	4	3	3	4	4
3	3	3	3	2	2	2	2	1	1
3	3	3	3	2	2	3	3	4	4
3	3	2	2	4	4	3	3	4	4
3	3	3	3	2	2	2	2	1	1
3	3	3	3	2	2	3	3	4	4
3	3	3	3	2	2	2	2	1	1
3	3	3	3	2	2	2	2	1	1
3	3	3	3	2	2	2	2	1	1
3	3	3	3	2	2	2	2	1	1
3	3	3	3	2	2	2	2	4	4
3	3	2	2	4	4	3	3	4	4
3	3	3	3	2	2	2	2	4	4
3	3	3	3	2	2	2	2	1	1
3	3	3	3	2	2	2	2	1	1
2	2	3	3	2	2	2	2	4	4
3	3	3	3	2	2	2	2	1	1
3	3	3	3	2	2	2	2	1	1
3	3	3	3	2	2	2	2	1	1
3	3	3	3	2	2	2	2	1	1
3	3	3	3	4	4	2	2	4	4
3	3	3	3	2	2	2	2	4	4
3	3	3	3	2	2	2	2	1	1
3	3	2	2	4	4	3	3	4	4
3	3	3	3	2	2	3	3	4	4
3	3	3	3	4	4	3	3	4	4
3	3	2	2	4	4	3	3	4	4
3	3	2	2	2	2	3	3	1	1
2	2	3	3	2	2	2	2	1	1
3	3	3	3	4	4	3	3	4	4
3	3	3	3	4	4	3	3	4	4
3	3	3	3	2	2	2	2	1	1
3	3	2	2	4	4	3	3	4	4

For Review Only

3	3	2	2	2	2	3	3	4	4
3	3	3	3	2	2	3	3	4	4
3	3	2	2	2	2	3	3	4	4
3	3	3	3	4	4	3	3	4	4
3	3	2	2	2	2	3	3	4	4
3	3	3	3	2	2	2	2	1	1
3	3	3	3	4	4	2	2	1	1
3	3	3	3	4	4	3	3	1	1
3	3	2	2	2	2	3	3	4	4
3	3	3	3	4	4	3	3	4	4
3	3	3	3	2	2	3	3	4	4
3	3	3	3	2	2	2	2	1	1
3	3	2	2	2	2	3	3	1	1
3	3	3	3	2	2	2	2	1	1
3	3	3	3	2	2	2	2	1	1
3	3	3	3	2	2	3	3	4	4
3	3	3	3	4	4	2	2	1	1
3	3	2	2	2	2	3	3	4	4
3	3	3	3	4	4	2	2	1	1
3	3	3	3	2	2	2	2	1	1
3	3	2	2	4	4	3	3	4	4
3	3	3	3	2	2	2	2	1	1
3	3	3	3	2	2	2	2	4	4
3	3	3	3	2	2	2	2	1	1
3	3	3	3	4	4	3	3	4	4
3	3	3	3	2	2	2	2	1	1
3	3	3	3	4	4	3	3	4	4
3	3	3	3	2	2	2	2	1	1
3	3	3	3	2	2	2	2	4	4
3	3	2	2	2	2	3	3	4	4
3	3	2	2	2	2	3	3	4	4
3	3	3	3	2	2	2	2	1	1
3	3	3	3	2	2	2	2	1	1
3	3	3	3	2	2	2	2	1	1
3	3	3	3	4	4	3	3	4	4
3	3	3	3	4	4	3	3	4	4
3	3	2	2	2	2	3	3	4	4
3	3	3	3	2	2	2	2	1	1
3	3	3	3	2	2	2	2	1	1
3	3	3	3	4	4	3	3	4	4
3	3	3	3	2	2	2	2	1	1
3	3	3	3	4	4	2	2	1	1
3	3	3	3	2	2	2	2	1	1
3	3	3	3	2	2	2	2	4	4
3	3	3	3	2	2	2	2	1	1
3	3	2	2	2	2	3	3	1	1
3	3	3	3	2	2	2	2	1	1
3	3	3	3	2	2	2	2	1	1
3	3	3	3	2	2	2	2	1	1
3	3	3	3	4	4	3	3	4	4
3	3	3	3	2	2	2	2	4	4
3	3	3	3	4	4	3	3	4	4
3	3	3	3	2	2	2	2	1	1
3	3	3	3	2	2	2	2	1	1
3	3	3	3	2	2	3	3	4	4

For Review Only

3	3	3	3	2	2	3	3	4	4
3	3	3	3	2	2	2	2	1	1

For Review Only

SNP1572	SNP1572	SNP1573	SNP1573	SNP1574	SNP1574	SNP1575	SNP1575	SNP1576	SNP1576
3	3	1	1	3	3	3	3	1	1
3	3	1	1	3	3	3	3	4	4
3	3	1	1	3	3	3	3	1	1
3	3	1	1	3	3	3	3	1	1
3	3	1	1	3	3	3	3	4	4
3	3	1	1	3	3	3	3	1	1
3	3	1	1	2	2	1	1	1	1
3	3	4	4	3	3	3	3	1	1
3	3	1	1	3	3	3	3	1	1
3	3	1	1	3	3	3	3	1	1
3	3	4	4	2	2	1	1	1	1
3	3	1	1	3	3	3	3	1	1
3	3	1	1	3	3	3	3	1	1
3	3	1	1	3	3	3	3	1	1
3	3	1	1	3	3	3	3	1	1
3	3	1	1	2	2	1	1	1	1
3	3	4	4	3	3	3	3	1	1
3	3	1	1	2	2	1	1	1	1
3	3	1	1	3	3	3	3	1	1
3	3	1	1	2	2	1	1	1	1
3	3	4	4	3	3	3	3	4	4
3	3	4	4	3	3	3	3	1	1
3	3	1	1	3	3	3	3	1	1
3	3	1	1	3	3	3	3	1	1
3	3	1	1	2	2	1	1	1	1
3	3	1	1	3	3	3	3	1	1
3	3	1	1	3	3	3	3	1	1
3	3	1	1	3	3	3	3	1	1
3	3	1	1	3	3	3	3	1	1
3	3	1	1	2	2	1	1	1	1
3	3	1	1	3	3	3	3	1	1
3	3	1	1	2	2	1	1	1	1
3	3	1	1	2	2	1	1	1	1
3	3	1	1	3	3	3	3	1	1
3	3	1	1	3	3	3	3	1	1
3	3	1	1	2	2	1	1	1	1
2	2	1	1	3	3	3	3	1	1
3	3	1	1	3	3	3	3	1	1
3	3	4	4	2	2	1	1	1	1
2	2	1	1	3	3	3	3	1	1
3	3	1	1	2	2	1	1	1	1
3	3	4	4	3	3	3	3	4	4
3	3	1	1	3	3	3	3	1	1
3	3	4	4	3	3	3	3	1	1
3	3	1	1	3	3	3	3	1	1
3	3	1	1	3	3	3	3	1	1
3	3	1	1	3	3	3	3	1	1
3	3	1	1	3	3	3	3	1	1
3	3	4	4	2	2	1	1	1	1
3	3	1	1	3	3	3	3	1	1
3	3	1	1	3	3	3	3	1	1
2	2	1	1	3	3	3	3	1	1

For Review Only

3	3	1	1	3	3	3	3	1	1
3	3	1	1	2	2	1	1	1	1
3	3	1	1	2	2	1	1	1	1
3	3	4	4	2	2	1	1	1	1
3	3	1	1	3	3	3	3	1	1
3	3	1	1	3	3	3	3	1	1
3	3	1	1	3	3	3	3	1	1
3	3	4	4	2	2	1	1	4	4
2	2	4	4	3	3	3	3	1	1
3	3	1	1	3	3	3	3	1	1
3	3	1	1	3	3	3	3	1	1
3	3	1	1	3	3	3	3	1	1
3	3	1	1	3	3	3	3	1	1
3	3	1	1	3	3	3	3	1	1
3	3	1	1	3	3	3	3	1	1
2	2	1	1	3	3	3	3	1	1
3	3	1	1	3	3	3	3	1	1
3	3	1	1	3	3	3	3	1	1
3	3	1	1	3	3	3	3	1	1
3	3	1	1	3	3	3	3	1	1
3	3	1	1	3	3	3	3	1	1
3	3	1	1	3	3	3	3	1	1
3	3	1	1	3	3	3	3	1	1
3	3	1	1	2	2	1	1	1	1
3	3	1	1	3	3	3	3	1	1
3	3	1	1	3	3	3	3	1	1
3	3	1	1	3	3	1	1	1	1
3	3	4	4	3	3	3	3	1	1
3	3	1	1	3	3	3	3	1	1
3	3	1	1	3	3	3	3	1	1
3	3	1	1	3	3	3	3	1	1
3	3	1	1	3	3	3	3	1	1
3	3	4	4	3	3	3	3	1	1
3	3	1	1	3	3	3	3	1	1
3	3	1	1	3	3	3	3	1	1
3	3	1	1	3	3	3	3	1	1
3	3	1	1	3	3	3	3	1	1
3	3	4	4	2	2	1	1	4	4
3	3	1	1	2	2	1	1	1	1
3	3	4	4	3	3	1	1	1	1
3	3	1	1	3	3	3	3	1	1
3	3	1	1	3	3	3	3	1	1
3	3	1	1	3	3	3	3	1	1
3	3	1	1	3	3	3	3	1	1
3	3	4	4	3	3	3	3	1	1
3	3	1	1	3	3	3	3	1	1
3	3	1	1	3	3	3	3	1	1
3	3	1	1	3	3	3	3	1	1
3	3	1	1	3	3	3	3	1	1
3	3	1	1	3	3	3	3	1	1
3	3	1	1	3	3	3	3	1	1
3	3	4	4	2	2	1	1	1	1
3	3	4	4	2	2	1	1	4	4
3	3	1	1	3	3	3	3	1	1
3	3	1	1	3	3	3	3	1	1

For Review Only

3	3	1	1	3	3	3	3	1	1
3	3	1	1	3	3	3	3	1	1
3	3	1	1	3	3	3	3	1	1
3	3	1	1	3	3	3	3	1	1
3	3	1	1	3	3	3	3	1	1
3	3	1	1	3	3	3	3	1	1
3	3	1	1	3	3	3	3	1	1
3	3	1	1	3	3	3	3	1	1
3	3	1	1	3	3	3	3	1	1
3	3	1	1	3	3	1	1	1	1
3	3	4	4	3	3	3	3	1	1
3	3	1	1	3	3	3	3	1	1
3	3	1	1	3	3	3	3	1	1
3	3	1	1	3	3	3	3	1	1
3	3	1	1	3	3	3	3	1	1
3	3	1	1	3	3	3	3	1	1
3	3	1	1	3	3	3	3	1	1
3	3	1	1	3	3	1	1	1	1
3	3	1	1	3	3	3	3	1	1
3	3	1	1	3	3	3	3	1	1
3	3	1	1	3	3	3	3	1	1
3	3	1	1	3	3	3	3	1	1
3	3	1	1	3	3	3	3	1	1
3	3	4	4	3	3	3	3	1	1
3	3	1	1	3	3	3	3	1	1
3	3	1	1	3	3	3	3	1	1
3	3	1	1	3	3	3	3	1	1
3	3	1	1	3	3	3	3	1	1
3	3	1	1	3	3	3	3	1	1
3	3	1	1	3	3	3	3	1	1
3	3	1	1	3	3	3	3	1	1
3	3	1	1	3	3	3	3	1	1
3	3	1	1	3	3	3	3	1	1
3	3	1	1	3	3	3	3	1	1
3	3	1	1	3	3	3	3	1	1
3	3	1	1	3	3	3	3	1	1
3	3	1	1	3	3	3	3	1	1
3	3	1	1	3	3	3	3	1	1
3	3	1	1	3	3	3	3	1	1
3	3	4	4	3	3	1	1	1	1
3	3	1	1	3	3	3	3	1	1
3	3	1	1	3	3	3	3	1	1
3	3	1	1	3	3	3	3	1	1
3	3	1	1	3	3	3	3	4	4
3	3	1	1	3	3	3	3	1	1
3	3	1	1	3	3	3	3	1	1
3	3	1	1	3	3	3	3	1	1
3	3	1	1	3	3	3	3	1	1
3	3	1	1	3	3	1	1	1	1
3	3	1	1	3	3	3	3	1	1
3	3	1	1	3	3	3	3	1	1
3	3	4	4	2	2	1	1	1	1
3	3	1	1	3	3	3	3	1	1
3	3	1	1	3	3	3	3	1	1
3	3	1	1	2	2	3	3	4	4

For Review Only

3	3	1	1	3	3	3	3	1	1
3	3	1	1	3	3	3	3	1	1
3	3	1	1	2	2	1	1	1	1
3	3	1	1	3	3	3	3	1	1
3	3	1	1	3	3	3	3	1	1
3	3	1	1	3	3	3	3	1	1
3	3	1	1	3	3	3	3	1	1
3	3	1	1	3	3	3	3	1	1
3	3	1	1	3	3	3	3	1	1
3	3	1	1	3	3	3	3	1	1
3	3	1	1	3	3	3	3	1	1
3	3	1	1	3	3	3	3	1	1
3	3	1	1	3	3	3	3	1	1
3	3	1	1	3	3	3	3	1	1
3	3	1	1	3	3	3	3	1	1
3	3	4	4	2	2	1	1	1	1
3	3	1	1	3	3	3	3	1	1
3	3	1	1	3	3	3	3	1	1
3	3	1	1	3	3	3	3	1	1
3	3	1	1	3	3	3	3	1	1
3	3	1	1	3	3	3	3	1	1
3	3	1	1	2	2	3	3	1	1
3	3	1	1	3	3	3	3	1	1
3	3	1	1	3	3	3	3	1	1
3	3	1	1	3	3	3	3	1	1
3	3	1	1	3	3	3	3	1	1
3	3	1	1	3	3	3	3	1	1
3	3	1	1	3	3	3	3	1	1
3	3	1	1	3	3	3	3	1	1
3	3	1	1	3	3	3	3	1	1
3	3	4	4	3	3	3	3	1	1
3	3	1	1	3	3	3	3	1	1
2	2	1	1	3	3	3	3	1	1
3	3	1	1	3	3	3	3	1	1
3	3	1	1	2	2	1	1	1	1
3	3	1	1	3	3	3	3	1	1
3	3	1	1	3	3	3	3	1	1
2	2	1	1	3	3	3	3	1	1
3	3	1	1	3	3	3	3	1	1
3	3	1	1	3	3	3	3	1	1
3	3	1	1	3	3	3	3	1	1
3	3	1	1	3	3	3	3	1	1
3	3	1	1	2	2	1	1	1	1
3	3	1	1	3	3	3	3	1	1
3	3	1	1	3	3	3	3	1	1
3	3	1	1	3	3	3	3	1	1
3	3	4	4	2	2	1	1	1	1
3	3	1	1	3	3	3	3	1	1
3	3	1	1	3	3	3	3	1	1
3	3	1	1	3	3	3	3	1	1
3	3	1	1	3	3	3	3	1	1
3	3	1	1	3	3	3	3	1	1
3	3	1	1	3	3	3	3	1	1
3	3	1	1	3	3	3	3	1	1
3	3	4	4	2	2	1	1	1	1

For Review Only

3	3	1	1	3	3	3	3	1	1
3	3	1	1	3	3	3	3	4	4
3	3	1	1	3	3	3	3	1	1
3	3	1	1	3	3	3	3	1	1
3	3	1	1	3	3	3	3	1	1
3	3	1	1	3	3	3	3	1	1
3	3	4	4	2	2	1	1	1	1
3	3	1	1	3	3	3	3	1	1
3	3	1	1	3	3	3	3	1	1
3	3	1	1	3	3	3	3	1	1
3	3	1	1	3	3	3	3	1	1
3	3	1	1	3	3	3	3	1	1
3	3	1	1	2	2	1	1	1	1
3	3	1	1	3	3	3	3	1	1
3	3	1	1	3	3	3	3	1	1
3	3	1	1	3	3	3	3	1	1
3	3	4	4	2	2	1	1	1	1
3	3	4	4	3	3	3	3	1	1
3	3	1	1	3	3	3	3	1	1
3	3	1	1	3	3	3	3	1	1
3	3	1	1	3	3	3	3	1	1
3	3	1	1	3	3	3	3	1	1
3	3	1	1	3	3	3	3	1	1
3	3	1	1	3	3	3	3	1	1
3	3	4	4	2	2	1	1	4	4
3	3	1	1	3	3	3	3	1	1
3	3	4	4	3	3	3	3	1	1
3	3	1	1	3	3	3	3	1	1
3	3	1	1	3	3	3	3	1	1
3	3	1	1	3	3	3	3	1	1
3	3	1	1	2	2	1	1	1	1
3	3	1	1	3	3	3	3	1	1
3	3	1	1	3	3	3	3	1	1
3	3	1	1	3	3	3	3	1	1
3	3	1	1	3	3	3	3	1	1
3	3	1	1	3	3	3	3	1	1
3	3	1	1	3	3	3	3	1	1
3	3	1	1	3	3	3	3	1	1
3	3	1	1	2	2	1	1	1	1
3	3	4	4	2	2	1	1	1	1
2	2	1	1	3	3	3	3	1	1
3	3	1	1	3	3	3	3	1	1
3	3	1	1	3	3	3	3	1	1
3	3	1	1	3	3	3	3	1	1
3	3	1	1	3	3	3	3	1	1
3	3	1	1	2	2	1	1	1	1
3	3	1	1	2	2	1	1	1	1
3	3	1	1	3	3	3	3	1	1
3	3	1	1	3	3	3	3	1	1
3	3	4	4	3	3	3	3	1	1
2	2	1	1	3	3	3	3	1	1
3	3	4	4	2	2	1	1	1	1
3	3	1	1	3	3	3	3	1	1
3	3	1	1	3	3	3	3	1	1
2	2	1	1	3	3	3	3	1	1

For Review Only

3	3	1	1	3	3	3	3	1	1
3	3	1	1	3	3	3	3	1	1

For Review Only

SNP1577	SNP1577	SNP1578	SNP1578	SNP1579	SNP1579	SNP158	SNP158	SNP1580	SNP1580
4	4	2	2	2	2	1	1	4	4
1	1	2	2	2	2	4	4	3	3
1	1	2	2	2	2	1	1	3	3
1	1	2	2	2	2	1	1	3	3
1	1	2	2	2	2	1	1	3	3
4	4	2	2	2	2	1	1	4	4
1	1	2	2	2	2	1	1	3	3
1	1	2	2	2	2	1	1	3	3
1	1	2	2	2	2	1	1	3	3
1	1	2	2	2	2	4	4	3	3
1	1	2	2	3	3	1	1	3	3
1	1	2	2	2	2	1	1	3	3
1	1	2	2	2	2	1	1	3	3
1	1	2	2	2	2	1	1	3	3
1	1	2	2	2	2	1	1	3	3
1	1	2	2	2	2	1	1	3	3
1	1	2	2	2	2	1	1	3	3
4	4	2	2	2	2	1	1	4	4
1	1	2	2	2	2	4	4	3	3
1	1	2	2	2	2	4	4	3	3
1	1	2	2	2	2	4	4	3	3
1	1	2	2	2	2	1	1	3	3
1	1	2	2	2	2	1	1	3	3
1	1	2	2	3	3	1	1	3	3
4	4	2	2	2	2	1	1	4	4
1	1	2	2	2	2	1	1	3	3
1	1	2	2	2	2	1	1	3	3
1	1	2	2	2	2	1	1	3	3
4	4	2	2	2	2	1	1	4	4
1	1	4	4	2	2	4	4	3	3
1	1	2	2	2	2	1	1	3	3
1	1	2	2	2	2	1	1	4	4
1	1	2	2	2	2	1	1	4	4
1	1	4	4	3	3	1	1	3	3
1	1	2	2	2	2	1	1	3	3
1	1	2	2	3	3	1	1	3	3
1	1	2	2	2	2	1	1	3	3
1	1	4	4	2	2	1	1	3	3
1	1	2	2	3	3	1	1	3	3
1	1	2	2	2	2	1	1	3	3
1	1	2	2	2	2	1	1	3	3
1	1	2	2	2	2	1	1	3	3
1	1	2	2	2	2	4	4	3	3
1	1	2	2	2	2	1	1	3	3
1	1	2	2	2	2	4	4	3	3
1	1	2	2	2	2	1	1	3	3
1	1	2	2	2	2	1	1	3	3
1	1	2	2	2	2	1	1	3	3
1	1	2	2	2	2	1	1	3	3
1	1	2	2	2	2	1	1	3	3
1	1	2	2	2	2	1	1	3	3
1	1	2	2	2	2	1	1	3	3

For Review Only

4	4	2	2	2	2	1	1	4	4
1	1	2	2	2	2	1	1	4	4
1	1	2	2	2	2	4	4	4	4
1	1	2	2	2	2	1	1	3	3
1	1	2	2	2	2	4	4	3	3
1	1	2	2	2	2	1	1	3	3
1	1	2	2	2	2	1	1	3	3
1	1	2	2	3	3	4	4	3	3
1	1	2	2	3	3	1	1	3	3
1	1	2	2	2	2	1	1	3	3
1	1	2	2	2	2	1	1	3	3
1	1	2	2	2	2	1	1	3	3
4	4	2	2	2	2	1	1	4	4
4	4	2	2	2	2	1	1	4	4
1	1	2	2	2	2	1	1	3	3
1	1	2	2	2	2	1	1	3	3
1	1	2	2	2	2	1	1	3	3
1	1	2	2	2	2	1	1	3	3
1	1	2	2	2	2	1	1	3	3
1	1	2	2	2	2	1	1	3	3
1	1	2	2	2	2	1	1	3	3
1	1	2	2	2	2	1	1	3	3
1	1	2	2	2	2	1	1	3	3
1	1	2	2	2	2	1	1	4	4
1	1	2	2	2	2	4	4	3	3
1	1	2	2	2	2	4	4	3	3
1	1	2	2	2	2	1	1	4	4
1	1	2	2	3	3	1	1	3	3
1	1	2	2	2	2	1	1	3	3
1	1	2	2	2	2	1	1	3	3
1	1	2	2	2	2	1	1	3	3
1	1	4	4	2	2	1	1	3	3
1	1	2	2	3	3	1	1	3	3
4	4	2	2	2	2	1	1	4	4
1	1	2	2	2	2	1	1	3	3
1	1	2	2	2	2	4	4	3	3
1	1	2	2	2	2	1	1	3	3
1	1	2	2	2	2	1	1	3	3
1	1	2	2	2	2	1	1	3	3
1	1	2	2	3	3	1	1	3	3
1	1	2	2	2	2	4	4	3	3
1	1	2	2	2	2	1	1	3	3
1	1	2	2	2	2	1	1	3	3
1	1	2	2	2	2	1	1	3	3
1	1	2	2	3	3	1	1	3	3
4	4	2	2	2	2	4	4	4	4
1	1	4	4	2	2	1	1	3	3
1	1	2	2	2	2	1	1	3	3
1	1	2	2	2	2	1	1	3	3
1	1	2	2	2	2	1	1	3	3
1	1	2	2	2	2	1	1	3	3
1	1	2	2	3	3	1	1	3	3
1	1	2	2	3	3	4	4	3	3
1	1	2	2	2	2	1	1	3	3
1	1	2	2	2	2	1	1	3	3

For Review Only

1	1	2	2	2	2	1	1	3	3
1	1	2	2	2	2	1	1	3	3
1	1	2	2	2	2	1	1	3	3
1	1	4	4	2	2	1	1	3	3
1	1	2	2	2	2	1	1	3	3
1	1	2	2	2	2	4	4	3	3
1	1	2	2	2	2	1	1	3	3
1	1	2	2	3	3	1	1	3	3
1	1	2	2	2	2	1	1	3	3
1	1	2	2	3	3	1	1	3	3
1	1	2	2	2	2	1	1	4	4
1	1	2	2	2	2	1	1	3	3
1	1	2	2	2	2	1	1	3	3
1	1	2	2	2	2	1	1	3	3
4	4	2	2	2	2	1	1	3	3
1	1	2	2	2	2	1	1	3	3
1	1	2	2	2	2	1	1	3	3
1	1	2	2	2	2	1	1	3	3
1	1	4	4	3	3	1	1	3	3
1	1	2	2	2	2	1	1	3	3
1	1	2	2	2	2	1	1	3	3
1	1	2	2	2	2	1	1	3	3
1	1	2	2	2	2	1	1	3	3
1	1	2	2	3	3	1	1	3	3
1	1	2	2	2	2	4	4	3	3
1	1	2	2	2	2	1	1	3	3
1	1	2	2	2	2	1	1	3	3
1	1	2	2	2	2	1	1	3	3
1	1	2	2	2	2	1	1	3	3
1	1	2	2	2	2	1	1	3	3
1	1	2	2	3	3	1	1	3	3
1	1	2	2	2	2	1	1	3	3
1	1	2	2	2	2	4	4	3	3
1	1	2	2	2	2	1	1	3	3
1	1	4	4	3	3	1	1	3	3
1	1	4	4	2	2	1	1	3	3
1	1	4	4	3	3	1	1	3	3
1	1	2	2	2	2	1	1	3	3
1	1	2	2	2	2	1	1	3	3
1	1	2	2	3	3	1	1	3	3
1	1	2	2	2	2	1	1	3	3
1	1	4	4	3	3	1	1	3	3
1	1	2	2	2	2	1	1	3	3
1	1	2	2	3	3	1	1	3	3
1	1	2	2	2	2	1	1	3	3
1	1	2	2	2	2	1	1	3	3
1	1	2	2	2	2	1	1	3	3
1	1	2	2	2	2	1	1	3	3
1	1	2	2	2	2	1	1	3	3
1	1	2	2	2	2	1	1	3	3
1	1	2	2	2	2	1	1	3	3
1	1	2	2	3	3	4	4	3	3
1	1	2	2	2	2	1	1	3	3
1	1	2	2	2	2	1	1	3	3
1	1	2	2	2	2	1	1	3	3

For Review Only

1	1	2	2	2	2	4	4	4	4
1	1	2	2	2	2	1	1	3	3
1	1	2	2	2	2	1	1	3	3
1	1	2	2	2	2	1	1	3	3
4	4	2	2	2	2	1	1	4	4
1	1	4	4	2	2	1	1	3	3
1	1	2	2	2	2	1	1	3	3
1	1	2	2	2	2	1	1	3	3
1	1	2	2	2	2	1	1	3	3
1	1	2	2	2	2	1	1	3	3
1	1	2	2	2	2	1	1	3	3
1	1	2	2	2	2	1	1	3	3
1	1	2	2	2	2	1	1	3	3
1	1	2	2	2	2	1	1	3	3
1	1	4	4	2	2	1	1	3	3
1	1	2	2	3	3	1	1	3	3
1	1	2	2	2	2	1	1	3	3
1	1	2	2	3	3	1	1	3	3
1	1	2	2	2	2	1	1	3	3
1	1	2	2	2	2	1	1	3	3
1	1	2	2	2	2	1	1	3	3
1	1	2	2	3	3	1	1	3	3
1	1	2	2	2	2	4	4	3	3
1	1	2	2	2	2	1	1	3	3
1	1	2	2	2	2	1	1	3	3
1	1	4	4	3	3	1	1	3	3
1	1	2	2	2	2	1	1	3	3
1	1	2	2	2	2	1	1	3	3
1	1	2	2	2	2	1	1	3	3
1	1	2	2	2	2	1	1	3	3
1	1	2	2	3	3	1	1	3	3
1	1	2	2	2	2	1	1	3	3
1	1	2	2	2	2	1	1	3	3
1	1	4	4	2	2	1	1	3	3
1	1	2	2	2	2	1	1	3	3
1	1	2	2	2	2	1	1	3	3
1	1	2	2	2	2	1	1	3	3
1	1	2	2	2	2	1	1	3	3
1	1	2	2	2	2	1	1	3	3
1	1	2	2	2	2	1	1	3	3
1	1	2	2	2	2	1	1	3	3
1	1	2	2	2	2	4	4	3	3
1	1	2	2	3	3	1	1	3	3
1	1	2	2	2	2	1	1	3	3
1	1	2	2	3	3	1	1	3	3
1	1	2	2	2	2	1	1	3	3
1	1	2	2	3	3	1	1	3	3
1	1	2	2	3	3	1	1	3	3
1	1	2	2	2	2	1	1	3	3
1	1	2	2	2	2	4	4	3	3
1	1	2	2	2	2	1	1	3	3
1	1	4	4	2	2	1	1	3	3
1	1	2	2	2	2	1	1	3	3
1	1	2	2	2	2	1	1	3	3
1	1	2	2	3	3	1	1	3	3

For Review Only

1	1	4	4	2	2	1	1	3	3
1	1	2	2	3	3	1	1	3	3
1	1	2	2	2	2	1	1	3	3
1	1	2	2	2	2	1	1	3	3
1	1	2	2	2	2	1	1	3	3
1	1	2	2	2	2	1	1	3	3
1	1	2	2	3	3	1	1	3	3
1	1	2	2	2	2	1	1	3	3
1	1	2	2	2	2	1	1	3	3
1	1	2	2	3	3	1	1	3	3
1	1	2	2	2	2	1	1	3	3
1	1	2	2	2	2	1	1	3	3
1	1	2	2	2	2	1	1	3	3
1	1	2	2	2	2	1	1	4	4
1	1	2	2	2	2	1	1	3	3
1	1	2	2	2	2	1	1	3	3
1	1	2	2	3	3	1	1	3	3
1	1	4	4	2	2	1	1	3	3
1	1	2	2	2	2	1	1	3	3
1	1	4	4	2	2	1	1	3	3
1	1	2	2	2	2	1	1	3	3
1	1	2	2	2	2	1	1	3	3
1	1	2	2	2	2	1	1	3	3
1	1	2	2	2	2	1	1	3	3
1	1	2	2	3	3	4	4	3	3
4	4	2	2	2	2	1	1	3	3
1	1	2	2	3	3	1	1	3	3
1	1	2	2	2	2	1	1	3	3
4	4	2	2	2	2	1	1	4	4
1	1	2	2	2	2	1	1	3	3
1	1	2	2	3	3	1	1	3	3
1	1	2	2	2	2	1	1	3	3
1	1	4	4	2	2	1	1	3	3
1	1	2	2	2	2	1	1	3	3
1	1	2	2	2	2	1	1	3	3
1	1	2	2	3	3	1	1	3	3
1	1	2	2	2	2	4	4	4	4
1	1	2	2	2	2	1	1	3	3
1	1	2	2	3	3	1	1	3	3
1	1	2	2	3	3	1	1	3	3
1	1	2	2	2	2	1	1	3	3
1	1	2	2	2	2	1	1	3	3
1	1	2	2	2	2	1	1	3	3
1	1	2	2	2	2	1	1	3	3
1	1	2	2	2	2	1	1	3	3
1	1	2	2	2	2	1	1	3	3
1	1	2	2	2	2	1	1	3	3
1	1	2	2	2	2	1	1	3	3
1	1	2	2	2	2	1	1	3	3
1	1	2	2	2	2	1	1	3	3
1	1	2	2	2	2	1	1	3	3
1	1	2	2	3	3	1	1	3	3
1	1	2	2	2	2	1	1	3	3
1	1	2	2	2	2	1	1	3	3
1	1	2	2	2	2	1	1	3	3

For Review Only

1	1	2	2	2	2	1	1	3	3
1	1	2	2	2	2	4	4	3	3

For Review Only

SNP1581	SNP1581	SNP1582	SNP1582	SNP1583	SNP1583	SNP1584	SNP1584	SNP1585	SNP1585
4	4	4	4	4	4	3	3	1	1
4	4	4	4	4	4	3	3	4	4
4	4	4	4	4	4	3	3	4	4
4	4	4	4	4	4	3	3	4	4
4	4	4	4	4	4	3	3	4	4
4	4	4	4	4	4	3	3	4	4
4	4	4	4	4	4	3	3	1	1
4	4	4	4	4	4	3	3	4	4
4	4	4	4	4	4	3	3	1	1
4	4	4	4	4	4	3	3	4	4
4	4	4	4	4	4	4	4	4	4
4	4	4	4	4	4	3	3	4	4
4	4	4	4	4	4	4	4	1	1
4	4	4	4	4	4	3	3	4	4
4	4	4	4	4	4	3	3	4	4
4	4	4	4	4	4	3	3	4	4
4	4	4	4	4	4	3	3	1	1
4	4	4	4	4	4	3	3	4	4
4	4	4	4	4	4	3	3	4	4
4	4	4	4	4	4	3	3	4	4
4	4	4	4	4	4	3	3	4	4
4	4	4	4	4	4	3	3	1	1
4	4	4	4	4	4	3	3	4	4
3	3	3	3	4	4	4	4	4	4
4	4	4	4	4	4	3	3	4	4
4	4	4	4	4	4	3	3	4	4
4	4	4	4	4	4	3	3	4	4
4	4	4	4	4	4	3	3	4	4
4	4	4	4	4	4	3	3	4	4
4	4	4	4	4	4	3	3	4	4
4	4	4	4	4	4	3	3	4	4
4	4	4	4	4	4	4	4	4	4
4	4	4	4	4	4	4	4	1	1
3	3	3	3	3	3	3	3	4	4
4	4	3	3	4	4	3	3	4	4
4	4	4	4	4	4	3	3	4	4
4	4	4	4	4	4	3	3	4	4
4	4	4	4	4	4	3	3	4	4
4	4	3	3	3	3	3	3	4	4
4	4	4	4	4	4	3	3	4	4
4	4	4	4	4	4	3	3	4	4
4	4	4	4	4	4	3	3	4	4
4	4	4	4	4	4	3	3	4	4
4	4	4	4	4	4	3	3	4	4
4	4	4	4	4	4	3	3	4	4
4	4	4	4	4	4	3	3	4	4
4	4	4	4	4	4	3	3	4	4
4	4	4	4	4	4	3	3	1	1
4	4	4	4	4	4	4	4	1	1
4	4	4	4	4	4	3	3	1	1
4	4	4	4	4	4	3	3	4	4
4	4	4	4	4	4	3	3	4	4

For Review Only

4	4	4	4	4	4	3	3	4	4
4	4	4	4	4	4	4	4	1	1
4	4	4	4	4	4	3	3	4	4
4	4	4	4	4	4	3	3	1	1
4	4	4	4	4	4	3	3	4	4
4	4	4	4	4	4	3	3	4	4
4	4	4	4	4	4	3	3	4	4
3	3	3	3	3	3	3	3	4	4
3	3	3	3	4	4	3	3	4	4
4	4	4	4	4	4	3	3	4	4
4	4	4	4	4	4	3	3	4	4
4	4	4	4	4	4	3	3	4	4
4	4	4	4	4	4	3	3	4	4
4	4	4	4	4	4	3	3	4	4
4	4	4	4	4	4	3	3	4	4
4	4	4	4	4	4	3	3	4	4
4	4	4	4	4	4	3	3	4	4
4	4	4	4	4	4	3	3	4	4
4	4	4	4	4	4	3	3	1	1
4	4	4	4	4	4	3	3	1	1
4	4	4	4	4	4	3	3	4	4
4	4	4	4	4	4	3	3	4	4
4	4	4	4	4	4	3	3	1	1
4	4	4	4	4	4	4	4	4	4
4	4	4	4	4	4	3	3	4	4
4	4	4	4	4	4	4	4	4	4
4	4	4	4	4	4	4	4	4	4
3	3	3	3	3	3	3	3	4	4
4	4	4	4	4	4	3	3	4	4
4	4	4	4	4	4	3	3	4	4
4	4	4	4	4	4	3	3	1	1
4	4	3	3	3	3	3	3	4	4
3	3	3	3	3	3	3	3	4	4
4	4	4	4	4	4	3	3	4	4
4	4	4	4	4	4	3	3	4	4
4	4	4	4	4	4	3	3	4	4
4	4	4	4	4	4	3	3	4	4
4	4	4	4	4	4	3	3	4	4
4	4	4	4	4	4	4	4	4	4
3	3	3	3	3	3	3	3	4	4
4	4	4	4	4	4	3	3	1	1
4	4	4	4	4	4	3	3	4	4
4	4	4	4	4	4	3	3	4	4
4	4	4	4	4	4	3	3	4	4
3	3	3	3	3	3	4	4	1	1
4	4	4	4	4	4	3	3	4	4
3	3	3	3	3	3	3	3	4	4
4	4	4	4	4	4	3	3	4	4
4	4	4	4	4	4	3	3	4	4
4	4	4	4	4	4	3	3	4	4
4	4	4	4	4	4	3	3	4	4
4	4	3	3	3	3	3	3	4	4
3	3	3	3	3	3	3	3	4	4
4	4	4	4	4	4	3	3	4	4
4	4	4	4	4	4	3	3	4	4

For Review Only

4	4	4	4	4	4	3	3	4	4
4	4	4	4	4	4	3	3	4	4
4	4	4	4	4	4	3	3	4	4
4	4	3	3	3	3	3	3	4	4
4	4	4	4	4	4	3	3	4	4
4	4	4	4	4	4	3	3	4	4
4	4	4	4	4	4	3	3	4	4
4	4	4	4	4	4	3	3	4	4
4	4	3	3	3	3	3	3	4	4
4	4	4	4	4	4	3	3	4	4
3	3	3	3	4	4	3	3	4	4
4	4	4	4	4	4	3	3	4	4
4	4	4	4	4	4	3	3	4	4
4	4	4	4	4	4	4	4	4	4
4	4	4	4	4	4	3	3	4	4
4	4	4	4	4	4	3	3	4	4
4	4	4	4	4	4	3	3	4	4
4	4	4	4	4	4	3	3	4	4
3	3	3	3	3	3	3	3	4	4
4	4	4	4	4	4	3	3	4	4
4	4	4	4	4	4	3	3	4	4
4	4	4	4	4	4	3	3	4	4
4	4	4	4	4	4	3	3	4	4
4	4	4	4	4	4	3	3	4	4
4	4	4	4	4	4	3	3	4	4
4	4	4	4	4	4	3	3	4	4
4	4	4	4	4	4	3	3	4	4
4	4	4	4	4	4	3	3	4	4
4	4	4	4	4	4	3	3	4	4
4	4	4	4	4	4	3	3	4	4
3	3	3	3	3	3	3	3	4	4
4	4	4	4	4	4	3	3	4	4
4	4	4	4	4	4	3	3	1	1
4	4	4	4	4	4	3	3	4	4
3	3	3	3	3	3	3	3	4	4
4	4	4	4	4	4	3	3	4	4
3	3	3	3	3	3	3	3	4	4
4	4	4	4	4	4	3	3	4	4
4	4	4	4	4	4	3	3	4	4
4	4	4	4	4	4	4	4	4	4
4	4	4	4	4	4	3	3	4	4
3	3	3	3	3	3	3	3	4	4
4	4	4	4	4	4	3	3	4	4
4	4	4	4	4	4	3	3	4	4
4	4	4	4	4	4	3	3	4	4
4	4	4	4	4	4	3	3	4	4
4	4	4	4	4	4	3	3	4	4
4	4	4	4	4	4	3	3	4	4
3	3	4	4	4	4	3	3	4	4
4	4	4	4	4	4	3	3	4	4
4	4	4	4	4	4	3	3	4	4
4	4	3	3	3	3	3	3	4	4
4	4	4	4	4	4	4	4	4	4
3	3	3	3	3	3	3	3	4	4
4	4	4	4	4	4	3	3	4	4

4	4	4	4	4	4	3	3	4	4
4	4	4	4	4	4	3	3	4	4
4	4	4	4	4	4	3	3	4	4
4	4	4	4	4	4	4	4	4	4
4	4	4	4	4	4	3	3	4	4
4	4	4	4	4	4	3	3	4	4
4	4	4	4	3	3	3	3	4	4
4	4	4	4	4	4	3	3	4	4
4	4	3	3	3	3	3	3	4	4
4	4	4	4	4	4	3	3	4	4
4	4	4	4	4	4	3	3	4	4
4	4	3	3	3	3	4	4	4	4
4	4	4	4	4	4	3	3	4	4
4	4	4	4	4	4	3	3	4	4
3	3	3	3	3	3	3	3	4	4
4	4	4	4	4	4	4	4	4	4
4	4	4	4	4	4	4	4	4	4
4	4	4	4	4	4	3	3	4	4
3	3	3	3	3	3	3	3	4	4
4	4	4	4	4	4	3	3	4	4
4	4	4	4	4	4	3	3	4	4
3	3	3	3	3	3	3	3	4	4
4	4	4	4	4	4	3	3	4	4
4	4	4	4	4	4	3	3	4	4
4	4	4	4	4	4	3	3	4	4
3	3	3	3	3	3	3	3	4	4
4	4	4	4	4	4	3	3	4	4
4	4	4	4	4	4	3	3	4	4
4	4	4	4	4	4	3	3	4	4
4	4	4	4	4	4	3	3	1	1
3	3	3	3	3	3	3	3	4	4
4	4	4	4	4	4	3	3	4	4
4	4	4	4	4	4	3	3	4	4
3	3	3	3	3	3	3	3	4	4
4	4	4	4	4	4	3	3	1	1
4	4	4	4	4	4	3	3	4	4
4	4	4	4	4	4	3	3	4	4
4	4	4	4	4	4	3	3	4	4
4	4	4	4	4	4	3	3	4	4
4	4	4	4	4	4	3	3	4	4
4	4	4	4	4	4	3	3	4	4
4	4	4	4	4	4	3	3	4	4
4	4	3	3	3	3	3	3	4	4
4	4	4	4	4	4	3	3	1	1
4	4	3	3	4	4	3	3	4	4
4	4	4	4	4	4	3	3	4	4
4	4	4	4	4	4	4	4	4	4
4	4	3	3	3	3	3	3	4	4
4	4	3	3	3	3	3	3	4	4
4	4	4	4	4	4	3	3	4	4
3	3	3	3	3	3	3	3	4	4
4	4	4	4	4	4	3	3	4	4
4	4	4	4	4	4	3	3	4	4
4	4	4	4	4	4	3	3	4	4
3	3	3	3	3	3	3	3	4	4

For Review Only

4	4	4	4	4	4	3	3	4	4
4	4	4	4	4	4	3	3	4	4
3	3	3	3	3	3	3	3	4	4
4	4	4	4	4	4	3	3	4	4
4	4	4	4	4	4	3	3	4	4
4	4	4	4	4	4	3	3	1	1
4	4	3	3	3	3	3	3	4	4
4	4	4	4	4	4	3	3	4	4
4	4	4	4	4	4	3	3	4	4
4	4	3	3	3	3	3	3	4	4
4	4	4	4	4	4	3	3	4	4
4	4	4	4	4	4	3	3	4	4
4	4	4	4	4	4	3	3	4	4
4	4	4	4	4	4	3	3	4	4
3	3	3	3	3	3	3	3	4	4
4	4	4	4	4	4	4	4	4	4
4	4	3	3	3	3	3	3	4	4
3	3	3	3	4	4	3	3	4	4
4	4	4	4	4	4	3	3	4	4
4	4	4	4	4	4	3	3	4	4
3	3	3	3	4	4	3	3	4	4
4	4	4	4	4	4	3	3	4	4
4	4	4	4	4	4	3	3	1	1
4	4	4	4	4	4	3	3	4	4
3	3	3	3	3	3	3	3	4	4
4	4	4	4	4	4	4	4	4	4
3	3	3	3	3	3	3	3	4	4
4	4	4	4	4	4	3	3	4	4
4	4	4	4	4	4	3	3	4	4
3	3	3	3	3	3	3	3	4	4
4	4	3	3	3	3	3	3	4	4
4	4	4	4	4	4	3	3	4	4
4	4	4	4	4	4	3	3	4	4
4	4	4	4	4	4	3	3	4	4
4	4	4	4	4	4	3	3	4	4
4	4	3	3	3	3	3	3	4	4
4	4	4	4	4	4	4	4	1	1
4	4	4	4	4	4	3	3	4	4
4	4	4	4	4	4	3	3	4	4
4	4	3	3	3	3	3	3	4	4
4	4	4	4	4	4	3	3	4	4
4	4	4	4	4	4	3	3	4	4
4	4	4	4	4	4	3	3	4	4
4	4	4	4	4	4	3	3	1	1
4	4	4	4	4	4	3	3	4	4
4	4	4	4	4	4	3	3	4	4
3	3	4	4	4	4	3	3	4	4
4	4	4	4	4	4	3	3	4	4
4	4	4	4	4	4	3	3	4	4
3	3	3	3	3	3	3	3	4	4
4	4	4	4	4	4	3	3	1	1
4	4	3	3	3	3	3	3	4	4
4	4	4	4	4	4	3	3	4	4
4	4	4	4	4	4	3	3	4	4
4	4	4	4	4	4	3	3	4	4

For Review Only

4	4	4	4	4	4	4	4	1	1
4	4	4	4	4	4	3	3	4	4

For Review Only

SNP1586	SNP1586	SNP1587	SNP1587	SNP1588	SNP1588	SNP1589	SNP1589	SNP159	SNP159
2	2	4	4	4	4	2	2	1	1
2	2	4	4	4	4	2	2	1	1
2	2	4	4	4	4	2	2	1	1
2	2	4	4	4	4	2	2	1	1
2	2	4	4	4	4	2	2	1	1
2	2	4	4	4	4	2	2	1	1
3	3	1	1	3	3	3	3	1	1
2	2	1	1	3	3	3	3	1	1
2	2	4	4	4	4	3	3	1	1
2	2	4	4	4	4	2	2	1	1
2	2	4	4	3	3	2	2	1	1
2	2	4	4	4	4	2	2	1	1
2	2	4	4	4	4	3	3	1	1
2	2	4	4	4	4	2	2	1	1
2	2	4	4	4	4	2	2	1	1
2	2	4	4	4	4	2	2	1	1
3	3	1	1	3	3	3	3	2	2
2	2	4	4	4	4	2	2	1	1
2	2	4	4	4	4	2	2	2	2
2	2	1	1	3	3	3	3	1	1
2	2	4	4	4	4	2	2	1	1
3	3	1	1	3	3	3	3	1	1
2	2	4	4	4	4	2	2	1	1
2	2	4	4	4	4	2	2	1	1
2	2	4	4	4	4	2	2	1	1
2	2	4	4	4	4	2	2	1	1
2	2	4	4	4	4	2	2	1	1
2	2	4	4	4	4	2	2	1	1
2	2	4	4	4	4	2	2	1	1
2	2	4	4	4	4	2	2	1	1
2	2	4	4	4	4	2	2	2	2
2	2	4	4	3	3	2	2	1	1
2	2	4	4	3	3	2	2	1	1
2	2	4	4	4	4	2	2	1	1
2	2	4	4	4	4	2	2	1	1
2	2	4	4	4	4	2	2	1	1
2	2	4	4	4	4	2	2	1	1
2	2	4	4	4	4	2	2	1	1
2	2	4	4	4	4	2	2	1	1
2	2	4	4	4	4	2	2	1	1
2	2	4	4	4	4	2	2	1	1
2	2	4	4	4	4	2	2	1	1
2	2	4	4	4	4	2	2	1	1
2	2	4	4	4	4	2	2	1	1
2	2	4	4	4	4	2	2	1	1
2	2	4	4	4	4	2	2	1	1
2	2	4	4	4	4	2	2	1	1
2	2	4	4	3	3	2	2	1	1
3	3	1	1	3	3	3	3	1	1
2	2	4	4	4	4	2	2	1	1
2	2	4	4	4	4	2	2	1	1
2	2	4	4	4	4	2	2	1	1

For Review Only

2	2	4	4	4	4	2	2	1	1
2	2	4	4	3	3	2	2	1	1
2	2	4	4	4	4	2	2	2	2
3	3	1	1	3	3	3	3	2	2
2	2	4	4	4	4	2	2	2	2
2	2	4	4	4	4	2	2	1	1
2	2	4	4	4	4	2	2	1	1
2	2	4	4	4	4	2	2	1	1
2	2	4	4	4	4	2	2	1	1
2	2	4	4	4	4	2	2	1	1
2	2	4	4	4	4	2	2	1	1
2	2	4	4	4	4	2	2	1	1
2	2	4	4	4	4	2	2	1	1
2	2	4	4	4	4	2	2	1	1
2	2	4	4	4	4	2	2	1	1
2	2	4	4	4	4	2	2	1	1
2	2	4	4	4	4	2	2	2	2
2	2	4	4	4	4	2	2	1	1
3	3	1	1	3	3	3	3	1	1
3	3	1	1	3	3	3	3	1	1
2	2	4	4	4	4	2	2	1	1
2	2	4	4	4	4	2	2	1	1
3	3	4	4	3	3	2	2	1	1
2	2	4	4	3	3	2	2	1	1
2	2	4	4	4	4	2	2	1	1
2	2	4	4	3	3	2	2	2	2
2	2	4	4	3	3	2	2	1	1
2	2	4	4	4	4	2	2	1	1
2	2	4	4	4	4	2	2	1	1
2	2	4	4	4	4	2	2	1	1
2	2	1	1	3	3	3	3	1	1
2	2	4	4	4	4	2	2	1	1
2	2	4	4	4	4	2	2	1	1
2	2	4	4	4	4	2	2	1	1
2	2	4	4	4	4	2	2	1	1
2	2	4	4	4	4	2	2	1	1
2	2	4	4	3	3	3	3	1	1
2	2	4	4	4	4	2	2	1	1
2	2	4	4	3	3	2	2	1	1
2	2	4	4	4	4	2	2	1	1
3	3	1	1	3	3	3	3	2	2
2	2	4	4	4	4	2	2	1	1
2	2	4	4	4	4	2	2	1	1
2	2	4	4	4	4	2	2	1	1
2	2	1	1	3	3	3	3	2	2
2	2	4	4	4	4	2	2	2	2
2	2	4	4	4	4	2	2	1	1
2	2	4	4	4	4	2	2	1	1
2	2	4	4	4	4	2	2	1	1
2	2	4	4	4	4	2	2	1	1
2	2	4	4	4	4	2	2	2	2
2	2	4	4	4	4	2	2	1	1
2	2	4	4	4	4	2	2	1	1
2	2	4	4	4	4	2	2	1	1
2	2	4	4	4	4	2	2	1	1

For Review Only

2	2	4	4	4	4	2	2	1	1
2	2	4	4	4	4	2	2	1	1
2	2	4	4	4	4	2	2	1	1
2	2	4	4	4	4	2	2	1	1
2	2	4	4	4	4	2	2	1	1
2	2	4	4	4	4	2	2	2	2
2	2	4	4	4	4	2	2	1	1
2	2	4	4	4	4	2	2	2	2
2	2	4	4	4	4	2	2	1	1
2	2	4	4	4	4	2	2	1	1
2	2	4	4	4	4	2	2	1	1
2	2	4	4	4	4	2	2	1	1
2	2	4	4	4	4	2	2	1	1
2	2	4	4	4	4	2	2	1	1
2	2	4	4	4	4	2	2	1	1
2	2	4	4	4	4	2	2	2	2
2	2	4	4	4	4	2	2	1	1
2	2	4	4	4	4	2	2	1	1
2	2	4	4	4	4	2	2	1	1
2	2	4	4	4	4	2	2	1	1
2	2	4	4	4	4	2	2	1	1
2	2	4	4	4	4	2	2	1	1
2	2	4	4	4	4	2	2	1	1
2	2	4	4	4	4	2	2	1	1
2	2	4	4	4	4	2	2	2	2
2	2	4	4	4	4	2	2	1	1
2	2	4	4	4	4	2	2	1	1
2	2	4	4	4	4	2	2	1	1
2	2	4	4	4	4	2	2	1	1
2	2	4	4	4	4	2	2	1	1
2	2	4	4	4	4	2	2	1	1
2	2	4	4	4	4	2	2	1	1
3	3	1	1	3	3	3	3	2	2
2	2	4	4	4	4	2	2	1	1
2	2	4	4	4	4	2	2	1	1
2	2	4	4	4	4	2	2	1	1
2	2	4	4	4	4	2	2	1	1
2	2	4	4	4	4	2	2	1	1
2	2	4	4	4	4	2	2	1	1
2	2	4	4	4	4	2	2	1	1
2	2	4	4	4	4	2	2	1	1
2	2	4	4	4	4	2	2	1	1
2	2	4	4	4	4	2	2	1	1
2	2	4	4	4	4	2	2	1	1
2	2	4	4	4	4	2	2	1	1
2	2	4	4	4	4	2	2	1	1
2	2	4	4	4	4	2	2	2	2
2	2	4	4	4	4	2	2	1	1
2	2	4	4	4	4	2	2	1	1
2	2	4	4	4	4	2	2	1	1
2	2	4	4	4	4	2	2	1	1
2	2	4	4	4	4	2	2	1	1
2	2	4	4	4	4	2	2	1	1
2	2	4	4	4	4	2	2	1	1
2	2	4	4	4	4	2	2	1	1

For Review Only

2	2	4	4	4	4	2	2	2	2
2	2	4	4	4	4	2	2	2	2
2	2	4	4	4	4	2	2	1	1
2	2	4	4	4	4	2	2	1	1
2	2	4	4	4	4	2	2	1	1
2	2	4	4	4	4	2	2	1	1
2	2	4	4	4	4	2	2	1	1
2	2	4	4	4	4	2	2	1	1
2	2	4	4	4	4	2	2	1	1
2	2	4	4	4	4	2	2	1	1
2	2	4	4	4	4	2	2	1	1
2	2	4	4	4	4	2	2	1	1
2	2	4	4	4	4	2	2	1	1
2	2	4	4	4	4	2	2	1	1
2	2	4	4	4	4	2	2	1	1
2	2	4	4	3	3	2	2	1	1
2	2	4	4	4	4	2	2	1	1
2	2	4	4	4	4	2	2	1	1
2	2	4	4	4	4	2	2	1	1
2	2	4	4	4	4	2	2	1	1
2	2	4	4	4	4	2	2	1	1
2	2	4	4	4	4	2	2	1	1
2	2	4	4	4	4	2	2	2	2
2	2	4	4	4	4	2	2	1	1
2	2	4	4	4	4	2	2	1	1
2	2	4	4	4	4	2	2	1	1
2	2	4	4	4	4	2	2	1	1
2	2	4	4	4	4	2	2	1	1
2	2	4	4	4	4	2	2	1	1
3	3	1	1	3	3	3	3	1	1
2	2	4	4	4	4	2	2	2	2
2	2	4	4	4	4	2	2	1	1
2	2	4	4	4	4	2	2	1	1
2	2	4	4	4	4	2	2	1	1
2	2	4	4	4	4	2	2	1	1
2	2	4	4	4	4	2	2	1	1
2	2	4	4	4	4	2	2	2	2
2	2	4	4	4	4	2	2	1	1
2	2	4	4	4	4	2	2	1	1
2	2	4	4	4	4	2	2	1	1
2	2	4	4	4	4	2	2	1	1
2	2	4	4	4	4	2	2	2	2
2	2	4	4	4	4	2	2	1	1
2	2	4	4	4	4	2	2	1	1
2	2	4	4	4	4	2	2	1	1
2	2	4	4	4	4	2	2	1	1
2	2	4	4	3	3	2	2	1	1
2	2	4	4	4	4	2	2	1	1
2	2	4	4	4	4	2	2	1	1
2	2	4	4	4	4	2	2	1	1
2	2	4	4	4	4	2	2	1	1
2	2	4	4	4	4	2	2	1	1
2	2	4	4	4	4	2	2	1	1
2	2	4	4	4	4	2	2	1	1
2	2	4	4	4	4	2	2	1	1

For Review Only

2	2	4	4	4	4	2	2	1	1
2	2	4	4	4	4	2	2	1	1
2	2	4	4	4	4	2	2	1	1
2	2	4	4	4	4	2	2	1	1
2	2	1	1	3	3	3	3	2	2
3	3	4	4	4	4	2	2	1	1
2	2	4	4	4	4	2	2	1	1
2	2	4	4	4	4	2	2	1	1
2	2	4	4	4	4	2	2	1	1
2	2	4	4	4	4	2	2	1	1
2	2	4	4	4	4	2	2	1	1
2	2	4	4	4	4	2	2	1	1
2	2	4	4	4	4	2	2	2	2
2	2	4	4	4	4	2	2	1	1
2	2	4	4	4	4	2	2	1	1
2	2	4	4	3	3	2	2	1	1
2	2	4	4	4	4	2	2	1	1
2	2	4	4	4	4	2	2	1	1
2	2	4	4	4	4	2	2	1	1
2	2	4	4	4	4	2	2	1	1
2	2	4	4	4	4	2	2	1	1
2	2	4	4	4	4	2	2	1	1
3	3	1	1	3	3	3	3	1	1
2	2	4	4	4	4	2	2	1	1
2	2	4	4	4	4	2	2	1	1
2	2	4	4	4	4	2	2	2	2
2	2	4	4	4	4	2	2	1	1
2	2	4	4	4	4	2	2	1	1
2	2	4	4	4	4	2	2	1	1
2	2	4	4	4	4	2	2	1	1
2	2	4	4	4	4	2	2	1	1
2	2	4	4	4	4	2	2	1	1
2	2	4	4	4	4	2	2	1	1
2	2	4	4	4	4	2	2	1	1
2	2	4	4	4	4	2	2	2	2
2	2	4	4	4	4	2	2	1	1
2	2	4	4	3	3	2	2	2	2
2	2	4	4	4	4	2	2	1	1
2	2	4	4	4	4	2	2	1	1
2	2	4	4	4	4	2	2	1	1
2	2	4	4	4	4	2	2	1	1
2	2	4	4	4	4	2	2	1	1
2	2	4	4	4	4	2	2	1	1
3	3	1	1	3	3	3	3	2	2
2	2	4	4	4	4	2	2	1	1
2	2	4	4	4	4	2	2	1	1
2	2	4	4	4	4	2	2	1	1
2	2	4	4	4	4	2	2	1	1
2	2	4	4	4	4	2	2	1	1
2	2	4	4	4	4	2	2	1	1
3	3	4	4	3	3	3	3	2	2
2	2	4	4	4	4	2	2	1	1
2	2	4	4	4	4	2	2	1	1
2	2	4	4	4	4	2	2	1	1
2	2	4	4	4	4	2	2	1	1

For Review Only

2	2	4	4	3	3	2	2	1	1
2	2	4	4	4	4	2	2	2	2

For Review Only

SNP1590	SNP1590	SNP1591	SNP1591	SNP1592	SNP1592	SNP1593	SNP1593	SNP1594	SNP1594
4	4	3	3	3	3	3	3	2	2
3	3	3	3	3	3	2	2	2	2
4	4	2	2	3	3	2	2	2	2
4	4	2	2	3	3	2	2	2	2
4	4	2	2	3	3	2	2	2	2
4	4	3	3	3	3	2	2	2	2
4	4	3	3	3	3	2	2	4	4
4	4	2	2	3	3	2	2	4	4
4	4	3	3	3	3	2	2	2	2
4	4	2	2	3	3	2	2	4	4
3	3	2	2	4	4	2	2	2	2
4	4	2	2	3	3	2	2	2	2
4	4	3	3	3	3	2	2	2	2
4	4	2	2	3	3	2	2	2	2
4	4	2	2	3	3	2	2	2	2
4	4	2	2	3	3	2	2	2	2
4	4	3	3	3	3	2	2	2	2
4	4	3	3	3	3	2	2	2	2
4	4	2	2	3	3	3	3	2	2
4	4	2	2	3	3	2	2	2	2
3	3	3	3	3	3	2	2	4	4
4	4	3	3	3	3	2	2	4	4
4	4	2	2	4	4	3	3	2	2
4	4	2	2	3	3	2	2	4	4
4	4	3	3	3	3	2	2	2	2
4	4	2	2	3	3	2	2	2	2
4	4	2	2	3	3	2	2	2	2
4	4	2	2	3	3	2	2	2	2
4	4	3	3	3	3	2	2	2	2
4	4	2	2	3	3	2	2	4	4
4	4	2	2	3	3	3	3	2	2
4	4	2	2	3	3	2	2	2	2
4	4	2	2	3	3	2	2	2	2
4	4	2	2	3	3	2	2	4	4
4	4	2	2	3	3	3	3	2	2
4	4	2	2	3	3	2	2	2	2
4	4	2	2	3	3	3	3	2	2
4	4	2	2	3	3	2	2	2	2
4	4	2	2	3	3	2	2	2	2
4	4	2	2	3	3	3	3	2	2
4	4	2	2	3	3	2	2	4	4
3	3	3	3	3	3	2	2	4	4
4	4	2	2	3	3	2	2	2	2
4	4	2	2	3	3	2	2	4	4
4	4	2	2	3	3	2	2	2	2
4	4	2	2	3	3	2	2	2	2
4	4	2	2	3	3	3	3	2	2
4	4	2	2	3	3	2	2	2	2
4	4	3	3	3	3	2	2	4	4
4	4	2	2	3	3	3	3	2	2
4	4	2	2	4	4	2	2	2	2
4	4	2	2	3	3	3	3	2	2

For Review Only

4	4	3	3	3	3	2	2	2	2
4	4	2	2	3	3	2	2	2	2
4	4	3	3	3	3	2	2	2	2
4	4	3	3	3	3	2	2	4	4
4	4	2	2	3	3	3	3	2	2
4	4	2	2	3	3	3	3	2	2
4	4	2	2	3	3	2	2	2	2
3	3	3	3	3	3	2	2	4	4
4	4	2	2	3	3	3	3	4	4
4	4	2	2	3	3	2	2	2	2
4	4	2	2	3	3	3	3	2	2
4	4	2	2	3	3	3	3	2	2
4	4	3	3	3	3	2	2	2	2
4	4	3	3	3	3	2	2	2	2
4	4	3	3	3	3	2	2	2	2
4	4	2	2	3	3	3	3	2	2
4	4	2	2	4	4	2	2	2	2
4	4	2	2	3	3	2	2	2	2
4	4	3	3	3	3	3	3	2	2
4	4	3	3	4	4	2	2	2	2
4	4	2	2	3	3	3	3	2	2
4	4	2	2	3	3	3	3	2	2
4	4	2	2	3	3	2	2	2	2
4	4	2	2	3	3	2	2	4	4
4	4	2	2	3	3	2	2	2	2
3	3	3	3	3	3	2	2	2	2
4	4	2	2	3	3	2	2	4	4
4	4	2	2	3	3	2	2	4	4
4	4	2	2	3	3	2	2	2	2
4	4	2	2	3	3	3	3	2	2
4	4	3	3	3	3	2	2	2	2
4	4	2	2	3	3	2	2	2	2
4	4	2	2	3	3	2	2	4	4
4	4	3	3	3	3	2	2	2	2
4	4	2	2	4	4	2	2	2	2
4	4	2	2	3	3	2	2	2	2
4	4	2	2	3	3	2	2	4	4
3	3	3	3	3	3	2	2	4	4
3	3	3	3	3	3	2	2	2	2
4	4	2	2	3	3	2	2	4	4
4	4	3	3	3	3	2	2	2	2
4	4	2	2	3	3	3	3	2	2
4	4	2	2	4	4	2	2	2	2
4	4	2	2	3	3	3	3	2	2
4	4	3	3	3	3	3	3	4	4
4	4	3	3	4	4	2	2	2	2
4	4	2	2	3	3	2	2	4	4
4	4	2	2	3	3	2	2	2	2
4	4	2	2	3	3	2	2	2	2
4	4	2	2	3	3	3	3	2	2
4	4	3	3	3	3	3	3	2	2
4	4	2	2	3	3	2	2	4	4
3	3	3	3	3	3	2	2	4	4
4	4	2	2	3	3	2	2	2	2
4	4	2	2	3	3	3	3	2	2

For Review Only

4	4	2	2	3	3	3	3	2	2
3	3	2	2	3	3	3	3	2	2
4	4	2	2	3	3	2	2	2	2
4	4	2	2	3	3	2	2	2	2
4	4	2	2	3	3	2	2	2	2
4	4	2	2	3	3	2	2	2	2
4	4	2	2	3	3	2	2	2	2
4	4	2	2	3	3	2	2	2	2
4	4	2	2	3	3	2	2	4	4
4	4	2	2	3	3	2	2	2	2
4	4	3	3	3	3	3	3	2	2
4	4	2	2	3	3	2	2	2	2
4	4	2	2	3	3	2	2	2	2
3	3	3	3	3	3	2	2	2	2
3	3	3	3	3	3	2	2	2	2
4	4	2	2	3	3	2	2	2	2
4	4	2	2	3	3	2	2	2	2
4	4	2	2	3	3	2	2	2	2
4	4	2	2	3	3	2	2	4	4
4	4	2	2	3	3	2	2	2	2
4	4	2	2	3	3	3	3	2	2
4	4	2	2	3	3	3	3	2	2
4	4	2	2	3	3	3	3	2	2
4	4	2	2	3	3	2	2	2	2
4	4	2	2	3	3	3	3	2	2
4	4	2	2	3	3	2	2	2	2
4	4	2	2	3	3	2	2	2	2
4	4	2	2	3	3	2	2	2	2
4	4	2	2	3	3	3	3	2	2
4	4	2	2	3	3	2	2	2	2
4	4	2	2	3	3	2	2	4	4
4	4	2	2	3	3	2	2	2	2
4	4	3	3	3	3	2	2	2	2
4	4	2	2	3	3	2	2	2	2
4	4	2	2	3	3	2	2	4	4
4	4	2	2	4	4	2	2	4	4
4	4	2	2	3	3	2	2	4	4
4	4	2	2	3	3	3	3	2	2
4	4	2	2	3	3	2	2	2	2
3	3	2	2	3	3	2	2	2	2
4	4	2	2	3	3	2	2	2	2
4	4	2	2	3	3	2	2	4	4
4	4	2	2	4	4	3	3	2	2
4	4	2	2	3	3	2	2	4	4
4	4	2	2	3	3	2	2	2	2
4	4	2	2	3	3	2	2	2	2
4	4	2	2	3	3	2	2	2	2
4	4	2	2	3	3	3	3	2	2
4	4	2	2	3	3	2	2	2	2
4	4	2	2	3	3	2	2	2	2
4	4	2	2	3	3	2	2	2	2
4	4	2	2	3	3	2	2	4	4
4	4	2	2	4	4	3	3	2	2
4	4	2	2	3	3	3	3	2	2
4	4	2	2	3	3	3	3	2	2

For Review Only

4	4	3	3	3	3	3	3	2	2
4	4	2	2	4	4	2	2	2	2
4	4	2	2	3	3	2	2	2	2
4	4	2	2	3	3	2	2	2	2
4	4	3	3	3	3	2	2	2	2
4	4	2	2	4	4	2	2	2	2
4	4	2	2	3	3	2	2	4	4
4	4	2	2	3	3	2	2	2	2
4	4	2	2	3	3	3	3	4	4
4	4	2	2	3	3	2	2	2	2
4	4	2	2	3	3	2	2	2	2
4	4	2	2	3	3	3	3	4	4
4	4	2	2	3	3	3	3	2	2
4	4	2	2	3	3	2	2	2	2
4	4	2	2	3	3	2	2	4	4
3	3	3	3	4	4	2	2	2	2
4	4	2	2	3	3	3	3	2	2
4	4	2	2	3	3	2	2	4	4
4	4	2	2	3	3	2	2	2	2
4	4	2	2	3	3	2	2	2	2
4	4	2	2	3	3	3	3	2	2
4	4	2	2	3	3	2	2	4	4
4	4	2	2	3	3	3	3	2	2
4	4	2	2	3	3	3	3	2	2
3	3	3	3	3	3	2	2	4	4
4	4	2	2	3	3	2	2	4	4
4	4	2	2	3	3	3	3	2	2
4	4	2	2	3	3	3	3	2	2
4	4	2	2	4	4	2	2	2	2
4	4	3	3	3	3	2	2	2	2
4	4	2	2	3	3	2	2	4	4
4	4	2	2	3	3	3	3	2	2
4	4	3	3	3	3	3	3	2	2
4	4	2	2	3	3	2	2	2	2
4	4	2	2	3	3	3	3	2	2
4	4	2	2	3	3	3	3	2	2
4	4	2	2	3	3	3	3	2	2
4	4	2	2	3	3	3	3	2	2
4	4	2	2	3	3	3	3	2	2
4	4	2	2	3	3	2	2	2	2
4	4	2	2	3	3	2	2	2	2
4	4	2	2	3	3	2	2	2	2
4	4	2	2	3	3	2	2	2	2
4	4	2	2	3	3	3	3	2	2
4	4	2	2	4	4	2	2	2	2
4	4	2	2	3	3	3	3	2	2
3	3	3	3	4	4	2	2	2	2
4	4	2	2	3	3	2	2	4	4
4	4	2	2	3	3	2	2	4	4
4	4	2	2	4	4	2	2	4	4
4	4	2	2	3	3	3	3	2	2
4	4	2	2	3	3	2	2	2	2
4	4	2	2	3	3	3	3	2	2
4	4	2	2	3	3	3	3	2	2
4	4	2	2	3	3	2	2	4	4

For Review Only

4	4	2	2	3	3	3	3	4	4
3	3	3	3	3	3	2	2	2	2
4	4	2	2	3	3	3	3	2	2
4	4	2	2	3	3	3	3	2	2
4	4	2	2	4	4	2	2	2	2
4	4	2	2	3	3	2	2	2	2
4	4	2	2	3	3	2	2	2	2
4	4	2	2	3	3	2	2	4	4
4	4	2	2	3	3	2	2	4	4
4	4	3	3	3	3	2	2	4	4
4	4	2	2	3	3	3	3	2	2
4	4	2	2	3	3	2	2	2	2
4	4	2	2	4	4	2	2	2	2
4	4	3	3	3	3	2	2	2	2
4	4	2	2	4	4	2	2	2	2
3	3	3	3	4	4	2	2	2	2
4	4	2	2	3	3	2	2	4	4
4	4	2	2	3	3	2	2	4	4
4	4	2	2	3	3	3	3	2	2
4	4	2	2	3	3	2	2	2	2
4	4	2	2	3	3	3	3	2	2
4	4	2	2	3	3	2	2	2	2
4	4	3	3	3	3	3	3	2	2
4	4	2	2	3	3	2	2	2	2
3	3	3	3	3	3	2	2	4	4
4	4	3	3	3	3	3	3	2	2
4	4	2	2	3	3	2	2	4	4
4	4	2	2	3	3	2	2	2	2
4	4	3	3	3	3	2	2	2	2
4	4	2	2	3	3	3	3	2	2
4	4	2	2	3	3	2	2	4	4
4	4	2	2	3	3	2	2	2	2
4	4	2	2	3	3	2	2	2	2
4	4	2	2	3	3	2	2	2	2
4	4	2	2	4	4	3	3	2	2
4	4	2	2	3	3	3	3	4	4
4	4	2	2	4	4	3	3	2	2
4	4	2	2	3	3	3	3	2	2
4	4	2	2	3	3	2	2	2	2
4	4	2	2	3	3	2	2	4	4
4	4	2	2	3	3	3	3	2	2
4	4	3	3	3	3	2	2	2	2
4	4	2	2	3	3	3	3	2	2
4	4	2	2	3	3	2	2	2	2
4	4	2	2	3	3	2	2	2	2
4	4	2	2	3	3	2	2	2	2
4	4	2	2	3	3	2	2	2	2
4	4	2	2	4	4	3	3	2	2
4	4	3	3	3	3	2	2	2	2
4	4	2	2	3	3	2	2	4	4
4	4	3	3	3	3	3	3	2	2
4	4	2	2	3	3	2	2	2	2
4	4	2	2	3	3	2	2	2	2
4	4	2	2	3	3	3	3	2	2
4	4	2	2	3	3	3	3	2	2

For Review Only

3	3	3	3	4	4	3	3	2	2
4	4	2	2	3	3	2	2	2	2

For Review Only

SNP1595	SNP1595	SNP1596	SNP1596	SNP1597	SNP1597	SNP1598	SNP1598	SNP1599	SNP1599
3	3	2	2	4	4	3	3	2	2
3	3	2	2	4	4	1	1	2	2
3	3	2	2	4	4	3	3	2	2
3	3	2	2	4	4	1	1	2	2
3	3	2	2	4	4	1	1	2	2
3	3	2	2	4	4	3	3	2	2
3	3	2	2	4	4	1	1	2	2
3	3	3	3	3	3	1	1	2	2
3	3	2	2	4	4	1	1	2	2
2	2	3	3	3	3	1	1	1	1
3	3	2	2	4	4	3	3	2	2
3	3	2	2	4	4	3	3	2	2
3	3	2	2	4	4	1	1	2	2
3	3	2	2	4	4	1	1	1	1
3	3	2	2	4	4	1	1	2	2
3	3	2	2	4	4	3	3	2	2
3	3	3	3	3	3	1	1	2	2
3	3	2	2	4	4	1	1	2	2
3	3	2	2	4	4	1	1	2	2
3	3	3	3	3	3	1	1	2	2
3	3	3	3	3	3	1	1	2	2
3	3	2	2	4	4	1	1	2	2
2	2	2	2	4	4	3	3	1	1
3	3	2	2	4	4	1	1	2	2
3	3	2	2	4	4	1	1	2	2
3	3	2	2	4	4	1	1	2	2
2	2	2	2	4	4	1	1	1	1
3	3	2	2	4	4	1	1	2	2
3	3	2	2	4	4	1	1	2	2
2	2	3	3	3	3	1	1	1	1
3	3	2	2	4	4	1	1	2	2
3	3	2	2	4	4	1	1	2	2
3	3	2	2	4	4	1	1	2	2
2	2	2	2	4	4	1	1	1	1
2	2	2	2	4	4	1	1	1	1
3	3	2	2	4	4	1	1	2	2
3	3	2	2	4	4	1	1	2	2
3	3	2	2	4	4	1	1	2	2
2	2	2	2	4	4	1	1	1	1
2	2	2	2	4	4	3	3	1	1
2	2	3	3	3	3	1	1	1	1
3	3	3	3	3	3	1	1	2	2
2	2	2	2	4	4	1	1	2	2
2	2	2	2	4	4	1	1	1	1
2	2	3	3	3	3	1	1	1	1
3	3	2	2	4	4	3	3	2	2
3	3	2	2	4	4	3	3	2	2
3	3	2	2	4	4	1	1	2	2
3	3	2	2	4	4	1	1	2	2
3	3	2	2	4	4	1	1	2	2
3	3	2	2	4	4	3	3	2	2
2	2	2	2	4	4	3	3	1	1

For Review Only

3	3	2	2	4	4	1	1	2	2
3	3	2	2	4	4	1	1	2	2
3	3	2	2	4	4	3	3	2	2
3	3	2	2	4	4	1	1	2	2
3	3	3	3	3	3	1	1	2	2
3	3	2	2	4	4	3	3	2	2
3	3	2	2	4	4	3	3	2	2
3	3	2	2	4	4	1	1	2	2
3	3	2	2	4	4	1	1	2	2
3	3	2	2	4	4	1	1	2	2
3	3	2	2	4	4	3	3	2	2
3	3	2	2	4	4	3	3	2	2
3	3	2	2	4	4	1	1	2	2
3	3	2	2	4	4	1	1	2	2
3	3	2	2	4	4	1	1	2	2
3	3	2	2	4	4	3	3	2	2
3	3	2	2	4	4	3	3	2	2
3	3	2	2	4	4	3	3	2	2
3	3	2	2	4	4	1	1	2	2
3	3	2	2	4	4	3	3	2	2
3	3	2	2	4	4	1	1	2	2
2	2	2	2	4	4	1	1	1	1
3	3	2	2	4	4	1	1	2	2
3	3	3	3	3	3	1	1	2	2
2	2	3	3	3	3	1	1	1	1
3	3	2	2	4	4	3	3	2	2
3	3	2	2	4	4	1	1	2	2
2	2	2	2	4	4	1	1	1	1
3	3	2	2	4	4	3	3	2	2
3	3	2	2	4	4	1	1	2	2
3	3	2	2	4	4	1	1	2	2
3	3	2	2	4	4	3	3	2	2
2	2	2	2	4	4	1	1	1	1
3	3	2	2	4	4	1	1	2	2
3	3	2	2	4	4	1	1	2	2
2	2	3	3	3	3	1	1	1	1
3	3	3	3	3	3	1	1	2	2
3	3	2	2	4	4	1	1	2	2
3	3	2	2	4	4	3	3	2	2
3	3	3	3	3	3	1	1	2	2
3	3	2	2	4	4	1	1	2	2
3	3	2	2	4	4	3	3	2	2
3	3	2	2	4	4	3	3	1	1
3	3	2	2	4	4	3	3	2	2
3	3	2	2	4	4	1	1	2	2
3	3	2	2	4	4	3	3	2	2
2	2	2	2	4	4	1	1	1	1
3	3	2	2	4	4	3	3	2	2
3	3	2	2	4	4	3	3	2	2
3	3	2	2	4	4	1	1	2	2
3	3	2	2	4	4	3	3	2	2
2	2	2	2	4	4	1	1	1	1
3	3	2	2	4	4	1	1	2	2
3	3	2	2	4	4	1	1	2	2
3	3	2	2	4	4	1	1	2	2

For Review Only

3	3	2	2	4	4	3	3	2	2
3	3	3	3	3	3	1	1	2	2
3	3	2	2	4	4	1	1	2	2
3	3	2	2	4	4	1	1	1	1
3	3	2	2	4	4	1	1	2	2
3	3	2	2	4	4	1	1	2	2
3	3	2	2	4	4	3	3	2	2
3	3	2	2	4	4	3	3	2	2
3	3	2	2	4	4	1	1	2	2
3	3	2	2	4	4	1	1	2	2
3	3	2	2	4	4	1	1	2	2
3	3	2	2	4	4	1	1	2	2
2	2	2	2	4	4	3	3	1	1
3	3	2	2	4	4	1	1	2	2
3	3	2	2	4	4	1	1	2	2
3	3	2	2	4	4	1	1	2	2
3	3	2	2	4	4	3	3	2	2
3	3	2	2	4	4	3	3	2	2
2	2	2	2	4	4	1	1	1	1
3	3	2	2	4	4	1	1	2	2
3	3	2	2	4	4	1	1	2	2
3	3	2	2	4	4	1	1	2	2
3	3	2	2	4	4	1	1	2	2
2	2	2	2	4	4	1	1	1	1
3	3	2	2	4	4	1	1	1	1
3	3	2	2	4	4	1	1	2	2
3	3	2	2	4	4	1	1	2	2
2	2	2	2	4	4	1	1	1	1
3	3	2	2	4	4	1	1	2	2
3	3	2	2	4	4	3	3	1	1
2	2	2	2	4	4	1	1	1	1
3	3	2	2	4	4	1	1	2	2
3	3	2	2	4	4	1	1	2	2
3	3	2	2	4	4	1	1	2	2
2	2	2	2	4	4	1	1	1	1
3	3	2	2	4	4	1	1	2	2
2	2	2	2	4	4	1	1	1	1
3	3	2	2	4	4	1	1	2	2
3	3	2	2	4	4	3	3	2	2
3	3	2	2	4	4	1	1	2	2
3	3	2	2	4	4	1	1	2	2
2	2	2	2	4	4	1	1	1	1
3	3	2	2	4	4	1	1	2	2
3	3	3	3	3	3	1	1	2	2
3	3	2	2	4	4	3	3	1	1
3	3	2	2	4	4	1	1	2	2
3	3	2	2	4	4	3	3	2	2
3	3	2	2	4	4	3	3	2	2
3	3	2	2	4	4	1	1	2	2
3	3	2	2	4	4	1	1	2	2
3	3	2	2	4	4	1	1	2	2
3	3	2	2	4	4	1	1	2	2
3	3	2	2	4	4	1	1	2	2
3	3	2	2	4	4	3	3	2	2
3	3	2	2	4	4	1	1	2	2

For Review Only

3	3	2	2	4	4	3	3	2	2
3	3	2	2	4	4	3	3	2	2
2	2	3	3	3	3	1	1	1	1
2	2	2	2	4	4	1	1	2	2
3	3	2	2	4	4	1	1	2	2
2	2	2	2	4	4	3	3	1	1
3	3	2	2	4	4	1	1	1	1
3	3	2	2	4	4	1	1	2	2
3	3	2	2	4	4	1	1	2	2
3	3	2	2	4	4	1	1	2	2
3	3	2	2	4	4	3	3	2	2
3	3	2	2	4	4	1	1	2	2
3	3	2	2	4	4	3	3	2	2
3	3	2	2	4	4	1	1	2	2
2	2	2	2	4	4	1	1	1	1
3	3	2	2	4	4	3	3	2	2
3	3	2	2	4	4	1	1	2	2
3	3	2	2	4	4	1	1	2	2
3	3	2	2	4	4	3	3	2	2
3	3	2	2	4	4	3	3	2	2
3	3	2	2	4	4	1	1	2	2
2	2	2	2	4	4	1	1	1	1
2	2	2	2	4	4	1	1	1	1
3	3	2	2	4	4	3	3	2	2
3	3	3	3	3	3	1	1	2	2
2	2	2	2	4	4	1	1	1	1
3	3	2	2	4	4	3	3	2	2
3	3	2	2	4	4	1	1	2	2
2	2	2	2	4	4	3	3	1	1
3	3	2	2	4	4	1	1	2	2
3	3	2	2	4	4	1	1	2	2
3	3	2	2	4	4	3	3	2	2
3	3	2	2	4	4	1	1	2	2
2	2	2	2	4	4	1	1	1	1
3	3	2	2	4	4	1	1	2	2
3	3	2	2	4	4	3	3	2	2
3	3	2	2	4	4	1	1	2	2
3	3	2	2	4	4	1	1	2	2
3	3	2	2	4	4	3	3	2	2
3	3	2	2	4	4	1	1	2	2
3	3	2	2	4	4	3	3	2	2
3	3	2	2	4	4	3	3	2	2
2	2	2	2	4	4	1	1	1	1
3	3	2	2	4	4	3	3	2	2
3	3	2	2	4	4	1	1	2	2
2	2	2	2	4	4	1	1	1	1
3	3	2	2	4	4	3	3	2	2
2	2	2	2	4	4	1	1	1	1
2	2	2	2	4	4	1	1	1	1
3	3	2	2	4	4	3	3	2	2
3	3	2	2	4	4	1	1	2	2
2	2	2	2	4	4	1	1	1	1
2	2	2	2	4	4	1	1	1	1
3	3	2	2	4	4	3	3	2	2
2	2	2	2	4	4	1	1	1	1

For Review Only

2	2	2	2	4	4	1	1	1	1
3	3	2	2	4	4	3	3	2	2
3	3	2	2	4	4	1	1	2	2
3	3	2	2	4	4	1	1	2	2
3	3	3	3	3	3	1	1	2	2
3	3	2	2	4	4	3	3	2	2
3	3	2	2	4	4	1	1	2	2
2	2	3	3	3	3	1	1	1	1
3	3	2	2	4	4	1	1	2	2
2	2	2	2	4	4	1	1	1	1
3	3	2	2	4	4	1	1	2	2
3	3	2	2	4	4	1	1	2	2
3	3	2	2	4	4	3	3	2	2
3	3	2	2	4	4	1	1	2	2
3	3	2	2	4	4	3	3	2	2
3	3	2	2	4	4	3	3	2	2
3	3	2	2	4	4	1	1	2	2
3	3	2	2	4	4	1	1	2	2
3	3	2	2	4	4	3	3	2	2
3	3	2	2	4	4	3	3	2	2
3	3	2	2	4	4	1	1	2	2
3	3	2	2	4	4	3	3	2	2
3	3	2	2	4	4	1	1	2	2
3	3	2	2	4	4	1	1	2	2
3	3	2	2	4	4	1	1	2	2
3	3	3	3	3	3	1	1	2	2
2	2	2	2	4	4	1	1	1	1
3	3	2	2	4	4	3	3	2	2
3	3	3	3	3	3	1	1	2	2
3	3	2	2	4	4	1	1	2	2
2	2	2	2	4	4	1	1	1	1
3	3	2	2	4	4	1	1	2	2
3	3	2	2	4	4	1	1	2	2
3	3	2	2	4	4	1	1	1	1
2	2	2	2	4	4	3	3	1	1
2	2	2	2	4	4	1	1	1	1
3	3	2	2	4	4	1	1	2	2
3	3	2	2	4	4	3	3	2	2
3	3	2	2	4	4	1	1	2	2
2	2	2	2	4	4	1	1	1	1
3	3	2	2	4	4	1	1	2	2
3	3	2	2	4	4	1	1	2	2
3	3	2	2	4	4	1	1	2	2
3	3	2	2	4	4	1	1	2	2
3	3	2	2	4	4	1	1	2	2
3	3	2	2	4	4	3	3	2	2
3	3	2	2	4	4	3	3	2	2
3	3	2	2	4	4	3	3	2	2
3	3	3	3	3	3	1	1	2	2
2	2	2	2	4	4	1	1	1	1
3	3	2	2	4	4	3	3	2	2
2	2	2	2	4	4	1	1	1	1
3	3	2	2	4	4	3	3	2	2
3	3	2	2	4	4	3	3	2	2
2	2	2	2	4	4	1	1	1	1

For Review Only

3	3	2	2	4	4	1	1	2	2
3	3	2	2	4	4	1	1	2	2

For Review Only

SNP160	SNP160	SNP1600	SNP1600	SNP1601	SNP1601	SNP1602	SNP1602	SNP1603	SNP1603
3	3	1	1	2	2	2	2	4	4
2	2	1	1	2	2	2	2	4	4
3	3	1	1	2	2	3	3	3	3
3	3	1	1	4	4	3	3	3	3
3	3	1	1	4	4	2	2	4	4
3	3	1	1	4	4	2	2	4	4
3	3	1	1	4	4	2	2	4	4
3	3	1	1	2	2	2	2	4	4
3	3	1	1	4	4	2	2	4	4
3	3	1	1	4	4	2	2	4	4
3	3	1	1	4	4	2	2	4	4
3	3	1	1	2	2	3	3	3	3
3	3	1	1	2	2	2	2	4	4
3	3	1	1	4	4	2	2	4	4
3	3	1	1	4	4	2	2	4	4
3	3	1	1	4	4	2	2	4	4
3	3	1	1	4	4	2	2	4	4
3	3	1	1	2	2	2	2	4	4
3	3	1	1	2	2	2	2	4	4
3	3	1	1	4	4	2	2	4	4
3	3	1	1	4	4	2	2	4	4
3	3	1	1	4	4	2	2	4	4
3	3	1	1	4	4	2	2	4	4
3	3	1	1	4	4	2	2	4	4
3	3	1	1	2	2	2	2	4	4
3	3	4	4	4	4	3	3	3	3
3	3	1	1	4	4	3	3	3	3
3	3	1	1	4	4	2	2	4	4
3	3	1	1	4	4	2	2	4	4
3	3	1	1	4	4	2	2	4	4
3	3	1	1	4	4	2	2	4	4
3	3	1	1	2	2	2	2	4	4
3	3	1	1	2	2	2	2	4	4
3	3	1	1	4	4	2	2	4	4
3	3	1	1	4	4	2	2	4	4
3	3	1	1	4	4	3	3	3	3
3	3	1	1	4	4	2	2	4	4
3	3	1	1	4	4	2	2	3	3
2	2	1	1	4	4	2	2	4	4
3	3	1	1	4	4	2	2	4	4
3	3	1	1	4	4	2	2	4	4
3	3	1	1	4	4	2	2	4	4
3	3	1	1	4	4	2	2	4	4
3	3	1	1	4	4	2	2	4	4
3	3	1	1	4	4	2	2	4	4
3	3	1	1	4	4	2	2	4	4
3	3	4	4	4	4	2	2	4	4
3	3	1	1	4	4	2	2	4	4
3	3	1	1	4	4	2	2	4	4
3	3	1	1	4	4	2	2	4	4
3	3	4	4	4	4	2	2	4	4
3	3	1	1	4	4	3	3	4	4

For Review Only

3	3	1	1	4	4	2	2	4	4
3	3	1	1	2	2	2	2	4	4
3	3	1	1	4	4	2	2	4	4
3	3	1	1	4	4	2	2	4	4
3	3	1	1	2	2	2	2	4	4
3	3	1	1	4	4	2	2	4	4
3	3	1	1	4	4	3	3	3	3
2	2	1	1	4	4	2	2	4	4
2	2	1	1	4	4	2	2	4	4
3	3	1	1	4	4	2	2	4	4
3	3	1	1	2	2	3	3	3	3
3	3	1	1	4	4	2	2	4	4
3	3	1	1	4	4	2	2	4	4
3	3	1	1	4	4	2	2	4	4
3	3	1	1	4	4	2	2	4	4
3	3	1	1	4	4	2	2	4	4
3	3	1	1	4	4	2	2	4	4
3	3	1	1	4	4	2	2	4	4
3	3	1	1	4	4	2	2	4	4
3	3	1	1	4	4	2	2	4	4
3	3	1	1	4	4	2	2	4	4
3	3	1	1	4	4	2	2	4	4
3	3	1	1	4	4	2	2	4	4
3	3	1	1	4	4	2	2	4	4
3	3	1	1	4	4	2	2	4	4
3	3	1	1	2	2	2	2	4	4
3	3	1	1	4	4	2	2	4	4
2	2	1	1	4	4	2	2	4	4
3	3	4	4	4	4	3	3	3	3
3	3	1	1	4	4	3	3	3	3
3	3	1	1	2	2	2	2	4	4
3	3	1	1	4	4	2	2	4	4
2	2	1	1	4	4	2	2	4	4
3	3	1	1	4	4	2	2	4	4
3	3	1	1	4	4	2	2	4	4
3	3	1	1	4	4	2	2	4	4
3	3	1	1	2	2	2	2	4	4
3	3	1	1	4	4	2	2	4	4
3	3	1	1	4	4	2	2	4	4
3	3	1	1	4	4	2	2	4	4
3	3	1	1	4	4	2	2	4	4
3	3	1	1	2	2	3	3	3	3
3	3	1	1	4	4	2	2	4	4
3	3	1	1	2	2	3	3	3	3
3	3	1	1	4	4	2	2	4	4
3	3	1	1	2	2	2	2	4	4
3	3	1	1	4	4	2	2	4	4
3	3	4	4	4	4	2	2	4	4
3	3	4	4	4	4	2	2	4	4
3	3	1	1	4	4	2	2	4	4
3	3	1	1	2	2	2	2	4	4
2	2	1	1	4	4	2	2	4	4
3	3	1	1	4	4	2	2	4	4
3	3	1	1	4	4	2	2	4	4
3	3	1	1	4	4	2	2	4	4

For Review Only

3	3	1	1	4	4	3	3	3	3
3	3	1	1	4	4	3	3	3	3
3	3	4	4	4	4	3	3	3	3
3	3	1	1	4	4	2	2	4	4
3	3	4	4	4	4	2	2	4	4
3	3	4	4	4	4	3	3	3	3
3	3	1	1	2	2	2	2	4	4
3	3	1	1	4	4	2	2	4	4
3	3	1	1	4	4	2	2	4	4
3	3	4	4	4	4	2	2	4	4
3	3	1	1	4	4	2	2	4	4
3	3	1	1	4	4	2	2	4	4
3	3	1	1	4	4	2	2	4	4
3	3	1	1	4	4	2	2	4	4
3	3	1	1	4	4	2	2	3	3
3	3	4	4	4	4	2	2	4	4
3	3	1	1	4	4	2	2	4	4
3	3	1	1	4	4	2	2	4	4
3	3	1	1	4	4	2	2	4	4
3	3	1	1	4	4	2	2	4	4
3	3	1	1	4	4	2	2	4	4
3	3	1	1	4	4	3	3	4	4
3	3	1	1	2	2	3	3	4	4
2	2	1	1	4	4	2	2	4	4
3	3	1	1	4	4	2	2	4	4
3	3	1	1	4	4	2	2	4	4
3	3	1	1	4	4	2	2	4	4
3	3	1	1	4	4	2	2	4	4
3	3	1	1	2	2	3	3	3	3
3	3	1	1	4	4	2	2	4	4
2	2	1	1	4	4	2	2	4	4
3	3	1	1	4	4	2	2	4	4
3	3	1	1	4	4	2	2	4	4
3	3	1	1	4	4	2	2	4	4
3	3	1	1	4	4	2	2	4	4
3	3	1	1	4	4	2	2	4	4
3	3	1	1	4	4	2	2	4	4
3	3	1	1	2	2	3	3	4	4
3	3	4	4	4	4	2	2	4	4
2	2	1	1	4	4	2	2	4	4
3	3	1	1	4	4	2	2	4	4
3	3	1	1	4	4	2	2	4	4
3	3	1	1	4	4	2	2	4	4
3	3	1	1	4	4	2	2	4	4
3	3	1	1	4	4	3	3	3	3
3	3	1	1	4	4	2	2	4	4
3	3	4	4	4	4	3	3	3	3
3	3	4	4	4	4	3	3	3	3
2	2	1	1	4	4	2	2	4	4
3	3	4	4	4	4	2	2	4	4
3	3	4	4	4	4	3	3	3	3
3	3	1	1	4	4	2	2	4	4
3	3	1	1	2	2	2	2	4	4
3	3	1	1	4	4	3	3	3	3
2	2	1	1	4	4	2	2	4	4

For Review Only

3	3	1	1	4	4	3	3	3	3
3	3	1	1	4	4	2	2	4	4
3	3	1	1	4	4	2	2	4	4
3	3	1	1	4	4	2	2	4	4
3	3	1	1	2	2	2	2	4	4
3	3	1	1	4	4	2	2	4	4
3	3	1	1	4	4	2	2	4	4
3	3	1	1	4	4	2	2	4	4
3	3	1	1	4	4	2	2	4	4
3	3	1	1	4	4	2	2	4	4
3	3	4	4	4	4	2	2	4	4
3	3	1	1	4	4	2	2	4	4
3	3	1	1	4	4	2	2	4	4
3	3	1	1	4	4	2	2	4	4
3	3	1	1	4	4	2	2	4	4
3	3	1	1	4	4	2	2	4	4
3	3	1	1	4	4	2	2	4	4
3	3	4	4	4	4	2	2	4	4
3	3	4	4	4	4	3	3	3	3
3	3	1	1	2	2	2	2	4	4
3	3	1	1	4	4	2	2	4	4
3	3	1	1	4	4	2	2	4	4
3	3	1	1	4	4	2	2	4	4
3	3	1	1	4	4	2	2	4	4
3	3	1	1	4	4	2	2	4	4
3	3	1	1	4	4	2	2	4	4
3	3	1	1	4	4	2	2	4	4
3	3	1	1	4	4	2	2	4	4
2	2	1	1	4	4	2	2	4	4
3	3	1	1	4	4	2	2	4	4
3	3	1	1	4	4	2	2	4	4
3	3	1	1	4	4	2	2	4	4
3	3	1	1	4	4	2	2	4	4
3	3	1	1	4	4	2	2	4	4
3	3	1	1	4	4	2	2	4	4
3	3	1	1	4	4	2	2	4	4
3	3	1	1	4	4	3	3	3	3
3	3	1	1	4	4	2	2	4	4
3	3	4	4	4	4	3	3	3	3
3	3	1	1	4	4	2	2	4	4
3	3	1	1	4	4	3	3	3	3
3	3	4	4	4	4	3	3	3	3
3	3	1	1	4	4	2	2	4	4
3	3	1	1	4	4	2	2	4	4
3	3	1	1	4	4	2	2	4	4
3	3	1	1	4	4	2	2	4	4
3	3	1	1	4	4	2	2	4	4
3	3	1	1	4	4	2	2	4	4
3	3	1	1	4	4	2	2	4	4
3	3	1	1	4	4	3	3	3	3
3	3	4	4	2	2	2	2	4	4
3	3	1	1	4	4	2	2	4	4
3	3	1	1	4	4	2	2	4	4
3	3	1	1	2	2	3	3	3	3
3	3	1	1	4	4	2	2	4	4

For Review Only

3	3	1	1	4	4	2	2	4	4
3	3	1	1	4	4	2	2	4	4
3	3	1	1	2	2	3	3	3	3
3	3	1	1	4	4	2	2	4	4
3	3	1	1	4	4	2	2	4	4
3	3	1	1	4	4	2	2	4	4
3	3	1	1	4	4	2	2	4	4
2	2	1	1	4	4	2	2	4	4
3	3	1	1	4	4	2	2	4	4
2	2	1	1	4	4	2	2	4	4
3	3	1	1	4	4	2	2	4	4
3	3	1	1	4	4	2	2	4	4
3	3	1	1	4	4	2	2	4	4
3	3	1	1	4	4	2	2	4	4
3	3	1	1	4	4	3	3	3	3
3	3	1	1	4	4	2	2	4	4
3	3	1	1	4	4	2	2	4	4
3	3	1	1	4	4	2	2	4	4
3	3	1	1	4	4	2	2	4	4
3	3	1	1	4	4	3	3	3	3
3	3	1	1	4	4	2	2	4	4
3	3	4	4	4	4	3	3	3	3
3	3	1	1	4	4	2	2	4	4
3	3	1	1	4	4	2	2	4	4
2	2	1	1	4	4	2	2	4	4
3	3	1	1	4	4	2	2	4	4
2	2	1	1	4	4	2	2	4	4
3	3	1	1	4	4	3	3	3	3
3	3	1	1	4	4	2	2	4	4
3	3	1	1	2	2	2	2	4	4
3	3	1	1	4	4	2	2	4	4
3	3	1	1	4	4	2	2	4	4
3	3	1	1	4	4	2	2	4	4
3	3	1	1	4	4	3	3	3	3
3	3	1	1	4	4	2	2	4	4
3	3	1	1	4	4	2	2	4	4
3	3	1	1	2	2	2	2	4	4
3	3	1	1	4	4	2	2	4	4
3	3	1	1	4	4	3	3	3	3
2	2	1	1	4	4	2	2	4	4
3	3	1	1	4	4	2	2	4	4
3	3	1	1	4	4	2	2	4	4
3	3	1	1	4	4	2	2	4	4
3	3	1	1	4	4	2	2	4	4
3	3	1	1	4	4	2	2	4	4
3	3	1	1	4	4	2	2	4	4
3	3	4	4	4	4	3	3	3	3
2	2	1	1	4	4	2	2	4	4
3	3	1	1	2	2	2	2	4	4
2	2	1	1	4	4	2	2	4	4
3	3	1	1	4	4	2	2	4	4
2	2	1	1	4	4	2	2	4	4
3	3	1	1	2	2	3	3	3	3
3	3	1	1	4	4	3	3	3	3
3	3	1	1	4	4	2	2	4	4

For Review Only

3	3	1	1	2	2	2	2	4	4
3	3	4	4	4	4	2	2	4	4

For Review Only

SNP1604	SNP1604	SNP1605	SNP1605	SNP1606	SNP1606	SNP1607	SNP1607	SNP1608	SNP1608
4	4	1	1	1	1	2	2	1	1
4	4	1	1	1	1	2	2	1	1
3	3	4	4	1	1	2	2	1	1
3	3	4	4	1	1	2	2	1	1
4	4	1	1	1	1	2	2	1	1
4	4	1	1	1	1	2	2	1	1
4	4	1	1	2	2	2	2	1	1
4	4	1	1	1	1	2	2	1	1
4	4	1	1	1	1	2	2	1	1
4	4	1	1	2	2	4	4	1	1
4	4	1	1	2	2	2	2	2	2
3	3	4	4	1	1	2	2	1	1
4	4	1	1	1	1	4	4	1	1
4	4	1	1	1	1	2	2	1	1
4	4	1	1	1	1	2	2	1	1
4	4	1	1	1	1	2	2	1	1
4	4	1	1	1	1	2	2	1	1
4	4	1	1	1	1	2	2	1	1
4	4	1	1	1	1	2	2	1	1
4	4	1	1	1	1	2	2	1	1
4	4	1	1	2	2	4	4	1	1
4	4	1	1	1	1	2	2	1	1
4	4	1	1	1	1	2	2	1	1
4	4	1	1	1	1	2	2	1	1
4	4	1	1	1	1	2	2	1	1
3	3	4	4	1	1	2	2	1	1
3	3	4	4	1	1	2	2	1	1
4	4	1	1	1	1	2	2	1	1
4	4	1	1	1	1	2	2	1	1
4	4	1	1	1	1	2	2	1	1
4	4	1	1	1	1	2	2	1	1
4	4	1	1	1	1	2	2	1	1
4	4	1	1	1	1	2	2	1	1
4	4	1	1	1	1	2	2	1	1
4	4	1	1	1	1	2	2	1	1
3	3	4	4	1	1	2	2	1	1
4	4	1	1	1	1	2	2	1	1
3	3	4	4	1	1	2	2	1	1
4	4	1	1	2	2	4	4	1	1
4	4	1	1	1	1	2	2	1	1
4	4	1	1	1	1	2	2	1	1
4	4	1	1	1	1	2	2	1	1
4	4	1	1	1	1	2	2	1	1
4	4	1	1	1	1	2	2	1	1
4	4	1	1	1	1	2	2	1	1
4	4	1	1	1	1	2	2	1	1
4	4	1	1	1	1	2	2	1	1
4	4	1	1	1	1	2	2	1	1
4	4	1	1	1	1	2	2	1	1
4	4	1	1	1	1	2	2	1	1
4	4	1	1	1	1	2	2	1	1
3	3	4	4	1	1	2	2	1	1

For Review Only

4	4	1	1	1	1	2	2	1	1
4	4	1	1	1	1	2	2	1	1
4	4	1	1	1	1	2	2	1	1
4	4	1	1	2	2	4	4	1	1
4	4	1	1	1	1	2	2	1	1
4	4	1	1	1	1	2	2	1	1
3	3	4	4	1	1	2	2	1	1
4	4	1	1	2	2	2	2	1	1
4	4	1	1	1	1	2	2	1	1
4	4	1	1	1	1	2	2	1	1
3	3	4	4	1	1	2	2	1	1
4	4	1	1	1	1	2	2	1	1
4	4	1	1	1	1	2	2	1	1
4	4	1	1	1	1	2	2	1	1
4	4	1	1	1	1	2	2	1	1
4	4	1	1	1	1	2	2	1	1
4	4	1	1	1	1	2	2	1	1
4	4	1	1	1	1	2	2	1	1
4	4	1	1	1	1	2	2	1	1
4	4	1	1	1	1	2	2	1	1
4	4	1	1	1	1	2	2	1	1
4	4	1	1	1	1	2	2	1	1
4	4	1	1	1	1	2	2	1	1
4	4	1	1	1	1	2	2	1	1
4	4	1	1	1	1	2	2	1	1
4	4	1	1	1	1	2	2	1	1
4	4	1	1	1	1	2	2	1	1
4	4	1	1	2	2	4	4	1	1
3	3	4	4	1	1	2	2	1	1
3	3	4	4	1	1	2	2	2	2
4	4	1	1	1	1	2	2	1	1
4	4	1	1	1	1	2	2	1	1
4	4	1	1	2	2	2	2	1	1
4	4	1	1	1	1	2	2	1	1
4	4	1	1	1	1	2	2	2	2
4	4	1	1	1	1	2	2	1	1
4	4	1	1	1	1	2	2	1	1
4	4	1	1	2	2	4	4	1	1
4	4	1	1	1	1	2	2	2	2
4	4	1	1	2	2	2	2	1	1
4	4	1	1	1	1	2	2	1	1
3	3	4	4	1	1	2	2	2	2
4	4	1	1	1	1	2	2	1	1
3	3	4	4	1	1	2	2	1	1
4	4	1	1	2	2	2	2	2	2
4	4	1	1	1	1	2	2	1	1
4	4	1	1	1	1	2	2	1	1
4	4	1	1	1	1	2	2	1	1
4	4	1	1	1	1	2	2	1	1
4	4	1	1	1	1	2	2	1	1
4	4	1	1	1	1	2	2	1	1
4	4	1	1	2	2	2	2	1	1
4	4	1	1	1	1	2	2	1	1
4	4	1	1	1	1	2	2	1	1
4	4	1	1	1	1	2	2	1	1

For Review Only

3	3	4	4	1	1	2	2	1	1
3	3	4	4	1	1	2	2	1	1
3	3	4	4	1	1	2	2	1	1
4	4	1	1	1	1	2	2	1	1
4	4	1	1	1	1	2	2	1	1
3	3	4	4	1	1	2	2	1	1
4	4	1	1	1	1	2	2	1	1
4	4	1	1	1	1	2	2	1	1
4	4	1	1	2	2	2	2	1	1
4	4	4	4	1	1	2	2	1	1
4	4	1	1	1	1	2	2	1	1
4	4	4	4	1	1	2	2	1	1
4	4	1	1	1	1	2	2	1	1
4	4	1	1	1	1	2	2	1	1
4	4	1	1	1	1	2	2	1	1
4	4	1	1	1	1	2	2	1	1
4	4	1	1	1	1	2	2	1	1
3	3	1	1	1	1	2	2	1	1
4	4	1	1	1	1	2	2	1	1
4	4	1	1	1	1	2	2	1	1
4	4	1	1	1	1	2	2	1	1
3	3	4	4	1	1	2	2	2	2
3	3	4	4	1	1	2	2	1	1
4	4	1	1	2	2	2	2	1	1
4	4	1	1	1	1	2	2	1	1
4	4	1	1	1	1	2	2	1	1
4	4	1	1	1	1	2	2	1	1
4	4	1	1	1	1	2	2	1	1
3	3	4	4	1	1	2	2	2	2
4	4	1	1	1	1	2	2	1	1
4	4	1	1	1	1	2	2	1	1
4	4	1	1	1	1	2	2	1	1
4	4	1	1	1	1	2	2	1	1
4	4	1	1	1	1	2	2	1	1
4	4	1	1	1	1	2	2	1	1
4	4	4	4	1	1	2	2	1	1
4	4	1	1	1	1	2	2	1	1
3	3	4	4	1	1	2	2	1	1
4	4	1	1	1	1	2	2	1	1
4	4	1	1	2	2	2	2	2	2
4	4	1	1	1	1	2	2	1	1
4	4	1	1	1	1	2	2	1	1
4	4	1	1	1	1	2	2	1	1
4	4	1	1	1	1	2	2	1	1
3	3	4	4	1	1	2	2	1	1
4	4	1	1	1	1	2	2	1	1
3	3	4	4	1	1	2	2	1	1
3	3	4	4	1	1	2	2	1	1
4	4	1	1	1	1	2	2	1	1
4	4	1	1	1	1	2	2	1	1
3	3	4	4	1	1	2	2	1	1
4	4	1	1	2	2	4	4	1	1
4	4	1	1	1	1	2	2	2	2
3	3	4	4	1	1	2	2	1	1
4	4	1	1	1	1	2	2	1	1

For Review Only

3	3	4	4	1	1	2	2	2	2
4	4	1	1	1	1	2	2	1	1
4	4	1	1	1	1	2	2	1	1
4	4	1	1	1	1	2	2	2	2
4	4	1	1	1	1	2	2	1	1
4	4	1	1	1	1	2	2	1	1
4	4	1	1	1	1	2	2	1	1
4	4	1	1	1	1	2	2	1	1
4	4	1	1	1	1	2	2	1	1
4	4	1	1	1	1	2	2	2	2
4	4	1	1	1	1	2	2	1	1
4	4	1	1	1	1	2	2	1	1
4	4	1	1	1	1	2	2	2	2
4	4	1	1	1	1	2	2	1	1
4	4	1	1	1	1	2	2	1	1
4	4	1	1	2	2	2	2	2	2
4	4	1	1	1	1	2	2	2	2
4	4	1	1	1	1	2	2	1	1
3	3	4	4	1	1	2	2	1	1
4	4	1	1	1	1	2	2	1	1
4	4	1	1	1	1	2	2	1	1
4	4	1	1	1	1	2	2	1	1
4	4	1	1	1	1	2	2	1	1
4	4	1	1	1	1	2	2	1	1
4	4	1	1	1	1	2	2	1	1
4	4	1	1	1	1	2	2	1	1
4	4	1	1	1	1	2	2	1	1
4	4	1	1	1	1	2	2	1	1
4	4	1	1	1	1	2	2	1	1
4	4	1	1	1	1	2	2	1	1
4	4	1	1	2	2	2	2	1	1
4	4	1	1	1	1	2	2	2	2
4	4	1	1	1	1	2	2	1	1
4	4	1	1	1	1	2	2	1	1
4	4	1	1	1	1	2	2	1	1
4	4	1	1	1	1	2	2	1	1
3	3	4	4	1	1	2	2	1	1
4	4	1	1	1	1	2	2	1	1
3	3	4	4	1	1	2	2	1	1
4	4	1	1	1	1	2	2	1	1
3	3	4	4	1	1	2	2	1	1
3	3	4	4	1	1	2	2	1	1
4	4	1	1	2	2	2	2	1	1
4	4	1	1	1	1	2	2	1	1
4	4	1	1	1	1	2	2	1	1
4	4	1	1	1	1	2	2	2	2
4	4	1	1	2	2	2	2	2	2
4	4	1	1	2	2	4	4	1	1
4	4	1	1	1	1	2	2	1	1
3	3	4	4	1	1	2	2	1	1
4	4	1	1	1	1	2	2	2	2
4	4	1	1	1	1	2	2	1	1
4	4	1	1	1	1	2	2	1	1
3	3	4	4	1	1	2	2	1	1
4	4	1	1	2	2	2	2	1	1

For Review Only

4	4	1	1	1	1	2	2	2	2
4	4	1	1	1	1	2	2	1	1
3	3	4	4	1	1	2	2	2	2
4	4	1	1	1	1	2	2	1	1
4	4	1	1	1	1	2	2	1	1
4	4	1	1	1	1	2	2	1	1
4	4	1	1	2	2	2	2	1	1
4	4	1	1	1	1	2	2	1	1
4	4	1	1	1	1	2	2	1	1
4	4	1	1	2	2	4	4	1	1
4	4	1	1	1	1	2	2	1	1
4	4	1	1	1	1	2	2	1	1
4	4	1	1	1	1	2	2	2	2
4	4	1	1	1	1	2	2	1	1
3	3	4	4	1	1	2	2	1	1
4	4	1	1	1	1	2	2	2	2
4	4	1	1	2	2	2	2	1	1
4	4	1	1	1	1	2	2	1	1
4	4	4	4	1	1	2	2	1	1
3	3	4	4	1	1	2	2	1	1
4	4	1	1	1	1	2	2	1	1
3	3	4	4	1	1	2	2	1	1
4	4	1	1	1	1	2	2	1	1
4	4	1	1	1	1	2	2	1	1
4	4	1	1	2	2	2	2	1	1
4	4	1	1	1	1	2	2	1	1
4	4	1	1	2	2	2	2	1	1
4	4	4	4	1	1	2	2	1	1
4	4	1	1	1	1	2	2	1	1
4	4	1	1	1	1	2	2	2	2
4	4	4	4	2	2	4	4	1	1
4	4	1	1	1	1	2	2	1	1
4	4	1	1	1	1	2	2	1	1
3	3	4	4	1	1	2	2	1	1
4	4	1	1	1	1	2	2	1	1
4	4	4	4	2	2	2	2	1	1
4	4	1	1	1	1	2	2	2	2
4	4	1	1	1	1	2	2	1	1
3	3	4	4	1	1	2	2	1	1
4	4	1	1	2	2	2	2	1	1
4	4	1	1	1	1	2	2	1	1
4	4	1	1	1	1	2	2	1	1
4	4	1	1	1	1	2	2	1	1
4	4	1	1	1	1	2	2	1	1
4	4	1	1	1	1	2	2	1	1
4	4	1	1	1	1	2	2	2	2
3	3	4	4	1	1	2	2	1	1
4	4	1	1	1	1	2	2	1	1
4	4	1	1	1	1	2	2	1	1
4	4	1	1	2	2	2	2	1	1
4	4	1	1	1	1	2	2	1	1
4	4	1	1	2	2	2	2	1	1
3	3	4	4	1	1	2	2	1	1
3	3	4	4	1	1	2	2	1	1
4	4	1	1	1	1	2	2	1	1

4	4	1	1	1	1	2	2	2	2
4	4	1	1	1	1	2	2	1	1

For Review Only

SNP1609	SNP1609	SNP161	SNP161	SNP1610	SNP1610	SNP1611	SNP1611	SNP1612	SNP1612
3	3	2	2	1	1	1	1	4	4
2	2	4	4	4	4	1	1	2	2
3	3	2	2	4	4	3	3	4	4
2	2	4	4	4	4	1	1	4	4
2	2	2	2	4	4	1	1	2	2
3	3	4	4	1	1	1	1	4	4
3	3	2	2	4	4	1	1	4	4
3	3	2	2	1	1	1	1	4	4
2	2	2	2	1	1	1	1	4	4
2	2	2	2	4	4	1	1	4	4
2	2	2	2	4	4	1	1	4	4
3	3	4	4	4	4	3	3	4	4
3	3	2	2	4	4	1	1	4	4
2	2	2	2	1	1	1	1	4	4
2	2	2	2	4	4	1	1	4	4
3	3	2	2	4	4	1	1	4	4
3	3	2	2	4	4	1	1	4	4
3	3	2	2	4	4	1	1	4	4
3	3	2	2	4	4	1	1	4	4
3	3	2	2	4	4	1	1	4	4
3	3	2	2	4	4	1	1	2	2
2	2	2	2	4	4	1	1	4	4
2	2	4	4	4	4	1	1	2	2
2	2	2	2	4	4	1	1	4	4
3	3	2	2	4	4	1	1	4	4
2	2	2	2	4	4	1	1	4	4
2	2	4	4	4	4	1	1	2	2
2	2	2	2	4	4	1	1	4	4
2	2	2	2	4	4	1	1	4	4
2	2	2	2	4	4	1	1	2	2
3	3	2	2	1	1	1	1	4	4
3	3	4	4	1	1	1	1	4	4
3	3	2	2	1	1	1	1	4	4
2	2	2	2	4	4	1	1	4	4
2	2	2	2	4	4	1	1	2	2
2	2	2	2	1	1	3	3	2	2
3	3	2	2	1	1	1	1	4	4
3	3	2	2	4	4	1	1	4	4
2	2	4	4	4	4	1	1	2	2
3	3	2	2	4	4	1	1	2	2
2	2	2	2	4	4	1	1	2	2
2	2	2	2	4	4	1	1	2	2
3	3	2	2	1	1	1	1	2	2
2	2	2	2	4	4	1	1	2	2
3	3	2	2	1	1	1	1	2	2
2	2	2	2	4	4	1	1	4	4
2	2	2	2	4	4	1	1	4	4
2	2	2	2	4	4	1	1	4	4
3	3	2	2	4	4	1	1	4	4
2	2	2	2	4	4	1	1	4	4
2	2	2	2	4	4	1	1	4	4
2	2	4	4	4	4	1	1	2	2

For Review Only

3	3	2	2	4	4	1	1	4	4
3	3	2	2	1	1	1	1	4	4
3	3	4	4	4	4	1	1	4	4
2	2	2	2	4	4	1	1	4	4
3	3	2	2	4	4	1	1	4	4
2	2	2	2	4	4	1	1	4	4
2	2	4	4	4	4	1	1	4	4
2	2	2	2	4	4	1	1	2	2
2	2	2	2	4	4	1	1	4	4
2	2	2	2	4	4	1	1	4	4
2	2	2	2	4	4	1	1	4	4
2	2	2	2	4	4	1	1	4	4
3	3	2	2	4	4	1	1	4	4
2	2	2	2	4	4	1	1	4	4
2	2	2	2	4	4	1	1	4	4
3	3	2	2	1	1	1	1	4	4
2	2	4	4	4	4	1	1	4	4
3	3	2	2	4	4	1	1	4	4
2	2	2	2	1	1	1	1	4	4
3	3	2	2	1	1	1	1	4	4
2	2	2	2	4	4	1	1	4	4
2	2	4	4	4	4	1	1	2	2
2	2	2	2	4	4	1	1	4	4
2	2	2	2	4	4	1	1	4	4
3	3	2	2	1	1	1	1	2	2
3	3	4	4	4	4	1	1	4	4
3	3	2	2	1	1	1	1	4	4
2	2	2	2	4	4	1	1	2	2
2	2	2	2	4	4	1	1	4	4
2	2	2	2	1	1	3	3	4	4
3	3	2	2	4	4	1	1	4	4
2	2	2	2	1	1	1	1	4	4
2	2	2	2	4	4	1	1	2	2
3	3	2	2	4	4	1	1	4	4
3	3	2	2	1	1	1	1	4	4
3	3	2	2	1	1	1	1	2	2
3	3	2	2	4	4	1	1	4	4
3	3	2	2	4	4	1	1	2	2
2	2	2	2	4	4	1	1	4	4
2	2	2	2	4	4	1	1	4	4
2	2	2	2	4	4	1	1	4	4
2	2	2	2	1	1	3	3	4	4
3	3	2	2	4	4	1	1	4	4
2	2	2	2	4	4	1	1	4	4
2	2	2	2	4	4	1	1	4	4
2	2	4	4	4	4	1	1	4	4
2	2	2	2	4	4	1	1	4	4
2	2	4	4	4	4	1	1	4	4
2	2	2	2	4	4	1	1	4	4
2	2	2	2	4	4	1	1	4	4
2	2	2	2	1	1	1	1	4	4
2	2	4	4	4	4	1	1	2	2
2	2	2	2	4	4	1	1	2	2
3	3	2	2	4	4	1	1	4	4
3	3	2	2	1	1	1	1	4	4

For Review Only

2	2	4	4	4	4	1	1	4	4
2	2	4	4	1	1	1	1	4	4
2	2	2	2	4	4	1	1	4	4
2	2	2	2	4	4	1	1	4	4
2	2	4	4	4	4	1	1	4	4
2	2	2	2	4	4	1	1	4	4
3	3	2	2	4	4	1	1	4	4
3	3	4	4	4	4	1	1	4	4
2	2	2	2	4	4	1	1	4	4
2	2	2	2	1	1	3	3	4	4
3	3	2	2	4	4	1	1	4	4
2	2	2	2	4	4	1	1	4	4
2	2	2	2	4	4	1	1	2	2
2	2	4	4	4	4	1	1	4	4
2	2	2	2	4	4	1	1	2	2
2	2	2	2	4	4	1	1	4	4
3	3	2	2	4	4	1	1	4	4
2	2	2	2	4	4	1	1	4	4
2	2	2	2	4	4	1	1	4	4
3	3	2	2	4	4	1	1	4	4
2	2	2	2	4	4	1	1	4	4
2	2	2	2	4	4	3	3	4	4
3	3	4	4	4	4	1	1	4	4
2	2	2	2	4	4	1	1	2	2
2	2	2	2	4	4	1	1	4	4
2	2	4	4	4	4	1	1	4	4
2	2	2	2	4	4	1	1	4	4
3	3	2	2	1	1	1	1	2	2
2	2	4	4	4	4	1	1	4	4
2	2	2	2	4	4	1	1	4	4
2	2	2	2	4	4	1	1	4	4
2	2	4	4	4	4	1	1	4	4
2	2	2	2	4	4	1	1	4	4
2	2	2	2	4	4	1	1	4	4
2	2	2	2	4	4	1	1	4	4
2	2	2	2	4	4	1	1	2	2
2	2	2	2	4	4	1	1	4	4
2	2	2	2	1	1	1	1	4	4
2	2	2	2	4	4	1	1	4	4
2	2	2	2	4	4	1	1	4	4
2	2	2	2	4	4	1	1	4	4
2	2	2	2	4	4	1	1	4	4
2	2	2	2	1	1	1	1	4	4
2	2	2	2	4	4	1	1	4	4
2	2	2	2	4	4	1	1	4	4
3	3	2	2	1	1	1	1	4	4
2	2	2	2	4	4	1	1	4	4
2	2	2	2	4	4	1	1	4	4
2	2	2	2	4	4	1	1	4	4
2	2	2	2	4	4	1	1	4	4
2	2	2	2	4	4	3	3	4	4
2	2	2	2	4	4	1	1	4	4
2	2	2	2	4	4	1	1	4	4
2	2	2	2	1	1	3	3	4	4
2	2	4	4	4	4	1	1	4	4

For Review Only

3	3	2	2	4	4	1	1	4	4
2	2	4	4	4	4	1	1	4	4
2	2	2	2	1	1	1	1	2	2
2	2	2	2	4	4	1	1	2	2
2	2	4	4	4	4	1	1	4	4
2	2	2	2	1	1	1	1	2	2
2	2	2	2	4	4	1	1	4	4
2	2	2	2	4	4	1	1	4	4
2	2	4	4	4	4	1	1	4	4
2	2	2	2	1	1	1	1	4	4
2	2	2	2	4	4	1	1	4	4
2	2	2	2	4	4	1	1	4	4
2	2	2	2	4	4	1	1	4	4
2	2	2	2	4	4	1	1	4	4
2	2	2	2	4	4	1	1	4	4
2	2	2	2	4	4	1	1	4	4
2	2	2	2	4	4	1	1	4	4
2	2	4	4	4	4	1	1	4	4
2	2	2	2	4	4	1	1	4	4
3	3	2	2	4	4	1	1	4	4
2	2	2	2	4	4	1	1	4	4
2	2	2	2	4	4	1	1	2	2
2	2	4	4	4	4	1	1	2	2
2	2	2	2	4	4	1	1	4	4
2	2	2	2	4	4	1	1	2	2
2	2	2	2	4	4	1	1	4	4
2	2	4	4	4	4	1	1	4	4
2	2	2	2	4	4	1	1	4	4
3	3	4	4	4	4	1	1	2	2
2	2	2	2	4	4	1	1	4	4
3	3	2	2	4	4	1	1	4	4
3	3	2	2	1	1	1	1	4	4
2	2	2	2	4	4	1	1	4	4
2	2	2	2	4	4	1	1	4	4
2	2	2	2	4	4	1	1	4	4
2	2	2	2	4	4	1	1	4	4
3	3	2	2	4	4	1	1	2	2
3	3	2	2	1	1	1	1	4	4
2	2	2	2	4	4	1	1	4	4
2	2	2	2	4	4	1	1	4	4
3	3	2	2	4	4	3	3	4	4
2	2	2	2	4	4	1	1	4	4
2	2	2	2	4	4	1	1	2	2
2	2	2	2	4	4	3	3	4	4
2	2	4	4	4	4	1	1	4	4
2	2	2	2	1	1	1	1	4	4
2	2	2	2	4	4	1	1	4	4
2	2	2	2	4	4	1	1	2	2
2	2	2	2	4	4	1	1	2	2
2	2	4	4	4	4	1	1	4	4
2	2	4	4	4	4	1	1	4	4
3	3	2	2	1	1	1	1	4	4
2	2	2	2	4	4	1	1	2	2
2	2	2	2	4	4	1	1	4	4
2	2	2	2	4	4	1	1	2	2

For Review Only

2	2	2	2	4	4	1	1	4	4
2	2	2	2	1	1	1	1	2	2
2	2	4	4	4	4	3	3	4	4
2	2	2	2	4	4	1	1	4	4
3	3	2	2	1	1	1	1	4	4
2	2	2	2	4	4	1	1	4	4
2	2	2	2	4	4	1	1	4	4
2	2	2	2	4	4	1	1	2	2
2	2	2	2	4	4	1	1	4	4
2	2	2	2	4	4	1	1	2	2
2	2	2	2	4	4	1	1	4	4
3	3	2	2	1	1	1	1	4	4
2	2	2	2	4	4	1	1	4	4
2	2	2	2	4	4	1	1	4	4
3	3	2	2	4	4	3	3	4	4
2	2	2	2	4	4	1	1	4	4
2	2	4	4	4	4	1	1	4	4
3	3	2	2	4	4	1	1	4	4
3	3	2	2	4	4	1	1	4	4
3	3	2	2	4	4	1	1	4	4
2	2	4	4	4	4	1	1	4	4
2	2	2	2	4	4	1	1	4	4
3	3	2	2	1	1	1	1	4	4
2	2	2	2	4	4	1	1	4	4
2	2	2	2	4	4	1	1	2	2
3	3	2	2	4	4	1	1	4	4
2	2	2	2	4	4	1	1	2	2
2	2	4	4	4	4	1	1	4	4
3	3	2	2	4	4	1	1	4	4
2	2	4	4	4	4	1	1	4	4
2	2	2	2	4	4	1	1	2	2
2	2	2	2	4	4	1	1	4	4
2	2	2	2	4	4	1	1	4	4
2	2	2	2	4	4	1	1	4	4
2	2	2	2	4	4	1	1	2	2
2	2	2	2	4	4	1	1	2	2
2	2	2	2	4	4	1	1	4	4
2	2	2	2	4	4	1	1	4	4
2	2	2	2	1	1	3	3	2	2
2	2	2	2	4	4	1	1	2	2
3	3	2	2	1	1	1	1	4	4
3	3	2	2	4	4	1	1	2	2
2	2	2	2	4	4	1	1	4	4
2	2	2	2	4	4	1	1	4	4
2	2	2	2	4	4	1	1	4	4
2	2	2	2	4	4	1	1	4	4
2	2	2	2	4	4	1	1	4	4
2	2	2	2	4	4	1	1	4	4
2	2	4	4	4	4	1	1	4	4
2	2	2	2	4	4	1	1	2	2
3	3	2	2	4	4	1	1	4	4
2	2	2	2	4	4	1	1	2	2
3	3	2	2	4	4	3	3	4	4
2	2	2	2	4	4	1	1	4	4
2	2	2	2	4	4	1	1	2	2

For Review Only

2	2	2	2	4	4	1	1	4	4
3	3	2	2	4	4	1	1	4	4

For Review Only

SNP1613	SNP1613	SNP1614	SNP1614	SNP1615	SNP1615	SNP1616	SNP1616	SNP1617	SNP1617
2	2	3	3	1	1	4	4	1	1
2	2	3	3	1	1	4	4	4	4
2	2	2	2	1	1	3	3	4	4
2	2	2	2	1	1	3	3	1	1
2	2	3	3	1	1	4	4	4	4
2	2	3	3	1	1	4	4	1	1
2	2	3	3	3	3	4	4	4	4
2	2	3	3	3	3	4	4	1	1
2	2	3	3	1	1	4	4	4	4
2	2	3	3	1	1	4	4	1	1
2	2	3	3	3	3	4	4	4	4
2	2	2	2	1	1	3	3	1	1
2	2	3	3	1	1	4	4	1	1
2	2	3	3	1	1	4	4	1	1
3	3	3	3	1	1	4	4	1	1
3	3	3	3	3	3	4	4	4	4
3	3	3	3	3	3	4	4	4	4
2	2	3	3	1	1	4	4	4	4
3	3	3	3	1	1	4	4	4	4
3	3	3	3	3	3	4	4	1	1
2	2	3	3	1	1	4	4	4	4
2	2	3	3	3	3	4	4	4	4
3	3	3	3	3	3	4	4	1	1
2	2	3	3	1	1	4	4	4	4
3	3	3	3	1	1	4	4	4	4
2	2	2	2	1	1	3	3	1	1
2	2	2	2	3	3	4	4	1	1
2	2	3	3	1	1	4	4	1	1
3	3	3	3	1	1	4	4	4	4
2	2	3	3	3	3	4	4	1	1
2	2	3	3	3	3	4	4	1	1
2	2	3	3	1	1	3	3	1	1
2	2	3	3	1	1	3	3	1	1
2	2	3	3	1	1	4	4	1	1
3	3	2	2	1	1	4	4	4	4
2	2	2	2	1	1	4	4	1	1
2	2	3	3	1	1	4	4	1	1
3	3	3	3	1	1	4	4	1	1
2	2	3	3	1	1	4	4	1	1
3	3	3	3	1	1	4	4	1	1
2	2	3	3	3	3	4	4	1	1
2	2	3	3	1	1	4	4	1	1
2	2	3	3	3	3	4	4	1	1
2	2	3	3	1	1	4	4	4	4
2	2	3	3	3	3	4	4	1	1
2	2	2	2	1	1	3	3	1	1
3	3	2	2	1	1	3	3	4	4
2	2	2	2	1	1	4	4	1	1
2	2	3	3	3	3	4	4	4	4
3	3	3	3	1	1	4	4	1	1
2	2	2	2	1	1	3	3	1	1
2	2	3	3	1	1	4	4	1	1

For Review Only

3	3	3	3	1	1	4	4	4	4
2	2	3	3	1	1	3	3	1	1
3	3	2	2	1	1	3	3	4	4
2	2	3	3	3	3	4	4	4	4
3	3	3	3	1	1	4	4	4	4
2	2	2	2	1	1	3	3	4	4
2	2	2	2	1	1	3	3	1	1
2	2	3	3	1	1	4	4	4	4
2	2	3	3	1	1	4	4	4	4
3	3	3	3	3	3	4	4	1	1
3	3	2	2	1	1	3	3	4	4
3	3	2	2	1	1	3	3	4	4
3	3	3	3	1	1	4	4	4	4
3	3	3	3	1	1	4	4	4	4
2	2	3	3	1	1	4	4	1	1
2	2	3	3	1	1	4	4	1	1
3	3	3	3	1	1	4	4	1	1
3	3	3	3	1	1	4	4	1	1
2	2	3	3	3	3	4	4	4	4
2	2	3	3	3	3	4	4	4	4
3	3	2	2	1	1	3	3	4	4
3	3	3	3	1	1	4	4	4	4
3	3	3	3	3	3	4	4	4	4
2	2	3	3	1	1	3	3	1	1
2	2	3	3	3	3	4	4	1	1
2	2	2	2	1	1	4	4	4	4
2	2	3	3	1	1	3	3	4	4
2	2	3	3	1	1	4	4	4	4
2	2	2	2	1	1	3	3	1	1
2	2	3	3	3	3	4	4	4	4
2	2	3	3	3	3	4	4	4	4
2	2	3	3	1	1	3	3	1	1
2	2	3	3	3	3	4	4	1	1
3	3	3	3	1	1	4	4	4	4
2	2	2	2	1	1	3	3	1	1
2	2	3	3	3	3	4	4	1	1
2	2	3	3	3	3	4	4	4	4
2	2	3	3	3	3	4	4	4	4
2	2	3	3	3	3	4	4	1	1
2	2	3	3	1	1	4	4	1	1
3	3	3	3	3	3	4	4	4	4
2	2	3	3	3	3	4	4	4	4
2	2	3	3	3	3	4	4	1	1
3	3	2	2	1	1	3	3	4	4
2	2	3	3	3	3	4	4	4	4
2	2	3	3	1	1	4	4	4	4
2	2	3	3	1	1	4	4	1	1
2	2	2	2	1	1	3	3	1	1
2	2	2	2	1	1	3	3	1	1
3	3	3	3	1	1	4	4	4	4
2	2	3	3	1	1	4	4	1	1
2	2	3	3	3	3	4	4	4	4
2	2	3	3	1	1	4	4	4	4
2	2	3	3	1	1	4	4	1	1
2	2	3	3	1	1	4	4	4	4

For Review Only

2	2	3	3	1	1	4	4	1	1
2	2	3	3	1	1	4	4	1	1
3	3	2	2	1	1	3	3	1	1
2	2	3	3	1	1	4	4	1	1
2	2	2	2	1	1	3	3	4	4
3	3	2	2	1	1	3	3	1	1
3	3	3	3	3	3	4	4	1	1
2	2	3	3	1	1	4	4	1	1
2	2	3	3	1	1	4	4	1	1
2	2	2	2	1	1	4	4	1	1
2	2	3	3	1	1	4	4	1	1
3	3	2	2	1	1	4	4	1	1
3	3	3	3	3	3	4	4	1	1
2	2	2	2	1	1	4	4	1	1
3	3	3	3	1	1	4	4	1	1
3	3	2	2	1	1	3	3	1	1
3	3	3	3	1	1	4	4	1	1
3	3	2	2	3	3	4	4	1	1
2	2	3	3	1	1	4	4	1	1
3	3	3	3	1	1	4	4	1	1
2	2	2	2	1	1	4	4	1	1
3	3	3	3	3	3	4	4	4	4
2	2	3	3	3	3	4	4	4	4
2	2	3	3	1	1	4	4	1	1
3	3	2	2	1	1	4	4	4	4
3	3	3	3	1	1	4	4	1	1
3	3	3	3	3	3	4	4	1	1
2	2	3	3	1	1	4	4	1	1
2	2	3	3	3	3	4	4	1	1
3	3	2	2	1	1	3	3	4	4
2	2	3	3	1	1	4	4	1	1
2	2	3	3	1	1	4	4	1	1
3	3	3	3	3	3	4	4	4	4
3	3	3	3	1	1	4	4	1	1
2	2	3	3	1	1	4	4	1	1
2	2	3	3	1	1	4	4	4	4
2	2	3	3	1	1	4	4	1	1
2	2	3	3	1	1	4	4	4	4
2	2	2	2	3	3	4	4	1	1
2	2	3	3	3	3	4	4	1	1
3	3	3	3	1	1	4	4	4	4
2	2	3	3	1	1	4	4	1	1
2	2	3	3	1	1	4	4	4	4
2	2	3	3	1	1	4	4	1	1
3	3	2	2	1	1	4	4	1	1
2	2	3	3	1	1	4	4	1	1
2	2	2	2	1	1	3	3	1	1
2	2	2	2	1	1	3	3	4	4
2	2	3	3	1	1	4	4	1	1
3	3	2	2	1	1	3	3	1	1
2	2	2	2	1	1	3	3	1	1
2	2	3	3	1	1	4	4	4	4
3	3	3	3	1	1	4	4	1	1
2	2	2	2	1	1	3	3	4	4
2	2	3	3	1	1	4	4	1	1

For Review Only

3	3	3	3	3	3	4	4	1	1
3	3	3	3	1	1	4	4	1	1
2	2	3	3	3	3	4	4	1	1
3	3	3	3	1	1	4	4	1	1
3	3	3	3	1	1	4	4	4	4
2	2	3	3	1	1	4	4	4	4
2	2	3	3	1	1	4	4	4	4
2	2	2	2	1	1	3	3	1	1
2	2	3	3	1	1	4	4	1	1
2	2	2	2	1	1	3	3	4	4
2	2	2	2	1	1	3	3	1	1
2	2	3	3	1	1	4	4	4	4
2	2	3	3	3	3	4	4	1	1
3	3	3	3	1	1	4	4	1	1
2	2	3	3	1	1	4	4	1	1
2	2	3	3	3	3	4	4	4	4
3	3	3	3	1	1	4	4	1	1
2	2	2	2	1	1	3	3	1	1
3	3	2	2	1	1	3	3	1	1
3	3	3	3	3	3	4	4	1	1
3	3	3	3	1	1	4	4	1	1
2	2	3	3	1	1	4	4	1	1
3	3	3	3	1	1	4	4	1	1
3	3	2	2	1	1	3	3	4	4
2	2	3	3	1	1	4	4	4	4
2	2	3	3	1	1	4	4	1	1
2	2	3	3	1	1	4	4	1	1
3	3	3	3	1	1	4	4	1	1
3	3	3	3	1	1	4	4	1	1
3	3	3	3	3	3	4	4	4	4
2	2	3	3	1	1	4	4	1	1
2	2	3	3	3	3	4	4	4	4
3	3	3	3	1	1	4	4	1	1
2	2	3	3	1	1	4	4	1	1
2	2	3	3	1	1	4	4	1	1
3	3	2	2	1	1	3	3	4	4
3	3	2	2	1	1	4	4	1	1
2	2	2	2	1	1	3	3	1	1
2	2	2	2	1	1	3	3	4	4
2	2	3	3	1	1	4	4	1	1
2	2	2	2	1	1	3	3	4	4
2	2	2	2	1	1	3	3	1	1
2	2	3	3	3	3	4	4	1	1
3	3	3	3	1	1	4	4	4	4
2	2	2	2	1	1	4	4	4	4
2	2	3	3	3	3	4	4	4	4
2	2	3	3	3	3	4	4	4	4
2	2	3	3	1	1	4	4	4	4
2	2	3	3	3	3	4	4	1	1
2	2	3	3	3	3	4	4	1	1
2	2	2	2	1	1	3	3	1	1
2	2	3	3	1	1	4	4	1	1
3	3	3	3	1	1	4	4	4	4
3	3	2	2	1	1	3	3	4	4
2	2	3	3	3	3	4	4	1	1

For Review Only

2	2	3	3	1	1	4	4	1	1
2	2	3	3	3	3	4	4	1	1
2	2	2	2	1	1	3	3	1	1
3	3	3	3	1	1	4	4	1	1
2	2	3	3	3	3	4	4	1	1
2	2	2	2	1	1	4	4	1	1
2	2	2	2	1	1	3	3	1	1
2	2	3	3	3	3	4	4	1	1
2	2	2	2	1	1	4	4	1	1
2	2	3	3	3	3	4	4	1	1
2	2	3	3	1	1	4	4	1	1
2	2	3	3	1	1	4	4	1	1
2	2	3	3	3	3	4	4	1	1
3	3	2	2	1	1	3	3	1	1
2	2	2	2	1	1	3	3	1	1
3	3	3	3	3	3	4	4	1	1
2	2	3	3	1	1	4	4	1	1
2	2	3	3	3	3	4	4	1	1
3	3	3	3	1	1	4	4	1	1
3	3	3	3	1	1	4	4	1	1
2	2	3	3	1	1	4	4	1	1
2	2	2	2	1	1	3	3	1	1
2	2	3	3	3	3	4	4	1	1
3	3	3	3	1	1	4	4	4	4
2	2	3	3	1	1	4	4	4	4
3	3	3	3	1	1	4	4	1	1
2	2	3	3	1	1	4	4	1	1
2	2	2	2	1	1	3	3	1	1
3	3	3	3	1	1	4	4	1	1
2	2	3	3	3	3	4	4	1	1
2	2	3	3	3	3	4	4	1	1
2	2	2	2	1	1	4	4	4	4
3	3	3	3	1	1	4	4	1	1
2	2	2	2	1	1	4	4	1	1
3	3	3	3	1	1	4	4	4	4
2	2	3	3	1	1	4	4	1	1
3	3	3	3	1	1	3	3	4	4
3	3	2	2	1	1	3	3	4	4
2	2	2	2	1	1	4	4	1	1
2	2	3	3	1	1	4	4	4	4
2	2	3	3	1	1	4	4	1	1
3	3	3	3	3	3	4	4	1	1
3	3	3	3	1	1	4	4	4	4
3	3	3	3	1	1	4	4	4	4
3	3	2	2	1	1	3	3	4	4
2	2	3	3	3	3	4	4	1	1
2	2	2	2	1	1	3	3	1	1
2	2	3	3	1	1	4	4	1	1
2	2	3	3	1	1	4	4	4	4
2	2	3	3	1	1	4	4	1	1
3	3	3	3	1	1	3	3	1	1
2	2	3	3	1	1	4	4	4	4
2	2	2	2	1	1	3	3	4	4
2	2	3	3	1	1	4	4	1	1
3	3	3	3	1	1	4	4	4	4

For Review Only

3	3	3	3	3	3	4	4	4	4
3	3	2	2	1	1	3	3	4	4

For Review Only

SNP1618	SNP1618	SNP1619	SNP1619	SNP162	SNP162	SNP1620	SNP1620	SNP1621	SNP1621
1	1	4	4	4	4	1	1	4	4
1	1	4	4	4	4	1	1	4	4
1	1	4	4	1	1	1	1	4	4
1	1	4	4	4	4	1	1	4	4
1	1	1	1	1	1	1	1	4	4
1	1	4	4	4	4	1	1	1	1
1	1	4	4	4	4	1	1	4	4
3	3	4	4	4	4	1	1	4	4
3	3	1	1	4	4	3	3	1	1
3	3	4	4	4	4	1	1	4	4
1	1	4	4	1	1	1	1	4	4
1	1	4	4	4	4	1	1	4	4
1	1	4	4	4	4	1	1	4	4
1	1	4	4	4	4	1	1	4	4
1	1	4	4	4	4	1	1	4	4
1	1	4	4	1	1	1	1	4	4
1	1	1	1	4	4	3	3	1	1
3	3	4	4	4	4	1	1	4	4
1	1	4	4	4	4	1	1	1	1
3	3	4	4	4	4	3	3	4	4
3	3	4	4	4	4	1	1	4	4
1	1	1	1	4	4	1	1	4	4
1	1	4	4	4	4	1	1	1	1
1	1	4	4	1	1	1	1	4	4
1	1	4	4	4	4	1	1	1	1
1	1	4	4	1	1	1	1	4	4
1	1	4	4	1	1	1	1	4	4
1	1	4	4	4	4	1	1	4	4
3	3	4	4	4	4	3	3	1	1
1	1	4	4	4	4	3	3	4	4
1	1	4	4	4	4	1	1	4	4
1	1	4	4	4	4	1	1	4	4
1	1	1	1	4	4	1	1	1	1
1	1	4	4	1	1	1	1	4	4
1	1	4	4	1	1	1	1	1	1
1	1	4	4	4	4	1	1	4	4
1	1	1	1	4	4	1	1	1	1
3	3	4	4	4	4	1	1	4	4
1	1	4	4	4	4	1	1	4	4
1	1	1	1	4	4	1	1	1	1
3	3	4	4	1	1	1	1	4	4
1	1	4	4	4	4	3	3	4	4
3	3	4	4	4	4	1	1	4	4
1	1	4	4	4	4	1	1	4	4
3	3	4	4	4	4	3	3	4	4
1	1	4	4	4	4	1	1	4	4
1	1	4	4	1	1	1	1	4	4
1	1	4	4	4	4	1	1	4	4
1	1	4	4	4	4	1	1	4	4
1	1	4	4	4	4	1	1	1	1
1	1	4	4	4	4	1	1	4	4
1	1	4	4	4	4	1	1	4	4

1	1	1	1	4	4	1	1	1	1
1	1	1	1	4	4	1	1	1	1
1	1	4	4	4	4	1	1	4	4
1	1	1	1	4	4	1	1	4	4
1	1	4	4	4	4	1	1	1	1
1	1	4	4	4	4	1	1	4	4
1	1	4	4	4	4	1	1	4	4
1	1	4	4	4	4	1	1	4	4
1	1	1	1	4	4	1	1	4	4
1	1	4	4	4	4	1	1	4	4
1	1	4	4	4	4	1	1	1	1
1	1	4	4	4	4	1	1	1	1
1	1	1	1	4	4	1	1	1	1
3	3	4	4	4	4	3	3	1	1
1	1	4	4	4	4	1	1	4	4
1	1	1	1	4	4	1	1	4	4
1	1	4	4	4	4	1	1	4	4
1	1	4	4	4	4	1	1	1	1
1	1	4	4	4	4	1	1	4	4
1	1	1	1	4	4	1	1	4	4
1	1	4	4	4	4	1	1	4	4
1	1	4	4	4	4	1	1	1	1
1	1	4	4	4	4	3	3	1	1
1	1	4	4	4	4	1	1	4	4
3	3	4	4	4	4	3	3	4	4
1	1	4	4	4	4	1	1	4	4
1	1	4	4	1	1	3	3	4	4
1	1	4	4	4	4	1	1	4	4
1	1	4	4	1	1	1	1	4	4
1	1	4	4	1	1	1	1	4	4
3	3	4	4	4	4	3	3	4	4
1	1	4	4	4	4	1	1	4	4
1	1	4	4	4	4	1	1	4	4
1	1	1	1	4	4	1	1	1	1
1	1	4	4	1	1	1	1	4	4
3	3	4	4	4	4	3	3	4	4
3	3	4	4	4	4	1	1	4	4
1	1	1	1	4	4	1	1	4	4
1	1	4	4	4	4	1	1	4	4
3	3	4	4	1	1	1	1	4	4
1	1	1	1	4	4	1	1	1	1
1	1	4	4	4	4	1	1	4	4
1	1	4	4	4	4	1	1	4	4
1	1	4	4	4	4	1	1	1	1
1	1	4	4	4	4	1	1	4	4
1	1	4	4	4	4	1	1	4	4
1	1	4	4	4	4	1	1	4	4
1	1	4	4	4	4	1	1	4	4
1	1	4	4	1	1	1	1	4	4
1	1	4	4	4	4	1	1	4	4
1	1	4	4	4	4	1	1	4	4
1	1	4	4	4	4	1	1	4	4
1	1	4	4	4	4	1	1	4	4
1	1	4	4	4	4	1	1	4	4
3	3	4	4	4	4	1	1	4	4

For Review Only

1	1	4	4	4	4	1	1	4	4
3	3	4	4	4	4	1	1	4	4
1	1	4	4	4	4	1	1	4	4
1	1	4	4	4	4	1	1	4	4
1	1	4	4	1	1	1	1	4	4
1	1	4	4	4	4	1	1	4	4
1	1	4	4	4	4	1	1	4	4
1	1	4	4	4	4	1	1	4	4
1	1	4	4	1	1	1	1	4	4
1	1	4	4	4	4	1	1	4	4
1	1	4	4	4	4	1	1	4	4
1	1	4	4	4	4	1	1	4	4
1	1	4	4	4	4	1	1	4	4
1	1	4	4	4	4	1	1	4	4
1	1	4	4	1	1	1	1	4	4
1	1	4	4	4	4	1	1	4	4
1	1	4	4	4	4	1	1	4	4
1	1	4	4	4	4	1	1	4	4
1	1	4	4	4	4	1	1	4	4
1	1	4	4	4	4	1	1	1	1
1	1	4	4	4	4	1	1	4	4
1	1	4	4	4	4	1	1	4	4
1	1	4	4	4	4	1	1	4	4
1	1	4	4	4	4	1	1	4	4
1	1	4	4	4	4	1	1	1	1
1	1	4	4	4	4	1	1	1	1
1	1	4	4	4	4	1	1	4	4
1	1	4	4	1	1	1	1	1	1
1	1	4	4	4	4	1	1	4	4
1	1	4	4	4	4	1	1	4	4
1	1	4	4	4	4	1	1	4	4
1	1	4	4	4	4	1	1	4	4
1	1	1	1	4	4	1	1	1	1
1	1	4	4	4	4	1	1	1	1
1	1	4	4	1	1	1	1	4	4
1	1	4	4	4	4	1	1	4	4
1	1	4	4	4	4	1	1	4	4
1	1	4	4	4	4	1	1	4	4
1	1	4	4	4	4	1	1	1	1
1	1	4	4	4	4	1	1	4	4
1	1	4	4	4	4	1	1	4	4
1	1	4	4	4	4	1	1	4	4
1	1	4	4	4	4	1	1	4	4
1	1	4	4	4	4	1	1	4	4
1	1	4	4	4	4	1	1	1	1
1	1	1	1	4	4	1	1	1	1
1	1	4	4	4	4	1	1	4	4
1	1	4	4	1	1	1	1	4	4
1	1	4	4	4	4	1	1	4	4
1	1	4	4	4	4	1	1	4	4
1	1	4	4	4	4	1	1	4	4
1	1	4	4	4	4	1	1	4	4
1	1	4	4	4	4	1	1	1	1
1	1	4	4	1	1	1	1	4	4
1	1	4	4	4	4	1	1	4	4

For Review Only

1	1	4	4	4	4	1	1	4	4
1	1	4	4	4	4	1	1	1	1
1	1	4	4	4	4	3	3	4	4
1	1	4	4	4	4	1	1	4	4
1	1	4	4	4	4	1	1	4	4
1	1	4	4	4	4	1	1	1	1
1	1	4	4	4	4	1	1	4	4
1	1	4	4	4	4	1	1	4	4
1	1	4	4	4	4	1	1	4	4
1	1	4	4	1	1	1	1	1	1
1	1	4	4	4	4	1	1	4	4
1	1	4	4	4	4	1	1	4	4
1	1	4	4	4	4	1	1	4	4
1	1	1	1	4	4	1	1	4	4
1	1	4	4	4	4	1	1	4	4
1	1	4	4	1	1	1	1	4	4
1	1	4	4	4	4	1	1	4	4
1	1	4	4	1	1	1	1	4	4
1	1	4	4	1	1	1	1	4	4
1	1	4	4	4	4	1	1	4	4
1	1	4	4	4	4	1	1	4	4
1	1	4	4	4	4	1	1	4	4
1	1	4	4	4	4	1	1	1	1
1	1	4	4	4	4	1	1	1	1
1	1	4	4	4	4	3	3	4	4
1	1	4	4	1	1	1	1	4	4
1	1	4	4	4	4	1	1	4	4
1	1	4	4	4	4	1	1	4	4
1	1	4	4	4	4	1	1	1	1
1	1	1	1	4	4	1	1	1	1
1	1	4	4	4	4	1	1	4	4
1	1	4	4	4	4	1	1	4	4
1	1	4	4	4	4	1	1	4	4
1	1	4	4	4	4	1	1	4	4
1	1	1	1	4	4	1	1	4	4
1	1	4	4	4	4	1	1	1	1
1	1	4	4	4	4	1	1	1	1
1	1	1	1	4	4	1	1	1	1
1	1	4	4	1	1	1	1	4	4
1	1	4	4	4	4	1	1	4	4
1	1	4	4	1	1	1	1	4	4
1	1	4	4	4	4	1	1	4	4
1	1	4	4	4	4	1	1	4	4
1	1	4	4	4	4	1	1	1	1
1	1	4	4	4	4	1	1	4	4
1	1	4	4	4	4	1	1	1	1
1	1	4	4	1	1	1	1	4	4
1	1	4	4	4	4	1	1	4	4
1	1	4	4	4	4	1	1	4	4
1	1	4	4	4	4	1	1	4	4
1	1	4	4	4	4	1	1	4	4
1	1	4	4	4	4	1	1	1	1
1	1	4	4	4	4	1	1	1	1
1	1	4	4	4	4	1	1	1	1
1	1	4	4	4	4	1	1	4	4

For Review Only

1	1	4	4	4	4	1	1	4	4
3	3	4	4	1	1	1	1	4	4
1	1	4	4	4	4	1	1	4	4
1	1	4	4	4	4	1	1	4	4
3	3	4	4	4	4	3	3	4	4
1	1	4	4	4	4	1	1	4	4
1	1	4	4	1	1	1	1	4	4
3	3	4	4	1	1	1	1	4	4
1	1	4	4	4	4	1	1	4	4
1	1	4	4	4	4	1	1	4	4
1	1	4	4	4	4	1	1	4	4
1	1	4	4	4	4	1	1	1	1
1	1	4	4	4	4	1	1	4	4
1	1	4	4	1	1	1	1	1	1
1	1	4	4	4	4	1	1	4	4
1	1	4	4	1	1	1	1	1	1
1	1	4	4	4	4	1	1	4	4
1	1	4	4	4	4	1	1	4	4
1	1	4	4	4	4	1	1	4	4
3	3	4	4	4	4	1	1	1	1
1	1	4	4	4	4	1	1	4	4
1	1	4	4	4	4	1	1	4	4
1	1	4	4	1	1	1	1	4	4
1	1	4	4	4	4	1	1	1	1
1	1	4	4	4	4	1	1	4	4
1	1	4	4	4	4	1	1	4	4
1	1	4	4	4	4	1	1	4	4
1	1	4	4	4	4	1	1	4	4
1	1	4	4	4	4	3	3	4	4
1	1	4	4	1	1	1	1	4	4
1	1	4	4	4	4	1	1	4	4
1	1	4	4	4	4	1	1	4	4
3	3	4	4	4	4	1	1	4	4
1	1	4	4	1	1	1	1	4	4
1	1	4	4	4	4	1	1	1	1
1	1	4	4	4	4	1	1	4	4
1	1	4	4	4	4	1	1	1	1
1	1	4	4	4	4	1	1	1	1
1	1	4	4	4	4	1	1	4	4
1	1	4	4	4	4	1	1	4	4
1	1	1	1	4	4	1	1	4	4
1	1	4	4	4	4	1	1	4	4
1	1	4	4	4	4	1	1	1	1
1	1	1	1	4	4	1	1	1	1
1	1	4	4	4	4	1	1	1	1
1	1	4	4	4	4	1	1	4	4
1	1	4	4	1	1	1	1	4	4
1	1	4	4	4	4	1	1	4	4
3	3	4	4	4	4	1	1	4	4
1	1	4	4	4	4	1	1	4	4
1	1	4	4	4	4	1	1	4	4
1	1	4	4	4	4	1	1	4	4
1	1	4	4	1	1	1	1	4	4
1	1	4	4	4	4	1	1	4	4
1	1	1	1	4	4	1	1	1	1

For Review Only

1	1	4	4	1	1	1	1	1	1
1	1	4	4	4	4	1	1	4	4

For Review Only

SNP1622	SNP1622	SNP1623	SNP1623	SNP1624	SNP1624	SNP1625	SNP1625	SNP1626	SNP1626
3	3	4	4	4	4	2	2	3	3
2	2	4	4	4	4	4	4	3	3
2	2	4	4	4	4	4	4	3	3
3	3	4	4	4	4	4	4	3	3
3	3	4	4	4	4	4	4	2	2
3	3	4	4	4	4	4	4	2	2
3	3	4	4	4	4	2	2	2	2
2	2	4	4	4	4	4	4	3	3
3	3	4	4	4	4	4	4	3	3
3	3	4	4	4	4	4	4	2	2
2	2	1	1	4	4	4	4	3	3
2	2	4	4	4	4	4	4	3	3
3	3	4	4	4	4	4	4	3	3
3	3	4	4	4	4	4	4	3	3
3	3	4	4	4	4	4	4	3	3
3	3	4	4	4	4	4	4	3	3
3	3	4	4	4	4	4	4	2	2
2	2	4	4	4	4	2	2	3	3
3	3	4	4	4	4	2	2	2	2
3	3	4	4	4	4	2	2	2	2
3	3	4	4	4	4	2	2	2	2
3	3	4	4	4	4	4	4	2	2
3	3	4	4	4	4	4	4	2	2
2	2	4	4	4	4	4	4	3	3
3	3	4	4	4	4	2	2	2	2
2	2	4	4	4	4	4	4	3	3
2	2	4	4	4	4	4	4	3	3
3	3	4	4	4	4	4	4	3	3
3	3	4	4	4	4	4	4	2	2
2	2	4	4	4	4	4	4	3	3
3	3	4	4	4	4	4	4	3	3
3	3	4	4	4	4	4	4	3	3
3	3	4	4	4	4	4	4	2	2
3	3	4	4	4	4	4	4	2	2
3	3	4	4	4	4	4	4	2	2
2	2	4	4	4	4	4	4	3	3
3	3	4	4	1	1	4	4	2	2
3	3	4	4	1	1	4	4	3	3
2	2	4	4	4	4	4	4	3	3
3	3	4	4	1	1	4	4	2	2
2	2	4	4	4	4	4	4	3	3
3	3	4	4	4	4	4	4	2	2
2	2	4	4	4	4	2	2	3	3
2	2	4	4	4	4	4	4	3	3
2	2	4	4	4	4	2	2	3	3
2	2	4	4	4	4	4	4	3	3
3	3	4	4	4	4	4	4	2	2
2	2	4	4	4	4	4	4	3	3
3	3	4	4	1	1	4	4	2	2
3	3	4	4	1	1	4	4	3	3
2	2	4	4	4	4	4	4	3	3
2	2	4	4	4	4	4	4	3	3

For Review Only

3	3	4	4	4	4	2	2	2	2
3	3	4	4	4	4	4	4	2	2
3	3	4	4	4	4	4	4	3	3
3	3	4	4	4	4	4	4	2	2
3	3	4	4	4	4	2	2	2	2
2	2	4	4	4	4	4	4	3	3
2	2	4	4	4	4	4	4	3	3
2	2	4	4	4	4	4	4	3	3
2	2	4	4	1	1	4	4	3	3
3	3	4	4	4	4	4	4	3	3
3	3	4	4	4	4	4	4	2	2
3	3	4	4	4	4	4	4	2	2
3	3	4	4	4	4	2	2	2	2
3	3	4	4	4	4	4	4	2	2
3	3	4	4	4	4	4	4	3	3
3	3	4	4	4	4	2	2	2	2
3	3	4	4	4	4	4	4	2	2
3	3	4	4	4	4	4	4	2	2
3	3	4	4	4	4	4	4	3	3
3	3	4	4	4	4	2	2	3	3
3	3	4	4	4	4	4	4	2	2
3	3	1	1	4	4	4	4	2	2
3	3	4	4	4	4	4	4	2	2
2	2	4	4	4	4	4	4	3	3
2	2	4	4	4	4	2	2	3	3
2	2	4	4	4	4	2	2	3	3
3	3	4	4	4	4	4	4	3	3
2	2	4	4	4	4	4	4	3	3
2	2	4	4	4	4	4	4	3	3
3	3	4	4	4	4	4	4	3	3
2	2	4	4	4	4	2	2	3	3
2	2	4	4	4	4	4	4	3	3
2	2	4	4	4	4	4	4	3	3
3	3	4	4	4	4	2	2	2	2
2	2	4	4	1	1	4	4	3	3
2	2	4	4	4	4	2	2	3	3
2	2	4	4	4	4	2	2	3	3
3	3	4	4	4	4	2	2	2	2
2	2	4	4	4	4	4	4	3	3
2	2	4	4	4	4	4	4	3	3
3	3	4	4	4	4	4	4	2	2
3	3	4	4	4	4	4	4	3	3
2	2	4	4	1	1	4	4	3	3
3	3	4	4	4	4	4	4	2	2
2	2	4	4	4	4	4	4	3	3
2	2	4	4	4	4	4	4	3	3
3	3	4	4	4	4	4	4	2	2
2	2	4	4	4	4	4	4	3	3
2	2	4	4	4	4	4	4	3	3
3	3	4	4	4	4	4	4	3	3
2	2	4	4	4	4	4	4	3	3
2	2	1	1	4	4	4	4	3	3
2	2	4	4	4	4	4	4	3	3
3	3	1	1	4	4	4	4	3	3
2	2	4	4	4	4	4	4	3	3

For Review Only

2	2	4	4	4	4	4	4	3	3
2	2	4	4	4	4	4	4	3	3
3	3	4	4	4	4	4	4	3	3
3	3	4	4	4	4	4	4	2	2
3	3	4	4	1	1	4	4	3	3
3	3	4	4	4	4	4	4	3	3
3	3	4	4	4	4	2	2	3	3
3	3	4	4	4	4	2	2	3	3
2	2	4	4	4	4	4	4	3	3
3	3	4	4	4	4	4	4	3	3
3	3	4	4	1	1	4	4	2	2
3	3	4	4	4	4	4	4	3	3
3	3	4	4	4	4	4	4	3	3
3	3	4	4	4	4	4	4	3	3
3	3	4	4	4	4	4	4	3	3
3	3	4	4	4	4	4	4	3	3
3	3	4	4	4	4	4	4	3	3
3	3	4	4	4	4	4	4	3	3
3	3	4	4	4	4	4	4	2	2
3	3	4	4	4	4	4	4	3	3
3	3	4	4	4	4	4	4	3	3
3	3	4	4	4	4	4	4	3	3
2	2	4	4	1	1	4	4	3	3
2	2	4	4	4	4	4	4	3	3
3	3	4	4	4	4	4	4	3	3
3	3	4	4	4	4	4	4	2	2
3	3	4	4	4	4	4	4	3	3
3	3	4	4	1	1	4	4	2	2
3	3	4	4	4	4	4	4	3	3
2	2	4	4	4	4	4	4	3	3
2	2	4	4	4	4	4	4	3	3
3	3	4	4	4	4	4	4	3	3
3	3	4	4	4	4	4	4	2	2
3	3	4	4	4	4	4	4	3	3
3	3	4	4	4	4	4	4	2	2
2	2	4	4	4	4	4	4	3	3
3	3	4	4	4	4	4	4	2	2
3	3	4	4	4	4	4	4	3	3
3	3	4	4	4	4	4	4	3	3
2	2	4	4	4	4	4	4	3	3
3	3	1	1	4	4	4	4	3	3
3	3	4	4	4	4	4	4	2	2
2	2	4	4	4	4	4	4	3	3
2	2	4	4	4	4	4	4	2	2
3	3	4	4	4	4	4	4	2	2
3	3	4	4	1	1	4	4	2	2
2	2	4	4	4	4	4	4	3	3
3	3	4	4	4	4	4	4	3	3
3	3	4	4	4	4	4	4	3	3
3	3	4	4	4	4	4	4	3	3
2	2	4	4	4	4	4	4	3	3
2	2	1	1	4	4	4	4	3	3
3	3	4	4	4	4	4	4	3	3
3	3	4	4	4	4	4	4	3	3
3	3	4	4	4	4	4	4	3	3

For Review Only

3	3	4	4	4	4	4	4	3	3
3	3	4	4	4	4	4	4	2	2
3	3	4	4	4	4	4	4	3	3
3	3	4	4	4	4	4	4	3	3
3	3	4	4	4	4	4	4	3	3
3	3	4	4	4	4	4	4	2	2
3	3	1	1	4	4	4	4	3	3
3	3	4	4	4	4	4	4	3	3
3	3	4	4	4	4	4	4	3	3
3	3	4	4	4	4	4	4	2	2
2	2	4	4	4	4	4	4	3	3
3	3	4	4	4	4	4	4	3	3
2	2	4	4	4	4	4	4	3	3
3	3	4	4	1	1	4	4	3	3
2	2	4	4	4	4	4	4	3	3
2	2	1	1	4	4	4	4	3	3
3	3	4	4	4	4	4	4	3	3
2	2	4	4	4	4	4	4	3	3
3	3	4	4	4	4	4	4	3	3
3	3	4	4	4	4	4	4	3	3
3	3	4	4	4	4	4	4	3	3
2	2	4	4	4	4	4	4	3	3
3	3	4	4	4	4	4	4	2	2
3	3	4	4	4	4	4	4	2	2
2	2	4	4	4	4	4	4	3	3
3	3	4	4	4	4	4	4	2	2
2	2	4	4	4	4	4	4	3	3
3	3	4	4	4	4	4	4	3	3
3	3	4	4	1	1	4	4	2	2
3	3	4	4	4	4	4	4	2	2
2	2	4	4	1	1	4	4	3	3
3	3	4	4	4	4	4	4	3	3
3	3	4	4	4	4	4	4	3	3
3	3	4	4	4	4	4	4	3	3
3	3	4	4	4	4	4	4	2	2
3	3	4	4	4	4	4	4	2	2
3	3	4	4	1	1	4	4	2	2
3	3	4	4	4	4	2	2	2	2
2	2	4	4	4	4	4	4	3	3
3	3	4	4	4	4	4	4	3	3
2	2	4	4	4	4	4	4	3	3
2	2	4	4	4	4	4	4	3	3
2	2	4	4	4	4	4	4	3	3
3	3	4	4	4	4	4	4	2	2
3	3	4	4	4	4	4	4	3	3
3	3	4	4	4	4	4	4	2	2
3	3	1	1	4	4	4	4	3	3
2	2	4	4	4	4	4	4	3	3
3	3	4	4	4	4	4	4	3	3
2	2	4	4	4	4	4	4	3	3
3	3	4	4	4	4	4	4	3	3
3	3	4	4	1	1	4	4	2	2
3	3	1	1	4	4	4	4	2	2
3	3	4	4	4	4	4	4	2	2
3	3	4	4	4	4	4	4	3	3

For Review Only

2	2	4	4	4	4	4	4	3	3
3	3	4	4	4	4	4	4	3	3
3	3	4	4	4	4	4	4	3	3
3	3	4	4	4	4	4	4	3	3
2	2	4	4	4	4	2	2	3	3
2	2	4	4	4	4	4	4	3	3
3	3	4	4	1	1	4	4	3	3
2	2	4	4	4	4	4	4	3	3
2	2	4	4	4	4	4	4	3	3
2	2	4	4	4	4	4	4	3	3
3	3	4	4	4	4	4	4	3	3
3	3	4	4	1	1	4	4	2	2
2	2	4	4	4	4	4	4	3	3
3	3	4	4	4	4	4	4	2	2
2	2	4	4	4	4	4	4	3	3
3	3	4	4	4	4	4	4	2	2
2	2	4	4	4	4	4	4	3	3
3	3	4	4	4	4	2	2	2	2
3	3	4	4	4	4	2	2	3	3
3	3	4	4	4	4	4	4	2	2
3	3	4	4	4	4	4	4	3	3
2	2	4	4	4	4	4	4	3	3
3	3	4	4	4	4	2	2	3	3
3	3	1	1	4	4	4	4	2	2
2	2	4	4	4	4	4	4	3	3
3	3	4	4	1	1	4	4	3	3
2	2	4	4	4	4	4	4	3	3
2	2	4	4	4	4	4	4	3	3
3	3	4	4	4	4	2	2	3	3
3	3	4	4	4	4	4	4	3	3
2	2	4	4	4	4	4	4	3	3
2	2	1	1	4	4	4	4	3	3
3	3	4	4	4	4	4	4	3	3
3	3	4	4	4	4	4	4	3	3
3	3	1	1	4	4	4	4	2	2
2	2	4	4	4	4	4	4	3	3
3	3	4	4	4	4	4	4	2	2
3	3	4	4	4	4	4	4	2	2
2	2	4	4	4	4	4	4	3	3
2	2	1	1	4	4	4	4	3	3
2	2	4	4	4	4	4	4	3	3
3	3	4	4	1	1	4	4	3	3
3	3	1	1	4	4	4	4	2	2
3	3	4	4	4	4	4	4	2	2
3	3	4	4	4	4	4	4	2	2
2	2	4	4	4	4	4	4	3	3
2	2	1	1	4	4	4	4	3	3
3	3	4	4	4	4	4	4	3	3
2	2	4	4	4	4	4	4	3	3
2	2	4	4	4	4	4	4	3	3
3	3	4	4	1	1	4	4	3	3
2	2	1	1	4	4	4	4	3	3
2	2	4	4	4	4	4	4	3	3
2	2	4	4	4	4	4	4	3	3
3	3	1	1	4	4	4	4	2	2

For Review Only

3	3	4	4	4	4	4	4	2	2
3	3	4	4	1	1	4	4	3	3

For Review Only

SNP1627	SNP1627	SNP1628	SNP1628	SNP1629	SNP1629	SNP163	SNP163	SNP1630	SNP1630
3	3	3	3	2	2	3	3	2	2
2	2	3	3	2	2	3	3	2	2
3	3	3	3	3	3	3	3	2	2
2	2	3	3	2	2	3	3	2	2
2	2	2	2	2	2	2	2	2	2
3	3	3	3	2	2	2	2	2	2
3	3	3	3	2	2	3	3	3	3
3	3	3	3	2	2	2	2	2	2
2	2	3	3	2	2	2	2	3	3
2	2	3	3	2	2	2	2	3	3
2	2	3	3	2	2	2	2	2	2
3	3	3	3	2	2	3	3	2	2
3	3	3	3	2	2	2	2	2	2
2	2	3	3	2	2	2	2	2	2
2	2	3	3	2	2	2	2	2	2
3	3	3	3	2	2	3	3	2	2
3	3	2	2	2	2	2	2	3	3
3	3	3	3	2	2	3	3	2	2
3	3	3	3	2	2	2	2	2	2
3	3	3	3	3	3	3	3	2	2
3	3	3	3	2	2	2	2	3	3
2	2	3	3	2	2	2	2	3	3
2	2	3	3	2	2	2	2	3	3
2	2	3	3	2	2	2	2	2	2
3	3	3	3	2	2	2	2	3	3
2	2	3	3	2	2	3	3	2	2
2	2	3	3	2	2	2	2	2	2
2	2	3	3	2	2	3	3	2	2
2	2	3	3	2	2	3	3	3	3
2	2	3	3	2	2	2	2	2	2
2	2	3	3	2	2	2	2	2	2
3	3	3	3	2	2	3	3	2	2
3	3	2	2	2	2	2	2	2	2
2	2	3	3	2	2	2	2	2	2
2	2	3	3	2	2	3	3	2	2
2	2	3	3	2	2	2	2	2	2
2	2	2	2	2	2	2	2	2	2
2	2	3	3	2	2	3	3	3	3
2	2	3	3	2	2	2	2	3	3
2	2	2	2	2	2	3	3	3	3
2	2	3	3	2	2	2	2	2	2
2	2	3	3	2	2	3	3	3	3
3	3	3	3	2	2	2	2	2	2
2	2	3	3	2	2	3	3	2	2
3	3	3	3	2	2	2	2	2	2
2	2	3	3	2	2	3	3	2	2
2	2	3	3	2	2	3	3	2	2
2	2	3	3	2	2	3	3	2	2
2	2	3	3	2	2	2	2	2	2
2	2	3	3	2	2	2	2	2	2
2	2	3	3	2	2	3	3	2	2
2	2	3	3	3	3	3	3	2	2

For Review Only

3	3	2	2	2	2	2	2	3	3
3	3	2	2	2	2	2	2	2	2
3	3	3	3	2	2	2	2	2	2
2	2	3	3	2	2	2	2	2	2
3	3	3	3	2	2	2	2	2	2
2	2	3	3	2	2	3	3	2	2
2	2	3	3	2	2	2	2	2	2
2	2	3	3	2	2	2	2	2	2
2	2	2	2	2	2	3	3	2	2
2	2	3	3	2	2	3	3	2	2
2	2	3	3	2	2	3	3	2	2
2	2	3	3	2	2	3	3	2	2
3	3	2	2	2	2	2	2	3	3
2	2	3	3	2	2	3	3	3	3
2	2	3	3	2	2	3	3	2	2
3	3	2	2	2	2	3	3	2	2
2	2	3	3	2	2	3	3	3	3
3	3	3	3	2	2	3	3	2	2
2	2	3	3	2	2	2	2	2	2
3	3	2	2	2	2	2	2	2	2
2	2	3	3	2	2	3	3	2	2
2	2	3	3	2	2	2	2	3	3
2	2	3	3	2	2	2	2	3	3
2	2	3	3	2	2	2	2	2	2
3	3	3	3	2	2	2	2	2	2
3	3	3	3	2	2	3	3	2	2
3	3	3	3	2	2	2	2	2	2
2	2	3	3	3	3	2	2	3	3
2	2	3	3	2	2	3	3	2	2
2	2	3	3	2	2	3	3	2	2
3	3	3	3	2	2	2	2	2	2
2	2	3	3	2	2	3	3	2	2
2	2	3	3	2	2	2	2	3	3
3	3	2	2	2	2	2	2	3	3
2	2	3	3	2	2	2	2	2	2
3	3	3	3	2	2	2	2	2	2
3	3	3	3	2	2	2	2	2	2
3	3	3	3	2	2	2	2	2	2
2	2	3	3	2	2	2	2	2	2
2	2	3	3	2	2	2	2	3	3
2	2	2	2	2	2	2	2	2	2
2	2	3	3	2	2	2	2	2	2
2	2	3	3	2	2	2	2	2	2
2	2	3	3	2	2	3	3	2	2
2	2	3	3	2	2	3	3	3	3
2	2	3	3	2	2	3	3	2	2
2	2	3	3	2	2	2	2	2	2
2	2	3	3	2	2	2	2	2	2
2	2	3	3	2	2	3	3	2	2
2	2	3	3	2	2	2	2	2	2
2	2	3	3	2	2	3	3	2	2
2	2	3	3	2	2	2	2	3	3
2	2	3	3	2	2	3	3	2	2
2	2	3	3	2	2	2	2	2	2
3	3	3	3	2	2	2	2	2	2

For Review Only

2	2	3	3	2	2	3	3	2	2
2	2	3	3	2	2	2	2	2	2
2	2	3	3	2	2	2	2	2	2
2	2	3	3	2	2	2	2	2	2
2	2	3	3	2	2	2	2	2	2
2	2	3	3	2	2	3	3	2	2
3	3	3	3	2	2	3	3	2	2
2	2	3	3	2	2	2	2	2	2
2	2	3	3	2	2	3	3	3	3
2	2	3	3	2	2	3	3	2	2
2	2	3	3	2	2	2	2	2	2
2	2	3	3	2	2	3	3	2	2
2	2	3	3	2	2	3	3	2	2
2	2	3	3	2	2	3	3	2	2
2	2	3	3	2	2	2	2	2	2
2	2	3	3	2	2	3	3	2	2
3	3	3	3	2	2	3	3	2	2
2	2	3	3	2	2	3	3	2	2
2	2	3	3	2	2	2	2	2	2
3	3	3	3	2	2	2	2	3	3
2	2	3	3	2	2	3	3	2	2
2	2	3	3	2	2	3	3	2	2
2	2	3	3	2	2	2	2	2	2
2	2	3	3	2	2	2	2	2	2
2	2	3	3	2	2	3	3	3	3
2	2	3	3	2	2	2	2	3	3
2	2	2	2	2	2	3	3	2	2
2	2	3	3	2	2	2	2	2	2
2	2	3	3	2	2	2	2	2	2
2	2	3	3	2	2	3	3	2	2
3	3	3	3	2	2	2	2	2	2
2	2	3	3	2	2	3	3	2	2
2	2	2	2	2	2	2	2	3	3
2	2	3	3	2	2	3	3	2	2
2	2	3	3	2	2	2	2	2	2
2	2	3	3	2	2	2	2	2	2
2	2	3	3	2	2	2	2	2	2
3	3	3	3	2	2	3	3	2	2
2	2	3	3	2	2	2	2	2	2
2	2	3	3	2	2	2	2	2	2
2	2	3	3	2	2	3	3	2	2
3	3	3	3	2	2	2	2	2	2
2	2	3	3	2	2	2	2	2	2
2	2	3	3	2	2	2	2	2	2
2	2	3	3	2	2	3	3	2	2
2	2	2	2	2	2	2	2	2	2
2	2	3	3	2	2	3	3	2	2
2	2	3	3	2	2	3	3	2	2
2	2	3	3	2	2	3	3	2	2
2	2	3	3	2	2	3	3	2	2
2	2	3	3	2	2	3	3	2	2
2	2	3	3	2	2	2	2	3	3
2	2	3	3	2	2	2	2	2	2
2	2	3	3	2	2	3	3	2	2
2	2	3	3	2	2	2	2	2	2

For Review Only

3	3	3	3	2	2	3	3	2	2
2	2	3	3	2	2	2	2	3	3
2	2	3	3	2	2	3	3	2	2
2	2	3	3	2	2	2	2	2	2
2	2	3	3	2	2	3	3	2	2
2	2	3	3	2	2	3	3	2	2
2	2	3	3	2	2	2	2	2	2
2	2	3	3	2	2	3	3	2	2
2	2	3	3	2	2	2	2	3	3
2	2	3	3	2	2	3	3	2	2
2	2	3	3	2	2	3	3	2	2
2	2	3	3	2	2	2	2	2	2
2	2	3	3	2	2	3	3	2	2
2	2	3	3	2	2	2	2	2	2
3	3	3	3	2	2	2	2	2	2
2	2	3	3	2	2	2	2	2	2
2	2	3	3	2	2	2	2	2	2
2	2	3	3	2	2	3	3	2	2
2	2	3	3	2	2	3	3	2	2
2	2	3	3	2	2	3	3	2	2
2	2	3	3	2	2	3	3	2	2
2	2	3	3	2	2	2	2	2	2
2	2	3	3	2	2	2	2	3	3
2	2	3	3	2	2	3	3	2	2
2	2	3	3	2	2	3	3	2	2
2	2	3	3	2	2	2	2	2	2
2	2	3	3	2	2	2	2	2	2
2	2	3	3	2	2	3	3	2	2
2	2	3	3	2	2	2	2	2	2
2	2	2	2	2	2	3	3	3	3
2	2	3	3	2	2	2	2	3	3
3	3	3	3	2	2	3	3	2	2
2	2	3	3	2	2	3	3	2	2
3	3	3	3	2	2	2	2	2	2
2	2	3	3	2	2	2	2	2	2
2	2	3	3	2	2	3	3	2	2
2	2	3	3	2	2	2	2	3	3
3	3	2	2	2	2	2	2	2	2
2	2	3	3	2	2	3	3	2	2
2	2	3	3	2	2	2	2	2	2
3	3	3	3	2	2	3	3	2	2
2	2	3	3	2	2	3	3	2	2
2	2	3	3	2	2	2	2	3	3
2	2	3	3	2	2	3	3	3	3
2	2	3	3	2	2	2	2	2	2
2	2	3	3	2	2	3	3	2	2
2	2	3	3	2	2	2	2	2	2
3	3	3	3	2	2	2	2	2	2
2	2	3	3	2	2	2	2	2	2
2	2	3	3	2	2	2	2	2	2
2	2	3	3	2	2	2	2	2	2
2	2	3	3	2	2	2	2	2	2
2	2	3	3	2	2	2	2	3	3
2	2	3	3	2	2	3	3	2	2
2	2	3	3	2	2	2	2	3	3

For Review Only

2	2	3	3	2	2	2	2	2	2
2	2	3	3	2	2	2	2	2	2
2	2	3	3	2	2	3	3	2	2
2	2	3	3	2	2	2	2	2	2
3	3	3	3	2	2	2	2	2	2
2	2	3	3	2	2	3	3	2	2
2	2	3	3	2	2	2	2	3	3
2	2	3	3	2	2	2	2	2	2
2	2	3	3	2	2	3	3	2	2
2	2	3	3	2	2	2	2	2	2
2	2	3	3	2	2	3	3	2	2
2	2	3	3	2	2	2	2	2	2
2	2	3	3	2	2	2	2	2	2
2	2	3	3	2	2	3	3	3	3
3	3	3	3	2	2	3	3	2	2
2	2	3	3	2	2	2	2	3	3
2	2	3	3	2	2	2	2	2	2
3	3	3	3	2	2	2	2	2	2
3	3	3	3	2	2	3	3	2	2
3	3	3	3	2	2	2	2	3	3
2	2	3	3	2	2	2	2	2	2
2	2	3	3	2	2	3	3	2	2
3	3	3	3	2	2	2	2	2	2
2	2	3	3	2	2	2	2	3	3
2	2	3	3	2	2	2	2	2	2
2	2	3	3	2	2	3	3	2	2
2	2	3	3	2	2	2	2	2	2
2	2	3	3	2	2	2	2	2	2
3	3	3	3	2	2	2	2	2	2
2	2	3	3	2	2	2	2	2	2
2	2	3	3	2	2	2	2	2	2
2	2	3	3	2	2	2	2	2	2
2	2	3	3	3	3	3	3	2	2
2	2	3	3	2	2	3	3	2	2
2	2	3	3	2	2	2	2	3	3
2	2	3	3	3	3	2	2	2	2
2	2	3	3	2	2	2	2	2	2
2	2	3	3	2	2	3	3	2	2
2	2	3	3	2	2	2	2	2	2
2	2	3	3	2	2	2	2	3	3
3	3	2	2	2	2	2	2	2	2
2	2	3	3	2	2	2	2	2	2
2	2	3	3	2	2	2	2	3	3
2	2	2	2	2	2	2	2	3	3
2	2	3	3	2	2	2	2	2	2
2	2	3	3	2	2	2	2	2	2
2	2	3	3	2	2	2	2	2	2
2	2	3	3	2	2	3	3	2	2
2	2	3	3	2	2	3	3	2	2
2	2	3	3	2	2	2	2	2	2
2	2	3	3	2	2	2	2	2	2
2	2	3	3	2	2	2	2	3	3
3	3	3	3	2	2	3	3	2	2
2	2	3	3	2	2	2	2	2	2
2	2	2	2	2	2	3	3	3	3

For Review Only

2	2	3	3	2	2	2	2	3	3
2	2	3	3	2	2	2	2	2	2

For Review Only

SNP1631	SNP1631	SNP1632	SNP1632	SNP1633	SNP1633	SNP1634	SNP1634	SNP1635	SNP1635
4	4	1	1	2	2	3	3	2	2
4	4	1	1	2	2	3	3	4	4
4	4	1	1	2	2	3	3	4	4
4	4	1	1	2	2	3	3	4	4
4	4	1	1	2	2	3	3	4	4
4	4	1	1	2	2	3	3	4	4
4	4	1	1	2	2	3	3	4	4
4	4	1	1	2	2	3	3	4	4
4	4	1	1	2	2	3	3	2	2
4	4	1	1	2	2	3	3	4	4
4	4	1	1	2	2	3	3	4	4
4	4	1	1	2	2	3	3	4	4
4	4	1	1	2	2	3	3	4	4
3	3	1	1	3	3	2	2	2	2
4	4	1	1	2	2	2	2	4	4
3	3	1	1	3	3	2	2	2	2
4	4	1	1	2	2	3	3	4	4
4	4	1	1	2	2	3	3	2	2
4	4	1	1	2	2	3	3	4	4
4	4	1	1	2	2	3	3	4	4
4	4	1	1	2	2	3	3	2	2
4	4	1	1	2	2	3	3	4	4
3	3	1	1	3	3	2	2	2	2
4	4	1	1	2	2	3	3	4	4
4	4	1	1	2	2	3	3	4	4
4	4	1	1	2	2	3	3	4	4
4	4	1	1	2	2	3	3	4	4
4	4	1	1	2	2	3	3	4	4
4	4	1	1	2	2	3	3	2	2
4	4	1	1	2	2	3	3	4	4
4	4	1	1	2	2	3	3	4	4
4	4	1	1	2	2	3	3	4	4
4	4	1	1	2	2	3	3	4	4
4	4	1	1	2	2	3	3	2	2
3	3	1	1	3	3	2	2	4	4
4	4	1	1	2	2	3	3	4	4
4	4	1	1	2	2	3	3	4	4
4	4	1	1	2	2	3	3	2	2
3	3	1	1	3	3	2	2	2	2
3	3	1	1	3	3	2	2	2	2
4	4	1	1	2	2	3	3	4	4
4	4	1	1	2	2	3	3	2	2
4	4	1	1	2	2	3	3	2	2
4	4	1	1	2	2	3	3	4	4
4	4	1	1	2	2	3	3	2	2
4	4	1	1	2	2	3	3	4	4
4	4	1	1	2	2	3	3	4	4
4	4	1	1	2	2	3	3	4	4
4	4	1	1	2	2	3	3	4	4
4	4	1	1	2	2	2	2	4	4
4	4	1	1	2	2	3	3	4	4
4	4	1	1	2	2	3	3	4	4

4	4	1	1	2	2	3	3	4	4
4	4	1	1	2	2	3	3	4	4
4	4	1	1	2	2	3	3	4	4
4	4	1	1	2	2	3	3	4	4
4	4	1	1	2	2	3	3	4	4
4	4	1	1	2	2	3	3	4	4
4	4	1	1	2	2	3	3	4	4
4	4	1	1	2	2	3	3	2	2
4	4	1	1	2	2	3	3	4	4
4	4	1	1	2	2	3	3	4	4
4	4	1	1	3	3	3	3	4	4
4	4	1	1	2	2	3	3	4	4
4	4	1	1	2	2	3	3	4	4
4	4	1	1	2	2	3	3	2	2
4	4	1	1	2	2	3	3	4	4
4	4	1	1	2	2	3	3	4	4
4	4	1	1	2	2	3	3	4	4
4	4	1	1	2	2	3	3	4	4
4	4	1	1	2	2	3	3	4	4
4	4	1	1	2	2	3	3	4	4
4	4	1	1	2	2	3	3	4	4
4	4	1	1	2	2	3	3	4	4
4	4	1	1	2	2	3	3	4	4
4	4	1	1	2	2	3	3	4	4
4	4	1	1	2	2	3	3	2	2
4	4	1	1	2	2	3	3	4	4
4	4	1	1	3	3	2	2	4	4
3	3	1	1	3	3	2	2	2	2
4	4	1	1	2	2	3	3	4	4
4	4	1	1	2	2	3	3	4	4
4	4	1	1	3	3	2	2	2	2
4	4	1	1	2	2	3	3	2	2
3	3	1	1	3	3	2	2	2	2
4	4	1	1	2	2	3	3	4	4
4	4	1	1	2	2	3	3	4	4
4	4	1	1	2	2	3	3	2	2
4	4	1	1	3	3	2	2	2	2
4	4	1	1	2	2	3	3	4	4
4	4	1	1	2	2	3	3	4	4
4	4	1	1	2	2	3	3	4	4
4	4	1	1	2	2	3	3	4	4
4	4	1	1	2	2	3	3	4	4
3	3	1	1	3	3	2	2	2	2
4	4	1	1	2	2	3	3	4	4
4	4	1	1	2	2	3	3	4	4
4	4	1	1	2	2	3	3	4	4
3	3	1	1	3	3	2	2	2	2
4	4	1	1	2	2	3	3	4	4
4	4	1	1	2	2	3	3	2	2
4	4	1	1	2	2	3	3	4	4
4	4	1	1	2	2	3	3	4	4
3	3	1	1	3	3	2	2	2	2
4	4	1	1	2	2	3	3	2	2
4	4	1	1	2	2	3	3	4	4
4	4	1	1	2	2	3	3	2	2

For Review Only

4	4	1	1	2	2	3	3	4	4
4	4	1	1	2	2	3	3	2	2
4	4	1	1	2	2	3	3	4	4
3	3	1	1	3	3	2	2	2	2
4	4	1	1	2	2	3	3	4	4
4	4	1	1	2	2	3	3	2	2
4	4	1	1	2	2	3	3	4	4
4	4	1	1	2	2	3	3	4	4
4	4	1	1	2	2	3	3	4	4
4	4	1	1	2	2	3	3	4	4
4	4	1	1	2	2	2	2	2	2
4	4	1	1	2	2	3	3	2	2
4	4	1	1	3	3	2	2	2	2
4	4	1	1	2	2	3	3	4	4
4	4	1	1	2	2	3	3	4	4
4	4	1	1	2	2	3	3	4	4
4	4	1	1	2	2	3	3	4	4
4	4	1	1	2	2	3	3	4	4
3	3	1	1	3	3	2	2	2	2
4	4	1	1	2	2	3	3	2	2
4	4	1	1	2	2	3	3	4	4
4	4	1	1	2	2	3	3	4	4
4	4	1	1	2	2	3	3	2	2
3	3	1	1	3	3	2	2	2	2
4	4	1	1	2	2	3	3	4	4
4	4	1	1	2	2	3	3	4	4
4	4	1	1	2	2	3	3	4	4
4	4	1	1	2	2	3	3	4	4
4	4	1	1	2	2	3	3	4	4
4	4	1	1	2	2	3	3	4	4
3	3	1	1	3	3	2	2	2	2
4	4	1	1	2	2	2	2	4	4
4	4	1	1	2	2	3	3	4	4
4	4	1	1	2	2	3	3	2	2
4	4	1	1	2	2	3	3	2	2
3	3	1	1	3	3	2	2	2	2
3	3	1	1	3	3	2	2	2	2
4	4	1	1	2	2	3	3	2	2
4	4	1	1	2	2	3	3	2	2
3	3	1	1	3	3	2	2	2	2
4	4	1	1	2	2	3	3	4	4
4	4	1	1	3	3	2	2	2	2
4	4	1	1	2	2	3	3	4	4
4	4	1	1	2	2	3	3	4	4
4	4	1	1	2	2	3	3	4	4
4	4	1	1	2	2	3	3	2	2
4	4	1	1	2	2	2	2	4	4
4	4	1	1	2	2	3	3	4	4
4	4	1	1	3	3	2	2	4	4
4	4	1	1	2	2	3	3	4	4
4	4	1	1	2	2	3	3	4	4
3	3	1	1	3	3	2	2	2	2
4	4	1	1	2	2	3	3	4	4
4	4	1	1	2	2	3	3	4	4
4	4	1	1	2	2	3	3	2	2

For Review Only

4	4	1	1	2	2	3	3	4	4
3	3	1	1	3	3	2	2	2	2
4	4	1	1	3	3	2	2	4	4
4	4	1	1	3	3	2	2	4	4
4	4	1	1	2	2	3	3	4	4
4	4	1	1	2	2	3	3	4	4
3	3	1	1	3	3	2	2	2	2
4	4	1	1	2	2	3	3	4	4
3	3	1	1	3	3	2	2	2	2
4	4	1	1	2	2	3	3	4	4
4	4	1	1	2	2	3	3	4	4
4	4	1	1	3	3	2	2	2	2
4	4	1	1	2	2	3	3	4	4
4	4	1	1	2	2	3	3	2	2
3	3	1	1	3	3	2	2	2	2
3	3	1	1	3	3	2	2	2	2
4	4	1	1	2	2	3	3	4	4
4	4	1	1	2	2	3	3	4	4
4	4	1	1	2	2	3	3	4	4
4	4	1	1	2	2	3	3	4	4
4	4	1	1	2	2	2	2	4	4
3	3	1	1	3	3	2	2	2	2
4	4	1	1	2	2	3	3	4	4
4	4	1	1	2	2	3	3	4	4
4	4	1	1	2	2	3	3	4	4
4	4	1	1	2	2	3	3	2	2
4	4	1	1	2	2	3	3	4	4
4	4	1	1	2	2	3	3	4	4
3	3	1	1	2	2	2	2	2	2
4	4	1	1	2	2	3	3	4	4
4	4	1	1	2	2	3	3	4	4
4	4	1	1	2	2	3	3	4	4
4	4	1	1	2	2	3	3	4	4
3	3	1	1	3	3	2	2	2	2
4	4	1	1	2	2	3	3	4	4
4	4	1	1	2	2	3	3	4	4
4	4	1	1	2	2	3	3	4	4
4	4	1	1	2	2	3	3	4	4
4	4	1	1	2	2	3	3	4	4
4	4	1	1	2	2	3	3	2	2
4	4	1	1	2	2	3	3	4	4
4	4	1	1	2	2	3	3	4	4
4	4	1	1	2	2	3	3	2	2
4	4	1	1	2	2	3	3	4	4
4	4	1	1	3	3	2	2	4	4
4	4	1	1	2	2	3	3	4	4
3	3	1	1	3	3	2	2	2	2
3	3	1	1	3	3	2	2	2	2
3	3	1	1	3	3	2	2	2	2
4	4	1	1	2	2	3	3	4	4
4	4	1	1	2	2	3	3	4	4
4	4	1	1	2	2	3	3	4	4
4	4	1	1	2	2	3	3	4	4
4	4	1	1	2	2	3	3	4	4
3	3	1	1	3	3	2	2	2	2

For Review Only

3	3	1	1	3	3	2	2	2	2
4	4	1	1	2	2	3	3	2	2
4	4	1	1	2	2	3	3	4	4
4	4	1	1	2	2	3	3	4	4
4	4	1	1	2	2	3	3	4	4
4	4	1	1	2	2	3	3	4	4
3	3	1	1	3	3	2	2	2	2
4	4	1	1	2	2	3	3	4	4
4	4	1	1	3	3	2	2	2	2
3	3	1	1	3	3	2	2	2	2
4	4	1	1	2	2	3	3	4	4
4	4	1	1	2	2	3	3	4	4
4	4	1	1	2	2	3	3	4	4
4	4	1	1	2	2	3	3	4	4
4	4	1	1	2	2	3	3	4	4
3	3	1	1	3	3	2	2	2	2
3	3	1	1	3	3	2	2	2	2
4	4	1	1	3	3	2	2	2	2
3	3	1	1	3	3	2	2	2	2
4	4	1	1	2	2	3	3	2	2
3	3	1	1	3	3	2	2	2	2
4	4	1	1	2	2	3	3	4	4
4	4	1	1	2	2	3	3	4	4
3	3	1	1	3	3	2	2	2	2
4	4	1	1	2	2	3	3	2	2
4	4	1	1	2	2	3	3	4	4
3	3	1	1	3	3	2	2	2	2
4	4	1	1	2	2	3	3	4	4
4	4	1	1	2	2	3	3	4	4
4	4	1	1	2	2	3	3	4	4
3	3	1	1	3	3	2	2	2	2
4	4	1	1	2	2	3	3	4	4
4	4	1	1	2	2	3	3	2	2
4	4	1	1	2	2	3	3	4	4
4	4	1	1	2	2	3	3	4	4
4	4	1	1	2	2	3	3	2	2
4	4	1	1	2	2	3	3	4	4
4	4	1	1	2	2	3	3	4	4
4	4	1	1	2	2	3	3	4	4
3	3	1	1	3	3	2	2	2	2
4	4	1	1	2	2	3	3	4	4
4	4	1	1	2	2	3	3	4	4
3	3	1	1	3	3	2	2	2	2
4	4	1	1	2	2	3	3	4	4
4	4	1	1	2	2	2	2	4	4
4	4	1	1	2	2	3	3	4	4
4	4	1	1	3	3	2	2	2	2
4	4	1	1	3	3	2	2	2	2
4	4	1	1	2	2	3	3	4	4
3	3	1	1	3	3	2	2	2	2
4	4	1	1	2	2	3	3	4	4
3	3	1	1	3	3	2	2	2	2
4	4	1	1	2	2	3	3	4	4
4	4	1	1	2	2	3	3	4	4
3	3	1	1	3	3	2	2	2	2

For Review Only

4	4	1	1	2	2	3	3	4	4
4	4	1	1	2	2	3	3	4	4

For Review Only

SNP1636	SNP1636	SNP1637	SNP1637	SNP1638	SNP1638	SNP1639	SNP1639	SNP164	SNP164
1	1	4	4	1	1	1	1	4	4
1	1	4	4	1	1	3	3	4	4
1	1	4	4	1	1	3	3	4	4
1	1	4	4	3	3	3	3	4	4
1	1	1	1	3	3	3	3	4	4
1	1	1	1	3	3	3	3	4	4
1	1	1	1	3	3	1	1	4	4
1	1	1	1	3	3	1	1	1	1
1	1	4	4	3	3	1	1	4	4
1	1	1	1	3	3	1	1	4	4
1	1	4	4	3	3	3	3	1	1
1	1	4	4	1	1	3	3	4	4
1	1	4	4	1	1	3	3	1	1
1	1	1	1	3	3	1	1	1	1
1	1	4	4	1	1	3	3	1	1
1	1	4	4	3	3	3	3	1	1
1	1	1	1	3	3	1	1	4	4
1	1	4	4	1	1	3	3	1	1
1	1	4	4	3	3	3	3	1	1
1	1	1	1	3	3	1	1	4	4
1	1	1	1	3	3	1	1	4	4
1	1	1	1	3	3	3	3	4	4
1	1	4	4	3	3	3	3	1	1
1	1	4	4	3	3	3	3	1	1
1	1	4	4	3	3	3	3	4	4
1	1	4	4	3	3	3	3	4	4
1	1	4	4	1	1	3	3	4	4
1	1	4	4	3	3	3	3	1	1
1	1	1	1	3	3	3	3	1	1
1	1	1	1	3	3	1	1	4	4
1	1	4	4	1	1	3	3	4	4
1	1	4	4	1	1	1	1	4	4
1	1	4	4	1	1	1	1	4	4
1	1	1	1	3	3	3	3	1	1
1	1	4	4	3	3	3	3	1	1
1	1	4	4	3	3	3	3	4	4
1	1	4	4	3	3	3	3	4	4
1	1	4	4	3	3	3	3	1	1
3	3	4	4	3	3	3	3	1	1
1	1	4	4	3	3	3	3	1	1
1	1	1	1	3	3	1	1	4	4
1	1	4	4	3	3	1	1	1	1
1	1	1	1	3	3	3	3	4	4
1	1	1	1	3	3	3	3	4	4
1	1	1	1	3	3	1	1	4	4
1	1	4	4	3	3	3	3	4	4
1	1	4	4	1	1	3	3	4	4
1	1	4	4	3	3	3	3	1	1
1	1	1	1	3	3	3	3	4	4
1	1	4	4	3	3	3	3	1	1
1	1	4	4	3	3	3	3	4	4
1	1	4	4	3	3	3	3	1	1

For Review Only

1	1	4	4	3	3	3	3	4	4
1	1	4	4	1	1	1	1	4	4
1	1	4	4	1	1	3	3	1	1
1	1	1	1	3	3	3	3	4	4
1	1	4	4	3	3	3	3	1	1
1	1	4	4	1	1	3	3	4	4
1	1	4	4	3	3	3	3	4	4
3	3	1	1	3	3	3	3	4	4
3	3	1	1	3	3	3	3	1	1
1	1	4	4	3	3	3	3	1	1
1	1	4	4	1	1	3	3	4	4
1	1	4	4	1	1	3	3	4	4
1	1	1	1	3	3	3	3	4	4
1	1	1	1	3	3	3	3	1	1
1	1	4	4	3	3	3	3	1	1
1	1	4	4	3	3	3	3	1	1
1	1	4	4	3	3	3	3	1	1
1	1	4	4	3	3	3	3	1	1
1	1	1	1	3	3	3	3	1	1
1	1	1	1	3	3	1	1	4	4
1	1	4	4	1	1	3	3	4	4
1	1	4	4	3	3	3	3	1	1
1	1	4	4	3	3	3	3	1	1
1	1	1	1	3	3	1	1	1	1
1	1	1	1	3	3	1	1	4	4
1	1	4	4	1	1	3	3	1	1
1	1	1	1	3	3	3	3	4	4
3	3	4	4	3	3	1	1	1	1
1	1	4	4	3	3	3	3	4	4
1	1	4	4	3	3	3	3	1	1
1	1	4	4	3	3	3	3	1	1
1	1	1	1	3	3	1	1	1	1
1	1	4	4	3	3	3	3	1	1
1	1	1	1	3	3	3	3	4	4
1	1	4	4	3	3	1	1	1	1
1	1	1	1	3	3	1	1	4	4
1	1	4	4	3	3	1	1	1	1
1	1	1	1	3	3	3	3	4	4
1	1	4	4	3	3	3	3	1	1
3	3	1	1	3	3	1	1	4	4
1	1	1	1	3	3	3	3	4	4
1	1	4	4	1	1	3	3	4	4
1	1	4	4	3	3	3	3	1	1
1	1	4	4	1	1	3	3	4	4
3	3	4	4	3	3	3	3	4	4
1	1	4	4	1	1	3	3	4	4
1	1	4	4	3	3	3	3	1	1
1	1	4	4	3	3	3	3	4	4
1	1	4	4	3	3	3	3	4	4
1	1	4	4	1	1	3	3	1	1
1	1	4	4	1	1	3	3	4	4
3	3	4	4	3	3	3	3	1	1
3	3	1	1	3	3	1	1	4	4
1	1	4	4	3	3	3	3	1	1
1	1	4	4	1	1	3	3	1	1

For Review Only

1	1	4	4	3	3	3	3	1	1
1	1	4	4	3	3	3	3	1	1
1	1	4	4	3	3	3	3	4	4
1	1	1	1	3	3	3	3	1	1
1	1	4	4	1	1	3	3	4	4
1	1	4	4	3	3	1	1	4	4
1	1	4	4	1	1	3	3	4	4
1	1	4	4	3	3	3	3	1	1
1	1	4	4	3	3	3	3	4	4
1	1	4	4	3	3	3	3	1	1
1	1	4	4	3	3	1	1	1	1
1	1	4	4	3	3	3	3	1	1
1	1	4	4	3	3	3	3	1	1
1	1	4	4	1	1	3	3	1	1
1	1	1	1	3	3	3	3	4	4
1	1	4	4	3	3	3	3	1	1
1	1	4	4	3	3	3	3	1	1
1	1	4	4	3	3	3	3	1	1
1	1	1	1	3	3	3	3	1	1
1	1	4	4	3	3	3	3	1	1
1	1	4	4	3	3	3	3	1	1
1	1	4	4	3	3	3	3	1	1
1	1	4	4	1	1	3	3	1	1
3	3	1	1	3	3	3	3	1	1
1	1	4	4	3	3	3	3	4	4
1	1	4	4	3	3	3	3	1	1
1	1	4	4	3	3	3	3	1	1
1	1	4	4	3	3	3	3	1	1
1	1	4	4	1	1	3	3	1	1
1	1	4	4	3	3	3	3	1	1
1	1	4	4	3	3	1	1	1	1
1	1	4	4	3	3	3	3	1	1
1	1	1	1	3	3	3	3	4	4
1	1	4	4	3	3	3	3	1	1
1	1	1	1	3	3	3	3	1	1
1	1	1	1	3	3	3	3	1	1
1	1	1	1	3	3	3	3	1	1
1	1	4	4	1	1	3	3	1	1
1	1	4	4	3	3	3	3	1	1
1	1	1	1	3	3	3	3	1	1
1	1	4	4	3	3	3	3	1	1
1	1	1	1	3	3	3	3	4	4
1	1	4	4	1	1	3	3	1	1
1	1	4	4	3	3	3	3	4	4
1	1	4	4	3	3	3	3	1	1
1	1	4	4	3	3	3	3	4	4
1	1	4	4	3	3	3	3	4	4
1	1	4	4	1	1	3	3	4	4
1	1	4	4	3	3	3	3	1	1
1	1	4	4	3	3	3	3	4	4
1	1	4	4	3	3	3	3	4	4
1	1	4	4	3	3	3	3	4	4
1	1	1	1	3	3	3	3	1	1
1	1	4	4	1	1	1	1	4	4
3	3	4	4	3	3	3	3	1	1

For Review Only

1	1	4	4	3	3	3	3	4	4
1	1	4	4	3	3	3	3	4	4
1	1	1	1	3	3	3	3	1	1
1	1	4	4	3	3	3	3	1	1
1	1	4	4	1	1	3	3	4	4
1	1	1	1	3	3	3	3	1	1
1	1	4	4	3	3	3	3	1	1
1	1	4	4	3	3	3	3	1	1
1	1	4	4	3	3	3	3	1	1
1	1	4	4	3	3	3	3	1	1
1	1	4	4	3	3	3	3	4	4
1	1	4	4	3	3	3	3	1	1
1	1	1	1	3	3	3	3	4	4
1	1	4	4	3	3	3	3	1	1
1	1	1	1	3	3	1	1	1	1
1	1	4	4	3	3	3	3	1	1
1	1	4	4	3	3	3	3	1	1
1	1	4	4	1	1	3	3	4	4
1	1	4	4	3	3	3	3	1	1
1	1	4	4	1	1	3	3	4	4
1	1	4	4	3	3	3	3	1	1
1	1	4	4	3	3	1	1	1	1
1	1	1	1	3	3	3	3	4	4
1	1	4	4	1	1	3	3	4	4
1	1	1	1	3	3	1	1	4	4
1	1	1	1	3	3	3	3	1	1
1	1	4	4	1	1	3	3	1	1
1	1	4	4	3	3	3	3	1	1
1	1	1	1	3	3	3	3	1	1
1	1	1	1	3	3	3	3	1	1
3	3	4	4	3	3	3	3	4	4
1	1	4	4	3	3	3	3	1	1
1	1	4	4	3	3	3	3	1	1
1	1	1	1	3	3	1	1	1	1
1	1	4	4	3	3	3	3	4	4
1	1	4	4	1	1	3	3	4	4
1	1	4	4	1	1	3	3	4	4
1	1	4	4	3	3	3	3	4	4
1	1	4	4	1	1	3	3	4	4
1	1	4	4	3	3	3	3	4	4
1	1	4	4	1	1	3	3	4	4
1	1	4	4	3	3	3	3	4	4
3	3	1	1	3	3	3	3	1	1
1	1	4	4	3	3	3	3	1	1
1	1	4	4	1	1	3	3	4	4
1	1	4	4	1	1	3	3	1	1
1	1	4	4	3	3	3	3	1	1
3	3	4	4	3	3	3	3	1	1
1	1	1	1	3	3	3	3	4	4
1	1	4	4	3	3	3	3	4	4
1	1	4	4	3	3	3	3	1	1
1	1	4	4	3	3	3	3	1	1
1	1	4	4	3	3	3	3	1	1
1	1	4	4	1	1	3	3	4	4
3	3	4	4	3	3	3	3	1	1

For Review Only

1	1	1	1	3	3	3	3	1	1
1	1	1	1	3	3	3	3	4	4
1	1	4	4	3	3	3	3	1	1
1	1	1	1	3	3	3	3	4	4
1	1	1	1	3	3	3	3	1	1
1	1	4	4	3	3	3	3	1	1
1	1	4	4	3	3	3	3	4	4
1	1	1	1	3	3	1	1	4	4
1	1	4	4	3	3	3	3	4	4
1	1	4	4	3	3	1	1	1	1
1	1	4	4	3	3	3	3	1	1
1	1	4	4	3	3	3	3	4	4
1	1	4	4	3	3	3	3	4	4
1	1	4	4	3	3	3	3	1	1
1	1	4	4	1	1	3	3	4	4
1	1	4	4	3	3	3	3	1	1
1	1	4	4	3	3	3	3	1	1
1	1	4	4	3	3	3	3	1	1
1	1	4	4	3	3	3	3	1	1
1	1	1	1	3	3	3	3	1	1
1	1	4	4	3	3	3	3	1	1
1	1	4	4	3	3	3	3	1	1
1	1	4	4	3	3	3	3	1	1
1	1	4	4	3	3	3	3	1	1
3	3	1	1	3	3	3	3	4	4
1	1	4	4	1	1	1	1	4	4
3	3	4	4	3	3	1	1	1	1
1	1	4	4	1	1	3	3	4	4
1	1	1	1	3	3	3	3	1	1
1	1	4	4	3	3	3	3	1	1
3	3	1	1	3	3	3	3	1	1
1	1	4	4	3	3	3	3	1	1
1	1	4	4	3	3	1	1	1	1
1	1	4	4	3	3	3	3	4	4
1	1	4	4	3	3	3	3	4	4
3	3	1	1	3	3	3	3	1	1
1	1	1	1	3	3	3	3	1	1
1	1	4	4	1	1	3	3	4	4
1	1	4	4	3	3	3	3	4	4
1	1	4	4	3	3	3	3	1	1
1	1	4	4	3	3	3	3	4	4
1	1	4	4	3	3	3	3	1	1
1	1	4	4	3	3	3	3	1	1
1	1	1	1	3	3	3	3	4	4
1	1	4	4	3	3	3	3	1	1
1	1	4	4	1	1	3	3	1	1
1	1	4	4	3	3	3	3	4	4
1	1	4	4	1	1	3	3	1	1
1	1	4	4	1	1	3	3	4	4
3	3	4	4	3	3	1	1	1	1
1	1	4	4	3	3	3	3	1	1
3	3	4	4	3	3	3	3	1	1
1	1	4	4	1	1	3	3	4	4
1	1	4	4	3	3	3	3	4	4
1	1	4	4	3	3	3	3	1	1

For Review Only

1	1	4	4	3	3	3	3	1	1
1	1	4	4	1	1	3	3	4	4

For Review Only

SNP1640	SNP1640	SNP1641	SNP1641	SNP1642	SNP1642	SNP1643	SNP1643	SNP1644	SNP1644
1	1	1	1	1	1	3	3	1	1
1	1	1	1	1	1	2	2	1	1
1	1	1	1	1	1	2	2	1	1
1	1	1	1	1	1	3	3	2	2
1	1	1	1	1	1	3	3	2	2
1	1	1	1	1	1	3	3	2	2
1	1	1	1	1	1	3	3	2	2
1	1	1	1	3	3	3	3	2	2
1	1	1	1	1	1	3	3	2	2
1	1	1	1	3	3	3	3	2	2
4	4	3	3	1	1	3	3	2	2
1	1	1	1	1	1	2	2	1	1
1	1	1	1	1	1	3	3	2	2
1	1	1	1	1	1	3	3	2	2
1	1	1	1	1	1	3	3	2	2
1	1	1	1	1	1	3	3	2	2
1	1	1	1	1	1	3	3	2	2
1	1	1	1	3	3	2	2	1	1
1	1	1	1	1	1	3	3	2	2
1	1	1	1	3	3	3	3	2	2
1	1	1	1	3	3	3	3	2	2
1	1	1	1	1	1	3	3	2	2
1	1	3	3	1	1	3	3	2	2
1	1	1	1	1	1	3	3	2	2
4	4	1	1	1	1	3	3	2	2
1	1	1	1	1	1	3	3	2	2
1	1	1	1	1	1	2	2	1	1
1	1	1	1	1	1	3	3	2	2
1	1	1	1	1	1	3	3	2	2
1	1	3	3	1	1	3	3	2	2
1	1	1	1	1	1	2	2	2	2
1	1	1	1	1	1	2	2	1	1
1	1	1	1	1	1	2	2	1	1
1	1	1	1	1	1	3	3	2	2
1	1	3	3	1	1	2	2	1	1
1	1	1	1	1	1	3	3	2	2
1	1	1	1	1	1	3	3	2	2
1	1	1	1	1	1	3	3	2	2
1	1	3	3	1	1	3	3	2	2
1	1	3	3	1	1	3	3	2	2
1	1	1	1	3	3	3	3	2	2
1	1	1	1	1	1	3	3	2	2
1	1	1	1	3	3	3	3	2	2
1	1	1	1	1	1	3	3	2	2
1	1	1	1	3	3	3	3	2	2
1	1	1	1	1	1	3	3	2	2
1	1	1	1	1	1	2	2	1	1
4	4	1	1	1	1	3	3	2	2
1	1	1	1	3	3	3	3	2	2
1	1	1	1	1	1	3	3	1	1
1	1	1	1	1	1	3	3	2	2
1	1	1	1	1	1	3	3	2	2

For Review Only

1	1	1	1	1	1	3	3	2	2
1	1	1	1	1	1	2	2	1	1
1	1	1	1	1	1	2	2	1	1
1	1	1	1	3	3	3	3	2	2
1	1	1	1	1	1	3	3	2	2
1	1	1	1	1	1	2	2	1	1
1	1	1	1	1	1	3	3	2	2
4	4	1	1	1	1	3	3	2	2
1	1	1	1	3	3	3	3	2	2
1	1	1	1	1	1	3	3	2	2
1	1	1	1	1	1	2	2	1	1
1	1	1	1	1	1	2	2	1	1
1	1	1	1	1	1	3	3	2	2
1	1	1	1	1	1	3	3	2	2
1	1	1	1	1	1	3	3	2	2
1	1	1	1	1	1	3	3	2	2
1	1	1	1	1	1	3	3	2	2
1	1	1	1	1	1	3	3	2	2
1	1	1	1	1	1	3	3	2	2
1	1	1	1	1	1	3	3	2	2
1	1	1	1	1	1	3	3	2	2
1	1	3	3	1	1	3	3	2	2
1	1	1	1	1	1	3	3	2	2
1	1	1	1	3	3	3	3	2	2
1	1	1	1	3	3	3	3	2	2
1	1	1	1	1	1	2	2	1	1
4	4	1	1	1	1	3	3	2	2
1	1	3	3	1	1	3	3	2	2
1	1	1	1	1	1	3	3	2	2
1	1	1	1	1	1	3	3	2	2
1	1	1	1	1	1	3	3	2	2
1	1	1	1	1	1	3	3	2	2
1	1	3	3	3	3	3	3	2	2
1	1	1	1	1	1	3	3	2	2
1	1	1	1	1	1	3	3	2	2
1	1	1	1	3	3	3	3	2	2
1	1	1	1	3	3	3	3	2	2
1	1	1	1	3	3	3	3	2	2
1	1	1	1	3	3	3	3	2	2
4	4	1	1	1	1	3	3	2	2
1	1	1	1	1	1	3	3	2	2
1	1	1	1	1	1	2	2	1	1
4	4	1	1	1	1	3	3	2	2
1	1	1	1	1	1	2	2	1	1
1	1	1	1	1	1	3	3	2	2
1	1	1	1	1	1	2	2	1	1
1	1	1	1	1	1	3	3	2	2
1	1	1	1	1	1	3	3	2	2
1	1	1	1	1	1	3	3	2	2
1	1	1	1	1	1	2	2	1	1
1	1	1	1	1	1	2	2	1	1
4	4	3	3	1	1	3	3	2	2
4	4	1	1	1	1	3	3	2	2
4	4	1	1	1	1	3	3	2	2
1	1	1	1	1	1	2	2	1	1

For Review Only

1	1	1	1	1	1	3	3	2	2
1	1	3	3	1	1	3	3	2	2
1	1	1	1	1	1	3	3	2	2
1	1	3	3	1	1	3	3	2	2
1	1	1	1	1	1	2	2	1	1
1	1	1	1	1	1	3	3	2	2
1	1	1	1	1	1	2	2	1	1
4	4	1	1	1	1	3	3	2	2
1	1	1	1	1	1	3	3	2	2
1	1	1	1	1	1	3	3	2	2
1	1	1	1	3	3	3	3	2	2
1	1	1	1	1	1	3	3	2	2
4	4	1	1	1	1	3	3	2	2
1	1	1	1	1	1	3	3	1	1
1	1	1	1	1	1	3	3	2	2
1	1	1	1	1	1	3	3	2	2
1	1	1	1	1	1	3	3	2	2
1	1	1	1	1	1	3	3	2	2
1	1	3	3	1	1	3	3	2	2
4	4	1	1	1	1	3	3	2	2
1	1	1	1	1	1	3	3	2	2
1	1	1	1	1	1	3	3	2	2
1	1	1	1	1	1	2	2	1	1
1	1	3	3	1	1	3	3	2	2
1	1	1	1	1	1	3	3	2	2
1	1	3	3	1	1	3	3	2	2
1	1	1	1	1	1	3	3	2	2
1	1	1	1	1	1	3	3	2	2
1	1	1	1	1	1	2	2	1	1
4	4	1	1	1	1	3	3	2	2
1	1	1	1	1	1	3	3	2	2
1	1	1	1	1	1	3	3	2	2
1	1	1	1	1	1	3	3	2	2
1	1	1	1	1	1	3	3	2	2
1	1	1	1	1	1	3	3	2	2
1	1	1	1	1	1	3	3	2	2
1	1	1	1	1	1	3	3	2	2
1	1	1	1	1	1	2	2	1	1
1	1	1	1	1	1	3	3	2	2
4	4	3	3	1	1	3	3	2	2
1	1	3	3	1	1	3	3	2	2
1	1	3	3	1	1	3	3	2	2
1	1	1	1	1	1	2	2	1	1
1	1	1	1	1	1	3	3	2	2
1	1	1	1	1	1	3	3	2	2
1	1	1	1	1	1	3	3	2	2
1	1	1	1	1	1	3	3	2	2
1	1	1	1	1	1	2	2	1	1
1	1	1	1	3	3	3	3	2	2
1	1	1	1	1	1	3	3	2	2
1	1	1	1	1	1	3	3	2	2
1	1	1	1	1	1	3	3	2	2
1	1	1	1	1	1	3	3	2	2
4	4	1	1	1	1	2	2	1	1
1	1	1	1	1	1	3	3	2	2

1	1	1	1	1	1	3	3	2	2
4	4	3	3	1	1	3	3	2	2
1	1	1	1	1	1	3	3	2	2
1	1	3	3	1	1	3	3	2	2
1	1	1	1	1	1	2	2	1	1
1	1	1	1	1	1	3	3	2	2
1	1	3	3	1	1	3	3	2	2
1	1	1	1	1	1	3	3	2	2
1	1	1	1	1	1	3	3	2	2
1	1	1	1	1	1	3	3	2	2
1	1	1	1	1	1	3	3	2	2
1	1	1	1	1	1	3	3	2	2
1	1	1	1	1	1	3	3	2	2
4	4	1	1	1	1	3	3	2	2
1	1	1	1	1	1	3	3	2	2
4	4	3	3	1	1	3	3	2	2
1	1	1	1	1	1	3	3	2	2
4	4	1	1	1	1	2	2	1	1
1	1	1	1	1	1	3	3	2	2
1	1	1	1	1	1	2	2	1	1
1	1	1	1	1	1	3	3	2	2
1	1	3	3	1	1	3	3	2	2
1	1	3	3	1	1	3	3	2	2
1	1	1	1	1	1	2	2	1	1
1	1	1	1	1	1	3	3	2	2
1	1	1	1	1	1	3	3	2	2
1	1	1	1	1	1	2	2	1	1
1	1	1	1	1	1	3	3	2	2
1	1	1	1	1	1	3	3	2	2
1	1	1	1	1	1	3	3	2	2
1	1	1	1	1	1	3	3	2	2
1	1	1	1	1	1	3	3	2	2
1	1	1	1	1	1	3	3	2	2
1	1	1	1	1	1	3	3	2	2
1	1	1	1	1	1	3	3	2	2
1	1	1	1	1	1	2	2	1	1
1	1	1	1	1	1	2	2	1	1
1	1	1	1	1	1	3	3	2	2
1	1	1	1	1	1	2	2	1	1
1	1	1	1	1	1	3	3	2	2
1	1	1	1	1	1	2	2	1	1
1	1	1	1	1	1	3	3	2	2
4	4	3	3	1	1	3	3	2	2
1	1	1	1	1	1	3	3	2	2
1	1	1	1	1	1	3	3	2	2
1	1	1	1	1	1	2	2	1	1
4	4	3	3	1	1	3	3	2	2
1	1	3	3	1	1	3	3	2	2
1	1	1	1	1	1	3	3	2	2
1	1	1	1	3	3	2	2	1	1
1	1	1	1	1	1	3	3	2	2
1	1	1	1	1	1	3	3	2	2
1	1	3	3	1	1	3	3	2	2
1	1	1	1	1	1	2	2	1	1
1	1	3	3	1	1	3	3	2	2

For Review Only

1	1	1	1	1	1	3	3	2	2
1	1	1	1	1	1	3	3	2	2
1	1	1	1	1	1	3	3	2	2
1	1	3	3	1	1	3	3	2	2
1	1	1	1	3	3	3	3	2	2
1	1	1	1	1	1	3	3	2	2
1	1	3	3	1	1	3	3	2	2
1	1	1	1	1	1	3	3	2	2
1	1	1	1	1	1	3	3	2	2
1	1	3	3	1	1	3	3	2	2
1	1	1	1	1	1	3	3	2	2
1	1	3	3	1	1	3	3	2	2
1	1	1	1	3	3	3	3	2	2
1	1	1	1	1	1	3	3	2	2
1	1	1	1	1	1	2	2	1	1
1	1	1	1	1	1	3	3	2	2
1	1	3	3	1	1	3	3	2	2
1	1	1	1	1	1	3	3	2	2
1	1	3	3	1	1	3	3	2	2
1	1	1	1	1	1	3	3	2	2
1	1	3	3	1	1	3	3	2	2
1	1	1	1	1	1	3	3	2	2
1	1	1	1	1	1	3	3	2	2
1	1	3	3	1	1	3	3	2	2
4	4	1	1	1	1	3	3	2	2
1	1	1	1	1	1	2	2	2	2
1	1	3	3	1	1	3	3	2	2
1	1	1	1	1	1	2	2	1	1
1	1	1	1	1	1	3	3	2	2
1	1	1	1	1	1	3	3	2	2
1	1	3	3	1	1	3	3	2	2
1	1	1	1	1	1	3	3	2	2
4	4	1	1	1	1	3	3	2	2
1	1	1	1	1	1	3	3	2	2
1	1	1	1	1	1	3	3	2	2
1	1	3	3	1	1	3	3	2	2
1	1	1	1	1	1	3	3	2	2
1	1	1	1	1	1	2	2	1	1
1	1	1	1	1	1	3	3	2	2
1	1	3	3	1	1	3	3	2	2
1	1	1	1	1	1	3	3	2	2
1	1	1	1	1	1	3	3	2	2
1	1	3	3	1	1	3	3	2	2
1	1	1	1	1	1	3	3	2	2
1	1	1	1	1	1	3	3	2	2
1	1	1	1	3	3	2	2	1	1
4	4	1	1	1	1	3	3	2	2
1	1	1	1	1	1	2	2	1	1
1	1	1	1	1	1	2	2	1	1
1	1	3	3	1	1	3	3	2	2
1	1	1	1	1	1	3	3	2	2
4	4	3	3	1	1	3	3	2	2
1	1	1	1	1	1	2	2	1	1
1	1	1	1	1	1	3	3	2	2
1	1	3	3	1	1	3	3	2	2

For Review Only

1	1	1	1	1	1	3	3	2	2
1	1	1	1	1	1	2	2	1	1

For Review Only

SNP1645	SNP1645	SNP1646	SNP1646	SNP1647	SNP1647	SNP1648	SNP1648	SNP1649	SNP1649
4	4	4	4	4	4	4	4	1	1
4	4	4	4	3	3	3	3	1	1
1	1	4	4	3	3	4	4	1	1
4	4	4	4	4	4	4	4	1	1
4	4	4	4	4	4	4	4	1	1
4	4	4	4	4	4	4	4	1	1
4	4	4	4	4	4	4	4	1	1
4	4	4	4	4	4	3	3	1	1
4	4	4	4	4	4	4	4	1	1
4	4	4	4	4	4	3	3	1	1
4	4	4	4	4	4	4	4	1	1
4	4	4	4	3	3	4	4	1	1
4	4	4	4	4	4	4	4	1	1
4	4	4	4	4	4	3	3	3	3
4	4	4	4	4	4	4	4	1	1
4	4	4	4	4	4	3	3	1	1
4	4	4	4	4	4	4	4	1	1
4	4	4	4	3	3	4	4	1	1
4	4	4	4	4	4	4	4	1	1
4	4	4	4	4	4	3	3	1	1
4	4	4	4	4	4	4	4	1	1
4	4	4	4	4	4	4	4	1	1
4	4	1	1	4	4	3	3	1	1
4	4	4	4	4	4	4	4	1	1
4	4	4	4	4	4	4	4	1	1
4	4	4	4	4	4	4	4	1	1
4	4	4	4	3	3	4	4	1	1
4	4	4	4	4	4	4	4	1	1
4	4	4	4	4	4	4	4	1	1
4	4	4	4	4	4	3	3	1	1
1	1	4	4	3	3	4	4	1	1
1	1	4	4	3	3	4	4	1	1
1	1	4	4	3	3	4	4	1	1
4	4	4	4	4	4	4	4	1	1
4	4	4	4	4	4	4	4	1	1
4	4	4	4	4	4	4	4	1	1
1	1	4	4	4	4	4	4	3	3
1	1	4	4	4	4	4	4	1	1
4	4	1	1	4	4	3	3	1	1
1	1	4	4	4	4	3	3	1	1
4	4	4	4	4	4	3	3	1	1
4	4	4	4	4	4	4	4	1	1
4	4	4	4	4	4	4	4	1	1
4	4	4	4	4	4	4	4	1	1
4	4	4	4	4	4	4	4	1	1
4	4	4	4	4	4	4	4	1	1
4	4	4	4	3	3	4	4	1	1
4	4	4	4	4	4	4	4	1	1
1	1	4	4	4	4	4	4	1	1
4	4	4	4	4	4	4	4	1	1
4	4	4	4	4	4	4	4	1	1
4	4	4	4	4	4	4	4	1	1

For Review Only

4	4	4	4	4	4	4	4	1	1
1	1	4	4	3	3	4	4	1	1
4	4	4	4	3	3	4	4	1	1
4	4	4	4	4	4	4	4	1	1
4	4	4	4	4	4	4	4	1	1
4	4	4	4	3	3	4	4	1	1
4	4	4	4	4	4	4	4	1	1
4	4	4	4	4	4	3	3	1	1
1	1	4	4	4	4	4	4	1	1
4	4	4	4	4	4	4	4	1	1
4	4	4	4	3	3	4	4	1	1
4	4	4	4	3	3	4	4	1	1
4	4	4	4	4	4	4	4	1	1
4	4	4	4	4	4	4	4	1	1
4	4	4	4	4	4	4	4	1	1
4	4	4	4	4	4	4	4	1	1
4	4	4	4	4	4	4	4	1	1
1	1	4	4	4	4	4	4	1	1
4	4	4	4	4	4	4	4	1	1
4	4	4	4	4	4	4	4	1	1
4	4	4	4	3	3	4	4	1	1
4	4	4	4	4	4	4	4	1	1
4	4	4	4	4	4	4	4	1	1
4	4	4	4	4	4	4	4	1	1
4	4	4	4	4	4	4	4	1	1
4	4	4	4	3	3	4	4	1	1
1	1	4	4	4	4	3	3	1	1
4	4	1	1	4	4	3	3	1	1
4	4	4	4	4	4	4	4	1	1
4	4	4	4	4	4	4	4	1	1
4	4	4	4	4	4	4	4	1	1
4	4	4	4	4	4	4	4	1	1
4	4	1	1	4	4	4	4	1	1
4	4	4	4	4	4	4	4	1	1
1	1	4	4	4	4	4	4	1	1
4	4	4	4	4	4	4	4	1	1
4	4	4	4	4	4	4	4	1	1
4	4	4	4	4	4	4	4	1	1
4	4	4	4	4	4	4	4	1	1
4	4	4	4	4	4	3	3	1	1
4	4	4	4	4	4	4	4	1	1
4	4	4	4	3	3	4	4	1	1
4	4	4	4	4	4	3	3	1	1
4	4	4	4	3	3	4	4	1	1
4	4	4	4	4	4	4	4	1	1
4	4	4	4	3	3	4	4	1	1
4	4	4	4	4	4	3	3	1	1
4	4	4	4	4	4	4	4	1	1
4	4	4	4	4	4	4	4	1	1
4	4	4	4	3	3	4	4	3	3
4	4	4	4	3	3	4	4	1	1
4	4	1	1	4	4	3	3	1	1
4	4	1	1	4	4	3	3	1	1
4	4	4	4	4	4	4	4	1	1
1	1	4	4	3	3	4	4	3	3

For Review Only

4	4	4	4	4	4	4	4	1	1
4	4	4	4	4	4	3	3	1	1
4	4	4	4	4	4	4	4	1	1
4	4	4	4	4	4	3	3	1	1
4	4	4	4	3	3	4	4	1	1
4	4	4	4	4	4	4	4	1	1
4	4	4	4	3	3	4	4	1	1
4	4	4	4	4	4	4	4	1	1
4	4	1	1	4	4	4	4	1	1
4	4	4	4	4	4	4	4	1	1
1	1	4	4	4	4	3	3	3	3
4	4	4	4	4	4	4	4	1	1
4	4	4	4	4	4	3	3	1	1
4	4	4	4	4	4	4	4	1	1
4	4	4	4	4	4	4	4	1	1
4	4	4	4	4	4	4	4	1	1
1	1	4	4	4	4	4	4	1	1
4	4	4	4	4	4	4	4	1	1
4	4	4	4	4	4	3	3	1	1
4	4	4	4	4	4	3	3	1	1
4	4	4	4	4	4	4	4	1	1
4	4	4	4	4	4	4	4	1	1
1	1	4	4	3	3	4	4	1	1
4	4	1	1	4	4	3	3	1	1
4	4	4	4	4	4	4	4	1	1
4	4	4	4	4	4	4	4	1	1
4	4	4	4	4	4	4	4	1	1
1	1	4	4	4	4	4	4	3	3
4	4	4	4	3	3	4	4	1	1
4	4	4	4	4	4	4	4	1	1
4	4	4	4	4	4	3	3	1	1
4	4	4	4	4	4	4	4	1	1
4	4	4	4	4	4	4	4	1	1
4	4	4	4	4	4	4	4	1	1
4	4	4	4	4	4	4	4	1	1
4	4	1	1	4	4	3	3	1	1
4	4	4	4	4	4	3	3	1	1
4	4	4	4	4	4	4	4	1	1
4	4	4	4	4	4	3	3	1	1
4	4	4	4	4	4	3	3	1	1
4	4	4	4	4	4	4	4	1	1
4	4	4	4	4	4	3	3	3	3
4	4	4	4	3	3	4	4	1	1
4	4	4	4	4	4	3	3	1	1
4	4	4	4	4	4	4	4	1	1
1	1	4	4	4	4	3	3	3	3
4	4	4	4	4	4	4	4	1	1
4	4	4	4	3	3	4	4	1	1
4	4	4	4	4	4	4	4	1	1
4	4	4	4	4	4	4	4	1	1
4	4	4	4	4	4	4	4	1	1
4	4	4	4	4	4	3	3	1	1
4	4	4	4	4	4	4	4	1	1
4	4	4	4	3	3	4	4	1	1
4	4	4	4	4	4	4	4	1	1

For Review Only

1	1	4	4	4	4	4	4	1	1
4	4	4	4	4	4	3	3	1	1
4	4	4	4	4	4	4	4	1	1
4	4	4	4	4	4	4	4	1	1
4	4	4	4	3	3	4	4	3	3
4	4	4	4	4	4	4	4	1	1
4	4	4	4	4	4	4	4	1	1
4	4	4	4	4	4	4	4	1	1
4	4	4	4	4	4	3	3	1	1
4	4	4	4	4	4	4	4	1	1
4	4	4	4	4	4	4	4	1	1
4	4	4	4	4	4	3	3	1	1
4	4	4	4	4	4	4	4	1	1
4	4	4	4	4	4	3	3	1	1
4	4	4	4	4	4	3	3	1	1
4	4	4	4	4	4	3	3	1	1
4	4	4	4	4	4	4	4	1	1
4	4	4	4	3	3	4	4	1	1
4	4	4	4	4	4	4	4	1	1
4	4	4	4	3	3	4	4	1	1
4	4	4	4	4	4	4	4	1	1
4	4	1	1	4	4	4	4	1	1
4	4	4	4	4	4	4	4	1	1
4	4	4	4	3	3	4	4	1	1
4	4	4	4	4	4	4	4	1	1
4	4	4	4	4	4	4	4	1	1
4	4	4	4	3	3	4	4	1	1
4	4	4	4	4	4	4	4	1	1
1	1	4	4	4	4	3	3	1	1
4	4	4	4	4	4	4	4	1	1
1	1	4	4	4	4	4	4	3	3
1	1	4	4	4	4	4	4	1	1
4	4	4	4	4	4	4	4	3	3
4	4	4	4	4	4	3	3	1	1
4	4	4	4	4	4	4	4	3	3
4	4	4	4	3	3	4	4	1	1
4	4	4	4	3	3	4	4	1	1
4	4	4	4	4	4	4	4	3	3
4	4	4	4	3	3	4	4	1	1
4	4	4	4	4	4	4	4	1	1
1	1	4	4	3	3	4	4	1	1
4	4	4	4	4	4	4	4	1	1
4	4	1	1	4	4	3	3	1	1
4	4	4	4	4	4	4	4	1	1
4	4	4	4	4	4	4	4	1	1
4	4	4	4	3	3	4	4	1	1
4	4	4	4	4	4	3	3	1	1
4	4	4	4	4	4	4	4	1	1
4	4	4	4	4	4	3	3	1	1
4	4	4	4	3	3	4	4	1	1
4	4	4	4	4	4	4	4	1	1
1	1	4	4	4	4	4	4	1	1
4	4	4	4	4	4	4	4	1	1
4	4	4	4	3	3	4	4	1	1
4	4	4	4	4	4	3	3	1	1

For Review Only

4	4	4	4	4	4	3	3	1	1
4	4	4	4	4	4	3	3	1	1
4	4	4	4	4	4	4	4	3	3
4	4	4	4	4	4	4	4	1	1
4	4	4	4	4	4	3	3	1	1
4	4	4	4	4	4	4	4	1	1
4	4	4	4	4	4	3	3	1	1
4	4	4	4	4	4	3	3	1	1
4	4	4	4	4	4	4	4	3	3
4	4	1	1	4	4	3	3	1	1
4	4	4	4	4	4	4	4	1	1
1	1	4	4	4	4	4	4	1	1
4	4	4	4	4	4	4	4	1	1
4	4	4	4	4	4	4	4	1	1
1	1	4	4	3	3	4	4	1	1
4	4	4	4	4	4	3	3	1	1
4	4	1	1	4	4	3	3	3	3
4	4	4	4	4	4	3	3	1	1
4	4	4	4	4	4	4	4	1	1
1	1	4	4	4	4	4	4	1	1
4	4	4	4	4	4	3	3	1	1
4	4	4	4	4	4	4	4	1	1
4	4	4	4	4	4	4	4	1	1
4	4	4	4	4	4	3	3	1	1
4	4	4	4	4	4	3	3	1	1
4	4	4	4	4	4	4	4	1	1
4	4	1	1	4	4	3	3	1	1
4	4	4	4	3	3	4	4	1	1
4	4	4	4	4	4	4	4	1	1
4	4	4	4	4	4	4	4	3	3
4	4	1	1	4	4	3	3	1	1
4	4	4	4	4	4	4	4	1	1
4	4	4	4	4	4	4	4	1	1
4	4	4	4	4	4	4	4	1	1
4	4	4	4	4	4	4	4	1	1
4	4	1	1	4	4	3	3	1	1
4	4	4	4	4	4	4	4	1	1
4	4	4	4	3	3	4	4	1	1
4	4	4	4	4	4	4	4	1	1
4	4	1	1	4	4	3	3	1	1
1	1	4	4	4	4	4	4	3	3
1	1	4	4	4	4	4	4	1	1
4	4	4	4	4	4	3	3	1	1
4	4	4	4	4	4	4	4	1	1
4	4	4	4	4	4	4	4	1	1
4	4	4	4	3	3	4	4	1	1
4	4	4	4	4	4	3	3	1	1
4	4	4	4	3	3	3	3	3	3
4	4	4	4	3	3	3	3	1	1
4	4	1	1	4	4	3	3	1	1
4	4	4	4	4	4	4	4	1	1
4	4	4	4	4	4	3	3	1	1
1	1	4	4	3	3	4	4	1	1
4	4	4	4	4	4	4	4	1	1
4	4	4	4	4	4	3	3	1	1

For Review Only

4	4	4	4	4	4	4	4	1	1
1	1	4	4	3	3	4	4	1	1

For Review Only

SNP165	SNP165	SNP1650	SNP1650	SNP1651	SNP1651	SNP1652	SNP1652	SNP1653	SNP1653
1	1	2	2	3	3	4	4	3	3
4	4	2	2	3	3	4	4	1	1
4	4	2	2	3	3	4	4	1	1
1	1	2	2	3	3	4	4	1	1
4	4	4	4	3	3	4	4	1	1
1	1	2	2	3	3	4	4	1	1
1	1	2	2	3	3	4	4	3	3
4	4	2	2	3	3	4	4	3	3
1	1	2	2	3	3	4	4	3	3
4	4	2	2	3	3	4	4	3	3
4	4	2	2	3	3	4	4	1	1
1	1	2	2	3	3	4	4	1	1
1	1	2	2	3	3	4	4	1	1
4	4	2	2	3	3	4	4	1	1
1	1	2	2	3	3	4	4	1	1
4	4	4	4	3	3	4	4	1	1
1	1	4	4	3	3	4	4	3	3
1	1	2	2	3	3	4	4	1	1
4	4	2	2	3	3	4	4	1	1
4	4	2	2	3	3	4	4	3	3
1	1	2	2	3	3	4	4	3	3
4	4	2	2	3	3	4	4	1	1
4	4	2	2	2	2	1	1	1	1
4	4	4	4	3	3	4	4	1	1
1	1	2	2	3	3	4	4	1	1
4	4	2	2	3	3	4	4	1	1
4	4	2	2	3	3	4	4	1	1
1	1	2	2	3	3	4	4	1	1
4	4	2	2	3	3	4	4	1	1
4	4	2	2	2	2	1	1	3	3
1	1	2	2	3	3	4	4	1	1
1	1	2	2	3	3	4	4	3	3
1	1	2	2	3	3	4	4	3	3
4	4	2	2	3	3	4	4	1	1
4	4	2	2	3	3	4	4	1	1
1	1	2	2	3	3	4	4	1	1
1	1	2	2	3	3	4	4	1	1
4	4	2	2	3	3	4	4	1	1
4	4	2	2	3	3	4	4	1	1
1	1	2	2	3	3	4	4	1	1
4	4	2	2	2	2	4	4	3	3
4	4	2	2	3	3	4	4	3	3
4	4	2	2	3	3	4	4	3	3
1	1	4	4	3	3	4	4	1	1
4	4	2	2	3	3	4	4	3	3
4	4	2	2	3	3	4	4	1	1
4	4	4	4	3	3	4	4	1	1
4	4	2	2	3	3	4	4	1	1
1	1	2	2	3	3	4	4	1	1
4	4	2	2	3	3	4	4	1	1
1	1	2	2	3	3	4	4	1	1
4	4	2	2	3	3	4	4	1	1

For Review Only

1	1	2	2	3	3	4	4	1	1
1	1	2	2	3	3	4	4	3	3
1	1	2	2	3	3	4	4	1	1
1	1	2	2	3	3	4	4	1	1
4	4	2	2	3	3	4	4	1	1
4	4	4	4	3	3	4	4	1	1
1	1	2	2	3	3	4	4	1	1
4	4	2	2	2	2	1	1	1	1
4	4	2	2	3	3	4	4	1	1
1	1	2	2	3	3	4	4	1	1
1	1	2	2	3	3	4	4	1	1
4	4	4	4	3	3	4	4	1	1
1	1	2	2	3	3	4	4	1	1
4	4	2	2	3	3	4	4	1	1
1	1	2	2	3	3	4	4	1	1
4	4	2	2	3	3	4	4	1	1
4	4	2	2	3	3	4	4	1	1
4	4	2	2	3	3	4	4	1	1
4	4	2	2	3	3	4	4	1	1
1	1	2	2	3	3	4	4	3	3
4	4	4	4	3	3	4	4	1	1
1	1	2	2	3	3	4	4	1	1
4	4	2	2	3	3	4	4	1	1
4	4	2	2	3	3	4	4	3	3
4	4	2	2	3	3	4	4	3	3
1	1	2	2	3	3	4	4	1	1
4	4	4	4	3	3	4	4	1	1
4	4	2	2	3	3	4	4	1	1
4	4	2	2	3	3	4	4	1	1
4	4	2	2	3	3	4	4	1	1
4	4	4	4	3	3	4	4	1	1
4	4	2	2	3	3	4	4	3	3
4	4	2	2	3	3	4	4	1	1
1	1	2	2	3	3	4	4	1	1
4	4	2	2	3	3	4	4	3	3
4	4	2	2	3	3	4	4	3	3
4	4	2	2	3	3	4	4	3	3
1	1	2	2	2	2	1	1	1	1
4	4	2	2	3	3	4	4	1	1
1	1	2	2	3	3	4	4	3	3
1	1	2	2	3	3	4	4	1	1
4	4	2	2	3	3	4	4	1	1
4	4	2	2	2	2	1	1	1	1
1	1	2	2	3	3	4	4	1	1
1	1	2	2	3	3	4	4	1	1
1	1	2	2	3	3	4	4	1	1
4	4	2	2	3	3	4	4	1	1
1	1	2	2	3	3	4	4	1	1
4	4	2	2	3	3	4	4	1	1
4	4	2	2	3	3	4	4	1	1
1	1	2	2	3	3	4	4	1	1
4	4	2	2	2	2	1	1	1	1
4	4	2	2	2	2	1	1	3	3
4	4	2	2	3	3	4	4	1	1
4	4	2	2	3	3	4	4	1	1

For Review Only

1	1	2	2	3	3	4	4	1	1
4	4	2	2	3	3	4	4	1	1
1	1	2	2	3	3	4	4	1	1
4	4	2	2	3	3	4	4	1	1
1	1	2	2	3	3	4	4	1	1
1	1	2	2	3	3	4	4	3	3
1	1	2	2	3	3	4	4	1	1
1	1	2	2	3	3	4	4	1	1
4	4	2	2	3	3	4	4	1	1
4	4	2	2	3	3	4	4	1	1
1	1	2	2	3	3	4	4	3	3
1	1	2	2	3	3	4	4	1	1
4	4	2	2	2	2	1	1	1	1
1	1	2	2	3	3	4	4	1	1
1	1	2	2	3	3	4	4	3	3
1	1	2	2	3	3	4	4	1	1
4	4	2	2	3	3	4	4	1	1
1	1	2	2	3	3	4	4	1	1
4	4	2	2	3	3	4	4	1	1
1	1	2	2	3	3	4	4	1	1
1	1	2	2	3	3	4	4	1	1
4	4	2	2	3	3	4	4	1	1
4	4	2	2	3	3	4	4	1	1
4	4	2	2	2	2	1	1	1	1
1	1	2	2	3	3	4	4	1	1
4	4	2	2	3	3	4	4	1	1
4	4	2	2	3	3	4	4	1	1
4	4	4	4	3	3	4	4	1	1
1	1	2	2	3	3	4	4	1	1
4	4	2	2	3	3	4	4	1	1
4	4	2	2	3	3	4	4	1	1
4	4	2	2	3	3	4	4	1	1
1	1	2	2	3	3	4	4	1	1
4	4	2	2	3	3	4	4	1	1
4	4	2	2	3	3	4	4	1	1
4	4	2	2	3	3	4	4	1	1
4	4	2	2	3	3	4	4	1	1
4	4	2	2	3	3	4	4	1	1
4	4	2	2	3	3	4	4	1	1
4	4	2	2	3	3	4	4	1	1
4	4	2	2	3	3	4	4	1	1
4	4	2	2	3	3	4	4	1	1
4	4	2	2	3	3	4	4	1	1
4	4	4	4	3	3	4	4	1	1
4	4	2	2	3	3	4	4	1	1
1	1	2	2	3	3	4	4	1	1
1	1	2	2	3	3	4	4	1	1
4	4	2	2	3	3	4	4	1	1
4	4	2	2	2	2	4	4	1	1
1	1	2	2	3	3	4	4	1	1
1	1	2	2	3	3	4	4	1	1
4	4	2	2	3	3	4	4	1	1
4	4	2	2	3	3	4	4	1	1
4	4	2	2	3	3	4	4	3	3
4	4	2	2	3	3	4	4	1	1

For Review Only

1	1	2	2	3	3	4	4	1	1
1	1	2	2	3	3	4	4	1	1
4	4	4	4	3	3	4	4	1	1
4	4	2	2	3	3	4	4	1	1
1	1	2	2	3	3	4	4	1	1
4	4	2	2	3	3	4	4	1	1
4	4	2	2	3	3	4	4	1	1
1	1	2	2	3	3	4	4	1	1
4	4	2	2	3	3	4	4	1	1
4	4	2	2	3	3	4	4	1	1
1	1	2	2	3	3	4	4	1	1
4	4	2	2	3	3	4	4	1	1
1	1	2	2	3	3	4	4	1	1
1	1	2	2	3	3	4	4	1	1
4	4	2	2	3	3	4	4	1	1
4	4	2	2	3	3	4	4	1	1
4	4	2	2	3	3	4	4	1	1
4	4	2	2	3	3	4	4	1	1
4	4	2	2	3	3	4	4	1	1
1	1	2	2	3	3	4	4	1	1
1	1	2	2	3	3	4	4	1	1
4	4	2	2	3	3	4	4	3	3
1	1	2	2	3	3	4	4	1	1
4	4	4	4	3	3	4	4	1	1
1	1	2	2	3	3	4	4	3	3
4	4	2	2	3	3	4	4	1	1
1	1	2	2	3	3	4	4	1	1
4	4	2	2	3	3	4	4	1	1
4	4	2	2	3	3	4	4	1	1
1	1	2	2	3	3	4	4	1	1
1	1	2	2	3	3	4	4	1	1
4	4	2	2	3	3	4	4	1	1
4	4	2	2	3	3	4	4	1	1
4	4	2	2	3	3	4	4	1	1
1	1	2	2	3	3	4	4	1	1
4	4	4	4	3	3	4	4	1	1
1	1	2	2	3	3	4	4	1	1
1	1	2	2	3	3	4	4	1	1
4	4	2	2	3	3	4	4	1	1
4	4	2	2	3	3	4	4	1	1
4	4	2	2	3	3	4	4	1	1
1	1	2	2	3	3	4	4	1	1
4	4	2	2	3	3	4	4	1	1
4	4	2	2	3	3	4	4	1	1
1	1	2	2	3	3	4	4	1	1
4	4	2	2	3	3	4	4	1	1
4	4	2	2	3	3	4	4	1	1
4	4	2	2	3	3	4	4	1	1
4	4	2	2	3	3	4	4	1	1
1	1	2	2	3	3	4	4	1	1
1	1	2	2	3	3	4	4	1	1
4	4	2	2	3	3	4	4	1	1
4	4	2	2	3	3	4	4	1	1
1	1	2	2	3	3	4	4	1	1
4	4	2	2	3	3	4	4	1	1

For Review Only

4	4	2	2	3	3	4	4	1	1
4	4	2	2	3	3	4	4	1	1
4	4	2	2	3	3	4	4	1	1
1	1	2	2	3	3	4	4	1	1
4	4	2	2	3	3	4	4	1	1
4	4	2	2	3	3	4	4	1	1
4	4	2	2	3	3	4	4	1	1
4	4	2	2	2	2	1	1	3	3
4	4	2	2	3	3	4	4	1	1
4	4	2	2	3	3	4	4	1	1
1	1	2	2	3	3	4	4	1	1
4	4	2	2	3	3	4	4	1	1
1	1	2	2	3	3	4	4	1	1
4	4	2	2	3	3	4	4	1	1
1	1	2	2	3	3	4	4	1	1
4	4	2	2	3	3	4	4	1	1
4	4	2	2	3	3	4	4	1	1
4	4	2	2	3	3	4	4	1	1
4	4	2	2	3	3	4	4	1	1
4	4	2	2	3	3	4	4	1	1
4	4	2	2	3	3	4	4	1	1
4	4	2	2	3	3	4	4	1	1
4	4	2	2	3	3	4	4	1	1
4	4	2	2	3	3	4	4	1	1
4	4	2	2	2	2	1	1	1	1
1	1	2	2	3	3	4	4	3	3
4	4	2	2	3	3	4	4	1	1
1	1	2	2	3	3	4	4	1	1
1	1	2	2	3	3	4	4	1	1
4	4	2	2	3	3	4	4	1	1
4	4	2	2	3	3	4	4	1	1
4	4	2	2	3	3	4	4	1	1
4	4	2	2	3	3	4	4	1	1
4	4	2	2	3	3	4	4	1	1
1	1	2	2	3	3	4	4	1	1
4	4	2	2	3	3	4	4	1	1
1	1	2	2	3	3	4	4	1	1
4	4	4	4	3	3	4	4	1	1
1	1	2	2	3	3	4	4	1	1
4	4	2	2	3	3	4	4	1	1
1	1	2	2	3	3	4	4	1	1
4	4	2	2	2	2	1	1	1	1
4	4	2	2	3	3	4	4	1	1
1	1	2	2	3	3	4	4	1	1
4	4	2	2	3	3	4	4	1	1
4	4	2	2	3	3	4	4	1	1
4	4	2	2	3	3	4	4	1	1
4	4	2	2	3	3	4	4	1	1
4	4	2	2	3	3	4	4	1	1
4	4	2	2	3	3	4	4	1	1
4	4	2	2	3	3	4	4	1	1
4	4	2	2	3	3	4	4	1	1
4	4	2	2	3	3	4	4	1	1
1	1	2	2	3	3	4	4	1	1
4	4	2	2	3	3	4	4	1	1

For Review Only

4	4	2	2	3	3	4	4	1	1
1	1	4	4	3	3	4	4	1	1

For Review Only

SNP1654	SNP1654	SNP1655	SNP1655	SNP1656	SNP1656	SNP1657	SNP1657	SNP1658	SNP1658
4	4	4	4	2	2	1	1	4	4
4	4	1	1	2	2	4	4	4	4
4	4	4	4	2	2	1	1	4	4
1	1	4	4	2	2	1	1	4	4
4	4	4	4	3	3	4	4	4	4
4	4	4	4	2	2	1	1	4	4
4	4	4	4	2	2	1	1	4	4
1	1	1	1	2	2	1	1	4	4
4	4	4	4	2	2	1	1	1	1
4	4	1	1	2	2	4	4	4	4
1	1	4	4	2	2	1	1	4	4
4	4	4	4	2	2	1	1	4	4
4	4	4	4	2	2	1	1	4	4
1	1	4	4	2	2	4	4	4	4
1	1	4	4	2	2	1	1	4	4
1	1	4	4	2	2	1	1	4	4
4	4	4	4	3	3	1	1	4	4
4	4	4	4	2	2	1	1	1	1
4	4	4	4	2	2	1	1	4	4
4	4	1	1	2	2	4	4	4	4
4	4	4	4	2	2	4	4	1	1
4	4	4	4	2	2	1	1	4	4
4	4	4	4	2	2	4	4	4	4
4	4	4	4	2	2	4	4	4	4
4	4	4	4	2	2	1	1	4	4
4	4	4	4	2	2	1	1	4	4
4	4	4	4	2	2	1	1	4	4
1	1	4	4	2	2	1	1	4	4
4	4	4	4	2	2	1	1	1	1
4	4	1	1	2	2	4	4	4	4
4	4	4	4	2	2	1	1	4	4
4	4	4	4	2	2	1	1	4	4
4	4	4	4	3	3	1	1	4	4
4	4	4	4	2	2	4	4	4	4
4	4	4	4	2	2	1	1	4	4
1	1	4	4	2	2	1	1	4	4
4	4	4	4	2	2	1	1	4	4
1	1	4	4	2	2	1	1	1	1
4	4	4	4	2	2	4	4	4	4
4	4	4	4	2	2	1	1	4	4
4	4	1	1	2	2	4	4	4	4
4	4	4	4	2	2	4	4	1	1
4	4	4	4	2	2	1	1	1	1
4	4	4	4	2	2	1	1	4	4
4	4	4	4	2	2	1	1	1	1
4	4	4	4	2	2	1	1	4	4
4	4	4	4	2	2	1	1	4	4
4	4	4	4	2	2	1	1	4	4
4	4	4	4	2	2	1	1	4	4
1	1	4	4	3	3	1	1	4	4
4	4	4	4	2	2	1	1	4	4
4	4	4	4	3	3	4	4	4	4

For Review Only

4	4	1	1	3	3	1	1	4	4
4	4	4	4	3	3	1	1	4	4
4	4	4	4	2	2	1	1	4	4
4	4	4	4	2	2	1	1	4	4
4	4	4	4	2	2	1	1	4	4
4	4	4	4	2	2	4	4	4	4
4	4	4	4	2	2	1	1	4	4
1	1	4	4	2	2	4	4	4	4
1	1	4	4	2	2	1	1	4	4
1	1	4	4	2	2	1	1	4	4
4	4	4	4	2	2	4	4	4	4
1	1	4	4	2	2	4	4	4	4
4	4	4	4	3	3	1	1	4	4
4	4	4	4	2	2	1	1	1	1
4	4	4	4	2	2	1	1	4	4
4	4	4	4	2	2	4	4	4	4
4	4	4	4	2	2	1	1	4	4
4	4	4	4	2	2	1	1	4	4
4	4	4	4	2	2	1	1	4	4
4	4	4	4	3	3	1	1	4	4
1	1	4	4	2	2	1	1	4	4
4	4	4	4	2	2	4	4	4	4
4	4	4	4	2	2	1	1	1	1
4	4	4	4	2	2	1	1	4	4
4	4	4	4	2	2	1	1	1	1
4	4	4	4	2	2	1	1	4	4
4	4	1	1	2	2	1	1	4	4
4	4	4	4	2	2	4	4	4	4
4	4	4	4	2	2	1	1	4	4
1	1	4	4	2	2	1	1	4	4
4	4	4	4	2	2	1	1	1	1
1	1	4	4	2	2	1	1	4	4
4	4	4	4	2	2	4	4	4	4
4	4	4	4	3	3	1	1	4	4
1	1	4	4	2	2	1	1	4	4
4	4	4	4	2	2	1	1	1	1
4	4	4	4	2	2	1	1	1	1
4	4	4	4	2	2	4	4	4	4
1	1	4	4	2	2	1	1	4	4
4	4	1	1	2	2	1	1	4	4
4	4	4	4	3	3	1	1	4	4
1	1	4	4	2	2	1	1	4	4
1	1	4	4	2	2	4	4	4	4
4	4	4	4	2	2	4	4	4	4
1	1	4	4	2	2	1	1	4	4
4	4	4	4	2	2	1	1	4	4
1	1	4	4	2	2	4	4	4	4
4	4	4	4	2	2	1	1	4	4
1	1	4	4	2	2	1	1	4	4
4	4	4	4	2	2	1	1	1	1
1	1	4	4	2	2	1	1	4	4
4	4	4	4	2	2	4	4	4	4
4	4	4	4	2	2	4	4	4	4
1	1	4	4	2	2	4	4	4	4
4	4	4	4	2	2	1	1	1	1

For Review Only

CSSA 5585 Guilford Rd., Madison, WI 53711

4	4	4	4	2	2	1	1	4	4
4	4	1	1	2	2	1	1	4	4
4	4	4	4	2	2	1	1	4	4
4	4	4	4	2	2	1	1	4	4
4	4	4	4	2	2	1	1	4	4
4	4	4	4	2	2	1	1	4	4
1	1	4	4	2	2	1	1	4	4
4	4	4	4	2	2	1	1	4	4
4	4	4	4	2	2	1	1	4	4
1	1	4	4	2	2	1	1	4	4
1	1	4	4	2	2	1	1	4	4
4	4	4	4	2	2	1	1	4	4
4	4	4	4	2	2	4	4	4	4
4	4	4	4	2	2	1	1	4	4
4	4	4	4	2	2	1	1	4	4
1	1	4	4	2	2	1	1	4	4
4	4	4	4	2	2	1	1	4	4
1	1	4	4	2	2	1	1	4	4
1	1	4	4	2	2	4	4	4	4
4	4	4	4	2	2	1	1	4	4
4	4	4	4	2	2	1	1	4	4
1	1	4	4	2	2	1	1	4	4
1	1	4	4	2	2	1	1	4	4
4	4	4	4	2	2	4	4	4	4
4	4	4	4	2	2	1	1	4	4
1	1	4	4	2	2	1	1	4	4
1	1	4	4	3	3	1	1	4	4
1	1	4	4	2	2	1	1	4	4
1	1	4	4	2	2	1	1	4	4
1	1	4	4	2	2	1	1	4	4
1	1	4	4	2	2	4	4	4	4
1	1	4	4	2	2	1	1	4	4
4	4	4	4	3	3	1	1	4	4
4	4	4	4	2	2	1	1	4	4
4	4	4	4	2	2	4	4	4	4
4	4	4	4	2	2	4	4	4	4
1	1	4	4	2	2	4	4	4	4
4	4	4	4	2	2	1	1	4	4
4	4	4	4	2	2	1	1	4	4
1	1	4	4	2	2	1	1	4	4
4	4	4	4	2	2	1	1	4	4
1	1	4	4	2	2	4	4	4	4
1	1	4	4	2	2	1	1	4	4
4	4	1	1	2	2	1	1	4	4
4	4	4	4	2	2	1	1	4	4
4	4	4	4	2	2	1	1	4	4
1	1	4	4	2	2	1	1	4	4
4	4	4	4	2	2	1	1	4	4
4	4	4	4	2	2	1	1	4	4
4	4	4	4	2	2	1	1	4	4
4	4	4	4	2	2	1	1	4	4
4	4	4	4	2	2	1	1	4	4
1	1	4	4	2	2	1	1	4	4
1	1	4	4	2	2	1	1	4	4
1	1	4	4	2	2	4	4	4	4

For Review Only

4	4	4	4	2	2	1	1	4	4
4	4	4	4	2	2	1	1	4	4
1	1	4	4	2	2	1	1	4	4
1	1	4	4	2	2	1	1	4	4
4	4	4	4	2	2	1	1	4	4
4	4	4	4	2	2	1	1	4	4
1	1	4	4	2	2	1	1	4	4
1	1	4	4	2	2	1	1	4	4
4	4	4	4	2	2	1	1	4	4
1	1	4	4	2	2	1	1	4	4
4	4	4	4	2	2	1	1	4	4
1	1	4	4	2	2	1	1	4	4
1	1	4	4	2	2	1	1	4	4
4	4	4	4	2	2	1	1	1	1
1	1	4	4	2	2	4	4	4	4
1	1	4	4	2	2	1	1	4	4
1	1	4	4	2	2	1	1	4	4
4	4	4	4	2	2	1	1	4	4
4	4	4	4	2	2	1	1	4	4
1	1	4	4	2	2	1	1	4	4
1	1	4	4	2	2	1	1	4	4
4	4	4	4	2	2	4	4	4	4
4	4	4	4	2	2	4	4	4	4
1	1	4	4	2	2	4	4	4	4
4	4	4	4	2	2	1	1	4	4
4	4	4	4	2	2	4	4	4	4
4	4	4	4	2	2	1	1	4	4
4	4	4	4	2	2	1	1	4	4
4	4	4	4	2	2	1	1	4	4
4	4	4	4	3	3	1	1	4	4
4	4	4	4	2	2	1	1	4	4
4	4	4	4	2	2	1	1	4	4
1	1	4	4	2	2	1	1	4	4
1	1	4	4	2	2	4	4	4	4
1	1	4	4	2	2	1	1	4	4
1	1	4	4	2	2	4	4	4	4
4	4	4	4	2	2	1	1	4	4
4	4	4	4	2	2	1	1	4	4
4	4	4	4	2	2	1	1	4	4
1	1	4	4	2	2	1	1	4	4
4	4	4	4	2	2	1	1	4	4
4	4	4	4	2	2	1	1	4	4
4	4	4	4	2	2	4	4	4	4
4	4	4	4	2	2	1	1	4	4
1	1	4	4	2	2	1	1	4	4
1	1	4	4	2	2	4	4	4	4
1	1	4	4	2	2	1	1	4	4
4	4	4	4	2	2	4	4	4	4
1	1	4	4	2	2	1	1	4	4
4	4	4	4	2	2	1	1	4	4
1	1	4	4	2	2	1	1	1	1
1	1	4	4	2	2	4	4	4	4
4	4	4	4	2	2	4	4	4	4
4	4	4	4	2	2	4	4	4	4
4	4	4	4	2	2	1	1	4	4

For Review Only

1	1	4	4	2	2	4	4	4	4
4	4	1	1	2	2	1	1	4	4
1	1	4	4	2	2	1	1	4	4
4	4	4	4	2	2	1	1	4	4
4	4	1	1	2	2	1	1	4	4
4	4	4	4	2	2	1	1	4	4
4	4	4	4	2	2	1	1	4	4
4	4	1	1	2	2	4	4	4	4
1	1	4	4	2	2	1	1	4	4
4	4	4	4	2	2	4	4	4	4
1	1	4	4	2	2	1	1	4	4
1	1	4	4	2	2	1	1	4	4
1	1	4	4	2	2	1	1	4	4
4	4	4	4	2	2	1	1	4	4
4	4	4	4	2	2	1	1	4	4
1	1	4	4	2	2	1	1	4	4
4	4	4	4	2	2	1	1	4	4
4	4	4	4	2	2	1	1	4	4
4	4	4	4	2	2	1	1	4	4
4	4	4	4	2	2	1	1	1	1
4	4	4	4	2	2	1	1	4	4
4	4	4	4	2	2	1	1	4	4
4	4	4	4	2	2	1	1	4	4
1	1	4	4	2	2	1	1	4	4
1	1	4	4	2	2	4	4	4	4
4	4	4	4	2	2	1	1	4	4
4	4	4	4	2	2	4	4	4	4
4	4	4	4	2	2	1	1	4	4
4	4	4	4	2	2	1	1	4	4
1	1	4	4	2	2	1	1	4	4
4	4	4	4	2	2	4	4	4	4
1	1	4	4	2	2	1	1	4	4
4	4	4	4	2	2	1	1	1	1
4	4	4	4	2	2	1	1	4	4
4	4	4	4	2	2	4	4	4	4
4	4	4	4	2	2	4	4	4	4
1	1	4	4	2	2	1	1	4	4
1	1	4	4	2	2	4	4	4	4
1	1	4	4	2	2	1	1	4	4
4	4	1	1	2	2	4	4	4	4
4	4	4	4	3	3	1	1	4	4
4	4	4	4	2	2	4	4	4	4
1	1	4	4	2	2	1	1	4	4
4	4	4	4	3	3	1	1	4	4
4	4	4	4	2	2	1	1	4	4
1	1	4	4	2	2	1	1	4	4
4	4	4	4	2	2	1	1	4	4
4	4	4	4	2	2	1	1	4	4
4	4	1	1	2	2	1	1	4	4
4	4	4	4	2	2	4	4	4	4
1	1	4	4	3	3	1	1	4	4
4	4	4	4	2	2	4	4	4	4
4	4	4	4	2	2	1	1	4	4
4	4	4	4	2	2	1	1	4	4
4	4	4	4	2	2	1	1	4	4

For Review Only

1	1	4	4	2	2	1	1	4	4
1	1	4	4	2	2	1	1	4	4

For Review Only

SNP1659	SNP1659	SNP166	SNP166	SNP1660	SNP1660	SNP1661	SNP1661	SNP1662	SNP1662
1	1	2	2	2	2	2	2	2	2
1	1	2	2	2	2	2	2	2	2
1	1	2	2	2	2	2	2	2	2
1	1	2	2	2	2	2	2	2	2
2	2	2	2	3	3	2	2	2	2
1	1	2	2	2	2	2	2	2	2
1	1	2	2	2	2	2	2	2	2
1	1	1	1	2	2	2	2	2	2
1	1	2	2	2	2	2	2	2	2
1	1	2	2	2	2	2	2	2	2
1	1	1	1	2	2	2	2	2	2
1	1	2	2	2	2	2	2	2	2
1	1	2	2	2	2	2	2	2	2
1	1	2	2	2	2	2	2	3	3
1	1	2	2	2	2	2	2	2	2
1	1	2	2	2	2	3	3	2	2
1	1	2	2	2	2	2	2	2	2
1	1	2	2	2	2	2	2	2	2
1	1	2	2	2	2	2	2	2	2
1	1	2	2	2	2	2	2	2	2
1	1	2	2	2	2	2	2	2	2
1	1	2	2	2	2	2	2	2	2
2	2	1	1	3	3	2	2	3	3
1	1	1	1	2	2	3	3	2	2
1	1	2	2	2	2	2	2	2	2
1	1	2	2	2	2	2	2	2	2
1	1	2	2	2	2	2	2	2	2
1	1	2	2	2	2	2	2	2	2
1	1	2	2	2	2	2	2	2	2
2	2	2	2	3	3	2	2	2	2
1	1	2	2	2	2	2	2	2	2
1	1	2	2	2	2	2	2	2	2
1	1	2	2	2	2	2	2	2	2
1	1	1	1	2	2	2	2	2	2
1	1	2	2	2	2	2	2	2	2
1	1	2	2	2	2	2	2	2	2
1	1	2	2	2	2	2	2	3	3
1	1	2	2	2	2	2	2	2	2
1	1	1	1	2	2	2	2	2	2
1	1	1	1	2	2	2	2	2	2
2	2	2	2	3	3	2	2	2	2
1	1	2	2	2	2	2	2	2	2
1	1	2	2	2	2	2	2	2	2
1	1	2	2	2	2	2	2	2	2
1	1	2	2	2	2	2	2	2	2
1	1	2	2	2	2	2	2	2	2
1	1	2	2	2	2	3	3	2	2
1	1	2	2	2	2	2	2	2	2
1	1	2	2	2	2	2	2	2	2
1	1	2	2	2	2	2	2	2	2
1	1	2	2	2	2	2	2	2	2
1	1	1	1	2	2	2	2	2	2

For Review Only

1	1	2	2	2	2	2	2	2	2
1	1	2	2	2	2	2	2	2	2
1	1	2	2	2	2	2	2	2	2
1	1	2	2	2	2	2	2	2	2
1	1	2	2	2	2	2	2	2	2
1	1	2	2	2	2	3	3	2	2
1	1	2	2	2	2	2	2	2	2
2	2	2	2	3	3	2	2	2	2
1	1	2	2	2	2	2	2	2	2
1	1	2	2	2	2	2	2	2	2
1	1	2	2	2	2	2	2	2	2
1	1	2	2	2	2	3	3	2	2
1	1	2	2	2	2	2	2	2	2
1	1	2	2	2	2	2	2	2	2
1	1	2	2	2	2	2	2	2	2
1	1	2	2	2	2	2	2	3	3
1	1	1	1	2	2	2	2	2	2
1	1	2	2	2	2	2	2	2	2
1	1	2	2	2	2	2	2	2	2
1	1	2	2	2	2	2	2	2	2
1	1	2	2	2	2	2	2	2	2
1	1	2	2	2	2	2	2	2	2
1	1	2	2	2	2	2	2	2	2
1	1	2	2	2	2	2	2	2	2
1	1	2	2	2	2	2	2	2	2
1	1	2	2	2	2	2	2	2	2
1	1	2	2	2	2	2	2	2	2
1	1	1	1	2	2	2	2	2	2
1	1	2	2	2	2	2	2	2	2
1	1	1	1	2	2	2	2	2	2
1	1	2	2	2	2	2	2	2	2
1	1	2	2	2	2	2	2	2	2
1	1	1	1	2	2	2	2	2	2
1	1	2	2	2	2	2	2	2	2
1	1	1	1	2	2	2	2	2	2
1	1	2	2	2	2	2	2	2	2
1	1	2	2	2	2	2	2	2	2
2	2	2	2	3	3	2	2	2	2
1	1	2	2	2	2	2	2	2	2
1	1	2	2	2	2	2	2	2	2
1	1	2	2	2	2	2	2	2	2
1	1	2	2	2	2	2	2	2	2
2	2	2	2	2	2	2	2	2	2
1	1	2	2	2	2	2	2	2	2
1	1	2	2	2	2	2	2	2	2
1	1	2	2	2	2	2	2	2	2
1	1	1	1	2	2	2	2	2	2
1	1	2	2	2	2	2	2	2	2
1	1	2	2	2	2	2	2	2	2
1	1	2	2	2	2	2	2	2	2
1	1	2	2	2	2	2	2	2	2
1	1	1	1	3	3	2	2	2	2
2	2	2	2	3	3	2	2	2	2
1	1	2	2	2	2	3	3	2	2
1	1	2	2	2	2	2	2	3	3

For Review Only

1	1	2	2	2	2	2	2	2	2
1	1	2	2	2	2	2	2	2	2
1	1	2	2	2	2	2	2	2	2
1	1	1	1	2	2	2	2	2	2
1	1	2	2	2	2	2	2	2	2
1	1	2	2	2	2	2	2	2	2
1	1	2	2	2	2	2	2	2	2
1	1	2	2	2	2	2	2	2	2
1	1	2	2	2	2	2	2	2	2
1	1	2	2	2	2	2	2	2	2
1	1	2	2	2	2	2	2	3	3
1	1	2	2	2	2	2	2	2	2
2	2	2	2	3	3	2	2	2	2
1	1	2	2	2	2	2	2	2	2
1	1	2	2	2	2	2	2	2	2
1	1	2	2	2	2	2	2	2	2
1	1	2	2	2	2	2	2	2	2
1	1	2	2	2	2	2	2	2	2
1	1	1	1	2	2	2	2	2	2
1	1	2	2	2	2	2	2	2	2
1	1	2	2	2	2	2	2	2	2
1	1	1	1	2	2	2	2	3	3
1	1	2	2	2	2	2	2	2	2
2	2	1	1	3	3	2	2	2	2
1	1	2	2	2	2	2	2	2	2
1	1	2	2	2	2	2	2	2	2
1	1	2	2	2	2	2	2	2	2
1	1	1	1	2	2	3	3	3	3
1	1	2	2	2	2	2	2	2	2
1	1	2	2	2	2	2	2	2	2
1	1	1	1	2	2	2	2	2	2
1	1	2	2	2	2	2	2	2	2
1	1	2	2	2	2	2	2	2	2
1	1	1	1	2	2	2	2	2	2
1	1	1	1	2	2	2	2	2	2
1	1	2	2	2	2	2	2	2	2
1	1	1	1	2	2	2	2	2	2
1	1	2	2	2	2	2	2	2	2
1	1	1	1	2	2	2	2	2	2
1	1	1	1	2	2	2	2	2	2
1	1	2	2	2	2	2	2	2	2
1	1	2	2	2	2	2	2	3	3
1	1	2	2	2	2	2	2	2	2
1	1	2	2	2	2	2	2	2	2
1	1	2	2	2	2	2	2	2	2
1	1	2	2	2	2	2	2	3	3
1	1	2	2	2	2	2	2	2	2
1	1	2	2	2	2	2	2	2	2
2	2	2	2	2	2	2	2	2	2
1	1	2	2	2	2	2	2	2	2
1	1	2	2	2	2	2	2	2	2
1	1	2	2	2	2	2	2	2	2
1	1	2	2	2	2	2	2	2	2
1	1	2	2	2	2	2	2	2	2
1	1	2	2	2	2	2	2	2	2

For Review Only

1	1	2	2	2	2	2	2	3	3
1	1	2	2	2	2	2	2	2	2
1	1	2	2	2	2	2	2	2	2
1	1	2	2	2	2	2	2	2	2
1	1	2	2	2	2	2	2	2	2
1	1	1	1	2	2	2	2	2	2
1	1	1	1	2	2	2	2	3	3
1	1	2	2	2	2	2	2	2	2
1	1	1	1	2	2	2	2	2	2
1	1	1	1	2	2	2	2	2	2
1	1	2	2	2	2	2	2	2	2
1	1	1	1	2	2	3	3	3	3
1	1	2	2	2	2	2	2	2	2
1	1	2	2	2	2	2	2	3	3
1	1	1	1	2	2	2	2	2	2
1	1	1	1	2	2	2	2	2	2
1	1	2	2	2	2	2	2	2	2
1	1	2	2	2	2	2	2	2	2
1	1	1	1	2	2	2	2	2	2
1	1	2	2	2	2	2	2	2	2
1	1	2	2	2	2	2	2	2	2
1	1	2	2	2	2	2	2	3	3
1	1	2	2	2	2	2	2	2	2
1	1	2	2	2	2	3	3	2	2
1	1	2	2	2	2	2	2	2	2
1	1	1	1	2	2	2	2	2	2
1	1	2	2	2	2	2	2	2	2
1	1	2	2	2	2	2	2	2	2
1	1	2	2	2	2	2	2	2	2
1	1	2	2	2	2	2	2	2	2
1	1	2	2	2	2	2	2	3	3
1	1	2	2	2	2	2	2	2	2
1	1	2	2	2	2	2	2	2	2
1	1	1	1	2	2	2	2	2	2
1	1	2	2	2	2	2	2	3	3
1	1	2	2	2	2	3	3	2	2
1	1	2	2	2	2	2	2	2	2
1	1	2	2	2	2	2	2	3	3
1	1	2	2	2	2	2	2	2	2
1	1	2	2	2	2	2	2	2	2
1	1	2	2	2	2	2	2	2	2
1	1	2	2	2	2	2	2	2	2
1	1	1	1	2	2	2	2	2	2
1	1	2	2	2	2	2	2	2	2
1	1	2	2	2	2	2	2	2	2
1	1	2	2	2	2	2	2	2	2
1	1	1	1	2	2	2	2	2	2
1	1	1	1	2	2	2	2	3	3
1	1	2	2	2	2	2	2	2	2
1	1	2	2	2	2	2	2	2	2
1	1	2	2	2	2	2	2	2	2
1	1	2	2	2	2	2	2	2	2
1	1	1	1	2	2	2	2	2	2
1	1	2	2	2	2	2	2	2	2
1	1	1	1	2	2	2	2	3	3

1	1	2	2	2	2	2	2	2	2
1	1	2	2	2	2	2	2	2	2
1	1	2	2	2	2	2	2	2	2
1	1	2	2	2	2	2	2	2	2
1	1	2	2	2	2	2	2	2	2
1	1	2	2	2	2	2	2	2	2
1	1	2	2	2	2	2	2	2	2
2	2	2	2	3	3	2	2	2	2
1	1	2	2	2	2	2	2	3	3
1	1	1	1	2	2	2	2	2	2
1	1	2	2	2	2	2	2	2	2
1	1	2	2	2	2	2	2	2	2
1	1	2	2	2	2	2	2	2	2
1	1	1	1	2	2	2	2	2	2
1	1	2	2	2	2	2	2	2	2
1	1	1	1	2	2	2	2	2	2
1	1	2	2	2	2	2	2	3	3
1	1	1	1	2	2	2	2	2	2
1	1	1	1	2	2	2	2	2	2
1	1	2	2	2	2	2	2	2	2
1	1	2	2	2	2	2	2	3	3
1	1	2	2	2	2	2	2	2	2
1	1	1	1	2	2	2	2	2	2
1	1	2	2	2	2	2	2	2	2
2	2	2	2	3	3	2	2	2	2
1	1	2	2	2	2	2	2	2	2
1	1	1	1	2	2	2	2	2	2
1	1	2	2	2	2	2	2	2	2
1	1	2	2	2	2	2	2	2	2
1	1	1	1	2	2	2	2	2	2
1	1	1	1	2	2	2	2	3	3
1	1	1	1	2	2	2	2	2	2
1	1	2	2	2	2	2	2	3	3
1	1	2	2	2	2	2	2	2	2
1	1	2	2	2	2	2	2	2	2
1	1	1	1	2	2	2	2	2	2
1	1	2	2	2	2	2	2	2	2
1	1	2	2	2	2	3	3	2	2
1	1	2	2	2	2	2	2	2	2
1	1	1	1	2	2	2	2	2	2
1	1	2	2	2	2	2	2	3	3
2	2	2	2	2	2	2	2	2	2
1	1	1	1	2	2	2	2	2	2
1	1	2	2	2	2	2	2	2	2
1	1	2	2	2	2	2	2	2	2
1	1	2	2	2	2	2	2	2	2
1	1	2	2	2	2	2	2	2	2
1	1	2	2	2	2	2	2	2	2
1	1	2	2	2	2	2	2	2	2
1	1	1	1	2	2	2	2	2	2
1	1	2	2	2	2	2	2	2	2
1	1	1	1	2	2	2	2	2	2
1	1	2	2	2	2	2	2	2	2
1	1	2	2	2	2	2	2	2	2
1	1	1	1	2	2	2	2	2	2

For Review Only

1	1	1	1	2	2	2	2	2	2
1	1	2	2	2	2	3	3	2	2

For Review Only

SNP1663	SNP1663	SNP1664	SNP1664	SNP1665	SNP1665	SNP1666	SNP1666	SNP1667	SNP1667
2	2	1	1	2	2	1	1	4	4
2	2	1	1	4	4	1	1	4	4
3	3	2	2	4	4	4	4	4	4
3	3	2	2	2	2	4	4	4	4
2	2	2	2	2	2	4	4	4	4
2	2	1	1	4	4	1	1	4	4
3	3	2	2	4	4	4	4	4	4
2	2	2	2	4	4	4	4	2	2
2	2	2	2	4	4	4	4	4	4
2	2	2	2	4	4	4	4	2	2
3	3	2	2	4	4	4	4	4	4
3	3	2	2	4	4	4	4	4	4
2	2	2	2	2	2	4	4	4	4
2	2	2	2	4	4	4	4	4	4
2	2	2	2	4	4	4	4	4	4
3	3	1	1	4	4	1	1	4	4
3	3	2	2	4	4	4	4	2	2
2	2	2	2	4	4	4	4	4	4
3	3	2	2	2	2	4	4	4	4
2	2	2	2	4	4	4	4	2	2
2	2	2	2	4	4	4	4	2	2
3	3	2	2	4	4	4	4	4	4
2	2	2	2	2	2	4	4	4	4
2	2	2	2	4	4	4	4	4	4
2	2	2	2	4	4	4	4	4	4
3	3	2	2	4	4	4	4	4	4
3	3	2	2	4	4	4	4	4	4
2	2	2	2	4	4	4	4	4	4
2	2	2	2	4	4	4	4	4	4
2	2	2	2	4	4	4	4	2	2
3	3	2	2	2	2	4	4	4	4
2	2	1	1	4	4	4	4	2	2
2	2	1	1	4	4	1	1	4	4
2	2	2	2	4	4	4	4	4	4
2	2	1	1	4	4	1	1	4	4
3	3	2	2	4	4	4	4	4	4
2	2	1	1	4	4	1	1	4	4
3	3	2	2	4	4	4	4	4	4
2	2	2	2	4	4	4	4	4	4
3	3	2	2	4	4	4	4	4	4
2	2	2	2	4	4	4	4	2	2
2	2	2	2	4	4	4	4	2	2
2	2	2	2	4	4	4	4	4	4
2	2	2	2	4	4	4	4	4	4
2	2	2	2	4	4	4	4	2	2
3	3	2	2	4	4	4	4	4	4
3	3	1	1	4	4	1	1	4	4
3	3	2	2	4	4	4	4	4	4
3	3	2	2	4	4	4	4	4	4
3	3	1	1	4	4	1	1	4	4
3	3	2	2	4	4	4	4	4	4
2	2	2	2	4	4	4	4	4	4

For Review Only

2	2	2	2	4	4	4	4	4	4
2	2	1	1	4	4	1	1	4	4
3	3	2	2	4	4	4	4	4	4
3	3	2	2	4	4	4	4	4	4
3	3	2	2	2	2	4	4	4	4
2	2	2	2	4	4	4	4	4	4
3	3	2	2	4	4	4	4	4	4
2	2	2	2	4	4	4	4	4	4
2	2	2	2	4	4	4	4	4	4
2	2	2	2	4	4	4	4	4	4
2	2	1	1	4	4	1	1	4	4
2	2	1	1	4	4	1	1	4	4
2	2	2	2	4	4	4	4	4	4
2	2	2	2	4	4	4	4	4	4
2	2	2	2	4	4	4	4	4	4
2	2	1	1	4	4	1	1	4	4
3	3	2	2	4	4	4	4	4	4
3	3	1	1	4	4	1	1	4	4
3	3	2	2	4	4	4	4	4	4
3	3	2	2	4	4	4	4	4	4
2	2	1	1	4	4	1	1	4	4
2	2	2	2	4	4	4	4	4	4
3	3	2	2	4	4	4	4	4	4
2	2	2	2	4	4	4	4	2	2
2	2	2	2	4	4	4	4	2	2
3	3	1	1	4	4	1	1	4	4
2	2	1	1	4	4	1	1	4	4
2	2	2	2	4	4	4	4	4	4
3	3	2	2	2	2	4	4	4	4
3	3	2	2	4	4	4	4	4	4
2	2	2	2	4	4	4	4	4	4
2	2	2	2	4	4	4	4	4	4
2	2	2	2	2	2	4	4	4	4
2	2	2	2	4	4	4	4	4	4
3	3	1	1	4	4	1	1	2	2
2	2	2	2	4	4	4	4	2	2
2	2	2	2	4	4	4	4	2	2
2	2	2	2	4	4	4	4	4	4
3	3	2	2	4	4	4	4	4	4
2	2	2	2	4	4	4	4	2	2
3	3	2	2	4	4	4	4	4	4
3	3	2	2	4	4	4	4	4	4
2	2	2	2	4	4	4	4	4	4
2	2	1	1	4	4	1	1	4	4
3	3	2	2	4	4	4	4	4	4
2	2	1	1	4	4	1	1	4	4
2	2	2	2	2	2	4	4	4	4
3	3	2	2	2	2	4	4	4	4
3	3	2	2	4	4	4	4	4	4
3	3	2	2	4	4	4	4	4	4
2	2	2	2	4	4	4	4	4	4
2	2	2	2	4	4	4	4	4	4
2	2	2	2	4	4	4	4	2	2
2	2	2	2	4	4	1	1	4	4
3	3	2	2	4	4	4	4	4	4

For Review Only

3	3	2	2	4	4	4	4	4	4
2	2	2	2	4	4	4	4	4	4
3	3	2	2	4	4	4	4	4	4
3	3	2	2	2	2	4	4	4	4
2	2	1	1	4	4	1	1	4	4
3	3	2	2	4	4	4	4	2	2
3	3	1	1	4	4	1	1	4	4
2	2	2	2	4	4	4	4	4	4
3	3	2	2	4	4	4	4	4	4
3	3	2	2	4	4	4	4	4	4
2	2	2	2	4	4	4	4	4	4
2	2	2	2	4	4	4	4	4	4
2	2	2	2	4	4	4	4	4	4
3	3	2	2	4	4	4	4	4	4
2	2	1	1	4	4	1	1	4	4
2	2	2	2	4	4	4	4	4	4
3	3	2	2	4	4	4	4	4	4
2	2	2	2	4	4	4	4	4	4
2	2	2	2	2	2	4	4	4	4
3	3	2	2	4	4	4	4	4	4
2	2	2	2	4	4	4	4	4	4
2	2	1	1	4	4	4	4	4	4
2	2	2	2	4	4	4	4	4	4
2	2	2	2	4	4	4	4	4	4
2	2	2	2	4	4	4	4	4	4
2	2	2	2	4	4	4	4	4	4
2	2	2	2	4	4	4	4	4	4
3	3	1	1	4	4	1	1	4	4
2	2	2	2	4	4	4	4	4	4
2	2	1	1	2	2	1	1	4	4
2	2	2	2	4	4	4	4	4	4
2	2	2	2	4	4	4	4	4	4
3	3	2	2	4	4	4	4	4	4
2	2	2	2	2	2	4	4	4	4
2	2	2	2	4	4	4	4	4	4
2	2	2	2	4	4	4	4	4	4
2	2	2	2	2	2	4	4	4	4
3	3	1	1	4	4	1	1	4	4
3	3	2	2	4	4	4	4	4	4
2	2	2	2	4	4	4	4	4	4
3	3	2	2	4	4	4	4	4	4
2	2	2	2	4	4	4	4	4	4
3	3	2	2	4	4	4	4	4	4
2	2	2	2	4	4	4	4	2	2
2	2	2	2	4	4	4	4	4	4
2	2	1	1	4	4	1	1	4	4
3	3	2	2	4	4	4	4	4	4
3	3	2	2	4	4	4	4	4	4
2	2	2	2	4	4	4	4	4	4
3	3	2	2	4	4	4	4	4	4
3	3	2	2	4	4	4	4	4	4
3	3	2	2	2	2	4	4	4	4
3	3	1	1	4	4	1	1	4	4
3	3	2	2	4	4	4	4	4	4
2	2	2	2	4	4	4	4	4	4

For Review Only

2	2	1	1	4	4	1	1	4	4
3	3	2	2	4	4	4	4	4	4
2	2	2	2	4	4	4	4	4	4
2	2	2	2	4	4	4	4	4	4
2	2	1	1	4	4	1	1	4	4
3	3	1	1	2	2	1	1	4	4
2	2	2	2	4	4	4	4	4	4
2	2	2	2	4	4	4	4	4	4
2	2	2	2	2	2	4	4	4	4
2	2	1	1	4	4	1	1	4	4
3	3	2	2	4	4	4	4	4	4
2	2	2	2	4	4	4	4	4	4
3	3	2	2	2	2	4	4	4	4
2	2	2	2	4	4	4	4	4	4
2	2	2	2	4	4	4	4	4	4
3	3	2	2	4	4	4	4	4	4
3	3	1	1	4	4	4	4	4	4
2	2	1	1	4	4	1	1	4	4
3	3	2	2	4	4	4	4	4	4
3	3	2	2	4	4	4	4	4	4
2	2	2	2	4	4	4	4	4	4
2	2	2	2	2	2	4	4	4	4
2	2	2	2	4	4	4	4	4	4
2	2	1	1	4	4	1	1	4	4
2	2	2	2	4	4	4	4	2	2
2	2	2	2	4	4	4	4	4	4
3	3	2	2	2	2	4	4	4	4
2	2	2	2	4	4	4	4	4	4
3	3	1	1	4	4	1	1	4	4
3	3	2	2	4	4	4	4	4	4
3	3	2	2	4	4	4	4	4	4
3	3	2	2	4	4	4	4	4	4
2	2	2	2	4	4	4	4	4	4
2	2	2	2	4	4	4	4	4	4
2	2	2	2	4	4	4	4	4	4
2	2	1	1	4	4	1	1	4	4
3	3	2	2	4	4	4	4	4	4
3	3	1	1	4	4	1	1	4	4
3	3	2	2	4	4	4	4	4	4
3	3	2	2	4	4	4	4	4	4
3	3	2	2	4	4	4	4	4	4
3	3	2	2	4	4	4	4	4	4
2	2	2	2	4	4	4	4	4	4
2	2	2	2	4	4	4	4	4	4
3	3	2	2	4	4	4	4	4	4
2	2	1	1	4	4	1	1	4	4
3	3	2	2	4	4	4	4	4	4
2	2	2	2	2	2	4	4	4	4
3	3	2	2	2	2	4	4	4	4
2	2	2	2	4	4	4	4	4	4
3	3	2	2	4	4	4	4	4	4
2	2	1	1	2	2	1	1	4	4
2	2	2	2	4	4	4	4	4	4
2	2	1	1	4	4	1	1	4	4
3	3	2	2	4	4	4	4	4	4

For Review Only

2	2	2	2	4	4	4	4	4	4
2	2	2	2	4	4	1	1	4	4
2	2	2	2	4	4	4	4	4	4
2	2	2	2	4	4	4	4	4	4
2	2	1	1	4	4	1	1	4	4
3	3	2	2	4	4	4	4	4	4
3	3	2	2	4	4	4	4	4	4
2	2	2	2	4	4	4	4	2	2
2	2	2	2	4	4	4	4	4	4
2	2	2	2	2	2	4	4	4	4
2	2	2	2	4	4	4	4	4	4
3	3	1	1	4	4	1	1	4	4
2	2	2	2	4	4	4	4	4	4
3	3	2	2	4	4	4	4	4	4
3	3	2	2	4	4	4	4	4	4
3	3	2	2	4	4	4	4	4	4
3	3	2	2	4	4	4	4	4	4
2	2	2	2	4	4	4	4	4	4
3	3	2	2	2	2	4	4	4	4
3	3	2	2	4	4	4	4	4	4
2	2	2	2	2	2	4	4	4	4
3	3	2	2	4	4	4	4	4	4
2	2	1	1	4	4	1	1	4	4
3	3	2	2	4	4	4	4	4	4
2	2	2	2	4	4	4	4	4	4
2	2	2	2	4	4	4	4	4	4
2	2	2	2	2	2	4	4	4	4
3	3	2	2	2	2	4	4	4	4
2	2	2	2	4	4	4	4	4	4
2	2	2	2	4	4	4	4	4	4
2	2	2	2	2	2	4	4	4	4
3	3	2	2	4	4	4	4	4	4
3	3	2	2	4	4	4	4	4	4
3	3	2	2	4	4	4	4	4	4
2	2	2	2	4	4	4	4	4	4
2	2	2	2	2	2	4	4	4	4
3	3	1	1	4	4	1	1	4	4
2	2	1	1	4	4	1	1	4	4
3	3	2	2	4	4	4	4	4	4
2	2	2	2	4	4	4	4	4	4
2	2	2	2	4	4	4	4	4	4
3	3	1	1	4	4	1	1	4	4
3	3	2	2	4	4	4	4	4	4
3	3	2	2	4	4	4	4	4	4
3	3	1	1	4	4	1	1	4	4
2	2	2	2	4	4	4	4	4	4
3	3	2	2	4	4	4	4	4	4
3	3	2	2	2	2	4	4	4	4
3	3	2	2	4	4	4	4	4	4
2	2	2	2	2	2	4	4	4	4
2	2	2	2	4	4	4	4	4	4
2	2	2	2	4	4	4	4	4	4
3	3	2	2	4	4	4	4	4	4
3	3	2	2	2	2	4	4	4	4
3	3	2	2	4	4	4	4	4	4

For Review Only

3	3	2	2	4	4	4	4	4	4
3	3	2	2	4	4	1	1	4	4

For Review Only

SNP1668	SNP1668	SNP1669	SNP1669	SNP167	SNP167	SNP1670	SNP1670	SNP1671	SNP1671
4	4	3	3	2	2	3	3	2	2
4	4	3	3	2	2	3	3	1	1
4	4	2	2	2	2	1	1	2	2
4	4	2	2	2	2	3	3	2	2
4	4	2	2	2	2	1	1	2	2
4	4	3	3	2	2	1	1	2	2
4	4	2	2	2	2	3	3	2	2
4	4	2	2	1	1	3	3	2	2
4	4	2	2	2	2	3	3	2	2
4	4	2	2	2	2	3	3	1	1
1	1	2	2	1	1	1	1	2	2
4	4	2	2	2	2	1	1	2	2
4	4	2	2	2	2	3	3	2	2
1	1	2	2	2	2	3	3	2	2
4	4	2	2	2	2	1	1	2	2
4	4	3	3	1	1	3	3	2	2
4	4	2	2	2	2	3	3	2	2
4	4	2	2	2	2	3	3	2	2
1	1	2	2	2	2	1	1	2	2
4	4	2	2	2	2	3	3	1	1
4	4	2	2	2	2	3	3	2	2
4	4	2	2	2	2	1	1	2	2
4	4	2	2	1	1	3	3	2	2
4	4	2	2	1	1	3	3	2	2
4	4	2	2	2	2	3	3	2	2
4	4	2	2	2	2	3	3	2	2
4	4	2	2	2	2	3	3	2	2
4	4	2	2	2	2	3	3	2	2
4	4	2	2	2	2	3	3	2	2
4	4	2	2	2	2	3	3	1	1
4	4	2	2	2	2	1	1	2	2
4	4	2	2	2	2	1	1	2	2
4	4	3	3	2	2	1	1	2	2
4	4	2	2	1	1	3	3	2	2
4	4	3	3	2	2	3	3	2	2
4	4	2	2	2	2	3	3	2	2
1	1	3	3	2	2	3	3	2	2
4	4	2	2	2	2	1	1	1	1
1	1	2	2	1	1	3	3	2	2
1	1	2	2	1	1	1	1	2	2
4	4	2	2	2	2	3	3	1	1
4	4	2	2	2	2	3	3	2	2
4	4	2	2	2	2	3	3	1	1
4	4	2	2	2	2	1	1	1	1
4	4	2	2	2	2	3	3	1	1
4	4	2	2	2	2	1	1	2	2
4	4	3	3	2	2	3	3	2	2
4	4	2	2	2	2	1	1	2	2
4	4	2	2	2	2	3	3	1	1
4	4	2	2	2	2	1	1	2	2
4	4	2	2	2	2	3	3	2	2
4	4	2	2	1	1	3	3	2	2

For Review Only

4	4	2	2	2	2	3	3	2	2
4	4	3	3	2	2	1	1	2	2
4	4	2	2	2	2	3	3	2	2
4	4	2	2	2	2	3	3	1	1
1	1	2	2	2	2	1	1	2	2
4	4	2	2	2	2	3	3	2	2
4	4	2	2	2	2	3	3	2	2
4	4	2	2	2	2	3	3	1	1
4	4	2	2	2	2	3	3	2	2
4	4	2	2	2	2	3	3	2	2
4	4	3	3	2	2	3	3	2	2
4	4	3	3	2	2	3	3	2	2
4	4	2	2	2	2	3	3	2	2
4	4	2	2	2	2	3	3	2	2
4	4	2	2	2	2	1	1	2	2
1	1	3	3	2	2	3	3	2	2
4	4	2	2	1	1	1	1	2	2
4	4	3	3	2	2	1	1	2	2
4	4	2	2	2	2	1	1	2	2
4	4	2	2	2	2	3	3	2	2
4	4	3	3	2	2	3	3	2	2
1	1	2	2	2	2	3	3	2	2
4	4	2	2	2	2	3	3	2	2
4	4	2	2	2	2	3	3	2	2
4	4	2	2	2	2	3	3	1	1
4	4	3	3	2	2	3	3	2	2
4	4	3	3	2	2	1	1	2	2
4	4	2	2	1	1	3	3	1	1
4	4	2	2	2	2	3	3	2	2
4	4	2	2	1	1	1	1	2	2
4	4	2	2	2	2	3	3	1	1
4	4	2	2	2	2	3	3	2	2
4	4	2	2	1	1	3	3	1	1
4	4	2	2	2	2	3	3	2	2
4	4	3	3	1	1	3	3	1	1
4	4	2	2	2	2	3	3	1	1
4	4	2	2	2	2	3	3	1	1
4	4	2	2	2	2	3	3	1	1
4	4	2	2	2	2	1	1	2	2
4	4	2	2	2	2	3	3	1	1
4	4	2	2	2	2	1	1	2	2
4	4	2	2	2	2	1	1	2	2
4	4	2	2	2	2	3	3	2	2
4	4	3	3	2	2	3	3	2	2
4	4	2	2	2	2	1	1	2	2
4	4	3	3	2	2	3	3	2	2
4	4	2	2	1	1	3	3	2	2
4	4	2	2	2	2	3	3	2	2
4	4	2	2	2	2	3	3	2	2
1	1	2	2	2	2	3	3	2	2
4	4	2	2	2	2	3	3	2	2
1	1	2	2	1	1	3	3	2	2
4	4	2	2	2	2	3	3	1	1
4	4	2	2	2	2	3	3	2	2
1	1	2	2	2	2	1	1	2	2

For Review Only

4	4	2	2	2	2	3	3	2	2
1	1	2	2	2	2	3	3	2	2
4	4	2	2	2	2	3	3	2	2
4	4	2	2	1	1	3	3	2	2
4	4	3	3	2	2	1	1	2	2
4	4	2	2	2	2	3	3	2	2
4	4	3	3	2	2	3	3	2	2
4	4	2	2	2	2	1	1	2	2
4	4	2	2	2	2	3	3	2	2
4	4	2	2	2	2	3	3	2	2
1	1	2	2	2	2	3	3	2	2
4	4	2	2	2	2	1	1	2	2
4	4	2	2	2	2	3	3	2	2
4	4	2	2	2	2	3	3	2	2
4	4	2	2	2	2	3	3	2	2
4	4	2	2	2	2	3	3	2	2
4	4	2	2	2	2	1	1	1	1
4	4	2	2	2	2	3	3	2	2
4	4	2	2	1	1	3	3	2	2
1	1	2	2	2	2	1	1	2	2
4	4	2	2	2	2	3	3	2	2
1	1	2	2	1	1	1	1	2	2
4	4	2	2	2	2	3	3	2	2
4	4	2	2	1	1	3	3	1	1
4	4	2	2	2	2	3	3	2	2
1	1	2	2	2	2	3	3	2	2
4	4	2	2	2	2	3	3	2	2
1	1	3	3	1	1	3	3	2	2
4	4	2	2	2	2	3	3	2	2
4	4	2	2	2	2	3	3	1	1
1	1	2	2	1	1	3	3	2	2
4	4	2	2	2	2	3	3	2	2
4	4	2	2	2	2	1	1	2	2
4	4	2	2	1	1	3	3	2	2
4	4	2	2	1	1	3	3	2	2
4	4	2	2	1	1	3	3	2	2
4	4	2	2	1	1	3	3	2	2
4	4	3	3	2	2	1	1	2	2
4	4	2	2	2	2	1	1	2	2
1	1	2	2	1	1	1	1	2	2
4	4	2	2	2	2	3	3	2	2
1	1	2	2	2	2	3	3	2	2
4	4	2	2	2	2	3	3	2	2
4	4	2	2	2	2	3	3	1	1
4	4	2	2	2	2	3	3	2	2
1	1	3	3	2	2	1	1	2	2
4	4	2	2	2	2	3	3	2	2
4	4	2	2	2	2	3	3	2	2
4	4	2	2	2	2	3	3	2	2
4	4	2	2	2	2	3	3	2	2
4	4	2	2	2	2	3	3	2	2
4	4	2	2	2	2	3	3	1	1
4	4	3	3	2	2	3	3	2	2
4	4	2	2	2	2	3	3	2	2
4	4	2	2	2	2	3	3	2	2

For Review Only

1	1	3	3	2	2	1	1	2	2
1	1	2	2	2	2	3	3	2	2
4	4	2	2	2	2	3	3	2	2
1	1	2	2	2	2	3	3	2	2
4	4	3	3	2	2	3	3	2	2
4	4	3	3	1	1	3	3	2	2
4	4	2	2	1	1	1	1	2	2
4	4	2	2	2	2	3	3	2	2
1	1	2	2	1	1	1	1	2	2
4	4	3	3	1	1	1	1	2	2
4	4	2	2	2	2	3	3	2	2
4	4	2	2	2	2	3	3	2	2
4	4	2	2	2	2	3	3	2	2
4	4	2	2	2	2	1	1	2	2
4	4	2	2	1	1	3	3	2	2
1	1	2	2	1	1	1	1	2	2
4	4	2	2	2	2	3	3	2	2
4	4	3	3	2	2	3	3	2	2
4	4	2	2	1	1	3	3	2	2
4	4	2	2	2	2	3	3	2	2
4	4	2	2	2	2	3	3	2	2
4	4	2	2	2	2	3	3	1	1
1	1	2	2	2	2	3	3	2	2
4	4	3	3	2	2	3	3	2	2
4	4	2	2	2	2	3	3	2	2
4	4	2	2	1	1	3	3	2	2
4	4	2	2	2	2	3	3	2	2
4	4	2	2	2	2	1	1	2	2
4	4	3	3	2	2	3	3	2	2
4	4	2	2	2	2	3	3	2	2
1	1	2	2	2	2	3	3	2	2
4	4	2	2	2	2	1	1	2	2
1	1	2	2	2	2	3	3	2	2
4	4	2	2	1	1	3	3	2	2
1	1	3	3	2	2	3	3	2	2
4	4	3	3	2	2	3	3	2	2
4	4	2	2	2	2	3	3	2	2
1	1	3	3	2	2	3	3	2	2
4	4	2	2	2	2	3	3	2	2
4	4	2	2	2	2	3	3	2	2
4	4	2	2	2	2	1	1	2	2
4	4	2	2	2	2	3	3	2	2
1	1	2	2	1	1	3	3	2	2
4	4	2	2	2	2	1	1	2	2
4	4	2	2	2	2	3	3	2	2
4	4	3	3	2	2	1	1	2	2
1	1	2	2	1	1	1	1	2	2
4	4	2	2	1	1	1	1	1	1
4	4	2	2	2	2	3	3	2	2
4	4	2	2	2	2	3	3	1	1
4	4	2	2	2	2	3	3	2	2
4	4	3	3	2	2	3	3	1	1
1	1	2	2	1	1	3	3	2	2
4	4	3	3	2	2	3	3	2	2
4	4	2	2	1	1	3	3	2	2

For Review Only

4	4	2	2	2	2	3	3	2	2
4	4	3	3	2	2	3	3	1	1
4	4	2	2	2	2	1	1	2	2
4	4	2	2	2	2	3	3	2	2
4	4	3	3	2	2	3	3	1	1
4	4	2	2	2	2	3	3	2	2
1	1	2	2	1	1	3	3	2	2
4	4	2	2	2	2	3	3	1	1
1	1	2	2	2	2	3	3	2	2
4	4	2	2	1	1	3	3	1	1
4	4	2	2	2	2	1	1	2	2
4	4	3	3	1	1	3	3	2	2
4	4	2	2	2	2	1	1	2	2
4	4	2	2	1	1	3	3	2	2
4	4	2	2	2	2	1	1	2	2
4	4	2	2	1	1	1	1	2	2
1	1	2	2	2	2	3	3	2	2
4	4	2	2	1	1	1	1	1	1
4	4	2	2	1	1	3	3	2	2
4	4	2	2	2	2	1	1	1	1
4	4	2	2	2	2	3	3	2	2
4	4	2	2	2	2	3	3	2	2
4	4	3	3	1	1	1	1	2	2
1	1	2	2	2	2	3	3	2	2
4	4	2	2	2	2	3	3	2	2
4	4	2	2	2	2	3	3	2	2
4	4	2	2	1	1	3	3	1	1
4	4	2	2	2	2	3	3	2	2
4	4	2	2	2	2	3	3	2	2
4	4	2	2	1	1	3	3	2	2
4	4	2	2	1	1	3	3	2	2
4	4	2	2	1	1	3	3	2	2
4	4	2	2	2	2	1	1	2	2
4	4	2	2	2	2	3	3	2	2
1	1	2	2	2	2	3	3	2	2
1	1	2	2	1	1	3	3	2	2
4	4	3	3	2	2	3	3	2	2
4	4	3	3	2	2	3	3	2	2
4	4	2	2	2	2	3	3	2	2
1	1	2	2	1	1	3	3	2	2
1	1	2	2	2	2	1	1	2	2
1	1	3	3	2	2	3	3	2	2
1	1	2	2	1	1	3	3	2	2
4	4	2	2	2	2	1	1	2	2
4	4	3	3	2	2	3	3	2	2
4	4	2	2	2	2	1	1	2	2
4	4	2	2	2	2	3	3	2	2
1	1	2	2	2	2	3	3	2	2
4	4	2	2	2	2	3	3	2	2
4	4	2	2	1	1	3	3	1	1
1	1	2	2	2	2	3	3	2	2
1	1	2	2	1	1	3	3	2	2
4	4	2	2	2	2	1	1	2	2
4	4	2	2	2	2	3	3	2	2
1	1	2	2	1	1	3	3	2	2

For Review Only

4	4	2	2	1	1	1	1	2	2
4	4	3	3	2	2	3	3	2	2

For Review Only

SNP1672	SNP1672	SNP1673	SNP1673	SNP1674	SNP1674	SNP1675	SNP1675	SNP1676	SNP1676
2	2	4	4	3	3	1	1	1	1
4	4	4	4	3	3	1	1	1	1
2	2	1	1	3	3	1	1	1	1
2	2	4	4	3	3	1	1	1	1
2	2	1	1	3	3	1	1	1	1
2	2	4	4	3	3	1	1	1	1
2	2	4	4	3	3	3	3	4	4
2	2	4	4	3	3	3	3	4	4
2	2	4	4	3	3	3	3	1	1
4	4	4	4	3	3	3	3	4	4
2	2	4	4	3	3	3	3	4	4
2	2	1	1	3	3	1	1	1	1
2	2	4	4	3	3	3	3	4	4
2	2	4	4	3	3	1	1	1	1
2	2	1	1	3	3	3	3	1	1
2	2	4	4	3	3	3	3	1	1
2	2	4	4	2	2	3	3	4	4
2	2	4	4	3	3	3	3	4	4
2	2	4	4	3	3	1	1	1	1
4	4	4	4	3	3	3	3	4	4
2	2	4	4	3	3	3	3	4	4
2	2	1	1	3	3	3	3	4	4
2	2	4	4	3	3	3	3	4	4
2	2	4	4	3	3	3	3	4	4
2	2	4	4	3	3	3	3	4	4
2	2	4	4	3	3	1	1	1	1
2	2	4	4	3	3	1	1	1	1
2	2	4	4	3	3	1	1	1	1
2	2	4	4	3	3	3	3	4	4
4	4	4	4	2	2	3	3	4	4
2	2	4	4	3	3	3	3	4	4
2	2	1	1	3	3	1	1	1	1
2	2	1	1	3	3	1	1	1	1
2	2	4	4	3	3	3	3	4	4
2	2	4	4	3	3	1	1	1	1
2	2	4	4	3	3	3	3	4	4
2	2	4	4	3	3	1	1	1	1
4	4	1	1	3	3	3	3	4	4
2	2	4	4	3	3	3	3	4	4
2	2	1	1	3	3	3	3	4	4
4	4	4	4	3	3	3	3	4	4
2	2	4	4	3	3	3	3	1	1
4	4	4	4	3	3	3	3	4	4
4	4	1	1	2	2	3	3	4	4
4	4	4	4	3	3	3	3	4	4
2	2	1	1	3	3	1	1	1	1
2	2	4	4	3	3	1	1	1	1
2	2	1	1	3	3	1	1	1	1
4	4	4	4	2	2	3	3	4	4
2	2	1	1	3	3	1	1	1	1
2	2	4	4	3	3	1	1	1	1
2	2	4	4	3	3	3	3	4	4

For Review Only

2	2	4	4	3	3	3	3	4	4
2	2	1	1	3	3	1	1	1	1
2	2	4	4	3	3	1	1	1	1
4	4	4	4	3	3	3	3	4	4
2	2	4	4	3	3	1	1	1	1
2	2	4	4	3	3	1	1	1	1
2	2	4	4	3	3	1	1	1	1
4	4	4	4	3	3	3	3	4	4
2	2	4	4	3	3	3	3	4	4
2	2	4	4	3	3	1	1	1	1
2	2	4	4	3	3	1	1	1	1
2	2	4	4	3	3	1	1	1	1
2	2	4	4	3	3	3	3	1	1
2	2	4	4	3	3	3	3	4	4
2	2	4	4	3	3	1	1	1	1
2	2	4	4	3	3	1	1	1	1
2	2	1	1	3	3	3	3	4	4
2	2	1	1	3	3	1	1	1	1
2	2	1	1	3	3	3	3	4	4
2	2	4	4	3	3	1	1	1	1
2	2	4	4	3	3	1	1	1	1
2	2	4	4	2	2	3	3	4	4
2	2	1	1	3	3	3	3	4	4
2	2	4	4	3	3	3	3	4	4
4	4	4	4	3	3	3	3	4	4
2	2	4	4	3	3	1	1	1	1
2	2	1	1	2	2	1	1	1	1
4	4	4	4	3	3	3	3	4	4
2	2	4	4	3	3	1	1	1	1
2	2	1	1	3	3	3	3	4	4
4	4	4	4	2	2	3	3	4	4
2	2	4	4	3	3	1	1	1	1
4	4	4	4	3	3	3	3	4	4
2	2	4	4	3	3	3	3	4	4
4	4	4	4	3	3	1	1	1	1
4	4	4	4	3	3	3	3	4	4
4	4	4	4	3	3	3	3	4	4
4	4	4	4	3	3	3	3	4	4
2	2	1	1	3	3	3	3	4	4
4	4	4	4	3	3	3	3	4	4
2	2	1	1	3	3	1	1	4	4
2	2	1	1	3	3	3	3	4	4
2	2	4	4	3	3	1	1	1	1
2	2	4	4	3	3	1	1	1	1
2	2	1	1	3	3	3	3	4	4
2	2	4	4	3	3	1	1	1	1
2	2	4	4	3	3	3	3	4	4
2	2	4	4	3	3	1	1	1	1
2	2	4	4	3	3	1	1	1	1
2	2	4	4	3	3	1	1	1	1
2	2	4	4	3	3	1	1	1	1
2	2	4	4	3	3	3	3	4	4
4	4	4	4	2	2	1	1	4	4
2	2	4	4	3	3	1	1	1	1
2	2	1	1	3	3	1	1	1	1

2	2	4	4	3	3	1	1	1	1
2	2	4	4	3	3	1	1	1	1
2	2	4	4	3	3	3	3	4	4
2	2	4	4	3	3	3	3	4	4
2	2	4	4	3	3	1	1	1	1
2	2	4	4	3	3	1	1	1	1
2	2	4	4	3	3	3	3	1	1
2	2	4	4	3	3	1	1	1	1
2	2	4	4	3	3	3	3	4	4
4	4	4	4	3	3	3	3	4	4
2	2	4	4	3	3	3	3	1	1
2	2	1	1	3	3	1	1	1	1
2	2	4	4	3	3	1	1	1	1
2	2	4	4	3	3	3	3	1	1
2	2	4	4	3	3	1	1	1	1
2	2	4	4	3	3	1	1	1	1
4	4	1	1	3	3	1	1	1	1
2	2	4	4	3	3	1	1	1	1
2	2	4	4	3	3	3	3	4	4
2	2	4	4	3	3	3	3	1	1
2	2	4	4	3	3	1	1	1	1
2	2	4	4	3	3	3	3	1	1
2	2	4	4	3	3	1	1	1	1
4	4	4	4	3	3	3	3	4	4
2	2	4	4	3	3	1	1	1	1
2	2	4	4	3	3	3	3	4	4
2	2	4	4	3	3	1	1	4	4
2	2	4	4	3	3	1	1	1	1
2	2	4	4	3	3	1	1	1	1
4	4	4	4	3	3	1	1	1	1
2	2	4	4	3	3	3	3	4	4
2	2	4	4	3	3	1	1	1	1
2	2	1	1	3	3	3	3	4	4
2	2	4	4	3	3	3	3	1	1
2	2	4	4	3	3	3	3	4	4
2	2	1	1	3	3	1	1	1	1
2	2	4	4	3	3	3	3	4	4
2	2	4	4	3	3	1	1	1	1
2	2	1	1	3	3	3	3	4	4
2	2	4	4	3	3	3	3	4	4
2	2	4	4	3	3	1	1	1	1
2	2	4	4	3	3	3	3	4	4
2	2	4	4	3	3	1	1	1	1
2	2	4	4	3	3	1	1	4	4
2	2	4	4	3	3	1	1	4	4
2	2	1	1	3	3	1	1	1	1
2	2	4	4	3	3	1	1	1	1
2	2	4	4	3	3	1	1	1	1
2	2	4	4	3	3	1	1	1	1
2	2	4	4	3	3	1	1	1	1
2	2	4	4	3	3	1	1	1	1
4	4	4	4	3	3	3	3	4	4
2	2	4	4	3	3	1	1	1	1
2	2	4	4	3	3	1	1	1	1
2	2	4	4	3	3	3	3	4	4

For Review Only

2	2	1	1	3	3	3	3	1	1
2	2	4	4	3	3	3	3	4	4
2	2	4	4	2	2	1	1	1	1
2	2	4	4	3	3	1	1	1	1
2	2	4	4	3	3	1	1	1	1
2	2	4	4	3	3	1	1	1	1
2	2	4	4	3	3	3	3	4	4
2	2	4	4	3	3	1	1	1	1
2	2	1	1	3	3	1	1	4	4
2	2	1	1	3	3	1	1	1	1
2	2	4	4	3	3	1	1	1	1
2	2	4	4	3	3	3	3	4	4
2	2	4	4	3	3	1	1	1	1
2	2	1	1	3	3	1	1	1	1
2	2	4	4	3	3	3	3	4	4
2	2	4	4	3	3	3	3	4	4
2	2	4	4	3	3	1	1	1	1
2	2	4	4	3	3	1	1	1	1
2	2	4	4	3	3	1	1	1	1
2	2	4	4	3	3	3	3	1	1
2	2	4	4	3	3	1	1	1	1
4	4	4	4	3	3	3	3	4	4
2	2	4	4	3	3	3	3	4	4
2	2	4	4	3	3	1	1	1	1
4	4	4	4	3	3	3	3	4	4
2	2	4	4	3	3	3	3	4	4
2	2	4	4	3	3	1	1	1	1
2	2	1	1	3	3	1	1	1	1
4	4	4	4	3	3	1	1	1	1
2	2	4	4	3	3	3	3	4	4
2	2	4	4	3	3	3	3	4	4
2	2	1	1	3	3	3	3	4	4
2	2	4	4	3	3	3	3	4	4
2	2	4	4	3	3	3	3	4	4
2	2	4	4	3	3	1	1	1	1
2	2	4	4	3	3	1	1	1	1
2	2	4	4	3	3	3	3	4	4
2	2	4	4	3	3	1	1	1	1
2	2	4	4	3	3	1	1	1	1
2	2	4	4	3	3	1	1	1	1
2	2	1	1	3	3	1	1	1	1
2	2	4	4	3	3	1	1	1	1
2	2	4	4	3	3	3	3	4	4
2	2	1	1	3	3	3	3	4	4
2	2	4	4	3	3	3	3	4	4
2	2	4	4	3	3	1	1	1	1
2	2	4	4	3	3	3	3	4	4
4	4	4	4	3	3	3	3	4	4
2	2	4	4	3	3	3	3	4	4
4	4	4	4	3	3	3	3	4	4
2	2	4	4	3	3	1	1	1	1
4	4	4	4	3	3	1	1	1	1
2	2	4	4	3	3	3	3	4	4
2	2	4	4	3	3	1	1	1	1
2	2	4	4	3	3	3	3	4	4

For Review Only

2	2	4	4	3	3	3	3	4	4
4	4	4	4	3	3	3	3	1	1
2	2	1	1	3	3	1	1	1	1
2	2	4	4	3	3	1	1	1	1
2	2	4	4	3	3	1	1	1	1
2	2	4	4	3	3	1	1	1	1
2	2	4	4	3	3	3	3	4	4
4	4	4	4	3	3	3	3	4	4
2	2	4	4	3	3	1	1	1	1
4	4	4	4	3	3	3	3	4	4
2	2	4	4	3	3	1	1	1	1
2	2	4	4	3	3	1	1	1	1
2	2	1	1	3	3	3	3	4	4
2	2	4	4	3	3	3	3	4	4
2	2	1	1	3	3	1	1	1	1
4	4	1	1	3	3	3	3	1	1
2	2	4	4	3	3	3	3	4	4
4	4	4	4	3	3	3	3	4	4
2	2	4	4	3	3	1	1	1	1
4	4	1	1	3	3	3	3	4	4
2	2	4	4	3	3	1	1	1	1
2	2	4	4	3	3	1	1	1	1
4	4	4	4	3	3	1	1	1	1
2	2	4	4	3	3	3	3	4	4
4	4	4	4	3	3	3	3	4	4
2	2	4	4	3	3	1	1	4	4
4	4	4	4	3	3	3	3	4	4
2	2	4	4	3	3	1	1	1	1
2	2	4	4	3	3	1	1	1	1
2	2	4	4	3	3	1	1	1	1
2	2	4	4	3	3	3	3	4	4
2	2	4	4	3	3	1	1	1	1
2	2	1	1	3	3	1	1	1	1
2	2	4	4	3	3	1	1	1	1
2	2	4	4	3	3	3	3	4	4
2	2	4	4	3	3	3	3	4	4
2	2	4	4	3	3	1	1	1	1
2	2	4	4	3	3	1	1	1	1
2	2	4	4	3	3	3	3	4	4
2	2	4	4	3	3	3	3	4	4
2	2	1	1	3	3	1	1	1	1
2	2	1	1	3	3	1	1	1	1
2	2	4	4	3	3	3	3	4	4
2	2	1	1	3	3	3	3	4	4
2	2	4	4	3	3	1	1	1	1
2	2	1	1	3	3	3	3	4	4
2	2	4	4	3	3	1	1	1	1
2	2	4	4	3	3	3	3	4	4
2	2	4	4	3	3	1	1	1	1
4	4	4	4	3	3	3	3	4	4
2	2	4	4	3	3	3	3	4	4
2	2	4	4	3	3	3	3	4	4
2	2	1	1	3	3	1	1	1	1
2	2	4	4	3	3	1	1	1	1
2	2	4	4	3	3	3	3	4	4

For Review Only

4	4	1	1	3	3	3	3	4	4
2	2	4	4	3	3	1	1	1	1

For Review Only

SNP1677	SNP1677	SNP1678	SNP1678	SNP1679	SNP1679	SNP168	SNP168	SNP1680	SNP1680
3	3	1	1	1	1	4	4	1	1
2	2	1	1	1	1	1	1	1	1
3	3	1	1	1	1	1	1	1	1
3	3	1	1	1	1	1	1	1	1
3	3	3	3	1	1	4	4	1	1
3	3	1	1	1	1	1	1	1	1
3	3	1	1	1	1	1	1	4	4
3	3	1	1	1	1	1	1	4	4
3	3	1	1	1	1	1	1	1	1
3	3	1	1	1	1	1	1	4	4
2	2	1	1	1	1	1	1	4	4
3	3	1	1	1	1	4	4	1	1
3	3	1	1	1	1	1	1	4	4
2	2	1	1	1	1	1	1	1	1
3	3	1	1	1	1	1	1	1	1
3	3	1	1	2	2	1	1	1	1
3	3	3	3	2	2	1	1	1	1
2	2	1	1	1	1	4	4	4	4
3	3	1	1	1	1	1	1	1	1
3	3	3	3	1	1	1	1	4	4
2	2	1	1	1	1	1	1	4	4
3	3	3	3	1	1	1	1	4	4
2	2	1	1	1	1	4	4	1	1
2	2	1	1	2	2	1	1	4	4
3	3	1	1	1	1	4	4	1	1
3	3	1	1	1	1	1	1	1	1
3	3	1	1	1	1	1	1	1	1
3	3	1	1	1	1	1	1	1	1
2	2	1	1	1	1	4	4	1	1
2	2	3	3	2	2	1	1	4	4
3	3	1	1	1	1	1	1	1	1
3	3	1	1	1	1	1	1	1	1
3	3	3	3	1	1	1	1	1	1
2	2	1	1	1	1	1	1	4	4
2	2	1	1	1	1	1	1	1	1
3	3	1	1	2	2	1	1	4	4
3	3	1	1	1	1	1	1	1	1
3	3	1	1	1	1	1	1	4	4
2	2	1	1	1	1	1	1	4	4
2	2	1	1	1	1	1	1	1	1
2	2	1	1	1	1	1	1	4	4
2	2	1	1	1	1	1	1	1	1
3	3	1	1	1	1	1	1	4	4
3	3	1	1	2	2	1	1	1	1
3	3	1	1	1	1	4	4	4	4
3	3	1	1	1	1	1	1	1	1
3	3	1	1	2	2	1	1	1	1
3	3	1	1	1	1	1	1	1	1
2	2	1	1	2	2	1	1	4	4
3	3	1	1	1	1	4	4	1	1
3	3	1	1	1	1	1	1	1	1
3	3	1	1	1	1	4	4	4	4

For Review Only

3	3	3	3	1	1	4	4	1	1
3	3	3	3	1	1	1	1	1	1
3	3	1	1	1	1	1	1	1	1
3	3	3	3	1	1	1	1	1	1
3	3	1	1	1	1	1	1	1	1
3	3	1	1	2	2	1	1	1	1
3	3	1	1	1	1	1	1	1	1
2	2	1	1	1	1	1	1	4	4
3	3	1	1	1	1	4	4	4	4
3	3	1	1	1	1	1	1	1	1
3	3	1	1	1	1	1	1	1	1
3	3	1	1	2	2	1	1	1	1
3	3	3	3	1	1	4	4	1	1
2	2	1	1	1	1	4	4	1	1
3	3	1	1	1	1	1	1	1	1
2	2	1	1	1	1	1	1	1	1
3	3	1	1	1	1	1	1	1	1
3	3	1	1	1	1	1	1	1	1
3	3	1	1	1	1	4	4	1	1
3	3	3	3	1	1	1	1	1	1
3	3	1	1	1	1	1	1	1	1
2	2	1	1	1	1	1	1	1	1
3	3	1	1	1	1	1	1	1	1
2	2	1	1	1	1	1	1	1	1
3	3	1	1	1	1	4	4	4	4
3	3	1	1	1	1	1	1	1	1
3	3	1	1	2	2	1	1	1	1
2	2	1	1	1	1	1	1	4	4
3	3	1	1	1	1	1	1	1	1
2	2	1	1	1	1	4	4	1	1
3	3	1	1	2	2	4	4	1	1
3	3	1	1	1	1	1	1	1	1
2	2	1	1	1	1	1	1	4	4
3	3	3	3	1	1	4	4	1	1
3	3	1	1	1	1	4	4	4	4
3	3	1	1	1	1	4	4	4	4
3	3	1	1	1	1	4	4	4	4
3	3	3	3	1	1	1	1	4	4
3	3	3	3	1	1	1	1	4	4
3	3	1	1	1	1	4	4	4	4
3	3	3	3	1	1	1	1	1	1
3	3	1	1	1	1	1	1	1	1
3	3	1	1	1	1	4	4	1	1
3	3	1	1	1	1	1	1	1	1
3	3	1	1	1	1	4	4	4	4
3	3	1	1	1	1	1	1	1	1
2	2	1	1	1	1	1	1	4	4
3	3	1	1	1	1	1	1	1	1
3	3	1	1	2	2	1	1	1	1
3	3	1	1	1	1	1	1	1	1
3	3	1	1	1	1	4	4	1	1
2	2	1	1	1	1	1	1	4	4
3	3	1	1	1	1	1	1	4	4
3	3	1	1	1	1	1	1	1	1
3	3	1	1	1	1	1	1	1	1

For Review Only

3	3	1	1	1	1	1	1	1	1
3	3	1	1	1	1	1	1	1	1
3	3	1	1	1	1	1	1	1	1
2	2	1	1	1	1	1	1	4	4
3	3	1	1	1	1	1	1	1	1
3	3	1	1	1	1	1	1	1	1
3	3	1	1	1	1	1	1	1	1
3	3	1	1	1	1	1	1	4	4
3	3	1	1	1	1	4	4	4	4
3	3	1	1	1	1	1	1	4	4
3	3	1	1	1	1	4	4	4	4
3	3	1	1	1	1	1	1	1	1
3	3	1	1	1	1	4	4	1	1
3	3	1	1	1	1	1	1	4	4
3	3	1	1	1	1	4	4	1	1
3	3	1	1	1	1	1	1	1	1
2	2	1	1	1	1	1	1	1	1
3	3	1	1	1	1	1	1	1	1
2	2	1	1	1	1	1	1	4	4
3	3	1	1	1	1	4	4	1	1
3	3	1	1	1	1	1	1	1	1
3	3	1	1	1	1	4	4	1	1
3	3	1	1	1	1	1	1	1	1
2	2	1	1	1	1	1	1	4	4
2	2	1	1	1	1	1	1	1	1
2	2	1	1	1	1	4	4	1	1
3	3	1	1	1	1	4	4	1	1
3	3	3	3	1	1	1	1	1	1
3	3	1	1	1	1	1	1	1	1
3	3	1	1	1	1	4	4	1	1
2	2	1	1	1	1	1	1	4	4
3	3	1	1	1	1	4	4	1	1
3	3	3	3	1	1	1	1	1	1
3	3	1	1	1	1	4	4	1	1
2	2	1	1	1	1	1	1	4	4
3	3	1	1	1	1	1	1	1	1
2	2	1	1	1	1	1	1	4	4
3	3	1	1	1	1	1	1	1	1
3	3	1	1	1	1	1	1	1	1
2	2	1	1	1	1	1	1	4	4
2	2	1	1	1	1	1	1	1	1
2	2	1	1	1	1	1	1	4	4
3	3	1	1	1	1	4	4	1	1
3	3	1	1	1	1	1	1	4	4
2	2	1	1	1	1	1	1	1	1
3	3	3	3	1	1	1	1	1	1
3	3	1	1	1	1	1	1	1	1
3	3	1	1	1	1	4	4	1	1
3	3	1	1	1	1	1	1	1	1
3	3	1	1	1	1	1	1	1	1
3	3	1	1	2	2	1	1	1	1
2	2	1	1	1	1	1	1	4	4
3	3	1	1	1	1	1	1	1	1
3	3	1	1	2	2	1	1	1	1
3	3	1	1	1	1	4	4	1	1

For Review Only

3	3	1	1	1	1	1	1	1	1
2	2	1	1	1	1	4	4	1	1
3	3	3	3	2	2	4	4	1	1
2	2	1	1	1	1	4	4	1	1
3	3	1	1	1	1	4	4	1	1
3	3	1	1	1	1	1	1	1	1
3	3	1	1	1	1	1	1	1	1
3	3	1	1	1	1	1	1	1	1
2	2	1	1	1	1	1	1	4	4
3	3	1	1	1	1	4	4	1	1
3	3	1	1	1	1	1	1	1	1
2	2	1	1	1	1	1	1	4	4
3	3	1	1	1	1	1	1	1	1
3	3	1	1	1	1	1	1	1	1
2	2	1	1	1	1	1	1	4	4
2	2	1	1	1	1	1	1	4	4
3	3	1	1	1	1	1	1	1	1
3	3	1	1	1	1	1	1	1	1
3	3	1	1	1	1	1	1	1	1
3	3	1	1	1	1	1	1	1	1
3	3	1	1	1	1	1	1	1	1
2	2	1	1	1	1	1	1	4	4
2	2	1	1	1	1	1	1	1	1
3	3	1	1	2	2	1	1	1	1
2	2	1	1	1	1	4	4	1	1
2	2	1	1	1	1	1	1	4	4
3	3	1	1	1	1	1	1	1	1
2	2	1	1	1	1	1	1	1	1
3	3	1	1	1	1	1	1	1	1
3	3	3	3	1	1	1	1	1	1
3	3	1	1	1	1	1	1	4	4
3	3	1	1	1	1	4	4	1	1
2	2	1	1	1	1	4	4	4	4
2	2	1	1	1	1	1	1	1	1
3	3	1	1	1	1	4	4	1	1
3	3	1	1	2	2	1	1	1	1
3	3	1	1	1	1	1	1	1	1
3	3	1	1	1	1	1	1	1	1
3	3	1	1	1	1	4	4	1	1
3	3	1	1	1	1	1	1	1	1
3	3	1	1	1	1	1	1	1	1
3	3	1	1	1	1	1	1	1	1
2	2	1	1	1	1	1	1	4	4
3	3	1	1	1	1	4	4	1	1
3	3	1	1	1	1	1	1	4	4
3	3	1	1	1	1	1	1	1	1
2	2	1	1	1	1	1	1	4	4
3	3	1	1	1	1	1	1	4	4
2	2	1	1	1	1	1	1	4	4
3	3	1	1	1	1	1	1	4	4
3	3	1	1	1	1	1	1	1	1
2	2	1	1	1	1	1	1	1	1
2	2	1	1	1	1	1	1	1	1
3	3	1	1	1	1	1	1	1	1
2	2	1	1	1	1	1	1	4	4

For Review Only

2	2	1	1	1	1	4	4	4	4
3	3	1	1	1	1	1	1	1	1
3	3	1	1	1	1	4	4	1	1
2	2	1	1	1	1	4	4	1	1
2	2	1	1	1	1	1	1	1	1
3	3	1	1	1	1	1	1	1	1
2	2	1	1	1	1	1	1	4	4
2	2	1	1	1	1	1	1	4	4
3	3	1	1	1	1	1	1	1	1
2	2	1	1	1	1	1	1	4	4
3	3	1	1	1	1	1	1	1	1
2	2	1	1	1	1	1	1	1	1
3	3	3	3	1	1	1	1	4	4
3	3	1	1	2	2	1	1	1	1
3	3	1	1	1	1	1	1	1	1
3	3	1	1	1	1	4	4	1	1
3	3	1	1	1	1	1	1	4	4
2	2	1	1	1	1	1	1	4	4
2	2	1	1	1	1	1	1	1	1
2	2	1	1	1	1	1	1	4	4
2	2	1	1	1	1	1	1	1	1
3	3	1	1	1	1	1	1	1	1
2	2	1	1	1	1	4	4	4	4
2	2	1	1	1	1	1	1	1	1
2	2	1	1	1	1	1	1	1	1
3	3	1	1	1	1	4	4	4	4
2	2	1	1	1	1	1	1	4	4
3	3	1	1	1	1	1	1	1	1
3	3	3	3	1	1	4	4	1	1
3	3	1	1	1	1	1	1	1	1
2	2	1	1	1	1	1	1	4	4
3	3	1	1	1	1	1	1	1	1
2	2	1	1	1	1	1	1	1	1
3	3	1	1	1	1	4	4	1	1
2	2	1	1	1	1	1	1	1	1
2	2	1	1	1	1	1	1	4	4
3	3	1	1	1	1	4	4	1	1
3	3	1	1	2	2	1	1	1	1
3	3	1	1	2	2	1	1	4	4
2	2	1	1	1	1	1	1	4	4
3	3	1	1	1	1	4	4	1	1
3	3	1	1	1	1	1	1	1	1
3	3	1	1	1	1	1	1	1	1
3	3	3	3	1	1	1	1	1	1
3	3	1	1	1	1	1	1	1	1
2	2	3	3	1	1	1	1	4	4
3	3	1	1	1	1	4	4	1	1
3	3	1	1	1	1	1	1	1	1
3	3	1	1	1	1	1	1	4	4
2	2	1	1	1	1	1	1	4	4
3	3	1	1	1	1	4	4	1	1
2	2	1	1	1	1	1	1	4	4
3	3	1	1	1	1	1	1	1	1
3	3	1	1	1	1	1	1	1	1
2	2	1	1	1	1	1	1	1	1

For Review Only

3	3	1	1	1	1	4	4	1	1
3	3	1	1	1	1	1	1	1	1

For Review Only

SNP1681	SNP1681	SNP1682	SNP1682	SNP1683	SNP1683	SNP1684	SNP1684	SNP1685	SNP1685
2	2	1	1	2	2	3	3	4	4
3	3	1	1	2	2	3	3	4	4
3	3	1	1	2	2	3	3	4	4
3	3	1	1	2	2	3	3	3	3
3	3	1	1	2	2	4	4	4	4
3	3	1	1	2	2	3	3	4	4
3	3	1	1	1	1	3	3	4	4
3	3	1	1	1	1	3	3	4	4
3	3	1	1	2	2	4	4	4	4
3	3	1	1	1	1	3	3	4	4
3	3	1	1	1	1	3	3	4	4
3	3	1	1	2	2	3	3	4	4
3	3	1	1	2	2	3	3	4	4
3	3	1	1	1	1	3	3	4	4
3	3	1	1	2	2	3	3	4	4
3	3	1	1	2	2	3	3	4	4
3	3	1	1	2	2	4	4	4	4
3	3	1	1	1	1	3	3	4	4
3	3	1	1	2	2	3	3	3	3
3	3	1	1	1	1	3	3	4	4
3	3	1	1	1	1	3	3	4	4
3	3	1	1	1	1	4	4	4	4
3	3	1	1	2	2	3	3	4	4
3	3	1	1	1	1	3	3	4	4
3	3	1	1	2	2	3	3	4	4
3	3	1	1	2	2	3	3	4	4
3	3	1	1	2	2	3	3	4	4
3	3	1	1	2	2	3	3	4	4
3	3	1	1	2	2	3	3	4	4
3	3	1	1	1	1	3	3	4	4
3	3	1	1	2	2	3	3	3	3
3	3	1	1	2	2	3	3	4	4
2	2	2	2	2	2	4	4	4	4
3	3	1	1	1	1	3	3	4	4
3	3	1	1	2	2	3	3	4	4
3	3	1	1	1	1	3	3	4	4
3	3	1	1	2	2	3	3	4	4
3	3	1	1	1	1	3	3	4	4
3	3	1	1	1	1	3	3	4	4
3	3	1	1	2	2	3	3	4	4
3	3	1	1	1	1	3	3	4	4
3	3	1	1	2	2	3	3	4	4
3	3	1	1	1	1	3	3	4	4
3	3	1	1	2	2	3	3	4	4
3	3	1	1	1	1	3	3	4	4
3	3	1	1	2	2	3	3	4	4
2	2	2	2	2	2	3	3	4	4
3	3	1	1	2	2	3	3	4	4
3	3	1	1	1	1	3	3	4	4
2	2	1	1	2	2	3	3	4	4
2	2	1	1	2	2	3	3	4	4
3	3	1	1	1	1	3	3	4	4

For Review Only

3	3	1	1	2	2	4	4	4	4
2	2	2	2	2	2	4	4	4	4
3	3	1	1	2	2	3	3	4	4
3	3	1	1	2	2	3	3	4	4
3	3	1	1	2	2	3	3	3	3
2	2	2	2	2	2	3	3	4	4
3	3	1	1	2	2	3	3	4	4
3	3	1	1	1	1	3	3	4	4
3	3	1	1	1	1	3	3	4	4
3	3	1	1	2	2	3	3	4	4
2	2	2	2	2	2	3	3	4	4
2	2	2	2	2	2	3	3	4	4
3	3	1	1	2	2	4	4	4	4
3	3	1	1	2	2	3	3	4	4
3	3	1	1	2	2	3	3	4	4
2	2	1	1	2	2	3	3	4	4
2	2	2	2	2	2	3	3	4	4
2	2	2	2	2	2	3	3	4	4
3	3	1	1	2	2	3	3	4	4
2	2	1	1	2	2	4	4	4	4
3	3	1	1	2	2	3	3	4	4
3	3	1	1	2	2	3	3	3	3
3	3	1	1	2	2	3	3	4	4
3	3	1	1	2	2	3	3	4	4
3	3	1	1	1	1	3	3	4	4
3	3	1	1	2	2	3	3	4	4
3	3	1	1	2	2	3	3	4	4
3	3	1	1	1	1	3	3	4	4
2	2	2	2	2	2	3	3	4	4
2	3	1	1	2	2	3	3	4	4
3	3	1	1	2	2	3	3	4	4
3	3	1	1	2	2	3	3	4	4
3	3	1	1	1	1	3	3	4	4
3	3	1	1	2	2	4	4	4	4
3	3	1	1	1	1	3	3	4	4
3	3	1	1	1	1	3	3	4	4
3	3	1	1	1	1	3	3	4	4
3	3	1	1	1	1	3	3	4	4
3	3	1	1	1	1	3	3	4	4
3	3	1	1	1	1	3	3	4	4
3	3	1	1	2	2	4	4	4	4
2	2	2	2	2	2	3	3	4	4
2	2	1	1	2	2	3	3	4	4
2	2	2	2	2	2	3	3	4	4
3	3	1	1	1	1	3	3	4	4
2	2	1	1	2	2	3	3	4	4
3	3	1	1	1	1	3	3	3	3
3	3	1	1	2	2	3	3	3	3
2	2	2	2	2	2	3	3	4	4
3	3	1	1	2	2	3	3	4	4
3	3	1	1	2	2	3	3	4	4
3	3	1	1	1	1	3	3	4	4
3	3	1	1	1	1	3	3	4	4
3	3	1	1	2	2	3	3	4	4
3	3	1	1	2	2	3	3	4	4

For Review Only

3	3	1	1	2	2	3	3	4	4
3	3	1	1	2	2	3	3	4	4
3	3	1	1	2	2	3	3	4	4
3	3	1	1	1	1	3	3	3	3
3	3	1	1	2	2	3	3	4	4
3	3	1	1	2	2	3	3	4	4
3	3	1	1	2	2	3	3	4	4
3	3	1	1	2	2	3	3	4	4
3	3	1	1	1	1	3	3	4	4
3	3	1	1	1	1	3	3	4	4
3	3	1	1	1	1	3	3	4	4
3	3	1	1	2	2	3	3	4	4
2	2	1	1	2	2	3	3	4	4
3	3	1	1	2	2	3	3	4	4
3	3	1	1	2	2	3	3	4	4
2	2	1	1	2	2	3	3	4	4
2	2	2	2	2	2	3	3	4	4
3	3	2	2	2	2	3	3	4	4
3	3	1	1	1	1	3	3	3	3
3	3	1	1	2	2	3	3	4	4
3	3	1	1	2	2	3	3	4	4
3	3	1	1	2	2	3	3	4	4
2	2	2	2	2	2	3	3	4	4
3	3	1	1	1	1	3	3	4	4
3	3	1	1	2	2	3	3	3	3
3	3	1	1	2	2	3	3	4	4
3	3	1	1	2	2	3	3	4	4
3	3	1	1	2	2	4	4	4	4
3	3	1	1	2	2	3	3	4	4
3	3	1	1	2	2	3	3	3	3
3	3	1	1	1	1	3	3	4	4
3	3	1	1	2	2	3	3	4	4
3	3	1	1	2	2	4	4	4	4
3	3	2	2	2	2	3	3	4	4
3	3	1	1	1	1	3	3	4	4
3	3	1	1	2	2	3	3	4	4
3	3	1	1	1	1	3	3	3	3
3	3	1	1	2	2	3	3	4	4
3	3	1	1	2	2	3	3	4	4
3	3	1	1	1	1	3	3	4	4
3	3	1	1	2	2	3	3	4	4
3	3	1	1	1	1	3	3	4	4
3	3	1	1	2	2	3	3	4	4
3	3	1	1	1	1	3	3	4	4
2	2	2	2	2	2	3	3	4	4
3	3	1	1	2	2	4	4	4	4
2	2	2	2	2	2	3	3	4	4
2	2	2	2	2	2	3	3	4	4
3	3	1	1	2	2	3	3	4	4
3	3	1	1	2	2	3	3	4	4
3	3	1	1	2	2	3	3	4	4
3	3	1	1	1	1	3	3	4	4
3	3	1	1	2	2	3	3	4	4
2	2	2	2	2	2	3	3	4	4
3	3	1	1	2	2	3	3	4	4

For Review Only

2	2	2	2	2	2	3	3	4	4
2	2	1	1	2	2	3	3	4	4
3	3	1	1	2	2	3	3	4	4
3	3	1	1	2	2	3	3	4	4
2	2	1	1	2	2	3	3	4	4
2	2	1	1	2	2	3	3	3	3
3	3	1	1	2	2	3	3	4	4
3	3	1	1	1	1	3	3	4	4
3	3	1	1	1	1	3	3	4	4
3	3	1	1	2	2	3	3	4	4
2	2	1	1	2	2	3	3	4	4
3	3	1	1	1	1	3	3	4	4
3	3	1	1	2	2	3	3	4	4
3	3	1	1	2	2	3	3	4	4
3	3	1	1	1	1	3	3	4	4
3	3	1	1	1	1	3	3	4	4
3	3	1	1	2	2	3	3	4	4
2	2	2	2	2	2	3	3	4	4
2	2	2	2	2	2	3	3	4	4
3	3	1	1	2	2	3	3	4	4
3	3	1	1	2	2	3	3	4	4
3	3	1	1	1	1	3	3	4	4
3	3	1	1	2	2	3	3	4	4
2	2	2	2	2	2	3	3	4	4
3	3	1	1	2	2	3	3	4	4
3	3	1	1	1	1	3	3	4	4
3	3	1	1	2	2	3	3	3	3
3	3	1	1	2	2	3	3	4	4
2	2	1	1	2	2	3	3	4	4
3	3	1	1	2	2	4	4	4	4
3	3	1	1	1	1	3	3	4	4
2	2	2	2	2	2	3	3	4	4
3	3	1	1	1	1	3	3	4	4
3	3	1	1	2	2	3	3	4	4
3	3	1	1	2	2	3	3	4	4
2	2	2	2	2	2	3	3	4	4
3	3	1	1	2	2	3	3	4	4
3	3	1	1	2	2	3	3	4	4
2	2	2	2	2	2	3	3	4	4
3	3	1	1	2	2	3	3	4	4
3	3	1	1	2	2	3	3	4	4
2	2	2	2	2	2	3	3	4	4
3	3	1	1	1	1	3	3	4	4
2	2	2	2	2	2	3	3	4	4
3	3	1	1	1	1	3	3	4	4
3	3	1	1	2	2	3	3	4	4
3	3	1	1	1	1	3	3	4	4
3	3	1	1	1	1	3	3	4	4
3	3	1	1	1	1	3	3	4	4
3	3	1	1	1	1	3	3	4	4
3	3	1	1	2	2	3	3	4	4
3	3	1	1	2	2	3	3	4	4
3	3	1	1	2	2	3	3	3	3
2	2	2	2	2	2	3	3	4	4
3	3	1	1	1	1	3	3	4	4

For Review Only

3	3	1	1	1	1	3	3	4	4
2	2	2	2	2	2	3	3	4	4
3	3	1	1	2	2	3	3	4	4
3	3	1	1	2	2	3	3	4	4
2	2	2	2	2	2	3	3	4	4
2	2	1	1	2	2	3	3	4	4
3	3	1	1	1	1	3	3	4	4
3	3	1	1	1	1	3	3	4	4
3	3	1	1	2	2	3	3	4	4
3	3	1	1	1	1	3	3	4	4
3	3	1	1	2	2	3	3	4	4
2	2	2	2	2	2	3	3	4	4
3	3	1	1	1	1	3	3	4	4
3	3	1	1	2	2	3	3	4	4
3	3	1	1	2	2	3	3	4	4
3	3	1	1	2	2	3	3	4	4
3	3	1	1	1	1	3	3	4	4
3	3	1	1	1	1	3	3	4	4
2	2	2	2	2	2	3	3	4	4
3	3	1	1	1	1	3	3	4	4
3	3	1	1	2	2	3	3	4	4
2	2	2	2	2	2	3	3	4	4
3	3	1	1	1	1	3	3	4	4
3	3	1	1	2	2	3	3	4	4
3	3	1	1	1	1	3	3	4	4
3	3	1	1	1	1	3	3	4	4
3	3	1	1	1	1	3	3	4	4
3	3	1	1	2	2	3	3	3	3
3	3	1	1	2	2	3	3	4	4
3	3	1	1	2	2	3	3	4	4
3	3	1	1	1	1	3	3	4	4
3	3	1	1	2	2	3	3	4	4
3	3	1	1	2	2	3	3	4	4
3	3	1	1	2	2	3	3	4	4
3	3	1	1	2	2	3	3	3	3
3	3	1	1	1	1	3	3	4	4
3	3	1	1	2	2	3	3	4	4
2	2	2	2	2	2	3	3	4	4
3	3	1	1	1	1	3	3	4	4
3	3	1	1	1	1	3	3	4	4
3	3	1	1	2	2	3	3	4	4
3	3	1	1	2	2	3	3	4	4
3	3	1	1	2	2	3	3	4	4
3	3	1	1	2	2	4	4	4	4
3	3	1	1	2	2	3	3	4	4
3	3	1	1	1	1	3	3	4	4
2	2	2	2	2	2	3	3	4	4
2	2	1	1	2	2	3	3	4	4
3	3	1	1	1	1	3	3	4	4
3	3	1	1	1	1	3	3	4	4
2	2	1	1	2	2	3	3	4	4
3	3	1	1	1	1	3	3	4	4
3	3	1	1	2	2	3	3	4	4
3	3	1	1	2	2	3	3	3	3
3	3	1	1	2	2	3	3	4	4

For Review Only

3	3	1	1	2	2	3	3	4	4
3	3	1	1	2	2	3	3	4	4

For Review Only

SNP1686	SNP1686	SNP1687	SNP1687	SNP1688	SNP1688	SNP1689	SNP1689	SNP169	SNP169
3	3	2	2	4	4	3	3	1	1
3	3	1	1	1	1	2	2	1	1
3	3	1	1	1	1	2	2	1	1
3	3	2	2	1	1	2	2	3	3
3	3	2	2	4	4	3	3	1	1
3	3	2	2	4	4	3	3	3	3
4	4	2	2	4	4	3	3	3	3
4	4	2	2	4	4	3	3	1	1
3	3	2	2	4	4	3	3	1	1
4	4	2	2	4	4	3	3	1	1
3	3	2	2	4	4	3	3	1	1
3	3	1	1	1	1	2	2	1	1
3	3	2	2	4	4	3	3	1	1
3	3	2	2	4	4	3	3	1	1
3	3	1	1	1	1	2	2	1	1
3	3	1	1	1	1	2	2	1	1
3	3	1	1	1	1	2	2	1	1
3	3	2	2	4	4	3	3	1	1
3	3	1	1	1	1	2	2	1	1
4	4	2	2	4	4	3	3	3	3
4	4	2	2	4	4	3	3	1	1
4	4	2	2	4	4	3	3	1	1
3	3	1	1	1	1	2	2	1	1
3	3	2	2	4	4	3	3	1	1
3	3	1	1	1	1	2	2	1	1
3	3	1	1	1	1	2	2	1	1
3	3	1	1	1	1	2	2	3	3
3	3	2	2	1	1	2	2	1	1
3	3	2	2	4	4	3	3	3	3
3	3	2	2	4	4	3	3	1	1
3	3	1	1	1	1	2	2	1	1
3	3	1	1	1	1	2	2	3	3
3	3	2	2	4	4	3	3	1	1
3	3	2	2	4	4	3	3	3	3
3	3	2	2	1	1	3	3	1	1
3	3	2	2	4	4	3	3	1	1
3	3	2	2	4	4	3	3	1	1
3	3	2	2	4	4	3	3	1	1
3	3	2	2	4	4	3	3	1	1
3	3	1	1	1	1	2	2	1	1
4	4	2	2	4	4	3	3	1	1
3	3	2	2	4	4	3	3	1	1
4	4	2	2	4	4	3	3	1	1
3	3	2	2	4	4	3	3	3	3
4	4	2	2	4	4	3	3	1	1
3	3	1	1	1	1	2	2	1	1
3	3	1	1	1	1	2	2	1	1
3	3	1	1	1	1	2	2	1	1
3	3	2	2	4	4	3	3	1	1
3	3	2	2	1	1	3	3	1	1
3	3	1	1	1	1	2	2	1	1
3	3	1	1	1	1	2	2	1	1

For Review Only

3	3	1	1	1	1	2	2	1	1
3	3	2	2	4	4	3	3	1	1
3	3	1	1	1	1	2	2	1	1
3	3	1	1	1	1	2	2	1	1
3	3	1	1	1	1	2	2	1	1
3	3	1	1	1	1	2	2	1	1
3	3	1	1	1	1	2	2	3	3
3	3	2	2	4	4	3	3	1	1
3	3	2	2	4	4	3	3	3	3
3	3	2	2	1	1	2	2	1	1
3	3	1	1	1	1	2	2	3	3
3	3	1	1	1	1	2	2	3	3
3	3	1	1	1	1	2	2	1	1
3	3	2	2	4	4	3	3	3	3
3	3	2	2	1	1	2	2	1	1
3	3	2	2	4	4	3	3	3	3
3	3	1	1	1	1	2	2	1	1
3	3	2	2	4	4	3	3	1	1
3	3	1	1	1	1	2	2	1	1
3	3	2	2	4	4	3	3	1	1
3	3	1	1	1	1	2	2	3	3
3	3	1	1	1	1	2	2	1	1
3	3	2	2	4	4	3	3	1	1
3	3	2	2	4	4	3	3	1	1
4	4	2	2	4	4	3	3	1	1
3	3	1	1	1	1	2	2	3	3
3	3	2	2	4	4	3	3	1	1
3	3	2	2	4	4	3	3	1	1
3	3	1	1	1	1	2	2	1	1
3	3	1	1	1	1	2	2	1	1
4	4	2	2	1	1	2	2	1	1
3	3	2	2	1	1	2	2	1	1
3	3	2	2	4	4	3	3	1	1
3	3	1	1	1	1	2	2	1	1
3	3	2	2	4	4	3	3	1	1
4	4	2	2	4	4	3	3	1	1
4	4	2	2	4	4	3	3	1	1
4	4	2	2	4	4	3	3	1	1
3	3	2	2	4	4	3	3	1	1
3	3	2	2	4	4	3	3	1	1
3	3	1	1	1	1	2	2	1	1
3	3	1	1	1	1	2	2	1	1
3	3	1	1	1	1	2	2	1	1
3	3	1	1	1	1	2	2	3	3
3	3	2	2	4	4	3	3	1	1
3	3	1	1	1	1	2	2	1	1
3	3	2	2	4	4	3	3	1	1
3	3	1	1	1	1	2	2	3	3
3	3	1	1	1	1	2	2	1	1
3	3	1	1	1	1	2	2	1	1
3	3	2	2	1	1	2	2	1	1
3	3	2	2	4	4	3	3	1	1
3	3	2	2	4	4	3	3	3	3
3	3	2	2	1	1	2	2	1	1
3	3	1	1	1	1	2	2	1	1

For Review Only

3	3	1	1	1	1	2	2	1	1
3	3	1	1	1	1	2	2	1	1
3	3	2	2	1	1	2	2	1	1
3	3	2	2	4	4	3	3	1	1
3	3	1	1	1	1	2	2	3	3
3	3	1	1	1	1	2	2	1	1
3	3	1	1	1	1	2	2	1	1
3	3	2	2	4	4	3	3	1	1
3	3	2	2	4	4	3	3	1	1
3	3	2	2	4	4	3	3	1	1
3	3	2	2	4	4	3	3	1	1
3	3	2	2	1	1	2	2	1	1
3	3	1	1	1	1	2	2	1	1
3	3	1	1	1	1	2	2	1	1
3	3	1	1	1	1	2	2	1	1
3	3	2	2	1	1	2	2	1	1
3	3	1	1	1	1	2	2	1	1
3	3	2	2	1	1	2	2	1	1
3	3	2	2	4	4	3	3	1	1
3	3	1	1	1	1	2	2	1	1
3	3	2	2	1	1	2	2	1	1
3	3	1	1	1	1	2	2	1	1
3	3	1	1	1	1	2	2	1	1
3	3	2	2	4	4	3	3	1	1
3	3	1	1	1	1	2	2	1	1
3	3	1	1	1	1	2	2	1	1
3	3	2	2	1	1	2	2	1	1
3	3	1	1	1	1	2	2	1	1
3	3	1	1	1	1	2	2	1	1
3	3	1	1	1	1	2	2	1	1
3	3	2	2	4	4	3	3	1	1
3	3	2	2	1	1	2	2	1	1
3	3	1	1	1	1	2	2	1	1
3	3	1	1	1	1	2	2	1	1
3	3	2	2	4	4	3	3	3	3
3	3	2	2	4	4	3	3	1	1
3	3	2	2	4	4	3	3	1	1
3	3	1	1	1	1	2	2	1	1
3	3	1	1	1	1	2	2	1	1
3	3	2	2	4	4	3	3	1	1
3	3	1	1	1	1	2	2	1	1
3	3	2	2	4	4	3	3	1	1
3	3	1	1	1	1	2	2	1	1
3	3	2	2	4	4	3	3	1	1
3	3	2	2	1	1	2	2	1	1
3	3	2	2	1	1	3	3	1	1
3	3	1	1	1	1	2	2	1	1
3	3	1	1	1	1	2	2	1	1
3	3	2	2	4	4	3	3	1	1
3	3	1	1	1	1	2	2	1	1
3	3	1	1	1	1	2	2	1	1
3	3	2	2	4	4	3	3	1	1
3	3	2	2	4	4	3	3	1	1
3	3	1	1	1	1	2	2	3	3
3	3	2	2	1	1	3	3	1	1

For Review Only

3	3	1	1	1	1	2	2	1	1
3	3	1	1	1	1	2	2	1	1
3	3	2	2	1	1	2	2	1	1
3	3	2	2	1	1	2	2	1	1
3	3	1	1	1	1	2	2	1	1
3	3	2	2	4	4	3	3	1	1
3	3	2	2	4	4	2	2	1	1
3	3	2	2	1	1	2	2	1	1
3	3	2	2	4	4	3	3	1	1
3	3	1	1	1	1	2	2	1	1
3	3	1	1	1	1	2	2	1	1
3	3	2	2	4	4	3	3	1	1
3	3	1	1	1	1	2	2	1	1
3	3	2	2	1	1	2	2	1	1
3	3	2	2	4	4	3	3	1	1
3	3	2	2	4	4	3	3	1	1
3	3	2	2	4	4	3	3	1	1
3	3	1	1	1	1	2	2	1	1
3	3	1	1	1	1	2	2	1	1
3	3	1	1	1	1	2	2	1	1
3	3	2	2	1	1	2	2	1	1
3	3	2	2	4	4	3	3	1	1
3	3	1	1	1	1	2	2	1	1
3	3	1	1	1	1	2	2	3	3
3	3	2	2	4	4	3	3	1	1
3	3	2	2	4	4	3	3	1	1
3	3	1	1	1	1	2	2	1	1
3	3	2	2	1	1	2	2	1	1
3	3	2	2	4	4	3	3	1	1
3	3	1	1	1	1	2	2	3	3
3	3	2	2	4	4	3	3	1	1
3	3	1	1	1	1	2	2	1	1
3	3	2	2	4	4	3	3	1	1
3	3	2	2	4	4	3	3	1	1
3	3	2	2	1	1	2	2	1	1
3	3	1	1	1	1	2	2	3	3
3	3	1	1	1	1	2	2	1	1
3	3	2	2	4	4	3	3	1	1
3	3	1	1	1	1	2	2	1	1
3	3	1	1	1	1	2	2	1	1
3	3	1	1	1	1	2	2	1	1
3	3	1	1	1	1	2	2	1	1
3	3	2	2	4	4	3	3	1	1
3	3	1	1	1	1	2	2	1	1
3	3	2	2	4	4	3	3	1	1
3	3	2	2	4	4	3	3	1	1
3	3	2	2	4	4	3	3	1	1
3	3	2	2	4	4	3	3	1	1
3	3	2	2	4	4	3	3	1	1
4	4	2	2	4	4	3	3	1	1
3	3	1	1	1	1	2	2	1	1
3	3	2	2	4	4	3	3	1	1
3	3	1	1	1	1	2	2	1	1
3	3	1	1	1	1	2	2	3	3
3	3	2	2	4	4	3	3	1	1

For Review Only

3	3	2	2	4	4	3	3	1	1
3	3	1	1	1	1	2	2	1	1
3	3	1	1	1	1	2	2	1	1
3	3	1	1	1	1	2	2	1	1
3	3	2	2	4	4	3	3	1	1
3	3	1	1	1	1	2	2	3	3
3	3	2	2	4	4	3	3	1	1
3	3	2	2	4	4	3	3	1	1
3	3	2	2	4	4	2	2	1	1
3	3	2	2	4	4	3	3	1	1
3	3	2	2	1	1	2	2	1	1
3	3	2	2	4	4	3	3	1	1
3	3	2	2	4	4	3	3	3	3
3	3	1	1	1	1	2	2	1	1
3	3	1	1	1	1	2	2	1	1
3	3	1	1	1	1	2	2	1	1
3	3	2	2	4	4	3	3	1	1
3	3	2	2	4	4	3	3	1	1
3	3	1	1	1	1	2	2	1	1
3	3	2	2	4	4	3	3	1	1
3	3	1	1	1	1	2	2	1	1
3	3	1	1	1	1	2	2	1	1
3	3	2	2	4	4	3	3	1	1
3	3	1	1	1	1	2	2	1	1
3	3	2	2	4	4	3	3	1	1
3	3	2	2	1	1	2	2	1	1
3	3	2	2	4	4	3	3	1	1
3	3	1	1	1	1	2	2	3	3
3	3	2	2	1	1	2	2	1	1
3	3	1	1	1	1	2	2	1	1
3	3	2	2	4	4	3	3	1	1
3	3	1	1	1	1	2	2	1	1
3	3	1	1	1	1	2	2	1	1
3	3	1	1	1	1	2	2	1	1
3	3	1	1	1	1	2	2	1	1
3	3	2	2	4	4	3	3	1	1
3	3	2	2	4	4	3	3	1	1
3	3	1	1	1	1	2	2	3	3
3	3	2	2	4	4	3	3	1	1
3	3	2	2	4	4	3	3	1	1
3	3	1	1	1	1	2	2	1	1
3	3	1	1	1	1	2	2	1	1
3	3	1	1	1	1	2	2	1	1
3	3	1	1	1	1	2	2	1	1
3	3	2	2	1	1	2	2	1	1
3	3	2	2	4	4	3	3	1	1
3	3	1	1	1	1	2	2	1	1
3	3	1	1	1	1	2	2	3	3
3	3	2	2	4	4	3	3	1	1
3	3	2	2	4	4	3	3	1	1
3	3	1	1	1	1	2	2	1	1
3	3	2	2	4	4	3	3	1	1
3	3	1	1	1	1	2	2	1	1
3	3	1	1	1	1	2	2	1	1
3	3	1	1	1	1	2	2	1	1

For Review Only

3	3	1	1	1	1	2	2	1	1
3	3	1	1	1	1	2	2	1	1

For Review Only

SNP1690	SNP1690	SNP1691	SNP1691	SNP1692	SNP1692	SNP1693	SNP1693	SNP1694	SNP1694
1	1	3	3	4	4	4	4	4	4
4	4	3	3	4	4	4	4	4	4
4	4	2	2	4	4	4	4	1	1
4	4	3	3	4	4	4	4	1	1
1	1	3	3	2	2	2	2	1	1
1	1	3	3	4	4	4	4	1	1
1	1	3	3	4	4	4	4	1	1
1	1	3	3	4	4	4	4	1	1
1	1	3	3	4	4	2	2	1	1
1	1	3	3	4	4	4	4	1	1
1	1	2	2	4	4	4	4	1	1
4	4	2	2	4	4	4	4	1	1
1	1	3	3	4	4	2	2	1	1
1	1	3	3	4	4	4	4	1	1
4	4	3	3	4	4	2	2	1	1
4	4	3	3	4	4	4	4	1	1
4	4	3	3	4	4	2	2	1	1
1	1	3	3	4	4	4	4	1	1
4	4	2	2	4	4	4	4	4	4
1	1	3	3	4	4	4	4	1	1
1	1	3	3	4	4	4	4	1	1
1	1	3	3	2	2	2	2	1	1
4	4	3	3	4	4	4	4	1	1
1	1	3	3	4	4	4	4	1	1
4	4	3	3	4	4	4	4	1	1
4	4	3	3	4	4	4	4	1	1
4	4	3	3	4	4	4	4	1	1
4	4	3	3	4	4	4	4	1	1
1	1	3	3	4	4	2	2	1	1
1	1	3	3	4	4	4	4	1	1
4	4	3	3	4	4	4	4	1	1
4	4	2	2	4	4	4	4	1	1
1	1	2	2	4	4	2	2	1	1
1	1	3	3	4	4	4	4	1	1
4	4	3	3	2	2	4	4	1	1
1	1	3	3	4	4	4	4	1	1
1	1	3	3	4	4	4	4	1	1
1	1	2	2	4	4	4	4	1	1
1	1	3	3	4	4	4	4	1	1
4	4	3	3	4	4	4	4	1	1
1	1	3	3	4	4	4	4	1	1
1	1	3	3	4	4	2	2	1	1
1	1	3	3	4	4	4	4	1	1
1	1	3	3	2	2	4	4	1	1
1	1	3	3	4	4	4	4	1	1
4	4	2	2	4	4	4	4	1	1
4	4	3	3	4	4	4	4	4	4
4	4	3	3	2	2	4	4	1	1
1	1	3	3	4	4	2	2	1	1
4	4	3	3	2	2	4	4	1	1
4	4	3	3	4	4	4	4	1	1
4	4	3	3	4	4	4	4	1	1

For Review Only

4	4	3	3	4	4	2	2	1	1
1	1	2	2	4	4	2	2	1	1
4	4	3	3	4	4	4	4	1	1
4	4	3	3	4	4	4	4	1	1
4	4	3	3	4	4	4	4	4	4
4	4	3	3	4	4	4	4	4	4
4	4	3	3	4	4	4	4	1	1
1	1	3	3	4	4	4	4	1	1
1	1	3	3	4	4	4	4	1	1
4	4	3	3	4	4	4	4	1	1
4	4	3	3	4	4	4	4	1	1
4	4	3	3	4	4	4	4	4	4
4	4	3	3	4	4	2	2	1	1
1	1	3	3	4	4	2	2	1	1
4	4	3	3	4	4	4	4	1	1
1	1	3	3	4	4	4	4	1	1
4	4	3	3	2	2	4	4	1	1
1	1	2	2	4	4	4	4	1	1
4	4	3	3	2	2	4	4	1	1
1	1	3	3	4	4	2	2	1	1
4	4	3	3	4	4	4	4	1	1
4	4	3	3	4	4	4	4	1	1
4	4	3	3	2	2	2	2	1	1
1	1	3	3	4	4	4	4	1	1
1	1	3	3	4	4	4	4	1	1
4	4	3	3	4	4	4	4	1	1
1	1	2	2	4	4	4	4	1	1
1	1	3	3	4	4	4	4	1	1
4	4	3	3	4	4	4	4	1	1
4	4	2	2	4	4	4	4	1	1
1	1	3	3	4	4	4	4	1	1
4	4	3	3	4	4	4	4	1	1
1	1	3	3	4	4	4	4	1	1
4	4	3	3	4	4	2	2	1	1
1	1	3	3	4	4	4	4	1	1
1	1	3	3	4	4	4	4	1	1
1	1	3	3	4	4	4	4	1	1
1	1	3	3	4	4	4	4	1	1
1	1	2	2	4	4	4	4	1	1
1	1	3	3	4	4	4	4	1	1
4	4	3	3	2	2	2	2	1	1
4	4	2	2	4	4	4	4	4	4
4	4	3	3	4	4	4	4	1	1
4	4	3	3	4	4	4	4	1	1
1	1	2	2	4	4	4	4	1	1
4	4	3	3	4	4	4	4	4	4
1	1	3	3	4	4	4	4	1	1
4	4	3	3	4	4	4	4	1	1
4	4	3	3	4	4	4	4	1	1
4	4	3	3	4	4	4	4	1	1
4	4	3	3	4	4	4	4	4	4
1	1	3	3	4	4	4	4	1	1
1	1	3	3	4	4	4	4	1	1
4	4	3	3	4	4	4	4	1	1
4	4	2	2	4	4	4	4	1	1

For Review Only

4	4	3	3	4	4	4	4	1	1
4	4	3	3	4	4	4	4	1	1
1	1	3	3	4	4	4	4	1	1
1	1	3	3	4	4	4	4	1	1
4	4	3	3	4	4	4	4	4	4
4	4	3	3	4	4	4	4	1	1
4	4	3	3	4	4	4	4	4	4
1	1	3	3	4	4	4	4	1	1
1	1	3	3	4	4	4	4	1	1
1	1	3	3	4	4	4	4	1	1
1	1	3	3	4	4	4	4	1	1
4	4	2	2	4	4	4	4	1	1
4	4	3	3	4	4	4	4	1	1
4	4	3	3	4	4	4	4	4	4
4	4	3	3	4	4	4	4	1	1
4	4	3	3	4	4	4	4	1	1
4	4	2	2	4	4	4	4	1	1
4	4	3	3	4	4	4	4	1	1
1	1	3	3	4	4	4	4	1	1
4	4	3	3	4	4	4	4	1	1
4	4	3	3	4	4	4	4	1	1
4	4	3	3	4	4	4	4	1	1
4	4	3	3	4	4	4	4	1	1
1	1	3	3	4	4	4	4	1	1
4	4	3	3	4	4	4	4	1	1
4	4	3	3	4	4	4	4	1	1
4	4	3	3	4	4	4	4	1	1
4	4	3	3	4	4	2	2	1	1
4	4	3	3	4	4	4	4	1	1
4	4	3	3	4	4	4	4	1	1
1	1	3	3	4	4	4	4	1	1
4	4	3	3	4	4	4	4	1	1
4	4	3	3	2	2	2	2	1	1
4	4	3	3	4	4	4	4	1	1
1	1	3	3	4	4	4	4	1	1
1	1	3	3	4	4	2	2	1	1
1	1	3	3	4	4	4	4	1	1
4	4	3	3	4	4	4	4	4	4
4	4	3	3	4	4	4	4	1	1
1	1	2	2	4	4	4	4	1	1
4	4	3	3	4	4	4	4	1	1
1	1	3	3	4	4	4	4	1	1
4	4	3	3	4	4	4	4	1	1
1	1	3	3	4	4	4	4	1	1
4	4	3	3	4	4	4	4	1	1
1	1	3	3	4	4	2	2	1	1
4	4	3	3	4	4	4	4	1	1
4	4	3	3	4	4	4	4	1	1
1	1	3	3	4	4	4	4	1	1
4	4	3	3	4	4	4	4	1	1
4	4	3	3	4	4	4	4	1	1
1	1	3	3	4	4	4	4	1	1
4	4	3	3	4	4	4	4	1	1
4	4	3	3	4	4	4	4	4	4
4	4	3	3	4	4	4	4	1	1

For Review Only

4	4	2	2	4	4	4	4	1	1
4	4	3	3	4	4	4	4	1	1
4	4	3	3	4	4	4	4	1	1
4	4	3	3	4	4	4	4	1	1
4	4	3	3	4	4	4	4	4	4
1	1	3	3	4	4	4	4	1	1
4	4	3	3	4	4	4	4	1	1
4	4	3	3	4	4	4	4	1	1
1	1	3	3	2	2	4	4	1	1
4	4	2	2	4	4	4	4	1	1
4	4	3	3	4	4	4	4	1	1
1	1	3	3	4	4	4	4	1	1
4	4	3	3	4	4	4	4	1	1
4	4	3	3	4	4	2	2	1	1
1	1	3	3	4	4	4	4	1	1
1	1	2	2	4	4	4	4	1	1
4	4	3	3	4	4	4	4	1	1
4	4	3	3	4	4	4	4	4	4
4	4	3	3	4	4	4	4	1	1
4	4	3	3	4	4	4	4	4	4
4	4	3	3	4	4	4	4	1	1
1	1	3	3	4	4	4	4	1	1
4	4	3	3	4	4	4	4	1	1
4	4	3	3	4	4	4	4	4	4
1	1	3	3	4	4	4	4	1	1
1	1	3	3	4	4	4	4	1	1
4	4	3	3	4	4	4	4	1	1
4	4	2	2	4	4	4	4	1	1
1	1	3	3	4	4	4	4	1	1
4	4	3	3	4	4	2	2	1	1
1	1	3	3	4	4	4	4	1	1
4	4	2	2	4	4	4	4	1	1
1	1	3	3	4	4	4	4	1	1
1	1	3	3	4	4	4	4	1	1
4	4	3	3	4	4	4	4	1	1
4	4	3	3	4	4	4	4	4	4
4	4	3	3	4	4	4	4	4	4
1	1	3	3	4	4	4	4	1	1
4	4	3	3	4	4	4	4	1	1
4	4	3	3	4	4	4	4	1	1
4	4	2	2	4	4	4	4	1	1
4	4	3	3	4	4	4	4	1	1
1	1	3	3	4	4	4	4	1	1
4	4	3	3	4	4	4	4	1	1
1	1	3	3	4	4	4	4	1	1
1	1	2	2	4	4	4	4	1	1
1	1	2	2	4	4	4	4	1	1
1	1	3	3	4	4	4	4	1	1
1	1	3	3	4	4	4	4	1	1
1	1	3	3	4	4	4	4	4	4
4	4	3	3	4	4	4	4	1	1
1	1	3	3	4	4	4	4	1	1
4	4	3	3	4	4	4	4	1	1
4	4	3	3	4	4	4	4	1	1
1	1	3	3	4	4	4	4	1	1

1	1	3	3	4	4	4	4	1	1
4	4	3	3	4	4	4	4	1	1
4	4	2	2	4	4	4	4	1	1
4	4	3	3	4	4	4	4	1	1
1	1	3	3	4	4	4	4	1	1
4	4	3	3	4	4	4	4	1	1
1	1	3	3	4	4	4	4	1	1
1	1	3	3	4	4	4	4	1	1
4	4	3	3	4	4	4	4	1	1
1	1	3	3	4	4	4	4	1	1
4	4	3	3	4	4	4	4	1	1
1	1	3	3	4	4	4	4	1	1
1	1	2	2	4	4	4	4	1	1
4	4	3	3	4	4	4	4	1	1
4	4	2	2	4	4	4	4	1	1
4	4	2	2	4	4	4	4	1	1
1	1	3	3	4	4	4	4	1	1
1	1	3	3	4	4	4	4	1	1
4	4	3	3	4	4	4	4	1	1
1	1	2	2	4	4	4	4	1	1
4	4	3	3	4	4	4	4	1	1
4	4	3	3	4	4	4	4	1	1
1	1	3	3	4	4	4	4	1	1
4	4	3	3	4	4	4	4	1	1
1	1	3	3	4	4	4	4	1	1
1	1	3	3	4	4	4	4	1	1
1	1	3	3	4	4	4	4	1	1
4	4	3	3	4	4	4	4	1	1
4	4	3	3	4	4	4	4	1	1
4	4	3	3	4	4	4	4	1	1
1	1	3	3	4	4	4	4	1	1
4	4	3	3	4	4	4	4	1	1
4	4	3	3	2	2	4	4	1	1
4	4	3	3	4	4	4	4	1	1
4	4	3	3	4	4	4	4	1	1
1	1	3	3	4	4	4	4	1	1
1	1	3	3	4	4	4	4	1	1
4	4	3	3	4	4	4	4	4	4
1	1	3	3	4	4	4	4	1	1
1	1	3	3	4	4	4	4	1	1
4	4	2	2	4	4	4	4	1	1
4	4	3	3	4	4	4	4	1	1
4	4	3	3	4	4	4	4	1	1
4	4	3	3	2	2	2	2	1	1
4	4	3	3	4	4	4	4	1	1
1	1	2	2	4	4	4	4	1	1
4	4	3	3	4	4	4	4	1	1
4	4	3	3	4	4	4	4	1	1
1	1	3	3	4	4	4	4	1	1
1	1	3	3	4	4	4	4	1	1
4	4	3	3	4	4	4	4	1	1
1	1	3	3	4	4	4	4	1	1
4	4	2	2	4	4	4	4	1	1
4	4	3	3	4	4	4	4	1	1
4	4	3	3	4	4	4	4	1	1

For Review Only

4	4	2	2	4	4	4	4	1	1
4	4	3	3	4	4	4	4	4	4

For Review Only

SNP1695	SNP1695	SNP1696	SNP1696	SNP1697	SNP1697	SNP1698	SNP1698	SNP1699	SNP1699
3	3	4	4	1	1	1	1	4	4
3	3	2	2	1	1	3	3	4	4
3	3	2	2	2	2	3	3	2	2
3	3	4	4	2	2	3	3	2	2
1	1	4	4	1	1	1	1	4	4
3	3	4	4	1	1	1	1	4	4
3	3	4	4	1	1	1	1	4	4
3	3	4	4	1	1	1	1	4	4
1	1	4	4	1	1	1	1	4	4
3	3	4	4	1	1	1	1	4	4
3	3	4	4	1	1	1	1	4	4
3	3	2	2	2	2	3	3	2	2
3	3	4	4	1	1	1	1	4	4
3	3	4	4	1	1	1	1	4	4
1	1	2	2	1	1	1	1	4	4
3	3	2	2	1	1	3	3	4	4
1	1	2	2	1	1	1	1	4	4
3	3	4	4	1	1	1	1	4	4
3	3	2	2	2	2	3	3	2	2
3	3	4	4	1	1	1	1	4	4
3	3	4	4	1	1	1	1	4	4
1	1	4	4	1	1	1	1	4	4
3	3	4	4	1	1	1	1	4	4
3	3	4	4	1	1	1	1	4	4
3	3	2	2	1	1	1	1	4	4
3	3	2	2	2	2	3	3	2	2
3	3	2	2	2	2	3	3	2	2
3	3	4	4	2	2	3	3	2	2
3	3	4	4	1	1	1	1	4	4
3	3	4	4	1	1	1	1	4	4
3	3	2	2	1	1	3	3	4	4
3	3	2	2	2	2	3	3	2	2
1	1	4	4	1	1	1	1	4	4
3	3	4	4	1	1	1	1	4	4
3	3	4	4	1	1	1	1	4	4
3	3	4	4	2	2	1	1	2	2
3	3	4	4	2	2	1	1	4	4
3	3	4	4	1	1	1	1	4	4
3	3	4	4	1	1	1	1	4	4
3	3	4	4	1	1	1	1	4	4
3	3	4	4	1	1	1	1	4	4
1	1	4	4	1	1	1	1	4	4
3	3	4	4	1	1	1	1	4	4
3	3	4	4	1	1	1	1	4	4
3	3	4	4	1	1	1	1	4	4
3	3	2	2	1	1	3	3	2	2
3	3	4	4	2	2	1	1	2	2
3	3	2	2	2	2	3	3	2	2
1	1	4	4	1	1	1	1	4	4
3	3	4	4	1	1	3	3	2	2
3	3	2	2	2	2	3	3	2	2
3	3	2	2	2	2	3	3	2	2

For Review Only

1	1	2	2	1	1	1	1	4	4
1	1	4	4	1	1	1	1	4	4
3	3	2	2	2	2	3	3	2	2
3	3	2	2	1	1	1	1	4	4
3	3	2	2	2	2	3	3	2	2
3	3	4	4	2	2	3	3	2	2
3	3	2	2	2	2	3	3	2	2
3	3	4	4	1	1	1	1	4	4
3	3	4	4	2	2	1	1	4	4
3	3	4	4	2	2	3	3	2	2
3	3	4	4	2	2	1	1	2	2
3	3	4	4	2	2	1	1	2	2
1	1	2	2	1	1	1	1	4	4
3	3	4	4	1	1	1	1	4	4
3	3	2	2	1	1	3	3	4	4
3	3	4	4	1	1	1	1	4	4
3	3	2	2	2	2	1	1	2	2
3	3	4	4	2	2	1	1	2	2
3	3	2	2	1	1	3	3	4	4
1	1	4	4	1	1	1	1	4	4
3	3	4	4	2	2	1	1	2	2
3	3	4	4	1	1	1	1	4	4
3	3	4	4	1	1	1	1	4	4
3	3	4	4	1	1	1	1	4	4
3	3	4	4	1	1	1	1	4	4
3	3	2	2	2	2	3	3	2	2
3	3	4	4	1	1	1	1	2	2
3	3	4	4	1	1	1	1	4	4
3	3	2	2	2	2	3	3	2	2
3	3	2	2	2	2	3	3	2	2
1	1	2	2	1	1	3	3	4	4
3	3	2	2	2	2	3	3	4	4
3	3	4	4	1	1	1	1	4	4
1	1	2	2	1	1	1	1	4	4
3	3	4	4	1	1	1	1	4	4
3	3	4	4	1	1	1	1	4	4
3	3	2	2	1	1	1	1	4	4
3	3	4	4	1	1	1	1	4	4
3	3	4	4	2	2	1	1	2	2
3	3	4	4	1	1	1	1	4	4
1	1	2	2	1	1	1	1	4	4
3	3	2	2	2	2	3	3	2	2
3	3	2	2	2	2	3	3	2	2
3	3	4	4	2	2	1	1	2	2
3	3	4	4	1	1	1	1	4	4
3	3	2	2	2	2	3	3	2	2
3	3	4	4	2	2	1	1	4	4
3	3	2	2	2	2	3	3	2	2
3	3	2	2	2	2	3	3	2	2
3	3	4	4	1	1	1	1	4	4
3	3	2	2	2	2	3	3	2	2
3	3	4	4	1	1	1	1	4	4
3	3	4	4	1	1	1	1	4	4
3	3	4	4	1	1	3	3	4	4
3	3	2	2	2	2	3	3	4	4

For Review Only

3	3	2	2	1	1	3	3	4	4
3	3	2	2	1	1	3	3	4	4
3	3	4	4	2	2	1	1	2	2
3	3	4	4	1	1	1	1	4	4
3	3	2	2	2	2	3	3	2	2
3	3	2	2	2	2	3	3	2	2
3	3	2	2	2	2	3	3	2	2
3	3	4	4	1	1	1	1	4	4
3	3	4	4	2	2	1	1	2	2
3	3	4	4	1	1	1	1	4	4
3	3	4	4	1	1	1	1	4	4
3	3	4	4	1	1	3	3	2	2
3	3	2	2	2	2	3	3	2	2
3	3	2	2	2	2	3	3	2	2
3	3	2	2	2	2	3	3	4	4
3	3	2	2	2	2	3	3	2	2
3	3	2	2	1	1	3	3	4	4
3	3	2	2	1	1	3	3	2	2
3	3	4	4	1	1	1	1	4	4
3	3	2	2	2	2	1	1	2	2
3	3	4	4	1	1	3	3	2	2
3	3	2	2	1	1	3	3	4	4
3	3	2	2	2	2	3	3	2	2
3	3	4	4	1	1	1	1	4	4
3	3	4	4	2	2	3	3	4	4
3	3	4	4	1	1	1	1	4	4
1	1	4	4	1	1	1	1	4	4
1	1	2	2	2	2	3	3	4	4
3	3	2	2	2	2	3	3	2	2
3	3	4	4	1	1	1	1	4	4
3	3	4	4	1	1	1	1	4	4
3	3	4	4	2	2	3	3	2	2
1	1	2	2	1	1	1	1	4	4
3	3	4	4	1	1	1	1	2	2
3	3	4	4	1	1	1	1	4	4
1	1	4	4	1	1	1	1	4	4
3	3	4	4	1	1	1	1	4	4
3	3	2	2	1	1	3	3	4	4
3	3	4	4	2	2	3	3	2	2
3	3	4	4	1	1	1	1	4	4
3	3	4	4	1	1	3	3	2	2
3	3	4	4	1	1	1	1	4	4
3	3	2	2	2	2	1	1	2	2
3	3	4	4	1	1	1	1	4	4
3	3	4	4	2	2	1	1	2	2
1	1	4	4	2	2	3	3	4	4
3	3	2	2	2	2	3	3	2	2
3	3	2	2	2	2	3	3	2	2
3	3	4	4	1	1	1	1	4	4
3	3	2	2	2	2	3	3	2	2
3	3	2	2	1	1	3	3	2	2
3	3	4	4	1	1	1	1	4	4
3	3	4	4	2	2	1	1	2	2
3	3	2	2	2	2	3	3	2	2
3	3	4	4	1	1	3	3	4	4

For Review Only

3	3	2	2	2	2	3	3	2	2
3	3	4	4	1	1	1	1	4	4
3	3	4	4	1	1	3	3	4	4
3	3	4	4	1	1	3	3	4	4
3	3	2	2	1	1	3	3	2	2
3	3	4	4	1	1	1	1	4	4
3	3	4	4	1	1	1	1	4	4
3	3	4	4	1	1	3	3	2	2
3	3	4	4	1	1	1	1	2	2
3	3	2	2	2	2	3	3	2	2
3	3	2	2	2	2	3	3	2	2
3	3	4	4	1	1	1	1	4	4
3	3	2	2	1	1	3	3	2	2
1	1	4	4	1	1	3	3	4	4
3	3	4	4	2	2	1	1	4	4
3	3	4	4	1	1	1	1	4	4
3	3	4	4	2	2	1	1	2	2
3	3	2	2	1	1	1	1	4	4
3	3	2	2	2	2	3	3	2	2
3	3	2	2	2	2	3	3	2	2
3	3	4	4	1	1	1	1	2	2
3	3	4	4	1	1	1	1	4	4
3	3	4	4	1	1	1	1	4	4
3	3	4	4	2	2	1	1	2	2
3	3	4	4	1	1	1	1	4	4
3	3	4	4	1	1	1	1	4	4
3	3	2	2	2	2	3	3	2	2
3	3	4	4	1	1	3	3	4	4
3	3	4	4	1	1	1	1	4	4
1	1	2	2	2	2	1	1	4	4
3	3	4	4	2	2	1	1	4	4
3	3	2	2	2	2	3	3	2	2
3	3	4	4	1	1	1	1	4	4
3	3	4	4	2	2	1	1	4	4
3	3	4	4	2	2	1	1	4	4
3	3	4	4	2	2	1	1	2	2
3	3	4	4	2	2	1	1	4	4
3	3	4	4	2	2	1	1	4	4
3	3	2	2	2	2	3	3	2	2
3	3	2	2	2	2	1	1	2	2
3	3	2	2	2	2	3	3	2	2
3	3	2	2	2	2	3	3	2	2
3	3	4	4	1	1	1	1	4	4
3	3	4	4	1	1	1	1	2	2
3	3	4	4	2	2	1	1	2	2
3	3	4	4	1	1	1	1	4	4
3	3	4	4	1	1	1	1	4	4
3	3	4	4	1	1	1	1	4	4
3	3	4	4	1	1	1	1	4	4
3	3	4	4	1	1	1	1	4	4
3	3	2	2	2	2	3	3	2	2
3	3	4	4	1	1	1	1	4	4
3	3	4	4	1	1	1	1	4	4
3	3	4	4	2	2	1	1	2	2
3	3	4	4	1	1	1	1	4	4

For Review Only

3	3	4	4	1	1	1	1	4	4
3	3	2	2	1	1	1	1	4	4
3	3	2	2	2	2	3	3	2	2
1	1	2	2	1	1	3	3	4	4
3	3	4	4	1	1	1	1	4	4
3	3	2	2	2	2	3	3	2	2
3	3	4	4	1	1	1	1	4	4
3	3	4	4	1	1	1	1	4	4
3	3	4	4	1	1	3	3	4	4
3	3	4	4	1	1	1	1	4	4
3	3	4	4	1	1	3	3	2	2
3	3	4	4	1	1	1	1	4	4
3	3	4	4	2	2	1	1	2	2
3	3	2	2	1	1	1	1	4	4
3	3	2	2	2	2	1	1	2	2
3	3	4	4	2	2	1	1	4	4
3	3	4	4	2	2	1	1	4	4
3	3	4	4	1	1	1	1	4	4
3	3	4	4	1	1	1	1	4	4
3	3	4	4	1	1	1	1	4	4
3	3	2	2	1	1	3	3	4	4
3	3	2	2	2	2	3	3	2	2
3	3	4	4	2	2	1	1	4	4
3	3	4	4	1	1	1	1	4	4
3	3	4	4	1	1	1	1	4	4
3	3	4	4	2	2	3	3	2	2
3	3	4	4	1	1	1	1	4	4
3	3	2	2	2	2	3	3	2	2
1	1	4	4	1	1	1	1	4	4
3	3	2	2	2	2	3	3	2	2
3	3	4	4	1	1	1	1	4	4
3	3	2	2	2	2	3	3	2	2
3	3	2	2	1	1	3	3	4	4
3	3	2	2	1	1	3	3	4	4
3	3	4	4	1	1	1	1	4	4
3	3	4	4	1	1	1	1	4	4
3	3	4	4	2	2	1	1	2	2
3	3	4	4	2	2	1	1	2	2
3	3	4	4	2	2	1	1	2	2
3	3	4	4	1	1	1	1	4	4
3	3	2	2	2	2	3	3	4	4
3	3	4	4	1	1	3	3	4	4
3	3	4	4	1	1	1	1	4	4
1	1	2	2	1	1	1	1	4	4
3	3	4	4	1	1	1	1	2	2
3	3	4	4	1	1	1	1	4	4
3	3	4	4	1	1	3	3	2	2
3	3	2	2	1	1	1	1	4	4
3	3	4	4	2	2	1	1	2	2
3	3	4	4	1	1	1	1	4	4
3	3	2	2	2	2	3	3	4	4
3	3	4	4	1	1	1	1	4	4
3	3	2	2	2	2	3	3	2	2
3	3	2	2	2	2	1	1	2	2
3	3	4	4	1	1	1	1	4	4

For Review Only

3	3	4	4	2	2	1	1	4	4
3	3	2	2	2	2	3	3	2	2

For Review Only

SNP170	SNP170	SNP1700	SNP1700	SNP1701	SNP1701	SNP1702	SNP1702	SNP1703	SNP1703
4	4	1	1	4	4	1	1	4	4
3	3	1	1	1	1	1	1	2	2
4	4	1	1	4	4	1	1	4	4
4	4	1	1	1	1	1	1	2	2
4	4	1	1	1	1	2	2	4	4
4	4	1	1	4	4	1	1	4	4
4	4	1	1	4	4	1	1	4	4
3	3	1	1	1	1	1	1	2	2
4	4	1	1	1	1	1	1	2	2
3	3	1	1	1	1	1	1	2	2
4	4	1	1	4	4	1	1	2	2
4	4	1	1	4	4	1	1	4	4
3	3	1	1	1	1	1	1	2	2
4	4	1	1	1	1	1	1	4	4
3	3	1	1	1	1	1	1	2	2
4	4	1	1	1	1	1	1	4	4
4	4	1	1	4	4	1	1	4	4
3	3	1	1	1	1	1	1	2	2
4	4	1	1	4	4	1	1	4	4
3	3	1	1	1	1	1	1	2	2
3	3	1	1	4	4	1	1	4	4
4	4	1	1	1	1	2	2	4	4
4	4	1	1	1	1	1	1	2	2
4	4	1	1	1	1	1	1	2	2
4	4	1	1	4	4	1	1	2	2
4	4	1	1	1	1	1	1	4	4
4	4	1	1	1	1	1	1	2	2
4	4	1	1	1	1	1	1	2	2
4	4	1	1	1	1	1	1	2	2
3	3	1	1	1	1	1	1	2	2
4	4	1	1	4	4	1	1	2	2
4	4	1	1	4	4	1	1	4	4
4	4	1	1	4	4	1	1	4	4
4	4	1	1	1	1	1	1	2	2
4	4	1	1	1	1	1	1	2	2
4	4	4	4	1	1	1	1	2	2
4	4	1	1	1	1	1	1	2	2
4	4	1	1	1	1	1	1	4	4
4	4	1	1	1	1	1	1	2	2
4	4	1	1	1	1	1	1	2	2
3	3	1	1	1	1	1	1	2	2
3	3	1	1	1	1	1	1	2	2
3	3	1	1	1	1	1	1	2	2
4	4	1	1	1	1	2	2	4	4
4	4	1	1	1	1	1	1	2	2
3	3	1	1	1	1	1	1	4	4
4	4	4	4	1	1	1	1	4	4
3	3	1	1	1	1	2	2	2	2
3	3	1	1	1	1	1	1	4	4
3	3	1	1	1	1	2	2	4	4
4	4	1	1	1	1	1	1	4	4
4	4	1	1	1	1	1	1	4	4

For Review Only

4	4	1	1	4	4	1	1	4	4
4	4	1	1	4	4	1	1	4	4
4	4	1	1	1	1	1	1	4	4
4	4	1	1	1	1	1	1	4	4
4	4	1	1	4	4	1	1	4	4
4	4	4	4	1	1	1	1	4	4
4	4	1	1	1	1	1	1	4	4
4	4	1	1	1	1	1	1	2	2
4	4	1	1	1	1	1	1	2	2
4	4	1	1	1	1	1	1	2	2
4	4	1	1	1	1	1	1	4	4
4	4	4	4	1	1	1	1	4	4
4	4	1	1	4	4	1	1	4	4
4	4	1	1	1	1	1	1	2	2
4	4	1	1	1	1	1	1	2	2
4	4	1	1	1	1	1	1	2	2
4	4	1	1	1	1	2	2	4	4
3	3	1	1	1	1	1	1	4	4
3	3	1	1	1	1	2	2	4	4
4	4	1	1	4	4	1	1	4	4
4	4	1	1	1	1	1	1	2	2
4	4	1	1	1	1	1	1	2	2
4	4	1	1	1	1	2	2	2	2
3	3	1	1	1	1	1	1	2	2
4	4	1	1	1	1	1	1	2	2
4	4	1	1	1	1	1	1	4	4
3	3	1	1	4	4	1	1	4	4
3	3	1	1	1	1	1	1	2	2
4	4	1	1	1	1	1	1	4	4
4	4	1	1	4	4	1	1	4	4
4	4	1	1	1	1	1	1	2	2
4	4	1	1	1	1	1	1	4	4
4	4	1	1	1	1	1	1	2	2
4	4	1	1	4	4	1	1	4	4
4	4	1	1	1	1	1	1	2	2
4	4	1	1	1	1	1	1	2	2
3	3	1	1	1	1	1	1	2	2
3	3	1	1	4	4	1	1	2	2
3	3	1	1	4	4	1	1	4	4
3	3	1	1	1	1	1	1	2	2
4	4	1	1	1	1	2	2	4	4
4	4	1	1	4	4	1	1	4	4
3	3	1	1	1	1	1	1	4	4
4	4	1	1	1	1	1	1	4	4
4	4	1	1	4	4	1	1	2	2
4	4	1	1	1	1	1	1	4	4
4	4	1	1	1	1	1	1	2	2
4	4	1	1	1	1	1	1	4	4
4	4	4	4	1	1	1	1	4	4
3	3	1	1	1	1	1	1	4	4
4	4	1	1	1	1	1	1	4	4
4	4	1	1	1	1	1	1	2	2
4	4	1	1	1	1	1	1	2	2
4	4	1	1	1	1	1	1	2	2
3	3	1	1	4	4	1	1	4	4

For Review Only

4	4	1	1	1	1	1	1	2	2
3	3	1	1	1	1	1	1	2	2
3	3	1	1	1	1	1	1	2	2
4	4	1	1	1	1	1	1	2	2
4	4	1	1	1	1	1	1	4	4
3	3	1	1	1	1	1	1	4	4
4	4	1	1	1	1	1	1	4	4
4	4	1	1	1	1	1	1	2	2
4	4	1	1	1	1	1	1	2	2
4	4	1	1	1	1	1	1	2	2
4	4	1	1	1	1	1	1	4	4
4	4	1	1	1	1	1	1	4	4
3	3	1	1	1	1	1	1	4	4
4	4	1	1	1	1	1	1	4	4
3	3	1	1	1	1	1	1	2	2
4	4	1	1	1	1	1	1	2	2
3	3	1	1	4	4	1	1	4	4
4	4	1	1	1	1	1	1	2	2
4	4	1	1	1	1	1	1	2	2
4	4	1	1	4	4	1	1	4	4
4	4	1	1	1	1	1	1	2	2
4	4	1	1	1	1	1	1	2	2
4	4	1	1	1	1	1	1	4	4
3	3	4	4	1	1	1	1	2	2
3	3	1	1	1	1	1	1	2	2
4	4	1	1	1	1	1	1	2	2
4	4	1	1	1	1	1	1	2	2
3	3	4	4	1	1	1	1	2	2
3	3	1	1	1	1	1	1	4	4
4	4	1	1	1	1	1	1	4	4
4	4	1	1	1	1	1	1	2	2
4	4	1	1	1	1	1	1	2	2
4	4	1	1	1	1	2	2	4	4
4	4	1	1	1	1	1	1	2	2
4	4	1	1	1	1	1	1	2	2
3	3	1	1	1	1	1	1	2	2
4	4	1	1	1	1	1	1	2	2
4	4	1	1	4	4	1	1	4	4
4	4	1	1	1	1	1	1	2	2
3	3	1	1	4	4	1	1	2	2
4	4	1	1	1	1	1	1	2	2
3	3	1	1	1	1	1	1	2	2
3	3	1	1	1	1	1	1	2	2
4	4	1	1	1	1	1	1	2	2
4	4	1	1	1	1	1	1	4	4
4	4	1	1	1	1	1	1	2	2
4	4	1	1	1	1	1	1	4	4
4	4	1	1	1	1	1	1	4	4
4	4	1	1	1	1	1	1	2	2
4	4	1	1	1	1	1	1	4	4
3	3	4	4	1	1	1	1	2	2
4	4	1	1	1	1	1	1	2	2
3	3	1	1	1	1	1	1	2	2
4	4	4	4	1	1	1	1	4	4
4	4	1	1	1	1	1	1	2	2

For Review Only

4	4	1	1	4	4	1	1	4	4
4	4	1	1	1	1	1	1	4	4
4	4	1	1	1	1	1	1	2	2
3	3	1	1	1	1	1	1	2	2
4	4	1	1	1	1	1	1	4	4
4	4	1	1	1	1	1	1	4	4
4	4	1	1	1	1	1	1	2	2
3	3	1	1	1	1	1	1	2	2
4	4	1	1	1	1	2	2	2	2
4	4	1	1	4	4	1	1	4	4
4	4	1	1	1	1	1	1	4	4
4	4	1	1	1	1	1	1	2	2
4	4	1	1	1	1	1	1	4	4
4	4	1	1	1	1	1	1	2	2
4	4	1	1	1	1	1	1	2	2
4	4	1	1	4	4	1	1	2	2
3	3	1	1	1	1	1	1	2	2
4	4	1	1	1	1	1	1	4	4
4	4	1	1	1	1	1	1	4	4
4	4	1	1	1	1	1	1	4	4
4	4	1	1	1	1	1	1	2	2
4	4	1	1	1	1	1	1	2	2
4	4	1	1	1	1	1	1	2	2
4	4	4	4	1	1	1	1	4	4
3	3	1	1	1	1	1	1	2	2
4	4	1	1	1	1	1	1	2	2
3	3	1	1	1	1	1	1	4	4
4	4	1	1	1	1	1	1	2	2
3	3	1	1	1	1	1	1	4	4
3	3	1	1	1	1	1	1	4	4
4	4	1	1	1	1	1	1	2	2
3	3	1	1	4	4	1	1	4	4
4	4	1	1	1	1	1	1	2	2
4	4	1	1	1	1	1	1	2	2
4	4	1	1	1	1	1	1	2	2
4	4	4	4	1	1	1	1	4	4
4	4	1	1	4	4	1	1	2	2
4	4	1	1	4	4	1	1	2	2
4	4	1	1	1	1	1	1	4	4
3	3	1	1	1	1	1	1	2	2
4	4	1	1	4	4	1	1	4	4
4	4	1	1	1	1	1	1	4	4
4	4	1	1	1	1	1	1	4	4
4	4	1	1	1	1	1	1	4	4
3	3	1	1	1	1	1	1	4	4
4	4	1	1	1	1	1	1	2	2
4	4	1	1	4	4	1	1	2	2
4	4	1	1	1	1	1	1	2	2
4	4	1	1	1	1	1	1	2	2
4	4	1	1	1	1	1	1	2	2
3	3	1	1	1	1	1	1	4	4
4	4	1	1	1	1	1	1	2	2
4	4	1	1	1	1	1	1	2	2
4	4	1	1	1	1	1	1	4	4
4	4	1	1	1	1	1	1	2	2

For Review Only

3	3	1	1	1	1	1	1	2	2
4	4	1	1	1	1	1	1	4	4
4	4	1	1	1	1	1	1	4	4
4	4	1	1	1	1	1	1	2	2
3	3	1	1	4	4	1	1	4	4
3	3	1	1	1	1	1	1	4	4
4	4	1	1	1	1	1	1	4	4
3	3	1	1	1	1	1	1	2	2
4	4	1	1	1	1	1	1	2	2
4	4	1	1	1	1	1	1	2	2
4	4	1	1	1	1	1	1	4	4
3	3	1	1	1	1	1	1	2	2
3	3	1	1	4	4	1	1	2	2
4	4	4	4	1	1	1	1	2	2
3	3	1	1	4	4	1	1	4	4
3	3	1	1	4	4	1	1	2	2
4	4	1	1	1	1	1	1	2	2
4	4	1	1	4	4	1	1	2	2
4	4	1	1	4	4	1	1	4	4
4	4	1	1	4	4	1	1	4	4
3	3	1	1	1	1	1	1	2	2
4	4	1	1	1	1	1	1	4	4
4	4	1	1	4	4	1	1	2	2
4	4	1	1	1	1	1	1	4	4
4	4	1	1	1	1	1	1	2	2
4	4	1	1	1	1	1	1	2	2
4	4	1	1	1	1	1	1	2	2
4	4	1	1	1	1	1	1	4	4
4	4	1	1	4	4	1	1	4	4
3	3	1	1	1	1	1	1	4	4
4	4	1	1	1	1	1	1	2	2
4	4	1	1	1	1	1	1	2	2
4	4	1	1	1	1	2	2	2	2
3	3	1	1	1	1	1	1	2	2
4	4	1	1	1	1	1	1	2	2
4	4	1	1	1	1	1	1	2	2
3	3	1	1	1	1	1	1	2	2
4	4	4	4	1	1	1	1	4	4
4	4	4	4	1	1	1	1	2	2
4	4	1	1	1	1	1	1	2	2
4	4	1	1	4	4	1	1	2	2
4	4	1	1	1	1	1	1	4	4
4	4	1	1	1	1	1	1	2	2
4	4	1	1	1	1	2	2	4	4
4	4	1	1	1	1	1	1	2	2
3	3	1	1	4	4	1	1	4	4
3	3	1	1	1	1	1	1	4	4
3	3	1	1	1	1	1	1	4	4
4	4	1	1	1	1	1	1	2	2
4	4	1	1	1	1	1	1	2	2
4	4	1	1	1	1	1	1	4	4
4	4	1	1	1	1	1	1	2	2
4	4	1	1	4	4	1	1	4	4
3	3	1	1	1	1	1	1	4	4
4	4	1	1	1	1	1	1	2	2

For Review Only

4	4	1	1	4	4	1	1	2	2
4	4	1	1	1	1	1	1	2	2

For Review Only

SNP1704	SNP1704	SNP1705	SNP1705	SNP1706	SNP1706	SNP1707	SNP1707	SNP1708	SNP1708
4	4	4	4	4	4	4	4	2	2
1	1	4	4	4	4	4	4	2	2
1	1	3	3	4	4	4	4	2	2
1	1	3	3	4	4	4	4	2	2
4	4	4	4	4	4	4	4	2	2
1	1	4	4	4	4	4	4	2	2
4	4	4	4	4	4	4	4	2	2
1	1	4	4	4	4	4	4	2	2
4	4	4	4	4	4	4	4	2	2
1	1	4	4	4	4	4	4	2	2
1	1	4	4	4	4	4	4	2	2
1	1	3	3	4	4	4	4	2	2
1	1	4	4	4	4	4	4	2	2
4	4	4	4	4	4	4	4	2	2
1	1	4	4	4	4	4	4	2	2
1	1	3	3	4	4	4	4	2	2
4	4	4	4	4	4	4	4	2	2
1	1	4	4	4	4	4	4	2	2
4	4	3	3	4	4	4	4	2	2
1	1	4	4	4	4	4	4	2	2
4	4	4	4	4	4	4	4	2	2
4	4	4	4	4	4	4	4	2	2
1	1	4	4	4	4	1	1	3	3
1	1	4	4	4	4	4	4	2	2
1	1	4	4	4	4	4	4	2	2
4	4	3	3	4	4	4	4	2	2
1	1	3	3	4	4	4	4	2	2
1	1	3	3	4	4	4	4	2	2
1	1	4	4	4	4	4	4	3	3
1	1	4	4	4	4	4	4	2	2
1	1	4	4	4	4	1	1	2	2
1	1	3	3	4	4	4	4	2	2
1	1	4	4	4	4	4	4	2	2
1	1	4	4	4	4	4	4	2	2
1	1	3	3	4	4	4	4	2	2
1	1	3	3	1	1	4	4	2	2
1	1	3	3	1	1	4	4	2	2
1	1	4	4	4	4	4	4	2	2
1	1	4	4	4	4	4	4	2	2
1	1	4	4	4	4	1	1	3	3
1	1	4	4	4	4	4	4	2	2
1	1	4	4	4	4	4	4	3	3
1	1	4	4	4	4	4	4	2	2
4	4	4	4	4	4	4	4	2	2
1	1	4	4	4	4	4	4	2	2
1	1	3	3	4	4	4	4	2	2
1	1	3	3	4	4	4	4	2	2
1	1	3	3	1	1	4	4	3	3
4	4	4	4	4	4	4	4	2	2
1	1	4	4	4	4	4	4	2	2
4	4	3	3	4	4	4	4	2	2
1	1	3	3	4	4	4	4	2	2

For Review Only

4	4	4	4	4	4	4	4	2	2
1	1	4	4	4	4	4	4	2	2
1	1	3	3	4	4	4	4	2	2
4	4	4	4	4	4	4	4	2	2
4	4	3	3	4	4	4	4	2	2
1	1	3	3	4	4	4	4	2	2
1	1	3	3	4	4	4	4	2	2
1	1	4	4	4	4	4	4	2	2
1	1	3	3	1	1	4	4	2	2
1	1	3	3	4	4	4	4	2	2
1	1	3	3	4	4	4	4	2	2
1	1	3	3	4	4	4	4	2	2
4	4	4	4	4	4	4	4	2	2
1	1	4	4	4	4	4	4	3	3
1	1	3	3	4	4	4	4	2	2
1	1	4	4	1	1	4	4	2	2
1	1	3	3	4	4	4	4	2	2
1	1	3	3	4	4	4	4	2	2
4	4	4	4	4	4	1	1	2	2
4	4	4	4	4	4	4	4	2	2
1	1	3	3	4	4	4	4	2	2
1	1	4	4	4	4	1	1	2	2
1	1	4	4	4	4	4	4	2	2
1	1	4	4	4	4	4	4	2	2
1	1	4	4	4	4	4	4	2	2
1	1	3	3	4	4	4	4	2	2
1	1	4	4	4	4	4	4	2	2
1	1	4	4	4	4	4	4	3	3
1	1	3	3	4	4	4	4	2	2
1	1	3	3	4	4	4	4	2	2
1	1	4	4	4	4	4	4	2	2
1	1	3	3	1	1	1	1	2	2
1	1	4	4	4	4	4	4	3	3
4	4	4	4	4	4	4	4	2	2
1	1	4	4	4	4	4	4	2	2
1	1	4	4	4	4	4	4	2	2
1	1	4	4	4	4	4	4	2	2
1	1	4	4	4	4	4	4	2	2
1	1	3	3	4	4	4	4	2	2
1	1	4	4	4	4	4	4	2	2
4	4	4	4	4	4	4	4	2	2
1	1	3	3	4	4	4	4	2	2
4	4	3	3	4	4	4	4	2	2
1	1	3	3	4	4	4	4	2	2
1	1	3	3	4	4	4	4	2	2
4	4	3	3	4	4	4	4	2	2
1	1	3	3	1	1	4	4	2	2
1	1	3	3	4	4	4	4	2	2
1	1	3	3	4	4	4	4	2	2
1	1	3	3	4	4	4	4	2	2
1	1	3	3	4	4	4	4	2	2
1	1	4	4	4	4	4	4	3	3
1	1	4	4	4	4	4	4	2	2
1	1	3	3	4	4	4	4	2	2
4	4	3	3	1	1	4	4	2	2

For Review Only

1	1	3	3	4	4	4	4	2	2
1	1	4	4	4	4	4	4	2	2
1	1	3	3	4	4	4	4	2	2
1	1	4	4	4	4	4	4	2	2
1	1	3	3	4	4	4	4	2	2
4	4	3	3	4	4	1	1	2	2
1	1	3	3	4	4	4	4	2	2
1	1	4	4	4	4	4	4	2	2
1	1	3	3	4	4	4	4	2	2
1	1	3	3	1	1	4	4	2	2
4	4	4	4	4	4	4	4	2	2
1	1	3	3	4	4	4	4	2	2
4	4	3	3	4	4	4	4	3	3
1	1	3	3	4	4	4	4	2	2
1	1	3	3	4	4	4	4	2	2
1	1	3	3	4	4	4	4	2	2
1	1	4	4	4	4	4	4	2	2
1	1	3	3	4	4	4	4	2	2
1	1	4	4	4	4	4	4	2	2
4	4	3	3	4	4	4	4	2	2
1	1	3	3	4	4	4	4	2	2
1	1	4	4	4	4	4	4	2	2
1	1	3	3	4	4	4	4	2	2
1	1	4	4	4	4	4	4	3	3
1	1	3	3	4	4	4	4	2	2
1	1	4	4	4	4	1	1	2	2
1	1	4	4	4	4	4	4	2	2
1	1	3	3	1	1	4	4	2	2
4	4	3	3	4	4	4	4	2	2
1	1	4	4	4	4	4	4	2	2
1	1	4	4	4	4	4	4	3	3
1	1	3	3	4	4	4	4	2	2
4	4	4	4	4	4	4	4	2	2
1	1	3	3	4	4	4	4	2	2
1	1	4	4	4	4	4	4	2	2
1	1	4	4	4	4	4	4	2	2
1	1	4	4	4	4	4	4	2	2
1	1	4	4	4	4	4	4	2	2
1	1	3	3	4	4	4	4	3	3
1	1	4	4	4	4	4	4	2	2
1	1	3	3	4	4	4	4	2	2
1	1	4	4	4	4	4	4	3	3
1	1	3	3	4	4	4	4	2	2
1	1	4	4	4	4	4	4	2	2
1	1	3	3	4	4	4	4	2	2
1	1	3	3	1	1	4	4	2	2
1	1	3	3	4	4	4	4	2	2
1	1	3	3	4	4	4	4	2	2
1	1	3	3	1	1	4	4	2	2
4	4	3	3	4	4	4	4	2	2
1	1	3	3	4	4	4	4	2	2
1	1	4	4	4	4	4	4	3	3
1	1	3	3	4	4	4	4	2	2
1	1	3	3	1	1	1	1	2	2
1	1	3	3	4	4	4	4	2	2

For Review Only

1	1	3	3	4	4	4	4	2	2
4	4	4	4	4	4	1	1	2	2
1	1	3	3	4	4	4	4	2	2
1	1	4	4	4	4	4	4	2	2
4	4	3	3	4	4	4	4	2	2
4	4	4	4	4	4	4	4	2	2
1	1	4	4	4	4	4	4	3	3
1	1	3	3	4	4	4	4	2	2
1	1	3	3	4	4	4	4	3	3
1	1	3	3	4	4	4	4	2	2
1	1	3	3	4	4	4	4	2	2
1	1	4	4	4	4	4	4	2	2
1	1	3	3	4	4	4	4	2	2
1	1	3	3	4	4	4	4	2	2
1	1	3	3	1	1	4	4	3	3
1	1	4	4	4	4	4	4	2	2
1	1	3	3	4	4	4	4	2	2
1	1	4	4	4	4	4	4	2	2
1	1	3	3	4	4	1	1	2	2
1	1	3	3	4	4	4	4	2	2
1	1	3	3	4	4	4	4	2	2
1	1	4	4	4	4	4	4	3	3
1	1	4	4	4	4	1	1	2	2
1	1	3	3	4	4	4	4	2	2
1	1	4	4	4	4	4	4	2	2
1	1	4	4	4	4	4	4	2	2
1	1	3	3	4	4	4	4	2	2
1	1	4	4	4	4	4	4	2	2
4	4	4	4	4	4	4	4	2	2
4	4	3	3	1	1	4	4	2	2
1	1	3	3	1	1	4	4	2	2
1	1	3	3	4	4	4	4	2	2
1	1	4	4	4	4	4	4	2	2
1	1	3	3	1	1	4	4	3	3
1	1	3	3	1	1	4	4	2	2
1	1	3	3	4	4	4	4	2	2
1	1	4	4	4	4	1	1	2	2
1	1	3	3	1	1	4	4	2	2
1	1	3	3	4	4	4	4	2	2
1	1	3	3	4	4	4	4	2	2
1	1	3	3	4	4	4	4	2	2
1	1	3	3	4	4	4	4	2	2
1	1	4	4	4	4	4	4	3	3
1	1	3	3	4	4	1	1	2	2
4	4	3	3	4	4	4	4	2	2
1	1	4	4	4	4	4	4	2	2
1	1	4	4	4	4	4	4	2	2
1	1	4	4	4	4	4	4	3	3
1	1	4	4	4	4	4	4	3	3
1	1	4	4	4	4	4	4	2	2
4	4	3	3	4	4	4	4	2	2
1	1	4	4	4	4	4	4	2	2
1	1	4	4	4	4	1	1	2	2
1	1	3	3	4	4	4	4	2	2
1	1	4	4	4	4	4	4	3	3

For Review Only

1	1	4	4	4	4	4	4	3	3
1	1	4	4	4	4	4	4	2	2
1	1	3	3	4	4	4	4	2	2
1	1	4	4	4	4	4	4	2	2
1	1	4	4	4	4	4	4	2	2
1	1	3	3	4	4	4	4	2	2
4	4	4	4	4	4	4	4	3	3
1	1	4	4	4	4	4	4	2	2
1	1	4	4	4	4	4	4	2	2
1	1	4	4	4	4	4	4	3	3
1	1	3	3	4	4	4	4	2	2
1	1	4	4	4	4	4	4	2	2
1	1	3	3	4	4	4	4	2	2
1	1	4	4	4	4	4	4	2	2
1	1	3	3	4	4	4	4	2	2
1	1	3	3	1	1	1	1	3	3
1	1	3	3	1	1	4	4	2	2
1	1	4	4	4	4	4	4	2	2
1	1	4	4	4	4	4	4	3	3
1	1	4	4	4	4	4	4	2	2
1	1	4	4	4	4	4	4	3	3
1	1	3	3	4	4	4	4	2	2
1	1	3	3	1	1	4	4	2	2
4	4	4	4	4	4	1	1	3	3
1	1	4	4	4	4	4	4	2	2
1	1	4	4	4	4	4	4	2	2
1	1	4	4	4	4	4	4	3	3
1	1	3	3	4	4	4	4	2	2
1	1	4	4	4	4	4	4	2	2
4	4	3	3	4	4	4	4	2	2
1	1	4	4	4	4	4	4	3	3
1	1	3	3	4	4	4	4	2	2
1	1	4	4	4	4	4	4	2	2
1	1	3	3	4	4	4	4	2	2
1	1	4	4	4	4	1	1	2	2
1	1	4	4	4	4	4	4	3	3
1	1	3	3	4	4	4	4	2	2
1	1	3	3	4	4	4	4	2	2
1	1	3	3	1	1	4	4	2	2
1	1	4	4	4	4	4	4	3	3
1	1	3	3	1	1	4	4	2	2
1	1	4	4	4	4	4	4	2	2
1	1	4	4	4	4	1	1	3	3
4	4	4	4	4	4	4	4	2	2
1	1	3	3	4	4	4	4	2	2
1	1	4	4	4	4	4	4	2	2
1	1	3	3	4	4	4	4	2	2
4	4	4	4	4	4	4	4	3	3
1	1	3	3	4	4	4	4	2	2
1	1	4	4	4	4	4	4	3	3
4	4	3	3	1	1	4	4	2	2
1	1	4	4	4	4	4	4	3	3
1	1	3	3	4	4	4	4	2	2
1	1	3	3	4	4	4	4	2	2
1	1	4	4	4	4	1	1	3	3

For Review Only

1	1	3	3	1	1	1	1	2	2
1	1	3	3	4	4	4	4	2	2

For Review Only

SNP1709	SNP1709	SNP171	SNP171	SNP1710	SNP1710	SNP1711	SNP1711	SNP1712	SNP1712
1	1	4	4	3	3	2	2	3	3
4	4	4	4	1	1	1	1	2	2
4	4	4	4	1	1	2	2	3	3
4	4	4	4	1	1	1	1	2	2
1	1	4	4	3	3	2	2	2	2
4	4	4	4	1	1	2	2	3	3
1	1	4	4	3	3	2	2	3	3
1	1	4	4	1	1	1	1	2	2
4	4	4	4	3	3	2	2	2	2
4	4	4	4	1	1	1	1	2	2
4	4	1	1	1	1	2	2	3	3
4	4	4	4	1	1	2	2	2	2
4	4	4	4	1	1	2	2	3	3
1	1	4	4	3	3	1	1	2	2
4	4	4	4	1	1	2	2	2	2
4	4	1	1	1	1	1	1	2	2
1	1	4	4	3	3	2	2	3	3
4	4	4	4	1	1	1	1	2	2
1	1	1	1	3	3	2	2	3	3
4	4	4	4	1	1	2	2	2	2
1	1	4	4	3	3	2	2	3	3
1	1	4	4	3	3	2	2	2	2
4	4	4	4	1	1	1	1	2	2
4	4	4	4	1	1	1	1	2	2
4	4	4	4	1	1	2	2	3	3
1	1	4	4	3	3	1	1	2	2
4	4	4	4	1	1	1	1	2	2
4	4	4	4	1	1	1	1	2	2
4	4	4	4	1	1	1	1	2	2
4	4	4	4	1	1	1	1	2	2
4	4	1	1	1	1	2	2	3	3
4	4	4	4	1	1	2	2	3	3
1	1	4	4	3	3	2	2	3	3
4	4	4	4	1	1	1	1	2	2
4	4	4	4	1	1	2	2	2	2
4	4	4	4	1	1	1	1	2	2
4	4	4	4	1	1	1	1	2	2
4	4	4	4	1	1	1	1	2	2
4	4	4	4	1	1	1	1	2	2
4	4	1	1	1	1	1	1	2	2
4	4	4	4	1	1	1	1	2	2
4	4	4	4	1	1	1	1	2	2
4	4	4	4	1	1	1	1	2	2
1	1	4	4	3	3	2	2	2	2
4	4	4	4	1	1	1	1	2	2
4	4	4	4	1	1	2	2	2	2
1	1	4	4	3	3	1	1	2	2
4	4	4	4	1	1	2	2	2	2
1	1	4	4	3	3	1	1	2	2
4	4	4	4	1	1	2	2	2	2
4	4	4	4	1	1	1	1	2	2
4	4	4	4	1	1	1	1	2	2

For Review Only

1	1	4	4	3	3	2	2	3	3
1	1	4	4	3	3	2	2	3	3
4	4	4	4	1	1	1	1	2	2
1	1	4	4	3	3	1	1	2	2
1	1	1	1	3	3	2	2	3	3
1	1	4	4	3	3	1	1	2	2
4	4	4	4	1	1	1	1	2	2
4	4	4	4	1	1	1	1	2	2
4	4	4	4	1	1	1	1	2	2
4	4	4	4	1	1	1	1	2	2
4	4	4	4	1	1	1	1	2	2
4	4	4	4	1	1	1	1	2	2
1	1	4	4	3	3	2	2	3	3
4	4	4	4	1	1	1	1	2	2
4	4	4	4	1	1	2	2	2	2
1	1	4	4	3	3	1	1	2	2
4	4	4	4	3	3	2	2	2	2
1	1	4	4	3	3	2	2	3	3
1	1	4	4	3	3	2	2	2	2
1	1	4	4	3	3	2	2	3	3
4	4	4	4	1	1	2	2	2	2
4	4	1	1	1	1	1	1	2	2
4	4	4	4	1	1	2	2	2	2
4	4	4	4	1	1	1	1	2	2
4	4	4	4	1	1	1	1	2	2
4	4	4	4	1	1	1	1	2	2
4	4	4	4	1	1	2	2	3	3
4	4	4	4	1	1	1	1	2	2
1	1	4	4	3	3	1	1	2	2
4	4	4	4	1	1	2	2	3	3
4	4	4	4	1	1	1	1	2	2
4	4	4	4	1	1	1	1	2	2
4	4	4	4	1	1	1	1	2	2
1	1	4	4	3	3	2	2	3	3
4	4	4	4	1	1	1	1	2	2
4	4	4	4	1	1	1	1	2	2
4	4	4	4	1	1	1	1	2	2
4	4	4	4	1	1	2	2	3	3
4	4	4	4	1	1	2	2	3	3
4	4	4	4	1	1	1	1	2	2
1	1	4	4	3	3	2	2	2	2
4	4	4	4	1	1	2	2	3	3
4	4	4	4	1	1	1	1	2	2
4	4	4	4	1	1	1	1	2	2
4	4	4	4	1	1	2	2	3	3
4	4	4	4	1	1	1	1	2	2
4	4	4	4	1	1	1	1	2	2
4	4	4	4	1	1	1	1	2	2
1	1	4	4	3	3	1	1	2	2
4	4	4	4	3	3	1	1	2	2
4	4	4	4	1	1	1	1	2	2
1	1	4	4	1	1	1	1	2	2
4	4	4	4	1	1	1	1	2	2
4	4	4	4	1	1	1	1	2	2
1	1	4	4	3	3	2	2	3	3

For Review Only

4	4	4	4	1	1	1	1	2	2
1	1	1	1	1	1	1	1	2	2
4	4	4	4	1	1	1	1	2	2
4	4	4	4	1	1	1	1	2	2
1	1	4	4	3	3	1	1	2	2
1	1	4	4	3	3	1	1	2	2
4	4	1	1	1	1	1	1	2	2
4	4	4	4	1	1	1	1	2	2
4	4	4	4	1	1	1	1	2	2
4	4	4	4	1	1	1	1	2	2
1	1	4	4	3	3	1	1	2	2
4	4	4	4	1	1	2	2	2	2
4	4	4	4	1	1	1	1	2	2
4	4	4	4	1	1	1	1	2	2
4	4	4	4	1	1	2	2	2	2
4	4	4	4	1	1	1	1	2	2
1	1	4	4	3	3	2	2	3	3
4	4	1	1	1	1	2	2	2	2
4	4	4	4	1	1	1	1	2	2
4	4	4	4	1	1	2	2	3	3
4	4	4	4	1	1	2	2	2	2
4	4	4	4	1	1	1	1	2	2
1	1	1	1	3	3	1	1	2	2
4	4	4	4	1	1	1	1	2	2
4	4	4	4	1	1	1	1	2	2
4	4	1	1	1	1	1	1	2	2
4	4	4	4	1	1	1	1	2	2
4	4	4	4	1	1	1	1	2	2
1	1	4	4	3	3	1	1	2	2
4	4	4	4	1	1	1	1	2	2
4	4	4	4	1	1	1	1	2	2
1	1	4	4	1	1	1	1	2	2
1	1	4	4	3	3	2	2	2	2
4	4	4	4	1	1	1	1	2	2
4	4	4	4	1	1	1	1	2	2
4	4	4	4	1	1	2	2	2	2
4	4	4	4	1	1	1	1	2	2
4	4	1	1	1	1	2	2	3	3
4	4	4	4	1	1	2	2	2	2
4	4	4	4	1	1	2	2	2	2
4	4	4	4	1	1	1	1	2	2
4	4	4	4	1	1	1	1	2	2
4	4	4	4	1	1	1	1	2	2
4	4	4	4	1	1	1	1	2	2
1	1	4	4	3	3	1	1	2	2
4	4	4	4	1	1	1	1	2	2
1	1	4	4	3	3	1	1	2	2
1	1	4	4	3	3	1	1	2	2
4	4	4	4	1	1	1	1	2	2
1	1	4	4	3	3	1	1	2	2
4	4	4	4	1	1	1	1	2	2
4	4	4	4	1	1	1	1	2	2
4	4	1	1	1	1	1	1	2	2
4	4	4	4	1	1	1	1	2	2
4	4	4	4	1	1	1	1	2	2

For Review Only

1	1	4	4	3	3	2	2	3	3
4	4	4	4	1	1	1	1	2	2
4	4	4	4	1	1	1	1	2	2
4	4	1	1	1	1	1	1	2	2
1	1	4	4	3	3	2	2	2	2
1	1	4	4	3	3	1	1	2	2
4	4	4	4	1	1	1	1	2	2
4	4	4	4	1	1	1	1	2	2
4	4	4	4	1	1	2	2	2	2
4	4	4	4	1	1	2	2	3	3
4	4	4	4	3	3	1	1	2	2
4	4	4	4	1	1	1	1	2	2
4	4	4	4	1	1	1	1	2	2
4	4	4	4	1	1	2	2	2	2
4	4	4	4	1	1	1	1	2	2
4	4	1	1	1	1	2	2	3	3
4	4	4	4	1	1	1	1	2	2
1	1	4	4	3	3	1	1	2	2
1	1	4	4	3	3	1	1	2	2
4	4	1	1	1	1	1	1	2	2
4	4	4	4	1	1	2	2	2	2
4	4	4	4	1	1	1	1	2	2
4	4	1	1	1	1	1	1	2	2
4	4	4	4	1	1	1	1	2	2
4	4	4	4	1	1	1	1	2	2
4	4	4	4	1	1	1	1	2	2
4	4	4	4	1	1	1	1	2	2
4	4	4	4	1	1	2	2	2	2
1	1	4	4	3	3	1	1	2	2
1	1	4	4	3	3	1	1	2	2
4	4	4	4	1	1	1	1	2	2
1	1	4	4	3	3	2	2	3	3
4	4	4	4	1	1	1	1	2	2
4	4	4	4	1	1	1	1	2	2
4	4	4	4	1	1	1	1	2	2
4	4	4	4	1	1	1	1	2	2
4	4	4	4	1	1	2	2	3	3
4	4	4	4	1	1	2	2	3	3
1	1	4	4	3	3	1	1	2	2
4	4	4	4	1	1	1	1	2	2
4	4	4	4	1	1	2	2	3	3
1	1	4	4	3	3	1	1	2	2
4	4	4	4	1	1	1	1	2	2
1	1	4	4	3	3	2	2	2	2
4	4	4	4	1	1	1	1	2	2
1	1	4	4	1	1	2	2	2	2
4	4	1	1	1	1	2	2	3	3
4	4	4	4	1	1	1	1	2	2
4	4	4	4	1	1	1	1	2	2
4	4	4	4	1	1	1	1	2	2
1	1	4	4	3	3	1	1	2	2
4	4	4	4	1	1	1	1	2	2
4	4	4	4	1	1	1	1	2	2
4	4	4	4	1	1	1	1	2	2
4	4	4	4	1	1	1	1	2	2

For Review Only

4	4	1	1	1	1	1	1	2	2
1	1	4	4	3	3	1	1	2	2
1	1	4	4	3	3	2	2	2	2
4	4	1	1	1	1	1	1	2	2
1	1	4	4	3	3	2	2	3	3
1	1	4	4	3	3	1	1	2	2
1	1	4	4	3	3	1	1	2	2
4	4	4	4	1	1	1	1	2	2
4	4	4	4	1	1	1	1	2	2
4	4	4	4	1	1	1	1	2	2
4	4	4	4	1	1	1	1	2	2
4	4	4	4	1	1	1	1	2	2
4	4	1	1	1	1	2	2	3	3
4	4	4	4	1	1	1	1	2	2
4	4	4	4	1	1	2	2	3	3
4	4	1	1	1	1	2	2	3	3
1	1	4	4	1	1	1	1	2	2
4	4	4	4	1	1	2	2	3	3
1	1	4	4	3	3	2	2	3	3
4	4	4	4	1	1	2	2	3	3
4	4	4	4	1	1	2	2	2	2
1	1	4	4	3	3	1	1	2	2
4	4	4	4	1	1	2	2	3	3
1	1	4	4	3	3	1	1	2	2
4	4	4	4	1	1	1	1	2	2
4	4	4	4	1	1	2	2	3	3
4	4	4	4	1	1	1	1	2	2
4	4	4	4	1	1	1	1	2	2
1	1	4	4	3	3	2	2	3	3
1	1	4	4	3	3	1	1	2	2
4	4	4	4	1	1	1	1	2	2
4	4	4	4	3	3	1	1	2	2
4	4	4	4	1	1	2	2	2	2
4	4	4	4	1	1	1	1	2	2
4	4	4	4	1	1	1	1	2	2
4	4	4	4	1	1	1	1	2	2
4	4	1	1	1	1	1	1	2	2
4	4	4	4	1	1	1	1	2	2
4	4	4	4	1	1	1	1	2	2
4	4	4	4	1	1	1	1	2	2
4	4	4	4	1	1	2	2	3	3
1	1	4	4	1	1	1	1	2	2
4	4	1	1	1	1	1	1	2	2
1	1	4	4	3	3	2	2	2	2
4	4	4	4	1	1	1	1	2	2
4	4	4	4	1	1	2	2	3	3
1	1	4	4	3	3	1	1	2	2
4	4	4	4	1	1	1	1	2	2
4	4	4	4	1	1	1	1	2	2
4	4	4	4	1	1	1	1	2	2
4	4	4	4	1	1	1	1	2	2
4	4	4	4	1	1	1	1	2	2
4	4	4	4	1	1	2	2	3	3
4	4	4	4	1	1	1	1	2	2
4	4	1	1	1	1	1	1	2	2

4	4	4	4	1	1	2	2	3	3
4	4	4	4	1	1	1	1	2	2

For Review Only

SNP1713	SNP1713	SNP1714	SNP1714	SNP1715	SNP1715	SNP1716	SNP1716	SNP1717	SNP1717
4	4	1	1	1	1	2	2	4	4
4	4	1	1	1	1	2	2	4	4
4	4	1	1	4	4	2	2	4	4
4	4	1	1	4	4	1	1	4	4
4	4	1	1	1	1	2	2	4	4
4	4	1	1	1	1	2	2	4	4
1	1	1	1	1	1	2	2	4	4
1	1	1	1	1	1	2	2	4	4
4	4	1	1	1	1	2	2	4	4
1	1	1	1	1	1	2	2	4	4
4	4	1	1	1	1	2	2	4	4
4	4	1	1	4	4	2	2	4	4
1	1	1	1	1	1	2	2	3	3
4	4	1	1	1	1	1	1	4	4
1	1	1	1	1	1	2	2	4	4
1	1	4	4	1	1	1	1	4	4
1	1	4	4	1	1	2	2	4	4
4	4	1	1	1	1	2	2	4	4
4	4	1	1	4	4	1	1	4	4
1	1	1	1	1	1	2	2	4	4
4	4	1	1	1	1	2	2	4	4
4	4	1	1	1	1	2	2	4	4
1	1	4	4	1	1	2	2	4	4
4	4	1	1	1	1	1	1	4	4
1	1	4	4	1	1	1	1	3	3
4	4	1	1	4	4	1	1	4	4
4	4	1	1	4	4	1	1	4	4
1	1	1	1	4	4	2	2	4	4
4	4	1	1	1	1	2	2	4	4
4	4	1	1	1	1	2	2	4	4
1	1	1	1	1	1	1	1	4	4
1	1	4	4	4	4	2	2	4	4
4	4	1	1	1	1	2	2	3	3
4	4	1	1	1	1	1	1	4	4
4	4	1	1	1	1	2	2	4	4
4	4	1	1	1	1	1	1	4	4
4	4	1	1	1	1	2	2	4	4
1	1	1	1	1	1	2	2	4	4
4	4	1	1	1	1	2	2	4	4
1	1	4	4	1	1	2	2	4	4
1	1	1	1	1	1	2	2	4	4
4	4	1	1	1	1	2	2	4	4
4	4	1	1	1	1	2	2	4	4
4	4	1	1	1	1	2	2	4	4
4	4	1	1	1	1	2	2	4	4
4	4	1	1	4	4	1	1	4	4
4	4	1	1	4	4	1	1	4	4
4	4	1	1	1	1	1	1	4	4
1	1	1	1	1	1	2	2	4	4
1	1	1	1	4	4	2	2	4	4
4	4	1	1	4	4	1	1	4	4
4	4	1	1	4	4	2	2	4	4

For Review Only

1	1	4	4	1	1	2	2	3	3
4	4	1	1	1	1	2	2	3	3
4	4	1	1	4	4	1	1	4	4
1	1	4	4	1	1	2	2	4	4
4	4	1	1	4	4	1	1	4	4
4	4	1	1	4	4	1	1	4	4
4	4	1	1	4	4	1	1	4	4
4	4	1	1	1	1	1	1	4	4
4	4	1	1	1	1	2	2	4	4
1	1	1	1	4	4	2	2	4	4
4	4	1	1	4	4	1	1	4	4
4	4	1	1	4	4	1	1	4	4
1	1	4	4	1	1	2	2	3	3
4	4	1	1	1	1	2	2	4	4
4	4	1	1	1	1	2	2	4	4
4	4	1	1	1	1	2	2	4	4
1	1	4	4	4	4	2	2	4	4
1	1	4	4	4	4	2	2	4	4
1	1	1	1	1	1	2	2	4	4
4	4	1	1	1	1	2	2	4	4
4	4	1	1	4	4	1	1	4	4
1	1	4	4	1	1	2	2	4	4
4	4	1	1	1	1	2	2	4	4
4	4	1	1	1	1	2	2	4	4
4	4	1	1	1	1	2	2	4	4
4	4	1	1	4	4	1	1	4	4
4	4	1	1	4	4	2	2	4	4
4	4	1	1	1	1	1	1	4	4
4	4	1	1	4	4	1	1	4	4
1	1	4	4	4	4	2	2	4	4
1	1	1	1	1	1	2	2	4	4
1	1	1	1	1	1	1	1	4	4
4	4	1	1	1	1	1	1	4	4
1	1	4	4	1	1	2	2	3	3
4	4	1	1	1	1	2	2	3	3
4	4	1	1	1	1	2	2	4	4
4	4	1	1	1	1	2	2	4	4
1	1	1	1	1	1	2	2	3	3
4	4	1	1	4	4	2	2	4	4
4	4	1	1	1	1	2	2	4	4
1	1	4	4	1	1	2	2	4	4
1	1	4	4	4	4	1	1	4	4
4	4	1	1	4	4	1	1	4	4
4	4	1	1	4	4	1	1	4	4
4	4	1	1	1	1	2	2	4	4
4	4	1	1	4	4	2	2	4	4
4	4	1	1	1	1	1	1	4	4
4	4	1	1	4	4	1	1	4	4
4	4	1	1	4	4	2	2	4	4
4	4	1	1	1	1	1	1	4	4
4	4	1	1	4	4	1	1	4	4
4	4	1	1	1	1	2	2	4	4
4	4	1	1	1	1	2	2	4	4
1	1	1	1	4	4	2	2	4	4
4	4	1	1	1	1	1	1	4	4

For Review Only

4	4	1	1	4	4	2	2	4	4
4	4	1	1	1	1	2	2	4	4
1	1	4	4	4	4	1	1	4	4
4	4	1	1	1	1	1	1	4	4
4	4	1	1	4	4	2	2	4	4
1	1	1	1	4	4	2	2	4	4
1	1	4	4	4	4	2	2	4	4
4	4	1	1	1	1	1	1	4	4
4	4	1	1	4	4	2	2	4	4
1	1	1	1	1	1	2	2	4	4
4	4	1	1	1	1	2	2	3	3
4	4	1	1	4	4	2	2	4	4
4	4	1	1	4	4	2	2	4	4
4	4	1	1	4	4	2	2	4	4
1	1	1	1	4	4	2	2	4	4
4	4	1	1	4	4	2	2	4	4
1	1	1	1	1	1	1	1	4	4
1	1	1	1	4	4	2	2	4	4
4	4	1	1	1	1	1	1	4	4
1	1	4	4	4	4	1	1	4	4
1	1	1	1	4	4	2	2	4	4
1	1	4	4	1	1	2	2	4	4
4	4	1	1	4	4	2	2	4	4
4	4	1	1	1	1	1	1	3	3
1	1	1	1	4	4	2	2	4	4
1	1	4	4	1	1	2	2	4	4
1	1	1	1	1	1	2	2	4	4
4	4	1	1	1	1	2	2	4	4
4	4	1	1	4	4	2	2	4	4
1	1	4	4	1	1	1	1	4	4
4	4	1	1	1	1	1	1	4	4
4	4	1	1	4	4	2	2	4	4
1	1	4	4	1	1	2	2	4	4
1	1	1	1	4	4	2	2	4	4
4	4	1	1	1	1	1	1	4	4
1	1	1	1	1	1	2	2	4	4
4	4	1	1	1	1	1	1	4	4
1	1	1	1	1	1	2	2	4	4
1	1	4	4	4	4	1	1	4	4
4	4	1	1	1	1	1	1	4	4
1	1	1	1	4	4	2	2	4	4
4	4	1	1	1	1	1	1	4	4
4	4	1	1	4	4	1	1	4	4
4	4	1	1	1	1	2	2	4	4
4	4	4	4	4	4	1	1	4	4
4	4	1	1	1	1	2	2	4	4
4	4	1	1	4	4	1	1	4	4
4	4	1	1	4	4	1	1	4	4
4	4	1	1	1	1	2	2	3	3
1	1	1	1	4	4	2	2	4	4
4	4	1	1	4	4	1	1	4	4
4	4	1	1	1	1	2	2	4	4
1	1	1	1	4	4	1	1	4	4
1	1	1	1	4	4	1	1	4	4
1	1	1	1	1	1	2	2	4	4

For Review Only

1	1	4	4	4	4	2	2	4	4
1	1	4	4	1	1	2	2	4	4
1	1	1	1	1	1	2	2	4	4
1	1	4	4	1	1	2	2	4	4
4	4	1	1	4	4	2	2	4	4
4	4	1	1	1	1	1	1	4	4
1	1	1	1	1	1	2	2	4	4
4	4	1	1	4	4	2	2	4	4
1	1	1	1	4	4	2	2	4	4
4	4	1	1	4	4	2	2	4	4
4	4	1	1	4	4	1	1	4	4
4	4	1	1	1	1	2	2	4	4
4	4	1	1	4	4	1	1	4	4
4	4	1	1	1	1	2	2	4	4
4	4	1	1	1	1	1	1	4	4
4	4	1	1	1	1	2	2	4	4
4	4	1	1	4	4	1	1	4	4
4	4	1	1	1	1	2	2	4	4
1	1	1	1	4	4	1	1	4	4
1	1	4	4	4	4	2	2	4	4
1	1	1	1	4	4	2	2	4	4
4	4	1	1	1	1	1	1	4	4
1	1	4	4	1	1	1	1	4	4
4	4	1	1	4	4	1	1	4	4
4	4	1	1	1	1	2	2	4	4
4	4	1	1	1	1	1	1	4	4
4	4	1	1	4	4	1	1	4	4
4	4	1	1	1	1	2	2	4	4
1	1	4	4	1	1	1	1	4	4
1	1	4	4	1	1	2	2	4	4
4	4	1	1	1	1	2	2	4	4
1	1	4	4	4	4	2	2	4	4
4	4	1	1	1	1	2	2	4	4
1	1	1	1	1	1	1	1	4	4
1	1	1	1	1	1	2	2	3	3
4	4	1	1	4	4	1	1	4	4
1	1	4	4	1	1	1	1	4	4
4	4	1	1	1	1	2	2	4	4
4	4	1	1	4	4	1	1	4	4
4	4	1	1	4	4	1	1	4	4
4	4	1	1	4	4	2	2	4	4
4	4	1	1	4	4	1	1	4	4
4	4	1	1	1	1	1	1	4	4
1	1	4	4	4	4	2	2	4	4
4	4	1	1	4	4	1	1	4	4
4	4	1	1	1	1	1	1	4	4
4	4	1	1	1	1	2	2	4	4
4	4	1	1	1	1	2	2	4	4
4	4	1	1	1	1	1	1	4	4
1	1	1	1	1	1	2	2	4	4
4	4	1	1	4	4	2	2	4	4
4	4	1	1	1	1	1	1	4	4
1	1	4	4	1	1	2	2	4	4
4	4	1	1	4	4	1	1	4	4
4	4	1	1	1	1	2	2	4	4

For Review Only

4	4	1	1	1	1	1	1	4	4
1	1	4	4	1	1	2	2	4	4
4	4	1	1	4	4	2	2	4	4
1	1	1	1	1	1	1	1	4	4
4	4	1	1	1	1	2	2	4	4
4	4	1	1	4	4	2	2	4	4
4	4	1	1	1	1	1	1	3	3
4	4	1	1	1	1	2	2	4	4
4	4	1	1	1	1	2	2	4	4
4	4	1	1	1	1	1	1	4	4
1	1	1	1	4	4	2	2	4	4
4	4	1	1	1	1	1	1	4	4
4	4	1	1	4	4	2	2	4	4
1	1	4	4	1	1	1	1	4	4
4	4	4	4	4	4	2	2	4	4
1	1	4	4	1	1	2	2	4	4
4	4	1	1	1	1	2	2	4	4
4	4	1	1	1	1	2	2	4	4
1	1	1	1	1	1	1	1	4	4
1	1	1	1	1	1	2	2	4	4
4	4	1	1	1	1	2	2	4	4
4	4	1	1	4	4	1	1	4	4
1	1	4	4	1	1	2	2	4	4
1	1	4	4	1	1	2	2	4	4
4	4	1	1	1	1	1	1	4	4
1	1	1	1	1	1	1	1	4	4
4	4	1	1	1	1	1	1	4	4
4	4	1	1	4	4	2	2	4	4
1	1	1	1	1	1	2	2	3	3
4	4	1	1	4	4	2	2	4	4
4	4	1	1	1	1	1	1	4	4
4	4	1	1	4	4	2	2	4	4
4	4	1	1	1	1	2	2	4	4
4	4	1	1	1	1	2	2	4	4
1	1	4	4	1	1	2	2	4	4
4	4	1	1	1	1	1	1	4	4
1	1	4	4	4	4	2	2	4	4
4	4	1	1	4	4	1	1	4	4
4	4	1	1	1	1	1	1	4	4
4	4	1	1	1	1	2	2	4	4
4	4	1	1	1	1	1	1	4	4
4	4	1	1	1	1	2	2	4	4
1	1	4	4	1	1	2	2	4	4
1	1	4	4	1	1	2	2	4	4
1	1	1	1	4	4	2	2	4	4
4	4	1	1	1	1	1	1	4	4
4	4	1	1	4	4	1	1	4	4
1	1	4	4	1	1	2	2	4	4
4	4	1	1	4	4	2	2	4	4
4	4	1	1	1	1	1	1	4	4
1	1	4	4	1	1	2	2	4	4
4	4	1	1	1	1	2	2	4	4
4	4	1	1	4	4	2	2	4	4
1	1	4	4	4	4	1	1	4	4
1	1	4	4	1	1	2	2	4	4

For Review Only

1	1	4	4	1	1	2	2	4	4
4	4	1	1	4	4	1	1	4	4

For Review Only

SNP1718	SNP1718	SNP1719	SNP1719	SNP172	SNP172	SNP1720	SNP1720	SNP1721	SNP1721
3	3	4	4	2	2	2	2	4	4
3	3	4	4	3	3	4	4	4	4
3	3	4	4	3	3	2	2	4	4
3	3	4	4	2	2	2	2	4	4
3	3	4	4	3	3	2	2	4	4
3	3	4	4	2	2	2	2	4	4
3	3	4	4	2	2	2	2	4	4
3	3	2	2	3	3	2	2	4	4
3	3	4	4	2	2	2	2	4	4
3	3	2	2	3	3	2	2	4	4
3	3	4	4	3	3	2	2	4	4
3	3	4	4	3	3	2	2	4	4
3	3	4	4	2	2	2	2	4	4
3	3	4	4	3	3	2	2	4	4
3	3	4	4	2	2	2	2	4	4
3	3	4	4	3	3	4	4	4	4
3	3	2	2	2	2	2	2	4	4
3	3	4	4	3	3	2	2	4	4
3	3	4	4	3	3	4	4	4	4
3	3	2	2	3	3	2	2	4	4
3	3	2	2	3	3	2	2	4	4
3	3	2	2	3	3	2	2	4	4
3	3	4	4	3	3	2	2	1	1
3	3	4	4	3	3	2	2	4	4
3	3	4	4	2	2	2	2	4	4
3	3	4	4	3	3	4	4	4	4
3	3	4	4	3	3	2	2	4	4
3	3	4	4	3	3	2	2	4	4
3	3	4	4	3	3	2	2	4	4
3	3	2	2	3	3	2	2	4	4
3	3	4	4	2	2	4	4	4	4
3	3	2	2	3	3	4	4	4	4
3	3	2	2	2	2	2	2	4	4
3	3	4	4	3	3	2	2	4	4
3	3	2	2	3	3	2	2	4	4
2	2	4	4	3	3	2	2	4	4
3	3	4	4	2	2	2	2	4	4
3	3	4	4	3	3	2	2	1	1
3	3	4	4	3	3	2	2	4	4
3	3	2	2	3	3	2	2	1	1
3	3	2	2	3	3	2	2	4	4
3	3	2	2	3	3	2	2	4	4
3	3	4	4	3	3	2	2	1	1
3	3	4	4	2	2	2	2	4	4
3	3	2	2	3	3	2	2	1	1
3	3	4	4	2	2	2	2	4	4
2	2	4	4	3	3	2	2	4	4
3	3	4	4	3	3	2	2	4	4
3	3	4	4	2	2	2	2	4	4
3	3	4	4	3	3	2	2	4	4
3	3	4	4	3	3	4	4	4	4
3	3	4	4	3	3	4	4	4	4

For Review Only

3	3	4	4	2	2	2	2	4	4
3	3	2	2	2	2	2	2	4	4
3	3	4	4	2	2	4	4	4	4
3	3	4	4	2	2	2	2	4	4
3	3	4	4	3	3	4	4	4	4
2	2	4	4	3	3	4	4	4	4
3	3	4	4	2	2	2	2	4	4
3	3	4	4	3	3	2	2	4	4
3	3	4	4	2	2	2	2	4	4
3	3	4	4	2	2	2	2	4	4
3	3	4	4	3	3	2	2	4	4
2	2	4	4	3	3	2	2	4	4
3	3	4	4	2	2	2	2	4	4
3	3	4	4	3	3	2	2	4	4
3	3	4	4	2	2	2	2	4	4
3	3	4	4	3	3	2	2	4	4
3	3	4	4	3	3	2	2	4	4
3	3	4	4	3	3	2	2	4	4
3	3	4	4	3	3	4	4	4	4
3	3	4	4	2	2	2	2	4	4
3	3	4	4	3	3	2	2	4	4
3	3	2	2	2	2	2	2	4	4
3	3	4	4	3	3	2	2	4	4
3	3	2	2	3	3	2	2	4	4
3	3	2	2	3	3	2	2	1	1
3	3	4	4	2	2	2	2	4	4
3	3	4	4	3	3	2	2	4	4
3	3	2	2	3	3	2	2	1	1
3	3	4	4	3	3	4	4	4	4
3	3	4	4	3	3	2	2	4	4
3	3	4	4	3	3	2	2	4	4
3	3	4	4	3	3	4	4	4	4
3	3	4	4	3	3	2	2	1	1
3	3	4	4	2	2	2	2	4	4
3	3	4	4	3	3	2	2	4	4
3	3	2	2	3	3	2	2	1	1
3	3	2	2	3	3	2	2	4	4
3	3	4	4	2	2	2	2	4	4
3	3	2	2	3	3	2	2	4	4
3	3	2	2	2	2	2	2	4	4
3	3	4	4	2	2	2	2	4	4
3	3	4	4	3	3	2	2	4	4
3	3	4	4	3	3	2	2	4	4
3	3	4	4	3	3	2	2	4	4
3	3	4	4	2	2	2	2	4	4
3	3	4	4	2	2	4	4	4	4
3	3	4	4	3	3	2	2	4	4
3	3	4	4	2	2	4	4	4	4
2	2	4	4	3	3	4	4	4	4
3	3	2	2	3	3	2	2	4	4
3	3	4	4	3	3	2	2	4	4
3	3	4	4	3	3	2	2	1	1
3	3	4	4	3	3	2	2	1	1
3	3	4	4	3	3	2	2	4	4
3	3	4	4	3	3	2	2	1	1

For Review Only

3	3	4	4	2	2	4	4	4	4
3	3	2	2	3	3	2	2	4	4
3	3	4	4	3	3	2	2	4	4
3	3	4	4	3	3	2	2	4	4
3	3	4	4	3	3	4	4	4	4
3	3	4	4	2	2	4	4	4	4
3	3	4	4	2	2	4	4	4	4
3	3	4	4	2	2	2	2	4	4
3	3	4	4	3	3	2	2	4	4
2	2	2	2	3	3	2	2	4	4
3	3	4	4	2	2	2	2	4	4
3	3	4	4	3	3	2	2	4	4
3	3	4	4	3	3	2	2	4	4
3	3	4	4	2	2	2	2	4	4
3	3	4	4	3	3	2	2	4	4
3	3	4	4	2	2	2	2	4	4
3	3	4	4	3	3	2	2	4	4
3	3	4	4	2	2	2	2	4	4
3	3	4	4	3	3	2	2	4	4
3	3	4	4	3	3	2	2	4	4
3	3	4	4	3	3	2	2	4	4
3	3	4	4	3	3	4	4	4	4
3	3	4	4	2	2	2	2	4	4
3	3	2	2	3	3	2	2	1	1
3	3	2	2	2	2	2	2	4	4
3	3	2	2	3	3	2	2	4	4
3	3	4	4	3	3	2	2	4	4
2	2	4	4	3	3	2	2	4	4
3	3	4	4	2	2	4	4	4	4
3	3	4	4	3	3	2	2	4	4
3	3	4	4	3	3	2	2	4	4
3	3	4	4	3	3	2	2	4	4
3	3	4	4	2	2	2	2	4	4
3	3	4	4	2	2	2	2	4	4
3	3	4	4	3	3	2	2	4	4
3	3	4	4	3	3	2	2	4	4
3	3	4	4	3	3	2	2	4	4
3	3	4	4	3	3	2	2	4	4
3	3	4	4	3	3	2	2	4	4
3	3	4	4	3	3	2	2	4	4
3	3	2	2	3	3	2	2	4	4
3	3	4	4	3	3	2	2	4	4
3	3	4	4	3	3	2	2	4	4
3	3	2	2	3	3	2	2	4	4
3	3	4	4	3	3	2	2	4	4
3	3	4	4	2	2	2	2	4	4
3	3	4	4	2	2	4	4	4	4
3	3	4	4	3	3	4	4	4	4
3	3	4	4	3	3	2	2	4	4
3	3	4	4	2	2	2	2	4	4
2	2	4	4	3	3	4	4	4	4
3	3	4	4	3	3	2	2	1	1
3	3	2	2	3	3	2	2	4	4
2	2	4	4	3	3	2	2	4	4
3	3	4	4	3	3	2	2	4	4

For Review Only

3	3	4	4	2	2	4	4	4	4
3	3	2	2	3	3	2	2	4	4
3	3	2	2	3	3	2	2	4	4
3	3	2	2	3	3	2	2	4	4
3	3	4	4	3	3	4	4	4	4
3	3	4	4	3	3	2	2	4	4
3	3	2	2	3	3	2	2	1	1
3	3	4	4	3	3	2	2	4	4
3	3	2	2	3	3	2	2	1	1
3	3	4	4	3	3	2	2	4	4
3	3	4	4	2	2	2	2	4	4
3	3	2	2	3	3	2	2	4	4
3	3	4	4	2	2	2	2	4	4
3	3	4	4	2	2	2	2	1	1
3	3	4	4	3	3	2	2	4	4
3	3	4	4	3	3	2	2	4	4
3	3	4	4	3	3	2	2	4	4
3	3	4	4	3	3	4	4	4	4
3	3	4	4	3	3	4	4	4	4
3	3	4	4	2	2	4	4	4	4
3	3	4	4	2	2	2	2	4	4
3	3	4	4	3	3	2	2	4	4
3	3	2	2	2	2	2	2	4	4
2	2	4	4	3	3	2	2	4	4
3	3	2	2	2	2	2	2	4	4
3	3	4	4	3	3	2	2	4	4
3	3	4	4	2	2	4	4	4	4
3	3	4	4	2	2	2	2	4	4
3	3	4	4	3	3	2	2	4	4
3	3	4	4	2	2	2	2	4	4
3	3	4	4	3	3	2	2	4	4
3	3	4	4	3	3	2	2	4	4
3	3	4	4	3	3	4	4	4	4
3	3	4	4	3	3	2	2	4	4
3	3	4	4	3	3	2	2	4	4
2	2	4	4	3	3	2	2	4	4
3	3	4	4	2	2	2	2	4	4
3	3	4	4	2	2	2	2	4	4
3	3	4	4	3	3	4	4	4	4
3	3	4	4	3	3	2	2	4	4
3	3	4	4	3	3	2	2	4	4
3	3	4	4	2	2	2	2	4	4
3	3	4	4	3	3	2	2	1	1
3	3	4	4	2	2	2	2	4	4
3	3	4	4	3	3	2	2	4	4
3	3	4	4	3	3	2	2	4	4
3	3	4	4	3	3	2	2	4	4
3	3	4	4	3	3	2	2	1	1
3	3	4	4	3	3	2	2	1	1
3	3	4	4	2	2	2	2	4	4
3	3	4	4	3	3	4	4	4	4
3	3	4	4	3	3	2	2	4	4
3	3	2	2	3	3	2	2	4	4
3	3	4	4	3	3	2	2	4	4
3	3	4	4	3	3	2	2	1	1

For Review Only

3	3	4	4	3	3	2	2	4	4
3	3	4	4	3	3	2	2	4	4
3	3	4	4	3	3	2	2	4	4
3	3	2	2	2	2	2	2	4	4
3	3	4	4	3	3	2	2	4	4
3	3	4	4	3	3	4	4	4	4
3	3	2	2	3	3	2	2	1	1
3	3	2	2	3	3	2	2	4	4
3	3	4	4	3	3	2	2	4	4
3	3	4	4	3	3	2	2	1	1
3	3	4	4	3	3	2	2	4	4
3	3	4	4	3	3	2	2	4	4
3	3	2	2	2	2	2	2	4	4
2	2	4	4	3	3	2	2	4	4
3	3	4	4	3	3	2	2	4	4
3	3	4	4	3	3	2	2	1	1
3	3	4	4	3	3	2	2	4	4
3	3	2	2	3	3	2	2	4	4
3	3	4	4	3	3	2	2	4	4
3	3	4	4	3	3	2	2	1	1
3	3	4	4	3	3	4	4	1	1
3	3	4	4	3	3	4	4	4	4
3	3	4	4	3	3	2	2	4	4
3	3	2	2	3	3	2	2	1	1
3	3	4	4	3	3	2	2	4	4
3	3	2	2	3	3	2	2	4	4
3	3	4	4	3	3	2	2	1	1
3	3	4	4	2	2	4	4	4	4
3	3	4	4	2	2	2	2	4	4
3	3	4	4	3	3	4	4	4	4
3	3	4	4	3	3	2	2	1	1
3	3	4	4	3	3	4	4	4	4
3	3	4	4	2	2	2	2	1	1
3	3	4	4	3	3	2	2	4	4
3	3	2	2	2	2	2	2	4	4
3	3	4	4	3	3	2	2	1	1
3	3	4	4	2	2	2	2	4	4
2	2	4	4	3	3	2	2	4	4
2	2	4	4	3	3	2	2	4	4
3	3	4	4	3	3	2	2	1	1
3	3	4	4	3	3	2	2	4	4
3	3	4	4	3	3	4	4	4	4
3	3	2	2	3	3	2	2	1	1
3	3	4	4	2	2	2	2	4	4
3	3	4	4	3	3	2	2	4	4
3	3	2	2	3	3	2	2	4	4
3	3	4	4	3	3	2	2	4	4
3	3	4	4	3	3	2	2	1	1
3	3	2	2	3	3	2	2	4	4
3	3	2	2	3	3	2	2	1	1
3	3	4	4	3	3	2	2	4	4
3	3	4	4	3	3	2	2	1	1
3	3	4	4	3	3	2	2	4	4
3	3	4	4	3	3	2	2	4	4
3	3	4	4	3	3	2	2	1	1

For Review Only

3	3	4	4	3	3	2	2	4	4
3	3	4	4	2	2	4	4	4	4

For Review Only

SNP1722	SNP1722	SNP1723	SNP1723	SNP1724	SNP1724	SNP1725	SNP1725	SNP1726	SNP1726
2	2	4	4	1	1	3	3	1	1
2	2	3	3	4	4	2	2	1	1
2	2	4	4	1	1	3	3	1	1
2	2	3	3	1	1	3	3	1	1
2	2	4	4	1	1	3	3	1	1
2	2	3	3	1	1	3	3	1	1
2	2	3	3	1	1	3	3	1	1
2	2	4	4	1	1	3	3	1	1
2	2	3	3	1	1	3	3	1	1
2	2	4	4	1	1	3	3	1	1
1	1	4	4	1	1	3	3	1	1
2	2	3	3	1	1	2	2	1	1
2	2	3	3	1	1	3	3	1	1
2	2	3	3	1	1	3	3	1	1
2	2	3	3	4	4	3	3	1	1
2	2	3	3	1	1	3	3	1	1
2	2	3	3	1	1	3	3	1	1
2	2	4	4	1	1	3	3	1	1
2	2	3	3	4	4	2	2	1	1
1	1	4	4	1	1	3	3	1	1
2	2	3	3	1	1	3	3	1	1
1	1	3	3	1	1	3	3	1	1
2	2	4	4	1	1	3	3	1	1
1	1	3	3	1	1	3	3	1	1
2	2	4	4	1	1	3	3	1	1
2	2	3	3	4	4	2	2	1	1
2	2	4	4	4	4	2	2	4	4
2	2	3	3	4	4	3	3	1	1
1	1	3	3	1	1	3	3	1	1
1	1	4	4	1	1	3	3	1	1
2	2	4	4	4	4	3	3	1	1
2	2	4	4	4	4	3	3	1	1
2	2	4	4	1	1	3	3	1	1
2	2	3	3	1	1	3	3	1	1
2	2	3	3	1	1	3	3	1	1
2	2	3	3	1	1	3	3	1	1
2	2	4	4	1	1	3	3	1	1
2	2	4	4	1	1	3	3	1	1
2	2	4	4	1	1	3	3	1	1
2	2	3	3	1	1	3	3	1	1
2	2	4	4	1	1	3	3	1	1
1	1	4	4	1	1	3	3	1	1
2	2	4	4	1	1	3	3	1	1
2	2	3	3	1	1	3	3	1	1
2	2	4	4	1	1	3	3	1	1
2	2	3	3	4	4	3	3	1	1
2	2	4	4	4	4	2	2	1	1
2	2	3	3	1	1	3	3	1	1
1	1	3	3	1	1	3	3	1	1
2	2	3	3	4	4	2	2	1	1
2	2	3	3	4	4	2	2	4	4
2	2	3	3	4	4	2	2	1	1

For Review Only

2	2	3	3	1	1	3	3	1	1
2	2	4	4	1	1	3	3	1	1
2	2	3	3	4	4	2	2	1	1
1	1	3	3	1	1	3	3	1	1
2	2	3	3	4	4	2	2	1	1
2	2	4	4	4	4	2	2	4	4
2	2	3	3	4	4	2	2	1	1
2	2	3	3	1	1	3	3	1	1
2	2	4	4	1	1	3	3	1	1
2	2	3	3	1	1	3	3	1	1
2	2	3	3	4	4	2	2	1	1
2	2	4	4	4	4	2	2	1	1
2	2	3	3	1	1	3	3	1	1
1	1	3	3	1	1	3	3	1	1
2	2	3	3	4	4	3	3	1	1
2	2	4	4	1	1	3	3	1	1
2	2	4	4	1	1	3	3	1	1
2	2	4	4	4	4	2	2	1	1
2	2	3	3	4	4	2	2	1	1
2	2	3	3	1	1	3	3	1	1
2	2	3	3	4	4	3	3	1	1
2	2	3	3	1	1	3	3	1	1
1	1	3	3	1	1	3	3	1	1
1	1	4	4	1	1	3	3	1	1
2	2	4	4	1	1	3	3	1	1
2	2	3	3	1	1	3	3	1	1
2	2	3	3	1	1	3	3	1	1
1	1	3	3	1	1	3	3	1	1
2	2	3	3	4	4	2	2	1	1
2	2	3	3	1	1	3	3	1	1
2	2	3	3	1	1	3	3	1	1
1	1	3	3	4	4	2	2	4	4
1	1	3	3	1	1	3	3	1	1
2	2	3	3	1	1	3	3	1	1
2	2	4	4	1	1	3	3	1	1
2	2	4	4	1	1	3	3	1	1
2	2	3	3	1	1	3	3	1	1
1	1	3	3	1	1	3	3	1	1
1	1	4	4	1	1	3	3	1	1
2	2	4	4	1	1	3	3	1	1
2	2	3	3	1	1	3	3	1	1
2	2	4	4	1	1	3	3	1	1
2	2	3	3	4	4	2	2	1	1
2	2	3	3	4	4	2	2	1	1
2	2	3	3	1	1	3	3	1	1
2	2	3	3	4	4	2	2	1	1
1	1	3	3	1	1	3	3	1	1
2	2	3	3	4	4	2	2	1	1
2	2	4	4	4	4	2	2	1	1
2	2	4	4	4	4	2	2	1	1
2	2	4	4	4	4	2	2	1	1
1	1	4	4	1	1	3	3	1	1
1	1	4	4	1	1	3	3	1	1
1	1	3	3	4	4	3	3	1	1
2	2	4	4	4	4	2	2	1	1

For Review Only

2	2	3	3	4	4	2	2	1	1
2	2	3	3	4	4	2	2	1	1
2	2	3	3	1	1	3	3	1	1
2	2	3	3	1	1	3	3	1	1
2	2	3	3	4	4	2	2	1	1
2	2	3	3	4	4	2	2	1	1
2	2	3	3	1	1	3	3	1	1
2	2	3	3	4	4	3	3	1	1
2	2	4	4	1	1	3	3	1	1
1	1	3	3	1	1	3	3	1	1
2	2	4	4	1	1	3	3	1	1
2	2	3	3	4	4	3	3	1	1
2	2	3	3	4	4	3	3	1	1
2	2	3	3	4	4	2	2	4	4
1	1	3	3	4	4	2	2	1	1
2	2	3	3	4	4	3	3	1	1
2	2	3	3	1	1	3	3	1	1
2	2	3	3	4	4	3	3	1	1
2	2	3	3	1	1	3	3	1	1
2	2	3	3	1	1	3	3	1	1
2	2	3	3	4	4	3	3	1	1
2	2	3	3	1	1	3	3	1	1
2	2	4	4	1	1	3	3	1	1
1	1	3	3	1	1	3	3	1	1
2	2	3	3	4	4	3	3	4	4
2	2	3	3	1	1	3	3	1	1
2	2	3	3	1	1	3	3	1	1
2	2	4	4	1	1	3	3	1	1
2	2	3	3	4	4	2	2	1	1
2	2	3	3	4	4	2	2	1	1
1	1	3	3	1	1	3	3	1	1
2	2	3	3	4	4	3	3	1	1
2	2	3	3	1	1	3	3	1	1
2	2	3	3	4	4	3	3	1	1
2	2	3	3	1	1	3	3	1	1
2	2	3	3	1	1	2	2	1	1
2	2	3	3	1	1	3	3	1	1
2	2	3	3	4	4	2	2	1	1
2	2	3	3	1	1	3	3	1	1
1	1	3	3	1	1	3	3	1	1
2	2	3	3	4	4	3	3	1	1
1	1	3	3	1	1	3	3	1	1
2	2	4	4	4	4	2	2	1	1
2	2	4	4	1	1	3	3	1	1
2	2	3	3	1	1	3	3	1	1
2	2	4	4	1	1	3	3	1	1
2	2	3	3	4	4	2	2	1	1
2	2	3	3	4	4	2	2	1	1
1	1	3	3	1	1	3	3	1	1
2	2	3	3	4	4	3	3	1	1
2	2	3	3	4	4	2	2	1	1
2	2	4	4	1	1	3	3	1	1
2	2	3	3	4	4	2	2	1	1
2	2	3	3	4	4	2	2	1	1
2	2	3	3	1	1	3	3	1	1

For Review Only

2	2	3	3	1	1	3	3	1	1
2	2	3	3	1	1	3	3	1	1
2	2	4	4	4	4	3	3	1	1
2	2	4	4	4	4	3	3	1	1
2	2	3	3	4	4	2	2	1	1
1	1	3	3	1	1	3	3	1	1
1	1	3	3	1	1	3	3	1	1
2	2	3	3	1	1	3	3	1	1
2	2	3	3	1	1	3	3	1	1
2	2	3	3	1	1	3	3	1	1
2	2	3	3	4	4	3	3	1	1
2	2	3	3	1	1	3	3	1	1
2	2	3	3	4	4	3	3	1	1
1	1	4	4	1	1	3	3	1	1
1	1	3	3	1	1	3	3	1	1
1	1	3	3	1	1	3	3	1	1
2	2	3	3	4	4	3	3	1	1
2	2	3	3	1	1	3	3	1	1
2	2	3	3	4	4	2	2	1	1
2	2	3	3	1	1	3	3	1	1
2	2	3	3	4	4	3	3	1	1
1	1	3	3	1	1	3	3	1	1
2	2	3	3	1	1	3	3	1	1
2	2	4	4	4	4	2	2	1	1
1	1	3	3	1	1	3	3	1	1
2	2	3	3	1	1	3	3	1	1
2	2	4	4	4	4	2	2	1	1
2	2	3	3	1	1	3	3	1	1
2	2	3	3	4	4	2	2	1	1
2	2	3	3	1	1	3	3	1	1
2	2	4	4	1	1	3	3	1	1
2	2	3	3	1	1	3	3	1	1
2	2	4	4	4	4	2	2	4	4
1	1	3	3	1	1	3	3	1	1
1	1	3	3	1	1	3	3	1	1
2	2	4	4	4	4	2	2	1	1
2	2	4	4	1	1	3	3	1	1
2	2	4	4	1	1	3	3	1	1
2	2	3	3	4	4	2	2	1	1
2	2	3	3	4	4	2	2	1	1
2	2	4	4	1	1	3	3	1	1
2	2	3	3	4	4	2	2	1	1
2	2	4	4	1	1	3	3	1	1
2	2	3	3	4	4	2	2	1	1
1	1	3	3	1	1	3	3	1	1
1	1	3	3	1	1	3	3	1	1
1	1	3	3	1	1	3	3	1	1
1	1	4	4	1	1	3	3	1	1
1	1	3	3	1	1	3	3	1	1
2	2	3	3	1	1	3	3	1	1
2	2	3	3	4	4	2	2	1	1
1	1	3	3	1	1	3	3	1	1
2	2	3	3	1	1	3	3	1	1
2	2	3	3	4	4	2	2	1	1
2	2	4	4	1	1	3	3	1	1

For Review Only

2	2	3	3	1	1	3	3	1	1
2	2	4	4	1	1	3	3	1	1
2	2	3	3	1	1	2	2	1	1
2	2	3	3	4	4	2	2	1	1
2	2	4	4	1	1	3	3	1	1
2	2	4	4	4	4	2	2	1	1
2	2	3	3	1	1	3	3	1	1
2	2	4	4	1	1	3	3	1	1
2	2	3	3	4	4	2	2	4	4
1	1	3	3	1	1	3	3	1	1
2	2	3	3	4	4	3	3	1	1
2	2	3	3	1	1	3	3	1	1
1	1	4	4	1	1	3	3	1	1
2	2	3	3	1	1	3	3	1	1
2	2	4	4	1	1	3	3	1	1
2	2	4	4	1	1	3	3	1	1
2	2	4	4	1	1	3	3	1	1
1	1	3	3	1	1	3	3	1	1
2	2	3	3	4	4	3	3	1	1
2	2	4	4	1	1	3	3	1	1
2	2	4	4	4	4	2	2	1	1
2	2	3	3	4	4	2	2	1	1
2	2	3	3	1	1	3	3	1	1
2	2	3	3	1	1	3	3	1	1
2	2	3	3	1	1	3	3	1	1
2	2	3	3	1	1	3	3	1	1
1	1	4	4	1	1	3	3	1	1
2	2	3	3	4	4	2	2	1	1
2	2	4	4	1	1	3	3	1	1
2	2	3	3	4	4	2	2	1	1
1	1	3	3	1	1	3	3	1	1
2	2	3	3	4	4	3	3	1	1
1	1	4	4	1	1	3	3	1	1
2	2	3	3	4	4	2	2	1	1
2	2	3	3	1	1	3	3	1	1
1	1	3	3	1	1	3	3	1	1
2	2	3	3	4	4	2	2	1	1
2	2	4	4	4	4	2	2	1	1
2	2	3	3	1	1	3	3	1	1
2	2	4	4	1	1	3	3	1	1
2	2	3	3	4	4	2	2	1	1
2	2	3	3	4	4	2	2	1	1
2	2	3	3	1	1	3	3	1	1
2	2	3	3	1	1	3	3	1	1
2	2	3	3	1	1	3	3	1	1
1	1	4	4	1	1	3	3	1	1
1	1	3	3	4	4	3	3	4	4
2	2	4	4	4	4	2	2	4	4
2	2	4	4	1	1	3	3	1	1
1	1	3	3	1	1	3	3	1	1
2	2	3	3	4	4	2	2	1	1
1	1	3	3	1	1	3	3	1	1
2	2	4	4	1	1	3	3	1	1
2	2	3	3	1	1	3	3	1	1
2	2	3	3	1	1	3	3	1	1

For Review Only

2	2	4	4	4	4	3	3	1	1
2	2	4	4	4	4	2	2	1	1

For Review Only

SNP1727	SNP1727	SNP1728	SNP1728	SNP1729	SNP1729	SNP173	SNP173	SNP1730	SNP1730
3	3	4	4	1	1	3	3	4	4
2	2	4	4	1	1	1	1	3	3
3	3	4	4	1	1	3	3	4	4
3	3	4	4	1	1	1	1	4	4
2	2	4	4	1	1	3	3	4	4
3	3	4	4	1	1	1	1	4	4
3	3	4	4	3	3	3	3	4	4
3	3	4	4	1	1	3	3	4	4
3	3	4	4	3	3	1	1	4	4
2	2	4	4	1	1	1	1	4	4
3	3	4	4	1	1	3	3	4	4
3	3	4	4	1	1	3	3	4	4
3	3	4	4	1	1	3	3	4	4
3	3	4	4	1	1	3	3	4	4
3	3	4	4	1	1	3	3	4	4
3	3	4	4	1	1	1	1	4	4
3	3	4	4	3	3	1	1	4	4
3	3	4	4	1	1	3	3	4	4
3	3	4	4	1	1	3	3	3	3
3	3	4	4	1	1	3	3	4	4
2	2	4	4	3	3	3	3	4	4
3	3	4	4	3	3	1	1	4	4
3	3	4	4	1	1	3	3	4	4
3	3	4	4	1	1	1	1	4	4
3	3	4	4	1	1	1	1	4	4
3	3	4	4	1	1	1	1	3	3
3	3	4	4	1	1	1	1	3	3
3	3	4	4	1	1	3	3	4	4
3	3	4	4	3	3	1	1	4	4
2	2	2	2	1	1	3	3	4	4
3	3	4	4	1	1	3	3	4	4
3	3	4	4	1	1	1	1	3	3
3	3	4	4	1	1	1	1	4	4
3	3	4	4	1	1	1	1	4	4
3	3	4	4	1	1	3	3	4	4
3	3	4	4	1	1	1	1	4	4
3	3	4	4	1	1	3	3	4	4
3	3	4	4	1	1	3	3	4	4
3	3	4	4	1	1	3	3	4	4
3	3	4	4	1	1	3	3	4	4
2	2	4	4	1	1	3	3	4	4
2	2	4	4	3	3	3	3	4	4
3	3	4	4	1	1	1	1	4	4
3	3	4	4	3	3	3	3	4	4
3	3	4	4	1	1	1	1	4	4
3	3	4	4	1	1	3	3	4	4
3	3	4	4	1	1	1	1	3	3
3	3	4	4	1	1	3	3	4	4
3	3	2	2	3	3	3	3	4	4
3	3	4	4	1	1	1	1	4	4
3	3	4	4	1	1	1	1	3	3
3	3	4	4	1	1	3	3	3	3

For Review Only

3	3	4	4	3	3	1	1	4	4
3	3	4	4	1	1	1	1	4	4
3	3	4	4	1	1	1	1	3	3
3	3	2	2	3	3	1	1	4	4
3	3	4	4	1	1	3	3	3	3
3	3	4	4	1	1	1	1	3	3
3	3	4	4	1	1	1	1	3	3
3	3	4	4	1	1	3	3	4	4
3	3	4	4	1	1	3	3	4	4
3	3	4	4	1	1	3	3	4	4
3	3	4	4	1	1	3	3	3	3
3	3	4	4	1	1	1	1	3	3
3	3	4	4	3	3	1	1	4	4
3	3	4	4	3	3	1	1	4	4
3	3	4	4	1	1	3	3	4	4
3	3	4	4	1	1	3	3	4	4
3	3	4	4	1	1	3	3	4	4
3	3	4	4	1	1	1	1	3	3
3	3	4	4	3	3	1	1	3	3
3	3	4	4	3	3	1	1	4	4
3	3	4	4	1	1	3	3	4	4
3	3	4	4	1	1	3	3	4	4
3	3	4	4	3	3	1	1	4	4
3	3	4	4	1	1	3	3	4	4
3	3	4	4	1	1	1	1	4	4
3	3	4	4	1	1	3	3	4	4
3	3	4	4	3	3	3	3	4	4
3	3	2	2	1	1	3	3	4	4
3	3	4	4	1	1	1	1	3	3
3	3	4	4	3	3	3	3	4	4
3	3	4	4	3	3	3	3	4	4
2	2	4	4	1	1	3	3	3	3
3	3	4	4	1	1	3	3	4	4
3	3	4	4	3	3	1	1	4	4
3	3	4	4	1	1	3	3	4	4
3	3	4	4	1	1	1	1	4	4
3	3	4	4	3	3	3	3	4	4
2	2	2	2	3	3	3	3	4	4
3	3	2	2	1	1	3	3	4	4
3	3	4	4	1	1	3	3	4	4
3	3	4	4	3	3	1	1	4	4
3	3	4	4	1	1	3	3	4	4
3	3	4	4	1	1	3	3	3	3
3	3	4	4	1	1	3	3	3	3
3	3	4	4	3	3	3	3	4	4
3	3	4	4	1	1	1	1	3	3
2	2	2	2	1	1	3	3	4	4
3	3	4	4	1	1	1	1	3	3
3	3	4	4	1	1	1	1	3	3
3	3	4	4	1	1	1	1	3	3
3	3	4	4	1	1	3	3	3	3
3	3	4	4	1	1	3	3	4	4
3	3	2	2	1	1	3	3	4	4
3	3	4	4	1	1	3	3	4	4
3	3	4	4	1	1	1	1	3	3

For Review Only

3	3	4	4	1	1	1	1	3	3
3	3	4	4	1	1	3	3	3	3
3	3	4	4	1	1	1	1	4	4
3	3	4	4	1	1	1	1	4	4
3	3	4	4	1	1	3	3	3	3
3	3	4	4	1	1	1	1	3	3
3	3	4	4	1	1	1	1	4	4
3	3	4	4	1	1	3	3	4	4
3	3	4	4	1	1	1	1	4	4
3	3	4	4	1	1	3	3	4	4
3	3	4	4	1	1	3	3	4	4
3	3	4	4	1	1	3	3	4	4
3	3	4	4	1	1	3	3	4	4
3	3	4	4	1	1	3	3	4	4
3	3	4	4	1	1	1	1	3	3
3	3	4	4	1	1	3	3	4	4
3	3	4	4	1	1	1	1	4	4
3	3	4	4	1	1	3	3	4	4
3	3	4	4	1	1	1	1	4	4
3	3	4	4	1	1	3	3	4	4
3	3	4	4	1	1	3	3	4	4
3	3	4	4	1	1	1	1	4	4
3	3	4	4	1	1	3	3	4	4
3	3	2	2	1	1	3	3	4	4
3	3	4	4	1	1	1	1	4	4
3	3	4	4	1	1	1	1	4	4
3	3	4	4	1	1	3	3	4	4
3	3	4	4	1	1	1	1	4	4
3	3	4	4	3	3	3	3	3	3
3	3	4	4	1	1	1	1	3	3
3	3	2	2	1	1	3	3	4	4
3	3	4	4	1	1	3	3	4	4
3	3	4	4	3	3	1	1	4	4
3	3	4	4	1	1	3	3	4	4
3	3	4	4	1	1	1	1	4	4
3	3	4	4	3	3	1	1	3	3
3	3	4	4	1	1	3	3	4	4
3	3	4	4	1	1	1	1	3	3
3	3	4	4	1	1	3	3	4	4
3	3	4	4	1	1	3	3	4	4
3	3	4	4	1	1	3	3	4	4
3	3	2	2	1	1	3	3	4	4
3	3	4	4	1	1	3	3	3	3
2	2	4	4	1	1	3	3	4	4
3	3	4	4	1	1	3	3	4	4
3	3	4	4	1	1	3	3	4	4
3	3	4	4	1	1	1	1	3	3
3	3	4	4	1	1	1	1	3	3
3	3	4	4	1	1	3	3	4	4
3	3	4	4	1	1	1	1	4	4
3	3	4	4	1	1	1	1	3	3
3	3	4	4	1	1	1	1	4	4
3	3	4	4	1	1	3	3	4	4
3	3	4	4	1	1	1	1	3	3
3	3	4	4	1	1	3	3	4	4

For Review Only

3	3	4	4	3	3	3	3	4	4
3	3	4	4	1	1	3	3	4	4
3	3	4	4	1	1	1	1	4	4
3	3	4	4	1	1	3	3	4	4
3	3	4	4	1	1	1	1	4	4
3	3	2	2	1	1	3	3	4	4
3	3	4	4	1	1	3	3	4	4
3	3	4	4	1	1	3	3	4	4
3	3	4	4	1	1	3	3	4	4
3	3	4	4	3	3	1	1	4	4
3	3	4	4	1	1	3	3	4	4
3	3	4	4	1	1	3	3	4	4
3	3	4	4	1	1	3	3	4	4
3	3	4	4	1	1	3	3	4	4
3	3	2	2	1	1	3	3	4	4
3	3	4	4	1	1	3	3	4	4
3	3	4	4	1	1	3	3	4	4
3	3	4	4	1	1	1	1	4	4
3	3	4	4	1	1	1	1	3	3
3	3	4	4	1	1	1	1	4	4
3	3	4	4	1	1	3	3	4	4
3	3	4	4	1	1	3	3	4	4
3	3	4	4	1	1	3	3	4	4
3	3	4	4	1	1	1	1	3	3
3	3	4	4	3	3	3	3	4	4
3	3	4	4	1	1	1	1	4	4
3	3	4	4	1	1	3	3	3	3
3	3	4	4	1	1	1	1	4	4
3	3	4	4	1	1	1	1	3	3
3	3	4	4	3	3	1	1	4	4
3	3	4	4	1	1	1	1	4	4
3	3	4	4	3	3	3	3	4	4
3	3	4	4	1	1	1	1	3	3
3	3	2	2	1	1	3	3	4	4
3	3	2	2	3	3	3	3	4	4
3	3	4	4	1	1	1	1	3	3
3	3	4	4	1	1	1	1	4	4
3	3	4	4	1	1	1	1	4	4
3	3	4	4	1	1	1	1	3	3
3	3	4	4	1	1	3	3	3	3
3	3	4	4	1	1	3	3	4	4
3	3	4	4	1	1	1	1	3	3
3	3	4	4	1	1	3	3	4	4
3	3	4	4	1	1	1	1	4	4
3	3	4	4	1	1	3	3	4	4
2	2	4	4	1	1	3	3	4	4
3	3	4	4	1	1	3	3	4	4
3	3	4	4	1	1	3	3	4	4
3	3	2	2	1	1	1	1	4	4
3	3	4	4	1	1	1	1	4	4
3	3	4	4	3	3	1	1	3	3
3	3	2	2	1	1	3	3	4	4
3	3	4	4	1	1	3	3	4	4
3	3	4	4	1	1	3	3	3	3
3	3	4	4	1	1	3	3	4	4

For Review Only

2	2	4	4	1	1	3	3	4	4
3	3	4	4	1	1	1	1	4	4
3	3	4	4	3	3	1	1	4	4
3	3	4	4	1	1	3	3	4	4
3	3	4	4	1	1	1	1	4	4
3	3	4	4	1	1	1	1	3	3
3	3	4	4	1	1	3	3	4	4
3	3	4	4	1	1	3	3	4	4
3	3	4	4	1	1	3	3	4	4
3	3	2	2	1	1	3	3	4	4
3	3	4	4	1	1	3	3	4	4
3	3	4	4	1	1	3	3	4	4
3	3	2	2	1	1	3	3	4	4
3	3	4	4	1	1	1	1	4	4
3	3	4	4	1	1	3	3	4	4
3	3	4	4	1	1	3	3	4	4
3	3	4	4	1	1	3	3	4	4
3	3	4	4	1	1	3	3	4	4
3	3	4	4	1	1	1	1	4	4
3	3	4	4	1	1	3	3	4	4
3	3	4	4	1	1	3	3	3	3
3	3	4	4	1	1	1	1	3	3
3	3	4	4	3	3	3	3	4	4
3	3	4	4	1	1	3	3	4	4
3	3	4	4	1	1	3	3	4	4
3	3	4	4	1	1	3	3	4	4
3	3	4	4	1	1	3	3	4	4
3	3	4	4	1	1	3	3	3	3
3	3	4	4	1	1	1	1	4	4
3	3	4	4	3	3	1	1	3	3
3	3	4	4	1	1	3	3	4	4
3	3	4	4	1	1	3	3	4	4
3	3	4	4	1	1	1	1	4	4
3	3	4	4	1	1	3	3	4	4
3	3	4	4	1	1	1	1	4	4
3	3	4	4	1	1	3	3	4	4
3	3	4	4	3	3	1	1	3	3
3	3	4	4	1	1	1	1	3	3
3	3	4	4	1	1	1	1	4	4
3	3	4	4	1	1	3	3	4	4
3	3	4	4	1	1	3	3	3	3
3	3	4	4	1	1	1	1	4	4
3	3	4	4	1	1	3	3	4	4
3	3	4	4	3	3	1	1	4	4
3	3	4	4	1	1	3	3	4	4
3	3	2	2	1	1	3	3	4	4
3	3	4	4	1	1	1	1	4	4
3	3	4	4	1	1	3	3	3	3
3	3	4	4	1	1	3	3	4	4
3	3	4	4	1	1	3	3	4	4
3	3	4	4	3	3	1	1	3	3
3	3	4	4	1	1	3	3	4	4
3	3	4	4	1	1	3	3	4	4
3	3	4	4	1	1	3	3	4	4
3	3	4	4	1	1	3	3	4	4

3	3	4	4	1	1	3	3	4	4
3	3	4	4	1	1	3	3	3	3

For Review Only

SNP1731	SNP1731	SNP1732	SNP1732	SNP1733	SNP1733	SNP1734	SNP1734	SNP1735	SNP1735
4	4	4	4	3	3	4	4	1	1
1	1	4	4	3	3	1	1	3	3
4	4	4	4	3	3	4	4	1	1
1	1	4	4	3	3	1	1	3	3
4	4	4	4	3	3	4	4	1	1
1	1	4	4	3	3	4	4	1	1
4	4	4	4	3	3	1	1	1	1
4	4	4	4	3	3	1	1	1	1
4	4	4	4	3	3	4	4	1	1
4	4	4	4	3	3	1	1	1	1
4	4	4	4	3	3	4	4	1	1
1	1	4	4	3	3	1	1	3	3
4	4	4	4	3	3	1	1	1	1
1	1	1	1	3	3	4	4	1	1
4	4	4	4	3	3	4	4	3	3
1	1	4	4	3	3	4	4	1	1
4	4	4	4	3	3	4	4	1	1
4	4	4	4	3	3	4	4	1	1
1	1	4	4	3	3	1	1	3	3
4	4	4	4	3	3	4	4	1	1
4	4	4	4	3	3	4	4	1	1
4	4	4	4	3	3	4	4	1	1
4	4	4	4	3	3	1	1	3	3
1	1	4	4	3	3	4	4	1	1
4	4	4	4	3	3	4	4	1	1
1	1	4	4	3	3	1	1	3	3
4	4	4	4	3	3	1	1	3	3
1	1	4	4	3	3	1	1	3	3
4	4	4	4	3	3	4	4	1	1
4	4	4	4	3	3	4	4	1	1
4	4	4	4	3	3	1	1	3	3
4	4	4	4	3	3	1	1	3	3
4	4	4	4	3	3	4	4	1	1
1	1	4	4	3	3	4	4	1	1
1	1	4	4	3	3	4	4	1	1
1	1	4	4	3	3	4	4	1	1
4	4	4	4	3	3	4	4	1	1
4	4	4	4	3	3	4	4	1	1
1	1	4	4	3	3	4	4	1	1
1	1	4	4	3	3	1	1	3	3
4	4	4	4	3	3	1	1	1	1
4	4	4	4	3	3	4	4	1	1
4	4	4	4	3	3	4	4	1	1
4	4	4	4	3	3	4	4	1	1
4	4	4	4	3	3	4	4	1	1
1	1	4	4	3	3	1	1	1	1
4	4	4	4	3	3	1	1	3	3
1	1	4	4	3	3	4	4	3	3
4	4	4	4	3	3	4	4	1	1
1	1	4	4	2	2	1	1	3	3
1	1	4	4	3	3	1	1	3	3
1	1	4	4	3	3	1	1	3	3

For Review Only

4	4	4	4	3	3	4	4	1	1
4	4	4	4	3	3	4	4	1	1
4	4	4	4	3	3	1	1	3	3
4	4	4	4	3	3	4	4	1	1
1	1	4	4	3	3	1	1	3	3
4	4	4	4	3	3	1	1	3	3
1	1	4	4	3	3	1	1	3	3
1	1	4	4	3	3	4	4	1	1
4	4	4	4	3	3	4	4	1	1
1	1	4	4	3	3	1	1	1	1
1	1	4	4	3	3	1	1	3	3
4	4	4	4	3	3	1	1	3	3
4	4	4	4	3	3	4	4	1	1
4	4	4	4	3	3	4	4	1	1
1	1	4	4	3	3	1	1	3	3
4	4	4	4	3	3	1	1	1	1
4	4	4	4	3	3	4	4	1	1
4	4	4	4	3	3	4	4	1	1
4	4	4	4	2	2	1	1	3	3
4	4	4	4	3	3	4	4	1	1
4	4	4	4	3	3	1	1	3	3
1	1	4	4	3	3	1	1	3	3
4	4	4	4	3	3	4	4	1	1
4	4	4	4	3	3	4	4	1	1
4	4	4	4	3	3	4	4	1	1
1	1	4	4	3	3	4	4	1	1
4	4	4	4	3	3	4	4	1	1
1	1	4	4	3	3	1	1	1	1
1	1	4	4	3	3	1	1	3	3
4	4	4	4	3	3	1	1	3	3
4	4	4	4	3	3	1	1	1	1
1	1	4	4	3	3	4	4	1	1
1	1	4	4	3	3	1	1	1	1
4	4	4	4	3	3	4	4	1	1
4	4	4	4	3	3	4	4	1	1
4	4	4	4	3	3	4	4	1	1
4	4	4	4	3	3	1	1	1	1
4	4	4	4	3	3	4	4	1	1
4	4	4	4	3	3	4	4	1	1
4	4	4	4	3	3	4	4	1	1
4	4	4	4	3	3	4	4	1	1
4	4	4	4	3	3	1	1	3	3
1	1	4	4	2	2	1	1	3	3
1	1	4	4	3	3	1	1	3	3
4	4	4	4	3	3	4	4	1	1
1	1	4	4	3	3	1	1	3	3
1	1	4	4	3	3	4	4	1	1
1	1	4	4	3	3	1	1	3	3
4	4	4	4	3	3	1	1	3	3
4	4	4	4	3	3	1	1	3	3
4	4	4	4	3	3	1	1	3	3
4	4	4	4	3	3	4	4	1	1
4	4	1	1	3	3	4	4	1	1
1	1	4	4	3	3	1	1	1	1
4	4	4	4	3	3	1	1	3	3

For Review Only

1	1	4	4	3	3	1	1	3	3
1	1	4	4	3	3	1	1	3	3
1	1	4	4	3	3	4	4	1	1
1	1	4	4	3	3	4	4	1	1
1	1	4	4	3	3	1	1	3	3
1	1	4	4	3	3	4	4	1	1
1	1	4	4	3	3	4	4	1	1
1	1	4	4	3	3	4	4	1	1
4	4	4	4	3	3	4	4	1	1
1	1	4	4	3	3	4	4	1	1
4	4	4	4	3	3	1	1	1	1
1	1	4	4	3	3	1	1	3	3
1	1	4	4	2	2	4	4	1	1
1	1	4	4	3	3	1	1	3	3
1	1	4	4	3	3	1	1	3	3
1	1	4	4	3	3	1	1	3	3
1	1	4	4	2	2	4	4	1	1
1	1	4	4	3	3	1	1	3	3
1	1	4	4	3	3	4	4	1	1
1	1	4	4	3	3	4	4	1	1
1	1	4	4	3	3	1	1	3	3
1	1	4	4	3	3	1	1	1	1
4	4	4	4	3	3	4	4	1	1
1	1	1	1	3	3	4	4	1	1
1	1	4	4	3	3	1	1	1	1
1	1	4	4	3	3	1	1	3	3
1	1	4	4	3	3	1	1	1	1
4	4	4	4	3	3	4	4	1	1
4	4	4	4	3	3	1	1	3	3
1	1	4	4	3	3	1	1	3	3
1	1	4	4	3	3	4	4	1	1
1	1	4	4	3	3	1	1	3	3
4	4	4	4	3	3	4	4	1	1
1	1	4	4	3	3	1	1	3	3
1	1	4	4	3	3	4	4	1	1
4	4	4	4	3	3	4	4	1	1
1	1	1	1	3	3	4	4	1	1
1	1	4	4	3	3	1	1	3	3
1	1	4	4	3	3	1	1	1	1
1	1	4	4	3	3	1	1	1	1
1	1	4	4	3	3	1	1	3	3
1	1	4	4	3	3	4	4	1	1
4	4	4	4	3	3	1	1	3	3
4	4	4	4	3	3	1	1	1	1
1	1	4	4	3	3	4	4	1	1
4	4	4	4	3	3	4	4	1	1
1	1	4	4	3	3	1	1	3	3
1	1	4	4	3	3	1	1	3	3
4	4	4	4	3	3	4	4	1	1
1	1	4	4	3	3	1	1	3	3
1	1	4	4	3	3	1	1	3	3
4	4	1	1	3	3	4	4	1	1
1	1	4	4	3	3	4	4	1	1
4	4	4	4	3	3	4	4	1	1
4	4	4	4	3	3	1	1	1	1

For Review Only

CSSA 5585 Guilford Rd., Madison, WI 53711

4	4	4	4	3	3	4	4	1	1
4	4	4	4	2	2	1	1	3	3
4	4	4	4	3	3	1	1	3	3
4	4	4	4	3	3	4	4	1	1
1	1	4	4	3	3	4	4	1	1
1	1	4	4	3	3	4	4	1	1
1	1	4	4	3	3	1	1	3	3
1	1	4	4	3	3	1	1	3	3
1	1	4	4	3	3	4	4	1	1
4	4	4	4	3	3	4	4	1	1
1	1	4	4	3	3	1	1	3	3
1	1	4	4	3	3	1	1	1	1
1	1	4	4	3	3	1	1	3	3
4	4	4	4	3	3	4	4	1	1
1	1	1	1	3	3	4	4	1	1
1	1	4	4	3	3	4	4	1	1
1	1	4	4	3	3	4	4	3	3
1	1	4	4	3	3	4	4	1	1
1	1	4	4	3	3	4	4	1	1
1	1	4	4	3	3	4	4	1	1
1	1	4	4	3	3	1	1	3	3
1	1	4	4	3	3	4	4	1	1
1	1	4	4	3	3	4	4	1	1
4	4	4	4	3	3	1	1	3	3
4	4	4	4	3	3	4	4	1	1
1	1	4	4	3	3	4	4	1	1
4	4	4	4	3	3	1	1	3	3
1	1	4	4	3	3	4	4	1	1
1	1	4	4	2	2	1	1	3	3
4	4	4	4	3	3	4	4	1	1
4	4	4	4	3	3	4	4	1	1
4	4	4	4	2	2	1	1	3	3
4	4	4	4	3	3	1	1	3	3
1	1	1	1	3	3	4	4	1	1
4	4	4	4	2	2	4	4	1	1
4	4	4	4	3	3	1	1	3	3
4	4	4	4	3	3	1	1	3	3
4	4	4	4	3	3	4	4	1	1
1	1	4	4	3	3	1	1	3	3
1	1	4	4	3	3	1	1	3	3
1	1	4	4	3	3	4	4	1	1
1	1	4	4	3	3	1	1	3	3
4	4	4	4	3	3	4	4	1	1
1	1	4	4	3	3	1	1	3	3
1	1	4	4	3	3	4	4	1	1
1	1	4	4	3	3	4	4	1	1
1	1	4	4	3	3	4	4	1	1
4	4	4	4	3	3	4	4	1	1
1	1	4	4	3	3	4	4	1	1
1	1	4	4	3	3	1	1	1	1
4	4	4	4	3	3	4	4	1	1
1	1	1	1	3	3	4	4	1	1
1	1	4	4	3	3	4	4	1	1
1	1	4	4	3	3	1	1	3	3
4	4	4	4	3	3	4	4	1	1

For Review Only

1	1	4	4	3	3	4	4	1	1
4	4	4	4	3	3	4	4	3	3
4	4	4	4	3	3	1	1	3	3
1	1	4	4	3	3	1	1	3	3
4	4	4	4	3	3	4	4	1	1
4	4	4	4	3	3	1	1	3	3
1	1	4	4	3	3	4	4	1	1
4	4	4	4	3	3	4	4	1	1
1	1	4	4	3	3	1	1	1	1
1	1	1	1	3	3	1	1	1	1
1	1	4	4	3	3	1	1	3	3
1	1	4	4	3	3	4	4	1	1
4	4	4	4	3	3	4	4	1	1
1	1	4	4	3	3	4	4	1	1
4	4	4	4	3	3	4	4	1	1
4	4	4	4	3	3	1	1	3	3
4	4	4	4	3	3	4	4	1	1
1	1	4	4	3	3	4	4	1	1
1	1	4	4	3	3	1	1	3	3
4	4	4	4	3	3	4	4	1	1
4	4	4	4	3	3	1	1	3	3
1	1	4	4	3	3	1	1	3	3
4	4	4	4	3	3	4	4	1	1
1	1	4	4	3	3	1	1	3	3
1	1	4	4	3	3	4	4	1	1
1	1	4	4	3	3	4	4	1	1
1	1	1	1	3	3	1	1	1	1
1	1	4	4	3	3	1	1	3	3
4	4	4	4	3	3	4	4	1	1
4	4	4	4	3	3	4	4	1	1
1	1	4	4	3	3	4	4	1	1
1	1	4	4	3	3	4	4	1	1
4	4	1	1	3	3	4	4	1	1
1	1	4	4	3	3	4	4	1	1
1	1	4	4	3	3	4	4	1	1
1	1	4	4	3	3	4	4	1	1
4	4	4	4	3	3	4	4	1	1
4	4	4	4	3	3	1	1	3	3
1	1	4	4	3	3	4	4	1	1
4	4	4	4	3	3	4	4	1	1
1	1	4	4	2	2	1	1	3	3
1	1	4	4	3	3	4	4	1	1
1	1	4	4	3	3	1	1	3	3
4	4	4	4	3	3	4	4	1	1
1	1	4	4	3	3	4	4	1	1
4	4	4	4	3	3	4	4	1	1
1	1	4	4	3	3	1	1	3	3
4	4	4	4	3	3	4	4	1	1
4	4	4	4	3	3	4	4	1	1
1	1	4	4	3	3	1	1	1	1
4	4	4	4	3	3	1	1	3	3
1	1	4	4	3	3	4	4	1	1
4	4	4	4	3	3	4	4	1	1
1	1	4	4	3	3	4	4	1	1
1	1	4	4	3	3	1	1	3	3

For Review Only

4	4	4	4	3	3	1	1	3	3
4	4	4	4	3	3	1	1	3	3

For Review Only

SNP1736	SNP1736	SNP1737	SNP1737	SNP1738	SNP1738	SNP1739	SNP1739	SNP174	SNP174
3	3	1	1	4	4	3	3	4	4
2	2	1	1	1	1	2	2	1	1
3	3	1	1	4	4	3	3	1	1
3	3	1	1	1	1	2	2	1	1
3	3	1	1	4	4	3	3	1	1
2	2	1	1	4	4	3	3	1	1
3	3	1	1	4	4	2	2	4	4
2	2	1	1	1	1	3	3	4	4
3	3	1	1	1	1	2	2	1	1
2	2	1	1	1	1	3	3	4	4
2	2	1	1	1	1	3	3	1	1
3	3	1	1	4	4	3	3	1	1
3	3	1	1	1	1	2	2	4	4
3	3	1	1	4	4	3	3	4	4
3	3	1	1	1	1	2	2	1	1
3	3	1	1	4	4	3	3	1	1
3	3	1	1	4	4	2	2	4	4
2	2	1	1	1	1	3	3	4	4
2	2	1	1	4	4	2	2	1	1
2	2	1	1	1	1	3	3	4	4
3	3	4	4	4	4	2	2	1	1
3	3	1	1	4	4	2	2	4	4
2	2	1	1	1	1	3	3	4	4
2	2	4	4	1	1	3	3	4	4
2	2	1	1	1	1	3	3	4	4
3	3	1	1	4	4	3	3	1	1
2	2	1	1	1	1	3	3	4	4
3	3	1	1	1	1	2	2	1	1
2	2	1	1	1	1	2	2	4	4
2	2	1	1	1	1	3	3	4	4
2	2	1	1	1	1	3	3	1	1
2	2	1	1	1	1	3	3	4	4
2	2	1	1	4	4	3	3	4	4
2	2	1	1	1	1	3	3	1	1
2	2	1	1	1	1	3	3	1	1
2	2	1	1	1	1	3	3	1	1
2	2	1	1	1	1	3	3	1	1
3	3	1	1	4	4	3	3	1	1
3	3	1	1	4	4	3	3	4	4
3	3	1	1	1	1	3	3	1	1
2	2	1	1	1	1	3	3	4	4
2	2	4	4	1	1	2	2	4	4
2	2	1	1	1	1	3	3	4	4
3	3	1	1	4	4	2	2	1	1
2	2	1	1	1	1	3	3	4	4
3	3	1	1	4	4	3	3	1	1
2	2	1	1	1	1	3	3	1	1
3	3	1	1	4	4	3	3	1	1
3	3	1	1	4	4	2	2	1	1
3	3	1	1	1	1	2	2	4	4
3	3	1	1	4	4	3	3	1	1
3	3	1	1	4	4	2	2	4	4

For Review Only

3	3	1	1	4	4	2	2	4	4
2	2	1	1	4	4	3	3	4	4
2	2	1	1	4	4	3	3	1	1
3	3	1	1	4	4	2	2	4	4
2	2	1	1	4	4	2	2	1	1
2	2	4	4	1	1	3	3	1	1
3	3	1	1	4	4	3	3	4	4
2	2	1	1	1	1	3	3	4	4
2	2	1	1	1	1	3	3	1	1
3	3	1	1	1	1	2	2	1	1
2	2	4	4	1	1	2	2	4	4
2	2	4	4	1	1	3	3	4	4
3	3	1	1	4	4	2	2	4	4
2	2	1	1	1	1	2	2	4	4
3	3	1	1	1	1	2	2	1	1
3	3	1	1	4	4	3	3	4	4
2	2	1	1	1	1	3	3	1	1
2	2	1	1	1	1	3	3	1	1
3	3	1	1	4	4	2	2	1	1
3	3	1	1	4	4	2	2	4	4
2	2	4	4	1	1	3	3	4	4
2	2	1	1	1	1	3	3	1	1
2	2	1	1	1	1	2	2	1	1
2	2	1	1	1	1	3	3	4	4
2	2	1	1	1	1	3	3	4	4
3	3	1	1	4	4	2	2	4	4
2	2	1	1	1	1	2	2	4	4
3	3	1	1	4	4	3	3	4	4
2	2	1	1	1	1	3	3	1	1
3	3	1	1	4	4	2	2	4	4
3	3	1	1	1	1	2	2	1	1
3	3	4	4	4	4	3	3	4	4
3	3	1	1	4	4	3	3	4	4
3	3	1	1	4	4	2	2	4	4
2	2	1	1	1	1	3	3	4	4
2	2	1	1	1	1	3	3	4	4
2	2	1	1	1	1	2	2	4	4
3	3	1	1	1	1	2	2	1	1
3	3	1	1	4	4	3	3	4	4
2	2	1	1	1	1	3	3	1	1
3	3	1	1	1	1	2	2	1	1
3	3	1	1	4	4	3	3	1	1
3	3	1	1	4	4	2	2	4	4
2	2	4	4	1	1	2	2	4	4
2	2	1	1	1	1	2	2	1	1
2	2	1	1	4	4	2	2	1	1
3	3	1	1	1	1	3	3	4	4
3	3	1	1	1	1	3	3	4	4
2	2	1	1	1	1	3	3	1	1
3	3	1	1	4	4	3	3	1	1
3	3	4	4	4	4	3	3	4	4
2	2	1	1	1	1	3	3	4	4
2	2	1	1	1	1	3	3	1	1
3	3	1	1	1	1	3	3	4	4
3	3	1	1	4	4	3	3	1	1

For Review Only

2	2	1	1	1	1	3	3	1	1
2	2	1	1	1	1	2	2	1	1
2	2	1	1	1	1	3	3	1	1
3	3	1	1	1	1	3	3	4	4
3	3	1	1	4	4	2	2	1	1
3	3	1	1	4	4	3	3	1	1
3	3	1	1	4	4	2	2	1	1
2	2	4	4	4	4	3	3	4	4
2	2	1	1	1	1	3	3	1	1
2	2	1	1	1	1	3	3	4	4
3	3	1	1	4	4	3	3	4	4
3	3	1	1	1	1	2	2	1	1
3	3	1	1	4	4	2	2	1	1
3	3	1	1	4	4	2	2	4	4
3	3	1	1	4	4	2	2	1	1
2	2	1	1	1	1	3	3	1	1
2	2	1	1	1	1	2	2	1	1
3	3	1	1	1	1	2	2	1	1
3	3	1	1	1	1	3	3	4	4
3	3	1	1	4	4	2	2	1	1
3	3	1	1	1	1	2	2	1	1
2	2	1	1	1	1	2	2	1	1
3	3	1	1	4	4	3	3	1	1
2	2	1	1	1	1	3	3	4	4
2	2	1	1	1	1	3	3	1	1
2	2	1	1	1	1	3	3	1	1
2	2	1	1	1	1	2	2	1	1
2	2	1	1	1	1	3	3	4	4
3	3	1	1	4	4	2	2	1	1
2	2	1	1	4	4	2	2	1	1
3	3	1	1	4	4	3	3	4	4
3	3	1	1	1	1	3	3	1	1
3	3	1	1	4	4	2	2	1	1
2	2	1	1	1	1	3	3	1	1
2	2	1	1	1	1	3	3	1	1
2	2	1	1	1	1	3	3	1	1
2	2	1	1	1	1	3	3	4	4
3	3	1	1	4	4	2	2	1	1
2	2	1	1	1	1	3	3	4	4
2	2	1	1	1	1	3	3	1	1
2	2	1	1	1	1	3	3	4	4
3	3	1	1	1	1	3	3	4	4
2	2	1	1	1	1	2	2	4	4
2	2	4	4	1	1	3	3	4	4
2	2	1	1	1	1	2	2	4	4
3	3	1	1	1	1	3	3	1	1
2	2	1	1	1	1	3	3	1	1
2	2	1	1	1	1	3	3	1	1
2	2	1	1	1	1	3	3	1	1
3	3	1	1	4	4	2	2	1	1
2	2	1	1	1	1	3	3	1	1
2	2	1	1	1	1	3	3	1	1
3	3	1	1	4	4	3	3	4	4
2	2	1	1	1	1	3	3	4	4
2	2	1	1	1	1	3	3	1	1

For Review Only

2	2	1	1	1	1	2	2	1	1
3	3	1	1	4	4	3	3	1	1
2	2	1	1	1	1	3	3	4	4
2	2	1	1	1	1	3	3	1	1
3	3	1	1	4	4	2	2	1	1
3	3	1	1	4	4	3	3	1	1
3	3	1	1	1	1	3	3	1	1
3	3	1	1	1	1	2	2	4	4
3	3	1	1	4	4	3	3	1	1
2	2	1	1	1	1	2	2	4	4
2	2	1	1	1	1	3	3	1	1
2	2	1	1	1	1	3	3	1	1
3	3	1	1	1	1	3	3	1	1
3	3	1	1	1	1	3	3	1	1
2	2	1	1	1	1	3	3	4	4
2	2	1	1	1	1	3	3	1	1
3	3	1	1	1	1	2	2	4	4
3	3	1	1	1	1	2	2	1	1
2	2	1	1	1	1	3	3	1	1
3	3	1	1	4	4	2	2	1	1
3	3	1	1	1	1	2	2	1	1
2	2	1	1	1	1	3	3	4	4
2	2	1	1	1	1	3	3	1	1
2	2	4	4	1	1	3	3	4	4
2	2	4	4	1	1	2	2	1	1
2	2	1	1	1	1	3	3	1	1
3	3	1	1	4	4	3	3	1	1
2	2	1	1	1	1	2	2	1	1
3	3	1	1	4	4	3	3	4	4
3	3	1	1	4	4	2	2	4	4
2	2	1	1	4	4	3	3	4	4
2	2	1	1	1	1	2	2	4	4
2	2	1	1	1	1	3	3	4	4
2	2	1	1	1	1	3	3	4	4
2	2	4	4	1	1	2	2	1	1
2	2	4	4	1	1	3	3	4	4
2	2	1	1	1	1	3	3	1	1
2	2	1	1	1	1	3	3	4	4
2	2	1	1	1	1	3	3	1	1
3	3	1	1	4	4	2	2	1	1
3	3	1	1	4	4	3	3	1	1
2	2	1	1	1	1	3	3	1	1
2	2	1	1	1	1	3	3	4	4
2	2	1	1	1	1	3	3	1	1
3	3	1	1	1	1	3	3	1	1
2	2	4	4	1	1	2	2	1	1
2	2	1	1	1	1	3	3	1	1
3	3	1	1	4	4	3	3	4	4
2	2	1	1	1	1	3	3	4	4
2	2	1	1	1	1	2	2	4	4
3	3	1	1	4	4	2	2	4	4
2	2	1	1	1	1	3	3	1	1
3	3	1	1	4	4	3	3	1	1
2	2	4	4	1	1	2	2	4	4
3	3	1	1	4	4	3	3	4	4

For Review Only

2	2	1	1	1	1	3	3	4	4
2	2	1	1	1	1	3	3	4	4
3	3	1	1	4	4	2	2	1	1
3	3	1	1	1	1	2	2	1	1
2	2	1	1	1	1	3	3	4	4
2	2	1	1	1	1	3	3	1	1
2	2	1	1	4	4	3	3	1	1
2	2	1	1	1	1	3	3	4	4
2	2	1	1	1	1	3	3	1	1
3	3	1	1	4	4	3	3	4	4
3	3	1	1	1	1	2	2	1	1
2	2	1	1	1	1	2	2	4	4
2	2	1	1	1	1	3	3	4	4
3	3	1	1	1	1	3	3	1	1
3	3	1	1	4	4	3	3	1	1
2	2	1	1	1	1	3	3	1	1
2	2	1	1	1	1	3	3	1	1
2	2	1	1	1	1	2	2	1	1
2	2	1	1	1	1	3	3	1	1
3	3	1	1	4	4	3	3	1	1
3	3	1	1	1	1	3	3	4	4
2	2	1	1	1	1	3	3	1	1
2	2	1	1	1	1	2	2	1	1
3	3	1	1	4	4	3	3	4	4
2	2	1	1	1	1	3	3	4	4
2	2	1	1	4	4	2	2	1	1
3	3	1	1	4	4	3	3	4	4
3	3	1	1	4	4	2	2	4	4
3	3	1	1	1	1	3	3	4	4
3	3	1	1	4	4	2	2	1	1
3	3	1	1	4	4	3	3	4	4
2	2	1	1	1	1	3	3	1	1
2	2	1	1	1	1	3	3	1	1
2	2	1	1	1	1	3	3	1	1
2	2	1	1	1	1	3	3	1	1
3	3	1	1	4	4	3	3	4	4
2	2	1	1	1	1	2	2	4	4
2	2	4	4	1	1	3	3	4	4
2	2	1	1	1	1	3	3	1	1
2	2	1	1	1	1	3	3	4	4
2	2	4	4	1	1	2	2	1	1
2	2	1	1	1	1	3	3	4	4
2	2	1	1	1	1	3	3	1	1
3	3	1	1	4	4	2	2	1	1
2	2	1	1	1	1	3	3	4	4
3	3	1	1	4	4	3	3	4	4
2	2	4	4	1	1	3	3	4	4
3	3	1	1	4	4	3	3	1	1
2	2	1	1	1	1	3	3	1	1
3	3	1	1	4	4	3	3	4	4
3	3	1	1	4	4	2	2	1	1
3	3	1	1	4	4	3	3	4	4
3	3	1	1	4	4	3	3	1	1
3	3	1	1	4	4	3	3	1	1
2	2	1	1	1	1	3	3	1	1

For Review Only

2	2	1	1	1	1	3	3	1	1
3	3	1	1	4	4	3	3	4	4

For Review Only

SNP1740	SNP1740	SNP1741	SNP1741	SNP1742	SNP1742	SNP1743	SNP1743	SNP1744	SNP1744
3	3	1	1	3	3	3	3	1	1
3	3	1	1	1	1	3	3	1	1
2	2	1	1	3	3	3	3	1	1
3	3	1	1	3	3	3	3	1	1
3	3	3	3	3	3	3	3	1	1
3	3	1	1	3	3	3	3	1	1
3	3	1	1	1	1	4	4	1	1
3	3	3	3	3	3	3	3	1	1
3	3	3	3	3	3	4	4	1	1
3	3	3	3	3	3	3	3	1	1
3	3	1	1	3	3	3	3	4	4
2	2	1	1	3	3	3	3	1	1
3	3	1	1	3	3	4	4	1	1
3	3	3	3	3	3	3	3	1	1
3	3	3	3	3	3	4	4	1	1
3	3	3	3	3	3	3	3	1	1
3	3	1	1	1	1	4	4	1	1
3	3	1	1	3	3	3	3	1	1
3	3	1	1	1	1	3	3	1	1
3	3	3	3	3	3	3	3	1	1
3	3	1	1	1	1	4	4	1	1
3	3	3	3	1	1	4	4	1	1
3	3	3	3	3	3	3	3	1	1
3	3	3	3	3	3	3	3	1	1
3	3	1	1	3	3	3	3	1	1
3	3	1	1	3	3	3	3	1	1
3	3	1	1	3	3	3	3	1	1
3	3	1	1	3	3	3	3	1	1
3	3	3	3	1	1	4	4	1	1
3	3	3	3	3	3	3	3	1	1
3	3	1	1	3	3	3	3	1	1
2	2	1	1	3	3	3	3	1	1
2	2	1	1	3	3	3	3	1	1
3	3	3	3	3	3	3	3	1	1
3	3	3	3	3	3	3	3	1	1
3	3	3	3	3	3	3	3	1	1
3	3	3	3	3	3	3	3	1	1
3	3	1	1	3	3	3	3	1	1
3	3	3	3	3	3	3	3	1	1
3	3	3	3	3	3	3	3	1	1
3	3	3	3	3	3	3	3	1	1
3	3	3	3	1	1	4	4	1	1
3	3	1	1	3	3	3	3	1	1
3	3	3	3	1	1	4	4	1	1
3	3	1	1	3	3	3	3	1	1
3	3	1	1	3	3	3	3	1	1
3	3	1	1	3	3	3	3	1	1
3	3	3	3	3	3	4	4	1	1
3	3	3	3	1	1	4	4	1	1
3	3	1	1	1	1	3	3	4	4
3	3	1	1	3	3	3	3	1	1
3	3	1	1	1	1	4	4	1	1

For Review Only

3	3	1	1	1	1	4	4	1	1
2	2	1	1	3	3	3	3	1	1
3	3	3	3	3	3	3	3	1	1
3	3	3	3	1	1	4	4	1	1
3	3	1	1	1	1	3	3	1	1
3	3	1	1	3	3	3	3	1	1
3	3	1	1	3	3	3	3	1	1
3	3	3	3	3	3	3	3	1	1
3	3	3	3	3	3	3	3	1	1
3	3	1	1	3	3	3	3	1	1
3	3	1	1	1	1	3	3	1	1
3	3	3	3	3	3	3	3	1	1
3	3	1	1	1	1	4	4	1	1
3	3	3	3	1	1	4	4	1	1
3	3	1	1	3	3	3	3	1	1
3	3	1	1	3	3	3	3	1	1
3	3	3	3	3	3	3	3	1	1
2	2	1	1	3	3	3	3	1	1
3	3	3	3	1	1	4	4	1	1
3	3	1	1	1	1	4	4	1	1
3	3	1	1	3	3	3	3	1	1
3	3	3	3	3	3	3	3	1	1
3	3	3	3	1	1	4	4	1	1
3	3	3	3	3	3	3	3	1	1
3	3	1	1	3	3	3	3	1	1
3	3	3	3	1	1	3	3	1	1
2	2	1	1	1	1	4	4	1	1
3	3	3	3	3	3	3	3	1	1
3	3	1	1	3	3	3	3	1	1
3	3	1	1	1	1	4	4	4	4
3	3	3	3	3	3	4	4	1	1
3	3	3	3	3	3	3	3	1	1
3	3	3	3	3	3	3	3	1	1
3	3	1	1	1	1	4	4	1	1
3	3	3	3	3	3	3	3	1	1
3	3	1	1	3	3	3	3	1	1
3	3	3	3	1	1	4	4	1	1
3	3	1	1	1	1	4	4	1	1
3	3	1	1	3	3	3	3	4	4
3	3	3	3	3	3	3	3	1	1
3	3	3	3	1	1	4	4	1	1
3	3	1	1	3	3	3	3	4	4
3	3	1	1	1	1	3	3	4	4
3	3	1	1	1	1	3	3	1	1
3	3	1	1	1	1	4	4	4	4
3	3	1	1	1	1	3	3	1	1
3	3	3	3	3	3	3	3	1	1
3	3	3	3	3	3	3	3	1	1
3	3	3	3	3	3	3	3	1	1
3	3	3	3	3	3	3	3	1	1
3	3	3	3	3	3	3	3	1	1
3	3	3	3	3	3	3	3	1	1
3	3	3	3	3	3	3	3	1	1
3	3	3	3	3	3	3	3	1	1
2	2	1	1	3	3	3	3	1	1

For Review Only

3	3	1	1	3	3	3	3	1	1
3	3	1	1	3	3	3	3	1	1
3	3	1	1	3	3	3	3	1	1
3	3	3	3	3	3	3	3	1	1
3	3	3	3	1	1	3	3	1	1
3	3	1	1	3	3	3	3	1	1
3	3	3	3	1	1	3	3	1	1
3	3	1	1	3	3	3	3	1	1
3	3	3	3	3	3	3	3	1	1
3	3	1	1	3	3	3	3	1	1
3	3	3	3	3	3	4	4	1	1
3	3	1	1	3	3	3	3	1	1
3	3	1	1	1	1	3	3	1	1
3	3	1	1	3	3	4	4	1	1
3	3	1	1	3	3	3	3	1	1
3	3	1	1	3	3	3	3	1	1
2	2	1	1	1	1	3	3	1	1
3	3	1	1	3	3	3	3	1	1
3	3	3	3	3	3	3	3	1	1
3	3	1	1	1	1	4	4	1	1
3	3	1	1	3	3	3	3	1	1
3	3	1	1	1	1	3	3	4	4
3	3	3	3	3	3	3	3	1	1
3	3	3	3	3	3	3	3	1	1
3	3	1	1	3	3	3	3	1	1
3	3	3	3	3	3	3	3	1	1
3	3	3	3	3	3	3	3	1	1
3	3	3	3	3	3	3	3	1	1
3	3	1	1	1	1	4	4	1	1
3	3	3	3	1	1	3	3	1	1
3	3	3	3	3	3	3	3	4	4
3	3	1	1	3	3	3	3	1	1
3	3	3	3	1	1	4	4	1	1
3	3	1	1	3	3	3	3	1	1
3	3	3	3	3	3	3	3	1	1
3	3	3	3	1	1	3	3	1	1
3	3	3	3	3	3	3	3	1	1
3	3	1	1	1	1	3	3	1	1
3	3	1	1	3	3	4	4	1	1
3	3	1	1	3	3	3	3	4	4
3	3	3	3	3	3	3	3	1	1
3	3	3	3	3	3	3	3	1	1
3	3	3	3	3	3	3	3	1	1
3	3	3	3	3	3	3	3	1	1
3	3	3	3	3	3	3	3	1	1
3	3	3	3	3	3	3	3	1	1
3	3	1	1	3	3	3	3	1	1
3	3	1	1	3	3	3	3	1	1
3	3	1	1	3	3	3	3	1	1
3	3	1	1	3	3	4	4	1	1
3	3	1	1	3	3	3	3	1	1
3	3	3	3	3	3	3	3	1	1
3	3	3	3	3	3	3	3	1	1
3	3	3	3	3	3	3	3	1	1
3	3	1	1	3	3	3	3	1	1

For Review Only

2	2	1	1	1	1	4	4	1	1
3	3	3	3	3	3	3	3	1	1
3	3	3	3	3	3	3	3	1	1
3	3	3	3	3	3	3	3	1	1
3	3	1	1	1	1	3	3	1	1
3	3	3	3	3	3	3	3	1	1
3	3	3	3	3	3	3	3	1	1
3	3	1	1	3	3	3	3	1	1
3	3	3	3	3	3	3	3	1	1
3	3	1	1	1	1	4	4	4	4
3	3	1	1	3	3	3	3	1	1
3	3	3	3	3	3	3	3	1	1
3	3	1	1	3	3	3	3	4	4
3	3	3	3	3	3	3	3	1	1
3	3	3	3	3	3	3	3	1	1
3	3	1	1	3	3	3	3	4	4
3	3	3	3	3	3	3	3	1	1
3	3	1	1	1	1	3	3	1	1
3	3	1	1	3	3	3	3	1	1
3	3	3	3	1	1	3	3	1	1
3	3	1	1	3	3	3	3	1	1
3	3	3	3	3	3	3	3	1	1
3	3	3	3	3	3	3	3	1	1
3	3	3	3	3	3	3	3	1	1
3	3	3	3	1	1	4	4	1	1
3	3	3	3	3	3	3	3	1	1
3	3	3	3	3	3	3	3	1	1
3	3	1	1	3	3	3	3	1	1
3	3	3	3	3	3	3	3	1	1
3	3	1	1	1	1	4	4	1	1
3	3	3	3	3	3	3	3	1	1
2	2	1	1	1	1	4	4	1	1
3	3	3	3	3	3	3	3	1	1
3	3	3	3	3	3	3	3	1	1
3	3	3	3	1	1	4	4	1	1
3	3	3	3	3	3	3	3	1	1
3	3	1	1	3	3	3	3	1	1
3	3	1	1	3	3	3	3	1	1
3	3	1	1	3	3	3	3	1	1
3	3	1	1	1	1	3	3	1	1
2	2	1	1	3	3	3	3	1	1
3	3	1	1	3	3	3	3	1	1
3	3	3	3	3	3	3	3	1	1
3	3	3	3	3	3	3	3	1	1
3	3	3	3	3	3	3	3	1	1
3	3	1	1	1	1	3	3	4	4
3	3	1	1	3	3	3	3	4	4
3	3	3	3	3	3	3	3	1	1
3	3	3	3	3	3	3	3	1	1
3	3	3	3	1	1	3	3	1	1
3	3	1	1	1	1	4	4	1	1
3	3	3	3	3	3	3	3	1	1
3	3	3	3	3	3	3	3	1	1
3	3	1	1	1	1	3	3	1	1
3	3	3	3	3	3	3	3	1	1

For Review Only

3	3	3	3	3	3	3	3	1	1
3	3	3	3	3	3	3	3	1	1
2	2	1	1	1	1	4	4	1	1
3	3	3	3	3	3	3	3	1	1
3	3	1	1	3	3	3	3	1	1
3	3	1	1	3	3	3	3	1	1
3	3	3	3	3	3	3	3	1	1
3	3	3	3	3	3	3	3	1	1
3	3	3	3	3	3	3	3	1	1
3	3	3	3	3	3	3	3	1	1
3	3	1	1	3	3	3	3	1	1
3	3	3	3	1	1	3	3	1	1
3	3	1	1	3	3	3	3	4	4
3	3	1	1	3	3	3	3	1	1
2	2	1	1	3	3	3	3	4	4
3	3	1	1	3	3	3	3	4	4
3	3	3	3	3	3	3	3	1	1
3	3	1	1	3	3	4	4	1	1
3	3	3	3	3	3	3	3	1	1
2	2	1	1	3	3	3	3	1	1
3	3	1	1	3	3	3	3	1	1
3	3	1	1	3	3	3	3	1	1
3	3	1	1	1	1	4	4	1	1
3	3	3	3	3	3	3	3	1	1
3	3	3	3	3	3	3	3	1	1
3	3	1	1	1	1	3	3	1	1
3	3	3	3	3	3	3	3	1	1
3	3	1	1	1	1	3	3	1	1
3	3	1	1	3	3	3	3	1	1
3	3	1	1	1	1	4	4	1	1
3	3	3	3	3	3	3	3	1	1
3	3	3	3	3	3	3	3	1	1
3	3	3	3	3	3	3	3	1	1
3	3	1	1	3	3	3	3	1	1
3	3	3	3	3	3	3	3	1	1
3	3	3	3	3	3	3	3	1	1
3	3	3	3	1	1	4	4	1	1
3	3	3	3	3	3	3	3	1	1
3	3	3	3	3	3	3	3	1	1
3	3	3	3	3	3	3	3	1	1
2	2	1	1	1	1	3	3	1	1
3	3	3	3	3	3	3	3	1	1
3	3	3	3	3	3	3	3	1	1
3	3	3	3	1	1	4	4	1	1
3	3	1	1	3	3	3	3	1	1
3	3	1	1	3	3	3	3	4	4
3	3	1	1	3	3	3	3	1	1
3	3	3	3	3	3	3	3	1	1
3	3	1	1	3	3	3	3	1	1
3	3	3	3	3	3	3	3	1	1
3	3	3	3	1	1	4	4	1	1
3	3	3	3	3	3	3	3	1	1
2	2	1	1	3	3	3	3	1	1
3	3	1	1	3	3	3	3	1	1
3	3	3	3	3	3	3	3	1	1

For Review Only

3	3	1	1	3	3	3	3	4	4
3	3	3	3	3	3	3	3	1	1

For Review Only

SNP1745	SNP1745	SNP1746	SNP1746	SNP1747	SNP1747	SNP1748	SNP1748	SNP1749	SNP1749
3	3	3	3	3	3	3	3	2	2
2	2	4	4	2	2	4	4	2	2
2	2	3	3	3	3	4	4	2	2
2	2	3	3	3	3	4	4	2	2
3	3	3	3	3	3	3	3	2	2
2	2	3	3	3	3	4	4	2	2
3	3	3	3	3	3	3	3	3	3
2	2	3	3	2	2	4	4	2	2
3	3	3	3	3	3	3	3	3	3
2	2	3	3	3	3	3	3	2	2
2	2	3	3	3	3	4	4	2	2
2	2	4	4	3	3	4	4	2	2
2	2	3	3	2	2	4	4	2	2
3	3	3	3	3	3	3	3	2	2
2	2	3	3	3	3	3	3	2	2
2	2	3	3	3	3	4	4	2	2
3	3	3	3	3	3	3	3	3	3
2	2	3	3	2	2	4	4	2	2
2	2	4	4	3	3	4	4	2	2
2	2	3	3	2	2	4	4	2	2
3	3	3	3	3	3	3	3	3	3
2	2	3	3	3	3	3	3	3	3
2	2	3	3	3	3	4	4	2	2
2	2	3	3	2	2	4	4	2	2
2	2	3	3	2	2	4	4	2	2
2	2	3	3	3	3	4	4	2	2
2	2	3	3	2	2	4	4	2	2
2	2	3	3	2	2	4	4	2	2
2	2	3	3	2	2	4	4	3	3
2	2	3	3	2	2	4	4	2	2
2	2	3	3	2	2	4	4	2	2
2	2	3	3	3	3	4	4	2	2
2	2	3	3	3	3	4	4	2	2
2	2	3	3	2	2	4	4	2	2
2	2	3	3	3	3	4	4	2	2
2	2	3	3	2	2	4	4	2	2
2	2	3	3	2	2	4	4	2	2
2	2	3	3	3	3	4	4	2	2
2	2	3	3	2	2	4	4	2	2
2	2	3	3	3	3	4	4	2	2
2	2	3	3	3	3	3	3	2	2
2	2	3	3	2	2	4	4	3	3
2	2	3	3	3	3	4	4	2	2
3	3	3	3	3	3	3	3	3	3
2	2	3	3	3	3	3	3	2	2
2	2	3	3	2	2	4	4	2	2
2	2	3	3	2	2	4	4	2	2
2	2	3	3	2	2	4	4	2	2
3	3	3	3	3	3	3	3	3	3
2	2	3	3	2	2	4	4	2	2
3	3	3	3	3	3	3	3	2	2
2	2	3	3	3	3	4	4	2	2

For Review Only

3	3	3	3	3	3	3	3	3	3
2	2	3	3	3	3	4	4	2	2
2	2	3	3	3	3	4	4	2	2
3	3	3	3	3	3	3	3	3	3
2	2	4	4	3	3	4	4	2	2
2	2	3	3	2	2	4	4	2	2
2	2	3	3	3	3	4	4	2	2
2	2	3	3	2	2	4	4	2	2
2	2	3	3	3	3	4	4	2	2
2	2	4	4	2	2	4	4	2	2
2	2	4	4	2	2	4	4	2	2
2	2	3	3	2	2	4	4	2	2
3	3	3	3	3	3	3	3	3	3
2	2	3	3	2	2	4	4	3	3
2	2	3	3	3	3	4	4	2	2
2	2	3	3	2	2	4	4	2	2
2	2	3	3	2	2	4	4	2	2
2	2	3	3	2	2	4	4	2	2
3	3	3	3	3	3	3	3	3	3
3	3	3	3	3	3	3	3	3	3
2	2	3	3	2	2	4	4	2	2
2	2	3	3	2	2	4	4	2	2
2	2	3	3	2	2	4	4	3	3
2	2	3	3	3	3	3	3	2	2
2	2	3	3	3	3	3	3	2	2
2	2	4	4	3	3	4	4	2	2
2	2	3	3	3	3	4	4	3	3
2	2	3	3	3	3	3	3	2	2
2	2	3	3	2	2	4	4	2	2
2	2	3	3	3	3	4	4	3	3
2	2	3	3	2	2	4	4	3	3
2	2	3	3	3	3	4	4	2	2
2	2	3	3	2	2	4	4	2	2
3	3	3	3	3	3	3	3	3	3
2	2	3	3	2	2	4	4	2	2
2	2	3	3	3	3	3	3	2	2
2	2	3	3	2	2	4	4	3	3
2	2	3	3	2	2	4	4	3	3
2	2	3	3	3	3	4	4	2	2
2	2	3	3	3	3	3	3	2	2
3	3	3	3	3	3	3	3	3	3
2	2	3	3	3	3	4	4	2	2
3	3	4	4	3	3	3	3	2	2
2	2	4	4	2	2	4	4	2	2
2	2	3	3	2	2	4	4	3	3
2	2	4	4	3	3	4	4	2	2
2	2	3	3	2	2	4	4	2	2
2	2	3	3	2	2	4	4	2	2
2	2	3	3	2	2	4	4	2	2
2	2	3	3	3	3	4	4	2	2
2	2	3	3	3	3	4	4	2	2
2	2	3	3	2	2	4	4	2	2
2	2	3	3	2	2	4	4	2	2
2	2	3	3	2	2	4	4	2	2
3	3	3	3	3	3	3	3	2	2

For Review Only

2	2	3	3	3	3	4	4	2	2
2	2	4	4	2	2	4	4	2	2
2	2	3	3	2	2	4	4	2	2
2	2	3	3	2	2	4	4	2	2
2	2	4	4	3	3	4	4	2	2
2	2	3	3	3	3	4	4	2	2
2	2	4	4	3	3	4	4	2	2
2	2	3	3	3	3	4	4	2	2
2	2	3	3	3	3	3	3	2	2
2	2	3	3	2	2	4	4	2	2
3	3	3	3	3	3	3	3	2	2
2	2	3	3	2	2	4	4	2	2
3	3	4	4	3	3	3	3	2	2
2	2	4	4	3	3	4	4	2	2
2	2	3	3	2	2	4	4	2	2
2	2	3	3	2	2	4	4	2	2
2	2	4	4	2	2	4	4	2	2
2	2	3	3	2	2	4	4	2	2
2	2	3	3	2	2	4	4	2	2
3	3	4	4	3	3	3	3	2	2
2	2	3	3	2	2	4	4	2	2
2	2	4	4	3	3	4	4	2	2
2	2	3	3	3	3	4	4	2	2
2	2	3	3	2	2	4	4	2	2
2	2	3	3	2	2	4	4	2	2
2	2	3	3	2	2	4	4	2	2
2	2	3	3	2	2	4	4	2	2
2	2	3	3	2	2	4	4	2	2
3	3	3	3	3	3	3	3	3	3
2	2	4	4	3	3	4	4	2	2
2	2	3	3	3	3	3	3	2	2
2	2	3	3	3	3	4	4	2	2
3	3	3	3	3	3	3	3	3	3
2	2	3	3	2	2	4	4	2	2
2	2	3	3	2	2	4	4	2	2
2	2	3	3	2	2	4	4	2	2
2	2	3	3	2	2	4	4	2	2
2	2	4	4	3	3	4	4	2	2
2	2	3	3	2	2	4	4	2	2
2	2	3	3	2	2	4	4	2	2
2	2	3	3	2	2	4	4	2	2
2	2	3	3	2	2	4	4	2	2
2	2	3	3	2	2	4	4	2	2
2	2	3	3	2	2	4	4	2	2
2	2	3	3	2	2	4	4	2	2
2	2	3	3	3	3	4	4	2	2
2	2	3	3	2	2	4	4	2	2
2	2	3	3	3	3	4	4	2	2
2	2	3	3	2	2	4	4	2	2
2	2	3	3	3	3	4	4	2	2
2	2	3	3	2	2	4	4	2	2
2	2	3	3	2	2	4	4	2	2
2	2	3	3	2	2	4	4	2	2
2	2	3	3	2	2	4	4	2	2
2	2	3	3	2	2	3	3	2	2

For Review Only

2	2	3	3	2	2	4	4	3	3
3	3	3	3	3	3	3	3	2	2
2	2	3	3	2	2	4	4	2	2
2	2	3	3	2	2	4	4	2	2
2	2	4	4	3	3	4	4	2	2
3	3	3	3	3	3	3	3	2	2
2	2	3	3	2	2	4	4	2	2
2	2	3	3	2	2	4	4	2	2
2	2	3	3	3	3	4	4	2	2
2	2	3	3	3	3	4	4	3	3
2	2	3	3	2	2	4	4	2	2
2	2	3	3	2	2	4	4	2	2
2	2	3	3	2	2	4	4	2	2
2	2	3	3	2	2	4	4	2	2
2	2	3	3	2	2	4	4	2	2
2	2	3	3	3	3	4	4	2	2
2	2	3	3	2	2	4	4	2	2
2	2	4	4	3	3	4	4	2	2
2	2	3	3	2	2	4	4	2	2
2	2	4	4	3	3	4	4	2	2
2	2	3	3	2	2	4	4	2	2
2	2	3	3	2	2	4	4	2	2
2	2	3	3	2	2	4	4	2	2
2	2	3	3	2	2	4	4	2	2
2	2	3	3	2	2	4	4	3	3
2	2	3	3	2	2	4	4	2	2
2	2	3	3	3	3	4	4	2	2
2	2	3	3	2	2	4	4	2	2
3	3	3	3	3	3	3	3	2	2
3	3	3	3	3	3	3	3	3	3
2	2	3	3	3	3	4	4	2	2
2	2	3	3	2	2	4	4	3	3
2	2	3	3	2	2	4	4	2	2
2	2	3	3	2	2	4	4	2	2
2	2	3	3	2	2	4	4	3	3
2	2	3	3	2	2	4	4	2	2
2	2	3	3	2	2	4	4	2	2
2	2	3	3	2	2	4	4	2	2
2	2	3	3	3	3	4	4	2	2
2	2	4	4	3	3	4	4	2	2
2	2	3	3	3	3	4	4	2	2
2	2	3	3	2	2	4	4	2	2
2	2	3	3	2	2	4	4	2	2
2	2	3	3	3	3	4	4	2	2
2	2	3	3	3	3	4	4	2	2
2	2	4	4	3	3	3	3	2	2
2	2	3	3	3	3	4	4	2	2
2	2	3	3	2	2	4	4	2	2
2	2	3	3	2	2	4	4	2	2
2	2	4	4	3	3	4	4	2	2
3	3	3	3	3	3	3	3	3	3
2	2	3	3	2	2	4	4	2	2
2	2	3	3	3	3	3	3	2	2
2	2	4	4	2	2	4	4	2	2
2	2	3	3	2	2	4	4	2	2

For Review Only

2	2	3	3	2	2	4	4	2	2
2	2	3	3	2	2	4	4	2	2
3	3	3	3	3	3	3	3	3	3
2	2	4	4	2	2	4	4	2	2
2	2	3	3	2	2	4	4	2	2
2	2	3	3	3	3	3	3	2	2
2	2	3	3	3	3	4	4	2	2
2	2	3	3	3	3	3	3	2	2
2	2	3	3	2	2	4	4	2	2
2	2	3	3	3	3	3	3	2	2
2	2	3	3	2	2	4	4	2	2
2	2	4	4	2	2	4	4	2	2
2	2	3	3	3	3	4	4	2	2
2	2	3	3	3	3	4	4	2	2
2	2	3	3	3	3	4	4	2	2
2	2	3	3	3	3	4	4	2	2
2	2	3	3	3	3	3	3	2	2
2	2	3	3	2	2	4	4	2	2
2	2	3	3	2	2	4	4	2	2
2	2	3	3	3	3	4	4	2	2
2	2	3	3	2	2	4	4	2	2
2	2	3	3	2	2	4	4	2	2
2	2	3	3	2	2	4	4	3	3
3	3	3	3	3	3	3	3	2	2
2	2	3	3	2	2	4	4	2	2
2	2	4	4	3	3	4	4	2	2
2	2	3	3	2	2	4	4	2	2
2	2	4	4	3	3	4	4	2	2
2	2	3	3	2	2	4	4	2	2
3	3	3	3	3	3	3	3	3	3
2	2	3	3	2	2	4	4	2	2
2	2	3	3	2	2	4	4	2	2
2	2	3	3	2	2	4	4	2	2
2	2	3	3	2	2	4	4	2	2
2	2	3	3	3	3	4	4	2	2
2	2	3	3	2	2	4	4	2	2
2	2	3	3	2	2	4	4	3	3
2	2	3	3	2	2	4	4	2	2
2	2	3	3	2	2	4	4	2	2
2	2	3	3	2	2	4	4	2	2
2	2	4	4	2	2	4	4	2	2
2	2	3	3	2	2	4	4	2	2
2	2	3	3	2	2	4	4	2	2
3	3	3	3	3	3	3	3	3	3
2	2	3	3	2	2	4	4	2	2
2	2	3	3	3	3	4	4	2	2
2	2	3	3	3	3	4	4	2	2
3	3	3	3	3	3	3	3	2	2
2	2	3	3	3	3	3	3	2	2
2	2	3	3	2	2	4	4	2	2
3	3	3	3	3	3	3	3	3	3
2	2	3	3	2	2	4	4	2	2
2	2	3	3	3	3	4	4	2	2
2	2	3	3	3	3	4	4	2	2
2	2	3	3	2	2	4	4	2	2

For Review Only

2	2	3	3	3	3	4	4	2	2
2	2	3	3	3	3	4	4	2	2

For Review Only

SNP175	SNP175	SNP1750	SNP1750	SNP1751	SNP1751	SNP1752	SNP1752	SNP1753	SNP1753
3	3	4	4	2	2	2	2	4	4
3	3	4	4	2	2	2	2	3	3
3	3	4	4	2	2	2	2	3	3
3	3	4	4	2	2	2	2	3	3
2	2	4	4	2	2	2	2	4	4
3	3	4	4	4	4	2	2	3	3
3	3	4	4	4	4	2	2	4	4
2	2	4	4	2	2	2	2	4	4
3	3	2	2	2	2	2	2	4	4
2	2	2	2	2	2	2	2	4	4
3	3	2	2	2	2	2	2	4	4
3	3	4	4	2	2	2	2	3	3
3	3	4	4	2	2	2	2	4	4
3	3	4	4	2	2	2	2	4	4
3	3	4	4	2	2	2	2	3	3
3	3	4	4	2	2	2	2	4	4
3	3	4	4	4	4	2	2	4	4
3	3	2	2	4	4	2	2	4	4
3	3	2	2	2	2	2	2	3	3
2	2	2	2	2	2	2	2	4	4
3	3	4	4	2	2	2	2	4	4
2	2	4	4	4	4	2	2	4	4
3	3	4	4	2	2	2	2	4	4
3	3	4	4	2	2	2	2	4	4
3	3	4	4	2	2	2	2	3	3
3	3	4	4	2	2	2	2	3	3
3	3	2	2	2	2	2	2	3	3
3	3	4	4	2	2	2	2	3	3
3	3	2	2	2	2	2	2	4	4
3	3	4	4	2	2	2	2	4	4
3	3	4	4	2	2	2	2	3	3
3	3	4	4	4	4	2	2	4	4
3	3	4	4	4	4	2	2	4	4
3	3	4	4	2	2	2	2	4	4
3	3	2	2	2	2	1	1	4	4
3	3	2	2	2	2	1	1	4	4
3	3	4	4	2	2	2	2	4	4
3	3	4	4	2	2	2	2	4	4
3	3	4	4	2	2	2	2	4	4
3	3	4	4	2	2	2	2	4	4
2	2	4	4	2	2	2	2	4	4
3	3	2	2	2	2	2	2	4	4
2	2	2	2	4	4	2	2	4	4
3	3	4	4	2	2	2	2	4	4
2	2	2	2	2	2	2	2	4	4
3	3	4	4	2	2	2	2	3	3
3	3	2	2	2	2	1	1	3	3
3	3	4	4	2	2	2	2	3	3
3	3	4	4	4	4	2	2	4	4
3	3	4	4	2	2	2	2	3	3
3	3	4	4	2	2	2	2	3	3
3	3	4	4	2	2	2	2	3	3

For Review Only

3	3	4	4	2	2	2	2	4	4
3	3	4	4	4	4	2	2	4	4
3	3	2	2	2	2	2	2	3	3
3	3	4	4	4	4	2	2	4	4
3	3	2	2	2	2	2	2	3	3
3	3	2	2	2	2	1	1	3	3
3	3	4	4	2	2	2	2	3	3
2	2	2	2	2	2	2	2	4	4
3	3	4	4	2	2	2	2	4	4
3	3	4	4	2	2	2	2	3	3
3	3	4	4	2	2	2	2	3	3
3	3	2	2	2	2	1	1	3	3
3	3	4	4	2	2	2	2	4	4
3	3	2	2	2	2	2	2	4	4
3	3	4	4	2	2	2	2	3	3
3	3	4	4	2	2	2	2	4	4
3	3	4	4	2	2	2	2	3	3
3	3	4	4	2	2	2	2	4	4
2	2	4	4	4	4	2	2	3	3
3	3	4	4	4	4	2	2	4	4
3	3	2	2	2	2	1	1	3	3
3	3	4	4	2	2	2	2	4	4
3	3	2	2	4	4	2	2	4	4
3	3	4	4	4	4	2	2	4	4
2	2	2	2	2	2	2	2	4	4
3	3	2	2	4	4	2	2	4	4
2	2	4	4	4	4	2	2	4	4
3	3	4	4	2	2	2	2	4	4
3	3	4	4	2	2	2	2	3	3
3	3	4	4	2	2	2	2	4	4
3	3	4	4	4	4	2	2	4	4
3	3	2	2	2	2	1	1	4	4
3	3	4	4	2	2	2	2	4	4
3	3	4	4	2	2	2	2	4	4
3	3	4	4	2	2	2	2	4	4
2	2	2	2	2	2	2	2	4	4
3	3	2	2	2	2	2	2	4	4
3	3	4	4	2	2	2	2	4	4
3	3	4	4	2	2	2	2	4	4
3	3	4	4	2	2	2	2	4	4
3	3	4	4	4	4	2	2	4	4
3	3	2	2	2	2	2	2	4	4
2	2	4	4	2	2	2	2	3	3
3	3	4	4	2	2	2	2	3	3
3	3	2	2	2	2	2	2	4	4
3	3	4	4	4	4	2	2	3	3
3	3	4	4	2	2	2	2	4	4
3	3	4	4	2	2	2	2	3	3
3	3	2	2	2	2	1	1	3	3
3	3	4	4	2	2	2	2	4	4
3	3	4	4	2	2	1	1	3	3
3	3	2	2	2	2	2	2	4	4
3	3	4	4	2	2	2	2	4	4
3	3	4	4	2	2	2	2	4	4
3	3	4	4	2	2	2	2	4	4

For Review Only

3	3	4	4	2	2	2	2	3	3
3	3	4	4	2	2	2	2	4	4
3	3	4	4	2	2	1	1	3	3
3	3	4	4	4	4	2	2	4	4
3	3	4	4	2	2	2	2	3	3
3	3	4	4	2	2	2	2	3	3
3	3	4	4	2	2	2	2	3	3
3	3	4	4	2	2	2	2	3	3
3	3	2	2	2	2	2	2	3	3
3	3	2	2	2	2	1	1	4	4
3	3	4	4	2	2	2	2	4	4
3	3	4	4	2	2	2	2	3	3
2	2	4	4	2	2	2	2	3	3
3	3	4	4	2	2	2	2	3	3
3	3	4	4	2	2	2	2	3	3
3	3	4	4	2	2	2	2	3	3
3	3	4	4	2	2	2	2	4	4
3	3	4	4	2	2	2	2	3	3
3	3	4	4	2	2	2	2	4	4
2	2	4	4	2	2	2	2	4	4
3	3	4	4	2	2	2	2	3	3
3	3	4	4	2	2	2	2	3	3
3	3	2	2	2	2	2	2	4	4
3	3	2	2	2	2	1	1	4	4
3	3	4	4	2	2	2	2	3	3
3	3	4	4	2	2	2	2	4	4
3	3	4	4	2	2	2	2	4	4
3	3	4	4	2	2	2	2	3	3
3	3	4	4	2	2	2	2	3	3
3	3	2	2	2	2	2	2	4	4
3	3	4	4	2	2	2	2	4	4
3	3	4	4	2	2	2	2	3	3
3	3	4	4	4	4	2	2	4	4
3	3	4	4	2	2	2	2	3	3
3	3	4	4	2	2	2	2	4	4
3	3	2	2	2	2	1	1	4	4
3	3	4	4	2	2	2	2	4	4
3	3	4	4	2	2	2	2	3	3
3	3	4	4	2	2	2	2	3	3
3	3	4	4	2	2	2	2	4	4
3	3	4	4	2	2	2	2	3	3
3	3	4	4	2	2	2	2	4	4
3	3	4	4	2	2	2	2	4	4
3	3	4	4	2	2	2	2	4	4
3	3	4	4	2	2	2	2	4	4
3	3	4	4	2	2	2	2	4	4
3	3	4	4	2	2	2	2	3	3
3	3	4	4	2	2	2	2	3	3
3	3	4	4	2	2	2	2	4	4
3	3	4	4	2	2	2	2	3	3
3	3	2	2	2	2	1	1	3	3
3	3	4	4	2	2	2	2	4	4
3	3	4	4	2	2	2	2	3	3
3	3	2	2	2	2	1	1	3	3
3	3	4	4	2	2	2	2	4	4

For Review Only

3	3	4	4	2	2	2	2	3	3
3	3	4	4	2	2	2	2	3	3
3	3	4	4	2	2	2	2	4	4
3	3	4	4	2	2	2	2	4	4
3	3	4	4	4	4	2	2	4	4
3	3	4	4	2	2	2	2	4	4
3	3	4	4	2	2	2	2	4	4
3	3	4	4	2	2	2	2	4	4
3	3	2	2	2	2	2	2	3	3
3	3	4	4	4	4	2	2	4	4
3	3	4	4	2	2	2	2	3	3
3	3	2	2	2	2	1	1	4	4
3	3	4	4	2	2	2	2	3	3
3	3	4	4	2	2	2	2	4	4
3	3	4	4	2	2	2	2	4	4
3	3	2	2	2	2	2	2	4	4
3	3	4	4	2	2	2	2	3	3
3	3	4	4	4	4	2	2	3	3
2	2	4	4	2	2	2	2	3	3
3	3	4	4	2	2	2	2	3	3
3	3	4	4	2	2	2	2	3	3
3	3	4	4	2	2	2	2	4	4
3	3	4	4	2	2	2	2	4	4
3	3	2	2	2	2	1	1	3	3
3	3	2	2	2	2	2	2	4	4
3	3	4	4	2	2	2	2	4	4
3	3	4	4	2	2	2	2	3	3
3	3	4	4	2	2	2	2	3	3
3	3	4	4	2	2	2	2	3	3
3	3	4	4	4	4	2	2	4	4
3	3	4	4	4	4	2	2	3	3
3	3	4	4	2	2	2	2	4	4
3	3	4	4	2	2	2	2	4	4
3	3	4	4	2	2	2	2	4	4
3	3	4	4	4	4	2	2	4	4
3	3	2	2	2	2	1	1	3	3
3	3	4	4	2	2	2	2	4	4
3	3	4	4	2	2	2	2	4	4
3	3	4	4	2	2	2	2	3	3
3	3	4	4	2	2	2	2	3	3
3	3	4	4	2	2	2	2	3	3
3	3	4	4	2	2	2	2	3	3
3	3	4	4	2	2	2	2	4	4
3	3	4	4	2	2	2	2	4	4
3	3	4	4	2	2	2	2	4	4
3	3	4	4	2	2	2	2	3	3
3	3	2	2	2	2	2	2	3	3
3	3	2	2	2	2	2	2	4	4
3	3	4	4	2	2	2	2	4	4
3	3	4	4	2	2	2	2	3	3
3	3	4	4	2	2	2	2	3	3
3	3	2	2	2	2	2	2	4	4
3	3	2	2	2	2	2	2	4	4
3	3	4	4	2	2	2	2	3	3
3	3	2	2	2	2	1	1	4	4

For Review Only

3	3	4	4	2	2	2	2	3	3
2	2	2	2	4	4	2	2	4	4
3	3	4	4	2	2	2	2	4	4
3	3	4	4	2	2	2	2	4	4
3	3	2	2	2	2	2	2	4	4
3	3	4	4	2	2	2	2	3	3
2	2	4	4	2	2	2	2	3	3
2	2	2	2	2	2	2	2	4	4
3	3	4	4	2	2	1	1	4	4
3	3	4	4	2	2	2	2	4	4
3	3	4	4	2	2	2	2	3	3
3	3	4	4	4	4	2	2	4	4
3	3	4	4	2	2	2	2	4	4
3	3	4	4	2	2	2	2	3	3
3	3	4	4	2	2	2	2	3	3
3	3	4	4	2	2	2	2	4	4
3	3	2	2	2	2	2	2	4	4
3	3	4	4	2	2	2	2	3	3
3	3	4	4	2	2	2	2	3	3
3	3	4	4	2	2	2	2	4	4
3	3	4	4	4	4	2	2	4	4
3	3	4	4	2	2	2	2	3	3
3	3	4	4	2	2	2	2	3	3
3	3	4	4	2	2	2	2	4	4
2	2	2	2	2	2	2	2	4	4
3	3	2	2	2	2	2	2	4	4
3	3	4	4	2	2	2	2	4	4
3	3	4	4	2	2	2	2	3	3
3	3	4	4	2	2	2	2	4	4
3	3	4	4	2	2	2	2	3	3
3	3	4	4	2	2	2	2	4	4
3	3	2	2	2	2	2	2	3	3
3	3	4	4	2	2	2	2	4	4
3	3	4	4	2	2	2	2	3	3
3	3	4	4	2	2	2	2	4	4
3	3	2	2	2	2	2	2	4	4
3	3	4	4	2	2	2	2	3	3
3	3	2	2	2	2	1	1	3	3
3	3	2	2	2	2	1	1	4	4
3	3	4	4	2	2	2	2	4	4
3	3	4	4	4	4	2	2	3	3
3	3	4	4	2	2	2	2	4	4
3	3	4	4	2	2	2	2	4	4
3	3	4	4	4	4	2	2	4	4
3	3	2	2	2	2	1	1	3	3
3	3	4	4	2	2	2	2	4	4
3	3	4	4	2	2	2	2	4	4
2	2	4	4	2	2	2	2	3	3
3	3	4	4	2	2	2	2	3	3
3	3	4	4	2	2	2	2	4	4
3	3	4	4	4	4	2	2	3	3
3	3	4	4	2	2	2	2	4	4
3	3	4	4	2	2	2	2	3	3
3	3	4	4	2	2	2	2	3	3
3	3	4	4	2	2	2	2	4	4

For Review Only

3	3	4	4	2	2	2	2	3	3
3	3	4	4	2	2	2	2	3	3

For Review Only

SNP1754	SNP1754	SNP1755	SNP1755	SNP1756	SNP1756	SNP1757	SNP1757	SNP1758	SNP1758
1	1	1	1	1	1	4	4	2	2
1	1	1	1	1	1	1	1	2	2
1	1	1	1	1	1	4	4	2	2
1	1	1	1	1	1	4	4	2	2
1	1	1	1	1	1	4	4	2	2
1	1	1	1	2	2	4	4	2	2
1	1	1	1	2	2	4	4	2	2
1	1	1	1	1	1	4	4	2	2
4	4	1	1	1	1	4	4	2	2
4	4	1	1	1	1	4	4	2	2
4	4	1	1	1	1	4	4	4	4
1	1	1	1	1	1	4	4	2	2
1	1	1	1	1	1	4	4	2	2
1	1	1	1	1	1	4	4	2	2
1	1	1	1	1	1	4	4	2	2
1	1	1	1	1	1	4	4	2	2
1	1	1	1	2	2	4	4	2	2
4	4	1	1	2	2	1	1	2	2
1	1	4	4	1	1	4	4	2	2
4	4	1	1	1	1	4	4	2	2
1	1	1	1	1	1	4	4	2	2
1	1	1	1	2	2	4	4	2	2
1	1	1	1	1	1	4	4	2	2
1	1	1	1	1	1	4	4	2	2
1	1	1	1	1	1	4	4	2	2
1	1	1	1	1	1	1	1	2	2
4	4	4	4	1	1	1	1	2	2
1	1	1	1	1	1	4	4	2	2
4	4	1	1	1	1	4	4	2	2
1	1	1	1	1	1	4	4	2	2
1	1	1	1	1	1	4	4	2	2
1	1	1	1	2	2	4	4	2	2
1	1	1	1	2	2	4	4	2	2
1	1	1	1	1	1	4	4	2	2
4	4	1	1	1	1	4	4	2	2
4	4	4	4	1	1	4	4	2	2
1	1	1	1	1	1	4	4	2	2
1	1	1	1	1	1	4	4	2	2
1	1	1	1	1	1	4	4	2	2
1	1	1	1	1	1	4	4	2	2
1	1	1	1	1	1	4	4	2	2
1	1	1	1	1	1	4	4	2	2
4	4	1	1	2	2	1	1	2	2
1	1	1	1	1	1	4	4	2	2
4	4	1	1	1	1	1	1	2	2
1	1	1	1	1	1	4	4	2	2
4	4	4	4	1	1	1	1	2	2
4	4	1	1	1	1	4	4	2	2
1	1	1	1	2	2	4	4	2	2
1	1	1	1	1	1	4	4	4	4
1	1	1	1	1	1	1	1	2	2
1	1	1	1	1	1	1	1	2	2

For Review Only

1	1	1	1	1	1	4	4	2	2
1	1	1	1	2	2	4	4	2	2
1	1	4	4	1	1	4	4	2	2
1	1	1	1	2	2	4	4	2	2
1	1	4	4	1	1	4	4	2	2
1	1	4	4	1	1	1	1	2	2
1	1	1	1	1	1	1	1	2	2
4	4	1	1	1	1	4	4	2	2
1	1	1	1	1	1	4	4	2	2
1	1	1	1	1	1	4	4	2	2
1	1	1	1	1	1	1	1	2	2
1	1	4	4	1	1	4	4	2	2
1	1	1	1	1	1	4	4	2	2
4	4	1	1	1	1	4	4	2	2
1	1	1	1	1	1	4	4	2	2
1	1	1	1	1	1	1	1	2	2
1	1	1	1	1	1	4	4	2	2
1	1	1	1	1	1	4	4	2	2
1	1	1	1	2	2	4	4	2	2
1	1	1	1	2	2	4	4	2	2
1	1	4	4	1	1	4	4	2	2
1	1	1	1	1	1	4	4	2	2
1	1	1	1	2	2	4	4	2	2
1	1	1	1	2	2	4	4	2	2
4	4	1	1	1	1	1	1	2	2
4	4	1	1	2	2	4	4	2	2
1	1	1	1	2	2	4	4	2	2
1	1	1	1	1	1	4	4	2	2
1	1	1	1	1	1	1	1	2	2
1	1	1	1	1	1	4	4	4	4
1	1	1	1	2	2	4	4	2	2
4	4	4	4	1	1	4	4	2	2
1	1	1	1	1	1	4	4	2	2
1	1	1	1	1	1	4	4	2	2
1	1	1	1	1	1	4	4	2	2
4	4	1	1	1	1	1	1	2	2
4	4	1	1	1	1	4	4	2	2
1	1	1	1	1	1	4	4	2	2
1	1	1	1	1	1	4	4	4	4
1	1	1	1	1	1	4	4	2	2
1	1	1	1	2	2	4	4	2	2
1	1	4	4	1	1	4	4	4	4
1	1	1	1	1	1	4	4	4	4
1	1	1	1	1	1	1	1	2	2
4	4	1	1	1	1	4	4	4	4
1	1	1	1	2	2	1	1	2	2
1	1	1	1	1	1	4	4	2	2
1	1	1	1	1	1	4	4	2	2
1	1	4	4	1	1	4	4	2	2
1	1	1	1	1	1	4	4	2	2
1	1	1	1	1	1	4	4	2	2
4	4	1	1	1	1	4	4	2	2
1	1	1	1	1	1	4	4	2	2
1	1	1	1	1	1	4	4	2	2
1	1	1	1	1	1	4	4	2	2

For Review Only

1	1	1	1	1	1	1	1	2	2
1	1	1	1	1	1	4	4	2	2
1	1	1	1	1	1	1	1	2	2
1	1	1	1	2	2	4	4	2	2
1	1	4	4	1	1	1	1	2	2
1	1	1	1	1	1	4	4	2	2
1	1	1	1	1	1	4	4	2	2
1	1	1	1	1	1	4	4	2	2
4	4	1	1	1	1	4	4	2	2
1	1	1	1	1	1	4	4	2	2
1	1	1	1	1	1	4	4	2	2
1	1	1	1	1	1	4	4	2	2
1	1	1	1	1	1	4	4	2	2
1	1	1	1	2	2	4	4	2	2
1	1	1	1	1	1	4	4	2	2
1	1	1	1	1	1	4	4	2	2
1	1	1	1	1	1	4	4	2	2
1	1	1	1	1	1	4	4	2	2
1	1	1	1	1	1	4	4	2	2
1	1	1	1	1	1	4	4	2	2
1	1	1	1	1	1	4	4	2	2
1	1	1	1	1	1	4	4	4	4
1	1	4	4	1	1	4	4	2	2
1	1	4	4	1	1	4	4	2	2
1	1	1	1	1	1	1	1	2	2
1	1	1	1	1	1	4	4	2	2
1	1	1	1	1	1	4	4	2	2
1	1	1	1	1	1	4	4	2	2
1	1	1	1	1	1	4	4	2	2
4	4	1	1	1	1	4	4	2	2
1	1	1	1	1	1	4	4	2	2
1	1	1	1	1	1	4	4	2	2
1	1	1	1	2	2	4	4	2	2
1	1	1	1	1	1	4	4	2	2
1	1	1	1	1	1	4	4	2	2
4	4	4	4	1	1	4	4	2	2
1	1	1	1	1	1	4	4	2	2
1	1	1	1	1	1	4	4	2	2
4	4	1	1	1	1	4	4	2	2
1	1	1	1	1	1	4	4	4	4
1	1	1	1	1	1	4	4	2	2
1	1	1	1	1	1	4	4	2	2
1	1	1	1	1	1	4	4	2	2
1	1	1	1	1	1	4	4	2	2
1	1	1	1	1	1	4	4	2	2
1	1	1	1	1	1	4	4	2	2
1	1	1	1	1	1	1	1	2	2
1	1	1	1	1	1	1	1	2	2
1	1	1	1	1	1	4	4	2	2
1	1	1	1	1	1	1	1	2	2
1	1	4	4	1	1	1	1	2	2
1	1	1	1	1	1	4	4	2	2
1	1	1	1	1	1	4	4	2	2
4	4	4	4	1	1	4	4	2	2
1	1	1	1	1	1	4	4	2	2

For Review Only

1	1	1	1	1	1	4	4	2	2
1	1	1	1	1	1	4	4	2	2
1	1	1	1	1	1	4	4	2	2
1	1	1	1	1	1	4	4	2	2
1	1	1	1	1	1	4	4	2	2
1	1	1	1	1	1	4	4	2	2
1	1	1	1	1	1	4	4	2	2
1	1	1	1	1	1	4	4	2	2
4	4	4	4	1	1	4	4	2	2
1	1	1	1	2	2	4	4	4	4
1	1	1	1	1	1	4	4	2	2
1	1	4	4	1	1	4	4	2	2
1	1	1	1	1	1	4	4	4	4
1	1	1	1	1	1	4	4	2	2
1	1	1	1	1	1	4	4	2	2
4	4	1	1	1	1	4	4	4	4
1	1	1	1	1	1	4	4	2	2
1	1	4	4	2	2	1	1	2	2
1	1	1	1	1	1	1	1	2	2
1	1	1	1	1	1	4	4	2	2
1	1	1	1	1	1	4	4	2	2
1	1	1	1	1	1	4	4	2	2
1	1	1	1	1	1	4	4	2	2
1	1	4	4	1	1	4	4	2	2
1	1	1	1	1	1	4	4	2	2
1	1	1	1	1	1	4	4	2	2
1	1	1	1	1	1	4	4	2	2
1	1	1	1	1	1	4	4	2	2
1	1	1	1	1	1	4	4	2	2
1	1	1	1	2	2	4	4	2	2
1	1	1	1	2	2	4	4	2	2
1	1	1	1	1	1	4	4	4	4
1	1	1	1	1	1	4	4	2	2
1	1	1	1	1	1	4	4	2	2
1	1	1	1	2	2	4	4	2	2
1	1	4	4	1	1	4	4	2	2
1	1	1	1	1	1	4	4	2	2
1	1	1	1	1	1	4	4	2	2
1	1	1	1	1	1	4	4	2	2
1	1	1	1	1	1	4	4	2	2
1	1	1	1	1	1	4	4	2	2
1	1	1	1	1	1	1	1	2	2
1	1	1	1	1	1	4	4	2	2
1	1	1	1	1	1	4	4	2	2
1	1	1	1	1	1	4	4	2	2
1	1	1	1	1	1	4	4	4	4
4	4	1	1	1	1	4	4	4	4
4	4	1	1	1	1	4	4	2	2
1	1	1	1	1	1	4	4	2	2
1	1	1	1	1	1	4	4	2	2
1	1	1	1	1	1	4	4	2	2
4	4	1	1	1	1	4	4	2	2
4	4	1	1	1	1	4	4	2	2
1	1	1	1	1	1	1	1	2	2
4	4	4	4	1	1	4	4	2	2

For Review Only

1	1	1	1	1	1	4	4	2	2
4	4	1	1	2	2	4	4	2	2
1	1	1	1	1	1	4	4	2	2
1	1	1	1	1	1	4	4	2	2
4	4	1	1	1	1	1	1	2	2
1	1	1	1	1	1	1	1	2	2
1	1	1	1	1	1	4	4	2	2
4	4	1	1	1	1	4	4	2	2
1	1	1	1	1	1	4	4	2	2
1	1	1	1	1	1	4	4	2	2
1	1	1	1	1	1	4	4	2	2
1	1	1	1	2	2	4	4	2	2
1	1	1	1	1	1	4	4	4	4
1	1	1	1	1	1	1	1	2	2
1	1	1	1	1	1	4	4	2	2
1	1	1	1	1	1	4	4	4	4
4	4	1	1	1	1	4	4	2	2
1	1	1	1	1	1	4	4	2	2
1	1	1	1	1	1	4	4	2	2
1	1	1	1	1	1	4	4	2	2
1	1	1	1	2	2	4	4	2	2
1	1	1	1	1	1	1	1	2	2
1	1	1	1	1	1	4	4	4	4
1	1	1	1	1	1	4	4	2	2
4	4	1	1	1	1	4	4	2	2
4	4	1	1	1	1	4	4	2	2
1	1	1	1	1	1	4	4	2	2
1	1	1	1	1	1	1	1	2	2
1	1	1	1	1	1	4	4	2	2
1	1	1	1	1	1	4	4	2	2
1	1	1	1	1	1	4	4	2	2
4	4	1	1	1	1	4	4	2	2
1	1	1	1	1	1	4	4	2	2
1	1	1	1	1	1	1	1	2	2
1	1	1	1	1	1	4	4	2	2
1	1	4	4	1	1	4	4	2	2
1	1	1	1	1	1	4	4	2	2
1	1	4	4	1	1	4	4	2	2
1	1	4	4	1	1	4	4	2	2
1	1	1	1	1	1	4	4	2	2
1	1	1	1	2	2	4	4	4	4
1	1	1	1	1	1	4	4	2	2
1	1	1	1	1	1	4	4	2	2
1	1	1	1	2	2	4	4	2	2
1	1	4	4	1	1	1	1	2	2
1	1	1	1	1	1	4	4	4	4
1	1	1	1	1	1	1	1	2	2
1	1	1	1	1	1	4	4	2	2
1	1	1	1	1	1	4	4	2	2
1	1	1	1	1	1	4	4	2	2
1	1	1	1	2	2	4	4	2	2
1	1	1	1	1	1	4	4	2	2
1	1	1	1	1	1	4	4	2	2
1	1	1	1	1	1	1	1	2	2
1	1	1	1	1	1	4	4	2	2

For Review Only

1	1	1	1	1	1	4	4	4	4
1	1	1	1	1	1	4	4	2	2

For Review Only

SNP1759	SNP1759	SNP176	SNP176	SNP1760	SNP1760	SNP1761	SNP1761	SNP1762	SNP1762
3	3	2	2	2	2	4	4	3	3
3	3	2	2	3	3	4	4	3	3
1	1	2	2	3	3	4	4	3	3
3	3	3	3	3	3	4	4	3	3
3	3	2	2	2	2	4	4	3	3
3	3	2	2	2	2	2	2	3	3
3	3	2	2	2	2	2	2	2	2
3	3	2	2	2	2	4	4	2	2
3	3	2	2	2	2	2	2	3	3
3	3	2	2	3	3	4	4	2	2
3	3	2	2	3	3	4	4	2	2
1	1	2	2	3	3	4	4	3	3
3	3	2	2	3	3	2	2	3	3
3	3	2	2	2	2	4	4	2	2
3	3	2	2	3	3	4	4	3	3
3	3	2	2	2	2	4	4	3	3
3	3	2	2	2	2	2	2	3	3
3	3	2	2	3	3	2	2	2	2
3	3	2	2	2	2	4	4	3	3
3	3	2	2	3	3	4	4	2	2
3	3	2	2	2	2	4	4	2	2
3	3	2	2	2	2	2	2	3	3
3	3	2	2	2	2	4	4	2	2
3	3	2	2	2	2	4	4	2	2
3	3	2	2	2	2	4	4	3	3
3	3	2	2	3	3	4	4	3	3
3	3	3	3	3	3	4	4	3	3
3	3	2	2	3	3	4	4	3	3
3	3	2	2	2	2	4	4	2	2
3	3	2	2	3	3	4	4	2	2
3	3	2	2	3	3	4	4	3	3
1	1	3	3	3	3	2	2	2	2
1	1	2	2	3	3	2	2	3	3
3	3	2	2	2	2	4	4	2	2
3	3	2	2	3	3	4	4	3	3
3	3	2	2	2	2	4	4	2	2
1	1	2	2	2	2	4	4	2	2
1	1	2	2	2	2	4	4	2	2
3	3	2	2	2	2	4	4	2	2
3	3	2	2	2	2	4	4	2	2
3	3	2	2	2	2	4	4	2	2
3	3	2	2	2	2	4	4	2	2
3	3	2	2	3	3	2	2	2	2
3	3	2	2	2	2	4	4	2	2
3	3	2	2	3	3	4	4	2	2
1	1	2	2	3	3	4	4	3	3
3	3	2	2	3	3	4	4	3	3
3	3	2	2	2	2	4	4	3	3
3	3	2	2	2	2	2	2	2	2
3	3	2	2	3	3	4	4	3	3
3	3	2	2	3	3	4	4	3	3
3	3	2	2	3	3	4	4	3	3

For Review Only

3	3	2	2	2	2	4	4	3	3
1	1	2	2	3	3	2	2	3	3
3	3	2	2	2	2	4	4	3	3
3	3	2	2	3	3	2	2	3	3
3	3	2	2	2	2	4	4	3	3
3	3	2	2	3	3	4	4	3	3
3	3	3	3	3	3	4	4	3	3
3	3	2	2	3	3	4	4	2	2
3	3	2	2	2	2	4	4	2	2
3	3	2	2	3	3	4	4	3	3
3	3	2	2	3	3	4	4	3	3
3	3	2	2	2	2	4	4	3	3
3	3	2	2	2	2	4	4	3	3
3	3	2	2	2	2	4	4	2	2
3	3	2	2	3	3	4	4	3	3
3	3	2	2	3	3	4	4	2	2
3	3	2	2	2	2	4	4	3	3
1	1	2	2	3	3	4	4	3	3
3	3	2	2	2	2	2	2	3	3
3	3	2	2	2	2	2	2	3	3
3	3	2	2	3	3	4	4	3	3
3	3	2	2	2	2	4	4	2	2
3	3	2	2	3	3	4	4	3	3
3	3	2	2	2	2	2	2	2	2
3	3	2	2	3	3	4	4	2	2
3	3	3	3	3	3	2	2	2	2
1	1	2	2	3	3	2	2	2	2
3	3	2	2	3	3	4	4	2	2
3	3	2	2	3	3	4	4	3	3
1	1	2	2	3	3	4	4	2	2
3	3	2	2	3	3	4	4	2	2
3	3	2	2	2	2	4	4	2	2
3	3	2	2	2	2	4	4	2	2
3	3	2	2	2	2	4	4	3	3
3	3	2	2	2	2	4	4	2	2
3	3	2	2	3	3	4	4	2	2
3	3	2	2	3	3	4	4	2	2
3	3	2	2	2	2	4	4	2	2
3	3	2	2	3	3	4	4	2	2
3	3	2	2	3	3	4	4	2	2
3	3	2	2	2	2	2	2	3	3
1	1	2	2	3	3	4	4	2	2
3	3	2	2	3	3	4	4	3	3
3	3	2	2	3	3	4	4	3	3
1	1	2	2	3	3	4	4	2	2
3	3	3	3	3	3	2	2	3	3
3	3	2	2	3	3	4	4	2	2
3	3	2	2	2	2	4	4	3	3
3	3	2	2	2	2	4	4	3	3
3	3	2	2	2	2	4	4	2	2
3	3	2	2	2	2	4	4	3	3
3	3	2	2	2	2	4	4	2	2
3	3	2	2	2	2	4	4	2	2
3	3	2	2	3	3	4	4	3	3
1	1	2	2	3	3	4	4	2	2

For Review Only

3	3	2	2	3	3	4	4	3	3
3	3	2	2	3	3	4	4	2	2
3	3	2	2	3	3	4	4	3	3
3	3	2	2	3	3	2	2	2	2
3	3	3	3	3	3	4	4	3	3
3	3	2	2	3	3	4	4	3	3
3	3	2	2	2	2	4	4	3	3
3	3	3	3	3	3	4	4	3	3
3	3	2	2	3	3	4	4	3	3
3	3	2	2	3	3	4	4	2	2
3	3	2	2	2	2	4	4	2	2
1	1	2	2	3	3	4	4	3	3
3	3	2	2	3	3	4	4	3	3
3	3	2	2	3	3	4	4	3	3
3	3	2	2	3	3	4	4	3	3
3	3	2	2	3	3	4	4	3	3
1	1	2	2	3	3	4	4	2	2
3	3	2	2	3	3	4	4	3	3
3	3	2	2	2	2	4	4	2	2
1	1	2	2	3	3	4	4	3	3
3	3	2	2	3	3	4	4	3	3
1	1	2	2	3	3	4	4	3	3
3	3	2	2	2	2	4	4	3	3
3	3	2	2	2	2	4	4	2	2
3	3	2	2	3	3	4	4	3	3
3	3	2	2	2	2	4	4	2	2
3	3	2	2	2	2	4	4	3	3
3	3	2	2	2	2	4	4	3	3
3	3	3	3	3	3	4	4	3	3
3	3	2	2	2	2	4	4	2	2
3	3	2	2	3	3	4	4	2	2
3	3	2	2	3	3	4	4	3	3
3	3	2	2	2	2	2	2	3	3
3	3	2	2	3	3	4	4	3	3
3	3	2	2	2	2	4	4	2	2
3	3	2	2	3	3	4	4	2	2
3	3	2	2	2	2	4	4	2	2
1	1	2	2	3	3	4	4	3	3
3	3	2	2	3	3	4	4	2	2
3	3	2	2	3	3	4	4	2	2
3	3	2	2	2	2	4	4	2	2
3	3	2	2	3	3	4	4	2	2
3	3	2	2	3	3	4	4	2	2
3	3	2	2	3	3	4	4	2	2
3	3	2	2	2	2	4	4	3	3
3	3	2	2	2	2	4	4	3	3
3	3	2	2	3	3	4	4	3	3
3	3	2	2	3	3	4	4	3	3
3	3	2	2	3	3	4	4	3	3
3	3	2	2	3	3	4	4	3	3
3	3	2	2	3	3	4	4	3	3
3	3	2	2	2	2	4	4	2	2
3	3	2	2	3	3	4	4	3	3
3	3	2	2	2	2	4	4	3	3
3	3	2	2	3	3	4	4	3	3

For Review Only

1	1	2	2	3	3	4	4	3	3
3	3	2	2	2	2	4	4	3	3
3	3	2	2	2	2	4	4	2	2
3	3	2	2	2	2	4	4	2	2
3	3	3	3	3	3	4	4	2	2
3	3	2	2	2	2	4	4	2	2
3	3	2	2	2	2	4	4	2	2
3	3	2	2	3	3	4	4	3	3
3	3	2	2	2	2	4	4	3	3
1	1	2	2	3	3	4	4	2	2
3	3	2	2	3	3	4	4	3	3
3	3	2	2	3	3	4	4	2	2
1	1	2	2	3	3	4	4	3	3
3	3	2	2	2	2	4	4	3	3
3	3	2	2	3	3	4	4	2	2
3	3	2	2	3	3	4	4	2	2
3	3	2	2	3	3	4	4	3	3
3	3	2	2	3	3	2	2	3	3
3	3	2	2	3	3	4	4	3	3
3	3	2	2	2	2	4	4	3	3
3	3	2	2	3	3	4	4	3	3
3	3	2	2	3	3	4	4	2	2
3	3	2	2	2	2	4	4	2	2
3	3	2	2	2	2	4	4	3	3
3	3	2	2	3	3	4	4	3	3
3	3	2	2	2	2	4	4	2	2
3	3	2	2	3	3	4	4	3	3
1	1	2	2	3	3	4	4	3	3
3	3	2	2	2	2	4	4	3	3
3	3	2	2	3	3	2	2	3	3
3	3	2	2	2	2	2	2	3	3
1	1	2	2	3	3	4	4	2	2
3	3	2	2	2	2	4	4	2	2
3	3	2	2	3	3	4	4	2	2
3	3	2	2	2	2	2	2	2	2
3	3	2	2	2	2	4	4	3	3
1	1	2	2	3	3	4	4	2	2
3	3	2	2	2	2	4	4	2	2
3	3	2	2	3	3	4	4	3	3
1	1	2	2	3	3	4	4	3	3
1	1	2	2	3	3	4	4	3	3
3	3	2	2	3	3	4	4	3	3
3	3	2	2	3	3	4	4	2	2
3	3	2	2	2	2	4	4	2	2
3	3	2	2	3	3	4	4	2	2
3	3	2	2	3	3	4	4	3	3
3	3	2	2	3	3	4	4	2	2
3	3	2	2	2	2	4	4	2	2
3	3	2	2	2	2	4	4	3	3
3	3	3	3	3	3	4	4	3	3
3	3	2	2	3	3	4	4	3	3
3	3	2	2	3	3	4	4	2	2
3	3	2	2	2	2	4	4	2	2
3	3	2	2	3	3	4	4	3	3
3	3	2	2	2	2	4	4	2	2

For Review Only

3	3	2	2	2	2	4	4	3	3
3	3	2	2	2	2	2	2	2	2
1	1	2	2	3	3	4	4	3	3
3	3	2	2	2	2	4	4	3	3
3	3	2	2	3	3	4	4	2	2
3	3	2	2	3	3	4	4	3	3
3	3	2	2	2	2	4	4	3	3
3	3	2	2	3	3	4	4	2	2
3	3	2	2	3	3	4	4	2	2
3	3	2	2	3	3	4	4	2	2
3	3	2	2	3	3	4	4	3	3
3	3	2	2	2	2	2	2	2	2
3	3	2	2	3	3	4	4	2	2
3	3	2	2	3	3	4	4	3	3
1	1	2	2	3	3	4	4	3	3
3	3	2	2	3	3	4	4	2	2
3	3	2	2	2	2	4	4	2	2
1	1	2	2	3	3	2	2	3	3
3	3	2	2	3	3	4	4	3	3
1	1	2	2	3	3	4	4	2	2
3	3	2	2	3	3	2	2	2	2
3	3	2	2	3	3	4	4	3	3
1	1	2	2	3	3	4	4	3	3
3	3	2	2	3	3	4	4	2	2
3	3	2	2	3	3	4	4	2	2
3	3	2	2	2	2	4	4	2	2
3	3	2	2	2	2	4	4	2	2
3	3	3	3	3	3	4	4	3	3
3	3	2	2	3	3	4	4	3	3
3	3	2	2	3	3	4	4	3	3
3	3	2	2	2	2	4	4	2	2
3	3	2	2	3	3	4	4	3	3
3	3	2	2	2	2	4	4	2	2
3	3	2	2	3	3	4	4	3	3
3	3	2	2	2	2	4	4	2	2
3	3	2	2	3	3	4	4	2	2
3	3	2	2	2	2	4	4	3	3
3	3	2	2	2	2	4	4	3	3
3	3	2	2	2	2	4	4	2	2
3	3	2	2	2	2	4	4	2	2
1	1	2	2	3	3	2	2	3	3
3	3	2	2	2	2	4	4	2	2
3	3	2	2	3	3	4	4	2	2
3	3	2	2	2	2	2	2	3	3
3	3	2	2	3	3	4	4	3	3
3	3	2	2	3	3	4	4	2	2
3	3	2	2	3	3	4	4	2	2
3	3	2	2	3	3	4	4	3	3
3	3	2	2	3	3	4	4	3	3
3	3	2	2	2	2	4	4	2	2
3	3	2	2	2	2	2	2	3	3
3	3	2	2	2	2	4	4	2	2
1	1	2	2	3	3	4	4	3	3
3	3	2	2	3	3	4	4	3	3
3	3	2	2	2	2	4	4	2	2

For Review Only

3	3	2	2	3	3	4	4	2	2
3	3	2	2	2	2	4	4	3	3

For Review Only

SNP1763	SNP1763	SNP1764	SNP1764	SNP1765	SNP1765	SNP1766	SNP1766	SNP1767	SNP1767
3	3	1	1	1	1	1	1	4	4
3	3	1	1	3	3	3	3	4	4
3	3	1	1	3	3	3	3	3	3
3	3	1	1	3	3	1	1	3	3
3	3	1	1	3	3	1	1	4	4
3	3	1	1	3	3	3	3	4	4
3	3	1	1	1	1	1	1	4	4
3	3	1	1	1	1	3	3	3	3
4	4	1	1	1	1	1	1	4	4
3	3	1	1	1	1	3	3	4	4
3	3	1	1	1	1	1	1	4	4
3	3	1	1	3	3	3	3	3	3
3	3	1	1	1	1	3	3	4	4
3	3	1	1	1	1	1	1	4	4
3	3	1	1	1	1	1	1	3	3
3	3	1	1	1	1	3	3	3	3
3	3	1	1	3	3	1	1	4	4
4	4	1	1	1	1	1	1	4	4
3	3	1	1	3	3	3	3	4	4
3	3	3	3	3	3	1	1	4	4
4	4	1	1	3	3	1	1	4	4
3	3	1	1	1	1	1	1	4	4
4	4	1	1	1	1	1	1	4	4
3	3	1	1	1	1	1	1	4	4
3	3	1	1	3	3	3	3	4	4
3	3	1	1	3	3	3	3	3	3
3	3	1	1	3	3	3	3	4	4
3	3	1	1	1	1	1	1	3	3
4	4	1	1	1	1	3	3	4	4
3	3	3	3	3	3	3	3	4	4
3	3	1	1	3	3	3	3	4	4
3	3	1	1	1	1	3	3	3	3
3	3	1	1	3	3	1	1	4	4
3	3	1	1	1	1	3	3	4	4
3	3	1	1	1	1	1	1	4	4
4	4	1	1	1	1	1	1	4	4
3	3	1	1	1	1	3	3	4	4
4	4	1	1	1	1	3	3	4	4
3	3	1	1	1	1	3	3	4	4
4	4	1	1	1	1	3	3	4	4
3	3	1	1	1	1	3	3	4	4
4	4	1	1	1	1	1	1	4	4
3	3	1	1	1	1	3	3	4	4
3	3	1	1	1	1	1	1	4	4
4	4	1	1	1	1	3	3	4	4
3	3	1	1	1	1	3	3	3	3
3	3	1	1	1	1	1	1	4	4
3	3	1	1	3	3	3	3	4	4
3	3	3	3	3	3	1	1	4	4
3	3	1	1	1	1	1	1	3	3
3	3	1	1	3	3	1	1	4	4
3	3	1	1	3	3	1	1	3	3

For Review Only

3	3	1	1	3	3	1	1	4	4
3	3	1	1	3	3	1	1	4	4
3	3	1	1	3	3	3	3	4	4
3	3	3	3	3	3	1	1	4	4
3	3	1	1	3	3	3	3	4	4
3	3	1	1	3	3	1	1	4	4
3	3	1	1	3	3	3	3	3	3
3	3	1	1	1	1	1	1	4	4
3	3	1	1	1	1	1	1	4	4
3	3	1	1	1	1	3	3	3	3
3	3	1	1	3	3	3	3	3	3
3	3	1	1	3	3	3	3	4	4
3	3	1	1	3	3	1	1	4	4
4	4	1	1	1	1	3	3	4	4
3	3	1	1	1	1	1	1	3	3
3	3	1	1	1	1	3	3	4	4
3	3	1	1	3	3	1	1	4	4
3	3	1	1	1	1	1	1	3	3
3	3	1	1	3	3	1	1	4	4
3	3	1	1	3	3	1	1	4	4
3	3	1	1	3	3	3	3	4	4
3	3	1	1	1	1	3	3	4	4
3	3	1	1	1	1	3	3	4	4
3	3	1	1	1	1	3	3	4	4
4	4	1	1	1	1	3	3	4	4
3	3	1	1	1	1	3	3	4	4
4	4	1	1	1	1	3	3	4	4
4	4	1	1	1	1	3	3	4	4
3	3	1	1	3	3	1	1	3	3
3	3	1	1	1	1	3	3	4	4
4	4	1	1	1	1	1	1	4	4
3	3	1	1	3	3	3	3	4	4
4	4	1	1	1	1	3	3	4	4
3	3	1	1	3	3	1	1	4	4
3	3	1	1	1	1	1	1	4	4
4	4	1	1	1	1	3	3	4	4
4	4	1	1	1	1	1	1	3	3
3	3	3	3	3	3	1	1	4	4
3	3	3	3	3	3	3	3	4	4
3	3	1	1	1	1	3	3	3	3
3	3	1	1	3	3	1	1	4	4
3	3	1	1	1	1	3	3	3	3
3	3	1	1	3	3	1	1	3	3
3	3	1	1	3	3	3	3	3	3
3	3	1	1	1	1	1	1	4	4
3	3	1	1	3	3	1	1	4	4
4	4	1	1	3	3	3	3	4	4
3	3	1	1	3	3	1	1	3	3
3	3	1	1	3	3	1	1	4	4
4	4	1	1	1	1	1	1	4	4
3	3	1	1	3	3	1	1	4	4
4	4	1	1	1	1	3	3	4	4
4	4	1	1	3	3	1	1	4	4
3	3	1	1	1	1	1	1	4	4
4	4	1	1	1	1	1	1	4	4

For Review Only

3	3	1	1	3	3	1	1	3	3
3	3	1	1	1	1	1	1	4	4
3	3	1	1	3	3	1	1	3	3
4	4	1	1	1	1	3	3	4	4
3	3	1	1	3	3	3	3	4	4
3	3	1	1	3	3	3	3	3	3
3	3	1	1	3	3	3	3	3	3
3	3	1	1	3	3	3	3	4	4
3	3	1	1	3	3	1	1	4	4
3	3	1	1	1	1	1	1	4	4
3	3	1	1	1	1	1	1	4	4
3	3	1	1	1	1	1	1	3	3
4	4	1	1	3	3	1	1	3	3
3	3	1	1	3	3	1	1	3	3
3	3	1	1	3	3	3	3	4	4
3	3	1	1	1	1	1	1	3	3
3	3	1	1	1	1	1	1	3	3
3	3	1	1	1	1	1	1	3	3
4	4	1	1	1	1	3	3	4	4
3	3	1	1	3	3	1	1	4	4
3	3	1	1	1	1	1	1	3	3
3	3	1	1	3	3	3	3	4	4
3	3	1	1	1	1	3	3	3	3
4	4	1	1	1	1	3	3	4	4
3	3	1	1	3	3	3	3	3	3
3	3	1	1	1	1	1	1	4	4
3	3	1	1	3	3	3	3	4	4
3	3	1	1	3	3	1	1	4	4
3	3	1	1	3	3	1	1	3	3
3	3	1	1	1	1	3	3	4	4
4	4	3	3	3	3	3	3	4	4
3	3	1	1	1	1	1	1	3	3
3	3	1	1	3	3	1	1	4	4
3	3	1	1	1	1	1	1	3	3
3	3	1	1	1	1	3	3	4	4
4	4	1	1	1	1	1	1	4	4
4	4	1	1	1	1	3	3	4	4
3	3	1	1	1	1	3	3	4	4
4	4	1	1	1	1	1	1	4	4
3	3	1	1	3	3	1	1	4	4
3	3	1	1	1	1	1	1	4	4
4	4	1	1	3	3	3	3	4	4
3	3	1	1	1	1	3	3	4	4
3	3	1	1	1	1	1	1	4	4
3	3	1	1	1	1	1	1	3	3
3	3	1	1	3	3	3	3	4	4
3	3	1	1	3	3	1	1	3	3
3	3	1	1	3	3	3	3	3	3
3	3	1	1	1	1	1	1	4	4
3	3	1	1	3	3	3	3	3	3
3	3	1	1	1	1	1	1	4	4
4	4	1	1	1	1	1	1	4	4
3	3	1	1	1	1	3	3	4	4
3	3	1	1	3	3	3	3	4	4
3	3	1	1	1	1	1	1	4	4

For Review Only

3	3	1	1	3	3	1	1	3	3
3	3	1	1	3	3	1	1	3	3
3	3	1	1	1	1	3	3	4	4
3	3	1	1	1	1	3	3	4	4
3	3	1	1	1	1	1	1	4	4
3	3	1	1	1	1	1	1	3	3
4	4	1	1	1	1	3	3	4	4
3	3	1	1	1	1	1	1	4	4
4	4	1	1	1	1	3	3	4	4
3	3	1	1	1	1	1	1	4	4
3	3	1	1	1	1	1	1	3	3
4	4	1	1	1	1	1	1	4	4
3	3	1	1	1	1	3	3	3	3
3	3	1	1	3	3	3	3	4	4
4	4	1	1	3	3	3	3	4	4
3	3	1	1	1	1	1	1	4	4
3	3	1	1	1	1	1	1	4	4
3	3	1	1	3	3	3	3	3	3
3	3	1	1	3	3	1	1	3	3
3	3	1	1	3	3	3	3	3	3
3	3	1	1	3	3	1	1	3	3
4	4	1	1	3	3	1	1	4	4
3	3	1	1	1	1	3	3	4	4
3	3	1	1	3	3	3	3	4	4
3	3	1	1	1	1	1	1	4	4
3	3	1	1	1	1	3	3	4	4
3	3	1	1	1	1	3	3	3	3
3	3	1	1	1	1	1	1	3	3
3	3	1	1	3	3	1	1	3	3
3	3	1	1	3	3	1	1	3	3
3	3	1	1	3	3	3	3	4	4
3	3	1	1	1	1	1	1	3	3
3	3	1	1	1	1	3	3	4	4
4	4	1	1	3	3	3	3	4	4
3	3	3	3	3	3	1	1	4	4
3	3	1	1	3	3	3	3	4	4
3	3	1	1	1	1	3	3	3	3
3	3	1	1	1	1	3	3	4	4
3	3	1	1	3	3	3	3	3	3
3	3	1	1	3	3	3	3	4	4
3	3	1	1	3	3	3	3	3	3
3	3	1	1	3	3	1	1	3	3
4	4	1	1	1	1	3	3	4	4
3	3	1	1	1	1	1	1	4	4
4	4	1	1	1	1	1	1	4	4
3	3	1	1	1	1	1	1	3	3
3	3	1	1	1	1	1	1	4	4
4	4	1	1	1	1	3	3	4	4
4	4	1	1	3	3	3	3	4	4
3	3	1	1	3	3	1	1	3	3
3	3	1	1	3	3	1	1	3	3
3	3	1	1	1	1	3	3	4	4
3	3	1	1	1	1	3	3	4	4
3	3	1	1	3	3	3	3	3	3
4	4	1	1	1	1	3	3	4	4

For Review Only

For Review Only

3	3	1	1	3	3	3	3	3	3
3	3	1	1	1	1	1	1	4	4
3	3	1	1	1	1	1	1	3	3
3	3	1	1	1	1	1	1	4	4
3	3	1	1	1	1	1	1	4	4
3	3	1	1	3	3	1	1	3	3
4	4	1	1	3	3	1	1	3	3
3	3	1	1	1	1	3	3	4	4
3	3	1	1	1	1	3	3	4	4
4	4	3	3	3	3	3	3	4	4
3	3	1	1	1	1	1	1	3	3
3	3	1	1	1	1	1	1	4	4
3	3	3	3	3	3	1	1	4	4
3	3	1	1	3	3	3	3	3	3
3	3	1	1	3	3	3	3	3	3
4	4	1	1	1	1	1	1	4	4
3	3	1	1	1	1	1	1	4	4
3	3	1	1	1	1	1	1	4	4
4	4	1	1	3	3	1	1	4	4
4	4	1	1	1	1	3	3	4	4
4	4	1	1	1	1	3	3	4	4
3	3	1	1	3	3	1	1	3	3
3	3	1	1	3	3	1	1	4	4
4	4	1	1	1	1	1	1	4	4
3	3	1	1	1	1	1	1	4	4
3	3	1	1	1	1	3	3	4	4
4	4	1	1	1	1	3	3	4	4
3	3	1	1	3	3	3	3	3	3
3	3	1	1	1	1	1	1	4	4
3	3	1	1	3	3	1	1	3	3
4	4	1	1	1	1	3	3	4	4
3	3	1	1	3	3	1	1	4	4
4	4	3	3	3	3	3	3	4	4
3	3	1	1	3	3	3	3	3	3
3	3	1	1	1	1	1	1	4	4
3	3	1	1	1	1	3	3	4	4
3	3	1	1	3	3	3	3	3	3
3	3	1	1	3	3	3	3	4	4
4	4	1	1	1	1	1	1	4	4
4	4	1	1	1	1	3	3	4	4
3	3	1	1	3	3	1	1	3	3
3	3	1	1	1	1	1	1	4	4
4	4	1	1	1	1	3	3	4	4
3	3	1	1	3	3	1	1	4	4
3	3	1	1	3	3	1	1	4	4
3	3	3	3	3	3	3	3	4	4
3	3	1	1	1	1	3	3	3	3
3	3	1	1	3	3	1	1	4	4
3	3	1	1	1	1	3	3	3	3
4	4	1	1	1	1	3	3	4	4
3	3	1	1	3	3	1	1	3	3
4	4	3	3	3	3	3	3	4	4
3	3	1	1	3	3	3	3	3	3
3	3	1	1	3	3	3	3	3	3
4	4	1	1	1	1	3	3	4	4

3	3	1	1	1	1	1	1	3	3
3	3	1	1	3	3	3	3	4	4

For Review Only

SNP1768	SNP1768	SNP1769	SNP1769	SNP177	SNP177	SNP1770	SNP1770	SNP1771	SNP1771
1	1	4	4	3	3	3	3	2	2
4	4	4	4	3	3	2	2	2	2
1	1	4	4	3	3	3	3	2	2
1	1	4	4	4	4	3	3	2	2
1	1	4	4	4	4	3	3	2	2
1	1	4	4	4	4	3	3	2	2
1	1	4	4	3	3	3	3	2	2
1	1	4	4	3	3	3	3	2	2
1	1	4	4	3	3	3	3	2	2
1	1	4	4	3	3	3	3	2	2
1	1	2	2	4	4	3	3	4	4
4	4	4	4	3	3	2	2	2	2
1	1	4	4	3	3	3	3	2	2
1	1	4	4	3	3	3	3	2	2
1	1	4	4	3	3	3	3	2	2
1	1	4	4	3	3	3	3	2	2
1	1	4	4	3	3	3	3	2	2
1	1	4	4	3	3	2	2	2	2
1	1	4	4	3	3	3	3	2	2
1	1	4	4	3	3	3	3	2	2
1	1	4	4	4	4	3	3	2	2
1	1	4	4	3	3	3	3	2	2
1	1	2	2	3	3	3	3	4	4
1	1	4	4	3	3	3	3	2	2
1	1	4	4	3	3	3	3	2	2
1	1	4	4	3	3	2	2	2	2
1	1	4	4	3	3	2	2	2	2
1	1	4	4	3	3	3	3	2	2
1	1	4	4	3	3	3	3	2	2
1	1	4	4	3	3	3	3	2	2
1	1	4	4	3	3	3	3	2	2
1	1	4	4	3	3	3	3	2	2
1	1	4	4	3	3	3	3	2	2
1	1	2	2	4	4	3	3	4	4
1	1	4	4	3	3	3	3	4	4
1	1	4	4	3	3	3	3	2	2
1	1	4	4	4	4	3	3	2	2
1	1	4	4	3	3	3	3	2	2
1	1	2	2	3	3	3	3	4	4
1	1	2	2	3	3	3	3	4	4
1	1	4	4	3	3	3	3	2	2
1	1	4	4	3	3	3	3	2	2
1	1	4	4	3	3	2	2	2	2
1	1	4	4	4	4	3	3	2	2
1	1	4	4	3	3	2	2	2	2
1	1	4	4	3	3	3	3	2	2
1	1	4	4	3	3	2	2	2	2
1	1	4	4	3	3	3	3	2	2
1	1	4	4	4	4	3	3	2	2
1	1	4	4	3	3	3	3	2	2
1	1	4	4	3	3	2	2	2	2
1	1	4	4	3	3	2	2	4	4

For Review Only

1	1	4	4	3	3	3	3	2	2
1	1	4	4	3	3	3	3	2	2
1	1	4	4	3	3	3	3	2	2
1	1	4	4	3	3	3	3	2	2
1	1	4	4	3	3	3	3	2	2
1	1	4	4	3	3	2	2	2	2
1	1	4	4	3	3	2	2	2	2
1	1	4	4	3	3	3	3	2	2
1	1	4	4	4	4	3	3	2	2
1	1	4	4	3	3	3	3	2	2
1	1	4	4	3	3	2	2	2	2
1	1	4	4	3	3	3	3	2	2
1	1	4	4	3	3	3	3	2	2
1	1	4	4	3	3	3	3	2	2
1	1	4	4	3	3	3	3	2	2
1	1	4	4	3	3	3	3	2	2
1	1	4	4	4	4	3	3	2	2
1	1	4	4	3	3	3	3	2	2
1	1	4	4	4	4	3	3	2	2
1	1	4	4	3	3	3	3	2	2
1	1	4	4	3	3	3	3	2	2
1	1	4	4	4	4	3	3	2	2
1	1	4	4	4	4	3	3	2	2
1	1	4	4	3	3	3	3	2	2
1	1	4	4	3	3	2	2	2	2
1	1	4	4	3	3	3	3	2	2
1	1	4	4	3	3	3	3	2	2
1	1	2	2	3	3	3	3	4	4
1	1	4	4	3	3	2	2	2	2
1	1	4	4	3	3	3	3	2	2
1	1	4	4	4	4	3	3	2	2
1	1	4	4	3	3	3	3	2	2
1	1	2	2	3	3	3	3	4	4
1	1	4	4	3	3	3	3	2	2
1	1	4	4	3	3	3	3	2	2
1	1	4	4	3	3	2	2	2	2
1	1	4	4	3	3	3	3	2	2
1	1	4	4	4	4	3	3	2	2
1	1	4	4	3	3	3	3	2	2
1	1	4	4	4	4	3	3	2	2
1	1	4	4	4	4	3	3	2	2
1	1	4	4	3	3	3	3	2	2
4	4	4	4	3	3	3	3	2	2
1	1	4	4	3	3	2	2	2	2
1	1	4	4	3	3	3	3	2	2
4	4	4	4	3	3	2	2	2	2
1	1	2	2	3	3	3	3	4	4
1	1	4	4	3	3	3	3	2	2
1	1	4	4	3	3	3	3	2	2
1	1	4	4	4	4	3	3	2	2
1	1	4	4	3	3	3	3	2	2
1	1	2	2	3	3	3	3	4	4
1	1	4	4	4	4	3	3	2	2
1	1	4	4	3	3	3	3	4	4
1	1	4	4	3	3	3	3	2	2

For Review Only

1	1	4	4	3	3	2	2	2	2
1	1	4	4	3	3	3	3	2	2
1	1	4	4	3	3	2	2	2	2
1	1	2	2	3	3	3	3	4	4
1	1	4	4	4	4	3	3	2	2
1	1	4	4	3	3	3	3	2	2
4	4	4	4	3	3	3	3	2	2
1	1	4	4	3	3	3	3	2	2
1	1	4	4	3	3	3	3	2	2
1	1	4	4	3	3	3	3	2	2
1	1	4	4	3	3	3	3	4	4
1	1	4	4	3	3	3	3	2	2
4	4	4	4	3	3	3	3	2	2
1	1	4	4	3	3	3	3	2	2
1	1	4	4	3	3	3	3	2	2
1	1	4	4	3	3	3	3	2	2
1	1	4	4	3	3	3	3	2	2
1	1	4	4	3	3	3	3	2	2
1	1	4	4	3	3	3	3	2	2
4	4	4	4	3	3	3	3	2	2
1	1	4	4	3	3	3	3	2	2
1	1	4	4	3	3	3	3	2	2
1	1	4	4	3	3	3	3	2	2
1	1	2	2	3	3	3	3	4	4
1	1	4	4	4	4	3	3	2	2
1	1	4	4	4	4	3	3	2	2
1	1	4	4	3	3	3	3	2	2
1	1	4	4	3	3	3	3	2	2
1	1	4	4	3	3	3	3	2	2
1	1	4	4	3	3	3	3	2	2
1	1	2	2	3	3	3	3	4	4
1	1	4	4	3	3	3	3	2	2
1	1	4	4	4	4	3	3	2	2
1	1	4	4	3	3	2	2	2	2
1	1	2	2	4	4	3	3	4	4
1	1	4	4	3	3	3	3	4	4
1	1	2	2	3	3	3	3	4	4
4	4	4	4	3	3	3	3	2	2
1	1	4	4	3	3	2	2	2	2
1	1	2	2	3	3	3	3	4	4
1	1	4	4	3	3	3	3	2	2
1	1	2	2	3	3	3	3	4	4
1	1	4	4	3	3	3	3	2	2
1	1	4	4	3	3	3	3	2	2
1	1	4	4	3	3	3	3	2	2
1	1	4	4	3	3	3	3	2	2
1	1	4	4	3	3	2	2	2	2
1	1	4	4	3	3	2	2	2	2
1	1	4	4	3	3	3	3	2	2
1	1	4	4	3	3	2	2	2	2
1	1	4	4	3	3	2	2	2	2
1	1	4	4	4	4	3	3	2	2
1	1	4	4	3	3	3	3	2	2
1	1	4	4	3	3	3	3	2	2
1	1	4	4	3	3	3	3	2	2

For Review Only

1	1	4	4	4	4	3	3	2	2
1	1	4	4	4	4	3	3	2	2
1	1	4	4	3	3	3	3	2	2
1	1	4	4	3	3	3	3	2	2
4	4	4	4	3	3	3	3	2	2
1	1	4	4	3	3	3	3	2	2
1	1	2	2	3	3	3	3	4	4
1	1	4	4	3	3	3	3	2	2
1	1	4	4	3	3	3	3	4	4
1	1	4	4	3	3	3	3	2	2
1	1	4	4	3	3	3	3	2	2
1	1	4	4	3	3	3	3	2	2
1	1	4	4	4	4	3	3	4	4
1	1	4	4	3	3	3	3	4	4
1	1	2	2	3	3	3	3	4	4
1	1	2	2	4	4	3	3	4	4
1	1	4	4	3	3	3	3	4	4
1	1	4	4	3	3	2	2	2	2
1	1	4	4	3	3	2	2	2	2
4	4	4	4	3	3	3	3	2	2
1	1	4	4	3	3	3	3	2	2
1	1	2	2	3	3	3	3	4	4
1	1	4	4	4	4	3	3	2	2
1	1	4	4	3	3	3	3	2	2
1	1	4	4	3	3	3	3	2	2
1	1	2	2	4	4	3	3	4	4
1	1	4	4	3	3	3	3	2	2
1	1	4	4	3	3	3	3	4	4
1	1	4	4	3	3	3	3	2	2
1	1	4	4	3	3	3	3	2	2
1	1	4	4	3	3	3	3	2	2
1	1	4	4	3	3	3	3	2	2
1	1	4	4	3	3	3	3	2	2
1	1	2	2	3	3	3	3	4	4
1	1	4	4	4	4	3	3	2	2
1	1	4	4	3	3	3	3	2	2
1	1	4	4	4	4	3	3	2	2
1	1	4	4	3	3	3	3	2	2
1	1	4	4	3	3	2	2	2	2
4	4	4	4	3	3	3	3	2	2
1	1	4	4	3	3	3	3	2	2
1	1	4	4	3	3	2	2	2	2
1	1	2	2	3	3	3	3	4	4
1	1	4	4	3	3	3	3	2	2
1	1	4	4	3	3	3	3	2	2
4	4	4	4	3	3	3	3	2	2
1	1	2	2	4	4	3	3	4	4
1	1	2	2	3	3	3	3	4	4
1	1	2	2	3	3	3	3	4	4
4	4	4	4	3	3	3	3	2	2
1	1	4	4	3	3	3	3	2	2
1	1	4	4	4	4	3	3	2	2
1	1	4	4	4	4	3	3	2	2
1	1	4	4	3	3	2	2	2	2
1	1	2	2	3	3	3	3	4	4

For Review Only

1	1	2	2	3	3	3	3	4	4
1	1	4	4	3	3	3	3	2	2
1	1	4	4	3	3	3	3	2	2
1	1	4	4	3	3	3	3	2	2
1	1	4	4	3	3	2	2	2	2
1	1	4	4	4	4	2	2	2	2
1	1	2	2	3	3	3	3	4	4
1	1	4	4	3	3	3	3	2	2
1	1	4	4	3	3	3	3	2	2
1	1	2	2	3	3	3	3	4	4
1	1	4	4	3	3	3	3	2	2
4	4	4	4	3	3	3	3	2	2
1	1	4	4	3	3	3	3	2	2
1	1	4	4	3	3	2	2	2	2
1	1	4	4	3	3	3	3	2	2
1	1	4	4	4	4	3	3	2	2
1	1	4	4	4	4	3	3	2	2
1	1	4	4	4	4	3	3	2	2
1	1	4	4	3	3	3	3	4	4
1	1	4	4	4	4	3	3	2	2
1	1	2	2	3	3	3	3	4	4
1	1	4	4	3	3	2	2	2	2
1	1	4	4	4	4	3	3	2	2
1	1	4	4	3	3	3	3	2	2
1	1	4	4	3	3	3	3	2	2
4	4	4	4	3	3	3	3	4	4
1	1	2	2	3	3	3	3	4	4
4	4	4	4	3	3	2	2	2	2
1	1	4	4	3	3	3	3	2	2
1	1	4	4	3	3	3	3	2	2
1	1	2	2	3	3	3	3	4	4
1	1	4	4	3	3	3	3	2	2
1	1	2	2	3	3	3	3	4	4
1	1	4	4	3	3	2	2	2	2
1	1	4	4	4	4	3	3	2	2
1	1	2	2	3	3	3	3	4	4
1	1	4	4	3	3	3	3	2	2
1	1	4	4	3	3	3	3	2	2
1	1	4	4	3	3	3	3	2	2
1	1	2	2	3	3	3	3	4	4
4	4	4	4	3	3	3	3	2	2
1	1	4	4	3	3	3	3	2	2
1	1	2	2	4	4	3	3	4	4
1	1	4	4	4	4	3	3	2	2
1	1	4	4	3	3	2	2	2	2
1	1	4	4	3	3	3	3	2	2
1	1	4	4	3	3	2	2	2	2
1	1	4	4	4	4	3	3	2	2
1	1	4	4	3	3	3	3	2	2
1	1	2	2	3	3	3	3	4	4
1	1	4	4	4	4	3	3	2	2
1	1	2	2	3	3	3	3	4	4
1	1	4	4	3	3	3	3	2	2
1	1	4	4	4	4	2	2	2	2
1	1	2	2	3	3	3	3	4	4

For Review Only

1	1	4	4	4	4	3	3	2	2
1	1	4	4	3	3	3	3	2	2

For Review Only

SNP1772	SNP1772	SNP1773	SNP1773	SNP1774	SNP1774	SNP1775	SNP1775	SNP1776	SNP1776
4	4	3	3	4	4	1	1	3	3
3	3	3	3	4	4	4	4	1	1
3	3	3	3	4	4	4	4	3	3
3	3	3	3	4	4	4	4	3	3
3	3	3	3	4	4	1	1	1	1
3	3	3	3	4	4	4	4	3	3
3	3	3	3	4	4	4	4	3	3
4	4	3	3	4	4	4	4	3	3
3	3	3	3	4	4	4	4	3	3
4	4	3	3	4	4	1	1	3	3
4	4	3	3	4	4	4	4	3	3
3	3	3	3	4	4	4	4	3	3
4	4	3	3	4	4	4	4	3	3
4	4	3	3	4	4	4	4	1	1
4	4	3	3	4	4	4	4	3	3
4	4	3	3	4	4	4	4	3	3
3	3	3	3	4	4	4	4	3	3
4	4	3	3	4	4	4	4	1	1
3	3	3	3	1	1	1	1	3	3
4	4	3	3	4	4	1	1	3	3
3	3	3	3	4	4	1	1	3	3
4	4	3	3	4	4	4	4	1	1
3	3	3	3	4	4	1	1	1	1
4	4	3	3	4	4	4	4	1	1
4	4	3	3	4	4	4	4	1	1
3	3	3	3	4	4	1	1	3	3
3	3	3	3	4	4	4	4	3	3
4	4	2	2	4	4	4	4	3	3
3	3	3	3	4	4	4	4	3	3
4	4	3	3	4	4	1	1	1	1
3	3	3	3	4	4	4	4	3	3
3	3	3	3	4	4	4	4	3	3
3	3	3	3	4	4	1	1	1	1
4	4	3	3	4	4	4	4	3	3
3	3	3	3	4	4	1	1	3	3
4	4	3	3	4	4	4	4	3	3
3	3	3	3	4	4	4	4	3	3
4	4	3	3	4	4	4	4	3	3
4	4	3	3	4	4	1	1	1	1
4	4	3	3	4	4	4	4	3	3
4	4	3	3	4	4	4	4	3	3
3	3	3	3	4	4	4	4	3	3
4	4	3	3	4	4	4	4	3	3
4	4	3	3	4	4	4	4	3	3
4	4	3	3	4	4	4	4	3	3
3	3	3	3	4	4	4	4	3	3
3	3	3	3	4	4	1	1	3	3
3	3	3	3	4	4	4	4	3	3
4	4	3	3	4	4	4	4	3	3
4	4	3	3	4	4	4	4	3	3
3	3	3	3	1	1	1	1	3	3
3	3	3	3	4	4	1	1	3	3

For Review Only

3	3	3	3	4	4	4	4	1	1
3	3	3	3	4	4	4	4	1	1
3	3	3	3	4	4	4	4	3	3
4	4	3	3	4	4	4	4	3	3
3	3	3	3	1	1	1	1	3	3
3	3	3	3	4	4	1	1	3	3
3	3	3	3	4	4	1	1	3	3
4	4	3	3	4	4	4	4	3	3
4	4	3	3	4	4	4	4	1	1
4	4	3	3	4	4	4	4	3	3
3	3	3	3	4	4	4	4	3	3
3	3	3	3	4	4	4	4	3	3
3	3	3	3	4	4	4	4	1	1
3	3	3	3	4	4	4	4	3	3
4	4	3	3	4	4	4	4	3	3
3	3	3	3	4	4	1	1	3	3
4	4	3	3	4	4	4	4	3	3
4	4	2	2	4	4	4	4	3	3
3	3	3	3	4	4	4	4	1	1
3	3	3	3	4	4	4	4	3	3
4	4	3	3	4	4	4	4	3	3
4	4	3	3	4	4	1	1	3	3
3	3	3	3	4	4	4	4	3	3
4	4	3	3	1	1	4	4	3	3
4	4	3	3	4	4	4	4	3	3
4	4	3	3	4	4	1	1	3	3
4	4	3	3	4	4	4	4	1	1
4	4	3	3	4	4	4	4	1	1
3	3	3	3	4	4	1	1	3	3
4	4	2	2	4	4	4	4	3	3
4	4	3	3	4	4	4	4	1	1
4	4	3	3	4	4	4	4	3	3
4	4	3	3	4	4	4	4	1	1
3	3	3	3	4	4	4	4	1	1
4	4	3	3	4	4	1	1	1	1
4	4	3	3	4	4	4	4	3	3
4	4	3	3	4	4	4	4	3	3
4	4	3	3	4	4	1	1	1	1
4	4	3	3	4	4	4	4	3	3
4	4	3	3	4	4	4	4	3	3
3	3	3	3	4	4	4	4	3	3
4	4	2	2	4	4	4	4	3	3
3	3	3	3	4	4	1	1	3	3
3	3	3	3	4	4	4	4	3	3
4	4	3	3	4	4	4	4	1	1
3	3	3	3	4	4	4	4	3	3
4	4	3	3	4	4	4	4	1	1
3	3	3	3	4	4	1	1	3	3
3	3	3	3	4	4	1	1	3	3
4	4	3	3	4	4	4	4	3	3
3	3	3	3	1	1	4	4	3	3
4	4	3	3	4	4	1	1	3	3
4	4	3	3	1	1	1	1	3	3
4	4	3	3	4	4	4	4	3	3
4	4	3	3	4	4	4	4	1	1

For Review Only

4	4	3	3	4	4	4	4	3	3
4	4	3	3	4	4	4	4	3	3
3	3	3	3	4	4	1	1	3	3
4	4	3	3	4	4	4	4	3	3
3	3	3	3	4	4	1	1	3	3
3	3	3	3	4	4	1	1	3	3
3	3	3	3	4	4	4	4	3	3
3	3	3	3	1	1	4	4	3	3
3	3	3	3	4	4	4	4	3	3
4	4	3	3	4	4	4	4	3	3
4	4	3	3	4	4	4	4	1	1
4	4	3	3	4	4	4	4	3	3
3	3	3	3	4	4	4	4	3	3
3	3	3	3	4	4	1	1	3	3
3	3	3	3	1	1	4	4	3	3
4	4	3	3	4	4	4	4	3	3
4	4	3	3	4	4	4	4	3	3
4	4	3	3	4	4	4	4	3	3
4	4	3	3	4	4	4	4	3	3
3	3	3	3	4	4	4	4	3	3
4	4	3	3	4	4	4	4	3	3
3	3	3	3	4	4	4	4	3	3
4	4	3	3	4	4	4	4	3	3
4	4	3	3	4	4	4	4	1	1
3	3	3	3	4	4	1	1	3	3
4	4	3	3	4	4	4	4	3	3
3	3	3	3	4	4	4	4	3	3
3	3	3	3	4	4	4	4	3	3
3	3	3	3	4	4	4	4	3	3
3	3	3	3	4	4	4	4	3	3
4	4	3	3	4	4	1	1	1	1
4	4	3	3	4	4	4	4	3	3
3	3	3	3	4	4	4	4	3	3
4	4	3	3	4	4	1	1	3	3
4	4	3	3	4	4	4	4	3	3
4	4	3	3	4	4	4	4	3	3
4	4	3	3	4	4	4	4	1	1
3	3	3	3	4	4	4	4	3	3
4	4	3	3	4	4	4	4	3	3
4	4	3	3	4	4	1	1	1	1
4	4	3	3	4	4	4	4	3	3
4	4	3	3	4	4	4	4	1	1
4	4	2	2	4	4	4	4	1	1
4	4	3	3	4	4	4	4	3	3
4	4	3	3	4	4	4	4	3	3
3	3	3	3	4	4	4	4	3	3
3	3	3	3	4	4	1	1	3	3
3	3	3	3	4	4	1	1	3	3
4	4	3	3	4	4	4	4	3	3
3	3	3	3	4	4	1	1	3	3
4	4	3	3	4	4	1	1	3	3
3	3	3	3	4	4	4	4	1	1
4	4	2	2	4	4	1	1	3	3
3	3	3	3	4	4	1	1	3	3
4	4	3	3	4	4	4	4	3	3

For Review Only

3	3	3	3	4	4	4	4	3	3
3	3	3	3	4	4	4	4	3	3
4	4	3	3	4	4	4	4	1	1
4	4	3	3	4	4	4	4	1	1
4	4	3	3	4	4	4	4	3	3
4	4	3	3	4	4	4	4	3	3
4	4	3	3	4	4	1	1	3	3
4	4	3	3	4	4	4	4	3	3
4	4	3	3	4	4	4	4	3	3
4	4	2	2	4	4	4	4	3	3
3	3	3	3	4	4	1	1	3	3
4	4	3	3	4	4	4	4	1	1
3	3	2	2	4	4	4	4	3	3
4	4	3	3	4	4	4	4	3	3
4	4	3	3	4	4	4	4	3	3
4	4	3	3	4	4	4	4	3	3
4	4	2	2	4	4	4	4	3	3
3	3	3	3	4	4	1	1	3	3
3	3	3	3	4	4	1	1	3	3
3	3	3	3	4	4	4	4	3	3
4	4	3	3	4	4	4	4	3	3
4	4	3	3	4	4	1	1	3	3
3	3	3	3	4	4	1	1	1	1
3	3	3	3	4	4	4	4	3	3
3	3	3	3	4	4	4	4	3	3
4	4	3	3	4	4	4	4	3	3
4	4	3	3	4	4	4	4	3	3
4	4	3	3	4	4	4	4	3	3
3	3	3	3	4	4	4	4	3	3
3	3	3	3	4	4	4	4	3	3
3	3	3	3	1	1	4	4	3	3
4	4	2	2	4	4	4	4	3	3
4	4	3	3	4	4	4	4	3	3
4	4	3	3	4	4	4	4	3	3
4	4	3	3	4	4	4	4	3	3
3	3	3	3	4	4	4	4	3	3
4	4	3	3	4	4	4	4	3	3
3	3	3	3	4	4	1	1	3	3
3	3	3	3	4	4	1	1	3	3
3	3	3	3	4	4	1	1	3	3
3	3	3	3	4	4	4	4	3	3
3	3	3	3	4	4	1	1	3	3
4	4	3	3	4	4	1	1	1	1
4	4	3	3	4	4	4	4	3	3
4	4	3	3	4	4	4	4	1	1
4	4	3	3	4	4	1	1	3	3
4	4	3	3	4	4	4	4	3	3
4	4	3	3	4	4	1	1	3	3
4	4	2	2	4	4	1	1	1	1
3	3	3	3	4	4	4	4	3	3
3	3	3	3	4	4	1	1	3	3
4	4	3	3	4	4	4	4	3	3
3	3	3	3	4	4	1	1	3	3
3	3	3	3	4	4	4	4	3	3
4	4	3	3	4	4	1	1	3	3

For Review Only

3	3	3	3	4	4	4	4	3	3
4	4	3	3	4	4	4	4	1	1
4	4	3	3	4	4	4	4	3	3
3	3	3	3	4	4	4	4	3	3
4	4	3	3	4	4	4	4	3	3
4	4	3	3	4	4	1	1	3	3
3	3	3	3	4	4	4	4	3	3
4	4	3	3	4	4	4	4	3	3
4	4	3	3	4	4	1	1	3	3
4	4	3	3	4	4	4	4	1	1
4	4	3	3	4	4	4	4	3	3
4	4	3	3	1	1	4	4	3	3
4	4	3	3	4	4	4	4	3	3
3	3	3	3	4	4	1	1	3	3
3	3	3	3	4	4	1	1	3	3
4	4	3	3	4	4	4	4	3	3
4	4	3	3	4	4	1	1	3	3
4	4	3	3	4	4	1	1	1	1
3	3	3	3	4	4	1	1	1	1
4	4	3	3	4	4	4	4	3	3
4	4	3	3	1	1	1	1	3	3
3	3	3	3	4	4	1	1	3	3
3	3	3	3	4	4	4	4	3	3
4	4	3	3	4	4	4	4	1	1
4	4	3	3	4	4	4	4	3	3
4	4	3	3	4	4	4	4	3	3
4	4	3	3	4	4	4	4	1	1
3	3	3	3	4	4	4	4	3	3
4	4	3	3	4	4	4	4	3	3
3	3	3	3	4	4	4	4	3	3
4	4	3	3	4	4	4	4	1	1
3	3	3	3	4	4	4	4	3	3
4	4	2	2	4	4	4	4	3	3
3	3	3	3	4	4	4	4	3	3
3	3	3	3	4	4	1	1	3	3
4	4	3	3	4	4	1	1	1	1
3	3	2	2	4	4	4	4	3	3
3	3	3	3	4	4	4	4	3	3
4	4	3	3	4	4	4	4	3	3
4	4	3	3	4	4	1	1	1	1
3	3	3	3	4	4	4	4	3	3
4	4	3	3	4	4	4	4	1	1
4	4	3	3	4	4	4	4	1	1
3	3	3	3	4	4	4	4	3	3
3	3	3	3	4	4	4	4	3	3
4	4	3	3	4	4	4	4	3	3
4	4	3	3	4	4	4	4	3	3
4	4	3	3	4	4	4	4	1	1
4	4	3	3	4	4	4	4	3	3
4	4	3	3	4	4	1	1	1	1
3	3	3	3	4	4	4	4	3	3
4	4	3	3	4	4	1	1	3	3
3	3	3	3	4	4	4	4	3	3
3	3	3	3	4	4	4	4	3	3
3	3	3	3	4	4	4	4	1	1

For Review Only

3	3	3	3	4	4	4	4	3	3
3	3	3	3	1	1	1	1	3	3

For Review Only

SNP1777	SNP1777	SNP1778	SNP1778	SNP1779	SNP1779	SNP178	SNP178	SNP1780	SNP1780
2	2	4	4	2	2	3	3	4	4
3	3	4	4	2	2	3	3	4	4
2	2	4	4	2	2	2	2	4	4
2	2	4	4	2	2	3	3	4	4
2	2	4	4	3	3	3	3	4	4
2	2	4	4	2	2	3	3	4	4
3	3	1	1	3	3	2	2	1	1
2	2	4	4	2	2	3	3	4	4
3	3	1	1	3	3	2	2	1	1
2	2	4	4	2	2	3	3	4	4
2	2	4	4	3	3	3	3	1	1
2	2	4	4	2	2	3	3	4	4
2	2	4	4	2	2	3	3	4	4
2	2	4	4	2	2	3	3	4	4
2	2	4	4	3	3	3	3	4	4
2	2	4	4	2	2	3	3	4	4
3	3	1	1	3	3	3	3	4	4
2	2	4	4	2	2	3	3	4	4
2	2	4	4	2	2	2	2	4	4
2	2	4	4	3	3	3	3	1	1
3	3	1	1	2	2	2	2	4	4
2	2	4	4	3	3	2	2	4	4
2	2	4	4	3	3	3	3	1	1
2	2	4	4	3	3	2	2	1	1
2	2	4	4	2	2	3	3	4	4
2	2	4	4	2	2	2	2	4	4
2	2	4	4	2	2	2	2	4	4
2	2	4	4	2	2	3	3	4	4
3	3	1	1	3	3	3	3	1	1
2	2	4	4	2	2	3	3	4	4
2	2	4	4	2	2	2	2	4	4
2	2	4	4	3	3	3	3	4	4
2	2	4	4	3	3	2	2	4	4
2	2	4	4	2	2	3	3	4	4
2	2	4	4	2	2	2	2	1	1
2	2	4	4	3	3	3	3	1	1
2	2	4	4	2	2	3	3	4	4
2	2	4	4	3	3	3	3	1	1
2	2	4	4	3	3	3	3	1	1
2	2	4	4	3	3	3	3	1	1
2	2	4	4	3	3	3	3	1	1
2	2	4	4	3	3	3	3	1	1
2	2	4	4	2	2	3	3	4	4
3	3	1	1	2	2	3	3	4	4
2	2	4	4	2	2	3	3	4	4
2	2	4	4	2	2	3	3	4	4
2	2	4	4	2	2	3	3	4	4
2	2	4	4	2	2	3	3	4	4
3	3	1	1	2	2	3	3	4	4
2	2	4	4	2	2	3	3	4	4
2	2	4	4	2	2	2	2	4	4
2	2	4	4	2	2	3	3	4	4

3	3	1	1	3	3	3	3	4	4
2	2	4	4	3	3	2	2	4	4
2	2	4	4	2	2	2	2	4	4
3	3	1	1	2	2	3	3	4	4
2	2	4	4	2	2	2	2	4	4
2	2	4	4	2	2	3	3	4	4
2	2	4	4	2	2	3	3	4	4
2	2	4	4	3	3	3	3	1	1
2	2	4	4	2	2	3	3	4	4
2	2	4	4	2	2	3	3	4	4
2	2	4	4	2	2	3	3	4	4
2	2	4	4	2	2	3	3	4	4
3	3	1	1	3	3	3	3	4	4
3	3	1	1	3	3	3	3	1	1
2	2	4	4	2	2	3	3	4	4
2	2	4	4	2	2	3	3	4	4
2	2	4	4	2	2	3	3	4	4
2	2	4	4	2	2	3	3	4	4
3	3	1	1	3	3	3	3	4	4
3	3	1	1	2	2	2	2	4	4
2	2	4	4	2	2	3	3	4	4
2	2	4	4	3	3	3	3	1	1
3	3	1	1	3	3	2	2	1	1
2	2	4	4	2	2	3	3	4	4
2	2	4	4	2	2	3	3	4	4
2	2	4	4	2	2	3	3	4	4
3	3	1	1	3	3	3	3	1	1
2	2	4	4	2	2	3	3	4	4
2	2	4	4	2	2	2	2	4	4
2	2	4	4	2	2	3	3	4	4
2	2	4	4	3	3	3	3	4	4
2	2	4	4	2	2	2	2	1	1
2	2	4	4	2	2	3	3	4	4
3	3	1	1	3	3	3	3	4	4
2	2	4	4	2	2	3	3	4	4
2	2	4	4	2	2	3	3	4	4
2	2	4	4	3	3	3	3	1	1
3	3	1	1	2	2	2	2	4	4
2	2	4	4	2	2	3	3	4	4
2	2	4	4	2	2	3	3	4	4
3	3	1	1	3	3	2	2	4	4
2	2	4	4	2	2	2	2	4	4
3	3	4	4	2	2	3	3	4	4
2	2	4	4	2	2	3	3	4	4
2	2	4	4	2	2	3	3	4	4
3	3	4	4	2	2	3	3	4	4
2	2	4	4	2	2	3	3	4	4
2	2	4	4	2	2	3	3	1	1
2	2	4	4	2	2	3	3	4	4
2	2	4	4	2	2	3	3	1	1
2	2	4	4	2	2	3	3	4	4
2	2	4	4	3	3	3	3	1	1
2	2	4	4	2	2	3	3	4	4
2	2	4	4	2	2	3	3	1	1
2	2	4	4	2	2	3	3	4	4

For Review Only

2	2	4	4	2	2	2	2	1	1
2	2	4	4	3	3	3	3	1	1
2	2	4	4	2	2	3	3	4	4
2	2	4	4	2	2	2	2	4	4
2	2	4	4	2	2	3	3	4	4
2	2	4	4	2	2	3	3	4	4
3	3	4	4	2	2	3	3	4	4
2	2	4	4	2	2	3	3	1	1
2	2	4	4	2	2	3	3	4	4
2	2	4	4	2	2	3	3	1	1
2	2	4	4	2	2	3	3	4	4
2	2	4	4	2	2	3	3	1	1
3	3	4	4	2	2	3	3	4	4
2	2	4	4	2	2	3	3	4	4
2	2	1	1	2	2	3	3	4	4
2	2	4	4	2	2	3	3	4	4
3	3	4	4	2	2	3	3	4	4
2	2	4	4	2	2	3	3	4	4
2	2	4	4	2	2	3	3	4	4
2	2	4	4	2	2	3	3	4	4
2	2	4	4	2	2	3	3	4	4
2	2	4	4	2	2	3	3	4	4
2	2	4	4	2	2	3	3	4	4
2	2	4	4	3	3	2	2	1	1
2	2	4	4	2	2	3	3	1	1
2	2	4	4	3	3	3	3	1	1
2	2	4	4	2	2	3	3	4	4
2	2	4	4	2	2	3	3	4	4
2	2	4	4	2	2	3	3	4	4
2	2	4	4	3	3	3	3	1	1
2	2	4	4	2	2	2	2	1	1
2	2	4	4	2	2	3	3	4	4
3	3	1	1	3	3	2	2	4	4
2	2	4	4	2	2	3	3	1	1
2	2	4	4	2	2	3	3	4	4
3	3	1	1	2	2	3	3	4	4
2	2	4	4	2	2	2	2	4	4
3	3	4	4	3	3	3	3	4	4
2	2	4	4	2	2	3	3	4	4
2	2	4	4	2	2	2	2	1	1
2	2	4	4	2	2	3	3	1	1
2	2	4	4	2	2	2	2	4	4
2	2	4	4	2	2	3	3	4	4
2	2	4	4	3	3	3	3	1	1
2	2	4	4	2	2	3	3	4	4
2	2	4	4	2	2	2	2	4	4
2	2	4	4	2	2	2	2	4	4
2	2	4	4	2	2	3	3	4	4
2	2	4	4	2	2	3	3	4	4
2	2	4	4	2	2	3	3	4	4
2	2	4	4	2	2	3	3	4	4
2	2	4	4	2	2	3	3	4	4
2	2	4	4	2	2	3	3	4	4
2	2	4	4	2	2	2	2	4	4
2	2	4	4	2	2	3	3	1	1

2	2	4	4	3	3	2	2	4	4
2	2	4	4	3	3	3	3	1	1
2	2	4	4	2	2	3	3	1	1
2	2	4	4	3	3	3	3	1	1
3	3	4	4	2	2	3	3	4	4
2	2	4	4	2	2	3	3	4	4
2	2	4	4	2	2	3	3	1	1
2	2	4	4	2	2	3	3	4	4
2	2	4	4	2	2	3	3	1	1
2	2	4	4	2	2	3	3	4	4
2	2	4	4	2	2	3	3	4	4
2	2	4	4	2	2	2	2	1	1
2	2	4	4	2	2	2	2	4	4
2	2	4	4	2	2	2	2	4	4
2	2	4	4	2	2	2	2	4	4
2	2	4	4	3	3	3	3	1	1
2	2	4	4	2	2	3	3	4	4
2	2	4	4	2	2	3	3	4	4
2	2	4	4	2	2	2	2	4	4
3	3	4	4	2	2	3	3	4	4
2	2	4	4	2	2	3	3	4	4
2	2	4	4	2	2	3	3	4	4
2	2	4	4	3	3	3	3	1	1
2	2	4	4	2	2	3	3	4	4
3	3	1	1	2	2	3	3	4	4
2	2	4	4	2	2	2	2	4	4
2	2	4	4	2	2	3	3	4	4
2	2	4	4	2	2	3	3	4	4
2	2	4	4	2	2	3	3	4	4
3	3	1	1	2	2	3	3	4	4
2	2	4	4	2	2	3	3	4	4
2	2	4	4	2	2	3	3	4	4
2	2	4	4	2	2	3	3	4	4
2	2	4	4	2	2	2	2	4	4
2	2	4	4	2	2	3	3	4	4
2	2	4	4	2	2	3	3	4	4
2	2	4	4	3	3	3	3	1	1
2	2	4	4	2	2	3	3	4	4
2	2	4	4	2	2	3	3	4	4
3	3	4	4	2	2	3	3	4	4
2	2	4	4	2	2	2	2	4	4
2	2	4	4	2	2	2	2	4	4
2	2	4	4	3	3	2	2	1	1
2	2	4	4	2	2	3	3	4	4
2	2	4	4	2	2	2	2	4	4
3	3	4	4	2	2	3	3	4	4
2	2	4	4	3	3	3	3	1	1
2	2	4	4	2	2	3	3	4	4
2	2	4	4	3	3	3	3	1	1
3	3	4	4	2	2	3	3	4	4
2	2	4	4	2	2	3	3	4	4
2	2	4	4	3	3	2	2	1	1
2	2	4	4	3	3	3	3	1	1
2	2	4	4	2	2	3	3	4	4
2	2	4	4	3	3	3	3	1	1

For Review Only

2	2	4	4	3	3	3	3	1	1
2	2	4	4	3	3	3	3	1	1
2	2	4	4	2	2	3	3	4	4
2	2	4	4	2	2	3	3	1	1
2	2	4	4	2	2	3	3	4	4
2	2	4	4	2	2	2	2	4	4
2	2	4	4	3	3	2	2	1	1
2	2	4	4	2	2	3	3	4	4
2	2	4	4	2	2	3	3	4	4
2	2	4	4	2	2	2	2	1	1
2	2	4	4	2	2	3	3	1	1
3	3	4	4	3	3	3	3	1	1
2	2	4	4	2	2	3	3	4	4
2	2	4	4	2	2	3	3	4	4
2	2	4	4	2	2	3	3	4	4
2	2	4	4	2	2	3	3	4	4
2	2	4	4	2	2	3	3	4	4
2	2	4	4	3	3	2	2	1	1
2	2	4	4	2	2	3	3	1	1
2	2	4	4	3	3	3	3	1	1
2	2	4	4	3	3	3	3	1	1
2	2	4	4	2	2	2	2	4	4
2	2	4	4	2	2	3	3	4	4
2	2	4	4	2	2	3	3	4	4
2	2	4	4	3	3	3	3	1	1
3	3	4	4	2	2	3	3	4	4
2	2	4	4	2	2	2	2	4	4
3	3	4	4	2	2	3	3	4	4
2	2	4	4	3	3	3	3	4	4
2	2	4	4	2	2	3	3	4	4
2	2	4	4	2	2	3	3	4	4
2	2	4	4	2	2	3	3	4	4
2	2	4	4	3	3	2	2	4	4
2	2	4	4	2	2	3	3	4	4
2	2	4	4	3	3	3	3	1	1
2	2	4	4	2	2	3	3	4	4
2	2	4	4	2	2	3	3	4	4
2	2	4	4	2	2	3	3	4	4
2	2	4	4	3	3	3	3	1	1
2	2	4	4	3	3	3	3	1	1
3	3	4	4	2	2	3	3	4	4
2	2	4	4	2	2	3	3	4	4
2	2	4	4	3	3	2	2	1	1
3	3	1	1	3	3	2	2	4	4
2	2	4	4	2	2	3	3	4	4
2	2	4	4	3	3	3	3	4	4
2	2	4	4	2	2	3	3	4	4
2	2	4	4	2	2	2	2	4	4
2	2	4	4	2	2	3	3	4	4
2	2	4	4	2	2	3	3	4	4
2	2	4	4	2	2	2	2	4	4
2	2	4	4	3	3	3	3	1	1
2	2	4	4	2	2	2	2	4	4
2	2	4	4	2	2	3	3	4	4
2	2	4	4	3	3	3	3	1	1

For Review Only

2	2	4	4	2	2	3	3	4	4
2	2	4	4	2	2	3	3	4	4

For Review Only

SNP1781	SNP1781	SNP1782	SNP1782	SNP1783	SNP1783	SNP1784	SNP1784	SNP1785	SNP1785
2	2	1	1	3	3	1	1	1	1
2	2	4	4	4	4	3	3	1	1
2	2	1	1	3	3	1	1	1	1
2	2	4	4	3	3	1	1	1	1
4	4	1	1	4	4	3	3	1	1
2	2	1	1	3	3	1	1	1	1
2	2	1	1	3	3	1	1	1	1
2	2	4	4	3	3	1	1	1	1
4	4	1	1	4	4	3	3	1	1
2	2	4	4	4	4	3	3	1	1
2	2	4	4	4	4	3	3	1	1
2	2	1	1	3	3	1	1	1	1
2	2	4	4	4	4	3	3	1	1
2	2	1	1	4	4	3	3	1	1
2	2	1	1	3	3	1	1	1	1
2	2	1	1	3	3	1	1	1	1
4	4	1	1	3	3	1	1	1	1
2	2	4	4	4	4	3	3	1	1
2	2	1	1	4	4	3	3	1	1
2	2	4	4	3	3	1	1	1	1
2	2	1	1	3	3	1	1	1	1
4	4	1	1	4	4	1	1	1	1
2	2	4	4	4	4	3	3	1	1
2	2	4	4	4	4	3	3	1	1
2	2	1	1	4	4	3	3	1	1
2	2	4	4	3	3	1	1	1	1
2	2	1	1	4	4	1	1	1	1
2	2	4	4	4	4	1	1	1	1
2	2	4	4	4	4	3	3	1	1
2	2	4	4	4	4	3	3	1	1
2	2	1	1	4	4	3	3	1	1
4	4	1	1	3	3	1	1	1	1
4	4	4	4	4	4	3	3	1	1
2	2	4	4	4	4	3	3	1	1
2	2	4	4	4	4	3	3	1	1
2	2	4	4	4	4	3	3	1	1
2	2	4	4	3	3	1	1	1	1
2	2	1	1	4	4	1	1	1	1
2	2	4	4	4	4	3	3	1	1
2	2	1	1	3	3	1	1	1	1
2	2	4	4	4	4	3	3	1	1
2	2	4	4	4	4	3	3	1	1
2	2	4	4	4	4	3	3	1	1
2	2	1	1	4	4	1	1	1	1
2	2	1	1	4	4	3	3	1	1
2	2	1	1	3	3	1	1	1	1
2	2	4	4	3	3	1	1	4	4
2	2	1	1	4	4	3	3	1	1
2	2	1	1	4	4	3	3	1	1
2	2	4	4	3	3	1	1	1	1
2	2	1	1	4	4	3	3	4	4
2	2	1	1	3	3	1	1	1	1

For Review Only

4	4	1	1	4	4	3	3	1	1
4	4	4	4	4	4	3	3	1	1
2	2	1	1	4	4	1	1	1	1
2	2	1	1	3	3	1	1	1	1
2	2	1	1	4	4	3	3	1	1
2	2	4	4	3	3	3	3	4	4
2	2	1	1	3	3	1	1	1	1
2	2	4	4	4	4	3	3	1	1
2	2	4	4	4	4	3	3	1	1
2	2	4	4	4	4	1	1	1	1
2	2	1	1	3	3	1	1	1	1
2	2	1	1	3	3	1	1	4	4
4	4	1	1	4	4	3	3	1	1
2	2	4	4	4	4	3	3	1	1
2	2	4	4	4	4	1	1	1	1
2	2	4	4	3	3	3	3	1	1
2	2	4	4	3	3	1	1	1	1
2	2	4	4	3	3	1	1	1	1
4	4	1	1	4	4	3	3	1	1
2	2	1	1	3	3	1	1	1	1
2	2	1	1	3	3	1	1	1	1
2	2	4	4	4	4	3	3	1	1
2	2	4	4	3	3	3	3	1	1
2	2	1	1	4	4	1	1	1	1
2	2	1	1	4	4	3	3	1	1
2	2	1	1	3	3	1	1	1	1
2	2	1	1	4	4	3	3	1	1
2	2	4	4	4	4	3	3	1	1
2	2	4	4	3	3	1	1	1	1
2	2	1	1	3	3	1	1	1	1
4	4	1	1	4	4	3	3	1	1
2	2	4	4	4	4	3	3	1	1
2	2	4	4	4	4	3	3	1	1
4	4	1	1	4	4	3	3	1	1
2	2	4	4	4	4	3	3	1	1
2	2	1	1	4	4	3	3	1	1
2	2	1	1	4	4	3	3	1	1
2	2	4	4	4	4	3	3	1	1
2	2	1	1	3	3	1	1	1	1
2	2	1	1	3	3	1	1	1	1
4	4	1	1	3	3	1	1	1	1
2	2	1	1	3	3	1	1	1	1
2	2	1	1	3	3	1	1	1	1
2	2	1	1	3	3	1	1	1	1
2	2	4	4	4	4	3	3	1	1
2	2	4	4	3	3	1	1	1	1
2	2	4	4	4	4	3	3	1	1
2	2	4	4	3	3	1	1	1	1
2	2	4	4	4	4	3	3	1	1
2	2	1	1	3	3	1	1	1	1
2	2	1	1	4	4	3	3	1	1
2	2	4	4	4	4	3	3	1	1
2	2	4	4	3	3	3	3	1	1
2	2	4	4	4	4	3	3	1	1
2	2	1	1	3	3	1	1	1	1

For Review Only

2	2	4	4	3	3	1	1	1	1
2	2	1	1	4	4	1	1	1	1
2	2	4	4	4	4	1	1	1	1
2	2	4	4	4	4	3	3	1	1
2	2	1	1	4	4	1	1	1	1
2	2	4	4	3	3	1	1	1	1
2	2	1	1	3	3	1	1	1	1
2	2	1	1	4	4	3	3	1	1
2	2	4	4	4	4	3	3	1	1
2	2	4	4	4	4	3	3	1	1
2	2	1	1	4	4	3	3	1	1
2	2	4	4	4	4	1	1	1	1
2	2	1	1	3	3	1	1	1	1
2	2	1	1	3	3	1	1	1	1
2	2	4	4	4	4	1	1	1	1
2	2	4	4	4	4	1	1	1	1
2	2	4	4	3	3	1	1	1	1
2	2	4	4	4	4	1	1	1	1
2	2	4	4	4	4	3	3	1	1
2	2	1	1	3	3	1	1	1	1
2	2	4	4	3	3	1	1	1	1
2	2	4	4	3	3	1	1	1	1
2	2	1	1	3	3	1	1	1	1
2	2	4	4	4	4	3	3	1	1
2	2	4	4	3	3	1	1	1	1
2	2	4	4	3	3	1	1	1	1
2	2	4	4	3	3	1	1	1	1
2	2	4	4	3	3	1	1	4	4
2	2	4	4	3	3	1	1	1	1
2	2	1	1	4	4	3	3	1	1
2	2	4	4	4	4	3	3	1	1
2	2	1	1	4	4	1	1	1	1
4	4	1	1	3	3	1	1	1	1
2	2	4	4	4	4	1	1	1	1
2	2	4	4	4	4	3	3	1	1
2	2	4	4	4	4	3	3	1	1
2	2	4	4	4	4	3	3	1	1
4	4	1	1	3	3	1	1	1	1
2	2	4	4	4	4	3	3	1	1
2	2	4	4	3	3	1	1	4	4
2	2	4	4	3	3	1	1	1	1
2	2	4	4	4	4	3	3	1	1
2	2	4	4	3	3	1	1	1	1
2	2	4	4	4	4	3	3	1	1
2	2	4	4	3	3	1	1	1	1
2	2	1	1	3	3	1	1	1	1
2	2	4	4	3	3	1	1	1	1
2	2	1	1	3	3	1	1	1	1
2	2	4	4	4	4	3	3	1	1
2	2	4	4	4	4	1	1	1	1
2	2	4	4	4	4	3	3	1	1
2	2	4	4	3	3	3	3	1	1
2	2	4	4	3	3	1	1	1	1
2	2	1	1	4	4	3	3	1	1
2	2	4	4	4	4	1	1	1	1

For Review Only

4	4	4	4	3	3	1	1	1	1
2	2	1	1	3	3	1	1	1	1
2	2	4	4	4	4	3	3	1	1
2	2	4	4	4	4	3	3	1	1
2	2	1	1	3	3	1	1	1	1
2	2	1	1	3	3	1	1	1	1
2	2	4	4	4	4	1	1	1	1
2	2	4	4	4	4	1	1	1	1
2	2	4	4	4	4	1	1	1	1
2	2	4	4	3	3	1	1	1	1
2	2	4	4	3	3	1	1	1	1
2	2	4	4	4	4	3	3	1	1
2	2	1	1	3	3	1	1	1	1
2	2	4	4	3	3	1	1	1	1
2	2	4	4	3	3	1	1	4	4
2	2	4	4	4	4	3	3	1	1
2	2	4	4	4	4	1	1	1	1
2	2	1	1	3	3	1	1	1	1
2	2	4	4	3	3	1	1	1	1
2	2	1	1	3	3	1	1	1	1
2	2	4	4	4	4	1	1	1	1
2	2	4	4	4	4	3	3	1	1
2	2	4	4	4	4	1	1	1	1
2	2	1	1	3	3	1	1	4	4
2	2	4	4	4	4	3	3	1	1
2	2	4	4	4	4	3	3	1	1
2	2	1	1	3	3	1	1	1	1
2	2	4	4	4	4	1	1	1	1
2	2	1	1	3	3	1	1	1	1
2	2	1	1	3	3	1	1	1	1
2	2	1	1	4	4	1	1	1	1
2	2	4	4	3	3	1	1	1	1
2	2	4	4	3	3	1	1	1	1
2	2	4	4	3	3	1	1	4	4
2	2	4	4	4	4	1	1	1	1
2	2	1	1	3	3	1	1	4	4
2	2	4	4	3	3	1	1	1	1
2	2	4	4	3	3	1	1	1	1
2	2	1	1	3	3	1	1	1	1
2	2	1	1	4	4	1	1	1	1
2	2	1	1	3	3	1	1	1	1
2	2	4	4	3	3	1	1	1	1
2	2	4	4	4	4	3	3	1	1
2	2	4	4	4	4	3	3	1	1
2	2	4	4	4	4	3	3	1	1
2	2	4	4	3	3	1	1	1	1
2	2	4	4	4	4	3	3	1	1
2	2	4	4	4	4	3	3	1	1
2	2	4	4	4	4	3	3	1	1
2	2	4	4	3	3	1	1	1	1
2	2	4	4	3	3	1	1	1	1
2	2	4	4	4	4	3	3	1	1
2	2	4	4	4	4	3	3	1	1
2	2	1	1	3	3	1	1	1	1
2	2	4	4	3	3	1	1	1	1

For Review Only

2	2	4	4	3	3	1	1	1	1
2	2	4	4	4	4	3	3	1	1
2	2	4	4	3	3	1	1	1	1
2	2	4	4	3	3	1	1	1	1
2	2	4	4	4	4	3	3	1	1
2	2	4	4	3	3	1	1	1	1
2	2	4	4	3	3	1	1	1	1
2	2	4	4	4	4	3	3	1	1
2	2	4	4	4	4	3	3	1	1
2	2	4	4	4	4	3	3	1	1
2	2	4	4	4	4	1	1	1	1
2	2	4	4	4	4	1	1	1	1
2	2	4	4	3	3	1	1	1	1
2	2	1	1	3	3	1	1	1	1
2	2	1	1	3	3	1	1	1	1
2	2	4	4	3	3	1	1	4	4
2	2	4	4	4	4	3	3	1	1
2	2	4	4	4	4	1	1	1	1
2	2	4	4	4	4	3	3	1	1
2	2	1	1	4	4	1	1	1	1
2	2	4	4	4	4	1	1	1	1
2	2	4	4	3	3	1	1	1	1
2	2	1	1	3	3	1	1	1	1
2	2	1	1	4	4	3	3	1	1
2	2	4	4	4	4	3	3	1	1
2	2	1	1	4	4	3	3	1	1
2	2	4	4	4	4	3	3	1	1
2	2	1	1	3	3	1	1	1	1
4	4	4	4	4	4	3	3	1	1
2	2	4	4	3	3	1	1	1	1
2	2	4	4	4	4	3	3	1	1
2	2	4	4	4	4	3	3	1	1
2	2	4	4	3	3	1	1	1	1
2	2	4	4	3	3	1	1	1	1
2	2	4	4	3	3	1	1	1	1
2	2	4	4	4	4	3	3	1	1
2	2	4	4	3	3	1	1	1	1
2	2	1	1	3	3	1	1	4	4
2	2	4	4	4	4	3	3	1	1
2	2	4	4	4	4	3	3	1	1
2	2	4	4	3	3	1	1	1	1
2	2	4	4	3	3	1	1	1	1
2	2	4	4	4	4	3	3	1	1
4	4	1	1	3	3	1	1	1	1
2	2	4	4	3	3	1	1	4	4
4	4	1	1	3	3	1	1	1	1
2	2	1	1	3	3	1	1	1	1
2	2	1	1	3	3	1	1	1	1
2	2	1	1	4	4	1	1	1	1
2	2	4	4	4	4	3	3	1	1
2	2	1	1	3	3	1	1	1	1
2	2	4	4	3	3	1	1	4	4
2	2	1	1	3	3	1	1	1	1
2	2	1	1	3	3	1	1	1	1
2	2	4	4	4	4	3	3	1	1

For Review Only

2	2	4	4	3	3	1	1	1	1
2	2	1	1	3	3	1	1	1	1

For Review Only

SNP1786	SNP1786	SNP1787	SNP1787	SNP1788	SNP1788	SNP1789	SNP1789	SNP179	SNP179
1	1	2	2	4	4	3	3	4	4
4	4	2	2	1	1	1	1	4	4
4	4	2	2	1	1	3	3	4	4
4	4	2	2	1	1	3	3	4	4
1	1	2	2	4	4	1	1	4	4
4	4	2	2	1	1	3	3	1	1
1	1	2	2	4	4	3	3	4	4
4	4	2	2	1	1	3	3	4	4
1	1	2	2	4	4	1	1	4	4
4	4	2	2	1	1	1	1	4	4
4	4	3	3	1	1	1	1	4	4
4	4	2	2	1	1	3	3	4	4
4	4	2	2	1	1	1	1	4	4
1	1	2	2	4	4	1	1	4	4
4	4	2	2	4	4	3	3	1	1
4	4	2	2	1	1	3	3	4	4
1	1	2	2	4	4	3	3	4	4
4	4	2	2	1	1	1	1	1	1
4	4	2	2	1	1	3	3	4	4
4	4	2	2	1	1	3	3	4	4
1	1	2	2	4	4	3	3	4	4
1	1	2	2	4	4	1	1	4	4
4	4	3	3	1	1	1	1	1	1
4	4	3	3	1	1	1	1	4	4
4	4	2	2	1	1	1	1	4	4
4	4	2	2	1	1	3	3	4	4
4	4	2	2	1	1	3	3	4	4
4	4	2	2	1	1	3	3	4	4
4	4	2	2	1	1	1	1	4	4
4	4	2	2	1	1	1	1	4	4
4	4	2	2	1	1	3	3	4	4
1	1	2	2	4	4	3	3	1	1
4	4	2	2	1	1	1	1	4	4
4	4	2	2	1	1	1	1	4	4
4	4	2	2	1	1	3	3	4	4
4	4	2	2	1	1	3	3	4	4
4	4	2	2	1	1	3	3	4	4
4	4	2	2	1	1	3	3	4	4
4	4	3	3	1	1	1	1	4	4
4	4	3	3	1	1	3	3	4	4
4	4	2	2	1	1	1	1	4	4
4	4	2	2	1	1	1	1	4	4
4	4	2	2	1	1	1	1	4	4
1	1	2	2	4	4	1	1	4	4
1	1	2	2	4	4	1	1	1	1
4	4	2	2	1	1	3	3	1	1
4	4	2	2	1	1	3	3	4	4
4	4	2	2	1	1	1	1	1	1
1	1	2	2	4	4	1	1	4	4
4	4	2	2	1	1	3	3	1	1
4	4	2	2	4	4	3	3	4	4
4	4	2	2	1	1	3	3	4	4

For Review Only

1	1	2	2	4	4	1	1	4	4
4	4	2	2	1	1	1	1	4	4
4	4	2	2	1	1	3	3	4	4
1	1	2	2	4	4	3	3	4	4
4	4	2	2	1	1	3	3	4	4
4	4	2	2	1	1	3	3	4	4
4	4	2	2	1	1	3	3	4	4
4	4	2	2	1	1	1	1	4	4
4	4	2	2	1	1	1	1	4	4
4	4	2	2	1	1	3	3	4	4
4	4	2	2	1	1	3	3	4	4
4	4	2	2	1	1	3	3	4	4
1	1	2	2	4	4	1	1	4	4
4	4	2	2	1	1	1	1	4	4
4	4	2	2	1	1	3	3	1	1
1	1	2	2	1	1	1	1	4	4
4	4	2	2	1	1	1	1	4	4
4	4	2	2	1	1	3	3	1	1
1	1	2	2	4	4	1	1	1	1
1	1	2	2	4	4	3	3	4	4
4	4	2	2	1	1	3	3	4	4
4	4	3	3	1	1	1	1	4	4
4	4	2	2	1	1	1	1	4	4
4	4	2	2	1	1	3	3	1	1
1	1	2	2	4	4	1	1	1	1
4	4	2	2	1	1	3	3	4	4
1	1	2	2	4	4	1	1	4	4
4	4	2	2	1	1	1	1	4	4
4	4	2	2	1	1	3	3	4	4
4	4	2	2	1	1	1	1	1	1
1	1	2	2	4	4	1	1	1	1
4	4	2	2	1	1	3	3	4	4
4	4	2	2	1	1	1	1	4	4
1	1	2	2	4	4	1	1	4	4
4	4	2	2	1	1	1	1	4	4
1	1	2	2	4	4	1	1	1	1
1	1	2	2	4	4	1	1	1	1
4	4	2	2	1	1	1	1	4	4
4	4	2	2	1	1	3	3	4	4
1	1	2	2	4	4	3	3	4	4
1	1	2	2	4	4	3	3	4	4
4	4	2	2	1	1	3	3	1	1
1	1	2	2	4	4	3	3	4	4
4	4	2	2	1	1	3	3	4	4
4	4	2	2	1	1	1	1	1	1
4	4	2	2	1	1	3	3	4	4
4	4	2	2	1	1	1	1	4	4
4	4	2	2	1	1	3	3	4	4
4	4	2	2	1	1	3	3	4	4
4	4	2	2	1	1	3	3	1	1
4	4	2	2	1	1	3	3	4	4
4	4	3	3	1	1	1	1	4	4
4	4	2	2	1	1	3	3	4	4
4	4	2	2	1	1	3	3	4	4
1	1	2	2	4	4	3	3	1	1

For Review Only

4	4	2	2	1	1	3	3	4	4
4	4	3	3	1	1	3	3	4	4
4	4	2	2	1	1	3	3	1	1
4	4	2	2	1	1	3	3	4	4
4	4	2	2	1	1	3	3	4	4
4	4	2	2	1	1	3	3	1	1
4	4	2	2	1	1	3	3	4	4
4	4	2	2	1	1	3	3	4	4
4	4	2	2	1	1	1	1	4	4
4	4	2	2	1	1	3	3	4	4
1	1	2	2	4	4	1	1	4	4
4	4	2	2	1	1	3	3	4	4
1	1	2	2	4	4	3	3	1	1
4	4	2	2	1	1	3	3	4	4
4	4	2	2	1	1	3	3	1	1
4	4	2	2	1	1	3	3	4	4
4	4	2	2	1	1	3	3	1	1
4	4	2	2	1	1	3	3	4	4
4	4	2	2	1	1	3	3	4	4
1	1	2	2	4	4	3	3	4	4
4	4	2	2	1	1	3	3	4	4
4	4	2	2	1	1	1	1	1	1
4	4	2	2	1	1	3	3	4	4
4	4	2	2	1	1	1	1	4	4
4	4	2	2	1	1	3	3	1	1
4	4	3	3	1	1	3	3	1	1
4	4	2	2	1	1	3	3	4	4
4	4	2	2	1	1	3	3	4	4
4	4	2	2	1	1	3	3	1	1
4	4	2	2	1	1	1	1	4	4
4	4	2	2	1	1	1	1	4	4
4	4	2	2	1	1	3	3	4	4
1	1	2	2	4	4	3	3	4	4
4	4	2	2	1	1	3	3	4	4
4	4	2	2	1	1	1	1	4	4
1	1	2	2	1	1	1	1	1	1
4	4	2	2	1	1	1	1	4	4
1	1	2	2	4	4	3	3	4	4
4	4	2	2	1	1	3	3	4	4
4	4	2	2	1	1	3	3	4	4
4	4	2	2	1	1	3	3	4	4
4	4	2	2	1	1	1	1	4	4
4	4	2	2	1	1	3	3	1	1
4	4	2	2	1	1	1	1	4	4
4	4	2	2	1	1	3	3	1	1
1	1	2	2	1	1	3	3	4	4
4	4	2	2	1	1	3	3	4	4
4	4	2	2	1	1	3	3	4	4
4	4	2	2	1	1	1	1	4	4
4	4	2	2	1	1	3	3	4	4
4	4	2	2	1	1	3	3	1	1
4	4	2	2	1	1	3	3	4	4
4	4	2	2	1	1	3	3	1	1
4	4	2	2	1	1	3	3	4	4
4	4	3	3	1	1	3	3	4	4

For Review Only

4	4	2	2	1	1	3	3	4	4
1	1	3	3	4	4	3	3	4	4
4	4	2	2	1	1	1	1	1	1
4	4	3	3	1	1	1	1	1	1
4	4	2	2	1	1	3	3	1	1
4	4	2	2	1	1	3	3	4	4
4	4	3	3	1	1	1	1	4	4
4	4	2	2	1	1	3	3	1	1
4	4	2	2	1	1	3	3	4	4
4	4	2	2	1	1	1	1	1	1
4	4	2	2	1	1	3	3	4	4
4	4	3	3	1	1	1	1	4	4
4	4	2	2	1	1	3	3	1	1
4	4	2	2	1	1	3	3	4	4
4	4	2	2	1	1	3	3	4	4
4	4	3	3	1	1	1	1	4	4
4	4	2	2	1	1	3	3	1	1
4	4	2	2	1	1	3	3	4	4
4	4	2	2	1	1	3	3	4	4
4	4	2	2	1	1	3	3	4	4
4	4	2	2	1	1	3	3	4	4
4	4	3	3	1	1	1	1	4	4
4	4	3	3	1	1	3	3	4	4
4	4	2	2	1	1	3	3	4	4
4	4	2	2	1	1	1	1	4	4
4	4	2	2	1	1	1	1	4	4
4	4	2	2	1	1	3	3	1	1
4	4	2	2	1	1	3	3	1	1
1	1	2	2	4	4	3	3	1	1
1	1	2	2	4	4	3	3	1	1
1	1	2	2	1	1	3	3	4	4
4	4	2	2	1	1	3	3	1	1
4	4	2	2	1	1	3	3	4	4
4	4	2	2	1	1	3	3	4	4
4	4	2	2	1	1	3	3	4	4
4	4	2	2	1	1	3	3	4	4
4	4	3	3	1	1	3	3	4	4
4	4	2	2	1	1	3	3	4	4
4	4	2	2	1	1	3	3	4	4
4	4	2	2	1	1	3	3	1	1
4	4	2	2	1	1	3	3	4	4
4	4	2	2	1	1	3	3	4	4
4	4	2	2	1	1	1	1	4	4
4	4	2	2	1	1	3	3	4	4
4	4	2	2	1	1	1	1	4	4
4	4	2	2	1	1	3	3	4	4
4	4	3	3	1	1	1	1	4	4
4	4	3	3	1	1	1	1	4	4
4	4	2	2	1	1	1	1	4	4
4	4	2	2	1	1	3	3	4	4
4	4	2	2	1	1	3	3	1	1
4	4	2	2	1	1	1	1	4	4
4	4	3	3	1	1	1	1	4	4
4	4	2	2	1	1	3	3	4	4
4	4	3	3	1	1	3	3	4	4

For Review Only

4	4	2	2	1	1	3	3	1	1
4	4	2	2	1	1	1	1	4	4
4	4	2	2	1	1	3	3	1	1
4	4	2	2	1	1	3	3	4	4
4	4	2	2	1	1	1	1	4	4
4	4	2	2	1	1	3	3	1	1
4	4	3	3	1	1	3	3	4	4
4	4	2	2	1	1	1	1	4	4
4	4	2	2	1	1	3	3	4	4
4	4	2	2	1	1	1	1	4	4
4	4	2	2	1	1	3	3	4	4
4	4	2	2	1	1	3	3	1	1
4	4	2	2	1	1	3	3	4	4
4	4	2	2	1	1	3	3	4	4
4	4	2	2	1	1	3	3	1	1
4	4	2	2	1	1	3	3	1	1
4	4	2	2	1	1	1	1	4	4
4	4	3	3	1	1	3	3	4	4
4	4	3	3	1	1	1	1	4	4
4	4	2	2	1	1	3	3	1	1
4	4	3	3	1	1	3	3	4	4
4	4	2	2	1	1	3	3	4	4
1	1	2	2	4	4	1	1	4	4
1	1	2	2	4	4	1	1	4	4
4	4	2	2	1	1	1	1	4	4
4	4	2	2	1	1	1	1	4	4
4	4	2	2	1	1	1	1	4	4
4	4	2	2	1	1	3	3	4	4
4	4	2	2	1	1	1	1	4	4
4	4	2	2	1	1	3	3	1	1
4	4	3	3	1	1	1	1	4	4
4	4	2	2	1	1	1	1	4	4
4	4	2	2	1	1	3	3	4	4
4	4	2	2	1	1	3	3	1	1
4	4	3	3	1	1	3	3	4	4
4	4	2	2	1	1	1	1	4	4
4	4	2	2	1	1	3	3	1	1
4	4	2	2	1	1	3	3	4	4
4	4	2	2	1	1	3	3	4	4
4	4	3	3	1	1	1	1	4	4
4	4	2	2	1	1	3	3	4	4
4	4	2	2	1	1	3	3	4	4
4	4	3	3	1	1	1	1	4	4
1	1	2	2	4	4	3	3	4	4
4	4	2	2	1	1	3	3	4	4
4	4	2	2	1	1	3	3	4	4
4	4	2	2	1	1	3	3	1	1
1	1	2	2	1	1	3	3	4	4
1	1	2	2	4	4	1	1	4	4
4	4	2	2	1	1	1	1	4	4
1	1	2	2	4	4	3	3	4	4
4	4	3	3	1	1	3	3	4	4
4	4	2	2	1	1	3	3	4	4
4	4	2	2	1	1	3	3	1	1
4	4	3	3	1	1	1	1	4	4

4	4	2	2	1	1	3	3	1	1
4	4	2	2	1	1	3	3	4	4

For Review Only

SNP1790	SNP1790	SNP1791	SNP1791	SNP1792	SNP1792	SNP1793	SNP1793	SNP1794	SNP1794
4	4	1	1	2	2	4	4	3	3
4	4	4	4	2	2	4	4	4	4
3	3	1	1	2	2	4	4	3	3
4	4	1	1	2	2	4	4	3	3
4	4	4	4	2	2	4	4	3	3
4	4	1	1	2	2	4	4	3	3
3	3	1	1	2	2	4	4	3	3
4	4	1	1	2	2	4	4	3	3
4	4	4	4	2	2	4	4	4	4
4	4	4	4	2	2	1	1	3	3
4	4	4	4	3	3	4	4	4	4
3	3	1	1	2	2	4	4	3	3
4	4	1	1	2	2	4	4	3	3
4	4	4	4	2	2	4	4	4	4
3	3	1	1	2	2	4	4	3	3
4	4	1	1	2	2	4	4	3	3
3	3	1	1	2	2	4	4	3	3
4	4	4	4	2	2	4	4	4	4
4	4	1	1	2	2	4	4	3	3
4	4	1	1	2	2	1	1	3	3
3	3	1	1	2	2	4	4	3	3
3	3	1	1	2	2	4	4	3	3
4	4	4	4	2	2	1	1	4	4
4	4	4	4	2	2	4	4	4	4
4	4	1	1	2	2	4	4	3	3
4	4	1	1	3	3	4	4	3	3
4	4	4	4	3	3	4	4	4	4
4	4	1	1	2	2	4	4	3	3
3	3	4	4	2	2	4	4	4	4
4	4	4	4	2	2	1	1	4	4
3	3	1	1	2	2	4	4	3	3
3	3	1	1	3	3	4	4	3	3
3	3	4	4	3	3	4	4	3	3
4	4	4	4	2	2	4	4	3	3
4	4	4	4	2	2	4	4	3	3
4	4	4	4	2	2	4	4	4	4
4	4	1	1	2	2	4	4	3	3
4	4	4	4	2	2	4	4	4	4
4	4	4	4	2	2	4	4	4	4
4	4	1	1	2	2	4	4	3	3
4	4	4	4	2	2	4	4	4	4
4	4	4	4	2	2	4	4	4	4
4	4	1	1	2	2	4	4	3	3
3	3	1	1	2	2	4	4	3	3
4	4	4	4	2	2	4	4	4	4
4	4	1	1	2	2	4	4	3	3
4	4	4	4	3	3	4	4	3	3
4	4	1	1	2	2	4	4	4	4
3	3	4	4	2	2	4	4	4	4
3	3	1	1	2	2	4	4	3	3
4	4	1	1	3	3	4	4	3	3
4	4	1	1	2	2	4	4	3	3

For Review Only

3	3	4	4	2	2	4	4	3	3
3	3	4	4	3	3	4	4	3	3
3	3	4	4	2	2	4	4	4	4
3	3	1	1	2	2	1	1	3	3
4	4	1	1	2	2	4	4	3	3
4	4	4	4	3	3	4	4	3	3
4	4	1	1	3	3	4	4	3	3
4	4	4	4	2	2	4	4	4	4
4	4	4	4	2	2	4	4	4	4
4	4	1	1	2	2	4	4	3	3
4	4	1	1	3	3	4	4	3	3
4	4	4	4	2	2	4	4	3	3
3	3	4	4	2	2	4	4	3	3
3	3	4	4	2	2	4	4	4	4
4	4	1	1	2	2	4	4	3	3
3	3	4	4	2	2	4	4	3	3
4	4	1	1	2	2	4	4	3	3
3	3	1	1	3	3	4	4	3	3
3	3	4	4	2	2	4	4	3	3
3	3	1	1	2	2	4	4	3	3
4	4	4	4	2	2	4	4	3	3
4	4	1	1	2	2	4	4	3	3
3	3	1	1	2	2	4	4	3	3
4	4	1	1	2	2	4	4	3	3
4	4	4	4	2	2	4	4	4	4
4	4	1	1	2	2	1	1	3	3
3	3	4	4	3	3	4	4	3	3
4	4	4	4	2	2	1	1	3	3
4	4	1	1	3	3	4	4	3	3
4	4	4	4	2	2	4	4	3	3
4	4	4	4	2	2	1	1	3	3
4	4	4	4	2	2	4	4	4	4
4	4	4	4	2	2	1	1	3	3
3	3	4	4	2	2	4	4	3	3
4	4	4	4	2	2	4	4	3	3
4	4	4	4	2	2	4	4	4	4
4	4	4	4	2	2	1	1	4	4
3	3	4	4	2	2	4	4	3	3
4	4	1	1	3	3	4	4	3	3
4	4	1	1	2	2	1	1	3	3
3	3	1	1	2	2	4	4	3	3
4	4	1	1	2	2	4	4	3	3
3	3	1	1	2	2	4	4	3	3
4	4	1	1	3	3	4	4	3	3
4	4	4	4	2	2	4	4	4	4
4	4	1	1	2	2	4	4	3	3
4	4	4	4	2	2	4	4	4	4
4	4	1	1	2	2	4	4	3	3
4	4	4	4	2	2	4	4	4	4
3	3	1	1	2	2	4	4	3	3
4	4	1	1	2	2	4	4	3	3
4	4	4	4	2	2	1	1	4	4
4	4	4	4	2	2	4	4	3	3
4	4	4	4	2	2	4	4	3	3
3	3	1	1	3	3	4	4	3	3

For Review Only

4	4	1	1	3	3	4	4	3	3
4	4	4	4	2	2	4	4	4	4
4	4	1	1	3	3	4	4	3	3
4	4	4	4	2	2	4	4	3	3
4	4	1	1	2	2	4	4	3	3
4	4	1	1	2	2	4	4	3	3
3	3	1	1	2	2	4	4	3	3
4	4	1	1	2	2	4	4	3	3
4	4	4	4	2	2	4	4	4	4
4	4	1	1	2	2	4	4	3	3
4	4	4	4	2	2	4	4	3	3
4	4	1	1	2	2	4	4	3	3
3	3	1	1	2	2	1	1	3	3
4	4	1	1	2	2	4	4	3	3
4	4	1	1	2	2	4	4	3	3
4	4	1	1	2	2	4	4	3	3
3	3	1	1	3	3	4	4	3	3
4	4	1	1	3	3	4	4	3	3
4	4	4	4	2	2	4	4	3	3
3	3	1	1	3	3	4	4	3	3
4	4	1	1	2	2	4	4	3	3
4	4	4	4	2	2	4	4	3	3
3	3	1	1	3	3	4	4	3	3
4	4	4	4	2	2	4	4	3	3
4	4	1	1	2	2	4	4	3	3
4	4	1	1	2	2	4	4	3	3
4	4	1	1	2	2	4	4	3	3
4	4	4	4	2	2	4	4	3	3
4	4	1	1	2	2	4	4	3	3
4	4	1	1	3	3	4	4	3	3
4	4	4	4	2	2	4	4	4	4
4	4	1	1	2	2	4	4	3	3
3	3	1	1	2	2	4	4	3	3
4	4	1	1	2	2	4	4	3	3
4	4	4	4	2	2	4	4	3	3
4	4	4	4	2	2	4	4	3	3
4	4	4	4	2	2	4	4	4	4
4	4	1	1	3	3	4	4	3	3
4	4	1	1	3	3	4	4	3	3
4	4	4	4	3	3	4	4	3	3
4	4	1	1	2	2	4	4	3	3
4	4	4	4	2	2	4	4	3	3
3	3	1	1	2	2	4	4	3	3
4	4	4	4	2	2	4	4	4	4
4	4	1	1	2	2	4	4	3	3
4	4	1	1	2	2	4	4	3	3
4	4	1	1	3	3	4	4	3	3
4	4	1	1	3	3	4	4	3	3
4	4	4	4	2	2	4	4	3	3
4	4	1	1	3	3	4	4	3	3
4	4	4	4	3	3	4	4	4	4
4	4	4	4	2	2	4	4	3	3
4	4	1	1	2	2	4	4	3	3
4	4	4	4	2	2	4	4	4	4
4	4	1	1	2	2	4	4	4	4

For Review Only

4	4	1	1	3	3	4	4	3	3
4	4	1	1	2	2	4	4	3	3
4	4	4	4	2	2	4	4	4	4
4	4	4	4	2	2	4	4	4	4
4	4	1	1	2	2	4	4	3	3
4	4	1	1	2	2	4	4	3	3
4	4	1	1	2	2	4	4	3	3
4	4	1	1	2	2	4	4	3	3
4	4	4	4	2	2	4	4	4	4
4	4	4	4	2	2	4	4	3	3
4	4	1	1	3	3	4	4	3	3
4	4	4	4	2	2	4	4	4	4
4	4	1	1	2	2	4	4	3	3
4	4	1	1	2	2	4	4	3	3
4	4	4	4	2	2	4	4	3	3
4	4	4	4	3	3	4	4	4	4
4	4	1	1	2	2	4	4	3	3
4	4	1	1	2	2	4	4	3	3
4	4	1	1	3	3	4	4	3	3
3	3	1	1	2	2	4	4	3	3
4	4	1	1	2	2	4	4	3	3
4	4	4	4	2	2	4	4	4	4
4	4	1	1	2	2	4	4	3	3
4	4	4	4	2	2	4	4	3	3
3	3	4	4	2	2	4	4	4	4
4	4	4	4	2	2	4	4	3	3
3	3	1	1	2	2	1	1	3	3
3	3	1	1	2	2	4	4	3	3
4	4	1	1	2	2	1	1	3	3
3	3	1	1	2	2	4	4	3	3
3	3	1	1	2	2	4	4	3	3
4	4	1	1	3	3	4	4	3	3
4	4	1	1	2	2	4	4	3	3
4	4	4	4	2	2	4	4	3	3
4	4	1	1	2	2	4	4	3	3
4	4	4	4	2	2	4	4	3	3
4	4	1	1	3	3	4	4	3	3
4	4	1	1	2	2	4	4	3	3
4	4	1	1	3	3	4	4	3	3
3	3	4	4	2	2	4	4	4	4
3	3	1	1	2	2	4	4	3	3
4	4	1	1	3	3	4	4	3	3
4	4	4	4	2	2	4	4	4	4
4	4	4	4	2	2	4	4	4	4
4	4	4	4	2	2	4	4	3	3
4	4	1	1	2	2	4	4	3	3
4	4	4	4	3	3	4	4	4	4
4	4	4	4	2	2	4	4	4	4
4	4	4	4	2	2	4	4	4	4
3	3	1	1	2	2	1	1	3	3
4	4	1	1	2	2	4	4	3	3
4	4	4	4	2	2	4	4	4	4
4	4	4	4	2	2	4	4	4	4
4	4	1	1	3	3	4	4	3	3
4	4	4	4	2	2	4	4	4	4

For Review Only

4	4	1	1	2	2	4	4	3	3
4	4	4	4	2	2	4	4	4	4
4	4	1	1	2	2	4	4	3	3
4	4	1	1	2	2	4	4	3	3
4	4	4	4	2	2	4	4	4	4
3	3	1	1	2	2	4	4	3	3
4	4	1	1	2	2	4	4	3	3
4	4	4	4	2	2	1	1	4	4
4	4	4	4	2	2	4	4	3	3
4	4	4	4	2	2	1	1	4	4
4	4	1	1	2	2	4	4	3	3
3	3	1	1	2	2	4	4	3	3
4	4	1	1	3	3	4	4	3	3
4	4	1	1	3	3	4	4	3	3
3	3	1	1	2	2	4	4	3	3
4	4	4	4	3	3	4	4	3	3
4	4	4	4	2	2	1	1	4	4
4	4	1	1	2	2	4	4	3	3
4	4	4	4	2	2	4	4	4	4
4	4	4	4	3	3	4	4	4	4
4	4	1	1	2	2	4	4	3	3
4	4	1	1	3	3	4	4	3	3
4	4	4	4	2	2	4	4	3	3
4	4	4	4	2	2	4	4	4	4
4	4	4	4	2	2	4	4	4	4
3	3	4	4	2	2	4	4	4	4
4	4	4	4	2	2	1	1	4	4
3	3	1	1	3	3	4	4	3	3
4	4	4	4	2	2	4	4	3	3
4	4	1	1	2	2	4	4	3	3
4	4	4	4	2	2	1	1	4	4
4	4	4	4	2	2	4	4	4	4
4	4	1	1	2	2	4	4	3	3
4	4	1	1	3	3	4	4	3	3
4	4	1	1	2	2	4	4	3	3
4	4	4	4	2	2	1	1	3	3
3	3	1	1	2	2	4	4	3	3
4	4	4	4	2	2	4	4	3	3
4	4	4	4	2	2	4	4	4	4
4	4	4	4	2	2	4	4	4	4
3	3	1	1	3	3	4	4	3	3
3	3	1	1	2	2	4	4	3	3
4	4	4	4	2	2	4	4	4	4
3	3	1	1	2	2	4	4	3	3
4	4	4	4	3	3	4	4	3	3
4	4	1	1	3	3	4	4	3	3
4	4	1	1	3	3	4	4	3	3
4	4	1	1	2	2	1	1	3	3
3	3	1	1	2	2	4	4	3	3
4	4	4	4	2	2	1	1	4	4
4	4	1	1	2	2	4	4	3	3
4	4	4	4	2	2	4	4	3	3
3	3	1	1	2	2	4	4	3	3
4	4	1	1	3	3	4	4	3	3
4	4	4	4	2	2	4	4	4	4

For Review Only

4	4	1	1	3	3	4	4	3	3
4	4	1	1	2	2	4	4	3	3

For Review Only

SNP1795	SNP1795	SNP1796	SNP1796	SNP1797	SNP1797	SNP1798	SNP1798	SNP1799	SNP1799
1	1	3	3	4	4	4	4	4	4
2	2	3	3	4	4	4	4	4	4
1	1	3	3	4	4	4	4	4	4
2	2	3	3	4	4	4	4	4	4
1	1	3	3	4	4	4	4	4	4
1	1	3	3	4	4	4	4	4	4
2	2	3	3	4	4	4	4	4	4
1	1	3	3	4	4	4	4	4	4
2	2	3	3	4	4	4	4	4	4
2	2	3	3	4	4	4	4	4	4
2	2	3	3	4	4	4	4	4	4
2	2	3	3	4	4	4	4	4	4
1	1	3	3	4	4	4	4	4	4
2	2	3	3	4	4	4	4	4	4
2	2	3	3	4	4	4	4	4	4
1	1	3	3	4	4	4	4	4	4
1	1	3	3	4	4	4	4	4	4
2	2	3	3	4	4	4	4	4	4
1	1	3	3	4	4	4	4	4	4
2	2	3	3	4	4	4	4	4	4
1	1	3	3	4	4	4	4	4	4
1	1	3	3	4	4	4	4	2	2
2	2	3	3	4	4	4	4	4	4
1	1	3	3	1	1	3	3	4	4
1	1	3	3	4	4	4	4	4	4
2	2	3	3	4	4	4	4	4	4
2	2	3	3	4	4	4	4	4	4
2	2	3	3	4	4	4	4	4	4
2	2	3	3	4	4	4	4	4	4
2	2	3	3	1	1	4	4	4	4
1	1	3	3	4	4	4	4	4	4
2	2	3	3	4	4	4	4	4	4
2	2	3	3	4	4	4	4	4	4
2	2	3	3	4	4	3	3	4	4
2	2	3	3	4	4	4	4	4	4
2	2	3	3	4	4	4	4	4	4
2	2	3	3	4	4	4	4	4	4
2	2	3	3	4	4	4	4	4	4
1	1	3	3	1	1	3	3	4	4
1	1	3	3	4	4	4	4	4	4
2	2	3	3	1	1	3	3	4	4
2	2	3	3	4	4	4	4	4	4
2	2	3	3	4	4	4	4	4	4
1	1	3	3	4	4	4	4	2	2
2	2	3	3	4	4	4	4	4	4
2	2	3	3	4	4	4	4	4	4
2	2	2	2	4	4	4	4	4	4
1	1	3	3	4	4	4	4	4	4
2	2	3	3	4	4	4	4	4	4
1	1	3	3	4	4	4	4	4	4
2	2	3	3	4	4	4	4	4	4
2	2	3	3	4	4	4	4	4	4

For Review Only

1	1	3	3	4	4	4	4	4	4
2	2	3	3	4	4	4	4	4	4
1	1	3	3	4	4	4	4	4	4
2	2	3	3	1	1	4	4	4	4
1	1	3	3	4	4	4	4	4	4
2	2	2	2	4	4	4	4	4	4
2	2	3	3	4	4	4	4	4	4
1	1	3	3	1	1	3	3	4	4
2	2	3	3	4	4	3	3	4	4
2	2	3	3	4	4	4	4	4	4
2	2	3	3	4	4	4	4	4	4
1	1	2	2	4	4	4	4	4	4
1	1	3	3	4	4	4	4	4	4
2	2	3	3	4	4	4	4	4	4
2	2	3	3	4	4	4	4	4	4
2	2	3	3	4	4	4	4	4	4
1	1	3	3	4	4	4	4	4	4
2	2	3	3	4	4	4	4	4	4
1	1	3	3	4	4	4	4	4	4
1	1	3	3	4	4	4	4	4	4
1	1	2	2	4	4	4	4	4	4
2	2	3	3	4	4	4	4	4	4
2	2	3	3	4	4	4	4	4	4
2	2	3	3	4	4	3	3	4	4
2	2	3	3	4	4	4	4	4	4
2	2	3	3	4	4	4	4	4	4
2	2	3	3	4	4	4	4	4	4
2	2	3	3	1	1	3	3	4	4
2	2	3	3	4	4	4	4	4	4
2	2	3	3	4	4	4	4	4	4
2	2	3	3	4	4	4	4	4	4
2	2	3	3	1	1	3	3	4	4
2	2	3	3	1	1	3	3	4	4
1	1	3	3	4	4	4	4	4	4
2	2	3	3	1	1	3	3	4	4
2	2	3	3	4	4	4	4	4	4
2	2	3	3	4	4	4	4	4	4
1	1	3	3	1	1	4	4	4	4
2	2	3	3	4	4	4	4	4	4
2	2	3	3	1	1	4	4	4	4
1	1	3	3	4	4	4	4	4	4
2	2	3	3	4	4	4	4	4	4
1	1	3	3	4	4	4	4	4	4
2	2	3	3	4	4	4	4	4	4
1	1	3	3	4	4	4	4	4	4
2	2	3	3	4	4	4	4	4	4
2	2	3	3	4	4	4	4	4	4
2	2	3	3	4	4	4	4	4	4
2	2	3	3	4	4	4	4	4	4
2	2	3	3	4	4	4	4	4	4
2	2	3	3	4	4	4	4	4	4
2	2	3	3	1	1	3	3	4	4
2	2	3	3	1	1	3	3	4	4
2	2	3	3	4	4	4	4	4	4
2	2	3	3	4	4	4	4	4	4

For Review Only

2	2	3	3	4	4	4	4	4	4
2	2	3	3	4	4	4	4	4	4
2	2	3	3	4	4	4	4	4	4
2	2	2	2	4	4	4	4	4	4
2	2	3	3	4	4	4	4	4	4
2	2	3	3	4	4	4	4	4	4
1	1	3	3	4	4	4	4	4	4
1	1	3	3	4	4	4	4	4	4
2	2	3	3	4	4	4	4	4	4
2	2	3	3	1	1	4	4	4	4
2	2	3	3	4	4	3	3	4	4
2	2	3	3	4	4	4	4	4	4
2	2	3	3	4	4	4	4	2	2
2	2	3	3	4	4	4	4	4	4
2	2	3	3	4	4	4	4	4	4
2	2	3	3	4	4	4	4	4	4
2	2	3	3	4	4	4	4	4	4
2	2	3	3	4	4	4	4	4	4
2	2	2	2	4	4	4	4	4	4
2	2	3	3	4	4	4	4	4	4
2	2	3	3	4	4	4	4	4	4
2	2	3	3	4	4	4	4	4	4
2	2	3	3	4	4	4	4	4	4
2	2	3	3	1	1	3	3	4	4
2	2	3	3	1	1	4	4	4	4
2	2	3	3	4	4	4	4	4	4
1	1	3	3	4	4	4	4	4	4
1	1	2	2	4	4	4	4	4	4
2	2	3	3	4	4	4	4	4	4
2	2	3	3	4	4	4	4	4	4
2	2	3	3	1	1	4	4	4	4
2	2	3	3	4	4	4	4	4	4
1	1	3	3	4	4	4	4	4	4
2	2	3	3	4	4	4	4	4	4
2	2	3	3	4	4	3	3	4	4
1	1	3	3	4	4	4	4	4	4
2	2	3	3	4	4	4	4	4	4
2	2	3	3	4	4	4	4	4	4
1	1	3	3	4	4	4	4	4	4
2	2	2	2	4	4	4	4	4	4
2	2	3	3	4	4	4	4	4	4
2	2	3	3	4	4	3	3	4	4
2	2	3	3	4	4	4	4	4	4
2	2	3	3	4	4	3	3	4	4
2	2	3	3	4	4	4	4	4	4
1	1	3	3	4	4	4	4	4	4
2	2	3	3	4	4	4	4	4	4
2	2	3	3	4	4	4	4	4	4
2	2	3	3	4	4	4	4	4	4
2	2	3	3	4	4	4	4	4	4
2	2	3	3	4	4	4	4	4	4
2	2	3	3	4	4	4	4	4	4
1	1	3	3	4	4	4	4	4	4
2	2	3	3	4	4	4	4	4	4
2	2	3	3	4	4	4	4	4	4

For Review Only

2	2	3	3	4	4	4	4	4	4
2	2	3	3	4	4	4	4	4	4
1	1	3	3	4	4	4	4	4	4
2	2	3	3	4	4	4	4	4	4
2	2	3	3	4	4	4	4	4	4
2	2	3	3	1	1	3	3	4	4
2	2	3	3	4	4	4	4	4	4
2	2	3	3	4	4	4	4	4	4
1	1	3	3	4	4	4	4	4	4
2	2	3	3	4	4	4	4	4	4
2	2	3	3	4	4	4	4	4	4
1	1	3	3	4	4	4	4	4	4
2	2	3	3	4	4	4	4	4	4
1	1	3	3	4	4	4	4	4	4
2	2	2	2	4	4	4	4	4	4
2	2	3	3	4	4	4	4	4	4
2	2	3	3	4	4	4	4	4	4
2	2	3	3	4	4	4	4	4	4
2	2	3	3	4	4	4	4	4	4
1	1	3	3	4	4	4	4	2	2
2	2	3	3	4	4	4	4	4	4
2	2	3	3	1	1	3	3	4	4
2	2	3	3	4	4	4	4	4	4
1	1	2	2	4	4	4	4	4	4
2	2	3	3	4	4	4	4	4	4
2	2	3	3	4	4	3	3	4	4
2	2	2	2	4	4	4	4	4	4
2	2	3	3	4	4	4	4	4	4
2	2	3	3	4	4	4	4	4	4
2	2	3	3	4	4	4	4	4	4
2	2	3	3	4	4	4	4	4	4
2	2	3	3	4	4	4	4	4	4
1	1	3	3	4	4	4	4	4	4
2	2	2	2	4	4	4	4	4	4
1	1	3	3	4	4	4	4	4	4
1	1	2	2	4	4	4	4	4	4
2	2	3	3	4	4	4	4	4	4
1	1	3	3	4	4	4	4	4	4
2	2	3	3	4	4	4	4	4	4
2	2	3	3	4	4	4	4	4	4
1	1	3	3	4	4	4	4	4	4
2	2	3	3	4	4	4	4	4	4
2	2	3	3	1	1	3	3	4	4
2	2	3	3	4	4	4	4	4	4
2	2	3	3	4	4	4	4	4	4
2	2	3	3	4	4	4	4	4	4
2	2	3	3	4	4	4	4	4	4
1	1	3	3	4	4	4	4	4	4
1	1	3	3	4	4	4	4	4	4
2	2	3	3	4	4	4	4	4	4
2	2	3	3	4	4	4	4	4	4
2	2	3	3	4	4	3	3	4	4
2	2	3	3	4	4	4	4	4	4
2	2	3	3	4	4	4	4	4	4
2	2	3	3	4	4	3	3	4	4

For Review Only

1	1	3	3	4	4	4	4	4	4
2	2	3	3	4	4	4	4	4	4
2	2	3	3	4	4	4	4	4	4
2	2	3	3	4	4	4	4	4	4
2	2	3	3	4	4	4	4	4	4
2	2	3	3	4	4	4	4	4	4
2	2	3	3	4	4	4	4	4	4
2	2	3	3	4	4	4	4	4	4
2	2	3	3	4	4	4	4	4	4
2	2	3	3	1	1	3	3	4	4
2	2	3	3	4	4	4	4	4	4
2	2	3	3	4	4	4	4	4	4
2	2	3	3	4	4	3	3	4	4
2	2	3	3	4	4	4	4	4	4
1	1	3	3	4	4	4	4	4	4
2	2	2	2	4	4	4	4	4	4
2	2	3	3	4	4	3	3	4	4
1	1	3	3	4	4	4	4	4	4
2	2	3	3	4	4	4	4	4	4
2	2	3	3	4	4	4	4	4	4
2	2	3	3	4	4	4	4	4	4
2	2	3	3	4	4	4	4	4	4
1	1	3	3	4	4	4	4	4	4
2	2	3	3	4	4	4	4	4	4
1	1	3	3	1	1	3	3	4	4
2	2	3	3	4	4	4	4	4	4
2	2	3	3	1	1	3	3	4	4
2	2	3	3	4	4	4	4	4	4
2	2	3	3	4	4	4	4	4	4
2	2	3	3	4	4	4	4	4	4
2	2	3	3	1	1	3	3	4	4
2	2	3	3	4	4	4	4	4	4
1	1	3	3	4	4	4	4	4	4
2	2	3	3	4	4	4	4	4	4
2	2	3	3	4	4	4	4	4	4
2	2	3	3	1	1	3	3	4	4
1	1	3	3	4	4	4	4	4	4
1	1	2	2	4	4	4	4	4	4
2	2	3	3	4	4	4	4	4	4
1	1	3	3	1	1	3	3	4	4
2	2	3	3	4	4	4	4	4	4
1	1	3	3	4	4	4	4	4	4
2	2	3	3	4	4	4	4	4	4
1	1	3	3	4	4	4	4	4	4
2	2	2	2	4	4	4	4	4	4
2	2	3	3	4	4	4	4	4	4
2	2	3	3	1	1	3	3	4	4
2	2	3	3	4	4	4	4	4	4
2	2	3	3	4	4	4	4	4	4
2	2	3	3	1	1	3	3	4	4
2	2	3	3	4	4	4	4	4	4
1	1	2	2	1	1	3	3	4	4
1	1	3	3	4	4	4	4	4	4
2	2	3	3	4	4	4	4	4	4
2	2	3	3	4	4	4	4	4	4

For Review Only

2	2	3	3	4	4	4	4	4	4
1	1	3	3	4	4	4	4	4	4

For Review Only

SNP180	SNP180	SNP1800	SNP1800	SNP1801	SNP1801	SNP1802	SNP1802	SNP1803	SNP1803
2	2	1	1	4	4	1	1	1	1
4	4	1	1	4	4	1	1	1	1
4	4	1	1	1	1	3	3	1	1
4	4	1	1	4	4	1	1	1	1
2	2	1	1	1	1	1	1	1	1
4	4	1	1	1	1	3	3	1	1
4	4	1	1	4	4	1	1	1	1
4	4	1	1	4	4	1	1	1	1
2	2	1	1	4	4	1	1	1	1
4	4	1	1	4	4	1	1	1	1
4	4	1	1	4	4	1	1	1	1
4	4	1	1	4	4	1	1	1	1
4	4	1	1	4	4	1	1	1	1
4	4	1	1	4	4	1	1	1	1
4	4	1	1	4	4	1	1	1	1
2	2	1	1	4	4	1	1	1	1
4	4	1	1	1	1	1	1	1	1
2	2	1	1	1	1	3	3	1	1
4	4	2	2	4	4	1	1	4	4
4	4	1	1	4	4	1	1	1	1
4	4	1	1	4	4	1	1	1	1
4	4	1	1	1	1	1	1	1	1
4	4	1	1	4	4	1	1	1	1
4	4	1	1	4	4	1	1	1	1
2	2	1	1	1	1	3	3	1	1
4	4	1	1	1	1	1	1	1	1
2	2	1	1	1	1	1	1	1	1
4	4	1	1	4	4	1	1	1	1
2	2	1	1	4	4	1	1	1	1
4	4	1	1	4	4	1	1	1	1
2	2	1	1	1	1	3	3	1	1
4	4	1	1	1	1	3	3	1	1
4	4	1	1	1	1	1	1	1	1
2	2	1	1	1	1	3	3	1	1
2	2	1	1	4	4	1	1	1	1
4	4	1	1	4	4	1	1	1	1
4	4	2	2	4	4	1	1	4	4
4	4	1	1	4	4	1	1	1	1
4	4	1	1	4	4	1	1	1	1
4	4	1	1	4	4	1	1	1	1
4	4	1	1	4	4	1	1	1	1
4	4	1	1	4	4	1	1	1	1
4	4	1	1	4	4	1	1	1	1
4	4	1	1	4	4	1	1	1	1
4	4	1	1	4	4	1	1	1	1
4	4	1	1	1	1	1	1	1	1
4	4	1	1	1	1	3	3	1	1
4	4	1	1	1	1	1	1	1	1
4	4	1	1	4	4	1	1	1	1
2	2	2	2	1	1	1	1	1	1
4	4	1	1	1	1	1	1	1	1
2	2	1	1	4	4	1	1	1	1

For Review Only

2	2	1	1	1	1	1	1	1	1
4	4	1	1	1	1	1	1	1	1
4	4	1	1	4	4	1	1	1	1
4	4	1	1	4	4	1	1	1	1
4	4	1	1	4	4	1	1	4	4
4	4	1	1	1	1	3	3	1	1
4	4	1	1	1	1	1	1	1	1
4	4	1	1	4	4	1	1	1	1
2	2	1	1	1	1	3	3	1	1
4	4	1	1	4	4	1	1	1	1
4	4	1	1	4	4	1	1	1	1
4	4	1	1	1	1	3	3	1	1
2	2	1	1	1	1	1	1	1	1
2	2	1	1	4	4	1	1	1	1
4	4	1	1	4	4	3	3	1	1
4	4	1	1	4	4	1	1	1	1
4	4	1	1	1	1	3	3	1	1
4	4	2	2	1	1	1	1	4	4
2	2	1	1	1	1	1	1	1	1
4	4	1	1	1	1	1	1	1	1
4	4	1	1	1	1	3	3	1	1
4	4	2	2	4	4	1	1	4	4
4	4	1	1	4	4	1	1	1	1
4	4	2	2	1	1	3	3	4	4
4	4	1	1	4	4	1	1	1	1
4	4	1	1	4	4	1	1	1	1
4	4	1	1	4	4	1	1	1	1
4	4	1	1	4	4	1	1	1	1
4	4	1	1	1	1	1	1	1	1
4	4	1	1	1	1	1	1	1	1
4	4	1	1	4	4	1	1	1	1
2	2	1	1	4	4	1	1	1	1
4	4	1	1	4	4	1	1	1	1
2	2	1	1	1	1	1	1	1	1
2	2	1	1	1	1	3	3	1	1
4	4	1	1	4	4	1	1	1	1
4	4	1	1	4	4	1	1	1	1
4	4	1	1	4	4	1	1	1	1
4	4	1	1	1	1	1	1	1	1
4	4	1	1	4	4	1	1	1	1
4	4	1	1	1	1	1	1	1	1
4	4	1	1	1	1	1	1	1	1
4	4	1	1	4	4	1	1	1	1
4	4	1	1	4	4	1	1	1	1
4	4	1	1	1	1	1	1	1	1
4	4	1	1	4	4	1	1	1	1
4	4	1	1	4	4	3	3	1	1
4	4	1	1	4	4	1	1	1	1
4	4	1	1	4	4	1	1	1	1
4	4	1	1	4	4	1	1	1	1
2	2	1	1	1	1	3	3	1	1
4	4	1	1	4	4	1	1	1	1
4	4	1	1	4	4	1	1	1	1
2	2	2	2	4	4	1	1	1	1
4	4	1	1	1	1	3	3	1	1

For Review Only

4	4	1	1	4	4	1	1	1	1
4	4	1	1	4	4	1	1	1	1
4	4	1	1	1	1	1	1	1	1
4	4	1	1	4	4	1	1	1	1
4	4	2	2	4	4	1	1	4	4
2	2	1	1	1	1	1	1	1	1
2	2	1	1	4	4	1	1	1	1
4	4	1	1	4	4	1	1	1	1
4	4	1	1	1	1	1	1	1	1
4	4	1	1	4	4	1	1	1	1
2	2	1	1	1	1	3	3	1	1
4	4	1	1	4	4	1	1	1	1
2	2	1	1	1	1	1	1	1	1
4	4	1	1	4	4	1	1	1	1
4	4	1	1	4	4	1	1	1	1
4	4	1	1	4	4	1	1	1	1
4	4	1	1	4	4	1	1	1	1
4	4	1	1	4	4	1	1	1	1
4	4	1	1	1	1	3	3	1	1
4	4	1	1	4	4	1	1	1	1
4	4	1	1	4	4	1	1	1	1
4	4	1	1	4	4	1	1	1	1
4	4	1	1	1	1	3	3	1	1
4	4	1	1	4	4	1	1	1	1
4	4	1	1	4	4	1	1	1	1
4	4	1	1	4	4	1	1	1	1
2	2	2	2	1	1	3	3	4	4
4	4	2	2	1	1	3	3	4	4
2	2	1	1	4	4	1	1	1	1
2	2	1	1	4	4	1	1	1	1
4	4	1	1	4	4	1	1	1	1
4	4	1	1	4	4	1	1	1	1
4	4	1	1	1	1	1	1	1	1
4	4	1	1	4	4	1	1	1	1
2	2	1	1	1	1	3	3	1	1
2	2	1	1	1	1	1	1	1	1
4	4	1	1	4	4	1	1	1	1
4	4	1	1	4	4	1	1	1	1
4	4	1	1	4	4	1	1	1	1
4	4	1	1	4	4	1	1	1	1
4	4	1	1	4	4	1	1	1	1
4	4	1	1	4	4	1	1	1	1
4	4	2	2	1	1	1	1	4	4
4	4	1	1	4	4	1	1	1	1
4	4	1	1	4	4	1	1	1	1
2	2	1	1	4	4	1	1	1	1
4	4	1	1	1	1	1	1	1	1
2	2	1	1	4	4	1	1	1	1
4	4	1	1	4	4	1	1	1	1
4	4	1	1	1	1	1	1	1	1
4	4	1	1	4	4	1	1	1	1
4	4	1	1	4	4	1	1	1	1
4	4	2	2	4	4	1	1	4	4
4	4	1	1	1	1	1	1	1	1
4	4	1	1	4	4	1	1	1	1

For Review Only

4	4	1	1	1	1	1	1	1	1
4	4	1	1	4	4	1	1	1	1
4	4	1	1	4	4	1	1	1	1
4	4	1	1	4	4	1	1	1	1
4	4	1	1	4	4	1	1	1	1
4	4	1	1	1	1	3	3	1	1
4	4	1	1	4	4	1	1	1	1
4	4	1	1	4	4	1	1	1	1
4	4	1	1	4	4	1	1	1	1
4	4	1	1	1	1	1	1	1	1
4	4	1	1	4	4	1	1	1	1
2	2	1	1	4	4	1	1	1	1
4	4	1	1	4	4	1	1	1	1
2	2	1	1	4	4	1	1	1	1
4	4	1	1	1	1	3	3	1	1
4	4	1	1	4	4	1	1	1	1
4	4	1	1	4	4	1	1	4	4
4	4	2	2	1	1	3	3	4	4
4	4	1	1	1	1	1	1	1	1
2	2	1	1	4	4	1	1	1	1
4	4	1	1	4	4	1	1	1	1
4	4	1	1	4	4	1	1	1	1
4	4	1	1	4	4	1	1	1	1
4	4	1	1	1	1	3	3	1	1
2	2	1	1	4	4	1	1	1	1
4	4	1	1	1	1	3	3	1	1
4	4	1	1	4	4	1	1	1	1
2	2	1	1	4	4	1	1	1	1
4	4	1	1	1	1	1	1	1	1
4	4	1	1	1	1	3	3	1	1
4	4	1	1	4	4	1	1	1	1
4	4	1	1	1	1	1	1	1	1
2	2	1	1	1	1	3	3	1	1
4	4	1	1	1	1	3	3	1	1
2	2	1	1	1	1	3	3	1	1
4	4	1	1	1	1	3	3	1	1
4	4	1	1	4	4	1	1	1	1
4	4	2	2	1	1	1	1	4	4
2	2	1	1	4	4	1	1	1	1
4	4	1	1	1	1	1	1	1	1
4	4	1	1	1	1	1	1	1	1
4	4	1	1	1	1	1	1	1	1
4	4	1	1	4	4	1	1	1	1
2	2	1	1	4	4	1	1	1	1
4	4	1	1	1	1	1	1	1	1
4	4	1	1	1	1	3	3	1	1
4	4	1	1	4	4	1	1	1	1
4	4	1	1	4	4	1	1	1	1
4	4	1	1	1	1	3	3	1	1
4	4	1	1	4	4	1	1	1	1
2	2	1	1	1	1	1	1	1	1
4	4	1	1	4	4	1	1	1	1
4	4	2	2	4	4	1	1	4	4
4	4	1	1	4	4	1	1	1	1
4	4	1	1	1	1	1	1	1	1

For Review Only

4	4	1	1	4	4	1	1	1	1
4	4	1	1	4	4	1	1	1	1
4	4	2	2	1	1	1	1	4	4
4	4	1	1	4	4	1	1	1	1
4	4	1	1	4	4	1	1	1	1
2	2	1	1	1	1	1	1	1	1
4	4	1	1	4	4	1	1	1	1
4	4	1	1	4	4	1	1	1	1
4	4	1	1	4	4	1	1	1	1
4	4	1	1	4	4	1	1	1	1
4	4	1	1	4	4	1	1	1	1
4	4	1	1	4	4	1	1	1	1
4	4	1	1	1	1	1	1	1	1
4	4	1	1	4	4	1	1	1	1
4	4	1	1	1	1	3	3	1	1
4	4	1	1	1	1	1	1	1	1
4	4	1	1	4	4	1	1	1	1
4	4	1	1	4	4	1	1	1	1
4	4	1	1	4	4	1	1	1	1
4	4	1	1	4	4	1	1	1	1
4	4	1	1	4	4	1	1	1	1
4	4	1	1	1	1	1	1	1	1
2	2	1	1	1	1	1	1	1	1
4	4	1	1	4	4	1	1	1	1
4	4	1	1	4	4	1	1	1	1
2	2	1	1	4	4	1	1	1	1
4	4	1	1	4	4	1	1	1	1
4	4	1	1	4	4	1	1	1	1
2	2	1	1	1	1	1	1	1	1
2	2	1	1	1	1	1	1	1	1
4	4	1	1	4	4	1	1	1	1
4	4	1	1	1	1	1	1	1	1
4	4	1	1	4	4	1	1	1	1
4	4	1	1	4	4	1	1	1	1
4	4	2	2	4	4	1	1	4	4
4	4	1	1	4	4	1	1	1	1
4	4	1	1	1	1	1	1	1	1
4	4	1	1	1	1	3	3	1	1
4	4	1	1	4	4	1	1	1	1
4	4	1	1	4	4	1	1	1	1
2	2	1	1	1	1	3	3	1	1
4	4	1	1	1	1	3	3	1	1
2	2	1	1	4	4	1	1	1	1
4	4	1	1	1	1	1	1	1	1
4	4	1	1	1	1	3	3	1	1
4	4	1	1	1	1	1	1	1	1
4	4	1	1	4	4	1	1	1	1
2	2	1	1	1	1	3	3	1	1
4	4	1	1	1	1	3	3	1	1
4	4	1	1	4	4	1	1	1	1
4	4	2	2	4	4	1	1	4	4
4	4	1	1	4	4	1	1	1	1
4	4	1	1	1	1	3	3	1	1
4	4	1	1	4	4	1	1	1	1
4	4	1	1	4	4	1	1	1	1

For Review Only

4	4	1	1	1	1	1	1	1	1
4	4	2	2	4	4	1	1	4	4

For Review Only

SNP1804	SNP1804	SNP1805	SNP1805	SNP1806	SNP1806	SNP1807	SNP1807	SNP1808	SNP1808
1	1	1	1	3	3	4	4	1	1
4	4	2	2	3	3	2	2	4	4
1	1	1	1	3	3	4	4	1	1
1	1	1	1	3	3	4	4	1	1
1	1	2	2	2	2	4	4	4	4
1	1	1	1	3	3	4	4	1	1
1	1	1	1	3	3	4	4	1	1
1	1	1	1	3	3	4	4	1	1
1	1	2	2	3	3	2	2	4	4
1	1	2	2	3	3	2	2	4	4
1	1	2	2	2	2	4	4	1	1
1	1	1	1	3	3	4	4	1	1
1	1	1	1	3	3	4	4	1	1
1	1	2	2	3	3	2	2	4	4
1	1	1	1	3	3	4	4	1	1
1	1	1	1	3	3	4	4	1	1
1	1	1	1	3	3	4	4	1	1
1	1	1	1	3	3	4	4	1	1
4	4	1	1	3	3	4	4	1	1
1	1	2	2	3	3	2	2	4	4
1	1	1	1	3	3	4	4	1	1
1	1	1	1	3	3	2	2	1	1
1	1	2	2	2	2	4	4	1	1
1	1	2	2	3	3	2	2	4	4
1	1	2	2	2	2	4	4	4	4
1	1	1	1	3	3	4	4	1	1
1	1	1	1	3	3	4	4	1	1
1	1	1	1	3	3	4	4	1	1
1	1	2	2	3	3	2	2	4	4
1	1	2	2	3	3	2	2	4	4
1	1	1	1	3	3	4	4	1	1
1	1	1	1	3	3	4	4	1	1
1	1	2	2	2	2	4	4	4	4
1	1	2	2	3	3	2	2	4	4
1	1	2	2	3	3	4	4	4	4
1	1	2	2	3	3	2	2	1	1
4	4	1	1	3	3	4	4	1	1
1	1	1	1	3	3	4	4	1	1
1	1	2	2	3	3	2	2	4	4
1	1	1	1	3	3	4	4	1	1
1	1	2	2	3	3	2	2	4	4
1	1	2	2	3	3	4	4	4	4
1	1	2	2	3	3	2	2	4	4
1	1	1	1	3	3	4	4	4	4
1	1	2	2	3	3	2	2	4	4
1	1	1	1	3	3	4	4	1	1
1	1	2	2	3	3	4	4	4	4
1	1	2	2	3	3	2	2	4	4
1	1	2	2	3	3	4	4	4	4
1	1	1	1	3	3	4	4	1	1
1	1	1	1	3	3	4	4	1	1
1	1	2	2	2	2	4	4	1	1

For Review Only

1	1	2	2	2	2	4	4	4	4
1	1	2	2	2	2	4	4	4	4
1	1	1	1	3	3	4	4	1	1
1	1	1	1	3	3	4	4	1	1
4	4	1	1	3	3	4	4	1	1
1	1	2	2	2	2	4	4	4	4
1	1	1	1	3	3	4	4	1	1
1	1	2	2	3	3	2	2	4	4
1	1	2	2	2	2	4	4	4	4
1	1	1	1	3	3	4	4	1	1
1	1	1	1	3	3	4	4	1	1
1	1	2	2	2	2	4	4	4	4
1	1	2	2	2	2	4	4	4	4
1	1	2	2	3	3	2	2	4	4
1	1	1	1	3	3	4	4	1	1
1	1	2	2	2	2	4	4	4	4
4	4	1	1	3	3	4	4	1	1
4	4	1	1	3	3	4	4	1	1
1	1	2	2	2	2	4	4	1	1
1	1	1	1	3	3	4	4	1	1
1	1	2	2	3	3	4	4	4	4
4	4	1	1	3	3	4	4	1	1
1	1	2	2	3	3	2	2	4	4
4	4	1	1	3	3	4	4	1	1
1	1	2	2	3	3	2	2	4	4
1	1	2	2	2	2	4	4	1	1
1	1	2	2	3	3	2	2	4	4
1	1	2	2	2	2	4	4	1	1
1	1	1	1	3	3	4	4	1	1
1	1	1	1	3	3	4	4	1	1
1	1	2	2	2	2	4	4	1	1
1	1	2	2	2	2	4	4	4	4
1	1	2	2	3	3	2	2	4	4
1	1	2	2	2	2	4	4	4	4
1	1	2	2	2	2	4	4	4	4
1	1	2	2	3	3	2	2	4	4
1	1	2	2	3	3	2	2	4	4
1	1	2	2	2	2	4	4	4	4
1	1	1	1	3	3	4	4	1	1
1	1	1	1	3	3	4	4	1	1
1	1	1	1	3	3	4	4	1	1
1	1	1	1	3	3	4	4	1	1
4	4	1	1	3	3	4	4	1	1
1	1	1	1	3	3	4	4	1	1
1	1	2	2	3	3	2	2	4	4
1	1	1	1	3	3	4	4	1	1
1	1	2	2	3	3	2	2	4	4
1	1	1	1	3	3	4	4	1	1
1	1	2	2	3	3	2	2	1	1
1	1	1	1	3	3	4	4	1	1
1	1	1	1	3	3	4	4	1	1
1	1	2	2	2	2	4	4	1	1
1	1	1	1	3	3	4	4	1	1
4	4	1	1	3	3	4	4	1	1
1	1	1	1	3	3	4	4	1	1

For Review Only

1	1	1	1	3	3	4	4	1	1
1	1	1	1	3	3	4	4	1	1
1	1	1	1	3	3	2	2	1	1
1	1	2	2	3	3	2	2	4	4
4	4	1	1	3	3	4	4	1	1
1	1	1	1	3	3	4	4	1	1
1	1	1	1	3	3	4	4	1	1
1	1	1	1	3	3	4	4	1	1
1	1	2	2	3	3	2	2	4	4
1	1	1	1	3	3	2	2	1	1
4	4	1	1	2	2	4	4	4	4
1	1	1	1	3	3	4	4	1	1
1	1	1	1	3	3	4	4	1	1
1	1	1	1	3	3	4	4	1	1
1	1	1	1	3	3	4	4	1	1
1	1	1	1	3	3	4	4	1	1
1	1	1	1	3	3	4	4	4	4
1	1	1	1	3	3	4	4	1	1
1	1	2	2	3	3	2	2	4	4
1	1	1	1	3	3	4	4	1	1
1	1	1	1	3	3	4	4	1	1
1	1	1	1	3	3	4	4	1	1
1	1	1	1	3	3	4	4	1	1
1	1	2	2	3	3	2	2	4	4
4	4	1	1	3	3	4	4	1	1
1	1	1	1	3	3	4	4	1	1
4	4	1	1	3	3	4	4	1	1
4	4	2	2	3	3	2	2	1	1
1	1	1	1	3	3	4	4	1	1
1	1	2	2	3	3	2	2	4	4
1	1	2	2	3	3	2	2	4	4
1	1	1	1	2	2	4	4	1	1
1	1	1	1	3	3	4	4	1	1
1	1	1	1	3	3	4	4	1	1
1	1	2	2	3	3	2	2	4	4
1	1	2	2	3	3	4	4	1	1
1	1	2	2	2	2	4	4	4	4
1	1	1	1	3	3	4	4	1	1
1	1	2	2	3	3	2	2	4	4
1	1	2	2	3	3	2	2	4	4
1	1	1	1	3	3	4	4	1	1
1	1	2	2	2	2	4	4	4	4
4	4	1	1	3	3	4	4	1	1
1	1	2	2	3	3	2	2	4	4
1	1	1	1	3	3	4	4	1	1
4	4	1	1	3	3	4	4	1	1
1	1	1	1	3	3	4	4	1	1
1	1	1	1	3	3	4	4	1	1
1	1	1	1	3	3	4	4	1	1
1	1	1	1	3	3	4	4	1	1
1	1	2	2	3	3	2	2	1	1
1	1	2	2	3	3	2	2	4	4
4	4	1	1	3	3	4	4	1	1
1	1	2	2	3	3	2	2	1	1
1	1	1	1	3	3	4	4	1	1

For Review Only

1	1	1	1	3	3	4	4	1	1
4	4	1	1	3	3	4	4	1	1
1	1	2	2	3	3	2	2	4	4
1	1	1	1	3	3	4	4	1	1
1	1	1	1	3	3	4	4	1	1
1	1	1	1	3	3	4	4	1	1
1	1	1	1	3	3	4	4	1	1
1	1	1	1	3	3	4	4	1	1
1	1	1	1	3	3	4	4	1	1
1	1	1	1	3	3	4	4	1	1
1	1	1	1	3	3	4	4	1	1
1	1	2	2	3	3	2	2	4	4
1	1	1	1	3	3	4	4	1	1
1	1	1	1	3	3	4	4	1	1
1	1	2	2	2	2	4	4	4	4
1	1	2	2	2	2	4	4	1	1
1	1	1	1	3	3	4	4	1	1
4	4	1	1	3	3	4	4	1	1
1	1	1	1	3	3	4	4	1	1
1	1	1	1	3	3	4	4	1	1
1	1	1	1	3	3	4	4	1	1
1	1	2	2	3	3	2	2	1	1
1	1	1	1	3	3	4	4	1	1
1	1	2	2	2	2	4	4	4	4
1	1	2	2	3	3	2	2	4	4
1	1	2	2	3	3	2	2	4	4
1	1	1	1	3	3	4	4	4	4
1	1	2	2	3	3	4	4	1	1
4	4	1	1	3	3	4	4	1	1
1	1	1	1	3	3	4	4	1	1
1	1	1	1	3	3	4	4	1	1
1	1	1	1	3	3	4	4	1	1
1	1	1	1	3	3	4	4	1	1
1	1	2	2	2	2	4	4	4	4
1	1	1	1	2	2	4	4	1	1
1	1	2	2	2	2	4	4	4	4
1	1	1	1	3	3	4	4	1	1
4	4	1	1	3	3	4	4	1	1
1	1	1	1	3	3	4	4	1	1
1	1	1	1	3	3	4	4	1	1
1	1	1	1	3	3	4	4	1	1
1	1	1	1	3	3	4	4	1	1
1	1	2	2	3	3	2	2	4	4
1	1	1	1	3	3	4	4	1	1
1	1	2	2	3	3	4	4	4	4
1	1	1	1	3	3	4	4	1	1
1	1	2	2	2	2	4	4	1	1
1	1	2	2	2	2	4	4	1	1
1	1	2	2	2	2	4	4	4	4
1	1	1	1	3	3	4	4	1	1
1	1	1	1	3	3	4	4	1	1
4	4	2	2	3	3	2	2	4	4
4	4	2	2	3	3	2	2	4	4
1	1	1	1	3	3	4	4	1	1
1	1	2	2	2	2	4	4	1	1

For Review Only

1	1	1	1	3	3	4	4	1	1
1	1	2	2	3	3	2	2	4	4
4	4	1	1	3	3	4	4	1	1
1	1	1	1	3	3	4	4	1	1
1	1	2	2	3	3	2	2	4	4
1	1	1	1	3	3	4	4	1	1
1	1	1	1	3	3	4	4	1	1
1	1	2	2	3	3	2	2	4	4
1	1	2	2	3	3	4	4	1	1
1	1	2	2	3	3	2	2	4	4
1	1	1	1	3	3	4	4	1	1
1	1	1	1	3	3	4	4	1	1
1	1	1	1	3	3	4	4	1	1
1	1	1	1	3	3	4	4	1	1
1	1	1	1	3	3	4	4	1	1
1	1	2	2	3	3	4	4	1	1
1	1	2	2	3	3	2	2	4	4
1	1	1	1	3	3	4	4	1	1
1	1	2	2	3	3	2	2	4	4
1	1	1	1	3	3	4	4	1	1
1	1	1	1	3	3	4	4	1	1
1	1	1	1	3	3	4	4	1	1
1	1	1	1	3	3	4	4	1	1
1	1	2	2	3	3	2	2	4	4
1	1	2	2	3	3	2	2	4	4
1	1	2	2	3	3	2	2	1	1
1	1	2	2	3	3	2	2	4	4
1	1	1	1	3	3	4	4	1	1
1	1	2	2	3	3	4	4	4	4
1	1	1	1	3	3	4	4	1	1
1	1	2	2	3	3	2	2	4	4
1	1	2	2	3	3	2	2	4	4
1	1	1	1	3	3	4	4	1	1
1	1	1	1	3	3	4	4	1	1
4	4	1	1	3	3	4	4	1	1
1	1	2	2	2	2	4	4	1	1
1	1	1	1	3	3	4	4	1	1
1	1	2	2	2	2	4	4	4	4
1	1	2	2	3	3	2	2	1	1
1	1	2	2	3	3	2	2	4	4
1	1	1	1	3	3	4	4	1	1
1	1	1	1	3	3	4	4	1	1
1	1	2	2	3	3	2	2	4	4
1	1	1	1	3	3	4	4	1	1
4	4	2	2	2	2	4	4	1	1
1	1	1	1	3	3	4	4	1	1
1	1	1	1	3	3	4	4	1	1
1	1	1	1	3	3	4	4	1	1
1	1	1	1	3	3	4	4	1	1
1	1	2	2	3	3	2	2	4	4
4	4	1	1	3	3	4	4	1	1
1	1	2	2	3	3	2	2	4	4
1	1	1	1	3	3	4	4	1	1
1	1	1	1	3	3	4	4	1	1
1	1	2	2	3	3	2	2	4	4

For Review Only

1	1	1	1	3	3	4	4	1	1
4	4	1	1	3	3	4	4	1	1

For Review Only

SNP1809	SNP1809	SNP181	SNP181	SNP1810	SNP1810	SNP1811	SNP1811	SNP1812	SNP1812
4	4	1	1	3	3	1	1	2	2
4	4	1	1	2	2	4	4	2	2
4	4	1	1	2	2	4	4	2	2
4	4	1	1	2	2	4	4	3	3
4	4	1	1	3	3	1	1	2	2
4	4	1	1	3	3	4	4	2	2
4	4	1	1	3	3	1	1	2	2
4	4	1	1	3	3	1	1	2	2
4	4	1	1	3	3	1	1	2	2
4	4	1	1	3	3	1	1	2	2
4	4	1	1	3	3	4	4	2	2
4	4	1	1	2	2	4	4	2	2
4	4	1	1	2	2	4	4	2	2
1	1	1	1	2	2	1	1	2	2
4	4	1	1	3	3	4	4	2	2
4	4	1	1	2	2	4	4	2	2
1	1	1	1	3	3	1	1	2	2
4	4	1	1	3	3	4	4	2	2
4	4	1	1	2	2	4	4	2	2
4	4	1	1	2	2	4	4	2	2
4	4	1	1	3	3	1	1	2	2
4	4	1	1	3	3	1	1	2	2
4	4	1	1	3	3	4	4	2	2
1	1	1	1	2	2	4	4	2	2
1	1	1	1	3	3	1	1	2	2
4	4	1	1	3	3	4	4	2	2
4	4	1	1	2	2	4	4	2	2
4	4	1	1	2	2	4	4	2	2
4	4	1	1	3	3	4	4	2	2
4	4	1	1	2	2	1	1	2	2
4	4	1	1	2	2	4	4	2	2
4	4	1	1	3	3	1	1	2	2
4	4	1	1	3	3	4	4	2	2
1	1	1	1	2	2	4	4	2	2
4	4	1	1	2	2	4	4	2	2
4	4	1	1	2	2	4	4	2	2
4	4	1	1	3	3	4	4	2	2
4	4	1	1	3	3	4	4	2	2
4	4	3	3	2	2	4	4	2	2
4	4	1	1	3	3	4	4	2	2
4	4	1	1	2	2	1	1	2	2
4	4	1	1	3	3	4	4	2	2
4	4	1	1	3	3	1	1	2	2
4	4	1	1	3	3	1	1	2	2
4	4	1	1	3	3	1	1	2	2
4	4	1	1	3	3	4	4	2	2
4	4	1	1	2	2	4	4	2	2
4	4	1	1	3	3	4	4	2	2
4	4	1	1	2	2	1	1	2	2
4	4	1	1	3	3	4	4	2	2
4	4	1	1	2	2	4	4	2	2
4	4	1	1	3	3	4	4	2	2

For Review Only

1	1	1	1	3	3	1	1	2	2
4	4	1	1	3	3	4	4	2	2
4	4	1	1	3	3	4	4	2	2
1	1	1	1	2	2	1	1	2	2
4	4	1	1	2	2	4	4	2	2
4	4	1	1	2	2	4	4	2	2
4	4	1	1	3	3	4	4	3	3
4	4	3	3	2	2	4	4	2	2
4	4	3	3	2	2	4	4	2	2
4	4	1	1	2	2	4	4	2	2
4	4	1	1	2	2	4	4	2	2
4	4	1	1	2	2	4	4	2	2
1	1	1	1	3	3	1	1	2	2
4	4	1	1	3	3	4	4	2	2
4	4	1	1	2	2	4	4	2	2
4	4	1	1	3	3	4	4	2	2
1	1	1	1	2	2	4	4	2	2
4	4	1	1	3	3	4	4	2	2
4	4	1	1	3	3	1	1	2	2
4	4	1	1	3	3	1	1	2	2
4	4	1	1	2	2	4	4	2	2
4	4	1	1	3	3	4	4	2	2
4	4	1	1	3	3	4	4	2	2
4	4	1	1	2	2	4	4	2	2
4	4	1	1	3	3	1	1	2	2
4	4	1	1	3	3	4	4	2	2
4	4	1	1	2	2	1	1	2	2
4	4	3	3	2	2	4	4	2	2
4	4	1	1	3	3	4	4	2	2
4	4	1	1	2	2	4	4	2	2
4	4	1	1	3	3	4	4	2	2
1	1	1	1	2	2	4	4	2	2
4	4	3	3	2	2	4	4	2	2
1	1	1	1	3	3	1	1	2	2
4	4	1	1	2	2	4	4	2	2
4	4	1	1	3	3	1	1	2	2
4	4	1	1	3	3	4	4	2	2
4	4	1	1	2	2	4	4	2	2
4	4	1	1	2	2	4	4	2	2
4	4	1	1	2	2	1	1	2	2
1	1	1	1	3	3	1	1	2	2
4	4	1	1	2	2	4	4	2	2
4	4	1	1	2	2	4	4	2	2
4	4	1	1	2	2	4	4	2	2
4	4	1	1	3	3	4	4	2	2
4	4	1	1	2	2	4	4	2	2
4	4	1	1	2	2	4	4	2	2
4	4	1	1	3	3	4	4	3	3
4	4	1	1	2	2	4	4	2	2
4	4	1	1	3	3	4	4	2	2
4	4	1	1	2	2	4	4	2	2
4	4	3	3	2	2	4	4	2	2
4	4	1	1	2	2	4	4	3	3
4	4	3	3	2	2	4	4	2	2
4	4	1	1	3	3	1	1	3	3

For Review Only

4	4	1	1	2	2	4	4	2	2
4	4	1	1	3	3	4	4	2	2
1	1	1	1	3	3	4	4	2	2
4	4	1	1	2	2	4	4	2	2
4	4	1	1	2	2	4	4	2	2
1	1	1	1	3	3	4	4	2	2
1	1	1	1	2	2	4	4	2	2
4	4	1	1	2	2	4	4	2	2
4	4	1	1	2	2	4	4	2	2
4	4	1	1	2	2	4	4	2	2
4	4	1	1	2	2	4	4	2	2
4	4	1	1	2	2	4	4	2	2
4	4	1	1	2	2	4	4	2	2
4	4	1	1	2	2	4	4	2	2
4	4	1	1	2	2	4	4	2	2
4	4	1	1	2	2	4	4	2	2
4	4	1	1	3	3	4	4	2	2
4	4	1	1	2	2	4	4	2	2
4	4	1	1	2	2	4	4	2	2
4	4	1	1	2	2	4	4	2	2
4	4	1	1	2	2	4	4	2	2
1	1	1	1	2	2	4	4	2	2
4	4	1	1	3	3	4	4	2	2
1	1	3	3	2	2	4	4	2	2
4	4	1	1	2	2	4	4	2	2
4	4	1	1	3	3	4	4	2	2
1	1	1	1	3	3	4	4	2	2
1	1	1	1	3	3	4	4	2	2
4	4	1	1	2	2	4	4	2	2
4	4	1	1	3	3	4	4	2	2
4	4	3	3	2	2	4	4	2	2
4	4	1	1	2	2	4	4	2	2
1	1	1	1	3	3	1	1	2	2
4	4	1	1	2	2	4	4	2	2
1	1	1	1	2	2	4	4	2	2
4	4	1	1	2	2	4	4	2	2
4	4	1	1	2	2	4	4	2	2
4	4	1	1	3	3	4	4	2	2
4	4	1	1	2	2	4	4	2	2
4	4	3	3	2	2	4	4	2	2
4	4	1	1	3	3	4	4	2	2
1	1	1	1	2	2	4	4	2	2
4	4	1	1	3	3	4	4	3	3
4	4	1	1	2	2	4	4	2	2
1	1	1	1	3	3	4	4	2	2
1	1	1	1	3	3	4	4	2	2
4	4	1	1	3	3	4	4	2	2
4	4	1	1	3	3	4	4	2	2
4	4	3	3	2	2	4	4	2	2
4	4	1	1	3	3	4	4	2	2
4	4	1	1	2	2	4	4	2	2
4	4	1	1	3	3	4	4	2	2
4	4	1	1	3	3	4	4	2	2
1	1	1	1	2	2	4	4	2	2
4	4	1	1	2	2	4	4	2	2

For Review Only

4	4	1	1	3	3	4	4	2	2
4	4	1	1	2	2	4	4	2	2
4	4	1	1	2	2	4	4	2	2
4	4	1	1	2	2	4	4	2	2
4	4	1	1	3	3	4	4	2	2
4	4	1	1	2	2	4	4	2	2
4	4	1	1	3	3	4	4	2	2
4	4	1	1	2	2	4	4	2	2
1	1	1	1	2	2	4	4	2	2
4	4	1	1	2	2	4	4	2	2
4	4	1	1	2	2	4	4	2	2
1	1	1	1	3	3	4	4	2	2
4	4	1	1	2	2	4	4	3	3
4	4	1	1	2	2	4	4	2	2
4	4	1	1	2	2	4	4	2	2
4	4	1	1	3	3	4	4	2	2
4	4	1	1	2	2	4	4	2	2
4	4	1	1	2	2	4	4	2	2
1	1	1	1	3	3	4	4	2	2
1	1	1	1	2	2	4	4	2	2
4	4	1	1	2	2	4	4	2	2
1	1	1	1	2	2	4	4	2	2
4	4	1	1	3	3	4	4	2	2
4	4	1	1	2	2	4	4	2	2
4	4	1	1	3	3	4	4	2	2
1	1	1	1	2	2	4	4	2	2
4	4	1	1	3	3	4	4	2	2
4	4	1	1	3	3	4	4	2	2
4	4	3	3	2	2	1	1	2	2
1	1	1	1	3	3	1	1	2	2
4	4	1	1	3	3	4	4	2	2
4	4	1	1	2	2	4	4	2	2
4	4	1	1	3	3	4	4	2	2
4	4	1	1	2	2	4	4	2	2
4	4	1	1	2	2	4	4	2	2
4	4	1	1	2	2	4	4	2	2
4	4	1	1	3	3	4	4	2	2
4	4	1	1	3	3	4	4	2	2
4	4	1	1	3	3	4	4	2	2
4	4	1	1	2	2	4	4	2	2
4	4	1	1	2	2	4	4	2	2
4	4	1	1	3	3	4	4	2	2
4	4	1	1	2	2	4	4	2	2
4	4	1	1	3	3	4	4	2	2
4	4	1	1	2	2	4	4	2	2
4	4	1	1	2	2	4	4	2	2
4	4	1	1	3	3	4	4	2	2
4	4	1	1	3	3	4	4	2	2
1	1	1	1	2	2	4	4	2	2
4	4	1	1	2	2	4	4	3	3
4	4	1	1	2	2	4	4	2	2
4	4	1	1	2	2	4	4	2	2
4	4	1	1	3	3	4	4	2	2
4	4	1	1	2	2	4	4	2	2
4	4	1	1	2	2	4	4	2	2

For Review Only

4	4	1	1	3	3	4	4	2	2
4	4	1	1	2	2	4	4	2	2
4	4	1	1	2	2	4	4	2	2
4	4	1	1	3	3	4	4	2	2
4	4	1	1	3	3	4	4	2	2
4	4	1	1	3	3	4	4	2	2
4	4	1	1	2	2	4	4	2	2
4	4	3	3	3	3	1	1	2	2
4	4	1	1	2	2	4	4	2	2
4	4	3	3	2	2	4	4	2	2
4	4	1	1	2	2	4	4	2	2
4	4	1	1	3	3	4	4	2	2
4	4	1	1	2	2	4	4	2	2
1	1	1	1	2	2	4	4	2	2
1	1	1	1	2	2	4	4	2	2
4	4	1	1	3	3	4	4	2	2
4	4	1	1	2	2	4	4	2	2
1	1	1	1	2	2	4	4	2	2
4	4	1	1	3	3	4	4	2	2
4	4	1	1	3	3	4	4	2	2
4	4	1	1	3	3	4	4	2	2
4	4	1	1	3	3	4	4	2	2
4	4	1	1	2	2	4	4	2	2
4	4	1	1	3	3	1	1	2	2
4	4	3	3	2	2	4	4	2	2
4	4	1	1	2	2	4	4	2	2
4	4	3	3	2	2	4	4	2	2
4	4	1	1	2	2	4	4	3	3
4	4	1	1	3	3	4	4	2	2
4	4	1	1	2	2	4	4	2	2
4	4	1	1	2	2	4	4	2	2
4	4	1	1	3	3	4	4	2	2
4	4	1	1	2	2	4	4	2	2
1	1	1	1	3	3	4	4	2	2
4	4	1	1	3	3	4	4	2	2
4	4	1	1	2	2	4	4	2	2
4	4	1	1	3	3	4	4	2	2
4	4	1	1	2	2	4	4	2	2
4	4	1	1	2	2	4	4	2	2
4	4	3	3	2	2	4	4	2	2
4	4	1	1	3	3	4	4	2	2
4	4	1	1	3	3	4	4	3	3
4	4	1	1	3	3	4	4	2	2
1	1	1	1	3	3	1	1	2	2
4	4	1	1	2	2	4	4	2	2
4	4	1	1	2	2	4	4	2	2
4	4	1	1	2	2	4	4	2	2
4	4	1	1	3	3	4	4	2	2
4	4	1	1	2	2	1	1	2	2
4	4	3	3	2	2	4	4	2	2
4	4	1	1	3	3	4	4	2	2
4	4	3	3	2	2	4	4	2	2
4	4	1	1	2	2	4	4	2	2
1	1	1	1	2	2	4	4	3	3
4	4	1	1	3	3	4	4	2	2

For Review Only

4	4	1	1	3	3	4	4	2	2
4	4	1	1	2	2	4	4	2	2

For Review Only

SNP1813	SNP1813	SNP1814	SNP1814	SNP1815	SNP1815	SNP1816	SNP1816	SNP1817	SNP1817
1	1	4	4	3	3	3	3	2	2
1	1	3	3	3	3	3	3	3	3
1	1	3	3	3	3	3	3	3	3
1	1	3	3	3	3	3	3	3	3
1	1	4	4	3	3	1	1	2	2
1	1	3	3	3	3	3	3	3	3
1	1	3	3	3	3	3	3	2	2
1	1	3	3	3	3	3	3	2	2
1	1	4	4	3	3	3	3	2	2
1	1	3	3	3	3	3	3	2	2
4	4	3	3	1	1	3	3	2	2
1	1	3	3	3	3	3	3	3	3
1	1	4	4	3	3	3	3	2	2
1	1	3	3	3	3	3	3	3	3
1	1	4	4	3	3	1	1	3	3
1	1	3	3	3	3	3	3	3	3
1	1	4	4	3	3	1	1	2	2
1	1	3	3	3	3	3	3	2	2
1	1	3	3	3	3	3	3	2	2
1	1	3	3	3	3	3	3	3	3
1	1	3	3	3	3	3	3	2	2
1	1	4	4	3	3	1	1	3	3
1	1	3	3	3	3	3	3	2	2
1	1	3	3	3	3	3	3	3	3
1	1	3	3	3	3	3	3	2	2
1	1	4	4	3	3	3	3	2	2
1	1	4	4	3	3	3	3	3	3
1	1	4	4	3	3	3	3	3	3
1	1	3	3	3	3	3	3	2	2
1	1	3	3	3	3	3	3	3	3
1	1	3	3	3	3	3	3	3	3
1	1	3	3	3	3	1	1	2	2
1	1	4	4	3	3	1	1	2	2
1	1	3	3	3	3	3	3	3	3
1	1	3	3	3	3	3	3	3	3
1	1	3	3	3	3	3	3	3	3
1	1	3	3	3	3	3	3	2	2
1	1	3	3	3	3	3	3	2	2
1	1	3	3	3	3	3	3	3	3
1	1	3	3	3	3	3	3	2	2
1	1	3	3	3	3	3	3	3	3
1	1	3	3	3	3	3	3	2	2
1	1	3	3	3	3	3	3	3	3
1	1	3	3	3	3	3	3	2	2
1	1	3	3	3	3	3	3	2	2
1	1	4	4	3	3	3	3	2	2
1	1	3	3	3	3	3	3	2	2
1	1	4	4	3	3	3	3	3	3
1	1	3	3	3	3	3	3	3	3
1	1	4	4	3	3	1	1	3	3
1	1	4	4	3	3	3	3	3	3
1	1	3	3	3	3	3	3	2	2

For Review Only

1	1	4	4	3	3	1	1	2	2
1	1	4	4	3	3	1	1	2	2
1	1	3	3	3	3	3	3	3	3
1	1	3	3	3	3	3	3	3	3
1	1	3	3	3	3	3	3	2	2
1	1	3	3	3	3	3	3	3	3
1	1	4	4	3	3	3	3	2	2
1	1	3	3	3	3	3	3	3	3
1	1	3	3	3	3	3	3	3	3
1	1	4	4	3	3	3	3	3	3
1	1	3	3	3	3	3	3	3	3
1	1	3	3	3	3	3	3	3	3
1	1	4	4	3	3	1	1	2	2
1	1	3	3	3	3	3	3	2	2
1	1	3	3	3	3	3	3	3	3
1	1	3	3	3	3	3	3	3	3
1	1	3	3	1	1	3	3	3	3
1	1	4	4	3	3	1	1	3	3
1	1	4	4	3	3	1	1	3	3
1	1	4	4	3	3	1	1	2	2
1	1	4	4	3	3	3	3	3	3
1	1	3	3	3	3	3	3	2	2
1	1	3	3	3	3	3	3	2	2
1	1	3	3	3	3	3	3	3	3
1	1	3	3	3	3	3	3	2	2
1	1	3	3	3	3	3	3	2	2
1	1	3	3	3	3	3	3	3	3
1	1	3	3	3	3	3	3	3	3
1	1	4	4	3	3	3	3	2	2
1	1	4	4	3	3	1	1	2	2
1	1	4	4	3	3	1	1	2	2
1	1	3	3	3	3	3	3	3	3
1	1	3	3	3	3	3	3	3	3
1	1	4	4	3	3	1	1	2	2
1	1	3	3	3	3	3	3	3	3
1	1	3	3	3	3	3	3	2	2
1	1	3	3	3	3	3	3	2	2
1	1	3	3	3	3	3	3	3	3
1	1	4	4	3	3	3	3	3	3
1	1	3	3	3	3	3	3	3	3
1	1	4	4	3	3	1	1	2	2
1	1	4	4	3	3	1	1	3	3
1	1	4	4	3	3	3	3	3	3
1	1	3	3	3	3	3	3	3	3
1	1	4	4	1	1	1	1	2	2
1	1	3	3	3	3	3	3	3	3
4	4	3	3	3	3	3	3	3	3
1	1	4	4	3	3	3	3	2	2
1	1	4	4	3	3	3	3	3	3
1	1	4	4	3	3	3	3	2	2
1	1	3	3	3	3	3	3	3	3
4	4	3	3	3	3	3	3	3	3
4	4	3	3	3	3	3	3	3	3
1	1	3	3	3	3	3	3	3	3
1	1	3	3	3	3	3	3	2	2

1	1	4	4	3	3	3	3	3	3
1	1	3	3	3	3	3	3	2	2
1	1	4	4	3	3	3	3	2	2
4	4	3	3	3	3	3	3	3	3
1	1	4	4	3	3	3	3	3	3
1	1	4	4	3	3	3	3	2	2
1	1	3	3	3	3	3	3	3	3
1	1	3	3	3	3	3	3	3	3
4	4	4	4	3	3	3	3	3	3
1	1	3	3	3	3	3	3	3	3
4	4	3	3	3	3	3	3	3	3
1	1	4	4	3	3	3	3	3	3
1	1	4	4	3	3	3	3	3	3
1	1	3	3	3	3	3	3	3	3
1	1	3	3	3	3	3	3	3	3
1	1	4	4	3	3	3	3	3	3
1	1	3	3	3	3	3	3	3	3
1	1	4	4	3	3	3	3	3	3
4	4	3	3	3	3	3	3	3	3
1	1	3	3	3	3	3	3	3	3
1	1	4	4	3	3	3	3	3	3
1	1	3	3	1	1	3	3	3	3
1	1	3	3	3	3	3	3	3	3
1	1	3	3	3	3	3	3	3	3
1	1	4	4	3	3	3	3	2	2
1	1	3	3	3	3	3	3	2	2
1	1	4	4	3	3	3	3	2	2
1	1	3	3	3	3	3	3	3	3
1	1	3	3	3	3	3	3	3	3
1	1	3	3	3	3	3	3	3	3
1	1	3	3	3	3	3	3	3	3
1	1	3	3	3	3	3	3	3	3
1	1	4	4	3	3	1	1	2	2
1	1	4	4	3	3	3	3	3	3
1	1	3	3	3	3	3	3	3	3
1	1	3	3	3	3	3	3	3	3
4	4	3	3	3	3	3	3	3	3
1	1	4	4	3	3	1	1	3	3
4	4	4	4	3	3	3	3	2	2
4	4	3	3	3	3	3	3	3	3
1	1	4	4	3	3	3	3	3	3
1	1	3	3	3	3	3	3	3	3
1	1	4	4	3	3	1	1	2	2
1	1	3	3	3	3	3	3	3	3
1	1	4	4	3	3	3	3	3	3
1	1	3	3	3	3	3	3	3	3
1	1	4	4	3	3	3	3	2	2
1	1	3	3	3	3	3	3	3	3
1	1	3	3	3	3	3	3	3	3
1	1	4	4	3	3	3	3	2	2
1	1	4	4	3	3	3	3	3	3
1	1	3	3	3	3	3	3	2	2
1	1	4	4	3	3	1	1	3	3
1	1	4	4	3	3	3	3	3	3
1	1	3	3	3	3	3	3	3	3

For Review Only

1	1	4	4	3	3	1	1	3	3
1	1	3	3	3	3	3	3	3	3
1	1	3	3	3	3	3	3	3	3
1	1	3	3	3	3	3	3	3	3
1	1	3	3	3	3	3	3	2	2
1	1	3	3	3	3	3	3	3	3
1	1	3	3	3	3	3	3	2	2
1	1	4	4	3	3	3	3	3	3
1	1	4	4	3	3	3	3	2	2
1	1	4	4	1	1	1	1	2	2
1	1	4	4	3	3	3	3	3	3
1	1	3	3	3	3	3	3	3	3
1	1	4	4	1	1	1	1	3	3
1	1	3	3	3	3	3	3	3	3
1	1	3	3	3	3	3	3	3	3
4	4	3	3	1	1	3	3	2	2
1	1	4	4	3	3	3	3	3	3
1	1	3	3	3	3	3	3	3	3
1	1	4	4	3	3	3	3	2	2
1	1	3	3	3	3	3	3	3	3
1	1	3	3	3	3	3	3	3	3
1	1	3	3	3	3	3	3	3	3
1	1	3	3	3	3	3	3	2	2
1	1	3	3	3	3	3	3	3	3
1	1	3	3	3	3	3	3	2	2
1	1	3	3	3	3	3	3	3	3
1	1	3	3	3	3	3	3	3	3
1	1	3	3	3	3	3	3	3	3
1	1	4	4	3	3	1	1	3	3
1	1	3	3	3	3	3	3	3	3
1	1	3	3	3	3	3	3	3	3
1	1	4	4	3	3	1	1	2	2
1	1	4	4	3	3	3	3	2	2
1	1	3	3	3	3	3	3	3	3
1	1	3	3	3	3	3	3	3	3
1	1	3	3	3	3	3	3	3	3
1	1	4	4	3	3	3	3	2	2
1	1	4	4	3	3	1	1	2	2
1	1	3	3	3	3	3	3	3	3
1	1	4	4	3	3	3	3	3	3
1	1	3	3	3	3	3	3	3	3
1	1	4	4	3	3	3	3	2	2
4	4	3	3	3	3	3	3	3	3
1	1	4	4	3	3	3	3	2	2
1	1	4	4	3	3	3	3	3	3
1	1	3	3	3	3	3	3	3	3
4	4	3	3	1	1	3	3	2	2
1	1	3	3	3	3	3	3	2	2
1	1	3	3	3	3	3	3	3	3
1	1	3	3	3	3	3	3	3	3
1	1	4	4	3	3	3	3	3	3
1	1	3	3	3	3	3	3	3	3
1	1	3	3	3	3	3	3	2	2
1	1	3	3	3	3	3	3	3	3
1	1	4	4	3	3	3	3	3	3

For Review Only

1	1	3	3	3	3	3	3	3	3
1	1	3	3	3	3	3	3	3	3
1	1	4	4	3	3	3	3	3	3
1	1	3	3	3	3	3	3	2	2
1	1	3	3	3	3	3	3	2	2
1	1	4	4	3	3	3	3	2	2
4	4	3	3	3	3	3	3	3	3
1	1	3	3	3	3	3	3	2	2
1	1	3	3	3	3	3	3	3	3
4	4	3	3	3	3	3	3	3	3
1	1	3	3	3	3	3	3	3	3
1	1	3	3	3	3	3	3	3	3
1	1	4	4	3	3	3	3	3	3
1	1	3	3	3	3	3	3	3	3
1	1	3	3	3	3	3	3	3	3
1	1	4	4	3	3	1	1	2	2
1	1	3	3	3	3	3	3	3	3
1	1	3	3	3	3	3	3	3	3
1	1	3	3	3	3	3	3	3	3
1	1	3	3	3	3	3	3	2	2
1	1	3	3	3	3	3	3	3	3
1	1	4	4	3	3	3	3	2	2
1	1	4	4	3	3	1	1	2	2
1	1	3	3	3	3	3	3	2	2
1	1	3	3	3	3	3	3	3	3
1	1	3	3	3	3	3	3	2	2
1	1	3	3	3	3	3	3	3	3
1	1	3	3	3	3	3	3	3	3
1	1	4	4	3	3	1	1	2	2
1	1	4	4	3	3	1	1	3	3
1	1	3	3	3	3	3	3	3	3
1	1	4	4	3	3	3	3	2	2
1	1	3	3	3	3	3	3	3	3
1	1	3	3	3	3	3	3	3	3
1	1	3	3	3	3	3	3	2	2
1	1	3	3	3	3	3	3	3	3
1	1	4	4	3	3	1	1	3	3
1	1	3	3	3	3	3	3	3	3
1	1	3	3	3	3	3	3	3	3
1	1	3	3	3	3	3	3	3	3
1	1	3	3	3	3	3	3	2	2
1	1	3	3	3	3	3	3	2	2
1	1	3	3	3	3	3	3	3	3
1	1	4	4	3	3	1	1	2	2
1	1	3	3	3	3	3	3	3	3
1	1	4	4	3	3	1	1	3	3
1	1	3	3	3	3	3	3	3	3
1	1	3	3	3	3	3	3	3	3
1	1	3	3	3	3	3	3	3	3
1	1	3	3	3	3	3	3	3	3
1	1	3	3	3	3	3	3	2	2
4	4	4	4	3	3	3	3	3	3
1	1	3	3	3	3	3	3	3	3
1	1	3	3	3	3	3	3	3	3
1	1	3	3	3	3	3	3	2	2

For Review Only

1	1	4	4	1	1	1	1	2	2
1	1	3	3	3	3	3	3	3	3

For Review Only

SNP1818	SNP1818	SNP1819	SNP1819	SNP182	SNP182	SNP1820	SNP1820	SNP1821	SNP1821
1	1	1	1	2	2	4	4	1	1
1	1	1	1	4	4	4	4	3	3
1	1	1	1	2	2	4	4	3	3
1	1	1	1	2	2	4	4	3	3
2	2	2	2	2	2	4	4	3	3
1	1	1	1	2	2	4	4	3	3
1	1	1	1	2	2	4	4	3	3
1	1	1	1	2	2	4	4	3	3
2	2	1	1	2	2	4	4	3	3
1	1	1	1	2	2	4	4	3	3
1	1	1	1	4	4	4	4	3	3
1	1	1	1	2	2	4	4	3	3
2	2	1	1	2	2	4	4	3	3
1	1	1	1	4	4	4	4	1	1
2	2	1	1	2	2	4	4	3	3
1	1	2	2	2	2	4	4	3	3
2	2	2	2	4	4	4	4	3	3
1	1	1	1	2	2	4	4	3	3
1	1	1	1	2	2	4	4	3	3
1	1	1	1	4	4	4	4	3	3
1	1	1	1	2	2	4	4	3	3
2	2	2	2	2	2	4	4	3	3
1	1	1	1	2	2	4	4	1	1
1	1	1	1	2	2	4	4	3	3
1	1	1	1	2	2	4	4	3	3
1	1	1	1	2	2	4	4	3	3
1	1	1	1	2	2	4	4	3	3
1	1	1	1	2	2	4	4	3	3
1	1	1	1	2	2	4	4	3	3
1	1	1	1	4	4	4	4	3	3
1	1	1	1	2	2	4	4	3	3
2	2	2	2	2	2	4	4	3	3
2	2	2	2	2	2	4	4	3	3
1	1	2	2	2	2	1	1	1	1
1	1	1	1	2	2	4	4	3	3
1	1	1	1	2	2	4	4	3	3
1	1	1	1	4	4	4	4	3	3
1	1	1	1	4	4	4	4	3	3
1	1	1	1	2	2	4	4	3	3
1	1	1	1	2	2	4	4	3	3
1	1	1	1	4	4	4	4	3	3
1	1	1	1	2	2	4	4	3	3
1	1	1	1	2	2	4	4	3	3
1	1	1	1	4	4	4	4	3	3
1	1	1	1	2	2	4	4	3	3
1	1	1	1	2	2	4	4	3	3
1	1	1	1	2	2	4	4	3	3
1	1	1	1	2	2	4	4	3	3
1	1	1	1	2	2	4	4	3	3
2	2	2	2	2	2	4	4	3	3
1	1	1	1	2	2	4	4	3	3
1	1	1	1	2	2	4	4	3	3

2	2	2	2	2	2	4	4	3	3
2	2	2	2	2	2	4	4	3	3
1	1	1	1	2	2	4	4	3	3
1	1	1	1	4	4	4	4	3	3
1	1	1	1	2	2	4	4	3	3
1	1	1	1	2	2	4	4	3	3
1	1	1	1	2	2	4	4	3	3
1	1	1	1	4	4	4	4	3	3
1	1	1	1	2	2	4	4	3	3
1	1	1	1	2	2	4	4	3	3
1	1	1	1	2	2	4	4	3	3
1	1	1	1	2	2	4	4	3	3
2	2	2	2	2	2	4	4	3	3
1	1	1	1	2	2	4	4	3	3
1	1	1	1	2	2	4	4	3	3
1	1	1	1	4	4	4	4	3	3
1	1	1	1	2	2	4	4	3	3
2	2	2	2	2	2	4	4	3	3
2	2	2	2	2	2	4	4	3	3
2	2	2	2	2	2	4	4	3	3
1	1	1	1	2	2	4	4	3	3
1	1	1	1	4	4	4	4	1	1
1	1	1	1	2	2	4	4	3	3
1	1	1	1	2	2	4	4	3	3
1	1	1	1	2	2	4	4	3	3
1	1	1	1	2	2	4	4	3	3
2	2	1	1	4	4	4	4	3	3
1	1	2	2	2	2	1	1	1	1
1	1	1	1	2	2	4	4	3	3
2	2	2	2	2	2	4	4	3	3
2	2	1	1	2	2	4	4	3	3
1	1	1	1	2	2	4	4	3	3
1	1	2	2	4	4	1	1	3	3
2	2	2	2	2	2	4	4	3	3
1	1	1	1	2	2	4	4	3	3
1	1	1	1	2	2	4	4	3	3
1	1	1	1	2	2	4	4	3	3
1	1	1	1	2	2	4	4	3	3
1	1	1	1	4	4	4	4	3	3
1	1	1	1	2	2	4	4	3	3
2	2	2	2	2	2	4	4	3	3
2	2	2	2	2	2	4	4	3	3
1	1	1	1	2	2	4	4	3	3
1	1	1	1	2	2	4	4	3	3
2	2	2	2	2	2	4	4	3	3
1	1	1	1	2	2	4	4	3	3
1	1	1	1	2	2	4	4	1	1
1	1	1	1	2	2	4	4	3	3
1	1	1	1	2	2	4	4	3	3
1	1	1	1	2	2	4	4	3	3
1	1	1	1	2	2	4	4	3	3
1	1	2	2	4	4	1	1	3	3
1	1	1	1	4	4	4	4	3	3
1	1	1	1	2	2	4	4	3	3
1	1	1	1	2	2	4	4	3	3

For Review Only

1	1	1	1	2	2	1	1	3	3
1	1	1	1	4	4	4	4	3	3
1	1	1	1	2	2	4	4	3	3
1	1	1	1	2	2	4	4	1	1
1	1	1	1	2	2	4	4	3	3
1	1	1	1	2	2	4	4	3	3
1	1	2	2	2	2	1	1	3	3
1	1	1	1	2	2	4	4	3	3
1	1	1	1	2	2	4	4	3	3
1	1	1	1	2	2	4	4	3	3
1	1	1	1	2	2	4	4	3	3
1	1	1	1	2	2	4	4	3	3
1	1	1	1	2	2	4	4	3	3
1	1	1	1	2	2	4	4	3	3
1	1	1	1	2	2	4	4	3	3
1	1	1	1	2	2	4	4	3	3
1	1	1	1	2	2	4	4	3	3
1	1	1	1	2	2	4	4	3	3
1	1	1	1	4	4	4	4	1	1
1	1	1	1	2	2	4	4	3	3
1	1	1	1	2	2	4	4	3	3
1	1	1	1	2	2	4	4	3	3
1	1	1	1	2	2	4	4	3	3
1	1	2	2	2	2	1	1	1	1
1	1	1	1	2	2	4	4	3	3
1	1	1	1	2	2	4	4	3	3
2	2	2	2	2	2	4	4	3	3
1	1	1	1	2	2	4	4	3	3
1	1	2	2	2	2	1	1	3	3
1	1	1	1	2	2	4	4	3	3
1	1	1	1	2	2	4	4	1	1
1	1	1	1	2	2	4	4	3	3
2	2	2	2	2	2	4	4	3	3
1	1	1	1	2	2	4	4	3	3
1	1	2	2	2	2	1	1	1	1
2	2	2	2	2	2	4	4	1	1
1	1	1	1	2	2	4	4	3	3
2	2	2	2	2	2	4	4	3	3
1	1	1	1	2	2	4	4	3	3
1	1	1	1	2	2	4	4	1	1
2	2	1	1	2	2	4	4	3	3
1	1	1	1	2	2	4	4	3	3
2	2	2	2	2	2	4	4	3	3
1	1	1	1	2	2	4	4	3	3
1	1	1	1	2	2	4	4	3	3
1	1	1	1	2	2	4	4	3	3
1	1	1	1	2	2	4	4	3	3
1	1	1	1	2	2	4	4	3	3
1	1	1	1	4	4	4	4	3	3
1	1	1	1	2	2	4	4	3	3
1	1	1	1	2	2	4	4	3	3
1	1	1	1	2	2	4	4	1	1
2	2	1	1	2	2	4	4	1	1
1	1	1	1	2	2	4	4	3	3
1	1	1	1	4	4	4	4	3	3

For Review Only

2	2	2	2	2	2	4	4	3	3
1	1	1	1	2	2	4	4	3	3
1	1	1	1	2	2	4	4	3	3
1	1	1	1	2	2	4	4	3	3
1	1	1	1	2	2	4	4	3	3
1	1	1	1	4	4	4	4	3	3
1	1	1	1	2	2	4	4	3	3
1	1	1	1	2	2	4	4	3	3
1	1	1	1	2	2	4	4	1	1
2	2	2	2	2	2	4	4	3	3
1	1	1	1	2	2	4	4	3	3
1	1	1	1	2	2	4	4	3	3
1	1	2	2	2	2	4	4	3	3
1	1	1	1	2	2	4	4	3	3
1	1	1	1	2	2	4	4	1	1
1	1	1	1	4	4	4	4	3	3
2	2	1	1	2	2	4	4	3	3
1	1	1	1	2	2	4	4	3	3
1	1	1	1	2	2	4	4	3	3
1	1	2	2	2	2	1	1	3	3
1	1	1	1	2	2	4	4	3	3
1	1	2	2	2	2	1	1	1	1
1	1	1	1	4	4	4	4	1	1
1	1	1	1	2	2	4	4	3	3
1	1	1	1	2	2	4	4	3	3
1	1	2	2	2	2	1	1	1	1
1	1	1	1	2	2	4	4	3	3
1	1	1	1	2	2	4	4	3	3
2	2	2	2	2	2	4	4	3	3
1	1	1	1	2	2	4	4	3	3
1	1	1	1	2	2	4	4	3	3
2	2	2	2	2	2	4	4	3	3
2	2	2	2	2	2	4	4	3	3
1	1	1	1	2	2	4	4	1	1
1	1	1	1	2	2	4	4	3	3
1	1	1	1	2	2	4	4	3	3
2	2	1	1	2	2	4	4	3	3
2	2	2	2	4	4	4	4	3	3
1	1	1	1	2	2	4	4	3	3
1	1	1	1	2	2	4	4	3	3
1	1	1	1	2	2	4	4	3	3
1	1	1	1	2	2	4	4	3	3
1	1	2	2	2	2	1	1	3	3
2	2	1	1	2	2	4	4	3	3
1	1	1	1	2	2	4	4	3	3
1	1	1	1	2	2	4	4	3	3
1	1	1	1	4	4	4	4	3	3
1	1	2	2	2	2	1	1	3	3
1	1	1	1	2	2	4	4	3	3
1	1	1	1	4	4	4	4	3	3
1	1	1	1	2	2	4	4	3	3
1	1	1	1	2	2	4	4	3	3
1	1	1	1	4	4	4	4	3	3
1	1	1	1	2	2	4	4	3	3
1	1	2	2	2	2	1	1	1	1

2	2	1	1	2	2	4	4	3	3
1	1	1	1	4	4	4	4	3	3
2	2	1	1	2	2	4	4	3	3
1	1	1	1	2	2	4	4	3	3
1	1	1	1	4	4	4	4	3	3
1	1	1	1	2	2	4	4	3	3
1	1	2	2	2	2	1	1	3	3
1	1	1	1	4	4	4	4	3	3
1	1	1	1	2	2	4	4	3	3
1	1	2	2	2	2	1	1	1	1
1	1	1	1	2	2	4	4	3	3
1	1	1	1	2	2	4	4	3	3
1	1	1	1	4	4	4	4	3	3
1	1	1	1	2	2	4	4	3	3
1	1	1	1	2	2	4	4	3	3
2	2	2	2	2	2	4	4	3	3
1	1	1	1	2	2	4	4	1	1
1	1	1	1	2	2	4	4	1	1
1	1	2	2	2	2	1	1	1	1
1	1	1	1	4	4	4	4	3	3
1	1	1	1	2	2	4	4	3	3
1	1	1	1	2	2	4	4	3	3
2	2	2	2	2	2	4	4	3	3
1	1	1	1	2	2	4	4	3	3
1	1	1	1	4	4	4	4	3	3
1	1	1	1	2	2	4	4	3	3
1	1	2	2	2	2	1	1	1	1
1	1	2	2	2	2	1	1	3	3
2	2	2	2	2	2	4	4	3	3
2	2	2	2	2	2	4	4	3	3
1	1	2	2	2	2	1	1	1	1
1	1	1	1	2	2	4	4	3	3
1	1	1	1	2	2	4	4	3	3
1	1	1	1	2	2	4	4	3	3
1	1	1	1	4	4	4	4	3	3
1	1	2	2	2	2	1	1	1	1
2	2	2	2	2	2	4	4	3	3
1	1	1	1	2	2	4	4	3	3
1	1	1	1	2	2	4	4	3	3
1	1	1	1	2	2	4	4	3	3
1	1	1	1	2	2	4	4	3	3
1	1	1	1	2	2	4	4	3	3
1	1	1	1	2	2	4	4	3	3
2	2	2	2	2	2	4	4	3	3
1	1	1	1	2	2	4	4	3	3
2	2	2	2	4	4	4	4	3	3
1	1	1	1	2	2	4	4	3	3
1	1	1	1	2	2	4	4	3	3
1	1	1	1	2	2	4	4	3	3
1	1	1	1	2	2	4	4	1	1
1	1	1	1	2	2	4	4	3	3
1	1	2	2	4	4	1	1	3	3
1	1	1	1	2	2	4	4	3	3
1	1	2	2	2	2	1	1	3	3
1	1	1	1	4	4	4	4	3	3

For Review Only

2	2	2	2	2	2	4	4	3	3
1	1	1	1	2	2	4	4	3	3

For Review Only

SNP1822	SNP1822	SNP1823	SNP1823	SNP1824	SNP1824	SNP1825	SNP1825	SNP1826	SNP1826
4	4	1	1	1	1	2	2	3	3
1	1	1	1	1	1	2	2	3	3
1	1	1	1	4	4	2	2	3	3
1	1	1	1	1	1	2	2	3	3
4	4	1	1	4	4	2	2	2	2
4	4	1	1	4	4	2	2	2	2
1	1	2	2	1	1	1	1	3	3
1	1	1	1	4	4	2	2	3	3
1	1	1	1	1	1	1	1	3	3
4	4	1	1	4	4	2	2	2	2
4	4	1	1	4	4	2	2	3	3
1	1	1	1	4	4	2	2	3	3
1	1	2	2	4	4	2	2	3	3
1	1	1	1	4	4	2	2	3	3
1	1	2	2	4	4	1	1	3	3
4	4	1	1	4	4	2	2	2	2
1	1	2	2	1	1	1	1	3	3
4	4	2	2	4	4	2	2	2	2
4	4	2	2	4	4	2	2	3	3
4	4	1	1	4	4	2	2	2	2
4	4	1	1	1	1	1	1	2	2
4	4	2	2	1	1	2	2	2	2
1	1	1	1	4	4	2	2	3	3
1	1	1	1	4	4	2	2	2	2
1	1	2	2	4	4	1	1	3	3
1	1	1	1	4	4	2	2	3	3
1	1	1	1	1	1	1	1	3	3
1	1	1	1	4	4	2	2	3	3
1	1	1	1	1	1	1	1	3	3
4	4	1	1	4	4	2	2	2	2
4	4	1	1	1	1	1	1	3	3
4	4	1	1	4	4	2	2	3	3
4	4	2	2	4	4	2	2	2	2
1	1	1	1	4	4	2	2	3	3
4	4	1	1	1	1	1	1	2	2
1	1	1	1	4	4	2	2	3	3
4	4	2	2	4	4	2	2	2	2
1	1	1	1	4	4	2	2	3	3
4	4	1	1	4	4	2	2	3	3
1	1	2	2	4	4	2	2	3	3
4	4	1	1	4	4	2	2	2	2
1	1	1	1	4	4	2	2	3	3
4	4	1	1	4	4	2	2	2	2
1	1	2	2	1	1	1	1	3	3
4	4	1	1	4	4	2	2	2	2
1	1	1	1	4	4	2	2	3	3
1	1	1	1	1	1	1	1	2	2
1	1	2	2	4	4	2	2	3	3
4	4	2	2	4	4	1	1	2	2
1	1	2	2	4	4	2	2	3	3
1	1	1	1	4	4	2	2	3	3
1	1	1	1	4	4	2	2	3	3

For Review Only

1	1	2	2	1	1	1	1	3	3
4	4	2	2	4	4	2	2	2	2
4	4	2	2	4	4	2	2	3	3
1	1	2	2	1	1	1	1	3	3
4	4	2	2	4	4	2	2	3	3
1	1	1	1	1	1	1	1	3	3
1	1	1	1	4	4	2	2	3	3
1	1	1	1	4	4	2	2	3	3
4	4	1	1	4	4	2	2	2	2
1	1	2	2	4	4	2	2	3	3
4	4	1	1	1	1	2	2	2	2
4	4	1	1	1	1	1	1	2	2
1	1	2	2	1	1	1	1	3	3
1	1	1	1	1	1	1	1	3	3
4	4	1	1	4	4	2	2	3	3
4	4	2	2	4	4	2	2	2	2
1	1	1	1	4	4	2	2	3	3
4	4	1	1	4	4	2	2	2	2
4	4	1	1	1	1	1	1	3	3
4	4	2	2	1	1	1	1	2	2
4	4	1	1	4	4	1	1	2	2
1	1	2	2	4	4	2	2	3	3
1	1	2	2	1	1	1	1	3	3
4	4	2	2	4	4	2	2	2	2
4	4	1	1	4	4	2	2	2	2
1	1	1	1	1	1	2	2	3	3
4	4	1	1	1	1	1	1	3	3
4	4	1	1	4	4	2	2	3	3
1	1	1	1	4	4	2	2	3	3
1	1	2	2	4	4	2	2	3	3
4	4	2	2	4	4	2	2	3	3
1	1	1	1	4	4	2	2	3	3
4	4	1	1	4	4	2	2	3	3
1	1	2	2	1	1	1	1	3	3
4	4	2	2	4	4	2	2	2	2
4	4	1	1	4	4	2	2	2	2
1	1	1	1	4	4	2	2	3	3
1	1	2	2	1	1	1	1	3	3
1	1	1	1	4	4	2	2	3	3
4	4	1	1	4	4	2	2	2	2
1	1	2	2	1	1	1	1	3	3
1	1	1	1	1	1	1	1	3	3
1	1	1	1	4	4	2	2	3	3
4	4	1	1	1	1	2	2	2	2
1	1	1	1	4	4	2	2	3	3
1	1	1	1	1	1	2	2	3	3
1	1	1	1	4	4	2	2	3	3
1	1	1	1	4	4	2	2	3	3
1	1	1	1	4	4	2	2	3	3
1	1	2	2	1	1	1	1	3	3
4	4	1	1	1	1	2	2	3	3
4	4	1	1	4	4	2	2	3	3
1	1	1	1	4	4	2	2	3	3
1	1	2	2	4	4	2	2	3	3
1	1	1	1	1	1	1	1	3	3

For Review Only

1	1	1	1	4	4	2	2	3	3
1	1	1	1	4	4	2	2	3	3
1	1	2	2	4	4	2	2	3	3
1	1	1	1	4	4	2	2	3	3
1	1	1	1	1	1	2	2	3	3
1	1	1	1	4	4	2	2	3	3
4	4	2	2	1	1	2	2	2	2
1	1	2	2	4	4	2	2	3	3
4	4	1	1	4	4	2	2	3	3
1	1	1	1	4	4	2	2	3	3
1	1	1	1	4	4	2	2	3	3
1	1	1	1	4	4	2	2	3	3
4	4	1	1	4	4	2	2	3	3
1	1	1	1	1	1	2	2	3	3
4	4	2	2	1	1	2	2	3	3
1	1	1	1	4	4	2	2	3	3
4	4	1	1	4	4	2	2	3	3
1	1	2	2	4	4	2	2	3	3
1	1	1	1	4	4	2	2	3	3
1	1	1	1	4	4	2	2	3	3
1	1	1	1	4	4	2	2	3	3
4	4	2	2	4	4	2	2	2	2
1	1	1	1	4	4	2	2	3	3
1	1	1	1	4	4	2	2	3	3
1	1	2	2	4	4	2	2	3	3
1	1	1	1	4	4	1	1	3	3
1	1	2	2	4	4	2	2	3	3
1	1	2	2	4	4	2	2	3	3
4	4	1	1	4	4	2	2	3	3
1	1	2	2	4	4	2	2	2	2
1	1	1	1	4	4	2	2	3	3
1	1	1	1	4	4	2	2	3	3
1	1	2	2	1	1	1	1	3	3
1	1	2	2	4	4	2	2	3	3
1	1	1	1	4	4	2	2	3	3
1	1	1	1	4	4	2	2	2	2
1	1	1	1	4	4	2	2	3	3
4	4	1	1	1	1	2	2	3	3
1	1	1	1	4	4	2	2	3	3
4	4	1	1	4	4	2	2	3	3
1	1	1	1	4	4	2	2	3	3
1	1	1	1	4	4	2	2	3	3
1	1	1	1	4	4	2	2	3	3
1	1	1	1	4	4	2	2	3	3
1	1	1	1	4	4	2	2	3	3
1	1	1	1	4	4	2	2	3	3
1	1	1	1	4	4	2	2	3	3
1	1	1	1	1	1	2	2	3	3
4	4	2	2	4	4	2	2	3	3
1	1	1	1	4	4	2	2	3	3
1	1	1	1	4	4	2	2	3	3
4	4	1	1	4	4	2	2	2	2
4	4	1	1	4	4	2	2	3	3
1	1	1	1	1	1	1	1	3	3
1	1	1	1	4	4	2	2	3	3

For Review Only

4	4	1	1	4	4	2	2	2	2
1	1	1	1	4	4	2	2	3	3
1	1	2	2	4	4	2	2	2	2
4	4	1	1	4	4	2	2	3	3
4	4	1	1	1	1	2	2	2	2
1	1	1	1	4	4	2	2	3	3
4	4	1	1	4	4	2	2	3	3
1	1	2	2	4	4	2	2	3	3
1	1	2	2	4	4	2	2	3	3
1	1	1	1	4	4	2	2	3	3
1	1	2	2	4	4	2	2	3	3
1	1	1	1	4	4	2	2	3	3
1	1	2	2	4	4	2	2	3	3
1	1	1	1	4	4	2	2	3	3
1	1	1	1	4	4	2	2	3	3
4	4	1	1	4	4	2	2	3	3
4	4	1	1	4	4	2	2	3	3
4	4	1	1	1	1	2	2	2	2
1	1	1	1	4	4	2	2	3	3
4	4	2	2	1	1	2	2	2	2
1	1	1	1	4	4	2	2	3	3
1	1	1	1	4	4	2	2	2	2
4	4	2	2	4	4	2	2	2	2
4	4	1	1	1	1	1	1	2	2
1	1	2	2	1	1	1	1	3	3
1	1	1	1	4	4	2	2	3	3
1	1	1	1	1	1	1	1	3	3
1	1	1	1	4	4	2	2	3	3
1	1	1	1	4	4	2	2	3	3
1	1	1	1	1	1	1	1	3	3
1	1	1	1	1	1	1	1	3	3
1	1	1	1	4	4	2	2	3	3
1	1	1	1	4	4	2	2	3	3
1	1	1	1	4	4	2	2	3	3
1	1	2	2	1	1	2	2	3	3
4	4	1	1	1	1	1	1	2	2
1	1	1	1	1	1	1	1	3	3
4	4	2	2	4	4	2	2	2	2
1	1	1	1	1	1	2	2	3	3
1	1	1	1	4	4	2	2	3	3
1	1	1	1	4	4	2	2	3	3
1	1	1	1	4	4	2	2	3	3
4	4	1	1	4	4	2	2	3	3
1	1	1	1	1	1	2	2	3	3
1	1	2	2	4	4	2	2	3	3
1	1	2	2	1	1	1	1	3	3
4	4	1	1	4	4	2	2	3	3
4	4	1	1	4	4	2	2	3	3
1	1	1	1	4	4	2	2	3	3
1	1	2	2	1	1	2	2	3	3
4	4	1	1	4	4	2	2	2	2
1	1	1	1	4	4	2	2	3	3
4	4	2	2	4	4	2	2	2	2
4	4	1	1	1	1	2	2	2	2
4	4	1	1	4	4	2	2	3	3

For Review Only

1	1	1	1	4	4	2	2	3	3
4	4	1	1	4	4	2	2	3	3
1	1	2	2	4	4	2	2	3	3
1	1	2	2	4	4	1	1	3	3
4	4	1	1	4	4	2	2	2	2
1	1	1	1	4	4	2	2	3	3
4	4	1	1	4	4	2	2	3	3
1	1	1	1	4	4	2	2	3	3
1	1	1	1	4	4	2	2	3	3
4	4	1	1	4	4	2	2	3	3
1	1	1	1	4	4	2	2	3	3
4	4	1	1	4	4	2	2	2	2
1	1	1	1	4	4	2	2	3	3
4	4	1	1	4	4	2	2	2	2
1	1	1	1	4	4	2	2	3	3
1	1	1	1	4	4	2	2	3	3
4	4	1	1	4	4	2	2	3	3
1	1	1	1	4	4	2	2	3	3
4	4	1	1	4	4	2	2	3	3
1	1	1	1	4	4	2	2	3	3
1	1	1	1	4	4	2	2	3	3
1	1	1	1	4	4	2	2	3	3
1	1	1	1	4	4	2	2	3	3
1	1	1	1	4	4	2	2	3	3
1	1	1	1	4	4	2	2	3	3
1	1	1	1	4	4	2	2	3	3
4	4	1	1	4	4	2	2	3	3
1	1	1	1	1	1	2	2	3	3
1	1	2	2	4	4	2	2	3	3
4	4	1	1	4	4	2	2	2	2
4	4	1	1	4	4	2	2	3	3
1	1	1	1	4	4	2	2	3	3
4	4	1	1	4	4	2	2	3	3
1	1	1	1	4	4	2	2	2	2
4	4	2	2	4	4	2	2	2	2
4	4	1	1	4	4	2	2	3	3
4	4	2	2	4	4	2	2	2	2
4	4	1	1	1	1	1	1	2	2
1	1	1	1	4	4	2	2	3	3
4	4	1	1	4	4	2	2	3	3
1	1	1	1	4	4	2	2	3	3
4	4	1	1	1	1	1	1	2	2
1	1	1	1	4	4	2	2	3	3
1	1	2	2	1	1	1	1	3	3
1	1	1	1	4	4	2	2	3	3
1	1	1	1	4	4	2	2	3	3
1	1	1	1	4	4	2	2	3	3
4	4	1	1	1	1	1	1	2	2
4	4	1	1	4	4	2	2	3	3
4	4	1	1	4	4	2	2	3	3
1	1	1	1	4	4	2	2	3	3
4	4	1	1	4	4	2	2	3	3
1	1	1	1	4	4	2	2	3	3
1	1	1	1	1	1	1	1	3	3
1	1	1	1	4	4	2	2	3	3

For Review Only

1	1	1	1	4	4	2	2	3	3
1	1	2	2	1	1	1	1	3	3

For Review Only

SNP1827	SNP1827	SNP1828	SNP1828	SNP1829	SNP1829	SNP183	SNP183	SNP1830	SNP1830
1	1	4	4	2	2	3	3	4	4
3	3	1	1	3	3	3	3	3	3
3	3	1	1	3	3	3	3	3	3
3	3	1	1	2	2	3	3	3	3
1	1	4	4	3	3	3	3	4	4
3	3	1	1	2	2	3	3	3	3
1	1	4	4	2	2	3	3	4	4
3	3	1	1	2	2	3	3	3	3
1	1	4	4	2	2	3	3	4	4
3	3	1	1	2	2	3	3	3	3
3	3	4	4	2	2	3	3	4	4
3	3	1	1	3	3	3	3	3	3
3	3	1	1	2	2	3	3	3	3
1	1	4	4	2	2	3	3	4	4
1	1	4	4	2	2	3	3	4	4
3	3	1	1	2	2	3	3	3	3
1	1	4	4	3	3	3	3	4	4
1	1	4	4	2	2	3	3	4	4
3	3	4	4	3	3	3	3	4	4
3	3	1	1	2	2	3	3	3	3
1	1	4	4	3	3	3	3	4	4
1	1	4	4	3	3	3	3	4	4
3	3	4	4	3	3	4	4	4	4
3	3	1	1	3	3	3	3	3	3
3	3	1	1	2	2	4	4	3	3
3	3	1	1	2	2	3	3	3	3
3	3	1	1	2	2	3	3	3	3
3	3	4	4	2	2	3	3	4	4
3	3	1	1	2	2	4	4	3	3
3	3	1	1	3	3	3	3	3	3
3	3	1	1	2	2	3	3	3	3
1	1	4	4	3	3	3	3	4	4
1	1	4	4	3	3	3	3	4	4
3	3	1	1	3	3	3	3	3	3
3	3	1	1	2	2	3	3	3	3
3	3	1	1	3	3	3	3	3	3
1	1	4	4	2	2	3	3	4	4
1	1	4	4	2	2	3	3	4	4
3	3	1	1	3	3	3	3	3	3
1	1	4	4	3	3	3	3	4	4
3	3	1	1	2	2	3	3	3	3
3	3	1	1	2	2	3	3	3	3
3	3	1	1	2	2	3	3	3	3
1	1	4	4	3	3	3	3	4	4
3	3	1	1	2	2	3	3	3	3
3	3	1	1	2	2	3	3	3	3
3	3	1	1	3	3	3	3	3	3
3	3	1	1	2	2	3	3	3	3
1	1	4	4	3	3	3	3	4	4
1	1	4	4	2	2	3	3	4	4
1	1	4	4	2	2	3	3	4	4
3	3	4	4	2	2	3	3	4	4

For Review Only

1	1	4	4	3	3	4	4	4	4
1	1	4	4	3	3	3	3	4	4
3	3	4	4	3	3	3	3	4	4
1	1	4	4	3	3	3	3	4	4
3	3	4	4	3	3	3	3	4	4
1	1	4	4	3	3	3	3	4	4
1	1	4	4	2	2	3	3	4	4
3	3	1	1	2	2	3	3	3	3
3	3	1	1	2	2	3	3	3	3
3	3	4	4	2	2	3	3	4	4
3	3	1	1	2	2	3	3	3	3
3	3	1	1	3	3	3	3	3	3
1	1	4	4	3	3	4	4	4	4
3	3	1	1	2	2	4	4	3	3
1	1	4	4	2	2	3	3	4	4
3	3	1	1	2	2	3	3	4	4
1	1	4	4	3	3	3	3	4	4
1	1	4	4	2	2	4	4	4	4
1	1	4	4	3	3	3	3	4	4
1	1	4	4	3	3	3	3	4	4
3	3	1	1	2	2	3	3	3	3
3	3	1	1	2	2	3	3	3	3
3	3	4	4	2	2	3	3	3	3
3	3	1	1	2	2	3	3	3	3
3	3	1	1	2	2	3	3	3	3
3	3	1	1	2	2	3	3	3	3
3	3	1	1	2	2	3	3	4	4
3	3	1	1	3	3	3	3	3	3
1	1	4	4	2	2	3	3	4	4
1	1	4	4	2	2	3	3	4	4
1	1	4	4	3	3	3	3	4	4
3	3	1	1	3	3	3	3	3	3
3	3	1	1	3	3	3	3	3	3
1	1	4	4	3	3	4	4	4	4
1	1	4	4	2	2	3	3	4	4
3	3	1	1	2	2	3	3	3	3
1	1	4	4	2	2	3	3	4	4
3	3	4	4	3	3	3	3	4	4
3	3	1	1	2	2	3	3	3	3
3	3	1	1	2	2	3	3	3	3
1	1	4	4	3	3	3	3	4	4
3	3	1	1	2	2	3	3	3	3
1	1	4	4	2	2	3	3	4	4
3	3	1	1	2	2	3	3	3	3
3	3	1	1	2	2	3	3	3	3
1	1	4	4	2	2	3	3	4	4
3	3	1	1	2	2	3	3	3	3
3	3	4	4	2	2	3	3	4	4
1	1	4	4	2	2	3	3	4	4
1	1	4	4	2	2	4	4	4	4
3	3	1	1	2	2	3	3	3	3
3	3	1	1	3	3	3	3	3	3
3	3	1	1	2	2	3	3	3	3
3	3	4	4	2	2	3	3	4	4
1	1	4	4	2	2	4	4	4	4

For Review Only

1	1	4	4	2	2	3	3	4	4
3	3	1	1	2	2	3	3	3	3
3	3	1	1	2	2	4	4	3	3
3	3	1	1	2	2	3	3	3	3
3	3	4	4	2	2	3	3	4	4
3	3	1	1	2	2	3	3	3	3
3	3	1	1	2	2	3	3	3	3
3	3	1	1	2	2	3	3	4	4
3	3	1	1	2	2	3	3	3	3
3	3	1	1	2	2	3	3	4	4
1	1	4	4	2	2	3	3	4	4
3	3	4	4	2	2	3	3	4	4
1	1	4	4	2	2	4	4	4	4
3	3	4	4	2	2	3	3	4	4
3	3	1	1	3	3	3	3	3	3
1	1	4	4	2	2	3	3	4	4
1	1	4	4	2	2	4	4	4	4
3	3	4	4	2	2	3	3	4	4
3	3	1	1	2	2	3	3	3	3
1	1	4	4	3	3	3	3	4	4
3	3	4	4	2	2	3	3	4	4
1	1	4	4	2	2	3	3	4	4
3	3	1	1	2	2	3	3	3	3
3	3	1	1	3	3	3	3	3	3
3	3	1	1	2	2	3	3	3	3
3	3	1	1	2	2	3	3	3	3
3	3	1	1	3	3	4	4	3	3
1	1	4	4	2	2	3	3	4	4
1	1	4	4	2	2	3	3	4	4
1	1	4	4	2	2	4	4	4	4
3	3	1	1	2	2	3	3	3	3
3	3	4	4	2	2	3	3	4	4
1	1	4	4	3	3	3	3	4	4
1	1	4	4	2	2	3	3	4	4
3	3	1	1	3	3	3	3	3	3
1	1	4	4	3	3	3	3	4	4
3	3	1	1	2	2	3	3	3	3
1	1	4	4	3	3	3	3	4	4
3	3	1	1	2	2	3	3	4	4
3	3	1	1	3	3	3	3	3	3
3	3	1	1	2	2	3	3	3	3
3	3	1	1	2	2	3	3	3	3
1	1	4	4	2	2	4	4	4	4
3	3	1	1	2	2	3	3	4	4
1	1	4	4	2	2	3	3	4	4
3	3	1	1	2	2	3	3	3	3
1	1	4	4	2	2	3	3	4	4
3	3	1	1	2	2	3	3	3	3
3	3	4	4	2	2	3	3	3	3
3	3	1	1	2	2	3	3	3	3
3	3	4	4	2	2	3	3	4	4
3	3	1	1	3	3	3	3	3	3
3	3	1	1	2	2	4	4	3	3
3	3	1	1	2	2	3	3	3	3
3	3	1	1	2	2	3	3	4	4

For Review Only

1	1	4	4	2	2	3	3	4	4
1	1	4	4	2	2	3	3	4	4
3	3	1	1	2	2	3	3	3	3
3	3	1	1	2	2	4	4	3	3
3	3	4	4	2	2	3	3	4	4
1	1	4	4	2	2	3	3	4	4
3	3	1	1	3	3	3	3	3	3
3	3	4	4	2	2	3	3	4	4
3	3	1	1	3	3	3	3	3	3
3	3	1	1	2	2	3	3	3	3
3	3	4	4	2	2	3	3	4	4
3	3	1	1	3	3	3	3	4	4
3	3	1	1	2	2	3	3	3	3
3	3	1	1	2	2	3	3	3	3
3	3	1	1	2	2	3	3	3	3
3	3	4	4	2	2	3	3	4	4
1	1	4	4	2	2	3	3	4	4
3	3	1	1	2	2	3	3	3	3
1	1	4	4	2	2	3	3	4	4
3	3	1	1	2	2	3	3	3	3
3	3	4	4	2	2	3	3	4	4
3	3	1	1	3	3	3	3	3	3
3	3	1	1	2	2	3	3	3	3
3	3	1	1	3	3	3	3	3	3
3	3	1	1	3	3	3	3	3	3
3	3	1	1	3	3	3	3	3	3
3	3	1	1	2	2	3	3	3	3
3	3	4	4	2	2	3	3	4	4
1	1	4	4	3	3	4	4	4	4
1	1	4	4	2	2	4	4	4	4
3	3	1	1	2	2	3	3	3	3
1	1	4	4	2	2	4	4	4	4
3	3	1	1	3	3	4	4	3	3
3	3	1	1	2	2	3	3	3	3
3	3	4	4	2	2	4	4	4	4
3	3	1	1	3	3	3	3	3	3
3	3	1	1	2	2	3	3	3	3
1	1	4	4	3	3	3	3	4	4
3	3	1	1	2	2	3	3	3	3
3	3	1	1	2	2	4	4	3	3
3	3	1	1	3	3	3	3	3	3
1	1	4	4	2	2	3	3	4	4
3	3	1	1	3	3	3	3	3	3
3	3	4	4	2	2	3	3	4	4
3	3	4	4	2	2	3	3	4	4
3	3	1	1	2	2	3	3	3	3
3	3	4	4	2	2	3	3	4	4
3	3	1	1	3	3	3	3	3	3
3	3	1	1	2	2	3	3	3	3
3	3	4	4	3	3	3	3	4	4
1	1	4	4	2	2	3	3	4	4
3	3	1	1	2	2	3	3	3	3
3	3	1	1	2	2	3	3	3	3
3	3	1	1	2	2	3	3	3	3
3	3	1	1	3	3	3	3	3	3

For Review Only

1	1	4	4	2	2	4	4	4	4
1	1	4	4	2	2	3	3	4	4
1	1	4	4	2	2	3	3	4	4
3	3	4	4	2	2	3	3	4	4
1	1	4	4	2	2	3	3	4	4
1	1	4	4	2	2	3	3	4	4
1	1	4	4	3	3	3	3	4	4
3	3	1	1	3	3	3	3	3	3
3	3	1	1	2	2	3	3	3	3
3	3	1	1	3	3	3	3	3	3
3	3	4	4	2	2	3	3	4	4
1	1	4	4	2	2	4	4	4	4
3	3	4	4	2	2	3	3	4	4
3	3	1	1	2	2	3	3	3	3
3	3	1	1	3	3	4	4	3	3
3	3	4	4	2	2	3	3	4	4
3	3	1	1	2	2	3	3	3	3
3	3	4	4	3	3	3	3	4	4
1	1	4	4	3	3	3	3	4	4
1	1	4	4	2	2	3	3	4	4
3	3	1	1	3	3	3	3	3	3
1	1	4	4	2	2	3	3	4	4
1	1	4	4	2	2	3	3	4	4
1	1	4	4	2	2	3	3	4	4
3	3	1	1	2	2	3	3	3	3
3	3	1	1	2	2	3	3	3	3
3	3	1	1	3	3	3	3	3	3
3	3	1	1	2	2	3	3	3	3
1	1	4	4	3	3	3	3	4	4
1	1	4	4	2	2	3	3	4	4
3	3	1	1	3	3	3	3	3	3
3	3	1	1	2	2	3	3	3	3
3	3	1	1	2	2	3	3	3	3
3	3	1	1	3	3	4	4	3	3
3	3	4	4	2	2	3	3	4	4
3	3	1	1	3	3	3	3	3	3
1	1	4	4	2	2	4	4	4	4
3	3	1	1	3	3	3	3	3	3
3	3	1	1	3	3	3	3	3	3
3	3	1	1	3	3	3	3	3	3
1	1	4	4	2	2	4	4	4	4
3	3	1	1	2	2	3	3	3	3
3	3	1	1	3	3	3	3	3	3
1	1	4	4	3	3	3	3	4	4
3	3	1	1	3	3	3	3	3	3
3	3	1	1	3	3	3	3	3	3
3	3	1	1	2	2	4	4	3	3
3	3	4	4	2	2	3	3	4	4
3	3	1	1	3	3	3	3	3	3
3	3	1	1	2	2	3	3	3	3
1	1	4	4	2	2	3	3	4	4
3	3	1	1	3	3	3	3	3	3
3	3	1	1	3	3	3	3	3	3
3	3	1	1	2	2	4	4	3	3
3	3	1	1	3	3	3	3	3	3

For Review Only

3	3	4	4	2	2	3	3	4	4
3	3	1	1	2	2	3	3	3	3

For Review Only

SNP1831	SNP1831	SNP1832	SNP1832	SNP1833	SNP1833	SNP1834	SNP1834	SNP1835	SNP1835
4	4	1	1	2	2	4	4	2	2
1	1	3	3	2	2	4	4	2	2
4	4	1	1	2	2	4	4	2	2
1	1	1	1	2	2	4	4	2	2
1	1	1	1	3	3	4	4	2	2
4	4	1	1	2	2	4	4	2	2
4	4	1	1	2	2	4	4	3	3
4	4	3	3	2	2	4	4	2	2
1	1	1	1	2	2	4	4	3	3
1	1	3	3	2	2	4	4	2	2
1	1	3	3	3	3	1	1	2	2
4	4	1	1	2	2	4	4	2	2
1	1	1	1	3	3	4	4	2	2
1	1	1	1	2	2	4	4	2	2
4	4	1	1	2	2	4	4	2	2
4	4	1	1	2	2	4	4	2	2
4	4	1	1	2	2	4	4	3	3
4	4	1	1	2	2	4	4	2	2
4	4	1	1	2	2	4	4	2	2
1	1	3	3	3	3	4	4	2	2
4	4	1	1	2	2	4	4	3	3
4	4	1	1	2	2	4	4	2	2
1	1	3	3	2	2	4	4	2	2
1	1	1	1	2	2	4	4	2	2
1	1	1	1	3	3	4	4	3	3
4	4	3	3	2	2	4	4	2	2
4	4	3	3	2	2	4	4	3	3
4	4	1	1	2	2	4	4	2	2
1	1	1	1	2	2	4	4	3	3
1	1	3	3	2	2	4	4	2	2
4	4	1	1	2	2	4	4	3	3
4	4	3	3	2	2	4	4	2	2
1	1	3	3	3	3	4	4	2	2
1	1	3	3	2	2	4	4	2	2
1	1	3	3	2	2	4	4	2	2
1	1	3	3	2	2	4	4	2	2
4	4	3	3	2	2	4	4	2	2
4	4	1	1	2	2	4	4	2	2
1	1	3	3	3	3	4	4	2	2
4	4	1	1	2	2	1	1	2	2
1	1	3	3	2	2	4	4	2	2
1	1	3	3	2	2	4	4	2	2
1	1	1	1	3	3	4	4	2	2
4	4	3	3	2	2	4	4	3	3
1	1	3	3	3	3	4	4	2	2
4	4	1	1	2	2	4	4	2	2
1	1	3	3	2	2	4	4	3	3
1	1	3	3	2	2	1	1	2	2
1	1	1	1	2	2	4	4	3	3
4	4	1	1	2	2	4	4	2	2
4	4	3	3	2	2	4	4	2	2
1	1	3	3	2	2	4	4	2	2

For Review Only

1	1	1	1	3	3	4	4	3	3
1	1	3	3	3	3	4	4	2	2
1	1	3	3	2	2	4	4	3	3
4	4	1	1	2	2	4	4	3	3
4	4	1	1	2	2	4	4	2	2
1	1	3	3	2	2	4	4	3	3
1	1	3	3	2	2	4	4	2	2
1	1	3	3	3	3	4	4	2	2
1	1	3	3	3	3	4	4	2	2
4	4	1	1	2	2	4	4	2	2
4	4	3	3	2	2	4	4	2	2
1	1	3	3	2	2	4	4	3	3
1	1	1	1	3	3	4	4	3	3
1	1	1	1	2	2	4	4	3	3
4	4	1	1	2	2	4	4	2	2
1	1	1	1	2	2	4	4	3	3
4	4	1	1	2	2	4	4	2	2
4	4	1	1	2	2	4	4	2	2
1	1	3	3	2	2	4	4	3	3
4	4	1	1	2	2	4	4	3	3
1	1	3	3	2	2	4	4	2	2
4	4	1	1	2	2	1	1	2	2
1	1	1	1	2	2	4	4	3	3
4	4	3	3	2	2	4	4	2	2
1	1	3	3	3	3	4	4	2	2
1	1	1	1	2	2	4	4	2	2
1	1	3	3	2	2	4	4	3	3
1	1	3	3	2	2	4	4	2	2
4	4	3	3	2	2	4	4	2	2
4	4	3	3	2	2	4	4	2	2
1	1	1	1	2	2	4	4	2	2
1	1	1	1	2	2	4	4	2	2
1	1	3	3	3	3	4	4	2	2
1	1	1	1	3	3	4	4	3	3
1	1	1	1	2	2	4	4	2	2
1	1	3	3	3	3	4	4	2	2
1	1	3	3	2	2	4	4	2	2
1	1	3	3	3	3	4	4	3	3
4	4	1	1	2	2	4	4	2	2
4	4	1	1	2	2	4	4	2	2
4	4	1	1	2	2	4	4	3	3
4	4	3	3	2	2	4	4	2	2
4	4	3	3	2	2	4	4	2	2
4	4	3	3	2	2	4	4	2	2
1	1	1	1	3	3	4	4	2	2
4	4	3	3	2	2	4	4	2	2
1	1	3	3	2	2	4	4	2	2
1	1	3	3	2	2	4	4	2	2
1	1	3	3	2	2	4	4	2	2
4	4	1	1	2	2	4	4	3	3
4	4	3	3	2	2	4	4	2	2
1	1	3	3	3	3	1	1	2	2
1	1	3	3	2	2	4	4	2	2
1	1	3	3	2	2	4	4	2	2
1	1	1	1	2	2	4	4	3	3

For Review Only

4	4	1	1	2	2	4	4	2	2
4	4	1	1	2	2	4	4	2	2
1	1	3	3	2	2	4	4	2	2
1	1	3	3	2	2	4	4	2	2
4	4	3	3	2	2	4	4	2	2
4	4	3	3	2	2	4	4	2	2
4	4	1	1	2	2	4	4	2	2
4	4	1	1	2	2	4	4	2	2
1	1	1	1	3	3	4	4	2	2
1	1	3	3	2	2	4	4	2	2
1	1	1	1	3	3	4	4	2	2
4	4	1	1	2	2	4	4	2	2
4	4	3	3	2	2	4	4	2	2
4	4	1	1	2	2	4	4	2	2
4	4	1	1	2	2	4	4	2	2
4	4	3	3	2	2	4	4	2	2
1	1	1	1	2	2	4	4	2	2
4	4	1	1	2	2	4	4	2	2
1	1	3	3	2	2	4	4	2	2
4	4	1	1	2	2	4	4	2	2
4	4	1	1	2	2	4	4	2	2
4	4	1	1	2	2	4	4	2	2
4	4	1	1	2	2	4	4	2	2
1	1	3	3	2	2	4	4	2	2
4	4	1	1	2	2	4	4	2	2
4	4	1	1	2	2	4	4	2	2
4	4	3	3	2	2	4	4	3	3
4	4	3	3	2	2	4	4	2	2
4	4	1	1	2	2	4	4	2	2
1	1	1	1	3	3	4	4	2	2
1	1	3	3	2	2	4	4	2	2
4	4	1	1	2	2	4	4	2	2
4	4	1	1	2	2	4	4	3	3
4	4	1	1	2	2	4	4	2	2
1	1	3	3	2	2	4	4	2	2
1	1	3	3	3	3	4	4	2	2
1	1	3	3	3	3	4	4	2	2
4	4	1	1	2	2	4	4	2	2
1	1	1	1	2	2	4	4	2	2
1	1	3	3	2	2	4	4	2	2
4	4	1	1	2	2	1	1	2	2
1	1	3	3	3	3	4	4	2	2
1	1	1	1	2	2	4	4	2	2
1	1	3	3	2	2	4	4	2	2
4	4	3	3	2	2	4	4	2	2
4	4	3	3	2	2	4	4	2	2
4	4	3	3	2	2	4	4	2	2
4	4	1	1	2	2	4	4	2	2
1	1	1	1	3	3	4	4	2	2
4	4	3	3	2	2	4	4	2	2
1	1	3	3	2	2	4	4	2	2
1	1	1	1	3	3	1	1	2	2
4	4	1	1	2	2	4	4	2	2
1	1	3	3	2	2	4	4	3	3
1	1	1	1	3	3	4	4	2	2

For Review Only

4	4	1	1	2	2	4	4	2	2
4	4	1	1	2	2	4	4	2	2
1	1	3	3	2	2	4	4	2	2
1	1	1	1	2	2	4	4	2	2
4	4	1	1	2	2	4	4	2	2
4	4	1	1	2	2	4	4	2	2
1	1	1	1	2	2	1	1	2	2
4	4	1	1	2	2	4	4	2	2
4	4	1	1	2	2	4	4	2	2
4	4	1	1	2	2	4	4	2	2
4	4	3	3	2	2	4	4	2	2
1	1	1	1	2	2	4	4	2	2
1	1	1	1	2	2	4	4	2	2
4	4	1	1	2	2	4	4	2	2
1	1	3	3	3	3	4	4	2	2
1	1	3	3	3	3	1	1	2	2
4	4	1	1	2	2	4	4	2	2
4	4	1	1	2	2	4	4	2	2
4	4	3	3	2	2	4	4	2	2
4	4	1	1	2	2	4	4	2	2
4	4	1	1	2	2	4	4	2	2
1	1	3	3	2	2	4	4	2	2
4	4	1	1	2	2	1	1	2	2
1	1	3	3	2	2	4	4	3	3
1	1	1	1	2	2	4	4	3	3
1	1	3	3	2	2	4	4	2	2
1	1	1	1	2	2	4	4	3	3
1	1	1	1	2	2	4	4	2	2
4	4	3	3	2	2	4	4	2	2
4	4	1	1	2	2	4	4	3	3
4	4	3	3	2	2	4	4	3	3
4	4	1	1	2	2	4	4	2	2
4	4	3	3	2	2	4	4	2	2
1	1	3	3	3	3	4	4	2	2
4	4	1	1	2	2	4	4	2	2
1	1	3	3	2	2	4	4	3	3
4	4	1	1	2	2	4	4	3	3
4	4	1	1	2	2	4	4	2	2
4	4	3	3	2	2	4	4	2	2
4	4	3	3	2	2	4	4	2	2
4	4	1	1	2	2	4	4	2	2
4	4	3	3	2	2	4	4	2	2
1	1	3	3	3	3	4	4	2	2
1	1	1	1	2	2	4	4	2	2
1	1	1	1	3	3	4	4	2	2
4	4	3	3	2	2	4	4	3	3
1	1	3	3	3	3	1	1	2	2
1	1	3	3	3	3	1	1	2	2
1	1	3	3	2	2	4	4	2	2
1	1	1	1	2	2	4	4	2	2
4	4	3	3	2	2	4	4	2	2
1	1	3	3	3	3	4	4	2	2
1	1	1	1	2	2	1	1	2	2
4	4	3	3	2	2	4	4	2	2
1	1	3	3	2	2	4	4	2	2

For Review Only

4	4	1	1	2	2	4	4	2	2
1	1	3	3	2	2	4	4	2	2
4	4	1	1	2	2	4	4	2	2
4	4	1	1	2	2	4	4	2	2
1	1	3	3	3	3	4	4	2	2
4	4	3	3	2	2	4	4	2	2
4	4	1	1	2	2	4	4	2	2
1	1	3	3	3	3	4	4	2	2
1	1	3	3	2	2	4	4	2	2
1	1	3	3	2	2	4	4	2	2
4	4	1	1	2	2	4	4	2	2
4	4	3	3	2	2	4	4	2	2
4	4	1	1	2	2	4	4	2	2
4	4	3	3	2	2	4	4	2	2
4	4	3	3	2	2	4	4	2	2
1	1	3	3	2	2	4	4	2	2
1	1	3	3	2	2	4	4	2	2
1	1	1	1	2	2	4	4	3	3
1	1	3	3	2	2	4	4	2	2
4	4	1	1	2	2	4	4	2	2
4	4	3	3	2	2	4	4	2	2
4	4	3	3	2	2	4	4	2	2
4	4	3	3	2	2	4	4	2	2
1	1	1	1	3	3	1	1	2	2
1	1	3	3	3	3	4	4	2	2
1	1	1	1	2	2	4	4	2	2
1	1	3	3	2	2	4	4	2	2
1	1	1	1	2	2	4	4	2	2
1	1	1	1	3	3	4	4	2	2
4	4	3	3	2	2	4	4	2	2
1	1	1	1	2	2	4	4	2	2
1	1	3	3	2	2	1	1	2	2
4	4	1	1	2	2	4	4	2	2
4	4	1	1	2	2	1	1	2	2
4	4	1	1	2	2	1	1	2	2
1	1	3	3	2	2	4	4	2	2
4	4	1	1	2	2	4	4	2	2
1	1	3	3	2	2	4	4	3	3
1	1	3	3	2	2	4	4	2	2
1	1	3	3	3	3	1	1	2	2
4	4	1	1	2	2	4	4	2	2
1	1	3	3	2	2	4	4	3	3
1	1	1	1	3	3	1	1	2	2
4	4	1	1	2	2	4	4	3	3
1	1	1	1	2	2	4	4	2	2
4	4	1	1	2	2	4	4	2	2
4	4	1	1	2	2	1	1	2	2
1	1	1	1	3	3	4	4	3	3
1	1	1	1	2	2	4	4	2	2
1	1	3	3	2	2	4	4	2	2
4	4	1	1	2	2	4	4	2	2
1	1	3	3	3	3	1	1	2	2
4	4	1	1	2	2	4	4	2	2
1	1	1	1	2	2	4	4	3	3
1	1	1	1	3	3	1	1	2	2

For Review Only

4	4	3	3	2	2	4	4	2	2
4	4	3	3	2	2	4	4	3	3

For Review Only

SNP1836	SNP1836	SNP1837	SNP1837	SNP1838	SNP1838	SNP1839	SNP1839	SNP184	SNP184
3	3	4	4	3	3	4	4	4	4
3	3	4	4	1	1	4	4	1	1
3	3	4	4	3	3	4	4	1	1
3	3	4	4	3	3	4	4	1	1
3	3	4	4	1	1	4	4	1	1
3	3	3	3	3	3	4	4	1	1
3	3	4	4	3	3	4	4	1	1
3	3	4	4	3	3	4	4	4	4
3	3	4	4	3	3	4	4	1	1
3	3	4	4	1	1	4	4	4	4
3	3	3	3	1	1	4	4	1	1
3	3	4	4	3	3	4	4	1	1
3	3	4	4	3	3	4	4	4	4
3	3	4	4	1	1	4	4	4	4
3	3	4	4	3	3	4	4	1	1
3	3	4	4	3	3	4	4	1	1
3	3	4	4	3	3	1	1	4	4
3	3	4	4	3	3	4	4	4	4
3	3	4	4	3	3	1	1	1	1
3	3	3	3	1	1	4	4	1	1
3	3	4	4	3	3	4	4	1	1
4	4	4	4	3	3	4	4	4	4
3	3	4	4	1	1	4	4	4	4
3	3	4	4	1	1	4	4	4	4
3	3	4	4	1	1	4	4	4	4
3	3	4	4	3	3	4	4	1	1
3	3	4	4	3	3	4	4	1	1
3	3	4	4	3	3	4	4	1	1
3	3	4	4	3	3	4	4	4	4
3	3	4	4	1	1	4	4	4	4
3	3	4	4	3	3	4	4	4	4
3	3	4	4	3	3	4	4	4	4
3	3	4	4	1	1	1	1	4	4
3	3	4	4	1	1	4	4	4	4
3	3	4	4	1	1	4	4	1	1
3	3	4	4	1	1	4	4	1	1
3	3	4	4	3	3	1	1	1	1
3	3	3	3	3	3	4	4	1	1
4	4	4	4	1	1	4	4	4	4
3	3	3	3	3	3	4	4	1	1
3	3	3	3	1	1	4	4	4	4
3	3	4	4	3	3	4	4	4	4
3	3	3	3	1	1	4	4	4	4
3	3	4	4	3	3	1	1	1	1
3	3	3	3	1	1	4	4	4	4
3	3	4	4	3	3	4	4	1	1
3	3	4	4	1	1	4	4	1	1
3	3	3	3	1	1	4	4	1	1
4	4	4	4	1	1	1	1	1	1
3	3	4	4	3	3	4	4	1	1
3	3	4	4	3	3	4	4	1	1
3	3	4	4	3	3	4	4	1	1

3	3	4	4	1	1	1	1	4	4
3	3	4	4	1	1	1	1	4	4
3	3	4	4	3	3	4	4	4	4
3	3	4	4	3	3	1	1	4	4
3	3	4	4	3	3	1	1	1	1
3	3	4	4	1	1	4	4	1	1
3	3	4	4	3	3	4	4	1	1
4	4	4	4	1	1	4	4	4	4
3	3	3	3	1	1	4	4	1	1
3	3	4	4	3	3	4	4	1	1
3	3	4	4	3	3	4	4	1	1
3	3	4	4	1	1	4	4	1	1
3	3	4	4	1	1	1	1	4	4
3	3	4	4	3	3	4	4	4	4
3	3	3	3	3	3	4	4	1	1
3	3	4	4	1	1	4	4	1	1
3	3	4	4	3	3	4	4	1	1
3	3	4	4	3	3	4	4	1	1
3	3	4	4	1	1	4	4	1	1
3	3	4	4	3	3	1	1	4	4
3	3	4	4	3	3	4	4	1	1
4	4	3	3	3	3	1	1	1	1
3	3	4	4	3	3	1	1	1	1
4	4	4	4	3	3	1	1	4	4
3	3	3	3	1	1	4	4	4	4
3	3	4	4	3	3	4	4	1	1
3	3	4	4	1	1	4	4	4	4
3	3	3	3	1	1	4	4	4	4
3	3	4	4	3	3	4	4	1	1
3	3	4	4	3	3	1	1	4	4
3	3	4	4	1	1	4	4	1	1
3	3	4	4	1	1	4	4	4	4
3	3	4	4	1	1	4	4	4	4
3	3	4	4	1	1	1	1	4	4
3	3	4	4	1	1	1	1	4	4
3	3	3	3	1	1	4	4	4	4
3	3	4	4	1	1	4	4	4	4
4	4	4	4	1	1	1	1	1	1
3	3	4	4	3	3	4	4	4	4
3	3	3	3	3	3	4	4	1	1
3	3	4	4	3	3	1	1	4	4
3	3	4	4	3	3	4	4	1	1
3	3	4	4	3	3	4	4	4	4
3	3	4	4	3	3	4	4	1	1
3	3	4	4	1	1	4	4	1	1
3	3	4	4	3	3	4	4	1	1
3	3	4	4	1	1	4	4	4	4
3	3	4	4	3	3	4	4	1	1
3	3	4	4	1	1	4	4	1	1
3	3	4	4	3	3	4	4	1	1
3	3	4	4	3	3	4	4	4	4
3	3	3	3	1	1	4	4	4	4
3	3	3	3	1	1	4	4	1	1
3	3	4	4	3	3	4	4	1	1
3	3	4	4	1	1	4	4	1	1

For Review Only

3	3	4	4	3	3	4	4	1	1
3	3	4	4	3	3	4	4	1	1
3	3	4	4	3	3	4	4	1	1
3	3	3	3	1	1	4	4	4	4
3	3	4	4	3	3	4	4	4	4
3	3	4	4	3	3	4	4	1	1
3	3	4	4	3	3	1	1	1	1
3	3	4	4	3	3	4	4	1	1
3	3	4	4	1	1	4	4	1	1
3	3	4	4	1	1	4	4	1	1
3	3	4	4	1	1	4	4	4	4
3	3	4	4	3	3	4	4	1	1
3	3	4	4	3	3	4	4	1	1
3	3	4	4	3	3	4	4	1	1
3	3	4	4	3	3	1	1	1	1
3	3	4	4	3	3	4	4	1	1
3	3	4	4	3	3	4	4	1	1
3	3	4	4	3	3	4	4	1	1
3	3	3	3	1	1	4	4	4	4
3	3	4	4	3	3	4	4	4	4
3	3	4	4	3	3	4	4	1	1
3	3	4	4	3	3	4	4	1	1
3	3	4	4	3	3	4	4	4	4
3	3	3	3	1	1	4	4	4	4
3	3	3	3	3	3	4	4	1	1
3	3	4	4	3	3	4	4	1	1
3	3	4	4	3	3	1	1	4	4
3	3	4	4	3	3	1	1	4	4
3	3	4	4	3	3	4	4	4	4
3	3	4	4	3	3	4	4	1	1
3	3	3	3	1	1	4	4	4	4
3	3	4	4	3	3	4	4	1	1
3	3	4	4	3	3	1	1	4	4
3	3	4	4	3	3	4	4	1	1
3	3	4	4	1	1	4	4	4	4
3	3	4	4	3	3	4	4	1	1
3	3	3	3	1	1	4	4	4	4
3	3	4	4	3	3	4	4	1	1
3	3	3	3	3	3	4	4	1	1
3	3	3	3	1	1	4	4	1	1
3	3	4	4	3	3	4	4	4	4
3	3	3	3	1	1	4	4	4	4
3	3	4	4	1	1	4	4	4	4
3	3	4	4	1	1	4	4	4	4
3	3	4	4	3	3	4	4	1	1
3	3	3	3	3	3	4	4	1	1
3	3	4	4	3	3	4	4	1	1
3	3	4	4	3	3	4	4	1	1
3	3	4	4	1	1	4	4	1	1
3	3	4	4	3	3	4	4	1	1
3	3	4	4	1	1	4	4	1	1
3	3	3	3	1	1	4	4	1	1
3	3	4	4	3	3	4	4	4	4
3	3	4	4	1	1	4	4	1	1
3	3	4	4	3	3	4	4	1	1

For Review Only

3	3	4	4	3	3	4	4	4	4
3	3	4	4	3	3	4	4	1	1
4	4	4	4	1	1	1	1	4	4
3	3	4	4	3	3	4	4	1	1
3	3	4	4	3	3	4	4	1	1
3	3	4	4	1	1	4	4	1	1
3	3	3	3	1	1	4	4	1	1
3	3	4	4	3	3	4	4	1	1
3	3	4	4	3	3	4	4	4	4
3	3	4	4	3	3	4	4	4	4
3	3	4	4	3	3	1	1	1	1
3	3	4	4	1	1	4	4	1	1
3	3	4	4	3	3	4	4	1	1
3	3	4	4	3	3	4	4	1	1
3	3	4	4	1	1	4	4	4	4
3	3	3	3	1	1	4	4	1	1
3	3	4	4	3	3	4	4	1	1
3	3	4	4	1	1	4	4	1	1
3	3	4	4	3	3	4	4	1	1
3	3	4	4	3	3	4	4	1	1
3	3	4	4	3	3	4	4	1	1
3	3	4	4	1	1	4	4	4	4
3	3	3	3	3	3	4	4	1	1
3	3	4	4	1	1	4	4	1	1
3	3	4	4	3	3	1	1	1	1
3	3	4	4	1	1	4	4	1	1
3	3	4	4	3	3	4	4	1	1
3	3	4	4	1	1	4	4	1	1
3	3	4	4	3	3	4	4	4	4
3	3	4	4	3	3	4	4	1	1
3	3	4	4	3	3	4	4	1	1
3	3	4	4	3	3	4	4	4	4
3	3	4	4	1	1	4	4	4	4
3	3	4	4	1	1	4	4	4	4
3	3	4	4	3	3	1	1	1	1
3	3	4	4	1	1	4	4	1	1
3	3	4	4	3	3	4	4	1	1
3	3	4	4	3	3	1	1	4	4
3	3	4	4	3	3	4	4	1	1
3	3	4	4	3	3	4	4	4	4
3	3	4	4	3	3	4	4	1	1
3	3	4	4	3	3	4	4	1	1
3	3	4	4	1	1	4	4	4	4
3	3	4	4	1	1	4	4	1	1
3	3	4	4	1	1	4	4	4	4
3	3	4	4	1	1	4	4	4	4
3	3	3	3	1	1	4	4	1	1
3	3	3	3	1	1	4	4	4	4
3	3	4	4	1	1	4	4	4	4
3	3	4	4	3	3	4	4	1	1
3	3	4	4	3	3	4	4	4	4
4	4	3	3	1	1	4	4	1	1
4	4	3	3	1	1	1	1	1	1
3	3	4	4	3	3	4	4	1	1
3	3	4	4	3	3	4	4	4	4

3	3	4	4	3	3	4	4	4	4
3	3	4	4	1	1	4	4	4	4
3	3	4	4	3	3	4	4	1	1
3	3	4	4	3	3	4	4	1	1
3	3	3	3	1	1	4	4	4	4
3	3	4	4	3	3	4	4	1	1
3	3	4	4	3	3	4	4	4	4
3	3	3	3	1	1	4	4	4	4
3	3	4	4	1	1	4	4	1	1
3	3	4	4	1	1	4	4	4	4
3	3	4	4	3	3	4	4	1	1
3	3	4	4	3	3	4	4	1	1
3	3	4	4	3	3	4	4	1	1
3	3	4	4	3	3	4	4	1	1
3	3	4	4	3	3	4	4	1	1
3	3	4	4	1	1	4	4	1	1
3	3	4	4	1	1	4	4	4	4
3	3	4	4	1	1	4	4	1	1
3	3	4	4	1	1	4	4	1	1
3	3	3	3	3	3	4	4	1	1
3	3	4	4	3	3	4	4	4	4
3	3	4	4	3	3	4	4	1	1
3	3	4	4	3	3	4	4	1	1
3	3	3	3	1	1	4	4	4	4
4	4	4	4	1	1	4	4	4	4
3	3	4	4	3	3	4	4	4	4
3	3	4	4	1	1	4	4	4	4
3	3	4	4	3	3	4	4	1	1
3	3	4	4	1	1	1	1	4	4
3	3	4	4	3	3	4	4	4	4
3	3	4	4	1	1	4	4	4	4
3	3	3	3	1	1	4	4	1	1
3	3	4	4	3	3	4	4	1	1
3	3	4	4	3	3	4	4	1	1
4	4	3	3	3	3	1	1	1	1
3	3	3	3	1	1	4	4	4	4
3	3	4	4	3	3	1	1	4	4
3	3	4	4	1	1	4	4	1	1
3	3	4	4	1	1	4	4	1	1
3	3	3	3	1	1	4	4	4	4
3	3	4	4	3	3	4	4	1	1
3	3	4	4	1	1	4	4	4	4
3	3	3	3	1	1	4	4	1	1
3	3	4	4	3	3	1	1	1	1
3	3	4	4	1	1	4	4	4	4
3	3	4	4	3	3	4	4	4	4
3	3	3	3	3	3	4	4	4	4
3	3	4	4	1	1	4	4	1	1
3	3	4	4	3	3	4	4	1	1
3	3	4	4	1	1	4	4	4	4
3	3	4	4	3	3	4	4	1	1
3	3	3	3	1	1	4	4	4	4
3	3	4	4	3	3	4	4	1	1
3	3	4	4	3	3	4	4	1	1
3	3	3	3	1	1	4	4	1	1

For Review Only

3	3	4	4	3	3	4	4	1	1
3	3	4	4	1	1	1	1	4	4

For Review Only

SNP1840	SNP1840	SNP1841	SNP1841	SNP1842	SNP1842	SNP1843	SNP1843	SNP1844	SNP1844
2	2	4	4	1	1	4	4	4	4
2	2	4	4	4	4	1	1	1	1
2	2	4	4	1	1	4	4	4	4
2	2	4	4	1	1	1	1	4	4
2	2	4	4	4	4	1	1	1	1
2	2	4	4	1	1	1	1	4	4
2	2	4	4	1	1	1	1	1	1
2	2	4	4	1	1	1	1	1	1
2	2	4	4	1	1	1	1	4	4
2	2	4	4	4	4	1	1	1	1
2	2	4	4	4	4	1	1	1	1
2	2	4	4	1	1	4	4	4	4
2	2	4	4	4	4	1	1	1	1
2	2	4	4	4	4	1	1	4	4
2	2	4	4	1	1	1	1	1	1
2	2	4	4	1	1	1	1	4	4
2	2	4	4	1	1	1	1	1	1
2	2	4	4	1	1	1	1	4	4
1	1	4	4	1	1	4	4	4	4
2	2	4	4	4	4	1	1	1	1
2	2	4	4	1	1	1	1	1	1
2	2	4	4	1	1	1	1	1	1
2	2	4	4	4	4	1	1	1	1
2	2	4	4	4	4	1	1	1	1
1	1	4	4	4	4	4	4	4	4
1	1	4	4	1	1	4	4	1	1
2	2	4	4	1	1	1	1	4	4
1	1	4	4	1	1	4	4	1	1
2	2	4	4	1	1	1	1	4	4
2	2	4	4	4	4	1	1	1	1
1	1	4	4	1	1	4	4	4	4
2	2	4	4	1	1	1	1	1	1
2	2	4	4	4	4	1	1	1	1
2	2	4	4	4	4	4	4	1	1
2	2	4	4	4	4	4	4	1	1
2	2	4	4	4	4	1	1	1	1
1	1	1	1	1	1	4	4	1	1
2	2	4	4	1	1	1	1	1	1
2	2	4	4	4	4	1	1	1	1
1	1	4	4	1	1	1	1	1	1
2	2	4	4	4	4	1	1	1	1
2	2	4	4	1	1	1	1	1	1
2	2	4	4	4	4	1	1	4	4
2	2	4	4	4	4	1	1	1	1
2	2	4	4	4	4	1	1	1	1
1	1	4	4	1	1	4	4	4	4
2	2	4	4	4	4	4	4	1	1
2	2	4	4	4	4	1	1	1	1
2	2	4	4	4	4	1	1	1	1
1	1	4	4	1	1	4	4	1	1
1	1	4	4	1	1	4	4	1	1
2	2	4	4	1	1	1	1	4	4

For Review Only

2	2	4	4	4	4	1	1	4	4
2	2	4	4	4	4	1	1	1	1
1	1	4	4	1	1	4	4	1	1
2	2	4	4	1	1	1	1	1	1
1	1	4	4	1	1	4	4	4	4
2	2	4	4	4	4	4	4	1	1
1	1	4	4	1	1	4	4	1	1
2	2	4	4	4	4	1	1	4	4
2	2	4	4	4	4	1	1	1	1
1	1	4	4	1	1	4	4	1	1
1	1	4	4	1	1	4	4	1	1
2	2	4	4	4	4	4	4	1	1
2	2	4	4	4	4	1	1	4	4
2	2	4	4	1	1	1	1	4	4
2	2	4	4	1	1	4	4	1	1
2	2	4	4	4	4	1	1	1	1
2	2	4	4	1	1	4	4	1	1
1	1	4	4	1	1	1	1	1	1
2	2	4	4	1	1	1	1	1	1
1	1	1	1	1	1	4	4	1	1
2	2	4	4	4	4	4	4	1	1
2	2	4	4	4	4	1	1	1	1
2	2	4	4	1	1	1	1	1	1
1	1	4	4	1	1	4	4	1	1
2	2	4	4	4	4	1	1	1	1
2	2	4	4	1	1	1	1	1	1
2	2	4	4	4	4	1	1	1	1
2	2	4	4	4	4	1	1	4	4
1	1	4	4	1	1	4	4	1	1
1	1	1	1	1	1	4	4	1	1
2	2	4	4	4	4	1	1	1	1
2	2	4	4	4	4	1	1	1	1
2	2	4	4	4	4	1	1	1	1
2	2	4	4	4	4	1	1	4	4
1	1	1	1	4	4	4	4	4	4
2	2	4	4	4	4	1	1	1	1
2	2	4	4	4	4	1	1	1	1
2	2	4	4	4	4	1	1	1	1
2	2	4	4	1	1	1	1	1	1
2	2	4	4	1	1	1	1	4	4
2	2	4	4	1	1	1	1	1	1
1	1	1	1	1	1	4	4	1	1
1	1	4	4	1	1	1	1	1	1
1	1	4	4	1	1	4	4	1	1
1	1	4	4	4	4	4	4	1	1
1	1	4	4	1	1	4	4	1	1
2	2	4	4	4	4	1	1	1	1
1	1	4	4	1	1	4	4	1	1
1	1	4	4	4	4	4	4	1	1
2	2	4	4	1	1	1	1	1	1
2	2	4	4	1	1	4	4	1	1
2	2	4	4	4	4	1	1	1	1
2	2	4	4	1	1	1	1	4	4
2	2	4	4	1	1	4	4	1	1
2	2	4	4	4	4	1	1	1	1

For Review Only

1	1	4	4	1	1	4	4	1	1
2	2	4	4	1	1	1	1	1	1
2	2	4	4	4	4	1	1	1	1
2	2	4	4	4	4	1	1	4	4
2	2	4	4	1	1	4	4	1	1
1	1	4	4	1	1	4	4	1	1
2	2	4	4	1	1	1	1	4	4
2	2	4	4	1	1	4	4	1	1
1	1	4	4	4	4	4	4	1	1
2	2	4	4	4	4	1	1	1	1
2	2	4	4	4	4	1	1	1	1
1	1	4	4	1	1	4	4	1	1
2	2	4	4	1	1	1	1	1	1
1	1	4	4	1	1	1	1	1	1
2	2	4	4	1	1	4	4	1	1
1	1	4	4	1	1	4	4	1	1
2	2	4	4	1	1	1	1	1	1
1	1	4	4	1	1	4	4	1	1
2	2	4	4	4	4	1	1	4	4
1	1	4	4	1	1	4	4	1	1
2	2	4	4	1	1	4	4	1	1
2	2	4	4	1	1	1	1	1	1
2	2	4	4	1	1	4	4	1	1
2	2	4	4	4	4	1	1	1	1
1	1	4	4	1	1	4	4	1	1
2	2	4	4	1	1	1	1	1	1
1	1	4	4	1	1	4	4	1	1
1	1	1	1	1	1	4	4	1	1
2	2	4	4	1	1	1	1	1	1
2	2	4	4	4	4	1	1	1	1
2	2	4	4	4	4	1	1	4	4
2	2	4	4	1	1	1	1	1	1
2	2	4	4	1	1	1	1	1	1
1	1	4	4	1	1	4	4	1	1
2	2	4	4	4	4	4	4	1	1
2	2	4	4	4	4	4	4	1	1
2	2	4	4	4	4	1	1	4	4
1	1	4	4	1	1	4	4	1	1
1	1	4	4	1	1	4	4	1	1
2	2	4	4	4	4	1	1	4	4
1	1	4	4	1	1	4	4	1	1
2	2	4	4	4	4	1	1	1	1
2	2	4	4	4	4	1	1	1	1
2	2	4	4	4	4	1	1	1	1
2	2	4	4	1	1	1	1	1	1
2	2	4	4	1	1	1	1	1	1
1	1	4	4	1	1	4	4	1	1
2	2	4	4	1	1	4	4	4	4
2	2	4	4	4	4	1	1	1	1
1	1	4	4	1	1	4	4	1	1
1	1	4	4	4	4	4	4	1	1
2	2	4	4	4	4	1	1	1	1
2	2	4	4	1	1	1	1	1	1
1	1	4	4	4	4	4	4	1	1
2	2	4	4	4	4	1	1	1	1

For Review Only

2	2	4	4	1	1	1	1	1	1
2	2	4	4	1	1	1	1	1	1
2	2	4	4	4	4	1	1	1	1
2	2	4	4	1	1	4	4	1	1
1	1	4	4	1	1	4	4	1	1
2	2	4	4	4	4	1	1	1	1
2	2	4	4	4	4	1	1	1	1
2	2	4	4	1	1	1	1	1	1
2	2	4	4	1	1	1	1	1	1
1	1	1	1	1	1	4	4	1	1
2	2	4	4	1	1	1	1	1	1
2	2	4	4	4	4	1	1	1	1
1	1	4	4	1	1	4	4	1	1
2	2	4	4	1	1	1	1	1	1
2	2	4	4	4	4	1	1	1	1
2	2	4	4	4	4	1	1	1	1
2	2	4	4	1	1	1	1	1	1
2	2	4	4	1	1	1	1	1	1
1	1	4	4	1	1	4	4	1	1
2	2	4	4	1	1	1	1	4	4
1	1	4	4	1	1	4	4	1	1
2	2	4	4	4	4	1	1	1	1
2	2	4	4	1	1	1	1	1	1
2	2	4	4	4	4	4	4	1	1
2	2	4	4	1	1	1	1	1	1
2	2	4	4	4	4	4	4	1	1
1	1	4	4	1	1	4	4	1	1
2	2	4	4	4	4	4	4	4	4
1	1	4	4	1	1	4	4	1	1
2	2	4	4	1	1	1	1	1	1
1	1	1	1	1	1	4	4	1	1
1	1	1	1	1	1	4	4	1	1
1	1	1	1	4	4	4	4	1	1
2	2	4	4	4	4	1	1	1	1
1	1	1	1	1	1	4	4	1	1
2	2	4	4	4	4	4	4	1	1
1	1	4	4	1	1	4	4	1	1
1	1	1	1	1	1	4	4	4	4
2	2	4	4	1	1	4	4	1	1
2	2	4	4	1	1	1	1	1	1
2	2	4	4	1	1	4	4	4	4
1	1	4	4	1	1	4	4	1	1
2	2	4	4	4	4	1	1	4	4
2	2	4	4	1	1	4	4	1	1
2	2	4	4	4	4	1	1	1	1
1	1	1	1	4	4	4	4	1	1
2	2	4	4	4	4	1	1	1	1
2	2	4	4	4	4	1	1	1	1
2	2	4	4	4	4	1	1	1	1
2	2	4	4	1	1	1	1	1	1
1	1	4	4	1	1	4	4	1	1
2	2	4	4	4	4	1	1	1	1
2	2	4	4	4	4	1	1	1	1
1	1	4	4	1	1	4	4	1	1
2	2	4	4	4	4	1	1	1	1

For Review Only

2	2	4	4	1	1	1	1	1	1
2	2	4	4	4	4	1	1	1	1
2	2	4	4	1	1	4	4	1	1
1	1	4	4	1	1	4	4	1	1
2	2	4	4	4	4	1	1	1	1
1	1	4	4	1	1	4	4	1	1
2	2	4	4	1	1	1	1	4	4
2	2	4	4	4	4	1	1	1	1
2	2	4	4	4	4	1	1	1	1
2	2	4	4	4	4	1	1	4	4
2	2	4	4	1	1	4	4	1	1
2	2	4	4	1	1	1	1	1	1
2	2	4	4	1	1	1	1	1	1
2	2	4	4	1	1	1	1	1	1
2	2	4	4	1	1	4	4	1	1
2	2	4	4	4	4	1	1	1	1
2	2	4	4	4	4	1	1	1	1
2	2	4	4	4	4	1	1	4	4
2	2	4	4	4	4	1	1	1	1
2	2	4	4	1	1	1	1	1	1
2	2	4	4	1	1	1	1	1	1
1	1	4	4	1	1	4	4	1	1
2	2	4	4	1	1	1	1	1	1
2	2	4	4	4	4	1	1	1	1
2	2	4	4	4	4	1	1	4	4
1	1	4	4	1	1	4	4	4	4
2	2	4	4	4	4	1	1	4	4
2	2	4	4	1	1	1	1	4	4
2	2	4	4	4	4	1	1	1	1
2	2	4	4	1	1	1	1	1	1
2	2	4	4	4	4	1	1	1	1
1	1	4	4	4	4	4	4	1	1
2	2	4	4	1	1	1	1	1	1
2	2	4	4	1	1	1	1	1	1
2	2	4	4	1	1	1	1	1	1
2	2	4	4	4	4	1	1	1	1
2	2	4	4	1	1	1	1	1	1
2	2	4	4	4	4	4	4	1	1
2	2	4	4	4	4	1	1	1	1
2	2	4	4	4	4	1	1	1	1
1	1	1	1	1	1	4	4	1	1
1	1	4	4	4	4	1	1	1	1
2	2	4	4	4	4	1	1	1	1
2	2	4	4	1	1	1	1	1	1
2	2	4	4	4	4	4	4	4	4
2	2	4	4	1	1	1	1	1	1
2	2	4	4	1	1	4	4	4	4
2	2	4	4	4	4	1	1	1	1
2	2	4	4	1	1	4	4	4	4
2	2	4	4	4	4	1	1	4	4
2	2	4	4	1	1	1	1	1	1
2	2	4	4	4	4	1	1	1	1
2	2	4	4	1	1	4	4	4	4
2	2	4	4	1	1	1	1	1	1
2	2	4	4	4	4	1	1	1	1

For Review Only

2	2	4	4	1	1	1	1	1	1
1	1	4	4	4	4	4	4	1	1

For Review Only

SNP1845	SNP1845	SNP1846	SNP1846	SNP1847	SNP1847	SNP1848	SNP1848	SNP1849	SNP1849
1	1	3	3	1	1	3	3	2	2
1	1	3	3	1	1	3	3	2	2
1	1	3	3	1	1	3	3	2	2
1	1	3	3	1	1	3	3	2	2
1	1	3	3	1	1	2	2	2	2
2	2	3	3	1	1	3	3	2	2
1	1	3	3	1	1	3	3	2	2
1	1	3	3	1	1	3	3	2	2
1	1	3	3	1	1	3	3	2	2
1	1	4	4	1	1	2	2	2	2
1	1	3	3	1	1	3	3	2	2
1	1	3	3	1	1	3	3	2	2
1	1	3	3	1	1	2	2	2	2
1	1	3	3	1	1	2	2	2	2
1	1	3	3	1	1	3	3	2	2
1	1	3	3	1	1	3	3	2	2
1	1	3	3	1	1	3	3	2	2
1	1	3	3	1	1	3	3	2	2
1	1	3	3	1	1	3	3	2	2
2	2	4	4	1	1	3	3	2	2
1	1	3	3	1	1	3	3	2	2
1	1	3	3	1	1	3	3	2	2
1	1	3	3	1	1	2	2	2	2
1	1	3	3	1	1	2	2	2	2
1	1	3	3	1	1	3	3	2	2
1	1	3	3	1	1	3	3	2	2
1	1	3	3	1	1	3	3	2	2
1	1	3	3	1	1	3	3	2	2
1	1	3	3	1	1	3	3	2	2
1	1	4	4	1	1	2	2	2	2
1	1	3	3	1	1	3	3	2	2
1	1	3	3	1	1	3	3	2	2
1	1	3	3	1	1	2	2	2	2
1	1	3	3	1	1	2	2	2	2
1	1	3	3	1	1	3	3	2	2
1	1	3	3	1	1	2	2	2	2
1	1	3	3	1	1	3	3	2	2
2	2	3	3	1	1	3	3	2	2
1	1	3	3	1	1	3	3	2	2
1	1	4	4	1	1	3	3	2	2
2	2	3	3	1	1	2	2	2	2
1	1	3	3	1	1	3	3	2	2
2	2	4	4	1	1	2	2	2	2
1	1	3	3	1	1	3	3	2	2
2	2	4	4	1	1	3	3	2	2
1	1	3	3	1	1	3	3	2	2
1	1	3	3	1	1	2	2	2	2
1	1	3	3	1	1	3	3	2	2
1	1	3	3	1	1	2	2	2	2
1	1	3	3	1	1	3	3	2	2
1	1	3	3	1	1	3	3	2	2
1	1	3	3	1	1	3	3	2	2

For Review Only

1	1	3	3	1	1	2	2	2	2
1	1	3	3	1	1	2	2	2	2
1	1	3	3	1	1	3	3	2	2
1	1	4	4	1	1	3	3	2	2
1	1	3	3	1	1	3	3	2	2
1	1	3	3	1	1	2	2	2	2
1	1	3	3	1	1	3	3	2	2
1	1	4	4	1	1	3	3	2	2
2	2	3	3	1	1	2	2	2	2
1	1	3	3	1	1	3	3	2	2
1	1	3	3	1	1	3	3	2	2
1	1	3	3	1	1	2	2	2	2
1	1	3	3	1	1	2	2	2	2
1	1	3	3	1	1	3	3	2	2
1	1	3	3	1	1	3	3	2	2
1	1	3	3	1	1	2	2	2	2
1	1	3	3	1	1	3	3	2	2
1	1	3	3	1	1	3	3	2	2
1	1	3	3	1	1	3	3	2	2
1	1	3	3	1	1	3	3	2	2
1	1	3	3	1	1	2	2	2	2
1	1	3	3	1	1	3	3	2	2
1	1	3	3	1	1	3	3	2	2
1	1	3	3	4	4	3	3	2	2
2	2	4	4	1	1	3	3	2	2
1	1	4	4	1	1	3	3	2	2
1	1	3	3	1	1	2	2	2	2
1	1	3	3	1	1	2	2	4	4
1	1	3	3	1	1	3	3	2	2
1	1	3	3	4	4	3	3	2	2
1	1	4	4	1	1	3	3	2	2
2	2	3	3	1	1	2	2	2	2
1	1	3	3	1	1	3	3	4	4
1	1	3	3	1	1	2	2	2	2
2	2	3	3	1	1	2	2	2	2
2	2	4	4	1	1	3	3	2	2
1	1	4	4	1	1	2	2	2	2
1	1	3	3	1	1	2	2	2	2
1	1	3	3	1	1	3	3	2	2
2	2	4	4	1	1	3	3	2	2
1	1	3	3	1	1	3	3	2	2
1	1	3	3	1	1	3	3	2	2
1	1	3	3	1	1	3	3	2	2
1	1	3	3	1	1	3	3	2	2
1	1	3	3	4	4	3	3	2	2
1	1	3	3	1	1	3	3	2	2
1	1	3	3	1	1	2	2	4	4
1	1	3	3	1	1	3	3	2	2
1	1	3	3	1	1	3	3	2	2
1	1	3	3	1	1	3	3	2	2
1	1	3	3	1	1	3	3	2	2
1	1	3	3	1	1	3	3	2	2
2	2	3	3	1	1	3	3	4	4
1	1	3	3	1	1	3	3	2	2
1	1	3	3	1	1	3	3	2	2

For Review Only

1	1	3	3	1	1	3	3	2	2
1	1	3	3	1	1	3	3	2	2
1	1	3	3	1	1	2	2	2	2
2	2	3	3	1	1	2	2	4	4
1	1	4	4	1	1	3	3	2	2
1	1	3	3	1	1	3	3	2	2
1	1	3	3	1	1	3	3	2	2
1	1	3	3	1	1	3	3	2	2
1	1	3	3	1	1	3	3	2	2
1	1	3	3	4	4	2	2	2	2
1	1	3	3	1	1	2	2	4	4
1	1	3	3	1	1	3	3	2	2
1	1	4	4	1	1	3	3	2	2
1	1	4	4	1	1	3	3	2	2
1	1	3	3	1	1	3	3	2	2
1	1	3	3	1	1	3	3	2	2
1	1	3	3	1	1	3	3	2	2
1	1	3	3	1	1	3	3	2	2
2	2	3	3	1	1	2	2	4	4
1	1	3	3	1	1	3	3	2	2
1	1	3	3	1	1	3	3	2	2
1	1	3	3	4	4	3	3	2	2
1	1	3	3	1	1	3	3	2	2
2	2	3	3	1	1	2	2	4	4
1	1	3	3	1	1	3	3	2	2
1	1	3	3	1	1	3	3	2	2
1	1	4	4	4	4	3	3	2	2
1	1	3	3	1	1	3	3	2	2
1	1	3	3	4	4	3	3	2	2
1	1	3	3	1	1	2	2	2	2
1	1	3	3	1	1	2	2	2	2
1	1	4	4	1	1	3	3	2	2
1	1	3	3	1	1	3	3	2	2
1	1	3	3	1	1	3	3	2	2
1	1	3	3	1	1	2	2	2	2
2	2	3	3	1	1	3	3	2	2
2	2	3	3	1	1	2	2	4	4
1	1	3	3	1	1	3	3	2	2
1	1	3	3	1	1	3	3	2	2
2	2	3	3	1	1	2	2	2	2
1	1	3	3	1	1	3	3	2	2
2	2	3	3	1	1	2	2	2	2
1	1	3	3	1	1	3	3	2	2
1	1	3	3	1	1	2	2	2	2
1	1	3	3	1	1	3	3	2	2
2	2	4	4	1	1	3	3	2	2
1	1	3	3	1	1	3	3	2	2
1	1	3	3	1	1	3	3	2	2
1	1	3	3	1	1	2	2	2	2
1	1	3	3	1	1	3	3	2	2
1	1	3	3	1	1	2	2	2	2
1	1	3	3	1	1	3	3	4	4
1	1	3	3	1	1	3	3	2	2
1	1	3	3	1	1	2	2	2	2
1	1	4	4	1	1	3	3	2	2

For Review Only

1	1	3	3	4	4	3	3	2	2
1	1	3	3	1	1	3	3	2	2
1	1	4	4	1	1	2	2	2	2
1	1	3	3	1	1	3	3	2	2
1	1	3	3	1	1	3	3	2	2
1	1	3	3	1	1	2	2	2	2
1	1	3	3	1	1	3	3	2	2
1	1	3	3	1	1	3	3	2	2
1	1	3	3	1	1	3	3	2	2
1	1	3	3	4	4	3	3	2	2
1	1	3	3	1	1	3	3	2	2
1	1	3	3	1	1	3	3	2	2
1	1	3	3	1	1	3	3	2	2
1	1	3	3	1	1	3	3	2	2
1	1	3	3	1	1	2	2	2	2
1	1	3	3	1	1	3	3	2	2
1	1	3	3	1	1	3	3	2	2
1	1	3	3	1	1	3	3	2	2
1	1	3	3	1	1	3	3	2	2
1	1	3	3	1	1	3	3	2	2
1	1	3	3	1	1	3	3	2	2
1	1	3	3	1	1	3	3	2	2
1	1	3	3	1	1	3	3	2	2
1	1	3	3	1	1	2	2	2	2
1	1	3	3	1	1	3	3	2	2
1	1	3	3	1	1	2	2	2	2
1	1	4	4	1	1	3	3	2	2
1	1	3	3	1	1	3	3	2	2
1	1	4	4	1	1	3	3	2	2
1	1	3	3	1	1	3	3	2	2
1	1	3	3	1	1	3	3	2	2
1	1	3	3	4	4	3	3	2	2
1	1	4	4	1	1	2	2	2	2
1	1	3	3	1	1	2	2	2	2
1	1	4	4	1	1	3	3	2	2
1	1	3	3	1	1	2	2	2	2
1	1	3	3	1	1	3	3	2	2
1	1	3	3	1	1	3	3	2	2
1	1	3	3	1	1	3	3	2	2
1	1	3	3	1	1	3	3	2	2
1	1	3	3	1	1	3	3	2	2
1	1	3	3	1	1	3	3	2	2
1	1	3	3	1	1	3	3	2	2
1	1	3	3	1	1	3	3	2	2
1	1	3	3	1	1	3	3	2	2
1	1	3	3	1	1	3	3	2	2
1	1	3	3	1	1	3	3	2	2
1	1	3	3	1	1	3	3	4	4
1	1	3	3	1	1	3	3	2	2
1	1	4	4	1	1	3	3	2	2
1	1	3	3	4	4	3	3	2	2
2	2	3	3	1	1	2	2	2	2
1	1	3	3	1	1	3	3	2	2
1	1	3	3	1	1	3	3	2	2
1	1	3	3	1	1	2	2	2	2

For Review Only

1	1	3	3	1	1	3	3	2	2
1	1	3	3	1	1	2	2	2	2
1	1	3	3	4	4	3	3	2	2
1	1	3	3	1	1	3	3	2	2
2	2	3	3	1	1	3	3	2	2
1	1	3	3	1	1	3	3	2	2
1	1	4	4	1	1	3	3	2	2
2	2	4	4	1	1	2	2	2	2
1	1	3	3	1	1	3	3	2	2
1	1	3	3	1	1	2	2	2	2
1	1	3	3	1	1	3	3	2	2
1	1	3	3	1	1	3	3	2	2
1	1	3	3	1	1	3	3	2	2
1	1	3	3	1	1	3	3	2	2
1	1	3	3	1	1	3	3	2	2
1	1	3	3	1	1	3	3	2	2
1	1	4	4	1	1	2	2	2	2
1	1	3	3	4	4	3	3	4	4
1	1	3	3	1	1	3	3	2	2
2	2	3	3	1	1	3	3	2	2
1	1	3	3	1	1	3	3	2	2
1	1	3	3	1	1	3	3	2	2
1	1	3	3	1	1	3	3	2	2
1	1	3	3	1	1	3	3	2	2
1	1	4	4	1	1	3	3	2	2
1	1	3	3	1	1	3	3	2	2
1	1	3	3	1	1	2	2	4	4
1	1	3	3	1	1	3	3	2	2
1	1	3	3	1	1	3	3	2	2
1	1	3	3	4	4	3	3	2	2
1	1	3	3	1	1	3	3	4	4
1	1	3	3	1	1	3	3	2	2
1	1	3	3	1	1	3	3	2	2
1	1	3	3	1	1	3	3	2	2
1	1	3	3	1	1	3	3	2	2
2	2	4	4	1	1	2	2	4	4
1	1	3	3	1	1	3	3	2	2
1	1	3	3	1	1	2	2	2	2
1	1	3	3	1	1	2	2	2	2
1	1	3	3	1	1	3	3	2	2
1	1	3	3	1	1	3	3	2	2
1	1	4	4	1	1	3	3	2	2
1	1	3	3	1	1	3	3	2	2
1	1	3	3	1	1	3	3	2	2
1	1	3	3	1	1	2	2	2	2
1	1	3	3	1	1	3	3	2	2
1	1	3	3	1	1	3	3	2	2
1	1	4	4	1	1	2	2	2	2
1	1	3	3	1	1	3	3	2	2
1	1	3	3	1	1	2	2	4	4
1	1	3	3	1	1	3	3	2	2
1	1	3	3	1	1	3	3	2	2
1	1	3	3	1	1	3	3	2	2
1	1	3	3	1	1	3	3	2	2
1	1	3	3	1	1	3	3	2	2

For Review Only

1	1	3	3	1	1	3	3	2	2
1	1	3	3	1	1	2	2	2	2

For Review Only

SNP185	SNP185	SNP1850	SNP1850	SNP1851	SNP1851	SNP1852	SNP1852	SNP1853	SNP1853
1	1	4	4	2	2	1	1	2	2
3	3	4	4	2	2	1	1	3	3
1	1	4	4	2	2	1	1	2	2
3	3	4	4	2	2	1	1	2	2
3	3	4	4	2	2	1	1	2	2
3	3	4	4	2	2	1	1	2	2
3	3	4	4	2	2	1	1	2	2
3	3	4	4	2	2	1	1	2	2
3	3	4	4	2	2	1	1	2	2
3	3	4	4	2	2	1	1	2	2
3	3	4	4	2	2	3	3	3	3
1	1	4	4	2	2	1	1	2	2
3	3	4	4	2	2	1	1	2	2
1	1	4	4	2	2	1	1	2	2
1	1	4	4	2	2	1	1	2	2
3	3	4	4	2	2	1	1	2	2
3	3	4	4	2	2	1	1	2	2
3	3	4	4	2	2	1	1	2	2
3	3	4	4	1	1	1	1	3	3
3	3	4	4	2	2	1	1	2	2
3	3	4	4	2	2	1	1	2	2
3	3	4	4	2	2	1	1	2	2
1	1	4	4	2	2	3	3	3	3
1	1	4	4	2	2	1	1	2	2
1	1	4	4	2	2	1	1	2	2
1	1	4	4	2	2	1	1	2	2
3	3	4	4	2	2	1	1	2	2
1	1	4	4	2	2	1	1	2	2
3	3	4	4	2	2	1	1	2	2
3	3	4	4	2	2	3	3	3	3
1	1	4	4	2	2	1	1	2	2
3	3	4	4	2	2	1	1	2	2
1	1	4	4	2	2	1	1	2	2
1	1	4	4	2	2	1	1	3	3
3	3	4	4	2	2	1	1	3	3
3	3	4	4	2	2	1	1	2	2
3	3	4	4	1	1	1	1	2	2
1	1	4	4	2	2	1	1	2	2
1	1	4	4	2	2	1	1	2	2
3	3	4	4	2	2	1	1	2	2
3	3	4	4	2	2	1	1	2	2
3	3	4	4	2	2	1	1	2	2
3	3	4	4	2	2	1	1	2	2
3	3	4	4	2	2	1	1	2	2
3	3	4	4	2	2	1	1	2	2
1	1	4	4	2	2	1	1	2	2
1	1	4	4	2	2	1	1	2	2
1	1	4	4	2	2	1	1	2	2
3	3	4	4	2	2	3	3	3	3
3	3	4	4	2	2	1	1	2	2
1	1	4	4	2	2	1	1	2	2
1	1	4	4	2	2	1	1	2	2

For Review Only

1	1	4	4	2	2	1	1	2	2
1	1	4	4	2	2	1	1	2	2
3	3	4	4	1	1	1	1	2	2
3	3	4	4	2	2	3	3	3	3
3	3	4	4	1	1	1	1	3	3
1	1	4	4	2	2	1	1	2	2
3	3	4	4	2	2	1	1	2	2
1	1	4	4	2	2	3	3	3	3
3	3	4	4	2	2	1	1	2	2
1	1	4	4	2	2	1	1	2	2
3	3	4	4	2	2	1	1	2	2
3	3	4	4	2	2	1	1	2	2
1	1	4	4	2	2	1	1	2	2
3	3	4	4	2	2	1	1	2	2
3	3	4	4	2	2	1	1	2	2
3	3	4	4	2	2	1	1	2	2
3	3	4	4	2	2	1	1	2	2
1	1	4	4	1	1	1	1	2	2
3	3	4	4	2	2	1	1	2	2
1	1	4	4	1	1	1	1	2	2
3	3	4	4	2	2	1	1	2	2
3	3	4	4	2	2	3	3	3	3
3	3	4	4	2	2	1	1	2	2
3	3	4	4	2	2	1	1	2	2
3	3	4	4	2	2	1	1	2	2
3	3	4	4	2	2	1	1	2	2
1	1	4	4	2	2	1	1	3	3
1	1	1	1	2	2	1	1	2	2
1	1	4	4	2	2	1	1	2	2
1	1	4	4	1	1	1	1	2	2
3	3	4	4	2	2	1	1	2	2
1	1	4	4	2	2	1	1	2	2
1	1	1	1	2	2	1	1	2	2
1	1	4	4	2	2	1	1	2	2
1	1	4	4	1	1	1	1	2	2
3	3	4	4	2	2	1	1	2	2
3	3	4	4	2	2	1	1	2	2
3	3	4	4	2	2	3	3	3	3
3	3	4	4	2	2	1	1	2	2
3	3	4	4	2	2	1	1	2	2
1	1	4	4	2	2	1	1	2	2
3	3	4	4	1	1	1	1	2	2
1	1	4	4	2	2	1	1	2	2
3	3	4	4	2	2	1	1	2	2
3	3	4	4	2	2	1	1	2	2
3	3	4	4	2	2	1	1	2	2
3	3	1	1	2	2	1	1	2	2
3	3	4	4	2	2	1	1	2	2
1	1	4	4	2	2	1	1	2	2
3	3	4	4	2	2	1	1	3	3
1	1	4	4	2	2	1	1	2	2
1	1	4	4	2	2	1	1	2	2
3	3	1	1	2	2	3	3	3	3
1	1	4	4	2	2	1	1	3	3
3	3	4	4	2	2	1	1	2	2

For Review Only

1	1	4	4	2	2	1	1	2	2
3	3	4	4	2	2	3	3	3	3
3	3	4	4	2	2	1	1	2	2
3	3	1	1	2	2	1	1	2	2
1	1	4	4	2	2	1	1	2	2
1	1	4	4	2	2	1	1	2	2
3	3	4	4	2	2	1	1	2	2
1	1	4	4	2	2	1	1	2	2
1	1	4	4	2	2	1	1	2	2
1	1	4	4	2	2	1	1	2	2
1	1	1	1	2	2	1	1	2	2
3	3	4	4	2	2	1	1	2	2
3	3	4	4	2	2	1	1	2	2
3	3	4	4	2	2	1	1	2	2
1	1	4	4	2	2	1	1	3	3
1	1	4	4	2	2	1	1	2	2
3	3	4	4	2	2	3	3	3	3
1	1	4	4	2	2	1	1	2	2
3	3	1	1	2	2	1	1	2	2
3	3	4	4	2	2	1	1	2	2
1	1	4	4	2	2	1	1	2	2
1	1	4	4	2	2	1	1	2	2
1	1	4	4	2	2	1	1	2	2
1	1	1	1	2	2	1	1	2	2
1	1	4	4	2	2	1	1	3	3
3	3	4	4	2	2	3	3	3	3
3	3	1	1	2	2	1	1	2	2
1	1	4	4	1	1	1	1	2	2
1	1	4	4	2	2	1	1	2	2
1	1	4	4	2	2	1	1	2	2
3	3	4	4	2	2	1	1	2	2
1	1	4	4	2	2	1	1	3	3
1	1	4	4	2	2	1	1	2	2
1	1	4	4	2	2	1	1	2	2
1	1	4	4	2	2	1	1	3	3
1	1	4	4	2	2	1	1	3	3
3	3	1	1	2	2	1	1	2	2
3	3	4	4	1	1	1	1	2	2
1	1	4	4	2	2	1	1	2	2
3	3	4	4	2	2	3	3	3	3
1	1	4	4	2	2	3	3	3	3
3	3	4	4	2	2	1	1	2	2
1	1	4	4	1	1	1	1	2	2
3	3	4	4	2	2	1	1	3	3
3	3	4	4	2	2	1	1	2	2
3	3	4	4	2	2	1	1	2	2
1	1	4	4	2	2	1	1	2	2
1	1	4	4	2	2	1	1	2	2
3	3	4	4	2	2	1	1	3	3
1	1	4	4	2	2	1	1	2	2
1	1	4	4	2	2	1	1	2	2
3	3	1	1	2	2	1	1	2	2
1	1	4	4	1	1	1	1	2	2
3	3	4	4	2	2	1	1	2	2
1	1	4	4	2	2	1	1	3	3

For Review Only

1	1	4	4	1	1	1	1	2	2
3	3	4	4	2	2	1	1	3	3
3	3	4	4	2	2	1	1	3	3
1	1	4	4	2	2	1	1	3	3
3	3	4	4	2	2	1	1	2	2
3	3	4	4	2	2	1	1	2	2
3	3	1	1	2	2	1	1	3	3
1	1	4	4	2	2	1	1	2	2
1	1	4	4	2	2	1	1	2	2
1	1	4	4	1	1	1	1	2	2
1	1	4	4	2	2	1	1	2	2
1	1	4	4	2	2	1	1	2	2
3	3	4	4	2	2	1	1	2	2
3	3	4	4	2	2	1	1	2	2
3	3	4	4	2	2	1	1	2	2
3	3	4	4	2	2	3	3	3	3
1	1	4	4	2	2	1	1	2	2
3	3	4	4	2	2	1	1	2	2
1	1	4	4	2	2	1	1	2	2
3	3	4	4	2	2	1	1	2	2
1	1	4	4	2	2	1	1	2	2
1	1	4	4	2	2	3	3	3	3
3	3	4	4	2	2	3	3	3	3
3	3	4	4	2	2	1	1	2	2
3	3	4	4	2	2	1	1	2	2
3	3	4	4	2	2	1	1	3	3
3	3	4	4	2	2	1	1	2	2
1	1	4	4	2	2	1	1	3	3
1	1	4	4	1	1	1	1	3	3
3	3	4	4	2	2	1	1	2	2
3	3	4	4	1	1	1	1	2	2
1	1	4	4	1	1	1	1	2	2
3	3	4	4	2	2	1	1	3	3
3	3	4	4	2	2	1	1	2	2
3	3	4	4	1	1	1	1	2	2
3	3	4	4	2	2	1	1	2	2
3	3	4	4	2	2	1	1	3	3
1	1	4	4	1	1	1	1	2	2
1	1	4	4	2	2	1	1	2	2
1	1	4	4	2	2	1	1	2	2
1	1	4	4	2	2	1	1	2	2
1	1	4	4	2	2	1	1	2	2
1	1	4	4	2	2	3	3	3	3
1	1	4	4	2	2	1	1	2	2
1	1	1	1	2	2	1	1	2	2
1	1	4	4	2	2	1	1	2	2
3	3	4	4	2	2	3	3	3	3
1	1	1	1	2	2	3	3	3	3
3	3	4	4	2	2	1	1	2	2
3	3	4	4	2	2	1	1	3	3
1	1	4	4	2	2	1	1	2	2
3	3	4	4	2	2	3	3	3	3
3	3	4	4	2	2	1	1	3	3
3	3	4	4	2	2	1	1	2	2
1	1	4	4	2	2	3	3	3	3

For Review Only

1	1	4	4	2	2	1	1	2	2
3	3	4	4	2	2	1	1	2	2
1	1	4	4	2	2	1	1	2	2
3	3	4	4	2	2	1	1	3	3
3	3	4	4	2	2	3	3	3	3
3	3	4	4	2	2	1	1	2	2
1	1	4	4	2	2	3	3	3	3
3	3	4	4	2	2	3	3	3	3
1	1	1	1	2	2	1	1	2	2
1	1	4	4	2	2	1	1	2	2
1	1	4	4	2	2	1	1	2	2
3	3	4	4	2	2	3	3	3	3
3	3	4	4	2	2	1	1	2	2
1	1	4	4	2	2	1	1	2	2
1	1	4	4	2	2	1	1	2	2
3	3	4	4	2	2	1	1	2	2
1	1	4	4	2	2	1	1	2	2
3	3	1	1	2	2	1	1	3	3
3	3	1	1	2	2	1	1	3	3
3	3	4	4	2	2	1	1	2	2
3	3	4	4	2	2	1	1	2	2
3	3	4	4	2	2	1	1	2	2
3	3	4	4	2	2	1	1	2	2
1	1	4	4	2	2	1	1	2	2
1	1	4	4	2	2	3	3	3	3
3	3	1	1	2	2	1	1	3	3
1	1	1	1	2	2	1	1	2	2
3	3	4	4	2	2	1	1	2	2
1	1	4	4	2	2	1	1	2	2
1	1	4	4	2	2	1	1	2	2
1	1	1	1	2	2	3	3	3	3
3	3	4	4	2	2	1	1	2	2
3	3	4	4	2	2	1	1	2	2
1	1	4	4	2	2	1	1	2	2
3	3	4	4	2	2	3	3	3	3
1	1	1	1	2	2	1	1	2	2
1	1	4	4	1	1	1	1	2	2
3	3	4	4	2	2	1	1	2	2
3	3	4	4	2	2	1	1	2	2
1	1	4	4	2	2	1	1	2	2
3	3	4	4	1	1	1	1	2	2
1	1	4	4	2	2	1	1	2	2
3	3	4	4	2	2	1	1	2	2
3	3	4	4	2	2	1	1	2	2
1	1	4	4	2	2	1	1	2	2
3	3	4	4	2	2	1	1	2	2
1	1	4	4	2	2	1	1	2	2
1	1	4	4	2	2	1	1	2	2
1	1	1	1	2	2	1	1	2	2
1	1	1	1	2	2	1	1	2	2
3	3	4	4	2	2	1	1	2	2
3	3	4	4	2	2	1	1	2	2
1	1	4	4	2	2	1	1	2	2
3	3	4	4	2	2	1	1	2	2
3	3	4	4	2	2	1	1	2	2

For Review Only

3	3	4	4	2	2	1	1	2	2
3	3	4	4	2	2	1	1	2	2

For Review Only

SNP1854	SNP1854	SNP1855	SNP1855	SNP1856	SNP1856	SNP1857	SNP1857	SNP1858	SNP1858
2	2	2	2	2	2	4	4	4	4
2	2	2	2	2	2	2	2	4	4
2	2	2	2	2	2	4	4	4	4
2	2	2	2	2	2	4	4	4	4
2	2	2	2	2	2	4	4	4	4
2	2	2	2	2	2	4	4	4	4
2	2	2	2	3	3	4	4	4	4
3	3	2	2	2	2	4	4	4	4
2	2	2	2	3	3	4	4	4	4
2	2	2	2	2	2	2	2	4	4
2	2	2	2	2	2	4	4	4	4
2	2	2	2	2	2	4	4	4	4
2	2	2	2	2	2	4	4	4	4
2	2	2	2	2	2	4	4	1	1
2	2	2	2	2	2	4	4	4	4
2	2	2	2	2	2	4	4	4	4
2	2	2	2	3	3	4	4	4	4
2	2	2	2	2	2	4	4	4	4
2	2	2	2	2	2	4	4	4	4
2	2	2	2	2	2	4	4	1	1
2	2	2	2	3	3	4	4	4	4
2	2	2	2	2	2	4	4	4	4
3	3	2	2	2	2	2	2	1	1
2	2	2	2	2	2	4	4	1	1
2	2	2	2	3	3	4	4	4	4
2	2	2	2	2	2	4	4	4	4
2	2	2	2	3	3	4	4	4	4
2	2	2	2	2	2	4	4	4	4
2	2	2	2	3	3	4	4	4	4
3	3	2	2	2	2	2	2	1	1
2	2	2	2	3	3	4	4	4	4
2	2	2	2	2	2	4	4	4	4
2	2	2	2	2	2	4	4	4	4
2	2	2	2	2	2	4	4	1	1
2	2	2	2	2	2	4	4	4	4
2	2	2	2	2	2	4	4	1	1
2	2	2	2	2	2	4	4	4	4
2	2	2	2	2	2	4	4	4	4
3	3	2	2	2	2	2	2	1	1
2	2	2	2	2	2	2	2	4	4
3	3	2	2	2	2	2	2	1	1
2	2	2	2	2	2	4	4	4	4
2	2	2	2	2	2	2	2	1	1
3	3	2	2	3	3	4	4	1	1
2	2	2	2	2	2	4	4	1	1
2	2	2	2	2	2	4	4	4	4
2	2	2	2	3	3	4	4	4	4
2	2	2	2	2	2	2	2	4	4
2	2	2	2	3	3	4	4	1	1
2	2	2	2	2	2	4	4	4	4
2	2	2	2	2	2	4	4	4	4
3	3	2	2	2	2	4	4	4	4

For Review Only

2	2	2	2	3	3	4	4	4	4
2	2	2	2	2	2	4	4	4	4
2	2	2	2	3	3	4	4	4	4
2	2	2	2	3	3	4	4	1	1
2	2	2	2	2	2	4	4	4	4
2	2	2	2	3	3	4	4	4	4
2	2	2	2	2	2	4	4	4	4
3	3	2	2	2	2	4	4	1	1
2	2	2	2	2	2	4	4	1	1
2	2	2	2	2	2	4	4	4	4
3	3	2	2	2	2	4	4	4	4
3	3	2	2	3	3	4	4	4	4
2	2	2	2	3	3	4	4	4	4
2	2	2	2	3	3	4	4	4	4
2	2	2	2	2	2	4	4	4	4
2	2	2	2	2	2	2	2	4	4
2	2	2	2	2	2	4	4	4	4
2	2	2	2	2	2	4	4	4	4
2	2	2	2	3	3	4	4	4	4
2	2	2	2	3	3	4	4	4	4
2	2	2	2	2	2	4	4	4	4
2	2	2	2	2	2	4	4	4	4
2	2	2	2	3	3	4	4	4	4
2	2	3	3	2	2	4	4	1	1
2	2	2	2	2	2	4	4	1	1
3	3	2	2	2	2	4	4	1	1
2	2	2	2	3	3	2	2	1	1
2	2	2	2	2	2	2	2	1	1
2	2	2	2	2	2	4	4	4	4
2	2	3	3	2	2	4	4	4	4
2	2	2	2	2	2	4	4	4	4
2	2	2	2	2	2	4	4	1	1
2	2	2	2	2	2	2	2	1	1
2	2	2	2	3	3	4	4	4	4
2	2	2	2	2	2	4	4	1	1
2	2	2	2	2	2	4	4	1	1
2	2	2	2	2	2	4	4	4	4
2	2	2	2	3	3	4	4	1	1
2	2	2	2	2	2	4	4	1	1
2	2	2	2	2	2	4	4	1	1
2	2	2	2	3	3	4	4	4	4
2	2	2	2	2	2	4	4	4	4
2	2	2	2	2	2	4	4	4	4
3	3	2	2	2	2	4	4	4	4
2	2	3	3	2	2	4	4	1	1
2	2	2	2	2	2	4	4	4	4
2	2	2	2	2	2	4	4	1	1
2	2	2	2	2	2	4	4	4	4
2	2	2	2	2	2	4	4	1	1
2	2	2	2	3	3	4	4	1	1
2	2	2	2	2	2	4	4	4	4
2	2	2	2	2	2	4	4	1	1
3	3	2	2	2	2	4	4	1	1
2	2	2	2	2	2	4	4	4	4
2	2	2	2	3	3	4	4	1	1

For Review Only

2	2	2	2	2	2	4	4	4	4
3	3	2	2	2	2	4	4	4	4
2	2	2	2	2	2	4	4	4	4
2	2	2	2	2	2	4	4	1	1
2	2	2	2	2	2	4	4	4	4
2	2	2	2	2	2	4	4	4	4
2	2	2	2	2	2	4	4	4	4
2	2	2	2	2	2	4	4	1	1
2	2	2	2	2	2	2	2	4	4
2	2	2	2	2	2	4	4	1	1
2	2	2	2	2	2	4	4	4	4
2	2	2	2	2	2	4	4	4	4
2	2	2	2	2	2	4	4	4	4
3	3	2	2	3	3	4	4	4	4
2	2	2	2	2	2	4	4	1	1
2	2	2	2	2	2	4	4	4	4
2	2	2	2	2	2	4	4	4	4
2	2	2	2	2	2	4	4	4	4
2	2	2	2	2	2	4	4	1	1
2	2	2	2	2	2	4	4	4	4
2	2	2	2	2	2	4	4	4	4
2	2	3	3	2	2	4	4	4	4
2	2	2	2	2	2	4	4	4	4
2	2	2	2	2	2	2	2	1	1
2	2	2	2	2	2	4	4	1	1
2	2	2	2	2	2	4	4	4	4
2	2	3	3	2	2	4	4	4	4
2	2	2	2	2	2	4	4	4	4
2	2	3	3	2	2	4	4	4	4
2	2	2	2	2	2	4	4	1	1
2	2	2	2	2	2	2	2	1	1
2	2	2	2	2	2	4	4	4	4
2	2	2	2	3	3	4	4	4	4
2	2	2	2	2	2	4	4	4	4
2	2	2	2	2	2	4	4	1	1
2	2	2	2	2	2	4	4	4	4
2	2	2	2	2	2	4	4	1	1
2	2	2	2	2	2	4	4	4	4
2	2	2	2	2	2	4	4	4	4
2	2	2	2	2	2	4	4	4	4
2	2	2	2	2	2	4	4	4	4
2	2	2	2	2	2	4	4	1	1
2	2	2	2	2	2	4	4	1	1
2	2	2	2	2	2	2	2	4	4
2	2	2	2	2	2	4	4	4	4
2	2	2	2	2	2	4	4	4	4
2	2	2	2	2	2	4	4	4	4
2	2	2	2	2	2	4	4	4	4
2	2	2	2	2	2	4	4	1	1
2	2	2	2	2	2	4	4	4	4
2	2	2	2	2	2	4	4	4	4
2	2	2	2	2	2	4	4	4	4
2	2	2	2	2	2	4	4	4	4
3	3	2	2	3	3	4	4	4	4
2	2	2	2	2	2	4	4	4	4

For Review Only

2	2	3	3	2	2	4	4	4	4
2	2	2	2	2	2	4	4	4	4
2	2	2	2	2	2	2	2	1	1
2	2	2	2	2	2	4	4	4	4
2	2	2	2	2	2	4	4	1	1
2	2	2	2	2	2	4	4	1	1
2	2	2	2	2	2	4	4	4	4
2	2	2	2	2	2	4	4	4	4
3	3	2	2	2	2	4	4	4	4
2	2	3	3	2	2	4	4	4	4
2	2	2	2	2	2	4	4	4	4
2	2	2	2	2	2	4	4	1	1
2	2	2	2	2	2	4	4	4	4
2	2	2	2	2	2	4	4	4	4
2	2	2	2	2	2	4	4	1	1
2	2	2	2	2	2	4	4	4	4
2	2	2	2	2	2	4	4	4	4
2	2	2	2	2	2	4	4	4	4
2	2	2	2	2	2	4	4	4	4
2	2	2	2	2	2	4	4	4	4
2	2	2	2	2	2	4	4	4	4
2	2	2	2	2	2	4	4	1	1
2	2	2	2	2	2	4	4	1	1
3	3	2	2	3	3	4	4	4	4
2	2	2	2	2	2	4	4	4	4
2	2	2	2	2	2	4	4	1	1
2	2	2	2	3	3	4	4	4	4
2	2	2	2	2	2	4	4	4	4
2	2	2	2	2	2	4	4	4	4
2	2	2	2	3	3	4	4	4	4
2	2	2	2	2	2	4	4	4	4
2	2	3	3	2	2	4	4	4	4
3	3	2	2	2	2	4	4	4	4
2	2	2	2	2	2	4	4	1	1
2	2	2	2	2	2	4	4	1	1
3	3	2	2	3	3	4	4	4	4
2	2	2	2	3	3	4	4	4	4
2	2	2	2	2	2	4	4	4	4
2	2	2	2	2	2	4	4	4	4
2	2	2	2	2	2	4	4	4	4
2	2	2	2	2	2	4	4	4	4
2	2	2	2	2	2	4	4	4	4
3	3	2	2	2	2	4	4	1	1
2	2	2	2	2	2	2	2	4	4
2	2	2	2	2	2	4	4	4	4
2	2	2	2	3	3	4	4	4	4
2	2	2	2	2	2	4	4	4	4
3	3	2	2	2	2	4	4	4	4
2	2	2	2	2	2	4	4	1	1
2	2	2	2	2	2	4	4	4	4
2	2	3	3	2	2	4	4	4	4
2	2	2	2	2	2	2	2	1	1
2	2	2	2	2	2	4	4	1	1
3	3	2	2	2	2	4	4	4	4
2	2	2	2	2	2	2	2	1	1

For Review Only

2	2	2	2	2	2	4	4	4	4
2	2	2	2	2	2	2	2	4	4
2	2	3	3	2	2	4	4	4	4
2	2	2	2	2	2	4	4	4	4
2	2	2	2	2	2	4	4	4	4
2	2	2	2	2	2	4	4	4	4
2	2	2	2	2	2	4	4	4	4
2	2	2	2	2	2	2	2	4	4
2	2	2	2	2	2	4	4	1	1
2	2	2	2	2	2	4	4	1	1
2	2	2	2	2	2	4	4	4	4
2	2	2	2	2	2	4	4	4	4
2	2	2	2	2	2	4	4	1	1
2	2	2	2	2	2	4	4	4	4
2	2	2	2	2	2	4	4	4	4
2	2	2	2	2	2	4	4	1	1
2	2	2	2	2	2	4	4	1	1
2	2	3	3	2	2	2	2	1	1
3	3	2	2	2	2	4	4	4	4
2	2	2	2	2	2	4	4	4	4
3	3	2	2	2	2	4	4	4	4
2	2	2	2	2	2	4	4	4	4
2	2	2	2	2	2	4	4	4	4
2	2	2	2	2	2	2	2	4	4
3	3	2	2	2	2	4	4	1	1
2	2	2	2	2	2	4	4	4	4
2	2	2	2	2	2	2	2	1	1
2	2	2	2	2	2	4	4	4	4
2	2	2	2	2	2	4	4	4	4
2	2	3	3	2	2	4	4	4	4
2	2	2	2	2	2	4	4	1	1
2	2	2	2	2	2	2	2	4	4
2	2	2	2	2	2	4	4	1	1
2	2	2	2	2	2	4	4	4	4
2	2	2	2	2	2	4	4	1	1
3	3	2	2	2	2	2	2	1	1
2	2	2	2	2	2	4	4	4	4
3	3	2	2	3	3	4	4	4	4
2	2	2	2	2	2	4	4	1	1
3	3	2	2	2	2	2	2	1	1
2	2	2	2	2	2	4	4	4	4
2	2	2	2	3	3	4	4	1	1
2	2	2	2	2	2	2	2	1	1
2	2	2	2	3	3	4	4	4	4
2	2	2	2	2	2	4	4	1	1
2	2	2	2	2	2	4	4	1	1
2	2	2	2	2	2	4	4	1	1
2	2	2	2	3	3	4	4	4	4
2	2	2	2	2	2	4	4	4	4
2	2	2	2	2	2	2	2	1	1
2	2	2	2	2	2	4	4	4	4
3	3	2	2	2	2	2	2	1	1
2	2	2	2	2	2	4	4	4	4
2	2	2	2	3	3	4	4	4	4
2	2	2	2	2	2	2	2	4	4

For Review Only

2	2	2	2	2	2	4	4	4	4
2	2	2	2	3	3	4	4	4	4

For Review Only

SNP1859	SNP1859	SNP186	SNP186	SNP1860	SNP1860	SNP1861	SNP1861	SNP1862	SNP1862
4	4	3	3	1	1	3	3	2	2
4	4	2	2	1	1	3	3	2	2
4	4	3	3	1	1	3	3	2	2
4	4	2	2	1	1	3	3	2	2
4	4	2	2	1	1	3	3	2	2
4	4	2	2	1	1	3	3	2	2
4	4	2	2	1	1	2	2	2	2
4	4	3	3	1	1	3	3	2	2
4	4	2	2	1	1	3	3	2	2
4	4	3	3	1	1	3	3	2	2
4	4	2	2	1	1	3	3	2	2
4	4	3	3	1	1	3	3	2	2
4	4	3	3	1	1	3	3	2	2
1	1	3	3	1	1	3	3	1	1
4	4	2	2	1	1	3	3	2	2
1	1	2	2	1	1	3	3	1	1
4	4	3	3	1	1	2	2	2	2
4	4	2	2	1	1	3	3	2	2
4	4	2	2	1	1	3	3	2	2
1	1	2	2	1	1	3	3	1	1
4	4	2	2	1	1	2	2	2	2
4	4	3	3	1	1	3	3	2	2
1	1	3	3	1	1	3	3	1	1
1	1	3	3	1	1	3	3	1	1
4	4	3	3	1	1	2	2	2	2
4	4	3	3	1	1	3	3	2	2
4	4	2	2	1	1	2	2	2	2
4	4	2	2	1	1	3	3	2	2
4	4	2	2	1	1	2	2	2	2
1	1	3	3	1	1	3	3	1	1
4	4	3	3	1	1	2	2	2	2
4	4	3	3	1	1	3	3	2	2
4	4	3	3	1	1	3	3	2	2
1	1	3	3	1	1	3	3	1	1
4	4	2	2	1	1	3	3	2	2
1	1	2	2	1	1	3	3	1	1
4	4	2	2	1	1	3	3	2	2
4	4	2	2	1	1	3	3	2	2
1	1	3	3	1	1	3	3	1	1
4	4	2	2	1	1	3	3	2	2
1	1	3	3	1	1	3	3	1	1
4	4	2	2	1	1	3	3	2	2
1	1	3	3	1	1	3	3	1	1
1	1	2	2	1	1	2	2	1	1
1	1	3	3	1	1	3	3	1	1
4	4	3	3	1	1	3	3	2	2
4	4	2	2	1	1	2	2	2	2
4	4	3	3	1	1	3	3	2	2
1	1	2	2	1	1	2	2	1	1
4	4	2	2	1	1	3	3	2	2
4	4	3	3	1	1	3	3	2	2
4	4	2	2	1	1	3	3	2	2

For Review Only

4	4	3	3	1	1	2	2	2	2
4	4	3	3	1	1	3	3	2	2
4	4	2	2	1	1	2	2	2	2
1	1	3	3	1	1	2	2	1	1
4	4	2	2	1	1	3	3	2	2
4	4	2	2	1	1	2	2	2	2
1	1	2	2	1	1	3	3	2	2
1	1	3	3	1	1	3	3	1	1
1	1	2	2	1	1	3	3	1	1
4	4	2	2	1	1	3	3	2	2
4	4	2	2	1	1	3	3	2	2
4	4	2	2	1	1	2	2	2	2
4	4	3	3	1	1	2	2	2	2
4	4	2	2	1	1	2	2	2	2
4	4	2	2	1	1	3	3	2	2
4	4	2	2	1	1	3	3	2	2
4	4	2	2	1	1	3	3	2	2
4	4	2	2	1	1	3	3	2	2
4	4	2	2	1	1	2	2	2	2
4	4	3	3	1	1	2	2	2	2
4	4	2	2	1	1	3	3	2	2
4	4	2	2	1	1	3	3	2	2
4	4	2	2	1	1	2	2	2	2
1	1	3	3	4	4	3	3	1	1
1	1	3	3	1	1	3	3	1	1
1	1	2	2	1	1	3	3	1	1
1	1	3	3	1	1	2	2	1	1
1	1	3	3	1	1	3	3	1	1
4	4	3	3	1	1	3	3	2	2
4	4	3	3	4	4	3	3	2	2
4	4	2	2	1	1	3	3	2	2
1	1	3	3	1	1	3	3	1	1
1	1	3	3	1	1	3	3	1	1
4	4	3	3	1	1	2	2	2	2
1	1	3	3	1	1	3	3	1	1
1	1	3	3	1	1	3	3	1	1
4	4	2	2	1	1	3	3	2	2
1	1	2	2	1	1	2	2	1	1
1	1	3	3	1	1	3	3	1	1
1	1	2	2	1	1	3	3	1	1
4	4	3	3	1	1	2	2	2	2
4	4	2	2	1	1	3	3	2	2
4	4	3	3	1	1	3	3	2	2
4	4	2	2	1	1	3	3	2	2
1	1	2	2	4	4	3	3	1	1
4	4	2	2	1	1	3	3	2	2
1	1	3	3	1	1	3	3	1	1
4	4	2	2	1	1	3	3	2	2
1	1	3	3	1	1	3	3	1	1
1	1	2	2	1	1	2	2	1	1
4	4	2	2	1	1	3	3	2	2
1	1	3	3	1	1	3	3	1	1
1	1	2	2	1	1	3	3	1	1
4	4	3	3	1	1	3	3	2	2
1	1	2	2	1	1	2	2	1	1

For Review Only

1	1	3	3	1	1	3	3	2	2
4	4	2	2	1	1	3	3	2	2
4	4	2	2	1	1	3	3	2	2
1	1	3	3	1	1	3	3	1	1
4	4	3	3	1	1	3	3	2	2
4	4	3	3	1	1	3	3	2	2
4	4	2	2	1	1	3	3	2	2
1	1	2	2	1	1	3	3	1	1
4	4	3	3	1	1	3	3	2	2
1	1	3	3	1	1	3	3	1	1
1	1	3	3	1	1	3	3	1	1
4	4	2	2	1	1	3	3	2	2
4	4	2	2	1	1	3	3	2	2
4	4	2	2	1	1	2	2	2	2
4	4	2	2	1	1	3	3	2	2
4	4	2	2	1	1	3	3	2	2
4	4	2	2	1	1	3	3	2	2
4	4	2	2	1	1	3	3	2	2
1	1	3	3	1	1	3	3	1	1
4	4	2	2	1	1	3	3	2	2
4	4	2	2	1	1	2	2	2	2
4	4	2	2	1	1	3	3	2	2
4	4	3	3	1	1	3	3	2	2
1	1	3	3	1	1	3	3	1	1
1	1	3	3	1	1	3	3	2	2
4	4	2	2	1	1	3	3	2	2
4	4	2	2	4	4	3	3	2	2
4	4	3	3	1	1	3	3	2	2
4	4	3	3	4	4	3	3	2	2
1	1	2	2	1	1	3	3	1	1
1	1	3	3	1	1	3	3	1	1
4	4	3	3	1	1	3	3	2	2
4	4	3	3	1	1	2	2	2	2
4	4	2	2	1	1	3	3	1	1
1	1	3	3	1	1	3	3	1	1
1	1	2	2	1	1	3	3	2	2
1	1	3	3	1	1	3	3	1	1
4	4	2	2	1	1	3	3	2	2
1	1	3	3	1	1	3	3	1	1
4	4	2	2	1	1	3	3	2	2
4	4	3	3	1	1	3	3	2	2
1	1	3	3	1	1	3	3	1	1
1	1	3	3	1	1	3	3	1	1
4	4	3	3	1	1	3	3	2	2
4	4	2	2	1	1	3	3	2	2
4	4	2	2	1	1	3	3	2	2
4	4	3	3	1	1	3	3	2	2
4	4	3	3	1	1	3	3	2	2
1	1	2	2	1	1	3	3	1	1
4	4	3	3	1	1	3	3	2	2
1	1	3	3	1	1	3	3	2	2
4	4	2	2	1	1	3	3	2	2
4	4	3	3	1	1	3	3	2	2
4	4	2	2	1	1	2	2	2	2
4	4	2	2	1	1	3	3	2	2

For Review Only

CSSA 5585 Guilford Rd., Madison, WI 53711

4	4	3	3	4	4	3	3	2	2
4	4	2	2	1	1	3	3	2	2
1	1	2	2	1	1	3	3	1	1
4	4	2	2	1	1	3	3	2	2
1	1	2	2	1	1	3	3	1	1
1	1	2	2	1	1	3	3	1	1
4	4	2	2	1	1	3	3	2	2
4	4	2	2	1	1	3	3	2	2
4	4	3	3	1	1	3	3	2	2
4	4	3	3	4	4	3	3	2	2
4	4	3	3	1	1	3	3	1	1
1	1	2	2	1	1	3	3	1	1
4	4	2	2	1	1	3	3	2	2
4	4	2	2	1	1	3	3	2	2
1	1	3	3	1	1	3	3	1	1
4	4	2	2	1	1	3	3	2	2
4	4	2	2	1	1	3	3	2	2
4	4	2	2	1	1	3	3	2	2
4	4	3	3	1	1	3	3	2	2
4	4	2	2	1	1	3	3	2	2
4	4	2	2	1	1	3	3	2	2
1	1	3	3	1	1	3	3	1	1
1	1	2	2	1	1	3	3	1	1
4	4	2	2	1	1	2	2	2	2
4	4	2	2	1	1	3	3	2	2
1	1	2	2	1	1	3	3	1	1
4	4	2	2	1	1	2	2	2	2
4	4	2	2	1	1	3	3	2	2
4	4	3	3	1	1	3	3	2	2
4	4	2	2	1	1	2	2	2	2
4	4	2	2	1	1	3	3	2	2
4	4	3	3	4	4	3	3	2	2
4	4	2	2	1	1	3	3	2	2
1	1	3	3	1	1	3	3	1	1
1	1	2	2	1	1	3	3	1	1
4	4	2	2	1	1	2	2	2	2
4	4	2	2	1	1	2	2	2	2
4	4	3	3	1	1	3	3	2	2
4	4	3	3	1	1	3	3	2	2
4	4	3	3	1	1	3	3	2	2
4	4	3	3	1	1	3	3	2	2
4	4	3	3	1	1	3	3	2	2
1	1	3	3	1	1	3	3	1	1
4	4	2	2	1	1	3	3	2	2
1	1	2	2	1	1	3	3	2	2
4	4	3	3	1	1	2	2	2	2
4	4	2	2	1	1	3	3	2	2
4	4	3	3	1	1	3	3	2	2
1	1	3	3	1	1	3	3	1	1
4	4	2	2	1	1	3	3	2	2
4	4	3	3	4	4	3	3	2	2
1	1	2	2	1	1	3	3	1	1
1	1	2	2	1	1	3	3	1	1
4	4	2	2	1	1	3	3	2	2
1	1	3	3	1	1	3	3	1	1

For Review Only

4	4	3	3	1	1	3	3	2	2
1	1	3	3	1	1	3	3	1	1
4	4	3	3	4	4	3	3	2	2
4	4	2	2	1	1	3	3	2	2
4	4	3	3	1	1	3	3	2	2
4	4	2	2	1	1	3	3	2	2
4	4	3	3	1	1	3	3	2	2
4	4	3	3	1	1	3	3	2	2
1	1	3	3	1	1	3	3	1	1
1	1	3	3	1	1	3	3	1	1
4	4	2	2	1	1	3	3	2	2
4	4	2	2	1	1	3	3	2	2
1	1	2	2	1	1	3	3	1	1
4	4	3	3	1	1	3	3	2	2
4	4	3	3	1	1	3	3	2	2
1	1	2	2	1	1	3	3	1	1
1	1	3	3	1	1	3	3	1	1
1	1	2	2	4	4	3	3	1	1
4	4	2	2	1	1	3	3	2	2
4	4	2	2	1	1	3	3	2	2
4	4	2	2	1	1	3	3	2	2
4	4	2	2	1	1	3	3	2	2
4	4	2	2	1	1	3	3	2	2
4	4	3	3	1	1	3	3	2	2
1	1	3	3	1	1	3	3	1	1
4	4	3	3	1	1	3	3	2	2
1	1	3	3	1	1	3	3	1	1
4	4	2	2	1	1	3	3	2	2
4	4	3	3	1	1	3	3	2	2
4	4	3	3	4	4	3	3	2	2
1	1	3	3	1	1	3	3	1	1
4	4	2	2	1	1	3	3	2	2
1	1	2	2	1	1	3	3	1	1
4	4	3	3	1	1	3	3	2	2
1	1	2	2	1	1	3	3	1	1
1	1	3	3	1	1	3	3	1	1
4	4	3	3	1	1	3	3	2	2
4	4	2	2	1	1	2	2	2	2
1	1	2	2	1	1	3	3	1	1
1	1	3	3	1	1	3	3	1	1
4	4	2	2	1	1	3	3	2	2
1	1	3	3	1	1	2	2	1	1
1	1	2	2	1	1	3	3	1	1
4	4	2	2	1	1	2	2	2	2
1	1	3	3	1	1	3	3	1	1
1	1	3	3	1	1	3	3	1	1
1	1	3	3	1	1	3	3	1	1
4	4	2	2	1	1	2	2	2	2
1	1	3	3	1	1	3	3	2	2
1	1	3	3	1	1	3	3	1	1
4	4	2	2	1	1	3	3	2	2
1	1	3	3	1	1	3	3	1	1
4	4	3	3	1	1	3	3	2	2
4	4	2	2	1	1	2	2	2	2
4	4	2	2	1	1	3	3	2	2

For Review Only

4	4	2	2	1	1	3	3	2	2
4	4	2	2	1	1	2	2	2	2

For Review Only

SNP1863	SNP1863	SNP1864	SNP1864	SNP1865	SNP1865	SNP1866	SNP1866	SNP1867	SNP1867
1	1	4	4	3	3	4	4	1	1
1	1	4	4	3	3	4	4	1	1
1	1	4	4	3	3	4	4	1	1
1	1	4	4	1	1	4	4	1	1
1	1	4	4	3	3	4	4	1	1
1	1	1	1	3	3	4	4	1	1
1	1	4	4	1	1	4	4	1	1
1	1	1	1	1	1	4	4	1	1
1	1	4	4	3	3	4	4	1	1
1	1	1	1	1	1	4	4	1	1
1	1	4	4	3	3	4	4	1	1
1	1	4	4	3	3	4	4	1	1
1	1	4	4	3	3	4	4	1	1
2	2	4	4	3	3	4	4	1	1
1	1	4	4	3	3	3	3	1	1
2	2	4	4	1	1	4	4	1	1
1	1	4	4	3	3	4	4	1	1
1	1	4	4	3	3	4	4	1	1
1	1	4	4	3	3	4	4	1	1
2	2	1	1	1	1	4	4	1	1
1	1	4	4	3	3	4	4	1	1
2	2	4	4	3	3	4	4	1	1
2	2	1	1	3	3	4	4	1	1
2	2	1	1	1	1	4	4	1	1
1	1	4	4	1	1	3	3	4	4
1	1	4	4	3	3	4	4	1	1
1	1	4	4	3	3	3	3	4	4
1	1	4	4	1	1	4	4	1	1
1	1	4	4	1	1	4	4	1	1
2	2	1	1	3	3	4	4	1	1
1	1	4	4	3	3	3	3	4	4
1	1	4	4	3	3	4	4	1	1
1	1	4	4	3	3	4	4	1	1
2	2	4	4	1	1	4	4	1	1
1	1	1	1	3	3	4	4	4	4
2	2	4	4	1	1	4	4	1	1
1	1	4	4	1	1	4	4	1	1
1	1	1	1	1	1	4	4	1	1
2	2	4	4	1	1	4	4	1	1
1	1	1	1	1	1	4	4	1	1
2	2	1	1	1	1	4	4	1	1
1	1	4	4	1	1	4	4	1	1
2	2	1	1	1	1	4	4	1	1
2	2	4	4	1	1	4	4	1	1
2	2	1	1	1	1	4	4	1	1
1	1	4	4	3	3	4	4	1	1
1	1	4	4	3	3	3	3	4	4
1	1	4	4	3	3	4	4	1	1
2	2	4	4	3	3	4	4	1	1
1	1	4	4	1	1	4	4	1	1
1	1	4	4	3	3	4	4	1	1
1	1	4	4	3	3	4	4	1	1

For Review Only

1	1	4	4	3	3	4	4	1	1
1	1	4	4	3	3	4	4	1	1
1	1	1	1	3	3	3	3	4	4
2	2	4	4	3	3	4	4	1	1
1	1	4	4	3	3	4	4	1	1
1	1	4	4	3	3	4	4	4	4
1	1	4	4	3	3	4	4	1	1
2	2	4	4	3	3	4	4	1	1
2	2	4	4	1	1	4	4	1	1
1	1	4	4	3	3	4	4	1	1
1	1	4	4	3	3	4	4	1	1
1	1	4	4	3	3	3	3	4	4
1	1	4	4	3	3	4	4	1	1
1	1	4	4	1	1	4	4	1	1
1	1	4	4	1	1	4	4	1	1
1	1	4	4	3	3	4	4	1	1
1	1	4	4	3	3	4	4	1	1
1	1	4	4	3	3	4	4	1	1
1	1	4	4	3	3	4	4	1	1
1	1	4	4	3	3	4	4	1	1
1	1	4	4	3	3	3	3	4	4
1	1	4	4	1	1	4	4	1	1
1	1	4	4	1	1	4	4	1	1
2	2	4	4	1	1	3	3	1	1
2	2	1	1	1	1	4	4	1	1
2	2	4	4	3	3	4	4	1	1
2	2	4	4	3	3	4	4	1	1
2	2	1	1	3	3	4	4	1	1
1	1	4	4	3	3	4	4	1	1
1	1	4	4	1	1	3	3	1	1
1	1	1	1	3	3	4	4	1	1
2	2	4	4	1	1	4	4	1	1
2	2	4	4	1	1	4	4	1	1
1	1	4	4	3	3	4	4	1	1
2	2	4	4	1	1	4	4	1	1
2	2	1	1	1	1	4	4	1	1
1	1	1	1	1	1	4	4	1	1
2	2	4	4	3	3	4	4	1	1
2	2	4	4	3	3	4	4	1	1
2	2	1	1	1	1	4	4	1	1
1	1	4	4	3	3	4	4	1	1
1	1	4	4	1	1	4	4	1	1
1	1	4	4	3	3	4	4	1	1
1	1	4	4	3	3	4	4	1	1
1	1	4	4	3	3	3	3	1	1
1	1	4	4	3	3	4	4	1	1
2	2	4	4	1	1	4	4	1	1
1	1	4	4	1	1	4	4	1	1
2	2	4	4	3	3	4	4	1	1
2	2	4	4	1	1	3	3	4	4
1	1	4	4	3	3	4	4	1	1
2	2	4	4	1	1	4	4	1	1
2	2	4	4	3	3	4	4	1	1
1	1	4	4	1	1	4	4	1	1
2	2	1	1	1	1	3	3	4	4

For Review Only

1	1	4	4	1	1	4	4	1	1
1	1	1	1	1	1	4	4	1	1
1	1	4	4	3	3	4	4	1	1
2	2	4	4	1	1	4	4	1	1
1	1	4	4	3	3	4	4	1	1
1	1	1	1	3	3	4	4	1	1
1	1	4	4	3	3	4	4	1	1
2	2	4	4	1	1	4	4	1	1
1	1	4	4	1	1	4	4	1	1
2	2	4	4	1	1	4	4	1	1
2	2	4	4	3	3	4	4	1	1
1	1	4	4	1	1	4	4	1	1
1	1	4	4	3	3	4	4	1	1
1	1	4	4	3	3	3	3	4	4
1	1	4	4	3	3	4	4	1	1
1	1	4	4	1	1	4	4	1	1
1	1	4	4	3	3	4	4	1	1
1	1	4	4	3	3	4	4	1	1
2	2	4	4	3	3	4	4	1	1
1	1	4	4	3	3	4	4	1	1
1	1	4	4	1	1	4	4	1	1
1	1	4	4	1	1	4	4	1	1
1	1	4	4	3	3	4	4	1	1
2	2	1	1	3	3	4	4	1	1
1	1	4	4	1	1	4	4	1	1
1	1	4	4	1	1	4	4	1	1
1	1	4	4	1	1	3	3	1	1
1	1	4	4	1	1	4	4	1	1
1	1	4	4	3	3	3	3	1	1
2	2	4	4	1	1	4	4	1	1
1	1	1	1	3	3	4	4	1	1
1	1	4	4	1	1	4	4	1	1
1	1	4	4	3	3	4	4	1	1
1	1	4	4	1	1	4	4	1	1
2	2	1	1	1	1	4	4	1	1
1	1	4	4	1	1	4	4	1	1
2	2	4	4	1	1	4	4	1	1
1	1	4	4	3	3	4	4	1	1
2	2	4	4	3	3	4	4	1	1
1	1	4	4	3	3	4	4	1	1
1	1	4	4	1	1	4	4	1	1
2	2	4	4	1	1	4	4	1	1
2	2	4	4	3	3	4	4	1	1
1	1	4	4	1	1	4	4	1	1
1	1	4	4	3	3	4	4	1	1
1	1	4	4	3	3	4	4	1	1
1	1	4	4	3	3	4	4	1	1
1	1	4	4	1	1	4	4	1	1
1	1	4	4	1	1	4	4	1	1
1	1	4	4	3	3	4	4	1	1
1	1	4	4	1	1	4	4	1	1
1	1	4	4	1	1	4	4	1	1
1	1	4	4	3	3	4	4	1	1
1	1	4	4	3	3	3	3	4	4
1	1	4	4	3	3	4	4	1	1

For Review Only

1	1	4	4	1	1	3	3	1	1
1	1	4	4	1	1	4	4	1	1
2	2	4	4	1	1	4	4	1	1
1	1	4	4	1	1	4	4	1	1
2	2	4	4	1	1	4	4	1	1
2	2	4	4	3	3	4	4	1	1
1	1	4	4	1	1	4	4	1	1
1	1	4	4	3	3	4	4	1	1
1	1	4	4	1	1	4	4	1	1
1	1	4	4	1	1	3	3	1	1
1	1	4	4	3	3	4	4	1	1
2	2	4	4	1	1	4	4	1	1
1	1	4	4	1	1	4	4	1	1
1	1	4	4	1	1	4	4	1	1
2	2	4	4	1	1	4	4	1	1
1	1	4	4	3	3	4	4	1	1
1	1	4	4	1	1	4	4	1	1
1	1	4	4	1	1	4	4	1	1
1	1	4	4	3	3	4	4	1	1
1	1	4	4	3	3	4	4	1	1
1	1	4	4	1	1	4	4	1	1
2	2	4	4	1	1	4	4	1	1
2	2	4	4	1	1	4	4	1	1
1	1	4	4	3	3	3	3	4	4
1	1	4	4	1	1	4	4	1	1
2	2	1	1	1	1	4	4	1	1
1	1	4	4	3	3	3	3	4	4
1	1	4	4	3	3	4	4	1	1
1	1	4	4	1	1	4	4	1	1
1	1	4	4	1	1	4	4	1	1
1	1	4	4	1	1	4	4	1	1
1	1	4	4	1	1	3	3	1	1
1	1	4	4	1	1	4	4	1	1
2	2	4	4	1	1	4	4	1	1
2	2	4	4	1	1	4	4	1	1
1	1	4	4	3	3	3	3	4	4
1	1	4	4	1	1	3	3	4	4
1	1	4	4	1	1	4	4	1	1
1	1	4	4	1	1	4	4	1	1
1	1	4	4	3	3	4	4	1	1
1	1	4	4	3	3	4	4	1	1
1	1	4	4	3	3	4	4	1	1
2	2	1	1	3	3	4	4	1	1
1	1	4	4	3	3	4	4	1	1
2	2	4	4	3	3	4	4	1	1
1	1	4	4	3	3	3	3	4	4
1	1	4	4	3	3	4	4	1	1
1	1	4	4	1	1	4	4	1	1
2	2	4	4	1	1	4	4	1	1
1	1	4	4	3	3	4	4	1	1
1	1	1	1	3	3	3	3	1	1
2	2	4	4	3	3	4	4	1	1
2	2	4	4	1	1	4	4	1	1
1	1	4	4	3	3	4	4	1	1
2	2	1	1	3	3	4	4	1	1

For Review Only

1	1	1	1	1	1	4	4	1	1
2	2	1	1	1	1	4	4	1	1
1	1	4	4	3	3	3	3	1	1
1	1	4	4	1	1	4	4	1	1
1	1	4	4	3	3	4	4	1	1
1	1	4	4	3	3	4	4	1	1
1	1	1	1	1	1	4	4	1	1
1	1	1	1	3	3	4	4	1	1
2	2	4	4	1	1	4	4	1	1
2	2	1	1	3	3	4	4	1	1
1	1	4	4	1	1	4	4	1	1
1	1	4	4	1	1	4	4	1	1
2	2	4	4	3	3	4	4	1	1
1	1	4	4	1	1	4	4	1	1
1	1	4	4	3	3	4	4	1	1
2	2	1	1	3	3	4	4	1	1
2	2	4	4	3	3	4	4	1	1
2	2	4	4	3	3	3	3	1	1
1	1	4	4	1	1	4	4	1	1
1	1	1	1	3	3	4	4	1	1
1	1	1	1	1	1	4	4	1	1
1	1	4	4	3	3	4	4	1	1
1	1	4	4	3	3	4	4	1	1
1	1	1	1	3	3	4	4	1	1
2	2	4	4	3	3	4	4	1	1
1	1	4	4	3	3	4	4	1	1
2	2	1	1	1	1	4	4	1	1
1	1	4	4	3	3	4	4	1	1
1	1	4	4	3	3	4	4	1	1
1	1	4	4	3	3	3	3	1	1
2	2	1	1	1	1	4	4	1	1
1	1	4	4	3	3	4	4	1	1
2	2	1	1	1	1	4	4	1	1
1	1	4	4	1	1	4	4	1	1
2	2	4	4	1	1	4	4	1	1
2	2	4	4	1	1	4	4	1	1
1	1	4	4	3	3	4	4	1	1
1	1	4	4	3	3	3	3	4	4
2	2	4	4	1	1	4	4	1	1
2	2	4	4	1	1	4	4	1	1
1	1	4	4	1	1	4	4	1	1
2	2	1	1	1	1	3	3	4	4
2	2	1	1	1	1	4	4	1	1
1	1	4	4	3	3	4	4	1	1
2	2	4	4	3	3	4	4	1	1
2	2	4	4	3	3	4	4	1	1
2	2	1	1	1	1	4	4	1	1
1	1	4	4	1	1	3	3	4	4
1	1	1	1	3	3	4	4	1	1
2	2	1	1	3	3	4	4	1	1
1	1	4	4	3	3	4	4	1	1
2	2	4	4	1	1	4	4	1	1
1	1	4	4	3	3	4	4	1	1
1	1	4	4	3	3	3	3	4	4
1	1	1	1	1	1	4	4	1	1

For Review Only

1	1	4	4	3	3	4	4	1	1
1	1	4	4	3	3	3	3	4	4

For Review Only

SNP1868	SNP1868	SNP1869	SNP1869	SNP187	SNP187	SNP1870	SNP1870	SNP1871	SNP1871
4	4	3	3	4	4	4	4	2	2
4	4	3	3	4	4	4	4	3	3
4	4	3	3	4	4	4	4	2	2
4	4	2	2	4	4	4	4	3	3
4	4	3	3	4	4	4	4	3	3
4	4	3	3	4	4	4	4	2	2
4	4	3	3	4	4	4	4	3	3
4	4	3	3	1	1	4	4	3	3
4	4	3	3	4	4	4	4	2	2
4	4	3	3	1	1	4	4	2	2
4	4	3	3	1	1	4	4	3	3
4	4	3	3	4	4	4	4	2	2
4	4	3	3	4	4	4	4	3	3
4	4	3	3	4	4	1	1	2	2
4	4	2	2	4	4	4	4	3	3
4	4	3	3	4	4	1	1	2	2
4	4	3	3	4	4	4	4	3	3
4	4	3	3	1	1	4	4	2	2
4	4	2	2	4	4	4	4	3	3
4	4	3	3	1	1	1	1	2	2
4	4	3	3	1	1	4	4	3	3
4	4	3	3	1	1	4	4	3	3
4	4	3	3	4	4	1	1	3	3
4	4	3	3	4	4	1	1	3	3
1	1	2	2	4	4	4	4	2	2
4	4	3	3	4	4	4	4	2	2
1	1	2	2	4	4	4	4	2	2
4	4	3	3	4	4	4	4	3	3
4	4	3	3	4	4	4	4	2	2
4	4	3	3	1	1	1	1	2	2
1	1	2	2	4	4	4	4	3	3
4	4	3	3	4	4	4	4	3	3
4	4	3	3	4	4	4	4	3	3
4	4	3	3	4	4	1	1	3	3
4	4	2	2	4	4	4	4	3	3
4	4	3	3	4	4	1	1	2	2
4	4	3	3	4	4	4	4	3	3
4	4	3	3	4	4	4	4	3	3
4	4	3	3	4	4	1	1	2	2
4	4	3	3	1	1	4	4	3	3
4	4	3	3	1	1	1	1	2	2
4	4	3	3	1	1	4	4	2	2
4	4	3	3	1	1	1	1	2	2
4	4	3	3	1	1	1	1	3	3
4	4	3	3	1	1	1	1	2	2
4	4	3	3	4	4	4	4	2	2
1	1	2	2	4	4	4	4	2	2
4	4	3	3	4	4	4	4	3	3
4	4	3	3	4	4	1	1	3	3
4	4	3	3	4	4	4	4	3	3
4	4	3	3	4	4	4	4	2	2
4	4	2	2	4	4	4	4	3	3

For Review Only

4	4	3	3	4	4	4	4	2	2
4	4	3	3	4	4	4	4	3	3
1	1	2	2	4	4	4	4	3	3
4	4	3	3	1	1	1	1	3	3
4	4	2	2	4	4	4	4	3	3
1	1	2	2	4	4	4	4	2	2
4	4	3	3	4	4	4	4	2	2
4	4	3	3	1	1	1	1	2	2
4	4	3	3	4	4	1	1	3	3
4	4	3	3	4	4	4	4	3	3
4	4	3	3	4	4	4	4	3	3
1	1	2	2	4	4	4	4	3	3
4	4	3	3	4	4	4	4	2	2
4	4	3	3	4	4	4	4	2	2
4	4	3	3	4	4	4	4	3	3
4	4	3	3	4	4	4	4	3	3
4	4	3	3	4	4	4	4	2	2
4	4	3	3	4	4	4	4	3	3
4	4	3	3	4	4	4	4	3	3
4	4	3	3	4	4	4	4	3	3
4	4	2	2	4	4	4	4	3	3
4	4	3	3	1	1	4	4	3	3
4	4	3	3	4	4	4	4	3	3
4	4	3	3	4	4	1	1	3	3
4	4	3	3	1	1	1	1	2	2
4	4	3	3	4	4	4	4	3	3
4	4	3	3	4	4	1	1	3	3
4	4	3	3	1	1	1	1	3	3
4	4	3	3	4	4	4	4	2	2
4	4	3	3	4	4	4	4	3	3
4	4	3	3	4	4	4	4	3	3
4	4	3	3	4	4	1	1	3	3
4	4	3	3	1	1	1	1	2	2
4	4	3	3	4	4	4	4	2	2
4	4	3	3	4	4	1	1	3	3
4	4	3	3	1	1	1	1	2	2
4	4	3	3	1	1	4	4	2	2
4	4	3	3	4	4	1	1	3	3
4	4	3	3	4	4	1	1	3	3
4	4	3	3	1	1	1	1	2	2
4	4	3	3	4	4	4	4	3	3
4	4	3	3	4	4	4	4	3	3
4	4	3	3	1	1	4	4	3	3
4	4	3	3	4	4	4	4	3	3
4	4	3	3	4	4	1	1	3	3
4	4	3	3	4	4	4	4	3	3
4	4	3	3	4	4	1	1	3	3
4	4	3	3	4	4	4	4	2	2
4	4	3	3	4	4	4	4	2	2
1	1	2	2	4	4	1	1	3	3
4	4	3	3	4	4	4	4	3	3
4	4	3	3	4	4	1	1	3	3
4	4	3	3	1	1	1	1	2	2
4	4	3	3	4	4	1	1	3	3
1	1	2	2	4	4	1	1	3	3

For Review Only

4	4	3	3	4	4	4	4	3	3
4	4	3	3	4	4	4	4	2	2
4	4	3	3	4	4	4	4	2	2
4	4	3	3	4	4	1	1	2	2
4	4	3	3	4	4	4	4	2	2
4	4	3	3	4	4	4	4	2	2
4	4	3	3	4	4	4	4	2	2
4	4	3	3	4	4	4	4	3	3
4	4	3	3	4	4	4	4	2	2
4	4	3	3	4	4	1	1	2	2
4	4	3	3	1	1	1	1	3	3
4	4	3	3	4	4	4	4	3	3
4	4	3	3	1	1	4	4	3	3
1	1	2	2	4	4	4	4	3	3
4	4	3	3	4	4	4	4	3	3
4	4	3	3	4	4	4	4	3	3
4	4	3	3	4	4	4	4	3	3
4	4	3	3	4	4	4	4	3	3
4	4	3	3	4	4	1	1	3	3
4	4	2	2	4	4	4	4	3	3
4	4	3	3	4	4	4	4	3	3
4	4	3	3	4	4	4	4	3	3
4	4	3	3	4	4	4	4	3	3
4	4	3	3	4	4	1	1	3	3
4	4	3	3	4	4	4	4	2	2
4	4	2	2	4	4	4	4	3	3
4	4	3	3	4	4	4	4	3	3
4	4	3	3	4	4	4	4	3	3
4	4	3	3	4	4	4	4	3	3
4	4	3	3	4	4	1	1	3	3
4	4	3	3	1	1	1	1	2	2
4	4	3	3	4	4	4	4	3	3
4	4	3	3	4	4	4	4	3	3
4	4	3	3	4	4	4	4	3	3
4	4	3	3	4	4	4	4	3	3
4	4	3	3	4	4	1	1	3	3
4	4	3	3	4	4	1	1	3	3
4	4	3	3	4	4	4	4	3	3
4	4	3	3	4	4	1	1	3	3
4	4	3	3	4	4	4	4	2	2
4	4	3	3	4	4	4	4	3	3
4	4	3	3	4	4	1	1	3	3
4	4	3	3	4	4	1	1	3	3
4	4	3	3	1	1	1	1	3	3
4	4	3	3	4	4	4	4	3	3
4	4	3	3	1	1	4	4	3	3
4	4	3	3	4	4	4	4	2	2
4	4	3	3	4	4	4	4	2	2
4	4	3	3	4	4	1	1	3	3
4	4	3	3	4	4	4	4	2	2
4	4	3	3	4	4	4	4	2	2
4	4	3	3	1	1	4	4	3	3
4	4	3	3	4	4	4	4	3	3
1	1	2	2	4	4	4	4	3	3
4	4	3	3	4	4	4	4	3	3

For Review Only

4	4	3	3	4	4	4	4	3	3
4	4	3	3	1	1	4	4	3	3
4	4	3	3	4	4	1	1	2	2
4	4	3	3	4	4	4	4	3	3
4	4	3	3	4	4	4	4	2	2
4	4	3	3	1	1	1	1	3	3
4	4	3	3	1	1	4	4	3	3
4	4	3	3	4	4	4	4	3	3
4	4	3	3	4	4	4	4	3	3
4	4	3	3	4	4	4	4	3	3
4	4	3	3	4	4	4	4	2	2
4	4	3	3	4	4	1	1	2	2
4	4	3	3	4	4	4	4	3	3
4	4	3	3	4	4	4	4	3	3
4	4	3	3	4	4	1	1	3	3
4	4	3	3	4	4	4	4	3	3
4	4	3	3	4	4	4	4	3	3
4	4	2	2	4	4	4	4	2	2
4	4	3	3	4	4	4	4	2	2
4	4	3	3	4	4	4	4	2	2
4	4	3	3	4	4	4	4	3	3
4	4	3	3	1	1	1	1	3	3
4	4	3	3	4	4	1	1	3	3
1	1	2	2	4	4	4	4	3	3
4	4	3	3	4	4	4	4	2	2
4	4	3	3	4	4	1	1	3	3
1	1	2	2	4	4	4	4	3	3
4	4	3	3	4	4	4	4	2	2
4	4	3	3	4	4	4	4	3	3
4	4	3	3	4	4	4	4	3	3
4	4	3	3	1	1	4	4	2	2
4	4	3	3	4	4	4	4	3	3
4	4	3	3	4	4	4	4	3	3
4	4	3	3	4	4	1	1	3	3
4	4	3	3	4	4	1	1	3	3
1	1	2	2	4	4	4	4	3	3
1	1	2	2	4	4	4	4	3	3
4	4	3	3	4	4	4	4	3	3
4	4	3	3	4	4	4	4	2	2
4	4	3	3	4	4	4	4	2	2
4	4	3	3	4	4	4	4	2	2
4	4	3	3	4	4	4	4	2	2
4	4	3	3	4	4	1	1	2	2
4	4	3	3	4	4	4	4	3	3
4	4	3	3	4	4	4	4	3	3
1	1	2	2	4	4	1	1	3	3
4	4	3	3	4	4	4	4	3	3
4	4	3	3	1	1	4	4	3	3
4	4	3	3	4	4	1	1	3	3
4	4	3	3	4	4	4	4	3	3
4	4	3	3	4	4	4	4	2	2
4	4	3	3	1	1	1	1	3	3
4	4	3	3	4	4	1	1	3	3
4	4	3	3	4	4	4	4	3	3
4	4	3	3	4	4	1	1	2	2

For Review Only

4	4	3	3	4	4	4	4	3	3
4	4	3	3	4	4	1	1	3	3
4	4	3	3	4	4	4	4	3	3
4	4	2	2	4	4	4	4	3	3
4	4	3	3	4	4	4	4	2	2
4	4	3	3	4	4	4	4	2	2
4	4	3	3	4	4	4	4	2	2
4	4	3	3	1	1	4	4	2	2
4	4	3	3	4	4	1	1	3	3
4	4	3	3	1	1	1	1	2	2
4	4	3	3	4	4	4	4	3	3
4	4	3	3	1	1	4	4	3	3
4	4	3	3	4	4	1	1	3	3
4	4	3	3	4	4	4	4	2	2
4	4	3	3	4	4	4	4	2	2
4	4	3	3	4	4	1	1	3	3
4	4	3	3	4	4	1	1	2	2
4	4	3	3	4	4	1	1	2	2
4	4	3	3	1	1	4	4	3	3
4	4	3	3	4	4	4	4	3	3
4	4	2	2	1	1	4	4	2	2
4	4	3	3	4	4	4	4	2	2
4	4	3	3	4	4	4	4	2	2
4	4	3	3	4	4	4	4	3	3
4	4	3	3	1	1	1	1	2	2
4	4	3	3	4	4	4	4	2	2
4	4	3	3	1	1	1	1	3	3
4	4	2	2	4	4	4	4	2	2
4	4	3	3	4	4	4	4	2	2
4	4	3	3	4	4	4	4	3	3
4	4	3	3	4	4	1	1	3	3
4	4	3	3	4	4	4	4	2	2
4	4	3	3	4	4	1	1	3	3
4	4	3	3	4	4	4	4	2	2
4	4	3	3	4	4	1	1	3	3
4	4	3	3	1	1	1	1	3	3
4	4	3	3	4	4	4	4	3	3
1	1	2	2	4	4	4	4	3	3
4	4	3	3	4	4	1	1	2	2
4	4	3	3	4	4	1	1	3	3
4	4	3	3	4	4	4	4	3	3
1	1	2	2	4	4	4	4	3	3
4	4	3	3	4	4	1	1	3	3
4	4	3	3	4	4	4	4	3	3
4	4	3	3	4	4	1	1	2	2
4	4	3	3	4	4	1	1	3	3
4	4	3	3	1	1	1	1	2	2
1	1	2	2	4	4	4	4	3	3
4	4	3	3	4	4	4	4	2	2
4	4	3	3	1	1	1	1	3	3
4	4	3	3	4	4	4	4	2	2
4	4	3	3	4	4	1	1	3	3
4	4	3	3	4	4	4	4	2	2
1	1	2	2	4	4	4	4	3	3
4	4	3	3	1	1	4	4	3	3

For Review Only

4	4	3	3	4	4	4	4	3	3
1	1	2	2	4	4	4	4	2	2

For Review Only

SNP1872	SNP1872	SNP1873	SNP1873	SNP1874	SNP1874	SNP1875	SNP1875	SNP1876	SNP1876
1	1	2	2	2	2	1	1	4	4
2	2	2	2	2	2	4	4	4	4
2	2	1	1	2	2	1	1	4	4
2	2	2	2	2	2	1	1	4	4
1	1	2	2	2	2	1	1	4	4
2	2	1	1	2	2	1	1	1	1
2	2	2	2	2	2	4	4	4	4
2	2	2	2	2	2	4	4	4	4
1	1	1	1	2	2	1	1	4	4
2	2	2	2	2	2	4	4	1	1
1	1	2	2	2	2	4	4	4	4
2	2	1	1	2	2	1	1	4	4
1	1	2	2	2	2	1	1	4	4
2	2	1	1	1	1	1	1	4	4
1	1	2	2	2	2	1	1	4	4
1	1	1	1	1	1	1	1	4	4
2	2	2	2	2	2	1	1	1	1
1	1	1	1	2	2	1	1	4	4
2	2	2	2	2	2	1	1	4	4
2	2	1	1	1	1	4	4	4	4
1	1	2	2	2	2	4	4	4	4
2	2	2	2	1	1	1	1	4	4
2	2	2	2	1	1	4	4	4	4
2	2	2	2	1	1	4	4	4	4
2	2	1	1	2	2	1	1	4	4
2	2	1	1	2	2	1	1	4	4
1	1	1	1	2	2	1	1	4	4
1	1	2	2	2	2	1	1	4	4
1	1	1	1	2	2	4	4	4	4
2	2	2	2	1	1	4	4	4	4
2	2	2	2	2	2	1	1	4	4
2	2	2	2	2	2	1	1	4	4
2	2	2	2	2	2	1	1	4	4
2	2	2	2	1	1	4	4	1	1
1	1	2	2	2	2	1	1	4	4
2	2	1	1	1	1	4	4	4	4
2	2	2	2	2	2	4	4	4	4
1	1	2	2	2	2	1	1	4	4
1	1	2	2	1	1	4	4	4	4
1	1	2	2	2	2	4	4	4	4
1	1	2	2	1	1	4	4	4	4
1	1	1	1	2	2	1	1	4	4
1	1	1	1	1	1	4	4	4	4
2	2	2	2	1	1	4	4	1	1
1	1	1	1	1	1	4	4	4	4
1	1	1	1	2	2	1	1	4	4
2	2	1	1	2	2	1	1	4	4
2	2	2	2	2	2	1	1	4	4
2	2	2	2	1	1	4	4	1	1
2	2	2	2	2	2	1	1	4	4
2	2	1	1	2	2	1	1	1	1
1	1	2	2	2	2	1	1	1	1

For Review Only

2	2	1	1	2	2	1	1	4	4
2	2	2	2	2	2	1	1	4	4
1	1	1	1	2	2	1	1	1	1
2	2	2	2	1	1	4	4	4	4
2	2	2	2	2	2	1	1	4	4
2	2	1	1	2	2	1	1	4	4
2	2	1	1	2	2	1	1	4	4
2	2	2	2	1	1	4	4	4	4
2	2	2	2	1	1	4	4	4	4
2	2	2	2	2	2	1	1	4	4
2	2	2	2	2	2	1	1	4	4
2	2	2	2	2	2	1	1	4	4
2	2	1	1	2	2	1	1	4	4
1	1	1	1	2	2	4	4	4	4
1	1	2	2	2	2	1	1	4	4
2	2	2	2	2	2	1	1	1	1
2	2	1	1	2	2	1	1	4	4
2	2	2	2	2	2	1	1	4	4
2	2	2	2	2	2	1	1	4	4
2	2	2	2	2	2	1	1	4	4
2	2	2	2	2	2	1	1	4	4
2	2	2	2	2	2	4	4	4	4
2	2	2	2	2	2	4	4	4	4
2	2	2	2	1	1	1	1	4	4
1	1	1	1	1	1	4	4	4	4
1	1	2	2	2	2	1	1	4	4
2	2	2	2	1	1	1	1	1	1
1	1	2	2	1	1	4	4	4	4
2	2	1	1	2	2	1	1	4	4
2	2	2	2	2	2	4	4	4	4
2	2	2	2	2	2	1	1	1	1
2	2	2	2	1	1	4	4	4	4
2	2	2	2	1	1	4	4	4	4
2	2	1	1	2	2	1	1	4	4
2	2	2	2	1	1	4	4	4	4
1	1	1	1	1	1	4	4	4	4
2	2	2	2	2	2	4	4	4	4
2	2	2	2	1	1	4	4	4	4
2	2	2	2	1	1	1	1	4	4
2	2	1	1	1	1	4	4	4	4
2	2	2	2	2	2	1	1	4	4
2	2	2	2	2	2	4	4	4	4
2	2	2	2	2	2	1	1	4	4
2	2	2	2	2	2	1	1	4	4
2	2	2	2	1	1	1	1	4	4
2	2	2	2	2	2	1	1	4	4
1	1	2	2	1	1	4	4	4	4
2	2	1	1	2	2	1	1	4	4
2	2	1	1	2	2	1	1	4	4
1	1	2	2	1	1	4	4	1	1
2	2	1	1	2	2	1	1	4	4
1	1	2	2	1	1	4	4	4	4
2	2	2	2	1	1	4	4	4	4
2	2	2	2	1	1	1	1	4	4
1	1	2	2	1	1	4	4	4	4

For Review Only

2	2	2	2	2	2	1	1	4	4
2	2	2	2	2	2	4	4	4	4
2	2	1	1	2	2	1	1	4	4
1	1	1	1	1	1	4	4	4	4
2	2	1	1	2	2	1	1	4	4
2	2	1	1	2	2	1	1	4	4
2	2	1	1	2	2	1	1	4	4
2	2	2	2	1	1	1	1	4	4
2	2	2	2	2	2	1	1	4	4
2	2	1	1	1	1	4	4	4	4
2	2	2	2	1	1	1	1	4	4
1	1	2	2	2	2	1	1	4	4
2	2	2	2	2	2	1	1	4	4
1	1	2	2	2	2	1	1	4	4
2	2	2	2	2	2	1	1	4	4
2	2	2	2	2	2	1	1	4	4
2	2	2	2	2	2	4	4	4	4
1	1	2	2	2	2	1	1	4	4
1	1	2	2	1	1	1	1	4	4
2	2	2	2	2	2	1	1	4	4
2	2	2	2	2	2	1	1	4	4
2	2	1	1	2	2	1	1	4	4
2	2	2	2	2	2	1	1	4	4
2	2	2	2	1	1	4	4	4	4
2	2	2	2	2	2	1	1	4	4
2	2	2	2	2	2	4	4	4	4
2	2	2	2	2	2	1	1	4	4
2	2	2	2	2	2	4	4	4	4
2	2	2	2	2	2	1	1	4	4
1	1	2	2	1	1	4	4	4	4
1	1	2	2	1	1	4	4	4	4
2	2	2	2	2	2	1	1	4	4
2	2	2	2	2	2	1	1	4	4
2	2	2	2	2	2	1	1	4	4
2	2	2	2	1	1	4	4	1	1
1	1	2	2	2	2	1	1	4	4
1	1	2	2	1	1	4	4	4	4
2	2	2	2	2	2	1	1	4	4
2	2	2	2	2	2	1	1	4	4
1	1	2	2	2	2	4	4	4	4
1	1	2	2	2	2	4	4	4	4
1	1	2	2	1	1	4	4	4	4
2	2	2	2	1	1	1	1	4	4
2	2	2	2	1	1	4	4	4	4
2	2	1	1	2	2	1	1	4	4
2	2	2	2	2	2	1	1	4	4
2	2	1	1	2	2	1	1	4	4
2	2	1	1	2	2	1	1	4	4
2	2	2	2	1	1	4	4	4	4
2	2	1	1	2	2	1	1	4	4
2	2	1	1	2	2	1	1	4	4
2	2	2	2	2	2	4	4	4	4
2	2	2	2	2	2	1	1	4	4
2	2	2	2	2	2	1	1	4	4
2	2	2	2	2	2	1	1	4	4

For Review Only

2	2	2	2	2	2	4	4	4	4
2	2	2	2	2	2	4	4	4	4
2	2	1	1	1	1	1	1	4	4
1	1	2	2	2	2	4	4	4	4
2	2	1	1	2	2	1	1	4	4
2	2	2	2	1	1	1	1	4	4
1	1	2	2	2	2	4	4	4	4
2	2	2	2	2	2	1	1	4	4
2	2	2	2	2	2	1	1	4	4
1	1	2	2	2	2	4	4	4	4
2	2	1	1	2	2	1	1	4	4
1	1	2	2	1	1	1	1	4	4
2	2	2	2	2	2	1	1	4	4
1	1	2	2	2	2	1	1	4	4
1	1	2	2	1	1	4	4	4	4
1	1	2	2	2	2	4	4	4	4
2	2	2	2	2	2	1	1	4	4
2	2	1	1	2	2	1	1	4	4
2	2	1	1	2	2	1	1	4	4
2	2	1	1	2	2	1	1	4	4
1	1	2	2	2	2	1	1	4	4
2	2	2	2	1	1	4	4	4	4
2	2	2	2	1	1	4	4	4	4
2	2	2	2	2	2	1	1	4	4
2	2	1	1	2	2	1	1	4	4
2	2	2	2	1	1	4	4	1	1
2	2	2	2	2	2	1	1	1	1
2	2	1	1	2	2	4	4	4	4
2	2	2	2	2	2	4	4	4	4
2	2	2	2	2	2	4	4	4	4
2	2	1	1	2	2	4	4	4	4
2	2	2	2	2	2	4	4	4	4
2	2	2	2	2	2	1	1	4	4
1	1	2	2	1	1	4	4	4	4
2	2	2	2	1	1	4	4	4	4
2	2	2	2	2	2	1	1	4	4
2	2	2	2	2	2	4	4	4	4
2	2	2	2	2	2	4	4	4	4
2	2	1	1	2	2	1	1	4	4
2	2	1	1	2	2	1	1	4	4
2	2	1	1	2	2	1	1	4	4
2	2	1	1	2	2	1	1	4	4
1	1	2	2	1	1	4	4	4	4
1	1	2	2	2	2	1	1	4	4
1	1	1	1	2	2	1	1	4	4
2	2	2	2	2	2	1	1	4	4
1	1	2	2	2	2	4	4	4	4
2	2	2	2	2	2	4	4	4	4
2	2	2	2	1	1	4	4	4	4
2	2	2	2	2	2	1	1	4	4
1	1	2	2	2	2	1	1	4	4
2	2	2	2	1	1	4	4	4	4
2	2	2	2	1	1	4	4	4	4
2	2	2	2	2	2	1	1	4	4
1	1	2	2	1	1	4	4	4	4

For Review Only

1	1	2	2	2	2	4	4	4	4
2	2	2	2	1	1	4	4	4	4
2	2	2	2	2	2	1	1	4	4
2	2	2	2	2	2	1	1	1	1
1	1	1	1	2	2	4	4	4	4
2	2	1	1	2	2	1	1	4	4
1	1	1	1	2	2	4	4	4	4
2	2	2	2	2	2	4	4	1	1
1	1	2	2	1	1	4	4	4	4
1	1	2	2	1	1	4	4	4	4
1	1	2	2	2	2	1	1	4	4
2	2	2	2	2	2	4	4	4	4
2	2	2	2	1	1	1	1	4	4
2	2	2	2	2	2	4	4	4	4
2	2	1	1	2	2	1	1	4	4
1	1	2	2	1	1	1	1	4	4
2	2	2	2	1	1	1	1	4	4
2	2	1	1	1	1	4	4	4	4
2	2	2	2	2	2	4	4	4	4
1	1	2	2	2	2	1	1	4	4
1	1	2	2	2	2	4	4	4	4
1	1	1	1	2	2	1	1	4	4
2	2	1	1	2	2	1	1	4	4
1	1	2	2	2	2	1	1	4	4
2	2	2	2	1	1	4	4	4	4
1	1	1	1	2	2	1	1	4	4
1	1	2	2	1	1	4	4	4	4
2	2	1	1	2	2	1	1	4	4
2	2	1	1	2	2	1	1	4	4
2	2	2	2	2	2	1	1	1	1
1	1	2	2	1	1	4	4	4	4
2	2	1	1	2	2	1	1	4	4
1	1	2	2	1	1	4	4	4	4
2	2	2	2	2	2	4	4	4	4
2	2	2	2	1	1	4	4	4	4
2	2	2	2	1	1	4	4	4	4
2	2	2	2	2	2	1	1	4	4
2	2	2	2	2	2	1	1	4	4
2	2	1	1	1	1	4	4	4	4
1	1	2	2	1	1	4	4	4	4
2	2	2	2	2	2	4	4	4	4
2	2	2	2	2	2	4	4	4	4
1	1	2	2	1	1	4	4	4	4
2	2	2	2	2	2	1	1	4	4
2	2	1	1	1	1	1	1	4	4
2	2	2	2	1	1	1	1	4	4
1	1	1	1	1	1	1	1	4	4
2	2	2	2	2	2	4	4	4	4
2	2	1	1	1	1	1	1	4	4
1	1	2	2	1	1	4	4	4	4
2	2	2	2	2	2	1	1	4	4
1	1	2	2	1	1	4	4	4	4
2	2	1	1	2	2	1	1	4	4
2	2	2	2	2	2	1	1	4	4
1	1	2	2	2	2	4	4	4	4

For Review Only

1	1	2	2	2	2	1	1	4	4
2	2	1	1	2	2	1	1	4	4

For Review Only

SNP1877	SNP1877	SNP1878	SNP1878	SNP1879	SNP1879	SNP188	SNP188	SNP1880	SNP1880
2	2	4	4	1	1	2	2	2	2
3	3	4	4	1	1	2	2	1	1
2	2	3	3	1	1	4	4	2	2
3	3	4	4	1	1	2	2	2	2
3	3	4	4	1	1	2	2	2	2
2	2	4	4	1	1	2	2	2	2
3	3	3	3	1	1	2	2	1	1
3	3	4	4	1	1	2	2	2	2
2	2	3	3	1	1	2	2	2	2
2	2	4	4	1	1	2	2	2	2
3	3	3	3	1	1	2	2	1	1
2	2	3	3	1	1	4	4	2	2
3	3	4	4	1	1	2	2	2	2
2	2	4	4	4	4	2	2	2	2
3	3	3	3	1	1	2	2	2	2
2	2	4	4	4	4	2	2	2	2
3	3	3	3	1	1	2	2	2	2
2	2	4	4	1	1	2	2	2	2
3	3	4	4	1	1	2	2	2	2
2	2	4	4	1	1	2	2	1	1
3	3	3	3	1	1	2	2	1	1
3	3	4	4	1	1	2	2	2	2
3	3	4	4	1	1	2	2	1	1
3	3	4	4	4	4	2	2	2	2
2	2	4	4	1	1	2	2	2	2
2	2	4	4	1	1	2	2	2	2
2	2	4	4	1	1	2	2	2	2
3	3	4	4	1	1	2	2	2	2
2	2	3	3	1	1	2	2	1	1
3	3	4	4	1	1	2	2	1	1
3	3	4	4	1	1	4	4	2	2
3	3	3	3	1	1	2	2	2	2
3	3	3	3	1	1	2	2	2	2
3	3	4	4	1	1	2	2	2	2
3	3	3	3	1	1	2	2	2	2
2	2	4	4	4	4	4	4	2	2
3	3	4	4	1	1	2	2	2	2
3	3	4	4	1	1	2	2	2	2
3	3	4	4	1	1	4	4	2	2
3	3	3	3	1	1	2	2	2	2
3	3	4	4	1	1	4	4	2	2
2	2	4	4	1	1	2	2	1	1
2	2	4	4	1	1	2	2	2	2
3	3	3	3	1	1	2	2	1	1
2	2	4	4	1	1	2	2	2	2
2	2	4	4	1	1	4	4	2	2
2	2	4	4	1	1	2	2	2	2
3	3	3	3	1	1	2	2	2	2
3	3	3	3	4	4	2	2	1	1
3	3	3	3	1	1	2	2	2	2
2	2	4	4	1	1	2	2	2	2
3	3	3	3	1	1	2	2	2	2

For Review Only

2	2	3	3	1	1	2	2	2	2
3	3	3	3	1	1	2	2	2	2
2	2	4	4	1	1	4	4	2	2
3	3	3	3	1	1	2	2	1	1
3	3	4	4	1	1	2	2	2	2
2	2	4	4	1	1	4	4	2	2
2	2	4	4	1	1	2	2	2	2
3	3	4	4	1	1	2	2	1	1
3	3	4	4	4	4	2	2	2	2
3	3	3	3	1	1	2	2	2	2
3	3	4	4	1	1	2	2	2	2
3	3	4	4	1	1	4	4	2	2
2	2	3	3	1	1	2	2	2	2
2	2	3	3	1	1	2	2	1	1
3	3	3	3	1	1	2	2	2	2
3	3	4	4	1	1	2	2	2	2
2	2	3	3	1	1	2	2	2	2
3	3	3	3	1	1	2	2	2	2
3	3	3	3	1	1	2	2	2	2
3	3	3	3	1	1	2	2	2	2
3	3	4	4	1	1	2	2	2	2
3	3	3	3	1	1	2	2	1	1
3	3	3	3	1	1	2	2	1	1
3	3	3	3	1	1	4	4	2	2
2	2	4	4	1	1	2	2	2	2
3	3	4	4	1	1	2	2	2	2
3	3	3	3	1	1	2	2	1	1
3	3	4	4	1	1	2	2	1	1
2	2	4	4	1	1	2	2	2	2
3	3	3	3	1	1	2	2	2	2
3	3	4	4	1	1	2	2	2	2
3	3	4	4	4	4	2	2	2	2
3	3	4	4	1	1	2	2	1	1
2	2	3	3	1	1	2	2	2	2
3	3	4	4	1	1	2	2	2	2
2	2	4	4	1	1	2	2	2	2
3	3	4	4	1	1	2	2	2	2
3	3	3	3	1	1	2	2	1	1
3	3	4	4	1	1	2	2	2	2
2	2	4	4	1	1	2	2	2	2
3	3	3	3	1	1	2	2	2	2
3	3	3	3	1	1	4	4	2	2
3	3	4	4	1	1	2	2	2	2
3	3	4	4	1	1	2	2	2	2
3	3	3	3	4	4	2	2	2	2
3	3	4	4	1	1	2	2	2	2
3	3	4	4	1	1	2	2	2	2
2	2	4	4	1	1	4	4	2	2
2	2	4	4	1	1	4	4	2	2
3	3	4	4	1	1	2	2	1	1
3	3	4	4	1	1	2	2	2	2
3	3	3	3	1	1	2	2	2	2
3	3	4	4	1	1	2	2	1	1
3	3	3	3	1	1	2	2	1	1
3	3	4	4	4	4	2	2	2	2

For Review Only

3	3	3	3	1	1	2	2	2	2
3	3	4	4	1	1	2	2	1	1
2	2	4	4	1	1	2	2	2	2
2	2	4	4	1	1	2	2	2	2
2	2	4	4	1	1	2	2	2	2
2	2	4	4	1	1	4	4	2	2
2	2	4	4	1	1	2	2	2	2
3	3	4	4	4	4	2	2	1	1
3	3	4	4	1	1	2	2	2	2
3	3	3	3	4	4	2	2	1	1
3	3	3	3	4	4	2	2	2	2
3	3	3	3	1	1	2	2	2	2
3	3	3	3	1	1	4	4	2	2
3	3	4	4	1	1	2	2	2	2
3	3	4	4	1	1	2	2	1	1
3	3	3	3	1	1	2	2	2	2
3	3	3	3	1	1	2	2	1	1
2	2	3	3	1	1	2	2	2	2
3	3	4	4	1	1	4	4	2	2
3	3	4	4	1	1	2	2	2	2
3	3	3	3	1	1	2	2	2	2
3	3	3	3	1	1	2	2	2	2
3	3	3	3	1	1	4	4	2	2
3	3	4	4	1	1	2	2	1	1
3	3	3	3	1	1	2	2	1	1
3	3	3	3	1	1	2	2	1	1
3	3	3	3	1	1	2	2	1	1
3	3	4	4	1	1	2	2	2	2
3	3	3	3	1	1	2	2	2	2
3	3	3	3	4	4	2	2	2	2
3	3	4	4	1	1	2	2	1	1
3	3	3	3	1	1	2	2	1	1
3	3	3	3	1	1	2	2	2	2
3	3	4	4	1	1	2	2	2	2
3	3	4	4	1	1	2	2	2	2
3	3	4	4	1	1	2	2	2	2
3	3	4	4	1	1	2	2	2	2
3	3	3	3	1	1	2	2	2	2
3	3	3	3	1	1	2	2	2	2
3	3	4	4	1	1	2	2	1	1
3	3	3	3	1	1	2	2	1	1
3	3	4	4	1	1	4	4	2	2
3	3	3	3	4	4	2	2	2	2
3	3	4	4	1	1	2	2	1	1
3	3	4	4	1	1	2	2	2	2
3	3	4	4	1	1	2	2	2	2
2	2	4	4	1	1	2	2	2	2
2	2	3	3	1	1	4	4	2	2
3	3	4	4	1	1	2	2	1	1
2	2	3	3	1	1	2	2	2	2
2	2	4	4	1	1	2	2	2	2
3	3	3	3	1	1	2	2	2	2
3	3	4	4	1	1	2	2	2	2
3	3	4	4	1	1	2	2	2	2
3	3	3	3	1	1	2	2	1	1

For Review Only

3	3	3	3	1	1	2	2	2	2
3	3	4	4	1	1	2	2	1	1
2	2	4	4	1	1	2	2	1	1
3	3	4	4	1	1	2	2	1	1
3	3	4	4	1	1	2	2	2	2
3	3	4	4	1	1	2	2	2	2
3	3	3	3	1	1	2	2	1	1
3	3	3	3	1	1	2	2	2	2
3	3	3	3	1	1	2	2	2	2
3	3	3	3	1	1	2	2	2	2
3	3	3	3	1	1	2	2	2	2
3	3	4	4	4	4	2	2	2	2
3	3	4	4	1	1	2	2	2	2
3	3	4	4	1	1	2	2	2	2
3	3	4	4	1	1	2	2	2	2
3	3	3	3	1	1	4	4	1	1
3	3	4	4	1	1	2	2	2	2
3	3	4	4	1	1	2	2	1	1
2	2	4	4	1	1	4	4	2	2
2	2	4	4	1	1	2	2	2	2
3	3	3	3	1	1	2	2	2	2
3	3	4	4	1	1	2	2	1	1
3	3	3	3	1	1	2	2	1	1
3	3	4	4	1	1	4	4	2	2
2	2	3	3	1	1	2	2	2	2
3	3	4	4	1	1	2	2	2	2
3	3	4	4	1	1	4	4	2	2
2	2	3	3	1	1	2	2	1	1
3	3	4	4	1	1	2	2	2	2
3	3	3	3	1	1	2	2	2	2
3	3	4	4	4	4	4	4	2	2
3	3	3	3	1	1	2	2	2	2
3	3	3	3	1	1	2	2	1	1
3	3	4	4	1	1	2	2	2	2
3	3	3	3	1	1	2	2	2	2
3	3	4	4	1	1	4	4	2	2
3	3	4	4	1	1	4	4	2	2
3	3	3	3	1	1	2	2	2	2
2	2	3	3	1	1	4	4	2	2
2	2	3	3	1	1	2	2	2	2
2	2	3	3	1	1	4	4	2	2
2	2	4	4	1	1	2	2	2	2
3	3	4	4	1	1	4	4	1	1
3	3	3	3	1	1	2	2	2	2
2	2	4	4	1	1	2	2	2	2
3	3	4	4	1	1	2	2	2	2
3	3	3	3	1	1	4	4	1	1
3	3	3	3	1	1	2	2	1	1
3	3	4	4	1	1	2	2	2	2
3	3	4	4	1	1	2	2	1	1
2	2	3	3	1	1	2	2	2	2
3	3	4	4	1	1	2	2	1	1
3	3	4	4	1	1	2	2	1	1
3	3	4	4	1	1	2	2	2	2
3	3	4	4	1	1	2	2	1	1

For Review Only

3	3	4	4	1	1	2	2	2	2
3	3	3	3	4	4	4	4	2	2
3	3	3	3	1	1	2	2	2	2
3	3	3	3	1	1	2	2	1	1
2	2	4	4	1	1	2	2	1	1
2	2	3	3	1	1	2	2	2	2
2	2	4	4	1	1	2	2	1	1
3	3	4	4	1	1	2	2	1	1
3	3	4	4	1	1	4	4	2	2
3	3	4	4	1	1	2	2	1	1
3	3	3	3	1	1	2	2	2	2
2	2	4	4	1	1	2	2	1	1
3	3	4	4	1	1	2	2	2	2
3	3	4	4	1	1	2	2	2	2
2	2	3	3	1	1	4	4	2	2
3	3	4	4	1	1	2	2	2	2
3	3	4	4	1	1	4	4	2	2
2	2	3	3	1	1	2	2	1	1
3	3	4	4	1	1	2	2	1	1
3	3	4	4	1	1	2	2	2	2
3	3	4	4	1	1	4	4	2	2
2	2	4	4	1	1	2	2	2	2
2	2	3	3	1	1	2	2	2	2
3	3	3	3	1	1	2	2	2	2
3	3	4	4	1	1	2	2	1	1
2	2	3	3	1	1	2	2	2	2
3	3	4	4	1	1	4	4	1	1
2	2	4	4	1	1	2	2	2	2
2	2	3	3	1	1	2	2	2	2
3	3	3	3	1	1	2	2	2	2
3	3	4	4	1	1	4	4	1	1
2	2	3	3	1	1	2	2	2	2
3	3	4	4	1	1	2	2	2	2
2	2	3	3	1	1	2	2	2	2
3	3	3	3	1	1	2	2	1	1
3	3	4	4	1	1	2	2	1	1
3	3	3	3	1	1	2	2	2	2
3	3	4	4	1	1	4	4	2	2
2	2	4	4	4	4	4	4	2	2
3	3	3	3	1	1	4	4	2	2
3	3	3	3	1	1	4	4	2	2
3	3	4	4	1	1	2	2	2	2
3	3	3	3	4	4	2	2	2	2
3	3	3	3	1	1	2	2	2	2
2	2	3	3	1	1	2	2	2	2
3	3	4	4	1	1	2	2	2	2
2	2	3	3	1	1	4	4	2	2
3	3	4	4	1	1	4	4	2	2
2	2	4	4	1	1	2	2	2	2
3	3	4	4	1	1	4	4	1	1
3	3	3	3	1	1	2	2	2	2
3	3	3	3	1	1	4	4	2	2
2	2	3	3	1	1	4	4	2	2
3	3	4	4	1	1	4	4	2	2
3	3	3	3	1	1	2	2	2	2

For Review Only

3	3	3	3	1	1	2	2	2	2
2	2	4	4	1	1	4	4	2	2

For Review Only

SNP1881	SNP1881	SNP1882	SNP1882	SNP1883	SNP1883	SNP1884	SNP1884	SNP1885	SNP1885
4	4	1	1	2	2	3	3	3	3
4	4	2	2	4	4	3	3	3	3
4	4	1	1	2	2	2	2	3	3
4	4	1	1	2	2	2	2	3	3
4	4	1	1	2	2	3	3	1	1
4	4	1	1	2	2	2	2	1	1
4	4	2	2	4	4	3	3	3	3
4	4	1	1	2	2	2	2	1	1
4	4	1	1	2	2	3	3	3	3
4	4	1	1	2	2	2	2	1	1
4	4	2	2	4	4	3	3	3	3
4	4	1	1	2	2	2	2	3	3
4	4	2	2	2	2	3	3	3	3
1	1	1	1	2	2	3	3	1	1
4	4	1	1	2	2	3	3	3	3
4	4	1	1	2	2	2	2	1	1
4	4	1	1	2	2	3	3	3	3
4	4	2	2	2	2	3	3	1	1
4	4	1	1	2	2	3	3	3	3
4	4	2	2	4	4	2	2	1	1
4	4	2	2	4	4	3	3	3	3
4	4	1	1	2	2	3	3	1	1
4	4	2	2	4	4	3	3	1	1
4	4	1	1	2	2	2	2	1	1
4	4	1	1	2	2	2	2	3	3
4	4	1	1	2	2	2	2	1	1
4	4	1	1	2	2	2	2	3	3
4	4	1	1	2	2	3	3	3	3
4	4	2	2	4	4	2	2	3	3
4	4	2	2	4	4	3	3	1	1
4	4	1	1	2	2	2	2	3	3
4	4	1	1	2	2	3	3	3	3
4	4	1	1	2	2	3	3	3	3
4	4	1	1	2	2	3	3	1	1
4	4	1	1	2	2	3	3	3	3
4	4	1	1	2	2	2	2	1	1
4	4	1	1	2	2	3	3	1	1
4	4	1	1	2	2	3	3	1	1
4	4	1	1	2	2	3	3	1	1
4	4	1	1	4	4	3	3	3	3
4	4	1	1	2	2	2	2	1	1
4	4	2	2	4	4	2	2	3	3
4	4	1	1	2	2	2	2	1	1
4	4	2	2	4	4	3	3	3	3
4	4	1	1	2	2	2	2	1	1
4	4	1	1	2	2	2	2	3	3
4	4	1	1	2	2	3	3	3	3
4	4	1	1	2	2	2	2	3	3
4	4	2	2	4	4	3	3	3	3
4	4	1	1	2	2	3	3	3	3
4	4	1	1	2	2	3	3	1	1
4	4	2	2	2	2	3	3	3	3

For Review Only

4	4	1	1	2	2	3	3	3	3
4	4	1	1	2	2	3	3	3	3
4	4	2	2	2	2	3	3	3	3
4	4	2	2	4	4	3	3	3	3
4	4	1	1	2	2	3	3	3	3
4	4	1	1	2	2	3	3	3	3
4	4	1	1	2	2	3	3	1	1
4	4	2	2	4	4	3	3	1	1
4	4	1	1	2	2	3	3	1	1
4	4	2	2	4	4	3	3	3	3
4	4	1	1	2	2	2	2	3	3
4	4	1	1	2	2	2	2	3	3
4	4	1	1	2	2	3	3	3	3
4	4	2	2	4	4	2	2	3	3
4	4	1	1	2	2	3	3	3	3
4	4	2	2	2	2	3	3	3	3
4	4	1	1	4	4	3	3	1	1
4	4	1	1	2	2	3	3	3	3
4	4	1	1	2	2	3	3	3	3
4	4	1	1	2	2	3	3	3	3
4	4	1	1	2	2	2	2	3	3
4	4	2	2	4	4	2	2	3	3
4	4	2	2	4	4	3	3	3	3
4	4	1	1	2	2	2	2	3	3
4	4	1	1	2	2	2	2	1	1
4	4	1	1	2	2	2	2	3	3
4	4	2	2	4	4	3	3	3	3
4	4	2	2	4	4	3	3	1	1
4	4	1	1	2	2	3	3	1	1
4	4	1	1	2	2	3	3	3	3
4	4	1	1	2	2	3	3	1	1
4	4	1	1	2	2	2	2	1	1
4	4	2	2	4	4	3	3	1	1
4	4	1	1	2	2	3	3	3	3
4	4	1	1	2	2	2	2	1	1
4	4	1	1	2	2	2	2	1	1
4	4	1	1	2	2	3	3	1	1
4	4	2	2	4	4	3	3	3	3
4	4	1	1	2	2	2	2	1	1
4	4	1	1	2	2	2	2	1	1
4	4	1	1	2	2	3	3	3	3
4	4	1	1	2	2	2	2	3	3
4	4	1	1	2	2	3	3	1	1
4	4	1	1	2	2	2	2	3	3
4	4	1	1	2	2	3	3	3	3
4	4	1	1	2	2	3	3	3	3
4	4	1	1	2	2	3	3	1	1
4	4	1	1	2	2	3	3	1	1
4	4	1	1	2	2	3	3	1	1
4	4	2	2	4	4	3	3	3	3
4	4	1	1	4	4	2	2	3	3
4	4	1	1	2	2	3	3	3	3
1	1	2	2	4	4	3	3	1	1
4	4	1	1	4	4	3	3	3	3
4	4	1	1	2	2	3	3	3	3

For Review Only

4	4	1	1	2	2	3	3	3	3
4	4	2	2	4	4	2	2	3	3
4	4	1	1	2	2	2	2	1	1
4	4	1	1	2	2	3	3	1	1
4	4	1	1	2	2	3	3	3	3
4	4	1	1	2	2	2	2	1	1
4	4	1	1	2	2	2	2	3	3
4	4	1	1	2	2	3	3	3	3
4	4	1	1	2	2	3	3	1	1
4	4	2	2	2	2	2	2	3	3
4	4	2	2	2	2	3	3	3	3
4	4	2	2	4	4	3	3	3	3
4	4	1	1	2	2	3	3	1	1
4	4	2	2	2	2	2	2	3	3
4	4	2	2	4	4	2	2	3	3
4	4	1	1	2	2	3	3	3	3
1	1	2	2	4	4	3	3	3	3
4	4	1	1	4	4	3	3	3	3
4	4	1	1	2	2	3	3	1	1
4	4	2	2	2	2	3	3	1	1
4	4	2	2	4	4	3	3	3	3
4	4	1	1	2	2	3	3	3	3
4	4	1	1	2	2	2	2	3	3
1	1	2	2	4	4	2	2	1	1
4	4	2	2	4	4	2	2	3	3
4	4	2	2	4	4	2	2	3	3
4	4	2	2	2	2	3	3	3	3
4	4	1	1	2	2	3	3	1	1
4	4	1	1	2	2	3	3	3	3
4	4	1	1	2	2	3	3	3	3
1	1	2	2	4	4	3	3	1	1
4	4	2	2	4	4	3	3	3	3
4	4	1	1	2	2	3	3	3	3
4	4	1	1	2	2	3	3	3	3
4	4	1	1	2	2	3	3	1	1
4	4	1	1	2	2	3	3	3	3
1	1	1	1	2	2	3	3	1	1
4	4	1	1	2	2	3	3	3	3
4	4	2	2	4	4	3	3	3	3
4	4	2	2	4	4	3	3	1	1
4	4	2	2	4	4	3	3	3	3
4	4	1	1	2	2	3	3	1	1
4	4	1	1	2	2	3	3	3	3
4	4	2	2	4	4	3	3	1	1
4	4	1	1	2	2	3	3	1	1
4	4	1	1	2	2	3	3	1	1
4	4	1	1	2	2	3	3	1	1
4	4	1	1	2	2	2	2	3	3
4	4	2	2	2	2	3	3	1	1
4	4	1	1	2	2	2	2	3	3
4	4	1	1	2	2	3	3	1	1
1	1	1	1	2	2	3	3	3	3
4	4	1	1	2	2	3	3	1	1
4	4	1	1	2	2	2	2	3	3
4	4	2	2	4	4	3	3	3	3

For Review Only

4	4	1	1	2	2	3	3	3	3
4	4	2	2	4	4	3	3	1	1
4	4	2	2	4	4	3	3	1	1
4	4	2	2	4	4	2	2	3	3
4	4	1	1	2	2	3	3	3	3
4	4	1	1	2	2	3	3	1	1
4	4	2	2	4	4	2	2	3	3
4	4	1	1	2	2	3	3	3	3
4	4	1	1	4	4	3	3	1	1
4	4	1	1	2	2	3	3	3	3
4	4	1	1	2	2	3	3	3	3
4	4	1	1	2	2	3	3	3	3
4	4	1	1	2	2	3	3	3	3
4	4	1	1	2	2	2	2	1	1
4	4	1	1	2	2	3	3	1	1
4	4	2	2	4	4	3	3	3	3
4	4	1	1	2	2	3	3	1	1
4	4	2	2	4	4	2	2	3	3
4	4	1	1	2	2	3	3	1	1
4	4	1	1	2	2	2	2	3	3
4	4	1	1	2	2	3	3	3	3
4	4	2	2	4	4	2	2	1	1
4	4	2	2	4	4	2	2	3	3
4	4	1	1	2	2	2	2	3	3
4	4	2	2	2	2	2	2	3	3
4	4	1	1	2	2	2	2	1	1
4	4	1	1	2	2	2	2	3	3
4	4	2	2	4	4	3	3	3	3
4	4	1	1	2	2	3	3	1	1
4	4	1	1	2	2	3	3	3	3
4	4	1	1	2	2	2	2	1	1
4	4	1	1	2	2	3	3	3	3
4	4	2	2	2	2	3	3	3	3
1	1	1	1	2	2	3	3	1	1
4	4	1	1	2	2	3	3	3	3
4	4	1	1	2	2	2	2	3	3
4	4	2	2	4	4	2	2	3	3
4	4	1	1	2	2	3	3	3	3
4	4	1	1	2	2	2	2	3	3
4	4	1	1	2	2	2	2	3	3
4	4	1	1	2	2	2	2	3	3
4	4	1	1	2	2	3	3	1	1
4	4	2	2	4	4	2	2	1	1
4	4	1	1	2	2	3	3	3	3
4	4	2	2	2	2	3	3	1	1
4	4	1	1	2	2	3	3	3	3
4	4	2	2	4	4	3	3	3	3
4	4	2	2	4	4	2	2	3	3
4	4	1	1	2	2	2	2	1	1
4	4	2	2	4	4	3	3	3	3
4	4	1	1	2	2	3	3	3	3
1	1	2	2	4	4	3	3	1	1
4	4	2	2	4	4	3	3	1	1
4	4	1	1	2	2	2	2	3	3
4	4	2	2	4	4	3	3	3	3

For Review Only

4	4	1	1	2	2	3	3	1	1
4	4	1	1	2	2	3	3	1	1
4	4	1	1	2	2	3	3	3	3
4	4	2	2	4	4	3	3	3	3
4	4	2	2	4	4	3	3	1	1
4	4	1	1	2	2	3	3	3	3
4	4	2	2	4	4	3	3	1	1
4	4	2	2	4	4	2	2	1	1
4	4	1	1	2	2	2	2	1	1
1	1	2	2	4	4	3	3	1	1
4	4	1	1	2	2	3	3	3	3
4	4	2	2	4	4	3	3	1	1
4	4	1	1	2	2	3	3	1	1
4	4	1	1	2	2	2	2	1	1
4	4	1	1	2	2	2	2	3	3
4	4	1	1	2	2	3	3	1	1
4	4	1	1	2	2	3	3	1	1
4	4	2	2	4	4	3	3	3	3
4	4	2	2	4	4	3	3	1	1
4	4	1	1	2	2	3	3	1	1
4	4	1	1	2	2	3	3	3	3
4	4	1	1	2	2	3	3	1	1
4	4	1	1	2	2	3	3	3	3
4	4	1	1	2	2	3	3	3	3
4	4	2	2	4	4	3	3	1	1
4	4	1	1	2	2	2	2	3	3
1	1	2	2	4	4	2	2	1	1
4	4	1	1	2	2	2	2	3	3
4	4	1	1	2	2	3	3	3	3
4	4	1	1	2	2	3	3	3	3
4	4	2	2	4	4	3	3	1	1
4	4	1	1	2	2	2	2	3	3
1	1	1	1	2	2	2	2	1	1
4	4	1	1	2	2	3	3	3	3
4	4	2	2	4	4	3	3	3	3
4	4	2	2	4	4	2	2	1	1
4	4	1	1	2	2	3	3	3	3
4	4	1	1	2	2	2	2	3	3
4	4	1	1	2	2	2	2	1	1
4	4	1	1	2	2	3	3	3	3
4	4	1	1	2	2	3	3	3	3
4	4	1	1	2	2	2	2	3	3
4	4	1	1	2	2	2	2	3	3
4	4	1	1	2	2	3	3	3	3
4	4	1	1	2	2	2	2	3	3
4	4	1	1	2	2	2	2	1	1
4	4	1	1	2	2	2	2	3	3
4	4	1	1	2	2	3	3	3	3
4	4	1	1	2	2	2	2	3	3
4	4	2	2	4	4	2	2	1	1
4	4	1	1	2	2	3	3	3	3
4	4	1	1	2	2	3	3	3	3
4	4	1	1	2	2	2	2	3	3
4	4	1	1	2	2	2	2	3	3
4	4	1	1	2	2	2	2	3	3

For Review Only

4	4	1	1	2	2	3	3	3	3
4	4	1	1	2	2	2	2	3	3

For Review Only

SNP1886	SNP1886	SNP1887	SNP1887	SNP1888	SNP1888	SNP1889	SNP1889	SNP189	SNP189
4	4	2	2	2	2	2	2	1	1
4	4	2	2	2	2	3	3	1	1
4	4	2	2	2	2	2	2	1	1
4	4	2	2	2	2	3	3	1	1
4	4	2	2	2	2	3	3	2	2
4	4	2	2	2	2	3	3	1	1
4	4	2	2	2	2	2	2	2	2
3	3	2	2	1	1	3	3	2	2
4	4	2	2	2	2	2	2	1	1
3	3	2	2	2	2	3	3	2	2
4	4	3	3	2	2	3	3	2	2
4	4	2	2	2	2	2	2	1	1
4	4	2	2	2	2	3	3	1	1
4	4	2	2	2	2	3	3	2	2
4	4	2	2	2	2	3	3	1	1
4	4	2	2	2	2	3	3	2	2
4	4	2	2	2	2	2	2	2	2
4	4	2	2	2	2	3	3	2	2
4	4	2	2	2	2	3	3	1	1
3	3	2	2	1	1	3	3	2	2
4	4	2	2	2	2	2	2	2	2
4	4	2	2	2	2	3	3	1	1
3	3	2	2	1	1	3	3	2	2
3	3	2	2	1	1	3	3	2	2
4	4	2	2	2	2	3	3	1	1
4	4	2	2	2	2	3	3	1	1
4	4	2	2	1	1	3	3	1	1
4	4	2	2	2	2	3	3	1	1
4	4	2	2	2	2	2	2	1	1
3	3	2	2	1	1	3	3	2	2
4	4	2	2	2	2	3	3	1	1
4	4	2	2	2	2	2	2	1	1
4	4	2	2	2	2	2	2	1	1
3	3	2	2	1	1	3	3	1	1
4	4	3	3	2	2	3	3	1	1
4	4	2	2	2	2	3	3	2	2
4	4	2	2	2	2	3	3	2	2
4	4	2	2	2	2	3	3	1	1
3	3	2	2	1	1	3	3	2	2
4	4	3	3	2	2	3	3	1	1
3	3	2	2	1	1	3	3	1	1
3	3	2	2	1	1	3	3	2	2
4	4	2	2	2	2	3	3	1	1
3	3	2	2	1	1	2	2	1	1
3	3	2	2	1	1	3	3	2	2
4	4	2	2	2	2	3	3	1	1
4	4	2	2	2	2	3	3	1	1
4	4	3	3	2	2	3	3	1	1
4	4	2	2	2	2	2	2	2	2
4	4	2	2	2	2	3	3	1	1
4	4	2	2	2	2	3	3	2	2
3	3	2	2	1	1	3	3	1	1

For Review Only

4	4	2	2	2	2	2	2	1	1
4	4	2	2	2	2	2	2	1	1
4	4	2	2	2	2	3	3	2	2
4	4	2	2	2	2	2	2	1	1
4	4	2	2	2	2	3	3	1	1
4	4	2	2	2	2	3	3	1	1
4	4	2	2	2	2	3	3	1	1
3	3	2	2	1	1	3	3	2	2
4	4	2	2	2	2	3	3	2	2
4	4	2	2	2	2	3	3	1	1
4	4	2	2	2	2	3	3	1	1
4	4	2	2	2	2	3	3	1	1
4	4	2	2	2	2	2	2	1	1
4	4	2	2	2	2	2	2	1	1
4	4	2	2	2	2	3	3	1	1
4	4	2	2	2	2	3	3	1	1
4	4	2	2	2	2	2	2	2	2
4	4	2	2	2	2	2	2	1	1
4	4	2	2	2	2	2	2	1	1
4	4	2	2	2	2	2	2	1	1
4	4	2	2	2	2	3	3	1	1
4	4	3	3	2	2	3	3	1	1
4	4	2	2	2	2	2	2	1	1
4	4	2	2	2	2	2	2	1	1
3	3	2	2	1	1	3	3	2	2
4	4	2	2	2	2	3	3	2	2
4	4	2	2	2	2	2	2	1	1
3	3	2	2	1	1	3	3	2	2
4	4	2	2	2	2	3	3	1	1
4	4	2	2	2	2	3	3	1	1
4	4	2	2	2	2	3	3	2	2
3	3	2	2	1	1	3	3	1	1
3	3	2	2	1	1	3	3	2	2
4	4	2	2	2	2	2	2	1	1
4	4	2	2	2	2	3	3	1	1
3	3	2	2	1	1	3	3	2	2
4	4	2	2	1	1	3	3	2	2
3	3	2	2	1	1	2	2	2	2
4	4	2	2	2	2	3	3	1	1
4	4	2	2	2	2	3	3	2	2
4	4	2	2	2	2	2	2	1	1
4	4	2	2	2	2	3	3	1	1
3	3	2	2	2	2	3	3	2	2
4	4	2	2	2	2	3	3	1	1
4	4	2	2	2	2	2	2	1	1
4	4	2	2	2	2	3	3	1	1
3	3	2	2	1	1	3	3	1	1
4	4	2	2	2	2	3	3	2	2
4	4	2	2	2	2	3	3	1	1
4	4	2	2	2	2	3	3	1	1
4	4	2	2	2	2	3	3	1	1
3	3	3	3	1	1	3	3	2	2
3	3	2	2	1	1	3	3	2	2
4	4	2	2	1	1	3	3	1	1
4	4	2	2	2	2	3	3	1	1

For Review Only

4	4	2	2	2	2	3	3	1	1
4	4	2	2	2	2	3	3	1	1
4	4	2	2	2	2	3	3	1	1
4	4	2	2	2	2	3	3	1	1
4	4	2	2	2	2	3	3	1	1
4	4	2	2	2	2	3	3	1	1
4	4	2	2	2	2	3	3	1	1
4	4	2	2	2	2	3	3	2	2
4	4	2	2	2	2	3	3	1	1
4	4	2	2	2	2	3	3	1	1
3	3	2	2	1	1	2	2	2	2
4	4	2	2	2	2	3	3	1	1
3	3	2	2	1	1	3	3	2	2
4	4	2	2	2	2	3	3	1	1
4	4	2	2	2	2	3	3	2	2
4	4	2	2	2	2	3	3	1	1
4	4	2	2	2	2	2	2	1	1
4	4	2	2	2	2	3	3	1	1
3	3	2	2	1	1	3	3	1	1
4	4	2	2	2	2	3	3	1	1
4	4	2	2	2	2	3	3	1	1
4	4	2	2	2	2	3	3	1	1
4	4	2	2	2	2	2	2	1	1
3	3	2	2	1	1	3	3	2	2
4	4	2	2	2	2	3	3	1	1
4	4	2	2	2	2	3	3	2	2
4	4	2	2	2	2	2	2	1	1
4	4	2	2	2	2	3	3	2	2
4	4	2	2	2	2	3	3	1	1
4	4	2	2	2	2	2	2	1	1
3	3	2	2	1	1	3	3	1	1
4	4	2	2	2	2	3	3	1	1
4	4	2	2	2	2	2	2	1	1
4	4	2	2	2	2	3	3	1	1
3	3	2	2	1	1	3	3	1	1
4	4	2	2	1	1	3	3	1	1
3	3	2	2	1	1	3	3	2	2
4	4	2	2	2	2	2	2	1	1
4	4	2	2	2	2	3	3	1	1
3	3	2	2	1	1	3	3	2	2
4	4	2	2	2	2	3	3	1	1
3	3	2	2	1	1	3	3	2	2
4	4	2	2	2	2	2	2	1	1
4	4	2	2	2	2	3	3	1	1
4	4	2	2	2	2	3	3	2	2
4	4	2	2	2	2	3	3	1	1
4	4	2	2	2	2	3	3	1	1
4	4	2	2	2	2	3	3	1	1
4	4	2	2	2	2	3	3	1	1
4	4	2	2	2	2	3	3	1	1
4	4	2	2	2	2	3	3	1	1
4	4	3	3	2	2	3	3	2	2
4	4	2	2	2	2	3	3	1	1
4	4	2	2	2	2	3	3	1	1
4	4	2	2	2	2	3	3	1	1

For Review Only

4	4	2	2	2	2	3	3	1	1
4	4	2	2	2	2	3	3	2	2
3	3	2	2	1	1	3	3	1	1
4	4	2	2	2	2	3	3	2	2
4	4	2	2	2	2	3	3	1	1
4	4	2	2	2	2	3	3	1	1
4	4	3	3	2	2	3	3	1	1
4	4	2	2	2	2	3	3	1	1
4	4	2	2	2	2	3	3	2	2
4	4	2	2	2	2	3	3	1	1
4	4	2	2	2	2	3	3	1	1
4	4	2	2	2	2	3	3	1	1
4	4	2	2	2	2	3	3	1	1
4	4	2	2	2	2	3	3	1	1
3	3	2	2	1	1	3	3	1	1
4	4	3	3	2	2	3	3	2	2
4	4	2	2	2	2	3	3	2	2
4	4	2	2	2	2	3	3	1	1
4	4	2	2	2	2	3	3	1	1
4	4	2	2	2	2	3	3	1	1
4	4	2	2	2	2	3	3	1	1
3	3	2	2	1	1	3	3	2	2
4	4	2	2	2	2	3	3	2	2
4	4	2	2	2	2	3	3	1	1
3	3	2	2	2	2	2	2	1	1
3	3	2	2	1	1	3	3	1	1
4	4	2	2	2	2	3	3	1	1
4	4	2	2	2	2	2	2	1	1
3	3	2	2	1	1	3	3	1	1
4	4	2	2	2	2	2	2	1	1
4	4	2	2	2	2	3	3	2	2
4	4	2	2	2	2	3	3	1	1
3	3	2	2	1	1	2	2	2	2
3	3	2	2	1	1	3	3	1	1
4	4	2	2	2	2	2	2	1	1
4	4	2	2	2	2	3	3	1	1
4	4	2	2	2	2	3	3	1	1
4	4	2	2	2	2	2	2	1	1
4	4	2	2	2	2	3	3	1	1
4	4	2	2	2	2	2	2	1	1
4	4	2	2	2	2	2	2	1	1
4	4	2	2	2	2	3	3	1	1
3	3	2	2	1	1	3	3	2	2
4	4	2	2	2	2	3	3	1	1
4	4	2	2	2	2	3	3	1	1
3	3	2	2	1	1	3	3	1	1
4	4	3	3	2	2	3	3	2	2
3	3	3	3	1	1	3	3	2	2
3	3	2	2	1	1	3	3	1	1
4	4	2	2	2	2	3	3	1	1
4	4	2	2	2	2	3	3	1	1
3	3	2	2	1	1	3	3	2	2
4	4	2	2	2	2	3	3	2	2
4	4	2	2	2	2	3	3	1	1
3	3	2	2	1	1	3	3	2	2

For Review Only

4	4	2	2	2	2	3	3	1	1
4	4	2	2	2	2	3	3	1	1
4	4	2	2	2	2	3	3	1	1
4	4	3	3	2	2	3	3	1	1
3	3	2	2	1	1	3	3	1	1
4	4	2	2	2	2	2	2	1	1
4	4	2	2	2	2	3	3	2	2
3	3	2	2	1	1	3	3	2	2
4	4	2	2	2	2	3	3	1	1
3	3	2	2	1	1	3	3	2	2
4	4	2	2	2	2	3	3	1	1
4	4	2	2	2	2	3	3	2	2
4	4	2	2	2	2	3	3	1	1
4	4	2	2	2	2	3	3	1	1
4	4	2	2	2	2	2	2	1	1
4	4	2	2	2	2	3	3	1	1
4	4	2	2	1	1	3	3	2	2
4	4	2	2	2	2	2	2	2	2
3	3	2	2	1	1	3	3	1	1
4	4	2	2	2	2	3	3	1	1
3	3	2	2	1	1	3	3	2	2
4	4	2	2	2	2	3	3	1	1
4	4	2	2	2	2	2	2	1	1
4	4	3	3	2	2	3	3	1	1
3	3	2	2	1	1	3	3	2	2
4	4	2	2	2	2	3	3	1	1
3	3	2	2	1	1	3	3	2	2
4	4	2	2	2	2	3	3	1	1
4	4	2	2	2	2	3	3	1	1
4	4	2	2	2	2	3	3	1	1
4	4	2	2	2	2	3	3	2	2
4	4	3	3	2	2	3	3	2	2
4	4	2	2	2	2	3	3	1	1
4	4	2	2	2	2	3	3	1	1
4	4	3	3	2	2	3	3	2	2
3	3	2	2	1	1	3	3	2	2
4	4	2	2	2	2	2	2	1	1
4	4	2	2	2	2	3	3	1	1
4	4	2	2	2	2	3	3	2	2
3	3	3	3	1	1	3	3	2	2
4	4	2	2	2	2	2	2	2	2
3	3	2	2	1	1	3	3	1	1
4	4	3	3	2	2	3	3	1	1
4	4	2	2	2	2	2	2	1	1
4	4	2	2	2	2	3	3	1	1
4	4	2	2	2	2	3	3	1	1
4	4	3	3	2	2	3	3	1	1
4	4	2	2	2	2	3	3	1	1
4	4	2	2	2	2	3	3	1	1
3	3	2	2	1	1	3	3	2	2
4	4	2	2	2	2	2	2	1	1
3	3	3	3	1	1	3	3	2	2
4	4	2	2	2	2	2	2	1	1
4	4	2	2	2	2	3	3	2	2
4	4	3	3	2	2	3	3	1	1

For Review Only

4	4	2	2	2	2	2	2	1	1
4	4	2	2	2	2	3	3	1	1

For Review Only

SNP1890	SNP1890	SNP1891	SNP1891	SNP1892	SNP1892	SNP1893	SNP1893	SNP1894	SNP1894
1	1	2	2	2	2	3	3	1	1
1	1	2	2	2	2	3	3	1	1
1	1	2	2	2	2	2	2	4	4
1	1	2	2	2	2	3	3	1	1
1	1	2	2	2	2	3	3	1	1
1	1	2	2	2	2	3	3	1	1
1	1	2	2	2	2	2	2	1	1
4	4	2	2	3	3	3	3	1	1
1	1	2	2	2	2	2	2	1	1
1	1	4	4	3	3	3	3	1	1
1	1	2	2	2	2	3	3	1	1
1	1	2	2	2	2	2	2	4	4
1	1	2	2	2	2	3	3	1	1
1	1	2	2	2	2	3	3	1	1
1	1	2	2	2	2	3	3	1	1
1	1	2	2	2	2	3	3	1	1
1	1	2	2	2	2	2	2	1	1
1	1	2	2	2	2	3	3	1	1
1	1	2	2	2	2	3	3	1	1
4	4	4	4	3	3	3	3	1	1
1	1	2	2	2	2	2	2	1	1
1	1	2	2	2	2	3	3	1	1
4	4	2	2	3	3	3	3	1	1
4	4	2	2	3	3	3	3	1	1
1	1	2	2	2	2	3	3	1	1
1	1	2	2	2	2	3	3	1	1
1	1	2	2	3	3	3	3	1	1
1	1	2	2	2	2	3	3	1	1
1	1	2	2	2	2	2	2	1	1
4	4	2	2	3	3	3	3	1	1
1	1	2	2	2	2	3	3	1	1
1	1	2	2	2	2	2	2	1	1
1	1	2	2	2	2	2	2	1	1
4	4	2	2	3	3	3	3	1	1
1	1	2	2	2	2	3	3	1	1
1	1	2	2	2	2	3	3	1	1
1	1	2	2	2	2	3	3	1	1
1	1	2	2	3	3	3	3	1	1
4	4	2	2	2	2	3	3	1	1
1	1	2	2	2	2	3	3	1	1
4	4	2	2	3	3	3	3	1	1
1	1	2	2	3	3	3	3	1	1
1	1	4	4	2	2	3	3	1	1
1	1	4	4	2	2	2	2	1	1
4	4	4	4	3	3	3	3	1	1
1	1	2	2	2	2	3	3	1	1
1	1	2	2	2	2	3	3	1	1
1	1	2	2	2	2	3	3	1	1
1	1	4	4	2	2	2	2	1	1
1	1	2	2	2	2	2	2	1	1
1	1	2	2	2	2	3	3	1	1
4	4	2	2	3	3	2	2	1	1

For Review Only

1	1	2	2	2	2	2	2	1	1
1	1	2	2	2	2	2	2	1	1
1	1	2	2	3	3	3	3	1	1
1	1	2	2	2	2	2	2	1	1
1	1	2	2	2	2	3	3	1	1
1	1	2	2	2	2	3	3	1	1
1	1	2	2	2	2	3	3	1	1
4	4	2	2	3	3	3	3	1	1
1	1	2	2	2	2	3	3	1	1
1	1	2	2	2	2	3	3	1	1
1	1	2	2	2	2	3	3	1	1
1	1	2	2	2	2	3	3	4	4
1	1	2	2	2	2	2	2	1	1
1	1	2	2	2	2	2	2	1	1
1	1	2	2	2	2	3	3	1	1
1	1	4	4	2	2	3	3	1	1
1	1	2	2	2	2	3	3	1	1
1	1	2	2	2	2	2	2	1	1
1	1	2	2	2	2	2	2	1	1
1	1	2	2	2	2	2	2	1	1
1	1	2	2	2	2	3	3	4	4
1	1	2	2	2	2	3	3	1	1
1	1	2	2	2	2	3	3	1	1
1	1	2	2	2	2	2	2	1	1
4	4	4	4	3	3	3	3	1	1
1	1	2	2	2	2	3	3	1	1
1	1	2	2	2	2	2	2	1	1
4	4	2	2	3	3	3	3	1	1
1	1	2	2	2	2	3	3	1	1
1	1	2	2	2	2	3	3	1	1
1	1	4	4	2	2	3	3	1	1
1	1	2	2	3	3	3	3	1	1
4	4	2	2	3	3	3	3	4	4
1	1	2	2	2	2	2	2	1	1
1	1	2	2	2	2	3	3	1	1
4	4	4	4	3	3	3	3	1	1
1	1	2	2	3	3	3	3	1	1
4	4	2	2	3	3	2	2	1	1
1	1	2	2	2	2	3	3	1	1
1	1	2	2	2	2	3	3	1	1
1	1	2	2	2	2	2	2	1	1
1	1	2	2	2	2	3	3	1	1
4	4	2	2	3	3	3	3	1	1
1	1	2	2	2	2	3	3	1	1
1	1	2	2	2	2	2	2	1	1
1	1	2	2	2	2	3	3	1	1
4	4	2	2	3	3	3	3	1	1
1	1	2	2	3	3	3	3	1	1
1	1	2	2	2	2	3	3	4	4
1	1	2	2	2	2	3	3	1	1
1	1	2	2	2	2	3	3	1	1
4	4	2	2	3	3	3	3	1	1
4	4	2	2	3	3	3	3	1	1
1	1	2	2	2	2	3	3	1	1
1	1	2	2	2	2	3	3	1	1

For Review Only

1	1	2	2	2	2	3	3	1	1
1	1	2	2	2	2	3	3	1	1
1	1	2	2	2	2	3	3	1	1
1	1	2	2	2	2	3	3	4	4
1	1	2	2	2	2	3	3	1	1
1	1	2	2	2	2	3	3	1	1
1	1	2	2	2	2	3	3	1	1
1	1	2	2	3	3	3	3	1	1
1	1	2	2	2	2	3	3	1	1
1	1	2	2	2	2	2	2	1	1
1	1	2	2	2	2	2	2	1	1
1	1	2	2	2	2	3	3	1	1
1	1	2	2	3	3	3	3	1	1
1	1	2	2	2	2	3	3	1	1
1	1	2	2	2	2	3	3	1	1
1	1	2	2	2	2	3	3	1	1
1	1	2	2	2	2	2	2	1	1
1	1	2	2	2	2	3	3	1	1
4	4	2	2	3	3	3	3	1	1
1	1	2	2	2	2	3	3	1	1
1	1	2	2	2	2	3	3	1	1
1	1	2	2	2	2	3	3	1	1
1	1	2	2	2	2	3	3	1	1
4	4	2	2	3	3	3	3	4	4
1	1	2	2	2	2	3	3	1	1
1	1	2	2	2	2	3	3	1	1
1	1	2	2	2	2	2	2	1	1
1	1	2	2	2	2	3	3	1	1
1	1	2	2	2	2	3	3	1	1
1	1	2	2	2	2	2	2	4	4
4	4	2	2	3	3	3	3	1	1
1	1	2	2	2	2	3	3	1	1
1	1	2	2	2	2	2	2	1	1
1	1	2	2	2	2	3	3	1	1
4	4	2	2	3	3	3	3	1	1
4	4	2	2	3	3	3	3	1	1
1	1	2	2	3	3	3	3	1	1
1	1	2	2	2	2	2	2	1	1
1	1	2	2	2	2	3	3	1	1
4	4	2	2	3	3	3	3	1	1
1	1	2	2	2	2	3	3	1	1
4	4	2	2	3	3	3	3	1	1
1	1	2	2	2	2	2	2	1	1
4	4	2	2	2	2	3	3	1	1
4	4	2	2	2	2	3	3	4	4
1	1	2	2	2	2	3	3	1	1
1	1	2	2	2	2	3	3	1	1
1	1	2	2	2	2	3	3	1	1
1	1	2	2	3	3	3	3	1	1
1	1	2	2	2	2	3	3	1	1
1	1	2	2	2	2	3	3	1	1
1	1	2	2	2	2	3	3	1	1
1	1	2	2	2	2	3	3	1	1
1	1	2	2	2	2	3	3	1	1
1	1	2	2	2	2	3	3	1	1

For Review Only

1	1	2	2	2	2	3	3	1	1
1	1	2	2	2	2	3	3	1	1
4	4	2	2	3	3	3	3	1	1
1	1	2	2	2	2	3	3	1	1
1	1	2	2	2	2	3	3	1	1
1	1	2	2	2	2	3	3	1	1
1	1	2	2	3	3	3	3	1	1
1	1	2	2	2	2	3	3	1	1
4	4	2	2	3	3	3	3	1	1
1	1	2	2	2	2	3	3	1	1
1	1	2	2	2	2	3	3	1	1
1	1	2	2	2	2	3	3	1	1
1	1	2	2	2	2	3	3	1	1
1	1	2	2	2	2	3	3	1	1
4	4	2	2	3	3	3	3	1	1
1	1	2	2	2	2	3	3	1	1
1	1	2	2	2	2	3	3	1	1
1	1	2	2	2	2	3	3	1	1
1	1	2	2	2	2	3	3	1	1
1	1	2	2	2	2	3	3	1	1
1	1	2	2	2	2	3	3	1	1
4	4	2	2	3	3	3	3	1	1
1	1	2	2	2	2	3	3	1	1
1	1	2	2	2	2	3	3	4	4
1	1	2	2	2	2	2	2	1	1
4	4	2	2	3	3	3	3	1	1
1	1	2	2	2	2	3	3	1	1
1	1	2	2	2	2	2	2	1	1
4	4	2	2	3	3	3	3	1	1
1	1	2	2	2	2	2	2	1	1
1	1	2	2	2	2	3	3	1	1
1	1	2	2	2	2	3	3	1	1
1	1	2	2	3	3	2	2	1	1
4	4	2	2	3	3	3	3	1	1
1	1	2	2	2	2	2	2	1	1
1	1	2	2	2	2	3	3	4	4
1	1	2	2	2	2	3	3	1	1
1	1	2	2	2	2	2	2	1	1
1	1	2	2	2	2	3	3	1	1
1	1	2	2	2	2	2	2	1	1
1	1	2	2	2	2	2	2	4	4
1	1	2	2	2	2	3	3	1	1
4	4	2	2	3	3	3	3	1	1
1	1	2	2	2	2	3	3	1	1
1	1	2	2	2	2	3	3	1	1
4	4	2	2	3	3	3	3	1	1
1	1	2	2	2	2	3	3	1	1
4	4	2	2	3	3	3	3	1	1
4	4	2	2	3	3	3	3	1	1
1	1	2	2	2	2	3	3	1	1
1	1	2	2	2	2	3	3	1	1
4	4	2	2	3	3	3	3	1	1
1	1	2	2	2	2	3	3	1	1
1	1	2	2	2	2	3	3	1	1
4	4	2	2	3	3	3	3	4	4

For Review Only

1	1	2	2	2	2	3	3	1	1
1	1	4	4	3	3	2	2	1	1
1	1	2	2	2	2	3	3	1	1
1	1	2	2	2	2	3	3	1	1
4	4	4	4	3	3	3	3	1	1
1	1	2	2	2	2	3	3	1	1
1	1	2	2	2	2	3	3	1	1
4	4	4	4	3	3	3	3	1	1
1	1	2	2	2	2	3	3	1	1
4	4	2	2	3	3	3	3	1	1
1	1	2	2	2	2	3	3	1	1
1	1	2	2	2	2	3	3	1	1
1	1	2	2	2	2	3	3	1	1
1	1	2	2	2	2	3	3	1	1
1	1	2	2	2	2	2	2	1	1
1	1	2	2	2	2	3	3	1	1
4	4	2	2	3	3	3	3	1	1
1	1	2	2	2	2	2	2	1	1
4	4	2	2	3	3	3	3	1	1
1	1	2	2	2	2	3	3	1	1
4	4	2	2	3	3	3	3	1	1
1	1	2	2	2	2	3	3	1	1
1	1	2	2	2	2	2	2	1	1
1	1	2	2	2	2	3	3	1	1
4	4	2	2	3	3	3	3	1	1
1	1	2	2	2	2	3	3	1	1
4	4	2	2	3	3	3	3	4	4
1	1	2	2	2	2	3	3	1	1
1	1	4	4	2	2	3	3	1	1
1	1	2	2	2	2	3	3	1	1
1	1	2	2	2	2	3	3	1	1
1	1	2	2	2	2	3	3	1	1
1	1	2	2	2	2	3	3	1	1
1	1	2	2	2	2	3	3	1	1
1	1	2	2	2	2	3	3	1	1
4	4	2	2	3	3	3	3	1	1
1	1	2	2	2	2	2	2	1	1
1	1	2	2	2	2	3	3	4	4
1	1	2	2	2	2	3	3	1	1
4	4	2	2	3	3	3	3	1	1
1	1	2	2	2	2	2	2	1	1
4	4	2	2	3	3	3	3	1	1
1	1	2	2	2	2	3	3	1	1
1	1	2	2	2	2	2	2	1	1
1	1	2	2	2	2	3	3	1	1
1	1	2	2	2	2	3	3	1	1
1	1	2	2	2	2	3	3	1	1
1	1	2	2	2	2	3	3	1	1
1	1	2	2	2	2	3	3	1	1
4	4	2	2	3	3	3	3	4	4
1	1	2	2	2	2	2	2	1	1
4	4	2	2	3	3	3	3	1	1
1	1	2	2	2	2	2	2	4	4
1	1	2	2	2	2	3	3	1	1
1	1	2	2	2	2	3	3	1	1

For Review Only

1	1	2	2	2	2	2	2	1	1
1	1	2	2	2	2	3	3	1	1

For Review Only

SNP1895	SNP1895	SNP1896	SNP1896	SNP1897	SNP1897	SNP1898	SNP1898	SNP1899	SNP1899
1	1	2	2	4	4	2	2	1	1
1	1	3	3	4	4	2	2	1	1
4	4	2	2	4	4	2	2	1	1
4	4	2	2	4	4	2	2	1	1
1	1	3	3	4	4	2	2	1	1
1	1	2	2	4	4	1	1	1	1
1	1	3	3	4	4	2	2	1	1
4	4	2	2	4	4	2	2	1	1
1	1	2	2	4	4	2	2	1	1
4	4	2	2	4	4	2	2	1	1
1	1	3	3	4	4	2	2	1	1
4	4	2	2	4	4	2	2	1	1
1	1	2	2	4	4	2	2	1	1
1	1	2	2	1	1	2	2	1	1
1	1	2	2	4	4	2	2	1	1
4	4	2	2	1	1	2	2	1	1
1	1	3	3	4	4	2	2	1	1
1	1	2	2	4	4	2	2	1	1
1	1	2	2	4	4	2	2	1	1
4	4	2	2	1	1	2	2	1	1
1	1	3	3	4	4	2	2	1	1
1	1	3	3	4	4	2	2	1	1
4	4	2	2	1	1	2	2	1	1
4	4	2	2	4	4	2	2	1	1
1	1	2	2	4	4	2	2	1	1
1	1	2	2	4	4	2	2	1	1
1	1	2	2	4	4	2	2	1	1
1	1	2	2	4	4	2	2	1	1
1	1	2	2	4	4	2	2	1	1
4	4	2	2	1	1	2	2	1	1
1	1	2	2	4	4	2	2	1	1
1	1	3	3	4	4	1	1	1	1
1	1	3	3	4	4	2	2	1	1
4	4	2	2	1	1	2	2	1	1
1	1	3	3	4	4	2	2	1	1
4	4	2	2	1	1	2	2	1	1
1	1	3	3	4	4	2	2	1	1
1	1	2	2	4	4	2	2	1	1
4	4	2	2	4	4	2	2	1	1
1	1	2	2	4	4	2	2	1	1
4	4	2	2	1	1	2	2	1	1
4	4	2	2	4	4	2	2	1	1
1	1	2	2	1	1	2	2	1	1
4	4	2	2	1	1	2	2	1	1
4	4	2	2	1	1	2	2	1	1
4	4	2	2	4	4	2	2	1	1
1	1	2	2	4	4	2	2	1	1
1	1	3	3	4	4	2	2	1	1
1	1	3	3	1	1	2	2	1	1
1	1	2	2	4	4	1	1	1	1
1	1	2	2	4	4	2	2	1	1
4	4	2	2	4	4	1	1	1	1

For Review Only

1	1	2	2	4	4	2	2	1	1
1	1	3	3	4	4	2	2	1	1
4	4	2	2	4	4	2	2	1	1
1	1	3	3	1	1	2	2	1	1
1	1	2	2	4	4	2	2	1	1
1	1	2	2	4	4	2	2	1	1
1	1	2	2	4	4	2	2	1	1
4	4	2	2	1	1	2	2	1	1
1	1	3	3	1	1	2	2	1	1
1	1	2	2	4	4	2	2	1	1
4	4	2	2	4	4	2	2	1	1
4	4	2	2	4	4	2	2	4	4
1	1	2	2	4	4	2	2	1	1
1	1	2	2	4	4	2	2	1	1
1	1	2	2	4	4	2	2	1	1
1	1	2	2	4	4	2	2	1	1
1	1	2	2	4	4	2	2	1	1
1	1	3	3	4	4	1	1	1	1
1	1	3	3	4	4	2	2	1	1
1	1	3	3	4	4	1	1	1	1
1	1	2	2	4	4	2	2	4	4
1	1	3	3	4	4	2	2	1	1
1	1	2	2	4	4	2	2	1	1
1	1	3	3	1	1	2	2	1	1
4	4	2	2	1	1	2	2	1	1
1	1	2	2	4	4	2	2	1	1
1	1	3	3	1	1	2	2	1	1
4	4	2	2	4	4	2	2	1	1
1	1	2	2	4	4	2	2	1	1
1	1	3	3	4	4	2	2	1	1
1	1	2	2	4	4	2	2	1	1
4	4	2	2	4	4	2	2	1	1
4	4	2	2	1	1	2	2	1	1
1	1	2	2	4	4	2	2	1	1
1	1	2	2	1	1	2	2	1	1
4	4	2	2	1	1	2	2	1	1
4	4	2	2	1	1	2	2	1	1
4	4	2	2	1	1	2	2	1	1
1	1	3	3	4	4	2	2	1	1
1	1	2	2	1	1	2	2	1	1
1	1	3	3	4	4	2	2	1	1
1	1	3	3	4	4	2	2	1	1
4	4	2	2	4	4	2	2	1	1
4	4	2	2	4	4	2	2	1	1
1	1	3	3	4	4	2	2	1	1
1	1	2	2	4	4	2	2	1	1
4	4	2	2	1	1	2	2	1	1
4	4	2	2	4	4	2	2	1	1
1	1	2	2	4	4	2	2	4	4
1	1	2	2	1	1	2	2	1	1
1	1	2	2	4	4	2	2	1	1
4	4	2	2	4	4	2	2	1	1
4	4	2	2	1	1	2	2	1	1
1	1	2	2	4	4	2	2	1	1
1	1	2	2	4	4	2	2	1	1

For Review Only

1	1	2	2	4	4	2	2	1	1
1	1	2	2	4	4	2	2	1	1
1	1	2	2	4	4	2	2	1	1
4	4	2	2	1	1	2	2	4	4
1	1	2	2	4	4	2	2	1	1
1	1	2	2	4	4	2	2	1	1
4	4	2	2	4	4	2	2	1	1
1	1	2	2	1	1	2	2	1	1
1	1	2	2	4	4	2	2	1	1
1	1	2	2	1	1	2	2	1	1
4	4	2	2	4	4	1	1	1	1
1	1	2	2	4	4	2	2	1	1
4	4	2	2	4	4	2	2	1	1
1	1	2	2	4	4	1	1	1	1
1	1	2	2	4	4	2	2	1	1
1	1	2	2	4	4	2	2	1	1
1	1	2	2	4	4	2	2	1	1
1	1	2	2	4	4	2	2	1	1
4	4	2	2	1	1	1	1	1	1
1	1	2	2	4	4	2	2	1	1
1	1	2	2	4	4	2	2	1	1
1	1	2	2	4	4	2	2	1	1
1	1	3	3	4	4	2	2	1	1
4	4	2	2	4	4	2	2	4	4
1	1	2	2	4	4	2	2	1	1
1	1	2	2	4	4	2	2	1	1
1	1	2	2	4	4	1	1	1	1
1	1	3	3	4	4	2	2	1	1
1	1	2	2	4	4	2	2	1	1
1	1	2	2	4	4	2	2	1	1
4	4	2	2	4	4	2	2	1	1
1	1	2	2	4	4	1	1	1	1
1	1	3	3	4	4	2	2	1	1
1	1	2	2	4	4	2	2	1	1
4	4	2	2	1	1	2	2	1	1
1	1	3	3	1	1	2	2	1	1
4	4	2	2	1	1	2	2	1	1
1	1	2	2	4	4	2	2	1	1
1	1	3	3	4	4	2	2	1	1
4	4	2	2	4	4	2	2	1	1
1	1	2	2	4	4	2	2	1	1
4	4	2	2	1	1	2	2	1	1
1	1	2	2	4	4	1	1	1	1
1	1	2	2	1	1	2	2	1	1
1	1	2	2	4	4	2	2	4	4
1	1	3	3	4	4	2	2	1	1
1	1	2	2	4	4	2	2	1	1
4	4	2	2	4	4	2	2	1	1
1	1	2	2	4	4	2	2	4	4
1	1	2	2	4	4	2	2	1	1
1	1	2	2	4	4	2	2	1	1
1	1	2	2	4	4	2	2	1	1
1	1	2	2	4	4	1	1	1	1
4	4	2	2	4	4	2	2	1	1
1	1	2	2	4	4	2	2	1	1

For Review Only

1	1	2	2	4	4	2	2	1	1
1	1	2	2	4	4	2	2	1	1
4	4	2	2	1	1	2	2	1	1
1	1	2	2	4	4	2	2	1	1
1	1	2	2	4	4	2	2	1	1
1	1	2	2	1	1	1	1	1	1
1	1	2	2	4	4	2	2	1	1
1	1	2	2	4	4	2	2	1	1
1	1	2	2	4	4	2	2	1	1
1	1	3	3	4	4	2	2	1	1
1	1	2	2	4	4	2	2	1	1
1	1	2	2	4	4	2	2	1	1
1	1	3	3	4	4	2	2	1	1
1	1	3	3	4	4	2	2	1	1
4	4	2	2	1	1	2	2	1	1
1	1	3	3	4	4	2	2	1	1
1	1	2	2	4	4	2	2	1	1
1	1	2	2	4	4	1	1	1	1
1	1	2	2	4	4	2	2	1	1
4	4	2	2	4	4	2	2	1	1
1	1	2	2	4	4	2	2	1	1
4	4	2	2	4	4	2	2	1	1
1	1	3	3	1	1	2	2	1	1
4	4	2	2	4	4	2	2	4	4
4	4	2	2	4	4	2	2	1	1
4	4	2	2	1	1	2	2	1	1
1	1	2	2	4	4	2	2	1	1
1	1	2	2	4	4	2	2	1	1
4	4	2	2	4	4	2	2	1	1
1	1	3	3	4	4	2	2	1	1
1	1	2	2	4	4	2	2	1	1
1	1	2	2	4	4	2	2	1	1
4	4	2	2	4	4	1	1	1	1
4	4	2	2	1	1	2	2	1	1
1	1	2	2	1	1	2	2	1	1
4	4	2	2	4	4	2	2	4	4
1	1	2	2	4	4	2	2	1	1
1	1	2	2	4	4	2	2	1	1
4	4	2	2	4	4	2	2	1	1
1	1	2	2	4	4	2	2	1	1
4	4	2	2	4	4	2	2	1	1
1	1	2	2	4	4	2	2	1	1
4	4	2	2	4	4	2	2	1	1
1	1	3	3	4	4	2	2	1	1
4	4	2	2	4	4	2	2	1	1
4	4	2	2	4	4	2	2	1	1
1	1	3	3	4	4	2	2	1	1
4	4	2	2	4	4	2	2	1	1
4	4	2	2	1	1	2	2	1	1
1	1	3	3	4	4	2	2	1	1
1	1	2	2	4	4	2	2	1	1
4	4	2	2	1	1	2	2	4	4
1	1	3	3	1	1	2	2	1	1
4	4	2	2	4	4	2	2	1	1
4	4	2	2	4	4	2	2	4	4

For Review Only

1	1	2	2	4	4	2	2	1	1
1	1	2	2	1	1	2	2	1	1
1	1	2	2	4	4	2	2	1	1
1	1	2	2	4	4	2	2	1	1
4	4	2	2	4	4	2	2	1	1
1	1	2	2	4	4	2	2	1	1
4	4	2	2	4	4	2	2	1	1
4	4	2	2	4	4	2	2	1	1
1	1	2	2	1	1	2	2	1	1
4	4	2	2	4	4	2	2	1	1
1	1	2	2	4	4	2	2	1	1
4	4	2	2	4	4	2	2	1	1
1	1	3	3	4	4	2	2	1	1
1	1	2	2	4	4	2	2	1	1
1	1	2	2	4	4	2	2	1	1
1	1	3	3	1	1	2	2	1	1
4	4	2	2	4	4	2	2	1	1
1	1	2	2	1	1	2	2	1	1
4	4	2	2	4	4	2	2	1	1
1	1	3	3	4	4	2	2	1	1
4	4	2	2	4	4	2	2	1	1
1	1	2	2	4	4	2	2	1	1
1	1	3	3	4	4	2	2	1	1
1	1	2	2	4	4	2	2	1	1
4	4	2	2	1	1	2	2	1	1
1	1	2	2	4	4	2	2	1	1
4	4	2	2	4	4	2	2	1	1
4	4	2	2	4	4	2	2	1	1
1	1	2	2	4	4	2	2	1	1
1	1	3	3	4	4	2	2	1	1
1	1	2	2	4	4	2	2	1	1
1	1	2	2	4	4	2	2	1	1
1	1	3	3	1	1	2	2	1	1
1	1	2	2	4	4	2	2	1	1
1	1	3	3	1	1	2	2	1	1
4	4	2	2	4	4	2	2	1	1
1	1	3	3	4	4	1	1	1	1
4	4	2	2	4	4	2	2	4	4
4	4	2	2	1	1	2	2	1	1
4	4	2	2	4	4	2	2	1	1
1	1	2	2	4	4	2	2	1	1
4	4	2	2	4	4	2	2	1	1
1	1	2	2	4	4	2	2	1	1
1	1	3	3	4	4	2	2	1	1
4	4	2	2	1	1	2	2	1	1
1	1	3	3	4	4	2	2	1	1
4	4	2	2	4	4	2	2	1	1
1	1	3	3	4	4	2	2	1	1
4	4	2	2	4	4	2	2	1	1
4	4	2	2	4	4	2	2	1	1
1	1	2	2	4	4	2	2	1	1
4	4	2	2	4	4	2	2	1	1
4	4	2	2	4	4	2	2	1	1
1	1	2	2	4	4	2	2	1	1
1	1	2	2	4	4	2	2	1	1

For Review Only

1	1	3	3	4	4	2	2	1	1
1	1	2	2	4	4	2	2	1	1

For Review Only

SNP190	SNP190	SNP1900	SNP1900	SNP1901	SNP1901	SNP1902	SNP1902	SNP1903	SNP1903
3	3	1	1	3	3	3	3	2	2
3	3	1	1	3	3	4	4	4	4
2	2	2	2	4	4	4	4	4	4
3	3	2	2	4	4	4	4	4	4
3	3	1	1	3	3	3	3	4	4
2	2	1	1	3	3	4	4	2	2
2	2	1	1	3	3	3	3	4	4
3	3	2	2	4	4	3	3	4	4
2	2	1	1	3	3	3	3	4	4
2	2	2	2	4	4	3	3	4	4
3	3	1	1	3	3	3	3	4	4
3	3	2	2	4	4	4	4	4	4
3	3	1	1	3	3	4	4	4	4
3	3	1	1	3	3	3	3	4	4
3	3	1	1	3	3	3	3	4	4
2	2	2	2	4	4	4	4	4	4
3	3	1	1	3	3	3	3	4	4
3	3	1	1	3	3	3	3	4	4
2	2	1	1	3	3	4	4	4	4
3	3	1	1	3	3	3	3	4	4
2	2	1	1	3	3	3	3	4	4
2	2	1	1	3	3	3	3	4	4
2	2	2	2	3	3	3	3	4	4
2	2	2	2	3	3	3	3	4	4
3	3	1	1	3	3	4	4	4	4
2	2	1	1	3	3	4	4	4	4
2	2	2	2	3	3	3	3	4	4
3	3	1	1	3	3	3	3	4	4
3	3	1	1	3	3	4	4	4	4
3	3	2	2	4	4	4	4	4	4
2	2	1	1	3	3	4	4	4	4
2	2	1	1	3	3	3	3	2	2
2	2	1	1	3	3	3	3	4	4
3	3	2	2	3	3	3	3	4	4
3	3	1	1	3	3	3	3	4	4
2	2	2	2	4	4	3	3	4	4
3	3	1	1	3	3	3	3	4	4
3	3	1	1	3	3	3	3	4	4
3	3	2	2	3	3	4	4	4	4
3	3	1	1	3	3	3	3	4	4
3	3	2	2	4	4	4	4	4	4
3	3	1	1	3	3	4	4	4	4
2	2	1	1	3	3	3	3	4	4
3	3	1	1	3	3	3	3	4	4
3	3	1	1	3	3	4	4	4	4
3	3	2	2	4	4	4	4	4	4
3	3	1	1	3	3	4	4	4	4
3	3	1	1	3	3	4	4	4	4
3	3	1	1	3	3	3	3	4	4
3	3	1	1	3	3	3	3	2	2
2	2	1	1	3	3	3	3	4	4
2	2	2	2	3	3	3	3	2	2

For Review Only

3	3	1	1	3	3	3	3	4	4
2	2	1	1	3	3	3	3	4	4
2	2	2	2	3	3	3	3	4	4
3	3	1	1	3	3	3	3	4	4
2	2	1	1	3	3	4	4	4	4
3	3	1	1	3	3	3	3	4	4
3	3	1	1	3	3	3	3	4	4
3	3	2	2	4	4	4	4	4	4
3	3	1	1	3	3	3	3	4	4
3	3	1	1	4	4	3	3	4	4
3	3	2	2	4	4	4	4	4	4
3	3	2	2	4	4	4	4	4	4
3	3	1	1	3	3	3	3	4	4
3	3	1	1	3	3	4	4	4	4
3	3	1	1	3	3	3	3	2	2
3	3	1	1	3	3	3	3	4	4
3	3	1	1	3	3	3	3	4	4
3	3	1	1	3	3	3	3	2	2
3	3	1	1	3	3	3	3	4	4
2	2	1	1	3	3	3	3	2	2
3	3	2	2	4	4	3	3	4	4
3	3	1	1	3	3	3	3	4	4
2	2	1	1	3	3	4	4	4	4
3	3	1	1	3	3	3	3	2	2
3	3	1	1	3	3	4	4	4	4
2	2	1	1	3	3	4	4	4	4
3	3	1	1	3	3	3	3	2	2
3	3	2	2	4	4	4	4	4	4
3	3	1	1	3	3	3	3	4	4
3	3	1	1	3	3	3	3	4	4
3	3	1	1	3	3	3	3	4	4
2	2	2	2	3	3	4	4	4	4
2	2	2	2	3	3	4	4	4	4
3	3	1	1	3	3	3	3	4	4
3	3	1	1	3	3	4	4	4	4
3	3	1	1	3	3	4	4	4	4
3	3	1	1	3	3	3	3	4	4
2	2	2	2	4	4	3	3	4	4
3	3	1	1	3	3	4	4	4	4
2	2	1	1	3	3	4	4	4	4
2	2	1	1	3	3	3	3	4	4
2	2	1	1	3	3	3	3	4	4
2	2	2	2	3	3	3	3	4	4
3	3	2	2	4	4	4	4	4	4
3	3	1	1	3	3	4	4	4	4
3	3	1	1	3	3	3	3	4	4
3	3	2	2	3	3	3	3	4	4
3	3	2	2	3	3	3	3	4	4
2	2	1	1	3	3	3	3	4	4
3	3	1	1	3	3	3	3	4	4
2	2	1	1	3	3	3	3	4	4
3	3	2	2	3	3	4	4	4	4
3	3	2	2	4	4	4	4	4	4
3	3	1	1	4	4	3	3	4	4
3	3	1	1	3	3	3	3	4	4

For Review Only

3	3	1	1	3	3	3	3	4	4
3	3	2	2	3	3	3	3	4	4
3	3	1	1	3	3	3	3	4	4
3	3	2	2	3	3	3	3	4	4
2	2	1	1	3	3	3	3	4	4
3	3	1	1	3	3	4	4	4	4
2	2	2	2	4	4	4	4	4	4
2	2	1	1	4	4	3	3	4	4
2	2	1	1	3	3	4	4	4	4
3	3	1	1	3	3	3	3	4	4
3	3	1	1	3	3	3	3	2	2
3	3	1	1	3	3	3	3	4	4
2	2	2	2	3	3	3	3	4	4
2	2	1	1	3	3	4	4	2	2
3	3	1	1	3	3	3	3	4	4
3	3	1	1	3	3	3	3	4	4
3	3	1	1	3	3	3	3	4	4
3	3	1	1	3	3	3	3	4	4
3	3	2	2	3	3	3	3	2	2
2	2	1	1	3	3	3	3	4	4
3	3	1	1	3	3	3	3	4	4
3	3	1	1	3	3	3	3	4	4
2	2	1	1	3	3	3	3	4	4
3	3	2	2	3	3	3	3	4	4
3	3	1	1	3	3	3	3	4	4
2	2	1	1	3	3	3	3	4	4
2	2	1	1	3	3	3	3	4	4
2	2	1	1	3	3	3	3	4	4
3	3	1	1	3	3	3	3	4	4
3	3	1	1	3	3	4	4	4	4
3	3	2	2	3	3	4	4	4	4
3	3	1	1	3	3	4	4	4	4
2	2	1	1	3	3	3	3	4	4
3	3	1	1	3	3	3	3	4	4
3	3	2	2	3	3	3	3	4	4
3	3	1	1	3	3	3	3	4	4
3	3	2	2	3	3	3	3	4	4
2	2	1	1	3	3	3	3	4	4
3	3	1	1	3	3	3	3	4	4
3	3	2	2	3	3	3	3	4	4
3	3	1	1	3	3	3	3	4	4
3	3	2	2	4	4	3	3	4	4
3	3	1	1	3	3	3	3	2	2
3	3	1	1	4	4	4	4	4	4
3	3	2	2	3	3	3	3	4	4
2	2	1	1	3	3	3	3	4	4
2	2	1	1	3	3	3	3	4	4
2	2	2	2	4	4	3	3	4	4
3	3	1	1	3	3	3	3	4	4
3	3	1	1	3	3	4	4	4	4
3	3	1	1	3	3	3	3	4	4
3	3	1	1	3	3	4	4	4	4
3	3	1	1	3	3	3	3	2	2
2	2	2	2	4	4	4	4	4	4
3	3	1	1	3	3	3	3	4	4

For Review Only

2	2	1	1	3	3	3	3	4	4
2	2	1	1	3	3	3	3	4	4
3	3	1	1	3	3	3	3	4	4
3	3	1	1	3	3	3	3	4	4
3	3	1	1	3	3	4	4	4	4
3	3	1	1	3	3	3	3	2	2
3	3	1	1	3	3	4	4	4	4
3	3	1	1	3	3	3	3	4	4
3	3	1	1	3	3	4	4	4	4
3	3	1	1	3	3	3	3	4	4
2	2	1	1	3	3	3	3	4	4
3	3	1	1	3	3	3	3	4	4
3	3	1	1	3	3	3	3	4	4
3	3	1	1	3	3	3	3	4	4
3	3	2	2	4	4	3	3	4	4
3	3	1	1	3	3	3	3	4	4
3	3	1	1	3	3	3	3	2	2
3	3	1	1	3	3	4	4	4	4
2	2	1	1	3	3	3	3	4	4
3	3	2	2	4	4	3	3	4	4
3	3	1	1	3	3	3	3	4	4
3	3	2	2	3	3	3	3	4	4
3	3	1	1	3	3	3	3	4	4
3	3	2	2	4	4	4	4	4	4
3	3	1	1	3	3	3	3	4	4
3	3	2	2	3	3	3	3	4	4
3	3	1	1	3	3	4	4	4	4
3	3	1	1	3	3	3	3	4	4
3	3	2	2	4	4	3	3	4	4
3	3	1	1	3	3	3	3	4	4
2	2	1	1	3	3	3	3	4	4
3	3	1	1	3	3	3	3	4	4
3	3	2	2	3	3	3	3	4	4
3	3	2	2	4	4	3	3	4	4
3	3	1	1	3	3	3	3	4	4
3	3	2	2	4	4	4	4	4	4
2	2	1	1	3	3	3	3	4	4
3	3	1	1	3	3	3	3	4	4
2	2	2	2	4	4	3	3	4	4
3	3	1	1	3	3	3	3	4	4
2	2	2	2	4	4	4	4	4	4
2	2	1	1	3	3	3	3	4	4
3	3	2	2	3	3	4	4	4	4
3	3	1	1	3	3	3	3	4	4
3	3	2	2	4	4	3	3	4	4
3	3	2	2	4	4	4	4	2	2
3	3	1	1	3	3	3	3	4	4
3	3	2	2	3	3	4	4	4	4
3	3	2	2	4	4	3	3	4	4
3	3	1	1	3	3	3	3	4	4
3	3	1	1	3	3	3	3	4	4
3	3	2	2	4	4	4	4	4	4
3	3	1	1	3	3	4	4	4	4
3	3	2	2	4	4	4	4	4	4
3	3	2	2	3	3	4	4	4	4

3	3	1	1	3	3	3	3	4	4
3	3	1	1	3	3	3	3	4	4
3	3	1	1	3	3	3	3	4	4
3	3	1	1	3	3	3	3	4	4
2	2	1	1	3	3	3	3	4	4
3	3	1	1	3	3	3	3	4	4
2	2	2	2	4	4	3	3	4	4
3	3	2	2	4	4	4	4	4	4
3	3	1	1	3	3	4	4	4	4
3	3	2	2	4	4	4	4	4	4
3	3	1	1	3	3	3	3	4	4
3	3	2	2	3	3	3	3	4	4
3	3	1	1	3	3	3	3	4	4
2	2	1	1	3	3	3	3	4	4
3	3	1	1	3	3	4	4	4	4
3	3	1	1	3	3	3	3	4	4
3	3	2	2	3	3	4	4	4	4
3	3	1	1	3	3	3	3	4	4
3	3	2	2	3	3	3	3	4	4
3	3	1	1	3	3	3	3	4	4
2	2	2	2	4	4	4	4	4	4
2	2	1	1	3	3	3	3	4	4
3	3	1	1	3	3	3	3	4	4
3	3	1	1	3	3	3	3	4	4
3	3	2	2	4	4	4	4	4	4
3	3	1	1	3	3	3	3	4	4
3	3	2	2	3	3	4	4	4	4
3	3	2	2	4	4	4	4	4	4
3	3	1	1	3	3	3	3	4	4
3	3	1	1	3	3	3	3	4	4
3	3	1	1	3	3	4	4	4	4
3	3	1	1	3	3	3	3	4	4
3	3	1	1	3	3	3	3	4	4
3	3	1	1	3	3	3	3	4	4
3	3	1	1	3	3	3	3	4	4
3	3	2	2	3	3	4	4	4	4
3	3	1	1	3	3	3	3	2	2
3	3	2	2	3	3	4	4	4	4
2	2	2	2	4	4	3	3	4	4
3	3	2	2	3	3	4	4	4	4
3	3	1	1	3	3	3	3	4	4
2	2	2	2	3	3	3	3	4	4
2	2	1	1	3	3	3	3	4	4
2	2	1	1	3	3	3	3	4	4
3	3	2	2	4	4	4	4	4	4
3	3	1	1	3	3	4	4	4	4
3	3	2	2	4	4	3	3	4	4
2	2	1	1	3	3	3	3	4	4
2	2	2	2	4	4	4	4	4	4
3	3	2	2	3	3	4	4	4	4
3	3	1	1	3	3	3	3	4	4
3	3	2	2	3	3	4	4	4	4
2	2	2	2	4	4	4	4	4	4
3	3	1	1	3	3	4	4	4	4
3	3	1	1	3	3	3	3	4	4

For Review Only

3	3	1	1	3	3	3	3	4	4
2	2	1	1	3	3	3	3	4	4

For Review Only

SNP1904	SNP1904	SNP1905	SNP1905	SNP1906	SNP1906	SNP1907	SNP1907	SNP1908	SNP1908
3	3	2	2	2	2	4	4	1	1
2	2	2	2	2	2	1	1	2	2
3	3	2	2	2	2	4	4	1	1
3	3	2	2	2	2	1	1	2	2
3	3	2	2	2	2	1	1	2	2
3	3	2	2	2	2	1	1	2	2
3	3	2	2	2	2	1	1	2	2
3	3	1	1	1	1	1	1	2	2
3	3	2	2	2	2	1	1	2	2
3	3	1	1	1	1	1	1	2	2
3	3	2	2	2	2	1	1	2	2
3	3	2	2	2	2	4	4	2	2
3	3	2	2	1	1	1	1	2	2
2	2	2	2	2	2	1	1	2	2
3	3	2	2	2	2	1	1	2	2
3	3	2	2	2	2	4	4	1	1
3	3	2	2	2	2	4	4	1	1
3	3	2	2	2	2	1	1	2	2
3	3	2	2	2	2	1	1	2	2
3	3	2	2	2	2	1	1	2	2
3	3	2	2	2	2	1	1	2	2
3	3	2	2	2	2	1	1	2	2
3	3	2	2	2	2	1	1	2	2
2	2	2	2	2	2	1	1	2	2
3	3	2	2	2	2	1	1	2	2
3	3	2	2	2	2	1	1	2	2
3	3	2	2	2	2	1	1	2	2
3	3	2	2	2	2	1	1	2	2
3	3	2	2	2	2	1	1	2	2
3	3	1	1	1	1	1	1	2	2
3	3	2	2	2	2	1	1	2	2
3	3	2	2	2	2	4	4	1	1
3	3	2	2	2	2	1	1	2	2
2	2	2	2	2	2	1	1	2	2
3	3	2	2	2	2	1	1	2	2
3	3	2	2	2	2	1	1	2	2
3	3	2	2	2	2	1	1	2	2
3	3	2	2	2	2	1	1	2	2
3	3	2	2	2	2	1	1	2	2
3	3	2	2	2	2	1	1	2	2
2	2	1	1	1	1	1	1	2	2
3	3	2	2	2	2	1	1	2	2
3	3	2	2	2	2	1	1	2	2
3	3	2	2	2	2	1	1	2	2
3	3	2	2	2	2	1	1	2	2
3	3	2	2	2	2	4	4	1	1
3	3	2	2	2	2	1	1	2	2
3	3	2	2	2	2	4	4	2	2
3	3	2	2	2	2	1	1	2	2
3	3	2	2	2	2	4	4	1	1
3	3	2	2	2	2	1	1	2	2
3	3	2	2	2	2	1	1	2	2

3	3	2	2	2	2	4	4	2	2
3	3	2	2	2	2	1	1	2	2
3	3	2	2	2	2	1	1	2	2
3	3	2	2	2	2	1	1	2	2
3	3	2	2	2	2	1	1	2	2
3	3	2	2	2	2	1	1	2	2
3	3	2	2	2	2	1	1	2	2
3	3	1	1	1	1	1	1	2	2
3	3	2	2	2	2	1	1	2	2
3	3	2	2	2	2	1	1	2	2
3	3	2	2	2	2	1	1	2	2
3	3	2	2	2	2	1	1	2	2
3	3	2	2	2	2	1	1	2	2
3	3	2	2	2	2	1	1	2	2
3	3	2	2	2	2	1	1	2	2
3	3	2	2	2	2	1	1	2	2
3	3	2	2	2	2	1	1	2	2
3	3	2	2	2	2	4	4	1	1
3	3	2	2	2	2	1	1	2	2
3	3	2	2	2	2	4	4	1	1
3	3	2	2	2	2	1	1	2	2
3	3	2	2	2	2	1	1	2	2
3	3	2	2	2	2	1	1	2	2
3	3	2	2	2	2	1	1	2	2
3	3	2	2	2	2	1	1	2	2
3	3	2	2	2	2	1	1	2	2
3	3	2	2	2	2	1	1	2	2
3	3	2	2	2	2	1	1	2	2
3	3	2	2	2	2	1	1	2	2
3	3	2	2	2	2	1	1	2	2
3	3	2	2	1	1	1	1	2	2
3	3	2	2	2	2	1	1	2	2
3	3	2	2	2	2	1	1	2	2
3	3	2	2	2	2	1	1	2	2
3	3	2	2	2	2	1	1	2	2
3	3	2	2	2	2	1	1	2	2
3	3	1	1	1	1	1	1	2	2
3	3	1	1	1	1	1	1	2	2
3	3	2	2	2	2	1	1	2	2
3	3	2	2	2	2	1	1	2	2
3	3	2	2	2	2	1	1	2	2
3	3	2	2	2	2	4	4	1	1
3	3	2	2	2	2	1	1	2	2
3	3	2	2	2	2	1	1	2	2
3	3	2	2	2	2	1	1	2	2
3	3	2	2	2	2	1	1	2	2
2	2	2	2	2	2	1	1	2	2
3	3	2	2	2	2	1	1	2	2
2	2	2	2	2	2	1	1	2	2
3	3	2	2	2	2	4	4	1	1
3	3	2	2	2	2	1	1	2	2
3	3	2	2	2	2	1	1	2	2
3	3	1	1	1	1	1	1	2	2
3	3	2	2	2	2	1	1	2	2
3	3	2	2	2	2	1	1	2	2

For Review Only

3	3	2	2	2	2	1	1	2	2
2	2	2	2	2	2	1	1	2	2
3	3	2	2	2	2	1	1	2	2
2	2	2	2	2	2	1	1	2	2
3	3	2	2	2	2	1	1	2	2
3	3	2	2	2	2	1	1	2	2
3	3	2	2	2	2	4	4	1	1
3	3	2	2	1	1	4	4	2	2
3	3	2	2	2	2	1	1	2	2
3	3	2	2	2	2	1	1	2	2
3	3	2	2	2	2	1	1	2	2
3	3	2	2	2	2	1	1	2	2
3	3	2	2	2	2	1	1	2	2
3	3	2	2	2	2	1	1	2	2
3	3	2	2	2	2	1	1	2	2
3	3	2	2	2	2	1	1	2	2
3	3	2	2	2	2	1	1	2	2
3	3	2	2	2	2	1	1	2	2
2	2	2	2	2	2	1	1	2	2
3	3	2	2	2	2	4	4	1	1
3	3	2	2	2	2	1	1	2	2
3	3	2	2	2	2	1	1	2	2
3	3	2	2	2	2	4	4	1	1
3	3	2	2	2	2	1	1	2	2
3	3	2	2	2	2	1	1	2	2
3	3	2	2	2	2	1	1	2	2
3	3	2	2	2	2	4	4	2	2
3	3	2	2	2	2	1	1	2	2
3	3	2	2	2	2	1	1	2	2
3	3	2	2	2	2	1	1	2	2
3	3	2	2	2	2	1	1	2	2
3	3	2	2	2	2	1	1	2	2
3	3	2	2	2	2	1	1	2	2
3	3	2	2	2	2	1	1	2	2
2	2	2	2	2	2	1	1	2	2
3	3	2	2	2	2	4	4	2	2
2	2	2	2	2	2	1	1	2	2
2	2	2	2	2	2	1	1	2	2
3	3	2	2	2	2	1	1	2	2
3	3	2	2	2	2	1	1	2	2
3	3	2	2	2	2	1	1	2	2
2	2	2	2	1	1	1	1	2	2
3	3	2	2	2	2	1	1	2	2
3	3	2	2	2	2	1	1	2	2
3	3	2	2	2	2	1	1	2	2
3	3	2	2	2	2	1	1	2	2
3	3	2	2	2	2	1	1	2	2
3	3	2	2	2	2	1	1	2	2
3	3	2	2	2	2	1	1	2	2
3	3	2	2	2	2	1	1	2	2
3	3	2	2	2	2	1	1	2	2
3	3	2	2	2	2	1	1	2	2
3	3	2	2	2	2	1	1	2	2
3	3	2	2	2	2	1	1	2	2
3	3	2	2	2	2	1	1	2	2

For Review Only

3	3	2	2	2	2	1	1	2	2
3	3	2	2	2	2	1	1	2	2
3	3	2	2	2	2	1	1	2	2
3	3	2	2	2	2	4	4	2	2
3	3	2	2	2	2	1	1	2	2
3	3	2	2	2	2	1	1	2	2
3	3	2	2	2	2	1	1	2	2
3	3	2	2	2	2	1	1	2	2
3	3	2	2	2	2	1	1	2	2
3	3	2	2	2	2	1	1	2	2
3	3	2	2	2	2	1	1	2	2
3	3	2	2	2	2	1	1	2	2
3	3	2	2	2	2	1	1	2	2
3	3	2	2	2	2	1	1	2	2
2	2	1	1	1	1	1	1	2	2
3	3	2	2	2	2	1	1	2	2
3	3	2	2	2	2	4	4	1	1
3	3	2	2	2	2	1	1	2	2
3	3	2	2	2	2	1	1	2	2
3	3	2	2	2	2	4	4	1	1
3	3	2	2	2	2	1	1	2	2
3	3	2	2	2	2	1	1	2	2
3	3	2	2	2	2	1	1	2	2
3	3	2	2	2	2	1	1	2	2
3	3	1	1	1	1	1	1	2	2
2	2	2	2	2	2	1	1	2	2
3	3	2	2	2	2	1	1	2	2
3	3	2	2	2	2	1	1	2	2
3	3	1	1	1	1	1	1	2	2
2	2	2	2	2	2	4	4	1	1
3	3	2	2	2	2	1	1	2	2
3	3	2	2	2	2	1	1	2	2
3	3	2	2	2	2	1	1	2	2
2	2	1	1	1	1	1	1	2	2
3	3	2	2	2	2	1	1	2	2
3	3	2	2	2	2	1	1	2	2
3	3	2	2	2	2	1	1	2	2
3	3	2	2	2	2	1	1	2	2
3	3	2	2	2	2	1	1	2	2
2	2	2	2	2	2	1	1	2	2
3	3	2	2	2	2	4	4	1	1
3	3	2	2	2	2	1	1	2	2
3	3	2	2	2	2	1	1	2	2
3	3	2	2	2	2	1	1	2	2
2	2	2	2	2	2	1	1	2	2
3	3	1	1	2	2	1	1	2	2
3	3	2	2	2	2	1	1	2	2
3	3	2	2	2	2	1	1	2	2
2	2	1	1	1	1	1	1	2	2
3	3	2	2	2	2	1	1	2	2
3	3	2	2	2	2	1	1	2	2
3	3	1	1	1	1	1	1	2	2
3	3	2	2	2	2	1	1	2	2
3	3	2	2	2	2	1	1	2	2
3	3	2	2	2	2	1	1	2	2

For Review Only

3	3	2	2	2	2	4	4	1	1
2	2	2	2	2	2	1	1	2	2
3	3	2	2	2	2	4	4	1	1
3	3	2	2	2	2	4	4	2	2
3	3	2	2	2	2	1	1	2	2
3	3	2	2	2	2	1	1	2	2
3	3	2	2	2	2	1	1	2	2
2	2	2	2	2	2	1	1	2	2
2	2	2	2	2	2	1	1	2	2
3	3	2	2	2	2	1	1	2	2
3	3	2	2	2	2	1	1	2	2
3	3	2	2	2	2	1	1	2	2
3	3	2	2	2	2	1	1	2	2
3	3	2	2	2	2	1	1	2	2
3	3	2	2	2	2	4	4	1	1
3	3	2	2	2	2	1	1	2	2
3	3	2	2	2	2	1	1	2	2
3	3	2	2	2	2	1	1	2	2
3	3	2	2	2	2	1	1	2	2
3	3	2	2	2	2	1	1	2	2
3	3	1	1	1	1	1	1	2	2
3	3	2	2	2	2	1	1	2	2
3	3	2	2	2	2	1	1	2	2
3	3	2	2	2	2	1	1	2	2
3	3	1	1	1	1	1	1	2	2
3	3	2	2	2	2	1	1	2	2
3	3	2	2	2	2	1	1	2	2
3	3	2	2	2	2	1	1	2	2
3	3	2	2	2	2	1	1	2	2
3	3	2	2	2	2	1	1	2	2
3	3	2	2	2	2	1	1	2	2
3	3	2	2	2	2	1	1	2	2
3	3	2	2	2	2	1	1	2	2
3	3	2	2	2	2	1	1	2	2
3	3	2	2	2	2	1	1	2	2
3	3	2	2	2	2	1	1	2	2
3	3	2	2	2	2	4	4	1	1
3	3	2	2	2	2	1	1	2	2
3	3	2	2	2	2	1	1	2	2
3	3	2	2	2	2	1	1	2	2
2	2	2	2	2	2	1	1	2	2
2	2	2	2	2	2	1	1	2	2
3	3	2	2	2	2	1	1	2	2
3	3	2	2	2	2	1	1	2	2
3	3	2	2	2	2	1	1	2	2
3	3	2	2	2	2	1	1	2	2
3	3	2	2	2	2	1	1	2	2
3	3	2	2	2	2	1	1	2	2
3	3	2	2	2	2	4	4	2	2
3	3	2	2	2	2	1	1	2	2
3	3	2	2	2	2	4	4	1	1
3	3	2	2	2	2	1	1	2	2
3	3	2	2	2	2	4	4	1	1
3	3	2	2	2	2	1	1	2	2
3	3	2	2	2	2	1	1	2	2

For Review Only

3	3	2	2	2	2	1	1	2	2
3	3	2	2	2	2	1	1	2	2

For Review Only

SNP1909	SNP1909	SNP191	SNP191	SNP1910	SNP1910	SNP1911	SNP1911	SNP1912	SNP1912
2	2	2	2	3	3	1	1	4	4
2	2	2	2	3	3	1	1	4	4
2	2	2	2	2	2	1	1	4	4
2	2	3	3	3	3	1	1	4	4
2	2	2	2	3	3	1	1	4	4
2	2	2	2	3	3	1	1	4	4
2	2	2	2	3	3	1	1	4	4
4	4	2	2	3	3	1	1	1	1
2	2	2	2	3	3	1	1	4	4
4	4	2	2	3	3	1	1	4	4
2	2	3	3	3	3	1	1	4	4
2	2	2	2	3	3	1	1	4	4
4	4	2	2	3	3	1	1	4	4
2	2	2	2	3	3	1	1	4	4
2	2	2	2	3	3	1	1	4	4
2	2	2	2	2	2	1	1	4	4
2	2	2	2	2	2	1	1	4	4
2	2	2	2	3	3	1	1	4	4
2	2	2	2	3	3	1	1	4	4
2	2	2	2	3	3	1	1	4	4
2	2	2	2	3	3	1	1	4	4
2	2	2	2	3	3	1	1	4	4
4	4	2	2	3	3	1	1	1	1
4	4	2	2	3	3	1	1	1	1
2	2	2	2	3	3	1	1	4	4
2	2	2	2	3	3	1	1	4	4
4	4	2	2	3	3	1	1	1	1
2	2	2	2	3	3	1	1	4	4
2	2	2	2	3	3	1	1	4	4
4	4	2	2	3	3	1	1	1	1
2	2	3	3	3	3	1	1	4	4
2	2	2	2	2	2	1	1	4	4
2	2	2	2	3	3	1	1	4	4
4	4	2	2	3	3	1	1	1	1
2	2	2	2	3	3	1	1	4	4
4	4	2	2	3	3	1	1	1	1
2	2	2	2	3	3	1	1	4	4
2	2	2	2	3	3	1	1	4	4
4	4	2	2	3	3	1	1	1	1
2	2	2	2	3	3	1	1	4	4
2	2	3	3	3	3	1	1	4	4
2	2	2	2	3	3	1	1	4	4
2	2	2	2	3	3	1	1	4	4
2	2	2	2	3	3	1	1	4	4
4	4	2	2	3	3	1	1	4	4
2	2	2	2	2	2	1	1	4	4
2	2	2	2	3	3	1	1	4	4
2	2	2	2	3	3	1	1	4	4
2	2	2	2	3	3	1	1	4	4
2	2	2	2	3	3	1	1	4	4
2	2	2	2	3	3	1	1	4	4
4	4	2	2	3	3	1	1	4	4

For Review Only

2	2	2	2	3	3	1	1	4	4
2	2	2	2	3	3	1	1	4	4
4	4	3	3	3	3	1	1	4	4
2	2	2	2	3	3	1	1	4	4
2	2	2	2	3	3	1	1	4	4
2	2	2	2	3	3	1	1	4	4
2	2	2	2	3	3	1	1	4	4
4	4	2	2	3	3	1	1	1	1
2	2	2	2	3	3	1	1	4	4
2	2	2	2	3	3	1	1	4	4
4	4	2	2	3	3	1	1	1	1
4	4	2	2	3	3	3	3	1	1
2	2	2	2	3	3	1	1	4	4
2	2	2	2	3	3	1	1	4	4
2	2	2	2	3	3	1	1	4	4
2	2	2	2	3	3	1	1	4	4
4	4	2	2	3	3	1	1	1	1
2	2	2	2	2	2	1	1	4	4
2	2	2	2	3	3	1	1	1	1
2	2	2	2	2	2	1	1	4	4
4	4	2	2	3	3	1	1	1	1
4	4	3	3	3	3	1	1	1	1
2	2	2	2	3	3	1	1	4	4
2	2	2	2	3	3	1	1	4	4
4	4	2	2	3	3	1	1	4	4
2	2	2	2	3	3	1	1	4	4
2	2	2	2	3	3	1	1	4	4
4	4	2	2	3	3	1	1	1	1
2	2	2	2	3	3	1	1	4	4
2	2	2	2	3	3	1	1	1	1
4	4	2	2	3	3	1	1	4	4
2	2	2	2	3	3	1	1	4	4
4	4	2	2	3	3	1	1	1	1
2	2	2	2	3	3	1	1	4	4
2	2	2	2	3	3	1	1	4	4
4	4	2	2	3	3	1	1	4	4
4	4	2	2	3	3	1	1	1	1
4	4	2	2	3	3	1	1	1	1
2	2	2	2	3	3	1	1	4	4
2	2	2	2	3	3	1	1	4	4
2	2	2	2	3	3	1	1	4	4
2	2	2	2	2	2	1	1	1	1
2	2	2	2	3	3	1	1	1	1
4	4	2	2	3	3	1	1	1	1
2	2	2	2	3	3	1	1	4	4
2	2	2	2	3	3	1	1	4	4
4	4	2	2	3	3	1	1	1	1
4	4	2	2	3	3	1	1	1	1
2	2	2	2	3	3	3	3	4	4
2	2	2	2	2	2	1	1	4	4
4	4	2	2	3	3	1	1	1	1
4	4	2	2	3	3	1	1	1	1
4	4	2	2	3	3	1	1	1	1
2	2	2	2	3	3	1	1	4	4
2	2	2	2	3	3	1	1	4	4

For Review Only

2	2	2	2	3	3	1	1	4	4
2	2	2	2	3	3	1	1	4	4
2	2	2	2	3	3	1	1	4	4
4	4	2	2	3	3	3	3	1	1
4	4	2	2	3	3	1	1	1	1
2	2	2	2	3	3	1	1	4	4
2	2	2	2	2	2	1	1	4	4
2	2	3	3	3	3	1	1	4	4
2	2	2	2	3	3	1	1	4	4
2	2	2	2	3	3	1	1	4	4
2	2	3	3	3	3	1	1	4	4
2	2	2	2	3	3	1	1	4	4
2	2	2	2	3	3	1	1	1	1
2	2	2	2	3	3	1	1	4	4
2	2	2	2	3	3	1	1	4	4
2	2	2	2	3	3	1	1	4	4
2	2	2	2	3	3	1	1	4	4
2	2	2	2	3	3	1	1	4	4
4	4	2	2	3	3	1	1	1	1
2	2	2	2	2	2	3	3	4	4
2	2	2	2	3	3	1	1	4	4
2	2	2	2	3	3	1	1	4	4
2	2	2	2	2	2	1	1	1	1
4	4	2	2	3	3	1	1	1	1
2	2	2	2	3	3	1	1	1	1
2	2	3	3	3	3	1	1	4	4
2	2	3	3	3	3	3	3	1	1
4	4	3	3	3	3	1	1	1	1
2	2	2	2	3	3	1	1	4	4
2	2	2	2	3	3	1	1	4	4
4	4	2	2	3	3	1	1	1	1
2	2	2	2	3	3	1	1	4	4
2	2	2	2	3	3	1	1	4	4
2	2	2	2	3	3	1	1	4	4
4	4	2	2	3	3	1	1	1	1
4	4	2	2	3	3	1	1	4	4
4	4	2	2	3	3	1	1	1	1
2	2	2	2	3	3	1	1	4	4
2	2	2	2	3	3	1	1	4	4
4	4	2	2	3	3	1	1	1	1
2	2	3	3	3	3	1	1	4	4
4	4	2	2	3	3	1	1	1	1
2	2	2	2	3	3	1	1	4	4
2	2	2	2	3	3	1	1	4	4
2	2	2	2	3	3	3	3	1	1
2	2	2	2	3	3	3	3	4	4
2	2	2	2	3	3	1	1	4	4
4	4	2	2	3	3	1	1	1	1
2	2	2	2	3	3	3	3	4	4
2	2	2	2	3	3	1	1	4	4
2	2	2	2	3	3	1	1	4	4
2	2	2	2	3	3	1	1	4	4
2	2	2	2	3	3	1	1	4	4
4	4	2	2	3	3	1	1	1	1
2	2	2	2	3	3	1	1	4	4

For Review Only

2	2	2	2	3	3	1	1	4	4
2	2	2	2	3	3	1	1	4	4
4	4	2	2	3	3	3	3	4	4
2	2	2	2	2	2	1	1	4	4
2	2	2	2	3	3	1	1	4	4
2	2	2	2	3	3	1	1	4	4
2	2	2	2	3	3	1	1	4	4
2	2	2	2	3	3	1	1	4	4
2	2	2	2	3	3	1	1	4	4
2	2	3	3	3	3	1	1	4	4
4	4	2	2	3	3	1	1	4	4
2	2	2	2	3	3	1	1	4	4
2	2	2	2	3	3	1	1	1	1
2	2	2	2	3	3	1	1	4	4
4	4	2	2	3	3	1	1	1	1
2	2	2	2	3	3	1	1	4	4
2	2	2	2	3	3	1	1	4	4
4	4	2	2	3	3	1	1	4	4
2	2	2	2	3	3	1	1	4	4
2	2	2	2	2	2	1	1	4	4
2	2	2	2	3	3	1	1	4	4
4	4	2	2	3	3	1	1	4	4
4	4	3	3	3	3	1	1	1	1
4	4	2	2	3	3	3	3	1	1
2	2	2	2	3	3	1	1	4	4
4	4	2	2	3	3	1	1	1	1
2	2	2	2	3	3	1	1	4	4
2	2	2	2	3	3	1	1	4	4
4	4	2	2	3	3	1	1	1	1
2	2	2	2	2	2	1	1	4	4
2	2	2	2	3	3	1	1	4	4
2	2	2	2	3	3	1	1	4	4
4	4	2	2	3	3	3	3	1	1
4	4	2	2	3	3	1	1	1	1
2	2	2	2	3	3	1	1	4	4
4	4	2	2	3	3	3	3	1	1
2	2	2	2	3	3	1	1	4	4
2	2	2	2	3	3	1	1	4	4
4	4	2	2	3	3	1	1	1	1
4	4	2	2	3	3	1	1	1	1
2	2	2	2	2	2	1	1	4	4
2	2	2	2	3	3	1	1	4	4
4	4	2	2	3	3	1	1	1	1
2	2	2	2	3	3	1	1	4	4
2	2	2	2	3	3	1	1	4	4
4	4	2	2	3	3	1	1	4	4
2	2	2	2	3	3	1	1	4	4
4	4	2	2	3	3	1	1	1	1
4	4	2	2	3	3	1	1	1	1
2	2	3	3	3	3	1	1	4	4
2	2	2	2	3	3	1	1	4	4
4	4	2	2	3	3	1	1	1	1
4	4	3	3	3	3	1	1	1	1
4	4	2	2	3	3	1	1	1	1
4	4	2	2	3	3	1	1	1	1

For Review Only

2	2	2	2	2	2	1	1	4	4
2	2	3	3	3	3	1	1	4	4
2	2	2	2	2	2	1	1	4	4
2	2	2	2	3	3	1	1	4	4
2	2	2	2	3	3	1	1	4	4
2	2	2	2	3	3	1	1	4	4
2	2	2	2	3	3	1	1	4	4
4	4	2	2	3	3	1	1	1	1
2	2	2	2	3	3	1	1	4	4
4	4	2	2	3	3	1	1	1	1
2	2	2	2	3	3	1	1	4	4
4	4	2	2	3	3	1	1	1	1
2	2	2	2	3	3	1	1	4	4
2	2	2	2	3	3	1	1	4	4
4	4	2	2	2	2	1	1	1	1
2	2	2	2	3	3	1	1	4	4
4	4	2	2	3	3	1	1	1	1
2	2	2	2	3	3	1	1	4	4
4	4	2	2	3	3	1	1	1	1
2	2	2	2	3	3	1	1	4	4
4	4	2	2	3	3	1	1	1	1
2	2	2	2	3	3	1	1	4	4
2	2	2	2	3	3	1	1	4	4
2	2	2	2	3	3	1	1	4	4
4	4	2	2	3	3	1	1	1	1
2	2	2	2	3	3	1	1	4	4
4	4	2	2	3	3	1	1	1	1
2	2	2	2	3	3	1	1	4	4
2	2	2	2	3	3	1	1	4	4
2	2	3	3	3	3	1	1	1	1
2	2	2	2	3	3	1	1	4	4
2	2	2	2	3	3	1	1	4	4
2	2	2	2	3	3	1	1	4	4
2	2	2	2	3	3	1	1	4	4
4	4	3	3	3	3	1	1	1	1
4	4	2	2	3	3	1	1	1	1
2	2	2	2	2	2	1	1	4	4
4	4	2	2	3	3	3	3	1	1
4	4	2	2	3	3	1	1	1	1
4	4	2	2	3	3	1	1	1	1
2	2	2	2	3	3	1	1	4	4
4	4	2	2	3	3	1	1	1	1
2	2	2	2	3	3	1	1	4	4
2	2	2	2	3	3	1	1	4	4
2	2	2	2	3	3	1	1	4	4
2	2	2	2	3	3	1	1	4	4
4	4	2	2	3	3	1	1	1	1
2	2	2	2	3	3	1	1	4	4
2	2	2	2	3	3	1	1	4	4
4	4	2	2	3	3	1	1	1	1
2	2	2	2	2	2	1	1	4	4
4	4	2	2	3	3	1	1	1	1
2	2	2	2	2	2	1	1	4	4
2	2	2	2	3	3	1	1	4	4
2	2	2	2	3	3	1	1	4	4

For Review Only

2	2	3	3	3	3	1	1	4	4
4	4	2	2	3	3	1	1	1	1

For Review Only

SNP1913	SNP1913	SNP1914	SNP1914	SNP1915	SNP1915	SNP1916	SNP1916	SNP1917	SNP1917
2	2	1	1	2	2	1	1	4	4
2	2	1	1	1	1	3	3	1	1
2	2	1	1	2	2	1	1	4	4
2	2	1	1	1	1	1	1	4	4
2	2	1	1	1	1	1	1	4	4
2	2	1	1	1	1	1	1	4	4
2	2	1	1	1	1	1	1	4	4
3	3	1	1	1	1	3	3	1	1
2	2	1	1	1	1	1	1	4	4
3	3	1	1	1	1	3	3	1	1
2	2	1	1	1	1	1	1	4	4
2	2	1	1	1	1	1	1	4	4
2	2	1	1	1	1	1	1	4	4
2	2	1	1	1	1	1	1	4	4
2	2	1	1	1	1	1	1	4	4
2	2	1	1	2	2	1	1	4	4
2	2	1	1	2	2	1	1	4	4
3	3	1	1	1	1	3	3	1	1
2	2	1	1	1	1	1	1	4	4
2	2	1	1	1	1	1	1	4	4
2	2	1	1	1	1	1	1	4	4
2	2	1	1	1	1	1	1	4	4
2	2	3	3	1	1	1	1	4	4
2	2	3	3	1	1	1	1	4	4
2	2	1	1	1	1	1	1	4	4
3	3	1	1	1	1	1	1	4	4
2	2	3	3	1	1	1	1	4	4
2	2	1	1	1	1	1	1	4	4
2	2	1	1	1	1	1	1	4	4
2	2	1	1	1	1	3	3	1	1
2	2	1	1	1	1	1	1	4	4
2	2	1	1	2	2	1	1	4	4
2	2	1	1	1	1	1	1	4	4
2	2	3	3	1	1	3	3	4	4
2	2	1	1	1	1	1	1	4	4
2	2	3	3	1	1	1	1	4	4
2	2	1	1	1	1	1	1	4	4
2	2	1	1	1	1	3	3	1	1
2	2	3	3	1	1	1	1	4	4
2	2	1	1	1	1	1	1	4	4
2	2	1	1	1	1	1	1	4	4
2	2	1	1	1	1	1	1	4	4
2	2	1	1	1	1	1	1	1	1
2	2	1	1	1	1	1	1	4	4
2	2	1	1	1	1	3	3	1	1
3	3	1	1	2	2	1	1	4	4
3	3	1	1	1	1	1	1	4	4
3	3	1	1	1	1	1	1	4	4
2	2	1	1	1	1	1	1	4	4
2	2	1	1	2	2	1	1	4	4
2	2	1	1	1	1	1	1	4	4
2	2	3	3	1	1	1	1	4	4

For Review Only

2	2	1	1	1	1	1	1	4	4
2	2	1	1	1	1	1	1	4	4
2	2	3	3	1	1	1	1	4	4
2	2	1	1	1	1	1	1	4	4
2	2	1	1	1	1	1	1	4	4
2	2	1	1	1	1	1	1	4	4
3	3	1	1	1	1	1	1	4	4
2	2	3	3	1	1	1	1	4	4
2	2	1	1	1	1	1	1	4	4
2	2	1	1	1	1	1	1	4	4
2	2	1	1	1	1	1	1	4	4
2	2	3	3	1	1	1	1	4	4
2	2	1	1	1	1	1	1	4	4
2	2	1	1	1	1	1	1	4	4
2	2	1	1	1	1	1	1	4	4
2	2	1	1	1	1	1	1	4	4
2	2	3	3	1	1	1	1	4	4
2	2	1	1	2	2	1	1	4	4
2	2	3	3	1	1	1	1	4	4
2	2	1	1	2	2	1	1	4	4
2	2	1	1	1	1	1	1	4	4
2	2	3	3	1	1	1	1	4	4
2	2	1	1	1	1	1	1	4	4
2	2	1	1	1	1	1	1	4	4
2	2	1	1	1	1	3	3	1	1
2	2	1	1	1	1	1	1	4	4
2	2	1	1	1	1	1	1	4	4
2	2	3	3	1	1	1	1	4	4
3	3	1	1	1	1	1	1	4	4
2	2	3	3	1	1	1	1	4	4
2	2	1	1	1	1	3	3	1	1
2	2	1	1	1	1	1	1	4	4
2	2	3	3	1	1	1	1	4	4
2	2	1	1	1	1	1	1	4	4
2	2	1	1	1	1	1	1	4	4
2	2	1	1	1	1	3	3	1	1
3	3	3	3	1	1	3	3	1	1
2	2	3	3	1	1	1	1	4	4
2	2	1	1	1	1	1	1	4	4
2	2	1	1	1	1	3	3	4	4
2	2	1	1	1	1	1	1	4	4
2	2	3	3	2	2	1	1	4	4
2	2	3	3	1	1	1	1	4	4
2	2	3	3	1	1	1	1	4	4
2	2	1	1	1	1	1	1	4	4
2	2	1	1	1	1	1	1	4	4
2	2	1	1	1	1	3	3	4	4
3	3	3	3	1	1	1	1	4	4
2	2	1	1	1	1	1	1	4	4
2	2	1	1	2	2	1	1	4	4
2	2	1	1	1	1	1	1	4	4
2	2	3	3	1	1	1	1	4	4
2	2	3	3	1	1	1	1	4	4
2	2	1	1	1	1	1	1	4	4
2	2	1	1	1	1	3	3	1	1

For Review Only

2	2	1	1	1	1	1	1	4	4
2	2	1	1	1	1	1	1	4	4
3	3	1	1	1	1	1	1	4	4
2	2	3	3	1	1	3	3	4	4
2	2	3	3	1	1	1	1	4	4
3	3	1	1	1	1	1	1	4	4
2	2	1	1	2	2	1	1	4	4
2	2	1	1	1	1	1	1	4	4
2	2	1	1	1	1	1	1	4	4
2	2	1	1	1	1	1	1	4	4
2	2	3	3	1	1	1	1	4	4
2	2	1	1	1	1	1	1	4	4
2	2	3	3	1	1	1	1	4	4
2	2	1	1	1	1	1	1	4	4
2	2	1	1	1	1	1	1	4	4
3	3	1	1	1	1	1	1	4	4
2	2	1	1	1	1	1	1	4	4
2	2	1	1	1	1	1	1	4	4
2	2	3	3	1	1	3	3	4	4
2	2	1	1	2	2	1	1	4	4
2	2	1	1	1	1	1	1	4	4
2	2	1	1	1	1	1	1	4	4
2	2	1	1	2	2	1	1	4	4
2	2	3	3	1	1	1	1	4	4
3	3	3	3	1	1	1	1	4	4
2	2	1	1	1	1	1	1	4	4
2	2	3	3	1	1	1	1	4	4
2	2	3	3	1	1	1	1	4	4
2	2	1	1	1	1	1	1	4	4
2	2	1	1	1	1	1	1	4	4
2	2	3	3	1	1	1	1	4	4
2	2	1	1	1	1	1	1	4	4
2	2	1	1	1	1	1	1	4	4
2	2	1	1	1	1	1	1	4	4
2	2	3	3	1	1	3	3	4	4
2	2	1	1	1	1	1	1	4	4
2	2	3	3	1	1	3	3	4	4
2	2	1	1	1	1	1	1	4	4
2	2	1	1	1	1	1	1	4	4
2	2	3	3	1	1	1	1	4	4
2	2	1	1	1	1	1	1	4	4
2	2	3	3	1	1	3	3	4	4
2	2	1	1	1	1	1	1	4	4
2	2	1	1	1	1	3	3	4	4
2	2	1	1	1	1	1	1	4	4
2	2	1	1	1	1	1	1	4	4
3	3	1	1	1	1	1	1	4	4
2	2	3	3	1	1	1	1	4	4
2	2	1	1	1	1	1	1	4	4
3	3	1	1	1	1	1	1	4	4
2	2	1	1	1	1	1	1	4	4
2	2	1	1	1	1	1	1	4	4
2	2	1	1	1	1	1	1	4	4
2	2	3	3	1	1	1	1	4	4
2	2	1	1	1	1	1	1	4	4

For Review Only

2	2	1	1	1	1	1	1	4	4
2	2	1	1	1	1	1	1	4	4
2	2	1	1	1	1	1	1	4	4
2	2	1	1	1	1	1	1	4	4
2	2	1	1	1	1	1	1	4	4
2	2	1	1	1	1	1	1	4	4
2	2	1	1	1	1	1	1	4	4
2	2	1	1	1	1	1	1	4	4
2	2	1	1	1	1	1	1	4	4
2	2	1	1	1	1	1	1	4	4
3	3	1	1	1	1	1	1	4	4
2	2	1	1	1	1	1	1	4	4
2	2	3	3	1	1	1	1	4	4
2	2	1	1	1	1	1	1	4	4
2	2	3	3	1	1	3	3	4	4
2	2	1	1	1	1	1	1	4	4
2	2	1	1	1	1	1	1	4	4
2	2	1	1	1	1	1	1	4	4
3	3	1	1	1	1	1	1	4	4
2	2	1	1	2	2	1	1	4	4
2	2	1	1	1	1	1	1	4	4
2	2	1	1	1	1	1	1	4	4
2	2	3	3	1	1	1	1	4	4
2	2	3	3	1	1	1	1	4	4
2	2	1	1	1	1	1	1	4	4
2	2	3	3	1	1	3	3	4	4
2	2	1	1	1	1	1	1	4	4
2	2	1	1	1	1	1	1	4	4
2	2	1	1	1	1	1	1	4	4
2	2	1	1	2	2	1	1	4	4
2	2	1	1	1	1	1	1	4	4
2	2	1	1	1	1	1	1	4	4
2	2	3	3	1	1	1	1	4	4
2	2	3	3	1	1	3	3	4	4
2	2	1	1	1	1	1	1	4	4
2	2	3	3	1	1	1	1	4	4
2	2	1	1	1	1	1	1	4	4
2	2	1	1	1	1	1	1	4	4
2	2	3	3	1	1	1	1	4	4
2	2	3	3	1	1	1	1	4	4
2	2	1	1	2	2	1	1	4	4
3	3	1	1	1	1	1	1	4	4
2	2	3	3	1	1	1	1	4	4
2	2	1	1	1	1	1	1	4	4
2	2	1	1	1	1	1	1	4	4
2	2	1	1	1	1	1	1	4	4
2	2	1	1	1	1	1	1	4	4
2	2	3	3	1	1	1	1	4	4
2	2	3	3	1	1	1	1	4	4
2	2	1	1	1	1	1	1	4	4
2	2	1	1	1	1	1	1	4	4
2	2	3	3	1	1	1	1	4	4
2	2	3	3	1	1	1	1	4	4
2	2	3	3	1	1	1	1	4	4
2	2	3	3	1	1	1	1	4	4

For Review Only

2	2	1	1	2	2	3	3	4	4
2	2	1	1	1	1	1	1	4	4
2	2	1	1	2	2	1	1	4	4
2	2	1	1	1	1	1	1	4	4
3	3	1	1	1	1	1	1	4	4
3	3	1	1	1	1	1	1	4	4
2	2	1	1	1	1	1	1	4	4
3	3	1	1	1	1	1	1	4	4
2	2	1	1	1	1	1	1	4	4
2	2	3	3	1	1	1	1	4	4
2	2	1	1	1	1	1	1	4	4
2	2	1	1	1	1	1	1	4	4
2	2	1	1	1	1	1	1	4	4
2	2	1	1	1	1	1	1	4	4
2	2	3	3	2	2	1	1	4	4
2	2	1	1	1	1	1	1	4	4
2	2	3	3	1	1	1	1	4	4
2	2	1	1	1	1	1	1	4	4
2	2	3	3	1	1	1	1	4	4
2	2	1	1	1	1	3	3	1	1
2	2	3	3	1	1	1	1	4	4
3	3	1	1	1	1	1	1	4	4
2	2	1	1	1	1	1	1	4	4
2	2	1	1	1	1	1	1	4	4
2	2	3	3	1	1	1	1	4	4
2	2	1	1	1	1	1	1	4	4
2	2	3	3	1	1	1	1	4	4
2	2	1	1	1	1	1	1	4	4
2	2	1	1	1	1	1	1	4	4
2	2	3	3	1	1	1	1	4	4
2	2	1	1	1	1	1	1	4	4
3	3	1	1	1	1	1	1	4	4
2	2	1	1	1	1	3	3	1	1
2	2	1	1	1	1	1	1	4	4
2	2	3	3	1	1	1	1	4	4
2	2	3	3	1	1	1	1	4	4
2	2	1	1	2	2	1	1	4	4
2	2	3	3	1	1	1	1	4	4
2	2	3	3	1	1	1	1	4	4
2	2	3	3	1	1	1	1	4	4
2	2	1	1	1	1	1	1	4	4
2	2	1	1	1	1	1	1	4	4
2	2	1	1	1	1	1	1	4	4
2	2	1	1	1	1	1	1	4	4
2	2	1	1	1	1	1	1	4	4
2	2	1	1	1	1	1	1	4	4
2	2	3	3	1	1	1	1	4	4
2	2	1	1	1	1	1	1	4	4
2	2	1	1	1	1	3	3	4	4
2	2	3	3	1	1	1	1	4	4
2	2	1	1	2	2	1	1	4	4
2	2	3	3	1	1	1	1	4	4
2	2	1	1	2	2	1	1	4	4
2	2	1	1	1	1	1	1	4	4
2	2	1	1	1	1	1	1	4	4

For Review Only

2	2	1	1	1	1	1	1	4	4
2	2	3	3	1	1	1	1	4	4

For Review Only

SNP1918	SNP1918	SNP1919	SNP1919	SNP192	SNP192	SNP1920	SNP1920	SNP1921	SNP1921
3	3	4	4	4	4	4	4	3	3
4	4	3	3	4	4	4	4	3	3
4	4	4	4	4	4	4	4	3	3
4	4	3	3	1	1	4	4	3	3
3	3	4	4	4	4	4	4	3	3
4	4	4	4	4	4	4	4	3	3
4	4	3	3	4	4	4	4	3	3
4	4	4	4	4	4	4	4	3	3
3	3	4	4	4	4	4	4	3	3
4	4	4	4	4	4	4	4	3	3
3	3	3	3	4	4	4	4	3	3
4	4	4	4	4	4	4	4	3	3
3	3	4	4	4	4	4	4	3	3
4	4	4	4	4	4	4	4	3	3
4	4	3	3	4	4	4	4	3	3
4	4	4	4	4	4	4	4	3	3
3	3	4	4	4	4	4	4	3	3
4	4	4	4	4	4	4	4	2	2
4	4	4	4	4	4	4	4	3	3
3	3	4	4	4	4	4	4	3	3
3	3	3	3	4	4	4	4	3	3
3	3	4	4	4	4	4	4	3	3
3	3	3	3	4	4	4	4	3	3
4	4	4	4	4	4	4	4	3	3
4	4	4	4	4	4	4	4	3	3
4	4	4	4	4	4	4	4	3	3
4	4	4	4	4	4	4	4	3	3
4	4	4	4	4	4	4	4	3	3
3	3	4	4	4	4	4	4	3	3
3	3	4	4	4	4	4	4	3	3
4	4	4	4	1	1	4	4	3	3
4	4	4	4	4	4	4	4	2	2
3	3	4	4	4	4	4	4	2	2
3	3	4	4	4	4	4	4	3	3
4	4	4	4	4	4	4	4	3	3
4	4	3	3	4	4	4	4	3	3
3	3	4	4	1	1	4	4	3	3
4	4	3	3	4	4	4	4	3	3
4	4	3	3	4	4	1	1	3	3
3	3	3	3	4	4	4	4	3	3
3	3	3	3	4	4	4	4	3	3
3	3	4	4	4	4	4	4	3	3
3	3	4	4	4	4	4	4	3	3
3	3	3	3	4	4	4	4	3	3
3	3	4	4	4	4	4	4	3	3
4	4	4	4	4	4	4	4	3	3
4	4	4	4	4	4	4	4	3	3
4	4	4	4	4	4	4	4	3	3
3	3	4	4	4	4	4	4	3	3
4	4	4	4	4	4	4	4	3	3
4	4	4	4	4	4	4	4	3	3
4	4	4	4	4	4	4	4	3	3

For Review Only

3	3	4	4	4	4	4	4	3	3
3	3	4	4	4	4	4	4	2	2
4	4	4	4	4	4	4	4	3	3
3	3	3	3	4	4	4	4	3	3
4	4	4	4	4	4	4	4	3	3
4	4	4	4	4	4	4	4	2	2
4	4	3	3	4	4	4	4	2	2
3	3	4	4	4	4	4	4	3	3
4	4	4	4	1	1	4	4	3	3
4	4	4	4	4	4	4	4	3	3
4	4	3	3	4	4	4	4	3	3
4	4	4	4	4	4	4	4	3	3
3	3	4	4	4	4	4	4	3	3
3	3	4	4	4	4	4	4	3	3
4	4	4	4	4	4	4	4	3	3
3	3	3	3	4	4	4	4	3	3
4	4	3	3	4	4	4	4	3	3
4	4	4	4	4	4	4	4	2	2
4	4	3	3	4	4	4	4	3	3
3	3	4	4	4	4	4	4	3	3
4	4	4	4	4	4	4	4	3	3
4	4	4	4	1	1	4	4	3	3
3	3	4	4	4	4	4	4	3	3
3	3	3	3	4	4	4	4	2	2
3	3	4	4	4	4	4	4	3	3
4	4	4	4	4	4	4	4	3	3
3	3	4	4	4	4	4	4	2	2
4	4	3	3	4	4	4	4	3	3
4	4	4	4	4	4	4	4	2	2
3	3	4	4	4	4	4	4	3	3
4	4	4	4	4	4	4	4	3	3
4	4	4	4	4	4	4	4	3	3
4	4	3	3	4	4	4	4	3	3
3	3	4	4	4	4	4	4	3	3
3	3	4	4	4	4	4	4	3	3
3	3	4	4	4	4	4	4	3	3
4	4	4	4	4	4	4	4	3	3
3	3	4	4	4	4	4	4	3	3
4	4	3	3	4	4	1	1	3	3
3	3	4	4	4	4	4	4	3	3
3	3	3	3	4	4	4	4	3	3
3	3	4	4	4	4	4	4	3	3
4	4	4	4	4	4	4	4	3	3
4	4	3	3	4	4	4	4	3	3
4	4	4	4	4	4	4	4	3	3
4	4	4	4	4	4	4	4	2	2
4	4	4	4	4	4	4	4	3	3
4	4	3	3	4	4	4	4	3	3
4	4	3	3	4	4	1	1	2	2
3	3	4	4	4	4	4	4	3	3
4	4	3	3	4	4	4	4	3	3
4	4	3	3	4	4	4	4	3	3
3	3	4	4	4	4	4	4	3	3
4	4	4	4	4	4	4	4	3	3
4	4	4	4	4	4	4	4	2	2

For Review Only

4	4	4	4	4	4	4	4	3	3
4	4	3	3	4	4	4	4	3	3
4	4	4	4	4	4	4	4	3	3
3	3	4	4	4	4	4	4	3	3
4	4	4	4	4	4	4	4	3	3
4	4	4	4	4	4	4	4	3	3
4	4	4	4	4	4	4	4	3	3
4	4	4	4	4	4	4	4	3	3
4	4	4	4	4	4	1	1	3	3
4	4	4	4	4	4	4	4	3	3
4	4	4	4	4	4	4	4	3	3
4	4	4	4	4	4	4	4	3	3
4	4	4	4	4	4	4	4	3	3
4	4	4	4	4	4	4	4	3	3
4	4	4	4	4	4	4	4	3	3
4	4	4	4	4	4	4	4	2	2
4	4	4	4	4	4	4	4	2	2
4	4	4	4	4	4	4	4	3	3
3	3	4	4	4	4	4	4	3	3
4	4	4	4	4	4	4	4	3	3
4	4	4	4	4	4	4	4	3	3
4	4	4	4	4	4	4	4	2	2
4	4	4	4	4	4	4	4	3	3
4	4	3	3	4	4	4	4	3	3
4	4	3	3	4	4	4	4	3	3
3	3	3	3	1	1	4	4	3	3
4	4	3	3	4	4	4	4	3	3
4	4	4	4	1	1	4	4	3	3
4	4	4	4	4	4	4	4	2	2
4	4	4	4	4	4	4	4	3	3
4	4	3	3	4	4	4	4	3	3
4	4	4	4	4	4	4	4	3	3
3	3	3	3	4	4	4	4	3	3
4	4	4	4	4	4	4	4	3	3
3	3	4	4	4	4	4	4	3	3
3	3	4	4	4	4	4	4	3	3
3	3	4	4	4	4	4	4	3	3
4	4	3	3	4	4	4	4	3	3
4	4	4	4	4	4	4	4	2	2
4	4	3	3	4	4	4	4	3	3
4	4	3	3	1	1	4	4	3	3
4	4	4	4	4	4	4	4	3	3
4	4	4	4	4	4	4	4	2	2
4	4	4	4	4	4	4	4	3	3
4	4	4	4	4	4	4	4	2	2
3	3	4	4	4	4	4	4	3	3
4	4	4	4	4	4	4	4	2	2
4	4	4	4	4	4	4	4	3	3
4	4	4	4	4	4	4	4	3	3
4	4	4	4	4	4	4	4	3	3
4	4	3	3	4	4	4	4	3	3
3	3	4	4	4	4	4	4	3	3
4	4	4	4	4	4	4	4	3	3
4	4	3	3	4	4	4	4	3	3
4	4	4	4	4	4	4	4	3	3

For Review Only

4	4	4	4	4	4	4	4	2	2
4	4	4	4	4	4	1	1	3	3
3	3	3	3	4	4	4	4	3	3
4	4	4	4	4	4	4	4	3	3
4	4	4	4	4	4	4	4	3	3
3	3	4	4	4	4	4	4	3	3
4	4	3	3	4	4	4	4	3	3
4	4	4	4	4	4	4	4	3	3
4	4	4	4	4	4	4	4	3	3
3	3	4	4	4	4	4	4	2	2
4	4	4	4	4	4	4	4	2	2
4	4	3	3	4	4	4	4	3	3
4	4	3	3	4	4	4	4	3	3
3	3	4	4	4	4	4	4	3	3
3	3	4	4	4	4	4	4	3	3
3	3	3	3	4	4	4	4	3	3
4	4	4	4	4	4	4	4	3	3
4	4	4	4	4	4	4	4	3	3
4	4	4	4	4	4	4	4	2	2
4	4	4	4	4	4	4	4	3	3
4	4	4	4	4	4	4	4	3	3
4	4	3	3	4	4	4	4	3	3
3	3	4	4	1	1	4	4	3	3
4	4	4	4	4	4	4	4	3	3
3	3	4	4	4	4	4	4	3	3
3	3	4	4	4	4	4	4	3	3
4	4	3	3	4	4	4	4	3	3
4	4	4	4	4	4	4	4	3	3
4	4	4	4	4	4	4	4	3	3
4	4	4	4	4	4	4	4	3	3
4	4	3	3	4	4	4	4	3	3
3	3	4	4	4	4	4	4	2	2
3	3	3	3	4	4	4	4	3	3
3	3	4	4	4	4	4	4	3	3
3	3	4	4	4	4	4	4	3	3
4	4	4	4	4	4	4	4	3	3
3	3	4	4	4	4	4	4	3	3
3	3	4	4	1	1	4	4	3	3
4	4	4	4	4	4	4	4	3	3
4	4	3	3	4	4	4	4	3	3
4	4	4	4	4	4	4	4	3	3
4	4	4	4	4	4	4	4	2	2
4	4	3	3	4	4	4	4	3	3
4	4	4	4	4	4	4	4	3	3
4	4	4	4	4	4	4	4	3	3
4	4	4	4	4	4	4	4	3	3
3	3	3	3	4	4	4	4	3	3
3	3	3	3	4	4	4	4	3	3
4	4	4	4	4	4	4	4	3	3
4	4	3	3	1	1	4	4	3	3
4	4	4	4	4	4	4	4	2	2
3	3	4	4	4	4	4	4	3	3
3	3	3	3	1	1	1	1	3	3
4	4	3	3	4	4	4	4	3	3
4	4	3	3	4	4	4	4	3	3

For Review Only

4	4	4	4	4	4	4	4	2	2
4	4	4	4	1	1	4	4	2	2
3	3	4	4	4	4	4	4	2	2
3	3	4	4	4	4	4	4	3	3
4	4	4	4	4	4	4	4	2	2
4	4	4	4	4	4	4	4	3	3
4	4	4	4	4	4	4	4	2	2
4	4	4	4	4	4	4	4	3	3
4	4	4	4	4	4	1	1	3	3
4	4	3	3	4	4	4	4	3	3
4	4	4	4	4	4	4	4	3	3
4	4	4	4	4	4	4	4	3	3
4	4	3	3	4	4	1	1	3	3
4	4	4	4	4	4	4	4	3	3
4	4	4	4	4	4	4	4	3	3
3	3	3	3	4	4	1	1	3	3
4	4	3	3	4	4	4	4	3	3
4	4	4	4	4	4	4	4	3	3
4	4	3	3	4	4	4	4	2	2
4	4	3	3	4	4	4	4	3	3
4	4	3	3	4	4	4	4	3	3
4	4	4	4	4	4	4	4	2	2
3	3	4	4	4	4	4	4	3	3
4	4	3	3	4	4	4	4	3	3
3	3	4	4	4	4	4	4	3	3
3	3	4	4	4	4	4	4	3	3
4	4	3	3	4	4	4	4	3	3
4	4	3	3	4	4	4	4	3	3
4	4	4	4	4	4	4	4	3	3
4	4	4	4	1	1	4	4	2	2
4	4	3	3	4	4	1	1	3	3
4	4	4	4	4	4	4	4	3	3
3	3	3	3	4	4	4	4	3	3
4	4	4	4	4	4	4	4	3	3
3	3	3	3	1	1	4	4	3	3
3	3	3	3	4	4	4	4	3	3
4	4	4	4	4	4	4	4	2	2
4	4	4	4	4	4	4	4	3	3
4	4	3	3	4	4	4	4	3	3
4	4	3	3	4	4	4	4	3	3
4	4	4	4	4	4	4	4	2	2
4	4	4	4	4	4	4	4	3	3
4	4	3	3	4	4	4	4	3	3
3	3	3	3	4	4	4	4	3	3
3	3	4	4	4	4	4	4	3	3
4	4	3	3	4	4	1	1	3	3
4	4	4	4	4	4	4	4	3	3
4	4	4	4	4	4	4	4	3	3
4	4	4	4	4	4	4	4	3	3
4	4	3	3	4	4	4	4	3	3
4	4	4	4	4	4	4	4	3	3
4	4	3	3	4	4	4	4	3	3
4	4	4	4	4	4	4	4	3	3
4	4	3	3	4	4	4	4	3	3
3	3	3	3	4	4	4	4	3	3

For Review Only

3	3	4	4	4	4	4	4	3	3
4	4	3	3	4	4	4	4	3	3

For Review Only

SNP1922	SNP1922	SNP1923	SNP1923	SNP1924	SNP1924	SNP1925	SNP1925	SNP1926	SNP1926
4	4	1	1	2	2	1	1	1	1
3	3	1	1	2	2	4	4	3	3
3	3	1	1	2	2	4	4	1	1
3	3	1	1	2	2	1	1	1	1
4	4	1	1	2	2	1	1	1	1
3	3	1	1	2	2	4	4	1	1
3	3	1	1	2	2	1	1	1	1
3	3	1	1	2	2	1	1	1	1
4	4	1	1	3	3	1	1	1	1
3	3	1	1	2	2	1	1	3	3
4	4	4	4	2	2	1	1	1	1
3	3	1	1	2	2	4	4	1	1
4	4	1	1	2	2	4	4	1	1
3	3	1	1	2	2	1	1	1	1
4	4	1	1	2	2	1	1	1	1
3	3	1	1	2	2	4	4	1	1
4	4	1	1	3	3	1	1	3	3
4	4	1	1	2	2	1	1	1	1
3	3	1	1	2	2	4	4	1	1
4	4	1	1	2	2	1	1	3	3
4	4	1	1	3	3	1	1	3	3
4	4	1	1	2	2	1	1	1	1
4	4	1	1	2	2	1	1	1	1
3	3	1	1	2	2	4	4	1	1
3	3	1	1	2	2	4	4	1	1
3	3	1	1	2	2	4	4	1	1
3	3	1	1	2	2	4	4	1	1
4	4	1	1	2	2	1	1	1	1
3	3	1	1	3	3	1	1	1	1
4	4	1	1	2	2	1	1	3	3
3	3	1	1	2	2	1	1	3	3
3	3	1	1	2	2	1	1	3	3
4	4	1	1	2	2	1	1	1	1
3	3	1	1	2	2	1	1	3	3
3	3	4	4	2	2	1	1	3	3
3	3	1	1	2	2	4	4	3	3
3	3	1	1	2	2	1	1	3	3
3	3	1	1	2	2	1	1	3	3
3	3	1	1	2	2	1	1	1	1
3	3	4	4	2	2	1	1	1	1
4	4	1	1	2	2	1	1	3	3
3	3	1	1	2	2	1	1	1	1
4	4	1	1	2	2	1	1	3	3
4	4	1	1	3	3	1	1	3	3
4	4	1	1	2	2	1	1	3	3
3	3	1	1	2	2	4	4	1	1
3	3	1	1	2	2	1	1	1	1
3	3	4	4	2	2	1	1	1	1
4	4	1	1	3	3	1	1	3	3
3	3	4	4	2	2	1	1	1	1
3	3	1	1	2	2	1	1	1	1
3	3	1	1	2	2	4	4	1	1

For Review Only

4	4	1	1	3	3	1	1	1	1
4	4	1	1	2	2	1	1	1	1
3	3	1	1	2	2	1	1	1	1
4	4	1	1	3	3	1	1	3	3
3	3	1	1	2	2	4	4	1	1
3	3	1	1	2	2	1	1	1	1
3	3	1	1	2	2	1	1	1	1
4	4	1	1	2	2	1	1	3	3
3	3	1	1	2	2	1	1	1	1
3	3	1	1	2	2	4	4	1	1
3	3	1	1	2	2	1	1	1	1
3	3	1	1	2	2	1	1	1	1
4	4	1	1	3	3	1	1	1	1
3	3	1	1	3	3	1	1	1	1
4	4	1	1	2	2	4	4	1	1
3	3	1	1	3	3	1	1	1	1
3	3	1	1	2	2	1	1	1	1
3	3	1	1	3	3	1	1	1	1
3	3	1	1	3	3	1	1	3	3
4	4	1	1	3	3	1	1	1	1
3	3	1	1	2	2	1	1	1	1
4	4	4	4	2	2	1	1	1	1
3	3	1	1	3	3	1	1	1	1
4	4	1	1	3	3	1	1	1	1
4	4	1	1	2	2	1	1	3	3
3	3	1	1	2	2	4	4	1	1
4	4	1	1	3	3	1	1	3	3
3	3	1	1	2	2	1	1	1	1
3	3	1	1	2	2	1	1	1	1
4	4	1	1	2	2	1	1	1	1
3	3	1	1	2	2	1	1	1	1
3	3	1	1	2	2	4	4	3	3
3	3	1	1	2	2	1	1	3	3
4	4	1	1	3	3	1	1	1	1
4	4	1	1	2	2	4	4	1	1
4	4	1	1	2	2	1	1	3	3
3	3	1	1	2	2	1	1	1	1
4	4	1	1	3	3	1	1	3	3
3	3	1	1	2	2	4	4	3	3
4	4	1	1	2	2	1	1	3	3
4	4	1	1	3	3	1	1	1	1
4	4	1	1	2	2	4	4	1	1
3	3	1	1	2	2	1	1	3	3
3	3	1	1	2	2	1	1	1	1
3	3	1	1	2	2	1	1	1	1
3	3	1	1	2	2	1	1	1	1
3	3	1	1	2	2	1	1	3	3
3	3	1	1	2	2	4	4	1	1
3	3	1	1	2	2	1	1	1	1
4	4	1	1	2	2	4	4	1	1
3	3	1	1	2	2	4	4	1	1
3	3	4	4	2	2	1	1	1	1
4	4	1	1	2	2	1	1	3	3
3	3	1	1	2	2	1	1	1	1
3	3	1	1	2	2	1	1	1	1

For Review Only

3	3	4	4	2	2	1	1	1	1
3	3	1	1	2	2	1	1	1	1
3	3	1	1	2	2	4	4	3	3
3	3	1	1	2	2	1	1	3	3
3	3	1	1	2	2	4	4	1	1
3	3	1	1	2	2	4	4	3	3
3	3	1	1	2	2	4	4	1	1
3	3	1	1	2	2	4	4	3	3
3	3	1	1	2	2	1	1	1	1
3	3	1	1	2	2	1	1	1	1
3	3	1	1	3	3	1	1	1	1
4	4	1	1	2	2	4	4	1	1
3	3	1	1	2	2	1	1	3	3
3	3	1	1	2	2	4	4	1	1
3	3	1	1	2	2	4	4	1	1
3	3	1	1	2	2	1	1	1	1
3	3	1	1	3	3	1	1	3	3
4	4	1	1	2	2	1	1	1	1
3	3	1	1	2	2	1	1	1	1
3	3	1	1	2	2	1	1	3	3
3	3	1	1	2	2	1	1	1	1
3	3	1	1	2	2	1	1	1	1
3	3	1	1	2	2	4	4	1	1
3	3	1	1	2	2	1	1	1	1
4	4	1	1	2	2	4	4	1	1
4	4	4	4	2	2	1	1	1	1
4	4	1	1	2	2	1	1	1	1
3	3	1	1	2	2	1	1	3	3
3	3	1	1	2	2	1	1	1	1
3	3	1	1	3	3	4	4	3	3
3	3	1	1	2	2	1	1	3	3
3	3	1	1	2	2	4	4	1	1
4	4	1	1	3	3	1	1	1	1
4	4	1	1	2	2	1	1	1	1
3	3	1	1	2	2	1	1	1	1
4	4	1	1	2	2	4	4	1	1
3	3	1	1	2	2	1	1	3	3
3	3	1	1	3	3	1	1	1	1
4	4	1	1	2	2	1	1	1	1
4	4	1	1	2	2	1	1	3	3
3	3	4	4	2	2	1	1	1	1
3	3	1	1	2	2	1	1	3	3
3	3	1	1	3	3	1	1	1	1
4	4	1	1	2	2	1	1	3	3
3	3	1	1	2	2	1	1	1	1
4	4	1	1	2	2	1	1	1	1
3	3	1	1	2	2	1	1	1	1
3	3	1	1	2	2	4	4	1	1
4	4	1	1	2	2	1	1	1	1
3	3	1	1	2	2	4	4	1	1
3	3	1	1	2	2	4	4	1	1
3	3	4	4	2	2	1	1	3	3
3	3	1	1	2	2	4	4	3	3
3	3	1	1	2	2	1	1	1	1
4	4	1	1	2	2	4	4	1	1

For Review Only

CSSA 5585 Guilford Rd., Madison, WI 53711

3	3	1	1	2	2	1	1	1	1
3	3	1	1	2	2	1	1	3	3
4	4	1	1	2	2	1	1	1	1
3	3	1	1	2	2	1	1	1	1
3	3	1	1	2	2	4	4	1	1
3	3	1	1	2	2	1	1	3	3
4	4	4	4	2	2	1	1	1	1
3	3	1	1	2	2	1	1	1	1
4	4	4	4	2	2	1	1	1	1
4	4	1	1	2	2	1	1	3	3
3	3	1	1	2	2	4	4	1	1
3	3	1	1	2	2	4	4	1	1
3	3	1	1	2	2	4	4	1	1
4	4	1	1	2	2	1	1	1	1
3	3	1	1	2	2	1	1	3	3
4	4	4	4	2	2	1	1	3	3
3	3	1	1	2	2	1	1	3	3
3	3	4	4	2	2	4	4	1	1
3	3	1	1	2	2	1	1	3	3
3	3	1	1	2	2	4	4	1	1
4	4	1	1	2	2	1	1	1	1
3	3	1	1	2	2	4	4	1	1
4	4	4	4	2	2	1	1	1	1
3	3	1	1	2	2	1	1	1	1
4	4	1	1	3	3	1	1	1	1
3	3	1	1	2	2	1	1	1	1
3	3	1	1	2	2	4	4	1	1
3	3	1	1	3	3	1	1	1	1
3	3	1	1	2	2	1	1	1	1
3	3	1	1	3	3	1	1	1	1
3	3	1	1	3	3	1	1	1	1
4	4	1	1	2	2	1	1	1	1
4	4	1	1	3	3	1	1	1	1
3	3	1	1	2	2	1	1	3	3
4	4	1	1	2	2	1	1	1	1
3	3	1	1	2	2	1	1	1	1
4	4	1	1	2	2	4	4	1	1
3	3	1	1	3	3	1	1	1	1
3	3	1	1	2	2	4	4	1	1
3	3	1	1	3	3	4	4	1	1
3	3	1	1	2	2	4	4	1	1
3	3	1	1	2	2	1	1	1	1
3	3	1	1	2	2	1	1	3	3
4	4	4	4	2	2	4	4	1	1
3	3	1	1	2	2	1	1	1	1
3	3	1	1	2	2	1	1	3	3
4	4	4	4	2	2	1	1	3	3
4	4	4	4	2	2	1	1	1	1
3	3	1	1	2	2	1	1	1	1
3	3	1	1	2	2	1	1	1	1
3	3	1	1	2	2	1	1	3	3
4	4	1	1	2	2	1	1	3	3
4	4	1	1	2	2	1	1	1	1
3	3	1	1	2	2	1	1	1	1
3	3	1	1	2	2	1	1	1	1

For Review Only

3	3	1	1	2	2	1	1	1	1
3	3	1	1	2	2	1	1	1	1
4	4	1	1	2	2	1	1	1	1
4	4	4	4	2	2	1	1	1	1
3	3	1	1	2	2	1	1	1	1
3	3	1	1	2	2	1	1	1	1
3	3	1	1	2	2	1	1	3	3
3	3	1	1	2	2	1	1	3	3
3	3	1	1	2	2	1	1	1	1
3	3	1	1	2	2	1	1	1	1
4	4	1	1	2	2	4	4	1	1
3	3	1	1	2	2	1	1	3	3
3	3	1	1	2	2	1	1	1	1
3	3	1	1	2	2	1	1	1	1
3	3	1	1	2	2	4	4	1	1
4	4	1	1	2	2	1	1	1	1
3	3	1	1	2	2	1	1	1	1
4	4	1	1	2	2	1	1	3	3
3	3	1	1	2	2	1	1	1	1
3	3	1	1	2	2	1	1	3	3
3	3	1	1	2	2	1	1	1	1
3	3	1	1	2	2	1	1	1	1
4	4	1	1	3	3	1	1	1	1
3	3	4	4	2	2	1	1	1	1
4	4	1	1	2	2	1	1	3	3
3	3	1	1	2	2	1	1	1	1
3	3	1	1	2	2	1	1	1	1
3	3	1	1	2	2	4	4	1	1
4	4	1	1	2	2	1	1	1	1
3	3	1	1	2	2	1	1	1	1
3	3	1	1	2	2	1	1	1	1
3	3	4	4	2	2	4	4	3	3
4	4	1	1	2	2	1	1	3	3
3	3	1	1	2	2	4	4	1	1
4	4	4	4	2	2	1	1	1	1
3	3	1	1	2	2	1	1	1	1
3	3	1	1	3	3	1	1	1	1
3	3	1	1	2	2	1	1	1	1
3	3	1	1	2	2	4	4	3	3
3	3	4	4	2	2	1	1	1	1
4	4	1	1	3	3	1	1	3	3
3	3	1	1	2	2	4	4	3	3
3	3	4	4	2	2	1	1	1	1
4	4	1	1	3	3	1	1	1	1
3	3	1	1	2	2	1	1	1	1
3	3	1	1	2	2	4	4	3	3
3	3	4	4	2	2	4	4	1	1
3	3	1	1	2	2	1	1	1	1
4	4	1	1	2	2	1	1	3	3
3	3	1	1	2	2	1	1	3	3
3	3	1	1	3	3	1	1	1	1
3	3	4	4	2	2	1	1	1	1
3	3	1	1	2	2	4	4	1	1
3	3	1	1	2	2	4	4	1	1
3	3	4	4	2	2	1	1	1	1

For Review Only

4	4	1	1	3	3	1	1	1	1
3	3	1	1	2	2	4	4	3	3

For Review Only

SNP1927	SNP1927	SNP1928	SNP1928	SNP1929	SNP1929	SNP193	SNP193	SNP1930	SNP1930
2	2	2	2	2	2	4	4	3	3
2	2	2	2	2	2	3	3	4	4
2	2	2	2	2	2	3	3	3	3
2	2	2	2	2	2	4	4	4	4
1	1	2	2	1	1	3	3	4	4
2	2	2	2	2	2	4	4	4	4
2	2	2	2	2	2	4	4	4	4
2	2	2	2	2	2	3	3	4	4
2	2	2	2	1	1	4	4	4	4
2	2	2	2	2	2	3	3	4	4
1	1	3	3	1	1	3	3	4	4
2	2	2	2	2	2	3	3	4	4
2	2	2	2	2	2	4	4	4	4
2	2	2	2	2	2	3	3	4	4
2	2	2	2	2	2	4	4	4	4
2	2	2	2	2	2	3	3	3	3
1	1	2	2	2	2	3	3	3	3
2	2	2	2	2	2	4	4	4	4
2	2	2	2	2	2	4	4	4	4
1	1	2	2	2	2	3	3	4	4
1	1	2	2	1	1	3	3	4	4
1	1	2	2	2	2	3	3	4	4
1	1	2	2	2	2	3	3	4	4
2	2	2	2	2	2	3	3	4	4
2	2	2	2	2	2	4	4	4	4
2	2	2	2	2	2	3	3	4	4
2	2	2	2	2	2	3	3	4	4
2	2	2	2	2	2	4	4	4	4
2	2	2	2	2	2	3	3	4	4
1	1	2	2	2	2	3	3	4	4
2	2	2	2	2	2	3	3	4	4
2	2	2	2	2	2	3	3	3	3
1	1	3	3	1	1	4	4	4	4
2	2	2	2	2	2	3	3	4	4
2	2	2	2	2	2	3	3	4	4
2	2	2	2	2	2	3	3	4	4
2	2	2	2	2	2	4	4	4	4
2	2	2	2	2	2	3	3	4	4
2	2	2	2	2	2	3	3	4	4
2	2	2	2	2	2	3	3	4	4
1	1	2	2	2	2	3	3	4	4
2	2	2	2	2	2	3	3	4	4
1	1	2	2	2	2	3	3	4	4
1	1	2	2	2	2	4	4	4	4
1	1	2	2	2	2	3	3	4	4
2	2	2	2	2	2	3	3	3	3
2	2	2	2	2	2	3	3	4	4
2	2	2	2	2	2	3	3	4	4
1	1	2	2	2	2	4	4	4	4
2	2	2	2	2	2	4	4	3	3
2	2	2	2	2	2	3	3	4	4
2	2	2	2	2	2	3	3	4	4

For Review Only

1	1	2	2	1	1	4	4	4	4
1	1	3	3	1	1	4	4	4	4
2	2	2	2	2	2	4	4	4	4
1	1	2	2	2	2	3	3	4	4
2	2	2	2	2	2	4	4	4	4
2	2	2	2	2	2	3	3	4	4
2	2	2	2	2	2	3	3	4	4
1	1	2	2	2	2	3	3	4	4
2	2	2	2	2	2	3	3	4	4
2	2	2	2	2	2	3	3	4	4
2	2	2	2	2	2	3	3	4	4
2	2	2	2	2	2	3	3	4	4
1	1	2	2	1	1	4	4	4	4
2	2	2	2	2	2	3	3	4	4
2	2	2	2	2	2	4	4	4	4
2	2	2	2	2	2	3	3	4	4
2	2	2	2	2	2	3	3	4	4
2	2	2	2	2	2	4	4	3	3
2	2	2	2	2	2	3	3	4	4
1	1	2	2	1	1	4	4	3	3
2	2	2	2	2	2	3	3	4	4
2	2	2	2	2	2	3	3	4	4
2	2	2	2	2	2	4	4	4	4
1	1	2	2	2	2	3	3	4	4
1	1	2	2	2	2	3	3	4	4
2	2	2	2	2	2	3	3	4	4
1	1	2	2	2	2	3	3	4	4
2	2	2	2	2	2	3	3	4	4
2	2	2	2	2	2	3	3	4	4
1	1	2	2	1	1	4	4	4	4
1	1	2	2	2	2	4	4	4	4
2	2	2	2	2	2	4	4	4	4
2	2	2	2	2	2	3	3	4	4
1	1	2	2	1	1	4	4	4	4
2	2	2	2	2	2	3	3	4	4
1	1	2	2	2	2	3	3	4	4
2	2	2	2	2	2	4	4	4	4
1	1	2	2	2	2	4	4	4	4
2	2	2	2	2	2	3	3	4	4
1	1	2	2	2	2	4	4	4	4
1	1	2	2	1	1	3	3	4	4
1	1	2	2	1	1	3	3	4	4
2	2	2	2	2	2	3	3	4	4
2	2	2	2	2	2	3	3	4	4
2	2	2	2	2	2	3	3	4	4
2	2	2	2	2	2	3	3	4	4
2	2	2	2	2	2	3	3	4	4
2	2	2	2	2	2	3	3	4	4
2	2	2	2	2	2	3	3	4	4
1	1	2	2	2	2	3	3	3	3
2	2	2	2	2	2	3	3	4	4
2	2	2	2	2	2	3	3	4	4
1	1	2	2	2	2	3	3	4	4
2	2	2	2	2	2	4	4	4	4
2	2	2	2	2	2	3	3	4	4

For Review Only

2	2	2	2	2	2	3	3	4	4
2	2	2	2	2	2	3	3	4	4
2	2	2	2	2	2	3	3	4	4
2	2	2	2	2	2	3	3	4	4
2	2	2	2	2	2	3	3	4	4
2	2	2	2	2	2	3	3	4	4
2	2	2	2	2	2	3	3	3	3
2	2	2	2	2	2	4	4	4	4
2	2	2	2	2	2	3	3	4	4
2	2	2	2	2	2	4	4	4	4
2	2	2	2	2	2	4	4	4	4
2	2	2	2	2	2	4	4	4	4
2	2	2	2	2	2	3	3	4	4
2	2	2	2	2	2	3	3	4	4
2	2	2	2	2	2	4	4	4	4
2	2	2	2	2	2	3	3	4	4
2	2	2	2	2	2	3	3	4	4
2	2	2	2	2	2	4	4	4	4
2	2	2	2	2	2	3	3	4	4
2	2	2	2	2	2	4	4	4	4
2	2	2	2	2	2	4	4	4	4
2	2	2	2	2	2	4	4	4	4
2	2	2	2	2	2	3	3	4	4
2	2	2	2	2	2	3	3	4	4
2	2	2	2	2	2	3	3	4	4
1	1	2	2	2	2	3	3	4	4
2	2	2	2	2	2	4	4	3	3
2	2	2	2	2	2	4	4	4	4
2	2	2	2	2	2	4	4	4	4
2	2	2	2	2	2	4	4	4	4
2	2	2	2	2	2	3	3	4	4
2	2	2	2	2	2	3	3	4	4
1	1	2	2	1	1	3	3	4	4
2	2	2	2	2	2	4	4	4	4
2	2	2	2	2	2	3	3	4	4
1	1	2	2	2	2	3	3	4	4
2	2	2	2	2	2	3	3	4	4
2	2	2	2	2	2	3	3	4	4
2	2	2	2	2	2	3	3	4	4
1	1	2	2	2	2	3	3	4	4
2	2	2	2	2	2	3	3	4	4
2	2	2	2	2	2	3	3	4	4
2	2	2	2	2	2	4	4	4	4
2	2	2	2	2	2	3	3	4	4
2	2	2	2	2	2	3	3	4	4
1	1	2	2	1	1	3	3	4	4
2	2	2	2	2	2	3	3	4	4
2	2	2	2	2	2	3	3	4	4
2	2	2	2	2	2	4	4	4	4
2	2	2	2	2	2	3	3	4	4
2	2	2	2	2	2	3	3	4	4
2	2	2	2	2	2	3	3	4	4
2	2	2	2	2	2	4	4	4	4
2	2	2	2	2	2	4	4	4	4
2	2	2	2	2	2	3	3	4	4

For Review Only

2	2	2	2	2	2	3	3	4	4
2	2	2	2	2	2	3	3	4	4
1	1	2	2	2	2	4	4	4	4
2	2	2	2	2	2	3	3	4	4
2	2	2	2	2	2	4	4	4	4
2	2	2	2	2	2	3	3	4	4
2	2	2	2	2	2	3	3	4	4
2	2	2	2	2	2	4	4	4	4
2	2	2	2	2	2	3	3	4	4
1	1	2	2	2	2	4	4	4	4
2	2	2	2	2	2	3	3	4	4
2	2	2	2	2	2	3	3	4	4
2	2	2	2	2	2	3	3	4	4
1	1	2	2	2	2	3	3	4	4
2	2	2	2	2	2	3	3	4	4
1	1	2	2	1	1	3	3	4	4
2	2	2	2	2	2	4	4	4	4
2	2	2	2	2	2	4	4	4	4
2	2	2	2	2	2	3	3	4	4
2	2	2	2	2	2	3	3	4	4
2	2	2	2	2	2	4	4	4	4
2	2	2	2	2	2	3	3	4	4
1	1	2	2	2	2	3	3	4	4
2	2	2	2	2	2	3	3	4	4
2	2	2	2	2	2	4	4	4	4
2	2	2	2	2	2	3	3	4	4
2	2	2	2	2	2	3	3	4	4
2	2	2	2	2	2	3	3	4	4
2	2	2	2	2	2	3	3	4	4
2	2	2	2	2	2	3	3	3	3
2	2	2	2	2	2	3	3	4	4
1	1	2	2	1	1	3	3	4	4
1	1	2	2	1	1	4	4	4	4
2	2	2	2	2	2	3	3	4	4
1	1	2	2	2	2	4	4	4	4
2	2	2	2	2	2	3	3	4	4
1	1	2	2	1	1	3	3	4	4
2	2	2	2	2	2	4	4	4	4
2	2	2	2	2	2	3	3	4	4
2	2	2	2	2	2	3	3	4	4
2	2	2	2	2	2	3	3	3	3
2	2	2	2	2	2	3	3	4	4
2	2	2	2	2	2	3	3	4	4
2	2	2	2	2	2	4	4	4	4
2	2	2	2	2	2	3	3	4	4
2	2	2	2	2	2	3	3	4	4
1	1	2	2	1	1	3	3	4	4
1	1	2	2	1	1	3	3	4	4
2	2	2	2	2	2	3	3	4	4
2	2	2	2	2	2	3	3	4	4
2	2	2	2	2	2	4	4	4	4
1	1	2	2	2	2	3	3	4	4
1	1	2	2	2	2	3	3	4	4
2	2	2	2	2	2	3	3	4	4
2	2	2	2	2	2	3	3	4	4

For Review Only

2	2	2	2	2	2	4	4	3	3
2	2	2	2	2	2	3	3	4	4
2	2	2	2	2	2	4	4	3	3
1	1	2	2	2	2	4	4	4	4
2	2	2	2	2	2	4	4	4	4
2	2	2	2	2	2	4	4	4	4
2	2	2	2	2	2	3	3	4	4
2	2	2	2	2	2	3	3	4	4
2	2	2	2	2	2	3	3	4	4
2	2	2	2	2	2	3	3	4	4
2	2	2	2	2	2	3	3	4	4
2	2	2	2	2	2	4	4	4	4
2	2	2	2	2	2	4	4	4	4
2	2	2	2	2	2	4	4	4	4
2	2	2	2	2	2	3	3	3	3
1	1	2	2	2	2	3	3	4	4
2	2	2	2	2	2	3	3	4	4
2	2	2	2	2	2	3	3	4	4
2	2	2	2	2	2	3	3	4	4
2	2	2	2	2	2	3	3	4	4
2	2	2	2	2	2	3	3	4	4
2	2	2	2	2	2	3	3	4	4
1	1	2	2	1	1	4	4	4	4
2	2	2	2	2	2	3	3	4	4
1	1	2	2	2	2	3	3	4	4
2	2	2	2	2	2	3	3	4	4
2	2	2	2	2	2	3	3	4	4
2	2	2	2	2	2	3	3	4	4
2	2	2	2	2	2	4	4	4	4
2	2	2	2	2	2	4	4	4	4
2	2	2	2	2	2	3	3	4	4
2	2	2	2	2	2	3	3	4	4
1	1	2	2	2	2	3	3	4	4
2	2	2	2	2	2	4	4	4	4
1	1	2	2	2	2	3	3	4	4
2	2	2	2	2	2	3	3	4	4
2	2	2	2	2	2	3	3	3	3
2	2	2	2	2	2	3	3	4	4
2	2	2	2	2	2	3	3	4	4
2	2	2	2	2	2	3	3	4	4
1	1	2	2	2	2	4	4	4	4
2	2	2	2	2	2	4	4	4	4
2	2	2	2	2	2	3	3	4	4
1	1	2	2	1	1	3	3	4	4
2	2	2	2	2	2	3	3	4	4
2	2	2	2	2	2	3	3	4	4
2	2	2	2	2	2	3	3	4	4
2	2	2	2	2	2	3	3	4	4
2	2	2	2	2	2	3	3	4	4
2	2	2	2	2	2	3	3	4	4
2	2	2	2	2	2	3	3	3	3
2	2	2	2	2	2	3	3	4	4
2	2	2	2	2	2	3	3	3	3
2	2	2	2	2	2	3	3	4	4
2	2	2	2	2	2	3	3	4	4

For Review Only

1	1	2	2	1	1	3	3	4	4
2	2	2	2	2	2	3	3	4	4

For Review Only

SNP1931	SNP1931	SNP1932	SNP1932	SNP1933	SNP1933	SNP1934	SNP1934	SNP1935	SNP1935
3	3	2	2	2	2	2	2	4	4
3	3	2	2	2	2	2	2	2	2
3	3	2	2	2	2	2	2	4	4
3	3	2	2	2	2	2	2	4	4
2	2	4	4	3	3	2	2	4	4
3	3	2	2	2	2	2	2	2	2
3	3	4	4	2	2	1	1	4	4
3	3	2	2	2	2	2	2	2	2
2	2	4	4	3	3	1	1	2	2
3	3	2	2	2	2	2	2	4	4
2	2	2	2	3	3	2	2	4	4
3	3	2	2	2	2	2	2	4	4
3	3	2	2	2	2	1	1	2	2
3	3	2	2	2	2	2	2	4	4
2	2	2	2	3	3	1	1	2	2
3	3	2	2	2	2	2	2	4	4
2	2	2	2	3	3	1	1	4	4
3	3	2	2	2	2	2	2	4	4
3	3	2	2	2	2	2	2	2	2
3	3	2	2	2	2	2	2	4	4
2	2	2	2	3	3	1	1	4	4
3	3	4	4	2	2	2	2	2	2
3	3	4	4	2	2	1	1	2	2
3	3	2	2	2	2	1	1	2	2
3	3	2	2	2	2	1	1	4	4
3	3	2	2	2	2	1	1	4	4
3	3	2	2	2	2	2	2	2	2
3	3	2	2	2	2	1	1	2	2
3	3	2	2	2	2	1	1	4	4
3	3	2	2	2	2	1	1	2	2
3	3	2	2	2	2	1	1	4	4
3	3	2	2	2	2	2	2	4	4
2	2	2	2	3	3	2	2	4	4
3	3	2	2	2	2	2	2	2	2
3	3	4	4	2	2	1	1	2	2
3	3	2	2	2	2	1	1	4	4
3	3	2	2	2	2	2	2	2	2
3	3	2	2	2	2	2	2	2	2
3	3	2	2	2	2	1	1	2	2
3	3	2	2	2	2	1	1	4	4
3	3	2	2	2	2	1	1	2	2
3	3	2	2	2	2	2	2	4	4
3	3	2	2	2	2	2	2	4	4
3	3	4	4	2	2	1	1	2	2
3	3	2	2	2	2	2	2	4	4
3	3	2	2	2	2	1	1	4	4
3	3	2	2	2	2	1	1	4	4
3	3	4	4	2	2	1	1	2	2
3	3	2	2	2	2	1	1	4	4
3	3	4	4	2	2	2	2	2	2
3	3	2	2	2	2	1	1	4	4
3	3	2	2	2	2	2	2	2	2

For Review Only

2	2	2	2	3	3	1	1	4	4
2	2	2	2	3	3	2	2	4	4
3	3	2	2	2	2	2	2	4	4
3	3	2	2	2	2	1	1	4	4
3	3	4	4	2	2	2	2	2	2
3	3	2	2	2	2	1	1	4	4
3	3	2	2	2	2	1	1	4	4
3	3	2	2	2	2	2	2	2	2
3	3	2	2	2	2	2	2	2	2
3	3	2	2	2	2	1	1	2	2
3	3	2	2	2	2	2	2	4	4
3	3	2	2	2	2	1	1	4	4
2	2	2	2	3	3	1	1	4	4
3	3	2	2	2	2	1	1	4	4
3	3	2	2	2	2	2	2	2	2
3	3	2	2	2	2	2	2	2	2
3	3	4	4	2	2	2	2	4	4
3	3	2	2	2	2	2	2	4	4
3	3	4	4	2	2	1	1	2	2
2	2	2	2	3	3	1	1	4	4
3	3	2	2	2	2	1	1	4	4
3	3	2	2	2	2	1	1	2	2
3	3	4	4	2	2	1	1	2	2
3	3	2	2	2	2	2	2	4	4
3	3	2	2	2	2	2	2	4	4
3	3	2	2	2	2	2	2	4	4
3	3	2	2	2	2	1	1	4	4
3	3	2	2	2	2	1	1	2	2
3	3	2	2	2	2	1	1	4	4
2	2	2	2	3	3	2	2	2	2
3	3	2	2	2	2	2	2	4	4
3	3	2	2	2	2	2	2	2	2
3	3	2	2	2	2	2	2	2	2
2	2	2	2	3	3	1	1	4	4
3	3	2	2	2	2	2	2	4	4
3	3	2	2	2	2	2	2	4	4
3	3	2	2	2	2	2	2	2	2
3	3	2	2	2	2	1	1	2	2
3	3	2	2	2	2	1	1	2	2
3	3	2	2	2	2	2	2	4	4
2	2	4	4	3	3	1	1	4	4
2	2	2	2	3	3	1	1	2	2
3	3	2	2	2	2	2	2	2	2
3	3	2	2	2	2	2	2	4	4
3	3	4	4	2	2	1	1	4	4
3	3	2	2	2	2	2	2	4	4
3	3	2	2	2	2	2	2	2	2
3	3	2	2	2	2	1	1	4	4
3	3	4	4	2	2	1	1	4	4
3	3	2	2	2	2	1	1	4	4
3	3	2	2	2	2	1	1	4	4
3	3	2	2	2	2	2	2	2	2
3	3	2	2	2	2	2	2	2	2
3	3	2	2	2	2	2	2	2	2
3	3	2	2	2	2	2	2	4	4

For Review Only

3	3	2	2	2	2	1	1	4	4
3	3	2	2	2	2	2	2	2	2
3	3	2	2	2	2	1	1	4	4
3	3	2	2	2	2	2	2	2	2
3	3	2	2	2	2	2	2	4	4
3	3	2	2	2	2	1	1	4	4
3	3	2	2	2	2	2	2	4	4
3	3	2	2	2	2	1	1	2	2
3	3	2	2	2	2	1	1	2	2
3	3	2	2	2	2	1	1	4	4
3	3	2	2	2	2	2	2	2	2
3	3	2	2	2	2	2	2	2	2
3	3	2	2	2	2	2	2	2	2
3	3	2	2	2	2	2	2	2	2
3	3	2	2	2	2	1	1	4	4
3	3	2	2	2	2	1	1	2	2
3	3	2	2	2	2	2	2	4	4
3	3	2	2	2	2	1	1	2	2
3	3	2	2	2	2	2	2	4	4
3	3	4	4	2	2	2	2	4	4
3	3	2	2	2	2	1	1	2	2
3	3	2	2	2	2	2	2	4	4
3	3	2	2	2	2	2	2	2	2
3	3	2	2	2	2	1	1	2	2
3	3	2	2	2	2	1	1	2	2
2	2	2	2	3	3	1	1	4	4
3	3	2	2	2	2	1	1	2	2
3	3	2	2	2	2	2	2	2	2
3	3	2	2	2	2	2	2	2	2
3	3	2	2	2	2	2	2	4	4
3	3	2	2	2	2	2	2	2	2
3	3	2	2	2	2	1	1	2	2
2	2	4	4	3	3	1	1	4	4
3	3	2	2	2	2	1	1	2	2
3	3	2	2	2	2	2	2	2	2
3	3	2	2	2	2	1	1	2	2
3	3	2	2	2	2	2	2	2	2
3	3	2	2	2	2	2	2	4	4
3	3	4	4	2	2	2	2	4	4
3	3	2	2	2	2	2	2	2	2
3	3	2	2	2	2	1	1	4	4
3	3	2	2	2	2	2	2	2	2
3	3	2	2	2	2	2	2	4	4
3	3	2	2	2	2	1	1	2	2
3	3	4	4	2	2	2	2	2	2
3	3	4	4	3	3	2	2	2	2
3	3	2	2	2	2	1	1	4	4
3	3	2	2	2	2	1	1	4	4
3	3	2	2	2	2	1	1	2	2
3	3	2	2	2	2	1	1	4	4
3	3	2	2	2	2	1	1	4	4
3	3	2	2	2	2	2	2	4	4
3	3	2	2	2	2	2	2	4	4
3	3	2	2	2	2	1	1	4	4
3	3	2	2	2	2	1	1	2	2

For Review Only

3	3	2	2	2	2	2	2	4	4
3	3	2	2	2	2	1	1	2	2
3	3	2	2	2	2	2	2	4	4
3	3	2	2	2	2	1	1	4	4
3	3	2	2	2	2	2	2	4	4
3	3	2	2	2	2	2	2	4	4
2	2	2	2	2	2	1	1	2	2
3	3	2	2	2	2	1	1	2	2
3	3	4	4	3	3	2	2	2	2
2	2	2	2	3	3	2	2	4	4
3	3	2	2	2	2	1	1	2	2
3	3	2	2	2	2	1	1	4	4
3	3	2	2	2	2	1	1	2	2
3	3	4	4	2	2	2	2	2	2
3	3	2	2	2	2	2	2	2	2
3	3	2	2	3	3	2	2	4	4
3	3	2	2	2	2	2	2	2	2
3	3	2	2	2	2	2	2	4	4
3	3	2	2	2	2	2	2	4	4
3	3	2	2	2	2	2	2	4	4
3	3	2	2	2	2	1	1	2	2
3	3	2	2	2	2	1	1	2	2
3	3	2	2	2	2	1	1	2	2
3	3	2	2	2	2	1	1	4	4
3	3	2	2	2	2	1	1	2	2
3	3	2	2	2	2	2	2	2	2
3	3	2	2	2	2	1	1	4	4
3	3	2	2	2	2	2	2	4	4
3	3	2	2	2	2	2	2	2	2
3	3	2	2	2	2	1	1	2	2
3	3	4	4	2	2	2	2	4	4
2	2	2	2	3	3	2	2	4	4
2	2	2	2	3	3	2	2	4	4
3	3	2	2	2	2	2	2	2	2
3	3	2	2	2	2	1	1	4	4
3	3	2	2	2	2	1	1	4	4
2	2	2	2	3	3	2	2	4	4
3	3	2	2	2	2	2	2	4	4
3	3	2	2	2	2	1	1	4	4
3	3	4	4	2	2	2	2	4	4
3	3	2	2	2	2	2	2	4	4
3	3	2	2	2	2	1	1	4	4
3	3	2	2	2	2	1	1	2	2
3	3	4	4	2	2	1	1	2	2
3	3	2	2	2	2	2	2	4	4
3	3	2	2	2	2	2	2	2	2
2	2	2	2	3	3	2	2	4	4
2	2	2	2	3	3	2	2	2	2
3	3	2	2	2	2	2	2	2	2
3	3	2	2	2	2	2	2	4	4
3	3	2	2	2	2	2	2	4	4
3	3	2	2	2	2	2	2	2	2
3	3	2	2	2	2	2	2	2	2
3	3	2	2	2	2	2	2	4	4
3	3	2	2	2	2	1	1	2	2

For Review Only

3	3	2	2	2	2	2	2	4	4
3	3	2	2	2	2	1	1	4	4
3	3	2	2	2	2	2	2	2	2
3	3	2	2	2	2	1	1	2	2
3	3	2	2	2	2	2	2	2	2
3	3	2	2	2	2	2	2	4	4
3	3	2	2	2	2	1	1	4	4
3	3	2	2	2	2	2	2	2	2
3	3	2	2	2	2	1	1	2	2
3	3	2	2	2	2	1	1	2	2
3	3	2	2	2	2	1	1	2	2
3	3	4	4	2	2	2	2	2	2
3	3	2	2	2	2	1	1	4	4
3	3	2	2	2	2	2	2	4	4
3	3	2	2	2	2	2	2	4	4
3	3	2	2	2	2	2	2	4	4
3	3	2	2	2	2	1	1	2	2
3	3	2	2	2	2	1	1	4	4
3	3	2	2	2	2	2	2	2	2
3	3	2	2	2	2	2	2	2	2
3	3	2	2	2	2	1	1	2	2
3	3	2	2	2	2	1	1	4	4
2	2	2	2	3	3	2	2	4	4
3	3	2	2	2	2	1	1	4	4
3	3	2	2	2	2	2	2	2	2
3	3	2	2	2	2	2	2	4	4
3	3	2	2	2	2	2	2	2	2
3	3	2	2	2	2	2	2	4	4
3	3	2	2	2	2	1	1	2	2
3	3	2	2	2	2	2	2	2	2
3	3	2	2	2	2	1	1	4	4
3	3	2	2	2	2	1	1	4	4
3	3	4	4	2	2	2	2	2	2
3	3	2	2	2	2	1	1	4	4
3	3	2	2	2	2	1	1	2	2
3	3	2	2	2	2	1	1	2	2
3	3	2	2	2	2	2	2	4	4
3	3	2	2	2	2	1	1	4	4
3	3	2	2	2	2	1	1	4	4
3	3	2	2	2	2	2	2	2	2
3	3	2	2	2	2	2	2	4	4
3	3	2	2	2	2	2	2	2	2
3	3	2	2	2	2	1	1	4	4
2	2	4	4	3	3	1	1	4	4
3	3	2	2	2	2	2	2	4	4
3	3	2	2	2	2	1	1	4	4
3	3	2	2	2	2	2	2	4	4
3	3	2	2	2	2	1	1	2	2
3	3	2	2	2	2	2	2	4	4
3	3	2	2	2	2	2	2	2	2
3	3	2	2	2	2	1	1	4	4
3	3	2	2	2	2	2	2	2	2
3	3	2	2	2	2	2	2	4	4
3	3	2	2	2	2	1	1	4	4
3	3	2	2	2	2	1	1	4	4

For Review Only

2	2	2	2	3	3	2	2	4	4
3	3	2	2	2	2	2	2	4	4

For Review Only

SNP1936	SNP1936	SNP1937	SNP1937	SNP1938	SNP1938	SNP1939	SNP1939	SNP194	SNP194
1	1	2	2	4	4	4	4	2	2
1	1	2	2	4	4	4	4	3	3
1	1	2	2	4	4	1	1	2	2
1	1	2	2	4	4	4	4	3	3
1	1	2	2	4	4	1	1	3	3
1	1	2	2	4	4	1	1	2	2
1	1	2	2	4	4	4	4	3	3
1	1	2	2	4	4	4	4	3	3
4	4	2	2	4	4	1	1	3	3
1	1	2	2	4	4	4	4	3	3
1	1	2	2	4	4	4	4	3	3
1	1	2	2	4	4	1	1	2	2
1	1	2	2	4	4	4	4	3	3
1	1	2	2	4	4	1	1	3	3
1	1	2	2	4	4	4	4	3	3
1	1	2	2	1	1	4	4	3	3
4	4	2	2	4	4	1	1	3	3
1	1	2	2	4	4	4	4	2	2
1	1	2	2	4	4	4	4	3	3
1	1	2	2	4	4	4	4	2	2
4	4	2	2	4	4	4	4	3	3
1	1	2	2	4	4	4	4	3	3
4	4	2	2	1	1	4	4	3	3
4	4	2	2	4	4	4	4	3	3
4	4	2	2	4	4	1	1	3	3
4	4	2	2	4	4	1	1	3	3
1	1	2	2	4	4	1	1	3	3
1	1	2	2	4	4	4	4	3	3
4	4	2	2	4	4	4	4	3	3
4	4	2	2	4	4	4	4	3	3
4	4	2	2	4	4	1	1	3	3
1	1	2	2	4	4	1	1	3	3
1	1	2	2	4	4	1	1	3	3
1	1	1	1	4	4	4	4	3	3
4	4	2	2	1	1	1	1	3	3
4	4	2	2	4	4	4	4	3	3
1	1	2	2	4	4	4	4	3	3
1	1	2	2	4	4	4	4	2	2
1	1	2	2	1	1	4	4	3	3
4	4	2	2	1	1	4	4	3	3
4	4	2	2	4	4	4	4	3	3
1	1	2	2	4	4	4	4	3	3
1	1	2	2	4	4	4	4	3	3
4	4	2	2	4	4	4	4	3	3
1	1	2	2	4	4	4	4	3	3
4	4	2	2	4	4	1	1	3	3
4	4	2	2	4	4	1	1	3	3
4	4	2	2	4	4	1	1	3	3
4	4	2	2	4	4	4	4	3	3
1	1	2	2	4	4	4	4	3	3
4	4	2	2	4	4	1	1	3	3
1	1	2	2	4	4	4	4	2	2

For Review Only

4	4	2	2	4	4	1	1	3	3
1	1	2	2	4	4	1	1	3	3
1	1	2	2	4	4	1	1	3	3
4	4	2	2	4	4	4	4	3	3
1	1	2	2	4	4	4	4	3	3
4	4	2	2	4	4	1	1	3	3
4	4	2	2	4	4	1	1	3	3
1	1	2	2	4	4	4	4	3	3
1	1	2	2	4	4	4	4	3	3
1	1	2	2	4	4	4	4	3	3
1	1	2	2	4	4	1	1	3	3
4	4	1	1	4	4	1	1	2	2
4	4	2	2	4	4	1	1	3	3
4	4	2	2	4	4	4	4	3	3
1	1	2	2	4	4	4	4	3	3
1	1	2	2	4	4	4	4	3	3
1	1	2	2	4	4	1	1	3	3
1	1	2	2	4	4	4	4	3	3
4	4	2	2	4	4	1	1	3	3
4	4	2	2	4	4	4	4	2	2
1	1	2	2	4	4	4	4	2	2
4	4	2	2	4	4	4	4	3	3
1	1	2	2	4	4	4	4	3	3
1	1	2	2	4	4	1	1	3	3
1	1	2	2	4	4	4	4	3	3
1	1	2	2	4	4	1	1	3	3
4	4	2	2	4	4	1	1	3	3
4	4	2	2	1	1	4	4	3	3
4	4	2	2	4	4	1	1	3	3
1	1	2	2	4	4	4	4	3	3
1	1	2	2	4	4	1	1	3	3
1	1	2	2	4	4	4	4	3	3
1	1	2	2	1	1	4	4	3	3
4	4	2	2	4	4	1	1	3	3
1	1	2	2	4	4	1	1	2	2
1	1	2	2	4	4	4	4	3	3
1	1	2	2	4	4	4	4	3	3
4	4	2	2	4	4	4	4	3	3
4	4	2	2	4	4	1	1	3	3
1	1	2	2	4	4	4	4	3	3
4	4	2	2	4	4	1	1	3	3
4	4	2	2	4	4	4	4	3	3
1	1	2	2	4	4	1	1	3	3
1	1	2	2	4	4	1	1	3	3
4	4	2	2	4	4	1	1	3	3
1	1	2	2	4	4	1	1	3	3
1	1	2	2	4	4	4	4	3	3
4	4	2	2	4	4	4	4	3	3
4	4	2	2	4	4	1	1	3	3
4	4	2	2	4	4	4	4	3	3
4	4	1	1	4	4	4	4	3	3
1	1	2	2	1	1	4	4	3	3
1	1	2	2	4	4	4	4	3	3
1	1	2	2	4	4	4	4	3	3
4	4	2	2	4	4	4	4	3	3

For Review Only

CSSA 5585 Guilford Rd., Madison, WI 53711

1	1	2	2	4	4	4	4	3	3
1	1	2	2	4	4	4	4	3	3
4	4	2	2	4	4	1	1	3	3
1	1	2	2	4	4	4	4	3	3
1	1	2	2	4	4	1	1	3	3
4	4	2	2	4	4	4	4	3	3
1	1	2	2	4	4	1	1	3	3
1	1	2	2	4	4	4	4	3	3
4	4	2	2	4	4	4	4	3	3
1	1	2	2	4	4	4	4	3	3
1	1	2	2	1	1	4	4	3	3
1	1	2	2	4	4	4	4	3	3
1	1	2	2	4	4	1	1	3	3
1	1	2	2	4	4	4	4	3	3
4	4	2	2	4	4	4	4	3	3
1	1	2	2	4	4	4	4	3	3
1	1	2	2	4	4	4	4	3	3
1	1	2	2	4	4	4	4	3	3
1	1	2	2	4	4	1	1	3	3
1	1	2	2	4	4	1	1	3	3
1	1	2	2	4	4	4	4	3	3
1	1	2	2	4	4	4	4	3	3
1	1	2	2	4	4	1	1	3	3
4	4	2	2	1	1	4	4	3	3
4	4	2	2	4	4	4	4	3	3
1	1	2	2	4	4	4	4	3	3
4	4	2	2	4	4	4	4	3	3
1	1	2	2	4	4	4	4	3	3
1	1	2	2	4	4	1	1	2	2
1	1	2	2	4	4	4	4	3	3
1	1	2	2	1	1	4	4	3	3
1	1	2	2	4	4	4	4	3	3
4	4	2	2	4	4	1	1	3	3
1	1	2	2	4	4	4	4	3	3
1	1	1	1	4	4	4	4	3	3
4	4	2	2	4	4	4	4	3	3
1	1	2	2	4	4	4	4	3	3
1	1	2	2	4	4	1	1	3	3
4	4	2	2	4	4	1	1	3	3
1	1	2	2	4	4	4	4	3	3
4	4	2	2	4	4	4	4	3	3
1	1	2	2	4	4	4	4	3	3
1	1	2	2	4	4	4	4	3	3
1	1	2	2	4	4	4	4	3	3
1	1	2	2	4	4	1	1	3	3
1	1	2	2	4	4	4	4	3	3
4	4	2	2	4	4	1	1	3	3
4	4	2	2	4	4	1	1	3	3
1	1	2	2	4	4	4	4	3	3
4	4	2	2	4	4	1	1	3	3
4	4	2	2	4	4	4	4	3	3
1	1	2	2	4	4	4	4	3	3
1	1	2	2	4	4	4	4	3	3
4	4	2	2	4	4	1	1	2	2
1	1	2	2	4	4	4	4	3	3

For Review Only

1	1	2	2	4	4	4	4	3	3
4	4	2	2	4	4	4	4	3	3
1	1	2	2	4	4	4	4	3	3
1	1	2	2	4	4	4	4	3	3
1	1	2	2	4	4	4	4	2	2
1	1	1	1	4	4	1	1	3	3
1	1	2	2	1	1	4	4	3	3
1	1	2	2	4	4	1	1	3	3
1	1	2	2	1	1	4	4	3	3
1	1	2	2	4	4	4	4	3	3
4	4	2	2	4	4	1	1	3	3
4	4	2	2	1	1	4	4	3	3
1	1	2	2	4	4	4	4	3	3
1	1	2	2	4	4	4	4	3	3
1	1	2	2	4	4	4	4	3	3
1	1	2	2	1	1	4	4	3	3
1	1	2	2	4	4	4	4	3	3
1	1	2	2	4	4	4	4	3	3
1	1	2	2	1	1	1	1	3	3
1	1	2	2	4	4	1	1	3	3
1	1	2	2	4	4	4	4	3	3
4	4	2	2	1	1	4	4	3	3
1	1	2	2	4	4	4	4	3	3
4	4	1	1	4	4	1	1	2	2
1	1	2	2	4	4	4	4	3	3
1	1	1	1	4	4	4	4	3	3
4	4	2	2	4	4	1	1	2	2
1	1	2	2	4	4	4	4	3	3
1	1	2	2	4	4	4	4	3	3
4	4	2	2	4	4	4	4	3	3
1	1	2	2	4	4	4	4	3	3
1	1	2	2	4	4	4	4	3	3
1	1	2	2	4	4	4	4	3	3
1	1	2	2	4	4	4	4	3	3
1	1	2	2	4	4	4	4	3	3
4	4	1	1	4	4	1	1	2	2
1	1	2	2	4	4	1	1	3	3
1	1	2	2	4	4	4	4	3	3
4	4	2	2	4	4	1	1	3	3
1	1	2	2	4	4	1	1	3	3
1	1	2	2	4	4	1	1	2	2
4	4	2	2	4	4	1	1	3	3
4	4	2	2	1	1	4	4	3	3
1	1	2	2	4	4	1	1	3	3
1	1	2	2	4	4	4	4	2	2
4	4	2	2	1	1	1	1	3	3
1	1	2	2	1	1	4	4	3	3
1	1	2	2	1	1	4	4	3	3
1	1	2	2	4	4	4	4	3	3
1	1	2	2	4	4	4	4	3	3
1	1	2	2	4	4	1	1	2	2
1	1	2	2	4	4	4	4	3	3
1	1	2	2	4	4	4	4	3	3
1	1	2	2	4	4	1	1	3	3
4	4	1	1	4	4	4	4	3	3

For Review Only

1	1	2	2	4	4	4	4	3	3
1	1	2	2	4	4	4	4	3	3
1	1	2	2	4	4	1	1	2	2
1	1	2	2	4	4	4	4	3	3
1	1	2	2	4	4	4	4	2	2
1	1	2	2	4	4	1	1	3	3
4	4	2	2	1	1	4	4	2	2
1	1	2	2	4	4	4	4	3	3
1	1	2	2	4	4	4	4	3	3
4	4	2	2	1	1	4	4	3	3
1	1	2	2	4	4	4	4	3	3
1	1	2	2	4	4	4	4	3	3
4	4	2	2	4	4	1	1	3	3
1	1	2	2	4	4	4	4	3	3
1	1	2	2	4	4	1	1	3	3
1	1	2	2	4	4	1	1	3	3
4	4	1	1	4	4	4	4	3	3
4	4	2	2	4	4	4	4	2	2
1	1	2	2	1	1	4	4	3	3
1	1	2	2	4	4	1	1	2	2
1	1	2	2	1	1	4	4	3	3
4	4	2	2	4	4	1	1	3	3
1	1	2	2	4	4	4	4	2	2
4	4	2	2	1	1	1	1	3	3
1	1	2	2	4	4	4	4	3	3
1	1	2	2	1	1	4	4	3	3
1	1	2	2	1	1	4	4	3	3
1	1	2	2	4	4	1	1	3	3
1	1	2	2	4	4	1	1	3	3
1	1	2	2	4	4	4	4	2	2
4	4	2	2	1	1	4	4	3	3
4	4	2	2	4	4	1	1	3	3
1	1	2	2	4	4	4	4	3	3
4	4	2	2	4	4	4	4	3	3
4	4	2	2	4	4	4	4	3	3
4	4	2	2	1	1	4	4	3	3
1	1	2	2	4	4	4	4	3	3
4	4	1	1	4	4	1	1	2	2
4	4	2	2	4	4	4	4	3	3
1	1	2	2	4	4	4	4	3	3
1	1	2	2	4	4	4	4	2	2
4	4	2	2	4	4	4	4	3	3
4	4	2	2	1	1	4	4	3	3
4	4	2	2	4	4	1	1	3	3
1	1	2	2	4	4	1	1	3	3
4	4	2	2	4	4	4	4	3	3
1	1	2	2	4	4	1	1	3	3
1	1	2	2	4	4	4	4	3	3
1	1	2	2	4	4	1	1	3	3
1	1	2	2	1	1	4	4	3	3
4	4	2	2	4	4	4	4	2	2
1	1	2	2	4	4	4	4	3	3
1	1	2	2	4	4	1	1	2	2
4	4	2	2	4	4	1	1	2	2
4	4	2	2	1	1	4	4	3	3

For Review Only

1	1	2	2	4	4	1	1	3	3
1	1	2	2	4	4	4	4	3	3

For Review Only

SNP1940	SNP1940	SNP1941	SNP1941	SNP1942	SNP1942	SNP1943	SNP1943	SNP1944	SNP1944
1	1	3	3	1	1	4	4	1	1
1	1	3	3	4	4	1	1	4	4
2	2	3	3	4	4	4	4	4	4
1	1	3	3	4	4	4	4	4	4
1	1	3	3	1	1	4	4	1	1
1	1	3	3	4	4	4	4	1	1
1	1	3	3	4	4	1	1	4	4
1	1	3	3	4	4	1	1	1	1
1	1	3	3	1	1	4	4	1	1
1	1	3	3	4	4	1	1	1	1
1	1	3	3	4	4	1	1	4	4
2	2	3	3	4	4	4	4	4	4
1	1	3	3	4	4	4	4	1	1
1	1	3	3	4	4	4	4	1	1
1	1	3	3	4	4	4	4	4	4
2	2	3	3	4	4	1	1	4	4
2	2	3	3	1	1	4	4	4	4
1	1	3	3	4	4	1	1	1	1
1	1	3	3	4	4	1	1	1	1
1	1	3	3	4	4	1	1	1	1
1	1	3	3	4	4	1	1	4	4
1	1	3	3	1	1	4	4	4	4
1	1	3	3	4	4	1	1	4	4
1	1	3	3	4	4	1	1	1	1
1	1	1	1	1	1	4	4	1	1
1	1	3	3	4	4	4	4	4	4
1	1	3	3	4	4	4	4	4	4
1	1	3	3	4	4	4	4	4	4
1	1	3	3	4	4	1	1	1	1
1	1	3	3	4	4	1	1	1	1
1	1	3	3	4	4	4	4	1	1
2	2	3	3	4	4	4	4	4	4
1	1	3	3	1	1	4	4	1	1
1	1	3	3	4	4	1	1	1	1
1	1	3	3	4	4	4	4	4	4
1	1	3	3	4	4	1	1	4	4
1	1	3	3	1	1	4	4	1	1
1	1	3	3	4	4	4	4	4	4
1	1	3	3	4	4	1	1	4	4
1	1	3	3	4	4	1	1	4	4
1	1	3	3	4	4	1	1	4	4
1	1	3	3	4	4	1	1	1	1
1	1	3	3	4	4	1	1	1	1
1	1	3	3	4	4	4	4	4	4
1	1	3	3	1	1	4	4	1	1
2	2	3	3	4	4	4	4	4	4
1	1	1	1	4	4	4	4	4	4
1	1	3	3	4	4	4	4	4	4
1	1	3	3	4	4	4	4	1	1
2	2	3	3	1	1	4	4	4	4
1	1	3	3	4	4	4	4	4	4
1	1	3	3	1	1	4	4	1	1

For Review Only

1	1	3	3	1	1	4	4	1	1
1	1	3	3	1	1	4	4	1	1
1	1	3	3	4	4	4	4	1	1
1	1	3	3	4	4	1	1	4	4
1	1	3	3	4	4	1	1	1	1
1	1	1	1	4	4	4	4	1	1
1	1	3	3	4	4	4	4	4	4
1	1	3	3	4	4	1	1	1	1
1	1	3	3	4	4	1	1	1	1
1	1	3	3	4	4	4	4	4	4
1	1	3	3	4	4	4	4	4	4
1	1	1	1	4	4	4	4	1	1
1	1	3	3	1	1	4	4	1	1
1	1	3	3	4	4	1	1	1	1
1	1	3	3	4	4	4	4	4	4
1	1	3	3	4	4	4	4	4	4
1	1	3	3	4	4	4	4	4	4
2	2	3	3	4	4	4	4	4	4
1	1	3	3	1	1	4	4	4	4
2	2	3	3	1	1	4	4	1	1
1	1	3	3	4	4	4	4	1	1
1	1	3	3	4	4	1	1	4	4
1	1	3	3	4	4	1	1	4	4
1	1	3	3	4	4	4	4	4	4
1	1	3	3	1	1	4	4	1	1
1	1	3	3	4	4	4	4	1	1
1	1	3	3	4	4	4	4	1	1
1	1	3	3	4	4	1	1	4	4
1	1	3	3	4	4	4	4	4	4
1	1	3	3	4	4	4	4	4	4
1	1	3	3	4	4	4	4	4	4
1	1	3	3	4	4	1	1	4	4
1	1	3	3	4	4	1	1	4	4
1	1	3	3	1	1	4	4	1	1
1	1	3	3	4	4	4	4	1	1
1	1	3	3	1	1	4	4	1	1
1	1	3	3	1	1	4	4	1	1
1	1	3	3	4	4	1	1	1	1
1	1	3	3	4	4	4	4	4	4
1	1	3	3	1	1	4	4	1	1
1	1	3	3	1	1	4	4	4	4
2	2	3	3	4	4	4	4	4	4
1	1	3	3	4	4	4	4	1	1
1	1	3	3	4	4	4	4	4	4
1	1	1	1	4	4	4	4	1	1
1	1	3	3	4	4	4	4	4	4
1	1	3	3	4	4	1	1	1	1
1	1	3	3	4	4	4	4	4	4
1	1	3	3	4	4	4	4	4	4
2	2	1	1	4	4	1	1	4	4
1	1	3	3	4	4	4	4	4	4
1	1	3	3	4	4	1	1	4	4
1	1	3	3	4	4	1	1	1	1
1	1	3	3	4	4	4	4	4	4
1	1	1	1	4	4	1	1	1	1

For Review Only

1	1	3	3	4	4	4	4	4	4
1	1	3	3	4	4	1	1	4	4
1	1	3	3	4	4	4	4	4	4
1	1	3	3	4	4	1	1	1	1
1	1	3	3	4	4	4	4	1	1
1	1	3	3	4	4	4	4	4	4
2	2	3	3	4	4	4	4	4	4
2	2	3	3	4	4	4	4	4	4
1	1	3	3	4	4	4	4	1	1
1	1	3	3	4	4	1	1	4	4
1	1	3	3	4	4	4	4	1	1
1	1	3	3	4	4	4	4	4	4
1	1	3	3	4	4	4	4	1	1
1	1	3	3	1	1	4	4	4	4
1	1	3	3	4	4	4	4	4	4
1	1	3	3	4	4	4	4	4	4
1	1	3	3	4	4	1	1	4	4
1	1	3	3	4	4	4	4	4	4
1	1	3	3	4	4	1	1	1	1
2	2	3	3	4	4	4	4	4	4
1	1	3	3	4	4	4	4	4	4
1	1	3	3	4	4	4	4	1	1
2	2	3	3	4	4	4	4	4	4
1	1	3	3	4	4	1	1	4	4
1	1	3	3	4	4	4	4	4	4
1	1	3	3	4	4	1	1	4	4
1	1	3	3	1	1	4	4	4	4
1	1	3	3	1	1	4	4	1	1
1	1	3	3	4	4	4	4	4	4
1	1	1	1	4	4	1	1	1	1
1	1	3	3	4	4	1	1	4	4
1	1	3	3	4	4	4	4	4	4
1	1	3	3	1	1	4	4	4	4
1	1	3	3	4	4	4	4	4	4
1	1	3	3	4	4	1	1	1	1
1	1	3	3	4	4	4	4	4	4
1	1	3	3	4	4	1	1	1	1
1	1	3	3	4	4	4	4	4	4
1	1	3	3	4	4	4	4	4	4
1	1	3	3	4	4	1	1	4	4
1	1	3	3	4	4	4	4	4	4
1	1	3	3	4	4	1	1	1	1
1	1	3	3	1	1	4	4	1	1
1	1	3	3	4	4	1	1	4	4
1	1	3	3	4	4	4	4	4	4
1	1	3	3	1	1	4	4	1	1
1	1	3	3	4	4	4	4	4	4
1	1	3	3	4	4	4	4	4	4
1	1	3	3	4	4	1	1	4	4
1	1	3	3	4	4	4	4	4	4
1	1	3	3	4	4	4	4	4	4
1	1	3	3	4	4	1	1	1	1
1	1	3	3	1	1	4	4	1	1
1	1	1	1	4	4	4	4	4	4
1	1	3	3	4	4	1	1	4	4

For Review Only

1	1	3	3	4	4	4	4	4	4
1	1	3	3	4	4	1	1	1	1
1	1	3	3	4	4	1	1	4	4
1	1	3	3	4	4	1	1	1	1
1	1	3	3	4	4	1	1	4	4
1	1	3	3	4	4	4	4	1	1
1	1	3	3	4	4	1	1	4	4
1	1	3	3	4	4	4	4	4	4
1	1	3	3	4	4	4	4	1	1
1	1	3	3	1	1	4	4	1	1
1	1	3	3	4	4	4	4	4	4
1	1	3	3	4	4	1	1	4	4
1	1	3	3	4	4	4	4	4	4
1	1	3	3	4	4	4	4	4	4
1	1	3	3	4	4	1	1	1	1
1	1	3	3	4	4	1	1	4	4
1	1	3	3	4	4	4	4	4	4
1	1	3	3	1	1	4	4	4	4
1	1	3	3	4	4	4	4	4	4
2	2	3	3	4	4	4	4	4	4
1	1	3	3	4	4	4	4	4	4
1	1	3	3	4	4	1	1	4	4
1	1	3	3	4	4	1	1	4	4
1	1	1	1	4	4	4	4	1	1
1	1	3	3	1	1	4	4	1	1
1	1	3	3	4	4	1	1	1	1
1	1	1	1	4	4	4	4	4	4
1	1	3	3	4	4	1	1	1	1
1	1	3	3	1	1	4	4	1	1
2	2	3	3	4	4	1	1	4	4
1	1	3	3	1	1	4	4	4	4
1	1	3	3	4	4	4	4	1	1
1	1	3	3	1	1	4	4	4	4
1	1	3	3	4	4	1	1	1	1
1	1	3	3	4	4	4	4	1	1
1	1	1	1	4	4	4	4	1	1
1	1	3	3	4	4	4	4	4	4
1	1	3	3	1	1	4	4	1	1
1	1	3	3	4	4	4	4	4	4
1	1	3	3	4	4	4	4	4	4
2	2	3	3	4	4	4	4	4	4
1	1	3	3	4	4	4	4	4	4
1	1	3	3	4	4	1	1	4	4
1	1	3	3	4	4	4	4	4	4
1	1	3	3	4	4	4	4	4	4
1	1	1	1	4	4	4	4	1	1
1	1	3	3	4	4	1	1	4	4
1	1	3	3	4	4	1	1	4	4
1	1	3	3	4	4	1	1	1	1
1	1	3	3	4	4	4	4	4	4
1	1	3	3	4	4	4	4	1	1
1	1	3	3	4	4	1	1	1	1
1	1	3	3	4	4	1	1	4	4
1	1	3	3	4	4	4	4	4	4
1	1	3	3	4	4	1	1	4	4

For Review Only

2	2	3	3	4	4	1	1	4	4
1	1	3	3	4	4	1	1	4	4
2	2	3	3	4	4	4	4	4	4
1	1	3	3	4	4	4	4	4	4
1	1	3	3	4	4	4	4	1	1
1	1	3	3	4	4	4	4	4	4
1	1	3	3	4	4	1	1	1	1
1	1	3	3	4	4	1	1	1	1
1	1	3	3	4	4	1	1	4	4
1	1	3	3	4	4	1	1	4	4
1	1	3	3	4	4	4	4	4	4
1	1	3	3	1	1	4	4	4	4
1	1	3	3	4	4	4	4	4	4
1	1	3	3	4	4	1	1	4	4
2	2	3	3	4	4	4	4	4	4
1	1	3	3	4	4	4	4	4	4
1	1	3	3	1	1	4	4	4	4
1	1	3	3	4	4	1	1	1	1
1	1	3	3	4	4	1	1	4	4
1	1	3	3	4	4	4	4	4	4
1	1	3	3	4	4	1	1	4	4
1	1	3	3	4	4	4	4	4	4
1	1	3	3	4	4	4	4	1	1
1	1	3	3	4	4	4	4	4	4
1	1	3	3	4	4	1	1	1	1
1	1	3	3	4	4	4	4	4	4
1	1	3	3	4	4	1	1	4	4
1	1	3	3	4	4	4	4	4	4
1	1	3	3	1	1	4	4	1	1
1	1	3	3	4	4	1	1	1	1
1	1	3	3	4	4	1	1	4	4
1	1	3	3	4	4	4	4	1	1
1	1	3	3	4	4	1	1	4	4
1	1	3	3	4	4	1	1	4	4
1	1	3	3	4	4	1	1	4	4
1	1	3	3	4	4	1	1	4	4
2	2	3	3	1	1	4	4	4	4
1	1	1	1	4	4	4	4	1	1
1	1	3	3	4	4	1	1	4	4
1	1	3	3	4	4	1	1	4	4
1	1	3	3	4	4	1	1	1	1
1	1	1	1	4	4	1	1	1	1
1	1	3	3	4	4	1	1	4	4
1	1	3	3	1	1	4	4	4	4
1	1	3	3	4	4	4	4	4	4
1	1	3	3	4	4	1	1	4	4
1	1	3	3	4	4	4	4	4	4
1	1	3	3	4	4	1	1	1	1
1	1	3	3	4	4	4	4	4	4
1	1	3	3	4	4	1	1	4	4
2	2	3	3	1	1	4	4	4	4
1	1	3	3	4	4	1	1	4	4
2	2	3	3	4	4	4	4	4	4
1	1	3	3	4	4	4	4	4	4
1	1	3	3	4	4	1	1	4	4

For Review Only

1	1	3	3	4	4	4	4	4	4
1	1	3	3	1	1	4	4	4	4

For Review Only

SNP1945	SNP1945	SNP1946	SNP1946	SNP1947	SNP1947	SNP1948	SNP1948	SNP1949	SNP1949
4	4	2	2	4	4	2	2	2	2
2	2	2	2	4	4	4	4	2	2
2	2	3	3	4	4	4	4	2	2
2	2	2	2	1	1	2	2	2	2
4	4	3	3	4	4	2	2	2	2
4	4	2	2	4	4	2	2	2	2
2	2	2	2	4	4	2	2	2	2
4	4	2	2	4	4	2	2	2	2
4	4	2	2	4	4	2	2	2	2
2	2	2	2	4	4	2	2	2	2
2	2	3	3	1	1	2	2	2	2
2	2	2	2	4	4	4	4	2	2
4	4	2	2	4	4	2	2	2	2
4	4	3	3	4	4	2	2	2	2
2	2	3	3	4	4	2	2	1	1
2	2	2	2	4	4	2	2	2	2
2	2	2	2	4	4	2	2	2	2
4	4	2	2	4	4	4	4	2	2
4	4	2	2	4	4	2	2	2	2
4	4	3	3	1	1	2	2	2	2
2	2	2	2	1	1	2	2	2	2
2	2	3	3	1	1	2	2	2	2
2	2	3	3	1	1	2	2	2	2
4	4	2	2	1	1	2	2	2	2
4	4	2	2	4	4	2	2	1	1
2	2	2	2	1	1	4	4	2	2
2	2	2	2	1	1	2	2	1	1
4	4	2	2	4	4	2	2	2	2
4	4	2	2	4	4	2	2	2	2
4	4	3	3	1	1	4	4	2	2
4	4	2	2	1	1	2	2	1	1
2	2	3	3	4	4	2	2	2	2
4	4	3	3	4	4	2	2	2	2
4	4	2	2	4	4	4	4	2	2
4	4	2	2	1	1	2	2	2	2
2	2	2	2	1	1	2	2	2	2
4	4	2	2	4	4	2	2	2	2
2	2	2	2	4	4	2	2	2	2
2	2	2	2	1	1	2	2	2	2
2	2	2	2	1	1	2	2	2	2
2	2	2	2	1	1	2	2	2	2
4	4	2	2	4	4	2	2	2	2
4	4	2	2	1	1	4	4	2	2
2	2	3	3	1	1	2	2	2	2
4	4	2	2	1	1	2	2	2	2
2	2	2	2	4	4	4	4	2	2
4	4	2	2	1	1	4	4	1	1
4	4	3	3	1	1	2	2	2	2
4	4	2	2	1	1	2	2	2	2
4	4	2	2	4	4	2	2	2	2
2	2	2	2	1	1	4	4	2	2
4	4	2	2	4	4	2	2	2	2

For Review Only

4	4	2	2	4	4	2	2	2	2
4	4	3	3	4	4	2	2	2	2
4	4	2	2	4	4	2	2	1	1
2	2	3	3	1	1	4	4	2	2
4	4	2	2	4	4	2	2	2	2
4	4	2	2	1	1	4	4	1	1
2	2	2	2	1	1	4	4	2	2
4	4	2	2	1	1	4	4	2	2
4	4	2	2	1	1	2	2	2	2
4	4	2	2	4	4	2	2	2	2
2	2	2	2	4	4	2	2	2	2
4	4	2	2	1	1	4	4	1	1
4	4	2	2	4	4	2	2	2	2
4	4	2	2	4	4	2	2	2	2
4	4	2	2	4	4	2	2	2	2
4	4	2	2	1	1	2	2	2	2
2	2	3	3	1	1	4	4	2	2
4	4	3	3	4	4	2	2	2	2
2	2	3	3	4	4	2	2	2	2
4	4	2	2	4	4	2	2	2	2
4	4	2	2	4	4	2	2	1	1
4	4	2	2	4	4	2	2	2	2
4	4	2	2	4	4	2	2	2	2
2	2	2	2	1	1	2	2	2	2
4	4	2	2	1	1	2	2	2	2
4	4	3	3	4	4	4	4	2	2
4	4	3	3	1	1	2	2	2	2
2	2	2	2	1	1	2	2	2	2
2	2	2	2	1	1	4	4	2	2
4	4	2	2	4	4	2	2	2	2
2	2	3	3	1	1	2	2	2	2
2	2	2	2	1	1	2	2	2	2
2	2	2	2	1	1	2	2	2	2
4	4	2	2	4	4	2	2	2	2
4	4	2	2	4	4	2	2	2	2
4	4	2	2	1	1	2	2	2	2
4	4	3	3	4	4	2	2	2	2
4	4	2	2	4	4	4	4	2	2
2	2	3	3	1	1	2	2	2	2
4	4	3	3	4	4	2	2	2	2
2	2	3	3	1	1	2	2	2	2
2	2	2	2	4	4	2	2	2	2
4	4	2	2	4	4	2	2	2	2
2	2	2	2	4	4	2	2	2	2
4	4	3	3	4	4	2	2	1	1
2	2	2	2	1	1	2	2	2	2
4	4	3	3	4	4	2	2	2	2
2	2	2	2	1	1	2	2	2	2
2	2	2	2	1	1	4	4	2	2
2	2	2	2	4	4	2	2	1	1
2	2	2	2	4	4	4	4	2	2
2	2	2	2	1	1	2	2	2	2
4	4	2	2	4	4	2	2	2	2
2	2	2	2	4	4	2	2	2	2
4	4	3	3	4	4	2	2	1	1

For Review Only

2	2	2	2	1	1	2	2	2	2
2	2	2	2	1	1	2	2	2	2
2	2	2	2	1	1	4	4	2	2
4	4	2	2	4	4	2	2	2	2
4	4	2	2	1	1	2	2	1	1
2	2	2	2	1	1	2	2	2	2
2	2	2	2	4	4	2	2	2	2
4	4	2	2	4	4	2	2	2	2
4	4	2	2	1	1	2	2	2	2
2	2	2	2	1	1	2	2	2	2
4	4	2	2	4	4	2	2	2	2
4	4	3	3	4	4	2	2	2	2
4	4	2	2	4	4	2	2	2	2
4	4	2	2	4	4	2	2	2	2
4	4	2	2	4	4	2	2	2	2
4	4	2	2	1	1	2	2	2	2
4	4	3	3	1	1	2	2	2	2
2	2	2	2	4	4	2	2	2	2
4	4	3	3	4	4	4	4	2	2
2	2	3	3	1	1	2	2	2	2
4	4	2	2	4	4	2	2	2	2
4	4	2	2	4	4	2	2	2	2
2	2	2	2	4	4	4	4	2	2
2	2	2	2	4	4	2	2	2	2
2	2	2	2	1	1	2	2	2	2
2	2	2	2	4	4	2	2	2	2
2	2	2	2	4	4	2	2	2	2
4	4	2	2	4	4	2	2	2	2
4	4	2	2	4	4	2	2	2	2
4	4	2	2	4	4	2	2	1	1
2	2	2	2	1	1	2	2	2	2
4	4	2	2	4	4	2	2	2	2
2	2	3	3	1	1	2	2	2	2
4	4	2	2	4	4	2	2	2	2
4	4	2	2	4	4	4	4	2	2
2	2	2	2	1	1	2	2	2	2
4	4	3	3	1	1	2	2	2	2
2	2	2	2	1	1	2	2	2	2
2	2	2	2	1	1	2	2	2	2
2	2	2	2	1	1	2	2	2	2
2	2	2	2	4	4	2	2	2	2
4	4	2	2	1	1	4	4	2	2
4	4	3	3	4	4	2	2	2	2
4	4	2	2	1	1	2	2	2	2
2	2	2	2	1	1	2	2	2	2
4	4	2	2	4	4	2	2	2	2
2	2	2	2	1	1	4	4	2	2
2	2	2	2	1	1	4	4	2	2
4	4	2	2	1	1	2	2	2	2
4	4	2	2	1	1	4	4	2	2
2	2	2	2	1	1	2	2	2	2
4	4	3	3	1	1	2	2	2	2
2	2	2	2	4	4	2	2	2	2
2	2	2	2	1	1	4	4	1	1
4	4	2	2	4	4	2	2	2	2

For Review Only

2	2	3	3	1	1	2	2	2	2
4	4	2	2	4	4	2	2	2	2
2	2	2	2	1	1	2	2	2	2
4	4	2	2	4	4	2	2	2	2
2	2	2	2	1	1	2	2	2	2
4	4	2	2	4	4	2	2	2	2
2	2	2	2	1	1	2	2	2	2
4	4	2	2	4	4	2	2	2	2
4	4	3	3	4	4	2	2	2	2
4	4	2	2	4	4	2	2	2	2
2	2	2	2	1	1	2	2	2	2
4	4	3	3	1	1	2	2	2	2
2	2	2	2	4	4	2	2	2	2
4	4	2	2	4	4	2	2	2	2
4	4	2	2	4	4	4	4	2	2
2	2	3	3	1	1	2	2	2	2
4	4	2	2	4	4	2	2	2	2
4	4	2	2	4	4	4	4	2	2
2	2	2	2	1	1	4	4	1	1
2	2	2	2	4	4	2	2	2	2
4	4	2	2	4	4	2	2	2	2
2	2	2	2	1	1	2	2	2	2
2	2	2	2	4	4	2	2	2	2
4	4	2	2	1	1	4	4	1	1
4	4	2	2	4	4	2	2	2	2
4	4	2	2	4	4	4	4	2	2
2	2	2	2	1	1	2	2	1	1
4	4	2	2	1	1	2	2	2	2
4	4	2	2	4	4	2	2	2	2
4	4	2	2	4	4	2	2	2	2
2	2	2	2	1	1	2	2	2	2
4	4	3	3	4	4	2	2	2	2
2	2	2	2	4	4	2	2	2	2
4	4	2	2	4	4	4	4	2	2
4	4	2	2	1	1	2	2	2	2
4	4	2	2	1	1	4	4	1	1
2	2	2	2	1	1	2	2	2	2
4	4	2	2	4	4	2	2	2	2
2	2	2	2	1	1	4	4	2	2
2	2	3	3	4	4	2	2	2	2
2	2	3	3	4	4	4	4	2	2
2	2	2	2	1	1	4	4	2	2
2	2	2	2	1	1	2	2	2	2
4	4	2	2	4	4	2	2	2	2
4	4	2	2	1	1	2	2	2	2
4	4	2	2	4	4	2	2	1	1
2	2	3	3	1	1	2	2	2	2
2	2	2	2	1	1	2	2	2	2
4	4	2	2	1	1	2	2	2	2
2	2	3	3	1	1	2	2	2	2
4	4	2	2	4	4	4	4	2	2
4	4	2	2	1	1	4	4	2	2
2	2	2	2	1	1	2	2	2	2
2	2	2	2	4	4	2	2	2	2
2	2	2	2	4	4	2	2	2	2

For Review Only

4	4	2	2	4	4	2	2	2	2
2	2	2	2	1	1	2	2	2	2
2	2	2	2	4	4	4	4	2	2
2	2	2	2	4	4	2	2	2	2
4	4	2	2	1	1	2	2	2	2
2	2	2	2	4	4	4	4	2	2
4	4	2	2	4	4	2	2	2	2
4	4	3	3	1	1	4	4	2	2
4	4	2	2	1	1	2	2	2	2
2	2	2	2	1	1	2	2	2	2
4	4	2	2	4	4	2	2	2	2
2	2	2	2	1	1	2	2	2	2
2	2	3	3	4	4	2	2	2	2
2	2	2	2	1	1	2	2	1	1
2	2	2	2	4	4	4	4	2	2
2	2	3	3	1	1	2	2	2	2
2	2	2	2	4	4	2	2	2	2
4	4	2	2	1	1	2	2	2	2
2	2	2	2	1	1	2	2	2	2
2	2	3	3	1	1	2	2	2	2
2	2	2	2	1	1	2	2	2	2
2	2	2	2	1	1	4	4	2	2
4	4	2	2	4	4	2	2	2	2
2	2	3	3	1	1	2	2	2	2
4	4	2	2	1	1	4	4	2	2
4	4	2	2	4	4	4	4	2	2
2	2	2	2	4	4	2	2	2	2
2	2	2	2	1	1	2	2	2	2
4	4	2	2	4	4	2	2	2	2
4	4	2	2	4	4	4	4	2	2
2	2	3	3	1	1	2	2	2	2
4	4	3	3	1	1	4	4	2	2
2	2	3	3	4	4	2	2	2	2
2	2	2	2	1	1	2	2	2	2
2	2	2	2	4	4	2	2	2	2
2	2	2	2	1	1	2	2	2	2
4	4	2	2	4	4	2	2	2	2
4	4	2	2	1	1	4	4	1	1
2	2	2	2	1	1	2	2	2	2
2	2	2	2	1	1	2	2	2	2
4	4	2	2	4	4	2	2	2	2
4	4	2	2	4	4	2	2	1	1
2	2	3	3	1	1	2	2	2	2
2	2	3	3	1	1	2	2	2	2
4	4	2	2	1	1	4	4	2	2
2	2	3	3	4	4	2	2	2	2
2	2	2	2	1	1	4	4	2	2
4	4	2	2	4	4	2	2	2	2
4	4	2	2	1	1	4	4	2	2
2	2	2	2	4	4	2	2	2	2
2	2	2	2	4	4	2	2	2	2
2	2	2	2	1	1	2	2	2	2
2	2	3	3	4	4	4	4	2	2
2	2	2	2	1	1	2	2	2	2
2	2	2	2	1	1	2	2	2	2

For Review Only

4	4	3	3	4	4	2	2	2	2
2	2	2	2	1	1	2	2	2	2

For Review Only

SNP195	SNP195	SNP1950	SNP1950	SNP1951	SNP1951	SNP1952	SNP1952	SNP1953	SNP1953
1	1	2	2	4	4	1	1	4	4
2	2	2	2	4	4	1	1	4	4
1	1	2	2	4	4	1	1	1	1
1	1	2	2	4	4	1	1	4	4
2	2	2	2	4	4	4	4	4	4
1	1	2	2	4	4	1	1	4	4
2	2	3	3	4	4	1	1	1	1
2	2	2	2	4	4	1	1	4	4
2	2	2	2	4	4	1	1	4	4
2	2	2	2	4	4	1	1	4	4
2	2	2	2	4	4	1	1	4	4
1	1	2	2	4	4	1	1	4	4
2	2	2	2	4	4	1	1	4	4
2	2	2	2	4	4	1	1	4	4
2	2	2	2	4	4	1	1	4	4
2	2	2	2	4	4	1	1	4	4
2	2	2	2	4	4	1	1	4	4
1	1	2	2	4	4	1	1	4	4
2	2	2	2	4	4	1	1	4	4
1	1	3	3	4	4	1	1	4	4
2	2	2	2	4	4	1	1	4	4
2	2	2	2	4	4	4	4	4	4
2	2	3	3	4	4	1	1	4	4
2	2	2	2	1	1	1	1	4	4
2	2	2	2	4	4	1	1	4	4
2	2	2	2	4	4	1	1	4	4
2	2	2	2	4	4	1	1	4	4
2	2	2	2	4	4	1	1	4	4
2	2	2	2	4	4	1	1	4	4
2	2	3	3	4	4	1	1	4	4
2	2	2	2	4	4	1	1	4	4
2	2	3	3	4	4	1	1	4	4
2	2	2	2	4	4	1	1	4	4
2	2	2	2	1	1	1	1	4	4
2	2	2	2	4	4	1	1	4	4
2	2	2	2	1	1	1	1	4	4
2	2	2	2	4	4	1	1	4	4
1	1	2	2	4	4	1	1	4	4
2	2	2	2	4	4	1	1	4	4
2	2	2	2	4	4	1	1	4	4
2	2	3	3	4	4	1	1	4	4
2	2	2	2	4	4	1	1	4	4
2	2	3	3	4	4	1	1	4	4
2	2	3	3	4	4	4	4	4	4
2	2	3	3	4	4	1	1	4	4
2	2	2	2	4	4	1	1	4	4
2	2	2	2	4	4	1	1	4	4
1	1	2	2	4	4	4	4	4	4
2	2	3	3	4	4	1	1	4	4
2	2	2	2	4	4	1	1	4	4
2	2	2	2	4	4	1	1	4	4
1	1	2	2	4	4	1	1	4	4

For Review Only

2	2	2	2	4	4	1	1	4	4
2	2	2	2	4	4	1	1	4	4
2	2	2	2	4	4	1	1	4	4
2	2	3	3	4	4	1	1	4	4
2	2	2	2	4	4	1	1	4	4
2	2	2	2	4	4	1	1	4	4
2	2	2	2	4	4	1	1	4	4
2	2	3	3	4	4	1	1	4	4
2	2	2	2	4	4	1	1	4	4
2	2	2	2	4	4	1	1	4	4
2	2	2	2	4	4	1	1	4	4
1	1	2	2	4	4	1	1	4	4
2	2	2	2	4	4	1	1	4	4
2	2	2	2	4	4	1	1	4	4
2	2	2	2	4	4	1	1	4	4
2	2	2	2	4	4	1	1	1	1
2	2	2	2	4	4	4	4	4	4
2	2	2	2	4	4	1	1	4	4
2	2	2	2	4	4	4	4	4	4
1	1	2	2	4	4	1	1	4	4
1	1	2	2	4	4	1	1	4	4
2	2	2	2	4	4	1	1	4	4
2	2	2	2	4	4	1	1	1	1
2	2	2	2	4	4	1	1	4	4
2	2	3	3	4	4	1	1	4	4
2	2	2	2	4	4	1	1	4	4
2	2	3	3	4	4	1	1	4	4
2	2	2	2	4	4	1	1	4	4
2	2	2	2	4	4	1	1	4	4
2	2	3	3	4	4	1	1	4	4
2	2	2	2	4	4	1	1	4	4
2	2	2	2	1	1	1	1	4	4
2	2	2	2	4	4	1	1	4	4
2	2	2	2	4	4	1	1	4	4
1	1	3	3	4	4	1	1	4	4
2	2	3	3	4	4	1	1	4	4
2	2	2	2	4	4	1	1	4	4
2	2	3	3	4	4	1	1	4	4
2	2	2	2	4	4	1	1	4	4
2	2	3	3	4	4	1	1	4	4
2	2	2	2	4	4	4	4	4	4
2	2	3	3	4	4	1	1	4	4
2	2	2	2	4	4	1	1	4	4
2	2	2	2	4	4	1	1	4	4
2	2	2	2	4	4	4	4	4	4
2	2	2	2	4	4	1	1	4	4
2	2	2	2	4	4	1	1	4	4
2	2	2	2	4	4	1	1	4	4
2	2	2	2	4	4	1	1	1	1
1	1	3	3	4	4	1	1	4	4
2	2	2	2	4	4	1	1	4	4
2	2	2	2	4	4	1	1	4	4
2	2	3	3	4	4	1	1	4	4
2	2	2	2	4	4	1	1	4	4
2	2	2	2	4	4	1	1	4	4

For Review Only

2	2	2	2	4	4	1	1	4	4
1	1	2	2	4	4	1	1	4	4
2	2	2	2	4	4	1	1	4	4
2	2	2	2	4	4	1	1	4	4
2	2	2	2	4	4	1	1	4	4
2	2	2	2	4	4	1	1	4	4
2	2	2	2	4	4	1	1	4	4
2	2	2	2	4	4	1	1	4	4
2	2	2	2	4	4	1	1	4	4
2	2	2	2	4	4	1	1	4	4
2	2	2	2	4	4	1	1	4	4
2	2	2	2	4	4	1	1	4	4
2	2	2	2	4	4	1	1	4	4
2	2	2	2	4	4	1	1	4	4
2	2	2	2	4	4	1	1	4	4
2	2	2	2	4	4	1	1	4	4
2	2	2	2	4	4	1	1	4	4
2	2	2	2	4	4	1	1	4	4
2	2	2	2	1	1	1	1	4	4
2	2	2	2	4	4	4	4	4	4
2	2	2	2	4	4	1	1	4	4
2	2	2	2	4	4	1	1	4	4
2	2	2	2	4	4	1	1	4	4
2	2	2	2	4	4	1	1	4	4
2	2	2	2	4	4	1	1	4	4
2	2	2	2	4	4	1	1	4	4
2	2	3	3	4	4	1	1	4	4
2	2	2	2	4	4	1	1	4	4
1	1	2	2	4	4	1	1	4	4
1	1	2	2	4	4	1	1	4	4
2	2	2	2	4	4	1	1	4	4
2	2	2	2	4	4	1	1	4	4
2	2	2	2	4	4	4	4	4	4
2	2	2	2	4	4	1	1	4	4
2	2	2	2	1	1	1	1	4	4
2	2	2	2	1	1	1	1	4	4
2	2	2	2	4	4	1	1	4	4
2	2	2	2	4	4	1	1	4	4
1	1	2	2	4	4	1	1	4	4
2	2	2	2	4	4	1	1	4	4
2	2	2	2	4	4	1	1	4	4
2	2	2	2	4	4	1	1	4	4
2	2	2	2	4	4	1	1	4	4
2	2	2	2	4	4	1	1	4	4
2	2	2	2	4	4	1	1	1	1
2	2	2	2	4	4	1	1	4	4
2	2	2	2	4	4	1	1	4	4
2	2	2	2	4	4	1	1	4	4
2	2	2	2	4	4	1	1	4	4
2	2	2	2	4	4	1	1	4	4
2	2	2	2	4	4	1	1	4	4
2	2	2	2	4	4	1	1	4	4
2	2	2	2	4	4	1	1	4	4
1	1	2	2	4	4	1	1	4	4
2	2	2	2	4	4	1	1	4	4

For Review Only

2	2	2	2	4	4	1	1	4	4
2	2	2	2	4	4	1	1	4	4
2	2	3	3	4	4	1	1	4	4
2	2	2	2	4	4	1	1	4	4
1	1	2	2	4	4	1	1	4	4
2	2	2	2	4	4	1	1	4	4
2	2	2	2	4	4	1	1	4	4
2	2	2	2	4	4	1	1	4	4
2	2	2	2	4	4	4	4	4	4
2	2	3	3	4	4	1	1	4	4
2	2	2	2	4	4	1	1	4	4
2	2	2	2	1	1	1	1	4	4
2	2	2	2	4	4	1	1	4	4
2	2	2	2	4	4	1	1	4	4
2	2	2	2	4	4	1	1	4	4
2	2	2	2	4	4	1	1	4	4
2	2	2	2	4	4	1	1	4	4
2	2	2	2	4	4	1	1	4	4
2	2	2	2	4	4	1	1	4	4
2	2	2	2	4	4	1	1	4	4
2	2	2	2	4	4	1	1	4	4
2	2	2	2	4	4	1	1	4	4
2	2	3	3	4	4	1	1	4	4
1	1	2	2	4	4	1	1	4	4
2	2	3	3	4	4	1	1	4	4
2	2	2	2	1	1	1	1	4	4
1	1	2	2	4	4	1	1	4	4
2	2	2	2	4	4	1	1	4	4
2	2	2	2	4	4	1	1	4	4
2	2	2	2	4	4	1	1	4	4
2	2	2	2	4	4	1	1	1	1
2	2	3	3	4	4	1	1	4	4
2	2	3	3	4	4	1	1	4	4
2	2	2	2	4	4	1	1	4	4
2	2	3	3	4	4	1	1	4	4
1	1	2	2	4	4	1	1	4	4
2	2	2	2	4	4	1	1	4	4
2	2	2	2	4	4	1	1	4	4
2	2	2	2	4	4	1	1	4	4
2	2	2	2	4	4	1	1	1	1
1	1	2	2	4	4	1	1	1	1
2	2	2	2	4	4	1	1	4	4
2	2	2	2	4	4	1	1	4	4
2	2	2	2	4	4	1	1	4	4
2	2	2	2	4	4	1	1	4	4
2	2	2	2	4	4	1	1	4	4
2	2	2	2	4	4	1	1	4	4
2	2	2	2	4	4	1	1	4	4
2	2	2	2	4	4	1	1	4	4
2	2	2	2	4	4	1	1	4	4
1	1	2	2	4	4	1	1	4	4
2	2	3	3	4	4	1	1	4	4
2	2	3	3	4	4	1	1	4	4
2	2	2	2	4	4	1	1	4	4
2	2	2	2	4	4	1	1	4	4

For Review Only

2	2	2	2	4	4	1	1	4	4
2	2	2	2	4	4	1	1	4	4
1	1	2	2	4	4	1	1	4	4
2	2	2	2	4	4	1	1	4	4
1	1	2	2	4	4	1	1	4	4
2	2	2	2	4	4	1	1	4	4
1	1	2	2	4	4	1	1	4	4
2	2	2	2	4	4	1	1	4	4
2	2	2	2	4	4	1	1	4	4
2	2	2	2	4	4	1	1	4	4
2	2	2	2	4	4	1	1	4	4
2	2	2	2	4	4	1	1	1	1
2	2	2	2	4	4	1	1	4	4
2	2	2	2	4	4	1	1	4	4
2	2	2	2	4	4	1	1	4	4
2	2	2	2	4	4	1	1	4	4
2	2	2	2	4	4	1	1	4	4
2	2	2	2	4	4	1	1	4	4
2	2	2	2	4	4	1	1	4	4
1	1	2	2	4	4	1	1	4	4
2	2	2	2	4	4	1	1	4	4
2	2	2	2	4	4	1	1	4	4
1	1	2	2	4	4	1	1	4	4
2	2	2	2	4	4	1	1	4	4
2	2	3	3	4	4	1	1	4	4
2	2	2	2	4	4	1	1	4	4
2	2	2	2	4	4	1	1	4	4
1	1	2	2	4	4	1	1	4	4
2	2	2	2	4	4	1	1	4	4
1	1	2	2	4	4	1	1	4	4
2	2	2	2	4	4	1	1	4	4
2	2	2	2	4	4	1	1	4	4
2	2	3	3	4	4	4	4	4	4
2	2	2	2	4	4	1	1	4	4
2	2	3	3	4	4	1	1	4	4
2	2	2	2	4	4	1	1	4	4
2	2	2	2	4	4	1	1	4	4
1	1	2	2	4	4	1	1	4	4
2	2	2	2	1	1	1	1	4	4
2	2	2	2	4	4	1	1	4	4
1	1	2	2	4	4	1	1	4	4
2	2	2	2	4	4	1	1	4	4
2	2	2	2	4	4	1	1	4	4
2	2	2	2	4	4	4	4	4	4
2	2	2	2	4	4	1	1	4	4
2	2	2	2	4	4	1	1	4	4
2	2	2	2	4	4	1	1	4	4
2	2	2	2	4	4	1	1	4	4
2	2	2	2	4	4	1	1	4	4
2	2	2	2	4	4	1	1	4	4
1	1	2	2	4	4	1	1	4	4
2	2	2	2	4	4	1	1	4	4
1	1	2	2	4	4	1	1	4	4
1	1	2	2	4	4	1	1	4	4
2	2	2	2	4	4	1	1	4	4

For Review Only

2	2	2	2	4	4	1	1	4	4
2	2	2	2	4	4	1	1	4	4

For Review Only

SNP1954	SNP1954	SNP1955	SNP1955	SNP1956	SNP1956	SNP1957	SNP1957	SNP1958	SNP1958
4	4	4	4	3	3	1	1	1	1
1	1	4	4	3	3	1	1	1	1
1	1	4	4	1	1	1	1	1	1
1	1	4	4	3	3	1	1	1	1
4	4	4	4	3	3	1	1	1	1
1	1	4	4	3	3	1	1	1	1
4	4	4	4	3	3	1	1	1	1
1	1	4	4	3	3	1	1	1	1
4	4	4	4	3	3	1	1	1	1
1	1	4	4	3	3	1	1	1	1
4	4	4	4	3	3	1	1	1	1
1	1	4	4	3	3	1	1	1	1
4	4	4	4	3	3	1	1	1	1
4	4	4	4	3	3	4	4	1	1
4	4	4	4	3	3	1	1	1	1
4	4	4	4	3	3	1	1	1	1
4	4	4	4	3	3	1	1	1	1
4	4	2	2	3	3	1	1	1	1
1	1	4	4	3	3	1	1	1	1
1	1	4	4	3	3	4	4	4	4
4	4	4	4	3	3	1	1	1	1
4	4	4	4	3	3	1	1	1	1
4	4	4	4	3	3	4	4	4	4
1	1	4	4	3	3	1	1	1	1
4	4	4	4	3	3	1	1	1	1
1	1	4	4	1	1	1	1	1	1
1	1	4	4	3	3	1	1	1	1
4	4	4	4	3	3	1	1	1	1
1	1	4	4	3	3	1	1	1	1
1	1	4	4	3	3	4	4	4	4
4	4	4	4	3	3	1	1	1	1
4	4	2	2	3	3	4	4	1	1
4	4	2	2	3	3	1	1	1	1
1	1	4	4	3	3	1	1	1	1
1	1	4	4	1	1	1	1	1	1
1	1	4	4	3	3	1	1	1	1
4	4	4	4	3	3	4	4	1	1
4	4	4	4	3	3	1	1	1	1
1	1	4	4	3	3	1	1	1	1
4	4	4	4	3	3	1	1	1	1
1	1	4	4	3	3	4	4	1	1
1	1	4	4	3	3	1	1	1	1
1	1	4	4	3	3	4	4	1	1
4	4	4	4	3	3	4	4	1	1
1	1	4	4	3	3	4	4	1	1
1	1	4	4	3	3	1	1	1	1
1	1	4	4	1	1	1	1	1	1
4	4	4	4	3	3	1	1	1	1
4	4	4	4	3	3	4	4	1	1
4	4	4	4	3	3	1	1	1	1
4	4	4	4	1	1	1	1	1	1
4	4	4	4	3	3	1	1	1	1

For Review Only

4	4	4	4	3	3	1	1	1	1
4	4	2	2	3	3	1	1	1	1
4	4	4	4	3	3	1	1	1	1
4	4	4	4	3	3	4	4	4	4
1	1	4	4	3	3	1	1	1	1
1	1	2	2	1	1	1	1	1	1
1	1	2	2	1	1	1	1	1	1
1	1	4	4	3	3	4	4	1	1
1	1	4	4	3	3	1	1	1	1
4	4	4	4	3	3	1	1	1	1
1	1	4	4	3	3	1	1	1	1
1	1	4	4	1	1	1	1	1	1
4	4	4	4	3	3	1	1	1	1
1	1	4	4	3	3	1	1	1	1
4	4	4	4	3	3	1	1	1	1
1	1	4	4	3	3	1	1	1	1
4	4	2	2	3	3	1	1	1	1
4	4	4	4	3	3	1	1	1	1
4	4	4	4	3	3	1	1	1	1
4	4	4	4	3	3	1	1	1	1
1	1	4	4	3	3	1	1	1	1
1	1	4	4	3	3	1	1	1	1
4	4	4	4	3	3	4	4	1	1
4	4	2	2	3	3	4	4	1	1
1	1	4	4	3	3	4	4	1	1
1	1	4	4	1	1	1	1	4	4
1	1	2	2	3	3	4	4	1	1
1	1	4	4	3	3	1	1	1	1
1	1	2	2	1	1	1	1	1	1
4	4	4	4	3	3	4	4	1	1
1	1	4	4	3	3	1	1	1	1
1	1	4	4	3	3	1	1	1	1
1	1	4	4	3	3	1	1	1	1
4	4	4	4	3	3	1	1	1	1
1	1	4	4	3	3	4	4	1	1
1	1	4	4	3	3	4	4	1	1
1	1	4	4	3	3	1	1	4	4
4	4	4	4	3	3	4	4	1	1
4	4	4	4	3	3	1	1	1	1
1	1	4	4	3	3	4	4	4	4
4	4	4	4	3	3	1	1	1	1
4	4	4	4	3	3	4	4	1	1
4	4	4	4	3	3	1	1	1	1
1	1	4	4	3	3	4	4	1	1
1	1	4	4	3	3	1	1	1	1
4	4	2	2	3	3	1	1	1	1
1	1	4	4	3	3	1	1	4	4
1	1	4	4	3	3	1	1	1	1
4	4	2	2	1	1	1	1	1	1
4	4	4	4	3	3	4	4	1	1
1	1	4	4	1	1	1	1	1	1
1	1	4	4	3	3	1	1	1	1
1	1	4	4	3	3	4	4	1	1
1	1	4	4	3	3	1	1	1	1
4	4	2	2	3	3	1	1	1	1

For Review Only

4	4	4	4	3	3	1	1	1	1
4	4	4	4	3	3	1	1	1	1
1	1	4	4	3	3	1	1	1	1
1	1	4	4	3	3	1	1	1	1
4	4	4	4	3	3	1	1	1	1
1	1	4	4	3	3	1	1	1	1
4	4	4	4	3	3	1	1	1	1
4	4	4	4	3	3	1	1	1	1
1	1	4	4	1	1	1	1	1	1
1	1	4	4	3	3	1	1	1	1
4	4	4	4	3	3	1	1	1	1
4	4	4	4	3	3	4	4	1	1
4	4	4	4	3	3	1	1	1	1
1	1	4	4	3	3	1	1	1	1
4	4	4	4	3	3	1	1	1	1
4	4	4	4	3	3	1	1	1	1
4	4	2	2	3	3	4	4	1	1
4	4	4	4	3	3	4	4	1	1
1	1	4	4	3	3	1	1	4	4
4	4	4	4	3	3	1	1	1	1
4	4	4	4	3	3	1	1	1	1
4	4	4	4	3	3	1	1	1	1
4	4	2	2	1	1	1	1	1	1
1	1	4	4	3	3	1	1	1	1
1	1	4	4	3	3	4	4	1	1
4	4	4	4	3	3	1	1	1	1
4	4	4	4	3	3	4	4	1	1
4	4	4	4	3	3	1	1	1	1
4	4	4	4	3	3	1	1	1	1
4	4	4	4	3	3	1	1	1	1
4	4	4	4	3	3	1	1	1	1
4	4	4	4	3	3	1	1	1	1
4	4	4	4	3	3	1	1	1	1
4	4	4	4	3	3	1	1	1	1
1	1	4	4	3	3	1	1	1	1
4	4	4	4	3	3	1	1	1	1
1	1	4	4	3	3	1	1	4	4
4	4	4	4	3	3	1	1	1	1
1	1	2	2	3	3	1	1	1	1
4	4	4	4	3	3	1	1	1	1
4	4	4	4	3	3	1	1	1	1
1	1	4	4	3	3	1	1	1	1
4	4	4	4	3	3	1	1	1	1
1	1	4	4	3	3	4	4	1	1
4	4	4	4	3	3	1	1	1	1
1	1	4	4	3	3	1	1	1	1
4	4	2	2	1	1	1	1	1	1
4	4	4	4	1	1	1	1	1	1
1	1	4	4	3	3	1	1	1	1
1	1	4	4	1	1	1	1	1	1
1	1	4	4	3	3	1	1	1	1
1	1	4	4	3	3	1	1	4	4
1	1	4	4	3	3	1	1	1	1
1	1	4	4	1	1	1	1	1	1
1	1	4	4	3	3	1	1	1	1

For Review Only

4	4	2	2	3	3	1	1	1	1
4	4	4	4	3	3	1	1	1	1
4	4	4	4	3	3	4	4	1	1
4	4	4	4	3	3	1	1	1	1
4	4	4	4	3	3	1	1	1	1
4	4	4	4	3	3	1	1	1	1
1	1	4	4	3	3	1	1	1	1
4	4	4	4	3	3	1	1	1	1
1	1	4	4	3	3	1	1	1	1
1	1	4	4	3	3	4	4	1	1
4	4	4	4	3	3	1	1	1	1
1	1	4	4	3	3	1	1	1	1
1	1	4	4	3	3	1	1	1	1
4	4	4	4	3	3	4	4	1	1
4	4	4	4	3	3	1	1	1	1
4	4	4	4	3	3	1	1	1	1
4	4	4	4	3	3	1	1	1	1
4	4	4	4	3	3	1	1	1	1
4	4	2	2	1	1	1	1	1	1
4	4	4	4	3	3	1	1	1	1
4	4	4	4	3	3	1	1	1	1
1	1	4	4	3	3	1	1	1	1
1	1	4	4	3	3	4	4	1	1
1	1	4	4	1	1	1	1	1	1
1	1	4	4	3	3	1	1	1	1
1	1	4	4	3	3	1	1	1	1
1	1	4	4	3	3	1	1	1	1
1	1	4	4	3	3	1	1	1	1
4	4	4	4	3	3	1	1	1	1
4	4	4	4	3	3	1	1	1	1
1	1	4	4	3	3	1	1	1	1
4	4	2	2	3	3	4	4	1	1
4	4	4	4	3	3	4	4	1	1
1	1	4	4	3	3	1	1	1	1
1	1	4	4	3	3	4	4	1	1
1	1	4	4	1	1	1	1	1	1
4	4	4	4	1	1	1	1	1	1
4	4	4	4	3	3	1	1	1	1
4	4	4	4	1	1	1	1	1	1
4	4	4	4	3	3	1	1	1	1
1	1	4	4	1	1	1	1	1	1
4	4	2	2	1	1	1	1	1	1
1	1	4	4	3	3	4	4	1	1
4	4	4	4	3	3	4	4	1	1
4	4	2	2	3	3	1	1	1	1
1	1	4	4	3	3	1	1	1	1
4	4	4	4	3	3	1	1	1	1
1	1	4	4	3	3	1	1	1	1
1	1	4	4	3	3	1	1	1	1
4	4	4	4	3	3	1	1	4	4
4	4	4	4	1	1	1	1	1	1
1	1	4	4	3	3	4	4	1	1
1	1	4	4	3	3	4	4	1	1
1	1	4	4	3	3	1	1	1	1
1	1	4	4	3	3	1	1	1	1

For Review Only

4	4	4	4	3	3	1	1	1	1
4	4	2	2	3	3	1	1	1	1
4	4	4	4	1	1	4	4	1	1
4	4	4	4	3	3	4	4	1	1
4	4	4	4	3	3	1	1	1	1
4	4	4	4	1	1	1	1	1	1
4	4	2	2	3	3	1	1	1	1
1	1	4	4	3	3	1	1	4	4
1	1	4	4	3	3	1	1	1	1
1	1	4	4	3	3	1	1	1	1
4	4	4	4	3	3	1	1	1	1
4	4	4	4	3	3	1	1	1	1
4	4	4	4	3	3	1	1	1	1
1	1	4	4	3	3	1	1	1	1
1	1	4	4	1	1	1	1	1	1
4	4	4	4	3	3	4	4	1	1
1	1	4	4	3	3	1	1	1	1
4	4	4	4	3	3	1	1	1	1
1	1	2	2	3	3	1	1	1	1
4	4	4	4	3	3	1	1	1	1
1	1	4	4	3	3	1	1	1	1
1	1	2	2	1	1	1	1	1	1
4	4	4	4	3	3	4	4	1	1
4	4	4	4	3	3	1	1	1	1
1	1	4	4	3	3	4	4	1	1
1	1	4	4	1	1	1	1	1	1
1	1	4	4	3	3	1	1	1	1
1	1	4	4	3	3	1	1	1	1
4	4	4	4	3	3	1	1	1	1
4	4	4	4	1	1	1	1	1	1
4	4	4	4	3	3	4	4	4	4
1	1	4	4	1	1	1	1	1	1
4	4	4	4	3	3	4	4	1	1
1	1	4	4	3	3	1	1	1	1
4	4	4	4	3	3	4	4	1	1
1	1	4	4	3	3	1	1	1	1
4	4	4	4	3	3	1	1	1	1
1	1	4	4	1	1	1	1	1	1
1	1	4	4	3	3	1	1	1	1
1	1	4	4	3	3	1	1	1	1
4	4	2	2	3	3	1	1	1	1
1	1	4	4	3	3	1	1	1	1
1	1	4	4	3	3	1	1	1	1
4	4	4	4	3	3	1	1	1	1
1	1	4	4	1	1	4	4	1	1
1	1	4	4	3	3	1	1	1	1
4	4	4	4	1	1	1	1	1	1
4	4	4	4	3	3	1	1	1	1
1	1	4	4	1	1	4	4	1	1
1	1	4	4	3	3	1	1	1	1
4	4	4	4	3	3	1	1	1	1
1	1	4	4	3	3	1	1	1	1
1	1	4	4	1	1	1	1	1	1
1	1	4	4	3	3	1	1	1	1
1	1	4	4	3	3	1	1	1	1

For Review Only

4	4	4	4	3	3	1	1	1	1
4	4	4	4	3	3	1	1	1	1

For Review Only

SNP1959	SNP1959	SNP196	SNP196	SNP1960	SNP1960	SNP1961	SNP1961	SNP1962	SNP1962
4	4	1	1	4	4	3	3	4	4
3	3	1	1	1	1	3	3	4	4
3	3	1	1	4	4	3	3	4	4
3	3	1	1	4	4	3	3	4	4
4	4	4	4	4	4	3	3	4	4
4	4	1	1	4	4	3	3	1	1
4	4	1	1	4	4	3	3	4	4
3	3	1	1	1	1	3	3	4	4
4	4	1	1	4	4	3	3	4	4
3	3	4	4	1	1	3	3	4	4
4	4	1	1	4	4	3	3	4	4
3	3	1	1	4	4	3	3	4	4
3	3	1	1	4	4	3	3	4	4
4	4	1	1	4	4	3	3	4	4
4	4	1	1	4	4	3	3	4	4
4	4	4	4	4	4	3	3	4	4
4	4	4	4	4	4	3	3	4	4
4	4	1	1	4	4	3	3	4	4
4	4	1	1	4	4	3	3	4	4
3	3	4	4	4	4	3	3	4	4
4	4	4	4	4	4	3	3	4	4
4	4	4	4	4	4	3	3	4	4
4	4	1	1	4	4	3	3	4	4
3	3	4	4	4	4	3	3	4	4
4	4	1	1	4	4	3	3	4	4
3	3	1	1	4	4	3	3	4	4
3	3	1	1	4	4	3	3	4	4
4	4	1	1	4	4	3	3	4	4
3	3	4	4	4	4	3	3	4	4
3	3	4	4	1	1	3	3	4	4
4	4	1	1	4	4	3	3	4	4
4	4	1	1	4	4	2	2	4	4
4	4	1	1	4	4	3	3	4	4
3	3	4	4	4	4	3	3	4	4
4	4	1	1	4	4	3	3	4	4
3	3	1	1	4	4	3	3	4	4
4	4	1	1	4	4	3	3	4	4
4	4	1	1	1	1	3	3	4	4
3	3	4	4	4	4	3	3	4	4
4	4	1	1	4	4	3	3	4	4
3	3	4	4	1	1	3	3	4	4
3	3	4	4	4	4	3	3	4	4
3	3	4	4	1	1	3	3	4	4
4	4	1	1	4	4	3	3	4	4
3	3	1	1	1	1	3	3	4	4
3	3	1	1	4	4	3	3	4	4
3	3	1	1	4	4	3	3	4	4
4	4	1	1	4	4	3	3	4	4
4	4	4	4	4	4	3	3	4	4
4	4	1	1	4	4	3	3	4	4
4	4	1	1	4	4	3	3	4	4
4	4	1	1	4	4	3	3	4	4

For Review Only

4	4	1	1	4	4	3	3	4	4
4	4	1	1	4	4	3	3	4	4
4	4	1	1	4	4	3	3	1	1
4	4	1	1	4	4	3	3	4	4
4	4	1	1	4	4	3	3	4	4
4	4	1	1	4	4	2	2	4	4
4	4	1	1	4	4	3	3	4	4
3	3	4	4	1	1	3	3	4	4
3	3	1	1	4	4	3	3	4	4
4	4	1	1	4	4	3	3	4	4
3	3	1	1	4	4	3	3	1	1
3	3	1	1	4	4	2	2	4	4
4	4	1	1	4	4	3	3	4	4
3	3	4	4	4	4	3	3	4	4
4	4	1	1	4	4	3	3	1	1
3	3	1	1	4	4	3	3	4	4
4	4	1	1	4	4	3	3	4	4
4	4	1	1	4	4	3	3	4	4
4	4	1	1	4	4	3	3	4	4
4	4	1	1	4	4	3	3	4	4
3	3	1	1	4	4	2	2	4	4
3	3	4	4	4	4	3	3	4	4
4	4	4	4	4	4	3	3	4	4
4	4	4	4	4	4	3	3	4	4
3	3	1	1	1	1	3	3	4	4
3	3	1	1	4	4	3	3	4	4
4	4	1	1	1	1	3	3	4	4
3	3	4	4	4	4	3	3	4	4
4	4	1	1	4	4	3	3	4	4
4	4	1	1	4	4	3	3	1	1
3	3	1	1	4	4	3	3	4	4
3	3	4	4	4	4	3	3	4	4
3	3	4	4	1	1	3	3	4	4
4	4	1	1	4	4	3	3	4	4
3	3	1	1	4	4	3	3	1	1
3	3	1	1	1	1	3	3	4	4
3	3	1	1	4	4	3	3	4	4
3	3	4	4	4	4	3	3	4	4
3	3	1	1	4	4	3	3	4	4
3	3	1	1	1	1	3	3	1	1
4	4	1	1	4	4	3	3	4	4
3	3	1	1	4	4	3	3	1	1
4	4	1	1	1	1	3	3	4	4
3	3	1	1	4	4	3	3	1	1
3	3	1	1	4	4	3	3	1	1
4	4	1	1	4	4	3	3	1	1
3	3	4	4	4	4	2	2	4	4
4	4	1	1	4	4	3	3	4	4
4	4	1	1	4	4	3	3	4	4
4	4	1	1	4	4	3	3	4	4
3	3	1	1	4	4	2	2	1	1
3	3	4	4	4	4	3	3	4	4
3	3	4	4	1	1	3	3	4	4
4	4	1	1	4	4	3	3	4	4
4	4	1	1	1	1	3	3	4	4

For Review Only

4	4	1	1	4	4	3	3	4	4
4	4	1	1	4	4	3	3	4	4
3	3	1	1	4	4	3	3	4	4
3	3	4	4	4	4	2	2	4	4
4	4	1	1	4	4	3	3	1	1
3	3	1	1	4	4	3	3	4	4
4	4	1	1	4	4	3	3	4	4
4	4	1	1	4	4	3	3	1	1
3	3	4	4	4	4	3	3	4	4
4	4	1	1	4	4	3	3	4	4
4	4	1	1	4	4	3	3	1	1
4	4	1	1	4	4	3	3	4	4
4	4	1	1	1	1	3	3	4	4
4	4	1	1	4	4	3	3	4	4
3	3	1	1	4	4	2	2	4	4
4	4	1	1	4	4	3	3	4	4
4	4	1	1	4	4	3	3	4	4
4	4	1	1	4	4	2	2	4	4
3	3	4	4	4	4	2	2	4	4
4	4	1	1	1	1	3	3	4	4
4	4	1	1	4	4	3	3	4	4
4	4	1	1	4	4	3	3	1	1
4	4	1	1	4	4	3	3	4	4
3	3	4	4	4	4	3	3	4	4
3	3	1	1	4	4	3	3	4	4
4	4	1	1	4	4	3	3	4	4
4	4	1	1	4	4	3	3	4	4
4	4	1	1	4	4	3	3	4	4
4	4	1	1	4	4	3	3	4	4
4	4	1	1	4	4	3	3	4	4
3	3	4	4	4	4	3	3	4	4
4	4	1	1	4	4	3	3	4	4
4	4	1	1	4	4	3	3	4	4
4	4	1	1	4	4	3	3	4	4
3	3	4	4	4	4	3	3	4	4
4	4	1	1	1	1	3	3	4	4
3	3	4	4	4	4	2	2	4	4
4	4	1	1	4	4	3	3	1	1
3	3	1	1	4	4	3	3	4	4
4	4	4	4	4	4	3	3	4	4
4	4	1	1	4	4	3	3	1	1
3	3	4	4	4	4	2	2	4	4
4	4	1	1	4	4	3	3	4	4
3	3	4	4	1	1	3	3	4	4
4	4	1	1	4	4	3	3	4	4
3	3	1	1	4	4	3	3	4	4
4	4	1	1	4	4	3	3	4	4
3	3	1	1	4	4	3	3	4	4
3	3	1	1	4	4	3	3	4	4
3	3	1	1	4	4	3	3	4	4
4	4	1	1	4	4	3	3	4	4
3	3	4	4	4	4	3	3	4	4
3	3	1	1	4	4	3	3	4	4
3	3	1	1	4	4	2	2	4	4
4	4	1	1	4	4	3	3	4	4

For Review Only

4	4	1	1	4	4	3	3	1	1
4	4	1	1	1	1	3	3	4	4
4	4	4	4	4	4	3	3	4	4
4	4	4	4	4	4	3	3	4	4
4	4	1	1	4	4	3	3	1	1
4	4	4	4	4	4	3	3	1	1
3	3	1	1	4	4	3	3	4	4
4	4	1	1	4	4	3	3	4	4
3	3	1	1	4	4	3	3	4	4
3	3	1	1	4	4	3	3	1	1
4	4	1	1	4	4	3	3	4	4
3	3	1	1	4	4	3	3	4	4
4	4	1	1	4	4	3	3	1	1
4	4	1	1	1	1	3	3	4	4
3	3	4	4	4	4	2	2	4	4
4	4	1	1	4	4	3	3	4	4
4	4	1	1	4	4	3	3	4	4
4	4	1	1	4	4	3	3	4	4
4	4	1	1	4	4	3	3	4	4
4	4	1	1	4	4	3	3	4	4
4	4	1	1	4	4	3	3	4	4
3	3	4	4	1	1	3	3	4	4
3	3	4	4	4	4	3	3	4	4
3	3	1	1	4	4	2	2	4	4
3	3	1	1	4	4	2	2	4	4
3	3	4	4	4	4	3	3	4	4
3	3	1	1	4	4	3	3	4	4
4	4	1	1	4	4	3	3	4	4
4	4	1	1	1	1	3	3	4	4
4	4	1	1	4	4	3	3	4	4
3	3	1	1	4	4	3	3	4	4
4	4	1	1	4	4	3	3	1	1
4	4	1	1	4	4	3	3	4	4
3	3	4	4	4	4	2	2	4	4
4	4	1	1	4	4	3	3	4	4
3	3	1	1	4	4	2	2	4	4
4	4	1	1	4	4	3	3	4	4
4	4	1	1	4	4	3	3	4	4
3	3	1	1	4	4	3	3	4	4
3	3	1	1	4	4	2	2	4	4
3	3	1	1	4	4	3	3	4	4
4	4	1	1	4	4	3	3	4	4
3	3	4	4	4	4	3	3	4	4
4	4	1	1	4	4	3	3	4	4
4	4	1	1	1	1	3	3	4	4
3	3	4	4	4	4	3	3	1	1
4	4	1	1	4	4	3	3	4	4
3	3	4	4	4	4	3	3	4	4
3	3	4	4	1	1	3	3	4	4
4	4	1	1	4	4	3	3	4	4
4	4	1	1	4	4	3	3	4	4
3	3	4	4	4	4	3	3	4	4
3	3	4	4	4	4	3	3	4	4
3	3	1	1	4	4	3	3	1	1
3	3	4	4	4	4	3	3	4	4

For Review Only

4	4	1	1	4	4	3	3	4	4
4	4	1	1	4	4	3	3	4	4
4	4	1	1	4	4	3	3	4	4
4	4	1	1	1	1	3	3	4	4
4	4	1	1	4	4	3	3	4	4
4	4	1	1	4	4	3	3	4	4
4	4	4	4	4	4	3	3	4	4
3	3	4	4	1	1	3	3	4	4
3	3	1	1	1	1	3	3	4	4
3	3	4	4	4	4	3	3	4	4
4	4	1	1	4	4	3	3	4	4
4	4	1	1	4	4	3	3	4	4
4	4	1	1	4	4	3	3	4	4
3	3	1	1	4	4	3	3	4	4
3	3	1	1	4	4	2	2	4	4
4	4	1	1	4	4	3	3	1	1
3	3	4	4	4	4	3	3	4	4
4	4	1	1	4	4	3	3	4	4
4	4	4	4	1	1	3	3	4	4
4	4	1	1	1	1	3	3	4	4
3	3	4	4	4	4	3	3	4	4
4	4	1	1	4	4	3	3	4	4
4	4	1	1	4	4	3	3	1	1
4	4	1	1	4	4	3	3	4	4
3	3	4	4	1	1	3	3	4	4
3	3	1	1	4	4	3	3	1	1
3	3	4	4	4	4	3	3	4	4
3	3	1	1	4	4	3	3	4	4
4	4	1	1	4	4	3	3	4	4
4	4	1	1	4	4	3	3	4	4
3	3	1	1	4	4	3	3	4	4
3	3	1	1	4	4	3	3	4	4
4	4	1	1	1	1	3	3	4	4
3	3	1	1	4	4	3	3	4	4
4	4	4	4	4	4	3	3	4	4
3	3	4	4	4	4	3	3	4	4
4	4	1	1	4	4	3	3	4	4
3	3	1	1	4	4	2	2	4	4
3	3	1	1	4	4	3	3	4	4
3	3	4	4	4	4	3	3	4	4
4	4	1	1	4	4	3	3	4	4
3	3	4	4	4	4	3	3	4	4
3	3	1	1	4	4	3	3	4	4
4	4	1	1	4	4	3	3	4	4
3	3	1	1	4	4	2	2	4	4
4	4	1	1	4	4	3	3	4	4
3	3	1	1	4	4	3	3	4	4
4	4	1	1	1	1	3	3	4	4
3	3	1	1	1	1	3	3	4	4
3	3	4	4	4	4	3	3	4	4
4	4	1	1	4	4	3	3	4	4
3	3	4	4	4	4	3	3	4	4
3	3	1	1	4	4	3	3	4	4
3	3	1	1	4	4	3	3	4	4
3	3	1	1	4	4	3	3	4	4

For Review Only

4	4	1	1	4	4	3	3	1	1
3	3	1	1	4	4	3	3	4	4

For Review Only

SNP1963	SNP1963	SNP1964	SNP1964	SNP1965	SNP1965	SNP1966	SNP1966	SNP1967	SNP1967
3	3	1	1	2	2	3	3	2	2
4	4	1	1	2	2	3	3	2	2
3	3	1	1	2	2	3	3	2	2
4	4	1	1	2	2	3	3	2	2
3	3	1	1	2	2	4	4	2	2
4	4	1	1	3	3	3	3	2	2
4	4	1	1	2	2	3	3	2	2
4	4	1	1	2	2	3	3	2	2
3	3	1	1	2	2	4	4	2	2
4	4	1	1	2	2	3	3	2	2
4	4	1	1	2	2	4	4	2	2
3	3	1	1	2	2	3	3	2	2
3	3	1	1	2	2	3	3	2	2
3	3	1	1	2	2	3	3	2	2
3	3	1	1	2	2	3	3	2	2
4	4	1	1	2	2	3	3	2	2
3	3	1	1	2	2	3	3	2	2
4	4	1	1	2	2	3	3	2	2
3	3	1	1	2	2	3	3	2	2
4	4	1	1	2	2	3	3	2	2
4	4	1	1	2	2	3	3	2	2
3	3	1	1	2	2	4	4	2	2
4	4	1	1	2	2	3	3	2	2
4	4	1	1	2	2	3	3	2	2
3	3	1	1	2	2	3	3	2	2
3	3	1	1	2	2	3	3	2	2
3	3	1	1	2	2	3	3	2	2
4	4	1	1	2	2	3	3	2	2
4	4	1	1	2	2	3	3	2	2
4	4	1	1	3	3	3	3	2	2
3	3	1	1	2	2	3	3	2	2
3	3	1	1	2	2	3	3	2	2
3	3	1	1	2	2	3	3	2	2
4	4	1	1	2	2	3	3	2	2
3	3	1	1	2	2	4	4	2	2
4	4	1	1	2	2	3	3	2	2
3	3	1	1	2	2	3	3	2	2
3	3	1	1	2	2	3	3	2	2
4	4	3	3	2	2	3	3	3	3
4	4	1	1	2	2	3	3	2	2
4	4	1	1	3	3	3	3	2	2
4	4	1	1	2	2	3	3	2	2
3	3	1	1	2	2	3	3	2	2
4	4	1	1	2	2	4	4	2	2
3	3	1	1	2	2	3	3	2	2
3	3	1	1	2	2	3	3	2	2
3	3	1	1	2	2	3	3	2	2
3	3	1	1	2	2	4	4	2	2
4	4	1	1	2	2	3	3	2	2
3	3	1	1	2	2	4	4	2	2
3	3	1	1	2	2	3	3	2	2
3	3	1	1	2	2	3	3	2	2

For Review Only

3	3	1	1	2	2	3	3	2	2
3	3	1	1	2	2	3	3	2	2
4	4	1	1	2	2	3	3	2	2
4	4	1	1	2	2	3	3	2	2
3	3	1	1	2	2	3	3	2	2
3	3	1	1	2	2	3	3	2	2
3	3	1	1	2	2	3	3	2	2
4	4	1	1	2	2	3	3	2	2
4	4	1	1	2	2	3	3	2	2
3	3	1	1	2	2	3	3	2	2
4	4	1	1	2	2	3	3	2	2
3	3	1	1	2	2	3	3	2	2
3	3	1	1	2	2	3	3	2	2
4	4	1	1	2	2	3	3	2	2
4	4	1	1	2	2	3	3	2	2
3	3	1	1	2	2	3	3	2	2
3	3	1	1	2	2	4	4	2	2
3	3	1	1	2	2	3	3	2	2
3	3	1	1	2	2	4	4	2	2
3	3	1	1	2	2	3	3	2	2
4	4	1	1	2	2	3	3	2	2
4	4	1	1	2	2	3	3	2	2
4	4	1	1	2	2	3	3	2	2
3	3	1	1	2	2	3	3	2	2
3	3	1	1	2	2	3	3	2	2
3	3	1	1	2	2	3	3	2	2
4	4	1	1	2	2	3	3	2	2
4	4	3	3	2	2	4	4	3	3
3	3	1	1	2	2	3	3	2	2
4	4	1	1	2	2	4	4	2	2
3	3	1	1	2	2	3	3	2	2
4	4	1	1	2	2	3	3	2	2
4	4	3	3	2	2	3	3	3	3
3	3	1	1	2	2	3	3	2	2
4	4	1	1	3	3	3	3	2	2
3	3	1	1	2	2	3	3	2	2
3	3	1	1	2	2	3	3	2	2
4	4	1	1	2	2	3	3	2	2
3	3	1	1	2	2	4	4	2	2
3	3	1	1	3	3	3	3	2	2
3	3	1	1	2	2	4	4	2	2
4	4	1	1	3	3	4	4	2	2
3	3	1	1	2	2	3	3	2	2
4	4	1	1	2	2	3	3	2	2
4	4	1	1	3	3	4	4	2	2
4	4	1	1	2	2	3	3	2	2
4	4	1	1	2	2	3	3	2	2
4	4	1	1	2	2	3	3	2	2
3	3	1	1	2	2	3	3	2	2
4	4	1	1	2	2	3	3	2	2
3	3	1	1	2	2	3	3	2	2
4	4	3	3	2	2	3	3	3	3
4	4	1	1	2	2	3	3	2	2
4	4	1	1	2	2	3	3	2	2
4	4	1	1	2	2	3	3	2	2

4	4	1	1	2	2	3	3	2	2
4	4	1	1	2	2	3	3	2	2
3	3	1	1	2	2	3	3	2	2
4	4	1	1	2	2	3	3	2	2
4	4	1	1	2	2	3	3	2	2
4	4	1	1	2	2	3	3	2	2
3	3	1	1	2	2	3	3	2	2
4	4	1	1	2	2	3	3	2	2
4	4	1	1	2	2	3	3	2	2
4	4	1	1	2	2	3	3	2	2
4	4	1	1	2	2	3	3	2	2
4	4	1	1	2	2	3	3	2	2
3	3	1	1	2	2	3	3	2	2
3	3	1	1	2	2	3	3	2	2
4	4	1	1	2	2	3	3	2	2
3	3	1	1	2	2	3	3	2	2
4	4	1	1	2	2	3	3	2	2
4	4	1	1	2	2	3	3	2	2
4	4	1	1	2	2	3	3	2	2
3	3	1	1	2	2	4	4	2	2
4	4	1	1	2	2	3	3	2	2
4	4	1	1	2	2	4	4	2	2
4	4	1	1	2	2	3	3	2	2
4	4	3	3	2	2	3	3	3	3
4	4	1	1	2	2	3	3	2	2
4	4	1	1	2	2	3	3	2	2
3	3	1	1	2	2	3	3	2	2
3	3	1	1	3	3	3	3	2	2
3	3	1	1	2	2	3	3	2	2
4	4	1	1	2	2	3	3	2	2
4	4	3	3	2	2	3	3	3	3
4	4	1	1	2	2	3	3	2	2
3	3	1	1	2	2	4	4	2	2
4	4	1	1	2	2	3	3	2	2
4	4	1	1	2	2	3	3	2	2
4	4	1	1	2	2	3	3	2	2
4	4	1	1	2	2	3	3	2	2
4	4	1	1	2	2	3	3	2	2
3	3	1	1	2	2	4	4	2	2
4	4	1	1	2	2	4	4	2	2
4	4	1	1	2	2	3	3	2	2
4	4	1	1	2	2	3	3	2	2
3	3	1	1	2	2	3	3	2	2
4	4	1	1	2	2	3	3	2	2
3	3	1	1	2	2	3	3	2	2
3	3	1	1	2	2	3	3	2	2
3	3	1	1	2	2	3	3	2	2
3	3	1	1	2	2	3	3	2	2
4	4	1	1	2	2	3	3	2	2
3	3	1	1	2	2	3	3	2	2
4	4	1	1	2	2	3	3	2	2
4	4	1	1	2	2	3	3	2	2
3	3	1	1	2	2	3	3	2	2
3	3	1	1	2	2	3	3	2	2
4	4	1	1	2	2	3	3	2	2

For Review Only

4	4	1	1	2	2	3	3	2	2
4	4	1	1	2	2	3	3	2	2
4	4	1	1	2	2	3	3	2	2
4	4	1	1	2	2	3	3	3	3
4	4	1	1	2	2	3	3	2	2
4	4	1	1	2	2	4	4	2	2
4	4	1	1	2	2	3	3	2	2
4	4	1	1	2	2	3	3	2	2
4	4	1	1	2	2	4	4	2	2
4	4	1	1	2	2	4	4	2	2
3	3	1	1	2	2	3	3	2	2
4	4	1	1	2	2	3	3	2	2
4	4	1	1	2	2	3	3	2	2
4	4	1	1	2	2	4	4	2	2
4	4	1	1	2	2	3	3	2	2
4	4	1	1	2	2	4	4	2	2
3	3	1	1	2	2	3	3	2	2
3	3	1	1	2	2	3	3	2	2
3	3	1	1	2	2	3	3	2	2
3	3	1	1	2	2	3	3	2	2
4	4	1	1	2	2	3	3	2	2
4	4	1	1	2	2	3	3	2	2
4	4	1	1	2	2	3	3	2	2
3	3	1	1	2	2	3	3	2	2
3	3	1	1	2	2	3	3	2	2
4	4	1	1	2	2	4	4	2	2
3	3	1	1	2	2	3	3	2	2
4	4	1	1	2	2	3	3	2	2
3	3	1	1	2	2	3	3	2	2
4	4	1	1	2	2	3	3	2	2
3	3	1	1	2	2	3	3	2	2
4	4	1	1	2	2	3	3	2	2
3	3	1	1	2	2	3	3	2	2
4	4	1	1	2	2	3	3	2	2
4	4	1	1	2	2	3	3	2	2
3	3	1	1	2	2	3	3	2	2
4	4	1	1	2	2	3	3	2	2
3	3	1	1	3	3	3	3	2	2
3	3	1	1	2	2	3	3	2	2
3	3	1	1	2	2	3	3	2	2
3	3	1	1	2	2	3	3	2	2
3	3	1	1	2	2	3	3	2	2
4	4	3	3	2	2	3	3	3	3
3	3	1	1	2	2	4	4	2	2
3	3	3	3	2	2	3	3	2	2
4	4	1	1	2	2	4	4	2	2
4	4	1	1	2	2	4	4	2	2
4	4	1	1	2	2	3	3	2	2
4	4	1	1	2	2	3	3	2	2
4	4	1	1	2	2	3	3	2	2
3	3	1	1	2	2	3	3	2	2
4	4	1	1	2	2	3	3	2	2
4	4	1	1	2	2	3	3	2	2
4	4	1	1	2	2	3	3	2	2
4	4	3	3	2	2	3	3	3	3

4	4	1	1	2	2	3	3	2	2
4	4	1	1	2	2	3	3	2	2
3	3	1	1	2	2	3	3	2	2
4	4	1	1	2	2	3	3	2	2
3	3	1	1	2	2	3	3	2	2
3	3	1	1	2	2	3	3	2	2
4	4	1	1	2	2	3	3	2	2
4	4	1	1	2	2	3	3	2	2
4	4	3	3	2	2	3	3	3	3
4	4	3	3	2	2	3	3	3	3
4	4	1	1	2	2	3	3	2	2
3	3	1	1	2	2	3	3	2	2
3	3	1	1	2	2	4	4	2	2
4	4	1	1	2	2	3	3	2	2
3	3	1	1	2	2	3	3	2	2
4	4	1	1	2	2	4	4	2	2
3	3	3	3	2	2	3	3	3	3
4	4	3	3	2	2	3	3	3	3
4	4	1	1	2	2	3	3	2	2
3	3	1	1	2	2	3	3	2	2
4	4	1	1	2	2	3	3	2	2
3	3	1	1	2	2	3	3	2	2
4	4	1	1	3	3	3	3	2	2
3	3	1	1	2	2	3	3	2	2
4	4	1	1	2	2	3	3	2	2
4	4	1	1	2	2	3	3	2	2
4	4	3	3	2	2	3	3	3	3
3	3	1	1	2	2	3	3	2	2
3	3	1	1	2	2	3	3	2	2
3	3	1	1	2	2	3	3	2	2
4	4	3	3	2	2	3	3	3	3
3	3	1	1	2	2	3	3	2	2
4	4	1	1	2	2	4	4	2	2
4	4	1	1	2	2	3	3	2	2
4	4	1	1	2	2	3	3	2	2
4	4	3	3	2	2	3	3	3	3
3	3	1	1	2	2	3	3	2	2
3	3	1	1	2	2	3	3	2	2
4	4	1	1	2	2	3	3	2	2
4	4	3	3	2	2	3	3	3	3
4	4	1	1	2	2	3	3	2	2
4	4	1	1	2	2	3	3	2	2
4	4	1	1	2	2	3	3	2	2
3	3	1	1	2	2	4	4	2	2
3	3	1	1	2	2	3	3	2	2
4	4	1	1	2	2	4	4	2	2
3	3	3	3	2	2	3	3	3	3
4	4	1	1	2	2	3	3	2	2
3	3	1	1	2	2	3	3	2	2
4	4	3	3	2	2	3	3	3	3
3	3	1	1	2	2	3	3	2	2
4	4	3	3	2	2	3	3	3	3
3	3	1	1	2	2	3	3	2	2
3	3	1	1	3	3	3	3	2	2
4	4	1	1	2	2	3	3	2	2

For Review Only

4	4	1	1	3	3	4	4	2	2
3	3	1	1	2	2	3	3	2	2

For Review Only

SNP1968	SNP1968	SNP1969	SNP1969	SNP197	SNP197	SNP1970	SNP1970	SNP1971	SNP1971
4	4	1	1	3	3	4	4	1	1
1	1	3	3	1	1	3	3	1	1
4	4	1	1	1	1	4	4	1	1
4	4	1	1	1	1	4	4	1	1
4	4	1	1	3	3	4	4	1	1
4	4	1	1	3	3	4	4	1	1
1	1	3	3	1	1	4	4	1	1
1	1	3	3	1	1	3	3	1	1
4	4	1	1	1	1	4	4	1	1
1	1	3	3	1	1	3	3	1	1
1	1	3	3	3	3	4	4	1	1
4	4	1	1	3	3	4	4	1	1
4	4	1	1	1	1	4	4	1	1
4	4	1	1	1	1	3	3	1	1
4	4	1	1	3	3	4	4	1	1
4	4	1	1	1	1	4	4	1	1
4	4	1	1	3	3	4	4	1	1
1	1	3	3	3	3	4	4	1	1
4	4	1	1	1	1	4	4	1	1
1	1	3	3	3	3	4	4	1	1
1	1	3	3	1	1	4	4	1	1
4	4	1	1	1	1	4	4	1	1
1	1	3	3	3	3	4	4	4	4
4	4	1	1	1	1	3	3	1	1
4	4	1	1	3	3	4	4	1	1
4	4	1	1	1	1	4	4	1	1
4	4	1	1	1	1	4	4	1	1
4	4	1	1	3	3	4	4	1	1
1	1	3	3	3	3	4	4	1	1
1	1	1	1	3	3	3	3	1	1
4	4	1	1	1	1	4	4	1	1
4	4	1	1	3	3	4	4	1	1
4	4	1	1	1	1	4	4	1	1
4	4	1	1	3	3	3	3	1	1
4	4	1	1	1	1	3	3	1	1
4	4	1	1	1	1	4	4	1	1
4	4	1	1	3	3	4	4	1	1
4	4	1	1	1	1	3	3	1	1
1	1	3	3	1	1	4	4	4	4
1	1	3	3	3	3	4	4	4	4
1	1	3	3	3	3	3	3	1	1
1	1	3	3	1	1	4	4	1	1
4	4	1	1	1	1	3	3	1	1
1	1	3	3	1	1	4	4	1	1
4	4	1	1	1	1	3	3	1	1
4	4	1	1	1	1	4	4	1	1
4	4	1	1	1	1	4	4	1	1
4	4	1	1	1	1	3	3	1	1
1	1	3	3	1	1	4	4	1	1
4	4	1	1	3	3	4	4	1	1
4	4	1	1	1	1	4	4	1	1
4	4	1	1	1	1	4	4	1	1

For Review Only

4	4	1	1	3	3	4	4	1	1
4	4	1	1	1	1	4	4	1	1
4	4	1	1	1	1	3	3	1	1
1	1	3	3	1	1	4	4	1	1
4	4	1	1	1	1	4	4	1	1
4	4	1	1	3	3	4	4	1	1
4	4	1	1	3	3	4	4	1	1
1	1	3	3	1	1	3	3	1	1
1	1	3	3	3	3	4	4	4	4
4	4	1	1	3	3	4	4	1	1
4	4	1	1	3	3	4	4	1	1
4	4	1	1	3	3	4	4	1	1
4	4	1	1	3	3	4	4	1	1
1	1	3	3	3	3	4	4	1	1
4	4	1	1	1	1	4	4	1	1
4	4	1	1	1	1	4	4	1	1
4	4	1	1	1	1	4	4	1	1
4	4	1	1	3	3	4	4	1	1
4	4	1	1	3	3	4	4	1	1
4	4	1	1	1	1	4	4	1	1
4	4	1	1	3	3	4	4	1	1
1	1	3	3	3	3	4	4	4	4
1	1	3	3	1	1	4	4	1	1
4	4	1	1	3	3	4	4	1	1
4	4	1	1	1	1	3	3	1	1
4	4	1	1	1	1	4	4	1	1
4	4	1	1	1	1	3	3	1	1
1	1	3	3	1	1	3	3	4	4
4	4	1	1	1	1	4	4	1	1
4	4	1	1	1	1	4	4	1	1
4	4	1	1	1	1	4	4	1	1
4	4	1	1	1	1	4	4	1	1
1	1	3	3	1	1	3	3	4	4
4	4	1	1	3	3	4	4	1	1
4	4	1	1	3	3	4	4	1	1
4	4	1	1	1	1	3	3	1	1
4	4	1	1	3	3	4	4	1	1
1	1	3	3	1	1	4	4	1	1
4	4	1	1	1	1	4	4	1	1
4	4	1	1	1	1	3	3	1	1
4	4	1	1	1	1	4	4	1	1
4	4	1	1	3	3	4	4	1	1
4	4	1	1	1	1	3	3	1	1
4	4	1	1	3	3	4	4	1	1
4	4	1	1	1	1	4	4	1	1
4	4	1	1	3	3	4	4	1	1
1	1	3	3	1	1	4	4	1	1
4	4	1	1	1	1	4	4	1	1
4	4	1	1	1	1	3	3	1	1
1	1	3	3	3	3	4	4	4	4
4	4	1	1	1	1	4	4	1	1
1	1	3	3	3	3	4	4	4	4
1	1	3	3	1	1	3	3	1	1
4	4	1	1	1	1	4	4	1	1
1	1	3	3	3	3	3	3	4	4

For Review Only

1	1	1	1	3	3	4	4	1	1
1	1	3	3	3	3	4	4	4	4
4	4	1	1	3	3	4	4	1	1
1	1	3	3	1	1	4	4	1	1
4	4	1	1	1	1	4	4	1	1
4	4	1	1	3	3	4	4	1	1
4	4	1	1	1	1	4	4	1	1
4	4	1	1	1	1	4	4	1	1
4	4	1	1	1	1	4	4	1	1
4	4	1	1	1	1	4	4	1	1
4	4	1	1	3	3	3	3	1	1
4	4	1	1	3	3	4	4	1	1
4	4	1	1	3	3	3	3	1	1
4	4	1	1	1	1	4	4	1	1
4	4	1	1	3	3	4	4	1	1
4	4	1	1	3	3	4	4	1	1
1	1	3	3	3	3	4	4	1	1
4	4	1	1	3	3	4	4	1	1
4	4	1	1	1	1	4	4	1	1
4	4	1	1	1	1	4	4	1	1
4	4	1	1	3	3	4	4	1	1
4	4	1	1	3	3	4	4	1	1
4	4	1	1	1	1	4	4	1	1
1	1	3	3	1	1	4	4	4	4
4	4	3	3	3	3	4	4	1	1
1	1	3	3	3	3	4	4	4	4
4	4	1	1	3	3	3	3	1	1
4	4	1	1	1	1	4	4	1	1
4	4	1	1	3	3	4	4	1	1
1	1	3	3	3	3	4	4	1	1
1	1	3	3	1	1	4	4	4	4
4	4	1	1	1	1	4	4	1	1
4	4	1	1	1	1	4	4	1	1
4	4	1	1	1	1	4	4	1	1
4	4	1	1	3	3	3	3	1	1
4	4	1	1	3	3	3	3	1	1
1	1	3	3	1	1	4	4	1	1
4	4	1	1	1	1	4	4	1	1
4	4	1	1	1	1	4	4	1	1
1	1	3	3	1	1	4	4	1	1
4	4	1	1	3	3	4	4	1	1
1	1	3	3	1	1	4	4	1	1
4	4	1	1	1	1	4	4	1	1
4	4	1	1	3	3	4	4	1	1
4	4	1	1	3	3	4	4	1	1
4	4	1	1	1	1	3	3	1	1
4	4	1	1	1	1	4	4	1	1
4	4	1	1	1	1	4	4	1	1
1	1	1	1	1	1	4	4	1	1
4	4	1	1	1	1	4	4	1	1
4	4	1	1	3	3	4	4	1	1
1	1	3	3	1	1	4	4	4	4
4	4	1	1	3	3	4	4	1	1
4	4	1	1	1	1	4	4	1	1
4	4	1	1	3	3	4	4	1	1

For Review Only

4	4	1	1	1	1	4	4	1	1
1	1	3	3	3	3	3	3	4	4
4	4	1	1	1	1	4	4	1	1
4	4	1	1	3	3	4	4	1	1
4	4	1	1	3	3	3	3	1	1
4	4	1	1	1	1	4	4	1	1
1	1	3	3	1	1	4	4	4	4
4	4	1	1	3	3	4	4	1	1
4	4	1	1	3	3	4	4	1	1
4	4	1	1	1	1	4	4	1	1
4	4	1	1	1	1	4	4	1	1
4	4	1	1	1	1	4	4	1	1
4	4	1	1	3	3	4	4	1	1
4	4	3	3	1	1	3	3	1	1
1	1	3	3	1	1	4	4	1	1
1	1	3	3	3	3	4	4	1	1
4	4	1	1	3	3	4	4	1	1
4	4	1	1	3	3	4	4	1	1
4	4	1	1	1	1	3	3	1	1
4	4	1	1	1	1	4	4	1	1
4	4	1	1	3	3	4	4	1	1
1	1	3	3	1	1	3	3	4	4
1	1	3	3	3	3	3	3	4	4
4	4	1	1	3	3	4	4	1	1
1	1	1	1	3	3	4	4	1	1
4	4	1	1	1	1	3	3	1	1
4	4	1	1	3	3	4	4	1	1
4	4	1	1	3	3	4	4	1	1
4	4	1	1	3	3	3	3	1	1
1	1	3	3	3	3	4	4	4	4
4	4	1	1	3	3	4	4	1	1
4	4	1	1	3	3	4	4	1	1
4	4	1	1	1	1	4	4	1	1
1	1	3	3	1	1	4	4	1	1
4	4	1	1	3	3	4	4	1	1
4	4	1	1	3	3	4	4	1	1
1	1	1	1	1	1	4	4	1	1
4	4	1	1	3	3	4	4	1	1
4	4	1	1	1	1	4	4	1	1
4	4	1	1	3	3	4	4	1	1
4	4	1	1	1	1	4	4	1	1
4	4	1	1	1	1	4	4	1	1
1	1	3	3	1	1	4	4	1	1
4	4	1	1	1	1	4	4	1	1
4	4	1	1	1	1	3	3	1	1
4	4	1	1	3	3	4	4	1	1
1	1	3	3	3	3	4	4	1	1
1	1	3	3	1	1	3	3	4	4
4	4	1	1	1	1	3	3	1	1
4	4	1	1	1	1	4	4	1	1
4	4	1	1	3	3	4	4	1	1
1	1	3	3	1	1	4	4	1	1
1	1	3	3	1	1	4	4	4	4
4	4	1	1	3	3	4	4	1	1
1	1	3	3	1	1	4	4	1	1

For Review Only

1	1	3	3	3	3	3	3	4	4
1	1	3	3	3	3	4	4	1	1
4	4	1	1	1	1	4	4	1	1
4	4	1	1	3	3	4	4	1	1
4	4	1	1	1	1	4	4	1	1
4	4	1	1	3	3	4	4	1	1
1	1	3	3	1	1	4	4	4	4
1	1	3	3	3	3	3	3	1	1
4	4	1	1	1	1	3	3	1	1
1	1	3	3	1	1	4	4	4	4
4	4	1	1	3	3	4	4	1	1
4	4	1	1	3	3	4	4	1	1
4	4	1	1	1	1	4	4	1	1
4	4	1	1	1	1	4	4	1	1
4	4	1	1	3	3	4	4	1	1
4	4	1	1	1	1	4	4	1	1
4	4	1	1	1	1	4	4	1	1
1	1	3	3	1	1	4	4	1	1
1	1	3	3	1	1	3	3	4	4
4	4	1	1	1	1	3	3	1	1
1	1	3	3	3	3	4	4	4	4
4	4	1	1	1	1	4	4	1	1
4	4	1	1	3	3	4	4	1	1
4	4	1	1	1	1	4	4	1	1
1	1	3	3	1	1	3	3	1	1
4	4	1	1	3	3	4	4	1	1
1	1	3	3	1	1	3	3	1	1
4	4	1	1	3	3	4	4	1	1
4	4	1	1	3	3	4	4	1	1
4	4	1	1	3	3	4	4	1	1
1	1	3	3	1	1	4	4	4	4
4	4	1	1	1	1	4	4	1	1
1	1	3	3	1	1	3	3	1	1
4	4	1	1	3	3	4	4	1	1
1	1	3	3	3	3	4	4	4	4
1	1	3	3	1	1	4	4	4	4
4	4	1	1	3	3	4	4	1	1
4	4	1	1	3	3	4	4	1	1
4	4	1	1	1	1	4	4	1	1
1	1	3	3	1	1	4	4	4	4
4	4	1	1	3	3	3	3	1	1
4	4	1	1	1	1	3	3	1	1
1	1	3	3	1	1	4	4	4	4
4	4	1	1	1	1	4	4	1	1
4	4	1	1	1	1	4	4	1	1
1	1	3	3	1	1	4	4	4	4
4	4	1	1	1	1	4	4	1	1
1	1	3	3	1	1	3	3	4	4
4	4	1	1	1	1	3	3	1	1
1	1	3	3	1	1	3	3	4	4
4	4	1	1	1	1	4	4	1	1
1	1	3	3	1	1	4	4	4	4
4	4	1	1	1	1	4	4	1	1
4	4	1	1	3	3	4	4	1	1
1	1	3	3	1	1	4	4	4	4

4	4	1	1	1	1	4	4	1	1
4	4	1	1	1	1	4	4	1	1

For Review Only

SNP1972	SNP1972	SNP1973	SNP1973	SNP1974	SNP1974	SNP1975	SNP1975	SNP1976	SNP1976
2	2	4	4	2	2	4	4	3	3
2	2	1	1	2	2	4	4	1	1
3	3	4	4	2	2	4	4	3	3
3	3	4	4	2	2	4	4	3	3
3	3	4	4	2	2	4	4	3	3
2	2	4	4	2	2	4	4	3	3
3	3	4	4	2	2	4	4	3	3
2	2	4	4	2	2	4	4	3	3
2	2	4	4	2	2	4	4	3	3
3	3	4	4	2	2	4	4	3	3
3	3	1	1	2	2	4	4	1	1
3	3	4	4	2	2	4	4	3	3
2	2	4	4	2	2	4	4	3	3
3	3	4	4	2	2	4	4	1	1
2	2	4	4	2	2	4	4	3	3
3	3	4	4	2	2	4	4	3	3
2	2	4	4	2	2	4	4	3	3
2	2	4	4	4	4	4	4	3	3
2	2	4	4	2	2	4	4	3	3
3	3	4	4	2	2	4	4	3	3
2	2	4	4	2	2	4	4	3	3
3	3	1	1	2	2	4	4	3	3
2	2	4	4	2	2	2	2	3	3
2	2	4	4	2	2	4	4	3	3
2	2	4	4	2	2	4	4	3	3
3	3	4	4	2	2	4	4	3	3
2	2	4	4	2	2	4	4	3	3
2	2	4	4	2	2	4	4	3	3
2	2	4	4	2	2	4	4	3	3
2	2	1	1	2	2	4	4	1	1
3	3	4	4	2	2	4	4	3	3
3	3	4	4	2	2	4	4	3	3
3	3	4	4	2	2	4	4	3	3
2	2	1	1	2	2	4	4	1	1
2	2	4	4	2	2	4	4	1	1
3	3	4	4	2	2	4	4	3	3
2	2	4	4	2	2	4	4	3	3
2	2	4	4	2	2	4	4	3	3
2	2	4	4	2	2	4	4	3	3
3	3	4	4	2	2	2	2	3	3
2	2	1	1	2	2	4	4	1	1
3	3	4	4	2	2	4	4	3	3
2	2	1	1	2	2	4	4	1	1
3	3	4	4	2	2	4	4	3	3
3	3	1	1	2	2	4	4	1	1
2	2	4	4	2	2	4	4	3	3
3	3	4	4	2	2	4	4	3	3
3	3	1	1	2	2	4	4	1	1
3	3	1	1	2	2	4	4	1	1
3	3	4	4	2	2	4	4	3	3
3	3	4	4	2	2	4	4	3	3
2	2	4	4	2	2	4	4	1	1

For Review Only

2	2	4	4	2	2	4	4	3	3
3	3	4	4	2	2	4	4	3	3
2	2	4	4	2	2	4	4	3	3
3	3	4	4	2	2	4	4	3	3
2	2	4	4	2	2	4	4	3	3
3	3	4	4	4	4	4	4	3	3
3	3	4	4	4	4	4	4	3	3
2	2	1	1	2	2	4	4	1	1
2	2	1	1	2	2	4	4	1	1
2	2	4	4	2	2	4	4	3	3
3	3	4	4	2	2	4	4	3	3
3	3	4	4	2	2	4	4	3	3
2	2	4	4	2	2	4	4	3	3
2	2	4	4	2	2	4	4	3	3
2	2	4	4	2	2	4	4	3	3
2	2	4	4	2	2	4	4	1	1
3	3	4	4	2	2	2	2	3	3
3	3	4	4	4	4	4	4	3	3
2	2	4	4	2	2	4	4	3	3
3	3	4	4	2	2	4	4	3	3
3	3	4	4	2	2	4	4	3	3
2	2	4	4	2	2	4	4	3	3
2	2	4	4	2	2	4	4	3	3
3	3	4	4	2	2	4	4	3	3
3	3	1	1	2	2	4	4	1	1
3	3	4	4	2	2	4	4	3	3
2	2	1	1	2	2	4	4	1	1
2	2	4	4	2	2	4	4	3	3
3	3	4	4	4	4	4	4	3	3
2	2	4	4	2	2	4	4	3	3
3	3	4	4	2	2	4	4	3	3
3	3	4	4	2	2	4	4	3	3
2	2	4	4	2	2	4	4	1	1
2	2	4	4	2	2	4	4	3	3
2	2	4	4	2	2	4	4	1	1
3	3	1	1	2	2	4	4	1	1
2	2	4	4	2	2	4	4	3	3
2	2	4	4	2	2	4	4	3	3
3	3	4	4	2	2	2	2	3	3
2	2	4	4	2	2	4	4	3	3
3	3	4	4	2	2	4	4	3	3
2	2	4	4	2	2	4	4	3	3
2	2	4	4	2	2	4	4	3	3
3	3	4	4	2	2	4	4	3	3
2	2	4	4	2	2	4	4	3	3
3	3	4	4	4	4	4	4	3	3
2	2	1	1	2	2	4	4	1	1
3	3	4	4	2	2	4	4	3	3
3	3	4	4	4	4	4	4	3	3
3	3	4	4	2	2	4	4	3	3
3	3	4	4	2	2	4	4	3	3
2	2	4	4	2	2	2	2	3	3
2	2	4	4	2	2	4	4	3	3
2	2	4	4	2	2	4	4	3	3
3	3	4	4	4	4	4	4	3	3

For Review Only

3	3	4	4	2	2	4	4	3	3
2	2	4	4	2	2	4	4	3	3
3	3	4	4	2	2	4	4	3	3
2	2	1	1	2	2	4	4	1	1
2	2	4	4	2	2	4	4	3	3
3	3	4	4	2	2	4	4	3	3
3	3	4	4	2	2	4	4	3	3
2	2	4	4	2	2	4	4	3	3
3	3	1	1	2	2	4	4	1	1
2	2	4	4	2	2	4	4	3	3
2	2	4	4	2	2	4	4	3	3
2	2	4	4	2	2	4	4	3	3
2	2	4	4	2	2	4	4	3	3
2	2	4	4	4	4	4	4	3	3
3	3	4	4	2	2	4	4	3	3
2	2	4	4	2	2	4	4	3	3
2	2	4	4	4	4	4	4	1	1
2	2	4	4	2	2	4	4	3	3
2	2	1	1	2	2	4	4	1	1
3	3	4	4	2	2	4	4	3	3
2	2	4	4	2	2	4	4	3	3
2	2	4	4	2	2	4	4	3	3
2	2	4	4	4	4	4	4	3	3
2	2	4	4	2	2	4	4	1	1
2	2	4	4	2	2	4	4	3	3
3	3	4	4	2	2	2	2	3	3
3	3	4	4	2	2	4	4	3	3
2	2	4	4	2	2	4	4	3	3
2	2	4	4	2	2	4	4	3	3
2	2	4	4	2	2	4	4	3	3
2	2	4	4	2	2	2	2	1	1
2	2	4	4	2	2	4	4	3	3
3	3	4	4	2	2	4	4	3	3
2	2	4	4	2	2	4	4	3	3
2	2	1	1	2	2	4	4	1	1
2	2	4	4	2	2	4	4	3	3
2	2	1	1	2	2	4	4	1	1
3	3	4	4	2	2	4	4	3	3
2	2	4	4	2	2	2	2	3	3
2	2	4	4	2	2	4	4	1	1
3	3	4	4	2	2	2	2	3	3
2	2	1	1	2	2	4	4	1	1
2	2	4	4	2	2	4	4	3	3
2	2	1	1	2	2	4	4	3	3
2	2	4	4	2	2	4	4	3	3
2	2	4	4	2	2	4	4	3	3
3	3	4	4	4	4	4	4	3	3
3	3	4	4	2	2	4	4	3	3
2	2	4	4	2	2	4	4	1	1
3	3	4	4	2	2	4	4	3	3
3	3	4	4	2	2	4	4	3	3
3	3	1	1	2	2	4	4	1	1
2	2	4	4	2	2	4	4	3	3
3	3	4	4	2	2	4	4	3	3
2	2	4	4	2	2	4	4	1	1

For Review Only

3	3	4	4	4	4	4	4	3	3
3	3	4	4	2	2	2	2	3	3
3	3	4	4	2	2	4	4	3	3
3	3	4	4	2	2	4	4	3	3
3	3	4	4	2	2	4	4	3	3
3	3	4	4	2	2	4	4	3	3
3	3	4	4	2	2	4	4	3	3
2	2	4	4	2	2	4	4	3	3
2	2	4	4	2	2	4	4	3	3
3	3	4	4	2	2	4	4	3	3
2	2	4	4	2	2	4	4	3	3
3	3	4	4	2	2	4	4	3	3
2	2	4	4	2	2	4	4	3	3
2	2	4	4	2	2	4	4	3	3
2	2	1	1	2	2	4	4	1	1
3	3	1	1	2	2	4	4	1	1
2	2	4	4	2	2	4	4	3	3
3	3	4	4	2	2	4	4	3	3
3	3	4	4	4	4	4	4	3	3
3	3	4	4	2	2	4	4	3	3
2	2	4	4	2	2	4	4	3	3
2	2	4	4	2	2	4	4	3	3
2	2	4	4	2	2	4	4	3	3
3	3	4	4	2	2	4	4	3	3
2	2	4	4	2	2	4	4	3	3
2	2	1	1	2	2	4	4	1	1
3	3	4	4	2	2	4	4	3	3
2	2	4	4	2	2	4	4	1	1
2	2	4	4	2	2	4	4	3	3
3	3	4	4	2	2	4	4	3	3
3	3	4	4	2	2	4	4	3	3
3	3	4	4	4	4	4	4	3	3
3	3	4	4	2	2	4	4	3	3
2	2	1	1	2	2	4	4	1	1
2	2	4	4	2	2	4	4	3	3
3	3	4	4	2	2	4	4	3	3
3	3	4	4	2	2	2	2	3	3
2	2	4	4	2	2	4	4	3	3
3	3	4	4	2	2	4	4	3	3
3	3	4	4	2	2	4	4	3	3
3	3	4	4	2	2	4	4	3	3
3	3	4	4	4	4	4	4	3	3
2	2	4	4	2	2	2	2	1	1
2	2	4	4	2	2	4	4	3	3
3	3	4	4	2	2	4	4	3	3
2	2	4	4	2	2	4	4	3	3
3	3	1	1	2	2	4	4	1	1
2	2	4	4	2	2	4	4	3	3
2	2	4	4	2	2	4	4	3	3
3	3	4	4	2	2	4	4	3	3
3	3	4	4	2	2	4	4	3	3
2	2	1	1	2	2	4	4	1	1
2	2	1	1	2	2	4	4	1	1
3	3	4	4	2	2	4	4	3	3
2	2	4	4	2	2	4	4	3	3

3	3	4	4	2	2	4	4	3	3
3	3	1	1	4	4	4	4	1	1
2	2	4	4	2	2	4	4	3	3
2	2	4	4	2	2	4	4	3	3
2	2	1	1	2	2	4	4	1	1
3	3	4	4	4	4	4	4	3	3
2	2	4	4	4	4	2	2	3	3
2	2	1	1	2	2	4	4	1	1
2	2	1	1	2	2	4	4	3	3
2	2	4	4	2	2	4	4	1	1
2	2	4	4	2	2	4	4	3	3
2	2	4	4	2	2	4	4	3	3
3	3	4	4	2	2	2	2	3	3
3	3	4	4	2	2	4	4	3	3
3	3	4	4	2	2	4	4	3	3
3	3	1	1	2	2	2	2	1	1
2	2	1	1	2	2	4	4	1	1
2	2	4	4	2	2	4	4	1	1
2	2	4	4	4	4	4	4	3	3
2	2	4	4	2	2	4	4	3	3
2	2	4	4	2	2	4	4	3	3
3	3	4	4	4	4	4	4	3	3
3	3	4	4	2	2	4	4	3	3
3	3	4	4	2	2	2	2	3	3
2	2	1	1	2	2	4	4	1	1
2	2	4	4	2	2	4	4	3	3
2	2	4	4	2	2	4	4	1	1
3	3	4	4	2	2	4	4	3	3
2	2	4	4	2	2	4	4	3	3
2	2	4	4	2	2	4	4	3	3
3	3	4	4	2	2	4	4	3	3
2	2	1	1	2	2	4	4	1	1
2	2	4	4	2	2	4	4	3	3
3	3	4	4	2	2	4	4	3	3
2	2	4	4	2	2	4	4	3	3
2	2	4	4	2	2	4	4	3	3
3	3	4	4	2	2	4	4	3	3
3	3	4	4	2	2	4	4	3	3
3	3	4	4	2	2	4	4	3	3
2	2	4	4	2	2	4	4	3	3
2	2	4	4	4	4	4	4	3	3
2	2	4	4	2	2	4	4	3	3
3	3	4	4	2	2	2	2	3	3
3	3	4	4	2	2	4	4	3	3
3	3	4	4	2	2	4	4	3	3
3	3	4	4	2	2	2	2	3	3
3	3	4	4	2	2	4	4	3	3
2	2	4	4	2	2	4	4	3	3
3	3	4	4	2	2	4	4	1	1
2	2	4	4	2	2	4	4	1	1
3	3	4	4	2	2	4	4	3	3
2	2	1	1	2	2	4	4	1	1
3	3	4	4	2	2	4	4	3	3
3	3	4	4	2	2	4	4	3	3
3	3	4	4	2	2	2	2	3	3

For Review Only

3	3	4	4	2	2	4	4	3	3
3	3	4	4	2	2	4	4	3	3

For Review Only

SNP1977	SNP1977	SNP1978	SNP1978	SNP1979	SNP1979	SNP198	SNP198	SNP1980	SNP1980
1	1	2	2	4	4	1	1	1	1
1	1	2	2	4	4	4	4	1	1
4	4	2	2	3	3	1	1	1	1
1	1	2	2	3	3	1	1	1	1
1	1	2	2	3	3	4	4	1	1
1	1	2	2	4	4	1	1	1	1
1	1	2	2	3	3	1	1	1	1
1	1	2	2	4	4	4	4	1	1
1	1	2	2	3	3	1	1	1	1
1	1	2	2	3	3	4	4	1	1
1	1	2	2	3	3	1	1	3	3
4	4	2	2	3	3	1	1	1	1
1	1	2	2	4	4	1	1	1	1
1	1	2	2	3	3	4	4	1	1
1	1	2	2	3	3	1	1	1	1
4	4	3	3	3	3	4	4	1	1
1	1	2	2	3	3	1	1	1	1
1	1	2	2	4	4	1	1	1	1
1	1	2	2	4	4	1	1	1	1
1	1	2	2	3	3	4	4	1	1
1	1	2	2	4	4	4	4	1	1
1	1	2	2	3	3	4	4	3	3
1	1	2	2	4	4	4	4	1	1
1	1	3	3	4	4	4	4	1	1
1	1	3	3	4	4	1	1	1	1
4	4	2	2	3	3	1	1	1	1
4	4	3	3	4	4	1	1	1	1
1	1	2	2	3	3	1	1	1	1
1	1	2	2	4	4	4	4	1	1
1	1	2	2	4	4	4	4	3	3
1	1	3	3	3	3	1	1	1	1
1	1	2	2	3	3	1	1	1	1
1	1	2	2	3	3	1	1	1	1
1	1	2	2	4	4	4	4	1	1
1	1	2	2	4	4	1	1	1	1
1	1	3	3	3	3	1	1	1	1
1	1	2	2	4	4	1	1	1	1
1	1	2	2	3	3	1	1	1	1
1	1	2	2	4	4	4	4	1	1
1	1	2	2	3	3	4	4	1	1
1	1	2	2	4	4	4	4	3	3
1	1	2	2	3	3	4	4	1	1
1	1	2	2	4	4	4	4	3	3
1	1	2	2	3	3	1	1	1	1
1	1	2	2	3	3	4	4	3	3
4	4	2	2	3	3	1	1	1	1
1	1	2	2	3	3	1	1	1	1
4	4	2	2	3	3	1	1	1	1
1	1	2	2	3	3	1	1	3	3
1	1	2	2	3	3	1	1	1	1
4	4	2	2	3	3	1	1	1	1
1	1	3	3	4	4	4	4	1	1

For Review Only

1	1	2	2	4	4	1	1	1	1
1	1	2	2	3	3	1	1	1	1
4	4	3	3	3	3	1	1	1	1
1	1	2	2	3	3	1	1	1	1
1	1	2	2	4	4	1	1	1	1
1	1	2	2	3	3	1	1	1	1
4	4	2	2	3	3	1	1	1	1
1	1	2	2	4	4	4	4	3	3
1	1	2	2	4	4	4	4	1	1
1	1	2	2	3	3	1	1	1	1
1	1	2	2	3	3	1	1	1	1
1	1	2	2	3	3	1	1	1	1
1	1	2	2	4	4	1	1	1	1
1	1	2	2	4	4	4	4	1	1
1	1	2	2	3	3	1	1	1	1
1	1	2	2	4	4	1	1	3	3
1	1	2	2	3	3	4	4	1	1
1	1	2	2	3	3	1	1	1	1
1	1	2	2	4	4	4	4	1	1
1	1	2	2	3	3	1	1	1	1
1	1	2	2	3	3	1	1	1	1
1	1	2	2	4	4	1	1	1	1
1	1	2	2	4	4	1	1	1	1
1	1	2	2	3	3	1	1	1	1
1	1	2	2	3	3	4	4	3	3
1	1	3	3	3	3	1	1	1	1
1	1	2	2	4	4	4	4	3	3
1	1	2	2	4	4	4	4	1	1
4	4	2	2	3	3	1	1	1	1
1	1	2	2	4	4	1	1	1	1
1	1	2	2	3	3	1	1	1	1
1	1	2	2	3	3	1	1	1	1
1	1	2	2	4	4	4	4	1	1
1	1	2	2	4	4	1	1	1	1
1	1	2	2	4	4	4	4	1	1
1	1	2	2	3	3	4	4	3	3
1	1	2	2	4	4	1	1	1	1
1	1	2	2	4	4	1	1	1	1
1	1	2	2	3	3	1	1	1	1
1	1	2	2	4	4	1	1	1	1
1	1	2	2	3	3	1	1	1	1
1	1	2	2	4	4	1	1	1	1
1	1	2	2	4	4	4	4	1	1
1	1	2	2	3	3	1	1	1	1
1	1	2	2	3	3	1	1	1	1
1	1	2	2	3	3	1	1	1	1
1	1	2	2	4	4	4	4	1	1
1	1	2	2	3	3	1	1	1	1
1	1	2	2	3	3	1	1	1	1
1	1	2	2	3	3	1	1	1	1
1	1	2	2	3	3	1	1	1	1
1	1	2	2	4	4	4	4	1	1
1	1	2	2	4	4	4	4	1	1
1	1	2	2	4	4	1	1	1	1
1	1	2	2	3	3	4	4	1	1

For Review Only

1	1	2	2	3	3	1	1	1	1
1	1	2	2	3	3	4	4	1	1
1	1	2	2	3	3	1	1	1	1
1	1	2	2	4	4	4	4	1	1
4	4	3	3	3	3	1	1	1	1
1	1	2	2	3	3	1	1	1	1
4	4	2	2	3	3	1	1	1	1
1	1	2	2	4	4	1	1	1	1
1	1	2	2	3	3	1	1	3	3
1	1	2	2	3	3	1	1	1	1
1	1	2	2	4	4	1	1	1	1
1	1	2	2	3	3	1	1	1	1
1	1	2	2	4	4	4	4	1	1
1	1	2	2	3	3	1	1	1	1
1	1	2	2	3	3	1	1	1	1
1	1	2	2	3	3	1	1	1	1
1	1	2	2	3	3	1	1	1	1
1	1	2	2	3	3	1	1	1	1
1	1	2	2	4	4	4	4	1	1
1	1	2	2	3	3	4	4	1	1
1	1	2	2	3	3	1	1	1	1
1	1	2	2	4	4	1	1	1	1
1	1	2	2	4	4	1	1	1	1
1	1	2	2	4	4	4	4	1	1
1	1	2	2	3	3	1	1	1	1
1	1	2	2	3	3	1	1	1	1
1	1	2	2	3	3	1	1	1	1
1	1	2	2	4	4	1	1	1	1
1	1	2	2	3	3	1	1	1	1
1	1	2	2	3	3	1	1	1	1
1	1	2	2	4	4	4	4	1	1
1	1	2	2	3	3	1	1	1	1
1	1	2	2	3	3	1	1	1	1
1	1	2	2	3	3	1	1	1	1
1	1	2	2	4	4	4	4	1	1
1	1	2	2	3	3	4	4	1	1
1	1	2	2	4	4	4	4	1	1
1	1	2	2	3	3	1	1	1	1
1	1	2	2	3	3	1	1	1	1
1	1	2	2	4	4	4	4	1	1
1	1	2	2	3	3	1	1	1	1
1	1	2	2	4	4	4	4	1	1
1	1	2	2	3	3	1	1	1	1
1	1	2	2	4	4	1	1	3	3
1	1	2	2	3	3	4	4	1	1
1	1	2	2	4	4	1	1	1	1
4	4	2	2	3	3	1	1	1	1
4	4	2	2	3	3	1	1	1	1
1	1	2	2	4	4	1	1	1	1
4	4	2	2	3	3	1	1	1	1
4	4	3	3	3	3	1	1	1	1
1	1	2	2	3	3	4	4	3	3
1	1	2	2	3	3	1	1	1	1
1	1	2	2	3	3	1	1	1	1
1	1	2	2	4	4	1	1	3	3

4	4	2	2	3	3	1	1	1	1
1	1	2	2	3	3	1	1	1	1
1	1	2	2	3	3	1	1	1	1
1	1	2	2	3	3	1	1	1	1
1	1	2	2	3	3	1	1	1	1
1	1	2	2	3	3	4	4	1	1
1	1	2	2	3	3	4	4	1	1
4	4	2	2	3	3	1	1	1	1
1	1	2	2	4	4	4	4	1	1
1	1	2	2	3	3	1	1	1	1
1	1	2	2	3	3	1	1	1	1
1	1	3	3	3	3	1	1	1	1
1	1	2	2	4	4	1	1	1	1
1	1	2	2	4	4	1	1	1	1
1	1	2	2	4	4	4	4	1	1
1	1	2	2	3	3	1	1	3	3
1	1	2	2	3	3	1	1	1	1
1	1	2	2	3	3	1	1	1	1
1	1	2	2	3	3	4	4	1	1
4	4	2	2	3	3	1	1	1	1
1	1	2	2	3	3	1	1	1	1
1	1	3	3	4	4	4	4	1	1
1	1	2	2	4	4	1	1	1	1
1	1	2	2	3	3	1	1	1	1
1	1	2	2	4	4	1	1	1	1
1	1	2	2	4	4	4	4	1	1
1	1	2	2	3	3	1	1	1	1
1	1	2	2	4	4	1	1	1	1
1	1	2	2	4	4	1	1	1	1
1	1	2	2	3	3	1	1	1	1
1	1	2	2	3	3	1	1	1	1
1	1	2	2	3	3	1	1	1	1
1	1	2	2	3	3	1	1	1	1
1	1	2	2	4	4	4	4	1	1
1	1	2	2	3	3	1	1	1	1
1	1	2	2	3	3	1	1	1	1
4	4	2	2	3	3	1	1	1	1
1	1	2	2	3	3	1	1	1	1
4	4	2	2	3	3	1	1	1	1
1	1	2	2	3	3	1	1	1	1
4	4	2	2	3	3	1	1	1	1
4	4	2	2	3	3	1	1	1	1
1	1	2	2	4	4	4	4	1	1
1	1	2	2	3	3	1	1	1	1
1	1	2	2	3	3	1	1	1	1
1	1	2	2	4	4	1	1	1	1
1	1	2	2	3	3	1	1	3	3
1	1	2	2	4	4	4	4	1	1
1	1	2	2	4	4	4	4	1	1
1	1	2	2	3	3	1	1	1	1
1	1	2	2	3	3	1	1	1	1
1	1	2	2	4	4	4	4	3	3
1	1	2	2	4	4	4	4	3	3
1	1	2	2	3	3	1	1	1	1
1	1	2	2	4	4	4	4	1	1

For Review Only

1	1	2	2	3	3	1	1	1	1
1	1	2	2	3	3	4	4	3	3
4	4	2	2	3	3	1	1	1	1
1	1	2	2	3	3	1	1	1	1
1	1	2	2	4	4	1	1	3	3
1	1	2	2	3	3	1	1	1	1
1	1	2	2	3	3	4	4	1	1
1	1	2	2	4	4	4	4	3	3
1	1	2	2	3	3	4	4	1	1
1	1	2	2	4	4	4	4	1	1
1	1	2	2	3	3	1	1	1	1
1	1	2	2	4	4	4	4	1	1
4	4	2	2	3	3	1	1	1	1
1	1	2	2	3	3	1	1	1	1
4	4	2	2	3	3	1	1	1	1
1	1	2	2	3	3	1	1	3	3
1	1	2	2	4	4	1	1	3	3
1	1	2	2	4	4	4	4	1	1
1	1	2	2	4	4	4	4	1	1
1	1	2	2	4	4	1	1	1	1
1	1	2	2	4	4	1	1	1	1
4	4	2	2	3	3	1	1	1	1
1	1	2	2	3	3	1	1	1	1
1	1	2	2	3	3	1	1	1	1
1	1	2	2	4	4	4	4	3	3
1	1	2	2	4	4	1	1	1	1
1	1	2	2	4	4	4	4	1	1
1	1	2	2	3	3	1	1	1	1
1	1	2	2	4	4	1	1	1	1
1	1	2	2	4	4	1	1	1	1
1	1	2	2	3	3	4	4	1	1
1	1	2	2	3	3	4	4	1	1
1	1	2	2	4	4	4	4	1	1
4	4	2	2	3	3	1	1	1	1
1	1	2	2	4	4	1	1	1	1
1	1	2	2	4	4	4	4	1	1
1	1	2	2	3	3	1	1	1	1
1	1	2	2	3	3	1	1	1	1
1	1	3	3	3	3	1	1	1	1
1	1	2	2	4	4	4	4	1	1
1	1	2	2	4	4	1	1	1	1
1	1	2	2	4	4	4	4	1	1
1	1	2	2	3	3	4	4	1	1
1	1	2	2	3	3	1	1	1	1
4	4	2	2	3	3	1	1	1	1
1	1	2	2	3	3	1	1	1	1
4	4	2	2	3	3	1	1	1	1
1	1	2	2	4	4	4	4	1	1
1	1	2	2	3	3	1	1	1	1
1	1	2	2	4	4	4	4	1	1
1	1	2	2	3	3	1	1	1	1
1	1	2	2	4	4	4	4	3	3
4	4	2	2	3	3	1	1	1	1
1	1	2	2	3	3	1	1	1	1
1	1	2	2	3	3	4	4	1	1

For Review Only

1	1	2	2	3	3	1	1	1	1
1	1	2	2	3	3	1	1	1	1

For Review Only

SNP1981	SNP1981	SNP1982	SNP1982	SNP1983	SNP1983	SNP1984	SNP1984	SNP1985	SNP1985
3	3	4	4	2	2	1	1	2	2
1	1	4	4	2	2	1	1	2	2
3	3	2	2	2	2	1	1	2	2
3	3	2	2	2	2	1	1	3	3
3	3	2	2	3	3	1	1	2	2
3	3	4	4	2	2	1	1	2	2
3	3	2	2	2	2	1	1	3	3
1	1	4	4	2	2	4	4	3	3
3	3	4	4	2	2	1	1	2	2
1	1	2	2	2	2	1	1	2	2
3	3	2	2	2	2	1	1	3	3
3	3	2	2	2	2	1	1	2	2
3	3	4	4	2	2	1	1	3	3
1	1	4	4	2	2	1	1	3	3
3	3	4	4	2	2	1	1	2	2
1	1	2	2	2	2	1	1	2	2
3	3	2	2	2	2	1	1	2	2
1	1	4	4	2	2	1	1	3	3
3	3	2	2	3	3	1	1	2	2
1	1	4	4	2	2	1	1	3	3
3	3	4	4	2	2	1	1	3	3
3	3	4	4	3	3	1	1	2	2
3	3	4	4	2	2	1	1	3	3
3	3	4	4	2	2	1	1	2	2
3	3	4	4	2	2	1	1	2	2
1	1	2	2	2	2	1	1	2	2
3	3	4	4	2	2	1	1	2	2
3	3	4	4	2	2	1	1	3	3
1	1	4	4	2	2	1	1	2	2
3	3	4	4	2	2	4	4	2	2
3	3	2	2	2	2	1	1	2	2
1	1	4	4	2	2	1	1	2	2
3	3	2	2	3	3	1	1	2	2
1	1	4	4	2	2	4	4	3	3
3	3	4	4	2	2	1	1	3	3
3	3	2	2	2	2	1	1	2	2
3	3	4	4	2	2	1	1	2	2
3	3	4	4	2	2	1	1	2	2
3	3	4	4	2	2	1	1	3	3
3	3	4	4	2	2	1	1	3	3
3	3	4	4	2	2	1	1	3	3
3	3	2	2	2	2	1	1	2	2
1	1	4	4	2	2	1	1	3	3
3	3	2	2	2	2	1	1	3	3
1	1	2	2	2	2	1	1	3	3
3	3	2	2	2	2	1	1	2	2
1	1	2	2	2	2	1	1	2	2
3	3	2	2	2	2	1	1	3	3
3	3	2	2	2	2	1	1	3	3
1	1	4	4	3	3	1	1	2	2
3	3	4	4	2	2	1	1	2	2
3	3	4	4	2	2	1	1	2	2

For Review Only

3	3	4	4	3	3	1	1	2	2
3	3	2	2	3	3	1	1	2	2
1	1	4	4	2	2	1	1	2	2
3	3	2	2	2	2	4	4	2	2
3	3	2	2	3	3	1	1	2	2
3	3	2	2	2	2	1	1	2	2
3	3	2	2	2	2	1	1	2	2
3	3	4	4	2	2	1	1	3	3
1	1	4	4	2	2	4	4	2	2
3	3	4	4	2	2	1	1	3	3
1	1	2	2	2	2	1	1	2	2
1	1	2	2	2	2	1	1	2	2
3	3	4	4	3	3	1	1	2	2
1	1	4	4	2	2	1	1	2	2
3	3	4	4	2	2	1	1	2	2
1	1	4	4	2	2	1	1	2	2
3	3	4	4	2	2	1	1	3	3
1	1	2	2	2	2	1	1	2	2
3	3	4	4	3	3	1	1	2	2
3	3	2	2	2	2	1	1	2	2
3	3	4	4	2	2	1	1	2	2
3	3	4	4	2	2	1	1	2	2
1	1	2	2	2	2	1	1	2	2
3	3	2	2	2	2	1	1	2	2
1	1	2	2	2	2	1	1	3	3
3	3	2	2	2	2	1	1	2	2
3	3	4	4	2	2	1	1	2	2
3	3	4	4	2	2	1	1	3	3
1	1	2	2	2	2	1	1	2	2
3	3	4	4	2	2	1	1	2	2
3	3	2	2	3	3	1	1	2	2
3	3	2	2	2	2	1	1	2	2
3	3	4	4	2	2	1	1	3	3
3	3	4	4	3	3	1	1	2	2
1	1	4	4	2	2	1	1	2	2
1	1	2	2	2	2	1	1	3	3
1	1	4	4	2	2	1	1	2	2
3	3	4	4	2	2	4	4	3	3
3	3	2	2	2	2	1	1	3	3
3	3	4	4	2	2	1	1	3	3
3	3	2	2	3	3	1	1	2	2
3	3	4	4	2	2	1	1	2	2
3	3	4	4	2	2	1	1	2	2
1	1	2	2	2	2	1	1	2	2
3	3	4	4	2	2	1	1	2	2
1	1	4	4	2	2	1	1	2	2
1	1	4	4	2	2	1	1	3	3
3	3	2	2	2	2	1	1	2	2
3	3	2	2	2	2	1	1	2	2
1	1	4	4	2	2	1	1	2	2
3	3	4	4	2	2	1	1	3	3
3	3	4	4	2	2	1	1	3	3
3	3	4	4	2	2	1	1	3	3
3	3	4	4	2	2	1	1	2	2
1	1	4	4	2	2	4	4	2	2

For Review Only

1	1	4	4	2	2	1	1	2	2
3	3	4	4	2	2	1	1	2	2
3	3	2	2	2	2	1	1	2	2
1	1	4	4	2	2	4	4	3	3
3	3	4	4	2	2	1	1	2	2
3	3	2	2	2	2	1	1	2	2
1	1	2	2	2	2	1	1	2	2
3	3	4	4	2	2	1	1	3	3
1	1	4	4	2	2	1	1	2	2
3	3	4	4	2	2	1	1	3	3
3	3	4	4	2	2	1	1	3	3
3	3	4	4	2	2	1	1	3	3
3	3	4	4	2	2	1	1	2	2
3	3	4	4	2	2	1	1	2	2
3	3	2	2	2	2	1	1	3	3
3	3	2	2	2	2	1	1	2	2
3	3	4	4	2	2	1	1	3	3
3	3	4	4	2	2	1	1	3	3
1	1	4	4	2	2	1	1	3	3
3	3	4	4	2	2	1	1	2	2
3	3	4	4	2	2	1	1	3	3
3	3	4	4	2	2	1	1	2	2
3	3	4	4	2	2	1	1	2	2
3	3	4	4	2	2	1	1	3	3
1	1	4	4	2	2	1	1	2	2
3	3	4	4	2	2	1	1	3	3
3	3	4	4	2	2	1	1	2	2
3	3	4	4	2	2	1	1	2	2
3	3	4	4	2	2	1	1	2	2
3	3	4	4	2	2	1	1	3	3
3	3	4	4	2	2	1	1	3	3
3	3	4	4	2	2	1	1	3	3
3	3	2	2	3	3	1	1	2	2
3	3	4	4	2	2	1	1	2	2
1	1	4	4	2	2	4	4	3	3
3	3	2	2	2	2	1	1	3	3
1	1	4	4	2	2	4	4	3	3
3	3	4	4	2	2	1	1	2	2
3	3	4	4	2	2	1	1	3	3
3	3	4	4	2	2	1	1	3	3
3	3	4	4	2	2	1	1	3	3
1	1	4	4	2	2	1	1	3	3
1	1	4	4	2	2	1	1	2	2
3	3	4	4	2	2	1	1	3	3
1	1	4	4	2	2	1	1	3	3
1	1	4	4	2	2	1	1	2	2
3	3	2	2	2	2	1	1	2	2
1	1	2	2	2	2	1	1	2	2
3	3	4	4	2	2	4	4	3	3
3	3	2	2	2	2	1	1	2	2
3	3	2	2	2	2	1	1	2	2
3	3	4	4	2	2	1	1	3	3
1	1	4	4	2	2	1	1	3	3
3	3	2	2	2	2	1	1	2	2
3	3	4	4	2	2	1	1	3	3

For Review Only

3	3	4	4	3	3	1	1	2	2
3	3	4	4	2	2	1	1	3	3
3	3	2	2	2	2	1	1	3	3
3	3	4	4	2	2	1	1	3	3
1	1	2	2	2	2	1	1	2	2
3	3	4	4	2	2	1	1	3	3
3	3	4	4	2	2	1	1	3	3
3	3	4	4	2	2	1	1	3	3
1	1	4	4	2	2	1	1	3	3
3	3	2	2	2	2	1	1	2	2
3	3	4	4	2	2	1	1	2	2
3	3	4	4	2	2	1	1	3	3
3	3	4	4	2	2	1	1	2	2
1	1	4	4	2	2	1	1	2	2
1	1	4	4	2	2	1	1	3	3
3	3	2	2	2	2	1	1	3	3
3	3	4	4	2	2	1	1	3	3
3	3	2	2	2	2	1	1	2	2
1	1	2	2	2	2	1	1	2	2
3	3	2	2	2	2	1	1	2	2
3	3	4	4	2	2	1	1	3	3
3	3	4	4	2	2	1	1	2	2
3	3	4	4	2	2	1	1	2	2
1	1	2	2	2	2	1	1	2	2
3	3	4	4	2	2	1	1	3	3
1	1	4	4	2	2	4	4	3	3
3	3	4	4	2	2	1	1	2	2
3	3	4	4	2	2	1	1	3	3
3	3	4	4	3	3	1	1	2	2
1	1	2	2	2	2	4	4	2	2
3	3	4	4	2	2	1	1	2	2
3	3	4	4	2	2	1	1	2	2
3	3	2	2	2	2	1	1	2	2
1	1	4	4	2	2	1	1	3	3
3	3	4	4	2	2	1	1	2	2
1	1	2	2	2	2	1	1	2	2
3	3	4	4	2	2	1	1	3	3
3	3	2	2	2	2	1	1	2	2
1	1	2	2	2	2	1	1	2	2
3	3	4	4	2	2	1	1	2	2
3	3	2	2	2	2	1	1	2	2
3	3	2	2	2	2	1	1	2	2
1	1	4	4	2	2	1	1	3	3
3	3	2	2	2	2	1	1	2	2
3	3	2	2	2	2	1	1	3	3
3	3	4	4	2	2	1	1	3	3
3	3	2	2	2	2	1	1	3	3
3	3	4	4	2	2	1	1	3	3
3	3	4	4	2	2	4	4	3	3
3	3	2	2	2	2	1	1	3	3
3	3	2	2	2	2	1	1	2	2
3	3	4	4	2	2	1	1	2	2
3	3	4	4	2	2	1	1	2	2
1	1	2	2	2	2	1	1	2	2
3	3	4	4	2	2	1	1	3	3

For Review Only

1	1	2	2	2	2	4	4	3	3
1	1	2	2	2	2	4	4	2	2
3	3	4	4	2	2	1	1	2	2
3	3	4	4	2	2	1	1	2	2
3	3	4	4	2	2	1	1	2	2
1	1	2	2	2	2	1	1	2	2
1	1	4	4	2	2	1	1	3	3
1	1	4	4	2	2	1	1	2	2
1	1	4	4	2	2	4	4	3	3
3	3	4	4	2	2	1	1	3	3
3	3	4	4	2	2	1	1	3	3
3	3	4	4	2	2	1	1	2	2
3	3	2	2	2	2	1	1	3	3
3	3	2	2	2	2	4	4	2	2
3	3	4	4	2	2	1	1	2	2
3	3	2	2	2	2	1	1	3	3
3	3	4	4	2	2	1	1	3	3
1	1	4	4	2	2	4	4	3	3
3	3	4	4	2	2	1	1	3	3
3	3	4	4	2	2	1	1	2	2
3	3	4	4	2	2	1	1	2	2
1	1	2	2	2	2	1	1	2	2
3	3	4	4	2	2	1	1	2	2
3	3	4	4	2	2	1	1	3	3
3	3	4	4	2	2	1	1	3	3
3	3	4	4	2	2	1	1	3	3
3	3	4	4	2	2	1	1	3	3
3	3	2	2	2	2	1	1	2	2
3	3	4	4	3	3	1	1	2	2
3	3	4	4	2	2	1	1	2	2
3	3	4	4	2	2	1	1	3	3
3	3	4	4	2	2	1	1	3	3
1	1	4	4	2	2	4	4	2	2
1	1	2	2	2	2	1	1	2	2
3	3	4	4	2	2	1	1	2	2
3	3	4	4	2	2	1	1	3	3
1	1	2	2	2	2	1	1	2	2
1	1	2	2	2	2	1	1	2	2
3	3	2	2	2	2	1	1	2	2
3	3	4	4	2	2	1	1	3	3
3	3	4	4	2	2	1	1	2	2
1	1	4	4	2	2	1	1	2	2
3	3	4	4	2	2	1	1	3	3
3	3	2	2	3	3	1	1	2	2
3	3	2	2	2	2	1	1	2	2
3	3	2	2	2	2	1	1	3	3
3	3	2	2	2	2	1	1	2	2
1	1	4	4	2	2	4	4	2	2
1	1	2	2	2	2	1	1	3	3
3	3	4	4	2	2	1	1	3	3
3	3	2	2	2	2	1	1	2	2
3	3	4	4	2	2	1	1	3	3
3	3	2	2	2	2	1	1	2	2
3	3	4	4	2	2	1	1	2	2
3	3	4	4	2	2	1	1	3	3

For Review Only

3	3	2	2	2	2	1	1	2	2
3	3	4	4	2	2	1	1	2	2

For Review Only

SNP1986	SNP1986	SNP1987	SNP1987	SNP1988	SNP1988	SNP1989	SNP1989	SNP199	SNP199
2	2	1	1	2	2	3	3	2	2
1	1	1	1	2	2	2	2	2	2
2	2	1	1	3	3	3	3	1	1
2	2	1	1	2	2	3	3	1	1
2	2	4	4	2	2	3	3	2	2
2	2	4	4	2	2	2	2	2	2
2	2	1	1	2	2	3	3	2	2
2	2	1	1	2	2	2	2	2	2
2	2	1	1	2	2	2	2	2	2
2	2	1	1	2	2	3	3	2	2
2	2	1	1	3	3	3	3	2	2
2	2	1	1	3	3	3	3	1	1
2	2	1	1	2	2	2	2	2	2
1	1	4	4	2	2	3	3	2	2
2	2	1	1	2	2	3	3	2	2
2	2	4	4	2	2	3	3	1	1
2	2	1	1	2	2	3	3	2	2
2	2	1	1	2	2	2	2	2	2
2	2	1	1	2	2	3	3	2	2
2	2	4	4	2	2	2	2	2	2
2	2	1	1	2	2	2	2	2	2
1	1	1	1	3	3	3	3	2	2
2	2	1	1	3	3	2	2	2	2
2	2	4	4	2	2	2	2	1	1
2	2	1	1	2	2	2	2	2	2
2	2	1	1	2	2	3	3	1	1
2	2	1	1	2	2	2	2	1	1
2	2	1	1	2	2	3	3	1	1
2	2	1	1	2	2	2	2	2	2
1	1	1	1	3	3	2	2	2	2
2	2	1	1	2	2	3	3	2	2
2	2	1	1	2	2	3	3	2	2
2	2	1	1	2	2	3	3	2	2
1	1	1	1	2	2	2	2	2	2
2	2	1	1	3	3	3	3	2	2
2	2	4	4	2	2	3	3	1	1
2	2	4	4	2	2	2	2	2	2
2	2	1	1	2	2	3	3	2	2
2	2	1	1	2	2	2	2	2	2
2	2	1	1	3	3	2	2	2	2
1	1	1	1	3	3	2	2	2	2
2	2	1	1	2	2	3	3	2	2
1	1	1	1	3	3	2	2	2	2
2	2	1	1	2	2	3	3	2	2
1	1	1	1	3	3	2	2	2	2
2	2	4	4	2	2	3	3	1	1
2	2	1	1	2	2	3	3	1	1
2	2	1	1	3	3	3	3	1	1
1	1	1	1	2	2	3	3	2	2
2	2	1	1	3	3	3	3	2	2
2	2	1	1	2	2	3	3	1	1
2	2	1	1	2	2	2	2	1	1

For Review Only

2	2	1	1	2	2	2	2	2	2
2	2	1	1	2	2	3	3	2	2
2	2	1	1	2	2	3	3	2	2
2	2	1	1	2	2	3	3	2	2
2	2	1	1	2	2	3	3	2	2
2	2	1	1	2	2	3	3	1	1
2	2	1	1	2	2	3	3	1	1
1	1	4	4	2	2	2	2	2	2
1	1	4	4	2	2	2	2	2	2
2	2	1	1	2	2	3	3	2	2
2	2	1	1	2	2	3	3	1	1
2	2	1	1	2	2	3	3	1	1
2	2	1	1	2	2	2	2	2	2
2	2	1	1	2	2	2	2	2	2
2	2	4	4	2	2	3	3	2	2
2	2	1	1	2	2	3	3	2	2
2	2	1	1	2	2	3	3	1	1
2	2	1	1	2	2	3	3	2	2
2	2	1	1	2	2	3	3	2	2
2	2	1	1	2	2	3	3	2	2
2	2	1	1	2	2	3	3	2	2
2	2	1	1	2	2	2	2	2	2
2	2	1	1	2	2	3	3	2	2
2	2	4	4	2	2	3	3	1	1
1	1	1	1	3	3	2	2	2	2
2	2	1	1	2	2	3	3	2	2
1	1	4	4	2	2	2	2	2	2
2	2	1	1	3	3	2	2	2	2
2	2	1	1	2	2	3	3	1	1
2	2	1	1	2	2	3	3	2	2
2	2	4	4	2	2	3	3	2	2
2	2	1	1	2	2	3	3	1	1
1	1	4	4	2	2	2	2	2	2
2	2	1	1	2	2	2	2	2	2
1	1	1	1	3	3	3	3	2	2
1	1	1	1	3	3	2	2	2	2
2	2	4	4	2	2	3	3	2	2
2	2	1	1	2	2	2	2	2	2
2	2	4	4	2	2	3	3	2	2
2	2	1	1	3	3	2	2	2	2
2	2	1	1	2	2	3	3	2	2
2	2	1	1	2	2	3	3	1	1
2	2	1	1	2	2	2	2	2	2
2	2	1	1	2	2	3	3	1	1
2	2	1	1	2	2	2	2	2	2
2	2	1	1	2	2	3	3	2	2
1	1	4	4	2	2	2	2	2	2
2	2	1	1	2	2	3	3	1	1
2	2	1	1	2	2	3	3	1	1
2	2	1	1	2	2	3	3	2	2
2	2	1	1	2	2	3	3	1	1
2	2	1	1	3	3	2	2	2	2
2	2	4	4	2	2	2	2	2	2
2	2	1	1	2	2	2	2	2	2
2	2	1	1	2	2	3	3	2	2

For Review Only

2	2	1	1	2	2	2	2	1	1
2	2	1	1	2	2	3	3	2	2
2	2	1	1	2	2	3	3	1	1
1	1	4	4	2	2	2	2	2	2
2	2	1	1	2	2	3	3	1	1
2	2	1	1	2	2	3	3	1	1
2	2	1	1	2	2	3	3	1	1
2	2	1	1	2	2	3	3	2	2
1	1	1	1	2	2	3	3	1	1
2	2	1	1	2	2	3	3	2	2
2	2	1	1	2	2	2	2	2	2
2	2	1	1	2	2	3	3	1	1
2	2	1	1	3	3	2	2	2	2
2	2	1	1	2	2	3	3	1	1
2	2	1	1	2	2	3	3	2	2
2	2	4	4	2	2	3	3	2	2
1	1	1	1	2	2	3	3	2	2
2	2	1	1	2	2	3	3	2	2
1	1	4	4	2	2	2	2	2	2
2	2	1	1	2	2	2	2	2	2
2	2	1	1	2	2	3	3	2	2
2	2	1	1	2	2	3	3	2	2
2	2	1	1	3	3	2	2	2	2
1	1	1	1	3	3	2	2	2	2
2	2	1	1	2	2	3	3	2	2
2	2	1	1	3	3	2	2	2	2
2	2	1	1	2	2	2	2	2	2
2	2	1	1	2	2	2	2	2	2
2	2	1	1	2	2	3	3	2	2
2	2	1	1	3	3	3	3	2	2
1	1	1	1	2	2	2	2	2	2
2	2	1	1	2	2	3	3	2	2
2	2	1	1	2	2	3	3	2	2
2	2	1	1	2	2	3	3	2	2
1	1	1	1	2	2	2	2	2	2
2	2	1	1	2	2	3	3	2	2
1	1	4	4	2	2	2	2	2	2
2	2	1	1	2	2	3	3	2	2
2	2	1	1	2	2	3	3	2	2
1	1	1	1	3	3	2	2	2	2
2	2	1	1	2	2	2	2	2	2
1	1	4	4	2	2	2	2	2	2
2	2	1	1	2	2	3	3	2	2
2	2	1	1	2	2	3	3	2	2
2	2	1	1	3	3	3	3	1	1
2	2	1	1	2	2	3	3	2	2
2	2	1	1	2	2	3	3	1	1
2	2	1	1	2	2	3	3	1	1
2	2	1	1	2	2	3	3	2	2
2	2	1	1	2	2	3	3	1	1
2	2	4	4	2	2	3	3	1	1
1	1	1	1	3	3	2	2	2	2
2	2	1	1	3	3	3	3	1	1
2	2	1	1	2	2	3	3	1	1
2	2	4	4	2	2	3	3	2	2

For Review Only

2	2	1	1	2	2	3	3	2	2
2	2	4	4	2	2	2	2	2	2
2	2	4	4	2	2	2	2	2	2
2	2	1	1	2	2	3	3	2	2
2	2	1	1	2	2	3	3	2	2
2	2	1	1	3	3	2	2	2	2
2	2	1	1	3	3	2	2	2	2
2	2	1	1	2	2	3	3	2	2
2	2	1	1	2	2	3	3	1	1
2	2	1	1	2	2	3	3	2	2
2	2	1	1	2	2	3	3	1	1
2	2	4	4	2	2	3	3	2	2
2	2	1	1	2	2	3	3	1	1
2	2	1	1	2	2	3	3	2	2
1	1	1	1	2	2	2	2	2	2
2	2	1	1	3	3	3	3	2	2
2	2	1	1	3	3	3	3	2	2
2	2	1	1	2	2	3	3	2	2
2	2	1	1	2	2	3	3	1	1
2	2	1	1	2	2	3	3	1	1
2	2	1	1	2	2	3	3	2	2
2	2	1	1	2	2	2	2	2	2
2	2	4	4	2	2	2	2	2	2
2	2	1	1	2	2	3	3	1	1
2	2	1	1	2	2	2	2	2	2
1	1	1	1	2	2	2	2	2	2
2	2	1	1	2	2	3	3	1	1
2	2	1	1	2	2	2	2	2	2
2	2	1	1	3	3	2	2	2	2
2	2	1	1	2	2	3	3	1	1
2	2	1	1	2	2	3	3	2	2
2	2	1	1	2	2	3	3	2	2
2	2	1	1	2	2	3	3	2	2
1	1	1	1	2	2	2	2	2	2
2	2	1	1	2	2	3	3	1	1
2	2	1	1	2	2	3	3	1	1
2	2	1	1	2	2	2	2	1	1
2	2	1	1	2	2	3	3	2	2
2	2	1	1	2	2	3	3	1	1
2	2	1	1	2	2	3	3	1	1
2	2	1	1	3	3	3	3	1	1
2	2	1	1	2	2	3	3	1	1
1	1	1	1	3	3	2	2	2	2
2	2	1	1	2	2	3	3	2	2
1	1	1	1	2	2	3	3	2	2
2	2	1	1	2	2	2	2	2	2
2	2	1	1	3	3	3	3	2	2
2	2	1	1	3	3	2	2	2	2
2	2	4	4	2	2	2	2	2	2
2	2	1	1	2	2	3	3	1	1
2	2	1	1	2	2	3	3	1	1
1	1	4	4	2	2	2	2	2	2
2	2	4	4	2	2	2	2	2	2
2	2	1	1	2	2	3	3	1	1
2	2	4	4	2	2	2	2	2	2

For Review Only

2	2	1	1	3	3	3	3	1	1
1	1	1	1	2	2	3	3	2	2
2	2	1	1	2	2	3	3	2	2
2	2	1	1	2	2	2	2	2	2
1	1	1	1	2	2	2	2	2	2
2	2	1	1	3	3	3	3	1	1
2	2	1	1	3	3	3	3	2	2
1	1	1	1	2	2	2	2	2	2
1	1	1	1	2	2	3	3	2	2
1	1	1	1	2	2	2	2	2	2
2	2	1	1	2	2	3	3	2	2
2	2	1	1	3	3	3	3	2	2
2	2	4	4	2	2	3	3	2	2
2	2	1	1	2	2	3	3	2	2
2	2	1	1	3	3	3	3	1	1
1	1	4	4	2	2	3	3	2	2
2	2	1	1	3	3	2	2	2	2
2	2	1	1	2	2	2	2	2	2
2	2	4	4	2	2	2	2	2	2
2	2	1	1	2	2	3	3	2	2
2	2	1	1	2	2	2	2	2	2
2	2	1	1	2	2	3	3	1	1
2	2	1	1	2	2	2	2	2	2
2	2	1	1	3	3	3	3	2	2
1	1	4	4	2	2	2	2	2	2
2	2	1	1	2	2	2	2	2	2
1	1	4	4	2	2	2	2	2	2
2	2	1	1	2	2	3	3	1	1
2	2	1	1	2	2	2	2	2	2
2	2	1	1	2	2	3	3	2	2
2	2	4	4	2	2	2	2	2	2
2	2	1	1	3	3	3	3	1	1
2	2	1	1	2	2	2	2	2	2
2	2	1	1	2	2	3	3	2	2
2	2	1	1	3	3	2	2	2	2
2	2	4	4	2	2	2	2	2	2
2	2	1	1	2	2	3	3	1	1
2	2	1	1	2	2	3	3	1	1
2	2	4	4	2	2	3	3	1	1
2	2	1	1	3	3	2	2	2	2
2	2	1	1	2	2	3	3	2	2
2	2	1	1	2	2	2	2	2	2
2	2	1	1	3	3	2	2	2	2
2	2	1	1	2	2	3	3	2	2
2	2	1	1	2	2	3	3	1	1
2	2	4	4	2	2	3	3	1	1
2	2	1	1	3	3	3	3	2	2
2	2	1	1	3	3	3	3	1	1
2	2	1	1	2	2	3	3	2	2
2	2	4	4	2	2	2	2	2	2
2	2	1	1	2	2	3	3	2	2
1	1	1	1	3	3	2	2	2	2
2	2	1	1	3	3	3	3	1	1
2	2	1	1	2	2	3	3	1	1
2	2	1	1	3	3	2	2	2	2

For Review Only

2	2	1	1	2	2	3	3	2	2
2	2	1	1	2	2	3	3	1	1

For Review Only

SNP1990	SNP1990	SNP1991	SNP1991	SNP1992	SNP1992	SNP1993	SNP1993	SNP1994	SNP1994
3	3	3	3	2	2	1	1	2	2
3	3	3	3	2	2	1	1	2	2
2	2	2	2	2	2	1	1	1	1
2	2	2	2	2	2	1	1	1	1
2	2	2	2	2	2	1	1	1	1
3	3	3	3	2	2	1	1	2	2
2	2	2	2	2	2	1	1	1	1
3	3	3	3	2	2	1	1	2	2
3	3	2	2	2	2	1	1	2	2
2	2	2	2	2	2	1	1	1	1
2	2	2	2	2	2	1	1	1	1
2	2	2	2	2	2	1	1	1	1
3	3	3	3	2	2	1	1	2	2
2	2	2	2	3	3	3	3	1	1
2	2	2	2	2	2	1	1	1	1
2	2	2	2	2	2	1	1	1	1
2	2	2	2	2	2	1	1	1	1
3	3	3	3	2	2	1	1	2	2
2	2	2	2	2	2	1	1	1	1
3	3	3	3	2	2	1	1	2	2
3	3	3	3	2	2	1	1	2	2
2	2	2	2	2	2	1	1	1	1
3	3	3	3	2	2	1	1	2	2
3	3	3	3	2	2	1	1	2	2
3	3	3	3	2	2	1	1	2	2
2	2	2	2	2	2	1	1	1	1
3	3	3	3	2	2	1	1	2	2
2	2	2	2	2	2	1	1	1	1
3	3	3	3	2	2	1	1	2	2
3	3	3	3	2	2	1	1	2	2
2	2	2	2	2	2	1	1	1	1
2	2	2	2	2	2	1	1	1	1
2	2	2	2	2	2	1	1	1	1
3	3	3	3	2	2	1	1	2	2
3	3	2	2	2	2	1	1	2	2
2	2	2	2	2	2	1	1	1	1
3	3	3	3	2	2	1	1	2	2
2	2	2	2	2	2	1	1	1	1
3	3	3	3	2	2	1	1	2	2
3	3	3	3	2	2	1	1	2	2
3	3	3	3	2	2	1	1	2	2
2	2	2	2	2	2	1	1	1	1
3	3	3	3	2	2	1	1	2	2
2	2	2	2	2	2	1	1	1	1
3	3	3	3	2	2	1	1	2	2
2	2	2	2	2	2	1	1	1	1
2	2	2	2	2	2	1	1	1	1
2	2	2	2	2	2	1	1	1	1
2	2	2	2	2	2	1	1	1	1
2	2	2	2	2	2	1	1	1	1
2	2	2	2	2	2	1	1	1	1
3	3	3	3	2	2	1	1	2	2

For Review Only

3	3	3	3	2	2	1	1	2	2
2	2	2	2	2	2	1	1	1	1
2	2	2	2	2	2	1	1	1	1
2	2	2	2	2	2	1	1	1	1
2	2	2	2	2	2	1	1	1	1
2	2	2	2	2	2	1	1	1	1
2	2	2	2	2	2	1	1	1	1
3	3	3	3	2	2	1	1	2	2
3	3	3	3	2	2	1	1	2	2
2	2	2	2	2	2	1	1	1	1
2	2	2	2	2	2	1	1	1	1
2	2	2	2	2	2	1	1	1	1
3	3	3	3	2	2	1	1	2	2
3	3	3	3	2	2	1	1	2	2
2	2	2	2	2	2	1	1	1	1
2	2	2	2	2	2	1	1	1	1
2	2	2	2	2	2	1	1	1	1
2	2	2	2	2	2	1	1	1	1
2	2	2	2	2	2	1	1	1	1
2	2	2	2	2	2	1	1	1	1
2	2	2	2	2	2	1	1	1	1
3	3	3	3	2	2	1	1	2	2
2	2	2	2	2	2	1	1	1	1
2	2	2	2	2	2	1	1	1	1
3	3	3	3	2	2	1	1	2	2
2	2	2	2	2	2	1	1	1	1
3	3	3	3	2	2	1	1	2	2
3	3	3	3	2	2	1	1	2	2
2	2	2	2	2	2	1	1	1	1
2	2	2	2	2	2	1	1	1	1
2	2	2	2	2	2	1	1	1	1
2	2	2	2	2	2	1	1	1	1
3	3	3	3	2	2	1	1	2	2
3	3	3	3	2	2	1	1	2	2
2	2	2	2	2	2	1	1	1	1
3	3	3	3	2	2	1	1	2	2
2	2	2	2	2	2	1	1	1	1
3	3	3	3	2	2	1	1	2	2
2	2	2	2	2	2	1	1	1	1
3	3	3	3	2	2	1	1	2	2
2	2	2	2	2	2	1	1	1	1
2	2	2	2	2	2	1	1	1	1
3	3	3	3	2	2	1	1	2	2
2	2	2	2	2	2	1	1	1	1
3	3	3	3	2	2	1	1	2	2
2	2	2	2	2	2	1	1	1	1
3	3	3	3	2	2	1	1	2	2
2	2	2	2	2	2	1	1	1	1
2	2	2	2	2	2	1	1	1	1
2	2	2	2	2	2	1	1	1	1
2	2	2	2	2	2	1	1	1	1
3	3	3	3	2	2	1	1	2	2
3	3	3	3	3	3	3	3	2	2
2	2	2	2	2	2	1	1	1	1
2	2	2	2	2	2	1	1	1	1

For Review Only

3	3	3	3	2	2	1	1	2	2
2	2	2	2	2	2	1	1	1	1
2	2	2	2	2	2	1	1	1	1
3	3	3	3	3	3	1	1	2	2
2	2	2	2	2	2	1	1	1	1
2	2	2	2	2	2	1	1	1	1
2	2	2	2	2	2	1	1	1	1
2	2	2	2	2	2	1	1	1	1
2	2	2	2	2	2	1	1	1	1
2	2	2	2	2	2	1	1	1	1
3	3	3	3	2	2	1	1	2	2
2	2	2	2	2	2	1	1	1	1
3	3	3	3	2	2	1	1	2	2
2	2	2	2	2	2	1	1	1	1
2	2	2	2	3	3	1	1	1	1
2	2	2	2	2	2	1	1	1	1
2	2	2	2	3	3	3	3	1	1
2	2	2	2	2	2	1	1	1	1
3	3	3	3	2	2	1	1	2	2
3	3	3	3	2	2	1	1	2	2
2	2	2	2	2	2	1	1	1	1
2	2	2	2	2	2	1	1	1	1
3	3	3	3	2	2	1	1	2	2
3	3	3	3	3	3	3	3	2	2
2	2	2	2	2	2	1	1	2	2
3	3	3	3	2	2	1	1	2	2
2	2	2	2	2	2	1	1	1	1
3	3	3	3	2	2	1	1	2	2
2	2	2	2	2	2	1	1	1	1
2	2	2	2	2	2	1	1	1	1
3	3	3	3	3	3	3	3	2	2
2	2	2	2	2	2	1	1	1	1
2	2	2	2	2	2	1	1	1	1
2	2	2	2	2	2	1	1	1	1
3	3	3	3	2	2	1	1	2	2
2	2	2	2	2	2	1	1	2	2
3	3	3	3	3	3	3	3	2	2
2	2	2	2	2	2	1	1	1	1
2	2	2	2	2	2	1	1	1	1
3	3	3	3	3	3	1	1	2	2
3	3	3	3	2	2	1	1	2	2
3	3	3	3	2	2	1	1	2	2
2	2	2	2	2	2	1	1	1	1
2	2	2	2	2	2	1	1	1	1
2	2	2	2	2	2	1	1	1	1
2	2	2	2	2	2	1	1	1	1
2	2	2	2	2	2	1	1	1	1
2	2	2	2	2	2	1	1	1	1
2	2	2	2	2	2	1	1	1	1
2	2	2	2	2	2	1	1	1	1
2	2	2	2	2	2	1	1	1	1
3	3	3	3	3	3	3	3	2	2
2	2	2	2	2	2	1	1	1	1
2	2	2	2	2	2	1	1	1	1
2	2	2	2	2	2	1	1	1	1

For Review Only

2	2	2	2	2	2	1	1	1	1
3	3	3	3	2	2	1	1	2	2
3	3	3	3	2	2	1	1	2	2
2	2	2	2	2	2	1	1	1	1
2	2	2	2	2	2	1	1	1	1
3	3	3	3	2	2	1	1	2	2
3	3	3	3	2	2	1	1	2	2
2	2	2	2	2	2	1	1	1	1
3	3	2	2	2	2	1	1	1	1
2	2	2	2	2	2	1	1	1	1
2	2	2	2	2	2	1	1	1	1
2	2	2	2	2	2	1	1	2	2
2	2	2	2	2	2	1	1	1	1
2	2	2	2	3	3	1	1	1	1
3	3	3	3	3	3	1	1	2	2
2	2	2	2	2	2	1	1	1	1
2	2	2	2	2	2	1	1	1	1
2	2	2	2	2	2	1	1	1	1
2	2	2	2	2	2	1	1	1	1
2	2	2	2	2	2	1	1	1	1
2	2	2	2	2	2	1	1	1	1
3	3	3	3	2	2	1	1	2	2
3	3	3	3	2	2	1	1	2	2
2	2	2	2	2	2	1	1	1	1
3	3	3	3	2	2	1	1	2	2
3	3	3	3	2	2	1	1	2	2
2	2	2	2	2	2	1	1	1	1
3	3	3	3	3	3	1	1	2	2
3	3	3	3	2	2	1	1	2	2
2	2	2	2	2	2	1	1	1	1
2	2	2	2	2	2	1	1	1	1
2	2	2	2	2	2	1	1	1	1
2	2	2	2	2	2	1	1	1	1
3	3	3	3	3	3	3	3	2	2
2	2	2	2	2	2	1	1	1	1
2	2	2	2	2	2	1	1	1	1
3	3	2	2	2	2	1	1	1	1
2	2	2	2	2	2	1	1	1	1
2	2	2	2	2	2	1	1	1	1
2	2	2	2	2	2	1	1	1	1
2	2	2	2	2	2	1	1	1	1
2	2	2	2	2	2	1	1	1	1
3	3	3	3	2	2	1	1	2	2
2	2	2	2	2	2	1	1	1	1
2	2	2	2	3	3	1	1	1	1
3	3	3	3	2	2	1	1	2	2
2	2	2	2	2	2	1	1	1	1
3	3	3	3	2	2	1	1	2	2
3	3	3	3	2	2	1	1	2	2
2	2	2	2	2	2	1	1	1	1
2	2	2	2	2	2	1	1	1	1
3	3	3	3	3	3	3	3	2	2
3	3	3	3	2	2	1	1	2	2
2	2	2	2	2	2	1	1	1	1
3	3	3	3	2	2	1	1	2	2

For Review Only

2	2	2	2	2	2	1	1	1	1
2	2	2	2	2	2	1	1	1	1
2	2	2	2	2	2	1	1	1	1
3	3	3	3	2	2	1	1	2	2
3	3	3	3	2	2	1	1	2	2
2	2	2	2	2	2	1	1	1	1
2	2	2	2	2	2	1	1	1	1
3	3	3	3	2	2	1	1	2	2
2	2	2	2	3	3	1	1	2	2
3	3	3	3	3	3	3	3	2	2
2	2	2	2	2	2	1	1	1	1
2	2	2	2	2	2	1	1	1	1
2	2	2	2	2	2	1	1	1	1
2	2	2	2	2	2	1	1	1	1
2	2	2	2	2	2	1	1	1	1
2	2	2	2	2	2	1	1	1	1
3	3	3	3	2	2	1	1	2	2
3	3	3	3	2	2	1	1	2	2
3	3	3	3	2	2	1	1	2	2
2	2	2	2	2	2	1	1	1	1
3	3	3	3	2	2	1	1	2	2
2	2	2	2	2	2	1	1	1	1
3	3	3	3	2	2	1	1	2	2
2	2	2	2	2	2	1	1	1	1
3	3	3	3	2	2	1	1	2	2
3	3	3	3	2	2	1	1	2	2
3	3	3	3	3	3	3	3	2	2
2	2	2	2	2	2	1	1	1	1
3	3	3	3	2	2	1	1	2	2
2	2	2	2	2	2	1	1	1	1
3	3	3	3	2	2	1	1	2	2
2	2	2	2	2	2	1	1	1	1
3	3	3	3	3	3	3	3	2	2
2	2	2	2	2	2	1	1	1	1
3	3	3	3	2	2	1	1	2	2
3	3	3	3	2	2	1	1	2	2
2	2	2	2	2	2	1	1	1	1
2	2	2	2	2	2	1	1	1	1
2	2	2	2	2	2	1	1	1	1
3	3	3	3	2	2	1	1	2	2
2	2	2	2	2	2	1	1	1	1
3	3	3	3	2	2	1	1	2	2
3	3	3	3	2	2	1	1	2	2
2	2	2	2	2	2	1	1	1	1
2	2	2	2	2	2	1	1	1	1
2	2	2	2	2	2	1	1	1	1
2	2	2	2	2	2	1	1	1	1
2	2	2	2	2	2	1	1	1	1
2	2	2	2	2	2	1	1	1	1
3	3	3	3	2	2	1	1	2	2
2	2	2	2	2	2	1	1	1	1
3	3	3	3	2	2	1	1	2	2
2	2	2	2	2	2	1	1	1	1
2	2	2	2	2	2	1	1	1	1
3	3	3	3	2	2	1	1	2	2

For Review Only

2	2	2	2	2	2	1	1	1	1
2	2	2	2	2	2	1	1	1	1

For Review Only

SNP1995	SNP1995	SNP1996	SNP1996	SNP1997	SNP1997	SNP1998	SNP1998	SNP1999	SNP1999
3	3	3	3	2	2	2	2	3	3
2	2	4	4	2	2	2	2	3	3
3	3	4	4	2	2	2	2	3	3
3	3	4	4	2	2	2	2	3	3
3	3	4	4	2	2	2	2	3	3
3	3	4	4	2	2	2	2	3	3
3	3	4	4	2	2	2	2	3	3
3	3	4	4	2	2	2	2	3	3
3	3	4	4	2	2	2	2	3	3
3	3	4	4	2	2	2	2	3	3
2	2	4	4	3	3	2	2	3	3
3	3	4	4	2	2	2	2	3	3
3	3	4	4	2	2	2	2	1	1
3	3	4	4	2	2	2	2	1	1
3	3	4	4	2	2	2	2	3	3
3	3	4	4	2	2	2	2	3	3
3	3	4	4	2	2	2	2	3	3
3	3	4	4	2	2	2	2	3	3
3	3	4	4	2	2	2	2	3	3
3	3	4	4	2	2	2	2	1	1
3	3	4	4	2	2	2	2	1	1
3	3	4	4	2	2	2	2	3	3
2	2	4	4	2	2	3	3	1	1
3	3	4	4	2	2	2	2	3	3
3	3	4	4	2	2	2	2	3	3
3	3	4	4	2	2	2	2	3	3
3	3	4	4	2	2	2	2	3	3
3	3	4	4	2	2	2	2	3	3
3	3	4	4	2	2	2	2	3	3
3	3	4	4	2	2	3	3	1	1
3	3	4	4	2	2	2	2	3	3
3	3	4	4	2	2	2	2	3	3
3	3	4	4	2	2	2	2	3	3
3	3	4	4	2	2	2	2	3	3
3	3	4	4	2	2	2	2	1	1
3	3	4	4	2	2	2	2	3	3
3	3	4	4	2	2	2	2	3	3
3	3	4	4	2	2	2	2	3	3
2	2	4	4	2	2	2	2	1	1
2	2	4	4	2	2	2	2	3	3
3	3	4	4	2	2	2	2	1	1
3	3	4	4	2	2	2	2	3	3
3	3	4	4	2	2	2	2	3	3
3	3	4	4	2	2	2	2	3	3
3	3	4	4	2	2	2	2	3	3
3	3	4	4	2	2	2	2	3	3
3	3	4	4	2	2	2	2	3	3
3	3	4	4	2	2	2	2	3	3
3	3	4	4	2	2	2	2	1	1
3	3	4	4	2	2	2	2	3	3
3	3	4	4	2	2	2	2	3	3
3	3	4	4	2	2	2	2	3	3

For Review Only

3	3	4	4	2	2	2	2	3	3
3	3	4	4	2	2	2	2	3	3
3	3	4	4	2	2	2	2	3	3
3	3	4	4	2	2	2	2	3	3
3	3	4	4	2	2	2	2	3	3
3	3	4	4	2	2	2	2	3	3
3	3	4	4	2	2	2	2	3	3
3	3	4	4	2	2	2	2	3	3
3	3	4	4	2	2	2	2	3	3
3	3	4	4	2	2	2	2	3	3
3	3	3	3	2	2	2	2	3	3
3	3	4	4	2	2	2	2	3	3
3	3	4	4	2	2	2	2	3	3
3	3	4	4	2	2	2	2	3	3
3	3	4	4	2	2	2	2	3	3
3	3	4	4	2	2	2	2	3	3
2	2	4	4	2	2	2	2	1	1
3	3	4	4	2	2	2	2	3	3
3	3	4	4	2	2	2	2	3	3
3	3	4	4	3	3	2	2	3	3
3	3	3	3	2	2	2	2	3	3
2	2	4	4	2	2	2	2	1	1
3	3	4	4	2	2	2	2	3	3
3	3	4	4	2	2	2	2	1	1
3	3	4	4	2	2	2	2	3	3
3	3	4	4	2	2	2	2	3	3
3	3	4	4	2	2	2	2	3	3
2	2	4	4	3	3	3	3	3	3
3	3	4	4	2	2	2	2	3	3
3	3	4	4	2	2	2	2	3	3
3	3	4	4	2	2	2	2	3	3
3	3	4	4	2	2	2	2	1	1
2	2	4	4	3	3	3	3	3	3
3	3	4	4	2	2	2	2	3	3
3	3	4	4	2	2	2	2	3	3
3	3	4	4	2	2	2	2	3	3
3	3	4	4	2	2	3	3	1	1
3	3	4	4	2	2	2	2	1	1
3	3	4	4	2	2	2	2	1	1
3	3	4	4	2	2	2	2	3	3
3	3	4	4	2	2	2	2	3	3
3	3	4	4	2	2	2	2	3	3
3	3	4	4	2	2	2	2	3	3
3	3	4	4	2	2	2	2	3	3
3	3	4	4	2	2	2	2	3	3
3	3	4	4	2	2	2	2	3	3
3	3	4	4	2	2	2	2	1	1
3	3	4	4	2	2	2	2	3	3
3	3	4	4	2	2	2	2	3	3
3	3	4	4	2	2	2	2	3	3
3	3	4	4	2	2	2	2	3	3
2	2	4	4	3	3	2	2	3	3
3	3	4	4	3	3	2	2	3	3
3	3	4	4	2	2	2	2	3	3
3	3	4	4	2	2	2	2	3	3

For Review Only

3	3	4	4	2	2	2	2	3	3
3	3	4	4	2	2	3	3	1	1
3	3	4	4	2	2	2	2	3	3
3	3	4	4	2	2	2	2	1	1
3	3	4	4	2	2	2	2	3	3
3	3	4	4	2	2	2	2	1	1
3	3	4	4	2	2	2	2	3	3
3	3	3	3	3	3	2	2	3	3
3	3	4	4	2	2	2	2	3	3
3	3	4	4	2	2	2	2	3	3
3	3	4	4	2	2	2	2	1	1
3	3	3	3	2	2	2	2	3	3
3	3	4	4	2	2	2	2	3	3
3	3	4	4	2	2	2	2	3	3
3	3	4	4	2	2	2	2	3	3
3	3	4	4	2	2	2	2	3	3
3	3	4	4	2	2	2	2	3	3
3	3	4	4	2	2	2	2	3	3
3	3	4	4	2	2	2	2	1	1
3	3	4	4	2	2	2	2	3	3
3	3	4	4	2	2	2	2	3	3
3	3	4	4	2	2	2	2	3	3
3	3	4	4	2	2	2	2	3	3
2	2	4	4	2	2	3	3	1	1
3	3	4	4	2	2	2	2	3	3
3	3	4	4	2	2	2	2	1	1
3	3	4	4	2	2	2	2	3	3
3	3	4	4	2	2	2	2	3	3
3	3	4	4	2	2	2	2	3	3
3	3	4	4	2	2	2	2	3	3
2	2	4	4	3	3	2	2	1	1
3	3	4	4	2	2	2	2	3	3
3	3	4	4	2	2	2	2	3	3
3	3	4	4	2	2	2	2	3	3
3	3	4	4	2	2	2	2	3	3
2	2	4	4	3	3	2	2	3	3
3	3	4	4	2	2	2	2	1	1
3	3	4	4	2	2	2	2	3	3
2	2	4	4	2	2	2	2	1	1
2	2	4	4	3	3	2	2	3	3
3	3	4	4	2	2	2	2	1	1
3	3	4	4	2	2	2	2	3	3
3	3	4	4	2	2	2	2	3	3
3	3	4	4	2	2	2	2	3	3
3	3	4	4	2	2	2	2	3	3
3	3	4	4	2	2	2	2	3	3
3	3	4	4	2	2	2	2	3	3
3	3	4	4	2	2	2	2	3	3
3	3	4	4	2	2	2	2	3	3
3	3	4	4	2	2	2	2	3	3
2	2	4	4	2	2	2	2	3	3
3	3	4	4	2	2	2	2	1	1
3	3	4	4	3	3	2	2	3	3
3	3	4	4	2	2	2	2	3	3
3	3	4	4	2	2	2	2	3	3

For Review Only

3	3	4	4	2	2	2	2	3	3
3	3	4	4	2	2	2	2	1	1
3	3	4	4	2	2	2	2	3	3
3	3	4	4	2	2	2	2	3	3
3	3	4	4	2	2	2	2	3	3
3	3	4	4	2	2	2	2	3	3
2	2	4	4	3	3	2	2	3	3
3	3	4	4	2	2	2	2	3	3
3	3	4	4	3	3	2	2	3	3
3	3	4	4	2	2	2	2	3	3
3	3	4	4	2	2	2	2	3	3
3	3	4	4	2	2	2	2	3	3
3	3	4	4	2	2	2	2	3	3
3	3	4	4	2	2	2	2	3	3
3	3	4	4	2	2	2	2	1	1
2	2	4	4	3	3	3	3	3	3
3	3	4	4	3	3	2	2	3	3
3	3	4	4	2	2	2	2	3	3
3	3	4	4	2	2	2	2	1	1
3	3	4	4	2	2	2	2	3	3
3	3	4	4	2	2	2	2	3	3
2	2	4	4	2	2	3	3	1	1
3	3	4	4	2	2	2	2	3	3
3	3	4	4	2	2	2	2	3	3
3	3	4	4	2	2	2	2	3	3
3	3	4	4	2	2	2	2	3	3
3	3	4	4	2	2	2	2	3	3
3	3	4	4	3	3	2	2	3	3
3	3	4	4	2	2	2	2	3	3
3	3	4	4	2	2	2	2	3	3
3	3	4	4	2	2	2	2	3	3
3	3	4	4	2	2	2	2	3	3
3	3	4	4	2	2	2	2	3	3
3	3	4	4	2	2	2	2	1	1
3	3	4	4	2	2	2	2	3	3
3	3	4	4	2	2	2	2	3	3
3	3	4	4	2	2	2	2	1	1
3	3	4	4	2	2	2	2	3	3
3	3	4	4	2	2	2	2	3	3
3	3	4	4	2	2	2	2	3	3
3	3	4	4	2	2	2	2	3	3
3	3	4	4	2	2	2	2	3	3
2	2	4	4	3	3	3	3	3	3
2	2	4	4	2	2	2	2	3	3
3	3	4	4	3	3	2	2	3	3
3	3	4	4	2	2	2	2	3	3
2	2	4	4	3	3	3	3	3	3
2	2	4	4	3	3	3	3	3	3
3	3	4	4	2	2	2	2	3	3
3	3	4	4	2	2	2	2	3	3
3	3	4	4	2	2	2	2	3	3
3	3	4	4	2	2	2	2	3	3
3	3	4	4	2	2	2	2	3	3
3	3	4	4	2	2	2	2	3	3
2	2	4	4	2	2	2	2	1	1

3	3	4	4	3	3	2	2	3	3
3	3	4	4	2	2	3	3	3	3
3	3	4	4	2	2	2	2	3	3
3	3	4	4	2	2	2	2	3	3
3	3	4	4	2	2	2	2	3	3
3	3	4	4	2	2	2	2	3	3
3	3	4	4	2	2	2	2	1	1
3	3	4	4	2	2	2	2	1	1
3	3	4	4	2	2	2	2	3	3
2	2	4	4	3	3	2	2	1	1
3	3	4	4	2	2	2	2	3	3
3	3	4	4	2	2	2	2	3	3
3	3	4	4	2	2	2	2	1	1
3	3	4	4	2	2	2	2	3	3
3	3	4	4	2	2	2	2	3	3
2	2	4	4	2	2	2	2	1	1
2	2	4	4	3	3	2	2	3	3
3	3	4	4	2	2	2	2	1	1
2	2	4	4	3	3	3	3	3	3
3	3	4	4	2	2	2	2	3	3
3	3	4	4	2	2	2	2	3	3
3	3	4	4	2	2	2	2	3	3
3	3	4	4	2	2	2	2	3	3
2	2	4	4	2	2	2	2	1	1
3	3	4	4	2	2	2	2	3	3
3	3	4	4	3	3	2	2	3	3
2	2	4	4	2	2	2	2	1	1
3	3	4	4	2	2	2	2	3	3
3	3	4	4	2	2	2	2	3	3
3	3	4	4	2	2	2	2	3	3
2	2	4	4	3	3	3	3	3	3
3	3	4	4	3	3	2	2	3	3
3	3	4	4	2	2	2	2	3	3
3	3	4	4	2	2	2	2	3	3
3	3	4	4	2	2	2	2	3	3
2	2	4	4	3	3	3	3	3	3
3	3	4	4	2	2	2	2	3	3
3	3	4	4	2	2	2	2	3	3
3	3	4	4	2	2	2	2	3	3
2	2	4	4	3	3	2	2	3	3
3	3	4	4	2	2	2	2	3	3
3	3	4	4	2	2	2	2	3	3
2	2	4	4	2	2	2	2	1	1
3	3	4	4	2	2	2	2	3	3
2	2	4	4	2	2	2	2	3	3
3	3	4	4	2	2	2	2	1	1
2	2	4	4	2	2	2	2	3	3
3	3	4	4	2	2	2	2	3	3
3	3	4	4	3	3	2	2	3	3
2	2	4	4	3	3	2	2	3	3
3	3	4	4	2	2	2	2	3	3
2	2	4	4	2	2	2	2	1	1
3	3	4	4	2	2	2	2	3	3
3	3	4	4	2	2	2	2	3	3
2	2	4	4	2	2	2	2	1	1

For Review Only

3	3	4	4	2	2	2	2	3	3
3	3	4	4	2	2	2	2	3	3

For Review Only

SNP200	SNP200	SNP2000	SNP2000	SNP2001	SNP2001	SNP2002	SNP2002	SNP2003	SNP2003
2	2	2	2	4	4	1	1	4	4
2	2	3	3	4	4	1	1	4	4
2	2	2	2	4	4	4	4	4	4
2	2	2	2	4	4	1	1	4	4
2	2	2	2	3	3	4	4	1	1
2	2	2	2	3	3	1	1	1	1
2	2	2	2	4	4	1	1	4	4
2	2	2	2	3	3	1	1	1	1
2	2	2	2	4	4	1	1	4	4
2	2	2	2	3	3	1	1	1	1
2	2	3	3	3	3	4	4	1	1
2	2	2	2	4	4	4	4	4	4
2	2	2	2	4	4	1	1	4	4
2	2	3	3	3	3	1	1	1	1
2	2	2	2	4	4	4	4	1	1
2	2	2	2	3	3	1	1	1	1
2	2	2	2	4	4	1	1	4	4
2	2	2	2	3	3	1	1	4	4
2	2	2	2	4	4	1	1	4	4
2	2	2	2	3	3	1	1	1	1
2	2	2	2	4	4	1	1	4	4
2	2	2	2	4	4	4	4	4	4
2	2	3	3	3	3	1	1	1	1
2	2	2	2	3	3	1	1	1	1
2	2	2	2	4	4	1	1	4	4
3	3	2	2	3	3	1	1	1	1
2	2	2	2	3	3	1	1	1	1
2	2	2	2	4	4	1	1	4	4
2	2	2	2	4	4	1	1	4	4
2	2	2	2	3	3	1	1	1	1
2	2	2	2	4	4	1	1	4	4
3	3	2	2	4	4	1	1	4	4
3	3	2	2	4	4	1	1	4	4
2	2	3	3	3	3	1	1	1	1
2	2	2	2	4	4	4	4	1	1
2	2	2	2	3	3	1	1	1	1
2	2	2	2	3	3	1	1	1	1
2	2	2	2	4	4	1	1	4	4
2	2	3	3	3	3	1	1	1	1
2	2	2	2	3	3	1	1	1	1
2	2	2	2	3	3	1	1	1	1
2	2	2	2	4	4	1	1	4	4
2	2	2	2	3	3	1	1	1	1
2	2	2	2	4	4	4	4	4	4
2	2	2	2	3	3	1	1	1	1
3	3	2	2	3	3	1	1	4	4
3	3	2	2	4	4	1	1	4	4
2	2	2	2	3	3	4	4	1	1
2	2	2	2	4	4	1	1	4	4
2	2	2	2	4	4	1	1	4	4
3	3	2	2	3	3	1	1	1	1
2	2	2	2	4	4	1	1	4	4

For Review Only

2	2	2	2	4	4	1	1	4	4
3	3	2	2	4	4	1	1	4	4
2	2	2	2	4	4	1	1	4	4
2	2	2	2	4	4	1	1	4	4
2	2	2	2	4	4	1	1	4	4
3	3	2	2	4	4	1	1	4	4
3	3	2	2	3	3	1	1	1	1
2	2	2	2	3	3	1	1	1	1
2	2	3	3	3	3	1	1	1	1
2	2	2	2	4	4	1	1	4	4
2	2	2	2	4	4	1	1	4	4
2	2	2	2	4	4	1	1	4	4
2	2	2	2	4	4	1	1	4	4
2	2	2	2	4	4	1	1	4	4
2	2	2	2	3	3	1	1	4	4
2	2	2	2	4	4	1	1	4	4
2	2	3	3	4	4	4	4	1	1
3	3	2	2	4	4	4	4	4	4
2	2	2	2	4	4	1	1	4	4
2	2	2	2	4	4	1	1	4	4
2	2	2	2	4	4	1	1	4	4
2	2	2	2	3	3	1	1	1	1
2	2	2	2	4	4	1	1	4	4
2	2	2	2	3	3	1	1	1	1
2	2	2	2	3	3	1	1	1	1
2	2	2	2	4	4	1	1	4	4
2	2	2	2	3	3	1	1	1	1
2	2	3	3	3	3	1	1	1	1
3	3	2	2	3	3	1	1	1	1
2	2	2	2	4	4	1	1	4	4
2	2	2	2	3	3	1	1	1	1
2	2	2	2	3	3	1	1	1	1
2	2	3	3	3	3	1	1	1	1
2	2	2	2	4	4	1	1	4	4
2	2	2	2	3	3	1	1	1	1
2	2	2	2	3	3	1	1	1	1
2	2	2	2	3	3	1	1	4	4
2	2	2	2	4	4	1	1	4	4
2	2	2	2	3	3	4	4	1	1
2	2	2	2	3	3	1	1	1	1
3	3	2	2	4	4	4	4	4	4
2	2	2	2	4	4	1	1	4	4
2	2	2	2	3	3	4	4	1	1
2	2	2	2	4	4	1	1	4	4
2	2	2	2	4	4	4	4	4	4
3	3	2	2	4	4	1	1	4	4
2	2	3	3	3	3	1	1	1	1
2	2	2	2	3	3	1	1	1	1
3	3	2	2	3	3	1	1	1	1
2	2	2	2	3	3	1	1	1	1
2	2	2	2	4	4	1	1	4	4
2	2	3	3	3	3	1	1	1	1
2	2	2	2	3	3	1	1	1	1
2	2	2	2	4	4	1	1	1	1
2	2	2	2	3	3	4	4	1	1

2	2	2	2	3	3	1	1	1	1
2	2	2	2	3	3	1	1	1	1
2	2	2	2	3	3	1	1	1	1
2	2	3	3	3	3	1	1	1	1
2	2	2	2	3	3	1	1	4	4
3	3	2	2	3	3	4	4	1	1
2	2	2	2	4	4	1	1	4	4
2	2	2	2	3	3	1	1	1	1
2	2	2	2	3	3	1	1	1	1
2	2	2	2	4	4	1	1	1	1
2	2	2	2	3	3	1	1	4	4
2	2	2	2	4	4	1	1	4	4
2	2	2	2	3	3	1	1	1	1
2	2	2	2	4	4	1	1	4	4
2	2	2	2	3	3	1	1	1	1
2	2	2	2	4	4	1	1	4	4
3	3	2	2	4	4	4	4	4	4
2	2	2	2	4	4	1	1	4	4
2	2	3	3	3	3	1	1	1	1
2	2	2	2	4	4	4	4	4	4
2	2	2	2	4	4	1	1	4	4
2	2	2	2	4	4	1	1	4	4
2	2	2	2	4	4	1	1	4	4
2	2	3	3	3	3	1	1	1	1
2	2	2	2	3	3	1	1	1	1
2	2	2	2	3	3	1	1	1	1
3	3	2	2	4	4	1	1	4	4
2	2	2	2	4	4	1	1	4	4
2	2	2	2	4	4	4	4	4	4
2	2	2	2	4	4	4	4	4	4
2	2	3	3	3	3	1	1	1	1
2	2	2	2	4	4	1	1	4	4
3	3	2	2	4	4	4	4	4	4
2	2	2	2	3	3	1	1	1	1
2	2	3	3	3	3	1	1	1	1
3	3	2	2	3	3	1	1	4	4
2	2	3	3	3	3	1	1	1	1
2	2	2	2	4	4	1	1	4	4
2	2	2	2	3	3	4	4	1	1
2	2	3	3	3	3	1	1	1	1
2	2	2	2	3	3	1	1	1	1
2	2	3	3	3	3	1	1	1	1
2	2	2	2	4	4	4	4	4	4
2	2	2	2	3	3	1	1	1	1
2	2	2	2	3	3	1	1	4	4
2	2	2	2	4	4	1	1	4	4
3	3	2	2	3	3	1	1	1	1
3	3	2	2	3	3	1	1	1	1
2	2	2	2	4	4	1	1	4	4
3	3	2	2	3	3	1	1	1	1
3	3	2	2	3	3	1	1	1	1
3	3	2	2	3	3	1	1	1	1
2	2	2	2	3	3	1	1	1	1
2	2	2	2	4	4	4	4	4	4
2	2	2	2	4	4	1	1	4	4

For Review Only

2	2	2	2	4	4	4	4	4	4
2	2	2	2	3	3	1	1	1	1
2	2	2	2	3	3	1	1	1	1
2	2	2	2	4	4	1	1	4	4
2	2	2	2	4	4	1	1	4	4
3	3	2	2	3	3	1	1	1	1
2	2	3	3	3	3	1	1	1	1
2	2	2	2	4	4	1	1	4	4
2	2	2	2	3	3	4	4	1	1
2	2	2	2	4	4	1	1	4	4
2	2	2	2	3	3	1	1	1	1
2	2	2	2	3	3	4	4	1	1
2	2	2	2	4	4	1	1	4	4
2	2	2	2	4	4	1	1	4	4
2	2	2	2	3	3	1	1	1	1
2	2	3	3	3	3	4	4	1	1
2	2	2	2	3	3	1	1	1	1
2	2	2	2	4	4	1	1	4	4
2	2	2	2	4	4	1	1	4	4
2	2	2	2	4	4	1	1	4	4
2	2	2	2	4	4	1	1	4	4
2	2	2	2	3	3	1	1	1	1
2	2	2	2	3	3	1	1	1	1
2	2	2	2	4	4	1	1	4	4
2	2	2	2	4	4	1	1	4	4
2	2	3	3	3	3	1	1	1	1
2	2	2	2	4	4	1	1	4	4
2	2	2	2	4	4	1	1	4	4
2	2	2	2	4	4	1	1	4	4
2	2	2	2	4	4	1	1	4	4
2	2	2	2	3	3	1	1	4	4
3	3	2	2	4	4	4	4	4	4
3	3	2	2	4	4	1	1	4	4
2	2	2	2	3	3	1	1	1	1
2	2	2	2	4	4	4	4	4	4
2	2	2	2	4	4	1	1	4	4
2	2	2	2	4	4	1	1	4	4
2	2	2	2	4	4	1	1	4	4
3	3	2	2	3	3	1	1	1	1
2	2	2	2	4	4	1	1	4	4
2	2	2	2	4	4	4	4	4	4
2	2	2	2	3	3	1	1	1	1
2	2	3	3	3	3	1	1	1	1
3	3	2	2	4	4	1	1	4	4
3	3	2	2	3	3	1	1	4	4
2	2	2	2	4	4	1	1	4	4
2	2	3	3	3	3	4	4	1	1
2	2	3	3	3	3	1	1	1	1
2	2	2	2	3	3	1	1	1	1
2	2	2	2	4	4	1	1	4	4
2	2	2	2	4	4	4	4	4	4
2	2	2	2	3	3	1	1	1	1
2	2	2	2	3	3	1	1	1	1
2	2	2	2	4	4	1	1	4	4
2	2	3	3	3	3	1	1	1	1

For Review Only

2	2	2	2	3	3	1	1	1	1
2	2	2	2	4	4	1	1	4	4
2	2	2	2	4	4	4	4	4	4
2	2	2	2	4	4	1	1	4	4
2	2	2	2	3	3	1	1	1	1
3	3	2	2	4	4	1	1	4	4
2	2	2	2	3	3	1	1	1	1
2	2	2	2	3	3	1	1	1	1
2	2	2	2	3	3	1	1	1	1
2	2	3	3	3	3	1	1	1	1
2	2	2	2	4	4	1	1	4	4
2	2	2	2	3	3	1	1	1	1
2	2	2	2	3	3	4	4	1	1
2	2	2	2	4	4	1	1	4	4
3	3	2	2	4	4	1	1	4	4
2	2	3	3	3	3	4	4	1	1
2	2	3	3	3	3	1	1	1	1
2	2	2	2	4	4	1	1	4	4
2	2	3	3	3	3	1	1	1	1
2	2	2	2	4	4	4	4	4	4
2	2	2	2	4	4	1	1	4	4
3	3	2	2	3	3	1	1	1	1
2	2	2	2	4	4	1	1	4	4
2	2	3	3	3	3	4	4	1	1
2	2	2	2	3	3	1	1	1	1
2	2	2	2	4	4	1	1	1	1
2	2	3	3	3	3	1	1	1	1
2	2	2	2	4	4	1	1	4	4
2	2	2	2	4	4	1	1	4	4
2	2	2	2	4	4	4	4	4	4
2	2	3	3	3	3	1	1	1	1
2	2	2	2	3	3	4	4	1	1
2	2	3	3	3	3	4	4	1	1
3	3	2	2	3	3	1	1	1	1
2	2	2	2	3	3	1	1	1	1
2	2	3	3	3	3	1	1	1	1
2	2	2	2	4	4	1	1	4	4
2	2	2	2	4	4	1	1	4	4
2	2	2	2	3	3	1	1	1	1
2	2	3	3	3	3	1	1	1	1
2	2	2	2	4	4	4	4	4	4
2	2	2	2	3	3	1	1	1	1
2	2	3	3	3	3	4	4	1	1
2	2	2	2	4	4	4	4	4	4
2	2	2	2	4	4	1	1	4	4
2	2	2	2	3	3	4	4	1	1
3	3	2	2	3	3	1	1	1	1
2	2	2	2	3	3	1	1	1	1
2	2	2	2	4	4	1	1	4	4
2	2	3	3	3	3	1	1	1	1
2	2	2	2	4	4	4	4	4	4
2	2	3	3	3	3	1	1	1	1
2	2	2	2	4	4	4	4	4	4
2	2	2	2	4	4	1	1	4	4
2	2	3	3	3	3	1	1	1	1

For Review Only

2	2	2	2	4	4	4	4	4	4
2	2	2	2	4	4	1	1	4	4

For Review Only

SNP2004	SNP2004	SNP2005	SNP2005	SNP2006	SNP2006	SNP2007	SNP2007	SNP2008	SNP2008
3	3	1	1	4	4	3	3	2	2
4	4	1	1	4	4	3	3	2	2
4	4	3	3	4	4	4	4	4	4
4	4	3	3	4	4	3	3	2	2
4	4	3	3	3	3	3	3	2	2
4	4	1	1	4	4	3	3	2	2
4	4	3	3	4	4	3	3	2	2
4	4	1	1	3	3	3	3	2	2
4	4	3	3	4	4	3	3	2	2
4	4	3	3	3	3	3	3	2	2
4	4	3	3	3	3	3	3	2	2
4	4	3	3	4	4	4	4	4	4
4	4	3	3	4	4	3	3	2	2
4	4	3	3	3	3	3	3	2	2
4	4	3	3	4	4	3	3	4	4
4	4	3	3	3	3	4	4	2	2
3	3	3	3	4	4	3	3	2	2
4	4	1	1	4	4	3	3	2	2
4	4	3	3	4	4	3	3	2	2
4	4	1	1	3	3	3	3	2	2
4	4	1	1	4	4	3	3	2	2
3	3	3	3	4	4	3	3	2	2
4	4	3	3	3	3	4	4	2	2
4	4	3	3	3	3	4	4	2	2
4	4	1	1	4	4	3	3	2	2
4	4	3	3	3	3	3	3	2	2
4	4	1	1	3	3	3	3	2	2
4	4	3	3	4	4	3	3	2	2
4	4	1	1	4	4	3	3	2	2
4	4	1	1	3	3	3	3	2	2
4	4	3	3	4	4	3	3	2	2
4	4	3	3	3	3	3	3	2	2
4	4	3	3	3	3	3	3	2	2
4	4	1	1	3	3	3	3	2	2
4	4	3	3	3	3	4	4	4	4
4	4	3	3	3	3	3	3	2	2
4	4	1	1	4	4	3	3	2	2
4	4	3	3	4	4	3	3	2	2
4	4	1	1	3	3	4	4	2	2
4	4	1	1	3	3	4	4	2	2
3	3	3	3	3	3	3	3	2	2
4	4	3	3	3	3	3	3	2	2
3	3	1	1	3	3	3	3	2	2
4	4	3	3	4	4	3	3	2	2
3	3	1	1	3	3	3	3	2	2
4	4	3	3	3	3	4	4	4	4
4	4	3	3	4	4	3	3	2	2
4	4	3	3	3	3	3	3	2	2
4	4	3	3	4	4	3	3	2	2
4	4	3	3	4	4	3	3	2	2
4	4	3	3	3	3	3	3	2	2
4	4	1	1	4	4	3	3	2	2

For Review Only

4	4	1	1	4	4	3	3	2	2
4	4	3	3	3	3	3	3	2	2
4	4	3	3	4	4	3	3	2	2
4	4	3	3	4	4	3	3	2	2
4	4	3	3	4	4	3	3	2	2
4	4	3	3	4	4	4	4	2	2
4	4	3	3	3	3	3	3	2	2
3	3	1	1	3	3	3	3	2	2
4	4	1	1	3	3	3	3	2	2
4	4	3	3	4	4	3	3	2	2
4	4	3	3	4	4	4	4	2	2
4	4	3	3	4	4	4	4	2	2
4	4	1	1	4	4	3	3	2	2
4	4	1	1	4	4	3	3	2	2
4	4	3	3	4	4	3	3	2	2
4	4	3	3	4	4	3	3	2	2
4	4	3	3	4	4	4	4	2	2
4	4	3	3	4	4	3	3	4	4
4	4	3	3	4	4	3	3	2	2
3	3	3	3	4	4	3	3	2	2
4	4	3	3	4	4	4	4	2	2
4	4	1	1	3	3	3	3	2	2
4	4	3	3	4	4	3	3	2	2
4	4	3	3	3	3	3	3	2	2
3	3	1	1	3	3	3	3	2	2
4	4	3	3	4	4	3	3	2	2
3	3	1	1	3	3	3	3	2	2
4	4	1	1	3	3	3	3	2	2
4	4	3	3	3	3	3	3	2	2
4	4	3	3	4	4	3	3	2	2
4	4	3	3	3	3	3	3	2	2
4	4	3	3	3	3	3	3	2	2
4	4	1	1	3	3	3	3	2	2
4	4	1	1	4	4	3	3	2	2
3	3	3	3	3	3	3	3	2	2
3	3	1	1	3	3	3	3	2	2
4	4	3	3	3	3	3	3	2	2
4	4	3	3	4	4	3	3	2	2
4	4	3	3	3	3	3	3	2	2
4	4	1	1	3	3	3	3	2	2
4	4	3	3	4	4	3	3	2	2
4	4	3	3	4	4	3	3	2	2
4	4	1	1	4	4	3	3	2	2
4	4	3	3	4	4	4	4	2	2
4	4	3	3	4	4	3	3	2	2
4	4	3	3	4	4	3	3	2	2
4	4	1	1	3	3	4	4	2	2
4	4	3	3	3	3	3	3	2	2
4	4	3	3	3	3	3	3	2	2
4	4	3	3	3	3	3	3	2	2
4	4	3	3	4	4	3	3	2	2
4	4	1	1	3	3	3	3	2	2
4	4	1	1	3	3	3	3	2	2
4	4	3	3	4	4	3	3	2	2
4	4	3	3	3	3	3	3	4	4

For Review Only

4	4	1	1	3	3	3	3	2	2
4	4	3	3	3	3	3	3	2	2
4	4	3	3	3	3	3	3	2	2
4	4	1	1	3	3	4	4	2	2
4	4	3	3	4	4	4	4	2	2
4	4	3	3	3	3	3	3	2	2
4	4	3	3	4	4	4	4	2	2
4	4	3	3	3	3	3	3	2	2
3	3	3	3	3	3	3	3	2	2
4	4	3	3	4	4	3	3	2	2
4	4	1	1	4	4	3	3	2	2
4	4	3	3	4	4	3	3	2	2
4	4	1	1	3	3	3	3	2	2
4	4	3	3	4	4	3	3	2	2
4	4	3	3	3	3	3	3	2	2
4	4	3	3	3	3	3	3	2	2
4	4	3	3	4	4	3	3	4	4
4	4	3	3	4	4	4	4	2	2
4	4	1	1	3	3	4	4	2	2
4	4	1	1	4	4	3	3	2	2
4	4	3	3	4	4	3	3	2	2
4	4	3	3	4	4	3	3	2	2
4	4	1	1	4	4	4	4	2	2
4	4	1	1	3	3	4	4	2	2
4	4	3	3	3	3	3	3	2	2
4	4	1	1	3	3	3	3	2	2
4	4	3	3	4	4	3	3	2	2
4	4	1	1	4	4	3	3	2	2
4	4	3	3	4	4	3	3	2	2
4	4	1	1	4	4	3	3	2	2
4	4	1	1	3	3	3	3	2	2
4	4	3	3	4	4	3	3	2	2
4	4	3	3	4	4	3	3	2	2
4	4	3	3	4	4	3	3	2	2
4	4	1	1	3	3	3	3	2	2
4	4	3	3	4	4	3	3	2	2
4	4	1	1	3	3	4	4	2	2
4	4	3	3	4	4	3	3	2	2
4	4	3	3	3	3	4	4	2	2
4	4	1	1	3	3	3	3	2	2
4	4	1	1	3	3	3	3	2	2
4	4	1	1	3	3	4	4	2	2
4	4	3	3	4	4	3	3	4	4
4	4	3	3	4	4	3	3	2	2
4	4	3	3	4	4	3	3	2	2
4	4	3	3	4	4	3	3	2	2
4	4	3	3	3	3	3	3	2	2
4	4	3	3	3	3	3	3	2	2
4	4	3	3	4	4	3	3	2	2
4	4	3	3	3	3	3	3	2	2
4	4	3	3	3	3	3	3	2	2
3	3	1	1	3	3	3	3	2	2
4	4	3	3	3	3	3	3	2	2
4	4	3	3	4	4	4	4	2	2
4	4	3	3	4	4	3	3	2	2

For Review Only

4	4	3	3	4	4	3	3	4	4
4	4	1	1	3	3	3	3	2	2
3	3	1	1	3	3	3	3	2	2
3	3	3	3	4	4	4	4	2	2
4	4	3	3	4	4	4	4	2	2
3	3	1	1	3	3	3	3	2	2
4	4	1	1	3	3	4	4	4	4
4	4	3	3	4	4	3	3	2	2
4	4	3	3	3	3	4	4	2	2
4	4	3	3	4	4	3	3	2	2
4	4	3	3	3	3	3	3	2	2
4	4	3	3	3	3	4	4	2	2
4	4	3	3	4	4	3	3	2	2
4	4	3	3	4	4	3	3	2	2
4	4	1	1	3	3	4	4	2	2
4	4	3	3	3	3	4	4	2	2
4	4	3	3	3	3	3	3	2	2
4	4	3	3	4	4	4	4	2	2
4	4	3	3	4	4	4	4	2	2
4	4	3	3	4	4	3	3	2	2
4	4	3	3	4	4	3	3	2	2
4	4	3	3	3	3	3	3	2	2
4	4	1	1	3	3	3	3	2	2
4	4	3	3	4	4	4	4	2	2
4	4	3	3	4	4	3	3	2	2
4	4	1	1	3	3	3	3	2	2
4	4	3	3	3	3	3	3	2	2
4	4	1	1	4	4	3	3	4	4
4	4	1	1	4	4	3	3	2	2
4	4	3	3	4	4	3	3	2	2
4	4	3	3	4	4	3	3	2	2
4	4	3	3	4	4	3	3	4	4
4	4	3	3	4	4	3	3	2	2
4	4	1	1	3	3	4	4	2	2
4	4	3	3	4	4	3	3	2	2
4	4	3	3	4	4	4	4	2	2
4	4	1	1	4	4	4	4	2	2
4	4	3	3	4	4	3	3	2	2
4	4	3	3	3	3	3	3	2	2
4	4	3	3	4	4	3	3	2	2
4	4	3	3	4	4	4	4	4	4
4	4	3	3	3	3	3	3	2	2
4	4	1	1	3	3	3	3	2	2
4	4	3	3	3	3	3	3	2	2
4	4	3	3	4	4	3	3	2	2
4	4	1	1	4	4	3	3	2	2
4	4	3	3	3	3	4	4	2	2
4	4	1	1	3	3	3	3	2	2
4	4	1	1	3	3	3	3	2	2
4	4	3	3	4	4	3	3	2	2
4	4	3	3	4	4	3	3	2	2
3	3	1	1	3	3	3	3	2	2
4	4	1	1	3	3	3	3	2	2
4	4	3	3	4	4	4	4	2	2
4	4	1	1	3	3	4	4	2	2

For Review Only

4	4	3	3	3	3	3	3	2	2
4	4	3	3	4	4	3	3	2	2
4	4	3	3	4	4	3	3	4	4
4	4	1	1	4	4	3	3	2	2
3	3	1	1	3	3	3	3	2	2
4	4	3	3	4	4	3	3	2	2
4	4	3	3	3	3	4	4	2	2
3	3	1	1	3	3	3	3	2	2
4	4	1	1	3	3	4	4	2	2
4	4	1	1	3	3	3	3	2	2
4	4	3	3	4	4	3	3	2	2
4	4	3	3	3	3	3	3	2	2
4	4	3	3	3	3	3	3	2	2
4	4	3	3	4	4	3	3	2	2
4	4	3	3	4	4	3	3	2	2
4	4	3	3	3	3	3	3	2	2
4	4	1	1	3	3	3	3	2	2
4	4	1	1	4	4	4	4	2	2
4	4	1	1	3	3	3	3	2	2
4	4	3	3	4	4	3	3	2	2
4	4	1	1	4	4	4	4	2	2
4	4	3	3	3	3	3	3	2	2
4	4	3	3	4	4	3	3	2	2
4	4	3	3	3	3	4	4	4	4
3	3	1	1	3	3	3	3	2	2
4	4	3	3	4	4	4	4	2	2
4	4	1	1	3	3	3	3	2	2
4	4	3	3	4	4	3	3	2	2
4	4	1	1	4	4	3	3	2	2
4	4	3	3	4	4	3	3	2	2
4	4	1	1	3	3	4	4	2	2
4	4	3	3	3	3	3	3	4	4
4	4	1	1	3	3	3	3	2	2
4	4	3	3	3	3	3	3	2	2
4	4	1	1	3	3	3	3	2	2
4	4	1	1	3	3	4	4	2	2
4	4	3	3	4	4	3	3	2	2
4	4	3	3	4	4	4	4	2	2
4	4	3	3	3	3	3	3	2	2
4	4	1	1	3	3	4	4	2	2
4	4	3	3	4	4	3	3	4	4
4	4	1	1	3	3	3	3	2	2
4	4	1	1	3	3	4	4	4	4
4	4	3	3	4	4	3	3	2	2
4	4	3	3	4	4	4	4	2	2
4	4	3	3	3	3	3	3	2	2
4	4	3	3	3	3	4	4	2	2
4	4	3	3	3	3	3	3	2	2
4	4	3	3	4	4	3	3	2	2
4	4	1	1	3	3	3	3	2	2
4	4	3	3	4	4	3	3	2	2
4	4	1	1	3	3	4	4	2	2
4	4	3	3	4	4	4	4	4	4
4	4	3	3	4	4	3	3	2	2
4	4	1	1	3	3	4	4	2	2

For Review Only

4	4	3	3	4	4	3	3	2	2
4	4	3	3	4	4	3	3	2	2

For Review Only

SNP2009	SNP2009	SNP201	SNP201	SNP2010	SNP2010	SNP2011	SNP2011	SNP2012	SNP2012
4	4	4	4	4	4	1	1	3	3
4	4	1	1	4	4	4	4	4	4
4	4	4	4	4	4	1	1	4	4
4	4	4	4	4	4	1	1	4	4
4	4	1	1	4	4	1	1	4	4
4	4	4	4	4	4	1	1	4	4
4	4	1	1	4	4	4	4	4	4
4	4	1	1	4	4	1	1	3	3
4	4	1	1	4	4	1	1	4	4
4	4	1	1	4	4	1	1	4	4
1	1	1	1	1	1	4	4	4	4
4	4	4	4	4	4	1	1	4	4
4	4	1	1	4	4	1	1	4	4
4	4	1	1	4	4	1	1	3	3
4	4	1	1	4	4	1	1	4	4
4	4	4	4	4	4	1	1	4	4
4	4	1	1	4	4	1	1	3	3
4	4	4	4	4	4	4	4	4	4
4	4	4	4	4	4	1	1	4	4
4	4	4	4	4	4	4	4	3	3
4	4	1	1	4	4	1	1	4	4
4	4	1	1	4	4	1	1	4	4
1	1	1	1	1	1	4	4	4	4
4	4	1	1	4	4	1	1	4	4
4	4	1	1	4	4	1	1	4	4
4	4	1	1	4	4	1	1	4	4
4	4	1	1	4	4	1	1	4	4
4	4	1	1	4	4	1	1	4	4
4	4	1	1	4	4	4	4	4	4
4	4	1	1	4	4	4	4	4	4
4	4	1	1	4	4	1	1	4	4
4	4	1	1	4	4	1	1	4	4
4	4	1	1	4	4	1	1	4	4
4	4	4	4	4	4	4	4	3	3
4	4	1	1	4	4	1	1	4	4
4	4	1	1	4	4	4	4	4	4
4	4	1	1	4	4	1	1	4	4
4	4	4	4	4	4	1	1	4	4
1	1	1	1	1	1	4	4	4	4
1	1	1	1	1	1	4	4	4	4
4	4	1	1	4	4	4	4	4	4
4	4	1	1	4	4	4	4	4	4
4	4	1	1	4	4	4	4	3	3
4	4	1	1	4	4	4	4	4	4
4	4	1	1	4	4	4	4	3	3
4	4	1	1	4	4	1	1	4	4
4	4	1	1	4	4	1	1	4	4
4	4	1	1	4	4	1	1	4	4
4	4	1	1	4	4	4	4	4	4
4	4	1	1	4	4	1	1	4	4
4	4	1	1	4	4	1	1	4	4
4	4	4	4	4	4	1	1	4	4

For Review Only

4	4	1	1	4	4	1	1	4	4
4	4	1	1	4	4	1	1	4	4
4	4	4	4	4	4	1	1	4	4
4	4	1	1	4	4	4	4	4	4
4	4	4	4	4	4	1	1	4	4
4	4	1	1	4	4	1	1	4	4
4	4	1	1	4	4	1	1	4	4
4	4	1	1	4	4	4	4	3	3
4	4	1	1	4	4	4	4	3	3
4	4	1	1	4	4	1	1	4	4
4	4	1	1	4	4	1	1	4	4
4	4	4	4	4	4	1	1	4	4
4	4	1	1	4	4	1	1	4	4
4	4	1	1	4	4	4	4	4	4
4	4	1	1	4	4	1	1	4	4
4	4	1	1	4	4	1	1	4	4
1	1	1	1	1	1	1	1	4	4
4	4	1	1	4	4	1	1	4	4
4	4	1	1	4	4	1	1	4	4
4	4	4	4	4	4	1	1	3	3
4	4	4	4	4	4	1	1	4	4
1	1	1	1	4	4	4	4	4	4
4	4	1	1	4	4	4	4	4	4
4	4	1	1	4	4	1	1	4	4
4	4	1	1	4	4	4	4	3	3
4	4	1	1	4	4	1	1	4	4
4	4	1	1	4	4	1	1	3	3
1	1	1	1	1	1	4	4	4	4
4	4	1	1	4	4	1	1	4	4
4	4	1	1	4	4	1	1	4	4
4	4	1	1	4	4	1	1	4	4
4	4	1	1	4	4	1	1	4	4
1	1	1	1	1	1	4	4	4	4
4	4	1	1	4	4	1	1	4	4
4	4	4	4	4	4	1	1	4	4
4	4	1	1	4	4	4	4	3	3
4	4	1	1	4	4	1	1	4	4
4	4	1	1	4	4	4	4	4	4
1	1	1	1	1	1	1	1	4	4
4	4	1	1	4	4	4	4	4	4
4	4	1	1	4	4	1	1	4	4
4	4	1	1	4	4	1	1	4	4
4	4	1	1	4	4	1	1	4	4
4	4	1	1	4	4	1	1	4	4
4	4	1	1	4	4	1	1	4	4
4	4	1	1	4	4	1	1	4	4
4	4	4	4	4	4	1	1	3	3
4	4	1	1	4	4	1	1	4	4
4	4	1	1	4	4	1	1	4	4
4	4	1	1	4	4	4	4	4	4
4	4	1	1	4	4	1	1	4	4
1	1	1	1	1	1	4	4	4	4
1	1	1	1	1	1	4	4	4	4
4	4	1	1	4	4	1	1	4	4
4	4	1	1	4	4	4	4	4	4

4	4	1	1	4	4	1	1	4	4
4	4	1	1	4	4	4	4	4	4
4	4	1	1	4	4	1	1	4	4
4	4	1	1	4	4	1	1	3	3
4	4	1	1	4	4	1	1	4	4
4	4	1	1	4	4	1	1	4	4
4	4	4	4	4	4	1	1	4	4
4	4	1	1	4	4	1	1	4	4
4	4	1	1	4	4	1	1	3	3
4	4	1	1	4	4	4	4	4	4
4	4	1	1	4	4	1	1	4	4
4	4	1	1	4	4	1	1	4	4
4	4	1	1	4	4	1	1	4	4
4	4	1	1	4	4	1	1	4	4
4	4	1	1	4	4	1	1	4	4
4	4	1	1	4	4	1	1	4	4
4	4	1	1	4	4	4	4	4	4
4	4	1	1	4	4	1	1	4	4
4	4	1	1	4	4	4	4	3	3
4	4	1	1	4	4	1	1	4	4
4	4	1	1	4	4	1	1	4	4
4	4	4	4	4	4	1	1	4	4
4	4	1	1	4	4	1	1	4	4
4	4	4	4	4	4	4	4	4	4
4	4	1	1	4	4	1	1	4	4
1	1	1	1	1	1	4	4	4	4
4	4	1	1	4	4	1	1	4	4
4	4	1	1	4	4	1	1	4	4
4	4	4	4	4	4	1	1	4	4
4	4	1	1	4	4	4	4	4	4
4	4	1	1	4	4	4	4	4	4
4	4	1	1	4	4	1	1	4	4
4	4	1	1	4	4	1	1	4	4
4	4	1	1	4	4	1	1	4	4
4	4	4	4	4	4	4	4	3	3
4	4	1	1	4	4	1	1	4	4
4	4	1	1	4	4	1	1	3	3
4	4	4	4	4	4	1	1	4	4
4	4	1	1	4	4	1	1	4	4
4	4	1	1	4	4	4	4	4	4
1	1	1	1	1	1	1	1	4	4
1	1	1	1	1	1	1	1	3	3
4	4	1	1	4	4	1	1	4	4
4	4	1	1	4	4	4	4	4	4
4	4	1	1	4	4	1	1	4	4
4	4	1	1	4	4	1	1	4	4
4	4	1	1	4	4	1	1	4	4
4	4	1	1	4	4	1	1	4	4
4	4	1	1	4	4	4	4	4	4
4	4	1	1	4	4	1	1	4	4
4	4	1	1	4	4	1	1	4	4
4	4	1	1	4	4	4	4	3	3
4	4	1	1	4	4	1	1	4	4
4	4	4	4	4	4	1	1	4	4
4	4	1	1	4	4	1	1	4	4

For Review Only

4	4	1	1	4	4	1	1	4	4
1	1	1	1	1	1	4	4	4	4
4	4	1	1	4	4	1	1	4	4
4	4	1	1	4	4	4	4	3	3
4	4	4	4	4	4	1	1	4	4
4	4	1	1	4	4	1	1	3	3
1	1	1	1	1	1	4	4	4	4
4	4	1	1	4	4	1	1	4	4
4	4	1	1	4	4	1	1	4	4
4	4	1	1	4	4	1	1	4	4
4	4	1	1	4	4	1	1	4	4
4	4	1	1	4	4	4	4	4	4
4	4	1	1	4	4	1	1	4	4
4	4	1	1	4	4	1	1	4	4
4	4	1	1	4	4	1	1	4	4
4	4	1	1	1	1	4	4	4	4
4	4	1	1	4	4	1	1	4	4
4	4	1	1	4	4	1	1	4	4
4	4	1	1	4	4	1	1	4	4
4	4	4	4	4	4	1	1	4	4
4	4	1	1	4	4	1	1	4	4
4	4	1	1	4	4	4	4	4	4
4	4	1	1	4	4	4	4	4	4
4	4	4	4	4	4	1	1	4	4
4	4	1	1	4	4	1	1	4	4
4	4	4	4	4	4	4	4	3	3
4	4	4	4	4	4	1	1	4	4
4	4	1	1	4	4	4	4	4	4
4	4	1	1	4	4	1	1	4	4
4	4	1	1	4	4	4	4	4	4
4	4	1	1	4	4	1	1	4	4
4	4	1	1	4	4	1	1	4	4
4	4	1	1	4	4	1	1	4	4
4	4	1	1	4	4	1	1	4	4
4	4	1	1	4	4	1	1	4	4
4	4	4	4	4	4	1	1	4	4
1	1	1	1	1	1	1	1	4	4
4	4	1	1	4	4	1	1	4	4
4	4	1	1	4	4	1	1	4	4
4	4	1	1	4	4	1	1	4	4
4	4	4	4	4	4	1	1	4	4
4	4	1	1	4	4	1	1	4	4
1	1	1	1	1	1	4	4	4	4
4	4	1	1	4	4	1	1	4	4
4	4	1	1	4	4	1	1	4	4
4	4	4	4	4	4	1	1	4	4
1	1	1	1	1	1	4	4	4	4
1	1	1	1	1	1	4	4	4	4
4	4	1	1	4	4	4	4	4	4
4	4	1	1	4	4	1	1	4	4
4	4	4	4	4	4	1	1	4	4
4	4	1	1	4	4	4	4	3	3
4	4	1	1	4	4	4	4	4	4
4	4	1	1	4	4	1	1	4	4
4	4	1	1	4	4	4	4	4	4

For Review Only

4	4	4	4	4	4	4	4	4	4
4	4	1	1	4	4	4	4	3	3
4	4	4	4	4	4	1	1	4	4
4	4	1	1	4	4	1	1	4	4
4	4	4	4	4	4	1	1	3	3
4	4	1	1	4	4	1	1	4	4
4	4	4	4	4	4	4	4	4	4
4	4	1	1	4	4	4	4	3	3
4	4	1	1	4	4	4	4	4	4
4	4	1	1	4	4	4	4	4	4
4	4	1	1	4	4	1	1	4	4
4	4	1	1	4	4	1	1	4	4
1	1	1	1	1	1	1	1	4	4
4	4	1	1	4	4	1	1	4	4
4	4	1	1	4	4	1	1	4	4
1	1	1	1	1	1	1	1	4	4
1	1	4	4	1	1	1	1	4	4
4	4	1	1	4	4	4	4	4	4
4	4	1	1	4	4	4	4	4	4
4	4	4	4	4	4	1	1	4	4
4	4	1	1	4	4	4	4	4	4
4	4	1	1	4	4	1	1	4	4
4	4	4	4	4	4	1	1	4	4
1	1	1	1	1	1	1	1	4	4
4	4	1	1	4	4	4	4	3	3
4	4	1	1	4	4	1	1	4	4
4	4	4	4	4	4	4	4	4	4
4	4	4	4	4	4	1	1	4	4
4	4	1	1	4	4	1	1	4	4
4	4	4	4	4	4	1	1	4	4
1	1	4	4	1	1	4	4	4	4
4	4	1	1	4	4	1	1	4	4
4	4	1	1	4	4	1	1	4	4
4	4	1	1	4	4	1	1	4	4
4	4	1	1	4	4	4	4	4	4
1	1	4	4	1	1	4	4	4	4
4	4	4	4	4	4	1	1	4	4
4	4	4	4	4	4	1	1	4	4
4	4	1	1	4	4	4	4	4	4
1	1	4	4	1	1	4	4	4	4
4	4	4	4	4	4	1	1	4	4
4	4	1	1	4	4	1	1	4	4
1	1	1	1	1	1	4	4	4	4
4	4	1	1	4	4	1	1	4	4
4	4	4	4	4	4	1	1	4	4
1	1	1	1	1	1	4	4	4	4
4	4	1	1	4	4	1	1	4	4
4	4	1	1	4	4	4	4	4	4
4	4	1	1	4	4	1	1	4	4
1	1	4	4	4	4	4	4	4	4
4	4	4	4	4	4	1	1	4	4
1	1	4	4	1	1	4	4	4	4
4	4	4	4	4	4	1	1	4	4
4	4	4	4	4	4	1	1	4	4
1	1	1	1	1	1	4	4	4	4

For Review Only

4	4	1	1	4	4	1	1	4	4
4	4	1	1	4	4	1	1	4	4

For Review Only

SNP2013	SNP2013	SNP2014	SNP2014	SNP2015	SNP2015	SNP2016	SNP2016	SNP2017	SNP2017
1	1	2	2	1	1	2	2	3	3
4	4	2	2	1	1	3	3	2	2
4	4	2	2	2	2	3	3	3	3
4	4	2	2	2	2	2	2	3	3
4	4	2	2	2	2	3	3	3	3
4	4	2	2	2	2	2	2	3	3
4	4	2	2	2	2	2	2	3	3
1	1	2	2	1	1	3	3	3	3
4	4	2	2	1	1	2	2	3	3
4	4	3	3	2	2	3	3	2	2
4	4	3	3	2	2	3	3	3	3
4	4	2	2	2	2	3	3	3	3
4	4	2	2	2	2	2	2	3	3
1	1	2	2	2	2	2	2	3	3
4	4	2	2	2	2	2	2	3	3
4	4	2	2	2	2	3	3	3	3
4	4	2	2	2	2	2	2	3	3
4	4	2	2	1	1	2	2	2	2
4	4	2	2	2	2	3	3	3	3
1	1	2	2	1	1	3	3	3	3
4	4	2	2	1	1	2	2	3	3
4	4	2	2	2	2	3	3	3	3
4	4	3	3	1	1	3	3	3	3
4	4	2	2	1	1	2	2	3	3
4	4	2	2	1	1	2	2	3	3
4	4	2	2	2	2	2	2	3	3
4	4	2	2	2	2	2	2	3	3
4	4	2	2	2	2	2	2	3	3
4	4	2	2	1	1	2	2	3	3
4	4	2	2	1	1	3	3	2	2
4	4	2	2	2	2	3	3	3	3
4	4	2	2	2	2	2	2	3	3
4	4	2	2	2	2	2	2	3	3
1	1	2	2	1	1	2	2	3	3
4	4	3	3	2	2	2	2	3	3
4	4	2	2	2	2	3	3	3	3
4	4	2	2	1	1	2	2	3	3
4	4	2	2	2	2	3	3	3	3
4	4	3	3	1	1	2	2	3	3
4	4	3	3	1	1	3	3	3	3
1	1	2	2	1	1	2	2	3	3
4	4	2	2	2	2	2	2	3	3
1	1	2	2	1	1	3	3	3	3
4	4	3	3	2	2	2	2	3	3
1	1	2	2	1	1	3	3	3	3
4	4	2	2	2	2	3	3	3	3
4	4	2	2	2	2	2	2	3	3
4	4	2	2	2	2	3	3	3	3
4	4	2	2	2	2	2	2	2	2
4	4	2	2	2	2	3	3	3	3
4	4	2	2	2	2	2	2	3	3
4	4	2	2	1	1	3	3	3	3

For Review Only

4	4	2	2	1	1	2	2	3	3
4	4	2	2	2	2	2	2	3	3
4	4	2	2	2	2	2	2	3	3
4	4	2	2	2	2	3	3	3	3
4	4	2	2	2	2	3	3	3	3
4	4	2	2	2	2	2	2	3	3
4	4	2	2	2	2	2	2	3	3
1	1	2	2	1	1	3	3	3	3
1	1	2	2	1	1	2	2	3	3
4	4	2	2	2	2	2	2	3	3
4	4	2	2	2	2	3	3	3	3
4	4	2	2	2	2	3	3	3	3
4	4	2	2	1	1	2	2	3	3
4	4	2	2	1	1	2	2	3	3
4	4	2	2	2	2	2	2	3	3
4	4	2	2	2	2	2	2	3	3
4	4	3	3	2	2	3	3	3	3
4	4	2	2	2	2	3	3	3	3
4	4	2	2	2	2	3	3	3	3
1	1	2	2	2	2	2	2	3	3
4	4	2	2	2	2	2	2	3	3
4	4	3	3	1	1	2	2	3	3
4	4	2	2	2	2	2	2	3	3
4	4	2	2	2	2	2	2	3	3
1	1	2	2	1	1	3	3	3	3
4	4	2	2	2	2	3	3	3	3
1	1	3	3	1	1	2	2	3	3
4	4	2	2	2	2	3	3	3	3
4	4	2	2	2	2	2	2	3	3
4	4	2	2	2	2	2	2	3	3
4	4	2	2	2	2	2	2	3	3
4	4	2	2	2	2	2	2	3	3
4	4	2	2	1	1	3	3	3	3
4	4	2	2	1	1	2	2	3	3
4	4	2	2	2	2	3	3	3	3
1	1	2	2	1	1	3	3	3	3
4	4	2	2	2	2	2	2	3	3
4	4	2	2	1	1	2	2	3	3
4	4	3	3	2	2	3	3	3	3
4	4	2	2	1	1	3	3	3	3
4	4	2	2	2	2	3	3	3	3
4	4	2	2	2	2	2	2	3	3
4	4	2	2	1	1	3	3	3	3
4	4	2	2	2	2	3	3	3	3
4	4	2	2	2	2	3	3	3	3
4	4	2	2	2	2	3	3	3	3
1	1	2	2	1	1	2	2	3	3
4	4	2	2	2	2	2	2	3	3
4	4	2	2	2	2	2	2	3	3
4	4	2	2	2	2	2	2	3	3
4	4	2	2	2	2	2	2	3	3
4	4	3	3	2	2	2	2	3	3
4	4	3	3	1	1	3	3	3	3
4	4	2	2	2	2	2	2	3	3
4	4	2	2	2	2	3	3	3	3

For Review Only

4	4	2	2	1	1	2	2	3	3
4	4	2	2	2	2	2	2	3	3
4	4	2	2	2	2	2	2	3	3
1	1	2	2	1	1	2	2	3	3
4	4	2	2	2	2	3	3	3	3
4	4	2	2	2	2	3	3	3	3
4	4	2	2	2	2	3	3	3	3
4	4	2	2	2	2	2	2	3	3
4	4	2	2	2	2	2	2	3	3
4	4	2	2	2	2	2	2	3	3
4	4	2	2	1	1	2	2	3	3
4	4	2	2	2	2	2	2	3	3
4	4	2	2	1	1	3	3	3	3
4	4	2	2	2	2	2	2	3	3
4	4	2	2	2	2	2	2	3	3
4	4	2	2	2	2	2	2	3	3
1	1	2	2	2	2	3	3	3	3
4	4	3	3	2	2	2	2	3	3
1	1	2	2	1	1	2	2	3	3
4	4	2	2	1	1	3	3	3	3
4	4	2	2	2	2	2	2	3	3
4	4	2	2	2	2	2	2	3	3
4	4	2	2	1	1	3	3	3	3
4	4	2	2	1	1	3	3	3	3
4	4	2	2	1	1	2	2	3	3
4	4	3	3	1	1	2	2	3	3
4	4	2	2	2	2	3	3	3	3
4	4	2	2	1	1	2	2	3	3
4	4	2	2	2	2	3	3	3	3
4	4	2	2	2	2	3	3	3	3
4	4	3	3	1	1	2	2	3	3
4	4	2	2	2	2	3	3	3	3
4	4	2	2	2	2	3	3	3	3
4	4	2	2	2	2	2	2	3	3
1	1	2	2	1	1	2	2	3	3
4	4	2	2	1	1	2	2	3	3
1	1	2	2	1	1	2	2	3	3
4	4	2	2	2	2	3	3	3	3
4	4	2	2	2	2	2	2	3	3
4	4	3	3	1	1	3	3	3	3
4	4	3	3	1	1	2	2	3	3
1	1	3	3	1	1	3	3	3	3
4	4	2	2	2	2	3	3	3	3
4	4	2	2	1	1	2	2	3	3
4	4	2	2	2	2	2	2	3	3
4	4	2	2	1	1	3	3	3	3
4	4	2	2	2	2	2	2	3	3
4	4	2	2	2	2	2	2	3	3
4	4	2	2	2	2	2	2	3	3
4	4	2	2	2	2	2	2	3	3
4	4	2	2	2	2	2	2	3	3
1	1	2	2	1	1	3	3	3	3
4	4	2	2	2	2	2	2	3	3
4	4	2	2	2	2	3	3	3	3
4	4	2	2	2	2	3	3	3	3

For Review Only

4	4	2	2	2	2	3	3	3	3
4	4	3	3	1	1	2	2	3	3
4	4	2	2	1	1	3	3	2	2
1	1	3	3	2	2	2	2	3	3
4	4	2	2	2	2	2	2	3	3
1	1	2	2	1	1	2	2	3	3
4	4	3	3	1	1	2	2	3	3
4	4	2	2	2	2	2	2	3	3
4	4	3	3	2	2	3	3	3	3
4	4	2	2	2	2	2	2	3	3
4	4	2	2	2	2	2	2	3	3
4	4	3	3	1	1	2	2	3	3
4	4	2	2	2	2	2	2	3	3
4	4	2	2	1	1	3	3	3	3
4	4	2	2	1	1	3	3	3	3
4	4	3	3	2	2	3	3	3	3
4	4	2	2	2	2	2	2	3	3
4	4	2	2	2	2	2	2	3	3
4	4	2	2	2	2	2	2	3	3
4	4	2	2	2	2	3	3	3	3
4	4	2	2	2	2	2	2	3	3
4	4	2	2	1	1	2	2	3	3
4	4	2	2	1	1	2	2	3	3
4	4	2	2	2	2	3	3	3	3
4	4	3	3	2	2	2	2	3	3
1	1	2	2	1	1	2	2	3	3
4	4	2	2	2	2	3	3	3	3
4	4	2	2	1	1	2	2	3	3
4	4	2	2	1	1	3	3	3	3
4	4	2	2	2	2	3	3	3	3
4	4	2	2	2	2	2	2	3	3
4	4	2	2	2	2	3	3	3	3
4	4	2	2	2	2	3	3	3	3
4	4	2	2	1	1	2	2	3	3
4	4	2	2	2	2	2	2	3	3
4	4	2	2	2	2	3	3	3	3
4	4	3	3	2	2	2	2	3	3
4	4	2	2	2	2	2	2	3	3
4	4	2	2	2	2	2	2	3	3
4	4	2	2	2	2	3	3	3	3
4	4	2	2	2	2	3	3	3	3
4	4	2	2	2	2	2	2	3	3
4	4	3	3	2	2	2	2	3	3
4	4	2	2	2	2	2	2	3	3
4	4	2	2	2	2	3	3	3	3
4	4	2	2	2	2	2	2	3	3
4	4	3	3	2	2	3	3	3	3
4	4	2	2	1	1	2	2	3	3
4	4	2	2	1	1	2	2	3	3
4	4	2	2	2	2	3	3	3	3
4	4	2	2	2	2	3	3	3	3
1	1	2	2	1	1	3	3	3	3
4	4	2	2	1	1	2	2	3	3
4	4	2	2	2	2	3	3	3	3
4	4	2	2	1	1	3	3	3	3

For Review Only

4	4	2	2	2	2	3	3	3	3
1	1	2	2	2	2	2	2	3	3
4	4	2	2	2	2	3	3	3	3
4	4	2	2	1	1	2	2	3	3
1	1	2	2	1	1	3	3	3	3
4	4	2	2	2	2	2	2	3	3
4	4	2	2	2	2	2	2	3	3
1	1	2	2	1	1	3	3	2	2
4	4	2	2	2	2	2	2	2	2
4	4	2	2	2	2	3	3	3	3
4	4	2	2	2	2	2	2	3	3
4	4	2	2	2	2	2	2	3	3
4	4	3	3	2	2	3	3	3	3
4	4	2	2	2	2	2	2	3	3
4	4	2	2	2	2	2	2	3	3
4	4	3	3	2	2	3	3	3	3
4	4	2	2	1	1	3	3	3	3
4	4	2	2	1	1	3	3	3	3
4	4	2	2	1	1	2	2	3	3
4	4	2	2	2	2	3	3	3	3
4	4	2	2	1	1	2	2	3	3
4	4	2	2	2	2	2	2	3	3
4	4	2	2	2	2	3	3	3	3
4	4	3	3	2	2	3	3	3	3
1	1	2	2	1	1	3	3	3	3
4	4	3	3	2	2	2	2	3	3
4	4	2	2	1	1	3	3	3	3
4	4	2	2	2	2	3	3	3	3
4	4	2	2	1	1	2	2	3	3
4	4	2	2	2	2	3	3	3	3
4	4	2	2	1	1	3	3	3	3
4	4	3	3	2	2	3	3	2	2
4	4	2	2	1	1	3	3	3	3
4	4	2	2	2	2	2	2	3	3
4	4	2	2	1	1	2	2	3	3
4	4	2	2	1	1	2	2	3	3
4	4	2	2	2	2	3	3	3	3
4	4	2	2	2	2	3	3	3	3
4	4	2	2	2	2	3	3	3	3
4	4	3	3	1	1	2	2	3	3
4	4	2	2	2	2	3	3	3	3
4	4	2	2	2	2	3	3	3	3
4	4	3	3	1	1	3	3	3	3
4	4	2	2	2	2	3	3	3	3
4	4	2	2	2	2	3	3	3	3
4	4	3	3	2	2	3	3	3	3
4	4	2	2	2	2	2	2	3	3
4	4	2	2	2	2	3	3	3	3
4	4	2	2	2	2	2	2	2	2
4	4	2	2	1	1	3	3	3	3
4	4	2	2	2	2	3	3	3	3
4	4	2	2	1	1	3	3	3	3
4	4	2	2	2	2	3	3	3	3
4	4	2	2	2	2	3	3	3	3
4	4	3	3	1	1	2	2	3	3

For Review Only

4	4	2	2	2	2	3	3	3	3
4	4	2	2	2	2	2	2	3	3

For Review Only

SNP2018	SNP2018	SNP2019	SNP2019	SNP202	SNP202	SNP2020	SNP2020	SNP2021	SNP2021
1	1	2	2	4	4	4	4	2	2
3	3	2	2	3	3	3	3	2	2
3	3	2	2	3	3	4	4	2	2
3	3	2	2	4	4	4	4	2	2
3	3	1	1	4	4	4	4	2	2
3	3	2	2	4	4	4	4	2	2
3	3	2	2	3	3	4	4	2	2
1	1	2	2	3	3	4	4	2	2
3	3	2	2	4	4	4	4	2	2
3	3	2	2	3	3	3	3	4	4
3	3	1	1	4	4	4	4	2	2
3	3	2	2	3	3	4	4	2	2
3	3	2	2	3	3	4	4	4	4
1	1	2	2	3	3	4	4	2	2
3	3	2	2	4	4	4	4	2	2
3	3	2	2	4	4	4	4	2	2
1	1	2	2	3	3	3	3	2	2
3	3	2	2	4	4	4	4	2	2
1	1	2	2	3	3	4	4	2	2
1	1	2	2	3	3	3	3	4	4
3	3	2	2	3	3	3	3	2	2
3	3	1	1	3	3	4	4	2	2
3	3	2	2	3	3	4	4	4	4
3	3	2	2	3	3	4	4	2	2
3	3	2	2	4	4	4	4	2	2
3	3	2	2	3	3	4	4	2	2
3	3	2	2	3	3	4	4	2	2
3	3	2	2	4	4	4	4	2	2
3	3	2	2	3	3	4	4	2	2
3	3	2	2	3	3	3	3	4	4
3	3	2	2	3	3	4	4	2	2
3	3	2	2	3	3	4	4	2	2
3	3	2	2	3	3	4	4	2	2
1	1	2	2	3	3	4	4	2	2
3	3	2	2	4	4	4	4	2	2
3	3	2	2	3	3	3	3	2	2
3	3	2	2	4	4	3	3	2	2
3	3	2	2	3	3	3	3	2	2
3	3	2	2	3	3	4	4	4	4
3	3	2	2	3	3	4	4	4	4
1	1	2	2	3	3	3	3	4	4
3	3	2	2	3	3	4	4	2	2
1	1	2	2	3	3	3	3	4	4
3	3	2	2	4	4	3	3	4	4
1	1	2	2	3	3	3	3	4	4
3	3	2	2	3	3	4	4	2	2
3	3	2	2	3	3	4	4	2	2
3	3	1	1	3	3	4	4	2	2
3	3	2	2	4	4	3	3	4	4
3	3	2	2	4	4	4	4	2	2
3	3	2	2	3	3	4	4	2	2
3	3	2	2	3	3	4	4	2	2

For Review Only

3	3	2	2	4	4	4	4	2	2
3	3	2	2	3	3	4	4	2	2
1	1	2	2	4	4	4	4	2	2
3	3	2	2	3	3	3	3	2	2
1	1	2	2	3	3	4	4	2	2
3	3	2	2	3	3	4	4	2	2
3	3	2	2	3	3	4	4	2	2
1	1	2	2	3	3	3	3	4	4
3	3	2	2	4	4	4	4	2	2
3	3	2	2	4	4	4	4	2	2
3	3	2	2	3	3	4	4	2	2
3	3	2	2	3	3	4	4	2	2
3	3	2	2	4	4	4	4	2	2
3	3	2	2	3	3	4	4	2	2
3	3	2	2	4	4	4	4	2	2
3	3	2	2	4	4	4	4	2	2
3	3	1	1	4	4	4	4	2	2
3	3	2	2	3	3	3	3	2	2
3	3	1	1	4	4	4	4	2	2
1	1	2	2	4	4	4	4	2	2
3	3	2	2	3	3	4	4	2	2
3	3	2	2	4	4	4	4	4	4
3	3	2	2	4	4	4	4	2	2
3	3	2	2	3	3	4	4	2	2
1	1	2	2	3	3	3	3	4	4
3	3	2	2	3	3	4	4	2	2
1	1	2	2	3	3	3	3	4	4
3	3	2	2	3	3	4	4	4	4
3	3	2	2	3	3	4	4	2	2
3	3	2	2	3	3	4	4	2	2
3	3	2	2	4	4	4	4	2	2
3	3	2	2	3	3	3	3	2	2
1	1	2	2	3	3	3	3	4	4
3	3	2	2	4	4	4	4	2	2
3	3	2	2	4	4	4	4	2	2
1	1	2	2	3	3	3	3	4	4
3	3	2	2	3	3	4	4	2	2
3	3	2	2	4	4	3	3	2	2
3	3	1	1	3	3	3	3	4	4
3	3	2	2	4	4	3	3	4	4
3	3	1	1	3	3	4	4	2	2
3	3	2	2	3	3	4	4	2	2
3	3	2	2	4	4	3	3	2	2
3	3	2	2	3	3	4	4	2	2
3	3	1	1	4	4	4	4	2	2
3	3	2	2	3	3	4	4	2	2
1	1	2	2	3	3	4	4	2	2
3	3	2	2	3	3	4	4	2	2
3	3	2	2	3	3	4	4	2	2
1	1	2	2	4	4	4	4	4	4
3	3	2	2	3	3	3	3	2	2
3	3	2	2	3	3	4	4	4	4
3	3	2	2	4	4	3	3	4	4
3	3	2	2	4	4	4	4	4	4
3	3	2	2	3	3	4	4	2	2

For Review Only

3	3	2	2	3	3	4	4	4	4
3	3	2	2	4	4	4	4	2	2
3	3	2	2	3	3	3	3	2	2
1	1	2	2	3	3	4	4	2	2
3	3	2	2	3	3	4	4	2	2
3	3	2	2	3	3	3	3	2	2
3	3	2	2	4	4	4	4	2	2
3	3	2	2	3	3	3	3	2	2
1	1	2	2	3	3	4	4	2	2
3	3	2	2	3	3	4	4	2	2
3	3	2	2	4	4	4	4	2	2
3	3	2	2	4	4	4	4	2	2
3	3	2	2	4	4	3	3	2	2
3	3	2	2	4	4	4	4	2	2
3	3	2	2	3	3	4	4	4	4
3	3	2	2	3	3	4	4	2	2
3	3	2	2	4	4	3	3	2	2
3	3	2	2	4	4	4	4	2	2
1	1	2	2	3	3	4	4	2	2
3	3	1	1	3	3	3	3	2	2
3	3	2	2	4	4	4	4	4	4
3	3	2	2	3	3	4	4	2	2
3	3	2	2	3	3	4	4	2	2
1	1	2	2	3	3	4	4	4	4
3	3	2	2	3	3	4	4	4	4
3	3	2	2	4	4	4	4	4	4
3	3	2	2	4	4	4	4	2	2
3	3	2	2	3	3	3	3	2	2
3	3	2	2	3	3	4	4	2	2
3	3	2	2	3	3	3	3	2	2
3	3	2	2	4	4	3	3	4	4
3	3	2	2	4	4	4	4	2	2
3	3	1	1	3	3	4	4	2	2
3	3	2	2	4	4	4	4	2	2
1	1	2	2	3	3	3	3	2	2
3	3	2	2	4	4	4	4	2	2
1	1	2	2	3	3	4	4	2	2
3	3	2	2	4	4	4	4	2	2
3	3	2	2	4	4	4	4	4	4
1	1	1	1	4	4	3	3	4	4
3	3	2	2	3	3	4	4	2	2
1	1	2	2	4	4	4	4	2	2
3	3	2	2	3	3	3	3	2	2
3	3	2	2	4	4	4	4	4	4
3	3	2	2	4	4	4	4	2	2
3	3	2	2	3	3	4	4	2	2
3	3	2	2	3	3	4	4	2	2
3	3	2	2	3	3	4	4	2	2
3	3	2	2	4	4	4	4	2	2
3	3	2	2	3	3	4	4	2	2
3	3	2	2	3	3	4	4	2	2
1	1	2	2	4	4	3	3	2	2
3	3	2	2	3	3	4	4	2	2
3	3	2	2	3	3	4	4	2	2
3	3	2	2	4	4	4	4	4	4

For Review Only

3	3	2	2	3	3	4	4	2	2
3	3	2	2	4	4	3	3	2	2
3	3	2	2	4	4	4	4	2	2
1	1	2	2	4	4	4	4	2	2
3	3	2	2	3	3	4	4	2	2
1	1	2	2	3	3	3	3	2	2
3	3	2	2	4	4	4	4	2	2
3	3	2	2	4	4	4	4	4	4
3	3	1	1	3	3	4	4	4	4
3	3	2	2	3	3	3	3	2	2
3	3	2	2	4	4	4	4	2	2
3	3	2	2	3	3	4	4	2	2
3	3	2	2	4	4	4	4	2	2
3	3	2	2	3	3	4	4	2	2
3	3	2	2	3	3	4	4	2	2
3	3	1	1	4	4	3	3	2	2
3	3	2	2	4	4	4	4	2	2
3	3	2	2	3	3	4	4	2	2
3	3	2	2	3	3	3	3	2	2
3	3	2	2	4	4	4	4	2	2
3	3	2	2	4	4	4	4	2	2
3	3	2	2	3	3	4	4	4	4
3	3	2	2	4	4	4	4	4	4
3	3	2	2	3	3	4	4	2	2
3	3	2	2	4	4	3	3	4	4
1	1	2	2	4	4	4	4	2	2
3	3	2	2	3	3	4	4	2	2
3	3	2	2	3	3	4	4	2	2
3	3	2	2	3	3	4	4	2	2
3	3	2	2	4	4	3	3	2	2
3	3	2	2	4	4	4	4	2	2
3	3	2	2	3	3	4	4	2	2
3	3	2	2	4	4	4	4	2	2
3	3	2	2	3	3	4	4	2	2
3	3	2	2	3	3	4	4	2	2
3	3	2	2	3	3	4	4	2	2
3	3	2	2	4	4	4	4	4	4
3	3	2	2	4	4	4	4	2	2
3	3	2	2	3	3	4	4	2	2
1	1	2	2	3	3	4	4	2	2
3	3	2	2	3	3	4	4	2	2
3	3	2	2	3	3	4	4	2	2
1	1	2	2	3	3	3	3	4	4
3	3	2	2	4	4	4	4	4	4
3	3	2	2	4	4	4	4	2	2
3	3	2	2	3	3	3	3	2	2
3	3	1	1	4	4	3	3	2	2
1	1	2	2	3	3	4	4	2	2
3	3	2	2	3	3	4	4	2	2
3	3	2	2	3	3	4	4	2	2
3	3	2	2	3	3	3	3	2	2
1	1	2	2	4	4	3	3	4	4
3	3	2	2	4	4	4	4	4	4
3	3	2	2	3	3	4	4	2	2
3	3	2	2	3	3	4	4	4	4

For Review Only

3	3	2	2	3	3	3	3	2	2
1	1	2	2	3	3	4	4	2	2
3	3	2	2	3	3	4	4	2	2
3	3	2	2	4	4	4	4	4	4
1	1	2	2	3	3	4	4	2	2
3	3	2	2	4	4	3	3	2	2
3	3	2	2	3	3	3	3	2	2
3	3	2	2	3	3	3	3	2	2
3	3	2	2	3	3	4	4	2	2
1	1	2	2	3	3	4	4	4	4
3	3	2	2	4	4	4	4	2	2
3	3	2	2	4	4	3	3	2	2
3	3	1	1	3	3	4	4	4	4
3	3	2	2	3	3	4	4	2	2
3	3	2	2	3	3	4	4	2	2
3	3	1	1	4	4	4	4	4	4
3	3	2	2	3	3	4	4	4	4
3	3	2	2	4	4	3	3	4	4
1	1	2	2	3	3	4	4	2	2
3	3	1	1	4	4	3	3	2	2
3	3	2	2	3	3	4	4	2	2
3	3	2	2	3	3	4	4	2	2
3	3	2	2	4	4	4	4	2	2
3	3	2	2	4	4	4	4	2	2
1	1	2	2	3	3	3	3	4	4
3	3	2	2	3	3	4	4	2	2
1	1	2	2	3	3	4	4	4	4
3	3	2	2	3	3	4	4	2	2
3	3	2	2	4	4	4	4	2	2
3	3	2	2	3	3	4	4	2	2
1	1	2	2	3	3	4	4	4	4
3	3	2	2	3	3	3	3	2	2
3	3	1	1	4	4	4	4	2	2
3	3	2	2	4	4	4	4	4	4
3	3	2	2	4	4	4	4	4	4
1	1	2	2	3	3	4	4	4	4
3	3	2	2	3	3	3	3	2	2
3	3	2	2	3	3	4	4	2	2
3	3	2	2	3	3	3	3	2	2
3	3	2	2	3	3	4	4	4	4
3	3	2	2	3	3	3	3	2	2
3	3	2	2	3	3	3	3	2	2
3	3	2	2	4	4	4	4	2	2
3	3	1	1	4	4	4	4	2	2
3	3	2	2	3	3	4	4	2	2
3	3	1	1	3	3	3	3	2	2
3	3	2	2	3	3	4	4	4	4
3	3	2	2	3	3	4	4	2	2
3	3	2	2	3	3	4	4	4	4
1	1	2	2	3	3	3	3	4	4
3	3	2	2	4	4	4	4	2	2
3	3	2	2	3	3	4	4	4	4
3	3	2	2	3	3	4	4	2	2
3	3	2	2	4	4	4	4	2	2
3	3	2	2	3	3	4	4	4	4

For Review Only

3	3	1	1	4	4	4	4	2	2
3	3	2	2	3	3	3	3	2	2

For Review Only

SNP2022	SNP2022	SNP2023	SNP2023	SNP2024	SNP2024	SNP2025	SNP2025	SNP2026	SNP2026
2	2	4	4	1	1	2	2	1	1
3	3	4	4	3	3	2	2	1	1
2	2	4	4	3	3	2	2	1	1
2	2	4	4	1	1	2	2	1	1
2	2	4	4	3	3	1	1	2	2
3	3	4	4	1	1	2	2	1	1
2	2	4	4	1	1	2	2	1	1
3	3	4	4	3	3	1	1	2	2
2	2	4	4	1	1	2	2	1	1
2	2	4	4	3	3	2	2	1	1
3	3	4	4	3	3	2	2	1	1
2	2	4	4	1	1	2	2	1	1
2	2	4	4	1	1	2	2	1	1
2	2	4	4	3	3	1	1	2	2
2	2	4	4	1	1	2	2	1	1
2	2	4	4	3	3	2	2	1	1
2	2	4	4	1	1	1	1	1	1
2	2	4	4	1	1	2	2	1	1
2	2	4	4	1	1	1	1	2	2
3	3	3	3	3	3	2	2	1	1
3	3	4	4	1	1	2	2	1	1
2	2	4	4	1	1	2	2	1	1
3	3	3	3	3	3	2	2	1	1
2	2	4	4	3	3	2	2	1	1
2	2	4	4	1	1	2	2	1	1
2	2	4	4	3	3	2	2	1	1
2	2	4	4	1	1	2	2	1	1
2	2	4	4	1	1	2	2	1	1
2	2	4	4	1	1	2	2	1	1
3	3	3	3	3	3	2	2	1	1
2	2	4	4	1	1	2	2	1	1
2	2	4	4	1	1	2	2	1	1
2	2	4	4	1	1	1	1	2	2
2	2	4	4	3	3	1	1	2	2
2	2	4	4	3	3	2	2	1	1
2	2	4	4	3	3	2	2	1	1
2	2	4	4	3	3	2	2	1	1
2	2	4	4	1	1	2	2	1	1
3	3	4	4	3	3	2	2	1	1
3	3	3	3	3	3	2	2	1	1
2	2	3	3	3	3	2	2	1	1
3	3	4	4	3	3	2	2	1	1
2	2	4	4	1	1	1	1	2	2
2	2	3	3	1	1	2	2	1	1
2	2	3	3	1	1	2	2	1	1
2	2	4	4	3	3	2	2	1	1
2	2	4	4	1	1	2	2	1	1
3	3	4	4	3	3	1	1	2	2
2	2	3	3	1	1	2	2	1	1
2	2	4	4	1	1	2	2	1	1
2	2	4	4	3	3	2	2	1	1
3	3	4	4	1	1	2	2	1	1

For Review Only

2	2	4	4	1	1	1	1	2	2
2	2	4	4	1	1	1	1	2	2
2	2	4	4	1	1	2	2	1	1
2	2	4	4	1	1	2	2	1	1
2	2	4	4	1	1	1	1	2	2
2	2	4	4	1	1	2	2	1	1
2	2	4	4	3	3	2	2	1	1
3	3	3	3	3	3	1	1	1	1
3	3	4	4	3	3	1	1	2	2
2	2	4	4	1	1	2	2	1	1
2	2	4	4	3	3	2	2	1	1
2	2	4	4	1	1	2	2	1	1
2	2	4	4	1	1	1	1	2	2
2	2	4	4	1	1	2	2	1	1
3	3	4	4	1	1	2	2	1	1
2	2	4	4	1	1	2	2	1	1
2	2	4	4	3	3	2	2	1	1
2	2	4	4	1	1	2	2	1	1
2	2	4	4	1	1	2	2	1	1
2	2	4	4	1	1	1	1	2	2
2	2	4	4	1	1	2	2	1	1
3	3	4	4	3	3	2	2	1	1
3	3	4	4	1	1	2	2	1	1
2	2	4	4	3	3	2	2	1	1
2	2	3	3	1	1	2	2	1	1
2	2	4	4	3	3	2	2	1	1
2	2	3	3	3	3	1	1	2	2
3	3	4	4	3	3	2	2	1	1
2	2	4	4	3	3	2	2	1	1
2	2	4	4	1	1	2	2	1	1
2	2	4	4	3	3	2	2	1	1
2	2	4	4	3	3	2	2	1	1
3	3	3	3	3	3	1	1	2	2
2	2	4	4	1	1	1	1	2	2
2	2	3	3	1	1	2	2	1	1
2	2	3	3	1	1	2	2	1	1
2	2	4	4	3	3	2	2	1	1
3	3	4	4	1	1	1	1	2	2
2	2	3	3	3	3	2	2	1	1
2	2	3	3	3	3	2	2	1	1
2	2	4	4	1	1	2	2	1	1
2	2	4	4	1	1	2	2	1	1
2	2	4	4	3	3	2	2	1	1
2	2	4	4	3	3	2	2	1	1
2	2	4	4	1	1	2	2	1	1
3	3	4	4	3	3	2	2	1	1
2	2	4	4	3	3	1	1	2	2
2	2	4	4	3	3	2	2	1	1
2	2	4	4	3	3	2	2	1	1
2	2	3	3	1	1	1	1	2	2
2	2	4	4	3	3	2	2	1	1
3	3	4	4	3	3	2	2	1	1
3	3	3	3	3	3	2	2	1	1
3	3	4	4	1	1	2	2	1	1
2	2	4	4	1	1	2	2	1	1

For Review Only

3	3	4	4	3	3	2	2	1	1
3	3	4	4	3	3	2	2	1	1
2	2	4	4	3	3	2	2	1	1
2	2	3	3	3	3	1	1	2	2
2	2	4	4	1	1	2	2	1	1
2	2	4	4	3	3	2	2	1	1
2	2	4	4	1	1	2	2	1	1
2	2	4	4	1	1	2	2	1	1
2	2	4	4	3	3	1	1	2	2
2	2	4	4	3	3	2	2	1	1
2	2	4	4	1	1	1	1	2	2
3	3	4	4	1	1	2	2	1	1
2	2	4	4	1	1	2	2	1	1
2	2	4	4	1	1	2	2	1	1
2	2	4	4	3	3	2	2	1	1
2	2	4	4	3	3	2	2	1	1
2	2	4	4	1	1	2	2	1	1
2	2	4	4	1	1	2	2	1	1
2	2	4	4	3	3	1	1	2	2
3	3	4	4	1	1	2	2	1	1
2	2	4	4	1	1	2	2	1	1
2	2	4	4	1	1	2	2	1	1
2	2	4	4	1	1	2	2	1	1
3	3	4	4	3	3	2	2	1	1
3	3	4	4	3	3	2	2	1	1
3	3	3	3	3	3	2	2	1	1
2	2	4	4	1	1	2	2	1	1
2	2	4	4	1	1	2	2	1	1
2	2	4	4	1	1	2	2	1	1
2	2	4	4	1	1	2	2	1	1
3	3	4	4	3	3	2	2	1	1
3	3	4	4	1	1	2	2	1	1
2	2	4	4	1	1	2	2	1	1
2	2	4	4	3	3	2	2	1	1
2	2	4	4	3	3	1	1	2	2
2	2	4	4	1	1	2	2	1	1
3	3	4	4	3	3	1	1	2	2
2	2	4	4	3	3	2	2	1	1
3	3	4	4	3	3	2	2	1	1
3	3	4	4	3	3	2	2	1	1
3	3	4	4	3	3	2	2	1	1
3	3	4	4	3	3	1	1	1	1
2	2	4	4	1	1	2	2	1	1
3	3	3	3	3	3	2	2	1	1
2	2	4	4	3	3	2	2	1	1
2	2	4	4	1	1	2	2	1	1
2	2	4	4	3	3	2	2	1	1
2	2	4	4	3	3	2	2	1	1
2	2	4	4	1	1	2	2	1	1
2	2	4	4	3	3	2	2	1	1
2	2	4	4	3	3	2	2	1	1
3	3	4	4	3	3	1	1	2	2
2	2	4	4	1	1	2	2	1	1
2	2	4	4	1	1	2	2	1	1
3	3	4	4	1	1	2	2	1	1

For Review Only

2	2	4	4	1	1	2	2	1	1
3	3	4	4	3	3	2	2	1	1
2	2	4	4	3	3	2	2	1	1
3	3	4	4	1	1	1	1	2	2
2	2	4	4	3	3	2	2	1	1
3	3	4	4	3	3	1	1	2	2
3	3	4	4	3	3	2	2	1	1
2	2	4	4	1	1	2	2	1	1
3	3	4	4	3	3	2	2	1	1
2	2	4	4	1	1	2	2	1	1
3	3	4	4	3	3	2	2	1	1
3	3	4	4	3	3	2	2	1	1
2	2	4	4	1	1	2	2	1	1
2	2	4	4	1	1	2	2	1	1
2	2	4	4	3	3	2	2	1	1
3	3	4	4	3	3	2	2	1	1
2	2	4	4	1	1	2	2	1	1
2	2	4	4	3	3	2	2	1	1
2	2	4	4	3	3	2	2	1	1
2	2	4	4	1	1	2	2	1	1
3	3	4	4	1	1	2	2	1	1
3	3	4	4	3	3	2	2	1	1
3	3	3	3	3	3	2	2	1	1
2	2	4	4	1	1	2	2	1	1
3	3	4	4	1	1	2	2	1	1
2	2	4	4	3	3	1	1	2	2
2	2	4	4	1	1	2	2	1	1
2	2	4	4	1	1	2	2	1	1
3	3	4	4	1	1	2	2	1	1
2	2	4	4	1	1	2	2	1	1
2	2	4	4	1	1	2	2	1	1
2	2	4	4	1	1	2	2	1	1
2	2	4	4	1	1	2	2	1	1
2	2	4	4	3	3	2	2	1	1
2	2	4	4	1	1	2	2	1	1
2	2	4	4	1	1	2	2	1	1
3	3	3	3	3	3	2	2	1	1
2	2	4	4	1	1	2	2	1	1
2	2	4	4	3	3	2	2	1	1
2	2	4	4	1	1	1	1	2	2
2	2	4	4	3	3	2	2	1	1
2	2	4	4	3	3	2	2	1	1
3	3	3	3	3	3	1	1	2	2
2	2	4	4	1	1	2	2	1	1
2	2	4	4	3	3	2	2	1	1
3	3	4	4	1	1	2	2	1	1
3	3	4	4	3	3	2	2	1	1
3	3	4	4	3	3	1	1	2	2
3	3	4	4	3	3	2	2	1	1
2	2	4	4	1	1	2	2	1	1
2	2	4	4	1	1	2	2	1	1
3	3	3	3	3	3	1	1	2	2
3	3	3	3	3	3	2	2	1	1
2	2	4	4	3	3	2	2	1	1
3	3	4	4	3	3	2	2	1	1

For Review Only

2	2	4	4	3	3	2	2	1	1
2	2	4	4	1	1	2	2	1	1
2	2	4	4	1	1	2	2	1	1
3	3	4	4	1	1	2	2	1	1
3	3	4	4	3	3	2	2	1	1
2	2	4	4	1	1	2	2	1	1
3	3	4	4	3	3	2	2	1	1
3	3	4	4	3	3	2	2	1	1
3	3	4	4	3	3	2	2	1	1
2	2	3	3	3	3	2	2	1	1
2	2	4	4	1	1	2	2	1	1
2	2	4	4	3	3	2	2	1	1
2	2	3	3	3	3	2	2	1	1
2	2	4	4	3	3	2	2	1	1
2	2	4	4	3	3	2	2	1	1
3	3	3	3	3	3	2	2	1	1
3	3	3	3	3	3	2	2	1	1
2	2	3	3	1	1	2	2	1	1
3	3	4	4	3	3	1	1	2	2
2	2	4	4	1	1	2	2	1	1
3	3	4	4	3	3	2	2	1	1
2	2	4	4	3	3	2	2	1	1
3	3	4	4	1	1	2	2	1	1
2	2	4	4	3	3	2	2	1	1
3	3	3	3	3	3	1	1	1	1
2	2	4	4	1	1	2	2	1	1
3	3	3	3	3	3	2	2	1	1
2	2	4	4	1	1	2	2	1	1
3	3	4	4	1	1	2	2	1	1
2	2	4	4	1	1	2	2	1	1
3	3	4	4	3	3	1	1	2	2
3	3	4	4	3	3	1	1	1	1
2	2	4	4	3	3	2	2	1	1
2	2	4	4	3	3	2	2	1	1
3	3	3	3	3	3	2	2	1	1
3	3	3	3	3	3	2	2	1	1
2	2	4	4	1	1	2	2	1	1
2	2	4	4	1	1	2	2	1	1
2	2	4	4	3	3	2	2	1	1
3	3	3	3	3	3	2	2	1	1
2	2	4	4	1	1	2	2	1	1
2	2	4	4	1	1	2	2	1	1
3	3	4	4	3	3	2	2	1	1
2	2	4	4	1	1	2	2	1	1
2	2	4	4	1	1	2	2	1	1
2	2	4	4	3	3	2	2	1	1
2	2	4	4	3	3	2	2	1	1
2	2	4	4	3	3	1	1	2	2
2	2	3	3	3	3	2	2	1	1
3	3	3	3	3	3	2	2	1	1
2	2	4	4	1	1	2	2	1	1
3	3	3	3	3	3	2	2	1	1
2	2	4	4	3	3	2	2	1	1
2	2	4	4	1	1	2	2	1	1
3	3	3	3	3	3	2	2	1	1

For Review Only

3	3	4	4	1	1	2	2	1	1
2	2	4	4	3	3	2	2	1	1

For Review Only

SNP2027	SNP2027	SNP2028	SNP2028	SNP2029	SNP2029	SNP203	SNP203	SNP2030	SNP2030
2	2	2	2	3	3	1	1	3	3
1	1	1	1	3	3	3	3	1	1
1	1	1	1	3	3	3	3	1	1
1	1	1	1	3	3	3	3	3	3
2	2	2	2	3	3	1	1	1	1
1	1	1	1	3	3	1	1	3	3
1	1	1	1	3	3	1	1	3	3
2	2	2	2	3	3	1	1	1	1
1	1	1	1	3	3	1	1	3	3
1	1	1	1	3	3	3	3	1	1
1	1	1	1	3	3	1	1	1	1
1	1	1	1	3	3	1	1	1	1
2	2	1	1	2	2	1	1	3	3
2	2	2	2	3	3	3	3	1	1
1	1	1	1	3	3	1	1	3	3
1	1	1	1	3	3	3	3	1	1
2	2	1	1	2	2	3	3	3	3
1	1	1	1	3	3	1	1	3	3
2	2	2	2	3	3	1	1	3	3
1	1	1	1	2	2	3	3	1	1
1	1	1	1	3	3	1	1	3	3
1	1	1	1	2	2	1	1	3	3
1	1	1	1	3	3	1	1	1	1
1	1	1	1	3	3	3	3	1	1
1	1	1	1	3	3	1	1	3	3
1	1	1	1	3	3	1	1	1	1
1	1	1	1	3	3	1	1	3	3
1	1	1	1	3	3	1	1	3	3
1	1	1	1	3	3	1	1	3	3
1	1	1	1	3	3	3	3	1	1
1	1	1	1	3	3	1	1	1	1
1	1	1	1	3	3	1	1	3	3
2	2	2	2	3	3	1	1	3	3
2	2	2	2	2	2	1	1	1	1
1	1	1	1	3	3	1	1	1	1
1	1	1	1	3	3	1	1	1	1
1	1	1	1	3	3	3	3	1	1
1	1	1	1	3	3	1	1	3	3
1	1	1	1	3	3	3	3	1	1
1	1	1	1	3	3	1	1	1	1
1	1	1	1	3	3	3	3	1	1
1	1	1	1	3	3	3	3	1	1
2	2	2	2	3	3	1	1	3	3
1	1	1	1	3	3	3	3	3	3
1	1	1	1	3	3	3	3	3	3
1	1	1	1	3	3	3	3	3	3
1	1	1	1	3	3	3	3	1	1
2	2	2	2	3	3	1	1	1	1
2	2	1	1	3	3	1	1	3	3
1	1	1	1	3	3	1	1	3	3
1	1	1	1	3	3	1	1	1	1
1	1	1	1	3	3	1	1	3	3

For Review Only

2	2	2	2	3	3	1	1	3	3
2	2	2	2	3	3	1	1	3	3
1	1	1	1	3	3	1	1	1	1
1	1	1	1	3	3	1	1	3	3
2	2	2	2	3	3	1	1	3	3
1	1	1	1	3	3	1	1	1	1
1	1	1	1	3	3	1	1	1	1
2	2	1	1	3	3	3	3	1	1
2	2	2	2	3	3	1	1	1	1
1	1	1	1	3	3	1	1	3	3
1	1	1	1	3	3	1	1	1	1
1	1	1	1	3	3	1	1	1	1
2	2	2	2	3	3	1	1	3	3
1	1	1	1	3	3	1	1	3	3
1	1	1	1	3	3	1	1	3	3
1	1	1	1	3	3	1	1	3	3
1	1	1	1	3	3	3	3	1	1
1	1	1	1	3	3	1	1	1	1
1	1	1	1	3	3	1	1	3	3
2	2	2	2	3	3	1	1	3	3
1	1	1	1	3	3	1	1	1	1
1	1	1	1	3	3	1	1	1	1
1	1	1	1	3	3	1	1	3	3
1	1	1	1	3	3	1	1	1	1
1	1	1	1	3	3	3	3	3	3
1	1	1	1	3	3	1	1	1	1
2	2	2	2	3	3	3	3	1	1
1	1	1	1	3	3	1	1	1	1
1	1	1	1	3	3	3	3	1	1
1	1	1	1	3	3	1	1	3	3
1	1	1	1	3	3	1	1	1	1
1	1	1	1	3	3	1	1	1	1
2	2	2	2	3	3	3	3	1	1
2	2	2	2	3	3	1	1	3	3
1	1	1	1	3	3	1	1	3	3
1	1	1	1	3	3	3	3	3	3
1	1	1	1	3	3	1	1	1	1
2	2	2	2	2	2	1	1	3	3
1	1	1	1	3	3	1	1	1	1
1	1	1	1	3	3	1	1	1	1
1	1	1	1	3	3	1	1	3	3
1	1	1	1	3	3	1	1	1	1
1	1	1	1	3	3	1	1	1	1
1	1	1	1	3	3	1	1	1	1
1	1	1	1	3	3	1	1	3	3
1	1	1	1	3	3	1	1	1	1
2	2	2	2	3	3	3	3	1	1
1	1	1	1	3	3	1	1	1	1
1	1	1	1	3	3	1	1	1	1
2	2	2	2	3	3	1	1	3	3
1	1	1	1	3	3	3	3	1	1
1	1	1	1	3	3	1	1	1	1
1	1	1	1	3	3	1	1	1	1
2	2	1	1	3	3	1	1	3	3
1	1	1	1	3	3	1	1	3	3

For Review Only

1	1	1	1	3	3	1	1	1	1
1	1	1	1	3	3	3	3	3	3
1	1	1	1	3	3	1	1	1	1
2	2	2	2	3	3	3	3	1	1
1	1	1	1	3	3	1	1	1	1
1	1	1	1	3	3	1	1	1	1
1	1	1	1	3	3	3	3	1	1
1	1	1	1	3	3	3	3	1	1
2	2	2	2	3	3	1	1	1	1
2	2	1	1	3	3	1	1	1	1
2	2	2	2	3	3	1	1	3	3
1	1	1	1	3	3	1	1	3	3
1	1	1	1	3	3	1	1	1	1
1	1	1	1	3	3	1	1	3	3
2	2	1	1	3	3	1	1	1	1
1	1	1	1	3	3	1	1	1	1
2	2	1	1	3	3	1	1	3	3
1	1	1	1	3	3	1	1	1	1
2	2	2	2	3	3	3	3	1	1
1	1	1	1	3	3	1	1	1	1
1	1	1	1	3	3	1	1	1	1
1	1	1	1	3	3	1	1	3	3
1	1	1	1	3	3	3	3	1	1
1	1	1	1	3	3	3	3	1	1
1	1	1	1	3	3	1	1	1	1
1	1	1	1	3	3	1	1	1	1
1	1	1	1	3	3	1	1	3	3
1	1	1	1	3	3	1	1	3	3
1	1	1	1	3	3	1	1	1	1
1	1	1	1	3	3	1	1	1	1
1	1	1	1	3	3	1	1	1	1
1	1	1	1	3	3	1	1	3	3
1	1	1	1	3	3	1	1	3	3
1	1	1	1	3	3	1	1	1	1
2	2	2	2	2	2	1	1	1	1
1	1	1	1	3	3	1	1	3	3
2	2	2	2	3	3	1	1	1	1
1	1	1	1	3	3	1	1	1	1
1	1	1	1	3	3	1	1	1	1
1	1	1	1	3	3	3	3	1	1
1	1	1	1	3	3	1	1	1	1
2	2	1	1	3	3	3	3	1	1
1	1	1	1	3	3	1	1	1	1
2	2	1	1	3	3	1	1	3	3
1	1	1	1	3	3	1	1	1	1
1	1	1	1	3	3	1	1	3	3
1	1	1	1	3	3	1	1	1	1
1	1	1	1	3	3	1	1	1	1
2	2	1	1	3	3	3	3	3	3
1	1	1	1	3	3	1	1	1	1
1	1	1	1	3	3	1	1	1	1
2	2	2	2	2	2	3	3	1	1
1	1	1	1	3	3	1	1	1	1
1	1	1	1	3	3	1	1	1	1
1	1	1	1	3	3	1	1	3	3

For Review Only

1	1	1	1	3	3	1	1	1	1
1	1	1	1	3	3	1	1	1	1
1	1	1	1	3	3	3	3	1	1
2	2	2	2	3	3	1	1	3	3
1	1	1	1	3	3	1	1	1	1
2	2	2	2	3	3	3	3	1	1
2	2	1	1	3	3	1	1	1	1
1	1	1	1	3	3	1	1	1	1
2	2	1	1	3	3	1	1	1	1
1	1	1	1	3	3	1	1	1	1
1	1	1	1	3	3	1	1	3	3
1	1	1	1	3	3	1	1	1	1
1	1	1	1	3	3	1	1	3	3
1	1	1	1	3	3	1	1	3	3
1	1	1	1	3	3	1	1	1	1
1	1	1	1	3	3	1	1	1	1
1	1	1	1	3	3	1	1	1	1
1	1	1	1	3	3	1	1	3	3
1	1	1	1	3	3	1	1	1	1
1	1	1	1	3	3	3	3	1	1
1	1	1	1	3	3	1	1	3	3
2	2	1	1	3	3	1	1	1	1
1	1	1	1	3	3	1	1	1	1
1	1	1	1	3	3	1	1	1	1
1	1	1	1	3	3	1	1	1	1
2	2	2	2	2	2	3	3	1	1
1	1	1	1	3	3	1	1	3	3
2	2	1	1	3	3	1	1	3	3
1	1	1	1	3	3	1	1	3	3
1	1	1	1	3	3	1	1	3	3
1	1	1	1	3	3	1	1	3	3
1	1	1	1	3	3	1	1	3	3
1	1	1	1	3	3	1	1	3	3
2	2	1	1	3	3	1	1	1	1
2	2	1	1	3	3	1	1	3	3
1	1	1	1	3	3	1	1	1	1
1	1	1	1	3	3	1	1	1	1
1	1	1	1	3	3	3	3	3	3
1	1	1	1	3	3	1	1	1	1
2	2	2	2	3	3	1	1	3	3
1	1	1	1	3	3	3	3	1	1
1	1	1	1	3	3	1	1	1	1
2	2	2	2	3	3	3	3	1	1
1	1	1	1	3	3	1	1	1	1
2	2	1	1	3	3	3	3	1	1
2	2	1	1	3	3	1	1	3	3
1	1	1	1	3	3	1	1	1	1
2	2	2	2	3	3	1	1	1	1
1	1	1	1	3	3	3	3	1	1
1	1	1	1	3	3	1	1	1	1
1	1	1	1	3	3	1	1	1	1
2	2	2	2	3	3	1	1	1	1
1	1	1	1	3	3	1	1	1	1
1	1	1	1	3	3	1	1	1	1
1	1	1	1	3	3	3	3	1	1

For Review Only

1	1	1	1	3	3	1	1	1	1
2	2	2	2	3	3	3	3	1	1
1	1	1	1	3	3	1	1	1	1
1	1	1	1	3	3	1	1	1	1
1	1	1	1	3	3	1	1	1	1
1	1	1	1	3	3	1	1	3	3
2	2	1	1	3	3	1	1	1	1
1	1	1	1	2	2	3	3	1	1
1	1	1	1	3	3	1	1	1	1
1	1	1	1	3	3	3	3	1	1
1	1	1	1	3	3	1	1	3	3
1	1	1	1	3	3	1	1	1	1
1	1	1	1	3	3	1	1	1	1
1	1	1	1	3	3	1	1	1	1
1	1	1	1	3	3	1	1	1	1
1	1	1	1	3	3	1	1	1	1
1	1	1	1	3	3	3	3	1	1
2	2	1	1	3	3	3	3	3	3
2	2	2	2	3	3	1	1	1	1
1	1	1	1	3	3	1	1	3	3
1	1	1	1	3	3	1	1	1	1
1	1	1	1	3	3	1	1	1	1
1	1	1	1	3	3	1	1	3	3
1	1	1	1	3	3	3	3	1	1
2	2	2	2	3	3	3	3	1	1
1	1	1	1	3	3	1	1	1	1
1	1	1	1	3	3	3	3	1	1
1	1	1	1	3	3	1	1	1	1
2	2	1	1	3	3	1	1	3	3
1	1	1	1	3	3	1	1	1	1
2	2	2	2	3	3	1	1	1	1
2	2	1	1	3	3	1	1	1	1
1	1	1	1	3	3	3	3	1	1
1	1	1	1	3	3	1	1	1	1
1	1	1	1	3	3	1	1	1	1
1	1	1	1	3	3	3	3	1	1
1	1	1	1	3	3	1	1	1	1
1	1	1	1	3	3	1	1	1	1
1	1	1	1	3	3	1	1	1	1
1	1	1	1	3	3	3	3	1	1
1	1	1	1	3	3	1	1	3	3
1	1	1	1	3	3	3	3	3	3
1	1	1	1	3	3	1	1	1	1
1	1	1	1	3	3	1	1	3	3
1	1	1	1	3	3	3	3	3	3
1	1	1	1	3	3	1	1	3	3
1	1	1	1	3	3	1	1	1	1
2	2	2	2	3	3	1	1	1	1
1	1	1	1	3	3	3	3	1	1
1	1	1	1	3	3	1	1	1	1
1	1	1	1	3	3	1	1	3	3
1	1	1	1	3	3	1	1	1	1
1	1	1	1	3	3	3	3	1	1
1	1	1	1	3	3	1	1	1	1
1	1	1	1	3	3	1	1	1	1

For Review Only

1	1	1	1	3	3	1	1	3	3
1	1	1	1	3	3	1	1	1	1

For Review Only

SNP2031	SNP2031	SNP2032	SNP2032	SNP2033	SNP2033	SNP2034	SNP2034	SNP2035	SNP2035
2	2	1	1	3	3	3	3	1	1
2	2	1	1	3	3	2	2	1	1
2	2	1	1	3	3	3	3	1	1
2	2	1	1	3	3	3	3	1	1
2	2	1	1	3	3	3	3	1	1
2	2	1	1	3	3	3	3	1	1
2	2	4	4	1	1	3	3	4	4
2	2	4	4	3	3	3	3	1	1
2	2	1	1	1	1	3	3	1	1
2	2	4	4	1	1	2	2	4	4
3	3	1	1	3	3	3	3	1	1
2	2	1	1	3	3	3	3	1	1
2	2	1	1	3	3	3	3	1	1
2	2	1	1	3	3	3	3	1	1
2	2	1	1	3	3	3	3	1	1
2	2	1	1	3	3	3	3	1	1
2	2	1	1	3	3	3	3	1	1
2	2	4	4	3	3	3	3	1	1
2	2	1	1	3	3	3	3	1	1
2	2	4	4	1	1	2	2	4	4
2	2	1	1	3	3	3	3	1	1
2	2	1	1	3	3	3	3	1	1
3	3	1	1	3	3	2	2	1	1
2	2	1	1	3	3	3	3	1	1
2	2	4	4	1	1	3	3	4	4
2	2	1	1	3	3	3	3	1	1
2	2	1	1	3	3	3	3	1	1
2	2	1	1	3	3	3	3	1	1
2	2	4	4	1	1	3	3	4	4
2	2	4	4	3	3	3	3	1	1
2	2	1	1	3	3	3	3	1	1
2	2	1	1	3	3	3	3	1	1
2	2	1	1	3	3	3	3	1	1
2	2	1	1	3	3	3	3	1	1
2	2	1	1	3	3	3	3	1	1
2	2	1	1	3	3	3	3	1	1
2	2	1	1	3	3	3	3	1	1
2	2	1	1	3	3	2	2	1	1
3	3	1	1	3	3	3	3	1	1
3	3	1	1	3	3	3	3	1	1
2	2	4	4	3	3	3	3	1	1
2	2	4	4	1	1	3	3	4	4
2	2	4	4	1	1	3	3	4	4
2	2	4	4	1	1	3	3	4	4
2	2	4	4	1	1	2	2	4	4
2	2	1	1	3	3	3	3	1	1
2	2	1	1	3	3	3	3	1	1
2	2	1	1	3	3	3	3	1	1
2	2	4	4	3	3	3	3	1	1
2	2	1	1	3	3	3	3	1	1
2	2	1	1	3	3	3	3	1	1
2	2	1	1	3	3	3	3	1	1

For Review Only

2	2	1	1	3	3	3	3	1	1
2	2	1	1	3	3	3	3	1	1
2	2	1	1	3	3	3	3	1	1
2	2	4	4	3	3	3	3	1	1
2	2	1	1	3	3	3	3	1	1
2	2	1	1	3	3	3	3	1	1
2	2	1	1	3	3	3	3	1	1
2	2	4	4	3	3	3	3	1	1
2	2	4	4	3	3	3	3	4	4
2	2	1	1	3	3	3	3	1	1
2	2	1	1	3	3	3	3	1	1
2	2	1	1	3	3	3	3	1	1
2	2	1	1	3	3	3	3	1	1
2	2	4	4	1	1	3	3	4	4
2	2	1	1	3	3	3	3	1	1
2	2	1	1	3	3	3	3	1	1
2	2	1	1	3	3	3	3	1	1
2	2	1	1	3	3	3	3	1	1
2	2	1	1	3	3	3	3	1	1
2	2	1	1	3	3	3	3	1	1
2	2	1	1	3	3	3	3	1	1
3	3	1	1	3	3	3	3	1	1
2	2	4	4	1	1	3	3	1	1
2	2	1	1	3	3	3	3	1	1
2	2	4	4	1	1	2	2	4	4
2	2	1	1	3	3	3	3	1	1
2	2	1	1	3	3	3	3	1	1
2	2	4	4	3	3	3	3	1	1
2	2	1	1	3	3	3	3	1	1
2	2	1	1	3	3	3	3	1	1
2	2	1	1	3	3	2	2	1	1
2	2	1	1	3	3	3	3	1	1
2	2	4	4	3	3	3	3	1	1
2	2	1	1	3	3	3	3	1	1
2	2	1	1	3	3	3	3	1	1
2	2	4	4	1	1	2	2	4	4
2	2	4	4	1	1	2	2	4	4
2	2	4	4	3	3	3	3	1	1
2	2	1	1	3	3	3	3	1	1
2	2	4	4	1	1	3	3	4	4
2	2	1	1	3	3	3	3	1	1
2	2	1	1	3	3	3	3	1	1
2	2	1	1	3	3	2	2	1	1
2	2	1	1	3	3	3	3	1	1
2	2	1	1	3	3	3	3	1	1
2	2	1	1	3	3	3	3	1	1
2	2	1	1	3	3	3	3	1	1
2	2	1	1	3	3	3	3	1	1
2	2	1	1	3	3	3	3	1	1
2	2	1	1	3	3	3	3	1	1
2	2	1	1	3	3	3	3	1	1
3	3	1	1	3	3	3	3	1	1
2	2	4	4	3	3	3	3	1	1
2	2	1	1	3	3	3	3	1	1
2	2	1	1	3	3	2	2	1	1

For Review Only

2	2	1	1	3	3	3	3	1	1
3	3	1	1	3	3	3	3	1	1
2	2	1	1	3	3	3	3	1	1
2	2	1	1	3	3	3	3	1	1
2	2	1	1	3	3	3	3	1	1
2	2	1	1	3	3	3	3	1	1
2	2	1	1	3	3	3	3	1	1
2	2	1	1	3	3	3	3	1	1
2	2	1	1	3	3	3	3	1	1
2	2	1	1	3	3	3	3	1	1
2	2	1	1	3	3	3	3	1	1
2	2	1	1	3	3	3	3	1	1
2	2	1	1	3	3	3	3	1	1
2	2	1	1	3	3	3	3	1	1
2	2	1	1	3	3	3	3	1	1
2	2	1	1	3	3	3	3	1	1
2	2	4	4	3	3	3	3	1	1
2	2	1	1	3	3	3	3	1	1
2	2	1	1	3	3	3	3	1	1
2	2	1	1	3	3	2	2	1	1
2	2	1	1	3	3	3	3	1	1
2	2	1	1	3	3	3	3	1	1
2	2	1	1	3	3	3	3	1	1
2	2	4	4	3	3	3	3	1	1
3	3	1	1	3	3	3	3	1	1
3	3	1	1	3	3	3	3	1	1
2	2	1	1	3	3	3	3	1	1
2	2	1	1	3	3	3	3	1	1
2	2	1	1	3	3	3	3	1	1
2	2	1	1	3	3	3	3	1	1
2	2	4	4	3	3	3	3	1	1
2	2	1	1	3	3	3	3	1	1
2	2	1	1	3	3	3	3	1	1
2	2	1	1	3	3	3	3	1	1
2	2	1	1	3	3	3	3	1	1
2	2	1	1	3	3	3	3	1	1
2	2	4	4	3	3	3	3	4	4
2	2	1	1	3	3	3	3	1	1
2	2	1	1	3	3	3	3	1	1
2	2	1	1	3	3	3	3	1	1
2	2	1	1	3	3	3	3	1	1
2	2	4	4	3	3	3	3	1	1
2	2	1	1	3	3	3	3	1	1
2	2	4	4	3	3	3	3	1	1
2	2	1	1	3	3	3	3	1	1
2	2	1	1	3	3	3	3	1	1
2	2	1	1	3	3	3	3	1	1
2	2	1	1	3	3	3	3	1	1
2	2	1	1	3	3	3	3	1	1
2	2	1	1	3	3	3	3	1	1
2	2	1	1	3	3	3	3	1	1
2	2	4	4	3	3	3	3	1	1
2	2	1	1	3	3	3	3	1	1
2	2	1	1	3	3	3	3	1	1
2	2	4	4	3	3	3	3	1	1

For Review Only

2	2	1	1	3	3	3	3	1	1
3	3	1	1	3	3	2	2	1	1
2	2	1	1	3	3	3	3	1	1
2	2	1	1	3	3	3	3	1	1
2	2	1	1	3	3	3	3	1	1
2	2	1	1	3	3	3	3	1	1
2	2	1	1	3	3	3	3	1	1
2	2	1	1	3	3	3	3	1	1
2	2	1	1	3	3	3	3	1	1
2	2	1	1	3	3	3	3	1	1
2	2	1	1	3	3	3	3	1	1
2	2	1	1	3	3	3	3	1	1
2	2	1	1	3	3	3	3	1	1
2	2	1	1	3	3	3	3	1	1
2	2	4	4	3	3	3	3	4	4
3	3	1	1	3	3	3	3	1	1
2	2	1	1	3	3	3	3	1	1
2	2	1	1	3	3	3	3	1	1
2	2	1	1	3	3	3	3	1	1
2	2	1	1	3	3	3	3	1	1
2	2	1	1	3	3	3	3	1	1
2	2	1	1	3	3	2	2	1	1
3	3	1	1	3	3	3	3	1	1
2	2	1	1	3	3	3	3	1	1
2	2	1	1	3	3	3	3	1	1
2	2	1	1	3	3	3	3	1	1
2	2	1	1	3	3	3	3	1	1
2	2	1	1	3	3	3	3	1	1
2	2	1	1	3	3	3	3	1	1
2	2	1	1	3	3	3	3	1	1
2	2	4	4	3	3	3	3	4	4
2	2	1	1	3	3	3	3	1	1
2	2	1	1	3	3	3	3	1	1
2	2	4	4	3	3	3	3	4	4
2	2	1	1	3	3	3	3	1	1
2	2	1	1	3	3	3	3	1	1
3	3	1	1	3	3	3	3	1	1
2	2	1	1	3	3	3	3	1	1
2	2	1	1	3	3	3	3	1	1
2	2	1	1	3	3	3	3	1	1
2	2	1	1	3	3	3	3	1	1
2	2	1	1	3	3	3	3	1	1
2	2	4	4	3	3	3	3	1	1
2	2	1	1	3	3	3	3	1	1
2	2	1	1	3	3	3	3	1	1
2	2	1	1	3	3	3	3	1	1
3	3	1	1	3	3	3	3	1	1
2	2	4	4	3	3	2	2	1	1
2	2	1	1	3	3	3	3	1	1
2	2	1	1	3	3	3	3	1	1
2	2	1	1	3	3	3	3	1	1
2	2	4	4	3	3	3	3	1	1
3	3	1	1	3	3	3	3	1	1
2	2	1	1	3	3	3	3	1	1
3	3	1	1	3	3	3	3	1	1

For Review Only

2	2	4	4	3	3	3	3	1	1
2	2	4	4	3	3	3	3	1	1
2	2	1	1	3	3	3	3	1	1
2	2	1	1	3	3	3	3	1	1
2	2	4	4	1	1	3	3	4	4
2	2	1	1	3	3	3	3	1	1
3	3	1	1	3	3	3	3	1	1
2	2	4	4	3	3	3	3	1	1
2	2	1	1	3	3	3	3	1	1
3	3	1	1	3	3	3	3	1	1
2	2	1	1	3	3	3	3	1	1
2	2	4	4	3	3	3	3	4	4
2	2	1	1	3	3	3	3	1	1
2	2	1	1	3	3	3	3	1	1
2	2	1	1	3	3	3	3	1	1
2	2	1	1	3	3	3	3	1	1
2	2	1	1	3	3	3	3	1	1
2	2	4	4	3	3	3	3	1	1
3	3	1	1	3	3	2	2	1	1
2	2	1	1	3	3	2	2	1	1
3	3	1	1	3	3	3	3	1	1
2	2	1	1	3	3	3	3	1	1
2	2	1	1	3	3	3	3	1	1
2	2	1	1	3	3	3	3	1	1
2	2	4	4	3	3	3	3	1	1
2	2	1	1	3	3	3	3	1	1
2	2	4	4	3	3	3	3	1	1
2	2	1	1	3	3	3	3	1	1
2	2	1	1	3	3	3	3	1	1
2	2	1	1	3	3	3	3	1	1
2	2	4	4	3	3	2	2	1	1
2	2	1	1	3	3	3	3	1	1
2	2	1	1	3	3	2	2	1	1
2	2	1	1	3	3	3	3	1	1
3	3	1	1	3	3	3	3	1	1
2	2	4	4	3	3	3	3	1	1
2	2	1	1	3	3	3	3	1	1
2	2	1	1	3	3	3	3	1	1
2	2	1	1	3	3	3	3	1	1
3	3	1	1	3	3	3	3	1	1
2	2	1	1	3	3	3	3	1	1
2	2	1	1	3	3	3	3	1	1
3	3	1	1	3	3	3	3	1	1
2	2	1	1	3	3	3	3	1	1
2	2	1	1	3	3	3	3	1	1
3	3	1	1	3	3	3	3	1	1
2	2	1	1	3	3	3	3	1	1
2	2	1	1	3	3	3	3	1	1
2	2	1	1	3	3	3	3	1	1
2	2	4	4	3	3	3	3	1	1
2	2	1	1	3	3	3	3	1	1
3	3	1	1	3	3	3	3	1	1
2	2	1	1	3	3	3	3	1	1
2	2	1	1	3	3	3	3	1	1
2	2	1	1	3	3	3	3	1	1

For Review Only

2	2	1	1	3	3	3	3	1	1
2	2	1	1	3	3	3	3	1	1

For Review Only

SNP2036	SNP2036	SNP2037	SNP2037	SNP2038	SNP2038	SNP2039	SNP2039	SNP204	SNP204
4	4	3	3	3	3	2	2	3	3
4	4	3	3	3	3	2	2	3	3
4	4	3	3	3	3	2	2	3	3
4	4	3	3	3	3	2	2	3	3
4	4	3	3	3	3	1	1	3	3
4	4	3	3	3	3	1	1	3	3
4	4	2	2	3	3	2	2	3	3
4	4	3	3	2	2	2	2	2	2
4	4	3	3	3	3	2	2	3	3
1	1	2	2	2	2	2	2	2	2
4	4	3	3	3	3	2	2	3	3
4	4	3	3	3	3	2	2	3	3
4	4	3	3	3	3	2	2	2	2
4	4	3	3	3	3	2	2	3	3
4	4	3	3	3	3	2	2	3	3
4	4	3	3	3	3	1	1	3	3
4	4	3	3	3	3	2	2	2	2
4	4	3	3	3	3	2	2	3	3
4	4	3	3	3	3	1	1	3	3
4	4	2	2	2	2	2	2	3	3
4	4	3	3	3	3	2	2	2	2
4	4	3	3	3	3	2	2	2	2
4	4	3	3	3	3	2	2	3	3
4	4	3	3	3	3	2	2	3	3
4	4	2	2	3	3	1	1	3	3
4	4	3	3	3	3	2	2	3	3
4	4	3	3	3	3	2	2	3	3
4	4	3	3	3	3	1	1	3	3
4	4	2	2	3	3	2	2	3	3
4	4	3	3	2	2	2	2	2	2
4	4	3	3	3	3	1	1	3	3
4	4	3	3	3	3	2	2	2	2
4	4	3	3	3	3	1	1	3	3
4	4	3	3	3	3	2	2	3	3
4	4	3	3	3	3	2	2	3	3
4	4	3	3	3	3	2	2	3	3
4	4	3	3	2	2	2	2	3	3
1	1	3	3	2	2	2	2	3	3
4	4	3	3	3	3	2	2	3	3
4	4	3	3	2	2	2	2	3	3
4	4	3	3	3	3	2	2	3	3
4	4	2	2	3	3	2	2	3	3
4	4	2	2	3	3	2	2	2	2
4	4	3	3	3	3	2	2	3	3
1	1	2	2	2	2	2	2	2	2
4	4	3	3	3	3	2	2	3	3
4	4	3	3	3	3	2	2	3	3
4	4	3	3	3	3	2	2	3	3
4	4	3	3	3	3	2	2	3	3
4	4	3	3	3	3	2	2	3	3
4	4	3	3	3	3	2	2	3	3
4	4	3	3	3	3	2	2	3	3

For Review Only

4	4	3	3	3	3	1	1	3	3
4	4	3	3	3	3	1	1	3	3
4	4	3	3	3	3	1	1	3	3
4	4	3	3	3	3	1	1	2	2
4	4	3	3	3	3	1	1	3	3
4	4	3	3	3	3	2	2	3	3
4	4	3	3	3	3	2	2	3	3
4	4	3	3	3	3	2	2	3	3
4	4	3	3	3	3	1	1	3	3
4	4	3	3	3	3	2	2	3	3
4	4	3	3	3	3	2	2	3	3
4	4	3	3	3	3	2	2	3	3
4	4	3	3	3	3	1	1	3	3
4	4	2	2	3	3	2	2	3	3
4	4	3	3	3	3	1	1	3	3
4	4	3	3	2	2	1	1	3	3
4	4	3	3	3	3	2	2	3	3
4	4	3	3	3	3	2	2	3	3
4	4	3	3	3	3	1	1	3	3
4	4	3	3	3	3	2	2	3	3
4	4	3	3	3	3	2	2	3	3
4	4	3	3	3	3	2	2	3	3
4	4	2	2	3	3	2	2	3	3
4	4	3	3	3	3	2	2	2	2
1	1	2	2	2	2	2	2	2	2
4	4	3	3	3	3	1	1	3	3
4	4	3	3	3	3	2	2	3	3
4	4	3	3	3	3	2	2	3	3
4	4	3	3	3	3	2	2	3	3
4	4	3	3	2	2	2	2	3	3
1	1	3	3	2	2	2	2	3	3
4	4	3	3	3	3	2	2	3	3
4	4	3	3	3	3	2	2	3	3
4	4	3	3	3	3	1	1	3	3
4	4	3	3	3	3	1	1	3	3
1	1	2	2	2	2	2	2	2	2
1	1	2	2	2	2	2	2	3	3
4	4	3	3	3	3	2	2	3	3
4	4	3	3	3	3	2	2	2	2
4	4	2	2	2	2	2	2	3	3
4	4	3	3	3	3	2	2	3	3
4	4	3	3	2	2	2	2	3	3
1	1	3	3	3	3	2	2	3	3
4	4	3	3	3	3	2	2	3	3
4	4	3	3	3	3	1	1	3	3
4	4	3	3	3	3	2	2	3	3
4	4	3	3	2	2	2	2	3	3
4	4	3	3	3	3	2	2	3	3
4	4	3	3	3	3	2	2	3	3
4	4	3	3	3	3	2	2	3	3
4	4	3	3	3	3	2	2	3	3
4	4	3	3	3	3	2	2	3	3
4	4	3	3	3	3	2	2	3	3
4	4	3	3	3	3	1	1	3	3
1	1	3	3	2	2	1	1	3	3

For Review Only

4	4	3	3	3	3	2	2	3	3
4	4	3	3	3	3	1	1	3	3
4	4	3	3	3	3	1	1	3	3
4	4	3	3	3	3	2	2	3	3
4	4	3	3	3	3	2	2	3	3
4	4	3	3	3	3	2	2	3	3
4	4	3	3	3	3	1	1	3	3
4	4	3	3	3	3	1	1	3	3
4	4	3	3	3	3	2	2	3	3
4	4	3	3	3	3	2	2	3	3
4	4	3	3	3	3	2	2	3	3
1	1	3	3	3	3	2	2	3	3
4	4	3	3	3	3	2	2	3	3
4	4	3	3	3	3	2	2	3	3
4	4	3	3	3	3	2	2	3	3
4	4	3	3	3	3	2	2	3	3
4	4	3	3	3	3	2	2	3	3
4	4	3	3	3	3	2	2	3	3
4	4	3	3	3	3	2	2	3	3
1	1	3	3	2	2	1	1	3	3
4	4	3	3	3	3	2	2	3	3
4	4	3	3	3	3	2	2	3	3
4	4	3	3	3	3	2	2	3	3
4	4	3	3	3	3	2	2	3	3
4	4	3	3	3	3	2	2	3	3
4	4	3	3	3	3	2	2	3	3
4	4	3	3	3	3	1	1	3	3
4	4	3	3	3	3	1	1	3	3
4	4	3	3	3	3	2	2	3	3
4	4	3	3	3	3	2	2	3	3
4	4	3	3	3	3	2	2	3	3
4	4	3	3	3	3	2	2	2	2
4	4	3	3	3	3	1	1	3	3
4	4	3	3	3	3	1	1	3	3
4	4	3	3	3	3	2	2	3	3
4	4	3	3	3	3	2	2	3	3
4	4	3	3	3	3	2	2	3	3
4	4	3	3	2	2	2	2	3	3
4	4	3	3	3	3	2	2	3	3
4	4	3	3	3	3	2	2	3	3
4	4	3	3	3	3	2	2	3	3
4	4	3	3	3	3	2	2	3	3
4	4	3	3	3	3	1	1	3	3
1	1	3	3	3	3	2	2	3	3
4	4	3	3	3	3	2	2	3	3
4	4	3	3	3	3	2	2	3	3
4	4	3	3	3	3	2	2	3	3
4	4	3	3	3	3	2	2	3	3
4	4	3	3	3	3	2	2	3	3
4	4	3	3	3	3	2	2	3	3
4	4	3	3	3	3	2	2	3	3
4	4	3	3	3	3	2	2	3	3
4	4	3	3	3	3	2	2	3	3
4	4	3	3	3	3	2	2	3	3
4	4	3	3	3	3	2	2	3	3

For Review Only

4	4	3	3	3	3	2	2	3	3
1	1	3	3	2	2	2	2	3	3
4	4	3	3	3	3	2	2	3	3
4	4	3	3	3	3	2	2	3	3
4	4	3	3	3	3	1	1	3	3
4	4	3	3	2	2	2	2	3	3
4	4	3	3	3	3	2	2	3	3
4	4	3	3	3	3	2	2	3	3
4	4	3	3	3	3	2	2	3	3
4	4	3	3	3	3	1	1	3	3
4	4	3	3	3	3	2	2	3	3
4	4	3	3	2	2	2	2	3	3
4	4	3	3	3	3	2	2	3	3
4	4	3	3	3	3	2	2	3	3
4	4	3	3	3	3	2	2	3	3
4	4	3	3	3	3	2	2	3	3
4	4	3	3	3	3	2	2	3	3
4	4	3	3	3	3	2	2	3	3
4	4	3	3	3	3	2	2	3	3
4	4	3	3	3	3	2	2	3	3
4	4	3	3	3	3	2	2	3	3
4	4	3	3	3	3	2	2	3	3
4	4	3	3	3	3	2	2	3	3
4	4	3	3	3	3	2	2	3	3
4	4	3	3	3	3	2	2	3	3
4	4	3	3	3	3	2	2	3	3
4	4	3	3	3	3	2	2	3	3
4	4	3	3	3	3	2	2	3	3
4	4	3	3	2	2	1	1	3	3
4	4	3	3	3	3	1	1	3	3
4	4	3	3	3	3	1	1	3	3
4	4	3	3	3	3	1	1	3	3
4	4	3	3	3	3	2	2	3	3
4	4	3	3	3	3	2	2	3	3
4	4	3	3	3	3	2	2	3	3
4	4	3	3	3	3	2	2	3	3
4	4	3	3	3	3	2	2	3	3
4	4	3	3	2	2	2	2	3	3
4	4	3	3	3	3	2	2	3	3
4	4	3	3	3	3	2	2	3	3
4	4	3	3	3	3	2	2	3	3
4	4	3	3	3	3	2	2	3	3
4	4	3	3	3	3	2	2	3	3
4	4	3	3	3	3	1	1	3	3
4	4	3	3	3	3	2	2	3	3
4	4	3	3	3	3	2	2	3	3
4	4	3	3	3	3	2	2	3	3
4	4	3	3	3	3	2	2	3	3
4	4	3	3	3	3	2	2	3	3
4	4	3	3	3	3	2	2	3	3
4	4	3	3	3	3	1	1	3	3
4	4	3	3	3	3	1	1	3	3
4	4	3	3	3	3	2	2	3	3
4	4	3	3	3	3	2	2	3	3
4	4	3	3	3	3	2	2	3	3

For Review Only

4	4	3	3	3	3	2	2	3	3
4	4	3	3	3	3	2	2	2	2
4	4	3	3	3	3	2	2	3	3
4	4	3	3	3	3	2	2	3	3
4	4	2	2	3	3	2	2	3	3
4	4	3	3	3	3	2	2	3	3
4	4	3	3	3	3	2	2	3	3
4	4	3	3	3	3	2	2	2	2
4	4	3	3	3	3	2	2	3	3
4	4	3	3	3	3	2	2	3	3
4	4	3	3	3	3	2	2	3	3
4	4	3	3	3	3	1	1	3	3
4	4	3	3	3	3	2	2	3	3
4	4	3	3	3	3	2	2	3	3
4	4	3	3	3	3	2	2	3	3
4	4	3	3	3	3	2	2	3	3
4	4	3	3	3	3	2	2	3	3
4	4	3	3	3	3	2	2	3	3
4	4	3	3	3	3	2	2	3	3
1	1	3	3	2	2	2	2	3	3
4	4	3	3	3	3	2	2	3	3
4	4	3	3	3	3	2	2	3	3
4	4	3	3	3	3	1	1	3	3
4	4	3	3	3	3	2	2	3	3
4	4	3	3	3	3	2	2	3	3
4	4	3	3	3	3	2	2	3	3
4	4	3	3	3	3	2	2	3	3
4	4	3	3	3	3	2	2	3	3
4	4	3	3	3	3	2	2	3	3
4	4	3	3	3	3	1	1	3	3
4	4	3	3	3	3	2	2	3	3
4	4	3	3	3	3	2	2	3	3
1	1	3	3	3	3	2	2	3	3
4	4	3	3	3	3	2	2	3	3
4	4	3	3	3	3	2	2	3	3
4	4	3	3	3	3	2	2	3	3
4	4	3	3	3	3	2	2	3	3
4	4	3	3	3	3	2	2	3	3
4	4	3	3	3	3	2	2	3	3
4	4	3	3	3	3	2	2	3	3
4	4	3	3	3	3	1	1	3	3
4	4	3	3	3	3	1	1	3	3
4	4	3	3	3	3	2	2	3	3
4	4	3	3	3	3	1	1	3	3
4	4	3	3	3	3	2	2	3	3
4	4	3	3	3	3	2	2	2	2
4	4	3	3	3	3	2	2	3	3
4	4	3	3	3	3	1	1	3	3
4	4	3	3	2	2	2	2	3	3
4	4	3	3	3	3	2	2	3	3
4	4	3	3	3	3	1	1	3	3
4	4	3	3	3	3	2	2	3	3
4	4	3	3	3	3	2	2	3	3
4	4	3	3	3	3	1	1	3	3
4	4	3	3	2	2	2	2	3	3

For Review Only

4	4	3	3	3	3	2	2	3	3
4	4	3	3	3	3	1	1	3	3

For Review Only

SNP2040	SNP2040	SNP2041	SNP2041	SNP2042	SNP2042	SNP2043	SNP2043	SNP2044	SNP2044
3	3	3	3	4	4	4	4	2	2
3	3	3	3	4	4	4	4	1	1
3	3	3	3	4	4	1	1	2	2
3	3	3	3	4	4	4	4	1	1
3	3	3	3	4	4	4	4	2	2
3	3	3	3	4	4	4	4	2	2
2	2	3	3	4	4	4	4	1	1
3	3	3	3	4	4	4	4	2	2
3	3	3	3	4	4	4	4	2	2
2	2	3	3	4	4	4	4	2	2
3	3	3	3	4	4	4	4	2	2
3	3	3	3	4	4	4	4	2	2
3	3	3	3	4	4	4	4	2	2
3	3	3	3	4	4	4	4	2	2
3	3	3	3	4	4	4	4	2	2
3	3	3	3	1	1	4	4	1	1
3	3	3	3	4	4	4	4	2	2
3	3	3	3	4	4	4	4	2	2
3	3	3	3	4	4	4	4	2	2
2	2	3	3	4	4	4	4	2	2
3	3	3	3	4	4	4	4	2	2
3	3	3	3	4	4	4	4	2	2
3	3	2	2	4	4	4	4	1	1
3	3	3	3	4	4	4	4	1	1
3	3	3	3	4	4	4	4	2	2
3	3	3	3	4	4	4	4	2	2
3	3	3	3	4	4	4	4	2	2
3	3	3	3	4	4	4	4	2	2
2	2	3	3	4	4	4	4	2	2
2	2	3	3	4	4	4	4	1	1
3	3	3	3	4	4	4	4	2	2
3	3	3	3	1	1	4	4	2	2
3	3	3	3	1	1	4	4	2	2
3	3	3	3	4	4	4	4	1	1
3	3	3	3	4	4	4	4	2	2
3	3	3	3	4	4	4	4	2	2
3	3	3	3	4	4	4	4	2	2
3	3	3	3	4	4	4	4	2	2
3	3	2	2	4	4	4	4	1	1
3	3	3	3	4	4	4	4	1	1
3	3	3	3	4	4	4	4	2	2
2	2	3	3	4	4	4	4	2	2
2	2	3	3	4	4	4	4	2	2
3	3	3	3	4	4	4	4	2	2
2	2	3	3	4	4	4	4	2	2
3	3	3	3	4	4	4	4	2	2
3	3	3	3	4	4	4	4	2	2
3	3	3	3	1	1	4	4	2	2
3	3	3	3	4	4	4	4	2	2
3	3	3	3	4	4	4	4	2	2
3	3	3	3	4	4	4	4	2	2
3	3	3	3	4	4	4	4	1	1

For Review Only

3	3	3	3	4	4	4	4	2	2
3	3	3	3	1	1	4	4	2	2
3	3	3	3	4	4	4	4	2	2
3	3	3	3	4	4	4	4	2	2
3	3	3	3	4	4	4	4	2	2
3	3	3	3	4	4	4	4	2	2
3	3	3	3	4	4	4	4	2	2
3	3	3	3	4	4	4	4	2	2
3	3	3	3	4	4	4	4	2	2
3	3	3	3	4	4	4	4	2	2
3	3	3	3	4	4	4	4	2	2
3	3	3	3	4	4	4	4	2	2
3	3	3	3	4	4	4	4	2	2
2	2	3	3	4	4	4	4	2	2
3	3	3	3	4	4	4	4	2	2
3	3	3	3	4	4	4	4	2	2
3	3	2	2	4	4	1	1	2	2
3	3	3	3	1	1	4	4	2	2
3	3	3	3	4	4	4	4	2	2
3	3	3	3	4	4	4	4	2	2
3	3	3	3	4	4	4	4	2	2
3	3	3	3	4	4	4	4	1	1
2	2	3	3	4	4	4	4	2	2
3	3	3	3	1	1	4	4	2	2
2	2	3	3	4	4	4	4	2	2
3	3	3	3	4	4	4	4	1	1
3	3	3	3	1	1	4	4	2	2
3	3	2	2	4	4	4	4	2	2
3	3	3	3	4	4	4	4	2	2
3	3	3	3	4	4	4	4	2	2
3	3	3	3	4	4	4	4	2	2
3	3	3	3	4	4	4	4	2	2
3	3	3	3	4	4	4	4	1	1
3	3	3	3	4	4	4	4	2	2
3	3	3	3	4	4	4	4	2	2
2	2	3	3	4	4	4	4	2	2
2	2	3	3	4	4	4	4	2	2
3	3	3	3	4	4	4	4	2	2
3	3	3	3	4	4	4	4	1	1
2	2	3	3	4	4	4	4	2	2
2	2	3	3	4	4	4	4	2	2
3	3	3	3	4	4	1	1	2	2
3	3	3	3	4	4	4	4	2	2
2	2	3	3	4	4	4	4	2	2
3	3	3	3	4	4	4	4	2	2
3	3	3	3	1	1	4	4	2	2
3	3	3	3	4	4	4	4	1	1
3	3	3	3	4	4	4	4	1	1
3	3	3	3	4	4	4	4	2	2
3	3	3	3	4	4	4	4	2	2
3	3	3	3	4	4	4	4	2	2
3	3	2	2	4	4	4	4	1	1
3	3	3	3	4	4	4	4	1	1
3	3	3	3	4	4	4	4	2	2
3	3	3	3	4	4	4	4	2	2

3	3	3	3	4	4	4	4	1	1
3	3	3	3	4	4	4	4	2	2
3	3	3	3	4	4	4	4	2	2
3	3	3	3	4	4	4	4	1	1
3	3	3	3	4	4	4	4	2	2
3	3	3	3	4	4	4	4	2	2
3	3	3	3	1	1	4	4	1	1
3	3	3	3	4	4	4	4	2	2
3	3	3	3	4	4	4	4	2	2
3	3	3	3	4	4	4	4	2	2
3	3	3	3	4	4	4	4	2	2
3	3	3	3	4	4	4	4	2	2
3	3	3	3	4	4	4	4	2	2
3	3	3	3	4	4	4	4	2	2
3	3	3	3	4	4	4	4	2	2
3	3	3	3	4	4	4	4	2	2
3	3	3	3	4	4	4	4	2	2
3	3	3	3	4	4	4	4	2	2
3	3	3	3	4	4	4	4	1	1
3	3	3	3	4	4	4	4	1	1
3	3	3	3	4	4	4	4	2	2
3	3	3	3	1	1	4	4	2	2
3	3	3	3	4	4	4	4	2	2
3	3	2	2	4	4	4	4	1	1
3	3	3	3	4	4	4	4	2	2
3	3	2	2	4	4	4	4	1	1
3	3	3	3	4	4	4	4	2	2
3	3	3	3	1	1	4	4	2	2
3	3	3	3	1	1	4	4	2	2
3	3	3	3	4	4	4	4	2	2
3	3	3	3	4	4	4	4	1	1
3	3	3	3	4	4	4	4	2	2
3	3	3	3	4	4	4	4	2	2
3	3	3	3	4	4	4	4	2	2
3	3	3	3	4	4	4	4	1	1
3	3	3	3	4	4	4	4	2	2
3	3	3	3	4	4	4	4	1	1
3	3	3	3	4	4	4	4	2	2
3	3	3	3	4	4	4	4	2	2
3	3	3	3	4	4	4	4	1	1
3	3	2	2	4	4	4	4	1	1
3	3	3	3	4	4	4	4	1	1
3	3	3	3	1	1	4	4	2	2
3	3	3	3	4	4	4	4	2	2
3	3	3	3	4	4	4	4	2	2
3	3	3	3	4	4	4	4	2	2
3	3	3	3	4	4	4	4	2	2
3	3	3	3	4	4	1	1	2	2
3	3	3	3	4	4	4	4	2	2
3	3	3	3	4	4	4	4	2	2
3	3	3	3	4	4	4	4	2	2
3	3	3	3	4	4	4	4	1	1
3	3	3	3	4	4	4	4	2	2
3	3	3	3	4	4	4	4	2	2
3	3	3	3	4	4	4	4	2	2

3	3	3	3	4	4	4	4	2	2
3	3	3	3	4	4	4	4	1	1
3	3	3	3	1	1	4	4	2	2
3	3	3	3	4	4	4	4	2	2
3	3	3	3	4	4	4	4	2	2
3	3	3	3	4	4	4	4	1	1
3	3	2	2	4	4	4	4	1	1
3	3	3	3	4	4	4	4	2	2
3	3	3	3	4	4	4	4	2	2
3	3	3	3	4	4	4	4	1	1
3	3	3	3	4	4	4	4	2	2
3	3	3	3	4	4	4	4	1	1
3	3	3	3	4	4	4	4	2	2
3	3	3	3	4	4	4	4	2	2
3	3	3	3	4	4	4	4	1	1
3	3	3	3	4	4	4	4	2	2
3	3	3	3	4	4	4	4	2	2
3	3	3	3	4	4	4	4	2	2
3	3	3	3	4	4	4	4	2	2
3	3	3	3	4	4	4	4	1	1
3	3	3	3	4	4	4	4	2	2
3	3	2	2	4	4	4	4	2	2
3	3	3	3	4	4	4	4	1	1
3	3	3	3	4	4	4	4	2	2
3	3	3	3	4	4	4	4	2	2
3	3	3	3	4	4	4	4	1	1
3	3	3	3	4	4	4	4	1	1
3	3	3	3	4	4	4	4	2	2
3	3	3	3	4	4	4	4	2	2
3	3	3	3	4	4	4	4	2	2
3	3	3	3	4	4	4	4	2	2
3	3	3	3	4	4	4	4	2	2
3	3	3	3	1	1	4	4	1	1
3	3	3	3	4	4	4	4	1	1
3	3	3	3	4	4	4	4	2	2
3	3	3	3	4	4	4	4	2	2
3	3	2	2	4	4	4	4	2	2
3	3	3	3	4	4	4	4	2	2
3	3	3	3	4	4	1	1	2	2
3	3	3	3	4	4	4	4	2	2
3	3	3	3	4	4	1	1	2	2
3	3	3	3	4	4	4	4	2	2
3	3	3	3	4	4	4	4	2	2
3	3	3	3	4	4	4	4	2	2
3	3	2	2	4	4	4	4	1	1
3	3	3	3	4	4	4	4	2	2
3	3	3	3	4	4	4	4	2	2
3	3	2	2	4	4	4	4	1	1
3	3	3	3	4	4	4	4	1	1
3	3	3	3	4	4	4	4	1	1
3	3	3	3	1	1	4	4	2	2
3	3	3	3	4	4	4	4	1	1
2	2	3	3	4	4	4	4	1	1
3	3	3	3	4	4	4	4	2	2
3	3	2	2	4	4	4	4	1	1

For Review Only

3	3	3	3	1	1	4	4	2	2
3	3	3	3	1	1	4	4	2	2
3	3	3	3	1	1	4	4	2	2
3	3	3	3	4	4	4	4	1	1
2	2	3	3	4	4	4	4	2	2
3	3	3	3	1	1	4	4	2	2
3	3	3	3	4	4	4	4	1	1
2	2	3	3	4	4	4	4	2	2
3	3	3	3	4	4	4	4	2	2
3	3	2	2	4	4	4	4	1	1
3	3	3	3	4	4	4	4	2	2
3	3	3	3	4	4	4	4	2	2
3	3	3	3	4	4	4	4	1	1
3	3	3	3	4	4	4	4	2	2
3	3	3	3	4	4	1	1	2	2
3	3	3	3	4	4	4	4	2	2
3	3	2	2	4	4	4	4	1	1
3	3	2	2	4	4	4	4	2	2
3	3	3	3	4	4	4	4	1	1
3	3	3	3	4	4	4	4	2	2
3	3	3	3	4	4	4	4	1	1
3	3	3	3	4	4	4	4	2	2
3	3	3	3	1	1	4	4	2	2
3	3	3	3	4	4	4	4	2	2
3	3	3	3	4	4	4	4	2	2
3	3	3	3	4	4	4	4	2	2
3	3	2	2	4	4	4	4	1	1
3	3	3	3	4	4	4	4	1	1
3	3	3	3	4	4	4	4	2	2
3	3	3	3	1	1	4	4	2	2
3	3	2	2	4	4	4	4	1	1
3	3	3	3	4	4	4	4	2	2
3	3	3	3	4	4	4	4	2	2
3	3	3	3	4	4	4	4	2	2
3	3	3	3	4	4	4	4	1	1
3	3	2	2	4	4	4	4	1	1
3	3	3	3	4	4	4	4	2	2
3	3	3	3	4	4	4	4	2	2
3	3	3	3	4	4	4	4	2	2
3	3	3	3	4	4	4	4	1	1
3	3	3	3	1	1	4	4	2	2
3	3	3	3	4	4	4	4	2	2
3	3	3	3	4	4	4	4	1	1
3	3	3	3	4	4	4	4	2	2
3	3	3	3	4	4	4	4	2	2
3	3	3	3	4	4	4	4	1	1
3	3	3	3	4	4	1	1	2	2
3	3	2	2	4	4	4	4	2	2
3	3	3	3	4	4	1	1	2	2
3	3	3	3	4	4	4	4	1	1
3	3	3	3	4	4	4	4	2	2
3	3	3	3	4	4	4	4	1	1
3	3	3	3	4	4	1	1	2	2
3	3	3	3	4	4	4	4	2	2
3	3	3	3	4	4	4	4	1	1

For Review Only

3	3	3	3	4	4	4	4	2	2
3	3	3	3	4	4	4	4	2	2

For Review Only

SNP2045	SNP2045	SNP2046	SNP2046	SNP2047	SNP2047	SNP2048	SNP2048	SNP2049	SNP2049
1	1	4	4	3	3	1	1	1	1
1	1	4	4	3	3	1	1	1	1
1	1	4	4	3	3	1	1	1	1
1	1	4	4	3	3	1	1	1	1
1	1	4	4	2	2	4	4	1	1
1	1	4	4	3	3	1	1	1	1
1	1	3	3	2	2	4	4	1	1
1	1	4	4	3	3	1	1	1	1
1	1	4	4	3	3	4	4	1	1
3	3	4	4	3	3	1	1	1	1
1	1	4	4	3	3	1	1	4	4
1	1	4	4	3	3	1	1	1	1
1	1	4	4	3	3	4	4	1	1
3	3	4	4	2	2	4	4	1	1
1	1	4	4	3	3	4	4	1	1
1	1	4	4	2	2	4	4	1	1
1	1	4	4	2	2	4	4	1	1
1	1	4	4	2	2	4	4	1	1
1	1	4	4	3	3	1	1	1	1
1	1	4	4	3	3	1	1	1	1
1	1	4	4	2	2	4	4	1	1
1	1	4	4	2	2	4	4	1	1
3	3	4	4	3	3	1	1	1	1
3	3	4	4	3	3	1	1	1	1
1	1	4	4	2	2	4	4	1	1
1	1	4	4	3	3	1	1	1	1
1	1	4	4	3	3	1	1	1	1
1	1	4	4	3	3	4	4	1	1
1	1	4	4	3	3	1	1	1	1
1	1	4	4	3	3	1	1	1	1
1	1	4	4	3	3	1	1	1	1
1	1	4	4	2	2	4	4	1	1
1	1	4	4	2	2	4	4	1	1
3	3	3	3	3	3	1	1	1	1
1	1	4	4	3	3	1	1	1	1
1	1	4	4	3	3	1	1	1	1
1	1	4	4	2	2	4	4	1	1
1	1	4	4	2	2	4	4	1	1
3	3	3	3	3	3	1	1	1	1
1	1	4	4	2	2	4	4	1	1
1	1	4	4	3	3	1	1	1	1
1	1	4	4	3	3	1	1	1	1
1	1	4	4	3	3	1	1	1	1
1	1	4	4	2	2	4	4	1	1
3	3	4	4	3	3	1	1	1	1
1	1	4	4	3	3	1	1	1	1
1	1	4	4	3	3	1	1	1	1
1	1	4	4	2	2	4	4	1	1
1	1	4	4	2	2	4	4	1	1
1	1	4	4	3	3	4	4	1	1
1	1	4	4	2	2	4	4	1	1
1	1	3	3	3	3	4	4	1	1

For Review Only

1	1	4	4	2	2	4	4	1	1
1	1	4	4	2	2	4	4	1	1
1	1	4	4	3	3	1	1	1	1
1	1	4	4	2	2	4	4	1	1
1	1	4	4	3	3	1	1	1	1
1	1	4	4	3	3	1	1	1	1
1	1	4	4	3	3	1	1	1	1
1	1	4	4	3	3	1	1	1	1
1	1	4	4	3	3	1	1	1	1
1	1	4	4	3	3	4	4	1	1
1	1	4	4	3	3	1	1	1	1
1	1	4	4	3	3	1	1	1	1
1	1	4	4	2	2	4	4	1	1
1	1	4	4	3	3	1	1	1	1
1	1	4	4	3	3	1	1	1	1
1	1	4	4	3	3	1	1	1	1
3	3	4	4	2	2	4	4	4	4
1	1	4	4	2	2	4	4	1	1
1	1	4	4	2	2	4	4	1	1
1	1	4	4	3	3	1	1	1	1
1	1	4	4	3	3	1	1	1	1
1	1	4	4	3	3	1	1	1	1
1	1	4	4	2	2	4	4	1	1
1	1	4	4	2	2	4	4	1	1
3	3	4	4	3	3	1	1	1	1
1	1	4	4	3	3	1	1	1	1
1	1	4	4	2	2	4	4	1	1
3	3	4	4	3	3	1	1	1	1
1	1	4	4	3	3	1	1	1	1
1	1	4	4	2	2	4	4	1	1
1	1	4	4	3	3	1	1	1	1
1	1	4	4	3	3	1	1	1	1
1	1	3	3	3	3	1	1	1	1
1	1	4	4	2	2	4	4	1	1
1	1	4	4	2	2	4	4	1	1
3	3	4	4	3	3	1	1	1	1
1	1	4	4	3	3	1	1	1	1
1	1	4	4	3	3	4	4	1	1
3	3	4	4	3	3	1	1	4	4
3	3	4	4	3	3	4	4	1	1
1	1	4	4	2	2	4	4	1	1
1	1	4	4	2	2	4	4	1	1
3	3	4	4	2	2	4	4	1	1
1	1	4	4	3	3	1	1	1	1
1	1	4	4	3	3	1	1	1	1
1	1	4	4	3	3	1	1	1	1
3	3	4	4	3	3	1	1	1	1
3	3	4	4	3	3	1	1	1	1
3	3	4	4	3	3	1	1	1	1
1	1	4	4	3	3	4	4	1	1
1	1	4	4	3	3	1	1	1	1
3	3	4	4	3	3	1	1	1	1
1	1	4	4	3	3	1	1	1	1
1	1	4	4	3	3	1	1	1	1
1	1	4	4	3	3	1	1	1	1

For Review Only

1	1	4	4	3	3	1	1	1	1
1	1	4	4	3	3	1	1	1	1
1	1	4	4	3	3	1	1	1	1
3	3	4	4	3	3	1	1	1	1
1	1	4	4	3	3	4	4	1	1
1	1	4	4	3	3	1	1	1	1
1	1	4	4	2	2	4	4	1	1
1	1	4	4	3	3	1	1	1	1
3	3	4	4	2	2	4	4	1	1
1	1	4	4	3	3	4	4	1	1
1	1	4	4	2	2	4	4	1	1
1	1	4	4	3	3	4	4	1	1
3	3	4	4	2	2	4	4	1	1
1	1	4	4	3	3	1	1	1	1
1	1	4	4	3	3	1	1	1	1
1	1	4	4	3	3	4	4	1	1
1	1	4	4	3	3	1	1	1	1
1	1	4	4	3	3	4	4	1	1
3	3	4	4	3	3	1	1	1	1
3	3	4	4	3	3	4	4	1	1
1	1	4	4	3	3	4	4	1	1
1	1	4	4	2	2	4	4	1	1
1	1	4	4	3	3	1	1	1	1
3	3	4	4	3	3	1	1	1	1
1	1	4	4	3	3	1	1	1	1
3	3	4	4	3	3	1	1	1	1
1	1	4	4	2	2	4	4	1	1
1	1	4	4	2	2	4	4	1	1
1	1	4	4	2	2	4	4	1	1
3	3	4	4	3	3	1	1	1	1
3	3	4	4	3	3	1	1	1	1
1	1	4	4	3	3	4	4	1	1
1	1	4	4	2	2	4	4	1	1
1	1	4	4	3	3	1	1	1	1
1	1	3	3	3	3	1	1	1	1
1	1	4	4	3	3	1	1	1	1
3	3	3	3	3	3	1	1	1	1
1	1	4	4	2	2	4	4	1	1
1	1	4	4	3	3	1	1	1	1
1	1	3	3	3	3	1	1	1	1
1	1	4	4	3	3	1	1	1	1
3	3	4	4	3	3	1	1	1	1
1	1	4	4	2	2	4	4	1	1
1	1	4	4	3	3	1	1	1	1
1	1	4	4	3	3	4	4	1	1
1	1	4	4	3	3	1	1	4	4
1	1	4	4	3	3	1	1	1	1
1	1	4	4	3	3	1	1	1	1
1	1	4	4	3	3	1	1	1	1
1	1	4	4	3	3	1	1	1	1
1	1	4	4	3	3	1	1	1	1
1	1	4	4	3	3	1	1	1	1
3	3	4	4	3	3	4	4	1	1
1	1	4	4	3	3	1	1	1	1
1	1	4	4	3	3	1	1	1	1

For Review Only

1	1	4	4	3	3	1	1	1	1
3	3	4	4	3	3	1	1	1	1
1	1	4	4	2	2	4	4	1	1
1	1	4	4	3	3	4	4	1	1
1	1	4	4	3	3	1	1	1	1
1	1	4	4	2	2	4	4	1	1
1	1	4	4	3	3	4	4	1	1
1	1	4	4	3	3	4	4	1	1
1	1	4	4	3	3	1	1	1	1
1	1	4	4	2	2	4	4	1	1
1	1	4	4	3	3	1	1	1	1
3	3	4	4	3	3	1	1	1	1
1	1	4	4	3	3	1	1	1	1
1	1	4	4	3	3	1	1	4	4
3	3	3	3	3	3	1	1	1	1
1	1	4	4	3	3	1	1	4	4
1	1	4	4	3	3	4	4	1	1
1	1	4	4	3	3	1	1	1	1
1	1	4	4	3	3	1	1	1	1
1	1	4	4	2	2	4	4	1	1
1	1	4	4	3	3	1	1	1	1
1	1	4	4	3	3	1	1	1	1
1	1	4	4	3	3	1	1	1	1
1	1	4	4	3	3	1	1	1	1
1	1	4	4	2	2	4	4	1	1
1	1	3	3	3	3	1	1	1	1
1	1	4	4	3	3	1	1	1	1
1	1	4	4	3	3	1	1	1	1
1	1	4	4	3	3	1	1	1	1
1	1	4	4	2	2	4	4	1	1
1	1	4	4	3	3	1	1	1	1
1	1	4	4	3	3	1	1	1	1
1	1	4	4	2	2	4	4	1	1
3	3	3	3	3	3	1	1	1	1
1	1	4	4	3	3	4	4	4	4
1	1	4	4	3	3	1	1	1	1
1	1	4	4	3	3	1	1	1	1
1	1	4	4	2	2	4	4	1	1
1	1	4	4	3	3	1	1	1	1
1	1	4	4	3	3	1	1	1	1
1	1	4	4	3	3	1	1	1	1
1	1	4	4	3	3	1	1	1	1
1	1	4	4	3	3	1	1	1	1
1	1	4	4	3	3	4	4	1	1
1	1	4	4	3	3	1	1	1	1
1	1	4	4	3	3	1	1	1	1
1	1	4	4	3	3	1	1	4	4
3	3	4	4	3	3	1	1	1	1
3	3	3	3	3	3	1	1	1	1
1	1	4	4	3	3	1	1	1	1
1	1	4	4	2	2	4	4	1	1
1	1	4	4	3	3	1	1	1	1
1	1	4	4	3	3	1	1	1	1
1	1	4	4	3	3	1	1	1	1
3	3	3	3	3	3	1	1	1	1

For Review Only

1	1	4	4	2	2	4	4	1	1
1	1	4	4	2	2	4	4	1	1
1	1	4	4	2	2	4	4	1	1
1	1	4	4	3	3	1	1	1	1
1	1	4	4	3	3	1	1	1	1
1	1	4	4	2	2	4	4	1	1
3	3	4	4	2	2	4	4	1	1
1	1	4	4	3	3	1	1	1	1
1	1	4	4	3	3	1	1	1	1
3	3	4	4	3	3	1	1	1	1
1	1	4	4	3	3	4	4	1	1
3	3	4	4	2	2	4	4	1	1
1	1	4	4	3	3	1	1	4	4
3	3	4	4	3	3	1	1	1	1
1	1	4	4	3	3	1	1	1	1
1	1	4	4	3	3	1	1	4	4
3	3	3	3	3	3	1	1	1	1
3	3	4	4	3	3	4	4	1	1
1	1	4	4	3	3	1	1	1	1
1	1	4	4	2	2	4	4	1	1
1	1	4	4	3	3	1	1	1	1
1	1	4	4	3	3	1	1	1	1
1	1	4	4	2	2	4	4	1	1
1	1	4	4	2	2	4	4	1	1
1	1	4	4	3	3	1	1	1	1
1	1	4	4	3	3	1	1	1	1
3	3	4	4	3	3	1	1	1	1
1	1	4	4	3	3	1	1	1	1
1	1	4	4	3	3	4	4	1	1
1	1	4	4	2	2	4	4	1	1
3	3	4	4	3	3	1	1	1	1
1	1	4	4	3	3	1	1	1	1
3	3	4	4	3	3	1	1	4	4
1	1	4	4	3	3	1	1	1	1
1	1	4	4	3	3	1	1	1	1
3	3	4	4	3	3	1	1	1	1
1	1	4	4	2	2	4	4	1	1
1	1	4	4	3	3	1	1	1	1
1	1	4	4	3	3	1	1	1	1
1	1	3	3	3	3	1	1	1	1
1	1	4	4	2	2	4	4	1	1
1	1	4	4	3	3	1	1	4	4
1	1	3	3	2	2	4	4	1	1
1	1	4	4	2	2	4	4	1	1
1	1	4	4	3	3	1	1	1	1
3	3	4	4	3	3	1	1	4	4
1	1	4	4	3	3	1	1	1	1
1	1	4	4	2	2	4	4	1	1
1	1	4	4	3	3	1	1	1	1
1	1	4	4	3	3	1	1	1	1
1	1	4	4	2	2	4	4	1	1
1	1	3	3	3	3	1	1	1	1
1	1	4	4	3	3	1	1	1	1
1	1	4	4	3	3	1	1	1	1
1	1	4	4	2	2	4	4	1	1

For Review Only

1	1	4	4	3	3	1	1	4	4
1	1	4	4	3	3	1	1	1	1

For Review Only

SNP205	SNP205	SNP2050	SNP2050	SNP2051	SNP2051	SNP2052	SNP2052	SNP2053	SNP2053
4	4	1	1	2	2	1	1	4	4
4	4	1	1	3	3	4	4	4	4
1	1	1	1	2	2	1	1	1	1
4	4	1	1	2	2	4	4	4	4
4	4	1	1	2	2	1	1	1	1
4	4	1	1	2	2	1	1	4	4
4	4	1	1	2	2	1	1	4	4
4	4	1	1	2	2	1	1	4	4
4	4	1	1	2	2	1	1	1	1
4	4	1	1	2	2	1	1	4	4
1	1	3	3	2	2	1	1	4	4
1	1	1	1	2	2	1	1	4	4
4	4	1	1	2	2	1	1	4	4
4	4	1	1	3	3	4	4	4	4
4	4	1	1	2	2	1	1	4	4
4	4	1	1	3	3	1	1	4	4
4	4	1	1	2	2	1	1	1	1
4	4	1	1	2	2	1	1	4	4
4	4	3	3	2	2	1	1	4	4
4	4	3	3	2	2	1	1	4	4
4	4	1	1	2	2	1	1	1	1
4	4	1	1	2	2	1	1	1	1
4	4	3	3	2	2	1	1	4	4
4	4	1	1	3	3	1	1	4	4
4	4	1	1	2	2	1	1	4	4
4	4	3	3	2	2	1	1	4	4
4	4	1	1	2	2	1	1	4	4
4	4	3	3	2	2	1	1	4	4
4	4	1	1	2	2	1	1	4	4
4	4	3	3	2	2	1	1	4	4
4	4	1	1	2	2	1	1	4	4
1	1	1	1	2	2	1	1	4	4
1	1	1	1	2	2	1	1	1	1
4	4	1	1	3	3	4	4	4	4
4	4	3	3	2	2	4	4	4	4
4	4	1	1	3	3	1	1	4	4
4	4	1	1	2	2	1	1	4	4
1	1	1	1	2	2	4	4	4	4
4	4	1	1	2	2	4	4	4	4
1	1	1	1	2	2	1	1	4	4
4	4	3	3	2	2	1	1	4	4
4	4	1	1	2	2	1	1	4	4
4	4	1	1	2	2	1	1	4	4
4	4	1	1	2	2	1	1	1	1
4	4	1	1	2	2	1	1	4	4
1	1	3	3	2	2	1	1	4	4
4	4	1	1	3	3	4	4	4	4
4	4	3	3	2	2	1	1	4	4
1	1	3	3	2	2	1	1	4	4
4	4	1	1	2	2	1	1	4	4
4	4	1	1	2	2	4	4	4	4
4	4	1	1	2	2	1	1	4	4

4	4	1	1	2	2	1	1	1	1
1	1	1	1	2	2	1	1	1	1
4	4	1	1	3	3	4	4	4	4
4	4	1	1	2	2	1	1	4	4
4	4	3	3	2	2	1	1	4	4
4	4	1	1	2	2	1	1	4	4
4	4	3	3	2	2	1	1	4	4
4	4	3	3	2	2	1	1	4	4
4	4	1	1	2	2	1	1	4	4
4	4	3	3	2	2	1	1	4	4
4	4	1	1	3	3	4	4	4	4
4	4	1	1	3	3	4	4	4	4
4	4	1	1	2	2	1	1	1	1
4	4	1	1	2	2	1	1	4	4
4	4	1	1	2	2	1	1	4	4
4	4	1	1	2	2	1	1	4	4
4	4	1	1	2	2	1	1	1	1
4	4	1	1	2	2	1	1	4	4
4	4	1	1	2	2	1	1	1	1
4	4	1	1	2	2	1	1	4	4
4	4	1	1	3	3	4	4	4	4
4	4	3	3	2	2	1	1	4	4
4	4	1	1	2	2	1	1	4	4
4	4	3	3	2	2	1	1	4	4
4	4	1	1	2	2	1	1	4	4
4	4	1	1	2	2	1	1	4	4
1	1	3	3	2	2	1	1	4	4
4	4	1	1	2	2	1	1	4	4
4	4	3	3	2	2	1	1	4	4
4	4	3	3	2	2	1	1	4	4
4	4	1	1	2	2	1	1	1	1
4	4	1	1	2	2	1	1	4	4
4	4	1	1	3	3	4	4	4	4
4	4	1	1	2	2	1	1	1	1
1	1	1	1	2	2	1	1	4	4
4	4	1	1	2	2	1	1	4	4
4	4	1	1	2	2	1	1	4	4
4	4	1	1	2	2	1	1	4	4
1	1	3	3	2	2	1	1	4	4
4	4	3	3	3	3	1	1	4	4
4	4	1	1	2	2	1	1	1	1
4	4	3	3	3	3	4	4	1	1
4	4	1	1	2	2	1	1	4	4
4	4	1	1	3	3	4	4	4	4
1	1	3	3	2	2	1	1	4	4
1	1	1	1	3	3	4	4	4	4
4	4	1	1	3	3	4	4	4	4
4	4	1	1	2	2	1	1	4	4
4	4	1	1	2	2	1	1	4	4
4	4	3	3	2	2	1	1	4	4
4	4	1	1	2	2	4	4	4	4
4	4	1	1	2	2	1	1	4	4
4	4	3	3	2	2	1	1	4	4
4	4	3	3	2	2	1	1	4	4
1	1	3	3	2	2	1	1	4	4

For Review Only

4	4	3	3	2	2	1	1	4	4
4	4	1	1	2	2	1	1	4	4
4	4	3	3	2	2	1	1	4	4
4	4	1	1	3	3	4	4	4	4
4	4	1	1	3	3	4	4	4	4
4	4	3	3	2	2	1	1	4	4
4	4	1	1	2	2	1	1	4	4
4	4	1	1	2	2	4	4	4	4
4	4	1	1	2	2	1	1	4	4
4	4	3	3	2	2	1	1	4	4
1	1	3	3	2	2	1	1	4	4
4	4	3	3	2	2	1	1	4	4
4	4	1	1	2	2	1	1	4	4
4	4	1	1	2	2	1	1	4	4
4	4	3	3	2	2	1	1	4	4
4	4	3	3	2	2	1	1	4	4
4	4	1	1	2	2	1	1	4	4
4	4	3	3	3	3	4	4	4	4
4	4	1	1	3	3	4	4	4	4
4	4	1	1	2	2	1	1	4	4
4	4	3	3	2	2	1	1	4	4
4	4	1	1	2	2	1	1	4	4
4	4	1	1	2	2	1	1	4	4
4	4	1	1	2	2	1	1	4	4
4	4	3	3	2	2	1	1	4	4
4	4	3	3	2	2	1	1	4	4
1	1	3	3	2	2	1	1	4	4
4	4	1	1	2	2	1	1	4	4
1	1	1	1	2	2	1	1	4	4
4	4	3	3	2	2	1	1	4	4
4	4	1	1	2	2	4	4	4	4
4	4	1	1	2	2	1	1	4	4
4	4	1	1	2	2	1	1	1	1
4	4	1	1	2	2	1	1	4	4
4	4	1	1	3	3	4	4	4	4
4	4	1	1	2	2	4	4	4	4
4	4	1	1	3	3	4	4	4	4
4	4	1	1	2	2	1	1	4	4
4	4	3	3	2	2	1	1	4	4
1	1	1	1	3	3	4	4	4	4
4	4	1	1	2	2	1	1	4	4
4	4	1	1	3	3	4	4	4	4
1	1	3	3	2	2	1	1	4	4
4	4	3	3	2	2	1	1	4	4
4	4	1	1	2	2	1	1	4	4
4	4	1	1	2	2	1	1	4	4
4	4	3	3	2	2	4	4	4	4
4	4	3	3	2	2	1	1	1	1
4	4	1	1	2	2	1	1	4	4
4	4	3	3	2	2	1	1	4	4
4	4	3	3	3	3	1	1	4	4
4	4	1	1	2	2	1	1	4	4
1	1	1	1	3	3	4	4	4	4
4	4	1	1	3	3	4	4	4	4
4	4	3	3	2	2	1	1	4	4

For Review Only

1	1	1	1	2	2	1	1	4	4
4	4	1	1	2	2	1	1	4	4
1	1	3	3	2	2	1	1	4	4
1	1	1	1	2	2	1	1	4	4
4	4	1	1	3	3	4	4	4	4
4	4	1	1	2	2	1	1	4	4
4	4	3	3	2	2	1	1	4	4
4	4	3	3	2	2	1	1	4	4
4	4	3	3	2	2	4	4	4	4
4	4	1	1	2	2	1	1	4	4
4	4	3	3	2	2	1	1	1	1
4	4	3	3	2	2	1	1	4	4
4	4	1	1	2	2	1	1	4	4
1	1	1	1	2	2	4	4	4	4
4	4	1	1	3	3	4	4	4	4
1	1	3	3	3	3	4	4	4	4
4	4	3	3	3	3	4	4	4	4
4	4	1	1	2	2	4	4	4	4
4	4	3	3	2	2	1	1	4	4
4	4	1	1	2	2	1	1	4	4
4	4	3	3	2	2	4	4	4	4
4	4	1	1	2	2	1	1	4	4
4	4	3	3	2	2	4	4	4	4
4	4	1	1	3	3	4	4	4	4
4	4	1	1	2	2	1	1	4	4
4	4	1	1	3	3	4	4	4	4
4	4	1	1	2	2	1	1	4	4
1	1	1	1	2	2	1	1	4	4
4	4	1	1	2	2	1	1	4	4
4	4	1	1	2	2	1	1	1	1
1	1	1	1	2	2	1	1	4	4
4	4	3	3	2	2	1	1	4	4
1	1	1	1	2	2	1	1	4	4
4	4	3	3	3	3	4	4	4	4
1	1	1	1	2	2	1	1	4	4
4	4	1	1	3	3	4	4	4	4
4	4	3	3	2	2	1	1	4	4
4	4	1	1	2	2	1	1	4	4
4	4	3	3	2	2	1	1	1	1
4	4	1	1	2	2	1	1	4	4
1	1	1	1	2	2	1	1	1	1
4	4	3	3	2	2	1	1	4	4
4	4	1	1	3	3	4	4	4	4
4	4	3	3	2	2	1	1	1	1
1	1	3	3	3	3	4	4	4	4
4	4	1	1	2	2	1	1	4	4
1	1	3	3	3	3	4	4	4	4
4	4	3	3	2	2	1	1	4	4
4	4	1	1	2	2	1	1	4	4
4	4	1	1	2	2	1	1	4	4
1	1	3	3	2	2	1	1	4	4
1	1	3	3	2	2	1	1	4	4
4	4	3	3	2	2	1	1	4	4
4	4	1	1	3	3	4	4	4	4
4	4	1	1	2	2	1	1	4	4

For Review Only

1	1	1	1	3	3	4	4	4	4
4	4	1	1	2	2	1	1	4	4
1	1	3	3	2	2	1	1	4	4
4	4	3	3	2	2	1	1	4	4
4	4	1	1	2	2	1	1	4	4
4	4	3	3	2	2	1	1	4	4
1	1	1	1	2	2	1	1	4	4
4	4	1	1	2	2	1	1	4	4
4	4	1	1	2	2	4	4	4	4
4	4	1	1	2	2	1	1	4	4
4	4	3	3	2	2	1	1	4	4
1	1	1	1	2	2	4	4	4	4
1	1	3	3	2	2	1	1	4	4
4	4	1	1	2	2	1	1	4	4
1	1	1	1	2	2	1	1	1	1
1	1	3	3	2	2	1	1	4	4
4	4	1	1	2	2	1	1	4	4
4	4	1	1	2	2	1	1	4	4
4	4	1	1	3	3	4	4	4	4
4	4	1	1	2	2	1	1	4	4
4	4	1	1	2	2	1	1	4	4
4	4	3	3	2	2	1	1	4	4
4	4	3	3	2	2	1	1	4	4
4	4	3	3	2	2	1	1	4	4
4	4	3	3	2	2	1	1	4	4
1	1	3	3	3	3	4	4	4	4
4	4	1	1	3	3	4	4	4	4
4	4	1	1	2	2	1	1	4	4
4	4	1	1	2	2	1	1	1	1
1	1	1	1	2	2	1	1	4	4
4	4	1	1	2	2	1	1	4	4
4	4	3	3	2	2	1	1	4	4
4	4	1	1	3	3	4	4	4	4
4	4	3	3	2	2	1	1	4	4
4	4	3	3	2	2	1	1	4	4
4	4	1	1	3	3	4	4	4	4
1	1	1	1	2	2	1	1	4	4
4	4	1	1	3	3	4	4	4	4
4	4	1	1	3	3	1	1	4	4
4	4	1	1	2	2	1	1	4	4
1	1	1	1	2	2	1	1	4	4
1	1	3	3	2	2	1	1	4	4
4	4	3	3	2	2	1	1	4	4
4	4	1	1	2	2	1	1	1	1
4	4	1	1	2	2	4	4	4	4
1	1	1	1	2	2	1	1	4	4
4	4	1	1	2	2	1	1	1	1
1	1	1	1	2	2	1	1	4	4
4	4	3	3	2	2	1	1	1	1
4	4	1	1	2	2	4	4	4	4
4	4	1	1	2	2	1	1	4	4
4	4	1	1	2	2	1	1	4	4
1	1	1	1	2	2	1	1	1	1
4	4	1	1	3	3	4	4	4	4
4	4	1	1	2	2	1	1	4	4

For Review Only

1	1	3	3	2	2	1	1	4	4
4	4	3	3	2	2	1	1	4	4

For Review Only

SNP2054	SNP2054	SNP2055	SNP2055	SNP2056	SNP2056	SNP2057	SNP2057	SNP2058	SNP2058
4	4	1	1	1	1	1	1	1	1
4	4	1	1	1	1	1	1	1	1
4	4	1	1	1	1	1	1	1	1
4	4	1	1	1	1	1	1	1	1
4	4	1	1	1	1	1	1	1	1
4	4	1	1	1	1	1	1	1	1
4	4	1	1	1	1	1	1	1	1
4	4	1	1	2	2	1	1	1	1
4	4	1	1	1	1	1	1	1	1
4	4	4	4	2	2	1	1	1	1
1	1	1	1	2	2	1	1	4	4
4	4	1	1	1	1	1	1	1	1
4	4	1	1	1	1	1	1	1	1
4	4	1	1	1	1	1	1	4	4
4	4	1	1	1	1	1	1	1	1
4	4	1	1	2	2	1	1	4	4
4	4	1	1	1	1	1	1	1	1
4	4	1	1	2	2	1	1	1	1
4	4	1	1	1	1	1	1	1	1
4	4	1	1	2	2	1	1	1	1
4	4	1	1	1	1	1	1	1	1
4	4	1	1	1	1	1	1	1	1
4	4	1	1	1	1	1	1	4	4
4	4	1	1	1	1	1	1	4	4
4	4	1	1	1	1	3	3	1	1
4	4	1	1	2	2	1	1	1	1
4	4	1	1	1	1	1	1	1	1
4	4	1	1	1	1	1	1	1	1
4	4	1	1	1	1	1	1	1	1
4	4	1	1	2	2	1	1	1	1
4	4	1	1	1	1	1	1	1	1
4	4	1	1	1	1	1	1	1	1
4	4	1	1	1	1	1	1	1	1
4	4	1	1	1	1	1	1	4	4
4	4	1	1	2	2	1	1	4	4
4	4	1	1	2	2	1	1	4	4
4	4	1	1	1	1	1	1	1	1
4	4	1	1	2	2	1	1	4	4
4	4	1	1	2	2	1	1	4	4
4	4	1	1	1	1	1	1	4	4
4	4	1	1	2	2	1	1	1	1
4	4	1	1	1	1	1	1	1	1
4	4	1	1	1	1	1	1	1	1
4	4	1	1	1	1	1	1	1	1
4	4	4	4	2	2	1	1	1	1
4	4	1	1	1	1	1	1	1	1
4	4	1	1	2	2	1	1	1	1
4	4	1	1	2	2	1	1	4	4
4	4	1	1	1	1	1	1	1	1
4	4	1	1	1	1	1	1	1	1
4	4	1	1	2	2	1	1	1	1
4	4	1	1	1	1	1	1	1	1

4	4	1	1	1	1	1	1	1	1
4	4	1	1	1	1	1	1	1	1
4	4	1	1	1	1	1	1	1	1
4	4	1	1	1	1	1	1	1	1
4	4	1	1	1	1	1	1	1	1
4	4	1	1	1	1	1	1	1	1
4	4	1	1	2	2	1	1	1	1
4	4	1	1	2	2	1	1	1	1
4	4	1	1	1	1	1	1	1	1
4	4	1	1	1	1	1	1	1	1
4	4	1	1	1	1	1	1	1	1
4	4	1	1	1	1	1	1	1	1
4	4	1	1	1	1	1	1	1	1
4	4	1	1	1	1	1	1	1	1
4	4	1	1	1	1	1	1	1	1
4	4	1	1	1	1	1	1	1	1
1	1	1	1	1	1	1	1	4	4
4	4	1	1	1	1	3	3	1	1
4	4	1	1	1	1	1	1	1	1
4	4	1	1	1	1	1	1	1	1
4	4	1	1	1	1	1	1	1	1
4	4	1	1	1	1	1	1	4	4
4	4	1	1	1	1	1	1	1	1
4	4	1	1	1	1	1	1	1	1
4	4	4	4	2	2	1	1	1	1
4	4	1	1	1	1	1	1	1	1
4	4	1	1	2	2	3	3	1	1
4	4	1	1	2	2	1	1	4	4
4	4	1	1	2	2	1	1	1	1
4	4	1	1	1	1	1	1	1	1
4	4	1	1	2	2	1	1	1	1
4	4	1	1	2	2	1	1	1	1
4	4	1	1	2	2	1	1	4	4
4	4	1	1	1	1	1	1	1	1
4	4	1	1	1	1	1	1	1	1
4	4	4	4	2	2	1	1	1	1
4	4	1	1	2	2	1	1	1	1
4	4	1	1	1	1	1	1	1	1
1	1	4	4	1	1	1	1	4	4
4	4	4	4	2	2	1	1	4	4
4	4	1	1	1	1	1	1	1	1
4	4	1	1	1	1	3	3	1	1
4	4	4	4	2	2	1	1	1	1
4	4	1	1	1	1	1	1	1	1
4	4	1	1	1	1	3	3	1	1
4	4	1	1	1	1	1	1	1	1
4	4	1	1	1	1	1	1	4	4
4	4	1	1	2	2	1	1	1	1
4	4	1	1	1	1	1	1	1	1
4	4	1	1	1	1	1	1	1	1
4	4	1	1	1	1	1	1	1	1
4	4	1	1	2	2	1	1	4	4
4	4	1	1	1	1	1	1	1	1
4	4	1	1	1	1	1	1	1	1
4	4	1	1	2	2	1	1	1	1

For Review Only

4	4	1	1	1	1	1	1	4	4
4	4	1	1	2	2	1	1	4	4
4	4	1	1	1	1	1	1	1	1
4	4	1	1	1	1	1	1	4	4
4	4	1	1	1	1	1	1	1	1
4	4	1	1	2	2	1	1	1	1
4	4	1	1	1	1	1	1	1	1
4	4	1	1	1	1	1	1	1	1
4	4	1	1	1	1	1	1	1	1
4	4	1	1	2	2	1	1	1	1
4	4	1	1	1	1	1	1	1	1
4	4	1	1	1	1	1	1	4	4
4	4	4	4	1	1	1	1	1	1
4	4	1	1	1	1	1	1	1	1
4	4	1	1	1	1	1	1	1	1
4	4	1	1	1	1	1	1	1	1
4	4	1	1	1	1	1	1	1	1
4	4	1	1	1	1	1	1	1	1
4	4	1	1	1	1	1	1	1	1
4	4	4	4	2	2	1	1	1	1
4	4	1	1	1	1	1	1	1	1
4	4	1	1	1	1	1	1	1	1
4	4	1	1	1	1	1	1	1	1
4	4	1	1	2	2	1	1	4	4
4	4	1	1	1	1	1	1	1	1
4	4	1	1	1	1	1	1	4	4
4	4	1	1	1	1	1	1	1	1
4	4	1	1	1	1	1	1	1	1
4	4	1	1	1	1	1	1	1	1
4	4	4	4	1	1	1	1	1	1
4	4	1	1	2	2	1	1	1	1
4	4	1	1	1	1	1	1	4	4
4	4	1	1	1	1	1	1	1	1
4	4	1	1	2	2	1	1	4	4
4	4	1	1	1	1	1	1	4	4
4	4	1	1	1	1	1	1	4	4
4	4	1	1	1	1	1	1	1	1
4	4	1	1	1	1	1	1	1	1
4	4	1	1	1	1	1	1	4	4
4	4	1	1	2	2	1	1	4	4
4	4	1	1	1	1	1	1	4	4
4	4	1	1	1	1	1	1	4	4
4	4	1	1	1	1	1	1	1	1
4	4	1	1	2	2	1	1	1	1
4	4	1	1	1	1	1	1	1	1
1	1	1	1	1	1	1	1	1	1
4	4	1	1	2	2	1	1	1	1
4	4	1	1	2	2	1	1	1	1
4	4	1	1	1	1	1	1	4	4
4	4	1	1	1	1	1	1	1	1
4	4	1	1	2	2	1	1	4	4
4	4	1	1	1	1	1	1	4	4
4	4	1	1	1	1	1	1	4	4
4	4	1	1	1	1	1	1	1	1
4	4	1	1	2	2	1	1	4	4

For Review Only

4	4	1	1	2	2	1	1	1	1
4	4	4	4	2	2	1	1	4	4
4	4	1	1	1	1	1	1	1	1
4	4	1	1	1	1	1	1	4	4
4	4	1	1	1	1	1	1	1	1
4	4	1	1	1	1	1	1	1	1
4	4	1	1	1	1	1	1	4	4
4	4	1	1	1	1	1	1	1	1
4	4	1	1	1	1	1	1	4	4
4	4	1	1	1	1	1	1	1	1
4	4	1	1	1	1	1	1	1	1
4	4	1	1	1	1	1	1	4	4
4	4	1	1	1	1	1	1	1	1
1	1	1	1	1	1	1	1	1	1
4	4	1	1	1	1	1	1	1	1
1	1	1	1	2	2	1	1	4	4
4	4	1	1	1	1	1	1	4	4
4	4	1	1	1	1	1	1	1	1
4	4	1	1	2	2	1	1	1	1
4	4	1	1	1	1	1	1	1	1
4	4	1	1	1	1	1	1	1	1
4	4	1	1	2	2	1	1	1	1
4	4	1	1	1	1	1	1	4	4
4	4	1	1	1	1	3	3	1	1
4	4	1	1	1	1	3	3	1	1
4	4	1	1	1	1	1	1	4	4
4	4	1	1	1	1	1	1	1	1
4	4	1	1	1	1	1	1	1	1
4	4	1	1	1	1	1	1	1	1
4	4	1	1	1	1	1	1	1	1
4	4	1	1	1	1	1	1	1	1
4	4	1	1	1	1	1	1	1	1
4	4	1	1	1	1	1	1	1	1
4	4	1	1	1	1	1	1	4	4
1	1	1	1	1	1	1	1	1	1
4	4	1	1	1	1	1	1	1	1
4	4	1	1	1	1	3	3	1	1
4	4	1	1	1	1	1	1	1	1
4	4	1	1	2	2	1	1	1	1
4	4	1	1	1	1	1	1	1	1
4	4	1	1	1	1	1	1	1	1
4	4	1	1	2	2	1	1	1	1
4	4	1	1	2	2	1	1	4	4
4	4	1	1	1	1	1	1	1	1
4	4	1	1	1	1	1	1	1	1
4	4	1	1	1	1	1	1	1	1
1	1	1	1	2	2	1	1	4	4
4	4	1	1	2	2	1	1	4	4
4	4	1	1	1	1	1	1	1	1
4	4	1	1	1	1	1	1	1	1
4	4	1	1	1	1	1	1	1	1
4	4	1	1	1	1	1	1	1	1
4	4	1	1	1	1	1	1	4	4
4	4	1	1	1	1	1	1	1	1
4	4	1	1	1	1	1	1	4	4

For Review Only

4	4	1	1	1	1	1	1	1	1
4	4	1	1	1	1	1	1	4	4
4	4	1	1	1	1	1	1	1	1
4	4	1	1	1	1	1	1	4	4
4	4	1	1	1	1	1	1	4	4
4	4	1	1	1	1	3	3	1	1
4	4	4	4	1	1	1	1	4	4
4	4	1	1	2	2	1	1	1	1
4	4	1	1	1	1	1	1	1	1
4	4	1	1	2	2	1	1	4	4
4	4	1	1	1	1	1	1	1	1
4	4	4	4	1	1	1	1	1	1
1	1	1	1	1	1	1	1	4	4
4	4	1	1	2	2	1	1	1	1
4	4	1	1	1	1	1	1	1	1
1	1	1	1	1	1	1	1	1	1
4	4	1	1	1	1	1	1	4	4
4	4	1	1	1	1	1	1	1	1
4	4	1	1	2	2	1	1	4	4
4	4	1	1	2	2	1	1	4	4
4	4	1	1	1	1	1	1	1	1
4	4	1	1	2	2	1	1	1	1
4	4	1	1	1	1	1	1	1	1
4	4	1	1	1	1	1	1	4	4
4	4	1	1	2	2	1	1	1	1
4	4	1	1	1	1	1	1	1	1
4	4	1	1	2	2	1	1	4	4
4	4	1	1	1	1	1	1	1	1
4	4	1	1	1	1	1	1	4	4
4	4	1	1	1	1	1	1	1	1
4	4	1	1	1	1	1	1	4	4
4	4	1	1	2	2	1	1	4	4
1	1	1	1	2	2	1	1	1	1
4	4	1	1	2	2	1	1	1	1
4	4	1	1	1	1	1	1	4	4
4	4	1	1	2	2	1	1	4	4
4	4	1	1	1	1	1	1	1	1
4	4	1	1	1	1	1	1	1	1
4	4	1	1	2	2	1	1	4	4
4	4	1	1	1	1	1	1	4	4
4	4	1	1	1	1	1	1	1	1
1	1	1	1	1	1	1	1	1	1
4	4	1	1	1	1	1	1	4	4
4	4	1	1	1	1	1	1	1	1
4	4	1	1	1	1	1	1	1	1
4	4	4	4	1	1	1	1	4	4
4	4	1	1	1	1	1	1	4	4
4	4	1	1	1	1	1	1	1	1
4	4	1	1	2	2	1	1	1	1
4	4	1	1	2	2	1	1	4	4
4	4	1	1	1	1	1	1	1	1
4	4	1	1	2	2	1	1	4	4
4	4	1	1	1	1	1	1	1	1
4	4	1	1	1	1	1	1	1	1
4	4	1	1	1	1	1	1	4	4

For Review Only

1	1	1	1	1	1	1	1	1	1
4	4	1	1	1	1	1	1	1	1

For Review Only

SNP2059	SNP2059	SNP206	SNP206	SNP2060	SNP2060	SNP2061	SNP2061	SNP2062	SNP2062
3	3	3	3	1	1	4	4	1	1
3	3	2	2	1	1	4	4	1	1
3	3	2	2	4	4	4	4	4	4
3	3	3	3	1	1	4	4	1	1
3	3	3	3	1	1	4	4	1	1
3	3	3	3	1	1	4	4	1	1
3	3	3	3	1	1	4	4	1	1
3	3	2	2	1	1	4	4	1	1
4	4	3	3	1	1	4	4	1	1
3	3	2	2	1	1	4	4	1	1
3	3	3	3	1	1	4	4	1	1
3	3	2	2	1	1	4	4	1	1
3	3	2	2	1	1	4	4	1	1
4	4	2	2	1	1	4	4	1	1
3	3	2	2	1	1	4	4	1	1
4	4	3	3	1	1	4	4	1	1
3	3	2	2	1	1	4	4	1	1
3	3	2	2	1	1	4	4	1	1
3	3	2	2	1	1	4	4	1	1
3	3	3	3	1	1	4	4	1	1
3	3	2	2	1	1	4	4	1	1
3	3	2	2	1	1	4	4	1	1
4	4	2	2	1	1	4	4	1	1
4	4	2	2	1	1	1	1	1	1
3	3	2	2	1	1	4	4	1	1
3	3	2	2	1	1	4	4	1	1
3	3	2	2	1	1	4	4	1	1
3	3	2	2	1	1	4	4	1	1
3	3	2	2	1	1	4	4	1	1
3	3	2	2	1	1	4	4	1	1
3	3	2	2	1	1	4	4	1	1
3	3	2	2	4	4	4	4	4	4
3	3	2	2	1	1	4	4	1	1
4	4	2	2	1	1	4	4	1	1
4	4	3	3	1	1	4	4	1	1
4	4	3	3	1	1	4	4	1	1
3	3	3	3	1	1	4	4	1	1
3	3	2	2	1	1	4	4	1	1
4	4	2	2	1	1	4	4	1	1
4	4	3	3	1	1	1	1	1	1
3	3	2	2	1	1	4	4	1	1
3	3	2	2	1	1	4	4	1	1
3	3	2	2	1	1	4	4	1	1
3	3	3	3	1	1	4	4	1	1
3	3	2	2	1	1	4	4	1	1
3	3	2	2	1	1	4	4	1	1
3	3	3	3	1	1	4	4	1	1
3	3	2	2	1	1	4	4	1	1
3	3	3	3	1	1	4	4	1	1
3	3	3	3	1	1	4	4	1	1
3	3	2	2	1	1	4	4	1	1
3	3	2	2	1	1	4	4	1	1

For Review Only

3	3	3	3	1	1	4	4	1	1
3	3	2	2	1	1	4	4	1	1
3	3	2	2	1	1	4	4	1	1
3	3	2	2	1	1	4	4	1	1
3	3	2	2	1	1	4	4	1	1
3	3	2	2	1	1	4	4	1	1
3	3	2	2	1	1	4	4	1	1
3	3	2	2	1	1	4	4	1	1
3	3	3	3	1	1	4	4	1	1
3	3	2	2	1	1	4	4	1	1
3	3	2	2	1	1	4	4	1	1
3	3	2	2	1	1	4	4	1	1
3	3	3	3	1	1	4	4	1	1
3	3	2	2	1	1	4	4	1	1
4	4	2	2	1	1	4	4	1	1
3	3	3	3	1	1	4	4	1	1
4	4	3	3	4	4	4	4	4	4
3	3	3	3	1	1	4	4	1	1
3	3	3	3	1	1	4	4	1	1
3	3	3	3	1	1	4	4	1	1
3	3	2	2	1	1	4	4	1	1
4	4	2	2	1	1	4	4	1	1
3	3	2	2	1	1	4	4	1	1
3	3	2	2	1	1	4	4	1	1
3	3	2	2	1	1	4	4	1	1
3	3	3	3	1	1	1	1	1	1
3	3	2	2	4	4	4	4	4	4
4	4	2	2	1	1	4	4	1	1
3	3	2	2	1	1	4	4	1	1
3	3	2	2	1	1	4	4	1	1
3	3	3	3	1	1	4	4	1	1
3	3	2	2	1	1	4	4	1	1
4	4	2	2	1	1	1	1	1	1
3	3	3	3	1	1	4	4	1	1
3	3	3	3	1	1	4	4	1	1
3	3	2	2	1	1	4	4	1	1
3	3	2	2	1	1	4	4	1	1
3	3	3	3	1	1	4	4	1	1
4	4	2	2	1	1	4	4	1	1
4	4	3	3	1	1	4	4	1	1
3	3	2	2	1	1	4	4	1	1
3	3	3	3	4	4	4	4	4	4
3	3	3	3	1	1	4	4	1	1
3	3	2	2	1	1	4	4	1	1
3	3	3	3	1	1	4	4	1	1
3	3	2	2	1	1	4	4	1	1
4	4	2	2	1	1	1	1	1	1
3	3	2	2	1	1	1	1	1	1
3	3	2	2	1	1	4	4	1	1
3	3	3	3	1	1	4	4	1	1
3	3	2	2	1	1	4	4	1	1
3	3	2	2	1	1	4	4	1	1
3	3	3	3	1	1	4	4	1	1
3	3	2	2	1	1	4	4	1	1
3	3	2	2	1	1	4	4	1	1

For Review Only

4	4	2	2	1	1	1	1	1	1
4	4	2	2	1	1	4	4	1	1
3	3	3	3	1	1	4	4	1	1
4	4	2	2	1	1	1	1	1	1
3	3	2	2	1	1	4	4	1	1
3	3	2	2	1	1	4	4	1	1
3	3	2	2	1	1	4	4	1	1
4	4	2	2	1	1	4	4	1	1
3	3	2	2	1	1	4	4	1	1
4	4	2	2	1	1	4	4	1	1
4	4	3	3	1	1	4	4	1	1
3	3	2	2	1	1	4	4	1	1
3	3	2	2	1	1	4	4	1	1
3	3	3	3	1	1	4	4	1	1
3	3	2	2	1	1	4	4	1	1
3	3	2	2	1	1	4	4	1	1
3	3	2	2	1	1	4	4	1	1
3	3	2	2	1	1	4	4	1	1
4	4	2	2	1	1	1	1	1	1
3	3	2	2	1	1	1	1	1	1
3	3	2	2	1	1	4	4	1	1
3	3	2	2	1	1	4	4	1	1
3	3	2	2	1	1	4	4	4	4
3	3	2	2	1	1	4	4	1	1
4	4	2	2	1	1	4	4	1	1
4	4	3	3	1	1	1	1	1	1
3	3	2	2	1	1	4	4	1	1
3	3	2	2	1	1	4	4	1	1
3	3	2	2	1	1	4	4	1	1
3	3	2	2	1	1	4	4	1	1
4	4	2	2	1	1	1	1	1	1
3	3	2	2	1	1	1	1	1	1
3	3	2	2	1	1	4	4	1	1
4	4	2	2	1	1	4	4	1	1
4	4	2	2	1	1	4	4	1	1
4	4	2	2	1	1	4	4	1	1
3	3	2	2	1	1	1	1	1	1
3	3	3	3	1	1	4	4	1	1
4	4	2	2	1	1	4	4	1	1
4	4	3	3	1	1	4	4	1	1
4	4	2	2	1	1	1	1	1	1
4	4	2	2	1	1	4	4	1	1
3	3	3	3	1	1	4	4	1	1
3	3	2	2	1	1	4	4	1	1
3	3	2	2	1	1	4	4	1	1
3	3	2	2	1	1	4	4	1	1
3	3	2	2	1	1	4	4	1	1
3	3	2	2	4	4	4	4	4	4
4	4	2	2	1	1	4	4	1	1
3	3	2	2	1	1	4	4	1	1
4	4	2	2	1	1	4	4	1	1
3	3	3	3	1	1	1	1	1	1
3	3	3	3	1	1	4	4	1	1
3	3	2	2	1	1	4	4	1	1
3	3	2	2	1	1	4	4	1	1

For Review Only

3	3	2	2	1	1	4	4	1	1
4	4	3	3	1	1	1	1	1	1
3	3	3	3	1	1	4	4	1	1
4	4	3	3	1	1	4	4	1	1
3	3	2	2	1	1	4	4	1	1
3	3	2	2	1	1	4	4	1	1
4	4	2	2	1	1	1	1	1	1
3	3	2	2	1	1	4	4	1	1
4	4	2	2	1	1	4	4	1	1
3	3	2	2	1	1	4	4	1	1
3	3	2	2	1	1	4	4	1	1
4	4	2	2	1	1	4	4	1	1
4	4	3	3	1	1	4	4	1	1
3	3	3	3	1	1	4	4	1	1
3	3	2	2	1	1	4	4	1	1
4	4	3	3	1	1	4	4	1	1
3	3	2	2	1	1	4	4	1	1
3	3	2	2	1	1	4	4	1	1
3	3	2	2	1	1	4	4	1	1
3	3	2	2	1	1	4	4	1	1
3	3	2	2	1	1	4	4	1	1
3	3	2	2	1	1	4	4	1	1
4	4	3	3	1	1	4	4	1	1
3	3	2	2	1	1	4	4	1	1
3	3	3	3	1	1	4	4	1	1
4	4	3	3	1	1	4	4	1	1
3	3	2	2	1	1	4	4	1	1
3	3	2	2	1	1	4	4	1	1
3	3	2	2	1	1	4	4	1	1
3	3	3	3	1	1	4	4	1	1
3	3	3	3	1	1	4	4	1	1
3	3	2	2	1	1	4	4	1	1
3	3	3	3	1	1	4	4	1	1
4	4	2	2	1	1	4	4	1	1
3	3	3	3	1	1	4	4	1	1
3	3	2	2	1	1	4	4	1	1
3	3	3	3	1	1	4	4	1	1
3	3	3	3	1	1	4	4	1	1
3	3	2	2	4	4	4	4	4	4
3	3	2	2	1	1	4	4	1	1
3	3	2	2	4	4	4	4	4	4
3	3	2	2	1	1	4	4	1	1
4	4	2	2	1	1	4	4	1	1
3	3	2	2	1	1	4	4	1	1
3	3	3	3	1	1	4	4	1	1
3	3	2	2	1	1	4	4	1	1
4	4	3	3	1	1	4	4	1	1
3	3	2	2	1	1	4	4	1	1
3	3	2	2	1	1	1	1	1	1
3	3	2	2	1	1	4	4	1	1
3	3	2	2	1	1	4	4	1	1
3	3	3	3	1	1	4	4	1	1
4	4	3	3	1	1	4	4	1	1
3	3	2	2	1	1	4	4	1	1
4	4	2	2	1	1	4	4	1	1

For Review Only

3	3	3	3	1	1	4	4	1	1
4	4	2	2	1	1	4	4	1	1
3	3	2	2	1	1	4	4	1	1
4	4	2	2	1	1	4	4	1	1
4	4	2	2	1	1	4	4	1	1
3	3	3	3	1	1	4	4	1	1
4	4	2	2	1	1	4	4	1	1
3	3	2	2	1	1	4	4	1	1
3	3	2	2	1	1	4	4	1	1
3	3	2	2	1	1	4	4	1	1
3	3	2	2	1	1	4	4	1	1
3	3	3	3	1	1	4	4	1	1
4	4	2	2	1	1	4	4	1	1
3	3	2	2	1	1	4	4	1	1
3	3	2	2	4	4	4	4	4	4
3	3	3	3	1	1	4	4	1	1
4	4	2	2	1	1	4	4	1	1
3	3	3	3	1	1	4	4	1	1
4	4	2	2	1	1	4	4	1	1
3	3	3	3	1	1	4	4	1	1
3	3	2	2	1	1	1	1	1	1
3	3	3	3	1	1	4	4	1	1
3	3	3	3	1	1	4	4	1	1
4	4	3	3	1	1	4	4	1	1
3	3	2	2	1	1	4	4	1	1
3	3	2	2	1	1	4	4	1	1
4	4	2	2	1	1	4	4	1	1
3	3	2	2	1	1	4	4	1	1
4	4	2	2	1	1	4	4	1	1
3	3	2	2	1	1	4	4	1	1
4	4	2	2	1	1	4	4	1	1
3	3	3	3	1	1	4	4	1	1
3	3	3	3	1	1	4	4	1	1
3	3	2	2	1	1	4	4	1	1
4	4	3	3	1	1	4	4	1	1
4	4	2	2	1	1	1	1	1	1
3	3	3	3	1	1	4	4	1	1
3	3	2	2	1	1	4	4	1	1
4	4	3	3	1	1	4	4	1	1
3	3	2	2	1	1	1	1	1	1
3	3	2	2	1	1	4	4	1	1
3	3	2	2	1	1	4	4	1	1
4	4	3	3	1	1	1	1	1	1
3	3	3	3	1	1	4	4	1	1
3	3	2	2	1	1	4	4	1	1
4	4	2	2	1	1	4	4	1	1
4	4	2	2	4	4	4	4	4	4
3	3	2	2	1	1	4	4	1	1
3	3	2	2	1	1	4	4	1	1
4	4	2	2	1	1	4	4	1	1
3	3	3	3	1	1	4	4	1	1
4	4	2	2	1	1	4	4	1	1
3	3	2	2	4	4	4	4	4	4
3	3	2	2	1	1	4	4	1	1
4	4	2	2	1	1	1	1	1	1

For Review Only

3	3	3	3	1	1	4	4	1	1
3	3	2	2	1	1	4	4	1	1

For Review Only

SNP2063	SNP2063	SNP2064	SNP2064	SNP2065	SNP2065	SNP2066	SNP2066	SNP2067	SNP2067
2	2	1	1	3	3	3	3	2	2
2	2	3	3	3	3	3	3	1	1
3	3	3	3	3	3	3	3	1	1
2	2	3	3	3	3	3	3	1	1
2	2	1	1	3	3	3	3	2	2
2	2	3	3	3	3	3	3	2	2
2	2	1	1	3	3	3	3	1	1
2	2	3	3	3	3	3	3	1	1
2	2	3	3	3	3	3	3	1	1
2	2	3	3	3	3	3	3	1	1
2	2	3	3	2	2	3	3	1	1
2	2	3	3	3	3	3	3	1	1
2	2	3	3	3	3	3	3	1	1
2	2	1	1	3	3	3	3	1	1
2	2	3	3	3	3	3	3	1	1
2	2	3	3	3	3	3	3	1	1
2	2	1	1	3	3	3	3	2	2
3	3	1	1	3	3	3	3	1	1
2	2	3	3	3	3	3	3	1	1
2	2	3	3	3	3	3	3	1	1
2	2	1	1	3	3	3	3	1	1
2	2	1	1	3	3	3	3	1	1
2	2	3	3	3	3	3	3	2	2
2	2	3	3	3	3	4	4	1	1
2	2	1	1	3	3	3	3	1	1
2	2	3	3	3	3	3	3	1	1
2	2	3	3	3	3	3	3	1	1
2	2	3	3	3	3	3	3	1	1
2	2	3	3	3	3	3	3	1	1
2	2	3	3	3	3	3	3	2	2
2	2	3	3	3	3	3	3	1	1
2	2	3	3	3	3	3	3	1	1
2	2	3	3	3	3	3	3	2	2
2	2	3	3	3	3	3	3	1	1
2	2	3	3	2	2	3	3	1	1
2	2	3	3	3	3	3	3	1	1
2	2	3	3	3	3	3	3	1	1
2	2	3	3	3	3	3	3	1	1
2	2	3	3	3	3	4	4	1	1
2	2	3	3	3	3	4	4	1	1
3	3	3	3	3	3	3	3	1	1
2	2	3	3	3	3	3	3	1	1
2	2	3	3	3	3	3	3	1	1
2	2	1	1	3	3	3	3	2	2
2	2	3	3	3	3	3	3	1	1
2	2	3	3	3	3	3	3	1	1
2	2	3	3	3	3	3	3	1	1
2	2	3	3	3	3	3	3	1	1
2	2	1	1	3	3	3	3	2	2
2	2	3	3	3	3	3	3	1	1
2	2	3	3	3	3	3	3	1	1
2	2	3	3	3	3	3	3	1	1

For Review Only

2	2	1	1	3	3	3	3	2	2
2	2	3	3	3	3	3	3	2	2
2	2	3	3	3	3	4	4	1	1
2	2	1	1	3	3	3	3	1	1
2	2	3	3	3	3	3	3	1	1
2	2	3	3	3	3	3	3	1	1
2	2	3	3	3	3	3	3	1	1
3	3	3	3	3	3	3	3	2	2
2	2	3	3	3	3	3	3	1	1
2	2	3	3	3	3	3	3	1	1
2	2	3	3	3	3	3	3	1	1
2	2	3	3	3	3	3	3	1	1
2	2	1	1	3	3	3	3	2	2
2	2	3	3	3	3	3	3	1	1
2	2	3	3	3	3	3	3	1	1
2	2	3	3	3	3	3	3	1	1
3	3	3	3	2	2	3	3	1	1
2	2	3	3	3	3	3	3	1	1
2	2	1	1	3	3	3	3	1	1
2	2	1	1	3	3	3	3	2	2
2	2	3	3	3	3	3	3	1	1
2	2	3	3	3	3	3	3	1	1
2	2	1	1	3	3	3	3	1	1
2	2	3	3	2	2	3	3	1	1
2	2	3	3	3	3	3	3	1	1
2	2	3	3	3	3	4	4	1	1
2	2	3	3	3	3	3	3	2	2
3	3	3	3	3	3	3	3	1	1
2	2	3	3	3	3	3	3	1	1
2	2	1	1	3	3	3	3	1	1
2	2	1	1	3	3	3	3	1	1
2	2	1	1	3	3	4	4	1	1
3	3	3	3	3	3	4	4	2	2
2	2	1	1	3	3	3	3	2	2
2	2	3	3	3	3	3	3	1	1
2	2	3	3	3	3	3	3	1	1
2	2	1	1	3	3	3	3	1	1
2	2	3	3	3	3	3	3	2	2
2	2	3	3	2	2	3	3	1	1
2	2	3	3	3	3	3	3	1	1
2	2	1	1	2	2	3	3	1	1
3	3	1	1	3	3	3	3	1	1
2	2	3	3	3	3	3	3	1	1
2	2	3	3	3	3	3	3	1	1
2	2	3	3	3	3	3	3	1	1
2	2	1	1	3	3	3	3	1	1
2	2	3	3	3	3	4	4	1	1
2	2	3	3	3	3	4	4	1	1
2	2	3	3	3	3	3	3	1	1
2	2	3	3	3	3	3	3	1	1
2	2	3	3	3	3	3	3	1	1
2	2	1	1	3	3	3	3	2	2
3	3	3	3	3	3	3	3	2	2
2	2	3	3	3	3	3	3	1	1
2	2	3	3	3	3	3	3	1	1

For Review Only

2	2	3	3	3	3	4	4	1	1
2	2	3	3	3	3	3	3	1	1
2	2	3	3	3	3	3	3	1	1
2	2	1	1	3	3	4	4	1	1
2	2	3	3	3	3	3	3	1	1
2	2	3	3	3	3	3	3	1	1
2	2	3	3	3	3	3	3	1	1
2	2	3	3	2	2	3	3	1	1
2	2	3	3	3	3	3	3	2	2
2	2	3	3	3	3	3	3	1	1
3	3	1	1	3	3	3	3	2	2
2	2	3	3	3	3	3	3	1	1
2	2	3	3	3	3	3	3	1	1
2	2	3	3	3	3	3	3	1	1
2	2	3	3	3	3	3	3	1	1
2	2	3	3	3	3	3	3	1	1
3	3	1	1	3	3	3	3	1	1
2	2	3	3	3	3	3	3	1	1
2	2	3	3	3	3	4	4	1	1
3	3	3	3	3	3	4	4	1	1
2	2	3	3	3	3	3	3	1	1
2	2	3	3	3	3	3	3	1	1
3	3	3	3	3	3	3	3	1	1
2	2	3	3	3	3	3	3	1	1
2	2	3	3	3	3	3	3	1	1
2	2	3	3	3	3	4	4	1	1
2	2	3	3	3	3	3	3	1	1
2	2	3	3	3	3	3	3	1	1
2	2	3	3	2	2	3	3	1	1
2	2	3	3	3	3	3	3	1	1
3	3	3	3	3	3	3	3	2	2
2	2	3	3	3	3	3	3	1	1
2	2	1	1	3	3	3	3	1	1
2	2	3	3	3	3	3	3	1	1
2	2	3	3	3	3	3	3	1	1
2	2	3	3	3	3	3	3	1	1
2	2	3	3	3	3	4	4	1	1
2	2	1	1	3	3	3	3	1	1
2	2	3	3	3	3	3	3	1	1
2	2	3	3	2	2	3	3	2	2
2	2	3	3	3	3	4	4	1	1
2	2	3	3	3	3	3	3	1	1
2	2	3	3	3	3	3	3	1	1
2	2	3	3	3	3	4	4	1	1
2	2	3	3	3	3	3	3	1	1
2	2	3	3	2	2	3	3	2	2
2	2	3	3	3	3	3	3	1	1
3	3	3	3	3	3	3	3	1	1
2	2	3	3	3	3	3	3	1	1
2	2	3	3	3	3	3	3	1	1
2	2	3	3	3	3	3	3	1	1
3	3	3	3	3	3	4	4	1	1
2	2	3	3	3	3	3	3	1	1
2	2	3	3	3	3	3	3	1	1
2	2	3	3	3	3	3	3	1	1

For Review Only

2	2	3	3	3	3	3	3	1	1
2	2	3	3	3	3	4	4	1	1
2	2	1	1	3	3	3	3	1	1
2	2	3	3	3	3	3	3	1	1
2	2	3	3	3	3	3	3	1	1
2	2	3	3	3	3	3	3	2	2
2	2	1	1	3	3	3	3	1	1
2	2	3	3	3	3	3	3	1	1
2	2	3	3	2	2	3	3	1	1
2	2	1	1	3	3	3	3	1	1
2	2	3	3	3	3	3	3	1	1
2	2	3	3	3	3	3	3	1	1
2	2	3	3	3	3	3	3	1	1
2	2	3	3	2	2	3	3	1	1
2	2	3	3	3	3	3	3	1	1
2	2	3	3	2	2	3	3	1	1
2	2	3	3	3	3	3	3	1	1
2	2	3	3	3	3	4	4	1	1
2	2	1	1	3	3	3	3	1	1
2	2	3	3	3	3	3	3	1	1
2	2	3	3	3	3	3	3	1	1
2	2	3	3	3	3	3	3	1	1
2	2	3	3	3	3	3	3	1	1
2	2	3	3	3	3	3	3	1	1
2	2	1	1	3	3	3	3	1	1
2	2	3	3	3	3	3	3	1	1
2	2	3	3	3	3	3	3	1	1
2	2	3	3	3	3	3	3	1	1
2	2	3	3	3	3	3	3	2	2
2	2	1	1	3	3	3	3	1	1
3	3	1	1	3	3	3	3	1	1
2	2	1	1	3	3	3	3	1	1
2	2	1	1	3	3	3	3	1	1
2	2	3	3	3	3	3	3	1	1
2	2	3	3	2	2	3	3	1	1
2	2	3	3	3	3	3	3	1	1
2	2	3	3	3	3	3	3	1	1
2	2	3	3	3	3	3	3	1	1
3	3	3	3	3	3	3	3	1	1
2	2	3	3	3	3	3	3	1	1
3	3	3	3	3	3	3	3	1	1
2	2	3	3	3	3	3	3	1	1
2	2	3	3	3	3	3	3	1	1
2	2	3	3	3	3	3	3	1	1
2	2	3	3	3	3	3	3	1	1
2	2	3	3	3	3	3	3	2	2
2	2	3	3	2	2	3	3	1	1
3	3	1	1	3	3	3	3	2	2
2	2	3	3	3	3	4	4	1	1
2	2	3	3	3	3	3	3	1	1
2	2	3	3	3	3	3	3	1	1
2	2	3	3	3	3	3	3	2	2
2	2	1	1	3	3	3	3	1	1
2	2	3	3	3	3	3	3	1	1
2	2	3	3	3	3	3	3	2	2

For Review Only

2	2	3	3	3	3	3	3	1	1
3	3	1	1	2	2	3	3	1	1
2	2	3	3	3	3	3	3	1	1
2	2	3	3	3	3	3	3	1	1
2	2	3	3	3	3	3	3	1	1
2	2	3	3	3	3	3	3	1	1
2	2	1	1	2	2	3	3	1	1
2	2	3	3	3	3	3	3	1	1
2	2	3	3	3	3	3	3	1	1
3	3	3	3	3	3	3	3	2	2
2	2	3	3	3	3	3	3	1	1
3	3	1	1	3	3	3	3	1	1
2	2	3	3	2	2	3	3	1	1
2	2	3	3	3	3	3	3	1	1
3	3	3	3	3	3	3	3	1	1
2	2	3	3	2	2	3	3	1	1
2	2	3	3	3	3	3	3	1	1
2	2	3	3	3	3	3	3	1	1
2	2	3	3	3	3	3	3	2	2
2	2	3	3	3	3	3	3	1	1
2	2	3	3	3	3	4	4	1	1
2	2	3	3	3	3	3	3	1	1
2	2	3	3	3	3	3	3	1	1
2	2	1	1	3	3	3	3	1	1
3	3	3	3	3	3	3	3	2	2
2	2	1	1	3	3	3	3	1	1
3	3	3	3	3	3	3	3	2	2
2	2	3	3	3	3	3	3	1	1
2	2	3	3	3	3	3	3	1	1
2	2	3	3	3	3	3	3	1	1
2	2	3	3	3	3	3	3	2	2
2	2	3	3	3	3	3	3	1	1
2	2	3	3	2	2	3	3	1	1
2	2	3	3	3	3	3	3	1	1
2	2	3	3	3	3	3	3	1	1
2	2	1	1	3	3	4	4	2	2
2	2	3	3	3	3	3	3	1	1
2	2	3	3	3	3	3	3	1	1
2	2	3	3	3	3	3	3	1	1
2	2	3	3	3	3	4	4	1	1
2	2	1	1	2	2	3	3	1	1
2	2	3	3	2	2	3	3	1	1
2	2	1	1	3	3	4	4	1	1
2	2	1	1	3	3	3	3	1	1
2	2	3	3	3	3	3	3	1	1
2	2	3	3	2	2	3	3	1	1
3	3	3	3	3	3	3	3	1	1
2	2	1	1	3	3	3	3	1	1
3	3	3	3	3	3	3	3	1	1
3	3	3	3	3	3	3	3	2	2
2	2	1	1	3	3	3	3	1	1
2	2	3	3	2	2	3	3	1	1
3	3	3	3	3	3	3	3	1	1
2	2	3	3	3	3	3	3	1	1
2	2	3	3	3	3	3	3	1	1

For Review Only

2	2	3	3	2	2	3	3	1	1
2	2	3	3	2	2	3	3	1	1

For Review Only

SNP2068	SNP2068	SNP2069	SNP2069	SNP207	SNP207	SNP2070	SNP2070	SNP2071	SNP2071
1	1	1	1	4	4	1	1	4	4
1	1	2	2	4	4	1	1	4	4
1	1	2	2	2	2	1	1	2	2
1	1	2	2	2	2	1	1	4	4
1	1	1	1	4	4	1	1	4	4
1	1	2	2	4	4	1	1	4	4
1	1	1	1	4	4	1	1	4	4
1	1	2	2	4	4	1	1	4	4
1	1	1	1	4	4	1	1	4	4
1	1	2	2	4	4	1	1	4	4
1	1	2	2	2	2	1	1	2	2
1	1	2	2	2	2	1	1	4	4
1	1	2	2	4	4	1	1	4	4
1	1	1	1	4	4	1	1	4	4
1	1	2	2	2	2	1	1	4	4
1	1	2	2	4	4	1	1	4	4
1	1	1	1	2	2	1	1	4	4
1	1	1	1	2	2	1	1	4	4
1	1	2	2	2	2	1	1	2	2
1	1	2	2	4	4	1	1	4	4
1	1	1	1	4	4	1	1	4	4
1	1	1	1	4	4	1	1	4	4
1	1	2	2	4	4	1	1	4	4
1	1	2	2	4	4	1	1	4	4
1	1	1	1	4	4	1	1	4	4
1	1	2	2	2	2	1	1	4	4
1	1	2	2	2	2	1	1	4	4
4	4	1	1	2	2	1	1	4	4
1	1	2	2	4	4	1	1	4	4
1	1	2	2	4	4	1	1	4	4
1	1	2	2	2	2	1	1	2	2
1	1	1	1	2	2	1	1	2	2
1	1	1	1	4	4	1	1	2	2
1	1	2	2	4	4	1	1	4	4
1	1	2	2	2	2	1	1	2	2
1	1	2	2	2	2	1	1	4	4
4	4	1	1	4	4	1	1	2	2
4	4	2	2	2	2	1	1	4	4
1	1	1	1	4	4	1	1	4	4
4	4	2	2	4	4	1	1	4	4
1	1	2	2	4	4	1	1	4	4
1	1	2	2	4	4	1	1	4	4
1	1	2	2	4	4	1	1	4	4
1	1	1	1	4	4	1	1	2	2
1	1	2	2	4	4	1	1	4	4
1	1	2	2	2	2	1	1	4	4
1	1	2	2	2	2	1	1	4	4
1	1	2	2	2	2	1	1	4	4
1	1	1	1	4	4	1	1	2	2
1	1	2	2	2	2	1	1	4	4
1	1	2	2	2	2	1	1	4	4
1	1	2	2	4	4	1	1	4	4

For Review Only

1	1	1	1	4	4	1	1	4	4
1	1	1	1	4	4	1	1	2	2
1	1	2	2	2	2	1	1	4	4
1	1	1	1	2	2	4	4	4	4
1	1	2	2	2	2	1	1	2	2
1	1	2	2	2	2	1	1	4	4
1	1	2	2	2	2	1	1	4	4
1	1	2	2	4	4	1	1	4	4
1	1	2	2	4	4	1	1	2	2
1	1	2	2	2	2	1	1	4	4
1	1	2	2	2	2	1	1	4	4
1	1	2	2	2	2	1	1	4	4
1	1	1	1	4	4	1	1	4	4
1	1	2	2	4	4	1	1	4	4
1	1	2	2	2	2	1	1	4	4
1	1	2	2	4	4	1	1	4	4
1	1	2	2	4	4	1	1	2	2
1	1	2	2	2	2	1	1	2	2
1	1	1	1	4	4	1	1	4	4
1	1	1	1	4	4	1	1	4	4
1	1	2	2	2	2	1	1	4	4
1	1	2	2	2	2	1	1	4	4
1	1	2	2	4	4	1	1	4	4
1	1	1	1	2	2	1	1	2	2
1	1	2	2	4	4	1	1	4	4
1	1	2	2	2	2	1	1	4	4
1	1	1	1	4	4	1	1	2	2
1	1	2	2	4	4	1	1	2	2
1	1	2	2	2	2	1	1	4	4
1	1	1	1	2	2	1	1	4	4
1	1	2	2	2	2	1	1	4	4
1	1	1	1	2	2	1	1	4	4
1	1	1	1	4	4	1	1	2	2
1	1	1	1	4	4	1	1	4	4
4	4	1	1	4	4	1	1	4	4
1	1	2	2	4	4	1	1	4	4
1	1	2	2	2	2	1	1	4	4
1	1	2	2	4	4	1	1	4	4
1	1	2	2	2	2	1	1	2	2
4	4	2	2	4	4	1	1	4	4
1	1	1	1	2	2	1	1	4	4
1	1	2	2	2	2	1	1	4	4
1	1	2	2	4	4	1	1	4	4
1	1	2	2	2	2	1	1	4	4
1	1	2	2	2	2	1	1	2	2
1	1	1	1	2	2	1	1	4	4
1	1	2	2	4	4	1	1	4	4
1	1	2	2	2	2	1	1	4	4
1	1	2	2	2	2	1	1	4	4
1	1	2	2	2	2	1	1	2	2
1	1	2	2	2	2	1	1	4	4
1	1	2	2	4	4	1	1	4	4
1	1	2	2	4	4	1	1	4	4
1	1	2	2	2	2	1	1	2	2
1	1	2	2	4	4	1	1	2	2

For Review Only

1	1	2	2	2	2	1	1	4	4
1	1	2	2	4	4	1	1	4	4
1	1	2	2	2	2	1	1	4	4
1	1	2	2	4	4	1	1	4	4
1	1	2	2	2	2	1	1	4	4
1	1	2	2	2	2	1	1	2	2
1	1	2	2	2	2	1	1	4	4
1	1	2	2	2	2	1	1	4	4
1	1	2	2	2	2	1	1	4	4
1	1	2	2	2	2	1	1	4	4
1	1	1	1	4	4	1	1	4	4
1	1	2	2	2	2	1	1	4	4
1	1	2	2	4	4	1	1	4	4
1	1	2	2	2	2	1	1	4	4
1	1	2	2	2	2	1	1	4	4
1	1	2	2	2	2	1	1	4	4
1	1	2	2	2	2	1	1	2	2
1	1	2	2	2	2	1	1	4	4
1	1	2	2	4	4	1	1	4	4
1	1	2	2	4	4	1	1	4	4
1	1	2	2	2	2	1	1	4	4
1	1	2	2	2	2	1	1	4	4
1	1	2	2	4	4	1	1	2	2
1	1	1	1	4	4	1	1	2	2
1	1	2	2	2	2	1	1	4	4
4	4	1	1	2	2	1	1	2	2
1	1	1	1	2	2	1	1	4	4
4	4	1	1	2	2	1	1	2	2
1	1	2	2	2	2	1	1	2	2
1	1	2	2	2	2	1	1	4	4
1	1	2	2	4	4	1	1	4	4
1	1	2	2	2	2	1	1	4	4
1	1	1	1	2	2	1	1	4	4
1	1	2	2	2	2	1	1	4	4
1	1	2	2	4	4	1	1	4	4
1	1	2	2	4	4	1	1	4	4
1	1	2	2	4	4	1	1	2	2
1	1	1	1	2	2	1	1	4	4
1	1	2	2	2	2	1	1	4	4
1	1	2	2	4	4	1	1	4	4
1	1	2	2	2	2	1	1	2	2
1	1	1	1	4	4	1	1	2	2
1	1	2	2	2	2	1	1	2	2
1	1	2	2	2	2	1	1	4	4
1	1	2	2	4	4	1	1	4	4
1	1	2	2	2	2	1	1	2	2
1	1	2	2	2	2	1	1	4	4
1	1	2	2	2	2	1	1	4	4
1	1	2	2	4	4	1	1	4	4
1	1	2	2	2	2	1	1	4	4
1	1	2	2	2	2	1	1	4	4
1	1	2	2	4	4	1	1	4	4
1	1	2	2	2	2	1	1	2	2
1	1	2	2	2	2	1	1	2	2
1	1	2	2	2	2	1	1	4	4

For Review Only

1	1	2	2	2	2	1	1	2	2
1	1	2	2	2	2	1	1	2	2
1	1	1	1	2	2	1	1	4	4
1	1	2	2	2	2	1	1	2	2
1	1	2	2	2	2	1	1	4	4
4	4	1	1	4	4	1	1	2	2
1	1	1	1	4	4	1	1	4	4
1	1	2	2	2	2	1	1	4	4
1	1	2	2	4	4	1	1	4	4
1	1	1	1	2	2	1	1	4	4
1	1	2	2	2	2	1	1	4	4
1	1	2	2	4	4	1	1	4	4
1	1	2	2	2	2	1	1	4	4
1	1	2	2	2	2	1	1	2	2
1	1	1	1	4	4	1	1	2	2
1	1	2	2	2	2	1	1	2	2
1	1	2	2	2	2	1	1	2	2
1	1	2	2	2	2	1	1	4	4
1	1	1	1	4	4	1	1	4	4
1	1	2	2	2	2	1	1	4	4
1	1	2	2	2	2	1	1	4	4
1	1	2	2	4	4	1	1	4	4
1	1	2	2	2	2	1	1	2	2
1	1	2	2	2	2	1	1	4	4
1	1	1	1	4	4	1	1	4	4
1	1	2	2	4	4	1	1	4	4
1	1	2	2	2	2	1	1	4	4
1	1	2	2	2	2	1	1	4	4
1	1	2	2	2	2	1	1	4	4
1	1	1	1	2	2	1	1	2	2
1	1	2	2	2	2	1	1	4	4
1	1	1	1	2	2	1	1	4	4
1	1	1	1	2	2	1	1	4	4
1	1	1	1	4	4	1	1	4	4
1	1	2	2	2	2	1	1	2	2
1	1	2	2	2	2	1	1	4	4
1	1	2	2	2	2	1	1	4	4
4	4	1	1	4	4	1	1	2	2
1	1	2	2	2	2	1	1	4	4
1	1	2	2	2	2	1	1	4	4
1	1	2	2	2	2	1	1	2	2
1	1	2	2	2	2	1	1	4	4
1	1	2	2	4	4	1	1	4	4
1	1	2	2	2	2	1	1	4	4
1	1	2	2	2	2	1	1	4	4
4	4	2	2	2	2	1	1	4	4
1	1	2	2	2	2	1	1	2	2
1	1	1	1	4	4	1	1	4	4
1	1	2	2	4	4	1	1	4	4
1	1	2	2	2	2	1	1	4	4
1	1	2	2	2	2	1	1	2	2
1	1	2	2	4	4	1	1	4	4
1	1	1	1	4	4	1	1	2	2
1	1	2	2	2	2	1	1	4	4
1	1	2	2	4	4	1	1	4	4

For Review Only

1	1	2	2	2	2	1	1	2	2
1	1	1	1	4	4	1	1	2	2
1	1	2	2	2	2	1	1	2	2
1	1	2	2	2	2	1	1	2	2
4	4	1	1	2	2	1	1	4	4
4	4	1	1	2	2	1	1	4	4
1	1	1	1	4	4	1	1	4	4
1	1	2	2	4	4	1	1	4	4
1	1	2	2	4	4	1	1	4	4
1	1	2	2	4	4	1	1	4	4
1	1	2	2	2	2	1	1	4	4
1	1	1	1	4	4	1	1	4	4
1	1	2	2	2	2	1	1	2	2
1	1	2	2	2	2	1	1	4	4
1	1	2	2	2	2	1	1	4	4
1	1	2	2	2	2	1	1	2	2
1	1	2	2	2	2	1	1	4	4
1	1	1	1	4	4	1	1	2	2
1	1	1	1	4	4	1	1	4	4
4	4	1	1	2	2	1	1	2	2
1	1	1	1	2	2	1	1	4	4
1	1	2	2	2	2	1	1	4	4
1	1	2	2	2	2	1	1	2	2
1	1	1	1	2	2	4	4	2	2
1	1	2	2	4	4	1	1	4	4
1	1	2	2	2	2	1	1	4	4
1	1	2	2	4	4	1	1	2	2
1	1	2	2	2	2	1	1	4	4
4	4	1	1	2	2	1	1	4	4
1	1	2	2	2	2	1	1	2	2
1	1	2	2	4	4	1	1	4	4
1	1	2	2	4	4	1	1	2	2
1	1	2	2	4	4	1	1	2	2
1	1	2	2	2	2	1	1	4	4
1	1	2	2	2	2	1	1	2	2
1	1	2	2	4	4	1	1	4	4
1	1	2	2	2	2	1	1	2	2
1	1	2	2	2	2	1	1	4	4
1	1	2	2	2	2	1	1	4	4
1	1	1	1	4	4	1	1	4	4
1	1	1	1	2	2	1	1	2	2
1	1	2	2	4	4	1	1	2	2
1	1	1	1	4	4	1	1	2	2
1	1	1	1	2	2	1	1	4	4
1	1	2	2	2	2	1	1	4	4
1	1	2	2	2	2	1	1	2	2
1	1	2	2	2	2	1	1	4	4
1	1	2	2	4	4	1	1	2	2
1	1	2	2	2	2	1	1	4	4
1	1	2	2	4	4	1	1	2	2
1	1	1	1	2	2	1	1	4	4
1	1	1	1	4	4	1	1	4	4
1	1	2	2	2	2	1	1	2	2
1	1	2	2	2	2	1	1	4	4
4	4	1	1	4	4	1	1	2	2

For Review Only

1	1	2	2	2	2	1	1	2	2
1	1	2	2	2	2	1	1	4	4

For Review Only

SNP2072	SNP2072	SNP2073	SNP2073	SNP2074	SNP2074	SNP2075	SNP2075	SNP2076	SNP2076
2	2	1	1	1	1	4	4	2	2
2	2	1	1	1	1	4	4	4	4
2	2	1	1	1	1	4	4	4	4
2	2	1	1	1	1	4	4	4	4
2	2	1	1	1	1	4	4	4	4
2	2	1	1	1	1	4	4	2	2
2	2	1	1	1	1	4	4	2	2
2	2	1	1	1	1	4	4	4	4
2	2	1	1	1	1	4	4	2	2
2	2	1	1	1	1	4	4	4	4
2	2	1	1	1	1	4	4	4	4
2	2	1	1	1	1	4	4	4	4
2	2	1	1	1	1	4	4	2	2
2	2	1	1	1	1	4	4	4	4
2	2	4	4	1	1	4	4	2	2
2	2	1	1	1	1	4	4	4	4
2	2	1	1	1	1	4	4	2	2
2	2	1	1	1	1	4	4	2	2
2	2	1	1	1	1	4	4	2	2
2	2	1	1	1	1	4	4	2	2
2	2	1	1	1	1	4	4	2	2
2	2	1	1	1	1	4	4	2	2
2	2	1	1	1	1	4	4	4	4
3	3	1	1	2	2	2	2	4	4
2	2	1	1	1	1	4	4	2	2
2	2	1	1	1	1	4	4	2	2
2	2	1	1	1	1	4	4	4	4
2	2	1	1	1	1	4	4	2	2
2	2	1	1	1	1	4	4	2	2
2	2	1	1	1	1	4	4	4	4
2	2	1	1	1	1	4	4	2	2
2	2	4	4	1	1	4	4	2	2
2	2	1	1	1	1	4	4	2	2
2	2	1	1	1	1	4	4	4	4
2	2	1	1	1	1	4	4	4	4
2	2	1	1	1	1	4	4	4	4
2	2	1	1	1	1	4	4	2	2
2	2	4	4	1	1	4	4	4	4
2	2	1	1	2	2	2	2	4	4
3	3	1	1	2	2	2	2	4	4
2	2	1	1	1	1	4	4	4	4
2	2	1	1	1	1	4	4	2	2
2	2	1	1	1	1	4	4	4	4
3	3	1	1	2	2	2	2	2	2
3	3	1	1	2	2	2	2	4	4
2	2	1	1	1	1	4	4	4	4
2	2	1	1	1	1	4	4	4	4
2	2	1	1	1	1	4	4	4	4
2	2	1	1	1	1	4	4	2	2
2	2	1	1	1	1	4	4	2	2
2	2	1	1	1	1	4	4	2	2
2	2	1	1	1	1	4	4	2	2

For Review Only

2	2	1	1	1	1	4	4	2	2
2	2	1	1	1	1	4	4	2	2
2	2	1	1	1	1	4	4	2	2
2	2	1	1	1	1	4	4	2	2
2	2	1	1	1	1	4	4	2	2
2	2	1	1	1	1	4	4	2	2
2	2	1	1	1	1	4	4	2	2
2	2	1	1	1	1	4	4	4	4
2	2	1	1	1	1	4	4	4	4
2	2	1	1	1	1	4	4	4	4
2	2	1	1	1	1	4	4	4	4
2	2	1	1	1	1	4	4	4	4
2	2	1	1	1	1	4	4	2	2
2	2	1	1	1	1	4	4	2	2
2	2	1	1	1	1	4	4	2	2
2	2	1	1	1	1	4	4	2	2
2	2	4	4	1	1	4	4	4	4
2	2	1	1	1	1	4	4	2	2
2	2	1	1	1	1	4	4	2	2
2	2	1	1	1	1	4	4	2	2
2	2	1	1	1	1	4	4	4	4
2	2	1	1	1	1	4	4	4	4
2	2	1	1	1	1	4	4	2	2
2	2	1	1	1	1	4	4	2	2
3	3	1	1	2	2	2	2	4	4
2	2	1	1	1	1	4	4	4	4
2	2	1	1	1	1	4	4	4	4
2	2	1	1	1	1	4	4	4	4
2	2	1	1	1	1	4	4	2	2
2	2	1	1	1	1	4	4	2	2
2	2	1	1	1	1	4	4	4	4
3	3	1	1	2	2	2	2	2	2
3	3	1	1	2	2	2	2	4	4
2	2	1	1	1	1	4	4	2	2
2	2	1	1	1	1	4	4	2	2
3	3	1	1	2	2	2	2	4	4
2	2	1	1	1	1	4	4	4	4
2	2	1	1	1	1	4	4	2	2
2	2	1	1	1	1	4	4	4	4
2	2	1	1	1	1	4	4	4	4
2	2	1	1	1	1	4	4	2	2
2	2	1	1	1	1	4	4	4	4
2	2	1	1	1	1	4	4	4	4
2	2	1	1	1	1	4	4	4	4
2	2	1	1	1	1	4	4	2	2
2	2	1	1	1	1	4	4	4	4
3	3	1	1	1	1	2	2	4	4
2	2	1	1	1	1	4	4	2	2
2	2	1	1	1	1	4	4	2	2
2	2	4	4	1	1	4	4	4	4
2	2	1	1	1	1	4	4	4	4
2	2	1	1	1	1	4	4	4	4
2	2	1	1	1	1	4	4	4	4
2	2	1	1	1	1	4	4	2	2
2	2	4	4	1	1	4	4	4	4

For Review Only

3	3	1	1	2	2	4	4	2	2
2	2	1	1	1	1	4	4	4	4
2	2	1	1	1	1	4	4	2	2
3	3	1	1	2	2	2	2	4	4
2	2	1	1	1	1	4	4	4	4
2	2	1	1	1	1	4	4	4	4
2	2	1	1	1	1	4	4	4	4
2	2	1	1	1	1	4	4	2	2
2	2	1	1	1	1	4	4	2	2
2	2	1	1	1	1	4	4	2	2
2	2	1	1	1	1	4	4	2	2
2	2	1	1	1	1	4	4	2	2
2	2	1	1	1	1	4	4	4	4
2	2	1	1	1	1	4	4	2	2
2	2	1	1	1	1	4	4	2	2
2	2	1	1	1	1	4	4	2	2
2	2	4	4	1	1	4	4	4	4
2	2	1	1	1	1	4	4	2	2
3	3	1	1	2	2	2	2	4	4
3	3	1	1	2	2	2	2	2	2
2	2	1	1	1	1	4	4	2	2
2	2	1	1	1	1	4	4	2	2
2	2	1	1	1	1	4	4	2	2
2	2	1	1	1	1	4	4	4	4
2	2	1	1	1	1	4	4	2	2
3	3	1	1	2	2	2	2	4	4
2	2	1	1	1	1	4	4	2	2
2	2	1	1	1	1	4	4	2	2
2	2	1	1	1	1	4	4	2	2
2	2	1	1	1	1	4	4	2	2
2	2	1	1	1	1	2	2	2	2
2	2	1	1	1	1	4	4	2	2
2	2	1	1	1	1	4	4	2	2
2	2	1	1	1	1	4	4	2	2
2	2	1	1	1	1	4	4	4	4
2	2	1	1	1	1	4	4	4	4
3	3	1	1	2	2	4	4	4	4
2	2	4	4	1	1	4	4	2	2
2	2	1	1	1	1	4	4	4	4
3	3	1	1	2	2	2	2	4	4
3	3	1	1	2	2	2	2	4	4
2	2	1	1	1	1	4	4	4	4
2	2	4	4	1	1	4	4	2	2
2	2	1	1	1	1	4	4	2	2
2	2	1	1	1	1	4	4	4	4
2	2	1	1	1	1	4	4	2	2
2	2	1	1	1	1	4	4	2	2
2	2	1	1	1	1	4	4	2	2
2	2	1	1	1	1	4	4	4	4
2	2	1	1	1	1	4	4	2	2
2	2	1	1	1	1	4	4	4	4
3	3	1	1	2	2	2	2	4	4
2	2	1	1	1	1	4	4	2	2
2	2	1	1	1	1	4	4	4	4
2	2	1	1	1	1	2	2	2	2

For Review Only

2	2	4	4	1	1	4	4	2	2
3	3	1	1	2	2	2	2	4	4
3	3	1	1	2	2	2	2	4	4
2	2	1	1	1	1	4	4	2	2
2	2	1	1	1	1	4	4	4	4
2	2	1	1	1	1	4	4	4	4
2	2	1	1	1	1	4	4	4	4
2	2	1	1	1	1	4	4	2	2
2	2	1	1	1	1	4	4	4	4
2	2	1	1	1	1	4	4	2	2
2	2	1	1	1	1	4	4	2	2
2	2	1	1	1	1	4	4	4	4
2	2	1	1	1	1	4	4	2	2
2	2	1	1	1	1	4	4	2	2
2	2	1	1	1	1	4	4	4	4
2	2	1	1	1	1	4	4	4	4
2	2	1	1	1	1	4	4	4	4
2	2	1	1	1	1	4	4	4	4
2	2	1	1	1	1	4	4	4	4
2	2	1	1	1	1	4	4	4	4
2	2	1	1	1	1	4	4	2	2
2	2	1	1	1	1	4	4	2	2
2	2	1	1	1	1	4	4	4	4
2	2	1	1	1	1	4	4	4	4
2	2	1	1	1	1	4	4	2	2
2	2	1	1	1	1	4	4	4	4
2	2	1	1	1	1	4	4	4	4
2	2	1	1	1	1	4	4	4	4
2	2	1	1	1	1	4	4	2	2
2	2	1	1	1	1	4	4	2	2
2	2	1	1	1	1	4	4	2	2
2	2	1	1	1	1	4	4	4	4
2	2	1	1	1	1	4	4	2	2
2	2	1	1	1	1	4	4	4	4
2	2	1	1	1	1	4	4	2	2
2	2	1	1	1	1	4	4	4	4
2	2	1	1	1	1	4	4	4	4
2	2	1	1	1	1	4	4	2	2
2	2	1	1	1	1	4	4	2	2
2	2	4	4	1	1	4	4	4	4
2	2	1	1	1	1	4	4	4	4
2	2	1	1	1	1	4	4	2	2
2	2	1	1	1	1	4	4	4	4
2	2	1	1	1	1	4	4	2	2
2	2	1	1	1	1	4	4	4	4
2	2	1	1	1	1	4	4	4	4
2	2	1	1	1	1	4	4	4	4
2	2	1	1	1	1	4	4	4	4
3	3	1	1	2	2	2	2	4	4
2	2	1	1	1	1	4	4	4	4
2	2	1	1	1	1	4	4	2	2
2	2	1	1	1	1	4	4	4	4
2	2	1	1	1	1	4	4	4	4
2	2	1	1	1	1	4	4	4	4
2	2	1	1	1	1	4	4	4	4

For Review Only

2	2	1	1	1	1	4	4	4	4
2	2	1	1	1	1	4	4	2	2
2	2	1	1	1	1	4	4	2	2
2	2	1	1	1	1	4	4	2	2
2	2	1	1	1	1	4	4	4	4
2	2	1	1	1	1	4	4	2	2
2	2	1	1	1	1	4	4	4	4
2	2	1	1	1	1	4	4	4	4
2	2	1	1	1	1	4	4	2	2
3	3	1	1	2	2	2	2	4	4
2	2	1	1	1	1	4	4	2	2
2	2	1	1	1	1	4	4	4	4
2	2	1	1	1	1	4	4	4	4
2	2	1	1	1	1	4	4	4	4
2	2	4	4	1	1	4	4	4	4
2	2	1	1	1	1	4	4	4	4
2	2	1	1	1	1	4	4	4	4
2	2	1	1	1	1	4	4	2	2
2	2	1	1	1	1	4	4	4	4
2	2	4	4	1	1	4	4	4	4
3	3	1	1	2	2	2	2	4	4
2	2	1	1	1	1	4	4	2	2
2	2	1	1	1	1	4	4	2	2
2	2	4	4	1	1	4	4	4	4
2	2	1	1	1	1	4	4	4	4
2	2	1	1	1	1	4	4	4	4
2	2	1	1	1	1	4	4	4	4
2	2	1	1	1	1	4	4	2	2
2	2	1	1	1	1	4	4	2	2
2	2	1	1	1	1	4	4	2	2
2	2	1	1	1	1	4	4	4	4
2	2	1	1	1	1	4	4	4	4
2	2	1	1	1	1	4	4	4	4
2	2	1	1	1	1	4	4	2	2
2	2	1	1	1	1	4	4	4	4
3	3	1	1	2	2	2	2	4	4
2	2	1	1	1	1	4	4	2	2
2	2	1	1	1	1	4	4	4	4
2	2	1	1	1	1	4	4	4	4
3	3	1	1	2	2	2	2	4	4
2	2	1	1	1	1	4	4	4	4
2	2	1	1	1	1	4	4	2	2
3	3	1	1	2	2	2	2	4	4
2	2	1	1	1	1	4	4	2	2
2	2	1	1	1	1	4	4	4	4
2	2	1	1	1	1	4	4	4	4
2	2	1	1	1	1	4	4	4	4
2	2	4	4	1	1	4	4	4	4
2	2	1	1	1	1	4	4	2	2
2	2	1	1	1	1	4	4	4	4
3	3	1	1	1	1	2	2	2	2
2	2	1	1	1	1	4	4	4	4
2	2	1	1	1	1	4	4	4	4
2	2	4	4	1	1	4	4	4	4
3	3	1	1	2	2	2	2	4	4

For Review Only

2	2	1	1	1	1	4	4	2	2
2	2	1	1	1	1	4	4	4	4

For Review Only

SNP2077	SNP2077	SNP2078	SNP2078	SNP2079	SNP2079	SNP208	SNP208	SNP2080	SNP2080
1	1	1	1	4	4	3	3	2	2
4	4	1	1	4	4	2	2	2	2
4	4	1	1	4	4	2	2	2	2
1	1	1	1	4	4	3	3	2	2
4	4	1	1	4	4	3	3	3	3
1	1	1	1	4	4	3	3	2	2
1	1	1	1	4	4	3	3	3	3
4	4	1	1	4	4	2	2	2	2
1	1	1	1	4	4	3	3	3	3
4	4	1	1	4	4	2	2	2	2
1	1	1	1	4	4	3	3	2	2
4	4	1	1	4	4	3	3	2	2
1	1	1	1	4	4	3	3	2	2
1	1	1	1	4	4	3	3	2	2
1	1	1	1	4	4	2	2	2	2
1	1	1	1	4	4	3	3	2	2
1	1	1	1	4	4	2	2	3	3
1	1	1	1	4	4	3	3	2	2
1	1	1	1	4	4	3	3	2	2
1	1	1	1	4	4	3	3	2	2
1	1	1	1	4	4	2	2	3	3
1	1	1	1	4	4	2	2	3	3
1	1	1	1	4	4	2	2	2	2
1	1	1	1	4	4	2	2	2	2
1	1	1	1	4	4	3	3	2	2
1	1	1	1	4	4	2	2	2	2
1	1	2	2	4	4	3	3	2	2
1	1	1	1	4	4	3	3	2	2
1	1	1	1	4	4	3	3	3	3
1	1	1	1	4	4	2	2	2	2
1	1	1	1	4	4	3	3	2	2
1	1	1	1	4	4	2	2	2	2
1	1	1	1	4	4	2	2	3	3
4	4	1	1	3	3	2	2	2	2
1	1	1	1	4	4	3	3	3	3
1	1	1	1	4	4	3	3	2	2
1	1	1	1	4	4	3	3	3	3
1	1	1	1	4	4	3	3	2	2
1	1	1	1	4	4	2	2	2	2
1	1	1	1	4	4	3	3	2	2
1	1	1	1	4	4	2	2	2	2
1	1	1	1	4	4	3	3	3	3
4	4	1	1	4	4	2	2	2	2
1	1	1	1	4	4	3	3	2	2
4	4	1	1	4	4	2	2	2	2
1	1	2	2	4	4	2	2	2	2
4	4	1	1	4	4	3	3	2	2
1	1	1	1	4	4	2	2	2	2
1	1	1	1	4	4	3	3	3	3
1	1	1	1	4	4	3	3	3	3
1	1	1	1	4	4	2	2	2	2
1	1	1	1	4	4	3	3	2	2

For Review Only

1	1	1	1	4	4	3	3	2	2
1	1	1	1	4	4	2	2	3	3
1	1	1	1	4	4	3	3	2	2
1	1	1	1	4	4	2	2	3	3
1	1	1	1	4	4	3	3	2	2
1	1	1	1	4	4	2	2	2	2
1	1	1	1	4	4	3	3	2	2
4	4	1	1	4	4	2	2	2	2
4	4	1	1	4	4	3	3	3	3
1	1	1	1	4	4	3	3	2	2
1	1	2	2	4	4	2	2	2	2
4	4	1	1	4	4	2	2	2	2
1	1	1	1	4	4	3	3	2	2
1	1	1	1	4	4	3	3	3	3
1	1	1	1	4	4	3	3	2	2
1	1	1	1	4	4	3	3	3	3
1	1	1	1	4	4	3	3	2	2
1	1	1	1	4	4	3	3	2	2
1	1	1	1	4	4	3	3	2	2
1	1	1	1	4	4	3	3	3	3
1	1	1	1	4	4	2	2	2	2
1	1	1	1	4	4	3	3	2	2
1	1	1	1	4	4	3	3	3	3
1	1	1	1	4	4	2	2	2	2
4	4	1	1	4	4	2	2	2	2
1	1	2	2	4	4	3	3	2	2
4	4	1	1	4	4	2	2	3	3
1	1	1	1	3	3	2	2	2	2
1	1	1	1	4	4	2	2	2	2
1	1	1	1	4	4	2	2	3	3
4	4	1	1	4	4	3	3	2	2
1	1	1	1	4	4	2	2	2	2
1	1	1	1	3	3	2	2	2	2
1	1	1	1	4	4	3	3	2	2
1	1	1	1	4	4	3	3	2	2
4	4	1	1	4	4	2	2	2	2
4	4	1	1	4	4	3	3	2	2
1	1	1	1	4	4	3	3	2	2
1	1	1	1	4	4	2	2	2	2
4	4	1	1	4	4	3	3	2	2
1	1	1	1	4	4	2	2	3	3
4	4	1	1	4	4	3	3	3	3
4	4	1	1	4	4	3	3	2	2
1	1	2	2	4	4	2	2	2	2
1	1	1	1	4	4	3	3	2	2
4	4	1	1	4	4	2	2	2	2
4	4	1	1	3	3	3	3	2	2
1	1	1	1	4	4	3	3	2	2
1	1	1	1	4	4	2	2	2	2
1	1	2	2	4	4	3	3	2	2
4	4	1	1	4	4	3	3	2	2
1	1	1	1	4	4	2	2	2	2
4	4	1	1	4	4	3	3	2	2
1	1	1	1	4	4	3	3	2	2
1	1	2	2	4	4	2	2	2	2

For Review Only

1	1	1	1	4	4	2	2	2	2
1	1	1	1	4	4	3	3	2	2
1	1	1	1	4	4	3	3	2	2
4	4	1	1	4	4	3	3	2	2
1	1	2	2	4	4	3	3	2	2
1	1	2	2	4	4	2	2	2	2
4	4	1	1	4	4	3	3	2	2
4	4	1	1	4	4	2	2	2	2
1	1	1	1	4	4	2	2	2	2
1	1	1	1	4	4	3	3	2	2
1	1	1	1	4	4	3	3	2	2
1	1	1	1	4	4	3	3	2	2
4	4	1	1	4	4	3	3	2	2
1	1	1	1	4	4	3	3	2	2
1	1	1	1	4	4	3	3	2	2
1	1	1	1	4	4	3	3	2	2
1	1	2	2	4	4	3	3	2	2
1	1	1	1	4	4	3	3	2	2
4	4	1	1	4	4	3	3	2	2
1	1	1	1	4	4	3	3	2	2
1	1	1	1	4	4	3	3	2	2
1	1	1	1	4	4	3	3	2	2
1	1	1	1	4	4	3	3	2	2
1	1	1	1	3	3	2	2	2	2
1	1	1	1	4	4	3	3	2	2
1	1	1	1	4	4	3	3	2	2
1	1	1	1	4	4	3	3	2	2
1	1	1	1	4	4	2	2	2	2
1	1	1	1	4	4	3	3	2	2
1	1	1	1	4	4	3	3	2	2
4	4	1	1	4	4	3	3	2	2
1	1	2	2	4	4	3	3	2	2
1	1	1	1	4	4	2	2	3	3
1	1	1	1	4	4	3	3	2	2
4	4	1	1	3	3	2	2	2	2
1	1	1	1	4	4	3	3	2	2
4	4	1	1	3	3	3	3	2	2
1	1	2	2	4	4	3	3	2	2
1	1	1	1	4	4	3	3	2	2
1	1	1	1	4	4	3	3	2	2
1	1	1	1	4	4	2	2	2	2
4	4	1	1	4	4	3	3	2	2
1	1	1	1	4	4	3	3	2	2
1	1	1	1	4	4	2	2	2	2
4	4	1	1	4	4	3	3	2	2
1	1	1	1	4	4	3	3	3	3
1	1	1	1	4	4	2	2	2	2
1	1	1	1	4	4	2	2	2	2
1	1	1	1	4	4	3	3	2	2
1	1	1	1	4	4	3	3	2	2
1	1	1	1	4	4	2	2	2	2
1	1	1	1	3	3	3	3	2	2
1	1	1	1	4	4	3	3	2	2
4	4	1	1	4	4	2	2	2	2
1	1	1	1	4	4	3	3	2	2

For Review Only

1	1	1	1	4	4	2	2	2	2
1	1	1	1	4	4	3	3	2	2
1	1	1	1	4	4	3	3	2	2
1	1	1	1	4	4	3	3	2	2
4	4	1	1	4	4	2	2	2	2
4	4	1	1	4	4	3	3	2	2
1	1	1	1	4	4	3	3	2	2
1	1	1	1	4	4	3	3	2	2
1	1	1	1	4	4	3	3	2	2
1	1	1	1	4	4	2	2	2	2
1	1	1	1	4	4	2	2	2	2
1	1	1	1	4	4	3	3	2	2
1	1	1	1	4	4	3	3	3	3
1	1	1	1	4	4	3	3	3	3
4	4	1	1	4	4	3	3	2	2
1	1	1	1	4	4	3	3	2	2
4	4	1	1	4	4	3	3	2	2
4	4	1	1	4	4	2	2	2	2
4	4	1	1	4	4	2	2	2	2
4	4	1	1	4	4	3	3	2	2
1	1	1	1	4	4	3	3	2	2
1	1	1	1	4	4	2	2	3	3
1	1	1	1	4	4	3	3	2	2
4	4	1	1	4	4	2	2	2	2
1	1	1	1	4	4	3	3	2	2
4	4	1	1	3	3	3	3	2	2
1	1	1	1	4	4	2	2	2	2
1	1	2	2	4	4	3	3	2	2
1	1	1	1	4	4	2	2	2	2
1	1	1	1	4	4	3	3	3	3
1	1	1	1	4	4	3	3	3	3
1	1	2	2	4	4	2	2	3	3
1	1	1	1	4	4	3	3	3	3
4	4	1	1	4	4	3	3	2	2
1	1	1	1	4	4	3	3	3	3
4	4	1	1	4	4	2	2	2	2
4	4	1	1	4	4	3	3	2	2
1	1	1	1	4	4	3	3	2	2
1	1	1	1	4	4	2	2	2	2
1	1	2	2	4	4	3	3	2	2
4	4	1	1	4	4	2	2	2	2
1	1	1	1	4	4	2	2	2	2
4	4	1	1	3	3	2	2	2	2
1	1	1	1	4	4	3	3	2	2
4	4	1	1	4	4	3	3	2	2
1	1	2	2	4	4	2	2	2	2
1	1	1	1	4	4	3	3	2	2
1	1	1	1	3	3	2	2	2	2
4	4	1	1	4	4	2	2	2	2
4	4	1	1	4	4	3	3	2	2
1	1	1	1	4	4	2	2	2	2
4	4	1	1	4	4	3	3	2	2
1	1	1	1	4	4	3	3	2	2
1	1	2	2	4	4	2	2	2	2
1	1	1	1	4	4	2	2	2	2

For Review Only

4	4	1	1	4	4	3	3	2	2
1	1	1	1	4	4	2	2	2	2
1	1	1	1	4	4	2	2	2	2
1	1	1	1	4	4	3	3	2	2
1	1	2	2	4	4	3	3	2	2
1	1	1	1	4	4	3	3	2	2
1	1	1	1	4	4	2	2	2	2
4	4	1	1	4	4	2	2	2	2
1	1	1	1	4	4	3	3	2	2
1	1	1	1	4	4	2	2	2	2
1	1	1	1	4	4	3	3	2	2
4	4	1	1	4	4	3	3	3	3
1	1	1	1	4	4	3	3	2	2
4	4	1	1	4	4	2	2	2	2
4	4	1	1	4	4	2	2	2	2
4	4	1	1	4	4	3	3	2	2
1	1	1	1	3	3	2	2	2	2
1	1	1	1	4	4	3	3	2	2
1	1	1	1	3	3	3	3	2	2
1	1	1	1	4	4	3	3	2	2
4	4	1	1	4	4	3	3	2	2
1	1	1	1	4	4	3	3	2	2
1	1	1	1	4	4	3	3	2	2
1	1	1	1	4	4	3	3	2	2
4	4	1	1	4	4	2	2	2	2
4	4	1	1	4	4	3	3	2	2
1	1	1	1	4	4	2	2	2	2
1	1	1	1	4	4	3	3	2	2
1	1	1	1	4	4	3	3	2	2
1	1	1	1	4	4	3	3	3	3
1	1	1	1	3	3	2	2	2	2
1	1	1	1	4	4	3	3	2	2
4	4	1	1	4	4	3	3	3	3
1	1	1	1	4	4	2	2	2	2
1	1	1	1	4	4	3	3	2	2
1	1	1	1	3	3	2	2	2	2
1	1	1	1	4	4	3	3	2	2
4	4	1	1	4	4	2	2	2	2
1	1	1	1	4	4	3	3	2	2
1	1	1	1	4	4	2	2	2	2
1	1	2	2	4	4	2	2	2	2
1	1	1	1	4	4	2	2	2	2
1	1	1	1	4	4	3	3	2	2
1	1	1	1	4	4	3	3	3	3
4	4	1	1	4	4	2	2	2	2
1	1	1	1	4	4	2	2	2	2
1	1	1	1	4	4	2	2	2	2
1	1	2	2	4	4	2	2	2	2
1	1	1	1	4	4	2	2	2	2
1	1	1	1	3	3	2	2	2	2
1	1	1	1	4	4	3	3	2	2
1	1	1	1	4	4	3	3	2	2
4	4	1	1	4	4	2	2	2	2
4	4	1	1	4	4	3	3	2	2
1	1	1	1	4	4	3	3	2	2

For Review Only

1	1	1	1	4	4	3	3	2	2
4	4	1	1	4	4	3	3	2	2

For Review Only

SNP2081	SNP2081	SNP2082	SNP2082	SNP2083	SNP2083	SNP2084	SNP2084	SNP2085	SNP2085
3	3	4	4	1	1	2	2	3	3
3	3	2	2	3	3	1	1	1	1
3	3	4	4	1	1	2	2	3	3
3	3	4	4	3	3	1	1	1	1
3	3	4	4	1	1	2	2	3	3
3	3	4	4	1	1	2	2	3	3
3	3	4	4	3	3	1	1	1	1
2	2	4	4	1	1	2	2	3	3
3	3	4	4	1	1	2	2	3	3
2	2	4	4	1	1	2	2	3	3
3	3	4	4	1	1	2	2	3	3
3	3	4	4	1	1	2	2	3	3
3	3	4	4	3	3	1	1	3	3
2	2	2	2	1	1	2	2	3	3
3	3	2	2	1	1	2	2	3	3
3	3	2	2	1	1	2	2	3	3
2	2	4	4	3	3	1	1	1	1
3	3	4	4	1	1	2	2	3	3
3	3	4	4	1	1	2	2	3	3
2	2	4	4	3	3	1	1	1	1
2	2	4	4	3	3	1	1	1	1
2	2	4	4	3	3	1	1	1	1
3	3	2	2	1	1	2	2	3	3
3	3	4	4	3	3	1	1	1	1
3	3	4	4	1	1	2	2	3	3
3	3	2	2	1	1	2	2	3	3
3	3	2	2	1	1	2	2	3	3
3	3	4	4	3	3	1	1	3	3
3	3	4	4	1	1	2	2	3	3
2	2	4	4	3	3	1	1	1	1
3	3	4	4	1	1	2	2	3	3
2	2	2	2	3	3	1	1	1	1
3	3	4	4	1	1	2	2	3	3
2	2	2	2	1	1	1	1	1	1
2	2	2	2	1	1	2	2	3	3
3	3	2	2	1	1	2	2	3	3
3	3	4	4	3	3	1	1	1	1
3	3	2	2	3	3	1	1	1	1
3	3	2	2	1	1	2	2	3	3
2	2	2	2	1	1	2	2	3	3
3	3	4	4	3	3	1	1	1	1
2	2	4	4	1	1	2	2	3	3
2	2	2	2	1	1	2	2	3	3
3	3	4	4	3	3	1	1	1	1
3	3	2	2	1	1	2	2	3	3
3	3	4	4	1	1	2	2	3	3
3	3	2	2	1	1	2	2	3	3
3	3	4	4	3	3	1	1	1	1
3	3	4	4	3	3	1	1	1	1
3	3	4	4	3	3	1	1	3	3
3	3	2	2	1	1	2	2	3	3
3	3	4	4	1	1	2	2	3	3

For Review Only

3	3	4	4	1	1	2	2	3	3
3	3	4	4	1	1	2	2	3	3
3	3	4	4	1	1	2	2	3	3
3	3	4	4	1	1	2	2	3	3
3	3	4	4	1	1	2	2	3	3
3	3	4	4	1	1	2	2	3	3
3	3	4	4	1	1	2	2	3	3
3	3	2	2	3	3	1	1	1	1
3	3	4	4	3	3	1	1	1	1
3	3	4	4	3	3	1	1	3	3
3	3	4	4	1	1	2	2	3	3
3	3	4	4	1	1	2	2	3	3
3	3	4	4	1	1	2	2	3	3
3	3	4	4	1	1	2	2	3	3
3	3	4	4	1	1	1	1	3	3
3	3	4	4	1	1	2	2	3	3
3	3	4	4	1	1	2	2	3	3
3	3	2	2	1	1	2	2	3	3
3	3	4	4	1	1	2	2	3	3
3	3	4	4	3	3	1	1	1	1
3	3	4	4	1	1	2	2	3	3
2	2	4	4	1	1	2	2	3	3
3	3	4	4	1	1	1	1	3	3
2	2	4	4	3	3	1	1	1	1
3	3	2	2	1	1	2	2	3	3
3	3	4	4	1	1	2	2	3	3
3	3	4	4	1	1	2	2	3	3
2	2	2	2	1	1	1	1	1	1
3	3	2	2	1	1	2	2	3	3
3	3	4	4	1	1	2	2	3	3
3	3	4	4	3	3	1	1	3	3
3	3	4	4	3	3	1	1	1	1
2	2	2	2	1	1	1	1	1	1
3	3	4	4	1	1	2	2	3	3
3	3	4	4	3	3	1	1	1	1
3	3	2	2	1	1	2	2	3	3
2	2	4	4	3	3	1	1	1	1
3	3	4	4	3	3	1	1	1	1
2	2	4	4	1	1	2	2	3	3
2	2	4	4	1	1	2	2	3	3
3	3	4	4	1	1	2	2	3	3
3	3	4	4	1	1	2	2	3	3
3	3	2	2	1	1	2	2	3	3
3	3	4	4	1	1	2	2	3	3
3	3	2	2	1	1	2	2	3	3
3	3	2	2	3	3	1	1	1	1
2	2	4	4	1	1	1	1	1	1
3	3	4	4	1	1	2	2	3	3
3	3	4	4	1	1	2	2	3	3
3	3	4	4	1	1	2	2	3	3
3	3	4	4	1	1	2	2	3	3
3	3	4	4	1	1	2	2	3	3
3	3	4	4	3	3	1	1	1	1
3	3	4	4	3	3	1	1	3	3
3	3	2	2	1	1	2	2	3	3

For Review Only

3	3	2	2	3	3	1	1	1	1
2	2	4	4	3	3	1	1	1	1
3	3	4	4	1	1	2	2	3	3
2	2	4	4	1	1	1	1	1	1
3	3	4	4	3	3	1	1	3	3
3	3	4	4	3	3	1	1	1	1
3	3	4	4	1	1	2	2	3	3
3	3	4	4	1	1	2	2	3	3
3	3	4	4	1	1	2	2	3	3
2	2	4	4	3	3	1	1	3	3
3	3	2	2	3	3	1	1	3	3
3	3	4	4	3	3	1	1	3	3
3	3	2	2	1	1	2	2	3	3
3	3	4	4	1	1	2	2	3	3
3	3	4	4	1	1	2	2	3	3
3	3	4	4	1	1	1	1	3	3
3	3	4	4	3	3	1	1	1	1
3	3	4	4	3	3	1	1	3	3
2	2	4	4	3	3	1	1	1	1
3	3	2	2	1	1	2	2	3	3
3	3	4	4	3	3	2	2	3	3
3	3	4	4	3	3	2	2	3	3
3	3	4	4	3	3	1	1	3	3
2	2	2	2	1	1	1	1	1	1
3	3	4	4	1	1	2	2	3	3
2	2	4	4	1	1	2	2	3	3
3	3	2	2	1	1	2	2	3	3
3	3	4	4	1	1	2	2	3	3
3	3	4	4	3	3	1	1	1	1
3	3	2	2	3	3	1	1	3	3
2	2	2	2	1	1	2	2	3	3
3	3	4	4	1	1	2	2	3	3
3	3	4	4	1	1	2	2	3	3
3	3	4	4	1	1	1	1	3	3
2	2	2	2	1	1	1	1	1	1
3	3	4	4	3	3	1	1	3	3
2	2	4	4	1	1	1	1	1	1
3	3	2	2	1	1	2	2	3	3
3	3	2	2	3	3	2	2	3	3
3	3	2	2	1	1	2	2	3	3
2	2	4	4	1	1	2	2	3	3
2	2	2	2	3	3	1	1	1	1
3	3	2	2	1	1	2	2	3	3
3	3	4	4	3	3	1	1	3	3
3	3	4	4	1	1	2	2	3	3
2	2	4	4	3	3	1	1	1	1
3	3	2	2	1	1	2	2	3	3
3	3	2	2	1	1	2	2	3	3
3	3	4	4	3	3	1	1	3	3
3	3	4	4	1	1	2	2	3	3
3	3	2	2	1	1	2	2	3	3
3	3	4	4	1	1	1	1	1	1
3	3	2	2	1	1	2	2	3	3
3	3	4	4	1	1	2	2	3	3
3	3	4	4	3	3	2	2	3	3

For Review Only

3	3	2	2	1	1	2	2	3	3
3	3	2	2	1	1	2	2	3	3
3	3	4	4	1	1	2	2	3	3
3	3	2	2	1	1	2	2	3	3
3	3	2	2	1	1	2	2	3	3
3	3	4	4	3	3	1	1	1	1
3	3	2	2	1	1	1	1	1	1
3	3	4	4	3	3	1	1	3	3
2	2	4	4	1	1	2	2	3	3
3	3	4	4	1	1	2	2	3	3
3	3	4	4	1	1	2	2	3	3
3	3	4	4	3	3	1	1	3	3
3	3	4	4	3	3	2	2	3	3
2	2	4	4	3	3	1	1	1	1
2	2	4	4	3	3	1	1	1	1
3	3	2	2	1	1	2	2	3	3
3	3	4	4	3	3	1	1	3	3
3	3	2	2	3	3	1	1	1	1
3	3	4	4	3	3	1	1	3	3
3	3	4	4	1	1	2	2	3	3
3	3	4	4	3	3	1	1	3	3
3	3	4	4	3	3	1	1	1	1
2	2	4	4	1	1	2	2	1	1
3	3	4	4	1	1	2	2	3	3
2	2	4	4	3	3	1	1	1	1
2	2	2	2	1	1	1	1	1	1
3	3	4	4	1	1	2	2	3	3
3	3	4	4	3	3	1	1	1	1
3	3	4	4	1	1	2	2	3	3
3	3	2	2	1	1	2	2	3	3
3	3	4	4	3	3	1	1	1	1
3	3	2	2	1	1	2	2	3	3
3	3	4	4	3	3	1	1	1	1
2	2	4	4	3	3	1	1	3	3
3	3	4	4	3	3	1	1	1	1
3	3	4	4	1	1	2	2	3	3
3	3	4	4	3	3	1	1	3	3
3	3	4	4	3	3	1	1	1	1
3	3	2	2	1	1	2	2	3	3
3	3	4	4	1	1	2	2	3	3
3	3	4	4	1	1	2	2	3	3
3	3	2	2	1	1	2	2	3	3
3	3	2	2	1	1	1	1	1	1
3	3	4	4	1	1	2	2	3	3
3	3	4	4	3	3	1	1	3	3
3	3	4	4	3	3	1	1	1	1
3	3	2	2	1	1	2	2	3	3
3	3	2	2	1	1	1	1	1	1
3	3	4	4	3	3	1	1	1	1
3	3	4	4	3	3	1	1	1	1
3	3	4	4	3	3	1	1	1	1
2	2	4	4	3	3	1	1	1	1
2	2	4	4	1	1	1	1	1	1
3	3	4	4	1	1	2	2	3	3
3	3	2	2	1	1	2	2	3	3

For Review Only

3	3	4	4	3	3	1	1	1	1
3	3	2	2	3	3	1	1	1	1
3	3	4	4	3	3	1	1	3	3
2	2	4	4	1	1	2	2	3	3
3	3	4	4	3	3	1	1	1	1
3	3	2	2	1	1	2	2	3	3
2	2	2	2	1	1	2	2	3	3
2	2	4	4	1	1	2	2	3	3
3	3	4	4	3	3	1	1	1	1
2	2	2	2	1	1	2	2	3	3
3	3	4	4	3	3	1	1	3	3
2	2	4	4	3	3	1	1	3	3
2	2	4	4	1	1	2	2	3	3
3	3	4	4	3	3	1	1	1	1
3	3	4	4	1	1	2	2	3	3
3	3	4	4	1	1	2	2	3	3
3	3	4	4	1	1	1	1	1	1
3	3	4	4	3	3	1	1	1	1
3	3	2	2	1	1	1	1	1	1
3	3	2	2	3	3	1	1	1	1
3	3	2	2	3	3	1	1	1	1
3	3	2	2	1	1	2	2	3	3
3	3	4	4	3	3	1	1	1	1
2	2	2	2	1	1	2	2	3	3
3	3	4	4	3	3	1	1	1	1
2	2	4	4	3	3	1	1	1	1
2	2	2	2	1	1	2	2	3	3
3	3	4	4	1	1	2	2	3	3
3	3	4	4	3	3	1	1	3	3
3	3	4	4	3	3	1	1	1	1
3	3	2	2	1	1	1	1	1	1
3	3	4	4	1	1	2	2	3	3
2	2	2	2	3	3	1	1	1	1
3	3	2	2	3	3	1	1	3	3
2	2	4	4	3	3	1	1	1	1
2	2	2	2	1	1	1	1	1	1
3	3	4	4	1	1	2	2	3	3
3	3	4	4	1	1	2	2	3	3
3	3	2	2	1	1	2	2	3	3
3	3	2	2	1	1	2	2	3	3
3	3	2	2	1	1	2	2	3	3
3	3	2	2	1	1	2	2	3	3
2	2	2	2	1	1	2	2	3	3
3	3	4	4	1	1	2	2	3	3
3	3	4	4	1	1	2	2	3	3
2	2	4	4	1	1	2	2	3	3
3	3	2	2	1	1	2	2	3	3
3	3	2	2	1	1	2	2	3	3
3	3	4	4	1	1	2	2	3	3
2	2	2	2	1	1	1	1	1	1
3	3	4	4	1	1	2	2	3	3
3	3	2	2	1	1	2	2	3	3
3	3	4	4	1	1	2	2	3	3
3	3	4	4	1	1	2	2	3	3
3	3	2	2	1	1	2	2	3	3

For Review Only

3	3	4	4	1	1	2	2	3	3
3	3	4	4	1	1	2	2	3	3

For Review Only

SNP2086	SNP2086	SNP2087	SNP2087	SNP2088	SNP2088	SNP2089	SNP2089	SNP209	SNP209
2	2	2	2	3	3	3	3	2	2
4	4	2	2	3	3	4	4	2	2
2	2	2	2	3	3	4	4	2	2
4	4	1	1	2	2	4	4	2	2
2	2	2	2	3	3	4	4	2	2
4	4	2	2	3	3	4	4	2	2
4	4	1	1	2	2	4	4	2	2
2	2	2	2	3	3	4	4	4	4
2	2	2	2	3	3	4	4	2	2
2	2	2	2	3	3	3	3	2	2
2	2	2	2	3	3	4	4	2	2
2	2	2	2	3	3	4	4	2	2
2	2	2	2	3	3	4	4	2	2
2	2	2	2	3	3	3	3	2	2
2	2	2	2	3	3	4	4	2	2
2	2	2	2	3	3	3	3	2	2
4	4	1	1	2	2	4	4	2	2
2	2	2	2	3	3	4	4	2	2
2	2	2	2	3	3	4	4	2	2
4	4	1	1	2	2	3	3	4	4
4	4	1	1	2	2	4	4	4	4
4	4	1	1	2	2	4	4	2	2
2	2	2	2	3	3	3	3	2	2
4	4	1	1	2	2	3	3	2	2
2	2	2	2	3	3	4	4	2	2
2	2	2	2	3	3	4	4	2	2
2	2	2	2	3	3	4	4	2	2
2	2	2	2	3	3	4	4	2	2
2	2	2	2	3	3	4	4	2	2
4	4	1	1	2	2	3	3	2	2
2	2	2	2	3	3	4	4	2	2
4	4	1	1	2	2	4	4	2	2
2	2	2	2	3	3	4	4	2	2
2	2	2	2	3	3	3	3	2	2
2	2	2	2	3	3	4	4	2	2
2	2	2	2	3	3	3	3	2	2
4	4	1	1	2	2	4	4	2	2
4	4	1	1	2	2	4	4	2	2
2	2	2	2	3	3	3	3	2	2
2	2	2	2	3	3	4	4	4	4
4	4	1	1	2	2	3	3	4	4
2	2	2	2	3	3	4	4	4	4
2	2	2	2	3	3	4	4	2	2
4	4	1	1	2	2	4	4	2	2
2	2	2	2	3	3	3	3	4	4
2	2	2	2	3	3	4	4	2	2
2	2	2	2	3	3	4	4	2	2
4	4	1	1	2	2	4	4	2	2
4	4	1	1	2	2	3	3	2	2
2	2	2	2	3	3	4	4	2	2
2	2	2	2	3	3	4	4	2	2
2	2	2	2	3	3	4	4	2	2

For Review Only

2	2	2	2	3	3	4	4	2	2
2	2	2	2	3	3	4	4	2	2
2	2	2	2	3	3	4	4	2	2
2	2	2	2	3	3	4	4	2	2
2	2	2	2	3	3	4	4	2	2
2	2	2	2	3	3	4	4	2	2
2	2	2	2	3	3	4	4	2	2
4	4	1	1	2	2	3	3	2	2
4	4	1	1	2	2	3	3	2	2
2	2	2	2	3	3	4	4	2	2
2	2	2	2	3	3	4	4	2	2
2	2	2	2	3	3	4	4	2	2
2	2	2	2	3	3	4	4	2	2
2	2	2	2	3	3	4	4	2	2
2	2	2	2	3	3	4	4	2	2
2	2	2	2	3	3	4	4	2	2
2	2	2	2	3	3	4	4	2	2
2	2	2	2	3	3	4	4	2	2
2	2	2	2	3	3	4	4	2	2
2	2	2	2	3	3	4	4	2	2
2	2	2	2	3	3	4	4	2	2
2	2	2	2	3	3	4	4	2	2
2	2	2	2	3	3	4	4	2	2
4	4	1	1	2	2	4	4	2	2
2	2	2	2	3	3	3	3	4	4
2	2	2	2	3	3	3	3	2	2
2	2	2	2	3	3	3	3	2	2
2	2	2	2	3	3	3	3	2	2
2	2	2	2	3	3	4	4	2	2
2	2	2	2	3	3	4	4	2	2
4	4	2	2	3	3	4	4	2	2
4	4	1	1	2	2	3	3	2	2
2	2	2	2	3	3	3	3	2	2
2	2	2	2	3	3	4	4	2	2
4	4	1	1	2	2	4	4	2	2
2	2	2	2	3	3	3	3	4	4
4	4	1	1	2	2	4	4	2	2
4	4	1	1	2	2	4	4	2	2
2	2	2	2	3	3	3	3	2	2
2	2	2	2	3	3	3	3	2	2
2	2	2	2	3	3	4	4	2	2
2	2	2	2	3	3	4	4	2	2
2	2	2	2	3	3	4	4	4	4
2	2	2	2	3	3	4	4	2	2
2	2	2	2	3	3	4	4	2	2
4	4	2	2	3	3	4	4	2	2
2	2	2	2	3	3	3	3	2	2
2	2	2	2	3	3	4	4	2	2
2	2	2	2	3	3	4	4	2	2
2	2	2	2	3	3	3	3	2	2
2	2	2	2	3	3	4	4	2	2
2	2	2	2	3	3	3	3	2	2
4	4	1	1	2	2	3	3	2	2
2	2	2	2	3	3	4	4	2	2
2	2	2	2	3	3	4	4	2	2

For Review Only

2	2	2	2	3	3	3	3	2	2
4	4	1	1	2	2	4	4	2	2
2	2	2	2	3	3	4	4	2	2
2	2	2	2	3	3	3	3	2	2
4	4	2	2	3	3	4	4	2	2
4	4	1	1	2	2	4	4	2	2
2	2	2	2	3	3	4	4	2	2
2	2	2	2	3	3	4	4	2	2
2	2	2	2	3	3	4	4	2	2
2	2	2	2	2	2	4	4	2	2
4	4	2	2	2	2	4	4	2	2
2	2	2	2	3	3	4	4	2	2
2	2	2	2	3	3	4	4	2	2
2	2	2	2	3	3	4	4	2	2
2	2	2	2	3	3	4	4	2	2
2	2	2	2	3	3	4	4	2	2
4	4	2	2	2	2	4	4	2	2
2	2	2	2	3	3	4	4	2	2
4	4	1	1	2	2	3	3	2	2
2	2	2	2	3	3	4	4	2	2
2	2	2	2	3	3	4	4	2	2
2	2	2	2	3	3	4	4	2	2
4	4	2	2	3	3	4	4	2	2
2	2	2	2	3	3	3	3	2	2
2	2	2	2	3	3	3	3	2	2
2	2	2	2	3	3	3	3	2	2
2	2	2	2	3	3	4	4	2	2
2	2	2	2	3	3	4	4	2	2
2	2	2	2	3	3	4	4	2	2
2	2	2	2	3	3	4	4	2	2
2	2	2	2	3	3	3	3	2	2
2	2	2	2	3	3	4	4	2	2
2	2	2	2	3	3	4	4	2	2
2	2	2	2	3	3	4	4	2	2
2	2	2	2	3	3	3	3	2	2
2	2	2	2	3	3	4	4	2	2
2	2	1	1	2	2	3	3	2	2
2	2	2	2	3	3	4	4	2	2
2	2	2	2	2	2	3	3	2	2
2	2	2	2	3	3	3	3	2	2
2	2	2	2	3	3	4	4	2	2
4	4	1	1	2	2	3	3	2	2
2	2	2	2	3	3	4	4	2	2
4	4	2	2	2	2	3	3	2	2
2	2	2	2	3	3	4	4	2	2
4	4	1	1	2	2	4	4	2	2
2	2	2	2	3	3	3	3	2	2
2	2	2	2	3	3	4	4	2	2
2	2	2	2	3	3	4	4	2	2
2	2	2	2	3	3	4	4	2	2
2	2	2	2	3	3	4	4	2	2
2	2	2	2	3	3	4	4	2	2
2	2	2	2	3	3	4	4	2	2
2	2	2	2	3	3	4	4	2	2
2	2	2	2	3	3	4	4	2	2

For Review Only

2	2	2	2	3	3	4	4	2	2
2	2	2	2	3	3	4	4	4	4
2	2	2	2	3	3	4	4	2	2
2	2	2	2	3	3	4	4	2	2
2	2	2	2	3	3	3	3	2	2
4	4	1	1	2	2	4	4	2	2
2	2	2	2	3	3	4	4	2	2
2	2	2	2	3	3	4	4	2	2
2	2	2	2	3	3	4	4	2	2
2	2	2	2	3	3	4	4	2	2
2	2	2	2	3	3	4	4	2	2
4	4	2	2	2	2	4	4	2	2
2	2	2	2	3	3	4	4	2	2
4	4	2	2	3	3	4	4	2	2
4	4	1	1	3	3	3	3	2	2
2	2	2	2	3	3	4	4	2	2
2	2	2	2	3	3	4	4	2	2
4	4	2	2	3	3	4	4	2	2
4	4	1	1	2	2	4	4	2	2
2	2	2	2	3	3	4	4	2	2
2	2	2	2	3	3	4	4	2	2
2	2	2	2	3	3	3	3	2	2
2	2	2	2	3	3	3	3	2	2
2	2	2	2	3	3	4	4	2	2
4	4	1	1	2	2	4	4	2	2
2	2	2	2	3	3	3	3	2	2
2	2	2	2	3	3	4	4	2	2
2	2	2	2	3	3	4	4	2	2
2	2	2	2	3	3	4	4	2	2
2	2	2	2	3	3	4	4	2	2
4	4	1	1	2	2	4	4	2	2
2	2	2	2	3	3	4	4	2	2
2	2	2	2	3	3	4	4	2	2
2	2	2	2	3	3	3	3	2	2
4	4	1	1	2	2	4	4	2	2
2	2	2	2	3	3	4	4	2	2
4	4	1	1	2	2	4	4	2	2
4	4	2	2	3	3	4	4	2	2
2	2	2	2	3	3	4	4	2	2
2	2	2	2	3	3	4	4	2	2
2	2	2	2	3	3	4	4	2	2
2	2	2	2	3	3	4	4	2	2
2	2	2	2	3	3	3	3	2	2
2	2	2	2	3	3	4	4	2	2
2	2	2	2	3	3	4	4	2	2
4	4	1	1	2	2	4	4	2	2
2	2	2	2	3	3	4	4	2	2
2	2	2	2	3	3	4	4	2	2
4	4	1	1	2	2	3	3	2	2
4	4	1	1	2	2	4	4	2	2
4	4	1	1	2	2	4	4	2	2
4	4	1	1	2	2	3	3	2	2
2	2	2	2	3	3	3	3	2	2
2	2	2	2	3	3	4	4	2	2
2	2	2	2	3	3	3	3	2	2

For Review Only

4	4	1	1	2	2	4	4	2	2
4	4	1	1	2	2	4	4	2	2
2	2	2	2	3	3	4	4	2	2
2	2	2	2	3	3	4	4	2	2
4	4	1	1	2	2	4	4	2	2
2	2	2	2	3	3	4	4	2	2
2	2	2	2	3	3	4	4	2	2
2	2	2	2	3	3	4	4	4	4
4	4	1	1	2	2	3	3	2	2
2	2	2	2	3	3	3	3	2	2
2	2	2	2	3	3	4	4	2	2
4	4	1	1	2	2	4	4	2	2
2	2	2	2	3	3	3	3	2	2
4	4	1	1	2	2	4	4	2	2
2	2	2	2	3	3	4	4	2	2
2	2	2	2	3	3	3	3	2	2
2	2	2	2	3	3	3	3	2	2
4	4	2	2	3	3	3	3	2	2
2	2	2	2	3	3	4	4	4	4
4	4	1	1	2	2	4	4	2	2
4	4	1	1	2	2	4	4	4	4
2	2	2	2	3	3	4	4	2	2
4	4	2	2	3	3	4	4	2	2
2	2	2	2	3	3	4	4	2	2
4	4	1	1	2	2	3	3	2	2
4	4	2	2	3	3	4	4	2	2
2	2	2	2	3	3	3	3	2	2
2	2	2	2	3	3	4	4	2	2
2	2	2	2	3	3	4	4	2	2
4	4	1	1	2	2	4	4	2	2
2	2	2	2	3	3	3	3	2	2
2	2	2	2	3	3	4	4	2	2
4	4	2	2	3	3	3	3	2	2
4	4	1	1	3	3	4	4	2	2
2	2	2	2	3	3	3	3	2	2
2	2	2	2	3	3	3	3	2	2
2	2	2	2	3	3	4	4	2	2
2	2	2	2	3	3	4	4	2	2
2	2	2	2	3	3	3	3	2	2
2	2	2	2	3	3	3	3	2	2
2	2	2	2	3	3	4	4	2	2
2	2	2	2	3	3	4	4	2	2
2	2	2	2	3	3	4	4	2	2
2	2	2	2	3	3	4	4	2	2
2	2	2	2	3	3	4	4	2	2
2	2	2	2	3	3	4	4	2	2
2	2	2	2	3	3	3	3	2	2
2	2	2	2	3	3	4	4	2	2
2	2	2	2	3	3	3	3	2	2
2	2	2	2	3	3	3	3	2	2
2	2	2	2	3	3	4	4	2	2
2	2	2	2	3	3	3	3	2	2
2	2	2	2	3	3	4	4	2	2
2	2	2	2	3	3	4	4	2	2
2	2	2	2	3	3	4	4	2	2

2	2	2	2	3	3	4	4	2	2
2	2	2	2	3	3	4	4	2	2

For Review Only

SNP2090	SNP2090	SNP2091	SNP2091	SNP2092	SNP2092	SNP2093	SNP2093	SNP2094	SNP2094
3	3	1	1	2	2	4	4	2	2
2	2	1	1	2	2	3	3	2	2
3	3	2	2	1	1	3	3	2	2
3	3	2	2	2	2	3	3	2	2
2	2	2	2	1	1	4	4	2	2
3	3	1	1	2	2	3	3	2	2
2	2	1	1	2	2	3	3	2	2
2	2	2	2	1	1	3	3	2	2
2	2	1	1	2	2	3	3	2	2
2	2	2	2	1	1	4	4	2	2
3	3	2	2	1	1	3	3	2	2
3	3	2	2	1	1	3	3	2	2
2	2	1	1	2	2	3	3	2	2
2	2	2	2	1	1	4	4	2	2
2	2	2	2	2	2	3	3	2	2
3	3	2	2	1	1	4	4	2	2
2	2	1	1	2	2	3	3	2	2
3	3	1	1	2	2	3	3	2	2
3	3	1	1	2	2	3	3	2	2
2	2	2	2	2	2	4	4	2	2
2	2	1	1	2	2	3	3	2	2
2	2	1	1	2	2	3	3	2	2
2	2	2	2	1	1	4	4	4	4
2	2	2	2	1	1	4	4	2	2
3	3	1	1	2	2	3	3	2	2
3	3	2	2	1	1	3	3	2	2
2	2	1	1	1	1	3	3	4	4
2	2	1	1	2	2	3	3	2	2
2	2	1	1	2	2	3	3	2	2
2	2	2	2	1	1	4	4	2	2
3	3	2	2	1	1	3	3	2	2
2	2	1	1	2	2	3	3	2	2
2	2	1	1	2	2	3	3	2	2
2	2	2	2	1	1	4	4	4	4
2	2	1	1	1	1	3	3	2	2
3	3	2	2	1	1	4	4	2	2
2	2	1	1	2	2	3	3	2	2
2	2	2	2	2	2	3	3	2	2
2	2	2	2	1	1	4	4	4	4
2	2	2	2	1	1	3	3	2	2
2	2	2	2	1	1	4	4	2	2
2	2	1	1	2	2	3	3	2	2
2	2	2	2	1	1	3	3	2	2
2	2	1	1	2	2	4	4	2	2
2	2	2	2	1	1	4	4	2	2
3	3	2	2	1	1	3	3	2	2
3	3	1	1	2	2	3	3	2	2
3	3	2	2	1	1	3	3	2	2
2	2	1	1	2	2	4	4	2	2
2	2	1	1	1	1	3	3	2	2
3	3	2	2	1	1	3	3	2	2
2	2	1	1	2	2	3	3	4	4

For Review Only

3	3	1	1	2	2	3	3	2	2
2	2	1	1	2	2	3	3	2	2
3	3	1	1	2	2	3	3	2	2
2	2	1	1	2	2	3	3	2	2
3	3	1	1	2	2	3	3	2	2
3	3	2	2	1	1	3	3	2	2
3	3	2	2	1	1	3	3	2	2
2	2	2	2	1	1	4	4	2	2
2	2	2	2	1	1	4	4	2	2
2	2	2	2	1	1	3	3	2	2
3	3	2	2	1	1	3	3	2	2
3	3	1	1	2	2	3	3	2	2
3	3	1	1	2	2	3	3	2	2
2	2	1	1	2	2	3	3	2	2
2	2	1	1	2	2	3	3	2	2
2	2	1	1	2	2	3	3	2	2
2	2	1	1	1	1	3	3	2	2
2	2	1	1	2	2	3	3	2	2
3	3	1	1	2	2	3	3	2	2
2	2	1	1	2	2	3	3	2	2
2	2	1	1	2	2	3	3	2	2
2	2	2	2	1	1	4	4	2	2
2	2	1	1	2	2	3	3	2	2
2	2	1	1	2	2	3	3	2	2
2	2	2	2	1	1	4	4	2	2
3	3	2	2	1	1	3	3	2	2
2	2	2	2	1	1	4	4	2	2
2	2	2	2	1	1	4	4	4	4
3	3	2	2	1	1	3	3	2	2
2	2	2	2	2	2	3	3	2	2
2	2	2	2	2	2	3	3	2	2
2	2	2	2	1	1	4	4	2	2
2	2	2	2	1	1	4	4	4	4
3	3	1	1	2	2	3	3	2	2
3	3	1	1	2	2	3	3	2	2
2	2	2	2	1	1	4	4	2	2
2	2	2	2	1	1	3	3	2	2
2	2	1	1	2	2	3	3	2	2
2	2	2	2	1	1	4	4	4	4
3	3	2	2	1	1	4	4	2	2
2	2	1	1	2	2	3	3	2	2
2	2	1	1	2	2	3	3	2	2
2	2	2	2	1	1	3	3	2	2
3	3	2	2	1	1	3	3	2	2
3	3	1	1	2	2	4	4	2	2
2	2	1	1	2	2	3	3	2	2
2	2	2	2	1	1	4	4	4	4
3	3	2	2	1	1	3	3	2	2
3	3	2	2	1	1	3	3	2	2
2	2	2	2	1	1	4	4	2	2
2	2	1	1	2	2	3	3	2	2
2	2	2	2	1	1	4	4	4	4
2	2	2	2	1	1	4	4	2	2
2	2	1	1	2	2	4	4	2	2
2	2	2	2	1	1	3	3	2	2

For Review Only

2	2	2	2	1	1	3	3	2	2
2	2	2	2	1	1	3	3	2	2
3	3	2	2	1	1	3	3	2	2
2	2	2	2	1	1	4	4	2	2
3	3	1	1	2	2	3	3	2	2
3	3	2	2	1	1	3	3	2	2
3	3	2	2	1	1	3	3	2	2
3	3	2	2	1	1	4	4	2	2
2	2	2	2	1	1	3	3	2	2
2	2	1	1	2	2	4	4	2	2
3	3	1	1	2	2	4	4	2	2
2	2	2	2	2	2	3	3	2	2
2	2	2	2	1	1	3	3	2	2
2	2	1	1	2	2	3	3	2	2
3	3	2	2	2	2	3	3	2	2
2	2	2	2	2	2	3	3	2	2
2	2	2	2	1	1	3	3	2	2
2	2	2	2	2	2	3	3	2	2
2	2	2	2	1	1	4	4	4	4
2	2	1	1	2	2	3	3	2	2
2	2	2	2	2	2	4	4	2	2
3	3	2	2	2	2	3	3	2	2
2	2	2	2	2	2	3	3	2	2
2	2	2	2	1	1	4	4	2	2
2	2	2	2	1	1	3	3	2	2
2	2	2	2	1	1	4	4	2	2
2	2	1	1	2	2	3	3	2	2
2	2	1	1	2	2	3	3	2	2
2	2	2	2	2	2	3	3	2	2
3	3	1	1	2	2	3	3	2	2
2	2	2	2	1	1	4	4	4	4
2	2	2	2	2	2	4	4	2	2
2	2	1	1	2	2	3	3	2	2
2	2	2	2	1	1	4	4	2	2
2	2	2	2	1	1	4	4	4	4
2	2	2	2	2	2	3	3	4	4
2	2	2	2	1	1	4	4	4	4
2	2	2	2	2	2	3	3	2	2
3	3	2	2	1	1	3	3	4	4
2	2	2	2	1	1	4	4	4	4
2	2	2	2	1	1	3	3	2	2
2	2	2	2	1	1	4	4	2	2
2	2	1	1	2	2	3	3	2	2
2	2	2	2	2	2	4	4	2	2
2	2	2	2	1	1	3	3	2	2
2	2	1	1	1	1	3	3	2	2
3	3	2	2	1	1	4	4	2	2
3	3	2	2	1	1	3	3	2	2
2	2	1	1	2	2	4	4	4	4
3	3	2	2	1	1	3	3	2	2
3	3	2	2	1	1	4	4	2	2
2	2	2	2	1	1	3	3	2	2
2	2	2	2	2	2	3	3	2	2
3	3	1	1	2	2	3	3	2	2
2	2	2	2	1	1	4	4	2	2

For Review Only

2	2	2	2	2	2	3	3	2	2
2	2	2	2	1	1	3	3	2	2
2	2	2	2	1	1	4	4	2	2
2	2	1	1	2	2	3	3	2	2
3	3	1	1	2	2	4	4	2	2
2	2	2	2	1	1	3	3	4	4
2	2	2	2	1	1	3	3	4	4
2	2	2	2	2	2	3	3	2	2
2	2	2	2	1	1	3	3	4	4
3	3	1	1	2	2	3	3	2	2
3	3	2	2	1	1	4	4	2	2
2	2	2	2	1	1	3	3	4	4
2	2	2	2	2	2	4	4	2	2
2	2	1	1	1	1	4	4	2	2
2	2	2	2	1	1	4	4	4	4
3	3	2	2	1	1	3	3	2	2
2	2	2	2	2	2	3	3	2	2
3	3	1	1	2	2	3	3	2	2
3	3	2	2	1	1	3	3	2	2
3	3	2	2	1	1	3	3	2	2
2	2	1	1	2	2	4	4	2	2
2	2	2	2	1	1	4	4	4	4
2	2	2	2	1	1	4	4	2	2
3	3	1	1	2	2	3	3	2	2
2	2	2	2	2	2	3	3	2	2
2	2	2	2	1	1	4	4	4	4
2	2	2	2	1	1	3	3	2	2
3	3	2	2	1	1	3	3	2	2
2	2	1	1	2	2	3	3	2	2
2	2	1	1	2	2	3	3	2	2
2	2	1	1	2	2	3	3	2	2
2	2	2	2	1	1	3	3	2	2
2	2	1	1	2	2	3	3	2	2
2	2	2	2	1	1	4	4	4	4
2	2	1	1	2	2	3	3	2	2
3	3	1	1	2	2	3	3	2	2
2	2	1	1	2	2	4	4	2	2
3	3	1	1	2	2	3	3	2	2
3	3	2	2	1	1	3	3	2	2
2	2	2	2	1	1	3	3	2	2
3	3	2	2	1	1	3	3	2	2
3	3	2	2	1	1	3	3	2	2
2	2	2	2	1	1	4	4	4	4
2	2	1	1	2	2	4	4	2	2
3	3	2	2	2	2	4	4	2	2
2	2	2	2	1	1	3	3	2	2
3	3	2	2	1	1	3	3	2	2
2	2	2	2	1	1	3	3	4	4
2	2	2	2	1	1	4	4	2	2
2	2	2	2	1	1	3	3	4	4
3	3	2	2	2	2	3	3	2	2
2	2	2	2	1	1	4	4	2	2
2	2	2	2	1	1	4	4	2	2
3	3	2	2	1	1	3	3	2	2
2	2	2	2	1	1	4	4	4	4

For Review Only

2	2	2	2	1	1	3	3	2	2
2	2	1	1	2	2	3	3	2	2
2	2	2	2	2	2	3	3	2	2
2	2	1	1	2	2	4	4	2	2
2	2	2	2	1	1	3	3	2	2
3	3	1	1	2	2	3	3	2	2
2	2	2	2	1	1	3	3	4	4
2	2	2	2	1	1	3	3	2	2
2	2	2	2	1	1	4	4	2	2
2	2	2	2	1	1	4	4	4	4
2	2	1	1	2	2	4	4	2	2
2	2	1	1	2	2	3	3	2	2
2	2	2	2	1	1	4	4	4	4
3	3	2	2	1	1	3	3	2	2
3	3	2	2	1	1	3	3	2	2
3	3	2	2	1	1	4	4	2	2
2	2	2	2	1	1	4	4	2	2
3	3	1	1	2	2	4	4	2	2
2	2	2	2	1	1	3	3	4	4
2	2	2	2	1	1	3	3	2	2
2	2	2	2	1	1	3	3	2	2
3	3	2	2	1	1	3	3	2	2
3	3	1	1	2	2	3	3	2	2
2	2	2	2	1	1	3	3	2	2
2	2	2	2	1	1	4	4	2	2
2	2	2	2	1	1	3	3	2	2
2	2	2	2	1	1	4	4	4	4
3	3	2	2	2	2	3	3	2	2
2	2	1	1	2	2	3	3	2	2
2	2	2	2	2	2	3	3	2	2
2	2	2	2	1	1	4	4	4	4
2	2	2	2	1	1	3	3	2	2
2	2	2	2	1	1	4	4	2	2
3	3	2	2	1	1	3	3	2	2
2	2	2	2	1	1	4	4	2	2
2	2	2	2	1	1	4	4	4	4
2	2	1	1	2	2	3	3	2	2
3	3	1	1	2	2	3	3	2	2
3	3	2	2	1	1	4	4	2	2
2	2	2	2	1	1	4	4	4	4
2	2	2	2	1	1	3	3	2	2
2	2	1	1	2	2	3	3	2	2
2	2	2	2	1	1	3	3	4	4
2	2	1	1	2	2	3	3	2	2
3	3	1	1	2	2	3	3	2	2
2	2	2	2	1	1	3	3	4	4
3	3	2	2	1	1	4	4	2	2
2	2	1	1	2	2	3	3	2	2
2	2	2	2	2	2	4	4	2	2
2	2	2	2	1	1	4	4	4	4
3	3	1	1	2	2	3	3	2	2
2	2	2	2	1	1	4	4	2	2
3	3	2	2	1	1	3	3	2	2
2	2	1	1	2	2	3	3	2	2
2	2	2	2	1	1	3	3	2	2

For Review Only

3	3	1	1	2	2	3	3	2	2
3	3	1	1	2	2	3	3	2	2

For Review Only

SNP2095	SNP2095	SNP2096	SNP2096	SNP2097	SNP2097	SNP2098	SNP2098	SNP2099	SNP2099
1	1	1	1	2	2	1	1	4	4
1	1	1	1	3	3	4	4	4	4
1	1	1	1	2	2	4	4	4	4
1	1	1	1	2	2	4	4	4	4
4	4	1	1	3	3	4	4	4	4
1	1	1	1	2	2	4	4	4	4
4	4	1	1	3	3	4	4	4	4
4	4	1	1	3	3	4	4	4	4
4	4	1	1	3	3	4	4	4	4
4	4	1	1	3	3	4	4	2	2
1	1	1	1	2	2	4	4	4	4
1	1	1	1	2	2	4	4	4	4
1	1	1	1	2	2	4	4	4	4
1	1	1	1	2	2	4	4	2	2
1	1	1	1	2	2	4	4	4	4
1	1	1	1	2	2	4	4	2	2
4	4	1	1	3	3	4	4	4	4
1	1	1	1	2	2	4	4	4	4
1	1	1	1	2	2	4	4	4	4
4	4	2	2	3	3	4	4	2	2
4	4	1	1	3	3	4	4	4	4
4	4	1	1	3	3	4	4	4	4
4	4	1	1	3	3	4	4	2	2
4	4	1	1	3	3	4	4	2	2
1	1	1	1	2	2	4	4	4	4
1	1	1	1	2	2	4	4	4	4
4	4	1	1	3	3	4	4	4	4
1	1	1	1	2	2	4	4	4	4
4	4	1	1	3	3	4	4	4	4
4	4	1	1	3	3	1	1	2	2
1	1	1	1	2	2	4	4	4	4
1	1	1	1	2	2	4	4	4	4
4	4	1	1	3	3	4	4	4	4
4	4	1	1	3	3	4	4	2	2
1	1	1	1	2	2	4	4	4	4
1	1	1	1	2	2	4	4	2	2
4	4	1	1	3	3	1	1	4	4
1	1	1	1	2	2	1	1	4	4
4	4	1	1	3	3	4	4	2	2
1	1	1	1	2	2	1	1	4	4
4	4	1	1	3	3	4	4	2	2
4	4	1	1	3	3	4	4	4	4
4	4	1	1	3	3	4	4	4	4
4	4	1	1	3	3	4	4	4	4
4	4	1	1	3	3	4	4	2	2
1	1	1	1	2	2	4	4	4	4
1	1	1	1	2	2	4	4	4	4
1	1	1	1	2	2	4	4	4	4
1	1	1	1	2	2	4	4	2	2
4	4	1	1	3	3	4	4	4	4
1	1	1	1	2	2	4	4	4	4
4	4	1	1	3	3	1	1	4	4

For Review Only

1	1	1	1	2	2	4	4	4	4
4	4	1	1	3	3	4	4	4	4
1	1	1	1	2	2	4	4	4	4
4	4	1	1	3	3	4	4	4	4
1	1	1	1	2	2	4	4	4	4
1	1	1	1	2	2	4	4	4	4
1	1	1	1	2	2	4	4	4	4
4	4	1	1	3	3	4	4	2	2
4	4	1	1	3	3	1	1	2	2
1	1	1	1	2	2	4	4	4	4
1	1	1	1	2	2	4	4	4	4
1	1	1	1	2	2	4	4	4	4
1	1	1	1	2	2	4	4	4	4
4	4	1	1	3	3	4	4	4	4
1	1	1	1	2	2	4	4	4	4
4	4	1	1	3	3	4	4	4	4
1	1	1	1	2	2	4	4	4	4
1	1	2	2	2	2	4	4	4	4
1	1	1	1	2	2	4	4	4	4
4	4	1	1	3	3	1	1	4	4
1	1	1	1	2	2	4	4	4	4
1	1	1	1	2	2	1	1	4	4
4	4	1	1	3	3	4	4	4	4
4	4	1	1	3	3	4	4	4	4
4	4	1	1	3	3	4	4	2	2
1	1	1	1	2	2	4	4	4	4
4	4	1	1	3	3	4	4	2	2
4	4	1	1	3	3	4	4	2	2
1	1	1	1	2	2	4	4	4	4
4	4	2	2	3	3	4	4	4	4
4	4	1	1	3	3	1	1	4	4
1	1	1	1	3	3	1	1	2	2
4	4	1	1	3	3	4	4	2	2
1	1	1	1	2	2	4	4	4	4
1	1	1	1	2	2	1	1	4	4
4	4	1	1	3	3	4	4	2	2
4	4	1	1	3	3	1	1	4	4
4	4	1	1	3	3	4	4	4	4
4	4	1	1	3	3	4	4	2	2
1	1	1	1	2	2	4	4	2	2
4	4	1	1	3	3	4	4	4	4
1	1	1	1	2	2	4	4	4	4
1	1	1	1	3	3	4	4	4	4
1	1	1	1	2	2	4	4	4	4
1	1	1	1	2	2	4	4	4	4
4	4	1	1	3	3	4	4	4	4
4	4	1	1	3	3	4	4	2	2
1	1	1	1	2	2	4	4	4	4
1	1	1	1	2	2	4	4	4	4
1	1	1	1	2	2	4	4	2	2
1	1	1	1	2	2	4	4	4	4
4	4	1	1	3	3	4	4	2	2
1	1	1	1	3	3	4	4	2	2
1	1	1	1	2	2	4	4	4	4
1	1	1	1	2	2	4	4	4	4

For Review Only

4	4	1	1	3	3	4	4	4	4
1	1	1	1	2	2	1	1	4	4
1	1	1	1	2	2	4	4	4	4
4	4	1	1	3	3	1	1	2	2
1	1	1	1	2	2	1	1	4	4
1	1	1	1	3	3	4	4	4	4
1	1	1	1	2	2	4	4	4	4
1	1	1	1	2	2	4	4	2	2
1	1	1	1	2	2	4	4	4	4
1	1	1	1	2	2	4	4	2	2
1	1	1	1	2	2	4	4	2	2
1	1	1	1	2	2	4	4	4	4
4	4	1	1	3	3	4	4	4	4
1	1	1	1	2	2	1	1	4	4
1	1	1	1	2	2	4	4	2	2
1	1	1	1	2	2	4	4	4	4
1	1	1	1	2	2	1	1	4	4
1	1	1	1	2	2	4	4	4	4
4	4	1	1	3	3	4	4	2	2
4	4	1	1	3	3	4	4	4	4
1	1	1	1	2	2	4	4	4	4
1	1	1	1	2	2	4	4	4	4
4	4	1	1	3	3	4	4	4	4
4	4	1	1	3	3	4	4	2	2
4	4	1	1	2	2	4	4	2	2
4	4	1	1	3	3	4	4	2	2
1	1	1	1	2	2	4	4	4	4
4	4	1	1	3	3	4	4	4	4
1	1	2	2	2	2	4	4	4	4
1	1	1	1	2	2	4	4	4	4
4	4	1	1	3	3	1	1	2	2
1	1	1	1	2	2	4	4	4	4
4	4	1	1	3	3	4	4	4	4
1	1	1	1	2	2	4	4	4	4
4	4	1	1	3	3	4	4	2	2
4	4	1	1	2	2	4	4	4	4
4	4	1	1	3	3	4	4	2	2
1	1	1	1	2	2	4	4	4	4
1	1	1	1	2	2	4	4	4	4
4	4	1	1	3	3	4	4	2	2
4	4	1	1	3	3	4	4	4	4
4	4	1	1	3	3	4	4	2	2
1	1	2	2	2	2	4	4	4	4
1	1	1	1	2	2	4	4	2	2
1	1	1	1	2	2	4	4	4	4
4	4	1	1	3	3	1	1	4	4
1	1	1	1	2	2	4	4	2	2
1	1	1	1	2	2	4	4	4	4
1	1	1	1	2	2	4	4	4	4
1	1	1	1	2	2	4	4	4	4
1	1	1	1	2	2	4	4	4	4
4	4	1	1	3	3	4	4	4	4
1	1	1	1	2	2	4	4	4	4
1	1	1	1	2	2	4	4	4	4
1	1	1	1	3	3	4	4	4	4

For Review Only

1	1	2	2	2	2	4	4	4	4
4	4	1	1	3	3	4	4	4	4
4	4	1	1	3	3	1	1	4	4
1	1	1	1	2	2	4	4	4	4
1	1	1	1	2	2	4	4	4	4
4	4	1	1	3	3	1	1	4	4
4	4	1	1	3	3	4	4	4	4
1	1	1	1	2	2	4	4	4	4
4	4	1	1	3	3	1	1	4	4
1	1	1	1	2	2	4	4	4	4
1	1	1	1	2	2	4	4	4	4
4	4	1	1	3	3	4	4	4	4
1	1	1	1	2	2	4	4	4	4
4	4	1	1	3	3	4	4	4	4
4	4	1	1	3	3	4	4	2	2
1	1	1	1	2	2	4	4	4	4
1	1	1	1	2	2	4	4	4	4
1	1	1	1	2	2	4	4	4	4
1	1	1	1	2	2	4	4	4	4
1	1	1	1	2	2	4	4	4	4
1	1	1	1	2	2	4	4	4	4
4	4	1	1	3	3	4	4	2	2
1	1	1	1	2	2	1	1	2	2
1	1	1	1	2	2	4	4	4	4
1	1	2	2	3	3	4	4	4	4
4	4	1	1	3	3	4	4	2	2
1	1	1	1	2	2	4	4	4	4
1	1	1	1	2	2	4	4	4	4
4	4	1	1	3	3	4	4	4	4
4	4	1	1	3	3	4	4	4	4
4	4	1	1	3	3	1	1	4	4
4	4	1	1	3	3	4	4	4	4
4	4	1	1	3	3	1	1	4	4
4	4	1	1	3	3	4	4	2	2
4	4	1	1	3	3	4	4	4	4
1	1	1	1	2	2	4	4	4	4
1	1	1	1	2	2	4	4	2	2
1	1	1	1	2	2	1	1	4	4
1	1	1	1	2	2	4	4	4	4
1	1	1	1	2	2	4	4	4	4
1	1	1	1	2	2	4	4	4	4
1	1	1	1	2	2	4	4	4	4
4	4	1	1	3	3	4	4	2	2
1	1	1	1	2	2	4	4	4	4
1	1	1	1	2	2	1	1	2	2
4	4	1	1	3	3	4	4	4	4
1	1	1	1	2	2	4	4	4	4
4	4	1	1	3	3	4	4	4	4
4	4	1	1	3	3	1	1	2	2
4	4	1	1	3	3	4	4	4	4
1	1	2	2	2	2	4	4	4	4
4	4	1	1	3	3	4	4	2	2
4	4	1	1	3	3	4	4	2	2
1	1	1	1	2	2	4	4	4	4
4	4	1	1	3	3	1	1	2	2

For Review Only

1	1	1	1	2	2	4	4	4	4
1	1	1	1	2	2	4	4	4	4
1	1	2	2	2	2	4	4	4	4
4	4	1	1	3	3	4	4	4	4
4	4	1	1	3	3	1	1	4	4
1	1	1	1	2	2	4	4	4	4
4	4	1	1	3	3	1	1	4	4
4	4	1	1	3	3	4	4	4	4
1	1	1	1	2	2	4	4	2	2
4	4	1	1	3	3	1	1	2	2
1	1	1	1	2	2	4	4	4	4
4	4	1	1	3	3	4	4	4	4
4	4	1	1	3	3	4	4	2	2
1	1	1	1	2	2	4	4	4	4
1	1	1	1	2	2	4	4	4	4
1	1	1	1	2	2	4	4	2	2
4	4	1	1	3	3	1	1	2	2
1	1	1	1	2	2	1	1	2	2
4	4	1	1	3	3	4	4	4	4
1	1	1	1	2	2	1	1	4	4
1	1	1	1	2	2	4	4	4	4
1	1	1	1	2	2	4	4	4	4
1	1	1	1	2	2	4	4	4	4
1	1	1	1	2	2	4	4	4	4
4	4	1	1	3	3	4	4	2	2
1	1	1	1	2	2	1	1	4	4
4	4	1	1	3	3	4	4	2	2
1	1	1	1	2	2	4	4	4	4
4	4	1	1	3	3	4	4	4	4
4	4	2	2	3	3	4	4	4	4
4	4	1	1	3	3	1	1	2	2
1	1	1	1	2	2	4	4	4	4
1	1	1	1	3	3	4	4	2	2
1	1	1	1	2	2	4	4	4	4
4	4	1	1	3	3	4	4	2	2
4	4	1	1	3	3	1	1	2	2
1	1	1	1	2	2	4	4	4	4
1	1	1	1	2	2	4	4	4	4
1	1	1	1	2	2	4	4	2	2
4	4	1	1	3	3	4	4	2	2
4	4	1	1	3	3	1	1	4	4
4	4	1	1	3	3	4	4	4	4
4	4	1	1	3	3	4	4	4	4
4	4	1	1	3	3	4	4	4	4
1	1	1	1	2	2	4	4	4	4
4	4	1	1	3	3	4	4	4	4
1	1	1	1	2	2	4	4	2	2
1	1	1	1	2	2	4	4	4	4
1	1	2	2	2	2	4	4	2	2
4	4	1	1	3	3	4	4	2	2
1	1	1	1	2	2	1	1	4	4
4	4	1	1	3	3	4	4	2	2
1	1	1	1	2	2	4	4	4	4
1	1	1	1	2	2	4	4	4	4
4	4	1	1	3	3	1	1	4	4

For Review Only

1	1	1	1	2	2	4	4	4	4
1	1	1	1	2	2	4	4	4	4

For Review Only

SNP210	SNP210	SNP2100	SNP2100	SNP2101	SNP2101	SNP2102	SNP2102	SNP2103	SNP2103
3	3	4	4	2	2	3	3	3	3
4	4	4	4	3	3	3	3	3	3
4	4	1	1	2	2	3	3	3	3
3	3	4	4	2	2	2	2	3	3
3	3	4	4	3	3	3	3	3	3
3	3	4	4	2	2	3	3	3	3
3	3	4	4	3	3	3	3	3	3
4	4	4	4	3	3	3	3	1	1
3	3	4	4	3	3	3	3	3	3
4	4	4	4	3	3	3	3	1	1
3	3	4	4	2	2	3	3	3	3
4	4	4	4	2	2	2	2	3	3
4	4	4	4	2	2	3	3	3	3
4	4	4	4	2	2	3	3	3	3
4	4	4	4	2	2	2	2	3	3
3	3	4	4	2	2	2	2	3	3
4	4	4	4	3	3	3	3	3	3
4	4	4	4	2	2	3	3	1	1
3	3	4	4	2	2	2	2	3	3
4	4	4	4	3	3	2	2	1	1
4	4	4	4	3	3	3	3	3	3
4	4	4	4	3	3	3	3	3	3
4	4	4	4	3	3	3	3	1	1
4	4	4	4	3	3	2	2	1	1
3	3	4	4	2	2	2	2	3	3
4	4	4	4	2	2	3	3	3	3
3	3	4	4	3	3	2	2	3	3
4	4	4	4	3	3	3	3	3	3
4	4	4	4	3	3	3	3	3	3
4	4	1	1	3	3	3	3	1	1
4	4	4	4	2	2	2	2	3	3
4	4	4	4	2	2	2	2	3	3
3	3	4	4	3	3	2	2	3	3
4	4	4	4	3	3	3	3	3	3
4	4	4	4	3	3	3	3	3	3
3	3	4	4	2	2	2	2	1	1
3	3	4	4	3	3	3	3	3	3
3	3	4	4	2	2	3	3	3	3
4	4	4	4	3	3	3	3	1	1
3	3	4	4	3	3	3	3	1	1
4	4	1	1	3	3	3	3	1	1
4	4	4	4	3	3	3	3	3	3
4	4	4	4	3	3	3	3	1	1
3	3	4	4	3	3	3	3	3	3
4	4	4	4	3	3	3	3	1	1
4	4	4	4	2	2	2	2	3	3
4	4	4	4	2	2	3	3	3	3
4	4	4	4	2	2	3	3	3	3
3	3	1	1	3	3	3	3	3	3
3	3	4	4	3	3	3	3	3	3
4	4	4	4	2	2	3	3	3	3
4	4	4	4	3	3	2	2	3	3

For Review Only

3	3	4	4	2	2	3	3	3	3
3	3	4	4	3	3	2	2	3	3
4	4	4	4	2	2	3	3	3	3
4	4	1	1	3	3	3	3	1	1
3	3	4	4	2	2	2	2	3	3
4	4	4	4	2	2	3	3	3	3
3	3	4	4	2	2	3	3	3	3
4	4	4	4	3	3	3	3	1	1
3	3	4	4	3	3	3	3	3	3
3	3	4	4	3	3	3	3	3	3
4	4	4	4	2	2	3	3	3	3
4	4	4	4	2	2	3	3	3	3
3	3	4	4	2	2	3	3	3	3
4	4	4	4	3	3	3	3	3	3
4	4	4	4	3	3	3	3	3	3
4	4	4	4	3	3	3	3	3	3
3	3	1	1	2	2	3	3	3	3
4	4	4	4	2	2	2	2	3	3
3	3	4	4	2	2	3	3	3	3
3	3	4	4	3	3	3	3	3	3
4	4	4	4	2	2	3	3	3	3
3	3	4	4	3	3	3	3	3	3
3	3	4	4	3	3	3	3	3	3
4	4	4	4	3	3	3	3	3	3
4	4	4	4	3	3	3	3	1	1
4	4	4	4	2	2	2	2	3	3
3	3	4	4	3	3	3	3	3	3
4	4	4	4	3	3	3	3	1	1
4	4	4	4	2	2	3	3	3	3
4	4	4	4	3	3	2	2	3	3
3	3	4	4	3	3	3	3	1	1
4	4	4	4	3	3	3	3	3	3
4	4	4	4	3	3	3	3	1	1
3	3	4	4	2	2	3	3	3	3
3	3	4	4	2	2	3	3	3	3
4	4	4	4	3	3	3	3	1	1
4	4	4	4	3	3	3	3	1	1
3	3	1	1	3	3	3	3	3	3
4	4	4	4	3	3	3	3	3	3
3	3	4	4	2	2	3	3	1	1
4	4	4	4	3	3	3	3	3	3
3	3	1	1	3	3	2	2	3	3
3	3	4	4	3	3	3	3	3	3
4	4	4	4	2	2	3	3	3	3
3	3	4	4	2	2	3	3	3	3
4	4	4	4	3	3	3	3	3	3
4	4	4	4	3	3	3	3	3	3
3	3	4	4	2	2	3	3	1	1
4	4	4	4	2	2	3	3	1	1
3	3	4	4	2	2	2	2	3	3
4	4	4	4	2	2	3	3	1	1
4	4	4	4	3	3	3	3	3	3
3	3	4	4	3	3	3	3	1	1
4	4	4	4	3	3	2	2	3	3
4	4	4	4	2	2	2	2	3	3

For Review Only

4	4	4	4	3	3	3	3	3	3
4	4	4	4	3	3	2	2	3	3
3	3	4	4	2	2	3	3	3	3
4	4	4	4	3	3	3	3	3	3
4	4	4	4	2	2	2	2	3	3
4	4	4	4	3	3	2	2	3	3
3	3	4	4	2	2	3	3	3	3
4	4	4	4	2	2	3	3	3	3
4	4	4	4	2	2	3	3	1	1
4	4	4	4	3	3	2	2	3	3
3	3	4	4	2	2	3	3	3	3
3	3	4	4	3	3	3	3	3	3
4	4	4	4	3	3	3	3	1	1
4	4	4	4	2	2	2	2	3	3
4	4	4	4	2	2	3	3	3	3
4	4	4	4	3	3	2	2	3	3
4	4	4	4	2	2	2	2	3	3
4	4	4	4	3	3	2	2	3	3
4	4	4	4	3	3	3	3	3	3
3	3	4	4	3	3	3	3	3	3
4	4	4	4	3	3	3	3	3	3
3	3	4	4	2	2	2	2	3	3
4	4	4	4	3	3	2	2	3	3
4	4	4	4	3	3	3	3	3	3
3	3	4	4	3	3	3	3	3	3
3	3	4	4	3	3	3	3	3	3
3	3	4	4	3	3	3	3	3	3
4	4	4	4	3	3	3	3	3	3
4	4	4	4	3	3	2	2	3	3
3	3	4	4	2	2	2	2	3	3
4	4	4	4	3	3	3	3	3	3
4	4	4	4	3	3	3	3	3	3
4	4	4	4	3	3	3	3	3	3
4	4	4	4	2	2	3	3	3	3
4	4	4	4	3	3	3	3	3	3
4	4	4	4	3	3	3	3	3	3
4	4	4	4	3	3	3	3	3	3
3	3	4	4	2	2	2	2	3	3
3	3	4	4	2	2	3	3	3	3
4	4	4	4	3	3	3	3	3	3
4	4	4	4	3	3	3	3	3	3
4	4	4	4	3	3	3	3	3	3
4	4	4	4	2	2	2	2	3	3
4	4	4	4	3	3	3	3	1	1
3	3	4	4	3	3	3	3	1	1
4	4	4	4	3	3	3	3	3	3
4	4	4	4	2	2	3	3	3	3
4	4	1	1	2	2	3	3	3	3
4	4	4	4	3	3	3	3	3	3
4	4	4	4	2	2	3	3	3	3
4	4	4	4	2	2	2	2	3	3
3	3	4	4	3	3	3	3	1	1
4	4	4	4	2	2	3	3	3	3
4	4	4	4	2	2	3	3	3	3
4	4	4	4	3	3	3	3	3	3

For Review Only

3	3	4	4	2	2	2	2	3	3
3	3	4	4	3	3	3	3	3	3
3	3	4	4	3	3	3	3	1	1
4	4	4	4	3	3	3	3	3	3
4	4	4	4	2	2	3	3	3	3
3	3	4	4	3	3	3	3	3	3
4	4	4	4	3	3	3	3	3	3
4	4	4	4	3	3	3	3	3	3
4	4	4	4	3	3	3	3	3	3
4	4	4	4	2	2	3	3	3	3
4	4	4	4	2	2	2	2	3	3
4	4	4	4	3	3	3	3	3	3
3	3	4	4	3	3	2	2	3	3
4	4	4	4	3	3	3	3	3	3
4	4	4	4	3	3	3	3	3	3
3	3	4	4	2	2	3	3	3	3
4	4	4	4	2	2	3	3	3	3
4	4	1	1	2	2	3	3	3	3
4	4	4	4	2	2	3	3	3	3
3	3	4	4	2	2	3	3	3	3
4	4	4	4	3	3	3	3	3	3
4	4	4	4	3	3	3	3	1	1
3	3	4	4	3	3	3	3	3	3
4	4	4	4	2	2	3	3	3	3
4	4	4	4	3	3	2	2	3	3
3	3	4	4	3	3	3	3	3	3
4	4	4	4	3	3	2	2	1	1
4	4	4	4	2	2	2	2	3	3
4	4	4	4	3	3	3	3	1	1
3	3	4	4	3	3	3	3	3	3
3	3	4	4	3	3	3	3	3	3
4	4	4	4	3	3	2	2	3	3
3	3	1	1	3	3	3	3	3	3
4	4	4	4	3	3	3	3	3	3
3	3	4	4	3	3	3	3	3	3
4	4	4	4	2	2	3	3	3	3
3	3	4	4	3	3	2	2	3	3
3	3	4	4	2	2	3	3	3	3
4	4	1	1	2	2	3	3	3	3
3	3	4	4	2	2	2	2	3	3
4	4	1	1	2	2	3	3	3	3
4	4	4	4	2	2	3	3	3	3
4	4	4	4	3	3	3	3	1	1
4	4	4	4	3	3	3	3	3	3
4	4	4	4	2	2	3	3	1	1
4	4	4	4	3	3	2	2	3	3
3	3	4	4	2	2	3	3	3	3
4	4	4	4	3	3	3	3	1	1
4	4	4	4	3	3	3	3	3	3
4	4	4	4	3	3	3	3	1	1
4	4	4	4	2	2	2	2	3	3
3	3	4	4	3	3	3	3	1	1
3	3	4	4	3	3	3	3	3	3
4	4	4	4	2	2	3	3	3	3
4	4	4	4	3	3	3	3	1	1

For Review Only

4	4	4	4	2	2	3	3	3	3
4	4	4	4	2	2	3	3	3	3
4	4	4	4	3	3	2	2	3	3
3	3	4	4	3	3	3	3	3	3
4	4	4	4	3	3	2	2	1	1
3	3	4	4	2	2	3	3	3	3
4	4	4	4	3	3	3	3	3	3
4	4	1	1	3	3	3	3	1	1
4	4	4	4	3	3	3	3	1	1
4	4	4	4	3	3	3	3	1	1
4	4	4	4	3	3	3	3	3	3
3	3	4	4	3	3	3	3	3	3
3	3	4	4	3	3	3	3	3	3
4	4	4	4	2	2	3	3	3	3
4	4	4	4	2	2	3	3	3	3
3	3	4	4	3	3	3	3	3	3
4	4	4	4	3	3	3	3	1	1
3	3	1	1	2	2	3	3	3	3
4	4	4	4	3	3	3	3	1	1
3	3	4	4	2	2	3	3	3	3
3	3	4	4	2	2	2	2	3	3
4	4	4	4	2	2	3	3	3	3
3	3	4	4	2	2	3	3	3	3
3	3	4	4	2	2	3	3	3	3
4	4	4	4	3	3	3	3	1	1
4	4	4	4	2	2	3	3	3	3
4	4	4	4	3	3	3	3	1	1
3	3	4	4	2	2	2	2	3	3
4	4	4	4	3	3	2	2	3	3
4	4	4	4	3	3	2	2	3	3
4	4	4	4	3	3	3	3	1	1
3	3	4	4	3	3	3	3	3	3
4	4	4	4	3	3	3	3	3	3
4	4	4	4	2	2	3	3	3	3
3	3	4	4	3	3	3	3	3	3
4	4	4	4	3	3	3	3	1	1
4	4	4	4	2	2	3	3	3	3
4	4	4	4	2	2	3	3	3	3
3	3	4	4	2	2	2	2	1	1
4	4	4	4	3	3	3	3	1	1
4	4	4	4	3	3	2	2	3	3
4	4	4	4	3	3	3	3	3	3
3	3	4	4	3	3	3	3	3	3
3	3	4	4	3	3	3	3	3	3
4	4	4	4	2	2	3	3	3	3
4	4	4	4	3	3	3	3	3	3
4	4	4	4	2	2	3	3	3	3
4	4	4	4	2	2	3	3	3	3
4	4	1	1	3	3	2	2	3	3
4	4	4	4	3	3	3	3	1	1
3	3	4	4	2	2	3	3	3	3
4	4	4	4	3	3	3	3	1	1
4	4	1	1	2	2	3	3	3	3
3	3	4	4	2	2	2	2	3	3
3	3	4	4	3	3	3	3	3	3

For Review Only

3	3	4	4	2	2	3	3	3	3
3	3	4	4	2	2	2	2	3	3

For Review Only

SNP2104	SNP2104	SNP2105	SNP2105	SNP2106	SNP2106	SNP2107	SNP2107	SNP2108	SNP2108
1	1	1	1	3	3	1	1	2	2
3	3	1	1	2	2	1	1	4	4
1	1	4	4	3	3	1	1	2	2
1	1	1	1	3	3	2	2	2	2
3	3	1	1	2	2	1	1	4	4
1	1	4	4	3	3	1	1	2	2
3	3	1	1	2	2	1	1	4	4
3	3	1	1	2	2	1	1	4	4
3	3	1	1	2	2	1	1	4	4
3	3	1	1	2	2	1	1	4	4
1	1	1	1	3	3	1	1	2	2
1	1	4	4	3	3	1	1	2	2
1	1	1	1	3	3	1	1	4	4
1	1	1	1	3	3	1	1	2	2
1	1	1	1	3	3	1	1	2	2
1	1	1	1	3	3	1	1	2	2
3	3	1	1	2	2	1	1	4	4
1	1	1	1	3	3	1	1	2	2
1	1	1	1	3	3	1	1	2	2
3	3	1	1	3	3	1	1	4	4
3	3	1	1	2	2	1	1	4	4
3	3	1	1	2	2	1	1	4	4
3	3	1	1	2	2	1	1	4	4
3	3	4	4	2	2	1	1	4	4
1	1	1	1	3	3	1	1	2	2
1	1	1	1	3	3	2	2	2	2
3	3	4	4	2	2	2	2	4	4
1	1	1	1	3	3	2	2	4	4
3	3	1	1	2	2	1	1	4	4
3	3	1	1	2	2	1	1	4	4
1	1	1	1	3	3	1	1	2	2
1	1	4	4	3	3	2	2	2	2
3	3	1	1	2	2	1	1	4	4
3	3	1	1	2	2	1	1	4	4
1	1	1	1	2	2	1	1	4	4
1	1	1	1	3	3	1	1	2	2
3	3	1	1	2	2	1	1	4	4
1	1	1	1	3	3	2	2	2	2
3	3	1	1	2	2	1	1	4	4
3	3	1	1	2	2	1	1	4	4
3	3	1	1	2	2	1	1	4	4
3	3	1	1	2	2	1	1	4	4
3	3	1	1	2	2	1	1	4	4
3	3	1	1	2	2	1	1	4	4
3	3	1	1	2	2	1	1	4	4
1	1	4	4	3	3	2	2	2	2
1	1	1	1	3	3	1	1	2	2
1	1	1	1	3	3	2	2	2	2
3	3	1	1	2	2	1	1	4	4
3	3	4	4	2	2	1	1	4	4
1	1	1	1	3	3	2	2	2	2
3	3	1	1	2	2	2	2	4	4

For Review Only

For Review Only

1	1	1	1	3	3	1	1	2	2
3	3	1	1	2	2	1	1	4	4
1	1	1	1	3	3	1	1	4	4
3	3	1	1	2	2	1	1	4	4
1	1	1	1	3	3	1	1	2	2
1	1	4	4	3	3	1	1	2	2
1	1	1	1	3	3	2	2	2	2
3	3	1	1	2	2	1	1	4	4
3	3	1	1	2	2	1	1	4	4
1	1	1	1	3	3	2	2	4	4
1	1	4	4	3	3	1	1	2	2
1	1	4	4	3	3	1	1	2	2
1	1	1	1	3	3	1	1	2	2
3	3	1	1	2	2	1	1	4	4
1	1	4	4	3	3	1	1	2	2
3	3	1	1	2	2	1	1	4	4
1	1	1	1	3	3	2	2	4	4
1	1	4	4	3	3	1	1	4	4
1	1	1	1	3	3	1	1	2	2
3	3	1	1	2	2	1	1	4	4
1	1	4	4	3	3	1	1	4	4
1	1	4	4	3	3	1	1	4	4
3	3	1	1	2	2	1	1	4	4
3	3	4	4	2	2	1	1	4	4
3	3	1	1	2	2	1	1	4	4
1	1	1	1	3	3	1	1	4	4
3	3	1	1	2	2	1	1	4	4
3	3	1	1	2	2	1	1	4	4
1	1	1	1	3	3	2	2	2	2
3	3	1	1	2	2	1	1	4	4
3	3	1	1	2	2	1	1	4	4
3	3	1	1	2	2	1	1	4	4
3	3	1	1	2	2	1	1	4	4
1	1	1	1	3	3	1	1	2	2
1	1	1	1	3	3	1	1	2	2
3	3	1	1	2	2	1	1	4	4
3	3	1	1	2	2	1	1	4	4
3	3	1	1	2	2	1	1	4	4
3	3	1	1	2	2	2	2	4	4
1	1	1	1	3	3	1	1	2	2
3	3	1	1	2	2	1	1	4	4
3	3	1	1	2	2	1	1	4	4
3	3	1	1	2	2	2	2	4	4
1	1	4	4	3	3	1	1	2	2
1	1	4	4	3	3	1	1	4	4
3	3	1	1	2	2	1	1	4	4
3	3	1	1	2	2	1	1	4	4
1	1	1	1	3	3	2	2	4	4
1	1	4	4	3	3	1	1	2	2
1	1	4	4	3	3	1	1	2	2
1	1	4	4	3	3	1	1	4	4
3	3	1	1	2	2	1	1	4	4
3	3	1	1	2	2	1	1	4	4
1	1	4	4	3	3	2	2	4	4
1	1	1	1	3	3	1	1	2	2

3	3	1	1	2	2	2	2	4	4
1	1	1	1	3	3	1	1	4	4
1	1	4	4	3	3	2	2	2	2
3	3	1	1	2	2	1	1	4	4
1	1	4	4	3	3	2	2	4	4
3	3	1	1	2	2	2	2	4	4
1	1	1	1	3	3	2	2	2	2
1	1	4	4	3	3	1	1	4	4
1	1	1	1	3	3	1	1	2	2
1	1	1	1	3	3	1	1	4	4
1	1	1	1	3	3	1	1	2	2
1	1	1	1	3	3	2	2	4	4
3	3	1	1	2	2	2	2	4	4
1	1	1	1	3	3	2	2	4	4
1	1	1	1	3	3	2	2	4	4
1	1	1	1	3	3	2	2	4	4
1	1	4	4	3	3	1	1	4	4
1	1	1	1	3	3	2	2	4	4
3	3	1	1	2	2	1	1	4	4
3	3	1	1	2	2	1	1	4	4
1	1	1	1	3	3	1	1	4	4
1	1	1	1	3	3	1	1	4	4
3	3	4	4	2	2	1	1	4	4
3	3	1	1	3	3	1	1	4	4
1	1	4	4	3	3	2	2	4	4
3	3	1	1	2	2	1	1	4	4
3	3	1	1	2	2	1	1	2	2
3	3	1	1	2	2	1	1	4	4
1	1	1	1	3	3	2	2	4	4
1	1	4	4	3	3	1	1	2	2
3	3	1	1	2	2	1	1	4	4
1	1	4	4	3	3	1	1	4	4
3	3	1	1	2	2	1	1	4	4
1	1	1	1	3	3	2	2	4	4
1	1	1	1	2	2	1	1	4	4
3	3	1	1	2	2	1	1	4	4
3	3	1	1	2	2	1	1	4	4
1	1	1	1	3	3	1	1	2	2
1	1	1	1	3	3	2	2	4	4
3	3	1	1	2	2	1	1	4	4
3	3	1	1	2	2	1	1	4	4
3	3	1	1	2	2	1	1	4	4
1	1	1	1	3	3	1	1	2	2
3	3	1	1	2	2	1	1	4	4
3	3	1	1	3	3	2	2	2	2
3	3	1	1	2	2	1	1	4	4
1	1	1	1	3	3	2	2	2	2
1	1	1	1	3	3	1	1	4	4
1	1	1	1	3	3	1	1	4	4
1	1	1	1	3	3	2	2	2	2
1	1	1	1	3	3	2	2	2	2
3	3	1	1	2	2	1	1	4	4
1	1	4	4	3	3	1	1	4	4
1	1	4	4	3	3	2	2	4	4
1	1	1	1	3	3	1	1	4	4

For Review Only

1	1	1	1	3	3	1	1	2	2
3	3	1	1	2	2	1	1	4	4
3	3	4	4	2	2	1	1	4	4
1	1	4	4	3	3	1	1	4	4
1	1	4	4	3	3	1	1	4	4
3	3	1	1	2	2	1	1	4	4
3	3	1	1	2	2	1	1	4	4
1	1	1	1	3	3	1	1	4	4
3	3	1	1	2	2	2	2	4	4
1	1	1	1	3	3	1	1	2	2
1	1	4	4	3	3	2	2	4	4
3	3	1	1	2	2	1	1	4	4
3	3	1	1	2	2	1	1	4	4
3	3	1	1	2	2	1	1	4	4
3	3	1	1	2	2	1	1	4	4
1	1	1	1	3	3	1	1	2	2
1	1	4	4	3	3	1	1	2	2
1	1	1	1	3	3	1	1	4	4
1	1	1	1	3	3	1	1	2	2
1	1	1	1	3	3	2	2	2	2
1	1	1	1	3	3	1	1	4	4
3	3	1	1	2	2	1	1	4	4
1	1	4	4	3	3	1	1	4	4
1	1	4	4	3	3	1	1	2	2
3	3	1	1	2	2	1	1	4	4
3	3	1	1	2	2	1	1	4	4
1	1	1	1	3	3	1	1	4	4
1	1	1	1	3	3	1	1	2	2
3	3	4	4	2	2	1	1	4	4
3	3	4	4	2	2	1	1	4	4
3	3	1	1	2	2	1	1	4	4
3	3	4	4	2	2	1	1	4	4
3	3	1	1	2	2	1	1	4	4
3	3	1	1	2	2	1	1	4	4
3	3	1	1	2	2	1	1	4	4
1	1	4	4	3	3	1	1	2	2
1	1	4	4	3	3	2	2	2	2
1	1	1	1	3	3	1	1	2	2
1	1	1	1	3	3	1	1	4	4
1	1	1	1	3	3	2	2	2	2
1	1	4	4	3	3	1	1	2	2
1	1	1	1	3	3	2	2	2	2
3	3	1	1	2	2	1	1	4	4
1	1	4	4	3	3	1	1	4	4
1	1	4	4	3	3	1	1	4	4
3	3	4	4	2	2	1	1	4	4
1	1	1	1	3	3	1	1	2	2
3	3	1	1	2	2	1	1	4	4
3	3	1	1	2	2	1	1	4	4
3	3	1	1	2	2	2	2	4	4
1	1	1	1	3	3	2	2	2	2
3	3	1	1	2	2	1	1	4	4
3	3	4	4	2	2	1	1	4	4
1	1	4	4	3	3	1	1	2	2
3	3	1	1	2	2	1	1	4	4

For Review Only

1	1	1	1	3	3	1	1	2	2
1	1	1	1	3	3	1	1	2	2
1	1	1	1	3	3	1	1	4	4
3	3	4	4	2	2	1	1	4	4
3	3	1	1	2	2	1	1	4	4
1	1	1	1	3	3	2	2	2	2
3	3	1	1	2	2	1	1	4	4
3	3	1	1	2	2	1	1	4	4
1	1	4	4	3	3	1	1	4	4
3	3	1	1	2	2	1	1	4	4
1	1	1	1	3	3	1	1	4	4
3	3	1	1	2	2	1	1	4	4
3	3	1	1	2	2	2	2	4	4
1	1	1	1	3	3	1	1	2	2
1	1	1	1	3	3	1	1	2	2
1	1	4	4	3	3	1	1	2	2
3	3	1	1	2	2	1	1	4	4
1	1	1	1	3	3	1	1	2	2
3	3	1	1	2	2	1	1	4	4
1	1	1	1	3	3	2	2	2	2
1	1	1	1	3	3	1	1	2	2
1	1	1	1	3	3	2	2	2	2
1	1	1	1	3	3	1	1	2	2
1	1	1	1	3	3	1	1	2	2
3	3	1	1	2	2	1	1	4	4
1	1	1	1	3	3	2	2	2	2
3	3	1	1	2	2	1	1	4	4
1	1	1	1	3	3	2	2	2	2
3	3	1	1	2	2	1	1	4	4
3	3	1	1	2	2	1	1	4	4
3	3	1	1	2	2	1	1	4	4
1	1	1	1	3	3	2	2	4	4
3	3	1	1	2	2	1	1	4	4
1	1	1	1	3	3	2	2	4	4
3	3	4	4	2	2	1	1	4	4
3	3	1	1	2	2	1	1	4	4
1	1	1	1	3	3	1	1	2	2
1	1	4	4	3	3	1	1	2	2
1	1	1	1	3	3	1	1	2	2
3	3	1	1	2	2	1	1	4	4
3	3	1	1	2	2	1	1	4	4
3	3	1	1	2	2	1	1	4	4
3	3	1	1	2	2	1	1	4	4
3	3	1	1	2	2	1	1	4	4
1	1	4	4	3	3	1	1	2	2
3	3	1	1	2	2	1	1	4	4
1	1	1	1	3	3	1	1	4	4
1	1	1	1	3	3	2	2	2	2
1	1	1	1	3	3	1	1	4	4
3	3	1	1	2	2	1	1	4	4
1	1	1	1	3	3	1	1	2	2
3	3	1	1	2	2	1	1	4	4
1	1	4	4	3	3	1	1	2	2
1	1	1	1	3	3	2	2	2	2
3	3	1	1	2	2	1	1	4	4

For Review Only

1	1	4	4	3	3	1	1	2	2
1	1	1	1	3	3	2	2	2	2

For Review Only

SNP2109	SNP2109	SNP211	SNP211	SNP2110	SNP2110	SNP2111	SNP2111	SNP2112	SNP2112
3	3	1	1	1	1	3	3	4	4
4	4	1	1	1	1	4	4	4	4
3	3	1	1	1	1	4	4	1	1
3	3	1	1	4	4	4	4	1	1
4	4	1	1	1	1	4	4	4	4
3	3	1	1	1	1	3	3	4	4
4	4	1	1	1	1	4	4	4	4
4	4	1	1	1	1	4	4	4	4
4	4	1	1	1	1	4	4	4	4
4	4	2	2	1	1	4	4	4	4
3	3	2	2	4	4	3	3	4	4
3	3	1	1	1	1	4	4	1	1
4	4	1	1	4	4	3	3	4	4
3	3	1	1	1	1	4	4	4	4
3	3	1	1	1	1	3	3	4	4
3	3	1	1	1	1	4	4	1	1
4	4	1	1	1	1	4	4	4	4
3	3	1	1	1	1	3	3	4	4
3	3	1	1	1	1	3	3	4	4
4	4	1	1	1	1	3	3	4	4
4	4	1	1	4	4	4	4	4	4
4	4	1	1	1	1	4	4	4	4
4	4	1	1	1	1	4	4	4	4
4	4	1	1	1	1	4	4	4	4
3	3	1	1	1	1	3	3	4	4
3	3	1	1	1	1	4	4	1	1
4	4	1	1	1	1	4	4	4	4
3	3	1	1	1	1	3	3	4	4
4	4	1	1	1	1	4	4	4	4
4	4	2	2	4	4	4	4	4	4
3	3	1	1	1	1	3	3	4	4
3	3	1	1	1	1	3	3	4	4
4	4	1	1	1	1	4	4	4	4
4	4	1	1	1	1	4	4	4	4
3	3	1	1	1	1	4	4	4	4
3	3	1	1	1	1	4	4	1	1
4	4	1	1	1	1	4	4	4	4
3	3	1	1	4	4	3	3	4	4
4	4	2	2	1	1	4	4	4	4
4	4	1	1	4	4	4	4	4	4
4	4	1	1	4	4	4	4	4	4
4	4	1	1	1	1	4	4	4	4
4	4	2	2	1	1	4	4	4	4
4	4	2	2	1	1	4	4	4	4
4	4	1	1	1	1	4	4	4	4
3	3	1	1	1	1	4	4	1	1
3	3	1	1	1	1	4	4	1	1
3	3	1	1	1	1	4	4	4	4
4	4	1	1	4	4	4	4	4	4
4	4	1	1	1	1	4	4	4	4
3	3	1	1	1	1	4	4	1	1
3	3	1	1	1	1	4	4	4	4

For Review Only

3	3	1	1	1	1	3	3	4	4
4	4	1	1	1	1	4	4	4	4
4	4	1	1	1	1	3	3	4	4
4	4	1	1	1	1	4	4	4	4
3	3	1	1	1	1	3	3	4	4
3	3	1	1	1	1	4	4	1	1
3	3	1	1	1	1	4	4	1	1
4	4	2	2	4	4	4	4	4	4
4	4	1	1	1	1	4	4	4	4
3	3	1	1	1	1	3	3	4	4
3	3	1	1	1	1	4	4	1	1
3	3	1	1	1	1	4	4	1	1
3	3	1	1	1	1	3	3	4	4
4	4	1	1	1	1	4	4	4	4
3	3	1	1	1	1	3	3	4	4
4	4	1	1	1	1	4	4	4	4
4	4	1	1	1	1	3	3	4	4
3	3	1	1	1	1	3	3	4	4
3	3	1	1	1	1	4	4	4	4
4	4	1	1	1	1	4	4	4	4
3	3	1	1	1	1	4	4	4	4
3	3	1	1	1	1	4	4	4	4
4	4	1	1	1	1	4	4	4	4
4	4	1	1	1	1	4	4	4	4
4	4	1	1	1	1	4	4	4	4
4	4	1	1	1	1	4	4	1	1
4	4	1	1	1	1	4	4	4	4
4	4	2	2	1	1	4	4	4	4
3	3	1	1	1	1	4	4	1	1
4	4	1	1	1	1	4	4	4	4
4	4	1	1	1	1	4	4	4	4
4	4	1	1	1	1	4	4	4	4
4	4	2	2	1	1	4	4	4	4
3	3	1	1	1	1	3	3	4	4
3	3	1	1	1	1	3	3	4	4
4	4	1	1	1	1	4	4	4	4
4	4	2	2	4	4	4	4	4	4
4	4	1	1	1	1	4	4	4	4
4	4	1	1	1	1	3	3	4	4
3	3	2	2	1	1	3	3	4	4
4	4	1	1	1	1	4	4	4	4
4	4	1	1	1	1	4	4	4	4
4	4	1	1	1	1	3	3	4	4
3	3	1	1	1	1	4	4	1	1
3	3	1	1	1	1	4	4	4	4
4	4	1	1	1	1	3	3	4	4
4	4	1	1	1	1	4	4	4	4
4	4	1	1	1	1	3	3	4	4
3	3	1	1	1	1	4	4	1	1
3	3	1	1	1	1	3	3	4	4
4	4	1	1	1	1	3	3	4	4
4	4	2	2	1	1	4	4	4	4
4	4	2	2	4	4	4	4	4	4
4	4	1	1	1	1	3	3	4	4
3	3	1	1	1	1	3	3	4	4

For Review Only

4	4	1	1	1	1	4	4	4	4
3	3	1	1	1	1	3	3	4	4
3	3	1	1	1	1	4	4	1	1
4	4	1	1	1	1	4	4	4	4
4	4	1	1	1	1	4	4	1	1
4	4	1	1	1	1	4	4	4	4
3	3	1	1	1	1	4	4	1	1
3	3	1	1	1	1	3	3	4	4
3	3	1	1	1	1	3	3	4	4
3	3	1	1	1	1	4	4	4	4
3	3	1	1	1	1	3	3	4	4
3	3	1	1	1	1	3	3	4	4
4	4	1	1	1	1	4	4	4	4
3	3	1	1	1	1	4	4	1	1
4	4	1	1	1	1	3	3	4	4
3	3	1	1	1	1	4	4	1	1
3	3	1	1	4	4	3	3	4	4
3	3	1	1	1	1	4	4	4	4
4	4	1	1	1	1	4	4	4	4
4	4	1	1	1	1	4	4	4	4
3	3	1	1	1	1	3	3	4	4
3	3	1	1	1	1	3	3	4	4
4	4	1	1	1	1	4	4	4	4
4	4	1	1	4	4	4	4	4	4
3	3	1	1	1	1	4	4	4	4
4	4	1	1	1	1	4	4	4	4
3	3	1	1	1	1	3	3	4	4
4	4	1	1	1	1	4	4	4	4
3	3	1	1	1	1	4	4	4	4
3	3	1	1	4	4	3	3	4	4
4	4	2	2	1	1	4	4	4	4
4	4	1	1	1	1	3	3	4	4
4	4	1	1	1	1	4	4	4	4
3	3	1	1	1	1	3	3	4	4
4	4	1	1	1	1	4	4	4	4
4	4	1	1	1	1	4	4	4	4
4	4	1	1	1	1	4	4	4	4
3	3	1	1	1	1	3	3	4	4
3	3	1	1	1	1	3	3	4	4
4	4	2	2	4	4	4	4	4	4
4	4	1	1	1	1	4	4	4	4
4	4	1	1	1	1	4	4	4	4
3	3	1	1	1	1	3	3	4	4
4	4	1	1	4	4	3	3	4	4
3	3	1	1	1	1	4	4	4	4
4	4	1	1	1	1	4	4	4	4
3	3	1	1	1	1	4	4	1	1
4	4	1	1	1	1	3	3	4	4
4	4	1	1	4	4	3	3	4	4
3	3	1	1	1	1	4	4	1	1
3	3	1	1	1	1	4	4	1	1
4	4	2	2	1	1	4	4	4	4
3	3	1	1	1	1	3	3	4	4
3	3	1	1	1	1	4	4	1	1
4	4	1	1	4	4	4	4	4	4

For Review Only

3	3	1	1	1	1	3	3	4	4
4	4	1	1	1	1	4	4	4	4
4	4	1	1	1	1	4	4	4	4
3	3	1	1	1	1	3	3	4	4
3	3	1	1	1	1	3	3	4	4
4	4	2	2	4	4	4	4	4	4
4	4	1	1	1	1	4	4	4	4
3	3	1	1	1	1	3	3	4	4
4	4	1	1	1	1	4	4	4	4
3	3	1	1	1	1	4	4	4	4
3	3	1	1	1	1	4	4	1	1
4	4	1	1	1	1	4	4	4	4
4	4	1	1	1	1	3	3	4	4
4	4	1	1	1	1	4	4	4	4
4	4	1	1	1	1	4	4	4	4
3	3	2	2	4	4	3	3	4	4
3	3	1	1	1	1	3	3	4	4
4	4	1	1	1	1	4	4	1	1
3	3	1	1	1	1	4	4	1	1
3	3	1	1	1	1	4	4	1	1
3	3	1	1	1	1	3	3	4	4
4	4	2	2	1	1	4	4	4	4
3	3	1	1	1	1	4	4	1	1
3	3	1	1	1	1	4	4	1	1
4	4	1	1	1	1	4	4	4	4
4	4	1	1	1	1	4	4	4	4
3	3	1	1	1	1	4	4	4	4
3	3	1	1	1	1	3	3	4	4
4	4	1	1	1	1	4	4	4	4
4	4	1	1	1	1	4	4	4	4
4	4	2	2	1	1	4	4	4	4
4	4	1	1	1	1	4	4	4	4
4	4	1	1	1	1	4	4	4	4
4	4	1	1	1	1	4	4	4	4
4	4	1	1	1	1	4	4	4	4
3	3	1	1	1	1	4	4	1	1
3	3	1	1	1	1	4	4	4	4
3	3	1	1	1	1	3	3	4	4
4	4	1	1	1	1	3	3	4	4
3	3	1	1	1	1	3	3	4	4
3	3	1	1	1	1	4	4	1	1
3	3	1	1	1	1	4	4	1	1
4	4	2	2	4	4	4	4	4	4
4	4	1	1	1	1	3	3	4	4
4	4	1	1	1	1	4	4	1	1
4	4	1	1	4	4	4	4	4	4
3	3	2	2	4	4	3	3	4	4
4	4	2	2	1	1	4	4	4	4
4	4	1	1	4	4	4	4	4	4
4	4	1	1	1	1	3	3	4	4
3	3	1	1	1	1	4	4	1	1
4	4	2	2	1	1	4	4	4	4
4	4	1	1	1	1	4	4	4	4
3	3	1	1	1	1	4	4	1	1
4	4	2	2	1	1	4	4	4	4

For Review Only

3	3	1	1	1	1	3	3	4	4
3	3	2	2	1	1	3	3	4	4
3	3	1	1	1	1	4	4	4	4
4	4	1	1	1	1	4	4	4	4
4	4	1	1	1	1	4	4	4	4
3	3	1	1	1	1	4	4	1	1
4	4	2	2	1	1	4	4	4	4
4	4	1	1	1	1	4	4	4	4
3	3	1	1	1	1	4	4	4	4
4	4	2	2	4	4	4	4	4	4
3	3	1	1	1	1	3	3	4	4
4	4	2	2	1	1	4	4	4	4
4	4	1	1	1	1	3	3	4	4
3	3	1	1	1	1	3	3	4	4
3	3	1	1	1	1	4	4	1	1
3	3	1	1	1	1	3	3	4	4
4	4	2	2	1	1	4	4	4	4
3	3	1	1	1	1	4	4	4	4
4	4	1	1	1	1	4	4	4	4
3	3	1	1	4	4	3	3	4	4
3	3	1	1	1	1	3	3	4	4
3	3	1	1	1	1	4	4	1	1
3	3	1	1	1	1	3	3	4	4
3	3	1	1	4	4	3	3	4	4
4	4	2	2	4	4	4	4	4	4
3	3	1	1	1	1	3	3	4	4
4	4	2	2	4	4	4	4	4	4
3	3	1	1	1	1	4	4	1	1
4	4	1	1	1	1	4	4	4	4
4	4	1	1	1	1	4	4	4	4
4	4	2	2	4	4	4	4	4	4
4	4	1	1	1	1	4	4	4	4
4	4	1	1	1	1	4	4	4	4
4	4	1	1	1	1	3	3	4	4
4	4	1	1	1	1	4	4	4	4
4	4	2	2	4	4	4	4	4	4
3	3	1	1	1	1	3	3	4	4
3	3	1	1	1	1	4	4	1	1
3	3	1	1	1	1	4	4	1	1
4	4	2	2	4	4	4	4	4	4
4	4	1	1	1	1	4	4	4	4
4	4	1	1	1	1	4	4	4	4
4	4	1	1	4	4	4	4	4	4
4	4	1	1	1	1	4	4	4	4
3	3	1	1	1	1	3	3	4	4
4	4	1	1	1	1	3	3	4	4
4	4	2	2	1	1	3	3	4	4
3	3	1	1	1	1	3	3	4	4
3	3	1	1	1	1	4	4	4	4
4	4	2	2	1	1	4	4	4	4
3	3	1	1	1	1	3	3	4	4
4	4	2	2	1	1	4	4	4	4
3	3	1	1	1	1	4	4	1	1
3	3	1	1	1	1	3	3	4	4
4	4	2	2	4	4	4	4	4	4

For Review Only

3	3	1	1	1	1	3	3	4	4
3	3	1	1	1	1	3	3	4	4

For Review Only

SNP2113	SNP2113	SNP2114	SNP2114	SNP2115	SNP2115	SNP2116	SNP2116	SNP2117	SNP2117
1	1	4	4	4	4	1	1	3	3
1	1	4	4	4	4	1	1	4	4
1	1	4	4	1	1	2	2	3	3
4	4	3	3	4	4	2	2	3	3
1	1	4	4	4	4	1	1	4	4
1	1	4	4	1	1	2	2	3	3
1	1	4	4	1	1	2	2	4	4
1	1	4	4	4	4	1	1	4	4
1	1	3	3	1	1	2	2	4	4
1	1	4	4	4	4	1	1	4	4
4	4	3	3	4	4	1	1	3	3
1	1	4	4	4	4	1	1	3	3
1	1	3	3	1	1	2	2	4	4
1	1	4	4	4	4	1	1	4	4
1	1	4	4	4	4	1	1	4	4
1	1	3	3	4	4	1	1	3	3
1	1	4	4	4	4	2	2	4	4
1	1	4	4	4	4	1	1	3	3
1	1	4	4	1	1	2	2	3	3
1	1	4	4	4	4	1	1	3	3
4	4	3	3	4	4	1	1	4	4
4	4	3	3	1	1	2	2	4	4
1	1	4	4	4	4	1	1	4	4
1	1	4	4	4	4	1	1	4	4
1	1	4	4	1	1	2	2	3	3
1	1	4	4	4	4	1	1	3	3
1	1	4	4	1	1	2	2	4	4
1	1	4	4	4	4	1	1	4	4
4	4	3	3	4	4	1	1	4	4
4	4	3	3	4	4	1	1	4	4
1	1	4	4	1	1	2	2	3	3
1	1	4	4	1	1	2	2	3	3
1	1	4	4	1	1	2	2	4	4
1	1	4	4	4	4	1	1	4	4
1	1	4	4	4	4	1	1	4	4
1	1	4	4	4	4	1	1	3	3
1	1	4	4	1	1	2	2	4	4
4	4	3	3	4	4	1	1	3	3
1	1	4	4	4	4	1	1	4	4
1	1	4	4	4	4	1	1	4	4
4	4	3	3	4	4	1	1	4	4
4	4	3	3	4	4	1	1	4	4
1	1	4	4	4	4	1	1	4	4
1	1	4	4	1	1	2	2	4	4
1	1	4	4	4	4	1	1	4	4
1	1	4	4	4	4	1	1	3	3
1	1	4	4	4	4	1	1	3	3
1	1	4	4	4	4	1	1	3	3
4	4	3	3	1	1	2	2	4	4
1	1	4	4	1	1	2	2	4	4
1	1	4	4	4	4	1	1	3	3
1	1	4	4	4	4	1	1	4	4

1	1	4	4	1	1	2	2	3	3
1	1	4	4	1	1	2	2	4	4
1	1	4	4	1	1	2	2	3	3
1	1	4	4	1	1	2	2	4	4
1	1	4	4	1	1	2	2	3	3
1	1	4	4	4	4	1	1	3	3
1	1	4	4	4	4	1	1	3	3
4	4	3	3	4	4	1	1	4	4
1	1	4	4	1	1	2	2	4	4
1	1	4	4	4	4	1	1	4	4
1	1	4	4	4	4	1	1	3	3
1	1	4	4	4	4	1	1	3	3
1	1	4	4	1	1	2	2	3	3
4	4	3	3	4	4	1	1	4	4
1	1	4	4	4	4	1	1	4	4
1	1	4	4	1	1	2	2	4	4
1	1	4	4	4	4	1	1	3	3
1	1	4	4	1	1	2	2	3	3
1	1	4	4	4	4	1	1	3	3
1	1	4	4	1	1	2	2	4	4
1	1	4	4	4	4	1	1	4	4
1	1	4	4	1	1	2	2	4	4
4	4	3	3	4	4	1	1	4	4
1	1	4	4	1	1	2	2	4	4
1	1	4	4	4	4	1	1	4	4
1	1	4	4	4	4	1	1	3	3
1	1	4	4	4	4	1	1	4	4
1	1	4	4	4	4	1	1	4	4
1	1	4	4	4	4	1	1	3	3
1	1	4	4	4	4	1	1	3	3
1	1	4	4	4	4	1	1	4	4
1	1	4	4	4	4	1	1	4	4
1	1	4	4	4	4	1	1	4	4
1	1	4	4	1	1	2	2	3	3
1	1	4	4	1	1	2	2	3	3
1	1	4	4	4	4	1	1	4	4
4	4	3	3	4	4	1	1	4	4
1	1	4	4	4	4	1	1	4	4
1	1	4	4	4	4	1	1	3	3
1	1	4	4	4	4	1	1	3	3
1	1	4	4	1	1	2	2	4	4
1	1	4	4	1	1	2	2	3	3
1	1	4	4	4	4	1	1	4	4
1	1	4	4	4	4	1	1	3	3
1	1	4	4	1	1	2	2	3	3
1	1	4	4	1	1	2	2	3	3
1	1	4	4	4	4	1	1	4	4
1	1	4	4	4	4	1	1	3	3
1	1	4	4	1	1	2	2	3	3
1	1	4	4	4	4	1	1	3	3
1	1	4	4	4	4	1	1	3	3
4	4	3	3	4	4	1	1	4	4
4	4	3	3	4	4	1	1	4	4
1	1	3	3	4	4	1	1	4	4
1	1	4	4	4	4	1	1	3	3

For Review Only

1	1	4	4	4	4	1	1	4	4
1	1	4	4	1	1	2	2	4	4
1	1	4	4	4	4	1	1	3	3
1	1	4	4	4	4	1	1	4	4
1	1	4	4	4	4	1	1	3	3
1	1	4	4	4	4	1	1	4	4
1	1	4	4	4	4	1	1	3	3
1	1	4	4	1	1	2	2	4	4
1	1	4	4	1	1	2	2	3	3
1	1	3	3	4	4	1	1	4	4
1	1	4	4	4	4	1	1	3	3
1	1	3	3	4	4	1	1	4	4
1	1	4	4	4	4	1	1	4	4
1	1	4	4	4	4	1	1	4	4
1	1	3	3	1	1	1	1	3	3
1	1	4	4	4	4	1	1	4	4
4	4	3	3	1	1	2	2	3	3
1	1	4	4	4	4	1	1	4	4
1	1	4	4	4	4	1	1	4	4
1	1	4	4	4	4	1	1	4	4
1	1	4	4	4	4	1	1	4	4
1	1	4	4	4	4	1	1	3	3
1	1	4	4	1	1	2	2	4	4
4	4	3	3	4	4	1	1	4	4
1	1	4	4	4	4	1	1	4	4
1	1	4	4	4	4	1	1	4	4
1	1	4	4	4	4	1	1	4	4
1	1	4	4	1	1	2	2	4	4
1	1	4	4	4	4	1	1	3	3
1	1	4	4	1	1	2	2	3	3
1	1	3	3	4	4	1	1	4	4
1	1	4	4	4	4	1	1	4	4
1	1	4	4	1	1	2	2	4	4
1	1	4	4	4	4	1	1	4	4
1	1	4	4	4	4	1	1	4	4
1	1	4	4	1	1	1	1	4	4
1	1	4	4	4	4	1	1	4	4
1	1	4	4	4	4	1	1	3	3
1	1	3	3	4	4	2	2	3	3
4	4	3	3	4	4	1	1	4	4
1	1	4	4	4	4	1	1	4	4
1	1	4	4	4	4	1	1	4	4
1	1	4	4	4	4	1	1	3	3
1	1	3	3	4	4	1	1	4	4
1	1	4	4	1	1	2	2	4	4
1	1	4	4	1	1	2	2	4	4
1	1	4	4	4	4	1	1	3	3
1	1	4	4	4	4	1	1	3	3
4	4	3	3	4	4	2	2	4	4
1	1	4	4	4	4	1	1	3	3
1	1	4	4	4	4	1	1	3	3
1	1	4	4	4	4	1	1	4	4
1	1	4	4	1	1	1	1	3	3
1	1	4	4	4	4	1	1	3	3
1	1	3	3	4	4	1	1	4	4

1	1	4	4	4	4	1	1	3	3
1	1	4	4	4	4	1	1	4	4
1	1	4	4	1	1	2	2	4	4
1	1	4	4	1	1	2	2	4	4
1	1	4	4	4	4	1	1	3	3
4	4	3	3	1	1	2	2	4	4
1	1	4	4	4	4	1	1	4	4
1	1	4	4	4	4	1	1	4	4
1	1	4	4	4	4	1	1	4	4
1	1	4	4	4	4	1	1	3	3
1	1	4	4	1	1	2	2	4	4
1	1	4	4	4	4	1	1	4	4
1	1	4	4	4	4	1	1	3	3
1	1	4	4	4	4	1	1	4	4
1	1	4	4	4	4	1	1	4	4
4	4	3	3	4	4	1	1	3	3
1	1	4	4	4	4	1	1	3	3
1	1	4	4	4	4	1	1	3	3
1	1	4	4	4	4	1	1	3	3
1	1	4	4	4	4	1	1	3	3
1	1	4	4	4	4	1	1	4	4
1	1	4	4	4	4	1	1	4	4
1	1	4	4	1	1	2	2	4	4
1	1	4	4	4	4	1	1	3	3
1	1	3	3	1	1	2	2	4	4
1	1	4	4	4	4	1	1	4	4
1	1	4	4	4	4	1	1	4	4
1	1	3	3	4	4	1	1	3	3
1	1	4	4	1	1	2	2	4	4
1	1	4	4	1	1	2	2	4	4
1	1	4	4	4	4	1	1	4	4
1	1	4	4	1	1	2	2	3	3
1	1	4	4	4	4	1	1	4	4
1	1	4	4	4	4	1	1	4	4
1	1	4	4	1	1	2	2	4	4
1	1	4	4	4	4	1	1	3	3
1	1	4	4	1	1	2	2	4	4
1	1	4	4	1	1	2	2	3	3
1	1	4	4	4	4	1	1	4	4
1	1	4	4	4	4	1	1	3	3
1	1	4	4	1	1	2	2	3	3
1	1	4	4	4	4	1	1	3	3
4	4	3	3	4	4	1	1	4	4
1	1	4	4	4	4	1	1	4	4
1	1	4	4	4	4	1	1	3	3
4	4	3	3	1	1	2	2	4	4
4	4	3	3	4	4	1	1	3	3
1	1	4	4	4	4	1	1	4	4
1	1	4	4	4	4	1	1	4	4
1	1	4	4	4	4	1	1	3	3
1	1	4	4	1	1	2	2	3	3
1	1	4	4	1	1	2	2	4	4
1	1	4	4	1	1	2	2	4	4
1	1	4	4	4	4	1	1	3	3
4	4	3	3	4	4	1	1	4	4

For Review Only

1	1	4	4	4	4	1	1	3	3
1	1	4	4	1	1	2	2	3	3
1	1	4	4	1	1	2	2	3	3
1	1	4	4	4	4	1	1	4	4
1	1	4	4	4	4	1	1	4	4
1	1	4	4	4	4	1	1	3	3
4	4	3	3	4	4	1	1	4	4
1	1	4	4	4	4	1	1	4	4
1	1	4	4	4	4	1	1	4	4
4	4	3	3	4	4	1	1	4	4
1	1	4	4	4	4	1	1	4	4
1	1	4	4	4	4	1	1	4	4
1	1	4	4	4	4	1	1	3	3
1	1	4	4	4	4	1	1	3	3
1	1	4	4	4	4	1	1	3	3
1	1	4	4	1	1	2	2	3	3
1	1	4	4	1	1	2	2	4	4
1	1	4	4	4	4	1	1	4	4
1	1	4	4	4	4	1	1	4	4
4	4	3	3	4	4	1	1	3	3
1	1	4	4	4	4	1	1	3	3
1	1	4	4	4	4	1	1	3	3
1	1	4	4	4	4	1	1	3	3
1	1	4	4	4	4	1	1	3	3
4	4	3	3	4	4	1	1	4	4
1	1	4	4	4	4	1	1	4	4
4	4	3	3	4	4	1	1	4	4
1	1	4	4	4	4	1	1	3	3
1	1	4	4	1	1	2	2	4	4
1	1	4	4	1	1	2	2	3	3
4	4	3	3	4	4	1	1	4	4
1	1	4	4	4	4	1	1	4	4
1	1	4	4	4	4	1	1	4	4
1	1	4	4	4	4	1	1	4	4
1	1	4	4	1	1	2	2	4	4
4	4	3	3	4	4	1	1	4	4
1	1	4	4	1	1	2	2	3	3
1	1	4	4	4	4	1	1	3	3
1	1	4	4	4	4	1	1	3	3
4	4	3	3	4	4	1	1	4	4
1	1	4	4	4	4	1	1	4	4
1	1	4	4	4	4	1	1	4	4
1	1	4	4	4	4	1	1	4	4
1	1	4	4	1	1	2	2	4	4
1	1	4	4	4	4	1	1	3	3
1	1	4	4	1	1	2	2	3	3
1	1	4	4	4	4	1	1	3	3
1	1	4	4	4	4	1	1	3	3
1	1	4	4	4	4	1	1	4	4
1	1	4	4	4	4	1	1	4	4
1	1	4	4	1	1	2	2	3	3
4	4	3	3	4	4	1	1	4	4
1	1	4	4	1	1	2	2	3	3
1	1	4	4	4	4	1	1	3	3
1	1	4	4	4	4	1	1	4	4

For Review Only

1	1	4	4	1	1	2	2	3	3
1	1	4	4	1	1	2	2	3	3

For Review Only

SNP2118	SNP2118	SNP2119	SNP2119	SNP212	SNP212	SNP2120	SNP2120	SNP2121	SNP2121
3	3	1	1	2	2	3	3	4	4
2	2	1	1	2	2	3	3	4	4
3	3	1	1	3	3	3	3	4	4
3	3	1	1	3	3	3	3	4	4
2	2	1	1	2	2	3	3	3	3
3	3	1	1	2	2	3	3	4	4
2	2	1	1	2	2	3	3	4	4
2	2	1	1	3	3	3	3	4	4
2	2	1	1	2	2	3	3	3	3
2	2	1	1	2	2	3	3	3	3
3	3	4	4	3	3	1	1	4	4
3	3	1	1	3	3	3	3	4	4
3	3	1	1	2	2	3	3	4	4
2	2	1	1	3	3	3	3	3	3
3	3	1	1	2	2	3	3	4	4
3	3	1	1	3	3	3	3	4	4
2	2	1	1	3	3	3	3	3	3
3	3	1	1	2	2	3	3	4	4
3	3	1	1	3	3	3	3	4	4
3	3	1	1	2	2	3	3	4	4
2	2	1	1	2	2	3	3	3	3
2	2	1	1	2	2	3	3	3	3
2	2	4	4	3	3	1	1	4	4
2	2	1	1	3	3	3	3	3	3
3	3	1	1	2	2	3	3	4	4
3	3	1	1	3	3	3	3	4	4
2	2	1	1	3	3	3	3	3	3
3	3	1	1	3	3	3	3	4	4
2	2	1	1	3	3	3	3	3	3
2	2	1	1	2	2	3	3	4	4
3	3	1	1	3	3	3	3	4	4
3	3	1	1	2	2	3	3	4	4
2	2	1	1	2	2	3	3	3	3
2	2	1	1	3	3	3	3	3	3
3	3	1	1	2	2	1	1	4	4
3	3	1	1	3	3	3	3	4	4
2	2	1	1	2	2	3	3	3	3
3	3	1	1	3	3	3	3	4	4
2	2	4	4	3	3	1	1	3	3
2	2	4	4	3	3	1	1	4	4
2	2	1	1	2	2	3	3	4	4
2	2	1	1	2	2	3	3	3	3
2	2	1	1	2	2	3	3	3	3
2	2	1	1	2	2	3	3	4	4
2	2	1	1	2	2	3	3	3	3
3	3	1	1	3	3	3	3	4	4
3	3	1	1	3	3	3	3	4	4
3	3	1	1	3	3	3	3	4	4
2	2	1	1	2	2	3	3	3	3
2	2	1	1	2	2	3	3	4	4
3	3	1	1	3	3	3	3	4	4
2	2	1	1	3	3	3	3	3	3

For Review Only

3	3	1	1	2	2	3	3	4	4
2	2	1	1	2	2	3	3	3	3
3	3	1	1	3	3	3	3	4	4
2	2	1	1	3	3	3	3	3	3
3	3	1	1	3	3	3	3	4	4
3	3	1	1	3	3	3	3	4	4
3	3	1	1	3	3	3	3	4	4
2	2	1	1	3	3	3	3	4	4
2	2	1	1	3	3	3	3	3	3
3	3	1	1	3	3	3	3	4	4
3	3	1	1	3	3	3	3	4	4
3	3	1	1	3	3	3	3	4	4
3	3	1	1	2	2	3	3	4	4
2	2	1	1	3	3	3	3	3	3
3	3	1	1	2	2	3	3	4	4
2	2	1	1	2	2	1	1	3	3
3	3	1	1	3	3	3	3	4	4
3	3	1	1	3	3	3	3	4	4
3	3	1	1	2	2	3	3	4	4
2	2	1	1	2	2	3	3	3	3
3	3	1	1	3	3	3	3	4	4
3	3	1	1	3	3	3	3	4	4
2	2	1	1	2	2	1	1	3	3
2	2	1	1	3	3	3	3	3	3
2	2	1	1	2	2	3	3	3	3
3	3	1	1	3	3	3	3	4	4
2	2	1	1	2	2	3	3	3	3
2	2	1	1	3	3	1	1	3	3
3	3	1	1	3	3	3	3	4	4
3	3	1	1	3	3	3	3	4	4
2	2	1	1	3	3	3	3	3	3
2	2	1	1	2	2	3	3	4	4
2	2	1	1	3	3	3	3	3	3
3	3	1	1	2	2	3	3	4	4
3	3	1	1	3	3	3	3	4	4
2	2	1	1	2	2	3	3	3	3
2	2	1	1	3	3	3	3	3	3
2	2	1	1	2	2	3	3	4	4
3	3	1	1	3	3	3	3	4	4
3	3	1	1	2	2	3	3	4	4
2	2	1	1	3	3	3	3	3	3
3	3	1	1	3	3	3	3	4	4
2	2	1	1	2	2	3	3	3	3
3	3	1	1	3	3	3	3	4	4
3	3	1	1	3	3	3	3	4	4
3	3	1	1	3	3	3	3	4	4
2	2	1	1	2	2	3	3	3	3
3	3	1	1	3	3	3	3	4	4
3	3	1	1	3	3	3	3	4	4
3	3	1	1	3	3	3	3	4	4
3	3	1	1	3	3	3	3	4	4
2	2	4	4	3	3	1	1	4	4
2	2	1	1	3	3	3	3	3	3
3	3	1	1	3	3	3	3	4	4
3	3	1	1	3	3	3	3	4	4

For Review Only

2	2	1	1	3	3	1	1	4	4
3	3	1	1	2	2	1	1	4	4
3	3	1	1	3	3	3	3	4	4
2	2	1	1	2	2	3	3	3	3
3	3	1	1	3	3	3	3	4	4
2	2	1	1	3	3	3	3	4	4
3	3	1	1	3	3	3	3	4	4
3	3	1	1	3	3	3	3	4	4
3	3	1	1	3	3	1	1	4	4
3	3	1	1	3	3	3	3	4	4
3	3	1	1	2	2	3	3	4	4
3	3	1	1	3	3	1	1	4	4
2	2	1	1	2	2	3	3	3	3
3	3	1	1	3	3	3	3	4	4
3	3	1	1	3	3	3	3	4	4
3	3	1	1	3	3	3	3	4	4
3	3	1	1	3	3	3	3	4	4
3	3	1	1	3	3	3	3	4	4
2	2	1	1	2	2	3	3	3	3
2	2	1	1	3	3	3	3	3	3
3	3	1	1	3	3	3	3	4	4
3	3	1	1	3	3	3	3	4	4
2	2	1	1	2	2	3	3	3	3
2	2	1	1	3	3	3	3	3	3
2	2	1	1	3	3	3	3	4	4
2	2	4	4	3	3	1	1	3	3
3	3	1	1	3	3	3	3	4	4
2	2	1	1	3	3	3	3	3	3
3	3	1	1	3	3	3	3	4	4
3	3	1	1	3	3	3	3	4	4
2	2	1	1	2	2	3	3	3	3
3	3	1	1	2	2	3	3	4	4
2	2	1	1	3	3	3	3	3	3
3	3	1	1	3	3	3	3	4	4
2	2	1	1	3	3	3	3	3	3
2	2	1	1	2	2	3	3	3	3
2	2	1	1	3	3	3	3	3	3
3	3	1	1	3	3	3	3	4	4
3	3	1	1	3	3	3	3	4	4
2	2	4	4	3	3	1	1	3	3
2	2	1	1	3	3	1	1	3	3
2	2	1	1	2	2	3	3	3	3
3	3	1	1	3	3	3	3	4	4
3	3	1	1	2	2	3	3	4	4
2	2	1	1	3	3	3	3	4	4
2	2	1	1	3	3	3	3	3	3
3	3	1	1	3	3	3	3	4	4
3	3	1	1	3	3	3	3	4	4
3	3	1	1	2	2	3	3	4	4
3	3	1	1	3	3	3	3	4	4
3	3	1	1	3	3	3	3	4	4
2	2	4	4	3	3	1	1	3	3
3	3	1	1	3	3	3	3	4	4
3	3	1	1	3	3	3	3	4	4
3	3	1	1	2	2	3	3	4	4

For Review Only

3	3	1	1	3	3	3	3	4	4
2	2	4	4	3	3	1	1	3	3
2	2	1	1	2	2	3	3	3	3
3	3	1	1	3	3	1	1	4	4
3	3	1	1	3	3	3	3	4	4
2	2	1	1	3	3	3	3	4	4
2	2	1	1	3	3	1	1	3	3
3	3	1	1	3	3	3	3	4	4
2	2	1	1	3	3	1	1	4	4
3	3	1	1	3	3	3	3	4	4
3	3	1	1	3	3	3	3	4	4
2	2	1	1	3	3	1	1	4	4
3	3	1	1	3	3	3	3	4	4
2	2	1	1	3	3	3	3	3	3
2	2	1	1	2	2	3	3	3	3
3	3	4	4	3	3	1	1	4	4
3	3	1	1	2	2	3	3	4	4
3	3	1	1	3	3	3	3	4	4
3	3	1	1	3	3	3	3	4	4
3	3	1	1	3	3	3	3	4	4
3	3	1	1	2	2	3	3	4	4
2	2	1	1	2	2	3	3	3	3
3	3	1	1	3	3	3	3	4	4
3	3	1	1	3	3	3	3	4	4
2	2	1	1	2	2	3	3	4	4
2	2	1	1	3	3	3	3	3	3
3	3	1	1	3	3	3	3	4	4
3	3	1	1	3	3	3	3	4	4
2	2	1	1	3	3	3	3	4	4
2	2	1	1	2	2	3	3	3	3
2	2	1	1	3	3	3	3	3	3
3	3	1	1	3	3	3	3	4	4
2	2	1	1	3	3	3	3	3	3
2	2	1	1	2	2	3	3	3	3
2	2	1	1	3	3	3	3	4	4
3	3	1	1	3	3	3	3	4	4
3	3	1	1	3	3	3	3	4	4
3	3	1	1	2	2	3	3	4	4
3	3	1	1	3	3	3	3	4	4
3	3	1	1	3	3	3	3	4	4
3	3	1	1	3	3	3	3	4	4
3	3	1	1	3	3	3	3	4	4
2	2	4	4	3	3	1	1	4	4
3	3	1	1	3	3	1	1	3	3
3	3	1	1	3	3	3	3	4	4
2	2	1	1	2	2	3	3	3	3
3	3	4	4	3	3	1	1	4	4
2	2	1	1	3	3	3	3	3	3
2	2	1	1	2	2	3	3	3	3
3	3	1	1	3	3	3	3	4	4
3	3	1	1	3	3	3	3	4	4
2	2	1	1	3	3	3	3	4	4
2	2	1	1	3	3	3	3	3	3
3	3	1	1	3	3	3	3	4	4
2	2	4	4	3	3	1	1	4	4

For Review Only

3	3	1	1	3	3	3	3	4	4
3	3	1	1	2	2	3	3	4	4
3	3	1	1	3	3	3	3	4	4
2	2	1	1	2	2	3	3	3	3
2	2	1	1	3	3	3	3	4	4
3	3	1	1	3	3	3	3	4	4
2	2	4	4	3	3	1	1	4	4
2	2	1	1	2	2	3	3	3	3
3	3	1	1	2	2	3	3	4	4
2	2	4	4	3	3	1	1	3	3
3	3	1	1	3	3	3	3	4	4
2	2	1	1	3	3	3	3	3	3
3	3	1	1	3	3	3	3	4	4
3	3	1	1	3	3	3	3	4	4
3	3	1	1	3	3	3	3	4	4
3	3	1	1	3	3	3	3	4	4
2	2	1	1	3	3	3	3	3	3
2	2	4	4	3	3	1	1	4	4
2	2	4	4	3	3	1	1	3	3
3	3	1	1	3	3	3	3	4	4
3	3	4	4	3	3	1	1	4	4
3	3	1	1	3	3	3	3	4	4
3	3	1	1	3	3	3	3	4	4
3	3	1	1	3	3	3	3	4	4
2	2	1	1	3	3	3	3	4	4
3	3	1	1	3	3	3	3	4	4
2	2	4	4	3	3	1	1	3	3
3	3	1	1	3	3	3	3	4	4
2	2	1	1	2	2	3	3	4	4
3	3	1	1	3	3	3	3	4	4
2	2	4	4	3	3	1	1	3	3
3	3	1	1	3	3	3	3	4	4
2	2	1	1	3	3	3	3	3	3
3	3	1	1	3	3	3	3	4	4
2	2	1	1	3	3	3	3	3	3
2	2	4	4	3	3	1	1	4	4
3	3	1	1	3	3	3	3	4	4
3	3	1	1	3	3	3	3	4	4
3	3	1	1	3	3	3	3	4	4
2	2	4	4	3	3	1	1	3	3
2	2	1	1	3	3	3	3	4	4
2	2	1	1	2	2	3	3	3	3
2	2	4	4	3	3	1	1	3	3
2	2	1	1	3	3	3	3	3	3
3	3	1	1	3	3	3	3	4	4
3	3	4	4	3	3	1	1	4	4
3	3	1	1	3	3	3	3	4	4
3	3	4	4	2	2	3	3	4	4
3	3	1	1	3	3	3	3	4	4
2	2	4	4	3	3	1	1	3	3
3	3	1	1	3	3	3	3	4	4
2	2	1	1	3	3	3	3	3	3
3	3	1	1	3	3	3	3	4	4
3	3	1	1	3	3	3	3	4	4
2	2	4	4	3	3	1	1	3	3

For Review Only

3	3	1	1	3	3	3	3	4	4
3	3	1	1	3	3	3	3	4	4

For Review Only

SNP2122	SNP2122	SNP2123	SNP2123	SNP2124	SNP2124	SNP2125	SNP2125	SNP2126	SNP2126
4	4	2	2	3	3	1	1	3	3
1	1	2	2	4	4	1	1	2	2
1	1	3	3	3	3	2	2	2	2
1	1	3	3	4	4	2	2	3	3
1	1	2	2	4	4	1	1	3	3
4	4	3	3	3	3	2	2	2	2
1	1	3	3	4	4	2	2	2	2
1	1	2	2	4	4	1	1	3	3
1	1	3	3	4	4	2	2	2	2
1	1	2	2	4	4	1	1	2	2
1	1	2	2	3	3	1	1	3	3
1	1	3	3	4	4	2	2	2	2
1	1	3	3	3	3	2	2	2	2
1	1	2	2	4	4	1	1	3	3
1	1	3	3	3	3	2	2	2	2
1	1	2	2	4	4	1	1	3	3
1	1	3	3	4	4	2	2	2	2
4	4	2	2	3	3	1	1	3	3
1	1	3	3	3	3	2	2	2	2
1	1	2	2	3	3	1	1	2	2
1	1	2	2	4	4	1	1	2	2
1	1	3	3	4	4	2	2	2	2
1	1	2	2	4	4	1	1	2	2
1	1	2	2	4	4	1	1	2	2
1	1	3	3	3	3	2	2	2	2
1	1	2	2	4	4	1	1	2	2
1	1	3	3	4	4	2	2	2	2
1	1	3	3	3	3	2	2	2	2
1	1	2	2	4	4	1	1	3	3
1	1	2	2	4	4	1	1	3	3
1	1	3	3	3	3	2	2	2	2
1	1	3	3	3	3	2	2	2	2
1	1	3	3	4	4	2	2	2	2
1	1	2	2	4	4	1	1	3	3
1	1	2	2	4	4	1	1	2	2
1	1	2	2	3	3	1	1	2	2
4	4	3	3	4	4	2	2	3	3
1	1	2	2	3	3	1	1	3	3
1	1	2	2	4	4	1	1	3	3
1	1	2	2	4	4	1	1	3	3
1	1	2	2	4	4	1	1	3	3
1	1	2	2	4	4	1	1	2	2
1	1	2	2	4	4	1	1	3	3
1	1	3	3	4	4	2	2	2	2
1	1	2	2	4	4	1	1	3	3
1	1	3	3	3	3	2	2	2	2
1	1	2	2	4	4	1	1	2	2
1	1	2	2	3	3	2	2	2	2
1	1	3	3	4	4	2	2	2	2
1	1	3	3	4	4	2	2	2	2
1	1	2	2	4	4	1	1	3	3
4	4	3	3	4	4	2	2	2	2

For Review Only

1	1	3	3	3	3	2	2	2	2
1	1	3	3	4	4	2	2	2	2
1	1	3	3	4	4	2	2	2	2
1	1	3	3	4	4	2	2	2	2
1	1	3	3	3	3	2	2	2	2
1	1	2	2	4	4	1	1	3	3
1	1	2	2	4	4	1	1	3	3
1	1	2	2	4	4	1	1	3	3
1	1	3	3	4	4	2	2	2	2
1	1	3	3	3	3	2	2	2	2
1	1	2	2	3	3	1	1	3	3
1	1	2	2	3	3	1	1	3	3
1	1	3	3	3	3	2	2	2	2
1	1	2	2	4	4	1	1	2	2
4	4	3	3	3	3	2	2	2	2
4	4	3	3	4	4	2	2	2	2
4	4	2	2	3	3	1	1	2	2
1	1	3	3	3	3	2	2	2	2
1	1	3	3	3	3	2	2	2	2
1	1	3	3	4	4	2	2	2	2
1	1	2	2	3	3	1	1	2	2
1	1	3	3	4	4	2	2	2	2
1	1	2	2	4	4	1	1	3	3
4	4	3	3	4	4	2	2	2	2
1	1	2	2	4	4	1	1	3	3
1	1	3	3	4	4	2	2	2	2
1	1	2	2	4	4	1	1	2	2
1	1	2	2	4	4	1	1	2	2
1	1	2	2	4	4	1	1	2	2
1	1	2	2	3	3	1	1	3	3
1	1	2	2	4	4	1	1	3	3
1	1	2	2	4	4	1	1	3	3
1	1	2	2	4	4	1	1	3	3
1	1	3	3	3	3	2	2	2	2
4	4	3	3	3	3	2	2	2	2
1	1	2	2	4	4	1	1	2	2
1	1	2	2	4	4	1	1	2	2
1	1	2	2	4	4	1	1	2	2
1	1	2	2	3	3	1	1	2	2
1	1	2	2	3	3	1	1	2	2
1	1	3	3	4	4	2	2	2	2
1	1	3	3	3	3	2	2	2	2
1	1	2	2	4	4	1	1	3	3
1	1	2	2	3	3	1	1	3	3
1	1	3	3	3	3	2	2	2	2
1	1	3	3	3	3	2	2	2	2
1	1	2	2	4	4	1	1	3	3
1	1	2	2	4	4	1	1	3	3
1	1	3	3	4	4	2	2	2	2
1	1	3	3	3	3	2	2	2	2
1	1	2	2	3	3	1	1	3	3
1	1	2	2	4	4	1	1	3	3
1	1	2	2	4	4	1	1	3	3
1	1	3	3	4	4	2	2	2	2
1	1	2	2	3	3	1	1	3	3

For Review Only

1	1	3	3	4	4	1	1	2	2
1	1	3	3	3	3	2	2	2	2
1	1	3	3	4	4	2	2	2	2
1	1	2	2	4	4	1	1	2	2
1	1	2	2	3	3	1	1	3	3
1	1	2	2	4	4	1	1	3	3
1	1	2	2	4	4	1	1	3	3
1	1	3	3	3	3	2	2	2	2
1	1	3	3	4	4	2	2	2	2
1	1	2	2	3	3	1	1	3	3
4	4	2	2	3	3	1	1	3	3
1	1	3	3	4	4	2	2	2	2
1	1	2	2	4	4	1	1	3	3
1	1	3	3	3	3	2	2	2	2
1	1	3	3	3	3	2	2	2	2
1	1	2	2	4	4	1	1	3	3
1	1	3	3	3	3	2	2	2	2
1	1	3	3	3	3	2	2	2	2
1	1	2	2	4	4	1	1	3	3
1	1	2	2	4	4	1	1	3	3
1	1	3	3	3	3	2	2	2	2
1	1	2	2	3	3	1	1	3	3
1	1	3	3	4	4	2	2	2	2
1	1	2	2	4	4	1	1	3	3
1	1	3	3	4	4	2	2	3	3
1	1	2	2	4	4	1	1	3	3
4	4	2	2	4	4	1	1	3	3
4	4	3	3	4	4	2	2	2	2
1	1	3	3	3	3	2	2	2	2
1	1	3	3	3	3	2	2	2	2
1	1	2	2	4	4	1	1	3	3
1	1	3	3	3	3	2	2	2	2
1	1	3	3	4	4	2	2	2	2
1	1	2	2	3	3	2	2	3	3
1	1	2	2	4	4	1	1	3	3
1	1	2	2	4	4	2	2	3	3
1	1	2	2	4	4	1	1	2	2
1	1	2	2	3	3	1	1	3	3
1	1	2	2	4	4	1	1	3	3
1	1	2	2	4	4	1	1	3	3
1	1	3	3	4	4	2	2	2	2
1	1	2	2	4	4	1	1	2	2
1	1	2	2	3	3	1	1	3	3
1	1	2	2	3	3	1	1	3	3
1	1	3	3	4	4	2	2	2	2
1	1	3	3	4	4	2	2	2	2
1	1	2	2	4	4	1	1	3	3
1	1	2	2	3	3	1	1	3	3
1	1	3	3	3	3	2	2	2	2
1	1	3	3	4	4	2	2	2	2
1	1	2	2	4	4	1	1	3	3
1	1	2	2	4	4	1	1	2	2
4	4	3	3	3	3	2	2	2	2
1	1	2	2	3	3	1	1	3	3
1	1	3	3	4	4	2	2	3	3

For Review Only

1	1	2	2	3	3	1	1	3	3
4	4	2	2	4	4	1	1	3	3
1	1	3	3	4	4	2	2	2	2
4	4	3	3	3	3	2	2	2	2
1	1	2	2	3	3	1	1	3	3
4	4	3	3	4	4	2	2	2	2
1	1	2	2	4	4	1	1	3	3
1	1	3	3	3	3	2	2	2	2
1	1	2	2	4	4	1	1	3	3
1	1	3	3	3	3	2	2	2	2
1	1	3	3	4	4	2	2	2	2
1	1	2	2	4	4	1	1	3	3
1	1	2	2	3	3	2	2	3	3
1	1	3	3	4	4	2	2	2	2
1	1	2	2	4	4	1	1	3	3
1	1	2	2	3	3	1	1	3	3
1	1	3	3	3	3	2	2	2	2
4	4	2	2	3	3	1	1	3	3
1	1	2	2	4	4	1	1	3	3
1	1	2	2	4	4	1	1	2	2
1	1	3	3	3	3	2	2	2	2
1	1	2	2	4	4	1	1	3	3
1	1	3	3	4	4	2	2	2	2
1	1	2	2	3	3	1	1	3	3
1	1	3	3	4	4	2	2	2	2
1	1	3	3	4	4	1	1	3	3
1	1	2	2	4	4	1	1	3	3
1	1	3	3	3	3	2	2	2	2
4	4	3	3	4	4	2	2	2	2
1	1	3	3	4	4	2	2	2	2
4	4	2	2	4	4	1	1	2	2
1	1	3	3	3	3	2	2	2	2
4	4	2	2	4	4	1	1	3	3
1	1	2	2	4	4	1	1	3	3
4	4	3	3	4	4	2	2	2	2
1	1	2	2	3	3	1	1	3	3
1	1	3	3	4	4	2	2	2	2
4	4	3	3	3	3	2	2	2	2
1	1	2	2	3	3	1	1	3	3
1	1	2	2	3	3	1	1	3	3
1	1	3	3	3	3	2	2	2	2
1	1	2	2	4	4	1	1	2	2
1	1	2	2	4	4	1	1	3	3
1	1	3	3	3	3	2	2	2	2
1	1	3	3	4	4	2	2	2	2
1	1	3	3	4	4	2	2	2	2
1	1	2	2	3	3	1	1	2	2
1	1	2	2	4	4	1	1	3	3
1	1	2	2	4	4	1	1	3	3
1	1	3	3	3	3	2	2	2	2
1	1	3	3	4	4	2	2	2	2
1	1	3	3	4	4	2	2	2	2
1	1	3	3	4	4	2	2	2	2
1	1	2	2	3	3	1	1	3	3
1	1	2	2	4	4	1	1	2	2

For Review Only

4	4	2	2	3	3	1	1	2	2
1	1	3	3	3	3	2	2	2	2
1	1	3	3	3	3	2	2	2	2
1	1	3	3	4	4	2	2	2	2
1	1	2	2	4	4	1	1	3	3
1	1	2	2	4	4	2	2	3	3
4	4	2	2	4	4	1	1	2	2
1	1	2	2	4	4	1	1	3	3
1	1	3	3	4	4	2	2	2	2
1	1	2	2	4	4	1	1	3	3
1	1	3	3	3	3	2	2	2	2
4	4	2	2	4	4	1	1	3	3
1	1	2	2	3	3	1	1	3	3
1	1	2	2	4	4	1	1	3	3
1	1	2	2	3	3	1	1	2	2
1	1	3	3	3	3	2	2	2	2
1	1	3	3	4	4	2	2	2	2
4	4	2	2	4	4	1	1	3	3
4	4	2	2	4	4	1	1	2	2
1	1	2	2	3	3	1	1	2	2
1	1	2	2	4	4	1	1	3	3
1	1	2	2	4	4	1	1	3	3
4	4	3	3	3	3	2	2	2	2
1	1	2	2	3	3	1	1	3	3
1	1	2	2	4	4	1	1	3	3
1	1	3	3	3	3	2	2	2	2
1	1	2	2	4	4	1	1	2	2
1	1	2	2	4	4	1	1	3	3
1	1	3	3	4	4	2	2	2	2
1	1	3	3	3	3	2	2	2	2
1	1	2	2	4	4	1	1	2	2
1	1	2	2	4	4	1	1	2	2
1	1	2	2	4	4	1	1	3	3
1	1	2	2	3	3	1	1	3	3
1	1	3	3	4	4	2	2	2	2
1	1	2	2	4	4	1	1	3	3
1	1	3	3	3	3	2	2	2	2
1	1	2	2	3	3	1	1	3	3
1	1	2	2	3	3	1	1	2	2
1	1	2	2	4	4	1	1	3	3
1	1	2	2	4	4	1	1	3	3
1	1	2	2	4	4	1	1	3	3
1	1	2	2	4	4	1	1	2	2
1	1	3	3	4	4	2	2	2	2
1	1	2	2	3	3	1	1	2	2
1	1	3	3	3	3	2	2	2	2
1	1	2	2	3	3	1	1	3	3
4	4	2	2	3	3	1	1	3	3
1	1	2	2	3	3	1	1	3	3
1	1	2	2	4	4	1	1	3	3
1	1	3	3	3	3	2	2	2	2
1	1	2	2	4	4	1	1	3	3
1	1	3	3	3	3	2	2	2	2
1	1	2	2	3	3	1	1	3	3
1	1	2	2	4	4	1	1	2	2

For Review Only

4	4	3	3	3	3	2	2	2	2
1	1	3	3	4	4	2	2	2	2

For Review Only

SNP2127	SNP2127	SNP2128	SNP2128	SNP2129	SNP2129	SNP213	SNP213	SNP2130	SNP2130
3	3	3	3	4	4	1	1	2	2
3	3	3	3	4	4	4	4	2	2
3	3	3	3	2	2	4	4	2	2
3	3	3	3	2	2	4	4	4	4
3	3	3	3	4	4	1	1	2	2
3	3	3	3	4	4	1	1	2	2
3	3	3	3	2	2	1	1	2	2
3	3	3	3	2	2	4	4	2	2
3	3	3	3	4	4	1	1	2	2
3	3	1	1	2	2	1	1	4	4
3	3	3	3	2	2	4	4	2	2
3	3	3	3	2	2	4	4	2	2
3	3	3	3	4	4	1	1	2	2
3	3	3	3	4	4	4	4	2	2
3	3	3	3	4	4	4	4	2	2
2	2	3	3	2	2	4	4	2	2
3	3	3	3	4	4	4	4	2	2
3	3	3	3	2	2	4	4	2	2
3	3	3	3	4	4	4	4	2	2
3	3	1	1	2	2	1	1	2	2
3	3	3	3	4	4	1	1	2	2
3	3	3	3	4	4	1	1	2	2
3	3	3	3	2	2	4	4	2	2
3	3	3	3	4	4	4	4	2	2
3	3	3	3	4	4	1	1	2	2
3	3	3	3	2	2	4	4	2	2
3	3	3	3	2	2	4	4	2	2
3	3	3	3	4	4	4	4	2	2
3	3	3	3	4	4	4	4	2	2
3	3	1	1	4	4	1	1	2	2
3	3	3	3	4	4	4	4	2	2
3	3	3	3	4	4	4	4	4	4
3	3	3	3	4	4	1	1	2	2
3	3	3	3	4	4	4	4	2	2
3	3	3	3	4	4	4	4	2	2
2	2	3	3	4	4	4	4	2	2
3	3	3	3	4	4	1	1	2	2
3	3	3	3	2	2	4	4	4	4
3	3	1	1	2	2	4	4	2	2
3	3	3	3	4	4	4	4	2	2
3	3	3	3	4	4	1	1	2	2
3	3	3	3	2	2	4	4	2	2
3	3	3	3	2	2	1	1	2	2
3	3	3	3	4	4	4	4	2	2
3	3	1	1	2	2	1	1	2	2
3	3	3	3	2	2	4	4	2	2
3	3	3	3	4	4	4	4	2	2
3	3	3	3	2	2	4	4	2	2
3	3	3	3	4	4	1	1	2	2
3	3	3	3	4	4	4	4	2	2
3	3	3	3	2	2	4	4	2	2
3	3	3	3	2	2	4	4	2	2

For Review Only

3	3	3	3	4	4	1	1	2	2
3	3	3	3	4	4	1	1	2	2
3	3	3	3	2	2	4	4	2	2
3	3	3	3	2	2	4	4	2	2
3	3	3	3	4	4	4	4	2	2
3	3	3	3	4	4	4	4	2	2
3	3	3	3	2	2	4	4	2	2
3	3	1	1	2	2	4	4	4	4
3	3	1	1	4	4	4	4	2	2
3	3	3	3	4	4	4	4	2	2
3	3	3	3	4	4	4	4	2	2
2	2	3	3	4	4	4	4	2	2
3	3	3	3	4	4	1	1	2	2
3	3	3	3	4	4	4	4	2	2
3	3	3	3	4	4	4	4	2	2
3	3	3	3	2	2	4	4	2	2
3	3	3	3	2	2	4	4	2	2
3	3	3	3	4	4	4	4	2	2
3	3	3	3	4	4	4	4	2	2
3	3	3	3	4	4	1	1	2	2
2	2	3	3	4	4	4	4	2	2
3	3	1	1	4	4	4	4	4	4
3	3	3	3	4	4	4	4	2	2
3	3	3	3	4	4	4	4	2	2
3	3	1	1	2	2	1	1	2	2
3	3	3	3	2	2	4	4	2	2
3	3	3	3	4	4	1	1	2	2
3	3	1	1	2	2	4	4	2	2
3	3	3	3	2	2	4	4	2	2
3	3	3	3	4	4	4	4	2	2
3	3	3	3	4	4	4	4	2	2
2	2	3	3	4	4	4	4	2	2
3	3	1	1	2	2	4	4	2	2
3	3	3	3	4	4	1	1	2	2
3	3	3	3	4	4	4	4	2	2
3	3	1	1	2	2	1	1	2	2
3	3	3	3	2	2	4	4	4	4
3	3	3	3	2	2	1	1	4	4
3	3	3	3	2	2	4	4	2	2
3	3	3	3	2	2	1	1	2	2
3	3	3	3	4	4	4	4	2	2
2	2	3	3	2	2	4	4	2	2
3	3	3	3	2	2	1	1	2	2
3	3	3	3	4	4	4	4	2	2
3	3	3	3	4	4	4	4	2	2
3	3	3	3	4	4	4	4	2	2
3	3	3	3	4	4	4	4	2	2
3	3	3	3	4	4	4	4	2	2
3	3	3	3	4	4	4	4	2	2
3	3	3	3	4	4	4	4	2	2
2	2	3	3	4	4	4	4	2	2
3	3	1	1	4	4	4	4	2	2
3	3	1	1	2	2	4	4	4	4
3	3	3	3	4	4	4	4	2	2
3	3	3	3	4	4	4	4	2	2

For Review Only

3	3	3	3	4	4	4	4	2	2
3	3	3	3	4	4	1	1	2	2
3	3	3	3	2	2	4	4	2	2
3	3	3	3	4	4	4	4	2	2
3	3	3	3	4	4	4	4	2	2
3	3	3	3	4	4	4	4	2	2
3	3	3	3	2	2	4	4	2	2
3	3	3	3	4	4	4	4	2	2
3	3	3	3	4	4	4	4	2	2
3	3	3	3	4	4	4	4	2	2
3	3	3	3	4	4	1	1	2	2
3	3	3	3	4	4	4	4	2	2
3	3	3	3	2	2	1	1	2	2
3	3	3	3	2	2	4	4	2	2
3	3	3	3	2	2	4	4	2	2
3	3	3	3	4	4	4	4	2	2
3	3	3	3	2	2	4	4	2	2
3	3	3	3	4	4	4	4	2	2
3	3	3	3	4	4	4	4	2	2
3	3	3	3	4	4	4	4	2	2
3	3	3	3	4	4	4	4	2	2
3	3	3	3	4	4	4	4	2	2
3	3	3	3	4	4	1	1	2	2
3	3	1	1	2	2	4	4	2	2
3	3	3	3	4	4	4	4	2	2
3	3	3	3	4	4	4	4	2	2
3	3	3	3	4	4	4	4	2	2
3	3	3	3	4	4	4	4	2	2
3	3	3	3	2	2	4	4	2	2
3	3	3	3	4	4	4	4	4	4
3	3	1	1	2	2	4	4	2	2
3	3	3	3	4	4	4	4	4	4
3	3	3	3	4	4	4	4	2	2
3	3	3	3	4	4	4	4	2	2
3	3	3	3	4	4	4	4	2	2
3	3	3	3	4	4	4	4	2	2
3	3	3	3	4	4	4	4	2	2
3	3	3	3	4	4	4	4	2	2
3	3	3	3	2	2	4	4	2	2
3	3	1	1	4	4	4	4	2	2
3	3	3	3	4	4	4	4	2	2
3	3	3	3	4	4	1	1	2	2
3	3	3	3	4	4	4	4	2	2
3	3	3	3	4	4	4	4	2	2
3	3	3	3	2	2	4	4	2	2
3	3	3	3	2	2	4	4	2	2
3	3	3	3	2	2	4	4	2	2
3	3	3	3	2	2	4	4	2	2
3	3	3	3	4	4	4	4	4	4
3	3	3	3	2	2	4	4	2	2
2	2	3	3	2	2	4	4	2	2
3	3	3	3	4	4	4	4	2	2
3	3	3	3	4	4	4	4	2	2
2	2	3	3	2	2	4	4	2	2
3	3	3	3	4	4	4	4	4	4

For Review Only

3	3	3	3	4	4	4	4	2	2
3	3	3	3	4	4	4	4	2	2
3	3	3	3	4	4	1	1	2	2
3	3	3	3	4	4	4	4	2	2
3	3	3	3	4	4	4	4	2	2
3	3	3	3	4	4	4	4	4	4
3	3	3	3	2	2	4	4	2	2
3	3	3	3	4	4	4	4	2	2
3	3	3	3	4	4	4	4	2	2
3	3	3	3	4	4	4	4	2	2
3	3	3	3	4	4	4	4	2	2
3	3	3	3	2	2	4	4	2	2
3	3	3	3	4	4	4	4	2	2
3	3	3	3	4	4	4	4	2	2
3	3	3	3	4	4	1	1	2	2
3	3	3	3	2	2	4	4	2	2
3	3	3	3	4	4	4	4	2	2
3	3	3	3	2	2	4	4	2	2
3	3	3	3	4	4	4	4	2	2
3	3	3	3	2	2	4	4	2	2
3	3	3	3	4	4	4	4	2	2
2	2	3	3	2	2	1	1	2	2
3	3	3	3	4	4	4	4	4	4
2	2	3	3	4	4	4	4	2	2
3	3	3	3	4	4	1	1	2	2
3	3	3	3	4	4	4	4	2	2
3	3	3	3	4	4	4	4	2	2
3	3	3	3	4	4	4	4	2	2
3	3	3	3	2	2	4	4	2	2
3	3	3	3	4	4	4	4	2	2
3	3	3	3	2	2	4	4	2	2
3	3	3	3	4	4	4	4	2	2
3	3	3	3	4	4	4	4	2	2
3	3	3	3	4	4	4	4	2	2
3	3	3	3	4	4	4	4	2	2
2	2	3	3	4	4	4	4	2	2
2	2	3	3	4	4	4	4	2	2
3	3	3	3	4	4	1	1	2	2
3	3	3	3	2	2	4	4	2	2
3	3	3	3	2	2	4	4	2	2
3	3	3	3	2	2	4	4	2	2
3	3	3	3	2	2	4	4	2	2
3	3	1	1	4	4	4	4	2	2
3	3	3	3	4	4	4	4	4	4
3	3	3	3	2	2	4	4	2	2
3	3	3	3	4	4	4	4	4	4
3	3	3	3	2	2	4	4	2	2
3	3	3	3	2	2	4	4	2	2
3	3	3	3	4	4	1	1	2	2
3	3	3	3	2	2	4	4	2	2
3	3	3	3	2	2	4	4	2	2
3	3	1	1	4	4	4	4	2	2
3	3	3	3	4	4	4	4	4	4
3	3	3	3	4	4	4	4	2	2
3	3	1	1	2	2	4	4	2	2

For Review Only

3	3	3	3	4	4	4	4	2	2
3	3	3	3	2	2	1	1	2	2
3	3	3	3	2	2	4	4	2	2
3	3	3	3	4	4	4	4	4	4
3	3	1	1	4	4	4	4	2	2
3	3	3	3	2	2	4	4	2	2
3	3	3	3	2	2	4	4	2	2
3	3	3	3	2	2	1	1	4	4
3	3	3	3	2	2	4	4	2	2
3	3	1	1	4	4	4	4	2	2
3	3	3	3	4	4	4	4	2	2
3	3	3	3	2	2	4	4	2	2
3	3	3	3	2	2	4	4	2	2
3	3	3	3	4	4	4	4	2	2
3	3	3	3	2	2	4	4	2	2
3	3	3	3	4	4	4	4	2	2
3	3	1	1	2	2	4	4	2	2
3	3	3	3	4	4	4	4	2	2
3	3	3	3	4	4	4	4	2	2
3	3	3	3	2	2	4	4	4	4
3	3	3	3	2	2	4	4	2	2
3	3	3	3	2	2	4	4	2	2
3	3	3	3	4	4	4	4	2	2
3	3	3	3	4	4	4	4	2	2
3	3	1	1	2	2	4	4	4	4
3	3	3	3	4	4	4	4	4	4
3	3	3	3	4	4	4	4	2	2
3	3	3	3	2	2	4	4	2	2
3	3	3	3	4	4	4	4	2	2
3	3	3	3	2	2	4	4	2	2
3	3	1	1	4	4	4	4	2	2
3	3	3	3	2	2	4	4	2	2
3	3	3	3	4	4	4	4	2	2
3	3	3	3	2	2	4	4	2	2
3	3	3	3	4	4	4	4	4	4
3	3	1	1	4	4	4	4	2	2
3	3	3	3	4	4	4	4	2	2
2	2	3	3	4	4	4	4	2	2
2	2	3	3	4	4	4	4	2	2
3	3	1	1	2	2	4	4	2	2
3	3	3	3	4	4	4	4	2	2
3	3	3	3	4	4	1	1	2	2
3	3	3	3	2	2	4	4	2	2
3	3	3	3	4	4	4	4	2	2
2	2	3	3	2	2	4	4	2	2
3	3	3	3	4	4	4	4	2	2
3	3	1	1	2	2	4	4	2	2
3	3	3	3	2	2	4	4	2	2
3	3	3	3	2	2	4	4	2	2
3	3	3	3	4	4	4	4	2	2
3	3	3	3	4	4	4	4	2	2
3	3	1	1	2	2	4	4	2	2
3	3	3	3	2	2	4	4	2	2
2	2	3	3	2	2	4	4	2	2
3	3	3	3	2	2	4	4	2	2

For Review Only

3	3	3	3	4	4	4	4	2	2
2	2	3	3	2	2	4	4	2	2

For Review Only

SNP2131	SNP2131	SNP2132	SNP2132	SNP2133	SNP2133	SNP2134	SNP2134	SNP2135	SNP2135
4	4	1	1	3	3	1	1	4	4
4	4	1	1	3	3	1	1	4	4
1	1	1	1	2	2	1	1	1	1
1	1	4	4	3	3	3	3	4	4
4	4	1	1	3	3	1	1	4	4
4	4	1	1	2	2	1	1	1	1
1	1	1	1	3	3	1	1	4	4
1	1	1	1	3	3	1	1	4	4
4	4	1	1	3	3	1	1	4	4
1	1	4	4	3	3	1	1	4	4
1	1	1	1	2	2	1	1	1	1
1	1	1	1	2	2	1	1	1	1
4	4	1	1	3	3	1	1	4	4
4	4	1	1	3	3	1	1	4	4
4	4	1	1	2	2	1	1	1	1
1	1	1	1	3	3	1	1	4	4
4	4	1	1	3	3	1	1	1	1
1	1	1	1	2	2	1	1	1	1
4	4	1	1	2	2	1	1	1	1
1	1	1	1	2	2	1	1	1	1
4	4	1	1	3	3	1	1	4	4
4	4	1	1	3	3	1	1	4	4
1	1	1	1	3	3	1	1	4	4
4	4	1	1	3	3	1	1	4	4
4	4	1	1	3	3	1	1	4	4
1	1	1	1	3	3	1	1	4	4
1	1	1	1	3	3	1	1	4	4
4	4	1	1	3	3	1	1	1	1
4	4	1	1	3	3	1	1	4	4
1	1	1	1	3	3	1	1	4	4
4	4	1	1	2	2	1	1	1	1
4	4	1	1	2	2	1	1	1	1
4	4	1	1	3	3	1	1	4	4
4	4	1	1	3	3	1	1	4	4
4	4	1	1	3	3	1	1	4	4
4	4	1	1	2	2	1	1	1	1
4	4	1	1	3	3	1	1	4	4
1	1	4	4	3	3	3	3	1	1
1	1	1	1	3	3	1	1	4	4
1	1	1	1	3	3	1	1	4	4
1	1	1	1	3	3	1	1	4	4
1	1	1	1	3	3	1	1	4	4
1	1	1	1	3	3	1	1	4	4
4	4	1	1	3	3	1	1	4	4
1	1	1	1	3	3	1	1	4	4
1	1	1	1	3	3	1	1	1	1
4	4	1	1	3	3	1	1	4	4
1	1	1	1	2	2	1	1	1	1
4	4	1	1	3	3	1	1	4	4
4	4	1	1	3	3	1	1	1	1
1	1	1	1	3	3	1	1	1	1
1	1	1	1	3	3	1	1	4	4

For Review Only

4	4	1	1	3	3	1	1	4	4
4	4	1	1	3	3	1	1	4	4
1	1	1	1	3	3	1	1	4	4
1	1	1	1	3	3	1	1	1	1
4	4	1	1	2	2	1	1	1	1
4	4	1	1	3	3	1	1	4	4
1	1	1	1	3	3	1	1	4	4
1	1	4	4	3	3	1	1	4	4
4	4	4	4	3	3	3	3	4	4
4	4	1	1	3	3	1	1	1	1
4	4	1	1	2	2	1	1	1	1
4	4	1	1	2	2	1	1	1	1
4	4	1	1	3	3	1	1	4	4
4	4	1	1	3	3	1	1	4	4
4	4	1	1	3	3	1	1	1	1
1	1	1	1	3	3	1	1	1	1
1	1	1	1	2	2	1	1	1	1
4	4	1	1	2	2	1	1	1	1
4	4	1	1	2	2	1	1	1	1
4	4	1	1	3	3	1	1	4	4
4	4	1	1	2	2	1	1	1	1
1	1	1	1	3	3	1	1	4	4
4	4	1	1	3	3	1	1	4	4
4	4	1	1	3	3	1	1	4	4
1	1	1	1	3	3	1	1	4	4
1	1	1	1	3	3	1	1	4	4
4	4	1	1	3	3	1	1	1	1
1	1	1	1	3	3	1	1	4	4
1	1	1	1	3	3	1	1	4	4
4	4	1	1	2	2	1	1	1	1
4	4	1	1	3	3	1	1	4	4
4	4	1	1	3	3	1	1	4	4
1	1	1	1	3	3	1	1	4	4
4	4	1	1	3	3	1	1	4	4
4	4	1	1	2	2	1	1	1	1
1	1	1	1	3	3	1	1	4	4
1	1	1	1	3	3	1	1	4	4
1	1	4	4	3	3	3	3	4	4
1	1	1	1	2	2	1	1	1	1
1	1	1	1	3	3	1	1	4	4
4	4	1	1	3	3	1	1	1	1
1	1	1	1	2	2	1	1	1	1
1	1	1	1	3	3	1	1	4	4
4	4	1	1	2	2	1	1	1	1
4	4	1	1	2	2	1	1	1	1
4	4	1	1	2	2	1	1	1	1
4	4	1	1	3	3	1	1	4	4
4	4	1	1	2	2	1	1	1	1
4	4	1	1	3	3	1	1	4	4
4	4	1	1	2	2	1	1	1	1
4	4	1	1	2	2	1	1	1	1
1	1	1	1	3	3	1	1	4	4
1	1	4	4	3	3	3	3	4	4
4	4	1	1	3	3	1	1	1	1
4	4	1	1	2	2	1	1	1	1

For Review Only

1	1	1	1	3	3	1	1	4	4
1	1	1	1	3	3	1	1	1	1
1	1	1	1	3	3	1	1	4	4
4	4	1	1	3	3	1	1	4	4
4	4	1	1	3	3	1	1	1	1
4	4	1	1	3	3	1	1	4	4
1	1	1	1	3	3	1	1	4	4
4	4	1	1	3	3	1	1	1	1
4	4	1	1	3	3	1	1	1	1
4	4	1	1	3	3	1	1	1	1
4	4	1	1	2	2	1	1	1	1
4	4	1	1	3	3	1	1	1	1
1	1	1	1	3	3	1	1	4	4
1	1	1	1	3	3	1	1	4	4
1	1	1	1	2	2	1	1	1	1
4	4	1	1	3	3	1	1	4	4
1	1	1	1	2	2	1	1	1	1
4	4	1	1	3	3	1	1	1	1
4	4	1	1	3	3	1	1	4	4
1	1	1	1	3	3	1	1	4	4
4	4	1	1	3	3	1	1	1	1
4	4	1	1	2	2	1	1	1	1
4	4	1	1	3	3	1	1	4	4
1	1	1	1	3	3	3	3	4	4
1	1	1	1	3	3	3	3	4	4
1	1	1	1	3	3	1	1	4	4
4	4	1	1	3	3	1	1	1	1
4	4	1	1	3	3	1	1	4	4
1	1	1	1	3	3	1	1	1	1
4	4	1	1	2	2	1	1	1	1
1	1	1	1	3	3	1	1	4	4
4	4	4	4	3	3	1	1	1	1
4	4	1	1	3	3	1	1	1	1
4	4	1	1	3	3	1	1	4	4
4	4	1	1	3	3	1	1	4	4
4	4	1	1	3	3	1	1	4	4
4	4	1	1	3	3	1	1	4	4
4	4	1	1	2	2	1	1	1	1
1	1	1	1	3	3	1	1	1	1
1	1	1	1	3	3	1	1	4	4
4	4	1	1	3	3	1	1	4	4
4	4	1	1	3	3	1	1	4	4
4	4	1	1	2	2	1	1	1	1
1	1	1	1	3	3	1	1	4	4
1	1	1	1	3	3	1	1	4	4
1	1	1	1	3	3	1	1	4	4
1	1	1	1	3	3	1	1	4	4
1	1	1	1	2	2	1	1	1	1
4	4	4	4	3	3	1	1	1	1
1	1	1	1	3	3	1	1	4	4
1	1	1	1	3	3	1	1	4	4
1	1	1	1	3	3	1	1	4	4
4	4	1	1	3	3	1	1	1	1
1	1	1	1	2	2	1	1	1	1
4	4	1	1	3	3	1	1	1	1

For Review Only

4	4	1	1	2	2	1	1	1	1
1	1	1	1	3	3	1	1	4	4
4	4	1	1	3	3	1	1	4	4
1	1	1	1	3	3	1	1	1	1
4	4	1	1	2	2	1	1	1	1
4	4	4	4	3	3	3	3	4	4
1	1	1	1	3	3	1	1	4	4
4	4	1	1	3	3	1	1	1	1
1	1	1	1	3	3	1	1	1	1
4	4	1	1	2	2	1	1	1	1
4	4	1	1	3	3	1	1	4	4
1	1	1	1	3	3	1	1	4	4
4	4	1	1	2	2	1	1	1	1
1	1	1	1	3	3	1	1	4	4
4	4	1	1	3	3	1	1	4	4
1	1	1	1	2	2	1	1	1	1
4	4	1	1	3	3	1	1	1	1
1	1	1	1	3	3	1	1	1	1
4	4	1	1	3	3	1	1	4	4
1	1	1	1	3	3	1	1	4	4
4	4	1	1	3	3	1	1	1	1
1	1	1	1	3	3	1	1	4	4
1	1	4	4	3	3	3	3	4	4
4	4	1	1	2	2	1	1	1	1
4	4	1	1	3	3	1	1	4	4
4	4	1	1	3	3	1	1	4	4
4	4	1	1	3	3	1	1	4	4
1	1	1	1	3	3	1	1	1	1
1	1	1	1	3	3	1	1	4	4
4	4	1	1	3	3	1	1	1	1
1	1	1	1	3	3	1	1	1	1
4	4	1	1	2	2	1	1	1	1
4	4	1	1	3	3	1	1	4	4
4	4	1	1	3	3	1	1	4	4
4	4	1	1	3	3	1	1	1	1
4	4	1	1	2	2	1	1	1	1
1	1	1	1	3	3	1	1	4	4
4	4	1	1	2	2	1	1	1	1
1	1	1	1	2	2	1	1	1	1
1	1	1	1	2	2	1	1	1	1
1	1	1	1	2	2	1	1	1	1
1	1	1	1	3	3	1	1	4	4
1	1	1	1	3	3	1	1	4	4
4	4	1	1	3	3	1	1	1	1
1	1	1	1	2	2	1	1	1	1
4	4	4	4	3	3	3	3	4	4
1	1	1	1	2	2	1	1	1	1
1	1	1	1	3	3	1	1	4	4
4	4	1	1	3	3	1	1	4	4
1	1	1	1	2	2	1	1	1	1
1	1	1	1	3	3	1	1	4	4
4	4	1	1	3	3	1	1	4	4
4	4	4	4	3	3	3	3	4	4
4	4	1	1	2	2	1	1	1	1
1	1	1	1	3	3	1	1	4	4

4	4	1	1	2	2	1	1	1	1
1	1	1	1	2	2	1	1	1	1
1	1	1	1	2	2	1	1	1	1
1	1	1	1	3	3	1	1	4	4
4	4	1	1	3	3	1	1	4	4
1	1	1	1	3	3	1	1	4	4
1	1	1	1	3	3	1	1	4	4
1	1	4	4	3	3	1	1	4	4
1	1	1	1	3	3	1	1	4	4
1	1	1	1	3	3	1	1	4	4
4	4	1	1	3	3	1	1	1	1
1	1	1	1	3	3	1	1	1	1
1	1	1	1	2	2	1	1	1	1
4	4	1	1	3	3	1	1	4	4
1	1	1	1	2	2	1	1	1	1
4	4	1	1	2	2	1	1	1	1
1	1	1	1	3	3	1	1	4	4
1	1	1	1	3	3	1	1	1	1
1	1	1	1	3	3	1	1	4	4
1	1	4	4	2	2	3	3	1	1
1	1	1	1	2	2	1	1	1	1
1	1	1	1	3	3	1	1	4	4
4	4	1	1	2	2	1	1	1	1
4	4	1	1	2	2	1	1	1	1
1	1	4	4	3	3	1	1	4	4
4	4	1	1	3	3	1	1	4	4
1	1	1	1	3	3	1	1	4	4
1	1	1	1	3	3	1	1	4	4
4	4	1	1	3	3	1	1	4	4
1	1	1	1	2	2	1	1	1	1
1	1	1	1	3	3	1	1	4	4
1	1	1	1	3	3	1	1	4	4
1	1	1	1	3	3	1	1	4	4
1	1	1	1	3	3	1	1	1	1
4	4	4	4	3	3	3	3	4	4
1	1	1	1	3	3	1	1	4	4
4	4	1	1	2	2	1	1	1	1
4	4	1	1	2	2	1	1	1	1
4	4	1	1	2	2	1	1	1	1
1	1	1	1	3	3	1	1	4	4
4	4	1	1	3	3	1	1	4	4
4	4	1	1	3	3	1	1	4	4
1	1	1	1	3	3	1	1	4	4
4	4	1	1	3	3	1	1	1	1
1	1	1	1	2	2	1	1	1	1
1	1	1	1	2	2	1	1	1	1
1	1	1	1	2	2	1	1	1	1
1	1	1	1	2	2	1	1	1	1
1	1	1	1	3	3	1	1	1	1
4	4	1	1	3	3	1	1	4	4
4	4	1	1	2	2	1	1	1	1
1	1	1	1	3	3	1	1	4	4
1	1	1	1	2	2	1	1	1	1
1	1	1	1	2	2	1	1	1	1
1	1	1	1	3	3	1	1	4	4

For Review Only

4	4	1	1	2	2	1	1	1	1
1	1	1	1	3	3	1	1	4	4

For Review Only

SNP2136	SNP2136	SNP2137	SNP2137	SNP2138	SNP2138	SNP2139	SNP2139	SNP214	SNP214
4	4	2	2	2	2	4	4	4	4
4	4	4	4	2	2	4	4	4	4
4	4	4	4	2	2	4	4	4	4
1	1	4	4	3	3	1	1	4	4
4	4	4	4	2	2	4	4	1	1
4	4	4	4	2	2	4	4	4	4
1	1	4	4	3	3	1	1	4	4
4	4	4	4	2	2	4	4	4	4
4	4	4	4	2	2	4	4	4	4
1	1	4	4	2	2	1	1	1	1
4	4	4	4	2	2	4	4	4	4
4	4	4	4	2	2	4	4	4	4
4	4	4	4	2	2	4	4	4	4
4	4	4	4	2	2	4	4	4	4
4	4	4	4	2	2	4	4	4	4
4	4	2	2	2	2	4	4	4	4
4	4	4	4	2	2	4	4	4	4
4	4	4	4	2	2	4	4	4	4
4	4	4	4	2	2	4	4	4	4
4	4	4	4	2	2	4	4	4	4
4	4	2	2	2	2	4	4	4	4
4	4	4	4	2	2	4	4	4	4
4	4	2	2	2	2	4	4	4	4
4	4	4	4	2	2	4	4	4	4
4	4	4	4	2	2	4	4	4	4
4	4	4	4	2	2	4	4	4	4
4	4	4	4	2	2	4	4	4	4
4	4	4	4	2	2	4	4	4	4
4	4	2	2	2	2	4	4	4	4
4	4	4	4	2	2	4	4	4	4
4	4	4	4	2	2	4	4	4	4
4	4	4	4	2	2	4	4	4	4
4	4	4	4	2	2	4	4	4	4
4	4	4	4	2	2	4	4	4	4
4	4	4	4	2	2	4	4	4	4
4	4	4	4	2	2	4	4	4	4
1	1	4	4	3	3	1	1	4	4
4	4	4	4	2	2	1	1	4	4
4	4	2	2	2	2	4	4	4	4
4	4	4	4	2	2	4	4	4	4
4	4	4	4	2	2	4	4	1	1
4	4	4	4	2	2	4	4	4	4
4	4	4	4	2	2	4	4	4	4
4	4	4	4	2	2	4	4	4	4
4	4	4	4	2	2	4	4	1	1
4	4	4	4	2	2	4	4	4	4
4	4	4	4	2	2	4	4	4	4
4	4	4	4	2	2	4	4	4	4
4	4	4	4	2	2	4	4	4	4
4	4	4	4	2	2	4	4	4	4
4	4	4	4	2	2	4	4	4	4
4	4	4	4	2	2	4	4	4	4

4	4	4	4	2	2	4	4	4	4
4	4	4	4	2	2	4	4	4	4
4	4	4	4	2	2	4	4	4	4
4	4	4	4	2	2	4	4	4	4
4	4	4	4	2	2	4	4	4	4
4	4	4	4	2	2	4	4	4	4
4	4	4	4	2	2	4	4	4	4
4	4	2	2	3	3	1	1	4	4
4	4	4	4	2	2	4	4	4	4
4	4	4	4	2	2	4	4	4	4
4	4	4	4	2	2	4	4	4	4
4	4	4	4	2	2	4	4	4	4
4	4	4	4	2	2	4	4	4	4
4	4	2	2	2	2	4	4	4	4
4	4	4	4	2	2	4	4	4	4
4	4	4	4	2	2	4	4	4	4
4	4	4	4	2	2	4	4	4	4
4	4	4	4	2	2	4	4	4	4
4	4	4	4	2	2	4	4	4	4
4	4	4	4	2	2	4	4	4	4
4	4	4	4	2	2	4	4	4	4
4	4	4	4	2	2	4	4	4	4
4	4	2	2	2	2	4	4	4	4
4	4	4	4	2	2	4	4	4	4
4	4	4	4	2	2	4	4	1	1
4	4	4	4	2	2	4	4	4	4
4	4	4	4	2	2	4	4	4	4
4	4	2	2	2	2	4	4	4	4
4	4	4	4	2	2	4	4	4	4
4	4	4	4	2	2	4	4	4	4
4	4	4	4	2	2	4	4	1	1
4	4	4	4	2	2	4	4	4	4
4	4	2	2	2	2	4	4	4	4
4	4	4	4	2	2	4	4	4	4
4	4	4	4	2	2	4	4	4	4
4	4	4	4	2	2	4	4	1	1
4	4	4	4	2	2	4	4	1	1
1	1	4	4	3	3	1	1	4	4
4	4	2	2	2	2	4	4	4	4
4	4	4	4	2	2	4	4	4	4
4	4	4	4	2	2	4	4	4	4
4	4	4	4	2	2	4	4	4	4
4	4	4	4	2	2	4	4	4	4
4	4	4	4	2	2	4	4	4	4
4	4	4	4	2	2	4	4	1	1
4	4	4	4	2	2	4	4	4	4
4	4	4	4	2	2	4	4	4	4
4	4	4	4	2	2	4	4	4	4
4	4	4	4	2	2	4	4	4	4
4	4	4	4	2	2	4	4	4	4
4	4	4	4	2	2	4	4	4	4
4	4	4	4	2	2	4	4	4	4
1	1	2	2	3	3	1	1	4	4
4	4	4	4	2	2	1	1	4	4
4	4	4	4	2	2	4	4	4	4

For Review Only

4	4	4	4	2	2	4	4	4	4
4	4	4	4	2	2	4	4	4	4
4	4	4	4	2	2	4	4	4	4
4	4	4	4	2	2	4	4	4	4
4	4	4	4	2	2	4	4	4	4
4	4	4	4	2	2	4	4	4	4
4	4	4	4	2	2	4	4	4	4
4	4	4	4	2	2	4	4	4	4
4	4	4	4	2	2	4	4	4	4
4	4	4	4	2	2	4	4	4	4
4	4	2	2	2	2	1	1	4	4
4	4	4	4	2	2	4	4	4	4
4	4	4	4	2	2	4	4	4	4
4	4	4	4	2	2	4	4	4	4
4	4	4	4	2	2	4	4	4	4
4	4	4	4	2	2	4	4	4	4
4	4	4	4	2	2	4	4	1	1
4	4	4	4	2	2	4	4	4	4
4	4	4	4	2	2	4	4	4	4
4	4	4	4	2	2	4	4	4	4
4	4	4	4	2	2	4	4	4	4
4	4	4	4	2	2	4	4	4	4
4	4	4	4	2	2	4	4	4	4
4	4	2	2	2	2	4	4	1	1
4	4	4	4	2	2	4	4	4	4
4	4	2	2	2	2	4	4	4	4
4	4	4	4	2	2	4	4	4	4
4	4	4	4	2	2	4	4	4	4
4	4	4	4	2	2	4	4	4	4
4	4	4	4	2	2	4	4	1	1
4	4	2	2	2	2	4	4	4	4
4	4	4	4	2	2	4	4	4	4
4	4	4	4	2	2	4	4	4	4
4	4	4	4	2	2	4	4	4	4
4	4	4	4	2	2	4	4	4	4
4	4	4	4	2	2	4	4	4	4
4	4	4	4	2	2	4	4	4	4
4	4	4	4	2	2	4	4	4	4
4	4	2	2	2	2	4	4	4	4
4	4	4	4	2	2	4	4	4	4
4	4	4	4	2	2	4	4	4	4
4	4	4	4	2	2	4	4	4	4
4	4	4	4	2	2	4	4	4	4
4	4	4	4	2	2	4	4	1	1
4	4	4	4	3	3	4	4	4	4
4	4	4	4	2	2	4	4	4	4
4	4	4	4	2	2	4	4	4	4
4	4	4	4	2	2	4	4	4	4
4	4	4	4	2	2	1	1	4	4
4	4	4	4	2	2	4	4	4	4
4	4	4	4	2	2	4	4	4	4
4	4	4	4	2	2	4	4	1	1
4	4	4	4	2	2	4	4	4	4
4	4	4	4	2	2	4	4	4	4
4	4	2	2	2	2	4	4	4	4

For Review Only

4	4	4	4	2	2	4	4	4	4
4	4	4	4	2	2	4	4	4	4
4	4	4	4	2	2	4	4	4	4
4	4	4	4	2	2	4	4	4	4
4	4	4	4	2	2	4	4	4	4
1	1	4	4	3	3	1	1	4	4
4	4	4	4	2	2	4	4	4	4
4	4	4	4	2	2	4	4	4	4
4	4	4	4	2	2	4	4	4	4
4	4	4	4	2	2	4	4	4	4
4	4	4	4	2	2	4	4	4	4
4	4	4	4	2	2	4	4	4	4
4	4	4	4	2	2	4	4	4	4
4	4	4	4	2	2	4	4	4	4
4	4	4	4	2	2	4	4	4	4
4	4	2	2	2	2	4	4	4	4
4	4	4	4	2	2	4	4	4	4
4	4	4	4	2	2	4	4	4	4
4	4	4	4	2	2	4	4	4	4
4	4	4	4	2	2	4	4	4	4
4	4	4	4	2	2	4	4	4	4
4	4	4	4	2	2	4	4	1	1
4	4	4	4	2	2	4	4	4	4
4	4	4	4	2	2	4	4	4	4
4	4	4	4	2	2	4	4	4	4
4	4	4	4	2	2	4	4	4	4
4	4	4	4	2	2	4	4	4	4
4	4	4	4	2	2	4	4	4	4
4	4	4	4	2	2	4	4	4	4
4	4	4	4	2	2	4	4	4	4
1	1	2	2	3	3	1	1	4	4
4	4	4	4	2	2	4	4	4	4
4	4	4	4	2	2	4	4	4	4
4	4	4	4	2	2	4	4	4	4
4	4	4	4	2	2	4	4	4	4
4	4	4	4	2	2	4	4	4	4
4	4	4	4	2	2	4	4	4	4
4	4	4	4	2	2	4	4	4	4
4	4	4	4	2	2	4	4	4	4
4	4	4	4	2	2	4	4	4	4
4	4	4	4	2	2	4	4	4	4
4	4	4	4	2	2	4	4	4	4
4	4	2	2	2	2	4	4	4	4
4	4	4	4	2	2	4	4	4	4
4	4	4	4	2	2	4	4	4	4
1	1	4	4	3	3	1	1	4	4
4	4	2	2	2	2	4	4	4	4
4	4	4	4	2	2	4	4	1	1
4	4	4	4	2	2	4	4	4	4
4	4	4	4	2	2	4	4	4	4
4	4	4	4	2	2	4	4	4	4
1	1	4	4	2	2	1	1	1	1
4	4	4	4	2	2	4	4	4	4
4	4	4	4	2	2	4	4	4	4
4	4	4	4	2	2	4	4	4	4

For Review Only

4	4	4	4	2	2	4	4	4	4
4	4	4	4	2	2	4	4	4	4
4	4	4	4	2	2	4	4	4	4
4	4	4	4	2	2	4	4	4	4
4	4	4	4	2	2	4	4	4	4
4	4	4	4	2	2	4	4	4	4
4	4	4	4	2	2	4	4	4	4
1	1	4	4	3	3	1	1	4	4
4	4	4	4	2	2	4	4	4	4
4	4	2	2	2	2	4	4	4	4
4	4	4	4	2	2	4	4	4	4
4	4	2	2	3	3	1	1	4	4
4	4	2	2	2	2	4	4	4	4
4	4	4	4	2	2	4	4	4	4
4	4	4	4	2	2	4	4	4	4
4	4	4	4	2	2	4	4	4	4
4	4	4	4	2	2	4	4	4	4
4	4	2	2	2	2	4	4	4	4
4	4	4	4	2	2	4	4	4	4
1	1	4	4	3	3	1	1	4	4
4	4	4	4	2	2	4	4	1	1
4	4	4	4	2	2	4	4	4	4
4	4	4	4	2	2	4	4	4	4
4	4	4	4	2	2	4	4	4	4
4	4	2	2	3	3	1	1	4	4
4	4	4	4	2	2	4	4	4	4
4	4	2	2	2	2	4	4	1	1
4	4	4	4	2	2	4	4	4	4
4	4	4	4	2	2	4	4	4	4
4	4	4	4	2	2	4	4	4	4
4	4	4	4	2	2	4	4	4	4
4	4	4	4	2	2	4	4	4	4
4	4	4	4	2	2	4	4	4	4
4	4	4	4	2	2	4	4	4	4
4	4	4	4	2	2	4	4	4	4
4	4	2	2	2	2	4	4	1	1
4	4	4	4	2	2	4	4	4	4
4	4	4	4	2	2	4	4	4	4
4	4	4	4	2	2	4	4	4	4
4	4	4	4	2	2	4	4	4	4
4	4	4	4	2	2	4	4	4	4
4	4	4	4	2	2	4	4	4	4
4	4	4	4	2	2	4	4	4	4
4	4	4	4	2	2	4	4	4	4
4	4	4	4	2	2	4	4	4	4
4	4	4	4	2	2	4	4	4	4
4	4	4	4	2	2	4	4	4	4
4	4	4	4	2	2	4	4	4	4
4	4	4	4	2	2	4	4	4	4
4	4	2	2	2	2	4	4	1	1
4	4	4	4	2	2	4	4	4	4
4	4	4	4	2	2	4	4	4	4
4	4	4	4	2	2	4	4	4	4
4	4	4	4	2	2	4	4	4	4
4	4	4	4	2	2	4	4	4	4

4	4	4	4	2	2	4	4	4	4
4	4	4	4	2	2	4	4	4	4

For Review Only

SNP2140	SNP2140	SNP2141	SNP2141	SNP2142	SNP2142	SNP2143	SNP2143	SNP2144	SNP2144
4	4	1	1	4	4	2	2	2	2
4	4	1	1	4	4	2	2	2	2
4	4	2	2	1	1	2	2	2	2
4	4	2	2	1	1	2	2	2	2
4	4	1	1	4	4	2	2	2	2
4	4	1	1	4	4	2	2	2	2
1	1	2	2	1	1	2	2	2	2
4	4	2	2	1	1	2	2	1	1
4	4	1	1	4	4	2	2	2	2
4	4	2	2	1	1	2	2	1	1
1	1	2	2	1	1	2	2	2	2
4	4	2	2	1	1	2	2	2	2
4	4	1	1	1	1	2	2	2	2
4	4	2	2	1	1	2	2	1	1
4	4	1	1	4	4	2	2	2	2
4	4	2	2	1	1	2	2	1	1
4	4	1	1	4	4	2	2	2	2
1	1	2	2	1	1	2	2	2	2
4	4	1	1	4	4	2	2	2	2
1	1	2	2	1	1	2	2	2	2
4	4	1	1	4	4	2	2	2	2
4	4	1	1	4	4	2	2	2	2
1	1	2	2	1	1	2	2	2	2
4	4	1	1	4	4	2	2	2	2
4	4	2	2	1	1	2	2	1	1
4	4	2	2	1	1	2	2	1	1
4	4	2	2	1	1	2	2	1	1
4	4	1	1	1	1	2	2	1	1
4	4	1	1	4	4	2	2	2	2
4	4	2	2	1	1	2	2	2	2
4	4	2	2	1	1	2	2	1	1
4	4	1	1	4	4	2	2	2	2
4	4	1	1	4	4	2	2	2	2
4	4	1	1	4	4	2	2	2	2
4	4	1	1	4	4	2	2	2	2
4	4	1	1	4	4	2	2	2	2
4	4	1	1	4	4	2	2	2	2
4	4	2	2	1	1	2	2	1	1
4	4	2	2	1	1	2	2	2	2
4	4	2	2	1	1	2	2	2	2
4	4	2	2	1	1	1	1	2	2
1	1	2	2	1	1	2	2	2	2
4	4	2	2	1	1	2	2	1	1
4	4	1	1	4	4	2	2	2	2
4	4	2	2	1	1	2	2	1	1
4	4	2	2	1	1	2	2	1	1
4	4	1	1	4	4	2	2	2	2
4	4	2	2	1	1	2	2	1	1
4	4	1	1	1	1	2	2	2	2
4	4	1	1	4	4	2	2	2	2
4	4	2	2	1	1	2	2	1	1
4	4	2	2	1	1	2	2	1	1

For Review Only

4	4	1	1	4	4	2	2	2	2
4	4	1	1	4	4	2	2	2	2
4	4	2	2	1	1	2	2	1	1
4	4	2	2	1	1	2	2	2	2
4	4	1	1	4	4	2	2	2	2
4	4	1	1	4	4	2	2	2	2
4	4	2	2	1	1	2	2	1	1
1	1	2	2	1	1	1	1	2	2
4	4	1	1	4	4	2	2	2	2
4	4	1	1	1	1	2	2	2	2
4	4	1	1	4	4	2	2	2	2
4	4	1	1	4	4	2	2	2	2
4	4	1	1	4	4	2	2	2	2
4	4	1	1	4	4	2	2	2	2
4	4	1	1	1	1	2	2	2	2
4	4	2	2	1	1	2	2	1	1
4	4	2	2	1	1	2	2	2	2
4	4	1	1	4	4	2	2	2	2
4	4	1	1	4	4	2	2	2	2
4	4	1	1	4	4	2	2	2	2
4	4	1	1	4	4	2	2	2	2
4	4	1	1	4	4	2	2	2	2
4	4	1	1	4	4	2	2	2	2
4	4	2	2	1	1	2	2	1	1
4	4	2	2	1	1	2	2	1	1
4	4	2	2	1	1	2	2	2	2
4	4	1	1	4	4	2	2	2	2
1	1	2	2	1	1	1	1	2	2
4	4	2	2	1	1	2	2	1	1
4	4	1	1	4	4	2	2	2	2
4	4	1	1	4	4	2	2	2	2
4	4	1	1	4	4	2	2	2	2
1	1	2	2	1	1	1	1	2	2
4	4	1	1	4	4	2	2	2	2
4	4	1	1	4	4	2	2	2	2
4	4	2	2	1	1	2	2	1	1
4	4	2	2	1	1	2	2	1	1
4	4	2	2	1	1	2	2	2	2
4	4	2	2	1	1	2	2	1	1
4	4	2	2	1	1	2	2	1	1
4	4	1	1	4	4	2	2	2	2
4	4	2	2	1	1	2	2	2	2
4	4	2	2	1	1	2	2	1	1
4	4	1	1	4	4	2	2	2	2
4	4	1	1	4	4	2	2	2	2
4	4	1	1	4	4	2	2	2	2
4	4	1	1	4	4	2	2	2	2
4	4	1	1	1	1	2	2	2	2
4	4	1	1	4	4	2	2	2	2
4	4	1	1	4	4	2	2	2	2
4	4	1	1	4	4	2	2	2	2
4	4	2	2	1	1	2	2	2	2
1	1	2	2	1	1	1	1	2	2
4	4	1	1	1	1	2	2	2	2
4	4	1	1	4	4	2	2	2	2

For Review Only

4	4	2	2	1	1	2	2	2	2
4	4	2	2	1	1	2	2	2	2
4	4	2	2	1	1	2	2	1	1
4	4	1	1	4	4	1	1	2	2
4	4	2	2	1	1	2	2	2	2
4	4	1	1	1	1	2	2	2	2
4	4	2	2	1	1	2	2	1	1
4	4	1	1	4	4	2	2	2	2
4	4	1	1	1	1	2	2	2	2
4	4	1	1	1	1	2	2	2	2
4	4	1	1	4	4	2	2	2	2
4	4	1	1	1	1	2	2	2	2
4	4	2	2	1	1	2	2	1	1
4	4	2	2	1	1	2	2	2	2
4	4	2	2	1	1	2	2	2	2
4	4	1	1	1	1	2	2	2	2
1	1	2	2	1	1	2	2	2	2
4	4	1	1	4	4	2	2	2	2
4	4	1	1	4	4	2	2	2	2
4	4	2	2	1	1	2	2	1	1
4	4	1	1	1	1	2	2	2	2
4	4	1	1	4	4	2	2	2	2
4	4	2	2	1	1	2	2	2	2
1	1	2	2	1	1	2	2	2	2
4	4	1	1	4	4	2	2	2	2
4	4	2	2	1	1	2	2	2	2
4	4	1	1	4	4	2	2	2	2
4	4	1	1	4	4	2	2	2	2
4	4	2	2	1	1	2	2	1	1
4	4	1	1	4	4	2	2	2	2
1	1	2	2	1	1	2	2	2	2
4	4	1	1	1	1	2	2	2	2
4	4	1	1	4	4	2	2	2	2
4	4	1	1	1	1	2	2	2	2
4	4	1	1	4	4	2	2	2	2
4	4	1	1	4	4	2	2	2	2
4	4	1	1	4	4	2	2	2	2
4	4	1	1	1	1	2	2	1	1
4	4	2	2	1	1	2	2	1	1
4	4	2	2	1	1	1	1	2	2
4	4	1	1	1	1	2	2	2	2
4	4	1	1	4	4	2	2	2	2
4	4	1	1	4	4	2	2	2	2
4	4	2	2	1	1	2	2	2	2
4	4	2	2	1	1	2	2	1	1
4	4	2	2	1	1	2	2	1	1
4	4	2	2	1	1	2	2	1	1
4	4	2	2	1	1	2	2	2	2
4	4	1	1	1	1	2	2	2	2
4	4	2	2	1	1	2	2	1	1
4	4	2	2	1	1	2	2	1	1
4	4	2	2	1	1	2	2	2	2
4	4	1	1	4	4	2	2	2	2
4	4	2	2	1	1	2	2	1	1
4	4	1	1	1	1	1	1	2	2

For Review Only

4	4	1	1	4	4	2	2	2	2
4	4	2	2	1	1	2	2	2	2
4	4	1	1	1	1	1	1	2	2
4	4	1	1	1	1	2	2	2	2
4	4	1	1	4	4	2	2	2	2
4	4	1	1	4	4	2	2	2	2
4	4	2	2	1	1	2	2	2	2
4	4	1	1	1	1	2	2	2	2
4	4	2	2	1	1	2	2	2	2
4	4	1	1	4	4	2	2	2	2
4	4	1	1	4	4	2	2	2	2
4	4	2	2	1	1	2	2	2	2
4	4	1	1	4	4	2	2	2	2
4	4	1	1	1	1	2	2	2	2
4	4	1	1	4	4	2	2	2	2
1	1	2	2	1	1	2	2	2	2
4	4	1	1	1	1	2	2	2	2
4	4	2	2	1	1	2	2	2	2
4	4	1	1	4	4	2	2	2	2
4	4	2	2	1	1	2	2	1	1
4	4	1	1	1	1	2	2	2	2
1	1	2	2	1	1	2	2	2	2
4	4	1	1	4	4	1	1	2	2
4	4	1	1	4	4	2	2	2	2
4	4	1	1	4	4	2	2	2	2
4	4	1	1	4	4	2	2	2	2
4	4	1	1	4	4	2	2	2	2
4	4	1	1	1	1	2	2	2	2
4	4	2	2	1	1	2	2	1	1
4	4	1	1	4	4	2	2	2	2
1	1	2	2	1	1	2	2	2	2
4	4	1	1	4	4	2	2	2	2
4	4	1	1	4	4	2	2	2	2
4	4	1	1	4	4	2	2	2	2
4	4	1	1	4	4	2	2	2	2
4	4	1	1	4	4	2	2	2	2
4	4	2	2	1	1	2	2	2	2
4	4	1	1	4	4	2	2	2	2
4	4	2	2	1	1	2	2	2	2
4	4	2	2	1	1	2	2	1	1
4	4	2	2	1	1	2	2	2	2
4	4	2	2	1	1	2	2	1	1
4	4	2	2	1	1	1	1	2	2
4	4	1	1	1	1	2	2	2	2
4	4	2	2	1	1	1	1	2	2
4	4	1	1	4	4	2	2	2	2
1	1	2	2	1	1	2	2	2	2
1	1	2	2	1	1	2	2	2	2
4	4	1	1	4	4	2	2	2	2
4	4	2	2	1	1	2	2	1	1
4	4	2	2	1	1	2	2	1	1
4	4	1	1	4	4	1	1	2	2
4	4	1	1	4	4	2	2	2	2
4	4	1	1	4	4	2	2	2	2
1	1	2	2	1	1	2	2	2	2

For Review Only

4	4	1	1	4	4	2	2	2	2
1	1	2	2	1	1	2	2	2	2
4	4	2	2	1	1	2	2	2	2
4	4	1	1	1	1	1	1	2	2
4	4	1	1	4	4	2	2	2	2
4	4	2	2	1	1	2	2	1	1
1	1	2	2	1	1	1	1	2	2
4	4	2	2	1	1	2	2	1	1
4	4	2	2	1	1	2	2	2	2
4	4	2	2	1	1	2	2	2	2
4	4	1	1	1	1	2	2	2	2
1	1	2	2	1	1	2	2	2	2
4	4	2	2	1	1	2	2	1	1
4	4	1	1	4	4	2	2	2	2
4	4	2	2	1	1	2	2	2	2
4	4	1	1	4	4	1	1	2	2
4	4	2	2	1	1	2	2	1	1
4	4	2	2	1	1	2	2	2	2
4	4	2	2	1	1	1	1	2	2
4	4	2	2	1	1	2	2	1	1
4	4	2	2	1	1	2	2	2	2
4	4	2	2	1	1	2	2	1	1
4	4	1	1	4	4	2	2	2	2
4	4	1	1	4	4	2	2	2	2
1	1	2	2	1	1	1	1	2	2
4	4	1	1	1	1	2	2	2	2
4	4	2	2	1	1	1	1	2	2
4	4	2	2	1	1	2	2	1	1
4	4	1	1	4	4	2	2	2	2
4	4	2	2	1	1	2	2	1	1
4	4	2	2	1	1	2	2	2	2
4	4	2	2	1	1	2	2	1	1
4	4	1	1	4	4	2	2	2	2
4	4	2	2	1	1	2	2	1	1
4	4	1	1	4	4	2	2	2	2
4	4	2	2	1	1	1	1	2	2
4	4	1	1	4	4	2	2	2	2
4	4	1	1	4	4	2	2	2	2
4	4	1	1	4	4	2	2	2	2
4	4	2	2	1	1	2	2	2	2
4	4	1	1	4	4	2	2	2	2
4	4	1	1	4	4	2	2	2	2
4	4	2	2	1	1	2	2	2	2
4	4	1	1	4	4	2	2	2	2
4	4	2	2	1	1	2	2	2	2
4	4	2	2	1	1	2	2	2	2
4	4	2	2	1	1	2	2	2	2
4	4	2	2	1	1	2	2	1	1
4	4	2	2	1	1	2	2	2	2
4	4	2	2	1	1	1	1	2	2
4	4	1	1	4	4	2	2	2	2
1	1	2	2	1	1	2	2	2	2
4	4	2	2	1	1	2	2	2	2
4	4	2	2	1	1	2	2	1	1
4	4	2	2	1	1	2	2	2	2

For Review Only

4	4	1	1	4	4	2	2	2	2
4	4	2	2	1	1	2	2	1	1

For Review Only

SNP2145	SNP2145	SNP2146	SNP2146	SNP2147	SNP2147	SNP2148	SNP2148	SNP2149	SNP2149
4	4	3	3	2	2	1	1	4	4
4	4	2	2	2	2	1	1	4	4
4	4	2	2	2	2	1	1	4	4
3	3	2	2	3	3	1	1	4	4
4	4	3	3	2	2	4	4	3	3
4	4	2	2	3	3	1	1	4	4
3	3	3	3	3	3	4	4	3	3
3	3	2	2	2	2	1	1	4	4
4	4	3	3	3	3	4	4	3	3
3	3	2	2	2	2	1	1	4	4
3	3	2	2	2	2	4	4	3	3
3	3	2	2	2	2	1	1	4	4
4	4	3	3	2	2	1	1	4	4
3	3	2	2	2	2	4	4	3	3
4	4	3	3	2	2	1	1	4	4
3	3	2	2	2	2	1	1	4	4
4	4	3	3	3	3	4	4	3	3
3	3	2	2	2	2	1	1	4	4
4	4	2	2	2	2	1	1	4	4
3	3	2	2	2	2	1	1	4	4
4	4	2	2	2	2	4	4	3	3
4	4	2	2	3	3	4	4	3	3
3	3	3	3	2	2	4	4	3	3
4	4	2	2	2	2	1	1	4	4
3	3	2	2	2	2	4	4	3	3
3	3	3	3	2	2	4	4	3	3
3	3	2	2	2	2	1	1	4	4
3	3	2	2	2	2	4	4	4	4
4	4	2	2	2	2	1	1	4	4
3	3	2	2	2	2	1	1	4	4
3	3	2	2	3	3	1	1	4	4
4	4	2	2	3	3	4	4	3	3
4	4	3	3	3	3	4	4	3	3
4	4	2	2	2	2	1	1	4	4
4	4	2	2	2	2	4	4	4	4
4	4	2	2	2	2	1	1	4	4
4	4	2	2	2	2	1	1	4	4
3	3	2	2	2	2	1	1	4	4
4	4	3	3	2	2	1	1	4	4
4	4	2	2	2	2	1	1	4	4
3	3	2	2	2	2	1	1	4	4
3	3	2	2	2	2	1	1	4	4
3	3	2	2	2	2	1	1	4	4
4	4	2	2	3	3	4	4	3	3
3	3	2	2	2	2	1	1	4	4
3	3	2	2	2	2	1	1	4	4
4	4	2	2	2	2	1	1	4	4
3	3	2	2	2	2	1	1	4	4
3	3	2	2	3	3	4	4	3	3
4	4	2	2	3	3	1	1	4	4
3	3	2	2	2	2	1	1	4	4
3	3	2	2	3	3	4	4	3	3

For Review Only

4	4	3	3	3	3	4	4	3	3
4	4	3	3	3	3	4	4	3	3
4	4	2	2	2	2	1	1	4	4
3	3	2	2	3	3	4	4	3	3
4	4	2	2	2	2	1	1	4	4
4	4	2	2	2	2	1	1	4	4
3	3	2	2	3	3	1	1	4	4
3	3	3	3	2	2	1	1	4	4
4	4	2	2	2	2	1	1	4	4
4	4	2	2	2	2	4	4	4	4
4	4	2	2	2	2	1	1	4	4
4	4	2	2	2	2	1	1	4	4
4	4	3	3	3	3	4	4	3	3
4	4	2	2	2	2	1	1	4	4
4	4	2	2	3	3	4	4	4	4
4	4	2	2	2	2	1	1	4	4
4	4	2	2	2	2	1	1	4	4
4	4	2	2	3	3	1	1	4	4
4	4	2	2	3	3	4	4	3	3
4	4	3	3	3	3	1	1	4	4
4	4	2	2	2	2	1	1	4	4
4	4	3	3	3	3	4	4	3	3
4	4	2	2	2	2	4	4	4	4
3	3	2	2	3	3	4	4	3	3
3	3	2	2	2	2	1	1	4	4
4	4	2	2	3	3	1	1	4	4
4	4	3	3	2	2	4	4	3	3
3	3	3	3	2	2	1	1	4	4
3	3	2	2	2	2	1	1	4	4
4	4	2	2	2	2	4	4	3	3
4	4	2	2	2	2	1	1	4	4
4	4	2	2	2	2	1	1	4	4
3	3	3	3	2	2	1	1	4	4
4	4	3	3	3	3	4	4	3	3
4	4	2	2	3	3	1	1	4	4
3	3	2	2	2	2	1	1	4	4
3	3	2	2	2	2	1	1	4	4
3	3	2	2	2	2	4	4	4	4
3	3	2	2	2	2	4	4	3	3
3	3	2	2	2	2	1	1	4	4
4	4	2	2	3	3	4	4	3	3
4	4	2	2	2	2	4	4	3	3
3	3	3	3	2	2	1	1	4	4
4	4	2	2	2	2	1	1	4	4
4	4	3	3	3	3	1	1	4	4
4	4	2	2	3	3	4	4	3	3
4	4	2	2	2	2	1	1	4	4
4	4	2	2	3	3	1	1	4	4
4	4	2	2	2	2	1	1	4	4
4	4	2	2	2	2	1	1	4	4
4	4	2	2	2	2	1	1	4	4
4	4	3	3	2	2	1	1	4	4
3	3	3	3	2	2	1	1	4	4
3	3	2	2	2	2	4	4	4	4
4	4	3	3	2	2	1	1	4	4

For Review Only

4	4	2	2	2	2	4	4	3	3
4	4	2	2	2	2	4	4	3	3
3	3	2	2	2	2	1	1	4	4
4	4	2	2	2	2	1	1	4	4
4	4	2	2	2	2	1	1	4	4
4	4	2	2	2	2	4	4	3	3
3	3	2	2	2	2	1	1	4	4
4	4	2	2	2	2	1	1	4	4
4	4	2	2	2	2	1	1	4	4
4	4	2	2	2	2	1	1	4	4
4	4	2	2	2	2	1	1	4	4
4	4	2	2	2	2	4	4	4	4
3	3	3	3	2	2	1	1	4	4
3	3	2	2	3	3	4	4	4	4
3	3	2	2	2	2	1	1	4	4
4	4	2	2	2	2	1	1	4	4
3	3	3	3	2	2	4	4	3	3
4	4	2	2	2	2	1	1	4	4
4	4	2	2	2	2	1	1	4	4
4	4	2	2	2	2	4	4	4	4
4	4	2	2	2	2	4	4	4	4
4	4	2	2	2	2	4	4	3	3
4	4	2	2	2	2	1	1	4	4
3	3	3	3	2	2	1	1	4	4
4	4	2	2	2	2	4	4	4	4
4	4	2	2	2	2	4	4	3	3
4	4	2	2	2	2	4	4	4	4
4	4	2	2	3	3	1	1	4	4
3	3	2	2	2	2	1	1	4	4
4	4	2	2	3	3	4	4	3	3
3	3	3	3	2	2	1	1	4	4
4	4	2	2	2	2	4	4	4	4
4	4	2	2	3	3	4	4	3	3
4	4	2	2	2	2	1	1	4	4
4	4	2	2	2	2	1	1	4	4
4	4	2	2	2	2	1	1	4	4
4	4	2	2	2	2	1	1	4	4
3	3	2	2	2	2	1	1	4	4
3	3	2	2	2	2	1	1	4	4
3	3	3	3	2	2	4	4	3	3
4	4	2	2	2	2	4	4	3	3
4	4	2	2	2	2	1	1	4	4
4	4	3	3	2	2	1	1	4	4
3	3	2	2	2	2	4	4	4	4
3	3	3	3	2	2	4	4	4	4
3	3	3	3	2	2	1	1	4	4
3	3	2	2	2	2	1	1	4	4
4	4	2	2	2	2	1	1	4	4
4	4	2	2	2	2	1	1	4	4
3	3	2	2	2	2	1	1	4	4
3	3	2	2	2	2	1	1	4	4
4	4	3	3	2	2	4	4	3	3
4	4	2	2	3	3	1	1	4	4
3	3	2	2	2	2	1	1	4	4
4	4	2	2	2	2	1	1	3	3

For Review Only

4	4	2	2	2	2	1	1	4	4
4	4	3	3	2	2	1	1	4	4
4	4	3	3	3	3	4	4	3	3
4	4	2	2	2	2	4	4	4	4
4	4	2	2	2	2	1	1	4	4
4	4	3	3	3	3	1	1	4	4
4	4	3	3	2	2	4	4	4	4
4	4	2	2	2	2	1	1	4	4
4	4	2	2	2	2	1	1	4	4
4	4	2	2	3	3	4	4	3	3
4	4	2	2	3	3	1	1	4	4
4	4	2	2	2	2	1	1	4	4
4	4	2	2	2	2	1	1	4	4
4	4	2	2	2	2	4	4	4	4
4	4	2	2	2	2	1	1	4	4
3	3	2	2	2	2	4	4	3	3
4	4	2	2	2	2	1	1	4	4
3	3	2	2	2	2	4	4	3	3
4	4	3	3	2	2	1	1	4	4
3	3	2	2	2	2	1	1	4	4
4	4	2	2	2	2	4	4	4	4
3	3	2	2	2	2	1	1	4	4
4	4	3	3	3	3	4	4	3	3
4	4	2	2	2	2	1	1	4	4
4	4	2	2	3	3	4	4	3	3
4	4	2	2	2	2	1	1	4	4
4	4	2	2	2	2	1	1	4	4
4	4	2	2	2	2	4	4	4	4
3	3	3	3	3	3	1	1	4	4
4	4	2	2	3	3	4	4	3	3
3	3	2	2	2	2	1	1	4	4
4	4	2	2	3	3	4	4	3	3
4	4	2	2	2	2	4	4	3	3
4	4	2	2	2	2	1	1	4	4
4	4	2	2	3	3	4	4	4	4
4	4	2	2	2	2	1	1	4	4
4	4	2	2	2	2	1	1	4	4
4	4	2	2	2	2	1	1	4	4
4	4	2	2	2	2	1	1	4	4
3	3	2	2	2	2	1	1	4	4
3	3	2	2	2	2	1	1	4	4
3	3	2	2	2	2	1	1	4	4
4	4	3	3	2	2	1	1	4	4
4	4	2	2	2	2	4	4	4	4
3	3	2	2	2	2	4	4	4	4
4	4	3	3	3	3	4	4	3	3
3	3	2	2	2	2	4	4	3	3
3	3	3	3	2	2	1	1	4	4
4	4	2	2	2	2	1	1	4	4
3	3	2	2	2	2	4	4	3	3
3	3	2	2	2	2	1	1	4	4
4	4	3	3	2	2	1	1	4	4
4	4	3	3	3	3	1	1	4	4
4	4	2	2	2	2	1	1	4	4
3	3	3	3	2	2	1	1	4	4

For Review Only

4	4	2	2	2	2	1	1	4	4
3	3	2	2	3	3	4	4	3	3
3	3	2	2	2	2	1	1	4	4
4	4	2	2	2	2	4	4	4	4
4	4	2	2	2	2	4	4	3	3
3	3	2	2	2	2	1	1	4	4
3	3	2	2	2	2	1	1	4	4
3	3	2	2	2	2	1	1	4	4
3	3	2	2	2	2	1	1	4	4
4	4	3	3	2	2	1	1	4	4
4	4	2	2	2	2	4	4	4	4
3	3	2	2	2	2	4	4	4	4
3	3	2	2	2	2	4	4	3	3
4	4	2	2	2	2	1	1	4	4
4	4	2	2	2	2	1	1	4	4
4	4	3	3	2	2	4	4	3	3
3	3	2	2	2	2	1	1	4	4
4	4	2	2	2	2	4	4	4	4
4	4	3	3	2	2	1	1	4	4
3	3	2	2	2	2	1	1	4	4
4	4	2	2	2	2	1	1	4	4
3	3	2	2	2	2	1	1	4	4
4	4	2	2	3	3	1	1	4	4
4	4	3	3	2	2	4	4	3	3
3	3	3	3	2	2	1	1	4	4
4	4	2	2	3	3	1	1	4	4
4	4	3	3	2	2	1	1	4	4
3	3	2	2	2	2	1	1	4	4
4	4	2	2	3	3	4	4	3	3
3	3	2	2	2	2	1	1	4	4
4	4	3	3	2	2	1	1	4	4
3	3	2	2	2	2	1	1	4	4
4	4	2	2	2	2	4	4	3	3
3	3	2	2	2	2	1	1	4	4
4	4	2	2	3	3	4	4	3	3
4	4	3	3	2	2	1	1	4	4
4	4	2	2	3	3	1	1	4	4
4	4	2	2	2	2	1	1	4	4
4	4	2	2	2	2	1	1	4	4
4	4	3	3	2	2	1	1	4	4
4	4	2	2	2	2	4	4	3	3
4	4	2	2	2	2	1	1	4	4
4	4	3	3	2	2	4	4	3	3
4	4	2	2	3	3	4	4	3	3
3	3	2	2	2	2	1	1	4	4
4	4	2	2	2	2	1	1	4	4
4	4	2	2	2	2	1	1	4	4
3	3	2	2	2	2	4	4	4	4
4	4	2	2	2	2	1	1	4	4
4	4	3	3	2	2	1	1	4	4
4	4	2	2	3	3	4	4	3	3
3	3	2	2	2	2	1	1	4	4
4	4	2	2	2	2	1	1	4	4
3	3	2	2	2	2	1	1	4	4
4	4	3	3	2	2	1	1	4	4

For Review Only

4	4	2	2	3	3	4	4	3	3
3	3	2	2	3	3	1	1	4	4

For Review Only

SNP215	SNP215	SNP2150	SNP2150	SNP2151	SNP2151	SNP2152	SNP2152	SNP2153	SNP2153
3	3	4	4	1	1	2	2	1	1
3	3	3	3	1	1	2	2	4	4
3	3	4	4	4	4	2	2	1	1
3	3	3	3	4	4	2	2	4	4
1	1	4	4	1	1	3	3	4	4
3	3	4	4	1	1	2	2	1	1
3	3	4	4	1	1	3	3	4	4
3	3	4	4	4	4	2	2	4	4
3	3	4	4	1	1	3	3	4	4
1	1	4	4	4	4	2	2	4	4
1	1	4	4	1	1	3	3	1	1
3	3	4	4	4	4	2	2	1	1
3	3	4	4	1	1	2	2	1	1
3	3	4	4	4	4	3	3	4	4
3	3	4	4	1	1	3	3	1	1
3	3	4	4	4	4	2	2	4	4
1	1	4	4	1	1	3	3	4	4
3	3	4	4	1	1	2	2	1	1
3	3	4	4	1	1	2	2	4	4
1	1	4	4	1	1	2	2	4	4
1	1	4	4	1	1	3	3	4	4
1	1	4	4	1	1	3	3	4	4
3	3	4	4	1	1	3	3	4	4
1	1	4	4	1	1	2	2	4	4
3	3	4	4	4	4	3	3	4	4
3	3	4	4	4	4	3	3	4	4
3	3	3	3	4	4	2	2	4	4
3	3	4	4	4	4	2	2	1	1
1	1	4	4	1	1	2	2	4	4
1	1	4	4	1	1	2	2	4	4
3	3	4	4	4	4	2	2	1	1
1	1	4	4	1	1	3	3	1	1
3	3	4	4	1	1	3	3	4	4
1	1	4	4	1	1	2	2	4	4
3	3	4	4	1	1	3	3	4	4
3	3	4	4	1	1	2	2	1	1
1	1	4	4	1	1	2	2	4	4
1	1	4	4	4	4	2	2	1	1
1	1	4	4	1	1	2	2	4	4
1	1	4	4	1	1	2	2	4	4
1	1	4	4	1	1	2	2	4	4
1	1	4	4	1	1	2	2	4	4
1	1	4	4	4	4	2	2	4	4
1	1	4	4	1	1	3	3	4	4
3	3	4	4	4	4	2	2	4	4
3	3	3	3	4	4	2	2	1	1
3	3	4	4	1	1	2	2	4	4
3	3	4	4	4	4	2	2	1	1
1	1	4	4	1	1	3	3	4	4
3	3	4	4	1	1	3	3	1	1
3	3	4	4	4	4	2	2	4	4
3	3	4	4	4	4	3	3	4	4

For Review Only

1	1	4	4	1	1	3	3	4	4
3	3	4	4	1	1	3	3	4	4
1	1	4	4	4	4	2	2	4	4
1	1	4	4	1	1	3	3	4	4
3	3	4	4	1	1	2	2	4	4
3	3	4	4	1	1	2	2	4	4
3	3	4	4	4	4	2	2	4	4
1	1	4	4	1	1	2	2	4	4
1	1	4	4	1	1	2	2	4	4
3	3	4	4	4	4	2	2	1	1
3	3	3	3	1	1	2	2	1	1
3	3	4	4	1	1	2	2	1	1
1	1	4	4	1	1	3	3	4	4
1	1	4	4	1	1	2	2	4	4
3	3	4	4	4	4	3	3	1	1
1	1	4	4	4	4	2	2	4	4
3	3	4	4	4	4	2	2	4	4
3	3	4	4	1	1	2	2	1	1
1	1	4	4	1	1	3	3	1	1
3	3	4	4	1	1	2	2	4	4
3	3	4	4	1	1	2	2	1	1
1	1	4	4	1	1	3	3	4	4
1	1	4	4	1	1	3	3	4	4
1	1	4	4	4	4	3	3	4	4
3	3	4	4	4	4	2	2	4	4
3	3	3	3	1	1	2	2	4	4
1	1	4	4	1	1	3	3	4	4
1	1	4	4	1	1	2	2	4	4
3	3	4	4	4	4	2	2	4	4
3	3	3	3	1	1	3	3	1	1
3	3	4	4	1	1	2	2	4	4
3	3	4	4	1	1	2	2	4	4
1	1	4	4	1	1	2	2	4	4
1	1	4	4	1	1	3	3	4	4
3	3	4	4	1	1	2	2	1	1
3	3	4	4	4	4	2	2	4	4
3	3	4	4	4	4	2	2	4	4
1	1	4	4	1	1	3	3	4	4
3	3	3	3	4	4	3	3	1	1
3	3	4	4	4	4	2	2	4	4
1	1	4	4	1	1	3	3	4	4
3	3	4	4	4	4	3	3	1	1
3	3	4	4	4	4	2	2	4	4
3	3	3	3	1	1	2	2	1	1
1	1	4	4	1	1	2	2	1	1
3	3	3	3	1	1	3	3	4	4
1	1	4	4	1	1	2	2	4	4
3	3	4	4	4	4	2	2	1	1
3	3	4	4	1	1	2	2	4	4
3	3	4	4	1	1	2	2	1	1
3	3	4	4	1	1	2	2	1	1
1	1	4	4	1	1	2	2	4	4
1	1	4	4	1	1	2	2	4	4
3	3	4	4	4	4	3	3	4	4
3	3	4	4	1	1	2	2	1	1

For Review Only

3	3	4	4	4	4	3	3	4	4
3	3	4	4	4	4	3	3	1	1
3	3	4	4	4	4	2	2	4	4
1	1	4	4	1	1	2	2	4	4
3	3	3	3	4	4	2	2	1	1
3	3	3	3	4	4	3	3	4	4
3	3	4	4	4	4	2	2	4	4
3	3	4	4	4	4	2	2	1	1
1	1	4	4	1	1	2	2	1	1
1	1	4	4	1	1	2	2	1	1
3	3	4	4	1	1	2	2	1	1
3	3	4	4	4	4	2	2	1	1
3	3	4	4	4	4	3	3	4	4
3	3	4	4	4	4	3	3	4	4
3	3	4	4	4	4	2	2	1	1
3	3	3	3	4	4	2	2	4	4
1	1	4	4	1	1	3	3	1	1
3	3	4	4	4	4	3	3	4	4
1	1	4	4	1	1	2	2	4	4
3	3	4	4	4	4	3	3	4	4
3	3	4	4	4	4	3	3	4	4
3	3	3	3	1	1	3	3	1	1
3	3	3	3	4	4	2	2	4	4
1	1	4	4	1	1	2	2	4	4
1	1	4	4	4	4	3	3	4	4
3	3	4	4	1	1	3	3	4	4
3	3	4	4	1	1	2	2	4	4
3	3	4	4	1	1	2	2	4	4
1	1	3	3	4	4	2	2	1	1
3	3	4	4	4	4	3	3	1	1
1	1	4	4	1	1	2	2	4	4
3	3	4	4	4	4	3	3	1	1
1	1	4	4	1	1	3	3	4	4
3	3	4	4	4	4	3	3	1	1
1	1	4	4	1	1	2	2	4	4
3	3	4	4	1	1	2	2	4	4
1	1	4	4	1	1	2	2	4	4
3	3	3	3	4	4	2	2	1	1
3	3	4	4	4	4	2	2	1	1
1	1	4	4	1	1	3	3	4	4
3	3	4	4	1	1	3	3	4	4
1	1	4	4	1	1	2	2	4	4
1	1	4	4	1	1	2	2	1	1
1	1	4	4	1	1	3	3	4	4
3	3	4	4	4	4	3	3	4	4
3	3	4	4	4	4	2	2	4	4
3	3	4	4	4	4	2	2	4	4
3	3	4	4	4	4	2	2	4	4
3	3	4	4	4	4	2	2	4	4
3	3	4	4	4	4	2	2	4	4
3	3	4	4	4	4	2	2	4	4
1	1	4	4	1	1	3	3	4	4
1	1	4	4	4	4	2	2	1	1
3	3	4	4	4	4	2	2	1	1
3	3	4	4	1	1	2	2	4	4

For Review Only

3	3	3	3	1	1	2	2	1	1
3	3	4	4	1	1	2	2	1	1
1	1	4	4	1	1	3	3	4	4
1	1	4	4	1	1	3	3	1	1
3	3	3	3	1	1	2	2	1	1
1	1	4	4	1	1	2	2	4	4
3	3	4	4	1	1	3	3	4	4
3	3	4	4	4	4	2	2	1	1
1	1	4	4	4	4	2	2	1	1
1	1	4	4	1	1	3	3	1	1
3	3	4	4	1	1	2	2	4	4
3	3	4	4	1	1	2	2	4	4
3	3	4	4	1	1	2	2	1	1
3	3	4	4	4	4	3	3	4	4
1	1	4	4	1	1	2	2	4	4
1	1	4	4	1	1	3	3	1	1
1	1	4	4	4	4	2	2	1	1
3	3	3	3	4	4	3	3	4	4
3	3	3	3	1	1	2	2	4	4
3	3	4	4	4	4	2	2	4	4
3	3	3	3	4	4	3	3	1	1
1	1	4	4	1	1	2	2	4	4
1	1	4	4	1	1	3	3	4	4
3	3	4	4	1	1	2	2	1	1
1	1	4	4	1	1	3	3	4	4
1	1	4	4	1	1	2	2	4	4
3	3	4	4	1	1	2	2	4	4
3	3	4	4	1	1	3	3	1	1
3	3	4	4	4	4	2	2	4	4
3	3	4	4	1	1	3	3	4	4
3	3	4	4	1	1	2	2	4	4
3	3	4	4	1	1	3	3	1	1
3	3	4	4	1	1	3	3	4	4
1	1	4	4	1	1	2	2	4	4
1	1	4	4	1	1	3	3	4	4
3	3	4	4	1	1	2	2	1	1
3	3	4	4	1	1	2	2	4	4
1	1	4	4	1	1	2	2	1	1
3	3	4	4	4	4	2	2	1	1
3	3	4	4	4	4	2	2	4	4
3	3	4	4	4	4	2	2	1	1
3	3	4	4	4	4	2	2	4	4
1	1	4	4	1	1	2	2	4	4
3	3	4	4	1	1	3	3	1	1
1	1	4	4	4	4	2	2	1	1
1	1	4	4	1	1	3	3	4	4
1	1	4	4	1	1	3	3	1	1
1	1	4	4	1	1	2	2	4	4
1	1	4	4	1	1	2	2	4	4
3	3	4	4	4	4	3	3	1	1
1	1	3	3	4	4	2	2	4	4
1	1	4	4	1	1	2	2	4	4
1	1	4	4	1	1	2	2	4	4
3	3	3	3	1	1	2	2	1	1
1	1	4	4	1	1	2	2	4	4

For Review Only

1	1	4	4	1	1	2	2	1	1
3	3	4	4	1	1	3	3	1	1
3	3	3	3	4	4	2	2	1	1
3	3	4	4	1	1	3	3	4	4
3	3	4	4	1	1	3	3	4	4
3	3	4	4	4	4	2	2	4	4
1	1	4	4	1	1	2	2	4	4
1	1	4	4	4	4	2	2	4	4
3	3	4	4	1	1	2	2	4	4
1	1	4	4	1	1	2	2	4	4
3	3	4	4	4	4	3	3	1	1
3	3	4	4	1	1	3	3	4	4
3	3	4	4	4	4	3	3	1	1
3	3	3	3	1	1	2	2	4	4
3	3	4	4	4	4	2	2	1	1
1	1	4	4	1	1	3	3	1	1
1	1	4	4	4	4	2	2	4	4
1	1	4	4	1	1	3	3	4	4
1	1	4	4	1	1	2	2	4	4
1	1	4	4	4	4	2	2	1	1
1	1	4	4	1	1	2	2	1	1
3	3	4	4	4	4	2	2	4	4
1	1	4	4	1	1	2	2	1	1
3	3	4	4	1	1	3	3	1	1
1	1	4	4	1	1	2	2	4	4
3	3	4	4	1	1	2	2	1	1
1	1	4	4	1	1	2	2	4	4
3	3	4	4	4	4	2	2	4	4
3	3	4	4	1	1	3	3	4	4
1	1	3	3	4	4	2	2	1	1
3	3	4	4	1	1	2	2	4	4
3	3	4	4	4	4	2	2	4	4
3	3	4	4	1	1	3	3	4	4
3	3	4	4	4	4	2	2	4	4
1	1	4	4	1	1	3	3	4	4
1	1	4	4	1	1	2	2	4	4
1	1	4	4	1	1	2	2	1	1
3	3	4	4	1	1	2	2	1	1
3	3	4	4	1	1	2	2	1	1
1	1	4	4	1	1	2	2	4	4
1	1	4	4	1	1	3	3	4	4
1	1	4	4	1	1	2	2	4	4
3	3	4	4	1	1	3	3	4	4
1	1	4	4	1	1	3	3	4	4
3	3	4	4	4	4	2	2	1	1
3	3	4	4	1	1	2	2	1	1
3	3	4	4	4	4	2	2	1	1
3	3	4	4	4	4	3	3	1	1
3	3	3	3	4	4	2	2	1	1
1	1	4	4	1	1	2	2	4	4
1	1	4	4	1	1	3	3	4	4
1	1	4	4	1	1	2	2	4	4
3	3	4	4	4	4	2	2	1	1
3	3	4	4	4	4	2	2	4	4
1	1	4	4	1	1	2	2	4	4

For Review Only

1	1	4	4	1	1	3	3	1	1
3	3	4	4	4	4	2	2	4	4

For Review Only

SNP2154	SNP2154	SNP2155	SNP2155	SNP2156	SNP2156	SNP2157	SNP2157	SNP2158	SNP2158
3	3	3	3	3	3	2	2	4	4
3	3	3	3	3	3	3	3	4	4
3	3	3	3	2	2	2	2	4	4
3	3	3	3	3	3	3	3	4	4
3	3	3	3	3	3	2	2	4	4
3	3	3	3	3	3	2	2	4	4
3	3	2	2	3	3	2	2	4	4
3	3	2	2	2	2	2	2	4	4
3	3	2	2	3	3	2	2	4	4
3	3	3	3	2	2	2	2	4	4
4	4	2	2	3	3	3	3	4	4
3	3	3	3	2	2	2	2	4	4
3	3	3	3	3	3	3	3	4	4
4	4	3	3	3	3	2	2	4	4
3	3	3	3	3	3	2	2	4	4
3	3	3	3	2	2	3	3	4	4
3	3	3	3	3	3	2	2	4	4
3	3	2	2	3	3	2	2	4	4
3	3	3	3	3	3	2	2	4	4
3	3	3	3	3	3	3	3	1	1
3	3	3	3	3	3	2	2	4	4
3	3	3	3	3	3	2	2	4	4
3	3	2	2	3	3	2	2	4	4
4	4	3	3	3	3	3	3	4	4
3	3	2	2	3	3	2	2	4	4
3	3	3	3	2	2	3	3	4	4
3	3	3	3	2	2	3	3	4	4
3	3	3	3	3	3	3	3	4	4
3	3	2	2	3	3	2	2	4	4
4	4	3	3	3	3	2	2	4	4
3	3	3	3	3	3	2	2	1	1
3	3	2	2	2	2	2	2	1	1
3	3	2	2	3	3	2	2	4	4
4	4	3	3	3	3	2	2	4	4
3	3	3	3	3	3	3	3	4	4
3	3	3	3	3	3	3	3	4	4
3	3	3	3	3	3	2	2	4	4
3	3	3	3	2	2	3	3	4	4
3	3	2	2	3	3	2	2	4	4
3	3	2	2	3	3	2	2	4	4
3	3	3	3	3	3	2	2	4	4
3	3	2	2	3	3	2	2	4	4
3	3	2	2	2	2	2	2	4	4
3	3	2	2	3	3	2	2	4	4
3	3	2	2	2	2	2	2	4	4
3	3	3	3	2	2	3	3	4	4
3	3	3	3	3	3	2	2	4	4
3	3	3	3	2	2	3	3	4	4
3	3	3	3	3	3	2	2	4	4
3	3	3	3	3	3	2	2	4	4
3	3	3	3	2	2	3	3	4	4
3	3	3	3	2	2	3	3	4	4

For Review Only

3	3	2	2	3	3	2	2	4	4
3	3	2	2	3	3	2	2	4	4
3	3	3	3	3	3	3	3	4	4
3	3	2	2	3	3	2	2	4	4
3	3	3	3	3	3	2	2	4	4
3	3	3	3	3	3	3	3	4	4
3	3	3	3	2	2	3	3	4	4
4	4	3	3	3	3	3	3	4	4
3	3	3	3	3	3	2	2	4	4
3	3	3	3	3	3	3	3	4	4
3	3	3	3	3	3	3	3	4	4
3	3	3	3	3	3	2	2	4	4
3	3	2	2	3	3	2	2	4	4
3	3	2	2	3	3	2	2	4	4
3	3	3	3	3	3	2	2	4	4
3	3	2	2	2	2	2	2	4	4
4	4	2	2	3	3	2	2	4	4
3	3	3	3	3	3	3	3	4	4
3	3	3	3	3	3	2	2	1	1
3	3	2	2	3	3	2	2	4	4
3	3	3	3	3	3	2	2	4	4
3	3	2	2	3	3	2	2	1	1
3	3	2	2	3	3	2	2	4	4
3	3	3	3	3	3	2	2	4	4
3	3	2	2	2	2	2	2	4	4
3	3	3	3	3	3	3	3	4	4
3	3	2	2	2	2	2	2	4	4
3	3	3	3	3	3	3	3	4	4
3	3	3	3	2	2	3	3	4	4
3	3	3	3	3	3	3	3	4	4
3	3	3	3	3	3	2	2	4	4
3	3	2	2	3	3	3	3	4	4
4	4	2	2	3	3	3	3	4	4
3	3	2	2	3	3	2	2	4	4
3	3	3	3	3	3	2	2	1	1
3	3	2	2	2	2	2	2	4	4
3	3	3	3	2	2	2	2	4	4
3	3	3	3	3	3	2	2	4	4
4	4	2	2	2	2	3	3	4	4
4	4	2	2	2	2	2	2	4	4
3	3	2	2	3	3	2	2	4	4
3	3	3	3	2	2	2	2	4	4
3	3	3	3	2	2	2	2	4	4
3	3	3	3	3	3	3	3	4	4
3	3	2	2	3	3	2	2	4	4
3	3	3	3	3	3	3	3	1	1
3	3	3	3	3	3	2	2	4	4
4	4	3	3	3	3	3	3	4	4
4	4	3	3	3	3	3	3	4	4
3	3	2	2	3	3	2	2	1	1
3	3	3	3	3	3	3	3	4	4
3	3	2	2	3	3	3	3	4	4
3	3	3	3	3	3	3	3	4	4
3	3	3	3	3	3	3	3	4	4
3	3	2	2	3	3	2	2	4	4

For Review Only

3	3	3	3	3	3	3	3	4	4
3	3	3	3	3	3	3	3	4	4
3	3	2	2	2	2	3	3	4	4
3	3	3	3	3	3	3	3	4	4
3	3	3	3	2	2	3	3	4	4
3	3	2	2	3	3	3	3	4	4
3	3	3	3	2	2	2	2	4	4
3	3	3	3	3	3	3	3	4	4
3	3	3	3	3	3	3	3	4	4
3	3	3	3	3	3	3	3	4	4
3	3	3	3	3	3	2	2	4	4
3	3	3	3	3	3	3	3	4	4
3	3	3	3	2	2	2	2	4	4
3	3	3	3	3	3	3	3	4	4
3	3	2	2	3	3	2	2	4	4
3	3	3	3	3	3	3	3	4	4
3	3	3	3	3	3	2	2	4	4
3	3	3	3	3	3	3	3	4	4
4	4	3	3	3	3	2	2	4	4
3	3	3	3	3	3	2	2	4	4
3	3	3	3	3	3	3	3	4	4
3	3	3	3	3	3	3	3	4	4
3	3	3	3	3	3	3	3	4	4
4	4	2	2	3	3	3	3	4	4
3	3	3	3	3	3	3	3	1	1
3	3	2	2	3	3	2	2	4	4
3	3	2	2	3	3	2	2	1	1
3	3	2	2	3	3	2	2	4	4
3	3	3	3	2	2	3	3	4	4
3	3	3	3	3	3	3	3	1	1
3	3	2	2	3	3	2	2	4	4
3	3	3	3	3	3	3	3	4	4
3	3	2	2	3	3	2	2	4	4
3	3	2	2	3	3	3	3	4	4
4	4	3	3	3	3	2	2	4	4
3	3	3	3	3	3	3	3	4	4
4	4	3	3	3	3	2	2	4	4
3	3	3	3	2	2	3	3	4	4
3	3	3	3	2	2	3	3	4	4
3	3	2	2	3	3	3	3	4	4
3	3	3	3	3	3	2	2	4	4
4	4	3	3	3	3	2	2	4	4
3	3	2	2	3	3	3	3	4	4
3	3	3	3	3	3	2	2	4	4
4	4	3	3	2	2	3	3	4	4
3	3	3	3	2	2	2	2	4	4
3	3	3	3	2	2	3	3	4	4
3	3	3	3	2	2	3	3	4	4
3	3	3	3	3	3	3	3	4	4
3	3	3	3	2	2	3	3	4	4
3	3	3	3	2	2	3	3	4	4
3	3	2	2	3	3	3	3	4	4
3	3	3	3	3	3	2	2	4	4
3	3	3	3	2	2	2	2	4	4
3	3	3	3	3	3	3	3	4	4

For Review Only

3	3	3	3	3	3	3	3	4	4
3	3	2	2	3	3	2	2	4	4
4	4	2	2	3	3	3	3	1	1
3	3	3	3	3	3	3	3	4	4
3	3	2	2	3	3	3	3	4	4
3	3	3	3	3	3	2	2	4	4
3	3	2	2	3	3	3	3	4	4
3	3	3	3	3	3	3	3	4	4
3	3	3	3	3	3	2	2	4	4
3	3	3	3	3	3	3	3	4	4
3	3	3	3	3	3	3	3	4	4
3	3	3	3	3	3	3	3	4	4
3	3	3	3	3	3	2	2	4	4
3	3	3	3	3	3	2	2	4	4
3	3	3	3	3	3	2	2	4	4
4	4	2	2	3	3	3	3	4	4
3	3	3	3	3	3	2	2	4	4
3	3	3	3	3	3	3	3	4	4
3	3	2	2	3	3	3	3	4	4
3	3	3	3	2	2	2	2	4	4
3	3	3	3	3	3	3	3	4	4
3	3	3	3	3	3	3	3	4	4
3	3	2	2	3	3	3	3	1	1
3	3	3	3	3	3	2	2	4	4
3	3	2	2	3	3	3	3	4	4
4	4	3	3	3	3	2	2	4	4
3	3	3	3	3	3	2	2	4	4
3	3	3	3	3	3	2	2	4	4
3	3	3	3	3	3	2	2	4	4
3	3	3	3	3	3	2	2	4	4
3	3	3	3	3	3	2	2	4	4
3	3	3	3	3	3	2	2	1	1
3	3	3	3	3	3	2	2	4	4
3	3	3	3	3	3	2	2	4	4
3	3	3	3	3	3	2	2	4	4
3	3	3	3	3	3	2	2	4	4
3	3	3	3	3	3	2	2	4	4
3	3	3	3	3	3	2	2	4	4
3	3	3	3	2	2	3	3	4	4
3	3	3	3	2	2	2	2	4	4
3	3	3	3	2	2	2	2	4	4
3	3	3	3	2	2	3	3	4	4
4	4	3	3	3	3	3	3	4	4
3	3	2	2	3	3	3	3	1	1
3	3	3	3	3	3	3	3	4	4
3	3	3	3	3	3	2	2	1	1
4	4	2	2	3	3	3	3	4	4
3	3	2	2	3	3	3	3	4	4
3	3	3	3	3	3	2	2	4	4
4	4	3	3	2	2	2	2	4	4
3	3	3	3	2	2	3	3	4	4
3	3	3	3	3	3	3	3	4	4
3	3	2	2	3	3	2	2	1	1
3	3	3	3	3	3	3	3	4	4
3	3	2	2	3	3	2	2	4	4

For Review Only

3	3	3	3	3	3	2	2	4	4
3	3	3	3	3	3	2	2	1	1
3	3	3	3	2	2	3	3	4	4
3	3	3	3	3	3	3	3	1	1
3	3	3	3	3	3	3	3	4	4
3	3	3	3	2	2	2	2	4	4
3	3	2	2	3	3	3	3	4	4
4	4	3	3	2	2	2	2	4	4
4	4	3	3	3	3	3	3	4	4
3	3	2	2	3	3	3	3	4	4
3	3	3	3	3	3	3	3	4	4
3	3	3	3	3	3	2	2	4	4
4	4	2	2	2	2	2	2	4	4
3	3	2	2	3	3	3	3	4	4
4	4	3	3	2	2	2	2	4	4
4	4	2	2	3	3	3	3	4	4
3	3	2	2	2	2	2	2	4	4
3	3	2	2	3	3	2	2	4	4
3	3	2	2	3	3	3	3	4	4
3	3	3	3	2	2	3	3	4	4
3	3	3	3	3	3	2	2	4	4
3	3	3	3	2	2	3	3	4	4
3	3	2	2	3	3	2	2	4	4
3	3	2	2	3	3	2	2	4	4
4	4	3	3	3	3	3	3	4	4
3	3	2	2	3	3	3	3	4	4
4	4	2	2	3	3	3	3	4	4
3	3	3	3	2	2	2	2	4	4
3	3	3	3	3	3	3	3	4	4
3	3	3	3	2	2	3	3	4	4
3	3	2	2	3	3	2	2	4	4
3	3	3	3	2	2	3	3	4	4
4	4	3	3	3	3	2	2	4	4
3	3	3	3	2	2	3	3	4	4
3	3	2	2	3	3	2	2	1	1
3	3	2	2	3	3	2	2	4	4
3	3	3	3	3	3	2	2	4	4
3	3	3	3	3	3	2	2	4	4
3	3	3	3	3	3	3	3	4	4
3	3	2	2	3	3	3	3	4	4
3	3	3	3	3	3	2	2	4	4
3	3	2	2	3	3	2	2	4	4
3	3	2	2	3	3	2	2	4	4
3	3	2	2	3	3	2	2	4	4
3	3	3	3	2	2	2	2	4	4
4	4	2	2	3	3	2	2	4	4
3	3	3	3	2	2	2	2	4	4
3	3	2	2	2	2	2	2	4	4
3	3	3	3	2	2	3	3	4	4
4	4	2	2	3	3	3	3	4	4
3	3	3	3	3	3	2	2	4	4
3	3	2	2	3	3	2	2	4	4
3	3	3	3	2	2	2	2	4	4
3	3	2	2	2	2	2	2	4	4
3	3	2	2	3	3	2	2	4	4

For Review Only

4	4	2	2	3	3	2	2	4	4
3	3	3	3	2	2	2	2	4	4

For Review Only

SNP2159	SNP2159	SNP216	SNP216	SNP2160	SNP2160	SNP2161	SNP2161	SNP2162	SNP2162
2	2	1	1	3	3	1	1	4	4
2	2	1	1	3	3	1	1	1	1
2	2	3	3	3	3	1	1	4	4
2	2	3	3	3	3	2	2	4	4
2	2	1	1	3	3	1	1	4	4
2	2	1	1	3	3	1	1	4	4
2	2	1	1	3	3	2	2	4	4
2	2	1	1	3	3	2	2	4	4
2	2	1	1	3	3	1	1	4	4
2	2	1	1	3	3	1	1	4	4
1	1	3	3	1	1	1	1	4	4
2	2	3	3	3	3	1	1	4	4
2	2	1	1	3	3	1	1	4	4
1	1	1	1	1	1	1	1	4	4
2	2	1	1	3	3	1	1	4	4
2	2	1	1	3	3	1	1	4	4
2	2	3	3	3	3	1	1	4	4
2	2	1	1	1	1	2	2	4	4
2	2	3	3	1	1	2	2	4	4
2	2	1	1	3	3	1	1	4	4
2	2	1	1	3	3	1	1	4	4
2	2	1	1	3	3	1	1	4	4
2	2	1	1	3	3	1	1	4	4
2	2	1	1	1	1	1	1	4	4
2	2	1	1	3	3	1	1	4	4
2	2	3	3	3	3	1	1	4	4
2	2	3	3	3	3	1	1	4	4
2	2	3	3	3	3	1	1	4	4
2	2	1	1	3	3	1	1	4	4
1	1	1	1	1	1	1	1	4	4
2	2	3	3	3	3	1	1	4	4
2	2	1	1	3	3	1	1	4	4
2	2	1	1	3	3	1	1	4	4
1	1	1	1	1	1	1	1	4	4
2	2	3	3	3	3	1	1	4	4
2	2	3	3	3	3	1	1	4	4
2	2	1	1	3	3	1	1	4	4
2	2	3	3	3	3	1	1	4	4
2	2	1	1	3	3	1	1	4	4
2	2	1	1	3	3	1	1	4	4
1	1	1	1	1	1	1	1	4	4
2	2	1	1	3	3	1	1	4	4
2	2	1	1	3	3	2	2	4	4
2	2	1	1	3	3	1	1	4	4
2	2	1	1	3	3	1	1	4	4
2	2	3	3	3	3	1	1	4	4
2	2	3	3	3	3	1	1	4	4
2	2	3	3	3	3	1	1	4	4
2	2	1	1	3	3	1	1	4	4
2	2	3	3	3	3	1	1	4	4
2	2	3	3	3	3	1	1	4	4
2	2	1	1	3	3	1	1	1	1

For Review Only

2	2	1	1	3	3	1	1	4	4
2	2	1	1	3	3	1	1	4	4
2	2	3	3	3	3	1	1	4	4
1	1	3	3	1	1	1	1	4	4
2	2	3	3	1	1	2	2	4	4
2	2	3	3	3	3	1	1	1	1
2	2	3	3	3	3	1	1	4	4
1	1	1	1	1	1	1	1	4	4
2	2	1	1	3	3	1	1	4	4
2	2	3	3	3	3	1	1	4	4
2	2	3	3	3	3	1	1	1	1
2	2	3	3	3	3	1	1	1	1
2	2	1	1	3	3	1	1	4	4
2	2	1	1	3	3	1	1	4	4
2	2	3	3	3	3	1	1	4	4
2	2	1	1	3	3	1	1	4	4
1	1	3	3	1	1	1	1	4	4
2	2	3	3	3	3	1	1	4	4
2	2	1	1	3	3	1	1	4	4
2	2	1	1	3	3	1	1	4	4
2	2	3	3	3	3	1	1	1	1
2	2	3	3	3	3	1	1	4	4
2	2	1	1	3	3	1	1	4	4
2	2	1	1	3	3	1	1	4	4
2	2	1	1	3	3	1	1	4	4
2	2	3	3	3	3	2	2	1	1
2	2	1	1	3	3	1	1	4	4
2	2	1	1	3	3	1	1	4	4
2	2	3	3	3	3	1	1	4	4
2	2	3	3	3	3	2	2	4	4
2	2	3	3	3	3	1	1	4	4
2	2	3	3	3	3	1	1	4	4
1	1	1	1	1	1	1	1	4	4
2	2	1	1	3	3	1	1	4	4
2	2	1	1	3	3	1	1	4	4
2	2	1	1	3	3	1	1	4	4
2	2	3	3	3	3	2	2	4	4
2	2	1	1	3	3	1	1	4	4
1	1	3	3	1	1	1	1	4	4
1	1	1	1	1	1	1	1	4	4
2	2	3	3	3	3	2	2	4	4
2	2	3	3	3	3	1	1	4	4
2	2	1	1	3	3	1	1	4	4
2	2	3	3	3	3	1	1	1	1
2	2	3	3	3	3	2	2	4	4
2	2	3	3	3	3	2	2	4	4
1	1	1	1	1	1	1	1	4	4
2	2	3	3	1	1	1	1	4	4
2	2	3	3	1	1	1	1	4	4
2	2	3	3	3	3	2	2	4	4
2	2	3	3	3	3	1	1	1	1
2	2	1	1	3	3	1	1	4	4
2	2	1	1	3	3	1	1	4	4
2	2	3	3	3	3	1	1	4	4
2	2	1	1	3	3	2	2	4	4

For Review Only

2	2	3	3	3	3	1	1	4	4
2	2	1	1	3	3	1	1	4	4
2	2	3	3	3	3	1	1	4	4
2	2	1	1	3	3	1	1	4	4
2	2	3	3	3	3	1	1	1	1
2	2	3	3	3	3	1	1	4	4
2	2	3	3	3	3	1	1	4	4
2	2	3	3	3	3	1	1	4	4
2	2	3	3	3	3	1	1	4	4
2	2	3	3	3	3	2	2	4	4
2	2	1	1	3	3	1	1	4	4
2	2	3	3	3	3	1	1	4	4
2	2	1	1	3	3	1	1	4	4
2	2	3	3	3	3	2	2	1	1
2	2	3	3	3	3	1	1	4	4
2	2	3	3	3	3	1	1	4	4
2	2	1	1	3	3	1	1	4	4
2	2	3	3	3	3	2	2	4	4
1	1	1	1	1	1	1	1	4	4
2	2	1	1	3	3	1	1	4	4
2	2	3	3	3	3	1	1	4	4
2	2	3	3	3	3	2	2	4	4
2	2	1	1	3	3	2	2	4	4
1	1	1	1	3	3	1	1	4	4
2	2	3	3	3	3	2	2	4	4
2	2	3	3	3	3	1	1	4	4
2	2	3	3	3	3	2	2	4	4
2	2	3	3	3	3	1	1	4	4
2	2	3	3	3	3	1	1	4	4
2	2	3	3	3	3	1	1	4	4
2	2	1	1	3	3	1	1	4	4
2	2	3	3	3	3	1	1	4	4
2	2	3	3	3	3	2	2	4	4
2	2	3	3	3	3	1	1	4	4
1	1	1	1	1	1	1	1	4	4
2	2	1	1	3	3	2	2	4	4
1	1	1	1	1	1	1	1	4	4
2	2	1	1	3	3	2	2	4	4
2	2	3	3	3	3	1	1	4	4
1	1	1	1	1	1	1	1	4	4
2	2	1	1	3	3	1	1	4	4
1	1	1	1	1	1	1	1	4	4
2	2	3	3	3	3	1	1	4	4
1	1	3	3	3	3	1	1	4	4
2	2	1	1	1	1	1	1	4	4
2	2	3	3	3	3	1	1	4	4
2	2	3	3	3	3	1	1	4	4
2	2	3	3	3	3	1	1	4	4
2	2	1	1	3	3	1	1	4	4
2	2	3	3	3	3	1	1	4	4
2	2	3	3	3	3	1	1	4	4
2	2	1	1	3	3	1	1	4	4
2	2	3	3	3	3	1	1	4	4
2	2	3	3	3	3	1	1	1	1
2	2	3	3	3	3	1	1	4	4

For Review Only

2	2	3	3	3	3	1	1	4	4
2	2	3	3	3	3	1	1	4	4
1	1	1	1	1	1	2	2	4	4
2	2	3	3	3	3	1	1	4	4
2	2	1	1	3	3	2	2	1	1
2	2	1	1	3	3	1	1	4	4
2	2	1	1	3	3	1	1	4	4
2	2	3	3	3	3	1	1	4	4
2	2	1	1	3	3	1	1	4	4
2	2	3	3	3	3	1	1	4	4
2	2	3	3	3	3	1	1	4	4
2	2	1	1	3	3	1	1	4	4
2	2	3	3	3	3	2	2	4	4
2	2	3	3	3	3	2	2	4	4
2	2	1	1	3	3	1	1	4	4
1	1	3	3	1	1	1	1	4	4
2	2	1	1	3	3	1	1	4	4
2	2	3	3	3	3	2	2	1	1
2	2	1	1	3	3	1	1	4	4
2	2	3	3	3	3	1	1	4	4
2	2	3	3	3	3	1	1	4	4
2	2	1	1	3	3	1	1	4	4
2	2	3	3	3	3	1	1	4	4
2	2	3	3	3	3	1	1	1	1
2	2	1	1	3	3	1	1	4	4
1	1	1	1	1	1	1	1	4	4
2	2	3	3	3	3	1	1	4	4
2	2	3	3	3	3	2	2	4	4
2	2	3	3	3	3	1	1	4	4
2	2	3	3	3	3	1	1	4	4
2	2	1	1	3	3	1	1	4	4
2	2	1	1	3	3	2	2	4	4
2	2	3	3	3	3	2	2	4	4
2	2	1	1	3	3	1	1	4	4
2	2	3	3	1	1	2	2	4	4
2	2	3	3	3	3	1	1	1	1
2	2	3	3	3	3	1	1	4	4
2	2	1	1	3	3	1	1	4	4
2	2	3	3	3	3	1	1	4	4
2	2	3	3	3	3	2	2	4	4
2	2	3	3	3	3	1	1	4	4
2	2	3	3	3	3	1	1	4	4
1	1	1	1	1	1	1	1	4	4
2	2	3	3	3	3	1	1	4	4
2	2	3	3	3	3	1	1	4	4
2	2	3	3	3	3	1	1	4	4
1	1	3	3	1	1	1	1	4	4
2	2	1	1	3	3	1	1	4	4
2	2	1	1	3	3	1	1	4	4
1	1	3	3	1	1	1	1	4	4
2	2	3	3	3	3	1	1	4	4
2	2	1	1	3	3	1	1	4	4
2	2	1	1	3	3	1	1	4	4
2	2	3	3	3	3	1	1	1	1
2	2	1	1	3	3	1	1	4	4

For Review Only

2	2	3	3	3	3	1	1	4	4
2	2	1	1	3	3	1	1	4	4
2	2	3	3	3	3	1	1	4	4
2	2	3	3	3	3	1	1	4	4
2	2	3	3	3	3	2	2	4	4
2	2	3	3	3	3	1	1	4	4
2	2	1	1	3	3	1	1	4	4
1	1	1	1	1	1	1	1	4	4
1	1	1	1	1	1	1	1	4	4
2	2	1	1	3	3	1	1	4	4
2	2	3	3	3	3	1	1	4	4
2	2	1	1	3	3	1	1	4	4
1	1	3	3	1	1	1	1	4	4
2	2	3	3	3	3	1	1	4	4
1	1	3	3	1	1	1	1	4	4
1	1	3	3	1	1	1	1	4	4
2	2	3	3	3	3	1	1	4	4
2	2	1	1	3	3	1	1	4	4
2	2	1	1	3	3	1	1	4	4
2	2	1	1	3	3	1	1	4	4
2	2	1	1	3	3	1	1	4	4
2	2	1	1	3	3	1	1	4	4
2	2	3	3	3	3	1	1	4	4
2	2	1	1	3	3	1	1	4	4
1	1	1	1	1	1	1	1	4	4
2	2	1	1	3	3	1	1	4	4
1	1	1	1	1	1	1	1	4	4
2	2	3	3	3	3	1	1	4	4
2	2	1	1	3	3	2	2	4	4
2	2	3	3	3	3	1	1	4	4
2	2	1	1	3	3	1	1	4	4
2	2	1	1	3	3	1	1	4	4
1	1	1	1	1	1	1	1	4	4
2	2	3	3	3	3	1	1	4	4
2	2	3	3	3	3	1	1	4	4
2	2	1	1	3	3	1	1	4	4
2	2	3	3	3	3	1	1	4	4
2	2	3	3	3	3	1	1	1	1
2	2	3	3	3	3	1	1	4	4
2	2	1	1	3	3	1	1	4	4
2	2	3	3	1	1	1	1	4	4
2	2	1	1	3	3	1	1	4	4
2	2	1	1	3	3	1	1	4	4
2	2	3	3	3	3	1	1	4	4
2	2	3	3	3	3	1	1	1	1
1	1	3	3	1	1	1	1	4	4
2	2	3	3	3	3	1	1	4	4
2	2	1	1	3	3	2	2	4	4
2	2	3	3	3	3	2	2	4	4
1	1	1	1	1	1	1	1	4	4
2	2	3	3	3	3	1	1	4	4
2	2	1	1	3	3	1	1	4	4
2	2	3	3	3	3	1	1	4	4
2	2	3	3	3	3	1	1	4	4
2	2	1	1	3	3	1	1	4	4

For Review Only

1	1	3	3	1	1	1	1	4	4
2	2	3	3	3	3	1	1	4	4

For Review Only

SNP2163	SNP2163	SNP2164	SNP2164	SNP2165	SNP2165	SNP2166	SNP2166	SNP2167	SNP2167
1	1	4	4	1	1	2	2	3	3
1	1	1	1	1	1	2	2	3	3
1	1	4	4	1	1	2	2	3	3
1	1	1	1	1	1	2	2	3	3
1	1	4	4	1	1	2	2	3	3
1	1	4	4	2	2	2	2	3	3
1	1	4	4	1	1	2	2	3	3
1	1	4	4	1	1	3	3	3	3
1	1	4	4	1	1	2	2	3	3
1	1	4	4	1	1	3	3	3	3
1	1	4	4	1	1	2	2	3	3
1	1	4	4	1	1	2	2	3	3
1	1	4	4	1	1	2	2	3	3
1	1	4	4	1	1	2	2	3	3
1	1	4	4	1	1	2	2	3	3
1	1	4	4	1	1	2	2	3	3
1	1	4	4	1	1	2	2	3	3
1	1	4	4	1	1	2	2	3	3
4	4	4	4	1	1	2	2	3	3
1	1	1	1	1	1	2	2	3	3
1	1	4	4	1	1	2	2	3	3
1	1	4	4	1	1	2	2	3	3
1	1	4	4	1	1	3	3	3	3
1	1	4	4	1	1	2	2	3	3
4	4	4	4	1	1	2	2	3	3
1	1	4	4	2	2	2	2	3	3
4	4	4	4	1	1	2	2	3	3
1	1	4	4	1	1	2	2	3	3
1	1	4	4	1	1	2	2	3	3
1	1	4	4	1	1	2	2	3	3
1	1	4	4	1	1	2	2	1	1
1	1	4	4	2	2	2	2	1	1
1	1	4	4	1	1	2	2	3	3
1	1	4	4	1	1	2	2	3	3
1	1	4	4	1	1	2	2	3	3
1	1	4	4	1	1	3	3	3	3
1	1	4	4	1	1	2	2	3	3
1	1	4	4	1	1	2	2	3	3
1	1	4	4	1	1	3	3	3	3
1	1	4	4	1	1	3	3	3	3
1	1	4	4	1	1	2	2	3	3
1	1	4	4	1	1	3	3	3	3
1	1	4	4	1	1	3	3	3	3
1	1	4	4	1	1	2	2	3	3
1	1	4	4	1	1	3	3	3	3
1	1	4	4	1	1	2	2	1	1
1	1	4	4	1	1	2	2	3	3
1	1	4	4	1	1	3	3	3	3
1	1	4	4	1	1	2	2	3	3
1	1	4	4	1	1	2	2	3	3
1	1	4	4	1	1	2	2	3	3
1	1	4	4	1	1	2	2	3	3

1	1	4	4	1	1	2	2	3	3
1	1	4	4	1	1	2	2	3	3
4	4	1	1	1	1	2	2	3	3
1	1	4	4	1	1	2	2	3	3
4	4	4	4	1	1	2	2	3	3
1	1	4	4	1	1	2	2	3	3
1	1	4	4	1	1	2	2	1	1
1	1	4	4	1	1	2	2	3	3
1	1	4	4	1	1	2	2	3	3
1	1	4	4	1	1	2	2	3	3
1	1	1	1	1	1	2	2	3	3
1	1	4	4	1	1	2	2	3	3
1	1	4	4	1	1	2	2	3	3
1	1	4	4	1	1	2	2	3	3
1	1	4	4	2	2	2	2	3	3
1	1	4	4	1	1	3	3	3	3
1	1	4	4	1	1	2	2	3	3
1	1	1	1	1	1	2	2	3	3
1	1	4	4	1	1	2	2	1	1
1	1	4	4	1	1	3	3	3	3
1	1	4	4	1	1	2	2	3	3
1	1	4	4	2	2	3	3	1	1
1	1	4	4	1	1	2	2	3	3
1	1	1	1	2	2	2	2	3	3
1	1	4	4	1	1	3	3	3	3
1	1	4	4	1	1	2	2	3	3
1	1	4	4	2	2	2	2	3	3
1	1	4	4	1	1	2	2	3	3
1	1	4	4	1	1	2	2	3	3
1	1	1	1	1	1	2	2	3	3
1	1	4	4	1	1	2	2	3	3
1	1	4	4	1	1	3	3	3	3
1	1	4	4	1	1	2	2	3	3
1	1	4	4	1	1	2	2	3	3
1	1	4	4	1	1	2	2	3	3
1	1	4	4	1	1	3	3	3	3
1	1	4	4	1	1	3	3	3	3
1	1	4	4	1	1	2	2	3	3
1	1	4	4	1	1	2	2	3	3
1	1	4	4	1	1	3	3	3	3
1	1	4	4	1	1	2	2	3	3
1	1	4	4	1	1	2	2	3	3
1	1	4	4	1	1	2	2	3	3
1	1	1	1	1	1	2	2	3	3
1	1	1	1	1	1	3	3	3	3
1	1	1	1	2	2	2	2	3	3
1	1	4	4	1	1	2	2	3	3
1	1	4	4	1	1	2	2	1	1
1	1	4	4	1	1	3	3	3	3
1	1	4	4	1	1	2	2	3	3
1	1	4	4	1	1	2	2	3	3
1	1	4	4	1	1	3	3	3	3
1	1	4	4	1	1	2	2	3	3
1	1	4	4	1	1	2	2	3	3
1	1	4	4	1	1	2	2	3	3

For Review Only

1	1	4	4	1	1	3	3	3	3
1	1	4	4	2	2	2	2	3	3
1	1	4	4	1	1	2	2	3	3
1	1	4	4	1	1	2	2	3	3
1	1	1	1	1	1	2	2	3	3
1	1	4	4	2	2	2	2	3	3
1	1	4	4	1	1	2	2	3	3
4	4	4	4	2	2	3	3	3	3
1	1	4	4	1	1	3	3	3	3
1	1	4	4	1	1	2	2	3	3
1	1	4	4	1	1	2	2	3	3
1	1	4	4	1	1	2	2	3	3
1	1	4	4	1	1	2	2	3	3
1	1	4	4	1	1	2	2	3	3
1	1	4	4	1	1	2	2	3	3
1	1	4	4	1	1	2	2	3	3
1	1	4	4	2	2	2	2	3	3
1	1	4	4	1	1	2	2	3	3
1	1	4	4	1	1	2	2	3	3
1	1	4	4	1	1	2	2	3	3
1	1	4	4	1	1	2	2	3	3
1	1	1	1	1	1	2	2	3	3
1	1	1	1	1	1	2	2	3	3
1	1	4	4	1	1	2	2	3	3
1	1	4	4	2	2	2	2	3	3
1	1	4	4	1	1	3	3	3	3
1	1	4	4	1	1	2	2	3	3
1	1	4	4	1	1	2	2	3	3
1	1	1	1	1	1	2	2	3	3
1	1	1	1	1	1	3	3	3	3
1	1	4	4	1	1	2	2	3	3
1	1	4	4	1	1	2	2	3	3
1	1	4	4	1	1	2	2	3	3
1	1	4	4	1	1	2	2	3	3
1	1	4	4	1	1	2	2	3	3
1	1	4	4	1	1	3	3	3	3
1	1	4	4	1	1	2	2	3	3
1	1	4	4	1	1	2	2	3	3
1	1	4	4	1	1	2	2	3	3
1	1	4	4	1	1	2	2	3	3
1	1	4	4	1	1	2	2	3	3
1	1	4	4	1	1	2	2	3	3
1	1	1	1	1	1	2	2	3	3
1	1	4	4	1	1	2	2	3	3
1	1	4	4	1	1	2	2	3	3
1	1	4	4	1	1	2	2	3	3
1	1	4	4	1	1	2	2	3	3
1	1	4	4	1	1	2	2	3	3
1	1	4	4	1	1	2	2	3	3
1	1	4	4	1	1	2	2	3	3
1	1	4	4	1	1	2	2	3	3
1	1	4	4	1	1	2	2	3	3
1	1	4	4	1	1	2	2	3	3
1	1	4	4	1	1	2	2	3	3
1	1	4	4	1	1	2	2	3	3

1	1	1	1	1	1	2	2	3	3
1	1	4	4	1	1	3	3	3	3
1	1	4	4	1	1	2	2	1	1
1	1	4	4	2	2	2	2	3	3
1	1	1	1	1	1	3	3	3	3
1	1	4	4	1	1	2	2	3	3
1	1	4	4	1	1	2	2	3	3
1	1	4	4	1	1	2	2	3	3
1	1	4	4	1	1	2	2	3	3
1	1	1	1	1	1	2	2	3	3
1	1	4	4	1	1	2	2	3	3
1	1	4	4	1	1	3	3	3	3
1	1	4	4	1	1	2	2	1	1
1	1	4	4	1	1	2	2	3	3
1	1	4	4	1	1	2	2	3	3
1	1	4	4	1	1	2	2	3	3
1	1	4	4	1	1	2	2	3	3
1	1	4	4	1	1	2	2	3	3
1	1	4	4	1	1	3	3	3	3
1	1	4	4	1	1	2	2	3	3
1	1	4	4	1	1	2	2	3	3
1	1	4	4	1	1	2	2	3	3
1	1	4	4	2	2	3	3	1	1
1	1	4	4	1	1	2	2	3	3
1	1	1	1	1	1	2	2	3	3
1	1	4	4	1	1	2	2	3	3
1	1	4	4	1	1	2	2	3	3
1	1	4	4	1	1	2	2	3	3
1	1	4	4	1	1	3	3	3	3
1	1	4	4	1	1	2	2	3	3
1	1	4	4	1	1	2	2	3	3
1	1	4	4	1	1	2	2	3	3
1	1	4	4	1	1	2	2	3	3
1	1	4	4	1	1	2	2	3	3
1	1	4	4	1	1	2	2	3	3
1	1	4	4	1	1	2	2	3	3
1	1	4	4	1	1	3	3	3	3
1	1	4	4	1	1	2	2	3	3
1	1	4	4	1	1	2	2	3	3
1	1	4	4	1	1	2	2	3	3
1	1	4	4	1	1	2	2	3	3
1	1	4	4	1	1	2	2	3	3
1	1	4	4	1	1	3	3	3	3
1	1	4	4	2	2	2	2	3	3
1	1	4	4	1	1	2	2	3	3
1	1	4	4	2	2	2	2	3	3
1	1	4	4	1	1	2	2	3	3
1	1	4	4	1	1	2	2	3	3
1	1	4	4	1	1	2	2	3	3
1	1	4	4	1	1	2	2	3	3
4	4	1	1	1	1	2	2	3	3
1	1	4	4	1	1	2	2	3	3
1	1	4	4	1	1	3	3	1	1
1	1	1	1	1	1	2	2	3	3
1	1	4	4	1	1	3	3	3	3

For Review Only

1	1	4	4	1	1	3	3	3	3
1	1	4	4	1	1	2	2	3	3
4	4	1	1	1	1	2	2	3	3
1	1	4	4	1	1	2	2	1	1
1	1	1	1	2	2	2	2	3	3
1	1	1	1	1	1	2	2	3	3
1	1	4	4	1	1	3	3	3	3
1	1	4	4	1	1	3	3	3	3
1	1	4	4	1	1	3	3	3	3
1	1	4	4	1	1	2	2	3	3
1	1	4	4	1	1	2	2	3	3
1	1	4	4	1	1	2	2	3	3
1	1	4	4	1	1	2	2	3	3
1	1	4	4	1	1	2	2	3	3
1	1	4	4	1	1	2	2	3	3
1	1	4	4	1	1	2	2	3	3
1	1	4	4	1	1	2	2	3	3
1	1	4	4	1	1	2	2	3	3
1	1	4	4	1	1	3	3	3	3
1	1	4	4	1	1	2	2	3	3
1	1	4	4	1	1	3	3	3	3
1	1	4	4	1	1	2	2	3	3
1	1	4	4	1	1	2	2	3	3
1	1	4	4	1	1	2	2	3	3
1	1	4	4	1	1	2	2	3	3
1	1	4	4	2	2	2	2	3	3
1	1	4	4	1	1	2	2	3	3
1	1	4	4	1	1	2	2	3	3
1	1	4	4	1	1	2	2	3	3
4	4	1	1	1	1	2	2	3	3
1	1	4	4	1	1	3	3	3	3
1	1	4	4	1	1	2	2	3	3
1	1	4	4	1	1	2	2	3	3
1	1	4	4	1	1	2	2	3	3
1	1	4	4	1	1	3	3	1	1
1	1	4	4	1	1	2	2	3	3
1	1	4	4	1	1	2	2	3	3
1	1	4	4	1	1	2	2	3	3
1	1	4	4	1	1	3	3	3	3
1	1	4	4	1	1	3	3	3	3
1	1	4	4	2	2	2	2	3	3
1	1	1	1	1	1	3	3	3	3
1	1	4	4	1	1	3	3	3	3
1	1	4	4	1	1	2	2	3	3
1	1	4	4	1	1	2	2	3	3
4	4	4	4	1	1	3	3	3	3
1	1	4	4	1	1	2	2	3	3
1	1	4	4	1	1	2	2	3	3
1	1	1	1	1	1	2	2	3	3
1	1	4	4	1	1	2	2	3	3
1	1	4	4	2	2	3	3	3	3
1	1	4	4	1	1	2	2	3	3
1	1	4	4	1	1	2	2	3	3
1	1	4	4	1	1	2	2	3	3
1	1	4	4	1	1	3	3	3	3

For Review Only

4	4	4	4	1	1	2	2	3	3
1	1	4	4	1	1	2	2	3	3

For Review Only

SNP2168	SNP2168	SNP2169	SNP2169	SNP217	SNP217	SNP2170	SNP2170	SNP2171	SNP2171
3	3	2	2	1	1	1	1	1	1
3	3	2	2	3	3	2	2	1	1
3	3	2	2	1	1	2	2	4	4
3	3	2	2	1	1	2	2	1	1
3	3	3	3	1	1	2	2	1	1
3	3	2	2	1	1	2	2	4	4
3	3	2	2	1	1	2	2	1	1
3	3	2	2	3	3	2	2	1	1
3	3	2	2	1	1	2	2	1	1
3	3	2	2	1	1	1	1	1	1
3	3	2	2	1	1	2	2	1	1
3	3	2	2	1	1	2	2	4	4
3	3	2	2	1	1	2	2	1	1
3	3	2	2	1	1	2	2	4	4
3	3	2	2	1	1	1	1	1	1
3	3	2	2	1	1	2	2	1	1
3	3	2	2	1	1	2	2	1	1
3	3	2	2	1	1	2	2	1	1
1	1	2	2	1	1	1	1	1	1
3	3	2	2	1	1	2	2	1	1
3	3	3	3	1	1	2	2	1	1
3	3	2	2	1	1	2	2	1	1
3	3	2	2	3	3	2	2	1	1
3	3	2	2	1	1	2	2	1	1
1	1	2	2	1	1	2	2	4	4
3	3	2	2	1	1	2	2	4	4
1	1	2	2	1	1	2	2	4	4
3	3	2	2	1	1	2	2	1	1
3	3	2	2	3	3	2	2	1	1
3	3	2	2	1	1	1	1	1	1
3	3	2	2	1	1	2	2	4	4
3	3	2	2	1	1	2	2	4	4
3	3	2	2	1	1	2	2	1	1
3	3	2	2	1	1	2	2	1	1
3	3	2	2	1	1	2	2	1	1
3	3	2	2	1	1	2	2	1	1
3	3	2	2	1	1	2	2	1	1
3	3	2	2	1	1	2	2	4	4
3	3	3	3	3	3	2	2	1	1
3	3	2	2	3	3	2	2	1	1
3	3	2	2	1	1	2	2	1	1
3	3	2	2	3	3	2	2	1	1
3	3	2	2	1	1	1	1	1	1
3	3	2	2	1	1	2	2	1	1
3	3	2	2	1	1	1	1	1	1
3	3	2	2	1	1	2	2	4	4
3	3	2	2	1	1	2	2	1	1
3	3	2	2	1	1	2	2	1	1
3	3	3	3	1	1	2	2	1	1
3	3	2	2	1	1	2	2	1	1
3	3	2	2	1	1	2	2	4	4
3	3	2	2	3	3	2	2	1	1

For Review Only

3	3	2	2	1	1	2	2	1	1
3	3	2	2	1	1	2	2	1	1
1	1	2	2	1	1	2	2	4	4
3	3	2	2	1	1	2	2	1	1
1	1	2	2	1	1	1	1	1	1
3	3	2	2	1	1	2	2	1	1
3	3	2	2	1	1	2	2	4	4
3	3	2	2	3	3	2	2	1	1
3	3	2	2	3	3	2	2	1	1
3	3	2	2	1	1	2	2	1	1
3	3	2	2	1	1	2	2	1	1
3	3	2	2	1	1	2	2	1	1
3	3	2	2	1	1	2	2	1	1
3	3	2	2	3	3	2	2	1	1
1	1	2	2	1	1	2	2	1	1
3	3	2	2	1	1	2	2	1	1
3	3	2	2	3	3	2	2	4	4
3	3	2	2	1	1	2	2	1	1
3	3	2	2	1	1	2	2	1	1
3	3	2	2	1	1	1	1	1	1
3	3	2	2	1	1	2	2	1	1
3	3	3	3	1	1	2	2	1	1
3	3	2	2	3	3	2	2	1	1
3	3	2	2	1	1	2	2	4	4
3	3	2	2	1	1	1	1	1	1
3	3	2	2	1	1	2	2	1	1
3	3	2	2	1	1	2	2	4	4
3	3	3	3	3	3	2	2	1	1
3	3	2	2	1	1	2	2	1	1
3	3	2	2	1	1	2	2	1	1
3	3	2	2	1	1	2	2	1	1
3	3	2	2	1	1	2	2	1	1
3	3	3	3	3	3	2	2	1	1
3	3	2	2	1	1	2	2	1	1
3	3	2	2	3	3	2	2	1	1
3	3	2	2	1	1	1	1	1	1
3	3	2	2	1	1	2	2	1	1
3	3	2	2	1	1	2	2	1	1
1	1	2	2	1	1	2	2	4	4
3	3	2	2	1	1	2	2	1	1
3	3	2	2	1	1	2	2	1	1
3	3	2	2	1	1	2	2	4	4
3	3	2	2	1	1	2	2	4	4
3	3	2	2	1	1	2	2	1	1
3	3	2	2	1	1	2	2	1	1
3	3	2	2	1	1	2	2	1	1
3	3	2	2	1	1	2	2	1	1
3	3	2	2	1	1	2	2	1	1
3	3	2	2	1	1	2	2	1	1
3	3	2	2	3	3	2	2	1	1
3	3	2	2	1	1	2	2	1	1
3	3	3	3	3	3	2	2	1	1
3	3	2	2	3	3	2	2	1	1
3	3	3	3	1	1	2	2	4	4
3	3	2	2	3	3	2	2	1	1

3	3	3	3	1	1	2	2	1	1
3	3	2	2	1	1	2	2	1	1
3	3	2	2	1	1	2	2	4	4
3	3	2	2	1	1	2	2	1	1
3	3	2	2	1	1	2	2	4	4
3	3	2	2	1	1	2	2	1	1
3	3	2	2	1	1	2	2	4	4
1	1	2	2	1	1	2	2	1	1
3	3	2	2	1	1	2	2	1	1
3	3	2	2	3	3	2	2	1	1
3	3	2	2	1	1	2	2	1	1
3	3	2	2	1	1	2	2	1	1
3	3	2	2	1	1	2	2	1	1
3	3	2	2	1	1	2	2	1	1
1	1	2	2	1	1	2	2	1	1
3	3	2	2	1	1	2	2	4	4
3	3	2	2	1	1	2	2	1	1
3	3	2	2	1	1	2	2	1	1
3	3	2	2	1	1	2	2	1	1
3	3	2	2	3	3	2	2	4	4
3	3	2	2	1	1	2	2	1	1
3	3	2	2	1	1	2	2	1	1
3	3	2	2	1	1	2	2	4	4
3	3	3	3	3	3	2	2	1	1
3	3	2	2	1	1	2	2	1	1
1	1	2	2	1	1	2	2	1	1
3	3	2	2	1	1	2	2	1	1
3	3	2	2	1	1	2	2	1	1
3	3	2	2	1	1	2	2	4	4
3	3	2	2	1	1	1	1	1	1
3	3	2	2	3	3	2	2	1	1
3	3	2	2	1	1	2	2	1	1
3	3	2	2	1	1	2	2	1	1
3	3	2	2	1	1	2	2	1	1
3	3	2	2	1	1	2	2	1	1
3	3	3	3	1	1	2	2	1	1
3	3	2	2	1	1	2	2	1	1
3	3	2	2	1	1	2	2	1	1
3	3	2	2	1	1	2	2	1	1
3	3	2	2	3	3	2	2	1	1
3	3	2	2	1	1	2	2	1	1
3	3	2	2	1	1	2	2	1	1
3	3	2	2	1	1	2	2	1	1
3	3	2	2	1	1	2	2	1	1
3	3	2	2	1	1	2	2	4	4
3	3	2	2	1	1	2	2	1	1
3	3	2	2	1	1	2	2	4	4
3	3	2	2	1	1	2	2	4	4
3	3	2	2	3	3	2	2	1	1
3	3	2	2	1	1	2	2	4	4
3	3	2	2	1	1	2	2	4	4
3	3	2	2	3	3	2	2	1	1
3	3	2	2	1	1	2	2	1	1
3	3	2	2	1	1	2	2	4	4
3	3	2	2	1	1	2	2	1	1

For Review Only

3	3	2	2	1	1	2	2	1	1
3	3	2	2	1	1	2	2	1	1
3	3	2	2	1	1	2	2	1	1
3	3	2	2	1	1	2	2	1	1
3	3	2	2	1	1	2	2	1	1
3	3	2	2	3	3	2	2	4	4
3	3	2	2	3	3	2	2	1	1
3	3	2	2	1	1	2	2	1	1
3	3	3	3	3	3	2	2	1	1
3	3	2	2	1	1	2	2	1	1
3	3	2	2	1	1	2	2	1	1
3	3	2	2	1	1	2	2	1	1
3	3	2	2	1	1	2	2	1	1
3	3	2	2	1	1	2	2	1	1
3	3	2	2	1	1	2	2	1	1
3	3	2	2	1	1	2	2	1	1
3	3	2	2	1	1	2	2	1	1
3	3	2	2	1	1	2	2	1	1
3	3	2	2	1	1	2	2	1	1
3	3	2	2	1	1	2	2	4	4
3	3	2	2	1	1	2	2	1	1
3	3	2	2	1	1	2	2	1	1
3	3	2	2	1	1	2	2	1	1
3	3	2	2	1	1	2	2	1	1
3	3	2	2	1	1	2	2	1	1
3	3	2	2	1	1	2	2	1	1
3	3	2	2	1	1	2	2	1	1
3	3	2	2	1	1	2	2	1	1
3	3	2	2	1	1	2	2	1	1
3	3	2	2	1	1	2	2	1	1
3	3	2	2	1	1	2	2	1	1
3	3	2	2	1	1	2	2	1	1
3	3	2	2	1	1	2	2	4	4
3	3	2	2	1	1	2	2	1	1
3	3	2	2	1	1	2	2	1	1
3	3	2	2	1	1	2	2	1	1
3	3	2	2	1	1	2	2	1	1
3	3	2	2	1	1	2	2	1	1
3	3	2	2	1	1	2	2	4	4
3	3	2	2	1	1	2	2	4	4
3	3	2	2	1	1	2	2	4	4
3	3	2	2	1	1	2	2	4	4
3	3	2	2	3	3	2	2	1	1
3	3	2	2	1	1	2	2	1	1
3	3	3	3	1	1	2	2	4	4
3	3	2	2	1	1	1	1	4	4
3	3	2	2	1	1	2	2	1	1
3	3	2	2	3	3	2	2	1	1
3	3	3	3	1	1	2	2	1	1
3	3	2	2	1	1	2	2	4	4
1	1	2	2	1	1	2	2	4	4
3	3	2	2	3	3	1	1	1	1
3	3	2	2	3	3	1	1	1	1
3	3	2	2	1	1	2	2	1	1
3	3	3	3	3	3	2	2	1	1

For Review Only

3	3	2	2	1	1	2	2	1	1
3	3	2	2	1	1	2	2	1	1
1	1	2	2	1	1	2	2	1	1
3	3	2	2	1	1	2	2	1	1
3	3	2	2	1	1	2	2	1	1
3	3	2	2	1	1	2	2	4	4
3	3	2	2	3	3	2	2	1	1
3	3	2	2	1	1	2	2	1	1
3	3	3	3	1	1	2	2	1	1
3	3	3	3	3	3	2	2	1	1
3	3	2	2	1	1	2	2	1	1
3	3	2	2	3	3	2	2	1	1
1	1	2	2	1	1	2	2	4	4
3	3	2	2	1	1	2	2	1	1
3	3	2	2	1	1	2	2	4	4
3	3	2	2	1	1	2	2	1	1
3	3	3	3	1	1	2	2	1	1
3	3	3	3	3	3	2	2	1	1
3	3	2	2	3	3	2	2	1	1
3	3	2	2	1	1	2	2	4	4
3	3	2	2	1	1	1	1	1	1
3	3	2	2	1	1	2	2	4	4
3	3	2	2	1	1	2	2	1	1
3	3	2	2	1	1	2	2	1	1
3	3	2	2	3	3	2	2	1	1
3	3	2	2	1	1	2	2	1	1
3	3	3	3	3	3	2	2	1	1
3	3	2	2	1	1	2	2	4	4
3	3	2	2	1	1	2	2	1	1
1	1	2	2	1	1	2	2	1	1
3	3	3	3	3	3	2	2	1	1
3	3	2	2	1	1	2	2	4	4
3	3	2	2	3	3	2	2	1	1
3	3	2	2	1	1	2	2	4	4
3	3	2	2	1	1	2	2	1	1
3	3	3	3	3	3	2	2	1	1
3	3	2	2	1	1	2	2	1	1
3	3	2	2	1	1	2	2	1	1
3	3	2	2	1	1	2	2	1	1
3	3	3	3	3	3	1	1	1	1
3	3	2	2	1	1	2	2	1	1
3	3	2	2	1	1	2	2	1	1
3	3	2	2	3	3	2	2	1	1
3	3	2	2	1	1	2	2	1	1
3	3	2	2	1	1	2	2	4	4
1	1	2	2	1	1	2	2	1	1
3	3	3	3	1	1	2	2	4	4
3	3	2	2	1	1	2	2	4	4
3	3	2	2	1	1	1	1	4	4
3	3	3	3	3	3	2	2	1	1
3	3	2	2	1	1	2	2	1	1
3	3	3	3	3	3	2	2	1	1
3	3	2	2	1	1	2	2	4	4
3	3	2	2	1	1	2	2	4	4
3	3	2	2	3	3	2	2	1	1

For Review Only

3	3	2	2	1	1	2	2	1	1
3	3	2	2	1	1	2	2	4	4

For Review Only

SNP2172	SNP2172	SNP2173	SNP2173	SNP2174	SNP2174	SNP2175	SNP2175	SNP2176	SNP2176
4	4	1	1	2	2	3	3	1	1
4	4	1	1	3	3	3	3	1	1
4	4	1	1	3	3	3	3	1	1
4	4	1	1	3	3	3	3	1	1
4	4	4	4	2	2	1	1	4	4
4	4	1	1	2	2	1	1	4	4
4	4	1	1	2	2	1	1	4	4
4	4	1	1	3	3	3	3	1	1
4	4	1	1	2	2	1	1	4	4
4	4	1	1	3	3	3	3	1	1
4	4	1	1	2	2	1	1	4	4
4	4	1	1	3	3	1	1	1	1
4	4	1	1	3	3	3	3	1	1
4	4	1	1	2	2	1	1	4	4
4	4	1	1	3	3	3	3	1	1
4	4	1	1	3	3	3	3	1	1
4	4	1	1	2	2	1	1	4	4
4	4	1	1	2	2	3	3	1	1
4	4	1	1	2	2	1	1	4	4
4	4	1	1	3	3	3	3	1	1
4	4	4	4	2	2	1	1	4	4
4	4	1	1	2	2	1	1	4	4
4	4	1	1	2	2	1	1	4	4
4	4	1	1	3	3	3	3	1	1
4	4	1	1	2	2	1	1	4	4
4	4	1	1	3	3	1	1	4	4
4	4	1	1	3	3	3	3	1	1
4	4	1	1	3	3	3	3	1	1
4	4	1	1	3	3	3	3	1	1
4	4	1	1	3	3	3	3	1	1
4	4	1	1	3	3	1	1	4	4
4	4	1	1	3	3	3	3	1	1
4	4	1	1	2	2	1	1	4	4
4	4	1	1	3	3	3	3	1	1
4	4	1	1	3	3	3	3	1	1
4	4	1	1	3	3	3	3	1	1
4	4	1	1	3	3	3	3	1	1
4	4	1	1	3	3	3	3	1	1
4	4	1	1	3	3	3	3	1	1
4	4	1	1	3	3	3	3	1	1
4	4	1	1	3	3	3	3	1	1
4	4	1	1	3	3	3	3	1	1
4	4	1	1	3	3	3	3	1	1
4	4	1	1	2	2	1	1	4	4
4	4	1	1	3	3	3	3	1	1
4	4	1	1	3	3	3	3	1	1
1	1	1	1	2	2	3	3	4	4
4	4	1	1	3	3	3	3	1	1
4	4	4	4	2	2	1	1	4	4
4	4	1	1	3	3	3	3	1	1
4	4	1	1	3	3	3	3	1	1
4	4	1	1	3	3	3	3	1	1

For Review Only

4	4	1	1	2	2	1	1	4	4
4	4	1	1	2	2	1	1	4	4
4	4	1	1	2	2	1	1	4	4
4	4	1	1	2	2	1	1	4	4
4	4	1	1	2	2	1	1	4	4
4	4	1	1	3	3	3	3	1	1
4	4	1	1	3	3	3	3	1	1
4	4	1	1	3	3	3	3	1	1
4	4	1	1	3	3	3	3	1	1
4	4	1	1	3	3	3	3	1	1
4	4	1	1	2	2	1	1	4	4
1	1	1	1	2	2	1	1	4	4
4	4	1	1	2	2	1	1	4	4
4	4	1	1	3	3	3	3	1	1
4	4	1	1	3	3	1	1	4	4
4	4	1	1	3	3	3	3	1	1
4	4	1	1	3	3	3	3	1	1
4	4	1	1	3	3	3	3	1	1
4	4	1	1	2	2	1	1	4	4
4	4	1	1	2	2	3	3	1	1
1	1	1	1	2	2	1	1	4	4
4	4	1	1	3	3	3	3	1	1
4	4	1	1	3	3	1	1	4	4
4	4	1	1	3	3	3	3	1	1
4	4	1	1	3	3	3	3	1	1
4	4	1	1	3	3	3	3	1	1
4	4	1	1	3	3	3	3	1	1
4	4	4	4	3	3	3	3	1	1
4	4	1	1	3	3	3	3	1	1
4	4	1	1	2	2	1	1	4	4
4	4	1	1	3	3	3	3	1	1
4	4	1	1	3	3	3	3	1	1
4	4	4	4	3	3	3	3	1	1
4	4	1	1	2	2	1	1	4	4
4	4	1	1	3	3	3	3	1	1
4	4	1	1	3	3	3	3	1	1
4	4	1	1	3	3	3	3	1	1
4	4	1	1	3	3	3	3	1	1
4	4	1	1	2	2	1	1	4	4
4	4	1	1	3	3	3	3	1	1
4	4	1	1	2	2	1	1	1	1
1	1	1	1	2	2	1	1	4	4
4	4	1	1	3	3	3	3	1	1
4	4	1	1	2	2	1	1	4	4
4	4	1	1	3	3	3	3	1	1
4	4	1	1	2	2	3	3	1	1
4	4	1	1	3	3	3	3	1	1
4	4	1	1	3	3	3	3	1	1
4	4	1	1	3	3	3	3	1	1
4	4	1	1	3	3	3	3	1	1
1	1	1	1	2	2	1	1	4	4
4	4	1	1	3	3	3	3	1	1
4	4	1	1	3	3	3	3	1	1
1	1	1	1	3	3	3	3	1	1
4	4	1	1	3	3	3	3	1	1

For Review Only

4	4	1	1	3	3	3	3	1	1
4	4	1	1	3	3	3	3	1	1
4	4	1	1	3	3	3	3	1	1
4	4	1	1	3	3	3	3	1	1
4	4	1	1	3	3	3	3	1	1
4	4	1	1	3	3	1	1	4	4
4	4	1	1	3	3	3	3	1	1
4	4	1	1	3	3	3	3	1	1
4	4	1	1	3	3	3	3	1	1
4	4	1	1	3	3	3	3	1	1
4	4	1	1	3	3	3	3	1	1
4	4	1	1	3	3	3	3	1	1
4	4	1	1	3	3	3	3	1	1
4	4	1	1	3	3	3	3	1	1
4	4	1	1	3	3	3	3	1	1
4	4	1	1	3	3	3	3	1	1
4	4	1	1	3	3	3	3	1	1
4	4	1	1	3	3	3	3	1	1
4	4	1	1	3	3	3	3	1	1
4	4	1	1	3	3	3	3	1	1
4	4	1	1	3	3	3	3	1	1
4	4	1	1	2	2	1	1	4	4
4	4	1	1	3	3	1	1	4	4
4	4	4	4	3	3	3	3	1	1
4	4	1	1	3	3	1	1	1	1
4	4	1	1	3	3	3	3	1	1
4	4	1	1	3	3	3	3	1	1
4	4	1	1	3	3	3	3	1	1
4	4	1	1	3	3	3	3	1	1
4	4	1	1	2	2	1	1	4	4
4	4	4	4	3	3	3	3	1	1
4	4	1	1	3	3	3	3	1	1
4	4	1	1	2	2	1	1	4	4
4	4	1	1	3	3	3	3	1	1
4	4	1	1	3	3	3	3	1	1
4	4	1	1	3	3	3	3	1	1
4	4	1	1	3	3	3	3	1	1
4	4	1	1	3	3	3	3	1	1
4	4	1	1	3	3	3	3	1	1
4	4	4	4	3	3	3	3	1	1
4	4	1	1	2	2	3	3	1	1
4	4	1	1	3	3	3	3	1	1
4	4	1	1	3	3	3	3	1	1
4	4	1	1	3	3	3	3	1	1
4	4	1	1	3	3	3	3	1	1
4	4	1	1	2	2	1	1	4	4
4	4	1	1	3	3	3	3	1	1
4	4	1	1	2	2	1	1	4	4
4	4	1	1	3	3	3	3	1	1
4	4	1	1	3	3	3	3	1	1
4	4	1	1	3	3	3	3	1	1
4	4	4	4	2	2	1	1	4	4
4	4	1	1	3	3	3	3	1	1
1	1	1	1	2	2	1	1	4	4
4	4	1	1	3	3	3	3	1	1

For Review Only

4	4	1	1	3	3	3	3	1	1
4	4	1	1	3	3	3	3	1	1
4	4	1	1	3	3	3	3	1	1
4	4	1	1	3	3	3	3	1	1
4	4	1	1	3	3	3	3	1	1
4	4	1	1	3	3	3	3	1	1
4	4	1	1	3	3	3	3	1	1
4	4	1	1	3	3	3	3	1	1
4	4	1	1	3	3	3	3	1	1
4	4	1	1	2	2	1	1	4	4
4	4	1	1	3	3	3	3	1	1
4	4	1	1	3	3	3	3	1	1
4	4	1	1	3	3	1	1	4	4
4	4	1	1	2	2	1	1	4	4
4	4	1	1	3	3	3	3	1	1
4	4	1	1	2	2	1	1	4	4
4	4	1	1	3	3	3	3	1	1
4	4	1	1	3	3	3	3	1	1
4	4	1	1	3	3	3	3	1	1
4	4	1	1	3	3	3	3	1	1
4	4	1	1	3	3	3	3	1	1
4	4	4	4	3	3	3	3	1	1
4	4	1	1	3	3	3	3	1	1
1	1	1	1	2	2	1	1	4	4
4	4	1	1	3	3	3	3	4	4
4	4	1	1	3	3	3	3	1	1
4	4	1	1	3	3	1	1	4	4
4	4	1	1	3	3	3	3	1	1
4	4	1	1	3	3	3	3	1	1
4	4	1	1	2	2	1	1	4	4
4	4	1	1	3	3	3	3	1	1
4	4	1	1	2	2	1	1	4	4
4	4	1	1	3	3	3	3	1	1
4	4	1	1	3	3	3	3	1	1
4	4	1	1	3	3	3	3	1	1
1	1	1	1	2	2	1	1	4	4
1	1	1	1	3	3	1	1	4	4
4	4	1	1	3	3	3	3	1	1
4	4	1	1	2	2	1	1	4	4
4	4	1	1	3	3	1	1	4	4
4	4	1	1	3	3	3	3	1	1
4	4	1	1	3	3	3	3	1	1
4	4	1	1	3	3	3	3	1	1
4	4	1	1	3	3	3	3	1	1
4	4	1	1	3	3	3	3	1	1
4	4	1	1	3	3	3	3	1	1
4	4	1	1	2	2	1	1	4	4
4	4	1	1	3	3	3	3	1	1
4	4	1	1	3	3	3	3	1	1
4	4	1	1	2	2	1	1	4	4
4	4	1	1	3	3	3	3	1	1
4	4	1	1	3	3	3	3	1	1
4	4	1	1	3	3	3	3	1	1
4	4	1	1	2	2	1	1	4	4
4	4	1	1	3	3	3	3	1	1

For Review Only

4	4	1	1	3	3	3	3	1	1
4	4	1	1	3	3	3	3	1	1
4	4	1	1	3	3	3	3	1	1
4	4	1	1	3	3	3	3	1	1
1	1	1	1	2	2	3	3	1	1
4	4	1	1	3	3	3	3	1	1
4	4	1	1	3	3	3	3	1	1
4	4	1	1	3	3	3	3	1	1
4	4	1	1	3	3	3	3	1	1
4	4	4	4	3	3	3	3	1	1
4	4	1	1	3	3	3	3	1	1
4	4	1	1	3	3	3	3	1	1
4	4	1	1	2	2	1	1	4	4
4	4	1	1	3	3	3	3	1	1
4	4	1	1	3	3	3	3	1	1
4	4	1	1	2	2	1	1	4	4
4	4	1	1	3	3	3	3	1	1
4	4	4	4	3	3	3	3	1	1
4	4	1	1	3	3	3	3	1	1
4	4	1	1	3	3	3	3	1	1
4	4	1	1	3	3	3	3	1	1
4	4	1	1	3	3	3	3	1	1
4	4	1	1	3	3	3	3	1	1
4	4	1	1	2	2	1	1	4	4
4	4	1	1	3	3	3	3	1	1
4	4	1	1	3	3	3	3	1	1
4	4	4	4	3	3	3	3	1	1
4	4	1	1	3	3	1	1	4	4
4	4	1	1	2	2	3	3	1	1
4	4	1	1	3	3	3	3	1	1
4	4	1	1	3	3	3	3	1	1
4	4	1	1	3	3	3	3	1	1
4	4	1	1	2	2	1	1	4	4
4	4	1	1	3	3	3	3	1	1
4	4	1	1	2	2	1	1	4	4
4	4	4	4	3	3	3	3	1	1
4	4	1	1	3	3	3	3	1	1
1	1	1	1	2	2	1	1	4	4
4	4	1	1	3	3	3	3	1	1
4	4	1	1	3	3	3	3	1	1
4	4	1	1	3	3	3	3	1	1
4	4	1	1	3	3	3	3	1	1
4	4	1	1	3	3	1	1	4	4
4	4	1	1	2	2	1	1	4	4
1	1	1	1	3	3	3	3	1	1
4	4	1	1	3	3	3	3	1	1
4	4	1	1	2	2	1	1	4	4
4	4	1	1	2	2	1	1	4	4
4	4	1	1	3	3	3	3	1	1
4	4	4	4	3	3	3	3	1	1
4	4	1	1	3	3	3	3	1	1
4	4	1	1	3	3	3	3	1	1
4	4	1	1	3	3	3	3	1	1
1	1	1	1	3	3	1	1	4	4
4	4	1	1	3	3	3	3	1	1

For Review Only

4	4	1	1	2	2	1	1	4	4
1	1	1	1	3	3	1	1	4	4

For Review Only

SNP2177	SNP2177	SNP2178	SNP2178	SNP2179	SNP2179	SNP218	SNP218	SNP2180	SNP2180
3	3	2	2	4	4	4	4	1	1
3	3	3	3	4	4	4	4	4	4
2	2	2	2	4	4	4	4	1	1
2	2	3	3	4	4	1	1	4	4
2	2	2	2	4	4	1	1	1	1
3	3	3	3	4	4	1	1	4	4
3	3	2	2	4	4	4	4	4	4
2	2	2	2	4	4	4	4	1	1
3	3	2	2	4	4	1	1	1	1
2	2	2	2	4	4	4	4	1	1
2	2	2	2	4	4	1	1	1	1
2	2	2	2	4	4	4	4	1	1
2	2	2	2	4	4	4	4	1	1
2	2	2	2	4	4	4	4	1	1
2	2	2	2	4	4	4	4	1	1
2	2	2	2	4	4	4	4	1	1
2	2	2	2	4	4	4	4	1	1
2	2	2	2	4	4	4	4	1	1
3	3	3	3	1	1	4	4	4	4
2	2	2	2	4	4	4	4	1	1
2	2	2	2	4	4	4	4	1	1
3	3	2	2	4	4	4	4	1	1
2	2	2	2	4	4	4	4	1	1
2	2	2	2	1	1	4	4	1	1
3	3	2	2	4	4	1	1	1	1
3	3	2	2	4	4	4	4	1	1
2	2	2	2	4	4	4	4	1	1
2	2	2	2	4	4	4	4	1	1
3	3	2	2	4	4	4	4	1	1
3	3	2	2	4	4	4	4	1	1
3	3	2	2	4	4	4	4	1	1
3	3	2	2	4	4	4	4	1	1
3	3	2	2	4	4	1	1	1	1
2	2	2	2	4	4	4	4	1	1
2	2	2	2	4	4	4	4	1	1
2	2	2	2	4	4	1	1	1	1
2	2	3	3	4	4	1	1	4	4
2	2	3	3	4	4	4	4	4	4
2	2	2	2	4	4	4	4	1	1
2	2	2	2	4	4	4	4	1	1
2	2	2	2	4	4	4	4	1	1
3	3	2	2	4	4	4	4	1	1
2	2	2	2	4	4	4	4	1	1
2	2	2	2	4	4	1	1	1	1
2	2	2	2	4	4	4	4	1	1
2	2	2	2	4	4	4	4	1	1
2	2	2	2	4	4	4	4	1	1
3	3	2	2	4	4	4	4	1	1
2	2	2	2	4	4	1	1	1	1
2	2	2	2	4	4	1	1	1	1
3	3	2	2	4	4	4	4	1	1
2	2	2	2	4	4	4	4	1	1

For Review Only

3	3	2	2	4	4	1	1	1	1
3	3	2	2	4	4	1	1	1	1
2	2	2	2	4	4	4	4	1	1
3	3	2	2	4	4	4	4	1	1
3	3	3	3	1	1	4	4	4	4
3	3	2	2	4	4	4	4	1	1
3	3	2	2	4	4	4	4	1	1
2	2	2	2	4	4	4	4	1	1
2	2	2	2	1	1	1	1	1	1
2	2	2	2	4	4	4	4	1	1
2	2	2	2	4	4	4	4	1	1
2	2	2	2	4	4	4	4	1	1
3	3	2	2	4	4	1	1	1	1
3	3	2	2	4	4	4	4	1	1
2	2	2	2	4	4	4	4	1	1
2	2	2	2	4	4	4	4	1	1
3	3	2	2	4	4	1	1	1	1
2	2	2	2	4	4	4	4	1	1
3	3	2	2	4	4	1	1	1	1
3	3	2	2	4	4	1	1	1	1
2	2	2	2	4	4	4	4	1	1
2	2	2	2	4	4	4	4	1	1
3	3	2	2	4	4	1	1	1	1
3	3	2	2	4	4	4	4	1	1
2	2	2	2	4	4	4	4	1	1
3	3	2	2	4	4	4	4	1	1
2	2	2	2	4	4	1	1	1	1
2	2	2	2	4	4	4	4	1	1
3	3	2	2	4	4	4	4	1	1
3	3	2	2	4	4	4	4	1	1
2	2	2	2	4	4	1	1	1	1
2	2	2	2	4	4	4	4	1	1
3	3	2	2	4	4	4	4	1	1
3	3	2	2	4	4	1	1	1	1
3	3	2	2	4	4	1	1	1	1
2	2	2	2	4	4	4	4	1	1
2	2	2	2	4	4	4	4	1	1
3	3	2	2	4	4	1	1	1	1
2	2	2	2	4	4	4	4	1	1
2	2	2	2	4	4	1	1	1	1
3	3	2	2	4	4	4	4	1	1
3	3	2	2	4	4	4	4	1	1
2	2	3	3	4	4	1	1	4	4
2	2	2	2	4	4	4	4	1	1
3	3	2	2	4	4	1	1	1	1
3	3	3	3	4	4	4	4	4	4
2	2	2	2	1	1	4	4	1	1
2	2	2	2	4	4	4	4	1	1
2	2	2	2	4	4	4	4	1	1
2	2	2	2	4	4	1	1	1	1
2	2	3	3	4	4	4	4	4	4
2	2	2	2	4	4	4	4	1	1
2	2	3	3	4	4	1	1	4	4
2	2	2	2	4	4	4	4	1	1
2	2	2	2	4	4	1	1	1	1

For Review Only

2	2	2	2	4	4	4	4	1	1
3	3	2	2	4	4	4	4	1	1
3	3	2	2	4	4	1	1	1	1
2	2	2	2	1	1	4	4	1	1
2	2	3	3	4	4	4	4	4	4
3	3	2	2	4	4	4	4	1	1
2	2	2	2	4	4	4	4	1	1
2	2	2	2	4	4	1	1	1	1
2	2	3	3	4	4	4	4	4	4
2	2	2	2	4	4	4	4	1	1
2	2	3	3	1	1	1	1	4	4
2	2	2	2	4	4	4	4	1	1
2	2	3	3	4	4	4	4	4	4
2	2	2	2	4	4	4	4	1	1
2	2	2	2	4	4	4	4	1	1
2	2	2	2	4	4	4	4	1	1
2	2	2	2	4	4	4	4	1	1
2	2	2	2	4	4	4	4	1	1
2	2	2	2	1	1	4	4	1	1
3	3	2	2	4	4	4	4	4	4
2	2	2	2	4	4	4	4	1	1
2	2	2	2	4	4	4	4	1	1
3	3	2	2	4	4	4	4	1	1
3	3	2	2	4	4	4	4	1	1
3	3	2	2	4	4	4	4	1	1
2	2	2	2	4	4	1	1	1	1
3	3	2	2	4	4	4	4	1	1
2	2	2	2	4	4	4	4	1	1
3	3	2	2	4	4	4	4	1	1
2	2	2	2	4	4	4	4	1	1
3	3	2	2	4	4	4	4	1	1
2	2	2	2	4	4	4	4	1	1
3	3	2	2	4	4	4	4	1	1
2	2	2	2	4	4	4	4	1	1
2	2	2	2	4	4	4	4	1	1
3	3	2	2	4	4	4	4	1	1
2	2	2	2	1	1	4	4	1	1
2	2	3	3	4	4	1	1	4	4
2	2	2	2	4	4	1	1	1	1
3	3	2	2	4	4	4	4	1	1
2	2	2	2	4	4	4	4	1	1
2	2	2	2	1	1	4	4	1	1
2	2	2	2	4	4	4	4	1	1
3	3	2	2	4	4	4	4	4	4
3	3	3	3	4	4	1	1	1	1
3	3	2	2	4	4	4	4	1	1
2	2	2	2	4	4	4	4	1	1
3	3	2	2	4	4	4	4	1	1
2	2	2	2	4	4	1	1	4	4
3	3	2	2	4	4	4	4	1	1
2	2	2	2	4	4	4	4	1	1
2	2	2	2	4	4	1	1	1	1
2	2	2	2	4	4	4	4	1	1
2	2	2	2	4	4	4	4	1	1
3	3	2	2	4	4	4	4	1	1

For Review Only

2	2	2	2	4	4	1	1	1	1
2	2	2	2	4	4	1	1	1	1
2	2	2	2	4	4	1	1	1	1
3	3	2	2	4	4	4	4	1	1
2	2	2	2	4	4	4	4	1	1
2	2	3	3	4	4	4	4	4	4
2	2	2	2	4	4	4	4	1	1
2	2	2	2	4	4	4	4	1	1
2	2	2	2	4	4	4	4	1	1
2	2	2	2	4	4	4	4	1	1
2	2	2	2	4	4	4	4	1	1
2	2	2	2	4	4	4	4	1	1
2	2	2	2	4	4	1	1	4	4
2	2	2	2	4	4	4	4	1	1
2	2	2	2	1	1	4	4	1	1
2	2	2	2	4	4	1	1	1	1
2	2	2	2	4	4	4	4	1	1
3	3	3	3	4	4	4	4	1	1
3	3	2	2	4	4	4	4	1	1
2	2	2	2	4	4	4	4	1	1
2	2	2	2	4	4	4	4	4	4
3	3	2	2	4	4	4	4	1	1
2	2	2	2	4	4	1	1	1	1
2	2	2	2	4	4	4	4	1	1
3	3	2	2	4	4	1	1	1	1
2	2	2	2	4	4	1	1	1	1
2	2	2	2	4	4	1	1	1	1
2	2	2	2	4	4	4	4	1	1
2	2	2	2	1	1	4	4	1	1
3	3	2	2	4	4	1	1	1	1
3	3	3	3	4	4	1	1	4	4
3	3	2	2	4	4	4	4	1	1
3	3	2	2	4	4	1	1	1	1
2	2	2	2	1	1	4	4	1	1
3	3	2	2	1	1	1	1	1	1
2	2	2	2	4	4	4	4	1	1
2	2	2	2	4	4	4	4	4	4
2	2	3	3	4	4	1	1	4	4
3	3	2	2	4	4	4	4	1	1
3	3	3	3	4	4	4	4	4	4
2	2	2	2	4	4	4	4	1	1
3	3	2	2	4	4	4	4	1	1
3	3	2	2	4	4	4	4	1	1
2	2	2	2	4	4	4	4	1	1
2	2	2	2	4	4	4	4	1	1
2	2	3	3	4	4	4	4	4	4
2	2	2	2	4	4	1	1	1	1
2	2	2	2	4	4	4	4	1	1
3	3	2	2	1	1	4	4	1	1
2	2	3	3	4	4	4	4	4	4
3	3	2	2	4	4	4	4	1	1
2	2	3	3	1	1	1	1	4	4
2	2	2	2	4	4	1	1	1	1
2	2	2	2	4	4	4	4	1	1
2	2	2	2	4	4	4	4	1	1

For Review Only

2	2	2	2	1	1	4	4	1	1
2	2	2	2	4	4	4	4	1	1
2	2	2	2	4	4	4	4	1	1
2	2	2	2	4	4	4	4	1	1
2	2	3	3	4	4	4	4	4	4
3	3	2	2	4	4	1	1	1	1
2	2	2	2	4	4	4	4	1	1
2	2	2	2	4	4	4	4	1	1
2	2	2	2	4	4	4	4	1	1
3	3	2	2	4	4	4	4	1	1
2	2	2	2	4	4	4	4	1	1
2	2	3	3	4	4	1	1	4	4
2	2	2	2	4	4	4	4	1	1
3	3	2	2	4	4	4	4	1	1
2	2	3	3	4	4	4	4	4	4
2	2	2	2	4	4	1	1	1	1
2	2	2	2	4	4	4	4	1	1
3	3	2	2	4	4	1	1	1	1
2	2	2	2	4	4	4	4	1	1
3	3	3	3	4	4	1	1	4	4
2	2	2	2	4	4	4	4	1	1
3	3	2	2	4	4	4	4	1	1
2	2	2	2	4	4	1	1	1	1
2	2	2	2	4	4	1	1	1	1
2	2	2	2	4	4	4	4	1	1
3	3	2	2	4	4	4	4	1	1
3	3	2	2	4	4	4	4	1	1
2	2	2	2	4	4	4	4	1	1
2	2	3	3	4	4	4	4	1	1
3	3	2	2	4	4	4	4	1	1
2	2	2	2	4	4	4	4	1	1
3	3	2	2	4	4	1	1	1	1
2	2	2	2	4	4	4	4	1	1
3	3	2	2	4	4	4	4	1	1
2	2	2	2	4	4	1	1	1	1
3	3	2	2	4	4	4	4	1	1
2	2	2	2	4	4	4	4	1	1
2	2	2	2	4	4	4	4	1	1
2	2	2	2	4	4	1	1	1	1
2	2	2	2	4	4	4	4	1	1
2	2	2	2	1	1	4	4	1	1
2	2	2	2	4	4	1	1	1	1
2	2	2	2	4	4	1	1	1	1
3	3	2	2	4	4	1	1	1	1
2	2	2	2	4	4	4	4	1	1
3	3	2	2	4	4	4	4	1	1
2	2	2	2	4	4	4	4	1	1
2	2	2	2	4	4	4	4	1	1
3	3	2	2	4	4	4	4	1	1
2	2	2	2	4	4	4	4	1	1
3	3	2	2	4	4	1	1	1	1
2	2	2	2	4	4	4	4	1	1
2	2	2	2	4	4	4	4	1	1
3	3	2	2	4	4	1	1	1	1
2	2	2	2	4	4	4	4	1	1

For Review Only

2	2	2	2	4	4	1	1	1	1
2	2	2	2	4	4	4	4	1	1

For Review Only

SNP2181	SNP2181	SNP2182	SNP2182	SNP2183	SNP2183	SNP2184	SNP2184	SNP2185	SNP2185
2	2	2	2	2	2	2	2	1	1
2	2	3	3	2	2	1	1	1	1
2	2	3	3	2	2	1	1	1	1
2	2	2	2	1	1	1	1	4	4
2	2	2	2	2	2	2	2	1	1
2	2	3	3	2	2	1	1	4	4
2	2	2	2	1	1	2	2	4	4
2	2	2	2	1	1	2	2	1	1
2	2	2	2	2	2	2	2	1	1
2	2	2	2	2	2	2	2	1	1
2	2	2	2	2	2	2	2	1	1
2	2	3	3	2	2	1	1	1	1
2	2	2	2	2	2	2	2	1	1
2	2	2	2	2	2	2	2	1	1
2	2	2	2	2	2	2	2	1	1
2	2	2	2	2	2	2	2	1	1
2	2	2	2	2	2	2	2	1	1
2	2	2	2	1	1	2	2	1	1
2	2	2	2	1	1	1	1	4	4
2	2	2	2	2	2	2	2	1	1
2	2	2	2	2	2	2	2	1	1
2	2	2	2	2	2	2	2	1	1
3	3	2	2	2	2	2	2	1	1
2	2	2	2	2	2	2	2	1	1
2	2	2	2	2	2	2	2	1	1
2	2	3	3	2	2	1	1	1	1
2	2	3	3	2	2	1	1	1	1
2	2	2	2	2	2	1	1	1	1
2	2	2	2	2	2	2	2	1	1
2	2	2	2	2	2	2	2	1	1
2	2	2	2	2	2	2	2	1	1
2	2	2	2	2	2	2	2	1	1
2	2	2	2	2	2	2	2	1	1
2	2	2	2	2	2	2	2	1	1
2	2	2	2	2	2	1	1	1	1
2	2	2	2	2	2	2	2	1	1
2	2	2	2	2	2	2	2	4	4
2	2	3	3	2	2	1	1	4	4
2	2	2	2	2	2	2	2	1	1
3	3	2	2	2	2	2	2	1	1
2	2	2	2	2	2	2	2	1	1
2	2	2	2	2	2	2	2	1	1
2	2	2	2	1	1	2	2	1	1
2	2	2	2	2	2	2	2	1	1
2	2	2	2	2	2	2	2	1	1
2	2	3	3	2	2	1	1	1	1
2	2	3	3	2	2	1	1	1	1
2	2	2	2	2	2	2	2	1	1
2	2	2	2	2	2	2	2	1	1
2	2	2	2	2	2	2	2	1	1
2	2	3	3	2	2	1	1	1	1
2	2	3	3	2	2	1	1	1	1

For Review Only

2	2	2	2	2	2	2	2	1	1
2	2	2	2	2	2	2	2	1	1
2	2	3	3	2	2	1	1	1	1
2	2	2	2	2	2	2	2	1	1
2	2	2	2	1	1	1	1	4	4
2	2	3	3	2	2	1	1	1	1
2	2	3	3	2	2	1	1	1	1
2	2	2	2	2	2	2	2	1	1
2	2	2	2	2	2	2	2	1	1
2	2	3	3	2	2	1	1	1	1
2	2	3	3	2	2	1	1	1	1
2	2	3	3	2	2	1	1	1	1
2	2	2	2	2	2	2	2	1	1
2	2	2	2	2	2	2	2	1	1
2	2	2	2	2	2	1	1	1	1
2	2	2	2	2	2	2	2	1	1
2	2	3	3	2	2	1	1	1	1
2	2	2	2	2	2	2	2	1	1
2	2	2	2	2	2	2	2	1	1
2	2	2	2	2	2	2	2	1	1
2	2	3	3	2	2	1	1	1	1
2	2	2	2	2	2	2	2	1	1
2	2	2	2	2	2	2	2	1	1
2	2	2	2	2	2	2	2	1	1
2	2	2	2	2	2	2	2	1	1
2	2	3	3	1	1	1	1	1	1
2	2	2	2	2	2	2	2	1	1
2	2	2	2	2	2	2	2	1	1
2	2	2	2	2	2	2	2	1	1
2	2	2	2	1	1	2	2	1	1
2	2	2	2	2	2	2	2	1	1
2	2	2	2	2	2	2	2	1	1
3	3	2	2	2	2	2	2	1	1
2	2	2	2	2	2	2	2	1	1
2	2	2	2	2	2	2	2	1	1
2	2	2	2	2	2	2	2	1	1
2	2	2	2	1	1	2	2	1	1
2	2	2	2	2	2	2	2	1	1
2	2	3	3	2	2	1	1	1	1
2	2	2	2	2	2	2	2	1	1
2	2	2	2	1	1	2	2	1	1
2	2	3	3	2	2	1	1	1	1
2	2	3	3	2	2	1	1	4	4
2	2	3	3	2	2	1	1	1	1
2	2	2	2	1	1	1	1	1	1
2	2	3	3	1	1	1	1	1	1
2	2	2	2	2	2	2	2	1	1
2	2	2	2	2	2	1	1	1	1
2	2	2	2	2	2	2	2	1	1
2	2	2	2	1	1	2	2	1	1
2	2	3	3	2	2	1	1	1	1
2	2	2	2	2	2	2	2	1	1
3	3	2	2	2	2	2	2	4	4
2	2	2	2	2	2	2	2	1	1
2	2	2	2	1	1	2	2	1	1

For Review Only

3	3	2	2	2	2	1	1	1	1
2	2	2	2	2	2	1	1	1	1
2	2	3	3	2	2	1	1	1	1
2	2	2	2	2	2	2	2	1	1
2	2	3	3	2	2	1	1	1	1
2	2	2	2	2	2	1	1	1	1
2	2	3	3	2	2	1	1	1	1
2	2	2	2	2	2	2	2	1	1
2	2	2	2	2	2	2	2	4	4
2	2	2	2	1	1	2	2	1	1
2	2	2	2	2	2	2	2	4	4
2	2	2	2	2	2	1	1	1	1
2	2	3	3	2	2	1	1	1	1
2	2	3	3	1	1	1	1	1	1
2	2	2	2	2	2	1	1	1	1
2	2	3	3	2	2	1	1	1	1
2	2	2	2	1	1	2	2	1	1
2	2	2	2	2	2	1	1	1	1
2	2	2	2	2	2	2	2	1	1
2	2	3	3	2	2	1	1	4	4
2	2	2	2	2	2	1	1	1	1
2	2	2	2	1	1	2	2	1	1
2	2	3	3	1	1	1	1	1	1
2	2	2	2	2	2	2	2	1	1
2	2	2	2	2	2	2	2	1	1
3	3	2	2	2	2	2	2	1	1
2	2	2	2	2	2	1	1	1	1
2	2	2	2	2	2	2	2	1	1
2	2	3	3	2	2	1	1	1	1
2	2	2	2	2	2	1	1	1	1
2	2	2	2	2	2	2	2	1	1
2	2	2	2	2	2	1	1	1	1
2	2	2	2	1	1	2	2	1	1
2	2	2	2	2	2	2	2	1	1
2	2	2	2	2	2	2	2	1	1
2	2	2	2	2	2	2	2	1	1
2	2	2	2	2	2	2	2	1	1
2	2	3	3	1	1	1	1	1	1
2	2	3	3	2	2	2	2	1	1
3	3	2	2	2	2	2	2	1	1
3	3	2	2	2	2	2	2	1	1
3	3	2	2	2	2	2	2	1	1
2	2	2	2	2	2	2	2	1	1
2	2	2	2	2	2	2	2	1	1
2	2	3	3	2	2	1	1	1	1
2	2	2	2	2	2	2	2	1	1
2	2	3	3	2	2	1	1	1	1
2	2	3	3	2	2	1	1	1	1
2	2	2	2	2	2	2	2	1	1
2	2	3	3	2	2	1	1	1	1
2	2	3	3	2	2	1	1	1	1
3	3	2	2	2	2	2	2	1	1
2	2	2	2	2	2	2	2	1	1
2	2	3	3	2	2	1	1	1	1
2	2	2	2	2	2	1	1	1	1

2	2	2	2	2	2	2	2	1	1
3	3	2	2	2	2	2	2	1	1
2	2	2	2	1	1	1	1	1	1
2	2	2	2	2	2	2	2	1	1
2	2	2	2	1	1	2	2	1	1
3	3	2	2	2	2	2	2	4	4
3	3	2	2	2	2	2	2	1	1
2	2	2	2	2	2	1	1	1	1
2	2	2	2	2	2	1	1	1	1
2	2	2	2	2	2	2	2	1	1
2	2	2	2	2	2	1	1	1	1
2	2	2	2	2	2	2	2	1	1
2	2	2	2	1	1	2	2	4	4
2	2	2	2	1	1	2	2	1	1
2	2	2	2	2	2	2	2	1	1
2	2	2	2	2	2	2	2	1	1
2	2	2	2	2	2	2	2	1	1
2	2	2	2	1	1	1	1	1	1
2	2	2	2	2	2	2	2	1	1
2	2	3	3	2	2	1	1	1	1
2	2	2	2	2	2	1	1	1	1
2	2	2	2	2	2	2	2	1	1
2	2	2	2	2	2	2	2	1	1
2	2	3	3	2	2	1	1	1	1
2	2	2	2	2	2	2	2	1	1
2	2	2	2	2	2	2	2	1	1
2	2	2	2	2	2	2	2	1	1
2	2	2	2	1	1	2	2	1	1
2	2	2	2	2	2	1	1	1	1
2	2	2	2	2	2	2	2	1	1
2	2	2	2	2	2	2	2	4	4
2	2	2	2	1	1	2	2	1	1
2	2	2	2	1	1	1	1	1	1
2	2	2	2	2	2	2	2	1	1
2	2	2	2	1	1	1	1	1	1
2	2	3	3	2	2	1	1	1	1
2	2	2	2	2	2	2	2	1	1
2	2	2	2	2	2	2	2	4	4
2	2	3	3	2	2	1	1	1	1
2	2	3	3	1	1	1	1	4	4
2	2	3	3	2	2	1	1	1	1
2	2	3	3	2	2	1	1	1	1
2	2	2	2	2	2	2	2	1	1
2	2	2	2	2	2	2	2	1	1
2	2	2	2	2	2	1	1	1	1
2	2	2	2	2	2	2	2	4	4
2	2	2	2	2	2	2	2	1	1
2	2	2	2	2	2	2	2	1	1
2	2	2	2	2	2	2	2	1	1
2	2	3	3	2	2	1	1	4	4
2	2	3	3	2	2	1	1	1	1
2	2	2	2	2	2	2	2	4	4
2	2	2	2	2	2	2	2	1	1
2	2	3	3	2	2	1	1	1	1
3	3	2	2	2	2	2	2	1	1

For Review Only

2	2	2	2	2	2	2	2	1	1
2	2	2	2	2	2	2	2	1	1
2	2	2	2	2	2	1	1	1	1
2	2	2	2	2	2	1	1	1	1
2	2	2	2	1	1	2	2	1	1
2	2	2	2	2	2	1	1	1	1
3	3	2	2	2	2	2	2	1	1
2	2	2	2	2	2	2	2	1	1
2	2	2	2	2	2	2	2	1	1
2	2	2	2	2	2	2	2	1	1
2	2	2	2	2	2	1	1	1	1
2	2	2	2	2	2	2	2	4	4
2	2	3	3	2	2	1	1	1	1
2	2	2	2	2	2	2	2	1	1
2	2	3	3	2	2	1	1	4	4
2	2	2	2	2	2	2	2	1	1
2	2	2	2	2	2	2	2	1	1
2	2	2	2	2	2	2	2	1	1
2	2	2	2	2	2	2	2	1	1
2	2	3	3	2	2	1	1	4	4
2	2	2	2	2	2	2	2	1	1
2	2	3	3	2	2	1	1	1	1
2	2	2	2	2	2	2	2	1	1
2	2	2	2	2	2	2	2	1	1
2	2	2	2	2	2	2	2	1	1
2	2	2	2	2	2	1	1	1	1
3	3	2	2	2	2	2	2	1	1
2	2	3	3	2	2	1	1	1	1
2	2	2	2	1	1	2	2	1	1
2	2	2	2	2	2	2	2	1	1
2	2	2	2	2	2	2	2	1	1
2	2	3	3	2	2	1	1	1	1
2	2	2	2	2	2	2	2	1	1
2	2	3	3	2	2	1	1	1	1
2	2	2	2	2	2	2	2	1	1
3	3	2	2	2	2	2	2	1	1
2	2	2	2	2	2	2	2	1	1
2	2	3	3	2	2	1	1	1	1
2	2	2	2	2	2	2	2	1	1
2	2	2	2	2	2	2	2	1	1
2	2	2	2	2	2	1	1	1	1
2	2	2	2	2	2	2	2	1	1
3	3	2	2	2	2	2	2	1	1
2	2	2	2	2	2	2	2	1	1
2	2	3	3	2	2	1	1	1	1
2	2	3	3	2	2	1	1	1	1
2	2	3	3	2	2	1	1	1	1
2	2	3	3	1	1	1	1	1	1
2	2	3	3	1	1	1	1	1	1
3	3	2	2	2	2	2	2	1	1
2	2	2	2	2	2	1	1	1	1
2	2	2	2	2	2	2	2	1	1
2	2	3	3	2	2	1	1	1	1
2	2	3	3	2	2	1	1	1	1
3	3	2	2	2	2	2	2	1	1

For Review Only

2	2	2	2	2	2	2	2	1	1
2	2	3	3	2	2	1	1	1	1

For Review Only

SNP2186	SNP2186	SNP2187	SNP2187	SNP2188	SNP2188	SNP2189	SNP2189	SNP219	SNP219
3	3	2	2	2	2	1	1	3	3
3	3	2	2	2	2	1	1	2	2
3	3	2	2	2	2	1	1	2	2
3	3	2	2	2	2	1	1	2	2
3	3	4	4	2	2	1	1	3	3
3	3	4	4	2	2	1	1	2	2
3	3	4	4	2	2	1	1	3	3
3	3	2	2	2	2	1	1	2	2
3	3	4	4	2	2	1	1	3	3
3	3	2	2	2	2	1	1	2	2
3	3	4	4	2	2	1	1	2	2
3	3	2	2	2	2	1	1	2	2
3	3	2	2	2	2	1	1	3	3
3	3	4	4	2	2	1	1	2	2
3	3	2	2	2	2	1	1	3	3
3	3	2	2	2	2	1	1	2	2
3	3	4	4	2	2	1	1	2	2
3	3	2	2	2	2	1	1	3	3
3	3	2	2	2	2	1	1	2	2
3	3	2	2	2	2	1	1	2	2
3	3	2	2	2	2	1	1	3	3
3	3	2	2	2	2	1	1	3	3
3	3	4	4	4	4	1	1	3	3
3	3	2	2	2	2	1	1	2	2
3	3	2	2	2	2	1	1	3	3
1	1	4	4	4	4	1	1	2	2
3	3	2	2	2	2	1	1	3	3
3	3	2	2	2	2	1	1	2	2
3	3	2	2	2	2	1	1	3	3
3	3	2	2	2	2	1	1	2	2
3	3	2	2	2	2	1	1	3	3
1	1	2	2	4	4	1	1	2	2
3	3	4	4	2	2	1	1	3	3
3	3	2	2	2	2	1	1	3	3
3	3	2	2	2	2	1	1	2	2
3	3	2	2	2	2	1	1	2	2
3	3	2	2	2	2	1	1	2	2
3	3	2	2	2	2	1	1	3	3
3	3	4	4	2	2	1	1	2	2
3	3	2	2	4	4	4	4	2	2
3	3	2	2	2	2	1	1	2	2
3	3	2	2	2	2	1	1	2	2
3	3	2	2	2	2	1	1	2	2
3	3	2	2	2	2	1	1	2	2
3	3	2	2	2	2	1	1	2	2
3	3	2	2	2	2	1	1	2	2
1	1	2	2	4	4	4	4	2	2
3	3	2	2	2	2	1	1	2	2
3	3	2	2	2	2	1	1	2	2
3	3	2	2	2	2	1	1	3	3
1	1	2	2	4	4	1	1	2	2
3	3	2	2	2	2	1	1	2	2

For Review Only

3	3	4	4	2	2	1	1	2	2
3	3	4	4	2	2	1	1	3	3
3	3	2	2	2	2	1	1	3	3
3	3	2	2	2	2	1	1	2	2
3	3	2	2	2	2	1	1	2	2
1	1	2	2	4	4	1	1	3	3
1	1	2	2	4	4	1	1	3	3
3	3	4	4	2	2	1	1	2	2
3	3	4	4	2	2	1	1	3	3
3	3	2	2	2	2	1	1	2	2
3	3	2	2	2	2	1	1	3	3
3	3	2	2	2	2	1	1	3	3
3	3	4	4	2	2	1	1	3	3
3	3	2	2	2	2	1	1	3	3
3	3	2	2	2	2	1	1	2	2
3	3	2	2	2	2	1	1	3	3
3	3	4	4	2	2	1	1	2	2
3	3	2	2	2	2	1	1	3	3
3	3	2	2	2	2	1	1	3	3
3	3	4	4	2	2	1	1	3	3
3	3	2	2	2	2	1	1	3	3
3	3	2	2	2	2	1	1	2	2
3	3	2	2	2	2	1	1	3	3
3	3	2	2	2	2	1	1	3	3
3	3	2	2	2	2	1	1	2	2
3	3	2	2	2	2	1	1	2	2
3	3	4	4	2	2	1	1	2	2
3	3	4	4	2	2	1	1	2	2
1	1	2	2	4	4	1	1	2	2
3	3	2	2	2	2	1	1	2	2
3	3	2	2	2	2	1	1	3	3
3	3	2	2	2	2	1	1	3	3
3	3	4	4	4	4	1	1	2	2
3	3	4	4	2	2	1	1	3	3
3	3	4	4	2	2	1	1	3	3
3	3	2	2	2	2	1	1	2	2
3	3	2	2	2	2	1	1	3	3
3	3	2	2	2	2	1	1	3	3
3	3	2	2	2	2	1	1	3	3
3	3	2	2	2	2	1	1	2	2
3	3	2	2	2	2	1	1	3	3
3	3	2	2	2	2	1	1	3	3
1	1	4	4	2	2	1	1	2	2
3	3	2	2	2	2	1	1	3	3
3	3	4	4	2	2	1	1	3	3
3	3	2	2	2	2	1	1	3	3
3	3	4	4	2	2	1	1	2	2
3	3	2	2	2	2	1	1	2	2
1	1	2	2	4	4	1	1	2	2
3	3	2	2	2	2	1	1	3	3
3	3	2	2	2	2	1	1	2	2
3	3	4	4	2	2	4	4	2	2
3	3	4	4	2	2	1	1	2	2
3	3	2	2	2	2	1	1	3	3
3	3	4	4	2	2	4	4	3	3

For Review Only

1	1	2	2	4	4	1	1	2	2
3	3	2	2	2	2	1	1	2	2
1	1	2	2	4	4	1	1	3	3
3	3	4	4	2	2	1	1	2	2
3	3	2	2	2	2	1	1	3	3
3	3	2	2	2	2	1	1	3	3
3	3	2	2	2	2	1	1	2	2
3	3	2	2	2	2	1	1	2	2
3	3	2	2	2	2	1	1	2	2
3	3	2	2	2	2	1	1	3	3
3	3	2	2	2	2	1	1	3	3
3	3	2	2	2	2	1	1	2	2
1	1	4	4	4	4	1	1	3	3
3	3	2	2	2	2	1	1	2	2
3	3	2	2	2	2	1	1	3	3
1	1	2	2	2	2	1	1	2	2
3	3	4	4	2	2	1	1	3	3
3	3	2	2	2	2	1	1	2	2
3	3	4	4	2	2	1	1	2	2
3	3	2	2	4	4	1	1	2	2
3	3	2	2	2	2	1	1	2	2
3	3	2	2	2	2	1	1	3	3
3	3	2	2	2	2	1	1	2	2
3	3	4	4	2	2	1	1	2	2
3	3	2	2	2	2	1	1	3	3
3	3	2	2	4	4	1	1	3	3
1	1	2	2	4	4	1	1	3	3
3	3	2	2	2	2	1	1	2	2
3	3	2	2	2	2	1	1	3	3
3	3	2	2	2	2	1	1	3	3
3	3	4	4	2	2	4	4	2	2
3	3	2	2	2	2	1	1	2	2
3	3	2	2	2	2	1	1	3	3
3	3	2	2	4	4	1	1	2	2
3	3	2	2	2	2	1	1	3	3
1	1	2	2	2	2	1	1	3	3
3	3	4	4	2	2	1	1	3	3
3	3	2	2	2	2	1	1	2	2
3	3	2	2	2	2	1	1	2	2
3	3	4	4	4	4	1	1	2	2
3	3	2	2	4	4	1	1	2	2
3	3	4	4	2	2	1	1	2	2
3	3	4	4	2	2	1	1	3	3
3	3	2	2	2	2	1	1	2	2
1	1	4	4	2	2	1	1	2	2
3	3	4	4	2	2	1	1	3	3
1	1	2	2	4	4	1	1	2	2
1	1	2	2	4	4	1	1	2	2
3	3	2	2	2	2	1	1	2	2
1	1	2	2	4	4	1	1	2	2
1	1	2	2	4	4	1	1	2	2
1	1	4	4	4	4	1	1	2	2
3	3	4	4	2	2	1	1	3	3
3	3	2	2	2	2	1	1	2	2
3	3	4	4	2	2	1	1	2	2

For Review Only

For Review Only

3	3	2	2	2	2	1	1	2	2
3	3	4	4	4	4	4	4	2	2
3	3	4	4	2	2	1	1	2	2
3	3	2	2	2	2	1	1	3	3
3	3	2	2	2	2	1	1	3	3
3	3	4	4	4	4	4	4	2	2
3	3	4	4	2	2	4	4	2	2
3	3	2	2	2	2	1	1	3	3
3	3	2	2	2	2	1	1	2	2
3	3	2	2	2	2	1	1	3	3
1	1	2	2	2	2	1	1	2	2
3	3	2	2	2	2	4	4	3	3
3	3	2	2	2	2	1	1	3	3
3	3	4	4	2	2	1	1	2	2
3	3	4	4	2	2	4	4	2	2
3	3	4	4	2	2	1	1	2	2
3	3	2	2	2	2	1	1	2	2
3	3	2	2	2	2	1	1	3	3
1	1	4	4	4	4	1	1	2	2
3	3	2	2	2	2	1	1	2	2
3	3	2	2	2	2	1	1	2	2
3	3	2	2	2	2	4	4	2	2
3	3	2	2	2	2	1	1	2	2
3	3	2	2	2	2	1	1	3	3
3	3	2	2	2	2	1	1	3	3
3	3	2	2	2	2	1	1	2	2
3	3	2	2	2	2	1	1	3	3
3	3	4	4	2	2	1	1	3	3
3	3	4	4	2	2	1	1	3	3
3	3	2	2	2	2	1	1	3	3
3	3	2	2	2	2	1	1	3	3
3	3	2	2	2	2	1	1	2	2
1	1	2	2	4	4	1	1	2	2
3	3	4	4	2	2	1	1	2	2
3	3	2	2	2	2	1	1	3	3
3	3	2	2	2	2	1	1	3	3
1	1	2	2	4	4	1	1	2	2
3	3	2	2	2	2	1	1	2	2
3	3	2	2	2	2	1	1	2	2
3	3	2	2	2	2	1	1	3	3
3	3	2	2	2	2	1	1	2	2
1	1	2	2	4	4	1	1	2	2
3	3	4	4	2	2	1	1	2	2
3	3	2	2	2	2	1	1	2	2
3	3	2	2	2	2	1	1	2	2
3	3	4	4	2	2	1	1	3	3
3	3	4	4	2	2	1	1	2	2
3	3	4	4	2	2	4	4	2	2
3	3	4	4	2	2	1	1	2	2
3	3	2	2	2	2	1	1	2	2
3	3	2	2	2	2	1	1	3	3
3	3	4	4	2	2	1	1	3	3
3	3	4	4	2	2	1	1	2	2
3	3	2	2	2	2	1	1	3	3
3	3	4	4	4	4	4	4	2	2

3	3	2	2	2	2	1	1	3	3
3	3	2	2	2	2	1	1	2	2
3	3	2	2	2	2	1	1	2	2
3	3	2	2	2	2	1	1	2	2
3	3	2	2	2	2	1	1	2	2
1	1	2	2	4	4	1	1	2	2
3	3	4	4	4	4	4	4	2	2
3	3	2	2	2	2	1	1	2	2
3	3	2	2	2	2	1	1	2	2
3	3	4	4	2	2	1	1	2	2
3	3	2	2	2	2	1	1	2	2
3	3	2	2	2	2	1	1	3	3
3	3	2	2	2	2	1	1	3	3
3	3	2	2	2	2	1	1	2	2
3	3	2	2	2	2	1	1	3	3
3	3	4	4	2	2	1	1	2	2
3	3	4	4	2	2	1	1	2	2
1	1	4	4	4	4	4	4	2	2
3	3	4	4	2	2	4	4	2	2
3	3	2	2	2	2	1	1	3	3
3	3	4	4	2	2	4	4	3	3
1	1	2	2	4	4	1	1	2	2
3	3	2	2	2	2	1	1	3	3
3	3	4	4	2	2	1	1	2	2
3	3	4	4	2	2	1	1	2	2
3	3	2	2	2	2	1	1	2	2
3	3	4	4	4	4	1	1	2	2
3	3	2	2	2	2	1	1	2	2
3	3	2	2	2	2	1	1	2	2
3	3	2	2	2	2	1	1	2	2
3	3	4	4	2	2	4	4	2	2
3	3	2	2	2	2	1	1	2	2
3	3	4	4	2	2	1	1	2	2
3	3	2	2	2	2	1	1	3	3
3	3	2	2	2	2	1	1	2	2
3	3	4	4	4	4	4	4	2	2
3	3	2	2	2	2	1	1	3	3
3	3	2	2	2	2	1	1	3	3
3	3	2	2	2	2	1	1	2	2
3	3	4	4	2	2	4	4	2	2
1	1	2	2	4	4	1	1	3	3
3	3	2	2	2	2	1	1	2	2
3	3	4	4	4	4	1	1	3	3
3	3	2	2	2	2	1	1	3	3
3	3	2	2	2	2	1	1	2	2
3	3	2	2	2	2	1	1	2	2
3	3	4	4	2	2	1	1	2	2
1	1	4	4	2	2	1	1	3	3
3	3	2	2	2	2	1	1	2	2
3	3	4	4	4	4	1	1	2	2
3	3	2	2	2	2	1	1	3	3
3	3	2	2	2	2	1	1	2	2
3	3	2	2	2	2	1	1	2	2
3	3	2	2	2	2	1	1	3	3
3	3	4	4	4	4	4	4	2	2

For Review Only

3	3	2	2	2	2	1	1	2	2
3	3	2	2	2	2	1	1	3	3

For Review Only

SNP2190	SNP2190	SNP2191	SNP2191	SNP2192	SNP2192	SNP2193	SNP2193	SNP2194	SNP2194
4	4	1	1	4	4	2	2	2	2
4	4	1	1	4	4	2	2	2	2
4	4	1	1	4	4	2	2	1	1
4	4	4	4	1	1	2	2	2	2
4	4	1	1	4	4	2	2	2	2
4	4	1	1	4	4	2	2	2	2
4	4	4	4	1	1	2	2	2	2
4	4	4	4	4	4	2	2	2	2
4	4	1	1	4	4	2	2	2	2
4	4	4	4	4	4	2	2	2	2
4	4	1	1	4	4	2	2	2	2
4	4	1	1	4	4	2	2	1	1
4	4	1	1	4	4	2	2	2	2
4	4	1	1	4	4	2	2	2	2
4	4	1	1	4	4	2	2	2	2
4	4	1	1	4	4	3	3	1	1
4	4	1	1	4	4	2	2	2	2
4	4	4	4	4	4	2	2	1	1
4	4	4	4	4	4	2	2	2	2
4	4	1	1	4	4	2	2	2	2
4	4	1	1	4	4	2	2	2	2
4	4	1	1	4	4	2	2	2	2
3	3	1	1	4	4	2	2	2	2
4	4	1	1	4	4	2	2	2	2
4	4	1	1	4	4	2	2	2	2
3	3	1	1	4	4	2	2	1	1
4	4	1	1	4	4	2	2	1	1
4	4	1	1	4	4	2	2	2	2
4	4	1	1	4	4	2	2	2	2
4	4	1	1	4	4	2	2	2	2
4	4	4	4	4	4	2	2	2	2
3	3	1	1	4	4	2	2	2	2
4	4	1	1	4	4	2	2	2	2
4	4	1	1	4	4	3	3	2	2
4	4	1	1	4	4	2	2	2	2
4	4	1	1	4	4	3	3	2	2
4	4	1	1	1	1	2	2	1	1
4	4	1	1	1	1	2	2	1	1
4	4	1	1	4	4	3	3	2	2
3	3	1	1	4	4	3	3	2	2
4	4	1	1	4	4	2	2	2	2
4	4	1	1	4	4	2	2	2	2
4	4	4	4	4	4	2	2	2	2
4	4	1	1	4	4	2	2	2	2
4	4	4	4	4	4	2	2	2	2
4	4	1	1	4	4	2	2	1	1
3	3	1	1	4	4	2	2	1	1
4	4	1	1	4	4	2	2	2	2
4	4	1	1	4	4	2	2	2	2
4	4	1	1	4	4	2	2	2	2
3	3	1	1	4	4	2	2	1	1
4	4	1	1	4	4	2	2	2	2

For Review Only

4	4	1	1	4	4	2	2	2	2
4	4	1	1	4	4	2	2	2	2
4	4	1	1	4	4	2	2	2	2
4	4	1	1	4	4	2	2	2	2
4	4	4	4	4	4	2	2	2	2
3	3	1	1	4	4	2	2	1	1
3	3	1	1	4	4	2	2	1	1
4	4	1	1	4	4	2	2	2	2
4	4	1	1	4	4	3	3	2	2
4	4	1	1	4	4	2	2	2	2
4	4	1	1	4	4	2	2	1	1
4	4	1	1	4	4	2	2	1	1
4	4	1	1	4	4	2	2	2	2
4	4	1	1	4	4	2	2	2	2
4	4	1	1	4	4	2	2	2	2
4	4	1	1	4	4	2	2	2	2
4	4	1	1	4	4	2	2	1	1
4	4	1	1	4	4	2	2	1	1
4	4	1	1	4	4	2	2	2	2
4	4	1	1	4	4	2	2	2	2
4	4	1	1	4	4	2	2	2	2
4	4	1	1	4	4	2	2	2	2
4	4	1	1	4	4	2	2	2	2
4	4	1	1	4	4	2	2	2	2
4	4	4	4	4	4	2	2	2	2
4	4	4	4	4	4	2	2	2	2
4	4	1	1	4	4	2	2	2	2
4	4	1	1	4	4	2	2	2	2
3	3	1	1	4	4	2	2	1	1
4	4	4	4	4	4	2	2	2	2
4	4	1	1	4	4	2	2	1	1
4	4	1	1	4	4	2	2	2	2
3	3	1	1	4	4	2	2	2	2
4	4	1	1	4	4	2	2	2	2
4	4	1	1	4	4	3	3	1	1
4	4	4	4	4	4	2	2	2	2
4	4	4	4	4	4	2	2	1	1
4	4	1	1	4	4	2	2	2	2
4	4	1	1	4	4	2	2	2	2
4	4	4	4	4	4	2	2	2	2
4	4	4	4	4	4	2	2	2	2
4	4	1	1	4	4	2	2	2	2
3	3	4	4	1	1	2	2	1	1
4	4	1	1	4	4	2	2	1	1
4	4	4	4	4	4	2	2	1	1
4	4	4	4	4	4	2	2	2	2
4	4	1	1	4	4	3	3	2	2
4	4	1	1	4	4	2	2	2	2
3	3	1	1	4	4	2	2	1	1
4	4	4	4	4	4	2	2	2	2
4	4	1	1	4	4	2	2	1	1
4	4	1	1	4	4	3	3	2	2
4	4	1	1	4	4	2	2	1	1
4	4	1	1	4	4	3	3	2	2
4	4	4	4	4	4	2	2	1	1

For Review Only

3	3	1	1	4	4	2	2	2	2
4	4	1	1	4	4	2	2	2	2
3	3	1	1	4	4	3	3	2	2
4	4	1	1	4	4	3	3	2	2
4	4	1	1	4	4	2	2	2	2
4	4	1	1	4	4	2	2	1	1
4	4	1	1	4	4	2	2	1	1
4	4	1	1	4	4	2	2	2	2
4	4	1	1	1	1	2	2	1	1
4	4	4	4	4	4	3	3	2	2
4	4	1	1	1	1	2	2	2	2
4	4	1	1	4	4	2	2	2	2
3	3	4	4	1	1	2	2	1	1
4	4	4	4	4	4	2	2	2	2
4	4	1	1	4	4	2	2	2	2
4	4	1	1	4	4	2	2	1	1
4	4	1	1	4	4	2	2	2	2
4	4	1	1	4	4	2	2	2	2
4	4	1	1	4	4	3	3	2	2
3	3	1	1	1	1	2	2	2	2
4	4	1	1	4	4	2	2	2	2
4	4	4	4	4	4	2	2	2	2
4	4	4	4	4	4	2	2	1	1
4	4	1	1	4	4	3	3	2	2
4	4	4	4	4	4	2	2	2	2
3	3	1	1	4	4	2	2	2	2
3	3	1	1	4	4	2	2	2	2
4	4	1	1	4	4	2	2	1	1
4	4	1	1	4	4	2	2	2	2
4	4	1	1	4	4	2	2	1	1
4	4	1	1	4	4	3	3	2	2
4	4	1	1	4	4	2	2	2	2
4	4	4	4	4	4	2	2	2	2
4	4	1	1	4	4	2	2	2	2
4	4	1	1	4	4	3	3	2	2
4	4	4	4	4	4	2	2	1	1
4	4	1	1	4	4	3	3	2	2
4	4	4	4	4	4	2	2	2	2
4	4	1	1	4	4	3	3	2	2
3	3	1	1	4	4	3	3	2	2
3	3	1	1	4	4	2	2	2	2
4	4	1	1	4	4	3	3	2	2
4	4	1	1	4	4	2	2	1	1
4	4	4	4	1	1	2	2	2	2
4	4	1	1	1	1	2	2	1	1
4	4	1	1	4	4	2	2	2	2
3	3	1	1	4	4	2	2	1	1
3	3	1	1	4	4	2	2	1	1
4	4	1	1	4	4	2	2	2	2
3	3	1	1	4	4	2	2	1	1
3	3	1	1	4	4	2	2	1	1
3	3	1	1	4	4	2	2	2	2
4	4	1	1	4	4	2	2	1	1
4	4	1	1	4	4	2	2	1	1
4	4	1	1	4	4	2	2	2	2

For Review Only

4	4	1	1	4	4	2	2	1	1
3	3	1	1	4	4	2	2	1	1
4	4	4	4	4	4	2	2	1	1
4	4	1	1	4	4	2	2	2	2
4	4	4	4	4	4	2	2	1	1
3	3	1	1	1	1	3	3	2	2
4	4	1	1	4	4	3	3	2	2
4	4	1	1	4	4	2	2	2	2
3	3	1	1	4	4	2	2	2	2
4	4	1	1	4	4	2	2	2	2
3	3	1	1	4	4	2	2	1	1
4	4	1	1	4	4	2	2	2	2
4	4	4	4	4	4	2	2	1	1
4	4	4	4	4	4	2	2	2	2
4	4	1	1	4	4	2	2	2	2
4	4	1	1	4	4	2	2	2	2
4	4	1	1	4	4	3	3	2	2
4	4	4	4	4	4	2	2	2	2
3	3	1	1	4	4	2	2	2	2
4	4	1	1	4	4	2	2	1	1
4	4	1	1	4	4	2	2	2	2
4	4	1	1	4	4	3	3	2	2
4	4	1	1	4	4	2	2	2	2
4	4	1	1	4	4	2	2	1	1
4	4	1	1	4	4	2	2	2	2
4	4	1	1	4	4	3	3	2	2
3	3	1	1	4	4	2	2	1	1
4	4	4	4	4	4	3	3	2	2
4	4	1	1	4	4	2	2	2	2
4	4	1	1	4	4	2	2	2	2
4	4	1	1	1	1	2	2	1	1
4	4	4	4	4	4	2	2	2	2
3	3	4	4	4	4	2	2	2	2
4	4	1	1	4	4	3	3	2	2
4	4	4	4	4	4	2	2	2	2
4	4	1	1	4	4	2	2	1	1
3	3	1	1	4	4	2	2	1	1
4	4	1	1	1	1	2	2	1	1
4	4	1	1	4	4	2	2	1	1
4	4	4	4	1	1	2	2	2	2
4	4	1	1	4	4	2	2	1	1
3	3	1	1	4	4	2	2	1	1
4	4	1	1	4	4	3	3	2	2
4	4	1	1	4	4	2	2	1	1
4	4	1	1	4	4	3	3	2	2
4	4	1	1	1	1	2	2	2	2
4	4	1	1	4	4	2	2	2	2
4	4	1	1	4	4	3	3	2	2
4	4	1	1	4	4	2	2	2	2
4	4	1	1	1	1	2	2	2	2
4	4	1	1	4	4	2	2	2	2
4	4	1	1	1	1	2	2	2	2
4	4	1	1	4	4	3	3	2	2
4	4	1	1	4	4	2	2	1	1
3	3	1	1	4	4	3	3	2	2

For Review Only

4	4	1	1	4	4	3	3	1	1
4	4	1	1	4	4	2	2	1	1
4	4	1	1	4	4	2	2	1	1
4	4	1	1	4	4	2	2	2	2
4	4	4	4	4	4	2	2	2	2
4	4	1	1	4	4	2	2	1	1
4	4	1	1	4	4	3	3	2	2
4	4	1	1	4	4	2	2	2	2
4	4	1	1	4	4	2	2	2	2
4	4	1	1	4	4	3	3	2	2
4	4	1	1	4	4	2	2	2	2
4	4	1	1	1	1	2	2	1	1
4	4	1	1	4	4	2	2	2	2
4	4	1	1	4	4	2	2	2	2
4	4	1	1	1	1	2	2	1	1
4	4	1	1	4	4	2	2	2	2
4	4	1	1	4	4	3	3	2	2
3	3	1	1	4	4	3	3	2	2
4	4	1	1	4	4	3	3	2	2
4	4	1	1	1	1	2	2	1	1
4	4	1	1	4	4	3	3	2	2
3	3	1	1	4	4	2	2	1	1
4	4	1	1	4	4	3	3	1	1
4	4	1	1	4	4	2	2	2	2
4	4	1	1	4	4	2	2	2	2
4	4	1	1	4	4	2	2	2	2
3	3	1	1	4	4	3	3	2	2
4	4	1	1	4	4	2	2	1	1
4	4	4	4	4	4	2	2	2	2
4	4	1	1	4	4	2	2	2	2
4	4	1	1	4	4	3	3	2	2
3	3	1	1	4	4	3	3	2	2
4	4	1	1	4	4	2	2	2	2
4	4	1	1	4	4	2	2	2	2
4	4	1	1	4	4	2	2	2	2
3	3	1	1	4	4	3	3	2	2
4	4	1	1	4	4	2	2	1	1
4	4	1	1	4	4	2	2	1	1
4	4	1	1	4	4	3	3	2	2
4	4	1	1	4	4	3	3	2	2
3	3	1	1	4	4	2	2	2	2
4	4	1	1	4	4	2	2	2	2
3	3	1	1	4	4	2	2	2	2
4	4	1	1	4	4	2	2	2	2
4	4	1	1	4	4	2	2	2	2
4	4	1	1	4	4	2	2	1	1
4	4	1	1	4	4	2	2	2	2
3	3	4	4	4	4	2	2	2	2
4	4	4	4	4	4	2	2	2	2
3	3	1	1	4	4	3	3	2	2
4	4	1	1	4	4	2	2	2	2
4	4	1	1	4	4	3	3	2	2
4	4	1	1	4	4	2	2	1	1
4	4	1	1	4	4	2	2	1	1
3	3	1	1	4	4	3	3	2	2

For Review Only

4	4	1	1	4	4	2	2	2	2
4	4	1	1	4	4	2	2	1	1

For Review Only

SNP2195	SNP2195	SNP2196	SNP2196	SNP2197	SNP2197	SNP2198	SNP2198	SNP2199	SNP2199
4	4	4	4	3	3	2	2	3	3
4	4	4	4	3	3	2	2	3	3
4	4	4	4	3	3	2	2	3	3
4	4	4	4	3	3	2	2	3	3
4	4	4	4	3	3	2	2	3	3
4	4	4	4	3	3	2	2	3	3
1	1	4	4	1	1	2	2	3	3
4	4	4	4	3	3	2	2	1	1
1	1	4	4	1	1	4	4	3	3
4	4	4	4	3	3	2	2	3	3
4	4	4	4	3	3	2	2	3	3
4	4	4	4	3	3	2	2	3	3
4	4	4	4	1	1	2	2	3	3
4	4	4	4	3	3	2	2	3	3
4	4	4	4	3	3	2	2	3	3
4	4	4	4	3	3	2	2	3	3
1	1	4	4	1	1	4	4	3	3
4	4	4	4	3	3	2	2	3	3
4	4	4	4	3	3	2	2	3	3
4	4	4	4	3	3	4	4	3	3
1	1	4	4	1	1	4	4	3	3
1	1	4	4	1	1	2	2	3	3
4	4	4	4	3	3	2	2	3	3
4	4	4	4	3	3	2	2	3	3
4	4	4	4	3	3	2	2	3	3
4	4	4	4	3	3	2	2	1	1
4	4	4	4	3	3	2	2	1	1
4	4	4	4	3	3	2	2	3	3
4	4	4	4	3	3	4	4	3	3
4	4	4	4	3	3	2	2	3	3
4	4	4	4	3	3	4	4	3	3
4	4	4	4	3	3	2	2	1	1
1	1	4	4	1	1	2	2	1	1
4	4	4	4	3	3	2	2	3	3
4	4	4	4	3	3	2	2	3	3
4	4	4	4	3	3	2	2	3	3
4	4	4	4	3	3	2	2	3	3
4	4	4	4	3	3	2	2	3	3
4	4	4	4	3	3	2	2	3	3
4	4	2	2	3	3	2	2	3	3
4	4	4	4	3	3	2	2	3	3
4	4	4	4	3	3	2	2	3	3
4	4	4	4	3	3	2	2	3	3
1	1	4	4	1	1	4	4	3	3
4	4	4	4	3	3	2	2	3	3
4	4	4	4	3	3	2	2	3	3
4	4	4	4	3	3	2	2	1	1
4	4	4	4	3	3	2	2	3	3
1	1	4	4	1	1	4	4	3	3
4	4	4	4	3	3	2	2	3	3
4	4	4	4	3	3	2	2	1	1
4	4	4	4	3	3	4	4	3	3

For Review Only

1	1	4	4	1	1	4	4	3	3
1	1	4	4	1	1	2	2	1	1
1	1	4	4	1	1	4	4	3	3
1	1	4	4	1	1	4	4	3	3
4	4	4	4	3	3	2	2	3	3
4	4	4	4	3	3	2	2	1	1
4	4	4	4	3	3	2	2	1	1
4	4	4	4	3	3	2	2	3	3
4	4	4	4	3	3	2	2	3	3
4	4	4	4	3	3	2	2	3	3
4	4	4	4	3	3	2	2	1	1
4	4	4	4	3	3	2	2	1	1
1	1	4	4	1	1	4	4	3	3
4	4	4	4	3	3	4	4	3	3
4	4	4	4	3	3	2	2	3	3
4	4	4	4	3	3	2	2	3	3
4	4	4	4	3	3	2	2	3	3
4	4	4	4	3	3	4	4	3	3
1	1	4	4	1	1	4	4	3	3
1	1	4	4	1	1	2	2	3	3
4	4	4	4	3	3	2	2	3	3
4	4	4	4	1	1	2	2	3	3
1	1	4	4	3	3	4	4	3	3
4	4	4	4	3	3	2	2	3	3
4	4	4	4	3	3	2	2	3	3
4	4	4	4	3	3	2	2	3	3
4	4	4	4	3	3	4	4	3	3
4	4	4	4	3	3	2	2	3	3
4	4	4	4	3	3	2	2	1	1
4	4	4	4	3	3	2	2	3	3
4	4	4	4	3	3	2	2	1	1
4	4	4	4	3	3	2	2	3	3
4	4	2	2	3	3	2	2	3	3
1	1	4	4	1	1	4	4	3	3
4	4	4	4	3	3	2	2	3	3
4	4	4	4	3	3	2	2	3	3
4	4	4	4	3	3	2	2	1	1
4	4	4	4	3	3	4	4	3	3
1	1	4	4	1	1	2	2	3	3
4	4	4	4	3	3	2	2	3	3
1	1	4	4	1	1	2	2	3	3
4	4	4	4	3	3	2	2	3	3
4	4	4	4	3	3	2	2	3	3
4	4	4	4	3	3	2	2	1	1
1	1	4	4	1	1	4	4	3	3
4	4	4	4	3	3	2	2	3	3
4	4	4	4	3	3	2	2	3	3
4	4	4	4	3	3	2	2	3	3
4	4	4	4	3	3	2	2	3	3
4	4	4	4	3	3	2	2	3	3
4	4	4	4	3	3	2	2	3	3
4	4	4	4	3	3	2	2	3	3
4	4	4	4	3	3	2	2	3	3
4	4	4	4	3	3	2	2	3	3
4	4	4	4	3	3	2	2	3	3

For Review Only

4	4	2	2	3	3	2	2	1	1
4	4	2	2	3	3	2	2	3	3
4	4	4	4	3	3	2	2	3	3
4	4	4	4	3	3	2	2	3	3
4	4	4	4	3	3	2	2	3	3
4	4	4	4	3	3	2	2	3	3
4	4	4	4	3	3	2	2	3	3
4	4	4	4	3	3	2	2	3	3
4	4	4	4	3	3	2	2	3	3
4	4	4	4	3	3	4	4	3	3
4	4	4	4	3	3	2	2	3	3
4	4	4	4	3	3	2	2	3	3
4	4	4	4	3	3	2	2	3	3
4	4	4	4	3	3	2	2	3	3
4	4	4	4	3	3	2	2	3	3
4	4	4	4	3	3	2	2	3	3
1	1	4	4	1	1	2	2	3	3
4	4	4	4	3	3	2	2	3	3
4	4	4	4	3	3	2	2	3	3
4	4	2	2	3	3	2	2	3	3
4	4	4	4	3	3	2	2	3	3
4	4	4	4	3	3	2	2	3	3
4	4	4	4	3	3	2	2	3	3
4	4	4	4	3	3	2	2	3	3
4	4	4	4	3	3	2	2	3	3
1	1	2	2	1	1	2	2	3	3
4	4	4	4	3	3	4	4	3	3
4	4	4	4	3	3	2	2	3	3
4	4	4	4	3	3	2	2	3	3
4	4	4	4	3	3	4	4	1	1
4	4	4	4	3	3	2	2	3	3
4	4	4	4	3	3	2	2	3	3
1	1	4	4	1	1	4	4	3	3
4	4	4	4	3	3	2	2	1	1
4	4	4	4	3	3	2	2	3	3
4	4	4	4	1	1	2	2	3	3
4	4	4	4	3	3	2	2	3	3
4	4	4	4	3	3	2	2	3	3
4	4	4	4	3	3	2	2	3	3
4	4	2	2	3	3	2	2	3	3
4	4	2	2	3	3	2	2	3	3
4	4	4	4	3	3	2	2	3	3
4	4	4	4	3	3	4	4	3	3
4	4	4	4	3	3	2	2	3	3
1	1	4	4	1	1	2	2	3	3
4	4	4	4	3	3	4	4	3	3
4	4	4	4	3	3	2	2	1	1
4	4	4	4	3	3	2	2	1	1
4	4	4	4	3	3	2	2	3	3
4	4	4	4	3	3	2	2	3	3
4	4	4	4	3	3	2	2	1	1
4	4	2	2	3	3	2	2	3	3
4	4	4	4	3	3	2	2	1	1
4	4	4	4	3	3	2	2	3	3
4	4	4	4	1	1	2	2	3	3

For Review Only

4	4	4	4	3	3	2	2	3	3
4	4	2	2	3	3	2	2	3	3
4	4	4	4	1	1	2	2	3	3
4	4	4	4	3	3	2	2	3	3
4	4	4	4	3	3	2	2	3	3
4	4	2	2	3	3	2	2	3	3
4	4	4	4	3	3	2	2	3	3
4	4	4	4	3	3	2	2	3	3
4	4	4	4	3	3	2	2	3	3
4	4	4	4	3	3	4	4	3	3
4	4	4	4	3	3	2	2	1	1
4	4	4	4	3	3	2	2	3	3
4	4	4	4	3	3	2	2	3	3
4	4	4	4	3	3	2	2	3	3
4	4	4	4	3	3	2	2	3	3
4	4	4	4	3	3	2	2	3	3
4	4	4	4	3	3	2	2	3	3
4	4	4	4	3	3	2	2	3	3
4	4	4	4	3	3	2	2	3	3
4	4	4	4	3	3	2	2	3	3
4	4	4	4	3	3	2	2	3	3
4	4	4	4	3	3	2	2	3	3
4	4	4	4	1	1	2	2	3	3
4	4	4	4	3	3	2	2	1	1
1	1	4	4	1	1	4	4	3	3
4	4	4	4	3	3	2	2	3	3
4	4	4	4	3	3	2	2	3	3
4	4	4	4	3	3	2	2	3	3
4	4	4	4	3	3	2	2	3	3
4	4	4	4	3	3	2	2	1	1
4	4	4	4	3	3	2	2	3	3
1	1	4	4	1	1	2	2	3	3
4	4	4	4	3	3	4	4	3	3
4	4	4	4	3	3	2	2	3	3
1	1	4	4	1	1	4	4	3	3
4	4	4	4	3	3	2	2	1	1
4	4	4	4	3	3	2	2	3	3
4	4	4	4	3	3	2	2	3	3
4	4	4	4	3	3	2	2	1	1
4	4	4	4	3	3	2	2	3	3
4	4	4	4	3	3	2	2	3	3
4	4	4	4	3	3	2	2	1	1
4	4	4	4	3	3	2	2	3	3
4	4	4	4	3	3	2	2	3	3
4	4	4	4	3	3	2	2	3	3
4	4	4	4	3	3	2	2	3	3
4	4	4	4	3	3	2	2	3	3
4	4	4	4	3	3	2	2	3	3
4	4	4	4	3	3	2	2	3	3
4	4	4	4	1	1	2	2	3	3
4	4	4	4	3	3	2	2	3	3
4	4	4	4	3	3	2	2	3	3
4	4	4	4	1	1	2	2	3	3
4	4	4	4	3	3	2	2	1	1
4	4	2	2	3	3	2	2	3	3

For Review Only

4	4	4	4	3	3	2	2	1	1
4	4	4	4	3	3	2	2	3	3
4	4	4	4	3	3	2	2	3	3
4	4	4	4	3	3	2	2	3	3
4	4	4	4	3	3	2	2	1	1
4	4	4	4	3	3	2	2	3	3
4	4	2	2	3	3	2	2	3	3
4	4	4	4	3	3	2	2	3	3
4	4	4	4	3	3	2	2	3	3
4	4	4	4	3	3	2	2	3	3
4	4	4	4	3	3	2	2	3	3
4	4	4	4	3	3	2	2	3	3
1	1	4	4	1	1	2	2	3	3
4	4	4	4	3	3	2	2	3	3
4	4	4	4	3	3	2	2	3	3
4	4	4	4	3	3	2	2	3	3
4	4	4	4	3	3	2	2	3	3
4	4	4	4	3	3	4	4	3	3
4	4	4	4	3	3	2	2	3	3
4	4	4	4	3	3	2	2	3	3
4	4	4	4	3	3	2	2	3	3
4	4	4	4	3	3	2	2	1	1
1	1	4	4	1	1	2	2	3	3
4	4	4	4	3	3	2	2	3	3
4	4	4	4	3	3	2	2	3	3
1	1	4	4	3	3	2	2	3	3
4	4	4	4	3	3	2	2	3	3
4	4	4	4	3	3	2	2	3	3
1	1	4	4	1	1	2	2	3	3
4	4	4	4	3	3	2	2	3	3
4	4	4	4	3	3	2	2	3	3
4	4	4	4	3	3	2	2	3	3
4	4	4	4	3	3	2	2	3	3
4	4	4	4	3	3	2	2	3	3
4	4	4	4	1	1	2	2	3	3
4	4	2	2	3	3	2	2	3	3
1	1	4	4	1	1	4	4	1	1
4	4	4	4	3	3	2	2	1	1
4	4	4	4	3	3	2	2	3	3
4	4	4	4	3	3	2	2	3	3
4	4	4	4	3	3	2	2	3	3
4	4	4	4	3	3	2	2	3	3
4	4	2	2	3	3	2	2	3	3
1	1	4	4	1	1	4	4	3	3
4	4	4	4	3	3	2	2	3	3
4	4	4	4	3	3	2	2	3	3
4	4	4	4	3	3	2	2	3	3
4	4	4	4	3	3	2	2	3	3
4	4	4	4	3	3	2	2	3	3
4	4	4	4	3	3	2	2	3	3
4	4	4	4	3	3	4	4	3	3
4	4	4	4	3	3	2	2	3	3
4	4	4	4	3	3	2	2	3	3
4	4	4	4	3	3	2	2	3	3
4	4	2	2	3	3	2	2	3	3

For Review Only

1	1	4	4	1	1	4	4	3	3
4	4	4	4	3	3	2	2	3	3

For Review Only

SNP220	SNP220	SNP2200	SNP2200	SNP2201	SNP2201	SNP2202	SNP2202	SNP2203	SNP2203
2	2	4	4	3	3	1	1	1	1
1	1	4	4	3	3	1	1	1	1
1	1	4	4	2	2	1	1	1	1
1	1	4	4	2	2	4	4	1	1
2	2	1	1	3	3	1	1	1	1
1	1	4	4	2	2	1	1	1	1
2	2	4	4	2	2	1	1	1	1
1	1	1	1	3	3	1	1	1	1
2	2	4	4	2	2	1	1	1	1
1	1	4	4	3	3	1	1	1	1
1	1	1	1	3	3	1	1	1	1
1	1	4	4	2	2	1	1	1	1
2	2	4	4	2	2	1	1	1	1
1	1	4	4	3	3	1	1	1	1
2	2	4	4	2	2	1	1	1	1
1	1	1	1	3	3	1	1	1	1
1	1	4	4	2	2	1	1	1	1
2	2	4	4	3	3	1	1	1	1
1	1	4	4	2	2	1	1	1	1
1	1	1	1	3	3	1	1	1	1
2	2	4	4	2	2	1	1	1	1
2	2	4	4	2	2	1	1	1	1
2	2	4	4	3	3	1	1	1	1
1	1	1	1	2	2	1	1	1	1
2	2	4	4	2	2	1	1	1	1
1	1	1	1	3	3	1	1	3	3
2	2	4	4	2	2	1	1	1	1
2	2	4	4	2	2	1	1	1	1
2	2	4	4	3	3	1	1	1	1
1	1	1	1	3	3	4	4	1	1
2	2	1	1	2	2	1	1	1	1
1	1	4	4	2	2	1	1	1	1
2	2	4	4	2	2	1	1	1	1
2	2	4	4	3	3	4	4	1	1
2	2	4	4	3	3	1	1	1	1
1	1	1	1	3	3	1	1	1	1
1	1	4	4	3	3	1	1	1	1
2	2	4	4	3	3	4	4	1	1
1	1	1	1	2	2	1	1	1	1
1	1	4	4	2	2	4	4	1	1
1	1	1	1	3	3	1	1	1	1
1	1	4	4	3	3	1	1	1	1
1	1	1	1	3	3	1	1	1	1
2	2	4	4	2	2	1	1	1	1
1	1	1	1	3	3	1	1	1	1
2	2	4	4	2	2	1	1	3	3
2	2	4	4	3	3	1	1	1	1
2	2	1	1	3	3	1	1	1	1
1	1	4	4	2	2	1	1	1	1
2	2	4	4	2	2	1	1	1	1
1	1	1	1	3	3	1	1	3	3
1	1	4	4	2	2	1	1	1	1

For Review Only

2	2	4	4	2	2	1	1	1	1
2	2	4	4	2	2	1	1	1	1
2	2	4	4	2	2	1	1	1	1
1	1	4	4	2	2	1	1	1	1
1	1	4	4	2	2	1	1	1	1
2	2	1	1	3	3	1	1	1	1
2	2	1	1	2	2	1	1	3	3
1	1	1	1	3	3	1	1	1	1
2	2	1	1	3	3	1	1	1	1
2	2	4	4	2	2	1	1	1	1
2	2	4	4	3	3	1	1	1	1
2	2	4	4	3	3	1	1	1	1
2	2	4	4	2	2	1	1	1	1
2	2	4	4	3	3	1	1	1	1
2	2	4	4	2	2	1	1	1	1
2	2	4	4	3	3	1	1	1	1
1	1	4	4	2	2	1	1	1	1
2	2	1	1	2	2	1	1	1	1
2	2	4	4	2	2	1	1	1	1
2	2	4	4	2	2	1	1	1	1
2	2	4	4	3	3	1	1	1	1
1	1	4	4	2	2	4	4	1	1
2	2	4	4	2	2	1	1	1	1
2	2	1	1	2	2	1	1	1	1
1	1	1	1	3	3	1	1	1	1
1	1	4	4	2	2	1	1	1	1
1	1	4	4	3	3	1	1	1	1
1	1	4	4	3	3	1	1	1	1
1	1	1	1	3	3	1	1	1	1
1	1	4	4	2	2	1	1	1	1
2	2	4	4	3	3	1	1	1	1
2	2	1	1	3	3	1	1	1	1
1	1	4	4	3	3	1	1	1	1
2	2	4	4	2	2	1	1	1	1
2	2	4	4	2	2	1	1	1	1
1	1	1	1	3	3	1	1	1	1
2	2	4	4	3	3	1	1	1	1
2	2	4	4	3	3	4	4	1	1
1	1	4	4	2	2	1	1	1	1
1	1	1	1	3	3	1	1	1	1
2	2	4	4	2	2	1	1	1	1
2	2	4	4	2	2	1	1	1	1
1	1	4	4	3	3	4	4	1	1
2	2	4	4	3	3	1	1	1	1
2	2	1	1	2	2	1	1	1	1
2	2	4	4	3	3	1	1	3	3
1	1	4	4	3	3	1	1	1	1
1	1	1	1	2	2	1	1	3	3
1	1	1	1	2	2	1	1	1	1
2	2	4	4	2	2	1	1	1	1
2	2	4	4	2	2	1	1	1	1
1	1	1	1	3	3	1	1	1	1
1	1	1	1	3	3	4	4	1	1
2	2	4	4	3	3	1	1	1	1
2	2	4	4	3	3	1	1	1	1

For Review Only

1	1	1	1	3	3	1	1	1	1
1	1	4	4	3	3	1	1	1	1
2	2	1	1	2	2	1	1	3	3
1	1	4	4	3	3	1	1	1	1
1	1	4	4	3	3	1	1	1	1
2	2	1	1	2	2	1	1	1	1
1	1	4	4	2	2	1	1	1	1
1	1	1	1	2	2	1	1	1	1
1	1	1	1	3	3	4	4	1	1
1	1	4	4	3	3	1	1	1	1
2	2	4	4	3	3	4	4	1	1
2	2	4	4	2	2	1	1	1	1
2	2	4	4	3	3	4	4	1	1
1	1	4	4	2	2	1	1	1	1
2	2	4	4	3	3	1	1	1	1
2	2	4	4	3	3	1	1	1	1
2	2	4	4	2	2	1	1	1	1
2	2	1	1	2	2	1	1	1	1
1	1	4	4	3	3	1	1	1	1
1	1	4	4	3	3	4	4	1	1
2	2	4	4	2	2	1	1	1	1
2	2	4	4	2	2	1	1	1	1
1	1	4	4	2	2	1	1	1	1
1	1	1	1	3	3	4	4	1	1
2	2	1	1	2	2	1	1	1	1
2	2	4	4	2	2	1	1	1	1
2	2	4	4	3	3	1	1	1	1
1	1	4	4	3	3	1	1	1	1
2	2	4	4	2	2	1	1	1	1
2	2	1	1	3	3	1	1	1	1
1	1	4	4	3	3	1	1	1	1
2	2	4	4	2	2	1	1	1	1
2	2	4	4	2	2	1	1	1	1
2	2	1	1	2	2	1	1	1	1
2	2	4	4	3	3	4	4	1	1
2	2	4	4	2	2	1	1	1	1
1	1	4	4	3	3	1	1	1	1
1	1	4	4	3	3	1	1	1	1
1	1	1	1	3	3	1	1	1	1
1	1	1	1	3	3	4	4	1	1
2	2	4	4	2	2	1	1	1	1
1	1	4	4	3	3	1	1	1	1
2	2	1	1	3	3	1	1	1	1
1	1	4	4	3	3	1	1	1	1
2	2	4	4	2	2	1	1	3	3
2	2	4	4	3	3	1	1	1	1
1	1	1	1	3	3	1	1	3	3
1	1	1	1	3	3	1	1	1	1
2	2	4	4	2	2	1	1	1	1
1	1	1	1	2	2	1	1	3	3
2	2	1	1	3	3	1	1	3	3
1	1	1	1	3	3	1	1	1	1
2	2	4	4	2	2	1	1	1	1
1	1	4	4	3	3	1	1	3	3
1	1	1	1	3	3	1	1	1	1

For Review Only

1	1	4	4	3	3	1	1	1	1
1	1	1	1	3	3	1	1	1	1
1	1	1	1	2	2	1	1	1	1
2	2	1	1	2	2	1	1	1	1
2	2	4	4	3	3	1	1	1	1
1	1	1	1	3	3	4	4	1	1
1	1	4	4	3	3	1	1	1	1
2	2	1	1	2	2	1	1	1	1
1	1	4	4	2	2	1	1	1	1
2	2	1	1	2	2	1	1	1	1
1	1	1	1	2	2	1	1	1	1
2	2	1	1	2	2	1	1	1	1
2	2	4	4	2	2	4	4	1	1
2	2	4	4	3	3	1	1	1	1
1	1	4	4	3	3	1	1	1	1
1	1	1	1	3	3	1	1	1	1
2	2	4	4	2	2	1	1	1	1
2	2	4	4	2	2	1	1	3	3
1	1	4	4	2	2	1	1	1	1
1	1	4	4	2	2	1	1	1	1
2	2	4	4	2	2	1	1	1	1
1	1	1	1	3	3	1	1	1	1
1	1	4	4	2	2	4	4	1	1
2	2	4	4	3	3	1	1	1	1
2	2	1	1	2	2	1	1	1	1
1	1	4	4	3	3	4	4	1	1
2	2	4	4	2	2	1	1	1	1
2	2	4	4	3	3	1	1	1	1
2	2	4	4	2	2	1	1	1	1
2	2	4	4	2	2	1	1	1	1
2	2	4	4	3	3	1	1	1	1
1	1	4	4	2	2	1	1	1	1
1	1	4	4	3	3	1	1	3	3
1	1	4	4	3	3	1	1	1	1
2	2	4	4	2	2	1	1	1	1
2	2	4	4	3	3	1	1	1	1
1	1	4	4	2	2	1	1	1	1
1	1	4	4	3	3	1	1	1	1
1	1	1	1	3	3	1	1	1	1
2	2	4	4	3	3	4	4	1	1
1	1	4	4	2	2	1	1	1	1
1	1	1	1	3	3	1	1	3	3
1	1	4	4	3	3	1	1	1	1
1	1	4	4	2	2	1	1	1	1
1	1	4	4	3	3	1	1	3	3
2	2	4	4	3	3	4	4	1	1
1	1	1	1	3	3	1	1	1	1
1	1	1	1	3	3	1	1	1	1
1	1	4	4	3	3	1	1	1	1
1	1	4	4	2	2	4	4	1	1
2	2	4	4	2	2	1	1	3	3
2	2	1	1	3	3	1	1	1	1
1	1	1	1	2	2	4	4	1	1
2	2	4	4	3	3	1	1	1	1
1	1	4	4	3	3	4	4	1	1

For Review Only

2	2	4	4	3	3	1	1	1	1
1	1	4	4	3	3	1	1	1	1
1	1	4	4	2	2	1	1	1	1
1	1	4	4	2	2	4	4	1	1
1	1	1	1	3	3	1	1	1	1
2	2	4	4	2	2	1	1	1	1
1	1	4	4	3	3	1	1	1	1
1	1	1	1	3	3	1	1	1	1
1	1	4	4	2	2	1	1	1	1
1	1	1	1	3	3	1	1	1	1
2	2	4	4	2	2	1	1	1	1
2	2	4	4	3	3	1	1	1	1
2	2	1	1	2	2	1	1	1	1
1	1	4	4	3	3	1	1	1	1
2	2	4	4	3	3	4	4	1	1
1	1	1	1	3	3	1	1	1	1
1	1	4	4	3	3	1	1	1	1
1	1	4	4	3	3	1	1	1	1
1	1	4	4	3	3	1	1	1	1
2	2	1	1	3	3	4	4	1	1
2	2	4	4	3	3	1	1	1	1
1	1	1	1	3	3	1	1	3	3
2	2	4	4	2	2	1	1	1	1
1	1	4	4	3	3	4	4	1	1
1	1	1	1	3	3	1	1	1	1
2	2	4	4	2	2	1	1	1	1
1	1	4	4	3	3	1	1	1	1
2	2	4	4	2	2	1	1	1	1
2	2	4	4	2	2	1	1	1	1
2	2	4	4	2	2	1	1	1	1
1	1	4	4	3	3	4	4	1	1
1	1	1	1	3	3	1	1	3	3
1	1	4	4	3	3	1	1	1	1
2	2	1	1	3	3	1	1	3	3
1	1	4	4	2	2	4	4	1	1
1	1	4	4	3	3	4	4	1	1
2	2	4	4	2	2	1	1	1	1
2	2	4	4	3	3	1	1	1	1
1	1	1	1	3	3	1	1	1	1
1	1	1	1	3	3	1	1	1	1
2	2	4	4	2	2	1	1	1	1
1	1	4	4	2	2	1	1	1	1
2	2	4	4	3	3	4	4	1	1
2	2	4	4	2	2	1	1	1	1
1	1	4	4	3	3	1	1	1	1
2	2	1	1	2	2	1	1	1	1
1	1	1	1	3	3	1	1	1	1
2	2	4	4	3	3	1	1	1	1
1	1	4	4	3	3	1	1	1	1
1	1	4	4	3	3	4	4	1	1
2	2	4	4	2	2	1	1	1	1
1	1	4	4	3	3	4	4	1	1
1	1	4	4	2	2	1	1	1	1
2	2	4	4	2	2	1	1	1	1
1	1	4	4	3	3	4	4	1	1

For Review Only

2	2	1	1	2	2	1	1	1	1
2	2	4	4	3	3	1	1	1	1

For Review Only

SNP2204	SNP2204	SNP2205	SNP2205	SNP2206	SNP2206	SNP2207	SNP2207	SNP2208	SNP2208
2	2	3	3	3	3	4	4	3	3
2	2	3	3	3	3	4	4	3	3
3	3	3	3	3	3	1	1	3	3
2	2	3	3	3	3	1	1	3	3
2	2	3	3	3	3	4	4	3	3
3	3	3	3	3	3	1	1	3	3
3	3	3	3	3	3	1	1	3	3
2	2	2	2	2	2	1	1	3	3
3	3	2	2	2	2	1	1	3	3
2	2	3	3	3	3	1	1	3	3
2	2	2	2	2	2	1	1	3	3
3	3	3	3	3	3	1	1	3	3
3	3	3	3	3	3	1	1	3	3
2	2	3	3	3	3	4	4	3	3
3	3	3	3	3	3	1	1	3	3
2	2	2	2	2	2	4	4	3	3
3	3	2	2	2	2	1	1	3	3
2	2	3	3	3	3	1	1	3	3
3	3	3	3	3	3	4	4	3	3
2	2	2	2	2	2	4	4	3	3
3	3	2	2	2	2	1	1	3	3
3	3	3	3	3	3	4	4	3	3
2	2	3	3	3	3	4	4	3	3
2	2	2	2	2	2	4	4	3	3
3	3	3	3	3	3	1	1	3	3
2	2	2	2	2	2	1	1	3	3
3	3	3	3	3	3	1	1	3	3
3	3	3	3	3	3	1	1	3	3
2	2	2	2	2	2	1	1	3	3
2	2	2	2	2	2	1	1	3	3
3	3	2	2	2	2	1	1	3	3
3	3	3	3	3	3	1	1	2	2
3	3	3	3	3	3	1	1	3	3
2	2	3	3	3	3	4	4	3	3
2	2	3	3	3	3	1	1	3	3
2	2	2	2	2	2	4	4	3	3
2	2	3	3	3	3	1	1	3	3
2	2	2	2	2	2	4	4	3	3
2	2	2	2	2	2	1	1	3	3
3	3	3	3	3	3	1	1	3	3
2	2	2	2	2	2	4	4	3	3
2	2	2	2	2	2	1	1	3	3
2	2	3	3	3	3	1	1	3	3
3	3	2	2	2	2	1	1	3	3
2	2	2	2	2	2	1	1	3	3
3	3	3	3	3	3	1	1	3	3
2	2	2	2	3	3	1	1	3	3
2	2	2	2	2	2	1	1	3	3
3	3	2	2	2	2	1	1	3	3
3	3	3	3	2	2	1	1	3	3
2	2	2	2	2	2	1	1	3	3
3	3	2	2	2	2	1	1	3	3

For Review Only

3	3	2	2	2	2	1	1	3	3
3	3	3	3	3	3	1	1	3	3
3	3	3	3	3	3	1	1	3	3
3	3	3	3	2	2	1	1	3	3
3	3	3	3	3	3	4	4	3	3
2	2	2	2	2	2	1	1	3	3
3	3	2	2	2	2	1	1	3	3
2	2	2	2	2	2	4	4	3	3
2	2	2	2	2	2	1	1	3	3
3	3	3	3	3	3	1	1	3	3
2	2	3	3	3	3	1	1	3	3
2	2	2	2	3	3	1	1	3	3
3	3	2	2	2	2	1	1	3	3
2	2	2	2	2	2	1	1	3	3
3	3	3	3	3	3	1	1	3	3
2	2	3	3	3	3	1	1	3	3
3	3	3	3	3	3	1	1	3	3
3	3	2	2	2	2	1	1	3	3
3	3	2	2	2	2	4	4	3	3
3	3	3	3	3	3	1	1	3	3
2	2	2	2	3	3	1	1	3	3
3	3	3	3	3	3	1	1	2	2
2	2	3	3	3	3	1	1	3	3
3	3	2	2	2	2	1	1	2	2
2	2	2	2	2	2	1	1	3	3
3	3	3	3	3	3	1	1	3	3
2	2	2	2	2	2	1	1	2	2
2	2	3	3	3	3	1	1	3	3
2	2	2	2	2	2	1	1	3	3
3	3	3	3	3	3	1	1	3	3
2	2	3	3	3	3	1	1	3	3
2	2	2	2	2	2	4	4	3	3
2	2	3	3	3	3	1	1	3	3
3	3	2	2	2	2	1	1	3	3
3	3	3	3	3	3	4	4	3	3
2	2	2	2	2	2	1	1	3	3
2	2	3	3	3	3	1	1	3	3
2	2	2	2	2	2	1	1	3	3
3	3	3	3	3	3	1	1	3	3
2	2	2	2	2	2	4	4	3	3
3	3	2	2	2	2	4	4	3	3
3	3	2	2	3	3	1	1	3	3
2	2	3	3	3	3	1	1	3	3
2	2	3	3	3	3	1	1	3	3
3	3	2	2	2	2	1	1	3	3
3	3	3	3	3	3	4	4	3	3
2	2	3	3	3	3	4	4	3	3
3	3	2	2	2	2	1	1	3	3
3	3	2	2	2	2	4	4	3	3
3	3	3	3	3	3	1	1	3	3
3	3	2	2	3	3	4	4	3	3
2	2	2	2	2	2	4	4	3	3
2	2	2	2	2	2	4	4	3	3
3	3	2	2	3	3	4	4	3	3
2	2	3	3	3	3	1	1	3	3

For Review Only

2	2	2	2	2	2	1	1	3	3
2	2	3	3	3	3	1	1	2	2
3	3	2	2	2	2	1	1	3	3
2	2	3	3	3	3	4	4	3	3
3	3	3	3	3	3	4	4	3	3
3	3	2	2	2	2	1	1	3	3
3	3	3	3	3	3	4	4	3	3
3	3	2	2	2	2	1	1	3	3
2	2	2	2	2	2	4	4	3	3
2	2	2	2	2	2	1	1	3	3
2	2	3	3	3	3	4	4	2	2
2	2	3	3	3	3	1	1	3	3
2	2	3	3	3	3	1	1	3	3
3	3	3	3	3	3	1	1	3	3
2	2	3	3	3	3	1	1	3	3
2	2	3	3	2	2	1	1	3	3
3	3	3	3	3	3	1	1	2	2
3	3	3	3	3	3	1	1	3	3
2	2	3	3	3	3	4	4	3	3
2	2	3	3	3	3	4	4	3	3
3	3	3	3	3	3	1	1	3	3
3	3	3	3	3	3	1	1	3	3
3	3	2	2	3	3	4	4	3	3
2	2	2	2	2	2	1	1	3	3
3	3	2	2	2	2	1	1	3	3
3	3	3	3	3	3	1	1	3	3
2	2	2	2	2	2	4	4	3	3
2	2	3	3	3	3	1	1	3	3
3	3	3	3	3	3	4	4	3	3
2	2	2	2	2	2	1	1	3	3
2	2	3	3	3	3	4	4	3	3
2	2	3	3	3	3	4	4	3	3
3	3	2	2	2	2	4	4	3	3
2	2	2	2	2	2	1	1	3	3
2	2	3	3	3	3	4	4	3	3
3	3	3	3	2	2	1	1	3	3
2	2	3	3	3	3	1	1	3	3
2	2	3	3	3	3	1	1	3	3
2	2	2	2	2	2	1	1	3	3
2	2	2	2	2	2	1	1	3	3
3	3	3	3	3	3	1	1	3	3
2	2	3	3	3	3	1	1	3	3
2	2	2	2	2	2	1	1	3	3
2	2	2	2	3	3	4	4	3	3
3	3	3	3	3	3	4	4	3	3
2	2	2	2	2	2	4	4	3	3
2	2	2	2	2	2	1	1	3	3
2	2	2	2	2	2	1	1	3	3
3	3	3	3	3	3	4	4	3	3
3	3	2	2	2	2	1	1	3	3
2	2	2	2	2	2	1	1	3	3
2	2	2	2	2	2	1	1	3	3
3	3	3	3	3	3	1	1	3	3
2	2	2	2	3	3	1	1	3	3
2	2	2	2	2	2	4	4	3	3

For Review Only

2	2	3	3	3	3	4	4	3	3
2	2	2	2	2	2	1	1	3	3
3	3	2	2	2	2	1	1	3	3
3	3	2	2	2	2	1	1	2	2
2	2	3	3	3	3	1	1	3	3
2	2	2	2	2	2	4	4	3	3
2	2	3	3	3	3	1	1	3	3
3	3	3	3	3	3	1	1	3	3
3	3	3	3	3	3	1	1	3	3
3	3	2	2	2	2	1	1	3	3
3	3	2	2	2	2	1	1	3	3
2	2	3	3	2	2	1	1	3	3
3	3	3	3	3	3	1	1	3	3
2	2	3	3	3	3	1	1	3	3
2	2	3	3	3	3	4	4	3	3
2	2	3	3	3	3	1	1	3	3
3	3	3	3	3	3	1	1	3	3
2	2	3	3	3	3	4	4	3	3
3	3	3	3	3	3	1	1	3	3
3	3	3	3	3	3	4	4	3	3
3	3	3	3	3	3	1	1	3	3
2	2	3	3	3	3	1	1	3	3
3	3	3	3	3	3	1	1	2	2
2	2	2	2	3	3	1	1	3	3
3	3	2	2	2	2	1	1	3	3
2	2	3	3	3	3	4	4	3	3
3	3	3	3	3	3	1	1	3	3
2	2	3	3	3	3	1	1	3	3
3	3	3	3	3	3	1	1	3	3
3	3	3	3	3	3	1	1	3	3
2	2	3	3	3	3	4	4	3	3
3	3	3	3	3	3	1	1	3	3
2	2	2	2	2	2	4	4	3	3
2	2	3	3	3	3	4	4	3	3
3	3	2	2	2	2	4	4	3	3
2	2	2	2	3	3	1	1	3	3
3	3	2	2	3	3	1	1	3	3
2	2	3	3	3	3	1	1	3	3
2	2	2	2	2	2	1	1	3	3
2	2	3	3	3	3	4	4	3	3
3	3	3	3	3	3	1	1	3	3
2	2	2	2	2	2	1	1	3	3
2	2	3	3	2	2	4	4	3	3
3	3	2	2	2	2	1	1	3	3
2	2	2	2	3	3	4	4	2	2
2	2	3	3	3	3	1	1	3	3
2	2	3	3	3	3	1	1	3	3
2	2	2	2	2	2	1	1	3	3
2	2	3	3	3	3	4	4	3	3
3	3	3	3	3	3	4	4	3	3
3	3	3	3	3	3	4	4	3	3
2	2	2	2	2	2	4	4	3	3
3	3	2	2	2	2	1	1	3	3
2	2	3	3	3	3	1	1	3	3
2	2	3	3	3	3	4	4	3	3

For Review Only

2	2	3	3	3	3	1	1	3	3
2	2	2	2	2	2	1	1	3	3
3	3	3	3	3	3	1	1	3	3
3	3	3	3	3	3	1	1	3	3
2	2	2	2	2	2	1	1	2	2
2	2	2	2	2	2	1	1	3	3
2	2	3	3	3	3	4	4	3	3
2	2	2	2	2	2	4	4	3	3
3	3	3	3	3	3	4	4	3	3
2	2	2	2	2	2	4	4	3	3
3	3	2	2	3	3	1	1	3	3
2	2	3	3	3	3	4	4	3	3
3	3	2	2	2	2	1	1	3	3
2	2	3	3	3	3	1	1	3	3
2	2	3	3	3	3	4	4	3	3
2	2	2	2	2	2	1	1	3	3
2	2	3	3	3	3	4	4	3	3
2	2	2	2	2	2	4	4	3	3
2	2	3	3	3	3	1	1	3	3
2	2	2	2	2	2	1	1	3	3
2	2	3	3	3	3	4	4	3	3
2	2	2	2	2	2	1	1	3	3
3	3	3	3	3	3	4	4	3	3
2	2	3	3	3	3	1	1	3	3
2	2	2	2	2	2	4	4	3	3
3	3	3	3	2	2	1	1	3	3
2	2	3	3	3	3	1	1	3	3
3	3	3	3	3	3	4	4	3	3
3	3	2	2	2	2	1	1	3	3
3	3	3	3	3	3	4	4	3	3
2	2	3	3	3	3	4	4	3	3
2	2	3	3	2	2	4	4	3	3
2	2	3	3	3	3	1	1	3	3
2	2	2	2	2	2	1	1	3	3
3	3	3	3	3	3	1	1	3	3
2	2	3	3	2	2	4	4	3	3
3	3	2	2	2	2	1	1	3	3
2	2	2	2	3	3	1	1	3	3
2	2	2	2	2	2	4	4	3	3
2	2	2	2	2	2	4	4	3	3
2	2	3	3	3	3	4	4	2	2
3	3	3	3	3	3	4	4	3	3
2	2	3	3	3	3	1	1	3	3
3	3	2	2	2	2	4	4	3	3
2	2	3	3	3	3	4	4	3	3
3	3	2	2	2	2	1	1	3	3
2	2	2	2	2	2	1	1	3	3
2	2	3	3	3	3	1	1	3	3
2	2	2	2	3	3	1	1	3	3
2	2	3	3	3	3	1	1	3	3
3	3	2	2	2	2	1	1	3	3
2	2	3	3	2	2	4	4	3	3
3	3	3	3	3	3	1	1	3	3
3	3	2	2	3	3	4	4	3	3
2	2	3	3	3	3	1	1	3	3

For Review Only

3	3	2	2	2	2	1	1	3	3
2	2	2	2	3	3	1	1	3	3

For Review Only

SNP2209	SNP2209	SNP221	SNP221	SNP2210	SNP2210	SNP2211	SNP2211	SNP2212	SNP2212
3	3	3	3	3	3	2	2	2	2
3	3	3	3	1	1	3	3	3	3
3	3	3	3	1	1	3	3	2	2
3	3	3	3	1	1	3	3	2	2
3	3	2	2	3	3	3	3	3	3
4	4	3	3	1	1	2	2	2	2
3	3	2	2	3	3	3	3	3	3
3	3	3	3	1	1	2	2	2	2
3	3	2	2	3	3	3	3	3	3
3	3	3	3	3	3	2	2	2	2
4	4	3	3	3	3	3	3	3	3
3	3	2	2	1	1	3	3	2	2
3	3	2	2	3	3	3	3	2	2
3	3	3	3	1	1	3	3	2	2
4	4	2	2	1	1	2	2	2	2
3	3	3	3	3	3	2	2	3	3
3	3	3	3	3	3	3	3	3	3
4	4	2	2	1	1	2	2	2	2
3	3	3	3	1	1	2	2	2	2
3	3	3	3	3	3	2	2	2	2
3	3	2	2	3	3	3	3	2	2
3	3	2	2	3	3	3	3	2	2
4	4	2	2	3	3	3	3	2	2
3	3	3	3	1	1	2	2	2	2
3	3	3	3	1	1	3	3	2	2
3	3	3	3	1	1	3	3	3	3
3	3	2	2	1	1	3	3	2	2
4	4	3	3	1	1	3	3	2	2
3	3	3	3	3	3	2	2	2	2
4	4	3	3	3	3	2	2	2	2
3	3	2	2	1	1	3	3	2	2
4	4	3	3	1	1	3	3	2	2
3	3	2	2	3	3	3	3	3	3
3	3	2	2	1	1	3	3	2	2
3	3	2	2	1	1	3	3	2	2
3	3	3	3	3	3	2	2	2	2
3	3	3	3	1	1	3	3	2	2
4	4	3	3	3	3	3	3	2	2
4	4	3	3	3	3	3	3	3	3
4	4	3	3	1	1	3	3	2	2
4	4	3	3	3	3	2	2	2	2
3	3	3	3	1	1	2	2	2	2
3	3	3	3	3	3	2	2	2	2
3	3	3	3	1	1	3	3	2	2
3	3	3	3	3	3	2	2	2	2
3	3	3	3	1	1	3	3	2	2
3	3	3	3	1	1	3	3	2	2
3	3	2	2	1	1	3	3	2	2
3	3	3	3	3	3	3	3	2	2
4	4	2	2	3	3	3	3	2	2
3	3	3	3	1	1	3	3	3	3
4	4	3	3	3	3	2	2	3	3

For Review Only

3	3	3	3	3	3	3	3	2	2
3	3	2	2	3	3	3	3	3	3
3	3	2	2	1	1	3	3	2	2
3	3	3	3	3	3	3	3	2	2
3	3	3	3	1	1	2	2	2	2
3	3	2	2	1	1	3	3	3	3
3	3	2	2	1	1	3	3	2	2
3	3	3	3	3	3	3	3	3	3
3	3	2	2	3	3	2	2	2	2
4	4	2	2	1	1	3	3	2	2
3	3	2	2	1	1	3	3	2	2
3	3	2	2	1	1	3	3	2	2
3	3	3	3	3	3	3	3	2	2
3	3	3	3	3	3	2	2	2	2
4	4	2	2	1	1	3	3	2	2
3	3	2	2	1	1	2	2	2	2
3	3	3	3	1	1	3	3	3	3
3	3	2	2	1	1	2	2	2	2
3	3	2	2	3	3	3	3	2	2
3	3	2	2	3	3	2	2	3	3
4	4	2	2	1	1	3	3	2	2
4	4	3	3	3	3	3	3	2	2
3	3	2	2	3	3	3	3	2	2
4	4	2	2	1	1	3	3	2	2
3	3	3	3	3	3	2	2	2	2
3	3	3	3	1	1	2	2	3	3
4	4	3	3	3	3	3	3	3	3
3	3	3	3	3	3	2	2	3	3
3	3	3	3	1	1	3	3	3	3
4	4	3	3	1	1	3	3	2	2
3	3	2	2	1	1	3	3	2	2
4	4	2	2	3	3	2	2	2	2
4	4	3	3	3	3	2	2	3	3
3	3	3	3	3	3	3	3	2	2
3	3	2	2	1	1	3	3	2	2
3	3	3	3	3	3	2	2	2	2
3	3	2	2	1	1	3	3	2	2
3	3	2	2	3	3	3	3	2	2
3	3	3	3	3	3	3	3	2	2
3	3	3	3	3	3	3	3	2	2
3	3	2	2	3	3	3	3	2	2
4	4	2	2	1	1	3	3	2	2
4	4	3	3	1	1	3	3	3	3
3	3	2	2	1	1	3	3	2	2
3	3	2	2	1	1	3	3	3	3
4	4	2	2	1	1	3	3	3	3
3	3	3	3	3	3	2	2	2	2
3	3	3	3	1	1	2	2	2	2
3	3	3	3	1	1	3	3	2	2
3	3	2	2	3	3	3	3	2	2
3	3	3	3	1	1	3	3	2	2
4	4	3	3	3	3	3	3	2	2
3	3	3	3	3	3	3	3	3	3
3	3	2	2	1	1	3	3	2	2
3	3	2	2	1	1	3	3	3	3

For Review Only

4	4	3	3	1	1	3	3	3	3
4	4	3	3	3	3	3	3	2	2
3	3	2	2	1	1	2	2	2	2
4	4	3	3	3	3	3	3	2	2
3	3	2	2	1	1	3	3	2	2
4	4	2	2	1	1	3	3	2	2
3	3	3	3	1	1	2	2	3	3
4	4	3	3	1	1	3	3	2	2
3	3	3	3	1	1	3	3	2	2
3	3	3	3	3	3	2	2	2	2
4	4	2	2	1	1	3	3	2	2
4	4	3	3	1	1	3	3	2	2
4	4	2	2	1	1	3	3	3	3
4	4	3	3	1	1	2	2	3	3
3	3	2	2	1	1	3	3	3	3
3	3	3	3	1	1	3	3	3	3
3	3	2	2	1	1	3	3	3	3
4	4	2	2	1	1	3	3	2	2
3	3	3	3	1	1	2	2	2	2
4	4	3	3	3	3	3	3	2	2
4	4	2	2	1	1	3	3	2	2
4	4	2	2	1	1	3	3	2	2
4	4	3	3	1	1	3	3	2	2
4	4	3	3	3	3	2	2	3	3
3	3	2	2	1	1	3	3	2	2
4	4	2	2	3	3	2	2	2	2
4	4	2	2	1	1	2	2	2	2
4	4	3	3	1	1	3	3	2	2
4	4	2	2	1	1	3	3	2	2
4	4	2	2	1	1	2	2	2	2
4	4	3	3	3	3	3	3	2	2
3	3	3	3	1	1	3	3	2	2
3	3	2	2	3	3	3	3	2	2
3	3	3	3	1	1	3	3	2	2
3	3	2	2	1	1	3	3	2	2
4	4	2	2	1	1	2	2	2	2
3	3	3	3	3	3	2	2	2	2
3	3	3	3	1	1	2	2	2	2
4	4	3	3	3	3	2	2	2	2
4	4	3	3	3	3	3	3	2	2
4	4	2	2	1	1	3	3	2	2
3	3	3	3	3	3	2	2	2	2
3	3	2	2	1	1	2	2	3	3
4	4	3	3	1	1	2	2	2	2
3	3	2	2	1	1	3	3	3	3
3	3	2	2	3	3	3	3	3	3
3	3	3	3	3	3	3	3	3	3
3	3	3	3	1	1	3	3	3	3
3	3	2	2	3	3	3	3	2	2
3	3	3	3	1	1	3	3	3	3
3	3	2	2	1	1	3	3	2	2
4	4	3	3	3	3	3	3	3	3
3	3	2	2	1	1	2	2	2	2
3	3	3	3	1	1	3	3	2	2
4	4	3	3	1	1	3	3	2	2

For Review Only

3	3	3	3	3	3	3	3	3	3
4	4	3	3	1	1	3	3	3	3
3	3	3	3	1	1	3	3	3	3
4	4	2	2	3	3	3	3	2	2
3	3	2	2	1	1	3	3	3	3
4	4	3	3	1	1	3	3	3	3
3	3	3	3	1	1	3	3	3	3
3	3	2	2	1	1	3	3	2	2
3	3	3	3	1	1	3	3	2	2
3	3	2	2	3	3	3	3	2	2
3	3	3	3	1	1	3	3	2	2
4	4	2	2	1	1	2	2	3	3
4	4	2	2	1	1	3	3	2	2
3	3	2	2	1	1	3	3	2	2
3	3	3	3	1	1	2	2	2	2
4	4	3	3	3	3	3	3	3	3
3	3	2	2	3	3	2	2	2	2
3	3	2	2	1	1	2	2	2	2
3	3	3	3	1	1	2	2	3	3
3	3	3	3	1	1	2	2	3	3
4	4	2	2	1	1	3	3	2	2
3	3	3	3	1	1	3	3	2	2
4	4	3	3	3	3	3	3	2	2
3	3	2	2	1	1	3	3	2	2
4	4	2	2	1	1	3	3	2	2
3	3	3	3	1	1	3	3	2	2
4	4	2	2	1	1	2	2	2	2
4	4	2	2	1	1	3	3	2	2
4	4	2	2	1	1	3	3	3	3
3	3	2	2	3	3	3	3	2	2
3	3	2	2	1	1	3	3	2	2
4	4	3	3	1	1	3	3	2	2
4	4	3	3	1	1	2	2	2	2
3	3	3	3	1	1	3	3	2	2
4	4	2	2	3	3	3	3	2	2
3	3	2	2	1	1	3	3	2	2
4	4	3	3	3	3	3	3	3	3
3	3	3	3	1	1	3	3	2	2
3	3	3	3	1	1	3	3	3	3
3	3	2	2	1	1	3	3	2	2
3	3	3	3	1	1	3	3	2	2
3	3	3	3	1	1	3	3	3	3
3	3	3	3	3	3	3	3	3	3
3	3	3	3	3	3	3	3	2	2
3	3	3	3	1	1	3	3	2	2
4	4	2	2	1	1	2	2	3	3
4	4	3	3	3	3	3	3	3	3
3	3	3	3	3	3	3	3	3	3
3	3	3	3	1	1	3	3	2	2
3	3	3	3	1	1	3	3	2	2
4	4	2	2	1	1	3	3	2	2
3	3	3	3	3	3	3	3	3	3
4	4	3	3	3	3	3	3	3	3
3	3	2	2	1	1	3	3	2	2
4	4	3	3	3	3	3	3	2	2

For Review Only

3	3	2	2	3	3	2	2	2	2
4	4	3	3	1	1	3	3	2	2
4	4	3	3	1	1	3	3	2	2
4	4	3	3	3	3	3	3	2	2
3	3	3	3	1	1	3	3	2	2
3	3	2	2	1	1	3	3	2	2
4	4	3	3	1	1	2	2	2	2
4	4	3	3	1	1	2	2	2	2
4	4	3	3	3	3	3	3	2	2
4	4	3	3	3	3	3	3	3	3
4	4	2	2	1	1	3	3	2	2
3	3	2	2	1	1	3	3	2	2
3	3	3	3	3	3	3	3	2	2
3	3	3	3	1	1	2	2	3	3
3	3	2	2	1	1	3	3	2	2
3	3	3	3	3	3	3	3	3	3
4	4	3	3	3	3	3	3	3	3
4	4	3	3	3	3	3	3	2	2
3	3	3	3	1	1	3	3	3	3
4	4	3	3	3	3	3	3	2	2
3	3	2	2	1	1	3	3	3	3
3	3	3	3	1	1	3	3	3	3
3	3	2	2	3	3	2	2	2	2
3	3	3	3	1	1	3	3	3	3
3	3	3	3	3	3	3	3	3	3
4	4	3	3	1	1	3	3	2	2
4	4	3	3	3	3	2	2	2	2
3	3	2	2	1	1	3	3	2	2
3	3	3	3	1	1	3	3	2	2
4	4	2	2	1	1	3	3	2	2
4	4	3	3	3	3	3	3	3	3
4	4	3	3	1	1	2	2	2	2
3	3	3	3	1	1	3	3	2	2
3	3	2	2	1	1	2	2	3	3
4	4	3	3	3	3	3	3	2	2
3	3	3	3	3	3	3	3	3	3
3	3	2	2	3	3	2	2	2	2
3	3	2	2	1	1	3	3	2	2
3	3	3	3	3	3	2	2	2	2
4	4	3	3	3	3	3	3	3	3
4	4	2	2	3	3	3	3	2	2
3	3	3	3	3	3	3	3	3	3
4	4	2	2	1	1	3	3	3	3
3	3	2	2	3	3	3	3	2	2
3	3	3	3	1	1	3	3	2	2
3	3	3	3	3	3	3	3	2	2
3	3	3	3	3	3	3	3	3	3
4	4	2	2	1	1	3	3	2	2
4	4	3	3	3	3	2	2	3	3
4	4	3	3	3	3	2	2	2	2
4	4	2	2	3	3	3	3	2	2
4	4	3	3	3	3	3	3	3	3
3	3	3	3	1	1	3	3	2	2
3	3	2	2	1	1	2	2	2	2
4	4	3	3	1	1	3	3	3	3

For Review Only

3	3	3	3	3	3	3	3	2	2
3	3	2	2	1	1	3	3	3	3

For Review Only

SNP2213	SNP2213	SNP2214	SNP2214	SNP2215	SNP2215	SNP2216	SNP2216	SNP2217	SNP2217
3	3	4	4	3	3	4	4	2	2
2	2	1	1	3	3	4	4	3	3
3	3	1	1	3	3	4	4	2	2
3	3	1	1	3	3	4	4	3	3
2	2	4	4	3	3	1	1	2	2
3	3	1	1	3	3	4	4	3	3
2	2	4	4	3	3	1	1	3	3
3	3	1	1	3	3	4	4	2	2
2	2	4	4	3	3	1	1	3	3
3	3	4	4	3	3	1	1	3	3
2	2	4	4	3	3	4	4	2	2
3	3	1	1	3	3	4	4	2	2
3	3	1	1	3	3	4	4	3	3
3	3	4	4	3	3	1	1	3	3
3	3	1	1	2	2	1	1	2	2
2	2	1	1	3	3	4	4	3	3
2	2	4	4	3	3	1	1	3	3
3	3	1	1	3	3	4	4	2	2
3	3	1	1	3	3	4	4	3	3
3	3	4	4	3	3	4	4	3	3
3	3	4	4	3	3	1	1	2	2
3	3	4	4	3	3	1	1	2	2
3	3	4	4	3	3	4	4	3	3
3	3	1	1	3	3	4	4	3	3
3	3	1	1	3	3	4	4	3	3
2	2	1	1	2	2	4	4	2	2
3	3	1	1	3	3	4	4	2	2
3	3	1	1	2	2	4	4	2	2
3	3	4	4	3	3	4	4	3	3
3	3	4	4	3	3	4	4	3	3
3	3	1	1	3	3	4	4	2	2
3	3	1	1	3	3	4	4	2	2
2	2	4	4	3	3	1	1	2	2
3	3	4	4	3	3	1	1	3	3
3	3	1	1	3	3	4	4	2	2
3	3	4	4	3	3	4	4	3	3
3	3	1	1	3	3	4	4	2	2
3	3	4	4	3	3	1	1	2	2
2	2	4	4	3	3	4	4	3	3
3	3	1	1	3	3	1	1	3	3
3	3	4	4	3	3	4	4	3	3
3	3	1	1	3	3	4	4	3	3
3	3	4	4	3	3	4	4	3	3
3	3	4	4	3	3	4	4	2	2
3	3	4	4	3	3	4	4	3	3
3	3	1	1	2	2	4	4	2	2
3	3	1	1	3	3	4	4	2	2
3	3	1	1	3	3	4	4	2	2
3	3	4	4	3	3	1	1	2	2
2	2	4	4	3	3	1	1	2	2
2	2	1	1	2	2	4	4	2	2
2	2	4	4	3	3	4	4	3	3

For Review Only

3	3	4	4	3	3	4	4	3	3
2	2	4	4	3	3	1	1	2	2
3	3	1	1	3	3	4	4	3	3
3	3	4	4	3	3	1	1	3	3
3	3	1	1	3	3	4	4	3	3
2	2	1	1	3	3	4	4	2	2
3	3	1	1	2	2	4	4	2	2
2	2	4	4	3	3	4	4	3	3
3	3	4	4	3	3	4	4	2	2
3	3	1	1	3	3	4	4	2	2
3	3	1	1	3	3	4	4	3	3
3	3	1	1	3	3	4	4	3	3
3	3	4	4	3	3	4	4	3	3
3	3	4	4	3	3	4	4	3	3
3	3	1	1	3	3	4	4	2	2
3	3	1	1	3	3	4	4	2	2
2	2	1	1	3	3	4	4	2	2
3	3	1	1	2	2	1	1	2	2
3	3	4	4	3	3	4	4	2	2
2	2	4	4	3	3	1	1	3	3
3	3	1	1	3	3	4	4	3	3
3	3	4	4	3	3	4	4	2	2
3	3	4	4	3	3	4	4	2	2
3	3	1	1	3	3	1	1	2	2
3	3	4	4	3	3	4	4	3	3
2	2	1	1	3	3	4	4	2	2
2	2	4	4	3	3	4	4	2	2
2	2	4	4	3	3	4	4	3	3
2	2	1	1	3	3	4	4	2	2
3	3	1	1	3	3	4	4	2	2
3	3	1	1	3	3	4	4	3	3
3	3	4	4	3	3	4	4	3	3
2	2	4	4	3	3	4	4	3	3
3	3	4	4	3	3	4	4	3	3
3	3	1	1	3	3	4	4	2	2
3	3	4	4	3	3	4	4	3	3
3	3	1	1	3	3	4	4	3	3
3	3	4	4	3	3	4	4	2	2
3	3	4	4	3	3	1	1	2	2
3	3	4	4	3	3	4	4	3	3
3	3	4	4	3	3	1	1	2	2
3	3	1	1	3	3	4	4	2	2
2	2	1	1	3	3	1	1	3	3
3	3	1	1	3	3	4	4	3	3
2	2	4	4	3	3	1	1	2	2
2	2	1	1	3	3	1	1	3	3
3	3	4	4	3	3	1	1	3	3
3	3	1	1	2	2	4	4	3	3
3	3	1	1	3	3	4	4	3	3
3	3	4	4	3	3	1	1	2	2
3	3	1	1	3	3	1	1	3	3
2	2	4	4	3	3	4	4	2	2
2	2	4	4	3	3	1	1	2	2
3	3	1	1	3	3	4	4	2	2
2	2	1	1	3	3	1	1	2	2

For Review Only

2	2	1	1	2	2	4	4	2	2
3	3	4	4	3	3	1	1	2	2
3	3	1	1	2	2	4	4	2	2
3	3	4	4	3	3	1	1	3	3
3	3	1	1	3	3	4	4	2	2
3	3	1	1	2	2	4	4	2	2
2	2	1	1	3	3	4	4	3	3
3	3	1	1	3	3	4	4	2	2
3	3	1	1	3	3	4	4	3	3
3	3	4	4	3	3	4	4	2	2
3	3	1	1	3	3	1	1	2	2
3	3	1	1	2	2	1	1	2	2
2	2	1	1	3	3	1	1	3	3
2	2	1	1	2	2	1	1	2	2
2	2	1	1	3	3	4	4	3	3
3	3	1	1	2	2	1	1	2	2
2	2	1	1	3	3	4	4	2	2
3	3	1	1	2	2	1	1	2	2
3	3	4	4	3	3	1	1	3	3
3	3	4	4	3	3	1	1	2	2
3	3	1	1	3	3	1	1	2	2
3	3	4	4	3	3	1	1	2	2
3	3	1	1	3	3	1	1	2	2
2	2	4	4	3	3	4	4	3	3
3	3	1	1	2	2	4	4	2	2
3	3	4	4	3	3	1	1	2	2
3	3	1	1	3	3	1	1	2	2
3	3	1	1	3	3	1	1	2	2
3	3	1	1	3	3	4	4	3	3
3	3	1	1	3	3	1	1	2	2
3	3	4	4	3	3	4	4	3	3
3	3	1	1	3	3	1	1	2	2
3	3	4	4	3	3	1	1	2	2
3	3	1	1	3	3	1	1	2	2
3	3	4	4	3	3	1	1	3	3
3	3	1	1	3	3	1	1	3	3
3	3	4	4	3	3	1	1	3	3
3	3	1	1	3	3	1	1	2	2
3	3	4	4	2	2	4	4	2	2
3	3	4	4	3	3	4	4	3	3
3	3	1	1	3	3	1	1	2	2
3	3	1	1	3	3	4	4	3	3
2	2	1	1	3	3	1	1	2	2
3	3	1	1	3	3	4	4	3	3
2	2	1	1	2	2	4	4	3	3
2	2	4	4	3	3	1	1	3	3
2	2	4	4	2	2	4	4	2	2
2	2	1	1	3	3	4	4	2	2
3	3	1	1	3	3	4	4	2	2
2	2	1	1	2	2	4	4	2	2
3	3	1	1	2	2	4	4	2	2
2	2	4	4	3	3	4	4	2	2
3	3	1	1	3	3	4	4	2	2
3	3	1	1	2	2	4	4	2	2
3	3	1	1	3	3	1	1	2	2

For Review Only

2	2	4	4	3	3	1	1	3	3
2	2	1	1	3	3	4	4	2	2
2	2	1	1	3	3	4	4	3	3
3	3	4	4	3	3	1	1	2	2
2	2	1	1	3	3	4	4	3	3
2	2	1	1	3	3	4	4	3	3
2	2	1	1	3	3	1	1	2	2
3	3	1	1	3	3	1	1	2	2
3	3	4	4	3	3	4	4	2	2
3	3	4	4	3	3	1	1	2	2
2	2	1	1	2	2	4	4	2	2
3	3	1	1	3	3	4	4	3	3
3	3	1	1	3	3	4	4	2	2
3	3	4	4	3	3	1	1	3	3
3	3	4	4	3	3	4	4	3	3
2	2	4	4	3	3	4	4	2	2
3	3	1	1	3	3	4	4	2	2
3	3	1	1	3	3	4	4	3	3
2	2	1	1	3	3	4	4	2	2
2	2	1	1	3	3	4	4	3	3
3	3	1	1	3	3	1	1	2	2
3	3	1	1	2	2	4	4	3	3
3	3	4	4	3	3	4	4	2	2
3	3	1	1	3	3	4	4	3	3
3	3	1	1	2	2	4	4	3	3
3	3	4	4	3	3	1	1	3	3
3	3	1	1	3	3	1	1	3	3
3	3	1	1	3	3	4	4	2	2
2	2	1	1	3	3	4	4	3	3
3	3	4	4	3	3	1	1	2	2
3	3	1	1	3	3	4	4	2	2
3	3	1	1	3	3	4	4	2	2
3	3	1	1	3	3	1	1	2	2
3	3	4	4	3	3	4	4	3	3
3	3	1	1	3	3	1	1	2	2
3	3	1	1	3	3	4	4	3	3
2	2	4	4	3	3	1	1	2	2
3	3	1	1	3	3	4	4	2	2
2	2	1	1	3	3	4	4	2	2
3	3	1	1	3	3	4	4	3	3
3	3	1	1	3	3	4	4	2	2
2	2	1	1	2	2	4	4	2	2
2	2	4	4	3	3	4	4	3	3
3	3	4	4	3	3	1	1	2	2
3	3	4	4	3	3	4	4	3	3
2	2	1	1	3	3	4	4	2	2
2	2	4	4	3	3	4	4	2	2
2	2	4	4	3	3	4	4	3	3
3	3	1	1	3	3	4	4	3	3
3	3	1	1	3	3	1	1	3	3
3	3	1	1	2	2	1	1	3	3
2	2	4	4	3	3	4	4	2	2
2	2	4	4	3	3	4	4	2	2
3	3	1	1	3	3	4	4	3	3
3	3	4	4	3	3	4	4	3	3

For Review Only

3	3	1	1	3	3	4	4	2	2
3	3	1	1	3	3	1	1	2	2
3	3	1	1	3	3	4	4	2	2
3	3	4	4	3	3	1	1	2	2
3	3	1	1	3	3	1	1	2	2
3	3	1	1	2	2	4	4	2	2
3	3	1	1	3	3	4	4	2	2
3	3	1	1	3	3	4	4	3	3
3	3	4	4	3	3	1	1	3	3
2	2	4	4	3	3	4	4	3	3
3	3	1	1	3	3	1	1	2	2
3	3	1	1	3	3	4	4	2	2
3	3	4	4	3	3	1	1	2	2
2	2	1	1	3	3	4	4	2	2
3	3	1	1	3	3	4	4	2	2
2	2	4	4	3	3	1	1	3	3
2	2	4	4	3	3	4	4	3	3
3	3	4	4	3	3	1	1	3	3
2	2	1	1	3	3	4	4	3	3
3	3	4	4	3	3	1	1	2	2
2	2	1	1	3	3	4	4	3	3
2	2	1	1	2	2	4	4	2	2
3	3	4	4	3	3	1	1	3	3
2	2	1	1	3	3	1	1	2	2
2	2	1	1	3	3	4	4	3	3
3	3	1	1	3	3	4	4	2	2
3	3	4	4	3	3	4	4	3	3
3	3	1	1	3	3	4	4	3	3
3	3	1	1	3	3	1	1	2	2
3	3	1	1	3	3	4	4	3	3
2	2	4	4	3	3	4	4	3	3
3	3	1	1	2	2	4	4	2	2
3	3	4	4	3	3	4	4	3	3
3	3	1	1	2	2	4	4	2	2
3	3	4	4	3	3	4	4	2	2
2	2	4	4	3	3	4	4	3	3
3	3	4	4	3	3	4	4	2	2
3	3	1	1	3	3	4	4	3	3
3	3	4	4	3	3	4	4	3	3
2	2	4	4	3	3	4	4	3	3
3	3	4	4	3	3	1	1	2	2
2	2	1	1	3	3	4	4	3	3
2	2	1	1	3	3	1	1	2	2
3	3	4	4	3	3	1	1	2	2
3	3	1	1	3	3	4	4	3	3
3	3	4	4	3	3	1	1	2	2
2	2	4	4	3	3	4	4	3	3
3	3	1	1	3	3	1	1	2	2
2	2	4	4	3	3	1	1	2	2
3	3	4	4	3	3	4	4	3	3
3	3	4	4	3	3	1	1	2	2
2	2	4	4	3	3	4	4	3	3
3	3	1	1	3	3	4	4	2	2
3	3	1	1	3	3	4	4	3	3
2	2	1	1	3	3	1	1	2	2

For Review Only

3	3	4	4	3	3	1	1	2	2
2	2	1	1	3	3	4	4	2	2

For Review Only

SNP2218	SNP2218	SNP2219	SNP2219	SNP222	SNP222	SNP2220	SNP2220	SNP2221	SNP2221
4	4	1	1	3	3	2	2	2	2
3	3	4	4	3	3	2	2	2	2
3	3	4	4	3	3	2	2	2	2
3	3	4	4	3	3	2	2	2	2
3	3	4	4	3	3	2	2	2	2
3	3	4	4	3	3	2	2	2	2
3	3	4	4	4	4	2	2	2	2
3	3	4	4	3	3	4	4	2	2
3	3	4	4	4	4	2	2	2	2
3	3	4	4	3	3	2	2	2	2
3	3	4	4	3	3	2	2	2	2
3	3	4	4	3	3	2	2	2	2
3	3	4	4	3	3	2	2	2	2
4	4	1	1	3	3	2	2	2	2
3	3	4	4	4	4	2	2	2	2
3	3	4	4	3	3	2	2	2	2
3	3	4	4	3	3	2	2	2	2
3	3	4	4	4	4	2	2	2	2
3	3	4	4	3	3	2	2	2	2
3	3	4	4	3	3	2	2	2	2
3	3	4	4	4	4	2	2	2	2
3	3	4	4	4	4	2	2	2	2
3	3	4	4	4	4	2	2	2	2
3	3	4	4	3	3	2	2	2	2
3	3	4	4	3	3	2	2	2	2
3	3	4	4	3	3	2	2	2	2
3	3	4	4	4	4	2	2	2	2
3	3	4	4	3	3	2	2	2	2
3	3	4	4	3	3	2	2	2	2
3	3	4	4	3	3	4	4	2	2
3	3	4	4	4	4	2	2	2	2
4	4	1	1	3	3	2	2	2	2
3	3	4	4	4	4	2	2	2	2
4	4	1	1	4	4	2	2	2	2
3	3	4	4	3	3	2	2	2	2
4	4	1	1	3	3	2	2	2	2
3	3	4	4	3	3	2	2	2	2
4	4	1	1	3	3	2	2	2	2
3	3	4	4	3	3	2	2	1	1
3	3	4	4	3	3	2	2	2	2
3	3	4	4	3	3	4	4	2	2
3	3	4	4	3	3	2	2	2	2
3	3	4	4	3	3	4	4	2	2
3	3	4	4	3	3	4	4	2	2
3	3	4	4	3	3	4	4	2	2
3	3	4	4	3	3	2	2	2	2
3	3	4	4	3	3	2	2	2	2
3	3	4	4	4	4	2	2	2	2
3	3	4	4	3	3	4	4	2	2
3	3	4	4	4	4	2	2	1	1
3	3	4	4	3	3	2	2	2	2
4	4	1	1	3	3	2	2	2	2

For Review Only

3	3	4	4	3	3	2	2	2	2
3	3	4	4	4	4	2	2	2	2
3	3	4	4	4	4	2	2	2	2
3	3	4	4	3	3	2	2	2	2
3	3	4	4	3	3	2	2	2	2
3	3	4	4	4	4	2	2	2	2
3	3	4	4	4	4	2	2	2	2
3	3	4	4	3	3	2	2	2	2
3	3	4	4	4	4	2	2	2	2
3	3	4	4	3	3	2	2	2	2
3	3	4	4	4	4	2	2	2	2
3	3	4	4	4	4	2	2	2	2
3	3	4	4	3	3	2	2	2	2
3	3	4	4	3	3	2	2	2	2
3	3	4	4	3	3	2	2	2	2
3	3	4	4	3	3	2	2	2	2
3	3	4	4	3	3	2	2	2	2
3	3	4	4	4	4	2	2	2	2
3	3	4	4	4	4	2	2	2	2
3	3	4	4	4	4	2	2	2	2
3	3	4	4	4	4	2	2	2	2
3	3	4	4	3	3	2	2	1	1
3	3	4	4	4	4	2	2	2	2
3	3	4	4	4	4	2	2	2	2
3	3	4	4	3	3	4	4	2	2
3	3	4	4	3	3	2	2	2	2
3	3	4	4	3	3	2	2	2	2
3	3	4	4	3	3	2	2	2	2
3	3	4	4	3	3	2	2	2	2
3	3	4	4	3	3	2	2	2	2
3	3	4	4	4	4	2	2	2	2
4	4	1	1	4	4	2	2	2	2
3	3	4	4	3	3	2	2	2	2
3	3	4	4	3	3	2	2	2	2
4	4	1	1	4	4	2	2	2	2
3	3	4	4	3	3	4	4	2	2
3	3	4	4	4	4	2	2	2	2
3	3	4	4	4	4	4	4	2	2
4	4	1	1	3	3	2	2	2	2
3	3	4	4	3	3	2	2	2	2
3	3	4	4	4	4	2	2	2	2
3	3	4	4	4	4	2	2	2	2
3	3	4	4	3	3	2	2	2	2
3	3	4	4	4	4	2	2	2	2
4	4	1	1	4	4	2	2	2	2
3	3	4	4	4	4	2	2	2	2
4	4	1	1	3	3	2	2	2	2
3	3	4	4	3	3	2	2	2	2
3	3	4	4	3	3	2	2	2	2
3	3	4	4	4	4	2	2	2	2
3	3	4	4	4	4	2	2	2	2
3	3	4	4	3	3	2	2	2	2
3	3	4	4	3	3	2	2	2	2
3	3	4	4	4	4	2	2	2	2
3	3	4	4	4	4	2	2	2	2

For Review Only

3	3	4	4	3	3	2	2	2	2
4	4	1	1	3	3	2	2	1	1
3	3	4	4	3	3	2	2	2	2
3	3	4	4	3	3	2	2	2	2
3	3	4	4	3	3	2	2	2	2
4	4	1	1	4	4	2	2	2	2
3	3	4	4	3	3	2	2	2	2
4	4	1	1	3	3	2	2	2	2
3	3	4	4	3	3	4	4	2	2
4	4	1	1	3	3	2	2	2	2
3	3	4	4	4	4	2	2	2	2
3	3	4	4	3	3	2	2	2	2
3	3	4	4	4	4	2	2	2	2
3	3	4	4	3	3	2	2	2	2
3	3	4	4	4	4	2	2	2	2
3	3	4	4	3	3	2	2	2	2
3	3	4	4	4	4	2	2	2	2
3	3	4	4	3	3	2	2	2	2
4	4	1	1	3	3	2	2	2	2
3	3	4	4	3	3	2	2	2	2
3	3	4	4	3	3	2	2	2	2
3	3	4	4	4	4	2	2	2	2
3	3	4	4	3	3	2	2	2	2
3	3	4	4	3	3	2	2	2	2
3	3	4	4	3	3	2	2	2	2
3	3	4	4	4	4	2	2	2	2
3	3	4	4	4	4	2	2	1	1
4	4	1	1	3	3	2	2	2	2
3	3	4	4	4	4	2	2	2	2
3	3	4	4	4	4	2	2	2	2
3	3	4	4	3	3	2	2	2	2
3	3	4	4	3	3	2	2	2	2
3	3	4	4	4	4	2	2	2	2
3	3	4	4	3	3	2	2	2	2
4	4	1	1	4	4	2	2	2	2
3	3	4	4	4	4	2	2	2	2
3	3	4	4	3	3	2	2	2	2
3	3	4	4	3	3	2	2	2	2
3	3	4	4	3	3	2	2	2	2
3	3	4	4	3	3	2	2	2	2
3	3	4	4	3	3	2	2	2	2
4	4	1	1	3	3	2	2	2	2
3	3	4	4	4	4	2	2	2	2
3	3	4	4	3	3	2	2	2	2
3	3	4	4	3	3	2	2	2	2
3	3	4	4	4	4	2	2	2	2
4	4	1	1	3	3	2	2	2	2
3	3	4	4	3	3	2	2	2	2
4	4	1	1	3	3	2	2	2	2
3	3	4	4	3	3	2	2	2	2
3	3	4	4	3	3	2	2	2	2
4	4	1	1	3	3	2	2	2	2
3	3	4	4	4	4	2	2	2	2
3	3	4	4	3	3	2	2	2	2
3	3	4	4	3	3	2	2	2	2

For Review Only

3	3	4	4	3	3	2	2	2	2
3	3	4	4	3	3	2	2	2	2
3	3	4	4	3	3	2	2	2	2
4	4	1	1	4	4	2	2	1	1
3	3	4	4	4	4	2	2	2	2
3	3	4	4	3	3	2	2	2	2
3	3	4	4	3	3	2	2	2	2
3	3	4	4	3	3	2	2	2	2
3	3	4	4	3	3	2	2	2	2
3	3	4	4	4	4	2	2	2	2
3	3	4	4	3	3	2	2	2	2
3	3	4	4	4	4	2	2	2	2
3	3	4	4	4	4	2	2	2	2
3	3	4	4	3	3	2	2	2	2
3	3	4	4	3	3	2	2	2	2
3	3	4	4	3	3	2	2	2	2
3	3	4	4	4	4	2	2	2	2
3	3	4	4	4	4	2	2	2	2
3	3	4	4	3	3	2	2	2	2
3	3	4	4	3	3	2	2	2	2
3	3	4	4	3	3	2	2	2	2
3	3	4	4	3	3	2	2	2	2
3	3	4	4	3	3	2	2	1	1
3	3	4	4	4	4	2	2	2	2
3	3	4	4	4	4	2	2	2	2
4	4	1	1	3	3	2	2	2	2
3	3	4	4	4	4	2	2	2	2
3	3	4	4	4	4	2	2	2	2
3	3	4	4	4	4	2	2	1	1
3	3	4	4	4	4	2	2	2	2
3	3	4	4	4	4	2	2	2	2
3	3	4	4	3	3	2	2	2	2
4	4	1	1	3	3	2	2	2	2
3	3	4	4	3	3	2	2	2	2
3	3	4	4	4	4	2	2	2	2
3	3	4	4	4	4	2	2	2	2
3	3	4	4	3	3	2	2	2	2
3	3	4	4	3	3	2	2	2	2
3	3	4	4	3	3	2	2	2	2
3	3	4	4	4	4	2	2	2	2
3	3	4	4	3	3	2	2	2	2
3	3	4	4	3	3	2	2	2	2
3	3	4	4	3	3	2	2	2	2
3	3	4	4	3	3	2	2	2	2
3	3	4	4	3	3	2	2	2	2
3	3	4	4	4	4	2	2	2	2
3	3	4	4	3	3	2	2	2	2
3	3	4	4	3	3	2	2	2	2
3	3	4	4	3	3	2	2	2	2
3	3	4	4	3	3	2	2	2	2
3	3	4	4	4	4	2	2	2	2
3	3	4	4	3	3	2	2	2	2
3	3	4	4	3	3	2	2	2	2
3	3	4	4	4	4	2	2	2	2
3	3	4	4	3	3	2	2	2	2

For Review Only

3	3	4	4	4	4	2	2	2	2
3	3	4	4	3	3	2	2	2	2
3	3	4	4	3	3	2	2	2	2
3	3	4	4	3	3	2	2	2	2
3	3	4	4	3	3	4	4	1	1
3	3	4	4	3	3	2	2	2	2
3	3	4	4	3	3	2	2	2	2
3	3	4	4	3	3	4	4	2	2
3	3	4	4	3	3	2	2	2	2
3	3	4	4	3	3	2	2	2	2
3	3	4	4	3	3	2	2	2	2
3	3	4	4	4	4	2	2	2	2
4	4	1	1	3	3	2	2	2	2
3	3	4	4	3	3	2	2	2	2
3	3	4	4	4	4	2	2	2	2
3	3	4	4	3	3	2	2	2	2
3	3	4	4	3	3	2	2	2	2
4	4	1	1	3	3	2	2	2	2
3	3	4	4	3	3	2	2	2	2
4	4	1	1	3	3	2	2	2	2
3	3	4	4	4	4	2	2	2	2
3	3	4	4	3	3	2	2	2	2
3	3	4	4	4	4	2	2	2	2
3	3	4	4	3	3	2	2	2	2
3	3	4	4	3	3	2	2	2	2
3	3	4	4	3	3	2	2	2	2
3	3	4	4	3	3	2	2	2	2
3	3	4	4	4	4	2	2	2	2
3	3	4	4	3	3	2	2	2	2
3	3	4	4	4	4	2	2	2	2
3	3	4	4	3	3	2	2	2	2
3	3	4	4	3	3	2	2	2	2
4	4	1	1	3	3	2	2	2	2
3	3	4	4	4	4	2	2	2	2
3	3	4	4	3	3	2	2	2	2
3	3	4	4	3	3	2	2	2	2
3	3	4	4	4	4	2	2	2	2
3	3	4	4	4	4	2	2	2	2
4	4	1	1	3	3	2	2	2	2
3	3	4	4	3	3	2	2	2	2
4	4	1	1	4	4	2	2	1	1
4	4	1	1	3	3	2	2	2	2
3	3	4	4	4	4	2	2	2	2
3	3	4	4	4	4	2	2	2	2
3	3	4	4	3	3	2	2	2	2
4	4	4	4	3	3	2	2	2	2
3	3	4	4	3	3	2	2	2	2
3	3	4	4	4	4	2	2	2	2
3	3	4	4	3	3	2	2	2	2
3	3	4	4	3	3	2	2	2	2
4	4	1	1	4	4	2	2	2	2
3	3	4	4	3	3	2	2	2	2
3	3	4	4	3	3	2	2	2	2
3	3	4	4	4	4	2	2	2	2
3	3	4	4	3	3	2	2	2	2

For Review Only

3	3	4	4	3	3	2	2	2	2
3	3	4	4	4	4	2	2	2	2

For Review Only

SNP2222	SNP2222	SNP2223	SNP2223	SNP2224	SNP2224	SNP2225	SNP2225	SNP2226	SNP2226
4	4	2	2	2	2	4	4	3	3
4	4	2	2	2	2	4	4	2	2
4	4	3	3	2	2	4	4	2	2
4	4	2	2	2	2	4	4	2	2
4	4	2	2	2	2	4	4	2	2
4	4	2	2	2	2	4	4	2	2
4	4	2	2	2	2	4	4	2	2
4	4	2	2	2	2	4	4	2	2
4	4	2	2	2	2	4	4	2	2
4	4	2	2	2	2	4	4	2	2
3	3	2	2	3	3	2	2	3	3
4	4	2	2	2	2	4	4	2	2
4	4	2	2	2	2	4	4	2	2
3	3	2	2	2	2	4	4	2	2
4	4	2	2	2	2	4	4	2	2
4	4	2	2	2	2	2	2	2	2
4	4	2	2	2	2	4	4	2	2
4	4	2	2	2	2	4	4	2	2
4	4	2	2	2	2	4	4	2	2
4	4	2	2	2	2	4	4	2	2
4	4	2	2	2	2	4	4	2	2
4	4	2	2	2	2	4	4	2	2
3	3	2	2	3	3	4	4	2	2
4	4	2	2	3	3	4	4	2	2
4	4	2	2	2	2	4	4	2	2
4	4	2	2	2	2	4	4	2	2
4	4	3	3	2	2	4	4	2	2
4	4	2	2	2	2	4	4	2	2
4	4	2	2	2	2	4	4	2	2
4	4	2	2	3	3	4	4	2	2
4	4	2	2	2	2	4	4	2	2
4	4	2	2	2	2	2	2	3	3
4	4	2	2	2	2	4	4	2	2
4	4	2	2	2	2	2	2	3	3
4	4	2	2	2	2	4	4	2	2
4	4	2	2	3	3	2	2	3	3
4	4	2	2	2	2	4	4	2	2
3	3	2	2	2	2	2	2	2	2
3	3	2	2	3	3	4	4	2	2
3	3	2	2	3	3	4	4	2	2
4	4	2	2	3	3	4	4	2	2
4	4	2	2	2	2	4	4	2	2
4	4	2	2	2	2	4	4	2	2
4	4	2	2	2	2	4	4	2	2
4	4	2	2	2	2	4	4	2	2
4	4	3	3	2	2	4	4	2	2
4	4	3	3	2	2	4	4	2	2
3	3	2	2	3	3	4	4	2	2
4	4	2	2	3	3	4	4	2	2
4	4	3	3	2	2	2	2	2	2
4	4	3	3	2	2	4	4	2	2
4	4	2	2	2	2	2	2	3	3

For Review Only

4	4	2	2	2	2	4	4	2	2
4	4	2	2	2	2	4	4	2	2
4	4	2	2	2	2	4	4	2	2
4	4	2	2	3	3	4	4	2	2
4	4	2	2	2	2	4	4	2	2
4	4	3	3	2	2	4	4	2	2
4	4	3	3	2	2	4	4	2	2
4	4	2	2	3	3	4	4	2	2
4	4	2	2	2	2	4	4	2	2
4	4	2	2	2	2	4	4	2	2
4	4	3	3	2	2	4	4	2	2
4	4	3	3	2	2	4	4	2	2
4	4	2	2	2	2	4	4	2	2
4	4	2	2	2	2	4	4	2	2
4	4	2	2	2	2	2	2	2	2
4	4	2	2	2	2	2	2	3	3
4	4	3	3	2	2	4	4	2	2
4	4	2	2	2	2	4	4	2	2
4	4	2	2	2	2	4	4	2	2
4	4	2	2	2	2	4	4	2	2
4	4	2	2	2	2	4	4	2	2
4	4	2	2	3	3	4	4	2	2
4	4	2	2	2	2	4	4	2	2
4	4	2	2	2	2	4	4	2	2
4	4	2	2	2	2	4	4	2	2
4	4	2	2	2	2	4	4	2	2
4	4	2	2	2	2	4	4	2	2
3	3	2	2	3	3	4	4	2	2
4	4	3	3	2	2	4	4	2	2
4	4	2	2	2	2	4	4	2	2
4	4	2	2	2	2	4	4	2	2
4	4	3	3	3	3	2	2	2	2
3	3	2	2	3	3	2	2	2	2
4	4	2	2	2	2	4	4	2	2
4	4	2	2	2	2	2	2	2	2
4	4	2	2	2	2	4	4	2	2
4	4	2	2	3	3	4	4	2	2
4	4	2	2	3	3	4	4	2	2
4	4	2	2	2	2	2	2	3	3
4	4	2	2	2	2	4	4	2	2
4	4	2	2	2	2	4	4	2	2
4	4	2	2	2	2	4	4	2	2
4	4	3	3	2	2	4	4	2	2
4	4	3	3	2	2	4	4	2	2
4	4	2	2	2	2	2	2	3	3
4	4	2	2	2	2	4	4	2	2
3	3	2	2	3	3	2	2	2	2
4	4	2	2	2	2	4	4	2	2
4	4	3	3	3	3	4	4	2	2
4	4	2	2	2	2	4	4	2	2
4	4	3	3	2	2	4	4	2	2
3	3	2	2	3	3	4	4	2	2
4	4	2	2	3	3	4	4	2	2
4	4	2	2	2	2	2	2	2	2
4	4	2	2	2	2	4	4	2	2

For Review Only

4	4	2	2	2	2	4	4	2	2
4	4	2	2	2	2	2	2	2	2
4	4	2	2	2	2	4	4	2	2
3	3	2	2	3	3	4	4	2	2
4	4	2	2	2	2	4	4	2	2
4	4	2	2	2	2	2	2	3	3
4	4	2	2	2	2	4	4	2	2
4	4	2	2	2	2	2	2	3	3
4	4	3	3	3	3	4	4	2	2
4	4	2	2	2	2	2	2	3	3
4	4	2	2	2	2	4	4	2	2
4	4	2	2	2	2	4	4	2	2
4	4	3	3	2	2	4	4	2	2
4	4	2	2	2	2	2	2	2	2
4	4	3	3	2	2	4	4	2	2
4	4	2	2	2	2	4	4	2	2
4	4	2	2	2	2	4	4	2	2
4	4	2	2	2	2	2	2	2	2
3	3	2	2	3	3	4	4	2	2
4	4	2	2	2	2	4	4	2	2
4	4	2	2	2	2	4	4	2	2
4	4	2	2	2	2	2	2	2	2
4	4	2	2	2	2	2	2	2	2
4	4	2	2	3	3	4	4	2	2
4	4	2	2	2	2	4	4	2	2
4	4	2	2	3	3	4	4	2	2
4	4	3	3	2	2	4	4	2	2
4	4	2	2	2	2	2	2	3	3
4	4	3	3	2	2	4	4	2	2
4	4	2	2	2	2	4	4	2	2
4	4	2	2	3	3	2	2	2	2
4	4	2	2	2	2	4	4	2	2
4	4	2	2	2	2	4	4	2	2
4	4	2	2	2	2	2	2	3	3
4	4	2	2	2	2	2	2	3	3
3	3	2	2	2	2	4	4	2	2
3	3	2	2	2	2	4	4	2	2
4	4	2	2	2	2	4	4	2	2
4	4	2	2	2	2	4	4	2	2
3	3	2	2	3	3	2	2	2	2
3	3	2	2	2	2	2	2	2	2
3	3	2	2	2	2	2	2	2	2
4	4	2	2	2	2	4	4	2	2
4	4	2	2	2	2	4	4	2	2
4	4	3	3	2	2	4	4	2	2
4	4	2	2	2	2	4	4	2	2
4	4	3	3	2	2	4	4	2	2
4	4	3	3	2	2	4	4	2	2
4	4	2	2	2	2	2	2	3	3
4	4	3	3	2	2	4	4	2	2
4	4	3	3	2	2	2	2	2	2
3	3	2	2	3	3	2	2	2	2
3	3	2	2	2	2	4	4	2	2
4	4	3	3	2	2	4	4	2	2
4	4	2	2	2	2	2	2	2	2

For Review Only

4	4	2	2	2	2	4	4	2	2
3	3	2	2	3	3	4	4	2	2
4	4	2	2	2	2	4	4	2	2
4	4	2	2	2	2	2	2	2	2
4	4	2	2	2	2	2	2	2	2
4	4	2	2	2	2	4	4	2	2
3	3	2	2	3	3	4	4	2	2
4	4	2	2	2	2	4	4	2	2
4	4	2	2	3	3	4	4	2	2
4	4	2	2	2	2	4	4	2	2
4	4	2	2	2	2	4	4	2	2
4	4	2	2	2	2	4	4	2	2
4	4	2	2	2	2	4	4	2	2
4	4	2	2	2	2	4	4	2	2
3	3	2	2	3	3	4	4	2	2
3	3	2	2	3	3	2	2	3	3
4	4	2	2	2	2	4	4	2	2
4	4	2	2	2	2	4	4	2	2
4	4	3	3	3	3	4	4	2	2
4	4	2	2	2	2	4	4	2	2
4	4	2	2	2	2	4	4	2	2
4	4	2	2	3	3	2	2	2	2
3	3	2	2	3	3	4	4	2	2
4	4	3	3	2	2	4	4	2	2
4	4	2	2	2	2	4	4	2	2
4	4	2	2	2	2	2	2	3	3
4	4	2	2	2	2	4	4	2	2
4	4	2	2	2	2	4	4	2	2
4	4	3	3	2	2	4	4	2	2
4	4	2	2	2	2	4	4	2	2
4	4	2	2	2	2	4	4	2	2
4	4	2	2	2	2	4	4	2	2
4	4	2	2	2	2	4	4	2	2
3	3	2	2	3	3	4	4	2	2
4	4	2	2	2	2	4	4	2	2
4	4	3	3	2	2	4	4	2	2
4	4	2	2	2	2	4	4	2	2
4	4	2	2	2	2	4	4	2	2
4	4	3	3	2	2	4	4	2	2
4	4	3	3	2	2	4	4	2	2
4	4	3	3	2	2	4	4	2	2
4	4	3	3	2	2	4	4	2	2
3	3	2	2	3	3	2	2	2	2
4	4	3	3	2	2	4	4	2	2
4	4	2	2	2	2	2	2	2	2
4	4	2	2	2	2	4	4	2	2
3	3	2	2	3	3	2	2	3	3
3	3	2	2	3	3	2	2	3	3
4	4	2	2	3	3	4	4	2	2
4	4	2	2	2	2	4	4	2	2
4	4	3	3	2	2	4	4	2	2
4	4	2	2	2	2	4	4	2	2
4	4	2	2	3	3	4	4	2	2
4	4	3	3	2	2	4	4	2	2
3	3	2	2	3	3	4	4	2	2

For Review Only

4	4	2	2	3	3	4	4	2	2
4	4	2	2	3	3	4	4	2	2
4	4	3	3	2	2	4	4	2	2
4	4	2	2	2	2	4	4	2	2
4	4	2	2	2	2	4	4	2	2
4	4	2	2	2	2	4	4	2	2
3	3	2	2	3	3	4	4	2	2
4	4	2	2	2	2	4	4	2	2
4	4	2	2	2	2	2	2	2	2
4	4	2	2	3	3	4	4	2	2
4	4	2	2	2	2	4	4	2	2
4	4	3	3	2	2	4	4	2	2
3	3	2	2	2	2	2	2	3	3
4	4	3	3	3	3	4	4	2	2
4	4	3	3	2	2	4	4	2	2
4	4	2	2	2	2	4	4	2	2
3	3	2	2	2	2	4	4	2	2
4	4	2	2	3	3	2	2	2	2
3	3	2	2	3	3	4	4	2	2
3	3	2	2	2	2	2	2	2	2
4	4	2	2	3	3	4	4	2	2
4	4	3	3	2	2	4	4	2	2
4	4	2	2	2	2	4	4	2	2
3	3	2	2	2	2	4	4	2	2
4	4	2	2	3	3	4	4	2	2
4	4	2	2	2	2	4	4	2	2
3	3	2	2	3	3	2	2	2	2
4	4	2	2	2	2	4	4	2	2
4	4	2	2	2	2	4	4	2	2
4	4	3	3	2	2	4	4	2	2
3	3	2	2	3	3	2	2	2	2
3	3	2	2	2	2	4	4	2	2
4	4	2	2	2	2	4	4	2	2
4	4	2	2	2	2	4	4	2	2
3	3	2	2	3	3	4	4	2	2
3	3	2	2	3	3	4	4	2	2
4	4	2	2	2	2	4	4	2	2
4	4	3	3	2	2	4	4	2	2
4	4	2	2	3	3	2	2	3	3
3	3	2	2	3	3	4	4	2	2
4	4	2	2	2	2	2	2	3	3
4	4	2	2	2	2	2	2	3	3
3	3	2	2	3	3	4	4	2	2
4	4	2	2	2	2	4	4	2	2
4	4	2	2	2	2	4	4	2	2
3	3	2	2	2	2	2	2	3	3
4	4	3	3	2	2	4	4	2	2
4	4	2	2	2	2	4	4	2	2
4	4	2	2	2	2	4	4	2	2
3	3	2	2	3	3	2	2	2	2
4	4	3	3	2	2	2	2	3	3
3	3	2	2	3	3	4	4	2	2
4	4	3	3	2	2	4	4	2	2
4	4	2	2	2	2	4	4	2	2
3	3	2	2	3	3	4	4	2	2

For Review Only

4	4	2	2	2	2	2	2	3	3
4	4	2	2	2	2	4	4	2	2

For Review Only

SNP2227	SNP2227	SNP2228	SNP2228	SNP2229	SNP2229	SNP223	SNP223	SNP2230	SNP2230
4	4	3	3	2	2	3	3	4	4
1	1	3	3	2	2	4	4	2	2
4	4	2	2	2	2	3	3	2	2
1	1	3	3	2	2	3	3	2	2
1	1	3	3	2	2	4	4	4	4
1	1	3	3	2	2	3	3	2	2
4	4	3	3	2	2	4	4	2	2
1	1	3	3	2	2	4	4	4	4
4	4	3	3	2	2	4	4	4	4
1	1	3	3	2	2	4	4	4	4
4	4	3	3	2	2	4	4	2	2
4	4	3	3	2	2	3	3	4	4
4	4	3	3	2	2	4	4	4	4
4	4	3	3	2	2	4	4	2	2
4	4	3	3	2	2	4	4	2	2
4	4	3	3	2	2	4	4	4	4
4	4	3	3	2	2	3	3	2	2
4	4	3	3	2	2	4	4	4	4
1	1	3	3	2	2	4	4	2	2
1	1	3	3	2	2	3	3	4	4
1	1	3	3	2	2	4	4	2	2
1	1	3	3	2	2	3	3	2	2
1	1	3	3	2	2	3	3	4	4
1	1	3	3	2	2	4	4	2	2
4	4	3	3	2	2	4	4	2	2
4	4	3	3	3	3	4	4	2	2
4	4	2	2	2	2	4	4	2	2
4	4	3	3	2	2	4	4	4	4
4	4	3	3	2	2	4	4	4	4
1	1	3	3	2	2	4	4	4	4
4	4	3	3	2	2	4	4	2	2
4	4	3	3	2	2	4	4	2	2
4	4	3	3	2	2	4	4	2	2
1	1	3	3	2	2	4	4	4	4
4	4	3	3	2	2	4	4	4	4
1	1	3	3	2	2	4	4	2	2
4	4	3	3	2	2	4	4	2	2
1	1	3	3	2	2	4	4	4	4
1	1	3	3	2	2	4	4	4	4
4	4	3	3	2	2	4	4	2	2
1	1	3	3	2	2	4	4	4	4
4	4	3	3	2	2	4	4	4	4
1	1	3	3	2	2	4	4	4	4
1	1	3	3	2	2	4	4	2	2
1	1	3	3	2	2	4	4	4	4
4	4	2	2	2	2	3	3	2	2
4	4	2	2	2	2	4	4	2	2
1	1	2	2	3	3	3	3	2	2
4	4	3	3	2	2	4	4	2	2
4	4	3	3	2	2	4	4	4	4
4	4	2	2	2	2	4	4	2	2
1	1	3	3	2	2	4	4	2	2

For Review Only

4	4	3	3	2	2	3	3	2	2
4	4	3	3	2	2	4	4	2	2
4	4	3	3	2	2	4	4	2	2
1	1	3	3	2	2	3	3	2	2
1	1	3	3	2	2	4	4	2	2
4	4	2	2	2	2	4	4	2	2
4	4	2	2	3	3	4	4	2	2
1	1	3	3	2	2	4	4	4	4
4	4	3	3	2	2	4	4	2	2
4	4	3	3	2	2	4	4	4	4
4	4	2	2	2	2	4	4	2	2
4	4	2	2	2	2	4	4	2	2
4	4	3	3	2	2	3	3	2	2
4	4	3	3	2	2	4	4	4	4
4	4	3	3	2	2	4	4	2	2
4	4	3	3	2	2	4	4	4	4
4	4	2	2	2	2	4	4	2	2
4	4	3	3	2	2	4	4	2	2
1	1	3	3	2	2	3	3	2	2
4	4	3	3	2	2	3	3	2	2
4	4	3	3	2	2	4	4	2	2
1	1	3	3	2	2	4	4	2	2
4	4	3	3	2	2	4	4	4	4
4	4	3	3	2	2	4	4	2	2
1	1	3	3	2	2	4	4	4	4
1	1	3	3	2	2	4	4	2	2
1	1	3	3	2	2	4	4	4	4
4	4	3	3	2	2	4	4	4	4
4	4	2	2	3	3	4	4	2	2
4	4	3	3	2	2	4	4	2	2
1	1	3	3	2	2	3	3	4	4
4	4	2	2	2	2	4	4	2	2
1	1	3	3	2	2	4	4	4	4
4	4	3	3	2	2	3	3	2	2
4	4	3	3	2	2	4	4	2	2
1	1	3	3	2	2	4	4	4	4
1	1	3	3	2	2	4	4	2	2
1	1	3	3	2	2	4	4	4	4
4	4	3	3	2	2	4	4	2	2
4	4	3	3	2	2	4	4	4	4
1	1	3	3	2	2	4	4	2	2
4	4	3	3	2	2	4	4	2	2
1	1	2	2	2	2	4	4	4	4
4	4	2	2	2	2	4	4	2	2
1	1	3	3	2	2	4	4	2	2
1	1	3	3	2	2	4	4	4	4
4	4	3	3	2	2	4	4	4	4
1	1	3	3	2	2	4	4	2	2
4	4	2	2	2	2	4	4	2	2
4	4	3	3	2	2	4	4	4	4
1	1	3	3	2	2	4	4	2	2
1	1	3	3	2	2	4	4	4	4
4	4	3	3	2	2	4	4	4	4
1	1	3	3	2	2	4	4	4	4
4	4	3	3	2	2	4	4	4	4

For Review Only

4	4	3	3	2	2	4	4	4	4
4	4	3	3	2	2	3	3	2	2
4	4	3	3	2	2	4	4	2	2
1	1	3	3	2	2	4	4	4	4
1	1	3	3	2	2	4	4	2	2
4	4	3	3	2	2	4	4	2	2
1	1	3	3	2	2	4	4	2	2
4	4	3	3	2	2	4	4	2	2
1	1	2	2	2	2	4	4	2	2
1	1	3	3	2	2	4	4	4	4
4	4	3	3	2	2	4	4	2	2
4	4	3	3	2	2	4	4	4	4
1	1	3	3	2	2	4	4	2	2
4	4	3	3	2	2	4	4	2	2
4	4	3	3	2	2	4	4	4	4
4	4	3	3	2	2	4	4	4	4
4	4	3	3	2	2	4	4	2	2
4	4	3	3	2	2	4	4	4	4
1	1	3	3	2	2	4	4	4	4
1	1	3	3	2	2	4	4	4	4
4	4	3	3	2	2	4	4	4	4
4	4	3	3	2	2	4	4	2	2
4	4	3	3	2	2	4	4	2	2
1	1	3	3	2	2	4	4	4	4
4	4	3	3	2	2	4	4	2	2
4	4	3	3	2	2	4	4	2	2
4	4	3	3	2	2	4	4	2	2
4	4	3	3	2	2	4	4	2	2
1	1	2	2	2	2	3	3	2	2
4	4	3	3	2	2	3	3	4	4
4	4	3	3	2	2	4	4	4	4
1	1	3	3	2	2	4	4	4	4
1	1	3	3	2	2	4	4	2	2
4	4	3	3	2	2	4	4	4	4
1	1	3	3	2	2	4	4	4	4
1	1	3	3	2	2	4	4	4	4
1	1	3	3	2	2	4	4	4	4
4	4	3	3	2	2	4	4	4	4
1	1	3	3	2	2	4	4	4	4
4	4	3	3	2	2	4	4	4	4
4	4	3	3	2	2	4	4	2	2
1	1	3	3	2	2	4	4	4	4
4	4	3	3	2	2	4	4	2	2
1	1	3	3	2	2	4	4	4	4
4	4	2	2	2	2	4	4	2	2
4	4	3	3	2	2	4	4	2	2
4	4	2	2	2	2	4	4	2	2
4	4	2	2	3	3	4	4	2	2
4	4	3	3	2	2	4	4	4	4
4	4	2	2	2	2	4	4	2	2
4	4	2	2	2	2	4	4	2	2
4	4	3	3	2	2	4	4	2	2
1	1	3	3	2	2	4	4	2	2
4	4	2	2	2	2	4	4	2	2
1	1	3	3	2	2	4	4	4	4

For Review Only

1	1	2	2	2	2	4	4	2	2
4	4	3	3	2	2	4	4	4	4
4	4	3	3	2	2	4	4	2	2
4	4	3	3	2	2	4	4	2	2
4	4	3	3	2	2	3	3	2	2
1	1	3	3	2	2	4	4	2	2
4	4	3	3	2	2	4	4	4	4
4	4	3	3	3	3	4	4	2	2
4	4	3	3	2	2	4	4	4	4
4	4	2	2	2	2	4	4	2	2
4	4	2	2	2	2	3	3	2	2
4	4	3	3	2	2	4	4	2	2
4	4	3	3	2	2	4	4	2	2
4	4	3	3	2	2	4	4	4	4
1	1	3	3	2	2	4	4	4	4
4	4	3	3	2	2	4	4	4	4
4	4	3	3	2	2	3	3	2	2
1	1	3	3	2	2	4	4	2	2
4	4	2	2	2	2	4	4	2	2
1	1	3	3	2	2	4	4	2	2
4	4	3	3	2	2	4	4	4	4
1	1	3	3	2	2	4	4	4	4
4	4	3	3	2	2	4	4	2	2
4	4	2	2	2	2	4	4	2	2
4	4	3	3	2	2	4	4	2	2
1	1	3	3	2	2	4	4	4	4
1	1	3	3	2	2	3	3	2	2
4	4	3	3	2	2	4	4	4	4
1	1	2	2	2	2	4	4	2	2
1	1	3	3	2	2	3	3	2	2
4	4	3	3	2	2	4	4	4	4
4	4	3	3	2	2	4	4	2	2
4	4	3	3	2	2	4	4	2	2
1	1	3	3	2	2	4	4	4	4
4	4	3	3	2	2	4	4	2	2
4	4	2	2	2	2	4	4	2	2
4	4	3	3	2	2	4	4	2	2
4	4	3	3	2	2	4	4	2	2
4	4	2	2	3	3	4	4	2	2
1	1	2	2	2	2	4	4	2	2
4	4	2	2	2	2	3	3	2	2
4	4	2	2	2	2	4	4	2	2
4	4	3	3	2	2	4	4	4	4
4	4	3	3	2	2	4	4	4	4
1	1	3	3	2	2	4	4	4	4
4	4	3	3	2	2	3	3	2	2
4	4	3	3	2	2	4	4	4	4
1	1	3	3	2	2	4	4	4	4
1	1	3	3	2	2	4	4	4	4
1	1	3	3	2	2	4	4	2	2
1	1	2	2	2	2	3	3	2	2
4	4	3	3	2	2	4	4	4	4
4	4	3	3	2	2	3	3	2	2
4	4	2	2	2	2	4	4	2	2
1	1	3	3	2	2	4	4	2	2

For Review Only

1	1	3	3	2	2	3	3	4	4
4	4	3	3	2	2	4	4	4	4
4	4	2	2	2	2	3	3	2	2
4	4	3	3	2	2	4	4	4	4
1	1	3	3	2	2	3	3	2	2
4	4	3	3	3	3	4	4	2	2
4	4	3	3	2	2	3	3	4	4
1	1	3	3	2	2	4	4	2	2
1	1	3	3	2	2	4	4	4	4
1	1	3	3	2	2	4	4	4	4
1	1	3	3	2	2	4	4	4	4
4	4	2	2	2	2	4	4	4	4
4	4	3	3	2	2	4	4	2	2
4	4	2	2	2	2	4	4	2	2
1	1	2	2	2	2	4	4	2	2
4	4	3	3	2	2	4	4	2	2
1	1	3	3	2	2	3	3	2	2
1	1	3	3	2	2	4	4	4	4
1	1	3	3	2	2	4	4	4	4
4	4	3	3	2	2	3	3	4	4
1	1	3	3	2	2	4	4	4	4
4	4	2	2	3	3	4	4	2	2
1	1	3	3	2	2	4	4	2	2
4	4	3	3	2	2	4	4	2	2
1	1	3	3	2	2	4	4	4	4
4	4	3	3	2	2	3	3	2	2
1	1	3	3	2	2	4	4	4	4
1	1	3	3	2	2	3	3	2	2
4	4	3	3	2	2	3	3	2	2
1	1	2	2	2	2	3	3	2	2
4	4	3	3	2	2	4	4	2	2
1	1	3	3	2	2	4	4	2	2
4	4	3	3	2	2	4	4	4	4
4	4	2	2	3	3	4	4	2	2
4	4	3	3	2	2	4	4	2	2
4	4	3	3	2	2	4	4	4	4
1	1	3	3	2	2	3	3	2	2
4	4	2	2	2	2	4	4	2	2
1	1	3	3	2	2	4	4	2	2
1	1	3	3	2	2	3	3	2	2
4	4	3	3	2	2	4	4	2	2
4	4	3	3	2	2	4	4	2	2
4	4	3	3	2	2	4	4	2	2
1	1	3	3	2	2	3	3	2	2
1	1	3	3	2	2	4	4	2	2
4	4	3	3	2	2	4	4	2	2
4	4	2	2	2	2	4	4	2	2
4	4	3	3	2	2	4	4	2	2
4	4	3	3	2	2	4	4	4	4
1	1	3	3	2	2	4	4	4	4
4	4	2	2	2	2	3	3	2	2
1	1	3	3	2	2	3	3	2	2
4	4	2	2	2	2	3	3	2	2
1	1	2	2	2	2	3	3	2	2
4	4	3	3	2	2	4	4	2	2

For Review Only

4	4	3	3	2	2	4	4	2	2
4	4	3	3	2	2	4	4	2	2

For Review Only

SNP2231	SNP2231	SNP2232	SNP2232	SNP2233	SNP2233	SNP2234	SNP2234	SNP2235	SNP2235
3	3	2	2	1	1	1	1	3	3
1	1	2	2	3	3	4	4	3	3
1	1	2	2	3	3	4	4	3	3
3	3	2	2	1	1	1	1	3	3
3	3	3	3	1	1	4	4	3	3
1	1	3	3	1	1	1	1	3	3
1	1	3	3	1	1	1	1	3	3
3	3	2	2	3	3	4	4	3	3
1	1	2	2	3	3	1	1	3	3
3	3	2	2	1	1	1	1	2	2
1	1	2	2	3	3	4	4	3	3
3	3	2	2	3	3	4	4	3	3
1	1	2	2	1	1	1	1	3	3
1	1	3	3	3	3	4	4	3	3
3	3	2	2	1	1	4	4	3	3
3	3	2	2	1	1	4	4	3	3
1	1	3	3	1	1	1	1	3	3
3	3	2	2	1	1	1	1	3	3
1	1	2	2	3	3	4	4	3	3
3	3	2	2	3	3	1	1	3	3
1	1	2	2	1	1	1	1	2	2
1	1	2	2	1	1	1	1	3	3
3	3	2	2	3	3	4	4	3	3
1	1	3	3	1	1	4	4	3	3
1	1	2	2	1	1	1	1	3	3
1	1	3	3	1	1	4	4	3	3
1	1	2	2	1	1	4	4	3	3
3	3	2	2	1	1	4	4	3	3
3	3	2	2	3	3	1	1	3	3
3	3	2	2	3	3	4	4	3	3
1	1	2	2	3	3	1	1	3	3
1	1	2	2	3	3	1	1	3	3
1	1	3	3	1	1	1	1	3	3
3	3	2	2	3	3	4	4	3	3
3	3	2	2	1	1	4	4	3	3
1	1	3	3	3	3	4	4	3	3
1	1	3	3	1	1	1	1	3	3
3	3	2	2	1	1	4	4	3	3
1	1	2	2	3	3	4	4	3	3
3	3	2	2	1	1	4	4	3	3
3	3	2	2	1	1	4	4	3	3
3	3	2	2	3	3	1	1	3	3
3	3	2	2	3	3	4	4	2	2
1	1	2	2	1	1	1	1	3	3
3	3	2	2	3	3	4	4	2	2
1	1	2	2	3	3	4	4	3	3
1	1	2	2	1	1	4	4	3	3
3	3	3	3	1	1	4	4	3	3
1	1	2	2	1	1	1	1	2	2
3	3	3	3	1	1	4	4	3	3
1	1	2	2	1	1	4	4	3	3
1	1	2	2	3	3	1	1	3	3

For Review Only

1	1	2	2	1	1	1	1	3	3
1	1	3	3	1	1	1	1	3	3
1	1	2	2	3	3	4	4	3	3
1	1	2	2	1	1	1	1	3	3
1	1	2	2	3	3	4	4	3	3
1	1	2	2	1	1	4	4	3	3
1	1	2	2	1	1	4	4	3	3
3	3	3	3	1	1	4	4	3	3
1	1	2	2	1	1	4	4	3	3
3	3	2	2	1	1	4	4	3	3
1	1	2	2	3	3	4	4	3	3
1	1	2	2	3	3	4	4	3	3
1	1	2	2	1	1	1	1	3	3
3	3	2	2	3	3	1	1	3	3
1	1	2	2	1	1	4	4	3	3
3	3	2	2	1	1	1	1	3	3
1	1	2	2	1	1	4	4	3	3
1	1	3	3	1	1	4	4	3	3
1	1	2	2	3	3	1	1	3	3
1	1	2	2	1	1	1	1	3	3
1	1	2	2	3	3	4	4	3	3
1	1	3	3	3	3	4	4	3	3
1	1	2	2	3	3	1	1	3	3
1	1	2	2	1	1	4	4	3	3
3	3	2	2	3	3	4	4	2	2
1	1	2	2	1	1	4	4	3	3
3	3	3	3	3	3	1	1	3	3
3	3	2	2	3	3	4	4	3	3
1	1	2	2	1	1	4	4	3	3
1	1	3	3	1	1	1	1	3	3
1	1	2	2	3	3	4	4	3	3
3	3	3	3	1	1	4	4	3	3
3	3	3	3	3	3	4	4	3	3
1	1	2	2	1	1	1	1	3	3
1	1	2	2	3	3	1	1	3	3
3	3	2	2	3	3	4	4	2	2
1	1	2	2	3	3	4	4	3	3
3	3	2	2	1	1	1	1	3	3
1	1	2	2	1	1	4	4	3	3
3	3	3	3	1	1	4	4	3	3
1	1	2	2	1	1	1	1	3	3
1	1	2	2	1	1	4	4	3	3
3	3	3	3	1	1	1	1	3	3
1	1	2	2	3	3	4	4	3	3
3	3	3	3	1	1	1	1	3	3
3	3	2	2	1	1	4	4	3	3
1	1	2	2	1	1	4	4	3	3
1	1	3	3	1	1	4	4	3	3
1	1	3	3	1	1	4	4	3	3
3	3	3	3	1	1	4	4	3	3
1	1	3	3	3	3	4	4	3	3
1	1	2	2	3	3	4	4	3	3
3	3	2	2	3	3	4	4	3	3
3	3	2	2	1	1	4	4	3	3
3	3	3	3	1	1	4	4	3	3

For Review Only

3	3	2	2	3	3	4	4	3	3
1	1	2	2	1	1	4	4	3	3
1	1	3	3	1	1	4	4	3	3
3	3	2	2	3	3	4	4	3	3
1	1	2	2	1	1	4	4	3	3
1	1	3	3	1	1	4	4	3	3
1	1	2	2	1	1	4	4	3	3
3	3	2	2	1	1	4	4	3	3
1	1	2	2	1	1	4	4	3	3
3	3	2	2	1	1	4	4	3	3
1	1	3	3	1	1	1	1	3	3
3	3	2	2	1	1	4	4	3	3
1	1	3	3	1	1	1	1	3	3
3	3	2	2	1	1	4	4	3	3
1	1	2	2	1	1	4	4	3	3
3	3	2	2	1	1	4	4	3	3
1	1	3	3	1	1	4	4	3	3
3	3	2	2	1	1	4	4	3	3
3	3	2	2	3	3	4	4	3	3
3	3	2	2	3	3	4	4	3	3
3	3	2	2	1	1	4	4	3	3
1	1	3	3	1	1	1	1	3	3
3	3	2	2	1	1	4	4	3	3
3	3	3	3	3	3	4	4	3	3
1	1	2	2	1	1	4	4	3	3
3	3	2	2	1	1	4	4	3	3
1	1	2	2	1	1	1	1	3	3
1	1	3	3	1	1	1	1	3	3
1	1	2	2	1	1	1	1	3	3
3	3	2	2	1	1	1	1	3	3
3	3	2	2	1	1	4	4	3	3
3	3	2	2	1	1	4	4	3	3
1	1	2	2	1	1	1	1	3	3
3	3	2	2	1	1	4	4	3	3
3	3	2	2	3	3	4	4	3	3
3	3	2	2	1	1	4	4	3	3
3	3	2	2	3	3	4	4	3	3
1	1	2	2	1	1	4	4	3	3
3	3	2	2	1	1	4	4	3	3
3	3	2	2	3	3	4	4	3	3
1	1	3	3	1	1	4	4	3	3
3	3	2	2	1	1	4	4	3	3
1	1	3	3	1	1	1	1	3	3
3	3	2	2	1	1	4	4	3	3
1	1	3	3	1	1	4	4	3	3
1	1	3	3	3	3	1	1	3	3
1	1	2	2	1	1	4	4	3	3
1	1	2	2	1	1	4	4	3	3
3	3	2	2	1	1	1	1	3	3
1	1	2	2	1	1	4	4	3	3
1	1	3	3	1	1	4	4	3	3
1	1	3	3	3	3	4	4	3	3
3	3	3	3	3	3	4	4	3	3
1	1	3	3	3	3	4	4	3	3
3	3	2	2	1	1	4	4	3	3

For Review Only

1	1	2	2	1	1	1	1	3	3
3	3	3	3	1	1	4	4	3	3
1	1	3	3	3	3	4	4	3	3
1	1	2	2	1	1	4	4	3	3
1	1	2	2	3	3	4	4	3	3
1	1	2	2	3	3	4	4	3	3
3	3	3	3	1	1	4	4	3	3
3	3	2	2	1	1	4	4	3	3
1	1	2	2	3	3	4	4	3	3
1	1	3	3	1	1	1	1	3	3
1	1	2	2	1	1	4	4	3	3
1	1	2	2	1	1	4	4	3	3
1	1	2	2	1	1	4	4	3	3
3	3	3	3	1	1	4	4	3	3
3	3	2	2	1	1	4	4	3	3
3	3	2	2	3	3	4	4	3	3
3	3	3	3	1	1	4	4	3	3
1	1	3	3	1	1	1	1	3	3
3	3	3	3	1	1	4	4	3	3
1	1	2	2	1	1	4	4	3	3
3	3	2	2	1	1	4	4	3	3
3	3	3	3	3	3	4	4	3	3
1	1	3	3	3	3	4	4	3	3
1	1	2	2	3	3	4	4	3	3
3	3	2	2	1	1	1	1	3	3
3	3	2	2	3	3	4	4	3	3
1	1	2	2	1	1	4	4	3	3
3	3	3	3	1	1	4	4	3	3
1	1	3	3	3	3	4	4	2	2
1	1	3	3	1	1	4	4	3	3
3	3	2	2	1	1	1	1	3	3
1	1	2	2	1	1	4	4	3	3
1	1	2	2	1	1	1	1	3	3
3	3	2	2	1	1	4	4	3	3
1	1	2	2	1	1	1	1	3	3
1	1	2	2	3	3	4	4	3	3
1	1	2	2	1	1	4	4	3	3
1	1	3	3	3	3	1	1	3	3
1	1	2	2	3	3	4	4	3	3
1	1	2	2	3	3	4	4	3	3
1	1	2	2	3	3	4	4	3	3
1	1	2	2	1	1	4	4	3	3
3	3	2	2	3	3	4	4	3	3
3	3	2	2	1	1	4	4	3	3
3	3	2	2	1	1	4	4	3	3
1	1	2	2	3	3	4	4	3	3
3	3	2	2	3	3	4	4	3	3
3	3	3	3	3	3	4	4	3	3
3	3	3	3	1	1	4	4	3	3
1	1	2	2	1	1	4	4	3	3
1	1	3	3	1	1	1	1	3	3
3	3	2	2	1	1	1	1	3	3
1	1	2	2	3	3	4	4	3	3
1	1	2	2	3	3	4	4	3	3
1	1	2	2	3	3	4	4	3	3

For Review Only

3	3	3	3	3	3	4	4	3	3
3	3	2	2	1	1	4	4	3	3
1	1	2	2	1	1	1	1	3	3
3	3	2	2	3	3	4	4	3	3
1	1	2	2	3	3	1	1	3	3
1	1	2	2	1	1	1	1	3	3
3	3	2	2	3	3	4	4	3	3
1	1	2	2	1	1	4	4	3	3
3	3	2	2	1	1	4	4	3	3
3	3	2	2	3	3	4	4	3	3
3	3	2	2	1	1	4	4	3	3
3	3	2	2	1	1	1	1	3	3
1	1	3	3	1	1	4	4	3	3
1	1	2	2	1	1	4	4	3	3
1	1	2	2	3	3	4	4	3	3
1	1	2	2	1	1	4	4	3	3
1	1	2	2	3	3	4	4	3	3
3	3	2	2	1	1	1	1	3	3
3	3	3	3	3	3	4	4	3	3
3	3	2	2	1	1	4	4	3	3
3	3	3	3	3	3	4	4	3	3
1	1	2	2	1	1	4	4	3	3
1	1	3	3	1	1	1	1	3	3
1	1	2	2	1	1	4	4	3	3
3	3	3	3	1	1	4	4	3	3
1	1	2	2	3	3	4	4	3	3
3	3	2	2	3	3	4	4	3	3
1	1	2	2	1	1	4	4	3	3
1	1	2	2	3	3	1	1	3	3
1	1	3	3	1	1	1	1	3	3
1	1	3	3	3	3	4	4	3	3
1	1	3	3	1	1	4	4	3	3
3	3	3	3	1	1	4	4	3	3
1	1	2	2	3	3	4	4	3	3
1	1	3	3	1	1	4	4	3	3
3	3	3	3	3	3	4	4	3	3
1	1	3	3	3	3	1	1	3	3
1	1	2	2	3	3	4	4	3	3
1	1	3	3	3	3	4	4	3	3
1	1	2	2	3	3	4	4	3	3
1	1	2	2	1	1	4	4	3	3
1	1	2	2	1	1	4	4	3	3
1	1	2	2	1	1	4	4	3	3
1	1	2	2	1	1	1	1	3	3
1	1	2	2	3	3	4	4	3	3
1	1	2	2	1	1	4	4	3	3
1	1	2	2	3	3	4	4	3	3
1	1	2	2	1	1	4	4	3	3
3	3	2	2	1	1	4	4	2	2
3	3	2	2	3	3	4	4	3	3
1	1	3	3	1	1	4	4	3	3
1	1	2	2	3	3	4	4	3	3
1	1	2	2	3	3	4	4	3	3
1	1	2	2	1	1	4	4	3	3
1	1	2	2	1	1	4	4	3	3

For Review Only

1	1	3	3	1	1	1	1	3	3
1	1	2	2	1	1	4	4	3	3

For Review Only

SNP2236	SNP2236	SNP2237	SNP2237	SNP2238	SNP2238	SNP2239	SNP2239	SNP224	SNP224
2	2	3	3	3	3	1	1	2	2
2	2	4	4	2	2	4	4	2	2
2	2	4	4	2	2	4	4	2	2
2	2	4	4	2	2	4	4	2	2
2	2	3	3	3	3	1	1	2	2
2	2	3	3	2	2	4	4	2	2
2	2	4	4	2	2	4	4	2	2
2	2	4	4	3	3	1	1	2	2
2	2	3	3	2	2	4	4	2	2
2	2	4	4	3	3	1	1	2	2
2	2	4	4	2	2	4	4	2	2
2	2	4	4	2	2	4	4	2	2
2	2	4	4	2	2	4	4	2	2
3	3	4	4	2	2	4	4	4	4
3	3	4	4	2	2	4	4	4	4
2	2	4	4	2	2	4	4	2	2
2	2	3	3	2	2	4	4	2	2
2	2	4	4	2	2	4	4	2	2
2	2	4	4	2	2	4	4	2	2
3	3	4	4	3	3	1	1	2	2
2	2	4	4	2	2	4	4	2	2
2	2	4	4	2	2	4	4	2	2
3	3	4	4	2	2	4	4	2	2
2	2	4	4	2	2	4	4	2	2
2	2	4	4	2	2	4	4	2	2
2	2	4	4	2	2	4	4	2	2
2	2	4	4	2	2	4	4	2	2
2	2	4	4	2	2	4	4	2	2
2	2	4	4	3	3	1	1	2	2
3	3	4	4	3	3	1	1	2	2
2	2	4	4	2	2	4	4	2	2
3	3	4	4	2	2	4	4	2	2
2	2	3	3	2	2	4	4	2	2
2	2	4	4	3	3	1	1	2	2
2	2	4	4	2	2	4	4	2	2
2	2	3	3	2	2	4	4	2	2
2	2	3	3	2	2	4	4	2	2
2	2	4	4	2	2	4	4	2	2
3	3	4	4	2	2	4	4	2	2
2	2	4	4	2	2	4	4	2	2
2	2	4	4	3	3	1	1	2	2
2	2	4	4	3	3	1	1	2	2
2	2	4	4	3	3	1	1	2	2
2	2	4	4	2	2	4	4	2	2
2	2	4	4	2	2	4	4	2	2
2	2	4	4	2	2	4	4	2	2
2	2	4	4	2	2	4	4	2	2
2	2	4	4	2	2	4	4	2	2
2	2	4	4	2	2	4	4	2	2
2	2	4	4	2	2	4	4	2	2
2	2	4	4	2	2	4	4	2	2
2	2	4	4	2	2	4	4	2	2

For Review Only

2	2	4	4	2	2	4	4	2	2
2	2	3	3	2	2	4	4	2	2
2	2	3	3	2	2	4	4	2	2
2	2	4	4	2	2	4	4	2	2
2	2	4	4	2	2	4	4	2	2
2	2	4	4	2	2	4	4	2	2
2	2	4	4	2	2	4	4	2	2
3	3	3	3	3	3	1	1	2	2
3	3	4	4	2	2	4	4	4	4
2	2	4	4	2	2	4	4	2	2
3	3	4	4	2	2	4	4	2	2
3	3	4	4	2	2	4	4	2	2
2	2	4	4	2	2	4	4	2	2
2	2	4	4	3	3	1	1	2	2
2	2	4	4	2	2	4	4	2	2
2	2	4	4	3	3	1	1	4	4
2	2	4	4	2	2	4	4	2	2
2	2	4	4	2	2	4	4	2	2
2	2	4	4	2	2	4	4	2	2
2	2	4	4	2	2	4	4	2	2
3	3	4	4	2	2	4	4	2	2
3	3	4	4	2	2	4	4	2	2
2	2	4	4	2	2	4	4	2	2
2	2	4	4	2	2	4	4	2	2
2	2	4	4	2	2	4	4	2	2
2	2	4	4	2	2	4	4	2	2
3	3	3	3	3	3	1	1	2	2
3	3	3	3	3	3	1	1	2	2
2	2	4	4	2	2	4	4	2	2
2	2	4	4	2	2	4	4	2	2
2	2	4	4	2	2	4	4	2	2
2	2	3	3	2	2	4	4	2	2
3	3	3	3	3	3	1	1	2	2
2	2	4	4	2	2	4	4	2	2
3	3	4	4	2	2	4	4	2	2
2	2	4	4	2	2	4	4	2	2
2	2	4	4	2	2	4	4	2	2
2	2	4	4	3	3	1	1	2	2
2	2	4	4	2	2	4	4	2	2
3	3	3	3	3	3	1	1	2	2
2	2	4	4	2	2	4	4	2	2
2	2	3	3	2	2	4	4	2	2
2	2	3	3	2	2	4	4	2	2
3	3	4	4	2	2	4	4	2	2
2	2	3	3	2	2	4	4	2	2
2	2	4	4	2	2	4	4	2	2
3	3	4	4	2	2	4	4	2	2
2	2	3	3	2	2	4	4	2	2
3	3	3	3	2	2	4	4	2	2
3	3	3	3	2	2	4	4	4	4
2	2	3	3	2	2	4	4	2	2
3	3	4	4	2	2	4	4	2	2
3	3	4	4	3	3	1	1	2	2
2	2	3	3	3	3	1	1	2	2
3	3	3	3	2	2	4	4	4	4

For Review Only

2	2	4	4	3	3	1	1	2	2
3	3	4	4	2	2	4	4	2	2
2	2	3	3	2	2	4	4	2	2
2	2	4	4	3	3	1	1	4	4
2	2	4	4	2	2	4	4	2	2
2	2	3	3	2	2	4	4	2	2
2	2	4	4	2	2	4	4	2	2
2	2	4	4	2	2	4	4	2	2
2	2	4	4	2	2	4	4	4	4
2	2	4	4	3	3	1	1	2	2
2	2	3	3	2	2	4	4	2	2
2	2	4	4	2	2	4	4	2	2
2	2	3	3	2	2	4	4	2	2
2	2	4	4	2	2	4	4	2	2
2	2	4	4	2	2	4	4	2	2
2	2	4	4	2	2	4	4	2	2
2	2	3	3	2	2	4	4	2	2
2	2	4	4	2	2	4	4	2	2
3	3	4	4	3	3	1	1	2	2
2	2	4	4	2	2	4	4	2	2
2	2	4	4	2	2	4	4	2	2
2	2	4	4	2	2	4	4	2	2
2	2	4	4	2	2	4	4	2	2
3	3	4	4	3	3	1	1	2	2
3	3	4	4	2	2	4	4	2	2
3	3	4	4	2	2	4	4	2	2
2	2	3	3	2	2	4	4	2	2
2	2	3	3	2	2	4	4	2	2
2	2	4	4	2	2	4	4	2	2
2	2	4	4	2	2	4	4	2	2
3	3	3	3	3	3	1	1	2	2
2	2	4	4	2	2	4	4	2	2
2	2	4	4	2	2	4	4	2	2
2	2	4	4	2	2	4	4	2	2
2	2	4	4	3	3	1	1	2	2
2	2	4	4	3	3	1	1	2	2
3	3	4	4	3	3	1	1	2	2
3	3	4	4	2	2	4	4	2	2
2	2	4	4	3	3	1	1	2	2
3	3	3	3	3	3	1	1	2	2
3	3	4	4	2	2	4	4	2	2
3	3	4	4	3	3	1	1	2	2
3	3	3	3	2	2	4	4	4	4
2	2	4	4	3	3	1	1	2	2
2	2	4	4	2	2	4	4	2	2
2	2	3	3	2	2	4	4	4	4
2	2	4	4	2	2	4	4	2	2
2	2	4	4	2	2	4	4	2	2
2	2	4	4	2	2	4	4	2	2
2	2	4	4	2	2	4	4	2	2
2	2	3	3	2	2	4	4	2	2
2	2	4	4	2	2	4	4	2	2
2	2	4	4	2	2	4	4	2	2
3	3	3	3	2	2	4	4	2	2
2	2	4	4	2	2	4	4	2	2

For Review Only

2	2	4	4	2	2	4	4	2	2
2	2	3	3	2	2	4	4	2	2
2	2	3	3	2	2	4	4	2	2
3	3	4	4	2	2	4	4	2	2
2	2	4	4	2	2	4	4	2	2
2	2	4	4	2	2	4	4	2	2
2	2	4	4	3	3	1	1	2	2
2	2	4	4	2	2	4	4	2	2
2	2	4	4	2	2	4	4	2	2
2	2	3	3	2	2	4	4	2	2
2	2	4	4	2	2	4	4	2	2
2	2	3	3	2	2	4	4	2	2
2	2	4	4	2	2	4	4	2	2
2	2	3	3	2	2	4	4	2	2
3	3	4	4	3	3	1	1	2	2
2	2	4	4	2	2	4	4	2	2
2	2	4	4	3	3	1	1	2	2
2	2	3	3	2	2	4	4	2	2
2	2	4	4	2	2	4	4	2	2
2	2	4	4	2	2	4	4	2	2
2	2	4	4	2	2	4	4	2	2
2	2	3	3	2	2	4	4	2	2
3	3	4	4	2	2	4	4	2	2
3	3	4	4	2	2	4	4	2	2
2	2	4	4	2	2	4	4	2	2
2	2	4	4	3	3	1	1	2	2
2	2	4	4	2	2	4	4	2	2
2	2	4	4	3	3	1	1	2	2
2	2	3	3	2	2	4	4	2	2
2	2	4	4	2	2	4	4	2	2
2	2	4	4	3	3	1	1	4	4
2	2	3	3	2	2	4	4	2	2
3	3	3	3	2	2	4	4	2	2
3	3	4	4	3	3	1	1	2	2
2	2	4	4	2	2	4	4	2	2
3	3	4	4	2	2	4	4	2	2
2	2	4	4	2	2	4	4	2	2
2	2	3	3	2	2	4	4	2	2
2	2	4	4	2	2	4	4	2	2
2	2	4	4	2	2	4	4	2	2
2	2	4	4	2	2	4	4	2	2
2	2	4	4	2	2	4	4	2	2
3	3	4	4	3	3	1	1	2	2
3	3	4	4	2	2	4	4	2	2
2	2	3	3	3	3	1	1	2	2
2	2	4	4	2	2	4	4	2	2
2	2	4	4	2	2	4	4	2	2
2	2	3	3	3	3	1	1	2	2
3	3	3	3	3	3	1	1	2	2
2	2	4	4	2	2	4	4	4	4
2	2	3	3	2	2	4	4	2	2
3	3	4	4	3	3	1	1	2	2
3	3	4	4	2	2	4	4	2	2
3	3	4	4	2	2	4	4	2	2
3	3	4	4	2	2	4	4	2	2

For Review Only

2	2	4	4	3	3	1	1	2	2
2	2	4	4	2	2	4	4	2	2
2	2	4	4	2	2	4	4	2	2
2	2	4	4	2	2	4	4	2	2
2	2	4	4	2	2	4	4	2	2
2	2	4	4	2	2	4	4	2	2
2	2	4	4	3	3	1	1	2	2
2	2	4	4	2	2	4	4	2	2
2	2	4	4	3	3	1	1	2	2
3	3	3	3	3	3	1	1	2	2
2	2	4	4	2	2	4	4	2	2
3	3	4	4	3	3	1	1	2	2
2	2	4	4	2	2	4	4	2	2
2	2	4	4	2	2	4	4	2	2
2	2	4	4	2	2	4	4	2	2
3	3	4	4	2	2	4	4	2	2
3	3	4	4	2	2	4	4	2	2
3	3	4	4	3	3	1	1	2	2
2	2	3	3	3	3	1	1	2	2
3	3	4	4	2	2	4	4	2	2
2	2	3	3	3	3	1	1	2	2
2	2	4	4	2	2	4	4	2	2
2	2	3	3	2	2	4	4	2	2
3	3	4	4	2	2	4	4	4	4
3	3	3	3	3	3	1	1	2	2
2	2	4	4	2	2	4	4	2	2
3	3	3	3	3	3	1	1	2	2
2	2	4	4	2	2	4	4	2	2
2	2	4	4	2	2	4	4	2	2
2	2	3	3	2	2	4	4	2	2
3	3	3	3	2	2	4	4	2	2
2	2	4	4	2	2	4	4	2	2
3	3	4	4	2	2	4	4	2	2
2	2	4	4	2	2	4	4	2	2
3	3	4	4	2	2	4	4	2	2
3	3	4	4	3	3	1	1	2	2
2	2	4	4	2	2	4	4	2	2
3	3	4	4	2	2	4	4	2	2
2	2	3	3	2	2	4	4	2	2
3	3	4	4	2	2	4	4	2	2
2	2	3	3	2	2	4	4	2	2
2	2	4	4	2	2	4	4	2	2
3	3	4	4	2	2	4	4	2	2
2	2	4	4	2	2	4	4	2	2
2	2	4	4	2	2	4	4	2	2
2	2	4	4	2	2	4	4	2	2
3	3	4	4	2	2	4	4	2	2
2	2	4	4	2	2	4	4	2	2
2	2	4	4	3	3	1	1	2	2
3	3	3	3	3	3	1	1	2	2
2	2	4	4	2	2	4	4	2	2
3	3	4	4	2	2	4	4	2	2
2	2	4	4	2	2	4	4	2	2
2	2	4	4	2	2	4	4	2	2
2	2	4	4	2	2	4	4	2	2

For Review Only

2	2	3	3	2	2	4	4	2	2
2	2	3	3	2	2	4	4	2	2

For Review Only

SNP2240	SNP2240	SNP2241	SNP2241	SNP2242	SNP2242	SNP2243	SNP2243	SNP2244	SNP2244
1	1	1	1	4	4	3	3	2	2
1	1	1	1	4	4	4	4	2	2
1	1	1	1	4	4	4	4	2	2
1	1	1	1	4	4	4	4	2	2
2	2	4	4	4	4	3	3	2	2
2	2	4	4	4	4	3	3	2	2
2	2	4	4	4	4	3	3	2	2
1	1	1	1	4	4	4	4	2	2
2	2	4	4	3	3	3	3	4	4
1	1	1	1	4	4	4	4	2	2
2	2	1	1	4	4	4	4	2	2
1	1	1	1	4	4	4	4	2	2
2	2	1	1	4	4	4	4	2	2
2	2	1	1	4	4	4	4	2	2
2	2	4	4	4	4	3	3	2	2
2	2	1	1	4	4	4	4	2	2
2	2	4	4	4	4	3	3	2	2
1	1	1	1	4	4	4	4	2	2
2	2	4	4	4	4	3	3	2	2
1	1	1	1	3	3	4	4	4	4
2	2	1	1	4	4	4	4	2	2
2	2	1	1	4	4	4	4	2	2
2	2	1	1	4	4	4	4	2	2
1	1	1	1	4	4	4	4	2	2
2	2	1	1	4	4	4	4	2	2
2	2	1	1	4	4	4	4	2	2
1	1	1	1	4	4	4	4	2	2
2	2	1	1	4	4	4	4	2	2
1	1	1	1	3	3	4	4	4	4
1	1	1	1	4	4	4	4	2	2
2	2	1	1	4	4	4	4	2	2
2	2	1	1	3	3	4	4	4	4
2	2	4	4	3	3	3	3	4	4
1	1	1	1	4	4	4	4	2	2
2	2	1	1	4	4	4	4	2	2
1	1	4	4	4	4	3	3	2	2
1	1	4	4	4	4	3	3	2	2
1	1	1	1	4	4	4	4	2	2
1	1	1	1	4	4	4	4	2	2
1	1	1	1	4	4	4	4	2	2
1	1	1	1	4	4	4	4	2	2
1	1	1	1	3	3	4	4	4	4
1	1	1	1	4	4	4	4	2	2
2	2	1	1	4	4	4	4	2	2
1	1	1	1	4	4	4	4	2	2
1	1	1	1	4	4	4	4	2	2
2	2	1	1	4	4	4	4	2	2
2	2	1	1	4	4	4	4	2	2
2	2	1	1	4	4	4	4	2	2
1	1	1	1	4	4	4	4	2	2
1	1	1	1	4	4	4	4	2	2
2	2	1	1	4	4	4	4	2	2

For Review Only

2	2	1	1	4	4	4	4	2	2
2	2	4	4	3	3	3	3	4	4
2	2	4	4	4	4	3	3	2	2
2	2	1	1	4	4	4	4	2	2
2	2	4	4	4	4	3	3	2	2
2	2	1	1	4	4	4	4	2	2
2	2	1	1	4	4	4	4	2	2
1	1	4	4	4	4	3	3	2	2
1	1	4	4	4	4	3	3	2	2
2	2	1	1	4	4	4	4	2	2
2	2	1	1	4	4	4	4	2	2
2	2	1	1	4	4	4	4	2	2
2	2	1	1	4	4	4	4	2	2
1	1	1	1	3	3	4	4	4	4
2	2	4	4	4	4	4	4	2	2
1	1	1	1	4	4	4	4	2	2
1	1	1	1	4	4	4	4	2	2
2	2	1	1	3	3	4	4	4	4
2	2	1	1	4	4	4	4	2	2
1	1	1	1	4	4	4	4	2	2
2	2	1	1	4	4	4	4	2	2
1	1	1	1	4	4	4	4	2	2
2	2	1	1	3	3	4	4	4	4
2	2	1	1	4	4	4	4	2	2
1	1	1	1	4	4	4	4	2	2
1	1	1	1	4	4	4	4	2	2
2	2	1	1	4	4	3	3	2	2
2	2	4	4	4	4	3	3	2	2
2	2	1	1	4	4	4	4	2	2
2	2	1	1	4	4	4	4	2	2
1	1	1	1	4	4	4	4	2	2
2	2	4	4	4	4	3	3	2	2
1	1	4	4	4	4	3	3	2	2
2	2	1	1	4	4	4	4	2	2
1	1	1	1	4	4	4	4	2	2
1	1	1	1	4	4	4	4	2	2
1	1	1	1	4	4	4	4	2	2
2	2	1	1	4	4	4	4	2	2
2	2	1	1	4	4	4	4	2	2
1	1	4	4	4	4	3	3	2	2
2	2	1	1	4	4	4	4	2	2
2	2	4	4	4	4	3	3	2	2
1	1	4	4	4	4	3	3	2	2
2	2	1	1	4	4	4	4	2	2
2	2	4	4	4	4	3	3	2	2
2	2	1	1	4	4	4	4	2	2
1	1	4	4	4	4	3	3	2	2
1	1	4	4	4	4	3	3	2	2
2	2	4	4	4	4	3	3	2	2
2	2	4	4	4	4	3	3	2	2
2	2	4	4	4	4	3	3	2	2
1	1	1	1	4	4	4	4	2	2
1	1	1	1	4	4	4	4	2	2
2	2	1	1	4	4	4	4	2	2
2	2	4	4	4	4	3	3	2	2

For Review Only

2	2	1	1	4	4	4	4	2	2
1	1	1	1	4	4	4	4	2	2
1	1	4	4	4	4	3	3	2	2
1	1	1	1	4	4	4	4	2	2
1	1	1	1	4	4	4	4	2	2
2	2	4	4	4	4	3	3	2	2
1	1	1	1	4	4	4	4	2	2
2	2	4	4	4	4	3	3	2	2
1	1	1	1	4	4	4	4	2	2
2	2	1	1	4	4	4	4	2	2
1	1	4	4	4	4	3	3	2	2
2	2	1	1	4	4	4	4	2	2
1	1	4	4	4	4	3	3	2	2
2	2	1	1	4	4	4	4	2	2
2	2	1	1	4	4	4	4	2	2
2	2	1	1	4	4	4	4	2	2
1	1	4	4	4	4	3	3	2	2
2	2	1	1	4	4	4	4	2	2
1	1	1	1	4	4	4	4	2	2
2	2	1	1	4	4	4	4	2	2
2	2	1	1	4	4	4	4	2	2
2	2	1	1	4	4	4	4	2	2
2	2	1	1	4	4	4	4	2	2
1	1	1	1	4	4	4	4	2	2
2	2	1	1	4	4	4	4	2	2
1	1	1	1	4	4	4	4	2	2
1	1	4	4	3	3	4	4	4	4
1	1	4	4	4	4	3	3	2	2
1	1	1	1	4	4	4	4	2	2
2	2	1	1	4	4	4	4	2	2
2	2	4	4	4	4	3	3	2	2
2	2	1	1	4	4	4	4	2	2
2	2	1	1	4	4	4	4	2	2
2	2	1	1	4	4	4	4	2	2
1	1	1	1	4	4	4	4	2	2
2	2	1	1	4	4	4	4	2	2
1	1	4	4	4	4	3	3	2	2
1	1	1	1	4	4	4	4	2	2
1	1	1	1	4	4	4	4	2	2
2	2	4	4	4	4	3	3	2	2
2	2	1	1	4	4	4	4	2	2
2	2	4	4	4	4	3	3	2	2
1	1	4	4	3	3	3	3	4	4
1	1	1	1	4	4	4	4	2	2
2	2	1	1	4	4	4	4	2	2
2	2	4	4	4	4	3	3	2	2
2	2	1	1	4	4	4	4	2	2
2	2	1	1	4	4	4	4	2	2
2	2	4	4	4	4	4	4	2	2
2	2	1	1	4	4	4	4	2	2
2	2	4	4	4	4	3	3	2	2
2	2	1	1	4	4	4	4	2	2
1	1	1	1	4	4	4	4	2	2
2	2	4	4	4	4	3	3	2	2
2	2	4	4	4	4	4	4	2	2

For Review Only

2	2	1	1	4	4	4	4	2	2
1	1	4	4	4	4	3	3	2	2
1	1	4	4	4	4	3	3	2	2
1	1	1	1	4	4	4	4	2	2
1	1	1	1	4	4	4	4	2	2
1	1	1	1	4	4	4	4	2	2
2	2	1	1	4	4	4	4	2	2
2	2	1	1	4	4	4	4	2	2
1	1	1	1	4	4	4	4	2	2
2	2	4	4	4	4	3	3	2	2
2	2	1	1	4	4	4	4	2	2
2	2	1	1	4	4	4	4	2	2
2	2	1	1	4	4	4	4	2	2
2	2	1	1	4	4	4	4	2	2
1	1	1	1	4	4	3	3	2	2
2	2	1	1	4	4	4	4	2	2
1	1	1	1	4	4	4	4	2	2
1	1	4	4	4	4	3	3	4	4
2	2	1	1	4	4	4	4	2	2
1	1	1	1	4	4	4	4	2	2
2	2	1	1	4	4	4	4	2	2
1	1	4	4	4	4	3	3	2	2
1	1	1	1	4	4	4	4	2	2
2	2	1	1	4	4	4	4	2	2
2	2	1	1	3	3	4	4	4	4
1	1	1	1	4	4	4	4	2	2
1	1	1	1	4	4	4	4	2	2
1	1	1	1	4	4	4	4	2	2
1	1	4	4	4	4	3	3	2	2
2	2	1	1	4	4	4	4	2	2
1	1	1	1	4	4	4	4	2	2
2	2	4	4	4	4	3	3	2	2
1	1	4	4	4	4	3	3	2	2
1	1	1	1	4	4	3	3	2	2
2	2	1	1	3	3	4	4	4	4
2	2	1	1	4	4	4	4	2	2
1	1	1	1	4	4	4	4	2	2
1	1	4	4	4	4	3	3	2	2
2	2	1	1	4	4	4	4	2	2
2	2	1	1	4	4	4	4	2	2
1	1	1	1	4	4	4	4	2	2
2	2	1	1	4	4	4	4	2	2
2	2	1	1	4	4	4	4	2	2
2	2	1	1	4	4	4	4	2	2
2	2	4	4	4	4	3	3	2	2
1	1	1	1	4	4	4	4	2	2
2	2	1	1	4	4	4	4	2	2
1	1	4	4	4	4	3	3	2	2
1	1	4	4	4	4	3	3	2	2
2	2	1	1	4	4	4	4	2	2
1	1	4	4	4	4	3	3	2	2
1	1	1	1	4	4	4	4	2	2
1	1	1	1	4	4	4	4	2	2
2	2	1	1	4	4	4	4	2	2
2	2	1	1	4	4	4	4	2	2

For Review Only

For Review Only

1	1	1	1	4	4	4	4	2	2
2	2	1	1	4	4	4	4	2	2
1	1	1	1	4	4	4	4	2	2
1	1	1	1	4	4	4	4	2	2
1	1	1	1	4	4	4	4	2	2
1	1	1	1	3	3	4	4	4	4
1	1	1	1	4	4	4	4	2	2
1	1	1	1	4	4	4	4	2	2
1	1	1	1	4	4	4	4	2	2
1	1	4	4	4	4	3	3	2	2
2	2	1	1	4	4	4	4	2	2
1	1	1	1	4	4	4	4	2	2
2	2	1	1	4	4	4	4	2	2
2	2	1	1	4	4	4	4	2	2
1	1	1	1	4	4	4	4	2	2
2	2	1	1	4	4	4	4	2	2
1	1	1	1	4	4	4	4	2	2
1	1	1	1	3	3	4	4	4	4
1	1	4	4	4	4	3	3	2	2
1	1	1	1	4	4	4	4	2	2
1	1	4	4	4	4	3	3	2	2
2	2	1	1	4	4	4	4	2	2
1	1	4	4	4	4	3	3	2	2
2	2	1	1	4	4	4	4	2	2
1	1	4	4	4	4	3	3	2	2
1	1	1	1	4	4	4	4	2	2
1	1	4	4	4	4	3	3	2	2
1	1	1	1	4	4	4	4	2	2
2	2	1	1	4	4	4	4	2	2
1	1	4	4	4	4	3	3	2	2
1	1	4	4	4	4	3	3	2	2
1	1	1	1	4	4	4	4	2	2
2	2	1	1	4	4	4	4	2	2
2	2	1	1	4	4	4	4	2	2
2	2	1	1	4	4	4	4	2	2
2	2	1	1	4	4	4	4	2	2
1	1	1	1	4	4	4	4	2	2
2	2	1	1	4	4	4	4	2	2
1	1	4	4	4	4	3	3	2	2
1	1	1	1	4	4	4	4	2	2
1	1	4	4	4	4	3	3	2	2
1	1	1	1	3	3	4	4	4	4
2	2	1	1	4	4	4	4	2	2
2	2	1	1	4	4	4	4	2	2
2	2	1	1	4	4	4	4	2	2
1	1	1	1	4	4	4	4	2	2
2	2	1	1	4	4	4	4	2	2
2	2	1	1	4	4	4	4	2	2
1	1	1	1	4	4	4	4	2	2
1	1	4	4	4	4	3	3	2	2
2	2	1	1	3	3	4	4	4	4
1	1	1	1	4	4	4	4	2	2
1	1	1	1	4	4	4	4	2	2
1	1	1	1	4	4	4	4	2	2
1	1	1	1	4	4	4	4	2	2

2	2	4	4	3	3	3	3	4	4
1	1	4	4	4	4	3	3	2	2

For Review Only

SNP2245	SNP2245	SNP2246	SNP2246	SNP2247	SNP2247	SNP2248	SNP2248	SNP2249	SNP2249
2	2	4	4	3	3	3	3	2	2
4	4	4	4	1	1	3	3	3	3
4	4	4	4	1	1	3	3	3	3
2	2	4	4	1	1	3	3	3	3
2	2	1	1	3	3	2	2	2	2
4	4	1	1	3	3	3	3	3	3
2	2	1	1	3	3	3	3	2	2
2	2	4	4	1	1	3	3	3	3
2	2	1	1	3	3	2	2	3	3
2	2	4	4	1	1	3	3	3	3
2	2	1	1	1	1	3	3	3	3
2	2	4	4	1	1	3	3	3	3
2	2	4	4	1	1	3	3	3	3
2	2	1	1	1	1	3	3	3	3
2	2	4	4	1	1	2	2	3	3
2	2	4	4	1	1	2	2	3	3
2	2	1	1	3	3	3	3	2	2
2	2	4	4	1	1	3	3	3	3
4	4	4	4	3	3	3	3	3	3
2	2	4	4	1	1	3	3	3	3
2	2	1	1	1	1	3	3	3	3
2	2	1	1	1	1	3	3	3	3
2	2	1	1	1	1	2	2	3	3
2	2	4	4	1	1	2	2	3	3
4	4	1	1	1	1	2	2	3	3
2	2	1	1	1	1	3	3	3	3
4	4	4	4	1	1	3	3	3	3
2	2	4	4	1	1	2	2	3	3
2	2	4	4	1	1	2	2	3	3
2	2	4	4	1	1	2	2	3	3
4	4	1	1	1	1	3	3	3	3
2	2	1	1	1	1	3	3	3	3
2	2	1	1	3	3	3	3	2	2
2	2	4	4	1	1	3	3	3	3
2	2	1	1	1	1	3	3	3	3
2	2	4	4	3	3	2	2	2	2
2	2	4	4	3	3	3	3	3	3
2	2	4	4	1	1	3	3	3	3
2	2	4	4	1	1	2	2	3	3
2	2	4	4	1	1	3	3	3	3
2	2	4	4	1	1	3	3	3	3
2	2	4	4	1	1	2	2	3	3
2	2	4	4	1	1	3	3	3	3
2	2	1	1	1	1	3	3	3	3
2	2	4	4	1	1	3	3	3	3
4	4	4	4	1	1	3	3	3	3
2	2	1	1	1	1	3	3	3	3
2	2	4	4	1	1	3	3	3	3
2	2	1	1	1	1	3	3	3	3
2	2	4	4	1	1	3	3	2	2
2	2	4	4	1	1	3	3	3	3
2	2	4	4	1	1	2	2	3	3

For Review Only

2	2	1	1	1	1	3	3	3	3
2	2	1	1	3	3	3	3	2	2
2	2	4	4	1	1	3	3	3	3
2	2	1	1	1	1	3	3	3	3
4	4	4	4	3	3	3	3	3	3
4	4	1	1	1	1	3	3	3	3
2	2	1	1	1	1	3	3	3	3
2	2	4	4	3	3	3	3	2	2
2	2	4	4	3	3	3	3	2	2
2	2	4	4	1	1	3	3	3	3
4	4	1	1	1	1	3	3	3	3
4	4	1	1	1	1	3	3	3	3
2	2	1	1	1	1	3	3	3	3
2	2	4	4	1	1	2	2	3	3
2	2	1	1	1	1	3	3	3	3
2	2	4	4	1	1	2	2	3	3
4	4	4	4	1	1	2	2	3	3
2	2	4	4	1	1	3	3	3	3
2	2	1	1	1	1	3	3	3	3
2	2	4	4	1	1	3	3	3	3
2	2	1	1	1	1	3	3	3	3
2	2	1	1	1	1	3	3	3	3
2	2	4	4	1	1	2	2	3	3
4	4	1	1	1	1	3	3	3	3
2	2	4	4	1	1	3	3	3	3
2	2	4	4	1	1	3	3	3	3
2	2	1	1	3	3	3	3	2	2
2	2	4	4	3	3	3	3	2	2
2	2	1	1	1	1	3	3	3	3
2	2	1	1	1	1	3	3	3	3
2	2	4	4	1	1	3	3	3	3
2	2	1	1	3	3	2	2	2	2
2	2	4	4	3	3	3	3	2	2
2	2	1	1	1	1	3	3	3	3
2	2	4	4	1	1	3	3	3	3
2	2	4	4	1	1	3	3	3	3
2	2	4	4	1	1	3	3	3	3
2	2	1	1	1	1	3	3	3	3
2	2	1	1	1	1	3	3	3	3
2	2	4	4	3	3	2	2	3	3
2	2	1	1	1	1	3	3	3	3
2	2	1	1	3	3	3	3	3	3
2	2	4	4	3	3	3	3	2	2
4	4	1	1	1	1	3	3	3	3
2	2	4	4	3	3	3	3	2	2
2	2	1	1	1	1	3	3	3	3
2	2	4	4	3	3	3	3	3	3
2	2	4	4	3	3	3	3	2	2
4	4	1	1	3	3	3	3	2	2
2	2	4	4	3	3	2	2	3	3
2	2	1	1	1	1	3	3	2	2
2	2	4	4	1	1	3	3	3	3
2	2	4	4	1	1	3	3	3	3
2	2	4	4	1	1	2	2	3	3
2	2	1	1	3	3	2	2	2	2

For Review Only

2	2	1	1	1	1	3	3	3	3
2	2	1	1	1	1	3	3	3	3
2	2	4	4	3	3	3	3	2	2
2	2	4	4	1	1	3	3	3	3
2	2	4	4	1	1	3	3	3	3
2	2	1	1	3	3	3	3	2	2
2	2	4	4	1	1	3	3	3	3
2	2	1	1	1	1	3	3	3	3
2	2	4	4	1	1	3	3	3	3
2	2	4	4	1	1	2	2	3	3
2	2	4	4	3	3	3	3	2	2
2	2	4	4	1	1	3	3	3	3
2	2	4	4	3	3	3	3	2	2
2	2	1	1	1	1	3	3	3	3
2	2	4	4	1	1	2	2	3	3
2	2	4	4	1	1	3	3	3	3
2	2	1	1	3	3	2	2	2	2
2	2	1	1	1	1	3	3	3	3
2	2	4	4	1	1	3	3	3	3
2	2	4	4	1	1	3	3	3	3
2	2	4	4	1	1	2	2	3	3
2	2	1	1	1	1	3	3	3	3
2	2	4	4	1	1	2	2	3	3
2	2	4	4	1	1	3	3	3	3
2	2	1	1	1	1	3	3	3	3
2	2	1	1	1	1	3	3	3	3
2	2	1	1	1	1	2	2	3	3
2	2	4	4	3	3	3	3	3	3
2	2	4	4	1	1	2	2	3	3
2	2	4	4	1	1	2	2	3	3
2	2	4	4	3	3	3	3	2	2
2	2	4	4	1	1	3	3	3	3
2	2	1	1	1	1	3	3	3	3
2	2	1	1	1	1	3	3	3	3
2	2	4	4	1	1	3	3	3	3
2	2	4	4	1	1	3	3	3	3
2	2	4	4	3	3	3	3	2	2
2	2	4	4	1	1	3	3	3	3
2	2	1	1	1	1	3	3	3	3
2	2	4	4	3	3	3	3	2	2
2	2	1	1	1	1	2	2	3	3
2	2	4	4	3	3	3	3	2	2
2	2	4	4	3	3	3	3	2	2
2	2	4	4	1	1	2	2	3	3
2	2	1	1	1	1	3	3	3	3
2	2	1	1	3	3	3	3	2	2
2	2	1	1	1	1	3	3	3	3
2	2	1	1	1	1	3	3	3	3
2	2	4	4	1	1	3	3	3	3
2	2	1	1	1	1	3	3	3	3
2	2	1	1	3	3	2	2	2	2
2	2	1	1	1	1	3	3	3	3
2	2	4	4	1	1	3	3	3	3
2	2	1	1	3	3	3	3	2	2
2	2	4	4	1	1	2	2	3	3

For Review Only

2	2	1	1	1	1	2	2	3	3
2	2	4	4	3	3	2	2	2	2
2	2	4	4	3	3	3	3	2	2
2	2	1	1	1	1	2	2	3	3
2	2	4	4	1	1	3	3	3	3
2	2	4	4	1	1	3	3	3	3
2	2	4	4	1	1	3	3	3	3
2	2	4	4	1	1	3	3	3	3
2	2	4	4	1	1	2	2	3	3
2	2	1	1	3	3	3	3	3	3
2	2	1	1	1	1	3	3	3	3
2	2	1	1	1	1	2	2	2	2
2	2	4	4	1	1	2	2	3	3
2	2	1	1	1	1	3	3	3	3
2	2	4	4	3	3	3	3	2	2
2	2	1	1	1	1	3	3	3	3
2	2	4	4	1	1	3	3	3	3
2	2	1	1	3	3	2	2	3	3
2	2	1	1	1	1	3	3	3	3
2	2	4	4	1	1	3	3	3	3
2	2	4	4	1	1	3	3	3	3
2	2	4	4	3	3	3	3	2	2
2	2	1	1	1	1	3	3	3	3
4	4	1	1	1	1	3	3	3	3
2	2	1	1	1	1	3	3	3	3
2	2	4	4	1	1	3	3	3	3
2	2	4	4	1	1	2	2	3	3
2	2	4	4	1	1	2	2	3	3
2	2	4	4	3	3	2	2	2	2
2	2	1	1	1	1	2	2	3	3
2	2	4	4	1	1	3	3	3	3
2	2	1	1	3	3	2	2	3	3
2	2	1	1	3	3	3	3	3	3
2	2	4	4	3	3	3	3	2	2
2	2	1	1	1	1	2	2	3	3
4	4	1	1	1	1	3	3	3	3
4	4	4	4	1	1	3	3	3	3
2	2	4	4	3	3	3	3	3	3
2	2	1	1	1	1	3	3	3	3
2	2	1	1	1	1	2	2	3	3
4	4	4	4	1	1	3	3	3	3
2	2	1	1	1	1	3	3	3	3
2	2	4	4	1	1	3	3	3	3
2	2	1	1	1	1	2	2	3	3
2	2	4	4	3	3	3	3	2	2
2	2	4	4	1	1	2	2	3	3
2	2	1	1	1	1	3	3	3	3
2	2	4	4	3	3	3	3	2	2
2	2	4	4	3	3	3	3	2	2
2	2	1	1	1	1	3	3	3	3
4	4	4	4	3	3	2	2	3	3
2	2	4	4	1	1	3	3	2	2
2	2	4	4	1	1	3	3	3	3
4	4	1	1	1	1	3	3	3	3
2	2	4	4	1	1	3	3	3	3

For Review Only

2	2	4	4	1	1	3	3	3	3
2	2	1	1	1	1	2	2	3	3
4	4	4	4	1	1	2	2	3	3
2	2	4	4	1	1	3	3	3	3
2	2	1	1	1	1	3	3	3	3
2	2	4	4	1	1	2	2	3	3
2	2	4	4	1	1	3	3	2	2
2	2	4	4	1	1	3	3	3	3
2	2	4	4	1	1	3	3	3	3
2	2	4	4	3	3	2	2	2	2
2	2	4	4	1	1	2	2	3	3
2	2	1	1	1	1	3	3	3	3
2	2	1	1	1	1	2	2	3	3
2	2	1	1	1	1	3	3	3	3
4	4	4	4	1	1	3	3	3	3
2	2	1	1	1	1	3	3	3	3
2	2	4	4	1	1	2	2	3	3
2	2	4	4	1	1	2	2	3	3
2	2	4	4	3	3	3	3	2	2
2	2	4	4	1	1	3	3	3	3
2	2	4	4	3	3	3	3	2	2
2	2	1	1	1	1	3	3	3	3
2	2	4	4	3	3	3	3	3	3
2	2	1	1	1	1	3	3	3	3
2	2	4	4	3	3	3	3	2	2
2	2	4	4	1	1	3	3	2	2
2	2	4	4	3	3	3	3	2	2
2	2	4	4	1	1	3	3	3	3
2	2	1	1	1	1	3	3	3	3
4	4	4	4	3	3	2	2	3	3
2	2	4	4	3	3	2	2	2	2
2	2	4	4	1	1	3	3	3	3
2	2	1	1	1	1	3	3	3	3
2	2	4	4	1	1	2	2	3	3
2	2	1	1	1	1	3	3	3	3
2	2	4	4	1	1	3	3	3	3
2	2	4	4	1	1	3	3	3	3
4	4	1	1	1	1	3	3	3	3
2	2	4	4	3	3	2	2	2	2
2	2	4	4	1	1	3	3	3	3
2	2	1	1	3	3	2	2	3	3
2	2	4	4	1	1	3	3	3	3
2	2	1	1	1	1	3	3	3	3
2	2	1	1	1	1	3	3	3	3
4	4	4	4	1	1	2	2	3	3
4	4	4	4	1	1	2	2	3	3
2	2	1	1	1	1	3	3	3	3
2	2	1	1	1	1	2	2	2	2
2	2	4	4	1	1	2	2	3	3
2	2	4	4	3	3	2	2	2	2
2	2	1	1	1	1	2	2	3	3
2	2	4	4	1	1	3	3	3	3
4	4	4	4	1	1	3	3	3	3
4	4	4	4	1	1	3	3	3	3
2	2	4	4	1	1	3	3	3	3

For Review Only

2	2	4	4	3	3	2	2	3	3
2	2	4	4	3	3	3	3	3	3

For Review Only

SNP225	SNP225	SNP2250	SNP2250	SNP2251	SNP2251	SNP2252	SNP2252	SNP2253	SNP2253
1	1	3	3	2	2	4	4	2	2
1	1	3	3	2	2	4	4	2	2
1	1	3	3	2	2	4	4	2	2
1	1	3	3	2	2	1	1	2	2
1	1	3	3	2	2	4	4	2	2
1	1	3	3	2	2	1	1	2	2
1	1	3	3	2	2	4	4	2	2
1	1	2	2	2	2	4	4	2	2
1	1	3	3	2	2	4	4	2	2
1	1	2	2	2	2	1	1	1	1
1	1	3	3	2	2	4	4	2	2
1	1	3	3	2	2	4	4	2	2
1	1	3	3	2	2	4	4	2	2
1	1	3	3	2	2	4	4	2	2
1	1	3	3	2	2	1	1	2	2
1	1	3	3	2	2	1	1	2	2
1	1	3	3	2	2	4	4	2	2
1	1	3	3	2	2	1	1	2	2
1	1	3	3	2	2	4	4	2	2
1	1	3	3	2	2	4	4	2	2
1	1	3	3	2	2	4	4	2	2
1	1	2	2	2	2	4	4	2	2
3	3	2	2	2	2	4	4	2	2
1	1	3	3	2	2	4	4	2	2
1	1	3	3	2	2	4	4	2	2
1	1	3	3	2	2	4	4	2	2
1	1	3	3	2	2	1	1	2	2
1	1	3	3	2	2	1	1	2	2
1	1	3	3	3	3	4	4	2	2
1	1	3	3	2	2	4	4	2	2
1	1	3	3	2	2	4	4	2	2
1	1	3	3	2	2	1	1	2	2
1	1	3	3	2	2	4	4	2	2
1	1	3	3	2	2	4	4	2	2
1	1	3	3	2	2	1	1	2	2
1	1	3	3	2	2	4	4	2	2
1	1	3	3	2	2	1	1	2	2
1	1	3	3	2	2	1	1	2	2
1	1	2	2	2	2	4	4	2	2
1	1	2	2	2	2	1	1	2	2
1	1	3	3	2	2	4	4	2	2
1	1	3	3	3	3	4	4	2	2
1	1	3	3	2	2	4	4	2	2
1	1	3	3	2	2	4	4	2	2
1	1	3	3	2	2	4	4	2	2
1	1	3	3	2	2	4	4	2	2
1	1	3	3	2	2	4	4	2	2
1	1	3	3	2	2	4	4	2	2
1	1	3	3	2	2	4	4	2	2
1	1	3	3	2	2	4	4	2	2
3	3	3	3	2	2	4	4	2	2
1	1	3	3	2	2	4	4	2	2

For Review Only

1	1	3	3	2	2	4	4	2	2
1	1	3	3	2	2	4	4	2	2
1	1	3	3	2	2	4	4	2	2
1	1	3	3	2	2	4	4	2	2
1	1	3	3	2	2	4	4	2	2
1	1	3	3	2	2	4	4	2	2
1	1	3	3	2	2	4	4	2	2
1	1	3	3	2	2	4	4	2	2
1	1	3	3	2	2	1	1	2	2
1	1	3	3	2	2	1	1	2	2
1	1	3	3	2	2	4	4	2	2
1	1	3	3	2	2	4	4	2	2
1	1	3	3	2	2	4	4	2	2
1	1	3	3	3	3	4	4	2	2
1	1	3	3	2	2	1	1	2	2
1	1	3	3	2	2	1	1	2	2
1	1	3	3	2	2	4	4	2	2
1	1	3	3	2	2	1	1	2	2
3	3	3	3	2	2	4	4	2	2
1	1	3	3	2	2	4	4	2	2
1	1	3	3	2	2	1	1	2	2
1	1	2	2	2	2	4	4	2	2
1	1	3	3	3	3	4	4	2	2
1	1	3	3	2	2	1	1	2	2
1	1	3	3	2	2	4	4	2	2
1	1	3	3	2	2	4	4	2	2
1	1	3	3	2	2	4	4	2	2
1	1	2	2	2	2	4	4	2	2
1	1	3	3	2	2	4	4	2	2
1	1	3	3	2	2	1	1	2	2
1	1	3	3	2	2	4	4	1	1
1	1	3	3	2	2	4	4	2	2
1	1	2	2	2	2	4	4	2	2
1	1	3	3	2	2	4	4	2	2
1	1	3	3	2	2	4	4	2	2
1	1	3	3	2	2	4	4	2	2
1	1	2	2	2	2	4	4	1	1
1	1	3	3	2	2	4	4	2	2
1	1	3	3	2	2	4	4	1	1
1	1	3	3	2	2	4	4	2	2
1	1	3	3	2	2	4	4	2	2
1	1	3	3	2	2	1	1	2	2
1	1	3	3	2	2	1	1	2	2
1	1	3	3	2	2	4	4	2	2
3	3	3	3	2	2	4	4	2	2
1	1	3	3	2	2	1	1	1	1
1	1	3	3	2	2	4	4	2	2
1	1	3	3	2	2	4	4	2	2
1	1	3	3	2	2	4	4	2	2
1	1	3	3	3	3	1	1	2	2
1	1	3	3	2	2	4	4	2	2
1	1	3	3	2	2	4	4	2	2
1	1	3	3	2	2	4	4	2	2
1	1	3	3	2	2	1	1	2	2
1	1	3	3	3	3	1	1	2	2

For Review Only

1	1	3	3	2	2	4	4	2	2
1	1	3	3	2	2	1	1	2	2
1	1	3	3	2	2	4	4	2	2
1	1	3	3	2	2	4	4	2	2
1	1	3	3	2	2	4	4	2	2
1	1	3	3	2	2	4	4	2	2
1	1	3	3	2	2	1	1	2	2
1	1	3	3	2	2	4	4	2	2
1	1	3	3	2	2	4	4	2	2
1	1	3	3	2	2	4	4	2	2
1	1	3	3	2	2	1	1	2	2
1	1	3	3	2	2	1	1	2	2
1	1	3	3	2	2	1	1	1	1
1	1	3	3	2	2	4	4	2	2
1	1	2	2	2	2	4	4	2	2
1	1	3	3	2	2	4	4	2	2
1	1	3	3	3	3	4	4	1	1
1	1	3	3	2	2	4	4	2	2
1	1	3	3	2	2	4	4	2	2
1	1	3	3	2	2	4	4	2	2
1	1	3	3	2	2	4	4	2	2
1	1	3	3	2	2	4	4	2	2
1	1	3	3	2	2	1	1	2	2
1	1	3	3	2	2	4	4	2	2
1	1	3	3	2	2	4	4	2	2
3	3	2	2	2	2	1	1	1	1
3	3	3	3	2	2	1	1	2	2
1	1	3	3	2	2	1	1	2	2
1	1	3	3	2	2	1	1	2	2
3	3	3	3	2	2	1	1	1	1
1	1	2	2	2	2	4	4	2	2
1	1	3	3	2	2	1	1	2	2
1	1	3	3	2	2	4	4	2	2
1	1	3	3	2	2	1	1	2	2
1	1	3	3	2	2	4	4	2	2
1	1	3	3	2	2	1	1	2	2
1	1	3	3	2	2	4	4	2	2
1	1	3	3	2	2	1	1	2	2
1	1	3	3	2	2	4	4	2	2
1	1	3	3	2	2	4	4	2	2
1	1	3	3	2	2	1	1	2	2
1	1	3	3	2	2	1	1	2	2
1	1	3	3	2	2	1	1	2	2
1	1	3	3	2	2	1	1	2	2
1	1	3	3	2	2	1	1	2	2
1	1	3	3	2	2	4	4	2	2
3	3	3	3	2	2	4	4	2	2
1	1	3	3	2	2	4	4	2	2
1	1	3	3	2	2	1	1	2	2
1	1	3	3	2	2	4	4	2	2
3	3	3	3	2	2	4	4	2	2
1	1	2	2	2	2	4	4	2	2
1	1	3	3	2	2	1	1	2	2
3	3	3	3	2	2	4	4	2	2
1	1	2	2	2	2	4	4	2	2

For Review Only

3	3	3	3	2	2	1	1	2	2
1	1	2	2	2	2	4	4	2	2
1	1	3	3	2	2	4	4	2	2
1	1	3	3	2	2	1	1	2	2
1	1	3	3	2	2	4	4	2	2
1	1	3	3	2	2	4	4	2	2
1	1	2	2	2	2	1	1	2	2
1	1	3	3	2	2	4	4	2	2
1	1	3	3	2	2	4	4	2	2
3	3	3	3	2	2	4	4	2	2
1	1	3	3	2	2	4	4	2	2
1	1	3	3	2	2	4	4	2	2
1	1	3	3	2	2	1	1	2	2
1	1	3	3	2	2	4	4	2	2
1	1	3	3	2	2	1	1	2	2
1	1	3	3	2	2	4	4	2	2
1	1	3	3	2	2	1	1	2	2
1	1	3	3	3	3	4	4	2	2
3	3	3	3	2	2	4	4	2	2
1	1	3	3	2	2	1	1	2	2
1	1	3	3	2	2	1	1	2	2
1	1	2	2	2	2	4	4	2	2
1	1	2	2	2	2	4	4	2	2
1	1	3	3	2	2	4	4	2	2
1	1	3	3	2	2	4	4	2	2
1	1	3	3	2	2	4	4	2	2
1	1	3	3	2	2	1	1	2	2
1	1	3	3	2	2	1	1	2	2
1	1	3	3	3	3	4	4	2	2
1	1	3	3	3	3	4	4	2	2
1	1	3	3	2	2	1	1	2	2
1	1	3	3	3	3	1	1	1	1
1	1	3	3	2	2	1	1	2	2
1	1	3	3	2	2	1	1	2	2
1	1	3	3	3	3	1	1	2	2
1	1	3	3	2	2	4	4	2	2
1	1	3	3	2	2	1	1	2	2
1	1	3	3	2	2	4	4	2	2
1	1	3	3	2	2	1	1	2	2
1	1	3	3	3	3	4	4	2	2
1	1	3	3	2	2	4	4	2	2
3	3	3	3	2	2	4	4	2	2
1	1	2	2	2	2	4	4	2	2
1	1	3	3	2	2	4	4	2	2
1	1	3	3	2	2	1	1	2	2
1	1	3	3	3	3	4	4	2	2
1	1	3	3	2	2	4	4	2	2
1	1	2	2	2	2	4	4	2	2
1	1	3	3	2	2	1	1	2	2
1	1	3	3	2	2	4	4	2	2
1	1	3	3	2	2	1	1	2	2
1	1	3	3	2	2	4	4	2	2
1	1	2	2	2	2	4	4	2	2
1	1	3	3	2	2	4	4	2	2
1	1	2	2	2	2	4	4	2	2

For Review Only

1	1	3	3	2	2	1	1	2	2
1	1	2	2	2	2	1	1	1	1
1	1	3	3	2	2	1	1	2	2
1	1	3	3	2	2	4	4	2	2
1	1	3	3	2	2	1	1	1	1
3	3	3	3	2	2	4	4	2	2
3	3	2	2	2	2	4	4	2	2
1	1	2	2	2	2	1	1	1	1
1	1	3	3	2	2	1	1	2	2
1	1	2	2	2	2	4	4	2	2
1	1	3	3	2	2	1	1	2	2
1	1	3	3	2	2	1	1	2	2
1	1	3	3	2	2	4	4	1	1
1	1	3	3	2	2	4	4	2	2
1	1	3	3	2	2	4	4	2	2
1	1	3	3	2	2	4	4	2	2
1	1	3	3	2	2	4	4	2	2
1	1	2	2	3	3	1	1	2	2
1	1	2	2	2	2	4	4	2	2
1	1	3	3	2	2	1	1	2	2
1	1	2	2	2	2	4	4	2	2
3	3	3	3	2	2	4	4	2	2
1	1	3	3	2	2	4	4	2	2
1	1	3	3	2	2	1	1	2	2
1	1	3	3	2	2	4	4	2	2
1	1	3	3	2	2	4	4	2	2
1	1	2	2	2	2	4	4	2	2
1	1	3	3	2	2	1	1	2	2
1	1	3	3	2	2	1	1	2	2
1	1	3	3	2	2	1	1	2	2
1	1	2	2	2	2	4	4	2	2
3	3	3	3	2	2	4	4	2	2
1	1	3	3	2	2	1	1	2	2
1	1	3	3	2	2	4	4	2	2
1	1	2	2	2	2	1	1	2	2
1	1	2	2	2	2	4	4	2	2
1	1	3	3	2	2	1	1	2	2
1	1	3	3	2	2	4	4	2	2
1	1	3	3	2	2	4	4	2	2
1	1	2	2	2	2	4	4	2	2
3	3	3	3	3	3	1	1	2	2
1	1	3	3	2	2	1	1	2	2
1	1	2	2	2	2	1	1	2	2
1	1	3	3	2	2	4	4	2	2
1	1	3	3	2	2	4	4	2	2
1	1	3	3	2	2	4	4	2	2
1	1	3	3	2	2	4	4	2	2
1	1	3	3	2	2	1	1	1	1
1	1	3	3	2	2	1	1	2	2
1	1	2	2	2	2	4	4	2	2
3	3	3	3	2	2	1	1	2	2
1	1	2	2	2	2	4	4	2	2
1	1	3	3	2	2	4	4	2	2
1	1	3	3	2	2	4	4	2	2
1	1	2	2	2	2	1	1	2	2

For Review Only

1	1	3	3	2	2	4	4	2	2
1	1	3	3	2	2	4	4	2	2

For Review Only

SNP2254	SNP2254	SNP2255	SNP2255	SNP2256	SNP2256	SNP2257	SNP2257	SNP2258	SNP2258
4	4	2	2	4	4	1	1	2	2
4	4	2	2	4	4	1	1	2	2
4	4	4	4	4	4	1	1	3	3
4	4	4	4	4	4	1	1	2	2
4	4	2	2	4	4	4	4	2	2
4	4	4	4	4	4	1	1	3	3
4	4	4	4	4	4	1	1	3	3
4	4	4	4	1	1	1	1	3	3
1	1	4	4	4	4	4	4	3	3
4	4	4	4	4	4	1	1	2	2
4	4	4	4	1	1	1	1	3	3
4	4	4	4	4	4	1	1	3	3
4	4	2	2	4	4	1	1	3	3
4	4	4	4	4	4	1	1	2	2
1	1	2	2	4	4	1	1	3	3
4	4	2	2	4	4	4	4	3	3
4	4	2	2	4	4	1	1	3	3
4	4	4	4	4	4	1	1	3	3
4	4	4	4	4	4	1	1	3	3
4	4	4	4	4	4	1	1	3	3
4	4	2	2	4	4	1	1	3	3
4	4	4	4	4	4	4	4	3	3
4	4	4	4	4	4	4	4	3	3
4	4	4	4	1	1	4	4	3	3
4	4	4	4	4	4	4	4	3	3
4	4	2	2	4	4	4	4	3	3
4	4	4	4	4	4	4	4	3	3
4	4	4	4	4	4	1	1	2	2
1	1	2	2	4	4	4	4	2	2
4	4	4	4	4	4	1	1	3	3
4	4	4	4	4	4	1	1	3	3
4	4	4	4	1	1	4	4	3	3
4	4	4	4	4	4	1	1	3	3
4	4	4	4	4	4	1	1	2	2
4	4	4	4	4	4	1	1	2	2
4	4	4	4	1	1	1	1	3	3
4	4	2	2	4	4	1	1	2	2
4	4	4	4	4	4	1	1	3	3
4	4	4	4	4	4	4	4	3	3
4	4	4	4	4	4	4	4	2	2
4	4	4	4	4	4	1	1	2	2
1	1	4	4	4	4	4	4	2	2
4	4	4	4	4	4	1	1	3	3
4	4	2	2	4	4	1	1	3	3
4	4	4	4	4	4	1	1	3	3
4	4	4	4	4	4	1	1	3	3
4	4	4	4	4	4	1	1	3	3
4	4	4	4	1	1	4	4	3	3
4	4	4	4	4	4	1	1	3	3
4	4	4	4	4	4	1	1	3	3
4	4	2	2	4	4	4	4	3	3
4	4	4	4	4	4	1	1	2	2

For Review Only

4	4	2	2	4	4	4	4	3	3
4	4	4	4	4	4	1	1	3	3
4	4	2	2	4	4	1	1	2	2
4	4	4	4	4	4	1	1	3	3
4	4	4	4	4	4	1	1	3	3
4	4	4	4	4	4	1	1	3	3
4	4	2	2	4	4	4	4	3	3
4	4	4	4	4	4	1	1	3	3
4	4	4	4	4	4	1	1	3	3
4	4	4	4	4	4	1	1	3	3
4	4	4	4	4	4	1	1	2	2
4	4	4	4	4	4	1	1	3	3
4	4	2	2	4	4	4	4	3	3
1	1	2	2	4	4	4	4	2	2
4	4	2	2	4	4	1	1	3	3
4	4	4	4	4	4	1	1	2	2
1	1	4	4	4	4	4	4	3	3
4	4	4	4	4	4	1	1	3	3
4	4	4	4	4	4	1	1	3	3
4	4	2	2	4	4	1	1	3	3
4	4	4	4	4	4	1	1	3	3
4	4	4	4	4	4	1	1	3	3
1	1	2	2	4	4	1	1	3	3
4	4	4	4	4	4	1	1	3	3
4	4	4	4	4	4	1	1	3	3
4	4	4	4	4	4	4	4	2	2
4	4	4	4	1	1	1	1	3	3
4	4	4	4	4	4	4	4	3	3
4	4	2	2	4	4	4	4	3	3
4	4	4	4	4	4	1	1	3	3
4	4	4	4	4	4	1	1	2	2
4	4	4	4	4	4	1	1	3	3
4	4	4	4	4	4	1	1	3	3
4	4	2	2	4	4	4	4	3	3
4	4	2	2	4	4	1	1	2	2
4	4	4	4	4	4	1	1	3	3
4	4	4	4	4	4	1	1	2	2
4	4	4	4	4	4	1	1	2	2
4	4	4	4	4	4	1	1	2	2
4	4	4	4	4	4	1	1	2	2
4	4	2	2	4	4	1	1	3	3
4	4	4	4	4	4	1	1	3	3
4	4	4	4	4	4	1	1	3	3
4	4	4	4	4	4	1	1	2	2
4	4	4	4	4	4	1	1	3	3
4	4	2	2	4	4	1	1	2	2
4	4	4	4	4	4	1	1	2	2
4	4	2	2	4	4	4	4	3	3
4	4	4	4	4	4	4	4	2	2
1	1	4	4	4	4	1	1	3	3
4	4	4	4	4	4	1	1	2	2
4	4	4	4	4	4	4	4	3	3
4	4	4	4	4	4	1	1	2	2
1	1	4	4	4	4	4	4	2	2
1	1	4	4	4	4	1	1	3	3

For Review Only

4	4	4	4	4	4	1	1	3	3
4	4	4	4	4	4	1	1	3	3
4	4	2	2	4	4	1	1	3	3
4	4	4	4	4	4	1	1	2	2
4	4	4	4	4	4	4	4	2	2
4	4	4	4	4	4	1	1	3	3
4	4	4	4	4	4	4	4	3	3
4	4	4	4	4	4	1	1	3	3
4	4	4	4	1	1	4	4	2	2
4	4	2	2	4	4	4	4	3	3
4	4	4	4	4	4	1	1	3	3
4	4	2	2	4	4	1	1	3	3
1	1	4	4	4	4	1	1	2	2
4	4	4	4	4	4	4	4	2	2
4	4	2	2	4	4	1	1	3	3
4	4	2	2	4	4	1	1	3	3
1	1	2	2	4	4	1	1	2	2
4	4	4	4	4	4	1	1	3	3
4	4	4	4	4	4	1	1	2	2
4	4	4	4	4	4	1	1	3	3
4	4	4	4	4	4	1	1	3	3
4	4	4	4	4	4	1	1	3	3
4	4	4	4	4	4	1	1	3	3
4	4	4	4	4	4	4	4	3	3
4	4	4	4	4	4	1	1	3	3
4	4	4	4	4	4	4	4	2	2
1	1	4	4	4	4	1	1	3	3
4	4	4	4	4	4	1	1	3	3
4	4	4	4	4	4	1	1	3	3
4	4	4	4	4	4	1	1	2	2
4	4	4	4	4	4	4	4	2	2
4	4	4	4	4	4	1	1	2	2
4	4	2	2	4	4	1	1	3	3
4	4	4	4	4	4	4	4	3	3
4	4	4	4	4	4	4	4	2	2
4	4	4	4	4	4	1	1	2	2
4	4	4	4	4	4	1	1	2	2
4	4	4	4	4	4	1	1	2	2
4	4	2	2	4	4	4	4	2	2
4	4	4	4	4	4	1	1	3	3
4	4	4	4	4	4	1	1	2	2
4	4	4	4	1	1	1	1	2	2
4	4	4	4	4	4	1	1	2	2
4	4	4	4	4	4	1	1	2	2
4	4	2	2	4	4	1	1	3	3
4	4	2	2	4	4	4	4	3	3
4	4	2	2	4	4	4	4	3	3
4	4	4	4	4	4	4	4	3	3
4	4	4	4	1	1	1	1	3	3
4	4	2	2	4	4	4	4	3	3
4	4	2	2	4	4	4	4	3	3
4	4	4	4	4	4	1	1	3	3
4	4	4	4	1	1	1	1	2	2
4	4	2	2	4	4	1	1	3	3
4	4	4	4	4	4	1	1	3	3

For Review Only

4	4	2	2	1	1	4	4	3	3
4	4	4	4	4	4	1	1	3	3
4	4	4	4	4	4	1	1	3	3
4	4	4	4	4	4	1	1	3	3
4	4	2	2	4	4	1	1	2	2
4	4	4	4	4	4	1	1	2	2
4	4	4	4	4	4	1	1	2	2
4	4	4	4	4	4	1	1	2	2
4	4	4	4	4	4	4	4	3	3
4	4	2	2	4	4	4	4	3	3
4	4	4	4	4	4	4	4	3	3
4	4	4	4	4	4	4	4	2	2
1	1	4	4	4	4	1	1	2	2
4	4	4	4	4	4	1	1	3	3
4	4	4	4	4	4	1	1	2	2
4	4	4	4	1	1	1	1	3	3
4	4	4	4	4	4	1	1	2	2
1	1	2	2	4	4	1	1	2	2
4	4	4	4	4	4	1	1	3	3
4	4	4	4	4	4	4	4	3	3
4	4	4	4	4	4	1	1	2	2
4	4	4	4	4	4	1	1	3	3
4	4	4	4	4	4	1	1	3	3
4	4	4	4	4	4	1	1	3	3
4	4	2	2	4	4	1	1	3	3
4	4	4	4	4	4	1	1	2	2
1	1	4	4	4	4	1	1	3	3
1	1	4	4	4	4	1	1	2	2
1	1	2	2	1	1	1	1	3	3
1	1	2	2	4	4	1	1	3	3
4	4	4	4	4	4	1	1	2	2
1	1	4	4	4	4	1	1	2	2
4	4	2	2	4	4	1	1	3	3
4	4	4	4	4	4	1	1	2	2
1	1	4	4	4	4	1	1	3	3
4	4	4	4	4	4	1	1	3	3
4	4	4	4	4	4	4	4	3	3
4	4	2	2	4	4	1	1	3	3
4	4	4	4	4	4	4	4	3	3
1	1	4	4	4	4	1	1	3	3
4	4	4	4	4	4	1	1	3	3
4	4	2	2	4	4	4	4	3	3
4	4	4	4	4	4	4	4	3	3
1	1	4	4	4	4	1	1	3	3
4	4	4	4	4	4	1	1	2	2
1	1	4	4	1	1	1	1	2	2
4	4	4	4	1	1	1	1	3	3
4	4	4	4	4	4	1	1	3	3
4	4	4	4	1	1	1	1	2	2
4	4	4	4	4	4	1	1	3	3
4	4	4	4	4	4	1	1	3	3
4	4	4	4	4	4	1	1	3	3
4	4	4	4	4	4	1	1	3	3
4	4	4	4	4	4	1	1	2	2
4	4	4	4	4	4	4	4	3	3

For Review Only

4	4	4	4	4	4	1	1	2	2
4	4	4	4	4	4	1	1	2	2
4	4	4	4	4	4	1	1	3	3
4	4	4	4	4	4	1	1	3	3
4	4	2	2	4	4	1	1	2	2
1	1	4	4	4	4	1	1	3	3
4	4	4	4	4	4	1	1	2	2
4	4	4	4	1	1	1	1	2	2
4	4	4	4	4	4	1	1	2	2
4	4	4	4	4	4	4	4	3	3
4	4	4	4	4	4	1	1	3	3
4	4	4	4	4	4	1	1	2	2
4	4	4	4	4	4	1	1	2	2
4	4	4	4	1	1	4	4	3	3
4	4	4	4	4	4	1	1	3	3
4	4	4	4	4	4	1	1	3	3
4	4	2	2	4	4	4	4	3	3
1	1	4	4	4	4	4	4	3	3
4	4	4	4	4	4	1	1	3	3
4	4	4	4	4	4	1	1	3	3
4	4	4	4	4	4	1	1	2	2
4	4	2	2	4	4	1	1	3	3
4	4	4	4	4	4	1	1	3	3
4	4	4	4	4	4	4	4	2	2
4	4	4	4	4	4	1	1	3	3
4	4	4	4	4	4	4	4	3	3
4	4	4	4	4	4	4	4	3	3
4	4	4	4	4	4	1	1	3	3
4	4	2	2	4	4	1	1	3	3
4	4	4	4	4	4	1	1	3	3
4	4	4	4	4	4	4	4	3	3
4	4	2	2	4	4	1	1	3	3
4	4	4	4	4	4	1	1	3	3
4	4	2	2	4	4	4	4	3	3
4	4	4	4	4	4	1	1	3	3
4	4	4	4	4	4	4	4	3	3
4	4	2	2	4	4	4	4	3	3
4	4	4	4	4	4	1	1	3	3
4	4	4	4	1	1	1	1	3	3
4	4	4	4	4	4	4	4	3	3
1	1	4	4	4	4	1	1	2	2
4	4	2	2	4	4	1	1	3	3
4	4	4	4	4	4	4	4	2	2
4	4	2	2	4	4	4	4	3	3
4	4	4	4	4	4	1	1	3	3
4	4	4	4	4	4	1	1	3	3
4	4	4	4	4	4	4	4	3	3
1	1	4	4	4	4	1	1	2	2
4	4	4	4	4	4	1	1	2	2
4	4	4	4	4	4	1	1	3	3
4	4	4	4	4	4	1	1	3	3
4	4	4	4	4	4	4	4	3	3
4	4	4	4	4	4	1	1	3	3
4	4	4	4	4	4	1	1	2	2
4	4	4	4	4	4	4	4	2	2

For Review Only

4	4	4	4	4	4	1	1	3	3
4	4	2	2	4	4	1	1	3	3

For Review Only

For Review Only

SNP2259	SNP2259	SNP226	SNP226	SNP2260	SNP2260	SNP2261	SNP2261	SNP2262	SNP2262
2	2	1	1	3	3	2	2	2	2
3	3	1	1	3	3	3	3	2	2
2	2	1	1	3	3	2	2	2	2
3	3	1	1	3	3	2	2	2	2
3	3	1	1	3	3	2	2	2	2
2	2	1	1	3	3	3	3	2	2
2	2	1	1	3	3	2	2	2	2
3	3	1	1	3	3	2	2	2	2
2	2	1	1	3	3	2	2	2	2
3	3	1	1	3	3	3	3	3	3
3	3	1	1	3	3	2	2	2	2
2	2	1	1	3	3	2	2	2	2
3	3	1	1	3	3	2	2	2	2
3	3	1	1	3	3	2	2	2	2
2	2	1	1	3	3	2	2	2	2
2	2	1	1	3	3	3	3	2	2
2	2	1	1	3	3	2	2	2	2
3	3	1	1	3	3	2	2	2	2
2	2	1	1	2	2	2	2	2	2
3	3	1	1	3	3	2	2	2	2
3	3	1	1	3	3	2	2	2	2
3	3	1	1	3	3	3	3	3	3
3	3	4	4	3	3	3	3	2	2
3	3	1	1	3	3	2	2	2	2
2	2	1	1	3	3	2	2	2	2
2	2	4	4	2	2	3	3	2	2
2	2	1	1	3	3	2	2	2	2
3	3	1	1	3	3	2	2	2	2
2	2	1	1	3	3	2	2	2	2
3	3	1	1	3	3	3	3	2	2
3	3	1	1	2	2	2	2	2	2
2	2	1	1	3	3	2	2	2	2
2	2	1	1	3	3	3	3	2	2
3	3	1	1	3	3	3	3	2	2
2	2	1	1	3	3	2	2	2	2
3	3	1	1	3	3	2	2	2	2
2	2	1	1	3	3	3	3	2	2
3	3	1	1	3	3	2	2	2	2
2	2	1	1	3	3	3	3	2	2
2	2	1	1	3	3	3	3	2	2
3	3	1	1	3	3	3	3	2	2
2	2	1	1	3	3	2	2	2	2
3	3	1	1	3	3	3	3	2	2
3	3	1	1	3	3	2	2	2	2
3	3	1	1	3	3	3	3	2	2
2	2	1	1	3	3	2	2	2	2
2	2	1	1	2	2	2	2	2	2
3	3	1	1	3	3	2	2	2	2
3	3	1	1	3	3	3	3	2	2
3	3	4	4	3	3	2	2	2	2
2	2	4	4	2	2	3	3	2	2
2	2	1	1	3	3	2	2	2	2

2	2	1	1	3	3	2	2	2	2
2	2	1	1	3	3	3	3	2	2
2	2	1	1	3	3	3	3	3	3
3	3	1	1	3	3	3	3	2	2
2	2	1	1	2	2	2	2	2	2
2	2	1	1	2	2	2	2	2	2
2	2	1	1	2	2	3	3	2	2
3	3	1	1	3	3	3	3	2	2
2	2	1	1	3	3	3	3	2	2
3	3	1	1	3	3	2	2	2	2
2	2	1	1	3	3	2	2	2	2
2	2	1	1	2	2	2	2	2	2
2	2	1	1	3	3	2	2	2	2
2	2	1	1	3	3	2	2	2	2
3	3	1	1	3	3	3	3	2	2
2	2	1	1	3	3	3	3	2	2
3	3	1	1	3	3	2	2	2	2
2	2	1	1	3	3	2	2	2	2
3	3	4	4	3	3	2	2	2	2
2	2	1	1	3	3	2	2	2	2
2	2	1	1	3	3	2	2	2	2
2	2	1	1	3	3	3	3	3	3
2	2	1	1	3	3	2	2	2	2
2	2	1	1	3	3	2	2	2	2
3	3	1	1	3	3	3	3	2	2
3	3	1	1	3	3	3	3	2	2
3	3	1	1	3	3	2	2	2	2
2	2	1	1	3	3	3	3	2	2
2	2	1	1	2	2	3	3	2	2
2	2	1	1	3	3	2	2	2	2
3	3	1	1	3	3	3	3	3	3
3	3	1	1	3	3	2	2	2	2
2	2	1	1	3	3	3	3	2	2
2	2	1	1	3	3	2	2	2	2
3	3	1	1	3	3	2	2	2	2
3	3	1	1	3	3	3	3	2	2
3	3	1	1	3	3	3	3	3	3
3	3	1	1	3	3	3	3	2	2
3	3	1	1	3	3	2	2	3	3
2	2	1	1	3	3	3	3	2	2
3	3	1	1	3	3	2	2	2	2
2	2	1	1	2	2	2	2	2	2
3	3	1	1	3	3	2	2	3	3
2	2	1	1	3	3	2	2	2	2
3	3	4	4	3	3	2	2	2	2
3	3	1	1	3	3	2	2	2	2
3	3	1	1	3	3	3	3	2	2
3	3	1	1	2	2	3	3	2	2
2	2	1	1	3	3	2	2	3	3
2	2	1	1	3	3	3	3	3	3
3	3	1	1	3	3	2	2	2	2
2	2	1	1	3	3	3	3	2	2
2	2	1	1	3	3	3	3	2	2
2	2	1	1	3	3	2	2	2	2
2	2	1	1	3	3	3	3	2	2

For Review Only

2	2	1	1	3	3	2	2	2	2
2	2	1	1	3	3	2	2	2	2
3	3	1	1	2	2	3	3	2	2
3	3	1	1	3	3	3	3	2	2
3	3	1	1	3	3	3	3	2	2
2	2	1	1	3	3	2	2	2	2
2	2	1	1	3	3	2	2	2	2
3	3	1	1	3	3	2	2	2	2
3	3	1	1	3	3	2	2	3	3
2	2	1	1	3	3	3	3	2	2
2	2	1	1	3	3	3	3	2	2
3	3	1	1	3	3	2	2	2	2
3	3	1	1	3	3	2	2	3	3
2	2	1	1	3	3	2	2	2	2
2	2	1	1	3	3	3	3	2	2
2	2	1	1	2	2	2	2	2	2
2	2	1	1	3	3	3	3	2	2
2	2	1	1	3	3	2	2	2	2
3	3	1	1	3	3	3	3	2	2
3	3	1	1	2	2	2	2	3	3
3	3	1	1	3	3	2	2	2	2
2	2	1	1	3	3	2	2	2	2
2	2	1	1	3	3	2	2	2	2
3	3	1	1	3	3	3	3	2	2
2	2	1	1	3	3	3	3	2	2
2	2	4	4	3	3	3	3	3	3
2	2	4	4	3	3	2	2	2	2
2	2	1	1	3	3	2	2	2	2
2	2	1	1	3	3	2	2	2	2
3	3	4	4	3	3	2	2	2	2
3	3	1	1	3	3	3	3	2	2
3	3	1	1	3	3	2	2	2	2
3	3	1	1	3	3	2	2	2	2
2	2	1	1	3	3	2	2	2	2
3	3	1	1	3	3	3	3	2	2
3	3	1	1	3	3	2	2	2	2
3	3	1	1	3	3	3	3	2	2
2	2	1	1	3	3	2	2	2	2
3	3	1	1	2	2	3	3	3	3
3	3	1	1	3	3	2	2	2	2
2	2	1	1	3	3	2	2	3	3
2	2	1	1	3	3	2	2	2	2
2	2	1	1	3	3	2	2	2	2
3	3	1	1	3	3	3	3	2	2
2	2	1	1	3	3	3	3	2	2
2	2	1	1	3	3	3	3	2	2
2	2	4	4	2	2	3	3	2	2
2	2	1	1	2	2	2	2	2	2
2	2	1	1	3	3	3	3	2	2
2	2	1	1	2	2	3	3	2	2
2	2	1	1	3	3	3	3	2	2
3	3	1	1	3	3	3	3	2	2
3	3	1	1	3	3	2	2	2	2
2	2	1	1	2	2	3	3	2	2
3	3	1	1	3	3	2	2	2	2

For Review Only

2	2	4	4	3	3	2	2	2	2
2	2	1	1	3	3	3	3	3	3
2	2	1	1	3	3	2	2	2	2
3	3	1	1	3	3	3	3	2	2
3	3	1	1	3	3	3	3	2	2
3	3	1	1	3	3	3	3	2	2
2	2	1	1	3	3	3	3	2	2
3	3	1	1	3	3	2	2	2	2
3	3	1	1	3	3	2	2	2	2
2	2	4	4	3	3	2	2	2	2
2	2	4	4	2	2	3	3	2	2
2	2	1	1	3	3	2	2	2	2
2	2	1	1	3	3	2	2	3	3
2	2	1	1	3	3	2	2	2	2
2	2	1	1	3	3	3	3	2	2
3	3	1	1	3	3	2	2	2	2
3	3	1	1	3	3	2	2	2	2
3	3	1	1	3	3	3	3	2	2
2	2	4	4	2	2	2	2	2	2
2	2	1	1	3	3	2	2	2	2
3	3	1	1	3	3	2	2	2	2
2	2	1	1	2	2	3	3	2	2
2	2	1	1	3	3	3	3	3	3
2	2	1	1	2	2	2	2	2	2
2	2	1	1	3	3	2	2	2	2
3	3	1	1	3	3	3	3	2	2
3	3	1	1	3	3	3	3	2	2
3	3	1	1	3	3	3	3	2	2
3	3	1	1	3	3	2	2	2	2
3	3	1	1	3	3	2	2	2	2
2	2	1	1	3	3	3	3	2	2
2	2	1	1	3	3	2	2	3	3
2	2	1	1	3	3	3	3	2	2
3	3	1	1	3	3	2	2	2	2
3	3	1	1	3	3	2	2	2	2
2	2	1	1	2	2	2	2	2	2
2	2	1	1	2	2	3	3	2	2
3	3	1	1	3	3	3	3	2	2
2	2	1	1	2	2	2	2	2	2
3	3	1	1	3	3	2	2	2	2
2	2	1	1	3	3	2	2	2	2
2	2	4	4	2	2	3	3	2	2
2	2	1	1	3	3	3	3	2	2
3	3	1	1	2	2	2	2	2	2
2	2	1	1	3	3	2	2	2	2
3	3	1	1	3	3	3	3	2	2
3	3	1	1	3	3	2	2	2	2
2	2	1	1	3	3	3	3	2	2
2	2	1	1	3	3	2	2	2	2
3	3	1	1	3	3	2	2	2	2
2	2	1	1	3	3	2	2	2	2
2	2	1	1	3	3	3	3	2	2
2	2	1	1	3	3	3	3	3	3
2	2	1	1	3	3	2	2	2	2
2	2	1	1	3	3	3	3	2	2

For Review Only

3	3	1	1	3	3	2	2	2	2
2	2	1	1	3	3	3	3	3	3
2	2	1	1	3	3	2	2	2	2
2	2	1	1	3	3	3	3	3	3
3	3	1	1	3	3	3	3	3	3
2	2	1	1	3	3	2	2	2	2
2	2	4	4	3	3	3	3	3	3
3	3	1	1	3	3	2	2	3	3
3	3	1	1	3	3	2	2	2	2
3	3	1	1	3	3	3	3	2	2
3	3	1	1	3	3	2	2	2	2
2	2	1	1	3	3	3	3	2	2
3	3	1	1	3	3	2	2	3	3
2	2	1	1	2	2	2	2	2	2
2	2	1	1	3	3	2	2	2	2
3	3	1	1	3	3	2	2	2	2
3	3	1	1	3	3	3	3	2	2
2	2	1	1	3	3	3	3	2	2
2	2	1	1	3	3	3	3	2	2
2	2	1	1	3	3	2	2	2	2
3	3	1	1	3	3	3	3	2	2
2	2	4	4	2	2	3	3	2	2
2	2	1	1	3	3	2	2	2	2
2	2	1	1	3	3	3	3	2	2
3	3	1	1	3	3	3	3	2	2
2	2	1	1	3	3	2	2	2	2
2	2	1	1	3	3	3	3	2	2
2	2	1	1	3	3	2	2	2	2
3	3	1	1	3	3	2	2	3	3
2	2	1	1	3	3	2	2	2	2
2	2	1	1	3	3	3	3	2	2
3	3	4	4	3	3	3	3	2	2
2	2	1	1	3	3	2	2	2	2
2	2	1	1	3	3	3	3	2	2
2	2	1	1	3	3	3	3	3	3
2	2	1	1	3	3	3	3	2	2
3	3	1	1	3	3	2	2	2	2
2	2	1	1	2	2	2	2	2	2
3	3	1	1	3	3	2	2	2	2
3	3	1	1	3	3	3	3	2	2
2	2	4	4	3	3	2	2	2	2
2	2	1	1	3	3	3	3	3	3
2	2	1	1	3	3	3	3	2	2
3	3	1	1	3	3	2	2	2	2
3	3	1	1	3	3	2	2	2	2
2	2	1	1	3	3	2	2	2	2
2	2	1	1	3	3	2	2	2	2
3	3	1	1	3	3	2	2	3	3
2	2	1	1	3	3	2	2	2	2
2	2	1	1	3	3	3	3	2	2
2	2	1	1	3	3	2	2	2	2
2	2	1	1	3	3	3	3	2	2
2	2	1	1	3	3	2	2	2	2
2	2	1	1	2	2	2	2	3	3
2	2	1	1	3	3	3	3	2	2

For Review Only

3	3	1	1	3	3	2	2	2	2
2	2	1	1	2	2	3	3	2	2

For Review Only

SNP2263	SNP2263	SNP2264	SNP2264	SNP2265	SNP2265	SNP2266	SNP2266	SNP2267	SNP2267
2	2	3	3	4	4	1	1	3	3
2	2	3	3	4	4	4	4	3	3
2	2	3	3	1	1	4	4	2	2
2	2	3	3	4	4	1	1	2	2
2	2	3	3	4	4	1	1	2	2
3	3	3	3	4	4	1	1	2	2
2	2	3	3	4	4	1	1	2	2
2	2	1	1	4	4	1	1	3	3
3	3	3	3	4	4	1	1	2	2
2	2	1	1	4	4	1	1	2	2
2	2	3	3	4	4	1	1	2	2
2	2	3	3	1	1	1	1	2	2
2	2	3	3	4	4	1	1	3	3
2	2	1	1	4	4	1	1	2	2
2	2	3	3	4	4	1	1	3	3
3	3	3	3	4	4	1	1	2	2
2	2	3	3	4	4	1	1	2	2
2	2	3	3	4	4	1	1	2	2
2	2	1	1	4	4	4	4	2	2
2	2	3	3	4	4	4	4	2	2
2	2	3	3	4	4	1	1	2	2
2	2	1	1	4	4	1	1	2	2
3	3	3	3	1	1	1	1	3	3
2	2	3	3	4	4	1	1	3	3
2	2	1	1	4	4	1	1	2	2
2	2	1	1	4	4	4	4	3	3
2	2	3	3	4	4	4	4	3	3
2	2	1	1	4	4	1	1	3	3
2	2	3	3	1	1	1	1	2	2
3	3	3	3	4	4	1	1	3	3
2	2	1	1	4	4	4	4	3	3
2	2	3	3	4	4	1	1	2	2
3	3	3	3	4	4	1	1	2	2
2	2	1	1	1	1	1	1	2	2
3	3	3	3	4	4	1	1	3	3
2	2	3	3	4	4	1	1	2	2
2	2	1	1	4	4	1	1	2	2
2	2	3	3	4	4	1	1	3	3
2	2	1	1	4	4	1	1	3	3
2	2	1	1	4	4	1	1	2	2
3	3	3	3	4	4	1	1	3	3
2	2	3	3	1	1	1	1	2	2
2	2	1	1	4	4	1	1	2	2
2	2	1	1	4	4	1	1	2	2
2	2	1	1	4	4	1	1	2	2
2	2	3	3	4	4	1	1	2	2
2	2	3	3	4	4	4	4	3	3
3	3	3	3	4	4	1	1	2	2
3	3	3	3	4	4	1	1	3	3
2	2	1	1	4	4	1	1	3	3
2	2	1	1	4	4	4	4	3	3
2	2	3	3	4	4	1	1	2	2

For Review Only

2	2	3	3	4	4	1	1	2	2
3	3	3	3	4	4	1	1	2	2
2	2	1	1	4	4	4	4	2	2
3	3	3	3	4	4	1	1	3	3
2	2	1	1	4	4	1	1	2	2
2	2	3	3	1	1	4	4	3	3
2	2	1	1	4	4	4	4	3	3
3	3	3	3	4	4	1	1	3	3
3	3	3	3	4	4	1	1	2	2
2	2	1	1	4	4	4	4	3	3
2	2	3	3	1	1	4	4	3	3
2	2	3	3	1	1	4	4	3	3
2	2	3	3	4	4	1	1	2	2
2	2	3	3	1	1	1	1	2	2
2	2	1	1	4	4	1	1	3	3
2	2	1	1	4	4	1	1	3	3
2	2	3	3	4	4	1	1	2	2
2	2	1	1	4	4	4	4	3	3
2	2	1	1	4	4	1	1	3	3
2	2	3	3	4	4	1	1	2	2
2	2	3	3	4	4	4	4	3	3
2	2	1	1	4	4	1	1	3	3
2	2	3	3	1	1	1	1	3	3
2	2	1	1	4	4	1	1	2	2
2	2	1	1	4	4	1	1	2	2
2	2	3	3	4	4	4	4	3	3
2	2	3	3	4	4	1	1	2	2
2	2	1	1	4	4	1	1	2	2
2	2	1	1	4	4	4	4	3	3
2	2	1	1	4	4	1	1	3	3
2	2	3	3	4	4	1	1	2	2
2	2	3	3	4	4	1	1	2	2
2	2	1	1	1	1	1	1	3	3
2	2	3	3	4	4	1	1	2	2
2	2	3	3	4	4	1	1	3	3
2	2	1	1	4	4	1	1	2	2
2	2	1	1	4	4	1	1	2	2
3	3	3	3	4	4	1	1	2	2
2	2	3	3	4	4	4	4	2	2
2	2	1	1	1	1	1	1	2	2
2	2	3	3	4	4	1	1	2	2
2	2	3	3	4	4	4	4	3	3
2	2	3	3	4	4	1	1	3	3
2	2	3	3	1	1	4	4	3	3
2	2	3	3	4	4	4	4	3	3
2	2	1	1	4	4	4	4	3	3
3	3	3	3	1	1	1	1	2	2
2	2	1	1	4	4	4	4	2	2
2	2	3	3	4	4	1	1	2	2
2	2	1	1	4	4	1	1	3	3
2	2	3	3	4	4	4	4	3	3
2	2	3	3	4	4	1	1	2	2
3	3	3	3	1	1	1	1	3	3
2	2	3	3	4	4	1	1	2	2
3	3	3	3	4	4	1	1	3	3

For Review Only

2	2	1	1	4	4	1	1	3	3
2	2	3	3	4	4	1	1	3	3
2	2	1	1	4	4	4	4	3	3
3	3	3	3	1	1	1	1	2	2
2	2	3	3	4	4	4	4	3	3
2	2	1	1	4	4	4	4	3	3
2	2	3	3	4	4	4	4	2	2
2	2	3	3	4	4	1	1	2	2
3	3	3	3	4	4	1	1	3	3
2	2	1	1	4	4	1	1	3	3
2	2	3	3	4	4	1	1	2	2
2	2	1	1	4	4	1	1	3	3
2	2	3	3	4	4	1	1	3	3
2	2	1	1	4	4	4	4	3	3
2	2	3	3	4	4	1	1	2	2
2	2	1	1	4	4	4	4	3	3
3	3	3	3	4	4	1	1	3	3
2	2	1	1	4	4	1	1	2	2
3	3	3	3	1	1	1	1	2	2
2	2	1	1	4	4	4	4	2	2
2	2	1	1	4	4	1	1	3	3
2	2	1	1	4	4	1	1	3	3
2	2	3	3	4	4	4	4	3	3
3	3	3	3	4	4	1	1	3	3
2	2	1	1	4	4	1	1	3	3
3	3	3	3	4	4	1	1	3	3
2	2	3	3	4	4	1	1	3	3
2	2	3	3	4	4	1	1	2	2
2	2	3	3	4	4	1	1	2	2
2	2	1	1	4	4	1	1	3	3
3	3	3	3	1	1	1	1	2	2
2	2	3	3	4	4	1	1	3	3
2	2	3	3	4	4	1	1	2	2
2	2	1	1	4	4	4	4	3	3
2	2	1	1	1	1	1	1	2	2
2	2	3	3	4	4	1	1	3	3
3	3	3	3	1	1	1	1	3	3
2	2	3	3	4	4	1	1	3	3
2	2	1	1	4	4	1	1	3	3
2	2	1	1	4	4	1	1	3	3
2	2	1	1	4	4	1	1	3	3
2	2	3	3	1	1	1	1	3	3
2	2	1	1	4	4	4	4	3	3
2	2	1	1	4	4	1	1	3	3
2	2	1	1	4	4	1	1	2	2
3	3	3	3	4	4	4	4	2	2
2	2	1	1	4	4	4	4	3	3
2	2	3	3	4	4	4	4	3	3
3	3	3	3	4	4	1	1	3	3
2	2	1	1	4	4	4	4	3	3
2	2	1	1	4	4	1	1	3	3
2	2	1	1	4	4	1	1	3	3
2	2	1	1	4	4	1	1	3	3
2	2	1	1	4	4	4	4	3	3
2	2	3	3	4	4	1	1	3	3

For Review Only

2	2	3	3	4	4	1	1	2	2
3	3	3	3	4	4	1	1	3	3
2	2	1	1	4	4	1	1	2	2
2	2	1	1	4	4	1	1	3	3
2	2	3	3	4	4	4	4	3	3
2	2	1	1	4	4	1	1	2	2
2	2	1	1	1	1	1	1	3	3
2	2	1	1	4	4	1	1	3	3
2	2	3	3	4	4	1	1	2	2
2	2	3	3	4	4	4	4	3	3
2	2	1	1	4	4	4	4	3	3
2	2	1	1	4	4	1	1	3	3
2	2	1	1	1	1	1	1	3	3
2	2	3	3	4	4	1	1	2	2
3	3	3	3	1	1	1	1	2	2
2	2	3	3	4	4	1	1	2	2
2	2	1	1	4	4	1	1	3	3
2	2	1	1	4	4	1	1	3	3
2	2	3	3	4	4	4	4	3	3
2	2	3	3	4	4	4	4	2	2
2	2	1	1	4	4	1	1	3	3
2	2	1	1	4	4	4	4	3	3
2	2	1	1	4	4	1	1	3	3
2	2	3	3	1	1	4	4	3	3
2	2	1	1	4	4	4	4	2	2
2	2	1	1	1	1	1	1	2	2
3	3	3	3	4	4	1	1	3	3
2	2	1	1	4	4	1	1	3	3
2	2	1	1	4	4	1	1	3	3
2	2	3	3	4	4	1	1	3	3
2	2	3	3	4	4	1	1	3	3
2	2	1	1	4	4	1	1	3	3
2	2	1	1	4	4	1	1	3	3
2	2	3	3	1	1	1	1	2	2
2	2	1	1	4	4	1	1	3	3
2	2	3	3	1	1	4	4	3	3
2	2	1	1	1	1	1	1	3	3
2	2	3	3	4	4	1	1	2	2
2	2	3	3	4	4	4	4	3	3
2	2	3	3	4	4	1	1	3	3
2	2	3	3	1	1	4	4	2	2
2	2	1	1	4	4	4	4	3	3
2	2	1	1	1	1	1	1	3	3
2	2	1	1	4	4	1	1	3	3
2	2	1	1	4	4	4	4	2	2
2	2	1	1	4	4	1	1	3	3
2	2	3	3	4	4	1	1	2	2
2	2	1	1	1	1	1	1	3	3
2	2	1	1	1	1	1	1	2	2
2	2	3	3	4	4	1	1	3	3
2	2	1	1	4	4	1	1	2	2
3	3	3	3	4	4	1	1	3	3
2	2	1	1	4	4	1	1	3	3
2	2	3	3	1	1	4	4	3	3
2	2	1	1	4	4	1	1	2	2

For Review Only

2	2	3	3	1	1	1	1	3	3
2	2	1	1	4	4	1	1	3	3
2	2	3	3	4	4	1	1	2	2
2	2	1	1	4	4	1	1	3	3
2	2	1	1	4	4	1	1	3	3
2	2	3	3	4	4	1	1	3	3
2	2	1	1	4	4	1	1	3	3
2	2	1	1	4	4	1	1	2	2
2	2	3	3	1	1	1	1	2	2
3	3	3	3	1	1	1	1	3	3
2	2	1	1	4	4	1	1	3	3
3	3	3	3	1	1	1	1	3	3
2	2	3	3	4	4	1	1	2	2
2	2	3	3	4	4	4	4	3	3
2	2	3	3	4	4	4	4	2	2
2	2	3	3	4	4	1	1	2	2
3	3	3	3	4	4	1	1	2	2
3	3	3	3	1	1	1	1	2	2
2	2	1	1	1	1	1	1	3	3
2	2	3	3	4	4	1	1	3	3
2	2	1	1	4	4	4	4	3	3
2	2	1	1	4	4	4	4	3	3
2	2	3	3	4	4	1	1	2	2
3	3	3	3	4	4	1	1	3	3
3	3	3	3	4	4	1	1	3	3
2	2	3	3	4	4	4	4	2	2
2	2	1	1	4	4	1	1	3	3
2	2	3	3	4	4	1	1	2	2
2	2	3	3	4	4	1	1	3	3
2	2	3	3	4	4	1	1	2	2
2	2	1	1	4	4	1	1	2	2
2	2	1	1	4	4	1	1	2	2
2	2	3	3	4	4	1	1	2	2
2	2	1	1	4	4	1	1	3	3
2	2	1	1	4	4	1	1	3	3
2	2	1	1	1	1	1	1	2	2
2	2	3	3	4	4	1	1	3	3
2	2	3	3	1	1	4	4	3	3
2	2	3	3	4	4	1	1	2	2
2	2	1	1	4	4	1	1	3	3
2	2	1	1	4	4	1	1	3	3
2	2	1	1	4	4	4	4	2	2
2	2	1	1	4	4	1	1	3	3
2	2	3	3	4	4	1	1	2	2
2	2	3	3	1	1	4	4	3	3
2	2	3	3	4	4	1	1	2	2
2	2	3	3	4	4	1	1	3	3
2	2	1	1	4	4	1	1	3	3
2	2	3	3	1	1	4	4	3	3
2	2	1	1	1	1	1	1	3	3
2	2	3	3	4	4	1	1	2	2
2	2	1	1	4	4	1	1	2	2
2	2	3	3	1	1	4	4	2	2
2	2	3	3	4	4	4	4	2	2
2	2	1	1	4	4	1	1	3	3

For Review Only

2	2	3	3	4	4	1	1	2	2
2	2	3	3	4	4	4	4	2	2

For Review Only

SNP2268	SNP2268	SNP2269	SNP2269	SNP227	SNP227	SNP2270	SNP2270	SNP2271	SNP2271
1	1	4	4	1	1	4	4	4	4
1	1	2	2	1	1	4	4	4	4
1	1	2	2	1	1	4	4	4	4
1	1	4	4	1	1	4	4	4	4
1	1	4	4	1	1	4	4	4	4
1	1	4	4	1	1	4	4	4	4
1	1	4	4	1	1	4	4	4	4
1	1	4	4	3	3	4	4	4	4
1	1	4	4	1	1	4	4	4	4
1	1	4	4	1	1	4	4	4	4
1	1	4	4	1	1	4	4	4	4
1	1	2	2	1	1	4	4	4	4
1	1	4	4	1	1	4	4	4	4
1	1	4	4	3	3	4	4	4	4
1	1	4	4	1	1	4	4	4	4
1	1	4	4	1	1	4	4	4	4
1	1	2	2	1	1	4	4	4	4
1	1	4	4	1	1	1	1	2	2
1	1	2	2	1	1	4	4	4	4
1	1	2	2	1	1	4	4	4	4
1	1	2	2	1	1	4	4	4	4
1	1	2	2	1	1	4	4	4	4
1	1	4	4	1	1	4	4	2	2
1	1	4	4	1	1	4	4	4	4
1	1	4	4	1	1	4	4	4	4
1	1	2	2	1	1	4	4	4	4
1	1	2	2	1	1	4	4	4	4
1	1	4	4	1	1	4	4	4	4
1	1	4	4	1	1	4	4	2	2
1	1	4	4	1	1	4	4	4	4
1	1	2	2	1	1	4	4	4	4
1	1	4	4	1	1	4	4	4	4
1	1	4	4	1	1	4	4	4	4
1	1	4	4	1	1	4	4	2	2
1	1	4	4	3	3	4	4	4	4
1	1	4	4	1	1	4	4	4	4
1	1	4	4	1	1	4	4	4	4
1	1	4	4	1	1	1	1	2	2
4	4	4	4	1	1	4	4	4	4
1	1	4	4	1	1	4	4	2	2
1	1	4	4	1	1	1	1	2	2
1	1	4	4	1	1	4	4	2	2
1	1	4	4	1	1	4	4	4	4
1	1	2	2	1	1	4	4	4	4
1	1	4	4	3	3	1	1	2	2
1	1	2	2	1	1	4	4	4	4
1	1	2	2	1	1	4	4	4	4
1	1	4	4	1	1	4	4	4	4
1	1	2	2	1	1	4	4	4	4
1	1	4	4	1	1	4	4	4	4
1	1	2	2	1	1	4	4	4	4
1	1	4	4	3	3	4	4	4	4

For Review Only

1	1	2	2	1	1	4	4	4	4
1	1	4	4	1	1	4	4	4	4
1	1	2	2	1	1	4	4	4	4
1	1	2	2	1	1	4	4	4	4
1	1	2	2	1	1	4	4	4	4
1	1	2	2	1	1	4	4	4	4
1	1	2	2	1	1	4	4	4	4
1	1	4	4	1	1	4	4	4	4
1	1	4	4	1	1	4	4	4	4
1	1	4	4	1	1	4	4	4	4
1	1	2	2	1	1	4	4	4	4
1	1	2	2	1	1	4	4	4	4
1	1	2	2	1	1	4	4	4	4
1	1	4	4	1	1	4	4	2	2
1	1	4	4	1	1	4	4	4	4
1	1	4	4	1	1	4	4	4	4
1	1	4	4	1	1	4	4	4	4
1	1	2	2	1	1	4	4	4	4
1	1	2	2	1	1	4	4	4	4
1	1	4	4	1	1	4	4	4	4
1	1	2	2	1	1	4	4	4	4
1	1	4	4	1	1	4	4	4	4
1	1	4	4	1	1	4	4	4	4
1	1	4	4	1	1	4	4	4	4
1	1	4	4	3	3	1	1	2	2
1	1	2	2	3	3	4	4	4	4
1	1	2	2	1	1	4	4	4	4
1	1	4	4	1	1	1	1	2	2
1	1	2	2	1	1	4	4	4	4
1	1	4	4	1	1	4	4	4	4
1	1	4	4	1	1	4	4	4	4
1	1	4	4	1	1	4	4	4	4
4	4	4	4	1	1	1	1	2	2
1	1	2	2	1	1	4	4	4	4
1	1	4	4	3	3	4	4	4	4
1	1	4	4	3	3	1	1	2	2
1	1	4	4	1	1	4	4	4	4
1	1	2	2	1	1	1	1	2	2
1	1	2	2	1	1	4	4	4	4
1	1	4	4	1	1	1	1	2	2
1	1	2	2	1	1	4	4	4	4
1	1	2	2	1	1	4	4	4	4
1	1	4	4	1	1	4	4	4	4
1	1	2	2	1	1	4	4	4	4
1	1	2	2	1	1	4	4	4	4
1	1	2	2	1	1	4	4	4	4
1	1	4	4	3	3	4	4	4	4
1	1	2	2	1	1	4	4	4	4
1	1	4	4	1	1	4	4	4	4
1	1	4	4	1	1	4	4	4	4
1	1	2	2	1	1	4	4	4	4
1	1	4	4	1	1	4	4	2	2
4	4	4	4	3	3	4	4	2	2
1	1	4	4	1	1	4	4	4	4
1	1	4	4	1	1	1	1	2	2

For Review Only

1	1	4	4	1	1	4	4	4	4
1	1	4	4	1	1	4	4	4	4
1	1	2	2	1	1	4	4	4	4
1	1	4	4	1	1	4	4	2	2
1	1	2	2	1	1	4	4	4	4
1	1	2	2	1	1	4	4	4	4
1	1	2	2	1	1	4	4	4	4
1	1	4	4	1	1	4	4	4	4
1	1	4	4	1	1	4	4	4	4
1	1	4	4	1	1	4	4	4	4
1	1	4	4	1	1	4	4	4	4
1	1	4	4	1	1	4	4	4	4
1	1	4	4	1	1	4	4	4	4
1	1	2	2	1	1	4	4	4	4
1	1	4	4	1	1	4	4	4	4
1	1	4	4	1	1	4	4	4	4
1	1	4	4	1	1	4	4	4	4
1	1	4	4	1	1	4	4	4	4
1	1	4	4	1	1	4	4	2	2
1	1	2	2	1	1	4	4	4	4
1	1	4	4	1	1	4	4	4	4
1	1	4	4	1	1	4	4	4	4
1	1	2	2	1	1	4	4	4	4
1	1	4	4	1	1	4	4	4	4
1	1	2	2	1	1	4	4	4	4
1	1	4	4	1	1	4	4	4	4
1	1	4	4	1	1	4	4	4	4
1	1	4	4	1	1	4	4	4	4
1	1	4	4	1	1	4	4	4	4
1	1	4	4	1	1	4	4	4	4
1	1	4	4	1	1	1	1	2	2
1	1	4	4	1	1	4	4	4	4
1	1	2	2	1	1	4	4	4	4
1	1	4	4	1	1	4	4	4	4
1	1	4	4	1	1	4	4	2	2
1	1	4	4	1	1	4	4	2	2
1	1	4	4	3	3	4	4	2	2
1	1	2	2	1	1	4	4	4	4
1	1	4	4	3	3	4	4	2	2
1	1	4	4	1	1	4	4	4	4
1	1	4	4	1	1	4	4	4	4
1	1	4	4	1	1	4	4	2	2
1	1	2	2	1	1	4	4	4	4
1	1	4	4	1	1	1	1	4	4
1	1	4	4	1	1	4	4	4	4
1	1	2	2	1	1	4	4	4	4
1	1	2	2	1	1	4	4	4	4
1	1	2	2	1	1	4	4	4	4
1	1	4	4	1	1	4	4	4	4
1	1	2	2	1	1	4	4	4	4
1	1	4	4	1	1	4	4	4	4
1	1	4	4	1	1	4	4	4	4
1	1	4	4	1	1	4	4	4	4
1	1	2	2	1	1	4	4	4	4
1	1	4	4	1	1	4	4	4	4

For Review Only

1	1	4	4	1	1	4	4	4	4
1	1	4	4	1	1	4	4	4	4
1	1	4	4	1	1	1	1	2	2
1	1	4	4	1	1	4	4	4	4
1	1	2	2	1	1	4	4	4	4
1	1	4	4	1	1	4	4	4	4
1	1	4	4	1	1	4	4	4	4
1	1	4	4	1	1	4	4	4	4
1	1	4	4	1	1	4	4	4	4
1	1	2	2	1	1	4	4	4	4
1	1	2	2	1	1	4	4	4	4
4	4	4	4	1	1	4	4	4	4
1	1	4	4	1	1	4	4	4	4
1	1	4	4	1	1	4	4	4	4
1	1	4	4	3	3	4	4	2	2
1	1	4	4	1	1	4	4	4	4
1	1	4	4	1	1	4	4	4	4
1	1	4	4	1	1	4	4	4	4
1	1	2	2	1	1	4	4	4	4
1	1	2	2	1	1	4	4	4	4
1	1	4	4	1	1	4	4	4	4
4	4	2	2	1	1	4	4	4	4
1	1	4	4	1	1	4	4	4	4
1	1	2	2	1	1	4	4	4	4
1	1	2	2	1	1	4	4	4	4
1	1	4	4	1	1	4	4	2	2
1	1	4	4	1	1	4	4	4	4
1	1	4	4	1	1	4	4	4	4
1	1	4	4	1	1	4	4	4	4
1	1	4	4	1	1	4	4	4	4
1	1	4	4	1	1	4	4	2	2
1	1	4	4	1	1	4	4	4	4
1	1	4	4	1	1	4	4	4	4
1	1	4	4	3	3	4	4	2	2
1	1	4	4	1	1	4	4	4	4
1	1	2	2	1	1	4	4	4	4
1	1	4	4	1	1	4	4	4	4
1	1	4	4	1	1	4	4	4	4
1	1	2	2	1	1	4	4	4	4
1	1	4	4	1	1	4	4	4	4
1	1	2	2	1	1	4	4	4	4
1	1	2	2	1	1	4	4	4	4
4	4	4	4	3	3	4	4	2	2
1	1	4	4	1	1	4	4	4	4
1	1	2	2	1	1	4	4	4	4
1	1	4	4	1	1	4	4	4	4
1	1	4	4	1	1	4	4	4	4
4	4	4	4	1	1	1	1	2	2
1	1	4	4	1	1	4	4	4	4
1	1	4	4	1	1	4	4	4	4
1	1	4	4	1	1	4	4	4	4
4	4	4	4	1	1	4	4	4	4
1	1	4	4	1	1	4	4	4	4
1	1	2	2	1	1	4	4	4	4
1	1	4	4	1	1	4	4	4	4

For Review Only

1	1	4	4	1	1	4	4	2	2
1	1	4	4	1	1	4	4	4	4
1	1	4	4	1	1	4	4	4	4
1	1	4	4	1	1	4	4	4	4
1	1	4	4	1	1	4	4	4	4
1	1	4	4	1	1	4	4	4	4
1	1	4	4	1	1	4	4	4	4
1	1	4	4	1	1	4	4	4	4
1	1	4	4	1	1	4	4	4	4
1	1	4	4	1	1	4	4	2	2
1	1	4	4	1	1	4	4	4	4
1	1	4	4	1	1	4	4	2	2
1	1	4	4	1	1	4	4	4	4
1	1	2	2	1	1	4	4	4	4
1	1	2	2	1	1	4	4	4	4
1	1	4	4	1	1	4	4	4	4
1	1	4	4	1	1	4	4	4	4
1	1	4	4	1	1	1	1	2	2
4	4	4	4	1	1	1	1	2	2
4	4	4	4	1	1	1	1	2	2
1	1	2	2	1	1	4	4	2	2
1	1	2	2	1	1	4	4	4	4
1	1	4	4	1	1	4	4	4	4
1	1	4	4	1	1	4	4	2	2
1	1	4	4	1	1	4	4	4	4
1	1	2	2	1	1	4	4	4	4
4	4	4	4	1	1	1	1	2	2
1	1	2	2	1	1	4	4	4	4
1	1	4	4	1	1	4	4	4	4
1	1	4	4	1	1	4	4	4	4
1	1	4	4	1	1	4	4	4	4
1	1	4	4	1	1	4	4	4	4
1	1	4	4	3	3	1	1	2	2
1	1	4	4	1	1	4	4	4	4
1	1	4	4	1	1	4	4	4	4
1	1	4	4	1	1	1	1	2	2
1	1	2	2	1	1	4	4	4	4
1	1	2	2	1	1	4	4	4	4
1	1	4	4	1	1	4	4	4	4
1	1	4	4	1	1	4	4	4	4
1	1	4	4	1	1	4	4	4	4
1	1	2	2	3	3	4	4	4	4
1	1	4	4	1	1	4	4	4	4
1	1	2	2	1	1	4	4	4	4
1	1	2	2	1	1	4	4	4	4
1	1	4	4	1	1	4	4	4	4
4	4	4	4	1	1	4	4	4	4
1	1	4	4	1	1	4	4	4	4
1	1	2	2	1	1	1	1	2	2
4	4	4	4	1	1	1	1	2	2
1	1	4	4	1	1	4	4	4	4
1	1	4	4	3	3	4	4	4	4
1	1	2	2	1	1	4	4	4	4
1	1	2	2	1	1	4	4	4	4
1	1	4	4	1	1	4	4	2	2

For Review Only

1	1	2	2	1	1	4	4	4	4
1	1	2	2	1	1	4	4	4	4

For Review Only

SNP2272	SNP2272	SNP2273	SNP2273	SNP2274	SNP2274	SNP2275	SNP2275	SNP2276	SNP2276
1	1	1	1	1	1	2	2	3	3
4	4	3	3	4	4	3	3	3	3
4	4	3	3	4	4	3	3	3	3
1	1	1	1	1	1	3	3	3	3
1	1	1	1	1	1	3	3	3	3
4	4	3	3	4	4	3	3	3	3
1	1	1	1	1	1	3	3	3	3
4	4	3	3	4	4	3	3	3	3
1	1	1	1	1	1	3	3	3	3
4	4	3	3	4	4	3	3	3	3
1	1	1	1	1	1	3	3	3	3
1	1	3	3	1	1	3	3	3	3
1	1	1	1	1	1	3	3	3	3
4	4	3	3	4	4	3	3	3	3
1	1	1	1	1	1	3	3	3	3
1	1	3	3	4	4	3	3	3	3
1	1	1	1	1	1	3	3	3	3
4	4	3	3	4	4	3	3	3	3
1	1	1	1	1	1	3	3	2	2
4	4	3	3	4	4	3	3	3	3
1	1	1	1	1	1	3	3	3	3
1	1	1	1	1	1	3	3	3	3
1	1	1	1	1	1	2	2	2	2
1	1	1	1	1	1	3	3	3	3
1	1	1	1	1	1	3	3	3	3
4	4	3	3	4	4	3	3	2	2
1	1	1	1	1	1	3	3	3	3
1	1	1	1	1	1	3	3	2	2
4	4	3	3	4	4	2	2	3	3
1	1	1	1	1	1	3	3	3	3
1	1	1	1	1	1	3	3	2	2
4	4	3	3	4	4	3	3	3	3
1	1	1	1	1	1	3	3	3	3
4	4	3	3	4	4	2	2	3	3
1	1	1	1	1	1	3	3	3	3
1	1	1	1	1	1	3	3	3	3
4	4	3	3	4	4	3	3	3	3
1	1	1	1	4	4	3	3	3	3
1	1	1	1	1	1	3	3	3	3
1	1	1	1	1	1	3	3	3	3
1	1	1	1	1	1	3	3	3	3
1	1	3	3	4	4	2	2	3	3
4	4	3	3	4	4	3	3	3	3
1	1	1	1	1	1	3	3	3	3
4	4	3	3	4	4	3	3	3	3
4	4	3	3	4	4	3	3	2	2
4	4	3	3	4	4	3	3	3	3
1	1	1	1	1	1	3	3	3	3
1	1	1	1	1	1	3	3	3	3
1	1	1	1	1	1	3	3	2	2
1	1	1	1	1	1	3	3	2	2
1	1	1	1	1	1	3	3	3	3

For Review Only

1	1	1	1	1	1	3	3	3	3
1	1	1	1	1	1	3	3	3	3
4	4	3	3	4	4	3	3	2	2
1	1	1	1	1	1	3	3	3	3
1	1	1	1	1	1	3	3	2	2
1	1	1	1	1	1	3	3	3	3
1	1	1	1	1	1	3	3	2	2
1	1	1	1	1	1	3	3	3	3
4	4	3	3	4	4	3	3	3	3
1	1	1	1	1	1	3	3	3	3
4	4	3	3	4	4	3	3	3	3
4	4	3	3	4	4	3	3	3	3
1	1	1	1	1	1	3	3	3	3
4	4	3	3	4	4	2	2	3	3
1	1	1	1	1	1	3	3	3	3
4	4	3	3	4	4	3	3	3	3
1	1	1	1	1	1	3	3	3	3
4	4	3	3	4	4	3	3	3	3
1	1	1	1	1	1	3	3	2	2
1	1	1	1	1	1	3	3	3	3
4	4	1	1	1	1	3	3	3	3
4	4	3	3	4	4	3	3	3	3
4	4	3	3	4	4	2	2	3	3
4	4	3	3	4	4	3	3	3	3
4	4	3	3	4	4	3	3	3	3
1	1	1	1	1	1	3	3	3	3
1	1	1	1	1	1	3	3	3	3
1	1	1	1	1	1	3	3	3	3
4	4	3	3	4	4	3	3	2	2
1	1	1	1	1	1	3	3	3	3
1	1	1	1	1	1	3	3	3	3
1	1	1	1	1	1	3	3	3	3
1	1	1	1	1	1	3	3	3	3
1	1	1	1	1	1	3	3	3	3
4	4	3	3	4	4	3	3	3	3
4	4	3	3	4	4	3	3	3	3
4	4	1	1	4	4	3	3	3	3
1	1	1	1	1	1	3	3	3	3
4	4	3	3	4	4	3	3	3	3
4	4	3	3	4	4	3	3	3	3
1	1	1	1	1	1	3	3	3	3
4	4	3	3	4	4	3	3	3	3
1	1	1	1	1	1	3	3	3	3
4	4	3	3	4	4	3	3	3	3
1	1	1	1	1	1	3	3	3	3
4	4	3	3	4	4	3	3	3	3
4	4	3	3	4	4	3	3	3	3
1	1	1	1	1	1	3	3	2	2
1	1	1	1	1	1	3	3	2	2
4	4	3	3	4	4	3	3	3	3
1	1	1	1	1	1	3	3	3	3
1	1	1	1	1	1	3	3	3	3
1	1	1	1	1	1	2	2	3	3
1	1	1	1	1	1	3	3	2	2
4	4	3	3	4	4	3	3	3	3

For Review Only

4	4	3	3	4	4	3	3	2	2
1	1	1	1	1	1	3	3	3	3
1	1	1	1	1	1	3	3	2	2
4	4	3	3	4	4	2	2	3	3
1	1	1	1	1	1	3	3	3	3
1	1	1	1	1	1	3	3	2	2
4	4	3	3	4	4	3	3	3	3
4	4	1	1	4	4	3	3	3	3
4	4	3	3	4	4	3	3	3	3
1	1	1	1	1	1	3	3	2	2
1	1	1	1	1	1	3	3	3	3
1	1	1	1	1	1	3	3	3	3
1	1	1	1	1	1	3	3	3	3
1	1	1	1	1	1	3	3	3	3
1	1	1	1	1	1	3	3	3	3
1	1	1	1	1	1	3	3	2	2
1	1	1	1	1	1	3	3	3	3
1	1	1	1	1	1	3	3	3	3
4	4	3	3	4	4	2	2	3	3
1	1	1	1	1	1	3	3	2	2
1	1	1	1	1	1	3	3	2	2
1	1	1	1	1	1	3	3	3	3
4	4	3	3	4	4	3	3	3	3
4	4	3	3	4	4	3	3	3	3
4	4	3	3	4	4	3	3	2	2
1	1	1	1	1	1	3	3	3	3
1	1	1	1	1	1	3	3	3	3
4	4	3	3	4	4	3	3	2	2
1	1	1	1	1	1	3	3	3	3
4	4	3	3	4	4	3	3	2	2
1	1	1	1	1	1	3	3	3	3
1	1	1	1	1	1	3	3	3	3
1	1	1	1	1	1	3	3	3	3
1	1	1	1	1	1	3	3	2	2
4	4	3	3	4	4	2	2	2	2
1	1	1	1	1	1	3	3	3	3
4	4	3	3	4	4	2	2	3	3
1	1	1	1	1	1	3	3	3	3
4	4	1	1	1	1	3	3	2	2
1	1	1	1	1	1	3	3	3	3
1	1	1	1	1	1	3	3	3	3
4	4	3	3	4	4	2	2	3	3
4	4	3	3	4	4	3	3	3	3
1	1	1	1	1	1	3	3	3	3
4	4	3	3	4	4	3	3	2	2
4	4	3	3	4	4	3	3	3	3
1	1	1	1	1	1	3	3	2	2
4	4	3	3	4	4	3	3	3	3
4	4	3	3	4	4	3	3	3	3
1	1	1	1	1	1	3	3	2	2
1	1	1	1	1	1	3	3	2	2
1	1	1	1	1	1	3	3	3	3
4	4	3	3	4	4	3	3	2	2
1	1	1	1	1	1	3	3	2	2
1	1	1	1	1	1	3	3	3	3

For Review Only

1	1	1	1	1	1	3	3	2	2
1	1	1	1	1	1	3	3	3	3
1	1	1	1	1	1	3	3	3	3
1	1	1	1	1	1	3	3	3	3
4	4	3	3	4	4	3	3	3	3
1	1	1	1	1	1	3	3	3	3
1	1	1	1	1	1	3	3	3	3
1	1	3	3	1	1	3	3	3	3
1	1	1	1	4	4	3	3	3	3
1	1	1	1	1	1	3	3	2	2
1	1	1	1	1	1	3	3	2	2
1	1	1	1	1	1	3	3	3	3
1	1	1	1	1	1	3	3	3	3
4	4	3	3	4	4	3	3	3	3
4	4	3	3	4	4	2	2	3	3
1	1	1	1	1	1	3	3	3	3
4	4	3	3	4	4	3	3	2	2
1	1	1	1	1	1	3	3	3	3
4	4	3	3	4	4	3	3	2	2
4	4	3	3	4	4	3	3	3	3
1	1	1	1	1	1	3	3	3	3
1	1	1	1	1	1	3	3	3	3
1	1	1	1	1	1	3	3	3	3
4	4	3	3	4	4	3	3	3	3
1	1	1	1	1	1	3	3	3	3
4	4	3	3	4	4	2	2	3	3
1	1	1	1	1	1	3	3	3	3
1	1	1	1	1	1	3	3	3	3
1	1	1	1	1	1	3	3	3	3
4	4	3	3	4	4	3	3	3	3
4	4	3	3	4	4	2	2	3	3
1	1	1	1	1	1	3	3	3	3
1	1	1	1	1	1	3	3	3	3
4	4	3	3	4	4	2	2	3	3
1	1	1	1	1	1	3	3	2	2
4	4	3	3	4	4	3	3	3	3
1	1	1	1	1	1	3	3	3	3
4	4	3	3	4	4	3	3	3	3
4	4	3	3	4	4	3	3	3	3
1	1	1	1	1	1	3	3	3	3
4	4	3	3	4	4	3	3	3	3
1	1	1	1	1	1	3	3	2	2
4	4	3	3	4	4	3	3	3	3
1	1	1	1	1	1	3	3	3	3
1	1	1	1	1	1	3	3	3	3
1	1	1	1	1	1	3	3	3	3
1	1	1	1	1	1	3	3	3	3
1	1	1	1	1	1	3	3	3	3
1	1	1	1	1	1	3	3	3	3
1	1	1	1	1	1	3	3	3	3
4	4	3	3	4	4	3	3	2	2
1	1	1	1	1	1	3	3	3	3
1	1	1	1	1	1	3	3	3	3
4	4	3	3	4	4	3	3	3	3
1	1	1	1	1	1	3	3	3	3

For Review Only

4	4	3	3	4	4	2	2	3	3
4	4	3	3	4	4	3	3	3	3
4	4	3	3	4	4	3	3	2	2
1	1	1	1	1	1	3	3	2	2
1	1	1	1	1	1	3	3	3	3
4	4	3	3	4	4	3	3	2	2
4	4	3	3	4	4	3	3	3	3
4	4	3	3	4	4	3	3	3	3
4	4	3	3	4	4	2	2	3	3
1	1	1	1	1	1	3	3	3	3
1	1	1	1	1	1	3	3	3	3
4	4	1	1	1	1	2	2	2	2
1	1	1	1	1	1	3	3	3	3
1	1	1	1	1	1	3	3	3	3
4	4	3	3	4	4	3	3	3	3
1	1	1	1	1	1	3	3	3	3
1	1	1	1	1	1	3	3	3	3
4	4	3	3	4	4	2	2	3	3
1	1	1	1	1	1	3	3	2	2
1	1	1	1	1	1	3	3	3	3
1	1	1	1	1	1	2	2	3	3
4	4	3	3	4	4	3	3	2	2
1	1	1	1	1	1	3	3	3	3
1	1	1	1	1	1	3	3	3	3
1	1	1	1	1	1	3	3	3	3
1	1	1	1	1	1	3	3	2	2
1	1	1	1	1	1	2	2	3	3
1	1	1	1	1	1	3	3	3	3
1	1	1	1	1	1	3	3	3	3
4	4	3	3	4	4	3	3	2	2
1	1	1	1	1	1	3	3	3	3
1	1	1	1	1	1	3	3	2	2
4	4	3	3	4	4	3	3	3	3
4	4	1	1	4	4	3	3	2	2
4	4	3	3	4	4	3	3	3	3
4	4	3	3	4	4	2	2	3	3
4	4	3	3	4	4	3	3	3	3
4	4	3	3	4	4	3	3	3	3
1	1	1	1	1	1	3	3	3	3
1	1	1	1	1	1	3	3	3	3
4	4	3	3	4	4	3	3	2	2
4	4	3	3	4	4	3	3	3	3
1	1	1	1	1	1	3	3	3	3
1	1	1	1	1	1	3	3	3	3
1	1	1	1	1	1	3	3	3	3
4	4	3	3	4	4	3	3	3	3
1	1	1	1	1	1	3	3	3	3
4	4	3	3	4	4	3	3	3	3
4	4	3	3	4	4	3	3	3	3
1	1	1	1	1	1	3	3	3	3
4	4	3	3	4	4	3	3	3	3
1	1	1	1	1	1	3	3	3	3
4	4	3	3	4	4	3	3	3	3
1	1	1	1	1	1	3	3	3	3
1	1	1	1	1	1	3	3	3	3

For Review Only

1	1	1	1	1	1	3	3	2	2
1	1	1	1	1	1	3	3	3	3

For Review Only

SNP2277	SNP2277	SNP2278	SNP2278	SNP2279	SNP2279	SNP228	SNP228	SNP2280	SNP2280
1	1	2	2	1	1	4	4	3	3
4	4	1	1	4	4	4	4	3	3
4	4	2	2	1	1	4	4	3	3
1	1	2	2	1	1	4	4	3	3
1	1	2	2	1	1	1	1	3	3
4	4	1	1	1	1	4	4	3	3
1	1	1	1	1	1	1	1	2	2
4	4	1	1	1	1	4	4	3	3
1	1	1	1	1	1	4	4	3	3
4	4	1	1	1	1	4	4	3	3
1	1	1	1	1	1	1	1	3	3
4	4	2	2	1	1	4	4	3	3
1	1	2	2	1	1	1	1	3	3
4	4	1	1	1	1	4	4	3	3
1	1	1	1	1	1	4	4	3	3
4	4	2	2	1	1	4	4	3	3
1	1	2	2	1	1	4	4	3	3
4	4	2	2	1	1	1	1	3	3
1	1	1	1	1	1	4	4	3	3
4	4	1	1	1	1	4	4	2	2
1	1	2	2	1	1	1	1	3	3
1	1	1	1	1	1	4	4	3	3
1	1	1	1	1	1	1	1	2	2
1	1	2	2	1	1	4	4	3	3
1	1	1	1	1	1	4	4	3	3
4	4	1	1	4	4	4	4	2	2
1	1	2	2	1	1	4	4	3	3
1	1	1	1	1	1	4	4	3	3
4	4	2	2	1	1	4	4	3	3
1	1	1	1	1	1	1	1	3	3
1	1	1	1	1	1	4	4	3	3
4	4	1	1	1	1	4	4	3	3
1	1	1	1	1	1	4	4	3	3
4	4	1	1	1	1	4	4	3	3
1	1	1	1	1	1	4	4	3	3
1	1	2	2	1	1	4	4	3	3
4	4	2	2	1	1	1	1	3	3
1	1	2	2	1	1	4	4	3	3
1	1	1	1	1	1	1	1	3	3
1	1	1	1	1	1	1	1	3	3
1	1	1	1	1	1	1	1	3	3
4	4	1	1	1	1	1	1	2	2
4	4	1	1	1	1	1	1	3	3
1	1	2	2	1	1	1	1	3	3
4	4	1	1	1	1	1	1	3	3
4	4	1	1	1	1	4	4	3	3
4	4	2	2	1	1	4	4	3	3
1	1	1	1	1	1	1	1	3	3
1	1	1	1	1	1	1	1	3	3
4	4	1	1	1	1	4	4	3	3
1	1	1	1	4	4	1	1	2	2
1	1	2	2	1	1	4	4	3	3

For Review Only

1	1	2	2	1	1	4	4	3	3
1	1	1	1	1	1	4	4	3	3
4	4	1	1	4	4	4	4	3	3
1	1	1	1	1	1	4	4	3	3
1	1	1	1	1	1	4	4	3	3
1	1	2	2	1	1	4	4	3	3
1	1	1	1	4	4	4	4	2	2
1	1	1	1	1	1	1	1	3	3
4	4	1	1	1	1	1	1	3	3
1	1	1	1	1	1	4	4	3	3
4	4	2	2	1	1	4	4	3	3
4	4	2	2	1	1	4	4	3	3
1	1	2	2	1	1	4	4	3	3
4	4	2	2	1	1	4	4	3	3
1	1	1	1	1	1	4	4	3	3
4	4	1	1	1	1	4	4	2	2
1	1	2	2	1	1	4	4	3	3
4	4	1	1	1	1	4	4	3	3
1	1	1	1	1	1	4	4	3	3
1	1	2	2	1	1	4	4	3	3
1	1	2	2	1	1	4	4	3	3
4	4	1	1	1	1	1	1	3	3
4	4	2	2	1	1	4	4	3	3
4	4	1	1	4	4	4	4	3	3
4	4	1	1	1	1	1	1	3	3
1	1	2	2	1	1	1	1	3	3
1	1	1	1	1	1	1	1	3	3
1	1	1	1	1	1	1	1	3	3
4	4	1	1	4	4	4	4	2	2
1	1	2	2	1	1	4	4	3	3
1	1	1	1	1	1	1	1	3	3
1	1	1	1	1	1	4	4	3	3
1	1	1	1	1	1	1	1	3	3
1	1	2	2	1	1	4	4	3	3
4	4	2	2	1	1	4	4	3	3
4	4	1	1	1	1	1	1	3	3
4	4	1	1	1	1	1	1	3	3
1	1	1	1	1	1	1	1	3	3
4	4	2	2	1	1	4	4	3	3
4	4	1	1	1	1	1	1	3	3
1	1	2	2	1	1	4	4	3	3
4	4	2	2	1	1	4	4	3	3
1	1	2	2	1	1	1	1	3	3
4	4	2	2	1	1	4	4	3	3
1	1	2	2	1	1	4	4	3	3
4	4	1	1	1	1	4	4	3	3
4	4	1	1	1	1	4	4	3	3
1	1	1	1	4	4	1	1	2	2
1	1	2	2	1	1	4	4	3	3
4	4	1	1	1	1	4	4	3	3
1	1	2	2	1	1	4	4	3	3
1	1	1	1	1	1	1	1	3	3
1	1	1	1	1	1	1	1	3	3
1	1	1	1	1	1	4	4	3	3
4	4	1	1	1	1	4	4	3	3

For Review Only

4	4	1	1	1	1	4	4	2	2
1	1	1	1	1	1	4	4	3	3
1	1	1	1	4	4	4	4	2	2
4	4	1	1	1	1	4	4	3	3
1	1	1	1	1	1	4	4	3	3
1	1	1	1	1	1	4	4	3	3
4	4	2	2	1	1	4	4	3	3
4	4	2	2	1	1	4	4	3	3
4	4	1	1	1	1	4	4	3	3
1	1	1	1	1	1	4	4	3	3
1	1	1	1	1	1	4	4	3	3
1	1	1	1	1	1	4	4	3	3
1	1	2	2	1	1	1	1	3	3
1	1	2	2	1	1	4	4	3	3
1	1	1	1	1	1	4	4	3	3
1	1	1	1	1	1	4	4	3	3
1	1	1	1	1	1	4	4	3	3
1	1	1	1	1	1	4	4	3	3
4	4	1	1	1	1	4	4	3	3
1	1	1	1	4	4	4	4	2	2
1	1	1	1	4	4	4	4	3	3
1	1	2	2	1	1	4	4	3	3
4	4	2	2	1	1	4	4	3	3
4	4	1	1	1	1	1	1	3	3
4	4	1	1	1	1	1	1	2	2
1	1	1	1	1	1	1	1	3	3
1	1	2	2	1	1	4	4	3	3
4	4	1	1	1	1	4	4	3	3
1	1	2	2	1	1	4	4	3	3
4	4	1	1	1	1	4	4	3	3
1	1	1	1	1	1	4	4	3	3
1	1	1	1	1	1	4	4	3	3
1	1	2	2	1	1	4	4	3	3
1	1	2	2	1	1	4	4	3	3
4	4	1	1	1	1	4	4	3	3
1	1	2	2	1	1	4	4	3	3
4	4	1	1	1	1	4	4	3	3
1	1	2	2	1	1	4	4	3	3
1	1	1	1	4	4	4	4	3	3
1	1	1	1	1	1	4	4	3	3
1	1	1	1	1	1	4	4	3	3
4	4	1	1	1	1	4	4	3	3
4	4	1	1	1	1	4	4	3	3
1	1	1	1	1	1	4	4	3	3
4	4	1	1	4	4	4	4	2	2
4	4	2	2	1	1	4	4	3	3
1	1	1	1	4	4	4	4	2	2
4	4	2	2	1	1	4	4	3	3
1	1	1	1	1	1	4	4	3	3
1	1	1	1	4	4	4	4	2	2
1	1	1	1	4	4	4	4	2	2
1	1	1	1	1	1	1	1	3	3
1	1	1	1	1	1	4	4	3	3
1	1	1	1	4	4	4	4	3	3
1	1	1	1	1	1	1	1	3	3

For Review Only

1	1	1	1	1	1	4	4	3	3
1	1	2	2	1	1	1	1	3	3
1	1	1	1	1	1	4	4	3	3
1	1	1	1	1	1	1	1	3	3
4	4	2	2	1	1	4	4	3	3
1	1	1	1	1	1	4	4	3	3
1	1	1	1	1	1	4	4	3	3
1	1	1	1	4	4	4	4	3	3
1	1	1	1	1	1	4	4	3	3
1	1	1	1	1	1	4	4	3	3
1	1	1	1	4	4	4	4	2	2
1	1	1	1	1	1	4	4	3	3
1	1	2	2	1	1	4	4	3	3
4	4	2	2	1	1	4	4	3	3
4	4	1	1	1	1	4	4	3	3
1	1	1	1	1	1	1	1	3	3
1	1	1	1	1	1	4	4	3	3
1	1	1	1	1	1	4	4	3	3
4	4	2	2	1	1	4	4	3	3
4	4	2	2	1	1	4	4	3	3
1	1	1	1	1	1	4	4	3	3
1	1	1	1	1	1	4	4	3	3
1	1	1	1	1	1	1	1	3	3
4	4	2	2	1	1	4	4	3	3
1	1	2	2	1	1	4	4	3	3
4	4	1	1	1	1	4	4	3	3
1	1	1	1	1	1	1	1	3	3
1	1	1	1	1	1	4	4	3	3
1	1	1	1	1	1	4	4	3	3
4	4	2	2	1	1	4	4	3	3
4	4	1	1	1	1	1	1	3	3
1	1	2	2	1	1	4	4	3	3
1	1	1	1	1	1	4	4	3	3
4	4	1	1	1	1	4	4	3	3
1	1	1	1	1	1	4	4	3	3
4	4	2	2	1	1	4	4	3	3
1	1	1	1	1	1	4	4	3	3
4	4	2	2	1	1	4	4	3	3
4	4	2	2	1	1	4	4	3	3
1	1	2	2	1	1	4	4	3	3
4	4	2	2	1	1	4	4	3	3
1	1	1	1	4	4	4	4	2	2
4	4	1	1	1	1	1	1	3	3
1	1	1	1	1	1	4	4	3	3
4	4	1	1	1	1	4	4	3	3
1	1	1	1	1	1	4	4	3	3
1	1	1	1	1	1	1	1	3	3
1	1	1	1	1	1	1	1	3	3
1	1	2	2	1	1	4	4	3	3
1	1	2	2	1	1	4	4	3	3
4	4	1	1	1	1	4	4	3	3
1	1	1	1	1	1	4	4	3	3
1	1	1	1	1	1	1	1	3	3
4	4	2	2	1	1	4	4	3	3
1	1	1	1	1	1	1	1	2	2

For Review Only

4	4	2	2	1	1	4	4	3	3
4	4	1	1	1	1	4	4	3	3
4	4	1	1	1	1	4	4	3	3
1	1	1	1	1	1	4	4	3	3
1	1	2	2	1	1	4	4	3	3
4	4	1	1	1	1	4	4	3	3
4	4	1	1	1	1	4	4	2	2
4	4	1	1	1	1	1	1	3	3
4	4	1	1	1	1	4	4	3	3
1	1	1	1	1	1	1	1	3	3
1	1	1	1	1	1	4	4	3	3
1	1	1	1	1	1	4	4	3	3
1	1	2	2	1	1	4	4	3	3
1	1	2	2	1	1	4	4	3	3
4	4	2	2	1	1	4	4	3	3
1	1	1	1	1	1	4	4	3	3
1	1	2	2	1	1	1	1	3	3
4	4	1	1	1	1	4	4	3	3
1	1	1	1	1	1	4	4	3	3
1	1	2	2	1	1	4	4	3	3
1	1	1	1	1	1	1	1	3	3
4	4	1	1	4	4	4	4	2	2
1	1	1	1	1	1	4	4	3	3
1	1	1	1	1	1	4	4	3	3
1	1	1	1	1	1	1	1	3	3
1	1	1	1	1	1	4	4	3	3
1	1	1	1	1	1	1	1	3	3
1	1	2	2	1	1	4	4	3	3
1	1	2	2	1	1	4	4	3	3
4	4	1	1	1	1	4	4	3	3
1	1	1	1	1	1	1	1	3	3
1	1	1	1	4	4	1	1	2	2
4	4	2	2	1	1	4	4	3	3
4	4	1	1	4	4	4	4	2	2
4	4	1	1	1	1	1	1	3	3
4	4	1	1	1	1	1	1	3	3
4	4	2	2	1	1	4	4	3	3
4	4	2	2	1	1	4	4	3	3
1	1	2	2	1	1	4	4	3	3
1	1	1	1	1	1	1	1	3	3
4	4	1	1	1	1	4	4	3	3
4	4	2	2	1	1	4	4	3	3
1	1	1	1	1	1	4	4	3	3
1	1	2	2	1	1	4	4	3	3
1	1	2	2	1	1	4	4	3	3
4	4	2	2	1	1	4	4	3	3
1	1	2	2	1	1	4	4	3	3
4	4	2	2	1	1	4	4	3	3
4	4	2	2	1	1	1	1	3	3
1	1	1	1	1	1	1	1	3	3
4	4	1	1	1	1	4	4	3	3
1	1	1	1	1	1	1	1	3	3
4	4	2	2	1	1	4	4	3	3
1	1	2	2	1	1	4	4	3	3
1	1	1	1	1	1	1	1	3	3

For Review Only

1	1	1	1	1	1	4	4	3	3
1	1	2	2	1	1	4	4	3	3

For Review Only

SNP2281	SNP2281	SNP2282	SNP2282	SNP2283	SNP2283	SNP2284	SNP2284	SNP2285	SNP2285
3	3	3	3	3	3	4	4	1	1
3	3	3	3	2	2	1	1	4	4
3	3	3	3	2	2	4	4	1	1
3	3	3	3	3	3	4	4	1	1
3	3	3	3	2	2	4	4	1	1
3	3	3	3	3	3	4	4	1	1
2	2	3	3	3	3	4	4	1	1
3	3	3	3	2	2	4	4	1	1
3	3	3	3	2	2	4	4	1	1
3	3	3	3	3	3	4	4	1	1
3	3	3	3	3	3	4	4	1	1
3	3	3	3	2	2	4	4	1	1
3	3	3	3	3	3	4	4	1	1
2	2	3	3	2	2	1	1	4	4
3	3	3	3	3	3	4	4	1	1
3	3	3	3	3	3	4	4	1	1
3	3	3	3	2	2	4	4	1	1
3	3	3	3	2	2	4	4	1	1
3	3	3	3	3	3	1	1	4	4
2	2	3	3	2	2	4	4	1	1
3	3	3	3	2	2	4	4	1	1
3	3	3	3	2	2	4	4	1	1
2	2	3	3	3	3	4	4	1	1
3	3	3	3	2	2	4	4	1	1
2	2	3	3	3	3	1	1	4	4
2	2	3	3	2	2	1	1	4	4
3	3	3	3	2	2	4	4	1	1
3	3	3	3	3	3	4	4	1	1
3	3	3	3	2	2	4	4	1	1
3	3	3	3	2	2	4	4	1	1
2	2	3	3	2	2	1	1	4	4
3	3	3	3	3	3	1	1	4	4
3	3	3	3	3	3	4	4	1	1
3	3	3	3	2	2	4	4	1	1
3	3	3	3	2	2	4	4	1	1
3	3	3	3	2	2	4	4	1	1
3	3	3	3	3	3	4	4	1	1
3	3	3	3	3	3	4	4	1	1
3	3	4	4	3	3	4	4	1	1
3	3	4	4	2	2	4	4	1	1
3	3	3	3	2	2	4	4	1	1
2	2	3	3	2	2	4	4	1	1
3	3	3	3	3	3	4	4	1	1
3	3	3	3	2	2	4	4	1	1
3	3	3	3	2	2	4	4	1	1
2	2	3	3	2	2	4	4	1	1
3	3	3	3	2	2	4	4	1	1
3	3	3	3	3	3	1	1	4	4
3	3	3	3	2	2	4	4	1	1
3	3	3	3	3	3	1	1	4	4
2	2	3	3	2	2	4	4	4	4
3	3	3	3	3	3	4	4	1	1

For Review Only

3	3	3	3	2	2	4	4	1	1
3	3	3	3	3	3	4	4	1	1
2	2	3	3	3	3	1	1	4	4
3	3	3	3	2	2	4	4	1	1
3	3	3	3	3	3	1	1	4	4
3	3	3	3	2	2	4	4	1	1
2	2	3	3	2	2	1	1	4	4
3	3	3	3	2	2	4	4	1	1
3	3	3	3	3	3	4	4	1	1
3	3	3	3	2	2	4	4	1	1
3	3	3	3	2	2	4	4	1	1
3	3	3	3	2	2	4	4	1	1
3	3	3	3	2	2	4	4	1	1
3	3	3	3	2	2	4	4	1	1
3	3	3	3	3	3	4	4	1	1
2	2	3	3	3	3	4	4	1	1
3	3	3	3	3	3	4	4	1	1
3	3	3	3	2	2	1	1	4	4
3	3	3	3	2	2	1	1	4	4
3	3	3	3	3	3	4	4	1	1
3	3	3	3	2	2	4	4	1	1
3	3	3	3	2	2	1	1	4	4
3	3	3	3	3	3	4	4	1	1
3	3	3	3	3	3	1	1	4	4
3	3	3	3	2	2	4	4	1	1
3	3	3	3	2	2	4	4	1	1
3	3	3	3	2	2	4	4	1	1
3	3	3	3	2	2	4	4	1	1
2	2	3	3	2	2	1	1	4	4
3	3	3	3	3	3	4	4	1	1
3	3	3	3	2	2	4	4	1	1
3	3	4	4	2	2	4	4	1	1
3	3	3	3	2	2	4	4	1	1
3	3	3	3	2	2	4	4	1	1
3	3	3	3	3	3	4	4	1	1
3	3	3	3	2	2	4	4	1	1
3	3	3	3	2	2	4	4	1	1
3	3	3	3	2	2	4	4	1	1
3	3	3	3	2	2	4	4	1	1
3	3	3	3	2	2	4	4	1	1
3	3	3	3	2	2	4	4	1	1
3	3	3	3	2	2	4	4	1	1
3	3	3	3	2	2	4	4	1	1
3	3	3	3	2	2	4	4	1	1
3	3	3	3	2	2	4	4	1	1
3	3	3	3	2	2	1	1	4	4
3	3	3	3	3	3	4	4	1	1
2	2	3	3	2	2	1	1	4	4
3	3	3	3	2	2	4	4	1	1
3	3	3	3	3	3	4	4	1	1
3	3	3	3	2	2	4	4	1	1
3	3	3	3	2	2	4	4	1	1
3	3	3	3	2	2	4	4	1	1
3	3	3	3	3	3	4	4	1	1
3	3	3	3	3	3	4	4	1	1

For Review Only

2	2	3	3	2	2	1	1	4	4
3	3	3	3	2	2	4	4	1	1
2	2	3	3	2	2	1	1	4	4
3	3	3	3	3	3	4	4	1	1
3	3	3	3	2	2	4	4	4	4
2	2	4	4	2	2	4	4	1	1
3	3	3	3	2	2	4	4	1	1
3	3	3	3	3	3	4	4	1	1
3	3	3	3	2	2	4	4	1	1
2	2	3	3	3	3	1	1	4	4
3	3	3	3	3	3	4	4	1	1
2	2	3	3	2	2	4	4	1	1
3	3	3	3	2	2	4	4	1	1
3	3	3	3	2	2	4	4	1	1
3	3	3	3	3	3	4	4	1	1
2	2	3	3	2	2	1	1	4	4
3	3	3	3	3	3	4	4	1	1
2	2	3	3	3	3	4	4	1	1
3	3	3	3	3	3	4	4	1	1
2	2	3	3	2	2	1	1	4	4
3	3	3	3	3	3	1	1	1	1
3	3	3	3	3	3	4	4	1	1
3	3	3	3	2	2	4	4	1	1
3	3	3	3	2	2	4	4	1	1
2	2	3	3	2	2	1	1	4	4
3	3	3	3	2	2	4	4	1	1
3	3	3	3	3	3	4	4	1	1
3	3	3	3	3	3	1	1	4	4
3	3	3	3	3	3	4	4	1	1
3	3	3	3	3	3	1	1	4	4
3	3	4	4	3	3	4	4	1	1
3	3	3	3	2	2	4	4	1	1
3	3	3	3	2	2	4	4	1	1
3	3	3	3	2	2	4	4	1	1
3	3	3	3	2	2	4	4	1	1
3	3	3	3	3	3	4	4	1	1
3	3	3	3	2	2	4	4	1	1
3	3	3	3	2	2	4	4	1	1
2	2	3	3	2	2	1	1	4	4
3	3	3	3	3	3	4	4	1	1
3	3	3	3	2	2	4	4	1	1
3	3	4	4	2	2	4	4	1	1
3	3	3	3	2	2	1	1	4	4
3	3	3	3	2	2	4	4	1	1
2	2	3	3	2	2	1	1	4	4
3	3	3	3	2	2	4	4	1	1
2	2	3	3	2	2	1	1	4	4
3	3	3	3	2	2	4	4	1	1
3	3	3	3	3	3	4	4	1	1
2	2	3	3	2	2	1	1	4	4
2	2	3	3	2	2	1	1	4	4
3	3	3	3	3	3	4	4	1	1
3	3	3	3	3	3	4	4	4	4
2	2	3	3	2	2	1	1	4	4
3	3	3	3	3	3	4	4	1	1

For Review Only

3	3	3	3	3	3	1	1	4	4
3	3	3	3	2	2	4	4	1	1
2	2	3	3	2	2	4	4	1	1
3	3	3	3	2	2	4	4	1	1
3	3	3	3	2	2	4	4	1	1
3	3	3	3	2	2	1	1	4	4
3	3	4	4	3	3	4	4	1	1
2	2	3	3	2	2	4	4	1	1
3	3	3	3	3	3	4	4	1	1
3	3	3	3	2	2	1	1	4	4
2	2	3	3	2	2	1	1	4	4
3	3	3	3	2	2	4	4	1	1
3	3	3	3	3	3	4	4	1	1
3	3	3	3	3	3	4	4	1	1
3	3	4	4	3	3	4	4	1	1
3	3	3	3	3	3	4	4	1	1
3	3	3	3	3	3	1	1	4	4
2	2	3	3	3	3	1	1	4	4
2	2	3	3	2	2	4	4	1	1
3	3	3	3	2	2	4	4	1	1
3	3	3	3	3	3	4	4	1	1
3	3	3	3	2	2	4	4	1	1
3	3	3	3	2	2	1	1	4	4
3	3	3	3	2	2	4	4	1	1
3	3	3	3	2	2	4	4	1	1
3	3	3	3	2	2	4	4	1	1
3	3	3	3	3	3	4	4	1	1
3	3	3	3	3	3	4	4	1	1
3	3	3	3	3	3	1	1	4	4
3	3	3	3	3	3	4	4	1	1
3	3	3	3	3	3	4	4	1	1
3	3	3	3	3	3	4	4	1	1
2	2	3	3	3	3	1	1	4	4
3	3	4	4	3	3	4	4	1	1
3	3	3	3	3	3	1	1	4	4
3	3	3	3	2	2	4	4	1	1
2	2	3	3	2	2	4	4	1	1
3	3	3	3	3	3	4	4	1	1
3	3	3	3	2	2	4	4	1	1
3	3	3	3	3	3	4	4	1	1
3	3	3	3	2	2	4	4	1	1
2	2	3	3	2	2	1	1	4	4
3	3	3	3	3	3	4	4	1	1
3	3	4	4	3	3	4	4	1	1
2	2	3	3	2	2	4	4	1	1
3	3	3	3	3	3	1	1	4	4
3	3	3	3	3	3	4	4	1	1
3	3	3	3	3	3	4	4	1	1
3	3	3	3	3	3	4	4	1	1
3	3	3	3	3	3	4	4	1	1
2	2	3	3	3	3	1	1	1	1
3	3	3	3	2	2	4	4	1	1
3	3	3	3	2	2	1	1	4	4
3	3	3	3	2	2	4	4	1	1
2	2	3	3	3	3	4	4	1	1

For Review Only

3	3	3	3	3	3	4	4	1	1
3	3	3	3	3	3	4	4	1	1
3	3	3	3	3	3	4	4	1	1
2	2	3	3	2	2	1	1	4	4
3	3	3	3	3	3	4	4	1	1
2	2	3	3	3	3	1	1	4	4
2	2	3	3	3	3	4	4	1	1
3	3	3	3	2	2	4	4	1	1
3	3	3	3	2	2	4	4	1	1
3	3	4	4	2	2	4	4	1	1
3	3	3	3	3	3	4	4	1	1
2	2	3	3	3	3	4	4	1	1
3	3	3	3	2	2	4	4	1	1
3	3	3	3	2	2	4	4	1	1
3	3	3	3	2	2	4	4	1	1
3	3	3	3	2	2	1	1	4	4
3	3	4	4	3	3	4	4	1	1
3	3	3	3	3	3	4	4	1	1
3	3	3	3	2	2	1	1	4	4
3	3	3	3	3	3	4	4	1	1
3	3	4	4	2	2	4	4	1	1
2	2	3	3	2	2	1	1	4	4
3	3	3	3	3	3	1	1	4	4
3	3	3	3	2	2	4	4	1	1
3	3	3	3	2	2	4	4	1	1
3	3	3	3	2	2	4	4	1	1
3	3	3	3	2	2	4	4	1	1
3	3	3	3	3	3	4	4	1	1
3	3	3	3	3	3	4	4	1	1
3	3	3	3	3	3	1	1	4	4
3	3	3	3	3	3	4	4	1	1
2	2	3	3	3	3	1	1	4	4
3	3	3	3	3	3	4	4	1	1
2	2	3	3	2	2	1	1	1	1
3	3	3	3	2	2	1	1	4	4
3	3	3	3	3	3	1	1	4	4
3	3	3	3	2	2	4	4	1	1
3	3	3	3	2	2	4	4	1	1
3	3	3	3	2	2	4	4	1	1
3	3	4	4	2	2	4	4	1	1
3	3	3	3	3	3	1	1	4	4
3	3	3	3	2	2	4	4	1	1
3	3	4	4	2	2	4	4	1	1
3	3	3	3	2	2	4	4	1	1
3	3	3	3	2	2	4	4	1	1
3	3	3	3	2	2	4	4	1	1
3	3	3	3	2	2	4	4	1	1
3	3	3	3	3	3	4	4	1	1
3	3	3	3	2	2	4	4	1	1
3	3	3	3	2	2	4	4	1	1
3	3	3	3	3	3	1	1	1	1
3	3	4	4	2	2	4	4	1	1
3	3	3	3	2	2	4	4	1	1
3	3	3	3	2	2	4	4	1	1
3	3	4	4	2	2	4	4	1	1

For Review Only

3	3	3	3	2	2	1	1	4	4
3	3	3	3	2	2	4	4	1	1

For Review Only

SNP2286	SNP2286	SNP2287	SNP2287	SNP2288	SNP2288	SNP2289	SNP2289	SNP229	SNP229
4	4	1	1	3	3	2	2	4	4
1	1	1	1	3	3	3	3	2	2
4	4	1	1	3	3	3	3	4	4
4	4	1	1	3	3	3	3	2	2
4	4	1	1	3	3	2	2	4	4
4	4	1	1	3	3	2	2	4	4
4	4	1	1	3	3	2	2	4	4
1	1	1	1	3	3	2	2	2	2
4	4	1	1	3	3	2	2	4	4
4	4	1	1	3	3	2	2	4	4
4	4	1	1	3	3	2	2	2	2
4	4	1	1	3	3	3	3	4	4
4	4	1	1	3	3	3	3	4	4
1	1	1	1	1	1	2	2	2	2
4	4	1	1	3	3	3	3	4	4
4	4	1	1	3	3	2	2	2	2
4	4	1	1	3	3	3	3	2	2
1	1	4	4	3	3	2	2	4	4
1	1	4	4	3	3	2	2	4	4
4	4	1	1	3	3	3	3	2	2
4	4	1	1	3	3	3	3	4	4
4	4	1	1	3	3	3	3	4	4
4	4	1	1	3	3	2	2	4	4
4	4	1	1	3	3	2	2	2	2
1	1	1	1	1	1	2	2	4	4
1	1	1	1	1	1	3	3	4	4
4	4	1	1	3	3	3	3	4	4
1	1	1	1	1	1	3	3	4	4
4	4	1	1	3	3	2	2	4	4
4	4	1	1	3	3	2	2	2	2
1	1	1	1	1	1	3	3	4	4
1	1	4	4	3	3	2	2	4	4
4	4	1	1	3	3	2	2	4	4
4	4	1	1	3	3	2	2	4	4
4	4	1	1	3	3	2	2	4	4
4	4	1	1	3	3	2	2	2	2
4	4	1	1	3	3	2	2	2	2
4	4	1	1	3	3	2	2	4	4
4	4	1	1	3	3	2	2	2	2
4	4	1	1	3	3	2	2	2	2
4	4	1	1	3	3	2	2	2	2
4	4	1	1	3	3	2	2	2	2
4	4	1	1	3	3	2	2	2	2
4	4	1	1	3	3	3	3	2	2
4	4	1	1	3	3	2	2	2	2
1	1	1	1	1	1	3	3	4	4
4	4	1	1	3	3	3	3	2	2
1	1	1	1	1	1	2	2	4	4
4	4	1	1	3	3	3	3	2	2
1	1	4	4	3	3	3	3	4	4
1	1	1	1	1	1	3	3	4	4
4	4	1	1	3	3	2	2	2	2

For Review Only

4	4	1	1	3	3	3	3	4	4
4	4	1	1	3	3	2	2	4	4
1	1	1	1	1	1	3	3	4	4
4	4	1	1	3	3	3	3	2	2
1	1	4	4	3	3	2	2	4	4
4	4	1	1	3	3	3	3	2	2
1	1	1	1	1	1	3	3	2	2
4	4	1	1	3	3	2	2	2	2
4	4	1	1	3	3	2	2	4	4
4	4	1	1	3	3	3	3	4	4
4	4	1	1	3	3	3	3	2	2
4	4	1	1	3	3	3	3	2	2
4	4	1	1	3	3	3	3	4	4
4	4	1	1	3	3	2	2	4	4
4	4	1	1	3	3	2	2	4	4
4	4	1	1	3	3	2	2	2	2
4	4	1	1	3	3	3	3	2	2
1	1	4	4	3	3	3	3	4	4
1	1	4	4	3	3	3	3	4	4
4	4	1	1	3	3	2	2	4	4
4	4	1	1	3	3	3	3	2	2
1	1	1	1	1	1	2	2	2	2
1	1	1	1	3	3	2	2	4	4
1	1	1	1	3	3	2	2	4	4
4	4	1	1	3	3	2	2	2	2
4	4	1	1	3	3	3	3	4	4
4	4	1	1	3	3	3	3	2	2
4	4	1	1	3	3	2	2	2	2
1	1	1	1	1	1	3	3	2	2
4	4	1	1	3	3	3	3	2	2
4	4	1	1	3	3	2	2	4	4
4	4	1	1	3	3	2	2	4	4
4	4	1	1	3	3	2	2	2	2
4	4	1	1	3	3	3	3	4	4
4	4	1	1	3	3	2	2	4	4
4	4	1	1	3	3	2	2	2	2
4	4	1	1	3	3	2	2	4	4
4	4	1	1	3	3	3	3	4	4
4	4	1	1	3	3	3	3	2	2
4	4	1	1	3	3	2	2	2	2
4	4	1	1	3	3	3	3	4	4
4	4	1	1	3	3	3	3	4	4
4	4	1	1	3	3	2	2	2	2
4	4	1	1	3	3	3	3	2	2
4	4	1	1	3	3	3	3	4	4
1	1	4	4	3	3	3	3	4	4
4	4	1	1	3	3	2	2	2	2
1	1	1	1	1	1	3	3	2	2
1	1	1	1	3	3	2	2	2	2
1	1	1	1	1	1	2	2	4	4
4	4	1	1	3	3	3	3	2	2
4	4	1	1	3	3	2	2	2	2
4	4	1	1	3	3	2	2	2	2
4	4	1	1	3	3	3	3	4	4
4	4	1	1	3	3	2	2	4	4

For Review Only

1	1	1	1	1	1	3	3	2	2
4	4	1	1	3	3	3	3	2	2
1	1	1	1	1	1	3	3	4	4
4	4	1	1	3	3	2	2	2	2
1	1	1	1	3	3	3	3	4	4
1	1	1	1	1	1	3	3	4	4
4	4	1	1	3	3	3	3	2	2
4	4	1	1	3	3	2	2	2	2
1	1	1	1	1	1	2	2	2	2
4	4	1	1	1	1	3	3	4	4
4	4	1	1	3	3	2	2	4	4
1	1	1	1	3	3	3	3	4	4
4	4	1	1	3	3	2	2	4	4
4	4	1	1	3	3	3	3	4	4
1	1	1	1	3	3	2	2	4	4
1	1	1	1	1	1	3	3	2	2
4	4	1	1	3	3	2	2	4	4
4	4	1	1	1	1	3	3	4	4
4	4	1	1	3	3	2	2	2	2
1	1	1	1	1	1	3	3	4	4
1	1	1	1	1	1	3	3	4	4
4	4	1	1	3	3	3	3	4	4
4	4	1	1	3	3	3	3	4	4
4	4	1	1	3	3	2	2	2	2
1	1	1	1	1	1	3	3	2	2
4	4	1	1	3	3	2	2	4	4
4	4	1	1	3	3	2	2	4	4
1	1	4	4	3	3	2	2	2	2
4	4	1	1	3	3	3	3	4	4
1	1	4	4	3	3	3	3	4	4
4	4	1	1	3	3	2	2	2	2
1	1	1	1	3	3	3	3	4	4
4	4	1	1	3	3	3	3	4	4
1	1	1	1	3	3	3	3	4	4
4	4	1	1	3	3	2	2	4	4
4	4	1	1	3	3	2	2	4	4
4	4	1	1	3	3	2	2	2	2
4	4	1	1	3	3	3	3	2	2
1	1	1	1	1	1	2	2	4	4
4	4	1	1	3	3	2	2	2	2
4	4	1	1	3	3	2	2	2	2
4	4	1	1	3	3	2	2	2	2
1	1	4	4	3	3	3	3	4	4
4	4	1	1	3	3	2	2	2	2
1	1	1	1	1	1	2	2	4	4
4	4	1	1	3	3	3	3	4	4
1	1	1	1	1	1	3	3	4	4
4	4	1	1	3	3	3	3	2	2
4	4	1	1	3	3	2	2	2	2
1	1	1	1	1	1	3	3	4	4
1	1	1	1	1	1	2	2	4	4
4	4	1	1	3	3	2	2	2	2
1	1	4	4	1	1	2	2	4	4
1	1	1	1	1	1	3	3	4	4
4	4	1	1	3	3	2	2	2	2

For Review Only

1	1	4	4	1	1	3	3	4	4
4	4	1	1	3	3	2	2	2	2
1	1	1	1	1	1	2	2	2	2
4	4	1	1	3	3	3	3	4	4
4	4	1	1	3	3	3	3	2	2
1	1	4	4	1	1	2	2	2	2
4	4	1	1	3	3	2	2	2	2
1	1	1	1	3	3	3	3	4	4
4	4	1	1	3	3	2	2	2	2
1	1	4	4	3	3	3	3	4	4
1	1	1	1	1	1	3	3	4	4
4	4	1	1	3	3	2	2	4	4
4	4	1	1	3	3	2	2	4	4
4	4	1	1	3	3	3	3	4	4
4	4	1	1	3	3	2	2	2	2
4	4	1	1	3	3	2	2	2	2
1	1	1	1	1	1	2	2	4	4
1	1	1	1	1	1	2	2	4	4
4	4	1	1	3	3	3	3	4	4
4	4	1	1	3	3	3	3	2	2
1	1	1	1	3	3	3	3	4	4
4	4	1	1	3	3	3	3	2	2
1	1	1	1	1	1	2	2	2	2
1	1	1	1	3	3	3	3	2	2
4	4	1	1	3	3	3	3	4	4
4	4	1	1	3	3	2	2	2	2
4	4	1	1	3	3	3	3	4	4
4	4	1	1	3	3	2	2	4	4
1	1	4	4	3	3	2	2	4	4
4	4	1	1	3	3	2	2	4	4
4	4	1	1	3	3	2	2	4	4
4	4	1	1	3	3	2	2	2	2
1	1	1	1	1	1	2	2	4	4
4	4	1	1	3	3	2	2	2	2
1	1	4	4	3	3	3	3	4	4
4	4	1	1	3	3	3	3	2	2
1	1	1	1	1	1	2	2	4	4
1	1	1	1	3	3	2	2	2	2
4	4	1	1	3	3	3	3	2	2
4	4	1	1	3	3	3	3	4	4
4	4	1	1	3	3	3	3	4	4
1	1	1	1	1	1	3	3	4	4
4	4	1	1	3	3	2	2	2	2
4	4	1	1	3	3	3	3	2	2
1	1	1	1	1	1	3	3	4	4
1	1	4	4	3	3	2	2	2	2
4	4	1	1	3	3	2	2	2	2
4	4	1	1	3	3	2	2	2	2
4	4	1	1	3	3	2	2	2	2
4	4	1	1	3	3	3	3	2	2
1	1	4	4	1	1	3	3	4	4
4	4	1	1	3	3	2	2	2	2
1	1	1	1	1	1	2	2	2	2
4	4	1	1	3	3	3	3	2	2
4	4	1	1	3	3	2	2	2	2

For Review Only

4	4	1	1	3	3	2	2	4	4
4	4	1	1	3	3	2	2	2	2
1	1	4	4	3	3	3	3	2	2
1	1	1	1	1	1	3	3	2	2
1	1	1	1	3	3	2	2	2	2
1	1	1	1	1	1	2	2	4	4
4	4	1	1	3	3	2	2	4	4
1	1	1	1	3	3	2	2	2	2
4	4	1	1	3	3	2	2	2	2
4	4	1	1	3	3	2	2	2	2
4	4	1	1	3	3	3	3	4	4
4	4	1	1	3	3	2	2	4	4
4	4	1	1	3	3	2	2	2	2
4	4	1	1	3	3	3	3	2	2
4	4	1	1	3	3	3	3	4	4
1	1	4	4	3	3	2	2	2	2
4	4	1	1	3	3	2	2	2	2
4	4	1	1	3	3	2	2	2	2
1	1	1	1	1	1	2	2	2	2
4	4	1	1	3	3	2	2	4	4
4	4	1	1	3	3	3	3	4	4
1	1	1	1	1	1	3	3	4	4
1	1	4	4	3	3	3	3	4	4
4	4	1	1	3	3	2	2	2	2
4	4	1	1	3	3	2	2	2	2
1	1	1	1	1	1	3	3	4	4
4	4	1	1	3	3	2	2	2	2
4	4	1	1	3	3	3	3	2	2
4	4	1	1	3	3	3	3	4	4
1	1	4	4	3	3	3	3	4	4
4	4	1	1	3	3	2	2	2	2
1	1	1	1	1	1	2	2	4	4
4	4	1	1	3	3	2	2	2	2
1	1	1	1	1	1	3	3	4	4
1	1	1	1	1	1	2	2	2	2
1	1	1	1	1	1	2	2	2	2
4	4	1	1	3	3	3	3	4	4
4	4	1	1	3	3	3	3	2	2
4	4	1	1	3	3	2	2	2	2
4	4	1	1	3	3	2	2	2	2
1	1	4	4	1	1	2	2	4	4
4	4	1	1	3	3	3	3	2	2
4	4	1	1	3	3	2	2	4	4
4	4	1	1	3	3	3	3	4	4
4	4	1	1	3	3	3	3	4	4
4	4	1	1	3	3	2	2	2	2
4	4	1	1	3	3	2	2	2	2
4	4	1	1	3	3	2	2	4	4
4	4	1	1	3	3	3	3	4	4
4	4	1	1	3	3	2	2	2	2
1	1	4	4	3	3	2	2	4	4
4	4	1	1	3	3	2	2	2	2
4	4	1	1	3	3	3	3	4	4
4	4	1	1	3	3	3	3	4	4
4	4	1	1	3	3	2	2	2	2

For Review Only

1	1	4	4	3	3	3	3	2	2
4	4	1	1	3	3	3	3	4	4

For Review Only

SNP2290	SNP2290	SNP2291	SNP2291	SNP2292	SNP2292	SNP2293	SNP2293	SNP2294	SNP2294
3	3	1	1	2	2	2	2	1	1
3	3	1	1	2	2	3	3	3	3
3	3	4	4	3	3	3	3	3	3
3	3	4	4	2	2	3	3	3	3
3	3	1	1	2	2	2	2	1	1
3	3	1	1	2	2	2	2	1	1
3	3	1	1	2	2	2	2	1	1
1	1	4	4	3	3	2	2	1	1
3	3	1	1	2	2	2	2	1	1
3	3	4	4	3	3	2	2	1	1
3	3	1	1	2	2	2	2	1	1
3	3	4	4	3	3	3	3	3	3
3	3	1	1	2	2	2	2	3	3
1	1	4	4	3	3	2	2	1	1
3	3	1	1	2	2	2	2	1	1
3	3	1	1	2	2	2	2	1	1
3	3	1	1	2	2	2	2	3	3
3	3	4	4	3	3	2	2	1	1
3	3	1	1	2	2	3	3	1	1
3	3	1	1	2	2	2	2	3	3
3	3	1	1	2	2	2	2	3	3
3	3	1	1	2	2	2	2	3	3
3	3	1	1	2	2	2	2	1	1
3	3	4	4	3	3	2	2	1	1
1	1	1	1	2	2	2	2	1	1
3	3	4	4	3	3	2	2	3	3
3	3	4	4	2	2	3	3	3	3
3	3	1	1	2	2	2	2	3	3
3	3	1	1	2	2	2	2	1	1
3	3	1	1	2	2	2	2	1	1
1	1	1	1	2	2	2	2	3	3
3	3	4	4	3	3	2	2	1	1
3	3	1	1	2	2	2	2	1	1
3	3	4	4	3	3	2	2	1	1
3	3	1	1	2	2	2	2	1	1
3	3	4	4	3	3	2	2	1	1
3	3	4	4	3	3	2	2	1	1
3	3	1	1	2	2	2	2	1	1
3	3	4	4	3	3	2	2	1	1
3	3	1	1	2	2	2	2	1	1
3	3	1	1	2	2	2	2	1	1
3	3	1	1	2	2	2	2	1	1
3	3	4	4	3	3	2	2	1	1
3	3	1	1	2	2	2	2	3	3
3	3	4	4	3	3	2	2	1	1
3	3	4	4	3	3	3	3	3	3
3	3	1	1	2	2	2	2	3	3
3	3	4	4	3	3	2	2	1	1
3	3	1	1	2	2	2	2	3	3
3	3	1	1	2	2	2	2	1	1
3	3	1	1	2	2	3	3	3	3
3	3	1	1	2	2	2	2	1	1

For Review Only

3	3	1	1	2	2	2	2	3	3
3	3	1	1	2	2	2	2	1	1
1	1	1	1	2	2	2	2	3	3
3	3	1	1	2	2	2	2	3	3
3	3	1	1	2	2	3	3	1	1
3	3	1	1	2	2	3	3	3	3
3	3	4	4	3	3	3	3	3	3
3	3	1	1	2	2	2	2	1	1
3	3	1	1	2	2	2	2	1	1
3	3	1	1	2	2	2	2	3	3
3	3	1	1	2	2	3	3	3	3
3	3	1	1	2	2	3	3	3	3
3	3	1	1	2	2	2	2	3	3
3	3	1	1	2	2	2	2	1	1
3	3	1	1	2	2	2	2	1	1
1	1	4	4	3	3	2	2	1	1
3	3	1	1	2	2	2	2	1	1
3	3	1	1	2	2	2	2	3	3
3	3	1	1	2	2	2	2	3	3
3	3	1	1	2	2	2	2	1	1
3	3	1	1	2	2	2	2	3	3
3	3	1	1	2	2	2	2	1	1
3	3	1	1	2	2	2	2	1	1
1	1	4	4	3	3	3	3	1	1
3	3	4	4	3	3	2	2	1	1
3	3	4	4	3	3	2	2	3	3
3	3	1	1	2	2	2	2	3	3
3	3	4	4	3	3	2	2	1	1
3	3	4	4	3	3	2	2	3	3
3	3	1	1	2	2	2	2	3	3
3	3	1	1	2	2	2	2	1	1
3	3	4	4	3	3	2	2	1	1
3	3	1	1	2	2	2	2	1	1
3	3	1	1	2	2	2	2	3	3
3	3	1	1	2	2	2	2	1	1
3	3	4	4	3	3	2	2	1	1
3	3	4	4	3	3	2	2	1	1
3	3	1	1	2	2	2	2	3	3
3	3	1	1	2	2	2	2	3	3
3	3	1	1	2	2	2	2	1	1
3	3	1	1	2	2	2	2	3	3
3	3	1	1	2	2	3	3	3	3
3	3	4	4	3	3	2	2	1	1
3	3	1	1	2	2	3	3	3	3
3	3	1	1	2	2	2	2	3	3
3	3	1	1	2	2	3	3	3	3
3	3	4	4	3	3	2	2	1	1
3	3	4	4	3	3	2	2	3	3
3	3	1	1	2	2	2	2	1	1
3	3	1	1	2	2	2	2	1	1
3	3	1	1	2	2	2	2	3	3
3	3	1	1	2	2	2	2	1	1
3	3	1	1	2	2	2	2	1	1
3	3	1	1	2	2	2	2	1	1
3	3	1	1	2	2	2	2	1	1

For Review Only

CSSA 5585 Guilford Rd., Madison, WI 53711

3	3	1	1	2	2	2	2	3	3
3	3	1	1	2	2	3	3	3	3
3	3	1	1	2	2	2	2	3	3
3	3	4	4	2	2	2	2	1	1
3	3	1	1	2	2	3	3	3	3
3	3	4	4	3	3	2	2	3	3
3	3	1	1	2	2	2	2	1	1
3	3	1	1	2	2	2	2	1	1
3	3	4	4	3	3	2	2	1	1
3	3	1	1	2	2	2	2	1	1
3	3	4	4	3	3	2	2	1	1
3	3	1	1	2	2	2	2	3	3
3	3	4	4	3	3	2	2	1	1
3	3	4	4	2	2	2	2	3	3
3	3	1	1	2	2	2	2	1	1
3	3	1	1	2	2	2	2	3	3
3	3	1	1	2	2	2	2	1	1
3	3	1	1	2	2	2	2	3	3
3	3	4	4	3	3	2	2	1	1
3	3	1	1	2	2	2	2	3	3
3	3	1	1	2	2	2	2	3	3
3	3	1	1	2	2	2	2	3	3
3	3	1	1	2	2	3	3	3	3
3	3	4	4	3	3	2	2	1	1
3	3	1	1	2	2	2	2	3	3
3	3	1	1	2	2	2	2	1	1
3	3	4	4	3	3	2	2	1	1
3	3	1	1	2	2	2	2	1	1
3	3	1	1	2	2	3	3	3	3
3	3	1	1	2	2	2	2	3	3
3	3	1	1	2	2	2	2	1	1
3	3	1	1	2	2	2	2	3	3
3	3	1	1	2	2	2	2	3	3
3	3	1	1	3	3	2	2	3	3
3	3	4	4	3	3	2	2	1	1
3	3	1	1	2	2	2	2	1	1
3	3	4	4	3	3	2	2	1	1
3	3	1	1	2	2	2	2	3	3
3	3	1	1	2	2	2	2	1	1
3	3	4	4	3	3	2	2	1	1
3	3	1	1	2	2	2	2	1	1
3	3	4	4	3	3	2	2	1	1
3	3	1	1	2	2	2	2	3	3
3	3	1	1	2	2	2	2	1	1
3	3	4	4	2	2	2	2	1	1
3	3	1	1	2	2	2	2	3	3
3	3	4	4	3	3	3	3	3	3
3	3	4	4	3	3	2	2	3	3
3	3	1	1	2	2	2	2	1	1
3	3	4	4	3	3	2	2	3	3
3	3	1	1	2	2	2	2	1	1
3	3	4	4	2	2	2	2	1	1
3	3	1	1	2	2	2	2	1	1
3	3	1	1	2	2	2	2	3	3
3	3	1	1	2	2	2	2	1	1

For Review Only

1	1	1	1	2	2	3	3	3	3
3	3	4	4	3	3	2	2	1	1
3	3	1	1	2	2	2	2	1	1
3	3	1	1	2	2	2	2	3	3
3	3	4	4	3	3	3	3	3	3
3	3	1	1	2	2	2	2	1	1
3	3	1	1	2	2	2	2	1	1
3	3	1	1	2	2	2	2	3	3
3	3	1	1	2	2	2	2	1	1
3	3	1	1	2	2	2	2	3	3
3	3	4	4	2	2	3	3	3	3
3	3	1	1	2	2	2	2	1	1
3	3	1	1	3	3	3	3	1	1
3	3	1	1	2	2	2	2	3	3
3	3	1	1	2	2	2	2	1	1
3	3	1	1	2	2	2	2	1	1
3	3	1	1	2	2	2	2	1	1
1	1	4	4	3	3	2	2	1	1
3	3	1	1	2	2	2	2	3	3
3	3	1	1	2	2	2	2	1	1
3	3	1	1	2	2	2	2	3	3
3	3	1	1	2	2	2	2	3	3
3	3	1	1	2	2	2	2	1	1
3	3	1	1	2	2	3	3	3	3
3	3	1	1	2	2	2	2	3	3
3	3	4	4	3	3	2	2	1	1
3	3	4	4	3	3	3	3	1	1
3	3	1	1	2	2	2	2	1	1
3	3	1	1	2	2	2	2	1	1
3	3	1	1	2	2	2	2	1	1
3	3	4	4	3	3	3	3	1	1
3	3	1	1	2	2	2	2	1	1
1	1	1	1	2	2	2	2	1	1
3	3	1	1	2	2	2	2	1	1
1	1	1	1	2	2	2	2	1	1
3	3	1	1	2	2	3	3	3	3
3	3	1	1	2	2	3	3	1	1
3	3	4	4	3	3	2	2	1	1
3	3	4	4	3	3	2	2	3	3
3	3	1	1	2	2	2	2	3	3
3	3	4	4	3	3	3	3	3	3
3	3	4	4	3	3	3	3	3	3
3	3	1	1	2	2	2	2	1	1
3	3	1	1	2	2	2	2	3	3
3	3	4	4	2	2	2	2	3	3
3	3	1	1	2	2	2	2	1	1
3	3	1	1	2	2	2	2	1	1
3	3	1	1	2	2	2	2	1	1
3	3	1	1	2	2	2	2	1	1
3	3	4	4	3	3	3	3	3	3
3	3	1	1	2	2	2	2	3	3
3	3	1	1	2	2	2	2	1	1
3	3	1	1	2	2	2	2	1	1
3	3	1	1	2	2	3	3	3	3
3	3	1	1	2	2	2	2	1	1

For Review Only

3	3	1	1	2	2	2	2	1	1
3	3	4	4	3	3	2	2	1	1
3	3	1	1	2	2	3	3	3	3
3	3	1	1	2	2	2	2	3	3
3	3	1	1	2	2	2	2	1	1
3	3	4	4	3	3	2	2	1	1
3	3	4	4	3	3	2	2	1	1
1	1	4	4	3	3	2	2	1	1
3	3	1	1	2	2	2	2	1	1
3	3	1	1	2	2	2	2	1	1
3	3	1	1	2	2	2	2	3	3
3	3	1	1	2	2	3	3	1	1
3	3	1	1	2	2	2	2	1	1
3	3	1	1	2	2	2	2	3	3
3	3	4	4	3	3	3	3	3	3
3	3	1	1	2	2	2	2	1	1
3	3	1	1	2	2	2	2	1	1
3	3	1	1	2	2	2	2	1	1
3	3	1	1	2	2	2	2	1	1
3	3	1	1	2	2	3	3	1	1
3	3	1	1	2	2	2	2	3	3
3	3	4	4	3	3	2	2	3	3
3	3	1	1	2	2	2	2	3	3
3	3	1	1	2	2	2	2	1	1
3	3	1	1	2	2	2	2	1	1
3	3	1	1	2	2	2	2	3	3
3	3	1	1	2	2	2	2	1	1
3	3	4	4	3	3	3	3	3	3
3	3	1	1	2	2	2	2	3	3
3	3	1	1	2	2	2	2	3	3
3	3	1	1	2	2	2	2	1	1
3	3	1	1	2	2	2	2	1	1
3	3	4	4	3	3	2	2	1	1
3	3	4	4	2	2	2	2	1	1
3	3	1	1	2	2	2	2	1	1
3	3	4	4	3	3	2	2	1	1
3	3	1	1	2	2	2	2	3	3
3	3	1	1	2	2	3	3	3	3
3	3	4	4	3	3	2	2	1	1
3	3	1	1	2	2	2	2	1	1
1	1	4	4	3	3	3	3	1	1
3	3	4	4	3	3	2	2	3	3
3	3	1	1	2	2	2	2	1	1
3	3	1	1	2	2	2	2	3	3
3	3	1	1	2	2	2	2	3	3
3	3	1	1	2	2	2	2	1	1
3	3	1	1	2	2	2	2	1	1
3	3	1	1	2	2	2	2	1	1
3	3	1	1	2	2	2	2	3	3
3	3	1	1	2	2	2	2	1	1
3	3	1	1	2	2	2	2	1	1
3	3	1	1	2	2	2	2	1	1
3	3	4	4	3	3	3	3	3	3
3	3	1	1	2	2	3	3	3	3
3	3	4	4	3	3	2	2	1	1

For Review Only

3	3	1	1	2	2	2	2	3	3
3	3	4	4	3	3	3	3	3	3

For Review Only

SNP2295	SNP2295	SNP2296	SNP2296	SNP2297	SNP2297	SNP2298	SNP2298	SNP2299	SNP2299
2	2	2	2	4	4	3	3	1	1
2	2	3	3	4	4	3	3	1	1
2	2	2	2	1	1	3	3	4	4
2	2	3	3	4	4	3	3	4	4
2	2	2	2	4	4	3	3	1	1
2	2	2	2	1	1	3	3	1	1
2	2	2	2	1	1	3	3	1	1
2	2	2	2	4	4	3	3	1	1
2	2	2	2	1	1	3	3	1	1
2	2	2	2	4	4	3	3	1	1
2	2	2	2	4	4	3	3	1	1
2	2	3	3	1	1	3	3	4	4
2	2	2	2	1	1	3	3	1	1
2	2	2	2	4	4	3	3	1	1
2	2	2	2	4	4	3	3	1	1
2	2	2	2	1	1	3	3	1	1
2	2	2	2	1	1	3	3	1	1
3	3	2	2	1	1	3	3	1	1
2	2	3	3	4	4	3	3	1	1
2	2	2	2	4	4	3	3	1	1
2	2	2	2	4	4	3	3	1	1
2	2	2	2	4	4	3	3	1	1
2	2	2	2	1	1	3	3	1	1
2	2	2	2	4	4	3	3	1	1
2	2	2	2	1	1	3	3	4	4
2	2	2	2	4	4	3	3	1	1
2	2	3	3	4	4	3	3	4	4
2	2	2	2	4	4	3	3	1	1
2	2	2	2	1	1	3	3	1	1
2	2	2	2	4	4	3	3	1	1
2	2	2	2	1	1	3	3	4	4
2	2	2	2	4	4	1	1	1	1
2	2	2	2	4	4	1	1	1	1
2	2	2	2	4	4	3	3	1	1
2	2	3	3	4	4	3	3	1	1
2	2	2	2	1	1	3	3	1	1
2	2	2	2	4	4	3	3	1	1
2	2	3	3	4	4	3	3	1	1
2	2	2	2	4	4	3	3	1	1
2	2	2	2	1	1	3	3	1	1
3	3	2	2	4	4	3	3	1	1
2	2	2	2	1	1	3	3	1	1
2	2	2	2	4	4	3	3	1	1
2	2	2	2	4	4	3	3	1	1
2	2	2	2	4	4	3	3	1	1
2	2	2	2	4	4	3	3	1	1
2	2	3	3	4	4	3	3	1	1
2	2	2	2	4	4	3	3	1	1
2	2	2	2	4	4	3	3	1	1
2	2	2	2	4	4	3	3	1	1
2	2	3	3	4	4	3	3	1	1
2	2	2	2	1	1	3	3	1	1

For Review Only

2	2	2	2	1	1	3	3	1	1
2	2	2	2	4	4	1	1	1	1
2	2	2	2	4	4	3	3	1	1
2	2	2	2	4	4	3	3	1	1
2	2	3	3	4	4	3	3	1	1
2	2	3	3	4	4	3	3	1	1
2	2	3	3	4	4	3	3	1	1
3	3	2	2	4	4	3	3	1	1
2	2	2	2	4	4	3	3	1	1
2	2	2	2	4	4	3	3	1	1
2	2	3	3	4	4	3	3	1	1
2	2	3	3	4	4	3	3	1	1
2	2	2	2	1	1	3	3	1	1
2	2	2	2	1	1	3	3	1	1
2	2	2	2	4	4	3	3	1	1
2	2	2	2	4	4	3	3	1	1
2	2	2	2	4	4	3	3	1	1
2	2	2	2	4	4	1	1	1	1
2	2	2	2	4	4	3	3	1	1
2	2	2	2	1	1	3	3	1	1
2	2	3	3	4	4	3	3	1	1
2	2	2	2	4	4	3	3	1	1
2	2	2	2	4	4	3	3	1	1
2	2	3	3	4	4	3	3	1	1
2	2	2	2	4	4	3	3	1	1
2	2	2	2	1	1	3	3	1	1
2	2	2	2	4	4	3	3	1	1
2	2	2	2	1	1	3	3	4	4
2	2	2	2	4	4	3	3	1	1
2	2	2	2	4	4	3	3	1	1
2	2	2	2	4	4	3	3	1	1
2	2	2	2	4	4	3	3	1	1
2	2	2	2	1	1	3	3	1	1
2	2	2	2	1	1	3	3	1	1
2	2	2	2	4	4	3	3	1	1
2	2	2	2	4	4	3	3	1	1
2	2	2	2	4	4	3	3	1	1
2	2	2	2	4	4	3	3	1	1
2	2	2	2	4	4	3	3	1	1
3	3	2	2	4	4	3	3	1	1
2	2	2	2	4	4	3	3	1	1
2	2	2	2	4	4	3	3	1	1
2	2	2	2	4	4	3	3	4	4
2	2	3	3	4	4	3	3	1	1
2	2	2	2	4	4	3	3	1	1
2	2	3	3	4	4	3	3	1	1
2	2	2	2	4	4	3	3	1	1
2	2	2	2	1	1	3	3	4	4
2	2	2	2	1	1	3	3	1	1
2	2	2	2	4	4	1	1	1	1
2	2	2	2	4	4	3	3	1	1
2	2	2	2	4	4	3	3	1	1
3	3	2	2	4	4	3	3	1	1
2	2	2	2	4	4	3	3	1	1
2	2	2	2	4	4	1	1	1	1

2	2	2	2	4	4	3	3	1	1
2	2	3	3	1	1	3	3	1	1
2	2	2	2	4	4	3	3	1	1
2	2	2	2	4	4	3	3	1	1
2	2	3	3	4	4	3	3	4	4
2	2	2	2	4	4	1	1	1	1
2	2	2	2	1	1	3	3	1	1
2	2	2	2	4	4	3	3	1	1
2	2	2	2	4	4	3	3	4	4
2	2	2	2	1	1	3	3	1	1
2	2	2	2	1	1	3	3	1	1
2	2	2	2	4	4	3	3	1	1
2	2	2	2	4	4	3	3	4	4
2	2	2	2	4	4	3	3	1	1
2	2	2	2	1	1	3	3	1	1
2	2	3	3	4	4	3	3	1	1
2	2	2	2	4	4	3	3	1	1
2	2	3	3	4	4	3	3	1	1
2	2	2	2	4	4	3	3	1	1
2	2	2	2	4	4	3	3	1	1
2	2	2	2	4	4	3	3	1	1
2	2	2	2	4	4	3	3	1	1
2	2	2	2	1	1	3	3	1	1
2	2	2	2	4	4	3	3	4	4
2	2	2	2	4	4	3	3	1	1
3	3	2	2	4	4	1	1	4	4
2	2	2	2	4	4	3	3	1	1
2	2	2	2	4	4	3	3	4	4
2	2	3	3	1	1	3	3	1	1
2	2	2	2	4	4	3	3	1	1
2	2	2	2	4	4	3	3	1	1
2	2	2	2	4	4	3	3	1	1
2	2	2	2	4	4	3	3	1	1
2	2	3	3	4	4	3	3	1	1
2	2	2	2	4	4	3	3	1	1
2	2	2	2	4	4	3	3	1	1
2	2	2	2	4	4	3	3	1	1
2	2	2	2	4	4	3	3	1	1
2	2	2	2	4	4	3	3	1	1
2	2	2	2	4	4	3	3	4	4
2	2	2	2	4	4	1	1	1	1
2	2	2	2	4	4	3	3	1	1
2	2	2	2	4	4	1	1	1	1
2	2	2	2	4	4	3	3	1	1
2	2	2	2	1	1	3	3	1	1
2	2	2	2	4	4	3	3	1	1
2	2	3	3	4	4	3	3	1	1
2	2	2	2	4	4	3	3	1	1
2	2	2	2	4	4	3	3	1	1
2	2	3	3	1	1	3	3	1	1
2	2	2	2	4	4	3	3	1	1
2	2	2	2	4	4	3	3	1	1
2	2	2	2	4	4	3	3	1	1
2	2	2	2	4	4	1	1	1	1
2	2	2	2	4	4	3	3	1	1

For Review Only

2	2	3	3	1	1	3	3	1	1
2	2	2	2	4	4	1	1	1	1
3	3	2	2	4	4	3	3	1	1
2	2	3	3	4	4	3	3	1	1
2	2	3	3	4	4	3	3	1	1
2	2	2	2	4	4	3	3	4	4
2	2	2	2	1	1	3	3	4	4
2	2	2	2	4	4	3	3	1	1
2	2	2	2	4	4	3	3	1	1
2	2	2	2	4	4	3	3	1	1
2	2	3	3	4	4	3	3	1	1
2	2	2	2	4	4	3	3	1	1
2	2	3	3	4	4	3	3	1	1
2	2	2	2	4	4	3	3	1	1
2	2	2	2	4	4	3	3	1	1
2	2	2	2	4	4	3	3	1	1
2	2	2	2	4	4	3	3	1	1
2	2	2	2	4	4	3	3	1	1
2	2	2	2	1	1	3	3	1	1
2	2	2	2	1	1	3	3	1	1
2	2	2	2	4	4	3	3	1	1
2	2	2	2	1	1	3	3	1	1
2	2	2	2	4	4	1	1	1	1
2	2	3	3	4	4	3	3	1	1
2	2	2	2	1	1	3	3	1	1
2	2	2	2	4	4	3	3	1	1
2	2	3	3	4	4	3	3	4	4
2	2	2	2	4	4	3	3	1	1
2	2	2	2	4	4	3	3	1	1
2	2	2	2	4	4	3	3	1	1
3	3	3	3	4	4	3	3	1	1
2	2	2	2	4	4	3	3	1	1
2	2	2	2	4	4	3	3	1	1
2	2	2	2	4	4	3	3	1	1
2	2	3	3	4	4	3	3	1	1
2	2	3	3	4	4	3	3	1	1
2	2	3	3	4	4	3	3	4	4
2	2	2	2	4	4	3	3	1	1
2	2	2	2	4	4	3	3	1	1
2	2	2	2	4	4	3	3	1	1
2	2	2	2	1	1	3	3	4	4
2	2	3	3	4	4	3	3	1	1
2	2	2	2	4	4	3	3	4	4
2	2	2	2	4	4	3	3	1	1
2	2	2	2	4	4	3	3	1	1
2	2	2	2	4	4	3	3	1	1
2	2	2	2	4	4	3	3	1	1
2	2	2	2	1	1	3	3	4	4
2	2	2	2	4	4	3	3	1	1
2	2	3	3	4	4	3	3	4	4
2	2	2	2	1	1	3	3	1	1
3	3	2	2	4	4	3	3	1	1
2	2	2	2	4	4	1	1	1	1
2	2	3	3	4	4	3	3	1	1
2	2	2	2	4	4	3	3	1	1

For Review Only

2	2	2	2	4	4	3	3	1	1
2	2	2	2	4	4	3	3	1	1
2	2	2	2	1	1	3	3	1	1
2	2	2	2	4	4	1	1	1	1
2	2	2	2	4	4	3	3	1	1
2	2	2	2	4	4	3	3	1	1
2	2	2	2	4	4	3	3	4	4
2	2	2	2	4	4	3	3	1	1
2	2	2	2	4	4	3	3	1	1
2	2	2	2	1	1	3	3	4	4
2	2	2	2	4	4	3	3	1	1
3	3	3	3	4	4	3	3	1	1
3	3	2	2	4	4	3	3	1	1
2	2	2	2	1	1	3	3	1	1
2	2	2	2	1	1	3	3	4	4
3	3	2	2	4	4	3	3	1	1
2	2	2	2	4	4	3	3	1	1
2	2	2	2	1	1	3	3	4	4
2	2	2	2	1	1	3	3	1	1
2	2	3	3	4	4	3	3	1	1
2	2	2	2	4	4	3	3	1	1
2	2	2	2	4	4	3	3	1	1
2	2	2	2	1	1	3	3	1	1
2	2	2	2	4	4	1	1	1	1
3	3	2	2	4	4	3	3	1	1
2	2	2	2	1	1	3	3	1	1
2	2	2	2	1	1	3	3	4	4
2	2	3	3	1	1	3	3	4	4
2	2	2	2	4	4	3	3	1	1
2	2	2	2	1	1	3	3	1	1
2	2	2	2	1	1	3	3	1	1
2	2	2	2	4	4	3	3	1	1
2	2	2	2	4	4	3	3	1	1
2	2	2	2	4	4	3	3	1	1
2	2	2	2	4	4	1	1	1	1
2	2	2	2	1	1	3	3	4	4
2	2	2	2	4	4	3	3	1	1
2	2	3	3	4	4	3	3	1	1
2	2	2	2	1	1	3	3	1	1
2	2	2	2	4	4	3	3	4	4
2	2	3	3	4	4	3	3	1	1
2	2	2	2	4	4	3	3	1	1
2	2	2	2	4	4	1	1	1	1
2	2	2	2	4	4	3	3	1	1
2	2	2	2	4	4	3	3	4	4
3	3	2	2	4	4	3	3	1	1
2	2	2	2	4	4	3	3	4	4
2	2	2	2	4	4	1	1	1	1
2	2	2	2	4	4	3	3	1	1
2	2	2	2	1	1	3	3	4	4
2	2	2	2	4	4	3	3	1	1
2	2	2	2	4	4	3	3	1	1
2	2	2	2	1	1	3	3	4	4
2	2	3	3	1	1	3	3	1	1
2	2	2	2	4	4	1	1	1	1

For Review Only

2	2	2	2	4	4	3	3	1	1
2	2	2	2	4	4	3	3	1	1

For Review Only

SNP230	SNP230	SNP2300	SNP2300	SNP2301	SNP2301	SNP2302	SNP2302	SNP2303	SNP2303
2	2	3	3	2	2	4	4	4	4
3	3	3	3	2	2	1	1	4	4
2	2	3	3	2	2	4	4	1	1
2	2	3	3	2	2	1	1	4	4
3	3	3	3	2	2	4	4	4	4
2	2	3	3	3	3	4	4	4	4
2	2	3	3	2	2	4	4	1	1
2	2	3	3	2	2	4	4	4	4
3	3	3	3	2	2	4	4	1	1
3	3	3	3	2	2	4	4	4	4
2	2	4	4	2	2	4	4	4	4
2	2	3	3	2	2	4	4	4	4
3	3	3	3	2	2	4	4	1	1
3	3	3	3	3	3	4	4	4	4
2	2	3	3	2	2	4	4	4	4
3	3	3	3	3	3	4	4	4	4
2	2	3	3	2	2	4	4	1	1
2	2	3	3	2	2	4	4	4	4
2	2	4	4	2	2	4	4	4	4
2	2	3	3	2	2	4	4	4	4
3	3	3	3	2	2	4	4	4	4
3	3	3	3	2	2	4	4	4	4
3	3	3	3	3	3	4	4	1	1
3	3	3	3	2	2	4	4	4	4
3	3	3	3	2	2	4	4	1	1
2	2	3	3	3	3	4	4	4	4
3	3	3	3	2	2	1	1	4	4
2	2	3	3	2	2	4	4	4	4
3	3	3	3	2	2	4	4	1	1
3	3	3	3	2	2	4	4	4	4
3	3	3	3	3	3	4	4	4	4
2	2	3	3	2	2	4	4	4	4
2	2	3	3	3	3	4	4	4	4
3	3	3	3	2	2	4	4	4	4
2	2	3	3	2	2	4	4	4	4
3	3	3	3	2	2	4	4	1	1
3	3	3	3	2	2	4	4	4	4
2	2	3	3	2	2	1	1	4	4
3	3	3	3	2	2	4	4	4	4
2	2	3	3	2	2	4	4	1	1
3	3	3	3	2	2	4	4	4	4
3	3	3	3	2	2	4	4	1	1
2	2	3	3	2	2	4	4	4	4
3	3	3	3	2	2	4	4	4	4
3	3	3	3	2	2	4	4	4	4
2	2	3	3	2	2	4	4	4	4
2	2	3	3	2	2	1	1	4	4
2	2	3	3	3	3	4	4	4	4
3	3	3	3	2	2	4	4	4	4
2	2	3	3	2	2	4	4	4	4
2	2	3	3	3	3	1	1	4	4
2	2	3	3	2	2	4	4	4	4

For Review Only

3	3	3	3	2	2	4	4	1	1
2	2	3	3	3	3	4	4	4	4
3	3	3	3	3	3	4	4	4	4
3	3	3	3	2	2	4	4	4	4
2	2	4	4	2	2	4	4	4	4
2	2	3	3	2	2	1	1	4	4
2	2	3	3	3	3	1	1	4	4
2	2	3	3	2	2	4	4	4	4
3	3	3	3	3	3	4	4	4	4
2	2	3	3	2	2	4	4	4	4
3	3	4	4	2	2	1	1	4	4
3	3	4	4	2	2	1	1	4	4
3	3	3	3	2	2	4	4	1	1
3	3	3	3	2	2	4	4	1	1
2	2	3	3	2	2	4	4	4	4
3	3	3	3	2	2	4	4	4	4
3	3	4	4	2	2	4	4	4	4
2	2	3	3	2	2	4	4	4	4
3	3	3	3	2	2	4	4	4	4
2	2	3	3	2	2	4	4	1	1
3	3	4	4	2	2	1	1	4	4
2	2	3	3	3	3	4	4	4	4
2	2	3	3	2	2	4	4	4	4
3	3	3	3	2	2	4	4	4	4
3	3	3	3	2	2	4	4	4	4
3	3	3	3	2	2	4	4	4	4
2	2	3	3	2	2	4	4	4	4
3	3	3	3	2	2	4	4	1	1
2	2	3	3	3	3	4	4	4	4
2	2	3	3	3	3	4	4	4	4
2	2	3	3	2	2	4	4	4	4
3	3	3	3	2	2	4	4	4	4
3	3	3	3	3	3	4	4	1	1
3	3	3	3	2	2	4	4	1	1
2	2	3	3	2	2	4	4	4	4
3	3	3	3	2	2	4	4	4	4
2	2	3	3	2	2	4	4	4	4
3	3	3	3	2	2	4	4	4	4
2	2	4	4	2	2	4	4	4	4
3	3	3	3	2	2	4	4	4	4
2	2	3	3	2	2	4	4	4	4
2	2	3	3	2	2	4	4	4	4
2	2	3	3	2	2	4	4	4	4
3	3	4	4	2	2	1	1	4	4
3	3	3	3	2	2	4	4	4	4
2	2	3	3	2	2	1	1	4	4
3	3	3	3	2	2	4	4	4	4
3	3	3	3	3	3	4	4	1	1
2	2	3	3	2	2	4	4	4	4
2	2	3	3	3	3	4	4	4	4
3	3	3	3	2	2	4	4	4	4
3	3	3	3	3	3	4	4	4	4
3	3	4	4	2	2	4	4	4	4
2	2	3	3	3	3	4	4	4	4
3	3	3	3	3	3	4	4	4	4

For Review Only

2	2	3	3	3	3	4	4	4	4
2	2	3	3	2	2	1	1	4	4
3	3	3	3	3	3	4	4	4	4
3	3	3	3	3	3	4	4	4	4
2	2	3	3	2	2	1	1	4	4
2	2	3	3	2	2	4	4	4	4
3	3	3	3	2	2	4	4	4	4
2	2	3	3	2	2	4	4	4	4
3	3	3	3	2	2	4	4	4	4
3	3	3	3	2	2	1	1	1	1
2	2	3	3	3	3	4	4	1	1
2	2	3	3	2	2	4	4	4	4
3	3	3	3	2	2	4	4	4	4
2	2	3	3	2	2	4	4	4	4
3	3	3	3	2	2	4	4	4	4
2	2	3	3	3	3	1	1	4	4
2	2	3	3	2	2	4	4	4	4
2	2	3	3	2	2	4	4	4	4
3	3	3	3	3	3	4	4	4	4
2	2	3	3	2	2	4	4	4	4
2	2	3	3	2	2	4	4	4	4
2	2	3	3	3	3	4	4	4	4
3	3	3	3	2	2	4	4	4	4
3	3	4	4	3	3	4	4	4	4
2	2	3	3	3	3	4	4	4	4
2	2	3	3	2	2	4	4	4	4
2	2	3	3	2	2	4	4	4	4
3	3	3	3	2	2	4	4	4	4
2	2	3	3	2	2	1	1	4	4
3	3	3	3	2	2	4	4	4	4
2	2	3	3	3	3	4	4	4	4
2	2	3	3	2	2	4	4	4	4
2	2	3	3	2	2	4	4	4	4
2	2	3	3	2	2	4	4	4	4
3	3	3	3	2	2	4	4	4	4
3	3	3	3	2	2	4	4	4	4
3	3	3	3	3	3	4	4	4	4
3	3	3	3	2	2	4	4	4	4
2	2	3	3	3	3	4	4	4	4
2	2	3	3	3	3	4	4	4	4
2	2	3	3	2	2	4	4	4	4
3	3	3	3	3	3	4	4	4	4
3	3	3	3	2	2	4	4	4	4
3	3	3	3	2	2	4	4	4	4
3	3	3	3	3	3	4	4	4	4
3	3	3	3	2	2	4	4	4	4
2	2	3	3	2	2	1	1	4	4
2	2	3	3	3	3	4	4	4	4
3	3	3	3	2	2	4	4	4	4
2	2	3	3	3	3	4	4	4	4
2	2	3	3	3	3	4	4	4	4
2	2	3	3	3	3	4	4	4	4
2	2	3	3	2	2	4	4	4	4
3	3	3	3	3	3	4	4	4	4
2	2	3	3	2	2	4	4	4	4

For Review Only

3	3	3	3	3	3	1	1	4	4
2	2	3	3	2	2	4	4	4	4
3	3	3	3	2	2	4	4	4	4
2	2	3	3	2	2	4	4	4	4
2	2	3	3	2	2	1	1	4	4
2	2	3	3	2	2	4	4	4	4
2	2	3	3	3	3	4	4	4	4
2	2	3	3	2	2	4	4	4	4
3	3	3	3	2	2	4	4	4	4
2	2	3	3	2	2	4	4	4	4
2	2	3	3	3	3	1	1	4	4
2	2	3	3	2	2	4	4	4	4
2	2	3	3	2	2	1	1	4	4
3	3	3	3	2	2	4	4	4	4
2	2	3	3	2	2	4	4	4	4
2	2	4	4	3	3	4	4	4	4
3	3	3	3	2	2	4	4	4	4
2	2	3	3	3	3	4	4	4	4
3	3	3	3	2	2	4	4	4	4
3	3	3	3	2	2	4	4	4	4
2	2	3	3	2	2	4	4	4	4
3	3	3	3	3	3	4	4	1	1
2	2	3	3	3	3	4	4	4	4
3	3	4	4	2	2	1	1	4	4
3	3	3	3	2	2	4	4	1	1
3	3	3	3	2	2	4	4	4	4
2	2	3	3	3	3	1	1	4	4
2	2	3	3	2	2	4	4	4	4
3	3	3	3	2	2	4	4	4	4
3	3	3	3	2	2	4	4	4	4
3	3	3	3	3	3	4	4	4	4
2	2	3	3	3	3	4	4	4	4
2	2	3	3	3	3	4	4	4	4
2	2	3	3	2	2	4	4	4	4
2	2	3	3	2	2	4	4	4	4
3	3	4	4	2	2	1	1	4	4
2	2	3	3	3	3	1	1	4	4
3	3	3	3	2	2	4	4	4	4
2	2	3	3	2	2	4	4	4	4
3	3	4	4	2	2	4	4	4	4
2	2	3	3	2	2	4	4	4	4
2	2	3	3	3	3	1	1	4	4
3	3	3	3	3	3	4	4	4	4
2	2	3	3	2	2	4	4	4	4
2	2	3	3	2	2	4	4	4	4
3	3	3	3	2	2	4	4	4	4
2	2	4	4	3	3	4	4	4	4
2	2	3	3	3	3	4	4	1	1
3	3	4	4	2	2	4	4	4	4
3	3	3	3	2	2	1	1	4	4
2	2	3	3	2	2	4	4	4	4
3	3	4	4	3	3	4	4	4	4
3	3	3	3	3	3	4	4	4	4
3	3	4	4	2	2	1	1	4	4
3	3	3	3	3	3	4	4	4	4

For Review Only

3	3	3	3	2	2	4	4	4	4
2	2	3	3	2	2	4	4	4	4
2	2	3	3	2	2	4	4	1	1
2	2	3	3	2	2	4	4	4	4
2	2	3	3	2	2	4	4	4	4
2	2	3	3	2	2	4	4	4	4
2	2	3	3	3	3	4	4	4	4
2	2	3	3	2	2	4	4	4	4
3	3	3	3	2	2	4	4	4	4
3	3	3	3	3	3	4	4	1	1
2	2	3	3	2	2	4	4	4	4
2	2	3	3	3	3	4	4	4	4
2	2	4	4	2	2	4	4	4	4
3	3	3	3	2	2	4	4	4	4
2	2	3	3	2	2	4	4	4	4
2	2	4	4	2	2	4	4	4	4
3	3	3	3	3	3	4	4	4	4
3	3	3	3	2	2	4	4	4	4
3	3	3	3	3	3	4	4	1	1
2	2	4	4	2	2	1	1	4	4
3	3	3	3	2	2	4	4	4	4
2	2	3	3	3	3	4	4	4	4
3	3	3	3	2	2	4	4	4	4
3	3	3	3	3	3	4	4	4	4
3	3	3	3	2	2	4	4	4	4
3	3	3	3	2	2	4	4	4	4
3	3	3	3	3	3	4	4	1	1
2	2	3	3	2	2	1	1	4	4
2	2	3	3	2	2	4	4	4	4
2	2	3	3	2	2	4	4	4	4
3	3	3	3	3	3	4	4	1	1
2	2	3	3	3	3	4	4	4	4
3	3	4	4	2	2	4	4	4	4
2	2	3	3	3	3	4	4	4	4
2	2	3	3	3	3	4	4	4	4
3	3	3	3	3	3	4	4	1	1
3	3	3	3	2	2	4	4	4	4
3	3	4	4	2	2	1	1	4	4
3	3	3	3	2	2	4	4	1	1
3	3	3	3	2	2	4	4	4	4
2	2	3	3	2	2	4	4	4	4
3	3	3	3	2	2	4	4	4	4
2	2	3	3	3	3	4	4	4	4
3	3	3	3	2	2	4	4	4	4
3	3	3	3	2	2	4	4	4	4
2	2	4	4	2	2	4	4	4	4
2	2	3	3	3	3	4	4	4	4
3	3	3	3	2	2	4	4	4	4
3	3	3	3	2	2	4	4	4	4
3	3	3	3	3	3	4	4	1	1
2	2	3	3	2	2	4	4	4	4
3	3	3	3	2	2	4	4	4	4
2	2	3	3	2	2	4	4	1	1
2	2	3	3	2	2	1	1	4	4
2	2	3	3	2	2	4	4	4	4

For Review Only

2	2	4	4	2	2	4	4	4	4
3	3	3	3	2	2	4	4	4	4

For Review Only

SNP2304	SNP2304	SNP2305	SNP2305	SNP2306	SNP2306	SNP2307	SNP2307	SNP2308	SNP2308
1	1	4	4	2	2	2	2	3	3
1	1	1	1	3	3	3	3	3	3
1	1	1	1	3	3	3	3	3	3
1	1	1	1	3	3	3	3	3	3
1	1	4	4	2	2	2	2	2	2
4	4	1	1	2	2	2	2	2	2
1	1	4	4	3	3	3	3	3	3
1	1	4	4	2	2	2	2	2	2
1	1	4	4	3	3	3	3	3	3
1	1	4	4	2	2	2	2	2	2
1	1	4	4	2	2	2	2	2	2
1	1	1	1	3	3	3	3	3	3
1	1	1	1	3	3	3	3	3	3
4	4	4	4	2	2	2	2	2	2
1	1	1	1	3	3	3	3	3	3
4	4	1	1	2	2	2	2	2	2
1	1	4	4	3	3	3	3	3	3
1	1	1	1	2	2	2	2	2	2
1	1	1	1	2	2	2	2	2	2
1	1	1	1	3	3	3	3	3	3
1	1	4	4	3	3	3	3	3	3
4	4	4	4	3	3	3	3	3	3
1	1	4	4	2	2	2	2	2	2
4	4	1	1	2	2	2	2	2	2
1	1	4	4	2	2	3	3	3	3
1	1	1	1	3	3	3	3	3	3
1	1	1	1	3	3	3	3	3	3
1	1	1	1	3	3	3	3	3	3
1	1	4	4	3	3	3	3	3	3
4	4	4	4	2	2	2	2	2	2
4	4	1	1	2	2	2	2	2	2
1	1	1	1	2	2	2	2	2	2
4	4	4	4	2	2	2	2	2	2
1	1	1	1	2	2	2	2	2	2
1	1	1	1	3	3	3	3	3	3
4	4	1	1	2	2	2	2	2	2
1	1	1	1	2	2	2	2	2	2
1	1	1	1	2	2	2	2	2	2
1	1	1	1	2	2	2	2	2	2
1	1	4	4	2	2	2	2	2	2
1	1	4	4	2	2	2	2	2	2
1	1	4	4	3	3	3	3	3	3
1	1	4	4	2	2	2	2	2	2
1	1	4	4	3	3	3	3	3	3
1	1	1	1	2	2	2	2	2	2
1	1	1	1	3	3	3	3	3	3
1	1	1	1	3	3	3	3	3	3
1	1	1	1	2	2	3	3	2	2
1	1	4	4	3	3	3	3	3	3
1	1	4	4	3	3	3	3	2	2
1	1	1	1	3	3	3	3	3	3
1	1	1	1	2	2	2	2	2	2

For Review Only

1	1	4	4	3	3	3	3	3	3
4	4	4	4	2	2	2	2	2	2
4	4	1	1	3	3	3	3	3	3
1	1	4	4	3	3	3	3	3	3
1	1	1	1	2	2	2	2	2	2
1	1	1	1	3	3	3	3	3	3
1	1	1	1	3	3	3	3	3	3
1	1	4	4	2	2	2	2	2	2
4	4	1	1	2	2	2	2	2	2
1	1	1	1	3	3	3	3	3	3
1	1	1	1	3	3	3	3	3	3
1	1	1	1	3	3	3	3	3	3
1	1	4	4	3	3	3	3	3	3
1	1	4	4	3	3	3	3	3	3
1	1	1	1	2	2	2	2	3	3
1	1	1	1	2	2	2	2	2	2
1	1	1	1	2	2	3	3	3	3
1	1	1	1	3	3	3	3	3	3
1	1	4	4	3	3	3	3	3	3
1	1	4	4	3	3	3	3	3	3
1	1	1	1	3	3	3	3	3	3
1	1	1	1	2	2	2	2	2	2
1	1	4	4	3	3	3	3	3	3
1	1	1	1	2	2	2	2	2	2
1	1	1	1	2	2	2	2	2	2
1	1	1	1	3	3	3	3	3	3
1	1	1	1	3	3	3	3	3	3
1	1	1	1	2	2	2	2	2	2
1	1	1	1	3	3	3	3	3	3
1	1	4	4	3	3	3	3	3	3
4	4	4	4	2	2	2	2	2	2
4	4	1	1	2	2	2	2	2	2
1	1	4	4	2	2	2	2	2	2
1	1	4	4	3	3	3	3	3	3
1	1	1	1	2	2	2	2	2	2
1	1	1	1	2	2	2	2	2	2
1	1	4	4	2	2	2	2	2	2
1	1	4	4	3	3	3	3	3	3
1	1	4	4	2	2	2	2	2	2
1	1	1	1	2	2	2	2	2	2
1	1	4	4	3	3	3	3	3	3
1	1	4	4	3	3	3	3	3	3
1	1	1	1	2	2	2	2	2	2
1	1	1	1	3	3	3	3	3	3
1	1	4	4	3	3	2	2	2	2
1	1	1	1	3	3	3	3	3	3
1	1	1	1	2	2	2	2	2	2
1	1	1	1	3	3	3	3	3	3
1	1	1	1	2	2	2	2	2	2
1	1	1	1	2	2	3	3	3	3
1	1	1	1	3	3	3	3	3	3
1	1	1	1	2	2	2	2	2	2
1	1	4	4	2	2	2	2	2	2
1	1	1	1	2	2	3	3	3	3
4	4	1	1	2	2	3	3	3	3

For Review Only

1	1	1	1	3	3	3	3	3	3
1	1	1	1	3	3	3	3	3	3
1	1	1	1	3	3	3	3	3	3
4	4	1	1	2	2	2	2	2	2
1	1	1	1	3	3	3	3	3	3
1	1	1	1	3	3	3	3	3	3
1	1	1	1	3	3	3	3	3	3
1	1	1	1	2	2	3	3	2	2
1	1	1	1	2	2	2	2	2	2
1	1	1	1	2	2	2	2	2	2
1	1	1	1	2	2	3	3	2	2
1	1	1	1	3	3	3	3	3	3
1	1	1	1	2	2	2	2	2	2
1	1	1	1	3	3	3	3	3	3
1	1	1	1	2	2	2	2	2	2
1	1	1	1	3	3	3	3	3	3
1	1	1	1	2	2	3	3	3	3
1	1	1	1	3	3	3	3	3	3
4	4	1	1	2	2	2	2	2	2
1	1	4	4	3	3	3	3	3	3
1	1	1	1	3	3	3	3	3	3
1	1	4	4	3	3	3	3	3	3
1	1	1	1	3	3	3	3	3	3
1	1	1	1	2	2	2	2	2	2
1	1	1	1	3	3	3	3	3	3
1	1	4	4	2	2	2	2	2	2
4	4	1	1	2	2	2	2	2	2
1	1	1	1	2	2	2	2	2	2
1	1	1	1	3	3	3	3	3	3
1	1	1	1	3	3	3	3	3	3
1	1	1	1	2	2	2	2	2	2
1	1	1	1	2	2	3	3	3	3
1	1	4	4	3	3	3	3	3	3
4	4	1	1	3	3	3	3	3	3
1	1	1	1	2	2	2	2	2	2
1	1	1	1	2	2	3	3	3	3
4	4	1	1	2	2	2	2	2	2
1	1	1	1	3	3	3	3	3	3
1	1	1	1	2	2	3	3	3	3
1	1	4	4	2	2	2	2	2	2
1	1	1	1	2	2	2	2	2	2
4	4	1	1	2	2	2	2	2	2
1	1	1	1	3	3	3	3	3	3
1	1	1	1	2	2	2	2	2	2
1	1	1	1	3	3	3	3	3	3
1	1	4	4	3	3	3	3	3	3
1	1	1	1	3	3	3	3	3	3
1	1	1	1	3	3	3	3	3	3
4	4	1	1	2	2	2	2	2	2
1	1	1	1	3	3	3	3	3	3
1	1	1	1	2	2	2	2	2	2
1	1	4	4	2	2	2	2	2	2
1	1	1	1	2	2	2	2	2	2
1	1	1	1	3	3	3	3	3	3
1	1	1	1	2	2	2	2	3	3

For Review Only

4	4	1	1	3	3	3	3	3	3
1	1	4	4	2	2	2	2	2	2
1	1	1	1	2	2	2	2	2	2
1	1	1	1	3	3	3	3	3	3
1	1	1	1	3	3	3	3	3	3
1	1	4	4	2	2	2	2	2	2
1	1	4	4	2	2	2	2	2	2
1	1	1	1	3	3	3	3	3	3
1	1	1	1	2	2	2	2	2	2
1	1	1	1	3	3	3	3	3	3
1	1	1	1	3	3	3	3	3	3
1	1	1	1	2	2	2	2	2	2
1	1	1	1	3	3	3	3	3	3
1	1	4	4	3	3	3	3	3	3
1	1	1	1	2	2	2	2	2	2
1	1	4	4	2	2	2	2	2	2
1	1	1	1	2	2	2	2	2	2
1	1	1	1	2	2	2	2	2	2
4	4	1	1	3	3	3	3	3	3
1	1	1	1	3	3	3	3	3	3
1	1	1	1	3	3	3	3	3	3
1	1	1	1	3	3	3	3	2	2
1	1	1	1	2	2	2	2	2	2
1	1	1	1	3	3	3	3	3	3
1	1	4	4	3	3	3	3	3	3
1	1	1	1	2	2	2	2	2	2
4	4	1	1	3	3	3	3	3	3
1	1	1	1	2	2	3	3	3	3
1	1	4	4	2	2	3	3	3	3
1	1	4	4	2	2	3	3	3	3
4	4	1	1	2	2	2	2	2	2
1	1	4	4	2	2	3	3	3	3
4	4	1	1	2	2	2	2	2	2
1	1	1	1	2	2	2	2	2	2
1	1	1	1	2	2	2	2	2	2
1	1	1	1	3	3	3	3	3	3
1	1	1	1	2	2	2	2	2	2
1	1	1	1	2	2	2	2	2	2
1	1	1	1	3	3	3	3	3	3
1	1	1	1	3	3	3	3	3	3
1	1	1	1	3	3	3	3	3	3
1	1	1	1	3	3	3	3	3	3
1	1	1	1	2	2	2	2	2	2
1	1	1	1	2	2	2	2	2	2
1	1	1	1	3	3	3	3	3	3
1	1	4	4	2	2	3	3	3	3
1	1	4	4	2	2	2	2	2	2
1	1	4	4	2	2	2	2	2	2
1	1	1	1	2	2	2	2	2	2
1	1	4	4	3	3	3	3	3	3
1	1	1	1	3	3	3	3	3	3
4	4	4	4	2	2	2	2	2	2
1	1	4	4	2	2	2	2	2	2
1	1	1	1	3	3	3	3	3	3
1	1	4	4	2	2	2	2	2	2

1	1	1	1	2	2	2	2	2	2
1	1	1	1	2	2	2	2	2	2
1	1	1	1	3	3	3	3	3	3
1	1	1	1	3	3	3	3	3	3
1	1	1	1	2	2	2	2	2	2
1	1	1	1	2	2	2	2	3	3
1	1	1	1	2	2	2	2	2	2
1	1	1	1	2	2	2	2	2	2
1	1	1	1	2	2	2	2	2	2
1	1	1	1	2	2	2	2	2	2
1	1	1	1	3	3	3	3	3	3
4	4	1	1	2	2	2	2	2	2
1	1	4	4	2	2	2	2	2	2
1	1	1	1	3	3	3	3	3	3
1	1	1	1	3	3	3	3	3	3
1	1	4	4	2	2	2	2	2	2
4	4	1	1	2	2	2	2	2	2
1	1	1	1	2	2	2	2	2	2
1	1	4	4	2	2	2	2	2	2
1	1	1	1	2	2	2	2	2	2
1	1	4	4	3	3	3	3	3	3
1	1	1	1	3	3	3	3	3	3
1	1	1	1	3	3	2	2	2	2
1	1	4	4	2	2	2	2	2	2
1	1	4	4	2	2	2	2	2	2
1	1	1	1	3	3	3	3	3	3
1	1	1	1	2	2	2	2	2	2
1	1	1	1	3	3	3	3	3	3
1	1	1	1	3	3	3	3	3	3
1	1	1	1	3	3	3	3	3	3
1	1	1	1	2	2	2	2	2	2
1	1	1	1	2	2	2	2	2	2
1	1	4	4	2	2	2	2	2	2
1	1	1	1	2	2	2	2	3	3
1	1	4	4	2	2	2	2	2	2
1	1	1	1	2	2	2	2	2	2
1	1	1	1	3	3	3	3	3	3
1	1	1	1	3	3	3	3	3	3
4	4	1	1	2	2	2	2	2	2
1	1	4	4	2	2	2	2	2	2
1	1	1	1	2	2	3	3	3	3
1	1	1	1	3	3	3	3	3	3
4	4	4	4	2	2	2	2	2	2
1	1	4	4	3	3	3	3	3	3
1	1	1	1	3	3	3	3	3	3
1	1	1	1	2	2	2	2	2	2
1	1	1	1	2	2	3	3	2	2
1	1	4	4	2	2	3	3	3	3
1	1	1	1	3	3	3	3	3	3
1	1	1	1	2	2	2	2	2	2
1	1	1	1	2	2	2	2	2	2
1	1	4	4	2	2	2	2	2	2
1	1	1	1	3	3	3	3	3	3
1	1	1	1	3	3	3	3	3	3
1	1	4	4	2	2	2	2	2	2

For Review Only

1	1	1	1	2	2	2	2	2	2
1	1	1	1	3	3	3	3	3	3

For Review Only

SNP2309	SNP2309	SNP231	SNP231	SNP2310	SNP2310	SNP2311	SNP2311	SNP2312	SNP2312
1	1	2	2	3	3	2	2	1	1
3	3	3	3	3	3	4	4	3	3
3	3	3	3	3	3	4	4	3	3
3	3	2	2	3	3	4	4	3	3
1	1	3	3	2	2	2	2	1	1
3	3	3	3	3	3	4	4	3	3
3	3	3	3	3	3	4	4	1	1
1	1	3	3	2	2	2	2	3	3
3	3	3	3	3	3	4	4	1	1
1	1	3	3	2	2	2	2	3	3
1	1	3	3	2	2	2	2	1	1
3	3	3	3	3	3	4	4	3	3
3	3	3	3	3	3	4	4	3	3
1	1	3	3	2	2	2	2	3	3
3	3	3	3	3	3	4	4	3	3
1	1	3	3	2	2	2	2	3	3
3	3	3	3	3	3	4	4	1	1
1	1	3	3	2	2	2	2	3	3
1	1	3	3	2	2	2	2	3	3
3	3	3	3	3	3	4	4	3	3
3	3	3	3	3	3	4	4	3	3
3	3	3	3	3	3	4	4	3	3
1	1	3	3	2	2	2	2	1	1
1	1	3	3	2	2	2	2	3	3
3	3	2	2	3	3	4	4	3	3
3	3	3	3	3	3	4	4	3	3
3	3	3	3	3	3	4	4	3	3
3	3	3	3	3	3	4	4	3	3
3	3	3	3	3	3	4	4	3	3
1	1	3	3	2	2	2	2	3	3
1	1	3	3	2	2	2	2	3	3
1	1	3	3	2	2	2	2	3	3
1	1	3	3	2	2	2	2	1	1
1	1	3	3	2	2	2	2	3	3
3	3	3	3	3	3	4	4	3	3
1	1	2	2	2	2	2	2	3	3
1	1	2	2	2	2	2	2	3	3
1	1	3	3	2	2	2	2	3	3
1	1	3	3	2	2	2	2	1	1
1	1	3	3	2	2	2	2	1	1
1	1	3	3	2	2	2	2	3	3
3	3	3	3	3	3	4	4	3	3
1	1	3	3	2	2	2	2	3	3
3	3	2	2	3	3	4	4	3	3
1	1	3	3	2	2	2	2	1	1
3	3	3	3	3	3	4	4	3	3
3	3	3	3	3	3	4	4	3	3
1	1	3	3	2	2	2	2	1	1
3	3	3	3	3	3	4	4	3	3
1	1	3	3	2	2	4	4	3	3
3	3	3	3	3	3	4	4	3	3
1	1	3	3	2	2	4	4	3	3

For Review Only

3	3	2	2	3	3	4	4	3	3
1	1	3	3	2	2	2	2	1	1
3	3	3	3	3	3	4	4	3	3
3	3	3	3	3	3	4	4	3	3
1	1	3	3	2	2	2	2	3	3
3	3	3	3	3	3	4	4	3	3
3	3	3	3	3	3	4	4	3	3
1	1	3	3	2	2	2	2	1	1
1	1	2	2	2	2	2	2	3	3
3	3	3	3	3	3	4	4	3	3
3	3	3	3	3	3	4	4	3	3
3	3	3	3	3	3	4	4	3	3
3	3	2	2	3	3	4	4	3	3
3	3	3	3	3	3	4	4	3	3
3	3	3	3	3	3	4	4	3	3
1	1	3	3	2	2	2	2	3	3
3	3	3	3	3	3	4	4	1	1
3	3	3	3	3	3	4	4	3	3
3	3	3	3	3	3	4	4	3	3
3	3	2	2	3	3	4	4	1	1
3	3	3	3	3	3	4	4	3	3
1	1	3	3	2	2	2	2	1	1
3	3	3	3	3	3	4	4	3	3
1	1	3	3	2	2	2	2	3	3
1	1	3	3	2	2	2	2	1	1
3	3	3	3	3	3	4	4	3	3
3	3	3	3	3	3	4	4	1	1
1	1	3	3	2	2	2	2	1	1
3	3	3	3	3	3	4	4	3	3
3	3	3	3	3	3	4	4	3	3
1	1	3	3	2	2	2	2	3	3
1	1	2	2	2	2	2	2	3	3
1	1	3	3	2	2	2	2	1	1
3	3	2	2	3	3	4	4	3	3
1	1	2	2	2	2	2	2	3	3
1	1	3	3	2	2	2	2	1	1
1	1	3	3	2	2	2	2	3	3
3	3	3	3	3	3	4	4	3	3
1	1	3	3	2	2	2	2	3	3
1	1	3	3	2	2	2	2	3	3
3	3	3	3	3	3	4	4	3	3
3	3	2	2	3	3	4	4	3	3
1	1	2	2	2	2	4	4	1	1
3	3	3	3	3	3	4	4	3	3
1	1	3	3	3	3	4	4	1	1
3	3	3	3	3	3	4	4	3	3
1	1	3	3	2	2	2	2	3	3
3	3	3	3	3	3	4	4	3	3
1	1	3	3	2	2	2	2	3	3
3	3	3	3	3	3	4	4	3	3
3	3	3	3	3	3	4	4	3	3
1	1	3	3	2	2	2	2	1	1
1	1	2	2	2	2	2	2	1	1
3	3	3	3	3	3	4	4	3	3
3	3	2	2	3	3	4	4	1	1

For Review Only

1	1	3	3	3	3	4	4	3	3
1	1	3	3	3	3	4	4	3	3
3	3	2	2	3	3	4	4	3	3
1	1	3	3	2	2	2	2	3	3
3	3	3	3	3	3	4	4	3	3
3	3	3	3	3	3	4	4	3	3
3	3	3	3	3	3	4	4	3	3
1	1	3	3	3	3	2	2	3	3
1	1	3	3	2	2	2	2	1	1
1	1	3	3	3	3	2	2	3	3
1	1	2	2	3	3	2	2	3	3
3	3	3	3	3	3	4	4	3	3
1	1	3	3	2	2	2	2	1	1
3	3	3	3	3	3	4	4	3	3
1	1	3	3	2	2	2	2	3	3
3	3	3	3	3	3	4	4	3	3
3	3	3	3	3	3	4	4	1	1
3	3	3	3	3	3	4	4	3	3
1	1	3	3	2	2	2	2	3	3
3	3	3	3	3	3	4	4	3	3
3	3	3	3	3	3	4	4	3	3
3	3	2	2	3	3	4	4	3	3
3	3	3	3	3	3	4	4	3	3
1	1	3	3	2	2	2	2	1	1
3	3	3	3	3	3	4	4	3	3
1	1	3	3	2	2	2	2	3	3
1	1	3	3	2	2	2	2	3	3
1	1	2	2	2	2	2	2	3	3
3	3	3	3	3	3	4	4	3	3
3	3	3	3	3	3	4	4	3	3
1	1	3	3	3	3	2	2	3	3
3	3	3	3	3	3	4	4	3	3
3	3	3	3	3	3	4	4	3	3
3	3	3	3	3	3	4	4	3	3
1	1	3	3	2	2	2	2	3	3
3	3	3	3	3	3	2	2	3	3
1	1	3	3	2	2	2	2	3	3
3	3	3	3	3	3	4	4	3	3
1	1	3	3	2	2	4	4	3	3
1	1	3	3	2	2	2	2	1	1
1	1	3	3	2	2	2	2	1	1
1	1	3	3	2	2	2	2	3	3
3	3	3	3	3	3	4	4	1	1
1	1	3	3	3	3	2	2	3	3
3	3	3	3	3	3	4	4	3	3
3	3	3	3	3	3	4	4	1	1
3	3	3	3	3	3	4	4	3	3
3	3	3	3	3	3	4	4	3	3
1	1	3	3	3	3	2	2	3	3
3	3	3	3	3	3	4	4	3	3
1	1	3	3	2	2	2	2	3	3
1	1	3	3	2	2	2	2	1	1
1	1	3	3	3	3	2	2	3	3
3	3	3	3	3	3	4	4	3	3
1	1	3	3	2	2	4	4	3	3

For Review Only

3	3	3	3	3	3	4	4	3	3
1	1	3	3	2	2	2	2	1	1
1	1	2	2	2	2	2	2	3	3
3	3	3	3	3	3	4	4	3	3
3	3	3	3	3	3	4	4	3	3
1	1	2	2	2	2	2	2	1	1
1	1	3	3	2	2	2	2	1	1
3	3	3	3	3	3	4	4	3	3
1	1	3	3	2	2	2	2	3	3
3	3	3	3	3	3	4	4	3	3
3	3	3	3	3	3	4	4	3	3
1	1	3	3	2	2	2	2	1	1
3	3	3	3	3	3	4	4	3	3
3	3	3	3	2	2	4	4	3	3
1	1	3	3	2	2	2	2	3	3
1	1	3	3	2	2	2	2	1	1
1	1	3	3	2	2	2	2	3	3
1	1	3	3	2	2	2	2	3	3
3	3	3	3	3	3	4	4	3	3
3	3	3	3	3	3	4	4	3	3
3	3	3	3	3	3	4	4	3	3
3	3	3	3	3	3	2	2	3	3
1	1	3	3	2	2	2	2	1	1
3	3	3	3	3	3	4	4	3	3
3	3	3	3	3	3	4	4	3	3
1	1	3	3	2	2	2	2	3	3
3	3	2	2	3	3	4	4	3	3
3	3	3	3	3	3	4	4	3	3
3	3	2	2	3	3	4	4	1	1
3	3	2	2	3	3	4	4	3	3
1	1	2	2	2	2	2	2	3	3
3	3	3	3	3	3	4	4	3	3
1	1	2	2	2	2	2	2	3	3
1	1	3	3	2	2	2	2	3	3
1	1	3	3	2	2	2	2	3	3
3	3	3	3	3	3	4	4	3	3
1	1	3	3	2	2	4	4	3	3
1	1	2	2	2	2	2	2	3	3
3	3	3	3	3	3	4	4	3	3
3	3	3	3	3	3	4	4	3	3
3	3	3	3	3	3	4	4	3	3
3	3	3	3	3	3	4	4	3	3
1	1	3	3	2	2	2	2	1	1
1	1	3	3	2	2	2	2	3	3
3	3	3	3	3	3	4	4	3	3
3	3	3	3	3	3	4	4	3	3
1	1	3	3	2	2	2	2	1	1
1	1	3	3	2	2	2	2	1	1
1	1	3	3	2	2	2	2	3	3
3	3	3	3	3	3	4	4	3	3
3	3	3	3	3	3	4	4	3	3
1	1	2	2	2	2	2	2	1	1
1	1	3	3	2	2	2	2	1	1
3	3	3	3	3	3	4	4	3	3
1	1	3	3	2	2	2	2	1	1

For Review Only

1	1	3	3	2	2	2	2	3	3
1	1	3	3	2	2	2	2	3	3
3	3	3	3	3	3	4	4	3	3
3	3	3	3	3	3	4	4	3	3
1	1	3	3	2	2	2	2	3	3
3	3	3	3	2	2	2	2	3	3
1	1	3	3	2	2	2	2	3	3
1	1	3	3	2	2	2	2	3	3
1	1	3	3	3	3	2	2	3	3
1	1	3	3	2	2	2	2	1	1
3	3	3	3	3	3	4	4	3	3
1	1	2	2	2	2	2	2	3	3
1	1	3	3	2	2	2	2	3	3
3	3	3	3	3	3	4	4	3	3
3	3	3	3	3	3	4	4	3	3
1	1	3	3	2	2	2	2	1	1
1	1	3	3	2	2	2	2	1	1
1	1	3	3	2	2	2	2	3	3
1	1	3	3	2	2	2	2	1	1
1	1	3	3	2	2	2	2	3	3
3	3	3	3	3	3	4	4	1	1
3	3	3	3	3	3	4	4	3	3
1	1	3	3	2	2	2	2	3	3
1	1	2	2	2	2	2	2	1	1
1	1	3	3	2	2	2	2	1	1
3	3	3	3	3	3	4	4	3	3
1	1	3	3	2	2	2	2	1	1
3	3	3	3	3	3	4	4	3	3
3	3	3	3	3	3	4	4	3	3
3	3	3	3	3	3	4	4	3	3
1	1	3	3	2	2	2	2	1	1
1	1	2	2	2	2	2	2	1	1
1	1	3	3	2	2	2	2	3	3
1	1	3	3	2	2	2	2	3	3
1	1	3	3	2	2	2	2	1	1
1	1	3	3	2	2	2	2	1	1
3	3	3	3	3	3	4	4	3	3
3	3	3	3	3	3	4	4	3	3
1	1	2	2	2	2	2	2	3	3
1	1	3	3	2	2	2	2	1	1
3	3	3	3	3	3	4	4	3	3
3	3	3	3	3	3	4	4	3	3
1	1	3	3	2	2	2	2	1	1
3	3	3	3	3	3	4	4	3	3
3	3	3	3	3	3	4	4	3	3
1	1	3	3	2	2	2	2	3	3
1	1	3	3	3	3	2	2	1	1
3	3	3	3	3	3	4	4	1	1
3	3	3	3	3	3	4	4	3	3
1	1	3	3	2	2	2	2	1	1
1	1	3	3	2	2	2	2	3	3
1	1	3	3	2	2	2	2	1	1
3	3	3	3	3	3	4	4	3	3
3	3	3	3	3	3	4	4	3	3
1	1	2	2	2	2	2	2	1	1

For Review Only

1	1	3	3	2	2	2	2	3	3
3	3	3	3	3	3	4	4	3	3

For Review Only

SNP2313	SNP2313	SNP2314	SNP2314	SNP2315	SNP2315	SNP2316	SNP2316	SNP2317	SNP2317
1	1	4	4	3	3	1	1	1	1
3	3	1	1	1	1	1	1	1	1
1	1	1	1	1	1	1	1	1	1
1	1	1	1	1	1	1	1	1	1
1	1	4	4	3	3	1	1	4	4
1	1	1	1	1	1	4	4	4	4
3	3	4	4	3	3	1	1	1	1
3	3	4	4	3	3	1	1	1	1
1	1	4	4	3	3	1	1	1	1
3	3	4	4	3	3	1	1	1	1
1	1	4	4	3	3	1	1	1	1
1	1	1	1	1	1	1	1	1	1
1	1	4	4	1	1	1	1	1	1
1	1	1	1	1	1	1	1	1	1
1	1	4	4	1	1	1	1	1	1
1	1	4	4	1	1	1	1	1	1
3	3	4	4	3	3	1	1	1	1
1	1	1	1	1	1	1	1	1	1
1	1	1	1	1	1	1	1	4	4
3	3	4	4	3	3	1	1	1	1
3	3	1	1	1	1	1	1	1	1
1	1	1	1	1	1	1	1	1	1
1	1	4	4	3	3	4	4	4	4
1	1	1	1	1	1	1	1	4	4
1	1	1	1	1	1	1	1	4	4
1	1	1	1	1	1	1	1	1	1
1	1	1	1	1	1	1	1	1	1
1	1	4	4	1	1	1	1	1	1
1	1	4	4	3	3	1	1	1	1
3	3	1	1	1	1	1	1	4	4
1	1	1	1	1	1	4	4	4	4
1	1	1	1	1	1	4	4	4	4
1	1	4	4	3	3	1	1	1	1
1	1	1	1	1	1	1	1	1	1
1	1	4	4	3	3	1	1	1	1
1	1	1	1	1	1	1	1	1	1
3	3	1	1	1	1	1	1	1	1
3	3	1	1	1	1	1	1	1	1
1	1	4	4	3	3	1	1	1	1
1	1	4	4	3	3	1	1	4	4
3	3	1	1	1	1	1	1	4	4
1	1	4	4	3	3	1	1	1	1
1	1	4	4	3	3	1	1	1	1
3	3	1	1	1	1	1	1	1	1
3	3	4	4	3	3	1	1	1	1
1	1	1	1	1	1	1	1	1	1
1	1	1	1	1	1	1	1	1	1
1	1	4	4	3	3	1	1	1	1
3	3	1	1	1	1	1	1	1	1
1	1	4	4	1	1	1	1	1	1
1	1	1	1	1	1	4	4	4	4
1	1	1	1	1	1	1	1	1	1

For Review Only

1	1	1	1	1	1	1	1	1	1
1	1	4	4	3	3	1	1	1	1
1	1	1	1	1	1	4	4	4	4
3	3	1	1	1	1	1	1	1	1
1	1	1	1	1	1	1	1	4	4
1	1	1	1	1	1	1	1	4	4
1	1	1	1	1	1	1	1	1	1
3	3	4	4	3	3	1	1	4	4
1	1	1	1	1	1	1	1	1	1
1	1	4	4	1	1	1	1	1	1
1	1	1	1	1	1	4	4	4	4
1	1	1	1	1	1	1	1	4	4
1	1	1	1	1	1	1	1	1	1
1	1	4	4	3	3	1	1	1	1
1	1	4	4	1	1	1	1	1	1
1	1	4	4	3	3	1	1	1	1
1	1	4	4	3	3	1	1	1	1
1	1	1	1	1	1	1	1	1	1
1	1	1	1	1	1	1	1	1	1
1	1	4	4	3	3	1	1	1	1
1	1	1	1	1	1	1	1	1	1
1	1	4	4	3	3	1	1	1	1
1	1	4	4	3	3	1	1	1	1
1	1	1	1	1	1	1	1	1	1
3	3	4	4	3	3	1	1	1	1
1	1	1	1	1	1	1	1	1	1
3	3	4	4	3	3	1	1	1	1
1	1	4	4	3	3	1	1	4	4
1	1	1	1	1	1	1	1	1	1
1	1	1	1	1	1	1	1	1	1
1	1	1	1	1	1	1	1	1	1
1	1	1	1	1	1	1	1	1	1
1	1	4	4	3	3	1	1	4	4
1	1	1	1	1	1	1	1	1	1
1	1	1	1	1	1	1	1	1	1
3	3	4	4	3	3	1	1	1	1
3	3	4	4	3	3	1	1	1	1
3	3	1	1	1	1	1	1	4	4
3	3	1	1	1	1	4	4	4	4
3	3	1	1	1	1	1	1	4	4
1	1	1	1	1	1	1	1	1	1
1	1	1	1	1	1	1	1	1	1
1	1	4	4	3	3	1	1	4	4
1	1	1	1	1	1	4	4	4	4
1	1	4	4	3	3	1	1	1	1
1	1	1	1	1	1	1	1	1	1
1	1	1	1	1	1	1	1	1	1
1	1	1	1	1	1	1	1	1	1
1	1	1	1	1	1	1	1	1	1
1	1	1	1	1	1	1	1	1	1
1	1	1	1	1	1	1	1	1	1
1	1	4	4	3	3	1	1	1	1
3	3	4	4	3	3	1	1	1	1
1	1	4	4	1	1	1	1	1	1
1	1	4	4	3	3	1	1	1	1

For Review Only

1	1	1	1	1	1	1	1	1	1
1	1	1	1	1	1	1	1	4	4
1	1	1	1	1	1	1	1	1	1
1	1	4	4	1	1	1	1	1	1
1	1	1	1	1	1	1	1	1	1
1	1	1	1	1	1	1	1	1	1
1	1	1	1	1	1	1	1	1	1
1	1	1	1	1	1	4	4	4	4
1	1	4	4	3	3	1	1	1	1
1	1	4	4	1	1	1	1	1	1
1	1	4	4	3	3	1	1	1	1
1	1	4	4	1	1	1	1	1	1
1	1	4	4	3	3	1	1	1	1
1	1	1	1	1	1	1	1	1	1
1	1	1	1	1	1	1	1	4	4
1	1	4	4	1	1	1	1	1	1
1	1	4	4	1	1	1	1	4	4
1	1	4	4	1	1	1	1	4	4
1	1	1	1	1	1	1	1	1	1
3	3	1	1	1	1	1	1	4	4
1	1	4	4	1	1	1	1	1	1
1	1	1	1	1	1	1	1	1	1
1	1	1	1	1	1	1	1	1	1
1	1	4	4	3	3	1	1	4	4
1	1	1	1	3	3	1	1	4	4
1	1	1	1	1	1	4	4	4	4
1	1	1	1	1	1	1	1	1	1
1	1	1	1	1	1	1	1	1	1
1	1	1	1	1	1	1	1	1	1
3	3	1	1	1	1	1	1	1	1
1	1	4	4	1	1	1	1	1	1
1	1	4	4	1	1	1	1	1	1
1	1	1	1	1	1	1	1	1	1
1	1	4	4	1	1	1	1	1	1
1	1	1	1	1	1	1	1	1	1
1	1	1	1	1	1	1	1	1	1
1	1	1	1	1	1	1	1	1	1
1	1	1	1	1	1	1	1	1	1
1	1	4	4	1	1	1	1	1	1
1	1	4	4	3	3	1	1	1	1
1	1	4	4	3	3	1	1	4	4
1	1	1	1	1	1	1	1	1	1
1	1	4	4	3	3	1	1	1	1
1	1	4	4	1	1	1	1	1	1
3	3	1	1	1	1	1	1	1	1
1	1	4	4	3	3	1	1	4	4
1	1	1	1	1	1	1	1	4	4
1	1	1	1	1	1	1	1	1	1
1	1	4	4	1	1	1	1	1	1
1	1	1	1	1	1	1	1	1	1
1	1	1	1	1	1	1	1	1	1
3	3	4	4	3	3	1	1	1	1
1	1	4	4	1	1	1	1	1	1
1	1	1	1	1	1	1	1	4	4
1	1	4	4	1	1	1	1	1	1

For Review Only

1	1	1	1	1	1	1	1	1	1
3	3	4	4	3	3	4	4	4	4
3	3	1	1	1	1	1	1	1	1
1	1	4	4	1	1	1	1	4	4
1	1	1	1	1	1	1	1	1	1
1	1	4	4	3	3	1	1	4	4
1	1	4	4	3	3	1	1	1	1
1	1	4	4	1	1	1	1	1	1
1	1	1	1	1	1	1	1	1	1
1	1	1	1	1	1	1	1	1	1
1	1	1	1	1	1	1	1	1	1
1	1	1	1	1	1	1	1	4	4
1	1	1	1	1	1	1	1	1	1
1	1	4	4	1	1	1	1	1	1
1	1	1	1	1	1	1	1	1	1
3	3	4	4	3	3	1	1	1	1
1	1	4	4	1	1	1	1	1	1
1	1	1	1	1	1	1	1	1	1
1	1	1	1	1	1	1	1	1	1
1	1	1	1	1	1	1	1	1	1
1	1	4	4	1	1	1	1	1	1
1	1	4	4	1	1	1	1	1	1
1	1	4	4	3	3	1	1	1	1
1	1	1	1	1	1	1	1	1	1
3	3	1	1	1	1	1	1	1	1
1	1	1	1	1	1	1	1	1	1
1	1	1	1	1	1	1	1	1	1
1	1	4	4	1	1	1	1	1	1
3	3	4	4	3	3	1	1	1	1
1	1	1	1	1	1	1	1	1	1
1	1	1	1	1	1	1	1	1	1
1	1	1	1	1	1	4	4	4	4
1	1	1	1	1	1	1	1	1	1
1	1	1	1	1	1	1	1	1	1
1	1	4	4	1	1	1	1	1	1
1	1	1	1	1	1	1	1	4	4
1	1	1	1	1	1	1	1	1	1
1	1	1	1	1	1	1	1	1	1
1	1	1	1	1	1	1	1	1	1
1	1	1	1	1	1	1	1	1	1
1	1	1	1	1	1	1	1	1	1
1	1	1	1	1	1	1	1	1	1
1	1	4	4	3	3	1	1	4	4
1	1	4	4	1	1	1	1	1	1
1	1	4	4	3	3	1	1	1	1
3	3	1	1	1	1	1	1	4	4
3	3	4	4	3	3	1	1	1	1
3	3	4	4	3	3	1	1	4	4
1	1	1	1	1	1	1	1	1	1
1	1	1	1	1	1	1	1	1	1
3	3	1	1	1	1	1	1	1	1
3	3	4	4	3	3	1	1	4	4
1	1	4	4	3	3	1	1	4	4
1	1	1	1	1	1	4	4	4	4
1	1	4	4	3	3	1	1	4	4

For Review Only

1	1	1	1	1	1	1	1	4	4
1	1	4	4	3	3	1	1	1	1
1	1	1	1	1	1	1	1	1	1
1	1	4	4	1	1	1	1	1	1
1	1	4	4	3	3	1	1	4	4
1	1	1	1	1	1	1	1	1	1
3	3	1	1	1	1	1	1	4	4
3	3	4	4	3	3	1	1	4	4
1	1	1	1	1	1	1	1	1	1
1	1	4	4	3	3	1	1	4	4
1	1	4	4	1	1	1	1	1	1
1	1	1	1	1	1	1	1	1	1
3	3	1	1	1	1	4	4	4	4
1	1	1	1	1	1	1	1	1	1
1	1	1	1	1	1	1	1	1	1
1	1	4	4	3	3	1	1	4	4
1	1	4	4	3	3	1	1	1	1
1	1	4	4	1	1	1	1	4	4
1	1	4	4	3	3	1	1	4	4
3	3	1	1	1	1	1	1	1	1
1	1	4	4	3	3	1	1	4	4
1	1	1	1	1	1	1	1	1	1
1	1	1	1	1	1	1	1	1	1
1	1	4	4	3	3	1	1	4	4
3	3	4	4	3	3	1	1	4	4
1	1	1	1	1	1	1	1	1	1
1	1	4	4	3	3	1	1	4	4
1	1	1	1	1	1	1	1	1	1
1	1	4	4	1	1	1	1	1	1
1	1	1	1	1	1	1	1	1	1
1	1	4	4	3	3	1	1	4	4
1	1	4	4	3	3	4	4	4	4
1	1	1	1	1	1	1	1	1	1
1	1	1	1	1	1	1	1	1	1
1	1	4	4	3	3	1	1	4	4
1	1	4	4	3	3	1	1	4	4
1	1	1	1	1	1	1	1	1	1
1	1	1	1	1	1	1	1	4	4
1	1	1	1	1	1	1	1	1	1
1	1	4	4	3	3	1	1	1	1
1	1	1	1	1	1	1	1	1	1
1	1	1	1	1	1	1	1	1	1
1	1	4	4	3	3	1	1	1	1
1	1	1	1	1	1	1	1	1	1
1	1	1	1	1	1	1	1	4	4
3	3	1	1	1	1	1	1	1	1
1	1	4	4	3	3	1	1	1	1
1	1	4	4	3	3	1	1	1	1
1	1	1	1	1	1	1	1	4	4
1	1	4	4	3	3	1	1	4	4
1	1	1	1	1	1	1	1	1	1
1	1	4	4	3	3	1	1	1	1
1	1	1	1	1	1	1	1	1	1
1	1	1	1	1	1	1	1	1	1
1	1	4	4	3	3	1	1	4	4

For Review Only

1	1	1	1	1	1	1	1	1	1
1	1	1	1	1	1	1	1	1	1

For Review Only

SNP2318	SNP2318	SNP2319	SNP2319	SNP232	SNP232	SNP2320	SNP2320	SNP2321	SNP2321
4	4	2	2	3	3	2	2	3	3
4	4	2	2	3	3	2	2	3	3
4	4	2	2	3	3	2	2	3	3
4	4	2	2	3	3	2	2	3	3
4	4	1	1	3	3	2	2	3	3
4	4	2	2	2	2	2	2	3	3
4	4	2	2	2	2	2	2	3	3
4	4	1	1	3	3	2	2	3	3
4	4	2	2	2	2	2	2	3	3
4	4	2	2	3	3	2	2	3	3
4	4	2	2	3	3	2	2	3	3
4	4	2	2	3	3	2	2	3	3
4	4	2	2	3	3	2	2	3	3
4	4	2	2	3	3	2	2	2	2
4	4	2	2	3	3	2	2	3	3
4	4	2	2	2	2	2	2	3	3
4	4	2	2	2	2	2	2	3	3
4	4	2	2	2	2	2	2	3	3
4	4	2	2	3	3	2	2	3	3
4	4	2	2	2	2	2	2	3	3
4	4	2	2	3	3	2	2	3	3
4	4	2	2	2	2	2	2	3	3
4	4	2	2	3	3	2	2	3	3
4	4	2	2	3	3	2	2	3	3
4	4	2	2	3	3	2	2	3	3
4	4	2	2	3	3	2	2	3	3
4	4	2	2	3	3	2	2	3	3
4	4	2	2	3	3	2	2	3	3
4	4	2	2	3	3	2	2	3	3
4	4	2	2	3	3	3	3	3	3
4	4	2	2	3	3	2	2	3	3
4	4	2	2	3	3	2	2	3	3
4	4	2	2	2	2	2	2	3	3
4	4	2	2	3	3	2	2	2	2
1	1	2	2	3	3	3	3	3	3
4	4	2	2	3	3	2	2	3	3
4	4	2	2	3	3	2	2	3	3
4	4	2	2	3	3	2	2	3	3
4	4	2	2	3	3	2	2	3	3
1	1	2	2	3	3	3	3	3	3
4	4	1	1	3	3	2	2	3	3
4	4	2	2	3	3	2	2	3	3
4	4	1	1	3	3	2	2	3	3
4	4	2	2	2	2	2	2	3	3
4	4	1	1	3	3	2	2	3	3
4	4	2	2	3	3	2	2	3	3
4	4	2	2	3	3	2	2	3	3
4	4	2	2	3	3	2	2	3	3
4	4	2	2	3	3	2	2	3	3
4	4	2	2	2	2	2	2	3	3
4	4	2	2	3	3	2	2	3	3
4	4	2	2	3	3	2	2	3	3

For Review Only

4	4	2	2	3	3	2	2	3	3
4	4	2	2	2	2	2	2	3	3
4	4	2	2	3	3	2	2	3	3
4	4	2	2	2	2	2	2	3	3
4	4	2	2	3	3	2	2	3	3
4	4	2	2	3	3	2	2	3	3
4	4	2	2	3	3	2	2	3	3
4	4	1	1	3	3	3	3	3	3
4	4	2	2	2	2	2	2	2	2
4	4	2	2	3	3	2	2	3	3
4	4	2	2	3	3	3	3	3	3
4	4	2	2	3	3	2	2	3	3
4	4	2	2	3	3	2	2	3	3
4	4	2	2	3	3	2	2	3	3
4	4	2	2	3	3	2	2	3	3
1	1	2	2	3	3	2	2	3	3
4	4	2	2	3	3	2	2	3	3
4	4	2	2	2	2	2	2	3	3
4	4	2	2	2	2	2	2	3	3
4	4	2	2	2	2	2	2	3	3
4	4	2	2	3	3	2	2	3	3
1	1	2	2	3	3	2	2	3	3
4	4	2	2	3	3	2	2	3	3
4	4	2	2	3	3	2	2	3	3
4	4	1	1	3	3	2	2	3	3
4	4	2	2	3	3	2	2	3	3
4	4	2	2	3	3	2	2	3	3
4	4	2	2	3	3	3	3	3	3
4	4	2	2	3	3	2	2	3	3
4	4	2	2	2	2	2	2	3	3
4	4	2	2	3	3	2	2	3	3
4	4	2	2	3	3	2	2	3	3
4	4	2	2	3	3	3	3	3	3
4	4	2	2	3	3	2	2	3	3
4	4	2	2	2	2	2	2	3	3
4	4	1	1	3	3	2	2	3	3
4	4	2	2	3	3	2	2	3	3
4	4	2	2	2	2	3	3	3	3
4	4	1	1	2	2	2	2	3	3
4	4	1	1	3	3	3	3	3	3
4	4	2	2	2	2	2	2	3	3
4	4	2	2	2	2	2	2	3	3
4	4	1	1	3	3	2	2	3	3
4	4	2	2	3	3	3	3	3	3
4	4	2	2	2	2	2	2	3	3
4	4	2	2	3	3	2	2	3	3
4	4	2	2	3	3	2	2	2	2
4	4	2	2	3	3	2	2	3	3
4	4	2	2	2	2	2	2	3	3
1	1	2	2	3	3	2	2	3	3
4	4	2	2	3	3	2	2	3	3
4	4	2	2	3	3	2	2	3	3
4	4	1	1	2	2	2	2	3	3
4	4	2	2	3	3	2	2	3	3
4	4	2	2	3	3	2	2	3	3

For Review Only

4	4	2	2	3	3	3	3	3	3
4	4	2	2	3	3	3	3	3	3
4	4	2	2	3	3	2	2	3	3
4	4	2	2	3	3	3	3	3	3
4	4	2	2	3	3	2	2	3	3
4	4	2	2	3	3	2	2	3	3
4	4	2	2	2	2	2	2	3	3
4	4	2	2	3	3	2	2	3	3
4	4	2	2	2	2	2	2	3	3
4	4	2	2	3	3	2	2	3	3
4	4	2	2	3	3	2	2	3	3
4	4	2	2	3	3	2	2	3	3
4	4	1	1	3	3	2	2	3	3
4	4	2	2	3	3	2	2	3	3
1	1	1	1	3	3	2	2	2	2
4	4	2	2	3	3	2	2	3	3
4	4	2	2	3	3	3	3	3	3
4	4	2	2	3	3	2	2	3	3
4	4	2	2	3	3	3	3	2	2
4	4	2	2	3	3	2	2	3	3
4	4	2	2	3	3	2	2	3	3
4	4	2	2	2	2	3	3	3	3
4	4	2	2	3	3	2	2	3	3
4	4	2	2	3	3	3	3	3	3
4	4	2	2	3	3	2	2	3	3
4	4	1	1	3	3	2	2	3	3
4	4	2	2	2	2	2	2	3	3
4	4	2	2	3	3	2	2	3	3
4	4	2	2	3	3	2	2	3	3
4	4	2	2	3	3	2	2	3	3
4	4	2	2	3	3	3	3	3	3
4	4	2	2	3	3	2	2	3	3
4	4	2	2	2	2	2	2	3	3
4	4	2	2	3	3	2	2	3	3
4	4	2	2	3	3	2	2	2	2
4	4	2	2	3	3	2	2	3	3
4	4	2	2	3	3	3	3	2	2
1	1	2	2	3	3	2	2	3	3
4	4	2	2	3	3	3	3	3	3
4	4	2	2	3	3	2	2	3	3
1	1	2	2	3	3	2	2	3	3
4	4	2	2	3	3	3	3	2	2
4	4	2	2	3	3	2	2	3	3
4	4	1	1	3	3	2	2	3	3
4	4	2	2	3	3	2	2	3	3
4	4	2	2	3	3	3	3	3	3
4	4	2	2	3	3	2	2	3	3
4	4	2	2	3	3	2	2	3	3
4	4	2	2	3	3	2	2	3	3
4	4	2	2	3	3	2	2	3	3
4	4	2	2	3	3	2	2	3	3
4	4	2	2	3	3	2	2	3	3
1	1	2	2	2	2	2	2	3	3
4	4	2	2	3	3	2	2	3	3
4	4	2	2	3	3	3	3	3	3

For Review Only

4	4	2	2	3	3	2	2	3	3
4	4	1	1	3	3	2	2	3	3
4	4	1	1	2	2	2	2	3	3
4	4	2	2	3	3	3	3	3	3
4	4	2	2	3	3	2	2	3	3
4	4	2	2	2	2	3	3	3	3
1	1	2	2	3	3	2	2	3	3
4	4	2	2	3	3	2	2	3	3
4	4	2	2	3	3	2	2	3	3
4	4	2	2	2	2	2	2	3	3
4	4	2	2	3	3	2	2	3	3
1	1	2	2	3	3	2	2	3	3
4	4	2	2	2	2	2	2	3	3
4	4	2	2	3	3	3	3	3	3
4	4	2	2	3	3	3	3	2	2
4	4	2	2	3	3	2	2	3	3
4	4	2	2	3	3	2	2	3	3
4	4	2	2	3	3	2	2	3	3
4	4	2	2	3	3	2	2	3	3
4	4	2	2	2	2	2	2	3	3
4	4	2	2	3	3	2	2	3	3
4	4	2	2	3	3	2	2	3	3
4	4	2	2	3	3	2	2	3	3
4	4	2	2	3	3	2	2	3	3
4	4	2	2	2	2	2	2	3	3
4	4	2	2	3	3	2	2	2	2
4	4	2	2	3	3	2	2	3	3
4	4	2	2	3	3	2	2	3	3
4	4	2	2	3	3	2	2	3	3
4	4	2	2	2	2	2	2	3	3
4	4	2	2	3	3	2	2	3	3
4	4	2	2	2	2	2	2	3	3
4	4	2	2	2	2	2	2	3	3
4	4	2	2	3	3	3	3	2	2
4	4	2	2	2	2	3	3	3	3
4	4	2	2	3	3	2	2	3	3
1	1	2	2	3	3	2	2	3	3
4	4	2	2	2	2	2	2	3	3
4	4	2	2	3	3	2	2	3	3
4	4	2	2	3	3	2	2	3	3
4	4	2	2	3	3	2	2	3	3
4	4	2	2	3	3	2	2	3	3
4	4	2	2	3	3	3	3	3	3
1	1	2	2	3	3	2	2	3	3
4	4	2	2	3	3	3	3	3	3
4	4	2	2	3	3	2	2	3	3
4	4	2	2	3	3	2	2	3	3
4	4	2	2	3	3	3	3	3	3
4	4	2	2	3	3	2	2	2	2
4	4	2	2	2	2	2	2	3	3
4	4	2	2	2	2	2	2	3	3
4	4	2	2	3	3	3	3	3	3
4	4	2	2	3	3	3	3	3	3
4	4	2	2	3	3	3	3	3	3
4	4	2	2	3	3	3	3	3	3

4	4	2	2	3	3	2	2	3	3
4	4	2	2	3	3	2	2	3	3
4	4	2	2	2	2	2	2	3	3
4	4	2	2	3	3	2	2	3	3
4	4	2	2	3	3	3	3	3	3
4	4	2	2	2	2	2	2	3	3
4	4	2	2	3	3	3	3	3	3
4	4	1	1	3	3	2	2	3	3
4	4	2	2	3	3	2	2	3	3
4	4	2	2	3	3	3	3	3	3
4	4	2	2	3	3	2	2	3	3
4	4	2	2	3	3	2	2	3	3
4	4	1	1	2	2	2	2	3	3
4	4	2	2	3	3	2	2	3	3
4	4	2	2	2	2	2	2	3	3
4	4	1	1	2	2	3	3	3	3
4	4	2	2	3	3	2	2	3	3
1	1	2	2	3	3	2	2	3	3
4	4	2	2	3	3	3	3	3	3
4	4	2	2	3	3	2	2	3	3
4	4	2	2	3	3	3	3	3	3
4	4	2	2	3	3	2	2	3	3
4	4	2	2	2	2	2	2	3	3
1	1	2	2	3	3	3	3	3	3
4	4	1	1	3	3	3	3	3	3
4	4	2	2	3	3	2	2	3	3
4	4	2	2	3	3	3	3	3	3
4	4	2	2	3	3	2	2	3	3
4	4	2	2	3	3	2	2	3	3
4	4	2	2	2	2	2	2	3	3
4	4	2	2	3	3	2	2	3	3
4	4	2	2	3	3	2	2	3	3
4	4	2	2	2	2	2	2	2	2
4	4	2	2	3	3	2	2	3	3
1	1	2	2	3	3	3	3	3	3
4	4	2	2	3	3	3	3	3	3
4	4	2	2	2	2	2	2	3	3
4	4	2	2	3	3	2	2	3	3
4	4	2	2	3	3	2	2	3	3
4	4	2	2	3	3	2	2	3	3
4	4	2	2	2	2	2	2	3	3
4	4	2	2	3	3	2	2	2	2
1	1	2	2	3	3	2	2	3	3
4	4	2	2	2	2	2	2	3	3
4	4	2	2	3	3	2	2	3	3
4	4	1	1	3	3	2	2	3	3
4	4	2	2	3	3	2	2	3	3
4	4	2	2	3	3	2	2	3	3
4	4	2	2	3	3	3	3	3	3
4	4	2	2	3	3	3	3	3	3
4	4	2	2	3	3	2	2	3	3
4	4	2	2	3	3	2	2	3	3
4	4	2	2	3	3	2	2	3	3
4	4	2	2	3	3	2	2	3	3
1	1	2	2	3	3	3	3	3	3

For Review Only

4	4	2	2	2	2	2	2	3	3
4	4	2	2	3	3	2	2	3	3

For Review Only

SNP2322	SNP2322	SNP2323	SNP2323	SNP2324	SNP2324	SNP2325	SNP2325	SNP2326	SNP2326
1	1	1	1	3	3	3	3	4	4
1	1	1	1	3	3	3	3	2	2
1	1	1	1	3	3	2	2	4	4
1	1	1	1	3	3	2	2	4	4
4	4	1	1	3	3	3	3	4	4
4	4	1	1	3	3	3	3	4	4
4	4	1	1	3	3	3	3	2	2
4	4	1	1	3	3	2	2	2	2
4	4	1	1	3	3	3	3	4	4
4	4	1	1	2	2	3	3	2	2
4	4	1	1	2	2	3	3	4	4
1	1	1	1	3	3	2	2	4	4
4	4	1	1	3	3	3	3	4	4
1	1	1	1	2	2	2	2	4	4
4	4	1	1	3	3	3	3	4	4
4	4	1	1	3	3	3	3	4	4
4	4	1	1	3	3	3	3	2	2
4	4	1	1	3	3	2	2	4	4
1	1	1	1	3	3	2	2	4	4
4	4	1	1	3	3	3	3	2	2
4	4	1	1	3	3	3	3	2	2
4	4	1	1	3	3	2	2	4	4
4	4	1	1	2	2	2	2	4	4
4	4	1	1	2	2	3	3	4	4
1	1	1	1	3	3	3	3	4	4
4	4	1	1	3	3	3	3	4	4
4	4	1	1	3	3	3	3	4	4
1	1	1	1	3	3	2	2	4	4
4	4	1	1	3	3	3	3	4	4
4	4	2	2	3	3	3	3	2	2
1	1	1	1	3	3	3	3	4	4
4	4	1	1	3	3	2	2	4	4
4	4	1	1	3	3	3	3	4	4
4	4	1	1	2	2	3	3	4	4
4	4	1	1	3	3	2	2	4	4
4	4	1	1	3	3	3	3	4	4
4	4	1	1	3	3	3	3	2	2
1	1	1	1	3	3	2	2	2	2
4	4	1	1	2	2	2	2	4	4
4	4	1	1	2	2	2	2	4	4
4	4	2	2	2	2	2	2	2	2
4	4	1	1	3	3	3	3	4	4
4	4	1	1	3	3	3	3	4	4
4	4	1	1	3	3	3	3	2	2
4	4	1	1	3	3	3	3	2	2
1	1	1	1	3	3	2	2	4	4
4	4	1	1	3	3	2	2	4	4
4	4	1	1	3	3	2	2	4	4
4	4	1	1	3	3	3	3	2	2
4	4	1	1	3	3	2	2	4	4
4	4	1	1	3	3	3	3	4	4
4	4	2	2	3	3	2	2	4	4

For Review Only

4	4	1	1	3	3	3	3	4	4
4	4	1	1	3	3	3	3	4	4
1	1	1	1	3	3	2	2	4	4
4	4	1	1	3	3	2	2	2	2
1	1	1	1	3	3	2	2	4	4
4	4	1	1	3	3	2	2	4	4
4	4	1	1	3	3	3	3	4	4
4	4	2	2	3	3	3	3	2	2
4	4	1	1	2	2	3	3	4	4
1	1	1	1	3	3	2	2	4	4
4	4	2	2	3	3	2	2	4	4
4	4	1	1	3	3	2	2	4	4
4	4	1	1	3	3	3	3	4	4
4	4	1	1	3	3	3	3	4	4
4	4	1	1	3	3	3	3	4	4
4	4	1	1	3	3	3	3	4	4
1	1	1	1	2	2	2	2	4	4
4	4	1	1	3	3	3	3	4	4
1	1	1	1	3	3	2	2	4	4
4	4	1	1	3	3	3	3	4	4
4	4	1	1	3	3	2	2	4	4
4	4	1	1	2	2	2	2	4	4
4	4	1	1	3	3	3	3	4	4
4	4	1	1	3	3	3	3	4	4
4	4	1	1	3	3	3	3	2	2
4	4	2	2	3	3	2	2	4	4
4	4	1	1	3	3	3	3	2	2
4	4	2	2	2	2	2	2	4	4
4	4	1	1	3	3	3	3	4	4
4	4	1	1	3	3	3	3	4	4
4	4	1	1	3	3	3	3	4	4
4	4	1	1	3	3	3	3	4	4
4	4	2	2	2	2	3	3	4	4
4	4	1	1	3	3	3	3	4	4
4	4	1	1	3	3	3	3	4	4
4	4	1	1	3	3	3	3	2	2
4	4	1	1	3	3	2	2	4	4
4	4	2	2	3	3	2	2	2	2
1	1	1	1	3	3	2	2	2	2
4	4	2	2	3	3	3	3	2	2
4	4	1	1	3	3	2	2	4	4
1	1	1	1	3	3	2	2	4	4
1	1	1	1	3	3	2	2	2	2
4	4	2	2	3	3	2	2	4	4
4	4	1	1	3	3	3	3	4	4
4	4	1	1	3	3	2	2	4	4
4	4	1	1	2	2	3	3	4	4
4	4	1	1	3	3	3	3	4	4
4	4	1	1	3	3	3	3	4	4
4	4	1	1	3	3	2	2	4	4
4	4	1	1	3	3	2	2	4	4
4	4	1	1	2	2	2	2	4	4
4	4	1	1	3	3	3	3	2	2
4	4	1	1	3	3	2	2	4	4
4	4	1	1	2	2	3	3	4	4

For Review Only

4	4	1	1	3	3	3	3	4	4
1	1	2	2	3	3	2	2	4	4
4	4	1	1	3	3	3	3	4	4
4	4	2	2	2	2	3	3	4	4
1	1	1	1	3	3	3	3	4	4
4	4	1	1	2	2	2	2	4	4
1	1	1	1	3	3	2	2	4	4
4	4	1	1	3	3	3	3	4	4
4	4	1	1	3	3	2	2	4	4
4	4	1	1	3	3	2	2	4	4
4	4	1	1	3	3	3	3	4	4
4	4	1	1	3	3	2	2	4	4
1	1	1	1	3	3	2	2	4	4
4	4	2	2	3	3	2	2	4	4
1	1	1	1	3	3	3	3	4	4
4	4	1	1	3	3	2	2	4	4
4	4	1	1	3	3	3	3	4	4
4	4	1	1	3	3	2	2	4	4
4	4	2	2	2	2	3	3	4	4
1	1	1	1	3	3	2	2	2	2
1	1	1	1	3	3	2	2	2	2
4	4	1	1	3	3	3	3	4	4
1	1	1	1	3	3	2	2	4	4
4	4	1	1	2	2	2	2	4	4
4	4	1	1	3	3	2	2	4	4
4	4	1	1	3	3	2	2	4	4
4	4	1	1	3	3	2	2	4	4
4	4	1	1	3	3	3	3	4	4
1	1	1	1	3	3	2	2	4	4
4	4	1	1	3	3	3	3	2	2
4	4	2	2	2	2	2	2	4	4
4	4	1	1	3	3	2	2	4	4
4	4	1	1	3	3	3	3	4	4
4	4	1	1	3	3	3	3	4	4
4	4	1	1	2	2	3	3	4	4
4	4	1	1	3	3	3	3	4	4
4	4	2	2	2	2	3	3	4	4
4	4	1	1	3	3	2	2	4	4
4	4	1	1	3	3	2	2	4	4
4	4	1	1	2	2	2	2	4	4
4	4	1	1	2	2	2	2	4	4
4	4	2	2	2	2	3	3	4	4
4	4	1	1	2	2	3	3	4	4
4	4	1	1	2	2	3	3	4	4
4	4	1	1	3	3	3	3	4	4
4	4	1	1	2	2	3	3	4	4
4	4	1	1	3	3	3	3	4	4
4	4	1	1	3	3	3	3	4	4
4	4	1	1	3	3	2	2	4	4
4	4	1	1	3	3	3	3	4	4
4	4	1	1	3	3	3	3	4	4
4	4	1	1	3	3	2	2	2	2
4	4	1	1	3	3	3	3	4	4
4	4	1	1	3	3	3	3	4	4
1	1	1	1	3	3	3	3	4	4

For Review Only

1	1	1	1	3	3	2	2	4	4
1	1	1	1	3	3	2	2	2	2
1	1	1	1	3	3	2	2	2	2
4	4	2	2	3	3	2	2	4	4
4	4	1	1	3	3	3	3	4	4
4	4	1	1	3	3	3	3	4	4
4	4	1	1	2	2	2	2	4	4
4	4	1	1	3	3	2	2	4	4
4	4	1	1	3	3	2	2	4	4
1	1	1	1	3	3	2	2	4	4
4	4	1	1	3	3	3	3	4	4
4	4	1	1	3	3	2	2	4	4
1	1	1	1	3	3	2	2	4	4
4	4	1	1	2	2	3	3	4	4
4	4	2	2	2	2	3	3	4	4
4	4	1	1	2	2	3	3	2	2
1	1	1	1	3	3	2	2	4	4
4	4	1	1	3	3	2	2	4	4
4	4	1	1	3	3	3	3	4	4
1	1	1	1	3	3	2	2	4	4
4	4	1	1	3	3	2	2	4	4
4	4	1	1	2	2	2	2	4	4
4	4	1	1	2	2	2	2	4	4
4	4	1	1	3	3	2	2	4	4
4	4	1	1	3	3	3	3	2	2
4	4	1	1	2	2	3	3	4	4
4	4	1	1	3	3	3	3	4	4
4	4	1	1	3	3	3	3	4	4
4	4	1	1	3	3	3	3	2	2
4	4	1	1	3	3	3	3	4	4
4	4	1	1	3	3	3	3	4	4
4	4	1	1	3	3	3	3	4	4
4	4	1	1	3	3	2	2	4	4
4	4	2	2	2	2	3	3	4	4
4	4	2	2	3	3	2	2	4	4
4	4	1	1	3	3	2	2	4	4
4	4	1	1	3	3	3	3	4	4
4	4	1	1	3	3	3	3	4	4
1	1	1	1	3	3	2	2	4	4
1	1	1	1	3	3	2	2	4	4
1	1	1	1	3	3	2	2	4	4
4	4	1	1	3	3	3	3	4	4
4	4	2	2	3	3	2	2	4	4
4	4	1	1	3	3	2	2	4	4
1	1	1	1	3	3	2	2	4	4
4	4	1	1	3	3	3	3	2	2
4	4	1	1	2	2	3	3	2	2
4	4	2	2	2	2	2	2	2	2
4	4	1	1	2	2	3	3	4	4
1	1	1	1	3	3	2	2	4	4
4	4	1	1	3	3	3	3	2	2
4	4	2	2	3	3	3	3	2	2
4	4	1	1	2	2	2	2	4	4
4	4	2	2	3	3	2	2	4	4
4	4	1	1	2	2	2	2	4	4

For Review Only

4	4	1	1	3	3	3	3	4	4
4	4	1	1	3	3	3	3	4	4
1	1	1	1	3	3	2	2	4	4
1	1	1	1	3	3	2	2	4	4
4	4	2	2	3	3	3	3	4	4
4	4	1	1	3	3	3	3	4	4
4	4	1	1	3	3	2	2	2	2
4	4	1	1	3	3	3	3	2	2
4	4	1	1	2	2	3	3	4	4
4	4	1	1	2	2	2	2	4	4
4	4	1	1	3	3	2	2	4	4
4	4	1	1	3	3	3	3	2	2
1	1	1	1	3	3	2	2	2	2
4	4	1	1	3	3	3	3	4	4
1	1	1	1	3	3	2	2	4	4
4	4	2	2	2	2	3	3	4	4
4	4	1	1	2	2	3	3	4	4
4	4	1	1	2	2	2	2	4	4
4	4	2	2	2	2	2	2	4	4
1	1	1	1	3	3	2	2	2	2
4	4	1	1	3	3	2	2	4	4
4	4	1	1	3	3	3	3	4	4
1	1	1	1	3	3	2	2	4	4
4	4	1	1	2	2	3	3	4	4
4	4	2	2	3	3	3	3	2	2
1	1	1	1	3	3	2	2	4	4
4	4	1	1	2	2	3	3	4	4
1	1	1	1	3	3	2	2	4	4
4	4	1	1	3	3	3	3	4	4
4	4	1	1	3	3	3	3	4	4
4	4	1	1	2	2	2	2	4	4
4	4	1	1	3	3	3	3	4	4
4	4	1	1	2	2	3	3	4	4
4	4	1	1	3	3	3	3	4	4
4	4	2	2	2	2	2	2	4	4
4	4	2	2	3	3	2	2	4	4
4	4	1	1	3	3	3	3	4	4
4	4	1	1	3	3	2	2	4	4
4	4	1	1	3	3	3	3	4	4
4	4	1	1	2	2	2	2	4	4
4	4	1	1	3	3	2	2	4	4
4	4	1	1	3	3	3	3	4	4
4	4	1	1	2	2	3	3	4	4
4	4	1	1	3	3	3	3	4	4
1	1	1	1	3	3	2	2	4	4
1	1	1	1	3	3	2	2	2	2
4	4	1	1	2	2	3	3	4	4
1	1	1	1	3	3	2	2	4	4
1	1	2	2	3	3	2	2	4	4
4	4	2	2	2	2	3	3	4	4
4	4	1	1	3	3	3	3	4	4
4	4	1	1	2	2	2	2	4	4
1	1	1	1	3	3	2	2	4	4
1	1	1	1	3	3	2	2	4	4
4	4	1	1	2	2	2	2	4	4

For Review Only

4	4	1	1	3	3	3	3	4	4
1	1	1	1	3	3	2	2	4	4

For Review Only

SNP2327	SNP2327	SNP2328	SNP2328	SNP2329	SNP2329	SNP233	SNP233	SNP2330	SNP2330
1	1	1	1	2	2	2	2	4	4
1	1	2	2	2	2	1	1	1	1
1	1	2	2	2	2	1	1	1	1
1	1	1	1	2	2	2	2	4	4
1	1	2	2	2	2	1	1	4	4
1	1	2	2	2	2	2	2	4	4
1	1	2	2	2	2	1	1	4	4
1	1	1	1	1	1	1	1	4	4
1	1	2	2	2	2	1	1	4	4
1	1	1	1	1	1	1	1	4	4
1	1	2	2	1	1	1	1	4	4
1	1	2	2	2	2	2	2	4	4
1	1	2	2	2	2	2	2	4	4
1	1	1	1	2	2	2	2	4	4
1	1	2	2	2	2	2	2	4	4
1	1	2	2	1	1	1	1	4	4
1	1	2	2	2	2	2	2	4	4
1	1	1	1	2	2	2	2	4	4
1	1	1	1	2	2	1	1	4	4
1	1	1	1	2	2	1	1	4	4
1	1	2	2	2	2	1	1	4	4
1	1	2	2	2	2	1	1	4	4
1	1	1	1	2	2	2	2	4	4
1	1	1	1	2	2	2	2	4	4
1	1	2	2	2	2	2	2	4	4
1	1	2	2	1	1	1	1	1	1
1	1	2	2	2	2	2	2	1	1
1	1	2	2	2	2	2	2	4	4
1	1	2	2	2	2	2	2	4	4
4	4	1	1	2	2	2	2	4	4
1	1	2	2	2	2	2	2	4	4
1	1	1	1	2	2	1	1	4	4
1	1	1	1	2	2	2	2	4	4
1	1	1	1	1	1	2	2	4	4
1	1	2	2	2	2	2	2	4	4
1	1	1	1	1	1	1	1	4	4
1	1	1	1	2	2	2	2	4	4
1	1	1	1	1	1	2	2	4	4
1	1	1	1	1	1	1	1	4	4
1	1	2	2	2	2	2	2	4	4
4	4	2	2	2	2	1	1	4	4
1	1	2	2	2	2	2	2	4	4
1	1	1	1	1	1	1	1	4	4
1	1	2	2	2	2	2	2	4	4
1	1	1	1	1	1	1	1	4	4
1	1	2	2	2	2	1	1	1	1
1	1	2	2	2	2	2	2	1	1
1	1	2	2	2	2	1	1	4	4
1	1	1	1	2	2	1	1	4	4
1	1	2	2	2	2	2	2	4	4
1	1	2	2	2	2	1	1	1	1
1	1	1	1	2	2	2	2	4	4

For Review Only

1	1	2	2	2	2	2	2	4	4
1	1	1	1	2	2	2	2	4	4
1	1	1	1	2	2	2	2	4	4
1	1	1	1	2	2	1	1	4	4
1	1	1	1	2	2	1	1	4	4
1	1	2	2	2	2	1	1	1	1
1	1	2	2	1	1	2	2	1	1
4	4	1	1	2	2	2	2	4	4
1	1	1	1	2	2	2	2	4	4
1	1	2	2	2	2	2	2	4	4
1	1	1	1	2	2	1	1	1	1
1	1	1	1	2	2	1	1	1	1
1	1	2	2	2	2	2	2	4	4
1	1	2	2	2	2	2	2	4	4
1	1	2	2	2	2	2	2	4	4
1	1	1	1	2	2	1	1	4	4
1	1	2	2	2	2	1	1	4	4
1	1	2	2	2	2	2	2	4	4
1	1	1	1	2	2	1	1	4	4
1	1	2	2	2	2	2	2	4	4
1	1	1	1	2	2	1	1	1	1
1	1	2	2	2	2	2	2	4	4
1	1	2	2	2	2	1	1	4	4
1	1	1	1	2	2	2	2	4	4
1	1	1	1	1	1	1	1	4	4
1	1	1	1	2	2	1	1	1	1
1	1	2	2	2	2	2	2	4	4
1	1	1	1	2	2	2	2	4	4
1	1	2	2	1	1	1	1	1	1
1	1	2	2	2	2	1	1	4	4
1	1	1	1	1	1	1	1	4	4
1	1	1	1	2	2	2	2	4	4
1	1	1	1	2	2	2	2	4	4
1	1	2	2	2	2	2	2	4	4
1	1	2	2	2	2	2	2	4	4
1	1	1	1	1	1	1	1	4	4
1	1	1	1	1	1	2	2	4	4
4	4	2	2	2	2	1	1	4	4
1	1	2	2	2	2	1	1	4	4
4	4	1	1	2	2	1	1	4	4
1	1	2	2	2	2	2	2	4	4
1	1	2	2	2	2	1	1	1	1
1	1	1	1	2	2	2	2	4	4
1	1	1	1	2	2	1	1	1	1
1	1	2	2	2	2	1	1	4	4
1	1	2	2	2	2	1	1	1	1
1	1	1	1	2	2	2	2	4	4
1	1	1	1	1	1	2	2	1	1
1	1	1	1	2	2	1	1	4	4
1	1	2	2	2	2	1	1	1	1
1	1	2	2	2	2	2	2	1	1
1	1	1	1	1	1	2	2	4	4
1	1	1	1	2	2	2	2	4	4
1	1	1	1	2	2	2	2	4	4
1	1	2	2	1	1	2	2	1	1

For Review Only

1	1	2	2	1	1	1	1	4	4
1	1	2	2	2	2	2	2	4	4
1	1	1	1	1	1	1	1	1	1
1	1	1	1	2	2	2	2	4	4
1	1	2	2	2	2	2	2	1	1
1	1	2	2	2	2	1	1	1	1
1	1	2	2	2	2	1	1	4	4
1	1	2	2	2	2	2	2	4	4
1	1	1	1	1	1	1	1	4	4
1	1	2	2	2	2	1	1	4	4
1	1	2	2	2	2	2	2	4	4
1	1	2	2	2	2	2	2	4	4
1	1	1	1	1	1	2	2	4	4
1	1	2	2	2	2	1	1	1	1
1	1	2	2	2	2	1	1	1	1
1	1	2	2	2	2	2	2	4	4
1	1	2	2	2	2	2	2	4	4
1	1	2	2	2	2	1	1	4	4
1	1	1	1	2	2	2	2	4	4
1	1	2	2	2	2	1	1	4	4
1	1	2	2	2	2	2	2	4	4
1	1	2	2	2	2	2	2	4	4
1	1	2	2	2	2	2	2	1	1
4	4	1	1	2	2	2	2	4	4
1	1	2	2	2	2	1	1	4	4
1	1	2	2	2	2	1	1	4	4
1	1	2	2	2	2	1	1	4	4
1	1	1	1	2	2	2	2	4	4
1	1	2	2	2	2	2	2	4	4
1	1	1	1	2	2	2	2	4	4
1	1	1	1	2	2	2	2	4	4
1	1	2	2	2	2	2	2	4	4
1	1	2	2	2	2	1	1	4	4
1	1	2	2	2	2	2	2	4	4
1	1	1	1	1	1	2	2	4	4
1	1	1	1	2	2	2	2	4	4
1	1	1	1	2	2	2	2	4	4
1	1	2	2	2	2	2	2	1	1
1	1	1	1	2	2	2	2	4	4
1	1	1	1	2	2	2	2	4	4
1	1	2	2	2	2	1	1	4	4
1	1	1	1	2	2	2	2	4	4
1	1	2	2	2	2	1	1	4	4
1	1	2	2	2	2	1	1	4	4
1	1	1	1	2	2	2	2	1	1
4	4	1	1	2	2	2	2	1	1
1	1	2	2	2	2	1	1	1	1
1	1	2	2	1	1	1	1	1	1
1	1	1	1	2	2	2	2	4	4
1	1	2	2	2	2	1	1	1	1
1	1	2	2	2	2	1	1	1	1
1	1	1	1	1	1	1	1	4	4
1	1	2	2	2	2	1	1	4	4
1	1	2	2	2	2	1	1	1	1
1	1	2	2	2	2	2	2	4	4

For Review Only

1	1	2	2	1	1	2	2	4	4
1	1	2	2	2	2	1	1	4	4
1	1	1	1	1	1	1	1	4	4
1	1	2	2	2	2	1	1	4	4
1	1	2	2	2	2	1	1	1	1
1	1	1	1	2	2	2	2	4	4
1	1	1	1	2	2	2	2	4	4
1	1	2	2	2	2	2	2	4	4
1	1	2	2	2	2	2	2	4	4
1	1	2	2	2	2	1	1	4	4
1	1	2	2	2	2	1	1	1	1
1	1	2	2	2	2	2	2	4	4
1	1	2	2	2	2	1	1	4	4
1	1	2	2	2	2	2	2	4	4
1	1	1	1	2	2	2	2	4	4
1	1	2	2	1	1	1	1	4	4
1	1	2	2	2	2	1	1	4	4
1	1	2	2	2	2	1	1	4	4
1	1	1	1	2	2	1	1	1	1
1	1	2	2	2	2	1	1	4	4
1	1	2	2	2	2	2	2	4	4
1	1	1	1	2	2	2	2	4	4
1	1	2	2	2	2	1	1	4	4
1	1	1	1	2	2	1	1	1	1
1	1	2	2	2	2	1	1	4	4
1	1	1	1	1	1	1	1	4	4
1	1	1	1	2	2	2	2	4	4
1	1	2	2	2	2	2	2	4	4
1	1	1	1	2	2	2	2	1	1
1	1	2	2	2	2	2	2	4	4
1	1	1	1	2	2	2	2	4	4
1	1	2	2	2	2	1	1	4	4
1	1	2	2	2	2	1	1	4	4
1	1	1	1	2	2	2	2	4	4
4	4	1	1	2	2	2	2	4	4
1	1	1	1	2	2	1	1	1	1
1	1	2	2	2	2	1	1	4	4
1	1	1	1	2	2	2	2	4	4
1	1	2	2	1	1	1	1	1	1
1	1	2	2	2	2	1	1	1	1
1	1	2	2	2	2	2	2	1	1
1	1	2	2	1	1	1	1	1	1
4	4	1	1	2	2	2	2	4	4
1	1	2	2	2	2	2	2	4	4
1	1	1	1	2	2	1	1	4	4
1	1	1	1	2	2	2	2	4	4
1	1	2	2	1	1	1	1	4	4
1	1	1	1	2	2	2	2	4	4
1	1	1	1	2	2	1	1	4	4
1	1	1	1	2	2	2	2	4	4
1	1	2	2	2	2	2	2	4	4
4	4	1	1	2	2	2	2	4	4
1	1	2	2	2	2	1	1	4	4
1	1	1	1	2	2	1	1	1	1
1	1	1	1	1	1	2	2	4	4

1	1	2	2	2	2	1	1	4	4
1	1	2	2	2	2	2	2	4	4
1	1	2	2	2	2	2	2	4	4
1	1	2	2	2	2	1	1	4	4
1	1	1	1	2	2	1	1	4	4
1	1	2	2	1	1	1	1	4	4
1	1	2	2	2	2	2	2	4	4
4	4	1	1	2	2	1	1	4	4
1	1	1	1	2	2	2	2	4	4
4	4	1	1	2	2	2	2	4	4
1	1	2	2	2	2	2	2	4	4
1	1	1	1	2	2	2	2	4	4
1	1	2	2	2	2	2	2	4	4
1	1	1	1	1	1	1	1	1	1
1	1	1	1	2	2	1	1	1	1
1	1	2	2	2	2	1	1	4	4
1	1	1	1	1	1	2	2	4	4
4	4	2	2	2	2	1	1	4	4
1	1	1	1	2	2	1	1	4	4
1	1	1	1	1	1	1	1	4	4
1	1	1	1	2	2	2	2	1	1
1	1	2	2	1	1	1	1	1	1
1	1	2	2	1	1	1	1	4	4
1	1	2	2	2	2	2	2	4	4
4	4	1	1	2	2	2	2	4	4
1	1	2	2	2	2	1	1	1	1
4	4	1	1	2	2	2	2	4	4
1	1	1	1	2	2	2	2	4	4
1	1	2	2	2	2	2	2	4	4
1	1	2	2	2	2	2	2	4	4
4	4	1	1	2	2	2	2	4	4
1	1	2	2	2	2	1	1	4	4
1	1	2	2	1	1	2	2	4	4
1	1	2	2	1	1	2	2	4	4
1	1	2	2	2	2	1	1	4	4
1	1	1	1	2	2	2	2	4	4
1	1	2	2	2	2	1	1	4	4
1	1	1	1	2	2	1	1	1	1
1	1	1	1	1	1	1	1	4	4
1	1	1	1	1	1	1	1	4	4
1	1	1	1	2	2	1	1	4	4
1	1	1	1	2	2	1	1	1	1
1	1	2	2	2	2	1	1	4	4
1	1	2	2	2	2	1	1	4	4
1	1	1	1	2	2	2	2	1	1
1	1	2	2	2	2	2	2	4	4
1	1	1	1	1	1	2	2	4	4
1	1	2	2	2	2	1	1	4	4
4	4	2	2	2	2	1	1	1	1
1	1	1	1	2	2	2	2	4	4
1	1	2	2	2	2	1	1	4	4
1	1	1	1	1	1	2	2	4	4
1	1	2	2	2	2	1	1	1	1
1	1	1	1	2	2	1	1	1	1
1	1	2	2	2	2	2	2	4	4

For Review Only

1	1	2	2	2	2	1	1	4	4
1	1	1	1	2	2	1	1	1	1

For Review Only

SNP2331	SNP2331	SNP2332	SNP2332	SNP2333	SNP2333	SNP2334	SNP2334	SNP2335	SNP2335
2	2	4	4	1	1	2	2	2	2
2	2	4	4	1	1	2	2	2	2
2	2	4	4	1	1	3	3	1	1
2	2	4	4	4	4	3	3	1	1
2	2	4	4	1	1	3	3	1	1
2	2	4	4	1	1	3	3	1	1
2	2	4	4	1	1	2	2	2	2
4	4	1	1	4	4	2	2	2	2
2	2	4	4	1	1	2	2	2	2
4	4	1	1	4	4	2	2	2	2
4	4	1	1	1	1	2	2	2	2
2	2	4	4	1	1	3	3	1	1
2	2	4	4	1	1	2	2	1	1
2	2	4	4	1	1	2	2	1	1
2	2	4	4	1	1	2	2	1	1
4	4	1	1	1	1	2	2	2	2
2	2	4	4	1	1	2	2	2	2
4	4	1	1	4	4	2	2	2	2
2	2	4	4	1	1	3	3	1	1
2	2	4	4	4	4	2	2	1	1
2	2	4	4	1	1	2	2	2	2
2	2	4	4	1	1	2	2	1	1
2	2	4	4	1	1	3	3	1	1
2	2	4	4	4	4	3	3	1	1
2	2	4	4	1	1	3	3	1	1
4	4	1	1	4	4	2	2	2	2
2	2	4	4	1	1	2	2	2	2
2	2	4	4	1	1	2	2	1	1
2	2	4	4	1	1	2	2	2	2
2	2	4	4	4	4	3	3	1	1
2	2	4	4	4	4	2	2	1	1
2	2	4	4	1	1	3	3	1	1
2	2	4	4	1	1	2	2	2	2
4	4	1	1	1	1	2	2	2	2
2	2	4	4	1	1	2	2	1	1
4	4	1	1	4	4	2	2	2	2
2	2	4	4	1	1	2	2	2	2
4	4	4	4	1	1	2	2	2	2
4	4	1	1	4	4	2	2	2	2
2	2	4	4	4	4	3	3	1	1
2	2	4	4	1	1	3	3	1	1
2	2	4	4	1	1	2	2	2	2
4	4	1	1	4	4	2	2	1	1
2	2	4	4	1	1	2	2	1	1
4	4	1	1	4	4	2	2	2	2
2	2	4	4	1	1	3	3	1	1
4	4	1	1	1	1	2	2	1	1
2	2	4	4	4	4	3	3	1	1
2	2	4	4	1	1	2	2	2	2
2	2	4	4	1	1	3	3	1	1
2	2	4	4	1	1	3	3	1	1
2	2	4	4	1	1	2	2	2	2

For Review Only

2	2	4	4	1	1	2	2	2	2
2	2	4	4	1	1	2	2	2	2
2	2	4	4	4	4	3	3	1	1
2	2	4	4	1	1	2	2	2	2
2	2	4	4	1	1	3	3	1	1
2	2	4	4	4	4	3	3	1	1
4	4	1	1	4	4	2	2	2	2
2	2	4	4	1	1	3	3	1	1
2	2	4	4	1	1	2	2	2	2
2	2	4	4	1	1	2	2	1	1
2	2	4	4	1	1	2	2	2	2
2	2	4	4	1	1	3	3	1	1
2	2	4	4	1	1	2	2	2	2
2	2	4	4	1	1	2	2	2	2
2	2	4	4	4	4	2	2	1	1
2	2	4	4	4	4	3	3	1	1
2	2	4	4	1	1	2	2	1	1
2	2	4	4	4	4	3	3	1	1
2	2	4	4	1	1	3	3	1	1
2	2	4	4	1	1	2	2	2	2
2	2	4	4	1	1	2	2	1	1
2	2	4	4	1	1	2	2	1	1
2	2	4	4	1	1	2	2	2	2
2	2	4	4	1	1	3	3	1	1
4	4	1	1	4	4	2	2	2	2
2	2	4	4	1	1	2	2	2	2
2	2	4	4	1	1	2	2	2	2
2	2	4	4	4	4	3	3	1	1
4	4	1	1	4	4	2	2	2	2
2	2	4	4	1	1	3	3	1	1
4	4	4	4	1	1	2	2	1	1
2	2	4	4	4	4	3	3	1	1
2	2	4	4	4	4	3	3	1	1
2	2	4	4	1	1	2	2	2	2
2	2	4	4	1	1	2	2	2	2
4	4	1	1	4	4	2	2	2	2
4	4	4	4	1	1	2	2	2	2
2	2	4	4	1	1	3	3	1	1
2	2	4	4	1	1	3	3	1	1
2	2	4	4	1	1	3	3	1	1
2	2	4	4	1	1	2	2	2	2
2	2	4	4	1	1	3	3	1	1
2	2	4	4	1	1	3	3	1	1
2	2	4	4	1	1	2	2	2	2
2	2	4	4	1	1	2	2	2	2
2	2	4	4	1	1	3	3	1	1
2	2	4	4	1	1	2	2	1	1
4	4	1	1	4	4	2	2	2	2
2	2	4	4	4	4	2	2	2	2
2	2	4	4	1	1	2	2	1	1
2	2	4	4	1	1	2	2	1	1
4	4	1	1	4	4	2	2	2	2
2	2	4	4	1	1	2	2	2	2
2	2	4	4	1	1	3	3	1	1
4	4	1	1	1	1	2	2	2	2

For Review Only

2	2	1	1	1	1	2	2	1	1
2	2	4	4	1	1	3	3	1	1
4	4	1	1	4	4	2	2	2	2
2	2	4	4	4	4	3	3	1	1
2	2	4	4	1	1	2	2	1	1
2	2	4	4	4	4	3	3	1	1
2	2	4	4	1	1	2	2	2	2
2	2	4	4	4	4	3	3	1	1
2	2	4	4	1	1	2	2	2	2
2	2	4	4	1	1	2	2	1	1
2	2	4	4	4	4	2	2	2	2
2	2	4	4	1	1	2	2	1	1
4	4	1	1	1	1	2	2	2	2
2	2	4	4	4	4	2	2	1	1
2	2	4	4	1	1	2	2	1	1
2	2	4	4	1	1	2	2	1	1
2	2	4	4	1	1	3	3	1	1
2	2	4	4	1	1	2	2	1	1
2	2	4	4	4	4	3	3	1	1
2	2	4	4	1	1	3	3	1	1
2	2	4	4	1	1	2	2	1	1
2	2	4	4	1	1	2	2	1	1
2	2	4	4	1	1	3	3	1	1
2	2	4	4	4	4	3	3	1	1
4	4	4	4	1	1	2	2	2	2
2	2	4	4	1	1	3	3	1	1
2	2	4	4	1	1	2	2	2	2
2	2	4	4	4	4	3	3	1	1
2	2	4	4	1	1	3	3	1	1
2	2	4	4	1	1	3	3	1	1
2	2	4	4	4	4	3	3	1	1
2	2	4	4	1	1	2	2	1	1
2	2	4	4	1	1	2	2	2	2
2	2	4	4	1	1	2	2	1	1
4	4	1	1	1	1	2	2	2	2
2	2	4	4	4	4	3	3	1	1
2	2	4	4	4	4	3	3	1	1
2	2	4	4	1	1	2	2	2	2
2	2	4	4	1	1	3	3	1	1
4	4	4	4	1	1	2	2	1	1
2	2	4	4	1	1	3	3	1	1
2	2	4	4	1	1	3	3	1	1
2	2	4	4	4	4	2	2	2	2
2	2	4	4	1	1	2	2	1	1
2	2	4	4	4	4	2	2	2	2
2	2	4	4	1	1	3	3	1	1
2	2	4	4	4	4	3	3	1	1
4	4	1	1	1	1	2	2	2	2
2	2	4	4	1	1	3	3	1	1
2	2	4	4	4	4	2	2	1	1
2	2	4	4	4	4	2	2	1	1
4	4	1	1	1	1	2	2	2	2
2	2	4	4	4	4	3	3	1	1
2	2	4	4	1	1	2	2	1	1
2	2	4	4	1	1	2	2	1	1

4	4	1	1	4	4	2	2	2	2
2	2	4	4	4	4	3	3	1	1
4	4	1	1	4	4	2	2	2	2
2	2	4	4	1	1	3	3	1	1
2	2	4	4	1	1	2	2	2	2
2	2	4	4	4	4	3	3	1	1
2	2	4	4	1	1	3	3	1	1
2	2	4	4	4	4	2	2	1	1
2	2	4	4	1	1	2	2	2	2
2	2	4	4	1	1	3	3	1	1
2	2	4	4	4	4	3	3	1	1
2	2	4	4	4	4	3	3	1	1
2	2	4	4	4	4	3	3	1	1
2	2	4	4	1	1	2	2	1	1
2	2	4	4	1	1	3	3	1	1
4	4	1	1	1	1	2	2	2	2
2	2	4	4	1	1	3	3	1	1
2	2	4	4	1	1	2	2	1	1
2	2	4	4	4	4	3	3	1	1
2	2	4	4	1	1	2	2	2	2
2	2	4	4	1	1	2	2	1	1
2	2	4	4	4	4	2	2	1	1
2	2	4	4	1	1	2	2	2	2
2	2	4	4	1	1	3	3	1	1
2	2	4	4	1	1	2	2	2	2
4	4	1	1	1	1	2	2	2	2
2	2	4	4	1	1	3	3	1	1
2	2	4	4	1	1	2	2	1	1
2	2	4	4	4	4	2	2	2	2
2	2	4	4	1	1	3	3	1	1
2	2	4	4	1	1	2	2	2	2
2	2	4	4	1	1	3	3	1	1
2	2	4	4	1	1	2	2	2	2
2	2	4	4	1	1	3	3	1	1
2	2	4	4	1	1	3	3	1	1
2	2	4	4	1	1	3	3	1	1
4	4	1	1	1	1	3	3	1	1
2	2	4	4	1	1	2	2	2	2
4	4	1	1	1	1	2	2	2	2
2	2	4	4	1	1	2	2	2	2
2	2	4	4	1	1	3	3	1	1
4	4	1	1	4	4	2	2	2	2
2	2	4	4	1	1	3	3	1	1
2	2	4	4	1	1	2	2	1	1
2	2	4	4	4	4	2	2	1	1
2	2	4	4	4	4	2	2	1	1
4	4	1	1	1	1	2	2	2	2
2	2	4	4	4	4	3	3	1	1
2	2	4	4	4	4	3	3	1	1
2	2	4	4	1	1	2	2	2	2
2	2	4	4	1	1	3	3	1	1
2	2	4	4	1	1	3	3	1	1
2	2	4	4	4	4	3	3	1	1
2	2	4	4	1	1	2	2	2	2
4	4	1	1	4	4	2	2	2	2

For Review Only

2	2	4	4	4	4	3	3	1	1
2	2	4	4	1	1	3	3	1	1
2	2	4	4	1	1	3	3	1	1
2	2	4	4	1	1	2	2	1	1
2	2	4	4	1	1	3	3	1	1
4	4	1	1	4	4	2	2	2	2
2	2	4	4	4	4	3	3	1	1
2	2	4	4	4	4	2	2	1	1
2	2	4	4	4	4	2	2	1	1
2	2	4	4	1	1	3	3	1	1
2	2	4	4	1	1	2	2	1	1
2	2	4	4	4	4	3	3	1	1
2	2	4	4	1	1	3	3	1	1
4	4	1	1	4	4	3	3	1	1
2	2	4	4	1	1	2	2	2	2
2	2	4	4	1	1	3	3	1	1
4	4	1	1	1	1	2	2	2	2
2	2	4	4	1	1	3	3	1	1
2	2	4	4	4	4	3	3	1	1
4	4	1	1	1	1	2	2	2	2
2	2	4	4	4	4	3	3	1	1
4	4	1	1	4	4	2	2	2	2
4	4	1	1	4	4	2	2	2	2
2	2	4	4	1	1	2	2	2	2
2	2	4	4	1	1	3	3	1	1
2	2	4	4	1	1	2	2	2	2
2	2	4	4	4	4	3	3	1	1
2	2	4	4	1	1	3	3	1	1
2	2	4	4	1	1	2	2	2	2
2	2	4	4	1	1	3	3	1	1
2	2	4	4	1	1	3	3	1	1
2	2	4	4	4	4	3	3	1	1
4	4	1	1	1	1	2	2	2	2
4	4	1	1	4	4	2	2	1	1
2	2	4	4	1	1	3	3	1	1
2	2	4	4	4	4	3	3	1	1
2	2	4	4	4	4	2	2	2	2
2	2	4	4	1	1	3	3	1	1
4	4	1	1	4	4	2	2	2	2
4	4	1	1	4	4	2	2	2	2
2	2	4	4	1	1	2	2	2	2
2	2	4	4	1	1	3	3	1	1
2	2	4	4	1	1	2	2	2	2
2	2	4	4	1	1	2	2	2	2
2	2	4	4	1	1	3	3	1	1
2	2	4	4	1	1	2	2	2	2
4	4	1	1	1	1	2	2	1	1
4	4	1	1	1	1	2	2	1	1
2	2	4	4	4	4	3	3	1	1
2	2	4	4	4	4	3	3	1	1
2	2	4	4	1	1	2	2	1	1
4	4	1	1	4	4	2	2	2	2
2	2	4	4	1	1	3	3	1	1
2	2	4	4	1	1	2	2	2	2
2	2	4	4	4	4	3	3	1	1

For Review Only

2	2	4	4	1	1	2	2	1	1
2	2	4	4	1	1	2	2	2	2

For Review Only

SNP2336	SNP2336	SNP2337	SNP2337	SNP2338	SNP2338	SNP2339	SNP2339	SNP234	SNP234
1	1	1	1	3	3	1	1	2	2
1	1	4	4	3	3	1	1	1	1
4	4	4	4	2	2	4	4	1	1
4	4	1	1	2	2	4	4	2	2
4	4	1	1	2	2	1	1	1	1
4	4	4	4	2	2	1	1	2	2
1	1	1	1	3	3	1	1	1	1
1	1	4	4	3	3	1	1	1	1
1	1	4	4	3	3	1	1	2	2
1	1	4	4	3	3	4	4	1	1
1	1	1	1	3	3	1	1	1	1
4	4	4	4	2	2	4	4	2	2
1	1	1	1	3	3	1	1	2	2
1	1	1	1	3	3	1	1	2	2
1	1	1	1	2	2	1	1	2	2
1	1	4	4	3	3	1	1	1	1
1	1	1	1	3	3	1	1	2	2
1	1	4	4	2	2	1	1	2	2
4	4	4	4	2	2	4	4	1	1
4	4	1	1	2	2	1	1	1	1
1	1	1	1	3	3	1	1	1	1
1	1	1	1	3	3	1	1	1	1
4	4	1	1	2	2	1	1	2	2
1	1	4	4	2	2	1	1	2	2
4	4	1	1	2	2	4	4	2	2
1	1	4	4	3	3	1	1	1	1
1	1	4	4	3	3	1	1	2	2
1	1	1	1	2	2	1	1	2	2
1	1	4	4	3	3	1	1	2	2
4	4	4	4	2	2	1	1	2	2
4	4	1	1	2	2	4	4	2	2
4	4	1	1	2	2	4	4	1	1
1	1	1	1	3	3	4	4	2	2
1	1	1	1	3	3	1	1	2	2
4	4	1	1	2	2	1	1	2	2
1	1	4	4	3	3	1	1	1	1
1	1	1	1	3	3	1	1	2	2
1	1	1	1	3	3	1	1	2	2
1	1	4	4	3	3	1	1	1	1
4	4	4	4	2	2	1	1	2	2
4	4	4	4	2	2	1	1	1	1
1	1	4	4	3	3	1	1	2	2
1	1	4	4	3	3	1	1	1	1
1	1	1	1	3	3	1	1	2	2
1	1	1	1	3	3	1	1	1	1
4	4	4	4	2	2	1	1	1	1
1	1	4	4	3	3	1	1	2	2
4	4	1	1	2	2	1	1	1	1
1	1	1	1	3	3	1	1	1	1
4	4	1	1	2	2	1	1	2	2
4	4	4	4	2	2	1	1	1	1
1	1	4	4	3	3	1	1	2	2

For Review Only

1	1	1	1	3	3	1	1	2	2
1	1	1	1	3	3	4	4	2	2
4	4	1	1	2	2	4	4	2	2
1	1	1	1	3	3	1	1	1	1
4	4	4	4	2	2	4	4	1	1
4	4	1	1	2	2	1	1	1	1
1	1	1	1	3	3	1	1	2	2
4	4	1	1	2	2	1	1	2	2
1	1	1	1	3	3	1	1	2	2
1	1	1	1	2	2	1	1	2	2
1	1	4	4	3	3	1	1	1	1
4	4	4	4	2	2	1	1	1	1
1	1	1	1	3	3	1	1	2	2
1	1	4	4	3	3	1	1	2	2
1	1	1	1	2	2	4	4	2	2
1	1	1	1	2	2	1	1	1	1
1	1	4	4	3	3	1	1	1	1
4	4	1	1	2	2	1	1	2	2
4	4	1	1	2	2	1	1	1	1
1	1	1	1	3	3	1	1	2	2
1	1	4	4	2	2	1	1	1	1
1	1	1	1	2	2	1	1	2	2
1	1	1	1	3	3	1	1	1	1
4	4	4	4	2	2	1	1	2	2
1	1	1	1	3	3	1	1	1	1
1	1	4	4	3	3	1	1	1	1
1	1	1	1	3	3	1	1	2	2
4	4	4	4	2	2	1	1	2	2
1	1	1	1	3	3	1	1	1	1
4	4	1	1	2	2	4	4	1	1
1	1	1	1	3	3	4	4	1	1
4	4	4	4	2	2	1	1	2	2
4	4	4	4	2	2	1	1	2	2
1	1	1	1	3	3	1	1	2	2
1	1	1	1	3	3	1	1	2	2
1	1	1	1	3	3	1	1	1	1
1	1	1	1	3	3	4	4	2	2
4	4	1	1	2	2	1	1	1	1
4	4	1	1	2	2	1	1	1	1
4	4	1	1	2	2	1	1	1	1
1	1	1	1	3	3	1	1	1	1
4	4	1	1	2	2	1	1	1	1
4	4	4	4	2	2	4	4	2	2
1	1	4	4	3	3	1	1	1	1
1	1	1	1	3	3	1	1	1	1
4	4	1	1	2	2	1	1	1	1
1	1	1	1	3	3	1	1	2	2
1	1	4	4	3	3	1	1	2	2
1	1	1	1	3	3	1	1	1	1
1	1	4	4	2	2	1	1	1	1
1	1	4	4	3	3	1	1	2	2
1	1	4	4	3	3	1	1	2	2
1	1	1	1	3	3	1	1	2	2
4	4	1	1	2	2	1	1	2	2
1	1	1	1	3	3	1	1	2	2

For Review Only

1	1	1	1	2	2	1	1	1	1
4	4	1	1	2	2	1	1	2	2
1	1	4	4	3	3	1	1	1	1
4	4	4	4	2	2	1	1	2	2
1	1	4	4	3	3	1	1	2	2
4	4	4	4	2	2	1	1	1	1
1	1	4	4	3	3	4	4	1	1
1	1	1	1	2	2	1	1	2	2
1	1	4	4	3	3	1	1	1	1
1	1	1	1	2	2	1	1	1	1
1	1	1	1	3	3	1	1	2	2
1	1	1	1	2	2	1	1	1	1
1	1	4	4	3	3	4	4	2	2
1	1	4	4	2	2	1	1	1	1
1	1	1	1	2	2	1	1	1	1
1	1	1	1	2	2	1	1	2	2
4	4	1	1	2	2	1	1	2	2
1	1	1	1	2	2	1	1	2	2
4	4	4	4	2	2	1	1	2	2
4	4	1	1	2	2	1	1	1	1
1	1	1	1	2	2	1	1	2	2
4	4	1	1	2	2	4	4	2	2
4	4	4	4	2	2	1	1	2	2
4	4	4	4	2	2	1	1	2	2
1	1	1	1	2	2	1	1	1	1
4	4	1	1	2	2	1	1	1	1
1	1	1	1	3	3	4	4	1	1
4	4	4	4	2	2	1	1	2	2
4	4	4	4	2	2	4	4	2	2
4	4	4	4	2	2	4	4	2	2
4	4	4	4	2	2	1	1	2	2
1	1	1	1	2	2	1	1	2	2
1	1	1	1	3	3	1	1	1	1
1	1	1	1	3	3	1	1	2	2
1	1	1	1	3	3	1	1	2	2
1	1	1	1	3	3	1	1	1	1
4	4	4	4	2	2	1	1	2	2
1	1	1	1	3	3	1	1	2	2
4	4	1	1	2	2	1	1	2	2
1	1	1	1	2	2	1	1	2	2
4	4	1	1	2	2	1	1	1	1
4	4	4	4	2	2	1	1	2	2
1	1	4	4	3	3	1	1	1	1
4	4	1	1	2	2	1	1	2	2
1	1	1	1	3	3	1	1	2	2
4	4	1	1	2	2	1	1	2	2
4	4	1	1	2	2	1	1	1	1
1	1	4	4	3	3	1	1	1	1
4	4	1	1	2	2	1	1	2	2
1	1	1	1	2	2	1	1	1	1
1	1	1	1	2	2	1	1	1	1
1	1	1	1	3	3	1	1	1	1
4	4	4	4	2	2	1	1	1	1
4	4	4	4	2	2	1	1	1	1
1	1	1	1	2	2	1	1	2	2

For Review Only

1	1	1	1	3	3	4	4	2	2
4	4	4	4	2	2	4	4	1	1
1	1	1	1	3	3	1	1	1	1
4	4	1	1	2	2	1	1	1	1
1	1	4	4	3	3	1	1	1	1
4	4	4	4	2	2	1	1	2	2
1	1	4	4	2	2	1	1	2	2
1	1	1	1	2	2	1	1	2	2
1	1	1	1	3	3	1	1	2	2
4	4	1	1	2	2	1	1	1	1
1	1	1	1	2	2	1	1	1	1
1	1	1	1	2	2	1	1	2	2
1	1	1	1	2	2	1	1	1	1
1	1	1	1	2	2	1	1	2	2
4	4	4	4	2	2	1	1	2	2
1	1	1	1	3	3	1	1	1	1
4	4	4	4	2	2	1	1	1	1
1	1	4	4	3	3	1	1	1	1
4	4	4	4	2	2	1	1	1	1
1	1	4	4	3	3	4	4	1	1
1	1	1	1	2	2	1	1	2	2
1	1	4	4	3	3	1	1	2	2
1	1	1	1	3	3	1	1	1	1
4	4	4	4	2	2	1	1	1	1
1	1	1	1	3	3	1	1	1	1
1	1	1	1	3	3	1	1	1	1
4	4	4	4	2	2	1	1	2	2
1	1	1	1	2	2	1	1	2	2
1	1	4	4	3	3	1	1	2	2
4	4	1	1	2	2	1	1	2	2
1	1	4	4	3	3	1	1	2	2
4	4	1	1	2	2	1	1	1	1
1	1	4	4	3	3	1	1	1	1
4	4	4	4	2	2	1	1	2	2
4	4	1	1	2	2	1	1	2	2
4	4	4	4	2	2	1	1	1	1
4	4	1	1	2	2	1	1	1	1
1	1	1	1	3	3	1	1	2	2
1	1	4	4	3	3	1	1	1	1
1	1	4	4	3	3	1	1	1	1
4	4	4	4	2	2	4	4	1	1
1	1	1	1	3	3	1	1	1	1
4	4	4	4	2	2	1	1	2	2
1	1	1	1	2	2	1	1	2	2
4	4	4	4	2	2	1	1	1	1
4	4	1	1	2	2	1	1	2	2
1	1	1	1	3	3	1	1	1	1
4	4	4	4	2	2	1	1	2	2
4	4	4	4	2	2	1	1	1	1
1	1	1	1	2	2	1	1	2	2
4	4	4	4	2	2	4	4	2	2
4	4	1	1	2	2	1	1	2	2
4	4	1	1	2	2	1	1	1	1
1	1	4	4	3	3	1	1	1	1
1	1	4	4	3	3	1	1	2	2

For Review Only

4	4	4	4	2	2	1	1	1	1
4	4	1	1	2	2	1	1	2	2
4	4	4	4	2	2	4	4	2	2
1	1	1	1	2	2	1	1	1	1
4	4	4	4	3	3	4	4	1	1
1	1	1	1	3	3	1	1	1	1
4	4	1	1	2	2	1	1	2	2
4	4	1	1	2	2	1	1	1	1
1	1	4	4	2	2	1	1	1	1
4	4	4	4	2	2	1	1	2	2
1	1	1	1	2	2	1	1	2	2
4	4	1	1	2	2	1	1	2	2
4	4	1	1	2	2	1	1	2	2
4	4	1	1	2	2	1	1	1	1
1	1	4	4	3	3	4	4	1	1
4	4	1	1	2	2	1	1	1	1
1	1	4	4	3	3	1	1	2	2
4	4	1	1	2	2	4	4	1	1
4	4	4	4	2	2	1	1	1	1
1	1	1	1	3	3	1	1	1	1
4	4	4	4	2	2	4	4	2	2
1	1	1	1	3	3	1	1	1	1
1	1	4	4	3	3	1	1	1	1
1	1	1	1	3	3	1	1	2	2
4	4	1	1	2	2	1	1	2	2
1	1	1	1	2	2	1	1	2	2
4	4	4	4	2	2	1	1	2	2
4	4	1	1	2	2	4	4	2	2
1	1	4	4	3	3	1	1	2	2
4	4	4	4	2	2	4	4	2	2
4	4	4	4	2	2	1	1	2	2
4	4	4	4	2	2	1	1	1	1
1	1	1	1	3	3	1	1	2	2
1	1	4	4	3	3	1	1	2	2
4	4	1	1	2	2	1	1	1	1
4	4	4	4	2	2	1	1	2	2
1	1	4	4	3	3	1	1	1	1
4	4	4	4	2	2	1	1	1	1
1	1	4	4	3	3	1	1	1	1
1	1	4	4	3	3	1	1	1	1
1	1	1	1	3	3	1	1	1	1
4	4	4	4	2	2	1	1	1	1
1	1	1	1	3	3	1	1	1	1
1	1	1	1	3	3	1	1	1	1
4	4	1	1	2	2	1	1	2	2
1	1	1	1	3	3	1	1	2	2
1	1	4	4	3	3	1	1	2	2
1	1	1	1	3	3	1	1	1	1
4	4	1	1	2	2	1	1	1	1
4	4	4	4	2	2	1	1	2	2
1	1	1	1	3	3	1	1	1	1
1	1	4	4	3	3	1	1	2	2
4	4	4	4	2	2	4	4	1	1
1	1	4	4	3	3	1	1	1	1
4	4	4	4	2	2	1	1	2	2

For Review Only

1	1	1	1	3	3	1	1	1	1
1	1	1	1	3	3	1	1	1	1

For Review Only

SNP2340	SNP2340	SNP2341	SNP2341	SNP2342	SNP2342	SNP2343	SNP2343	SNP2344	SNP2344
1	1	1	1	1	1	3	3	3	3
1	1	2	2	1	1	1	1	3	3
1	1	2	2	1	1	1	1	3	3
1	1	2	2	1	1	1	1	3	3
4	4	2	2	1	1	3	3	3	3
1	1	2	2	1	1	3	3	3	3
4	4	2	2	4	4	3	3	3	3
4	4	1	1	1	1	3	3	3	3
1	1	2	2	4	4	3	3	3	3
1	1	1	1	1	1	3	3	3	3
4	4	2	2	1	1	3	3	3	3
1	1	2	2	1	1	1	1	3	3
1	1	2	2	1	1	1	1	3	3
4	4	1	1	4	4	3	3	2	2
1	1	1	1	1	1	1	1	3	3
4	4	1	1	1	1	3	3	3	3
1	1	2	2	4	4	3	3	3	3
1	1	2	2	1	1	3	3	3	3
1	1	2	2	1	1	1	1	3	3
1	1	1	1	1	1	3	3	3	3
4	4	2	2	4	4	3	3	3	3
1	1	2	2	4	4	3	3	3	3
4	4	2	2	1	1	3	3	2	2
4	4	1	1	4	4	3	3	3	3
1	1	2	2	4	4	3	3	3	3
1	1	2	2	1	1	1	1	3	3
1	1	2	2	1	1	1	1	3	3
1	1	1	1	1	1	1	1	3	3
4	4	2	2	1	1	3	3	3	3
4	4	1	1	1	1	3	3	3	3
1	1	2	2	1	1	1	1	3	3
1	1	2	2	1	1	3	3	3	3
1	1	2	2	4	4	3	3	3	3
4	4	2	2	1	1	3	3	3	3
4	4	2	2	1	1	1	1	3	3
4	4	2	2	1	1	3	3	3	3
1	1	2	2	1	1	3	3	3	3
4	4	1	1	1	1	3	3	3	3
4	4	2	2	1	1	3	3	3	3
4	4	2	2	1	1	3	3	3	3
4	4	2	2	1	1	3	3	3	3
4	4	2	2	1	1	3	3	3	3
4	4	1	1	1	1	3	3	3	3
1	1	2	2	4	4	3	3	3	3
4	4	1	1	1	1	3	3	3	3
1	1	2	2	1	1	1	1	3	3
1	1	2	2	1	1	1	1	3	3
4	4	2	2	1	1	3	3	3	3
1	1	2	2	4	4	3	3	3	3
1	1	2	2	1	1	3	3	3	3
1	1	2	2	1	1	1	1	3	3
1	1	2	2	1	1	1	1	3	3

For Review Only

1	1	2	2	4	4	3	3	3	3
1	1	2	2	4	4	3	3	3	3
1	1	2	2	1	1	3	3	3	3
1	1	2	2	4	4	3	3	3	3
1	1	2	2	1	1	1	1	3	3
1	1	2	2	1	1	1	1	3	3
1	1	2	2	1	1	1	1	3	3
4	4	2	2	1	1	3	3	2	2
1	1	2	2	1	1	3	3	3	3
1	1	1	1	1	1	1	1	3	3
1	1	1	1	1	1	1	1	3	3
1	1	1	1	1	1	1	1	3	3
1	1	2	2	4	4	3	3	3	3
4	4	2	2	1	1	3	3	3	3
1	1	2	2	1	1	1	1	3	3
1	1	2	2	1	1	3	3	3	3
1	1	2	2	1	1	1	1	3	3
1	1	2	2	1	1	1	1	3	3
1	1	1	1	4	4	3	3	3	3
4	4	2	2	1	1	3	3	3	3
1	1	1	1	1	1	1	1	3	3
4	4	2	2	1	1	3	3	3	3
4	4	1	1	4	4	3	3	3	3
4	4	2	2	1	1	1	1	3	3
4	4	1	1	1	1	3	3	3	3
1	1	2	2	1	1	1	1	3	3
1	1	2	2	1	1	3	3	2	2
4	4	1	1	1	1	3	3	3	3
1	1	2	2	1	1	1	1	3	3
1	1	2	2	1	1	1	1	3	3
4	4	1	1	1	1	3	3	3	3
4	4	1	1	1	1	3	3	3	3
4	4	1	1	1	1	3	3	3	3
1	1	2	2	4	4	3	3	3	3
1	1	1	1	1	1	3	3	3	3
4	4	1	1	1	1	3	3	3	3
4	4	1	1	1	1	3	3	3	3
1	1	2	2	4	4	3	3	3	3
1	1	2	2	1	1	3	3	3	3
4	4	1	1	1	1	3	3	2	2
1	1	2	2	4	4	3	3	3	3
1	1	2	2	1	1	1	1	3	3
1	1	1	1	1	1	3	3	3	3
1	1	1	1	1	1	1	1	3	3
1	1	2	2	4	4	3	3	3	3
1	1	2	2	1	1	1	1	3	3
4	4	1	1	1	1	3	3	3	3
1	1	2	2	1	1	1	1	3	3
4	4	1	1	1	1	3	3	3	3
1	1	2	2	1	1	1	1	2	2
1	1	1	1	1	1	1	1	3	3
4	4	2	2	1	1	3	3	3	3
4	4	2	2	1	1	3	3	3	3
4	4	1	1	1	1	1	1	3	3
1	1	2	2	1	1	1	1	3	3

For Review Only

1	1	2	2	1	1	1	1	3	3
1	1	2	2	1	1	1	1	3	3
1	1	2	2	1	1	1	1	3	3
4	4	1	1	1	1	3	3	2	2
1	1	2	2	4	4	1	1	3	3
1	1	2	2	1	1	1	1	3	3
1	1	1	1	1	1	1	1	3	3
1	1	2	2	1	1	3	3	3	3
4	4	1	1	1	1	3	3	3	3
1	1	2	2	1	1	3	3	3	3
1	1	2	2	1	1	3	3	3	3
1	1	2	2	1	1	1	1	3	3
1	1	1	1	1	1	3	3	3	3
1	1	2	2	1	1	1	1	3	3
1	1	1	1	1	1	1	1	3	3
1	1	2	2	1	1	1	1	3	3
1	1	2	2	1	1	1	1	3	3
1	1	1	1	1	1	1	1	3	3
4	4	1	1	1	1	3	3	2	2
1	1	2	2	1	1	3	3	3	3
1	1	2	2	1	1	1	1	3	3
1	1	2	2	1	1	1	1	3	3
1	1	2	2	1	1	1	1	3	3
4	4	1	1	1	1	3	3	3	3
4	4	2	2	1	1	1	1	3	3
4	4	2	2	1	1	3	3	3	3
1	1	2	2	1	1	1	1	3	3
1	1	2	2	4	4	3	3	3	3
1	1	1	1	1	1	1	1	3	3
1	1	1	1	1	1	1	1	3	3
4	4	2	2	1	1	3	3	3	3
1	1	2	2	1	1	1	1	3	3
1	1	2	2	4	4	3	3	3	3
1	1	2	2	1	1	1	1	3	3
4	4	2	2	1	1	3	3	3	3
1	1	1	1	1	1	3	3	3	3
4	4	1	1	1	1	3	3	2	2
1	1	2	2	1	1	1	1	3	3
1	1	2	2	1	1	1	1	3	3
4	4	2	2	1	1	3	3	3	3
1	1	2	2	1	1	3	3	3	3
4	4	1	1	1	1	3	3	2	2
1	1	2	2	1	1	1	1	3	3
4	4	2	2	1	1	3	3	3	3
1	1	1	1	1	1	1	1	3	3
1	1	2	2	4	4	1	1	3	3
1	1	2	2	1	1	1	1	3	3
1	1	2	2	1	1	1	1	3	3
1	1	2	2	1	1	1	1	3	3
1	1	2	2	1	1	1	1	3	3
1	1	2	2	1	1	1	1	3	3
4	4	2	2	1	1	3	3	3	3
1	1	2	2	1	1	3	3	3	3
1	1	2	2	1	1	1	1	3	3
1	1	2	2	1	1	1	1	3	3

For Review Only

1	1	1	1	1	1	1	1	3	3
1	1	2	2	1	1	3	3	3	3
4	4	1	1	1	1	3	3	3	3
1	1	2	2	1	1	1	1	3	3
1	1	2	2	1	1	1	1	3	3
1	1	2	2	1	1	3	3	3	3
4	4	2	2	1	1	3	3	3	3
1	1	2	2	1	1	1	1	3	3
4	4	2	2	1	1	3	3	3	3
1	1	2	2	1	1	3	3	3	3
1	1	2	2	1	1	1	1	3	3
4	4	2	2	1	1	3	3	3	3
1	1	2	2	1	1	1	1	3	3
1	1	2	2	1	1	1	1	3	3
4	4	2	2	1	1	3	3	2	2
4	4	2	2	1	1	3	3	3	3
1	1	2	2	1	1	1	1	3	3
1	1	2	2	1	1	1	1	3	3
1	1	2	2	1	1	1	1	3	3
1	1	1	1	1	1	1	1	3	3
1	1	2	2	1	1	1	1	3	3
4	4	2	2	1	1	3	3	3	3
4	4	2	2	1	1	3	3	2	2
1	1	1	1	1	1	1	1	3	3
1	1	2	2	4	4	3	3	3	3
4	4	2	2	1	1	3	3	3	3
1	1	2	2	1	1	1	1	3	3
1	1	2	2	1	1	1	1	3	3
1	1	1	1	4	4	1	1	3	3
1	1	2	2	4	4	1	1	3	3
4	4	1	1	1	1	3	3	3	3
1	1	2	2	1	1	1	1	3	3
1	1	2	2	1	1	1	1	3	3
4	4	2	2	1	1	3	3	2	2
1	1	2	2	1	1	3	3	3	3
1	1	1	1	1	1	1	1	3	3
4	4	2	2	4	4	3	3	3	3
1	1	2	2	1	1	3	3	3	3
1	1	2	2	1	1	1	1	3	3
1	1	1	1	1	1	1	1	3	3
1	1	2	2	1	1	1	1	3	3
1	1	2	2	1	1	1	1	3	3
4	4	1	1	1	1	3	3	3	3
4	4	2	2	1	1	1	1	3	3
1	1	1	1	1	1	1	1	3	3
1	1	2	2	1	1	1	1	3	3
4	4	2	2	1	1	3	3	3	3
4	4	1	1	1	1	3	3	3	3
4	4	1	1	1	1	3	3	3	3
1	1	2	2	1	1	1	1	3	3
1	1	1	1	1	1	1	1	3	3
4	4	1	1	1	1	3	3	2	2
4	4	2	2	1	1	3	3	2	2
1	1	1	1	1	1	1	1	3	3
4	4	2	2	1	1	3	3	3	3

For Review Only

4	4	2	2	1	1	3	3	3	3
4	4	2	2	1	1	1	1	2	2
1	1	2	2	1	1	1	1	3	3
1	1	2	2	1	1	3	3	3	3
1	1	1	1	1	1	3	3	3	3
1	1	2	2	1	1	1	1	3	3
4	4	2	2	1	1	3	3	3	3
4	4	2	2	1	1	3	3	3	3
4	4	2	2	1	1	3	3	3	3
4	4	1	1	1	1	3	3	3	3
1	1	1	1	1	1	1	1	3	3
4	4	1	1	1	1	3	3	3	3
1	1	2	2	1	1	3	3	3	3
1	1	2	2	1	1	1	1	3	3
1	1	2	2	1	1	1	1	3	3
1	1	2	2	1	1	3	3	2	2
4	4	2	2	1	1	3	3	3	3
1	1	1	1	1	1	1	1	3	3
4	4	2	2	1	1	3	3	3	3
4	4	2	2	1	1	3	3	3	3
1	1	2	2	1	1	1	1	3	3
1	1	2	2	1	1	1	1	3	3
1	1	1	1	1	1	3	3	3	3
4	4	2	2	1	1	3	3	3	3
4	4	2	2	1	1	3	3	2	2
1	1	2	2	1	1	1	1	3	3
4	4	1	1	1	1	3	3	3	3
1	1	2	2	1	1	1	1	3	3
1	1	1	1	1	1	3	3	3	3
1	1	1	1	1	1	1	1	3	3
4	4	1	1	1	1	3	3	3	3
4	4	2	2	1	1	3	3	3	3
1	1	2	2	1	1	1	1	2	2
1	1	2	2	4	4	1	1	3	3
4	4	2	2	1	1	3	3	2	2
4	4	1	1	1	1	3	3	3	3
1	1	2	2	1	1	3	3	3	3
1	1	1	1	1	1	1	1	3	3
4	4	2	2	1	1	3	3	3	3
4	4	2	2	1	1	3	3	3	3
1	1	2	2	1	1	1	1	3	3
1	1	2	2	1	1	1	1	3	3
4	4	2	2	4	4	3	3	3	3
1	1	2	2	4	4	3	3	3	3
1	1	2	2	1	1	1	1	3	3
1	1	2	2	1	1	3	3	3	3
4	4	2	2	1	1	3	3	3	3
1	1	2	2	4	4	1	1	3	3
1	1	2	2	1	1	1	1	3	3
4	4	1	1	1	1	3	3	3	3
1	1	2	2	1	1	3	3	3	3
4	4	2	2	1	1	3	3	3	3
1	1	2	2	1	1	1	1	3	3
1	1	2	2	1	1	1	1	3	3
4	4	2	2	1	1	3	3	3	3

For Review Only

1	1	2	2	1	1	3	3	3	3
1	1	1	1	4	4	1	1	3	3

For Review Only

SNP2345	SNP2345	SNP2346	SNP2346	SNP2347	SNP2347	SNP2348	SNP2348	SNP2349	SNP2349
2	2	2	2	4	4	2	2	1	1
1	1	3	3	1	1	2	2	1	1
1	1	2	2	4	4	2	2	1	1
1	1	2	2	4	4	2	2	1	1
2	2	2	2	4	4	4	4	1	1
2	2	2	2	4	4	2	2	1	1
2	2	2	2	4	4	2	2	1	1
2	2	2	2	4	4	2	2	1	1
2	2	2	2	4	4	2	2	1	1
2	2	2	2	4	4	4	4	1	1
2	2	2	2	4	4	2	2	1	1
1	1	2	2	4	4	2	2	1	1
2	2	2	2	4	4	2	2	1	1
1	1	2	2	4	4	2	2	1	1
2	2	2	2	4	4	2	2	1	1
2	2	2	2	4	4	2	2	1	1
2	2	2	2	4	4	2	2	1	1
2	2	2	2	4	4	4	4	1	1
1	1	2	2	4	4	2	2	1	1
2	2	2	2	4	4	2	2	1	1
2	2	2	2	4	4	2	2	1	1
2	2	3	3	4	4	2	2	1	1
2	2	2	2	4	4	2	2	1	1
2	2	2	2	4	4	2	2	1	1
1	1	2	2	4	4	2	2	1	1
2	2	2	2	4	4	2	2	1	1
2	2	2	2	4	4	2	2	1	1
1	1	2	2	4	4	2	2	1	1
2	2	2	2	4	4	2	2	1	1
1	1	3	3	4	4	4	4	1	1
1	1	2	2	4	4	2	2	1	1
2	2	2	2	4	4	2	2	1	1
2	2	2	2	4	4	2	2	1	1
2	2	2	2	4	4	4	4	1	1
2	2	2	2	4	4	2	2	1	1
2	2	2	2	4	4	2	2	1	1
2	2	2	2	4	4	2	2	1	1
2	2	2	2	4	4	2	2	1	1
2	2	2	2	4	4	2	2	1	1
2	2	2	2	4	4	2	2	1	1
1	1	3	3	4	4	4	4	1	1
1	1	3	3	4	4	2	2	1	1
2	2	2	2	4	4	2	2	1	1
2	2	2	2	4	4	2	2	1	1
2	2	2	2	4	4	4	4	1	1
1	1	2	2	4	4	2	2	1	1
1	1	3	3	1	1	2	2	1	1
2	2	2	2	4	4	2	2	1	1
1	1	3	3	4	4	2	2	1	1
2	2	2	2	4	4	2	2	1	1
1	1	2	2	4	4	2	2	1	1
2	2	2	2	4	4	2	2	1	1

For Review Only

2	2	2	2	4	4	2	2	1	1
2	2	2	2	4	4	2	2	1	1
2	2	2	2	4	4	2	2	1	1
1	1	3	3	4	4	2	2	1	1
1	1	2	2	4	4	2	2	1	1
1	1	3	3	1	1	2	2	1	1
2	2	2	2	4	4	2	2	1	1
2	2	2	2	4	4	4	4	3	3
2	2	2	2	4	4	2	2	1	1
1	1	2	2	4	4	2	2	1	1
1	1	3	3	1	1	2	2	1	1
1	1	3	3	1	1	2	2	1	1
2	2	2	2	4	4	2	2	1	1
2	2	2	2	4	4	2	2	1	1
2	2	2	2	4	4	2	2	1	1
2	2	2	2	4	4	2	2	1	1
1	1	2	2	4	4	2	2	1	1
2	2	2	2	4	4	2	2	1	1
2	2	2	2	4	4	2	2	1	1
2	2	2	2	4	4	2	2	1	1
1	1	3	3	4	4	2	2	1	1
2	2	2	2	4	4	2	2	1	1
2	2	2	2	4	4	2	2	1	1
2	2	2	2	4	4	4	4	1	1
2	2	2	2	4	4	4	4	1	1
1	1	3	3	1	1	2	2	1	1
2	2	2	2	4	4	2	2	1	1
2	2	2	2	4	4	2	2	3	3
2	2	2	2	4	4	2	2	1	1
2	2	2	2	4	4	2	2	1	1
2	2	2	2	4	4	4	4	1	1
2	2	2	2	4	4	2	2	1	1
2	2	2	2	4	4	2	2	3	3
2	2	2	2	4	4	2	2	1	1
2	2	2	2	4	4	2	2	1	1
2	2	2	2	4	4	4	4	1	1
2	2	2	2	4	4	4	4	1	1
1	1	3	3	4	4	2	2	1	1
1	1	2	2	4	4	2	2	1	1
2	2	2	2	4	4	2	2	1	1
2	2	2	2	4	4	2	2	1	1
1	1	2	2	4	4	2	2	1	1
1	1	2	2	4	4	2	2	1	1
1	1	3	3	1	1	2	2	1	1
2	2	2	2	4	4	2	2	1	1
1	1	2	2	4	4	2	2	1	1
2	2	2	2	4	4	4	4	1	1
2	2	2	2	4	4	2	2	1	1
2	2	2	2	4	4	2	2	1	1
2	2	2	2	4	4	2	2	1	1
1	1	3	3	1	1	2	2	1	1
2	2	2	2	4	4	2	2	1	1
2	2	2	2	4	4	4	4	1	1
2	2	2	2	4	4	2	2	1	1
2	2	2	2	4	4	2	2	1	1

For Review Only

2	2	2	2	4	4	2	2	1	1
2	2	2	2	4	4	2	2	3	3
2	2	2	2	4	4	2	2	1	1
2	2	2	2	4	4	4	4	3	3
1	1	2	2	4	4	2	2	1	1
2	2	2	2	4	4	2	2	1	1
1	1	2	2	4	4	2	2	1	1
2	2	2	2	4	4	2	2	1	1
2	2	2	2	4	4	2	2	1	1
2	2	2	2	4	4	2	2	3	3
2	2	2	2	4	4	2	2	1	1
2	2	2	2	4	4	2	2	1	1
1	1	2	2	4	4	2	2	1	1
1	1	2	2	4	4	2	2	1	1
1	1	2	2	4	4	2	2	3	3
2	2	2	2	4	4	2	2	1	1
2	2	2	2	4	4	2	2	3	3
2	2	2	2	4	4	2	2	1	1
2	2	2	2	4	4	4	4	3	3
2	2	2	2	4	4	2	2	1	1
2	2	2	2	4	4	2	2	1	1
2	2	2	2	4	4	2	2	1	1
2	2	2	2	4	4	2	2	1	1
2	2	2	2	4	4	2	2	3	3
2	2	2	2	4	4	2	2	1	1
2	2	2	2	4	4	2	2	1	1
2	2	2	2	4	4	2	2	1	1
2	2	2	2	4	4	2	2	1	1
1	1	2	2	4	4	2	2	1	1
2	2	2	2	4	4	2	2	1	1
2	2	2	2	4	4	2	2	3	3
2	2	2	2	4	4	2	2	1	1
2	2	2	2	4	4	2	2	1	1
2	2	2	2	4	4	2	2	1	1
2	2	2	2	4	4	4	4	1	1
2	2	2	2	4	4	2	2	1	1
2	2	2	2	4	4	4	4	3	3
2	2	2	2	4	4	2	2	1	1
2	2	2	2	4	4	2	2	1	1
2	2	2	2	4	4	2	2	1	1
2	2	2	2	4	4	2	2	1	1
2	2	2	2	4	4	2	2	3	3
2	2	2	2	4	4	2	2	1	1
2	2	2	2	4	4	2	2	1	1
2	2	2	2	4	4	2	2	1	1
2	2	2	2	4	4	2	2	3	3
2	2	2	2	4	4	2	2	1	1
2	2	2	2	4	4	2	2	1	1
2	2	2	2	4	4	2	2	1	1
2	2	2	2	4	4	2	2	1	1
2	2	2	2	4	4	2	2	1	1
2	2	2	2	4	4	2	2	1	1
2	2	2	2	4	4	2	2	1	1
2	2	2	2	4	4	2	2	1	1
2	2	2	2	4	4	2	2	1	1

For Review Only

1	1	2	2	4	4	2	2	1	1
1	1	2	2	4	4	4	4	1	1
2	2	2	2	4	4	4	4	1	1
2	2	2	2	4	4	2	2	3	3
2	2	2	2	4	4	2	2	1	1
2	2	2	2	4	4	2	2	1	1
2	2	2	2	4	4	2	2	1	1
2	2	2	2	4	4	2	2	1	1
2	2	2	2	4	4	2	2	1	1
1	1	2	2	4	4	2	2	1	1
2	2	2	2	4	4	2	2	1	1
2	2	2	2	4	4	2	2	1	1
1	1	2	2	4	4	2	2	1	1
2	2	2	2	4	4	2	2	1	1
2	2	2	2	4	4	2	2	3	3
2	2	2	2	4	4	2	2	1	1
2	2	2	2	4	4	2	2	1	1
1	1	2	2	4	4	2	2	1	1
2	2	2	2	4	4	2	2	1	1
1	1	2	2	4	4	2	2	1	1
2	2	2	2	4	4	2	2	1	1
2	2	2	2	4	4	2	2	1	1
2	2	2	2	4	4	2	2	1	1
1	1	3	3	1	1	2	2	1	1
2	2	2	2	4	4	2	2	1	1
2	2	2	2	4	4	2	2	1	1
1	1	2	2	4	4	2	2	1	1
2	2	2	2	4	4	2	2	1	1
2	2	2	2	4	4	2	2	1	1
2	2	2	2	4	4	2	2	1	1
2	2	2	2	4	4	2	2	1	1
2	2	2	2	4	4	2	2	1	1
2	2	2	2	4	4	2	2	1	1
2	2	2	2	4	4	2	2	3	3
2	2	2	2	4	4	2	2	1	1
1	1	3	3	1	1	2	2	1	1
2	2	2	2	4	4	2	2	1	1
2	2	2	2	4	4	2	2	1	1
1	1	2	2	4	4	2	2	1	1
1	1	2	2	4	4	2	2	1	1
1	1	2	2	4	4	2	2	1	1
2	2	2	2	4	4	2	2	1	1
2	2	2	2	4	4	2	2	3	3
2	2	2	2	4	4	2	2	1	1
1	1	2	2	4	4	2	2	1	1
2	2	2	2	4	4	2	2	1	1
2	2	2	2	4	4	2	2	1	1
2	2	2	2	4	4	2	2	3	3
2	2	2	2	4	4	4	4	3	3
1	1	2	2	4	4	2	2	1	1
2	2	2	2	4	4	2	2	1	1
2	2	2	2	4	4	4	4	3	3
2	2	2	2	4	4	2	2	3	3
1	1	3	3	1	1	2	2	1	1
2	2	2	2	4	4	2	2	1	1

For Review Only

2	2	2	2	4	4	2	2	3	3
2	2	2	2	4	4	2	2	1	1
1	1	2	2	4	4	2	2	1	1
2	2	2	2	4	4	2	2	1	1
2	2	2	2	4	4	2	2	1	1
2	2	2	2	4	4	2	2	1	1
2	2	2	2	4	4	2	2	1	1
2	2	2	2	4	4	2	2	1	1
2	2	2	2	4	4	2	2	1	1
2	2	2	2	4	4	4	4	3	3
1	1	2	2	4	4	2	2	1	1
2	2	2	2	4	4	2	2	1	1
1	1	2	2	4	4	2	2	1	1
2	2	2	2	4	4	2	2	1	1
1	1	2	2	4	4	2	2	1	1
2	2	2	2	4	4	2	2	3	3
2	2	2	2	4	4	2	2	1	1
2	2	2	2	4	4	2	2	3	3
2	2	2	2	4	4	2	2	3	3
2	2	2	2	4	4	2	2	1	1
2	2	2	2	4	4	2	2	1	1
2	2	2	2	4	4	2	2	1	1
1	1	2	2	4	4	2	2	1	1
2	2	2	2	4	4	2	2	1	1
2	2	2	2	4	4	4	4	3	3
1	1	2	2	4	4	2	2	1	1
2	2	2	2	4	4	2	2	3	3
1	1	2	2	4	4	2	2	1	1
2	2	2	2	4	4	2	2	1	1
2	2	2	2	4	4	2	2	1	1
2	2	2	2	4	4	2	2	3	3
2	2	2	2	4	4	2	2	1	1
2	2	2	2	4	4	2	2	1	1
2	2	2	2	4	4	2	2	1	1
2	2	2	2	4	4	2	2	3	3
2	2	2	2	4	4	2	2	1	1
2	2	2	2	4	4	2	2	1	1
1	1	3	3	1	1	2	2	1	1
2	2	2	2	4	4	2	2	1	1
2	2	2	2	4	4	2	2	1	1
2	2	2	2	4	4	2	2	1	1
2	2	2	2	4	4	2	2	1	1
2	2	2	2	4	4	2	2	1	1
2	2	2	2	4	4	2	2	1	1
2	2	2	2	4	4	2	2	1	1
1	1	2	2	4	4	2	2	1	1
2	2	2	2	4	4	2	2	1	1
1	1	2	2	4	4	2	2	1	1
1	1	2	2	4	4	4	4	1	1
2	2	2	2	4	4	2	2	3	3
2	2	2	2	4	4	2	2	1	1
2	2	2	2	4	4	2	2	1	1
1	1	2	2	4	4	2	2	1	1
1	1	2	2	4	4	2	2	1	1
2	2	2	2	4	4	2	2	1	1

For Review Only

2	2	2	2	4	4	2	2	1	1
1	1	3	3	1	1	2	2	1	1

For Review Only

SNP235	SNP235	SNP2350	SNP2350	SNP2351	SNP2351	SNP2352	SNP2352	SNP2353	SNP2353
1	1	2	2	3	3	4	4	3	3
1	1	2	2	3	3	4	4	3	3
1	1	2	2	4	4	1	1	2	2
1	1	2	2	4	4	4	4	3	3
1	1	2	2	4	4	1	1	2	2
3	3	2	2	4	4	4	4	3	3
1	1	2	2	4	4	1	1	2	2
1	1	2	2	3	3	1	1	2	2
1	1	2	2	4	4	1	1	2	2
1	1	2	2	3	3	1	1	2	2
1	1	2	2	4	4	1	1	2	2
3	3	2	2	4	4	1	1	3	3
1	1	2	2	4	4	4	4	3	3
1	1	2	2	4	4	1	1	2	2
1	1	2	2	4	4	4	4	3	3
1	1	2	2	4	4	1	1	2	2
1	1	2	2	3	3	4	4	3	3
1	1	2	2	4	4	4	4	3	3
1	1	2	2	4	4	4	4	3	3
1	1	2	2	3	3	4	4	3	3
1	1	2	2	4	4	1	1	2	2
1	1	2	2	4	4	4	4	3	3
1	1	2	2	4	4	1	1	2	2
1	1	2	2	3	3	1	1	3	3
1	1	2	2	4	4	4	4	3	3
1	1	2	2	4	4	4	4	3	3
3	3	2	2	4	4	1	1	2	2
1	1	2	2	4	4	4	4	3	3
1	1	2	2	4	4	1	1	2	2
1	1	2	2	3	3	1	1	2	2
3	3	2	2	4	4	4	4	3	3
1	1	2	2	4	4	4	4	3	3
3	3	2	2	4	4	4	4	3	3
1	1	2	2	4	4	4	4	3	3
3	3	4	4	4	4	1	1	2	2
1	1	2	2	4	4	1	1	2	2
1	1	2	2	3	3	4	4	3	3
1	1	2	2	3	3	1	1	2	2
1	1	2	2	3	3	1	1	2	2
3	3	4	4	4	4	1	1	2	2
1	1	2	2	4	4	1	1	2	2
3	3	2	2	4	4	1	1	2	2
1	1	2	2	3	3	1	1	2	2
1	1	2	2	4	4	4	4	3	3
1	1	2	2	3	3	1	1	2	2
1	1	2	2	4	4	1	1	2	2
3	3	2	2	4	4	1	1	2	2
1	1	4	4	4	4	1	1	2	2
1	1	2	2	3	3	4	4	3	3
1	1	2	2	4	4	4	4	3	3
1	1	2	2	4	4	1	1	2	2
1	1	2	2	4	4	1	1	2	2

For Review Only

1	1	2	2	4	4	4	4	3	3
3	3	2	2	4	4	4	4	3	3
1	1	2	2	4	4	4	4	3	3
1	1	2	2	3	3	4	4	3	3
1	1	2	2	4	4	4	4	3	3
1	1	2	2	4	4	1	1	2	2
3	3	2	2	4	4	1	1	2	2
1	1	2	2	3	3	1	1	2	2
1	1	2	2	3	3	4	4	3	3
1	1	2	2	4	4	4	4	3	3
1	1	2	2	4	4	1	1	2	2
1	1	2	2	4	4	1	1	2	2
1	1	2	2	4	4	4	4	3	3
1	1	2	2	4	4	1	1	2	2
3	3	2	2	4	4	4	4	3	3
1	1	2	2	3	3	4	4	3	3
1	1	2	2	4	4	4	4	3	3
3	3	2	2	4	4	4	4	3	3
1	1	2	2	3	3	4	4	3	3
1	1	2	2	4	4	1	1	2	2
1	1	2	2	4	4	1	1	2	2
1	1	2	2	4	4	4	4	3	3
1	1	2	2	3	3	4	4	2	2
1	1	2	2	4	4	4	4	3	3
1	1	2	2	3	3	1	1	2	2
1	1	2	2	4	4	1	1	2	2
3	3	2	2	4	4	4	4	3	3
1	1	4	4	3	3	4	4	3	3
1	1	2	2	4	4	1	1	2	2
1	1	2	2	4	4	4	4	3	3
1	1	2	2	3	3	1	1	2	2
1	1	2	2	4	4	1	1	2	2
1	1	4	4	4	4	4	4	3	3
1	1	2	2	4	4	4	4	3	3
1	1	2	2	4	4	4	4	3	3
1	1	2	2	3	3	1	1	2	2
1	1	2	2	3	3	1	1	2	2
1	1	2	2	4	4	4	4	3	3
1	1	2	2	4	4	4	4	3	3
1	1	2	2	3	3	1	1	2	2
1	1	2	2	4	4	4	4	3	3
1	1	2	2	4	4	1	1	2	2
1	1	2	2	3	3	4	4	3	3
1	1	2	2	4	4	1	1	2	2
1	1	2	2	4	4	4	4	3	3
1	1	2	2	3	3	4	4	3	3
1	1	2	2	4	4	1	1	2	2
1	1	2	2	4	4	1	1	2	2
1	1	2	2	4	4	1	1	2	2
1	1	2	2	4	4	1	1	2	2
3	3	2	2	3	3	1	1	2	2
1	1	2	2	3	3	1	1	2	2
1	1	2	2	3	3	1	1	2	2
1	1	2	2	3	3	4	4	3	3
1	1	2	2	4	4	1	1	2	2

For Review Only

1	1	2	2	4	4	1	1	2	2
1	1	4	4	4	4	4	4	3	3
1	1	2	2	4	4	1	1	2	2
1	1	2	2	4	4	4	4	3	3
1	1	2	2	3	3	1	1	2	2
1	1	2	2	4	4	1	1	2	2
1	1	2	2	4	4	4	4	3	3
3	3	2	2	4	4	4	4	3	3
1	1	4	4	3	3	1	1	2	2
1	1	2	2	4	4	4	4	2	2
1	1	2	2	4	4	4	4	3	3
1	1	2	2	3	3	4	4	3	3
3	3	2	2	3	3	4	4	3	3
1	1	2	2	4	4	1	1	2	2
1	1	2	2	4	4	1	1	2	2
1	1	2	2	4	4	4	4	3	3
3	3	2	2	4	4	4	4	3	3
1	1	2	2	4	4	1	1	3	3
1	1	2	2	4	4	4	4	3	3
1	1	2	2	4	4	4	4	3	3
1	1	2	2	4	4	4	4	3	3
1	1	2	2	4	4	4	4	3	3
3	3	2	2	4	4	4	4	3	3
1	1	4	4	3	3	4	4	3	3
1	1	2	2	4	4	1	1	2	2
1	1	4	4	4	4	1	1	2	2
1	1	2	2	4	4	4	4	3	3
1	1	2	2	3	3	4	4	3	3
3	3	2	2	4	4	4	4	3	3
3	3	2	2	3	3	4	4	3	3
1	1	4	4	4	4	4	4	3	3
1	1	2	2	3	3	4	4	2	2
1	1	2	2	4	4	4	4	3	3
1	1	2	2	3	3	4	4	3	3
1	1	2	2	4	4	4	4	3	3
1	1	2	2	3	3	4	4	3	3
1	1	2	2	3	3	1	1	2	2
3	3	2	2	4	4	1	1	2	2
1	1	4	4	4	4	1	1	2	2
1	1	2	2	4	4	1	1	2	2
1	1	4	4	4	4	4	4	3	3
1	1	2	2	4	4	1	1	2	2
1	1	2	2	4	4	4	4	3	3
1	1	2	2	3	3	4	4	3	3
1	1	2	2	4	4	4	4	3	3
3	3	2	2	4	4	1	1	2	2
1	1	2	2	4	4	1	1	2	2
1	1	2	2	4	4	1	1	2	2
1	1	2	2	3	3	4	4	3	3
1	1	2	2	4	4	1	1	2	2
1	1	2	2	4	4	1	1	2	2
1	1	2	2	3	3	1	1	2	2
1	1	2	2	4	4	4	4	3	3
1	1	2	2	4	4	1	1	2	2
1	1	2	2	3	3	4	4	3	3

For Review Only

3	3	2	2	4	4	4	4	3	3
1	1	2	2	4	4	4	4	3	3
1	1	2	2	3	3	1	1	2	2
1	1	2	2	4	4	4	4	3	3
1	1	2	2	3	3	4	4	3	3
1	1	2	2	4	4	4	4	3	3
1	1	4	4	4	4	1	1	2	2
1	1	2	2	4	4	4	4	3	3
3	3	4	4	4	4	1	1	2	2
1	1	2	2	4	4	4	4	3	3
1	1	2	2	4	4	1	1	2	2
1	1	4	4	4	4	1	1	2	2
1	1	2	2	4	4	4	4	3	3
3	3	2	2	4	4	4	4	3	3
1	1	2	2	4	4	1	1	2	2
1	1	2	2	4	4	1	1	2	2
1	1	2	2	4	4	4	4	3	3
1	1	2	2	3	3	4	4	3	3
1	1	2	2	4	4	1	1	2	2
1	1	2	2	4	4	4	4	3	3
1	1	2	2	3	3	4	4	3	3
1	1	4	4	4	4	4	4	3	3
1	1	2	2	4	4	4	4	3	3
1	1	2	2	4	4	1	1	2	2
1	1	2	2	4	4	4	4	3	3
1	1	2	2	4	4	4	4	3	3
1	1	2	2	4	4	4	4	3	3
3	3	2	2	4	4	4	4	3	3
1	1	2	2	3	3	1	1	2	2
1	1	2	2	4	4	4	4	3	3
1	1	2	2	3	3	1	1	2	2
1	1	2	2	4	4	4	4	3	3
1	1	2	2	4	4	4	4	3	3
1	1	2	2	4	4	1	1	2	2
1	1	2	2	3	3	4	4	3	3
1	1	2	2	4	4	1	1	2	2
1	1	4	4	4	4	1	1	2	2
1	1	2	2	3	3	4	4	3	3
1	1	2	2	4	4	1	1	2	2
1	1	2	2	4	4	1	1	2	2
1	1	2	2	3	3	1	1	2	2
1	1	2	2	4	4	1	1	2	2
1	1	2	2	4	4	4	4	3	3
1	1	4	4	4	4	4	4	2	2
1	1	2	2	4	4	4	4	2	2
1	1	2	2	4	4	4	4	3	3
1	1	2	2	4	4	1	1	2	2
1	1	4	4	4	4	1	1	2	2
1	1	2	2	4	4	4	4	3	3
3	3	2	2	3	3	4	4	3	3
1	1	2	2	4	4	4	4	3	3
1	1	2	2	3	3	4	4	3	3
1	1	2	2	4	4	4	4	3	3
1	1	2	2	4	4	1	1	2	2
1	1	2	2	3	3	1	1	2	2

For Review Only

1	1	2	2	4	4	4	4	3	3
1	1	2	2	4	4	4	4	3	3
3	3	2	2	4	4	4	4	3	3
1	1	2	2	4	4	4	4	3	3
1	1	2	2	3	3	4	4	3	3
1	1	2	2	4	4	4	4	3	3
1	1	2	2	3	3	1	1	2	2
1	1	2	2	3	3	1	1	2	2
1	1	2	2	4	4	1	1	2	2
1	1	2	2	3	3	4	4	3	3
1	1	2	2	3	3	4	4	3	3
1	1	2	2	3	3	1	1	2	2
3	3	2	2	4	4	4	4	3	3
1	1	2	2	4	4	1	1	2	2
1	1	2	2	4	4	1	1	2	2
1	1	2	2	4	4	4	4	3	3
3	3	2	2	3	3	1	1	2	2
1	1	4	4	4	4	4	4	3	3
1	1	2	2	4	4	4	4	3	3
1	1	2	2	3	3	1	1	2	2
1	1	4	4	3	3	1	1	2	2
1	1	2	2	4	4	1	1	2	2
1	1	2	2	4	4	4	4	3	3
1	1	2	2	4	4	1	1	2	2
1	1	2	2	3	3	1	1	2	2
1	1	2	2	4	4	4	4	3	3
1	1	4	4	4	4	4	4	3	3
3	3	2	2	4	4	4	4	3	3
1	1	2	2	3	3	4	4	3	3
3	3	2	2	4	4	4	4	3	3
1	1	4	4	3	3	1	1	2	2
1	1	2	2	4	4	1	1	2	2
3	3	2	2	4	4	4	4	3	3
1	1	2	2	4	4	1	1	2	2
1	1	2	2	4	4	1	1	2	2
1	1	4	4	3	3	4	4	3	3
1	1	2	2	4	4	4	4	3	3
1	1	2	2	4	4	1	1	2	2
1	1	2	2	4	4	1	1	2	2
1	1	4	4	3	3	1	1	2	2
1	1	2	2	3	3	4	4	3	3
1	1	2	2	4	4	1	1	2	2
1	1	2	2	4	4	1	1	2	2
1	1	2	2	4	4	4	4	3	3
1	1	2	2	4	4	1	1	2	2
3	3	2	2	4	4	4	4	3	3
1	1	2	2	3	3	4	4	3	3
1	1	2	2	4	4	4	4	3	3
1	1	2	2	4	4	4	4	3	3
1	1	4	4	4	4	4	4	3	3
1	1	2	2	4	4	4	4	3	3
1	1	2	2	3	3	1	1	2	2
1	1	2	2	4	4	1	1	2	2
1	1	2	2	4	4	1	1	2	2
1	1	4	4	4	4	1	1	2	2

For Review Only

1	1	2	2	4	4	4	4	3	3
1	1	2	2	4	4	1	1	2	2

For Review Only

SNP2354	SNP2354	SNP2355	SNP2355	SNP2356	SNP2356	SNP2357	SNP2357	SNP2358	SNP2358
2	2	4	4	3	3	1	1	3	3
2	2	4	4	3	3	1	1	3	3
2	2	4	4	3	3	1	1	3	3
2	2	4	4	3	3	1	1	3	3
2	2	4	4	1	1	1	1	2	2
2	2	4	4	3	3	1	1	3	3
2	2	4	4	3	3	1	1	2	2
2	2	2	2	3	3	1	1	2	2
2	2	4	4	3	3	1	1	2	2
2	2	2	2	3	3	1	1	2	2
2	2	4	4	3	3	1	1	2	2
2	2	4	4	3	3	1	1	3	3
2	2	4	4	3	3	1	1	3	3
3	3	4	4	3	3	1	1	3	3
2	2	4	4	3	3	1	1	3	3
2	2	4	4	3	3	1	1	3	3
2	2	4	4	3	3	1	1	2	2
2	2	4	4	3	3	1	1	3	3
3	3	4	4	3	3	1	1	2	2
2	2	4	4	1	1	1	1	2	2
2	2	4	4	3	3	1	1	2	2
2	2	4	4	3	3	1	1	2	2
2	2	4	4	3	3	1	1	3	3
2	2	4	4	1	1	2	2	3	3
2	2	4	4	3	3	1	1	2	2
2	2	4	4	1	1	2	2	3	3
2	2	4	4	3	3	1	1	3	3
2	2	4	4	3	3	1	1	3	3
2	2	4	4	3	3	1	1	2	2
2	2	4	4	1	1	2	2	3	3
2	2	4	4	3	3	1	1	3	3
2	2	4	4	3	3	1	1	3	3
2	2	4	4	3	3	1	1	3	3
2	2	4	4	3	3	1	1	3	3
2	2	2	2	1	1	2	2	3	3
2	2	4	4	3	3	1	1	3	3
2	2	4	4	3	3	1	1	3	3
2	2	4	4	3	3	1	1	2	2
2	2	4	4	3	3	1	1	3	3
2	2	4	4	1	1	2	2	2	2
2	2	4	4	1	1	1	1	3	3
2	2	2	2	3	3	1	1	3	3
2	2	4	4	1	1	1	1	2	2
2	2	4	4	3	3	1	1	2	2
2	2	2	2	3	3	1	1	2	2
2	2	4	4	3	3	1	1	3	3
2	2	4	4	1	1	2	2	3	3
2	2	4	4	1	1	2	2	2	2
2	2	4	4	3	3	1	1	2	2
2	2	4	4	1	1	2	2	2	2
3	3	4	4	3	3	1	1	3	3
2	2	4	4	3	3	1	1	3	3

For Review Only

2	2	4	4	3	3	1	1	2	2
2	2	4	4	3	3	1	1	3	3
2	2	4	4	3	3	1	1	2	2
2	2	4	4	3	3	1	1	3	3
3	3	4	4	3	3	1	1	2	2
3	3	4	4	3	3	1	1	3	3
2	2	4	4	1	1	2	2	3	3
2	2	4	4	1	1	2	2	3	3
2	2	4	4	3	3	1	1	3	3
2	2	4	4	3	3	1	1	3	3
3	3	4	4	3	3	1	1	2	2
3	3	4	4	3	3	1	1	2	2
2	2	4	4	3	3	1	1	2	2
2	2	4	4	3	3	1	1	2	2
2	2	4	4	3	3	1	1	3	3
2	2	4	4	1	1	1	1	3	3
2	2	4	4	3	3	1	1	2	2
2	2	4	4	1	1	2	2	3	3
2	2	4	4	1	1	2	2	2	2
2	2	4	4	3	3	1	1	2	2
2	2	4	4	3	3	1	1	2	2
2	2	4	4	3	3	2	2	3	3
2	2	4	4	3	3	1	1	2	2
2	2	4	4	3	3	1	1	3	3
2	2	2	2	3	3	1	1	2	2
3	3	4	4	3	3	1	1	3	3
2	2	4	4	3	3	1	1	2	2
2	2	4	4	1	1	2	2	2	2
2	2	4	4	1	1	2	2	3	3
2	2	4	4	3	3	1	1	3	3
2	2	4	4	3	3	1	1	3	3
2	2	4	4	1	1	2	2	3	3
2	2	4	4	1	1	2	2	3	3
2	2	4	4	3	3	1	1	2	2
2	2	4	4	3	3	1	1	3	3
2	2	2	2	3	3	1	1	2	2
2	2	4	4	3	3	1	1	2	2
2	2	4	4	1	1	1	1	2	2
3	3	2	2	3	3	1	1	2	2
2	2	2	2	1	1	1	1	2	2
2	2	4	4	3	3	1	1	2	2
2	2	4	4	3	3	1	1	3	3
3	3	2	2	3	3	1	1	2	2
3	3	4	4	3	3	1	1	2	2
2	2	4	4	3	3	1	1	2	2
2	2	4	4	3	3	1	1	3	3
2	2	4	4	3	3	1	1	3	3
2	2	4	4	1	1	2	2	2	2
2	2	4	4	3	3	1	1	2	2
2	2	4	4	3	3	1	1	3	3
3	3	4	4	3	3	1	1	3	3
2	2	4	4	3	3	1	1	2	2
2	2	4	4	3	3	1	1	3	3
2	2	4	4	3	3	1	1	3	3
2	2	4	4	3	3	1	1	3	3

For Review Only

2	2	4	4	1	1	2	2	3	3
2	2	4	4	1	1	2	2	3	3
2	2	4	4	3	3	1	1	3	3
2	2	4	4	1	1	2	2	3	3
2	2	4	4	3	3	1	1	3	3
2	2	4	4	1	1	2	2	3	3
3	3	4	4	3	3	1	1	3	3
2	2	4	4	3	3	1	1	3	3
2	2	4	4	3	3	1	1	2	2
2	2	4	4	3	3	1	1	3	3
2	2	4	4	1	1	2	2	2	2
2	2	4	4	3	3	1	1	3	3
3	3	4	4	3	3	1	1	2	2
2	2	4	4	3	3	1	1	3	3
2	2	4	4	3	3	1	1	2	2
2	2	4	4	1	1	1	1	3	3
2	2	4	4	1	1	2	2	3	3
2	2	4	4	3	3	1	1	3	3
2	2	4	4	1	1	2	2	3	3
2	2	2	2	3	3	1	1	2	2
2	2	4	4	3	3	1	1	3	3
2	2	4	4	1	1	2	2	3	3
2	2	4	4	3	3	1	1	3	3
2	2	4	4	1	1	2	2	2	2
2	2	4	4	1	1	2	2	3	3
2	2	4	4	3	3	1	1	3	3
2	2	4	4	3	3	1	1	3	3
2	2	4	4	3	3	1	1	3	3
3	3	4	4	3	3	1	1	2	2
2	2	4	4	1	1	2	2	3	3
2	2	4	4	1	1	2	2	3	3
2	2	4	4	3	3	1	1	3	3
2	2	4	4	3	3	1	1	2	2
2	2	4	4	3	3	1	1	3	3
2	2	4	4	3	3	1	1	3	3
2	2	4	4	3	3	1	1	3	3
2	2	4	4	1	1	2	2	2	2
2	2	4	4	3	3	1	1	3	3
2	2	4	4	1	1	2	2	3	3
2	2	4	4	3	3	1	1	3	3
2	2	4	4	3	3	1	1	3	3
2	2	4	4	1	1	2	2	3	3
2	2	4	4	3	3	1	1	3	3
2	2	4	4	1	1	1	1	3	3
2	2	2	2	3	3	1	1	2	2
2	2	4	4	1	1	2	2	3	3
2	2	4	4	3	3	1	1	3	3
2	2	4	4	1	1	2	2	3	3
2	2	4	4	1	1	1	1	3	3
2	2	4	4	3	3	1	1	3	3
2	2	4	4	3	3	1	1	3	3
2	2	4	4	3	3	1	1	2	2
2	2	4	4	1	1	2	2	3	3
2	2	4	4	3	3	1	1	3	3
2	2	4	4	3	3	1	1	3	3

For Review Only

3	3	4	4	1	1	2	2	2	2
3	3	2	2	3	3	1	1	3	3
2	2	2	2	3	3	1	1	2	2
2	2	4	4	1	1	2	2	3	3
2	2	4	4	3	3	1	1	3	3
2	2	4	4	1	1	2	2	3	3
2	2	4	4	3	3	1	1	3	3
2	2	4	4	3	3	1	1	3	3
2	2	4	4	3	3	1	1	2	2
3	3	4	4	3	3	1	1	3	3
2	2	4	4	3	3	1	1	3	3
2	2	4	4	3	3	2	2	3	3
3	3	4	4	3	3	1	1	3	3
2	2	4	4	3	3	1	1	3	3
2	2	4	4	1	1	2	2	3	3
2	2	4	4	3	3	1	1	2	2
2	2	4	4	1	1	2	2	3	3
2	2	4	4	3	3	1	1	3	3
2	2	4	4	1	1	2	2	3	3
2	2	4	4	3	3	1	1	3	3
2	2	4	4	3	3	1	1	3	3
2	2	4	4	3	3	1	1	3	3
2	2	4	4	3	3	1	1	3	3
3	3	4	4	3	3	1	1	2	2
2	2	4	4	3	3	1	1	2	2
2	2	4	4	3	3	1	1	3	3
3	3	4	4	3	3	1	1	3	3
2	2	4	4	3	3	1	1	3	3
2	2	4	4	3	3	1	1	2	2
2	2	4	4	1	1	2	2	3	3
2	2	4	4	3	3	1	1	3	3
2	2	4	4	3	3	1	1	3	3
2	2	4	4	3	3	1	1	3	3
2	2	4	4	1	1	2	2	3	3
2	2	4	4	1	1	1	1	3	3
3	3	4	4	3	3	1	1	2	2
2	2	4	4	3	3	1	1	3	3
2	2	4	4	3	3	1	1	3	3
2	2	4	4	1	1	2	2	3	3
3	3	4	4	3	3	1	1	2	2
2	2	4	4	3	3	1	1	3	3
2	2	4	4	1	1	2	2	3	3
2	2	2	2	1	1	2	2	3	3
2	2	4	4	3	3	1	1	3	3
2	2	4	4	1	1	1	1	3	3
2	2	4	4	3	3	1	1	3	3
2	2	4	4	3	3	1	1	2	2
2	2	4	4	1	1	2	2	3	3
2	2	4	4	1	1	2	2	3	3
3	3	4	4	3	3	1	1	3	3
2	2	4	4	3	3	1	1	2	2
2	2	4	4	1	1	2	2	2	2
2	2	4	4	1	1	2	2	3	3
3	3	4	4	3	3	1	1	2	2
2	2	4	4	3	3	1	1	3	3

For Review Only

2	2	4	4	1	1	2	2	3	3
2	2	4	4	1	1	2	2	3	3
2	2	4	4	3	3	1	1	3	3
2	2	4	4	3	3	1	1	3	3
2	2	2	2	3	3	1	1	2	2
2	2	4	4	1	1	2	2	3	3
2	2	2	2	1	1	2	2	3	3
2	2	2	2	1	1	2	2	2	2
2	2	4	4	1	1	1	1	2	2
2	2	4	4	1	1	2	2	2	2
2	2	4	4	3	3	1	1	3	3
2	2	2	2	3	3	1	1	3	3
3	3	2	2	3	3	1	1	2	2
2	2	4	4	1	1	2	2	3	3
2	2	4	4	3	3	1	1	3	3
2	2	4	4	1	1	2	2	2	2
2	2	4	4	3	3	1	1	2	2
2	2	4	4	1	1	2	2	2	2
2	2	4	4	1	1	2	2	3	3
2	2	4	4	3	3	1	1	2	2
2	2	4	4	1	1	2	2	3	3
2	2	4	4	1	1	2	2	3	3
3	3	4	4	3	3	1	1	2	2
2	2	4	4	3	3	1	1	3	3
2	2	4	4	1	1	2	2	3	3
2	2	4	4	3	3	1	1	3	3
2	2	4	4	1	1	2	2	3	3
3	3	4	4	3	3	1	1	3	3
2	2	4	4	3	3	1	1	2	2
2	2	4	4	3	3	1	1	2	2
2	2	4	4	1	1	2	2	2	2
2	2	4	4	3	3	1	1	2	2
2	2	4	4	3	3	1	1	2	2
2	2	4	4	1	1	2	2	3	3
2	2	4	4	1	1	2	2	3	3
2	2	4	4	1	1	1	1	2	2
2	2	4	4	3	3	1	1	3	3
3	3	4	4	3	3	1	1	2	2
2	2	4	4	3	3	1	1	3	3
2	2	4	4	3	3	1	1	3	3
2	2	4	4	3	3	1	1	3	3
2	2	4	4	1	1	2	2	3	3
2	2	4	4	3	3	1	1	3	3
2	2	4	4	3	3	1	1	2	2
2	2	4	4	3	3	1	1	2	2
3	3	2	2	3	3	1	1	2	2
2	2	4	4	1	1	2	2	2	2
3	3	4	4	3	3	1	1	3	3
2	2	4	4	1	1	1	1	3	3
2	2	4	4	1	1	2	2	3	3
2	2	4	4	3	3	1	1	3	3
2	2	4	4	3	3	1	1	3	3
2	2	4	4	3	3	1	1	3	3
3	3	4	4	3	3	1	1	3	3
2	2	4	4	1	1	2	2	3	3

For Review Only

2	2	4	4	3	3	1	1	2	2
2	2	4	4	3	3	1	1	3	3

For Review Only

SNP2359	SNP2359	SNP236	SNP236	SNP2360	SNP2360	SNP2361	SNP2361	SNP2362	SNP2362
3	3	3	3	2	2	2	2	2	2
3	3	3	3	4	4	2	2	2	2
3	3	3	3	4	4	2	2	2	2
3	3	3	3	4	4	2	2	2	2
3	3	3	3	2	2	2	2	2	2
3	3	3	3	4	4	2	2	2	2
3	3	3	3	4	4	2	2	2	2
3	3	3	3	4	4	2	2	2	2
3	3	3	3	2	2	2	2	2	2
3	3	3	3	4	4	2	2	2	2
4	4	3	3	2	2	1	1	2	2
3	3	3	3	2	2	2	2	2	2
3	3	3	3	4	4	2	2	2	2
4	4	4	4	4	4	1	1	2	2
3	3	3	3	2	2	2	2	2	2
3	3	3	3	4	4	2	2	2	2
3	3	3	3	4	4	2	2	2	2
4	4	4	4	2	2	2	2	2	2
4	4	3	3	2	2	2	2	4	4
3	3	3	3	4	4	2	2	2	2
3	3	3	3	4	4	2	2	2	2
3	3	3	3	2	2	2	2	2	2
3	3	4	4	4	4	1	1	2	2
3	3	3	3	2	2	1	1	2	2
3	3	3	3	2	2	2	2	2	2
3	3	3	3	4	4	1	1	2	2
3	3	3	3	2	2	2	2	2	2
3	3	3	3	2	2	2	2	2	2
3	3	4	4	2	2	2	2	2	2
3	3	3	3	4	4	2	2	2	2
3	3	3	3	2	2	2	2	2	2
3	3	3	3	2	2	2	2	2	2
3	3	3	3	4	4	2	2	2	2
3	3	4	4	2	2	2	2	2	2
3	3	3	3	4	4	2	2	2	2
3	3	3	3	2	2	1	1	2	2
3	3	3	3	4	4	2	2	2	2
3	3	3	3	4	4	2	2	2	2
3	3	3	3	4	4	1	1	2	2
3	3	3	3	4	4	1	1	2	2
3	3	3	3	4	4	1	1	2	2
3	3	4	4	4	4	2	2	2	2
3	3	3	3	4	4	2	2	2	2
3	3	3	3	4	4	2	2	2	2
3	3	3	3	4	4	2	2	2	2
3	3	3	3	4	4	2	2	2	2
3	3	3	3	4	4	2	2	2	2
3	3	3	3	2	2	1	1	2	2
3	3	3	3	4	4	1	1	2	2
3	3	3	3	2	2	2	2	2	2
3	3	3	3	4	4	2	2	2	2
3	3	3	3	2	2	1	1	2	2

For Review Only

3	3	3	3	2	2	2	2	2	2
3	3	3	3	4	4	2	2	2	2
3	3	3	3	2	2	2	2	2	2
3	3	3	3	4	4	2	2	2	2
4	4	3	3	2	2	2	2	2	2
3	3	3	3	4	4	2	2	2	2
3	3	3	3	4	4	2	2	2	2
3	3	4	4	4	4	1	1	2	2
3	3	3	3	4	4	2	2	2	2
3	3	3	3	2	2	2	2	2	2
4	4	3	3	4	4	2	2	2	2
4	4	3	3	4	4	2	2	2	2
3	3	3	3	2	2	2	2	2	2
3	3	4	4	2	2	2	2	2	2
3	3	3	3	2	2	2	2	2	2
3	3	3	3	4	4	2	2	2	2
4	4	3	3	4	4	1	1	2	2
3	3	3	3	2	2	2	2	2	2
3	3	3	3	2	2	2	2	2	2
3	3	3	3	2	2	2	2	2	2
4	4	3	3	4	4	2	2	2	2
3	3	3	3	2	2	1	1	2	2
3	3	3	3	4	4	2	2	2	2
3	3	3	3	2	2	2	2	2	2
3	3	3	3	4	4	2	2	2	2
3	3	3	3	2	2	2	2	2	2
3	3	3	3	4	4	1	1	2	2
4	4	4	4	4	4	1	1	2	2
3	3	3	3	4	4	1	1	2	2
3	3	3	3	2	2	2	2	4	4
3	3	3	3	4	4	2	2	2	2
3	3	3	3	2	2	1	1	2	2
3	3	4	4	4	4	1	1	2	2
3	3	3	3	2	2	2	2	2	2
3	3	3	3	2	2	2	2	2	2
3	3	3	3	4	4	2	2	2	2
3	3	4	4	2	2	2	2	2	2
3	3	3	3	4	4	2	2	2	2
4	4	3	3	4	4	2	2	2	2
3	3	3	3	4	4	1	1	2	2
3	3	3	3	2	2	2	2	2	2
3	3	3	3	2	2	2	2	4	4
3	3	3	3	4	4	1	1	2	2
4	4	3	3	4	4	2	2	2	2
3	3	3	3	2	2	2	2	2	2
3	3	3	3	2	2	2	2	2	2
3	3	4	4	2	2	2	2	2	2
4	4	4	4	2	2	1	1	2	2
4	4	3	3	4	4	2	2	2	2
3	3	3	3	2	2	2	2	2	2
3	3	3	3	2	2	2	2	2	2
3	3	4	4	4	4	1	1	2	2
3	3	3	3	4	4	1	1	2	2
3	3	3	3	2	2	2	2	2	2
3	3	3	3	2	2	2	2	2	2

For Review Only

3	3	3	3	2	2	1	1	2	2
3	3	3	3	4	4	2	2	2	2
3	3	3	3	4	4	1	1	2	2
3	3	4	4	2	2	2	2	2	2
3	3	3	3	4	4	2	2	2	2
3	3	3	3	2	2	1	1	2	2
3	3	3	3	4	4	2	2	2	2
3	3	3	3	2	2	2	2	2	2
3	3	3	3	4	4	1	1	2	2
3	3	3	3	2	2	2	2	2	2
3	3	3	3	4	4	2	2	2	2
3	3	3	3	2	2	2	2	2	2
3	3	3	3	2	2	1	1	2	2
3	3	3	3	2	2	2	2	2	2
3	3	3	3	2	2	2	2	2	2
3	3	3	3	4	4	2	2	2	2
3	3	3	3	2	2	1	1	2	2
3	3	3	3	4	4	2	2	2	2
3	3	4	4	4	4	2	2	2	2
3	3	3	3	4	4	2	2	2	2
3	3	3	3	2	2	2	2	2	2
3	3	3	3	2	2	2	2	4	4
3	3	3	3	2	2	2	2	2	2
4	4	4	4	4	4	1	1	2	2
3	3	3	3	2	2	1	1	2	2
3	3	3	3	4	4	2	2	2	2
3	3	3	3	2	2	2	2	2	2
3	3	3	3	2	2	2	2	4	4
4	4	3	3	2	2	2	2	2	2
3	3	3	3	4	4	2	2	2	2
3	3	4	4	4	4	1	1	2	2
3	3	3	3	2	2	2	2	2	2
3	3	3	3	2	2	2	2	2	2
3	3	3	3	4	4	2	2	2	2
3	3	4	4	2	2	2	2	2	2
3	3	3	3	2	2	2	2	2	2
3	3	4	4	2	2	2	2	2	2
3	3	3	3	4	4	2	2	2	2
3	3	3	3	2	2	1	1	2	2
3	3	3	3	4	4	1	1	2	2
3	3	3	3	2	2	1	1	2	2
3	3	4	4	2	2	1	1	2	2
3	3	3	3	4	4	2	2	2	2
3	3	3	3	2	2	2	2	2	2
4	4	3	3	4	4	1	1	2	2
3	3	3	3	2	2	2	2	2	2
3	3	3	3	4	4	2	2	2	2
3	3	3	3	4	4	2	2	2	2
3	3	3	3	2	2	2	2	2	2
3	3	3	3	4	4	2	2	2	2
3	3	3	3	4	4	1	1	2	2
3	3	3	3	4	4	1	1	2	2
3	3	3	3	2	2	2	2	2	2
3	3	3	3	4	4	1	1	2	2
3	3	3	3	4	4	2	2	2	2

For Review Only

4	4	3	3	4	4	2	2	2	2
3	3	3	3	4	4	1	1	2	2
3	3	3	3	4	4	2	2	2	2
3	3	3	3	2	2	1	1	2	2
3	3	3	3	2	2	2	2	2	2
3	3	3	3	4	4	1	1	2	2
3	3	3	3	4	4	1	1	2	2
3	3	3	3	4	4	2	2	2	2
3	3	3	3	4	4	2	2	2	2
3	3	3	3	2	2	2	2	2	2
3	3	3	3	4	4	2	2	2	2
3	3	3	3	2	2	1	1	2	2
3	3	3	3	2	2	2	2	2	2
3	3	3	3	2	2	2	2	2	2
3	3	4	4	4	4	2	2	2	2
4	4	3	3	4	4	1	1	2	2
3	3	3	3	2	2	2	2	2	2
3	3	3	3	2	2	2	2	2	2
3	3	3	3	2	2	1	1	2	2
3	3	3	3	4	4	2	2	2	2
3	3	3	3	2	2	2	2	2	2
3	3	4	4	2	2	1	1	2	2
3	3	3	3	2	2	1	1	2	2
4	4	3	3	4	4	2	2	2	2
3	3	3	3	4	4	2	2	2	2
3	3	3	3	2	2	2	2	2	2
3	3	3	3	2	2	2	2	2	2
3	3	3	3	2	2	2	2	2	2
3	3	3	3	4	4	1	1	4	4
3	3	3	3	4	4	1	1	2	2
3	3	3	3	2	2	2	2	2	2
3	3	3	3	2	2	2	2	4	4
3	3	3	3	2	2	2	2	2	2
3	3	4	4	4	4	2	2	2	2
3	3	4	4	4	4	2	2	2	2
4	4	3	3	4	4	2	2	2	2
3	3	3	3	4	4	2	2	2	2
3	3	3	3	2	2	2	2	2	2
3	3	3	3	4	4	2	2	2	2
3	3	3	3	2	2	2	2	2	2
3	3	3	3	4	4	2	2	2	2
3	3	3	3	4	4	2	2	2	2
3	3	4	4	4	4	2	2	2	2
3	3	3	3	2	2	2	2	2	2
3	3	3	3	4	4	1	1	2	2
3	3	3	3	4	4	1	1	2	2
4	4	3	3	4	4	1	1	2	2
3	3	4	4	4	4	1	1	2	2
3	3	3	3	2	2	1	1	2	2
3	3	3	3	2	2	2	2	2	2
3	3	4	4	4	4	2	2	2	2
3	3	3	3	4	4	2	2	2	2
3	3	3	3	2	2	1	1	2	2
4	4	3	3	4	4	2	2	2	2
3	3	4	4	4	4	1	1	2	2

For Review Only

3	3	3	3	4	4	2	2	2	2
3	3	3	3	2	2	2	2	2	2
3	3	3	3	4	4	2	2	2	2
3	3	3	3	2	2	2	2	2	2
3	3	3	3	4	4	2	2	2	2
3	3	3	3	2	2	2	2	2	2
3	3	3	3	4	4	1	1	2	2
3	3	3	3	4	4	2	2	2	2
4	4	3	3	2	2	1	1	2	2
4	4	4	4	4	4	1	1	2	2
3	3	3	3	2	2	2	2	2	2
3	3	3	3	4	4	2	2	2	2
4	4	3	3	4	4	2	2	2	2
3	3	3	3	2	2	2	2	2	2
3	3	3	3	4	4	2	2	2	2
4	4	3	3	2	2	1	1	2	2
3	3	3	3	4	4	1	1	2	2
4	4	3	3	2	2	2	2	2	2
3	3	3	3	2	2	1	1	2	2
3	3	3	3	4	4	1	1	2	2
3	3	4	4	2	2	1	1	2	2
3	3	3	3	4	4	1	1	2	2
4	4	3	3	2	2	2	2	2	2
3	3	3	3	4	4	1	1	2	2
3	3	4	4	4	4	1	1	2	2
3	3	3	3	2	2	2	2	2	2
3	3	4	4	4	4	1	1	2	2
3	3	3	3	2	2	2	2	2	2
3	3	3	3	2	2	2	2	2	2
4	4	3	3	2	2	2	2	2	2
4	4	4	4	4	4	1	1	2	2
3	3	3	3	2	2	1	1	2	2
4	4	3	3	4	4	2	2	2	2
3	3	4	4	4	4	2	2	2	2
3	3	3	3	2	2	1	1	2	2
4	4	4	4	4	4	1	1	2	2
3	3	3	3	2	2	2	2	2	2
4	4	3	3	4	4	2	2	2	2
3	3	3	3	2	2	1	1	2	2
3	3	3	3	4	4	1	1	2	2
3	3	3	3	4	4	2	2	2	2
3	3	3	3	2	2	2	2	2	2
3	3	3	3	4	4	1	1	2	2
3	3	3	3	2	2	2	2	2	2
4	4	4	4	4	4	2	2	2	2
4	4	3	3	4	4	2	2	2	2
3	3	4	4	4	4	2	2	2	2
3	3	3	3	2	2	1	1	2	2
3	3	3	3	4	4	1	1	2	2
3	3	4	4	4	4	1	1	2	2
3	3	3	3	2	2	2	2	2	2
3	3	4	4	4	4	2	2	2	2
3	3	3	3	4	4	2	2	2	2
3	3	3	3	2	2	2	2	2	2
3	3	3	3	4	4	1	1	2	2

For Review Only

4	4	3	3	2	2	2	2	2	2
3	3	3	3	2	2	2	2	2	2

For Review Only

SNP2363	SNP2363	SNP2364	SNP2364	SNP2365	SNP2365	SNP2366	SNP2366	SNP2367	SNP2367
1	1	2	2	4	4	3	3	1	1
2	2	3	3	4	4	1	1	3	3
2	2	3	3	4	4	1	1	3	3
1	1	2	2	4	4	1	1	1	1
1	1	2	2	4	4	1	1	1	1
1	1	2	2	4	4	1	1	3	3
1	1	2	2	4	4	3	3	1	1
1	1	2	2	1	1	1	1	1	1
1	1	2	2	4	4	3	3	1	1
2	2	2	2	1	1	1	1	3	3
1	1	2	2	4	4	1	1	3	3
2	2	3	3	4	4	1	1	3	3
1	1	2	2	4	4	1	1	3	3
2	2	3	3	4	4	3	3	1	1
1	1	2	2	4	4	3	3	3	3
1	1	2	2	4	4	1	1	3	3
1	1	2	2	4	4	3	3	1	1
1	1	2	2	4	4	1	1	3	3
2	2	3	3	4	4	1	1	3	3
1	1	2	2	4	4	1	1	1	1
1	1	2	2	4	4	3	3	1	1
1	1	2	2	4	4	3	3	3	3
1	1	2	2	4	4	1	1	3	3
1	1	2	2	4	4	1	1	3	3
1	1	3	3	4	4	3	3	3	3
1	1	2	2	4	4	1	1	1	1
1	1	2	2	4	4	1	1	1	1
1	1	2	2	4	4	1	1	3	3
1	1	2	2	4	4	1	1	1	1
1	1	2	2	4	4	1	1	3	3
1	1	3	3	4	4	1	1	3	3
1	1	2	2	4	4	1	1	1	1
1	1	2	2	4	4	3	3	1	1
1	1	2	2	4	4	1	1	1	1
1	1	2	2	4	4	1	1	3	3
1	1	2	2	4	4	1	1	3	3
1	1	2	2	4	4	3	3	1	1
1	1	2	2	4	4	3	3	1	1
1	1	2	2	4	4	1	1	3	3
1	1	2	2	4	4	1	1	3	3
1	1	2	2	4	4	1	1	3	3
1	1	2	2	1	1	1	1	1	1
2	2	2	2	1	1	1	1	3	3
1	1	2	2	4	4	3	3	3	3
2	2	2	2	1	1	1	1	3	3
1	1	2	2	4	4	1	1	3	3
2	2	3	3	4	4	1	1	3	3
1	1	2	2	4	4	1	1	3	3
2	2	2	2	4	4	3	3	3	3
1	1	2	2	4	4	1	1	1	1
2	2	3	3	4	4	1	1	3	3
1	1	2	2	4	4	1	1	1	1

For Review Only

1	1	2	2	4	4	3	3	1	1
1	1	2	2	4	4	3	3	1	1
1	1	2	2	4	4	1	1	1	1
1	1	2	2	4	4	3	3	3	3
2	2	3	3	4	4	1	1	3	3
2	2	3	3	4	4	1	1	3	3
1	1	2	2	4	4	1	1	1	1
1	1	2	2	4	4	1	1	3	3
1	1	2	2	4	4	3	3	3	3
1	1	2	2	4	4	1	1	3	3
2	2	3	3	4	4	1	1	3	3
2	2	3	3	4	4	1	1	3	3
1	1	2	2	4	4	3	3	1	1
1	1	2	2	4	4	1	1	1	1
1	1	2	2	4	4	1	1	3	3
1	1	2	2	4	4	1	1	3	3
2	2	3	3	4	4	1	1	3	3
1	1	2	2	4	4	1	1	1	1
1	1	2	2	4	4	3	3	1	1
1	1	2	2	4	4	3	3	1	1
1	1	3	3	4	4	1	1	3	3
1	1	2	2	4	4	1	1	3	3
1	1	2	2	4	4	1	1	1	1
1	1	2	2	4	4	1	1	1	1
2	2	2	2	1	1	1	1	3	3
2	2	3	3	4	4	1	1	3	3
2	2	2	2	4	4	1	1	3	3
1	1	2	2	4	4	1	1	3	3
1	1	2	2	4	4	1	1	1	1
1	1	2	2	4	4	1	1	1	1
1	1	2	2	4	4	1	1	1	1
1	1	2	2	4	4	1	1	1	1
1	1	2	2	4	4	1	1	3	3
1	1	2	2	4	4	3	3	1	1
1	1	2	2	4	4	3	3	1	1
2	2	2	2	1	1	1	1	3	3
1	1	2	2	4	4	1	1	1	1
1	1	2	2	4	4	3	3	3	3
2	2	3	3	1	1	1	1	3	3
1	1	2	2	1	1	3	3	3	3
1	1	2	2	4	4	3	3	1	1
2	2	3	3	4	4	1	1	3	3
2	2	3	3	4	4	1	1	3	3
2	2	3	3	4	4	1	1	3	3
1	1	2	2	4	4	1	1	1	1
1	1	2	2	4	4	1	1	1	1
1	1	2	2	4	4	1	1	3	3
1	1	2	2	4	4	1	1	1	1
1	1	2	2	4	4	1	1	1	1
1	1	2	2	4	4	1	1	1	1
2	2	3	3	4	4	1	1	3	3
1	1	2	2	4	4	1	1	3	3
1	1	2	2	4	4	1	1	3	3
2	2	2	2	4	4	1	1	3	3
1	1	2	2	4	4	1	1	1	1

For Review Only

1	1	2	2	4	4	1	1	1	1
1	1	2	2	4	4	1	1	3	3
1	1	2	2	4	4	1	1	1	1
1	1	2	2	4	4	1	1	3	3
1	1	2	2	4	4	1	1	3	3
1	1	2	2	4	4	1	1	1	1
2	2	3	3	4	4	1	1	3	3
1	1	2	2	4	4	1	1	3	3
1	1	2	2	4	4	1	1	3	3
1	1	2	2	4	4	1	1	3	3
1	1	2	2	4	4	1	1	3	3
1	1	2	2	4	4	1	1	3	3
2	2	3	3	4	4	1	1	3	3
1	1	2	2	4	4	1	1	3	3
2	2	3	3	4	4	3	3	3	3
1	1	2	2	4	4	1	1	3	3
1	1	2	2	4	4	3	3	3	3
1	1	2	2	4	4	1	1	3	3
1	1	2	2	4	4	1	1	3	3
1	1	3	3	4	4	3	3	3	3
1	1	2	2	4	4	1	1	3	3
1	1	2	2	4	4	1	1	3	3
2	2	3	3	4	4	1	1	3	3
1	1	2	2	4	4	1	1	3	3
1	1	2	2	4	4	1	1	3	3
1	1	2	2	4	4	1	1	3	3
1	1	2	2	4	4	1	1	1	1
1	1	2	2	4	4	1	1	1	1
2	2	3	3	4	4	1	1	3	3
1	1	2	2	4	4	1	1	3	3
1	1	2	2	4	4	1	1	3	3
1	1	2	2	4	4	1	1	3	3
1	1	2	2	4	4	3	3	1	1
1	1	2	2	4	4	1	1	3	3
1	1	2	2	4	4	1	1	1	1
1	1	2	2	4	4	1	1	3	3
1	1	2	2	4	4	1	1	3	3
1	1	2	2	4	4	1	1	1	1
1	1	2	2	4	4	1	1	3	3
1	1	2	2	4	4	1	1	3	3
1	1	2	2	4	4	1	1	3	3
1	1	2	2	4	4	1	1	3	3
1	1	2	2	4	4	1	1	1	1
1	1	2	2	4	4	1	1	3	3
1	1	2	2	4	4	1	1	1	1
1	1	2	2	4	4	3	3	1	1
1	1	2	2	4	4	1	1	1	1
1	1	2	2	4	4	1	1	1	1
1	1	2	2	4	4	1	1	3	3
1	1	2	2	4	4	1	1	1	1
1	1	2	2	4	4	1	1	1	1
1	1	2	2	4	4	1	1	3	3
1	1	2	2	4	4	1	1	3	3
1	1	2	2	4	4	1	1	1	1
1	1	2	2	4	4	1	1	3	3

For Review Only

2	2	3	3	4	4	1	1	3	3
2	2	3	3	4	4	1	1	3	3
1	1	2	2	1	1	1	1	3	3
1	1	2	2	4	4	1	1	3	3
1	1	2	2	4	4	1	1	1	1
2	2	3	3	4	4	1	1	3	3
1	1	2	2	4	4	1	1	1	1
1	1	2	2	4	4	1	1	3	3
1	1	2	2	4	4	1	1	3	3
2	2	3	3	4	4	1	1	3	3
1	1	2	2	4	4	1	1	3	3
1	1	2	2	4	4	3	3	3	3
2	2	3	3	4	4	1	1	3	3
1	1	2	2	4	4	1	1	1	1
1	1	2	2	4	4	1	1	3	3
1	1	2	2	4	4	1	1	3	3
1	1	2	2	4	4	1	1	3	3
1	1	2	2	4	4	1	1	1	1
1	1	2	2	4	4	1	1	1	1
1	1	3	3	4	4	1	1	3	3
1	1	2	2	4	4	1	1	3	3
1	1	2	2	4	4	1	1	3	3
1	1	2	2	4	4	1	1	3	3
2	2	3	3	4	4	1	1	3	3
1	1	2	2	4	4	3	3	1	1
1	1	2	2	4	4	1	1	1	1
2	2	3	3	4	4	1	1	3	3
1	1	2	2	4	4	1	1	3	3
2	2	3	3	4	4	1	1	3	3
1	1	2	2	4	4	3	3	1	1
1	1	2	2	4	4	1	1	1	1
1	1	2	2	4	4	1	1	1	1
1	1	2	2	4	4	1	1	1	1
1	1	2	2	4	4	1	1	3	3
1	1	3	3	4	4	1	1	3	3
2	2	3	3	4	4	1	1	3	3
1	1	2	2	4	4	1	1	3	3
1	1	2	2	4	4	3	3	1	1
2	2	3	3	4	4	1	1	3	3
2	2	3	3	4	4	1	1	3	3
2	2	3	3	4	4	1	1	3	3
1	1	2	2	4	4	1	1	1	1
1	1	2	2	4	4	1	1	3	3
1	1	2	2	4	4	1	1	3	3
1	1	3	3	4	4	1	1	3	3
2	2	3	3	4	4	1	1	3	3
1	1	2	2	4	4	1	1	3	3
1	1	2	2	4	4	1	1	3	3
1	1	2	2	4	4	1	1	1	1
2	2	3	3	4	4	1	1	3	3
1	1	2	2	4	4	1	1	1	1
1	1	2	2	4	4	1	1	1	1
1	1	2	2	4	4	1	1	3	3
2	2	3	3	4	4	1	1	3	3
1	1	2	2	4	4	1	1	3	3

For Review Only

1	1	2	2	4	4	1	1	3	3
2	2	2	2	4	4	1	1	3	3
1	1	2	2	4	4	1	1	3	3
1	1	2	2	4	4	1	1	3	3
1	1	2	2	1	1	1	1	1	1
1	1	2	2	4	4	1	1	1	1
1	1	2	2	1	1	1	1	3	3
2	2	2	2	1	1	1	1	3	3
1	1	2	2	4	4	1	1	3	3
1	1	2	2	1	1	1	1	3	3
1	1	2	2	4	4	1	1	3	3
1	1	2	2	4	4	1	1	1	1
2	2	3	3	1	1	1	1	3	3
1	1	2	2	4	4	1	1	1	1
2	2	3	3	4	4	1	1	3	3
1	1	2	2	4	4	1	1	1	1
1	1	2	2	4	4	1	1	3	3
1	1	2	2	1	1	1	1	3	3
1	1	2	2	4	4	1	1	3	3
1	1	2	2	4	4	3	3	1	1
1	1	2	2	4	4	1	1	3	3
1	1	2	2	4	4	1	1	1	1
2	2	3	3	4	4	1	1	3	3
1	1	2	2	4	4	1	1	1	1
1	1	2	2	4	4	1	1	3	3
2	2	2	2	4	4	1	1	3	3
1	1	2	2	4	4	1	1	3	3
2	2	3	3	4	4	1	1	3	3
1	1	2	2	4	4	1	1	1	1
1	1	2	2	4	4	1	1	1	1
1	1	2	2	4	4	1	1	3	3
1	1	2	2	4	4	1	1	1	1
1	1	2	2	4	4	1	1	1	1
1	1	2	2	4	4	1	1	1	1
1	1	2	2	4	4	1	1	3	3
1	1	2	2	4	4	1	1	3	3
1	1	2	2	4	4	1	1	1	1
2	2	3	3	4	4	1	1	3	3
1	1	2	2	4	4	1	1	3	3
1	1	2	2	4	4	1	1	3	3
1	1	3	3	4	4	1	1	3	3
1	1	2	2	4	4	3	3	1	1
1	1	2	2	4	4	3	3	1	1
1	1	2	2	4	4	3	3	1	1
2	2	3	3	4	4	1	1	3	3
2	2	3	3	1	1	3	3	3	3
1	1	2	2	4	4	1	1	1	1
2	2	3	3	4	4	3	3	3	3
2	2	3	3	4	4	1	1	3	3
1	1	2	2	4	4	1	1	3	3
1	1	2	2	4	4	1	1	1	1
1	1	2	2	4	4	1	1	3	3
2	2	3	3	4	4	1	1	3	3
2	2	3	3	4	4	1	1	3	3
1	1	2	2	4	4	1	1	3	3

For Review Only

1	1	2	2	4	4	1	1	1	1
1	1	2	2	4	4	1	1	3	3

For Review Only

SNP2368	SNP2368	SNP2369	SNP2369	SNP237	SNP237	SNP2370	SNP2370	SNP2371	SNP2371
3	3	4	4	2	2	3	3	2	2
4	4	1	1	2	2	3	3	2	2
4	4	1	1	2	2	3	3	2	2
4	4	4	4	2	2	3	3	2	2
3	3	4	4	2	2	3	3	2	2
3	3	4	4	2	2	3	3	2	2
3	3	4	4	2	2	3	3	2	2
3	3	4	4	2	2	1	1	4	4
3	3	4	4	2	2	3	3	2	2
4	4	1	1	2	2	1	1	4	4
3	3	4	4	4	4	3	3	2	2
4	4	1	1	2	2	3	3	2	2
3	3	4	4	2	2	3	3	2	2
4	4	4	4	2	2	3	3	2	2
3	3	4	4	2	2	3	3	2	2
3	3	4	4	2	2	3	3	2	2
3	3	4	4	2	2	3	3	2	2
3	3	4	4	2	2	3	3	2	2
4	4	1	1	2	2	3	3	2	2
3	3	4	4	2	2	3	3	2	2
3	3	4	4	2	2	3	3	2	2
3	3	4	4	2	2	3	3	2	2
3	3	4	4	2	2	3	3	2	2
3	3	4	4	2	2	1	1	4	4
4	4	1	1	2	2	1	1	4	4
3	3	4	4	2	2	3	3	2	2
3	3	4	4	2	2	3	3	2	2
4	4	4	4	2	2	3	3	2	2
3	3	4	4	2	2	3	3	2	2
3	3	4	4	2	2	3	3	2	2
4	4	1	1	2	2	3	3	2	2
3	3	4	4	2	2	3	3	2	2
3	3	4	4	2	2	3	3	2	2
3	3	4	4	2	2	3	3	2	2
3	3	4	4	2	2	3	3	2	2
3	3	4	4	2	2	3	3	2	2
3	3	4	4	2	2	3	3	2	2
3	3	4	4	2	2	3	3	2	2
3	3	1	1	4	4	3	3	2	2
3	3	1	1	2	2	3	3	2	2
3	3	4	4	2	2	3	3	2	2
3	3	4	4	2	2	1	1	4	4
4	4	1	1	2	2	3	3	2	2
3	3	4	4	2	2	3	3	2	2
4	4	1	1	2	2	1	1	4	4
4	4	4	4	2	2	3	3	2	2
4	4	1	1	2	2	3	3	2	2
3	3	1	1	2	2	3	3	2	2
4	4	1	1	2	2	3	3	2	2
3	3	4	4	2	2	3	3	2	2
4	4	1	1	2	2	3	3	2	2
3	3	4	4	2	2	3	3	2	2

For Review Only

3	3	4	4	2	2	3	3	2	2
3	3	4	4	2	2	3	3	2	2
3	3	4	4	2	2	3	3	2	2
3	3	4	4	2	2	3	3	2	2
4	4	1	1	2	2	3	3	2	2
3	3	4	4	2	2	3	3	2	2
3	3	4	4	2	2	3	3	2	2
3	3	4	4	2	2	3	3	2	2
3	3	4	4	2	2	3	3	2	2
4	4	4	4	2	2	3	3	2	2
4	4	1	1	2	2	3	3	2	2
4	4	1	1	2	2	3	3	2	2
3	3	4	4	2	2	3	3	2	2
3	3	4	4	2	2	3	3	2	2
3	3	4	4	2	2	3	3	2	2
3	3	4	4	2	2	3	3	2	2
4	4	4	4	2	2	3	3	2	2
3	3	4	4	2	2	3	3	2	2
3	3	4	4	2	2	3	3	2	2
3	3	4	4	2	2	3	3	2	2
4	4	1	1	2	2	3	3	2	2
3	3	4	4	2	2	3	3	2	2
3	3	4	4	2	2	3	3	2	2
3	3	4	4	2	2	3	3	2	2
4	4	1	1	2	2	1	1	4	4
4	4	1	1	2	2	3	3	2	2
4	4	1	1	2	2	3	3	2	2
3	3	4	4	2	2	3	3	2	2
3	3	4	4	2	2	3	3	2	2
3	3	4	4	2	2	3	3	2	2
3	3	4	4	2	2	3	3	2	2
3	3	4	4	2	2	3	3	2	2
3	3	4	4	2	2	3	3	2	2
3	3	4	4	2	2	3	3	2	2
3	3	4	4	2	2	3	3	2	2
4	4	1	1	2	2	1	1	4	4
3	3	4	4	2	2	1	1	4	4
3	3	4	4	2	2	3	3	2	2
4	4	1	1	2	2	3	3	2	2
3	3	4	4	2	2	3	3	2	2
3	3	4	4	2	2	3	3	2	2
4	4	1	1	2	2	3	3	2	2
4	4	1	1	2	2	3	3	2	2
4	4	1	1	2	2	3	3	2	2
3	3	4	4	2	2	3	3	2	2
3	3	4	4	2	2	3	3	2	2
3	3	4	4	2	2	3	3	2	2
3	3	4	4	2	2	3	3	2	2
3	3	4	4	2	2	3	3	2	2
3	3	4	4	2	2	3	3	2	2
4	4	1	1	2	2	3	3	2	2
3	3	1	1	4	4	3	3	2	2
3	3	4	4	2	2	3	3	2	2
3	3	4	4	2	2	3	3	2	2
3	3	4	4	2	2	3	3	2	2

For Review Only

3	3	4	4	2	2	3	3	2	2
3	3	4	4	2	2	1	1	2	2
3	3	4	4	2	2	3	3	2	2
3	3	4	4	2	2	3	3	2	2
4	4	4	4	2	2	3	3	2	2
3	3	4	4	2	2	3	3	2	2
4	4	4	4	2	2	3	3	2	2
3	3	4	4	2	2	3	3	2	2
3	3	1	1	2	2	3	3	2	2
3	3	4	4	2	2	3	3	2	2
3	3	4	4	2	2	3	3	2	2
3	3	4	4	2	2	3	3	2	2
4	4	1	1	2	2	3	3	2	2
4	4	4	4	2	2	3	3	2	2
4	4	1	1	2	2	3	3	2	2
3	3	4	4	2	2	3	3	2	2
3	3	4	4	2	2	3	3	2	2
3	3	4	4	2	2	3	3	2	2
3	3	4	4	2	2	3	3	2	2
4	4	1	1	2	2	3	3	2	2
4	4	4	4	2	2	3	3	2	2
3	3	4	4	2	2	3	3	2	2
4	4	4	4	2	2	3	3	2	2
3	3	4	4	4	4	3	3	2	2
3	3	4	4	2	2	3	3	2	2
3	3	1	1	2	2	3	3	2	2
3	3	4	4	2	2	1	1	4	4
3	3	4	4	2	2	3	3	2	2
4	4	1	1	2	2	3	3	2	2
4	4	4	4	2	2	3	3	2	2
3	3	4	4	4	4	3	3	2	2
3	3	4	4	2	2	3	3	2	2
3	3	4	4	2	2	3	3	2	2
3	3	4	4	2	2	3	3	2	2
3	3	4	4	2	2	3	3	2	2
3	3	4	4	2	2	3	3	2	2
3	3	4	4	2	2	3	3	2	2
3	3	4	4	2	2	3	3	2	2
3	3	4	4	4	4	3	3	2	2
3	3	4	4	4	4	3	3	2	2
3	3	4	4	2	2	3	3	2	2
3	3	4	4	2	2	3	3	2	2
3	3	4	4	2	2	3	3	2	2
3	3	4	4	2	2	3	3	2	2
3	3	4	4	2	2	3	3	2	2
3	3	4	4	2	2	3	3	2	2
3	3	4	4	2	2	3	3	2	2
3	3	4	4	2	2	3	3	2	2
3	3	4	4	2	2	3	3	2	2
3	3	4	4	2	2	3	3	2	2
3	3	4	4	2	2	3	3	2	2
3	3	4	4	2	2	3	3	2	2
3	3	4	4	2	2	3	3	2	2
3	3	4	4	2	2	3	3	2	2
3	3	4	4	2	2	3	3	2	2

For Review Only

4	4	4	4	2	2	3	3	2	2
4	4	1	1	2	2	3	3	2	2
4	4	4	4	2	2	3	3	2	2
3	3	4	4	2	2	3	3	2	2
3	3	4	4	2	2	3	3	2	2
4	4	1	1	2	2	3	3	2	2
3	3	4	4	2	2	3	3	2	2
3	3	4	4	2	2	3	3	2	2
3	3	4	4	2	2	3	3	2	2
4	4	1	1	2	2	3	3	2	2
3	3	4	4	2	2	3	3	2	2
3	3	4	4	2	2	3	3	2	2
4	4	4	4	2	2	3	3	2	2
3	3	4	4	2	2	3	3	2	2
3	3	4	4	2	2	3	3	2	2
3	3	4	4	4	4	3	3	2	2
3	3	4	4	2	2	3	3	2	2
3	3	4	4	2	2	3	3	2	2
3	3	4	4	2	2	3	3	2	2
4	4	4	4	2	2	3	3	2	2
4	4	4	4	2	2	3	3	2	2
3	3	4	4	4	4	3	3	2	2
3	3	4	4	2	2	3	3	2	2
4	4	1	1	2	2	3	3	2	2
3	3	4	4	2	2	3	3	4	4
3	3	4	4	2	2	3	3	2	2
4	4	1	1	2	2	3	3	2	2
3	3	4	4	2	2	3	3	2	2
4	4	1	1	2	2	3	3	2	2
3	3	4	4	2	2	3	3	2	2
3	3	4	4	2	2	3	3	2	2
3	3	4	4	2	2	3	3	2	2
3	3	4	4	2	2	3	3	2	2
3	3	4	4	2	2	3	3	2	2
4	4	4	4	2	2	3	3	2	2
4	4	1	1	2	2	3	3	2	2
3	3	4	4	2	2	3	3	2	2
3	3	4	4	2	2	3	3	2	2
4	4	1	1	2	2	3	3	2	2
4	4	1	1	2	2	3	3	2	2
4	4	1	1	2	2	3	3	2	2
3	3	4	4	2	2	3	3	2	2
3	3	4	4	4	4	3	3	2	2
3	3	4	4	2	2	3	3	2	2
4	4	4	4	2	2	3	3	2	2
4	4	1	1	2	2	3	3	2	2
3	3	4	4	4	4	3	3	2	2
3	3	4	4	4	4	3	3	2	2
3	3	4	4	4	4	1	1	4	4
4	4	1	1	2	2	3	3	2	2
3	3	4	4	2	2	3	3	2	2
3	3	4	4	2	2	3	3	2	2
3	3	4	4	2	2	3	3	2	2
4	4	1	1	2	2	3	3	2	2
3	3	4	4	4	4	1	1	4	4

For Review Only

3	3	4	4	2	2	1	1	4	4
4	4	1	1	2	2	3	3	2	2
4	4	4	4	2	2	3	3	2	2
4	4	4	4	2	2	3	3	2	2
3	3	4	4	2	2	3	3	2	2
3	3	4	4	2	2	3	3	2	2
3	3	4	4	4	4	3	3	2	2
4	4	1	1	2	2	3	3	2	2
3	3	4	4	2	2	3	3	2	2
3	3	4	4	4	4	3	3	2	2
4	4	4	4	2	2	3	3	2	2
3	3	4	4	2	2	3	3	2	2
4	4	1	1	2	2	3	3	2	2
3	3	4	4	2	2	3	3	2	2
4	4	1	1	2	2	3	3	2	2
3	3	4	4	2	2	3	3	2	2
3	3	4	4	4	4	3	3	2	2
3	3	4	4	2	2	3	3	2	2
3	3	4	4	2	2	3	3	2	2
3	3	4	4	2	2	3	3	2	2
3	3	1	1	2	2	3	3	2	2
3	3	4	4	2	2	3	3	2	2
4	4	1	1	2	2	3	3	2	2
3	3	4	4	2	2	3	3	2	2
3	3	4	4	2	2	3	3	2	2
4	4	1	1	2	2	1	1	2	2
3	3	4	4	4	4	3	3	2	2
4	4	1	1	2	2	3	3	2	2
3	3	4	4	2	2	3	3	2	2
3	3	4	4	2	2	3	3	2	2
3	3	4	4	4	4	3	3	2	2
3	3	4	4	2	2	3	3	2	2
3	3	4	4	2	2	1	1	4	4
3	3	4	4	2	2	3	3	2	2
3	3	4	4	2	2	3	3	2	2
3	3	4	4	4	4	3	3	2	2
3	3	4	4	2	2	3	3	2	2
4	4	1	1	2	2	3	3	2	2
3	3	4	4	2	2	3	3	2	2
3	3	1	1	4	4	3	3	2	2
4	4	4	4	2	2	3	3	2	2
3	3	4	4	2	2	3	3	2	2
3	3	4	4	2	2	3	3	2	2
3	3	4	4	2	2	3	3	2	2
4	4	1	1	2	2	3	3	2	2
4	4	1	1	2	2	3	3	2	2
3	3	4	4	4	4	3	3	2	2
4	4	4	4	2	2	3	3	2	2
4	4	1	1	2	2	3	3	2	2
3	3	4	4	2	2	3	3	2	2
3	3	4	4	2	2	3	3	2	2
3	3	1	1	4	4	3	3	2	2
4	4	1	1	2	2	3	3	2	2
4	4	1	1	2	2	3	3	4	4
3	3	1	1	2	2	3	3	2	2

For Review Only

3	3	4	4	2	2	3	3	2	2
3	3	4	4	2	2	3	3	2	2

For Review Only

SNP2372	SNP2372	SNP2373	SNP2373	SNP2374	SNP2374	SNP2375	SNP2375	SNP2376	SNP2376
3	3	4	4	4	4	4	4	4	4
3	3	4	4	1	1	4	4	2	2
3	3	4	4	1	1	4	4	2	2
3	3	4	4	4	4	1	1	4	4
3	3	4	4	4	4	4	4	4	4
3	3	4	4	4	4	1	1	4	4
3	3	4	4	4	4	4	4	4	4
3	3	4	4	4	4	4	4	4	4
3	3	4	4	4	4	4	4	4	4
3	3	4	4	4	4	4	4	4	4
3	3	4	4	4	4	4	4	4	4
3	3	4	4	1	1	4	4	2	2
3	3	4	4	4	4	4	4	4	4
3	3	4	4	4	4	1	1	4	4
3	3	4	4	4	4	4	4	4	4
3	3	4	4	4	4	4	4	4	4
3	3	4	4	4	4	4	4	4	4
3	3	4	4	4	4	4	4	4	4
3	3	1	1	1	1	1	1	2	2
3	3	4	4	4	4	1	1	4	4
3	3	4	4	4	4	4	4	4	4
3	3	4	4	4	4	4	4	4	4
3	3	4	4	4	4	1	1	4	4
3	3	4	4	4	4	4	4	4	4
3	3	4	4	4	4	1	1	4	4
3	3	4	4	1	1	1	1	2	2
3	3	4	4	4	4	4	4	4	4
3	3	4	4	1	1	4	4	4	4
3	3	4	4	4	4	4	4	4	4
3	3	4	4	4	4	1	1	4	4
3	3	4	4	1	1	1	1	2	2
4	4	1	1	4	4	1	1	4	4
3	3	4	4	4	4	4	4	4	4
3	3	4	4	4	4	4	4	4	4
3	3	4	4	4	4	1	1	4	4
3	3	4	4	4	4	4	4	4	4
3	3	4	4	4	4	4	4	4	4
3	3	4	4	4	4	4	4	4	4
3	3	4	4	4	4	4	4	4	4
3	3	4	4	4	4	1	1	4	4
3	3	4	4	4	4	4	4	4	4
3	3	4	4	4	4	4	4	4	4
3	3	4	4	4	4	4	4	4	4
3	3	4	4	4	4	4	4	4	4
3	3	4	4	4	4	4	4	4	4
3	3	4	4	1	1	4	4	2	2
3	3	4	4	1	1	4	4	2	2
3	3	4	4	4	4	1	1	4	4
3	3	4	4	4	4	4	4	4	4
3	3	1	1	1	1	4	4	2	2
3	3	4	4	1	1	4	4	2	2
4	4	1	1	1	1	4	4	2	2

For Review Only

3	3	4	4	4	4	4	4	4	4
3	3	4	4	4	4	4	4	4	4
4	4	1	1	1	1	1	1	2	2
3	3	4	4	4	4	4	4	4	4
3	3	1	1	1	1	1	1	2	2
3	3	4	4	1	1	4	4	2	2
3	3	4	4	1	1	1	1	2	2
3	3	4	4	4	4	1	1	4	4
3	3	4	4	4	4	4	4	4	4
3	3	4	4	1	1	4	4	4	4
3	3	4	4	1	1	4	4	2	2
3	3	4	4	1	1	4	4	2	2
3	3	4	4	4	4	4	4	4	4
3	3	4	4	4	4	4	4	4	4
3	3	4	4	1	1	4	4	4	4
3	3	4	4	4	4	4	4	4	4
3	3	4	4	1	1	4	4	2	2
4	4	1	1	1	1	1	1	2	2
3	3	4	4	4	4	1	1	4	4
3	3	4	4	4	4	4	4	4	4
3	3	4	4	1	1	4	4	2	2
3	3	4	4	4	4	4	4	4	4
3	3	4	4	4	4	4	4	4	4
3	3	4	4	1	1	4	4	2	2
3	3	4	4	4	4	4	4	4	4
3	3	4	4	1	1	4	4	2	2
3	3	4	4	4	4	4	4	4	4
3	3	4	4	4	4	1	1	4	4
3	3	4	4	1	1	1	1	2	2
3	3	4	4	4	4	4	4	4	4
3	3	4	4	4	4	4	4	4	4
3	3	4	4	4	4	4	4	4	4
3	3	4	4	4	4	1	1	4	4
3	3	4	4	4	4	4	4	4	4
3	3	4	4	4	4	4	4	4	4
3	3	4	4	4	4	4	4	4	4
3	3	4	4	4	4	4	4	4	4
3	3	4	4	4	4	4	4	4	4
3	3	4	4	1	1	1	1	2	2
3	3	4	4	4	4	1	1	4	4
3	3	4	4	4	4	4	4	4	4
3	3	4	4	1	1	4	4	2	2
3	3	4	4	1	1	1	1	2	2
3	3	4	4	1	1	4	4	2	2
3	3	4	4	4	4	4	4	4	4
3	3	4	4	4	4	4	4	4	4
3	3	4	4	4	4	1	1	4	4
3	3	4	4	1	1	4	4	2	2
3	3	4	4	4	4	1	1	4	4
4	4	1	1	4	4	4	4	4	4
3	3	4	4	1	1	4	4	2	2
3	3	4	4	4	4	4	4	4	4
3	3	4	4	4	4	4	4	4	4
3	3	4	4	4	4	4	4	4	4
4	4	1	1	4	4	4	4	4	4

For Review Only

3	3	4	4	1	1	1	1	2	2
3	3	4	4	4	4	1	1	4	4
3	3	4	4	1	1	4	4	2	2
3	3	4	4	4	4	1	1	4	4
3	3	4	4	1	1	4	4	2	2
3	3	4	4	1	1	4	4	4	4
3	3	4	4	4	4	4	4	4	4
3	3	4	4	4	4	4	4	4	4
3	3	4	4	4	4	4	4	4	4
4	4	4	4	4	4	4	4	4	4
3	3	4	4	4	4	1	1	4	4
3	3	4	4	1	1	4	4	4	4
3	3	4	4	1	1	4	4	2	2
3	3	4	4	1	1	4	4	2	2
3	3	4	4	1	1	4	4	2	2
3	3	4	4	1	1	4	4	4	4
3	3	4	4	4	4	1	1	4	4
3	3	4	4	1	1	4	4	4	4
3	3	4	4	4	4	1	1	4	4
3	3	4	4	1	1	1	1	2	2
3	3	4	4	1	1	4	4	4	4
3	3	4	4	4	4	4	4	4	4
3	3	4	4	1	1	4	4	2	2
3	3	4	4	4	4	1	1	4	4
4	4	1	1	1	1	1	1	2	2
3	3	4	4	4	4	1	1	4	4
3	3	4	4	4	4	4	4	4	4
3	3	4	4	4	4	4	4	4	4
3	3	4	4	4	4	1	1	4	4
3	3	4	4	1	1	1	1	4	4
3	3	4	4	4	4	1	1	4	4
3	3	4	4	1	1	1	1	4	4
3	3	4	4	4	4	4	4	4	4
3	3	4	4	1	1	4	4	4	4
3	3	4	4	4	4	4	4	4	4
4	4	1	1	4	4	4	4	4	4
3	3	4	4	4	4	1	1	4	4
3	3	4	4	1	1	4	4	2	2
4	4	1	1	1	1	1	1	4	4
3	3	4	4	4	4	4	4	4	4
3	3	4	4	4	4	1	1	4	4
3	3	4	4	4	4	1	1	4	4
4	4	1	1	1	1	1	1	2	2
3	3	4	4	4	4	4	4	4	4
3	3	4	4	1	1	4	4	2	2
3	3	4	4	4	4	1	1	4	4
3	3	4	4	1	1	4	4	2	2
3	3	4	4	1	1	1	1	2	2
3	3	4	4	4	4	1	1	4	4
3	3	4	4	1	1	4	4	2	2
3	3	4	4	1	1	4	4	2	2
3	3	4	4	4	4	4	4	4	4
3	3	4	4	1	1	1	1	4	4
3	3	4	4	1	1	4	4	2	2
3	3	4	4	4	4	1	1	4	4

For Review Only

3	3	4	4	1	1	1	1	2	2
3	3	4	4	1	1	1	1	2	2
3	3	4	4	4	4	4	4	4	4
4	4	1	1	1	1	1	1	4	4
3	3	4	4	4	4	4	4	4	4
3	3	4	4	1	1	1	1	2	2
3	3	4	4	4	4	1	1	4	4
3	3	4	4	1	1	4	4	4	4
3	3	4	4	4	4	4	4	4	4
3	3	4	4	1	1	4	4	2	2
3	3	4	4	1	1	4	4	4	4
3	3	4	4	4	4	1	1	4	4
3	3	4	4	4	4	4	4	4	4
4	4	4	4	4	4	4	4	4	4
3	3	4	4	4	4	1	1	4	4
3	3	4	4	4	4	4	4	4	4
3	3	4	4	1	1	1	1	4	4
3	3	4	4	1	1	4	4	2	2
3	3	4	4	4	4	4	4	4	4
3	3	4	4	4	4	4	4	4	4
3	3	4	4	1	1	4	4	4	4
3	3	4	4	4	4	4	4	4	4
3	3	4	4	4	4	4	4	4	4
3	3	4	4	1	1	4	4	2	2
3	3	4	4	4	4	4	4	4	4
3	3	4	4	4	4	4	4	4	4
3	3	4	4	1	1	1	1	2	2
4	4	1	1	4	4	4	4	4	4
3	3	4	4	1	1	4	4	2	2
4	4	1	1	4	4	4	4	4	4
3	3	4	4	1	1	4	4	2	2
4	4	1	1	4	4	1	1	4	4
3	3	4	4	4	4	4	4	4	4
3	3	4	4	4	4	1	1	4	4
3	3	4	4	4	4	4	4	4	4
3	3	4	4	1	1	4	4	2	2
3	3	4	4	4	4	4	4	2	2
3	3	4	4	4	4	4	4	4	4
3	3	4	4	1	1	1	1	2	2
3	3	4	4	1	1	4	4	2	2
3	3	4	4	1	1	4	4	2	2
3	3	4	4	1	1	1	1	2	2
3	3	4	4	4	4	1	1	4	4
3	3	4	4	1	1	4	4	4	4
3	3	4	4	1	1	4	4	2	2
3	3	4	4	1	1	4	4	2	2
3	3	4	4	4	4	4	4	4	4
3	3	4	4	4	4	1	1	4	4
3	3	4	4	4	4	1	1	4	4
3	3	4	4	4	4	4	4	4	4
3	3	4	4	4	4	1	1	4	4
3	3	4	4	1	1	1	1	4	4
3	3	4	4	4	4	1	1	4	4
3	3	4	4	1	1	4	4	2	2
3	3	4	4	4	4	4	4	4	4

For Review Only

4	4	1	1	4	4	1	1	4	4
3	3	4	4	4	4	1	1	4	4
3	3	4	4	4	4	1	1	4	4
3	3	4	4	1	1	4	4	4	4
3	3	4	4	4	4	4	4	4	4
3	3	4	4	4	4	1	1	2	2
3	3	4	4	4	4	1	1	4	4
3	3	4	4	1	1	4	4	4	4
3	3	4	4	4	4	1	1	4	4
3	3	4	4	4	4	1	1	4	4
3	3	4	4	1	1	4	4	4	4
3	3	4	4	1	1	4	4	2	2
3	3	4	4	1	1	1	1	2	2
3	3	4	4	4	4	1	1	4	4
3	3	4	4	1	1	4	4	2	2
3	3	4	4	4	4	1	1	4	4
3	3	4	4	4	4	4	4	4	4
3	3	4	4	4	4	1	1	4	4
3	3	4	4	4	4	1	1	4	4
3	3	4	4	4	4	4	4	4	4
3	3	4	4	4	4	1	1	4	4
3	3	4	4	1	1	1	1	2	2
3	3	4	4	1	1	4	4	2	2
3	3	4	4	4	4	1	1	4	4
3	3	4	4	4	4	1	1	4	4
3	3	4	4	1	1	4	4	4	4
3	3	4	4	4	4	1	1	4	4
3	3	4	4	4	4	1	1	4	4
3	3	4	4	4	4	4	4	4	4
3	3	4	4	4	4	1	1	4	4
3	3	4	4	4	4	1	1	4	4
3	3	4	4	1	1	4	4	2	2
3	3	4	4	4	4	4	4	4	4
3	3	4	4	1	1	1	1	2	2
3	3	4	4	4	4	1	1	4	4
3	3	4	4	4	4	1	1	4	4
3	3	4	4	4	4	4	4	4	4
3	3	4	4	1	1	4	4	2	2
3	3	4	4	4	4	4	4	4	4
3	3	4	4	4	4	4	4	4	4
3	3	4	4	4	4	1	1	4	4
3	3	4	4	4	4	4	4	4	4
3	3	4	4	4	4	1	1	4	4
3	3	4	4	4	4	4	4	4	4
3	3	4	4	1	1	4	4	2	2
3	3	4	4	1	1	4	4	2	2
3	3	4	4	1	1	1	1	2	2
4	4	1	1	1	1	4	4	2	2
3	3	4	4	1	1	1	1	2	2
3	3	4	4	4	4	1	1	4	4
4	4	1	1	4	4	4	4	4	4
3	3	4	4	4	4	4	4	4	4
3	3	4	4	1	1	4	4	2	2
3	3	4	4	1	1	1	1	2	2
3	3	4	4	4	4	1	1	4	4

For Review Only

3	3	4	4	4	4	4	4	4	4
3	3	4	4	1	1	4	4	2	2

For Review Only

SNP2377	SNP2377	SNP2378	SNP2378	SNP2379	SNP2379	SNP238	SNP238	SNP2380	SNP2380
4	4	4	4	3	3	3	3	1	1
4	4	4	4	3	3	3	3	1	1
4	4	4	4	3	3	3	3	4	4
4	4	2	2	3	3	3	3	1	1
4	4	2	2	3	3	4	4	1	1
4	4	2	2	3	3	3	3	1	1
4	4	4	4	3	3	4	4	1	1
4	4	2	2	3	3	4	4	1	1
4	4	4	4	3	3	4	4	1	1
4	4	2	2	1	1	3	3	1	1
4	4	4	4	3	3	4	4	1	1
4	4	4	4	3	3	3	3	1	1
1	1	2	2	3	3	4	4	1	1
4	4	2	2	3	3	3	3	1	1
4	4	2	2	3	3	4	4	1	1
4	4	4	4	3	3	3	3	1	1
1	1	4	4	3	3	4	4	4	4
4	4	2	2	3	3	3	3	1	1
4	4	2	2	3	3	3	3	1	1
1	1	2	2	3	3	3	3	4	4
4	4	4	4	3	3	4	4	1	1
1	1	4	4	3	3	3	3	4	4
4	4	2	2	3	3	4	4	1	1
4	4	2	2	3	3	3	3	1	1
4	4	2	2	3	3	3	3	1	1
4	4	2	2	3	3	3	3	1	1
4	4	4	4	3	3	3	3	1	1
4	4	2	2	3	3	3	3	1	1
4	4	4	4	3	3	4	4	1	1
4	4	2	2	3	3	3	3	1	1
4	4	2	2	3	3	3	3	1	1
4	4	2	2	3	3	3	3	1	1
4	4	4	4	3	3	4	4	1	1
4	4	4	4	3	3	3	3	1	1
4	4	2	2	3	3	3	3	1	1
4	4	4	4	3	3	3	3	1	1
4	4	4	4	3	3	3	3	1	1
4	4	2	2	3	3	4	4	1	1
4	4	4	4	1	1	4	4	1	1
4	4	2	2	1	1	4	4	1	1
4	4	2	2	3	3	4	4	1	1
4	4	4	4	3	3	4	4	1	1
4	4	2	2	1	1	3	3	1	1
1	1	4	4	3	3	3	3	4	4
4	4	2	2	1	1	3	3	1	1
4	4	4	4	3	3	3	3	1	1
4	4	4	4	3	3	3	3	1	1
4	4	2	2	1	1	3	3	1	1
1	1	4	4	3	3	4	4	4	4
4	4	2	2	3	3	3	3	1	1
4	4	4	4	3	3	3	3	1	1
4	4	4	4	3	3	3	3	1	1

For Review Only

1	1	4	4	3	3	4	4	4	4
4	4	4	4	3	3	4	4	1	1
4	4	2	2	3	3	3	3	1	1
1	1	4	4	3	3	4	4	4	4
4	4	2	2	3	3	3	3	1	1
4	4	4	4	3	3	3	3	1	1
4	4	2	2	3	3	3	3	1	1
4	4	2	2	3	3	3	3	1	1
4	4	4	4	3	3	3	3	1	1
4	4	2	2	3	3	4	4	1	1
4	4	4	4	3	3	3	3	1	1
4	4	4	4	3	3	3	3	1	1
1	1	4	4	3	3	4	4	4	4
4	4	4	4	3	3	4	4	1	1
4	4	2	2	3	3	4	4	1	1
4	4	2	2	3	3	3	3	1	1
4	4	4	4	3	3	3	3	1	1
4	4	2	2	3	3	3	3	1	1
1	1	2	2	3	3	3	3	4	4
4	4	4	4	3	3	4	4	1	1
4	4	2	2	3	3	3	3	1	1
4	4	2	2	3	3	4	4	1	1
4	4	2	2	3	3	4	4	1	1
1	1	4	4	3	3	3	3	4	4
4	4	2	2	1	1	3	3	1	1
4	4	4	4	3	3	3	3	1	1
1	1	4	4	1	1	3	3	4	4
4	4	2	2	1	1	3	3	1	1
4	4	2	2	3	3	3	3	1	1
4	4	4	4	3	3	4	4	1	1
4	4	2	2	3	3	4	4	1	1
4	4	4	4	3	3	3	3	1	1
4	4	2	2	1	1	3	3	1	1
1	1	4	4	3	3	4	4	4	4
4	4	4	4	3	3	3	3	1	1
4	4	2	2	1	1	3	3	1	1
4	4	2	2	3	3	3	3	1	1
1	1	2	2	3	3	4	4	4	4
4	4	2	2	3	3	4	4	1	1
4	4	2	2	3	3	3	3	1	1
1	1	4	4	3	3	4	4	4	4
4	4	4	4	3	3	3	3	1	1
4	4	2	2	3	3	4	4	1	1
4	4	4	4	3	3	3	3	1	1
1	1	4	4	3	3	4	4	4	4
4	4	2	2	3	3	3	3	1	1
4	4	2	2	3	3	3	3	1	1
4	4	2	2	3	3	3	3	1	1
4	4	2	2	3	3	3	3	1	1
4	4	2	2	3	3	4	4	1	1
4	4	4	4	3	3	3	3	1	1
4	4	4	4	1	1	4	4	1	1
4	4	4	4	3	3	3	3	1	1
4	4	2	2	3	3	3	3	1	1
4	4	2	2	3	3	3	3	1	1

For Review Only

4	4	2	2	3	3	3	3	1	1
4	4	2	2	3	3	4	4	1	1
4	4	4	4	3	3	3	3	1	1
4	4	2	2	3	3	3	3	1	1
4	4	4	4	3	3	3	3	1	1
4	4	4	4	3	3	3	3	1	1
4	4	4	4	3	3	3	3	1	1
4	4	4	4	3	3	3	3	1	1
4	4	2	2	3	3	3	3	1	1
4	4	2	2	3	3	3	3	1	1
4	4	2	2	3	3	4	4	1	1
4	4	2	2	3	3	4	4	1	1
4	4	2	2	3	3	3	3	1	1
4	4	2	2	3	3	3	3	1	1
4	4	2	2	3	3	3	3	1	1
4	4	2	2	3	3	3	3	1	1
4	4	2	2	3	3	3	3	1	1
4	4	4	4	3	3	3	3	1	1
4	4	2	2	3	3	3	3	1	1
4	4	2	2	3	3	3	3	1	1
4	4	2	2	3	3	3	3	1	1
4	4	2	2	3	3	4	4	1	1
4	4	4	4	3	3	3	3	1	1
4	4	2	2	3	3	4	4	1	1
4	4	2	2	3	3	4	4	1	1
4	4	2	2	1	1	3	3	1	1
4	4	4	4	3	3	3	3	1	1
4	4	4	4	3	3	3	3	1	1
4	4	2	2	3	3	3	3	1	1
4	4	2	2	3	3	3	3	1	1
4	4	2	2	3	3	4	4	1	1
4	4	2	2	3	3	3	3	1	1
1	1	4	4	3	3	4	4	4	4
4	4	2	2	3	3	3	3	1	1
4	4	4	4	3	3	3	3	1	1
4	4	2	2	3	3	3	3	1	1
4	4	2	2	3	3	3	3	1	1
4	4	4	4	3	3	3	3	1	1
4	4	2	2	3	3	4	4	1	1
4	4	2	2	3	3	4	4	1	1
4	4	2	2	3	3	4	4	1	1
4	4	2	2	3	3	3	3	1	1
4	4	2	2	3	3	3	3	1	1
4	4	2	2	3	3	4	4	1	1
4	4	4	4	3	3	3	3	1	1
4	4	2	2	3	3	3	3	1	1
4	4	4	4	3	3	3	3	1	1
4	4	2	2	3	3	3	3	1	1
4	4	2	2	3	3	4	4	1	1
4	4	4	4	3	3	3	3	1	1
4	4	2	2	3	3	3	3	1	1
4	4	4	4	3	3	3	3	1	1
4	4	2	2	3	3	3	3	1	1
4	4	4	4	3	3	3	3	1	1
4	4	2	2	3	3	3	3	1	1

For Review Only

4	4	2	2	3	3	3	3	1	1
4	4	2	2	3	3	4	4	1	1
4	4	2	2	3	3	3	3	1	1
4	4	2	2	3	3	4	4	1	1
4	4	4	4	3	3	3	3	1	1
4	4	2	2	3	3	3	3	1	1
4	4	2	2	3	3	3	3	1	1
4	4	2	2	3	3	3	3	1	1
4	4	2	2	3	3	3	3	1	1
1	1	4	4	3	3	3	3	4	4
4	4	4	4	3	3	3	3	1	1
4	4	2	2	3	3	3	3	1	1
4	4	2	2	3	3	4	4	1	1
4	4	2	2	3	3	3	3	1	1
4	4	2	2	3	3	3	3	1	1
4	4	4	4	3	3	4	4	1	1
4	4	2	2	3	3	3	3	1	1
4	4	2	2	3	3	3	3	1	1
4	4	4	4	3	3	3	3	1	1
4	4	2	2	3	3	3	3	1	1
4	4	2	2	3	3	4	4	1	1
4	4	4	4	3	3	4	4	1	1
4	4	4	4	3	3	4	4	1	1
4	4	4	4	3	3	3	3	1	1
4	4	4	4	3	3	3	3	1	1
4	4	4	4	3	3	3	3	1	1
4	4	2	2	3	3	3	3	1	1
4	4	2	2	3	3	3	3	1	1
4	4	4	4	3	3	4	4	1	1
4	4	4	4	3	3	3	3	1	1
4	4	4	4	3	3	3	3	1	1
4	4	2	2	3	3	3	3	1	1
4	4	4	4	3	3	3	3	1	1
4	4	2	2	3	3	3	3	1	1
4	4	2	2	3	3	3	3	1	1
4	4	4	4	3	3	3	3	1	1
4	4	4	4	3	3	3	3	1	1
4	4	4	4	3	3	3	3	1	1
4	4	2	2	3	3	3	3	1	1
4	4	4	4	3	3	3	3	1	1
4	4	4	4	3	3	3	3	4	4
4	4	2	2	3	3	3	3	1	1
4	4	2	2	3	3	4	4	1	1
4	4	4	4	3	3	4	4	1	1
4	4	2	2	3	3	3	3	1	1
4	4	4	4	3	3	3	3	1	1
4	4	4	4	3	3	4	4	1	1
4	4	2	2	3	3	4	4	1	1
4	4	2	2	3	3	4	4	1	1
4	4	4	4	3	3	3	3	1	1
4	4	2	2	3	3	3	3	1	1
4	4	2	2	3	3	3	3	1	1
4	4	2	2	3	3	4	4	1	1
4	4	4	4	3	3	3	3	1	1
4	4	4	4	3	3	4	4	1	1

For Review Only

4	4	2	2	3	3	3	3	1	1
1	1	2	2	1	1	3	3	4	4
4	4	2	2	3	3	3	3	1	1
4	4	2	2	3	3	4	4	1	1
4	4	2	2	3	3	3	3	1	1
4	4	2	2	3	3	3	3	1	1
4	4	2	2	3	3	4	4	1	1
4	4	2	2	1	1	4	4	1	1
4	4	2	2	3	3	3	3	1	1
4	4	2	2	3	3	4	4	1	1
4	4	2	2	3	3	3	3	1	1
4	4	2	2	3	3	3	3	1	1
4	4	2	2	3	3	4	4	1	1
4	4	2	2	3	3	3	3	1	1
4	4	4	4	3	3	3	3	4	4
4	4	2	2	3	3	4	4	1	1
4	4	4	4	3	3	4	4	1	1
4	4	2	2	1	1	4	4	1	1
4	4	2	2	3	3	4	4	1	1
4	4	2	2	3	3	4	4	1	1
4	4	2	2	1	1	4	4	4	4
4	4	2	2	3	3	3	3	1	1
1	1	4	4	3	3	4	4	4	4
4	4	2	2	3	3	3	3	1	1
4	4	2	2	3	3	3	3	1	1
4	4	4	4	3	3	3	3	1	1
4	4	2	2	1	1	4	4	1	1
4	4	2	2	3	3	3	3	1	1
4	4	4	4	3	3	4	4	1	1
4	4	2	2	3	3	3	3	1	1
4	4	2	2	3	3	4	4	1	1
4	4	4	4	3	3	3	3	1	1
4	4	2	2	3	3	3	3	1	1
4	4	2	2	3	3	3	3	1	1
4	4	2	2	3	3	4	4	1	1
4	4	2	2	3	3	4	4	1	1
1	1	4	4	3	3	3	3	4	4
4	4	4	4	3	3	3	3	1	1
4	4	4	4	3	3	3	3	1	1
4	4	4	4	1	1	4	4	1	1
4	4	2	2	3	3	3	3	1	1
4	4	4	4	3	3	4	4	1	1
4	4	2	2	3	3	3	3	1	1
1	1	4	4	3	3	4	4	4	4
4	4	4	4	3	3	3	3	4	4
4	4	4	4	3	3	3	3	1	1
4	4	2	2	3	3	4	4	1	1
4	4	2	2	3	3	3	3	1	1
4	4	2	2	3	3	3	3	1	1
4	4	2	2	1	1	3	3	1	1
4	4	4	4	3	3	3	3	1	1
4	4	4	4	1	1	4	4	1	1
4	4	4	4	3	3	3	3	4	4
4	4	2	2	3	3	3	3	1	1
4	4	2	2	1	1	3	3	1	1

For Review Only

4	4	4	4	3	3	4	4	4	4
4	4	4	4	3	3	3	3	1	1

For Review Only

SNP2381	SNP2381	SNP2382	SNP2382	SNP2383	SNP2383	SNP2384	SNP2384	SNP2385	SNP2385
4	4	3	3	3	3	4	4	1	1
4	4	3	3	3	3	1	1	1	1
4	4	3	3	3	3	1	1	1	1
4	4	3	3	3	3	4	4	4	4
4	4	3	3	3	3	4	4	1	1
4	4	3	3	3	3	4	4	1	1
1	1	3	3	3	3	4	4	1	1
4	4	3	3	3	3	4	4	1	1
4	4	3	3	3	3	4	4	1	1
4	4	3	3	3	3	4	4	4	4
4	4	3	3	3	3	4	4	1	1
4	4	3	3	3	3	4	4	1	1
4	4	3	3	3	3	4	4	1	1
4	4	3	3	3	3	4	4	1	1
4	4	3	3	3	3	4	4	1	1
4	4	3	3	3	3	4	4	1	1
4	4	3	3	3	3	4	4	1	1
4	4	3	3	3	3	4	4	1	1
4	4	3	3	3	3	1	1	4	4
4	4	3	3	3	3	4	4	1	1
4	4	3	3	3	3	4	4	1	1
4	4	3	3	3	3	4	4	1	1
4	4	4	4	1	1	4	4	1	1
4	4	3	3	3	3	4	4	1	1
4	4	3	3	3	3	4	4	1	1
4	4	3	3	3	3	4	4	1	1
4	4	3	3	3	3	4	4	1	1
4	4	3	3	3	3	4	4	1	1
4	4	3	3	3	3	4	4	1	1
4	4	4	4	1	1	4	4	4	4
4	4	3	3	3	3	4	4	1	1
4	4	3	3	3	3	4	4	1	1
4	4	3	3	3	3	4	4	1	1
4	4	3	3	3	3	4	4	1	1
1	1	3	3	3	3	4	4	1	1
1	1	3	3	3	3	4	4	1	1
4	4	3	3	3	3	4	4	1	1
4	4	3	3	3	3	4	4	1	1
1	1	3	3	3	3	4	4	1	1
1	1	4	4	1	1	4	4	1	1
4	4	4	4	1	1	4	4	1	1
4	4	3	3	3	3	4	4	1	1
4	4	3	3	3	3	4	4	4	4
4	4	3	3	3	3	4	4	1	1
4	4	3	3	3	3	4	4	4	4
4	4	3	3	3	3	4	4	1	1
4	4	3	3	3	3	1	1	1	1
4	4	3	3	3	3	4	4	1	1
4	4	3	3	3	3	4	4	1	1
4	4	3	3	3	3	1	1	1	1
4	4	3	3	3	3	1	1	4	4
4	4	3	3	3	3	1	1	1	1

For Review Only

4	4	3	3	3	3	4	4	1	1
4	4	3	3	3	3	4	4	1	1
4	4	3	3	3	3	1	1	4	4
4	4	3	3	3	3	4	4	1	1
4	4	3	3	3	3	1	1	4	4
4	4	3	3	3	3	4	4	1	1
4	4	3	3	3	3	4	4	1	1
4	4	4	4	1	1	4	4	1	1
4	4	3	3	3	3	4	4	1	1
4	4	3	3	3	3	4	4	1	1
4	4	3	3	3	3	1	1	1	1
4	4	3	3	3	3	1	1	1	1
4	4	3	3	3	3	4	4	1	1
4	4	3	3	3	3	4	4	1	1
4	4	3	3	3	3	4	4	1	1
4	4	3	3	3	3	4	4	1	1
4	4	3	3	3	3	1	1	1	1
4	4	3	3	3	3	1	1	4	4
4	4	3	3	3	3	4	4	1	1
4	4	3	3	3	3	4	4	1	1
4	4	3	3	3	3	1	1	1	1
4	4	3	3	3	3	4	4	1	1
4	4	3	3	3	3	4	4	1	1
4	4	3	3	3	3	1	1	4	4
4	4	3	3	3	3	4	4	4	4
4	4	3	3	3	3	1	1	1	1
1	1	4	4	1	1	4	4	4	4
4	4	3	3	3	3	4	4	1	1
4	4	3	3	3	3	4	4	1	1
4	4	3	3	3	3	4	4	1	1
4	4	3	3	3	3	4	4	1	1
1	1	3	3	3	3	4	4	1	1
4	4	3	3	3	3	4	4	1	1
4	4	3	3	3	3	4	4	1	1
4	4	3	3	3	3	4	4	1	1
4	4	3	3	3	3	4	4	4	4
4	4	4	4	1	1	4	4	1	1
4	4	3	3	3	3	4	4	1	1
4	4	3	3	3	3	1	1	4	4
4	4	4	4	1	1	4	4	1	1
4	4	3	3	3	3	4	4	1	1
4	4	3	3	3	3	1	1	1	1
4	4	3	3	3	3	1	1	4	4
4	4	3	3	3	3	1	1	4	4
4	4	3	3	3	3	1	1	1	1
4	4	3	3	3	3	4	4	1	1
4	4	3	3	3	3	4	4	1	1
1	1	3	3	3	3	4	4	1	1
4	4	3	3	3	3	4	4	1	1
4	4	4	4	1	1	4	4	1	1
4	4	3	3	3	3	1	1	1	1
1	1	3	3	3	3	4	4	1	1
1	1	4	4	1	1	4	4	1	1
4	4	3	3	3	3	4	4	1	1
4	4	3	3	3	3	4	4	1	1

4	4	3	3	3	3	4	4	1	1
1	1	3	3	3	3	4	4	1	1
1	1	3	3	3	3	4	4	1	1
1	1	3	3	3	3	4	4	1	1
4	4	3	3	3	3	1	1	1	1
4	4	3	3	3	3	4	4	1	1
4	4	3	3	3	3	4	4	1	1
4	4	3	3	3	3	4	4	1	1
4	4	3	3	3	3	4	4	1	1
4	4	3	3	3	3	4	4	1	1
4	4	3	3	3	3	4	4	1	1
4	4	3	3	3	3	4	4	1	1
4	4	3	3	3	3	1	1	4	4
4	4	3	3	3	3	1	1	1	1
1	1	3	3	3	3	1	1	1	1
4	4	3	3	3	3	4	4	1	1
4	4	3	3	3	3	4	4	1	1
4	4	3	3	3	3	4	4	1	1
4	4	3	3	3	3	4	4	1	1
4	4	3	3	3	3	1	1	1	1
4	4	3	3	3	3	4	4	1	1
4	4	3	3	3	3	4	4	1	1
4	4	3	3	3	3	1	1	1	1
1	1	3	3	3	3	4	4	1	1
4	4	3	3	1	1	4	4	1	1
4	4	3	3	3	3	4	4	1	1
4	4	3	3	3	3	4	4	1	1
4	4	3	3	3	3	4	4	1	1
4	4	3	3	3	3	4	4	1	1
4	4	3	3	3	3	4	4	1	1
1	1	3	3	3	3	4	4	1	1
4	4	3	3	3	3	4	4	1	1
4	4	3	3	3	3	4	4	1	1
4	4	3	3	3	3	4	4	1	1
4	4	3	3	3	3	4	4	1	1
4	4	3	3	3	3	4	4	1	1
4	4	3	3	3	3	4	4	1	1
4	4	3	3	3	3	1	1	1	1
4	4	4	4	3	3	4	4	1	1
4	4	3	3	3	3	4	4	1	1
4	4	3	3	3	3	4	4	1	1
1	1	3	3	3	3	4	4	1	1
4	4	3	3	3	3	4	4	4	4
4	4	3	3	3	3	4	4	1	1
4	4	3	3	3	3	4	4	1	1
4	4	3	3	3	3	4	4	1	1
4	4	3	3	3	3	4	4	1	1
4	4	3	3	3	3	4	4	1	1
4	4	3	3	3	3	4	4	1	1
4	4	3	3	3	3	4	4	1	1
4	4	3	3	3	3	4	4	1	1
4	4	3	3	3	3	4	4	1	1
4	4	3	3	3	3	4	4	4	4
4	4	3	3	3	3	4	4	1	1
4	4	4	4	3	3	4	4	1	1

For Review Only

4	4	3	3	3	3	1	1	4	4
4	4	3	3	3	3	1	1	4	4
4	4	3	3	3	3	4	4	1	1
1	1	3	3	3	3	4	4	1	1
4	4	3	3	3	3	4	4	1	1
1	1	3	3	3	3	1	1	1	1
4	4	3	3	3	3	4	4	1	1
4	4	3	3	3	3	4	4	1	1
4	4	3	3	1	1	4	4	1	1
4	4	3	3	3	3	1	1	4	4
4	4	3	3	3	3	4	4	1	1
4	4	3	3	3	3	4	4	1	1
4	4	3	3	3	3	4	4	1	1
4	4	3	3	3	3	4	4	1	1
1	1	3	3	3	3	4	4	1	1
4	4	3	3	3	3	4	4	1	1
4	4	3	3	3	3	4	4	1	1
4	4	3	3	3	3	1	1	1	1
1	1	3	3	3	3	4	4	1	1
4	4	3	3	3	3	4	4	1	1
4	4	3	3	3	3	4	4	1	1
4	4	3	3	3	3	4	4	1	1
4	4	4	4	1	1	4	4	1	1
4	4	3	3	3	3	1	1	1	1
4	4	3	3	3	3	4	4	1	1
4	4	3	3	3	3	4	4	1	1
4	4	3	3	3	3	4	4	1	1
4	4	3	3	3	3	4	4	1	1
4	4	3	3	3	3	4	4	1	1
4	4	3	3	3	3	4	4	1	1
4	4	3	3	3	3	4	4	1	1
4	4	3	3	3	3	4	4	1	1
4	4	3	3	3	3	4	4	1	1
1	1	3	3	3	3	4	4	1	1
4	4	3	3	3	3	4	4	1	1
4	4	3	3	3	3	1	1	1	1
4	4	3	3	3	3	4	4	1	1
4	4	3	3	3	3	4	4	1	1
4	4	3	3	3	3	1	1	1	1
4	4	3	3	3	3	1	1	4	4
4	4	3	3	3	3	1	1	1	1
4	4	3	3	3	3	4	4	1	1
1	1	3	3	3	3	4	4	1	1
4	4	3	3	3	3	4	4	1	1
4	4	4	4	3	3	4	4	1	1
4	4	3	3	3	3	1	1	1	1
4	4	3	3	3	3	4	4	1	1
1	1	3	3	3	3	4	4	1	1
4	4	3	3	3	3	4	4	1	1
4	4	3	3	3	3	4	4	1	1
4	4	3	3	3	3	4	4	1	1
4	4	4	4	1	1	4	4	1	1
4	4	4	4	1	1	4	4	1	1
4	4	3	3	3	3	1	1	1	1
1	1	3	3	3	3	4	4	1	1

4	4	3	3	3	3	4	4	1	1
4	4	3	3	3	3	4	4	1	1
4	4	3	3	3	3	4	4	1	1
4	4	3	3	3	3	4	4	1	1
4	4	3	3	3	3	4	4	1	1
4	4	3	3	3	3	4	4	1	1
1	1	3	3	3	3	4	4	1	1
4	4	3	3	3	3	4	4	4	4
4	4	3	3	3	3	4	4	1	1
1	1	4	4	1	1	4	4	1	1
4	4	3	3	3	3	4	4	1	1
4	4	3	3	3	3	4	4	1	1
4	4	3	3	3	3	1	1	4	4
1	1	3	3	3	3	4	4	1	1
4	4	3	3	3	3	1	1	1	1
4	4	4	4	1	1	1	1	1	1
1	1	3	3	3	3	4	4	1	1
4	4	3	3	3	3	4	4	1	1
1	1	3	3	3	3	4	4	1	1
4	4	3	3	3	3	4	4	1	1
1	1	3	3	3	3	4	4	1	1
4	4	3	3	3	3	4	4	1	1
4	4	3	3	3	3	1	1	4	4
1	1	3	3	3	3	4	4	1	1
4	4	4	4	1	1	4	4	1	1
4	4	3	3	3	3	4	4	4	4
4	4	4	4	1	1	4	4	1	1
4	4	3	3	3	3	4	4	1	1
4	4	3	3	3	3	4	4	1	1
4	4	3	3	3	3	4	4	1	1
1	1	3	3	3	3	4	4	1	1
1	1	3	3	3	3	4	4	1	1
4	4	3	3	3	3	4	4	1	1
1	1	3	3	3	3	4	4	1	1
4	4	4	4	1	1	4	4	1	1
1	1	3	3	3	3	4	4	1	1
4	4	3	3	3	3	4	4	1	1
4	4	3	3	3	3	1	1	1	1
1	1	3	3	3	3	4	4	1	1
1	1	3	3	3	3	4	4	1	1
4	4	3	3	3	3	4	4	1	1
4	4	3	3	3	3	4	4	1	1
4	4	3	3	3	3	4	4	1	1
4	4	3	3	3	3	4	4	1	1
4	4	3	3	3	3	1	1	4	4
4	4	3	3	3	3	1	1	4	4
4	4	4	4	1	1	4	4	1	1
4	4	4	4	1	1	1	1	4	4
4	4	4	4	1	1	4	4	1	1
1	1	4	4	1	1	4	4	1	1
4	4	3	3	3	3	4	4	1	1
1	1	3	3	3	3	4	4	1	1
4	4	3	3	3	3	1	1	1	1
4	4	3	3	3	3	4	4	4	4
1	1	3	3	3	3	4	4	1	1

For Review Only

4	4	3	3	3	3	1	1	1	1
4	4	3	3	3	3	1	1	1	1

For Review Only

SNP2386	SNP2386	SNP2387	SNP2387	SNP2388	SNP2388	SNP2389	SNP2389	SNP239	SNP239
1	1	4	4	3	3	2	2	4	4
1	1	3	3	3	3	2	2	4	4
1	1	3	3	3	3	2	2	4	4
1	1	4	4	3	3	2	2	4	4
1	1	4	4	4	4	2	2	1	1
1	1	4	4	3	3	2	2	4	4
1	1	4	4	3	3	2	2	4	4
1	1	4	4	4	4	3	3	4	4
1	1	4	4	3	3	2	2	1	1
1	1	3	3	4	4	2	2	4	4
1	1	4	4	3	3	2	2	4	4
1	1	3	3	3	3	2	2	4	4
1	1	4	4	3	3	2	2	4	4
1	1	4	4	3	3	2	2	4	4
1	1	4	4	3	3	2	2	4	4
1	1	4	4	3	3	2	2	4	4
1	1	4	4	3	3	2	2	1	1
1	1	4	4	3	3	2	2	4	4
1	1	3	3	3	3	2	2	4	4
1	1	4	4	3	3	3	3	4	4
1	1	4	4	3	3	2	2	4	4
1	1	4	4	3	3	2	2	4	4
1	1	4	4	3	3	2	2	1	1
1	1	4	4	3	3	2	2	4	4
1	1	4	4	3	3	2	2	4	4
4	4	3	3	3	3	2	2	4	4
4	4	4	4	3	3	2	2	4	4
1	1	3	3	3	3	2	2	4	4
1	1	4	4	3	3	3	3	1	1
1	1	4	4	3	3	2	2	4	4
1	1	3	3	3	3	2	2	4	4
1	1	4	4	3	3	2	2	4	4
1	1	4	4	3	3	2	2	1	1
1	1	4	4	3	3	2	2	4	4
1	1	3	3	3	3	2	2	4	4
1	1	4	4	3	3	2	2	4	4
1	1	4	4	3	3	2	2	4	4
1	1	4	4	4	4	2	2	1	1
1	1	4	4	3	3	2	2	4	4
1	1	4	4	3	3	2	2	4	4
1	1	4	4	4	4	2	2	4	4
1	1	4	4	3	3	3	3	4	4
1	1	3	3	4	4	3	3	4	4
1	1	4	4	3	3	2	2	4	4
1	1	3	3	4	4	3	3	4	4
1	1	3	3	3	3	2	2	4	4
4	4	3	3	3	3	2	2	4	4
1	1	4	4	3	3	2	2	4	4
1	1	4	4	3	3	2	2	1	1
1	1	4	4	3	3	2	2	4	4
4	4	3	3	3	3	2	2	4	4
1	1	3	3	3	3	2	2	4	4

For Review Only

1	1	4	4	3	3	2	2	1	1
1	1	4	4	3	3	2	2	1	1
1	1	3	3	3	3	2	2	4	4
1	1	4	4	3	3	2	2	4	4
1	1	3	3	3	3	2	2	4	4
4	4	3	3	3	3	2	2	4	4
4	4	3	3	3	3	2	2	4	4
1	1	4	4	3	3	2	2	4	4
1	1	4	4	3	3	2	2	4	4
1	1	3	3	3	3	2	2	4	4
1	1	3	3	3	3	2	2	4	4
1	1	3	3	3	3	2	2	4	4
1	1	4	4	3	3	2	2	1	1
1	1	4	4	3	3	3	3	1	1
1	1	4	4	3	3	2	2	4	4
1	1	4	4	4	4	2	2	4	4
1	1	3	3	3	3	2	2	4	4
1	1	3	3	3	3	2	2	4	4
1	1	4	4	3	3	2	2	4	4
1	1	4	4	3	3	2	2	1	1
1	1	3	3	3	3	2	2	4	4
1	1	3	3	3	3	2	2	4	4
1	1	4	4	3	3	2	2	1	1
1	1	3	3	3	3	3	3	4	4
1	1	3	3	4	4	3	3	4	4
1	1	3	3	3	3	2	2	4	4
1	1	3	3	3	3	2	2	4	4
1	1	4	4	3	3	2	2	4	4
4	4	3	3	3	3	2	2	4	4
1	1	4	4	3	3	3	3	1	1
1	1	4	4	3	3	3	3	4	4
1	1	4	4	3	3	2	2	4	4
1	1	4	4	3	3	2	2	4	4
1	1	4	4	3	3	2	2	1	1
1	1	4	4	3	3	2	2	4	4
1	1	3	3	4	4	3	3	4	4
1	1	4	4	3	3	3	3	4	4
1	1	4	4	4	4	2	2	1	1
1	1	3	3	3	3	2	2	1	1
1	1	4	4	3	3	2	2	4	4
1	1	4	4	3	3	2	2	1	1
1	1	3	3	3	3	2	2	4	4
1	1	3	3	3	3	2	2	4	4
1	1	3	3	3	3	2	2	4	4
1	1	3	3	3	3	2	2	1	1
1	1	4	4	3	3	2	2	4	4
1	1	4	4	3	3	3	3	4	4
1	1	3	3	3	3	2	2	4	4
1	1	4	4	3	3	2	2	4	4
1	1	3	3	3	3	2	2	4	4
1	1	3	3	3	3	2	2	4	4
1	1	4	4	3	3	2	2	4	4
1	1	4	4	3	3	2	2	4	4
1	1	3	3	3	3	2	2	4	4
1	1	4	4	3	3	2	2	4	4

For Review Only

4	4	3	3	3	3	2	2	4	4
1	1	4	4	3	3	2	2	1	1
4	4	3	3	3	3	2	2	4	4
1	1	4	4	3	3	2	2	4	4
1	1	3	3	3	3	2	2	4	4
1	1	3	3	3	3	2	2	4	4
1	1	4	4	3	3	2	2	4	4
1	1	4	4	3	3	2	2	4	4
1	1	4	4	3	3	2	2	4	4
1	1	3	3	3	3	2	2	4	4
1	1	4	4	3	3	2	2	4	4
1	1	3	3	3	3	2	2	4	4
1	1	3	3	3	3	2	2	4	4
1	1	3	3	3	3	2	2	4	4
1	1	3	3	3	3	2	2	4	4
1	1	3	3	3	3	2	2	4	4
1	1	4	4	3	3	2	2	4	4
1	1	3	3	3	3	2	2	4	4
1	1	4	4	3	3	2	2	4	4
1	1	3	3	3	3	2	2	4	4
4	4	3	3	3	3	2	2	4	4
1	1	4	4	3	3	2	2	1	1
1	1	3	3	3	3	2	2	4	4
1	1	4	4	3	3	3	3	4	4
1	1	3	3	3	3	2	2	4	4
1	1	4	4	3	3	2	2	4	4
1	1	4	4	3	3	2	2	4	4
1	1	4	4	3	3	2	2	4	4
1	1	4	4	3	3	2	2	4	4
1	1	3	3	3	3	2	2	4	4
1	1	4	4	3	3	2	2	4	4
1	1	3	3	3	3	2	2	4	4
1	1	4	4	3	3	2	2	1	1
4	4	3	3	3	3	2	2	4	4
1	1	4	4	3	3	2	2	4	4
1	1	4	4	3	3	2	2	4	4
1	1	4	4	3	3	2	2	4	4
1	1	3	3	3	3	3	3	4	4
1	1	3	3	3	3	2	2	4	4
1	1	4	4	3	3	2	2	4	4
1	1	4	4	3	3	2	2	1	1
1	1	4	4	3	3	2	2	4	4
1	1	3	3	3	3	2	2	4	4
1	1	4	4	3	3	2	2	4	4
1	1	3	3	3	3	2	2	4	4
1	1	4	4	3	3	2	2	4	4
4	4	3	3	3	3	2	2	4	4
4	4	3	3	3	3	2	2	4	4
1	1	4	4	3	3	2	2	4	4
4	4	3	3	3	3	2	2	4	4
4	4	3	3	3	3	2	2	4	4
1	1	4	4	3	3	2	2	4	4
1	1	3	3	3	3	3	3	4	4
1	1	3	3	3	3	2	2	4	4
1	1	4	4	3	3	2	2	4	4

For Review Only

1	1	3	3	3	3	2	2	4	4
1	1	3	3	3	3	2	2	4	4
1	1	3	3	4	4	3	3	4	4
1	1	3	3	3	3	2	2	4	4
1	1	4	4	3	3	2	2	4	4
1	1	3	3	3	3	2	2	4	4
1	1	3	3	3	3	2	2	4	4
1	1	3	3	3	3	2	2	4	4
1	1	4	4	3	3	2	2	4	4
1	1	3	3	3	3	2	2	4	4
4	4	3	3	3	3	2	2	4	4
1	1	4	4	3	3	2	2	4	4
1	1	3	3	3	3	2	2	4	4
1	1	4	4	3	3	2	2	4	4
1	1	4	4	3	3	2	2	4	4
1	1	4	4	3	3	2	2	4	4
1	1	3	3	3	3	3	3	4	4
1	1	3	3	3	3	2	2	4	4
4	4	4	4	3	3	2	2	4	4
1	1	4	4	3	3	2	2	4	4
1	1	3	3	3	3	2	2	4	4
1	1	3	3	3	3	2	2	4	4
1	1	3	3	3	3	2	2	1	1
1	1	3	3	3	3	2	2	4	4
1	1	4	4	3	3	2	2	4	4
1	1	4	4	3	3	2	2	4	4
1	1	4	4	3	3	2	2	4	4
1	1	4	4	3	3	2	2	4	4
1	1	4	4	3	3	2	2	1	1
1	1	4	4	3	3	2	2	4	4
1	1	4	4	3	3	2	2	4	4
1	1	4	4	3	3	3	3	4	4
1	1	4	4	3	3	2	2	4	4
1	1	4	4	3	3	2	2	4	4
1	1	4	4	4	4	2	2	4	4
1	1	3	3	3	3	2	2	4	4
4	4	4	4	3	3	2	2	4	4
1	1	4	4	3	3	2	2	4	4
4	4	3	3	3	3	2	2	4	4
1	1	3	3	3	3	2	2	4	4
1	1	3	3	3	3	2	2	4	4
4	4	3	3	3	3	2	2	4	4
1	1	4	4	3	3	2	2	4	4
1	1	3	3	3	3	2	2	4	4
1	1	3	3	3	3	2	2	4	4
1	1	3	3	3	3	2	2	4	4
1	1	4	4	3	3	2	2	4	4
1	1	3	3	3	3	2	2	4	4
1	1	4	4	3	3	2	2	4	4
1	1	4	4	3	3	2	2	4	4
1	1	4	4	3	3	2	2	4	4
1	1	4	4	3	3	2	2	4	4
1	1	3	3	3	3	2	2	1	1
1	1	3	3	3	3	2	2	4	4
1	1	4	4	3	3	2	2	4	4

For Review Only

1	1	4	4	3	3	2	2	4	4
1	1	3	3	3	3	2	2	4	4
1	1	3	3	3	3	2	2	4	4
1	1	3	3	3	3	2	2	1	1
1	1	4	4	3	3	2	2	4	4
4	4	4	4	3	3	2	2	4	4
1	1	4	4	3	3	3	3	4	4
1	1	3	3	4	4	2	2	4	4
1	1	4	4	3	3	2	2	4	4
1	1	4	4	3	3	2	2	4	4
1	1	3	3	3	3	2	2	4	4
1	1	4	4	3	3	2	2	4	4
1	1	3	3	3	3	2	2	1	1
4	4	3	3	3	3	2	2	4	4
1	1	3	3	3	3	2	2	4	4
1	1	3	3	3	3	2	2	1	1
1	1	4	4	3	3	2	2	4	4
1	1	4	4	3	3	2	2	1	1
1	1	3	3	3	3	2	2	4	4
1	1	4	4	4	4	2	2	1	1
1	1	4	4	3	3	2	2	4	4
4	4	3	3	3	3	2	2	4	4
1	1	3	3	3	3	2	2	1	1
1	1	4	4	3	3	2	2	4	4
1	1	4	4	3	3	2	2	4	4
1	1	3	3	3	3	2	2	4	4
1	1	4	4	3	3	2	2	4	4
1	1	4	4	3	3	2	2	4	4
1	1	4	4	3	3	2	2	4	4
1	1	4	4	3	3	2	2	4	4
1	1	3	3	3	3	2	2	4	4
1	1	3	3	3	3	2	2	4	4
1	1	4	4	3	3	2	2	4	4
4	4	3	3	3	3	2	2	4	4
1	1	3	3	3	3	2	2	1	1
1	1	4	4	3	3	2	2	4	4
1	1	4	4	3	3	2	2	4	4
1	1	3	3	3	3	2	2	4	4
1	1	4	4	3	3	2	2	4	4
1	1	4	4	3	3	2	2	4	4
1	1	4	4	3	3	2	2	4	4
1	1	4	4	3	3	2	2	1	1
1	1	4	4	3	3	2	2	4	4
1	1	4	4	3	3	2	2	1	1
1	1	3	3	3	3	2	2	4	4
1	1	3	3	3	3	2	2	4	4
1	1	3	3	3	3	2	2	4	4
1	1	3	3	3	3	2	2	4	4
1	1	3	3	3	3	3	3	4	4
1	1	4	4	3	3	2	2	4	4
1	1	4	4	3	3	2	2	4	4
1	1	4	4	3	3	2	2	4	4
1	1	3	3	3	3	2	2	4	4
1	1	3	3	3	3	2	2	4	4
1	1	4	4	3	3	2	2	4	4

For Review Only

1	1	3	3	3	3	2	2	1	1
1	1	3	3	3	3	2	2	4	4

For Review Only

SNP2390	SNP2390	SNP2391	SNP2391	SNP2392	SNP2392	SNP2393	SNP2393	SNP2394	SNP2394
4	4	4	4	1	1	1	1	4	4
4	4	4	4	1	1	1	1	1	1
4	4	4	4	1	1	4	4	1	1
4	4	4	4	4	4	1	1	4	4
1	1	3	3	1	1	4	4	4	4
4	4	4	4	4	4	1	1	4	4
4	4	4	4	1	1	4	4	4	4
1	1	3	3	1	1	1	1	4	4
4	4	4	4	1	1	4	4	4	4
1	1	3	3	1	1	1	1	1	1
4	4	4	4	1	1	1	1	4	4
4	4	4	4	1	1	4	4	1	1
4	4	4	4	1	1	4	4	4	4
4	4	4	4	1	1	1	1	4	4
4	4	4	4	1	1	1	1	4	4
4	4	4	4	1	1	1	1	4	4
4	4	4	4	1	1	4	4	4	4
4	4	4	4	4	4	1	1	4	4
4	4	4	4	4	4	1	1	4	4
1	1	4	4	1	1	1	1	4	4
4	4	4	4	1	1	1	1	4	4
4	4	4	4	1	1	1	1	4	4
4	4	4	4	1	1	1	1	4	4
1	1	3	3	1	1	4	4	4	4
4	4	4	4	4	4	1	1	4	4
1	1	3	3	1	1	4	4	1	1
4	4	4	4	4	4	1	1	4	4
4	4	4	4	1	1	1	1	4	4
4	4	4	4	1	1	1	1	4	4
1	1	3	3	1	1	1	1	4	4
4	4	4	4	1	1	1	1	4	4
4	4	4	4	4	4	1	1	4	4
4	4	4	4	4	4	1	1	4	4
4	4	4	4	1	1	4	4	4	4
1	1	3	3	1	1	1	1	4	4
4	4	4	4	1	1	4	4	4	4
4	4	4	4	1	1	1	1	4	4
1	1	3	3	1	1	1	1	4	4
4	4	4	4	1	1	1	1	4	4
1	1	3	3	1	1	1	1	4	4
1	1	3	3	1	1	1	1	4	4
4	4	4	4	1	1	1	1	4	4
1	1	4	4	1	1	1	1	1	1
4	4	4	4	1	1	1	1	4	4
1	1	3	3	1	1	1	1	1	1
4	4	4	4	1	1	4	4	1	1
4	4	4	4	1	1	1	1	1	1
1	1	4	4	1	1	1	1	4	4
4	4	4	4	1	1	1	1	4	4
4	4	4	4	1	1	4	4	4	4
4	4	4	4	1	1	1	1	1	1
4	4	4	4	4	4	1	1	1	1

For Review Only

4	4	4	4	1	1	1	1	4	4
4	4	4	4	4	4	1	1	4	4
4	4	4	4	4	4	1	1	1	1
4	4	4	4	4	4	1	1	4	4
4	4	4	4	4	4	1	1	4	4
4	4	4	4	1	1	1	1	1	1
1	1	3	3	1	1	1	1	1	1
1	1	3	3	1	1	1	1	4	4
4	4	4	4	1	1	4	4	4	4
4	4	4	4	4	4	1	1	4	4
4	4	4	4	1	1	1	1	1	1
4	4	4	4	1	1	1	1	1	1
4	4	4	4	1	1	1	1	4	4
4	4	4	4	1	1	1	1	4	4
4	4	4	4	4	4	1	1	4	4
1	1	4	4	1	1	1	1	4	4
4	4	4	4	1	1	1	1	1	1
4	4	4	4	1	1	4	4	1	1
4	4	4	4	1	1	1	1	4	4
4	4	4	4	1	1	4	4	4	4
4	4	4	4	1	1	1	1	1	1
4	4	3	3	1	1	1	1	4	4
4	4	4	4	1	1	1	1	4	4
4	4	4	4	1	1	1	1	4	4
1	1	3	3	1	1	1	1	1	1
4	4	4	4	4	4	1	1	1	1
4	4	4	4	1	1	1	1	1	1
1	1	3	3	1	1	1	1	4	4
1	1	3	3	1	1	4	4	1	1
4	4	4	4	4	4	1	1	4	4
4	4	4	4	1	1	1	1	4	4
4	4	4	4	1	1	4	4	4	4
1	1	3	3	1	1	1	1	4	4
4	4	4	4	1	1	1	1	4	4
4	4	4	4	1	1	4	4	4	4
1	1	3	3	1	1	1	1	1	1
4	4	4	4	1	1	1	1	4	4
1	1	3	3	1	1	1	1	4	4
4	4	4	4	1	1	1	1	1	1
1	1	3	3	1	1	1	1	4	4
4	4	4	4	1	1	1	1	4	4
4	4	4	4	1	1	4	4	1	1
4	4	4	4	1	1	1	1	1	1
4	4	4	4	1	1	1	1	1	1
4	4	4	4	1	1	4	4	4	4
1	1	3	3	1	1	1	1	4	4
4	4	4	4	1	1	4	4	4	4
1	1	3	3	1	1	4	4	1	1
4	4	4	4	1	1	4	4	4	4
4	4	4	4	4	4	1	1	4	4
4	4	4	4	1	1	1	1	1	1
4	4	4	4	1	1	1	1	4	4
4	4	4	4	1	1	1	1	4	4
1	1	4	4	1	1	1	1	4	4
4	4	4	4	4	4	4	4	4	4

For Review Only

1	1	3	3	1	1	4	4	1	1
1	1	3	3	1	1	1	1	4	4
4	4	4	4	1	1	4	4	1	1
1	1	3	3	1	1	1	1	4	4
4	4	4	4	4	4	1	1	1	1
4	4	4	4	1	1	4	4	4	4
4	4	4	4	4	4	1	1	4	4
4	4	4	4	4	4	1	1	4	4
4	4	4	4	1	1	1	1	4	4
4	4	3	3	1	1	1	1	4	4
4	4	3	3	4	4	1	1	4	4
4	4	4	4	1	1	1	1	4	4
4	4	4	4	1	1	1	1	1	1
4	4	4	4	4	4	1	1	1	1
4	4	4	4	1	1	1	1	4	4
4	4	3	3	1	1	1	1	4	4
4	4	4	4	1	1	1	1	4	4
4	4	4	4	1	1	1	1	4	4
1	1	3	3	1	1	4	4	4	4
4	4	4	4	4	4	1	1	4	4
4	4	4	4	1	1	1	1	4	4
1	1	4	4	4	4	1	1	4	4
4	4	4	4	1	1	4	4	1	1
1	1	3	3	1	1	1	1	4	4
1	1	3	3	1	1	1	1	4	4
4	4	4	4	1	1	1	1	4	4
4	4	4	4	1	1	1	1	4	4
4	4	4	4	1	1	1	1	4	4
4	4	4	4	4	4	1	1	4	4
1	1	3	3	1	1	1	1	4	4
1	1	4	4	1	1	1	1	4	4
4	4	4	4	1	1	1	1	1	1
4	4	4	4	1	1	1	1	4	4
4	4	4	4	1	1	4	4	4	4
4	4	4	4	1	1	4	4	4	4
4	4	3	3	1	1	1	1	4	4
1	1	3	3	1	1	4	4	4	4
4	4	4	4	1	1	1	1	1	1
4	4	4	4	1	1	4	4	1	1
4	4	4	4	1	1	1	1	4	4
4	4	4	4	1	1	1	1	4	4
1	1	3	3	1	1	4	4	4	4
4	4	4	4	1	1	4	4	1	1
4	4	4	4	1	1	1	1	4	4
4	4	4	4	1	1	4	4	1	1
1	1	3	3	4	4	4	4	4	4
4	4	4	4	1	1	4	4	1	1
1	1	3	3	1	1	1	1	1	1
1	1	4	4	1	1	1	1	4	4
4	4	4	4	1	1	1	1	1	1
4	4	4	4	1	1	4	4	1	1
4	4	4	4	1	1	1	1	4	4
4	4	4	4	1	1	4	4	4	4
4	4	4	4	1	1	4	4	1	1
4	4	4	4	1	1	1	1	4	4

For Review Only

1	1	3	3	1	1	1	1	1	1
4	4	4	4	1	1	1	1	1	1
1	1	3	3	1	1	1	1	4	4
1	1	3	3	1	1	1	1	4	4
4	4	4	4	1	1	1	1	4	4
1	1	3	3	1	1	1	1	4	4
4	4	4	4	1	1	1	1	4	4
4	4	4	4	1	1	1	1	4	4
4	4	4	4	1	1	1	1	4	4
4	4	4	4	1	1	1	1	4	4
4	4	4	4	1	1	1	1	1	1
4	4	4	4	1	1	1	1	4	4
4	4	4	4	4	4	1	1	4	4
4	4	4	4	4	4	4	4	4	4
1	1	3	3	1	1	4	4	4	4
4	4	4	4	1	1	1	1	4	4
1	1	4	4	1	1	4	4	4	4
4	4	4	4	1	1	1	1	1	1
4	4	4	4	1	1	4	4	4	4
4	4	4	4	4	4	1	1	4	4
4	4	3	3	1	1	1	1	4	4
4	4	4	4	1	1	1	1	4	4
4	4	4	4	1	1	1	1	4	4
4	4	4	4	1	1	1	1	1	1
4	4	4	4	1	1	1	1	4	4
4	4	4	4	1	1	4	4	4	4
4	4	4	4	4	4	1	1	4	4
4	4	4	4	1	1	1	1	4	4
4	4	4	4	4	4	1	1	4	4
4	4	4	4	1	1	1	1	4	4
4	4	4	4	1	1	1	1	4	4
4	4	4	4	1	1	1	1	4	4
4	4	4	4	1	1	1	1	4	4
1	1	3	3	1	1	4	4	4	4
1	1	3	3	1	1	4	4	4	4
4	4	4	4	1	1	1	1	1	1
4	4	4	4	1	1	1	1	4	4
4	4	4	4	1	1	1	1	4	4
1	1	3	3	1	1	1	1	1	1
4	4	4	4	1	1	1	1	1	1
4	4	4	4	1	1	4	4	1	1
1	1	3	3	1	1	1	1	1	1
1	1	3	3	1	1	1	1	4	4
4	4	4	4	1	1	1	1	4	4
4	4	3	3	4	4	4	4	4	4
4	4	4	4	1	1	1	1	4	4
4	4	4	4	1	1	1	1	4	4
1	1	3	3	1	1	1	1	4	4
1	1	3	3	1	1	1	1	4	4
4	4	4	4	4	4	1	1	4	4
4	4	4	4	4	4	1	1	4	4
1	1	3	3	1	1	1	1	4	4
1	1	3	3	1	1	1	1	4	4
4	4	4	4	1	1	1	1	1	1
4	4	4	4	1	1	1	1	4	4

For Review Only

1	1	3	3	1	1	4	4	4	4
1	1	4	4	1	1	1	1	4	4
4	4	4	4	4	4	4	4	4	4
4	4	4	4	4	4	1	1	4	4
4	4	4	4	1	1	1	1	4	4
1	1	3	3	1	1	1	1	4	4
1	1	3	3	1	1	4	4	4	4
1	1	3	3	1	1	1	1	1	1
4	4	4	4	1	1	1	1	4	4
1	1	3	3	1	1	1	1	4	4
4	4	4	4	1	1	1	1	4	4
4	4	4	4	1	1	1	1	4	4
4	4	4	4	1	1	1	1	1	1
1	1	3	3	1	1	1	1	1	1
4	4	4	4	1	1	4	4	1	1
1	1	3	3	1	1	1	1	4	4
4	4	4	4	1	1	1	1	4	4
1	1	3	3	1	1	4	4	4	4
1	1	3	3	1	1	1	1	4	4
1	1	3	3	1	1	1	1	4	4
1	1	3	3	1	1	1	1	4	4
1	1	3	3	1	1	4	4	1	1
4	4	4	4	1	1	1	1	4	4
4	4	4	4	4	4	1	1	4	4
1	1	3	3	1	1	1	1	4	4
4	4	4	4	1	1	1	1	4	4
1	1	3	3	1	1	1	1	4	4
4	4	4	4	4	4	1	1	4	4
4	4	4	4	1	1	1	1	4	4
4	4	4	4	4	4	1	1	4	4
1	1	3	3	1	1	1	1	4	4
4	4	4	4	1	1	1	1	1	1
4	4	4	4	1	1	4	4	4	4
1	1	3	3	1	1	1	1	1	1
1	1	3	3	4	4	1	1	4	4
1	1	3	3	1	1	1	1	4	4
4	4	4	4	1	1	4	4	4	4
4	4	4	4	1	1	1	1	1	1
4	4	4	4	1	1	4	4	4	4
4	4	4	4	1	1	1	1	4	4
4	4	4	4	1	1	4	4	4	4
4	4	4	4	4	4	4	4	4	4
4	4	4	4	1	1	1	1	4	4
4	4	4	4	1	1	1	1	4	4
4	4	4	4	1	1	1	1	1	1
4	4	4	4	1	1	1	1	1	1
1	1	3	3	1	1	1	1	1	1
4	4	4	4	1	1	1	1	1	1
1	1	3	3	1	1	4	4	1	1
1	1	3	3	1	1	1	1	4	4
4	4	4	4	1	1	1	1	4	4
4	4	4	4	1	1	1	1	4	4
4	4	4	4	1	1	4	4	1	1
4	4	4	4	1	1	1	1	1	1
1	1	3	3	1	1	1	1	4	4

For Review Only

4	4	4	4	1	1	1	1	4	4
4	4	4	4	1	1	1	1	1	1

For Review Only

SNP2395	SNP2395	SNP2396	SNP2396	SNP2397	SNP2397	SNP2398	SNP2398	SNP2399	SNP2399
2	2	1	1	4	4	4	4	1	1
2	2	1	1	4	4	4	4	1	1
2	2	1	1	4	4	4	4	4	4
2	2	2	2	1	1	4	4	1	1
4	4	2	2	1	1	1	1	1	1
4	4	2	2	1	1	1	1	1	1
4	4	2	2	1	1	1	1	4	4
4	4	2	2	1	1	1	1	4	4
4	4	2	2	1	1	1	1	4	4
2	2	2	2	4	4	4	4	4	4
2	2	1	1	4	4	4	4	1	1
2	2	1	1	1	1	4	4	4	4
2	2	2	2	1	1	4	4	1	1
4	4	2	2	1	1	1	1	4	4
4	4	2	2	1	1	1	1	1	1
2	2	2	2	4	4	4	4	4	4
4	4	2	2	1	1	1	1	1	1
4	4	2	2	1	1	1	1	1	1
2	2	1	1	4	4	4	4	1	1
4	4	2	2	1	1	1	1	1	1
2	2	1	1	4	4	4	4	4	4
4	4	2	2	1	1	1	1	1	1
2	2	1	1	4	4	4	4	1	1
4	4	2	2	1	1	1	1	4	4
2	2	2	2	1	1	1	1	1	1
4	4	2	2	1	1	1	1	1	1
2	2	1	1	4	4	4	4	1	1
2	2	2	2	1	1	4	4	1	1
4	4	2	2	1	1	1	1	4	4
4	4	2	2	1	1	1	1	1	1
2	2	2	2	4	4	4	4	1	1
2	2	1	1	4	4	4	4	1	1
4	4	2	2	1	1	1	1	1	1
2	2	1	1	4	4	4	4	4	4
2	2	2	2	1	1	4	4	1	1
2	2	1	1	4	4	1	1	4	4
2	2	1	1	4	4	4	4	4	4
2	2	2	2	4	4	4	4	1	1
2	2	2	2	4	4	4	4	1	1
4	4	2	2	1	1	1	1	4	4
2	2	2	2	4	4	4	4	1	1
2	2	1	1	4	4	4	4	4	4
2	2	2	2	4	4	4	4	4	4
4	4	2	2	1	1	1	1	1	1
2	2	2	2	4	4	1	1	4	4
2	2	1	1	4	4	4	4	4	4
4	4	2	2	1	1	1	1	1	1
4	4	2	2	1	1	1	1	4	4
4	4	2	2	1	1	1	1	1	1
4	4	2	2	1	1	1	1	1	1
4	4	2	2	1	1	1	1	4	4
2	2	1	1	4	4	4	4	1	1

For Review Only

4	4	2	2	1	1	1	1	1	1
4	4	2	2	1	1	1	1	1	1
4	4	2	2	1	1	1	1	1	1
4	4	2	2	1	1	1	1	1	1
2	2	1	1	4	4	4	4	1	1
4	4	2	2	1	1	4	4	4	4
4	4	2	2	1	1	1	1	4	4
4	4	2	2	1	1	1	1	4	4
4	4	2	2	1	1	1	1	4	4
2	2	2	2	1	1	4	4	1	1
4	4	2	2	1	1	4	4	1	1
4	4	2	2	1	1	4	4	1	1
4	4	2	2	1	1	1	1	1	1
4	4	2	2	1	1	1	1	4	4
2	2	2	2	1	1	1	1	1	1
2	2	2	2	4	4	1	1	4	4
4	4	2	2	1	1	1	1	4	4
4	4	2	2	1	1	1	1	4	4
4	4	2	2	1	1	1	1	1	1
4	4	2	2	1	1	1	1	4	4
4	4	2	2	1	1	4	4	1	1
2	2	1	1	1	1	4	4	1	1
4	4	2	2	1	1	1	1	4	4
2	2	1	1	4	4	4	4	4	4
2	2	2	2	4	4	1	1	4	4
2	2	1	1	4	4	4	4	1	1
4	4	2	2	1	1	1	1	1	1
4	4	2	2	1	1	1	1	4	4
4	4	2	2	1	1	1	1	4	4
4	4	2	2	1	1	1	1	1	1
4	4	2	2	1	1	1	1	1	1
4	4	2	2	1	1	1	1	4	4
4	4	2	2	1	1	1	1	1	1
4	4	2	2	1	1	1	1	1	1
2	2	1	1	4	4	4	4	1	1
2	2	2	2	4	4	1	1	4	4
4	4	2	2	1	1	1	1	1	1
2	2	2	2	4	4	4	4	1	1
2	2	1	1	4	4	4	4	4	4
4	4	2	2	1	1	1	1	4	4
4	4	2	2	1	1	1	1	1	1
2	2	2	2	1	1	1	1	4	4
4	4	2	2	1	1	1	1	4	4
4	4	2	2	1	1	4	4	1	1
2	2	1	1	4	4	4	4	1	1
2	2	1	1	4	4	4	4	1	1
2	2	2	2	4	4	4	4	4	4
4	4	2	2	1	1	1	1	4	4
4	4	2	2	1	1	1	1	4	4
4	4	2	2	1	1	1	1	1	1
2	2	2	2	1	1	1	1	1	1
2	2	1	1	4	4	4	4	1	1
2	2	1	1	4	4	4	4	1	1
4	4	2	2	1	1	4	4	4	4
4	4	2	2	1	1	1	1	1	1

For Review Only

4	4	2	2	1	1	1	1	4	4
4	4	2	2	1	1	1	1	4	4
2	2	1	1	4	4	1	1	4	4
4	4	2	2	1	1	1	1	1	1
2	2	2	2	1	1	4	4	1	1
4	4	2	2	1	1	1	1	1	1
4	4	2	2	1	1	4	4	1	1
2	2	2	2	1	1	1	1	4	4
4	4	2	2	1	1	1	1	4	4
2	2	2	2	1	1	1	1	4	4
4	4	2	2	1	1	1	1	1	1
2	2	2	2	1	1	4	4	1	1
4	4	2	2	1	1	1	1	4	4
2	2	2	2	1	1	4	4	1	1
2	2	2	2	1	1	1	1	1	1
2	2	2	2	1	1	4	4	1	1
4	4	2	2	1	1	1	1	4	4
2	2	2	2	1	1	4	4	1	1
4	4	2	2	1	1	1	1	1	1
4	4	2	2	1	1	1	1	1	1
2	2	2	2	1	1	1	1	1	1
4	4	2	2	1	1	4	4	1	1
2	2	2	2	4	4	4	4	1	1
4	4	2	2	1	1	1	1	1	1
4	4	2	2	1	1	1	1	4	4
2	2	1	1	4	4	4	4	4	4
2	2	1	1	4	4	4	4	4	4
2	2	1	1	4	4	4	4	4	4
4	4	2	2	1	1	1	1	1	1
2	2	1	1	4	4	4	4	1	1
4	4	2	2	1	1	1	1	4	4
2	2	2	2	1	1	4	4	1	1
4	4	2	2	1	1	1	1	1	1
2	2	2	2	1	1	1	1	4	4
2	2	1	1	4	4	4	4	4	4
4	4	2	2	1	1	1	1	1	1
4	4	2	2	1	1	1	1	4	4
2	2	1	1	4	4	4	4	1	1
2	2	1	1	4	4	4	4	1	1
2	2	2	2	4	4	4	4	1	1
2	2	2	2	1	1	4	4	4	4
4	4	2	2	1	1	1	1	4	4
4	4	2	2	1	1	1	1	4	4
2	2	2	2	1	1	4	4	1	1
4	4	2	2	1	1	1	1	1	1
4	4	2	2	1	1	1	1	1	1
4	4	2	2	1	1	1	1	4	4
4	4	2	2	1	1	1	1	4	4
2	2	2	2	1	1	4	4	4	4
4	4	2	2	1	1	1	1	4	4
4	4	2	2	1	1	1	1	4	4
2	2	1	1	4	4	4	4	4	4
4	4	2	2	1	1	1	1	4	4
4	4	2	2	1	1	4	4	1	1
2	2	2	2	1	1	4	4	4	4

For Review Only

4	4	2	2	1	1	1	1	4	4
4	4	2	2	1	1	1	1	4	4
2	2	2	2	4	4	1	1	4	4
4	4	2	2	1	1	1	1	4	4
2	2	2	2	1	1	4	4	1	1
4	4	2	2	1	1	1	1	4	4
4	4	2	2	1	1	1	1	1	1
2	2	2	2	1	1	4	4	1	1
2	2	2	2	4	4	4	4	4	4
2	2	1	1	4	4	4	4	1	1
4	4	2	2	1	1	1	1	4	4
4	4	2	2	1	1	1	1	4	4
4	4	2	2	1	1	1	1	4	4
4	4	2	2	1	1	1	1	1	1
4	4	2	2	1	1	1	1	4	4
2	2	1	1	4	4	4	4	4	4
2	2	2	2	1	1	1	1	1	1
2	2	1	1	4	4	4	4	1	1
4	4	2	2	1	1	1	1	4	4
4	4	2	2	1	1	4	4	1	1
2	2	2	2	1	1	4	4	1	1
4	4	2	2	1	1	1	1	1	1
2	2	1	1	4	4	4	4	1	1
4	4	2	2	1	1	4	4	1	1
2	2	2	2	1	1	1	1	1	1
2	2	1	1	4	4	4	4	4	4
4	4	2	2	1	1	1	1	1	1
2	2	2	2	1	1	4	4	1	1
2	2	1	1	4	4	4	4	1	1
4	4	2	2	1	1	1	1	4	4
4	4	2	2	1	1	1	1	4	4
4	4	2	2	1	1	1	1	4	4
2	2	1	1	4	4	4	4	4	4
4	4	2	2	1	1	1	1	4	4
2	2	2	2	4	4	4	4	4	4
4	4	2	2	1	1	4	4	1	1
4	4	2	2	1	1	1	1	4	4
2	2	1	1	4	4	4	4	4	4
4	4	2	2	1	1	1	1	4	4
4	4	2	2	1	1	1	1	4	4
2	2	1	1	4	4	4	4	4	4
4	4	2	2	1	1	1	1	4	4
2	2	2	2	4	4	4	4	1	1
2	2	1	1	4	4	4	4	4	4
2	2	2	2	1	1	1	1	1	1
4	4	2	2	1	1	4	4	4	4
2	2	1	1	4	4	4	4	4	4
4	4	2	2	1	1	1	1	1	1
4	4	2	2	1	1	1	1	4	4
4	4	2	2	1	1	1	1	1	1
4	4	2	2	1	1	1	1	1	1
4	4	2	2	1	1	1	1	4	4
4	4	2	2	1	1	1	1	1	1
4	4	2	2	1	1	4	4	1	1
2	2	1	1	4	4	4	4	4	4

For Review Only

4	4	2	2	1	1	1	1	4	4
2	2	1	1	1	1	4	4	4	4
4	4	2	2	1	1	1	1	1	1
2	2	2	2	1	1	4	4	1	1
4	4	2	2	1	1	1	1	4	4
4	4	2	2	1	1	1	1	4	4
4	4	2	2	1	1	1	1	4	4
2	2	2	2	4	4	4	4	4	4
4	4	2	2	1	1	1	1	4	4
2	2	2	2	4	4	4	4	1	1
2	2	2	2	1	1	4	4	1	1
4	4	2	2	1	1	1	1	4	4
2	2	1	1	4	4	4	4	4	4
4	4	2	2	1	1	1	1	4	4
4	4	2	2	1	1	1	1	4	4
4	4	2	2	1	1	1	1	4	4
2	2	1	1	4	4	4	4	1	1
4	4	2	2	1	1	1	1	4	4
4	4	2	2	1	1	1	1	1	1
2	2	2	2	4	4	4	4	1	1
4	4	2	2	1	1	1	1	4	4
4	4	2	2	1	1	1	1	4	4
2	2	1	1	4	4	4	4	1	1
4	4	2	2	1	1	1	1	4	4
4	4	2	2	1	1	1	1	4	4
4	4	2	2	1	1	4	4	1	1
4	4	2	2	1	1	1	1	1	1
4	4	2	2	1	1	1	1	1	1
4	4	2	2	1	1	1	1	1	1
4	4	2	2	1	1	1	1	1	1
4	4	2	2	1	1	1	1	4	4
4	4	2	2	1	1	1	1	1	1
4	4	2	2	1	1	1	1	4	4
4	4	2	2	1	1	1	1	4	4
4	4	2	2	1	1	1	1	4	4
2	2	2	2	4	4	4	4	1	1
4	4	2	2	1	1	1	1	1	1
4	4	2	2	1	1	4	4	1	1
2	2	1	1	4	4	1	1	4	4
2	2	2	2	4	4	4	4	4	4
4	4	2	2	1	1	1	1	4	4
2	2	1	1	4	4	4	4	1	1
4	4	2	2	1	1	1	1	4	4
4	4	2	2	1	1	1	1	1	1
4	4	2	2	1	1	4	4	4	4
2	2	1	1	4	4	4	4	4	4
4	4	2	2	1	1	1	1	4	4
4	4	2	2	1	1	1	1	4	4
4	4	2	2	1	1	1	1	1	1
4	4	2	2	1	1	1	1	1	1
2	2	1	1	4	4	4	4	1	1
2	2	2	2	4	4	4	4	4	4
2	2	1	1	4	4	4	4	4	4
4	4	2	2	1	1	1	1	4	4
4	4	2	2	1	1	1	1	4	4

For Review Only

2	2	1	1	4	4	4	4	1	1
2	2	1	1	4	4	4	4	1	1

For Review Only

SNP240	SNP240	SNP2400	SNP2400	SNP2401	SNP2401	SNP2402	SNP2402	SNP2403	SNP2403
3	3	3	3	4	4	4	4	2	2
3	3	3	3	1	1	4	4	1	1
3	3	2	2	1	1	4	4	2	2
3	3	3	3	1	1	4	4	1	1
3	3	2	2	1	1	4	4	1	1
3	3	2	2	1	1	4	4	1	1
3	3	3	3	4	4	4	4	2	2
3	3	3	3	4	4	1	1	2	2
3	3	3	3	4	4	4	4	2	2
3	3	2	2	4	4	4	4	2	2
3	3	2	2	1	1	4	4	2	2
3	3	2	2	1	1	4	4	2	2
3	3	3	3	1	1	4	4	1	1
3	3	2	2	4	4	1	1	2	2
3	3	2	2	1	1	4	4	1	1
3	3	2	2	4	4	4	4	2	2
3	3	2	2	1	1	4	4	1	1
3	3	2	2	1	1	4	4	1	1
3	3	3	3	1	1	1	1	1	1
3	3	2	2	1	1	1	1	1	1
3	3	2	2	4	4	4	4	2	2
3	3	2	2	1	1	4	4	1	1
3	3	2	2	1	1	4	4	1	1
3	3	2	2	4	4	1	1	2	2
3	3	2	2	1	1	4	4	1	1
3	3	2	2	1	1	4	4	1	1
3	3	2	2	1	1	4	4	1	1
3	3	2	2	1	1	4	4	2	2
3	3	2	2	4	4	4	4	2	2
3	3	2	2	1	1	4	4	1	1
3	3	3	3	1	1	4	4	1	1
3	3	2	2	1	1	4	4	1	1
3	3	3	3	1	1	4	4	1	1
3	3	2	2	4	4	1	1	2	2
3	3	3	3	4	4	4	4	2	2
2	2	2	2	4	4	4	4	2	2
3	3	3	3	4	4	4	4	2	2
3	3	3	3	1	1	4	4	2	2
3	3	2	2	4	4	4	4	1	1
3	3	3	3	4	4	4	4	2	2
3	3	2	2	1	1	4	4	1	1
3	3	2	2	4	4	4	4	2	2
3	3	2	2	4	4	1	1	2	2
3	3	2	2	1	1	4	4	1	1
3	3	2	2	4	4	1	1	2	2
3	3	2	2	1	1	4	4	1	1
3	3	2	2	1	1	4	4	1	1
3	3	2	2	1	1	4	4	2	2
3	3	2	2	1	1	4	4	1	1
3	3	2	2	1	1	4	4	2	2
3	3	2	2	1	1	4	4	1	1
3	3	2	2	1	1	4	4	1	1

For Review Only

3	3	2	2	1	1	4	4	1	1
3	3	3	3	1	1	4	4	1	1
3	3	2	2	1	1	1	1	1	1
3	3	2	2	1	1	4	4	1	1
3	3	3	3	1	1	1	1	1	1
2	2	2	2	1	1	4	4	1	1
2	2	2	2	1	1	4	4	1	1
3	3	2	2	4	4	4	4	2	2
3	3	3	3	4	4	4	4	2	2
3	3	3	3	1	1	4	4	2	2
2	2	2	2	1	1	4	4	1	1
2	2	2	2	1	1	4	4	1	1
3	3	2	2	1	1	4	4	1	1
3	3	2	2	4	4	4	4	2	2
3	3	3	3	1	1	4	4	2	2
3	3	3	3	4	4	4	4	2	2
3	3	2	2	1	1	4	4	1	1
3	3	2	2	1	1	4	4	2	2
3	3	2	2	1	1	4	4	1	1
3	3	2	2	4	4	4	4	2	2
2	2	2	2	1	1	4	4	1	1
3	3	3	3	4	4	4	4	2	2
3	3	3	3	4	4	4	4	2	2
3	3	2	2	4	4	4	4	2	2
3	3	2	2	4	4	1	1	2	2
3	3	2	2	1	1	4	4	1	1
3	3	3	3	1	1	4	4	1	1
3	3	2	2	4	4	1	1	1	1
3	3	2	2	1	1	4	4	1	1
3	3	2	2	1	1	4	4	1	1
3	3	2	2	1	1	4	4	1	1
2	2	2	2	4	4	4	4	2	2
3	3	2	2	1	1	1	1	1	1
3	3	2	2	1	1	4	4	1	1
3	3	2	2	1	1	4	4	1	1
3	3	2	2	4	4	1	1	2	2
3	3	2	2	1	1	4	4	1	1
3	3	2	2	1	1	4	4	1	1
3	3	2	2	4	4	4	4	2	2
3	3	3	3	4	4	4	4	2	2
3	3	2	2	1	1	4	4	1	1
2	2	2	2	4	4	4	4	2	2
3	3	2	2	4	4	4	4	2	2
2	2	2	2	1	1	4	4	1	1
3	3	2	2	1	1	4	4	1	1
3	3	2	2	1	1	4	4	1	1
3	3	2	2	4	4	1	1	2	2
2	2	2	2	1	1	4	4	1	1
3	3	2	2	4	4	4	4	2	2
3	3	3	3	1	1	4	4	1	1
3	3	3	3	1	1	4	4	2	2
2	2	2	2	4	4	4	4	2	2
3	3	2	2	4	4	4	4	1	1
3	3	3	3	4	4	4	4	2	2
3	3	3	3	1	1	1	1	1	1

For Review Only

3	3	3	3	1	1	4	4	2	2
3	3	3	3	4	4	4	4	2	2
3	3	2	2	1	1	4	4	1	1
3	3	2	2	1	1	1	1	1	1
3	3	3	3	1	1	4	4	1	1
3	3	2	2	1	1	4	4	1	1
3	3	2	2	1	1	4	4	1	1
2	2	2	2	4	4	4	4	2	2
3	3	2	2	4	4	4	4	2	2
3	3	3	3	4	4	4	4	2	2
3	3	3	3	1	1	4	4	1	1
3	3	3	3	1	1	4	4	2	2
3	3	2	2	4	4	4	4	2	2
3	3	2	2	1	1	4	4	1	1
3	3	2	2	4	4	4	4	2	2
3	3	3	3	1	1	4	4	2	2
3	3	2	2	1	1	4	4	1	1
3	3	3	3	1	1	4	4	2	2
3	3	2	2	1	1	1	1	1	1
3	3	2	2	1	1	4	4	2	2
3	3	3	3	1	1	4	4	2	2
3	3	2	2	1	1	4	4	1	1
2	2	2	2	1	1	4	4	2	2
3	3	2	2	1	1	1	1	1	1
3	3	3	3	1	1	4	4	1	1
3	3	3	3	4	4	4	4	2	2
3	3	2	2	4	4	4	4	2	2
3	3	2	2	4	4	4	4	2	2
3	3	2	2	1	1	4	4	1	1
3	3	3	3	1	1	1	1	1	1
3	3	2	2	4	4	1	1	2	2
3	3	3	3	1	1	4	4	2	2
3	3	2	2	1	1	4	4	1	1
3	3	2	2	1	1	4	4	2	2
3	3	2	2	4	4	1	1	2	2
3	3	2	2	1	1	4	4	1	1
3	3	2	2	4	4	1	1	2	2
3	3	2	2	1	1	4	4	1	1
3	3	2	2	1	1	4	4	1	1
3	3	2	2	4	4	4	4	1	1
2	2	3	3	4	4	4	4	2	2
3	3	2	2	4	4	1	1	2	2
3	3	2	2	1	1	1	1	1	1
3	3	3	3	1	1	1	1	2	2
3	3	2	2	1	1	4	4	2	2
3	3	2	2	1	1	4	4	1	1
3	3	2	2	1	1	4	4	1	1
3	3	2	2	1	1	4	4	1	1
3	3	2	2	4	4	4	4	2	2
3	3	2	2	1	1	4	4	1	1
3	3	2	2	1	1	4	4	1	1
3	3	2	2	4	4	4	4	2	2
3	3	2	2	4	4	4	4	2	2
3	3	3	3	1	1	4	4	1	1
3	3	3	3	4	4	4	4	2	2

For Review Only

3	3	2	2	1	1	4	4	1	1
3	3	2	2	4	4	4	4	2	2
3	3	2	2	4	4	4	4	2	2
3	3	3	3	1	1	4	4	2	2
3	3	3	3	1	1	4	4	1	1
3	3	3	3	1	1	1	1	1	1
3	3	2	2	4	4	4	4	2	2
3	3	2	2	1	1	4	4	2	2
3	3	2	2	4	4	4	4	2	2
3	3	2	2	1	1	4	4	1	1
3	3	3	3	4	4	4	4	2	2
3	3	2	2	4	4	1	1	2	2
3	3	3	3	1	1	4	4	2	2
3	3	2	2	1	1	4	4	1	1
3	3	2	2	4	4	1	1	2	2
3	3	2	2	4	4	4	4	2	2
3	3	2	2	4	4	4	4	2	2
3	3	3	3	4	4	4	4	2	2
3	3	2	2	1	1	4	4	2	2
3	3	2	2	1	1	4	4	2	2
3	3	2	2	1	1	4	4	2	2
3	3	2	2	4	4	1	1	2	2
3	3	3	3	1	1	4	4	1	1
2	2	2	2	1	1	4	4	1	1
3	3	2	2	1	1	4	4	2	2
3	3	2	2	4	4	1	1	2	2
2	2	2	2	1	1	4	4	1	1
3	3	2	2	1	1	4	4	1	1
3	3	2	2	1	1	4	4	1	1
3	3	2	2	4	4	4	4	2	2
3	3	2	2	4	4	4	4	2	2
3	3	2	2	1	1	4	4	1	1
3	3	3	3	4	4	4	4	2	2
3	3	2	2	4	4	1	1	2	2
3	3	2	2	4	4	4	4	2	2
2	2	2	2	1	1	4	4	1	1
3	3	2	2	4	4	4	4	2	2
3	3	3	3	4	4	4	4	2	2
3	3	2	2	1	1	4	4	1	1
3	3	2	2	4	4	1	1	2	2
3	3	2	2	4	4	4	4	2	2
3	3	2	2	1	1	4	4	1	1
3	3	2	2	1	1	4	4	1	1
3	3	2	2	4	4	4	4	2	2
3	3	2	2	1	1	4	4	1	1
2	2	3	3	1	1	4	4	1	1
3	3	2	2	4	4	4	4	2	2
3	3	2	2	1	1	4	4	1	1
3	3	2	2	4	4	4	4	2	2
3	3	2	2	1	1	4	4	1	1
3	3	2	2	1	1	4	4	1	1
3	3	2	2	4	4	4	4	2	2
3	3	3	3	1	1	4	4	1	1
2	2	2	2	1	1	4	4	1	1
3	3	3	3	4	4	4	4	2	2

For Review Only

3	3	2	2	4	4	1	1	2	2
3	3	2	2	4	4	4	4	2	2
3	3	2	2	1	1	4	4	1	1
3	3	3	3	1	1	4	4	1	1
3	3	2	2	4	4	1	1	2	2
3	3	2	2	4	4	4	4	2	2
3	3	2	2	4	4	4	4	1	1
3	3	2	2	4	4	1	1	2	2
3	3	2	2	4	4	4	4	2	2
3	3	2	2	1	1	4	4	1	1
3	3	2	2	1	1	4	4	2	2
3	3	2	2	4	4	4	4	2	2
3	3	2	2	4	4	4	4	2	2
3	3	2	2	1	1	4	4	1	1
3	3	2	2	4	4	4	4	2	2
3	3	2	2	4	4	4	4	2	2
3	3	2	2	1	1	4	4	1	1
3	3	2	2	4	4	4	4	2	2
3	3	3	3	1	1	4	4	1	1
3	3	3	3	1	1	4	4	2	2
3	3	3	3	4	4	4	4	2	2
3	3	2	2	1	1	4	4	1	1
3	3	2	2	1	1	4	4	1	1
3	3	3	3	4	4	4	4	2	2
3	3	2	2	4	4	4	4	2	2
3	3	2	2	1	1	4	4	2	2
3	3	2	2	1	1	1	1	1	1
2	2	2	2	1	1	4	4	1	1
3	3	2	2	1	1	4	4	2	2
3	3	2	2	1	1	4	4	1	1
3	3	2	2	4	4	4	4	2	2
3	3	2	2	1	1	4	4	2	2
3	3	2	2	1	1	1	1	1	1
3	3	2	2	1	1	4	4	1	1
3	3	3	3	4	4	4	4	2	2
3	3	2	2	1	1	1	1	1	1
3	3	2	2	1	1	1	1	1	1
2	2	2	2	1	1	4	4	1	1
2	2	2	2	4	4	4	4	2	2
3	3	3	3	4	4	4	4	2	2
3	3	2	2	4	4	4	4	2	2
3	3	2	2	1	1	4	4	1	1
3	3	2	2	4	4	4	4	2	2
3	3	2	2	1	1	4	4	1	1
3	3	3	3	1	1	4	4	2	2
3	3	3	3	4	4	4	4	2	2
3	3	2	2	4	4	4	4	2	2
3	3	2	2	1	1	1	1	1	1
3	3	2	2	1	1	4	4	1	1
3	3	2	2	4	4	4	4	1	1
3	3	2	2	1	1	1	1	1	1
3	3	2	2	4	4	4	4	2	2
3	3	2	2	4	4	4	4	2	2
3	3	2	2	4	4	4	4	2	2
3	3	3	3	4	4	4	4	2	2

For Review Only

3	3	2	2	1	1	4	4	1	1
3	3	2	2	1	1	1	1	1	1

For Review Only

SNP2404	SNP2404	SNP2405	SNP2405	SNP2406	SNP2406	SNP2407	SNP2407	SNP2408	SNP2408
3	3	2	2	1	1	2	2	2	2
3	3	2	2	3	3	4	4	2	2
3	3	2	2	3	3	4	4	2	2
2	2	2	2	1	1	2	2	2	2
3	3	2	2	1	1	2	2	3	3
2	2	2	2	1	1	2	2	2	2
3	3	2	2	1	1	2	2	2	2
3	3	2	2	1	1	2	2	2	2
3	3	2	2	1	1	2	2	2	2
3	3	2	2	1	1	2	2	2	2
3	3	2	2	1	1	2	2	2	2
3	3	2	2	1	1	2	2	2	2
3	3	2	2	1	1	2	2	2	2
3	3	2	2	1	1	2	2	2	2
3	3	2	2	1	1	2	2	2	2
3	3	2	2	1	1	2	2	2	2
2	2	2	2	1	1	2	2	2	2
3	3	2	2	1	1	2	2	3	3
2	2	4	4	1	1	2	2	2	2
2	2	2	2	1	1	2	2	2	2
3	3	2	2	1	1	2	2	2	2
2	2	2	2	1	1	2	2	2	2
3	3	2	2	1	1	2	2	2	2
3	3	2	2	1	1	2	2	2	2
2	2	2	2	1	1	2	2	2	2
3	3	2	2	1	1	2	2	2	2
2	2	2	2	1	1	2	2	2	2
3	3	2	2	1	1	2	2	2	2
3	3	2	2	1	1	2	2	2	2
3	3	2	2	1	1	2	2	2	2
2	2	2	2	1	1	2	2	2	2
2	2	4	4	1	1	2	2	2	2
2	2	2	2	1	1	2	2	2	2
3	3	2	2	1	1	2	2	2	2
3	3	2	2	1	1	2	2	2	2
3	3	2	2	1	1	2	2	2	2
3	3	2	2	1	1	2	2	2	2
3	3	2	2	1	1	2	2	2	2
3	3	2	2	1	1	2	2	2	2
3	3	2	2	1	1	2	2	2	2
3	3	2	2	1	1	2	2	3	3
3	3	2	2	1	1	2	2	2	2
3	3	2	2	1	1	2	2	2	2
2	2	2	2	1	1	2	2	2	2
3	3	2	2	1	1	2	2	3	3
3	3	2	2	1	1	2	2	2	2
3	3	2	2	1	1	2	2	2	2
3	3	2	2	1	1	2	2	2	2
2	2	2	2	1	1	2	2	2	2
2	2	2	2	1	1	2	2	2	2
3	3	2	2	3	3	4	4	3	3
3	3	2	2	3	3	4	4	2	2

For Review Only

2	2	2	2	1	1	2	2	2	2
2	2	2	2	1	1	2	2	2	2
2	2	2	2	3	3	4	4	2	2
2	2	2	2	1	1	2	2	2	2
2	2	4	4	1	1	2	2	2	2
3	3	2	2	1	1	2	2	2	2
3	3	2	2	1	1	2	2	2	2
3	3	2	2	1	1	2	2	2	2
3	3	2	2	1	1	2	2	2	2
3	3	2	2	1	1	2	2	2	2
3	3	2	2	1	1	2	2	2	2
3	3	2	2	1	1	2	2	2	2
2	2	2	2	1	1	2	2	2	2
3	3	2	2	1	1	2	2	2	2
3	3	2	2	1	1	2	2	2	2
3	3	2	2	1	1	2	2	2	2
3	3	2	2	3	3	4	4	3	3
3	3	4	4	1	1	2	2	2	2
2	2	2	2	1	1	2	2	2	2
3	3	2	2	1	1	2	2	2	2
3	3	2	2	1	1	2	2	2	2
3	3	2	2	1	1	2	2	2	2
3	3	2	2	1	1	2	2	2	2
3	3	2	2	3	3	4	4	3	3
3	3	2	2	1	1	2	2	3	3
3	3	2	2	1	1	4	4	2	2
2	2	2	2	1	1	2	2	2	2
3	3	2	2	1	1	2	2	2	2
3	3	2	2	1	1	2	2	2	2
2	2	2	2	1	1	2	2	2	2
2	2	2	2	1	1	2	2	3	3
3	3	2	2	1	1	2	2	2	2
3	3	2	2	1	1	2	2	2	2
2	2	2	2	1	1	2	2	2	2
3	3	2	2	1	1	2	2	2	2
3	3	2	2	1	1	2	2	3	3
3	3	2	2	1	1	2	2	3	3
3	3	2	2	1	1	2	2	2	2
3	3	2	2	3	3	4	4	3	3
3	3	2	2	1	1	2	2	2	2
2	2	4	4	1	1	2	2	2	2
3	3	2	2	3	3	4	4	2	2
3	3	2	2	3	3	4	4	3	3
3	3	2	2	1	1	2	2	2	2
2	2	2	2	1	1	2	2	2	2
3	3	2	2	1	1	2	2	2	2
3	3	2	2	1	1	2	2	2	2
3	3	2	2	1	1	2	2	2	2
3	3	2	2	1	1	2	2	2	2
3	3	2	2	1	1	2	2	2	2
3	3	2	2	3	3	4	4	2	2
3	3	2	2	1	1	2	2	2	2
3	3	2	2	1	1	2	2	2	2
3	3	2	2	1	1	2	2	2	2
3	3	4	4	1	1	2	2	2	2

For Review Only

3	3	2	2	1	1	2	2	2	2
3	3	2	2	1	1	2	2	2	2
3	3	2	2	3	3	4	4	2	2
3	3	2	2	1	1	2	2	2	2
2	2	2	2	3	3	4	4	2	2
2	2	2	2	1	1	2	2	2	2
2	2	2	2	1	1	2	2	2	2
3	3	2	2	1	1	2	2	2	2
3	3	2	2	1	1	2	2	2	2
3	3	2	2	1	1	2	2	2	2
3	3	2	2	1	1	2	2	2	2
3	3	2	2	1	1	2	2	2	2
3	3	2	2	3	3	4	4	3	3
3	3	2	2	1	1	4	4	2	2
3	3	2	2	1	1	4	4	2	2
3	3	2	2	1	1	2	2	2	2
3	3	2	2	1	1	2	2	2	2
3	3	2	2	1	1	2	2	2	2
3	3	2	2	1	1	2	2	2	2
3	3	2	2	1	1	2	2	2	2
3	3	2	2	1	1	2	2	2	2
2	2	2	2	1	1	2	2	2	2
3	3	2	2	3	3	4	4	2	2
3	3	2	2	1	1	2	2	2	2
3	3	2	2	1	1	2	2	2	2
3	3	2	2	1	1	2	2	2	2
3	3	2	2	1	1	2	2	2	2
3	3	2	2	1	1	2	2	2	2
2	2	2	2	1	1	2	2	2	2
3	3	2	2	1	1	2	2	2	2
3	3	2	2	1	1	2	2	2	2
3	3	2	2	1	1	2	2	2	2
2	2	4	4	1	1	2	2	2	2
3	3	2	2	1	1	2	2	2	2
3	3	2	2	1	1	2	2	2	2
3	3	4	4	1	1	2	2	2	2
3	3	2	2	1	1	2	2	2	2
3	3	2	2	3	3	4	4	2	2
3	3	2	2	3	3	2	2	2	2
3	3	2	2	1	1	2	2	2	2
3	3	2	2	1	1	2	2	2	2
3	3	2	2	1	1	2	2	2	2
3	3	4	4	1	1	2	2	2	2
3	3	2	2	1	1	2	2	2	2
3	3	2	2	1	1	2	2	2	2
3	3	2	2	1	1	2	2	2	2
3	3	2	2	1	1	2	2	2	2
3	3	2	2	1	1	2	2	2	2
3	3	2	2	1	1	2	2	2	2
3	3	2	2	1	1	2	2	2	2
3	3	2	2	1	1	2	2	2	2
3	3	2	2	1	1	2	2	2	2
3	3	2	2	1	1	2	2	2	2
3	3	2	2	1	1	2	2	2	2
3	3	2	2	1	1	2	2	2	2

For Review Only

2	2	2	2	1	1	2	2	2	2
3	3	2	2	3	3	4	4	3	3
3	3	2	2	1	1	2	2	3	3
3	3	2	2	1	1	2	2	2	2
3	3	2	2	1	1	2	2	2	2
3	3	2	2	1	1	2	2	2	2
3	3	2	2	1	1	2	2	2	2
3	3	2	2	1	1	2	2	2	2
3	3	2	2	1	1	2	2	2	2
2	2	2	2	1	1	2	2	2	2
3	3	2	2	3	3	2	2	2	2
3	3	2	2	1	1	2	2	2	2
3	3	2	2	1	1	2	2	2	2
3	3	2	2	1	1	2	2	2	2
3	3	2	2	1	1	2	2	2	2
3	3	2	2	1	1	2	2	2	2
3	3	2	2	1	1	2	2	2	2
3	3	2	2	3	3	4	4	2	2
3	3	2	2	1	1	2	2	2	2
3	3	2	2	1	1	2	2	2	2
3	3	2	2	1	1	2	2	2	2
3	3	2	2	1	1	2	2	2	2
3	3	2	2	1	1	2	2	2	2
3	3	2	2	1	1	2	2	2	2
2	2	2	2	1	1	2	2	2	2
3	3	2	2	1	1	2	2	2	2
3	3	2	2	1	1	2	2	2	2
3	3	2	2	1	1	2	2	2	2
3	3	2	2	1	1	2	2	2	2
3	3	4	4	1	1	2	2	2	2
3	3	2	2	1	1	2	2	2	2
3	3	4	4	1	1	2	2	2	2
3	3	2	2	1	1	2	2	2	2
3	3	2	2	1	1	2	2	2	2
3	3	2	2	1	1	2	2	2	2
3	3	2	2	1	1	2	2	2	2
3	3	2	2	1	1	2	2	2	2
3	3	2	2	1	1	2	2	2	2
3	3	2	2	3	3	4	4	2	2
3	3	2	2	3	3	4	4	3	3
3	3	2	2	3	3	4	4	2	2
3	3	2	2	1	1	2	2	2	2
3	3	2	2	1	1	2	2	2	2
3	3	2	2	1	1	2	2	2	2
2	2	2	2	1	1	2	2	2	2
3	3	2	2	1	1	2	2	2	2
3	3	2	2	1	1	2	2	2	2
3	3	2	2	1	1	2	2	2	2
3	3	2	2	1	1	2	2	2	2
2	2	2	2	1	1	2	2	2	2
2	2	2	2	1	1	2	2	2	2
3	3	2	2	1	1	2	2	2	2
3	3	2	2	1	1	2	2	2	2
3	3	2	2	1	1	2	2	2	2
3	3	2	2	1	1	2	2	2	2

For Review Only

3	3	4	4	1	1	2	2	2	2
3	3	2	2	1	1	2	2	2	2
2	2	2	2	1	1	2	2	2	2
3	3	2	2	1	1	2	2	2	2
3	3	2	2	1	1	2	2	2	2
3	3	2	2	1	1	2	2	2	2
3	3	2	2	1	1	2	2	2	2
3	3	2	2	1	1	2	2	2	2
3	3	2	2	1	1	2	2	3	3
3	3	2	2	1	1	2	2	3	3
3	3	2	2	1	1	2	2	2	2
3	3	2	2	1	1	2	2	2	2
3	3	2	2	3	3	4	4	3	3
3	3	2	2	1	1	2	2	2	2
3	3	2	2	3	3	4	4	2	2
3	3	2	2	1	1	2	2	2	2
3	3	2	2	1	1	2	2	2	2
3	3	2	2	1	1	2	2	2	2
3	3	2	2	1	1	2	2	2	2
3	3	2	2	1	1	2	2	2	2
3	3	2	2	1	1	2	2	2	2
3	3	2	2	1	1	2	2	2	2
2	2	2	2	1	1	2	2	2	2
3	3	2	2	1	1	2	2	2	2
3	3	2	2	1	1	2	2	2	2
3	3	2	2	1	1	4	4	2	2
3	3	2	2	1	1	2	2	2	2
2	2	2	2	1	1	2	2	2	2
3	3	2	2	1	1	2	2	2	2
2	2	2	2	1	1	2	2	2	2
3	3	2	2	1	1	2	2	2	2
3	3	2	2	3	3	4	4	2	2
3	3	2	2	1	1	2	2	2	2
3	3	2	2	1	1	2	2	2	2
3	3	2	2	1	1	2	2	2	2
3	3	2	2	1	1	2	2	2	2
2	2	2	2	1	1	2	2	2	2
3	3	2	2	1	1	2	2	2	2
3	3	2	2	1	1	2	2	2	2
3	3	2	2	1	1	2	2	2	2
3	3	2	2	1	1	2	2	2	2
3	3	4	4	1	1	2	2	2	2
3	3	2	2	1	1	2	2	2	2
2	2	2	2	1	1	2	2	2	2
3	3	2	2	3	3	4	4	2	2
3	3	2	2	3	3	4	4	2	2
3	3	2	2	1	1	2	2	2	2
3	3	4	4	3	3	4	4	2	2
3	3	2	2	3	3	4	4	3	3
3	3	2	2	1	1	2	2	2	2
3	3	4	4	1	1	2	2	2	2
3	3	2	2	1	1	2	2	2	2
3	3	2	2	3	3	4	4	2	2
3	3	2	2	3	3	4	4	3	3
3	3	2	2	1	1	2	2	2	2

For Review Only

3	3	2	2	1	1	2	2	2	2
3	3	2	2	3	3	4	4	2	2

For Review Only

SNP2409	SNP2409	SNP241	SNP241	SNP2410	SNP2410	SNP2411	SNP2411	SNP2412	SNP2412
2	2	1	1	4	4	2	2	1	1
2	2	2	2	4	4	2	2	1	1
2	2	2	2	4	4	2	2	1	1
2	2	2	2	4	4	2	2	4	4
2	2	1	1	4	4	2	2	1	1
2	2	2	2	4	4	2	2	4	4
2	2	1	1	4	4	2	2	1	1
2	2	2	2	4	4	2	2	1	1
2	2	1	1	4	4	2	2	1	1
2	2	2	2	4	4	3	3	1	1
2	2	2	2	1	1	2	2	1	1
2	2	2	2	4	4	2	2	1	1
2	2	1	1	4	4	2	2	1	1
2	2	1	1	4	4	3	3	4	4
2	2	1	1	4	4	2	2	1	1
2	2	1	1	1	1	2	2	1	1
2	2	2	2	4	4	2	2	4	4
2	2	1	1	4	4	2	2	1	1
2	2	1	1	1	1	3	3	4	4
2	2	2	2	1	1	3	3	1	1
2	2	1	1	1	1	3	3	1	1
2	2	1	1	4	4	2	2	1	1
2	2	1	1	1	1	3	3	1	1
2	2	1	1	4	4	3	3	4	4
2	2	1	1	4	4	2	2	1	1
2	2	1	1	4	4	2	2	4	4
2	2	1	1	4	4	2	2	4	4
2	2	2	2	4	4	2	2	1	1
2	2	1	1	4	4	2	2	1	1
2	2	2	2	4	4	2	2	1	1
2	2	1	1	1	1	2	2	1	1
2	2	2	2	4	4	3	3	1	1
2	2	1	1	4	4	2	2	1	1
2	2	1	1	4	4	2	2	4	4
2	2	1	1	4	4	3	3	1	1
2	2	2	2	4	4	2	2	4	4
2	2	2	2	1	1	2	2	1	1
2	2	1	1	4	4	2	2	1	1
2	2	1	1	1	1	3	3	1	1
2	2	2	2	4	4	2	2	1	1
2	2	2	2	4	4	3	3	1	1
2	2	1	1	4	4	2	2	1	1
2	2	2	2	4	4	3	3	1	1
2	2	2	2	4	4	3	3	1	1
2	2	2	2	4	4	3	3	1	1
2	2	2	2	4	4	2	2	1	1
2	2	1	1	4	4	2	2	1	1
2	2	1	1	4	4	2	2	1	1
2	2	1	1	4	4	2	2	1	1
2	2	1	1	4	4	2	2	1	1
2	2	1	1	4	4	2	2	4	4
2	2	2	2	4	4	2	2	1	1

For Review Only

2	2	1	1	4	4	2	2	1	1
2	2	1	1	4	4	2	2	1	1
2	2	1	1	4	4	2	2	1	1
2	2	2	2	4	4	2	2	1	1
2	2	1	1	1	1	3	3	4	4
2	2	2	2	1	1	2	2	4	4
2	2	2	2	4	4	2	2	4	4
2	2	2	2	4	4	3	3	1	1
2	2	1	1	4	4	3	3	4	4
2	2	2	2	4	4	2	2	1	1
2	2	2	2	1	1	2	2	1	1
2	2	2	2	1	1	2	2	1	1
2	2	1	1	4	4	2	2	1	1
2	2	1	1	4	4	2	2	1	1
2	2	2	2	4	4	2	2	1	1
2	2	1	1	4	4	2	2	1	1
2	2	1	1	1	1	3	3	1	1
2	2	1	1	4	4	2	2	1	1
2	2	1	1	4	4	2	2	4	4
2	2	1	1	4	4	2	2	1	1
2	2	2	2	4	4	2	2	1	1
2	2	2	2	4	4	3	3	1	1
2	2	1	1	4	4	3	3	1	1
2	2	1	1	4	4	2	2	1	1
2	2	2	2	4	4	3	3	1	1
2	2	1	1	4	4	2	2	1	1
2	2	2	2	4	4	2	2	1	1
1	1	2	2	4	4	2	2	1	1
2	2	1	1	4	4	2	2	4	4
2	2	2	2	4	4	2	2	1	1
2	2	2	2	4	4	3	3	1	1
2	2	1	1	4	4	2	2	1	1
1	1	2	2	4	4	2	2	1	1
2	2	1	1	4	4	2	2	1	1
1	1	1	1	1	1	2	2	1	1
2	2	2	2	4	4	3	3	1	1
2	2	2	2	4	4	3	3	1	1
2	2	1	1	4	4	2	2	1	1
2	2	1	1	1	1	3	3	4	4
2	2	2	2	4	4	3	3	1	1
2	2	1	1	4	4	2	2	1	1
2	2	2	2	4	4	2	2	1	1
2	2	2	2	4	4	2	2	1	1
2	2	2	2	4	4	2	2	1	1
2	2	1	1	4	4	2	2	1	1
2	2	1	1	1	1	2	2	1	1
2	2	2	2	4	4	3	3	4	4
2	2	2	2	4	4	2	2	1	1
2	2	1	1	4	4	2	2	1	1
2	2	1	1	4	4	3	3	4	4
2	2	1	1	4	4	3	3	1	1
2	2	2	2	1	1	3	3	1	1
2	2	2	2	1	1	3	3	1	1
2	2	1	1	4	4	2	2	4	4
2	2	1	1	4	4	3	3	1	1

For Review Only

2	2	1	1	4	4	2	2	4	4
2	2	2	2	4	4	2	2	1	1
2	2	1	1	4	4	2	2	4	4
1	1	1	1	4	4	3	3	4	4
2	2	1	1	4	4	2	2	1	1
2	2	1	1	4	4	2	2	1	1
2	2	1	1	1	1	2	2	1	1
2	2	2	2	4	4	2	2	1	1
2	2	2	2	4	4	3	3	1	1
2	2	2	2	4	4	2	2	1	1
2	2	1	1	4	4	2	2	1	1
2	2	2	2	4	4	2	2	1	1
2	2	1	1	4	4	2	2	1	1
2	2	2	2	4	4	2	2	1	1
2	2	2	2	4	4	2	2	1	1
2	2	2	2	4	4	2	2	4	4
2	2	1	1	4	4	3	3	1	1
2	2	2	2	4	4	2	2	1	1
1	1	2	2	4	4	3	3	4	4
2	2	2	2	4	4	3	3	1	1
2	2	2	2	4	4	2	2	1	1
2	2	1	1	1	1	2	2	1	1
2	2	2	2	1	1	2	2	1	1
1	1	2	2	4	4	2	2	1	1
2	2	2	2	4	4	2	2	1	1
2	2	1	1	1	1	3	3	1	1
2	2	1	1	4	4	2	2	1	1
2	2	2	2	1	1	3	3	1	1
2	2	1	1	4	4	2	2	1	1
2	2	1	1	1	1	3	3	1	1
2	2	2	2	4	4	2	2	1	1
2	2	2	2	4	4	2	2	1	1
2	2	1	1	4	4	2	2	1	1
2	2	1	1	4	4	2	2	1	1
2	2	1	1	4	4	2	2	4	4
1	1	1	1	4	4	2	2	1	1
2	2	1	1	4	4	3	3	4	4
2	2	1	1	4	4	3	3	1	1
2	2	2	2	1	1	2	2	4	4
1	1	2	2	4	4	2	2	1	1
2	2	2	2	4	4	3	3	1	1
2	2	2	2	4	4	3	3	4	4
2	2	1	1	4	4	3	3	1	1
2	2	2	2	4	4	2	2	1	1
2	2	1	1	4	4	2	2	4	4
2	2	1	1	4	4	2	2	1	1
2	2	1	1	4	4	2	2	4	4
2	2	2	2	4	4	2	2	1	1
2	2	2	2	4	4	2	2	1	1
2	2	1	1	4	4	2	2	4	4
2	2	1	1	4	4	2	2	4	4
2	2	1	1	1	1	3	3	1	1
2	2	1	1	4	4	2	2	1	1
2	2	1	1	1	1	2	2	4	4
2	2	2	2	4	4	2	2	1	1

For Review Only

2	2	1	1	4	4	2	2	4	4
2	2	2	2	4	4	2	2	1	1
2	2	2	2	4	4	2	2	1	1
2	2	1	1	4	4	3	3	1	1
2	2	2	2	4	4	2	2	1	1
2	2	2	2	4	4	3	3	1	1
1	1	2	2	4	4	2	2	1	1
2	2	2	2	4	4	2	2	1	1
2	2	2	2	1	1	2	2	1	1
2	2	1	1	1	1	2	2	1	1
2	2	1	1	4	4	2	2	4	4
2	2	1	1	4	4	3	3	1	1
2	2	1	1	4	4	2	2	4	4
2	2	1	1	4	4	2	2	1	1
2	2	2	2	4	4	2	2	4	4
2	2	2	2	1	1	2	2	1	1
2	2	1	1	4	4	2	2	1	1
2	2	2	2	4	4	2	2	1	1
2	2	1	1	4	4	2	2	4	4
2	2	1	1	1	1	2	2	1	1
2	2	2	2	4	4	2	2	1	1
2	2	2	2	4	4	2	2	1	1
1	1	2	2	1	1	3	3	1	1
2	2	2	2	1	1	2	2	1	1
2	2	1	1	4	4	3	3	1	1
2	2	2	2	4	4	2	2	4	4
2	2	2	2	4	4	3	3	4	4
2	2	1	1	4	4	3	3	1	1
2	2	1	1	1	1	2	2	1	1
2	2	1	1	4	4	2	2	1	1
2	2	1	1	4	4	3	3	1	1
2	2	2	2	4	4	2	2	1	1
2	2	1	1	1	1	2	2	1	1
2	2	2	2	4	4	2	2	4	4
2	2	1	1	4	4	2	2	1	1
2	2	2	2	1	1	2	2	1	1
2	2	2	2	4	4	2	2	4	4
2	2	2	2	1	1	2	2	1	1
2	2	2	2	4	4	2	2	1	1
2	2	1	1	1	1	3	3	4	4
2	2	2	2	4	4	2	2	1	1
2	2	1	1	4	4	2	2	4	4
2	2	2	2	4	4	3	3	1	1
2	2	2	2	4	4	2	2	1	1
2	2	2	2	4	4	3	3	1	1
2	2	2	2	1	1	2	2	1	1
2	2	2	2	1	1	2	2	1	1
1	1	2	2	4	4	2	2	1	1
2	2	2	2	4	4	3	3	4	4
2	2	2	2	4	4	3	3	4	4
2	2	1	1	4	4	2	2	1	1
2	2	1	1	4	4	3	3	4	4
1	1	2	2	4	4	3	3	1	1
2	2	2	2	1	1	2	2	1	1
2	2	2	2	1	1	3	3	1	1

For Review Only

2	2	1	1	4	4	2	2	1	1
2	2	2	2	1	1	3	3	1	1
2	2	2	2	4	4	3	3	1	1
2	2	2	2	4	4	3	3	1	1
2	2	2	2	4	4	3	3	1	1
2	2	1	1	4	4	2	2	1	1
2	2	1	1	4	4	3	3	1	1
2	2	2	2	4	4	2	2	1	1
2	2	2	2	4	4	2	2	1	1
1	1	2	2	4	4	3	3	1	1
2	2	1	1	4	4	2	2	1	1
2	2	1	1	4	4	3	3	1	1
2	2	1	1	1	1	3	3	4	4
2	2	2	2	4	4	3	3	1	1
2	2	1	1	1	1	2	2	1	1
2	2	2	2	4	4	2	2	1	1
2	2	1	1	1	1	3	3	1	1
2	2	2	2	4	4	2	2	1	1
1	1	2	2	4	4	3	3	1	1
2	2	1	1	4	4	2	2	1	1
2	2	2	2	4	4	3	3	1	1
2	2	1	1	4	4	2	2	4	4
2	2	1	1	4	4	2	2	1	1
2	2	1	1	4	4	3	3	1	1
2	2	2	2	4	4	3	3	1	1
2	2	1	1	4	4	2	2	4	4
1	1	2	2	4	4	2	2	1	1
2	2	2	2	4	4	2	2	4	4
2	2	2	2	4	4	2	2	1	1
2	2	1	1	4	4	2	2	1	1
2	2	2	2	4	4	3	3	1	1
2	2	1	1	4	4	2	2	1	1
2	2	1	1	4	4	3	3	4	4
2	2	1	1	4	4	2	2	1	1
2	2	2	2	4	4	3	3	1	1
2	2	2	2	4	4	2	2	1	1
2	2	1	1	4	4	3	3	1	1
2	2	2	2	1	1	2	2	1	1
2	2	2	2	4	4	2	2	4	4
2	2	1	1	4	4	3	3	1	1
2	2	1	1	4	4	2	2	1	1
2	2	2	2	1	1	2	2	1	1
2	2	1	1	4	4	3	3	1	1
2	2	1	1	4	4	2	2	1	1
2	2	2	2	4	4	2	2	1	1
2	2	1	1	4	4	3	3	4	4
2	2	2	2	4	4	3	3	1	1
2	2	1	1	4	4	2	2	1	1
2	2	2	2	4	4	2	2	4	4
1	1	2	2	4	4	3	3	1	1
2	2	1	1	4	4	2	2	1	1
2	2	2	2	4	4	3	3	1	1
2	2	2	2	4	4	2	2	1	1
2	2	1	1	4	4	2	2	4	4
2	2	2	2	4	4	2	2	1	1

For Review Only

2	2	2	2	4	4	2	2	1	1
2	2	1	1	4	4	2	2	4	4

For Review Only

SNP2413	SNP2413	SNP2414	SNP2414	SNP2415	SNP2415	SNP2416	SNP2416	SNP2417	SNP2417
1	1	1	1	1	1	1	1	4	4
1	1	1	1	1	1	1	1	4	4
1	1	1	1	1	1	1	1	4	4
1	1	1	1	1	1	3	3	4	4
1	1	1	1	1	1	1	1	4	4
3	3	1	1	2	2	1	1	4	4
3	3	1	1	1	1	1	1	4	4
1	1	1	1	1	1	1	1	4	4
1	1	1	1	1	1	1	1	4	4
1	1	1	1	1	1	3	3	4	4
1	1	1	1	1	1	1	1	4	4
1	1	1	1	1	1	1	1	4	4
1	1	1	1	1	1	1	1	4	4
1	1	1	1	1	1	1	1	4	4
1	1	1	1	1	1	1	1	4	4
1	1	2	2	1	1	1	1	4	4
3	3	1	1	1	1	1	1	4	4
1	1	1	1	1	1	1	1	4	4
1	1	1	1	1	1	1	1	4	4
1	1	1	1	1	1	1	1	4	4
1	1	1	1	1	1	1	1	4	4
1	1	1	1	1	1	1	1	4	4
1	1	1	1	1	1	3	3	4	4
1	1	1	1	1	1	1	1	4	4
3	3	1	1	2	2	1	1	4	4
1	1	2	2	1	1	1	1	4	4
1	1	2	2	1	1	1	1	4	4
1	1	1	1	1	1	1	1	4	4
3	3	1	1	1	1	1	1	4	4
1	1	1	1	1	1	1	1	4	4
1	1	1	1	1	1	1	1	4	4
1	1	1	1	1	1	1	1	4	4
1	1	1	1	1	1	1	1	4	4
1	1	1	1	1	1	1	1	4	4
1	1	1	1	1	1	1	1	4	4
1	1	1	1	1	1	1	1	4	4
1	1	1	1	1	1	1	1	4	4
1	1	1	1	1	1	1	1	4	4
1	1	1	1	1	1	3	3	4	4
3	3	1	1	1	1	1	1	2	2
1	1	1	1	1	1	3	3	4	4
3	3	1	1	1	1	1	1	4	4
1	1	1	1	1	1	1	1	4	4
1	1	1	1	1	1	1	1	4	4
1	1	1	1	1	1	1	1	4	4
1	1	1	1	2	2	1	1	4	4
1	1	2	2	1	1	1	1	4	4
1	1	1	1	1	1	1	1	4	4
1	1	1	1	1	1	1	1	4	4
1	1	1	1	1	1	1	1	4	4
1	1	2	2	2	2	1	1	4	4
1	1	1	1	1	1	1	1	4	4

For Review Only

3	3	1	1	1	1	1	1	4	4
1	1	1	1	1	1	1	1	4	4
3	3	1	1	2	2	1	1	4	4
1	1	1	1	1	1	1	1	4	4
1	1	1	1	1	1	1	1	4	4
1	1	2	2	2	2	1	1	4	4
1	1	2	2	2	2	1	1	4	4
1	1	1	1	1	1	1	1	4	4
1	1	1	1	1	1	1	1	4	4
1	1	2	2	1	1	1	1	4	4
1	1	1	1	1	1	1	1	4	4
1	1	1	1	1	1	1	1	4	4
3	3	1	1	1	1	1	1	4	4
3	3	1	1	1	1	1	1	4	4
1	1	1	1	1	1	1	1	4	4
1	1	1	1	1	1	1	1	4	4
1	1	1	1	2	2	1	1	4	4
1	1	1	1	2	2	1	1	4	4
1	1	1	1	1	1	1	1	4	4
3	3	1	1	1	1	1	1	4	4
1	1	1	1	1	1	1	1	4	4
1	1	1	1	1	1	1	1	4	4
1	1	1	1	1	1	1	1	4	4
1	1	1	1	1	1	1	1	4	4
1	1	1	1	1	1	1	1	4	4
1	1	1	1	1	1	1	1	4	4
1	1	1	1	1	1	1	1	2	2
1	1	1	1	1	1	3	3	4	4
1	1	2	2	2	2	1	1	4	4
1	1	1	1	1	1	1	1	4	4
1	1	1	1	1	1	3	3	4	4
1	1	1	1	1	1	1	1	4	4
1	1	1	1	1	1	3	3	4	4
3	3	1	1	1	1	1	1	4	4
1	1	1	1	1	1	1	1	4	4
1	1	1	1	1	1	1	1	4	4
1	1	1	1	1	1	3	3	4	4
1	1	1	1	1	1	1	1	4	4
1	1	1	1	1	1	1	1	4	4
1	1	1	1	1	1	1	1	4	4
1	1	1	1	1	1	1	1	4	4
1	1	1	1	1	1	1	1	4	4
1	1	1	1	1	1	3	3	4	4
1	1	1	1	1	1	1	1	4	4
1	1	1	1	1	1	1	1	4	4
1	1	1	1	1	1	1	1	4	4
1	1	1	1	1	1	3	3	4	4
1	1	1	1	2	2	1	1	4	4
1	1	1	1	1	1	1	1	4	4
1	1	1	1	1	1	1	1	4	4
1	1	1	1	1	1	1	1	4	4
1	1	1	1	1	1	1	1	2	2
1	1	1	1	1	1	1	1	4	4
1	1	1	1	1	1	1	1	4	4
1	1	1	1	1	1	1	1	4	4

For Review Only

1	1	2	2	2	2	1	1	4	4
1	1	1	1	1	1	1	1	2	2
1	1	1	1	2	2	1	1	4	4
1	1	1	1	1	1	3	3	4	4
1	1	1	1	1	1	1	1	4	4
1	1	1	1	1	1	1	1	4	4
1	1	2	2	1	1	1	1	4	4
1	1	1	1	1	1	1	1	4	4
1	1	1	1	1	1	1	1	4	4
1	1	1	1	1	1	1	1	4	4
3	3	1	1	1	1	1	1	4	4
1	1	1	1	1	1	1	1	4	4
1	1	1	1	1	1	3	3	4	4
1	1	1	1	1	1	1	1	4	4
1	1	1	1	1	1	1	1	4	4
1	1	1	1	1	1	1	1	4	4
1	1	1	1	2	2	1	1	4	4
1	1	1	1	1	1	1	1	4	4
1	1	1	1	1	1	1	1	4	4
1	1	1	1	2	2	1	1	4	4
1	1	1	1	1	1	1	1	4	4
1	1	1	1	1	1	1	1	4	4
1	1	1	1	1	1	1	1	4	4
1	1	1	1	1	1	1	1	4	4
1	1	2	2	1	1	1	1	4	4
1	1	1	1	1	1	1	1	4	4
1	1	1	1	2	2	1	1	4	4
1	1	1	1	2	2	1	1	4	4
1	1	1	1	1	1	1	1	4	4
1	1	1	1	2	2	1	1	4	4
1	1	1	1	1	1	3	3	4	4
1	1	1	1	1	1	1	1	4	4
1	1	1	1	1	1	1	1	4	4
1	1	1	1	1	1	3	3	4	4
1	1	1	1	1	1	1	1	4	4
1	1	1	1	1	1	1	1	4	4
1	1	1	1	1	1	1	1	4	4
1	1	1	1	1	1	1	1	4	4
1	1	1	1	1	1	1	1	4	4
1	1	1	1	1	1	3	3	4	4
1	1	1	1	1	1	1	1	4	4
1	1	1	1	1	1	3	3	4	4
1	1	1	1	2	2	1	1	4	4
1	1	1	1	1	1	1	1	4	4
1	1	1	1	1	1	1	1	4	4
1	1	1	1	1	1	1	1	4	4
1	1	2	2	2	2	1	1	4	4
1	1	2	2	2	2	1	1	4	4
1	1	1	1	1	1	1	1	4	4
1	1	1	1	2	2	1	1	4	4
1	1	2	2	2	2	1	1	4	4
1	1	1	1	2	2	1	1	4	4
1	1	2	2	1	1	1	1	4	4
1	1	1	1	1	1	1	1	4	4
1	1	1	1	1	1	1	1	4	4

For Review Only

1	1	1	1	1	1	1	1	4	4
1	1	1	1	1	1	1	1	4	4
1	1	1	1	1	1	1	1	4	4
1	1	1	1	1	1	1	1	2	2
3	3	1	1	1	1	1	1	4	4
1	1	1	1	2	2	1	1	2	2
1	1	1	1	1	1	1	1	4	4
1	1	1	1	1	1	1	1	4	4
1	1	1	1	1	1	1	1	4	4
1	1	1	1	1	1	1	1	4	4
1	1	1	1	1	1	1	1	4	4
1	1	1	1	1	1	1	1	4	4
1	1	1	1	2	2	1	1	4	4
1	1	1	1	2	2	1	1	4	4
1	1	1	1	1	1	3	3	4	4
1	1	1	1	1	1	1	1	4	4
1	1	1	1	1	1	1	1	4	4
1	1	1	1	2	2	1	1	4	4
1	1	1	1	2	2	1	1	4	4
1	1	2	2	1	1	1	1	4	4
1	1	1	1	1	1	1	1	4	4
1	1	1	1	1	1	3	3	4	4
1	1	1	1	1	1	1	1	4	4
1	1	1	1	1	1	1	1	4	4
3	3	1	1	1	1	1	1	4	4
1	1	1	1	1	1	1	1	4	4
1	1	1	1	1	1	1	1	4	4
1	1	1	1	1	1	1	1	4	4
1	1	1	1	1	1	1	1	4	4
1	1	2	2	1	1	1	1	4	4
1	1	1	1	1	1	1	1	4	4
1	1	1	1	2	2	1	1	4	4
1	1	1	1	2	2	1	1	4	4
1	1	1	1	1	1	3	3	4	4
1	1	1	1	1	1	1	1	4	4
1	1	1	1	1	1	1	1	4	4
1	1	1	1	1	1	1	1	4	4
1	1	1	1	1	1	1	1	4	4
1	1	2	2	2	2	1	1	4	4
1	1	1	1	2	2	1	1	4	4
1	1	1	1	1	1	1	1	4	4
1	1	2	2	2	2	1	1	4	4
1	1	1	1	1	1	3	3	4	4
1	1	1	1	1	1	1	1	4	4
1	1	1	1	1	1	1	1	4	4
1	1	1	1	2	2	1	1	4	4
1	1	1	1	1	1	1	1	4	4
1	1	1	1	1	1	3	3	4	4
1	1	1	1	1	1	3	3	4	4
1	1	1	1	1	1	1	1	4	4
1	1	1	1	1	1	1	1	4	4
1	1	1	1	1	1	1	1	4	4
1	1	1	1	1	1	1	1	4	4
1	1	1	1	1	1	1	1	4	4
1	1	1	1	1	1	3	3	2	2

For Review Only

1	1	2	2	1	1	1	1	4	4
1	1	1	1	1	1	3	3	4	4
1	1	1	1	1	1	1	1	4	4
1	1	1	1	1	1	1	1	4	4
1	1	1	1	1	1	1	1	4	4
1	1	2	2	2	2	1	1	4	4
1	1	1	1	2	2	1	1	4	4
1	1	1	1	1	1	1	1	4	4
1	1	1	1	1	1	3	3	4	4
1	1	1	1	1	1	3	3	4	4
1	1	1	1	1	1	1	1	4	4
1	1	1	1	1	1	1	1	4	4
1	1	1	1	1	1	1	1	4	4
1	1	1	1	2	2	1	1	4	4
1	1	1	1	1	1	1	1	4	4
1	1	1	1	1	1	1	1	4	4
1	1	1	1	1	1	3	3	4	4
1	1	1	1	1	1	1	1	4	4
1	1	1	1	1	1	1	1	4	4
1	1	1	1	1	1	1	1	4	4
1	1	1	1	1	1	3	3	2	2
1	1	2	2	2	2	1	1	4	4
1	1	1	1	1	1	1	1	4	4
1	1	1	1	1	1	1	1	2	2
1	1	1	1	1	1	1	1	4	4
3	3	1	1	1	1	1	1	4	4
1	1	1	1	1	1	3	3	4	4
1	1	1	1	1	1	1	1	4	4
1	1	1	1	1	1	1	1	4	4
1	1	1	1	1	1	1	1	4	4
1	1	1	1	1	1	3	3	4	4
1	1	1	1	1	1	1	1	4	4
1	1	1	1	2	2	1	1	4	4
1	1	1	1	2	2	1	1	4	4
1	1	1	1	1	1	1	1	4	4
1	1	1	1	1	1	3	3	4	4
1	1	2	2	1	1	1	1	4	4
1	1	1	1	1	1	1	1	4	4
1	1	1	1	1	1	1	1	4	4
1	1	1	1	1	1	3	3	2	2
1	1	1	1	1	1	1	1	4	4
1	1	1	1	1	1	1	1	4	4
1	1	1	1	1	1	1	1	4	4
1	1	1	1	1	1	1	1	4	4
1	1	1	1	1	1	1	1	4	4
1	1	1	1	1	1	1	1	4	4
1	1	1	1	1	1	3	3	4	4
1	1	1	1	2	2	1	1	4	4
3	3	2	2	1	1	1	1	4	4
1	1	1	1	1	1	3	3	4	4
1	1	1	1	1	1	1	1	4	4
1	1	1	1	1	1	3	3	4	4
1	1	1	1	1	1	1	1	4	4
1	1	1	1	2	2	1	1	4	4
1	1	1	1	1	1	1	1	2	2

For Review Only

1	1	1	1	1	1	1	1	4	4
1	1	1	1	1	1	1	1	4	4

For Review Only

SNP2418	SNP2418	SNP2419	SNP2419	SNP242	SNP242	SNP2420	SNP2420	SNP2421	SNP2421
2	2	2	2	1	1	2	2	2	2
2	2	3	3	1	1	2	2	2	2
2	2	2	2	1	1	2	2	2	2
2	2	2	2	1	1	2	2	2	2
2	2	2	2	3	3	2	2	2	2
2	2	2	2	1	1	2	2	2	2
2	2	2	2	3	3	2	2	2	2
2	2	2	2	3	3	2	2	2	2
2	2	2	2	3	3	2	2	2	2
2	2	2	2	3	3	2	2	2	2
1	1	2	2	3	3	3	3	3	3
2	2	2	2	3	3	2	2	2	2
2	2	2	2	1	1	2	2	2	2
2	2	2	2	1	1	2	2	2	2
2	2	2	2	1	1	2	2	2	2
2	2	2	2	3	3	2	2	2	2
2	2	3	3	1	1	2	2	2	2
2	2	2	2	3	3	2	2	2	2
1	1	2	2	3	3	3	3	3	3
2	2	3	3	1	1	2	2	2	2
2	2	3	3	1	1	2	2	2	2
2	2	2	2	1	1	2	2	2	2
2	2	2	2	1	1	2	2	2	2
2	2	2	2	3	3	2	2	2	2
2	2	2	2	1	1	2	2	2	2
2	2	2	2	1	1	2	2	2	2
2	2	2	2	3	3	2	2	2	2
2	2	2	2	3	3	2	2	2	2
2	2	2	2	3	3	2	2	2	2
2	2	3	3	1	1	2	2	2	2
2	2	2	2	1	1	2	2	2	2
2	2	2	2	3	3	2	2	2	2
2	2	2	2	1	1	2	2	2	2
2	2	2	2	3	3	2	2	2	2
2	2	2	2	3	3	2	2	2	2
2	2	2	2	3	3	2	2	2	2
1	1	3	3	1	1	2	2	3	3
2	2	2	2	1	1	2	2	3	3
1	1	3	3	1	1	3	3	3	3
2	2	3	3	1	1	2	2	2	2
2	2	3	3	3	3	2	2	2	2
2	2	2	2	3	3	2	2	2	2
2	2	2	2	3	3	2	2	2	2
2	2	3	3	1	1	2	2	2	2
2	2	2	2	3	3	2	2	2	2
2	2	2	2	1	1	2	2	2	2
2	2	2	2	3	3	2	2	2	2
2	2	2	2	1	1	2	2	2	2
2	2	3	3	3	3	2	2	2	2
2	2	2	2	1	1	2	2	3	3
2	2	2	2	1	1	2	2	2	2
2	2	2	2	3	3	2	2	2	2

For Review Only

2	2	2	2	1	1	2	2	2	2
2	2	2	2	1	1	2	2	2	2
2	2	2	2	3	3	2	2	2	2
2	2	3	3	3	3	2	2	2	2
1	1	2	2	3	3	3	3	3	3
2	2	2	2	3	3	2	2	2	2
2	2	2	2	3	3	2	2	2	2
2	2	3	3	1	1	2	2	2	2
2	2	2	2	1	1	2	2	2	2
2	2	2	2	3	3	2	2	2	2
2	2	2	2	3	3	2	2	2	2
2	2	2	2	3	3	2	2	2	2
2	2	2	2	1	1	2	2	2	2
2	2	2	2	3	3	2	2	2	2
2	2	3	3	3	3	2	2	2	2
2	2	3	3	3	3	2	2	2	2
2	2	3	3	3	3	2	2	2	2
2	2	2	2	3	3	2	2	2	2
2	2	2	2	3	3	2	2	2	2
2	2	2	2	1	1	2	2	2	2
2	2	2	2	3	3	2	2	2	2
2	2	3	3	3	3	3	3	2	2
2	2	2	2	3	3	2	2	2	2
2	2	2	2	1	1	2	2	2	2
2	2	2	2	3	3	2	2	2	2
2	2	2	2	3	3	2	2	2	2
2	2	3	3	3	3	2	2	2	2
2	2	2	2	3	3	2	2	2	2
2	2	2	2	1	1	2	2	2	2
1	1	2	2	3	3	3	3	3	3
2	2	2	2	3	3	2	2	2	2
2	2	2	2	3	3	2	2	2	2
2	2	2	2	1	1	2	2	2	2
2	2	2	2	1	1	2	2	2	2
2	2	2	2	1	1	2	2	2	2
2	2	2	2	3	3	2	2	2	2
2	2	2	2	3	3	2	2	2	2
2	2	3	3	3	3	2	2	2	2
2	2	2	2	1	1	2	2	2	2
2	2	2	2	3	3	2	2	2	2
2	2	2	2	3	3	2	2	2	2
1	1	2	2	3	3	3	3	3	3
2	2	3	3	1	1	2	2	2	2
2	2	2	2	3	3	2	2	2	2
1	1	2	2	3	3	3	3	3	3
2	2	2	2	1	1	2	2	2	2
2	2	2	2	1	1	2	2	2	2
2	2	2	2	3	3	2	2	2	2
2	2	2	2	1	1	2	2	2	2
2	2	2	2	3	3	2	2	2	2
2	2	3	3	3	3	2	2	2	2
1	1	3	3	1	1	3	3	2	2
2	2	3	3	1	1	2	2	2	2
2	2	3	3	3	3	2	2	2	2
2	2	2	2	1	1	2	2	2	2

For Review Only

2	2	2	2	1	1	2	2	2	2
1	1	3	3	3	3	3	3	3	3
2	2	2	2	3	3	2	2	2	2
2	2	2	2	1	1	2	2	2	2
2	2	2	2	3	3	2	2	2	2
2	2	2	2	1	1	2	2	2	2
2	2	2	2	3	3	2	2	2	2
1	1	2	2	3	3	3	3	3	3
1	1	3	3	1	1	3	3	3	3
2	2	2	2	3	3	2	2	2	2
2	2	2	2	1	1	2	2	2	2
2	2	3	3	3	3	2	2	2	2
2	2	2	2	3	3	2	2	2	2
2	2	2	2	3	3	2	2	2	2
2	2	3	3	1	1	3	3	2	2
2	2	3	3	3	3	2	2	2	2
2	2	3	3	3	3	2	2	2	2
2	2	2	2	3	3	2	2	2	2
2	2	2	2	1	1	2	2	2	2
2	2	3	3	3	3	2	2	2	2
2	2	3	3	3	3	2	2	2	2
2	2	2	2	1	1	2	2	2	2
2	2	2	2	1	1	2	2	2	2
2	2	2	2	3	3	2	2	2	2
2	2	2	2	1	1	2	2	2	2
2	2	2	2	3	3	2	2	2	2
2	2	2	2	3	3	2	2	2	2
1	1	2	2	1	1	3	3	3	3
2	2	2	2	1	1	2	2	2	2
1	1	3	3	3	3	3	3	3	3
1	1	3	3	1	1	2	2	2	2
2	2	2	2	3	3	2	2	2	2
2	2	2	2	3	3	2	2	2	2
2	2	2	2	1	1	2	2	2	2
2	2	2	2	3	3	2	2	2	2
2	2	2	2	3	3	2	2	2	2
2	2	2	2	1	1	2	2	2	2
1	1	2	2	3	3	3	3	3	3
2	2	2	2	1	1	3	3	2	2
1	1	3	3	3	3	3	3	3	3
2	2	2	2	3	3	2	2	2	2
2	2	2	2	1	1	2	2	2	2
2	2	2	2	3	3	2	2	2	2
2	2	3	3	3	3	2	2	2	2
1	1	3	3	1	1	3	3	3	3
2	2	2	2	1	1	2	2	2	2
2	2	2	2	1	1	2	2	2	2
2	2	2	2	1	1	2	2	2	2
2	2	3	3	1	1	2	2	2	2
2	2	2	2	3	3	2	2	2	2
2	2	2	2	1	1	2	2	2	2
2	2	3	3	3	3	2	2	2	2
2	2	2	2	3	3	2	2	2	2
2	2	2	2	3	3	2	2	2	2
1	1	2	2	3	3	2	2	2	2

For Review Only

1	1	2	2	1	1	3	3	3	3
2	2	3	3	3	3	2	2	2	2
2	2	2	2	3	3	2	2	2	2
1	1	2	2	3	3	3	3	3	3
2	2	2	2	1	1	2	2	2	2
2	2	2	2	1	1	2	2	2	2
2	2	3	3	3	3	2	2	2	2
2	2	2	2	3	3	2	2	2	2
2	2	3	3	1	1	2	2	2	2
1	1	2	2	3	3	3	3	3	3
2	2	2	2	1	1	2	2	2	2
2	2	3	3	3	3	2	2	2	2
2	2	2	2	3	3	2	2	3	3
2	2	2	2	3	3	2	2	2	2
2	2	2	2	1	1	2	2	2	2
1	1	2	2	3	3	3	3	3	3
2	2	2	2	3	3	2	2	2	2
2	2	2	2	1	1	2	2	2	2
2	2	2	2	1	1	2	2	2	2
2	2	2	2	3	3	2	2	2	2
2	2	2	2	3	3	2	2	2	2
2	2	3	3	3	3	2	2	2	2
2	2	2	2	3	3	2	2	2	2
2	2	2	2	3	3	2	2	2	2
2	2	3	3	3	3	2	2	2	2
2	2	2	2	3	3	2	2	2	2
2	2	2	2	1	1	2	2	2	2
2	2	2	2	3	3	2	2	2	2
2	2	3	3	1	1	2	2	2	2
2	2	2	2	1	1	2	2	2	2
2	2	2	2	1	1	2	2	2	2
2	2	2	2	3	3	2	2	2	2
2	2	2	2	3	3	2	2	2	2
2	2	2	2	1	1	2	2	2	2
2	2	2	2	3	3	2	2	2	2
2	2	2	2	3	3	2	2	2	2
2	2	2	2	3	3	2	2	2	2
1	1	2	2	1	1	3	3	3	3
2	2	2	2	1	1	2	2	2	2
2	2	2	2	3	3	2	2	2	2
2	2	2	2	1	1	2	2	2	2
2	2	2	2	1	1	2	2	2	2
2	2	3	3	3	3	2	2	2	2
2	2	2	2	1	1	2	2	2	2
2	2	2	2	3	3	2	2	2	2
2	2	2	2	3	3	2	2	2	2
1	1	2	2	3	3	3	3	3	3
2	2	3	3	3	3	2	2	2	2
1	1	2	2	3	3	3	3	3	3
2	2	2	2	3	3	2	2	2	2
2	2	2	2	3	3	2	2	2	2
2	2	3	3	1	1	2	2	2	2
2	2	2	2	3	3	2	2	2	2
2	2	2	2	3	3	2	2	2	2
1	1	2	2	1	1	3	3	3	3

1	1	2	2	3	3	3	3	3	3
2	2	3	3	1	1	2	2	2	2
2	2	2	2	1	1	2	2	2	2
2	2	3	3	3	3	2	2	2	2
2	2	2	2	1	1	2	2	2	2
2	2	2	2	3	3	2	2	2	2
2	2	2	2	1	1	2	2	2	2
2	2	2	2	3	3	2	2	2	2
2	2	2	2	3	3	2	2	2	2
1	1	2	2	3	3	3	3	3	3
2	2	2	2	3	3	2	2	2	2
1	1	3	3	1	1	3	3	3	3
2	2	2	2	3	3	2	2	2	2
2	2	2	2	1	1	2	2	2	2
2	2	2	2	1	1	2	2	2	2
2	2	2	2	3	3	2	2	2	2
1	1	2	2	1	1	3	3	3	3
2	2	3	3	3	3	2	2	2	2
2	2	2	2	3	3	2	2	2	2
1	1	2	2	3	3	3	3	3	3
2	2	2	2	1	1	2	2	2	2
2	2	2	2	1	1	2	2	2	2
1	1	2	2	3	3	2	2	2	2
2	2	3	3	1	1	2	2	2	2
2	2	3	3	1	1	2	2	2	2
2	2	2	2	3	3	2	2	2	2
1	1	2	2	3	3	3	3	3	3
2	2	2	2	3	3	2	2	2	2
2	2	2	2	1	1	2	2	2	2
2	2	2	2	1	1	2	2	2	2
1	1	2	2	1	1	3	3	3	3
2	2	2	2	3	3	2	2	2	2
2	2	2	2	3	3	2	2	2	2
2	2	2	2	3	3	2	2	2	2
2	2	2	2	3	3	2	2	2	2
2	2	2	2	3	3	2	2	2	2
1	1	2	2	3	3	3	3	3	3
2	2	2	2	3	3	2	2	2	2
2	2	2	2	3	3	2	2	2	2
1	1	3	3	1	1	3	3	3	3
1	1	2	2	3	3	3	3	3	3
2	2	2	2	3	3	2	2	2	2
2	2	3	3	3	3	2	2	2	2
2	2	2	2	3	3	2	2	2	2
2	2	2	2	3	3	2	2	2	2
2	2	2	2	3	3	2	2	2	2
2	2	2	2	1	1	2	2	2	2
2	2	2	2	3	3	2	2	2	2
2	2	3	3	1	1	2	2	2	2
1	1	2	2	1	1	3	3	3	3
2	2	2	2	3	3	2	2	2	2
1	1	2	2	1	1	3	3	2	2
2	2	2	2	1	1	2	2	2	2
2	2	2	2	3	3	2	2	2	2
1	1	3	3	1	1	3	3	3	3

For Review Only

1	1	2	2	3	3	3	3	3	3
2	2	2	2	3	3	2	2	2	2

For Review Only

SNP2422	SNP2422	SNP2423	SNP2423	SNP2424	SNP2424	SNP2425	SNP2425	SNP2426	SNP2426
1	1	1	1	1	1	4	4	1	1
1	1	4	4	4	4	2	2	4	4
1	1	1	1	1	1	4	4	1	1
1	1	1	1	4	4	2	2	4	4
1	1	1	1	4	4	2	2	4	4
1	1	1	1	4	4	2	2	4	4
1	1	1	1	1	1	4	4	1	1
1	1	1	1	4	4	2	2	4	4
1	1	1	1	4	4	4	4	1	1
1	1	1	1	4	4	2	2	4	4
4	4	1	1	4	4	2	2	4	4
1	1	1	1	4	4	2	2	1	1
1	1	1	1	4	4	4	4	1	1
1	1	1	1	1	1	4	4	1	1
1	1	1	1	1	1	4	4	1	1
1	1	1	1	4	4	2	2	4	4
1	1	1	1	1	1	4	4	1	1
1	1	1	1	4	4	2	2	4	4
4	4	1	1	4	4	2	2	4	4
1	1	1	1	1	1	4	4	1	1
1	1	1	1	1	1	4	4	1	1
1	1	1	1	1	1	4	4	1	1
1	1	1	1	4	4	2	2	4	4
1	1	1	1	4	4	2	2	4	4
1	1	1	1	1	1	4	4	1	1
1	1	4	4	4	4	2	2	4	4
1	1	1	1	1	1	4	4	1	1
1	1	1	1	4	4	4	4	1	1
1	1	1	1	4	4	2	2	4	4
1	1	1	1	4	4	2	2	4	4
1	1	1	1	4	4	2	2	4	4
1	1	1	1	1	1	4	4	1	1
1	1	1	1	1	1	4	4	1	1
1	1	1	1	4	4	2	2	4	4
1	1	1	1	4	4	2	2	4	4
1	1	1	1	4	4	2	2	4	4
4	4	1	1	1	1	4	4	1	1
4	4	1	1	1	1	4	4	1	1
4	4	1	1	4	4	2	2	4	4
1	1	1	1	4	4	2	2	4	4
1	1	1	1	4	4	2	2	4	4
1	1	1	1	4	4	2	2	4	4
1	1	1	1	4	4	2	2	4	4
1	1	1	1	1	1	4	4	1	1
1	1	1	1	4	4	2	2	4	4
1	1	1	1	1	1	4	4	1	1
1	1	1	1	4	4	2	2	4	4
1	1	1	1	4	4	2	2	4	4
1	1	1	1	1	1	4	4	1	1
1	1	1	1	1	1	4	4	1	1
1	1	1	1	1	1	4	4	1	1
1	1	4	4	4	4	4	4	1	1

For Review Only

1	1	1	1	1	1	4	4	1	1
1	1	1	1	1	1	4	4	1	1
1	1	4	4	4	4	2	2	4	4
1	1	1	1	1	1	4	4	1	1
4	4	1	1	4	4	2	2	4	4
1	1	4	4	4	4	2	2	4	4
1	1	4	4	4	4	2	2	4	4
1	1	1	1	1	1	4	4	1	1
1	1	1	1	1	1	4	4	1	1
1	1	1	1	4	4	4	4	1	1
1	1	1	1	4	4	2	2	4	4
1	1	1	1	4	4	2	2	4	4
1	1	1	1	1	1	4	4	1	1
1	1	1	1	4	4	2	2	4	4
1	1	1	1	4	4	4	4	1	1
1	1	1	1	4	4	2	2	4	4
1	1	1	1	4	4	4	4	1	1
1	1	1	1	1	1	4	4	1	1
1	1	1	1	1	1	4	4	1	1
1	1	1	1	1	1	4	4	1	1
1	1	1	1	4	4	2	2	4	4
1	1	1	1	4	4	2	2	1	1
1	1	1	1	1	1	4	4	1	1
1	1	4	4	4	4	2	2	4	4
1	1	1	1	4	4	2	2	4	4
1	1	4	4	1	1	4	4	1	1
1	1	1	1	1	1	4	4	1	1
1	1	1	1	4	4	2	2	4	4
1	1	4	4	4	4	2	2	4	4
1	1	1	1	4	4	2	2	4	4
1	1	1	1	4	4	2	2	4	4
1	1	1	1	4	4	2	2	4	4
1	1	1	1	4	4	2	2	4	4
1	1	1	1	1	1	4	4	1	1
1	1	1	1	4	4	2	2	4	4
1	1	1	1	4	4	2	2	4	4
1	1	1	1	4	4	2	2	4	4
1	1	1	1	1	1	4	4	1	1
1	1	1	1	4	4	2	2	4	4
1	1	1	1	4	4	4	4	1	1
1	1	1	1	1	1	4	4	1	1
4	4	1	1	1	1	4	4	1	1
1	1	1	1	1	1	4	4	1	1
1	1	1	1	4	4	2	2	4	4
4	4	1	1	4	4	4	4	4	4
1	1	1	1	1	1	4	4	1	1
1	1	1	1	4	4	4	4	4	4
1	1	4	4	4	4	2	2	4	4
1	1	1	1	4	4	2	2	4	4
1	1	1	1	4	4	4	4	1	1
1	1	1	1	4	4	2	2	4	4
4	4	1	1	4	4	2	2	4	4
1	1	1	1	1	1	4	4	1	1
1	1	1	1	4	4	4	4	1	1
1	1	1	1	1	1	4	4	1	1

For Review Only

1	1	4	4	4	4	2	2	4	4
4	4	1	1	4	4	2	2	4	4
1	1	4	4	4	4	2	2	4	4
1	1	1	1	4	4	2	2	4	4
1	1	4	4	4	4	4	4	1	1
1	1	1	1	4	4	2	2	4	4
1	1	1	1	4	4	2	2	4	4
4	4	1	1	4	4	4	4	1	1
4	4	1	1	1	1	4	4	1	1
1	1	1	1	1	1	4	4	1	1
1	1	1	1	4	4	2	2	4	4
1	1	1	1	1	1	4	4	1	1
1	1	1	1	1	1	4	4	1	1
1	1	1	1	4	4	4	4	1	1
1	1	1	1	4	4	2	2	1	1
1	1	1	1	4	4	4	4	1	1
1	1	1	1	1	1	4	4	1	1
1	1	1	1	4	4	2	2	1	1
1	1	1	1	4	4	2	2	4	4
1	1	1	1	1	1	4	4	1	1
1	1	1	1	4	4	4	4	1	1
1	1	1	1	4	4	2	2	4	4
1	1	4	4	4	4	4	4	4	4
1	1	1	1	1	1	4	4	1	1
1	1	1	1	1	1	4	4	1	1
1	1	1	1	1	1	4	4	1	1
1	1	1	1	1	1	4	4	1	1
4	4	1	1	1	1	4	4	1	1
1	1	1	1	1	1	4	4	1	1
4	4	1	1	4	4	2	2	4	4
4	4	1	1	4	4	4	4	4	4
1	1	1	1	4	4	4	4	1	1
1	1	1	1	1	1	4	4	1	1
1	1	1	1	1	1	4	4	1	1
1	1	1	1	4	4	2	2	4	4
1	1	1	1	4	4	2	2	1	1
1	1	1	1	4	4	4	4	4	4
4	4	4	4	1	1	4	4	1	1
1	1	1	1	4	4	4	4	4	4
4	4	1	1	4	4	2	2	4	4
1	1	1	1	4	4	2	2	4	4
1	1	1	1	1	1	4	4	1	1
1	1	1	1	1	1	4	4	1	1
1	1	1	1	4	4	4	4	1	1
4	4	4	4	4	4	4	4	1	1
1	1	1	1	4	4	2	2	4	4
1	1	4	4	4	4	2	2	4	4
1	1	4	4	4	4	2	2	4	4
1	1	1	1	4	4	4	4	1	1
1	1	4	4	4	4	2	2	4	4
1	1	4	4	4	4	2	2	4	4
1	1	1	1	4	4	2	2	4	4
1	1	1	1	4	4	2	2	1	1
1	1	4	4	4	4	2	2	4	4
1	1	1	1	4	4	4	4	1	1

For Review Only

4	4	1	1	4	4	2	2	4	4
1	1	1	1	1	1	4	4	1	1
1	1	1	1	4	4	2	2	4	4
4	4	1	1	4	4	2	2	4	4
1	1	1	1	4	4	2	2	4	4
1	1	1	1	4	4	2	2	4	4
1	1	1	1	1	1	4	4	1	1
1	1	4	4	4	4	2	2	1	1
4	4	1	1	4	4	4	4	1	1
4	4	1	1	1	1	4	4	1	1
1	1	1	1	4	4	2	2	4	4
1	1	1	1	4	4	4	4	1	1
1	1	1	1	4	4	2	2	1	1
1	1	1	1	4	4	2	2	4	4
1	1	1	1	1	1	4	4	1	1
4	4	1	1	4	4	2	2	4	4
1	1	1	1	4	4	4	4	1	1
1	1	4	4	1	1	4	4	1	1
1	1	1	1	4	4	2	2	4	4
1	1	1	1	4	4	2	2	4	4
1	1	1	1	4	4	4	4	1	1
1	1	1	1	4	4	2	2	4	4
1	1	1	1	1	1	4	4	1	1
1	1	1	1	4	4	2	2	4	4
1	1	1	1	1	1	4	4	1	1
1	1	1	1	4	4	2	2	4	4
1	1	1	1	4	4	2	2	4	4
1	1	1	1	4	4	4	4	1	1
1	1	1	1	1	1	4	4	1	1
1	1	1	1	1	1	4	4	1	1
1	1	1	1	4	4	2	2	4	4
1	1	1	1	4	4	2	2	4	4
1	1	1	1	1	1	4	4	1	1
1	1	1	1	1	1	4	4	1	1
1	1	1	1	1	1	4	4	1	1
1	1	1	1	4	4	2	2	4	4
1	1	1	1	1	1	4	4	1	1
4	4	1	1	1	1	4	4	1	1
1	1	1	1	4	4	2	2	4	4
1	1	1	1	1	1	4	4	1	1
1	1	1	1	1	1	4	4	1	1
1	1	4	4	4	4	2	2	4	4
1	1	1	1	4	4	2	2	4	4
1	1	1	1	4	4	4	4	1	1
1	1	1	1	4	4	2	2	1	1
1	1	1	1	4	4	4	4	1	1
4	4	1	1	4	4	2	2	4	4
1	1	1	1	1	1	4	4	1	1
4	4	1	1	4	4	2	2	4	4
1	1	1	1	4	4	2	2	4	4
1	1	1	1	1	1	4	4	1	1
1	1	1	1	1	1	4	4	1	1
1	1	1	1	1	1	4	4	1	1
1	1	1	1	4	4	2	2	4	4
4	4	1	1	4	4	2	2	4	4

For Review Only

4	4	1	1	1	1	4	4	1	1
1	1	1	1	1	1	4	4	1	1
1	1	1	1	1	1	4	4	1	1
1	1	1	1	1	1	4	4	1	1
1	1	1	1	1	1	4	4	1	1
1	1	1	1	1	1	4	4	1	1
1	1	1	1	1	1	4	4	1	1
1	1	1	1	4	4	2	2	4	4
1	1	1	1	1	1	4	4	1	1
4	4	1	1	4	4	2	2	4	4
1	1	1	1	4	4	4	4	1	1
4	4	1	1	1	1	4	4	1	1
1	1	1	1	4	4	2	2	4	4
1	1	4	4	4	4	2	2	4	4
1	1	1	1	1	1	4	4	1	1
1	1	1	1	4	4	2	2	4	4
4	4	1	1	4	4	2	2	4	4
1	1	1	1	4	4	4	4	1	1
1	1	1	1	4	4	2	2	4	4
4	4	1	1	1	1	4	4	1	1
1	1	1	1	4	4	2	2	4	4
1	1	4	4	4	4	2	2	4	4
1	1	1	1	1	1	4	4	1	1
1	1	1	1	4	4	2	2	4	4
1	1	1	1	1	1	4	4	1	1
1	1	1	1	4	4	2	2	1	1
4	4	1	1	4	4	2	2	4	4
1	1	1	1	4	4	2	2	4	4
1	1	1	1	1	1	4	4	1	1
1	1	1	1	1	1	4	4	1	1
4	4	1	1	4	4	2	2	4	4
1	1	4	4	4	4	2	2	4	4
1	1	1	1	4	4	2	2	4	4
1	1	4	4	1	1	4	4	1	1
1	1	1	1	1	1	4	4	1	1
1	1	1	1	4	4	2	2	4	4
4	4	1	1	1	1	4	4	1	1
1	1	1	1	4	4	2	2	4	4
1	1	1	1	4	4	2	2	4	4
4	4	1	1	4	4	2	2	4	4
4	4	1	1	1	1	4	4	1	1
1	1	1	1	4	4	2	2	4	4
1	1	1	1	1	1	4	4	1	1
1	1	1	1	1	1	4	4	1	1
1	1	4	4	4	4	4	4	1	1
1	1	1	1	1	1	4	4	1	1
1	1	4	4	4	4	2	2	4	4
1	1	1	1	1	1	4	4	1	1
1	1	1	1	4	4	2	2	4	4
4	4	1	1	4	4	2	2	4	4
1	1	1	1	1	1	4	4	1	1
4	4	1	1	4	4	2	2	4	4
1	1	1	1	1	1	4	4	1	1
1	1	1	1	1	1	4	4	1	1
4	4	1	1	4	4	2	2	4	4

For Review Only

4	4	1	1	4	4	2	2	4	4
1	1	1	1	4	4	2	2	4	4

For Review Only

SNP2427	SNP2427	SNP2428	SNP2428	SNP2429	SNP2429	SNP243	SNP243	SNP2430	SNP2430
4	4	1	1	1	1	1	1	3	3
4	4	1	1	1	1	1	1	3	3
4	4	4	4	1	1	1	1	3	3
4	4	1	1	1	1	1	1	3	3
1	1	1	1	4	4	1	1	1	1
4	4	1	1	1	1	1	1	3	3
4	4	1	1	1	1	1	1	3	3
1	1	4	4	4	4	1	1	1	1
4	4	1	1	1	1	1	1	3	3
4	4	4	4	1	1	1	1	3	3
4	4	4	4	1	1	1	1	3	3
4	4	4	4	1	1	1	1	3	3
4	4	1	1	1	1	1	1	3	3
4	4	4	4	1	1	1	1	3	3
4	4	1	1	1	1	1	1	3	3
4	4	4	4	1	1	1	1	3	3
4	4	1	1	1	1	1	1	3	3
1	1	4	4	4	4	1	1	1	1
4	4	1	1	1	1	1	1	3	3
4	4	4	4	1	1	1	1	3	3
4	4	1	1	1	1	1	1	3	3
4	4	4	4	1	1	1	1	3	3
4	4	4	4	1	1	1	1	3	3
4	4	4	4	1	1	1	1	3	3
4	4	1	1	4	4	1	1	3	3
4	4	1	1	1	1	1	1	3	3
4	4	4	4	1	1	1	1	3	3
4	4	1	1	1	1	1	1	3	3
4	4	1	1	1	1	1	1	3	3
4	4	4	4	1	1	1	1	3	3
4	4	1	1	1	1	1	1	3	3
4	4	1	1	1	1	1	1	3	3
4	4	1	1	1	1	1	1	3	3
4	4	4	4	1	1	1	1	3	3
4	4	4	4	1	1	1	1	3	3
4	4	4	4	1	1	1	1	3	3
4	4	1	1	1	1	1	1	3	3
4	4	1	1	1	1	1	1	3	3
4	4	4	4	1	1	1	1	3	3
4	4	4	4	1	1	1	1	3	3
1	1	4	4	4	4	1	1	1	1
4	4	1	1	1	1	1	1	3	3
1	1	4	4	4	4	1	1	1	1
4	4	4	4	1	1	1	1	3	3
1	1	4	4	4	4	1	1	1	1
4	4	4	4	1	1	1	1	3	3
4	4	4	4	1	1	1	1	3	3
4	4	1	1	1	1	1	1	3	3
4	4	4	4	1	1	1	1	3	3
4	4	1	1	1	1	1	1	3	3
4	4	4	4	1	1	1	1	3	3
4	4	1	1	1	1	1	1	3	3

For Review Only

4	4	1	1	1	1	1	1	3	3
4	4	1	1	1	1	1	1	3	3
4	4	1	1	4	4	1	1	3	3
4	4	4	4	1	1	1	1	3	3
4	4	1	1	1	1	1	1	3	3
4	4	1	1	1	1	1	1	3	3
4	4	1	1	1	1	1	1	3	3
4	4	4	4	1	1	1	1	3	3
4	4	4	4	1	1	1	1	3	3
4	4	1	1	1	1	1	1	3	3
4	4	4	4	1	1	1	1	3	3
4	4	4	4	1	1	1	1	3	3
4	4	1	1	1	1	1	1	3	3
4	4	1	1	1	1	1	1	3	3
4	4	1	1	1	1	1	1	3	3
4	4	4	4	1	1	1	1	1	1
4	4	4	4	1	1	1	1	3	3
4	4	1	1	1	1	1	1	3	3
4	4	1	1	1	1	1	1	3	3
4	4	1	1	1	1	1	1	3	3
4	4	4	4	1	1	1	1	3	3
4	4	4	4	1	1	1	1	3	3
4	4	1	1	1	1	1	1	3	3
4	4	1	1	1	1	1	1	3	3
1	1	4	4	4	4	1	1	1	1
4	4	1	1	1	1	1	1	3	3
1	1	4	4	4	4	1	1	1	1
4	4	4	4	1	1	1	1	3	3
4	4	1	1	1	1	1	1	3	3
4	4	1	1	1	1	1	1	3	3
1	1	1	1	4	4	1	1	1	1
4	4	4	4	1	1	1	1	3	3
4	4	4	4	1	1	1	1	3	3
4	4	1	1	1	1	1	1	3	3
4	4	1	1	1	1	1	1	3	3
1	1	4	4	4	4	1	1	1	1
1	1	4	4	4	4	1	1	1	1
4	4	4	4	1	1	1	1	3	3
4	4	4	4	1	1	1	1	3	3
1	1	4	4	4	4	1	1	1	1
4	4	1	1	1	1	1	1	3	3
4	4	4	4	1	1	1	1	3	3
4	4	4	4	1	1	1	1	3	3
4	4	4	4	1	1	1	1	3	3
4	4	1	1	4	4	1	1	3	3
4	4	1	1	1	1	1	1	3	3
4	4	4	4	1	1	1	1	3	3
4	4	1	1	1	1	1	1	3	3
4	4	4	4	1	1	1	1	3	3
4	4	1	1	1	1	1	1	3	3
4	4	1	1	1	1	1	1	3	3
4	4	4	4	1	1	1	1	3	3
4	4	4	4	1	1	1	1	3	3
4	4	1	1	1	1	1	1	3	3
4	4	1	1	1	1	1	1	3	3

For Review Only

4	4	1	1	1	1	1	1	3	3
4	4	1	1	1	1	1	1	3	3
4	4	1	1	1	1	1	1	3	3
4	4	4	4	1	1	1	1	3	3
4	4	1	1	1	1	1	1	3	3
4	4	1	1	1	1	1	1	3	3
4	4	4	4	1	1	1	1	3	3
4	4	1	1	1	1	1	1	3	3
4	4	4	4	1	1	1	1	1	1
4	4	4	4	1	1	1	1	3	3
4	4	1	1	1	1	1	1	3	3
4	4	1	1	1	1	1	1	3	3
4	4	4	4	1	1	1	1	3	3
4	4	1	1	1	1	1	1	3	3
4	4	1	1	1	1	1	1	3	3
4	4	1	1	1	1	1	1	3	3
4	4	4	4	1	1	1	1	3	3
4	4	1	1	1	1	1	1	3	3
4	4	4	4	1	1	1	1	3	3
4	4	1	1	1	1	1	1	3	3
4	4	1	1	1	1	1	1	3	3
4	4	1	1	1	1	1	1	3	3
4	4	1	1	1	1	1	1	3	3
4	4	4	4	1	1	1	1	3	3
4	4	1	1	1	1	1	1	3	3
4	4	4	4	1	1	1	1	3	3
4	4	1	1	1	1	1	1	3	3
4	4	1	1	1	1	1	1	3	3
4	4	1	1	1	1	1	1	3	3
4	4	1	1	1	1	1	1	3	3
4	4	4	4	1	1	1	1	3	3
4	4	1	1	1	1	1	1	3	3
4	4	1	1	1	1	1	1	3	3
4	4	1	1	1	1	1	1	3	3
4	4	4	4	1	1	1	1	3	3
4	4	1	1	1	1	1	1	3	3
4	4	4	4	1	1	1	1	3	3
4	4	1	1	1	1	1	1	3	3
4	4	1	1	1	1	1	1	3	3
4	4	4	4	1	1	1	1	3	3
4	4	1	1	1	1	1	1	3	3
4	4	4	4	1	1	1	1	3	3
4	4	1	1	1	1	1	1	3	3
1	1	4	4	4	4	1	1	1	1
4	4	1	1	1	1	1	1	3	3
4	4	1	1	1	1	1	1	3	3
4	4	1	1	1	1	1	1	3	3
4	4	1	1	1	1	1	1	3	3
4	4	1	1	1	1	1	1	3	3
4	4	1	1	1	1	1	1	3	3
4	4	1	1	1	1	1	1	3	3
4	4	4	4	1	1	1	1	3	3
4	4	1	1	1	1	1	1	3	3
4	4	1	1	1	1	1	1	3	3
4	4	1	1	1	1	1	1	3	3

For Review Only

4	4	1	1	1	1	1	1	3	3
4	4	4	4	1	1	1	1	3	3
1	1	1	1	4	4	1	1	1	1
4	4	1	1	1	1	1	1	3	3
4	4	1	1	1	1	1	1	3	3
4	4	1	1	1	1	1	1	3	3
4	4	4	4	1	1	1	1	3	3
4	4	1	1	1	1	1	1	3	3
4	4	4	4	1	1	1	1	3	3
4	4	1	1	1	1	1	1	3	3
4	4	1	1	1	1	1	1	3	3
4	4	4	4	1	1	1	1	3	3
4	4	1	1	1	1	1	1	3	3
4	4	1	1	1	1	1	1	3	3
4	4	4	4	1	1	1	1	3	3
4	4	4	4	1	1	1	1	3	3
4	4	1	1	1	1	1	1	3	3
4	4	1	1	1	1	1	1	3	3
4	4	4	4	1	1	1	1	3	3
4	4	4	4	1	1	1	1	3	3
4	4	1	1	1	1	1	1	3	3
4	4	4	4	1	1	1	1	3	3
4	4	4	4	1	1	1	1	3	3
4	4	4	4	1	1	1	1	3	3
4	4	1	1	1	1	1	1	3	3
4	4	4	4	1	1	1	1	3	3
4	4	1	1	1	1	1	1	3	3
4	4	4	4	1	1	1	1	3	3
4	4	1	1	4	4	1	1	3	3
4	4	1	1	1	1	1	1	3	3
4	4	4	4	1	1	4	4	3	3
4	4	1	1	1	1	1	1	3	3
4	4	4	4	1	1	1	1	3	3
4	4	4	4	1	1	1	1	3	3
4	4	1	1	1	1	4	4	3	3
4	4	4	4	1	1	1	1	3	3
4	4	1	1	4	4	1	1	3	3
4	4	1	1	1	1	1	1	3	3
4	4	4	4	1	1	1	1	3	3
4	4	4	4	1	1	1	1	3	3
4	4	4	4	1	1	1	1	3	3
4	4	1	1	1	1	1	1	3	3
4	4	4	4	1	1	1	1	3	3
4	4	1	1	1	1	1	1	3	3
4	4	4	4	1	1	1	1	3	3
4	4	1	1	1	1	1	1	3	3
4	4	4	4	1	1	1	1	3	3
4	4	4	4	1	1	1	1	3	3
4	4	4	4	1	1	1	1	3	3
4	4	4	4	1	1	1	1	3	3
4	4	1	1	1	1	1	1	3	3
4	4	4	4	1	1	1	1	3	3
4	4	4	4	1	1	1	1	3	3
4	4	4	4	1	1	1	1	3	3
4	4	1	1	1	1	1	1	3	3

For Review Only

4	4	4	4	1	1	1	1	3	3
4	4	4	4	1	1	1	1	3	3
4	4	1	1	1	1	1	1	3	3
4	4	1	1	1	1	1	1	3	3
4	4	4	4	1	1	1	1	3	3
4	4	1	1	1	1	1	1	3	3
4	4	4	4	1	1	1	1	3	3
1	1	4	4	4	4	1	1	1	1
4	4	4	4	1	1	1	1	3	3
4	4	4	4	1	1	1	1	3	3
4	4	1	1	1	1	1	1	3	3
4	4	4	4	1	1	1	1	3	3
4	4	4	4	1	1	1	1	3	3
4	4	1	1	1	1	1	1	3	3
4	4	4	4	1	1	1	1	3	3
4	4	1	1	1	1	1	1	3	3
4	4	4	4	1	1	1	1	3	3
4	4	4	4	1	1	1	1	3	3
4	4	4	4	1	1	1	1	3	3
4	4	1	1	1	1	1	1	3	3
4	4	4	4	1	1	4	4	3	3
4	4	1	1	1	1	1	1	3	3
4	4	1	1	1	1	1	1	3	3
4	4	4	4	1	1	1	1	3	3
4	4	4	4	1	1	1	1	3	3
4	4	4	4	1	1	1	1	3	3
4	4	4	4	1	1	1	1	3	3
4	4	4	4	1	1	1	1	3	3
4	4	1	1	1	1	1	1	3	3
4	4	1	1	1	1	1	1	3	3
4	4	4	4	1	1	1	1	3	3
4	4	1	1	1	1	1	1	3	3
4	4	1	1	1	1	1	1	3	3
4	4	1	1	1	1	1	1	3	3
4	4	4	4	1	1	1	1	3	3
4	4	4	4	1	1	1	1	3	3
4	4	1	1	1	1	1	1	3	3
4	4	4	4	1	1	1	1	3	3
4	4	4	4	1	1	1	1	3	3
4	4	4	4	1	1	1	1	3	3
4	4	1	1	1	1	1	1	3	3
4	4	1	1	1	1	1	1	3	3
4	4	4	4	1	1	1	1	3	3
4	4	1	1	1	1	1	1	3	3
4	4	1	1	1	1	1	1	3	3
4	4	4	4	1	1	1	1	3	3
4	4	1	1	1	1	1	1	3	3
4	4	4	4	1	1	1	1	3	3
1	1	1	1	4	4	1	1	1	1
4	4	4	4	1	1	1	1	3	3
4	4	1	1	4	4	1	1	3	3
4	4	4	4	1	1	1	1	3	3
4	4	4	4	1	1	1	1	3	3
4	4	4	4	1	1	1	1	3	3
4	4	4	4	1	1	1	1	3	3

For Review Only

4	4	1	1	1	1	1	1	3	3
4	4	4	4	1	1	1	1	3	3

For Review Only

SNP2431	SNP2431	SNP2432	SNP2432	SNP2433	SNP2433	SNP2434	SNP2434	SNP2435	SNP2435
3	3	2	2	2	2	4	4	2	2
3	3	2	2	1	1	2	2	2	2
1	1	2	2	1	1	4	4	2	2
1	1	2	2	2	2	4	4	2	2
1	1	2	2	2	2	4	4	2	2
1	1	2	2	2	2	4	4	2	2
1	1	2	2	1	1	4	4	2	2
1	1	2	2	1	1	2	2	2	2
1	1	2	2	2	2	4	4	2	2
1	1	2	2	1	1	2	2	2	2
1	1	2	2	1	1	4	4	2	2
1	1	2	2	1	1	4	4	2	2
1	1	2	2	2	2	4	4	2	2
1	1	2	2	1	1	4	4	3	3
1	1	2	2	2	2	4	4	2	2
1	1	2	2	1	1	4	4	2	2
1	1	3	3	2	2	4	4	2	2
1	1	2	2	1	1	4	4	2	2
1	1	2	2	2	2	4	4	2	2
1	1	3	3	1	1	4	4	2	2
1	1	2	2	2	2	4	4	2	2
1	1	3	3	1	1	4	4	2	2
1	1	2	2	1	1	4	4	2	2
1	1	2	2	1	1	4	4	2	2
1	1	2	2	2	2	4	4	2	2
3	3	2	2	1	1	2	2	2	2
1	1	2	2	1	1	4	4	2	2
1	1	2	2	2	2	4	4	2	2
1	1	2	2	2	2	4	4	2	2
1	1	2	2	1	1	4	4	2	2
1	1	2	2	2	2	4	4	2	2
1	1	2	2	2	2	4	4	2	2
1	1	2	2	2	2	4	4	2	2
1	1	2	2	1	1	4	4	2	2
1	1	2	2	1	1	4	4	2	2
1	1	2	2	1	1	4	4	3	3
1	1	3	3	2	2	4	4	3	3
1	1	2	2	2	2	4	4	2	2
1	1	2	2	1	1	4	4	2	2
1	1	2	2	1	1	4	4	2	2
1	1	2	2	1	1	4	4	2	2
1	1	2	2	2	2	4	4	2	2
1	1	2	2	1	1	4	4	2	2
1	1	3	3	1	1	4	4	2	2
1	1	2	2	1	1	4	4	2	2
3	3	2	2	1	1	2	2	2	2
3	3	2	2	1	1	4	4	2	2
1	1	2	2	1	1	4	4	2	2
1	1	3	3	1	1	4	4	2	2
1	1	3	3	2	2	4	4	2	2
3	3	2	2	1	1	4	4	2	2
1	1	2	2	1	1	2	2	2	2

For Review Only

1	1	3	3	2	2	4	4	2	2
1	1	2	2	2	2	4	4	2	2
1	1	2	2	1	1	2	2	2	2
1	1	3	3	1	1	4	4	2	2
1	1	2	2	2	2	4	4	2	2
3	3	2	2	1	1	2	2	2	2
3	3	2	2	1	1	2	2	2	2
1	1	2	2	1	1	4	4	2	2
1	1	2	2	1	1	4	4	3	3
1	1	2	2	1	1	4	4	2	2
3	3	2	2	1	1	4	4	2	2
3	3	2	2	1	1	4	4	2	2
1	1	3	3	2	2	4	4	2	2
1	1	2	2	2	2	4	4	2	2
1	1	2	2	2	2	4	4	2	2
1	1	2	2	1	1	4	4	2	2
3	3	2	2	1	1	4	4	2	2
3	3	2	2	2	2	4	4	3	3
1	1	3	3	2	2	4	4	3	3
3	3	2	2	2	2	4	4	2	2
3	3	2	2	1	1	4	4	2	2
1	1	2	2	1	1	4	4	2	2
1	1	2	2	2	2	4	4	2	2
1	1	2	2	1	1	2	2	3	3
1	1	2	2	1	1	4	4	2	2
1	1	2	2	1	1	2	2	2	2
1	1	3	3	1	1	2	2	2	2
1	1	2	2	1	1	4	4	2	2
3	3	2	2	1	1	2	2	2	2
1	1	2	2	2	2	4	4	2	2
1	1	2	2	2	2	4	4	2	2
1	1	2	2	1	1	4	4	2	2
1	1	2	2	1	1	4	4	2	2
1	1	3	3	2	2	4	4	2	2
1	1	2	2	2	2	4	4	2	2
1	1	2	2	1	1	4	4	2	2
1	1	2	2	1	1	4	4	2	2
1	1	2	2	1	1	4	4	2	2
1	1	3	3	1	1	4	4	2	2
1	1	2	2	1	1	4	4	2	2
1	1	3	3	2	2	4	4	2	2
1	1	3	3	1	1	4	4	2	2
3	3	2	2	1	1	4	4	2	2
3	3	2	2	1	1	4	4	2	2
1	1	2	2	2	2	4	4	3	3
3	3	2	2	2	2	4	4	2	2
1	1	2	2	1	1	4	4	2	2
3	3	2	2	1	1	2	2	2	2
1	1	2	2	1	1	4	4	2	2
1	1	3	3	2	2	4	4	3	3
1	1	2	2	2	2	4	4	3	3
1	1	2	2	1	1	4	4	2	2
1	1	2	2	1	1	4	4	2	2
1	1	2	2	2	2	4	4	2	2
1	1	3	3	2	2	4	4	3	3

For Review Only

1	1	2	2	1	1	2	2	2	2
1	1	3	3	2	2	4	4	2	2
3	3	2	2	1	1	2	2	2	2
1	1	2	2	1	1	4	4	2	2
1	1	2	2	1	1	2	2	2	2
1	1	2	2	1	1	2	2	2	2
1	1	2	2	1	1	4	4	2	2
1	1	2	2	1	1	4	4	2	2
1	1	2	2	1	1	4	4	2	2
1	1	2	2	1	1	4	4	2	2
1	1	2	2	2	2	4	4	2	2
3	3	2	2	2	2	2	2	2	2
1	1	2	2	1	1	4	4	2	2
1	1	2	2	1	1	2	2	2	2
1	1	2	2	2	2	2	2	2	2
3	3	2	2	1	1	2	2	2	2
3	3	2	2	2	2	4	4	3	3
3	3	2	2	1	1	2	2	2	2
1	1	2	2	1	1	4	4	3	3
1	1	2	2	1	1	4	4	2	2
1	1	2	2	2	2	2	2	2	2
1	1	2	2	2	2	4	4	2	2
3	3	2	2	1	1	2	2	2	2
1	1	2	2	1	1	4	4	3	3
1	1	2	2	1	1	2	2	2	2
1	1	3	3	1	1	4	4	3	3
1	1	3	3	1	1	4	4	2	2
1	1	3	3	2	2	4	4	3	3
1	1	2	2	2	2	4	4	2	2
1	1	2	2	2	2	4	4	2	2
1	1	2	2	1	1	4	4	2	2
1	1	2	2	2	2	4	4	2	2
1	1	3	3	2	2	4	4	2	2
3	3	2	2	2	2	4	4	2	2
1	1	2	2	1	1	4	4	2	2
1	1	2	2	2	2	4	4	2	2
1	1	2	2	1	1	4	4	3	3
3	3	2	2	1	1	2	2	3	3
1	1	2	2	1	1	2	2	2	2
1	1	2	2	1	1	4	4	2	2
1	1	2	2	2	2	4	4	3	3
1	1	2	2	1	1	4	4	2	2
3	3	2	2	2	2	4	4	3	3
1	1	2	2	1	1	4	4	2	2
1	1	2	2	1	1	2	2	2	2
3	3	3	3	2	2	4	4	2	2
3	3	2	2	1	1	2	2	2	2
3	3	2	2	1	1	2	2	2	2
1	1	2	2	2	2	4	4	2	2
3	3	2	2	1	1	2	2	2	2
3	3	2	2	1	1	2	2	2	2
3	3	2	2	1	1	4	4	2	2
1	1	2	2	2	2	4	4	2	2
3	3	2	2	1	1	2	2	2	2
1	1	2	2	2	2	4	4	2	2

For Review Only

1	1	2	2	2	2	4	4	2	2
1	1	2	2	1	1	4	4	3	3
1	1	2	2	1	1	4	4	2	2
1	1	2	2	2	2	4	4	2	2
3	3	2	2	2	2	4	4	2	2
3	3	2	2	2	2	4	4	2	2
1	1	2	2	1	1	4	4	2	2
1	1	2	2	1	1	2	2	2	2
1	1	2	2	1	1	4	4	2	2
1	1	3	3	2	2	4	4	2	2
1	1	2	2	1	1	2	2	2	2
1	1	2	2	1	1	4	4	2	2
3	3	2	2	2	2	4	4	2	2
3	3	2	2	2	2	4	4	2	2
1	1	2	2	1	1	4	4	2	2
1	1	2	2	1	1	4	4	2	2
1	1	2	2	2	2	4	4	2	2
1	1	3	3	1	1	2	2	2	2
3	3	2	2	1	1	4	4	2	2
1	1	2	2	1	1	4	4	2	2
1	1	2	2	2	2	4	4	2	2
1	1	2	2	1	1	4	4	2	2
1	1	3	3	1	1	4	4	3	3
3	3	2	2	1	1	4	4	2	2
1	1	3	3	2	2	4	4	2	2
1	1	2	2	1	1	4	4	2	2
1	1	2	2	2	2	4	4	2	2
1	1	2	2	2	2	4	4	2	2
1	1	3	3	2	2	4	4	3	3
1	1	2	2	2	2	4	4	2	2
1	1	2	2	1	1	4	4	2	2
3	3	2	2	2	2	4	4	2	2
1	1	3	3	1	1	4	4	2	2
1	1	2	2	1	1	4	4	2	2
3	3	2	2	1	1	4	4	3	3
3	3	2	2	1	1	4	4	2	2
1	1	2	2	1	1	2	2	2	2
1	1	3	3	2	2	4	4	3	3
3	3	2	2	1	1	4	4	2	2
1	1	3	3	1	1	4	4	2	2
1	1	2	2	1	1	4	4	2	2
3	3	2	2	1	1	2	2	2	2
1	1	2	2	1	1	4	4	2	2
1	1	2	2	2	2	4	4	2	2
1	1	2	2	1	1	4	4	3	3
3	3	2	2	2	2	4	4	2	2
1	1	2	2	1	1	4	4	2	2
1	1	2	2	1	1	4	4	2	2
1	1	2	2	1	1	4	4	2	2
1	1	2	2	1	1	4	4	2	2
1	1	2	2	2	2	4	4	2	2
1	1	2	2	1	1	4	4	2	2
1	1	3	3	1	1	4	4	3	3
3	3	2	2	1	1	4	4	2	2
1	1	2	2	1	1	4	4	2	2

For Review Only

1	1	2	2	1	1	4	4	2	2
1	1	2	2	1	1	4	4	2	2
1	1	2	2	2	2	4	4	2	2
1	1	2	2	1	1	4	4	3	3
1	1	2	2	1	1	4	4	2	2
1	1	3	3	2	2	2	2	2	2
3	3	2	2	1	1	4	4	2	2
1	1	2	2	1	1	4	4	2	2
1	1	2	2	1	1	4	4	2	2
1	1	2	2	1	1	4	4	2	2
1	1	2	2	2	2	4	4	2	2
1	1	2	2	1	1	4	4	2	2
1	1	3	3	1	1	4	4	2	2
3	3	2	2	1	1	2	2	2	2
1	1	2	2	1	1	4	4	2	2
1	1	3	3	1	1	2	2	2	2
1	1	2	2	1	1	4	4	2	2
1	1	3	3	1	1	4	4	2	2
1	1	2	2	1	1	4	4	2	2
1	1	2	2	2	2	4	4	2	2
1	1	2	2	1	1	4	4	2	2
3	3	2	2	1	1	2	2	2	2
1	1	3	3	2	2	4	4	2	2
1	1	2	2	1	1	4	4	3	3
1	1	2	2	1	1	4	4	2	2
1	1	2	2	1	1	2	2	2	2
1	1	2	2	1	1	4	4	2	2
1	1	2	2	1	1	4	4	2	2
1	1	2	2	2	2	4	4	2	2
1	1	2	2	2	2	4	4	2	2
1	1	2	2	1	1	4	4	2	2
1	1	2	2	1	1	2	2	2	2
3	3	2	2	2	2	4	4	2	2
3	3	2	2	1	1	2	2	2	2
1	1	2	2	1	1	4	4	3	3
1	1	2	2	1	1	4	4	2	2
1	1	3	3	2	2	4	4	2	2
3	3	2	2	1	1	4	4	2	2
1	1	2	2	1	1	4	4	3	3
1	1	2	2	1	1	4	4	2	2
3	3	2	2	1	1	4	4	3	3
1	1	3	3	2	2	4	4	2	2
1	1	2	2	1	1	4	4	3	3
1	1	3	3	2	2	4	4	2	2
1	1	2	2	1	1	2	2	2	2
1	1	3	3	1	1	4	4	2	2
1	1	2	2	1	1	2	2	2	2
3	3	2	2	1	1	4	4	3	3
3	3	2	2	1	1	2	2	2	2
1	1	2	2	1	1	4	4	2	2
1	1	2	2	2	2	4	4	2	2
1	1	2	2	1	1	4	4	2	2
1	1	2	2	1	1	4	4	2	2
1	1	3	3	1	1	4	4	2	2
1	1	2	2	1	1	4	4	3	3

For Review Only

1	1	2	2	1	1	2	2	2	2
3	3	2	2	1	1	4	4	2	2

For Review Only

SNP2436	SNP2436	SNP2437	SNP2437	SNP2438	SNP2438	SNP2439	SNP2439	SNP244	SNP244
1	1	4	4	2	2	1	1	2	2
1	1	4	4	2	2	3	3	2	2
1	1	4	4	2	2	3	3	2	2
1	1	4	4	2	2	3	3	2	2
1	1	4	4	2	2	1	1	2	2
3	3	1	1	2	2	1	1	2	2
3	3	1	1	2	2	3	3	2	2
1	1	4	4	2	2	3	3	2	2
3	3	4	4	2	2	1	1	2	2
1	1	4	4	2	2	3	3	2	2
3	3	1	1	2	2	3	3	2	2
1	1	4	4	2	2	3	3	2	2
1	1	4	4	2	2	1	1	2	2
1	1	4	4	2	2	3	3	2	2
1	1	4	4	2	2	1	1	2	2
1	1	4	4	2	2	3	3	2	2
3	3	1	1	2	2	1	1	2	2
1	1	4	4	2	2	3	3	2	2
3	3	1	1	2	2	1	1	2	2
1	1	4	4	2	2	3	3	2	2
1	1	4	4	2	2	1	1	2	2
1	1	4	4	2	2	3	3	2	2
1	1	4	4	2	2	3	3	1	1
1	1	4	4	2	2	3	3	2	2
3	3	1	1	2	2	1	1	2	2
1	1	4	4	2	2	3	3	2	2
1	1	4	4	2	2	3	3	2	2
1	1	4	4	2	2	3	3	2	2
3	3	1	1	2	2	1	1	2	2
1	1	4	4	2	2	3	3	2	2
1	1	1	1	2	2	1	1	2	2
1	1	4	4	2	2	3	3	2	2
1	1	4	4	2	2	1	1	2	2
1	1	4	4	2	2	3	3	2	2
1	1	4	4	2	2	3	3	2	2
1	1	4	4	2	2	3	3	2	2
1	1	4	4	2	2	1	1	2	2
1	1	4	4	2	2	3	3	2	2
1	1	4	4	3	3	3	3	1	1
3	3	1	1	2	2	3	3	2	2
1	1	4	4	2	2	3	3	2	2
3	3	1	1	2	2	1	1	2	2
1	1	4	4	2	2	3	3	2	2
1	1	4	4	2	2	3	3	2	2
1	1	4	4	2	2	3	3	2	2
1	1	4	4	2	2	3	3	2	2
1	1	4	4	2	2	3	3	2	2
1	1	4	4	2	2	3	3	2	2
1	1	4	4	2	2	3	3	2	2
1	1	4	4	2	2	1	1	2	2
1	1	4	4	2	2	1	1	2	2
1	1	4	4	2	2	3	3	2	2

For Review Only

3	3	1	1	2	2	1	1	2	2
1	1	4	4	2	2	1	1	2	2
1	1	4	4	2	2	3	3	2	2
1	1	4	4	2	2	3	3	2	2
3	3	1	1	2	2	1	1	2	2
1	1	4	4	2	2	3	3	2	2
1	1	4	4	2	2	3	3	2	2
1	1	4	4	3	3	3	3	2	2
1	1	4	4	2	2	3	3	2	2
1	1	4	4	2	2	3	3	2	2
1	1	4	4	2	2	3	3	2	2
1	1	4	4	2	2	3	3	2	2
3	3	1	1	2	2	1	1	2	2
3	3	1	1	2	2	1	1	2	2
1	1	4	4	2	2	3	3	2	2
1	1	4	4	2	2	3	3	2	2
3	3	4	4	2	2	3	3	1	1
1	1	4	4	2	2	3	3	2	2
1	1	4	4	2	2	1	1	2	2
3	3	1	1	2	2	1	1	2	2
1	1	4	4	2	2	3	3	2	2
1	1	4	4	2	2	3	3	2	2
1	1	4	4	2	2	1	1	2	2
1	1	4	4	2	2	3	3	2	2
1	1	4	4	2	2	3	3	2	2
3	3	1	1	2	2	3	3	2	2
1	1	4	4	2	2	3	3	2	2
1	1	4	4	2	2	3	3	1	1
1	1	4	4	2	2	3	3	2	2
1	1	4	4	2	2	3	3	2	2
1	1	4	4	2	2	1	1	2	2
1	1	4	4	2	2	3	3	2	2
1	1	4	4	3	3	3	3	1	1
3	3	1	1	2	2	1	1	2	2
1	1	4	4	2	2	1	1	2	2
1	1	4	4	2	2	3	3	2	2
1	1	4	4	3	3	3	3	2	2
1	1	4	4	2	2	3	3	2	2
3	3	1	1	2	2	1	1	2	2
1	1	4	4	2	2	3	3	2	2
1	1	4	4	2	2	1	1	2	2
1	1	4	4	2	2	3	3	2	2
1	1	4	4	2	2	1	1	2	2
1	1	4	4	2	2	3	3	2	2
1	1	4	4	2	2	3	3	2	2
1	1	4	4	2	2	1	1	2	2
1	1	4	4	2	2	3	3	2	2
1	1	4	4	2	2	3	3	2	2
1	1	4	4	2	2	3	3	2	2
1	1	4	4	2	2	3	3	2	2
1	1	4	4	2	2	3	3	2	2
1	1	4	4	3	3	3	3	2	2
1	1	4	4	2	2	3	3	2	2
1	1	4	4	2	2	3	3	1	1
1	1	4	4	2	2	1	1	2	2

For Review Only

1	1	4	4	2	2	3	3	2	2
3	3	1	1	2	2	3	3	2	2
1	1	1	1	2	2	3	3	2	2
1	1	4	4	2	2	3	3	2	2
1	1	4	4	2	2	3	3	2	2
1	1	4	4	2	2	3	3	2	2
1	1	4	4	2	2	3	3	2	2
1	1	4	4	2	2	3	3	2	2
1	1	4	4	3	3	3	3	2	2
1	1	4	4	2	2	3	3	2	2
3	3	4	4	2	2	3	3	2	2
1	1	4	4	2	2	3	3	2	2
1	1	4	4	2	2	1	1	2	2
1	1	4	4	2	2	3	3	2	2
1	1	4	4	2	2	3	3	2	2
1	1	4	4	2	2	3	3	2	2
1	1	4	4	2	2	3	3	2	2
1	1	4	4	2	2	3	3	2	2
1	1	4	4	2	2	3	3	2	2
1	1	4	4	2	2	1	1	1	1
1	1	4	4	2	2	3	3	2	2
1	1	4	4	2	2	3	3	2	2
1	1	4	4	2	2	3	3	2	2
1	1	4	4	2	2	3	3	1	1
1	1	4	4	2	2	3	3	2	2
1	1	4	4	2	2	3	3	2	2
1	1	4	4	2	2	3	3	2	2
1	1	4	4	2	2	1	1	2	2
1	1	4	4	2	2	1	1	2	2
1	1	4	4	2	2	3	3	2	2
1	1	4	4	2	2	3	3	2	2
1	1	4	4	2	2	3	3	2	2
1	1	4	4	2	2	1	1	2	2
1	1	4	4	2	2	3	3	2	2
1	1	4	4	2	2	3	3	2	2
1	1	4	4	2	2	3	3	2	2
1	1	4	4	2	2	3	3	2	2
1	1	4	4	2	2	3	3	2	2
1	1	4	4	2	2	3	3	2	2
1	1	4	4	2	2	3	3	1	1
1	1	4	4	2	2	3	3	2	2
1	1	4	4	2	2	3	3	2	2
1	1	4	4	2	2	3	3	2	2
1	1	4	4	2	2	3	3	2	2
1	1	4	4	2	2	3	3	2	2
1	1	4	4	2	2	1	1	2	2
1	1	4	4	2	2	3	3	2	2
1	1	4	4	2	2	3	3	2	2
1	1	4	4	2	2	3	3	2	2
1	1	4	4	2	2	3	3	2	2
1	1	4	4	2	2	3	3	2	2
1	1	4	4	2	2	3	3	2	2
1	1	4	4	2	2	3	3	2	2
1	1	4	4	2	2	3	3	2	2
1	1	4	4	2	2	3	3	2	2

For Review Only

1	1	4	4	2	2	3	3	2	2
1	1	4	4	2	2	1	1	2	2
1	1	4	4	2	2	3	3	2	2
1	1	4	4	2	2	3	3	2	2
3	3	1	1	2	2	1	1	2	2
1	1	4	4	2	2	1	1	1	1
1	1	4	4	2	2	3	3	2	2
1	1	4	4	2	2	3	3	2	2
1	1	4	4	3	3	3	3	2	2
1	1	4	4	2	2	1	1	2	2
1	1	4	4	2	2	3	3	2	2
1	1	4	4	2	2	3	3	2	2
1	1	4	4	2	2	3	3	2	2
1	1	4	4	2	2	1	1	2	2
1	1	4	4	2	2	3	3	2	2
3	3	1	1	2	2	3	3	2	2
1	1	4	4	2	2	3	3	2	2
1	1	4	4	2	2	3	3	2	2
1	1	1	1	2	2	3	3	2	2
1	1	4	4	2	2	3	3	2	2
1	1	4	4	2	2	3	3	2	2
1	1	4	4	2	2	3	3	2	2
1	1	4	4	2	2	3	3	2	2
1	1	4	4	2	2	3	3	2	2
3	3	1	1	2	2	1	1	2	2
1	1	4	4	2	2	3	3	2	2
1	1	4	4	2	2	3	3	2	2
1	1	4	4	2	2	3	3	2	2
1	1	4	4	2	2	1	1	2	2
1	1	4	4	2	2	1	1	2	2
1	1	4	4	2	2	3	3	2	2
1	1	4	4	2	2	3	3	2	2
1	1	4	4	2	2	3	3	2	2
1	1	4	4	2	2	3	3	2	2
1	1	4	4	2	2	3	3	2	2
1	1	4	4	2	2	3	3	2	2
1	1	4	4	2	2	3	3	2	2
1	1	4	4	2	2	1	1	2	2
1	1	4	4	2	2	3	3	2	2
1	1	4	4	2	2	1	1	2	2
1	1	4	4	2	2	3	3	2	2
1	1	4	4	2	2	3	3	2	2
1	1	4	4	2	2	3	3	2	2
1	1	4	4	2	2	3	3	2	2
1	1	4	4	2	2	3	3	2	2
1	1	4	4	2	2	1	1	2	2
3	3	1	1	2	2	3	3	2	2
1	1	4	4	2	2	3	3	2	2
1	1	4	4	2	2	3	3	2	2
1	1	4	4	2	2	3	3	2	2
1	1	4	4	2	2	1	1	2	2
1	1	4	4	2	2	3	3	2	2
1	1	4	4	2	2	3	3	1	1
1	1	4	4	2	2	3	3	2	2
1	1	4	4	3	3	3	3	2	2

For Review Only

1	1	4	4	2	2	3	3	2	2
1	1	4	4	2	2	3	3	2	2
1	1	4	4	2	2	3	3	2	2
1	1	4	4	2	2	3	3	2	2
1	1	4	4	2	2	3	3	2	2
1	1	4	4	2	2	3	3	2	2
1	1	4	4	2	2	3	3	2	2
1	1	4	4	2	2	3	3	2	2
1	1	4	4	2	2	3	3	2	2
1	1	4	4	3	3	3	3	1	1
1	1	4	4	2	2	3	3	2	2
1	1	4	4	2	2	3	3	2	2
3	3	1	1	2	2	1	1	2	2
1	1	1	1	2	2	3	3	2	2
1	1	4	4	2	2	3	3	2	2
3	3	1	1	2	2	3	3	2	2
1	1	4	4	3	3	3	3	2	2
1	1	4	4	2	2	3	3	1	1
1	1	4	4	2	2	3	3	2	2
1	1	4	4	2	2	1	1	2	2
1	1	4	4	2	2	3	3	2	2
1	1	4	4	2	2	3	3	2	2
1	1	4	4	2	2	1	1	2	2
1	1	4	4	2	2	3	3	2	2
1	1	4	4	3	3	3	3	2	2
1	1	4	4	2	2	3	3	2	2
1	1	4	4	3	3	3	3	1	1
3	3	1	1	2	2	3	3	2	2
1	1	4	4	2	2	1	1	2	2
1	1	4	4	2	2	1	1	2	2
1	1	4	4	3	3	3	3	1	1
1	1	4	4	2	2	3	3	2	2
3	3	1	1	2	2	1	1	2	2
1	1	4	4	2	2	3	3	2	2
1	1	4	4	2	2	3	3	2	2
1	1	4	4	2	2	3	3	1	1
1	1	4	4	2	2	1	1	2	2
1	1	4	4	2	2	3	3	2	2
1	1	4	4	2	2	3	3	2	2
1	1	4	4	3	3	3	3	1	1
1	1	4	4	2	2	3	3	2	2
1	1	4	4	2	2	1	1	2	2
1	1	4	4	2	2	3	3	1	1
1	1	4	4	2	2	1	1	2	2
1	1	1	1	2	2	3	3	2	2
3	3	1	1	2	2	1	1	2	2
1	1	4	4	3	3	3	3	2	2
1	1	4	4	2	2	1	1	2	2
1	1	4	4	2	2	3	3	2	2
1	1	4	4	3	3	3	3	1	1
3	3	1	1	2	2	1	1	2	2
1	1	4	4	3	3	3	3	1	1
1	1	4	4	2	2	3	3	2	2
3	3	1	1	2	2	1	1	2	2
1	1	4	4	2	2	3	3	2	2

For Review Only

3	3	1	1	2	2	3	3	2	2
3	3	1	1	2	2	3	3	2	2

For Review Only

SNP2440	SNP2440	SNP2441	SNP2441	SNP2442	SNP2442	SNP2443	SNP2443	SNP2444	SNP2444
2	2	4	4	2	2	4	4	3	3
2	2	4	4	2	2	4	4	3	3
2	2	4	4	2	2	4	4	3	3
2	2	4	4	2	2	4	4	3	3
2	2	4	4	3	3	2	2	1	1
2	2	4	4	2	2	4	4	3	3
2	2	4	4	2	2	4	4	3	3
2	2	4	4	2	2	4	4	1	1
2	2	4	4	2	2	4	4	3	3
2	2	4	4	3	3	2	2	1	1
2	2	4	4	2	2	4	4	3	3
2	2	4	4	2	2	4	4	3	3
2	2	4	4	2	2	4	4	3	3
2	2	4	4	2	2	4	4	3	3
2	2	4	4	2	2	4	4	3	3
2	2	4	4	2	2	4	4	1	1
2	2	2	2	2	2	4	4	3	3
2	2	4	4	3	3	2	2	1	1
2	2	4	4	2	2	4	4	3	3
2	2	2	2	2	2	4	4	3	3
2	2	4	4	2	2	4	4	3	3
2	2	2	2	2	2	4	4	3	3
2	2	4	4	2	2	4	4	3	3
2	2	4	4	3	3	4	4	3	3
2	2	4	4	2	2	4	4	3	3
2	2	4	4	2	2	4	4	3	3
2	2	4	4	2	2	4	4	3	3
2	2	4	4	2	2	4	4	3	3
2	2	4	4	3	3	2	2	3	3
2	2	4	4	3	3	2	2	3	3
2	2	4	4	2	2	4	4	3	3
2	2	4	4	2	2	4	4	3	3
2	2	4	4	2	2	4	4	3	3
2	2	4	4	2	2	4	4	3	3
2	2	4	4	2	2	4	4	3	3
2	2	4	4	2	2	4	4	3	3
2	2	2	2	2	2	4	4	3	3
2	2	4	4	2	2	4	4	1	1
3	3	4	4	3	3	4	4	1	1
2	2	4	4	3	3	4	4	3	3
2	2	4	4	3	3	2	2	1	1
2	2	4	4	3	3	2	2	3	3
2	2	4	4	3	3	2	2	1	1
2	2	2	2	2	2	4	4	3	3
2	2	4	4	3	3	2	2	1	1
2	2	4	4	2	2	4	4	3	3
2	2	4	4	2	2	4	4	3	3
2	2	4	4	2	2	4	4	3	3
2	2	2	2	2	2	4	4	3	3
2	2	4	4	2	2	4	4	3	3
2	2	4	4	2	2	4	4	3	3
2	2	4	4	2	2	4	4	3	3

For Review Only

2	2	2	2	2	2	4	4	3	3
2	2	4	4	2	2	4	4	3	3
2	2	4	4	2	2	4	4	3	3
2	2	2	2	2	2	4	4	3	3
2	2	4	4	2	2	4	4	3	3
2	2	4	4	2	2	4	4	3	3
2	2	4	4	2	2	4	4	3	3
3	3	4	4	2	2	4	4	3	3
2	2	2	2	2	2	4	4	3	3
2	2	4	4	2	2	4	4	3	3
2	2	4	4	2	2	4	4	3	3
2	2	4	4	2	2	4	4	3	3
2	2	2	2	2	2	4	4	3	3
2	2	4	4	3	3	2	2	3	3
2	2	4	4	2	2	4	4	3	3
2	2	4	4	3	3	2	2	3	3
2	2	4	4	2	2	4	4	3	3
2	2	4	4	2	2	4	4	3	3
2	2	2	2	2	2	4	4	3	3
2	2	4	4	2	2	4	4	3	3
2	2	4	4	2	2	4	4	3	3
2	2	4	4	2	2	4	4	3	3
2	2	4	4	2	2	4	4	3	3
2	2	4	4	3	3	2	2	3	3
2	2	4	4	3	3	2	2	1	1
2	2	4	4	2	2	4	4	3	3
2	2	2	2	2	2	4	4	1	1
2	2	4	4	2	2	4	4	3	3
2	2	4	4	2	2	4	4	3	3
2	2	4	4	2	2	4	4	3	3
2	2	4	4	3	3	2	2	1	1
2	2	4	4	2	2	4	4	3	3
3	3	4	4	2	2	4	4	3	3
2	2	2	2	2	2	4	4	3	3
2	2	4	4	2	2	4	4	3	3
2	2	4	4	3	3	2	2	1	1
2	2	4	4	3	3	2	2	3	3
2	2	4	4	2	2	4	4	3	3
2	2	2	2	3	3	2	2	3	3
2	2	4	4	2	2	4	4	1	1
2	2	2	2	2	2	4	4	3	3
2	2	2	2	2	2	4	4	3	3
2	2	4	4	2	2	4	4	3	3
2	2	4	4	2	2	4	4	3	3
2	2	2	2	2	2	4	4	3	3
2	2	4	4	2	2	4	4	3	3
2	2	4	4	2	2	4	4	3	3
2	2	4	4	2	2	4	4	3	3
2	2	4	4	2	2	4	4	3	3
2	2	2	2	2	2	4	4	3	3
2	2	4	4	2	2	4	4	3	3
3	3	4	4	3	3	4	4	1	1
2	2	4	4	2	2	4	4	3	3
2	2	2	2	2	2	4	4	3	3
2	2	2	2	2	2	4	4	1	1

For Review Only

2	2	4	4	2	2	4	4	3	3
2	2	2	2	3	3	4	4	3	3
2	2	4	4	2	2	4	4	3	3
2	2	4	4	2	2	4	4	3	3
2	2	4	4	2	2	4	4	3	3
2	2	4	4	2	2	4	4	3	3
2	2	4	4	2	2	4	4	3	3
2	2	4	4	2	2	4	4	3	3
3	3	4	4	2	2	4	4	1	1
2	2	4	4	2	2	4	4	3	3
2	2	4	4	2	2	4	4	1	1
2	2	4	4	2	2	4	4	3	3
2	2	4	4	2	2	4	4	3	3
2	2	4	4	2	2	4	4	3	3
2	2	4	4	2	2	4	4	3	3
2	2	4	4	2	2	4	4	3	3
2	2	4	4	2	2	4	4	3	3
2	2	4	4	2	2	4	4	3	3
2	2	4	4	2	2	4	4	3	3
2	2	4	4	2	2	4	4	3	3
2	2	4	4	2	2	4	4	3	3
2	2	4	4	2	2	4	4	3	3
2	2	4	4	2	2	4	4	3	3
2	2	4	4	2	2	4	4	3	3
2	2	4	4	2	2	4	4	3	3
2	2	2	2	2	2	4	4	3	3
2	2	2	2	2	2	4	4	3	3
2	2	4	4	2	2	4	4	3	3
2	2	4	4	2	2	4	4	3	3
2	2	4	4	2	2	4	4	3	3
3	3	4	4	2	2	4	4	3	3
2	2	4	4	2	2	4	4	3	3
2	2	2	2	2	2	4	4	3	3
2	2	4	4	2	2	4	4	3	3
2	2	4	4	2	2	4	4	3	3
2	2	4	4	2	2	4	4	3	3
2	2	4	4	2	2	4	4	3	3
2	2	4	4	2	2	4	4	3	3
2	2	4	4	2	2	4	4	3	3
2	2	4	4	2	2	4	4	1	1
2	2	2	2	2	2	4	4	3	3
2	2	4	4	2	2	4	4	3	3
2	2	4	4	2	2	4	4	3	3
2	2	4	4	2	2	4	4	3	3
2	2	4	4	2	2	4	4	3	3
2	2	2	2	2	2	4	4	3	3
2	2	4	4	2	2	4	4	3	3
2	2	4	4	2	2	4	4	3	3
2	2	4	4	2	2	4	4	3	3
2	2	4	4	2	2	4	4	3	3
2	2	4	4	2	2	4	4	3	3
2	2	4	4	2	2	4	4	3	3
2	2	4	4	2	2	4	4	3	3
2	2	4	4	2	2	4	4	3	3
3	3	4	4	2	2	4	4	3	3

For Review Only

2	2	4	4	2	2	4	4	3	3
2	2	4	4	2	2	4	4	3	3
2	2	4	4	3	3	2	2	1	1
2	2	4	4	3	3	4	4	3	3
2	2	4	4	2	2	4	4	3	3
2	2	4	4	3	3	4	4	3	3
2	2	4	4	2	2	4	4	3	3
2	2	4	4	2	2	4	4	3	3
2	2	4	4	2	2	4	4	3	3
2	2	2	2	2	2	4	4	3	3
2	2	4	4	2	2	4	4	3	3
2	2	4	4	2	2	4	4	3	3
2	2	4	4	2	2	4	4	3	3
2	2	4	4	2	2	4	4	3	3
2	2	4	4	2	2	4	4	3	3
2	2	4	4	2	2	4	4	1	1
2	2	4	4	2	2	4	4	3	3
2	2	2	2	2	2	4	4	3	3
2	2	4	4	2	2	4	4	3	3
2	2	4	4	2	2	4	4	3	3
2	2	4	4	2	2	4	4	3	3
2	2	4	4	2	2	4	4	3	3
2	2	2	2	2	2	4	4	3	3
2	2	4	4	2	2	4	4	3	3
2	2	2	2	2	2	4	4	3	3
2	2	4	4	2	2	4	4	3	3
2	2	4	4	2	2	4	4	3	3
2	2	4	4	2	2	4	4	3	3
2	2	2	2	2	2	4	4	3	3
2	2	4	4	2	2	4	4	3	3
2	2	4	4	2	2	4	4	1	1
2	2	4	4	2	2	4	4	3	3
2	2	2	2	2	2	4	4	3	3
2	2	4	4	2	2	4	4	3	3
2	2	4	4	2	2	4	4	3	3
2	2	4	4	2	2	4	4	3	3
2	2	4	4	2	2	4	4	3	3
2	2	2	2	2	2	4	4	3	3
2	2	4	4	2	2	4	4	3	3
2	2	2	2	2	2	4	4	3	3
2	2	4	4	2	2	4	4	3	3
2	2	4	4	2	2	4	4	3	3
2	2	4	4	2	2	4	4	3	3
2	2	4	4	2	2	4	4	3	3
2	2	4	4	2	2	4	4	3	3
2	2	4	4	2	2	4	4	3	3
2	2	4	4	2	2	4	4	1	1
2	2	4	4	2	2	4	4	3	3
2	2	4	4	2	2	4	4	3	3
2	2	4	4	2	2	4	4	3	3
2	2	4	4	2	2	4	4	3	3
2	2	4	4	2	2	4	4	3	3
2	2	2	2	2	2	4	4	3	3
2	2	4	4	2	2	4	4	3	3
3	3	4	4	3	3	4	4	3	3

For Review Only

2	2	4	4	2	2	4	4	3	3
2	2	4	4	2	2	4	4	3	3
2	2	4	4	2	2	4	4	3	3
2	2	4	4	2	2	4	4	3	3
2	2	4	4	2	2	4	4	3	3
2	2	4	4	2	2	4	4	3	3
2	2	4	4	2	2	4	4	3	3
2	2	4	4	3	3	2	2	1	1
2	2	4	4	2	2	4	4	3	3
3	3	4	4	2	2	4	4	3	3
2	2	4	4	2	2	4	4	3	3
2	2	4	4	2	2	4	4	1	1
2	2	2	2	3	3	2	2	3	3
2	2	4	4	2	2	4	4	3	3
2	2	4	4	2	2	4	4	3	3
2	2	2	2	2	2	4	4	3	3
3	3	4	4	2	2	4	4	1	1
2	2	2	2	2	2	4	4	3	3
2	2	4	4	2	2	4	4	3	3
2	2	4	4	2	2	4	4	1	1
2	2	4	4	3	3	4	4	3	3
2	2	4	4	2	2	4	4	3	3
2	2	2	2	2	2	4	4	3	3
2	2	4	4	3	3	4	4	3	3
3	3	4	4	2	2	4	4	3	3
2	2	4	4	2	2	4	4	3	3
3	3	4	4	2	2	4	4	3	3
2	2	4	4	2	2	4	4	3	3
2	2	4	4	2	2	4	4	3	3
2	2	4	4	2	2	4	4	3	3
3	3	4	4	2	2	4	4	3	3
2	2	4	4	2	2	4	4	3	3
2	2	4	4	2	2	4	4	1	1
2	2	4	4	2	2	4	4	3	3
2	2	4	4	2	2	4	4	3	3
2	2	4	4	2	2	4	4	3	3
2	2	2	2	2	2	4	4	3	3
2	2	4	4	2	2	4	4	3	3
2	2	4	4	2	2	4	4	3	3
3	3	4	4	3	3	4	4	1	1
2	2	4	4	2	2	4	4	3	3
2	2	2	2	2	2	4	4	3	3
2	2	4	4	2	2	4	4	3	3
2	2	2	2	2	2	4	4	3	3
2	2	4	4	2	2	4	4	3	3
2	2	2	2	2	2	4	4	3	3
3	3	4	4	2	2	4	4	3	3
2	2	4	4	2	2	4	4	1	1
2	2	4	4	3	3	2	2	1	1
3	3	4	4	2	2	4	4	3	3
2	2	4	4	2	2	4	4	3	3
3	3	4	4	3	3	4	4	1	1
2	2	4	4	2	2	4	4	3	3
2	2	2	2	2	2	4	4	3	3
2	2	4	4	3	3	4	4	3	3

For Review Only

2	2	2	2	2	2	4	4	3	3
2	2	4	4	2	2	4	4	3	3

For Review Only

SNP2445	SNP2445	SNP2446	SNP2446	SNP2447	SNP2447	SNP2448	SNP2448	SNP2449	SNP2449
1	1	2	2	1	1	3	3	1	1
1	1	2	2	2	2	2	2	4	4
1	1	2	2	1	1	3	3	1	1
1	1	2	2	1	1	3	3	4	4
4	4	2	2	1	1	2	2	4	4
1	1	2	2	1	1	2	2	4	4
1	1	2	2	1	1	2	2	4	4
4	4	2	2	1	1	3	3	1	1
1	1	2	2	1	1	2	2	4	4
4	4	4	4	1	1	3	3	1	1
1	1	2	2	1	1	2	2	4	4
1	1	2	2	1	1	2	2	1	1
1	1	2	2	2	2	2	2	4	4
1	1	2	2	1	1	3	3	1	1
1	1	2	2	2	2	3	3	1	1
4	4	2	2	1	1	3	3	1	1
1	1	2	2	2	2	2	2	4	4
4	4	2	2	1	1	2	2	4	4
1	1	2	2	1	1	2	2	4	4
1	1	2	2	2	2	3	3	1	1
1	1	2	2	1	1	2	2	4	4
1	1	2	2	2	2	2	2	4	4
1	1	4	4	1	1	3	3	1	1
1	1	2	2	1	1	3	3	1	1
1	1	2	2	1	1	2	2	4	4
1	1	2	2	2	2	2	2	4	4
1	1	2	2	2	2	2	2	4	4
1	1	2	2	2	2	2	2	4	4
1	1	2	2	1	1	2	2	4	4
1	1	2	2	1	1	3	3	1	1
1	1	2	2	2	2	2	2	4	4
1	1	4	4	1	1	2	2	4	4
1	1	2	2	1	1	2	2	4	4
1	1	2	2	1	1	3	3	1	1
1	1	2	2	1	1	2	2	4	4
1	1	2	2	2	2	2	2	4	4
1	1	2	2	2	2	3	3	1	1
4	4	2	2	1	1	3	3	1	1
4	4	4	4	1	1	3	3	1	1
1	1	2	2	1	1	2	2	4	4
4	4	4	4	1	1	3	3	1	1
1	1	2	2	1	1	2	2	4	4
4	4	2	2	1	1	3	3	1	1
1	1	4	4	2	2	2	2	4	4
4	4	2	2	1	1	2	2	4	4
1	1	2	2	2	2	2	2	4	4
1	1	2	2	2	2	2	2	4	4
1	1	2	2	1	1	2	2	4	4
1	1	2	2	2	2	2	2	4	4
1	1	2	2	2	2	2	2	4	4
1	1	2	2	2	2	2	2	4	4
1	1	2	2	1	1	2	2	4	4

For Review Only

1	1	2	2	2	2	2	2	4	4
1	1	2	2	1	1	2	2	4	4
1	1	2	2	1	1	2	2	4	4
1	1	2	2	2	2	3	3	1	1
1	1	2	2	1	1	2	2	4	4
1	1	2	2	2	2	2	2	4	4
1	1	2	2	2	2	2	2	4	4
1	1	4	4	1	1	3	3	1	1
1	1	4	4	2	2	3	3	1	1
1	1	2	2	2	2	2	2	4	4
1	1	2	2	2	2	3	3	1	1
1	1	2	2	2	2	3	3	1	1
1	1	2	2	2	2	2	2	4	4
1	1	2	2	1	1	2	2	4	4
1	1	4	4	2	2	2	2	4	4
1	1	4	4	1	1	3	3	1	1
1	1	4	4	2	2	2	2	4	4
1	1	2	2	2	2	3	3	1	1
1	1	2	2	2	2	3	3	1	1
1	1	2	2	1	1	2	2	4	4
1	1	2	2	2	2	3	3	1	1
1	1	2	2	1	1	2	2	4	4
1	1	2	2	1	1	3	3	1	1
1	1	2	2	1	1	3	3	1	1
4	4	2	2	1	1	2	2	4	4
1	1	2	2	1	1	2	2	4	4
1	1	2	2	2	2	2	2	4	4
1	1	4	4	1	1	3	3	1	1
1	1	2	2	2	2	2	2	4	4
1	1	2	2	1	1	2	2	4	4
4	4	4	4	2	2	3	3	1	1
1	1	4	4	1	1	3	3	1	1
1	1	2	2	1	1	3	3	1	1
1	1	2	2	2	2	2	2	4	4
1	1	2	2	2	2	2	2	4	4
4	4	2	2	1	1	2	2	4	4
4	4	4	4	1	1	3	3	1	1
1	1	2	2	1	1	2	2	4	4
1	1	2	2	2	2	2	2	4	4
4	4	4	4	1	1	3	3	1	1
1	1	2	2	2	2	2	2	4	4
1	1	2	2	2	2	2	2	4	4
1	1	2	2	2	2	3	3	1	1
1	1	2	2	2	2	3	3	1	1
1	1	2	2	2	2	2	2	4	4
1	1	2	2	2	2	2	2	4	4
1	1	4	4	1	1	3	3	1	1
1	1	4	4	2	2	3	3	1	1
1	1	2	2	1	1	3	3	1	1
1	1	2	2	2	2	3	3	1	1
1	1	2	2	2	2	3	3	1	1
4	4	4	4	1	1	3	3	1	1
1	1	4	4	1	1	3	3	1	1
1	1	2	2	1	1	3	3	1	1
4	4	2	2	2	2	3	3	1	1

For Review Only

1	1	2	2	2	2	2	2	4	4
1	1	2	2	2	2	2	2	4	4
1	1	2	2	2	2	2	2	4	4
1	1	2	2	1	1	3	3	1	1
1	1	2	2	2	2	2	2	4	4
1	1	2	2	1	1	2	2	4	4
1	1	2	2	1	1	3	3	1	1
1	1	4	4	1	1	2	2	4	4
4	4	4	4	1	1	3	3	1	1
1	1	2	2	2	2	2	2	4	4
4	4	2	2	1	1	2	2	4	4
1	1	2	2	2	2	2	2	4	4
1	1	2	2	1	1	3	3	1	1
1	1	2	2	2	2	2	2	4	4
1	1	4	4	2	2	2	2	4	4
1	1	2	2	2	2	2	2	4	4
1	1	4	4	2	2	3	3	1	1
1	1	2	2	2	2	2	2	4	4
1	1	2	2	1	1	3	3	1	1
1	1	2	2	1	1	2	2	4	4
1	1	2	2	2	2	2	2	4	4
1	1	2	2	1	1	2	2	4	4
1	1	2	2	2	2	2	2	1	1
1	1	2	2	1	1	3	3	1	1
1	1	2	2	1	1	3	3	1	1
1	1	2	2	2	2	3	3	1	1
1	1	2	2	2	2	3	3	1	1
1	1	2	2	1	1	3	3	1	1
1	1	2	2	1	1	2	2	4	4
1	1	2	2	2	2	2	2	4	4
1	1	2	2	1	1	3	3	1	1
1	1	2	2	2	2	2	2	1	1
1	1	2	2	2	2	2	2	4	4
1	1	2	2	2	2	2	2	4	4
1	1	2	2	1	1	3	3	1	1
1	1	4	4	1	1	3	3	1	1
1	1	2	2	1	1	3	3	1	1
1	1	2	2	2	2	3	3	1	1
1	1	2	2	2	2	2	2	4	4
1	1	4	4	1	1	3	3	1	1
1	1	2	2	2	2	3	3	1	1
1	1	2	2	1	1	3	3	1	1
1	1	2	2	2	2	3	3	1	1
4	4	2	2	2	2	2	2	4	4
1	1	4	4	2	2	3	3	1	1
1	1	2	2	2	2	3	3	1	1
1	1	2	2	2	2	2	2	4	4
1	1	2	2	2	2	2	2	1	1
1	1	2	2	2	2	3	3	4	4
1	1	2	2	2	2	2	2	4	4
1	1	2	2	2	2	2	2	4	4
1	1	4	4	2	2	2	2	4	4
1	1	4	4	1	1	3	3	1	1
1	1	2	2	2	2	3	3	1	1
1	1	2	2	1	1	2	2	4	4

For Review Only

1	1	2	2	1	1	2	2	4	4
1	1	2	2	1	1	3	3	1	1
4	4	2	2	1	1	2	2	4	4
1	1	4	4	2	2	3	3	1	1
1	1	2	2	2	2	2	2	4	4
1	1	2	2	2	2	2	2	4	4
1	1	2	2	1	1	3	3	1	1
1	1	2	2	2	2	2	2	4	4
1	1	4	4	1	1	2	2	4	4
1	1	2	2	2	2	2	2	4	4
1	1	2	2	1	1	2	2	4	4
1	1	2	2	2	2	3	3	1	1
1	1	2	2	2	2	2	2	4	4
1	1	2	2	2	2	2	2	4	4
1	1	2	2	1	1	3	3	1	1
4	4	2	2	1	1	2	2	4	4
1	1	4	4	1	1	3	3	1	1
1	1	2	2	2	2	2	2	4	4
1	1	2	2	2	2	2	2	4	4
1	1	2	2	1	1	3	3	1	1
1	1	2	2	2	2	2	2	4	4
1	1	4	4	1	1	3	3	1	1
1	1	2	2	2	2	3	3	1	1
1	1	2	2	2	2	3	3	1	1
1	1	4	4	2	2	2	2	4	4
1	1	2	2	1	1	3	3	1	1
1	1	2	2	1	1	2	2	4	4
1	1	4	4	2	2	3	3	1	1
1	1	2	2	2	2	3	3	1	1
1	1	2	2	1	1	3	3	1	1
4	4	2	2	1	1	3	3	1	1
1	1	2	2	2	2	2	2	4	4
1	1	2	2	2	2	3	3	1	1
1	1	2	2	1	1	3	3	1	1
1	1	4	4	2	2	3	3	1	1
1	1	2	2	2	2	3	3	1	1
1	1	2	2	2	2	2	2	4	4
1	1	2	2	2	2	3	3	1	1
1	1	2	2	2	2	3	3	1	1
1	1	2	2	2	2	3	3	1	1
1	1	2	2	1	1	3	3	1	1
1	1	2	2	2	2	2	2	4	4
1	1	4	4	1	1	3	3	1	1
1	1	2	2	1	1	2	2	4	4
1	1	2	2	2	2	2	2	4	4
1	1	2	2	2	2	2	2	4	4
4	4	2	2	1	1	2	2	4	4
1	1	2	2	1	1	3	3	1	1
1	1	2	2	1	1	3	3	1	1
1	1	4	4	1	1	3	3	1	1
1	1	2	2	1	1	2	2	4	4
1	1	4	4	1	1	3	3	1	1
1	1	4	4	2	2	3	3	1	1
1	1	2	2	2	2	3	3	1	1
1	1	2	2	1	1	3	3	1	1

For Review Only

1	1	4	4	1	1	3	3	1	1
1	1	2	2	1	1	3	3	4	4
1	1	2	2	1	1	3	3	1	1
1	1	4	4	2	2	3	3	1	1
1	1	2	2	1	1	3	3	1	1
1	1	2	2	1	1	2	2	1	1
1	1	2	2	2	2	2	2	1	1
4	4	2	2	1	1	3	3	1	1
1	1	2	2	1	1	3	3	1	1
4	4	2	2	1	1	3	3	1	1
1	1	2	2	2	2	2	2	4	4
4	4	4	4	1	1	3	3	1	1
1	1	2	2	2	2	2	2	4	4
1	1	2	2	2	2	2	2	4	4
1	1	2	2	1	1	3	3	1	1
1	1	2	2	2	2	2	2	4	4
4	4	4	4	1	1	3	3	1	1
1	1	2	2	2	2	2	2	4	4
1	1	4	4	1	1	2	2	4	4
4	4	2	2	1	1	3	3	1	1
1	1	4	4	1	1	3	3	1	1
1	1	2	2	2	2	2	2	4	4
1	1	2	2	2	2	2	2	4	4
1	1	4	4	1	1	3	3	1	1
1	1	4	4	1	1	3	3	1	1
1	1	4	4	1	1	2	2	4	4
1	1	4	4	1	1	3	3	1	1
1	1	2	2	1	1	2	2	4	4
1	1	2	2	2	2	2	2	4	4
1	1	2	2	1	1	2	2	4	4
1	1	2	2	1	1	3	3	1	1
1	1	2	2	1	1	2	2	4	4
4	4	2	2	2	2	2	2	4	4
1	1	2	2	2	2	3	3	1	1
1	1	2	2	1	1	3	3	1	1
1	1	4	4	1	1	3	3	1	1
1	1	2	2	2	2	3	3	1	1
1	1	2	2	2	2	3	3	1	1
1	1	2	2	2	2	2	2	4	4
4	4	4	4	1	1	3	3	1	1
1	1	2	2	2	2	3	3	1	1
1	1	2	2	2	2	3	3	1	1
1	1	2	2	1	1	3	3	1	1
1	1	2	2	2	2	2	2	4	4
1	1	2	2	2	2	3	3	1	1
1	1	2	2	2	2	2	2	4	4
1	1	4	4	1	1	3	3	1	1
4	4	2	2	2	2	3	3	1	1
4	4	2	2	2	2	2	2	4	4
1	1	4	4	1	1	3	3	1	1
1	1	2	2	2	2	2	2	4	4
4	4	4	4	1	1	3	3	1	1
1	1	2	2	1	1	3	3	1	1
1	1	4	4	2	2	2	2	4	4
1	1	2	2	1	1	3	3	1	1

For Review Only

1	1	2	2	2	2	2	2	4	4
1	1	2	2	2	2	2	2	4	4

For Review Only

SNP245	SNP245	SNP2450	SNP2450	SNP2451	SNP2451	SNP2452	SNP2452	SNP2453	SNP2453
1	1	4	4	3	3	3	3	4	4
2	2	3	3	3	3	1	1	4	4
2	2	4	4	3	3	3	3	4	4
1	1	4	4	3	3	3	3	4	4
1	1	4	4	3	3	3	3	4	4
1	1	4	4	3	3	3	3	4	4
1	1	4	4	3	3	3	3	4	4
1	1	4	4	3	3	3	3	4	4
1	1	4	4	3	3	3	3	4	4
1	1	4	4	3	3	3	3	4	4
1	1	4	4	3	3	3	3	4	4
2	2	4	4	3	3	3	3	4	4
1	1	4	4	3	3	3	3	4	4
1	1	4	4	3	3	3	3	4	4
1	1	4	4	3	3	3	3	3	3
1	1	4	4	3	3	3	3	4	4
1	1	4	4	3	3	1	1	4	4
1	1	4	4	3	3	3	3	4	4
1	1	4	4	3	3	1	1	4	4
1	1	4	4	3	3	1	1	4	4
1	1	4	4	3	3	3	3	4	4
1	1	4	4	3	3	1	1	4	4
1	1	4	4	3	3	3	3	4	4
1	1	4	4	2	2	3	3	4	4
1	1	4	4	3	3	3	3	4	4
2	2	3	3	3	3	1	1	4	4
1	1	3	3	3	3	1	1	4	4
1	1	4	4	3	3	3	3	4	4
1	1	4	4	3	3	3	3	4	4
1	1	4	4	3	3	3	3	4	4
1	1	4	4	3	3	3	3	4	4
1	1	4	4	3	3	3	3	4	4
1	1	4	4	3	3	3	3	4	4
1	1	4	4	3	3	3	3	4	4
1	1	4	4	3	3	3	3	4	4
1	1	4	4	3	3	3	3	4	4
1	1	4	4	3	3	1	1	3	3
1	1	4	4	3	3	3	3	4	4
1	1	4	4	3	3	3	3	4	4
1	1	4	4	3	3	3	3	4	4
2	2	4	4	3	3	3	3	4	4
1	1	4	4	3	3	3	3	4	4
1	1	4	4	3	3	3	3	4	4
1	1	4	4	3	3	1	1	4	4
1	1	4	4	2	2	3	3	4	4
2	2	4	4	3	3	1	1	4	4
1	1	3	3	3	3	1	1	4	4
2	2	4	4	3	3	3	3	4	4
1	1	4	4	3	3	1	1	4	4
1	1	4	4	3	3	3	3	4	4
2	2	3	3	3	3	1	1	4	4
1	1	3	3	3	3	3	3	4	4

For Review Only

1	1	4	4	3	3	1	1	4	4
1	1	4	4	3	3	3	3	4	4
1	1	4	4	3	3	3	3	4	4
1	1	4	4	3	3	1	1	4	4
1	1	4	4	3	3	1	1	4	4
2	2	3	3	3	3	1	1	4	4
1	1	3	3	3	3	1	1	4	4
1	1	4	4	2	2	3	3	4	4
1	1	4	4	2	2	1	1	3	3
1	1	4	4	3	3	3	3	4	4
1	1	4	4	3	3	1	1	4	4
1	1	4	4	3	3	1	1	4	4
1	1	4	4	3	3	1	1	4	4
1	1	4	4	3	3	3	3	4	4
1	1	4	4	2	2	3	3	4	4
1	1	4	4	2	2	3	3	4	4
1	1	4	4	3	3	1	1	4	4
1	1	4	4	3	3	1	1	4	4
1	1	4	4	3	3	1	1	4	4
1	1	4	4	3	3	3	3	4	4
1	1	4	4	3	3	1	1	4	4
1	1	4	4	3	3	3	3	4	4
1	1	4	4	3	3	3	3	4	4
1	1	4	4	3	3	3	3	4	4
1	1	4	4	2	2	3	3	4	4
1	1	4	4	3	3	3	3	4	4
1	1	4	4	3	3	1	1	4	4
1	1	4	4	3	3	3	3	4	4
2	2	3	3	3	3	1	1	4	4
1	1	4	4	3	3	3	3	4	4
1	1	4	4	3	3	3	3	4	4
1	1	4	4	3	3	3	3	4	4
1	1	4	4	3	3	3	3	4	4
1	1	4	4	3	3	1	1	4	4
1	1	4	4	3	3	3	3	4	4
1	1	4	4	2	2	3	3	4	4
1	1	4	4	3	3	3	3	4	4
1	1	4	4	3	3	3	3	4	4
1	1	4	4	3	3	1	1	3	3
1	1	4	4	3	3	3	3	4	4
1	1	4	4	3	3	1	1	4	4
1	1	4	4	3	3	1	1	4	4
1	1	4	4	3	3	1	1	4	4
1	1	4	4	3	3	1	1	4	4
1	1	4	4	3	3	1	1	4	4
1	1	3	3	3	3	1	1	4	4
1	1	4	4	3	3	3	3	4	4
1	1	4	4	2	2	1	1	4	4
2	2	4	4	3	3	3	3	4	4
1	1	4	4	3	3	1	1	3	3
1	1	4	4	3	3	1	1	4	4
1	1	4	4	3	3	3	3	4	4
1	1	4	4	3	3	3	3	4	4
1	1	4	4	2	2	3	3	4	4
1	1	4	4	3	3	1	1	3	3

For Review Only

2	2	3	3	3	3	3	3	4	4
1	1	4	4	3	3	3	3	4	4
1	1	4	4	3	3	1	1	4	4
1	1	4	4	3	3	3	3	4	4
1	1	3	3	3	3	1	1	4	4
2	2	4	4	3	3	3	3	4	4
1	1	4	4	3	3	3	3	4	4
1	1	4	4	3	3	3	3	4	4
2	2	4	4	3	3	3	3	4	4
1	1	4	4	3	3	1	1	4	4
1	1	4	4	3	3	3	3	4	4
1	1	4	4	3	3	3	3	4	4
1	1	4	4	3	3	3	3	4	4
1	1	4	4	3	3	3	3	4	4
1	1	4	4	3	3	3	3	4	4
2	2	4	4	3	3	1	1	4	4
1	1	4	4	2	2	1	1	4	4
1	1	4	4	3	3	3	3	4	4
1	1	4	4	3	3	3	3	4	4
1	1	4	4	3	3	3	3	4	4
1	1	4	4	3	3	3	3	4	4
1	1	4	4	3	3	3	3	4	4
1	1	4	4	3	3	1	1	4	4
1	1	4	4	3	3	3	3	4	4
2	2	3	3	3	3	3	3	4	4
1	1	4	4	3	3	1	1	3	3
1	1	4	4	3	3	1	1	4	4
2	2	4	4	3	3	3	3	4	4
1	1	4	4	3	3	3	3	4	4
1	1	4	4	3	3	1	1	4	4
1	1	4	4	3	3	3	3	4	4
1	1	4	4	3	3	3	3	4	4
1	1	4	4	3	3	1	1	4	4
2	2	4	4	3	3	1	1	4	4
1	1	4	4	3	3	3	3	4	4
1	1	4	4	3	3	3	3	4	4
1	1	4	4	3	3	3	3	4	4
1	1	4	4	3	3	1	1	4	4
1	1	4	4	3	3	3	3	4	4
1	1	4	4	3	3	3	3	4	4
1	1	4	4	3	3	3	3	4	4
1	1	4	4	3	3	3	3	4	4
1	1	4	4	3	3	1	1	4	4
1	1	4	4	3	3	3	3	4	4
1	1	4	4	3	3	3	3	4	4
1	1	4	4	3	3	1	1	3	3
2	2	3	3	3	3	1	1	4	4
2	2	3	3	3	3	1	1	4	4
1	1	4	4	3	3	3	3	4	4
2	2	4	4	3	3	1	1	4	4
2	2	3	3	3	3	1	1	4	4
1	1	4	4	3	3	1	1	4	4
1	1	4	4	3	3	3	3	4	4
1	1	4	4	3	3	1	1	4	4
1	1	4	4	3	3	3	3	4	4

For Review Only

1	1	4	4	3	3	3	3	4	4
1	1	4	4	3	3	3	3	4	4
1	1	4	4	3	3	3	3	4	4
1	1	4	4	3	3	3	3	4	4
2	2	4	4	3	3	1	1	4	4
1	1	4	4	3	3	1	1	4	4
1	1	4	4	3	3	3	3	4	4
1	1	4	4	3	3	3	3	4	4
1	1	4	4	3	3	3	3	4	4
1	1	4	4	3	3	1	1	4	4
2	2	4	4	3	3	3	3	4	4
1	1	4	4	3	3	3	3	4	4
1	1	4	4	3	3	1	1	4	4
1	1	4	4	3	3	1	1	4	4
1	1	4	4	3	3	3	3	4	4
1	1	4	4	3	3	3	3	4	4
1	1	4	4	3	3	3	3	4	4
1	1	3	3	3	3	1	1	4	4
2	2	4	4	3	3	1	1	4	4
1	1	4	4	3	3	3	3	4	4
1	1	4	4	3	3	3	3	4	4
1	1	4	4	3	3	3	3	4	4
1	1	4	4	3	3	1	1	3	3
1	1	4	4	3	3	1	1	4	4
1	1	4	4	3	3	1	1	4	4
1	1	4	4	3	3	3	3	4	4
1	1	4	4	3	3	3	3	4	4
1	1	4	4	3	3	3	3	4	4
2	2	4	4	3	3	1	1	3	3
1	1	4	4	3	3	3	3	4	4
1	1	4	4	3	3	3	3	4	4
1	1	4	4	3	3	1	1	4	4
1	1	4	4	3	3	1	1	4	4
1	1	4	4	3	3	3	3	4	4
1	1	4	4	3	3	1	1	4	4
1	1	4	4	3	3	1	1	4	4
1	1	4	4	3	3	3	3	4	4
1	1	4	4	3	3	1	1	3	3
2	2	4	4	3	3	1	1	4	4
1	1	4	4	3	3	1	1	4	4
2	2	4	4	3	3	3	3	4	4
2	2	3	3	3	3	1	1	4	4
1	1	4	4	3	3	3	3	4	4
2	2	4	4	3	3	3	3	4	4
1	1	4	4	3	3	1	1	4	4
1	1	4	4	3	3	1	1	4	4
1	1	4	4	3	3	3	3	4	4
1	1	4	4	3	3	3	3	4	4
1	1	4	4	3	3	3	3	4	4
1	1	4	4	3	3	3	3	4	4
1	1	4	4	3	3	3	3	4	4
1	1	4	4	2	2	3	3	4	4
1	1	4	4	3	3	1	1	3	3
1	1	4	4	3	3	1	1	4	4
1	1	4	4	3	3	3	3	4	4

For Review Only

1	1	4	4	3	3	3	3	4	4
1	1	4	4	3	3	3	3	4	4
2	2	4	4	3	3	3	3	4	4
1	1	4	4	3	3	3	3	4	4
1	1	4	4	3	3	3	3	4	4
1	1	3	3	3	3	3	3	4	4
1	1	4	4	3	3	1	1	4	4
1	1	4	4	3	3	3	3	4	4
1	1	4	4	3	3	3	3	4	4
1	1	4	4	3	3	3	3	4	4
1	1	4	4	3	3	3	3	4	4
1	1	4	4	3	3	3	3	4	4
1	1	4	4	3	3	1	1	3	3
2	2	4	4	3	3	1	1	4	4
2	2	4	4	3	3	3	3	4	4
1	1	4	4	3	3	3	3	3	3
1	1	4	4	3	3	3	3	4	4
1	1	4	4	3	3	1	1	4	4
1	1	4	4	3	3	3	3	4	4
1	1	4	4	3	3	3	3	4	4
1	1	4	4	3	3	3	3	4	4
2	2	3	3	3	3	1	1	4	4
1	1	4	4	3	3	1	1	4	4
1	1	4	4	3	3	3	3	4	4
1	1	4	4	2	2	3	3	4	4
1	1	4	4	3	3	3	3	4	4
1	1	4	4	3	3	3	3	4	4
1	1	4	4	3	3	3	3	4	4
1	1	4	4	3	3	3	3	4	4
1	1	4	4	3	3	3	3	4	4
1	1	4	4	3	3	3	3	4	4
1	1	4	4	3	3	3	3	4	4
1	1	4	4	3	3	1	1	4	4
2	2	4	4	3	3	1	1	4	4
1	1	4	4	3	3	3	3	4	4
1	1	4	4	3	3	3	3	4	4
1	1	4	4	3	3	1	1	4	4
1	1	4	4	3	3	1	1	4	4
1	1	4	4	3	3	3	3	4	4
1	1	4	4	3	3	3	3	4	4
1	1	4	4	3	3	1	1	4	4
1	1	4	4	3	3	1	1	3	3
1	1	4	4	3	3	3	3	4	4
1	1	4	4	3	3	1	1	4	4
1	1	4	4	3	3	3	3	4	4
1	1	4	4	3	3	1	1	3	3
1	1	4	4	3	3	3	3	4	4
1	1	4	4	3	3	1	1	4	4
2	2	4	4	3	3	1	1	4	4
1	1	4	4	2	2	3	3	4	4
1	1	4	4	3	3	1	1	4	4
1	1	4	4	3	3	3	3	4	4
2	2	4	4	3	3	3	3	4	4
1	1	4	4	3	3	1	1	4	4
1	1	4	4	3	3	3	3	4	4

For Review Only

1	1	4	4	3	3	1	1	4	4
1	1	3	3	3	3	1	1	4	4

For Review Only

SNP2454	SNP2454	SNP2455	SNP2455	SNP2456	SNP2456	SNP2457	SNP2457	SNP2458	SNP2458
1	1	1	1	3	3	4	4	3	3
3	3	1	1	3	3	4	4	3	3
1	1	1	1	3	3	4	4	3	3
3	3	1	1	3	3	4	4	3	3
1	1	1	1	2	2	2	2	2	2
3	3	1	1	3	3	4	4	3	3
3	3	1	1	3	3	4	4	3	3
1	1	1	1	3	3	2	2	2	2
3	3	1	1	3	3	4	4	3	3
3	3	1	1	3	3	4	4	3	3
3	3	1	1	2	2	4	4	3	3
1	1	1	1	3	3	4	4	3	3
3	3	1	1	3	3	4	4	3	3
1	1	1	1	3	3	4	4	3	3
1	1	1	1	3	3	4	4	3	3
1	1	1	1	3	3	4	4	3	3
3	3	1	1	3	3	4	4	3	3
3	3	1	1	3	3	4	4	3	3
3	3	1	1	3	3	4	4	3	3
3	3	1	1	3	3	4	4	3	3
3	3	2	2	3	3	4	4	3	3
3	3	1	1	3	3	4	4	3	3
1	1	2	2	2	2	4	4	3	3
1	1	2	2	3	3	4	4	3	3
3	3	1	1	3	3	2	2	3	3
3	3	1	1	3	3	4	4	3	3
3	3	1	1	3	3	4	4	3	3
3	3	1	1	3	3	4	4	3	3
3	3	1	1	3	3	4	4	3	3
1	1	2	2	2	2	4	4	3	3
3	3	1	1	3	3	4	4	3	3
3	3	1	1	3	3	4	4	3	3
3	3	1	1	3	3	4	4	3	3
1	1	2	2	3	3	4	4	3	3
3	3	2	2	2	2	4	4	3	3
3	3	1	1	3	3	4	4	3	3
1	1	1	1	3	3	4	4	3	3
3	3	2	2	2	2	2	2	2	2
1	1	2	2	2	2	4	4	3	3
3	3	2	2	2	2	4	4	3	3
1	1	2	2	2	2	4	4	2	2
3	3	1	1	3	3	4	4	3	3
1	1	1	1	2	2	2	2	2	2
3	3	2	2	3	3	4	4	3	3
3	3	1	1	2	2	2	2	2	2
3	3	1	1	3	3	4	4	3	3
3	3	1	1	3	3	4	4	3	3
3	3	1	1	2	2	4	4	3	3
3	3	2	2	3	3	4	4	3	3
3	3	1	1	2	2	4	4	3	3
3	3	1	1	3	3	4	4	3	3
3	3	1	1	3	3	4	4	3	3

For Review Only

3	3	1	1	3	3	4	4	3	3
3	3	1	1	3	3	4	4	3	3
3	3	1	1	3	3	4	4	3	3
3	3	1	1	3	3	4	4	3	3
3	3	1	1	3	3	4	4	3	3
3	3	1	1	3	3	4	4	3	3
3	3	1	1	3	3	4	4	3	3
1	1	2	2	2	2	4	4	3	3
1	1	1	1	3	3	4	4	3	3
3	3	1	1	3	3	4	4	3	3
3	3	1	1	3	3	4	4	3	3
3	3	1	1	3	3	4	4	3	3
3	3	1	1	3	3	4	4	3	3
3	3	1	1	3	3	4	4	3	3
3	3	1	1	3	3	4	4	3	3
1	1	2	2	3	3	2	2	2	2
3	3	1	1	3	3	4	4	3	3
1	1	1	1	3	3	4	4	3	3
1	1	1	1	3	3	4	4	3	3
3	3	1	1	3	3	4	4	3	3
3	3	1	1	3	3	4	4	3	3
3	3	2	2	2	2	4	4	3	3
1	1	1	1	2	2	4	4	3	3
1	1	2	2	3	3	4	4	3	3
3	3	1	1	2	2	2	2	2	2
3	3	1	1	3	3	4	4	3	3
3	3	2	2	3	3	2	2	2	2
1	1	2	2	2	2	4	4	3	3
3	3	1	1	3	3	4	4	3	3
3	3	1	1	3	3	4	4	3	3
3	3	1	1	3	3	2	2	2	2
1	1	1	1	3	3	4	4	3	3
1	1	2	2	2	2	4	4	3	3
3	3	1	1	3	3	4	4	3	3
3	3	1	1	3	3	4	4	3	3
3	3	1	1	2	2	2	2	2	2
3	3	2	2	3	3	2	2	2	2
3	3	1	1	3	3	4	4	3	3
3	3	1	1	3	3	4	4	3	3
1	1	2	2	3	3	2	2	2	2
3	3	1	1	3	3	4	4	3	3
3	3	1	1	3	3	4	4	3	3
3	3	1	1	3	3	4	4	3	3
3	3	1	1	3	3	4	4	3	3
3	3	1	1	3	3	4	4	3	3
3	3	1	1	3	3	4	4	3	3
1	1	1	1	3	3	4	4	3	3
3	3	1	1	3	3	4	4	3	3
3	3	1	1	3	3	4	4	3	3
1	1	2	2	3	3	4	4	3	3
1	1	1	1	3	3	4	4	3	3
3	3	2	2	2	2	4	4	3	3
3	3	2	2	2	2	4	4	3	3
1	1	1	1	3	3	4	4	3	3
1	1	1	1	3	3	4	4	3	3

For Review Only

3	3	1	1	2	2	4	4	3	3
3	3	2	2	2	2	4	4	3	3
3	3	1	1	3	3	4	4	3	3
1	1	1	1	2	2	4	4	3	3
3	3	1	1	3	3	4	4	3	3
3	3	1	1	3	3	4	4	3	3
1	1	1	1	3	3	4	4	3	3
3	3	1	1	3	3	4	4	3	3
1	1	2	2	3	3	4	4	3	3
3	3	1	1	3	3	4	4	3	3
3	3	1	1	3	3	4	4	3	3
3	3	1	1	3	3	4	4	3	3
3	3	1	1	2	2	4	4	3	3
3	3	1	1	3	3	4	4	3	3
3	3	2	2	3	3	4	4	3	3
3	3	1	1	3	3	4	4	3	3
1	1	1	1	3	3	4	4	3	3
3	3	1	1	3	3	4	4	3	3
1	1	2	2	3	3	4	4	3	3
3	3	1	1	2	2	4	4	3	3
3	3	1	1	3	3	4	4	3	3
3	3	1	1	3	3	4	4	3	3
1	1	1	1	3	3	4	4	3	3
1	1	2	2	2	2	4	4	3	3
3	3	1	1	2	2	4	4	3	3
1	1	1	1	3	3	4	4	3	3
1	1	1	1	3	3	4	4	3	3
1	1	1	1	3	3	4	4	3	3
1	1	1	1	3	3	4	4	3	3
3	3	1	1	3	3	4	4	3	3
1	1	2	2	3	3	4	4	3	3
3	3	1	1	2	2	4	4	3	3
3	3	1	1	3	3	4	4	3	3
3	3	1	1	3	3	4	4	3	3
1	1	2	2	3	3	4	4	3	3
1	1	1	1	2	2	4	4	3	3
3	3	1	1	3	3	4	4	3	3
1	1	1	1	3	3	4	4	3	3
3	3	1	1	3	3	4	4	3	3
1	1	1	1	2	2	4	4	3	3
1	1	1	1	2	2	4	4	3	3
3	3	1	1	3	3	4	4	3	3
1	1	1	1	3	3	4	4	3	3
3	3	1	1	3	3	4	4	2	2
3	3	1	1	2	2	4	4	3	3
1	1	1	1	3	3	4	4	3	3
3	3	1	1	3	3	4	4	3	3
3	3	1	1	3	3	4	4	3	3
1	1	1	1	3	3	4	4	3	3
3	3	1	1	3	3	4	4	3	3
3	3	1	1	3	3	4	4	3	3
1	1	1	1	2	2	4	4	3	3
1	1	1	1	3	3	4	4	3	3
1	1	1	1	3	3	4	4	3	3
3	3	1	1	2	2	4	4	3	3

For Review Only

1	1	1	1	3	3	4	4	3	3
3	3	1	1	2	2	4	4	3	3
3	3	1	1	2	2	2	2	2	2
1	1	2	2	2	2	4	4	3	3
3	3	1	1	3	3	4	4	3	3
3	3	1	1	3	3	4	4	3	3
1	1	1	1	2	2	4	4	3	3
3	3	1	1	3	3	4	4	3	3
3	3	1	1	2	2	4	4	3	3
3	3	1	1	3	3	4	4	3	3
3	3	1	1	2	2	4	4	3	3
1	1	1	1	3	3	4	4	3	3
3	3	1	1	3	3	4	4	3	3
3	3	1	1	3	3	4	4	3	3
1	1	1	1	3	3	4	4	3	3
3	3	1	1	2	2	4	4	3	3
1	1	1	1	3	3	4	4	3	3
3	3	1	1	3	3	4	4	3	3
3	3	1	1	3	3	4	4	3	3
1	1	1	1	3	3	4	4	3	3
3	3	1	1	3	3	4	4	3	3
1	1	2	2	3	3	4	4	3	3
1	1	2	2	3	3	4	4	3	3
3	3	1	1	3	3	4	4	3	3
3	3	1	1	3	3	4	4	3	3
1	1	2	2	3	3	4	4	3	3
3	3	1	1	3	3	2	2	3	3
1	1	1	1	3	3	4	4	3	3
1	1	1	1	3	3	4	4	3	3
1	1	1	1	2	2	4	4	3	3
1	1	1	1	3	3	4	4	3	3
3	3	1	1	3	3	4	4	3	3
1	1	1	1	3	3	4	4	3	3
1	1	1	1	3	3	4	4	3	3
1	1	1	1	3	3	4	4	3	3
3	3	1	1	3	3	4	4	3	3
3	3	1	1	2	2	4	4	3	3
1	1	1	1	3	3	4	4	3	3
3	3	1	1	3	3	4	4	3	3
3	3	1	1	3	3	4	4	3	3
1	1	1	1	3	3	4	4	3	3
3	3	1	1	3	3	4	4	3	3
1	1	2	2	2	2	4	4	3	3
3	3	1	1	2	2	4	4	3	3
3	3	1	1	3	3	4	4	3	3
3	3	1	1	3	3	4	4	3	3
3	3	1	1	2	2	4	4	3	3
1	1	2	2	2	2	4	4	3	3
1	1	2	2	3	3	4	4	3	3
3	3	1	1	3	3	4	4	3	3
1	1	1	1	3	3	4	4	3	3
3	3	2	2	3	3	4	4	3	3
1	1	2	2	3	3	4	4	3	3
3	3	1	1	3	3	4	4	3	3
3	3	2	2	2	2	4	4	3	3

For Review Only

1	1	2	2	3	3	4	4	3	3
1	1	1	1	3	3	4	4	3	3
1	1	1	1	3	3	4	4	3	3
1	1	1	1	3	3	4	4	3	3
1	1	2	2	2	2	2	2	2	2
3	3	1	1	3	3	4	4	3	3
3	3	1	1	3	3	4	4	3	3
1	1	1	1	2	2	4	4	3	3
1	1	1	1	3	3	4	4	3	3
1	1	2	2	2	2	4	4	3	3
3	3	1	1	3	3	4	4	3	3
1	1	1	1	3	3	4	4	3	3
3	3	1	1	3	3	4	4	3	3
3	3	1	1	3	3	4	4	3	3
1	1	1	1	3	3	4	4	3	3
3	3	2	2	3	3	4	4	3	3
3	3	2	2	2	2	4	4	3	3
1	1	2	2	3	3	4	4	3	3
3	3	2	2	2	2	4	4	3	3
3	3	2	2	2	2	2	2	2	2
1	1	1	1	3	3	4	4	3	3
3	3	1	1	3	3	4	4	3	3
1	1	1	1	3	3	4	4	3	3
1	1	1	1	2	2	4	4	3	3
1	1	2	2	2	2	4	4	3	3
3	3	1	1	3	3	4	4	3	3
1	1	2	2	2	2	4	4	3	3
3	3	1	1	3	3	2	2	3	3
3	3	1	1	2	2	4	4	3	3
1	1	1	1	3	3	4	4	3	3
1	1	2	2	2	2	4	4	3	3
3	3	1	1	2	2	4	4	3	3
3	3	2	2	3	3	4	4	3	3
3	3	1	1	3	3	4	4	3	3
1	1	2	2	2	2	4	4	3	3
1	1	2	2	2	2	4	4	3	3
1	1	1	1	3	3	4	4	3	3
3	3	1	1	3	3	4	4	3	3
3	3	1	1	3	3	4	4	3	3
1	1	2	2	2	2	4	4	3	3
1	1	1	1	3	3	4	4	3	3
1	1	1	1	3	3	4	4	3	3
1	1	1	1	2	2	4	4	3	3
3	3	1	1	3	3	4	4	3	3
1	1	1	1	3	3	4	4	3	3
3	3	1	1	3	3	4	4	3	3
1	1	2	2	2	2	4	4	3	3
1	1	1	1	3	3	4	4	3	3
3	3	2	2	3	3	2	2	2	2
1	1	2	2	2	2	4	4	3	3
3	3	1	1	3	3	2	2	3	3
3	3	2	2	2	2	4	4	3	3
1	1	1	1	3	3	4	4	3	3
3	3	1	1	3	3	4	4	3	3
1	1	1	1	2	2	4	4	3	3

For Review Only

3	3	1	1	3	3	4	4	3	3
3	3	1	1	3	3	4	4	3	3

For Review Only

SNP2459	SNP2459	SNP246	SNP246	SNP2460	SNP2460	SNP2461	SNP2461	SNP2462	SNP2462
4	4	1	1	3	3	4	4	2	2
4	4	3	3	3	3	3	3	2	2
4	4	1	1	3	3	4	4	2	2
4	4	3	3	3	3	4	4	2	2
4	4	3	3	3	3	4	4	2	2
4	4	1	1	3	3	4	4	2	2
4	4	3	3	3	3	4	4	2	2
4	4	3	3	3	3	4	4	2	2
4	4	3	3	3	3	4	4	2	2
4	4	3	3	3	3	4	4	2	2
4	4	3	3	3	3	4	4	2	2
4	4	3	3	3	3	4	4	2	2
4	4	3	3	3	3	4	4	2	2
4	4	3	3	3	3	4	4	2	2
4	4	3	3	3	3	4	4	2	2
4	4	3	3	3	3	4	4	2	2
1	1	3	3	3	3	4	4	2	2
4	4	1	1	3	3	4	4	3	3
4	4	1	1	3	3	4	4	2	2
1	1	3	3	3	3	4	4	3	3
4	4	3	3	3	3	4	4	2	2
1	1	3	3	3	3	4	4	2	2
4	4	3	3	3	3	4	4	2	2
4	4	3	3	3	3	4	4	2	2
4	4	3	3	3	3	4	4	3	3
4	4	3	3	1	1	4	4	2	2
4	4	3	3	3	3	4	4	2	2
4	4	3	3	3	3	4	4	2	2
4	4	3	3	3	3	4	4	2	2
4	4	3	3	3	3	4	4	2	2
4	4	1	1	3	3	4	4	2	2
4	4	3	3	1	1	4	4	2	2
4	4	3	3	3	3	4	4	2	2
4	4	3	3	3	3	4	4	2	2
4	4	3	3	3	3	4	4	2	2
4	4	3	3	3	3	4	4	2	2
4	4	3	3	3	3	4	4	2	2
4	4	3	3	3	3	4	4	2	2
4	4	3	3	3	3	4	4	2	2
4	4	3	3	3	3	4	4	2	2
4	4	3	3	3	3	4	4	2	2
4	4	1	1	1	1	4	4	2	2
4	4	3	3	3	3	4	4	2	2
1	1	3	3	3	3	4	4	2	2
4	4	3	3	3	3	4	4	2	2
4	4	1	1	3	3	4	4	2	2
4	4	3	3	3	3	4	4	2	2
4	4	3	3	3	3	4	4	2	2
1	1	3	3	3	3	4	4	2	2
4	4	3	3	1	1	4	4	2	2
4	4	3	3	3	3	4	4	2	2
4	4	3	3	3	3	3	3	2	2

For Review Only

1	1	3	3	3	3	4	4	2	2
4	4	3	3	3	3	4	4	2	2
4	4	3	3	1	1	4	4	2	2
1	1	3	3	3	3	4	4	2	2
4	4	1	1	3	3	4	4	2	2
4	4	3	3	1	1	4	4	2	2
4	4	3	3	1	1	4	4	2	2
4	4	3	3	3	3	4	4	2	2
4	4	3	3	3	3	4	4	2	2
4	4	3	3	3	3	4	4	2	2
4	4	3	3	3	3	3	3	2	2
4	4	3	3	3	3	3	3	2	2
1	1	3	3	3	3	4	4	2	2
4	4	3	3	3	3	4	4	2	2
4	4	3	3	3	3	4	4	2	2
4	4	3	3	3	3	4	4	2	2
4	4	3	3	3	3	4	4	2	2
4	4	1	1	3	3	4	4	2	2
1	1	1	1	3	3	4	4	2	2
4	4	1	1	1	1	4	4	2	2
4	4	3	3	3	3	4	4	2	2
4	4	3	3	3	3	4	4	2	2
4	4	3	3	3	3	4	4	2	2
4	4	3	3	3	3	4	4	2	2
4	4	3	3	3	3	4	4	2	2
4	4	3	3	3	3	3	3	2	2
1	1	3	3	3	3	4	4	2	2
4	4	3	3	3	3	4	4	2	2
4	4	3	3	1	1	4	4	2	2
4	4	1	1	3	3	4	4	2	2
1	1	3	3	3	3	4	4	2	2
4	4	3	3	3	3	4	4	2	2
4	4	3	3	3	3	4	4	2	2
1	1	3	3	3	3	4	4	2	2
4	4	1	1	1	1	4	4	2	2
4	4	3	3	3	3	4	4	2	2
4	4	3	3	3	3	4	4	2	2
4	4	3	3	3	3	4	4	2	2
4	4	1	1	3	3	4	4	2	2
4	4	1	1	3	3	4	4	2	2
1	1	1	1	3	3	4	4	2	2
1	1	1	1	3	3	4	4	2	2
1	1	1	1	3	3	4	4	2	2
4	4	3	3	3	3	3	3	2	2
1	1	3	3	3	3	4	4	2	2
4	4	1	1	3	3	4	4	3	3
4	4	3	3	3	3	4	4	2	2
4	4	1	1	1	1	4	4	2	2
4	4	3	3	3	3	4	4	2	2
4	4	3	3	3	3	4	4	2	2
1	1	3	3	3	3	4	4	2	2
4	4	3	3	3	3	4	4	2	2
4	4	3	3	3	3	4	4	2	2
4	4	3	3	3	3	4	4	2	2
4	4	1	1	3	3	4	4	2	2

For Review Only

4	4	3	3	1	1	4	4	2	2
4	4	3	3	3	3	4	4	2	2
4	4	3	3	1	1	4	4	2	2
4	4	3	3	3	3	4	4	2	2
4	4	3	3	3	3	4	4	2	2
4	4	3	3	3	3	4	4	2	2
4	4	3	3	3	3	4	4	2	2
4	4	3	3	3	3	4	4	2	2
4	4	3	3	3	3	4	4	2	2
4	4	3	3	3	3	4	4	2	2
4	4	1	1	3	3	4	4	2	2
4	4	3	3	3	3	4	4	2	2
4	4	1	1	3	3	4	4	2	2
4	4	3	3	3	3	4	4	2	2
4	4	3	3	3	3	4	4	2	2
4	4	3	3	3	3	4	4	2	2
4	4	1	1	3	3	4	4	2	2
4	4	3	3	3	3	4	4	2	2
4	4	3	3	3	3	4	4	2	2
4	4	1	1	3	3	4	4	2	2
4	4	3	3	3	3	4	4	2	2
4	4	3	3	3	3	4	4	2	2
4	4	3	3	3	3	4	4	2	2
4	4	3	3	3	3	4	4	2	2
4	4	3	3	3	3	4	4	2	2
4	4	3	3	3	3	4	4	2	2
1	1	3	3	3	3	4	4	2	2
4	4	3	3	3	3	4	4	2	2
4	4	3	3	3	3	4	4	2	2
4	4	3	3	3	3	4	4	3	3
4	4	3	3	3	3	4	4	2	2
4	4	3	3	3	3	4	4	2	2
1	1	1	1	3	3	4	4	2	2
4	4	3	3	3	3	4	4	2	2
4	4	3	3	3	3	4	4	2	2
4	4	3	3	3	3	4	4	2	2
4	4	3	3	3	3	4	4	2	2
4	4	3	3	3	3	3	3	2	2
4	4	3	3	3	3	4	4	2	2
4	4	3	3	3	3	4	4	2	2
4	4	3	3	3	3	4	4	2	2
4	4	3	3	3	3	4	4	2	2
4	4	1	1	3	3	4	4	2	2
4	4	3	3	3	3	4	4	2	2
4	4	3	3	1	1	4	4	2	2
4	4	3	3	1	1	4	4	2	2
4	4	3	3	1	1	4	4	2	2
4	4	3	3	1	1	4	4	2	2
4	4	3	3	3	3	4	4	2	2
4	4	3	3	1	1	4	4	2	2
4	4	3	3	1	1	4	4	2	2
4	4	1	1	3	3	4	4	2	2
4	4	3	3	3	3	4	4	2	2
4	4	3	3	1	1	4	4	2	2
4	4	3	3	3	3	4	4	2	2

For Review Only

4	4	3	3	3	3	4	4	2	2
4	4	3	3	3	3	4	4	2	2
4	4	3	3	3	3	4	4	2	2
4	4	3	3	3	3	4	4	2	2
4	4	3	3	3	3	4	4	2	2
4	4	3	3	3	3	4	4	2	2
4	4	3	3	3	3	4	4	2	2
4	4	3	3	3	3	4	4	2	2
4	4	3	3	3	3	4	4	2	2
1	1	1	1	3	3	4	4	2	2
4	4	3	3	3	3	4	4	2	2
4	4	3	3	3	3	4	4	2	2
4	4	3	3	3	3	4	4	2	2
4	4	3	3	3	3	4	4	2	2
4	4	3	3	3	3	4	4	2	2
4	4	3	3	3	3	4	4	2	2
4	4	3	3	3	3	4	4	2	2
4	4	3	3	3	3	3	3	2	2
4	4	3	3	3	3	4	4	2	2
4	4	1	1	3	3	4	4	2	2
4	4	3	3	3	3	4	4	2	2
4	4	3	3	3	3	4	4	2	2
4	4	3	3	3	3	4	4	2	2
4	4	3	3	3	3	3	3	2	2
1	1	1	1	3	3	4	4	2	2
4	4	3	3	3	3	4	4	2	2
4	4	3	3	3	3	4	4	3	3
4	4	3	3	3	3	4	4	2	2
4	4	3	3	3	3	4	4	2	2
4	4	1	1	3	3	4	4	2	2
4	4	1	1	3	3	4	4	2	2
4	4	1	1	3	3	4	4	2	2
1	1	3	3	3	3	4	4	2	2
4	4	3	3	3	3	4	4	2	2
4	4	3	3	3	3	4	4	2	2
4	4	3	3	3	3	3	3	2	2
4	4	3	3	3	3	4	4	2	2
4	4	1	1	3	3	4	4	2	2
4	4	3	3	3	3	4	4	2	2
1	1	1	1	3	3	4	4	2	2
4	4	1	1	3	3	4	4	2	2
4	4	3	3	1	1	4	4	2	2
4	4	3	3	3	3	4	4	2	2
4	4	3	3	3	3	4	4	2	2
4	4	3	3	3	3	4	4	2	2
4	4	1	1	3	3	4	4	2	2
4	4	3	3	3	3	4	4	2	2
4	4	3	3	3	3	4	4	2	2
4	4	3	3	3	3	4	4	2	2
4	4	3	3	3	3	4	4	2	2
4	4	1	1	3	3	4	4	2	2
4	4	3	3	3	3	4	4	2	2
4	4	3	3	3	3	4	4	2	2
4	4	3	3	3	3	3	3	2	2
4	4	3	3	3	3	4	4	2	2

For Review Only

4	4	1	1	3	3	4	4	2	2
4	4	3	3	3	3	4	4	3	3
4	4	1	1	3	3	4	4	2	2
4	4	3	3	3	3	4	4	2	2
4	4	3	3	3	3	4	4	2	2
4	4	3	3	1	1	4	4	2	2
4	4	3	3	3	3	4	4	2	2
4	4	3	3	3	3	4	4	2	2
4	4	3	3	3	3	4	4	2	2
4	4	3	3	3	3	4	4	2	2
4	4	3	3	3	3	4	4	2	2
4	4	1	1	3	3	4	4	2	2
4	4	1	1	3	3	4	4	2	2
4	4	1	1	1	1	4	4	2	2
4	4	1	1	3	3	4	4	2	2
4	4	3	3	3	3	4	4	2	2
4	4	3	3	3	3	4	4	2	2
1	1	3	3	3	3	4	4	2	2
4	4	3	3	3	3	4	4	2	2
4	4	1	1	3	3	4	4	2	2
4	4	3	3	3	3	4	4	2	2
4	4	3	3	1	1	4	4	2	2
1	1	1	1	3	3	4	4	2	2
4	4	3	3	3	3	4	4	2	2
4	4	3	3	3	3	4	4	2	2
4	4	1	1	3	3	4	4	2	2
4	4	3	3	3	3	4	4	2	2
4	4	3	3	3	3	4	4	3	3
4	4	3	3	3	3	4	4	2	2
4	4	1	1	3	3	4	4	2	2
4	4	3	3	3	3	4	4	2	2
4	4	3	3	1	1	4	4	2	2
4	4	3	3	3	3	4	4	2	2
4	4	3	3	1	1	4	4	2	2
4	4	3	3	3	3	4	4	2	2
4	4	3	3	3	3	4	4	2	2
1	1	1	1	3	3	4	4	2	2
4	4	3	3	3	3	3	3	2	2
4	4	3	3	3	3	4	4	2	2
4	4	3	3	3	3	4	4	2	2
4	4	1	1	3	3	4	4	2	2
4	4	3	3	3	3	4	4	2	2
4	4	3	3	3	3	4	4	2	2
1	1	3	3	3	3	4	4	2	2
4	4	1	1	3	3	4	4	2	2
4	4	1	1	3	3	4	4	2	2
4	4	3	3	1	1	4	4	2	2
4	4	1	1	3	3	4	4	2	2
4	4	3	3	3	3	4	4	2	2
4	4	3	3	3	3	4	4	2	2
4	4	3	3	1	1	4	4	3	3
4	4	3	3	3	3	4	4	2	2
4	4	1	1	3	3	4	4	2	2
1	1	1	1	3	3	4	4	2	2
4	4	3	3	3	3	4	4	2	2

For Review Only

4	4	3	3	3	3	4	4	2	2
4	4	3	3	3	3	3	3	2	2

For Review Only

SNP2463	SNP2463	SNP2464	SNP2464	SNP2465	SNP2465	SNP2466	SNP2466	SNP2467	SNP2467
1	1	4	4	2	2	3	3	4	4
1	1	3	3	2	2	3	3	4	4
1	1	4	4	2	2	3	3	4	4
1	1	4	4	2	2	3	3	4	4
1	1	4	4	2	2	3	3	4	4
2	2	3	3	2	2	3	3	4	4
2	2	3	3	2	2	3	3	4	4
1	1	4	4	2	2	3	3	4	4
1	1	3	3	2	2	3	3	4	4
2	2	3	3	2	2	3	3	4	4
2	2	3	3	2	2	3	3	4	4
1	1	4	4	2	2	3	3	4	4
1	1	4	4	3	3	3	3	4	4
1	1	4	4	2	2	3	3	4	4
1	1	4	4	3	3	3	3	4	4
1	1	4	4	2	2	3	3	4	4
2	2	3	3	3	3	2	2	4	4
2	2	3	3	2	2	3	3	4	4
2	2	3	3	2	2	3	3	4	4
1	1	4	4	3	3	2	2	4	4
1	1	4	4	2	2	3	3	4	4
1	1	4	4	3	3	2	2	4	4
1	1	4	4	2	2	3	3	4	4
1	1	4	4	2	2	3	3	4	4
2	2	3	3	2	2	3	3	4	4
1	1	3	3	2	2	3	3	4	4
1	1	3	3	2	2	3	3	4	4
1	1	4	4	2	2	3	3	4	4
2	2	3	3	2	2	3	3	4	4
1	1	4	4	2	2	3	3	4	4
2	2	3	3	2	2	3	3	4	4
2	2	3	3	2	2	3	3	4	4
2	2	3	3	2	2	3	3	4	4
1	1	4	4	2	2	3	3	4	4
1	1	4	4	2	2	3	3	4	4
1	1	4	4	2	2	3	3	4	4
1	1	4	4	3	3	3	3	1	1
1	1	4	4	2	2	3	3	4	4
1	1	4	4	2	2	3	3	4	4
1	1	4	4	2	2	3	3	4	4
1	1	4	4	2	2	3	3	4	4
2	2	3	3	2	2	3	3	4	4
1	1	4	4	2	2	3	3	4	4
1	1	4	4	3	3	2	2	4	4
2	2	3	3	2	2	3	3	4	4
1	1	4	4	2	2	3	3	4	4
1	1	3	3	2	2	3	3	4	4
2	2	4	4	2	2	3	3	4	4
1	1	4	4	3	3	2	2	4	4
1	1	4	4	3	3	2	2	4	4
1	1	3	3	2	2	3	3	4	4
1	1	3	3	2	2	3	3	4	4

For Review Only

2	2	3	3	3	3	2	2	4	4
2	2	3	3	2	2	3	3	4	4
2	2	3	3	2	2	3	3	4	4
1	1	4	4	3	3	2	2	4	4
2	2	3	3	2	2	3	3	4	4
1	1	3	3	2	2	3	3	4	4
1	1	3	3	2	2	3	3	4	4
1	1	4	4	2	2	3	3	4	4
1	1	4	4	3	3	3	3	1	1
1	1	4	4	2	2	3	3	4	4
1	1	4	4	2	2	3	3	4	4
1	1	4	4	2	2	3	3	4	4
2	2	3	3	3	3	2	2	4	4
2	2	3	3	2	2	3	3	4	4
1	1	4	4	2	2	3	3	4	4
1	1	4	4	2	2	3	3	4	4
2	2	3	3	2	2	3	3	4	4
1	1	4	4	2	2	3	3	4	4
1	1	4	4	3	3	2	2	4	4
2	2	3	3	2	2	3	3	4	4
1	1	4	4	2	2	3	3	4	4
1	1	4	4	2	2	3	3	1	1
1	1	4	4	2	2	3	3	4	4
1	1	4	4	2	2	3	3	4	4
2	2	3	3	2	2	3	3	4	4
1	1	3	3	2	2	3	3	4	4
1	1	4	4	3	3	2	2	4	4
1	1	4	4	2	2	3	3	4	4
1	1	3	3	2	2	3	3	4	4
1	1	4	4	2	2	3	3	4	4
1	1	4	4	3	3	2	2	4	4
1	1	4	4	2	2	3	3	4	4
1	1	4	4	2	2	3	3	4	4
2	2	3	3	3	3	2	2	4	4
2	2	3	3	2	2	3	3	4	4
2	2	3	3	2	2	3	3	4	4
1	1	4	4	2	2	3	3	4	4
1	1	4	4	2	2	3	3	4	4
2	2	3	3	3	3	3	3	1	1
1	1	4	4	2	2	3	3	4	4
1	1	4	4	3	3	2	2	4	4
1	1	4	4	3	3	2	2	4	4
1	1	4	4	3	3	2	2	4	4
1	1	4	4	2	2	3	3	4	4
1	1	4	4	3	3	2	2	4	4
1	1	3	3	2	2	3	3	4	4
1	1	4	4	2	2	3	3	4	4
1	1	4	4	2	2	3	3	4	4
1	1	4	4	2	2	3	3	4	4
1	1	4	4	2	2	3	3	4	4
1	1	4	4	3	3	2	2	4	4
1	1	4	4	2	2	3	3	4	4
1	1	4	4	2	2	3	3	4	4
1	1	4	4	2	2	3	3	4	4
1	1	4	4	2	2	3	3	4	4

For Review Only

1	1	3	3	2	2	3	3	4	4
1	1	4	4	3	3	2	2	4	4
1	1	4	4	2	2	3	3	4	4
1	1	4	4	2	2	3	3	4	4
1	1	3	3	2	2	3	3	4	4
1	1	4	4	2	2	3	3	4	4
1	1	4	4	2	2	3	3	4	4
1	1	3	3	2	2	3	3	4	4
1	1	4	4	2	2	3	3	4	4
1	1	4	4	2	2	2	2	4	4
2	2	3	3	2	2	3	3	4	4
1	1	4	4	2	2	3	3	4	4
1	1	4	4	2	2	3	3	4	4
1	1	4	4	2	2	3	3	4	4
1	1	4	4	2	2	3	3	4	4
1	1	4	4	2	2	3	3	4	4
1	1	4	4	2	2	3	3	4	4
1	1	4	4	2	2	3	3	4	4
1	1	4	4	2	2	3	3	4	4
1	1	4	4	2	2	3	3	4	4
1	1	4	4	2	2	3	3	4	4
1	1	4	4	2	2	3	3	4	4
1	1	4	4	2	2	3	3	4	4
1	1	4	4	2	2	3	3	4	4
1	1	4	4	2	2	3	3	4	4
1	1	4	4	2	2	3	3	1	1
2	2	3	3	3	3	2	2	4	4
1	1	4	4	2	2	3	3	4	4
1	1	4	4	2	2	3	3	4	4
1	1	3	3	3	3	2	2	4	4
1	1	4	4	2	2	3	3	4	4
1	1	4	4	2	2	3	3	4	4
1	1	4	4	3	3	2	2	4	4
1	1	4	4	2	2	3	3	4	4
1	1	4	4	2	2	3	3	4	4
1	1	4	4	2	2	3	3	4	4
1	1	4	4	2	2	3	3	4	4
1	1	4	4	2	2	3	3	4	4
1	1	4	4	2	2	3	3	4	4
1	1	4	4	2	2	3	3	4	4
1	1	4	4	3	3	2	2	4	4
1	1	4	4	2	2	3	3	4	4
1	1	4	4	2	2	3	3	4	4
1	1	4	4	2	2	3	3	4	4
1	1	4	4	2	2	3	3	4	4
1	1	4	4	2	2	3	3	4	4
1	1	3	3	2	2	3	3	4	4
1	1	3	3	2	2	3	3	4	4
2	2	4	4	2	2	3	3	4	4
1	1	4	4	2	2	3	3	4	4
1	1	3	3	2	2	3	3	4	4
1	1	4	4	2	2	3	3	4	4
1	1	4	4	2	2	3	3	4	4
1	1	4	4	2	2	3	3	4	4
1	1	4	4	2	2	3	3	4	4

For Review Only

1	1	4	4	2	2	3	3	4	4
1	1	4	4	2	2	3	3	4	4
1	1	4	4	2	2	3	3	4	4
1	1	4	4	2	2	3	3	4	4
2	2	3	3	2	2	3	3	4	4
1	1	4	4	2	2	3	3	4	4
1	1	4	4	2	2	3	3	4	4
1	1	4	4	2	2	3	3	4	4
1	1	4	4	2	2	3	3	4	4
1	1	4	4	3	3	2	2	4	4
1	1	4	4	2	2	3	3	4	4
1	1	4	4	2	2	3	3	4	4
1	1	4	4	2	2	3	3	4	4
1	1	3	3	2	2	3	3	4	4
1	1	4	4	2	2	3	3	4	4
2	2	3	3	2	2	3	3	4	4
1	1	4	4	2	2	3	3	4	4
1	1	3	3	3	3	2	2	4	4
1	1	4	4	2	2	3	3	4	4
1	1	4	4	2	2	3	3	4	4
1	1	4	4	2	2	3	3	4	4
1	1	4	4	2	2	3	3	4	4
1	1	4	4	2	2	3	3	1	1
1	1	4	4	2	2	3	3	4	4
2	2	3	3	3	3	2	2	4	4
1	1	4	4	2	2	3	3	4	4
1	1	3	3	2	2	3	3	4	4
1	1	4	4	2	2	3	3	4	4
1	1	4	4	2	2	3	3	4	4
1	1	4	4	2	2	3	3	4	4
1	1	3	3	2	2	3	3	4	4
1	1	4	4	2	2	3	3	4	4
2	2	3	3	3	3	2	2	4	4
1	1	4	4	2	2	3	3	4	4
1	1	4	4	2	2	3	3	4	4
1	1	4	4	2	2	3	3	4	4
1	1	3	3	2	2	3	3	4	4
1	1	4	4	3	3	3	3	1	1
1	1	3	3	2	2	3	3	4	4
1	1	3	3	3	3	2	2	4	4
1	1	4	4	2	2	3	3	4	4
1	1	3	3	2	2	3	3	4	4
1	1	4	4	2	2	3	3	4	4
1	1	4	4	2	2	3	3	4	4
1	1	4	4	3	3	2	2	4	4
1	1	4	4	2	2	3	3	4	4
2	2	3	3	2	2	3	3	4	4
1	1	4	4	2	2	3	3	4	4
1	1	4	4	2	2	3	3	4	4
1	1	4	4	2	2	3	3	4	4
1	1	4	4	2	2	3	3	4	4
1	1	4	4	2	2	3	3	4	4
1	1	4	4	2	2	3	3	1	1
1	1	4	4	2	2	3	3	4	4
1	1	4	4	2	2	3	3	4	4

1	1	4	4	2	2	3	3	4	4
1	1	4	4	2	2	3	3	4	4
1	1	4	4	2	2	3	3	4	4
1	1	4	4	2	2	3	3	4	4
1	1	4	4	2	2	3	3	4	4
1	1	3	3	2	2	3	3	4	4
1	1	4	4	2	2	3	3	4	4
1	1	4	4	2	2	3	3	4	4
1	1	4	4	2	2	3	3	4	4
1	1	4	4	2	2	3	3	4	4
1	1	4	4	2	2	3	3	4	4
1	1	3	3	2	2	3	3	4	4
2	2	3	3	3	3	3	3	1	1
1	1	4	4	2	2	3	3	4	4
1	1	4	4	2	2	3	3	4	4
2	2	4	4	2	2	3	3	1	1
1	1	4	4	2	2	3	3	4	4
1	1	4	4	3	3	2	2	4	4
1	1	4	4	2	2	3	3	4	4
1	1	4	4	2	2	3	3	4	4
1	1	4	4	2	2	3	3	4	4
1	1	3	3	2	2	3	3	4	4
1	1	4	4	3	3	2	2	4	4
1	1	4	4	2	2	3	3	4	4
1	1	4	4	2	2	3	3	4	4
1	1	4	4	2	2	3	3	4	4
1	1	4	4	2	2	3	3	4	4
1	1	3	3	2	2	3	3	4	4
1	1	4	4	3	3	2	2	4	4
1	1	4	4	2	2	3	3	4	4
1	1	4	4	2	2	3	3	4	4
2	2	3	3	2	2	3	3	4	4
2	2	3	3	2	2	3	3	4	4
1	1	4	4	2	2	3	3	4	4
1	1	4	4	2	2	3	3	4	4
1	1	4	4	2	2	3	3	4	4
1	1	4	4	3	3	2	2	4	4
1	1	4	4	2	2	3	3	4	4
1	1	4	4	2	2	3	3	4	4
1	1	4	4	2	2	3	3	4	4
1	1	4	4	2	2	3	3	4	4
1	1	4	4	2	2	3	3	4	4
1	1	4	4	2	2	3	3	4	4
1	1	4	4	3	3	2	2	4	4
1	1	4	4	2	2	3	3	4	4
2	2	3	3	3	3	3	3	1	1
1	1	4	4	2	2	3	3	4	4
1	1	4	4	2	2	3	3	4	4
2	2	3	3	2	2	3	3	4	4
1	1	4	4	2	2	3	3	4	4
2	2	3	3	2	2	3	3	4	4
1	1	4	4	2	2	3	3	4	4
1	1	4	4	2	2	3	3	4	4
1	1	3	3	3	3	2	2	4	4
1	1	4	4	2	2	3	3	4	4

For Review Only

2	2	3	3	2	2	3	3	4	4
1	1	3	3	2	2	3	3	4	4

For Review Only

SNP2468	SNP2468	SNP2469	SNP2469	SNP247	SNP247	SNP2470	SNP2470	SNP2471	SNP2471
4	4	3	3	2	2	1	1	2	2
4	4	2	2	4	4	4	4	2	2
4	4	2	2	2	2	1	1	2	2
4	4	2	2	4	4	4	4	2	2
4	4	3	3	2	2	4	4	4	4
4	4	2	2	2	2	1	1	2	2
4	4	2	2	4	4	4	4	2	2
4	4	2	2	4	4	1	1	2	2
4	4	2	2	4	4	4	4	2	2
4	4	3	3	4	4	4	4	2	2
4	4	2	2	4	4	4	4	2	2
4	4	2	2	4	4	1	1	2	2
4	4	2	2	4	4	4	4	2	2
4	4	2	2	4	4	1	1	2	2
4	4	2	2	2	2	1	1	4	4
4	4	2	2	4	4	1	1	2	2
1	1	2	2	4	4	4	4	4	4
4	4	3	3	2	2	4	4	2	2
4	4	2	2	2	2	1	1	2	2
1	1	2	2	4	4	1	1	2	2
4	4	2	2	4	4	4	4	4	4
1	1	2	2	4	4	4	4	2	2
4	4	2	2	2	2	4	4	2	2
4	4	2	2	4	4	4	4	2	2
4	4	2	2	4	4	4	4	2	2
4	4	2	2	4	4	4	4	2	2
4	4	2	2	4	4	4	4	2	2
4	4	2	2	2	2	1	1	2	2
4	4	3	3	4	4	4	4	4	4
4	4	3	3	4	4	4	4	2	2
4	4	2	2	2	2	4	4	2	2
4	4	3	3	4	4	4	4	2	2
4	4	2	2	4	4	4	4	2	2
4	4	3	3	4	4	1	1	2	2
4	4	2	2	4	4	1	1	2	2
4	4	2	2	4	4	1	1	2	2
4	4	2	2	4	4	1	1	2	2
4	4	2	2	2	2	1	1	2	2
4	4	2	2	4	4	1	1	2	2
4	4	3	3	4	4	1	1	2	2
4	4	3	3	4	4	1	1	2	2
4	4	3	3	2	2	4	4	4	4
4	4	3	3	4	4	1	1	2	2
1	1	2	2	4	4	4	4	2	2
4	4	3	3	4	4	1	1	2	2
4	4	2	2	4	4	1	1	2	2
4	4	2	2	4	4	4	4	2	2
4	4	2	2	4	4	4	4	2	2
1	1	2	2	2	2	1	1	2	2
1	1	2	2	2	2	1	1	4	4
4	4	2	2	4	4	4	4	2	2
4	4	2	2	2	2	4	4	2	2

For Review Only

1	1	2	2	4	4	4	4	4	4
4	4	2	2	4	4	4	4	2	2
4	4	2	2	4	4	4	4	2	2
1	1	2	2	4	4	1	1	2	2
4	4	2	2	2	2	1	1	2	2
4	4	2	2	2	2	4	4	2	2
4	4	2	2	4	4	4	4	2	2
4	4	2	2	4	4	4	4	2	2
4	4	2	2	4	4	1	1	2	2
4	4	2	2	2	2	1	1	2	2
4	4	2	2	4	4	1	1	2	2
4	4	2	2	2	2	1	1	2	2
1	1	2	2	4	4	4	4	4	4
4	4	3	3	4	4	4	4	4	4
4	4	2	2	2	2	1	1	2	2
4	4	2	2	2	2	1	1	2	2
4	4	2	2	2	2	4	4	2	2
4	4	2	2	2	2	1	1	2	2
1	1	2	2	2	2	1	1	4	4
4	4	2	2	2	2	4	4	4	4
4	4	2	2	2	2	1	1	2	2
4	4	3	3	2	2	1	1	2	2
4	4	2	2	4	4	4	4	2	2
4	4	3	3	4	4	1	1	2	2
4	4	3	3	4	4	1	1	2	2
4	4	2	2	2	2	1	1	2	2
1	1	2	2	4	4	4	4	2	2
4	4	3	3	4	4	4	4	2	2
4	4	2	2	4	4	4	4	2	2
4	4	2	2	2	2	4	4	2	2
4	4	3	3	4	4	1	1	2	2
4	4	2	2	4	4	1	1	2	2
4	4	2	2	4	4	4	4	2	2
1	1	2	2	4	4	4	4	4	4
4	4	2	2	2	2	4	4	4	4
4	4	3	3	4	4	1	1	2	2
4	4	2	2	4	4	1	1	2	2
4	4	2	2	4	4	4	4	2	2
4	4	3	3	2	2	4	4	2	2
4	4	2	2	2	2	1	1	2	2
1	1	2	2	2	2	4	4	4	4
1	1	3	3	2	2	4	4	2	2
1	1	2	2	2	2	1	1	2	2
4	4	2	2	4	4	1	1	2	2
1	1	2	2	4	4	4	4	2	2
4	4	2	2	2	2	4	4	2	2
4	4	2	2	4	4	4	4	2	2
4	4	2	2	2	2	1	1	2	2
4	4	2	2	4	4	1	1	2	2
4	4	2	2	4	4	1	1	2	2
1	1	2	2	4	4	1	1	2	2
4	4	2	2	4	4	1	1	2	2
4	4	3	3	4	4	4	4	2	2
4	4	2	2	2	2	1	1	2	2
4	4	2	2	2	2	1	1	2	2

For Review Only

4	4	2	2	2	2	4	4	2	2
4	4	3	3	2	2	4	4	2	2
4	4	2	2	4	4	4	4	2	2
4	4	2	2	2	2	1	1	2	2
4	4	2	2	4	4	4	4	2	2
4	4	2	2	4	4	1	1	2	2
4	4	2	2	2	2	1	1	2	2
4	4	2	2	2	2	1	1	2	2
4	4	2	2	4	4	1	1	2	2
1	1	2	2	4	4	1	1	2	2
4	4	2	2	2	2	1	1	2	2
4	4	2	2	2	2	1	1	2	2
4	4	2	2	2	2	1	1	2	2
4	4	2	2	2	2	1	1	2	2
4	4	2	2	4	4	1	1	2	2
4	4	2	2	4	4	1	1	2	2
4	4	2	2	2	2	1	1	2	2
4	4	2	2	2	2	1	1	2	2
4	4	2	2	4	4	1	1	2	2
4	4	2	2	2	2	4	4	2	2
4	4	2	2	2	2	1	1	2	2
4	4	2	2	2	2	4	4	2	2
4	4	2	2	2	2	1	1	2	2
4	4	2	2	4	4	4	4	2	2
4	4	2	2	4	4	1	1	2	2
4	4	3	3	2	2	1	1	2	2
1	1	2	2	2	2	1	1	2	2
4	4	2	2	4	4	1	1	2	2
4	4	2	2	4	4	1	1	2	2
4	4	2	2	2	2	1	1	2	2
4	4	3	3	4	4	1	1	2	2
4	4	2	2	2	2	1	1	2	2
1	1	2	2	2	2	4	4	4	4
4	4	2	2	4	4	1	1	2	2
4	4	3	3	4	4	1	1	2	2
4	4	2	2	4	4	1	1	2	2
4	4	2	2	2	2	4	4	2	2
4	4	2	2	4	4	1	1	2	2
4	4	2	2	2	2	4	4	2	2
4	4	2	2	4	4	4	4	2	2
4	4	2	2	4	4	1	1	2	2
4	4	2	2	4	4	1	1	2	2
4	4	2	2	2	2	1	1	2	2
4	4	3	3	4	4	1	1	2	2
4	4	2	2	4	4	1	1	2	2
4	4	2	2	4	4	1	1	2	2
4	4	2	2	4	4	4	4	2	2
4	4	2	2	4	4	4	4	2	2
4	4	2	2	4	4	1	1	2	2
4	4	2	2	4	4	4	4	2	2
4	4	2	2	4	4	4	4	2	2
4	4	2	2	2	2	4	4	2	2
4	4	2	2	2	2	1	1	2	2
4	4	2	2	4	4	1	1	2	2
4	4	2	2	4	4	1	1	2	2

For Review Only

4	4	3	3	4	4	1	1	2	2
4	4	2	2	2	2	1	1	2	2
4	4	3	3	4	4	4	4	2	2
4	4	3	3	4	4	1	1	2	2
4	4	2	2	4	4	4	4	2	2
4	4	3	3	4	4	4	4	2	2
4	4	2	2	4	4	1	1	2	2
4	4	2	2	2	2	4	4	2	2
4	4	2	2	2	2	4	4	2	2
1	1	2	2	2	2	4	4	2	2
4	4	2	2	4	4	4	4	2	2
4	4	2	2	4	4	4	4	2	2
4	4	2	2	4	4	1	1	2	2
4	4	2	2	4	4	1	1	2	2
4	4	2	2	4	4	1	1	2	2
4	4	2	2	4	4	4	4	2	2
4	4	2	2	2	2	1	1	2	2
4	4	2	2	4	4	1	1	2	2
4	4	2	2	4	4	4	4	2	2
4	4	2	2	2	2	1	1	2	2
4	4	2	2	2	2	1	1	2	2
4	4	2	2	4	4	4	4	2	2
4	4	3	3	2	2	1	1	2	2
4	4	2	2	2	2	1	1	2	2
1	1	2	2	2	2	4	4	2	2
4	4	2	2	4	4	1	1	2	2
4	4	2	2	2	2	1	1	2	2
4	4	2	2	2	2	1	1	2	2
4	4	2	2	4	4	1	1	2	2
4	4	2	2	2	2	1	1	2	2
4	4	2	2	2	2	4	4	2	2
4	4	2	2	2	2	4	4	2	2
1	1	2	2	4	4	1	1	2	2
4	4	3	3	4	4	1	1	2	2
4	4	2	2	2	2	1	1	2	2
4	4	2	2	2	2	1	1	2	2
4	4	2	2	4	4	4	4	2	2
4	4	2	2	2	2	1	1	2	2
4	4	2	2	4	4	1	1	2	2
1	1	2	2	2	2	1	1	2	2
4	4	2	2	2	2	1	1	2	2
4	4	2	2	4	4	4	4	2	2
4	4	2	2	4	4	4	4	2	2
4	4	2	2	2	2	1	1	2	2
4	4	2	2	2	2	1	1	2	2
4	4	2	2	2	2	4	4	2	2
4	4	2	2	4	4	4	4	2	2
4	4	2	2	4	4	4	4	2	2
4	4	2	2	4	4	4	4	2	2
4	4	2	2	4	4	1	1	2	2
4	4	2	2	2	2	1	1	2	2
4	4	2	2	4	4	4	4	2	2
4	4	3	3	4	4	1	1	2	2
4	4	2	2	4	4	1	1	2	2
4	4	3	3	4	4	4	4	2	2

For Review Only

4	4	2	2	2	2	1	1	2	2
4	4	2	2	4	4	1	1	2	2
4	4	2	2	2	2	1	1	2	2
4	4	2	2	2	2	1	1	2	2
4	4	2	2	4	4	1	1	2	2
4	4	2	2	2	2	4	4	2	2
4	4	2	2	2	2	4	4	2	2
4	4	3	3	2	2	1	1	2	2
4	4	2	2	4	4	4	4	2	2
4	4	3	3	4	4	1	1	2	2
4	4	2	2	2	2	1	1	2	2
4	4	2	2	2	2	4	4	2	2
4	4	3	3	2	2	4	4	2	2
4	4	2	2	2	2	4	4	2	2
4	4	2	2	2	2	1	1	2	2
4	4	2	2	4	4	4	4	2	2
4	4	2	2	2	2	1	1	2	2
1	1	2	2	2	2	1	1	2	2
4	4	2	2	4	4	4	4	2	2
4	4	2	2	2	2	4	4	2	2
4	4	3	3	4	4	4	4	2	2
4	4	2	2	4	4	4	4	2	2
1	1	2	2	2	2	1	1	2	2
4	4	3	3	2	2	1	1	2	2
4	4	2	2	4	4	4	4	2	2
4	4	2	2	2	2	4	4	2	2
4	4	3	3	4	4	4	4	2	2
4	4	2	2	4	4	4	4	2	2
4	4	2	2	4	4	4	4	4	4
4	4	2	2	2	2	1	1	2	2
4	4	2	2	4	4	4	4	2	2
4	4	2	2	4	4	4	4	2	2
4	4	2	2	4	4	4	4	2	2
4	4	2	2	4	4	1	1	2	2
4	4	3	3	4	4	1	1	2	2
4	4	3	3	4	4	4	4	2	2
1	1	2	2	2	2	1	1	4	4
4	4	2	2	2	2	1	1	2	2
4	4	2	2	4	4	1	1	2	2
4	4	3	3	4	4	4	4	2	2
4	4	2	2	2	2	1	1	2	2
4	4	2	2	4	4	4	4	2	2
4	4	2	2	4	4	1	1	2	2
1	1	2	2	4	4	4	4	4	4
4	4	2	2	2	2	1	1	2	2
4	4	2	2	2	2	4	4	2	2
4	4	2	2	4	4	4	4	2	2
4	4	2	2	2	2	1	1	2	2
4	4	3	3	2	2	4	4	2	2
4	4	3	3	4	4	4	4	2	2
4	4	2	2	4	4	4	4	2	2
4	4	2	2	4	4	1	1	2	2
4	4	2	2	2	2	1	1	2	2
1	1	2	2	2	2	4	4	2	2
4	4	3	3	4	4	1	1	2	2

For Review Only

4	4	2	2	4	4	4	4	2	2
4	4	2	2	4	4	4	4	2	2

For Review Only

SNP2472	SNP2472	SNP2473	SNP2473	SNP2474	SNP2474	SNP2475	SNP2475	SNP2476	SNP2476
4	4	1	1	3	3	1	1	3	3
4	4	1	1	2	2	3	3	3	3
4	4	4	4	3	3	3	3	3	3
4	4	1	1	3	3	3	3	2	2
2	2	1	1	3	3	3	3	2	2
4	4	1	1	3	3	3	3	3	3
4	4	1	1	3	3	3	3	2	2
4	4	4	4	3	3	3	3	2	2
4	4	1	1	3	3	3	3	2	2
4	4	4	4	3	3	3	3	2	2
2	2	1	1	3	3	3	3	2	2
4	4	1	1	3	3	3	3	3	3
4	4	1	1	3	3	3	3	2	2
4	4	1	1	3	3	3	3	2	2
4	4	1	1	3	3	3	3	2	2
4	4	1	1	3	3	3	3	2	2
4	4	1	1	3	3	3	3	2	2
4	4	1	1	3	3	3	3	3	3
4	4	1	1	3	3	3	3	3	3
4	4	1	1	3	3	3	3	2	2
4	4	1	1	3	3	3	3	3	3
4	4	1	1	3	3	3	3	2	2
2	2	1	1	3	3	1	1	2	2
4	4	1	1	3	3	3	3	2	2
4	4	1	1	3	3	3	3	3	3
4	4	4	4	3	3	3	3	2	2
4	4	1	1	3	3	3	3	3	3
4	4	1	1	3	3	3	3	2	2
4	4	1	1	3	3	3	3	3	3
4	4	1	1	3	3	3	3	2	2
4	4	4	4	3	3	3	3	3	3
4	4	1	1	3	3	3	3	2	2
4	4	1	1	3	3	3	3	2	2
4	4	1	1	3	3	3	3	2	2
4	4	1	1	3	3	1	1	2	2
4	4	1	1	3	3	3	3	2	2
4	4	1	1	3	3	3	3	3	3
2	2	1	1	3	3	3	3	2	2
2	2	1	1	3	3	1	1	2	2
2	2	1	1	3	3	3	3	2	2
2	2	1	1	3	3	3	3	2	2
4	4	1	1	3	3	3	3	3	3
2	2	1	1	3	3	3	3	2	2
4	4	1	1	3	3	3	3	2	2
2	2	4	4	3	3	3	3	2	2
4	4	4	4	3	3	3	3	3	3
4	4	1	1	2	2	3	3	2	2
2	2	1	1	3	3	3	3	2	2
4	4	1	1	3	3	3	3	2	2
4	4	1	1	3	3	3	3	2	2
4	4	4	4	3	3	3	3	2	2
4	4	4	4	2	2	3	3	2	2

For Review Only

4	4	1	1	3	3	3	3	2	2
4	4	1	1	3	3	3	3	2	2
4	4	1	1	3	3	3	3	2	2
4	4	1	1	3	3	3	3	2	2
4	4	1	1	3	3	3	3	3	3
4	4	4	4	3	3	3	3	2	2
4	4	4	4	3	3	3	3	2	2
2	2	1	1	3	3	3	3	2	2
4	4	1	1	3	3	3	3	2	2
4	4	1	1	3	3	3	3	2	2
4	4	1	1	2	2	3	3	3	3
4	4	1	1	2	2	3	3	3	3
4	4	1	1	3	3	3	3	2	2
4	4	1	1	3	3	3	3	3	3
4	4	1	1	3	3	3	3	2	2
4	4	1	1	3	3	3	3	2	2
4	4	1	1	3	3	1	1	3	3
4	4	1	1	3	3	3	3	2	2
4	4	1	1	3	3	3	3	3	3
4	4	1	1	3	3	3	3	2	2
4	4	1	1	2	2	3	3	3	3
4	4	1	1	3	3	1	1	2	2
4	4	1	1	3	3	3	3	2	2
4	4	1	1	3	3	3	3	3	3
2	2	4	4	3	3	3	3	2	2
4	4	4	4	2	2	3	3	3	3
4	4	4	4	3	3	3	3	2	2
2	2	1	1	3	3	3	3	2	2
4	4	4	4	3	3	3	3	2	2
4	4	1	1	3	3	3	3	2	2
4	4	1	1	3	3	3	3	2	2
4	4	1	1	3	3	3	3	2	2
2	2	1	1	3	3	3	3	2	2
4	4	1	1	3	3	3	3	2	2
4	4	1	1	3	3	3	3	2	2
2	2	4	4	3	3	3	3	2	2
4	4	4	4	3	3	3	3	2	2
2	2	1	1	3	3	3	3	2	2
4	4	4	4	3	3	1	1	2	2
4	4	1	1	3	3	3	3	2	2
4	4	1	1	3	3	3	3	2	2
4	4	4	4	3	3	3	3	2	2
4	4	4	4	3	3	3	3	2	2
4	4	1	1	2	2	3	3	3	3
4	4	1	1	3	3	3	3	2	2
4	4	1	1	3	3	3	3	2	2
4	4	1	1	3	3	3	3	2	2
4	4	4	4	3	3	3	3	2	2
4	4	1	1	3	3	3	3	2	2
4	4	1	1	3	3	3	3	2	2
4	4	1	1	3	3	3	3	3	3
2	2	1	1	3	3	1	1	2	2
2	2	1	1	3	3	1	1	3	3
4	4	1	1	3	3	1	1	2	2
4	4	1	1	3	3	3	3	2	2

For Review Only

4	4	4	4	3	3	3	3	2	2
4	4	1	1	3	3	3	3	2	2
4	4	4	4	3	3	3	3	2	2
4	4	1	1	3	3	3	3	2	2
4	4	1	1	3	3	3	3	2	2
4	4	1	1	3	3	3	3	2	2
4	4	1	1	3	3	3	3	2	2
4	4	1	1	3	3	3	3	2	2
4	4	1	1	3	3	3	3	2	2
4	4	1	1	3	3	3	3	2	2
4	4	1	1	3	3	3	3	2	2
4	4	1	1	3	3	3	3	2	2
2	2	4	4	3	3	3	3	2	2
4	4	4	4	2	2	3	3	3	3
4	4	1	1	3	3	3	3	2	2
4	4	1	1	3	3	3	3	2	2
4	4	1	1	3	3	3	3	2	2
4	4	1	1	3	3	3	3	2	2
4	4	1	1	3	3	3	3	2	2
4	4	1	1	3	3	3	3	2	2
4	4	1	1	3	3	3	3	2	2
4	4	1	1	3	3	3	3	2	2
4	4	1	1	3	3	3	3	3	3
4	4	1	1	3	3	3	3	2	2
4	4	1	1	3	3	3	3	2	2
4	4	1	1	3	3	1	1	2	2
4	4	1	1	3	3	3	3	2	2
4	4	1	1	3	3	3	3	2	2
4	4	1	1	3	3	3	3	2	2
4	4	1	1	3	3	3	3	2	2
4	4	1	1	3	3	3	3	2	2
4	4	1	1	3	3	3	3	2	2
4	4	1	1	3	3	3	3	2	2
4	4	1	1	3	3	3	3	2	2
4	4	1	1	3	3	3	3	2	2
4	4	1	1	3	3	3	3	2	2
4	4	1	1	3	3	3	3	2	2
4	4	1	1	2	2	3	3	3	3
4	4	1	1	3	3	3	3	2	2
2	2	1	1	3	3	3	3	2	2
4	4	1	1	3	3	1	1	2	2
4	4	1	1	3	3	3	3	2	2
4	4	1	1	3	3	3	3	2	2
4	4	1	1	3	3	3	3	2	2
4	4	4	4	3	3	3	3	2	2
4	4	1	1	3	3	3	3	2	2
4	4	4	4	3	3	3	3	2	2
4	4	4	4	3	3	3	3	2	2
4	4	1	1	3	3	3	3	2	2
4	4	4	4	3	3	3	3	2	2
4	4	4	4	3	3	3	3	2	2
2	2	1	1	3	3	1	1	2	2
4	4	1	1	3	3	3	3	2	2
4	4	4	4	3	3	3	3	3	3
4	4	1	1	3	3	3	3	2	2

For Review Only

4	4	1	1	3	3	3	3	2	2
2	2	4	4	3	3	1	1	2	2
2	2	1	1	3	3	3	3	2	2
4	4	1	1	3	3	1	1	2	2
4	4	1	1	3	3	3	3	3	3
4	4	1	1	3	3	3	3	2	2
2	2	1	1	3	3	3	3	2	2
4	4	1	1	3	3	3	3	2	2
4	4	1	1	3	3	1	1	2	2
4	4	1	1	3	3	3	3	3	3
2	2	4	4	3	3	3	3	2	2
2	2	1	1	3	3	3	3	2	2
4	4	1	1	3	3	3	3	2	2
4	4	1	1	3	3	3	3	2	2
4	4	1	1	3	3	3	3	2	2
2	2	1	1	3	3	3	3	2	2
4	4	1	1	3	3	1	1	2	2
4	4	4	4	2	2	3	3	3	3
4	4	1	1	3	3	3	3	2	2
4	4	1	1	3	3	3	3	2	2
4	4	1	1	3	3	3	3	2	2
4	4	1	1	3	3	1	1	2	2
4	4	1	1	3	3	1	1	2	2
4	4	1	1	2	2	3	3	3	3
4	4	1	1	3	3	3	3	2	2
4	4	1	1	3	3	3	3	2	2
4	4	1	1	3	3	3	3	2	2
4	4	1	1	3	3	3	3	2	2
4	4	4	4	3	3	3	3	2	2
2	2	1	1	3	3	3	3	2	2
4	4	4	4	3	3	1	1	2	2
4	4	1	1	3	3	3	3	2	2
4	4	1	1	3	3	3	3	2	2
4	4	1	1	3	3	3	3	2	2
4	4	1	1	3	3	3	3	2	2
4	4	1	1	3	3	3	3	3	3
2	2	1	1	3	3	3	3	3	3
4	4	1	1	3	3	3	3	3	3
4	4	4	4	3	3	3	3	2	2
4	4	4	4	3	3	3	3	2	2
4	4	4	4	3	3	3	3	3	3
4	4	4	4	3	3	3	3	2	2
2	2	1	1	3	3	1	1	2	2
4	4	1	1	3	3	3	3	2	2
4	4	1	1	3	3	3	3	2	2
4	4	1	1	3	3	3	3	3	3
2	2	1	1	3	3	3	3	2	2
2	2	1	1	3	3	3	3	2	2
4	4	1	1	3	3	3	3	2	2
4	4	1	1	3	3	3	3	2	2
4	4	1	1	3	3	3	3	2	2
4	4	1	1	3	3	3	3	2	2
4	4	1	1	3	3	1	1	2	2
4	4	1	1	2	2	3	3	3	3
2	2	1	1	3	3	1	1	2	2

For Review Only

4	4	1	1	3	3	1	1	2	2
4	4	1	1	3	3	3	3	2	2
4	4	1	1	3	3	3	3	2	2
4	4	1	1	3	3	1	1	2	2
2	2	1	1	3	3	3	3	2	2
4	4	1	1	3	3	3	3	2	2
4	4	1	1	3	3	1	1	2	2
2	2	1	1	3	3	3	3	2	2
4	4	1	1	3	3	3	3	2	2
2	2	1	1	3	3	1	1	2	2
4	4	1	1	3	3	3	3	2	2
4	4	1	1	3	3	1	1	2	2
4	4	4	4	3	3	1	1	2	2
4	4	4	4	3	3	3	3	2	2
4	4	4	4	3	3	3	3	3	3
4	4	1	1	3	3	3	3	2	2
2	2	1	1	3	3	1	1	2	2
4	4	1	1	3	3	1	1	2	2
2	2	1	1	3	3	1	1	2	2
2	2	1	1	3	3	3	3	2	2
4	4	1	1	3	3	1	1	2	2
4	4	4	4	3	3	3	3	2	2
4	4	1	1	3	3	3	3	2	2
2	2	1	1	3	3	1	1	2	2
2	2	1	1	3	3	3	3	2	2
4	4	4	4	3	3	3	3	2	2
2	2	1	1	3	3	3	3	2	2
4	4	1	1	3	3	3	3	2	2
4	4	1	1	3	3	3	3	2	2
4	4	1	1	3	3	3	3	2	2
2	2	1	1	3	3	1	1	2	2
2	2	4	4	3	3	3	3	2	2
4	4	1	1	3	3	3	3	2	2
4	4	4	4	3	3	3	3	2	2
2	2	1	1	3	3	1	1	2	2
2	2	1	1	3	3	3	3	2	2
4	4	1	1	3	3	3	3	2	2
4	4	1	1	2	2	3	3	3	3
4	4	1	1	3	3	3	3	2	2
2	2	1	1	3	3	1	1	2	2
4	4	1	1	3	3	3	3	2	2
4	4	1	1	3	3	3	3	2	2
2	2	1	1	3	3	1	1	2	2
4	4	1	1	3	3	3	3	2	2
4	4	1	1	3	3	3	3	3	3
4	4	4	4	3	3	1	1	3	3
2	2	4	4	3	3	1	1	2	2
4	4	4	4	3	3	3	3	2	2
4	4	4	4	3	3	3	3	2	2
2	2	1	1	3	3	3	3	2	2
4	4	1	1	3	3	3	3	2	2
2	2	1	1	3	3	1	1	2	2
4	4	4	4	3	3	3	3	3	3
4	4	1	1	3	3	3	3	2	2
2	2	1	1	3	3	3	3	2	2

For Review Only

4	4	1	1	3	3	3	3	2	2
4	4	1	1	2	2	3	3	3	3

For Review Only

SNP2477	SNP2477	SNP2478	SNP2478	SNP2479	SNP2479	SNP248	SNP248	SNP2480	SNP2480
4	4	3	3	4	4	4	4	1	1
4	4	3	3	4	4	4	4	1	1
4	4	4	4	4	4	3	3	1	1
4	4	4	4	4	4	4	4	1	1
4	4	4	4	1	1	4	4	1	1
4	4	4	4	4	4	3	3	1	1
4	4	3	3	4	4	3	3	1	1
4	4	4	4	1	1	4	4	1	1
4	4	3	3	4	4	3	3	1	1
4	4	4	4	1	1	4	4	1	1
4	4	4	4	4	4	4	4	1	1
4	4	4	4	4	4	3	3	1	1
4	4	4	4	4	4	3	3	1	1
4	4	4	4	4	4	4	4	1	1
4	4	3	3	4	4	3	3	1	1
4	4	4	4	4	4	4	4	1	1
4	4	3	3	4	4	4	4	1	1
4	4	4	4	1	1	3	3	1	1
4	4	4	4	4	4	3	3	1	1
4	4	4	4	1	1	4	4	1	1
1	1	4	4	4	4	3	3	1	1
1	1	4	4	4	4	3	3	1	1
4	4	4	4	4	4	3	3	1	1
4	4	4	4	4	4	4	4	1	1
4	4	3	3	4	4	4	4	1	1
4	4	3	3	4	4	3	3	1	1
4	4	3	3	4	4	4	4	1	1
4	4	3	3	4	4	4	4	1	1
4	4	3	3	4	4	4	4	3	3
4	4	4	4	1	1	4	4	1	1
4	4	4	4	4	4	3	3	3	3
4	4	4	4	4	4	3	3	1	1
4	4	4	4	4	4	3	3	1	1
4	4	3	3	4	4	3	3	1	1
4	4	3	3	4	4	4	4	1	1
4	4	4	4	4	4	4	4	1	1
4	4	4	4	4	4	4	4	1	1
1	1	4	4	4	4	4	4	1	1
4	4	4	4	4	4	4	4	1	1
4	4	3	3	4	4	4	4	1	1
4	4	4	4	1	1	4	4	1	1
4	4	3	3	4	4	4	4	3	3
4	4	4	4	1	1	4	4	1	1
1	1	4	4	4	4	4	4	1	1
4	4	4	4	1	1	4	4	1	1
4	4	4	4	4	4	3	3	1	1
4	4	3	3	4	4	3	3	1	1
4	4	4	4	4	4	3	3	1	1
4	4	4	4	4	4	4	4	1	1
4	4	4	4	4	4	3	3	1	1
4	4	3	3	4	4	3	3	1	1
4	4	3	3	4	4	4	4	1	1

For Review Only

4	4	3	3	4	4	4	4	1	1
4	4	4	4	4	4	3	3	1	1
4	4	3	3	4	4	4	4	3	3
1	1	4	4	4	4	4	4	1	1
4	4	4	4	4	4	3	3	1	1
4	4	3	3	4	4	3	3	1	1
4	4	3	3	4	4	3	3	1	1
4	4	4	4	1	1	4	4	1	1
4	4	4	4	4	4	3	3	1	1
4	4	3	3	4	4	4	4	1	1
4	4	4	4	4	4	3	3	1	1
4	4	4	4	4	4	3	3	1	1
4	4	3	3	4	4	4	4	1	1
4	4	3	3	4	4	4	4	3	3
4	4	3	3	4	4	4	4	1	1
4	4	4	4	1	1	4	4	1	1
4	4	4	4	4	4	4	4	1	1
4	4	3	3	4	4	3	3	1	1
4	4	3	3	4	4	3	3	1	1
4	4	3	3	4	4	3	3	1	1
4	4	4	4	4	4	3	3	1	1
4	4	3	3	4	4	4	4	1	1
4	4	3	3	4	4	3	3	1	1
4	4	3	3	1	1	3	3	1	1
4	4	4	4	1	1	4	4	1	1
4	4	4	4	4	4	3	3	1	1
1	1	4	4	1	1	4	4	1	1
4	4	4	4	4	4	4	4	1	1
4	4	3	3	4	4	4	4	1	1
4	4	4	4	4	4	4	4	1	1
1	1	4	4	1	1	4	4	1	1
4	4	4	4	4	4	4	4	1	1
4	4	4	4	4	4	4	4	1	1
4	4	3	3	4	4	4	4	1	1
4	4	3	3	4	4	3	3	1	1
4	4	4	4	1	1	4	4	1	1
1	1	4	4	1	1	4	4	1	1
1	1	4	4	4	4	3	3	1	1
4	4	4	4	1	1	4	4	1	1
4	4	4	4	4	4	4	4	1	1
1	1	4	4	4	4	3	3	1	1
4	4	4	4	4	4	3	3	3	3
1	1	4	4	4	4	4	4	1	1
4	4	4	4	4	4	3	3	1	1
4	4	4	4	4	4	3	3	1	1
4	4	3	3	4	4	3	3	1	1
4	4	4	4	4	4	4	4	1	1
4	4	4	4	4	4	4	4	1	1
4	4	4	4	4	4	3	3	1	1
4	4	3	3	4	4	3	3	1	1
4	4	3	3	4	4	4	4	1	1
4	4	4	4	4	4	4	4	1	1
4	4	4	4	4	4	4	4	1	1
4	4	4	4	4	4	4	4	1	1
4	4	3	3	4	4	3	3	1	1

For Review Only

4	4	3	3	4	4	4	4	1	1
4	4	3	3	4	4	4	4	1	1
4	4	4	4	4	4	3	3	1	1
4	4	4	4	4	4	4	4	1	1
4	4	3	3	4	4	3	3	1	1
4	4	4	4	4	4	3	3	1	1
4	4	4	4	4	4	4	4	1	1
4	4	3	3	4	4	4	4	1	1
4	4	4	4	4	4	4	4	1	1
4	4	4	4	1	1	4	4	1	1
4	4	3	3	4	4	4	4	1	1
4	4	3	3	4	4	4	4	1	1
1	1	4	4	4	4	3	3	1	1
4	4	3	3	4	4	3	3	1	1
4	4	3	3	4	4	4	4	1	1
4	4	3	3	4	4	4	4	1	1
4	4	3	3	4	4	3	3	1	1
4	4	3	3	4	4	4	4	1	1
4	4	4	4	4	4	4	4	3	3
1	1	4	4	4	4	4	4	1	1
4	4	3	3	4	4	4	4	1	1
4	4	4	4	4	4	4	4	1	1
4	4	4	4	4	4	3	3	1	1
4	4	4	4	4	4	4	4	1	1
4	4	3	3	4	4	4	4	1	1
4	4	3	3	4	4	3	3	3	3
4	4	3	3	4	4	4	4	3	3
4	4	4	4	4	4	4	4	1	1
4	4	4	4	4	4	3	3	1	1
4	4	4	4	4	4	4	4	1	1
4	4	4	4	4	4	4	4	1	1
4	4	3	3	4	4	4	4	1	1
1	1	4	4	4	4	3	3	1	1
4	4	3	3	4	4	4	4	1	1
4	4	3	3	4	4	3	3	1	1
4	4	3	3	4	4	3	3	1	1
4	4	4	4	4	4	4	4	1	1
4	4	4	4	4	4	3	3	1	1
4	4	4	4	4	4	4	4	1	1
4	4	4	4	4	4	4	4	1	1
4	4	3	3	4	4	4	4	1	1
4	4	4	4	4	4	4	4	3	3
4	4	4	4	4	4	4	4	1	1
4	4	4	4	4	4	4	4	1	1
4	4	4	4	4	4	3	3	1	1
4	4	4	4	4	4	4	4	1	1
4	4	3	3	4	4	3	3	1	1
4	4	3	3	4	4	4	4	1	1
4	4	4	4	4	4	4	4	1	1
4	4	3	3	4	4	4	4	1	1
4	4	3	3	4	4	3	3	1	1
4	4	4	4	4	4	4	4	1	1
4	4	4	4	4	4	3	3	1	1
4	4	4	4	4	4	3	3	1	1
4	4	4	4	4	4	4	4	1	1

For Review Only

4	4	4	4	4	4	3	3	1	1
4	4	3	3	4	4	4	4	1	1
1	1	4	4	1	1	4	4	1	1
4	4	3	3	4	4	3	3	1	1
4	4	3	3	4	4	4	4	1	1
4	4	4	4	4	4	4	4	1	1
4	4	4	4	4	4	4	4	1	1
4	4	3	3	4	4	4	4	1	1
4	4	4	4	4	4	4	4	1	1
4	4	4	4	4	4	3	3	1	1
4	4	3	3	4	4	3	3	1	1
4	4	3	3	4	4	4	4	1	1
4	4	4	4	4	4	3	3	1	1
4	4	4	4	4	4	4	4	1	1
4	4	4	4	4	4	4	4	3	3
4	4	4	4	4	4	4	4	1	1
4	4	3	3	4	4	3	3	1	1
4	4	3	3	4	4	3	3	1	1
4	4	4	4	4	4	3	3	1	1
4	4	4	4	4	4	4	4	1	1
4	4	3	3	4	4	4	4	1	1
4	4	4	4	4	4	4	4	1	1
4	4	3	3	4	4	4	4	3	3
4	4	4	4	4	4	3	3	1	1
4	4	3	3	4	4	3	3	1	1
4	4	3	3	4	4	4	4	1	1
4	4	4	4	4	4	3	3	1	1
4	4	3	3	4	4	3	3	1	1
4	4	3	3	4	4	3	3	1	1
4	4	4	4	4	4	3	3	1	1
4	4	3	3	4	4	4	4	1	1
4	4	4	4	4	4	4	4	1	1
4	4	3	3	4	4	4	4	3	3
4	4	4	4	4	4	4	4	3	3
4	4	4	4	4	4	4	4	1	1
4	4	4	4	4	4	3	3	1	1
4	4	3	3	4	4	3	3	1	1
4	4	4	4	4	4	4	4	1	1
4	4	3	3	4	4	4	4	1	1
4	4	4	4	4	4	3	3	1	1
4	4	4	4	4	4	3	3	1	1
4	4	3	3	4	4	3	3	1	1
4	4	4	4	4	4	4	4	1	1
1	1	3	3	4	4	4	4	1	1
4	4	4	4	4	4	4	4	1	1
4	4	4	4	4	4	3	3	1	1
4	4	4	4	4	4	4	4	1	1
4	4	4	4	4	4	4	4	1	1
4	4	4	4	4	4	4	4	1	1
1	1	4	4	4	4	4	4	1	1
4	4	4	4	4	4	3	3	1	1
4	4	4	4	4	4	4	4	1	1
4	4	3	3	4	4	4	4	3	3
4	4	4	4	4	4	3	3	1	1
4	4	4	4	4	4	4	4	1	1

For Review Only

4	4	4	4	4	4	3	3	1	1
4	4	4	4	4	4	4	4	1	1
4	4	4	4	4	4	4	4	1	1
4	4	3	3	4	4	4	4	1	1
4	4	4	4	4	4	4	4	1	1
4	4	3	3	4	4	3	3	1	1
4	4	3	3	4	4	3	3	1	1
4	4	4	4	1	1	4	4	1	1
4	4	4	4	4	4	4	4	1	1
4	4	4	4	4	4	4	4	1	1
4	4	3	3	4	4	4	4	1	1
4	4	3	3	1	1	3	3	1	1
4	4	4	4	1	1	4	4	1	1
4	4	4	4	4	4	4	4	1	1
4	4	4	4	4	4	3	3	1	1
4	4	4	4	4	4	4	4	1	1
4	4	4	4	4	4	4	4	1	1
4	4	4	4	4	4	4	4	1	1
4	4	4	4	4	4	4	4	1	1
4	4	4	4	4	4	4	4	1	1
4	4	4	4	4	4	4	4	1	1
4	4	3	3	4	4	3	3	1	1
4	4	4	4	4	4	4	4	1	1
4	4	3	3	4	4	4	4	1	1
4	4	4	4	1	1	4	4	1	1
4	4	3	3	4	4	4	4	1	1
4	4	4	4	4	4	4	4	1	1
4	4	4	4	4	4	4	4	1	1
4	4	3	3	4	4	4	4	1	1
4	4	4	4	4	4	3	3	1	1
4	4	4	4	4	4	4	4	1	1
4	4	4	4	4	4	3	3	1	1
4	4	4	4	4	4	4	4	1	1
4	4	4	4	4	4	3	3	3	3
4	4	3	3	4	4	4	4	3	3
4	4	4	4	4	4	4	4	3	3
4	4	4	4	4	4	3	3	1	1
4	4	4	4	4	4	3	3	1	1
4	4	4	4	4	4	4	4	1	1
4	4	4	4	4	4	4	4	1	1
4	4	4	4	4	4	3	3	1	1
4	4	4	4	4	4	4	4	3	3
4	4	3	3	4	4	4	4	1	1
1	1	4	4	4	4	3	3	1	1
4	4	4	4	4	4	4	4	1	1
4	4	4	4	4	4	4	4	1	1
4	4	4	4	4	4	4	4	1	1
4	4	4	4	4	4	4	4	1	1
4	4	3	3	1	1	3	3	1	1
4	4	4	4	4	4	4	4	1	1
4	4	4	4	4	4	4	4	1	1
4	4	4	4	4	4	4	4	1	1
4	4	4	4	4	4	3	3	1	1
4	4	4	4	4	4	3	3	1	1
4	4	4	4	4	4	4	4	1	1

For Review Only

4	4	4	4	4	4	4	4	1	1
4	4	3	3	4	4	3	3	1	1

For Review Only

SNP2481	SNP2481	SNP2482	SNP2482	SNP2483	SNP2483	SNP2484	SNP2484	SNP2485	SNP2485
3	3	3	3	1	1	3	3	2	2
2	2	3	3	2	2	3	3	2	2
3	3	3	3	2	2	3	3	2	2
2	2	2	2	1	1	3	3	2	2
3	3	3	3	2	2	3	3	2	2
3	3	3	3	2	2	3	3	2	2
2	2	3	3	2	2	3	3	2	2
2	2	3	3	2	2	3	3	2	2
2	2	3	3	2	2	3	3	2	2
2	2	3	3	2	2	3	3	2	2
3	3	3	3	2	2	3	3	1	1
3	3	2	2	1	1	3	3	2	2
3	3	3	3	2	2	3	3	2	2
3	3	2	2	1	1	3	3	2	2
3	3	2	2	1	1	3	3	2	2
2	2	3	3	2	2	3	3	2	2
2	2	3	3	2	2	3	3	2	2
2	2	3	3	2	2	3	3	2	2
3	3	3	3	2	2	3	3	2	2
3	3	2	2	1	1	3	3	2	2
3	3	3	3	2	2	3	3	2	2
3	3	3	3	2	2	3	3	2	2
3	3	2	2	1	1	3	3	2	2
2	2	2	2	1	1	3	3	2	2
2	2	3	3	2	2	3	3	2	2
2	2	3	3	2	2	2	2	2	2
2	2	3	3	2	2	3	3	2	2
2	2	2	2	1	1	3	3	2	2
2	2	3	3	2	2	3	3	2	2
3	3	3	3	2	2	3	3	2	2
3	3	3	3	2	2	3	3	2	2
3	3	2	2	1	1	3	3	2	2
3	3	3	3	2	2	3	3	2	2
3	3	2	2	1	1	3	3	2	2
2	2	3	3	2	2	3	3	1	1
3	3	2	2	1	1	3	3	2	2
3	3	3	3	2	2	3	3	2	2
2	2	3	3	2	2	3	3	2	2
3	3	3	3	2	2	3	3	1	1
2	2	3	3	2	2	3	3	1	1
3	3	3	3	2	2	3	3	2	2
2	2	3	3	2	2	3	3	2	2
3	3	3	3	2	2	3	3	2	2
3	3	3	3	2	2	3	3	2	2
3	3	3	3	2	2	3	3	2	2
2	2	3	3	2	2	3	3	2	2
2	2	3	3	2	2	3	3	2	2
3	3	2	2	1	1	3	3	1	1
3	3	3	3	2	2	3	3	2	2
3	3	2	2	1	1	3	3	2	2
2	2	3	3	2	2	3	3	2	2
2	2	2	2	1	1	3	3	2	2

For Review Only

2	2	3	3	2	2	3	3	2	2
3	3	2	2	2	2	3	3	2	2
2	2	2	2	1	1	3	3	2	2
3	3	3	3	2	2	3	3	2	2
3	3	3	3	2	2	3	3	2	2
2	2	3	3	2	2	2	2	2	2
2	2	3	3	2	2	2	2	2	2
3	3	3	3	2	2	3	3	2	2
3	3	2	2	1	1	3	3	2	2
2	2	2	2	1	1	3	3	2	2
3	3	3	3	2	2	3	3	2	2
3	3	3	3	2	2	3	3	2	2
2	2	3	3	2	2	3	3	2	2
2	2	3	3	2	2	3	3	2	2
2	2	3	3	1	1	3	3	2	2
3	3	3	3	2	2	3	3	2	2
3	3	3	3	1	1	3	3	2	2
2	2	2	2	1	1	3	3	2	2
2	2	3	3	2	2	3	3	2	2
2	2	3	3	2	2	3	3	2	2
3	3	3	3	2	2	3	3	2	2
2	2	2	2	1	1	3	3	1	1
2	2	3	3	2	2	3	3	2	2
2	2	3	3	2	2	3	3	2	2
3	3	3	3	2	2	3	3	2	2
2	2	3	3	2	2	3	3	2	2
3	3	3	3	2	2	3	3	2	2
3	3	3	3	2	2	3	3	2	2
2	2	3	3	2	2	2	2	2	2
3	3	2	2	1	1	3	3	2	2
3	3	3	3	2	2	3	3	2	2
3	3	2	2	1	1	3	3	2	2
3	3	3	3	2	2	3	3	2	2
2	2	3	3	2	2	3	3	2	2
2	2	2	2	1	1	3	3	2	2
3	3	3	3	2	2	3	3	2	2
3	3	3	3	2	2	3	3	2	2
3	3	3	3	2	2	3	3	2	2
3	3	3	3	2	2	3	3	2	2
3	3	3	3	2	2	3	3	2	2
3	3	3	3	2	2	3	3	2	2
3	3	2	2	1	1	3	3	2	2
2	2	2	2	1	1	3	3	2	2
3	3	3	3	2	2	3	3	2	2
3	3	2	2	1	1	3	3	2	2
2	2	2	2	1	1	3	3	2	2
3	3	2	2	1	1	3	3	2	2
2	2	3	3	2	2	2	2	2	2
2	2	2	2	1	1	3	3	2	2
2	2	3	3	2	2	3	3	2	2
2	2	2	2	1	1	3	3	2	2
3	3	3	3	2	2	3	3	1	1
3	3	3	3	2	2	3	3	2	2
2	2	2	2	1	1	3	3	2	2
2	2	3	3	2	2	3	3	2	2

For Review Only

2	2	3	3	2	2	2	2	2	2
2	2	3	3	2	2	3	3	1	1
3	3	2	2	1	1	2	2	2	2
3	3	3	3	2	2	3	3	2	2
2	2	2	2	2	2	3	3	2	2
3	3	2	2	1	1	2	2	2	2
2	2	2	2	1	1	3	3	2	2
3	3	3	3	2	2	3	3	2	2
3	3	3	3	2	2	3	3	2	2
3	3	2	2	1	1	3	3	2	2
2	2	2	2	1	1	3	3	2	2
2	2	3	3	2	2	3	3	2	2
2	2	2	2	1	1	3	3	2	2
2	2	3	3	2	2	3	3	2	2
2	2	3	3	2	2	3	3	2	2
2	2	2	2	1	1	2	2	2	2
2	2	2	2	1	1	3	3	2	2
2	2	3	3	2	2	2	2	2	2
3	3	3	3	2	2	3	3	2	2
3	3	2	2	1	1	3	3	2	2
2	2	3	3	2	2	3	3	2	2
3	3	2	2	1	1	3	3	2	2
3	3	3	3	2	2	3	3	2	2
3	3	3	3	2	2	3	3	2	2
2	2	3	3	2	2	2	2	2	2
2	2	2	2	1	1	3	3	1	1
2	2	2	2	1	1	3	3	2	2
3	3	2	2	1	1	3	3	2	2
3	3	2	2	1	1	3	3	2	2
2	2	2	2	1	1	3	3	2	2
3	3	2	2	1	1	3	3	2	2
2	2	2	2	1	1	3	3	2	2
3	3	3	3	2	2	3	3	2	2
2	2	3	3	1	1	3	3	2	2
3	3	2	2	1	1	3	3	2	2
3	3	3	3	2	2	3	3	2	2
3	3	3	3	2	2	3	3	2	2
3	3	3	3	2	2	3	3	2	2
3	3	3	3	1	1	3	3	2	2
3	3	3	3	2	2	3	3	2	2
2	2	2	2	1	1	3	3	2	2
3	3	3	3	2	2	3	3	2	2
3	3	2	2	1	1	3	3	2	2
2	2	2	2	2	2	3	3	2	2
3	3	3	3	2	2	2	2	2	2
3	3	3	3	2	2	3	3	2	2
2	2	3	3	2	2	2	2	2	2
2	2	3	3	2	2	2	2	2	2
3	3	3	3	2	2	3	3	2	2
2	2	3	3	2	2	2	2	2	2
2	2	3	3	2	2	2	2	2	2
3	3	3	3	2	2	3	3	2	2
3	3	3	3	2	2	3	3	2	2
3	3	3	3	2	2	2	2	2	2
3	3	2	2	1	1	3	3	2	2

For Review Only

3	3	3	3	2	2	3	3	2	2
2	2	2	2	1	1	3	3	2	2
3	3	3	3	2	2	3	3	2	2
2	2	3	3	2	2	3	3	2	2
2	2	3	3	2	2	3	3	2	2
3	3	3	3	2	2	3	3	2	2
2	2	2	2	2	2	3	3	1	1
2	2	2	2	1	1	3	3	2	2
3	3	2	2	1	1	3	3	2	2
3	3	3	3	2	2	3	3	2	2
2	2	2	2	2	2	3	3	2	2
2	2	2	2	1	1	3	3	1	1
3	3	2	2	1	1	3	3	2	2
3	3	3	3	2	2	3	3	2	2
3	3	2	2	1	1	3	3	2	2
3	3	3	3	2	2	3	3	1	1
3	3	2	2	1	1	3	3	2	2
2	2	3	3	2	2	3	3	2	2
3	3	2	2	1	1	3	3	2	2
2	2	2	2	1	1	3	3	2	2
2	2	2	2	1	1	3	3	2	2
3	3	2	2	1	1	3	3	1	1
2	2	2	2	1	1	3	3	1	1
3	3	3	3	2	2	3	3	2	2
2	2	3	3	2	2	3	3	2	2
3	3	2	2	1	1	3	3	2	2
2	2	2	2	1	1	3	3	2	2
2	2	2	2	1	1	3	3	2	2
2	2	2	2	1	1	3	3	2	2
3	3	3	3	2	2	3	3	2	2
2	2	2	2	1	1	3	3	2	2
3	3	2	2	1	1	3	3	2	2
2	2	2	2	1	1	3	3	2	2
3	3	2	2	1	1	3	3	2	2
3	3	3	3	2	2	3	3	2	2
3	3	3	3	2	2	3	3	2	2
2	2	3	3	2	2	2	2	2	2
3	3	3	3	2	2	3	3	2	2
2	2	3	3	2	2	3	3	2	2
3	3	2	2	1	1	3	3	2	2
3	3	3	3	2	2	3	3	2	2
2	2	3	3	2	2	2	2	2	2
3	3	3	3	2	2	3	3	2	2
2	2	2	2	1	1	3	3	2	2
3	3	2	2	1	1	3	3	2	2
3	3	3	3	2	2	3	3	2	2
3	3	3	3	2	2	3	3	1	1
3	3	3	3	2	2	3	3	1	1
3	3	2	2	1	1	3	3	2	2
2	2	3	3	2	2	3	3	2	2
3	3	2	2	1	1	3	3	2	2
3	3	3	3	2	2	3	3	2	2
2	2	3	3	2	2	3	3	1	1
3	3	3	3	2	2	3	3	2	2
3	3	3	3	2	2	3	3	1	1

For Review Only

3	3	3	3	2	2	3	3	2	2
3	3	2	2	1	1	3	3	2	2
3	3	2	2	1	1	3	3	2	2
2	2	2	2	1	1	3	3	2	2
2	2	3	3	2	2	3	3	2	2
2	2	2	2	2	2	2	2	2	2
2	2	3	3	2	2	3	3	1	1
3	3	3	3	2	2	3	3	2	2
3	3	2	2	2	2	3	3	2	2
3	3	3	3	2	2	3	3	1	1
2	2	2	2	1	1	3	3	2	2
2	2	2	2	1	1	3	3	2	2
3	3	2	2	1	1	3	3	2	2
3	3	2	2	1	1	2	2	2	2
3	3	3	3	2	2	3	3	2	2
3	3	3	3	2	2	3	3	2	2
3	3	3	3	2	2	3	3	1	1
3	3	3	3	2	2	2	2	1	1
3	3	3	3	2	2	3	3	2	2
3	3	3	3	2	2	3	3	2	2
3	3	3	3	2	2	3	3	1	1
2	2	3	3	2	2	2	2	2	2
3	3	2	2	1	1	3	3	2	2
2	2	2	2	1	1	3	3	1	1
3	3	3	3	2	2	3	3	2	2
2	2	3	3	2	2	3	3	2	2
3	3	3	3	2	2	3	3	1	1
2	2	2	2	1	1	3	3	2	2
2	2	3	3	2	2	3	3	2	2
3	3	2	2	1	1	3	3	2	2
3	3	3	3	2	2	3	3	1	1
3	3	3	3	2	2	2	2	2	2
3	3	3	3	2	2	3	3	2	2
2	2	3	3	2	2	2	2	2	2
2	2	3	3	2	2	3	3	1	1
3	3	3	3	2	2	3	3	1	1
3	3	3	3	2	2	3	3	2	2
3	3	3	3	2	2	3	3	2	2
3	3	2	2	1	1	3	3	2	2
3	3	3	3	2	2	3	3	1	1
3	3	2	2	1	1	3	3	2	2
3	3	2	2	1	1	3	3	2	2
2	2	2	2	1	1	3	3	1	1
3	3	3	3	2	2	3	3	2	2
2	2	3	3	2	2	3	3	2	2
3	3	3	3	2	2	3	3	2	2
2	2	3	3	2	2	2	2	2	2
3	3	3	3	2	2	3	3	2	2
2	2	3	3	2	2	3	3	2	2
3	3	3	3	2	2	3	3	2	2
3	3	2	2	1	1	3	3	2	2
3	3	2	2	2	2	3	3	1	1
3	3	3	3	2	2	3	3	2	2
2	2	2	2	1	1	3	3	2	2
3	3	3	3	2	2	3	3	1	1

For Review Only

3	3	2	2	1	1	3	3	2	2
2	2	3	3	2	2	3	3	2	2

For Review Only

SNP2486	SNP2486	SNP2487	SNP2487	SNP2488	SNP2488	SNP2489	SNP2489	SNP249	SNP249
3	3	4	4	4	4	1	1	3	3
3	3	1	1	1	1	4	4	3	3
3	3	4	4	4	4	4	4	3	3
3	3	4	4	4	4	1	1	3	3
3	3	4	4	4	4	4	4	3	3
3	3	4	4	4	4	4	4	3	3
3	3	1	1	1	1	4	4	3	3
3	3	4	4	4	4	4	4	3	3
3	3	1	1	1	1	1	1	3	3
3	3	4	4	4	4	4	4	3	3
3	3	4	4	4	4	4	4	2	2
3	3	4	4	4	4	4	4	3	3
3	3	4	4	4	4	4	4	3	3
3	3	4	4	4	4	1	1	3	3
3	3	4	4	4	4	1	1	3	3
3	3	1	1	1	1	1	1	3	3
3	3	1	1	1	1	4	4	3	3
3	3	4	4	4	4	4	4	3	3
3	3	4	4	4	4	4	4	3	3
3	3	4	4	4	4	4	4	3	3
3	3	4	4	4	4	4	4	3	3
3	3	4	4	4	4	4	4	3	3
3	3	4	4	4	4	1	1	3	3
2	2	4	4	4	4	4	4	3	3
3	3	1	1	1	1	4	4	3	3
3	3	1	1	1	1	4	4	3	3
3	3	1	1	1	1	4	4	3	3
3	3	4	4	4	4	1	1	3	3
3	3	1	1	1	1	4	4	3	3
3	3	4	4	4	4	4	4	3	3
3	3	4	4	4	4	4	4	3	3
3	3	4	4	4	4	4	4	3	3
3	3	4	4	4	4	4	4	3	3
3	3	4	4	4	4	1	1	3	3
3	3	1	1	4	4	4	4	3	3
3	3	4	4	4	4	4	4	3	3
3	3	4	4	4	4	4	4	3	3
3	3	4	4	4	4	4	4	3	3
2	2	4	4	4	4	1	1	3	3
3	3	1	1	1	1	1	1	2	2
3	3	4	4	4	4	4	4	3	3
3	3	1	1	1	1	4	4	3	3
3	3	4	4	4	4	4	4	3	3
3	3	4	4	4	4	4	4	3	3
3	3	4	4	4	4	4	4	3	3
3	3	4	4	4	4	4	4	3	3
3	3	1	1	1	1	4	4	3	3
3	3	4	4	4	4	4	4	3	3
3	3	4	4	4	4	4	4	3	3
3	3	4	4	4	4	4	4	3	3
3	3	1	1	1	1	4	4	3	3
3	3	1	1	1	1	1	1	3	3

For Review Only

3	3	1	1	1	1	4	4	3	3
3	3	4	4	4	4	4	4	3	3
3	3	1	1	1	1	1	1	3	3
3	3	4	4	4	4	4	4	3	3
3	3	4	4	4	4	4	4	3	3
3	3	1	1	1	1	4	4	3	3
3	3	1	1	1	1	4	4	3	3
3	3	4	4	4	4	4	4	3	3
3	3	4	4	4	4	1	1	3	3
3	3	4	4	4	4	4	4	3	3
3	3	4	4	4	4	1	1	3	3
3	3	4	4	4	4	1	1	3	3
3	3	1	1	1	1	4	4	3	3
3	3	1	1	1	1	4	4	3	3
3	3	4	4	4	4	4	4	3	3
3	3	4	4	4	4	4	4	3	3
3	3	4	4	4	4	4	4	3	3
3	3	1	1	1	1	1	1	3	3
3	3	1	1	1	1	4	4	3	3
3	3	1	1	1	1	4	4	3	3
3	3	4	4	4	4	1	1	3	3
3	3	4	4	4	4	4	4	2	2
3	3	4	4	4	4	4	4	3	3
3	3	1	1	1	1	4	4	3	3
3	3	4	4	4	4	4	4	3	3
3	3	4	4	4	4	4	4	3	3
3	3	4	4	4	4	1	1	3	3
2	2	4	4	4	4	4	4	2	2
3	3	1	1	1	1	4	4	2	2
3	3	4	4	4	4	1	1	3	3
3	3	4	4	4	4	1	1	3	3
3	3	4	4	4	4	1	1	3	3
2	2	4	4	4	4	1	1	2	2
3	3	1	1	1	1	4	4	3	3
3	3	1	1	1	1	4	4	3	3
3	3	4	4	4	4	4	4	3	3
3	3	4	4	4	4	1	1	3	3
3	3	4	4	4	4	4	4	3	3
3	3	4	4	4	4	4	4	3	3
3	3	4	4	4	4	1	1	3	3
3	3	4	4	4	4	4	4	3	3
3	3	4	4	4	4	1	1	3	3
3	3	4	4	4	4	1	1	3	3
3	3	4	4	4	4	1	1	3	3
3	3	4	4	4	4	1	1	3	3
3	3	1	1	1	1	4	4	3	3
3	3	4	4	4	4	1	1	3	3
3	3	4	4	4	4	4	4	3	3
3	3	4	4	4	4	1	1	3	3
3	3	1	1	1	1	4	4	3	3
3	3	1	1	1	1	4	4	3	3
2	2	4	4	4	4	1	1	2	2
3	3	4	4	4	4	4	4	3	3
3	3	4	4	4	4	4	4	3	3
3	3	1	1	1	1	4	4	3	3

For Review Only

3	3	1	1	1	1	4	4	2	2
2	2	4	4	4	4	4	4	3	3
3	3	4	4	4	4	4	4	3	3
3	3	4	4	4	4	4	4	2	2
3	3	1	1	1	1	4	4	3	3
3	3	4	4	4	4	1	1	3	3
3	3	1	1	1	1	4	4	3	3
3	3	4	4	4	4	4	4	3	3
3	3	4	4	4	4	4	4	3	3
3	3	4	4	4	4	4	4	3	3
3	3	1	1	1	1	1	1	3	3
3	3	4	4	4	4	4	4	3	3
3	3	4	4	4	4	1	1	3	3
3	3	1	1	1	1	4	4	3	3
3	3	4	4	4	4	4	4	3	3
3	3	1	1	4	4	1	1	3	3
3	3	1	1	1	1	1	1	3	3
3	3	4	4	4	4	1	1	3	3
3	3	4	4	4	4	4	4	3	3
3	3	4	4	4	4	1	1	3	3
3	3	4	4	4	4	4	4	3	3
3	3	4	4	4	4	1	1	3	3
3	3	4	4	4	4	1	1	3	3
3	3	4	4	4	4	4	4	2	2
3	3	1	1	1	1	4	4	3	3
3	3	1	1	1	1	1	1	3	3
3	3	4	4	4	4	1	1	3	3
3	3	4	4	4	4	4	4	3	3
3	3	4	4	4	4	1	1	3	3
3	3	4	4	4	4	4	4	3	3
3	3	4	4	4	4	1	1	3	3
3	3	4	4	4	4	1	1	3	3
3	3	4	4	4	4	4	4	3	3
3	3	4	4	1	1	4	4	3	3
3	3	4	4	4	4	1	1	3	3
3	3	4	4	4	4	4	4	3	3
3	3	4	4	4	4	4	4	2	2
3	3	4	4	4	4	1	1	3	3
3	3	4	4	4	4	4	4	3	3
3	3	4	4	4	4	1	1	3	3
3	3	1	1	1	1	1	1	3	3
3	3	4	4	4	4	4	4	2	2
3	3	4	4	4	4	1	1	3	3
3	3	4	4	4	4	1	1	3	3
3	3	4	4	4	4	4	4	3	3
3	3	4	4	4	4	4	4	3	3
3	3	1	1	1	1	4	4	3	3
3	3	1	1	1	1	4	4	3	3
3	3	4	4	4	4	4	4	3	3
3	3	4	4	4	4	4	4	3	3
3	3	1	1	1	1	4	4	3	3
3	3	4	4	4	4	4	4	2	2
3	3	4	4	1	1	4	4	3	3
3	3	4	4	4	4	1	1	3	3
3	3	4	4	4	4	1	1	3	3

For Review Only

3	3	4	4	4	4	4	4	3	3
3	3	1	1	1	1	1	1	3	3
3	3	4	4	4	4	4	4	3	3
2	2	1	1	1	1	4	4	3	3
3	3	1	1	1	1	4	4	3	3
2	2	4	4	4	4	1	1	3	3
3	3	4	4	4	4	1	1	2	2
3	3	4	4	4	4	4	4	3	3
3	3	4	4	4	4	4	4	3	3
3	3	4	4	4	4	4	4	3	3
3	3	1	1	1	1	4	4	3	3
2	2	4	4	4	4	1	1	3	3
3	3	4	4	4	4	1	1	3	3
3	3	4	4	4	4	4	4	3	3
3	3	4	4	4	4	1	1	3	3
3	3	4	4	4	4	1	1	2	2
3	3	4	4	4	4	4	4	3	3
3	3	1	1	1	1	4	4	3	3
3	3	4	4	4	4	1	1	3	3
3	3	1	1	1	1	4	4	3	3
3	3	4	4	4	4	1	1	3	3
3	3	4	4	4	4	1	1	2	2
3	3	1	1	1	1	4	4	2	2
3	3	4	4	4	4	1	1	3	3
3	3	1	1	1	1	4	4	3	3
3	3	4	4	4	4	1	1	3	3
3	3	4	4	4	4	4	4	3	3
3	3	4	4	4	4	1	1	3	3
3	3	1	1	1	1	1	1	3	3
3	3	4	4	4	4	4	4	3	3
3	3	1	1	1	1	1	1	3	3
3	3	4	4	4	4	1	1	3	3
3	3	4	4	4	4	4	4	3	3
3	3	4	4	4	4	1	1	3	3
3	3	4	4	4	4	4	4	3	3
3	3	4	4	4	4	1	1	3	3
3	3	1	1	1	1	4	4	3	3
3	3	4	4	4	4	4	4	3	3
3	3	1	1	1	1	4	4	3	3
3	3	4	4	4	4	1	1	3	3
3	3	4	4	4	4	4	4	3	3
3	3	1	1	1	1	4	4	3	3
2	2	4	4	4	4	4	4	2	2
3	3	4	4	4	4	1	1	3	3
3	3	4	4	4	4	4	4	3	3
3	3	4	4	4	4	1	1	3	3
3	3	4	4	4	4	1	1	2	2
3	3	4	4	4	4	4	4	2	2
3	3	4	4	4	4	4	4	3	3
3	3	4	4	4	4	4	4	3	3
3	3	4	4	4	4	1	1	3	3
3	3	4	4	4	4	4	4	3	3
3	3	1	1	1	1	4	4	3	3
3	3	4	4	4	4	1	1	3	3
2	2	4	4	4	4	4	4	3	3

For Review Only

3	3	1	1	1	1	4	4	3	3
3	3	4	4	4	4	4	4	3	3
3	3	4	4	4	4	1	1	3	3
3	3	1	1	1	1	1	1	3	3
3	3	4	4	4	4	1	1	3	3
3	3	1	1	1	1	4	4	3	3
3	3	4	4	4	4	4	4	3	3
3	3	4	4	4	4	4	4	3	3
3	3	4	4	4	4	1	1	3	3
2	2	4	4	4	4	4	4	3	3
3	3	4	4	4	4	1	1	3	3
3	3	1	1	1	1	1	1	3	3
3	3	4	4	4	4	1	1	3	3
3	3	4	4	4	4	1	1	3	3
3	3	4	4	4	4	4	4	3	3
3	3	4	4	4	4	4	4	3	3
2	2	4	4	4	4	1	1	2	2
3	3	4	4	4	4	4	4	2	2
3	3	4	4	4	4	4	4	2	2
3	3	4	4	4	4	4	4	3	3
2	2	4	4	4	4	1	1	3	3
3	3	1	1	1	1	4	4	3	3
3	3	4	4	4	4	4	4	3	3
2	2	1	1	1	1	1	1	2	2
3	3	4	4	4	4	4	4	3	3
3	3	4	4	4	4	4	4	3	3
2	2	4	4	4	4	1	1	3	3
3	3	4	4	4	4	4	4	3	3
3	3	4	4	4	4	4	4	3	3
3	3	4	4	4	4	1	1	3	3
2	2	4	4	4	4	1	1	3	3
3	3	4	4	4	4	4	4	3	3
3	3	4	4	4	4	4	4	3	3
3	3	4	4	4	4	4	4	3	3
3	3	1	1	1	1	4	4	3	3
2	2	4	4	4	4	1	1	2	2
3	3	1	1	1	1	4	4	3	3
3	3	4	4	4	4	1	1	3	3
3	3	4	4	4	4	4	4	3	3
2	2	4	4	4	4	1	1	2	2
3	3	4	4	4	4	1	1	3	3
3	3	4	4	4	4	4	4	3	3
3	3	1	1	1	1	1	1	3	3
3	3	4	4	4	4	4	4	3	3
3	3	4	4	4	4	1	1	3	3
3	3	4	4	4	4	4	4	3	3
3	3	4	4	4	4	4	4	3	3
3	3	4	4	4	4	4	4	3	3
3	3	1	1	4	4	1	1	3	3
2	2	4	4	4	4	1	1	2	2
3	3	4	4	4	4	4	4	3	3
2	2	4	4	4	4	1	1	3	3
3	3	4	4	4	4	4	4	3	3
3	3	4	4	4	4	1	1	3	3
2	2	4	4	4	4	1	1	3	3

For Review Only

3	3	4	4	4	4	4	4	3	3
3	3	1	1	1	1	4	4	3	3

For Review Only

SNP2490	SNP2490	SNP2491	SNP2491	SNP2492	SNP2492	SNP2493	SNP2493	SNP2494	SNP2494
1	1	3	3	4	4	3	3	4	4
1	1	2	2	3	3	3	3	3	3
1	1	3	3	4	4	3	3	4	4
1	1	3	3	4	4	3	3	4	4
1	1	3	3	4	4	3	3	4	4
1	1	2	2	3	3	2	2	3	3
1	1	3	3	4	4	3	3	4	4
2	2	3	3	4	4	3	3	4	4
1	1	3	3	3	3	3	3	4	4
1	1	3	3	4	4	3	3	4	4
1	1	3	3	4	4	2	2	4	4
1	1	3	3	4	4	3	3	4	4
1	1	2	2	3	3	3	3	3	3
1	1	3	3	4	4	3	3	4	4
1	1	3	3	3	3	3	3	4	4
1	1	3	3	4	4	3	3	4	4
1	1	2	2	3	3	3	3	3	3
2	2	3	3	4	4	3	3	4	4
1	1	3	3	4	4	2	2	4	4
2	2	2	2	3	3	3	3	3	3
1	1	3	3	4	4	2	2	4	4
1	1	2	2	3	3	3	3	3	3
2	2	3	3	4	4	3	3	4	4
1	1	3	3	4	4	3	3	4	4
1	1	3	3	4	4	3	3	4	4
1	1	3	3	3	3	3	3	4	4
1	1	3	3	4	4	3	3	4	4
1	1	3	3	4	4	3	3	4	4
2	2	3	3	4	4	3	3	4	4
1	1	3	3	3	3	3	3	4	4
1	1	3	3	4	4	2	2	4	4
1	1	3	3	4	4	2	2	4	4
1	1	3	3	4	4	2	2	4	4
1	1	3	3	4	4	3	3	4	4
1	1	3	3	4	4	3	3	4	4
1	1	3	3	4	4	3	3	4	4
1	1	2	2	3	3	3	3	3	3
1	1	3	3	4	4	3	3	4	4
1	1	3	3	4	4	3	3	4	4
1	1	3	3	4	4	3	3	4	4
2	2	3	3	4	4	3	3	4	4
2	2	3	3	4	4	3	3	4	4
2	2	3	3	4	4	3	3	4	4
1	1	2	2	3	3	3	3	3	3
2	2	3	3	4	4	3	3	4	4
1	1	3	3	4	4	3	3	4	4
1	1	3	3	4	4	3	3	4	4
1	1	3	3	4	4	2	2	4	4
2	2	2	2	3	3	3	3	3	3
1	1	3	3	4	4	3	3	4	4
1	1	3	3	4	4	3	3	4	4
1	1	2	2	3	3	3	3	3	3

For Review Only

1	1	2	2	3	3	3	3	3	3
1	1	3	3	4	4	2	2	4	4
1	1	3	3	4	4	3	3	4	4
1	1	2	2	3	3	3	3	3	3
1	1	3	3	4	4	2	2	4	4
1	1	3	3	4	4	3	3	4	4
1	1	3	3	4	4	3	3	4	4
1	1	3	3	4	4	3	3	4	4
1	1	2	2	3	3	3	3	3	3
1	1	3	3	4	4	3	3	4	4
1	1	3	3	4	4	3	3	4	4
1	1	3	3	4	4	3	3	4	4
1	1	2	2	3	3	3	3	3	3
2	2	3	3	4	4	3	3	4	4
1	1	3	3	3	3	3	3	4	4
2	2	3	3	4	4	3	3	4	4
2	2	3	3	4	4	2	2	4	4
1	1	3	3	4	4	3	3	4	4
1	1	2	2	3	3	3	3	3	3
1	1	3	3	4	4	3	3	4	4
1	1	3	3	4	4	3	3	4	4
1	1	3	3	4	4	2	2	4	4
2	2	3	3	4	4	3	3	4	4
2	2	3	3	4	4	3	3	4	4
2	2	3	3	4	4	3	3	4	4
1	1	2	2	3	3	3	3	3	3
1	1	2	2	3	3	2	2	3	3
1	1	3	3	4	4	3	3	4	4
1	1	3	3	4	4	3	3	4	4
1	1	3	3	4	4	2	2	4	4
1	1	2	2	3	3	3	3	4	4
1	1	3	3	4	4	3	3	4	4
1	1	3	3	4	4	3	3	4	4
1	1	2	2	3	3	3	3	3	3
1	1	3	3	4	4	3	3	4	4
2	2	3	3	4	4	3	3	4	4
1	1	3	3	4	4	3	3	4	4
1	1	3	3	4	4	2	2	4	4
1	1	2	2	3	3	2	2	3	3
1	1	3	3	4	4	3	3	4	4
1	1	2	2	3	3	2	2	3	3
1	1	3	3	4	4	2	2	4	4
1	1	3	3	4	4	3	3	4	4
1	1	3	3	4	4	3	3	4	4
1	1	3	3	3	3	3	3	4	4
1	1	2	2	3	3	3	3	3	3
1	1	3	3	4	4	3	3	4	4
1	1	3	3	4	4	3	3	4	4
1	1	3	3	4	4	3	3	4	4
1	1	3	3	4	4	3	3	4	4
1	1	3	3	3	3	3	3	4	4
1	1	3	3	4	4	3	3	4	4
1	1	3	3	4	4	3	3	4	4
1	1	3	3	4	4	2	2	4	4
1	1	3	3	4	4	3	3	4	4

For Review Only

1	1	3	3	4	4	3	3	4	4
1	1	3	3	4	4	3	3	4	4
1	1	3	3	4	4	2	2	4	4
1	1	3	3	4	4	3	3	4	4
1	1	3	3	4	4	3	3	4	4
1	1	3	3	4	4	2	2	4	4
1	1	3	3	4	4	3	3	4	4
1	1	3	3	4	4	3	3	4	4
1	1	3	3	4	4	3	3	4	4
1	1	3	3	4	4	3	3	4	4
1	1	3	3	3	3	3	3	4	4
1	1	3	3	3	3	3	3	4	4
1	1	3	3	4	4	3	3	4	4
1	1	3	3	3	3	3	3	3	3
1	1	3	3	4	4	3	3	4	4
1	1	3	3	4	4	3	3	4	4
1	1	3	3	4	4	3	3	4	4
1	1	3	3	4	4	3	3	4	4
1	1	3	3	4	4	3	3	4	4
1	1	3	3	3	3	2	2	4	4
1	1	3	3	4	4	3	3	4	4
1	1	3	3	4	4	3	3	4	4
1	1	3	3	4	4	3	3	4	4
2	2	3	3	4	4	3	3	4	4
1	1	3	3	4	4	3	3	4	4
1	1	2	2	3	3	3	3	3	3
1	1	2	2	3	3	3	3	3	3
1	1	3	3	4	4	3	3	4	4
1	1	3	3	4	4	3	3	4	4
1	1	3	3	4	4	3	3	4	4
2	2	3	3	4	4	3	3	4	4
1	1	3	3	3	3	3	3	4	4
1	1	2	2	3	3	2	2	3	3
1	1	3	3	4	4	3	3	4	4
1	1	3	3	4	4	3	3	4	4
1	1	3	3	4	4	3	3	4	4
1	1	3	3	4	4	3	3	4	4
1	1	3	3	4	4	3	3	4	4
1	1	3	3	4	4	2	2	4	4
1	1	3	3	4	4	2	2	4	4
1	1	3	3	3	3	3	3	4	4
1	1	3	3	4	4	3	3	4	4
1	1	3	3	4	4	3	3	4	4
2	2	3	3	4	4	3	3	4	4
1	1	3	3	3	3	3	3	4	4
1	1	3	3	4	4	3	3	4	4
1	1	3	3	4	4	3	3	4	4
1	1	3	3	4	4	3	3	4	4
1	1	3	3	4	4	3	3	4	4
1	1	3	3	4	4	3	3	4	4
1	1	3	3	4	4	3	3	4	4
2	2	3	3	4	4	3	3	4	4
1	1	3	3	3	3	3	3	4	4
1	1	3	3	4	4	3	3	4	4
1	1	3	3	4	4	3	3	4	4

For Review Only

1	1	3	3	4	4	3	3	3	3
1	1	3	3	4	4	3	3	4	4
1	1	3	3	4	4	2	2	4	4
1	1	3	3	4	4	3	3	4	4
1	1	2	2	3	3	3	3	3	3
1	1	3	3	4	4	3	3	4	4
1	1	3	3	4	4	3	3	4	4
1	1	3	3	4	4	3	3	4	4
1	1	3	3	4	4	3	3	4	4
1	1	3	3	4	4	2	2	4	4
1	1	3	3	4	4	3	3	4	4
1	1	3	3	4	4	3	3	4	4
1	1	3	3	4	4	2	2	4	4
1	1	3	3	4	4	3	3	4	4
1	1	3	3	4	4	3	3	4	4
1	1	3	3	4	4	2	2	4	4
1	1	3	3	4	4	3	3	4	4
1	1	3	3	3	3	3	3	4	4
1	1	3	3	4	4	2	2	4	4
1	1	3	3	4	4	3	3	4	4
1	1	3	3	4	4	3	3	4	4
1	1	3	3	4	4	3	3	4	4
1	1	3	3	4	4	3	3	4	4
1	1	3	3	4	4	3	3	4	4
1	1	2	2	3	3	3	3	3	3
1	1	3	3	4	4	3	3	4	4
1	1	3	3	4	4	3	3	4	4
1	1	3	3	4	4	3	3	4	4
1	1	3	3	4	4	3	3	4	4
1	1	3	3	4	4	2	2	4	4
1	1	3	3	4	4	3	3	4	4
1	1	3	3	4	4	2	2	4	4
1	1	3	3	4	4	3	3	4	4
1	1	3	3	4	4	3	3	4	4
1	1	3	3	4	4	3	3	4	4
1	1	3	3	4	4	3	3	4	4
1	1	2	2	3	3	3	3	3	3
1	1	2	2	3	3	3	3	3	3
1	1	3	3	4	4	3	3	4	4
1	1	3	3	4	4	3	3	4	4
1	1	3	3	4	4	3	3	4	4
1	1	3	3	4	4	3	3	4	4
1	1	3	3	4	4	3	3	4	4
1	1	3	3	4	4	3	3	4	4
1	1	3	3	4	4	3	3	4	4
1	1	3	3	4	4	3	3	4	4
1	1	3	3	4	4	2	2	4	4
1	1	3	3	4	4	3	3	4	4
1	1	3	3	4	4	3	3	4	4
1	1	3	3	4	4	3	3	4	4
1	1	3	3	4	4	3	3	3	3
1	1	3	3	4	4	3	3	4	4
1	1	3	3	4	4	3	3	4	4
1	1	3	3	4	4	3	3	4	4
2	2	3	3	4	4	3	3	4	4

For Review Only

1	1	3	3	4	4	3	3	4	4
2	2	3	3	4	4	3	3	4	4
1	1	3	3	4	4	3	3	3	3
1	1	3	3	3	3	3	3	4	4
2	2	3	3	4	4	3	3	4	4
1	1	3	3	4	4	3	3	4	4
1	1	3	3	4	4	3	3	4	4
2	2	3	3	4	4	3	3	4	4
1	1	3	3	4	4	3	3	4	4
1	1	3	3	4	4	3	3	4	4
1	1	3	3	4	4	3	3	4	4
1	1	3	3	4	4	3	3	4	4
1	1	2	2	3	3	2	2	3	3
1	1	3	3	4	4	2	2	4	4
1	1	3	3	4	4	3	3	4	4
1	1	2	2	3	3	3	3	3	3
1	1	3	3	4	4	3	3	4	4
1	1	2	2	3	3	3	3	3	3
1	1	3	3	4	4	3	3	4	4
1	1	3	3	4	4	3	3	4	4
1	1	3	3	4	4	3	3	4	4
1	1	3	3	4	4	3	3	4	4
1	1	3	3	4	4	3	3	4	4
2	2	3	3	4	4	3	3	4	4
1	1	3	3	4	4	3	3	4	4
1	1	3	3	4	4	3	3	4	4
1	1	3	3	4	4	3	3	4	4
1	1	3	3	4	4	3	3	4	4
1	1	3	3	3	3	3	3	4	4
1	1	3	3	4	4	3	3	3	3
1	1	3	3	4	4	3	3	4	4
2	2	3	3	4	4	2	2	4	4
1	1	3	3	4	4	2	2	4	4
1	1	3	3	4	4	3	3	4	4
1	1	2	2	3	3	3	3	3	3
1	1	3	3	4	4	3	3	4	4
1	1	2	2	3	3	3	3	4	4
1	1	3	3	4	4	3	3	4	4
1	1	3	3	4	4	3	3	4	4
2	2	3	3	4	4	3	3	4	4
1	1	3	3	4	4	3	3	4	4
1	1	3	3	4	4	3	3	4	4
2	2	3	3	4	4	3	3	4	4
1	1	2	2	3	3	3	3	3	3
1	1	3	3	4	4	3	3	4	4
1	1	2	2	3	3	2	2	4	4
1	1	3	3	4	4	3	3	4	4
1	1	3	3	4	4	3	3	4	4
1	1	3	3	4	4	3	3	4	4
1	1	3	3	4	4	3	3	4	4
1	1	3	3	4	4	3	3	4	4
1	1	3	3	4	4	3	3	4	4
1	1	3	3	4	4	3	3	4	4
1	1	3	3	4	4	3	3	4	4
2	2	3	3	4	4	3	3	4	4

1	1	3	3	4	4	2	2	4	4
1	1	3	3	4	4	3	3	4	4

For Review Only

SNP2495	SNP2495	SNP2496	SNP2496	SNP2497	SNP2497	SNP2498	SNP2498	SNP2499	SNP2499
1	1	4	4	3	3	3	3	4	4
1	1	4	4	2	2	3	3	4	4
1	1	1	1	3	3	3	3	4	4
1	1	1	1	3	3	3	3	2	2
1	1	1	1	3	3	3	3	2	2
1	1	4	4	2	2	3	3	4	4
1	1	4	4	2	2	3	3	4	4
2	2	1	1	3	3	3	3	2	2
1	1	4	4	2	2	3	3	4	4
1	1	1	1	3	3	3	3	2	2
2	2	1	1	3	3	2	2	2	2
1	1	1	1	3	3	3	3	4	4
1	1	4	4	2	2	3	3	4	4
1	1	1	1	3	3	3	3	2	2
1	1	4	4	2	2	3	3	4	4
1	1	1	1	3	3	3	3	2	2
1	1	4	4	2	2	3	3	4	4
2	2	4	4	2	2	3	3	4	4
1	1	1	1	3	3	3	3	4	4
2	2	4	4	2	2	3	3	4	4
1	1	4	4	2	2	3	3	2	2
1	1	4	4	2	2	3	3	2	2
2	2	1	1	3	3	3	3	2	2
1	1	1	1	3	3	3	3	2	2
1	1	1	1	3	3	3	3	4	4
1	1	4	4	2	2	3	3	2	2
1	1	1	1	3	3	3	3	4	4
1	1	1	1	3	3	3	3	2	2
2	2	4	4	2	2	3	3	4	4
2	2	1	1	3	3	3	3	2	2
1	1	1	1	3	3	3	3	4	4
1	1	1	1	3	3	3	3	2	2
1	1	1	1	3	3	3	3	4	4
1	1	1	1	3	3	3	3	4	4
1	1	1	1	3	3	3	3	2	2
1	1	1	1	3	3	3	3	2	2
1	1	4	4	2	2	3	3	4	4
1	1	1	1	3	3	3	3	2	2
1	1	1	1	3	3	3	3	2	2
2	2	1	1	3	3	2	2	2	2
2	2	1	1	3	3	2	2	4	4
2	2	4	4	2	2	3	3	4	4
2	2	1	1	3	3	3	3	2	2
1	1	4	4	2	2	3	3	2	2
2	2	1	1	3	3	3	3	2	2
1	1	4	4	2	2	3	3	4	4
1	1	1	1	3	3	3	3	2	2
1	1	1	1	3	3	3	3	2	2
2	2	4	4	2	2	3	3	4	4
1	1	1	1	3	3	3	3	2	2
1	1	4	4	2	2	3	3	2	2
1	1	4	4	2	2	3	3	2	2

For Review Only

1	1	4	4	2	2	3	3	4	4
1	1	1	1	3	3	3	3	4	4
1	1	1	1	3	3	3	3	2	2
1	1	4	4	2	2	3	3	4	4
1	1	1	1	3	3	3	3	4	4
1	1	4	4	2	2	3	3	2	2
1	1	4	4	2	2	3	3	2	2
1	1	1	1	3	3	3	3	2	2
1	1	4	4	2	2	2	2	2	2
1	1	1	1	3	3	3	3	2	2
1	1	1	1	3	3	3	3	2	2
1	1	1	1	3	3	3	3	2	2
1	1	4	4	2	2	3	3	4	4
2	2	4	4	2	2	3	3	4	4
1	1	1	1	3	3	3	3	2	2
2	2	4	4	2	2	3	3	2	2
2	2	4	4	2	2	3	3	4	4
1	1	4	4	2	2	3	3	2	2
1	1	4	4	2	2	3	3	2	2
1	1	4	4	2	2	3	3	4	4
1	1	1	1	3	3	3	3	2	2
1	1	1	1	3	3	3	3	2	2
2	2	4	4	2	2	3	3	4	4
2	2	4	4	2	2	3	3	4	4
2	2	1	1	3	3	3	3	2	2
1	1	4	4	2	2	3	3	4	4
1	1	4	4	2	2	2	2	4	4
1	1	1	1	3	3	3	3	2	2
1	1	4	4	2	2	3	3	2	2
1	1	1	1	3	3	3	3	2	2
1	1	1	1	2	2	2	2	4	4
1	1	1	1	3	3	2	2	2	2
1	1	1	1	3	3	2	2	2	2
1	1	4	4	2	2	3	3	4	4
1	1	1	1	3	3	3	3	2	2
2	2	1	1	3	3	3	3	2	2
1	1	1	1	3	3	2	2	4	4
1	1	1	1	3	3	3	3	2	2
1	1	4	4	2	2	3	3	4	4
1	1	1	1	3	3	2	2	4	4
1	1	4	4	2	2	3	3	4	4
1	1	1	1	3	3	3	3	2	2
1	1	4	4	2	2	3	3	2	2
1	1	1	1	3	3	3	3	2	2
1	1	4	4	2	2	3	3	2	2
1	1	4	4	2	2	3	3	4	4
1	1	1	1	3	3	3	3	4	4
1	1	4	4	2	2	3	3	2	2
1	1	1	1	3	3	3	3	2	2
1	1	1	1	3	3	3	3	2	2
1	1	1	1	3	3	3	3	4	4
1	1	1	1	3	3	3	3	2	2
1	1	1	1	3	3	3	3	4	4
1	1	1	1	3	3	3	3	2	2
1	1	1	1	3	3	3	3	2	2

For Review Only

1	1	1	1	2	2	3	3	2	2
1	1	1	1	3	3	3	3	2	2
1	1	4	4	2	2	3	3	2	2
1	1	1	1	3	3	2	2	2	2
1	1	1	1	3	3	3	3	4	4
1	1	1	1	3	3	2	2	2	2
1	1	1	1	3	3	3	3	4	4
1	1	1	1	3	3	3	3	2	2
1	1	1	1	3	3	3	3	2	2
1	1	1	1	3	3	3	3	2	2
1	1	1	1	2	2	3	3	4	4
1	1	1	1	2	2	3	3	2	2
1	1	1	1	3	3	3	3	2	2
1	1	4	4	2	2	3	3	2	2
1	1	1	1	3	3	3	3	2	2
1	1	1	1	3	3	3	3	2	2
1	1	4	4	2	2	3	3	2	2
1	1	1	1	3	3	3	3	2	2
1	1	1	1	3	3	2	2	2	2
1	1	4	4	2	2	3	3	2	2
1	1	1	1	3	3	3	3	2	2
1	1	1	1	3	3	2	2	2	2
1	1	1	1	3	3	3	3	4	4
2	2	1	1	3	3	3	3	2	2
1	1	1	1	3	3	3	3	2	2
1	1	4	4	2	2	3	3	2	2
1	1	4	4	2	2	2	2	2	2
1	1	4	4	2	2	3	3	4	4
1	1	1	1	3	3	2	2	2	2
1	1	4	4	2	2	3	3	4	4
2	2	1	1	3	3	3	3	2	2
1	1	1	1	3	3	3	3	2	2
1	1	4	4	2	2	3	3	4	4
1	1	4	4	2	2	3	3	4	4
1	1	1	1	3	3	3	3	4	4
1	1	1	1	3	3	3	3	2	2
1	1	1	1	3	3	2	2	2	2
1	1	1	1	3	3	3	3	4	4
1	1	4	4	2	2	3	3	2	2
1	1	1	1	3	3	3	3	2	2
1	1	1	1	3	3	3	3	2	2
1	1	1	1	3	3	2	2	2	2
1	1	4	4	2	2	3	3	2	2
1	1	1	1	3	3	3	3	4	4
1	1	1	1	3	3	3	3	4	4
1	1	4	4	3	3	3	3	2	2
1	1	4	4	2	2	3	3	2	2
1	1	4	4	2	2	3	3	2	2
1	1	1	1	3	3	3	3	2	2
1	1	4	4	2	2	3	3	4	4
1	1	4	4	2	2	3	3	2	2
2	2	1	1	3	3	2	2	2	2
1	1	1	1	3	3	3	3	2	2
1	1	1	1	3	3	3	3	2	2
1	1	1	1	3	3	3	3	2	2

For Review Only

1	1	4	4	2	2	3	3	2	2
1	1	1	1	3	3	3	3	2	2
1	1	1	1	3	3	3	3	2	2
1	1	1	1	3	3	3	3	2	2
1	1	4	4	2	2	3	3	4	4
1	1	4	4	2	2	2	2	2	2
2	2	1	1	3	3	3	3	2	2
1	1	1	1	3	3	3	3	2	2
1	1	1	1	3	3	3	3	2	2
1	1	4	4	2	2	3	3	4	4
1	1	1	1	3	3	3	3	2	2
1	1	1	1	3	3	3	3	2	2
1	1	1	1	2	2	3	3	2	2
1	1	4	4	3	3	3	3	2	2
1	1	1	1	3	3	3	3	2	2
2	2	1	1	3	3	3	3	2	2
1	1	1	1	3	3	3	3	2	2
1	1	4	4	2	2	3	3	4	4
1	1	4	4	2	2	2	2	2	2
1	1	1	1	3	3	3	3	4	4
1	1	1	1	3	3	3	3	2	2
2	2	1	1	3	3	2	2	2	2
1	1	4	4	2	2	3	3	4	4
1	1	1	1	3	3	3	3	2	2
1	1	4	4	2	2	3	3	4	4
1	1	1	1	3	3	3	3	4	4
1	1	1	1	3	3	3	3	4	4
1	1	1	1	3	3	3	3	2	2
1	1	1	1	3	3	3	3	2	2
1	1	1	1	3	3	3	3	4	4
1	1	1	1	3	3	3	3	4	4
1	1	4	4	2	2	3	3	2	2
1	1	1	1	3	3	3	3	4	4
1	1	1	1	3	3	3	3	2	2
1	1	4	4	2	2	3	3	2	2
1	1	1	1	3	3	3	3	2	2
1	1	4	4	2	2	3	3	4	4
1	1	4	4	2	2	3	3	4	4
1	1	4	4	2	2	3	3	2	2
1	1	1	1	3	3	3	3	2	2
1	1	1	1	3	3	3	3	4	4
1	1	4	4	2	2	3	3	2	2
1	1	1	1	3	3	3	3	2	2
1	1	1	1	3	3	3	3	2	2
1	1	1	1	3	3	3	3	2	2
1	1	4	4	2	2	3	3	2	2
2	2	1	1	3	3	3	3	2	2
2	2	1	1	3	3	3	3	2	2
1	1	1	1	3	3	3	3	2	2
1	1	1	1	3	3	3	3	4	4
1	1	4	4	2	2	2	2	2	2
1	1	1	1	3	3	3	3	2	2
1	1	4	4	2	2	3	3	2	2
1	1	1	1	3	3	3	3	2	2
2	2	1	1	3	3	3	3	2	2

For Review Only

1	1	1	1	3	3	3	3	2	2
2	2	4	4	2	2	3	3	4	4
1	1	4	4	2	2	2	2	2	2
1	1	4	4	2	2	3	3	2	2
2	2	1	1	3	3	2	2	4	4
1	1	4	4	2	2	3	3	2	2
1	1	4	4	2	2	3	3	2	2
2	2	1	1	3	3	3	3	2	2
1	1	1	1	3	3	3	3	2	2
1	1	1	1	3	3	3	3	2	2
1	1	1	1	3	3	3	3	2	2
1	1	1	1	3	3	3	3	2	2
1	1	4	4	2	2	3	3	2	2
1	1	4	4	3	3	2	2	2	2
1	1	1	1	3	3	3	3	4	4
1	1	4	4	2	2	3	3	2	2
2	2	1	1	3	3	3	3	2	2
1	1	4	4	2	2	3	3	2	2
2	2	1	1	3	3	3	3	2	2
1	1	1	1	3	3	3	3	2	2
1	1	1	1	3	3	2	2	2	2
1	1	4	4	2	2	3	3	2	2
1	1	4	4	2	2	2	2	2	2
2	2	1	1	3	3	3	3	2	2
1	1	1	1	3	3	3	3	2	2
1	1	1	1	3	3	3	3	4	4
1	1	1	1	3	3	2	2	2	2
1	1	1	1	3	3	3	3	4	4
1	1	1	1	3	3	3	3	4	4
1	1	4	4	2	2	2	2	2	2
1	1	1	1	3	3	2	2	2	2
2	2	1	1	3	3	3	3	2	2
1	1	4	4	2	2	3	3	2	2
1	1	4	4	2	2	3	3	2	2
1	1	4	4	3	3	3	3	2	2
1	1	1	1	3	3	3	3	2	2
1	1	4	4	2	2	3	3	4	4
1	1	1	1	3	3	3	3	2	2
1	1	1	1	3	3	3	3	2	2
2	2	1	1	3	3	3	3	2	2
1	1	4	4	2	2	3	3	2	2
1	1	1	1	3	3	3	3	4	4
2	2	1	1	3	3	3	3	2	2
1	1	4	4	2	2	3	3	4	4
1	1	1	1	3	3	3	3	2	2
1	1	4	4	2	2	3	3	4	4
1	1	1	1	3	3	3	3	2	2
1	1	4	4	2	2	3	3	2	2
1	1	1	1	3	3	2	2	4	4
1	1	1	1	3	3	2	2	2	2
1	1	4	4	2	2	3	3	4	4
2	2	1	1	3	3	3	3	2	2
1	1	1	1	3	3	3	3	4	4
1	1	4	4	2	2	3	3	2	2
2	2	1	1	3	3	2	2	2	2

For Review Only

1	1	1	1	3	3	2	2	2	2
1	1	1	1	3	3	3	3	4	4

For Review Only

SNP250	SNP250	SNP2500	SNP2500	SNP2501	SNP2501	SNP2502	SNP2502	SNP2503	SNP2503
4	4	2	2	1	1	4	4	1	1
4	4	1	1	1	1	4	4	4	4
3	3	1	1	1	1	4	4	4	4
4	4	2	2	1	1	4	4	4	4
4	4	1	1	1	1	4	4	4	4
3	3	1	1	1	1	4	4	4	4
3	3	1	1	4	4	1	1	1	1
4	4	2	2	1	1	1	1	4	4
4	4	1	1	1	1	4	4	1	1
4	4	2	2	1	1	4	4	4	4
4	4	1	1	1	1	4	4	4	4
3	3	1	1	1	1	4	4	4	4
4	4	1	1	1	1	4	4	4	4
4	4	2	2	1	1	4	4	4	4
3	3	1	1	1	1	4	4	4	4
3	3	1	1	1	1	4	4	4	4
4	4	1	1	4	4	4	4	4	4
3	3	2	2	4	4	4	4	4	4
3	3	1	1	1	1	4	4	4	4
4	4	2	2	4	4	4	4	4	4
3	3	2	2	4	4	4	4	4	4
3	3	1	1	4	4	4	4	4	4
3	3	2	2	1	1	4	4	4	4
3	3	2	2	1	1	1	1	4	4
4	4	1	1	1	1	4	4	1	1
3	3	2	2	1	1	4	4	4	4
3	3	1	1	1	1	4	4	4	4
4	4	1	1	1	1	4	4	4	4
4	4	1	1	4	4	1	1	4	4
4	4	2	2	1	1	4	4	4	4
3	3	1	1	1	1	4	4	4	4
3	3	2	2	1	1	4	4	4	4
3	3	1	1	1	1	4	4	1	1
3	3	1	1	1	1	4	4	4	4
3	3	2	2	1	1	4	4	4	4
4	4	1	1	1	1	4	4	4	4
4	4	1	1	1	1	4	4	4	4
4	4	1	1	1	1	4	4	4	4
4	4	2	2	1	1	1	1	4	4
4	4	1	1	1	1	1	1	4	4
4	4	2	2	1	1	4	4	4	4
4	4	2	2	4	4	1	1	4	4
4	4	2	2	1	1	4	4	4	4
4	4	2	2	4	4	4	4	1	1
4	4	2	2	1	1	4	4	4	4
3	3	1	1	1	1	4	4	4	4
3	3	2	2	1	1	4	4	4	4
3	3	1	1	1	1	4	4	4	4
4	4	2	2	4	4	4	4	1	1
4	4	1	1	1	1	4	4	4	4
3	3	2	2	1	1	4	4	4	4
4	4	1	1	1	1	4	4	4	4

For Review Only

4	4	1	1	4	4	4	4	1	1
3	3	1	1	1	1	4	4	1	1
3	3	1	1	1	1	4	4	4	4
4	4	1	1	4	4	4	4	4	4
3	3	1	1	1	1	4	4	4	4
3	3	2	2	1	1	4	4	4	4
3	3	2	2	1	1	4	4	4	4
4	4	2	2	1	1	1	1	4	4
3	3	2	2	1	1	4	4	4	4
4	4	1	1	1	1	4	4	4	4
3	3	2	2	1	1	4	4	4	4
3	3	2	2	1	1	4	4	4	4
4	4	1	1	4	4	4	4	1	1
4	4	1	1	4	4	1	1	4	4
4	4	1	1	1	1	4	4	4	4
4	4	2	2	4	4	4	4	4	4
4	4	2	2	1	1	4	4	4	4
3	3	1	1	4	4	4	4	4	4
3	3	1	1	4	4	4	4	4	4
3	3	1	1	1	1	4	4	4	4
4	4	2	2	1	1	4	4	4	4
4	4	2	2	1	1	4	4	4	4
3	3	1	1	1	1	4	4	1	1
3	3	1	1	1	1	1	1	4	4
4	4	2	2	1	1	4	4	4	4
3	3	1	1	1	1	4	4	4	4
4	4	1	1	4	4	4	4	4	4
4	4	2	2	1	1	1	1	4	4
4	4	2	2	1	1	4	4	4	4
4	4	1	1	1	1	4	4	4	4
4	4	1	1	1	1	4	4	4	4
3	3	2	2	1	1	4	4	4	4
4	4	2	2	1	1	1	1	4	4
4	4	1	1	4	4	4	4	4	4
3	3	2	2	1	1	4	4	4	4
4	4	2	2	1	1	4	4	4	4
4	4	2	2	1	1	4	4	4	4
3	3	1	1	1	1	4	4	1	1
4	4	2	2	1	1	4	4	4	4
4	4	2	2	1	1	4	4	4	4
3	3	1	1	4	4	4	4	4	4
3	3	1	1	1	1	4	4	4	4
4	4	2	2	4	4	4	4	4	4
3	3	2	2	1	1	4	4	4	4
3	3	1	1	4	4	4	4	4	4
3	3	1	1	1	1	4	4	4	4
4	4	1	1	1	1	1	1	4	4
4	4	2	2	1	1	1	1	4	4
3	3	2	2	1	1	4	4	4	4
3	3	1	1	1	1	4	4	4	4
4	4	2	2	1	1	4	4	4	4
4	4	2	2	1	1	1	1	4	4
4	4	2	2	1	1	1	1	4	4
4	4	1	1	1	1	4	4	4	4
3	3	1	1	1	1	4	4	4	4

For Review Only

4	4	2	2	1	1	4	4	4	4
4	4	2	2	1	1	1	1	4	4
3	3	1	1	1	1	4	4	4	4
4	4	2	2	1	1	1	1	4	4
3	3	1	1	1	1	4	4	4	4
3	3	1	1	1	1	4	4	4	4
3	3	1	1	1	1	4	4	4	4
4	4	1	1	1	1	4	4	4	4
4	4	2	2	1	1	4	4	4	4
4	4	1	1	1	1	4	4	4	4
3	3	1	1	1	1	1	1	1	1
4	4	1	1	1	1	4	4	4	4
3	3	1	1	1	1	4	4	4	4
3	3	1	1	1	1	4	4	4	4
4	4	1	1	1	1	4	4	4	4
4	4	1	1	1	1	4	4	4	4
3	3	2	2	1	1	4	4	4	4
4	4	1	1	1	1	4	4	4	4
4	4	2	2	1	1	4	4	4	4
4	4	1	1	1	1	4	4	4	4
4	4	1	1	1	1	4	4	4	4
3	3	1	1	1	1	4	4	4	4
3	3	1	1	1	1	4	4	4	4
4	4	2	2	1	1	1	1	4	4
3	3	2	2	1	1	4	4	4	4
3	3	2	2	1	1	4	4	4	4
3	3	1	1	1	1	1	1	4	4
4	4	1	1	1	1	4	4	4	4
3	3	2	2	1	1	4	4	4	4
4	4	1	1	4	4	4	4	4	4
4	4	1	1	1	1	4	4	4	4
4	4	1	1	1	1	4	4	4	4
3	3	1	1	4	4	4	4	4	4
4	4	1	1	1	1	4	4	4	4
3	3	1	1	1	1	4	4	4	4
3	3	2	2	1	1	4	4	4	4
4	4	1	1	1	1	1	1	4	4
3	3	1	1	1	1	4	4	4	4
4	4	1	1	1	1	4	4	4	4
4	4	1	1	1	1	4	4	4	4
4	4	2	2	1	1	4	4	4	4
4	4	2	2	1	1	1	1	4	4
3	3	1	1	4	4	4	4	4	4
4	4	2	2	1	1	4	4	4	4
3	3	1	1	1	1	4	4	4	4
3	3	1	1	1	1	4	4	4	4
3	3	2	2	1	1	4	4	4	4
4	4	2	2	1	1	4	4	4	4
4	4	1	1	1	1	1	1	4	4
4	4	1	1	1	1	4	4	4	4
3	3	2	2	1	1	4	4	4	4
4	4	2	2	1	1	4	4	4	4
4	4	1	1	1	1	4	4	4	4
3	3	2	2	1	1	4	4	4	4
4	4	1	1	1	1	4	4	4	4

For Review Only

3	3	2	2	1	1	4	4	4	4
4	4	2	2	1	1	4	4	4	4
4	4	2	2	1	1	4	4	4	4
3	3	2	2	1	1	1	1	4	4
4	4	1	1	1	1	4	4	4	4
4	4	2	2	4	4	1	1	4	4
4	4	1	1	1	1	4	4	4	4
4	4	1	1	1	1	4	4	4	4
4	4	2	2	1	1	4	4	4	4
3	3	2	2	4	4	4	4	4	4
4	4	1	1	1	1	4	4	4	4
3	3	2	2	1	1	1	1	4	4
3	3	2	2	1	1	4	4	4	4
4	4	1	1	1	1	4	4	4	4
4	4	1	1	1	1	1	1	4	4
4	4	1	1	1	1	4	4	4	4
4	4	1	1	1	1	4	4	4	4
3	3	1	1	1	1	1	1	4	4
3	3	1	1	1	1	1	1	4	4
3	3	1	1	1	1	4	4	4	4
4	4	1	1	1	1	4	4	4	4
4	4	2	2	1	1	4	4	4	4
4	4	2	2	4	4	4	4	4	4
3	3	2	2	1	1	4	4	4	4
4	4	1	1	4	4	4	4	4	4
4	4	1	1	1	1	4	4	4	4
3	3	1	1	1	1	4	4	4	4
3	3	2	2	1	1	4	4	4	4
3	3	2	2	1	1	4	4	4	4
3	3	2	2	1	1	4	4	4	4
4	4	2	2	1	1	4	4	4	4
4	4	1	1	1	1	4	4	4	4
3	3	2	2	1	1	1	1	4	4
4	4	1	1	1	1	1	1	4	4
4	4	1	1	4	4	4	4	4	4
3	3	2	2	1	1	4	4	4	4
3	3	2	2	1	1	4	4	4	4
4	4	1	1	1	1	4	4	4	4
4	4	2	2	1	1	4	4	4	4
3	3	1	1	1	1	4	4	4	4
3	3	1	1	1	1	4	4	4	4
3	3	2	2	1	1	4	4	4	4
4	4	2	2	1	1	1	1	4	4
4	4	1	1	1	1	4	4	4	4
4	4	1	1	1	1	4	4	4	4
3	3	2	2	1	1	1	1	4	4
4	4	1	1	1	1	4	4	4	4
4	4	1	1	1	1	4	4	4	4
4	4	2	2	1	1	4	4	4	4
4	4	2	2	1	1	4	4	4	4
3	3	1	1	1	1	4	4	4	4
4	4	2	2	1	1	1	1	4	4
4	4	2	2	4	4	4	4	4	4
3	3	2	2	1	1	4	4	4	4
4	4	2	2	1	1	1	1	4	4

For Review Only

3	3	1	1	1	1	4	4	4	4
4	4	1	1	1	1	4	4	4	4
4	4	1	1	1	1	4	4	4	4
4	4	1	1	1	1	4	4	4	4
4	4	1	1	1	1	4	4	4	4
3	3	1	1	1	1	4	4	4	4
3	3	2	2	1	1	4	4	4	4
4	4	2	2	1	1	4	4	4	4
4	4	1	1	1	1	1	1	4	4
4	4	2	2	1	1	1	1	4	4
4	4	1	1	1	1	4	4	4	4
3	3	2	2	1	1	4	4	4	4
4	4	2	2	1	1	4	4	4	4
4	4	1	1	1	1	4	4	4	4
3	3	1	1	1	1	4	4	4	4
4	4	1	1	1	1	4	4	4	4
4	4	2	2	1	1	1	1	4	4
4	4	2	2	1	1	1	1	4	4
4	4	2	2	1	1	4	4	4	4
4	4	1	1	1	1	4	4	4	4
4	4	2	2	1	1	1	1	4	4
3	3	2	2	1	1	4	4	4	4
4	4	1	1	4	4	4	4	4	4
4	4	2	2	1	1	1	1	4	4
4	4	2	2	1	1	1	1	4	4
4	4	2	2	1	1	4	4	4	4
4	4	2	2	1	1	1	1	4	4
3	3	2	2	1	1	4	4	4	4
4	4	1	1	4	4	4	4	4	4
3	3	1	1	1	1	4	4	4	4
4	4	2	2	1	1	1	1	4	4
3	3	1	1	1	1	4	4	4	4
4	4	2	2	1	1	4	4	4	4
3	3	2	2	1	1	4	4	4	4
4	4	2	2	1	1	4	4	4	4
4	4	2	2	1	1	1	1	4	4
3	3	1	1	4	4	4	4	4	4
3	3	2	2	1	1	4	4	4	4
4	4	1	1	1	1	4	4	4	4
4	4	2	2	1	1	1	1	4	4
3	3	1	1	4	4	4	4	4	4
4	4	2	2	1	1	4	4	4	4
3	3	2	2	1	1	4	4	4	4
3	3	1	1	4	4	4	4	4	4
4	4	1	1	1	1	4	4	4	4
4	4	2	2	1	1	4	4	4	4
4	4	2	2	1	1	4	4	4	4
3	3	1	1	1	1	4	4	4	4
3	3	1	1	1	1	1	1	4	4
4	4	2	2	1	1	1	1	4	4
4	4	1	1	4	4	4	4	4	4
4	4	2	2	1	1	1	1	4	4
3	3	1	1	1	1	4	4	4	4
3	3	2	2	4	4	4	4	4	4
4	4	1	1	1	1	1	1	4	4

For Review Only

4	4	1	1	1	1	4	4	4	4
3	3	2	2	1	1	4	4	4	4

For Review Only

SNP2504	SNP2504	SNP2505	SNP2505	SNP2506	SNP2506	SNP2507	SNP2507	SNP2508	SNP2508
4	4	3	3	2	2	2	2	4	4
4	4	1	1	2	2	4	4	4	4
1	1	1	1	2	2	2	2	4	4
4	4	1	1	2	2	2	2	3	3
4	4	1	1	2	2	2	2	4	4
4	4	1	1	2	2	4	4	4	4
4	4	1	1	2	2	2	2	4	4
4	4	1	1	2	2	4	4	4	4
4	4	1	1	2	2	4	4	4	4
4	4	1	1	2	2	4	4	4	4
1	1	1	1	3	3	2	2	3	3
1	1	1	1	2	2	2	2	4	4
4	4	1	1	2	2	2	2	4	4
1	1	1	1	2	2	4	4	4	4
4	4	1	1	2	2	2	2	4	4
4	4	1	1	2	2	2	2	3	3
4	4	1	1	2	2	2	2	4	4
1	1	1	1	3	3	4	4	4	4
4	4	1	1	2	2	4	4	4	4
1	1	1	1	3	3	2	2	4	4
4	4	1	1	2	2	2	2	4	4
4	4	1	1	2	2	2	2	4	4
1	1	1	1	3	3	2	2	3	3
4	4	1	1	2	2	4	4	3	3
4	4	1	1	2	2	4	4	4	4
4	4	1	1	2	2	4	4	4	4
1	1	1	1	3	3	2	2	4	4
4	4	1	1	2	2	2	2	4	4
4	4	1	1	2	2	4	4	4	4
4	4	1	1	2	2	4	4	4	4
1	1	1	1	2	2	4	4	4	4
4	4	1	1	2	2	2	2	4	4
4	4	1	1	2	2	4	4	4	4
1	1	1	1	2	2	4	4	4	4
1	1	1	1	2	2	2	2	4	4
4	4	1	1	2	2	2	2	3	3
4	4	1	1	2	2	2	2	4	4
4	4	1	1	2	2	2	2	4	4
1	1	1	1	2	2	2	2	4	4
1	1	1	1	3	3	4	4	4	4
4	4	1	1	2	2	4	4	4	4
4	4	1	1	2	2	2	2	4	4
4	4	1	1	2	2	4	4	4	4
4	4	1	1	2	2	2	2	4	4
4	4	1	1	2	2	4	4	4	4
4	4	1	1	2	2	4	4	4	4
4	4	1	1	2	2	4	4	4	4
1	1	1	1	2	2	2	2	4	4
4	4	1	1	2	2	2	2	4	4
4	4	1	1	2	2	4	4	4	4
4	4	1	1	2	2	4	4	4	4
4	4	1	1	2	2	4	4	4	4

For Review Only

4	4	1	1	2	2	4	4	4	4
4	4	1	1	2	2	4	4	4	4
1	1	1	1	2	2	4	4	4	4
4	4	1	1	2	2	2	2	4	4
4	4	1	1	2	2	4	4	4	4
4	4	1	1	2	2	4	4	4	4
4	4	1	1	2	2	4	4	4	4
1	1	1	1	3	3	2	2	4	4
1	1	1	1	3	3	2	2	4	4
4	4	1	1	2	2	2	2	4	4
4	4	1	1	2	2	2	2	4	4
4	4	1	1	2	2	2	2	4	4
4	4	1	1	2	2	4	4	4	4
4	4	1	1	2	2	4	4	4	4
4	4	1	1	2	2	4	4	4	4
4	4	1	1	2	2	2	2	4	4
1	1	1	1	2	2	2	2	4	4
4	4	1	1	2	2	2	2	4	4
4	4	1	1	2	2	4	4	4	4
4	4	1	1	2	2	4	4	4	4
4	4	1	1	2	2	2	2	4	4
1	1	1	1	2	2	4	4	4	4
4	4	1	1	2	2	4	4	4	4
4	4	1	1	2	2	4	4	4	4
4	4	1	1	2	2	4	4	4	4
4	4	1	1	2	2	2	2	4	4
4	4	1	1	2	2	2	2	4	4
1	1	3	3	2	2	2	2	4	4
4	4	1	1	2	2	4	4	4	4
4	4	1	1	2	2	2	2	4	4
4	4	1	1	2	2	2	2	4	4
4	4	1	1	2	2	2	2	3	3
1	1	1	1	3	3	2	2	4	4
4	4	1	1	2	2	4	4	4	4
4	4	1	1	2	2	4	4	4	4
4	4	1	1	2	2	4	4	4	4
4	4	1	1	2	2	2	2	4	4
4	4	1	1	2	2	2	2	4	4
4	4	1	1	2	2	2	2	4	4
1	1	1	1	2	2	4	4	4	4
4	4	1	1	2	2	2	2	4	4
1	1	1	1	2	2	2	2	4	4
4	4	1	1	2	2	2	2	4	4
4	4	1	1	2	2	2	2	4	4
4	4	1	1	2	2	2	2	4	4
4	4	1	1	2	2	4	4	4	4
1	1	1	1	2	2	2	2	4	4
4	4	1	1	2	2	4	4	4	4
1	1	1	1	2	2	4	4	3	3
4	4	1	1	2	2	4	4	4	4
1	1	1	1	2	2	2	2	4	4
1	1	1	1	2	2	2	2	4	4
1	1	3	3	3	3	2	2	4	4
4	4	1	1	2	2	2	2	4	4
4	4	1	1	2	2	4	4	4	4

For Review Only

4	4	1	1	2	2	4	4	4	4
4	4	1	1	2	2	2	2	4	4
4	4	1	1	2	2	4	4	4	4
4	4	1	1	2	2	2	2	4	4
4	4	1	1	2	2	4	4	4	4
1	1	1	1	2	2	2	2	3	3
4	4	1	1	2	2	2	2	3	3
4	4	1	1	2	2	2	2	3	3
1	1	1	1	2	2	2	2	4	4
1	1	1	1	2	2	2	2	4	4
4	4	1	1	2	2	4	4	4	4
4	4	1	1	2	2	2	2	4	4
4	4	1	1	2	2	2	2	4	4
4	4	1	1	2	2	4	4	4	4
4	4	1	1	2	2	4	4	4	4
4	4	1	1	2	2	2	2	4	4
1	1	1	1	3	3	2	2	4	4
4	4	1	1	2	2	2	2	4	4
4	4	1	1	2	2	2	2	4	4
1	1	1	1	3	3	2	2	4	4
4	4	1	1	2	2	2	2	4	4
4	4	1	1	2	2	2	2	4	4
1	1	1	1	2	2	2	2	4	4
1	1	3	3	2	2	4	4	3	3
4	4	1	1	2	2	4	4	4	4
1	1	1	1	3	3	4	4	4	4
1	1	1	1	2	2	4	4	4	4
4	4	1	1	2	2	2	2	4	4
4	4	1	1	2	2	2	2	3	3
4	4	1	1	2	2	4	4	4	4
1	1	1	1	2	2	4	4	4	4
4	4	1	1	2	2	2	2	4	4
4	4	1	1	2	2	2	2	4	4
4	4	1	1	2	2	2	2	4	4
1	1	1	1	2	2	4	4	4	4
4	4	1	1	2	2	2	2	4	4
4	4	1	1	2	2	2	2	4	4
4	4	1	1	2	2	2	2	4	4
4	4	1	1	2	2	2	2	4	4
1	1	1	1	2	2	2	2	4	4
4	4	1	1	2	2	4	4	4	4
1	1	1	1	2	2	4	4	4	4
4	4	1	1	2	2	2	2	4	4
4	4	1	1	2	2	2	2	4	4
4	4	1	1	2	2	2	2	4	4
4	4	1	1	2	2	2	2	4	4
4	4	1	1	2	2	4	4	4	4
4	4	1	1	2	2	4	4	4	4
4	4	1	1	2	2	2	2	4	4
4	4	1	1	2	2	4	4	4	4
4	4	1	1	2	2	4	4	4	4
1	1	1	1	3	3	4	4	4	4
4	4	1	1	2	2	4	4	4	4
4	4	1	1	2	2	2	2	4	4
4	4	1	1	2	2	2	2	4	4

For Review Only

4	4	1	1	2	2	2	2	4	4
4	4	1	1	2	2	4	4	4	4
4	4	1	1	2	2	2	2	4	4
4	4	1	1	2	2	4	4	4	4
4	4	1	1	2	2	4	4	4	4
4	4	1	1	2	2	4	4	4	4
1	1	1	1	3	3	2	2	4	4
4	4	1	1	2	2	2	2	4	4
1	1	1	1	2	2	2	2	4	4
4	4	1	1	2	2	2	2	4	4
4	4	1	1	2	2	4	4	4	4
1	1	1	1	2	2	4	4	3	3
4	4	1	1	2	2	2	2	4	4
4	4	1	1	2	2	2	2	4	4
1	1	1	1	2	2	4	4	4	4
1	1	1	1	2	2	2	2	4	4
4	4	1	1	2	2	4	4	4	4
4	4	1	1	2	2	4	4	4	4
1	1	1	1	2	2	4	4	3	3
4	4	1	1	2	2	2	2	3	3
4	4	1	1	2	2	2	2	4	4
1	1	3	3	2	2	2	2	4	4
1	1	1	1	2	2	4	4	4	4
4	4	1	1	2	2	2	2	4	4
4	4	1	1	2	2	2	2	4	4
1	1	1	1	2	2	4	4	4	4
4	4	1	1	2	2	2	2	4	4
4	4	1	1	2	2	4	4	4	4
1	1	1	1	2	2	4	4	4	4
4	4	1	1	2	2	4	4	4	4
4	4	3	3	2	2	4	4	4	4
4	4	1	1	2	2	2	2	4	4
1	1	1	1	2	2	4	4	4	4
1	1	1	1	2	2	4	4	4	4
4	4	1	1	2	2	2	2	4	4
4	4	1	1	2	2	2	2	4	4
1	1	1	1	2	2	4	4	4	4
4	4	1	1	2	2	2	2	4	4
1	1	1	1	2	2	4	4	4	4
4	4	1	1	2	2	4	4	4	4
1	1	1	1	2	2	2	2	4	4
4	4	1	1	2	2	4	4	4	4
1	1	1	1	3	3	2	2	4	4
4	4	1	1	2	2	4	4	4	4
4	4	1	1	2	2	2	2	4	4
4	4	1	1	2	2	2	2	4	4
1	1	1	1	2	2	2	2	4	4
1	1	1	1	2	2	2	2	4	4
1	1	1	1	3	3	2	2	4	4
4	4	1	1	2	2	2	2	3	3
4	4	1	1	2	2	2	2	4	4
1	1	1	1	3	3	2	2	4	4
1	1	1	1	2	2	4	4	4	4
4	4	1	1	2	2	2	2	4	4
1	1	1	1	3	3	2	2	4	4

For Review Only

1	1	1	1	2	2	4	4	4	4
1	1	1	1	3	3	2	2	4	4
4	4	1	1	2	2	2	2	4	4
1	1	1	1	2	2	4	4	4	4
1	1	1	1	3	3	4	4	4	4
4	4	1	1	2	2	4	4	4	4
1	1	1	1	2	2	2	2	4	4
4	4	1	1	2	2	4	4	4	4
1	1	1	1	2	2	4	4	3	3
1	1	1	1	2	2	4	4	4	4
4	4	1	1	2	2	2	2	4	4
4	4	3	3	2	2	4	4	4	4
4	4	1	1	2	2	2	2	4	4
4	4	1	1	2	2	4	4	4	4
1	1	1	1	2	2	2	2	4	4
1	1	1	1	3	3	2	2	4	4
1	1	3	3	2	2	4	4	4	4
1	1	1	1	2	2	2	2	4	4
1	1	1	1	2	2	2	2	4	4
4	4	1	1	2	2	2	2	4	4
1	1	1	1	3	3	2	2	3	3
4	4	1	1	2	2	4	4	4	4
4	4	1	1	2	2	2	2	4	4
1	1	1	1	3	3	4	4	4	4
1	1	1	1	3	3	2	2	4	4
4	4	1	1	2	2	4	4	4	4
1	1	3	3	2	2	2	2	4	4
4	4	1	1	2	2	4	4	3	3
4	4	1	1	2	2	4	4	4	4
4	4	1	1	2	2	2	2	4	4
1	1	1	1	2	2	2	2	4	4
4	4	1	1	2	2	2	2	4	4
4	4	1	1	2	2	2	2	4	4
4	4	1	1	2	2	4	4	4	4
1	1	1	1	2	2	4	4	4	4
1	1	3	3	2	2	4	4	4	4
4	4	1	1	2	2	4	4	4	4
4	4	1	1	2	2	2	2	4	4
4	4	1	1	2	2	2	2	3	3
1	1	1	1	3	3	2	2	4	4
4	4	1	1	2	2	2	2	4	4
4	4	1	1	2	2	2	2	4	4
1	1	1	1	3	3	4	4	4	4
4	4	1	1	2	2	2	2	4	4
4	4	1	1	2	2	4	4	4	4
4	4	1	1	2	2	2	2	4	4
4	4	3	3	2	2	2	2	4	4
4	4	1	1	2	2	2	2	4	4
4	4	1	1	2	2	4	4	4	4
1	1	3	3	3	3	2	2	4	4
4	4	1	1	2	2	4	4	4	4
1	1	3	3	2	2	2	2	4	4
1	1	1	1	2	2	2	2	4	4
4	4	1	1	2	2	4	4	4	4
1	1	1	1	3	3	2	2	4	4

For Review Only

4	4	1	1	2	2	2	2	4	4
4	4	1	1	2	2	4	4	4	4

For Review Only

SNP2509	SNP2509	SNP251	SNP251	SNP2510	SNP2510	SNP2511	SNP2511	SNP2512	SNP2512
4	4	1	1	1	1	2	2	2	2
4	4	4	4	1	1	3	3	3	3
4	4	1	1	1	1	2	2	2	2
4	4	1	1	1	1	2	2	2	2
1	1	4	4	1	1	2	2	2	2
4	4	1	1	1	1	2	2	2	2
1	1	1	1	1	1	2	2	2	2
1	1	1	1	1	1	2	2	2	2
1	1	1	1	1	1	2	2	2	2
1	1	4	4	1	1	2	2	2	2
1	1	4	4	1	1	3	3	3	3
4	4	1	1	1	1	3	3	2	2
4	4	1	1	1	1	2	2	2	2
1	1	1	1	2	2	3	3	2	2
4	4	1	1	1	1	2	2	2	2
1	1	4	4	1	1	3	3	3	3
1	1	1	1	1	1	2	2	2	2
4	4	4	4	1	1	3	3	3	3
4	4	4	4	1	1	2	2	2	2
4	4	4	4	1	1	2	2	2	2
4	4	4	4	1	1	2	2	2	2
1	1	4	4	1	1	2	2	2	2
4	4	1	1	2	2	3	3	3	3
1	1	4	4	1	1	3	3	3	3
4	4	1	1	1	1	2	2	2	2
1	1	1	1	1	1	2	2	2	2
4	4	1	1	1	1	3	3	3	3
4	4	1	1	1	1	2	2	2	2
1	1	1	1	1	1	2	2	2	2
1	1	4	4	1	1	2	2	2	2
4	4	1	1	1	1	3	3	3	3
4	4	1	1	1	1	2	2	2	2
1	1	1	1	1	1	2	2	2	2
1	1	1	1	1	1	3	3	3	3
1	1	1	1	1	1	2	2	2	2
1	1	1	1	1	1	2	2	2	2
4	4	1	1	1	1	2	2	2	2
1	1	1	1	1	1	2	2	2	2
1	1	4	4	2	2	3	3	3	3
4	4	1	1	1	1	3	3	3	3
1	1	4	4	1	1	2	2	2	2
4	4	1	1	1	1	2	2	2	2
1	1	4	4	1	1	2	2	2	2
1	1	1	1	1	1	2	2	2	2
1	1	4	4	1	1	2	2	2	2
4	4	1	1	1	1	2	2	2	2
1	1	1	1	1	1	3	3	3	3
1	1	4	4	1	1	3	3	3	3
1	1	1	1	1	1	2	2	2	2
4	4	1	1	1	1	2	2	2	2
1	1	1	1	1	1	2	2	2	2
4	4	4	4	1	1	2	2	2	2

For Review Only

1	1	1	1	1	1	2	2	2	2
1	1	1	1	1	1	2	2	2	2
4	4	4	4	1	1	2	2	2	2
1	1	1	1	1	1	2	2	2	2
4	4	4	4	1	1	2	2	2	2
1	1	1	1	1	1	2	2	2	2
1	1	1	1	1	1	2	2	2	2
1	1	4	4	1	1	3	3	3	3
1	1	4	4	1	1	3	3	3	3
4	4	1	1	1	1	2	2	3	3
1	1	1	1	1	1	3	3	3	3
1	1	1	1	1	1	3	3	3	3
1	1	1	1	1	1	2	2	2	2
1	1	1	1	1	1	2	2	2	2
4	4	1	1	1	1	2	2	2	2
1	1	1	1	1	1	2	2	2	2
1	1	4	4	1	1	2	2	2	2
4	4	1	1	1	1	2	2	2	2
4	4	4	4	1	1	2	2	2	2
1	1	1	1	1	1	2	2	2	2
1	1	1	1	1	1	2	2	2	2
4	4	1	1	2	2	2	2	2	2
1	1	1	1	1	1	2	2	2	2
4	4	1	1	1	1	2	2	2	2
1	1	4	4	1	1	2	2	2	2
4	4	1	1	1	1	2	2	2	2
1	1	1	1	1	1	2	2	2	2
1	1	1	1	2	2	2	2	2	2
1	1	1	1	1	1	2	2	2	2
4	4	4	4	1	1	2	2	2	2
1	1	1	1	1	1	2	2	2	2
1	1	1	1	1	1	2	2	2	2
1	1	4	4	2	2	3	3	2	2
1	1	1	1	1	1	2	2	2	2
1	1	4	4	1	1	2	2	2	2
1	1	4	4	1	1	2	2	2	2
1	1	1	1	1	1	2	2	2	2
1	1	1	1	1	1	2	2	2	2
4	4	1	1	1	1	2	2	2	2
1	1	1	1	1	1	2	2	2	2
1	1	1	1	1	1	2	2	2	2
4	4	4	4	1	1	2	2	2	2
4	4	4	4	1	1	2	2	2	2
1	1	1	1	1	1	3	3	3	3
4	4	4	4	1	1	2	2	2	2
4	4	1	1	1	1	2	2	2	2
1	1	1	1	1	1	2	2	2	2
1	1	1	1	1	1	2	2	2	2
4	4	1	1	1	1	3	3	3	3
1	1	1	1	1	1	2	2	2	2
4	4	1	1	1	1	2	2	2	2
1	1	4	4	2	2	3	3	3	3
1	1	4	4	1	1	3	3	3	3
4	4	1	1	2	2	2	2	2	2
1	1	4	4	1	1	2	2	2	2

For Review Only

1	1	1	1	2	2	2	2	2	2
1	1	4	4	1	1	2	2	2	2
1	1	1	1	1	1	2	2	2	2
1	1	1	1	1	1	2	2	2	2
4	4	1	1	1	1	2	2	2	2
1	1	1	1	1	1	3	3	2	2
4	4	1	1	1	1	3	3	3	3
4	4	1	1	1	1	2	2	3	3
1	1	1	1	2	2	3	3	3	3
1	1	4	4	1	1	2	2	2	2
1	1	1	1	1	1	3	3	2	2
4	4	1	1	1	1	2	2	2	2
4	4	4	4	1	1	2	2	2	2
4	4	1	1	1	1	2	2	2	2
1	1	1	1	1	1	3	3	2	2
1	1	1	1	1	1	2	2	2	2
4	4	1	1	1	1	3	3	3	3
1	1	1	1	1	1	2	2	2	2
1	1	1	1	2	2	3	3	2	2
4	4	1	1	1	1	3	3	3	3
4	4	1	1	1	1	2	2	2	2
1	1	4	4	1	1	2	2	2	2
4	4	1	1	1	1	2	2	2	2
1	1	1	1	2	2	2	2	3	3
1	1	1	1	1	1	2	2	2	2
1	1	1	1	1	1	3	3	3	3
4	4	4	4	1	1	3	3	2	2
4	4	1	1	1	1	2	2	2	2
1	1	1	1	1	1	2	2	2	2
4	4	4	4	1	1	2	2	2	2
1	1	1	1	2	2	2	2	2	2
4	4	1	1	1	1	2	2	2	2
1	1	1	1	1	1	2	2	2	2
1	1	1	1	1	1	2	2	2	2
1	1	1	1	1	1	3	3	3	3
1	1	4	4	1	1	2	2	2	2
1	1	1	1	1	1	2	2	2	2
4	4	1	1	1	1	3	3	3	3
1	1	4	4	2	2	2	2	3	3
1	1	4	4	2	2	2	2	2	2
4	4	1	1	1	1	2	2	2	2
1	1	1	1	1	1	3	3	2	2
1	1	1	1	1	1	2	2	2	2
1	1	1	1	1	1	2	2	3	3
1	1	4	4	1	1	2	2	2	2
1	1	1	1	1	1	2	2	2	2
1	1	1	1	1	1	2	2	2	2
1	1	1	1	1	1	2	2	2	2
1	1	1	1	1	1	2	2	2	2
4	4	1	1	1	1	2	2	2	2
1	1	1	1	1	1	2	2	2	2
1	1	4	4	1	1	3	3	3	3
4	4	1	1	1	1	2	2	2	2
1	1	4	4	1	1	2	2	2	2
4	4	1	1	1	1	2	2	2	2

For Review Only

1	1	1	1	1	1	2	2	2	2
4	4	1	1	1	1	2	2	2	2
1	1	1	1	1	1	2	2	2	2
1	1	1	1	1	1	2	2	2	2
4	4	1	1	1	1	2	2	2	2
1	1	4	4	1	1	2	2	2	2
1	1	4	4	2	2	2	2	2	2
4	4	1	1	1	1	2	2	2	2
1	1	4	4	2	2	3	3	3	3
1	1	4	4	1	1	2	2	2	2
4	4	1	1	1	1	2	2	2	2
1	1	1	1	1	1	3	3	3	3
4	4	1	1	1	1	3	3	3	3
1	1	1	1	1	1	2	2	2	2
1	1	1	1	2	2	3	3	2	2
1	1	4	4	1	1	3	3	3	3
4	4	1	1	1	1	2	2	3	3
4	4	4	4	1	1	2	2	2	2
4	4	4	4	1	1	3	3	3	3
4	4	1	1	1	1	2	2	3	3
4	4	1	1	1	1	2	2	2	2
1	1	1	1	2	2	2	2	2	2
4	4	1	1	1	1	2	2	2	2
1	1	1	1	1	1	3	3	3	3
1	1	1	1	1	1	2	2	2	2
1	1	1	1	1	1	3	3	3	3
4	4	1	1	1	1	2	2	2	2
4	4	1	1	1	1	2	2	2	2
4	4	1	1	1	1	3	3	3	3
1	1	1	1	1	1	2	2	2	2
1	1	4	4	1	1	2	2	2	2
4	4	1	1	1	1	2	2	2	2
4	4	1	1	1	1	2	2	2	2
1	1	1	1	1	1	3	3	2	2
1	1	1	1	1	1	2	2	2	2
1	1	1	1	1	1	3	3	3	3
4	4	4	4	1	1	2	2	3	3
4	4	1	1	1	1	2	2	2	2
1	1	1	1	1	1	2	2	2	2
4	4	1	1	1	1	2	2	2	2
4	4	1	1	1	1	2	2	2	2
1	1	1	1	1	1	2	2	2	2
1	1	1	1	2	2	3	3	3	3
1	1	1	1	1	1	2	2	2	2
1	1	1	1	1	1	2	2	2	2
1	1	1	1	2	2	2	2	2	2
1	1	4	4	1	1	3	3	3	3
1	1	4	4	2	2	2	2	2	2
1	1	4	4	1	1	3	3	3	3
1	1	1	1	1	1	3	3	3	3
1	1	4	4	1	1	2	2	2	2
1	1	4	4	1	1	3	3	3	3
1	1	1	1	1	1	2	2	2	2
1	1	1	1	1	1	3	3	3	3
1	1	4	4	2	2	3	3	3	3

For Review Only

1	1	1	1	2	2	3	3	3	3
1	1	4	4	1	1	3	3	3	3
1	1	1	1	1	1	2	2	2	2
4	4	1	1	1	1	2	2	2	2
1	1	4	4	1	1	3	3	3	3
1	1	1	1	1	1	2	2	2	2
1	1	4	4	1	1	2	2	2	2
1	1	4	4	1	1	2	2	2	2
4	4	1	1	1	1	3	3	3	3
1	1	4	4	2	2	2	2	2	2
4	4	1	1	1	1	2	2	2	2
1	1	4	4	1	1	3	3	2	2
4	4	1	1	1	1	2	2	2	2
1	1	4	4	1	1	2	2	2	2
1	1	1	1	1	1	2	2	2	2
1	1	4	4	1	1	3	3	3	3
1	1	1	1	1	1	3	3	3	3
1	1	4	4	2	2	2	2	2	2
1	1	4	4	2	2	3	3	3	3
1	1	1	1	1	1	2	2	2	2
1	1	1	1	1	1	3	3	3	3
1	1	1	1	1	1	2	2	2	2
4	4	4	4	1	1	2	2	2	2
1	1	1	1	1	1	3	3	3	3
1	1	4	4	1	1	3	3	3	3
4	4	4	4	1	1	2	2	2	2
1	1	1	1	2	2	2	2	2	2
4	4	1	1	1	1	3	3	3	3
1	1	1	1	1	1	2	2	2	2
1	1	4	4	1	1	2	2	2	2
1	1	1	1	2	2	3	3	3	3
1	1	4	4	1	1	2	2	2	2
1	1	4	4	1	1	2	2	2	2
1	1	1	1	1	1	2	2	2	2
1	1	1	1	1	1	2	2	2	2
1	1	1	1	2	2	2	2	2	2
1	1	1	1	1	1	2	2	2	2
1	1	1	1	1	1	3	3	3	3
1	1	1	1	1	1	2	2	2	2
1	1	4	4	2	2	3	3	3	3
4	4	1	1	1	1	2	2	2	2
4	4	1	1	1	1	2	2	2	2
1	1	4	4	1	1	3	3	3	3
1	1	1	1	1	1	2	2	2	2
1	1	4	4	2	2	2	2	2	2
4	4	1	1	1	1	2	2	2	2
1	1	1	1	2	2	2	2	2	2
1	1	4	4	2	2	2	2	2	2
1	1	1	1	1	1	2	2	2	2
1	1	1	1	2	2	3	3	3	3
4	4	4	4	1	1	2	2	2	2
1	1	4	4	2	2	3	3	3	3
4	4	1	1	1	1	2	2	2	2
4	4	1	1	1	1	2	2	2	2
1	1	1	1	1	1	3	3	3	3

For Review Only

CSSA 5585 Guilford Rd., Madison, WI 53711

4	4	4	4	1	1	2	2	2	2
4	4	4	4	1	1	3	3	3	3

For Review Only

SNP2513	SNP2513	SNP2514	SNP2514	SNP2515	SNP2515	SNP2516	SNP2516	SNP2517	SNP2517
1	1	4	4	3	3	3	3	2	2
1	1	4	4	3	3	3	3	3	3
1	1	4	4	3	3	3	3	2	2
4	4	4	4	3	3	2	2	2	2
1	1	4	4	3	3	2	2	2	2
1	1	4	4	3	3	3	3	2	2
1	1	4	4	3	3	3	3	2	2
1	1	4	4	3	3	2	2	2	2
1	1	4	4	3	3	3	3	2	2
1	1	4	4	3	3	2	2	2	2
4	4	4	4	3	3	3	3	2	2
1	1	4	4	3	3	3	3	2	2
1	1	4	4	3	3	3	3	2	2
4	4	4	4	2	2	2	2	2	2
1	1	4	4	3	3	3	3	2	2
4	4	4	4	3	3	2	2	2	2
1	1	4	4	3	3	2	2	2	2
4	4	4	4	3	3	3	3	2	2
1	1	4	4	3	3	2	2	2	2
1	1	4	4	3	3	2	2	2	2
1	1	4	4	3	3	3	3	2	2
4	4	4	4	3	3	2	2	2	2
4	4	3	3	3	3	3	3	2	2
4	4	4	4	3	3	3	3	2	2
4	4	4	4	3	3	3	3	2	2
1	1	4	4	3	3	3	3	3	3
4	4	4	4	3	3	2	2	2	2
4	4	4	4	3	3	3	3	2	2
1	1	4	4	3	3	3	3	2	2
1	1	4	4	3	3	3	3	2	2
4	4	4	4	3	3	2	2	2	2
1	1	4	4	3	3	3	3	2	2
1	1	4	4	3	3	3	3	2	2
4	4	4	4	3	3	2	2	2	2
1	1	4	4	3	3	3	3	2	2
1	1	3	3	2	2	3	3	3	3
1	1	4	4	3	3	2	2	2	2
1	1	4	4	3	3	3	3	2	2
4	4	4	4	2	2	3	3	2	2
4	4	4	4	3	3	3	3	2	2
1	1	4	4	3	3	2	2	2	2
1	1	4	4	3	3	3	3	2	2
1	1	4	4	3	3	2	2	2	2
1	1	4	4	3	3	2	2	2	2
1	1	4	4	3	3	2	2	2	2
1	1	4	4	3	3	3	3	2	2
1	1	4	4	3	3	2	2	3	3
4	4	4	4	3	3	3	3	2	2
1	1	4	4	3	3	2	2	2	2
1	1	4	4	3	3	2	2	2	2
1	1	4	4	3	3	3	3	2	2
1	1	4	4	2	2	3	3	2	2

For Review Only

1	1	4	4	3	3	2	2	2	2
1	1	4	4	3	3	3	3	2	2
1	1	4	4	3	3	3	3	2	2
1	1	4	4	3	3	2	2	2	2
1	1	4	4	3	3	2	2	2	2
1	1	4	4	3	3	3	3	3	3
1	1	4	4	3	3	3	3	3	3
4	4	4	4	3	3	3	3	2	2
4	4	4	4	3	3	2	2	2	2
4	4	4	4	3	3	3	3	2	2
1	1	4	4	3	3	2	2	3	3
1	1	4	4	3	3	2	2	3	3
1	1	4	4	3	3	2	2	2	2
1	1	4	4	3	3	3	3	2	2
4	4	4	4	3	3	3	3	2	2
1	1	4	4	3	3	3	3	2	2
1	1	4	4	3	3	3	3	2	2
1	1	4	4	3	3	3	3	2	2
1	1	4	4	3	3	2	2	2	2
1	1	4	4	3	3	2	2	2	2
1	1	4	4	3	3	3	3	3	3
1	1	4	4	3	3	3	3	2	2
1	1	4	4	3	3	3	3	2	2
1	1	4	4	3	3	3	3	2	2
1	1	4	4	3	3	2	2	2	2
1	1	4	4	3	3	3	3	2	2
1	1	4	4	3	3	2	2	2	2
1	1	4	4	2	2	3	3	2	2
1	1	4	4	3	3	3	3	3	3
1	1	4	4	3	3	2	2	2	2
1	1	4	4	3	3	2	2	2	2
1	1	4	4	3	3	3	3	3	3
4	4	4	4	2	2	3	3	2	2
1	1	4	4	3	3	3	3	2	2
1	1	4	4	3	3	3	3	2	2
1	1	4	4	3	3	2	2	2	2
1	1	4	4	3	3	2	2	2	2
1	1	4	4	3	3	3	3	2	2
1	1	4	4	3	3	2	2	2	2
1	1	4	4	3	3	2	2	2	2
1	1	4	4	3	3	2	2	2	2
1	1	4	4	3	3	2	2	2	2
1	1	4	4	3	3	2	2	2	2
1	1	4	4	3	3	2	2	3	3
1	1	4	4	3	3	2	2	2	2
1	1	4	4	3	3	3	3	2	2
4	4	3	3	2	2	3	3	3	3
1	1	4	4	3	3	3	3	3	3
4	4	4	4	3	3	2	2	2	2
1	1	4	4	3	3	3	3	2	2
1	1	4	4	3	3	2	2	2	2
4	4	4	4	2	2	3	3	2	2
4	4	4	4	3	3	3	3	2	2
1	1	4	4	3	3	3	3	2	2
1	1	4	4	3	3	3	3	2	2

For Review Only

1	1	4	4	3	3	3	3	3	3
1	1	4	4	3	3	3	3	2	2
1	1	4	4	3	3	3	3	3	3
4	4	3	3	2	2	3	3	3	3
1	1	4	4	3	3	3	3	3	3
1	1	4	4	3	3	3	3	2	2
4	4	4	4	3	3	2	2	2	2
4	4	4	4	3	3	2	2	2	2
4	4	4	4	3	3	2	2	2	2
1	1	3	3	3	3	3	3	2	2
4	4	4	4	3	3	3	3	2	2
4	4	4	4	3	3	3	3	2	2
1	1	4	4	3	3	2	2	2	2
1	1	4	4	3	3	3	3	2	2
1	1	4	4	3	3	3	3	2	2
1	1	4	4	3	3	3	3	2	2
4	4	4	4	3	3	3	3	2	2
4	4	4	4	3	3	3	3	2	2
4	4	4	4	2	2	3	3	2	2
1	1	4	4	3	3	3	3	3	3
4	4	4	4	3	3	3	3	2	2
1	1	4	4	3	3	2	2	2	2
1	1	4	4	3	3	3	3	2	2
1	1	4	4	3	3	3	3	2	2
1	1	4	4	3	3	3	3	3	3
4	4	4	4	3	3	2	2	2	2
1	1	4	4	3	3	3	3	2	2
1	1	4	4	3	3	2	2	2	2
4	4	4	4	3	3	2	2	2	2
1	1	4	4	3	3	2	2	2	2
1	1	4	4	2	2	2	2	2	2
4	4	4	4	3	3	3	3	2	2
1	1	4	4	3	3	2	2	2	2
4	4	4	4	3	3	3	3	2	2
4	4	4	4	3	3	2	2	2	2
4	4	4	4	3	3	2	2	2	2
1	1	3	3	2	2	3	3	3	3
1	1	4	4	3	3	2	2	2	2
4	4	4	4	3	3	3	3	2	2
1	1	4	4	3	3	3	3	3	3
4	4	4	4	3	3	2	2	2	2
4	4	4	4	2	2	2	2	2	2
1	1	4	4	3	3	3	3	2	2
4	4	4	4	3	3	3	3	2	2
1	1	4	4	3	3	3	3	3	3
1	1	4	4	3	3	3	3	2	2
1	1	4	4	3	3	3	3	3	3
1	1	4	4	3	3	3	3	3	3
1	1	4	4	3	3	3	3	2	2
1	1	4	4	3	3	3	3	3	3
1	1	4	4	3	3	3	3	3	3
4	4	4	4	3	3	3	3	2	2
1	1	4	4	3	3	3	3	2	2
1	1	4	4	3	3	2	2	3	3
4	4	4	4	3	3	3	3	2	2

For Review Only

1	1	4	4	3	3	2	2	2	2
1	1	4	4	3	3	2	2	2	2
1	1	4	4	3	3	3	3	2	2
4	4	4	4	3	3	3	3	2	2
1	1	4	4	3	3	3	3	2	2
1	1	4	4	3	3	2	2	2	2
4	4	4	4	3	3	3	3	2	2
1	1	4	4	3	3	3	3	2	2
4	4	4	4	3	3	3	3	2	2
1	1	4	4	3	3	2	2	2	2
1	1	4	4	3	3	3	3	2	2
4	4	4	4	3	3	3	3	2	2
4	4	4	4	3	3	3	3	2	2
1	1	4	4	3	3	3	3	2	2
4	4	4	4	2	2	2	2	2	2
4	4	4	4	3	3	3	3	2	2
4	4	4	4	3	3	2	2	2	2
1	1	4	4	3	3	3	3	2	2
4	4	4	4	3	3	3	3	2	2
4	4	4	4	3	3	2	2	2	2
1	1	4	4	3	3	3	3	2	2
1	1	4	4	3	3	3	3	2	2
1	1	4	4	3	3	2	2	2	2
1	1	4	4	3	3	2	2	3	3
1	1	4	4	3	3	2	2	2	2
4	4	4	4	3	3	2	2	2	2
1	1	4	4	3	3	3	3	2	2
1	1	4	4	3	3	3	3	2	2
4	4	4	4	3	3	3	3	2	2
1	1	4	4	3	3	2	2	2	2
1	1	4	4	3	3	3	3	2	2
1	1	4	4	3	3	3	3	2	2
1	1	4	4	3	3	2	2	2	2
4	4	4	4	2	2	2	2	2	2
1	1	4	4	3	3	2	2	2	2
1	1	4	4	3	3	2	2	3	3
4	4	4	4	3	3	2	2	2	2
1	1	4	4	3	3	2	2	2	2
1	1	4	4	3	3	3	3	2	2
1	1	4	4	3	3	2	2	2	2
1	1	4	4	3	3	3	3	2	2
1	1	4	4	3	3	3	3	3	3
4	4	4	4	2	2	3	3	2	2
1	1	4	4	3	3	3	3	2	2
1	1	3	3	2	2	2	2	2	2
1	1	4	4	3	3	3	3	2	2
4	4	4	4	3	3	3	3	2	2
1	1	4	4	3	3	3	3	2	2
4	4	4	4	3	3	2	2	2	2
4	4	4	4	3	3	2	2	2	2
1	1	4	4	3	3	2	2	2	2
4	4	4	4	3	3	3	3	2	2
1	1	4	4	3	3	2	2	2	2
1	1	4	4	3	3	2	2	3	3
4	4	4	4	2	2	3	3	2	2

For Review Only

4	4	4	4	3	3	2	2	2	2
4	4	4	4	3	3	3	3	2	2
1	1	4	4	3	3	2	2	2	2
1	1	4	4	3	3	3	3	2	2
4	4	4	4	3	3	3	3	2	2
1	1	4	4	3	3	2	2	2	2
1	1	4	4	2	2	3	3	2	2
1	1	4	4	3	3	2	2	2	2
4	4	4	4	2	2	2	2	2	2
1	1	4	4	3	3	3	3	2	2
4	4	4	4	3	3	3	3	2	2
4	4	4	4	3	3	3	3	2	2
4	4	4	4	3	3	2	2	2	2
1	1	4	4	3	3	3	3	3	3
1	1	4	4	3	3	3	3	2	2
4	4	4	4	3	3	2	2	2	2
4	4	3	3	3	3	3	3	2	2
1	1	4	4	3	3	3	3	2	2
4	4	4	4	3	3	3	3	2	2
1	1	4	4	3	3	3	3	2	2
4	4	4	4	2	2	3	3	2	2
1	1	4	4	3	3	3	3	3	3
1	1	4	4	3	3	2	2	2	2
4	4	4	4	3	3	3	3	2	2
4	4	4	4	3	3	3	3	2	2
1	1	4	4	3	3	3	3	2	2
1	1	3	3	3	3	3	3	2	2
4	4	4	4	3	3	2	2	2	2
1	1	4	4	3	3	3	3	2	2
1	1	4	4	3	3	2	2	2	2
4	4	3	3	3	3	3	3	2	2
1	1	4	4	3	3	3	3	3	3
1	1	4	4	3	3	3	3	2	2
1	1	4	4	3	3	3	3	3	3
1	1	4	4	3	3	2	2	2	2
1	1	4	4	3	3	3	3	2	2
1	1	4	4	3	3	2	2	2	2
1	1	4	4	3	3	2	2	3	3
1	1	3	3	2	2	3	3	3	3
4	4	4	4	2	2	3	3	2	2
1	1	4	4	3	3	2	2	2	2
1	1	4	4	3	3	3	3	2	2
4	4	4	4	3	3	3	3	2	2
1	1	4	4	3	3	2	2	2	2
1	1	4	4	3	3	3	3	2	2
1	1	4	4	3	3	2	2	2	2
4	4	4	4	2	2	3	3	3	3
1	1	4	4	3	3	3	3	2	2
1	1	4	4	3	3	3	3	2	2
4	4	3	3	3	3	3	3	2	2
1	1	4	4	3	3	2	2	2	2
4	4	3	3	2	2	3	3	2	2
1	1	4	4	3	3	3	3	2	2
1	1	4	4	3	3	2	2	2	2
4	4	4	4	2	2	3	3	2	2

For Review Only

1	1	4	4	3	3	2	2	2	2
1	1	4	4	3	3	3	3	3	3

For Review Only

SNP2518	SNP2518	SNP2519	SNP2519	SNP252	SNP252	SNP2520	SNP2520	SNP2521	SNP2521
3	3	2	2	1	1	1	1	2	2
4	4	2	2	4	4	3	3	4	4
3	3	2	2	1	1	1	1	2	2
3	3	2	2	4	4	1	1	2	2
3	3	2	2	4	4	1	1	2	2
3	3	2	2	4	4	3	3	2	2
3	3	2	2	4	4	1	1	2	2
3	3	2	2	4	4	3	3	4	4
3	3	2	2	4	4	1	1	2	2
3	3	2	2	4	4	3	3	4	4
3	3	2	2	4	4	3	3	4	4
3	3	2	2	1	1	1	1	2	2
3	3	2	2	4	4	1	1	2	2
3	3	2	2	4	4	1	1	4	4
3	3	2	2	4	4	1	1	2	2
3	3	2	2	1	1	1	1	2	2
3	3	2	2	4	4	3	3	4	4
3	3	2	2	4	4	3	3	4	4
3	3	4	4	4	4	1	1	2	2
3	3	4	4	4	4	3	3	4	4
3	3	2	2	4	4	1	1	2	2
3	3	2	2	4	4	1	1	2	2
3	3	2	2	4	4	3	3	4	4
3	3	2	2	4	4	3	3	4	4
3	3	2	2	4	4	3	3	2	2
4	4	2	2	4	4	1	1	2	2
3	3	2	2	4	4	1	1	2	2
3	3	2	2	4	4	1	1	2	2
3	3	4	4	4	4	3	3	4	4
3	3	2	2	4	4	3	3	4	4
4	4	4	4	4	4	1	1	2	2
3	3	2	2	4	4	3	3	2	2
3	3	2	2	4	4	3	3	2	2
3	3	2	2	4	4	3	3	4	4
3	3	2	2	4	4	1	1	2	2
3	3	2	2	4	4	1	1	2	2
3	3	2	2	4	4	3	3	4	4
3	3	2	2	4	4	1	1	2	2
3	3	2	2	4	4	3	3	4	4
3	3	2	2	4	4	1	1	4	4
3	3	2	2	4	4	3	3	4	4
3	3	4	4	4	4	3	3	4	4
3	3	4	4	4	4	3	3	4	4
3	3	2	2	4	4	1	1	2	2
3	3	4	4	4	4	3	3	4	4
4	4	2	2	1	1	1	1	2	2
4	4	2	2	4	4	1	1	2	2
3	3	2	2	4	4	1	1	4	4
3	3	2	2	4	4	1	1	4	4
3	3	2	2	4	4	1	1	2	2
3	3	2	2	4	4	1	1	2	2
3	3	2	2	4	4	1	1	4	4

For Review Only

3	3	2	2	4	4	1	1	2	2
3	3	2	2	4	4	3	3	2	2
3	3	2	2	4	4	1	1	2	2
3	3	2	2	4	4	1	1	2	2
3	3	4	4	4	4	1	1	2	2
4	4	2	2	4	4	1	1	2	2
4	4	2	2	4	4	1	1	2	2
3	3	2	2	4	4	3	3	4	4
3	3	2	2	4	4	3	3	4	4
3	3	2	2	4	4	1	1	2	2
4	4	2	2	4	4	1	1	2	2
4	4	2	2	4	4	1	1	2	2
3	3	2	2	4	4	1	1	2	2
3	3	4	4	4	4	3	3	4	4
3	3	2	2	4	4	1	1	2	2
3	3	2	2	4	4	3	3	4	4
3	3	2	2	1	1	3	3	4	4
3	3	2	2	4	4	1	1	2	2
3	3	2	2	1	1	3	3	4	4
3	3	2	2	4	4	1	1	2	2
4	4	2	2	4	4	1	1	2	2
3	3	2	2	4	4	3	3	4	4
3	3	2	2	4	4	3	3	2	2
3	3	2	2	4	4	3	3	4	4
3	3	4	4	4	4	3	3	4	4
3	3	2	2	4	4	1	1	2	2
3	3	2	2	4	4	3	3	4	4
3	3	2	2	4	4	3	3	4	4
4	4	2	2	4	4	1	1	2	2
3	3	4	4	1	1	1	1	2	2
3	3	2	2	4	4	1	1	2	2
3	3	2	2	4	4	1	1	2	2
3	3	2	2	4	4	3	3	4	4
3	3	2	2	4	4	1	1	2	2
3	3	2	2	4	4	3	3	4	4
3	3	4	4	4	4	3	3	4	4
3	3	2	2	4	4	3	3	4	4
3	3	2	2	4	4	1	1	2	2
3	3	2	2	1	1	1	1	2	2
3	3	2	2	4	4	3	3	4	4
3	3	2	2	4	4	1	1	2	2
3	3	2	2	1	1	1	1	2	2
3	3	2	2	4	4	3	3	4	4
4	4	2	2	4	4	1	1	2	2
3	3	2	2	4	4	1	1	2	2
3	3	2	2	4	4	3	3	4	4
3	3	2	2	4	4	3	3	4	4
4	4	2	2	4	4	1	1	2	2
3	3	2	2	4	4	1	1	2	2
3	3	2	2	4	4	1	1	2	2
3	3	2	2	4	4	1	1	2	2
3	3	2	2	4	4	3	3	4	4
3	3	2	2	4	4	3	3	4	4
3	3	2	2	4	4	1	1	4	4
3	3	2	2	4	4	1	1	2	2

For Review Only

4	4	2	2	4	4	1	1	2	2
3	3	2	2	4	4	1	1	2	2
4	4	2	2	4	4	1	1	2	2
3	3	2	2	4	4	3	3	4	4
3	3	2	2	4	4	3	3	4	4
3	3	2	2	4	4	1	1	2	2
3	3	2	2	1	1	1	1	2	2
3	3	2	2	4	4	1	1	2	2
3	3	2	2	4	4	3	3	4	4
3	3	2	2	4	4	1	1	2	2
3	3	2	2	4	4	1	1	2	2
3	3	2	2	4	4	1	1	2	2
3	3	2	2	4	4	3	3	4	4
3	3	2	2	4	4	1	1	2	2
3	3	2	2	4	4	1	1	2	2
4	4	2	2	4	4	1	1	2	2
3	3	2	2	4	4	3	3	4	4
4	4	2	2	4	4	1	1	2	2
3	3	2	2	4	4	3	3	4	4
3	3	2	2	4	4	1	1	2	2
3	3	2	2	4	4	1	1	2	2
3	3	4	4	1	1	1	1	2	2
3	3	2	2	4	4	1	1	2	2
3	3	2	2	4	4	3	3	4	4
3	3	2	2	4	4	1	1	2	2
3	3	2	2	4	4	1	1	2	2
3	3	2	2	1	1	1	1	2	2
3	3	2	2	4	4	1	1	2	2
3	3	2	2	4	4	1	1	2	2
3	3	2	2	4	4	1	1	2	2
3	3	2	2	4	4	3	3	4	4
3	3	2	2	4	4	1	1	2	2
3	3	2	2	4	4	1	1	2	2
3	3	2	2	4	4	1	1	2	2
3	3	2	2	4	4	3	3	4	4
3	3	2	2	4	4	1	1	4	4
3	3	2	2	4	4	3	3	4	4
4	4	2	2	4	4	3	3	4	4
3	3	2	2	4	4	1	1	4	4
3	3	2	2	4	4	3	3	4	4
3	3	2	2	4	4	3	3	4	4
3	3	2	2	4	4	3	3	4	4
3	3	2	2	4	4	1	1	2	2
3	3	2	2	4	4	1	1	4	4
4	4	2	2	4	4	1	1	4	4
3	3	2	2	4	4	1	1	2	2
4	4	2	2	4	4	1	1	2	2
4	4	2	2	4	4	1	1	2	2
3	3	2	2	4	4	1	1	2	2
4	4	2	2	4	4	1	1	2	2
4	4	2	2	4	4	1	1	2	2
3	3	2	2	4	4	3	3	4	4
3	3	2	2	4	4	1	1	4	4
4	4	2	2	4	4	1	1	2	2
3	3	2	2	4	4	1	1	2	2

For Review Only

3	3	2	2	4	4	1	1	2	2
3	3	2	2	4	4	3	3	4	4
3	3	4	4	4	4	1	1	2	2
3	3	2	2	4	4	3	3	4	4
3	3	2	2	4	4	3	3	4	4
3	3	2	2	4	4	3	3	4	4
3	3	2	2	4	4	3	3	4	4
3	3	2	2	4	4	1	1	2	2
3	3	2	2	4	4	3	3	4	4
3	3	4	4	1	1	3	3	2	2
3	3	2	2	4	4	1	1	2	2
3	3	2	2	4	4	3	3	4	4
3	3	2	2	1	1	1	1	2	2
3	3	2	2	4	4	1	1	2	2
3	3	2	2	4	4	3	3	4	4
3	3	2	2	4	4	3	3	4	4
3	3	2	2	4	4	1	1	4	4
3	3	2	2	4	4	1	1	2	2
3	3	2	2	4	4	1	1	2	2
3	3	2	2	1	1	1	1	2	2
3	3	2	2	4	4	1	1	2	2
3	3	2	2	4	4	1	1	4	4
3	3	2	2	4	4	3	3	4	4
4	4	2	2	4	4	1	1	2	2
3	3	2	2	4	4	1	1	2	2
3	3	2	2	4	4	3	3	4	4
3	3	2	2	4	4	1	1	2	2
3	3	2	2	4	4	3	3	4	4
3	3	2	2	4	4	3	3	4	4
3	3	2	2	4	4	3	3	4	4
4	4	2	2	4	4	1	1	2	2
3	3	4	4	4	4	1	1	2	2
3	3	2	2	1	1	1	1	2	2
3	3	2	2	4	4	3	3	4	4
3	3	2	2	4	4	1	1	2	2
4	4	2	2	4	4	1	1	2	2
4	4	2	2	4	4	1	1	2	2
3	3	2	2	1	1	3	3	4	4
3	3	2	2	4	4	1	1	2	2
3	3	2	2	4	4	1	1	2	2
3	3	2	2	1	1	1	1	2	2
4	4	2	2	4	4	1	1	2	2
3	3	2	2	4	4	3	3	4	4
3	3	2	2	4	4	1	1	2	2
3	3	2	2	4	4	1	1	2	2
3	3	2	2	4	4	3	3	4	4
3	3	2	2	4	4	3	3	4	4
3	3	2	2	4	4	3	3	4	4
3	3	2	2	4	4	1	1	4	4
3	3	2	2	1	1	1	1	2	2
3	3	2	2	1	1	3	3	4	4
3	3	2	2	4	4	3	3	4	4
3	3	2	2	4	4	3	3	4	4
3	3	2	2	4	4	1	1	2	2
3	3	2	2	4	4	3	3	4	4

For Review Only

3	3	2	2	4	4	1	1	2	2
3	3	2	2	4	4	3	3	4	4
3	3	2	2	1	1	1	1	2	2
3	3	2	2	4	4	1	1	2	2
3	3	2	2	4	4	3	3	4	4
3	3	2	2	4	4	3	3	2	2
3	3	2	2	4	4	1	1	2	2
3	3	2	2	4	4	3	3	4	4
3	3	2	2	4	4	1	1	4	4
3	3	2	2	4	4	3	3	4	4
3	3	2	2	4	4	1	1	2	2
3	3	2	2	4	4	1	1	2	2
3	3	2	2	1	1	1	1	2	2
4	4	2	2	4	4	3	3	2	2
3	3	2	2	4	4	1	1	2	2
3	3	2	2	1	1	3	3	4	4
3	3	2	2	4	4	3	3	4	4
3	3	2	2	4	4	3	3	4	4
3	3	2	2	4	4	3	3	4	4
3	3	2	2	4	4	1	1	2	2
3	3	2	2	4	4	3	3	4	4
4	4	2	2	4	4	1	1	2	2
3	3	2	2	1	1	1	1	2	2
3	3	2	2	4	4	3	3	4	4
3	3	2	2	4	4	3	3	4	4
3	3	2	2	4	4	1	1	4	4
3	3	2	2	4	4	3	3	4	4
3	3	2	2	4	4	1	1	2	2
3	3	2	2	4	4	1	1	2	2
3	3	2	2	1	1	3	3	4	4
3	3	2	2	4	4	3	3	4	4
4	4	2	2	4	4	3	3	2	2
3	3	2	2	4	4	3	3	4	4
4	4	2	2	4	4	1	1	2	2
3	3	2	2	4	4	3	3	4	4
3	3	2	2	4	4	3	3	4	4
3	3	2	2	4	4	1	1	2	2
4	4	2	2	4	4	1	1	2	2
3	3	2	2	4	4	1	1	2	2
3	3	2	2	4	4	3	3	4	4
3	3	2	2	4	4	1	1	2	2
3	3	2	2	4	4	1	1	2	2
3	3	2	2	4	4	3	3	4	4
3	3	2	2	4	4	1	1	2	2
3	3	2	2	4	4	1	1	2	2
3	3	2	2	4	4	1	1	2	2
4	4	2	2	4	4	1	1	2	2
3	3	2	2	4	4	3	3	4	4
3	3	2	2	4	4	1	1	2	2
3	3	2	2	4	4	3	3	4	4
3	3	2	2	4	4	1	1	2	2
3	3	2	2	4	4	3	3	4	4
3	3	2	2	1	1	1	1	2	2
3	3	2	2	4	4	1	1	2	2
3	3	2	2	4	4	3	3	4	4

For Review Only

3	3	2	2	1	1	1	1	2	2
4	4	2	2	4	4	1	1	2	2

For Review Only

SNP2522	SNP2522	SNP2523	SNP2523	SNP2524	SNP2524	SNP2525	SNP2525	SNP2526	SNP2526
2	2	1	1	1	1	2	2	3	3
1	1	2	2	1	1	2	2	3	3
1	1	1	1	1	1	2	2	2	2
2	2	1	1	1	1	2	2	3	3
2	2	1	1	4	4	2	2	3	3
2	2	1	1	1	1	2	2	3	3
1	1	2	2	4	4	2	2	3	3
2	2	1	1	1	1	2	2	3	3
2	2	1	1	1	1	2	2	3	3
2	2	1	1	1	1	2	2	3	3
1	1	2	2	4	4	2	2	3	3
1	1	1	1	1	1	2	2	2	2
2	2	1	1	1	1	3	3	3	3
1	1	2	2	1	1	2	2	3	3
2	2	1	1	1	1	2	2	3	3
1	1	2	2	1	1	2	2	3	3
2	2	1	1	1	1	2	2	3	3
2	2	1	1	4	4	2	2	3	3
2	2	1	1	1	1	2	2	2	2
2	2	1	1	1	1	2	2	3	3
2	2	1	1	4	4	2	2	3	3
1	1	2	2	1	1	2	2	3	3
1	1	2	2	4	4	2	2	3	3
2	2	1	1	4	4	2	2	3	3
2	2	1	1	1	1	2	2	3	3
1	1	2	2	1	1	2	2	3	3
2	2	1	1	1	1	2	2	3	3
2	2	1	1	1	1	2	2	3	3
2	2	1	1	4	4	2	2	3	3
1	1	2	2	4	4	2	2	3	3
1	1	1	1	1	1	2	2	2	2
2	2	1	1	1	1	2	2	3	3
2	2	1	1	1	1	2	2	3	3
1	1	2	2	1	1	2	2	3	3
2	2	1	1	1	1	2	2	3	3
2	2	1	1	4	4	2	2	3	3
2	2	1	1	1	1	2	2	3	3
1	1	1	1	4	4	2	2	2	2
1	1	2	2	4	4	3	3	3	3
1	1	2	2	4	4	3	3	3	3
1	1	2	2	4	4	2	2	3	3
2	2	1	1	4	4	2	2	3	3
2	2	1	1	4	4	2	2	3	3
1	1	2	2	1	1	2	2	3	3
2	2	1	1	4	4	2	2	3	3
1	1	1	1	1	1	2	2	2	2
1	1	2	2	1	1	2	2	3	3
2	2	1	1	4	4	2	2	3	3
1	1	1	1	1	1	2	2	3	3
2	2	1	1	1	1	2	2	3	3
1	1	1	1	1	1	2	2	2	2
1	1	1	1	1	1	2	2	2	2

For Review Only

2	2	1	1	1	1	2	2	3	3
2	2	1	1	1	1	2	2	3	3
2	2	1	1	1	1	2	2	3	3
1	1	2	2	1	1	2	2	3	3
2	2	1	1	1	1	2	2	2	2
1	1	2	2	1	1	2	2	3	3
1	1	2	2	1	1	2	2	3	3
2	2	1	1	4	4	2	2	3	3
2	2	1	1	1	1	2	2	3	3
2	2	1	1	1	1	2	2	3	3
1	1	2	2	1	1	2	2	3	3
1	1	2	2	1	1	2	2	3	3
2	2	1	1	1	1	2	2	3	3
2	2	1	1	4	4	2	2	3	3
2	2	1	1	1	1	2	2	3	3
1	1	2	2	4	4	2	2	3	3
1	1	1	1	1	1	2	2	2	2
2	2	1	1	1	1	2	2	3	3
2	2	1	1	1	1	2	2	3	3
2	2	1	1	1	1	2	2	3	3
1	1	2	2	1	1	2	2	3	3
2	2	1	1	1	1	2	2	3	3
2	2	1	1	4	4	2	2	3	3
1	1	2	2	1	1	2	2	2	2
2	2	1	1	4	4	2	2	3	3
1	1	1	1	1	1	2	2	2	2
2	2	1	1	1	1	2	2	3	3
2	2	1	1	4	4	3	3	3	3
1	1	2	2	1	1	2	2	3	3
2	2	1	1	1	1	2	2	2	2
2	2	1	1	1	1	2	2	3	3
2	2	1	1	4	4	2	2	3	3
2	2	1	1	4	4	3	3	3	3
2	2	1	1	1	1	2	2	3	3
2	2	1	1	4	4	2	2	3	3
2	2	1	1	4	4	2	2	3	3
2	2	1	1	1	1	2	2	3	3
1	1	2	2	4	4	2	2	3	3
1	1	1	1	1	1	2	2	2	2
2	2	1	1	4	4	2	2	3	3
2	2	1	1	1	1	2	2	3	3
1	1	1	1	4	4	2	2	2	2
1	1	1	1	1	1	2	2	2	2
1	1	2	2	1	1	2	2	3	3
2	2	1	1	1	1	2	2	3	3
2	2	1	1	1	1	2	2	3	3
1	1	2	2	1	1	2	2	3	3
1	1	2	2	1	1	3	3	3	3
2	2	1	1	4	4	2	2	3	3
2	2	1	1	1	1	3	3	3	3
1	1	2	2	4	4	2	2	3	3
1	1	2	2	4	4	3	3	3	3
2	2	1	1	4	4	2	2	3	3
2	2	1	1	1	1	2	2	3	3
2	2	1	1	1	1	2	2	3	3

For Review Only

1	1	2	2	1	1	2	2	3	3
2	2	1	1	4	4	3	3	3	3
1	1	2	2	1	1	2	2	3	3
1	1	2	2	1	1	2	2	3	3
2	2	1	1	1	1	2	2	2	2
2	2	1	1	1	1	2	2	3	3
1	1	2	2	1	1	2	2	3	3
2	2	1	1	4	4	2	2	3	3
2	2	1	1	4	4	2	2	3	3
2	2	1	1	1	1	2	2	3	3
1	1	2	2	4	4	2	2	3	3
2	2	2	2	1	1	2	2	3	3
1	1	1	1	4	4	2	2	2	2
2	2	1	1	1	1	2	2	2	2
2	2	1	1	1	1	2	2	3	3
2	2	2	2	1	1	2	2	3	3
2	2	1	1	1	1	2	2	3	3
2	2	1	1	1	1	2	2	3	3
1	1	2	2	1	1	2	2	3	3
1	1	1	1	1	1	2	2	3	3
2	2	1	1	1	1	2	2	3	3
2	2	1	1	1	1	2	2	2	2
2	2	1	1	1	1	3	3	2	2
2	2	1	1	4	4	2	2	3	3
2	2	1	1	1	1	2	2	3	3
2	2	1	1	1	1	2	2	3	3
2	2	1	1	1	1	2	2	3	3
2	2	1	1	1	1	2	2	3	3
2	2	1	1	1	1	2	2	3	3
2	2	1	1	1	1	2	2	3	3
2	2	1	1	4	4	3	3	3	3
2	2	1	1	1	1	2	2	3	3
2	2	1	1	1	1	2	2	3	3
2	2	1	1	1	1	2	2	3	3
1	1	2	2	1	1	2	2	3	3
2	2	1	1	4	4	2	2	3	3
1	1	2	2	1	1	2	2	3	3
2	2	2	2	1	1	2	2	3	3
2	2	1	1	1	1	2	2	3	3
2	2	1	1	4	4	3	3	3	3
2	2	1	1	1	1	3	3	2	2
2	2	1	1	4	4	2	2	3	3
2	2	1	1	1	1	2	2	3	3
2	2	1	1	4	4	2	2	3	3
1	1	2	2	4	4	2	2	3	3
1	1	1	1	1	1	2	2	2	2
1	1	2	2	1	1	2	2	3	3
2	2	2	2	1	1	2	2	3	3
2	2	1	1	1	1	2	2	3	3
1	1	2	2	1	1	2	2	3	3
1	1	2	2	1	1	2	2	3	3
1	1	2	2	4	4	2	2	3	3
2	2	1	1	1	1	2	2	3	3
1	1	2	2	1	1	2	2	3	3
2	2	1	1	1	1	2	2	3	3

For Review Only

2	2	1	1	1	1	2	2	3	3
1	1	1	1	4	4	2	2	2	2
2	2	1	1	4	4	2	2	3	3
2	2	1	1	4	4	3	3	3	3
2	2	1	1	1	1	2	2	3	3
2	2	1	1	4	4	3	3	3	3
2	2	1	1	4	4	2	2	3	3
2	2	1	1	1	1	2	2	3	3
2	2	1	1	4	4	2	2	3	3
2	2	1	1	1	1	2	2	3	3
2	2	1	1	1	1	2	2	3	3
2	2	2	2	4	4	2	2	3	3
2	2	2	2	1	1	2	2	2	2
2	2	1	1	1	1	2	2	2	2
2	2	2	2	4	4	2	2	3	3
1	1	2	2	4	4	2	2	3	3
2	2	1	1	1	1	2	2	3	3
1	1	1	1	1	1	2	2	2	2
1	1	1	1	1	1	3	3	2	2
2	2	1	1	1	1	2	2	3	3
2	2	1	1	1	1	2	2	3	3
2	2	1	1	4	4	2	2	3	3
2	2	1	1	1	1	2	2	3	3
1	1	2	2	1	1	2	2	3	3
2	2	1	1	1	1	2	2	3	3
1	1	2	2	1	1	2	2	3	3
2	2	1	1	1	1	2	2	3	3
2	2	1	1	1	1	2	2	3	3
2	2	1	1	1	1	2	2	3	3
2	2	1	1	4	4	2	2	3	3
1	1	1	1	4	4	2	2	2	2
2	2	1	1	1	1	2	2	2	2
2	2	1	1	1	1	2	2	3	3
1	1	2	2	4	4	2	2	3	3
2	2	1	1	1	1	2	2	3	3
1	1	2	2	1	1	2	2	3	3
2	2	1	1	1	1	2	2	3	3
2	2	1	1	1	1	2	2	3	3
2	2	1	1	1	1	2	2	3	3
1	1	1	1	4	4	2	2	2	2
1	1	1	1	1	1	2	2	2	2
1	1	2	2	1	1	2	2	3	3
2	2	1	1	4	4	3	3	3	3
2	2	1	1	1	1	2	2	3	3
2	2	1	1	1	1	3	3	2	2
2	2	1	1	1	1	2	2	3	3
1	1	2	2	4	4	2	2	3	3
2	2	1	1	4	4	2	2	3	3
2	2	1	1	1	1	2	2	3	3
1	1	2	2	1	1	2	2	3	3
2	2	1	1	1	1	2	2	3	3
2	2	1	1	4	4	2	2	3	3
2	2	1	1	1	1	2	2	3	3
1	1	2	2	1	1	2	2	3	3
2	2	1	1	4	4	3	3	3	3

For Review Only

1	1	2	2	1	1	2	2	3	3
1	1	2	2	1	1	2	2	3	3
2	2	1	1	1	1	2	2	3	3
2	2	1	1	1	1	2	2	3	3
2	2	1	1	4	4	2	2	3	3
2	2	1	1	1	1	2	2	3	3
2	2	1	1	1	1	2	2	3	3
2	2	1	1	1	1	2	2	3	3
2	2	1	1	1	1	2	2	3	3
2	2	1	1	4	4	3	3	3	3
2	2	1	1	1	1	2	2	3	3
1	1	2	2	4	4	2	2	3	3
1	1	1	1	1	1	2	2	2	2
1	1	2	2	1	1	2	2	3	3
1	1	1	1	1	1	2	2	2	2
2	2	1	1	1	1	2	2	3	3
1	1	2	2	4	4	3	3	3	3
2	2	1	1	1	1	3	3	3	3
1	1	2	2	4	4	2	2	3	3
2	2	1	1	4	4	2	2	3	3
1	1	2	2	1	1	3	3	3	3
1	1	2	2	1	1	2	2	3	3
2	2	1	1	1	1	2	2	3	3
1	1	2	2	4	4	3	3	3	3
2	2	1	1	4	4	2	2	3	3
1	1	1	1	1	1	2	2	2	2
2	2	1	1	4	4	3	3	3	3
1	1	2	2	1	1	2	2	3	3
2	2	1	1	1	1	2	2	3	3
2	2	1	1	1	1	2	2	3	3
1	1	2	2	4	4	2	2	3	3
1	1	2	2	4	4	2	2	3	3
2	2	1	1	1	1	2	2	3	3
2	2	2	2	1	1	2	2	3	3
2	2	1	1	1	1	2	2	3	3
2	2	1	1	4	4	3	3	3	3
2	2	1	1	1	1	2	2	3	3
1	1	2	2	1	1	2	2	3	3
2	2	1	1	4	4	2	2	3	3
1	1	2	2	4	4	3	3	3	3
2	2	1	1	1	1	2	2	3	3
2	2	1	1	1	1	2	2	3	3
1	1	2	2	4	4	2	2	3	3
2	2	1	1	1	1	2	2	3	3
2	2	1	1	1	1	2	2	3	3
1	1	1	1	1	1	2	2	2	2
1	1	2	2	4	4	2	2	3	3
1	1	1	1	1	1	2	2	2	2
1	1	1	1	1	1	2	2	2	2
2	2	1	1	4	4	3	3	3	3
2	2	1	1	1	1	2	2	3	3
1	1	2	2	4	4	3	3	3	3
1	1	1	1	1	1	2	2	2	2
2	2	1	1	1	1	2	2	2	2
1	1	2	2	4	4	3	3	3	3

For Review Only

2	2	1	1	1	1	2	2	3	3
1	1	2	2	1	1	2	2	3	3

For Review Only

SNP2527	SNP2527	SNP2528	SNP2528	SNP2529	SNP2529	SNP253	SNP253	SNP2530	SNP2530
2	2	3	3	2	2	2	2	3	3
2	2	3	3	2	2	3	3	4	4
2	2	3	3	3	3	2	2	3	3
2	2	3	3	2	2	3	3	3	3
2	2	3	3	2	2	2	2	3	3
2	2	3	3	2	2	2	2	3	3
2	2	3	3	2	2	2	2	3	3
2	2	3	3	2	2	3	3	3	3
2	2	3	3	2	2	2	2	3	3
2	2	3	3	2	2	3	3	3	3
2	2	3	3	2	2	3	3	3	3
2	2	3	3	2	2	2	2	3	3
2	2	3	3	2	2	2	2	3	3
2	2	3	3	2	2	3	3	3	3
2	2	3	3	2	2	2	2	3	3
2	2	3	3	2	2	2	2	3	3
2	2	3	3	2	2	3	3	3	3
2	2	3	3	2	2	2	2	3	3
2	2	3	3	2	2	2	2	3	3
2	2	3	3	2	2	3	3	3	3
2	2	3	3	2	2	2	2	3	3
2	2	3	3	2	2	2	2	3	3
2	2	3	3	2	2	2	2	3	3
2	2	3	3	2	2	2	2	3	3
2	2	3	3	3	3	2	2	3	3
2	2	3	3	2	2	2	2	4	4
2	2	2	2	3	3	2	2	3	3
2	2	3	3	2	2	2	2	3	3
2	2	3	3	2	2	2	2	3	3
2	2	3	3	2	2	3	3	3	3
2	2	3	3	2	2	3	3	3	3
2	2	3	3	3	3	2	2	3	3
2	2	3	3	3	3	2	2	3	3
2	2	3	3	2	2	2	2	3	3
2	2	3	3	2	2	2	2	4	4
2	2	3	3	2	2	2	2	3	3
2	2	3	3	3	3	3	3	3	3
2	2	3	3	2	2	3	3	3	3
2	2	3	3	2	2	3	3	3	3
2	2	3	3	2	2	3	3	3	3
2	2	3	3	2	2	3	3	3	3
2	2	3	3	2	2	3	3	3	3
2	2	3	3	2	2	3	3	3	3
2	2	3	3	2	2	3	3	3	3
2	2	3	3	2	2	3	3	3	3
3	3	3	3	2	2	2	2	3	3
2	2	3	3	2	2	2	2	4	4
2	2	3	3	2	2	2	2	3	3
2	2	3	3	2	2	3	3	3	3
2	2	3	3	2	2	2	2	3	3
3	3	2	2	2	2	2	2	3	3
2	2	3	3	2	2	3	3	3	3

For Review Only

2	2	3	3	2	2	2	2	3	3
2	2	3	3	3	3	2	2	3	3
2	2	3	3	3	3	2	2	3	3
2	2	3	3	2	2	3	3	3	3
2	2	3	3	2	2	2	2	3	3
3	3	2	2	2	2	2	2	4	4
3	3	2	2	2	2	2	2	3	3
2	2	3	3	2	2	3	3	3	3
2	2	3	3	3	3	2	2	3	3
2	2	3	3	2	2	2	2	3	3
2	2	3	3	2	2	2	2	4	4
2	2	3	3	2	2	2	2	4	4
2	2	3	3	2	2	2	2	3	3
2	2	3	3	2	2	2	2	3	3
2	2	3	3	2	2	2	2	3	3
2	2	3	3	2	2	2	2	3	3
2	2	2	2	2	2	3	3	3	3
3	3	2	2	2	2	2	2	3	3
2	2	3	3	2	2	3	3	3	3
2	2	3	3	2	2	2	2	3	3
2	2	3	3	2	2	2	2	4	4
2	2	3	3	3	3	3	3	3	3
2	2	3	3	2	2	2	2	3	3
2	2	3	3	2	2	2	2	3	3
2	2	3	3	2	2	3	3	3	3
2	2	3	3	2	2	2	2	3	3
2	2	3	3	2	2	3	3	3	3
2	2	3	3	2	2	3	3	3	3
3	3	2	2	2	2	3	3	4	4
2	2	3	3	2	2	3	3	3	3
2	2	3	3	2	2	3	3	3	3
2	2	3	3	2	2	2	2	3	3
2	2	3	3	2	2	3	3	3	3
2	2	3	3	2	2	2	2	3	3
2	2	3	3	2	2	2	2	3	3
2	2	3	3	2	2	3	3	3	3
2	2	3	3	2	2	3	3	3	3
2	2	3	3	2	2	2	2	3	3
2	2	3	3	3	3	3	3	3	3
2	2	3	3	2	2	3	3	3	3
2	2	3	3	2	2	2	2	3	3
2	2	3	3	2	2	2	2	3	3
3	3	2	2	2	2	3	3	3	3
2	2	3	3	2	2	2	2	4	4
2	2	3	3	2	2	2	2	3	3
2	2	3	3	2	2	2	2	3	3
2	2	3	3	2	2	3	3	3	3
3	3	2	2	2	2	2	2	4	4
2	2	3	3	2	2	2	2	3	3
2	2	3	3	2	2	2	2	3	3
2	2	3	3	2	2	2	2	3	3
2	2	3	3	2	2	3	3	3	3
2	2	3	3	2	2	3	3	3	3
2	2	3	3	2	2	3	3	3	3
2	2	3	3	2	2	2	2	3	3

For Review Only

2	2	3	3	2	2	3	3	4	4
2	2	3	3	2	2	3	3	3	3
3	3	2	2	2	2	2	2	4	4
2	2	3	3	2	2	3	3	3	3
2	2	2	2	3	3	2	2	3	3
3	3	2	2	2	2	2	2	4	4
3	3	2	2	2	2	2	2	3	3
2	2	3	3	2	2	2	2	3	3
2	2	3	3	2	2	3	3	3	3
2	2	3	3	2	2	2	2	3	3
2	2	3	3	2	2	2	2	3	3
2	2	3	3	2	2	2	2	4	4
2	2	3	3	2	2	2	2	3	3
2	2	3	3	2	2	2	2	3	3
2	2	3	3	3	3	2	2	4	4
2	2	3	3	2	2	2	2	4	4
3	3	2	2	2	2	2	2	3	3
2	2	3	3	2	2	2	2	4	4
2	2	3	3	2	2	3	3	3	3
2	2	3	3	2	2	3	3	3	3
2	2	3	3	2	2	2	2	3	3
2	2	3	3	2	2	3	3	3	3
2	2	3	3	3	3	2	2	3	3
2	2	3	3	2	2	3	3	3	3
2	2	3	3	2	2	2	2	4	4
2	2	3	3	2	2	2	2	3	3
2	2	3	3	2	2	3	3	3	3
2	2	3	3	2	2	3	3	3	3
3	3	2	2	2	2	2	2	3	3
3	3	2	2	2	2	3	3	3	3
2	2	3	3	2	2	2	2	3	3
2	2	3	3	2	2	2	2	3	3
2	2	3	3	2	2	2	2	3	3
2	2	3	3	2	2	2	2	3	3
2	2	3	3	2	2	2	2	3	3
2	2	3	3	2	2	2	2	3	3
2	2	3	3	2	2	3	3	3	3
2	2	3	3	2	2	2	2	3	3
2	2	2	2	2	2	3	3	4	4
2	2	3	3	2	2	3	3	3	3
2	2	3	3	2	2	2	2	3	3
2	2	3	3	2	2	3	3	3	3
3	3	2	2	2	2	2	2	3	3
2	2	3	3	2	2	2	2	3	3
2	2	3	3	2	2	2	2	4	4
2	2	3	3	2	2	2	2	3	3
3	3	2	2	2	2	2	2	4	4
3	3	2	2	2	2	2	2	4	4
2	2	3	3	2	2	3	3	3	3
3	3	2	2	2	2	2	2	4	4
3	3	2	2	2	2	2	2	4	4
2	2	3	3	2	2	3	3	3	3
2	2	3	3	2	2	2	2	3	3
2	2	3	3	2	2	2	2	4	4
2	2	3	3	2	2	3	3	3	3

For Review Only

2	2	3	3	3	3	2	2	3	3
2	2	3	3	2	2	3	3	3	3
2	2	3	3	2	2	3	3	3	3
2	2	3	3	2	2	2	2	3	3
2	2	3	3	2	2	3	3	3	3
2	2	3	3	2	2	3	3	3	3
2	2	3	3	2	2	3	3	3	3
2	2	3	3	2	2	2	2	3	3
2	2	3	3	2	2	3	3	3	3
3	3	2	2	2	2	2	2	3	3
2	2	3	3	2	2	2	2	4	4
2	2	3	3	2	2	2	2	3	3
3	3	2	2	2	2	2	2	3	3
3	3	3	3	2	2	2	2	3	3
2	2	3	3	2	2	2	2	3	3
2	2	3	3	2	2	3	3	3	3
2	2	3	3	2	2	2	2	3	3
2	2	3	3	2	2	2	2	3	3
3	3	2	2	2	2	2	2	3	3
2	2	2	2	2	2	2	2	3	3
2	2	3	3	2	2	2	2	3	3
2	2	3	3	2	2	3	3	3	3
2	2	3	3	3	3	3	3	3	3
2	2	3	3	2	2	2	2	4	4
2	2	3	3	2	2	2	2	3	3
2	2	3	3	2	2	3	3	3	3
2	2	2	2	2	2	2	2	3	3
2	2	2	2	2	2	2	2	3	3
3	3	2	2	2	2	2	2	3	3
2	2	3	3	2	2	2	2	3	3
2	2	3	3	2	2	2	2	3	3
3	3	2	2	2	2	3	3	3	3
2	2	3	3	2	2	2	2	3	3
2	2	3	3	2	2	2	2	3	3
2	2	3	3	2	2	2	2	3	3
2	2	3	3	2	2	2	2	4	4
2	2	3	3	3	3	2	2	4	4
2	2	3	3	3	3	3	3	3	3
3	3	2	2	2	2	2	2	3	3
2	2	3	3	3	3	2	2	3	3
2	2	3	3	3	3	2	2	3	3
3	3	2	2	2	2	2	2	4	4
2	2	3	3	2	2	3	3	3	3
2	2	3	3	2	2	2	2	3	3
2	2	3	3	3	3	3	3	3	3
2	2	3	3	3	3	2	2	3	3
2	2	3	3	2	2	3	3	3	3
2	2	3	3	2	2	3	3	3	3
2	2	3	3	2	2	3	3	3	3
2	2	3	3	2	2	2	2	3	3
2	2	3	3	3	3	2	2	3	3
2	2	3	3	2	2	3	3	3	3
2	2	3	3	3	3	3	3	3	3
2	2	3	3	2	2	2	2	4	4
2	2	3	3	2	2	3	3	3	3

For Review Only

2	2	3	3	2	2	2	2	3	3
2	2	3	3	3	3	3	3	3	3
2	2	3	3	3	3	3	3	3	3
2	2	3	3	2	2	3	3	3	3
2	2	3	3	2	2	3	3	3	3
2	2	3	3	3	3	2	2	4	4
3	3	2	2	2	2	2	2	3	3
2	2	3	3	2	2	3	3	3	3
2	2	3	3	2	2	3	3	3	3
2	2	3	3	2	2	3	3	3	3
2	2	3	3	2	2	2	2	3	3
2	2	3	3	2	2	2	2	3	3
2	2	3	3	3	3	3	3	3	3
3	3	3	3	2	2	3	3	4	4
2	2	3	3	3	3	2	2	3	3
2	2	3	3	3	3	3	3	3	3
2	2	3	3	2	2	3	3	3	3
2	2	3	3	2	2	3	3	3	3
2	2	3	3	2	2	3	3	3	3
2	2	3	3	2	2	3	3	3	3
2	2	3	3	3	3	2	2	3	3
3	3	2	2	2	2	2	2	4	4
2	2	3	3	2	2	2	2	3	3
2	2	3	3	2	2	3	3	3	3
2	2	3	3	2	2	3	3	3	3
2	2	3	3	3	3	2	2	3	3
2	2	3	3	2	2	3	3	3	3
2	2	2	2	2	2	2	2	3	3
2	2	3	3	2	2	2	2	3	3
2	2	3	3	3	3	2	2	3	3
2	2	3	3	2	2	3	3	3	3
2	2	3	3	2	2	2	2	4	4
3	3	2	2	2	2	3	3	3	3
3	3	3	3	2	2	2	2	4	4
2	2	3	3	3	3	3	3	3	3
2	2	3	3	2	2	3	3	3	3
2	2	3	3	2	2	2	2	3	3
2	2	3	3	2	2	2	2	4	4
2	2	3	3	2	2	2	2	3	3
2	2	3	3	2	2	3	3	3	3
2	2	3	3	2	2	2	2	3	3
2	2	3	3	2	2	3	3	3	3
2	2	3	3	2	2	2	2	3	3
2	2	3	3	2	2	2	2	3	3
3	3	2	2	2	2	2	2	3	3
2	2	3	3	3	3	3	3	3	3
2	2	3	3	2	2	2	2	4	4
3	3	2	2	2	2	2	2	3	3
2	2	3	3	3	3	2	2	3	3
2	2	3	3	2	2	2	2	3	3
3	3	2	2	2	2	3	3	3	3
2	2	3	3	2	2	3	3	3	3
2	2	3	3	3	3	2	2	3	3
3	3	2	2	2	2	2	2	3	3
2	2	3	3	2	2	3	3	3	3

For Review Only

2	2	3	3	3	3	3	3	3	3
2	2	3	3	3	3	2	2	4	4

For Review Only

SNP2531	SNP2531	SNP2532	SNP2532	SNP2533	SNP2533	SNP2534	SNP2534	SNP2535	SNP2535
3	3	2	2	2	2	2	2	1	1
3	3	2	2	2	2	3	3	4	4
1	1	1	1	2	2	3	3	1	1
3	3	2	2	4	4	2	2	4	4
3	3	2	2	4	4	2	2	4	4
3	3	2	2	4	4	2	2	4	4
3	3	2	2	4	4	2	2	4	4
3	3	2	2	4	4	2	2	4	4
3	3	2	2	4	4	2	2	4	4
3	3	2	2	4	4	2	2	4	4
3	3	2	2	2	2	2	2	4	4
1	1	2	2	2	2	3	3	1	1
3	3	2	2	4	4	2	2	4	4
3	3	2	2	2	2	2	2	4	4
3	3	2	2	4	4	2	2	4	4
3	3	2	2	2	2	2	2	4	4
3	3	2	2	4	4	2	2	4	4
3	3	2	2	4	4	2	2	4	4
3	3	2	2	2	2	3	3	1	1
3	3	2	2	4	4	2	2	1	1
3	3	2	2	2	2	3	3	1	1
3	3	2	2	4	4	2	2	4	4
3	3	2	2	4	4	3	3	4	4
3	3	2	2	4	4	2	2	4	4
3	3	2	2	2	2	2	2	4	4
3	3	2	2	4	4	2	2	4	4
1	1	1	1	2	2	3	3	1	1
3	3	2	2	4	4	2	2	4	4
3	3	2	2	4	4	2	2	4	4
3	3	2	2	4	4	2	2	4	4
3	3	2	2	2	2	3	3	1	1
1	1	1	1	2	2	3	3	1	1
1	1	1	1	4	4	2	2	4	4
3	3	2	2	4	4	2	2	4	4
3	3	2	2	2	2	2	2	4	4
3	3	2	2	4	4	2	2	4	4
1	1	1	1	2	2	2	2	4	4
3	3	2	2	4	4	2	2	4	4
3	3	2	2	2	2	2	2	4	4
3	3	2	2	4	4	2	2	4	4
3	3	2	2	4	4	2	2	4	4
3	3	2	2	2	2	3	3	1	1
3	3	2	2	4	4	2	2	4	4
3	3	2	2	4	4	2	2	4	4
3	3	2	2	4	4	2	2	4	4
3	3	2	2	2	2	3	3	1	1
3	3	2	2	4	4	2	2	4	4
3	3	2	2	4	4	2	2	4	4
3	3	2	2	4	4	2	2	4	4
3	3	2	2	4	4	2	2	4	4
3	3	2	2	4	4	2	2	4	4
3	3	2	2	4	4	2	2	4	4

For Review Only

3	3	2	2	4	4	2	2	4	4
1	1	1	1	4	4	2	2	4	4
3	3	2	2	2	2	3	3	4	4
3	3	2	2	4	4	2	2	4	4
3	3	2	2	2	2	3	3	1	1
3	3	2	2	4	4	2	2	4	4
3	3	2	2	4	4	2	2	4	4
3	3	2	2	4	4	2	2	4	4
1	1	1	1	4	4	2	2	4	4
3	3	2	2	4	4	2	2	4	4
3	3	2	2	2	2	3	3	4	4
3	3	2	2	2	2	3	3	4	4
3	3	2	2	4	4	2	2	4	4
3	3	2	2	4	4	2	2	4	4
3	3	2	2	4	4	2	2	4	4
3	3	2	2	4	4	2	2	4	4
3	3	2	2	2	2	2	2	4	4
3	3	2	2	4	4	3	3	1	1
3	3	2	2	2	2	3	3	1	1
3	3	2	2	4	4	2	2	4	4
3	3	2	2	2	2	2	2	4	4
1	1	1	1	4	4	2	2	1	1
3	3	2	2	4	4	2	2	4	4
3	3	2	2	2	2	3	3	4	4
3	3	2	2	4	4	2	2	4	4
3	3	2	2	2	2	3	3	4	4
3	3	2	2	4	4	2	2	4	4
3	3	2	2	2	2	2	2	4	4
3	3	2	2	4	4	2	2	4	4
3	3	2	2	4	4	3	3	4	4
3	3	2	2	4	4	2	2	4	4
3	3	2	2	2	2	2	2	4	4
3	3	2	2	4	4	2	2	4	4
3	3	2	2	4	4	2	2	4	4
3	3	2	2	4	4	2	2	4	4
3	3	2	2	4	4	2	2	4	4
3	3	2	2	4	4	2	2	4	4
3	3	2	2	4	4	2	2	4	4
1	1	1	1	2	2	3	3	1	1
3	3	2	2	4	4	2	2	4	4
3	3	2	2	4	4	2	2	4	4
3	3	2	2	4	4	3	3	4	4
3	3	2	2	2	2	2	2	4	4
3	3	2	2	2	2	3	3	4	4
3	3	2	2	2	2	2	2	4	4
3	3	2	2	4	4	3	3	4	4
3	3	2	2	2	2	2	2	4	4
3	3	2	2	4	4	2	2	4	4
3	3	2	2	2	2	3	3	1	1
3	3	2	2	4	4	2	2	4	4
3	3	2	2	2	2	3	3	4	4
3	3	2	2	4	4	2	2	4	4
3	3	2	2	2	2	3	3	1	1
3	3	2	2	4	4	2	2	4	4
3	3	2	2	4	4	2	2	4	4

For Review Only

3	3	2	2	4	4	2	2	4	4
3	3	2	2	4	4	2	2	4	4
3	3	2	2	4	4	2	2	4	4
3	3	2	2	4	4	2	2	4	4
1	1	1	1	4	4	3	3	4	4
3	3	2	2	4	4	2	2	4	4
3	3	2	2	2	2	3	3	1	1
3	3	2	2	4	4	3	3	1	1
3	3	2	2	4	4	2	2	4	4
3	3	2	2	4	4	2	2	4	4
3	3	2	2	4	4	2	2	4	4
3	3	2	2	4	4	2	2	4	4
3	3	2	2	2	2	2	2	4	4
3	3	2	2	4	4	2	2	4	4
3	3	2	2	4	4	2	2	4	4
3	3	2	2	4	4	2	2	4	4
3	3	2	2	2	2	2	2	4	4
3	3	2	2	4	4	2	2	4	4
3	3	2	2	4	4	2	2	4	4
3	3	2	2	2	2	2	2	4	4
3	3	2	2	4	4	2	2	4	4
3	3	2	2	2	2	2	2	4	4
3	3	1	1	4	4	2	2	4	4
3	3	2	2	4	4	2	2	4	4
3	3	2	2	4	4	2	2	4	4
3	3	2	2	4	4	2	2	4	4
3	3	2	2	4	4	2	2	4	4
3	3	2	2	2	2	3	3	1	1
3	3	2	2	4	4	2	2	1	1
3	3	2	2	4	4	3	3	4	4
3	3	2	2	2	2	2	2	4	4
3	3	2	2	4	4	2	2	4	4
3	3	2	2	4	4	2	2	4	4
3	3	2	2	4	4	2	2	4	4
3	3	2	2	4	4	2	2	4	4
3	3	2	2	4	4	2	2	4	4
3	3	2	2	4	4	2	2	4	4
3	3	2	2	2	2	3	3	4	4
3	3	2	2	4	4	2	2	4	4
3	3	2	2	4	4	2	2	4	4
3	3	2	2	4	4	2	2	4	4
3	3	2	2	2	2	2	2	4	4
3	3	2	2	2	2	2	2	4	4
3	3	2	2	4	4	2	2	4	4
3	3	2	2	4	4	2	2	4	4
3	3	2	2	4	4	2	2	4	4
3	3	2	2	4	4	2	2	4	4
3	3	2	2	4	4	2	2	4	4
3	3	2	2	4	4	2	2	4	4
3	3	2	2	4	4	2	2	4	4
3	3	2	2	4	4	2	2	4	4
3	3	2	2	2	2	2	2	4	4
3	3	2	2	2	2	2	2	1	1
3	3	2	2	2	2	3	3	4	4
3	3	2	2	4	4	2	2	4	4

For Review Only

1	1	1	1	4	4	2	2	4	4
3	3	2	2	2	2	2	2	4	4
3	3	2	2	4	4	2	2	4	4
3	3	2	2	4	4	2	2	4	4
3	3	2	2	2	2	3	3	4	4
3	3	2	2	4	4	2	2	4	4
3	3	2	2	4	4	2	2	4	4
3	3	2	2	4	4	2	2	4	4
3	3	2	2	4	4	2	2	4	4
3	3	2	2	4	4	2	2	4	4
3	3	2	2	2	2	3	3	1	1
3	3	2	2	4	4	2	2	4	4
3	3	2	2	4	4	3	3	4	4
3	3	2	2	4	4	2	2	4	4
3	3	2	2	4	4	2	2	4	4
3	3	2	2	2	2	2	2	4	4
3	3	2	2	2	2	2	2	4	4
3	3	2	2	2	2	3	3	4	4
3	3	2	2	2	2	2	2	4	4
3	3	2	2	2	2	3	3	1	1
3	3	2	2	4	4	2	2	4	4
3	3	2	2	4	4	2	2	4	4
1	1	1	1	2	2	3	3	1	1
3	3	2	2	2	2	3	3	4	4
3	3	2	2	4	4	2	2	4	4
3	3	2	2	4	4	2	2	4	4
3	3	2	2	2	2	3	3	1	1
3	3	2	2	4	4	2	2	4	4
3	3	2	2	2	2	2	2	4	4
3	3	2	2	4	4	2	2	4	4
3	3	2	2	4	4	2	2	4	4
3	3	2	2	2	2	2	2	4	4
3	3	2	2	2	2	3	3	1	1
3	3	2	2	2	2	2	2	4	4
3	3	2	2	4	4	2	2	4	4
3	3	2	2	2	2	3	3	4	4
1	1	1	1	2	2	3	3	1	1
1	1	1	1	2	2	3	3	1	1
3	3	2	2	4	4	2	2	4	4
1	1	1	1	2	2	2	2	4	4
1	1	1	1	2	2	3	3	1	1
3	3	2	2	4	4	2	2	4	4
3	3	2	2	2	2	2	2	4	4
3	3	2	2	4	4	2	2	4	4
3	3	2	2	4	4	2	2	4	4
1	1	1	1	2	2	3	3	4	4
3	3	2	2	2	2	2	2	4	4
3	3	2	2	4	4	2	2	4	4
3	3	2	2	2	2	2	2	4	4
3	3	2	2	4	4	2	2	4	4
1	1	1	1	4	4	2	2	1	1
3	3	2	2	4	4	2	2	4	4
1	1	1	1	4	4	2	2	4	4
3	3	2	2	2	2	3	3	4	4
3	3	2	2	2	2	2	2	4	4

3	3	2	2	4	4	2	2	4	4
1	1	1	1	2	2	2	2	4	4
1	1	1	1	4	4	2	2	1	1
3	3	2	2	2	2	2	2	4	4
3	3	2	2	4	4	2	2	4	4
3	3	2	2	4	4	2	2	4	4
3	3	2	2	4	4	2	2	4	4
3	3	2	2	4	4	2	2	4	4
3	3	2	2	2	2	3	3	4	4
3	3	2	2	4	4	2	2	4	4
3	3	2	2	4	4	2	2	4	4
3	3	2	2	4	4	2	2	4	4
1	1	1	1	4	4	3	3	4	4
3	3	2	2	4	4	2	2	1	1
1	1	1	1	2	2	2	2	4	4
1	1	1	1	4	4	2	2	4	4
3	3	2	2	2	2	2	2	4	4
3	3	2	2	4	4	2	2	4	4
3	3	2	2	4	4	2	2	4	4
3	3	2	2	4	4	2	2	4	4
1	1	1	1	2	2	2	2	4	4
3	3	2	2	4	4	2	2	4	4
3	3	2	2	2	2	3	3	1	1
3	3	2	2	2	2	2	2	4	4
3	3	2	2	4	4	2	2	4	4
1	1	1	1	4	4	2	2	4	4
3	3	2	2	4	4	2	2	4	4
3	3	2	2	2	2	3	3	1	1
3	3	2	2	4	4	2	2	4	4
1	1	1	1	4	4	2	2	1	1
3	3	2	2	4	4	2	2	4	4
3	3	2	2	4	4	2	2	4	4
3	3	2	2	4	4	2	2	4	4
3	3	2	2	4	4	2	2	4	4
1	1	1	1	4	4	2	2	4	4
3	3	2	2	2	2	2	2	4	4
3	3	2	2	4	4	2	2	4	4
3	3	2	2	2	2	3	3	4	4
3	3	2	2	4	4	2	2	4	4
3	3	2	2	2	2	2	2	4	4
3	3	2	2	2	2	2	2	4	4
3	3	2	2	4	4	3	3	1	1
3	3	2	2	2	2	2	2	4	4
3	3	2	2	4	4	2	2	4	4
3	3	2	2	2	2	3	3	4	4
1	1	1	1	2	2	3	3	1	1
3	3	2	2	4	4	2	2	4	4
3	3	2	2	4	4	2	2	4	4
1	1	1	1	4	4	2	2	4	4
3	3	2	2	4	4	2	2	4	4
3	3	2	2	4	4	3	3	4	4
3	3	2	2	2	2	2	2	4	4
1	1	1	1	2	2	3	3	1	1
3	3	2	2	2	2	3	3	1	1
3	3	2	2	4	4	2	2	4	4

For Review Only

1	1	1	1	4	4	2	2	4	4
1	1	1	1	2	2	3	3	1	1

For Review Only

SNP2536	SNP2536	SNP2537	SNP2537	SNP2538	SNP2538	SNP2539	SNP2539	SNP254	SNP254
1	1	1	1	4	4	4	4	2	2
4	4	1	1	3	3	1	1	3	3
1	1	1	1	4	4	1	1	3	3
1	1	4	4	4	4	1	1	2	2
1	1	1	1	3	3	1	1	2	2
4	4	1	1	4	4	1	1	2	2
1	1	4	4	3	3	1	1	2	2
1	1	1	1	3	3	1	1	3	3
4	4	4	4	4	4	1	1	2	2
1	1	1	1	3	3	1	1	3	3
4	4	4	4	3	3	1	1	2	2
1	1	1	1	4	4	1	1	3	3
4	4	4	4	4	4	1	1	2	2
4	4	1	1	3	3	1	1	2	2
4	4	4	4	4	4	1	1	2	2
4	4	1	1	3	3	1	1	3	3
1	1	4	4	4	4	1	1	2	2
1	1	1	1	3	3	1	1	3	3
1	1	4	4	4	4	1	1	3	3
1	1	1	1	4	4	1	1	3	3
1	1	4	4	3	3	4	4	2	2
1	1	4	4	4	4	1	1	2	2
1	1	4	4	3	3	4	4	2	2
1	1	1	1	3	3	4	4	2	2
4	4	4	4	4	4	1	1	2	2
1	1	1	1	4	4	1	1	3	3
1	1	1	1	4	4	1	1	3	3
1	1	4	4	4	4	1	1	2	2
1	1	1	1	3	3	1	1	3	3
1	1	1	1	3	3	1	1	2	2
1	1	1	1	4	4	1	1	3	3
1	1	1	1	4	4	1	1	3	3
1	1	4	4	4	4	1	1	2	2
1	1	1	1	3	3	1	1	2	2
4	4	4	4	4	4	1	1	2	2
1	1	1	1	3	3	1	1	3	3
4	4	4	4	4	4	1	1	2	2
1	1	4	4	3	3	1	1	2	2
4	4	1	1	3	3	4	4	2	2
4	4	1	1	3	3	1	1	3	3
1	1	1	1	3	3	1	1	3	3
1	1	1	1	3	3	1	1	3	3
1	1	1	1	3	3	1	1	3	3
1	1	4	4	4	4	1	1	2	2
1	1	1	1	3	3	1	1	3	3
1	1	1	1	4	4	1	1	3	3
1	1	1	1	4	4	1	1	2	2
1	1	1	1	3	3	1	1	3	3
1	1	1	1	4	4	1	1	2	2
1	1	4	4	4	4	1	1	2	2
1	1	1	1	4	4	1	1	3	3
4	4	1	1	4	4	1	1	2	2

For Review Only

1	1	4	4	4	4	1	1	2	2
1	1	4	4	4	4	1	1	2	2
1	1	1	1	4	4	1	1	2	2
1	1	4	4	4	4	1	1	2	2
1	1	4	4	4	4	1	1	3	3
1	1	1	1	4	4	1	1	3	3
1	1	1	1	4	4	1	1	3	3
4	4	1	1	3	3	1	1	2	2
1	1	4	4	4	4	4	4	2	2
1	1	1	1	4	4	1	1	2	2
4	4	1	1	4	4	1	1	3	3
4	4	1	1	4	4	1	1	3	3
1	1	4	4	4	4	1	1	2	2
1	1	1	1	3	3	1	1	3	3
4	4	4	4	4	4	1	1	2	2
1	1	4	4	3	3	4	4	2	2
4	4	4	4	4	4	4	4	2	2
1	1	1	1	4	4	1	1	2	2
1	1	4	4	4	4	1	1	2	2
1	1	4	4	4	4	1	1	2	2
4	4	1	1	4	4	1	1	3	3
1	1	1	1	4	4	4	4	2	2
1	1	4	4	3	3	1	1	2	2
4	4	1	1	4	4	1	1	2	2
1	1	1	1	3	3	1	1	3	3
4	4	1	1	4	4	1	1	3	3
4	4	1	1	4	4	1	1	2	2
4	4	1	1	3	3	4	4	2	2
1	1	1	1	4	4	1	1	3	3
1	1	1	1	4	4	1	1	3	3
1	1	1	1	3	3	1	1	2	2
1	1	1	1	3	3	1	1	2	2
1	1	1	1	3	3	4	4	2	2
1	1	4	4	4	4	1	1	2	2
1	1	4	4	3	3	4	4	2	2
1	1	1	1	3	3	1	1	3	3
1	1	1	1	3	3	1	1	2	2
1	1	4	4	3	3	1	1	2	2
1	1	1	1	4	4	4	4	2	2
1	1	4	4	3	3	1	1	2	2
1	1	4	4	4	4	1	1	2	2
1	1	1	1	4	4	1	1	3	3
4	4	4	4	4	4	1	1	2	2
4	4	1	1	4	4	1	1	3	3
4	4	1	1	4	4	1	1	2	2
1	1	4	4	3	3	1	1	2	2
1	1	1	1	3	3	1	1	2	2
1	1	1	1	4	4	1	1	2	2
1	1	1	1	3	3	1	1	3	3
1	1	4	4	4	4	1	1	2	2
4	4	1	1	4	4	1	1	3	3
4	4	1	1	3	3	4	4	2	2
1	1	1	1	3	3	4	4	2	2
1	1	4	4	4	4	1	1	2	2
1	1	4	4	4	4	1	1	2	2

For Review Only

1	1	4	4	4	4	1	1	3	3
1	1	1	1	4	4	1	1	2	2
1	1	1	1	4	4	1	1	3	3
4	4	4	4	3	3	4	4	2	2
1	1	1	1	4	4	1	1	2	2
1	1	1	1	4	4	1	1	3	3
1	1	1	1	4	4	1	1	3	3
4	4	1	1	3	3	1	1	2	2
4	4	1	1	3	3	1	1	3	3
1	1	1	1	4	4	1	1	2	2
1	1	4	4	3	3	1	1	2	2
1	1	4	4	4	4	1	1	2	2
4	4	4	4	3	3	1	1	2	2
4	4	1	1	4	4	1	1	3	3
1	1	4	4	4	4	4	4	3	3
4	4	4	4	4	4	1	1	2	2
4	4	4	4	4	4	4	4	2	2
4	4	4	4	4	4	1	1	3	3
4	4	1	1	3	3	1	1	2	2
4	4	4	4	4	4	1	1	2	2
1	1	1	1	4	4	1	1	2	2
1	1	4	4	4	4	1	1	2	2
1	1	1	1	4	4	1	1	3	3
1	1	1	1	3	3	1	1	2	2
1	1	1	1	4	4	1	1	2	2
1	1	1	1	3	3	4	4	2	2
1	1	4	4	3	3	4	4	2	2
1	1	4	4	3	3	1	1	3	3
1	1	1	1	4	4	1	1	3	3
1	1	4	4	4	4	1	1	3	3
4	4	1	1	3	3	1	1	2	2
4	4	4	4	4	4	1	1	2	2
4	4	4	4	4	4	1	1	2	2
1	1	4	4	4	4	1	1	2	2
1	1	1	1	3	3	1	1	2	2
1	1	1	1	4	4	1	1	2	2
4	4	1	1	3	3	1	1	2	2
1	1	1	1	4	4	1	1	3	3
4	4	4	4	4	4	1	1	2	2
4	4	4	4	3	3	1	1	2	2
1	1	4	4	4	4	4	4	3	3
4	4	1	1	3	3	1	1	2	2
4	4	1	1	4	4	1	1	3	3
4	4	1	1	3	3	1	1	3	3
1	1	1	1	4	4	1	1	2	2
4	4	1	1	4	4	1	1	3	3
1	1	1	1	4	4	1	1	3	3
1	1	1	1	4	4	1	1	3	3
4	4	4	4	4	4	1	1	2	2
1	1	1	1	4	4	1	1	2	2
1	1	1	1	4	4	1	1	3	3
4	4	4	4	3	3	4	4	2	2
1	1	1	1	3	3	4	4	2	2
4	4	1	1	4	4	1	1	3	3
4	4	1	1	4	4	1	1	2	2

For Review Only

4	4	1	1	3	3	1	1	2	2
4	4	4	4	3	3	4	4	2	2
1	1	1	1	3	3	4	4	2	2
1	1	4	4	4	4	4	4	2	2
4	4	1	1	3	3	1	1	3	3
4	4	1	1	3	3	4	4	2	2
4	4	4	4	4	4	1	1	2	2
1	1	1	1	4	4	1	1	2	2
4	4	1	1	3	3	1	1	2	2
1	1	1	1	4	4	1	1	3	3
1	1	4	4	4	4	1	1	2	2
4	4	1	1	4	4	1	1	2	2
4	4	1	1	4	4	1	1	2	2
4	4	4	4	4	4	1	1	2	2
1	1	1	1	3	3	1	1	2	2
4	4	4	4	3	3	1	1	2	2
1	1	1	1	4	4	1	1	3	3
4	4	4	4	4	4	1	1	3	3
1	1	1	1	4	4	1	1	3	3
1	1	1	1	4	4	1	1	3	3
1	1	4	4	4	4	1	1	2	2
1	1	1	1	3	3	4	4	2	2
1	1	1	1	4	4	4	4	2	2
4	4	1	1	4	4	1	1	3	3
1	1	4	4	4	4	1	1	2	2
1	1	4	4	3	3	1	1	2	2
1	1	1	1	4	4	1	1	2	2
4	4	4	4	4	4	1	1	2	2
4	4	4	4	4	4	1	1	3	3
1	1	1	1	4	4	1	1	2	2
1	1	1	1	3	3	4	4	2	2
4	4	1	1	4	4	1	1	3	3
1	1	4	4	3	3	4	4	2	2
4	4	1	1	3	3	1	1	2	2
1	1	4	4	4	4	1	1	2	2
4	4	1	1	4	4	1	1	3	3
1	1	1	1	4	4	1	1	3	3
1	1	4	4	4	4	1	1	2	2
1	1	1	1	4	4	1	1	3	3
4	4	4	4	4	4	1	1	2	2
1	1	1	1	4	4	1	1	3	3
1	1	1	1	4	4	1	1	3	3
4	4	1	1	3	3	4	4	2	2
1	1	4	4	4	4	1	1	2	2
1	1	1	1	4	4	1	1	2	2
4	4	4	4	4	4	1	1	2	2
4	4	4	4	3	3	1	1	2	2
1	1	4	4	3	3	1	1	2	2
4	4	1	1	3	3	1	1	2	2
1	1	4	4	4	4	1	1	2	2
1	1	1	1	3	3	1	1	2	2
4	4	1	1	3	3	4	4	2	2
1	1	1	1	4	4	4	4	2	2
4	4	1	1	4	4	1	1	3	3
4	4	1	1	3	3	4	4	2	2

For Review Only

1	1	1	1	3	3	1	1	3	3
4	4	4	4	4	4	1	1	2	2
1	1	1	1	3	3	1	1	3	3
4	4	4	4	4	4	4	4	2	2
1	1	4	4	3	3	1	1	2	2
1	1	4	4	4	4	1	1	2	2
1	1	4	4	4	4	4	4	2	2
1	1	1	1	3	3	1	1	2	2
1	1	1	1	3	3	1	1	2	2
4	4	1	1	3	3	4	4	2	2
4	4	4	4	4	4	1	1	2	2
1	1	1	1	3	3	4	4	2	2
1	1	1	1	4	4	4	4	3	3
1	1	1	1	4	4	1	1	3	3
4	4	1	1	4	4	1	1	3	3
1	1	4	4	4	4	1	1	2	2
4	4	1	1	3	3	4	4	3	3
1	1	1	1	3	3	1	1	2	2
1	1	1	1	3	3	4	4	3	3
1	1	4	4	3	3	1	1	2	2
4	4	1	1	4	4	4	4	2	2
1	1	1	1	4	4	1	1	3	3
1	1	4	4	4	4	1	1	2	2
4	4	1	1	4	4	4	4	2	2
4	4	1	1	3	3	1	1	2	2
1	1	1	1	3	3	1	1	2	2
1	1	1	1	3	3	4	4	2	2
1	1	1	1	4	4	1	1	3	3
1	1	4	4	3	3	1	1	2	2
1	1	1	1	3	3	1	1	3	3
1	1	1	1	3	3	4	4	2	2
4	4	1	1	3	3	1	1	3	3
4	4	4	4	4	4	1	1	2	2
1	1	1	1	4	4	1	1	2	2
1	1	1	1	4	4	4	4	2	2
4	4	1	1	3	3	1	1	2	2
1	1	1	1	4	4	1	1	2	2
4	4	1	1	4	4	1	1	3	3
1	1	1	1	3	3	1	1	3	3
4	4	1	1	3	3	4	4	2	2
4	4	4	4	4	4	1	1	3	3
1	1	1	1	4	4	1	1	2	2
4	4	1	1	4	4	4	4	2	2
1	1	4	4	4	4	1	1	2	2
4	4	1	1	4	4	1	1	2	2
1	1	4	4	4	4	4	4	2	2
1	1	1	1	4	4	4	4	2	2
4	4	1	1	4	4	1	1	2	2
1	1	1	1	4	4	1	1	3	3
1	1	1	1	3	3	4	4	2	2
1	1	1	1	4	4	1	1	2	2
4	4	1	1	3	3	4	4	2	2
1	1	1	1	4	4	1	1	3	3
1	1	4	4	4	4	1	1	2	2
4	4	1	1	4	4	1	1	2	2

For Review Only

1	1	4	4	4	4	1	1	2	2
1	1	1	1	4	4	4	4	3	3

For Review Only

SNP2540	SNP2540	SNP2541	SNP2541	SNP2542	SNP2542	SNP2543	SNP2543	SNP2544	SNP2544
3	3	4	4	2	2	2	2	1	1
3	3	4	4	2	2	2	2	1	1
3	3	4	4	4	4	2	2	1	1
3	3	4	4	2	2	2	2	1	1
3	3	4	4	2	2	2	2	1	1
3	3	4	4	4	4	2	2	1	1
2	2	4	4	2	2	2	2	1	1
3	3	4	4	2	2	2	2	1	1
3	3	4	4	2	2	2	2	1	1
3	3	4	4	2	2	2	2	1	1
2	2	4	4	2	2	1	1	1	1
3	3	4	4	4	4	2	2	1	1
3	3	4	4	2	2	2	2	1	1
3	3	4	4	2	2	2	2	1	1
3	3	4	4	4	4	2	2	1	1
3	3	4	4	2	2	2	2	1	1
2	2	4	4	2	2	2	2	1	1
3	3	4	4	2	2	2	2	1	1
3	3	4	4	4	4	2	2	1	1
3	3	1	1	2	2	2	2	3	3
2	2	1	1	2	2	2	2	3	3
2	2	4	4	2	2	2	2	1	1
2	2	4	4	2	2	1	1	1	1
3	3	4	4	2	2	1	1	1	1
2	2	4	4	2	2	2	2	1	1
3	3	4	4	2	2	2	2	1	1
3	3	4	4	2	2	2	2	1	1
3	3	4	4	2	2	2	2	1	1
3	3	4	4	2	2	2	2	1	1
3	3	4	4	2	2	2	2	3	3
3	3	4	4	2	2	2	2	1	1
3	3	4	4	4	4	2	2	1	1
2	2	4	4	4	4	2	2	1	1
3	3	1	1	2	2	2	2	1	1
3	3	4	4	4	4	2	2	1	1
3	3	4	4	2	2	2	2	1	1
2	2	4	4	2	2	2	2	1	1
2	2	4	4	2	2	2	2	1	1
3	3	4	4	2	2	2	2	1	1
3	3	4	4	2	2	1	1	3	3
3	3	4	4	2	2	2	2	3	3
3	3	1	1	4	4	2	2	1	1
3	3	4	4	2	2	2	2	1	1
2	2	4	4	2	2	1	1	1	1
3	3	4	4	2	2	2	2	1	1
3	3	4	4	2	2	2	2	1	1
3	3	4	4	4	4	2	2	1	1
3	3	4	4	2	2	1	1	3	3
3	3	4	4	2	2	2	2	3	3
2	2	4	4	2	2	2	2	1	1
3	3	4	4	2	2	2	2	1	1
3	3	4	4	4	4	2	2	1	1

For Review Only

2	2	4	4	2	2	2	2	1	1
2	2	4	4	4	4	2	2	1	1
3	3	4	4	4	4	2	2	1	1
2	2	4	4	2	2	1	1	1	1
3	3	4	4	4	4	2	2	1	1
3	3	4	4	2	2	2	2	1	1
3	3	4	4	2	2	2	2	1	1
3	3	4	4	2	2	2	2	3	3
2	2	4	4	2	2	2	2	1	1
3	3	4	4	2	2	2	2	1	1
3	3	4	4	4	4	2	2	1	1
3	3	4	4	4	4	2	2	1	1
2	2	4	4	2	2	2	2	1	1
3	3	4	4	2	2	2	2	1	1
3	3	4	4	4	4	2	2	1	1
3	3	4	4	2	2	1	1	1	1
2	2	4	4	2	2	2	2	1	1
3	3	4	4	4	4	2	2	1	1
2	2	4	4	2	2	2	2	1	1
2	2	4	4	2	2	2	2	1	1
3	3	4	4	4	4	2	2	1	1
3	3	4	4	2	2	2	2	3	3
2	2	4	4	2	2	2	2	1	1
3	3	4	4	4	4	2	2	1	1
3	3	4	4	2	2	2	2	1	1
3	3	4	4	4	4	2	2	1	1
3	3	4	4	2	2	2	2	1	1
3	3	4	4	2	2	2	2	3	3
3	3	4	4	2	2	2	2	1	1
3	3	4	4	2	2	2	2	1	1
3	3	4	4	2	2	2	2	1	1
3	3	4	4	2	2	2	2	1	1
3	3	4	4	2	2	2	2	1	1
2	2	4	4	2	2	2	2	1	1
2	2	4	4	2	2	2	2	1	1
3	3	4	4	2	2	2	2	1	1
3	3	4	4	2	2	2	2	3	3
2	2	4	4	2	2	1	1	3	3
3	3	4	4	2	2	2	2	1	1
3	3	4	4	2	2	2	2	3	3
2	2	4	4	2	2	2	2	1	1
3	3	4	4	4	4	2	2	1	1
2	2	4	4	2	2	2	2	1	1
3	3	4	4	4	4	2	2	1	1
3	3	1	1	2	2	2	2	1	1
2	2	4	4	2	2	2	2	1	1
3	3	4	4	2	2	2	2	1	1
3	3	4	4	2	2	2	2	1	1
3	3	4	4	2	2	2	2	1	1
3	3	4	4	4	4	2	2	3	3
3	3	4	4	4	4	2	2	1	1
3	3	4	4	2	2	2	2	3	3
3	3	1	1	2	2	2	2	3	3
3	3	4	4	4	4	2	2	1	1
2	2	4	4	4	4	2	2	1	1

For Review Only

3	3	4	4	2	2	2	2	1	1
3	3	4	4	4	4	2	2	1	1
3	3	4	4	2	2	2	2	1	1
3	3	4	4	2	2	2	2	1	1
3	3	4	4	4	4	2	2	1	1
3	3	4	4	2	2	2	2	1	1
3	3	4	4	2	2	2	2	1	1
3	3	4	4	4	4	2	2	1	1
3	3	4	4	2	2	1	1	3	3
3	3	4	4	2	2	2	2	1	1
3	3	4	4	2	2	2	2	1	1
3	3	4	4	2	2	2	2	1	1
2	2	4	4	2	2	2	2	1	1
3	3	4	4	4	4	2	2	1	1
2	2	4	4	4	4	2	2	3	3
3	3	4	4	2	2	2	2	1	1
2	2	4	4	2	2	2	2	1	1
3	3	4	4	4	4	2	2	1	1
3	3	4	4	2	2	2	2	1	1
2	2	4	4	4	4	2	2	1	1
3	3	4	4	4	4	2	2	1	1
3	3	4	4	2	2	2	2	1	1
3	3	4	4	4	4	2	2	1	1
3	3	4	4	2	2	2	2	1	1
3	3	4	4	2	2	2	2	1	1
3	3	4	4	2	2	1	1	1	1
2	2	4	4	4	4	2	2	1	1
2	2	1	1	2	2	2	2	1	1
3	3	4	4	2	2	2	2	1	1
2	2	4	4	2	2	2	2	1	1
3	3	4	4	2	2	1	1	1	1
3	3	4	4	2	2	2	2	1	1
2	2	4	4	2	2	2	2	1	1
3	3	4	4	2	2	2	2	1	1
3	3	1	1	2	2	2	2	1	1
3	3	4	4	2	2	2	2	1	1
3	3	4	4	2	2	2	2	1	1
3	3	4	4	4	4	2	2	1	1
3	3	4	4	2	2	2	2	1	1
3	3	4	4	2	2	1	1	1	1
3	3	4	4	2	2	2	2	1	1
3	3	4	4	2	2	2	2	1	1
3	3	4	4	4	4	2	2	1	1
3	3	4	4	2	2	2	2	1	1
3	3	4	4	2	2	2	2	1	1
3	3	4	4	4	4	2	2	1	1
3	3	4	4	2	2	2	2	1	1
3	3	4	4	2	2	2	2	1	1
3	3	4	4	2	2	2	2	1	1
3	3	4	4	2	2	2	2	1	1
3	3	4	4	2	2	2	2	1	1
2	2	4	4	2	2	1	1	1	1
3	3	4	4	4	4	2	2	1	1
3	3	4	4	4	4	2	2	1	1
3	3	4	4	2	2	1	1	1	1

For Review Only

3	3	4	4	2	2	2	2	1	1
2	2	4	4	2	2	2	2	1	1
3	3	4	4	2	2	2	2	1	1
2	2	4	4	2	2	2	2	1	1
3	3	4	4	2	2	2	2	1	1
3	3	4	4	2	2	2	2	1	1
3	3	4	4	2	2	2	2	1	1
3	3	4	4	2	2	2	2	1	1
3	3	4	4	2	2	2	2	1	1
3	3	4	4	2	2	2	2	1	1
3	3	4	4	2	2	2	2	1	1
3	3	4	4	2	2	1	1	1	1
3	3	4	4	2	2	2	2	1	1
3	3	4	4	4	4	2	2	1	1
3	3	4	4	2	2	2	2	1	1
2	2	4	4	2	2	1	1	1	1
3	3	4	4	2	2	2	2	1	1
2	2	4	4	4	4	2	2	1	1
3	3	4	4	2	2	2	2	1	1
3	3	4	4	2	2	2	2	1	1
3	3	4	4	2	2	2	2	1	1
3	3	4	4	2	2	2	2	1	1
3	3	1	1	2	2	2	2	3	3
3	3	4	4	4	4	2	2	1	1
2	2	4	4	4	4	2	2	1	1
2	2	1	1	2	2	2	2	1	1
3	3	4	4	4	4	2	2	1	1
2	2	4	4	2	2	2	2	1	1
2	2	4	4	2	2	2	2	1	1
3	3	4	4	2	2	2	2	1	1
2	2	4	4	2	2	2	2	1	1
3	3	4	4	2	2	2	2	1	1
2	2	4	4	2	2	2	2	1	1
3	3	4	4	2	2	2	2	1	1
2	2	4	4	2	2	2	2	1	1
3	3	4	4	4	4	2	2	1	1
3	3	4	4	2	2	2	2	1	1
2	2	1	1	2	2	2	2	1	1
3	3	4	4	2	2	2	2	1	1
2	2	4	4	4	4	2	2	1	1
3	3	4	4	4	4	2	2	1	1
3	3	4	4	2	2	2	2	1	1
3	3	4	4	2	2	1	1	1	1
3	3	4	4	2	2	2	2	1	1
3	3	4	4	4	4	2	2	1	1
2	2	4	4	4	4	2	2	1	1
2	2	4	4	2	2	1	1	1	1
2	2	4	4	2	2	2	2	3	3
3	3	4	4	2	2	2	2	1	1
2	2	4	4	2	2	2	2	1	1
3	3	4	4	2	2	2	2	1	1
3	3	4	4	2	2	2	2	1	1
3	3	4	4	2	2	2	2	3	3
3	3	4	4	4	4	2	2	1	1
3	3	4	4	2	2	1	1	1	1

For Review Only

3	3	4	4	2	2	2	2	1	1
2	2	4	4	2	2	1	1	1	1
3	3	4	4	2	2	2	2	1	1
3	3	4	4	4	4	2	2	1	1
2	2	4	4	2	2	2	2	1	1
3	3	4	4	2	2	2	2	1	1
2	2	4	4	2	2	1	1	1	1
3	3	4	4	2	2	2	2	1	1
3	3	4	4	2	2	2	2	1	1
3	3	4	4	2	2	1	1	3	3
3	3	4	4	2	2	2	2	1	1
3	3	4	4	2	2	2	2	1	1
3	3	4	4	2	2	2	2	1	1
3	3	4	4	2	2	2	2	1	1
3	3	4	4	4	4	2	2	1	1
2	2	4	4	2	2	2	2	3	3
3	3	4	4	2	2	1	1	1	1
3	3	4	4	2	2	1	1	3	3
3	3	4	4	2	2	1	1	1	1
2	2	4	4	2	2	2	2	1	1
3	3	4	4	4	4	1	1	1	1
3	3	4	4	2	2	2	2	1	1
3	3	1	1	2	2	2	2	1	1
3	3	4	4	2	2	1	1	1	1
3	3	4	4	2	2	2	2	3	3
3	3	4	4	4	4	2	2	1	1
3	3	4	4	2	2	1	1	1	1
3	3	4	4	2	2	2	2	1	1
2	2	4	4	2	2	2	2	1	1
3	3	4	4	2	2	2	2	1	1
3	3	4	4	2	2	1	1	1	1
3	3	4	4	2	2	2	2	1	1
2	2	4	4	2	2	2	2	1	1
3	3	4	4	2	2	2	2	1	1
3	3	4	4	2	2	2	2	1	1
3	3	4	4	2	2	2	2	1	1
3	3	4	4	2	2	2	2	1	1
3	3	4	4	4	4	2	2	1	1
3	3	4	4	2	2	2	2	1	1
3	3	4	4	2	2	1	1	3	3
2	2	4	4	2	2	2	2	1	1
3	3	4	4	4	4	2	2	1	1
3	3	4	4	2	2	1	1	1	1
2	2	4	4	2	2	2	2	1	1
3	3	4	4	2	2	1	1	3	3
2	2	4	4	2	2	2	2	1	1
3	3	4	4	2	2	2	2	3	3
3	3	4	4	4	4	2	2	1	1
3	3	4	4	4	4	2	2	3	3
3	3	4	4	2	2	2	2	1	1
3	3	4	4	2	2	2	2	1	1
3	3	4	4	2	2	1	1	3	3
3	3	4	4	4	4	2	2	1	1
2	2	4	4	2	2	2	2	1	1
3	3	4	4	2	2	1	1	1	1

For Review Only

2	2	4	4	2	2	2	2	1	1
3	3	4	4	4	4	2	2	1	1

For Review Only

SNP2545	SNP2545	SNP2546	SNP2546	SNP2547	SNP2547	SNP2548	SNP2548	SNP2549	SNP2549
3	3	3	3	2	2	1	1	2	2
3	3	3	3	1	1	4	4	2	2
3	3	3	3	1	1	1	1	2	2
3	3	3	3	2	2	1	1	2	2
1	1	2	2	2	2	4	4	2	2
3	3	3	3	1	1	1	1	2	2
1	1	2	2	2	2	1	1	2	2
3	3	3	3	2	2	1	1	2	2
3	3	2	2	2	2	1	1	2	2
1	1	2	2	1	1	4	4	2	2
3	3	3	3	2	2	4	4	2	2
3	3	3	3	1	1	1	1	2	2
3	3	2	2	2	2	1	1	2	2
3	3	3	3	2	2	4	4	4	4
3	3	3	3	2	2	1	1	2	2
3	3	3	3	1	1	4	4	4	4
3	3	3	3	2	2	1	1	2	2
1	1	2	2	1	1	1	1	2	2
3	3	3	3	2	2	1	1	2	2
3	3	3	3	1	1	1	1	2	2
1	1	2	2	2	2	1	1	2	2
3	3	3	3	2	2	1	1	2	2
1	1	2	2	2	2	4	4	2	2
3	3	3	3	2	2	4	4	4	4
3	3	3	3	2	2	1	1	2	2
3	3	3	3	2	2	4	4	2	2
3	3	3	3	1	1	1	1	2	2
3	3	3	3	2	2	4	4	2	2
1	1	2	2	2	2	1	1	2	2
1	1	2	2	2	2	4	4	2	2
3	3	3	3	1	1	1	1	2	2
3	3	3	3	1	1	1	1	2	2
3	3	3	3	2	2	1	1	2	2
3	3	3	3	2	2	4	4	2	2
3	3	3	3	2	2	1	1	2	2
3	3	3	3	2	2	4	4	4	4
3	3	3	3	2	2	1	1	2	2
3	3	3	3	2	2	4	4	2	2
3	3	3	3	2	2	4	4	2	2
1	1	2	2	2	2	4	4	2	2
1	1	2	2	2	2	4	4	2	2
1	1	2	2	2	2	1	1	2	2
1	1	2	2	1	1	1	1	2	2
3	3	3	3	2	2	1	1	2	2
1	1	2	2	1	1	4	4	2	2
3	3	3	3	2	2	1	1	2	2
3	3	3	3	2	2	1	1	2	2
3	3	3	3	2	2	4	4	2	2
3	3	2	2	2	2	4	4	2	2
3	3	2	2	2	2	4	4	2	2
3	3	3	3	2	2	1	1	2	2
3	3	3	3	2	2	1	1	2	2

For Review Only

3	3	3	3	2	2	1	1	2	2
3	3	3	3	2	2	1	1	2	2
3	3	3	3	1	1	1	1	2	2
3	3	3	3	2	2	1	1	2	2
3	3	3	3	2	2	1	1	2	2
3	3	3	3	2	2	1	1	2	2
3	3	3	3	2	2	1	1	2	2
1	1	2	2	2	2	4	4	2	2
3	3	3	3	2	2	1	1	2	2
3	3	3	3	2	2	4	4	2	2
3	3	3	3	1	1	1	1	2	2
3	3	3	3	1	1	1	1	2	2
3	3	3	3	2	2	1	1	2	2
1	1	2	2	2	2	1	1	2	2
3	3	3	3	2	2	1	1	2	2
1	1	2	2	2	2	1	1	2	2
3	3	3	3	2	2	1	1	2	2
3	3	3	3	1	1	1	1	2	2
3	3	3	3	2	2	1	1	2	2
3	3	3	3	2	2	1	1	2	2
3	3	3	3	1	1	1	1	2	2
3	3	3	3	2	2	1	1	2	2
3	3	2	2	2	2	1	1	2	2
3	3	3	3	2	2	1	1	2	2
1	1	2	2	1	1	4	4	2	2
3	3	3	3	1	1	1	1	2	2
3	3	3	3	2	2	1	1	2	2
3	3	3	3	2	2	4	4	2	2
3	3	3	3	1	1	1	1	2	2
3	3	3	3	1	1	1	1	2	2
3	3	3	3	2	2	1	1	2	2
3	3	3	3	2	2	4	4	4	4
3	3	3	3	2	2	4	4	2	2
3	3	3	3	2	2	1	1	2	2
1	1	2	2	2	2	1	1	2	2
1	1	2	2	1	1	4	4	2	2
3	3	3	3	2	2	1	1	2	2
1	1	2	2	2	2	4	4	2	2
3	3	3	3	2	2	1	1	2	2
1	1	2	2	2	2	4	4	2	2
3	3	3	3	2	2	1	1	2	2
3	3	3	3	2	2	1	1	2	2
3	3	3	3	2	2	1	1	2	2
3	3	3	3	1	1	1	1	2	2
3	3	3	3	1	1	1	1	2	2
3	3	3	3	2	2	4	4	2	2
3	3	3	3	2	2	4	4	4	4
3	3	3	3	2	2	1	1	2	2
3	3	3	3	2	2	1	1	4	4
3	3	3	3	2	2	1	1	2	2
3	3	3	3	1	1	1	1	2	2
3	3	3	3	2	2	4	4	2	2
1	1	2	2	2	2	4	4	2	2
3	3	3	3	2	2	4	4	2	2
3	3	3	3	2	2	1	1	2	2

For Review Only

3	3	2	2	2	2	4	4	2	2
3	3	3	3	2	2	4	4	2	2
3	3	3	3	2	2	1	1	2	2
1	1	2	2	2	2	4	4	2	2
3	3	3	3	1	1	1	1	2	2
3	3	3	3	2	2	1	1	2	2
3	3	3	3	1	1	1	1	2	2
3	3	3	3	1	1	4	4	2	2
3	3	3	3	1	1	4	4	4	4
3	3	3	3	2	2	4	4	2	2
1	1	2	2	2	2	1	1	2	2
3	3	3	3	2	2	4	4	2	2
1	1	2	2	2	2	4	4	2	2
3	3	3	3	1	1	1	1	2	2
3	3	3	3	2	2	1	1	2	2
3	3	3	3	2	2	1	1	2	2
3	3	3	3	2	2	1	1	2	2
3	3	3	3	2	2	1	1	2	2
1	1	2	2	2	2	4	4	2	2
3	3	2	2	2	2	4	4	2	2
3	3	3	3	2	2	4	4	2	2
3	3	3	3	1	1	1	1	2	2
3	3	3	3	1	1	1	1	2	2
3	3	3	3	2	2	4	4	2	2
3	3	2	2	2	2	4	4	2	2
1	1	2	2	2	2	4	4	2	2
3	3	3	3	2	2	1	1	2	2
3	3	3	3	2	2	1	1	2	2
3	3	3	3	1	1	1	1	2	2
3	3	2	2	2	2	1	1	2	2
3	3	3	3	2	2	4	4	4	4
3	3	2	2	2	2	4	4	2	2
3	3	3	3	2	2	1	1	2	2
3	3	3	3	2	2	1	1	2	2
3	3	3	3	2	2	4	4	2	2
3	3	3	3	2	2	1	1	2	2
1	1	2	2	2	2	4	4	2	2
3	3	3	3	1	1	1	1	2	2
1	1	3	3	2	2	4	4	2	2
3	3	3	3	2	2	4	4	2	2
3	3	3	3	2	2	1	1	2	2
3	3	3	3	2	2	4	4	4	4
3	3	3	3	1	1	1	1	2	2
3	3	2	2	2	2	4	4	2	2
3	3	3	3	2	2	4	4	2	2
3	3	3	3	1	1	1	1	2	2
3	3	3	3	2	2	1	1	2	2
3	3	3	3	1	1	1	1	2	2
3	3	2	2	2	2	1	1	2	2
3	3	3	3	2	2	1	1	2	2
3	3	3	3	2	2	1	1	2	2
3	3	3	3	2	2	4	4	2	2
3	3	3	3	2	2	4	4	4	4
3	3	3	3	1	1	1	1	2	2
3	3	2	2	2	2	4	4	2	2

For Review Only

1	1	2	2	2	2	4	4	2	2
1	1	2	2	2	2	4	4	2	2
1	1	2	2	1	1	4	4	2	2
3	3	3	3	2	2	1	1	2	2
3	3	3	3	1	1	4	4	2	2
1	1	2	2	2	2	1	1	2	2
3	3	2	2	2	2	4	4	2	2
3	3	3	3	2	2	4	4	2	2
3	3	3	3	2	2	4	4	2	2
3	3	3	3	1	1	1	1	2	2
3	3	3	3	2	2	1	1	2	2
3	3	3	3	2	2	1	1	4	4
3	3	3	3	1	1	1	1	2	2
3	3	3	3	2	2	1	1	2	2
3	3	3	3	2	2	4	4	4	4
3	3	3	3	2	2	4	4	2	2
3	3	3	3	2	2	4	4	2	2
3	3	3	3	2	2	4	4	2	2
3	3	3	3	2	2	1	1	2	2
3	3	3	3	1	1	1	1	2	2
3	3	3	3	2	2	4	4	2	2
3	3	2	2	2	2	4	4	2	2
3	3	3	3	2	2	1	1	2	2
3	3	3	3	1	1	1	1	2	2
3	3	3	3	2	2	1	1	2	2
3	3	3	3	2	2	4	4	2	2
3	3	3	3	1	1	1	1	2	2
3	3	3	3	2	2	1	1	2	2
3	3	3	3	2	2	1	1	2	2
3	3	3	3	1	1	1	1	2	2
1	1	2	2	1	1	4	4	2	2
3	3	3	3	1	1	1	1	2	2
3	3	3	3	2	2	1	1	2	2
3	3	3	3	2	2	4	4	4	4
3	3	3	3	2	2	1	1	2	2
3	3	3	3	1	1	1	1	2	2
3	3	3	3	2	2	1	1	2	2
3	3	3	3	2	2	4	4	2	2
3	3	3	3	1	1	1	1	2	2
3	3	3	3	2	2	1	1	2	2
3	3	3	3	1	1	1	1	2	2
3	3	3	3	2	2	1	1	2	2
3	3	3	3	2	2	4	4	2	2
3	3	3	3	2	2	1	1	2	2
3	3	3	3	2	2	1	1	2	2
3	3	3	3	2	2	1	1	2	2
3	3	3	3	2	2	4	4	2	2
1	1	2	2	2	2	4	4	2	2
1	1	2	2	2	2	4	4	2	2
3	3	3	3	2	2	1	1	2	2
1	1	2	2	1	1	4	4	2	2
1	1	2	2	2	2	4	4	2	2
3	3	3	3	2	2	1	1	2	2
3	3	3	3	1	1	1	1	2	2
3	3	3	3	2	2	4	4	2	2

For Review Only

3	3	3	3	1	1	4	4	4	4
3	3	3	3	2	2	1	1	2	2
1	1	2	2	1	1	4	4	2	2
3	3	3	3	2	2	1	1	2	2
1	1	2	2	2	2	4	4	2	2
3	3	3	3	2	2	1	1	2	2
3	3	3	3	2	2	1	1	2	2
1	1	2	2	2	2	4	4	2	2
3	3	3	3	2	2	4	4	4	4
1	1	2	2	2	2	4	4	2	2
3	3	3	3	2	2	4	4	2	2
1	1	2	2	2	2	4	4	2	2
3	3	3	3	2	2	1	1	2	2
3	3	3	3	2	2	1	1	2	2
3	3	3	3	1	1	1	1	2	2
3	3	3	3	2	2	1	1	2	2
1	1	2	2	2	2	4	4	2	2
3	3	3	3	2	2	1	1	2	2
1	1	2	2	2	2	4	4	2	2
3	3	3	3	2	2	4	4	2	2
3	3	3	3	2	2	1	1	2	2
3	3	3	3	2	2	1	1	2	2
3	3	3	3	2	2	1	1	2	2
3	3	3	3	2	2	1	1	2	2
1	1	2	2	2	2	4	4	2	2
3	3	3	3	1	1	4	4	2	2
3	3	3	3	2	2	4	4	2	2
3	3	3	3	1	1	1	1	2	2
3	3	2	2	2	2	4	4	2	2
1	1	2	2	1	1	4	4	2	2
1	1	2	2	2	2	4	4	2	2
3	3	3	3	2	2	4	4	2	2
3	3	3	3	2	2	1	1	2	2
3	3	3	3	2	2	1	1	2	2
3	3	3	3	2	2	1	1	2	2
1	1	2	2	2	2	4	4	2	2
3	3	3	3	1	1	1	1	2	2
3	3	3	3	1	1	1	1	2	2
3	3	3	3	2	2	4	4	4	4
3	3	3	3	2	2	4	4	2	2
3	3	3	3	2	2	1	1	2	2
3	3	3	3	2	2	1	1	2	2
3	3	3	3	2	2	1	1	2	2
3	3	3	3	2	2	1	1	2	2
3	3	3	3	2	2	1	1	2	2
3	3	3	3	2	2	1	1	2	2
3	3	3	3	1	1	4	4	2	2
3	3	3	3	2	2	1	1	2	2
3	3	3	3	2	2	1	1	2	2
3	3	3	3	2	2	4	4	2	2
3	3	3	3	2	2	1	1	2	2
3	3	3	3	2	2	4	4	2	2
3	3	3	3	1	1	1	1	2	2
3	3	3	3	2	2	1	1	2	2
3	3	3	3	2	2	1	1	2	2

For Review Only

3	3	3	3	2	2	1	1	2	2
3	3	3	3	1	1	1	1	2	2

For Review Only

SNP255	SNP255	SNP2550	SNP2550	SNP2551	SNP2551	SNP2552	SNP2552	SNP2553	SNP2553
1	1	4	4	4	4	4	4	4	4
2	2	4	4	1	1	1	1	4	4
2	2	4	4	1	1	1	1	4	4
1	1	4	4	4	4	4	4	4	4
1	1	4	4	1	1	1	1	4	4
1	1	4	4	4	4	4	4	4	4
2	2	4	4	4	4	4	4	4	4
2	2	4	4	4	4	4	4	4	4
1	1	4	4	4	4	4	4	4	4
2	2	4	4	1	1	1	1	4	4
1	1	4	4	1	1	1	1	4	4
2	2	4	4	4	4	4	4	4	4
1	1	4	4	4	4	4	4	4	4
1	1	4	4	1	1	1	1	4	4
1	1	4	4	4	4	4	4	4	4
2	2	4	4	1	1	1	1	4	4
1	1	4	4	4	4	4	4	4	4
2	2	4	4	4	4	4	4	4	4
2	2	4	4	4	4	4	4	4	4
2	2	4	4	4	4	4	4	2	2
1	1	4	4	4	4	4	4	2	2
1	1	4	4	4	4	4	4	4	4
1	1	4	4	1	1	1	1	2	2
1	1	4	4	1	1	1	1	2	2
1	1	4	4	4	4	4	4	4	4
2	2	4	4	4	4	4	4	4	4
2	2	4	4	4	4	4	4	4	4
2	2	4	4	4	4	4	4	4	4
2	2	4	4	4	4	4	4	4	4
1	1	4	4	1	1	1	1	2	2
2	2	4	4	4	4	4	4	4	4
2	2	4	4	4	4	4	4	4	4
1	1	4	4	4	4	4	4	4	4
1	1	4	4	1	1	1	1	4	4
1	1	4	4	4	4	4	4	4	4
2	2	4	4	1	1	1	1	4	4
1	1	4	4	4	4	4	4	4	4
1	1	4	4	1	1	1	1	4	4
1	1	4	4	1	1	1	1	2	2
2	2	4	4	4	4	1	1	2	2
2	2	4	4	1	1	1	1	4	4
2	2	4	4	4	4	4	4	4	4
2	2	4	4	4	4	4	4	4	4
1	1	4	4	4	4	4	4	2	2
2	2	4	4	1	1	1	1	4	4
2	2	4	4	4	4	4	4	4	4
1	1	2	2	4	4	4	4	4	4
2	2	4	4	1	1	1	1	2	2
1	1	4	4	1	1	4	4	2	2
1	1	4	4	4	4	4	4	4	4
2	2	4	4	4	4	4	4	4	4
2	2	4	4	4	4	4	4	4	4

For Review Only

1	1	4	4	4	4	4	4	4	4
1	1	4	4	4	4	4	4	4	4
1	1	4	4	4	4	4	4	4	4
1	1	4	4	4	4	4	4	4	4
2	2	4	4	4	4	4	4	4	4
2	2	4	4	4	4	4	4	4	4
2	2	4	4	4	4	4	4	4	4
1	1	4	4	1	1	1	1	2	2
1	1	4	4	4	4	4	4	4	4
2	2	4	4	4	4	4	4	4	4
2	2	2	2	4	4	4	4	4	4
2	2	2	2	4	4	4	4	4	4
1	1	4	4	4	4	4	4	4	4
2	2	4	4	4	4	4	4	4	4
2	2	4	4	4	4	4	4	4	4
1	1	4	4	4	4	4	4	4	4
1	1	4	4	4	4	4	4	4	4
1	1	4	4	4	4	4	4	4	4
1	1	4	4	4	4	4	4	4	4
1	1	4	4	4	4	4	4	4	4
2	2	4	4	4	4	4	4	4	4
1	1	4	4	4	4	4	4	2	2
1	1	4	4	4	4	4	4	4	4
1	1	4	4	4	4	4	4	4	4
2	2	4	4	1	1	1	1	4	4
2	2	4	4	4	4	4	4	4	4
1	1	4	4	4	4	4	4	4	4
1	1	4	4	1	1	1	1	2	2
2	2	4	4	4	4	4	4	4	4
2	2	4	4	4	4	4	4	4	4
1	1	4	4	4	4	4	4	4	4
1	1	4	4	1	1	1	1	4	4
1	1	4	4	1	1	1	1	2	2
1	1	4	4	4	4	4	4	4	4
1	1	4	4	4	4	4	4	4	4
2	2	4	4	1	1	1	1	4	4
1	1	4	4	4	4	4	4	2	2
1	1	4	4	1	1	1	1	4	4
1	1	4	4	4	4	4	4	4	4
1	1	4	4	1	1	1	1	2	2
1	1	4	4	4	4	4	4	4	4
2	2	4	4	4	4	4	4	4	4
1	1	4	4	4	4	4	4	4	4
2	2	2	2	4	4	4	4	4	4
1	1	4	4	4	4	4	4	4	4
1	1	4	4	1	1	1	1	4	4
1	1	4	4	1	1	1	1	2	2
1	1	4	4	4	4	4	4	4	4
2	2	4	4	1	1	1	1	4	4
1	1	2	2	4	4	4	4	2	2
2	2	4	4	4	4	4	4	4	4
1	1	4	4	1	1	1	1	2	2
1	1	4	4	1	1	1	1	2	2
1	1	4	4	1	1	4	4	2	2
1	1	2	2	4	4	4	4	4	4

2	2	4	4	4	4	4	4	4	4
1	1	4	4	4	4	4	4	4	4
2	2	4	4	4	4	4	4	4	4
1	1	4	4	1	1	1	1	4	4
1	1	4	4	4	4	4	4	4	4
2	2	4	4	4	4	4	4	4	4
2	2	4	4	4	4	4	4	4	4
1	1	4	4	1	1	1	1	4	4
2	2	4	4	1	1	1	1	2	2
2	2	4	4	4	4	1	1	4	4
1	1	4	4	4	4	4	4	4	4
2	2	4	4	4	4	4	4	4	4
1	1	4	4	1	1	1	1	4	4
2	2	4	4	4	4	4	4	4	4
2	2	4	4	4	4	4	4	2	2
2	2	4	4	4	4	4	4	4	4
1	1	4	4	4	4	4	4	4	4
2	2	4	4	4	4	4	4	4	4
1	1	4	4	1	1	1	1	4	4
1	1	4	4	1	1	4	4	4	4
2	2	4	4	4	4	4	4	4	4
2	2	4	4	4	4	4	4	4	4
2	2	4	4	4	4	4	4	4	4
1	1	4	4	1	1	1	1	2	2
2	2	4	4	4	4	4	4	4	4
1	1	4	4	1	1	1	1	4	4
1	1	4	4	4	4	4	4	4	4
2	2	4	4	4	4	4	4	4	4
2	2	4	4	4	4	4	4	4	4
2	2	4	4	4	4	4	4	4	4
1	1	4	4	1	1	1	1	4	4
2	2	4	4	4	4	1	1	4	4
1	1	4	4	4	4	4	4	4	4
2	2	4	4	4	4	4	4	4	4
1	1	4	4	1	1	1	1	4	4
1	1	4	4	1	1	4	4	4	4
1	1	4	4	1	1	1	1	4	4
2	2	4	4	4	4	4	4	4	4
1	1	4	4	4	4	1	1	4	4
1	1	4	4	1	1	1	1	4	4
2	2	4	4	4	4	4	4	4	4
1	1	4	4	1	1	1	1	2	2
2	2	4	4	4	4	4	4	4	4
2	2	4	4	1	1	1	1	4	4
1	1	4	4	4	4	4	4	4	4
2	2	2	2	4	4	4	4	4	4
2	2	4	4	4	4	4	4	4	4
2	2	4	4	4	4	4	4	4	4
1	1	4	4	4	4	4	4	4	4
2	2	4	4	4	4	4	4	4	4
2	2	4	4	4	4	4	4	4	4
1	1	4	4	1	1	1	1	2	2
2	2	4	4	4	4	4	4	4	4
2	2	2	2	4	4	4	4	4	4
1	1	4	4	4	4	4	4	4	4

For Review Only

1	1	4	4	4	4	4	4	4	4
1	1	4	4	1	1	1	1	4	4
1	1	4	4	1	1	1	1	4	4
2	2	4	4	4	4	4	4	4	4
2	2	4	4	1	1	1	1	4	4
1	1	4	4	4	4	4	4	4	4
1	1	4	4	4	4	4	4	2	2
2	2	4	4	4	4	4	4	4	4
2	2	4	4	1	1	1	1	4	4
2	2	4	4	4	4	4	4	4	4
2	2	4	4	4	4	4	4	4	4
1	1	4	4	4	4	4	4	4	4
1	1	4	4	4	4	4	4	4	4
2	2	4	4	4	4	4	4	4	4
1	1	4	4	1	1	1	1	4	4
1	1	4	4	1	1	1	1	4	4
2	2	4	4	4	4	4	4	4	4
2	2	4	4	4	4	1	1	4	4
2	2	4	4	4	4	4	4	4	4
2	2	4	4	4	4	4	4	4	4
2	2	4	4	4	4	4	4	4	4
1	1	4	4	1	1	1	1	4	4
1	1	4	4	4	4	4	4	2	2
2	2	2	2	4	4	4	4	4	4
1	1	4	4	4	4	4	4	4	4
1	1	4	4	1	1	1	1	4	4
1	1	4	4	4	4	4	4	4	4
1	1	4	4	4	4	4	4	4	4
2	2	4	4	4	4	4	4	4	4
1	1	4	4	4	4	4	4	4	4
1	1	4	4	1	1	1	1	4	4
2	2	4	4	4	4	4	4	4	4
1	1	4	4	4	4	4	4	4	4
1	1	4	4	1	1	1	1	2	2
1	1	4	4	4	4	4	4	4	4
2	2	2	2	4	4	4	4	4	4
2	2	4	4	4	4	4	4	4	4
1	1	4	4	4	4	4	4	4	4
2	2	4	4	4	4	4	4	4	4
1	1	4	4	4	4	4	4	4	4
2	2	4	4	1	1	1	1	4	4
2	2	4	4	4	4	4	4	4	4
1	1	4	4	1	1	1	1	2	2
2	2	4	4	4	4	4	4	4	4
1	1	4	4	4	4	4	4	4	4
2	2	4	4	4	4	4	4	4	4
1	1	4	4	1	1	1	1	4	4
1	1	4	4	1	1	1	1	2	2
1	1	4	4	1	1	1	1	4	4
2	2	4	4	4	4	4	4	4	4
1	1	4	4	4	4	4	4	4	4
1	1	4	4	1	1	1	1	2	2
1	1	4	4	4	4	4	4	2	2
2	2	2	2	4	4	4	4	4	4
1	1	4	4	1	1	1	1	4	4

For Review Only

2	2	4	4	1	1	1	1	4	4
1	1	4	4	4	4	4	4	4	4
2	2	4	4	4	4	4	4	4	4
1	1	4	4	4	4	4	4	4	4
1	1	4	4	1	1	1	1	4	4
1	1	4	4	4	4	4	4	4	4
1	1	4	4	4	4	4	4	4	4
1	1	4	4	1	1	1	1	4	4
2	2	4	4	1	1	1	1	4	4
1	1	4	4	1	1	1	1	2	2
2	2	4	4	4	4	4	4	4	4
1	1	4	4	1	1	1	1	4	4
2	2	4	4	4	4	4	4	4	4
2	2	4	4	4	4	4	4	4	4
2	2	4	4	1	1	1	1	4	4
1	1	4	4	4	4	4	4	2	2
2	2	4	4	1	1	1	1	4	4
1	1	4	4	4	4	4	4	2	2
2	2	4	4	1	1	1	1	4	4
1	1	4	4	1	1	1	1	4	4
1	1	4	4	4	4	4	4	4	4
2	2	4	4	4	4	4	4	4	4
1	1	4	4	4	4	4	4	4	4
1	1	4	4	4	4	4	4	4	4
1	1	4	4	1	1	1	1	2	2
2	2	4	4	1	1	1	1	4	4
1	1	4	4	1	1	1	1	2	2
2	2	4	4	4	4	4	4	4	4
1	1	4	4	4	4	4	4	4	4
2	2	4	4	4	4	4	4	4	4
1	1	4	4	1	1	1	1	4	4
2	2	4	4	1	1	1	1	4	4
1	1	4	4	4	4	4	4	4	4
1	1	4	4	4	4	4	4	4	4
1	1	4	4	4	4	4	4	4	4
1	1	4	4	1	1	1	1	4	4
1	1	4	4	4	4	4	4	4	4
2	2	2	2	4	4	4	4	4	4
2	2	4	4	1	1	1	1	4	4
1	1	4	4	1	1	1	1	2	2
2	2	4	4	4	4	4	4	4	4
1	1	2	2	4	4	4	4	4	4
1	1	4	4	4	4	4	4	4	4
1	1	4	4	4	4	4	4	4	4
1	1	4	4	4	4	4	4	2	2
2	2	4	4	4	4	4	4	4	4
1	1	4	4	1	1	1	1	2	2
1	1	4	4	4	4	4	4	4	4
2	2	4	4	4	4	4	4	4	4
1	1	4	4	1	1	1	1	2	2
1	1	4	4	4	4	4	4	4	4
1	1	4	4	1	1	1	1	2	2
2	2	4	4	1	1	1	1	4	4
1	1	4	4	4	4	4	4	4	4
1	1	4	4	4	4	4	4	4	4

For Review Only

1	1	4	4	4	4	4	4	4	4
2	2	4	4	4	4	4	4	4	4

For Review Only

SNP2554	SNP2554	SNP2555	SNP2555	SNP2556	SNP2556	SNP2557	SNP2557	SNP2558	SNP2558
2	2	3	3	2	2	4	4	1	1
3	3	3	3	3	3	4	4	1	1
3	3	3	3	3	3	4	4	1	1
2	2	3	3	2	2	4	4	1	1
3	3	3	3	2	2	1	1	3	3
2	2	3	3	3	3	4	4	1	1
2	2	3	3	3	3	4	4	1	1
2	2	3	3	2	2	1	1	3	3
2	2	3	3	2	2	4	4	1	1
3	3	3	3	3	3	4	4	1	1
3	3	3	3	2	2	1	1	3	3
2	2	3	3	3	3	4	4	1	1
2	2	3	3	2	2	4	4	1	1
3	3	3	3	2	2	4	4	1	1
2	2	3	3	2	2	4	4	1	1
3	3	3	3	2	2	4	4	1	1
2	2	3	3	2	2	4	4	1	1
2	2	3	3	3	3	4	4	1	1
2	2	3	3	3	3	4	4	1	1
2	2	3	3	3	3	4	4	1	1
2	2	3	3	2	2	4	4	1	1
2	2	3	3	2	2	4	4	1	1
3	3	3	3	2	2	4	4	1	1
3	3	3	3	2	2	4	4	1	1
2	2	3	3	2	2	4	4	1	1
2	2	3	3	2	2	1	1	3	3
2	2	3	3	3	3	4	4	1	1
2	2	3	3	2	2	4	4	3	3
2	2	3	3	2	2	4	4	1	1
3	3	2	2	2	2	4	4	1	1
2	2	3	3	3	3	1	1	3	3
2	2	3	3	3	3	4	4	1	1
2	2	3	3	2	2	4	4	1	1
3	3	3	3	3	3	1	1	3	3
2	2	3	3	2	2	4	4	3	3
3	3	3	3	2	2	4	4	1	1
2	2	3	3	2	2	4	4	1	1
3	3	3	3	2	2	4	4	1	1
3	3	3	3	2	2	4	4	1	1
2	2	2	2	2	2	4	4	1	1
3	3	3	3	2	2	1	1	3	3
2	2	3	3	2	2	4	4	1	1
2	2	3	3	3	3	4	4	1	1
2	2	3	3	2	2	4	4	1	1
3	3	3	3	3	3	4	4	1	1
2	2	3	3	2	2	4	4	1	1
2	2	3	3	2	2	1	1	3	3
3	3	3	3	2	2	1	1	3	3
2	2	3	3	2	2	4	4	1	1
2	2	3	3	2	2	4	4	1	1
2	2	3	3	2	2	1	1	3	3
2	2	3	3	3	3	4	4	1	1

For Review Only

2	2	3	3	2	2	4	4	1	1
2	2	3	3	2	2	4	4	1	1
2	2	3	3	3	3	4	4	1	1
2	2	3	3	2	2	4	4	1	1
2	2	3	3	3	3	4	4	1	1
2	2	3	3	2	2	1	1	3	3
2	2	3	3	3	3	1	1	3	3
3	3	2	2	2	2	4	4	1	1
2	2	3	3	2	2	4	4	1	1
2	2	3	3	2	2	4	4	1	1
2	2	3	3	3	3	4	4	1	1
2	2	3	3	3	3	4	4	1	1
2	2	3	3	2	2	4	4	1	1
2	2	3	3	2	2	4	4	1	1
2	2	3	3	3	3	4	4	1	1
2	2	3	3	2	2	4	4	1	1
2	2	3	3	3	3	4	4	1	1
2	2	3	3	3	3	4	4	3	3
2	2	3	3	2	2	4	4	1	1
2	2	3	3	2	2	4	4	1	1
2	2	3	3	3	3	4	4	1	1
2	2	3	3	3	3	4	4	1	1
2	2	3	3	2	2	4	4	1	1
2	2	3	3	2	2	4	4	1	1
3	3	3	3	3	3	4	4	1	1
2	2	3	3	3	3	4	4	1	1
2	2	3	3	2	2	1	1	3	3
3	3	3	3	2	2	4	4	1	1
2	2	3	3	3	3	1	1	3	3
2	2	3	3	3	3	4	4	1	1
2	2	3	3	2	2	1	1	3	3
2	2	2	2	2	2	4	4	1	1
3	3	3	3	2	2	1	1	3	3
2	2	3	3	2	2	4	4	1	1
2	2	3	3	2	2	4	4	1	1
3	3	3	3	3	3	4	4	1	1
2	2	3	3	2	2	1	1	3	3
3	3	3	3	2	2	4	4	3	3
2	2	3	3	3	3	4	4	1	1
3	3	3	3	2	2	1	1	3	3
2	2	3	3	2	2	4	4	1	1
2	2	3	3	2	2	4	4	1	1
2	2	3	3	3	3	4	4	1	1
2	2	3	3	3	3	4	4	1	1
2	2	3	3	3	3	4	4	1	1
3	3	3	3	2	2	4	4	1	1
3	3	3	3	2	2	4	4	1	1
2	2	3	3	2	2	1	1	3	3
3	3	3	3	2	2	1	1	3	3
2	2	3	3	2	2	4	4	1	1
2	2	3	3	3	3	4	4	1	1
3	3	3	3	2	2	4	4	1	1
2	2	2	2	2	2	4	4	1	1
2	2	3	3	2	2	4	4	1	1
2	2	3	3	2	2	4	4	1	1

For Review Only

2	2	3	3	2	2	1	1	3	3
2	2	3	3	2	2	4	4	1	1
2	2	3	3	2	2	4	4	1	1
3	3	3	3	2	2	4	4	1	1
2	2	3	3	3	3	4	4	1	1
2	2	3	3	2	2	4	4	1	1
2	2	3	3	3	3	4	4	1	1
3	3	3	3	2	2	4	4	1	1
3	3	3	3	3	3	4	4	3	3
2	2	2	2	2	2	4	4	1	1
2	2	3	3	2	2	4	4	1	1
2	2	3	3	2	2	4	4	1	1
3	3	3	3	3	3	4	4	1	1
2	2	3	3	3	3	4	4	1	1
2	2	3	3	2	2	4	4	1	1
2	2	3	3	2	2	4	4	3	3
2	2	3	3	2	2	4	4	1	1
2	2	3	3	2	2	4	4	1	1
3	3	3	3	2	2	4	4	1	1
2	2	3	3	2	2	4	4	1	1
2	2	3	3	2	2	4	4	3	3
2	2	3	3	2	2	4	4	1	1
2	2	3	3	3	3	4	4	1	1
3	3	3	3	2	2	4	4	1	1
2	2	3	3	2	2	4	4	3	3
2	2	2	2	2	2	4	4	1	1
2	2	3	3	2	2	4	4	1	1
2	2	3	3	2	2	4	4	1	1
2	2	3	3	3	3	4	4	1	1
2	2	3	3	2	2	4	4	1	1
3	3	3	3	2	2	4	4	1	1
3	3	3	3	2	2	4	4	1	1
2	2	3	3	2	2	4	4	1	1
2	2	3	3	2	2	4	4	1	1
3	3	3	3	3	3	1	1	3	3
3	3	3	3	3	3	4	4	1	1
3	3	3	3	2	2	4	4	1	1
2	2	3	3	3	3	4	4	1	1
2	2	3	3	2	2	1	1	3	3
3	3	3	3	2	2	4	4	1	1
2	2	3	3	2	2	4	4	1	1
3	3	3	3	2	2	4	4	1	1
2	2	3	3	3	3	4	4	1	1
3	3	3	3	2	2	1	1	3	3
2	2	3	3	2	2	4	4	1	1
2	2	3	3	3	3	4	4	1	1
2	2	3	3	2	2	1	1	3	3
2	2	3	3	3	3	1	1	3	3
2	2	3	3	2	2	4	4	1	1
2	2	3	3	2	2	4	4	1	1
2	2	3	3	2	2	1	1	3	3
3	3	3	3	2	2	1	1	3	3
3	3	3	3	2	2	4	4	1	1
2	2	3	3	2	2	4	4	1	1
3	3	3	3	2	2	4	4	1	1

For Review Only

2	2	3	3	2	2	1	1	3	3
3	3	3	3	2	2	4	4	1	1
3	3	2	2	3	3	4	4	1	1
2	2	3	3	2	2	4	4	1	1
3	3	3	3	3	3	4	4	1	1
2	2	3	3	2	2	4	4	1	1
2	2	3	3	2	2	4	4	1	1
2	2	3	3	2	2	4	4	3	3
3	3	2	2	2	2	4	4	1	1
2	2	3	3	3	3	4	4	1	1
2	2	3	3	2	2	4	4	1	1
3	3	3	3	2	2	1	1	3	3
2	2	3	3	3	3	4	4	1	1
2	2	3	3	2	2	4	4	1	1
3	3	3	3	2	2	4	4	1	1
3	3	3	3	2	2	4	4	1	1
3	3	3	3	2	2	4	4	1	1
2	2	3	3	2	2	4	4	1	1
2	2	3	3	2	2	4	4	1	1
2	2	3	3	3	3	4	4	1	1
2	2	3	3	2	2	4	4	1	1
3	3	3	3	2	2	1	1	3	3
2	2	3	3	2	2	4	4	1	1
2	2	3	3	3	3	4	4	1	1
2	2	3	3	2	2	4	4	1	1
3	3	3	3	2	2	1	1	3	3
2	2	3	3	3	3	4	4	1	1
2	2	3	3	2	2	4	4	3	3
2	2	3	3	2	2	4	4	1	1
2	2	3	3	3	3	4	4	1	1
2	2	2	2	3	3	4	4	1	1
2	2	3	3	3	3	4	4	1	1
2	2	3	3	2	2	4	4	1	1
3	3	3	3	2	2	4	4	1	1
2	2	3	3	2	2	1	1	3	3
2	2	3	3	3	3	4	4	1	1
2	2	3	3	2	2	4	4	1	1
2	2	3	3	2	2	4	4	1	1
2	2	3	3	3	3	1	1	3	3
2	2	3	3	3	3	4	4	1	1
3	3	3	3	3	3	4	4	1	1
2	2	3	3	2	2	1	1	3	3
3	3	3	3	2	2	1	1	3	3
2	2	3	3	2	2	4	4	1	1
2	2	3	3	3	3	4	4	1	1
2	2	3	3	2	2	4	4	1	1
3	3	3	3	2	2	4	4	1	1
3	3	3	3	2	2	4	4	1	1
3	3	3	3	2	2	4	4	1	1
2	2	3	3	2	2	4	4	1	1
2	2	3	3	3	3	4	4	1	1
2	2	2	2	2	2	4	4	1	1
2	2	3	3	2	2	4	4	1	1
2	2	3	3	3	3	4	4	1	1
3	3	3	3	2	2	4	4	1	1

For Review Only

3	3	3	3	3	3	4	4	1	1
2	2	3	3	2	2	4	4	1	1
2	2	3	3	3	3	4	4	1	1
2	2	3	3	2	2	4	4	1	1
3	3	3	3	2	2	1	1	3	3
2	2	3	3	2	2	4	4	3	3
2	2	3	3	2	2	4	4	1	1
3	3	3	3	2	2	4	4	1	1
3	3	3	3	2	2	4	4	3	3
3	3	3	3	2	2	4	4	1	1
2	2	3	3	2	2	4	4	1	1
2	2	2	2	2	2	4	4	1	1
2	2	3	3	3	3	4	4	1	1
2	2	3	3	2	2	4	4	1	1
3	3	3	3	3	3	4	4	1	1
2	2	3	3	2	2	4	4	1	1
3	3	3	3	2	2	4	4	1	1
2	2	3	3	2	2	4	4	1	1
3	3	3	3	2	2	4	4	1	1
3	3	3	3	2	2	4	4	1	1
2	2	3	3	2	2	4	4	1	1
2	2	3	3	2	2	1	1	3	3
2	2	2	2	2	2	4	4	1	1
2	2	3	3	2	2	4	4	1	1
3	3	2	2	2	2	4	4	1	1
3	3	3	3	3	3	4	4	1	1
3	3	3	3	2	2	1	1	3	3
2	2	3	3	3	3	4	4	1	1
2	2	3	3	2	2	4	4	1	1
2	2	3	3	3	3	4	4	1	1
3	3	3	3	2	2	4	4	1	1
3	3	3	3	2	2	1	1	3	3
2	2	3	3	2	2	4	4	1	1
2	2	3	3	2	2	1	1	3	3
2	2	3	3	2	2	4	4	1	1
3	3	3	3	2	2	4	4	1	1
2	2	2	2	3	3	4	4	1	1
2	2	3	3	3	3	4	4	1	1
3	3	2	2	2	2	4	4	1	1
3	3	3	3	2	2	1	1	3	3
2	2	3	3	2	2	4	4	1	1
2	2	3	3	2	2	4	4	1	1
2	2	3	3	2	2	4	4	1	1
2	2	3	3	2	2	4	4	1	1
2	2	3	3	3	3	4	4	1	1
2	2	3	3	2	2	4	4	1	1
3	3	3	3	3	3	1	1	3	3
2	2	3	3	2	2	4	4	1	1
2	2	3	3	2	2	1	1	3	3
3	3	3	3	2	2	4	4	1	1
2	2	3	3	3	3	4	4	1	1
3	3	3	3	2	2	1	1	3	3
3	3	3	3	3	3	4	4	1	1
2	2	3	3	3	3	4	4	1	1
2	2	3	3	2	2	4	4	1	1

For Review Only

2	2	3	3	2	2	4	4	1	1
2	2	3	3	3	3	4	4	1	1

For Review Only

SNP2559	SNP2559	SNP256	SNP256	SNP2560	SNP2560	SNP2561	SNP2561	SNP2562	SNP2562
2	2	4	4	1	1	3	3	2	2
1	1	4	4	1	1	3	3	2	2
1	1	4	4	1	1	3	3	2	2
1	1	4	4	1	1	3	3	2	2
2	2	4	4	1	1	2	2	2	2
1	1	4	4	1	1	3	3	2	2
1	1	4	4	1	1	3	3	2	2
2	2	1	1	1	1	3	3	2	2
2	2	4	4	1	1	3	3	2	2
1	1	1	1	1	1	2	2	2	2
2	2	4	4	3	3	3	3	2	2
1	1	4	4	1	1	3	3	2	2
1	1	4	4	1	1	3	3	2	2
2	2	4	4	1	1	2	2	2	2
1	1	4	4	1	1	3	3	2	2
2	2	4	4	3	3	3	3	2	2
2	2	4	4	1	1	3	3	2	2
1	1	4	4	1	1	3	3	2	2
1	1	4	4	1	1	3	3	2	2
1	1	4	4	1	1	2	2	2	2
2	2	1	1	1	1	3	3	2	2
2	2	4	4	1	1	3	3	2	2
2	2	4	4	1	1	2	2	2	2
2	2	4	4	1	1	3	3	2	2
2	2	4	4	1	1	3	3	2	2
1	1	4	4	1	1	3	3	2	2
1	1	4	4	3	3	3	3	2	2
1	1	4	4	1	1	3	3	2	2
2	2	4	4	1	1	3	3	2	2
2	2	1	1	1	1	2	2	2	2
1	1	4	4	1	1	2	2	2	2
1	1	1	1	3	3	3	3	2	2
2	2	4	4	3	3	3	3	2	2
1	1	4	4	1	1	2	2	2	2
1	1	4	4	1	1	3	3	2	2
2	2	4	4	1	1	2	2	2	2
2	2	4	4	1	1	3	3	2	2
2	2	4	4	1	1	3	3	2	2
2	2	4	4	1	1	2	2	2	2
2	2	4	4	1	1	3	3	3	3
2	2	1	1	1	1	2	2	2	2
2	2	4	4	1	1	3	3	2	2
1	1	4	4	1	1	3	3	2	2
2	2	4	4	1	1	3	3	2	2
1	1	4	4	1	1	2	2	2	2
1	1	4	4	1	1	3	3	2	2
1	1	4	4	1	1	3	3	2	2
1	1	4	4	3	3	3	3	3	3
2	2	4	4	1	1	3	3	2	2
1	1	4	4	1	1	3	3	2	2
1	1	4	4	1	1	3	3	2	2
1	1	4	4	1	1	2	2	2	2

For Review Only

2	2	4	4	1	1	3	3	2	2
2	2	4	4	3	3	3	3	2	2
1	1	4	4	1	1	3	3	2	2
2	2	4	4	1	1	3	3	2	2
1	1	4	4	1	1	3	3	2	2
1	1	4	4	1	1	3	3	2	2
1	1	4	4	1	1	3	3	2	2
2	2	4	4	1	1	2	2	2	2
2	2	4	4	1	1	3	3	2	2
1	1	4	4	1	1	3	3	2	2
1	1	4	4	1	1	2	2	2	2
1	1	4	4	1	1	2	2	2	2
2	2	4	4	1	1	3	3	2	2
2	2	4	4	1	1	3	3	2	2
1	1	4	4	1	1	3	3	2	2
2	2	4	4	1	1	3	3	2	2
1	1	4	4	3	3	3	3	2	2
1	1	4	4	1	1	3	3	2	2
2	2	4	4	1	1	2	2	2	2
2	2	4	4	1	1	3	3	2	2
1	1	4	4	1	1	2	2	2	2
1	1	4	4	1	1	3	3	3	3
2	2	4	4	1	1	3	3	2	2
2	2	4	4	1	1	3	3	2	2
1	1	4	4	1	1	2	2	2	2
1	1	4	4	1	1	3	3	2	2
1	1	1	1	1	1	3	3	2	2
2	2	4	4	1	1	2	2	2	2
1	1	4	4	1	1	3	3	2	2
1	1	4	4	3	3	3	3	2	2
2	2	4	4	1	1	3	3	2	2
2	2	4	4	1	1	3	3	2	2
2	2	4	4	1	1	2	2	2	2
2	2	4	4	1	1	3	3	2	2
2	2	4	4	1	1	3	3	2	2
1	1	4	4	1	1	2	2	2	2
2	2	1	1	1	1	3	3	2	2
2	2	4	4	1	1	3	3	2	2
1	1	4	4	3	3	3	3	2	2
2	2	4	4	1	1	3	3	2	2
2	2	4	4	1	1	3	3	2	2
2	2	4	4	3	3	3	3	2	2
1	1	1	1	1	1	2	2	2	2
1	1	4	4	1	1	2	2	2	2
1	1	4	4	3	3	3	3	2	2
2	2	4	4	1	1	3	3	2	2
2	2	4	4	1	1	2	2	2	2
2	2	4	4	1	1	2	2	2	2
1	1	4	4	1	1	3	3	2	2
1	1	4	4	1	1	3	3	2	2
1	1	4	4	1	1	3	3	2	2
2	2	4	4	1	1	2	2	2	2
2	2	4	4	1	1	2	2	2	2
1	1	4	4	1	1	3	3	2	2
2	2	4	4	1	1	3	3	2	2

For Review Only

1	1	4	4	1	1	3	3	2	2
2	2	4	4	3	3	3	3	3	3
1	1	4	4	1	1	3	3	2	2
2	2	4	4	1	1	2	2	2	2
1	1	4	4	1	1	3	3	2	2
1	1	4	4	1	1	3	3	2	2
1	1	4	4	3	3	3	3	2	2
1	1	4	4	1	1	3	3	2	2
1	1	4	4	1	1	3	3	3	3
2	2	4	4	1	1	3	3	2	2
2	2	4	4	1	1	3	3	2	2
1	1	4	4	1	1	3	3	2	2
1	1	4	4	1	1	2	2	2	2
1	1	4	4	1	1	3	3	2	2
1	1	4	4	1	1	2	2	2	2
1	1	4	4	1	1	3	3	2	2
2	2	4	4	1	1	3	3	2	2
1	1	4	4	1	1	3	3	2	2
2	2	4	4	1	1	2	2	2	2
2	2	4	4	1	1	3	3	2	2
1	1	4	4	1	1	3	3	2	2
1	1	4	4	3	3	3	3	2	2
1	1	4	4	1	1	3	3	2	2
2	2	4	4	1	1	2	2	2	2
1	1	4	4	1	1	3	3	2	2
2	2	4	4	1	1	3	3	3	3
2	2	4	4	1	1	3	3	2	2
2	2	4	4	1	1	3	3	2	2
1	1	4	4	1	1	3	3	2	2
2	2	4	4	1	1	3	3	2	2
2	2	4	4	1	1	2	2	2	2
2	2	4	4	1	1	3	3	2	2
2	2	4	4	1	1	3	3	2	2
1	1	4	4	1	1	3	3	2	2
1	1	4	4	1	1	2	2	2	2
1	1	4	4	1	1	2	2	2	2
2	2	4	4	1	1	2	2	2	2
1	1	4	4	1	1	2	2	2	2
1	1	4	4	1	1	3	3	2	2
2	2	4	4	3	3	3	3	2	2
2	2	4	4	1	1	3	3	3	3
2	2	4	4	1	1	2	2	2	2
1	1	4	4	1	1	2	2	2	2
2	2	4	4	1	1	3	3	2	2
2	2	4	4	1	1	3	3	2	2
1	1	4	4	1	1	3	3	2	2
1	1	4	4	1	1	3	3	2	2
1	1	4	4	1	1	3	3	2	2
2	2	4	4	1	1	3	3	2	2
1	1	4	4	1	1	3	3	2	2
1	1	4	4	1	1	3	3	2	2
2	2	4	4	1	1	3	3	2	2
1	1	4	4	1	1	3	3	2	2
1	1	4	4	1	1	3	3	2	2
1	1	4	4	1	1	3	3	2	2

For Review Only

1	1	4	4	1	1	3	3	2	2
2	2	1	1	1	1	3	3	2	2
1	1	4	4	1	1	3	3	2	2
2	2	4	4	1	1	3	3	2	2
1	1	4	4	1	1	3	3	2	2
2	2	4	4	1	1	3	3	2	2
1	1	4	4	1	1	3	3	3	3
1	1	4	4	1	1	3	3	2	2
1	1	4	4	3	3	3	3	2	2
1	1	4	4	3	3	3	3	2	2
1	1	4	4	1	1	3	3	2	2
2	2	4	4	1	1	3	3	2	2
1	1	4	4	1	1	3	3	2	2
1	1	4	4	1	1	3	3	2	2
2	2	4	4	1	1	2	2	2	2
2	2	4	4	3	3	3	3	2	2
1	1	4	4	1	1	3	3	2	2
2	2	4	4	1	1	3	3	2	2
2	2	4	4	1	1	3	3	2	2
1	1	4	4	3	3	3	3	2	2
1	1	4	4	1	1	3	3	2	2
2	2	4	4	1	1	2	2	3	3
2	2	4	4	1	1	3	3	2	2
1	1	4	4	1	1	2	2	2	2
2	2	4	4	1	1	3	3	2	2
2	2	4	4	1	1	2	2	2	2
1	1	4	4	1	1	3	3	2	2
2	2	4	4	1	1	3	3	2	2
2	2	4	4	1	1	3	3	2	2
1	1	4	4	1	1	3	3	2	2
1	1	4	4	1	1	3	3	2	2
1	1	4	4	3	3	3	3	2	2
2	2	4	4	1	1	3	3	2	2
2	2	4	4	1	1	2	2	2	2
2	2	4	4	1	1	2	2	2	2
1	1	4	4	1	1	2	2	2	2
2	2	4	4	1	1	3	3	2	2
2	2	4	4	1	1	3	3	2	2
1	1	4	4	1	1	3	3	2	2
1	1	4	4	1	1	3	3	2	2
1	1	4	4	1	1	3	3	2	2
1	1	4	4	1	1	3	3	2	2
2	2	4	4	1	1	2	2	2	2
1	1	4	4	1	1	3	3	2	2
1	1	4	4	1	1	2	2	2	2
2	2	4	4	1	1	3	3	2	2
2	2	4	4	3	3	3	3	2	2
2	2	4	4	1	1	2	2	3	3
2	2	4	4	1	1	2	2	2	2
2	2	4	4	1	1	3	3	2	2
1	1	4	4	1	1	3	3	2	2
2	2	4	4	1	1	2	2	2	2
2	2	4	4	1	1	3	3	2	2
1	1	4	4	1	1	2	2	2	2
2	2	4	4	1	1	2	2	2	2

For Review Only

1	1	4	4	1	1	2	2	2	2
2	2	4	4	1	1	2	2	2	2
1	1	4	4	1	1	3	3	2	2
1	1	4	4	1	1	3	3	2	2
2	2	4	4	1	1	3	3	2	2
1	1	4	4	1	1	3	3	2	2
2	2	4	4	1	1	3	3	2	2
2	2	1	1	1	1	2	2	2	2
2	2	4	4	1	1	2	2	2	2
2	2	4	4	1	1	2	2	2	2
1	1	4	4	1	1	3	3	2	2
2	2	4	4	1	1	3	3	2	2
1	1	4	4	3	3	3	3	2	2
2	2	4	4	1	1	3	3	2	2
1	1	4	4	1	1	3	3	2	2
2	2	4	4	3	3	3	3	2	2
2	2	4	4	1	1	2	2	3	3
2	2	4	4	1	1	3	3	2	2
2	2	4	4	1	1	3	3	2	2
2	2	4	4	1	1	2	2	2	2
2	2	4	4	1	1	2	2	2	2
1	1	4	4	1	1	3	3	2	2
2	2	4	4	1	1	3	3	2	2
2	2	4	4	1	1	3	3	3	3
2	2	4	4	1	1	2	2	2	2
1	1	4	4	1	1	3	3	2	2
2	2	4	4	1	1	2	2	2	2
1	1	4	4	1	1	3	3	2	2
2	2	4	4	1	1	3	3	2	2
1	1	4	4	1	1	3	3	2	2
2	2	4	4	1	1	2	2	3	3
2	2	4	4	3	3	3	3	2	2
2	2	4	4	3	3	3	3	2	2
1	1	4	4	1	1	3	3	2	2
2	2	4	4	1	1	3	3	2	2
2	2	4	4	1	1	2	2	2	2
1	1	4	4	1	1	2	2	2	2
1	1	4	4	1	1	2	2	2	2
2	2	4	4	1	1	2	2	2	2
2	2	4	4	1	1	2	2	3	3
1	1	4	4	1	1	2	2	2	2
2	2	4	4	1	1	2	2	2	2
1	1	4	4	1	1	3	3	3	3
2	2	4	4	1	1	3	3	2	2
1	1	4	4	1	1	3	3	3	3
2	2	4	4	3	3	3	3	2	2
1	1	4	4	1	1	2	2	2	2
1	1	4	4	1	1	2	2	2	2
2	2	4	4	1	1	3	3	2	2
2	2	4	4	1	1	2	2	2	2
1	1	4	4	1	1	3	3	2	2
2	2	4	4	1	1	2	2	3	3
1	1	4	4	1	1	3	3	2	2
1	1	4	4	1	1	3	3	2	2
2	2	4	4	1	1	2	2	3	3

For Review Only

2	2	4	4	3	3	3	3	2	2
1	1	4	4	3	3	3	3	2	2

For Review Only

SNP2563	SNP2563	SNP2564	SNP2564	SNP2565	SNP2565	SNP2566	SNP2566	SNP2567	SNP2567
3	3	3	3	4	4	1	1	4	4
3	3	3	3	4	4	4	4	4	4
3	3	2	2	4	4	4	4	4	4
3	3	3	3	4	4	1	1	4	4
3	3	3	3	4	4	1	1	4	4
3	3	3	3	4	4	4	4	4	4
3	3	3	3	4	4	1	1	4	4
3	3	3	3	4	4	1	1	4	4
3	3	3	3	4	4	1	1	4	4
3	3	3	3	4	4	4	4	4	4
3	3	3	3	4	4	1	1	4	4
3	3	3	3	4	4	4	4	4	4
3	3	3	3	4	4	1	1	4	4
3	3	3	3	4	4	1	1	4	4
1	1	2	2	1	1	1	1	4	4
3	3	3	3	4	4	1	1	2	2
1	1	3	3	4	4	4	4	4	4
3	3	3	3	4	4	4	4	4	4
3	3	3	3	4	4	4	4	4	4
1	1	3	3	4	4	4	4	4	4
3	3	3	3	4	4	4	4	4	4
1	1	3	3	4	4	4	4	4	4
3	3	3	3	4	4	1	1	4	4
3	3	3	3	4	4	1	1	4	4
3	3	3	3	4	4	4	4	4	4
3	3	2	2	1	1	1	1	4	4
3	3	3	3	4	4	4	4	2	2
3	3	3	3	4	4	1	1	4	4
3	3	3	3	4	4	1	1	4	4
3	3	3	3	4	4	1	1	4	4
3	3	3	3	4	4	4	4	4	4
3	3	3	3	4	4	4	4	2	2
3	3	3	3	4	4	1	1	2	2
3	3	3	3	4	4	4	4	4	4
1	1	2	2	1	1	1	1	4	4
3	3	3	3	4	4	1	1	4	4
1	1	3	3	4	4	1	1	4	4
3	3	3	3	4	4	1	1	4	4
3	3	3	3	4	4	1	1	4	4
3	3	3	3	4	4	1	1	4	4
3	3	2	2	4	4	1	1	4	4
3	3	3	3	4	4	1	1	4	4
3	3	3	3	4	4	4	4	4	4
1	1	3	3	4	4	4	4	4	4
3	3	3	3	4	4	1	1	4	4
1	1	3	3	4	4	4	4	4	4
1	1	2	2	1	1	1	1	4	4
3	3	2	2	1	1	1	1	4	4
3	3	3	3	4	4	1	1	4	4
3	3	3	3	4	4	4	4	4	4
1	1	2	2	1	1	1	1	4	4
1	1	3	3	4	4	4	4	4	4

For Review Only

For Review Only

1	1	3	3	4	4	4	4	4	4
3	3	3	3	4	4	1	1	2	2
3	3	3	3	4	4	4	4	4	4
1	1	3	3	4	4	4	4	4	4
3	3	3	3	4	4	4	4	4	4
1	1	2	2	1	1	1	1	4	4
1	1	3	3	4	4	4	4	4	4
3	3	3	3	4	4	1	1	4	4
1	1	3	3	4	4	1	1	4	4
3	3	3	3	4	4	1	1	4	4
1	1	3	3	4	4	4	4	2	2
1	1	3	3	4	4	4	4	2	2
1	1	3	3	4	4	4	4	4	4
3	3	3	3	4	4	1	1	4	4
3	3	3	3	4	4	1	1	4	4
3	3	3	3	4	4	1	1	4	4
1	1	3	3	4	4	4	4	4	4
3	3	3	3	4	4	4	4	4	4
1	1	3	3	4	4	4	4	4	4
3	3	3	3	4	4	1	1	4	4
1	1	3	3	4	4	4	4	4	4
3	3	3	3	4	4	1	1	4	4
3	3	3	3	4	4	1	1	4	4
3	3	3	3	4	4	1	1	4	4
3	3	3	3	4	4	1	1	4	4
3	3	3	3	4	4	4	4	4	4
1	1	3	3	4	4	1	1	4	4
3	3	3	3	4	4	1	1	4	4
1	1	3	3	4	4	4	4	4	4
3	3	3	3	4	4	4	4	4	4
3	3	3	3	4	4	1	1	4	4
3	3	3	3	4	4	1	1	4	4
3	3	3	3	4	4	1	1	4	4
1	1	3	3	4	4	4	4	4	4
3	3	3	3	4	4	1	1	4	4
3	3	3	3	4	4	1	1	4	4
3	3	3	3	4	4	1	1	4	4
3	3	3	3	4	4	4	4	4	4
1	1	3	3	4	4	4	4	4	4
3	3	3	3	4	4	1	1	4	4
3	3	3	3	4	4	4	4	4	4
3	3	3	3	4	4	1	1	4	4
1	1	3	3	4	4	4	4	4	4
1	1	3	3	4	4	4	4	2	2
1	1	2	2	1	1	1	1	4	4
3	3	3	3	4	4	1	1	4	4
3	3	3	3	4	4	1	1	4	4
1	1	3	3	4	4	1	1	4	4
3	3	2	2	1	1	1	1	4	4
1	1	3	3	4	4	1	1	4	4
3	3	3	3	4	4	4	4	4	4
3	3	3	3	4	4	1	1	4	4
3	3	3	3	4	4	1	1	4	4
3	3	3	3	4	4	1	1	4	4
1	1	3	3	4	4	1	1	4	4

3	3	2	2	1	1	1	1	4	4
3	3	3	3	4	4	1	1	4	4
1	1	2	2	1	1	1	1	4	4
3	3	3	3	4	4	1	1	4	4
3	3	3	3	4	4	4	4	4	4
1	1	2	2	1	1	1	1	4	4
1	1	3	3	4	4	4	4	2	2
3	3	3	3	4	4	4	4	4	4
3	3	3	3	4	4	4	4	4	4
3	3	3	3	4	4	1	1	4	4
3	3	3	3	4	4	1	1	4	4
3	3	3	3	4	4	1	1	4	4
3	3	3	3	4	4	4	4	4	4
1	1	3	3	4	4	1	1	4	4
3	3	3	3	4	4	1	1	4	4
3	3	2	2	1	1	1	1	4	4
1	1	3	3	4	4	1	1	4	4
3	3	3	3	4	4	1	1	4	4
3	3	3	3	4	4	1	1	4	4
3	3	3	3	4	4	4	4	4	4
3	3	3	3	4	4	1	1	4	4
3	3	3	3	4	4	4	4	4	4
3	3	3	3	4	4	4	4	4	4
3	3	3	3	4	4	1	1	4	4
3	3	2	2	1	1	1	1	4	4
3	3	3	3	4	4	1	1	4	4
1	1	2	2	4	4	1	1	4	4
3	3	3	3	4	4	1	1	4	4
1	1	3	3	4	4	4	4	4	4
1	1	3	3	4	4	1	1	4	4
3	3	3	3	4	4	1	1	4	4
3	3	3	3	4	4	1	1	4	4
1	1	3	3	4	4	4	4	4	4
3	3	2	2	1	1	1	1	4	4
3	3	2	2	4	4	4	4	4	4
3	3	3	3	4	4	4	4	4	4
3	3	3	3	4	4	1	1	4	4
3	3	3	3	4	4	4	4	4	4
3	3	2	2	4	4	1	1	4	4
3	3	3	3	4	4	1	1	4	4
3	3	3	3	4	4	1	1	4	4
3	3	3	3	4	4	1	1	4	4
3	3	3	3	4	4	4	4	4	4
3	3	3	3	4	4	1	1	4	4
3	3	2	2	1	1	1	1	4	4
1	1	2	2	1	1	1	1	4	4
1	1	2	2	1	1	1	1	4	4
1	1	3	3	4	4	4	4	4	4
3	3	3	3	4	4	1	1	4	4
1	1	2	2	1	1	1	1	4	4
1	1	2	2	1	1	1	1	4	4
3	3	3	3	4	4	1	1	4	4
3	3	3	3	4	4	1	1	4	4
1	1	3	3	4	4	4	4	4	4
3	3	3	3	4	4	1	1	4	4

For Review Only

1	1	2	2	1	1	1	1	4	4
3	3	3	3	4	4	1	1	4	4
3	3	3	3	4	4	1	1	4	4
3	3	3	3	4	4	1	1	4	4
3	3	3	3	4	4	4	4	4	4
3	3	3	3	4	4	1	1	4	4
3	3	2	2	1	1	1	1	4	4
1	1	3	3	4	4	1	1	4	4
3	3	3	3	4	4	4	4	4	4
1	1	3	3	4	4	4	4	4	4
3	3	2	2	1	1	1	1	4	4
1	1	3	3	4	4	1	1	4	4
1	1	3	3	4	4	4	4	4	4
1	1	2	2	1	1	1	1	4	4
3	3	3	3	4	4	1	1	4	4
3	3	3	3	4	4	1	1	4	4
3	3	3	3	4	4	1	1	4	4
3	3	3	3	4	4	1	1	4	4
1	1	3	3	4	4	1	1	4	4
1	1	3	3	4	4	4	4	2	2
3	3	3	3	4	4	1	1	4	4
3	3	3	3	4	4	1	1	4	4
1	1	3	3	4	4	1	1	4	4
1	1	3	3	4	4	4	4	2	2
3	3	3	3	4	4	1	1	4	4
3	3	3	3	4	4	1	1	4	4
1	1	3	3	4	4	4	4	4	4
3	3	3	3	4	4	1	1	4	4
1	1	3	3	4	4	1	1	4	4
1	1	3	3	4	4	4	4	4	4
3	3	3	3	4	4	4	4	4	4
1	1	3	3	4	4	4	4	4	4
3	3	3	3	4	4	1	1	4	4
3	3	3	3	4	4	1	1	4	4
1	1	3	3	4	4	1	1	4	4
1	1	3	3	4	4	4	4	2	2
1	1	3	3	4	4	1	1	4	4
1	1	3	3	4	4	1	1	4	4
1	1	3	3	1	1	4	4	4	4
3	3	3	3	4	4	4	4	4	4
3	3	3	3	4	4	4	4	4	4
1	1	2	2	1	1	1	1	4	4
3	3	3	3	4	4	1	1	4	4
1	1	2	2	1	1	1	1	4	4
3	3	3	3	4	4	4	4	4	4
3	3	3	3	4	4	1	1	4	4
3	3	3	3	4	4	1	1	4	4
3	3	3	3	4	4	4	4	4	4
3	3	3	3	4	4	1	1	4	4
3	3	3	3	4	4	1	1	4	4
1	1	3	3	4	4	4	4	4	4
3	3	3	3	4	4	1	1	4	4
1	1	3	3	4	4	1	1	4	4
1	1	3	3	4	4	4	4	2	2
3	3	3	3	4	4	1	1	4	4

For Review Only

3	3	3	3	4	4	4	4	4	4
1	1	3	3	4	4	1	1	2	2
1	1	3	3	4	4	4	4	4	4
3	3	3	3	4	4	4	4	4	4
3	3	3	3	4	4	1	1	4	4
3	3	2	2	1	1	1	1	4	4
1	1	3	3	4	4	1	1	4	4
3	3	3	3	4	4	1	1	4	4
3	3	3	3	4	4	1	1	4	4
3	3	3	3	4	4	1	1	4	4
3	3	3	3	4	4	1	1	4	4
3	3	3	3	4	4	1	1	4	4
1	1	3	3	4	4	4	4	4	4
1	1	3	3	4	4	1	1	4	4
3	3	3	3	4	4	4	4	4	4
1	1	3	3	4	4	1	1	4	4
1	1	3	3	4	4	1	1	4	4
1	1	3	3	4	4	1	1	4	4
3	3	3	3	4	4	1	1	4	4
3	3	3	3	4	4	1	1	4	4
3	3	3	3	4	4	1	1	4	4
1	1	2	2	1	1	1	1	4	4
3	3	3	3	4	4	1	1	4	4
1	1	3	3	4	4	1	1	4	4
3	3	3	3	4	4	1	1	4	4
3	3	3	3	4	4	4	4	4	4
3	3	3	3	4	4	1	1	4	4
3	3	3	3	4	4	4	4	4	4
3	3	3	3	4	4	1	1	4	4
1	1	3	3	4	4	4	4	4	4
3	3	3	3	4	4	1	1	4	4
3	3	3	3	4	4	1	1	4	4
1	1	3	3	4	4	1	1	4	4
1	1	2	2	1	1	1	1	4	4
1	1	3	3	4	4	1	1	4	4
3	3	3	3	4	4	1	1	4	4
1	1	3	3	4	4	4	4	4	4
1	1	3	3	4	4	4	4	2	2
3	3	3	3	4	4	1	1	4	4
3	3	3	3	4	4	1	1	4	4
1	1	2	2	1	1	1	1	4	4
1	1	3	3	4	4	1	1	4	4
1	1	2	2	1	1	1	1	4	4
1	1	3	3	4	4	4	4	4	4
3	3	3	3	4	4	4	4	4	4
1	1	3	3	4	4	1	1	4	4
3	3	3	3	4	4	4	4	4	4
1	1	2	2	1	1	1	1	4	4
3	3	3	3	4	4	1	1	4	4
3	3	3	3	4	4	1	1	4	4
1	1	3	3	4	4	4	4	4	4
3	3	3	3	4	4	1	1	4	4
3	3	3	3	4	4	4	4	4	4
1	1	3	3	4	4	4	4	4	4
1	1	3	3	4	4	1	1	4	4

For Review Only

3	3	3	3	4	4	1	1	4	4
3	3	3	3	4	4	4	4	4	4

For Review Only

SNP2568	SNP2568	SNP2569	SNP2569	SNP257	SNP257	SNP2570	SNP2570	SNP2571	SNP2571
1	1	4	4	4	4	3	3	4	4
2	2	4	4	4	4	3	3	4	4
2	2	4	4	4	4	3	3	4	4
1	1	1	1	4	4	1	1	4	4
2	2	4	4	4	4	3	3	4	4
2	2	1	1	2	2	1	1	4	4
1	1	4	4	4	4	3	3	4	4
2	2	1	1	4	4	1	1	4	4
2	2	4	4	4	4	3	3	4	4
2	2	1	1	4	4	1	1	4	4
2	2	1	1	4	4	1	1	4	4
2	2	4	4	4	4	3	3	4	4
2	2	4	4	4	4	3	3	4	4
2	2	1	1	4	4	1	1	4	4
2	2	4	4	4	4	3	3	4	4
2	2	1	1	4	4	1	1	4	4
2	2	1	1	4	4	1	1	4	4
1	1	1	1	2	2	1	1	4	4
2	2	1	1	4	4	1	1	4	4
2	2	1	1	2	2	1	1	4	4
1	1	4	4	4	4	3	3	4	4
2	2	4	4	4	4	3	3	4	4
2	2	1	1	4	4	1	1	4	4
2	2	1	1	4	4	1	1	4	4
2	2	4	4	2	2	3	3	4	4
2	2	4	4	4	4	3	3	4	4
1	1	1	1	2	2	1	1	4	4
2	2	4	4	4	4	3	3	4	4
1	1	1	1	4	4	1	1	4	4
2	2	1	1	4	4	1	1	4	4
1	1	4	4	4	4	3	3	4	4
2	2	4	4	4	4	3	3	4	4
2	2	4	4	4	4	3	3	4	4
2	2	1	1	4	4	1	1	4	4
2	2	1	1	4	4	1	1	4	4
2	2	4	4	4	4	3	3	4	4
2	2	4	4	4	4	3	3	4	4
2	2	4	4	4	4	1	1	1	1
2	2	1	1	4	4	1	1	4	4
2	2	1	1	4	4	1	1	4	4
2	2	1	1	4	4	1	1	4	4
1	1	1	1	4	4	1	1	4	4
1	1	1	1	4	4	1	1	4	4
2	2	4	4	4	4	3	3	4	4
2	2	1	1	4	4	1	1	4	4
2	2	4	4	4	4	3	3	4	4
2	2	1	1	4	4	1	1	4	4
2	2	4	4	4	4	3	3	4	4
2	2	1	1	4	4	1	1	4	4
2	2	4	4	4	4	3	3	4	4
2	2	4	4	4	4	3	3	4	4
2	2	1	1	4	4	1	1	4	4

For Review Only

2	2	4	4	4	4	3	3	4	4
2	2	4	4	4	4	3	3	4	4
2	2	4	4	4	4	3	3	4	4
2	2	4	4	4	4	3	3	4	4
2	2	1	1	4	4	1	1	4	4
2	2	4	4	4	4	3	3	4	4
2	2	1	1	4	4	1	1	4	4
2	2	1	1	4	4	1	1	4	4
2	2	1	1	4	4	3	3	4	4
2	2	4	4	4	4	1	1	4	4
2	2	1	1	2	2	1	1	4	4
2	2	1	1	4	4	1	1	4	4
2	2	4	4	4	4	3	3	4	4
1	1	1	1	4	4	1	1	4	4
2	2	4	4	2	2	1	1	4	4
1	1	1	1	4	4	1	1	1	1
2	2	1	1	4	4	1	1	4	4
2	2	4	4	4	4	3	3	4	4
2	2	4	4	4	4	3	3	4	4
1	1	4	4	4	4	3	3	4	4
2	2	1	1	4	4	1	1	4	4
2	2	1	1	4	4	1	1	4	4
1	1	1	1	4	4	1	1	4	4
1	1	1	1	2	2	1	1	1	1
2	2	1	1	4	4	1	1	4	4
2	2	1	1	4	4	1	1	4	4
2	2	1	1	4	4	3	3	4	4
2	2	1	1	4	4	1	1	4	4
2	2	4	4	4	4	3	3	4	4
1	1	4	4	4	4	3	3	4	4
2	2	4	4	4	4	3	3	4	4
2	2	1	1	4	4	1	1	4	4
2	2	1	1	4	4	1	1	4	4
2	2	4	4	4	4	3	3	4	4
1	1	1	1	2	2	1	1	4	4
2	2	1	1	4	4	1	1	4	4
2	2	1	1	4	4	1	1	4	4
2	2	1	1	4	4	1	1	4	4
2	2	1	1	4	4	1	1	4	4
2	2	1	1	4	4	1	1	4	4
2	2	4	4	4	4	3	3	4	4
2	2	4	4	2	2	3	3	4	4
2	2	4	4	4	4	1	1	4	4
2	2	1	1	2	2	1	1	4	4
2	2	4	4	4	4	3	3	4	4
2	2	4	4	4	4	3	3	1	1
2	2	1	1	4	4	3	3	4	4
2	2	4	4	4	4	3	3	4	4
2	2	4	4	4	4	3	3	4	4
2	2	4	4	4	4	3	3	4	4
2	2	4	4	4	4	3	3	4	4
2	2	1	1	4	4	1	1	4	4
2	2	1	1	4	4	1	1	4	4
2	2	4	4	4	4	1	1	4	4
2	2	4	4	4	4	3	3	4	4

For Review Only

2	2	1	1	4	4	1	1	4	4
2	2	1	1	4	4	1	1	4	4
2	2	4	4	4	4	3	3	4	4
2	2	1	1	4	4	3	3	4	4
2	2	4	4	4	4	3	3	4	4
2	2	1	1	4	4	1	1	4	4
2	2	1	1	4	4	1	1	4	4
2	2	4	4	2	2	3	3	4	4
2	2	1	1	4	4	1	1	4	4
2	2	4	4	4	4	1	1	4	4
1	1	4	4	4	4	3	3	4	4
2	2	4	4	4	4	1	1	1	1
2	2	1	1	4	4	1	1	4	4
2	2	4	4	4	4	1	1	4	4
2	2	4	4	4	4	3	3	4	4
2	2	4	4	4	4	3	3	4	4
2	2	1	1	4	4	1	1	4	4
2	2	4	4	4	4	3	3	4	4
2	2	1	1	4	4	1	1	4	4
2	2	4	4	4	4	3	3	4	4
2	2	4	4	4	4	3	3	4	4
1	1	4	4	4	4	3	3	4	4
2	2	4	4	2	2	3	3	4	4
2	2	1	1	4	4	1	1	4	4
2	2	4	4	4	4	3	3	4	4
2	2	4	4	4	4	3	3	4	4
2	2	4	4	4	4	3	3	4	4
1	1	4	4	4	4	3	3	1	1
2	2	4	4	4	4	3	3	4	4
2	2	4	4	4	4	3	3	4	4
2	2	1	1	4	4	1	1	4	4
2	2	4	4	4	4	3	3	4	4
2	2	4	4	4	4	3	3	4	4
2	2	4	4	4	4	3	3	4	4
2	2	1	1	4	4	1	1	4	4
2	2	4	4	4	4	3	3	4	4
2	2	1	1	4	4	3	3	4	4
1	1	1	1	4	4	1	1	4	4
2	2	1	1	4	4	1	1	4	4
2	2	1	1	4	4	1	1	4	4
2	2	1	1	4	4	1	1	4	4
2	2	1	1	4	4	1	1	4	4
2	2	4	4	4	4	3	3	4	4
2	2	1	1	4	4	1	1	4	4
2	2	1	1	4	4	1	1	4	4
2	2	4	4	4	4	3	3	4	4
2	2	4	4	4	4	3	3	4	4
2	2	4	4	4	4	3	3	4	4
1	1	1	1	4	4	1	1	4	4
2	2	4	4	4	4	3	3	4	4
2	2	4	4	4	4	3	3	4	4
2	2	1	1	4	4	1	1	4	4
2	2	4	4	4	4	1	1	4	4
2	2	4	4	4	4	3	3	4	4
2	2	4	4	4	4	3	3	4	4

For Review Only

2	2	4	4	4	4	3	3	4	4
2	2	1	1	4	4	1	1	4	4
2	2	4	4	4	4	3	3	4	4
2	2	1	1	4	4	1	1	4	4
2	2	4	4	4	4	3	3	4	4
1	1	1	1	4	4	1	1	4	4
2	2	1	1	4	4	1	1	4	4
2	2	4	4	4	4	3	3	4	4
2	2	4	4	4	4	3	3	4	4
1	1	4	4	4	4	3	3	4	4
2	2	4	4	4	4	3	3	4	4
2	2	1	1	4	4	1	1	4	4
2	2	1	1	4	4	1	1	4	4
2	2	4	4	4	4	3	3	4	4
2	2	1	1	4	4	1	1	4	4
2	2	1	1	4	4	1	1	4	4
2	2	1	1	4	4	3	3	4	4
2	2	1	1	4	4	1	1	4	4
2	2	4	4	4	4	3	3	4	4
2	2	1	1	4	4	1	1	4	4
2	2	4	4	4	4	3	3	4	4
2	2	4	4	4	4	3	3	4	4
2	2	1	1	4	4	1	1	4	4
2	2	1	1	4	4	1	1	4	4
2	2	4	4	4	4	3	3	4	4
2	2	1	1	4	4	1	1	4	4
2	2	1	1	4	4	1	1	4	4
2	2	1	1	4	4	1	1	4	4
2	2	1	1	4	4	1	1	1	1
2	2	1	1	4	4	1	1	4	4
2	2	4	4	4	4	3	3	4	4
2	2	1	1	4	4	1	1	4	4
2	2	4	4	2	2	3	3	1	1
2	2	1	1	4	4	1	1	4	4
2	2	4	4	4	4	3	3	4	4
2	2	1	1	4	4	1	1	4	4
2	2	4	4	2	2	3	3	4	4
2	2	4	4	4	4	3	3	4	4
2	2	4	4	4	4	3	3	4	4
1	1	1	1	4	4	1	1	4	4
2	2	4	4	4	4	3	3	4	4
2	2	4	4	4	4	3	3	4	4
2	2	1	1	4	4	1	1	4	4
2	2	4	4	4	4	3	3	4	4
1	1	4	4	2	2	1	1	4	4
2	2	1	1	4	4	1	1	4	4
2	2	1	1	4	4	1	1	4	4
2	2	1	1	4	4	1	1	4	4
2	2	1	1	4	4	1	1	4	4
1	1	1	1	4	4	1	1	4	4
2	2	4	4	4	4	3	3	4	4
2	2	1	1	4	4	3	3	4	4
2	2	4	4	4	4	1	1	4	4
2	2	1	1	2	2	1	1	4	4
2	2	1	1	4	4	1	1	4	4

2	2	4	4	4	4	3	3	4	4
2	2	1	1	4	4	1	1	4	4
2	2	4	4	4	4	3	3	4	4
2	2	4	4	4	4	3	3	4	4
2	2	1	1	2	2	1	1	1	1
1	1	4	4	4	4	3	3	1	1
2	2	4	4	2	2	3	3	4	4
2	2	1	1	4	4	1	1	4	4
2	2	1	1	4	4	1	1	4	4
2	2	1	1	4	4	1	1	4	4
2	2	4	4	4	4	3	3	4	4
2	2	4	4	4	4	3	3	4	4
2	2	1	1	4	4	1	1	4	4
2	2	4	4	4	4	3	3	4	4
2	2	4	4	4	4	3	3	4	4
2	2	1	1	4	4	1	1	4	4
2	2	1	1	4	4	1	1	4	4
2	2	1	1	4	4	1	1	4	4
2	2	1	1	4	4	1	1	4	4
2	2	1	1	4	4	1	1	1	1
1	1	1	1	2	2	1	1	4	4
2	2	4	4	4	4	3	3	4	4
1	1	4	4	4	4	3	3	4	4
2	2	1	1	4	4	1	1	4	4
2	2	1	1	4	4	1	1	4	4
2	2	4	4	4	4	3	3	4	4
2	2	1	1	4	4	1	1	4	4
2	2	1	1	4	4	1	1	4	4
2	2	4	4	4	4	3	3	4	4
2	2	4	4	4	4	3	3	4	4
2	2	1	1	4	4	1	1	4	4
2	2	4	4	4	4	3	3	4	4
2	2	1	1	4	4	3	3	4	4
2	2	4	4	4	4	3	3	4	4
2	2	1	1	4	4	1	1	4	4
2	2	1	1	4	4	1	1	4	4
2	2	4	4	4	4	3	3	4	4
2	2	1	1	4	4	1	1	4	4
2	2	4	4	4	4	3	3	4	4
2	2	1	1	4	4	1	1	4	4
2	2	4	4	4	4	3	3	4	4
2	2	4	4	4	4	3	3	4	4
2	2	1	1	4	4	1	1	4	4
2	2	4	4	4	4	3	3	4	4
2	2	1	1	4	4	1	1	4	4
2	2	1	1	4	4	1	1	4	4
2	2	4	4	4	4	3	3	4	4
2	2	1	1	4	4	1	1	4	4
2	2	4	4	4	4	3	3	4	4
2	2	1	1	4	4	1	1	4	4
2	2	4	4	4	4	3	3	4	4
2	2	1	1	4	4	1	1	4	4
2	2	4	4	4	4	3	3	4	4
2	2	1	1	4	4	1	1	4	4
2	2	1	1	4	4	1	1	4	4

For Review Only

1	1	4	4	4	4	3	3	4	4
2	2	1	1	4	4	1	1	4	4

For Review Only

SNP2572	SNP2572	SNP2573	SNP2573	SNP2574	SNP2574	SNP2575	SNP2575	SNP2576	SNP2576
4	4	3	3	1	1	1	1	4	4
4	4	3	3	1	1	1	1	1	1
4	4	3	3	1	1	1	1	1	1
4	4	3	3	4	4	1	1	1	1
4	4	3	3	1	1	1	1	4	4
4	4	3	3	1	1	1	1	1	1
4	4	3	3	1	1	1	1	1	1
4	4	3	3	1	1	1	1	4	4
4	4	3	3	1	1	1	1	4	4
4	4	3	3	1	1	1	1	1	1
1	1	3	3	1	1	1	1	4	4
4	4	3	3	1	1	1	1	1	1
1	1	3	3	1	1	1	1	4	4
4	4	3	3	1	1	1	1	4	4
4	4	3	3	1	1	1	1	1	1
4	4	3	3	1	1	1	1	4	4
4	4	3	3	1	1	1	1	4	4
4	4	3	3	1	1	1	1	1	1
4	4	3	3	1	1	1	1	1	1
4	4	3	3	1	1	4	4	1	1
4	4	3	3	1	1	4	4	4	4
4	4	3	3	1	1	1	1	4	4
1	1	3	3	1	1	1	1	4	4
4	4	3	3	1	1	4	4	4	4
4	4	3	3	1	1	1	1	4	4
4	4	3	3	1	1	1	1	1	1
4	4	3	3	4	4	1	1	1	1
4	4	3	3	1	1	1	1	1	1
4	4	3	3	1	1	1	1	4	4
1	1	3	3	1	1	4	4	4	4
4	4	3	3	1	1	1	1	1	1
4	4	3	3	1	1	1	1	1	1
1	1	3	3	1	1	1	1	4	4
4	4	3	3	1	1	1	1	4	4
4	4	3	3	1	1	1	1	1	1
1	1	3	3	1	1	1	1	4	4
1	1	3	3	1	1	1	1	4	4
4	4	3	3	4	4	1	1	4	4
4	4	3	3	1	1	4	4	4	4
1	1	1	1	1	1	4	4	4	4
4	4	3	3	1	1	4	4	4	4
4	4	3	3	1	1	1	1	4	4
4	4	3	3	1	1	1	1	1	1
4	4	3	3	1	1	4	4	4	4
4	4	3	3	1	1	1	1	1	1
4	4	3	3	4	4	1	1	1	1
4	4	3	3	1	1	1	1	1	1
4	4	1	1	1	1	4	4	1	1
1	1	3	3	1	1	4	4	4	4
4	4	3	3	1	1	1	1	1	1
4	4	3	3	1	1	1	1	1	1
4	4	3	3	1	1	1	1	1	1

For Review Only

4	4	3	3	1	1	1	1	4	4
1	1	3	3	1	1	1	1	4	4
4	4	3	3	1	1	1	1	1	1
4	4	3	3	1	1	1	1	4	4
4	4	3	3	1	1	1	1	1	1
4	4	3	3	1	1	1	1	1	1
4	4	3	3	4	4	1	1	1	1
4	4	3	3	1	1	4	4	4	4
1	1	3	3	1	1	1	1	4	4
4	4	3	3	1	1	1	1	1	1
4	4	3	3	1	1	1	1	1	1
4	4	3	3	1	1	1	1	1	1
4	4	3	3	1	1	1	1	4	4
4	4	3	3	1	1	1	1	4	4
4	4	3	3	1	1	1	1	1	1
4	4	3	3	1	1	1	1	4	4
4	4	3	3	1	1	1	1	1	1
4	4	3	3	1	1	1	1	1	1
4	4	3	3	1	1	1	1	4	4
1	1	3	3	1	1	1	1	4	4
4	4	3	3	1	1	1	1	1	1
4	4	3	3	1	1	4	4	1	1
1	1	3	3	1	1	1	1	4	4
4	4	3	3	1	1	1	1	4	4
4	4	3	3	1	1	1	1	1	1
4	4	3	3	1	1	1	1	1	1
4	4	3	3	1	1	1	1	1	1
4	4	3	3	1	1	4	4	4	4
4	4	3	3	1	1	1	1	1	1
4	4	3	3	1	1	1	1	1	1
4	4	3	3	1	1	1	1	4	4
1	1	3	3	1	1	1	1	4	4
4	4	3	3	1	1	4	4	4	4
4	4	3	3	1	1	1	1	4	4
1	1	3	3	1	1	1	1	4	4
4	4	3	3	1	1	1	1	1	1
4	4	3	3	1	1	4	4	4	4
4	4	3	3	1	1	1	1	4	4
4	4	3	3	4	4	1	1	1	1
4	4	3	3	1	1	4	4	4	4
4	4	3	3	1	1	1	1	4	4
1	1	3	3	1	1	1	1	4	4
4	4	3	3	1	1	1	1	4	4
4	4	3	3	1	1	1	1	1	1
4	4	3	3	1	1	1	1	1	1
4	4	3	3	1	1	1	1	1	1
1	1	3	3	1	1	4	4	4	4
1	1	3	3	1	1	1	1	4	4
4	4	3	3	1	1	1	1	1	1
4	4	3	3	1	1	4	4	1	1
4	4	3	3	1	1	1	1	1	1
1	1	3	3	1	1	4	4	4	4
4	4	3	3	1	1	4	4	4	4
4	4	3	3	1	1	1	1	1	1
4	4	3	3	1	1	1	1	4	4

For Review Only

4	4	3	3	1	1	1	1	1	1
4	4	3	3	4	4	1	1	4	4
4	4	3	3	1	1	1	1	1	1
1	1	3	3	1	1	1	1	1	1
4	4	3	3	1	1	1	1	1	1
4	4	3	3	1	1	1	1	1	1
4	4	3	3	1	1	1	1	1	1
4	4	3	3	1	1	1	1	1	1
4	4	3	3	1	1	4	4	1	1
4	4	3	3	1	1	1	1	4	4
4	4	3	3	1	1	1	1	4	4
4	4	3	3	1	1	1	1	1	1
4	4	3	3	1	1	1	1	4	4
4	4	3	3	1	1	1	1	1	1
4	4	3	3	1	1	1	1	4	4
4	4	3	3	1	1	1	1	1	1
1	1	3	3	1	1	1	1	4	4
4	4	3	3	1	1	1	1	1	1
1	1	3	3	1	1	1	1	4	4
4	4	3	3	1	1	1	1	1	1
4	4	3	3	1	1	1	1	1	1
4	4	3	3	1	1	1	1	1	1
4	4	3	3	1	1	1	1	4	4
1	1	3	3	1	1	4	4	1	1
4	4	3	3	1	1	1	1	1	1
1	1	1	1	1	1	1	1	4	4
4	4	3	3	1	1	1	1	4	4
4	4	3	3	1	1	1	1	4	4
4	4	3	3	4	4	1	1	1	1
1	1	3	3	1	1	1	1	1	1
4	4	3	3	1	1	1	1	4	4
4	4	3	3	1	1	1	1	4	4
4	4	3	3	1	1	1	1	4	4
4	4	3	3	1	1	1	1	1	1
4	4	3	3	1	1	1	1	4	4
4	4	3	3	1	1	1	1	1	1
1	1	3	3	1	1	4	4	4	4
4	4	3	3	1	1	1	1	1	1
4	4	3	3	1	1	4	4	1	1
4	4	3	3	1	1	1	1	4	4
1	1	3	3	1	1	1	1	4	4
4	4	3	3	1	1	4	4	4	4
4	4	3	3	1	1	1	1	1	1
4	4	3	3	1	1	1	1	4	4
4	4	3	3	4	4	1	1	1	1
4	4	1	1	1	1	1	1	1	1
4	4	3	3	1	1	1	1	1	1
4	4	3	3	1	1	1	1	1	1
4	4	3	3	1	1	1	1	4	4
4	4	3	3	1	1	1	1	1	1
4	4	3	3	1	1	1	1	1	1
1	1	3	3	1	1	4	4	4	4
4	4	3	3	1	1	1	1	1	1
4	4	3	3	1	1	1	1	1	1
4	4	3	3	1	1	1	1	4	4

For Review Only

4	4	3	3	1	1	1	1	1	1
1	1	3	3	1	1	1	1	4	4
4	4	3	3	1	1	1	1	1	1
1	1	3	3	1	1	1	1	1	1
4	4	3	3	1	1	1	1	1	1
4	4	3	3	1	1	1	1	4	4
4	4	1	1	1	1	1	1	1	1
4	4	3	3	1	1	1	1	1	1
4	4	3	3	1	1	1	1	1	1
4	4	3	3	1	1	1	1	1	1
4	4	3	3	1	1	1	1	1	1
4	4	3	3	1	1	4	4	4	4
4	4	3	3	4	4	1	1	1	1
4	4	3	3	1	1	1	1	1	1
1	1	3	3	1	1	1	1	4	4
1	1	3	3	1	1	1	1	4	4
4	4	3	3	1	1	1	1	1	1
1	1	3	3	1	1	1	1	4	4
1	1	3	3	1	1	1	1	4	4
4	4	3	3	1	1	1	1	1	1
4	4	3	3	1	1	1	1	1	1
1	1	1	1	1	1	1	1	4	4
1	1	3	3	1	1	4	4	4	4
4	4	3	3	1	1	1	1	1	1
4	4	3	3	1	1	1	1	4	4
1	1	3	3	1	1	1	1	4	4
4	4	3	3	4	4	1	1	1	1
1	1	3	3	1	1	1	1	4	4
4	4	3	3	1	1	1	1	1	1
4	4	3	3	1	1	1	1	1	1
4	4	3	3	1	1	1	1	1	1
4	4	3	3	1	1	1	1	1	1
1	1	3	3	1	1	1	1	4	4
1	1	3	3	1	1	4	4	4	4
1	1	3	3	1	1	1	1	4	4
4	4	3	3	1	1	1	1	1	1
1	1	3	3	1	1	1	1	4	4
1	1	3	3	1	1	1	1	4	4
4	4	3	3	1	1	1	1	1	1
4	4	3	3	1	1	1	1	4	4
4	4	3	3	1	1	1	1	1	1
4	4	3	3	1	1	1	1	1	1
4	4	3	3	1	1	4	4	4	4
4	4	3	3	1	1	1	1	1	1
4	4	3	3	1	1	1	1	1	1
4	4	3	3	1	1	1	1	4	4
1	1	3	3	1	1	1	1	4	4
4	4	1	1	1	1	4	4	4	4
4	4	3	3	1	1	1	1	4	4
1	1	3	3	4	4	1	1	4	4
4	4	3	3	1	1	1	1	1	1
4	4	3	3	1	1	4	4	4	4
1	1	3	3	1	1	4	4	4	4
4	4	3	3	1	1	1	1	1	1
4	4	3	3	1	1	1	1	4	4

For Review Only

4	4	3	3	1	1	1	1	1	1
4	4	3	3	1	1	1	1	1	1
4	4	3	3	1	1	1	1	1	1
4	4	3	3	1	1	1	1	4	4
4	4	3	3	1	1	4	4	4	4
4	4	3	3	1	1	1	1	1	1
4	4	3	3	1	1	1	1	1	1
4	4	3	3	1	1	1	1	4	4
1	1	3	3	1	1	1	1	4	4
1	1	3	3	1	1	1	1	4	4
4	4	3	3	1	1	1	1	1	1
4	4	3	3	1	1	1	1	4	4
4	4	3	3	4	4	1	1	1	1
1	1	3	3	1	1	1	1	4	4
4	4	3	3	1	1	1	1	1	1
1	1	3	3	1	1	4	4	4	4
4	4	1	1	1	1	1	1	4	4
4	4	3	3	1	1	4	4	1	1
4	4	1	1	1	1	1	1	1	1
4	4	3	3	4	4	1	1	4	4
4	4	3	3	1	1	1	1	1	1
4	4	3	3	1	1	1	1	1	1
1	1	3	3	1	1	1	1	4	4
4	4	1	1	1	1	1	1	4	4
4	4	3	3	1	1	4	4	4	4
4	4	3	3	1	1	1	1	1	1
1	1	3	3	1	1	4	4	4	4
4	4	3	3	4	4	1	1	1	1
4	4	3	3	1	1	1	1	4	4
4	4	3	3	1	1	1	1	1	1
1	1	1	1	1	1	1	1	4	4
1	1	3	3	1	1	1	1	4	4
1	1	3	3	1	1	1	1	4	4
4	4	3	3	1	1	1	1	1	1
1	1	3	3	1	1	4	4	4	4
1	1	3	3	1	1	1	1	4	4
4	4	3	3	1	1	1	1	1	1
4	4	3	3	1	1	1	1	1	1
1	1	3	3	1	1	1	1	4	4
4	4	1	1	1	1	4	4	4	4
4	4	3	3	1	1	1	1	1	1
1	1	3	3	1	1	1	1	1	1
4	4	1	1	1	1	1	1	1	1
4	4	3	3	1	1	1	1	4	4
4	4	1	1	1	1	4	4	1	1
1	1	3	3	4	4	1	1	4	4
4	4	3	3	1	1	4	4	1	1
4	4	3	3	1	1	1	1	1	1
4	4	3	3	1	1	4	4	4	4
4	4	3	3	4	4	4	4	4	4
4	4	3	3	1	1	1	1	1	1
1	1	1	1	1	1	4	4	4	4
4	4	3	3	1	1	1	1	1	1
4	4	3	3	4	4	1	1	4	4
4	4	1	1	1	1	1	1	4	4

For Review Only

1	1	3	3	1	1	1	1	4	4
4	4	3	3	1	1	1	1	1	1

For Review Only

SNP2577	SNP2577	SNP2578	SNP2578	SNP2579	SNP2579	SNP258	SNP258	SNP2580	SNP2580
3	3	1	1	4	4	4	4	3	3
3	3	4	4	4	4	4	4	3	3
3	3	4	4	4	4	4	4	3	3
3	3	1	1	4	4	4	4	3	3
3	3	4	4	4	4	4	4	3	3
3	3	4	4	4	4	4	4	3	3
3	3	1	1	4	4	4	4	3	3
3	3	4	4	1	1	4	4	3	3
3	3	4	4	1	1	4	4	3	3
3	3	4	4	4	4	4	4	3	3
1	1	4	4	4	4	4	4	3	3
3	3	4	4	4	4	4	4	3	3
3	3	1	1	4	4	4	4	3	3
1	1	4	4	4	4	4	4	3	3
3	3	4	4	4	4	4	4	3	3
3	3	4	4	4	4	4	4	3	3
3	3	1	1	4	4	4	4	3	3
3	3	1	1	4	4	4	4	3	3
3	3	4	4	4	4	4	4	3	3
3	3	1	1	1	1	4	4	3	3
3	3	1	1	4	4	3	3	3	3
3	3	1	1	4	4	4	4	3	3
1	1	4	4	4	4	4	4	3	3
3	3	4	4	4	4	3	3	3	3
3	3	4	4	4	4	4	4	3	3
3	3	4	4	4	4	4	4	3	3
3	3	1	1	4	4	4	4	3	3
3	3	4	4	4	4	4	4	3	3
3	3	1	1	1	1	4	4	3	3
3	3	4	4	1	1	3	3	3	3
3	3	1	1	4	4	4	4	3	3
3	3	4	4	4	4	4	4	3	3
3	3	4	4	4	4	4	4	3	3
3	3	4	4	4	4	4	4	3	3
1	1	4	4	4	4	4	4	3	3
3	3	4	4	4	4	4	4	3	3
3	3	4	4	4	4	4	4	3	3
3	3	4	4	4	4	4	4	2	2
1	1	4	4	4	4	4	4	3	3
1	1	4	4	4	4	4	4	3	3
3	3	4	4	1	1	4	4	3	3
3	3	1	1	1	1	4	4	3	3
3	3	1	1	1	1	4	4	3	3
3	3	1	1	4	4	4	4	3	3
3	3	4	4	1	1	4	4	3	3
3	3	4	4	4	4	4	4	3	3
3	3	1	1	4	4	4	4	3	3
3	3	4	4	4	4	4	4	3	3
3	3	4	4	1	1	4	4	3	3
3	3	4	4	4	4	4	4	3	3
3	3	4	4	4	4	4	4	3	3
1	1	1	1	4	4	4	4	3	3

For Review Only

3	3	1	1	4	4	4	4	3	3
3	3	4	4	4	4	4	4	3	3
3	3	4	4	4	4	4	4	3	3
3	3	1	1	4	4	3	3	3	3
3	3	4	4	4	4	4	4	3	3
3	3	4	4	4	4	4	4	3	3
3	3	4	4	4	4	4	4	3	3
3	3	4	4	1	1	4	4	3	3
3	3	4	4	4	4	4	4	3	3
3	3	4	4	4	4	4	4	3	3
3	3	1	1	4	4	4	4	3	3
3	3	1	1	4	4	4	4	3	3
3	3	1	1	4	4	4	4	3	3
3	3	1	1	1	1	4	4	3	3
1	1	4	4	4	4	4	4	3	3
3	3	4	4	4	4	4	4	3	3
1	1	4	4	4	4	4	4	3	3
3	3	4	4	4	4	4	4	3	3
3	3	1	1	4	4	4	4	3	3
3	3	1	1	4	4	4	4	3	3
3	3	1	1	4	4	4	4	3	3
1	1	4	4	4	4	4	4	3	3
3	3	1	1	1	1	4	4	3	3
3	3	4	4	1	1	3	3	3	3
3	3	4	4	1	1	4	4	3	3
3	3	4	4	4	4	4	4	3	3
3	3	4	4	4	4	4	4	3	3
1	1	4	4	4	4	3	3	3	3
3	3	4	4	4	4	4	4	3	3
3	3	1	1	4	4	4	4	3	3
3	3	4	4	4	4	4	4	3	3
3	3	4	4	4	4	4	4	3	3
1	1	4	4	4	4	4	4	3	3
3	3	1	1	4	4	4	4	3	3
3	3	1	1	4	4	4	4	3	3
3	3	4	4	1	1	4	4	3	3
3	3	4	4	1	1	4	4	3	3
3	3	4	4	1	1	4	4	3	3
3	3	4	4	4	4	4	4	3	3
3	3	4	4	4	4	4	4	3	3
3	3	1	1	4	4	4	4	3	3
3	3	4	4	4	4	4	4	3	3
3	3	1	1	4	4	4	4	3	3
3	3	1	1	4	4	4	4	3	3
3	3	1	1	4	4	4	4	3	3
3	3	4	4	4	4	4	4	2	2
3	3	4	4	4	4	4	4	2	2
3	3	4	4	4	4	4	4	3	3
3	3	4	4	4	4	4	4	3	3
3	3	1	1	4	4	4	4	3	3
3	3	4	4	4	4	4	4	3	3
1	1	4	4	4	4	4	4	3	3
3	3	4	4	4	4	4	4	3	3
3	3	4	4	4	4	4	4	3	3
3	3	1	1	4	4	4	4	3	3

For Review Only

1	1	4	4	4	4	4	4	3	3
3	3	4	4	4	4	3	3	3	3
3	3	4	4	4	4	4	4	3	3
3	3	4	4	4	4	4	4	2	2
3	3	1	1	4	4	4	4	3	3
3	3	4	4	4	4	4	4	3	3
3	3	4	4	4	4	4	4	3	3
3	3	4	4	4	4	4	4	3	3
3	3	4	4	4	4	4	4	3	3
3	3	4	4	1	1	4	4	3	3
3	3	4	4	4	4	4	4	3	3
3	3	4	4	4	4	4	4	3	3
3	3	4	4	4	4	4	4	3	3
3	3	4	4	4	4	4	4	3	3
3	3	4	4	4	4	4	4	3	3
3	3	4	4	4	4	4	4	3	3
3	3	4	4	4	4	4	4	2	2
1	1	4	4	4	4	4	4	3	3
3	3	4	4	4	4	4	4	2	2
3	3	4	4	4	4	4	4	3	3
3	3	4	4	4	4	4	4	3	3
3	3	1	1	4	4	4	4	3	3
3	3	4	4	4	4	4	4	3	3
1	1	4	4	4	4	3	3	3	3
1	1	4	4	4	4	4	4	3	3
3	3	4	4	4	4	4	4	3	3
3	3	4	4	4	4	3	3	2	2
3	3	1	1	4	4	4	4	2	2
3	3	4	4	4	4	4	4	3	3
3	3	1	1	4	4	4	4	2	2
1	1	4	4	4	4	4	4	3	3
3	3	4	4	4	4	4	4	3	3
3	3	1	1	4	4	4	4	3	3
3	3	4	4	4	4	4	4	3	3
3	3	4	4	4	4	4	4	3	3
3	3	4	4	4	4	4	4	3	3
3	3	4	4	4	4	4	4	2	2
3	3	1	1	4	4	4	4	3	3
1	1	4	4	4	4	4	4	3	3
1	1	4	4	4	4	4	4	3	3
1	1	4	4	4	4	4	4	3	3
3	3	4	4	4	4	4	4	2	2
3	3	4	4	4	4	4	4	3	3
3	3	4	4	1	1	4	4	3	3
3	3	4	4	4	4	4	4	3	3
3	3	1	1	4	4	4	4	3	3
3	3	4	4	4	4	4	4	3	3
3	3	4	4	4	4	4	4	3	3
3	3	4	4	4	4	4	4	3	3
3	3	4	4	4	4	4	4	3	3
3	3	4	4	4	4	4	4	3	3
1	1	4	4	4	4	4	4	3	3
3	3	4	4	4	4	4	4	2	2
3	3	4	4	4	4	4	4	3	3
3	3	4	4	4	4	4	4	3	3

For Review Only

3	3	4	4	4	4	4	4	3	3
3	3	4	4	4	4	4	4	3	3
3	3	4	4	4	4	3	3	3	3
3	3	4	4	4	4	4	4	3	3
3	3	4	4	4	4	4	4	3	3
1	1	1	1	4	4	4	4	3	3
1	1	4	4	4	4	4	4	3	3
3	3	4	4	4	4	4	4	3	3
3	3	4	4	4	4	4	4	3	3
3	3	1	1	4	4	4	4	3	3
3	3	4	4	4	4	4	4	3	3
1	1	4	4	4	4	4	4	3	3
3	3	4	4	4	4	4	4	3	3
3	3	4	4	4	4	4	4	3	3
3	3	4	4	4	4	4	4	3	3
1	1	4	4	4	4	4	4	3	3
3	3	4	4	4	4	4	4	3	3
3	3	4	4	4	4	4	4	3	3
3	3	4	4	4	4	4	4	3	3
3	3	4	4	4	4	4	4	3	3
3	3	4	4	4	4	4	4	3	3
1	1	4	4	4	4	4	4	3	3
1	1	4	4	4	4	4	4	3	3
3	3	1	1	4	4	4	4	3	3
3	3	4	4	4	4	3	3	3	3
3	3	4	4	4	4	4	4	3	3
3	3	4	4	4	4	4	4	3	3
3	3	4	4	4	4	4	4	3	3
3	3	4	4	4	4	4	4	2	2
3	3	1	1	4	4	4	4	3	3
3	3	4	4	4	4	4	4	3	3
3	3	4	4	4	4	4	4	3	3
3	3	1	1	4	4	3	3	3	3
3	3	4	4	4	4	4	4	3	3
3	3	1	1	4	4	4	4	3	3
3	3	1	1	4	4	4	4	3	3
3	3	4	4	4	4	4	4	3	3
3	3	4	4	4	4	4	4	3	3
3	3	4	4	4	4	4	4	3	3
3	3	1	1	4	4	4	4	3	3
3	3	4	4	4	4	4	4	3	3
3	3	4	4	4	4	4	4	3	3
1	1	4	4	4	4	4	4	3	3
1	1	4	4	4	4	4	4	3	3
1	1	4	4	4	4	3	3	3	3
3	3	4	4	4	4	4	4	3	3
1	1	4	4	4	4	4	4	3	3
1	1	4	4	4	4	4	4	3	3
3	3	4	4	4	4	3	3	3	3
3	3	1	1	4	4	4	4	3	3
3	3	4	4	4	4	4	4	3	3
3	3	4	4	4	4	4	4	3	3
1	1	4	4	4	4	4	4	3	3
3	3	1	1	4	4	4	4	3	3
1	1	4	4	4	4	4	4	3	3

For Review Only

3	3	4	4	4	4	4	4	3	3
3	3	4	4	1	1	3	3	3	3
3	3	4	4	4	4	4	4	3	3
1	1	4	4	4	4	4	4	3	3
3	3	4	4	1	1	4	4	3	3
3	3	4	4	4	4	3	3	2	2
3	3	4	4	4	4	4	4	3	3
3	3	4	4	1	1	4	4	3	3
3	3	4	4	1	1	4	4	3	3
1	1	4	4	4	4	3	3	3	3
3	3	4	4	4	4	4	4	3	3
3	3	4	4	4	4	4	4	3	3
3	3	4	4	4	4	4	4	3	3
3	3	4	4	4	4	4	4	3	3
3	3	4	4	4	4	4	4	3	3
1	1	4	4	4	4	4	4	3	3
1	1	4	4	4	4	4	4	3	3
1	1	4	4	4	4	4	4	3	3
1	1	4	4	4	4	4	4	3	3
3	3	4	4	4	4	4	4	2	2
3	3	4	4	4	4	4	4	2	2
3	3	4	4	4	4	4	4	3	3
3	3	1	1	4	4	4	4	3	3
1	1	4	4	4	4	4	4	3	3
3	3	4	4	1	1	4	4	3	3
3	3	4	4	4	4	4	4	3	3
1	1	4	4	4	4	3	3	3	3
3	3	4	4	4	4	4	4	3	3
3	3	4	4	1	1	4	4	3	3
3	3	4	4	4	4	4	4	3	3
1	1	4	4	4	4	4	4	3	3
1	1	4	4	4	4	4	4	3	3
3	3	4	4	4	4	4	4	3	3
3	3	4	4	4	4	4	4	3	3
1	1	4	4	4	4	4	4	3	3
1	1	4	4	4	4	3	3	3	3
3	3	1	1	4	4	4	4	3	3
3	3	1	1	4	4	4	4	3	3
3	3	4	4	4	4	4	4	3	3
1	1	4	4	4	4	4	4	3	3
3	3	1	1	4	4	4	4	3	3
3	3	1	1	4	4	4	4	3	3
1	1	4	4	4	4	4	4	3	3
3	3	1	1	4	4	4	4	3	3
1	1	4	4	4	4	4	4	3	3
3	3	4	4	4	4	4	4	3	3
3	3	4	4	4	4	4	4	3	3
3	3	4	4	4	4	4	4	3	3
3	3	4	4	4	4	4	4	3	3
1	1	4	4	4	4	4	4	3	3
3	3	1	1	4	4	4	4	3	3
1	1	4	4	4	4	4	4	3	3
3	3	4	4	4	4	4	4	3	3
3	3	1	1	4	4	4	4	3	3
1	1	4	4	4	4	4	4	3	3

For Review Only

3	3	4	4	4	4	4	4	3	3
3	3	4	4	4	4	4	4	3	3

For Review Only

SNP2581	SNP2581	SNP2582	SNP2582	SNP2583	SNP2583	SNP2584	SNP2584	SNP2585	SNP2585
1	1	3	3	2	2	2	2	3	3
1	1	3	3	2	2	2	2	3	3
1	1	3	3	2	2	2	2	3	3
1	1	2	2	2	2	2	2	3	3
1	1	3	3	3	3	2	2	3	3
1	1	3	3	2	2	2	2	3	3
1	1	2	2	2	2	2	2	3	3
1	1	3	3	3	3	2	2	3	3
1	1	3	3	2	2	2	2	3	3
3	3	3	3	2	2	2	2	3	3
1	1	3	3	2	2	2	2	3	3
1	1	3	3	2	2	2	2	3	3
1	1	3	3	2	2	2	2	3	3
1	1	3	3	2	2	2	2	3	3
1	1	3	3	2	2	2	2	3	3
1	1	3	3	2	2	2	2	3	3
1	1	2	2	2	2	2	2	3	3
1	1	2	2	2	2	2	2	3	3
1	1	3	3	2	2	2	2	3	3
1	1	2	2	2	2	1	1	3	3
1	1	2	2	2	2	1	1	2	2
3	3	3	3	2	2	2	2	3	3
3	3	3	3	2	2	1	1	3	3
3	3	3	3	2	2	2	2	2	2
1	1	3	3	2	2	2	2	3	3
1	1	3	3	2	2	2	2	3	3
3	3	2	2	2	2	2	2	3	3
1	1	3	3	2	2	2	2	3	3
1	1	2	2	2	2	2	2	3	3
1	1	3	3	3	3	1	1	3	3
1	1	2	2	2	2	2	2	3	3
1	1	3	3	2	2	2	2	3	3
1	1	3	3	2	2	2	2	3	3
3	3	3	3	2	2	2	2	3	3
1	1	3	3	2	2	2	2	3	3
1	1	3	3	2	2	2	2	3	3
1	1	3	3	2	2	2	2	3	3
1	1	3	3	2	2	2	2	3	3
3	3	3	3	2	2	1	1	3	3
1	1	3	3	2	2	1	1	3	3
1	1	3	3	3	3	1	1	3	3
1	1	2	2	2	2	2	2	3	3
1	1	3	3	2	2	2	2	3	3
3	3	3	3	2	2	1	1	3	3
1	1	3	3	2	2	2	2	3	3
1	1	3	3	2	2	2	2	3	3
1	1	2	2	2	2	2	2	3	3
1	1	3	3	2	2	1	1	3	3
1	1	3	3	2	2	1	1	3	3
1	1	3	3	2	2	2	2	3	3
1	1	3	3	2	2	2	2	3	3
1	1	2	2	2	2	2	2	3	3

For Review Only

1	1	2	2	2	2	2	2	3	3
1	1	3	3	2	2	2	2	3	3
1	1	3	3	2	2	2	2	3	3
3	3	2	2	2	2	2	2	3	3
1	1	3	3	2	2	2	2	3	3
1	1	3	3	2	2	2	2	3	3
1	1	3	3	2	2	2	2	3	3
1	1	3	3	2	2	1	1	3	3
1	1	3	3	2	2	2	2	3	3
1	1	3	3	2	2	2	2	3	3
1	1	2	2	2	2	2	2	3	3
1	1	2	2	2	2	2	2	3	3
1	1	2	2	2	2	2	2	3	3
1	1	2	2	2	2	2	2	3	3
1	1	3	3	2	2	2	2	3	3
3	3	2	2	2	2	2	2	3	3
1	1	3	3	2	2	2	2	3	3
1	1	3	3	2	2	2	2	3	3
1	1	2	2	2	2	2	2	3	3
1	1	2	2	2	2	2	2	3	3
1	1	2	2	2	2	2	2	3	3
3	3	3	3	2	2	1	1	2	2
1	1	3	3	2	2	2	2	3	3
1	1	3	3	2	2	2	2	3	3
1	1	3	3	2	2	2	2	3	3
1	1	3	3	2	2	2	2	3	3
3	3	3	3	3	3	2	2	3	3
3	3	3	3	2	2	1	1	2	2
1	1	3	3	2	2	2	2	3	3
1	1	2	2	2	2	2	2	3	3
1	1	3	3	2	2	2	2	3	3
1	1	3	3	2	2	2	2	3	3
3	3	3	3	2	2	1	1	2	2
1	1	2	2	2	2	2	2	3	3
1	1	2	2	2	2	2	2	3	3
1	1	3	3	2	2	2	2	3	3
1	1	3	3	2	2	1	1	3	3
3	3	3	3	3	3	2	2	3	3
1	1	3	3	2	2	2	2	3	3
1	1	3	3	3	3	1	1	3	3
1	1	2	2	2	2	2	2	3	3
1	1	3	3	2	2	2	2	3	3
1	1	2	2	2	2	2	2	3	3
1	1	2	2	2	2	2	2	3	3
1	1	2	2	2	2	2	2	3	3
1	1	3	3	2	2	2	2	3	3
1	1	3	3	2	2	2	2	3	3
1	1	3	3	2	2	2	2	3	3
1	1	3	3	2	2	2	2	3	3
1	1	2	2	2	2	1	1	3	3
1	1	3	3	2	2	2	2	3	3
3	3	3	3	2	2	1	1	2	2
1	1	3	3	2	2	1	1	3	3
1	1	3	3	2	2	2	2	2	2
1	1	2	2	2	2	2	2	3	3

For Review Only

1	1	3	3	2	2	2	2	2	2
1	1	3	3	2	2	2	2	3	3
1	1	3	3	2	2	2	2	3	3
1	1	3	3	2	2	2	2	3	3
1	1	3	3	2	2	2	2	3	3
1	1	3	3	2	2	2	2	3	3
1	1	3	3	2	2	2	2	3	3
1	1	3	3	2	2	2	2	3	3
1	1	3	3	3	3	1	1	2	2
1	1	3	3	2	2	2	2	3	3
1	1	2	2	2	2	2	2	3	3
1	1	3	3	2	2	2	2	3	3
1	1	3	3	2	2	2	2	3	3
1	1	3	3	2	2	2	2	3	3
1	1	3	3	2	2	1	1	3	3
1	1	3	3	2	2	2	2	3	3
1	1	3	3	2	2	2	2	3	3
1	1	3	3	2	2	2	2	3	3
1	1	3	3	2	2	2	2	3	3
1	1	3	3	2	2	2	2	3	3
1	1	3	3	2	2	2	2	3	3
1	1	2	2	2	2	2	2	3	3
1	1	3	3	2	2	2	2	3	3
3	3	3	3	2	2	1	1	3	3
1	1	3	3	2	2	2	2	3	3
3	3	3	3	2	2	2	2	3	3
1	1	3	3	2	2	2	2	3	3
1	1	2	2	2	2	2	2	3	3
1	1	3	3	2	2	2	2	3	3
1	1	2	2	2	2	2	2	3	3
3	3	3	3	2	2	2	2	2	2
1	1	3	3	2	2	2	2	3	3
1	1	2	2	2	2	2	2	3	3
1	1	3	3	2	2	2	2	3	3
3	3	3	3	2	2	2	2	3	3
3	3	3	3	2	2	2	2	3	3
1	1	3	3	2	2	2	2	3	3
1	1	2	2	2	2	2	2	3	3
1	1	3	3	2	2	1	1	2	2
3	3	3	3	2	2	2	2	2	2
1	1	3	3	2	2	2	2	3	3
1	1	3	3	2	2	2	2	3	3
1	1	3	3	2	2	2	2	3	3
1	1	3	3	3	3	2	2	3	3
1	1	3	3	2	2	2	2	3	3
1	1	2	2	2	2	2	2	3	3
1	1	3	3	2	2	2	2	3	3
1	1	3	3	2	2	2	2	3	3
1	1	3	3	2	2	2	2	3	3
1	1	3	3	2	2	2	2	3	3
1	1	3	3	2	2	2	2	3	3
3	3	3	3	2	2	1	1	3	3
1	1	3	3	2	2	2	2	3	3
1	1	2	2	2	2	2	2	3	3
1	1	3	3	2	2	2	2	3	3

For Review Only

1	1	3	3	2	2	2	2	3	3
1	1	3	3	2	2	2	2	3	3
1	1	3	3	2	2	2	2	3	3
1	1	3	3	2	2	2	2	3	3
1	1	3	3	2	2	2	2	3	3
1	1	2	2	2	2	2	2	3	3
3	3	3	3	2	2	2	2	3	3
1	1	3	3	2	2	2	2	3	3
3	3	3	3	2	2	1	1	3	3
1	1	2	2	2	2	2	2	3	3
1	1	3	3	2	2	2	2	3	3
1	1	3	3	2	2	2	2	2	2
1	1	3	3	2	2	2	2	3	3
1	1	3	3	2	2	2	2	3	3
3	3	3	3	2	2	2	2	3	3
1	1	3	3	2	2	2	2	3	3
1	1	3	3	2	2	2	2	3	3
1	1	3	3	2	2	2	2	3	3
1	1	3	3	2	2	2	2	3	3
1	1	3	3	2	2	2	2	3	3
1	1	3	3	2	2	2	2	3	3
3	3	3	3	2	2	2	2	3	3
3	3	3	3	2	2	1	1	3	3
1	1	2	2	2	2	2	2	3	3
1	1	2	2	2	2	2	2	3	3
3	3	3	3	2	2	2	2	3	3
1	1	3	3	2	2	2	2	3	3
1	1	3	3	2	2	2	2	3	3
1	1	3	3	2	2	2	2	3	3
1	1	2	2	2	2	2	2	3	3
1	1	3	3	2	2	2	2	3	3
1	1	3	3	2	2	2	2	3	3
1	1	2	2	2	2	2	2	3	3
3	3	3	3	2	2	2	2	3	3
1	1	2	2	2	2	2	2	3	3
1	1	2	2	2	2	2	2	3	3
3	3	3	3	2	2	2	2	3	3
1	1	3	3	2	2	2	2	3	3
1	1	3	3	2	2	2	2	3	3
1	1	2	2	2	2	2	2	3	3
1	1	3	3	2	2	2	2	3	3
1	1	3	3	2	2	2	2	3	3
1	1	3	3	2	2	1	1	2	2
1	1	3	3	2	2	2	2	3	3
1	1	3	3	2	2	2	2	3	3
1	1	3	3	2	2	2	2	2	2
1	1	3	3	2	2	2	2	3	3
3	3	3	3	2	2	1	1	3	3
3	3	3	3	2	2	2	2	3	3
1	1	2	2	2	2	2	2	3	3
1	1	3	3	2	2	2	2	3	3
1	1	3	3	2	2	1	1	3	3
3	3	3	3	2	2	1	1	3	3
1	1	2	2	2	2	2	2	3	3
1	1	3	3	2	2	1	1	2	2

For Review Only

3	3	3	3	2	2	2	2	3	3
1	1	3	3	2	2	2	2	3	3
1	1	3	3	2	2	2	2	3	3
1	1	3	3	2	2	2	2	3	3
3	3	3	3	3	3	1	1	3	3
1	1	3	3	2	2	2	2	3	3
3	3	3	3	2	2	2	2	3	3
3	3	3	3	2	2	2	2	3	3
1	1	3	3	2	2	2	2	3	3
3	3	3	3	2	2	1	1	2	2
1	1	3	3	2	2	2	2	3	3
1	1	3	3	2	2	2	2	3	3
1	1	3	3	2	2	2	2	3	3
1	1	3	3	2	2	2	2	3	3
1	1	3	3	2	2	2	2	3	3
1	1	3	3	2	2	1	1	3	3
3	3	3	3	2	2	2	2	3	3
3	3	3	3	2	2	1	1	2	2
1	1	3	3	2	2	2	2	3	3
1	1	3	3	2	2	2	2	3	3
1	1	3	3	2	2	2	2	3	3
1	1	3	3	2	2	2	2	3	3
1	1	2	2	2	2	2	2	3	3
1	1	3	3	2	2	2	2	3	3
1	1	3	3	2	2	1	1	3	3
1	1	3	3	2	2	2	2	3	3
3	3	3	3	2	2	1	1	2	2
1	1	3	3	2	2	2	2	3	3
1	1	3	3	2	2	2	2	3	3
1	1	3	3	2	2	2	2	3	3
3	3	3	3	2	2	1	1	2	2
1	1	3	3	2	2	2	2	3	3
1	1	3	3	2	2	2	2	3	3
1	1	3	3	2	2	2	2	3	3
3	3	3	3	2	2	1	1	3	3
3	3	3	3	2	2	1	1	2	2
1	1	2	2	2	2	2	2	3	3
1	1	2	2	2	2	2	2	3	3
1	1	3	3	2	2	2	2	3	3
1	1	3	3	2	2	1	1	2	2
1	1	2	2	2	2	2	2	3	3
1	1	2	2	2	2	2	2	3	3
1	1	3	3	2	2	2	2	3	3
1	1	2	2	2	2	2	2	3	3
1	1	3	3	2	2	1	1	2	2
1	1	3	3	2	2	2	2	3	3
1	1	3	3	2	2	1	1	3	3
1	1	3	3	3	3	1	1	3	3
1	1	3	3	3	3	1	1	3	3
3	3	3	3	2	2	1	1	2	2
1	1	2	2	2	2	2	2	3	3
3	3	3	3	2	2	1	1	2	2
1	1	3	3	2	2	2	2	3	3
1	1	2	2	2	2	2	2	3	3
1	1	3	3	2	2	2	2	3	3

For Review Only

1	1	2	2	2	2	2	2	3	3
1	1	3	3	2	2	2	2	3	3

For Review Only

SNP2586	SNP2586	SNP2587	SNP2587	SNP2588	SNP2588	SNP2589	SNP2589	SNP259	SNP259
1	1	4	4	4	4	1	1	3	3
1	1	4	4	4	4	3	3	3	3
1	1	4	4	4	4	1	1	3	3
1	1	4	4	4	4	1	1	3	3
1	1	4	4	4	4	1	1	3	3
1	1	4	4	4	4	1	1	3	3
1	1	4	4	4	4	1	1	3	3
1	1	4	4	4	4	1	1	3	3
1	1	4	4	4	4	1	1	3	3
1	1	4	4	4	4	1	1	3	3
1	1	1	1	4	4	1	1	3	3
1	1	4	4	4	4	1	1	3	3
1	1	4	4	4	4	1	1	3	3
1	1	4	4	4	4	1	1	3	3
1	1	4	4	4	4	1	1	3	3
1	1	4	4	4	4	3	3	3	3
1	1	4	4	4	4	1	1	3	3
1	1	4	4	4	4	1	1	3	3
1	1	4	4	4	4	1	1	3	3
1	1	4	4	4	4	1	1	3	3
1	1	4	4	4	4	1	1	2	2
1	1	4	4	4	4	1	1	3	3
3	3	1	1	1	1	1	1	3	3
1	1	4	4	4	4	1	1	2	2
1	1	4	4	4	4	1	1	3	3
1	1	4	4	4	4	1	1	3	3
1	1	4	4	4	4	3	3	3	3
1	1	4	4	4	4	1	1	3	3
1	1	4	4	4	4	1	1	3	3
1	1	4	4	4	4	1	1	2	2
1	1	4	4	4	4	1	1	3	3
1	1	4	4	4	4	1	1	3	3
1	1	4	4	4	4	1	1	3	3
1	1	4	4	4	4	1	1	3	3
1	1	4	4	4	4	1	1	3	3
1	1	4	4	4	4	1	1	3	3
1	1	4	4	4	4	1	1	3	3
1	1	4	4	4	4	1	1	3	3
3	3	4	4	1	1	1	1	3	3
3	3	4	4	1	1	1	1	3	3
1	1	4	4	4	4	1	1	3	3
1	1	4	4	4	4	1	1	3	3
1	1	4	4	4	4	1	1	3	3
1	1	4	4	4	4	1	1	3	3
1	1	4	4	4	4	1	1	3	3
1	1	4	4	4	4	1	1	3	3
1	1	4	4	4	4	1	1	3	3
3	3	4	4	1	1	1	1	3	3
1	1	4	4	4	4	1	1	3	3
1	1	4	4	4	4	1	1	3	3
1	1	4	4	4	4	1	1	3	3
1	1	4	4	4	4	1	1	3	3

For Review Only

1	1	4	4	4	4	1	1	3	3
1	1	4	4	4	4	1	1	3	3
1	1	4	4	4	4	1	1	3	3
1	1	4	4	4	4	1	1	2	2
1	1	4	4	4	4	1	1	3	3
1	1	4	4	4	4	1	1	3	3
1	1	4	4	4	4	1	1	3	3
1	1	4	4	4	4	1	1	3	3
1	1	4	4	4	4	1	1	3	3
1	1	4	4	4	4	1	1	3	3
1	1	4	4	4	4	1	1	3	3
1	1	4	4	4	4	1	1	3	3
1	1	4	4	4	4	1	1	3	3
1	1	4	4	4	4	1	1	3	3
1	1	4	4	4	4	1	1	3	3
1	1	4	4	4	4	1	1	3	3
1	1	4	4	4	4	1	1	3	3
1	1	4	4	4	4	1	1	3	3
1	1	4	4	4	4	1	1	3	3
1	1	4	4	4	4	1	1	3	3
1	1	4	4	4	4	1	1	3	3
3	3	4	4	1	1	1	1	3	3
1	1	4	4	4	4	1	1	3	3
1	1	4	4	4	4	1	1	2	2
1	1	4	4	4	4	1	1	3	3
1	1	4	4	4	4	1	1	3	3
1	1	4	4	4	4	1	1	3	3
1	1	4	4	4	4	1	1	2	2
1	1	4	4	4	4	1	1	3	3
1	1	4	4	4	4	1	1	3	3
1	1	4	4	4	4	1	1	3	3
1	1	4	4	4	4	1	1	3	3
3	3	4	4	1	1	1	1	3	3
1	1	4	4	4	4	1	1	3	3
1	1	4	4	4	4	1	1	3	3
1	1	4	4	4	4	1	1	3	3
1	1	4	4	4	4	1	1	3	3
1	1	4	4	4	4	1	1	3	3
1	1	4	4	4	4	1	1	3	3
1	1	4	4	4	4	1	1	3	3
1	1	4	4	4	4	1	1	3	3
1	1	4	4	4	4	1	1	3	3
1	1	4	4	4	4	1	1	3	3
1	1	4	4	4	4	1	1	3	3
1	1	4	4	4	4	1	1	3	3
1	1	4	4	4	4	3	3	3	3
1	1	4	4	4	4	1	1	3	3
1	1	4	4	4	4	1	1	3	3
1	1	4	4	4	4	1	1	3	3
1	1	4	4	4	4	1	1	3	3
1	1	4	4	4	4	1	1	3	3
3	3	4	4	1	1	1	1	3	3
1	1	4	4	4	4	1	1	3	3
1	1	4	4	4	4	1	1	3	3
1	1	4	4	4	4	1	1	3	3

For Review Only

1	1	4	4	4	4	1	1	3	3
3	3	4	4	4	4	1	1	3	3
1	1	4	4	4	4	3	3	3	3
1	1	4	4	4	4	1	1	3	3
1	1	4	4	4	4	3	3	3	3
1	1	4	4	4	4	1	1	3	3
1	1	4	4	4	4	3	3	3	3
1	1	4	4	4	4	1	1	3	3
3	3	4	4	4	4	1	1	3	3
1	1	4	4	4	4	1	1	3	3
1	1	4	4	4	4	1	1	3	3
1	1	4	4	4	4	1	1	3	3
1	1	4	4	4	4	1	1	3	3
1	1	4	4	4	4	1	1	3	3
1	1	4	4	4	4	1	1	3	3
1	1	4	4	4	4	1	1	3	3
1	1	4	4	4	4	1	1	3	3
1	1	4	4	4	4	1	1	3	3
1	1	4	4	4	4	1	1	3	3
1	1	4	4	4	4	1	1	3	3
1	1	4	4	4	4	1	1	3	3
1	1	4	4	4	4	1	1	3	3
1	1	4	4	4	4	1	1	3	3
3	3	4	4	1	1	1	1	2	2
1	1	4	4	4	4	1	1	3	3
3	3	4	4	1	1	1	1	3	3
1	1	4	4	4	4	1	1	2	2
1	1	4	4	4	4	1	1	3	3
1	1	4	4	4	4	1	1	3	3
1	1	4	4	4	4	1	1	3	3
1	1	4	4	4	4	1	1	3	3
1	1	4	4	4	4	1	1	3	3
1	1	4	4	4	4	1	1	3	3
1	1	4	4	4	4	1	1	3	3
1	1	4	4	4	4	1	1	3	3
1	1	4	4	4	4	1	1	3	3
1	1	4	4	4	4	1	1	3	3
1	1	4	4	4	4	1	1	3	3
3	3	4	4	4	4	1	1	3	3
1	1	4	4	4	4	1	1	3	3
3	3	4	4	4	4	1	1	3	3
1	1	4	4	4	4	1	1	3	3
1	1	4	4	4	4	1	1	3	3
1	1	4	4	4	4	1	1	3	3
1	1	4	4	4	4	1	1	3	3
1	1	4	4	4	4	1	1	3	3
1	1	4	4	4	4	1	1	3	3
1	1	4	4	4	4	1	1	3	3
1	1	4	4	4	4	1	1	3	3
1	1	4	4	4	4	1	1	3	3
1	1	4	4	4	4	1	1	3	3
1	1	1	1	4	4	1	1	3	3
1	1	4	4	4	4	1	1	3	3
1	1	4	4	4	4	1	1	3	3
1	1	4	4	4	4	1	1	3	3

For Review Only

1	1	4	4	4	4	1	1	3	3
1	1	1	1	4	4	1	1	3	3
1	1	4	4	4	4	1	1	2	2
3	3	4	4	4	4	1	1	3	3
1	1	4	4	4	4	1	1	3	3
1	1	4	4	4	4	1	1	3	3
3	3	4	4	4	4	1	1	3	3
1	1	4	4	4	4	1	1	3	3
3	3	4	4	1	1	1	1	3	3
1	1	4	4	4	4	1	1	3	3
1	1	4	4	4	4	1	1	3	3
1	1	4	4	4	4	1	1	3	3
1	1	4	4	4	4	1	1	3	3
1	1	4	4	4	4	1	1	3	3
1	1	4	4	4	4	1	1	3	3
1	1	1	1	4	4	1	1	3	3
1	1	4	4	4	4	1	1	3	3
1	1	4	4	4	4	3	3	3	3
1	1	4	4	4	4	3	3	3	3
1	1	4	4	4	4	3	3	3	3
1	1	4	4	4	4	1	1	3	3
1	1	4	4	4	4	1	1	3	3
1	1	4	4	4	4	1	1	3	3
1	1	4	4	4	4	1	1	3	3
1	1	4	4	4	4	1	1	3	3
1	1	1	1	4	4	1	1	3	3
1	1	4	4	4	4	1	1	3	3
1	1	4	4	4	4	1	1	3	3
1	1	4	4	4	4	1	1	3	3
1	1	4	4	4	4	1	1	3	3
1	1	4	4	4	4	1	1	3	3
1	1	4	4	4	4	1	1	3	3
1	1	4	4	4	4	1	1	2	2
1	1	4	4	4	4	1	1	3	3
1	1	4	4	4	4	1	1	3	3
1	1	4	4	4	4	1	1	3	3
3	3	4	4	4	4	1	1	3	3
1	1	4	4	4	4	1	1	3	3
1	1	4	4	4	4	1	1	3	3
1	1	4	4	4	4	1	1	3	3
1	1	4	4	4	4	1	1	3	3
1	1	4	4	4	4	1	1	3	3
1	1	4	4	4	4	1	1	3	3
1	1	4	4	4	4	1	1	3	3
1	1	4	4	4	4	1	1	2	2
1	1	4	4	4	4	1	1	3	3
1	1	1	1	4	4	1	1	3	3
1	1	4	4	4	4	1	1	3	3
1	1	4	4	4	4	1	1	2	2
1	1	1	1	4	4	1	1	3	3
1	1	4	4	4	4	1	1	3	3
1	1	4	4	4	4	1	1	3	3
1	1	4	4	4	4	1	1	3	3
1	1	4	4	4	4	1	1	3	3
1	1	4	4	4	4	1	1	3	3

1	1	4	4	4	4	1	1	3	3
1	1	4	4	4	4	1	1	2	2
1	1	4	4	4	4	1	1	3	3
1	1	4	4	4	4	1	1	3	3
1	1	4	4	4	4	1	1	3	3
1	1	4	4	4	4	1	1	2	2
1	1	4	4	4	4	1	1	3	3
1	1	4	4	4	4	1	1	3	3
1	1	4	4	4	4	1	1	3	3
1	1	4	4	4	4	1	1	2	2
1	1	4	4	4	4	1	1	3	3
1	1	4	4	4	4	1	1	3	3
1	1	4	4	4	4	1	1	3	3
1	1	4	4	4	4	3	3	3	3
1	1	4	4	4	4	1	1	3	3
1	1	1	1	4	4	1	1	3	3
3	3	4	4	1	1	1	1	3	3
1	1	4	4	4	4	1	1	3	3
1	1	4	4	4	4	1	1	3	3
1	1	4	4	4	4	1	1	3	3
1	1	4	4	4	4	1	1	3	3
1	1	4	4	4	4	1	1	3	3
1	1	4	4	4	4	1	1	3	3
3	3	4	4	1	1	1	1	3	3
1	1	4	4	4	4	1	1	3	3
1	1	4	4	4	4	1	1	3	3
1	1	4	4	4	4	1	1	2	2
1	1	4	4	4	4	1	1	3	3
1	1	4	4	4	4	1	1	3	3
1	1	4	4	4	4	1	1	3	3
3	3	4	4	1	1	1	1	3	3
1	1	4	4	4	4	1	1	3	3
1	1	1	1	4	4	1	1	3	3
1	1	4	4	4	4	1	1	3	3
1	1	4	4	4	4	1	1	3	3
3	3	4	4	4	4	1	1	2	2
1	1	4	4	4	4	1	1	3	3
1	1	4	4	4	4	1	1	3	3
1	1	4	4	4	4	1	1	3	3
3	3	4	4	1	1	1	1	3	3
1	1	4	4	4	4	1	1	3	3
1	1	4	4	4	4	1	1	2	2
3	3	4	4	1	1	1	1	3	3
1	1	4	4	4	4	1	1	3	3
3	3	4	4	4	4	1	1	3	3
1	1	4	4	4	4	1	1	3	3
1	1	4	4	4	4	1	1	3	3
1	1	4	4	4	4	1	1	3	3
1	1	4	4	4	4	1	1	3	3
3	3	4	4	1	1	1	1	3	3
1	1	4	4	4	4	1	1	3	3
3	3	4	4	1	1	1	1	3	3
1	1	4	4	4	4	1	1	3	3
1	1	4	4	4	4	1	1	3	3
3	3	4	4	1	1	1	1	3	3

For Review Only

1	1	1	1	4	4	1	1	3	3
1	1	4	4	4	4	1	1	3	3

For Review Only

SNP2590	SNP2590	SNP2591	SNP2591	SNP2592	SNP2592	SNP2593	SNP2593	SNP2594	SNP2594
1	1	3	3	3	3	2	2	3	3
1	1	3	3	3	3	2	2	3	3
1	1	3	3	3	3	2	2	3	3
1	1	3	3	3	3	2	2	3	3
1	1	3	3	3	3	2	2	3	3
1	1	3	3	3	3	2	2	3	3
1	1	3	3	3	3	2	2	3	3
4	4	2	2	2	2	3	3	3	3
1	1	3	3	3	3	2	2	3	3
1	1	3	3	2	2	3	3	3	3
4	4	3	3	3	3	2	2	1	1
1	1	3	3	3	3	2	2	3	3
1	1	3	3	3	3	2	2	3	3
4	4	3	3	3	3	2	2	3	3
1	1	3	3	3	3	2	2	3	3
1	1	3	3	3	3	2	2	3	3
4	4	3	3	3	3	2	2	3	3
1	1	3	3	3	3	2	2	3	3
1	1	3	3	3	3	2	2	3	3
4	4	3	3	3	3	2	2	3	3
1	1	3	3	3	3	2	2	3	3
1	1	3	3	2	2	3	3	3	3
4	4	3	3	3	3	2	2	1	1
4	4	3	3	3	3	2	2	3	3
1	1	3	3	3	3	2	2	3	3
1	1	3	3	3	3	2	2	3	3
1	1	3	3	3	3	2	2	3	3
1	1	3	3	3	3	2	2	3	3
4	4	3	3	3	3	2	2	3	3
4	4	3	3	3	3	2	2	3	3
1	1	3	3	3	3	2	2	3	3
4	4	3	3	3	3	2	2	3	3
4	4	3	3	3	3	2	2	3	3
4	4	2	2	2	2	3	3	3	3
1	1	3	3	2	2	3	3	3	3
1	1	3	3	3	3	2	2	3	3
1	1	3	3	3	3	2	2	3	3
1	1	3	3	3	3	2	2	3	3
4	4	3	3	3	3	2	2	3	3
4	4	3	3	3	3	2	2	3	3
4	4	3	3	3	3	2	2	3	3
4	4	3	3	3	3	2	2	3	3
4	4	3	3	3	3	2	2	3	3
1	1	3	3	3	3	2	2	3	3
4	4	3	3	3	3	2	2	3	3
1	1	3	3	3	3	2	2	3	3
1	1	3	3	3	3	2	2	3	3
1	1	3	3	3	3	2	2	3	3
4	4	3	3	3	3	2	2	3	3
1	1	3	3	3	3	2	2	3	3
1	1	3	3	3	3	2	2	3	3
1	1	3	3	3	3	2	2	3	3

For Review Only

1	1	3	3	3	3	2	2	3	3
4	4	3	3	3	3	2	2	3	3
1	1	3	3	3	3	2	2	3	3
1	1	3	3	3	3	2	2	3	3
1	1	3	3	3	3	2	2	3	3
1	1	3	3	3	3	2	2	3	3
1	1	3	3	3	3	2	2	3	3
4	4	2	2	2	2	3	3	3	3
4	4	3	3	3	3	2	2	3	3
1	1	3	3	3	3	2	2	3	3
1	1	3	3	3	3	2	2	3	3
1	1	3	3	3	3	2	2	3	3
1	1	3	3	3	3	2	2	3	3
4	4	3	3	3	3	2	2	3	3
1	1	3	3	3	3	2	2	3	3
1	1	3	3	2	2	3	3	3	3
4	4	3	3	3	3	2	2	3	3
1	1	3	3	3	3	2	2	3	3
1	1	3	3	3	3	2	2	3	3
1	1	3	3	3	3	2	2	3	3
1	1	3	3	3	3	2	2	3	3
4	4	3	3	3	3	2	2	3	3
4	4	3	3	3	3	2	2	3	3
4	4	3	3	3	3	2	2	3	3
4	4	3	3	3	3	2	2	3	3
1	1	3	3	3	3	2	2	3	3
4	4	3	3	2	2	3	3	3	3
4	4	3	3	3	3	2	2	3	3
1	1	3	3	3	3	2	2	3	3
4	4	3	3	3	3	2	2	3	3
1	1	3	3	3	3	2	2	3	3
1	1	3	3	3	3	2	2	3	3
4	4	3	3	3	3	2	2	3	3
1	1	3	3	3	3	2	2	3	3
1	1	3	3	3	3	2	2	3	3
4	4	3	3	3	3	2	2	3	3
1	1	3	3	3	3	2	2	3	3
4	4	3	3	3	3	2	2	3	3
1	1	3	3	3	3	2	2	3	3
4	4	3	3	3	3	2	2	3	3
1	1	3	3	3	3	2	2	3	3
4	4	3	3	3	3	2	2	3	3
4	4	3	3	3	3	2	2	3	3
1	1	3	3	3	3	2	2	3	3
1	1	3	3	3	3	2	2	3	3
1	1	3	3	3	3	2	2	3	3
4	4	3	3	3	3	2	2	3	3
1	1	3	3	3	3	2	2	3	3
1	1	3	3	3	3	2	2	3	3
4	4	3	3	3	3	2	2	3	3
1	1	3	3	3	3	2	2	3	3
4	4	3	3	3	3	2	2	3	3
4	4	2	2	2	2	3	3	3	3
1	1	2	2	2	2	2	2	3	3
1	1	3	3	3	3	2	2	3	3

For Review Only

4	4	3	3	3	3	2	2	3	3
1	1	3	3	3	3	2	2	3	3
1	1	3	3	3	3	2	2	3	3
4	4	3	3	3	3	2	2	3	3
1	1	3	3	3	3	2	2	3	3
1	1	3	3	3	3	2	2	3	3
1	1	3	3	3	3	2	2	3	3
1	1	3	3	3	3	2	2	3	3
4	4	3	3	3	3	2	2	3	3
1	1	2	2	2	2	3	3	3	3
1	1	3	3	3	3	2	2	3	3
1	1	3	3	3	3	2	2	3	3
4	4	3	3	3	3	2	2	3	3
1	1	3	3	3	3	2	2	3	3
1	1	3	3	3	3	2	2	3	3
1	1	3	3	3	3	2	2	3	3
4	4	2	2	2	2	3	3	3	3
1	1	3	3	3	3	2	2	3	3
4	4	3	3	3	3	2	2	3	3
1	1	3	3	3	3	2	2	3	3
1	1	3	3	3	3	2	2	3	3
4	4	3	3	3	3	2	2	3	3
1	1	3	3	3	3	2	2	3	3
4	4	3	3	3	3	2	2	3	3
4	4	3	3	3	3	2	2	3	3
1	1	3	3	3	3	2	2	3	3
1	1	3	3	3	3	2	2	3	3
1	1	3	3	3	3	2	2	3	3
1	1	3	3	3	3	2	2	3	3
1	1	3	3	3	3	2	2	3	3
4	4	2	2	2	2	2	2	3	3
1	1	3	3	3	3	2	2	3	3
1	1	3	3	3	3	2	2	3	3
1	1	3	3	3	3	2	2	3	3
4	4	2	2	2	2	3	3	3	3
1	1	3	3	3	3	2	2	3	3
4	4	3	3	3	3	2	2	3	3
1	1	3	3	3	3	2	2	3	3
4	4	3	3	3	3	2	2	3	3
4	4	3	3	3	3	2	2	3	3
4	4	3	3	3	3	2	2	1	1
4	4	3	3	3	3	2	2	3	3
1	1	3	3	3	3	2	2	3	3
4	4	3	3	2	2	3	3	3	3
1	1	3	3	3	3	2	2	3	3
1	1	3	3	3	3	2	2	3	3
1	1	3	3	3	3	2	2	3	3
1	1	3	3	3	3	2	2	3	3
1	1	3	3	3	3	2	2	3	3
1	1	3	3	3	3	2	2	3	3
1	1	3	3	3	3	2	2	3	3
4	4	3	3	3	3	2	2	1	1
4	4	3	3	2	2	2	2	3	3
1	1	3	3	3	3	2	2	3	3
1	1	2	2	2	2	2	2	3	3

1	1	3	3	3	3	2	2	3	3
4	4	3	3	3	3	2	2	1	1
1	1	3	3	3	3	2	2	3	3
4	4	3	3	3	3	2	2	3	3
1	1	3	3	3	3	2	2	3	3
4	4	3	3	3	3	2	2	3	3
4	4	3	3	3	3	2	2	3	3
1	1	3	3	3	3	2	2	3	3
1	1	3	3	3	3	2	2	3	3
1	1	3	3	3	3	2	2	3	3
1	1	3	3	3	3	2	2	3	3
4	4	3	3	3	3	2	2	3	3
1	1	3	3	3	3	2	2	3	3
1	1	3	3	3	3	2	2	3	3
4	4	3	3	3	3	2	2	3	3
4	4	3	3	3	3	2	2	1	1
4	4	3	3	3	3	2	2	3	3
1	1	3	3	3	3	2	2	3	3
1	1	3	3	3	3	2	2	3	3
1	1	3	3	3	3	2	2	3	3
1	1	3	3	3	3	2	2	3	3
1	1	3	3	3	3	2	2	3	3
4	4	3	3	3	3	2	2	3	3
1	1	3	3	3	3	2	2	3	3
1	1	3	3	3	3	2	2	3	3
4	4	2	2	2	2	3	3	1	1
1	1	3	3	3	3	2	2	3	3
4	4	3	3	2	2	3	3	3	3
4	4	3	3	3	3	2	2	3	3
4	4	3	3	3	3	2	2	3	3
1	1	3	3	3	3	2	2	3	3
1	1	3	3	3	3	2	2	3	3
1	1	3	3	3	3	2	2	3	3
4	4	3	3	3	3	2	2	3	3
1	1	3	3	3	3	2	2	3	3
1	1	3	3	3	3	2	2	3	3
1	1	3	3	3	3	2	2	3	3
1	1	3	3	3	3	2	2	3	3
1	1	3	3	3	3	2	2	3	3
1	1	3	3	3	3	2	2	3	3
1	1	3	3	3	3	2	2	3	3
1	1	3	3	3	3	2	2	3	3
4	4	3	3	3	3	2	2	3	3
1	1	3	3	3	3	2	2	3	3
1	1	3	3	3	3	2	2	3	3
4	4	3	3	3	3	2	2	3	3
4	4	3	3	3	3	2	2	1	1
4	4	3	3	3	3	2	2	3	3
4	4	3	3	3	3	2	2	3	3
1	1	3	3	3	3	2	2	1	1
1	1	3	3	3	3	2	2	3	3
4	4	2	2	2	2	3	3	3	3
4	4	3	3	3	3	2	2	3	3
1	1	3	3	3	3	2	2	3	3
4	4	2	2	2	2	3	3	3	3

For Review Only

1	1	3	3	3	3	2	2	3	3
4	4	3	3	3	3	2	2	3	3
1	1	3	3	3	3	2	2	3	3
1	1	3	3	3	3	2	2	3	3
4	4	3	3	2	2	3	3	3	3
1	1	3	3	3	3	2	2	3	3
1	1	2	2	2	2	3	3	3	3
4	4	3	3	2	2	3	3	3	3
4	4	3	3	3	3	2	2	3	3
4	4	3	3	3	3	2	2	3	3
1	1	3	3	3	3	2	2	3	3
1	1	3	3	3	3	2	2	3	3
1	1	3	3	3	3	2	2	3	3
1	1	3	3	3	3	2	2	3	3
1	1	3	3	3	3	2	2	3	3
4	4	2	2	2	2	3	3	1	1
4	4	3	3	3	3	2	2	3	3
4	4	3	3	3	3	2	2	3	3
4	4	2	2	2	2	3	3	3	3
1	1	3	3	3	3	2	2	3	3
4	4	3	3	3	3	2	2	3	3
1	1	3	3	3	3	2	2	3	3
4	4	3	3	3	3	2	2	3	3
4	4	3	3	3	3	2	2	3	3
4	4	2	2	2	2	3	3	3	3
1	1	3	3	3	3	2	2	3	3
4	4	3	3	3	3	2	2	3	3
1	1	3	3	3	3	2	2	3	3
1	1	3	3	3	3	2	2	3	3
1	1	3	3	3	3	2	2	3	3
4	4	3	3	3	3	2	2	3	3
1	1	3	3	3	3	2	2	3	3
4	4	3	3	3	3	2	2	1	1
1	1	3	3	3	3	2	2	3	3
4	4	3	3	3	3	2	2	3	3
4	4	3	3	3	3	2	2	3	3
1	1	3	3	3	3	2	2	3	3
1	1	3	3	3	3	2	2	3	3
1	1	3	3	3	3	2	2	3	3
4	4	3	3	3	3	2	2	3	3
1	1	3	3	3	3	2	2	3	3
1	1	3	3	3	3	2	2	3	3
4	4	3	3	3	3	2	2	3	3
1	1	3	3	3	3	2	2	3	3
1	1	3	3	3	3	2	2	3	3
1	1	3	3	3	3	2	2	3	3
1	1	3	3	3	3	2	2	3	3
4	4	3	3	3	3	2	2	3	3
1	1	3	3	3	3	2	2	3	3
4	4	3	3	3	3	2	2	3	3
4	4	3	3	3	3	2	2	3	3
4	4	3	3	3	3	2	2	3	3
1	1	3	3	3	3	2	2	3	3
1	1	3	3	3	3	2	2	3	3
4	4	3	3	3	3	2	2	3	3

For Review Only

1	1	3	3	3	3	2	2	1	1
1	1	3	3	3	3	2	2	3	3

For Review Only

SNP2595	SNP2595	SNP2596	SNP2596	SNP2597	SNP2597	SNP2598	SNP2598	SNP2599	SNP2599
4	4	3	3	3	3	2	2	4	4
4	4	3	3	3	3	2	2	1	1
4	4	3	3	3	3	2	2	4	4
4	4	3	3	1	1	2	2	4	4
4	4	3	3	1	1	2	2	4	4
4	4	3	3	3	3	3	3	4	4
4	4	3	3	3	3	2	2	1	1
4	4	3	3	3	3	2	2	4	4
4	4	3	3	1	1	2	2	1	1
4	4	3	3	1	1	2	2	1	1
4	4	3	3	1	1	2	2	1	1
4	4	3	3	3	3	2	2	4	4
4	4	3	3	3	3	2	2	1	1
4	4	3	3	3	3	2	2	1	1
4	4	3	3	3	3	2	2	4	4
4	4	3	3	1	1	2	2	4	4
4	4	3	3	3	3	2	2	4	4
1	1	3	3	1	1	2	2	4	4
4	4	3	3	3	3	2	2	4	4
4	4	3	3	3	3	2	2	1	1
4	4	3	3	3	3	2	2	1	1
4	4	3	3	3	3	2	2	4	4
4	4	3	3	3	3	2	2	1	1
4	4	3	3	1	1	2	2	1	1
4	4	3	3	1	1	2	2	4	4
4	4	3	3	3	3	2	2	4	4
4	4	3	3	3	3	2	2	4	4
4	4	3	3	3	3	2	2	4	4
4	4	3	3	3	3	2	2	4	4
4	4	3	3	3	3	2	2	1	1
4	4	3	3	3	3	3	3	1	1
4	4	2	2	3	3	2	2	1	1
4	4	2	2	3	3	2	2	1	1
4	4	3	3	1	1	2	2	1	1
4	4	3	3	1	1	2	2	4	4
4	4	3	3	3	3	2	2	4	4
1	1	3	3	1	1	2	2	4	4
1	1	3	3	1	1	2	2	4	4
4	4	3	3	1	1	2	2	4	4
1	1	3	3	1	1	2	2	4	4
4	4	3	3	3	3	2	2	4	4
4	4	3	3	3	3	2	2	1	1
4	4	3	3	1	1	2	2	1	1
4	4	3	3	1	1	2	2	1	1
4	4	3	3	1	1	2	2	1	1
4	4	3	3	3	3	2	2	4	4
4	4	3	3	3	3	2	2	4	4
4	4	3	3	3	3	2	2	1	1
4	4	3	3	3	3	2	2	4	4
4	4	3	3	3	3	2	2	4	4
4	4	3	3	3	3	2	2	4	4
4	4	3	3	3	3	3	3	1	1

For Review Only

4	4	3	3	3	3	2	2	4	4
4	4	2	2	3	3	2	2	1	1
4	4	3	3	3	3	2	2	4	4
4	4	3	3	3	3	2	2	1	1
4	4	3	3	3	3	2	2	4	4
4	4	3	3	3	3	2	2	4	4
4	4	3	3	3	3	2	2	4	4
4	4	3	3	1	1	2	2	4	4
4	4	3	3	3	3	2	2	1	1
4	4	3	3	3	3	2	2	4	4
4	4	3	3	3	3	2	2	4	4
4	4	3	3	3	3	2	2	4	4
4	4	3	3	3	3	2	2	4	4
4	4	3	3	3	3	2	2	4	4
4	4	3	3	1	1	2	2	4	4
1	1	3	3	1	1	2	2	1	1
4	4	3	3	3	3	2	2	1	1
4	4	3	3	3	3	3	3	4	4
4	4	3	3	3	3	2	2	1	1
4	4	3	3	3	3	2	2	1	1
4	4	3	3	3	3	2	2	4	4
4	4	3	3	1	1	2	2	1	1
4	4	3	3	3	3	2	2	4	4
1	1	3	3	3	3	2	2	4	4
4	4	3	3	1	1	2	2	1	1
4	4	3	3	3	3	2	2	4	4
4	4	3	3	3	3	2	2	4	4
4	4	3	3	1	1	2	2	1	1
4	4	3	3	3	3	2	2	4	4
4	4	3	3	3	3	2	2	1	1
4	4	3	3	1	1	2	2	1	1
4	4	3	3	3	3	2	2	1	1
4	4	3	3	3	3	2	2	4	4
4	4	3	3	3	3	2	2	4	4
1	1	3	3	3	3	2	2	1	1
4	4	3	3	1	1	2	2	1	1
4	4	3	3	1	1	2	2	1	1
4	4	3	3	3	3	2	2	1	1
4	4	3	3	3	3	3	3	1	1
4	4	3	3	3	3	2	2	1	1
4	4	3	3	3	3	2	2	1	1
4	4	2	2	3	3	2	2	4	4
4	4	3	3	3	3	2	2	4	4
4	4	3	3	3	3	2	2	4	4
4	4	3	3	3	3	2	2	1	1
1	1	3	3	3	3	2	2	1	1
4	4	3	3	3	3	2	2	1	1
4	4	3	3	3	3	2	2	4	4
4	4	3	3	3	3	2	2	4	4
4	4	2	2	1	1	2	2	4	4
4	4	3	3	3	3	2	2	4	4
4	4	3	3	1	1	2	2	4	4
4	4	3	3	3	3	2	2	4	4
4	4	3	3	1	1	2	2	4	4
1	1	3	3	1	1	2	2	4	4

For Review Only

4	4	3	3	3	3	2	2	4	4
4	4	3	3	1	1	2	2	4	4
4	4	3	3	3	3	2	2	4	4
4	4	3	3	1	1	2	2	1	1
4	4	3	3	3	3	2	2	4	4
4	4	3	3	3	3	2	2	4	4
4	4	3	3	3	3	2	2	4	4
4	4	3	3	1	1	3	3	1	1
4	4	3	3	3	3	2	2	4	4
4	4	3	3	3	3	2	2	4	4
4	4	3	3	3	3	2	2	4	4
4	4	3	3	1	1	2	2	4	4
4	4	3	3	3	3	2	2	4	4
4	4	3	3	1	1	2	2	4	4
4	4	3	3	1	1	2	2	4	4
4	4	3	3	1	1	2	2	4	4
1	1	3	3	3	3	2	2	1	1
4	4	3	3	1	1	2	2	4	4
4	4	3	3	1	1	2	2	4	4
4	4	3	3	1	1	2	2	1	1
4	4	3	3	1	1	2	2	4	4
4	4	2	2	3	3	2	2	1	1
4	4	3	3	3	3	2	2	4	4
4	4	3	3	1	1	2	2	1	1
4	4	3	3	1	1	2	2	4	4
1	1	3	3	1	1	2	2	1	1
1	1	3	3	3	3	2	2	1	1
4	4	3	3	3	3	2	2	1	1
4	4	3	3	3	3	2	2	4	4
1	1	3	3	3	3	2	2	4	4
4	4	3	3	1	1	2	2	4	4
4	4	3	3	1	1	2	2	4	4
4	4	3	3	3	3	2	2	1	1
4	4	3	3	1	1	2	2	4	4
4	4	3	3	1	1	2	2	1	1
4	4	3	3	3	3	2	2	1	1
4	4	3	3	1	1	2	2	1	1
4	4	3	3	3	3	2	2	4	4
1	1	3	3	1	1	2	2	1	1
4	4	3	3	1	1	2	2	1	1
1	1	3	3	1	1	2	2	1	1
4	4	3	3	3	3	2	2	1	1
4	4	3	3	3	3	3	3	1	1
4	4	3	3	1	1	2	2	1	1
4	4	3	3	3	3	2	2	4	4
4	4	3	3	1	1	2	2	4	4
4	4	3	3	3	3	2	2	4	4
4	4	3	3	3	3	2	2	4	4
4	4	3	3	1	1	2	2	4	4
4	4	3	3	3	3	2	2	4	4
4	4	3	3	3	3	2	2	4	4
4	4	3	3	1	1	2	2	4	4
4	4	3	3	1	1	2	2	4	4
4	4	3	3	3	3	2	2	4	4
4	4	3	3	1	1	2	2	4	4

For Review Only

4	4	3	3	3	3	2	2	4	4
4	4	3	3	3	3	2	2	1	1
4	4	3	3	1	1	2	2	1	1
1	1	3	3	1	1	2	2	1	1
4	4	3	3	3	3	2	2	1	1
4	4	3	3	1	1	2	2	1	1
4	4	3	3	1	1	2	2	1	1
4	4	3	3	3	3	2	2	4	4
4	4	3	3	1	1	2	2	4	4
4	4	3	3	3	3	2	2	4	4
4	4	3	3	3	3	2	2	4	4
4	4	3	3	1	1	2	2	1	1
4	4	3	3	3	3	2	2	4	4
4	4	3	3	3	3	2	2	4	4
4	4	3	3	3	3	2	2	1	1
4	4	3	3	1	1	2	2	1	1
4	4	3	3	3	3	2	2	4	4
1	1	3	3	3	3	2	2	1	1
4	4	3	3	3	3	2	2	4	4
4	4	3	3	3	3	2	2	4	4
4	4	3	3	1	1	2	2	4	4
4	4	3	3	1	1	2	2	4	4
1	1	3	3	1	1	2	2	1	1
4	4	3	3	3	3	2	2	4	4
4	4	3	3	3	3	2	2	1	1
4	4	3	3	1	1	2	2	4	4
4	4	3	3	3	3	2	2	4	4
1	1	3	3	1	1	2	2	1	1
4	4	3	3	3	3	2	2	4	4
4	4	3	3	3	3	3	3	4	4
4	4	3	3	3	3	2	2	4	4
4	4	3	3	3	3	2	2	1	1
1	1	3	3	3	3	2	2	1	1
4	4	3	3	3	3	2	2	1	1
4	4	3	3	3	3	2	2	4	4
4	4	3	3	3	3	2	2	4	4
4	4	3	3	3	3	2	2	4	4
1	1	3	3	3	3	2	2	4	4
4	4	3	3	3	3	2	2	4	4
4	4	3	3	3	3	2	2	4	4
4	4	3	3	3	3	2	2	4	4
4	4	3	3	3	3	2	2	4	4
4	4	3	3	1	1	2	2	1	1
4	4	3	3	3	3	2	2	4	4
4	4	3	3	1	1	2	2	4	4
1	1	3	3	3	3	2	2	4	4
4	4	3	3	1	1	2	2	1	1
4	4	3	3	1	1	2	2	1	1
4	4	3	3	3	3	2	2	1	1
4	4	3	3	3	3	2	2	1	1
4	4	3	3	3	3	3	3	4	4
4	4	3	3	1	1	2	2	1	1
1	1	3	3	1	1	2	2	4	4
4	4	3	3	3	3	2	2	4	4
4	4	3	3	1	1	2	2	1	1

For Review Only

4	4	3	3	3	3	3	3	4	4
4	4	3	3	3	3	2	2	4	4
4	4	3	3	3	3	2	2	1	1
4	4	3	3	1	1	2	2	1	1
1	1	3	3	3	3	2	2	4	4
4	4	3	3	3	3	2	2	4	4
1	1	3	3	1	1	2	2	1	1
4	4	3	3	1	1	2	2	1	1
4	4	3	3	1	1	2	2	4	4
4	4	3	3	1	1	2	2	1	1
4	4	3	3	1	1	2	2	4	4
4	4	3	3	3	3	2	2	4	4
4	4	3	3	3	3	3	3	1	1
4	4	3	3	3	3	2	2	1	1
4	4	3	3	3	3	2	2	4	4
4	4	3	3	1	1	2	2	1	1
4	4	3	3	1	1	2	2	4	4
4	4	3	3	1	1	2	2	1	1
4	4	3	3	1	1	2	2	4	4
1	1	3	3	1	1	2	2	4	4
1	1	3	3	1	1	2	2	4	4
4	4	3	3	3	3	2	2	4	4
4	4	2	2	3	3	3	3	1	1
4	4	3	3	3	3	2	2	1	1
4	4	3	3	1	1	2	2	4	4
4	4	3	3	3	3	2	2	1	1
4	4	3	3	3	3	2	2	1	1
4	4	3	3	3	3	2	2	4	4
1	1	3	3	3	3	2	2	4	4
4	4	3	3	3	3	3	3	4	4
4	4	3	3	1	1	2	2	1	1
4	4	3	3	3	3	2	2	4	4
4	4	3	3	3	3	2	2	1	1
4	4	3	3	3	3	2	2	4	4
1	1	3	3	1	1	2	2	1	1
4	4	3	3	1	1	2	2	1	1
4	4	3	3	3	3	3	3	1	1
4	4	3	3	3	3	2	2	4	4
4	4	3	3	3	3	2	2	4	4
4	4	3	3	1	1	2	2	4	4
4	4	3	3	3	3	2	2	4	4
4	4	3	3	3	3	2	2	4	4
4	4	3	3	3	3	2	2	1	1
4	4	3	3	3	3	2	2	1	1
4	4	3	3	3	3	2	2	4	4
1	1	3	3	3	3	2	2	1	1
4	4	3	3	3	3	2	2	4	4
4	4	3	3	3	3	2	2	4	4
4	4	3	3	3	3	2	2	4	4
4	4	3	3	1	1	2	2	1	1
4	4	2	2	3	3	3	3	1	1
4	4	3	3	1	1	2	2	4	4
4	4	3	3	3	3	2	2	4	4
4	4	3	3	3	3	2	2	1	1
4	4	3	3	1	1	2	2	4	4

For Review Only

4	4	3	3	1	1	2	2	1	1
4	4	3	3	3	3	2	2	4	4

For Review Only

SNP260	SNP260	SNP2600	SNP2600	SNP2601	SNP2601	SNP2602	SNP2602	SNP2603	SNP2603
4	4	4	4	2	2	1	1	3	3
1	1	4	4	2	2	1	1	4	4
1	1	4	4	2	2	1	1	4	4
4	4	4	4	2	2	1	1	4	4
4	4	4	4	2	2	1	1	3	3
4	4	4	4	2	2	1	1	3	3
4	4	4	4	2	2	1	1	3	3
1	1	4	4	2	2	1	1	4	4
4	4	4	4	2	2	1	1	3	3
1	1	4	4	2	2	1	1	4	4
4	4	4	4	2	2	4	4	4	4
1	1	4	4	2	2	1	1	4	4
1	1	4	4	2	2	1	1	4	4
1	1	4	4	2	2	1	1	4	4
4	4	4	4	2	2	1	1	3	3
1	1	4	4	2	2	1	1	4	4
1	1	4	4	2	2	1	1	4	4
1	1	4	4	2	2	1	1	4	4
1	1	4	4	2	2	1	1	4	4
1	1	4	4	2	2	1	1	4	4
1	1	4	4	3	3	1	1	4	4
1	1	4	4	2	2	1	1	4	4
1	1	4	4	2	2	4	4	4	4
1	1	4	4	2	2	1	1	4	4
4	4	4	4	2	2	1	1	4	4
1	1	4	4	2	2	1	1	4	4
1	1	4	4	2	2	1	1	4	4
4	4	4	4	2	2	1	1	4	4
1	1	4	4	2	2	1	1	4	4
1	1	4	4	2	2	1	1	4	4
1	1	4	4	2	2	1	1	4	4
1	1	4	4	3	3	1	1	4	4
4	4	4	4	3	3	1	1	4	4
1	1	4	4	2	2	1	1	4	4
4	4	4	4	2	2	4	4	4	4
1	1	4	4	2	2	1	1	4	4
4	4	4	4	2	2	1	1	4	4
4	4	4	4	2	2	1	1	4	4
1	1	4	4	2	2	1	1	4	4
1	1	4	4	2	2	1	1	4	4
1	1	4	4	2	2	1	1	4	4
1	1	4	4	2	2	1	1	4	4
1	1	4	4	2	2	1	1	4	4
4	4	4	4	3	3	1	1	4	4
1	1	4	4	2	2	1	1	4	4
1	1	4	4	2	2	1	1	4	4
4	4	4	4	2	2	1	1	4	4
1	1	4	4	2	2	1	1	4	4
4	4	4	4	2	2	1	1	4	4
4	4	4	4	2	2	1	1	4	4
4	4	4	4	2	2	1	1	4	4
1	1	4	4	2	2	1	1	4	4

For Review Only

4	4	4	4	3	3	1	1	4	4
4	4	4	4	3	3	1	1	4	4
4	4	4	4	2	2	1	1	4	4
1	1	4	4	3	3	1	1	4	4
1	1	4	4	2	2	1	1	4	4
1	1	4	4	2	2	1	1	4	4
1	1	4	4	2	2	1	1	4	4
1	1	4	4	2	2	1	1	4	4
4	4	4	4	2	2	1	1	4	4
1	1	4	4	2	2	1	1	4	4
1	1	4	4	2	2	1	1	4	4
1	1	4	4	2	2	1	1	4	4
4	4	4	4	3	3	1	1	4	4
1	1	4	4	2	2	1	1	4	4
4	4	4	4	2	2	1	1	3	3
4	4	4	4	2	2	1	1	4	4
4	4	4	4	2	2	1	1	4	4
4	4	4	4	2	2	1	1	4	4
4	4	4	4	2	2	1	1	4	4
4	4	4	4	2	2	1	1	3	3
1	1	4	4	2	2	1	1	4	4
4	4	4	4	2	2	1	1	4	4
4	4	4	4	2	2	1	1	4	4
1	1	4	4	2	2	1	1	4	4
1	1	4	4	2	2	1	1	4	4
1	1	4	4	2	2	1	1	4	4
4	4	4	4	2	2	1	1	4	4
1	1	1	1	2	2	1	1	4	4
1	1	4	4	2	2	1	1	4	4
1	1	4	4	3	3	1	1	4	4
4	4	4	4	3	3	1	1	4	4
1	1	4	4	2	2	1	1	4	4
1	1	4	4	2	2	1	1	4	4
4	4	4	4	3	3	1	1	4	4
4	4	4	4	2	2	1	1	3	3
1	1	4	4	2	2	1	1	4	4
1	1	4	4	2	2	1	1	4	4
4	4	4	4	2	2	1	1	4	4
1	1	4	4	2	2	1	1	4	4
4	4	4	4	2	2	1	1	4	4
4	4	4	4	3	3	1	1	4	4
1	1	4	4	3	3	1	1	4	4
4	4	4	4	2	2	1	1	4	4
1	1	4	4	2	2	1	1	4	4
4	4	4	4	2	2	1	1	3	3
4	4	4	4	2	2	1	1	4	4
1	1	4	4	2	2	1	1	4	4
1	1	4	4	2	2	1	1	4	4
1	1	4	4	2	2	1	1	4	4
4	4	4	4	3	3	1	1	4	4
1	1	4	4	2	2	1	1	4	4
1	1	1	1	2	2	1	1	4	4
4	4	4	4	2	2	1	1	4	4
1	1	4	4	2	2	1	1	4	4
4	4	4	4	2	2	1	1	3	3

For Review Only

1	1	4	4	2	2	4	4	4	4
4	4	4	4	2	2	1	1	4	4
1	1	4	4	2	2	1	1	4	4
1	1	4	4	2	2	1	1	4	4
4	4	4	4	2	2	1	1	4	4
1	1	4	4	2	2	1	1	4	4
1	1	4	4	2	2	1	1	4	4
4	4	4	4	2	2	1	1	4	4
1	1	1	1	2	2	1	1	4	4
4	4	4	4	2	2	1	1	4	4
4	4	4	4	2	2	1	1	4	4
4	4	4	4	2	2	1	1	4	4
4	4	4	4	2	2	1	1	4	4
1	1	4	4	2	2	1	1	4	4
1	1	4	4	2	2	1	1	4	4
4	4	4	4	2	2	1	1	4	4
4	4	4	4	2	2	1	1	4	4
1	1	4	4	2	2	1	1	4	4
1	1	4	4	2	2	1	1	4	4
4	4	4	4	3	3	1	1	4	4
4	4	4	4	2	2	1	1	4	4
4	4	4	4	3	3	1	1	4	4
1	1	4	4	2	2	1	1	4	4
1	1	1	1	2	2	1	1	4	4
4	4	4	4	2	2	1	1	4	4
4	4	4	4	2	2	1	1	4	4
1	1	4	4	2	2	1	1	4	4
1	1	4	4	2	2	1	1	4	4
1	1	4	4	2	2	1	1	4	4
1	1	4	4	2	2	1	1	4	4
1	1	1	1	2	2	1	1	4	4
1	1	4	4	2	2	1	1	4	4
4	4	4	4	3	3	1	1	4	4
4	4	4	4	2	2	1	1	4	4
1	1	4	4	2	2	1	1	4	4
4	4	4	4	2	2	1	1	4	4
1	1	4	4	2	2	1	1	4	4
1	1	4	4	2	2	1	1	4	4
4	4	1	1	2	2	1	1	4	4
4	4	4	4	2	2	4	4	4	4
1	1	4	4	2	2	4	4	4	4
1	1	4	4	2	2	1	1	4	4
4	4	4	4	2	2	1	1	3	3
1	1	4	4	2	2	1	1	4	4
4	4	4	4	2	2	1	1	4	4
1	1	4	4	2	2	1	1	3	3
1	1	4	4	2	2	1	1	4	4
1	1	4	4	2	2	1	1	4	4
4	4	4	4	2	2	1	1	4	4
1	1	4	4	2	2	1	1	4	4
1	1	4	4	2	2	1	1	4	4
4	4	4	4	2	2	4	4	4	4
4	4	4	4	2	2	1	1	4	4
1	1	4	4	2	2	1	1	4	4
1	1	4	4	2	2	1	1	4	4

For Review Only

4	4	4	4	2	2	1	1	4	4
4	4	4	4	2	2	4	4	4	4
1	1	4	4	2	2	1	1	4	4
4	4	4	4	2	2	1	1	4	4
1	1	4	4	2	2	1	1	4	4
4	4	4	4	2	2	1	1	4	4
4	4	4	4	2	2	1	1	4	4
4	4	4	4	2	2	1	1	4	4
1	1	4	4	2	2	1	1	4	4
1	1	4	4	2	2	1	1	4	4
1	1	4	4	2	2	1	1	4	4
4	4	1	1	2	2	1	1	4	4
4	4	4	4	2	2	1	1	4	4
4	4	4	4	2	2	4	4	4	4
1	1	4	4	2	2	1	1	4	4
4	4	4	4	2	2	4	4	4	4
1	1	4	4	2	2	1	1	4	4
1	1	4	4	2	2	1	1	4	4
1	1	4	4	2	2	1	1	4	4
1	1	4	4	2	2	1	1	4	4
4	4	4	4	2	2	1	1	4	4
1	1	4	4	2	2	1	1	4	4
4	4	4	4	2	2	1	1	4	4
1	1	4	4	2	2	1	1	4	4
1	1	4	4	3	3	1	1	4	4
4	4	4	4	2	2	4	4	4	4
4	4	4	4	2	2	1	1	4	4
4	4	4	4	2	2	1	1	4	4
1	1	4	4	2	2	1	1	4	4
4	4	4	4	2	2	1	1	4	4
4	4	4	4	2	2	1	1	3	3
1	1	4	4	2	2	1	1	4	4
1	1	4	4	2	2	1	1	4	4
1	1	4	4	2	2	1	1	4	4
4	4	4	4	2	2	1	1	4	4
1	1	4	4	2	2	1	1	4	4
1	1	4	4	2	2	1	1	4	4
4	4	4	4	2	2	1	1	4	4
1	1	4	4	2	2	1	1	4	4
1	1	4	4	2	2	1	1	3	3
1	1	4	4	2	2	1	1	4	4
1	1	4	4	2	2	1	1	4	4
1	1	1	1	2	2	1	1	4	4
4	4	4	4	2	2	1	1	4	4
1	1	4	4	2	2	1	1	4	4
1	1	4	4	2	2	1	1	4	4
4	4	4	4	2	2	4	4	4	4
1	1	4	4	2	2	1	1	4	4
1	1	4	4	2	2	1	1	4	4
1	1	4	4	2	2	4	4	4	4
1	1	4	4	2	2	1	1	4	4
4	4	4	4	2	2	1	1	4	4
4	4	4	4	2	2	1	1	4	4
1	1	4	4	2	2	1	1	4	4
1	1	1	1	2	2	1	1	4	4

For Review Only

1	1	4	4	2	2	1	1	4	4
1	1	4	4	2	2	1	1	4	4
1	1	4	4	2	2	1	1	4	4
4	4	4	4	2	2	1	1	4	4
4	4	4	4	2	2	1	1	4	4
1	1	4	4	2	2	1	1	4	4
4	4	4	4	2	2	1	1	3	3
1	1	4	4	2	2	1	1	4	4
1	1	4	4	2	2	1	1	4	4
1	1	1	1	2	2	1	1	4	4
4	4	4	4	2	2	1	1	4	4
4	4	4	4	2	2	1	1	3	3
1	1	4	4	2	2	1	1	4	4
1	1	4	4	2	2	1	1	4	4
1	1	4	4	2	2	1	1	4	4
4	4	4	4	2	2	4	4	4	4
1	1	4	4	2	2	1	1	4	4
4	4	4	4	2	2	1	1	4	4
1	1	4	4	2	2	1	1	4	4
4	4	4	4	2	2	1	1	4	4
1	1	4	4	2	2	1	1	4	4
1	1	4	4	2	2	1	1	4	4
4	4	4	4	3	3	1	1	4	4
4	4	4	4	2	2	1	1	4	4
1	1	4	4	2	2	1	1	4	4
1	1	4	4	2	2	1	1	4	4
1	1	4	4	2	2	1	1	4	4
1	1	4	4	2	2	1	1	4	4
4	4	4	4	2	2	1	1	4	4
1	1	4	4	2	2	1	1	4	4
1	1	1	1	2	2	1	1	4	4
1	1	4	4	2	2	1	1	4	4
4	4	4	4	2	2	4	4	4	4
1	1	4	4	2	2	1	1	4	4
4	4	4	4	2	2	1	1	4	4
1	1	1	1	2	2	1	1	4	4
4	4	4	4	2	2	1	1	4	4
1	1	4	4	2	2	1	1	4	4
1	1	4	4	2	2	1	1	4	4
1	1	1	1	2	2	1	1	4	4
1	1	4	4	2	2	1	1	4	4
1	1	4	4	2	2	1	1	4	4
4	4	4	4	2	2	1	1	4	4
4	4	4	4	3	3	1	1	4	4
1	1	1	1	2	2	1	1	4	4
1	1	4	4	2	2	1	1	4	4
1	1	4	4	2	2	1	1	4	4
1	1	4	4	2	2	1	1	4	4
1	1	4	4	2	2	1	1	4	4
1	1	4	4	2	2	1	1	4	4
4	4	4	4	3	3	1	1	4	4
1	1	4	4	2	2	1	1	4	4
1	1	4	4	2	2	1	1	4	4
4	4	4	4	2	2	1	1	4	4
4	4	4	4	2	2	1	1	4	4

For Review Only

4	4	4	4	2	2	4	4	4	4
1	1	4	4	2	2	1	1	4	4

For Review Only

SNP2604	SNP2604	SNP2605	SNP2605	SNP2606	SNP2606	SNP2607	SNP2607	SNP2608	SNP2608
3	3	3	3	1	1	4	4	1	1
2	2	3	3	1	1	4	4	1	1
2	2	3	3	1	1	4	4	1	1
2	2	3	3	1	1	4	4	1	1
3	3	2	2	1	1	4	4	1	1
3	3	3	3	1	1	4	4	1	1
2	2	3	3	1	1	4	4	1	1
2	2	2	2	1	1	4	4	1	1
2	2	3	3	1	1	4	4	1	1
2	2	2	2	1	1	4	4	1	1
2	2	3	3	1	1	4	4	1	1
2	2	3	3	1	1	4	4	1	1
2	2	3	3	1	1	4	4	1	1
2	2	2	2	4	4	4	4	3	3
2	2	3	3	1	1	4	4	1	1
2	2	3	3	1	1	4	4	1	1
2	2	3	3	1	1	4	4	1	1
2	2	3	3	1	1	4	4	1	1
2	2	3	3	1	1	4	4	1	1
2	2	3	3	1	1	4	4	3	3
2	2	3	3	1	1	1	1	3	3
2	2	3	3	1	1	1	1	3	3
2	2	3	3	1	1	4	4	1	1
2	2	3	3	1	1	4	4	1	1
2	2	3	3	1	1	4	4	1	1
2	2	3	3	1	1	4	4	1	1
2	2	3	3	1	1	4	4	1	1
2	2	3	3	1	1	4	4	1	1
2	2	3	3	1	1	4	4	1	1
2	2	3	3	1	1	4	4	3	3
2	2	3	3	1	1	4	4	1	1
2	2	3	3	1	1	1	1	1	1
2	2	3	3	1	1	1	1	1	1
2	2	2	2	1	1	4	4	1	1
2	2	3	3	1	1	4	4	3	3
2	2	3	3	1	1	4	4	1	1
2	2	3	3	1	1	4	4	1	1
2	2	3	3	1	1	4	4	1	1
2	2	3	3	1	1	4	4	1	1
2	2	2	2	1	1	4	4	1	1
2	2	3	3	1	1	4	4	1	1
2	2	3	3	1	1	4	4	1	1
2	2	3	3	1	1	4	4	3	3
2	2	3	3	1	1	1	1	1	1
2	2	2	2	1	1	4	4	3	3
2	2	3	3	1	1	4	4	1	1
2	2	3	3	1	1	4	4	1	1
2	2	3	3	1	1	4	4	1	1
2	2	3	3	1	1	4	4	1	1
2	2	3	3	1	1	4	4	1	1
2	2	3	3	1	1	4	4	1	1
2	2	3	3	1	1	4	4	1	1

For Review Only

2	2	3	3	1	1	1	1	1	1
2	2	3	3	1	1	1	1	1	1
2	2	3	3	1	1	4	4	1	1
2	2	3	3	1	1	1	1	1	1
2	2	3	3	1	1	4	4	1	1
2	2	3	3	1	1	4	4	1	1
2	2	3	3	1	1	4	4	1	1
2	2	2	2	1	1	4	4	1	1
2	2	3	3	1	1	4	4	3	3
2	2	3	3	1	1	4	4	1	1
2	2	3	3	1	1	4	4	1	1
2	2	3	3	1	1	4	4	1	1
2	2	3	3	1	1	1	1	1	1
2	2	3	3	1	1	4	4	1	1
3	3	3	3	1	1	4	4	1	1
2	2	3	3	1	1	4	4	3	3
2	2	3	3	4	4	4	4	1	1
2	2	3	3	1	1	4	4	1	1
2	2	3	3	1	1	4	4	1	1
3	3	3	3	1	1	4	4	1	1
2	2	3	3	1	1	4	4	1	1
2	2	3	3	1	1	4	4	3	3
2	2	3	3	1	1	4	4	1	1
2	2	3	3	1	1	4	4	1	1
2	2	2	2	1	1	4	4	3	3
2	2	3	3	1	1	4	4	1	1
2	2	3	3	1	1	4	4	1	1
2	2	3	3	1	1	4	4	1	1
2	2	3	3	1	1	4	4	1	1
2	2	3	3	1	1	1	1	1	1
2	2	3	3	1	1	1	1	1	1
2	2	3	3	1	1	4	4	1	1
2	2	3	3	1	1	4	4	1	1
2	2	3	3	1	1	1	1	1	1
2	2	3	3	1	1	4	4	3	3
2	2	2	2	1	1	4	4	3	3
2	2	3	3	1	1	4	4	1	1
2	2	2	2	1	1	4	4	1	1
2	2	3	3	1	1	4	4	3	3
2	2	3	3	1	1	4	4	3	3
2	2	3	3	1	1	1	1	1	1
2	2	3	3	1	1	1	1	1	1
2	2	3	3	1	1	4	4	1	1
2	2	3	3	1	1	4	4	1	1
3	3	3	3	1	1	4	4	1	1
2	2	3	3	1	1	4	4	1	1
2	2	2	2	1	1	4	4	1	1
2	2	3	3	1	1	4	4	1	1
2	2	3	3	1	1	4	4	1	1
2	2	3	3	1	1	1	1	1	1
2	2	3	3	1	1	4	4	1	1
2	2	3	3	1	1	4	4	1	1
2	2	2	2	1	1	4	4	1	1
2	2	3	3	1	1	4	4	1	1
3	3	3	3	1	1	4	4	1	1

For Review Only

2	2	3	3	1	1	4	4	1	1
2	2	3	3	1	1	4	4	1	1
2	2	3	3	1	1	4	4	1	1
2	2	3	3	1	1	4	4	1	1
2	2	3	3	1	1	4	4	1	1
2	2	3	3	1	1	4	4	1	1
2	2	3	3	1	1	4	4	1	1
2	2	3	3	1	1	4	4	1	1
2	2	3	3	1	1	4	4	1	1
2	2	3	3	1	1	4	4	1	1
3	3	3	3	1	1	4	4	1	1
2	2	3	3	1	1	4	4	1	1
2	2	2	2	4	4	4	4	1	1
2	2	3	3	1	1	4	4	1	1
2	2	3	3	1	1	4	4	1	1
2	2	3	3	1	1	4	4	1	1
2	2	3	3	4	4	4	4	3	3
2	2	3	3	1	1	4	4	1	1
2	2	3	3	1	1	4	4	1	1
2	2	2	2	1	1	1	1	1	1
2	2	3	3	1	1	4	4	1	1
2	2	3	3	1	1	1	1	1	1
2	2	3	3	1	1	4	4	1	1
2	2	3	3	1	1	4	4	1	1
2	2	3	3	1	1	4	4	1	1
2	2	3	3	1	1	4	4	3	3
2	2	3	3	1	1	4	4	1	1
2	2	3	3	1	1	4	4	1	1
2	2	3	3	1	1	4	4	1	1
2	2	3	3	1	1	1	1	1	1
2	2	2	2	1	1	4	4	1	1
2	2	3	3	1	1	4	4	1	1
2	2	3	3	1	1	1	1	1	1
2	2	3	3	1	1	4	4	1	1
2	2	2	2	1	1	4	4	1	1
2	2	3	3	1	1	4	4	1	1
2	2	2	2	1	1	4	4	3	3
2	2	3	3	1	1	4	4	1	1
2	2	3	3	1	1	4	4	1	1
2	2	3	3	1	1	4	4	1	1
2	2	3	3	1	1	4	4	3	3
2	2	2	2	1	1	4	4	1	1
3	3	3	3	4	4	4	4	1	1
2	2	2	2	1	1	4	4	3	3
2	2	3	3	1	1	4	4	1	1
3	3	3	3	1	1	4	4	1	1
2	2	3	3	1	1	4	4	1	1
2	2	3	3	1	1	4	4	1	1
2	2	3	3	1	1	4	4	1	1
2	2	3	3	1	1	4	4	1	1
2	2	3	3	1	1	4	4	1	1
2	2	3	3	1	1	4	4	1	1
2	2	2	2	1	1	4	4	1	1
2	2	3	3	1	1	4	4	1	1
2	2	2	2	1	1	4	4	1	1

For Review Only

2	2	3	3	1	1	4	4	1	1
2	2	2	2	4	4	4	4	3	3
2	2	2	2	1	1	4	4	1	1
2	2	3	3	1	1	4	4	1	1
2	2	3	3	1	1	4	4	1	1
2	2	3	3	1	1	4	4	1	1
2	2	3	3	1	1	4	4	1	1
2	2	3	3	1	1	4	4	1	1
2	2	3	3	1	1	4	4	1	1
2	2	3	3	1	1	4	4	1	1
2	2	3	3	1	1	4	4	1	1
2	2	3	3	1	1	4	4	1	1
2	2	3	3	1	1	4	4	1	1
2	2	3	3	1	1	4	4	1	1
2	2	2	2	1	1	4	4	1	1
2	2	2	2	1	1	4	4	1	1
2	2	2	2	1	1	4	4	1	1
2	2	3	3	1	1	4	4	1	1
2	2	3	3	1	1	4	4	1	1
2	2	3	3	1	1	4	4	1	1
2	2	3	3	1	1	4	4	1	1
2	2	3	3	1	1	4	4	1	1
2	2	3	3	1	1	4	4	3	3
2	2	3	3	1	1	4	4	1	1
2	2	3	3	1	1	4	4	1	1
2	2	2	2	1	1	4	4	1	1
2	2	3	3	1	1	4	4	1	1
2	2	3	3	1	1	4	4	1	1
2	2	3	3	4	4	4	4	1	1
2	2	3	3	1	1	4	4	1	1
3	3	3	3	1	1	4	4	1	1
2	2	3	3	4	4	4	4	1	1
2	2	3	3	1	1	4	4	1	1
2	2	2	2	1	1	4	4	1	1
2	2	3	3	1	1	4	4	1	1
2	2	3	3	1	1	4	4	1	1
2	2	3	3	1	1	4	4	1	1
2	2	3	3	1	1	4	4	1	1
2	2	3	3	1	1	4	4	1	1
3	3	3	3	4	4	4	4	1	1
2	2	3	3	1	1	4	4	1	1
2	2	3	3	1	1	4	4	1	1
2	2	3	3	1	1	4	4	1	1
2	2	3	3	1	1	4	4	1	1
2	2	3	3	1	1	4	4	1	1
2	2	3	3	1	1	4	4	1	1
2	2	3	3	1	1	4	4	1	1
2	2	3	3	1	1	4	4	1	1
2	2	2	2	1	1	4	4	3	3
2	2	3	3	1	1	4	4	1	1
2	2	3	3	1	1	4	4	1	1
2	2	3	3	1	1	4	4	3	3
2	2	3	3	1	1	4	4	1	1
2	2	3	3	1	1	4	4	1	1
2	2	3	3	1	1	4	4	1	1

For Review Only

2	2	2	2	1	1	4	4	1	1
2	2	3	3	1	1	4	4	1	1
2	2	3	3	1	1	4	4	1	1
2	2	3	3	1	1	4	4	1	1
2	2	3	3	1	1	4	4	1	1
2	2	3	3	1	1	4	4	1	1
3	3	3	3	1	1	4	4	3	3
2	2	2	2	1	1	4	4	1	1
2	2	3	3	1	1	4	4	1	1
2	2	3	3	1	1	4	4	1	1
2	2	3	3	1	1	4	4	1	1
3	3	3	3	1	1	4	4	1	1
2	2	3	3	1	1	4	4	3	3
2	2	3	3	1	1	4	4	1	1
2	2	3	3	1	1	4	4	1	1
2	2	3	3	1	1	4	4	1	1
2	2	3	3	1	1	4	4	1	1
2	2	3	3	1	1	4	4	3	3
2	2	3	3	1	1	4	4	1	1
2	2	3	3	1	1	4	4	1	1
2	2	3	3	1	1	4	4	1	1
2	2	3	3	1	1	4	4	1	1
2	2	3	3	1	1	1	1	1	1
2	2	3	3	4	4	4	4	3	3
2	2	2	2	1	1	4	4	1	1
2	2	3	3	1	1	4	4	1	1
2	2	3	3	1	1	4	4	1	1
2	2	3	3	1	1	4	4	1	1
2	2	3	3	1	1	4	4	1	1
2	2	3	3	1	1	4	4	1	1
2	2	3	3	1	1	4	4	1	1
2	2	3	3	1	1	4	4	1	1
2	2	3	3	1	1	4	4	3	3
2	2	3	3	1	1	4	4	1	1
2	2	3	3	1	1	4	4	3	3
2	2	3	3	1	1	4	4	1	1
2	2	3	3	1	1	4	4	1	1
2	2	3	3	1	1	4	4	1	1
2	2	3	3	1	1	4	4	1	1
2	2	2	2	1	1	4	4	1	1
2	2	3	3	1	1	4	4	1	1
2	2	3	3	1	1	4	4	1	1
2	2	3	3	4	4	4	4	3	3
2	2	3	3	1	1	1	1	1	1
2	2	3	3	1	1	4	4	1	1
2	2	3	3	1	1	4	4	3	3
2	2	3	3	1	1	4	4	1	1
2	2	3	3	1	1	4	4	1	1
2	2	3	3	1	1	4	4	1	1
2	2	3	3	1	1	4	4	3	3
2	2	3	3	1	1	1	1	1	1
2	2	2	2	1	1	4	4	1	1
2	2	3	3	1	1	4	4	1	1
2	2	3	3	1	1	4	4	1	1
2	2	3	3	1	1	4	4	1	1

For Review Only

2	2	3	3	1	1	4	4	1	1
2	2	3	3	1	1	4	4	1	1

For Review Only

SNP2609	SNP2609	SNP261	SNP261	SNP2610	SNP2610	SNP2611	SNP2611	SNP2612	SNP2612
2	2	1	1	3	3	4	4	2	2
2	2	1	1	3	3	4	4	3	3
2	2	1	1	3	3	4	4	3	3
2	2	1	1	2	2	1	1	3	3
2	2	4	4	3	3	4	4	2	2
2	2	1	1	3	3	4	4	2	2
2	2	1	1	3	3	4	4	2	2
2	2	1	1	3	3	4	4	3	3
2	2	1	1	3	3	4	4	3	3
1	1	4	4	3	3	4	4	3	3
2	2	1	1	2	2	1	1	3	3
2	2	1	1	3	3	4	4	3	3
2	2	1	1	3	3	4	4	3	3
2	2	1	1	3	3	4	4	3	3
2	2	1	1	3	3	4	4	3	3
2	2	4	4	3	3	4	4	3	3
2	2	4	4	3	3	4	4	3	3
2	2	1	1	2	2	1	1	3	3
2	2	1	1	3	3	4	4	3	3
2	2	4	4	3	3	4	4	3	3
2	2	4	4	3	3	4	4	3	3
2	2	4	4	3	3	4	4	3	3
2	2	1	1	2	2	1	1	3	3
2	2	4	4	3	3	4	4	3	3
2	2	1	1	3	3	4	4	2	2
2	2	1	1	3	3	4	4	3	3
2	2	1	1	3	3	4	4	3	3
2	2	1	1	3	3	4	4	3	3
2	2	4	4	3	3	4	4	3	3
2	2	4	4	3	3	4	4	3	3
2	2	1	1	3	3	4	4	3	3
2	2	1	1	3	3	4	4	3	3
2	2	1	1	3	3	4	4	3	3
2	2	4	4	3	3	4	4	3	3
2	2	1	1	3	3	1	1	3	3
2	2	1	1	3	3	4	4	3	3
2	2	1	1	2	2	1	1	3	3
2	2	1	1	2	2	1	1	3	3
2	2	4	4	3	3	4	4	3	3
2	2	1	1	2	2	1	1	3	3
2	2	4	4	3	3	4	4	3	3
2	2	4	4	3	3	4	4	3	3
1	1	4	4	3	3	4	4	3	3
1	1	1	1	3	3	4	4	3	3
1	1	1	1	3	3	4	4	3	3
2	2	1	1	3	3	4	4	3	3
2	2	1	1	3	3	4	4	3	3
2	2	1	1	3	3	4	4	3	3
2	2	4	4	3	3	4	4	3	3
2	2	1	1	3	3	1	1	3	3
2	2	1	1	3	3	4	4	3	3
2	2	1	1	3	3	4	4	3	3

For Review Only

2	2	1	1	3	3	4	4	3	3
2	2	1	1	3	3	4	4	3	3
2	2	1	1	3	3	4	4	3	3
1	1	1	1	3	3	4	4	3	3
2	2	1	1	3	3	4	4	3	3
2	2	1	1	3	3	4	4	3	3
2	2	1	1	3	3	4	4	3	3
2	2	4	4	3	3	4	4	3	3
2	2	1	1	3	3	4	4	3	3
2	2	1	1	3	3	4	4	3	3
2	2	1	1	3	3	4	4	3	3
2	2	1	1	3	3	4	4	3	3
2	2	1	1	3	3	4	4	3	3
2	2	4	4	3	3	4	4	3	3
2	2	1	1	3	3	4	4	3	3
1	1	1	1	2	2	1	1	3	3
2	2	1	1	3	3	4	4	3	3
2	2	1	1	3	3	4	4	2	2
2	2	1	1	3	3	4	4	3	3
2	2	1	1	3	3	4	4	2	2
2	2	1	1	3	3	4	4	3	3
2	2	4	4	3	3	4	4	3	3
2	2	4	4	3	3	1	1	3	3
2	2	4	4	2	2	1	1	3	3
1	1	1	1	3	3	4	4	3	3
2	2	1	1	3	3	4	4	3	3
2	2	1	1	3	3	4	4	3	3
2	2	4	4	3	3	4	4	3	3
2	2	1	1	3	3	4	4	3	3
2	2	1	1	3	3	4	4	3	3
2	2	1	1	3	3	4	4	3	3
2	2	4	4	3	3	4	4	3	3
2	2	4	4	3	3	4	4	3	3
2	2	1	1	3	3	4	4	3	3
2	2	1	1	2	2	1	1	2	2
1	1	1	1	3	3	4	4	3	3
2	2	1	1	3	3	4	4	3	3
1	1	4	4	3	3	4	4	3	3
2	2	1	1	3	3	4	4	3	3
2	2	1	1	3	3	4	4	3	3
2	2	1	1	3	3	4	4	3	3
2	2	1	1	3	3	4	4	3	3
2	2	1	1	3	3	4	4	3	3
2	2	1	1	3	3	4	4	3	3
2	2	1	1	3	3	4	4	2	2
2	2	1	1	2	2	1	1	3	3
2	2	4	4	3	3	4	4	3	3
2	2	1	1	3	3	4	4	3	3
2	2	1	1	3	3	4	4	3	3
2	2	1	1	3	3	4	4	3	3
2	2	1	1	3	3	4	4	3	3
2	2	4	4	3	3	4	4	3	3
2	2	4	4	3	3	4	4	3	3
2	2	1	1	3	3	4	4	3	3
2	2	1	1	3	3	4	4	2	2

For Review Only

2	2	1	1	3	3	1	1	3	3
2	2	1	1	3	3	4	4	3	3
2	2	1	1	3	3	4	4	3	3
2	2	4	4	3	3	4	4	3	3
2	2	1	1	3	3	4	4	3	3
2	2	1	1	3	3	4	4	3	3
2	2	1	1	3	3	4	4	3	3
2	2	1	1	3	3	4	4	3	3
2	2	4	4	3	3	4	4	3	3
2	2	4	4	3	3	4	4	3	3
2	2	1	1	3	3	4	4	2	2
2	2	1	1	3	3	1	1	3	3
2	2	1	1	3	3	4	4	3	3
2	2	1	1	3	3	4	4	3	3
2	2	1	1	3	3	4	4	3	3
2	2	1	1	3	3	4	4	3	3
2	2	1	1	3	3	1	1	3	3
2	2	1	1	3	3	4	4	3	3
2	2	4	4	3	3	4	4	3	3
2	2	1	1	3	3	4	4	3	3
2	2	1	1	3	3	4	4	3	3
2	2	1	1	3	3	4	4	3	3
2	2	1	1	3	3	4	4	3	3
2	2	4	4	3	3	4	4	3	3
2	2	1	1	3	3	4	4	3	3
2	2	1	1	2	2	1	1	3	3
2	2	1	1	2	2	1	1	3	3
2	2	1	1	3	3	4	4	3	3
2	2	1	1	3	3	4	4	3	3
2	2	1	1	2	2	1	1	3	3
2	2	4	4	3	3	4	4	3	3
2	2	1	1	3	3	4	4	3	3
2	2	1	1	3	3	4	4	3	3
2	2	1	1	3	3	4	4	3	3
2	2	4	4	3	3	4	4	3	3
2	2	1	1	3	3	4	4	3	3
2	2	4	4	3	3	4	4	3	3
2	2	1	1	3	3	4	4	3	3
2	2	1	1	2	2	1	1	3	3
2	2	4	4	3	3	1	1	3	3
2	2	1	1	2	2	1	1	3	3
2	2	4	4	3	3	4	4	3	3
2	2	1	1	3	3	1	1	2	2
2	2	1	1	3	3	4	4	3	3
2	2	1	1	3	3	4	4	3	3
2	2	1	1	3	3	4	4	3	3
2	2	1	1	3	3	4	4	3	3
2	2	1	1	3	3	4	4	3	3
2	2	1	1	3	3	4	4	3	3
2	2	1	1	3	3	4	4	3	3
2	2	1	1	3	3	4	4	3	3
2	2	4	4	2	2	1	1	3	3
2	2	1	1	3	3	1	1	3	3
2	2	1	1	3	3	4	4	3	3
2	2	1	1	3	3	4	4	3	3

For Review Only

2	2	1	1	3	3	4	4	3	3
2	2	1	1	2	2	1	1	3	3
2	2	4	4	3	3	4	4	3	3
2	2	4	4	3	3	4	4	3	3
2	2	1	1	3	3	4	4	3	3
2	2	4	4	3	3	4	4	3	3
2	2	1	1	3	3	4	4	3	3
2	2	1	1	3	3	4	4	3	3
2	2	4	4	3	3	1	1	3	3
2	2	1	1	3	3	4	4	3	3
2	2	1	1	3	3	4	4	3	3
2	2	1	1	3	3	4	4	3	3
2	2	1	1	3	3	1	1	3	3
2	2	1	1	3	3	4	4	3	3
2	2	4	4	3	3	4	4	3	3
2	2	1	1	2	2	1	1	3	3
2	2	1	1	3	3	4	4	3	3
2	2	1	1	2	2	1	1	3	3
2	2	1	1	3	3	4	4	3	3
2	2	1	1	3	3	4	4	3	3
2	2	1	1	3	3	4	4	3	3
2	2	4	4	3	3	4	4	3	3
2	2	4	4	2	2	1	1	3	3
2	2	1	1	3	3	4	4	3	3
2	2	1	1	2	2	1	1	3	3
2	2	4	4	2	2	1	1	3	3
2	2	1	1	3	3	4	4	3	3
2	2	1	1	3	3	1	1	3	3
2	2	1	1	3	3	4	4	3	3
2	2	1	1	3	3	4	4	3	3
2	2	1	1	3	3	4	4	2	2
2	2	1	1	3	3	4	4	3	3
2	2	1	1	2	2	1	1	3	3
2	2	4	4	3	3	4	4	3	3
2	2	1	1	3	3	1	1	3	3
2	2	1	1	3	3	4	4	3	3
2	2	1	1	3	3	1	1	3	3
2	2	1	1	2	2	1	1	3	3
2	2	1	1	3	3	4	4	3	3
2	2	1	1	3	3	4	4	2	2
2	2	1	1	3	3	4	4	3	3
2	2	1	1	3	3	4	4	3	3
2	2	4	4	3	3	4	4	3	3
2	2	1	1	3	3	4	4	3	3
2	2	1	1	3	3	4	4	3	3
2	2	4	4	2	2	1	1	3	3
2	2	1	1	2	2	1	1	3	3
2	2	4	4	3	3	4	4	3	3
2	2	4	4	3	3	4	4	3	3
2	2	1	1	2	2	1	1	3	3
2	2	1	1	3	3	4	4	3	3
2	2	4	4	3	3	4	4	3	3
2	2	4	4	2	2	1	1	3	3
2	2	1	1	3	3	4	4	3	3
2	2	4	4	3	3	4	4	3	3

For Review Only

2	2	1	1	3	3	4	4	2	2
2	2	1	1	3	3	4	4	3	3
2	2	1	1	3	3	4	4	3	3
2	2	1	1	3	3	1	1	3	3
1	1	1	1	2	2	1	1	3	3
2	2	1	1	3	3	1	1	3	3
2	2	4	4	2	2	1	1	2	2
1	1	4	4	3	3	4	4	3	3
2	2	1	1	3	3	4	4	3	3
2	2	4	4	3	3	4	4	3	3
2	2	1	1	3	3	4	4	3	3
2	2	1	1	3	3	4	4	2	2
2	2	1	1	3	3	4	4	3	3
2	2	1	1	3	3	4	4	3	3
2	2	1	1	3	3	4	4	3	3
2	2	1	1	2	2	1	1	3	3
2	2	4	4	3	3	4	4	3	3
2	2	1	1	3	3	4	4	3	3
2	2	4	4	3	3	4	4	3	3
2	2	1	1	2	2	1	1	3	3
2	2	4	4	2	2	1	1	3	3
2	2	1	1	3	3	4	4	3	3
2	2	1	1	2	2	1	1	3	3
2	2	1	1	3	3	4	4	3	3
2	2	4	4	3	3	4	4	3	3
2	2	1	1	3	3	4	4	3	3
2	2	4	4	3	3	4	4	3	3
2	2	1	1	3	3	4	4	3	3
2	2	1	1	2	2	1	1	3	3
2	2	1	1	3	3	4	4	3	3
2	2	1	1	3	3	4	4	3	3
2	2	1	1	3	3	4	4	3	3
2	2	1	1	2	2	1	1	3	3
2	2	1	1	3	3	4	4	3	3
2	2	4	4	2	2	1	1	3	3
2	2	4	4	3	3	4	4	3	3
2	2	1	1	3	3	4	4	2	2
2	2	1	1	3	3	4	4	3	3
2	2	1	1	3	3	4	4	3	3
2	2	4	4	3	3	4	4	3	3
2	2	1	1	3	3	1	1	3	3
2	2	4	4	3	3	4	4	3	3
2	2	1	1	3	3	4	4	3	3
2	2	1	1	3	3	4	4	3	3
2	2	1	1	3	3	4	4	3	3
2	2	1	1	2	2	1	1	3	3
2	2	1	1	3	3	4	4	3	3
2	2	1	1	3	3	4	4	3	3
2	2	1	1	3	3	4	4	3	3
2	2	4	4	3	3	4	4	3	3
2	2	1	1	3	3	4	4	3	3
2	2	4	4	3	3	4	4	3	3
2	2	1	1	3	3	4	4	3	3
2	2	1	1	3	3	4	4	3	3
2	2	1	1	3	3	4	4	3	3

For Review Only

2	2	1	1	2	2	1	1	3	3
2	2	1	1	3	3	4	4	3	3

For Review Only

SNP2613	SNP2613	SNP2614	SNP2614	SNP2615	SNP2615	SNP2616	SNP2616	SNP2617	SNP2617
2	2	4	4	2	2	3	3	2	2
2	2	4	4	2	2	3	3	3	3
2	2	4	4	2	2	3	3	2	2
2	2	4	4	2	2	3	3	3	3
2	2	4	4	2	2	3	3	3	3
2	2	4	4	2	2	3	3	3	3
2	2	4	4	2	2	3	3	3	3
2	2	4	4	2	2	3	3	3	3
2	2	4	4	2	2	3	3	2	2
2	2	4	4	3	3	3	3	3	3
3	3	4	4	2	2	3	3	3	3
2	2	4	4	2	2	3	3	2	2
2	2	4	4	2	2	3	3	2	2
3	3	4	4	2	2	3	3	3	3
2	2	4	4	2	2	2	2	2	2
3	3	4	4	2	2	3	3	3	3
2	2	4	4	2	2	3	3	3	3
2	2	4	4	2	2	3	3	3	3
2	2	4	4	2	2	3	3	3	3
2	2	4	4	2	2	3	3	3	3
2	2	4	4	2	2	2	2	2	2
2	2	4	4	2	2	2	2	2	2
3	3	4	4	2	2	3	3	3	3
2	2	4	4	2	2	3	3	3	3
2	2	4	4	2	2	3	3	2	2
2	2	4	4	2	2	3	3	3	3
2	2	4	4	2	2	3	3	3	3
2	2	4	4	2	2	3	3	3	3
2	2	4	4	2	2	3	3	2	2
2	2	4	4	2	2	3	3	3	3
2	2	4	4	2	2	3	3	3	3
3	3	4	4	2	2	3	3	3	3
3	3	4	4	2	2	3	3	3	3
3	3	4	4	2	2	3	3	3	3
2	2	4	4	3	3	3	3	3	3
3	3	4	4	2	2	3	3	3	3
2	2	4	4	2	2	3	3	3	3
2	2	4	4	2	2	3	3	2	2
3	3	4	4	2	2	3	3	3	3
3	3	4	4	2	2	3	3	2	2
2	2	4	4	2	2	3	3	3	3
2	2	4	4	2	2	3	3	3	3
2	2	4	4	3	3	3	3	3	3
2	2	4	4	3	3	2	2	2	2
2	2	4	4	3	3	3	3	3	3
2	2	4	4	2	2	3	3	3	3
2	2	4	4	2	2	3	3	3	3
3	3	4	4	2	2	3	3	3	3
2	2	4	4	2	2	3	3	3	3
2	2	4	4	2	2	3	3	2	2
2	2	4	4	2	2	3	3	3	3
2	2	4	4	2	2	3	3	3	3

For Review Only

2	2	4	4	2	2	2	2	2	2
3	3	4	4	2	2	3	3	3	3
2	2	3	3	2	2	3	3	3	3
2	2	4	4	3	3	2	2	3	3
2	2	4	4	2	2	3	3	3	3
2	2	4	4	2	2	3	3	3	3
2	2	4	4	2	2	3	3	3	3
2	2	4	4	2	2	3	3	3	3
2	2	4	4	2	2	3	3	3	3
2	2	4	4	2	2	3	3	3	3
2	2	4	4	2	2	3	3	3	3
2	2	4	4	2	2	3	3	3	3
2	2	4	4	2	2	2	2	2	2
2	2	4	4	2	2	3	3	2	2
3	3	4	4	2	2	3	3	3	3
2	2	4	4	2	2	3	3	3	3
2	2	4	4	2	2	3	3	3	3
2	2	3	3	2	2	3	3	3	3
2	2	4	4	2	2	3	3	3	3
2	2	4	4	2	2	3	3	2	2
2	2	4	4	2	2	3	3	3	3
2	2	4	4	2	2	3	3	3	3
2	2	4	4	2	2	3	3	3	3
2	2	4	4	2	2	3	3	3	3
2	2	4	4	3	3	3	3	3	3
2	2	4	4	2	2	3	3	3	3
2	2	4	4	2	2	3	3	2	2
2	2	4	4	2	2	3	3	3	3
2	2	3	3	2	2	3	3	3	3
2	2	4	4	2	2	2	2	3	3
2	2	4	4	2	2	2	2	2	2
2	2	4	4	2	2	3	3	3	3
2	2	4	4	2	2	3	3	3	3
2	2	4	4	2	2	2	2	2	2
2	2	4	4	2	2	3	3	2	2
2	2	4	4	3	3	3	3	3	3
2	2	4	4	2	2	3	3	3	3
2	2	4	4	3	3	3	3	2	2
2	2	4	4	2	2	3	3	3	3
2	2	4	4	2	2	3	3	2	2
2	2	4	4	2	2	2	2	2	2
2	2	4	4	2	2	2	2	3	3
2	2	4	4	2	2	3	3	3	3
2	2	4	4	2	2	3	3	3	3
2	2	4	4	2	2	3	3	2	2
2	2	4	4	2	2	3	3	3	3
3	3	4	4	2	2	3	3	3	3
2	2	4	4	2	2	3	3	3	3
3	3	4	4	2	2	3	3	3	3
2	2	4	4	2	2	2	2	3	3
2	2	4	4	2	2	3	3	3	3
3	3	4	4	2	2	3	3	3	3
2	2	4	4	2	2	3	3	3	3
2	2	4	4	2	2	3	3	3	3
2	2	4	4	2	2	3	3	3	3

For Review Only

2	2	4	4	2	2	3	3	3	3
2	2	4	4	2	2	3	3	3	3
2	2	4	4	2	2	3	3	3	3
2	2	4	4	2	2	3	3	3	3
2	2	4	4	2	2	3	3	3	3
2	2	4	4	2	2	3	3	2	2
2	2	4	4	2	2	3	3	3	3
2	2	4	4	2	2	2	2	3	3
3	3	4	4	2	2	3	3	3	3
2	2	4	4	2	2	3	3	3	3
2	2	4	4	2	2	3	3	2	2
2	2	4	4	2	2	3	3	3	3
2	2	4	4	2	2	3	3	3	3
2	2	4	4	2	2	3	3	3	3
2	2	4	4	2	2	3	3	3	3
2	2	4	4	2	2	3	3	3	3
2	2	4	4	2	2	3	3	3	3
2	2	4	4	2	2	3	3	2	2
2	2	4	4	2	2	3	3	3	3
2	2	4	4	2	2	2	2	2	2
2	2	4	4	2	2	3	3	3	3
2	2	4	4	2	2	2	2	3	3
2	2	4	4	2	2	3	3	2	2
3	3	4	4	2	2	3	3	3	3
2	2	4	4	2	2	3	3	3	3
3	3	4	4	2	2	3	3	3	3
3	3	4	4	2	2	3	3	3	3
2	2	4	4	2	2	3	3	2	2
2	2	4	4	2	2	3	3	3	3
2	2	4	4	2	2	2	2	3	3
3	3	4	4	3	3	3	3	3	3
2	2	4	4	2	2	3	3	3	3
2	2	4	4	2	2	2	2	2	2
2	2	4	4	2	2	3	3	3	3
3	3	4	4	2	2	3	3	3	3
2	2	4	4	2	2	2	2	3	3
3	3	4	4	2	2	3	3	3	3
2	2	3	3	2	2	3	3	3	3
2	2	4	4	2	2	3	3	3	3
3	3	4	4	2	2	3	3	3	3
2	2	4	4	2	2	3	3	3	3
2	2	4	4	2	2	3	3	3	3
3	3	3	3	2	2	3	3	3	3
2	2	4	4	2	2	3	3	3	3
2	2	4	4	2	2	3	3	3	3
2	2	4	4	2	2	3	3	3	3
2	2	4	4	2	2	3	3	3	3
2	2	4	4	2	2	3	3	3	3
2	2	4	4	2	2	3	3	3	3
2	2	4	4	2	2	3	3	3	3
2	2	4	4	2	2	3	3	3	3
3	3	4	4	2	2	3	3	3	3
3	3	4	4	2	2	3	3	2	2
2	2	4	4	2	2	3	3	2	2
2	2	4	4	2	2	3	3	3	3

For Review Only

2	2	4	4	2	2	3	3	2	2
2	2	4	4	2	2	3	3	3	3
2	2	4	4	2	2	3	3	3	3
2	2	4	4	2	2	3	3	3	3
2	2	4	4	2	2	3	3	2	2
2	2	4	4	2	2	3	3	3	3
3	3	4	4	2	2	3	3	3	3
2	2	4	4	2	2	3	3	3	3
3	3	4	4	2	2	3	3	3	3
2	2	4	4	2	2	3	3	3	3
2	2	4	4	2	2	3	3	3	3
2	2	4	4	2	2	3	3	3	3
2	2	3	3	2	2	3	3	3	3
2	2	4	4	2	2	3	3	3	3
3	3	4	4	2	2	3	3	3	3
2	2	4	4	2	2	3	3	3	3
3	3	4	4	2	2	3	3	3	3
2	2	4	4	2	2	3	3	3	3
2	2	4	4	2	2	3	3	3	3
2	2	4	4	2	2	3	3	3	3
2	2	4	4	2	2	3	3	3	3
2	2	4	4	2	2	3	3	3	3
2	2	4	4	2	2	3	3	3	3
2	2	4	4	2	2	3	3	3	3
2	2	4	4	2	2	2	2	3	3
3	3	4	4	2	2	3	3	3	3
2	2	4	4	2	2	3	3	3	3
2	2	4	4	2	2	3	3	3	3
2	2	4	4	2	2	3	3	3	3
2	2	3	3	2	2	3	3	3	3
2	2	4	4	2	2	3	3	3	3
2	2	4	4	2	2	3	3	3	3
3	3	4	4	2	2	3	3	3	3
3	3	4	4	2	2	3	3	3	3
2	2	4	4	2	2	3	3	3	3
2	2	4	4	2	2	3	3	3	3
2	2	4	4	2	2	3	3	3	3
2	2	4	4	2	2	3	3	3	3
2	2	4	4	2	2	3	3	3	3
2	2	4	4	2	2	3	3	3	3
2	2	4	4	2	2	3	3	2	2
2	2	4	4	2	2	3	3	3	3
3	3	4	4	2	2	3	3	3	3
2	2	4	4	2	2	3	3	2	2
2	2	4	4	2	2	3	3	3	3
2	2	4	4	2	2	3	3	3	3
3	3	4	4	2	2	3	3	3	3
3	3	4	4	2	2	3	3	3	3
2	2	4	4	2	2	3	3	3	3
2	2	4	4	2	2	3	3	3	3
3	3	3	3	2	2	3	3	3	3
2	2	4	4	2	2	3	3	3	3
2	2	4	4	2	2	3	3	3	3
2	2	4	4	2	2	3	3	3	3
3	3	4	4	2	2	3	3	3	3

3	3	3	3	2	2	3	3	3	3
2	2	4	4	2	2	3	3	3	3
2	2	4	4	2	2	3	3	2	2
2	2	4	4	2	2	2	2	3	3
2	2	4	4	3	3	3	3	3	3
2	2	4	4	2	2	3	3	3	3
2	2	4	4	2	2	3	3	3	3
2	2	4	4	3	3	3	3	3	3
2	2	4	4	2	2	3	3	3	3
3	3	4	4	2	2	3	3	3	3
2	2	4	4	2	2	3	3	3	3
2	2	4	4	2	2	3	3	3	3
2	2	4	4	2	2	3	3	3	3
2	2	4	4	2	2	3	3	3	3
2	2	4	4	2	2	3	3	2	2
2	2	4	4	2	2	3	3	3	3
2	2	4	4	2	2	3	3	3	3
2	2	4	4	2	2	3	3	2	2
3	3	4	4	3	3	3	3	3	3
2	2	4	4	2	2	3	3	3	3
2	2	4	4	2	2	3	3	3	3
2	2	4	4	2	2	2	2	3	3
2	2	4	4	2	2	2	2	2	2
2	2	4	4	2	2	3	3	3	3
2	2	4	4	2	2	3	3	3	3
2	2	4	4	2	2	3	3	3	3
3	3	4	4	2	2	3	3	3	3
2	2	4	4	2	2	3	3	3	3
2	2	4	4	2	2	2	2	2	2
2	2	3	3	2	2	3	3	3	3
3	3	4	4	2	2	3	3	3	3
3	3	4	4	2	2	3	3	3	3
2	2	4	4	2	2	3	3	3	3
2	2	4	4	2	2	3	3	3	3
2	2	4	4	2	2	3	3	3	3
3	3	4	4	2	2	3	3	3	3
2	2	3	3	2	2	3	3	2	2
2	2	4	4	2	2	3	3	3	3
3	3	4	4	2	2	3	3	3	3
3	3	4	4	2	2	3	3	3	3
2	2	4	4	2	2	3	3	3	3
2	2	4	4	2	2	3	3	3	3
2	2	4	4	2	2	3	3	3	3
2	2	4	4	2	2	2	2	2	2
2	2	4	4	2	2	3	3	3	3
2	2	4	4	2	2	3	3	3	3
2	2	4	4	2	2	3	3	3	3
2	2	4	4	2	2	3	3	3	3
2	2	4	4	2	2	3	3	2	2
3	3	4	4	2	2	3	3	3	3
2	2	3	3	2	2	2	2	2	2
3	3	4	4	2	2	3	3	3	3
2	2	4	4	2	2	3	3	2	2
2	2	4	4	2	2	3	3	3	3
2	2	4	4	2	2	3	3	3	3

For Review Only

2	2	4	4	2	2	2	2	3	3
2	2	4	4	2	2	3	3	3	3

For Review Only

SNP2618	SNP2618	SNP2619	SNP2619	SNP262	SNP262	SNP2620	SNP2620	SNP2621	SNP2621
1	1	3	3	2	2	4	4	4	4
1	1	3	3	2	2	1	1	4	4
1	1	3	3	2	2	1	1	4	4
1	1	3	3	2	2	1	1	4	4
1	1	3	3	3	3	1	1	4	4
1	1	4	4	2	2	1	1	4	4
1	1	3	3	2	2	1	1	4	4
4	4	3	3	2	2	1	1	4	4
1	1	3	3	2	2	1	1	4	4
4	4	3	3	3	3	1	1	4	4
4	4	3	3	2	2	1	1	4	4
1	1	3	3	2	2	1	1	4	4
1	1	3	3	3	3	1	1	1	1
1	1	3	3	2	2	1	1	4	4
1	1	3	3	2	2	1	1	4	4
4	4	3	3	3	3	1	1	4	4
1	1	3	3	3	3	1	1	1	1
1	1	3	3	2	2	1	1	4	4
1	1	3	3	2	2	1	1	1	1
4	4	3	3	3	3	1	1	4	4
1	1	3	3	3	3	1	1	4	4
1	1	3	3	3	3	1	1	1	1
1	1	3	3	2	2	1	1	4	4
1	1	3	3	2	2	1	1	4	4
1	1	3	3	2	2	1	1	4	4
1	1	3	3	2	2	1	1	4	4
1	1	3	3	2	2	1	1	1	1
1	1	3	3	2	2	1	1	4	4
1	1	3	3	3	3	1	1	4	4
4	4	3	3	3	3	1	1	4	4
1	1	3	3	2	2	1	1	4	4
1	1	3	3	2	2	1	1	4	4
1	1	3	3	2	2	1	1	4	4
1	1	3	3	3	3	1	1	4	4
1	1	3	3	2	2	1	1	4	4
1	1	3	3	2	2	1	1	4	4
4	4	3	3	2	2	1	1	4	4
4	4	3	3	2	2	1	1	4	4
4	4	3	3	3	3	1	1	4	4
4	4	3	3	2	2	1	1	4	4
1	1	3	3	3	3	1	1	4	4
1	1	3	3	3	3	4	4	4	4
4	4	3	3	2	2	1	1	4	4
4	4	3	3	2	2	1	1	1	1
4	4	3	3	2	2	1	1	4	4
1	1	3	3	2	2	1	1	4	4
1	1	3	3	2	2	1	1	4	4
1	1	3	3	2	2	1	1	4	4
1	1	3	3	3	3	1	1	4	4
1	1	3	3	2	2	1	1	4	4
1	1	3	3	2	2	1	1	4	4
1	1	3	3	2	2	1	1	1	1

For Review Only

1	1	3	3	2	2	1	1	1	1
1	1	3	3	2	2	1	1	4	4
1	1	4	4	2	2	1	1	4	4
1	1	3	3	2	2	1	1	1	1
1	1	3	3	2	2	1	1	1	1
1	1	3	3	2	2	1	1	4	4
1	1	3	3	2	2	1	1	4	4
4	4	3	3	3	3	1	1	4	4
1	1	3	3	2	2	1	1	4	4
1	1	3	3	2	2	1	1	4	4
1	1	3	3	2	2	1	1	4	4
1	1	3	3	2	2	1	1	4	4
1	1	3	3	2	2	1	1	1	1
1	1	3	3	3	3	1	1	4	4
1	1	3	3	2	2	1	1	4	4
1	1	3	3	2	2	1	1	4	4
1	1	3	3	2	2	1	1	4	4
1	1	4	4	2	2	4	4	4	4
1	1	3	3	2	2	1	1	1	1
1	1	3	3	2	2	1	1	4	4
1	1	3	3	2	2	1	1	4	4
4	4	3	3	3	3	1	1	4	4
1	1	3	3	3	3	1	1	4	4
1	1	3	3	3	3	1	1	1	1
4	4	3	3	2	2	1	1	4	4
1	1	3	3	2	2	1	1	4	4
1	1	3	3	2	2	1	1	1	1
4	4	3	3	3	3	1	1	4	4
1	1	4	4	2	2	1	1	4	4
1	1	3	3	2	2	1	1	4	4
1	1	3	3	2	2	1	1	4	4
1	1	3	3	3	3	1	1	4	4
1	1	3	3	3	3	1	1	4	4
1	1	3	3	2	2	1	1	1	1
1	1	3	3	2	2	1	1	4	4
4	4	3	3	2	2	1	1	4	4
1	1	3	3	2	2	1	1	4	4
1	1	3	3	3	3	1	1	4	4
1	1	3	3	2	2	1	1	4	4
1	1	3	3	2	2	1	1	4	4
1	1	3	3	2	2	1	1	1	1
1	1	3	3	2	2	1	1	1	1
1	1	3	3	2	2	1	1	4	4
1	1	3	3	2	2	1	1	4	4
1	1	3	3	2	2	1	1	4	4
1	1	3	3	2	2	1	1	4	4
1	1	3	3	3	3	1	1	4	4
1	1	3	3	2	2	1	1	4	4
4	4	3	3	2	2	1	1	4	4
1	1	3	3	2	2	1	1	4	4
1	1	3	3	2	2	1	1	4	4
4	4	3	3	3	3	1	1	4	4
1	1	3	3	3	3	4	4	4	4
1	1	3	3	2	2	1	1	4	4
1	1	3	3	2	2	1	1	4	4

For Review Only

1	1	3	3	2	2	1	1	4	4
1	1	3	3	2	2	1	1	4	4
1	1	3	3	2	2	1	1	4	4
1	1	3	3	3	3	1	1	4	4
1	1	3	3	2	2	1	1	1	1
1	1	3	3	2	2	1	1	4	4
1	1	3	3	2	2	1	1	4	4
1	1	3	3	2	2	1	1	4	4
1	1	3	3	3	3	1	1	4	4
1	1	3	3	3	3	1	1	4	4
1	1	3	3	2	2	1	1	4	4
1	1	3	3	2	2	1	1	4	4
1	1	3	3	2	2	1	1	4	4
1	1	3	3	2	2	1	1	1	1
1	1	3	3	2	2	1	1	1	1
1	1	3	3	2	2	1	1	4	4
1	1	3	3	2	2	1	1	4	4
1	1	3	3	2	2	1	1	4	4
1	1	3	3	3	3	1	1	4	4
4	4	3	3	2	2	1	1	4	4
1	1	3	3	2	2	1	1	4	4
1	1	3	3	2	2	1	1	4	4
1	1	3	3	2	2	1	1	1	1
1	1	3	3	3	3	1	1	4	4
1	1	3	3	3	3	1	1	4	4
4	4	3	3	2	2	1	1	4	4
1	1	3	3	2	2	1	1	4	4
1	1	3	3	2	2	4	4	4	4
1	1	3	3	2	2	1	1	4	4
1	1	3	3	2	2	1	1	4	4
4	4	3	3	3	3	1	1	4	4
1	1	3	3	2	2	1	1	4	4
1	1	3	3	2	2	1	1	4	4
1	1	3	3	2	2	1	1	4	4
1	1	3	3	3	3	1	1	4	4
1	1	3	3	2	2	1	1	4	4
1	1	3	3	3	3	1	1	4	4
1	1	4	4	2	2	1	1	4	4
4	4	3	3	2	2	1	1	4	4
4	4	3	3	3	3	1	1	4	4
1	1	3	3	2	2	1	1	4	4
1	1	3	3	3	3	1	1	4	4
1	1	4	4	2	2	1	1	4	4
1	1	3	3	2	2	1	1	4	4
1	1	3	3	2	2	1	1	4	4
4	4	3	3	2	2	1	1	4	4
1	1	3	3	2	2	1	1	4	4
1	1	3	3	2	2	1	1	4	4
1	1	3	3	2	2	1	1	4	4
1	1	3	3	2	2	1	1	4	4
1	1	3	3	2	2	1	1	4	4
4	4	3	3	3	3	1	1	4	4
1	1	3	3	2	2	4	4	4	4
1	1	3	3	2	2	1	1	4	4
1	1	3	3	2	2	1	1	4	4

For Review Only

1	1	3	3	2	2	1	1	4	4
1	1	3	3	2	2	1	1	4	4
4	4	3	3	3	3	1	1	4	4
4	4	3	3	3	3	1	1	4	4
1	1	3	3	2	2	4	4	4	4
4	4	3	3	3	3	1	1	4	4
1	1	3	3	2	2	1	1	4	4
1	1	3	3	2	2	1	1	4	4
1	1	4	4	2	2	1	1	4	4
1	1	3	3	2	2	4	4	4	4
1	1	3	3	2	2	1	1	4	4
4	4	3	3	2	2	1	1	4	4
1	1	4	4	2	2	1	1	4	4
1	1	3	3	2	2	1	1	4	4
1	1	3	3	3	3	1	1	4	4
4	4	3	3	2	2	1	1	4	4
1	1	3	3	2	2	1	1	4	4
1	1	3	3	2	2	1	1	4	4
1	1	3	3	2	2	1	1	4	4
1	1	3	3	2	2	1	1	4	4
1	1	3	3	2	2	1	1	4	4
4	4	3	3	3	3	1	1	4	4
4	4	3	3	3	3	1	1	4	4
1	1	3	3	2	2	1	1	4	4
1	1	3	3	2	2	1	1	1	1
1	1	3	3	3	3	1	1	4	4
1	1	3	3	2	2	1	1	4	4
1	1	3	3	2	2	1	1	4	4
1	1	3	3	2	2	1	1	4	4
1	1	4	4	2	2	1	1	4	4
1	1	3	3	2	2	1	1	4	4
1	1	3	3	2	2	1	1	4	4
1	1	3	3	2	2	4	4	4	4
1	1	3	3	3	3	1	1	4	4
1	1	3	3	2	2	1	1	1	1
1	1	3	3	2	2	1	1	4	4
1	1	3	3	2	2	1	1	4	4
1	1	3	3	2	2	4	4	4	4
1	1	3	3	2	2	1	1	4	4
1	1	3	3	2	2	1	1	4	4
1	1	3	3	2	2	1	1	4	4
1	1	3	3	2	2	1	1	4	4
4	4	3	3	3	3	1	1	4	4
1	1	3	3	2	2	1	1	4	4
1	1	3	3	3	3	1	1	1	1
1	1	3	3	3	3	1	1	1	1
4	4	3	3	2	2	1	1	4	4
4	4	3	3	3	3	1	1	4	4
1	1	3	3	3	3	1	1	4	4
1	1	3	3	2	2	1	1	4	4
1	1	4	4	2	2	1	1	4	4
4	4	3	3	3	3	1	1	4	4
4	4	3	3	3	3	1	1	4	4
1	1	3	3	2	2	1	1	4	4
1	1	3	3	3	3	1	1	4	4

For Review Only

1	1	4	4	2	2	1	1	4	4
1	1	3	3	2	2	1	1	1	1
1	1	3	3	2	2	1	1	4	4
4	4	3	3	2	2	1	1	4	4
4	4	3	3	2	2	1	1	4	4
1	1	3	3	2	2	1	1	4	4
4	4	3	3	3	3	1	1	4	4
4	4	3	3	3	3	1	1	4	4
1	1	3	3	2	2	1	1	4	4
4	4	3	3	3	3	1	1	4	4
1	1	3	3	2	2	1	1	4	4
1	1	3	3	2	2	1	1	4	4
1	1	3	3	2	2	1	1	4	4
1	1	3	3	2	2	1	1	4	4
1	1	3	3	2	2	1	1	4	4
4	4	3	3	2	2	1	1	4	4
4	4	3	3	3	3	1	1	4	4
1	1	3	3	2	2	1	1	4	4
4	4	3	3	3	3	1	1	4	4
4	4	3	3	2	2	1	1	4	4
4	4	3	3	3	3	1	1	1	1
1	1	3	3	2	2	1	1	4	4
1	1	3	3	2	2	1	1	4	4
1	1	3	3	2	2	1	1	4	4
4	4	3	3	3	3	1	1	4	4
1	1	3	3	2	2	1	1	4	4
1	1	3	3	3	3	1	1	4	4
1	1	3	3	2	2	1	1	4	4
1	1	3	3	2	2	1	1	4	4
1	1	4	4	2	2	1	1	4	4
1	1	3	3	2	2	1	1	4	4
1	1	3	3	2	2	1	1	4	4
1	1	3	3	2	2	1	1	4	4
1	1	3	3	2	2	1	1	4	4
4	4	3	3	3	3	1	1	4	4
4	4	3	3	3	3	1	1	4	4
1	1	4	4	2	2	1	1	1	1
1	1	3	3	2	2	1	1	4	4
1	1	3	3	2	2	1	1	4	4
4	4	3	3	3	3	1	1	4	4
1	1	3	3	2	2	4	4	1	1
1	1	3	3	2	2	1	1	4	4
1	1	3	3	2	2	1	1	4	4
1	1	3	3	2	2	1	1	1	1
1	1	3	3	2	2	1	1	4	4
1	1	3	3	2	2	1	1	4	4
1	1	3	3	2	2	1	1	4	4
1	1	3	3	2	2	1	1	4	4
1	1	3	3	2	2	1	1	4	4
4	4	3	3	3	3	1	1	4	4
1	1	4	4	2	2	1	1	4	4
4	4	3	3	3	3	1	1	4	4
1	1	3	3	2	2	1	1	4	4
1	1	3	3	2	2	1	1	4	4
4	4	3	3	2	2	1	1	4	4

For Review Only

1	1	3	3	2	2	1	1	4	4
1	1	3	3	2	2	4	4	1	1

For Review Only

SNP2622	SNP2622	SNP2623	SNP2623	SNP2624	SNP2624	SNP2625	SNP2625	SNP2626	SNP2626
4	4	2	2	2	2	1	1	2	2
4	4	2	2	2	2	1	1	2	2
2	2	2	2	2	2	1	1	2	2
4	4	2	2	2	2	1	1	2	2
4	4	2	2	2	2	1	1	2	2
2	2	3	3	2	2	1	1	2	2
4	4	2	2	2	2	1	1	2	2
4	4	2	2	2	2	1	1	2	2
4	4	2	2	2	2	1	1	2	2
4	4	2	2	2	2	3	3	2	2
4	4	3	3	2	2	1	1	2	2
4	4	2	2	2	2	1	1	2	2
4	4	2	2	2	2	1	1	2	2
4	4	2	2	2	2	1	1	2	2
4	4	2	2	3	3	1	1	4	4
4	4	3	3	2	2	1	1	2	2
4	4	2	2	3	3	1	1	2	2
2	2	2	2	2	2	1	1	2	2
4	4	3	3	3	3	1	1	2	2
4	4	2	2	2	2	3	3	2	2
4	4	2	2	2	2	3	3	4	4
4	4	2	2	3	3	3	3	4	4
4	4	2	2	2	2	3	3	2	2
4	4	2	2	2	2	1	1	2	2
4	4	2	2	2	2	1	1	2	2
4	4	2	2	2	2	1	1	2	2
2	2	3	3	3	3	1	1	2	2
4	4	2	2	2	2	1	1	2	2
4	4	2	2	2	2	1	1	2	2
4	4	2	2	2	2	3	3	2	2
4	4	3	3	2	2	1	1	2	2
4	4	3	3	2	2	1	1	2	2
4	4	3	3	2	2	1	1	2	2
4	4	2	2	2	2	1	1	2	2
4	4	2	2	2	2	1	1	2	2
4	4	2	2	2	2	1	1	2	2
4	4	2	2	2	2	1	1	4	4
4	4	2	2	2	2	1	1	2	2
4	4	2	2	2	2	1	1	2	2
4	4	2	2	2	2	1	1	2	2
4	4	2	2	2	2	3	3	2	2
4	4	2	2	2	2	3	3	2	2
4	4	2	2	2	2	3	3	2	2
4	4	2	2	2	2	3	3	4	4
4	4	2	2	2	2	3	3	2	2
4	4	3	3	2	2	1	1	2	2
4	4	2	2	2	2	1	1	2	2
4	4	3	3	2	2	1	1	2	2
4	4	3	3	2	2	1	1	2	2
4	4	2	2	2	2	1	1	2	2
4	4	2	2	2	2	1	1	2	2
4	4	3	3	3	3	1	1	2	2

For Review Only

4	4	2	2	3	3	1	1	4	4
4	4	3	3	2	2	1	1	2	2
2	2	3	3	2	2	1	1	2	2
4	4	2	2	3	3	1	1	4	4
4	4	3	3	3	3	1	1	2	2
4	4	2	2	2	2	1	1	2	2
4	4	2	2	2	2	1	1	2	2
4	4	2	2	2	2	3	3	2	2
4	4	2	2	2	2	3	3	2	2
4	4	2	2	2	2	1	1	2	2
4	4	2	2	2	2	1	1	2	2
4	4	2	2	2	2	1	1	2	2
4	4	2	2	3	3	1	1	4	4
4	4	2	2	2	2	1	1	2	2
4	4	2	2	2	2	1	1	2	2
4	4	2	2	2	2	3	3	2	2
4	4	3	3	2	2	3	3	2	2
2	2	2	2	2	2	1	1	2	2
4	4	3	3	3	3	1	1	2	2
4	4	2	2	2	2	1	1	2	2
4	4	2	2	2	2	1	1	2	2
4	4	2	2	2	2	1	1	2	2
4	4	2	2	2	2	1	1	2	2
4	4	2	2	3	3	1	1	2	2
4	4	2	2	2	2	3	3	2	2
4	4	2	2	2	2	1	1	2	2
4	4	2	2	3	3	1	1	2	2
4	4	2	2	2	2	1	1	2	2
2	2	2	2	2	2	1	1	2	2
4	4	2	2	2	2	1	1	2	2
4	4	2	2	2	2	3	3	4	4
4	4	2	2	2	2	1	1	2	2
4	4	2	2	2	2	1	1	2	2
4	4	2	2	3	3	1	1	4	4
4	4	2	2	2	2	3	3	2	2
4	4	2	2	2	2	3	3	2	2
4	4	2	2	2	2	3	3	2	2
4	4	2	2	2	2	1	1	2	2
4	4	3	3	2	2	1	1	2	2
4	4	2	2	2	2	3	3	2	2
4	4	2	2	3	3	1	1	4	4
4	4	2	2	3	3	1	1	2	2
4	4	2	2	2	2	1	1	2	2
4	4	2	2	2	2	1	1	2	2
4	4	2	2	2	2	1	1	2	2
4	4	2	2	2	2	1	1	2	2
4	4	2	2	2	2	1	1	2	2
4	4	2	2	2	2	1	1	2	2
4	4	2	2	2	2	1	1	2	2
4	4	2	2	2	2	1	1	4	4
4	4	2	2	3	3	1	1	2	2
4	4	2	2	2	2	1	1	2	2
4	4	2	2	2	2	1	1	2	2
4	4	2	2	2	2	3	3	2	2
4	4	2	2	2	2	1	1	2	2

For Review Only

4	4	2	2	2	2	1	1	2	2
4	4	3	3	3	3	3	3	2	2
4	4	2	2	2	2	1	1	2	2
4	4	2	2	2	2	1	1	2	2
2	2	2	2	3	3	1	1	2	2
4	4	2	2	2	2	1	1	2	2
4	4	3	3	2	2	1	1	2	2
4	4	2	2	2	2	1	1	2	2
2	2	2	2	2	2	1	1	2	2
4	4	2	2	2	2	3	3	2	2
4	4	2	2	2	2	1	1	2	2
4	4	2	2	2	2	1	1	2	2
4	4	2	2	2	2	1	1	2	2
4	4	3	3	2	2	1	1	2	2
4	4	2	2	2	2	1	1	2	2
4	4	2	2	2	2	1	1	2	2
4	4	2	2	2	2	3	3	2	2
4	4	2	2	2	2	1	1	2	2
4	4	2	2	2	2	1	1	2	2
4	4	2	2	2	2	1	1	4	4
4	4	2	2	2	2	1	1	2	2
4	4	2	2	2	2	1	1	2	2
4	4	2	2	3	3	1	1	2	2
4	4	2	2	2	2	1	1	2	2
4	4	2	2	2	2	1	1	2	2
4	4	2	2	2	2	1	1	2	2
4	4	2	2	2	2	1	1	4	4
4	4	2	2	2	2	1	1	2	2
4	4	3	3	2	2	1	1	4	4
4	4	2	2	2	2	1	1	2	2
4	4	2	2	2	2	3	3	2	2
4	4	2	2	2	2	1	1	2	2
4	4	2	2	3	3	1	1	4	4
4	4	2	2	2	2	1	1	2	2
4	4	2	2	2	2	1	1	2	2
4	4	2	2	2	2	1	1	2	2
4	4	2	2	2	2	1	1	2	2
2	2	2	2	2	2	1	1	2	2
4	4	2	2	2	2	1	1	2	2
4	4	3	3	2	2	3	3	2	2
4	4	2	2	2	2	1	1	2	2
4	4	2	2	2	2	1	1	2	2
2	2	3	3	3	3	1	1	2	2
4	4	2	2	2	2	3	3	2	2
4	4	2	2	2	2	1	1	2	2
4	4	2	2	2	2	1	1	2	2
4	4	2	2	2	2	1	1	2	2
4	4	2	2	2	2	1	1	2	2
4	4	3	3	2	2	1	1	2	2
4	4	2	2	2	2	1	1	2	2
4	4	2	2	2	2	1	1	2	2
4	4	2	2	2	2	1	1	2	2
2	2	3	3	2	2	1	1	2	2
4	4	2	2	2	2	1	1	2	2
4	4	2	2	2	2	3	3	2	2

For Review Only

4	4	2	2	2	2	1	1	2	2
4	4	2	2	2	2	1	1	2	2
4	4	2	2	2	2	3	3	2	2
4	4	2	2	2	2	1	1	2	2
4	4	2	2	2	2	1	1	2	2
4	4	2	2	2	2	1	1	2	2
4	4	2	2	2	2	1	1	2	2
4	4	2	2	2	2	1	1	2	2
2	2	2	2	2	2	1	1	2	2
4	4	2	2	2	2	1	1	2	2
4	4	2	2	2	2	1	1	2	2
4	4	2	2	2	2	1	1	2	2
2	2	2	2	2	2	1	1	2	2
4	4	2	2	2	2	1	1	2	2
4	4	2	2	2	2	1	1	2	2
4	4	3	3	2	2	1	1	2	2
2	2	2	2	2	2	1	1	2	2
4	4	2	2	2	2	1	1	2	2
4	4	2	2	2	2	1	1	2	2
4	4	3	3	2	2	1	1	2	2
4	4	2	2	2	2	1	1	2	2
4	4	2	2	2	2	1	1	2	2
4	4	2	2	2	2	1	1	2	2
4	4	2	2	2	2	1	1	2	2
4	4	2	2	3	3	3	3	4	4
4	4	2	2	2	2	1	1	2	2
4	4	2	2	2	2	1	1	2	2
4	4	2	2	2	2	3	3	2	2
4	4	2	2	2	2	1	1	2	2
2	2	2	2	2	2	1	1	2	2
4	4	2	2	2	2	1	1	2	2
4	4	2	2	2	2	1	1	2	2
4	4	2	2	2	2	3	3	2	2
4	4	2	2	2	2	1	1	2	2
4	4	2	2	3	3	1	1	2	2
4	4	2	2	2	2	1	1	2	2
4	4	2	2	2	2	1	1	2	2
4	4	2	2	2	2	1	1	4	4
4	4	2	2	2	2	1	1	2	2
4	4	2	2	2	2	1	1	2	2
2	2	2	2	2	2	1	1	2	2
4	4	2	2	2	2	1	1	2	2
4	4	2	2	2	2	1	1	2	2
4	4	2	2	3	3	1	1	2	2
4	4	2	2	3	3	1	1	2	2
4	4	2	2	3	3	1	1	2	2
4	4	3	3	2	2	1	1	2	2
4	4	2	2	2	2	1	1	2	2
4	4	2	2	2	2	1	1	2	2
4	4	2	2	2	2	1	1	2	2
2	2	3	3	2	2	1	1	4	4
4	4	3	3	2	2	3	3	2	2
4	4	2	2	2	2	1	1	2	2
4	4	2	2	2	2	1	1	2	2
4	4	3	3	2	2	1	1	2	2

2	2	3	3	2	2	1	1	2	2
4	4	2	2	2	2	1	1	2	2
2	2	2	2	2	2	1	1	2	2
4	4	2	2	2	2	3	3	4	4
4	4	2	2	2	2	1	1	2	2
4	4	2	2	3	3	1	1	2	2
4	4	2	2	2	2	1	1	2	2
4	4	2	2	2	2	3	3	2	2
4	4	2	2	2	2	1	1	2	2
4	4	3	3	2	2	3	3	2	2
4	4	2	2	2	2	1	1	2	2
4	4	2	2	2	2	3	3	2	2
4	4	3	3	2	2	1	1	2	2
4	4	3	3	2	2	1	1	4	4
2	2	2	2	2	2	1	1	2	2
4	4	3	3	2	2	1	1	2	2
4	4	2	2	2	2	1	1	2	2
4	4	2	2	2	2	3	3	2	2
4	4	2	2	2	2	1	1	2	2
4	4	2	2	2	2	1	1	2	2
4	4	2	2	3	3	1	1	2	2
4	4	2	2	2	2	1	1	2	2
4	4	2	2	2	2	1	1	2	2
4	4	2	2	2	2	1	1	2	2
4	4	2	2	2	2	3	3	2	2
4	4	3	3	2	2	3	3	2	2
4	4	2	2	2	2	3	3	2	2
4	4	3	3	2	2	1	1	2	2
4	4	2	2	3	3	1	1	4	4
2	2	3	3	2	2	1	1	4	4
4	4	2	2	2	2	3	3	2	2
4	4	3	3	2	2	1	1	2	2
4	4	3	3	2	2	1	1	2	2
4	4	2	2	2	2	1	1	4	4
4	4	2	2	2	2	1	1	2	2
4	4	2	2	2	2	1	1	2	2
2	2	2	2	3	3	1	1	2	2
4	4	2	2	2	2	1	1	2	2
4	4	2	2	2	2	1	1	2	2
4	4	2	2	2	2	1	1	2	2
4	4	2	2	3	3	1	1	2	2
4	4	2	2	2	2	1	1	2	2
4	4	2	2	2	2	1	1	2	2
4	4	2	2	3	3	1	1	4	4
4	4	3	3	2	2	1	1	2	2
4	4	3	3	2	2	1	1	2	2
4	4	2	2	2	2	1	1	2	2
2	2	2	2	2	2	1	1	2	2
4	4	2	2	3	3	3	3	2	2
4	4	2	2	2	2	1	1	2	2
2	2	2	2	2	2	1	1	2	2
4	4	2	2	2	2	1	1	2	2
2	2	2	2	2	2	1	1	2	2
4	4	3	3	2	2	3	3	2	2
4	4	2	2	2	2	1	1	2	2

For Review Only

4	4	3	3	2	2	1	1	2	2
2	2	2	2	3	3	1	1	2	2

For Review Only

SNP2627	SNP2627	SNP2628	SNP2628	SNP2629	SNP2629	SNP263	SNP263	SNP2630	SNP2630
2	2	4	4	2	2	2	2	3	3
4	4	4	4	2	2	2	2	2	2
2	2	4	4	4	4	2	2	3	3
4	4	4	4	2	2	2	2	3	3
2	2	4	4	2	2	2	2	3	3
2	2	4	4	4	4	2	2	3	3
4	4	4	4	2	2	2	2	3	3
2	2	4	4	2	2	2	2	3	3
4	4	4	4	4	4	2	2	3	3
4	4	4	4	2	2	2	2	3	3
2	2	4	4	2	2	3	3	3	3
4	4	4	4	2	2	2	2	3	3
4	4	4	4	4	4	2	2	3	3
2	2	4	4	2	2	2	2	3	3
4	4	4	4	2	2	2	2	3	3
2	2	4	4	2	2	2	2	3	3
4	4	4	4	2	2	2	2	3	3
4	4	4	4	2	2	2	2	3	3
4	4	4	4	2	2	2	2	2	2
4	4	4	4	2	2	3	3	3	3
4	4	1	1	2	2	2	2	3	3
4	4	1	1	2	2	2	2	3	3
4	4	4	4	2	2	2	2	3	3
2	2	4	4	2	2	3	3	3	3
2	2	4	4	2	2	2	2	3	3
4	4	4	4	2	2	2	2	3	3
4	4	4	4	4	4	2	2	2	2
4	4	4	4	2	2	2	2	3	3
4	4	4	4	2	2	2	2	3	3
2	2	4	4	2	2	2	2	3	3
4	4	4	4	4	4	2	2	3	3
4	4	4	4	4	4	2	2	3	3
4	4	4	4	4	4	2	2	3	3
2	2	4	4	2	2	2	2	3	3
4	4	4	4	2	2	2	2	3	3
2	2	4	4	2	2	2	2	3	3
2	2	1	1	2	2	2	2	3	3
4	4	4	4	2	2	2	2	3	3
2	2	4	4	2	2	2	2	3	3
4	4	4	4	2	2	3	3	3	3
2	2	4	4	2	2	2	2	3	3
4	4	4	4	2	2	2	2	3	3
4	4	4	4	2	2	2	2	3	3
4	4	1	1	2	2	2	2	3	3
4	4	4	4	2	2	2	2	3	3
4	4	4	4	4	4	2	2	3	3
2	2	4	4	2	2	2	2	3	3
4	4	4	4	2	2	3	3	3	3
4	4	4	4	2	2	2	2	3	3
4	4	4	4	2	2	2	2	3	3
4	4	4	4	2	2	2	2	3	3
4	4	4	4	4	4	2	2	2	2

For Review Only

4	4	1	1	2	2	2	2	3	3
4	4	4	4	4	4	2	2	3	3
2	2	4	4	4	4	2	2	3	3
4	4	1	1	2	2	2	2	3	3
4	4	4	4	2	2	2	2	2	2
4	4	4	4	2	2	2	2	3	3
4	4	4	4	4	4	2	2	3	3
2	2	4	4	2	2	2	2	3	3
4	4	4	4	2	2	2	2	3	3
4	4	4	4	2	2	2	2	3	3
2	2	4	4	4	4	2	2	3	3
2	2	4	4	4	4	2	2	3	3
4	4	1	1	2	2	2	2	3	3
4	4	4	4	2	2	2	2	3	3
4	4	4	4	2	2	2	2	3	3
4	4	4	4	2	2	2	2	3	3
4	4	4	4	2	2	2	2	3	3
4	4	4	4	4	4	2	2	3	3
4	4	4	4	2	2	2	2	3	3
2	2	4	4	4	4	2	2	3	3
2	2	4	4	2	2	2	2	3	3
2	2	4	4	2	2	3	3	3	3
4	4	4	4	2	2	2	2	3	3
4	4	4	4	2	2	2	2	2	2
4	4	4	4	2	2	2	2	3	3
2	2	4	4	4	4	2	2	3	3
4	4	4	4	2	2	3	3	3	3
4	4	4	4	2	2	2	2	3	3
4	4	4	4	4	4	2	2	3	3
4	4	4	4	2	2	2	2	3	3
4	4	1	1	2	2	2	2	3	3
2	2	4	4	2	2	2	2	3	3
2	2	4	4	2	2	3	3	3	3
4	4	1	1	2	2	2	2	3	3
4	4	4	4	2	2	2	2	3	3
4	4	4	4	2	2	2	2	3	3
2	2	4	4	2	2	2	2	3	3
2	2	4	4	2	2	2	2	3	3
2	2	4	4	2	2	2	2	3	3
2	2	4	4	2	2	2	2	3	3
4	4	1	1	2	2	2	2	3	3
4	4	4	4	2	2	2	2	2	2
4	4	4	4	4	4	2	2	3	3
2	2	4	4	4	4	2	2	3	3
4	4	4	4	2	2	2	2	3	3
4	4	4	4	2	2	2	2	3	3
2	2	4	4	2	2	3	3	3	3
4	4	4	4	2	2	2	2	3	3
2	2	4	4	2	2	2	2	3	3
2	2	1	1	2	2	2	2	3	3
4	4	4	4	2	2	2	2	3	3
2	2	4	4	2	2	3	3	3	3
4	4	4	4	2	2	2	2	3	3
4	4	4	4	2	2	2	2	2	2
2	2	4	4	4	4	2	2	3	3

For Review Only

4	4	4	4	2	2	2	2	3	3
4	4	4	4	2	2	2	2	2	2
4	4	4	4	2	2	2	2	3	3
2	2	4	4	2	2	2	2	3	3
4	4	4	4	2	2	2	2	3	3
4	4	4	4	2	2	2	2	3	3
4	4	4	4	2	2	2	2	3	3
2	2	4	4	4	4	2	2	3	3
2	2	4	4	4	4	2	2	3	3
2	2	4	4	2	2	2	2	3	3
4	4	4	4	4	4	2	2	3	3
4	4	4	4	2	2	2	2	3	3
4	4	4	4	4	4	2	2	3	3
4	4	4	4	4	4	2	2	3	3
4	4	4	4	2	2	2	2	3	3
4	4	4	4	2	2	2	2	3	3
4	4	4	4	2	2	2	2	3	3
4	4	4	4	2	2	2	2	3	3
2	2	4	4	2	2	3	3	3	3
4	4	1	1	2	2	2	2	3	3
4	4	4	4	2	2	2	2	3	3
4	4	4	4	2	2	2	2	3	3
4	4	4	4	2	2	2	2	2	2
4	4	4	4	2	2	3	3	3	3
4	4	4	4	2	2	2	2	3	3
2	2	4	4	2	2	2	2	3	3
4	4	4	4	2	2	2	2	3	3
4	4	4	4	2	2	2	2	3	3
4	4	4	4	2	2	2	2	3	3
4	4	4	4	2	2	3	3	3	3
2	2	4	4	2	2	2	2	3	3
4	4	4	4	2	2	2	2	3	3
4	4	1	1	2	2	2	2	3	3
4	4	4	4	2	2	2	2	3	3
2	2	4	4	2	2	2	2	3	3
4	4	1	1	2	2	2	2	3	3
2	2	4	4	2	2	2	2	3	3
4	4	4	4	4	4	2	2	3	3
4	4	4	4	2	2	3	3	3	3
4	4	4	4	2	2	2	2	3	3
4	4	4	4	2	2	2	2	3	3
2	2	4	4	2	2	2	2	3	3
4	4	4	4	4	4	2	2	2	2
2	2	4	4	2	2	2	2	3	3
4	4	4	4	2	2	2	2	2	2
2	2	4	4	2	2	2	2	3	3
4	4	4	4	2	2	2	2	3	3
4	4	4	4	4	4	3	3	3	3
2	2	4	4	2	2	2	2	3	3
4	4	4	4	2	2	2	2	3	3
4	4	4	4	2	2	2	2	3	3
4	4	4	4	2	2	2	2	3	3
4	4	4	4	4	4	2	2	3	3
2	2	4	4	2	2	2	2	3	3
2	2	4	4	2	2	2	2	3	3

For Review Only

4	4	4	4	2	2	2	2	3	3
4	4	4	4	2	2	2	2	3	3
4	4	4	4	2	2	2	2	3	3
4	4	4	4	4	4	2	2	2	2
4	4	4	4	2	2	3	3	2	2
4	4	4	4	2	2	2	2	3	3
2	2	4	4	2	2	2	2	3	3
4	4	4	4	2	2	2	2	3	3
4	4	4	4	4	4	3	3	3	3
4	4	4	4	2	2	2	2	3	3
4	4	4	4	2	2	2	2	3	3
2	2	4	4	2	2	2	2	3	3
4	4	4	4	2	2	2	2	3	3
4	4	4	4	2	2	2	2	3	3
2	2	4	4	2	2	2	2	3	3
2	2	4	4	2	2	3	3	3	3
4	4	4	4	2	2	2	2	3	3
4	4	4	4	2	2	2	2	3	3
4	4	4	4	2	2	2	2	3	3
4	4	4	4	2	2	2	2	3	3
4	4	4	4	2	2	2	2	3	3
2	2	4	4	2	2	2	2	3	3
2	2	4	4	2	2	3	3	3	3
2	2	4	4	4	4	2	2	3	3
4	4	1	1	4	4	2	2	3	3
2	2	4	4	2	2	3	3	3	3
4	4	4	4	2	2	2	2	3	3
4	4	4	4	2	2	2	2	3	3
4	4	4	4	2	2	2	2	3	3
4	4	4	4	4	4	2	2	3	3
4	4	4	4	4	4	2	2	3	3
4	4	4	4	2	2	2	2	3	3
4	4	4	4	2	2	2	2	3	3
2	2	4	4	2	2	2	2	3	3
4	4	4	4	2	2	2	2	2	2
2	2	4	4	4	4	2	2	3	3
2	2	4	4	2	2	2	2	3	3
2	2	1	1	2	2	2	2	3	3
4	4	4	4	4	4	3	3	3	3
2	2	4	4	4	4	2	2	3	3
2	2	4	4	4	4	2	2	3	3
4	4	4	4	2	2	2	2	3	3
2	2	4	4	2	2	2	2	3	3
4	4	4	4	4	4	2	2	3	3
4	4	4	4	4	4	2	2	3	3
4	4	4	4	2	2	2	2	2	2
2	2	4	4	2	2	3	3	3	3
2	2	4	4	2	2	2	2	3	3
4	4	4	4	2	2	3	3	3	3
4	4	4	4	2	2	2	2	3	3
4	4	1	1	4	4	2	2	2	2
4	4	4	4	2	2	2	2	3	3
2	2	4	4	2	2	3	3	3	3
2	2	4	4	4	4	2	2	3	3
2	2	4	4	2	2	3	3	3	3

For Review Only

4	4	4	4	4	4	2	2	3	3
4	4	4	4	4	4	2	2	2	2
4	4	4	4	4	4	2	2	3	3
4	4	1	1	2	2	2	2	3	3
2	2	4	4	2	2	2	2	3	3
4	4	4	4	2	2	2	2	3	3
4	4	4	4	2	2	2	2	3	3
2	2	4	4	2	2	2	2	3	3
2	2	4	4	2	2	3	3	3	3
4	4	4	4	2	2	2	2	3	3
4	4	4	4	2	2	2	2	3	3
4	4	4	4	2	2	2	2	3	3
2	2	4	4	2	2	2	2	3	3
4	4	1	1	2	2	2	2	3	3
2	2	4	4	4	4	2	2	3	3
2	2	4	4	2	2	2	2	3	3
2	2	4	4	2	2	2	2	3	3
2	2	4	4	2	2	3	3	3	3
4	4	4	4	2	2	2	2	3	3
4	4	4	4	2	2	2	2	3	3
4	4	4	4	2	2	2	2	2	2
4	4	4	4	2	2	2	2	3	3
2	2	4	4	2	2	2	2	3	3
2	2	4	4	2	2	2	2	3	3
2	2	4	4	2	2	2	2	3	3
2	2	4	4	4	4	2	2	3	3
4	4	4	4	2	2	2	2	3	3
4	4	4	4	2	2	2	2	3	3
4	4	1	1	2	2	2	2	3	3
4	4	1	1	4	4	2	2	3	3
2	2	4	4	2	2	2	2	3	3
4	4	4	4	2	2	2	2	3	3
4	4	4	4	2	2	3	3	3	3
4	4	4	4	2	2	2	2	3	3
2	2	4	4	2	2	3	3	3	3
4	4	4	4	2	2	2	2	3	3
4	4	4	4	4	4	2	2	3	3
2	2	4	4	4	4	2	2	3	3
2	2	4	4	2	2	2	2	3	3
2	2	4	4	2	2	2	2	3	3
4	4	4	4	2	2	2	2	3	3
2	2	4	4	2	2	2	2	3	3
2	2	4	4	2	2	2	2	3	3
4	4	1	1	2	2	2	2	3	3
4	4	4	4	2	2	2	2	3	3
2	2	4	4	2	2	2	2	3	3
4	4	4	4	4	4	2	2	3	3
4	4	4	4	4	4	2	2	3	3
4	4	4	4	2	2	2	2	2	2
2	2	4	4	2	2	2	2	3	3
4	4	4	4	4	4	2	2	3	3
2	2	4	4	2	2	3	3	3	3
2	2	4	4	4	4	2	2	3	3
4	4	4	4	2	2	2	2	3	3
2	2	4	4	2	2	2	2	3	3

For Review Only

4	4	4	4	2	2	2	2	3	3
4	4	4	4	4	4	2	2	2	2

For Review Only

SNP2631	SNP2631	SNP2632	SNP2632	SNP2633	SNP2633	SNP2634	SNP2634	SNP2635	SNP2635
2	2	3	3	1	1	4	4	4	4
4	4	3	3	1	1	4	4	1	1
2	2	3	3	1	1	4	4	1	1
4	4	3	3	1	1	4	4	1	1
2	2	3	3	1	1	1	1	1	1
2	2	3	3	1	1	4	4	1	1
4	4	3	3	1	1	1	1	1	1
2	2	3	3	1	1	1	1	1	1
4	4	3	3	1	1	1	1	1	1
2	2	3	3	1	1	1	1	1	1
2	2	3	3	1	1	1	1	4	4
2	2	3	3	1	1	4	4	1	1
4	4	3	3	1	1	1	1	1	1
2	2	3	3	1	1	4	4	1	1
2	2	1	1	1	1	4	4	1	1
2	2	3	3	1	1	1	1	1	1
4	4	3	3	1	1	1	1	1	1
4	4	3	3	1	1	1	1	1	1
4	4	3	3	1	1	4	4	4	4
4	4	3	3	1	1	1	1	1	1
4	4	1	1	4	4	1	1	1	1
4	4	1	1	4	4	1	1	1	1
2	2	3	3	1	1	4	4	4	4
2	2	3	3	1	1	1	1	1	1
4	4	3	3	1	1	1	1	1	1
4	4	3	3	1	1	1	1	1	1
4	4	3	3	1	1	4	4	1	1
2	2	3	3	1	1	1	1	1	1
4	4	3	3	1	1	1	1	1	1
2	2	3	3	1	1	1	1	1	1
4	4	3	3	1	1	1	1	1	1
4	4	3	3	1	1	4	4	1	1
4	4	3	3	1	1	1	1	4	4
2	2	3	3	1	1	1	1	1	1
4	4	3	3	1	1	1	1	1	1
2	2	3	3	1	1	1	1	1	1
2	2	1	1	4	4	1	1	4	4
4	4	3	3	1	1	1	1	1	1
2	2	3	3	1	1	1	1	1	1
4	4	3	3	1	1	4	4	4	4
2	2	3	3	1	1	1	1	1	1
4	4	3	3	1	1	1	1	1	1
4	4	3	3	1	1	1	1	1	1
4	4	1	1	4	4	1	1	1	1
2	2	3	3	1	1	1	1	1	1
4	4	3	3	1	1	4	4	1	1
4	4	3	3	1	1	1	1	1	1
2	2	3	3	1	1	4	4	1	1
2	2	3	3	1	1	1	1	1	1
4	4	3	3	1	1	1	1	4	4
4	4	3	3	1	1	1	1	1	1
4	4	3	3	1	1	4	4	1	1

For Review Only

4	4	1	1	4	4	1	1	1	1
4	4	3	3	1	1	1	1	4	4
4	4	3	3	1	1	4	4	1	1
4	4	1	1	4	4	1	1	1	1
4	4	3	3	1	1	4	4	4	4
4	4	3	3	1	1	1	1	1	1
4	4	3	3	1	1	1	1	1	1
2	2	3	3	1	1	1	1	1	1
2	2	3	3	1	1	1	1	1	1
2	2	3	3	1	1	4	4	1	1
4	4	3	3	1	1	1	1	1	1
4	4	3	3	1	1	1	1	1	1
4	4	1	1	4	4	1	1	1	1
4	4	3	3	1	1	1	1	1	1
2	2	3	3	1	1	4	4	1	1
4	4	3	3	1	1	4	4	4	4
4	4	3	3	1	1	4	4	1	1
4	4	3	3	1	1	1	1	1	1
4	4	3	3	1	1	1	1	1	1
2	2	3	3	1	1	1	1	4	4
2	2	3	3	1	1	1	1	1	1
2	2	3	3	1	1	1	1	1	1
4	4	3	3	1	1	1	1	1	1
4	4	3	3	1	1	1	1	4	4
2	2	3	3	1	1	1	1	1	1
2	2	3	3	1	1	4	4	1	1
4	4	3	3	1	1	4	4	1	1
2	2	3	3	1	1	1	1	1	1
4	4	3	3	1	1	1	1	1	1
4	4	3	3	1	1	1	1	1	1
2	2	1	1	4	4	1	1	1	1
2	2	3	3	1	1	1	1	1	1
2	2	3	3	1	1	1	1	1	1
4	4	1	1	4	4	1	1	1	1
4	4	3	3	1	1	1	1	1	1
2	2	3	3	1	1	1	1	1	1
2	2	3	3	1	1	1	1	1	1
2	2	3	3	1	1	1	1	1	1
2	2	3	3	1	1	1	1	1	1
2	2	3	3	1	1	1	1	1	1
4	4	1	1	4	4	1	1	1	1
4	4	3	3	1	1	1	1	1	1
4	4	3	3	1	1	4	4	4	4
2	2	3	3	1	1	1	1	1	1
4	4	3	3	1	1	1	1	1	1
4	4	3	3	1	1	4	4	4	4
2	2	3	3	1	1	1	1	1	1
4	4	3	3	1	1	1	1	1	1
4	4	3	3	1	1	1	1	1	1
2	2	1	1	4	4	1	1	1	1
4	4	3	3	1	1	4	4	1	1
2	2	3	3	1	1	1	1	1	1
2	2	3	3	1	1	1	1	1	1
2	2	3	3	1	1	1	1	1	1
4	4	3	3	1	1	1	1	4	4

For Review Only

2	2	3	3	1	1	1	1	1	1
2	2	3	3	1	1	1	1	1	1
4	4	3	3	1	1	1	1	1	1
2	2	3	3	1	1	1	1	1	1
4	4	3	3	1	1	1	1	1	1
4	4	3	3	1	1	1	1	1	1
4	4	3	3	1	1	4	4	1	1
2	2	3	3	1	1	4	4	1	1
2	2	3	3	1	1	1	1	1	1
2	2	3	3	1	1	1	1	1	1
4	4	3	3	1	1	4	4	4	4
2	2	3	3	1	1	4	4	1	1
2	2	3	3	1	1	4	4	4	4
2	2	3	3	1	1	4	4	1	1
2	2	3	3	1	1	1	1	1	1
4	4	3	3	1	1	1	1	1	1
4	4	3	3	1	1	4	4	1	1
2	2	3	3	1	1	4	4	1	1
2	2	3	3	1	1	1	1	1	1
2	2	1	1	4	4	1	1	1	1
2	2	3	3	1	1	1	1	1	1
4	4	3	3	1	1	1	1	1	1
4	4	3	3	1	1	1	1	4	4
2	2	3	3	1	1	1	1	1	1
2	2	3	3	1	1	1	1	1	1
2	2	3	3	1	1	1	1	4	4
4	4	3	3	1	1	4	4	4	4
4	4	3	3	1	1	1	1	1	1
4	4	1	1	1	1	4	4	1	1
4	4	3	3	1	1	1	1	4	4
2	2	3	3	1	1	1	1	1	1
2	2	3	3	1	1	4	4	1	1
4	4	1	1	4	4	1	1	1	1
4	4	3	3	1	1	4	4	1	1
2	2	3	3	1	1	1	1	1	1
2	2	3	3	1	1	1	1	1	1
2	2	3	3	1	1	1	1	1	1
4	4	3	3	1	1	4	4	1	1
2	2	3	3	1	1	1	1	1	1
2	2	3	3	1	1	1	1	1	1
2	2	3	3	1	1	4	4	4	4
2	2	3	3	1	1	1	1	1	1
4	4	3	3	1	1	4	4	1	1
2	2	3	3	1	1	4	4	1	1
2	2	3	3	1	1	4	4	1	1
4	4	3	3	1	1	1	1	1	1
4	4	3	3	1	1	1	1	1	1
4	4	3	3	1	1	1	1	1	1
2	2	3	3	1	1	1	1	1	1
4	4	3	3	1	1	4	4	1	1
4	4	3	3	1	1	1	1	1	1
2	2	3	3	1	1	1	1	4	4
2	2	3	3	1	1	1	1	1	1
4	4	3	3	1	1	1	1	1	1
2	2	3	3	1	1	4	4	1	1

For Review Only

2	2	3	3	1	1	1	1	1	1
2	2	3	3	1	1	4	4	4	4
2	2	3	3	1	1	1	1	1	1
4	4	3	3	1	1	1	1	1	1
4	4	3	3	1	1	4	4	1	1
4	4	3	3	1	1	1	1	1	1
2	2	3	3	1	1	4	4	1	1
2	2	3	3	1	1	4	4	1	1
2	2	3	3	1	1	4	4	1	1
4	4	3	3	1	1	4	4	1	1
4	4	3	3	1	1	4	4	1	1
2	2	3	3	1	1	1	1	1	1
4	4	3	3	1	1	4	4	1	1
4	4	3	3	1	1	1	1	1	1
2	2	3	3	1	1	1	1	1	1
2	2	3	3	1	1	1	1	4	4
2	2	3	3	1	1	1	1	1	1
4	4	3	3	1	1	4	4	1	1
4	4	3	3	1	1	1	1	1	1
4	4	3	3	1	1	4	4	1	1
2	2	3	3	1	1	4	4	1	1
2	2	3	3	1	1	1	1	1	1
2	2	3	3	1	1	1	1	4	4
4	4	3	3	1	1	1	1	1	1
4	4	1	1	1	1	1	1	4	4
2	2	3	3	1	1	1	1	4	4
4	4	3	3	1	1	4	4	1	1
2	2	3	3	1	1	4	4	1	1
4	4	3	3	1	1	4	4	1	1
4	4	3	3	1	1	1	1	1	1
4	4	3	3	1	1	1	1	4	4
4	4	3	3	1	1	4	4	1	1
4	4	3	3	1	1	1	1	4	4
2	2	3	3	1	1	1	1	1	1
4	4	3	3	1	1	1	1	4	4
4	4	3	3	1	1	1	1	1	1
2	2	3	3	1	1	4	4	1	1
2	2	1	1	4	4	4	4	4	4
4	4	3	3	1	1	1	1	1	1
2	2	3	3	1	1	4	4	4	4
2	2	3	3	1	1	4	4	1	1
4	4	3	3	1	1	1	1	1	1
2	2	3	3	1	1	1	1	1	1
2	2	3	3	1	1	4	4	1	1
4	4	3	3	1	1	1	1	1	1
4	4	3	3	1	1	1	1	1	1
2	2	3	3	1	1	1	1	4	4
2	2	3	3	1	1	1	1	1	1
2	2	3	3	1	1	1	1	1	1
4	4	3	3	1	1	1	1	4	4
4	4	1	1	4	4	4	4	1	1
2	2	3	3	1	1	1	1	1	1
2	2	3	3	1	1	1	1	4	4
4	4	3	3	1	1	1	1	1	1
2	2	3	3	1	1	1	1	1	1

For Review Only

2	2	3	3	1	1	1	1	1	1
4	4	3	3	1	1	4	4	4	4
2	2	3	3	1	1	4	4	1	1
2	2	1	1	1	1	1	1	4	4
2	2	3	3	1	1	1	1	4	4
4	4	3	3	1	1	1	1	1	1
4	4	3	3	1	1	1	1	1	1
2	2	3	3	1	1	1	1	1	1
2	2	3	3	1	1	1	1	1	1
2	2	3	3	1	1	1	1	1	1
2	2	3	3	1	1	4	4	1	1
4	4	3	3	1	1	1	1	1	1
2	2	3	3	1	1	4	4	1	1
4	4	1	1	4	4	4	4	1	1
2	2	3	3	1	1	4	4	1	1
2	2	3	3	1	1	1	1	4	4
2	2	3	3	1	1	1	1	1	1
2	2	3	3	1	1	1	1	1	1
2	2	3	3	1	1	1	1	1	1
4	4	3	3	1	1	1	1	4	4
4	4	3	3	1	1	1	1	1	1
4	4	3	3	1	1	1	1	1	1
2	2	3	3	1	1	1	1	4	4
2	2	3	3	1	1	4	4	1	1
2	2	3	3	1	1	1	1	1	1
2	2	3	3	1	1	4	4	4	4
2	2	3	3	1	1	1	1	1	1
4	4	3	3	1	1	4	4	1	1
2	2	1	1	4	4	1	1	4	4
2	2	1	1	4	4	4	4	1	1
2	2	3	3	1	1	1	1	1	1
2	2	3	3	1	1	1	1	1	1
4	4	3	3	1	1	1	1	4	4
2	2	3	3	1	1	1	1	1	1
2	2	3	3	1	1	1	1	4	4
2	2	3	3	1	1	1	1	1	1
4	4	3	3	1	1	1	1	1	1
4	4	3	3	1	1	1	1	1	1
2	2	3	3	1	1	1	1	1	1
2	2	3	3	1	1	1	1	1	1
4	4	3	3	1	1	4	4	1	1
4	4	3	3	1	1	1	1	1	1
2	2	3	3	1	1	4	4	1	1
4	4	1	1	4	4	1	1	1	1
2	2	3	3	1	1	1	1	1	1
2	2	3	3	1	1	4	4	4	4
4	4	3	3	1	1	1	1	1	1
4	4	3	3	1	1	1	1	1	1
4	4	3	3	1	1	1	1	1	1
2	2	3	3	1	1	1	1	1	1
4	4	3	3	1	1	1	1	1	1
2	2	3	3	1	1	1	1	1	1
2	2	3	3	1	1	4	4	1	1
4	4	3	3	1	1	4	4	1	1
2	2	3	3	1	1	1	1	1	1

For Review Only

4	4	3	3	1	1	1	1	4	4
4	4	3	3	1	1	1	1	1	1

For Review Only

SNP2636	SNP2636	SNP2637	SNP2637	SNP2638	SNP2638	SNP2639	SNP2639	SNP264	SNP264
1	1	3	3	1	1	4	4	4	4
1	1	3	3	1	1	4	4	3	3
4	4	1	1	1	1	4	4	3	3
1	1	3	3	1	1	4	4	3	3
4	4	1	1	1	1	1	1	4	4
1	1	3	3	1	1	4	4	4	4
1	1	3	3	4	4	4	4	4	4
1	1	3	3	1	1	4	4	4	4
1	1	3	3	1	1	1	1	4	4
1	1	3	3	1	1	4	4	4	4
1	1	3	3	1	1	4	4	3	3
1	1	3	3	1	1	4	4	3	3
1	1	3	3	1	1	1	1	4	4
1	1	3	3	1	1	4	4	3	3
1	1	3	3	1	1	1	1	4	4
1	1	3	3	1	1	1	1	4	4
4	4	1	1	1	1	4	4	3	3
1	1	1	1	4	4	4	4	3	3
1	1	3	3	1	1	4	4	3	3
1	1	3	3	1	1	4	4	4	4
4	4	1	1	4	4	4	4	4	4
4	4	1	1	1	1	1	1	4	4
1	1	3	3	1	1	1	1	4	4
1	1	3	3	1	1	1	1	4	4
1	1	1	1	1	1	1	1	4	4
1	1	3	3	1	1	1	1	3	3
1	1	1	1	1	1	1	1	3	3
1	1	3	3	1	1	1	1	4	4
1	1	3	3	4	4	1	1	4	4
4	4	1	1	1	1	1	1	4	4
1	1	3	3	1	1	4	4	3	3
1	1	3	3	1	1	1	1	3	3
1	1	3	3	1	1	4	4	4	4
1	1	3	3	1	1	4	4	4	4
1	1	3	3	1	1	4	4	4	4
1	1	3	3	1	1	4	4	3	3
1	1	3	3	1	1	4	4	4	4
1	1	3	3	1	1	4	4	3	3
1	1	3	3	1	1	1	1	3	3
1	1	3	3	1	1	1	1	4	4
1	1	3	3	1	1	4	4	4	4
1	1	3	3	1	1	1	1	4	4
1	1	3	3	1	1	4	4	4	4
4	4	1	1	1	1	4	4	4	4
1	1	3	3	1	1	4	4	4	4
1	1	3	3	1	1	4	4	3	3
1	1	3	3	1	1	4	4	3	3
1	1	3	3	1	1	1	1	4	4
1	1	3	3	1	1	4	4	4	4
1	1	3	3	1	1	4	4	3	3
1	1	3	3	1	1	1	1	3	3
1	1	3	3	1	1	4	4	4	4

For Review Only

4	4	1	1	1	1	1	1	4	4
1	1	3	3	1	1	4	4	4	4
1	1	3	3	1	1	4	4	3	3
4	4	1	1	1	1	4	4	3	3
1	1	3	3	1	1	4	4	3	3
1	1	3	3	1	1	1	1	3	3
1	1	3	3	1	1	1	1	3	3
1	1	3	3	1	1	4	4	4	4
1	1	3	3	1	1	4	4	3	3
1	1	3	3	1	1	1	1	4	4
1	1	3	3	1	1	4	4	3	3
1	1	3	3	1	1	4	4	3	3
4	4	1	1	1	1	1	1	4	4
1	1	3	3	4	4	1	1	4	4
1	1	3	3	1	1	4	4	4	4
1	1	3	3	4	4	4	4	4	4
4	4	1	1	1	1	1	1	4	4
1	1	3	3	1	1	4	4	3	3
4	4	1	1	1	1	4	4	4	4
1	1	3	3	1	1	4	4	4	4
1	1	3	3	1	1	4	4	3	3
1	1	3	3	1	1	1	1	3	3
1	1	3	3	4	4	4	4	4	4
1	1	3	3	1	1	4	4	3	3
1	1	3	3	1	1	4	4	4	4
1	1	3	3	1	1	1	1	3	3
1	1	3	3	1	1	4	4	4	4
1	1	3	3	1	1	1	1	3	3
1	1	3	3	1	1	1	1	3	3
4	4	1	1	1	1	4	4	3	3
1	1	3	3	1	1	1	1	3	3
1	1	3	3	1	1	4	4	3	3
1	1	3	3	1	1	4	4	3	3
4	4	1	1	1	1	1	1	4	4
1	1	3	3	4	4	4	4	4	4
1	1	3	3	1	1	4	4	4	4
1	1	3	3	1	1	4	4	3	3
4	4	1	1	1	1	4	4	4	4
1	1	3	3	1	1	4	4	3	3
1	1	3	3	1	1	4	4	4	4
4	4	1	1	1	1	4	4	3	3
4	4	1	1	1	1	4	4	3	3
1	1	3	3	1	1	4	4	4	4
1	1	3	3	1	1	4	4	3	3
1	1	3	3	1	1	4	4	3	3
1	1	3	3	1	1	4	4	3	3
1	1	3	3	1	1	4	4	4	4
4	4	1	1	1	1	1	1	3	3
1	1	3	3	1	1	1	1	3	3
1	1	3	3	1	1	4	4	4	4
1	1	3	3	1	1	4	4	3	3
1	1	3	3	1	1	1	1	3	3
1	1	3	3	1	1	4	4	3	3
1	1	3	3	1	1	1	1	4	4
1	1	3	3	1	1	4	4	4	4

For Review Only

1	1	3	3	1	1	4	4	4	4
4	4	1	1	1	1	4	4	4	4
1	1	3	3	1	1	1	1	3	3
1	1	3	3	1	1	4	4	4	4
1	1	3	3	1	1	1	1	3	3
1	1	3	3	1	1	4	4	3	3
1	1	3	3	1	1	1	1	3	3
1	1	3	3	1	1	1	1	3	3
4	4	1	1	1	1	1	1	3	3
1	1	3	3	1	1	4	4	4	4
1	1	3	3	1	1	4	4	3	3
1	1	3	3	1	1	1	1	4	4
1	1	3	3	1	1	4	4	4	4
1	1	3	3	1	1	1	1	4	4
1	1	3	3	1	1	1	1	3	3
1	1	3	3	1	1	1	1	4	4
1	1	1	1	1	1	4	4	3	3
1	1	3	3	1	1	4	4	4	4
1	1	3	3	1	1	4	4	4	4
1	1	3	3	1	1	4	4	4	4
1	1	3	3	1	1	1	1	4	4
1	1	1	1	1	1	4	4	3	3
1	1	3	3	1	1	4	4	3	3
1	1	3	3	1	1	1	1	4	4
1	1	3	3	1	1	4	4	3	3
1	1	3	3	1	1	1	1	3	3
1	1	3	3	1	1	1	1	4	4
4	4	1	1	1	1	4	4	3	3
1	1	3	3	1	1	4	4	3	3
1	1	3	3	1	1	4	4	3	3
1	1	3	3	1	1	4	4	4	4
4	4	1	1	1	1	4	4	4	4
4	4	1	1	1	1	4	4	3	3
1	1	3	3	1	1	1	1	4	4
1	1	3	3	1	1	1	1	4	4
1	1	3	3	1	1	1	1	4	4
1	1	3	3	1	1	4	4	4	4
1	1	3	3	1	1	4	4	3	3
1	1	3	3	1	1	1	1	4	4
1	1	3	3	1	1	4	4	3	3
1	1	3	3	1	1	1	1	3	3
1	1	3	3	1	1	4	4	3	3
1	1	3	3	1	1	4	4	3	3
1	1	3	3	1	1	4	4	4	4
1	1	3	3	1	1	1	1	4	4
1	1	3	3	1	1	1	1	3	3
1	1	3	3	1	1	1	1	3	3
1	1	3	3	1	1	1	1	3	3
1	1	3	3	1	1	4	4	4	4
1	1	3	3	1	1	1	1	3	3
1	1	3	3	1	1	1	1	3	3
1	1	3	3	1	1	4	4	4	4
4	4	3	3	1	1	4	4	3	3
1	1	3	3	1	1	4	4	3	3
1	1	3	3	1	1	4	4	4	4

For Review Only

1	1	3	3	1	1	1	1	3	3
1	1	3	3	1	1	4	4	3	3
1	1	3	3	1	1	4	4	4	4
4	4	1	1	1	1	1	1	3	3
1	1	3	3	1	1	4	4	3	3
1	1	3	3	4	4	4	4	4	4
1	1	3	3	1	1	1	1	4	4
1	1	3	3	1	1	1	1	4	4
4	4	1	1	1	1	1	1	4	4
4	4	1	1	1	1	1	1	3	3
1	1	3	3	1	1	1	1	3	3
1	1	3	3	1	1	1	1	4	4
1	1	3	3	1	1	1	1	3	3
1	1	3	3	1	1	1	1	4	4
1	1	3	3	1	1	4	4	4	4
1	1	3	3	1	1	4	4	3	3
1	1	3	3	1	1	4	4	3	3
1	1	3	3	1	1	4	4	3	3
1	1	1	1	1	1	4	4	4	4
1	1	3	3	1	1	1	1	3	3
1	1	3	3	1	1	1	1	4	4
1	1	3	3	1	1	4	4	3	3
1	1	3	3	1	1	4	4	3	3
1	1	3	3	1	1	4	4	3	3
1	1	3	3	1	1	4	4	4	4
1	1	3	3	1	1	4	4	4	4
1	1	3	3	1	1	4	4	4	4
1	1	3	3	1	1	1	1	3	3
4	4	1	1	1	1	4	4	3	3
1	1	3	3	1	1	4	4	3	3
1	1	3	3	4	4	4	4	3	3
4	4	1	1	1	1	4	4	3	3
1	1	3	3	1	1	4	4	4	4
1	1	3	3	1	1	4	4	4	4
1	1	3	3	1	1	4	4	3	3
1	1	3	3	1	1	4	4	3	3
1	1	3	3	1	1	1	1	3	3
1	1	3	3	1	1	4	4	4	4
1	1	3	3	1	1	1	1	3	3
1	1	3	3	1	1	4	4	3	3
4	4	1	1	1	1	4	4	3	3
1	1	3	3	1	1	1	1	3	3
1	1	3	3	1	1	1	1	3	3
1	1	3	3	1	1	1	1	3	3
1	1	3	3	1	1	1	1	3	3
1	1	3	3	1	1	4	4	4	4
1	1	3	3	1	1	4	4	3	3
4	4	1	1	1	1	4	4	3	3
1	1	3	3	1	1	4	4	4	4
1	1	3	3	1	1	4	4	4	4
1	1	3	3	1	1	4	4	3	3
1	1	3	3	1	1	4	4	3	3
1	1	3	3	1	1	4	4	4	4
1	1	3	3	1	1	4	4	3	3
1	1	3	3	1	1	1	1	4	4

For Review Only

1	1	3	3	1	1	4	4	3	3
1	1	3	3	1	1	4	4	4	4
4	4	3	3	1	1	4	4	3	3
1	1	3	3	1	1	4	4	4	4
1	1	3	3	1	1	4	4	4	4
1	1	3	3	1	1	4	4	3	3
4	4	1	1	1	1	4	4	4	4
4	4	1	1	1	1	4	4	4	4
1	1	3	3	1	1	1	1	4	4
1	1	3	3	1	1	1	1	3	3
1	1	3	3	1	1	1	1	4	4
1	1	3	3	4	4	4	4	3	3
1	1	3	3	1	1	4	4	3	3
1	1	3	3	1	1	1	1	3	3
4	4	1	1	1	1	4	4	3	3
1	1	3	3	1	1	4	4	4	4
1	1	3	3	1	1	1	1	3	3
1	1	3	3	1	1	1	1	4	4
1	1	3	3	1	1	4	4	3	3
1	1	3	3	1	1	4	4	3	3
4	4	1	1	1	1	4	4	3	3
1	1	3	3	1	1	4	4	3	3
1	1	3	3	1	1	4	4	3	3
1	1	3	3	1	1	1	1	3	3
1	1	3	3	1	1	4	4	4	4
1	1	3	3	1	1	1	1	3	3
1	1	3	3	1	1	1	1	4	4
1	1	3	3	1	1	4	4	3	3
1	1	3	3	1	1	1	1	4	4
1	1	3	3	1	1	4	4	3	3
1	1	3	3	1	1	1	1	4	4
1	1	3	3	1	1	4	4	4	4
1	1	3	3	1	1	4	4	4	4
1	1	3	3	1	1	1	1	3	3
1	1	3	3	1	1	4	4	3	3
1	1	3	3	1	1	1	1	4	4
1	1	3	3	1	1	1	1	3	3
1	1	3	3	1	1	4	4	3	3
1	1	3	3	1	1	4	4	3	3
1	1	3	3	1	1	1	1	4	4
1	1	3	3	1	1	4	4	3	3
1	1	3	3	1	1	4	4	3	3
1	1	3	3	1	1	1	1	4	4
4	4	1	1	1	1	1	1	3	3
1	1	3	3	1	1	4	4	3	3
1	1	3	3	1	1	4	4	3	3
1	1	3	3	1	1	1	1	3	3
4	4	1	1	1	1	4	4	4	4
1	1	3	3	1	1	4	4	3	3
1	1	3	3	1	1	4	4	4	4
1	1	3	3	1	1	4	4	3	3
1	1	3	3	1	1	1	1	4	4
4	4	1	1	1	1	4	4	3	3
4	4	1	1	1	1	4	4	3	3
1	1	3	3	1	1	1	1	4	4

For Review Only

1	1	3	3	1	1	4	4	3	3
1	1	3	3	1	1	4	4	3	3

For Review Only

SNP2640	SNP2640	SNP2641	SNP2641	SNP2642	SNP2642	SNP2643	SNP2643	SNP2644	SNP2644
2	2	3	3	1	1	4	4	1	1
1	1	3	3	1	1	4	4	1	1
2	2	3	3	1	1	4	4	1	1
2	2	3	3	1	1	4	4	1	1
2	2	3	3	1	1	2	2	2	2
1	1	3	3	1	1	4	4	1	1
1	1	3	3	1	1	4	4	1	1
1	1	2	2	4	4	2	2	2	2
2	2	3	3	1	1	4	4	1	1
1	1	3	3	1	1	4	4	2	2
2	2	3	3	1	1	4	4	1	1
2	2	3	3	1	1	4	4	1	1
1	1	3	3	1	1	4	4	1	1
1	1	3	3	1	1	4	4	1	1
1	1	3	3	4	4	4	4	1	1
1	1	3	3	1	1	4	4	1	1
2	2	3	3	1	1	4	4	1	1
1	1	2	2	4	4	4	4	1	1
2	2	3	3	1	1	2	2	1	1
2	2	3	3	1	1	4	4	1	1
2	2	3	3	1	1	4	4	1	1
2	2	3	3	1	1	4	4	1	1
2	2	3	3	1	1	4	4	1	1
1	1	3	3	1	1	4	4	1	1
2	2	3	3	1	1	4	4	1	1
2	2	3	3	1	1	4	4	1	1
2	2	3	3	1	1	4	4	1	1
1	1	3	3	1	1	4	4	1	1
2	2	3	3	1	1	4	4	1	1
1	1	3	3	1	1	4	4	1	1
2	2	3	3	1	1	4	4	1	1
1	1	3	3	1	1	4	4	1	1
2	2	3	3	1	1	4	4	1	1
2	2	3	3	1	1	4	4	1	1
1	1	3	3	4	4	4	4	1	1
1	1	3	3	1	1	4	4	1	1
2	2	3	3	1	1	4	4	1	1
2	2	3	3	1	1	4	4	2	2
1	1	2	2	4	4	4	4	1	1
1	1	2	2	4	4	4	4	1	1
1	1	3	3	1	1	2	2	2	2
2	2	3	3	1	1	4	4	2	2
2	2	3	3	1	1	4	4	2	2
2	2	3	3	1	1	4	4	1	1
2	2	3	3	1	1	2	2	2	2
2	2	3	3	1	1	4	4	1	1
2	2	3	3	1	1	4	4	1	1
1	1	3	3	1	1	4	4	1	1
1	1	3	3	1	1	4	4	1	1
2	2	3	3	1	1	4	4	1	1
2	2	3	3	1	1	4	4	1	1
1	1	3	3	1	1	4	4	1	1

For Review Only

2	2	3	3	1	1	4	4	1	1
2	2	3	3	1	1	4	4	1	1
2	2	3	3	1	1	4	4	1	1
2	2	3	3	1	1	4	4	1	1
2	2	3	3	1	1	2	2	1	1
2	2	3	3	1	1	4	4	1	1
2	2	3	3	1	1	4	4	1	1
1	1	3	3	1	1	4	4	1	1
1	1	3	3	4	4	4	4	1	1
2	2	3	3	1	1	4	4	1	1
2	2	3	3	1	1	4	4	1	1
2	2	3	3	1	1	4	4	1	1
2	2	3	3	1	1	4	4	1	1
2	2	3	3	1	1	4	4	1	1
1	1	3	3	1	1	4	4	1	1
2	2	3	3	1	1	4	4	2	2
2	2	3	3	1	1	4	4	1	1
1	1	3	3	1	1	4	4	1	1
2	2	3	3	1	1	4	4	1	1
2	2	3	3	1	1	4	4	1	1
2	2	3	3	1	1	4	4	1	1
1	1	2	2	4	4	4	4	1	1
2	2	3	3	1	1	4	4	1	1
2	2	3	3	1	1	4	4	1	1
2	2	3	3	1	1	2	2	2	2
2	2	3	3	1	1	4	4	1	1
1	1	3	3	1	1	4	4	1	1
1	1	2	2	4	4	4	4	1	1
2	2	3	3	1	1	4	4	1	1
1	1	3	3	1	1	4	4	1	1
1	1	3	3	1	1	4	4	1	1
1	1	3	3	1	1	4	4	1	1
1	1	2	2	4	4	4	4	1	1
2	2	3	3	1	1	4	4	1	1
1	1	3	3	1	1	4	4	1	1
2	2	3	3	1	1	2	2	2	2
1	1	2	2	4	4	4	4	1	1
2	2	3	3	1	1	2	2	2	2
1	1	3	3	1	1	4	4	1	1
1	1	3	3	1	1	2	2	2	2
2	2	3	3	1	1	4	4	1	1
1	1	3	3	1	1	4	4	1	1
2	2	3	3	1	1	4	4	1	1
2	2	3	3	1	1	4	4	1	1
1	1	3	3	1	1	4	4	2	2
2	2	3	3	1	1	4	4	1	1
1	1	3	3	1	1	4	4	1	1
2	2	3	3	1	1	4	4	1	1
2	2	3	3	1	1	4	4	1	1
1	1	3	3	1	1	4	4	2	2
2	2	3	3	1	1	4	4	1	1
1	1	2	2	4	4	4	4	1	1
1	1	3	3	1	1	4	4	1	1
1	1	3	3	4	4	4	4	1	1
2	2	3	3	1	1	4	4	2	2

For Review Only

2	2	2	2	4	4	4	4	1	1
1	1	3	3	4	4	4	4	2	2
2	2	3	3	1	1	4	4	1	1
1	1	3	3	1	1	4	4	1	1
2	2	3	3	1	1	4	4	1	1
2	2	3	3	1	1	4	4	1	1
2	2	3	3	1	1	4	4	1	1
2	2	3	3	1	1	4	4	1	1
2	2	3	3	1	1	4	4	1	1
1	1	3	3	1	1	4	4	1	1
1	1	3	3	4	4	4	4	1	1
2	2	3	3	1	1	4	4	1	1
2	2	3	3	1	1	4	4	1	1
1	1	3	3	1	1	4	4	1	1
2	2	3	3	1	1	4	4	1	1
1	1	3	3	4	4	4	4	1	1
1	1	3	3	1	1	4	4	1	1
2	2	3	3	1	1	4	4	1	1
1	1	3	3	1	1	4	4	1	1
2	2	3	3	1	1	4	4	1	1
2	2	3	3	4	4	4	4	1	1
1	1	3	3	1	1	4	4	1	1
2	2	3	3	1	1	4	4	1	1
2	2	2	2	4	4	4	4	1	1
2	2	3	3	4	4	4	4	1	1
2	2	2	2	4	4	4	4	1	1
1	1	3	3	1	1	4	4	1	1
1	1	3	3	1	1	4	4	1	1
2	2	3	3	1	1	4	4	1	1
2	2	3	3	1	1	4	4	1	1
1	1	2	2	4	4	4	4	1	1
1	1	3	3	1	1	4	4	1	1
2	2	3	3	1	1	4	4	1	1
2	2	3	3	1	1	4	4	1	1
2	2	3	3	1	1	4	4	1	1
1	1	3	3	1	1	4	4	1	1
1	1	3	3	1	1	4	4	1	1
2	2	3	3	1	1	4	4	1	1
2	2	3	3	4	4	4	4	1	1
2	2	2	2	4	4	4	4	1	1
2	2	3	3	4	4	4	4	1	1
1	1	3	3	1	1	2	2	1	1
1	1	3	3	1	1	4	4	1	1
1	1	3	3	1	1	4	4	2	2
2	2	3	3	1	1	4	4	1	1
1	1	3	3	1	1	4	4	1	1
2	2	3	3	1	1	4	4	1	1
2	2	3	3	1	1	4	4	1	1
1	1	3	3	1	1	4	4	1	1
2	2	3	3	1	1	4	4	1	1
2	2	3	3	1	1	4	4	1	1
2	2	3	3	1	1	4	4	1	1
1	1	3	3	1	1	4	4	1	1
2	2	3	3	1	1	4	4	1	1
1	1	3	3	1	1	4	4	1	1

For Review Only

1	1	3	3	1	1	4	4	1	1
2	2	3	3	1	1	4	4	1	1
2	2	3	3	1	1	2	2	1	1
2	2	3	3	1	1	4	4	1	1
2	2	3	3	1	1	4	4	1	1
1	1	3	3	1	1	4	4	1	1
2	2	2	2	4	4	4	4	1	1
1	1	3	3	1	1	4	4	1	1
1	1	3	3	4	4	4	4	1	1
1	1	3	3	1	1	4	4	1	1
2	2	3	3	1	1	4	4	1	1
2	2	3	3	4	4	4	4	1	1
2	2	3	3	1	1	4	4	1	1
1	1	3	3	1	1	4	4	1	1
1	1	3	3	1	1	4	4	1	1
2	2	2	2	4	4	4	4	1	1
1	1	3	3	1	1	4	4	1	1
2	2	3	3	1	1	4	4	1	1
2	2	3	3	1	1	4	4	1	1
2	2	3	3	1	1	4	4	1	1
1	1	3	3	4	4	4	4	1	1
1	1	3	3	4	4	4	4	1	1
2	2	2	2	4	4	4	4	1	1
2	2	3	3	1	1	4	4	1	1
2	2	3	3	1	1	4	4	1	1
2	2	3	3	1	1	4	4	1	1
1	1	3	3	1	1	4	4	1	1
1	1	3	3	1	1	4	4	1	1
1	1	3	3	1	1	4	4	1	1
1	1	3	3	1	1	4	4	1	1
2	2	3	3	1	1	4	4	1	1
1	1	3	3	1	1	4	4	1	1
2	2	3	3	1	1	4	4	1	1
1	1	3	3	1	1	4	4	1	1
2	2	3	3	1	1	4	4	1	1
2	2	3	3	1	1	4	4	1	1
2	2	2	2	4	4	4	4	1	1
2	2	3	3	1	1	4	4	1	1
2	2	3	3	1	1	4	4	1	1
2	2	3	3	1	1	4	4	1	1
2	2	3	3	1	1	4	4	1	1
2	2	3	3	1	1	4	4	1	1
2	2	2	2	4	4	4	4	1	1
2	2	3	3	1	1	4	4	1	1
1	1	3	3	1	1	4	4	1	1
2	2	2	2	4	4	4	4	1	1
2	2	2	2	4	4	4	4	1	1
2	2	2	2	4	4	4	4	1	1
1	1	3	3	1	1	2	2	1	1
2	2	3	3	1	1	4	4	1	1
2	2	2	2	4	4	4	4	1	1
1	1	3	3	1	1	4	4	1	1
2	2	2	2	4	4	4	4	1	1
2	2	3	3	1	1	4	4	1	1
1	1	3	3	1	1	4	4	1	1

For Review Only

1	1	3	3	1	1	4	4	1	1
2	2	3	3	1	1	4	4	1	1
2	2	3	3	1	1	4	4	1	1
2	2	3	3	4	4	4	4	1	1
2	2	2	2	4	4	4	4	1	1
2	2	3	3	4	4	4	4	1	1
1	1	2	2	4	4	4	4	1	1
1	1	3	3	1	1	2	2	2	2
1	1	3	3	4	4	4	4	1	1
2	2	2	2	4	4	4	4	1	1
1	1	3	3	1	1	4	4	1	1
1	1	3	3	1	1	4	4	1	1
1	1	3	3	1	1	4	4	1	1
1	1	3	3	1	1	4	4	1	1
2	2	3	3	1	1	4	4	1	1
2	2	3	3	1	1	4	4	1	1
1	1	3	3	1	1	4	4	1	1
1	1	3	3	4	4	4	4	1	1
2	2	3	3	1	1	4	4	1	1
2	2	3	3	1	1	4	4	2	2
1	1	3	3	1	1	4	4	1	1
2	2	3	3	4	4	4	4	1	1
2	2	3	3	1	1	4	4	1	1
1	1	2	2	4	4	4	4	1	1
1	1	3	3	1	1	4	4	1	1
2	2	3	3	4	4	4	4	1	1
2	2	2	2	4	4	4	4	1	1
2	2	3	3	1	1	4	4	1	1
2	2	3	3	4	4	4	4	1	1
2	2	3	3	1	1	4	4	1	1
1	1	2	2	4	4	4	4	1	1
1	1	3	3	1	1	4	4	1	1
2	2	3	3	1	1	4	4	1	1
2	2	3	3	1	1	4	4	1	1
2	2	2	2	4	4	4	4	1	1
1	1	3	3	1	1	4	4	1	1
1	1	3	3	1	1	4	4	1	1
2	2	3	3	1	1	4	4	1	1
1	1	3	3	1	1	4	4	1	1
1	1	2	2	4	4	4	4	1	1
2	2	3	3	1	1	4	4	1	1
1	1	3	3	1	1	4	4	1	1
2	2	2	2	4	4	4	4	1	1
2	2	3	3	1	1	4	4	1	1
2	2	3	3	1	1	4	4	1	1
2	2	3	3	1	1	4	4	1	1
2	2	3	3	1	1	4	4	1	1
2	2	3	3	1	1	4	4	2	2
1	1	3	3	1	1	4	4	2	2
1	1	2	2	4	4	4	4	1	1
2	2	3	3	1	1	4	4	1	1
1	1	3	3	1	1	4	4	1	1
2	2	3	3	1	1	4	4	1	1
2	2	3	3	1	1	4	4	1	1
1	1	2	2	4	4	4	4	1	1

For Review Only

2	2	3	3	1	1	4	4	1	1
2	2	3	3	1	1	4	4	1	1

For Review Only

SNP2645	SNP2645	SNP2646	SNP2646	SNP2647	SNP2647	SNP2648	SNP2648	SNP2649	SNP2649
1	1	3	3	4	4	2	2	4	4
1	1	3	3	4	4	2	2	4	4
1	1	3	3	4	4	2	2	4	4
1	1	3	3	2	2	3	3	4	4
4	4	4	4	2	2	2	2	1	1
1	1	3	3	4	4	2	2	4	4
1	1	3	3	4	4	2	2	4	4
4	4	4	4	2	2	2	2	4	4
1	1	4	4	4	4	2	2	4	4
4	4	4	4	2	2	2	2	4	4
4	4	3	3	2	2	2	2	1	1
1	1	3	3	4	4	2	2	4	4
1	1	4	4	4	4	2	2	4	4
4	4	4	4	2	2	3	3	4	4
1	1	4	4	4	4	2	2	4	4
4	4	4	4	2	2	2	2	4	4
1	1	4	4	2	2	2	2	1	1
1	1	3	3	4	4	2	2	4	4
1	1	3	3	2	2	2	2	1	1
4	4	4	4	2	2	2	2	4	4
1	1	3	3	4	4	2	2	1	1
1	1	4	4	4	4	2	2	1	1
1	1	4	4	4	4	2	2	1	1
4	4	4	4	2	2	2	2	4	4
4	4	4	4	2	2	3	3	4	4
4	4	3	3	2	2	2	2	4	4
1	1	3	3	4	4	2	2	4	4
4	4	4	4	2	2	2	2	4	4
1	1	3	3	4	4	2	2	4	4
4	4	3	3	2	2	2	2	4	4
4	4	3	3	2	2	3	3	4	4
1	1	3	3	2	2	2	2	4	4
1	1	3	3	4	4	2	2	4	4
4	4	4	4	2	2	2	2	4	4
4	4	4	4	2	2	2	2	4	4
4	4	4	4	2	2	2	2	4	4
4	4	3	3	2	2	2	2	4	4
4	4	4	4	2	2	2	2	4	4
4	4	3	3	2	2	2	2	4	4
4	4	4	4	2	2	2	2	4	4
4	4	4	4	2	2	2	2	4	4
4	4	3	3	2	2	2	2	4	4
4	4	3	3	2	2	2	2	4	4
4	4	4	4	2	2	2	2	1	1
4	4	4	4	2	2	2	2	4	4
1	1	4	4	4	4	2	2	4	4
4	4	4	4	2	2	2	2	4	4
4	4	3	3	2	2	2	2	4	4
1	1	4	4	4	4	2	2	4	4
4	4	4	4	2	2	2	2	4	4
4	4	4	4	2	2	2	2	4	4
1	1	3	3	2	2	2	2	4	4

For Review Only

1	1	4	4	4	4	2	2	1	1
1	1	3	3	4	4	2	2	4	4
1	1	3	3	4	4	2	2	4	4
1	1	4	4	4	4	2	2	4	4
1	1	3	3	2	2	2	2	1	1
4	4	4	4	2	2	2	2	4	4
4	4	4	4	2	2	2	2	4	4
4	4	3	3	2	2	2	2	4	4
1	1	3	3	4	4	2	2	4	4
1	1	4	4	4	4	2	2	4	4
4	4	4	4	2	2	3	3	4	4
4	4	4	4	2	2	3	3	4	4
1	1	4	4	4	4	2	2	1	1
1	1	3	3	4	4	2	2	4	4
1	1	3	3	2	2	3	3	4	4
4	4	4	4	2	2	3	3	1	1
4	4	4	4	2	2	3	3	4	4
4	4	4	4	2	2	2	2	4	4
4	4	4	4	2	2	2	2	1	1
1	1	3	3	4	4	2	2	4	4
4	4	4	4	2	2	3	3	4	4
4	4	3	3	2	2	2	2	4	4
1	1	3	3	4	4	2	2	4	4
4	4	3	3	2	2	3	3	1	1
4	4	4	4	2	2	2	2	4	4
1	1	3	3	4	4	2	2	4	4
4	4	3	3	2	2	3	3	4	4
4	4	3	3	2	2	2	2	4	4
4	4	4	4	2	2	2	2	4	4
4	4	3	3	2	2	2	2	4	4
4	4	4	4	2	2	2	2	4	4
4	4	3	3	2	2	2	2	4	4
4	4	4	4	2	2	2	2	4	4
1	1	4	4	4	4	2	2	1	1
4	4	3	3	2	2	3	3	4	4
4	4	4	4	2	2	2	2	4	4
4	4	3	3	2	2	2	2	4	4
4	4	4	4	2	2	2	2	1	1
1	1	3	3	2	2	2	2	4	4
4	4	4	4	2	2	2	2	4	4
1	1	4	4	2	2	2	2	1	1
4	4	3	3	2	2	2	2	4	4
4	4	4	4	2	2	3	3	4	4
4	4	4	4	2	2	3	3	4	4
1	1	4	4	4	4	2	2	1	1
1	1	3	3	4	4	2	2	1	1
4	4	4	4	2	2	2	2	4	4
4	4	4	4	2	2	2	2	4	4
4	4	4	4	2	2	2	2	4	4
4	4	4	4	2	2	2	2	4	4
1	1	3	3	4	4	3	3	4	4
4	4	3	3	2	2	2	2	4	4
4	4	4	4	2	2	2	2	1	1
1	1	4	4	2	2	2	2	4	4
4	4	4	4	2	2	2	2	4	4

For Review Only

4	4	3	3	2	2	2	2	4	4
4	4	3	3	2	2	2	2	4	4
4	4	4	4	2	2	2	2	4	4
4	4	4	4	2	2	2	2	4	4
1	1	3	3	2	2	3	3	4	4
4	4	4	4	2	2	2	2	4	4
1	1	3	3	4	4	2	2	4	4
1	1	4	4	4	4	2	2	4	4
4	4	4	4	2	2	2	2	4	4
4	4	4	4	2	2	2	2	4	4
1	1	3	3	4	4	2	2	4	4
1	1	3	3	4	4	2	2	4	4
4	4	4	4	2	2	3	3	4	4
1	1	3	3	4	4	2	2	4	4
4	4	4	4	2	2	3	3	4	4
4	4	4	4	2	2	2	2	4	4
4	4	4	4	2	2	3	3	4	4
4	4	4	4	2	2	3	3	4	4
4	4	4	4	2	2	2	2	4	4
1	1	3	3	2	2	2	2	1	1
1	1	4	4	2	2	2	2	4	4
4	4	3	3	2	2	2	2	4	4
4	4	3	3	2	2	3	3	1	1
4	4	3	3	2	2	2	2	4	4
4	4	4	4	2	2	2	2	4	4
4	4	4	4	2	2	2	2	4	4
1	1	3	3	2	2	2	2	1	1
4	4	4	4	2	2	3	3	4	4
1	1	3	3	4	4	2	2	4	4
4	4	4	4	2	2	2	2	1	1
4	4	4	4	2	2	2	2	4	4
1	1	4	4	2	2	2	2	4	4
1	1	4	4	4	4	2	2	1	1
1	1	4	4	4	4	2	2	4	4
4	4	4	4	2	2	2	2	4	4
4	4	4	4	2	2	3	3	4	4
4	4	4	4	2	2	2	2	4	4
1	1	3	3	4	4	2	2	4	4
4	4	4	4	2	2	2	2	4	4
4	4	4	4	2	2	2	2	4	4
1	1	3	3	2	2	2	2	4	4
4	4	4	4	2	2	2	2	4	4
4	4	3	3	2	2	3	3	4	4
4	4	4	4	2	2	2	2	4	4
1	1	3	3	4	4	2	2	4	4
4	4	4	4	2	2	2	2	1	1
4	4	4	4	2	2	2	2	4	4
4	4	4	4	2	2	2	2	4	4
1	1	4	4	4	4	2	2	4	4
1	1	4	4	4	4	2	2	4	4
4	4	4	4	2	2	2	2	4	4
4	4	3	3	2	2	2	2	1	1
4	4	4	4	2	2	3	3	4	4
4	4	4	4	2	2	3	3	4	4
4	4	4	4	2	2	2	2	4	4

For Review Only

4	4	4	4	2	2	2	2	4	4
4	4	4	4	2	2	3	3	1	1
4	4	4	4	2	2	2	2	4	4
4	4	3	3	2	2	2	2	1	1
1	1	3	3	4	4	2	2	4	4
4	4	3	3	2	2	2	2	4	4
4	4	3	3	2	2	3	3	4	4
1	1	4	4	2	2	2	2	4	4
4	4	4	4	2	2	2	2	4	4
1	1	3	3	4	4	2	2	4	4
1	1	4	4	4	4	2	2	4	4
4	4	4	4	2	2	3	3	4	4
1	1	4	4	4	4	2	2	4	4
4	4	4	4	2	2	3	3	1	1
1	1	4	4	2	2	2	2	4	4
4	4	3	3	2	2	2	2	1	1
4	4	4	4	2	2	2	2	4	4
1	1	4	4	4	4	2	2	4	4
4	4	4	4	2	2	2	2	4	4
1	1	3	3	4	4	2	2	4	4
1	1	4	4	2	2	2	2	4	4
4	4	4	4	2	2	2	2	4	4
4	4	3	3	2	2	2	2	4	4
4	4	4	4	2	2	3	3	4	4
1	1	4	4	4	4	2	2	4	4
4	4	4	4	2	2	2	2	1	1
1	1	4	4	4	4	2	2	4	4
4	4	4	4	2	2	3	3	4	4
4	4	4	4	2	2	3	3	4	4
1	1	4	4	4	4	2	2	4	4
4	4	3	3	2	2	2	2	4	4
4	4	4	4	2	2	3	3	4	4
4	4	3	3	2	2	3	3	1	1
4	4	4	4	2	2	2	2	4	4
4	4	4	4	2	2	2	2	4	4
4	4	4	4	2	2	3	3	4	4
1	1	3	3	4	4	2	2	4	4
1	1	3	3	4	4	2	2	4	4
4	4	4	4	2	2	2	2	4	4
4	4	3	3	2	2	3	3	4	4
1	1	3	3	4	4	2	2	4	4
4	4	4	4	2	2	2	2	4	4
4	4	4	4	2	2	2	2	4	4
4	4	3	3	2	2	2	2	4	4
4	4	3	3	2	2	2	2	4	4
4	4	3	3	2	2	3	3	1	1
4	4	3	3	2	2	2	2	1	1
4	4	3	3	2	2	2	2	1	1
4	4	4	4	2	2	2	2	4	4
1	1	3	3	4	4	2	2	1	1
1	1	3	3	4	4	2	2	4	4
4	4	4	4	2	2	2	2	4	4
4	4	3	3	2	2	2	2	4	4
4	4	4	4	2	2	3	3	4	4
4	4	3	3	2	2	2	2	4	4

For Review Only

4	4	4	4	2	2	3	3	4	4
1	1	3	3	4	4	2	2	1	1
1	1	4	4	4	4	2	2	4	4
4	4	3	3	2	2	2	2	4	4
4	4	4	4	2	2	2	2	1	1
4	4	4	4	2	2	2	2	4	4
1	1	4	4	2	2	2	2	4	4
4	4	4	4	2	2	2	2	4	4
4	4	4	4	2	2	2	2	4	4
4	4	4	4	2	2	2	2	4	4
1	1	4	4	2	2	2	2	4	4
4	4	3	3	2	2	2	2	4	4
1	1	3	3	4	4	2	2	4	4
1	1	4	4	4	4	2	2	4	4
1	1	3	3	4	4	2	2	4	4
4	4	3	3	2	2	2	2	1	1
4	4	3	3	2	2	2	2	4	4
4	4	4	4	2	2	2	2	4	4
4	4	3	3	2	2	2	2	4	4
4	4	4	4	2	2	2	2	4	4
4	4	3	3	2	2	2	2	4	4
4	4	4	4	2	2	2	2	4	4
1	1	4	4	4	4	2	2	1	1
4	4	3	3	2	2	3	3	4	4
4	4	3	3	2	2	2	2	4	4
1	1	3	3	4	4	2	2	4	4
4	4	3	3	2	2	2	2	4	4
1	1	3	3	4	4	2	2	4	4
1	1	4	4	4	4	2	2	1	1
1	1	4	4	4	4	2	2	4	4
4	4	4	4	2	2	2	2	4	4
4	4	3	3	2	2	2	2	4	4
4	4	4	4	2	2	3	3	1	1
4	4	4	4	2	2	2	2	4	4
4	4	3	3	2	2	2	2	4	4
4	4	3	3	2	2	2	2	4	4
1	1	4	4	4	4	2	2	4	4
4	4	4	4	2	2	3	3	4	4
4	4	4	4	2	2	2	2	4	4
4	4	3	3	2	2	2	2	4	4
1	1	4	4	4	4	2	2	4	4
4	4	4	4	2	2	2	2	1	1
4	4	3	3	2	2	3	3	4	4
1	1	4	4	4	4	2	2	1	1
4	4	4	4	2	2	3	3	4	4
1	1	3	3	4	4	2	2	4	4
4	4	4	4	2	2	2	2	4	4
4	4	4	4	2	2	2	2	4	4
4	4	3	3	2	2	2	2	4	4
4	4	3	3	2	2	2	2	4	4
1	1	4	4	2	2	2	2	4	4
4	4	3	3	2	2	2	2	4	4
1	1	3	3	2	2	2	2	4	4
1	1	4	4	4	4	2	2	4	4
4	4	3	3	2	2	2	2	4	4

For Review Only

4	4	4	4	2	2	2	2	1	1
4	4	3	3	2	2	3	3	4	4

For Review Only

SNP265	SNP265	SNP2650	SNP2650	SNP2651	SNP2651	SNP2652	SNP2652	SNP2653	SNP2653
2	2	3	3	1	1	4	4	1	1
2	2	1	1	1	1	1	1	1	1
2	2	3	3	1	1	1	1	1	1
2	2	3	3	1	1	1	1	1	1
3	3	3	3	1	1	1	1	1	1
2	2	3	3	1	1	4	4	1	1
3	3	1	1	1	1	4	4	1	1
2	2	3	3	1	1	1	1	1	1
3	3	3	3	1	1	4	4	1	1
3	3	1	1	1	1	1	1	1	1
3	3	3	3	4	4	1	1	1	1
2	2	3	3	1	1	1	1	1	1
2	2	3	3	1	1	4	4	1	1
2	2	1	1	1	1	1	1	1	1
2	2	3	3	1	1	4	4	1	1
2	2	1	1	4	4	1	1	1	1
2	2	3	3	1	1	4	4	1	1
3	3	1	1	1	1	4	4	1	1
3	3	3	3	1	1	1	1	1	1
2	2	3	3	1	1	1	1	1	1
2	2	3	3	1	1	4	4	1	1
3	3	3	3	1	1	4	4	1	1
3	3	3	3	1	1	4	4	1	1
2	2	1	1	1	1	1	1	1	1
3	3	3	3	1	1	4	4	2	2
3	3	3	3	1	1	1	1	2	2
3	3	3	3	1	1	1	1	1	1
2	2	3	3	1	1	1	1	1	1
3	3	3	3	1	1	4	4	1	1
2	2	1	1	1	1	1	1	1	1
3	3	3	3	1	1	1	1	2	2
3	3	1	1	1	1	1	1	1	1
3	3	3	3	1	1	4	4	1	1
3	3	3	3	1	1	1	1	2	2
3	3	3	3	1	1	1	1	1	1
2	2	1	1	1	1	1	1	2	2
2	2	1	1	4	4	1	1	1	1
2	2	3	3	1	1	1	1	1	1
2	2	1	1	4	4	1	1	1	1
2	2	1	1	1	1	1	1	1	1
2	2	3	3	1	1	4	4	1	1
2	2	3	3	1	1	1	1	1	1
2	2	3	3	1	1	1	1	1	1
2	2	3	3	1	1	4	4	1	1
3	3	3	3	1	1	1	1	1	1
2	2	3	3	1	1	1	1	1	1
3	3	3	3	1	1	1	1	1	1
2	2	3	3	1	1	1	1	2	2
2	2	1	1	1	1	4	4	1	1
3	3	3	3	1	1	1	1	1	1
3	3	3	3	1	1	1	1	2	2
3	3	3	3	1	1	1	1	2	2

For Review Only

3	3	3	3	1	1	4	4	1	1
3	3	3	3	1	1	4	4	1	1
3	3	1	1	1	1	4	4	1	1
2	2	3	3	1	1	4	4	1	1
3	3	3	3	1	1	1	1	1	1
3	3	3	3	1	1	1	1	2	2
3	3	3	3	1	1	1	1	1	1
2	2	1	1	1	1	1	1	1	1
3	3	1	1	1	1	4	4	1	1
2	2	3	3	1	1	1	1	1	1
3	3	3	3	1	1	1	1	1	1
3	3	3	3	1	1	1	1	1	1
3	3	3	3	1	1	4	4	1	1
3	3	3	3	1	1	4	4	1	1
2	2	3	3	1	1	4	4	1	1
2	2	3	3	1	1	1	1	1	1
2	2	3	3	1	1	1	1	1	1
3	3	1	1	1	1	1	1	1	1
3	3	3	3	1	1	1	1	1	1
3	3	3	3	1	1	4	4	1	1
3	3	3	3	1	1	1	1	1	1
2	2	1	1	4	4	1	1	2	2
3	3	3	3	1	1	4	4	1	1
3	3	3	3	1	1	1	1	1	1
3	3	3	3	1	1	1	1	1	1
2	2	3	3	1	1	1	1	2	2
2	2	1	1	1	1	1	1	1	1
3	3	1	1	1	1	1	1	1	1
2	2	3	3	1	1	1	1	2	2
3	3	1	1	1	1	1	1	1	1
2	2	3	3	1	1	1	1	1	1
3	3	1	1	1	1	1	1	1	1
2	2	1	1	1	1	1	1	1	1
3	3	3	3	1	1	4	4	1	1
2	2	1	1	1	1	1	1	1	1
3	3	3	3	1	1	1	1	1	1
3	3	3	3	1	1	1	1	1	1
3	3	3	3	1	1	1	1	1	1
2	2	1	1	1	1	1	1	1	1
2	2	3	3	1	1	1	1	1	1
3	3	3	3	1	1	4	4	1	1
3	3	1	1	1	1	1	1	1	1
3	3	3	3	1	1	1	1	1	1
3	3	3	3	1	1	1	1	1	1
3	3	3	3	1	1	4	4	1	1
3	3	3	3	1	1	1	1	1	1
2	2	1	1	1	1	1	1	1	1
3	3	1	1	1	1	1	1	2	2
2	2	3	3	1	1	1	1	1	1
2	2	3	3	1	1	1	1	1	1
3	3	3	3	1	1	1	1	1	1
2	2	1	1	4	4	1	1	1	1
2	2	3	3	1	1	1	1	1	1
2	2	3	3	1	1	1	1	1	1
2	2	3	3	1	1	1	1	1	1

For Review Only

2	2	3	3	1	1	1	1	1	1
2	2	1	1	1	1	1	1	1	1
3	3	3	3	1	1	1	1	2	2
2	2	1	1	1	1	1	1	1	1
3	3	3	3	1	1	1	1	1	1
3	3	3	3	1	1	1	1	1	1
3	3	3	3	1	1	1	1	1	1
2	2	3	3	1	1	1	1	1	1
3	3	3	3	1	1	1	1	1	1
2	2	1	1	1	1	1	1	2	2
2	2	3	3	1	1	1	1	1	1
2	2	3	3	1	1	1	1	1	1
3	3	3	3	1	1	1	1	1	1
2	2	3	3	1	1	1	1	1	1
3	3	1	1	1	1	1	1	1	1
2	2	3	3	1	1	1	1	1	1
3	3	1	1	1	1	1	1	1	1
2	2	3	3	1	1	4	4	1	1
2	2	1	1	1	1	1	1	1	1
3	3	3	3	4	4	1	1	1	1
2	2	3	3	1	1	1	1	1	1
3	3	3	3	1	1	1	1	1	1
2	2	3	3	1	1	1	1	1	1
2	2	1	1	4	4	1	1	1	1
3	3	3	3	1	1	1	1	1	1
3	3	1	1	4	4	1	1	2	2
3	3	3	3	1	1	4	4	1	1
3	3	1	1	1	1	1	1	1	1
3	3	3	3	1	1	4	4	1	1
2	2	3	3	1	1	1	1	1	1
2	2	1	1	1	1	1	1	1	1
2	2	3	3	1	1	1	1	2	2
3	3	3	3	1	1	4	4	1	1
2	2	3	3	1	1	1	1	1	1
3	3	3	3	1	1	1	1	2	2
2	2	3	3	1	1	4	4	1	1
2	2	1	1	1	1	1	1	1	1
3	3	1	1	1	1	1	1	1	1
2	2	3	3	1	1	1	1	1	1
2	2	3	3	1	1	1	1	1	1
2	2	1	1	1	1	4	4	2	2
2	2	1	1	1	1	1	1	2	2
3	3	1	1	1	1	1	1	1	1
2	2	1	1	1	1	1	1	1	1
3	3	3	3	1	1	1	1	1	1
3	3	3	3	4	4	1	1	1	1
3	3	3	3	1	1	1	1	2	2
2	2	1	1	1	1	1	1	1	1
2	2	1	1	1	1	1	1	1	1
2	2	3	3	1	1	1	1	2	2
3	3	3	3	1	1	1	1	2	2
2	2	3	3	4	4	1	1	1	1
3	3	1	1	1	1	4	4	1	1
3	3	3	3	1	1	1	1	1	1
2	2	3	3	1	1	1	1	2	2

For Review Only

3	3	1	1	1	1	1	1	2	2
2	2	3	3	1	1	1	1	1	1
2	2	3	3	4	4	1	1	1	1
3	3	3	3	4	4	1	1	2	2
2	2	3	3	1	1	1	1	1	1
2	2	1	1	4	4	1	1	1	1
2	2	3	3	1	1	1	1	1	1
2	2	3	3	1	1	4	4	1	1
2	2	3	3	1	1	4	4	1	1
3	3	1	1	1	1	4	4	1	1
2	2	3	3	1	1	1	1	2	2
2	2	3	3	1	1	1	1	1	1
2	2	3	3	1	1	1	1	1	1
2	2	3	3	4	4	1	1	1	1
2	2	1	1	1	1	1	1	1	1
2	2	3	3	4	4	1	1	1	1
3	3	3	3	1	1	1	1	1	1
3	3	1	1	1	1	1	1	2	2
2	2	1	1	1	1	1	1	2	2
3	3	3	3	1	1	1	1	1	1
2	2	3	3	1	1	1	1	1	1
3	3	1	1	1	1	1	1	1	1
2	2	1	1	4	4	1	1	1	1
3	3	3	3	1	1	1	1	1	1
2	2	3	3	1	1	4	4	1	1
2	2	3	3	1	1	1	1	2	2
3	3	3	3	1	1	1	1	1	1
3	3	1	1	1	1	1	1	1	1
3	3	1	1	1	1	1	1	1	1
3	3	1	1	1	1	4	4	1	1
3	3	3	3	1	1	1	1	2	2
3	3	1	1	1	1	1	1	1	1
3	3	3	3	1	1	1	1	1	1
2	2	1	1	1	1	1	1	1	1
3	3	3	3	1	1	1	1	1	1
3	3	3	3	1	1	1	1	1	1
3	3	1	1	1	1	1	1	2	2
2	2	1	1	1	1	1	1	1	1
2	2	1	1	1	1	1	1	1	1
3	3	3	3	1	1	1	1	2	2
2	2	3	3	1	1	1	1	1	1
3	3	3	3	1	1	1	1	2	2
2	2	1	1	1	1	1	1	1	1
2	2	3	3	1	1	1	1	1	1
2	2	3	3	1	1	1	1	2	2
2	2	3	3	1	1	1	1	1	1
2	2	3	3	4	4	1	1	1	1
3	3	3	3	1	1	1	1	1	1
2	2	1	1	1	1	1	1	2	2
2	2	3	3	1	1	4	4	1	1
3	3	3	3	1	1	4	4	2	2
2	2	1	1	4	4	1	1	1	1
2	2	1	1	4	4	1	1	1	1
3	3	3	3	1	1	1	1	1	1
2	2	1	1	4	4	1	1	1	1

For Review Only

3	3	1	1	1	1	1	1	1	1
2	2	3	3	1	1	1	1	1	1
2	2	3	3	1	1	4	4	1	1
2	2	3	3	4	4	1	1	1	1
2	2	3	3	1	1	1	1	1	1
3	3	3	3	1	1	1	1	2	2
3	3	1	1	1	1	1	1	1	1
3	3	1	1	1	1	1	1	1	1
2	2	3	3	1	1	1	1	1	1
2	2	1	1	1	1	1	1	1	1
2	2	3	3	1	1	1	1	1	1
3	3	1	1	1	1	1	1	2	2
2	2	1	1	1	1	4	4	1	1
3	3	1	1	1	1	4	4	2	2
3	3	3	3	1	1	1	1	1	1
3	3	3	3	4	4	1	1	2	2
2	2	1	1	4	4	1	1	1	1
3	3	1	1	1	1	1	1	1	1
3	3	1	1	1	1	1	1	1	1
2	2	3	3	1	1	1	1	1	1
3	3	1	1	4	4	1	1	1	1
3	3	3	3	1	1	1	1	2	2
3	3	3	3	1	1	1	1	1	1
2	2	1	1	1	1	1	1	1	1
2	2	1	1	1	1	1	1	1	1
3	3	3	3	1	1	4	4	1	1
2	2	1	1	1	1	1	1	1	1
3	3	3	3	1	1	1	1	1	1
2	2	3	3	1	1	4	4	1	1
3	3	3	3	1	1	4	4	2	2
2	2	1	1	4	4	1	1	1	1
3	3	1	1	1	1	1	1	2	2
2	2	3	3	1	1	1	1	1	1
3	3	3	3	1	1	4	4	1	1
2	2	1	1	4	4	1	1	2	2
3	3	1	1	1	1	1	1	1	1
3	3	1	1	1	1	4	4	1	1
3	3	3	3	1	1	1	1	1	1
2	2	1	1	1	1	1	1	2	2
2	2	1	1	4	4	1	1	1	1
3	3	3	3	1	1	1	1	1	1
3	3	3	3	1	1	1	1	1	1
3	3	3	3	1	1	1	1	1	1
3	3	3	3	1	1	4	4	1	1
2	2	3	3	1	1	1	1	1	1
2	2	1	1	1	1	4	4	1	1
2	2	1	1	1	1	1	1	1	1
3	3	3	3	1	1	1	1	1	1
2	2	1	1	1	1	1	1	2	2
2	2	1	1	1	1	1	1	1	1
3	3	3	3	1	1	1	1	1	1
2	2	1	1	4	4	1	1	1	1
2	2	3	3	1	1	1	1	1	1
3	3	3	3	1	1	4	4	1	1
2	2	1	1	4	4	1	1	1	1

For Review Only

3	3	3	3	1	1	1	1	1	1
2	2	3	3	1	1	1	1	1	1

For Review Only

SNP2654	SNP2654	SNP2655	SNP2655	SNP2656	SNP2656	SNP2657	SNP2657	SNP2658	SNP2658
2	2	3	3	4	4	4	4	2	2
2	2	4	4	4	4	4	4	4	4
2	2	4	4	4	4	4	4	4	4
2	2	4	4	4	4	4	4	4	4
1	1	3	3	1	1	2	2	2	2
2	2	3	3	4	4	4	4	2	2
2	2	3	3	4	4	4	4	2	2
1	1	4	4	1	1	2	2	4	4
2	2	4	4	4	4	4	4	2	2
2	2	4	4	1	1	2	2	4	4
1	1	4	4	1	1	4	4	4	4
2	2	4	4	4	4	4	4	4	4
2	2	4	4	4	4	4	4	4	4
2	2	4	4	4	4	4	4	4	4
2	2	4	4	4	4	4	4	2	2
2	2	4	4	1	1	4	4	4	4
1	1	4	4	1	1	4	4	4	4
2	2	4	4	4	4	4	4	4	4
1	1	4	4	4	4	4	4	4	4
2	2	4	4	1	1	4	4	4	4
1	1	4	4	4	4	4	4	4	4
1	1	4	4	1	1	4	4	4	4
1	1	4	4	1	1	4	4	4	4
2	2	4	4	1	1	2	2	4	4
1	1	4	4	4	4	4	4	4	4
2	2	4	4	4	4	4	4	4	4
2	2	4	4	4	4	4	4	4	4
2	2	4	4	4	4	4	4	4	4
2	2	4	4	4	4	4	4	4	4
2	2	4	4	1	1	2	2	4	4
2	2	4	4	4	4	4	4	4	4
2	2	4	4	4	4	4	4	4	4
1	1	4	4	4	4	4	4	4	4
2	2	4	4	1	1	4	4	4	4
2	2	4	4	4	4	4	4	4	4
2	2	4	4	1	1	2	2	4	4
2	2	4	4	4	4	4	4	4	4
2	2	4	4	4	4	4	4	4	4
2	2	4	4	1	1	4	4	4	4
2	2	4	4	1	1	4	4	4	4
2	2	4	4	1	1	2	2	4	4
2	2	4	4	4	4	4	4	4	4
1	1	4	4	4	4	4	4	4	4
1	1	4	4	1	1	4	4	4	4
1	1	4	4	1	1	2	2	4	4
2	2	4	4	4	4	4	4	4	4
2	2	4	4	4	4	4	4	4	4
2	2	4	4	1	1	4	4	4	4
2	2	4	4	4	4	4	4	4	4
2	2	4	4	4	4	4	4	4	4
2	2	4	4	4	4	4	4	4	4
2	2	4	4	4	4	4	4	4	4

For Review Only

1	1	4	4	1	1	4	4	4	4
1	1	4	4	4	4	4	4	4	4
2	2	4	4	4	4	4	4	4	4
1	1	4	4	1	1	4	4	4	4
1	1	4	4	4	4	4	4	4	4
2	2	4	4	4	4	4	4	4	4
2	2	4	4	4	4	4	4	4	4
2	2	4	4	1	1	4	4	4	4
2	2	4	4	4	4	4	4	4	4
2	2	4	4	4	4	4	4	4	4
2	2	4	4	4	4	4	4	4	4
2	2	4	4	4	4	4	4	4	4
1	1	4	4	1	1	4	4	4	4
2	2	4	4	4	4	4	4	4	4
2	2	4	4	4	4	4	4	4	4
1	1	4	4	4	4	4	4	4	4
2	2	4	4	1	1	4	4	4	4
2	2	3	3	4	4	4	4	2	2
1	1	4	4	1	1	4	4	4	4
1	1	3	3	4	4	4	4	2	2
2	2	4	4	4	4	4	4	4	4
2	2	4	4	1	1	4	4	4	4
1	1	4	4	4	4	4	4	4	4
1	1	4	4	4	4	4	4	4	4
1	1	4	4	1	1	2	2	4	4
2	2	4	4	4	4	4	4	4	4
2	2	4	4	1	1	4	4	4	4
2	2	4	4	1	1	4	4	4	4
2	2	4	4	4	4	4	4	4	4
2	2	4	4	4	4	4	4	4	4
2	2	4	4	1	1	4	4	4	4
2	2	4	4	1	1	4	4	4	4
2	2	4	4	1	1	4	4	4	4
1	1	4	4	1	1	4	4	4	4
2	2	3	3	4	4	4	4	2	2
1	1	4	4	1	1	2	2	4	4
2	2	4	4	1	1	2	2	4	4
1	1	4	4	1	1	2	2	4	4
2	2	4	4	4	4	4	4	4	4
2	2	4	4	1	1	2	2	4	4
1	1	4	4	1	1	4	4	4	4
2	2	4	4	4	4	4	4	4	4
1	1	4	4	1	1	4	4	4	4
2	2	4	4	4	4	4	4	4	4
1	1	3	3	4	4	4	4	2	2
1	1	4	4	4	4	4	4	4	4
2	2	4	4	1	1	4	4	4	4
2	2	4	4	4	4	4	4	4	4
2	2	4	4	1	1	4	4	4	4
2	2	4	4	4	4	4	4	4	4
2	2	4	4	4	4	4	4	4	4
2	2	4	4	1	1	4	4	4	4
1	1	4	4	4	4	4	4	4	4
2	2	4	4	4	4	4	4	4	4
2	2	3	3	4	4	4	4	2	2

For Review Only

2	2	4	4	4	4	4	4	4	4
2	2	4	4	4	4	4	4	4	4
2	2	4	4	4	4	4	4	4	4
2	2	4	4	4	4	4	4	4	4
2	2	4	4	4	4	4	4	4	4
2	2	4	4	4	4	4	4	4	4
1	1	4	4	4	4	4	4	4	4
2	2	3	3	4	4	4	4	2	2
2	2	4	4	1	1	4	4	4	4
2	2	4	4	4	4	4	4	4	4
2	2	3	3	4	4	4	4	2	2
2	2	4	4	4	4	4	4	4	4
1	1	4	4	1	1	2	2	4	4
2	2	4	4	4	4	4	4	4	4
2	2	4	4	4	4	4	4	4	4
2	2	4	4	4	4	4	4	4	4
2	2	4	4	4	4	4	4	4	4
2	2	4	4	4	4	4	4	4	4
2	2	4	4	4	4	4	4	4	4
1	1	4	4	1	1	2	2	4	4
2	2	4	4	4	4	4	4	4	4
2	2	4	4	4	4	4	4	4	4
2	2	4	4	4	4	4	4	4	4
2	2	4	4	1	1	4	4	4	4
2	2	4	4	4	4	4	4	4	4
2	2	4	4	1	1	4	4	4	4
1	1	4	4	4	4	4	4	4	4
2	2	4	4	4	4	4	4	4	4
2	2	4	4	4	4	4	4	4	4
1	1	4	4	4	4	4	4	4	4
2	2	4	4	1	1	4	4	4	4
2	2	4	4	4	4	4	4	4	4
1	1	4	4	1	1	4	4	4	4
2	2	4	4	4	4	4	4	4	4
2	2	4	4	4	4	4	4	4	4
2	2	4	4	4	4	4	4	4	4
2	2	4	4	1	1	4	4	4	4
2	2	4	4	4	4	4	4	4	4
2	2	4	4	1	1	4	4	4	4
2	2	4	4	1	1	4	4	4	4
2	2	4	4	4	4	4	4	4	4
2	2	4	4	1	1	4	4	4	4
2	2	3	3	4	4	4	4	2	2
2	2	4	4	1	1	4	4	4	4
2	2	4	4	4	4	4	4	4	4
1	1	3	3	4	4	4	4	2	2
2	2	4	4	4	4	4	4	4	4
2	2	4	4	4	4	4	4	4	4
2	2	4	4	4	4	4	4	4	4
2	2	4	4	4	4	4	4	4	4
2	2	4	4	4	4	4	4	4	4
1	1	4	4	4	4	4	4	4	4
2	2	4	4	4	4	4	4	4	4
2	2	4	4	4	4	4	4	4	4
2	2	4	4	4	4	4	4	4	4

For Review Only

2	2	4	4	4	4	4	4	4	4
1	1	4	4	1	1	2	2	4	4
1	1	4	4	1	1	2	2	4	4
1	1	4	4	1	1	4	4	4	4
2	2	4	4	4	4	4	4	4	4
2	2	4	4	4	4	4	4	4	4
2	2	4	4	1	1	4	4	4	4
2	2	4	4	4	4	4	4	4	4
2	2	4	4	1	1	4	4	4	4
2	2	4	4	4	4	4	4	4	4
2	2	4	4	4	4	4	4	4	4
2	2	4	4	4	4	4	4	4	4
2	2	4	4	4	4	4	4	4	4
1	1	4	4	4	4	4	4	4	4
2	2	4	4	4	4	4	4	4	4
1	1	4	4	1	1	4	4	4	4
2	2	4	4	4	4	4	4	4	4
1	1	4	4	4	4	4	4	4	4
2	2	4	4	4	4	4	4	4	4
1	1	4	4	4	4	4	4	4	4
2	2	4	4	4	4	4	4	4	4
2	2	4	4	4	4	4	4	4	4
2	2	4	4	1	1	4	4	4	4
2	2	4	4	4	4	4	4	4	4
1	1	4	4	1	1	4	4	4	4
1	1	4	4	1	1	4	4	4	4
2	2	4	4	4	4	4	4	4	4
2	2	4	4	4	4	4	4	4	4
2	2	4	4	4	4	4	4	4	4
2	2	4	4	1	1	4	4	4	4
1	1	4	4	4	4	4	4	4	4
2	2	4	4	4	4	4	4	4	4
1	1	4	4	4	4	4	4	4	4
2	2	4	4	4	4	4	4	4	4
2	2	4	4	4	4	4	4	4	4
2	2	4	4	4	4	4	4	4	4
2	2	4	4	1	1	4	4	4	4
2	2	4	4	4	4	4	4	4	4
2	2	4	4	4	4	4	4	4	4
1	1	4	4	4	4	4	4	4	4
2	2	4	4	4	4	4	4	4	4
2	2	4	4	4	4	4	4	4	4
2	2	4	4	1	1	4	4	4	4
2	2	4	4	4	4	4	4	4	4
2	2	4	4	4	4	4	4	4	4
2	2	4	4	4	4	4	4	4	4
1	1	4	4	1	1	4	4	4	4
1	1	4	4	1	1	4	4	4	4
2	2	4	4	4	4	4	4	4	4
1	1	4	4	4	4	4	4	4	4
2	2	4	4	4	4	4	4	4	4
2	2	4	4	1	1	4	4	4	4
2	2	4	4	1	1	4	4	4	4
2	2	4	4	4	4	4	4	4	4
2	2	4	4	1	1	4	4	4	4

For Review Only

2	2	3	3	4	4	4	4	2	2
2	2	4	4	4	4	4	4	4	4
2	2	4	4	4	4	4	4	4	4
2	2	4	4	1	1	4	4	4	4
1	1	4	4	4	4	4	4	4	4
2	2	4	4	4	4	4	4	4	4
2	2	4	4	4	4	4	4	4	4
2	2	4	4	1	1	2	2	4	4
2	2	4	4	4	4	4	4	4	4
2	2	4	4	1	1	4	4	4	4
2	2	4	4	4	4	4	4	4	4
2	2	4	4	4	4	4	4	4	4
2	2	4	4	4	4	4	4	4	4
2	2	4	4	4	4	4	4	4	4
2	2	4	4	4	4	4	4	4	4
1	1	4	4	4	4	4	4	4	4
2	2	4	4	1	1	4	4	4	4
2	2	4	4	1	1	4	4	4	4
2	2	4	4	1	1	4	4	4	4
2	2	4	4	1	1	4	4	4	4
2	2	4	4	4	4	4	4	4	4
2	2	4	4	4	4	4	4	4	4
1	1	4	4	1	1	4	4	4	4
2	2	4	4	1	1	4	4	4	4
2	2	4	4	1	1	4	4	4	4
2	2	4	4	4	4	4	4	4	4
2	2	4	4	1	1	4	4	4	4
2	2	4	4	4	4	4	4	4	4
1	1	4	4	4	4	4	4	4	4
2	2	4	4	4	4	4	4	4	4
2	2	4	4	1	1	4	4	4	4
2	2	4	4	1	1	4	4	4	4
1	1	4	4	4	4	4	4	4	4
2	2	4	4	4	4	4	4	4	4
2	2	4	4	1	1	4	4	4	4
2	2	4	4	1	1	4	4	4	4
2	2	3	3	4	4	4	4	2	2
2	2	4	4	4	4	4	4	4	4
2	2	4	4	1	1	2	2	4	4
2	2	4	4	1	1	4	4	4	4
2	2	4	4	4	4	4	4	4	4
1	1	4	4	4	4	4	4	4	4
2	2	4	4	1	1	4	4	4	4
1	1	4	4	1	1	4	4	4	4
2	2	4	4	4	4	4	4	4	4
2	2	4	4	4	4	4	4	4	4
2	2	4	4	4	4	4	4	4	4
2	2	4	4	4	4	4	4	4	4
2	2	4	4	4	4	4	4	4	4
2	2	4	4	4	4	4	4	4	4
2	2	4	4	4	4	4	4	4	4
2	2	4	4	1	1	4	4	4	4
2	2	4	4	4	4	4	4	4	4
2	2	4	4	4	4	4	4	4	4
2	2	4	4	1	1	4	4	4	4

For Review Only

1	1	4	4	1	1	4	4	4	4
2	2	4	4	4	4	4	4	4	4

For Review Only

SNP2659	SNP2659	SNP266	SNP266	SNP2660	SNP2660	SNP2661	SNP2661	SNP2662	SNP2662
2	2	2	2	4	4	2	2	3	3
2	2	2	2	4	4	4	4	2	2
2	2	2	2	4	4	4	4	2	2
2	2	2	2	4	4	2	2	2	2
3	3	2	2	1	1	2	2	3	3
2	2	2	2	4	4	4	4	3	3
2	2	2	2	4	4	4	4	3	3
3	3	2	2	1	1	2	2	2	2
2	2	2	2	4	4	4	4	3	3
3	3	2	2	1	1	4	4	2	2
2	2	1	1	4	4	2	2	2	2
2	2	2	2	4	4	4	4	2	2
2	2	2	2	4	4	4	4	2	2
2	2	2	2	4	4	4	4	2	2
2	2	2	2	4	4	4	4	2	2
2	2	2	2	1	1	4	4	2	2
2	2	2	2	4	4	2	2	2	2
2	2	2	2	4	4	4	4	2	2
2	2	2	2	4	4	4	4	2	2
3	3	2	2	4	4	4	4	2	2
2	2	2	2	4	4	2	2	2	2
2	2	2	2	4	4	2	2	2	2
2	2	2	2	4	4	4	4	2	2
3	3	2	2	1	1	4	4	2	2
2	2	2	2	4	4	2	2	2	2
2	2	2	2	4	4	4	4	2	2
2	2	2	2	4	4	4	4	2	2
2	2	2	2	4	4	4	4	2	2
2	2	2	2	4	4	4	4	2	2
3	3	2	2	1	1	4	4	2	2
2	2	2	2	4	4	4	4	2	2
2	2	1	1	4	4	4	4	2	2
2	2	2	2	4	4	2	2	2	2
2	2	2	2	4	4	4	4	2	2
2	2	2	2	4	4	4	4	2	2
3	3	2	2	1	1	4	4	2	2
2	2	1	1	4	4	4	4	2	2
2	2	1	1	4	4	4	4	2	2
2	2	2	2	4	4	4	4	2	2
2	2	1	1	4	4	4	4	2	2
2	2	2	2	4	4	4	4	2	2
2	2	2	2	4	4	4	4	2	2
2	2	2	2	4	4	2	2	2	2
2	2	1	1	4	4	2	2	2	2
3	3	2	2	1	1	2	2	2	2
2	2	2	2	4	4	4	4	2	2
2	2	2	2	4	4	4	4	2	2
2	2	2	2	4	4	4	4	2	2
2	2	2	2	4	4	4	4	2	2
2	2	1	1	4	4	4	4	2	2
2	2	2	2	4	4	4	4	2	2
2	2	2	2	4	4	4	4	2	2

For Review Only

2	2	1	1	4	4	2	2	2	2
2	2	2	2	4	4	2	2	2	2
2	2	2	2	4	4	4	4	2	2
2	2	1	1	4	4	2	2	2	2
2	2	2	2	4	4	4	4	2	2
2	2	2	2	4	4	4	4	2	2
2	2	2	2	4	4	4	4	2	2
2	2	2	2	4	4	4	4	2	2
2	2	1	1	4	4	4	4	2	2
2	2	2	2	4	4	4	4	2	2
2	2	2	2	4	4	4	4	2	2
2	2	2	2	4	4	4	4	2	2
2	2	1	1	4	4	2	2	2	2
2	2	2	2	4	4	4	4	2	2
2	2	2	2	4	4	4	4	2	2
2	2	1	1	4	4	4	4	2	2
2	2	2	2	4	4	4	4	2	2
2	2	2	2	4	4	4	4	3	3
2	2	1	1	4	4	2	2	2	2
2	2	2	2	4	4	2	2	3	3
2	2	2	2	4	4	4	4	2	2
2	2	2	2	4	4	4	4	2	2
2	2	2	2	4	4	4	4	2	2
2	2	2	2	4	4	2	2	2	2
3	3	2	2	1	1	2	2	2	2
2	2	2	2	4	4	4	4	2	2
2	2	1	1	4	4	4	4	2	2
2	2	2	2	4	4	4	4	2	2
2	2	2	2	4	4	4	4	2	2
2	2	2	2	4	4	4	4	2	2
2	2	2	2	4	4	4	4	2	2
2	2	2	2	1	1	4	4	2	2
2	2	2	2	4	4	4	4	2	2
2	2	1	1	4	4	2	2	2	2
2	2	2	2	4	4	4	4	3	3
3	3	2	2	1	1	2	2	2	2
3	3	2	2	1	1	4	4	2	2
3	3	2	2	1	1	2	2	2	2
2	2	2	2	4	4	4	4	2	2
3	3	2	2	1	1	4	4	2	2
2	2	1	1	4	4	2	2	2	2
2	2	2	2	4	4	4	4	2	2
2	2	2	2	4	4	4	4	2	2
2	2	2	2	4	4	4	4	2	2
2	2	1	1	4	4	4	4	3	3
2	2	2	2	4	4	2	2	2	2
2	2	2	2	4	4	4	4	2	2
2	2	2	2	4	4	4	4	2	2
3	3	2	2	4	4	4	4	2	2
2	2	2	2	4	4	4	4	2	2
2	2	2	2	4	4	4	4	2	2
2	2	2	2	4	4	4	4	2	2
2	2	2	2	4	4	4	4	2	2
2	2	2	2	4	4	4	4	2	2
2	2	2	2	4	4	4	4	3	3

For Review Only

2	2	2	2	4	4	4	4	2	2
2	2	2	2	4	4	4	4	2	2
2	2	2	2	4	4	4	4	2	2
2	2	2	2	4	4	4	4	2	2
2	2	2	2	4	4	4	4	2	2
2	2	2	2	4	4	4	4	2	2
2	2	2	2	4	4	4	4	2	2
2	2	2	2	4	4	4	4	2	2
3	3	2	2	4	4	4	4	2	2
2	2	2	2	4	4	4	4	2	2
2	2	2	2	4	4	4	4	3	3
2	2	2	2	4	4	4	4	2	2
3	3	2	2	4	4	2	2	2	2
2	2	2	2	4	4	4	4	2	2
2	2	2	2	4	4	4	4	2	2
2	2	2	2	4	4	4	4	2	2
2	2	1	1	4	4	4	4	2	2
2	2	2	2	4	4	4	4	2	2
2	2	2	2	4	4	4	4	2	2
3	3	2	2	1	1	2	2	2	2
2	2	2	2	4	4	4	4	2	2
2	2	2	2	4	4	4	4	2	2
2	2	2	2	4	4	4	4	2	2
2	2	2	2	4	4	4	4	2	2
2	2	2	2	4	4	4	4	2	2
2	2	2	2	4	4	4	4	2	2
2	2	2	2	4	4	4	4	2	2
2	2	2	2	4	4	4	4	2	2
2	2	1	1	4	4	4	4	2	2
2	2	2	2	4	4	2	2	2	2
2	2	2	2	4	4	4	4	2	2
2	2	2	2	4	4	4	4	2	2
2	2	1	1	4	4	2	2	2	2
2	2	2	2	4	4	4	4	2	2
2	2	2	2	4	4	4	4	2	2
2	2	2	2	4	4	4	4	2	2
2	2	2	2	4	4	4	4	2	2
2	2	2	2	4	4	4	4	2	2
2	2	2	2	4	4	4	4	2	2
2	2	2	2	4	4	4	4	2	2
2	2	2	2	4	4	4	4	2	2
2	2	2	2	4	4	4	4	2	2
2	2	1	1	4	4	4	4	3	3
2	2	2	2	4	4	4	4	2	2
2	2	2	2	1	1	4	4	2	2
2	2	2	2	4	4	4	4	3	3
2	2	2	2	4	4	4	4	2	2
2	2	2	2	4	4	4	4	2	2
2	2	2	2	4	4	4	4	2	2
2	2	2	2	4	4	4	4	2	2
2	2	2	2	4	4	4	4	2	2
2	2	2	2	4	4	2	2	2	2
2	2	2	2	4	4	4	4	2	2
2	2	2	2	4	4	4	4	2	2
2	2	2	2	4	4	4	4	2	2

For Review Only

2	2	2	2	4	4	4	4	2	2
3	3	2	2	1	1	2	2	2	2
3	3	2	2	1	1	2	2	2	2
2	2	2	2	4	4	4	4	2	2
2	2	2	2	4	4	4	4	2	2
2	2	2	2	4	4	4	4	2	2
2	2	2	2	4	4	2	2	2	2
2	2	2	2	4	4	4	4	2	2
2	2	2	2	4	4	4	4	2	2
2	2	1	1	4	4	4	4	2	2
2	2	2	2	4	4	4	4	2	2
3	3	2	2	4	4	4	4	2	2
2	2	2	2	4	4	4	4	2	2
2	2	2	2	4	4	4	4	2	2
2	2	2	2	4	4	4	4	2	2
2	2	1	1	4	4	2	2	2	2
2	2	2	2	4	4	4	4	2	2
2	2	2	2	4	4	2	2	2	2
2	2	2	2	4	4	4	4	2	2
2	2	2	2	4	4	4	4	2	2
2	2	2	2	4	4	4	4	2	2
2	2	2	2	4	4	4	4	2	2
2	2	2	2	4	4	4	4	2	2
2	2	2	2	4	4	4	4	2	2
2	2	1	1	4	4	4	4	2	2
2	2	2	2	4	4	2	2	2	2
2	2	2	2	4	4	4	4	2	2
2	2	2	2	4	4	4	4	2	2
2	2	2	2	4	4	4	4	2	2
2	2	2	2	4	4	4	4	2	2
2	2	2	2	4	4	2	2	2	2
2	2	2	2	4	4	4	4	2	2
2	2	2	2	4	4	2	2	2	2
2	2	2	2	4	4	4	4	2	2
2	2	1	1	4	4	4	4	2	2
2	2	2	2	4	4	4	4	2	2
2	2	2	2	4	4	4	4	2	2
2	2	1	1	4	4	4	4	2	2
2	2	2	2	4	4	4	4	2	2
2	2	2	2	4	4	2	2	2	2
2	2	2	2	4	4	4	4	2	2
2	2	2	2	4	4	4	4	2	2
2	2	2	2	4	4	4	4	2	2
2	2	2	2	4	4	4	4	2	2
2	2	2	2	4	4	4	4	2	2
2	2	2	2	4	4	4	4	2	2
2	2	1	1	4	4	2	2	2	2
2	2	2	2	4	4	2	2	2	2
2	2	2	2	4	4	4	4	2	2
2	2	2	2	4	4	2	2	2	2
2	2	1	1	4	4	4	4	2	2
2	2	2	2	4	4	4	4	2	2
2	2	2	2	4	4	4	4	2	2
2	2	2	2	4	4	4	4	2	2
2	2	2	2	4	4	4	4	2	2

For Review Only

2	2	1	1	4	4	4	4	3	3
2	2	2	2	4	4	4	4	2	2
2	2	2	2	4	4	4	4	2	2
2	2	2	2	4	4	4	4	2	2
2	2	2	2	4	4	2	2	2	2
2	2	2	2	4	4	4	4	2	2
2	2	2	2	4	4	4	4	2	2
3	3	2	2	1	1	4	4	2	2
3	3	2	2	4	4	4	4	2	2
2	2	2	2	4	4	4	4	2	2
2	2	2	2	4	4	4	4	2	2
2	2	2	2	4	4	4	4	2	2
2	2	2	2	4	4	4	4	2	2
2	2	2	2	4	4	4	4	2	2
2	2	2	2	4	4	4	4	2	2
2	2	1	1	4	4	2	2	2	2
2	2	2	2	4	4	4	4	2	2
2	2	1	1	4	4	4	4	2	2
2	2	2	2	4	4	4	4	2	2
2	2	1	1	4	4	4	4	2	2
2	2	2	2	4	4	4	4	2	2
2	2	2	2	4	4	4	4	2	2
2	2	1	1	4	4	2	2	2	2
2	2	2	2	4	4	4	4	2	2
2	2	2	2	4	4	4	4	2	2
2	2	2	2	4	4	2	2	2	2
2	2	2	2	4	4	4	4	2	2
2	2	2	2	4	4	2	2	2	2
2	2	2	2	4	4	2	2	2	2
2	2	1	1	4	4	4	4	2	2
2	2	2	2	4	4	4	4	2	2
2	2	2	2	4	4	4	4	2	2
2	2	2	2	4	4	2	2	2	2
2	2	2	2	4	4	4	4	2	2
2	2	2	2	4	4	4	4	2	2
2	2	2	2	4	4	4	4	2	2
2	2	1	1	4	4	4	4	3	3
2	2	2	2	4	4	4	4	2	2
3	3	2	2	1	1	4	4	2	2
2	2	2	2	4	4	4	4	2	2
2	2	1	1	4	4	4	4	2	2
2	2	2	2	4	4	4	4	2	2
2	2	2	2	4	4	4	4	2	2
2	2	1	1	4	4	2	2	2	2
2	2	2	2	4	4	4	4	2	2
2	2	2	2	4	4	4	4	2	2
2	2	2	2	4	4	4	4	2	2
2	2	2	2	4	4	4	4	2	2
2	2	2	2	4	4	4	4	2	2
2	2	2	2	4	4	4	4	2	2
2	2	1	1	4	4	4	4	2	2
2	2	2	2	4	4	4	4	2	2
2	2	2	2	4	4	4	4	2	2
2	2	2	2	4	4	4	4	2	2
2	2	1	1	4	4	4	4	2	2

For Review Only

2	2	1	1	4	4	2	2	2	2
2	2	2	2	4	4	4	4	2	2

For Review Only

SNP2663	SNP2663	SNP2664	SNP2664	SNP2665	SNP2665	SNP2666	SNP2666	SNP2667	SNP2667
3	3	1	1	2	2	2	2	2	2
4	4	1	1	2	2	3	3	4	4
4	4	1	1	3	3	3	3	4	4
4	4	1	1	3	3	3	3	2	2
3	3	1	1	3	3	2	2	2	2
3	3	1	1	2	2	3	3	4	4
3	3	1	1	3	3	3	3	4	4
4	4	1	1	3	3	2	2	2	2
3	3	1	1	2	2	2	2	2	2
4	4	1	1	3	3	3	3	4	4
4	4	1	1	2	2	2	2	2	2
4	4	1	1	3	3	3	3	2	2
4	4	1	1	3	3	3	3	2	2
4	4	3	3	3	3	3	3	4	4
3	3	1	1	2	2	3	3	2	2
4	4	1	1	3	3	3	3	4	4
4	4	1	1	2	2	2	2	2	2
4	4	1	1	2	2	3	3	4	4
4	4	1	1	3	3	3	3	4	4
4	4	1	1	3	3	3	3	4	4
4	4	1	1	3	3	2	2	2	2
4	4	1	1	3	3	2	2	2	2
4	4	1	1	3	3	2	2	2	2
4	4	1	1	2	2	3	3	4	4
4	4	1	1	2	2	2	2	2	2
4	4	1	1	2	2	3	3	4	4
4	4	1	1	2	2	3	3	4	4
4	4	1	1	2	2	3	3	4	4
4	4	1	1	2	2	3	3	4	4
4	4	1	1	3	3	3	3	4	4
4	4	1	1	2	2	3	3	2	2
4	4	1	1	2	2	3	3	4	4
4	4	1	1	2	2	2	2	2	2
4	4	3	3	3	3	3	3	4	4
4	4	1	1	2	2	3	3	2	2
4	4	1	1	2	2	3	3	4	4
4	4	1	1	2	2	3	3	4	4
4	4	1	1	3	3	3	3	2	2
4	4	1	1	3	3	3	3	4	4
4	4	1	1	3	3	3	3	4	4
4	4	1	1	3	3	3	3	4	4
4	4	1	1	2	2	3	3	4	4
4	4	1	1	2	2	2	2	2	2
4	4	1	1	3	3	2	2	2	2
4	4	1	1	2	2	2	2	2	2
4	4	1	1	3	3	3	3	4	4
4	4	1	1	2	2	3	3	4	4
4	4	1	1	2	2	3	3	4	4
4	4	1	1	3	3	3	3	2	2
4	4	1	1	3	3	2	2	2	2
4	4	1	1	2	2	3	3	4	4
4	4	3	3	2	2	3	3	4	4

For Review Only

4	4	1	1	2	2	3	3	2	2
4	4	1	1	2	2	2	2	2	2
4	4	1	1	2	2	3	3	4	4
4	4	1	1	3	3	2	2	2	2
4	4	1	1	3	3	3	3	4	4
4	4	1	1	2	2	3	3	4	4
4	4	1	1	2	2	3	3	4	4
4	4	1	1	3	3	3	3	4	4
4	4	1	1	2	2	3	3	4	4
4	4	1	1	2	2	3	3	2	2
4	4	1	1	2	2	3	3	4	4
4	4	1	1	2	2	3	3	4	4
4	4	1	1	2	2	2	2	2	2
4	4	1	1	2	2	3	3	4	4
4	4	1	1	2	2	3	3	4	4
4	4	1	1	2	2	2	2	2	2
4	4	1	1	2	2	3	3	4	4
3	3	1	1	2	2	3	3	4	4
4	4	1	1	2	2	2	2	2	2
3	3	1	1	3	3	2	2	2	2
4	4	1	1	2	2	3	3	4	4
4	4	1	1	2	2	3	3	4	4
4	4	1	1	2	2	2	2	2	2
4	4	1	1	3	3	2	2	2	2
4	4	1	1	2	2	2	2	2	2
4	4	1	1	2	2	3	3	4	4
4	4	1	1	3	3	3	3	4	4
4	4	1	1	3	3	3	3	4	4
4	4	1	1	2	2	3	3	4	4
4	4	1	1	2	2	3	3	4	4
4	4	1	1	2	2	3	3	4	4
4	4	1	1	2	2	3	3	4	4
4	4	1	1	3	3	3	3	4	4
4	4	1	1	2	2	2	2	2	2
3	3	1	1	2	2	3	3	4	4
4	4	1	1	2	2	2	2	2	2
4	4	1	1	2	2	3	3	4	4
4	4	1	1	3	3	2	2	2	2
4	4	1	1	2	2	3	3	4	4
4	4	1	1	2	2	3	3	2	2
4	4	1	1	3	3	2	2	2	2
4	4	1	1	2	2	3	3	4	4
4	4	1	1	3	3	2	2	2	2
4	4	1	1	2	2	3	3	4	4
3	3	1	1	3	3	3	3	4	4
4	4	1	1	2	2	2	2	2	2
4	4	3	3	3	3	3	3	4	4
4	4	1	1	2	2	3	3	4	4
4	4	1	1	2	2	3	3	4	4
4	4	1	1	2	2	3	3	4	4
4	4	1	1	2	2	3	3	4	4
4	4	1	1	3	3	3	3	4	4
4	4	1	1	3	3	3	3	4	4
4	4	1	1	3	3	3	3	4	4
3	3	1	1	2	2	3	3	4	4

For Review Only

4	4	1	1	2	2	3	3	2	2
4	4	1	1	2	2	3	3	4	4
4	4	1	1	2	2	3	3	4	4
4	4	3	3	3	3	3	3	4	4
4	4	1	1	2	2	3	3	4	4
4	4	1	1	2	2	3	3	4	4
4	4	1	1	3	3	2	2	2	2
4	4	1	1	2	2	3	3	4	4
4	4	1	1	2	2	3	3	4	4
4	4	1	1	2	2	3	3	4	4
3	3	1	1	3	3	3	3	4	4
4	4	1	1	2	2	3	3	4	4
4	4	1	1	3	3	2	2	2	2
4	4	1	1	2	2	3	3	4	4
4	4	1	1	2	2	3	3	4	4
4	4	1	1	2	2	3	3	4	4
4	4	1	1	2	2	3	3	4	4
4	4	1	1	2	2	3	3	2	2
4	4	3	3	2	2	3	3	4	4
4	4	1	1	3	3	2	2	2	2
4	4	1	1	2	2	3	3	4	4
4	4	1	1	2	2	3	3	2	2
4	4	1	1	3	3	3	3	4	4
4	4	1	1	3	3	3	3	4	4
4	4	1	1	2	2	3	3	4	4
4	4	1	1	2	2	3	3	4	4
4	4	1	1	2	2	3	3	4	4
4	4	1	1	2	2	3	3	4	4
4	4	1	1	2	2	3	3	2	2
4	4	1	1	2	2	2	2	2	2
4	4	1	1	3	3	3	3	4	4
4	4	1	1	2	2	3	3	4	4
4	4	1	1	3	3	2	2	2	2
4	4	1	1	2	2	3	3	4	4
4	4	3	3	3	3	3	3	4	4
4	4	1	1	2	2	3	3	4	4
4	4	3	3	3	3	3	3	4	4
4	4	1	1	2	2	3	3	4	4
4	4	3	3	2	2	3	3	4	4
4	4	1	1	2	2	3	3	2	2
4	4	1	1	2	2	3	3	4	4
4	4	3	3	3	3	3	3	4	4
3	3	1	1	2	2	3	3	4	4
4	4	1	1	3	3	3	3	4	4
4	4	1	1	3	3	3	3	4	4
3	3	1	1	2	2	3	3	4	4
4	4	1	1	2	2	3	3	4	4
4	4	1	1	2	2	3	3	4	4
4	4	1	1	3	3	3	3	4	4
4	4	1	1	2	2	3	3	4	4
4	4	1	1	2	2	3	3	4	4
4	4	1	1	3	3	2	2	2	2
4	4	1	1	2	2	3	3	4	4
4	4	1	1	3	3	3	3	4	4
4	4	1	1	3	3	3	3	2	2

For Review Only

4	4	1	1	2	2	3	3	4	4
4	4	1	1	2	2	2	2	2	2
4	4	1	1	2	2	2	2	2	2
4	4	1	1	2	2	3	3	4	4
4	4	1	1	3	3	3	3	4	4
4	4	1	1	3	3	3	3	4	4
4	4	1	1	3	3	3	3	2	2
4	4	1	1	2	2	3	3	4	4
4	4	3	3	2	2	3	3	4	4
4	4	3	3	2	2	3	3	4	4
4	4	1	1	2	2	3	3	4	4
4	4	1	1	2	2	3	3	4	4
4	4	1	1	2	2	3	3	4	4
3	3	3	3	2	2	3	3	2	2
4	4	1	1	3	3	3	3	4	4
4	4	1	1	2	2	2	2	2	2
4	4	1	1	2	2	3	3	4	4
4	4	1	1	2	2	2	2	2	2
4	4	1	1	2	2	3	3	4	4
4	4	1	1	3	3	2	2	2	2
4	4	1	1	2	2	3	3	2	2
4	4	1	1	2	2	3	3	4	4
4	4	1	1	2	2	3	3	4	4
4	4	1	1	2	2	3	3	4	4
4	4	1	1	2	2	2	2	2	2
4	4	3	3	3	3	2	2	2	2
4	4	1	1	2	2	3	3	2	2
4	4	1	1	2	2	3	3	4	4
4	4	1	1	2	2	3	3	4	4
4	4	1	1	3	3	3	3	4	4
4	4	1	1	2	2	2	2	2	2
4	4	1	1	2	2	3	3	4	4
4	4	1	1	2	2	2	2	2	2
4	4	1	1	3	3	3	3	4	4
4	4	1	1	2	2	3	3	2	2
4	4	1	1	2	2	3	3	4	4
4	4	1	1	2	2	3	3	4	4
4	4	1	1	2	2	3	3	4	4
4	4	1	1	2	2	3	3	4	4
4	4	1	1	2	2	2	2	2	2
4	4	1	1	3	3	3	3	4	4
4	4	1	1	2	2	3	3	4	4
4	4	1	1	3	3	3	3	4	4
4	4	1	1	2	2	3	3	4	4
4	4	1	1	2	2	3	3	4	4
4	4	1	1	2	2	3	3	2	2
4	4	1	1	2	2	2	2	2	2
4	4	1	1	3	3	2	2	2	2
4	4	1	1	2	2	3	3	4	4
4	4	1	1	3	3	2	2	2	2
4	4	1	1	2	2	3	3	4	4
4	4	1	1	3	3	3	3	4	4
4	4	3	3	2	2	3	3	4	4
4	4	1	1	2	2	3	3	4	4
4	4	1	1	3	3	3	3	4	4

For Review Only

3	3	1	1	2	2	3	3	4	4
4	4	1	1	3	3	3	3	4	4
4	4	1	1	3	3	3	3	4	4
4	4	1	1	2	2	3	3	2	2
4	4	1	1	3	3	2	2	2	2
4	4	1	1	2	2	3	3	4	4
4	4	1	1	2	2	3	3	4	4
4	4	1	1	3	3	3	3	4	4
4	4	1	1	3	3	3	3	4	4
4	4	1	1	3	3	3	3	4	4
4	4	1	1	2	2	3	3	2	2
4	4	1	1	3	3	3	3	4	4
4	4	1	1	2	2	3	3	4	4
4	4	1	1	2	2	3	3	4	4
4	4	1	1	3	3	3	3	4	4
4	4	1	1	2	2	2	2	2	2
4	4	1	1	3	3	3	3	4	4
4	4	1	1	2	2	3	3	4	4
4	4	1	1	2	2	3	3	4	4
4	4	1	1	3	3	3	3	4	4
4	4	1	1	3	3	3	3	4	4
4	4	1	1	2	2	3	3	4	4
4	4	1	1	2	2	2	2	2	2
4	4	1	1	3	3	3	3	4	4
4	4	1	1	3	3	3	3	4	4
4	4	1	1	2	2	3	3	2	2
4	4	1	1	3	3	3	3	4	4
4	4	1	1	3	3	3	3	2	2
4	4	1	1	2	2	2	2	2	2
4	4	1	1	2	2	3	3	4	4
4	4	1	1	3	3	3	3	4	4
4	4	1	1	2	2	3	3	4	4
4	4	3	3	2	2	2	2	2	2
4	4	1	1	3	3	3	3	4	4
4	4	1	1	2	2	3	3	4	4
4	4	1	1	3	3	3	3	4	4
3	3	1	1	3	3	3	3	4	4
4	4	1	1	2	2	3	3	4	4
4	4	1	1	2	2	3	3	4	4
4	4	1	1	3	3	3	3	4	4
4	4	1	1	2	2	3	3	4	4
4	4	1	1	2	2	3	3	4	4
4	4	1	1	3	3	3	3	4	4
4	4	1	1	3	3	2	2	2	2
4	4	1	1	3	3	3	3	4	4
4	4	1	1	2	2	3	3	4	4
4	4	1	1	3	3	3	3	4	4
4	4	1	1	2	2	3	3	4	4
4	4	1	1	2	2	3	3	4	4
4	4	1	1	3	3	3	3	4	4
4	4	1	1	3	3	3	3	4	4
4	4	1	1	3	3	3	3	4	4
4	4	1	1	3	3	3	3	4	4
4	4	1	1	3	3	3	3	4	4
4	4	1	1	3	3	3	3	4	4

For Review Only

4	4	1	1	2	2	2	2	2	2
4	4	1	1	2	2	3	3	4	4

For Review Only

SNP2668	SNP2668	SNP2669	SNP2669	SNP267	SNP267	SNP2670	SNP2670	SNP2671	SNP2671
2	2	2	2	3	3	1	1	4	4
2	2	2	2	3	3	1	1	1	1
2	2	2	2	3	3	1	1	4	4
2	2	2	2	3	3	1	1	1	1
3	3	4	4	3	3	1	1	1	1
2	2	2	2	3	3	1	1	4	4
2	2	2	2	3	3	1	1	1	1
2	2	2	2	2	2	1	1	4	4
2	2	2	2	3	3	1	1	1	1
3	3	2	2	2	2	1	1	1	1
2	2	2	2	2	2	1	1	1	1
2	2	2	2	3	3	1	1	4	4
2	2	2	2	2	2	1	1	4	4
2	2	2	2	3	3	1	1	4	4
2	2	2	2	3	3	1	1	1	1
2	2	2	2	3	3	3	3	4	4
2	2	2	2	3	3	1	1	1	1
2	2	2	2	2	2	1	1	4	4
2	2	2	2	3	3	1	1	1	1
2	2	2	2	2	2	1	1	1	1
2	2	2	2	3	3	1	1	1	1
2	2	2	2	2	2	1	1	1	1
2	2	4	4	3	3	1	1	1	1
2	2	2	2	3	3	1	1	1	1
2	2	2	2	3	3	1	1	4	4
2	2	2	2	3	3	1	1	1	1
2	2	2	2	3	3	1	1	4	4
2	2	2	2	3	3	1	1	1	1
2	2	2	2	3	3	1	1	1	1
2	2	2	2	2	2	1	1	1	1
2	2	2	2	3	3	1	1	1	1
2	2	2	2	3	3	1	1	4	4
2	2	2	2	3	3	1	1	4	4
2	2	2	2	3	3	1	1	4	4
2	2	2	2	3	3	1	1	1	1
2	2	2	2	3	3	1	1	1	1
3	3	2	2	3	3	1	1	1	1
3	3	2	2	3	3	1	1	1	1
2	2	4	4	3	3	3	3	4	4
2	2	2	2	2	2	1	1	1	1
3	3	2	2	2	2	1	1	4	4
2	2	2	2	3	3	1	1	1	1
2	2	2	2	2	2	1	1	4	4
2	2	4	4	3	3	1	1	1	1
2	2	2	2	2	2	1	1	4	4
2	2	2	2	3	3	1	1	4	4
2	2	2	2	3	3	1	1	1	1
2	2	2	2	3	3	1	1	1	1
2	2	2	2	3	3	1	1	1	1
2	2	2	2	3	3	1	1	1	1
2	2	2	2	3	3	1	1	1	1
2	2	2	2	3	3	1	1	4	4

For Review Only

2	2	2	2	3	3	1	1	1	1
2	2	2	2	3	3	1	1	4	4
2	2	2	2	3	3	1	1	4	4
2	2	2	2	3	3	1	1	1	1
2	2	2	2	3	3	1	1	1	1
2	2	2	2	3	3	1	1	1	1
2	2	2	2	3	3	1	1	4	4
2	2	4	4	3	3	1	1	1	1
2	2	2	2	3	3	1	1	4	4
2	2	2	2	3	3	1	1	1	1
2	2	2	2	3	3	1	1	1	1
2	2	2	2	3	3	1	1	1	1
2	2	2	2	3	3	1	1	1	1
2	2	2	2	3	3	1	1	1	1
2	2	2	2	3	3	1	1	1	1
2	2	2	2	3	3	1	1	1	1
2	2	2	2	3	3	1	1	4	4
2	2	2	2	3	3	1	1	4	4
2	2	2	2	2	2	1	1	1	1
2	2	2	2	3	3	1	1	4	4
2	2	2	2	3	3	1	1	1	1
2	2	2	2	3	3	1	1	1	1
2	2	2	2	3	3	1	1	1	1
2	2	2	2	3	3	1	1	1	1
2	2	2	2	2	2	1	1	4	4
2	2	2	2	2	2	1	1	1	1
2	2	2	2	3	3	1	1	4	4
2	2	4	4	3	3	3	3	4	4
2	2	2	2	3	3	1	1	1	1
2	2	2	2	3	3	1	1	1	1
3	3	2	2	3	3	1	1	1	1
2	2	2	2	3	3	1	1	1	1
2	2	4	4	2	2	3	3	4	4
2	2	2	2	3	3	1	1	1	1
2	2	2	2	3	3	1	1	4	4
2	2	2	2	2	2	1	1	4	4
3	3	2	2	3	3	1	1	1	1
2	2	2	2	3	3	1	1	1	1
2	2	2	2	3	3	1	1	1	1
3	3	4	4	3	3	1	1	1	1
2	2	2	2	3	3	1	1	1	1
2	2	2	2	3	3	1	1	1	1
2	2	2	2	3	3	1	1	4	4
2	2	2	2	3	3	1	1	1	1
2	2	2	2	3	3	1	1	1	1
2	2	2	2	3	3	1	1	1	1
2	2	2	2	3	3	1	1	4	4
2	2	2	2	3	3	1	1	4	4
2	2	2	2	3	3	1	1	1	1
2	2	2	2	3	3	1	1	1	1
2	2	2	2	3	3	1	1	1	1
2	2	4	4	3	3	1	1	1	1
2	2	4	4	3	3	1	1	1	1
2	2	4	4	3	3	1	1	1	1
3	3	2	2	3	3	1	1	1	1

For Review Only

2	2	4	4	3	3	1	1	1	1
3	3	2	2	3	3	1	1	1	1
2	2	2	2	3	3	1	1	1	1
2	2	2	2	3	3	1	1	4	4
2	2	2	2	3	3	1	1	1	1
2	2	2	2	3	3	1	1	1	1
2	2	2	2	3	3	1	1	4	4
2	2	2	2	3	3	1	1	1	1
2	2	2	2	3	3	1	1	4	4
2	2	2	2	3	3	1	1	1	1
2	2	2	2	3	3	1	1	1	1
2	2	2	2	3	3	1	1	1	1
2	2	2	2	3	3	1	1	4	4
2	2	2	2	3	3	1	1	1	1
2	2	4	4	3	3	1	1	1	1
2	2	2	2	3	3	1	1	1	1
2	2	2	2	3	3	1	1	1	1
2	2	2	2	3	3	1	1	1	1
2	2	2	2	3	3	1	1	4	4
2	2	2	2	3	3	1	1	1	1
2	2	2	2	3	3	1	1	1	1
2	2	2	2	3	3	1	1	1	1
2	2	2	2	3	3	1	1	4	4
2	2	2	2	3	3	1	1	1	1
2	2	2	2	3	3	1	1	1	1
2	2	2	2	3	3	1	1	1	1
2	2	2	2	3	3	1	1	1	1
2	2	2	2	3	3	1	1	4	4
2	2	2	2	3	3	1	1	4	4
2	2	2	2	2	2	1	1	4	4
2	2	4	4	2	2	1	1	1	1
2	2	2	2	3	3	1	1	1	1
2	2	2	2	3	3	1	1	1	1
2	2	2	2	3	3	1	1	1	1
2	2	2	2	3	3	1	1	4	4
2	2	2	2	3	3	1	1	4	4
2	2	2	2	3	3	1	1	4	4
2	2	2	2	3	3	1	1	1	1
2	2	4	4	3	3	1	1	1	1
2	2	4	4	2	2	1	1	1	1
2	2	2	2	3	3	1	1	1	1
2	2	2	2	3	3	1	1	4	4
2	2	2	2	3	3	1	1	4	4
3	3	4	4	3	3	1	1	1	1
2	2	2	2	3	3	1	1	1	1
3	3	2	2	3	3	1	1	1	1
2	2	2	2	3	3	1	1	1	1
2	2	2	2	3	3	1	1	1	1
2	2	2	2	3	3	1	1	1	1
2	2	2	2	3	3	1	1	1	1
2	2	2	2	3	3	1	1	1	1
2	2	4	4	3	3	1	1	1	1
2	2	2	2	3	3	1	1	4	4
2	2	2	2	3	3	1	1	1	1
2	2	4	4	3	3	1	1	1	1

For Review Only

2	2	2	2	3	3	1	1	1	1
2	2	2	2	3	3	1	1	1	1
2	2	2	2	3	3	1	1	4	4
2	2	2	2	3	3	1	1	1	1
2	2	2	2	3	3	1	1	4	4
2	2	2	2	3	3	1	1	1	1
2	2	4	4	3	3	1	1	1	1
2	2	2	2	3	3	1	1	1	1
2	2	4	4	3	3	1	1	4	4
2	2	2	2	3	3	1	1	1	1
2	2	2	2	3	3	1	1	1	1
2	2	2	2	3	3	1	1	1	1
2	2	2	2	3	3	1	1	1	1
2	2	2	2	3	3	1	1	1	1
2	2	2	2	3	3	1	1	4	4
2	2	2	2	2	2	1	1	1	1
2	2	2	2	3	3	1	1	4	4
2	2	2	2	3	3	1	1	1	1
2	2	2	2	3	3	1	1	4	4
2	2	2	2	3	3	1	1	1	1
2	2	2	2	3	3	1	1	1	1
2	2	2	2	2	2	1	1	1	1
2	2	2	2	3	3	1	1	1	1
2	2	2	2	3	3	1	1	1	1
2	2	2	2	2	2	1	1	4	4
2	2	2	2	3	3	1	1	1	1
2	2	2	2	3	3	1	1	1	1
2	2	2	2	3	3	1	1	1	1
2	2	2	2	3	3	1	1	4	4
2	2	2	2	3	3	1	1	4	4
2	2	2	2	3	3	1	1	4	4
2	2	2	2	3	3	1	1	1	1
2	2	2	2	3	3	1	1	1	1
2	2	4	4	3	3	1	1	4	4
2	2	2	2	3	3	1	1	1	1
2	2	2	2	3	3	1	1	1	1
2	2	2	2	3	3	1	1	1	1
2	2	2	2	3	3	1	1	1	1
2	2	2	2	3	3	1	1	1	1
2	2	2	2	3	3	1	1	4	4
2	2	2	2	3	3	1	1	4	4
2	2	2	2	3	3	1	1	1	1
2	2	4	4	3	3	3	3	4	4
2	2	2	2	3	3	1	1	1	1
2	2	4	4	3	3	1	1	4	4
2	2	2	2	3	3	1	1	1	1
2	2	2	2	2	2	1	1	1	1
2	2	4	4	2	2	1	1	1	1
2	2	2	2	3	3	1	1	1	1
2	2	2	2	3	3	1	1	1	1
2	2	2	2	3	3	1	1	1	1
3	3	4	4	3	3	1	1	1	1
2	2	2	2	3	3	1	1	1	1
2	2	2	2	3	3	1	1	1	1
2	2	4	4	3	3	1	1	1	1

For Review Only

2	2	2	2	3	3	1	1	4	4
2	2	2	2	2	2	1	1	4	4
2	2	2	2	3	3	1	1	4	4
2	2	2	2	3	3	1	1	1	1
3	3	2	2	2	2	1	1	1	1
2	2	2	2	3	3	1	1	1	1
2	2	2	2	2	2	1	1	1	1
2	2	2	2	2	2	1	1	1	1
2	2	4	4	3	3	1	1	4	4
2	2	4	4	2	2	3	3	4	4
2	2	2	2	3	3	1	1	1	1
2	2	2	2	3	3	1	1	1	1
2	2	2	2	3	3	1	1	1	1
2	2	2	2	3	3	1	1	1	1
2	2	2	2	3	3	1	1	4	4
2	2	2	2	3	3	1	1	1	1
2	2	2	2	3	3	3	3	4	4
2	2	2	2	3	3	1	1	4	4
2	2	2	2	2	2	3	3	4	4
3	3	2	2	3	3	1	1	1	1
2	2	2	2	2	2	1	1	1	1
2	2	2	2	3	3	1	1	1	1
2	2	2	2	3	3	1	1	1	1
2	2	2	2	3	3	1	1	1	1
2	2	4	4	3	3	1	1	1	1
2	2	2	2	3	3	1	1	1	1
2	2	4	4	3	3	1	1	1	1
2	2	2	2	3	3	1	1	4	4
2	2	2	2	3	3	1	1	1	1
2	2	2	2	3	3	1	1	1	1
2	2	4	4	3	3	3	3	4	4
2	2	2	2	3	3	1	1	1	1
2	2	2	2	3	3	1	1	1	1
2	2	2	2	3	3	1	1	1	1
2	2	2	2	3	3	1	1	1	1
2	2	4	4	2	2	3	3	1	1
2	2	2	2	3	3	1	1	4	4
2	2	2	2	3	3	1	1	1	1
2	2	2	2	3	3	1	1	1	1
2	2	2	2	3	3	3	3	4	4
2	2	2	2	3	3	1	1	1	1
2	2	2	2	3	3	1	1	1	1
2	2	2	2	3	3	1	1	1	1
2	2	2	2	3	3	1	1	1	1
2	2	2	2	3	3	1	1	1	1
2	2	2	2	3	3	1	1	1	1
2	2	4	4	2	2	1	1	1	1
3	3	2	2	3	3	1	1	4	4
3	3	4	4	3	3	1	1	1	1
2	2	4	4	2	2	3	3	4	4
2	2	2	2	3	3	1	1	4	4
2	2	4	4	2	2	3	3	4	4
2	2	2	2	3	3	1	1	4	4
2	2	2	2	3	3	1	1	4	4
2	2	2	2	3	3	1	1	1	1

2	2	2	2	3	3	1	1	1	1
2	2	2	2	3	3	1	1	4	4

For Review Only

SNP2672	SNP2672	SNP2673	SNP2673	SNP2674	SNP2674	SNP2675	SNP2675	SNP2676	SNP2676
2	2	2	2	3	3	1	1	3	3
2	2	2	2	3	3	1	1	3	3
2	2	2	2	3	3	1	1	3	3
2	2	2	2	3	3	1	1	3	3
3	3	3	3	3	3	4	4	3	3
2	2	2	2	3	3	1	1	3	3
2	2	2	2	3	3	1	1	3	3
2	2	2	2	3	3	4	4	3	3
2	2	2	2	3	3	1	1	3	3
2	2	2	2	3	3	4	4	3	3
2	2	2	2	3	3	1	1	2	2
2	2	2	2	3	3	1	1	3	3
2	2	2	2	3	3	1	1	3	3
2	2	2	2	3	3	1	1	3	3
2	2	2	2	3	3	1	1	3	3
2	2	2	2	3	3	1	1	3	3
2	2	2	2	3	3	1	1	3	3
2	2	2	2	3	3	1	1	3	3
2	2	2	2	1	1	1	1	3	3
2	2	2	2	3	3	1	1	3	3
2	2	2	2	3	3	1	1	3	3
2	2	2	2	3	3	1	1	3	3
2	2	2	2	3	3	1	1	2	2
2	2	2	2	3	3	1	1	3	3
2	2	2	2	3	3	4	4	3	3
2	2	2	2	3	3	1	1	3	3
2	2	2	2	1	1	1	1	3	3
2	2	2	2	3	3	1	1	3	3
2	2	2	2	3	3	1	1	3	3
2	2	2	2	3	3	4	4	3	3
2	2	2	2	3	3	1	1	3	3
2	2	2	2	3	3	1	1	3	3
2	2	2	2	3	3	1	1	3	3
2	2	2	2	3	3	1	1	3	3
2	2	2	2	3	3	1	1	2	2
2	2	2	2	1	1	1	1	3	3
2	2	2	2	3	3	1	1	3	3
2	2	2	2	3	3	4	4	3	3
3	3	3	3	3	3	1	1	2	2
2	2	2	2	3	3	1	1	2	2
2	2	2	2	3	3	4	4	3	3
2	2	2	2	3	3	4	4	3	3
2	2	2	2	3	3	4	4	3	3
2	2	2	2	3	3	1	1	3	3
2	2	2	2	3	3	4	4	3	3
2	2	2	2	3	3	1	1	3	3
2	2	2	2	3	3	1	1	3	3
2	2	2	2	3	3	1	1	3	3
2	2	2	2	3	3	1	1	3	3
2	2	2	2	3	3	1	1	3	3
2	2	2	2	3	3	1	1	3	3
2	2	2	2	3	3	1	1	3	3

For Review Only

2	2	2	2	3	3	1	1	3	3
2	2	2	2	3	3	1	1	3	3
2	2	2	2	1	1	1	1	3	3
2	2	2	2	3	3	1	1	3	3
2	2	2	2	1	1	1	1	3	3
2	2	2	2	3	3	1	1	3	3
2	2	2	2	3	3	1	1	3	3
2	2	2	2	3	3	1	1	3	3
2	2	2	2	3	3	1	1	3	3
2	2	2	2	3	3	1	1	3	3
2	2	2	2	3	3	1	1	3	3
2	2	2	2	3	3	1	1	3	3
2	2	2	2	3	3	1	1	3	3
2	2	2	2	3	3	1	1	3	3
2	2	2	2	3	3	1	1	3	3
2	2	2	2	3	3	1	1	3	3
2	2	2	2	1	1	1	1	2	2
2	2	2	2	3	3	1	1	3	3
2	2	2	2	3	3	1	1	3	3
2	2	2	2	3	3	1	1	3	3
2	2	2	2	3	3	1	1	3	3
2	2	2	2	3	3	1	1	2	2
2	2	2	2	3	3	1	1	3	3
2	2	2	2	1	1	4	4	3	3
2	2	2	2	3	3	4	4	3	3
2	2	2	2	1	1	1	1	3	3
2	2	2	2	3	3	1	1	3	3
3	3	3	3	3	3	1	1	2	2
2	2	2	2	3	3	1	1	3	3
2	2	2	2	3	3	1	1	3	3
2	2	2	2	3	3	4	4	3	3
2	2	2	2	3	3	1	1	3	3
3	3	3	3	3	3	1	1	2	2
2	2	2	2	3	3	1	1	3	3
2	2	2	2	3	3	1	1	3	3
2	2	2	2	3	3	4	4	3	3
2	2	2	2	3	3	4	4	3	3
2	2	2	2	3	3	4	4	3	3
2	2	2	2	3	3	1	1	3	3
2	2	2	2	3	3	4	4	3	3
2	2	2	2	3	3	1	1	3	3
2	2	2	2	3	3	1	1	3	3
2	2	2	2	3	3	1	1	3	3
2	2	2	2	3	3	1	1	3	3
2	2	2	2	3	3	1	1	3	3
2	2	2	2	3	3	1	1	3	3
2	2	2	2	3	3	1	1	3	3
2	2	2	2	3	3	1	1	3	3
2	2	2	2	3	3	1	1	3	3
2	2	2	2	3	3	1	1	3	3
2	2	2	2	3	3	1	1	3	3
2	2	2	2	3	3	1	1	2	2
2	2	2	2	3	3	1	1	3	3
2	2	2	2	3	3	1	1	3	3
2	2	2	2	3	3	4	4	3	3

For Review Only

2	2	2	2	3	3	1	1	2	2
2	2	2	2	3	3	4	4	3	3
2	2	2	2	1	1	1	1	3	3
2	2	2	2	3	3	1	1	3	3
2	2	2	2	3	3	1	1	3	3
3	3	2	2	3	3	1	1	3	3
2	2	2	2	3	3	1	1	3	3
2	2	2	2	1	1	1	1	3	3
2	2	2	2	3	3	1	1	3	3
2	2	2	2	1	1	1	1	3	3
2	2	2	2	3	3	1	1	3	3
2	2	2	2	3	3	1	1	3	3
2	2	2	2	3	3	1	1	3	3
2	2	2	2	3	3	1	1	3	3
2	2	2	2	3	3	1	1	3	3
2	2	2	2	3	3	1	1	3	3
2	2	2	2	3	3	1	1	3	3
2	2	2	2	3	3	1	1	3	3
2	2	2	2	3	3	1	1	3	3
2	2	2	2	3	3	1	1	3	3
2	2	2	2	3	3	1	1	3	3
2	2	2	2	3	3	1	1	3	3
2	2	2	2	3	3	1	1	3	3
3	3	3	3	3	3	1	1	2	2
2	2	2	2	3	3	1	1	3	3
2	2	2	2	3	3	1	1	2	2
2	2	2	2	3	3	4	4	3	3
2	2	2	2	3	3	1	1	3	3
2	2	2	2	3	3	1	1	3	3
2	2	2	2	1	1	1	1	3	3
3	3	3	3	3	3	1	1	3	3
2	2	2	2	3	3	1	1	3	3
2	2	2	2	3	3	1	1	3	3
2	2	2	2	3	3	1	1	3	3
2	2	2	2	3	3	1	1	3	3
2	2	2	2	3	3	1	1	3	3
2	2	2	2	3	3	1	1	3	3
2	2	2	2	3	3	1	1	3	3
2	2	2	2	3	3	1	1	3	3
3	3	3	3	3	3	1	1	2	2
2	2	2	2	3	3	1	1	2	2
2	2	3	3	3	3	1	1	3	3
2	2	2	2	3	3	1	1	3	3
2	2	2	2	3	3	4	4	3	3
2	2	2	2	3	3	1	1	3	3
2	2	2	2	3	3	1	1	3	3
2	2	2	2	3	3	1	1	3	3
2	2	2	2	3	3	1	1	3	3
2	2	2	2	3	3	1	1	3	3
2	2	2	2	3	3	1	1	3	3
2	2	2	2	3	3	1	1	3	3
2	2	2	2	3	3	1	1	3	3
2	2	2	2	3	3	1	1	3	3
2	2	2	2	3	3	1	1	3	3
2	2	2	2	3	3	1	1	3	3

For Review Only

2	2	2	2	3	3	1	1	3	3
2	2	2	2	3	3	1	1	3	3
2	2	2	2	3	3	1	1	3	3
2	2	2	2	3	3	1	1	2	2
2	2	2	2	3	3	1	1	3	3
2	2	2	2	3	3	1	1	3	3
3	3	3	3	3	3	1	1	2	2
2	2	2	2	3	3	1	1	3	3
3	3	3	3	3	3	1	1	3	3
2	2	2	2	3	3	1	1	3	3
2	2	2	2	3	3	1	1	3	3
2	2	2	2	3	3	1	1	2	2
2	2	2	2	3	3	1	1	3	3
2	2	2	2	3	3	1	1	3	3
2	2	2	2	3	3	1	1	3	3
2	2	2	2	3	3	1	1	2	2
2	2	2	2	3	3	1	1	3	3
2	2	2	2	3	3	1	1	3	3
2	2	2	2	1	1	1	1	3	3
2	2	2	2	3	3	1	1	3	3
2	2	2	2	3	3	1	1	3	3
2	2	2	2	3	3	1	1	3	3
2	2	2	2	3	3	1	1	2	2
2	2	2	2	3	3	1	1	3	3
2	2	2	2	3	3	1	1	3	3
2	2	2	2	3	3	1	1	3	3
2	2	2	2	3	3	1	1	3	3
2	2	2	2	3	3	1	1	3	3
2	2	2	2	3	3	1	1	3	3
2	2	2	2	3	3	1	1	3	3
2	2	2	2	3	3	1	1	3	3
2	2	2	2	3	3	1	1	3	3
2	2	2	2	3	3	1	1	3	3
2	2	2	2	3	3	1	1	3	3
2	2	2	2	3	3	1	1	3	3
2	2	2	2	3	3	1	1	3	3
2	2	2	2	3	3	1	1	3	3
2	2	2	2	3	3	1	1	3	3
2	2	2	2	3	3	1	1	3	3
2	2	2	2	3	3	1	1	3	3
2	2	2	2	3	3	1	1	3	3
2	2	2	2	3	3	1	1	3	3
3	3	3	3	3	3	1	1	2	2
2	2	2	2	3	3	1	1	3	3
2	2	2	2	3	3	1	1	3	3
2	2	2	2	3	3	1	1	3	3
2	2	2	2	3	3	1	1	2	2
3	3	3	3	3	3	1	1	3	3
2	2	2	2	3	3	4	4	3	3
2	2	2	2	3	3	1	1	3	3
2	2	2	2	3	3	1	1	3	3
2	2	2	2	3	3	1	1	3	3
2	2	2	2	3	3	1	1	2	2
2	2	2	2	3	3	1	1	3	3
3	3	3	3	3	3	1	1	2	2

For Review Only

2	2	2	2	3	3	1	1	3	3
2	2	2	2	3	3	1	1	3	3
2	2	2	2	3	3	1	1	3	3
2	2	2	2	3	3	1	1	3	3
2	2	2	2	3	3	4	4	3	3
2	2	2	2	3	3	1	1	3	3
2	2	2	2	3	3	1	1	3	3
2	2	2	2	3	3	1	1	3	3
2	2	2	2	3	3	1	1	3	3
3	3	3	3	3	3	1	1	2	2
2	2	2	2	3	3	1	1	3	3
2	2	2	2	1	1	1	1	3	3
2	2	2	2	3	3	1	1	3	3
2	2	2	2	3	3	1	1	3	3
2	2	2	2	3	3	1	1	3	3
2	2	2	2	3	3	1	1	2	2
2	2	2	2	3	3	1	1	2	2
3	3	3	3	3	3	1	1	3	3
2	2	2	2	3	3	1	1	3	3
2	2	2	2	3	3	4	4	3	3
2	2	2	2	3	3	1	1	3	3
2	2	2	2	3	3	1	1	3	3
2	2	2	2	3	3	1	1	3	3
2	2	2	2	3	3	1	1	2	2
2	2	2	2	3	3	1	1	3	3
2	2	2	2	3	3	1	1	3	3
3	3	3	3	3	3	1	1	2	2
2	2	2	2	3	3	1	1	3	3
2	2	2	2	3	3	1	1	3	3
2	2	2	2	3	3	1	1	3	3
3	3	3	3	3	3	1	1	3	3
2	2	2	2	3	3	1	1	3	3
2	2	2	2	3	3	4	4	3	3
2	2	2	2	3	3	1	1	3	3
2	2	2	2	3	3	1	1	2	2
3	3	3	3	3	3	1	1	3	3
2	2	2	2	3	3	1	1	3	3
2	2	2	2	3	3	1	1	3	3
2	2	2	2	1	1	1	1	3	3
2	2	2	2	3	3	1	1	2	2
2	2	2	2	3	3	1	1	3	3
2	2	2	2	3	3	1	1	3	3
2	2	2	2	3	3	1	1	2	2
2	2	2	2	3	3	1	1	3	3
2	2	2	2	3	3	1	1	3	3
2	2	2	2	3	3	1	1	3	3
2	2	2	2	3	3	1	1	3	3
2	2	2	2	3	3	1	1	3	3
3	3	3	3	3	3	4	4	3	3
3	3	3	3	3	3	1	1	2	2
2	2	2	2	3	3	1	1	3	3
2	2	2	2	3	3	1	1	2	2
2	2	2	2	3	3	1	1	3	3
2	2	2	2	3	3	1	1	3	3
2	2	2	2	3	3	1	1	2	2

For Review Only

2	2	2	2	3	3	1	1	3	3
2	2	2	2	3	3	1	1	3	3

For Review Only

SNP2677	SNP2677	SNP2678	SNP2678	SNP2679	SNP2679	SNP268	SNP268	SNP2680	SNP2680
1	1	1	1	3	3	1	1	3	3
1	1	1	1	3	3	2	2	1	1
1	1	1	1	3	3	2	2	1	1
1	1	1	1	3	3	2	2	1	1
4	4	2	2	3	3	2	2	1	1
1	1	1	1	3	3	1	1	3	3
1	1	1	1	3	3	1	1	1	1
4	4	1	1	3	3	2	2	3	3
1	1	1	1	3	3	1	1	3	3
4	4	1	1	3	3	2	2	3	3
1	1	2	2	3	3	2	2	1	1
1	1	1	1	3	3	2	2	3	3
1	1	1	1	3	3	1	1	1	1
1	1	1	1	3	3	2	2	3	3
1	1	1	1	3	3	1	1	1	1
1	1	1	1	3	3	1	1	1	1
1	1	2	2	3	3	1	1	1	1
1	1	1	1	3	3	1	1	3	3
1	1	1	1	3	3	2	2	1	1
1	1	1	1	3	3	2	2	3	3
1	1	2	2	3	3	2	2	1	1
1	1	2	2	3	3	1	1	1	1
1	1	2	2	3	3	2	2	1	1
1	1	1	1	3	3	2	2	1	1
1	1	2	2	3	3	2	2	1	1
1	1	1	1	3	3	2	2	3	3
1	1	1	1	3	3	2	2	3	3
1	1	1	1	3	3	1	1	1	1
1	1	1	1	3	3	2	2	1	1
4	4	1	1	3	3	2	2	1	1
1	1	1	1	3	3	1	1	3	3
1	1	1	1	2	2	1	1	3	3
1	1	1	1	2	2	1	1	3	3
1	1	1	1	3	3	1	1	3	3
1	1	1	1	3	3	2	2	1	1
1	1	1	1	3	3	2	2	1	1
1	1	1	1	3	3	2	2	1	1
4	4	1	1	3	3	2	2	3	3
1	1	1	1	3	3	2	2	1	1
1	1	1	1	3	3	2	2	1	1
4	4	1	1	3	3	2	2	3	3
4	4	1	1	3	3	2	2	1	1
4	4	1	1	3	3	2	2	3	3
1	1	2	2	3	3	1	1	1	1
4	4	1	1	3	3	2	2	3	3
1	1	1	1	3	3	2	2	3	3
1	1	1	1	3	3	2	2	3	3
1	1	1	1	3	3	2	2	1	1
1	1	1	1	3	3	1	1	3	3
1	1	2	2	3	3	1	1	1	1
1	1	1	1	3	3	2	2	3	3
1	1	1	1	2	2	2	2	1	1

For Review Only

1	1	2	2	3	3	2	2	1	1
1	1	1	1	2	2	1	1	3	3
1	1	1	1	3	3	2	2	3	3
1	1	2	2	3	3	1	1	1	1
1	1	1	1	3	3	2	2	1	1
1	1	1	1	3	3	2	2	3	3
1	1	1	1	3	3	2	2	3	3
1	1	1	1	3	3	2	2	1	1
1	1	1	1	3	3	2	2	3	3
1	1	1	1	3	3	1	1	1	1
1	1	1	1	3	3	2	2	3	3
1	1	1	1	3	3	2	2	3	3
1	1	2	2	3	3	2	2	1	1
1	1	1	1	3	3	2	2	1	1
1	1	1	1	3	3	1	1	1	1
1	1	1	1	3	3	2	2	1	1
1	1	1	1	3	3	2	2	3	3
1	1	1	1	3	3	1	1	1	1
1	1	2	2	3	3	1	1	1	1
1	1	1	1	3	3	1	1	3	3
1	1	1	1	3	3	2	2	3	3
1	1	1	1	3	3	2	2	1	1
1	1	1	1	3	3	2	2	3	3
1	1	1	1	3	3	2	2	1	1
4	4	1	1	3	3	2	2	3	3
1	1	1	1	3	3	2	2	1	1
1	1	1	1	3	3	1	1	1	1
1	1	1	1	3	3	2	2	1	1
1	1	1	1	3	3	2	2	3	3
1	1	1	1	3	3	1	1	1	1
1	1	1	1	3	3	2	2	1	1
1	1	1	1	3	3	2	2	1	1
1	1	1	1	3	3	2	2	1	1
1	1	2	2	3	3	2	2	1	1
1	1	1	1	2	2	1	1	3	3
4	4	1	1	3	3	2	2	3	3
4	4	1	1	3	3	2	2	1	1
4	4	2	2	3	3	2	2	1	1
1	1	1	1	3	3	1	1	1	1
4	4	1	1	3	3	2	2	3	3
1	1	2	2	3	3	1	1	1	1
1	1	1	1	3	3	1	1	3	3
1	1	1	1	3	3	1	1	3	3
1	1	1	1	3	3	2	2	3	3
1	1	1	1	3	3	2	2	1	1
1	1	1	1	3	3	2	2	1	1
4	4	1	1	3	3	1	1	1	1
1	1	1	1	3	3	2	2	1	1
1	1	1	1	3	3	1	1	3	3
1	1	1	1	3	3	2	2	1	1
1	1	1	1	3	3	2	2	3	3
1	1	1	1	3	3	2	2	1	1
1	1	1	1	3	3	2	2	1	1
1	1	1	1	3	3	2	2	1	1
4	4	1	1	3	3	2	2	1	1

For Review Only

1	1	1	1	3	3	2	2	3	3
4	4	1	1	3	3	2	2	1	1
1	1	1	1	3	3	2	2	3	3
1	1	1	1	3	3	2	2	1	1
1	1	1	1	3	3	2	2	3	3
1	1	1	1	3	3	2	2	3	3
1	1	1	1	3	3	1	1	3	3
1	1	1	1	3	3	2	2	1	1
1	1	1	1	3	3	1	1	3	3
1	1	1	1	3	3	2	2	1	1
1	1	1	1	3	3	1	1	1	1
1	1	1	1	3	3	2	2	1	1
1	1	1	1	3	3	1	1	3	3
1	1	1	1	3	3	2	2	1	1
1	1	1	1	3	3	2	2	1	1
1	1	1	1	3	3	2	2	1	1
1	1	1	1	3	3	2	2	1	1
1	1	1	1	3	3	2	2	1	1
1	1	1	1	3	3	2	2	3	3
1	1	2	2	3	3	2	2	1	1
1	1	1	1	3	3	2	2	1	1
1	1	1	1	3	3	1	1	1	1
1	1	1	1	3	3	1	1	1	1
1	1	1	1	3	3	2	2	1	1
1	1	1	1	3	3	2	2	1	1
1	1	1	1	3	3	2	2	1	1
4	4	1	1	3	3	2	2	1	1
1	1	1	1	3	3	2	2	1	1
1	1	1	1	3	3	1	1	3	3
1	1	1	1	3	3	2	2	1	1
1	1	1	1	3	3	2	2	1	1
1	1	1	1	3	3	2	2	1	1
1	1	2	2	3	3	1	1	1	1
1	1	1	1	3	3	2	2	1	1
1	1	1	1	3	3	1	1	3	3
1	1	1	1	3	3	1	1	3	3
1	1	1	1	3	3	2	2	1	1
1	1	1	1	3	3	2	2	3	3
1	1	1	1	2	2	2	2	1	1
1	1	1	1	3	3	2	2	1	1
1	1	1	1	3	3	2	2	1	1
1	1	1	1	3	3	2	2	1	1
1	1	1	1	3	3	2	2	3	3
4	4	1	1	3	3	2	2	1	1
1	1	1	1	3	3	2	2	1	1
1	1	1	1	3	3	2	2	1	1
1	1	1	1	3	3	2	2	3	3
1	1	1	1	3	3	2	2	3	3
4	4	1	1	3	3	2	2	1	1
1	1	1	1	3	3	2	2	3	3
1	1	1	1	3	3	2	2	3	3
1	1	2	2	3	3	2	2	1	1
1	1	1	1	3	3	1	1	1	1
1	1	1	1	3	3	2	2	3	3
1	1	1	1	3	3	2	2	1	1

1	1	1	1	3	3	2	2	3	3
1	1	2	2	3	3	2	2	1	1
1	1	1	1	3	3	2	2	3	3
1	1	1	1	3	3	2	2	1	1
1	1	1	1	3	3	2	2	3	3
1	1	1	1	3	3	2	2	1	1
1	1	1	1	2	2	2	2	1	1
1	1	1	1	3	3	2	2	1	1
1	1	1	1	3	3	1	1	3	3
1	1	1	1	3	3	1	1	1	1
1	1	1	1	3	3	2	2	1	1
1	1	1	1	3	3	2	2	1	1
1	1	1	1	3	3	1	1	3	3
1	1	1	1	3	3	2	2	1	1
1	1	1	1	3	3	2	2	1	1
1	1	2	2	3	3	2	2	1	1
1	1	1	1	3	3	1	1	1	1
1	1	1	1	3	3	2	2	1	1
1	1	1	1	3	3	2	2	1	1
1	1	1	1	3	3	1	1	3	3
1	1	1	1	3	3	1	1	1	1
1	1	1	1	2	2	2	2	1	1
1	1	1	1	3	3	2	2	1	1
1	1	1	1	3	3	2	2	3	3
1	1	1	1	3	3	2	2	3	3
1	1	2	2	3	3	1	1	1	1
1	1	1	1	3	3	2	2	1	1
1	1	1	1	3	3	2	2	1	1
1	1	1	1	3	3	1	1	1	1
1	1	1	1	3	3	1	1	3	3
1	1	1	1	3	3	2	2	1	1
1	1	1	1	3	3	1	1	1	1
1	1	1	1	3	3	2	2	1	1
1	1	1	1	3	3	2	2	1	1
1	1	1	1	3	3	1	1	3	3
1	1	1	1	3	3	2	2	3	3
1	1	1	1	3	3	2	2	1	1
1	1	1	1	3	3	1	1	3	3
1	1	1	1	3	3	2	2	3	3
1	1	1	1	3	3	2	2	3	3
1	1	1	1	3	3	2	2	3	3
1	1	1	1	3	3	2	2	3	3
1	1	1	1	3	3	2	2	1	1
1	1	1	1	3	3	2	2	1	1
1	1	1	1	3	3	1	1	3	3
1	1	1	1	2	2	1	1	1	1
1	1	2	2	3	3	2	2	1	1
1	1	2	2	2	2	2	2	1	1
4	4	1	1	3	3	2	2	1	1
1	1	2	2	3	3	1	1	1	1
1	1	1	1	3	3	1	1	1	1
1	1	1	1	3	3	2	2	1	1
1	1	1	1	3	3	2	2	1	1
1	1	1	1	3	3	2	2	3	3
1	1	1	1	3	3	2	2	1	1

For Review Only

1	1	1	1	3	3	2	2	3	3
1	1	1	1	3	3	2	2	1	1
1	1	1	1	3	3	1	1	3	3
1	1	1	1	3	3	2	2	1	1
4	4	1	1	3	3	2	2	1	1
1	1	1	1	3	3	2	2	1	1
1	1	1	1	3	3	1	1	1	1
1	1	1	1	3	3	2	2	1	1
1	1	1	1	3	3	1	1	3	3
1	1	1	1	3	3	2	2	1	1
1	1	1	1	3	3	2	2	1	1
1	1	1	1	3	3	2	2	1	1
1	1	1	1	3	3	1	1	1	1
1	1	1	1	3	3	2	2	1	1
1	1	1	1	3	3	1	1	3	3
1	1	2	2	3	3	1	1	1	1
1	1	1	1	3	3	2	2	1	1
1	1	1	1	3	3	2	2	3	3
1	1	1	1	2	2	2	2	1	1
4	4	1	1	3	3	2	2	3	3
1	1	1	1	3	3	2	2	1	1
1	1	1	1	3	3	2	2	3	3
1	1	2	2	3	3	1	1	1	1
1	1	1	1	3	3	2	2	1	1
1	1	1	1	3	3	2	2	1	1
1	1	1	1	3	3	2	2	3	3
1	1	1	1	3	3	2	2	3	3
1	1	1	1	3	3	2	2	3	3
1	1	1	1	3	3	2	2	1	1
1	1	1	1	3	3	1	1	1	1
1	1	1	1	2	2	2	2	1	1
1	1	1	1	3	3	2	2	1	1
4	4	2	2	3	3	2	2	1	1
1	1	1	1	3	3	2	2	3	3
1	1	1	1	3	3	2	2	1	1
1	1	1	1	2	2	2	2	1	1
1	1	1	1	3	3	1	1	3	3
1	1	1	1	3	3	2	2	3	3
1	1	1	1	3	3	2	2	1	1
1	1	1	1	3	3	2	2	1	1
1	1	1	1	3	3	1	1	3	3
1	1	1	1	3	3	2	2	1	1
1	1	1	1	3	3	2	2	3	3
1	1	2	2	3	3	1	1	1	1
1	1	1	1	3	3	2	2	1	1
1	1	1	1	3	3	2	2	1	1
1	1	1	1	3	3	2	2	3	3
4	4	1	1	3	3	1	1	3	3
4	4	1	1	3	3	2	2	3	3
1	1	1	1	3	3	2	2	3	3
1	1	1	1	3	3	2	2	1	1
1	1	1	1	3	3	2	2	1	1
1	1	1	1	3	3	2	2	3	3
1	1	1	1	3	3	2	2	1	1
1	1	1	1	3	3	2	2	1	1

For Review Only

1	1	2	2	3	3	1	1	1	1
1	1	1	1	3	3	2	2	3	3

For Review Only

SNP2681	SNP2681	SNP2682	SNP2682	SNP2683	SNP2683	SNP2684	SNP2684	SNP2685	SNP2685
1	1	4	4	1	1	2	2	4	4
1	1	4	4	3	3	2	2	4	4
1	1	2	2	1	1	2	2	4	4
1	1	2	2	1	1	2	2	4	4
1	1	2	2	1	1	3	3	4	4
1	1	2	2	1	1	3	3	4	4
1	1	2	2	1	1	3	3	4	4
1	1	2	2	1	1	2	2	4	4
1	1	4	4	1	1	2	2	4	4
1	1	2	2	1	1	2	2	4	4
1	1	2	2	1	1	2	2	4	4
1	1	2	2	1	1	2	2	4	4
1	1	4	4	1	1	2	2	3	3
1	1	2	2	1	1	2	2	4	4
1	1	2	2	1	1	2	2	4	4
1	1	4	4	1	1	2	2	4	4
1	1	2	2	1	1	2	2	4	4
1	1	2	2	1	1	2	2	4	4
1	1	2	2	1	1	2	2	4	4
1	1	2	2	1	1	2	2	4	4
1	1	2	2	1	1	2	2	4	4
1	1	2	2	1	1	2	2	4	4
4	4	2	2	3	3	2	2	4	4
1	1	4	4	3	3	2	2	4	4
1	1	2	2	1	1	2	2	4	4
1	1	2	2	1	1	2	2	3	3
1	1	2	2	1	1	2	2	4	4
1	1	2	2	1	1	2	2	4	4
1	1	4	4	1	1	2	2	4	4
1	1	4	4	1	1	2	2	4	4
1	1	2	2	1	1	2	2	4	4
1	1	2	2	1	1	2	2	4	4
1	1	4	4	1	1	2	2	4	4
1	1	2	2	1	1	2	2	4	4
1	1	2	2	1	1	2	2	3	3
1	1	4	4	1	1	2	2	4	4
1	1	2	2	1	1	2	2	4	4
1	1	2	2	1	1	2	2	4	4
4	4	4	4	3	3	2	2	4	4
1	1	2	2	1	1	2	2	4	4
1	1	2	2	1	1	2	2	4	4
1	1	4	4	1	1	2	2	4	4
1	1	2	2	1	1	2	2	4	4
1	1	2	2	1	1	2	2	4	4
1	1	2	2	1	1	2	2	4	4
1	1	2	2	1	1	2	2	3	3
1	1	2	2	1	1	2	2	4	4
1	1	2	2	1	1	2	2	4	4
1	1	2	2	1	1	2	2	4	4
1	1	2	2	1	1	2	2	4	4
1	1	2	2	1	1	2	2	3	3
1	1	2	2	1	1	2	2	4	4

For Review Only

1	1	2	2	1	1	2	2	4	4
1	1	4	4	1	1	2	2	4	4
1	1	2	2	3	3	2	2	4	4
1	1	2	2	1	1	2	2	4	4
1	1	2	2	1	1	2	2	4	4
1	1	2	2	1	1	2	2	3	3
1	1	2	2	1	1	2	2	3	3
1	1	4	4	1	1	2	2	4	4
1	1	2	2	1	1	2	2	4	4
1	1	2	2	1	1	2	2	4	4
1	1	2	2	1	1	2	2	4	4
1	1	2	2	1	1	2	2	4	4
1	1	2	2	1	1	2	2	4	4
1	1	4	4	1	1	2	2	4	4
1	1	2	2	1	1	2	2	4	4
1	1	2	2	1	1	2	2	4	4
1	1	2	2	1	1	2	2	4	4
1	1	2	2	1	1	2	2	3	3
1	1	2	2	1	1	2	2	4	4
1	1	4	4	1	1	3	3	4	4
1	1	2	2	1	1	2	2	4	4
1	1	2	2	1	1	2	2	4	4
1	1	4	4	1	1	2	2	4	4
1	1	2	2	1	1	2	2	4	4
1	1	2	2	1	1	2	2	4	4
1	1	2	2	1	1	3	3	4	4
1	1	2	2	1	1	2	2	4	4
4	4	2	2	3	3	2	2	4	4
1	1	2	2	1	1	2	2	3	3
1	1	2	2	1	1	2	2	4	4
1	1	2	2	1	1	2	2	4	4
1	1	2	2	1	1	2	2	4	4
4	4	4	4	1	1	2	2	4	4
1	1	2	2	1	1	2	2	4	4
1	1	2	2	1	1	3	3	4	4
1	1	2	2	1	1	2	2	4	4
1	1	4	4	1	1	2	2	4	4
1	1	2	2	1	1	2	2	4	4
1	1	2	2	1	1	2	2	4	4
1	1	2	2	1	1	2	2	4	4
1	1	2	2	1	1	2	2	4	4
1	1	2	2	1	1	2	2	4	4
1	1	2	2	1	1	2	2	4	4
1	1	2	2	1	1	2	2	4	4
1	1	2	2	1	1	3	3	4	4
1	1	2	2	1	1	2	2	4	4
1	1	2	2	1	1	2	2	4	4
1	1	4	4	1	1	2	2	3	3
1	1	2	2	1	1	2	2	4	4
1	1	2	2	1	1	2	2	4	4
1	1	2	2	3	3	2	2	4	4
4	4	4	4	1	1	2	2	4	4
1	1	2	2	3	3	2	2	4	4
1	1	2	2	1	1	2	2	4	4
1	1	2	2	1	1	3	3	4	4

For Review Only

1	1	2	2	1	1	2	2	3	3
1	1	2	2	1	1	2	2	4	4
1	1	2	2	1	1	2	2	3	3
1	1	4	4	1	1	2	2	4	4
1	1	2	2	1	1	2	2	4	4
1	1	2	2	1	1	2	2	3	3
1	1	2	2	1	1	2	2	4	4
1	1	2	2	1	1	2	2	4	4
1	1	2	2	1	1	2	2	4	4
1	1	4	4	1	1	2	2	4	4
1	1	2	2	1	1	2	2	4	4
1	1	2	2	1	1	2	2	4	4
1	1	2	2	1	1	2	2	4	4
1	1	2	2	1	1	2	2	4	4
1	1	2	2	3	3	2	2	4	4
1	1	2	2	1	1	2	2	3	3
1	1	2	2	1	1	2	2	3	3
1	1	2	2	1	1	2	2	4	4
1	1	2	2	1	1	2	2	4	4
1	1	2	2	1	1	2	2	4	4
1	1	2	2	1	1	2	2	4	4
1	1	2	2	1	1	2	2	4	4
1	1	2	2	1	1	2	2	4	4
4	4	4	4	3	3	2	2	4	4
1	1	2	2	1	1	2	2	4	4
1	1	2	2	1	1	2	2	3	3
1	1	2	2	1	1	2	2	4	4
1	1	2	2	1	1	2	2	4	4
1	1	2	2	1	1	2	2	4	4
1	1	2	2	1	1	2	2	4	4
4	4	4	4	1	1	2	2	4	4
1	1	2	2	1	1	2	2	4	4
1	1	2	2	1	1	2	2	4	4
1	1	2	2	1	1	2	2	3	3
1	1	2	2	1	1	2	2	4	4
1	1	2	2	1	1	2	2	4	4
1	1	4	4	1	1	2	2	4	4
1	1	2	2	1	1	2	2	3	3
1	1	2	2	1	1	2	2	3	3
1	1	2	2	3	3	2	2	4	4
1	1	2	2	1	1	2	2	4	4
1	1	2	2	1	1	2	2	4	4
1	1	2	2	1	1	3	3	3	3
1	1	2	2	1	1	2	2	4	4
1	1	4	4	1	1	2	2	3	3
1	1	2	2	1	1	3	3	4	4
1	1	2	2	1	1	2	2	3	3
1	1	2	2	1	1	2	2	3	3
1	1	4	4	1	1	2	2	4	4
1	1	2	2	1	1	2	2	3	3
1	1	2	2	1	1	2	2	3	3
1	1	2	2	1	1	2	2	3	3
1	1	2	2	1	1	2	2	4	4
1	1	2	2	1	1	2	2	4	4
1	1	4	4	1	1	2	2	4	4

For Review Only

1	1	2	2	1	1	2	2	3	3
1	1	2	2	1	1	2	2	4	4
1	1	2	2	1	1	2	2	4	4
1	1	2	2	1	1	2	2	4	4
1	1	2	2	3	3	2	2	4	4
1	1	2	2	1	1	2	2	4	4
1	1	2	2	3	3	2	2	4	4
1	1	2	2	1	1	2	2	4	4
1	1	2	2	3	3	2	2	4	4
1	1	2	2	1	1	2	2	4	4
1	1	2	2	1	1	2	2	3	3
4	4	4	4	1	1	2	2	4	4
1	1	2	2	1	1	2	2	3	3
1	1	2	2	1	1	2	2	3	3
1	1	4	4	1	1	2	2	4	4
1	1	2	2	3	3	2	2	4	4
1	1	2	2	1	1	2	2	4	4
1	1	2	2	1	1	3	3	4	4
1	1	4	4	1	1	2	2	3	3
1	1	2	2	1	1	2	2	4	4
1	1	2	2	1	1	2	2	4	4
1	1	2	2	3	3	2	2	4	4
1	1	2	2	1	1	2	2	4	4
1	1	2	2	1	1	2	2	4	4
1	1	4	4	1	1	2	2	3	3
1	1	2	2	1	1	2	2	4	4
1	1	2	2	1	1	2	2	4	4
1	1	2	2	1	1	2	2	3	3
1	1	2	2	1	1	2	2	3	3
1	1	2	2	1	1	2	2	4	4
1	1	4	4	1	1	2	2	4	4
1	1	2	2	1	1	2	2	4	4
1	1	2	2	1	1	2	2	4	4
1	1	4	4	1	1	2	2	4	4
1	1	2	2	3	3	2	2	4	4
1	1	2	2	1	1	2	2	4	4
1	1	2	2	1	1	2	2	4	4
1	1	2	2	1	1	2	2	4	4
1	1	2	2	1	1	2	2	3	3
1	1	2	2	1	1	2	2	4	4
1	1	2	2	1	1	2	2	4	4
1	1	2	2	1	1	2	2	3	3
1	1	4	4	3	3	2	2	4	4
1	1	2	2	1	1	2	2	3	3
1	1	2	2	1	1	2	2	4	4
1	1	2	2	1	1	3	3	4	4
1	1	2	2	1	1	2	2	4	4
4	4	2	2	3	3	2	2	4	4
1	1	2	2	1	1	2	2	4	4
1	1	2	2	1	1	2	2	4	4
1	1	2	2	1	1	2	2	4	4
1	1	4	4	1	1	2	2	4	4
1	1	2	2	1	1	2	2	4	4
1	1	2	2	1	1	2	2	4	4
4	4	4	4	3	3	2	2	4	4

For Review Only

1	1	2	2	1	1	2	2	4	4
1	1	2	2	1	1	2	2	3	3
1	1	2	2	1	1	2	2	4	4
1	1	2	2	1	1	2	2	4	4
1	1	2	2	1	1	2	2	4	4
1	1	2	2	1	1	2	2	3	3
1	1	2	2	1	1	2	2	3	3
1	1	4	4	1	1	2	2	4	4
1	1	2	2	1	1	2	2	4	4
4	4	4	4	3	3	2	2	4	4
1	1	2	2	1	1	2	2	4	4
1	1	4	4	1	1	2	2	4	4
1	1	2	2	1	1	2	2	4	4
1	1	2	2	1	1	2	2	3	3
1	1	2	2	1	1	2	2	4	4
1	1	2	2	1	1	2	2	4	4
1	1	4	4	1	1	2	2	4	4
4	4	2	2	1	1	2	2	4	4
1	1	4	4	3	3	2	2	4	4
1	1	2	2	1	1	2	2	4	4
1	1	2	2	1	1	2	2	4	4
1	1	2	2	1	1	2	2	3	3
1	1	2	2	1	1	2	2	4	4
1	1	2	2	1	1	2	2	4	4
1	1	4	4	1	1	2	2	4	4
1	1	4	4	1	1	2	2	4	4
4	4	2	2	3	3	2	2	4	4
1	1	2	2	1	1	2	2	4	4
1	1	2	2	1	1	2	2	4	4
1	1	2	2	1	1	2	2	4	4
4	4	4	4	3	3	2	2	4	4
1	1	4	4	1	1	2	2	4	4
1	1	2	2	1	1	2	2	3	3
1	1	2	2	1	1	2	2	3	3
1	1	2	2	1	1	2	2	4	4
4	4	4	4	3	3	2	2	4	4
1	1	2	2	1	1	2	2	3	3
1	1	2	2	1	1	2	2	4	4
1	1	4	4	1	1	2	2	4	4
4	4	4	4	3	3	2	2	4	4
1	1	2	2	3	3	2	2	4	4
1	1	2	2	1	1	2	2	4	4
1	1	2	2	1	1	2	2	4	4
1	1	2	2	1	1	2	2	4	4
1	1	2	2	1	1	2	2	4	4
1	1	2	2	1	1	2	2	4	4
4	4	2	2	1	1	2	2	3	3
1	1	2	2	1	1	2	2	3	3
1	1	2	2	1	1	2	2	4	4
4	4	4	4	3	3	2	2	4	4
1	1	2	2	1	1	2	2	4	4
4	4	4	4	1	1	2	2	4	4
1	1	2	2	1	1	2	2	4	4
1	1	2	2	1	1	2	2	3	3
1	1	2	2	1	1	2	2	4	4

For Review Only

1	1	2	2	1	1	2	2	4	4
1	1	2	2	1	1	2	2	4	4

For Review Only

SNP2686	SNP2686	SNP2687	SNP2687	SNP2688	SNP2688	SNP2689	SNP2689	SNP269	SNP269
4	4	1	1	2	2	4	4	3	3
2	2	1	1	2	2	4	4	2	2
4	4	1	1	2	2	4	4	3	3
4	4	1	1	2	2	4	4	2	2
2	2	4	4	1	1	1	1	3	3
4	4	1	1	2	2	4	4	2	2
4	4	1	1	2	2	4	4	3	3
4	4	4	4	1	1	1	1	2	2
4	4	1	1	2	2	4	4	3	3
4	4	4	4	1	1	1	1	2	2
4	4	1	1	2	2	4	4	3	3
4	4	1	1	2	2	4	4	2	2
4	4	1	1	2	2	1	1	2	2
4	4	1	1	2	2	4	4	2	2
4	4	1	1	2	2	4	4	3	3
4	4	1	1	2	2	4	4	2	2
4	4	1	1	2	2	4	4	2	2
4	4	1	1	2	2	4	4	2	2
4	4	4	4	1	1	1	1	2	2
4	4	1	1	2	2	4	4	3	3
4	4	1	1	2	2	4	4	3	3
4	4	1	1	2	2	4	4	2	2
2	2	1	1	2	2	4	4	2	2
2	2	1	1	2	2	4	4	2	2
4	4	4	4	1	1	1	1	2	2
4	4	1	1	2	2	4	4	2	2
4	4	1	1	2	2	4	4	3	3
4	4	1	1	2	2	4	4	2	2
4	4	1	1	2	2	4	4	3	3
2	2	4	4	1	1	1	1	2	2
4	4	1	1	2	2	4	4	2	2
4	4	1	1	2	2	4	4	3	3
4	4	1	1	2	2	4	4	2	2
4	4	1	1	2	2	4	4	2	2
4	4	1	1	2	2	4	4	2	2
4	4	1	1	2	2	4	4	3	3
4	4	1	1	2	2	4	4	2	2
4	4	1	1	2	2	1	1	2	2
4	4	1	1	2	2	4	4	2	2
4	4	1	1	2	2	4	4	2	2
4	4	4	4	1	1	1	1	2	2
4	4	1	1	1	1	1	1	3	3
4	4	4	4	1	1	1	1	2	2
4	4	1	1	2	2	4	4	2	2
4	4	4	4	1	1	1	1	2	2
4	4	1	1	2	2	4	4	2	2
4	4	1	1	2	2	4	4	2	2
4	4	1	1	2	2	4	4	2	2
4	4	1	1	2	2	4	4	2	2
4	4	1	1	2	2	4	4	2	2
4	4	1	1	2	2	4	4	2	2
4	4	1	1	2	2	4	4	2	2

For Review Only

4	4	1	1	2	2	4	4	2	2
4	4	1	1	2	2	4	4	2	2
2	2	1	1	2	2	4	4	2	2
4	4	1	1	2	2	4	4	2	2
4	4	4	4	1	1	1	1	2	2
4	4	1	1	2	2	4	4	3	3
4	4	1	1	2	2	4	4	2	2
4	4	1	1	2	2	4	4	2	2
4	4	1	1	2	2	4	4	2	2
4	4	1	1	2	2	4	4	2	2
4	4	1	1	2	2	4	4	2	2
4	4	1	1	2	2	4	4	3	3
4	4	1	1	2	2	4	4	2	2
4	4	1	1	2	2	4	4	3	3
4	4	1	1	2	2	4	4	2	2
4	4	1	1	2	2	4	4	3	3
4	4	1	1	2	2	4	4	2	2
4	4	1	1	2	2	4	4	2	2
4	4	1	1	2	2	4	4	2	2
4	4	1	1	2	2	4	4	2	2
4	4	1	1	2	2	4	4	3	3
4	4	1	1	2	2	4	4	3	3
4	4	1	1	2	2	4	4	2	2
4	4	4	4	1	1	1	1	2	2
4	4	4	4	1	1	1	1	2	2
4	4	1	1	2	2	4	4	2	2
4	4	1	1	2	2	4	4	2	2
2	2	1	1	2	2	4	4	2	2
4	4	1	1	2	2	4	4	2	2
4	4	1	1	2	2	4	4	2	2
4	4	4	4	1	1	1	1	3	3
4	4	1	1	2	2	4	4	2	2
4	4	1	1	2	2	4	4	2	2
4	4	1	1	2	2	4	4	2	2
4	4	1	1	2	2	4	4	2	2
4	4	4	4	1	1	1	1	2	2
4	4	4	4	1	1	1	1	3	3
4	4	4	4	1	1	1	1	2	2
4	4	1	1	2	2	4	4	3	3
4	4	4	4	1	1	1	1	2	2
4	4	1	1	2	2	4	4	2	2
4	4	1	1	2	2	4	4	2	2
4	4	1	1	2	2	4	4	2	2
4	4	1	1	2	2	4	4	2	2
4	4	1	1	2	2	4	4	2	2
4	4	1	1	2	2	4	4	2	2
4	4	1	1	2	2	1	1	2	2
4	4	1	1	2	2	4	4	2	2
4	4	1	1	2	2	4	4	3	3
4	4	1	1	2	2	1	1	2	2
2	2	1	1	2	2	4	4	2	2
4	4	1	1	2	2	4	4	2	2
2	2	1	1	2	2	4	4	2	2
4	4	1	1	2	2	4	4	2	2
4	4	4	4	1	1	1	1	2	2

For Review Only

4	4	1	1	2	2	4	4	2	2
4	4	4	4	1	1	1	1	2	2
4	4	1	1	2	2	4	4	2	2
4	4	1	1	2	2	4	4	2	2
4	4	1	1	2	2	4	4	2	2
4	4	1	1	2	2	4	4	3	3
4	4	1	1	2	2	4	4	2	2
4	4	1	1	2	2	4	4	2	2
4	4	1	1	2	2	4	4	3	3
4	4	1	1	2	2	4	4	2	2
4	4	1	1	2	2	4	4	3	3
4	4	1	1	2	2	4	4	2	2
4	4	1	1	2	2	4	4	2	2
4	4	1	1	2	2	4	4	2	2
4	4	1	1	2	2	4	4	3	3
4	4	1	1	2	2	4	4	2	2
4	4	1	1	2	2	4	4	2	2
4	4	1	1	2	2	4	4	2	2
4	4	1	1	2	2	4	4	2	2
4	4	1	1	2	2	4	4	2	2
4	4	1	1	2	2	4	4	2	2
4	4	1	1	2	2	4	4	2	2
4	4	1	1	2	2	4	4	3	3
2	2	1	1	2	2	4	4	2	2
4	4	1	1	2	2	4	4	2	2
4	4	1	1	2	2	4	4	2	2
4	4	1	1	2	2	1	1	2	2
4	4	1	1	2	2	4	4	3	3
4	4	1	1	2	2	4	4	2	2
4	4	1	1	2	2	4	4	2	2
4	4	1	1	2	2	4	4	2	2
4	4	1	1	2	2	4	4	2	2
4	4	1	1	2	2	4	4	2	2
4	4	1	1	2	2	4	4	2	2
4	4	1	1	2	2	4	4	2	2
4	4	1	1	2	2	4	4	2	2
4	4	1	1	2	2	4	4	2	2
4	4	1	1	2	2	4	4	3	3
4	4	1	1	2	2	4	4	2	2
2	2	1	1	2	2	4	4	2	2
4	4	1	1	2	2	4	4	2	2
4	4	1	1	2	2	4	4	2	2
4	4	1	1	2	2	4	4	3	3
4	4	1	1	2	2	1	1	2	2
4	4	1	1	2	2	4	4	2	2
4	4	1	1	2	2	4	4	2	2
4	4	1	1	2	2	4	4	2	2
4	4	1	1	2	2	4	4	2	2
4	4	1	1	2	2	1	1	3	3
4	4	1	1	2	2	4	4	2	2
4	4	1	1	2	2	4	4	2	2
4	4	1	1	2	2	4	4	2	2
4	4	1	1	2	2	4	4	2	2
4	4	1	1	2	2	4	4	2	2
4	4	1	1	2	2	4	4	2	2

For Review Only

4	4	1	1	2	2	4	4	2	2
4	4	1	1	2	2	4	4	2	2
4	4	1	1	2	2	4	4	2	2
4	4	1	1	2	2	4	4	2	2
2	2	1	1	2	2	4	4	2	2
4	4	1	1	2	2	4	4	2	2
2	2	1	1	2	2	4	4	2	2
4	4	1	1	2	2	4	4	2	2
4	4	1	1	2	2	4	4	2	2
4	4	1	1	2	2	4	4	3	3
4	4	1	1	2	2	4	4	2	2
4	4	1	1	2	2	4	4	2	2
4	4	1	1	2	2	4	4	2	2
4	4	1	1	2	2	1	1	2	2
4	4	1	1	2	2	4	4	2	2
4	4	1	1	2	2	4	4	2	2
4	4	1	1	2	2	4	4	2	2
2	2	1	1	2	2	4	4	2	2
4	4	1	1	2	2	4	4	3	3
4	4	1	1	2	2	4	4	2	2
4	4	1	1	2	2	4	4	2	2
2	2	1	1	2	2	4	4	2	2
4	4	1	1	2	2	4	4	3	3
4	4	1	1	2	2	4	4	3	3
4	4	1	1	2	2	4	4	2	2
4	4	1	1	2	2	4	4	2	2
4	4	1	1	2	2	4	4	2	2
4	4	1	1	2	2	4	4	2	2
4	4	1	1	2	2	4	4	2	2
4	4	1	1	2	2	4	4	2	2
4	4	1	1	2	2	4	4	3	3
4	4	1	1	2	2	4	4	2	2
4	4	1	1	2	2	4	4	2	2
4	4	1	1	2	2	4	4	2	2
2	2	1	1	2	2	4	4	2	2
4	4	1	1	2	2	4	4	3	3
4	4	1	1	2	2	4	4	2	2
4	4	1	1	2	2	4	4	2	2
4	4	1	1	2	2	4	4	2	2
4	4	1	1	2	2	4	4	3	3
4	4	1	1	2	2	4	4	3	3
4	4	1	1	2	2	4	4	2	2
2	2	1	1	2	2	4	4	2	2
4	4	1	1	2	2	4	4	2	2
4	4	1	1	2	2	4	4	2	2
4	4	1	1	2	2	4	4	2	2
4	4	1	1	2	2	4	4	2	2
2	2	1	1	2	2	4	4	2	2
4	4	1	1	2	2	4	4	2	2
4	4	1	1	2	2	4	4	2	2
4	4	1	1	2	2	4	4	2	2
4	4	1	1	2	2	4	4	3	3
4	4	1	1	2	2	4	4	3	3
4	4	1	1	2	2	4	4	2	2
2	2	1	1	2	2	4	4	2	2

For Review Only

4	4	1	1	2	2	4	4	3	3
2	2	1	1	2	2	4	4	2	2
4	4	1	1	2	2	4	4	3	3
4	4	1	1	2	2	4	4	2	2
4	4	1	1	1	1	1	1	2	2
4	4	1	1	2	2	4	4	2	2
4	4	1	1	2	2	4	4	2	2
4	4	4	4	1	1	1	1	2	2
4	4	1	1	2	2	4	4	2	2
2	2	1	1	2	2	4	4	2	2
4	4	1	1	2	2	4	4	2	2
4	4	1	1	2	2	4	4	3	3
4	4	1	1	2	2	4	4	3	3
4	4	1	1	2	2	4	4	2	2
4	4	1	1	2	2	4	4	2	2
4	4	1	1	2	2	4	4	2	2
4	4	1	1	2	2	4	4	2	2
4	4	1	1	2	2	4	4	2	2
2	2	1	1	2	2	4	4	2	2
4	4	4	4	1	1	1	1	2	2
4	4	1	1	2	2	4	4	2	2
4	4	1	1	2	2	4	4	2	2
4	4	1	1	2	2	4	4	3	3
4	4	1	1	2	2	4	4	2	2
4	4	1	1	2	2	4	4	2	2
4	4	1	1	2	2	4	4	2	2
2	2	1	1	2	2	4	4	2	2
4	4	1	1	2	2	4	4	2	2
4	4	1	1	2	2	4	4	2	2
4	4	1	1	2	2	4	4	2	2
4	4	1	1	2	2	4	4	2	2
4	4	1	1	2	2	4	4	2	2
4	4	1	1	2	2	1	1	2	2
4	4	1	1	2	2	4	4	2	2
4	4	1	1	2	2	4	4	3	3
2	2	1	1	2	2	4	4	2	2
4	4	1	1	2	2	4	4	3	3
4	4	1	1	2	2	4	4	3	3
4	4	1	1	2	2	4	4	3	3
4	4	1	1	2	2	4	4	2	2
2	2	1	1	2	2	4	4	3	3
4	4	1	1	2	2	4	4	3	3
4	4	1	1	2	2	4	4	2	2
4	4	1	1	2	2	4	4	2	2
4	4	1	1	2	2	4	4	2	2
4	4	1	1	2	2	4	4	3	3
4	4	1	1	2	2	4	4	2	2
4	4	4	4	1	1	1	1	2	2
4	4	4	4	1	1	1	1	2	2
2	2	1	1	2	2	4	4	2	2
4	4	1	1	2	2	4	4	2	2
4	4	1	1	2	2	4	4	2	2
4	4	1	1	2	2	4	4	3	3
4	4	1	1	2	2	4	4	2	2
4	4	1	1	2	2	4	4	2	2

For Review Only

4	4	1	1	2	2	4	4	3	3
4	4	1	1	2	2	4	4	2	2

For Review Only

SNP2690	SNP2690	SNP2691	SNP2691	SNP2692	SNP2692	SNP2693	SNP2693	SNP2694	SNP2694
3	3	3	3	4	4	4	4	2	2
3	3	3	3	1	1	1	1	2	2
3	3	3	3	1	1	1	1	2	2
3	3	3	3	1	1	1	1	2	2
3	3	4	4	1	1	1	1	3	3
3	3	3	3	4	4	4	4	2	2
3	3	3	3	4	4	4	4	2	2
3	3	4	4	1	1	1	1	3	3
3	3	3	3	4	4	4	4	2	2
3	3	4	4	1	1	1	1	2	2
3	3	3	3	1	1	1	1	2	2
3	3	3	3	1	1	1	1	2	2
3	3	3	3	4	4	4	4	2	2
3	3	3	3	1	1	1	1	3	3
3	3	3	3	4	4	4	4	2	2
3	3	3	3	1	1	1	1	2	2
3	3	3	3	4	4	4	4	2	2
3	3	3	3	4	4	4	4	3	3
3	3	3	3	1	1	1	1	2	2
3	3	3	3	4	4	4	4	2	2
3	3	3	3	4	4	4	4	2	2
3	3	3	3	4	4	4	4	2	2
3	3	3	3	1	1	1	1	3	3
3	3	3	3	1	1	1	1	3	3
3	3	3	3	4	4	4	4	2	2
4	4	3	3	4	4	4	4	2	2
3	3	3	3	1	1	1	1	2	2
3	3	3	3	4	4	4	4	2	2
3	3	3	3	4	4	4	4	2	2
3	3	4	4	1	1	1	1	3	3
3	3	3	3	1	1	1	1	2	2
3	3	3	3	1	1	1	1	2	2
3	3	3	3	1	1	1	1	2	2
3	3	3	3	1	1	1	1	3	3
4	4	3	3	4	4	4	4	2	2
3	3	3	3	1	1	1	1	2	2
3	3	3	3	4	4	4	4	2	2
3	3	4	4	4	4	4	4	2	2
3	3	3	3	1	1	1	1	3	3
3	3	3	3	4	4	4	4	2	2
3	3	4	4	1	1	1	1	3	3
3	3	4	4	4	4	4	4	2	2
3	3	4	4	4	4	4	4	3	3
3	3	3	3	4	4	4	4	2	2
3	3	4	4	1	1	1	1	3	3
3	3	3	3	4	4	4	4	2	2
4	4	3	3	1	1	1	1	2	2
3	3	3	3	1	1	1	1	3	3
3	3	3	3	4	4	4	4	3	3
3	3	3	3	1	1	1	1	2	2
3	3	3	3	4	4	4	4	2	2
4	4	3	3	1	1	1	1	2	2

For Review Only

3	3	3	3	4	4	4	4	2	2
3	3	3	3	1	1	1	1	2	2
3	3	3	3	1	1	1	1	2	2
3	3	3	3	4	4	4	4	2	2
3	3	3	3	1	1	1	1	2	2
4	4	3	3	4	4	4	4	2	2
4	4	3	3	4	4	4	4	2	2
3	3	3	3	1	1	1	1	3	3
3	3	3	3	4	4	4	4	2	2
3	3	3	3	4	4	4	4	2	2
3	3	3	3	1	1	4	4	2	2
3	3	3	3	1	1	1	1	2	2
3	3	3	3	4	4	4	4	2	2
3	3	3	3	4	4	4	4	2	2
3	3	3	3	4	4	4	4	2	2
3	3	3	3	4	4	4	4	3	3
3	3	3	3	4	4	4	4	2	2
4	4	3	3	4	4	4	4	2	2
3	3	3	3	4	4	4	4	2	2
3	3	3	3	4	4	4	4	2	2
3	3	3	3	1	1	1	1	2	2
3	3	3	3	4	4	4	4	2	2
3	3	3	3	4	4	4	4	2	2
3	3	3	3	1	1	1	1	2	2
3	3	4	4	1	1	1	1	3	3
4	4	3	3	1	1	1	1	2	2
3	3	3	3	4	4	4	4	3	3
3	3	3	3	1	1	1	1	3	3
4	4	3	3	4	4	4	4	2	2
3	3	3	3	1	1	1	1	2	2
3	3	4	4	4	4	4	4	2	2
3	3	3	3	1	1	1	1	2	2
3	3	3	3	1	1	1	1	3	3
3	3	3	3	4	4	4	4	2	2
3	3	3	3	4	4	4	4	2	2
3	3	4	4	1	1	1	1	3	3
3	3	4	4	1	1	1	1	2	2
3	3	4	4	1	1	1	1	3	3
3	3	3	3	1	1	1	1	2	2
3	3	4	4	1	1	1	1	3	3
3	3	3	3	4	4	4	4	2	2
3	3	3	3	1	1	1	1	2	2
3	3	3	3	4	4	4	4	2	2
3	3	3	3	1	1	1	1	2	2
3	3	3	3	1	1	4	4	2	2
3	3	3	3	1	1	4	4	2	2
3	3	3	3	1	1	1	1	3	3
4	4	3	3	4	4	4	4	2	2
3	3	3	3	1	1	1	1	2	2
4	4	3	3	1	1	1	1	2	2
3	3	3	3	1	1	1	1	2	2
3	3	3	3	1	1	1	1	3	3
3	3	3	3	1	1	1	1	3	3
3	3	3	3	1	1	1	1	2	2
4	4	4	4	1	1	1	1	3	3

For Review Only

4	4	3	3	4	4	4	4	2	2
3	3	4	4	4	4	4	4	2	2
3	3	3	3	4	4	4	4	2	2
3	3	3	3	1	1	1	1	2	2
3	3	3	3	1	1	1	1	2	2
3	3	3	3	4	4	4	4	2	2
3	3	3	3	1	1	1	1	2	2
3	3	3	3	1	1	1	1	2	2
3	3	3	3	1	1	1	1	2	2
3	3	3	3	4	4	1	1	2	2
3	3	3	3	4	4	4	4	2	2
3	3	3	3	4	4	4	4	2	2
3	3	3	3	1	1	1	1	2	2
4	4	3	3	1	1	4	4	2	2
3	3	3	3	1	1	1	1	2	2
3	3	3	3	4	4	4	4	2	2
4	4	3	3	4	4	4	4	2	2
3	3	3	3	4	4	4	4	2	2
3	3	3	3	1	1	1	1	3	3
3	3	3	3	1	1	1	1	2	2
3	3	3	3	4	4	4	4	2	2
3	3	3	3	4	4	1	1	2	2
3	3	3	3	1	1	1	1	2	2
3	3	3	3	1	1	1	1	3	3
4	4	3	3	4	4	4	4	2	2
3	3	3	3	4	4	4	4	2	2
3	3	3	3	1	1	1	1	2	2
3	3	3	3	4	4	4	4	2	2
3	3	3	3	1	1	1	1	2	2
3	3	3	3	4	4	4	4	2	2
3	3	3	3	1	1	1	1	3	3
3	3	3	3	4	4	4	4	2	2
3	3	3	3	4	4	4	4	2	2
4	4	3	3	4	4	4	4	2	2
3	3	3	3	1	1	1	1	3	3
3	3	3	3	4	4	4	4	2	2
3	3	3	3	1	1	1	1	2	2
3	3	3	3	4	4	4	4	2	2
3	3	3	3	4	4	4	4	3	3
3	3	3	3	1	1	1	1	2	2
3	3	3	3	4	4	4	4	2	2
3	3	3	3	1	1	1	1	3	3
3	3	3	3	4	4	4	4	3	3
3	3	3	3	1	1	1	1	3	3
3	3	3	3	4	4	4	4	2	2
3	3	3	3	1	1	1	1	2	2
4	4	3	3	4	4	4	4	2	2
3	3	3	3	4	4	4	4	2	2
3	3	3	3	4	4	4	4	2	2
4	4	3	3	4	4	4	4	2	2
4	4	3	3	4	4	4	4	2	2
3	3	3	3	4	4	4	4	3	3
3	3	3	3	1	1	1	1	2	2
3	3	3	3	1	1	1	1	2	2
3	3	3	3	4	4	1	1	2	2

For Review Only

3	3	3	3	4	4	4	4	2	2
3	3	3	3	1	1	1	1	2	2
3	3	3	3	1	1	1	1	3	3
3	3	3	3	1	1	1	1	2	2
3	3	3	3	1	1	1	1	2	2
3	3	3	3	4	4	4	4	2	2
3	3	3	3	1	1	1	1	3	3
3	3	3	3	4	4	4	4	2	2
3	3	3	3	1	1	1	1	3	3
3	3	3	3	1	1	1	1	2	2
3	3	3	3	4	4	4	4	2	2
3	3	3	3	4	4	4	4	3	3
3	3	3	3	4	4	4	4	2	2
3	3	3	3	4	4	4	4	2	2
3	3	3	3	1	1	1	1	3	3
3	3	3	3	1	1	1	1	2	2
3	3	3	3	1	1	4	4	2	2
3	3	3	3	1	1	4	4	2	2
3	3	3	3	4	4	4	4	2	2
3	3	3	3	1	1	1	1	2	2
3	3	3	3	4	4	4	4	2	2
3	3	3	3	1	1	1	1	3	3
3	3	3	3	4	4	4	4	2	2
3	3	3	3	1	1	1	1	2	2
3	3	3	3	4	4	4	4	3	3
3	3	3	3	1	1	1	1	3	3
3	3	3	3	1	1	1	1	3	3
4	4	3	3	4	4	4	4	2	2
4	4	3	3	4	4	4	4	2	2
3	3	3	3	4	4	4	4	2	2
3	3	3	3	4	4	4	4	2	2
4	4	3	3	1	1	1	1	2	2
3	3	3	3	1	1	1	1	2	2
3	3	3	3	1	1	1	1	3	3
3	3	3	3	1	1	1	1	2	2
3	3	3	3	1	1	1	1	2	2
3	3	3	3	4	4	4	4	2	2
3	3	3	3	4	4	4	4	2	2
3	3	3	3	4	4	4	4	2	2
3	3	3	3	4	4	1	1	2	2
3	3	3	3	1	1	1	1	2	2
4	4	3	3	4	4	4	4	2	2
3	3	3	3	1	1	1	1	3	3
3	3	3	3	4	4	4	4	2	2
3	3	3	3	1	1	1	1	2	2
3	3	3	3	1	1	1	1	2	2
3	3	3	3	1	1	1	1	2	2
3	3	3	3	1	1	1	1	3	3
3	3	3	3	1	1	1	1	2	2
3	3	3	3	4	4	4	4	2	2
3	3	3	3	1	1	1	1	2	2
3	3	3	3	1	1	1	1	3	3
3	3	3	3	4	4	4	4	2	2
3	3	3	3	1	1	1	1	2	2
3	3	3	3	1	1	1	1	3	3

For Review Only

3	3	3	3	1	1	1	1	2	2
3	3	3	3	4	4	4	4	2	2
3	3	3	3	1	1	1	1	2	2
3	3	3	3	1	1	4	4	2	2
3	3	4	4	4	4	4	4	2	2
4	4	3	3	4	4	4	4	2	2
3	3	3	3	4	4	4	4	2	2
3	3	4	4	1	1	1	1	3	3
3	3	3	3	1	1	1	1	3	3
3	3	3	3	1	1	1	1	3	3
3	3	3	3	4	4	4	4	2	2
3	3	3	3	4	4	4	4	2	2
3	3	3	3	1	1	1	1	2	2
3	3	3	3	4	4	4	4	2	2
3	3	3	3	1	1	1	1	2	2
3	3	3	3	4	4	4	4	2	2
3	3	3	3	4	4	4	4	2	2
3	3	3	3	4	4	4	4	3	3
3	3	3	3	1	1	1	1	2	2
3	3	4	4	4	4	4	4	2	2
3	3	3	3	1	1	1	1	2	2
4	4	3	3	4	4	4	4	2	2
3	3	3	3	4	4	4	4	2	2
3	3	3	3	4	4	4	4	3	3
3	3	3	3	1	1	1	1	3	3
3	3	3	3	4	4	1	1	2	2
3	3	3	3	1	1	1	1	3	3
3	3	3	3	1	1	1	1	2	2
3	3	3	3	4	4	4	4	2	2
3	3	3	3	1	1	1	1	2	2
3	3	3	3	1	1	1	1	3	3
3	3	3	3	1	1	1	1	2	2
3	3	3	3	4	4	4	4	3	3
3	3	3	3	4	4	4	4	2	2
3	3	3	3	4	4	4	4	2	2
3	3	3	3	1	1	1	1	3	3
3	3	3	3	4	4	4	4	2	2
3	3	3	3	1	1	1	1	2	2
3	3	3	3	1	1	1	1	2	2
3	3	3	3	1	1	1	1	3	3
3	3	3	3	1	1	1	1	2	2
3	3	3	3	1	1	1	1	2	2
3	3	3	3	1	1	1	1	3	3
3	3	3	3	4	4	4	4	2	2
3	3	3	3	4	4	4	4	3	3
3	3	3	3	1	1	1	1	2	2
3	3	3	3	4	4	4	4	3	3
3	3	4	4	4	4	4	4	2	2
4	4	4	4	1	1	1	1	3	3
3	3	3	3	1	1	1	1	3	3
3	3	3	3	1	1	1	1	2	2
3	3	3	3	1	1	1	1	3	3
3	3	3	3	1	1	1	1	2	2
3	3	3	3	4	4	4	4	2	2
3	3	3	3	4	4	4	4	2	2

For Review Only

3	3	3	3	4	4	4	4	2	2
3	3	3	3	1	1	1	1	2	2

For Review Only

SNP2695	SNP2695	SNP2696	SNP2696	SNP2697	SNP2697	SNP2698	SNP2698	SNP2699	SNP2699
1	1	3	3	1	1	2	2	3	3
1	1	3	3	4	4	1	1	2	2
1	1	2	2	4	4	1	1	2	2
4	4	2	2	4	4	2	2	2	2
1	1	3	3	1	1	1	1	2	2
1	1	3	3	1	1	2	2	3	3
1	1	3	3	1	1	2	2	3	3
1	1	3	3	4	4	1	1	2	2
1	1	3	3	1	1	2	2	2	2
1	1	3	3	4	4	1	1	2	2
1	1	3	3	1	1	1	1	2	2
4	4	2	2	4	4	1	1	2	2
4	4	3	3	1	1	2	2	2	2
4	4	3	3	4	4	1	1	2	2
1	1	3	3	4	4	2	2	2	2
1	1	3	3	4	4	1	1	2	2
4	4	3	3	4	4	1	1	2	2
1	1	3	3	4	4	2	2	3	3
4	4	3	3	4	4	1	1	2	2
1	1	3	3	4	4	2	2	3	3
4	4	3	3	4	4	2	2	3	3
4	4	3	3	4	4	1	1	2	2
1	1	3	3	1	1	1	1	2	2
1	1	3	3	1	1	1	1	2	2
1	1	3	3	4	4	1	1	2	2
4	4	2	2	4	4	1	1	2	2
4	4	2	2	4	4	1	1	2	2
4	4	2	2	4	4	1	1	2	2
1	1	3	3	1	1	2	2	3	3
1	1	3	3	1	1	1	1	2	2
4	4	3	3	4	4	1	1	2	2
4	4	3	3	4	4	1	1	2	2
4	4	3	3	4	4	1	1	2	2
4	4	3	3	4	4	1	1	2	2
1	1	2	2	1	1	1	1	2	2
4	4	3	3	4	4	1	1	2	2
4	4	3	3	4	4	2	2	3	3
4	4	3	3	4	4	1	1	2	2
1	1	3	3	1	1	1	1	2	2
1	1	3	3	1	1	1	1	2	2
1	1	3	3	1	1	1	1	2	2
1	1	3	3	1	1	2	2	3	3
1	1	3	3	4	4	2	2	3	3
4	4	3	3	4	4	1	1	2	2
1	1	3	3	4	4	1	1	2	2
4	4	3	3	4	4	1	1	2	2
1	1	2	2	4	4	1	1	2	2
4	4	2	2	4	4	1	1	2	2
1	1	3	3	1	1	2	2	2	2
4	4	3	3	4	4	1	1	2	2
4	4	2	2	4	4	1	1	2	2
1	1	2	2	4	4	1	1	2	2

For Review Only

4	4	3	3	4	4	1	1	2	2
4	4	3	3	4	4	1	1	2	2
4	4	3	3	4	4	1	1	2	2
4	4	3	3	4	4	1	1	2	2
4	4	3	3	4	4	1	1	2	2
4	4	2	2	4	4	1	1	2	2
4	4	2	2	4	4	1	1	2	2
1	1	3	3	1	1	1	1	2	2
4	4	3	3	4	4	2	2	3	3
4	4	2	2	4	4	1	1	2	2
1	1	2	2	4	4	1	1	2	2
1	1	2	2	4	4	1	1	2	2
4	4	3	3	4	4	1	1	2	2
1	1	3	3	1	1	2	2	3	3
1	1	3	3	4	4	2	2	2	2
1	1	3	3	4	4	2	2	2	2
1	1	2	2	1	1	2	2	3	3
4	4	3	3	4	4	1	1	2	2
4	4	3	3	4	4	1	1	2	2
1	1	3	3	1	1	2	2	3	3
1	1	2	2	4	4	1	1	2	2
1	1	3	3	4	4	2	2	3	3
1	1	3	3	4	4	2	2	3	3
1	1	3	3	1	1	1	1	2	2
1	1	3	3	4	4	1	1	2	2
4	4	2	2	4	4	1	1	2	2
1	1	3	3	1	1	1	1	2	2
1	1	3	3	1	1	1	1	2	2
4	4	2	2	4	4	1	1	2	2
4	4	3	3	4	4	1	1	2	2
4	4	3	3	4	4	1	1	2	2
4	4	2	2	4	4	1	1	2	2
1	1	3	3	1	1	1	1	2	2
4	4	3	3	4	4	1	1	2	2
1	1	3	3	1	1	2	2	3	3
1	1	3	3	4	4	1	1	2	2
4	4	3	3	1	1	1	1	2	2
1	1	3	3	1	1	1	1	2	2
4	4	3	3	4	4	1	1	2	2
1	1	3	3	4	4	1	1	2	2
4	4	3	3	4	4	1	1	2	2
4	4	3	3	4	4	1	1	2	2
4	4	3	3	4	4	2	2	3	3
1	1	2	2	4	4	1	1	2	2
1	1	3	3	1	1	1	1	2	2
1	1	3	3	4	4	1	1	2	2
4	4	3	3	4	4	1	1	2	2
4	4	3	3	4	4	1	1	2	2
1	1	2	2	1	1	1	1	2	2
1	1	3	3	4	4	1	1	2	2
1	1	2	2	4	4	1	1	2	2
1	1	3	3	1	1	1	1	2	2
1	1	3	3	1	1	1	1	2	2
1	1	2	2	4	4	1	1	2	2
1	1	3	3	1	1	1	1	2	2

For Review Only

1	1	2	2	1	1	2	2	2	2
4	4	3	3	4	4	1	1	2	2
4	4	2	2	4	4	1	1	2	2
4	4	3	3	4	4	1	1	2	2
4	4	2	2	4	4	1	1	2	2
4	4	2	2	4	4	1	1	2	2
1	1	2	2	4	4	1	1	2	2
4	4	2	2	4	4	1	1	2	2
1	1	2	2	1	1	1	1	2	2
4	4	3	3	4	4	1	1	2	2
1	1	3	3	1	1	2	2	2	2
4	4	2	2	4	4	1	1	2	2
4	4	3	3	4	4	1	1	2	2
4	4	2	2	4	4	1	1	2	2
4	4	2	2	4	4	1	1	2	2
4	4	2	2	4	4	1	1	2	2
1	1	3	3	1	1	1	1	2	2
4	4	2	2	4	4	1	1	2	2
4	4	3	3	4	4	1	1	2	2
4	4	3	3	4	4	1	1	2	2
4	4	2	2	4	4	1	1	2	2
4	4	3	3	4	4	1	1	2	2
1	1	3	3	4	4	1	1	2	2
1	1	3	3	1	1	1	1	2	2
4	4	2	2	4	4	2	2	2	2
1	1	3	3	4	4	1	1	2	2
1	1	3	3	4	4	1	1	2	2
4	4	3	3	4	4	1	1	2	2
4	4	2	2	4	4	1	1	2	2
4	4	3	3	4	4	2	2	3	3
1	1	3	3	1	1	1	1	2	2
4	4	3	3	4	4	1	1	2	2
4	4	3	3	4	4	1	1	2	2
4	4	2	2	4	4	1	1	2	2
4	4	3	3	4	4	1	1	2	2
4	4	3	3	4	4	1	1	2	2
4	4	3	3	4	4	1	1	2	2
1	1	2	2	4	4	1	1	2	2
1	1	2	2	4	4	1	1	2	2
1	1	3	3	1	1	1	1	2	2
1	1	3	3	1	1	2	2	3	3
4	4	3	3	4	4	1	1	2	2
1	1	3	3	1	1	1	1	2	2
1	1	3	3	1	1	1	1	2	2
4	4	2	2	4	4	2	2	2	2
1	1	3	3	1	1	1	1	2	2
4	4	2	2	4	4	1	1	2	2
4	4	3	3	4	4	1	1	2	2
4	4	3	3	4	4	2	2	2	2
4	4	2	2	4	4	1	1	2	2
4	4	2	2	4	4	1	1	2	2
1	1	3	3	1	1	1	1	2	2
4	4	3	3	4	4	1	1	2	2
4	4	2	2	4	4	1	1	2	2
4	4	3	3	4	4	1	1	2	2

For Review Only

4	4	2	2	4	4	1	1	2	2
4	4	3	3	4	4	1	1	2	2
1	1	3	3	4	4	1	1	2	2
1	1	3	3	1	1	1	1	2	2
1	1	2	2	4	4	1	1	2	2
1	1	3	3	1	1	2	2	3	3
1	1	2	2	4	4	1	1	2	2
4	4	2	2	4	4	1	1	2	2
4	4	3	3	4	4	1	1	2	2
1	1	3	3	4	4	1	1	2	2
4	4	2	2	4	4	1	1	2	2
1	1	3	3	1	1	2	2	2	2
4	4	2	2	4	4	1	1	2	2
4	4	3	3	4	4	1	1	2	2
4	4	3	3	4	4	1	1	2	2
1	1	3	3	1	1	1	1	2	2
4	4	3	3	4	4	1	1	2	2
4	4	3	3	4	4	2	2	3	3
4	4	3	3	4	4	1	1	2	2
1	1	2	2	4	4	1	1	2	2
4	4	3	3	4	4	1	1	2	2
4	4	3	3	4	4	1	1	2	2
1	1	3	3	4	4	2	2	3	3
1	1	2	2	4	4	1	1	2	2
4	4	3	3	4	4	2	2	2	2
4	4	3	3	4	4	1	1	2	2
4	4	2	2	4	4	1	1	2	2
4	4	3	3	4	4	1	1	2	2
1	1	3	3	1	1	1	1	2	2
4	4	3	3	4	4	2	2	3	3
4	4	3	3	4	4	2	2	3	3
4	4	3	3	4	4	1	1	2	2
1	1	3	3	4	4	1	1	2	2
4	4	3	3	4	4	1	1	2	2
1	1	3	3	4	4	1	1	2	2
1	1	2	2	4	4	1	1	2	2
4	4	3	3	4	4	2	2	3	3
4	4	3	3	4	4	2	2	3	3
4	4	3	3	4	4	1	1	2	2
1	1	3	3	4	4	1	1	2	2
1	1	2	2	4	4	1	1	2	2
4	4	2	2	4	4	1	1	2	2
1	1	3	3	1	1	1	1	2	2
4	4	3	3	4	4	1	1	2	2
4	4	2	2	4	4	1	1	2	2
1	1	3	3	1	1	1	1	2	2
1	1	3	3	1	1	1	1	2	2
1	1	3	3	4	4	1	1	2	2
4	4	3	3	4	4	1	1	2	2
4	4	3	3	4	4	1	1	2	2
4	4	2	2	4	4	1	1	2	2
4	4	3	3	4	4	1	1	2	2
1	1	3	3	1	1	2	2	3	3
1	1	2	2	4	4	1	1	2	2
1	1	3	3	1	1	1	1	2	2

For Review Only

4	4	3	3	4	4	1	1	2	2
1	1	3	3	1	1	1	1	2	2
1	1	2	2	4	4	1	1	2	2
4	4	3	3	4	4	2	2	2	2
1	1	3	3	1	1	1	1	2	2
4	4	3	3	4	4	1	1	2	2
1	1	3	3	4	4	1	1	2	2
1	1	3	3	1	1	1	1	2	2
4	4	3	3	4	4	1	1	2	2
1	1	3	3	1	1	1	1	2	2
4	4	2	2	4	4	1	1	2	2
4	4	3	3	4	4	2	2	3	3
4	4	3	3	4	4	1	1	2	2
4	4	3	3	4	4	1	1	2	2
1	1	2	2	4	4	1	1	2	2
1	1	3	3	1	1	2	2	3	3
1	1	3	3	1	1	2	2	3	3
4	4	3	3	4	4	1	1	2	2
1	1	3	3	4	4	1	1	2	2
4	4	3	3	4	4	1	1	2	2
1	1	3	3	1	1	1	1	2	2
4	4	2	2	4	4	1	1	2	2
4	4	3	3	4	4	1	1	2	2
1	1	3	3	1	1	2	2	3	3
1	1	3	3	1	1	1	1	2	2
4	4	3	3	4	4	1	1	2	2
1	1	3	3	1	1	1	1	2	2
4	4	2	2	4	4	1	1	2	2
4	4	3	3	4	4	1	1	2	2
4	4	2	2	4	4	1	1	2	2
1	1	3	3	4	4	1	1	2	2
4	4	3	3	4	4	1	1	2	2
4	4	3	3	4	4	1	1	2	2
4	4	3	3	4	4	1	1	2	2
1	1	3	3	4	4	2	2	3	3
1	1	3	3	4	4	1	1	2	2
4	4	3	3	4	4	1	1	2	2
1	1	2	2	4	4	1	1	2	2
4	4	3	3	4	4	1	1	2	2
1	1	3	3	1	1	1	1	2	2
1	1	3	3	4	4	1	1	2	2
4	4	3	3	4	4	1	1	2	2
1	1	2	2	1	1	1	1	2	2
4	4	3	3	4	4	1	1	2	2
1	1	2	2	4	4	1	1	2	2
4	4	3	3	4	4	1	1	2	2
4	4	3	3	4	4	1	1	2	2
1	1	2	2	1	1	1	1	2	2
1	1	3	3	4	4	1	1	2	2
1	1	3	3	1	1	1	1	2	2
4	4	3	3	4	4	1	1	2	2
1	1	3	3	1	1	1	1	2	2
1	1	2	2	4	4	1	1	2	2
1	1	2	2	4	4	1	1	2	2
1	1	3	3	1	1	2	2	3	3

For Review Only

4	4	3	3	4	4	1	1	2	2
1	1	3	3	4	4	1	1	2	2

For Review Only

SNP270	SNP270	SNP2700	SNP2700	SNP2701	SNP2701	SNP2702	SNP2702	SNP2703	SNP2703
2	2	3	3	1	1	1	1	2	2
2	2	3	3	4	4	1	1	2	2
2	2	3	3	4	4	1	1	2	2
2	2	3	3	4	4	1	1	2	2
3	3	3	3	4	4	3	3	4	4
2	2	3	3	4	4	1	1	2	2
2	2	3	3	4	4	1	1	2	2
2	2	3	3	4	4	3	3	4	4
2	2	3	3	4	4	1	1	2	2
3	3	3	3	4	4	1	1	2	2
2	2	3	3	4	4	1	1	2	2
2	2	3	3	4	4	1	1	2	2
2	2	3	3	1	1	1	1	2	2
2	2	3	3	4	4	1	1	2	2
2	2	3	3	4	4	1	1	2	2
3	3	3	3	4	4	1	1	2	2
2	2	3	3	4	4	1	1	2	2
2	2	3	3	4	4	3	3	4	4
2	2	2	2	4	4	1	1	2	2
3	3	3	3	4	4	1	1	2	2
3	3	3	3	4	4	1	1	2	2
3	3	3	3	4	4	1	1	2	2
2	2	3	3	4	4	3	3	2	2
3	3	3	3	1	1	3	3	4	4
2	2	3	3	4	4	1	1	2	2
2	2	3	3	4	4	1	1	2	2
2	2	3	3	4	4	1	1	2	2
2	2	3	3	4	4	1	1	2	2
3	3	3	3	4	4	1	1	2	2
3	3	3	3	4	4	3	3	4	4
2	2	3	3	4	4	1	1	2	2
2	2	3	3	4	4	1	1	2	2
2	2	3	3	4	4	1	1	2	2
3	3	3	3	4	4	3	3	4	4
2	2	3	3	4	4	1	1	2	2
2	2	3	3	4	4	1	1	2	2
2	2	3	3	4	4	1	1	2	2
2	2	3	3	1	1	1	1	2	2
3	3	3	3	4	4	3	3	2	2
2	2	3	3	4	4	1	1	2	2
3	3	3	3	4	4	3	3	4	4
2	2	3	3	4	4	1	1	2	2
3	3	3	3	4	4	3	3	4	4
2	2	3	3	4	4	1	1	2	2
2	2	3	3	4	4	3	3	4	4
2	2	3	3	4	4	1	1	2	2
2	2	3	3	4	4	1	1	2	2
2	2	3	3	4	4	3	3	4	4
3	3	3	3	4	4	3	3	2	2
2	2	3	3	4	4	1	1	2	2
2	2	3	3	4	4	1	1	2	2
2	2	3	3	4	4	1	1	2	2

For Review Only

2	2	3	3	4	4	1	1	2	2
2	2	3	3	4	4	1	1	2	2
2	2	2	2	4	4	1	1	2	2
2	2	3	3	4	4	1	1	2	2
2	2	2	2	4	4	1	1	2	2
2	2	3	3	4	4	1	1	2	2
2	2	3	3	4	4	1	1	2	2
3	3	3	3	4	4	3	3	4	4
2	2	3	3	4	4	1	1	2	2
2	2	3	3	4	4	1	1	2	2
2	2	2	2	4	4	1	1	2	2
2	2	2	2	4	4	1	1	2	2
2	2	3	3	4	4	1	1	2	2
3	3	3	3	4	4	1	1	2	2
2	2	3	3	4	4	1	1	2	2
2	2	3	3	4	4	3	3	4	4
2	2	3	3	4	4	1	1	2	2
2	2	3	3	4	4	1	1	2	2
2	2	3	3	4	4	1	1	2	2
2	2	3	3	4	4	1	1	2	2
2	2	2	2	4	4	1	1	2	2
3	3	3	3	4	4	1	1	2	2
3	3	3	3	4	4	1	1	2	2
3	3	3	3	4	4	1	1	2	2
2	2	3	3	4	4	3	3	4	4
2	2	3	3	4	4	1	1	2	2
2	2	3	3	4	4	3	3	4	4
3	3	3	3	4	4	3	3	4	4
2	2	3	3	4	4	1	1	2	2
2	2	3	3	4	4	1	1	2	2
2	2	3	3	4	4	1	1	2	2
3	3	3	3	4	4	3	3	4	4
3	3	3	3	4	4	3	3	4	4
2	2	3	3	4	4	1	1	2	2
2	2	3	3	4	4	1	1	2	2
2	2	3	3	4	4	3	3	4	4
2	2	3	3	4	4	3	3	4	4
3	3	3	3	4	4	1	1	2	2
2	2	3	3	4	4	1	1	2	2
2	2	3	3	4	4	3	3	4	4
2	2	3	3	4	4	1	1	2	2
2	2	3	3	4	4	1	1	2	2
2	2	3	3	4	4	1	1	2	2
2	2	2	2	4	4	1	1	2	2
2	2	3	3	1	1	1	1	2	2
2	2	3	3	4	4	1	1	2	2
3	3	3	3	4	4	1	1	2	2
2	2	3	3	4	4	1	1	2	2
2	2	3	3	4	4	1	1	2	2
2	2	3	3	4	4	1	1	2	2
2	2	3	3	4	4	1	1	2	2
3	3	3	3	4	4	3	3	4	4
3	3	3	3	1	1	3	3	4	4
2	2	3	3	4	4	1	1	2	2
2	2	3	3	4	4	3	3	4	4

For Review Only

2	2	3	3	4	4	1	1	2	2
2	2	3	3	4	4	1	1	2	2
2	2	3	3	4	4	1	1	2	2
3	3	3	3	4	4	1	1	2	2
2	2	3	3	4	4	1	1	2	2
2	2	3	3	4	4	1	1	2	2
2	2	3	3	4	4	1	1	2	2
2	2	3	3	4	4	1	1	2	2
3	3	3	3	4	4	1	1	2	2
3	3	3	3	4	4	1	1	2	2
2	2	3	3	1	1	1	1	2	2
2	2	3	3	1	1	1	1	2	2
2	2	3	3	4	4	1	1	2	2
2	2	3	3	4	4	1	1	2	2
2	2	3	3	4	4	1	1	2	2
2	2	3	3	4	4	1	1	2	2
2	2	3	3	4	4	1	1	2	2
2	2	3	3	4	4	1	1	2	2
3	3	3	3	4	4	3	3	4	4
2	2	3	3	4	4	1	1	2	2
2	2	3	3	4	4	1	1	2	2
2	2	3	3	4	4	1	1	2	2
2	2	3	3	4	4	1	1	2	2
3	3	3	3	1	1	3	3	4	4
2	2	3	3	4	4	1	1	2	2
2	2	3	3	4	4	1	1	2	2
2	2	3	3	4	4	1	1	2	2
2	2	3	3	4	4	1	1	2	2
2	2	3	3	4	4	1	1	2	2
2	2	3	3	4	4	1	1	2	2
3	3	3	3	4	4	1	1	2	2
2	2	3	3	4	4	1	1	2	2
2	2	3	3	4	4	1	1	2	2
2	2	3	3	4	4	1	1	2	2
3	3	3	3	4	4	3	3	4	4
2	2	3	3	4	4	1	1	2	2
3	3	3	3	4	4	1	1	2	2
2	2	3	3	4	4	1	1	2	2
2	2	3	3	4	4	1	1	4	4
3	3	3	3	4	4	1	1	2	2
2	2	3	3	4	4	1	1	2	2
3	3	3	3	4	4	1	1	2	2
2	2	3	3	4	4	3	3	4	4
2	2	3	3	4	4	1	1	4	4
2	2	3	3	4	4	1	1	2	2
2	2	3	3	4	4	1	1	2	2
2	2	3	3	4	4	1	1	2	2
2	2	3	3	4	4	1	1	2	2
3	3	3	3	4	4	1	1	2	2
2	2	3	3	4	4	1	1	2	2
2	2	3	3	4	4	1	1	2	2
3	3	3	3	4	4	3	3	2	2
2	2	3	3	4	4	1	1	2	2
2	2	3	3	4	4	1	1	2	2
2	2	3	3	4	4	1	1	4	4

2	2	3	3	4	4	1	1	2	2
2	2	3	3	4	4	1	1	2	2
3	3	3	3	4	4	3	3	4	4
3	3	3	3	4	4	1	1	2	2
2	2	2	2	4	4	1	1	2	2
3	3	3	3	4	4	1	1	2	2
2	2	3	3	4	4	3	3	4	4
2	2	3	3	4	4	1	1	2	2
2	2	3	3	4	4	1	1	2	2
2	2	3	3	4	4	1	1	2	2
2	2	3	3	4	4	1	1	2	2
3	3	3	3	4	4	1	1	2	2
2	2	3	3	4	4	1	1	2	2
2	2	3	3	4	4	1	1	2	2
3	3	3	3	4	4	3	3	2	2
2	2	3	3	4	4	1	1	2	2
2	2	3	3	4	4	1	1	2	2
2	2	3	3	4	4	1	1	2	2
2	2	3	3	4	4	1	1	2	2
2	2	3	3	4	4	1	1	2	2
2	2	3	3	4	4	1	1	2	2
3	3	3	3	4	4	1	1	2	2
3	3	3	3	4	4	1	1	2	2
2	2	2	2	4	4	1	1	2	2
2	2	3	3	1	1	3	3	4	4
3	3	3	3	4	4	3	3	4	4
2	2	3	3	4	4	3	3	4	4
2	2	3	3	4	4	1	1	2	2
2	2	3	3	4	4	1	1	2	2
2	2	3	3	4	4	1	1	2	2
2	2	3	3	4	4	1	1	2	2
2	2	3	3	4	4	1	1	2	2
2	2	3	3	4	4	1	1	2	2
3	3	3	3	4	4	3	3	2	2
2	2	3	3	4	4	1	1	2	2
2	2	2	2	4	4	1	1	2	2
2	2	3	3	4	4	1	1	2	2
2	2	3	3	4	4	1	1	2	2
2	2	3	3	4	4	1	1	2	2
2	2	2	2	4	4	1	1	2	2
2	2	3	3	4	4	1	1	2	2
2	2	3	3	4	4	1	1	2	2
3	3	3	3	4	4	3	3	2	2
2	2	3	3	4	4	1	1	2	2
2	2	3	3	4	4	1	1	2	2
3	3	3	3	4	4	1	1	2	2
2	2	3	3	4	4	1	1	2	2
3	3	3	3	4	4	3	3	4	4
3	3	3	3	4	4	1	1	2	2
2	2	3	3	4	4	1	1	2	2
2	2	3	3	4	4	1	1	2	2
3	3	3	3	4	4	3	3	4	4
3	3	3	3	4	4	1	1	2	2
2	2	2	2	4	4	1	1	2	2
3	3	3	3	4	4	1	1	2	2

For Review Only

2	2	3	3	4	4	1	1	2	2
2	2	3	3	4	4	1	1	2	2
2	2	3	3	4	4	1	1	2	2
2	2	3	3	4	4	1	1	2	2
2	2	3	3	4	4	1	1	2	2
2	2	3	3	4	4	1	1	2	2
3	3	3	3	4	4	1	1	2	2
3	3	3	3	4	4	3	3	4	4
2	2	3	3	4	4	1	1	2	2
3	3	3	3	4	4	3	3	2	2
2	2	3	3	4	4	1	1	2	2
2	2	3	3	4	4	1	1	2	2
2	2	3	3	4	4	1	1	2	2
2	2	3	3	4	4	1	1	2	2
2	2	3	3	4	4	1	1	2	2
2	2	3	3	4	4	1	1	2	2
3	3	3	3	4	4	1	1	2	2
2	2	3	3	4	4	3	3	4	4
3	3	3	3	4	4	1	1	2	2
2	2	3	3	1	1	1	1	2	2
3	3	3	3	4	4	1	1	2	2
2	2	3	3	4	4	1	1	2	2
2	2	3	3	4	4	1	1	2	2
2	2	3	3	4	4	3	3	4	4
3	3	3	3	4	4	3	3	4	4
2	2	3	3	1	1	1	1	2	2
3	3	3	3	4	4	3	3	4	4
2	2	3	3	4	4	1	1	2	2
2	2	3	3	4	4	1	1	2	2
2	2	3	3	4	4	1	1	2	2
2	2	3	3	4	4	1	1	2	2
2	2	3	3	4	4	1	1	2	2
2	2	3	3	4	4	3	3	4	4
2	2	3	3	4	4	1	1	2	2
3	3	3	3	4	4	1	1	2	2
3	3	3	3	4	4	1	1	2	2
2	2	3	3	4	4	1	1	2	2
2	2	2	2	4	4	1	1	2	2
2	2	3	3	4	4	1	1	2	2
3	3	3	3	4	4	3	3	4	4
2	2	3	3	4	4	1	1	2	2
3	3	3	3	4	4	1	1	2	2
2	2	3	3	4	4	3	3	4	4
2	2	3	3	4	4	1	1	2	2
2	2	3	3	4	4	1	1	2	2
2	2	3	3	4	4	1	1	2	2
2	2	3	3	4	4	1	1	2	2
2	2	3	3	4	4	1	1	2	2
2	2	3	3	4	4	3	3	4	4
2	2	3	3	1	1	3	3	4	4
2	2	3	3	4	4	1	1	2	2
3	3	3	3	4	4	3	3	2	2
2	2	3	3	4	4	1	1	2	2
2	2	3	3	4	4	1	1	2	2
2	2	3	3	4	4	1	1	2	2

For Review Only

2	2	3	3	4	4	1	1	2	2
2	2	3	3	4	4	1	1	2	2

For Review Only

SNP2704	SNP2704	SNP2705	SNP2705	SNP2706	SNP2706	SNP2707	SNP2707	SNP2708	SNP2708
3	3	1	1	1	1	1	1	1	1
1	1	1	1	1	1	1	1	1	1
1	1	4	4	1	1	1	1	1	1
1	1	1	1	1	1	1	1	1	1
1	1	1	1	3	3	1	1	1	1
3	3	4	4	1	1	1	1	1	1
3	3	1	1	1	1	1	1	1	1
1	1	1	1	1	1	4	4	1	1
3	3	1	1	1	1	1	1	1	1
1	1	1	1	3	3	4	4	1	1
1	1	1	1	1	1	1	1	4	4
1	1	4	4	1	1	1	1	1	1
1	1	4	4	1	1	1	1	1	1
1	1	1	1	3	3	1	1	1	1
1	1	4	4	1	1	1	1	1	1
1	1	4	4	1	1	1	1	1	1
1	1	1	1	1	1	1	1	1	1
3	3	1	1	3	3	4	4	1	1
1	1	4	4	1	1	1	1	1	1
3	3	1	1	1	1	4	4	1	1
3	3	1	1	1	1	1	1	1	1
1	1	1	1	1	1	1	1	1	1
1	1	1	1	3	3	1	1	1	1
1	1	1	1	3	3	1	1	1	1
1	1	1	1	1	1	1	1	1	1
1	1	4	4	1	1	1	1	1	1
1	1	4	4	1	1	1	1	1	1
1	1	1	1	1	1	1	1	1	1
3	3	1	1	1	1	4	4	1	1
1	1	1	1	1	1	1	1	1	1
1	1	4	4	1	1	4	4	1	1
1	1	1	1	1	1	1	1	1	1
1	1	1	1	1	1	1	1	1	1
1	1	1	1	3	3	1	1	1	1
1	1	1	1	1	1	1	1	1	1
1	1	1	1	1	1	1	1	1	1
3	3	1	1	1	1	1	1	4	4
1	1	1	1	1	1	4	4	1	1
1	1	1	1	1	1	1	1	1	1
1	1	1	1	1	1	1	1	4	4
1	1	1	1	1	1	1	1	1	1
3	3	1	1	1	1	1	1	1	1
1	1	1	1	1	1	4	4	1	1
1	1	1	1	1	1	1	1	4	4
1	1	1	1	1	1	4	4	1	1
1	1	4	4	1	1	1	1	1	1
1	1	4	4	1	1	1	1	1	1
1	1	1	1	1	1	1	1	1	1
1	1	1	1	3	3	1	1	1	1
1	1	1	1	1	1	1	1	1	1
1	1	4	4	1	1	1	1	1	1
1	1	4	4	1	1	1	1	1	1

For Review Only

1	1	1	1	1	1	1	1	1	1
1	1	1	1	1	1	1	1	1	1
1	1	1	1	1	1	1	1	1	1
1	1	1	1	1	1	1	1	1	1
1	1	4	4	1	1	1	1	4	4
1	1	4	4	1	1	1	1	1	1
1	1	1	1	1	1	4	4	1	1
1	1	1	1	3	3	1	1	4	4
3	3	1	1	1	1	1	1	1	1
1	1	1	1	1	1	1	1	1	1
1	1	4	4	1	1	1	1	1	1
1	1	4	4	1	1	1	1	1	1
1	1	1	1	1	1	1	1	1	1
3	3	1	1	1	1	4	4	1	1
3	3	1	1	1	1	1	1	1	1
1	1	1	1	1	1	1	1	1	1
1	1	1	1	3	3	1	1	1	1
1	1	4	4	1	1	4	4	1	1
1	1	1	1	1	1	1	1	1	1
3	3	1	1	1	1	1	1	1	1
1	1	1	1	1	1	1	1	1	1
3	3	1	1	1	1	1	1	1	1
3	3	1	1	1	1	1	1	1	1
1	1	1	1	1	1	1	1	1	1
1	1	1	1	1	1	4	4	1	1
1	1	4	4	1	1	1	1	1	1
1	1	1	1	1	1	1	1	1	1
1	1	1	1	3	3	1	1	4	4
1	1	4	4	1	1	1	1	1	1
1	1	4	4	1	1	1	1	1	1
1	1	1	1	1	1	4	4	1	1
1	1	4	4	3	3	4	4	1	1
1	1	1	1	1	1	1	1	1	1
1	1	1	1	1	1	1	1	1	1
3	3	1	1	1	1	1	1	1	1
1	1	1	1	1	1	4	4	1	1
1	1	1	1	1	1	4	4	1	1
1	1	1	1	1	1	1	1	4	4
1	1	1	1	1	1	1	1	1	1
1	1	1	1	3	3	1	1	1	1
1	1	1	1	1	1	1	1	1	1
1	1	1	1	1	1	1	1	1	1
1	1	1	1	1	1	1	1	1	1
1	1	4	4	1	1	1	1	1	1
1	1	1	1	1	1	4	4	1	1
1	1	1	1	1	1	1	1	1	1
1	1	1	1	1	1	1	1	1	1
1	1	1	1	1	1	1	1	1	1
1	1	4	4	1	1	1	1	1	1
1	1	4	4	1	1	1	1	1	1
1	1	4	4	1	1	1	1	1	1
1	1	1	1	1	1	1	1	4	4
1	1	1	1	1	1	4	4	1	1
1	1	1	1	1	1	1	1	1	1
1	1	1	1	1	1	1	1	1	1

For Review Only

1	1	4	4	1	1	1	1	1	1
3	3	4	4	1	1	1	1	1	1
1	1	4	4	1	1	1	1	1	1
1	1	1	1	1	1	1	1	1	1
1	1	4	4	1	1	4	4	1	1
1	1	1	1	1	1	1	1	1	1
1	1	4	4	1	1	4	4	1	1
1	1	1	1	1	1	1	1	1	1
1	1	4	4	1	1	1	1	1	1
1	1	1	1	1	1	1	1	1	1
3	3	4	4	1	1	1	1	1	1
1	1	1	1	1	1	1	1	1	1
1	1	1	1	1	1	1	1	1	1
1	1	1	1	1	1	1	1	1	1
1	1	1	1	1	1	1	1	1	1
1	1	1	1	1	1	1	1	1	1
1	1	1	1	1	1	1	1	1	1
1	1	1	1	1	1	1	1	1	1
1	1	1	1	1	1	1	1	1	1
1	1	1	1	1	1	1	1	1	1
1	1	1	1	1	1	1	1	1	1
1	1	4	4	1	1	1	1	1	1
1	1	4	4	1	1	1	1	1	1
1	1	4	4	3	3	1	1	4	4
1	1	1	1	1	1	1	1	1	1
1	1	1	1	1	1	1	1	4	4
1	1	1	1	1	1	1	1	1	1
1	1	1	1	1	1	1	1	1	1
1	1	4	4	1	1	4	4	1	1
1	1	1	1	1	1	1	1	1	1
1	1	1	1	3	3	1	1	4	4
1	1	1	1	1	1	1	1	1	1
1	1	1	1	1	1	1	1	1	1
1	1	1	1	1	1	1	1	1	1
1	1	1	1	3	3	1	1	1	1
1	1	1	1	3	3	1	1	1	1
1	1	1	1	1	1	1	1	1	1
1	1	4	4	1	1	1	1	1	1
1	1	4	4	1	1	1	1	1	1
1	1	1	1	1	1	1	1	1	1
3	3	1	1	1	1	1	1	1	1
1	1	1	1	1	1	1	1	1	1
1	1	4	4	1	1	4	4	1	1
1	1	1	1	1	1	1	1	1	1
1	1	1	1	1	1	1	1	1	1
1	1	1	1	1	1	1	1	1	1
1	1	4	4	1	1	1	1	1	1
1	1	4	4	1	1	1	1	1	1
1	1	1	1	1	1	1	1	4	4
1	1	4	4	1	1	1	1	1	1
1	1	4	4	1	1	1	1	1	1
1	1	1	1	1	1	1	1	4	4
1	1	1	1	1	1	1	1	1	1
1	1	1	1	1	1	1	1	1	1
1	1	1	1	1	1	1	1	4	4

For Review Only

1	1	4	4	1	1	1	1	1	1
1	1	1	1	1	1	1	1	1	1
1	1	1	1	1	1	4	4	1	1
1	1	1	1	1	1	1	1	1	1
1	1	4	4	1	1	4	4	1	1
3	3	1	1	1	1	1	1	4	4
1	1	4	4	1	1	1	1	1	1
1	1	1	1	1	1	1	1	1	1
1	1	4	4	1	1	1	1	4	4
1	1	4	4	1	1	1	1	1	1
1	1	1	1	1	1	1	1	1	1
3	3	1	1	1	1	1	1	1	1
1	1	1	1	1	1	4	4	1	1
1	1	1	1	1	1	1	1	1	1
1	1	1	1	1	1	1	1	1	1
1	1	1	1	1	1	1	1	1	1
1	1	1	1	1	1	1	1	1	1
1	1	1	1	1	1	1	1	1	1
1	1	1	1	1	1	1	1	1	1
1	1	4	4	1	1	4	4	1	1
1	1	1	1	1	1	1	1	1	1
1	1	1	1	1	1	1	1	1	1
3	3	1	1	1	1	1	1	1	1
1	1	4	4	1	1	1	1	1	1
3	3	1	1	3	3	1	1	1	1
1	1	1	1	3	3	1	1	1	1
1	1	1	1	1	1	1	1	1	1
1	1	1	1	1	1	1	1	1	1
1	1	1	1	1	1	1	1	1	1
1	1	4	4	1	1	4	4	1	1
3	3	1	1	1	1	1	1	1	1
1	1	4	4	1	1	1	1	1	1
1	1	1	1	1	1	1	1	1	1
1	1	1	1	1	1	1	1	1	1
1	1	1	1	1	1	1	1	1	1
1	1	4	4	1	1	1	1	1	1
3	3	1	1	1	1	1	1	1	1
3	3	1	1	1	1	1	1	1	1
1	1	4	4	1	1	1	1	1	1
1	1	1	1	3	3	1	1	1	1
1	1	4	4	1	1	1	1	1	1
1	1	4	4	1	1	1	1	1	1
1	1	1	1	3	3	1	1	1	1
1	1	1	1	1	1	1	1	1	1
1	1	1	1	1	1	1	1	1	1
1	1	1	1	1	1	1	1	1	1
1	1	1	1	1	1	1	1	1	1
1	1	1	1	1	1	1	1	1	1
1	1	1	1	1	1	1	1	1	1
1	1	1	1	1	1	1	1	1	1
1	1	1	1	1	1	1	1	1	1
1	1	1	1	1	1	1	1	4	4
3	3	1	1	1	1	1	1	1	1
1	1	4	4	1	1	1	1	1	1
1	1	4	4	1	1	1	1	4	4

For Review Only

1	1	4	4	1	1	4	4	1	1
1	1	1	1	1	1	1	1	1	1
1	1	4	4	1	1	1	1	1	1
1	1	1	1	1	1	1	1	1	1
1	1	1	1	1	1	1	1	1	1
1	1	1	1	1	1	1	1	1	1
1	1	1	1	1	1	1	1	1	1
1	1	1	1	1	1	1	1	1	1
1	1	1	1	1	1	1	1	4	4
1	1	1	1	3	3	1	1	4	4
1	1	1	1	1	1	1	1	1	1
3	3	1	1	1	1	1	1	1	1
1	1	1	1	1	1	1	1	1	1
1	1	1	1	1	1	1	1	1	1
1	1	4	4	3	3	1	1	1	1
3	3	1	1	1	1	1	1	1	1
3	3	1	1	1	1	1	1	1	1
1	1	1	1	1	1	1	1	1	1
1	1	1	1	1	1	1	1	1	1
1	1	1	1	1	1	4	4	1	1
1	1	1	1	1	1	1	1	1	1
1	1	4	4	1	1	1	1	1	1
1	1	1	1	1	1	1	1	1	1
3	3	1	1	1	1	1	1	1	1
1	1	1	1	3	3	1	1	4	4
1	1	4	4	1	1	1	1	1	1
1	1	1	1	1	1	1	1	1	1
1	1	4	4	1	1	4	4	1	1
1	1	1	1	1	1	1	1	1	1
1	1	4	4	1	1	4	4	1	1
1	1	1	1	3	3	1	1	1	1
1	1	1	1	1	1	1	1	1	1
1	1	1	1	1	1	1	1	1	1
1	1	4	4	1	1	1	1	1	1
3	3	1	1	1	1	1	1	1	1
1	1	1	1	3	3	1	1	1	1
1	1	4	4	1	1	4	4	1	1
1	1	4	4	1	1	1	1	1	1
1	1	1	1	1	1	1	1	1	1
1	1	1	1	1	1	1	1	1	1
1	1	1	1	1	1	1	1	1	1
1	1	1	1	1	1	1	1	1	1
1	1	4	4	1	1	1	1	1	1
1	1	1	1	1	1	1	1	1	1
1	1	4	4	3	3	1	1	1	1
1	1	1	1	1	1	1	1	1	1
1	1	4	4	3	3	1	1	1	1
1	1	4	4	1	1	1	1	1	1
1	1	1	1	1	1	4	4	1	1
1	1	4	4	1	1	1	1	1	1
1	1	1	1	1	1	1	1	1	1
1	1	1	1	3	3	1	1	4	4
1	1	4	4	1	1	1	1	1	1
1	1	4	4	3	3	1	1	1	1
3	3	1	1	1	1	1	1	4	4

For Review Only

1	1	1	1	1	1	1	1	1	1
1	1	4	4	1	1	1	1	1	1

For Review Only

SNP2709	SNP2709	SNP271	SNP271	SNP2710	SNP2710	SNP2711	SNP2711	SNP2712	SNP2712
3	3	4	4	3	3	2	2	3	3
3	3	4	4	3	3	1	1	1	1
3	3	4	4	2	2	2	2	1	1
3	3	4	4	2	2	2	2	1	1
3	3	4	4	2	2	2	2	1	1
3	3	4	4	3	3	2	2	3	3
3	3	4	4	3	3	2	2	3	3
3	3	4	4	3	3	2	2	1	1
3	3	4	4	3	3	2	2	1	1
3	3	4	4	3	3	2	2	1	1
4	4	1	1	2	2	2	2	1	1
3	3	4	4	2	2	2	2	1	1
3	3	4	4	3	3	2	2	1	1
3	3	1	1	3	3	2	2	1	1
3	3	4	4	2	2	2	2	1	1
3	3	4	4	3	3	2	2	1	1
3	3	4	4	2	2	2	2	1	1
3	3	4	4	3	3	2	2	3	3
4	4	4	4	2	2	2	2	1	1
3	3	1	1	3	3	2	2	3	3
3	3	4	4	3	3	2	2	3	3
3	3	4	4	2	2	2	2	1	1
4	4	4	4	2	2	2	2	1	1
3	3	1	1	3	3	2	2	1	1
4	4	4	4	2	2	2	2	1	1
3	3	4	4	3	3	2	2	1	1
3	3	4	4	2	2	2	2	1	1
3	3	4	4	2	2	2	2	1	1
3	3	4	4	3	3	2	2	3	3
3	3	4	4	2	2	2	2	1	1
3	3	4	4	2	2	2	2	1	1
3	3	4	4	3	3	2	2	1	1
3	3	4	4	3	3	2	2	1	1
3	3	4	4	3	3	2	2	1	1
3	3	4	4	3	3	2	2	1	1
3	3	4	4	3	3	2	2	1	1
4	4	4	4	3	3	1	1	1	1
3	3	4	4	2	2	2	2	1	1
3	3	1	1	2	2	2	2	1	1
3	3	1	1	2	2	2	2	1	1
3	3	4	4	3	3	2	2	1	1
3	3	4	4	3	3	2	2	3	3
3	3	1	1	3	3	2	2	3	3
4	4	4	4	2	2	1	1	1	1
3	3	4	4	3	3	2	2	1	1
3	3	4	4	2	2	1	1	3	3
3	3	4	4	3	3	2	2	1	1
3	3	4	4	2	2	2	2	1	1
3	3	1	1	2	2	2	2	1	1
3	3	4	4	2	2	2	2	1	1
3	3	4	4	3	3	2	2	3	3
3	3	4	4	2	2	2	2	1	1

For Review Only

3	3	4	4	2	2	2	2	1	1
3	3	4	4	3	3	2	2	1	1
3	3	4	4	2	2	2	2	1	1
3	3	1	1	2	2	2	2	1	1
4	4	4	4	2	2	2	2	1	1
3	3	4	4	3	3	2	2	3	3
3	3	4	4	3	3	2	2	3	3
4	4	4	4	2	2	2	2	1	1
3	3	4	4	3	3	1	1	1	1
3	3	4	4	2	2	2	2	1	1
3	3	4	4	3	3	2	2	1	1
3	3	4	4	3	3	2	2	1	1
3	3	4	4	2	2	2	2	1	1
3	3	4	4	3	3	2	2	3	3
3	3	4	4	2	2	2	2	1	1
3	3	4	4	3	3	2	2	3	3
3	3	1	1	3	3	2	2	1	1
3	3	4	4	3	3	2	2	3	3
3	3	4	4	2	2	1	1	1	1
3	3	4	4	3	3	2	2	3	3
3	3	4	4	3	3	2	2	1	1
4	4	1	1	3	3	2	2	3	3
3	3	4	4	3	3	2	2	3	3
4	4	4	4	2	2	2	2	1	1
3	3	4	4	3	3	2	2	1	1
3	3	4	4	3	3	2	2	1	1
3	3	1	1	2	2	2	2	1	1
4	4	1	1	3	3	2	2	1	1
3	3	4	4	3	3	2	2	3	3
4	4	4	4	2	2	1	1	1	1
3	3	4	4	2	2	2	2	1	1
3	3	4	4	3	3	2	2	1	1
3	3	1	1	2	2	2	2	1	1
3	3	4	4	2	2	2	2	1	1
3	3	4	4	3	3	2	2	3	3
3	3	4	4	3	3	2	2	1	1
3	3	4	4	2	2	2	2	1	1
3	3	4	4	2	2	2	2	1	1
4	4	4	4	3	3	2	2	1	1
3	3	4	4	2	2	2	2	1	1
3	3	4	4	2	2	2	2	1	1
4	4	4	4	3	3	1	1	1	1
3	3	4	4	3	3	2	2	1	1
3	3	4	4	3	3	2	2	1	1
3	3	4	4	2	2	2	2	1	1
3	3	4	4	2	2	2	2	1	1
3	3	1	1	2	2	2	2	1	1
3	3	4	4	3	3	2	2	3	3
3	3	1	1	2	2	2	2	1	1
3	3	4	4	3	3	2	2	1	1
3	3	4	4	3	3	2	2	1	1
4	4	1	1	2	2	2	2	1	1
3	3	1	1	2	2	2	2	1	1
3	3	4	4	2	2	2	2	1	1
3	3	4	4	3	3	2	2	1	1

For Review Only

3	3	1	1	3	3	2	2	3	3
3	3	4	4	3	3	2	2	1	1
3	3	4	4	3	3	2	2	3	3
3	3	1	1	2	2	2	2	1	1
4	4	4	4	3	3	1	1	1	1
3	3	4	4	3	3	2	2	3	3
3	3	4	4	2	2	2	2	1	1
3	3	4	4	3	3	2	2	1	1
3	3	4	4	2	2	2	2	1	1
4	4	4	4	2	2	2	2	1	1
3	3	4	4	3	3	2	2	3	3
3	3	4	4	2	2	2	2	1	1
3	3	4	4	2	2	2	2	1	1
3	3	4	4	2	2	2	2	1	1
3	3	4	4	2	2	2	2	1	1
3	3	4	4	2	2	2	2	1	1
3	3	4	4	3	3	2	2	1	1
3	3	4	4	2	2	2	2	1	1
3	3	1	1	2	2	2	2	1	1
4	4	4	4	2	2	2	2	1	1
3	3	4	4	2	2	2	2	1	1
4	4	4	4	2	2	1	1	1	1
3	3	4	4	3	3	2	2	1	1
4	4	1	1	2	2	2	2	1	1
3	3	4	4	3	3	2	2	3	3
4	4	4	4	3	3	2	2	3	3
3	3	4	4	3	3	2	2	1	1
3	3	4	4	3	3	2	2	1	1
3	3	4	4	2	2	2	2	1	1
3	3	1	1	3	3	2	2	1	1
3	3	1	1	3	3	2	2	1	1
3	3	4	4	2	2	2	2	1	1
3	3	4	4	2	2	2	2	1	1
3	3	4	4	2	2	2	2	1	1
3	3	4	4	3	3	2	2	1	1
3	3	4	4	2	2	2	2	1	1
3	3	1	1	2	2	2	2	1	1
3	3	4	4	3	3	2	2	3	3
3	3	4	4	3	3	2	2	1	1
3	3	1	1	2	2	2	2	1	1
4	4	4	4	3	3	2	2	3	3
3	3	1	1	3	3	2	2	1	1
3	3	4	4	3	3	2	2	3	3
3	3	4	4	2	2	2	2	1	1
3	3	4	4	3	3	2	2	3	3
4	4	4	4	3	3	2	2	1	1
3	3	4	4	3	3	2	2	3	3
3	3	4	4	3	3	2	2	3	3
3	3	4	4	2	2	2	2	1	1
3	3	4	4	3	3	2	2	3	3
3	3	4	4	3	3	2	2	3	3
4	4	1	1	3	3	2	2	3	3
3	3	4	4	2	2	2	2	1	1
3	3	4	4	3	3	2	2	1	1
4	4	4	4	2	2	2	2	1	1

For Review Only

3	3	4	4	3	3	2	2	3	3
3	3	1	1	2	2	2	2	1	1
3	3	4	4	3	3	2	2	1	1
4	4	4	4	2	2	2	2	1	1
3	3	1	1	3	3	2	2	1	1
4	4	4	4	3	3	2	2	3	3
3	3	1	1	2	2	2	2	1	1
3	3	4	4	3	3	2	2	1	1
4	4	1	1	2	2	2	2	1	1
3	3	4	4	2	2	1	1	1	1
3	3	4	4	3	3	2	2	1	1
3	3	4	4	3	3	2	2	1	1
3	3	4	4	3	3	1	1	1	1
3	3	4	4	3	3	2	2	1	1
3	3	1	1	3	3	2	2	1	1
3	3	1	1	2	2	2	2	1	1
3	3	4	4	2	2	2	2	1	1
3	3	4	4	3	3	1	1	1	1
3	3	4	4	3	3	2	2	1	1
3	3	4	4	2	2	2	2	1	1
3	3	4	4	2	2	2	2	1	1
3	3	1	1	2	2	2	2	1	1
4	4	1	1	3	3	2	2	3	3
3	3	4	4	3	3	2	2	1	1
3	3	4	4	3	3	2	2	1	1
3	3	1	1	3	3	2	2	1	1
3	3	4	4	2	2	1	1	1	1
3	3	4	4	2	2	2	2	1	1
3	3	4	4	3	3	2	2	3	3
3	3	4	4	3	3	1	1	1	1
3	3	4	4	3	3	2	2	3	3
3	3	4	4	2	2	2	2	1	1
4	4	4	4	3	3	2	2	1	1
3	3	1	1	3	3	2	2	1	1
3	3	4	4	2	2	2	2	1	1
3	3	4	4	3	3	2	2	1	1
3	3	4	4	3	3	2	2	1	1
4	4	4	4	3	3	1	1	1	1
3	3	4	4	3	3	2	2	3	3
3	3	1	1	3	3	2	2	1	1
3	3	4	4	2	2	2	2	1	1
3	3	4	4	3	3	2	2	3	3
3	3	4	4	3	3	2	2	1	1
3	3	4	4	2	2	2	2	1	1
3	3	4	4	3	3	2	2	1	1
3	3	4	4	3	3	2	2	1	1
3	3	1	1	2	2	2	2	1	1
3	3	1	1	2	2	2	2	1	1
3	3	1	1	3	3	2	2	1	1
3	3	4	4	2	2	2	2	1	1
3	3	4	4	3	3	2	2	1	1
4	4	1	1	2	2	2	2	1	1
4	4	4	4	3	3	2	2	3	3
3	3	4	4	3	3	2	2	1	1
4	4	4	4	2	2	2	2	1	1

For Review Only

3	3	4	4	3	3	2	2	1	1
4	4	4	4	2	2	2	2	1	1
3	3	4	4	2	2	2	2	1	1
4	4	4	4	2	2	2	2	3	3
3	3	4	4	3	3	2	2	1	1
3	3	4	4	3	3	2	2	3	3
3	3	4	4	3	3	2	2	3	3
3	3	4	4	3	3	2	2	1	1
3	3	1	1	2	2	2	2	1	1
3	3	1	1	3	3	2	2	1	1
3	3	4	4	2	2	2	2	1	1
3	3	4	4	3	3	2	2	3	3
4	4	4	4	3	3	2	2	1	1
3	3	1	1	3	3	2	2	1	1
3	3	1	1	2	2	2	2	1	1
3	3	4	4	3	3	2	2	3	3
3	3	1	1	3	3	2	2	3	3
4	4	4	4	2	2	2	2	1	1
3	3	1	1	2	2	2	2	1	1
3	3	4	4	2	2	1	1	1	1
3	3	4	4	2	2	2	2	1	1
3	3	4	4	3	3	2	2	3	3
3	3	4	4	2	2	2	2	1	1
3	3	1	1	3	3	2	2	3	3
4	4	4	4	2	2	2	2	1	1
3	3	4	4	2	2	2	2	1	1
3	3	1	1	2	2	2	2	1	1
3	3	4	4	2	2	2	2	1	1
3	3	4	4	2	2	2	2	1	1
3	3	4	4	2	2	2	2	1	1
3	3	1	1	2	2	2	2	1	1
3	3	4	4	2	2	2	2	1	1
3	3	1	1	3	3	2	2	3	3
3	3	4	4	3	3	2	2	3	3
4	4	1	1	3	3	2	2	3	3
3	3	1	1	2	2	2	2	1	1
3	3	4	4	3	3	2	2	3	3
3	3	4	4	3	3	2	2	1	1
3	3	4	4	3	3	2	2	1	1
3	3	1	1	2	2	2	2	1	1
3	3	4	4	2	2	2	2	1	1
4	4	4	4	3	3	2	2	1	1
3	3	4	4	3	3	2	2	1	1
3	3	4	4	2	2	2	2	1	1
3	3	4	4	2	2	2	2	1	1
4	4	4	4	3	3	2	2	1	1
3	3	4	4	3	3	2	2	3	3
3	3	4	4	3	3	2	2	3	3
3	3	4	4	2	2	2	2	1	1
3	3	1	1	3	3	2	2	1	1
4	4	4	4	3	3	2	2	1	1
4	4	1	1	2	2	2	2	1	1
3	3	4	4	2	2	2	2	1	1
3	3	1	1	3	3	1	1	3	3
4	4	4	4	3	3	2	2	3	3

For Review Only

3	3	4	4	2	2	2	2	1	1
3	3	4	4	3	3	2	2	1	1

For Review Only

SNP2713	SNP2713	SNP2714	SNP2714	SNP2715	SNP2715	SNP2716	SNP2716	SNP2717	SNP2717
4	4	4	4	3	3	4	4	4	4
4	4	1	1	1	1	2	2	3	3
4	4	4	4	3	3	4	4	4	4
2	2	4	4	3	3	2	2	4	4
2	2	4	4	3	3	4	4	4	4
4	4	4	4	3	3	4	4	4	4
4	4	4	4	3	3	4	4	4	4
4	4	4	4	3	3	4	4	4	4
4	4	4	4	3	3	4	4	4	4
2	2	4	4	3	3	4	4	4	4
2	2	4	4	3	3	2	2	3	3
2	2	4	4	3	3	2	2	4	4
2	2	4	4	3	3	4	4	4	4
4	4	4	4	3	3	4	4	4	4
4	4	4	4	3	3	4	4	4	4
2	2	4	4	3	3	2	2	4	4
2	2	4	4	3	3	4	4	4	4
4	4	4	4	3	3	4	4	4	4
2	2	4	4	3	3	4	4	4	4
4	4	4	4	3	3	4	4	4	4
4	4	4	4	3	3	4	4	4	4
2	2	4	4	3	3	4	4	4	4
2	2	4	4	3	3	2	2	4	4
2	2	4	4	3	3	4	4	4	4
2	2	4	4	1	1	4	4	4	4
4	4	4	4	3	3	4	4	4	4
4	4	4	4	3	3	2	2	4	4
2	2	4	4	3	3	2	2	4	4
4	4	4	4	3	3	4	4	4	4
2	2	4	4	3	3	4	4	4	4
2	2	4	4	3	3	2	2	4	4
4	4	4	4	3	3	4	4	4	4
4	4	4	4	3	3	4	4	4	4
4	4	4	4	3	3	4	4	4	4
4	4	4	4	3	3	2	2	4	4
2	2	4	4	3	3	2	2	4	4
4	4	4	4	3	3	2	2	3	3
2	2	4	4	3	3	2	2	4	4
2	2	4	4	3	3	4	4	4	4
2	2	4	4	3	3	4	4	4	4
2	2	4	4	3	3	4	4	4	4
4	4	4	4	3	3	4	4	4	4
4	4	4	4	3	3	4	4	4	4
2	2	4	4	3	3	2	2	3	3
4	4	4	4	3	3	4	4	4	4
4	4	4	4	3	3	2	2	4	4
4	4	4	4	3	3	4	4	4	4
2	2	4	4	3	3	2	2	3	3
2	2	4	4	3	3	2	2	3	3
2	2	4	4	3	3	2	2	4	4
4	4	4	4	3	3	2	2	4	4
4	4	4	4	3	3	4	4	4	4

For Review Only

2	2	4	4	3	3	4	4	4	4
4	4	4	4	3	3	4	4	4	4
2	2	4	4	3	3	2	2	3	3
2	2	4	4	3	3	4	4	4	4
2	2	4	4	3	3	4	4	4	4
4	4	4	4	3	3	4	4	4	4
4	4	4	4	3	3	4	4	4	4
4	4	4	4	3	3	4	4	4	4
4	4	4	4	3	3	2	2	3	3
2	2	4	4	3	3	2	2	4	4
4	4	4	4	3	3	2	2	3	3
4	4	4	4	3	3	2	2	3	3
2	2	4	4	3	3	4	4	4	4
4	4	4	4	3	3	4	4	4	4
2	2	4	4	3	3	4	4	4	4
4	4	4	4	3	3	4	4	4	4
4	4	4	4	3	3	4	4	4	4
4	4	4	4	3	3	4	4	4	4
2	2	4	4	3	3	2	2	3	3
4	4	4	4	3	3	4	4	4	4
4	4	4	4	3	3	2	2	4	4
4	4	4	4	3	3	2	2	3	3
4	4	4	4	3	3	4	4	4	4
4	4	1	1	1	1	2	2	3	3
4	4	4	4	3	3	4	4	4	4
4	4	4	4	3	3	2	2	3	3
2	2	4	4	3	3	4	4	4	4
2	2	4	4	3	3	4	4	4	4
4	4	4	4	3	3	4	4	4	4
2	2	4	4	3	3	2	2	3	3
2	2	4	4	3	3	4	4	4	4
2	2	1	1	1	1	4	4	4	4
2	2	4	4	3	3	4	4	4	4
2	2	4	4	3	3	4	4	4	4
4	4	4	4	3	3	4	4	4	4
4	4	4	4	3	3	4	4	4	4
2	2	4	4	3	3	4	4	4	4
2	2	4	4	3	3	4	4	4	4
4	4	4	4	3	3	2	2	3	3
2	2	4	4	3	3	4	4	4	4
2	2	4	4	3	3	4	4	3	3
4	4	4	4	3	3	2	2	3	3
4	4	4	4	3	3	4	4	4	4
4	4	4	4	3	3	2	2	3	3
2	2	4	4	3	3	4	4	4	4
2	2	4	4	1	1	4	4	4	4
2	2	4	4	3	3	4	4	4	4
4	4	4	4	3	3	4	4	4	4
2	2	4	4	3	3	4	4	4	4
4	4	4	4	3	3	2	2	4	4
4	4	4	4	3	3	4	4	4	4
2	2	4	4	3	3	4	4	4	4
2	2	4	4	3	3	4	4	4	4
2	2	4	4	3	3	4	4	4	4
4	4	4	4	3	3	4	4	4	4

For Review Only

4	4	4	4	3	3	4	4	4	4
4	4	4	4	3	3	2	2	4	4
4	4	4	4	3	3	2	2	4	4
2	2	4	4	3	3	4	4	4	4
4	4	4	4	3	3	2	2	4	4
4	4	4	4	3	3	4	4	4	4
2	2	4	4	3	3	2	2	4	4
4	4	4	4	3	3	4	4	4	4
2	2	4	4	3	3	4	4	4	4
2	2	4	4	3	3	2	2	4	4
4	4	4	4	3	3	4	4	4	4
2	2	4	4	3	3	2	2	4	4
2	2	4	4	3	3	4	4	4	4
4	4	4	4	1	1	4	4	4	4
2	2	1	1	3	3	4	4	4	4
2	2	4	4	3	3	4	4	4	4
2	2	4	4	3	3	4	4	4	4
2	2	4	4	3	3	2	2	4	4
2	2	4	4	3	3	4	4	4	4
2	2	4	4	3	3	4	4	4	4
2	2	4	4	3	3	2	2	4	4
2	2	4	4	3	3	2	2	3	3
4	4	4	4	3	3	4	4	4	4
2	2	4	4	3	3	4	4	4	4
4	4	4	4	3	3	2	2	3	3
4	4	4	4	3	3	4	4	4	4
2	2	4	4	3	3	4	4	4	4
4	4	4	4	3	3	4	4	4	4
2	2	4	4	3	3	4	4	4	4
2	2	4	4	3	3	4	4	4	4
4	4	4	4	3	3	4	4	4	4
2	2	4	4	3	3	4	4	4	4
2	2	4	4	3	3	4	4	3	3
2	2	4	4	3	3	4	4	4	4
4	4	4	4	3	3	4	4	4	4
4	4	4	4	3	3	4	4	4	4
2	2	4	4	3	3	4	4	4	4
4	4	4	4	3	3	4	4	4	4
4	4	4	4	3	3	4	4	4	4
2	2	4	4	3	3	2	2	3	3
4	4	4	4	3	3	4	4	4	4
4	4	4	4	3	3	4	4	4	4
4	4	4	4	3	3	2	2	3	3
2	2	4	4	3	3	4	4	4	4
4	4	4	4	3	3	4	4	4	4
4	4	4	4	3	3	4	4	4	4
4	4	4	4	3	3	4	4	4	4
4	4	4	4	3	3	2	2	4	4
4	4	4	4	3	3	4	4	4	4
4	4	4	4	3	3	4	4	4	4
4	4	4	4	3	3	4	4	4	4
4	4	4	4	3	3	4	4	4	4
4	4	4	4	3	3	2	2	3	3
4	4	4	4	3	3	4	4	4	4
2	2	4	4	3	3	4	4	4	4

For Review Only

4	4	4	4	3	3	2	2	3	3
2	2	4	4	3	3	4	4	4	4
4	4	4	4	3	3	4	4	4	4
2	2	4	4	3	3	4	4	4	4
4	4	4	4	3	3	4	4	4	4
4	4	4	4	3	3	4	4	4	4
2	2	4	4	3	3	2	2	4	4
2	2	4	4	3	3	2	2	4	4
2	2	4	4	3	3	4	4	4	4
2	2	1	1	1	1	2	2	3	3
4	4	4	4	3	3	4	4	4	4
2	2	4	4	3	3	4	4	4	4
4	4	4	4	3	3	2	2	3	3
4	4	4	4	3	3	2	2	4	4
4	4	4	4	3	3	4	4	4	4
2	2	4	4	3	3	2	2	3	3
4	4	4	4	3	3	2	2	4	4
4	4	1	1	1	1	2	2	3	3
4	4	4	4	3	3	4	4	4	4
2	2	4	4	3	3	2	2	4	4
2	2	4	4	3	3	2	2	4	4
2	2	4	4	3	3	2	2	4	4
4	4	4	4	3	3	4	4	4	4
4	4	4	4	3	3	2	2	3	3
2	2	4	4	3	3	2	2	4	4
4	4	4	4	3	3	4	4	4	4
2	2	4	4	3	3	2	2	3	3
2	2	4	4	3	3	4	4	4	4
4	4	4	4	3	3	4	4	4	4
4	4	4	4	3	3	4	4	4	4
4	4	4	4	3	3	4	4	4	4
2	2	4	4	3	3	4	4	4	4
4	4	4	4	3	3	4	4	4	4
4	4	4	4	3	3	4	4	4	4
2	2	4	4	3	3	2	2	3	3
4	4	4	4	3	3	2	2	3	3
4	4	4	4	3	3	4	4	4	4
4	4	4	4	3	3	2	2	3	3
4	4	4	4	3	3	2	2	4	4
4	4	4	4	3	3	2	2	4	4
4	4	4	4	3	3	4	4	4	4
4	4	4	4	3	3	4	4	4	4
4	4	4	4	3	3	2	2	4	4
2	2	4	4	3	3	4	4	4	4
4	4	4	4	3	3	2	2	4	4
4	4	4	4	3	3	2	2	4	4
2	2	4	4	3	3	2	2	3	3
2	2	4	4	3	3	2	2	4	4
4	4	4	4	3	3	4	4	4	4
2	2	4	4	3	3	4	4	4	4
4	4	1	1	1	1	2	2	4	4
4	4	4	4	3	3	4	4	4	4
4	4	4	4	3	3	2	2	3	3
4	4	4	4	3	3	2	2	3	3
2	2	4	4	3	3	2	2	4	4

For Review Only

4	4	4	4	3	3	2	2	3	3
2	2	4	4	3	3	4	4	4	4
2	2	1	1	1	1	4	4	4	4
2	2	4	4	3	3	2	2	4	4
4	4	4	4	3	3	4	4	4	4
4	4	4	4	3	3	4	4	4	4
4	4	4	4	3	3	2	2	4	4
2	2	4	4	3	3	4	4	4	4
2	2	4	4	3	3	4	4	4	4
2	2	4	4	3	3	4	4	4	4
2	2	4	4	3	3	2	2	4	4
4	4	4	4	3	3	2	2	3	3
4	4	4	4	3	3	2	2	3	3
4	4	1	1	1	1	4	4	4	4
4	4	4	4	3	3	4	4	4	4
4	4	4	4	3	3	2	2	3	3
4	4	4	4	3	3	4	4	4	4
2	2	4	4	3	3	4	4	4	4
2	2	4	4	3	3	2	2	4	4
2	2	4	4	3	3	2	2	3	3
4	4	4	4	3	3	4	4	4	4
4	4	4	4	3	3	4	4	4	4
2	2	4	4	3	3	4	4	4	4
4	4	4	4	3	3	4	4	4	4
4	4	4	4	3	3	4	4	4	4
4	4	4	4	3	3	2	2	4	4
2	2	4	4	3	3	4	4	4	4
2	2	4	4	3	3	2	2	3	3
2	2	4	4	3	3	4	4	4	4
2	2	1	1	1	1	4	4	4	4
2	2	4	4	3	3	2	2	4	4
2	2	4	4	3	3	2	2	3	3
4	4	4	4	3	3	2	2	3	3
4	4	4	4	3	3	4	4	4	4
4	4	4	4	3	3	2	2	3	3
2	2	4	4	3	3	4	4	4	4
4	4	4	4	3	3	4	4	4	4
4	4	4	4	3	3	2	2	3	3
2	2	4	4	3	3	2	2	4	4
2	2	4	4	3	3	4	4	4	4
2	2	4	4	3	3	2	2	3	3
4	4	4	4	3	3	4	4	4	4
4	4	4	4	3	3	4	4	4	4
2	2	4	4	3	3	4	4	4	4
4	4	4	4	3	3	4	4	4	4
4	4	4	4	3	3	2	2	3	3
4	4	4	4	3	3	4	4	4	4
4	4	4	4	3	3	4	4	4	4
2	2	4	4	3	3	4	4	4	4
2	2	4	4	3	3	4	4	4	4
2	2	4	4	3	3	4	4	4	4
2	2	4	4	3	3	4	4	4	4
4	4	4	4	3	3	4	4	4	4
4	4	4	4	3	3	2	2	3	3
4	4	4	4	3	3	4	4	4	4

For Review Only

2	2	4	4	3	3	2	2	3	3
4	4	1	1	1	1	2	2	4	4

For Review Only

SNP2718	SNP2718	SNP2719	SNP2719	SNP272	SNP272	SNP2720	SNP2720	SNP2721	SNP2721
1	1	4	4	1	1	1	1	2	2
1	1	2	2	1	1	4	4	2	2
1	1	4	4	1	1	1	1	2	2
1	1	4	4	3	3	1	1	2	2
4	4	2	2	1	1	4	4	2	2
1	1	4	4	1	1	1	1	2	2
1	1	4	4	1	1	1	1	2	2
1	1	2	2	1	1	4	4	2	2
4	4	4	4	1	1	1	1	2	2
1	1	4	4	1	1	4	4	2	2
1	1	2	2	1	1	4	4	2	2
1	1	4	4	1	1	1	1	2	2
1	1	4	4	1	1	1	1	2	2
1	1	2	2	3	3	4	4	3	3
4	4	4	4	1	1	1	1	2	2
1	1	2	2	1	1	4	4	2	2
1	1	2	2	1	1	4	4	2	2
1	1	4	4	1	1	1	1	2	2
1	1	2	2	1	1	4	4	2	2
1	1	4	4	1	1	1	1	2	2
4	4	4	4	1	1	1	1	2	2
4	4	2	2	1	1	4	4	2	2
1	1	2	2	1	1	4	4	2	2
4	4	2	2	3	3	4	4	2	2
1	1	2	2	1	1	4	4	3	3
1	1	4	4	1	1	1	1	3	3
1	1	2	2	1	1	4	4	2	2
1	1	4	4	1	1	1	1	2	2
4	4	4	4	1	1	1	1	2	2
4	4	2	2	3	3	4	4	2	2
1	1	2	2	3	3	4	4	2	2
1	1	4	4	3	3	1	1	2	2
4	4	4	4	1	1	1	1	2	2
1	1	2	2	3	3	4	4	2	2
1	1	4	4	1	1	1	1	2	2
4	4	2	2	3	3	4	4	3	3
1	1	4	4	1	1	1	1	2	2
1	1	2	2	1	1	4	4	2	2
1	1	2	2	3	3	4	4	2	2
1	1	4	4	3	3	4	4	2	2
4	4	2	2	1	1	4	4	2	2
4	4	4	4	1	1	1	1	2	2
1	1	4	4	1	1	1	1	2	2
1	1	2	2	1	1	4	4	2	2
1	1	4	4	1	1	1	1	2	2
1	1	4	4	1	1	1	1	2	2
1	1	2	2	1	1	4	4	2	2
1	1	2	2	1	1	4	4	3	3
4	4	4	4	3	3	1	1	2	2
1	1	2	2	1	1	4	4	2	2
1	1	4	4	1	1	1	1	2	2
1	1	4	4	1	1	1	1	2	2

For Review Only

4	4	2	2	1	1	4	4	2	2
4	4	4	4	1	1	1	1	2	2
1	1	4	4	1	1	1	1	2	2
4	4	2	2	1	1	4	4	2	2
1	1	2	2	1	1	4	4	2	2
1	1	4	4	1	1	1	1	2	2
1	1	4	4	1	1	1	1	2	2
1	1	2	2	3	3	4	4	2	2
4	4	4	4	1	1	1	1	2	2
1	1	4	4	1	1	1	1	2	2
1	1	2	2	1	1	4	4	2	2
1	1	2	2	1	1	4	4	2	2
4	4	2	2	1	1	4	4	2	2
4	4	4	4	1	1	1	1	2	2
1	1	4	4	1	1	1	1	2	2
1	1	4	4	1	1	1	1	2	2
1	1	4	4	1	1	1	1	2	2
1	1	4	4	1	1	1	1	2	2
4	4	2	2	1	1	4	4	2	2
4	4	4	4	1	1	1	1	2	2
1	1	2	2	1	1	4	4	2	2
1	1	4	4	1	1	1	1	2	2
4	4	4	4	1	1	1	1	2	2
1	1	4	4	1	1	1	1	2	2
1	1	4	4	1	1	1	1	2	2
1	1	2	2	1	1	4	4	2	2
1	1	2	2	1	1	4	4	2	2
1	1	2	2	3	3	4	4	2	2
1	1	4	4	1	1	1	1	3	3
1	1	2	2	1	1	4	4	2	2
4	4	2	2	1	1	4	4	2	2
1	1	2	2	3	3	4	4	2	2
1	1	2	2	3	3	4	4	2	2
4	4	2	2	1	1	4	4	2	2
1	1	4	4	3	3	1	1	3	3
1	1	4	4	1	1	1	1	2	2
4	4	4	4	1	1	4	4	2	2
1	1	2	2	1	1	4	4	2	2
1	1	2	2	1	1	4	4	2	2
4	4	4	4	1	1	1	1	2	2
4	4	4	4	1	1	1	1	2	2
1	1	2	2	1	1	4	4	2	2
1	1	4	4	1	1	1	1	2	2
1	1	2	2	1	1	4	4	2	2
1	1	2	2	1	1	4	4	2	2
1	1	2	2	1	1	4	4	2	2
1	1	2	2	1	1	4	4	3	3
1	1	4	4	1	1	1	1	3	3
4	4	2	2	1	1	4	4	2	2
1	1	2	2	1	1	4	4	2	2
1	1	4	4	1	1	4	4	2	2
1	1	2	2	1	1	4	4	2	2
1	1	2	2	1	1	4	4	2	2
1	1	4	4	1	1	1	1	2	2
1	1	2	2	1	1	4	4	2	2

For Review Only

1	1	4	4	1	1	1	1	2	2
1	1	4	4	3	3	1	1	2	2
4	4	4	4	1	1	1	1	3	3
1	1	2	2	3	3	4	4	2	2
1	1	2	2	1	1	4	4	2	2
1	1	4	4	1	1	1	1	2	2
1	1	2	2	1	1	4	4	2	2
1	1	4	4	1	1	1	1	2	2
4	4	2	2	1	1	4	4	2	2
1	1	2	2	1	1	4	4	2	2
1	1	4	4	1	1	1	1	2	2
1	1	4	4	1	1	1	1	2	2
1	1	2	2	1	1	4	4	2	2
1	1	4	4	1	1	1	1	2	2
1	1	2	2	1	1	4	4	2	2
1	1	4	4	1	1	1	1	2	2
1	1	4	4	1	1	1	1	2	2
1	1	2	2	3	3	1	1	2	2
1	1	2	2	3	3	4	4	2	2
1	1	2	2	1	1	4	4	2	2
1	1	4	4	1	1	1	1	2	2
1	1	2	2	1	1	4	4	2	2
1	1	2	2	1	1	1	1	2	2
1	1	2	2	3	3	4	4	2	2
1	1	4	4	1	1	1	1	2	2
1	1	4	4	3	3	1	1	2	2
1	1	2	2	1	1	4	4	2	2
4	4	4	4	1	1	1	1	3	3
1	1	2	2	1	1	4	4	2	2
1	1	4	4	3	3	1	1	2	2
1	1	2	2	3	3	4	4	2	2
1	1	4	4	1	1	1	1	2	2
4	4	4	4	1	1	1	1	2	2
1	1	4	4	1	1	1	1	2	2
1	1	2	2	3	3	4	4	2	2
1	1	2	2	1	1	1	1	2	2
1	1	2	2	1	1	4	4	2	2
1	1	2	2	1	1	4	4	2	2
1	1	4	4	1	1	1	1	2	2
1	1	2	2	1	1	4	4	2	2
1	1	4	4	1	1	1	1	2	2
1	1	2	2	3	3	4	4	3	3
1	1	4	4	1	1	1	1	2	2
1	1	2	2	1	1	4	4	2	2
1	1	4	4	1	1	1	1	2	2
1	1	2	2	3	3	4	4	2	2
1	1	4	4	1	1	1	1	2	2
1	1	4	4	1	1	1	1	2	2
1	1	4	4	1	1	1	1	2	2
1	1	4	4	1	1	1	1	3	3
1	1	4	4	1	1	1	1	2	2
1	1	4	4	1	1	1	1	2	2
1	1	4	4	1	1	1	1	3	3
1	1	2	2	1	1	4	4	3	3
1	1	4	4	1	1	4	4	2	2

For Review Only

1	1	4	4	1	1	1	1	3	3
1	1	4	4	1	1	4	4	2	2
4	4	4	4	1	1	1	1	2	2
1	1	2	2	1	1	4	4	2	2
1	1	2	2	1	1	4	4	2	2
1	1	4	4	3	3	1	1	2	2
1	1	2	2	3	3	4	4	2	2
1	1	4	4	1	1	1	1	2	2
1	1	2	2	1	1	4	4	2	2
1	1	2	2	1	1	4	4	2	2
1	1	4	4	1	1	1	1	2	2
4	4	4	4	3	3	1	1	2	2
1	1	4	4	1	1	1	1	2	2
1	1	4	4	1	1	1	1	2	2
1	1	2	2	1	1	4	4	2	2
1	1	2	2	3	3	4	4	2	2
1	1	4	4	3	3	1	1	3	3
1	1	2	2	1	1	4	4	2	2
1	1	2	2	1	1	4	4	3	3
1	1	2	2	1	1	4	4	2	2
1	1	4	4	1	1	1	1	2	2
4	4	2	2	3	3	4	4	2	2
1	1	4	4	1	1	1	1	2	2
1	1	2	2	1	1	4	4	2	2
4	4	4	4	1	1	1	1	2	2
1	1	2	2	3	3	4	4	2	2
1	1	2	2	1	1	4	4	2	2
1	1	4	4	1	1	1	1	2	2
1	1	4	4	1	1	1	1	2	2
4	4	4	4	1	1	1	1	2	2
1	1	4	4	1	1	1	1	2	2
1	1	2	2	1	1	4	4	2	2
4	4	2	2	1	1	4	4	2	2
1	1	2	2	1	1	4	4	2	2
1	1	4	4	1	1	1	1	2	2
1	1	2	2	1	1	4	4	2	2
1	1	4	4	1	1	1	1	2	2
1	1	4	4	1	1	1	1	2	2
1	1	4	4	1	1	1	1	2	2
1	1	2	2	1	1	4	4	2	2
1	1	4	4	1	1	1	1	2	2
1	1	4	4	1	1	1	1	2	2
1	1	2	2	3	3	4	4	2	2
1	1	4	4	1	1	1	1	2	2
1	1	2	2	1	1	4	4	2	2
1	1	2	2	1	1	4	4	3	3
1	1	2	2	3	3	4	4	2	2
1	1	2	2	3	3	4	4	2	2
1	1	2	2	1	1	4	4	3	3
1	1	2	2	1	1	4	4	2	2
1	1	2	2	1	1	4	4	2	2
1	1	2	2	1	1	4	4	2	2
1	1	4	4	1	1	1	1	2	2
1	1	2	2	1	1	4	4	2	2
1	1	2	2	3	3	4	4	2	2

For Review Only

1	1	2	2	1	1	4	4	2	2
1	1	2	2	1	1	1	1	2	2
1	1	2	2	1	1	4	4	2	2
1	1	4	4	1	1	1	1	2	2
1	1	2	2	1	1	4	4	2	2
1	1	4	4	1	1	1	1	3	3
1	1	4	4	3	3	1	1	2	2
1	1	4	4	1	1	4	4	2	2
1	1	2	2	1	1	4	4	2	2
1	1	2	2	3	3	4	4	2	2
1	1	4	4	1	1	1	1	2	2
1	1	4	4	1	1	1	1	2	2
1	1	2	2	1	1	4	4	2	2
1	1	2	2	1	1	4	4	3	3
1	1	4	4	1	1	1	1	2	2
1	1	4	4	3	3	1	1	2	2
1	1	4	4	3	3	1	1	2	2
1	1	2	2	1	1	4	4	2	2
1	1	2	2	3	3	4	4	2	2
1	1	2	2	1	1	4	4	2	2
1	1	4	4	1	1	1	1	2	2
1	1	4	4	1	1	1	1	3	3
1	1	2	2	1	1	4	4	2	2
1	1	4	4	3	3	1	1	2	2
1	1	2	2	3	3	4	4	2	2
1	1	4	4	1	1	1	1	2	2
1	1	2	2	3	3	4	4	2	2
1	1	2	2	1	1	4	4	2	2
4	4	2	2	1	1	4	4	2	2
1	1	2	2	1	1	4	4	2	2
1	1	2	2	3	3	4	4	2	2
1	1	2	2	1	1	4	4	2	2
1	1	4	4	3	3	1	1	2	2
1	1	4	4	1	1	1	1	2	2
1	1	4	4	1	1	1	1	2	2
1	1	2	2	3	3	4	4	2	2
1	1	4	4	1	1	1	1	2	2
1	1	2	2	1	1	4	4	2	2
4	4	2	2	3	3	4	4	3	3
1	1	2	2	3	3	4	4	2	2
1	1	4	4	1	1	1	1	2	2
1	1	2	2	1	1	4	4	3	3
1	1	2	2	3	3	4	4	2	2
4	4	2	2	1	1	4	4	2	2
1	1	4	4	1	1	1	1	2	2
1	1	2	2	1	1	4	4	2	2
1	1	4	4	1	1	1	1	2	2
1	1	4	4	1	1	1	1	2	2
4	4	4	4	3	3	1	1	2	2
1	1	2	2	3	3	4	4	2	2
1	1	4	4	1	1	1	1	2	2
1	1	2	2	3	3	4	4	2	2
1	1	4	4	1	1	1	1	2	2
1	1	4	4	1	1	1	1	2	2
1	1	4	4	3	3	1	1	2	2

For Review Only

1	1	2	2	1	1	4	4	2	2
1	1	2	2	1	1	4	4	2	2

For Review Only

SNP2722	SNP2722	SNP2723	SNP2723	SNP2724	SNP2724	SNP2725	SNP2725	SNP2726	SNP2726
3	3	1	1	2	2	3	3	1	1
3	3	4	4	3	3	3	3	2	2
2	2	1	1	2	2	1	1	1	1
2	2	4	4	3	3	3	3	1	1
3	3	1	1	2	2	3	3	1	1
2	2	1	1	2	2	3	3	1	1
2	2	1	1	2	2	3	3	1	1
3	3	1	1	2	2	3	3	1	1
2	2	1	1	2	2	3	3	1	1
3	3	1	1	2	2	3	3	1	1
3	3	4	4	3	3	3	3	2	2
2	2	4	4	3	3	3	3	1	1
2	2	1	1	2	2	3	3	1	1
3	3	1	1	2	2	3	3	1	1
2	2	1	1	2	2	3	3	1	1
3	3	4	4	3	3	3	3	1	1
3	3	1	1	2	2	3	3	1	1
2	2	1	1	2	2	3	3	1	1
3	3	1	1	2	2	3	3	1	1
2	2	1	1	2	2	3	3	1	1
2	2	1	1	2	2	3	3	1	1
3	3	1	1	2	2	3	3	1	1
3	3	1	1	2	2	3	3	1	1
3	3	1	1	2	2	3	3	1	1
3	3	4	4	3	3	3	3	2	2
2	2	1	1	2	2	3	3	1	1
3	3	4	4	3	3	3	3	2	2
2	2	4	4	3	3	1	1	1	1
2	2	1	1	2	2	3	3	1	1
3	3	1	1	2	2	3	3	1	1
3	3	4	4	3	3	3	3	2	2
2	2	1	1	2	2	1	1	1	1
2	2	1	1	2	2	1	1	1	1
3	3	1	1	2	2	3	3	1	1
2	2	4	4	3	3	3	3	1	1
3	3	4	4	3	3	3	3	2	2
2	2	4	4	3	3	1	1	2	2
3	3	4	4	2	2	3	3	1	1
3	3	1	1	2	2	3	3	1	1
3	3	4	4	2	2	3	3	1	1
3	3	1	1	2	2	3	3	1	1
2	2	1	1	2	2	3	3	1	1
2	2	1	1	2	2	3	3	1	1
3	3	4	4	3	3	3	3	2	2
2	2	1	1	2	2	1	1	1	1
2	2	4	4	3	3	1	1	2	2
3	3	1	1	2	2	3	3	1	1
3	3	4	4	3	3	3	3	2	2
2	2	1	1	2	2	3	3	1	1
3	3	4	4	2	2	3	3	2	2
2	2	4	4	3	3	1	1	2	2
2	2	4	4	3	3	1	1	1	1

For Review Only

3	3	1	1	2	2	3	3	1	1
2	2	1	1	2	2	1	1	1	1
2	2	4	4	3	3	1	1	2	2
3	3	1	1	2	2	3	3	1	1
3	3	1	1	2	2	3	3	1	1
2	2	1	1	2	2	1	1	1	1
2	2	1	1	2	2	1	1	1	1
3	3	1	1	2	2	3	3	1	1
2	2	4	4	3	3	1	1	2	2
2	2	4	4	3	3	3	3	1	1
3	3	4	4	3	3	3	3	2	2
3	3	4	4	3	3	3	3	2	2
3	3	1	1	2	2	3	3	1	1
2	2	1	1	2	2	3	3	1	1
2	2	4	4	2	2	3	3	1	1
2	2	4	4	2	2	3	3	1	1
2	2	4	4	3	3	1	1	2	2
2	2	4	4	2	2	1	1	1	1
3	3	4	4	3	3	3	3	2	2
3	3	1	1	2	2	3	3	1	1
3	3	4	4	3	3	3	3	2	2
2	2	4	4	3	3	1	1	2	2
2	2	4	4	2	2	3	3	1	1
2	2	4	4	3	3	1	1	2	2
2	2	1	1	2	2	1	1	1	1
3	3	4	4	3	3	3	3	2	2
3	3	1	1	2	2	3	3	1	1
3	3	1	1	2	2	3	3	1	1
2	2	1	1	2	2	1	1	1	1
3	3	4	4	3	3	3	3	2	2
3	3	1	1	2	2	3	3	1	1
3	3	1	1	2	2	3	3	1	1
3	3	1	1	2	2	3	3	1	1
3	3	1	1	2	2	3	3	1	1
2	2	1	1	2	2	3	3	1	1
2	2	1	1	2	2	1	1	1	1
3	3	1	1	2	2	3	3	1	1
3	3	1	1	2	2	3	3	1	1
3	3	4	4	3	3	3	3	2	2
2	2	1	1	2	2	1	1	1	1
2	2	1	1	2	2	3	3	1	1
3	3	4	4	3	3	3	3	2	2
2	2	1	1	2	2	1	1	1	1
3	3	4	4	3	3	3	3	2	2
3	3	1	1	2	2	3	3	1	1
3	3	4	4	2	2	3	3	1	1
3	3	1	1	2	2	3	3	1	1
2	2	1	1	2	2	1	1	1	1
2	2	1	1	2	2	3	3	1	1
3	3	4	4	3	3	3	3	1	1
3	3	1	1	2	2	3	3	1	1
3	3	1	1	2	2	3	3	1	1
2	2	1	1	2	2	3	3	1	1
3	3	4	4	3	3	3	3	1	1
3	3	1	1	2	2	3	3	1	1

For Review Only

2	2	1	1	2	2	1	1	1	1
2	2	4	4	3	3	3	3	1	1
2	2	4	4	3	3	1	1	2	2
3	3	1	1	2	2	3	3	1	1
3	3	4	4	3	3	3	3	2	2
2	2	4	4	3	3	1	1	2	2
3	3	4	4	3	3	3	3	1	1
2	2	1	1	2	2	3	3	1	1
3	3	1	1	2	2	3	3	1	1
3	3	4	4	3	3	3	3	2	2
2	2	1	1	2	2	3	3	1	1
2	2	1	1	2	2	3	3	1	1
3	3	1	1	2	2	3	3	1	1
2	2	4	4	3	3	3	3	1	1
3	3	1	1	2	2	3	3	1	1
2	2	4	4	3	3	3	3	1	1
2	2	1	1	2	2	1	1	1	1
2	2	1	1	3	3	3	3	1	1
3	3	1	1	2	2	3	3	1	1
3	3	1	1	2	2	3	3	1	1
2	2	4	4	3	3	1	1	1	1
3	3	4	4	3	3	3	3	2	2
2	2	1	1	2	2	3	3	1	1
3	3	1	1	2	2	3	3	1	1
2	2	4	4	3	3	1	1	1	1
2	2	1	1	2	2	3	3	1	1
3	3	1	1	2	2	3	3	1	1
2	2	4	4	3	3	1	1	2	2
3	3	4	4	3	3	3	3	2	2
2	2	4	4	3	3	3	3	1	1
3	3	1	1	2	2	3	3	1	1
2	2	4	4	3	3	3	3	1	1
2	2	1	1	2	2	3	3	1	1
2	2	4	4	2	2	3	3	1	1
3	3	1	1	2	2	3	3	1	1
3	3	1	1	2	2	3	3	1	1
3	3	1	1	3	3	3	3	1	1
3	3	1	1	2	2	3	3	1	1
2	2	4	4	2	2	1	1	1	1
3	3	4	4	3	3	3	3	1	1
2	2	1	1	2	2	1	1	1	1
3	3	1	1	2	2	3	3	1	1
2	2	4	4	3	3	3	3	2	2
3	3	1	1	2	2	3	3	1	1
2	2	1	1	2	2	1	1	1	1
3	3	1	1	2	2	3	3	1	1
2	2	1	1	2	2	1	1	1	1
2	2	4	4	3	3	1	1	2	2
2	2	4	4	2	2	3	3	1	1
2	2	1	1	2	2	1	1	1	1
2	2	1	1	2	2	1	1	1	1
2	2	1	1	2	2	1	1	1	1
2	2	4	4	3	3	3	3	2	2
3	3	4	4	3	3	3	3	2	2
3	3	1	1	2	2	3	3	1	1

For Review Only

2	2	4	4	3	3	1	1	2	2
3	3	1	1	2	2	3	3	1	1
2	2	1	1	2	2	1	1	1	1
3	3	1	1	2	2	3	3	1	1
3	3	1	1	2	2	3	3	1	1
2	2	1	1	2	2	3	3	1	1
3	3	1	1	2	2	3	3	1	1
2	2	4	4	2	2	3	3	1	1
3	3	1	1	2	2	3	3	1	1
3	3	4	4	3	3	3	3	2	2
2	2	1	1	2	2	3	3	1	1
3	3	1	1	2	2	1	1	1	1
2	2	4	4	3	3	1	1	2	2
3	3	4	4	3	3	3	3	1	1
3	3	1	1	2	2	3	3	1	1
3	3	4	4	3	3	3	3	2	2
2	2	4	4	3	3	3	3	2	2
3	3	4	4	3	3	1	1	2	2
3	3	1	1	2	2	3	3	1	1
3	3	4	4	3	3	3	3	1	1
2	2	4	4	3	3	3	3	1	1
3	3	1	1	2	2	3	3	1	1
2	2	4	4	3	3	1	1	1	1
3	3	4	4	3	3	3	3	2	2
2	2	1	1	2	2	3	3	1	1
3	3	1	1	2	2	3	3	1	1
3	3	4	4	3	3	3	3	2	2
2	2	1	1	2	2	3	3	1	1
2	2	1	1	2	2	1	1	1	1
2	2	1	1	2	2	1	1	1	1
2	2	1	1	2	2	3	3	1	1
3	3	1	1	2	2	3	3	1	1
3	3	1	1	2	2	3	3	1	1
3	3	1	1	2	2	3	3	1	1
3	3	4	4	3	3	3	3	2	2
3	3	4	4	3	3	3	3	2	2
2	2	1	1	2	2	1	1	1	1
2	2	4	4	3	3	1	1	2	2
2	2	4	4	3	3	1	1	2	2
3	3	4	4	3	3	3	3	2	2
2	2	1	1	2	2	1	1	1	1
2	2	1	1	2	2	1	1	1	1
3	3	1	1	2	2	3	3	1	1
2	2	1	1	2	2	3	3	1	1
3	3	4	4	3	3	3	3	1	1
3	3	4	4	3	3	3	3	1	1
3	3	4	4	3	3	3	3	2	2
3	3	1	1	2	2	3	3	1	1
3	3	1	1	2	2	3	3	1	1
3	3	4	4	3	3	3	3	2	2
3	3	4	4	3	3	3	3	1	1
3	3	1	1	2	2	3	3	1	1
2	2	4	4	3	3	1	1	2	2
3	3	4	4	3	3	3	3	2	2
3	3	1	1	2	2	3	3	1	1

For Review Only

3	3	4	4	3	3	3	3	2	2
3	3	4	4	3	3	3	3	2	2
3	3	1	1	2	2	3	3	1	1
2	2	4	4	3	3	3	3	1	1
3	3	1	1	2	2	1	1	1	1
2	2	1	1	2	2	1	1	1	1
2	2	4	4	3	3	1	1	1	1
3	3	1	1	2	2	3	3	1	1
3	3	4	4	2	2	3	3	1	1
3	3	1	1	2	2	3	3	1	1
2	2	4	4	3	3	3	3	1	1
2	2	4	4	3	3	3	3	2	2
3	3	4	4	3	3	3	3	2	2
3	3	4	4	3	3	3	3	2	2
2	2	4	4	2	2	1	1	1	1
2	2	4	4	3	3	3	3	2	2
2	2	1	1	2	2	1	1	1	1
3	3	4	4	3	3	3	3	2	2
3	3	1	1	2	2	3	3	1	1
3	3	4	4	3	3	3	3	2	2
2	2	1	1	2	2	1	1	1	1
2	2	1	1	2	2	1	1	1	1
3	3	4	4	3	3	3	3	2	2
2	2	1	1	2	2	1	1	1	1
3	3	1	1	2	2	3	3	1	1
2	2	4	4	3	3	1	1	1	1
3	3	1	1	2	2	3	3	1	1
3	3	4	4	3	3	3	3	1	1
3	3	1	1	2	2	3	3	1	1
3	3	4	4	3	3	3	3	2	2
3	3	1	1	2	2	3	3	1	1
3	3	4	4	3	3	3	3	2	2
2	2	4	4	3	3	1	1	2	2
2	2	4	4	2	2	1	1	1	1
2	2	4	4	3	3	1	1	2	2
3	3	1	1	2	2	3	3	1	1
2	2	1	1	2	2	3	3	1	1
3	3	4	4	3	3	3	3	2	2
3	3	4	4	3	3	3	3	2	2
3	3	1	1	2	2	3	3	1	1
3	3	4	4	3	3	3	3	2	2
3	3	1	1	2	2	3	3	1	1
3	3	1	1	2	2	3	3	1	1
3	3	1	1	2	2	3	3	1	1
2	2	1	1	2	2	3	3	1	1
3	3	4	4	3	3	3	3	2	2
2	2	1	1	2	2	1	1	1	1
2	2	4	4	2	2	1	1	1	1
2	2	4	4	2	2	3	3	1	1
3	3	1	1	2	2	3	3	1	1
3	3	4	4	3	3	3	3	2	2
3	3	1	1	2	2	3	3	1	1
2	2	1	1	2	2	1	1	1	1
2	2	4	4	3	3	1	1	2	2
2	2	1	1	2	2	1	1	1	1

For Review Only

3	3	4	4	3	3	3	3	2	2
3	3	4	4	3	3	3	3	2	2

For Review Only

SNP2727	SNP2727	SNP2728	SNP2728	SNP2729	SNP2729	SNP273	SNP273	SNP2730	SNP2730
4	4	2	2	3	3	1	1	2	2
2	2	4	4	3	3	1	1	3	3
4	4	2	2	3	3	1	1	3	3
2	2	2	2	3	3	2	2	2	2
4	4	4	4	3	3	2	2	2	2
4	4	4	4	3	3	1	1	3	3
4	4	4	4	3	3	1	1	3	3
4	4	4	4	3	3	1	1	2	2
4	4	2	2	3	3	1	1	3	3
4	4	4	4	3	3	1	1	2	2
2	2	2	2	1	1	2	2	3	3
2	2	2	2	3	3	1	1	3	3
4	4	2	2	3	3	1	1	3	3
4	4	2	2	3	3	1	1	3	3
4	4	2	2	3	3	1	1	2	2
2	2	2	2	3	3	1	1	2	2
4	4	2	2	3	3	1	1	3	3
4	4	2	2	3	3	1	1	3	3
4	4	4	4	3	3	1	1	3	3
4	4	4	4	3	3	1	1	3	3
4	4	4	4	3	3	1	1	3	3
4	4	2	2	3	3	1	1	3	3
4	4	2	2	3	3	1	1	2	2
4	4	2	2	3	3	1	1	3	3
2	2	2	2	3	3	1	1	3	3
4	4	2	2	3	3	1	1	3	3
2	2	4	4	3	3	1	1	3	3
2	2	2	2	3	3	1	1	2	2
4	4	4	4	3	3	1	1	3	3
4	4	2	2	3	3	1	1	3	3
2	2	2	2	3	3	1	1	3	3
4	4	2	2	3	3	1	1	2	2
4	4	2	2	3	3	1	1	3	3
4	4	2	2	3	3	1	1	3	3
2	2	2	2	3	3	2	2	3	3
2	2	2	2	3	3	1	1	3	3
2	2	2	2	3	3	2	2	3	3
4	4	2	2	3	3	2	2	2	2
4	4	2	2	3	3	1	1	2	2
4	4	2	2	3	3	1	1	3	3
4	4	4	4	3	3	1	1	3	3
4	4	4	4	3	3	1	1	3	3
4	4	4	4	3	3	1	1	2	2
2	2	2	2	3	3	2	2	2	2
4	4	2	2	3	3	1	1	2	2
2	2	2	2	3	3	1	1	3	3
4	4	4	4	3	3	1	1	3	3
2	2	2	2	3	3	1	1	2	2
4	4	2	2	3	3	2	2	2	2
4	4	2	2	3	3	2	2	2	2
2	2	2	2	3	3	1	1	3	3
2	2	2	2	3	3	1	1	2	2

For Review Only

4	4	2	2	3	3	1	1	3	3
4	4	2	2	3	3	1	1	3	3
2	2	2	2	3	3	1	1	3	3
4	4	2	2	3	3	1	1	3	3
4	4	4	4	3	3	1	1	3	3
4	4	2	2	3	3	1	1	3	3
4	4	2	2	3	3	1	1	3	3
4	4	2	2	3	3	1	1	2	2
2	2	2	2	3	3	2	2	3	3
2	2	2	2	3	3	1	1	3	3
2	2	4	4	3	3	1	1	3	3
2	2	4	4	3	3	1	1	3	3
4	4	2	2	3	3	1	1	3	3
4	4	4	4	3	3	1	1	3	3
4	4	2	2	3	3	1	1	2	2
4	4	4	4	3	3	2	2	2	2
2	2	2	2	3	3	2	2	3	3
4	4	2	2	3	3	1	1	2	2
2	2	2	2	3	3	1	1	3	3
4	4	2	2	3	3	1	1	3	3
2	2	2	2	3	3	1	1	3	3
2	2	2	2	3	3	2	2	2	2
4	4	4	4	3	3	2	2	3	3
2	2	2	2	3	3	1	1	3	3
4	4	2	2	3	3	1	1	2	2
2	2	4	4	3	3	1	1	3	3
4	4	2	2	3	3	1	1	2	2
4	4	2	2	1	1	1	1	2	2
4	4	2	2	3	3	1	1	3	3
2	2	2	2	3	3	2	2	3	3
4	4	2	2	3	3	2	2	3	3
4	4	2	2	3	3	1	1	3	3
4	4	2	2	1	1	1	1	2	2
4	4	2	2	3	3	1	1	3	3
4	4	4	4	3	3	2	2	3	3
4	4	2	2	3	3	1	1	2	2
4	4	4	4	3	3	1	1	3	3
4	4	2	2	3	3	2	2	3	3
2	2	4	4	3	3	1	1	3	3
4	4	2	2	3	3	2	2	2	2
4	4	4	4	3	3	1	1	3	3
2	2	4	4	3	3	1	1	3	3
4	4	2	2	3	3	1	1	3	3
2	2	2	2	3	3	1	1	3	3
4	4	2	2	3	3	2	2	2	2
4	4	2	2	3	3	2	2	3	3
4	4	2	2	3	3	1	1	3	3
4	4	2	2	3	3	1	1	3	3
4	4	4	4	3	3	1	1	3	3
2	2	4	4	3	3	2	2	3	3
4	4	2	2	3	3	1	1	3	3
4	4	2	2	1	1	1	1	2	2
4	4	2	2	3	3	2	2	2	2
4	4	2	2	3	3	1	1	2	2
4	4	4	4	3	3	1	1	2	2

4	4	2	2	3	3	1	1	2	2
4	4	2	2	3	3	1	1	2	2
2	2	2	2	3	3	1	1	3	3
4	4	2	2	3	3	1	1	3	3
2	2	2	2	3	3	1	1	3	3
2	2	2	2	3	3	1	1	3	3
2	2	2	2	3	3	1	1	3	3
4	4	2	2	3	3	1	1	3	3
4	4	4	4	3	3	1	1	3	3
2	2	2	2	3	3	1	1	2	2
4	4	2	2	3	3	1	1	3	3
4	4	2	2	3	3	1	1	2	2
4	4	4	4	3	3	1	1	3	3
2	2	2	2	3	3	1	1	2	2
4	4	2	2	3	3	1	1	2	2
4	4	2	2	3	3	1	1	3	3
4	4	2	2	3	3	1	1	2	2
4	4	2	2	3	3	1	1	3	3
4	4	2	2	3	3	1	1	3	3
4	4	4	4	3	3	1	1	2	2
2	2	2	2	3	3	1	1	3	3
2	2	2	2	3	3	1	1	3	3
4	4	4	4	3	3	1	1	3	3
4	4	2	2	1	1	1	1	2	2
2	2	2	2	3	3	1	1	2	2
4	4	4	4	3	3	2	2	3	3
4	4	4	4	3	3	1	1	3	3
2	2	2	2	3	3	1	1	3	3
2	2	2	2	3	3	1	1	3	3
4	4	2	2	3	3	1	1	3	3
4	4	2	2	3	3	1	1	2	2
4	4	2	2	3	3	1	1	2	2
4	4	4	4	3	3	1	1	3	3
4	4	2	2	3	3	1	1	3	3
4	4	2	2	3	3	1	1	3	3
4	4	2	2	3	3	1	1	3	3
4	4	2	2	3	3	1	1	3	3
4	4	2	2	3	3	1	1	3	3
4	4	2	2	3	3	1	1	2	2
2	2	2	2	3	3	1	1	2	2
4	4	2	2	3	3	2	2	3	3
4	4	2	2	3	3	1	1	3	3
2	2	4	4	3	3	1	1	2	2
4	4	2	2	3	3	1	1	2	2
4	4	2	2	3	3	2	2	3	3
4	4	4	4	3	3	1	1	3	3
4	4	2	2	3	3	1	1	3	3
2	2	2	2	3	3	1	1	3	3
2	2	2	2	3	3	1	1	2	2
4	4	2	2	3	3	1	1	3	3
4	4	2	2	3	3	1	1	3	3
4	4	2	2	1	1	2	2	2	2
2	2	2	2	3	3	1	1	2	2
2	2	4	4	3	3	1	1	3	3
4	4	2	2	3	3	1	1	2	2

For Review Only

2	2	2	2	3	3	1	1	3	3
4	4	4	4	3	3	2	2	3	3
4	4	2	2	3	3	1	1	2	2
4	4	4	4	3	3	1	1	2	2
4	4	4	4	3	3	1	1	3	3
4	4	4	4	1	1	2	2	3	3
4	4	2	2	3	3	1	1	2	2
4	4	2	2	3	3	1	1	3	3
4	4	2	2	3	3	1	1	2	2
2	2	2	2	3	3	2	2	3	3
4	4	4	4	3	3	1	1	3	3
4	4	2	2	3	3	1	1	3	3
2	2	2	2	3	3	2	2	3	3
2	2	2	2	3	3	1	1	3	3
4	4	2	2	3	3	1	1	3	3
2	2	2	2	3	3	2	2	3	3
2	2	2	2	3	3	1	1	3	3
2	2	2	2	3	3	1	1	3	3
4	4	4	4	3	3	1	1	3	3
2	2	2	2	3	3	1	1	3	3
2	2	2	2	3	3	1	1	3	3
4	4	2	2	3	3	1	1	3	3
2	2	2	2	3	3	2	2	3	3
2	2	4	4	3	3	1	1	3	3
4	4	4	4	3	3	1	1	3	3
4	4	2	2	3	3	2	2	3	3
2	2	2	2	3	3	1	1	2	2
4	4	2	2	3	3	1	1	2	2
4	4	2	2	3	3	1	1	2	2
4	4	2	2	3	3	2	2	3	3
4	4	4	4	3	3	2	2	3	3
4	4	2	2	3	3	2	2	3	3
4	4	4	4	3	3	1	1	3	3
4	4	2	2	3	3	1	1	3	3
2	2	4	4	3	3	2	2	3	3
2	2	4	4	3	3	1	1	3	3
4	4	2	2	3	3	1	1	3	3
2	2	2	2	3	3	1	1	3	3
2	2	2	2	3	3	1	1	3	3
2	2	4	4	3	3	1	1	3	3
4	4	2	2	3	3	1	1	3	3
4	4	2	2	3	3	1	1	3	3
4	4	2	2	1	1	1	1	3	3
4	4	2	2	3	3	1	1	3	3
2	2	4	4	3	3	1	1	3	3
2	2	4	4	3	3	1	1	2	2
2	2	2	2	3	3	2	2	3	3
4	4	2	2	1	1	1	1	2	2
4	4	2	2	3	3	1	1	3	3
2	2	2	2	3	3	1	1	3	3
2	2	4	4	3	3	1	1	3	3
4	4	2	2	3	3	2	2	2	2
2	2	2	2	3	3	2	2	3	3
2	2	4	4	3	3	1	1	3	3
4	4	2	2	3	3	1	1	2	2

For Review Only

2	2	2	2	3	3	1	1	3	3
2	2	2	2	3	3	1	1	3	3
4	4	2	2	3	3	1	1	3	3
2	2	2	2	3	3	1	1	3	3
4	4	2	2	3	3	2	2	3	3
4	4	2	2	3	3	1	1	3	3
2	2	2	2	3	3	1	1	3	3
4	4	4	4	3	3	1	1	3	3
4	4	2	2	3	3	1	1	2	2
4	4	2	2	3	3	1	1	2	2
2	2	2	2	3	3	1	1	2	2
2	2	4	4	3	3	2	2	3	3
2	2	4	4	3	3	1	1	3	3
2	2	4	4	3	3	1	1	3	3
4	4	2	2	3	3	1	1	3	3
2	2	2	2	3	3	2	2	3	3
4	4	2	2	3	3	1	1	3	3
2	2	4	4	1	1	1	1	3	3
4	4	2	2	1	1	1	1	3	3
2	2	2	2	3	3	2	2	3	3
4	4	2	2	3	3	1	1	2	2
4	4	2	2	3	3	1	1	3	3
2	2	2	2	3	3	1	1	3	3
4	4	2	2	3	3	1	1	2	2
4	4	2	2	3	3	1	1	2	2
4	4	2	2	3	3	1	1	3	3
4	4	2	2	1	1	1	1	2	2
2	2	2	2	3	3	1	1	3	3
4	4	2	2	3	3	2	2	3	3
2	2	2	2	3	3	1	1	3	3
4	4	2	2	3	3	1	1	3	3
2	2	2	2	3	3	1	1	3	3
2	2	2	2	3	3	1	1	3	3
4	4	2	2	3	3	1	1	3	3
2	2	2	2	3	3	2	2	3	3
4	4	2	2	1	1	1	1	3	3
4	4	4	4	3	3	2	2	3	3
2	2	4	4	3	3	1	1	3	3
2	2	2	2	3	3	1	1	3	3
4	4	2	2	3	3	1	1	2	2
2	2	4	4	3	3	1	1	3	3
4	4	4	4	3	3	1	1	3	3
4	4	4	4	3	3	2	2	2	2
4	4	2	2	3	3	2	2	3	3
4	4	2	2	3	3	1	1	2	2
2	2	4	4	3	3	1	1	3	3
4	4	2	2	3	3	1	1	2	2
4	4	2	2	3	3	1	1	3	3
4	4	2	2	3	3	1	1	3	3
4	4	2	2	1	1	1	1	2	2
2	2	4	4	3	3	1	1	2	2
4	4	2	2	1	1	1	1	2	2
4	4	2	2	3	3	1	1	3	3
2	2	2	2	3	3	2	2	3	3
4	4	2	2	3	3	2	2	3	3

For Review Only

2	2	2	2	3	3	2	2	3	3
2	2	4	4	3	3	1	1	3	3

For Review Only

SNP2731	SNP2731	SNP2732	SNP2732	SNP2733	SNP2733	SNP2734	SNP2734	SNP2735	SNP2735
4	4	1	1	1	1	4	4	4	4
4	4	1	1	1	1	3	3	4	4
4	4	1	1	1	1	4	4	2	2
4	4	1	1	1	1	3	3	4	4
4	4	2	2	1	1	4	4	4	4
4	4	1	1	1	1	4	4	4	4
4	4	1	1	1	1	4	4	4	4
3	3	2	2	1	1	4	4	4	4
4	4	1	1	1	1	4	4	4	4
4	4	2	2	1	1	4	4	4	4
4	4	1	1	1	1	3	3	4	4
4	4	1	1	1	1	4	4	4	4
4	4	1	1	1	1	4	4	4	4
4	4	1	1	1	1	4	4	2	2
4	4	1	1	1	1	4	4	4	4
4	4	1	1	1	1	3	3	4	4
4	4	1	1	1	1	4	4	4	4
4	4	1	1	1	1	4	4	2	2
4	4	1	1	1	1	4	4	4	4
3	3	1	1	1	1	4	4	4	4
3	3	1	1	1	1	4	4	4	4
3	3	1	1	1	1	4	4	4	4
3	3	2	2	3	3	4	4	4	4
3	3	1	1	1	1	4	4	4	4
4	4	1	1	1	1	3	3	4	4
4	4	1	1	1	1	4	4	2	2
4	4	1	1	1	1	3	3	2	2
4	4	1	1	1	1	4	4	4	4
4	4	1	1	1	1	4	4	4	4
3	3	1	1	1	1	4	4	4	4
4	4	1	1	1	1	3	3	4	4
3	3	1	1	1	1	4	4	2	2
4	4	1	1	1	1	4	4	4	4
4	4	1	1	1	1	4	4	4	4
4	4	1	1	1	1	4	4	4	4
4	4	1	1	1	1	3	3	4	4
4	4	1	1	1	1	3	3	4	4
4	4	2	2	1	1	4	4	4	4
3	3	2	2	1	1	4	4	4	4
4	4	1	1	1	1	4	4	4	4
3	3	1	1	1	1	4	4	2	2
4	4	1	1	1	1	4	4	4	4
3	3	2	2	1	1	4	4	4	4
4	4	1	1	1	1	3	3	4	4
3	3	2	2	1	1	4	4	4	4
4	4	1	1	1	1	3	3	2	2
4	4	1	1	1	1	4	4	4	4
4	4	2	2	1	1	4	4	4	4
4	4	2	2	1	1	4	4	4	4
4	4	2	2	1	1	4	4	4	4
4	4	1	1	1	1	3	3	2	2
4	4	1	1	1	1	3	3	4	4

For Review Only

4	4	1	1	1	1	4	4	4	4
4	4	1	1	1	1	4	4	4	4
4	4	1	1	1	1	3	3	4	4
4	4	1	1	1	1	4	4	4	4
4	4	1	1	1	1	4	4	4	4
4	4	1	1	1	1	4	4	2	2
4	4	1	1	1	1	4	4	4	4
3	3	2	2	1	1	4	4	4	4
3	3	1	1	1	1	3	3	4	4
4	4	1	1	1	1	4	4	4	4
4	4	1	1	1	1	3	3	4	4
4	4	1	1	1	1	3	3	4	4
4	4	1	1	1	1	4	4	4	4
4	4	1	1	1	1	4	4	4	4
4	4	1	1	1	1	4	4	4	4
3	3	2	2	1	1	4	4	2	2
3	3	1	1	1	1	4	4	4	4
4	4	1	1	1	1	4	4	4	4
4	4	1	1	1	1	3	3	4	4
4	4	1	1	1	1	4	4	4	4
4	4	1	1	1	1	4	4	4	4
3	3	2	2	3	3	3	3	4	4
4	4	1	1	1	1	4	4	4	4
3	3	1	1	1	1	3	3	4	4
3	3	2	2	1	1	4	4	4	4
4	4	1	1	1	1	3	3	4	4
4	4	2	2	1	1	4	4	2	2
4	4	2	2	1	1	4	4	4	4
4	4	1	1	1	1	4	4	2	2
4	4	1	1	1	1	3	3	4	4
4	4	1	1	1	1	4	4	4	4
4	4	1	1	1	1	4	4	4	4
4	4	2	2	1	1	4	4	4	4
4	4	1	1	1	1	4	4	4	4
4	4	1	1	1	1	4	4	4	4
3	3	2	2	1	1	4	4	4	4
4	4	1	1	1	1	4	4	4	4
4	4	1	1	1	1	4	4	2	2
3	3	1	1	1	1	3	3	4	4
4	4	2	2	1	1	4	4	4	4
4	4	1	1	1	1	4	4	4	4
4	4	1	1	1	1	3	3	4	4
4	4	1	1	1	1	4	4	4	4
4	4	1	1	1	1	3	3	4	4
4	4	1	1	1	1	4	4	4	4
4	4	1	1	1	1	4	4	4	4
4	4	1	1	1	1	4	4	4	4
4	4	1	1	1	1	4	4	4	4
4	4	1	1	1	1	4	4	2	2
4	4	1	1	3	3	4	4	4	4
4	4	1	1	1	1	4	4	4	4
3	3	2	2	1	1	4	4	2	2
3	3	2	2	1	1	4	4	4	4
4	4	1	1	1	1	4	4	4	4
4	4	2	2	1	1	4	4	4	4

For Review Only

4	4	2	2	1	1	4	4	2	2
4	4	1	1	1	1	4	4	4	4
4	4	1	1	1	1	3	3	2	2
4	4	1	1	1	1	4	4	4	4
4	4	1	1	1	1	3	3	4	4
4	4	1	1	1	1	4	4	2	2
4	4	1	1	1	1	3	3	4	4
4	4	1	1	1	1	4	4	4	4
4	4	1	1	1	1	4	4	2	2
4	4	1	1	1	1	4	4	4	4
4	4	1	1	1	1	4	4	4	4
4	4	1	1	1	1	4	4	2	2
4	4	1	1	1	1	4	4	4	4
4	4	1	1	1	1	4	4	4	4
4	4	2	2	1	1	4	4	4	4
4	4	1	1	1	1	4	4	2	2
4	4	1	1	1	1	4	4	4	4
4	4	1	1	1	1	4	4	4	4
4	4	1	1	1	1	4	4	4	4
4	4	2	2	1	1	4	4	4	4
4	4	2	2	1	1	4	4	4	4
4	4	1	1	1	1	3	3	4	4
4	4	1	1	1	1	4	4	4	4
3	3	2	2	1	1	4	4	4	4
4	4	1	1	3	3	3	3	4	4
3	3	1	1	1	1	4	4	4	4
4	4	1	1	1	1	4	4	4	4
4	4	1	1	1	1	3	3	4	4
4	4	1	1	1	1	3	3	4	4
4	4	1	1	1	1	4	4	4	4
4	4	2	2	1	1	4	4	4	4
4	4	2	2	1	1	4	4	4	4
4	4	1	1	1	1	4	4	4	4
4	4	1	1	1	1	4	4	2	2
4	4	1	1	1	1	4	4	4	4
4	4	1	1	1	1	4	4	4	4
4	4	1	1	1	1	4	4	4	4
4	4	1	1	1	1	4	4	2	2
4	4	2	2	1	1	4	4	2	2
4	4	2	2	1	1	3	3	4	4
3	3	1	1	3	3	4	4	4	4
4	4	1	1	1	1	4	4	4	4
4	4	2	2	1	1	3	3	4	4
4	4	1	1	1	1	4	4	2	2
4	4	1	1	1	1	4	4	4	4
4	4	1	1	1	1	4	4	4	4
4	4	1	1	1	1	4	4	2	2
4	4	1	1	1	1	3	3	4	4
4	4	1	1	1	1	4	4	4	4
4	4	1	1	1	1	4	4	2	2
4	4	1	1	1	1	4	4	2	2
4	4	2	2	1	1	4	4	4	4
4	4	1	1	1	1	4	4	4	4
4	4	1	1	1	1	3	3	4	4
4	4	2	2	1	1	4	4	4	4

4	4	1	1	1	1	3	3	2	2
3	3	1	1	1	1	4	4	4	4
4	4	2	2	1	1	4	4	4	4
3	3	1	1	3	3	4	4	4	4
4	4	1	1	1	1	4	4	4	4
4	4	1	1	1	1	4	4	4	4
4	4	2	2	1	1	4	4	4	4
4	4	1	1	1	1	4	4	4	4
4	4	2	2	1	1	4	4	4	4
4	4	1	1	1	1	3	3	4	4
4	4	1	1	1	1	4	4	2	2
3	3	2	2	1	1	4	4	4	4
4	4	1	1	1	1	3	3	4	4
4	4	1	1	1	1	3	3	4	4
4	4	1	1	1	1	4	4	4	4
4	4	1	1	1	1	3	3	4	4
4	4	1	1	1	1	3	3	4	4
4	4	1	1	1	1	3	3	4	4
4	4	1	1	1	1	4	4	4	4
4	4	1	1	1	1	3	3	4	4
4	4	1	1	1	1	4	4	4	4
3	3	1	1	1	1	4	4	2	2
3	3	1	1	3	3	4	4	4	4
4	4	1	1	1	1	3	3	4	4
4	4	1	1	1	1	4	4	4	4
4	4	1	1	1	1	4	4	4	4
4	4	2	2	1	1	3	3	4	4
4	4	1	1	1	1	4	4	4	4
4	4	2	2	1	1	4	4	4	4
4	4	1	1	1	1	3	3	4	4
4	4	1	1	1	1	4	4	4	4
4	4	1	1	1	1	4	4	4	4
4	4	1	1	1	1	4	4	4	4
4	4	1	1	1	1	4	4	4	4
4	4	1	1	1	1	3	3	4	4
4	4	1	1	1	1	3	3	4	4
4	4	1	1	1	1	4	4	4	4
4	4	1	1	1	1	3	3	4	4
4	4	1	1	1	1	3	3	4	4
4	4	1	1	1	1	3	3	4	4
4	4	1	1	1	1	4	4	2	2
4	4	1	1	1	1	4	4	2	2
3	3	1	1	1	1	4	4	4	4
4	4	1	1	1	1	4	4	4	4
4	4	1	1	1	1	4	4	4	4
4	4	2	2	1	1	4	4	4	4
4	4	1	1	1	1	3	3	4	4
4	4	2	2	1	1	4	4	4	4
4	4	1	1	1	1	4	4	4	4
4	4	1	1	1	1	4	4	4	4
4	4	1	1	1	1	4	4	4	4
3	3	2	2	1	1	4	4	4	4
3	3	1	1	3	3	3	3	4	4
4	4	1	1	1	1	3	3	4	4
3	3	2	2	1	1	3	3	4	4

For Review Only

4	4	1	1	1	1	3	3	4	4
4	4	2	2	1	1	4	4	4	4
4	4	1	1	1	1	4	4	2	2
3	3	1	1	3	3	4	4	4	4
4	4	1	1	1	1	4	4	4	4
4	4	1	1	1	1	4	4	4	4
3	3	1	1	1	1	4	4	4	4
3	3	1	1	1	1	4	4	2	2
4	4	2	2	1	1	4	4	4	4
4	4	2	2	1	1	4	4	4	4
4	4	1	1	1	1	4	4	4	4
4	4	2	2	1	1	4	4	4	4
3	3	1	1	3	3	3	3	4	4
4	4	1	1	1	1	3	3	4	4
4	4	1	1	1	1	4	4	2	2
4	4	1	1	1	1	3	3	4	4
3	3	1	1	1	1	4	4	4	4
4	4	1	1	1	1	3	3	4	4
4	4	1	1	1	1	4	4	4	4
4	4	1	1	1	1	3	3	4	4
3	3	1	1	1	1	4	4	4	4
4	4	1	1	1	1	4	4	2	2
4	4	1	1	1	1	3	3	4	4
3	3	2	2	1	1	4	4	2	2
3	3	2	2	1	1	4	4	4	4
4	4	1	1	1	1	4	4	4	4
3	3	2	2	1	1	4	4	4	4
4	4	1	1	1	1	3	3	4	4
4	4	1	1	1	1	4	4	4	4
4	4	1	1	1	1	3	3	4	4
3	3	1	1	1	1	4	4	4	4
4	4	1	1	1	1	3	3	4	4
4	4	1	1	1	1	3	3	4	4
4	4	1	1	1	1	4	4	2	2
3	3	1	1	3	3	3	3	4	4
4	4	1	1	1	1	4	4	4	4
4	4	1	1	1	1	4	4	4	4
4	4	1	1	1	1	3	3	4	4
4	4	1	1	1	1	3	3	4	4
3	3	2	2	1	1	4	4	4	4
4	4	1	1	1	1	3	3	4	4
4	4	1	1	1	1	4	4	4	4
3	3	2	2	1	1	4	4	2	2
4	4	1	1	1	1	4	4	4	4
4	4	2	2	1	1	4	4	4	4
3	3	1	1	1	1	3	3	4	4
3	3	2	2	1	1	4	4	4	4
4	4	1	1	1	1	4	4	2	2
4	4	1	1	1	1	4	4	4	4
4	4	2	2	1	1	4	4	4	4
4	4	1	1	1	1	3	3	4	4
3	3	2	2	1	1	4	4	4	4
4	4	1	1	1	1	4	4	2	2
4	4	1	1	1	1	3	3	4	4
4	4	1	1	1	1	4	4	4	4

For Review Only

4	4	1	1	1	1	3	3	4	4
4	4	1	1	1	1	3	3	4	4

For Review Only

SNP2736	SNP2736	SNP2737	SNP2737	SNP2738	SNP2738	SNP2739	SNP2739	SNP274	SNP274
2	2	4	4	1	1	4	4	3	3
2	2	4	4	1	1	4	4	3	3
2	2	4	4	1	1	4	4	3	3
3	3	4	4	3	3	4	4	3	3
2	2	4	4	3	3	3	3	3	3
2	2	4	4	1	1	4	4	3	3
3	3	4	4	1	1	4	4	3	3
3	3	4	4	1	1	3	3	3	3
2	2	4	4	1	1	4	4	3	3
3	3	4	4	3	3	3	3	3	3
2	2	3	3	1	1	4	4	2	2
3	3	4	4	1	1	4	4	3	3
3	3	4	4	1	1	4	4	3	3
3	3	3	3	1	1	4	4	3	3
3	3	4	4	1	1	3	3	3	3
3	3	4	4	3	3	4	4	3	3
3	3	4	4	3	3	4	4	3	3
3	3	4	4	3	3	4	4	3	3
2	2	4	4	1	1	4	4	3	3
3	3	4	4	1	1	4	4	2	2
3	3	4	4	1	1	4	4	3	3
3	3	4	4	3	3	4	4	3	3
3	3	3	3	1	1	3	3	3	3
3	3	4	4	3	3	4	4	3	3
2	2	4	4	3	3	4	4	3	3
3	3	4	4	3	3	4	4	3	3
3	3	4	4	1	1	4	4	3	3
3	3	4	4	3	3	3	3	3	3
2	2	4	4	1	1	4	4	3	3
3	3	4	4	3	3	4	4	3	3
2	2	4	4	3	3	4	4	3	3
3	3	4	4	1	1	4	4	3	3
2	2	4	4	1	1	4	4	3	3
2	2	3	3	1	1	4	4	3	3
2	2	4	4	3	3	4	4	3	3
3	3	4	4	3	3	4	4	3	3
2	2	4	4	3	3	4	4	3	3
3	3	4	4	3	3	4	4	3	3
2	2	3	3	1	1	3	3	2	2
3	3	4	4	3	3	4	4	2	2
3	3	4	4	3	3	4	4	3	3
2	2	4	4	1	1	4	4	3	3
3	3	4	4	1	1	3	3	3	3
2	2	4	4	3	3	4	4	3	3
3	3	4	4	1	1	3	3	3	3
3	3	4	4	3	3	4	4	3	3
3	3	4	4	1	1	4	4	3	3
2	2	4	4	3	3	3	3	2	2
2	2	4	4	1	1	3	3	3	3
3	3	4	4	3	3	3	3	3	3
2	2	4	4	3	3	4	4	3	3
2	2	4	4	1	1	4	4	3	3

For Review Only

2	2	4	4	3	3	4	4	3	3
2	2	4	4	1	1	4	4	3	3
2	2	4	4	1	1	4	4	3	3
3	3	4	4	3	3	4	4	2	2
2	2	4	4	1	1	4	4	3	3
3	3	4	4	3	3	4	4	3	3
3	3	4	4	3	3	4	4	3	3
3	3	4	4	3	3	3	3	3	3
2	2	4	4	3	3	4	4	3	3
3	3	4	4	3	3	4	4	3	3
2	2	4	4	1	1	4	4	3	3
2	2	4	4	1	1	4	4	3	3
3	3	4	4	3	3	4	4	3	3
2	2	4	4	1	1	4	4	3	3
3	3	4	4	3	3	4	4	3	3
2	2	4	4	1	1	3	3	3	3
2	2	4	4	3	3	4	4	2	2
3	3	4	4	3	3	4	4	3	3
2	2	4	4	3	3	4	4	3	3
2	2	4	4	1	1	4	4	3	3
2	2	4	4	1	1	4	4	3	3
3	3	4	4	3	3	4	4	2	2
2	2	4	4	1	1	4	4	3	3
2	2	4	4	1	1	4	4	3	3
3	3	4	4	1	1	3	3	3	3
2	2	4	4	1	1	4	4	3	3
3	3	4	4	3	3	3	3	2	2
3	3	3	3	1	1	3	3	3	3
3	3	4	4	3	3	4	4	3	3
3	3	4	4	3	3	4	4	3	3
2	2	4	4	3	3	4	4	3	3
2	2	4	4	3	3	3	3	3	3
3	3	3	3	1	1	3	3	2	2
2	2	4	4	3	3	4	4	3	3
2	2	4	4	1	1	4	4	3	3
3	3	4	4	1	1	3	3	3	3
3	3	4	4	3	3	4	4	3	3
3	3	4	4	3	3	4	4	3	3
2	2	4	4	1	1	4	4	3	3
3	3	4	4	1	1	3	3	3	3
3	3	4	4	1	1	4	4	3	3
3	3	4	4	3	3	4	4	3	3
3	3	4	4	3	3	3	3	3	3
2	2	4	4	1	1	4	4	3	3
3	3	4	4	3	3	4	4	3	3
3	3	4	4	1	1	4	4	3	3
3	3	3	3	1	1	4	4	3	3
3	3	4	4	3	3	4	4	3	3
3	3	4	4	1	1	4	4	2	2
3	3	4	4	1	1	4	4	3	3
3	3	4	4	1	1	4	4	3	3
3	3	3	3	1	1	3	3	2	2
3	3	4	4	3	3	3	3	3	3
3	3	4	4	3	3	3	3	3	3
3	3	4	4	1	1	3	3	3	3

For Review Only

3	3	4	4	3	3	4	4	2	2
3	3	4	4	3	3	4	4	3	3
3	3	4	4	3	3	4	4	3	3
3	3	3	3	1	1	4	4	3	3
2	2	4	4	1	1	4	4	3	3
3	3	4	4	3	3	4	4	3	3
3	3	4	4	3	3	4	4	3	3
3	3	4	4	3	3	3	3	2	2
3	3	4	4	1	1	3	3	3	3
3	3	4	4	3	3	3	3	3	3
2	2	4	4	1	1	4	4	3	3
3	3	4	4	3	3	4	4	3	3
3	3	4	4	3	3	4	4	3	3
3	3	4	4	1	1	4	4	3	3
3	3	4	4	1	1	3	3	3	3
3	3	4	4	3	3	4	4	3	3
3	3	4	4	3	3	4	4	3	3
3	3	4	4	3	3	4	4	3	3
3	3	3	3	1	1	4	4	3	3
3	3	4	4	1	1	3	3	3	3
3	3	4	4	3	3	3	3	3	3
3	3	4	4	3	3	4	4	3	3
2	2	4	4	1	1	4	4	3	3
3	3	3	3	1	1	3	3	2	2
3	3	4	4	3	3	4	4	2	2
3	3	4	4	1	1	4	4	3	3
3	3	4	4	3	3	4	4	3	3
3	3	4	4	3	3	4	4	3	3
3	3	4	4	3	3	4	4	3	3
3	3	4	4	3	3	4	4	2	2
3	3	3	3	1	1	3	3	3	3
3	3	4	4	3	3	3	3	3	3
3	3	4	4	1	1	4	4	3	3
3	3	4	4	3	3	4	4	3	3
2	2	3	3	1	1	4	4	3	3
3	3	3	3	3	3	4	4	3	3
3	3	3	3	1	1	4	4	3	3
3	3	4	4	1	1	4	4	3	3
3	3	4	4	3	3	3	3	3	3
3	3	3	3	1	1	3	3	2	2
3	3	4	4	3	3	4	4	2	2
3	3	3	3	1	1	4	4	3	3
2	2	4	4	1	1	3	3	3	3
3	3	4	4	3	3	3	3	3	3
3	3	4	4	3	3	4	4	3	3
3	3	4	4	1	1	4	4	3	3
3	3	4	4	3	3	4	4	3	3
2	2	4	4	3	3	4	4	3	3
3	3	4	4	3	3	4	4	3	3
3	3	4	4	3	3	4	4	3	3
3	3	4	4	3	3	4	4	3	3
3	3	4	4	3	3	3	3	2	2
2	2	4	4	1	1	4	4	3	3
3	3	4	4	1	1	4	4	3	3
3	3	4	4	3	3	3	3	2	2

For Review Only

2	2	4	4	3	3	4	4	3	3
3	3	4	4	3	3	4	4	2	2
3	3	4	4	1	1	3	3	3	3
3	3	4	4	3	3	4	4	3	3
3	3	4	4	1	1	4	4	2	2
2	2	4	4	1	1	4	4	3	3
3	3	4	4	1	1	3	3	3	3
3	3	4	4	3	3	4	4	3	3
3	3	3	3	1	1	3	3	2	2
3	3	4	4	3	3	4	4	3	3
3	3	4	4	1	1	4	4	2	2
2	2	4	4	3	3	3	3	3	3
3	3	4	4	3	3	3	3	3	3
3	3	4	4	3	3	4	4	3	3
3	3	3	3	1	1	4	4	3	3
2	2	3	3	1	1	3	3	2	2
2	2	4	4	3	3	4	4	3	3
2	2	4	4	1	1	4	4	3	3
3	3	4	4	1	1	4	4	3	3
3	3	4	4	3	3	4	4	3	3
3	3	4	4	1	1	4	4	3	3
3	3	4	4	3	3	3	3	3	3
3	3	4	4	3	3	4	4	2	2
2	2	4	4	1	1	4	4	3	3
3	3	4	4	1	1	4	4	3	3
2	2	3	3	1	1	4	4	3	3
3	3	4	4	3	3	3	3	3	3
3	3	4	4	3	3	4	4	3	3
3	3	4	4	3	3	3	3	3	3
2	2	4	4	3	3	3	3	3	3
2	2	4	4	1	1	4	4	3	3
3	3	4	4	3	3	4	4	3	3
3	3	4	4	3	3	4	4	3	3
3	3	3	3	1	1	4	4	3	3
2	2	4	4	1	1	4	4	3	3
2	2	4	4	1	1	4	4	3	3
3	3	4	4	3	3	4	4	3	3
2	2	4	4	3	3	4	4	3	3
2	2	4	4	3	3	4	4	3	3
2	2	4	4	1	1	4	4	3	3
2	2	4	4	1	1	4	4	3	3
3	3	4	4	3	3	4	4	3	3
3	3	3	3	1	1	3	3	3	3
3	3	4	4	3	3	4	4	3	3
3	3	4	4	1	1	4	4	3	3
3	3	4	4	1	1	3	3	3	3
2	2	3	3	1	1	3	3	2	2
3	3	3	3	1	1	3	3	3	3
3	3	4	4	3	3	4	4	3	3
3	3	4	4	3	3	4	4	2	2
3	3	4	4	1	1	4	4	3	3
3	3	4	4	3	3	3	3	3	3
3	3	4	4	3	3	4	4	3	3
2	2	4	4	1	1	4	4	3	3
3	3	3	3	1	1	4	4	3	3

For Review Only

2	2	4	4	3	3	4	4	3	3
3	3	4	4	1	1	4	4	3	3
2	2	4	4	3	3	4	4	3	3
3	3	4	4	3	3	4	4	2	2
3	3	4	4	1	1	4	4	3	3
2	2	4	4	3	3	4	4	3	3
3	3	4	4	3	3	4	4	3	3
3	3	4	4	3	3	4	4	3	3
3	3	4	4	1	1	3	3	2	2
3	3	3	3	1	1	3	3	2	2
3	3	4	4	3	3	4	4	3	3
2	2	4	4	3	3	4	4	3	3
2	2	4	4	1	1	4	4	3	3
3	3	4	4	1	1	4	4	3	3
2	2	4	4	1	1	4	4	3	3
2	2	4	4	1	1	4	4	3	3
3	3	4	4	3	3	4	4	2	2
2	2	4	4	3	3	3	3	3	3
3	3	3	3	1	1	4	4	3	3
2	2	4	4	3	3	4	4	3	3
2	2	4	4	1	1	3	3	3	3
3	3	4	4	3	3	4	4	3	3
2	2	4	4	3	3	4	4	3	3
2	2	4	4	3	3	3	3	2	2
3	3	4	4	3	3	3	3	3	3
2	2	4	4	1	1	4	4	3	3
3	3	3	3	1	1	3	3	2	2
3	3	4	4	3	3	4	4	3	3
3	3	4	4	3	3	4	4	3	3
3	3	4	4	3	3	4	4	3	3
3	3	3	3	1	1	3	3	2	2
2	2	4	4	3	3	4	4	3	3
2	2	4	4	3	3	4	4	3	3
3	3	4	4	3	3	4	4	3	3
3	3	4	4	3	3	4	4	2	2
3	3	3	3	1	1	3	3	3	3
3	3	4	4	1	1	4	4	3	3
2	2	4	4	1	1	4	4	3	3
3	3	4	4	3	3	4	4	3	3
2	2	3	3	1	1	4	4	2	2
2	2	4	4	1	1	4	4	3	3
3	3	4	4	1	1	4	4	3	3
2	2	4	4	1	1	3	3	3	3
3	3	4	4	3	3	4	4	3	3
2	2	4	4	1	1	3	3	3	3
2	2	4	4	1	1	4	4	3	3
3	3	4	4	3	3	3	3	3	3
3	3	4	4	3	3	4	4	3	3
3	3	4	4	1	1	3	3	3	3
3	3	3	3	1	1	3	3	3	3
3	3	4	4	3	3	4	4	3	3
2	2	3	3	1	1	3	3	2	2
2	2	4	4	1	1	4	4	3	3
3	3	4	4	3	3	4	4	3	3
2	2	4	4	3	3	4	4	3	3

For Review Only

2	2	4	4	3	3	4	4	3	3
2	2	4	4	1	1	4	4	3	3

For Review Only

For Review Only

SNP2740	SNP2740	SNP2741	SNP2741	SNP2742	SNP2742	SNP2743	SNP2743	SNP2744	SNP2744
1	1	3	3	1	1	1	1	1	1
4	4	3	3	1	1	4	4	2	2
4	4	3	3	1	1	4	4	2	2
1	1	2	2	1	1	1	1	1	1
1	1	3	3	4	4	1	1	1	1
1	1	3	3	1	1	1	1	1	1
1	1	2	2	1	1	1	1	1	1
1	1	2	2	1	1	1	1	1	1
1	1	3	3	1	1	1	1	1	1
1	1	2	2	1	1	1	1	1	1
1	1	3	3	1	1	1	1	1	1
4	4	3	3	1	1	1	1	1	1
1	1	2	2	1	1	1	1	1	1
1	1	2	2	1	1	1	1	1	1
1	1	2	2	1	1	1	1	1	1
4	4	2	2	1	1	1	1	1	1
1	1	2	2	1	1	1	1	1	1
1	1	2	2	1	1	1	1	1	1
1	1	3	3	1	1	1	1	1	1
1	1	2	2	1	1	1	1	1	1
1	1	2	2	4	4	1	1	1	1
1	1	2	2	1	1	1	1	1	1
1	1	2	2	1	1	1	1	1	1
1	1	2	2	4	4	1	1	1	1
1	1	3	3	1	1	1	1	1	1
1	1	2	2	1	1	1	1	1	1
4	4	2	2	1	1	1	1	1	1
1	1	2	2	1	1	1	1	1	1
1	1	3	3	1	1	1	1	1	1
1	1	2	2	4	4	1	1	1	1
1	1	3	3	1	1	1	1	1	1
4	4	2	2	1	1	1	1	1	1
1	1	3	3	1	1	1	1	1	1
1	1	3	3	1	1	1	1	1	1
1	1	2	2	1	1	1	1	1	1
1	1	2	2	4	4	1	1	1	1
1	1	3	3	1	1	1	1	1	1
1	1	3	3	1	1	1	1	1	1
1	1	2	2	1	1	1	1	1	1
1	1	2	2	1	1	1	1	1	1
1	1	2	2	4	4	1	1	1	1
1	1	3	3	4	4	1	1	1	1
1	1	2	2	1	1	1	1	1	1
1	1	3	3	1	1	1	1	1	1
1	1	2	2	1	1	1	1	1	1
4	4	2	2	1	1	1	1	1	1
1	1	2	2	1	1	4	4	2	2
1	1	2	2	1	1	1	1	1	1
1	1	3	3	1	1	1	1	1	1
1	1	2	2	1	1	1	1	1	1
1	1	3	3	1	1	1	1	1	1
1	1	3	3	1	1	1	1	1	1

1	1	3	3	1	1	1	1	1	1
1	1	3	3	1	1	1	1	1	1
1	1	2	2	1	1	1	1	1	1
1	1	2	2	1	1	1	1	1	1
1	1	3	3	1	1	1	1	1	1
1	1	2	2	1	1	1	1	1	1
1	1	2	2	1	1	1	1	1	1
1	1	2	2	1	1	1	1	1	1
1	1	3	3	1	1	1	1	1	1
1	1	2	2	1	1	1	1	1	1
4	4	3	3	1	1	4	4	2	2
4	4	3	3	1	1	4	4	2	2
1	1	3	3	1	1	1	1	1	1
1	1	3	3	1	1	1	1	1	1
1	1	2	2	1	1	1	1	1	1
1	1	3	3	1	1	1	1	1	1
1	1	3	3	1	1	1	1	1	1
1	1	2	2	1	1	1	1	1	1
1	1	3	3	1	1	1	1	1	1
1	1	3	3	1	1	1	1	1	1
1	1	2	2	1	1	4	4	2	2
1	1	2	2	1	1	1	1	1	1
1	1	2	2	1	1	1	1	1	1
1	1	2	2	1	1	4	4	2	2
1	1	2	2	1	1	1	1	1	1
4	4	3	3	1	1	4	4	2	2
1	1	2	2	1	1	1	1	1	1
1	1	2	2	1	1	1	1	1	1
1	1	2	2	1	1	1	1	1	1
1	1	2	2	1	1	1	1	1	1
1	1	3	3	1	1	1	1	1	1
1	1	2	2	1	1	1	1	1	1
1	1	2	2	1	1	1	1	1	1
1	1	3	3	1	1	1	1	1	1
1	1	3	3	1	1	1	1	1	1
1	1	2	2	1	1	1	1	1	1
1	1	2	2	1	1	1	1	1	1
1	1	2	2	1	1	1	1	1	1
1	1	3	3	1	1	1	1	1	1
1	1	2	2	4	4	1	1	1	1
1	1	3	3	1	1	1	1	1	1
1	1	2	2	1	1	4	4	2	2
1	1	2	2	1	1	1	1	1	1
4	4	3	3	1	1	4	4	2	2
1	1	2	2	1	1	1	1	1	1
4	4	2	2	1	1	1	1	1	1
1	1	2	2	1	1	1	1	1	1
1	1	2	2	1	1	1	1	1	1
1	1	2	2	4	4	1	1	1	1
1	1	2	2	1	1	1	1	1	1
1	1	2	2	1	1	4	4	2	2
1	1	2	2	1	1	1	1	1	1
1	1	2	2	1	1	1	1	1	1
1	1	2	2	1	1	1	1	1	1
1	1	2	2	4	4	1	1	1	1

For Review Only

1	1	2	2	1	1	1	1	1	1
1	1	2	2	1	1	1	1	1	1
1	1	2	2	4	4	1	1	1	1
1	1	2	2	1	1	1	1	1	1
1	1	2	2	1	1	1	1	1	1
1	1	2	2	1	1	1	1	1	1
4	4	2	2	1	1	1	1	1	1
1	1	2	2	1	1	1	1	1	1
1	1	2	2	4	4	1	1	1	1
1	1	2	2	1	1	1	1	1	1
1	1	3	3	1	1	1	1	1	1
1	1	2	2	1	1	1	1	1	1
1	1	2	2	1	1	1	1	1	1
1	1	2	2	1	1	1	1	1	1
1	1	2	2	1	1	1	1	1	1
1	1	2	2	1	1	1	1	1	1
1	1	2	2	1	1	1	1	1	1
1	1	2	2	1	1	1	1	1	1
1	1	2	2	1	1	1	1	1	1
1	1	2	2	1	1	1	1	1	1
1	1	2	2	1	1	1	1	1	1
1	1	2	2	1	1	1	1	1	1
1	1	3	3	1	1	4	4	2	2
1	1	2	2	1	1	1	1	1	1
1	1	2	2	1	1	1	1	1	1
1	1	2	2	1	1	1	1	1	1
1	1	2	2	1	1	1	1	1	1
1	1	2	2	1	1	1	1	1	1
4	4	2	2	1	1	1	1	1	1
1	1	2	2	1	1	1	1	1	1
1	1	2	2	1	1	1	1	1	1
1	1	2	2	1	1	1	1	1	1
1	1	3	3	1	1	1	1	1	1
1	1	2	2	1	1	1	1	1	1
1	1	3	3	1	1	1	1	1	1
1	1	2	2	1	1	1	1	1	1
1	1	2	2	1	1	1	1	1	1
4	4	2	2	1	1	1	1	1	1
1	1	2	2	1	1	1	1	1	1
1	1	2	2	1	1	1	1	1	1
1	1	2	2	1	1	1	1	1	1
1	1	2	2	1	1	1	1	1	1
1	1	3	3	1	1	1	1	1	1
1	1	2	2	4	4	1	1	1	1
1	1	2	2	1	1	1	1	1	1
1	1	2	2	1	1	1	1	1	1
1	1	2	2	1	1	1	1	1	1
1	1	2	2	1	1	1	1	1	1
1	1	2	2	1	1	1	1	1	1
1	1	2	2	1	1	1	1	1	1
1	1	2	2	1	1	1	1	1	1
1	1	2	2	1	1	1	1	1	1
1	1	3	3	1	1	1	1	1	1
4	4	2	2	1	1	4	4	2	2
1	1	2	2	1	1	1	1	1	1

1	1	3	3	1	1	1	1	1	1
1	1	2	2	1	1	1	1	1	1
1	1	2	2	1	1	1	1	1	1
1	1	2	2	1	1	1	1	1	1
1	1	2	2	1	1	4	4	2	2
1	1	3	3	1	1	1	1	1	1
1	1	2	2	1	1	1	1	1	1
1	1	2	2	1	1	1	1	1	1
1	1	2	2	1	1	1	1	1	1
1	1	2	2	1	1	1	1	1	1
1	1	2	2	1	1	1	1	1	1
1	1	2	2	4	4	1	1	1	1
1	1	2	2	1	1	1	1	1	1
1	1	2	2	1	1	1	1	1	1
1	1	2	2	1	1	1	1	1	1
1	1	3	3	1	1	1	1	1	1
1	1	2	2	1	1	1	1	1	1
4	4	3	3	1	1	1	1	1	1
1	1	2	2	1	1	1	1	1	1
4	4	2	2	1	1	1	1	1	1
1	1	2	2	1	1	1	1	1	1
1	1	2	2	4	4	1	1	1	1
1	1	2	2	1	1	1	1	1	1
4	4	3	3	1	1	4	4	2	2
1	1	2	2	1	1	1	1	1	1
1	1	3	3	1	1	1	1	1	1
4	4	3	3	1	1	1	1	1	1
1	1	2	2	1	1	1	1	1	1
1	1	2	2	1	1	1	1	1	1
1	1	3	3	1	1	1	1	1	1
1	1	3	3	1	1	1	1	1	1
1	1	2	2	1	1	1	1	1	1
1	1	2	2	1	1	1	1	1	1
1	1	2	2	1	1	1	1	1	1
1	1	3	3	1	1	1	1	1	1
4	4	3	3	1	1	4	4	2	2
1	1	2	2	1	1	1	1	1	1
1	1	3	3	1	1	1	1	1	1
1	1	2	2	1	1	1	1	1	1
1	1	3	3	1	1	1	1	1	1
4	4	3	3	1	1	4	4	2	2
1	1	2	2	1	1	1	1	1	1
1	1	2	2	1	1	1	1	1	1
1	1	2	2	1	1	1	1	1	1
1	1	2	2	1	1	1	1	1	1
1	1	3	3	1	1	4	4	2	2
1	1	3	3	1	1	1	1	1	1
1	1	2	2	1	1	1	1	1	1
1	1	2	2	1	1	1	1	1	1
1	1	2	2	1	1	1	1	1	1
4	4	2	2	1	1	1	1	1	1
1	1	2	2	1	1	1	1	1	1
1	1	2	2	1	1	1	1	1	1
4	4	3	3	1	1	4	4	2	2
1	1	2	2	1	1	1	1	1	1

For Review Only

1	1	3	3	1	1	1	1	1	1
1	1	2	2	1	1	1	1	1	1
1	1	3	3	1	1	1	1	1	1
1	1	2	2	1	1	1	1	1	1
1	1	2	2	1	1	1	1	1	1
1	1	2	2	1	1	1	1	1	1
1	1	2	2	1	1	1	1	1	1
1	1	2	2	1	1	1	1	1	1
1	1	2	2	1	1	1	1	1	1
1	1	2	2	1	1	1	1	1	1
1	1	2	2	1	1	1	1	1	1
1	1	2	2	1	1	1	1	1	1
1	1	3	3	1	1	1	1	1	1
1	1	2	2	1	1	1	1	1	1
4	4	3	3	1	1	4	4	2	2
1	1	3	3	1	1	1	1	1	1
1	1	2	2	1	1	1	1	1	1
1	1	3	3	1	1	1	1	1	1
1	1	2	2	1	1	1	1	1	1
1	1	3	3	1	1	1	1	1	1
1	1	2	2	1	1	4	4	2	2
1	1	2	2	1	1	1	1	1	1
1	1	3	3	1	1	1	1	1	1
1	1	2	2	1	1	1	1	1	1
1	1	2	2	1	1	1	1	1	1
1	1	3	3	1	1	1	1	1	1
1	1	2	2	1	1	1	1	1	1
4	4	2	2	1	1	1	1	1	1
1	1	2	2	1	1	1	1	1	1
4	4	2	2	1	1	1	1	1	1
1	1	2	2	1	1	1	1	1	1
1	1	3	3	1	1	1	1	1	1
1	1	3	3	1	1	1	1	1	1
1	1	2	2	1	1	1	1	1	1
1	1	2	2	1	1	1	1	1	1
1	1	2	2	1	1	1	1	1	1
1	1	2	2	1	1	1	1	1	1
4	4	3	3	1	1	4	4	2	2
1	1	2	2	4	4	1	1	1	1
1	1	2	2	1	1	1	1	1	1
1	1	3	3	1	1	1	1	1	1
1	1	2	2	1	1	1	1	1	1
1	1	2	2	1	1	1	1	1	1
1	1	2	2	1	1	1	1	1	1
4	4	3	3	1	1	4	4	2	2
1	1	3	3	1	1	1	1	1	1
1	1	2	2	1	1	1	1	1	1
1	1	2	2	4	4	1	1	1	1
1	1	2	2	4	4	1	1	2	2
1	1	2	2	1	1	1	1	1	1
1	1	2	2	1	1	1	1	1	1
1	1	2	2	1	1	1	1	1	1
4	4	3	3	1	1	4	4	2	2
4	4	3	3	1	1	1	1	1	1
1	1	2	2	1	1	1	1	1	1

For Review Only

1	1	3	3	1	1	1	1	1	1
4	4	3	3	1	1	4	4	2	2

For Review Only

SNP2745	SNP2745	SNP2746	SNP2746	SNP2747	SNP2747	SNP2748	SNP2748	SNP2749	SNP2749
1	1	3	3	2	2	3	3	3	3
1	1	1	1	2	2	3	3	3	3
4	4	3	3	2	2	3	3	3	3
4	4	3	3	2	2	3	3	3	3
4	4	3	3	2	2	3	3	3	3
4	4	3	3	2	2	3	3	3	3
1	1	3	3	2	2	3	3	3	3
1	1	3	3	4	4	4	4	2	2
1	1	3	3	2	2	3	3	3	3
4	4	1	1	2	2	3	3	3	3
4	4	3	3	2	2	3	3	2	2
4	4	3	3	2	2	3	3	3	3
4	4	3	3	2	2	3	3	3	3
4	4	3	3	2	2	3	3	3	3
4	4	3	3	2	2	3	3	3	3
4	4	3	3	2	2	3	3	3	3
4	4	3	3	2	2	3	3	3	3
1	1	3	3	2	2	3	3	3	3
4	4	3	3	2	2	3	3	3	3
4	4	3	3	2	2	3	3	3	3
4	4	3	3	2	2	3	3	3	3
4	4	3	3	2	2	3	3	3	3
4	4	1	1	2	2	3	3	2	2
4	4	3	3	2	2	3	3	3	3
4	4	3	3	2	2	3	3	3	3
4	4	3	3	2	2	3	3	3	3
4	4	3	3	2	2	3	3	3	3
4	4	3	3	2	2	3	3	3	3
1	1	3	3	4	4	4	4	2	2
4	4	1	1	2	2	3	3	3	3
4	4	3	3	2	2	3	3	3	3
1	1	3	3	2	2	3	3	3	3
1	1	3	3	2	2	3	3	3	3
4	4	3	3	2	2	3	3	3	3
4	4	3	3	2	2	3	3	3	3
1	1	3	3	4	4	4	4	2	2
4	4	3	3	2	2	3	3	3	3
4	4	3	3	2	2	3	3	3	3
4	4	3	3	2	2	3	3	3	3
4	4	1	1	2	2	3	3	2	2
1	1	3	3	2	2	3	3	3	3
1	1	3	3	2	2	3	3	2	2
1	1	3	3	4	4	4	4	2	2
4	4	1	1	2	2	3	3	3	3
1	1	1	1	4	4	4	4	2	2
4	4	3	3	2	2	3	3	3	3
4	4	3	3	2	2	3	3	3	3
1	1	3	3	4	4	4	4	3	3
1	1	1	1	2	2	3	3	3	3
4	4	3	3	2	2	3	3	3	3
4	4	3	3	2	2	3	3	3	3
4	4	3	3	2	2	3	3	2	2

For Review Only

4	4	3	3	2	2	3	3	3	3
1	1	3	3	2	2	3	3	3	3
4	4	3	3	2	2	3	3	3	3
4	4	3	3	2	2	3	3	3	3
4	4	3	3	2	2	3	3	3	3
4	4	3	3	2	2	3	3	3	3
4	4	3	3	2	2	3	3	3	3
1	1	1	1	2	2	3	3	3	3
4	4	3	3	2	2	3	3	3	3
4	4	3	3	2	2	3	3	3	3
4	4	3	3	2	2	3	3	3	3
4	4	3	3	2	2	3	3	3	3
4	4	3	3	2	2	3	3	3	3
1	1	3	3	4	4	4	4	2	2
4	4	3	3	2	2	3	3	3	3
1	1	1	1	2	2	3	3	3	3
4	4	1	1	2	2	3	3	2	2
4	4	3	3	2	2	3	3	3	3
4	4	3	3	2	2	3	3	3	3
1	1	3	3	2	2	3	3	3	3
4	4	3	3	2	2	3	3	3	3
4	4	3	3	2	2	3	3	2	2
1	1	3	3	4	4	4	4	2	2
1	1	3	3	2	2	3	3	3	3
1	1	1	1	4	4	4	4	2	2
1	1	3	3	4	4	4	4	3	3
1	1	1	1	2	2	3	3	3	3
4	4	3	3	2	2	3	3	2	2
4	4	3	3	2	2	3	3	3	3
4	4	3	3	2	2	3	3	3	3
4	4	1	1	2	2	3	3	3	3
4	4	1	1	2	2	3	3	3	3
4	4	1	1	2	2	3	3	2	2
4	4	3	3	2	2	3	3	3	3
4	4	3	3	2	2	3	3	3	3
1	1	1	1	4	4	4	4	2	2
1	1	1	1	4	4	4	4	2	2
1	1	3	3	4	4	4	4	2	2
4	4	3	3	2	2	3	3	3	3
1	1	1	1	4	4	4	4	2	2
4	4	3	3	2	2	3	3	3	3
4	4	3	3	2	2	3	3	3	3
4	4	1	1	2	2	3	3	3	3
4	4	3	3	2	2	3	3	3	3
1	1	3	3	2	2	3	3	3	3
4	4	3	3	2	2	3	3	3	3
1	1	3	3	2	2	3	3	3	3
4	4	3	3	4	4	4	4	3	3
4	4	3	3	2	2	3	3	3	3
4	4	3	3	2	2	3	3	2	2
4	4	3	3	2	2	3	3	3	3
4	4	1	1	2	2	3	3	2	2
1	1	1	1	2	2	3	3	3	3
4	4	3	3	2	2	3	3	3	3
1	1	3	3	2	2	3	3	3	3

For Review Only

4	4	3	3	2	2	3	3	2	2
4	4	3	3	2	2	3	3	3	3
1	1	3	3	4	4	4	4	2	2
4	4	3	3	2	2	3	3	3	3
4	4	3	3	2	2	3	3	3	3
4	4	1	1	2	2	3	3	3	3
4	4	3	3	2	2	3	3	3	3
4	4	3	3	2	2	3	3	3	3
1	1	3	3	2	2	3	3	3	3
1	1	3	3	2	2	3	3	3	3
1	1	1	1	2	2	3	3	3	3
4	4	3	3	2	2	3	3	3	3
4	4	3	3	2	2	3	3	3	3
1	1	3	3	2	2	3	3	3	3
4	4	3	3	2	2	3	3	3	3
4	4	3	3	2	2	3	3	3	3
1	1	1	1	2	2	3	3	3	3
4	4	3	3	2	2	3	3	3	3
4	4	3	3	2	2	3	3	3	3
4	4	1	1	2	2	3	3	3	3
4	4	3	3	2	2	3	3	3	3
4	4	3	3	2	2	3	3	3	3
4	4	3	3	2	2	3	3	3	3
4	4	1	1	2	2	3	3	2	2
4	4	3	3	2	2	3	3	3	3
4	4	1	1	2	2	3	3	3	3
4	4	3	3	2	2	3	3	3	3
1	1	3	3	4	4	4	4	3	3
4	4	3	3	2	2	3	3	3	3
4	4	3	3	2	2	3	3	3	3
4	4	3	3	2	2	3	3	2	2
4	4	3	3	4	4	4	4	3	3
4	4	3	3	2	2	3	3	3	3
4	4	3	3	2	2	3	3	3	3
4	4	3	3	2	2	3	3	3	3
4	4	1	1	2	2	3	3	3	3
1	1	3	3	4	4	4	4	3	3
4	4	3	3	2	2	3	3	3	3
4	4	3	3	2	2	3	3	2	2
4	4	3	3	2	2	3	3	2	2
4	4	3	3	2	2	3	3	3	3
4	4	3	3	2	2	3	3	3	3
1	1	3	3	2	2	3	3	3	3
4	4	3	3	4	4	4	4	3	3
4	4	3	3	2	2	3	3	3	3
1	1	3	3	2	2	3	3	3	3
4	4	3	3	2	2	3	3	3	3
4	4	3	3	2	2	3	3	3	3
1	1	3	3	2	2	3	3	3	3
4	4	3	3	2	2	3	3	3	3
4	4	3	3	2	2	3	3	3	3
1	1	3	3	2	2	3	3	3	3
4	4	3	3	2	2	3	3	3	3
1	1	3	3	4	4	4	4	2	2
4	4	3	3	2	2	3	3	3	3

For Review Only

4	4	3	3	2	2	3	3	3	3
4	4	1	1	2	2	3	3	3	3
4	4	1	1	2	2	3	3	3	3
4	4	3	3	2	2	3	3	3	3
4	4	3	3	2	2	3	3	3	3
4	4	3	3	2	2	3	3	3	3
4	4	3	3	2	2	3	3	2	2
4	4	3	3	2	2	3	3	3	3
4	4	3	3	2	2	3	3	3	3
4	4	3	3	2	2	3	3	3	3
4	4	3	3	2	2	3	3	3	3
4	4	3	3	2	2	3	3	3	3
4	4	3	3	2	2	3	3	3	3
1	1	3	3	2	2	3	3	3	3
1	1	3	3	4	4	4	4	3	3
4	4	3	3	2	2	3	3	2	2
4	4	3	3	2	2	3	3	3	3
1	1	3	3	4	4	4	4	3	3
4	4	1	1	2	2	3	3	3	3
4	4	3	3	2	2	3	3	3	3
4	4	3	3	2	2	3	3	3	3
4	4	3	3	2	2	3	3	3	3
4	4	3	3	2	2	3	3	2	2
4	4	3	3	2	2	3	3	3	3
1	1	3	3	2	2	3	3	3	3
4	4	3	3	2	2	3	3	3	3
4	4	3	3	2	2	3	3	3	3
1	1	3	3	2	2	3	3	3	3
1	1	1	1	2	2	3	3	3	3
4	4	3	3	2	2	3	3	3	3
1	1	3	3	4	4	4	4	3	3
4	4	3	3	2	2	3	3	3	3
4	4	3	3	2	2	3	3	3	3
1	1	3	3	4	4	4	4	3	3
4	4	3	3	2	2	3	3	3	3
4	4	3	3	2	2	3	3	3	3
4	4	3	3	2	2	3	3	3	3
1	1	3	3	2	2	3	3	3	3
4	4	3	3	2	2	3	3	3	3
4	4	3	3	2	2	3	3	3	3
4	4	3	3	2	2	3	3	3	3
4	4	3	3	2	2	3	3	3	3
4	4	3	3	2	2	3	3	2	2
4	4	3	3	2	2	3	3	3	3
4	4	1	1	2	2	3	3	3	3
4	4	3	3	2	2	3	3	3	3
4	4	3	3	2	2	3	3	2	2
4	4	3	3	2	2	3	3	2	2
1	1	3	3	4	4	4	4	3	3
4	4	3	3	2	2	3	3	3	3
4	4	3	3	2	2	3	3	3	3
1	1	1	1	2	2	3	3	3	3
4	4	3	3	2	2	3	3	3	3
4	4	3	3	2	2	3	3	3	3
4	4	1	1	2	2	3	3	2	2

For Review Only

4	4	3	3	2	2	3	3	3	3
1	1	3	3	2	2	3	3	3	3
4	4	3	3	2	2	3	3	3	3
4	4	3	3	2	2	3	3	3	3
1	1	1	1	4	4	4	4	2	2
4	4	3	3	2	2	3	3	3	3
4	4	3	3	2	2	3	3	3	3
1	1	1	1	4	4	4	4	2	2
4	4	3	3	2	2	3	3	3	3
4	4	3	3	2	2	3	3	2	2
4	4	3	3	2	2	3	3	3	3
1	1	1	1	4	4	4	4	3	3
4	4	3	3	2	2	3	3	3	3
4	4	3	3	2	2	3	3	3	3
4	4	3	3	2	2	3	3	3	3
4	4	3	3	2	2	3	3	3	3
4	4	3	3	2	2	3	3	3	3
4	4	3	3	2	2	3	3	3	3
4	4	3	3	2	2	3	3	2	2
4	4	3	3	2	2	3	3	3	3
1	1	3	3	2	2	3	3	3	3
4	4	3	3	2	2	3	3	3	3
4	4	3	3	2	2	3	3	3	3
4	4	1	1	2	2	3	3	3	3
1	1	1	1	2	2	3	3	3	3
4	4	3	3	2	2	3	3	3	3
4	4	1	1	2	2	3	3	2	2
4	4	3	3	2	2	3	3	3	3
4	4	3	3	2	2	3	3	3	3
4	4	3	3	2	2	3	3	3	3
1	1	3	3	2	2	3	3	3	3
4	4	3	3	2	2	3	3	3	3
1	1	3	3	2	2	3	3	3	3
4	4	3	3	2	2	3	3	3	3
4	4	3	3	2	2	3	3	2	2
4	4	1	1	2	2	3	3	2	2
4	4	3	3	2	2	3	3	3	3
4	4	3	3	2	2	3	3	3	3
1	1	3	3	4	4	4	4	2	2
4	4	1	1	2	2	3	3	2	2
4	4	3	3	2	2	3	3	3	3
1	1	3	3	2	2	3	3	3	3
4	4	1	1	2	2	3	3	3	3
4	4	3	3	2	2	3	3	3	3
4	4	3	3	2	2	3	3	3	3
4	4	3	3	2	2	3	3	3	3
4	4	3	3	2	2	3	3	3	3
1	1	3	3	2	2	3	3	3	3
4	4	3	3	2	2	3	3	3	3
4	4	3	3	2	2	3	3	2	2
4	4	3	3	2	2	3	3	3	3
4	4	3	3	2	2	3	3	3	3
4	4	3	3	2	2	3	3	3	3
4	4	3	3	2	2	3	3	3	3
4	4	1	1	2	2	3	3	2	2

For Review Only

4	4	3	3	2	2	3	3	3	3
4	4	3	3	2	2	3	3	3	3

For Review Only

SNP275	SNP275	SNP2750	SNP2750	SNP2751	SNP2751	SNP2752	SNP2752	SNP2753	SNP2753
4	4	2	2	1	1	1	1	1	1
1	1	2	2	1	1	4	4	3	3
4	4	2	2	1	1	4	4	1	1
4	4	2	2	1	1	1	1	1	1
4	4	4	4	4	4	4	4	1	1
1	1	2	2	1	1	1	1	1	1
4	4	2	2	1	1	1	1	1	1
1	1	2	2	1	1	1	1	3	3
4	4	2	2	1	1	1	1	1	1
1	1	4	4	4	4	4	4	3	3
1	1	2	2	1	1	4	4	1	1
4	4	2	2	1	1	4	4	1	1
4	4	2	2	1	1	1	1	3	3
1	1	2	2	1	1	4	4	3	3
4	4	2	2	1	1	1	1	1	1
4	4	4	4	4	4	4	4	1	1
1	1	4	4	4	4	4	4	1	1
4	4	4	4	4	4	4	4	1	1
4	4	2	2	1	1	1	1	1	1
1	1	2	2	1	1	1	1	3	3
4	4	2	2	1	1	1	1	1	1
4	4	4	4	4	4	4	4	1	1
4	4	2	2	1	1	4	4	1	1
1	1	2	2	1	1	4	4	1	1
4	4	4	4	4	4	4	4	1	1
4	4	2	2	1	1	1	1	1	1
4	4	2	2	1	1	1	1	1	1
4	4	2	2	1	1	1	1	1	1
4	4	2	2	1	1	1	1	1	1
1	1	4	4	4	4	4	4	3	3
4	4	2	2	4	4	4	4	1	1
4	4	2	2	1	1	1	1	1	1
4	4	2	2	1	1	1	1	1	1
4	4	2	2	1	1	4	4	3	3
4	4	2	2	1	1	1	1	1	1
4	4	2	2	1	1	4	4	1	1
1	1	4	4	1	1	4	4	1	1
4	4	4	4	4	4	1	1	1	1
1	1	2	2	1	1	4	4	1	1
1	1	4	4	4	4	4	4	1	1
1	1	4	4	4	4	4	4	1	1
1	1	2	2	1	1	1	1	1	1
1	1	2	2	1	1	1	1	3	3
1	1	4	4	4	4	4	4	3	3
4	4	2	2	1	1	1	1	3	3
4	4	2	2	1	1	1	1	1	1
4	4	2	2	1	1	4	4	1	1
4	4	2	2	1	1	1	1	1	1
1	1	2	2	1	1	1	1	3	3
4	4	2	2	4	4	4	4	1	1
4	4	2	2	1	1	1	1	1	1
4	4	2	2	1	1	4	4	1	1

For Review Only

4	4	4	4	4	4	4	4	1	1
4	4	2	2	1	1	1	1	1	1
4	4	4	4	1	1	1	1	1	1
1	1	4	4	4	4	4	4	1	1
4	4	2	2	1	1	1	1	1	1
4	4	2	2	1	1	1	1	1	1
4	4	2	2	1	1	1	1	1	1
1	1	4	4	4	4	4	4	3	3
4	4	4	4	1	1	4	4	1	1
4	4	2	2	4	4	1	1	1	1
4	4	2	2	1	1	4	4	1	1
4	4	2	2	1	1	4	4	1	1
4	4	4	4	4	4	4	4	1	1
4	4	2	2	1	1	1	1	1	1
4	4	2	2	1	1	1	1	1	1
4	4	2	2	1	1	1	1	3	3
1	1	2	2	1	1	1	1	1	1
4	4	2	2	1	1	1	1	1	1
4	4	4	4	4	4	4	4	1	1
4	4	2	2	1	1	1	1	1	1
4	4	2	2	1	1	4	4	1	1
1	1	2	2	1	1	1	1	1	1
4	4	2	2	1	1	1	1	1	1
4	4	2	2	1	1	4	4	1	1
4	4	2	2	1	1	1	1	3	3
4	4	2	2	1	1	1	1	1	1
1	1	2	2	4	4	4	4	3	3
1	1	2	2	1	1	4	4	1	1
4	4	2	2	1	1	1	1	1	1
4	4	4	4	4	4	4	4	1	1
4	4	4	4	4	4	4	4	3	3
4	4	2	2	1	1	4	4	1	1
1	1	2	2	1	1	4	4	1	1
4	4	4	4	4	4	4	4	1	1
4	4	2	2	1	1	1	1	1	1
4	4	2	2	1	1	1	1	3	3
1	1	2	2	4	4	4	4	1	1
4	4	4	4	4	4	4	4	1	1
4	4	2	2	1	1	1	1	1	1
4	4	2	2	1	1	1	1	3	3
4	4	2	2	1	1	1	1	1	1
4	4	2	2	1	1	4	4	1	1
4	4	2	2	1	1	1	1	3	3
4	4	2	2	1	1	4	4	1	1
4	4	2	2	1	1	4	4	1	1
4	4	2	2	1	1	1	1	1	1
1	1	2	2	1	1	4	4	3	3
4	4	2	2	1	1	1	1	3	3
1	1	2	2	1	1	1	1	3	3
4	4	2	2	1	1	1	1	1	1
4	4	2	2	1	1	4	4	1	1
1	1	2	2	1	1	4	4	1	1
1	1	4	4	4	4	4	4	3	3
4	4	2	2	1	1	1	1	1	1
4	4	2	2	1	1	1	1	1	1

For Review Only

1	1	2	2	1	1	1	1	1	1
4	4	2	2	1	1	1	1	1	1
4	4	2	2	1	1	1	1	1	1
1	1	2	2	1	1	4	4	3	3
4	4	2	2	1	1	1	1	1	1
4	4	2	2	1	1	1	1	1	1
4	4	4	4	4	4	4	4	1	1
4	4	4	4	1	1	1	1	1	1
4	4	2	2	1	1	1	1	3	3
4	4	2	2	4	4	1	1	1	1
4	4	2	2	1	1	1	1	1	1
4	4	2	2	4	4	1	1	1	1
4	4	4	4	4	4	4	4	1	1
4	4	2	2	1	1	4	4	1	1
4	4	4	4	1	1	4	4	1	1
4	4	2	2	1	1	1	1	1	1
4	4	2	2	1	1	1	1	3	3
4	4	2	2	1	1	1	1	1	1
1	1	2	2	1	1	4	4	3	3
4	4	2	2	1	1	1	1	3	3
4	4	2	2	1	1	1	1	1	1
4	4	4	4	4	4	1	1	1	1
4	4	2	2	1	1	4	4	1	1
1	1	2	2	1	1	4	4	1	1
4	4	2	2	1	1	1	1	1	1
4	4	2	2	1	1	1	1	1	1
4	4	4	4	4	4	4	4	1	1
4	4	4	4	1	1	4	4	1	1
4	4	4	4	4	4	4	4	1	1
1	1	2	2	1	1	1	1	1	1
1	1	2	2	1	1	4	4	1	1
4	4	4	4	1	1	1	1	1	1
4	4	2	2	1	1	1	1	1	1
4	4	2	2	1	1	1	1	1	1
4	4	2	2	1	1	4	4	3	3
4	4	2	2	1	1	1	1	1	1
1	1	2	2	1	1	4	4	3	3
4	4	2	2	1	1	1	1	1	1
4	4	2	2	1	1	1	1	1	1
1	1	2	2	1	1	4	4	1	1
4	4	2	2	1	1	1	1	1	1
1	1	2	2	1	1	4	4	3	3
4	4	2	2	1	1	1	1	1	1
1	1	2	2	4	4	4	4	1	1
4	4	4	4	1	1	1	1	1	1
4	4	2	2	1	1	1	1	1	1
4	4	2	2	1	1	1	1	1	1
1	1	2	2	1	1	1	1	1	1
1	1	2	2	1	1	1	1	1	1
4	4	2	2	1	1	1	1	1	1
4	4	2	2	1	1	1	1	1	1
1	1	2	2	1	1	1	1	1	1
4	4	2	2	4	4	4	4	1	1
4	4	2	2	1	1	4	4	1	1
1	1	2	2	4	4	1	1	1	1

For Review Only

4	4	2	2	1	1	1	1	1	1
1	1	4	4	4	4	1	1	3	3
1	1	2	2	1	1	1	1	3	3
4	4	2	2	1	1	1	1	1	1
1	1	2	2	1	1	4	4	1	1
1	1	2	2	1	1	1	1	1	1
4	4	2	2	1	1	4	4	1	1
4	4	2	2	1	1	1	1	1	1
1	1	2	2	1	1	4	4	1	1
4	4	4	4	4	4	4	4	1	1
4	4	2	2	1	1	1	1	1	1
4	4	2	2	1	1	4	4	1	1
4	4	2	2	1	1	1	1	1	1
4	4	2	2	1	1	1	1	1	1
1	1	2	2	1	1	4	4	3	3
1	1	2	2	1	1	4	4	1	1
4	4	2	2	1	1	4	4	1	1
4	4	2	2	1	1	1	1	1	1
4	4	2	2	1	1	1	1	1	1
4	4	4	4	4	4	4	4	1	1
4	4	2	2	1	1	1	1	1	1
1	1	2	2	1	1	4	4	1	1
1	1	2	2	1	1	1	1	1	1
4	4	2	2	1	1	4	4	1	1
4	4	2	2	1	1	1	1	1	1
1	1	2	2	1	1	4	4	1	1
4	4	2	2	1	1	1	1	1	1
4	4	2	2	1	1	1	1	1	1
4	4	2	2	1	1	1	1	3	3
4	4	2	2	1	1	1	1	1	1
4	4	2	2	1	1	1	1	1	1
4	4	2	2	4	4	4	4	1	1
4	4	4	4	4	4	4	4	1	1
1	1	2	2	1	1	4	4	3	3
4	4	2	2	1	1	1	1	1	1
4	4	2	2	1	1	4	4	1	1
4	4	2	2	1	1	1	1	1	1
1	1	4	4	1	1	4	4	1	1
1	1	2	2	1	1	1	1	1	1
1	1	2	2	1	1	4	4	1	1
4	4	2	2	1	1	4	4	1	1
4	4	2	2	1	1	1	1	1	1
1	1	2	2	1	1	4	4	1	1
4	4	2	2	1	1	1	1	1	1
4	4	2	2	1	1	4	4	3	3
4	4	2	2	1	1	4	4	1	1
1	1	2	2	1	1	4	4	1	1
1	1	2	2	1	1	4	4	1	1
1	1	4	4	4	4	4	4	3	3
4	4	4	4	4	4	4	4	1	1
4	4	2	2	1	1	1	1	1	1
1	1	4	4	4	4	4	4	3	3
1	1	2	2	1	1	1	1	1	1
4	4	2	2	1	1	4	4	1	1
4	4	2	2	1	1	4	4	1	1

For Review Only

4	4	4	4	4	4	4	4	1	1
1	1	2	2	1	1	1	1	1	1
4	4	4	4	4	4	4	4	1	1
4	4	2	2	1	1	1	1	1	1
4	4	2	2	1	1	1	1	3	3
4	4	2	2	1	1	1	1	1	1
4	4	2	2	1	1	1	1	1	1
1	1	4	4	4	4	4	4	3	3
1	1	2	2	1	1	4	4	1	1
1	1	2	2	1	1	4	4	1	1
4	4	2	2	1	1	1	1	1	1
4	4	2	2	1	1	1	1	3	3
4	4	2	2	1	1	1	1	1	1
1	1	2	2	1	1	1	1	3	3
1	1	2	2	1	1	4	4	1	1
4	4	4	4	4	4	4	4	1	1
1	1	2	2	1	1	1	1	1	1
1	1	4	4	4	4	4	4	1	1
1	1	2	2	1	1	4	4	1	1
4	4	4	4	4	4	4	4	1	1
4	4	2	2	1	1	4	4	1	1
4	4	2	2	1	1	1	1	1	1
4	4	4	4	4	4	4	4	1	1
1	1	2	2	1	1	1	1	1	1
1	1	4	4	4	4	4	4	3	3
4	4	2	2	1	1	1	1	1	1
4	4	2	2	1	1	4	4	1	1
4	4	4	4	4	4	4	4	1	1
4	4	2	2	1	1	1	1	1	1
4	4	4	4	4	4	4	4	1	1
1	1	2	2	1	1	4	4	1	1
4	4	2	2	1	1	1	1	1	1
1	1	2	2	1	1	1	1	1	1
4	4	2	2	1	1	1	1	3	3
1	1	2	2	1	1	1	1	1	1
1	1	2	2	1	1	4	4	1	1
4	4	2	2	1	1	1	1	1	1
4	4	2	2	1	1	4	4	1	1
4	4	2	2	1	1	4	4	1	1
1	1	2	2	1	1	4	4	1	1
4	4	2	2	1	1	1	1	1	1
4	4	2	2	1	1	1	1	1	1
4	4	2	2	1	1	1	1	1	1
4	4	4	4	4	4	4	4	1	1
4	4	2	2	1	1	4	4	1	1
4	4	2	2	1	1	1	1	1	1
1	1	2	2	1	1	1	1	1	1
4	4	2	2	1	1	1	1	1	1
4	4	2	2	1	1	4	4	1	1
1	1	2	2	1	1	4	4	1	1
4	4	4	4	4	4	4	4	1	1
1	1	2	2	1	1	4	4	1	1
4	4	2	2	1	1	4	4	1	1
4	4	2	2	1	1	1	1	1	1
1	1	2	2	1	1	1	1	1	1

For Review Only

4	4	4	4	4	4	4	4	1	1
4	4	2	2	1	1	4	4	1	1

For Review Only

SNP2754	SNP2754	SNP2755	SNP2755	SNP2756	SNP2756	SNP2757	SNP2757	SNP2758	SNP2758
1	1	1	1	2	2	4	4	3	3
1	1	1	1	2	2	1	1	1	1
1	1	1	1	2	2	1	1	1	1
1	1	1	1	2	2	1	1	1	1
3	3	1	1	2	2	4	4	3	3
1	1	1	1	2	2	4	4	3	3
1	1	1	1	2	2	1	1	1	1
1	1	1	1	2	2	4	4	1	1
1	1	1	1	2	2	4	4	3	3
3	3	2	2	3	3	1	1	1	1
1	1	1	1	2	2	1	1	1	1
1	1	1	1	2	2	1	1	1	1
1	1	1	1	2	2	1	1	1	1
1	1	1	1	2	2	1	1	1	1
1	1	1	1	2	2	1	1	1	1
3	3	1	1	2	2	1	1	1	1
3	3	2	2	3	3	1	1	1	1
3	3	1	1	2	2	4	4	3	3
1	1	1	1	2	2	1	1	1	1
1	1	1	1	2	2	1	1	1	1
1	1	1	1	2	2	1	1	1	1
3	3	2	2	3	3	1	1	1	1
1	1	1	1	2	2	1	1	1	1
1	1	1	1	2	2	4	4	3	3
3	3	2	2	2	2	1	1	1	1
1	1	1	1	2	2	1	1	1	1
1	1	1	1	2	2	1	1	1	1
1	1	1	1	2	2	1	1	1	1
1	1	1	1	2	2	4	4	3	3
3	3	2	2	3	3	1	1	1	1
3	3	2	2	3	3	4	4	3	3
1	1	1	1	2	2	1	1	1	1
1	1	1	1	2	2	4	4	3	3
1	1	1	1	2	2	4	4	3	3
1	1	1	1	2	2	1	1	1	1
1	1	1	1	2	2	1	1	1	1
1	1	1	1	2	2	4	4	3	3
3	3	2	2	3	3	4	4	1	1
1	1	1	1	2	2	1	1	1	1
3	3	2	2	3	3	1	1	1	1
1	1	1	1	2	2	1	1	1	1
1	1	1	1	2	2	4	4	3	3
1	1	1	1	2	2	1	1	1	1
3	3	2	2	3	3	4	4	3	3
1	1	1	1	2	2	1	1	1	1
1	1	1	1	2	2	1	1	1	1
1	1	1	1	2	2	1	1	1	1
1	1	1	1	2	2	1	1	1	1
1	1	1	1	2	2	4	4	1	1
1	1	1	1	3	3	1	1	1	1
1	1	1	1	2	2	4	4	3	3
1	1	1	1	2	2	4	4	3	3

3	3	2	2	3	3	4	4	3	3
1	1	1	1	2	2	4	4	3	3
1	1	1	1	2	2	4	4	3	3
3	3	2	2	3	3	1	1	1	1
1	1	1	1	2	2	1	1	1	1
1	1	1	1	2	2	1	1	1	1
1	1	1	1	2	2	1	1	1	1
3	3	1	1	2	2	1	1	1	1
1	1	1	1	2	2	4	4	3	3
1	1	1	1	2	2	1	1	1	1
1	1	1	1	2	2	1	1	1	1
1	1	1	1	2	2	1	1	1	1
3	3	2	2	3	3	4	4	3	3
1	1	1	1	2	2	4	4	3	3
1	1	1	1	2	2	4	4	1	1
1	1	1	1	2	2	1	1	1	1
1	1	1	1	2	2	4	4	1	1
1	1	1	1	2	2	1	1	1	1
3	3	2	2	3	3	1	1	1	1
1	1	1	1	2	2	4	4	3	3
1	1	1	1	2	2	1	1	1	1
1	1	1	1	2	2	1	1	1	1
1	1	1	1	2	2	4	4	1	1
1	1	1	1	2	2	4	4	3	3
1	1	1	1	2	2	1	1	1	1
1	1	1	1	2	2	4	4	3	3
3	3	2	2	3	3	1	1	1	1
1	1	1	1	2	2	1	1	1	1
1	1	1	1	2	2	1	1	1	1
3	3	2	2	3	3	4	4	3	3
3	3	2	2	3	3	4	4	3	3
1	1	1	1	2	2	1	1	1	1
1	1	1	1	2	2	1	1	1	1
3	3	2	2	3	3	4	4	3	3
1	1	1	1	2	2	4	4	3	3
1	1	1	1	2	2	1	1	1	1
3	3	2	2	3	3	1	1	1	1
3	3	2	2	3	3	1	1	1	1
1	1	1	1	2	2	1	1	1	1
1	1	1	1	2	2	1	1	1	1
1	1	1	1	2	2	1	1	1	1
1	1	1	1	2	2	4	4	3	3
1	1	1	1	2	2	4	4	1	1
1	1	1	1	2	2	1	1	1	1
1	1	1	1	2	2	1	1	1	1
1	1	1	1	2	2	1	1	1	1
1	1	1	1	2	2	1	1	1	1
1	1	1	1	2	2	1	1	1	1
1	1	1	1	2	2	1	1	1	1
1	1	1	1	2	2	1	1	1	1
1	1	1	1	2	2	4	4	3	3
1	1	1	1	2	2	1	1	1	1
3	3	1	1	2	2	1	1	1	1
1	1	1	1	2	2	1	1	1	1
1	1	1	1	2	2	1	1	1	1

For Review Only

1	1	1	1	2	2	1	1	1	1
1	1	1	1	2	2	4	4	1	1
1	1	1	1	2	2	1	1	1	1
1	1	1	1	2	2	1	1	1	1
1	1	1	1	2	2	1	1	1	1
1	1	1	1	2	2	1	1	1	1
3	3	2	2	3	3	1	1	1	1
1	1	1	1	2	2	4	4	3	3
1	1	1	1	2	2	1	1	1	1
1	1	1	1	2	2	1	1	1	1
1	1	1	1	2	2	4	4	3	3
3	3	1	1	2	2	1	1	1	1
3	3	2	2	3	3	4	4	1	1
1	1	1	1	2	2	1	1	1	1
1	1	1	1	2	2	1	1	1	1
1	1	1	1	2	2	1	1	1	1
1	1	1	1	2	2	4	4	1	1
1	1	1	1	2	2	1	1	1	1
1	1	1	1	2	2	4	4	3	3
1	1	1	1	2	2	1	1	1	1
1	1	1	1	2	2	1	1	1	1
3	3	2	2	3	3	4	4	3	3
1	1	1	1	2	2	1	1	1	1
1	1	1	1	2	2	1	1	1	1
1	1	1	1	2	2	1	1	1	1
1	1	1	1	2	2	1	1	1	1
3	3	2	2	3	3	1	1	1	1
1	1	1	1	2	2	4	4	1	1
3	3	2	2	3	3	4	4	3	3
1	1	1	1	2	2	1	1	1	1
1	1	1	1	2	2	1	1	1	1
1	1	1	1	2	2	1	1	1	1
1	1	1	1	2	2	1	1	1	1
1	1	1	1	2	2	1	1	1	1
1	1	1	1	2	2	4	4	3	3
1	1	1	1	2	2	1	1	1	1
1	1	1	1	2	2	1	1	1	1
1	1	1	1	2	2	1	1	1	1
1	1	1	1	2	2	1	1	1	1
1	1	1	1	2	2	1	1	1	1
1	1	1	1	2	2	1	1	1	1
1	1	1	1	2	2	1	1	1	1
1	1	1	1	2	2	4	4	3	3
3	3	2	2	3	3	1	1	1	1
1	1	1	1	2	2	1	1	1	1
1	1	1	1	2	2	1	1	1	1
1	1	1	1	2	2	1	1	1	1
1	1	1	1	2	2	4	4	3	3
1	1	1	1	2	2	1	1	1	1
1	1	1	1	2	2	1	1	1	1
1	1	1	1	2	2	1	1	1	1
1	1	1	1	2	2	4	4	3	3
1	1	1	1	2	2	4	4	3	3
1	1	1	1	2	2	4	4	3	3
1	1	1	1	2	2	1	1	1	1

For Review Only

1	1	1	1	2	2	1	1	1	1
3	3	2	2	3	3	1	1	1	1
1	1	1	1	2	2	1	1	1	1
1	1	1	1	2	2	1	1	1	1
1	1	1	1	2	2	4	4	3	3
1	1	1	1	2	2	4	4	3	3
1	1	1	1	2	2	1	1	1	1
1	1	1	1	2	2	1	1	1	1
1	1	1	1	2	2	1	1	1	1
3	3	2	2	3	3	1	1	1	1
1	1	1	1	2	2	1	1	1	1
1	1	1	1	2	2	1	1	1	1
1	1	1	1	2	2	4	4	3	3
1	1	1	1	2	2	1	1	1	1
1	1	1	1	2	2	1	1	1	1
1	1	1	1	2	2	1	1	1	1
1	1	1	1	2	2	1	1	3	3
1	1	1	1	2	2	1	1	1	1
1	1	1	1	2	2	1	1	1	1
3	3	2	2	3	3	1	1	1	1
1	1	1	1	2	2	1	1	1	1
1	1	1	1	2	2	1	1	1	1
1	1	1	1	2	2	1	1	1	1
1	1	1	1	2	2	1	1	1	1
1	1	1	1	2	2	1	1	1	1
1	1	1	1	2	2	4	4	3	3
1	1	1	1	2	2	1	1	1	1
1	1	1	1	2	2	1	1	1	1
1	1	1	1	2	2	1	1	1	1
1	1	1	1	2	2	4	4	3	3
1	1	1	1	2	2	4	4	3	3
3	3	2	2	2	2	1	1	1	1
3	3	2	2	3	3	4	4	1	1
1	1	1	1	2	2	1	1	1	1
1	1	1	1	2	2	4	4	1	1
1	1	1	1	2	2	1	1	1	1
1	1	1	1	2	2	1	1	1	1
1	1	1	1	2	2	4	4	3	3
1	1	1	1	2	2	4	4	3	3
1	1	1	1	2	2	4	4	3	3
1	1	1	1	2	2	1	1	1	1
1	1	1	1	2	2	1	1	1	1
1	1	1	1	2	2	1	1	1	1
1	1	1	1	2	2	1	1	1	1
1	1	1	1	2	2	1	1	1	1
1	1	1	1	2	2	4	4	3	3
1	1	1	1	2	2	1	1	1	1
1	1	1	1	2	2	1	1	1	1
3	3	1	1	2	2	1	1	1	1
3	3	2	2	3	3	1	1	1	1
1	1	1	1	2	2	1	1	1	1
3	3	1	1	2	2	4	4	3	3
1	1	1	1	2	2	1	1	1	1
1	1	1	1	2	2	1	1	1	1
1	1	1	1	2	2	1	1	1	1

For Review Only

3	3	1	1	2	2	4	4	3	3
1	1	1	1	2	2	1	1	1	1
3	3	2	2	3	3	1	1	1	1
1	1	1	1	2	2	1	1	1	1
1	1	1	1	2	2	1	1	1	1
1	1	1	1	2	2	1	1	1	1
1	1	1	1	2	2	1	1	1	1
3	3	2	2	3	3	1	1	1	1
1	1	1	1	2	2	4	4	3	3
1	1	1	1	2	2	1	1	1	1
1	1	1	1	2	2	1	1	1	1
1	1	1	1	2	2	4	4	3	3
1	1	1	1	2	2	1	1	1	1
1	1	1	1	2	2	4	4	3	3
1	1	1	1	2	2	4	4	1	1
3	3	1	1	2	2	1	1	1	1
1	1	1	1	2	2	1	1	1	1
1	1	1	1	2	2	1	1	1	1
1	1	1	1	2	2	1	1	1	1
3	3	2	2	3	3	1	1	1	1
1	1	1	1	2	2	1	1	1	1
1	1	1	1	2	2	1	1	1	1
3	3	2	2	3	3	1	1	1	1
1	1	1	1	2	2	1	1	1	1
3	3	1	1	2	2	1	1	1	1
1	1	1	1	2	2	4	4	3	3
1	1	1	1	2	2	1	1	1	1
3	3	1	1	3	3	1	1	1	1
1	1	1	1	3	3	1	1	1	1
3	3	2	2	3	3	4	4	3	3
1	1	1	1	2	2	4	4	3	3
1	1	1	1	2	2	1	1	1	1
1	1	1	1	2	2	1	1	1	1
1	1	1	1	2	2	1	1	1	1
1	1	1	1	2	2	1	1	1	1
1	1	1	1	2	2	4	4	3	3
1	1	1	1	2	2	1	1	1	1
1	1	1	1	2	2	1	1	1	1
1	1	1	1	2	2	1	1	1	1
1	1	1	1	2	2	1	1	1	1
1	1	1	1	2	2	4	4	1	1
1	1	1	1	2	2	1	1	1	1
1	1	1	1	2	2	1	1	1	1
3	3	2	2	3	3	1	1	1	1
1	1	1	1	2	2	4	4	3	3
1	1	1	1	2	2	1	1	1	1
1	1	1	1	2	2	1	1	1	1
1	1	1	1	2	2	1	1	1	1
1	1	1	1	2	2	1	1	1	1
1	1	1	1	2	2	1	1	1	1
3	3	2	2	2	2	4	4	3	3
1	1	1	1	2	2	1	1	1	1
1	1	1	1	2	2	1	1	1	1
1	1	1	1	2	2	1	1	1	1
1	1	1	1	2	2	1	1	1	1

For Review Only

3	3	2	2	2	2	1	1	1	1
1	1	1	1	2	2	4	4	3	3

For Review Only

SNP2759	SNP2759	SNP276	SNP276	SNP2760	SNP2760	SNP2761	SNP2761	SNP2762	SNP2762
4	4	4	4	1	1	3	3	1	1
4	4	3	3	3	3	3	3	4	4
4	4	4	4	3	3	3	3	4	4
4	4	4	4	3	3	3	3	4	4
4	4	4	4	3	3	3	3	4	4
4	4	3	3	1	1	3	3	1	1
4	4	4	4	1	1	3	3	1	1
4	4	3	3	3	3	3	3	4	4
4	4	4	4	1	1	3	3	1	1
4	4	4	4	3	3	3	3	4	4
4	4	4	4	3	3	3	3	4	4
4	4	4	4	3	3	3	3	4	4
4	4	4	4	1	1	3	3	4	4
4	4	4	4	3	3	3	3	4	4
4	4	4	4	3	3	3	3	4	4
4	4	4	4	3	3	3	3	4	4
1	1	4	4	3	3	3	3	4	4
4	4	4	4	3	3	3	3	4	4
4	4	4	4	3	3	3	3	4	4
1	1	4	4	1	1	3	3	1	1
1	1	4	4	1	1	3	3	1	1
4	4	4	4	3	3	3	3	4	4
4	4	4	4	3	3	3	3	4	4
4	4	4	4	3	3	3	3	4	4
4	4	4	4	3	3	3	3	4	4
4	4	4	4	1	1	3	3	1	1
4	4	4	4	3	3	3	3	4	4
4	4	4	4	3	3	3	3	4	4
4	4	4	4	1	1	3	3	1	1
1	1	3	3	3	3	3	3	4	4
4	4	4	4	3	3	3	3	4	4
4	4	4	4	1	1	3	3	1	1
4	4	4	4	1	1	3	3	1	1
4	4	4	4	3	3	3	3	4	4
4	4	4	4	3	3	3	3	4	4
4	4	4	4	3	3	3	3	4	4
4	4	3	3	3	3	3	3	4	4
4	4	4	4	3	3	3	3	4	4
4	4	4	4	3	3	2	2	4	4
4	4	4	4	3	3	2	2	4	4
1	1	3	3	3	3	3	3	4	4
4	4	4	4	1	1	3	3	4	4
4	4	4	4	1	1	3	3	1	1
4	4	3	3	3	3	3	3	4	4
1	1	4	4	1	1	3	3	1	1
4	4	4	4	1	1	3	3	4	4
4	4	4	4	3	3	3	3	4	4
4	4	4	4	1	1	3	3	1	1
4	4	3	3	1	1	3	3	1	1
4	4	4	4	3	3	3	3	4	4
4	4	4	4	1	1	3	3	1	1
4	4	4	4	3	3	3	3	4	4

For Review Only

4	4	4	4	3	3	3	3	4	4
4	4	4	4	1	1	3	3	1	1
4	4	4	4	3	3	3	3	4	4
1	1	4	4	3	3	3	3	4	4
4	4	4	4	3	3	3	3	4	4
4	4	4	4	1	1	3	3	1	1
4	4	4	4	1	1	3	3	1	1
4	4	3	3	3	3	3	3	4	4
4	4	4	4	3	3	3	3	4	4
4	4	4	4	3	3	3	3	4	4
4	4	4	4	3	3	3	3	4	4
4	4	4	4	3	3	3	3	4	4
4	4	4	4	3	3	3	3	4	4
4	4	4	4	1	1	3	3	1	1
4	4	4	4	3	3	3	3	4	4
4	4	4	4	1	1	3	3	1	1
4	4	4	4	1	1	3	3	1	1
4	4	4	4	1	1	3	3	1	1
4	4	4	4	3	3	3	3	4	4
4	4	4	4	1	1	3	3	1	1
4	4	4	4	3	3	3	3	4	4
4	4	4	4	1	1	3	3	1	1
4	4	4	4	1	1	3	3	1	1
4	4	4	4	3	3	3	3	4	4
1	1	4	4	1	1	3	3	1	1
4	4	4	4	3	3	3	3	4	4
4	4	4	4	3	3	3	3	4	4
4	4	3	3	3	3	2	2	4	4
4	4	4	4	1	1	3	3	1	1
4	4	4	4	3	3	3	3	4	4
1	1	4	4	3	3	3	3	4	4
4	4	4	4	3	3	3	3	4	4
4	4	4	4	3	3	2	2	4	4
4	4	4	4	3	3	3	3	4	4
4	4	4	4	1	1	3	3	1	1
1	1	4	4	1	1	3	3	1	1
1	1	4	4	3	3	3	3	4	4
1	1	4	4	3	3	3	3	4	4
4	4	4	4	3	3	3	3	4	4
1	1	4	4	1	1	3	3	1	1
4	4	4	4	1	1	3	3	1	1
4	4	4	4	3	3	3	3	4	4
4	4	4	4	3	3	3	3	1	1
4	4	4	4	3	3	3	3	4	4
4	4	4	4	3	3	3	3	1	1
4	4	4	4	3	3	3	3	1	1
4	4	4	4	3	3	3	3	4	4
4	4	4	4	1	1	3	3	1	1
4	4	4	4	1	1	3	3	1	1
4	4	4	4	1	1	3	3	1	1
4	4	4	4	3	3	3	3	4	4
4	4	4	4	3	3	3	3	4	4
4	4	4	4	3	3	3	3	4	4
4	4	4	4	3	3	3	3	4	4
4	4	4	4	1	1	3	3	1	1

For Review Only

4	4	4	4	1	1	3	3	1	1
4	4	4	4	3	3	3	3	1	1
4	4	4	4	1	1	3	3	1	1
4	4	4	4	3	3	3	3	4	4
4	4	4	4	3	3	3	3	4	4
4	4	4	4	1	1	3	3	1	1
4	4	4	4	3	3	3	3	4	4
4	4	4	4	3	3	3	3	4	4
4	4	4	4	1	1	3	3	1	1
4	4	4	4	3	3	3	3	4	4
4	4	4	4	3	3	3	3	1	1
4	4	4	4	3	3	3	3	4	4
4	4	4	4	3	3	3	3	4	4
4	4	4	4	3	3	3	3	4	4
4	4	4	4	3	3	3	3	4	4
4	4	4	4	3	3	3	3	4	4
4	4	4	4	1	1	3	3	1	1
4	4	4	4	3	3	3	3	4	4
4	4	4	4	3	3	2	2	4	4
4	4	4	4	3	3	3	3	4	4
4	4	4	4	3	3	3	3	4	4
4	4	4	4	3	3	3	3	4	4
4	4	4	4	3	3	3	3	4	4
4	4	4	4	3	3	2	2	4	4
4	4	4	4	1	1	3	3	1	1
4	4	4	4	1	1	3	3	1	1
4	4	4	4	3	3	3	3	4	4
4	4	4	4	3	3	3	3	4	4
4	4	4	4	3	3	3	3	4	4
4	4	4	4	1	1	3	3	1	1
4	4	4	4	3	3	2	2	4	4
4	4	4	4	3	3	3	3	4	4
4	4	4	4	1	1	3	3	1	1
4	4	4	4	3	3	3	3	4	4
4	4	4	4	3	3	3	3	4	4
4	4	4	4	3	3	3	3	1	1
4	4	4	4	3	3	3	3	4	4
4	4	4	4	1	1	3	3	1	1
4	4	4	4	1	1	3	3	1	1
4	4	4	4	3	3	2	2	4	4
4	4	4	4	1	1	3	3	1	1
4	4	4	4	3	3	3	3	4	4
4	4	4	4	1	1	3	3	1	1
1	1	4	4	3	3	3	3	4	4
4	4	4	4	3	3	3	3	4	4
4	4	4	4	3	3	3	3	4	4
4	4	4	4	1	1	3	3	1	1
4	4	3	3	1	1	3	3	1	1
4	4	4	4	3	3	3	3	4	4
4	4	4	4	1	1	3	3	1	1
4	4	4	4	1	1	3	3	1	1
4	4	4	4	1	1	2	2	1	1
4	4	4	4	3	3	3	3	4	4
4	4	4	4	3	3	3	3	4	4
4	4	4	4	3	3	3	3	4	4

For Review Only

4	4	4	4	1	1	3	3	1	1
4	4	4	4	3	3	3	3	4	4
1	1	3	3	1	1	3	3	1	1
4	4	4	4	3	3	3	3	4	4
4	4	4	4	3	3	3	3	4	4
4	4	3	3	1	1	3	3	1	1
4	4	4	4	3	3	3	3	4	4
4	4	4	4	3	3	3	3	4	4
4	4	4	4	3	3	3	3	4	4
4	4	4	4	3	3	3	3	4	4
4	4	4	4	1	1	3	3	1	1
4	4	4	4	3	3	2	2	1	1
4	4	4	4	1	1	3	3	1	1
4	4	4	4	3	3	3	3	4	4
4	4	4	4	3	3	3	3	4	4
4	4	4	4	3	3	3	3	4	4
4	4	4	4	3	3	3	3	4	4
4	4	4	4	3	3	3	3	1	1
4	4	4	4	3	3	3	3	4	4
4	4	4	4	3	3	3	3	4	4
4	4	4	4	3	3	3	3	4	4
4	4	3	3	3	3	3	3	4	4
4	4	4	4	1	1	3	3	1	1
4	4	4	4	3	3	3	3	4	4
4	4	4	4	1	1	3	3	1	1
4	4	4	4	3	3	3	3	4	4
4	4	4	4	3	3	3	3	4	4
4	4	4	4	3	3	3	3	4	4
4	4	4	4	3	3	3	3	1	1
4	4	4	4	3	3	3	3	1	1
4	4	4	4	1	1	3	3	1	1
4	4	4	4	3	3	3	3	1	1
4	4	4	4	3	3	3	3	4	4
4	4	4	4	3	3	2	2	4	4
4	4	4	4	1	1	3	3	1	1
4	4	4	4	3	3	3	3	4	4
4	4	4	4	1	1	3	3	4	4
4	4	4	4	3	3	3	3	4	4
4	4	3	3	1	1	3	3	1	1
4	4	4	4	3	3	3	3	4	4
4	4	4	4	3	3	3	3	4	4
4	4	4	4	1	1	3	3	1	1
4	4	3	3	3	3	2	2	4	4
4	4	4	4	3	3	3	3	1	1
4	4	4	4	3	3	3	3	4	4
4	4	4	4	3	3	3	3	4	4
4	4	4	4	3	3	3	3	4	4
4	4	3	3	3	3	3	3	4	4
4	4	4	4	3	3	3	3	4	4
4	4	4	4	3	3	3	3	4	4
4	4	4	4	3	3	3	3	4	4
4	4	4	4	3	3	3	3	4	4
4	4	4	4	1	1	3	3	1	1
4	4	4	4	3	3	3	3	4	4
4	4	4	4	3	3	2	2	4	4

For Review Only

4	4	4	4	3	3	3	3	4	4
4	4	3	3	3	3	3	3	1	1
4	4	4	4	3	3	3	3	4	4
4	4	4	4	3	3	3	3	4	4
4	4	4	4	1	1	3	3	1	1
4	4	4	4	1	1	3	3	1	1
4	4	4	4	1	1	3	3	1	1
4	4	4	4	3	3	3	3	4	4
4	4	4	4	3	3	2	2	4	4
4	4	4	4	3	3	2	2	4	4
4	4	4	4	3	3	3	3	4	4
4	4	4	4	1	1	3	3	1	1
4	4	4	4	3	3	3	3	4	4
4	4	3	3	3	3	3	3	4	4
4	4	4	4	3	3	3	3	4	4
4	4	4	4	3	3	3	3	4	4
4	4	4	4	1	1	3	3	1	1
4	4	3	3	3	3	3	3	4	4
4	4	3	3	3	3	3	3	4	4
4	4	4	4	3	3	3	3	4	4
4	4	4	4	3	3	2	2	4	4
4	4	4	4	1	1	3	3	1	1
4	4	4	4	3	3	3	3	4	4
4	4	4	4	1	1	2	2	1	1
4	4	3	3	3	3	3	3	4	4
4	4	4	4	3	3	3	3	4	4
4	4	4	4	3	3	2	2	4	4
4	4	4	4	3	3	3	3	4	4
4	4	4	4	3	3	3	3	4	4
4	4	4	4	3	3	3	3	4	4
4	4	4	4	3	3	3	3	4	4
4	4	4	4	1	1	3	3	1	1
4	4	4	4	1	1	3	3	1	1
4	4	4	4	1	1	3	3	1	1
4	4	4	4	1	1	3	3	1	1
4	4	3	3	3	3	3	3	4	4
4	4	4	4	1	1	3	3	1	1
4	4	4	4	3	3	3	3	4	4
4	4	4	4	3	3	3	3	4	4
4	4	4	4	3	3	2	2	4	4
4	4	4	4	1	1	3	3	1	1
4	4	4	4	3	3	3	3	4	4
4	4	4	4	3	3	2	2	4	4
4	4	4	4	3	3	3	3	4	4
4	4	4	4	3	3	3	3	4	4
4	4	4	4	3	3	3	3	4	4
4	4	3	3	1	1	2	2	1	1
4	4	4	4	1	1	3	3	1	1
4	4	4	4	3	3	3	3	4	4
4	4	3	3	3	3	2	2	4	4
4	4	4	4	3	3	3	3	4	4
4	4	4	4	3	3	2	2	4	4
4	4	4	4	3	3	3	3	4	4
4	4	4	4	3	3	3	3	1	1
4	4	3	3	1	1	2	2	1	1

For Review Only

4	4	4	4	3	3	3	3	4	4
4	4	4	4	3	3	3	3	4	4

For Review Only

SNP2763	SNP2763	SNP2764	SNP2764	SNP2765	SNP2765	SNP2766	SNP2766	SNP2767	SNP2767
2	2	1	1	1	1	2	2	3	3
1	1	1	1	1	1	2	2	3	3
2	2	1	1	4	4	2	2	3	3
2	2	3	3	4	4	2	2	3	3
2	2	3	3	1	1	2	2	3	3
2	2	3	3	4	4	2	2	3	3
2	2	3	3	1	1	2	2	3	3
2	2	3	3	1	1	2	2	3	3
2	2	3	3	1	1	2	2	3	3
1	1	3	3	1	1	2	2	3	3
2	2	3	3	1	1	2	2	3	3
2	2	3	3	4	4	2	2	3	3
1	1	3	3	1	1	2	2	3	3
1	1	3	3	1	1	2	2	3	3
2	2	3	3	4	4	2	2	3	3
2	2	3	3	4	4	2	2	3	3
1	1	3	3	4	4	2	2	3	3
2	2	3	3	4	4	2	2	3	3
2	2	3	3	1	1	2	2	3	3
1	1	3	3	1	1	2	2	3	3
2	2	3	3	4	4	2	2	3	3
2	2	3	3	4	4	2	2	3	3
2	2	3	3	1	1	2	2	3	3
2	2	3	3	1	1	2	2	3	3
2	2	3	3	4	4	2	2	3	3
2	2	3	3	4	4	2	2	3	3
2	2	3	3	4	4	2	2	3	3
2	2	3	3	4	4	2	2	3	3
2	2	3	3	1	1	2	2	3	3
1	1	3	3	1	1	2	2	3	3
2	2	3	3	4	4	2	2	3	3
2	2	1	1	4	4	3	3	1	1
2	2	1	1	4	4	3	3	1	1
1	1	3	3	1	1	2	2	3	3
2	2	3	3	4	4	2	2	3	3
2	2	3	3	1	1	2	2	3	3
2	2	3	3	4	4	2	2	3	3
2	2	3	3	4	4	2	2	3	3
2	2	3	3	1	1	2	2	3	3
2	2	3	3	1	1	2	2	3	3
1	1	3	3	1	1	2	2	3	3
2	2	3	3	4	4	2	2	3	3
1	1	1	1	1	1	2	2	1	1
1	1	3	3	4	4	2	2	3	3
1	1	1	1	1	1	3	3	1	1
2	2	3	3	4	4	2	2	3	3
2	2	1	1	4	4	3	3	3	3
2	2	3	3	1	1	2	2	3	3
1	1	3	3	1	1	2	2	3	3
2	2	3	3	4	4	2	2	3	3
2	2	3	3	4	4	2	2	3	3
2	2	1	1	1	1	2	2	3	3

For Review Only

2	2	3	3	4	4	2	2	3	3
2	2	1	1	4	4	3	3	1	1
2	2	3	3	4	4	2	2	3	3
1	1	3	3	4	4	2	2	3	3
2	2	3	3	1	1	2	2	3	3
2	2	3	3	4	4	2	2	3	3
2	2	3	3	4	4	2	2	3	3
1	1	3	3	1	1	2	2	3	3
2	2	3	3	1	1	2	2	3	3
2	2	3	3	4	4	2	2	3	3
2	2	1	1	4	4	3	3	1	1
2	2	1	1	4	4	3	3	1	1
2	2	3	3	4	4	2	2	3	3
2	2	3	3	1	1	2	2	3	3
2	2	3	3	4	4	2	2	3	3
1	1	3	3	1	1	2	2	3	3
1	1	3	3	1	1	2	2	3	3
2	2	3	3	4	4	2	2	3	3
2	2	3	3	4	4	2	2	3	3
2	2	1	1	1	1	3	3	1	1
2	2	1	1	4	4	2	2	3	3
2	2	3	3	1	1	2	2	3	3
2	2	3	3	1	1	2	2	3	3
2	2	3	3	4	4	2	2	3	3
1	1	1	1	1	1	3	3	1	1
2	2	3	3	4	4	2	2	3	3
1	1	3	3	1	1	2	2	3	3
2	2	3	3	1	1	2	2	3	3
2	2	3	3	4	4	2	2	3	3
2	2	3	3	4	4	2	2	3	3
1	1	3	3	4	4	2	2	3	3
2	2	3	3	4	4	2	2	3	3
2	2	3	3	1	1	2	2	3	3
2	2	3	3	4	4	2	2	3	3
2	2	3	3	4	4	2	2	3	3
1	1	1	1	1	1	3	3	1	1
1	1	3	3	1	1	2	2	3	3
1	1	3	3	1	1	2	2	3	3
2	2	3	3	4	4	2	2	3	3
1	1	1	1	1	1	2	2	1	1
2	2	3	3	4	4	2	2	3	3
2	2	3	3	4	4	2	2	3	3
1	1	3	3	4	4	2	2	3	3
2	2	1	1	4	4	2	2	1	1
2	2	1	1	1	1	3	3	3	3
2	2	3	3	4	4	2	2	3	3
1	1	3	3	4	4	2	2	3	3
2	2	3	3	1	1	2	2	3	3
2	2	3	3	4	4	2	2	3	3
2	2	3	3	1	1	2	2	3	3
2	2	3	3	4	4	3	3	1	1
2	2	3	3	1	1	2	2	3	3
1	1	3	3	1	1	2	2	3	3
2	2	3	3	4	4	3	3	3	3
2	2	3	3	1	1	2	2	3	3

2	2	3	3	1	1	2	2	3	3
2	2	3	3	4	4	2	2	1	1
2	2	3	3	1	1	2	2	3	3
1	1	3	3	4	4	2	2	3	3
2	2	3	3	4	4	2	2	3	3
2	2	3	3	1	1	2	2	3	3
2	2	3	3	4	4	2	2	3	3
2	2	3	3	4	4	2	2	3	3
2	2	3	3	1	1	2	2	3	3
2	2	3	3	1	1	2	2	3	3
2	2	3	3	1	1	2	2	3	3
2	2	3	3	4	4	2	2	3	3
2	2	3	3	4	4	2	2	3	3
2	2	3	3	4	4	2	2	3	3
2	2	3	3	4	4	2	2	3	3
2	2	3	3	4	4	2	2	3	3
1	1	3	3	1	1	2	2	3	3
2	2	3	3	1	1	2	2	3	3
1	1	3	3	4	4	2	2	3	3
1	1	3	3	4	4	2	2	3	3
2	2	3	3	4	4	2	2	3	3
2	2	3	3	4	4	2	2	3	3
2	2	1	1	4	4	2	2	3	3
2	2	3	3	1	1	2	2	3	3
2	2	3	3	4	4	2	2	3	3
2	2	3	3	1	1	2	2	3	3
2	2	3	3	4	4	2	2	3	3
2	2	3	3	4	4	2	2	3	3
2	2	3	3	4	4	2	2	3	3
2	2	1	1	4	4	3	3	1	1
2	2	3	3	1	1	2	2	3	3
2	2	3	3	4	4	2	2	3	3
2	2	3	3	4	4	2	2	3	3
2	2	3	3	4	4	2	2	3	3
1	1	3	3	1	1	2	2	3	3
2	2	3	3	4	4	2	2	3	3
1	1	3	3	4	4	2	2	3	3
2	2	3	3	4	4	2	2	3	3
2	2	3	3	1	1	2	2	3	3
2	2	3	3	1	1	2	2	3	3
2	2	3	3	1	1	2	2	3	3
1	1	3	3	1	1	2	2	3	3
2	2	3	3	1	1	2	2	3	3
1	1	3	3	1	1	2	2	3	3
2	2	3	3	4	4	2	2	3	3
2	2	3	3	1	1	2	2	3	3
2	2	3	3	4	4	2	2	3	3
2	2	3	3	4	4	2	2	3	3
2	2	3	3	1	1	2	2	3	3
2	2	3	3	4	4	2	2	3	3
2	2	3	3	4	4	2	2	3	3
2	2	3	3	1	1	2	2	3	3
2	2	3	3	1	1	2	2	3	3
2	2	1	1	1	1	3	3	1	1
1	1	3	3	1	1	2	2	3	3

For Review Only

2	2	3	3	4	4	2	2	3	3
1	1	3	3	1	1	2	2	3	3
1	1	1	1	1	1	3	3	1	1
2	2	3	3	1	1	2	2	3	3
2	2	1	1	4	4	2	2	3	3
2	2	3	3	4	4	2	2	3	3
2	2	3	3	1	1	2	2	3	3
2	2	3	3	4	4	2	2	3	3
2	2	3	3	4	4	2	2	3	3
2	2	3	3	4	4	2	2	3	3
2	2	3	3	4	4	2	2	3	3
2	2	3	3	1	1	2	2	3	3
2	2	3	3	4	4	2	2	3	3
2	2	3	3	1	1	2	2	3	3
1	1	3	3	1	1	2	2	3	3
2	2	3	3	1	1	2	2	3	3
1	1	3	3	4	4	2	2	3	3
2	2	3	3	4	4	2	2	3	3
2	2	3	3	4	4	2	2	3	3
2	2	3	3	4	4	2	2	3	3
2	2	3	3	4	4	2	2	3	3
2	2	3	3	1	1	2	2	3	3
2	2	3	3	1	1	2	2	3	3
2	2	1	1	4	4	3	3	1	1
2	2	3	3	1	1	2	2	3	3
2	2	3	3	1	1	2	2	3	3
2	2	3	3	4	4	2	2	3	3
2	2	3	3	1	1	2	2	3	3
1	1	3	3	1	1	2	2	3	3
2	2	3	3	4	4	2	2	3	3
2	2	3	3	4	4	2	2	3	3
2	2	3	3	4	4	2	2	3	3
2	2	3	3	1	1	2	2	3	3
1	1	3	3	4	4	2	2	3	3
2	2	1	1	1	1	3	3	1	1
2	2	1	1	4	4	3	3	1	1
2	2	3	3	4	4	2	2	3	3
2	2	3	3	1	1	2	2	3	3
2	2	3	3	4	4	2	2	3	3
2	2	1	1	4	4	3	3	1	1
2	2	1	1	4	4	2	2	3	3
2	2	3	3	4	4	2	2	3	3
2	2	3	3	1	1	2	2	3	3
2	2	3	3	1	1	2	2	3	3
2	2	3	3	1	1	2	2	3	3
2	2	1	1	4	4	3	3	3	3
2	2	3	3	1	1	2	2	3	3
2	2	3	3	1	1	2	2	3	3
1	1	3	3	4	4	2	2	3	3
2	2	3	3	4	4	2	2	3	3
2	2	3	3	4	4	2	2	3	3
1	1	3	3	1	1	2	2	3	3
2	2	3	3	1	1	2	2	3	3
2	2	1	1	4	4	3	3	1	1
2	2	3	3	1	1	2	2	3	3

For Review Only

2	2	3	3	4	4	2	2	3	3
2	2	3	3	1	1	2	2	3	3
2	2	3	3	4	4	2	2	3	3
2	2	3	3	4	4	2	2	3	3
1	1	3	3	1	1	2	2	3	3
2	2	3	3	4	4	2	2	3	3
2	2	3	3	1	1	2	2	3	3
1	1	3	3	1	1	2	2	3	3
2	2	3	3	4	4	2	2	3	3
2	2	3	3	1	1	2	2	3	3
2	2	3	3	4	4	2	2	3	3
1	1	3	3	4	4	2	2	3	3
2	2	3	3	4	4	2	2	3	3
2	2	3	3	4	4	2	2	3	3
2	2	1	1	4	4	2	2	3	3
2	2	3	3	1	1	2	2	3	3
2	2	3	3	1	1	2	2	3	3
2	2	3	3	1	1	2	2	3	3
2	2	3	3	1	1	2	2	3	3
2	2	3	3	4	4	2	2	3	3
2	2	3	3	1	1	2	2	3	3
2	2	3	3	4	4	2	2	3	3
2	2	3	3	4	4	2	2	3	3
2	2	3	3	4	4	2	2	3	3
1	1	3	3	1	1	2	2	3	3
2	2	3	3	4	4	2	2	3	3
2	2	3	3	1	1	2	2	3	3
2	2	3	3	4	4	2	2	3	3
2	2	3	3	4	4	2	2	3	3
2	2	3	3	4	4	2	2	3	3
2	2	3	3	1	1	2	2	3	3
2	2	3	3	4	4	2	2	3	3
1	1	3	3	1	1	2	2	3	3
2	2	3	3	4	4	2	2	3	3
2	2	3	3	1	1	2	2	3	3
2	2	3	3	1	1	2	2	3	3
2	2	3	3	4	4	2	2	3	3
2	2	1	1	4	4	3	3	1	1
2	2	3	3	1	1	2	2	3	3
2	2	3	3	1	1	2	2	3	3
2	2	1	1	1	1	3	3	1	1
2	2	3	3	4	4	2	2	3	3
2	2	3	3	1	1	2	2	3	3
2	2	3	3	4	4	2	2	3	3
2	2	1	1	4	4	2	2	3	3
2	2	3	3	4	4	2	2	3	3
2	2	3	3	4	4	2	2	3	3
2	2	3	3	1	1	2	2	3	3
2	2	1	1	4	4	3	3	1	1
2	2	3	3	1	1	2	2	3	3
2	2	3	3	4	4	2	2	3	3
2	2	3	3	1	1	2	2	3	3
2	2	1	1	4	4	2	2	3	3
2	2	3	3	4	4	2	2	3	3
2	2	3	3	1	1	2	2	3	3

For Review Only

2	2	3	3	4	4	2	2	3	3
2	2	1	1	4	4	3	3	1	1

For Review Only

SNP2768	SNP2768	SNP2769	SNP2769	SNP277	SNP277	SNP2770	SNP2770	SNP2771	SNP2771
1	1	4	4	2	2	4	4	4	4
1	1	4	4	1	1	4	4	1	1
1	1	4	4	2	2	4	4	4	4
1	1	4	4	2	2	4	4	1	1
1	1	4	4	1	1	4	4	1	1
1	1	4	4	1	1	4	4	1	1
1	1	2	2	2	2	4	4	1	1
1	1	4	4	1	1	4	4	4	4
1	1	2	2	2	2	4	4	1	1
1	1	4	4	1	1	1	1	1	1
1	1	4	4	1	1	4	4	1	1
1	1	4	4	2	2	4	4	4	4
1	1	4	4	1	1	4	4	1	1
1	1	4	4	1	1	4	4	1	1
1	1	2	2	1	1	4	4	1	1
1	1	4	4	2	2	4	4	1	1
1	1	2	2	2	2	1	1	1	1
1	1	4	4	1	1	4	4	1	1
1	1	2	2	2	2	4	4	4	4
1	1	2	2	1	1	1	1	1	1
1	1	4	4	1	1	4	4	1	1
1	1	2	2	2	2	4	4	1	1
1	1	4	4	1	1	4	4	1	1
1	1	4	4	1	1	4	4	1	1
1	1	4	4	1	1	4	4	1	1
1	1	4	4	1	1	4	4	1	1
1	1	4	4	1	1	4	4	1	1
1	1	4	4	2	2	4	4	1	1
1	1	2	2	1	1	4	4	1	1
1	1	4	4	1	1	1	1	1	1
1	1	4	4	2	2	4	4	1	1
4	4	4	4	2	2	4	4	4	4
4	4	2	2	2	2	4	4	4	4
1	1	4	4	1	1	4	4	1	1
1	1	4	4	1	1	4	4	1	1
1	1	4	4	2	2	4	4	1	1
1	1	4	4	1	1	4	4	1	1
1	1	4	4	1	1	4	4	1	1
1	1	4	4	1	1	4	4	1	1
1	1	4	4	1	1	4	4	1	1
1	1	4	4	1	1	1	1	1	1
1	1	4	4	1	1	4	4	1	1
4	4	4	4	1	1	4	4	4	4
1	1	4	4	2	2	4	4	1	1
4	4	4	4	1	1	1	1	4	4
1	1	4	4	2	2	4	4	1	1
4	4	2	2	1	1	4	4	1	1
1	1	4	4	1	1	4	4	1	1
1	1	2	2	2	2	4	4	4	4
1	1	4	4	2	2	4	4	1	1
1	1	2	2	1	1	4	4	1	1
1	1	4	4	1	1	4	4	4	4

For Review Only

1	1	2	2	1	1	4	4	1	1
4	4	2	2	2	2	4	4	4	4
1	1	2	2	2	2	4	4	4	4
1	1	2	2	2	2	1	1	1	1
1	1	2	2	2	2	4	4	4	4
1	1	2	2	1	1	4	4	1	1
1	1	2	2	1	1	4	4	1	1
1	1	4	4	1	1	4	4	1	1
1	1	2	2	2	2	4	4	1	1
1	1	4	4	2	2	4	4	1	1
4	4	4	4	2	2	4	4	1	1
4	4	4	4	1	1	4	4	1	1
1	1	2	2	1	1	4	4	1	1
1	1	2	2	1	1	4	4	1	1
1	1	4	4	2	2	4	4	1	1
1	1	4	4	1	1	4	4	1	1
1	1	2	2	2	2	4	4	1	1
1	1	2	2	2	2	4	4	1	1
1	1	2	2	2	2	4	4	1	1
4	4	2	2	2	2	4	4	4	4
1	1	4	4	1	1	4	4	1	1
1	1	4	4	1	1	4	4	1	1
1	1	2	2	1	1	4	4	1	1
1	1	4	4	1	1	4	4	1	1
4	4	4	4	1	1	1	1	4	4
1	1	4	4	1	1	4	4	4	4
1	1	4	4	2	2	4	4	1	1
1	1	4	4	1	1	4	4	1	1
1	1	2	2	1	1	4	4	1	1
1	1	2	2	2	2	4	4	1	1
1	1	4	4	1	1	4	4	1	1
1	1	4	4	1	1	4	4	1	1
1	1	4	4	1	1	4	4	1	1
1	1	2	2	1	1	4	4	1	1
1	1	4	4	1	1	4	4	1	1
4	4	4	4	1	1	1	1	4	4
1	1	4	4	1	1	1	1	1	1
1	1	4	4	1	1	1	1	1	1
1	1	4	4	1	1	4	4	1	1
4	4	4	4	1	1	1	1	4	4
1	1	2	2	2	2	4	4	1	1
1	1	2	2	2	2	4	4	1	1
1	1	2	2	1	1	1	1	4	4
4	4	4	4	2	2	4	4	1	1
4	4	2	2	2	2	4	4	4	4
1	1	4	4	1	1	4	4	4	4
1	1	4	4	1	1	4	4	1	1
1	1	2	2	1	1	4	4	1	1
1	1	4	4	2	2	4	4	1	1
1	1	4	4	1	1	4	4	1	1
4	4	4	4	2	2	4	4	1	1
1	1	4	4	1	1	4	4	1	1
1	1	4	4	2	2	4	4	1	1
4	4	4	4	1	1	4	4	1	1
1	1	2	2	1	1	4	4	1	1

For Review Only

1	1	2	2	1	1	4	4	1	1
1	1	4	4	1	1	4	4	1	1
1	1	4	4	1	1	4	4	1	1
1	1	4	4	1	1	4	4	1	1
1	1	4	4	1	1	4	4	4	4
1	1	4	4	1	1	4	4	1	1
1	1	4	4	2	2	4	4	1	1
1	1	2	2	1	1	4	4	1	1
1	1	4	4	2	2	4	4	1	1
1	1	4	4	1	1	4	4	1	1
1	1	4	4	1	1	4	4	1	1
1	1	4	4	2	2	4	4	1	1
1	1	2	2	1	1	1	1	1	1
1	1	4	4	1	1	4	4	4	4
1	1	4	4	1	1	4	4	4	4
1	1	2	2	2	2	4	4	1	1
1	1	2	2	1	1	4	4	1	1
1	1	4	4	2	2	4	4	1	1
1	1	4	4	1	1	4	4	1	1
1	1	4	4	1	1	1	1	4	4
1	1	4	4	2	2	4	4	1	1
1	1	2	2	1	1	4	4	1	1
4	4	2	2	2	2	4	4	1	1
1	1	4	4	1	1	4	4	1	1
1	1	4	4	1	1	4	4	1	1
1	1	4	4	1	1	4	4	4	4
1	1	4	4	2	2	4	4	1	1
1	1	2	2	1	1	4	4	1	1
1	1	4	4	2	2	4	4	1	1
4	4	4	4	1	1	4	4	1	1
1	1	4	4	1	1	4	4	4	4
1	1	4	4	1	1	4	4	4	4
1	1	2	2	2	2	4	4	1	1
1	1	2	2	1	1	4	4	1	1
1	1	4	4	1	1	4	4	1	1
1	1	4	4	2	2	1	1	4	4
1	1	4	4	1	1	4	4	1	1
1	1	4	4	1	1	4	4	1	1
1	1	4	4	1	1	4	4	1	1
1	1	4	4	1	1	4	4	1	1
1	1	4	4	2	2	4	4	1	1
1	1	4	4	1	1	4	4	4	4
1	1	2	2	1	1	4	4	4	4
1	1	4	4	1	1	4	4	1	1
1	1	4	4	1	1	4	4	1	1
1	1	2	2	1	1	4	4	1	1
1	1	2	2	1	1	4	4	1	1
1	1	2	2	1	1	4	4	1	1
1	1	4	4	1	1	4	4	1	1
1	1	2	2	1	1	4	4	1	1
1	1	4	4	1	1	4	4	1	1
1	1	4	4	1	1	4	4	1	1
1	1	4	4	2	2	4	4	1	1
4	4	4	4	1	1	4	4	1	1
1	1	4	4	1	1	4	4	1	1

For Review Only

1	1	2	2	1	1	4	4	1	1
1	1	2	2	1	1	1	1	1	1
4	4	4	4	2	2	1	1	4	4
1	1	4	4	1	1	4	4	1	1
1	1	4	4	1	1	4	4	1	1
1	1	4	4	2	2	4	4	1	1
1	1	4	4	1	1	4	4	1	1
1	1	4	4	1	1	4	4	1	1
1	1	4	4	1	1	4	4	4	4
1	1	4	4	2	2	4	4	1	1
1	1	2	2	1	1	4	4	1	1
1	1	4	4	1	1	4	4	1	1
1	1	4	4	2	2	4	4	1	1
1	1	2	2	2	2	4	4	1	1
1	1	4	4	1	1	4	4	4	4
1	1	4	4	1	1	4	4	1	1
1	1	4	4	2	2	4	4	1	1
1	1	4	4	1	1	4	4	4	4
1	1	4	4	1	1	4	4	1	1
1	1	4	4	2	2	4	4	1	1
1	1	4	4	2	2	4	4	4	4
1	1	4	4	1	1	4	4	1	1
1	1	4	4	1	1	4	4	1	1
4	4	4	4	1	1	4	4	1	1
1	1	2	2	1	1	4	4	1	1
1	1	4	4	1	1	4	4	1	1
1	1	4	4	2	2	4	4	1	1
1	1	4	4	1	1	4	4	1	1
1	1	2	2	1	1	1	1	4	4
1	1	4	4	2	2	4	4	4	4
1	1	4	4	1	1	4	4	1	1
1	1	2	2	2	2	4	4	1	1
1	1	4	4	2	2	4	4	1	1
1	1	4	4	1	1	4	4	4	4
4	4	4	4	2	2	4	4	1	1
4	4	4	4	1	1	4	4	1	1
1	1	4	4	1	1	4	4	1	1
1	1	4	4	2	2	4	4	1	1
1	1	2	2	1	1	4	4	1	1
4	4	2	2	1	1	4	4	1	1
1	1	4	4	2	2	4	4	4	4
1	1	2	2	1	1	4	4	1	1
1	1	4	4	1	1	4	4	4	4
1	1	4	4	2	2	4	4	4	4
1	1	4	4	1	1	4	4	1	1
4	4	2	2	2	2	4	4	1	1
1	1	4	4	1	1	4	4	1	1
1	1	4	4	1	1	4	4	1	1
1	1	4	4	1	1	4	4	1	1
1	1	4	4	1	1	4	4	1	1
1	1	4	4	2	2	4	4	1	1
1	1	4	4	1	1	4	4	1	1
1	1	4	4	1	1	4	4	1	1
4	4	4	4	2	2	4	4	1	1
1	1	4	4	1	1	4	4	1	1

For Review Only

1	1	4	4	1	1	4	4	1	1
1	1	4	4	1	1	4	4	4	4
1	1	2	2	2	2	4	4	1	1
1	1	4	4	1	1	4	4	4	4
1	1	4	4	1	1	4	4	4	4
1	1	2	2	2	2	4	4	1	1
1	1	4	4	1	1	4	4	1	1
1	1	4	4	1	1	1	1	1	1
1	1	4	4	2	2	4	4	1	1
1	1	4	4	1	1	4	4	1	1
1	1	4	4	2	2	4	4	1	1
1	1	4	4	1	1	4	4	1	1
1	1	4	4	1	1	4	4	1	1
1	1	4	4	1	1	4	4	1	1
1	1	4	4	2	2	4	4	4	4
1	1	2	2	2	2	4	4	1	1
1	1	4	4	1	1	4	4	1	1
1	1	2	2	1	1	4	4	4	4
1	1	4	4	1	1	4	4	1	1
1	1	4	4	1	1	4	4	1	1
1	1	4	4	1	1	4	4	1	1
1	1	2	2	1	1	4	4	1	1
1	1	2	2	2	2	4	4	1	1
1	1	2	2	1	1	4	4	1	1
1	1	4	4	1	1	4	4	1	1
1	1	2	2	2	2	4	4	1	1
1	1	4	4	1	1	4	4	1	1
1	1	4	4	2	2	4	4	1	1
1	1	2	2	1	1	4	4	4	4
1	1	4	4	2	2	4	4	1	1
1	1	4	4	1	1	4	4	1	1
1	1	2	2	1	1	4	4	1	1
1	1	2	2	2	2	4	4	1	1
1	1	4	4	1	1	4	4	1	1
1	1	4	4	1	1	4	4	1	1
1	1	4	4	1	1	4	4	1	1
1	1	4	4	2	2	4	4	4	4
4	4	4	4	1	1	4	4	1	1
1	1	4	4	2	2	4	4	1	1
1	1	4	4	1	1	4	4	1	1
4	4	4	4	2	2	4	4	1	1
1	1	2	2	1	1	4	4	1	1
1	1	2	2	1	1	4	4	1	1
1	1	2	2	2	2	4	4	1	1
1	1	4	4	1	1	4	4	4	4
1	1	4	4	1	1	4	4	1	1
1	1	4	4	1	1	4	4	1	1
1	1	4	4	1	1	4	4	1	1
4	4	4	4	2	2	4	4	4	4
1	1	4	4	1	1	4	4	1	1
1	1	4	4	1	1	4	4	1	1
1	1	4	4	1	1	4	4	1	1
1	1	4	4	2	2	4	4	4	4
1	1	4	4	2	2	4	4	4	4
1	1	4	4	1	1	4	4	1	1

For Review Only

1	1	2	2	2	2	4	4	1	1
4	4	4	4	1	1	4	4	1	1

For Review Only

SNP2772	SNP2772	SNP2773	SNP2773	SNP2774	SNP2774	SNP2775	SNP2775	SNP2776	SNP2776
3	3	1	1	1	1	2	2	2	2
1	1	2	2	4	4	2	2	2	2
3	3	1	1	1	1	3	3	3	3
1	1	1	1	1	1	2	2	3	3
1	1	1	1	1	1	2	2	3	3
1	1	1	1	1	1	2	2	2	2
1	1	1	1	1	1	2	2	2	2
3	3	1	1	1	1	2	2	2	2
1	1	1	1	1	1	2	2	2	2
1	1	1	1	1	1	2	2	2	2
1	1	1	1	1	1	2	2	2	2
1	1	1	1	1	1	3	3	3	3
1	1	1	1	1	1	3	3	2	2
1	1	1	1	1	1	2	2	3	3
1	1	1	1	1	1	3	3	2	2
1	1	1	1	1	1	3	3	3	3
1	1	1	1	1	1	2	2	3	3
1	1	1	1	1	1	2	2	2	2
3	3	1	1	1	1	3	3	2	2
1	1	1	1	1	1	2	2	2	2
1	1	1	1	1	1	2	2	3	3
1	1	1	1	1	1	2	2	3	3
1	1	1	1	1	1	2	2	2	2
1	1	2	2	4	4	2	2	2	2
1	1	1	1	1	1	3	3	2	2
1	1	1	1	1	1	3	3	2	2
1	1	1	1	1	1	3	3	2	2
1	1	1	1	1	1	3	3	2	2
1	1	1	1	1	1	2	2	2	2
1	1	1	1	1	1	2	2	2	2
1	1	1	1	1	1	3	3	2	2
3	3	1	1	1	1	3	3	3	3
3	3	1	1	1	1	2	2	2	2
1	1	1	1	1	1	2	2	2	2
1	1	1	1	1	1	2	2	2	2
1	1	1	1	1	1	2	2	3	3
1	1	2	2	4	4	3	3	2	2
1	1	2	2	4	4	2	2	3	3
1	1	1	1	1	1	2	2	2	2
1	1	1	1	1	1	2	2	3	3
1	1	1	1	1	1	2	2	2	2
1	1	1	1	1	1	2	2	2	2
3	3	2	2	4	4	2	2	3	3
1	1	2	2	4	4	2	2	2	2
3	3	1	1	1	1	2	2	2	2
1	1	1	1	1	1	3	3	3	3
1	1	1	1	1	1	2	2	3	3
1	1	1	1	1	1	2	2	2	2
1	1	2	2	1	1	2	2	2	2
1	1	1	1	1	1	3	3	2	2
1	1	1	1	1	1	2	2	2	2
3	3	1	1	1	1	3	3	2	2

For Review Only

1	1	1	1	1	1	2	2	2	2
3	3	1	1	1	1	2	2	2	2
3	3	1	1	1	1	2	2	3	3
1	1	1	1	1	1	2	2	2	2
3	3	1	1	1	1	3	3	2	2
1	1	1	1	1	1	2	2	3	3
1	1	1	1	1	1	2	2	2	2
1	1	2	2	4	4	2	2	3	3
1	1	1	1	1	1	2	2	2	2
1	1	1	1	1	1	3	3	2	2
1	1	1	1	1	1	3	3	3	3
1	1	1	1	1	1	3	3	3	3
1	1	1	1	1	1	2	2	2	2
1	1	1	1	1	1	2	2	2	2
1	1	1	1	1	1	3	3	2	2
1	1	2	2	4	4	3	3	2	2
1	1	1	1	1	1	3	3	3	3
1	1	1	1	1	1	3	3	2	2
1	1	1	1	1	1	2	2	2	2
3	3	1	1	1	1	2	2	3	3
1	1	1	1	1	1	3	3	3	3
1	1	1	1	1	1	2	2	2	2
1	1	1	1	1	1	2	2	2	2
1	1	1	1	1	1	3	3	2	2
3	3	1	1	1	1	2	2	2	2
3	3	1	1	1	1	2	2	2	2
1	1	2	2	4	4	3	3	2	2
1	1	1	1	1	1	2	2	2	2
1	1	1	1	1	1	2	2	2	2
1	1	1	1	1	1	3	3	2	2
1	1	2	2	4	4	2	2	2	2
1	1	2	2	4	4	3	3	3	3
1	1	1	1	1	1	2	2	2	2
1	1	1	1	1	1	2	2	2	2
1	1	1	1	1	1	2	2	3	3
3	3	1	1	1	1	2	2	2	2
1	1	1	1	1	1	2	2	2	2
1	1	1	1	1	1	2	2	2	2
1	1	1	1	1	1	3	3	2	2
3	3	1	1	1	1	2	2	2	2
1	1	2	2	4	4	2	2	2	2
1	1	1	1	1	1	3	3	3	3
3	3	1	1	1	1	3	3	2	2
1	1	1	1	1	1	3	3	3	3
3	3	1	1	1	1	2	2	2	2
3	3	1	1	1	1	2	2	2	2
1	1	1	1	1	1	2	2	2	2
1	1	1	1	1	1	2	2	2	2
1	1	1	1	1	1	2	2	2	2
1	1	1	1	1	1	3	3	2	2
1	1	1	1	1	1	3	3	2	2
1	1	1	1	1	1	2	2	3	3
1	1	2	2	4	4	2	2	2	2
1	1	1	1	1	1	3	3	2	2
1	1	1	1	1	1	3	3	2	2

For Review Only

1	1	1	1	1	1	2	2	2	2
1	1	1	1	1	1	2	2	2	2
1	1	1	1	1	1	2	2	2	2
1	1	1	1	1	1	2	2	2	2
1	1	1	1	1	1	2	2	2	2
1	1	1	1	1	1	2	2	2	2
1	1	1	1	1	1	3	3	3	3
1	1	1	1	1	1	3	3	2	2
1	1	1	1	1	1	2	2	2	2
1	1	1	1	1	1	2	2	2	2
1	1	1	1	1	1	2	2	2	2
1	1	1	1	1	1	3	3	2	2
1	1	1	1	1	1	3	3	2	2
3	3	1	1	1	1	3	3	2	2
1	1	1	1	1	1	3	3	2	2
1	1	1	1	1	1	2	2	2	2
1	1	2	2	4	4	3	3	3	3
1	1	1	1	1	1	3	3	2	2
1	1	1	1	1	1	2	2	2	2
3	3	1	1	1	1	3	3	2	2
1	1	1	1	1	1	3	3	2	2
1	1	1	1	1	1	3	3	3	3
1	1	1	1	1	1	2	2	3	3
1	1	1	1	1	1	2	2	2	2
1	1	1	1	1	1	2	2	2	2
3	3	1	1	1	1	2	2	2	2
1	1	1	1	1	1	3	3	2	2
1	1	1	1	1	1	2	2	2	2
1	1	1	1	1	1	3	3	3	3
1	1	1	1	1	1	2	2	2	2
3	3	1	1	1	1	2	2	2	2
1	1	1	1	1	1	2	2	2	2
1	1	2	2	4	4	2	2	2	2
1	1	1	1	1	1	2	2	2	2
1	1	1	1	1	1	2	2	2	2
3	3	1	1	1	1	2	2	2	2
1	1	1	1	1	1	2	2	2	2
1	1	1	1	1	1	3	3	2	2
1	1	1	1	1	1	3	3	2	2
1	1	1	1	1	1	2	2	2	2
1	1	1	1	1	1	3	3	2	2
3	3	1	1	1	1	2	2	2	2
3	3	1	1	1	1	3	3	2	2
1	1	1	1	1	1	2	2	2	2
1	1	1	1	1	1	2	2	2	2
1	1	1	1	1	1	3	3	2	2
1	1	1	1	1	1	2	2	2	2
1	1	1	1	1	1	3	3	2	2
1	1	2	2	1	1	3	3	2	2
1	1	1	1	1	1	2	2	2	2
1	1	1	1	1	1	2	2	2	2
1	1	1	1	1	1	2	2	3	3
1	1	1	1	1	1	3	3	2	2
1	1	2	2	4	4	3	3	3	3
1	1	1	1	1	1	2	2	2	2

For Review Only

1	1	1	1	1	1	2	2	2	2
1	1	1	1	1	1	3	3	2	2
3	3	1	1	1	1	2	2	2	2
1	1	1	1	1	1	2	2	2	2
1	1	1	1	1	1	2	2	2	2
1	1	1	1	1	1	2	2	2	2
1	1	1	1	1	1	3	3	2	2
1	1	1	1	1	1	3	3	2	2
1	1	1	1	1	1	2	2	2	2
1	1	1	1	1	1	3	3	2	2
1	1	1	1	1	1	2	2	2	2
1	1	2	2	4	4	2	2	2	2
1	1	1	1	1	1	3	3	2	2
1	1	1	1	1	1	3	3	2	2
3	3	1	1	1	1	2	2	2	2
1	1	1	1	1	1	2	2	2	2
1	1	1	1	1	1	3	3	2	2
3	3	1	1	1	1	2	2	2	2
1	1	2	2	4	4	2	2	2	2
1	1	1	1	1	1	3	3	3	3
1	1	1	1	1	1	3	3	2	2
1	1	1	1	1	1	2	2	2	2
1	1	1	1	1	1	2	2	2	2
1	1	1	1	1	1	3	3	3	3
1	1	1	1	1	1	3	3	2	2
1	1	1	1	1	1	2	2	2	2
1	1	1	1	1	1	3	3	2	2
1	1	1	1	1	1	3	3	2	2
3	3	1	1	1	1	3	3	2	2
3	3	1	1	1	1	3	3	2	2
1	1	2	2	4	4	2	2	2	2
1	1	1	1	1	1	3	3	2	2
1	1	1	1	1	1	2	2	2	2
3	3	1	1	1	1	2	2	2	2
1	1	2	2	4	4	2	2	3	3
1	1	1	1	1	1	3	3	3	3
1	1	1	1	1	1	3	3	2	2
1	1	2	2	4	4	3	3	2	2
1	1	1	1	1	1	3	3	2	2
1	1	1	1	1	1	2	2	2	2
3	3	1	1	1	1	3	3	3	3
1	1	1	1	1	1	2	2	2	2
3	3	1	1	1	1	2	2	2	2
1	1	1	1	1	1	2	2	2	2
1	1	1	1	1	1	2	2	2	2
1	1	1	1	1	1	3	3	2	2
1	1	1	1	1	1	2	2	2	2
1	1	1	1	1	1	2	2	2	2
1	1	1	1	1	1	2	2	2	2
1	1	1	1	1	1	2	2	3	3
1	1	1	1	1	1	3	3	2	2
1	1	2	2	4	4	3	3	2	2
1	1	1	1	1	1	2	2	2	2
1	1	1	1	1	1	3	3	3	3
1	1	1	1	1	1	2	2	2	2

For Review Only

1	1	1	1	1	1	2	2	2	2
3	3	1	1	1	1	2	2	2	2
1	1	1	1	1	1	3	3	2	2
1	1	1	1	1	1	2	2	2	2
3	3	2	2	4	4	2	2	3	3
1	1	1	1	1	1	2	2	2	2
1	1	1	1	1	1	2	2	2	2
1	1	1	1	1	1	2	2	2	2
1	1	1	1	1	1	2	2	2	2
1	1	1	1	1	1	2	2	2	2
1	1	1	1	1	1	3	3	2	2
1	1	1	1	1	1	2	2	2	2
1	1	1	1	1	1	2	2	2	2
1	1	1	1	1	1	3	3	2	2
3	3	1	1	1	1	3	3	3	3
1	1	1	1	1	1	2	2	2	2
1	1	1	1	1	1	2	2	2	2
1	1	1	1	1	1	2	2	2	2
1	1	1	1	1	1	2	2	3	3
1	1	2	2	4	4	2	2	3	3
1	1	1	1	1	1	3	3	3	3
1	1	1	1	1	1	2	2	2	2
1	1	1	1	1	1	2	2	2	2
1	1	1	1	1	1	3	3	2	2
1	1	2	2	4	4	2	2	3	3
1	1	1	1	1	1	3	3	2	2
1	1	1	1	1	1	2	2	2	2
1	1	1	1	1	1	3	3	3	3
1	1	1	1	1	1	2	2	2	2
1	1	1	1	1	1	3	3	2	2
1	1	1	1	1	1	2	2	2	2
1	1	1	1	1	1	2	2	2	2
1	1	1	1	1	1	3	3	2	2
1	1	1	1	1	1	3	3	2	2
1	1	1	1	1	1	2	2	2	2
1	1	1	1	1	1	2	2	2	2
3	3	1	1	1	1	3	3	2	2
1	1	1	1	1	1	3	3	3	3
1	1	1	1	1	1	2	2	3	3
1	1	1	1	1	1	2	2	3	3
1	1	1	1	1	1	2	2	3	3
1	1	1	1	1	1	2	2	2	2
1	1	1	1	1	1	3	3	2	2
1	1	1	1	1	1	2	2	2	2
1	1	1	1	1	1	3	3	2	2
1	1	1	1	1	1	3	3	2	2
1	1	1	1	1	1	3	3	2	2
1	1	1	1	1	1	2	2	2	2
1	1	1	1	1	1	2	2	3	3
1	1	1	1	1	1	2	2	2	2
1	1	1	1	1	1	3	3	2	2
1	1	1	1	1	1	2	2	2	2
3	3	1	1	1	1	3	3	3	3
3	3	1	1	1	1	3	3	2	2
1	1	1	1	1	1	2	2	3	3

For Review Only

1	1	1	1	1	1	2	2	2	2
1	1	1	1	1	1	3	3	2	2

For Review Only

SNP2777	SNP2777	SNP2778	SNP2778	SNP2779	SNP2779	SNP278	SNP278	SNP2780	SNP2780
3	3	3	3	2	2	3	3	4	4
2	2	3	3	2	2	3	3	1	1
3	3	4	4	2	2	3	3	4	4
3	3	4	4	2	2	3	3	4	4
2	2	4	4	2	2	3	3	4	4
2	2	3	3	2	2	3	3	4	4
2	2	3	3	2	2	3	3	4	4
2	2	4	4	1	1	3	3	4	4
2	2	3	3	2	2	3	3	4	4
2	2	3	3	2	2	3	3	4	4
2	2	3	3	2	2	3	3	4	4
3	3	4	4	2	2	3	3	4	4
2	2	3	3	2	2	3	3	4	4
2	2	4	4	2	2	3	3	4	4
3	3	3	3	2	2	3	3	4	4
3	3	4	4	2	2	3	3	4	4
2	2	4	4	2	2	3	3	1	1
2	2	4	4	2	2	3	3	4	4
3	3	3	3	2	2	3	3	4	4
2	2	4	4	2	2	3	3	4	4
2	2	3	3	2	2	3	3	4	4
2	2	3	3	2	2	3	3	4	4
2	2	4	4	2	2	3	3	4	4
2	2	4	4	2	2	3	3	1	1
3	3	3	3	2	2	3	3	4	4
3	3	3	3	2	2	3	3	4	4
3	3	3	3	2	2	3	3	4	4
3	3	3	3	2	2	3	3	4	4
2	2	3	3	2	2	3	3	4	4
3	3	3	3	2	2	3	3	4	4
3	3	3	3	2	2	3	3	4	4
3	3	3	3	2	2	3	3	4	4
2	2	3	3	2	2	3	3	4	4
2	2	3	3	2	2	3	3	4	4
2	2	3	3	2	2	3	3	4	4
2	2	4	4	2	2	3	3	4	4
3	3	3	3	1	1	3	3	1	1
2	2	4	4	2	2	3	3	4	4
2	2	3	3	2	2	3	3	4	4
2	2	4	4	2	2	3	3	4	4
2	2	4	4	2	2	3	3	4	4
2	2	4	4	2	2	3	3	4	4
2	2	4	4	2	2	3	3	1	1
2	2	3	3	2	2	4	4	4	4
2	2	4	4	2	2	3	3	4	4
3	3	4	4	2	2	3	3	4	4
2	2	4	4	2	2	3	3	4	4
2	2	3	3	2	2	3	3	4	4
2	2	4	4	2	2	3	3	1	1
3	3	3	3	2	2	3	3	4	4
2	2	3	3	2	2	3	3	4	4
2	2	3	3	2	2	3	3	4	4

For Review Only

2	2	3	3	2	2	3	3	4	4
2	2	3	3	2	2	3	3	4	4
2	2	4	4	2	2	3	3	4	4
2	2	3	3	2	2	3	3	4	4
3	3	3	3	2	2	3	3	4	4
2	2	4	4	2	2	3	3	4	4
2	2	3	3	2	2	3	3	4	4
2	2	4	4	2	2	4	4	1	1
2	2	3	3	1	1	3	3	4	4
3	3	3	3	2	2	3	3	4	4
3	3	4	4	2	2	3	3	4	4
3	3	4	4	2	2	3	3	4	4
2	2	3	3	2	2	3	3	4	4
2	2	3	3	2	2	3	3	4	4
3	3	3	3	2	2	3	3	4	4
2	2	3	3	2	2	3	3	1	1
2	2	4	4	2	2	3	3	4	4
3	3	3	3	2	2	3	3	4	4
2	2	3	3	2	2	3	3	4	4
2	2	4	4	2	2	3	3	4	4
3	3	3	3	2	2	3	3	4	4
2	2	3	3	2	2	3	3	4	4
2	2	3	3	2	2	3	3	4	4
3	3	3	3	2	2	3	3	4	4
2	2	4	4	2	2	3	3	4	4
2	2	3	3	2	2	3	3	4	4
3	3	3	3	2	2	3	3	1	1
2	2	3	3	2	2	3	3	4	4
2	2	3	3	2	2	3	3	4	4
3	3	3	3	2	2	3	3	4	4
2	2	3	3	2	2	3	3	1	1
3	3	3	3	2	2	3	3	1	1
2	2	3	3	2	2	3	3	4	4
2	2	3	3	2	2	3	3	4	4
2	2	4	4	2	2	3	3	4	4
2	2	4	4	2	2	3	3	4	4
2	2	3	3	2	2	3	3	4	4
2	2	3	3	2	2	3	3	4	4
3	3	3	3	2	2	3	3	4	4
2	2	3	3	2	2	3	3	4	4
2	2	3	3	2	2	3	3	4	4
3	3	4	4	2	2	3	3	4	4
3	3	3	3	1	1	3	3	4	4
3	3	4	4	2	2	3	3	4	4
2	2	3	3	2	2	3	3	4	4
2	2	3	3	1	1	3	3	4	4
2	2	3	3	2	2	4	4	4	4
2	2	3	3	2	2	3	3	4	4
2	2	3	3	2	2	3	3	4	4
3	3	3	3	2	2	3	3	4	4
3	3	3	3	2	2	3	3	4	4
2	2	4	4	2	2	3	3	4	4
2	2	3	3	1	1	3	3	1	1
3	3	3	3	2	2	3	3	4	4
3	3	3	3	2	2	3	3	4	4

For Review Only

2	2	3	3	2	2	3	3	4	4
2	2	3	3	2	2	3	3	4	4
2	2	3	3	2	2	3	3	4	4
2	2	3	3	2	2	4	4	4	4
2	2	3	3	2	2	3	3	4	4
3	3	3	3	2	2	3	3	4	4
3	3	4	4	2	2	3	3	4	4
2	2	3	3	2	2	3	3	4	4
2	2	3	3	2	2	3	3	4	4
2	2	4	4	2	2	3	3	4	4
2	2	3	3	2	2	3	3	4	4
3	3	4	4	2	2	3	3	4	4
3	3	3	3	2	2	3	3	4	4
3	3	3	3	2	2	3	3	4	4
3	3	3	3	2	2	3	3	4	4
2	2	4	4	2	2	3	3	4	4
3	3	4	4	2	2	3	3	1	1
3	3	3	3	2	2	3	3	4	4
2	2	3	3	2	2	4	4	4	4
2	2	3	3	2	2	3	3	4	4
3	3	3	3	2	2	3	3	4	4
3	3	4	4	2	2	3	3	4	4
2	2	4	4	2	2	3	3	4	4
2	2	3	3	1	1	4	4	4	4
2	2	3	3	2	2	3	3	4	4
2	2	3	3	2	2	3	3	4	4
3	3	3	3	2	2	3	3	4	4
2	2	3	3	2	2	3	3	4	4
3	3	4	4	2	2	3	3	4	4
3	3	3	3	2	2	4	4	4	4
2	2	4	4	2	2	3	3	4	4
2	2	3	3	2	2	3	3	4	4
2	2	3	3	2	2	3	3	4	4
2	2	3	3	2	2	3	3	4	4
2	2	3	3	2	2	3	3	4	4
2	2	3	3	2	2	3	3	4	4
2	2	3	3	2	2	4	4	4	4
2	2	3	3	2	2	3	3	4	4
2	2	3	3	2	2	3	3	4	4
2	2	3	3	2	2	3	3	4	4
3	3	3	3	2	2	3	3	4	4
2	2	3	3	2	2	4	4	4	4
3	3	3	3	2	2	3	3	4	4
2	2	4	4	2	2	3	3	4	4
2	2	3	3	2	2	3	3	4	4
3	3	3	3	2	2	4	4	4	4
2	2	3	3	2	2	3	3	4	4
3	3	3	3	2	2	3	3	4	4
3	3	3	3	2	2	3	3	4	4
2	2	3	3	2	2	3	3	4	4
2	2	3	3	2	2	3	3	4	4
2	2	4	4	2	2	3	3	4	4
3	3	4	4	2	2	3	3	4	4
3	3	4	4	2	2	3	3	1	1
2	2	3	3	2	2	3	3	4	4

For Review Only

2	2	3	3	2	2	3	3	4	4
3	3	3	3	2	2	3	3	4	4
3	3	3	3	2	2	3	3	4	4
2	2	3	3	2	2	3	3	4	4
2	2	3	3	2	2	3	3	4	4
2	2	3	3	2	2	3	3	4	4
3	3	3	3	2	2	3	3	4	4
2	2	3	3	2	2	3	3	4	4
2	2	3	3	2	2	3	3	4	4
3	3	3	3	2	2	3	3	4	4
2	2	3	3	2	2	3	3	4	4
2	2	4	4	2	2	3	3	4	4
3	3	3	3	2	2	3	3	4	4
3	3	3	3	2	2	3	3	4	4
2	2	4	4	2	2	4	4	4	4
2	2	3	3	2	2	3	3	4	4
3	3	3	3	2	2	3	3	4	4
2	2	3	3	2	2	3	3	4	4
2	2	3	3	2	2	3	3	1	1
3	3	3	3	2	2	3	3	4	4
3	3	3	3	2	2	3	3	4	4
2	2	3	3	2	2	3	3	4	4
2	2	3	3	2	2	3	3	4	4
3	3	4	4	2	2	3	3	4	4
2	2	3	3	2	2	4	4	4	4
2	2	3	3	2	2	3	3	4	4
3	3	3	3	2	2	3	3	4	4
3	3	3	3	2	2	3	3	4	4
3	3	3	3	1	1	3	3	4	4
3	3	3	3	1	1	3	3	4	4
2	2	3	3	2	2	3	3	4	4
3	3	3	3	2	2	3	3	4	4
3	3	3	3	2	2	3	3	4	4
2	2	3	3	2	2	4	4	4	4
2	2	4	4	2	2	3	3	4	4
3	3	4	4	2	2	3	3	4	4
3	3	3	3	2	2	3	3	4	4
3	3	3	3	1	1	3	3	1	1
3	3	3	3	2	2	3	3	4	4
2	2	3	3	2	2	3	3	4	4
3	3	4	4	2	2	3	3	4	4
2	2	3	3	2	2	3	3	4	4
2	2	3	3	2	2	4	4	4	4
2	2	3	3	2	2	3	3	4	4
2	2	3	3	2	2	3	3	1	1
3	3	3	3	2	2	3	3	4	4
2	2	3	3	2	2	3	3	4	4
2	2	3	3	2	2	3	3	4	4
2	2	4	4	1	1	4	4	4	4
2	2	3	3	2	2	3	3	4	4
3	3	3	3	2	2	3	3	4	4
3	3	3	3	2	2	3	3	1	1
2	2	3	3	2	2	3	3	4	4
3	3	4	4	2	2	3	3	4	4
2	2	4	4	2	2	3	3	4	4

For Review Only

2	2	3	3	2	2	3	3	4	4
2	2	3	3	2	2	3	3	4	4
3	3	3	3	2	2	3	3	4	4
2	2	3	3	2	2	3	3	4	4
2	2	4	4	1	1	3	3	1	1
3	3	3	3	2	2	3	3	4	4
2	2	3	3	2	2	3	3	4	4
2	2	4	4	1	1	3	3	4	4
2	2	3	3	2	2	3	3	4	4
2	2	3	3	2	2	3	3	4	4
3	3	3	3	2	2	3	3	4	4
2	2	3	3	2	2	3	3	4	4
2	2	3	3	2	2	3	3	4	4
3	3	3	3	2	2	3	3	4	4
3	3	4	4	2	2	3	3	4	4
2	2	3	3	2	2	3	3	4	4
2	2	3	3	2	2	3	3	4	4
2	2	3	3	1	1	3	3	4	4
2	2	4	4	2	2	3	3	4	4
2	2	4	4	2	2	3	3	4	4
2	2	4	4	2	2	3	3	4	4
2	2	3	3	2	2	3	3	4	4
2	2	3	3	2	2	3	3	4	4
3	3	3	3	2	2	3	3	4	4
2	2	4	4	2	2	4	4	1	1
3	3	3	3	2	2	3	3	4	4
2	2	3	3	2	2	3	3	4	4
3	3	4	4	2	2	3	3	4	4
2	2	3	3	2	2	3	3	4	4
3	3	3	3	2	2	3	3	4	4
2	2	4	4	2	2	4	4	4	4
2	2	3	3	2	2	3	3	4	4
3	3	4	4	2	2	3	3	4	4
3	3	3	3	2	2	3	3	4	4
2	2	3	3	2	2	3	3	4	4
2	2	3	3	2	2	3	3	4	4
3	3	3	3	2	2	3	3	4	4
3	3	4	4	2	2	3	3	4	4
2	2	4	4	2	2	3	3	4	4
2	2	4	4	2	2	3	3	4	4
3	3	4	4	2	2	3	3	4	4
2	2	3	3	2	2	3	3	4	4
3	3	3	3	2	2	3	3	4	4
2	2	3	3	2	2	3	3	4	4
3	3	3	3	2	2	3	3	4	4
3	3	3	3	2	2	3	3	4	4
3	3	4	4	2	2	3	3	4	4
2	2	3	3	2	2	3	3	4	4
2	2	3	3	2	2	3	3	4	4
2	2	3	3	2	2	3	3	4	4
3	3	3	3	2	2	3	3	4	4
2	2	3	3	2	2	3	3	4	4
3	3	4	4	2	2	3	3	4	4
3	3	3	3	1	1	3	3	4	4
2	2	4	4	2	2	3	3	4	4

For Review Only

2	2	3	3	2	2	3	3	4	4
3	3	3	3	2	2	3	3	4	4

For Review Only

SNP2781	SNP2781	SNP2782	SNP2782	SNP2783	SNP2783	SNP2784	SNP2784	SNP2785	SNP2785
4	4	2	2	1	1	2	2	1	1
2	2	3	3	4	4	2	2	1	1
4	4	2	2	1	1	2	2	1	1
4	4	3	3	1	1	2	2	1	1
4	4	2	2	1	1	2	2	1	1
2	2	3	3	4	4	2	2	1	1
2	2	3	3	4	4	2	2	1	1
2	2	3	3	4	4	2	2	1	1
2	2	3	3	4	4	2	2	1	1
2	2	2	2	1	1	2	2	1	1
4	4	2	2	1	1	2	2	1	1
4	4	2	2	1	1	2	2	1	1
2	2	3	3	4	4	2	2	1	1
2	2	3	3	4	4	2	2	1	1
2	2	3	3	4	4	2	2	1	1
4	4	2	2	1	1	2	2	1	1
4	4	2	2	1	1	2	2	1	1
2	2	2	2	1	1	2	2	1	1
4	4	2	2	1	1	2	2	1	1
2	2	2	2	1	1	2	2	1	1
2	2	2	2	1	1	2	2	1	1
2	2	2	2	1	1	2	2	1	1
2	2	3	3	4	4	1	1	1	1
2	2	2	2	1	1	2	2	1	1
2	2	3	3	4	4	2	2	1	1
2	2	3	3	4	4	2	2	1	1
2	2	3	3	4	4	2	2	3	3
2	2	3	3	4	4	2	2	1	1
2	2	3	3	4	4	2	2	1	1
2	2	3	3	4	4	2	2	1	1
2	2	3	3	4	4	2	2	1	1
2	2	2	2	1	1	2	2	1	1
2	2	3	3	4	4	2	2	1	1
2	2	3	3	4	4	2	2	1	1
2	2	3	3	4	4	2	2	1	1
4	4	2	2	1	1	2	2	3	3
2	2	3	3	4	4	2	2	1	1
4	4	2	2	1	1	2	2	1	1
2	2	3	3	4	4	1	1	1	1
4	4	3	3	1	1	1	1	1	1
2	2	2	2	1	1	2	2	1	1
2	2	2	2	4	4	2	2	1	1
2	2	2	2	1	1	2	2	1	1
2	2	2	2	1	1	2	2	1	1
2	2	2	2	1	1	2	2	1	1
2	2	2	2	1	1	2	2	1	1
2	2	2	2	1	1	2	2	1	1
2	2	3	3	4	4	2	2	1	1
4	4	2	2	4	4	2	2	1	1
2	2	3	3	4	4	2	2	1	1
4	4	2	2	1	1	2	2	1	1
2	2	3	3	4	4	2	2	3	3

For Review Only

2	2	3	3	4	4	2	2	1	1
2	2	3	3	4	4	2	2	1	1
2	2	3	3	4	4	2	2	1	1
2	2	3	3	4	4	2	2	1	1
4	4	2	2	1	1	2	2	1	1
4	4	2	2	1	1	2	2	1	1
2	2	3	3	4	4	2	2	1	1
2	2	2	2	1	1	2	2	1	1
2	2	2	2	1	1	2	2	1	1
2	2	3	3	4	4	2	2	1	1
4	4	2	2	1	1	2	2	1	1
4	4	2	2	1	1	2	2	1	1
2	2	3	3	4	4	2	2	1	1
2	2	3	3	4	4	2	2	1	1
2	2	3	3	4	4	2	2	1	1
2	2	2	2	1	1	2	2	1	1
2	2	3	3	4	4	2	2	1	1
2	2	3	3	4	4	2	2	1	1
2	2	3	3	4	4	2	2	1	1
4	4	2	2	1	1	2	2	1	1
4	4	3	3	4	4	2	2	1	1
2	2	3	3	4	4	1	1	1	1
2	2	3	3	4	4	2	2	1	1
2	2	2	2	1	1	2	2	1	1
2	2	2	2	1	1	2	2	1	1
2	2	3	3	4	4	2	2	1	1
2	2	3	3	4	4	2	2	1	1
2	2	3	3	4	4	1	1	1	1
2	2	3	3	4	4	2	2	1	1
2	2	3	3	4	4	2	2	1	1
2	2	3	3	1	1	2	2	1	1
2	2	2	2	1	1	2	2	1	1
2	2	3	3	4	4	1	1	1	1
2	2	3	3	4	4	2	2	1	1
4	4	2	2	1	1	1	1	1	1
2	2	2	2	1	1	2	2	1	1
2	2	2	2	1	1	2	2	1	1
2	2	3	3	4	4	2	2	1	1
2	2	3	3	4	4	2	2	1	1
2	2	2	2	1	1	2	2	1	1
2	2	3	3	4	4	2	2	1	1
4	4	2	2	1	1	2	2	1	1
4	4	2	2	1	1	2	2	1	1
4	4	2	2	1	1	2	2	1	1
2	2	3	3	4	4	2	2	1	1
2	2	3	3	4	4	2	2	1	1
2	2	3	3	4	4	2	2	1	1
2	2	3	3	4	4	2	2	1	1
2	2	3	3	4	4	2	2	1	1
2	2	3	3	4	4	2	2	1	1
2	2	3	3	4	4	2	2	1	1
2	2	2	2	1	1	1	1	1	1
2	2	3	3	4	4	2	2	1	1
2	2	2	2	4	4	2	2	1	1
2	2	3	3	4	4	2	2	1	1

For Review Only

2	2	3	3	4	4	2	2	1	1
2	2	3	3	4	4	2	2	1	1
2	2	3	3	4	4	2	2	1	1
2	2	3	3	4	4	2	2	1	1
2	2	2	2	4	4	2	2	3	3
2	2	3	3	4	4	2	2	1	1
4	4	2	2	1	1	2	2	1	1
2	2	3	3	4	4	2	2	1	1
2	2	3	3	4	4	2	2	3	3
2	2	3	3	4	4	2	2	1	1
2	2	3	3	4	4	2	2	1	1
2	2	3	3	4	4	2	2	1	1
2	2	3	3	4	4	2	2	1	1
2	2	3	3	4	4	2	2	1	1
2	2	3	3	1	1	2	2	1	1
2	2	3	3	4	4	2	2	1	1
2	2	2	2	1	1	2	2	1	1
2	2	3	3	4	4	2	2	1	1
2	2	3	3	4	4	2	2	1	1
2	2	3	3	4	4	2	2	1	1
2	2	3	3	4	4	2	2	1	1
4	4	2	2	1	1	2	2	1	1
4	4	2	2	1	1	2	2	3	3
2	2	3	3	4	4	1	1	1	1
2	2	3	3	4	4	2	2	1	1
2	2	3	3	4	4	1	1	1	1
2	2	3	3	4	4	2	2	1	1
2	2	3	3	4	4	2	2	1	1
4	4	2	2	1	1	2	2	1	1
2	2	3	3	4	4	2	2	1	1
2	2	2	2	1	1	2	2	1	1
2	2	3	3	4	4	2	2	1	1
2	2	3	3	4	4	2	2	1	1
2	2	3	3	4	4	2	2	1	1
2	2	3	3	4	4	2	2	1	1
2	2	3	3	4	4	1	1	1	1
2	2	3	3	4	4	2	2	1	1
2	2	3	3	4	4	2	2	1	1
2	2	3	3	4	4	2	2	1	1
4	4	2	2	4	4	1	1	1	1
2	2	3	3	4	4	2	2	1	1
2	2	3	3	4	4	2	2	1	1
2	2	3	3	4	4	2	2	1	1
2	2	3	3	4	4	2	2	1	1
2	2	3	3	4	4	2	2	1	1
2	2	3	3	4	4	2	2	1	1
2	2	3	3	4	4	2	2	1	1
2	2	3	3	4	4	2	2	1	1
2	2	3	3	4	4	2	2	1	1
2	2	3	3	4	4	2	2	1	1
2	2	3	3	4	4	2	2	1	1
2	2	2	2	1	1	2	2	1	1
4	4	3	3	1	1	2	2	1	1
4	4	2	2	1	1	2	2	1	1
2	2	3	3	4	4	2	2	1	1

For Review Only

2	2	3	3	4	4	2	2	1	1
2	2	3	3	4	4	2	2	1	1
2	2	2	2	1	1	2	2	1	1
2	2	3	3	4	4	2	2	1	1
2	2	3	3	4	4	2	2	1	1
2	2	3	3	4	4	2	2	1	1
2	2	3	3	4	4	1	1	1	1
2	2	3	3	4	4	2	2	1	1
2	2	3	3	4	4	1	1	1	1
2	2	3	3	4	4	2	2	1	1
2	2	3	3	4	4	2	2	1	1
2	2	2	2	4	4	2	2	1	1
2	2	3	3	4	4	2	2	3	3
2	2	3	3	4	4	2	2	1	1
2	2	2	2	1	1	2	2	1	1
4	4	2	2	1	1	1	1	1	1
2	2	3	3	4	4	2	2	1	1
2	2	3	3	4	4	2	2	1	1
2	2	3	3	4	4	2	2	1	1
2	2	2	2	1	1	2	2	1	1
2	2	3	3	4	4	2	2	1	1
2	2	3	3	4	4	2	2	1	1
2	2	3	3	4	4	1	1	1	1
4	4	2	2	1	1	2	2	1	1
2	2	2	2	1	1	2	2	1	1
2	2	3	3	4	4	2	2	1	1
2	2	3	3	4	4	2	2	1	1
2	2	3	3	4	4	2	2	1	1
2	2	3	3	4	4	2	2	1	1
2	2	3	3	4	4	2	2	1	1
2	2	3	3	4	4	2	2	1	1
2	2	3	3	4	4	2	2	1	1
2	2	3	3	4	4	2	2	1	1
2	2	3	3	4	4	2	2	1	1
2	2	3	3	4	4	2	2	1	1
4	4	2	2	1	1	2	2	1	1
2	2	3	3	4	4	1	1	1	1
2	2	3	3	4	4	2	2	1	1
2	2	3	3	4	4	2	2	1	1
2	2	3	3	4	4	2	2	3	3
4	4	2	2	1	1	2	2	1	1
2	2	3	3	4	4	2	2	1	1
2	2	3	3	4	4	1	1	1	1
2	2	3	3	4	4	2	2	1	1
2	2	3	3	4	4	2	2	3	3
2	2	3	3	4	4	1	1	1	1
4	4	2	2	1	1	1	1	1	1
2	2	3	3	4	4	1	1	1	1
2	2	2	2	1	1	2	2	1	1
2	2	2	2	1	1	2	2	1	1
2	2	3	3	4	4	1	1	1	1
2	2	2	2	1	1	2	2	1	1
2	2	3	3	4	4	1	1	1	1
4	4	2	2	1	1	2	2	1	1
2	2	2	2	1	1	1	1	1	1

For Review Only

2	2	3	3	4	4	2	2	1	1
2	2	3	3	4	4	2	2	1	1
2	2	3	3	4	4	2	2	1	1
2	2	3	3	4	4	1	1	1	1
2	2	3	3	4	4	1	1	1	1
2	2	3	3	4	4	2	2	1	1
2	2	3	3	4	4	1	1	1	1
2	2	2	2	1	1	2	2	1	1
2	2	3	3	4	4	2	2	1	1
2	2	3	3	4	4	2	2	1	1
2	2	3	3	4	4	2	2	1	1
2	2	3	3	4	4	2	2	1	1
2	2	3	3	4	4	2	2	1	1
2	2	3	3	4	4	2	2	1	1
4	4	2	2	1	1	2	2	1	1
4	4	2	2	1	1	2	2	1	1
2	2	3	3	4	4	1	1	1	1
2	2	3	3	4	4	2	2	1	1
2	2	2	2	1	1	2	2	1	1
4	4	2	2	1	1	2	2	1	1
2	2	2	2	1	1	2	2	1	1
2	2	3	3	4	4	2	2	1	1
2	2	3	3	4	4	2	2	1	1
2	2	3	3	4	4	1	1	1	1
2	2	2	2	1	1	2	2	1	1
2	2	2	2	1	1	2	2	1	1
2	2	3	3	4	4	1	1	1	1
4	4	2	2	1	1	2	2	1	1
2	2	3	3	4	4	2	2	1	1
2	2	3	3	4	4	2	2	3	3
2	2	2	2	1	1	1	1	1	1
2	2	3	3	4	4	2	2	1	1
2	2	3	3	4	4	2	2	1	1
2	2	3	3	4	4	2	2	1	1
2	2	3	3	4	4	1	1	1	1
2	2	3	3	4	4	2	2	1	1
2	2	3	3	4	4	2	2	1	1
4	4	2	2	1	1	2	2	1	1
4	4	3	3	1	1	2	2	3	3
2	2	2	2	1	1	1	1	1	1
2	2	3	3	4	4	2	2	1	1
2	2	3	3	4	4	2	2	1	1
2	2	3	3	4	4	1	1	1	1
2	2	3	3	4	4	2	2	1	1
2	2	3	3	4	4	2	2	1	1
2	2	3	3	4	4	2	2	1	1
2	2	2	2	1	1	2	2	1	1
2	2	3	3	4	4	2	2	3	3
2	2	2	2	1	1	2	2	1	1
2	2	3	3	4	4	1	1	1	1
2	2	3	3	4	4	2	2	1	1
2	2	3	3	4	4	1	1	1	1
4	4	2	2	1	1	2	2	1	1
2	2	3	3	4	4	2	2	3	3
4	4	2	2	1	1	1	1	3	3

For Review Only

4	4	2	2	1	1	2	2	1	1
2	2	3	3	4	4	2	2	1	1

For Review Only

SNP2786	SNP2786	SNP2787	SNP2787	SNP2788	SNP2788	SNP2789	SNP2789	SNP279	SNP279
4	4	1	1	1	1	1	1	1	1
4	4	1	1	1	1	1	1	3	3
4	4	1	1	1	1	4	4	3	3
4	4	1	1	1	1	1	1	3	3
4	4	1	1	4	4	1	1	3	3
4	4	1	1	1	1	1	1	3	3
4	4	1	1	1	1	1	1	1	1
4	4	1	1	1	1	1	1	3	3
4	4	1	1	1	1	1	1	1	1
4	4	2	2	1	1	1	1	3	3
4	4	1	1	1	1	1	1	3	3
4	4	1	1	1	1	4	4	3	3
4	4	1	1	1	1	1	1	1	1
4	4	1	1	1	1	1	1	3	3
4	4	1	1	1	1	1	1	3	3
4	4	1	1	4	4	1	1	3	3
4	4	1	1	4	4	1	1	1	1
4	4	1	1	1	1	1	1	3	3
4	4	1	1	1	1	1	1	3	3
4	4	1	1	1	1	1	1	3	3
4	4	1	1	1	1	1	1	3	3
4	4	1	1	1	1	1	1	1	1
4	4	1	1	1	1	1	1	3	3
4	4	1	1	1	1	1	1	3	3
4	4	1	1	4	4	1	1	3	3
4	4	1	1	1	1	4	4	3	3
2	2	1	1	1	1	4	4	3	3
4	4	1	1	1	1	1	1	3	3
4	4	1	1	1	1	1	1	3	3
4	4	1	1	1	1	4	4	3	3
4	4	1	1	1	1	4	4	3	3
4	4	1	1	1	1	1	1	3	3
4	4	1	1	1	1	1	1	3	3
2	2	1	1	1	1	1	1	3	3
4	4	1	1	1	1	1	1	3	3
2	2	1	1	4	4	1	1	3	3
4	4	1	1	1	1	1	1	3	3
4	4	1	1	1	1	1	1	3	3
4	4	1	1	1	1	1	1	3	3
4	4	1	1	1	1	1	1	3	3
4	4	1	1	4	4	1	1	3	3
4	4	1	1	1	1	1	1	3	3
4	4	2	2	1	1	1	1	3	3
4	4	1	1	4	4	1	1	3	3
4	4	2	2	1	1	1	1	3	3
4	4	1	1	1	1	1	1	3	3
4	4	1	1	1	1	1	1	3	3
4	4	2	2	1	1	1	1	3	3
4	4	1	1	1	1	1	1	3	3
4	4	1	1	4	4	1	1	3	3
4	4	1	1	1	1	1	1	3	3
2	2	1	1	1	1	1	1	3	3

For Review Only

4	4	1	1	4	4	1	1	1	1
4	4	1	1	1	1	1	1	3	3
4	4	1	1	1	1	1	1	3	3
4	4	1	1	4	4	1	1	1	1
4	4	1	1	1	1	1	1	3	3
4	4	1	1	1	1	1	1	3	3
4	4	1	1	1	1	1	1	3	3
4	4	1	1	4	4	1	1	3	3
4	4	1	1	1	1	1	1	3	3
4	4	1	1	1	1	1	1	3	3
4	4	1	1	1	1	1	1	3	3
4	4	1	1	1	1	1	1	3	3
4	4	1	1	4	4	1	1	1	1
4	4	1	1	1	1	1	1	3	3
4	4	1	1	1	1	1	1	3	3
4	4	1	1	1	1	1	1	3	3
4	4	1	1	1	1	1	1	3	3
4	4	1	1	1	1	1	1	3	3
4	4	1	1	4	4	1	1	1	1
4	4	1	1	1	1	1	1	1	1
4	4	1	1	1	1	1	1	3	3
4	4	1	1	1	1	1	1	3	3
4	4	1	1	1	1	1	1	3	3
4	4	1	1	1	1	1	1	3	3
4	4	2	2	1	1	1	1	3	3
2	2	1	1	1	1	1	1	3	3
4	4	2	2	4	4	1	1	3	3
4	4	2	2	1	1	4	4	3	3
4	4	1	1	1	1	1	1	3	3
4	4	1	1	4	4	1	1	3	3
4	4	1	1	1	1	1	1	1	1
4	4	1	1	4	4	1	1	3	3
4	4	1	1	1	1	1	1	3	3
4	4	1	1	4	4	1	1	1	1
4	4	1	1	1	1	1	1	3	3
4	4	2	2	1	1	1	1	3	3
4	4	1	1	1	1	1	1	3	3
4	4	1	1	1	1	1	1	1	1
4	4	1	1	1	1	4	4	3	3
4	4	1	1	1	1	1	1	3	3
4	4	1	1	1	1	1	1	3	3
4	4	1	1	4	4	1	1	3	3
4	4	1	1	1	1	1	1	3	3
4	4	1	1	1	1	1	1	3	3
4	4	1	1	1	1	1	1	3	3
4	4	1	1	1	1	1	1	3	3
4	4	1	1	1	1	1	1	3	3
4	4	1	1	1	1	1	1	3	3
4	4	1	1	1	1	1	1	3	3
4	4	1	1	1	1	1	1	3	3
4	4	1	1	1	1	1	1	3	3
4	4	1	1	1	1	1	1	3	3
4	4	1	1	4	4	1	1	3	3
4	4	1	1	1	1	1	1	3	3
4	4	1	1	1	1	1	1	3	3

For Review Only

4	4	1	1	1	1	1	1	3	3
4	4	1	1	1	1	1	1	3	3
4	4	1	1	1	1	1	1	3	3
4	4	1	1	1	1	1	1	3	3
2	2	1	1	1	1	1	1	3	3
4	4	1	1	1	1	1	1	3	3
4	4	1	1	4	4	1	1	3	3
4	4	1	1	1	1	1	1	3	3
4	4	1	1	1	1	1	1	3	3
4	4	1	1	1	1	1	1	3	3
4	4	1	1	1	1	1	1	3	3
4	4	1	1	1	1	1	1	3	3
4	4	1	1	1	1	1	1	3	3
2	2	1	1	1	1	1	1	3	3
4	4	1	1	1	1	1	1	3	3
4	4	1	1	1	1	1	1	3	3
4	4	1	1	1	1	1	1	3	3
4	4	1	1	1	1	1	1	3	3
4	4	1	1	1	1	1	1	3	3
4	4	1	1	1	1	1	1	3	3
4	4	1	1	1	1	1	1	3	3
4	4	1	1	4	4	1	1	3	3
2	2	1	1	1	1	1	1	3	3
4	4	1	1	1	1	1	1	3	3
4	4	1	1	1	1	1	1	3	3
4	4	1	1	1	1	1	1	3	3
4	4	1	1	1	1	1	1	3	3
4	4	1	1	1	1	1	1	3	3
4	4	1	1	4	4	1	1	3	3
4	4	1	1	1	1	1	1	3	3
4	4	1	1	1	1	1	1	3	3
4	4	1	1	1	1	1	1	3	3
4	4	1	1	1	1	1	1	3	3
4	4	1	1	1	1	1	1	3	3
2	2	1	1	1	1	1	1	3	3
2	2	1	1	1	1	1	1	3	3
4	4	1	1	1	1	1	1	3	3
4	4	1	1	1	1	1	1	3	3
4	4	1	1	1	1	1	1	3	3
4	4	1	1	1	1	1	1	3	3
4	4	1	1	1	1	1	1	3	3
4	4	1	1	1	1	1	1	3	3
4	4	1	1	1	1	1	1	3	3
4	4	1	1	1	1	1	1	3	3
4	4	1	1	1	1	1	1	3	3
4	4	1	1	1	1	1	1	3	3
4	4	1	1	1	1	1	1	3	3
4	4	1	1	1	1	4	4	3	3
4	4	1	1	1	1	1	1	3	3
4	4	1	1	1	1	1	1	3	3
4	4	1	1	1	1	1	1	3	3
4	4	1	1	1	1	1	1	3	3
4	4	1	1	1	1	1	1	3	3
4	4	1	1	1	1	1	1	3	3
4	4	1	1	1	1	1	1	3	3

4	4	1	1	1	1	1	1	3	3
4	4	1	1	1	1	1	1	3	3
4	4	2	2	1	1	1	1	3	3
4	4	1	1	1	1	1	1	3	3
4	4	1	1	1	1	1	1	1	1
4	4	1	1	1	1	1	1	3	3
4	4	1	1	1	1	1	1	3	3
4	4	1	1	1	1	1	1	3	3
4	4	1	1	1	1	1	1	3	3
4	4	1	1	1	1	1	1	1	1
4	4	1	1	1	1	1	1	3	3
4	4	1	1	1	1	1	1	3	3
4	4	1	1	1	1	1	1	3	3
4	4	1	1	1	1	1	1	3	3
4	4	1	1	1	1	1	1	3	3
4	4	1	1	1	1	1	1	3	3
4	4	1	1	1	1	1	1	3	3
4	4	1	1	1	1	1	1	3	3
4	4	1	1	1	1	1	1	3	3
4	4	1	1	4	4	1	1	3	3
4	4	1	1	1	1	1	1	3	3
4	4	1	1	1	1	4	4	3	3
4	4	1	1	1	1	1	1	3	3
4	4	1	1	1	1	1	1	3	3
4	4	1	1	1	1	1	1	3	3
2	2	1	1	1	1	1	1	3	3
4	4	1	1	1	1	1	1	3	3
4	4	1	1	1	1	1	1	3	3
4	4	2	2	1	1	1	1	3	3
4	4	2	2	1	1	1	1	1	1
4	4	1	1	1	1	1	1	3	3
4	4	1	1	1	1	1	1	3	3
4	4	1	1	1	1	1	1	3	3
4	4	1	1	1	1	1	1	3	3
4	4	1	1	1	1	1	1	3	3
4	4	1	1	1	1	1	1	3	3
4	4	1	1	1	1	1	1	3	3
4	4	1	1	1	1	1	1	3	3
4	4	1	1	1	1	4	4	3	3
2	2	1	1	1	1	1	1	3	3
4	4	1	1	1	1	4	4	3	3
4	4	1	1	1	1	1	1	3	3
4	4	1	1	1	1	1	1	3	3
4	4	1	1	1	1	1	1	3	3
4	4	1	1	1	1	1	1	3	3
4	4	1	1	1	1	1	1	3	3
4	4	1	1	1	1	1	1	3	3
4	4	1	1	1	1	1	1	3	3
2	2	1	1	4	4	1	1	3	3
4	4	1	1	4	4	1	1	3	3
2	2	1	1	1	1	1	1	3	3
4	4	1	1	1	1	1	1	3	3
4	4	1	1	1	1	1	1	3	3
4	4	1	1	1	1	1	1	3	3
4	4	1	1	1	1	1	1	3	3

For Review Only

4	4	1	1	1	1	1	1	3	3
4	4	1	1	1	1	1	1	1	1
4	4	1	1	4	4	1	1	3	3
4	4	1	1	1	1	1	1	3	3
4	4	1	1	1	1	4	4	3	3
4	4	1	1	1	1	1	1	3	3
4	4	1	1	1	1	1	1	3	3
4	4	1	1	1	1	1	1	3	3
4	4	1	1	1	1	4	4	3	3
4	4	1	1	1	1	4	4	3	3
4	4	1	1	1	1	1	1	3	3
4	4	1	1	1	1	1	1	3	3
4	4	1	1	1	1	1	1	3	3
4	4	1	1	1	1	1	1	3	3
4	4	1	1	1	1	4	4	3	3
4	4	1	1	4	4	1	1	3	3
4	4	1	1	1	1	1	1	3	3
4	4	2	2	1	1	4	4	3	3
4	4	1	1	1	1	1	1	3	3
4	4	1	1	1	1	1	1	3	3
4	4	1	1	1	1	1	1	3	3
4	4	1	1	1	1	1	1	3	3
4	4	1	1	4	4	1	1	3	3
4	4	1	1	1	1	1	1	3	3
4	4	1	1	4	4	1	1	3	3
4	4	1	1	1	1	1	1	1	1
4	4	1	1	1	1	1	1	3	3
4	4	1	1	4	4	1	1	3	3
4	4	1	1	1	1	1	1	1	1
2	2	1	1	4	4	1	1	3	3
4	4	2	2	4	4	1	1	3	3
4	4	1	1	1	1	1	1	3	3
4	4	1	1	1	1	1	1	3	3
4	4	1	1	1	1	4	4	3	3
4	4	1	1	1	1	1	1	3	3
4	4	2	2	1	1	1	1	3	3
4	4	1	1	1	1	1	1	3	3
4	4	1	1	1	1	1	1	3	3
2	2	1	1	4	4	1	1	3	3
4	4	1	1	1	1	1	1	3	3
4	4	1	1	1	1	1	1	3	3
4	4	1	1	1	1	1	1	3	3
4	4	1	1	1	1	1	1	3	3
4	4	1	1	4	4	1	1	1	1
4	4	1	1	1	1	1	1	3	3
4	4	1	1	1	1	4	4	3	3
4	4	1	1	1	1	4	4	3	3
2	2	1	1	1	1	1	1	3	3
4	4	1	1	1	1	1	1	3	3
4	4	1	1	1	1	4	4	3	3
4	4	1	1	4	4	1	1	3	3
4	4	1	1	4	4	1	1	3	3
4	4	1	1	1	1	4	4	3	3
2	2	1	1	1	1	1	1	3	3
2	2	1	1	1	1	1	1	3	3

For Review Only

4	4	1	1	1	1	1	1	3	3
4	4	1	1	1	1	1	1	3	3

For Review Only

SNP2790	SNP2790	SNP2791	SNP2791	SNP2792	SNP2792	SNP2793	SNP2793	SNP2794	SNP2794
4	4	1	1	2	2	4	4	3	3
3	3	4	4	2	2	3	3	3	3
3	3	4	4	2	2	4	4	2	2
4	4	4	4	2	2	3	3	3	3
4	4	4	4	2	2	3	3	3	3
4	4	4	4	2	2	3	3	3	3
4	4	4	4	2	2	3	3	3	3
3	3	4	4	2	2	3	3	3	3
4	4	4	4	2	2	3	3	3	3
4	4	4	4	3	3	3	3	3	3
4	4	4	4	2	2	3	3	3	3
4	4	4	4	2	2	4	4	2	2
4	4	4	4	2	2	3	3	3	3
4	4	4	4	3	3	3	3	3	3
4	4	4	4	2	2	3	3	3	3
4	4	4	4	2	2	3	3	3	3
4	4	4	4	2	2	3	3	3	3
4	4	4	4	2	2	3	3	3	3
4	4	4	4	2	2	4	4	3	3
3	3	4	4	3	3	3	3	2	2
4	4	4	4	3	3	3	3	3	3
4	4	4	4	2	2	3	3	3	3
4	4	4	4	2	2	4	4	3	3
4	4	4	4	2	2	3	3	3	3
4	4	4	4	2	2	4	4	3	3
4	4	4	4	2	2	4	4	3	3
3	3	4	4	2	2	4	4	3	3
4	4	4	4	2	2	4	4	3	3
3	3	4	4	2	2	3	3	3	3
4	4	4	4	3	3	3	3	3	3
4	4	4	4	2	2	4	4	3	3
4	4	4	4	3	3	3	3	3	3
4	4	4	4	2	2	3	3	3	3
4	4	4	4	2	2	3	3	3	3
4	4	4	4	2	2	3	3	3	3
3	3	4	4	2	2	3	3	2	2
4	4	1	1	3	3	3	3	3	3
4	4	4	4	2	2	3	3	3	3
4	4	4	4	2	2	3	3	3	3
4	4	4	4	2	2	3	3	3	3
4	4	4	4	3	3	3	3	3	3
3	3	4	4	2	2	3	3	3	3
4	4	4	4	3	3	3	3	3	3
4	4	4	4	3	3	3	3	3	3
4	4	4	4	3	3	3	3	3	3
4	4	4	4	2	2	4	4	3	3
4	4	4	4	2	2	3	3	3	3
4	4	4	4	2	2	3	3	3	3
4	4	4	4	2	2	3	3	3	3
4	4	4	4	2	2	3	3	3	3
4	4	4	4	2	2	3	3	3	3
4	4	4	4	2	2	4	4	3	3

For Review Only

4	4	4	4	2	2	3	3	3	3
4	4	4	4	2	2	3	3	3	3
4	4	4	4	2	2	3	3	3	3
3	3	4	4	2	2	3	3	3	3
4	4	4	4	2	2	4	4	3	3
4	4	4	4	2	2	3	3	3	3
4	4	4	4	2	2	3	3	3	3
3	3	4	4	2	2	3	3	3	3
3	3	4	4	2	2	3	3	3	3
4	4	4	4	2	2	4	4	3	3
4	4	4	4	2	2	3	3	3	3
4	4	4	4	2	2	3	3	3	3
4	4	4	4	2	2	3	3	3	3
3	3	4	4	2	2	3	3	3	3
4	4	4	4	2	2	4	4	3	3
4	4	4	4	2	2	4	4	3	3
4	4	4	4	2	2	4	4	3	3
3	3	4	4	2	2	4	4	3	3
4	4	4	4	2	2	3	3	3	3
4	4	4	4	2	2	3	3	3	3
4	4	4	4	2	2	3	3	3	3
4	4	4	4	2	2	4	4	3	3
4	4	4	4	2	2	4	4	3	3
4	4	4	4	3	3	4	4	3	3
4	4	4	4	3	3	3	3	3	3
4	4	4	4	2	2	3	3	3	3
4	4	1	1	2	2	4	4	3	3
3	3	4	4	2	2	3	3	2	2
4	4	4	4	2	2	3	3	3	3
4	4	4	4	2	2	4	4	3	3
4	4	4	4	3	3	3	3	3	3
4	4	4	4	3	3	3	3	3	3
3	3	4	4	2	2	3	3	3	3
4	4	4	4	2	2	3	3	3	3
4	4	4	4	2	2	4	4	3	3
4	4	4	4	3	3	3	3	3	3
3	3	4	4	3	3	3	3	3	3
4	4	4	4	2	2	3	3	3	3
4	4	4	4	2	2	4	4	3	3
4	4	4	4	3	3	3	3	3	3
4	4	4	4	2	2	3	3	3	3
3	3	4	4	2	2	4	4	2	2
4	4	4	4	2	2	4	4	3	3
4	4	4	4	2	2	3	3	3	3
4	4	4	4	2	2	3	3	3	3
4	4	4	4	2	2	3	3	3	3
3	3	4	4	3	3	3	3	3	3
4	4	4	4	3	3	3	3	3	3
4	4	4	4	2	2	3	3	3	3
4	4	4	4	2	2	4	4	3	3
4	4	4	4	2	2	3	3	3	3
4	4	4	4	2	2	3	3	3	3
4	4	4	4	2	2	4	4	3	3
4	4	4	4	2	2	3	3	3	3
4	4	4	4	2	2	4	4	3	3

For Review Only

4	4	4	4	2	2	3	3	3	3
4	4	4	4	2	2	3	3	3	3
4	4	4	4	2	2	3	3	3	3
4	4	4	4	2	2	3	3	3	3
3	3	4	4	2	2	3	3	3	3
4	4	4	4	2	2	3	3	3	3
4	4	4	4	2	2	3	3	3	3
4	4	4	4	3	3	4	4	3	3
4	4	4	4	2	2	3	3	3	3
3	3	4	4	2	2	3	3	3	3
3	3	4	4	2	2	4	4	3	3
4	4	4	4	2	2	4	4	3	3
4	4	4	4	2	2	4	4	3	3
4	4	4	4	2	2	4	4	3	3
4	4	4	4	2	2	3	3	3	3
4	4	4	4	2	2	3	3	3	3
4	4	4	4	2	2	4	4	3	3
4	4	4	4	2	2	3	3	3	3
4	4	4	4	3	3	3	3	3	3
4	4	4	4	2	2	4	4	3	3
4	4	4	4	2	2	3	3	3	3
4	4	4	4	2	2	4	4	3	3
3	3	4	4	2	2	3	3	2	2
3	3	4	4	2	2	3	3	3	3
4	4	4	4	2	2	4	4	3	3
4	4	4	4	2	2	3	3	3	3
4	4	4	4	2	2	4	4	3	3
4	4	4	4	2	2	3	3	3	3
4	4	4	4	2	2	3	3	3	3
4	4	4	4	2	2	3	3	3	3
4	4	4	4	3	3	3	3	3	3
4	4	4	4	2	2	3	3	3	3
4	4	4	4	2	2	3	3	3	3
4	4	4	4	2	2	3	3	3	3
4	4	4	4	2	2	3	3	3	3
4	4	4	4	2	2	3	3	3	3
4	4	4	4	3	3	3	3	3	3
4	4	4	4	3	3	4	4	3	3
4	4	4	4	2	2	4	4	3	3
4	4	4	4	2	2	3	3	3	3
4	4	4	4	2	2	4	4	3	3
3	3	4	4	3	3	3	3	3	3
3	3	4	4	2	2	4	4	3	3
4	4	4	4	2	2	3	3	3	3
4	4	4	4	3	3	3	3	3	3
4	4	1	1	3	3	3	3	3	3
4	4	4	4	2	2	3	3	3	3
4	4	4	4	2	2	4	4	3	3
4	4	4	4	2	2	3	3	3	3
4	4	4	4	2	2	3	3	3	3
4	4	4	4	2	2	3	3	3	3
4	4	4	4	2	2	3	3	3	3
4	4	4	4	2	2	3	3	3	3
4	4	4	4	2	2	3	3	3	3
4	4	4	4	2	2	3	3	3	3

4	4	4	4	2	2	3	3	3	3
4	4	4	4	2	2	4	4	3	3
4	4	1	1	2	2	4	4	3	3
4	4	4	4	2	2	4	4	3	3
4	4	1	1	2	2	3	3	3	3
4	4	4	4	2	2	3	3	3	3
4	4	4	4	2	2	4	4	3	3
4	4	4	4	2	2	3	3	3	3
4	4	4	4	2	2	3	3	3	3
4	4	4	4	2	2	3	3	3	3
4	4	4	4	2	2	3	3	3	3
4	4	4	4	2	2	3	3	3	3
4	4	4	4	2	2	4	4	3	3
4	4	1	1	3	3	4	4	3	3
4	4	4	4	3	3	3	3	3	3
4	4	4	4	2	2	3	3	3	3
4	4	4	4	2	2	3	3	3	3
4	4	4	4	2	2	3	3	3	3
4	4	4	4	2	2	3	3	3	3
4	4	4	4	2	2	3	3	3	3
4	4	4	4	2	2	3	3	3	3
4	4	1	1	3	3	3	3	3	3
4	4	4	4	2	2	4	4	3	3
4	4	4	4	2	2	3	3	3	3
4	4	4	4	3	3	3	3	3	3
4	4	4	4	2	2	3	3	3	3
4	4	4	4	2	2	3	3	3	3
4	4	4	4	2	2	4	4	3	3
4	4	4	4	2	2	4	4	3	3
3	3	4	4	2	2	3	3	3	3
4	4	4	4	2	2	4	4	3	3
4	4	4	4	2	2	4	4	3	3
4	4	1	1	2	2	4	4	3	3
4	4	4	4	2	2	3	3	3	3
4	4	4	4	2	2	3	3	3	3
4	4	4	4	2	2	3	3	3	3
4	4	4	4	2	2	4	4	3	3
4	4	1	1	3	3	3	3	3	3
4	4	4	4	2	2	4	4	3	3
4	4	4	4	2	2	3	3	3	3
3	3	4	4	2	2	4	4	2	2
4	4	4	4	2	2	3	3	3	3
4	4	1	1	2	2	3	3	3	3
4	4	1	1	2	2	4	4	3	3
4	4	4	4	2	2	3	3	3	3
4	4	4	4	2	2	4	4	3	3
4	4	4	4	2	2	3	3	3	3
4	4	4	4	2	2	3	3	3	3
4	4	4	4	2	2	3	3	3	3
4	4	4	4	3	3	3	3	3	3
4	4	4	4	2	2	3	3	3	3
4	4	1	1	2	2	3	3	3	3
4	4	4	4	2	2	4	4	3	3
4	4	4	4	2	2	3	3	3	3
3	3	4	4	2	2	3	3	3	3

For Review Only

3	3	4	4	2	2	3	3	3	3
4	4	1	1	2	2	4	4	3	3
3	3	4	4	2	2	4	4	2	2
4	4	4	4	2	2	4	4	3	3
4	4	1	1	2	2	3	3	3	3
4	4	4	4	2	2	3	3	3	3
4	4	4	4	2	2	4	4	3	3
3	3	4	4	2	2	3	3	3	3
4	4	4	4	2	2	3	3	3	3
4	4	4	4	2	2	3	3	3	3
4	4	4	4	2	2	3	3	3	3
4	4	4	4	3	3	4	4	3	3
4	4	4	4	2	2	4	4	3	3
4	4	4	4	2	2	3	3	3	3
3	3	4	4	2	2	4	4	2	2
4	4	4	4	2	2	3	3	3	3
4	4	4	4	2	2	3	3	3	3
3	3	4	4	2	2	3	3	3	3
4	4	4	4	2	2	3	3	3	3
4	4	4	4	2	2	3	3	3	3
4	4	4	4	2	2	3	3	3	3
4	4	4	4	2	2	3	3	3	3
4	4	4	4	2	2	3	3	3	3
4	4	4	4	2	2	4	4	3	3
3	3	4	4	2	2	3	3	3	3
4	4	4	4	3	3	3	3	3	3
3	3	4	4	2	2	3	3	3	3
4	4	4	4	2	2	3	3	3	3
4	4	4	4	2	2	3	3	3	3
3	3	4	4	2	2	3	3	2	2
3	3	4	4	2	2	3	3	3	3
4	4	4	4	2	2	3	3	3	3
4	4	4	4	3	3	4	4	3	3
4	4	4	4	2	2	4	4	3	3
4	4	4	4	2	2	4	4	3	3
4	4	4	4	2	2	3	3	3	3
3	3	4	4	2	2	3	3	3	3
4	4	4	4	2	2	3	3	3	3
3	3	4	4	2	2	3	3	2	2
4	4	4	4	2	2	3	3	3	3
4	4	1	1	2	2	4	4	3	3
3	3	4	4	2	2	3	3	3	3
4	4	4	4	2	2	4	4	3	3
4	4	4	4	2	2	3	3	3	3
4	4	4	4	2	2	3	3	3	3
4	4	4	4	2	2	4	4	3	3
4	4	4	4	2	2	4	4	3	3
3	3	4	4	2	2	3	3	3	3
4	4	4	4	2	2	3	3	3	3
4	4	4	4	2	2	3	3	3	3
4	4	4	4	2	2	3	3	3	3
4	4	4	4	2	2	3	3	3	3
3	3	4	4	2	2	4	4	2	2
4	4	4	4	2	2	3	3	3	3
3	3	4	4	2	2	3	3	3	3

For Review Only

4	4	4	4	2	2	3	3	3	3
4	4	4	4	2	2	3	3	3	3

For Review Only

SNP2795	SNP2795	SNP2796	SNP2796	SNP2797	SNP2797	SNP2798	SNP2798	SNP2799	SNP2799
4	4	2	2	4	4	3	3	2	2
3	3	2	2	4	4	3	3	2	2
4	4	4	4	2	2	2	2	2	2
4	4	2	2	4	4	3	3	2	2
4	4	2	2	4	4	3	3	2	2
4	4	2	2	4	4	3	3	2	2
4	4	2	2	4	4	3	3	1	1
4	4	2	2	4	4	3	3	2	2
4	4	2	2	4	4	3	3	2	2
4	4	2	2	4	4	3	3	1	1
4	4	2	2	4	4	3	3	2	2
4	4	2	2	4	4	2	2	2	2
3	3	2	2	4	4	3	3	2	2
4	4	2	2	4	4	3	3	2	2
4	4	2	2	4	4	3	3	2	2
4	4	2	2	4	4	3	3	2	2
4	4	2	2	4	4	3	3	2	2
4	4	2	2	4	4	3	3	2	2
3	3	4	4	2	2	2	2	2	2
4	4	2	2	4	4	3	3	2	2
4	4	2	2	4	4	3	3	2	2
4	4	2	2	4	4	3	3	2	2
4	4	4	4	2	2	2	2	2	2
4	4	2	2	4	4	3	3	1	1
4	4	4	4	2	2	2	2	2	2
4	4	4	4	2	2	2	2	2	2
4	4	4	4	2	2	2	2	2	2
4	4	2	2	4	4	2	2	2	2
4	4	2	2	4	4	3	3	2	2
3	3	2	2	4	4	3	3	1	1
4	4	4	4	2	2	2	2	2	2
4	4	2	2	4	4	3	3	1	1
4	4	2	2	4	4	3	3	2	2
4	4	2	2	4	4	3	3	2	2
4	4	2	2	4	4	3	3	2	2
4	4	2	2	4	4	3	3	2	2
4	4	2	2	4	4	3	3	2	2
4	4	2	2	4	4	3	3	2	2
4	4	2	2	4	4	2	2	1	1
4	4	2	2	4	4	3	3	1	1
4	4	2	2	4	4	3	3	1	1
4	4	2	2	4	4	3	3	2	2
3	3	2	2	4	4	3	3	2	2
3	3	2	2	4	4	3	3	2	2
4	4	2	2	4	4	3	3	1	1
4	4	2	2	4	4	2	2	2	2
4	4	2	2	4	4	3	3	2	2
4	4	2	2	4	4	3	3	2	2
3	3	2	2	4	4	3	3	2	2
4	4	2	2	4	4	3	3	2	2
4	4	2	2	4	4	3	3	2	2
4	4	2	2	4	4	2	2	2	2

For Review Only

4	4	2	2	4	4	3	3	2	2
4	4	2	2	4	4	3	3	2	2
4	4	2	2	4	4	3	3	2	2
4	4	2	2	4	4	3	3	2	2
3	3	4	4	2	2	2	2	2	2
4	4	2	2	4	4	3	3	2	2
4	4	2	2	4	4	3	3	2	2
3	3	2	2	4	4	3	3	1	1
4	4	2	2	4	4	3	3	2	2
4	4	2	2	4	4	2	2	2	2
4	4	2	2	4	4	3	3	2	2
4	4	2	2	4	4	3	3	2	2
4	4	2	2	4	4	2	2	2	2
4	4	2	2	4	4	3	3	2	2
4	4	2	2	4	4	2	2	2	2
3	3	2	2	4	4	3	3	2	2
4	4	4	4	2	2	2	2	2	2
4	4	4	4	2	2	2	2	2	2
4	4	2	2	4	4	3	3	2	2
4	4	2	2	4	4	3	3	2	2
4	4	2	2	4	4	3	3	2	2
4	4	4	4	2	2	2	2	2	2
4	4	2	2	4	4	3	3	2	2
4	4	4	4	2	2	2	2	2	2
4	4	2	2	4	4	3	3	1	1
4	4	2	2	4	4	3	3	2	2
3	3	2	2	4	4	2	2	1	1
4	4	2	2	4	4	3	3	1	1
4	4	2	2	4	4	3	3	2	2
4	4	2	2	4	4	2	2	2	2
4	4	2	2	4	4	3	3	2	2
3	3	2	2	4	4	3	3	2	2
4	4	2	2	4	4	3	3	1	1
4	4	2	2	4	4	3	3	2	2
4	4	2	2	4	4	3	3	2	2
4	4	2	2	4	4	3	3	1	1
4	4	2	2	4	4	3	3	1	1
4	4	2	2	4	4	3	3	2	2
4	4	4	4	2	2	2	2	2	2
3	3	2	2	4	4	3	3	1	1
4	4	2	2	4	4	3	3	2	2
4	4	2	2	4	4	2	2	2	2
4	4	4	4	2	2	2	2	1	1
4	4	2	2	4	4	3	3	2	2
4	4	2	2	4	4	3	3	1	1
4	4	2	2	4	4	3	3	2	2
4	4	2	2	4	4	3	3	2	2
4	4	2	2	4	4	3	3	2	2
4	4	2	2	4	4	3	3	2	2
4	4	4	4	2	2	2	2	2	2
4	4	2	2	4	4	3	3	2	2
4	4	2	2	4	4	3	3	1	1
3	3	2	2	4	4	3	3	1	1
4	4	2	2	4	4	2	2	1	1
4	4	4	4	2	2	2	2	2	2

For Review Only

CSSA 5585 Guilford Rd., Madison, WI 53711

4	4	2	2	4	4	3	3	2	2
4	4	2	2	4	4	3	3	2	2
4	4	2	2	4	4	3	3	2	2
4	4	2	2	4	4	3	3	2	2
4	4	2	2	4	4	3	3	2	2
4	4	2	2	4	4	3	3	2	2
4	4	2	2	4	4	3	3	2	2
4	4	4	4	2	2	2	2	2	2
4	4	2	2	4	4	3	3	2	2
4	4	2	2	4	4	3	3	2	2
4	4	2	2	4	4	3	3	2	2
4	4	2	2	4	4	2	2	2	2
4	4	4	4	2	2	2	2	1	1
4	4	2	2	4	4	2	2	2	2
4	4	2	2	4	4	2	2	2	2
4	4	2	2	4	4	3	3	1	1
4	4	4	4	4	4	2	2	1	1
4	4	2	2	4	4	2	2	2	2
4	4	2	2	4	4	3	3	2	2
4	4	4	4	2	2	2	2	2	2
4	4	2	2	4	4	2	2	2	2
4	4	2	2	4	4	3	3	2	2
4	4	2	2	4	4	2	2	2	2
4	4	2	2	4	4	3	3	1	1
4	4	4	4	4	4	2	2	2	2
4	4	2	2	4	4	3	3	2	2
4	4	4	4	2	2	2	2	2	2
4	4	2	2	4	4	3	3	2	2
4	4	2	2	4	4	3	3	2	2
4	4	2	2	4	4	3	3	2	2
4	4	2	2	4	4	3	3	2	2
4	4	2	2	4	4	3	3	2	2
4	4	2	2	4	4	3	3	2	2
4	4	2	2	4	4	3	3	2	2
4	4	2	2	4	4	3	3	2	2
4	4	2	2	4	4	3	3	1	1
4	4	2	2	4	4	3	3	2	2
4	4	2	2	4	4	3	3	2	2
4	4	4	4	4	4	2	2	2	2
4	4	2	2	4	4	3	3	1	1
4	4	2	2	4	4	2	2	2	2
4	4	2	2	4	4	3	3	2	2
4	4	4	4	2	2	2	2	2	2
3	3	2	2	4	4	3	3	1	1
4	4	2	2	4	4	3	3	1	1
4	4	2	2	4	4	3	3	2	2
4	4	2	2	4	4	3	3	2	2
4	4	4	4	2	2	2	2	2	2
4	4	2	2	4	4	3	3	2	2
4	4	2	2	4	4	3	3	2	2
4	4	2	2	4	4	2	2	2	2
4	4	2	2	4	4	3	3	2	2
4	4	2	2	4	4	3	3	2	2
4	4	2	2	4	4	3	3	2	2
3	3	2	2	4	4	3	3	2	2

For Review Only

4	4	2	2	4	4	3	3	2	2
4	4	4	4	2	2	2	2	2	2
4	4	2	2	4	4	2	2	1	1
4	4	4	4	2	2	2	2	2	2
4	4	2	2	4	4	3	3	2	2
4	4	2	2	4	4	3	3	2	2
4	4	2	2	4	4	2	2	1	1
4	4	2	2	4	4	2	2	2	2
4	4	2	2	4	4	2	2	2	2
4	4	2	2	4	4	3	3	2	2
4	4	2	2	4	4	3	3	2	2
4	4	2	2	4	4	3	3	1	1
4	4	4	4	2	2	2	2	2	2
4	4	2	2	4	4	2	2	2	2
4	4	2	2	4	4	3	3	2	2
4	4	2	2	4	4	3	3	2	2
4	4	2	2	4	4	2	2	2	2
4	4	2	2	4	4	3	3	2	2
4	4	2	2	4	4	3	3	2	2
4	4	2	2	4	4	3	3	2	2
4	4	2	2	4	4	2	2	2	2
3	3	2	2	4	4	3	3	1	1
4	4	4	4	2	2	2	2	2	2
4	4	2	2	4	4	3	3	2	2
4	4	2	2	4	4	3	3	2	2
4	4	2	2	4	4	3	3	2	2
4	4	2	2	4	4	3	3	2	2
4	4	2	2	4	4	2	2	2	2
4	4	2	2	4	4	3	3	2	2
4	4	2	2	4	4	3	3	2	2
4	4	2	2	4	4	3	3	2	2
4	4	4	4	2	2	2	2	2	2
4	4	4	4	2	2	2	2	2	2
4	4	2	2	4	4	3	3	2	2
4	4	2	2	4	4	3	3	2	2
4	4	2	2	4	4	3	3	2	2
4	4	4	4	2	2	2	2	2	2
4	4	2	2	4	4	3	3	2	2
4	4	4	4	4	4	2	2	2	2
4	4	2	2	4	4	3	3	2	2
4	4	4	4	2	2	2	2	2	2
4	4	2	2	4	4	3	3	2	2
4	4	2	2	4	4	2	2	1	1
4	4	2	2	4	4	2	2	2	2
4	4	2	2	4	4	3	3	2	2
4	4	2	2	4	4	2	2	2	2
4	4	2	2	4	4	3	3	2	2
4	4	2	2	4	4	3	3	1	1
4	4	2	2	4	4	3	3	1	1
4	4	2	2	4	4	2	2	2	2
4	4	2	2	4	4	3	3	2	2
3	3	2	2	4	4	3	3	2	2
4	4	4	4	2	2	2	2	2	2
4	4	2	2	4	4	3	3	2	2
4	4	2	2	4	4	3	3	1	1

For Review Only

4	4	2	2	4	4	3	3	2	2
4	4	2	2	4	4	2	2	2	2
4	4	2	2	4	4	2	2	2	2
4	4	4	4	2	2	2	2	2	2
4	4	2	2	4	4	3	3	2	2
4	4	2	2	4	4	3	3	2	2
4	4	2	2	4	4	3	3	2	2
4	4	2	2	4	4	3	3	1	1
3	3	2	2	4	4	3	3	1	1
4	4	2	2	4	4	2	2	1	1
4	4	2	2	4	4	2	2	2	2
4	4	2	2	4	4	3	3	2	2
4	4	4	4	2	2	2	2	2	2
4	4	2	2	4	4	3	3	2	2
4	4	4	4	2	2	2	2	1	1
4	4	2	2	4	4	3	3	2	2
4	4	2	2	4	4	3	3	2	2
4	4	2	2	4	4	2	2	2	2
4	4	2	2	4	4	3	3	1	1
4	4	2	2	4	4	3	3	2	2
4	4	2	2	4	4	3	3	1	1
4	4	2	2	4	4	3	3	2	2
4	4	2	2	4	4	3	3	2	2
4	4	4	4	2	2	2	2	1	1
3	3	2	2	4	4	3	3	1	1
4	4	2	2	4	4	2	2	2	2
4	4	2	2	4	4	3	3	1	1
4	4	2	2	4	4	3	3	2	2
4	4	2	2	4	4	3	3	2	2
4	4	2	2	4	4	3	3	2	2
4	4	2	2	4	4	3	3	1	1
4	4	2	2	4	4	3	3	2	2
4	4	4	4	2	2	2	2	2	2
4	4	4	4	2	2	2	2	2	2
4	4	4	4	2	2	2	2	2	2
4	4	2	2	4	4	2	2	1	1
4	4	2	2	4	4	3	3	2	2
4	4	2	2	4	4	3	3	2	2
4	4	2	2	4	4	3	3	2	2
4	4	2	2	4	4	3	3	1	1
4	4	2	2	4	4	2	2	2	2
4	4	2	2	4	4	3	3	2	2
4	4	4	4	2	2	2	2	1	1
4	4	2	2	4	4	3	3	2	2
4	4	2	2	4	4	3	3	2	2
4	4	4	4	2	2	2	2	2	2
4	4	4	4	2	2	2	2	2	2
4	4	2	2	4	4	3	3	2	2
3	3	2	2	4	4	3	3	2	2
4	4	2	2	4	4	3	3	1	1
4	4	2	2	4	4	3	3	2	2
4	4	2	2	4	4	3	3	1	1
4	4	4	4	2	2	2	2	2	2
4	4	2	2	4	4	3	3	2	2
4	4	2	2	4	4	3	3	1	1

For Review Only

4	4	2	2	4	4	3	3	2	2
4	4	4	4	2	2	2	2	2	2

For Review Only

SNP280	SNP280	SNP2800	SNP2800	SNP2801	SNP2801	SNP2802	SNP2802	SNP2803	SNP2803
4	4	1	1	2	2	2	2	4	4
1	1	1	1	2	2	2	2	4	4
4	4	1	1	2	2	2	2	4	4
4	4	1	1	2	2	2	2	4	4
1	1	1	1	2	2	2	2	4	4
1	1	1	1	2	2	2	2	4	4
4	4	4	4	2	2	1	1	4	4
1	1	1	1	2	2	2	2	4	4
4	4	1	1	2	2	2	2	4	4
1	1	4	4	3	3	2	2	2	2
1	1	1	1	2	2	2	2	4	4
4	4	1	1	2	2	2	2	4	4
1	1	1	1	2	2	2	2	4	4
1	1	1	1	2	2	2	2	2	2
4	4	1	1	2	2	2	2	4	4
4	4	1	1	2	2	2	2	4	4
4	4	1	1	2	2	2	2	4	4
1	1	1	1	2	2	2	2	4	4
4	4	1	1	2	2	2	2	4	4
1	1	1	1	2	2	2	2	2	2
1	1	1	1	2	2	2	2	2	2
4	4	1	1	2	2	2	2	4	4
1	1	1	1	2	2	2	2	4	4
1	1	4	4	2	2	1	1	2	2
1	1	1	1	2	2	2	2	2	2
1	1	1	1	2	2	2	2	4	4
1	1	1	1	2	2	2	2	4	4
4	4	1	1	2	2	2	2	4	4
1	1	1	1	2	2	2	2	4	4
1	1	4	4	3	3	2	2	2	2
4	4	1	1	2	2	2	2	4	4
4	4	4	4	3	3	2	2	4	4
4	4	1	1	2	2	2	2	4	4
1	1	1	1	2	2	2	2	4	4
1	1	1	1	2	2	1	1	4	4
4	4	1	1	2	2	2	2	4	4
1	1	1	1	2	2	2	2	2	2
1	1	1	1	2	2	2	2	4	4
1	1	4	4	2	2	1	1	4	4
1	1	4	4	2	2	1	1	4	4
1	1	4	4	3	3	2	2	2	2
1	1	1	1	2	2	2	2	4	4
1	1	1	1	3	3	2	2	2	2
4	4	1	1	2	2	2	2	2	2
1	1	4	4	3	3	2	2	2	2
4	4	1	1	2	2	2	2	4	4
1	1	1	1	2	2	2	2	4	4
1	1	1	1	2	2	2	2	4	4
4	4	1	1	2	2	2	2	4	4
4	4	1	1	2	2	2	2	4	4
1	1	1	1	2	2	2	2	4	4
1	1	1	1	2	2	2	2	4	4

For Review Only

1	1	1	1	2	2	2	2	4	4
4	4	1	1	2	2	2	2	4	4
4	4	1	1	2	2	2	2	4	4
4	4	1	1	2	2	2	2	4	4
4	4	1	1	2	2	2	2	4	4
1	1	1	1	2	2	2	2	4	4
1	1	1	1	2	2	2	2	4	4
1	1	4	4	2	2	1	1	2	2
4	4	1	1	2	2	2	2	2	2
4	4	1	1	2	2	2	2	4	4
4	4	1	1	2	2	2	2	4	4
1	1	1	1	2	2	2	2	4	4
1	1	1	1	2	2	2	2	4	4
1	1	1	1	2	2	2	2	4	4
4	4	1	1	2	2	2	2	4	4
1	1	1	1	2	2	2	2	2	2
4	4	1	1	2	2	2	2	4	4
4	4	1	1	2	2	2	2	4	4
4	4	1	1	2	2	2	2	4	4
4	4	1	1	2	2	2	2	4	4
1	1	1	1	2	2	2	2	4	4
1	1	1	1	2	2	2	2	4	4
1	1	1	1	2	2	2	2	4	4
1	1	1	1	2	2	2	2	2	2
1	1	4	4	3	3	2	2	2	2
1	1	1	1	2	2	2	2	4	4
4	4	4	4	3	3	1	1	4	4
1	1	4	4	3	3	1	1	4	4
1	1	1	1	2	2	2	2	4	4
4	4	1	1	2	2	2	2	2	2
1	1	1	1	2	2	2	2	4	4
1	1	1	1	2	2	2	2	2	2
1	1	4	4	3	3	1	1	2	2
1	1	1	1	2	2	2	2	4	4
1	1	1	1	2	2	2	2	4	4
1	1	4	4	3	3	2	2	2	2
1	1	4	4	2	2	1	1	2	2
1	1	1	1	2	2	2	2	4	4
1	1	1	1	2	2	2	2	4	4
1	1	4	4	3	3	2	2	2	2
4	4	1	1	2	2	2	2	4	4
4	4	1	1	2	2	2	2	4	4
1	1	4	4	2	2	1	1	4	4
4	4	1	1	2	2	2	2	4	4
4	4	4	4	3	3	2	2	4	4
1	1	1	1	2	2	2	2	4	4
1	1	1	1	2	2	2	2	4	4
1	1	1	1	2	2	2	2	2	2
4	4	1	1	2	2	2	2	4	4
1	1	1	1	2	2	2	2	4	4
4	4	1	1	2	2	2	2	4	4
1	1	4	4	2	2	1	1	4	4
4	4	1	1	3	3	1	1	4	4
1	1	1	1	2	2	2	2	4	4
1	1	1	1	2	2	2	2	4	4

For Review Only

1	1	1	1	2	2	2	2	4	4
1	1	1	1	2	2	2	2	4	4
1	1	1	1	2	2	2	2	4	4
1	1	1	1	2	2	2	2	4	4
1	1	1	1	2	2	2	2	4	4
1	1	1	1	2	2	2	2	4	4
4	4	1	1	2	2	2	2	4	4
1	1	1	1	2	2	2	2	4	4
4	4	1	1	2	2	1	1	4	4
1	1	1	1	2	2	2	2	4	4
1	1	1	1	2	2	2	2	4	4
4	4	1	1	2	2	2	2	4	4
1	1	4	4	2	2	1	1	4	4
1	1	1	1	2	2	2	2	4	4
1	1	1	1	2	2	2	2	4	4
4	4	1	1	2	2	1	1	4	4
1	1	4	4	3	3	2	2	2	2
4	4	1	1	2	2	2	2	4	4
1	1	1	1	2	2	2	2	2	2
1	1	1	1	2	2	2	2	4	4
4	4	1	1	2	2	2	2	4	4
1	1	1	1	2	2	2	2	4	4
4	4	1	1	2	2	2	2	4	4
1	1	4	4	2	2	1	1	2	2
1	1	1	1	2	2	2	2	4	4
1	1	1	1	2	2	2	2	4	4
4	4	1	1	2	2	2	2	4	4
1	1	1	1	2	2	2	2	4	4
4	4	1	1	2	2	2	2	4	4
1	1	1	1	2	2	2	2	4	4
1	1	1	1	2	2	1	1	4	4
1	1	1	1	3	3	2	2	4	4
4	4	1	1	2	2	2	2	4	4
1	1	1	1	2	2	2	2	4	4
1	1	1	1	2	2	2	2	4	4
4	4	1	1	2	2	2	2	2	2
1	1	1	1	2	2	2	2	4	4
1	1	1	1	2	2	2	2	2	2
1	1	1	1	2	2	2	2	4	4
1	1	4	4	2	2	1	1	4	4
4	4	1	1	2	2	2	2	4	4
1	1	1	1	2	2	2	2	2	2
1	1	1	1	2	2	2	2	4	4
1	1	4	4	2	2	2	2	4	4
1	1	4	4	3	3	2	2	2	2
1	1	1	1	2	2	2	2	2	2
1	1	1	1	2	2	2	2	4	4
1	1	1	1	2	2	2	2	4	4
1	1	1	1	2	2	2	2	4	4
1	1	1	1	2	2	2	2	4	4
1	1	1	1	2	2	2	2	4	4
1	1	1	1	2	2	2	2	4	4
4	4	1	1	2	2	2	2	4	4
1	1	1	1	2	2	2	2	4	4
1	1	1	1	2	2	1	1	4	4

For Review Only

1	1	1	1	2	2	2	2	4	4
1	1	1	1	2	2	2	2	4	4
4	4	4	4	3	3	2	2	4	4
1	1	1	1	2	2	1	1	4	4
1	1	1	1	2	2	2	2	4	4
4	4	1	1	2	2	2	2	4	4
1	1	4	4	3	3	1	1	4	4
1	1	1	1	2	2	2	2	4	4
1	1	1	1	2	2	1	1	4	4
4	4	1	1	2	2	2	2	2	2
1	1	1	1	2	2	2	2	4	4
1	1	4	4	3	3	1	1	4	4
4	4	1	1	2	2	2	2	4	4
4	4	1	1	2	2	2	2	2	2
1	1	1	1	2	2	2	2	4	4
1	1	1	1	2	2	2	2	4	4
4	4	1	1	2	2	2	2	4	4
1	1	1	1	2	2	2	2	4	4
1	1	1	1	2	2	2	2	4	4
4	4	1	1	2	2	2	2	4	4
4	4	1	1	2	2	2	2	4	4
1	1	4	4	3	3	1	1	2	2
1	1	1	1	2	2	2	2	4	4
1	1	1	1	2	2	2	2	4	4
1	1	1	1	2	2	2	2	2	2
1	1	1	1	2	2	2	2	4	4
4	4	1	1	2	2	2	2	4	4
1	1	1	1	2	2	2	2	2	2
1	1	1	1	2	2	2	2	4	4
4	4	1	1	2	2	2	2	4	4
1	1	1	1	2	2	2	2	4	4
4	4	1	1	2	2	2	2	2	2
4	4	1	1	2	2	2	2	4	4
1	1	1	1	2	2	2	2	4	4
4	4	1	1	2	2	2	2	4	4
1	1	1	1	2	2	2	2	4	4
1	1	1	1	2	2	2	2	4	4
4	4	1	1	2	2	2	2	2	2
1	1	1	1	2	2	2	2	4	4
1	1	1	1	2	2	2	2	4	4
4	4	1	1	2	2	2	2	4	4
1	1	1	1	2	2	2	2	4	4
1	1	4	4	2	2	1	1	4	4
4	4	1	1	2	2	2	2	4	4
1	1	1	1	2	2	2	2	4	4
4	4	1	1	2	2	2	2	4	4
1	1	1	1	2	2	2	2	4	4
1	1	4	4	3	3	1	1	4	4
1	1	4	4	2	2	1	1	4	4
1	1	1	1	2	2	2	2	2	2
4	4	1	1	2	2	2	2	4	4
1	1	1	1	2	2	2	2	2	2
1	1	1	1	2	2	2	2	4	4
4	4	1	1	2	2	2	2	4	4
1	1	4	4	2	2	1	1	4	4

For Review Only

1	1	1	1	2	2	2	2	4	4
1	1	1	1	2	2	2	2	4	4
4	4	1	1	2	2	2	2	4	4
1	1	1	1	2	2	2	2	4	4
1	1	1	1	2	2	2	2	4	4
4	4	1	1	2	2	2	2	4	4
1	1	1	1	2	2	2	2	4	4
1	1	4	4	3	3	2	2	2	2
4	4	4	4	2	2	1	1	4	4
1	1	4	4	2	2	1	1	4	4
4	4	1	1	2	2	2	2	4	4
1	1	1	1	2	2	2	2	2	2
1	1	1	1	2	2	2	2	4	4
1	1	1	1	2	2	2	2	4	4
4	4	4	4	3	3	2	2	4	4
4	4	1	1	2	2	2	2	4	4
1	1	1	1	2	2	2	2	4	4
1	1	1	1	2	2	2	2	4	4
1	1	4	4	3	3	1	1	4	4
4	4	1	1	2	2	2	2	4	4
1	1	4	4	3	3	1	1	4	4
1	1	1	1	2	2	2	2	4	4
4	4	1	1	2	2	2	2	4	4
1	1	4	4	2	2	1	1	4	4
1	1	4	4	2	2	1	1	2	2
1	1	1	1	2	2	2	2	4	4
1	1	4	4	3	3	1	1	4	4
4	4	1	1	2	2	2	2	4	4
1	1	1	1	2	2	2	2	4	4
4	4	1	1	2	2	2	2	4	4
1	1	4	4	2	2	1	1	4	4
1	1	1	1	2	2	1	1	4	4
4	4	1	1	2	2	2	2	2	2
1	1	1	1	2	2	2	2	4	4
1	1	1	1	2	2	2	2	4	4
1	1	4	4	2	2	1	1	2	2
4	4	1	1	2	2	2	2	4	4
1	1	1	1	2	2	2	2	4	4
4	4	1	1	2	2	2	2	4	4
1	1	4	4	2	2	1	1	4	4
4	4	1	1	2	2	2	2	4	4
1	1	1	1	2	2	2	2	4	4
1	1	4	4	2	2	1	1	4	4
4	4	1	1	2	2	2	2	4	4
1	1	1	1	2	2	2	2	4	4
1	1	1	1	2	2	2	2	4	4
1	1	1	1	2	2	2	2	4	4
1	1	1	1	2	2	2	2	4	4
4	4	1	1	2	2	2	2	4	4
1	1	4	4	3	3	1	1	2	2
1	1	1	1	2	2	2	2	4	4
1	1	4	4	2	2	1	1	4	4
4	4	1	1	2	2	2	2	4	4
4	4	1	1	2	2	2	2	4	4
1	1	4	4	2	2	1	1	4	4

For Review Only

4	4	1	1	2	2	2	2	4	4
1	1	1	1	2	2	2	2	4	4

For Review Only

SNP2804	SNP2804	SNP2805	SNP2805	SNP2806	SNP2806	SNP2807	SNP2807	SNP2808	SNP2808
1	1	2	2	2	2	3	3	3	3
1	1	2	2	1	1	3	3	3	3
1	1	2	2	2	2	3	3	3	3
1	1	2	2	2	2	3	3	3	3
1	1	2	2	2	2	3	3	3	3
1	1	2	2	2	2	3	3	3	3
1	1	2	2	2	2	2	2	3	3
1	1	2	2	2	2	2	2	3	3
1	1	2	2	2	2	3	3	3	3
1	1	2	2	1	1	2	2	2	2
1	1	2	2	2	2	3	3	3	3
1	1	2	2	2	2	3	3	3	3
1	1	2	2	2	2	3	3	3	3
1	1	2	2	2	2	3	3	3	3
1	1	2	2	2	2	3	3	3	3
1	1	2	2	2	2	3	3	3	3
1	1	2	2	2	2	3	3	3	3
4	4	2	2	2	2	3	3	2	2
1	1	2	2	2	2	3	3	3	3
1	1	2	2	1	1	3	3	2	2
1	1	2	2	2	2	3	3	2	2
1	1	2	2	2	2	3	3	3	3
1	1	2	2	1	1	3	3	3	3
1	1	2	2	2	2	3	3	3	3
1	1	2	2	2	2	3	3	2	2
1	1	2	2	2	2	3	3	3	3
1	1	2	2	2	2	3	3	3	3
1	1	2	2	2	2	3	3	3	3
1	1	2	2	2	2	3	3	3	3
1	1	2	2	2	2	2	2	2	2
1	1	2	2	2	2	3	3	3	3
1	1	2	2	2	2	2	2	2	2
1	1	2	2	2	2	3	3	3	3
1	1	2	2	2	2	3	3	3	3
1	1	2	2	1	1	3	3	2	2
1	1	2	2	2	2	3	3	3	3
1	1	2	2	2	2	3	3	3	3
1	1	2	2	2	2	3	3	3	3
1	1	2	2	1	1	3	3	2	2
1	1	2	2	1	1	3	3	3	3
1	1	2	2	1	1	2	2	2	2
1	1	2	2	2	2	3	3	2	2
1	1	2	2	1	1	2	2	2	2
1	1	2	2	1	1	3	3	2	2
1	1	2	2	1	1	2	2	2	2
1	1	2	2	2	2	3	3	3	3
1	1	2	2	2	2	3	3	3	3
1	1	2	2	2	2	3	3	3	3
1	1	2	2	1	1	3	3	3	3
1	1	2	2	2	2	3	3	3	3
1	1	2	2	2	2	3	3	3	3
1	1	2	2	2	2	3	3	3	3

For Review Only

1	1	2	2	2	2	3	3	3	3
1	1	2	2	2	2	3	3	3	3
1	1	2	2	2	2	3	3	3	3
1	1	2	2	2	2	3	3	3	3
1	1	2	2	2	2	3	3	3	3
4	4	2	2	2	2	3	3	3	3
1	1	2	2	2	2	3	3	3	3
1	1	2	2	1	1	2	2	2	2
1	1	2	2	1	1	3	3	2	2
1	1	2	2	2	2	3	3	3	3
4	4	2	2	2	2	3	3	3	3
4	4	2	2	2	2	3	3	3	3
1	1	2	2	2	2	3	3	3	3
1	1	2	2	2	2	3	3	3	3
1	1	2	2	2	2	3	3	3	3
1	1	2	2	1	1	3	3	3	3
1	1	2	2	2	2	3	3	3	3
1	1	3	3	2	2	3	3	3	3
1	1	2	2	2	2	3	3	3	3
1	1	2	2	2	2	3	3	3	3
1	1	2	2	2	2	3	3	3	3
1	1	2	2	2	2	3	3	2	2
1	1	2	2	2	2	3	3	3	3
1	1	2	2	2	2	3	3	2	2
1	1	2	2	1	1	2	2	2	2
1	1	2	2	2	2	3	3	3	3
1	1	2	2	1	1	2	2	3	3
1	1	2	2	1	1	2	2	3	3
1	1	2	2	2	2	3	3	3	3
1	1	2	2	2	2	3	3	3	3
1	1	2	2	2	2	3	3	3	3
1	1	2	2	1	1	3	3	2	2
1	1	2	2	1	1	2	2	2	2
1	1	2	2	2	2	3	3	3	3
1	1	2	2	1	1	3	3	3	3
1	1	2	2	1	1	2	2	2	2
1	1	2	2	2	2	2	2	2	2
1	1	2	2	1	1	3	3	3	3
1	1	2	2	2	2	3	3	2	2
1	1	2	2	2	2	2	2	2	2
1	1	2	2	2	2	3	3	3	3
1	1	2	2	2	2	3	3	3	3
1	1	3	3	1	1	2	2	3	3
4	4	2	2	2	2	3	3	3	3
1	1	2	2	2	2	2	2	3	3
1	1	2	2	2	2	3	3	3	3
1	1	2	2	2	2	3	3	3	3
1	1	2	2	2	2	3	3	3	3
1	1	2	2	2	2	3	3	3	3
1	1	3	3	2	2	3	3	3	3
1	1	2	2	2	2	3	3	3	3
1	1	2	2	1	1	3	3	2	2
1	1	2	2	1	1	2	2	2	2
1	1	2	2	2	2	3	3	3	3
1	1	3	3	2	2	3	3	3	3

For Review Only

1	1	2	2	2	2	3	3	3	3
1	1	2	2	2	2	2	2	3	3
1	1	2	2	2	2	3	3	3	3
1	1	2	2	2	2	3	3	3	3
1	1	2	2	2	2	3	3	3	3
1	1	2	2	2	2	3	3	3	3
1	1	2	2	2	2	3	3	3	3
1	1	2	2	2	2	3	3	3	3
1	1	2	2	2	2	3	3	3	3
1	1	2	2	2	2	3	3	3	3
1	1	2	2	1	1	3	3	3	3
1	1	2	2	2	2	3	3	3	3
1	1	3	3	2	2	2	2	3	3
1	1	2	2	2	2	3	3	3	3
1	1	2	2	2	2	3	3	3	3
1	1	2	2	2	2	3	3	3	3
1	1	3	3	2	2	3	3	3	3
1	1	2	2	2	2	3	3	3	3
1	1	2	2	2	2	3	3	2	2
1	1	2	2	1	1	3	3	3	3
1	1	2	2	2	2	3	3	3	3
1	1	2	2	2	2	3	3	3	3
1	1	2	2	2	2	3	3	3	3
1	1	2	2	1	1	3	3	2	2
1	1	2	2	1	1	3	3	2	2
1	1	2	2	1	1	3	3	2	2
1	1	2	2	2	2	3	3	3	3
1	1	2	2	2	2	3	3	3	3
1	1	2	2	2	2	3	3	3	3
1	1	2	2	1	1	3	3	3	3
1	1	2	2	2	2	3	3	3	3
1	1	2	2	2	2	3	3	3	3
1	1	2	2	2	2	3	3	3	3
1	1	2	2	2	2	3	3	3	3
1	1	2	2	2	2	3	3	3	3
1	1	2	2	1	1	3	3	3	3
1	1	2	2	2	2	3	3	3	3
1	1	2	2	2	2	3	3	3	3
1	1	2	2	2	2	3	3	3	3
1	1	2	2	1	1	3	3	3	3
1	1	2	2	2	2	3	3	2	2
1	1	2	2	2	2	3	3	2	2
1	1	3	3	2	2	3	3	3	3
1	1	2	2	2	2	3	3	3	3
1	1	2	2	2	2	3	3	2	2
1	1	2	2	2	2	3	3	3	3
1	1	2	2	2	2	3	3	3	3
1	1	2	2	2	2	3	3	3	3
1	1	2	2	2	2	3	3	3	3
1	1	2	2	2	2	3	3	3	3
1	1	2	2	2	2	3	3	3	3
1	1	2	2	2	2	3	3	3	3
1	1	2	2	2	2	2	2	3	3
4	4	2	2	2	2	3	3	3	3
1	1	2	2	2	2	3	3	3	3

For Review Only

1	1	2	2	2	2	3	3	3	3
1	1	3	3	1	1	2	2	2	2
1	1	2	2	1	1	3	3	3	3
1	1	2	2	2	2	3	3	2	2
1	1	2	2	2	2	3	3	3	3
4	4	2	2	2	2	3	3	2	2
1	1	3	3	1	1	2	2	3	3
1	1	2	2	2	2	3	3	3	3
1	1	2	2	1	1	3	3	3	3
1	1	2	2	2	2	3	3	3	3
1	1	2	2	2	2	3	3	3	3
1	1	2	2	1	1	3	3	3	3
1	1	2	2	2	2	3	3	3	3
1	1	2	2	2	2	3	3	3	3
1	1	2	2	2	2	3	3	3	3
1	1	2	2	1	1	3	3	3	3
1	1	2	2	2	2	3	3	3	3
1	1	2	2	2	2	3	3	3	3
1	1	2	2	2	2	3	3	3	3
1	1	2	2	2	2	3	3	3	3
1	1	2	2	2	2	3	3	3	3
1	1	2	2	2	2	2	2	2	2
1	1	2	2	1	1	3	3	2	2
4	4	2	2	2	2	3	3	3	3
1	1	2	2	2	2	3	3	2	2
1	1	2	2	2	2	3	3	3	3
1	1	2	2	2	2	3	3	3	3
1	1	3	3	2	2	3	3	3	3
1	1	2	2	1	1	3	3	3	3
1	1	2	2	2	2	3	3	3	3
1	1	2	2	2	2	3	3	3	3
1	1	3	3	2	2	3	3	3	3
1	1	2	2	2	2	3	3	3	3
1	1	2	2	2	2	3	3	3	3
1	1	2	2	2	2	3	3	3	3
4	4	2	2	2	2	3	3	3	3
1	1	2	2	1	1	3	3	3	3
1	1	2	2	2	2	3	3	3	3
1	1	2	2	2	2	3	3	3	3
1	1	2	2	2	2	3	3	3	3
1	1	2	2	2	2	3	3	3	3
1	1	2	2	2	2	3	3	3	3
1	1	2	2	1	1	3	3	3	3
1	1	2	2	2	2	3	3	3	3
1	1	2	2	2	2	3	3	3	3
4	4	2	2	1	1	3	3	3	3
1	1	2	2	1	1	3	3	3	3
1	1	2	2	1	1	2	2	3	3
1	1	2	2	2	2	2	2	3	3
1	1	2	2	2	2	3	3	2	2
1	1	2	2	1	1	3	3	3	3
1	1	2	2	1	1	3	3	3	3
1	1	2	2	1	1	3	3	2	2
4	4	2	2	2	2	3	3	3	3
1	1	2	2	1	1	3	3	2	2

For Review Only

1	1	2	2	2	2	3	3	3	3
1	1	2	2	2	2	3	3	3	3
1	1	2	2	2	2	3	3	3	3
1	1	2	2	1	1	3	3	3	3
1	1	2	2	1	1	3	3	3	3
1	1	2	2	2	2	3	3	3	3
1	1	2	2	1	1	3	3	2	2
1	1	2	2	1	1	2	2	2	2
1	1	2	2	2	2	2	2	3	3
1	1	2	2	1	1	3	3	2	2
1	1	2	2	2	2	3	3	3	3
1	1	2	2	1	1	3	3	2	2
1	1	2	2	2	2	3	3	2	2
1	1	2	2	2	2	3	3	3	3
1	1	2	2	2	2	2	2	3	3
1	1	2	2	2	2	3	3	3	3
1	1	2	2	1	1	2	2	2	2
1	1	2	2	2	2	3	3	3	3
1	1	2	2	2	2	2	2	3	3
1	1	2	2	2	2	3	3	3	3
1	1	2	2	2	2	3	3	3	3
1	1	2	2	2	2	3	3	3	3
1	1	2	2	2	2	3	3	3	3
1	1	3	3	1	1	3	3	2	2
1	1	2	2	1	1	2	2	2	2
1	1	2	2	2	2	3	3	3	3
1	1	2	2	1	1	2	2	2	2
1	1	2	2	2	2	3	3	3	3
1	1	2	2	2	2	3	3	3	3
1	1	2	2	2	2	3	3	3	3
1	1	2	2	1	1	2	2	2	2
1	1	2	2	2	2	3	3	3	3
1	1	3	3	2	2	3	3	3	3
1	1	2	2	2	2	3	3	3	3
1	1	2	2	1	1	3	3	2	2
1	1	2	2	1	1	2	2	2	2
1	1	2	2	2	2	3	3	3	3
4	4	2	2	2	2	3	3	3	3
1	1	2	2	2	2	3	3	3	3
1	1	2	2	1	1	3	3	3	3
1	1	2	2	2	2	3	3	3	3
1	1	2	2	2	2	3	3	3	3
1	1	3	3	1	1	3	3	2	2
1	1	2	2	2	2	3	3	3	3
1	1	2	2	2	2	3	3	3	3
1	1	2	2	2	2	3	3	2	2
1	1	2	2	2	2	3	3	2	2
1	1	2	2	1	1	3	3	3	3
1	1	2	2	2	2	3	3	3	3
1	1	2	2	1	1	2	2	2	2
1	1	2	2	2	2	3	3	3	3
1	1	2	2	1	1	3	3	2	2
1	1	2	2	2	2	3	3	3	3
1	1	2	2	2	2	3	3	3	3
1	1	2	2	1	1	3	3	3	3

For Review Only

1	1	2	2	2	2	3	3	3	3
1	1	2	2	2	2	3	3	3	3

For Review Only

SNP2809	SNP2809	SNP281	SNP281	SNP2810	SNP2810	SNP2811	SNP2811	SNP2812	SNP2812
4	4	4	4	1	1	4	4	2	2
4	4	1	1	4	4	4	4	3	3
4	4	4	4	1	1	4	4	2	2
4	4	4	4	1	1	4	4	2	2
4	4	1	1	1	1	4	4	2	2
2	2	1	1	1	1	4	4	2	2
4	4	1	1	1	1	4	4	2	2
2	2	1	1	4	4	4	4	3	3
4	4	1	1	1	1	4	4	2	2
2	2	1	1	4	4	4	4	3	3
2	2	1	1	1	1	4	4	2	2
4	4	4	4	1	1	4	4	2	2
4	4	1	1	4	4	4	4	3	3
2	2	1	1	1	1	4	4	2	2
4	4	1	1	1	1	4	4	3	3
2	2	4	4	1	1	4	4	2	2
4	4	1	1	4	4	4	4	3	3
2	2	1	1	4	4	4	4	3	3
4	4	4	4	1	1	4	4	2	2
4	4	1	1	4	4	4	4	3	3
2	2	1	1	1	1	4	4	2	2
4	4	1	1	1	1	4	4	2	2
2	2	1	1	4	4	4	4	3	3
2	2	1	1	1	1	4	4	2	2
4	4	1	1	1	1	4	4	2	2
4	4	1	1	1	1	4	4	2	2
2	2	1	1	1	1	4	4	2	2
4	4	4	4	1	1	4	4	3	3
2	2	1	1	1	1	4	4	2	2
2	2	1	1	4	4	4	4	3	3
4	4	4	4	1	1	4	4	2	2
4	4	4	4	1	1	4	4	2	2
4	4	4	4	1	1	4	4	2	2
4	4	1	1	1	1	4	4	3	3
4	4	1	1	1	1	4	4	3	3
4	4	4	4	1	1	4	4	2	2
4	4	1	1	1	1	4	4	2	2
2	2	1	1	1	1	4	4	2	2
2	2	1	1	4	4	4	4	3	3
4	4	1	1	4	4	4	4	3	3
2	2	1	1	4	4	4	4	3	3
4	4	1	1	1	1	4	4	2	2
2	2	1	1	4	4	4	4	3	3
2	2	1	1	4	4	4	4	3	3
2	2	1	1	1	1	4	4	2	2
4	4	4	4	1	1	4	4	2	2
4	4	1	1	1	1	4	4	2	2
4	4	4	4	1	1	4	4	2	2
2	2	1	1	4	4	4	4	3	3
4	4	4	4	1	1	4	4	2	2
4	4	1	1	1	1	4	4	2	2
2	2	1	1	1	1	4	4	2	2

For Review Only

4	4	1	1	1	1	4	4	2	2
4	4	4	4	1	1	4	4	2	2
4	4	4	4	1	1	4	4	2	2
2	2	1	1	4	4	4	4	3	3
4	4	4	4	1	1	4	4	2	2
4	4	1	1	1	1	4	4	2	2
4	4	1	1	1	1	4	4	2	2
2	2	1	1	4	4	4	4	3	3
2	2	1	1	4	4	4	4	3	3
4	4	4	4	1	1	4	4	3	3
4	4	4	4	1	1	4	4	2	2
4	4	1	1	1	1	4	4	2	2
4	4	1	1	1	1	4	4	2	2
2	2	1	1	1	1	4	4	2	2
4	4	1	1	4	4	4	4	3	3
4	4	1	1	4	4	4	4	3	3
4	4	4	4	1	1	4	4	2	2
4	4	4	4	1	1	4	4	2	2
2	2	4	4	1	1	4	4	2	2
4	4	1	1	1	1	4	4	2	2
4	4	1	1	1	1	4	4	3	3
2	2	1	1	1	1	1	1	3	3
2	2	1	1	1	1	4	4	2	2
4	4	1	1	1	1	4	4	2	2
2	2	1	1	1	1	4	4	2	2
4	4	1	1	1	1	4	4	2	2
4	4	4	4	4	4	4	4	3	3
2	2	1	1	4	4	4	4	3	3
4	4	1	1	1	1	4	4	2	2
2	2	4	4	1	1	4	4	3	3
2	2	1	1	1	1	4	4	2	2
4	4	1	1	4	4	4	4	3	3
2	2	1	1	4	4	4	4	3	3
4	4	1	1	1	1	4	4	2	2
4	4	1	1	4	4	4	4	3	3
2	2	1	1	1	1	4	4	2	2
2	2	1	1	1	1	4	4	2	2
2	2	1	1	4	4	4	4	3	3
2	2	1	1	1	1	4	4	2	2
4	4	1	1	1	1	4	4	2	2
4	4	1	1	1	1	4	4	2	2
4	4	4	4	1	1	4	4	2	2
4	4	1	1	4	4	4	4	3	3
4	4	4	4	1	1	4	4	2	2
4	4	4	4	1	1	4	4	2	2
2	2	1	1	1	1	4	4	2	2
4	4	1	1	1	1	4	4	3	3
4	4	1	1	1	1	4	4	2	2
4	4	4	4	1	1	4	4	2	2
2	2	1	1	1	1	4	4	2	2
2	2	4	4	1	1	4	4	2	2
2	2	1	1	4	4	4	4	3	3
4	4	1	1	4	4	4	4	3	3
2	2	4	4	1	1	4	4	3	3
4	4	1	1	1	1	4	4	2	2

For Review Only

4	4	1	1	1	1	4	4	2	2
2	2	1	1	1	1	4	4	2	2
4	4	1	1	1	1	4	4	2	2
2	2	1	1	1	1	4	4	2	2
4	4	1	1	1	1	4	4	3	3
4	4	1	1	1	1	4	4	2	2
2	2	4	4	1	1	4	4	2	2
4	4	1	1	1	1	4	4	2	2
4	4	4	4	1	1	4	4	2	2
2	2	1	1	1	1	4	4	3	3
4	4	1	1	4	4	4	4	3	3
4	4	1	1	1	1	4	4	3	3
4	4	1	1	1	1	4	4	3	3
4	4	1	1	1	1	4	4	2	2
4	4	1	1	1	1	1	1	2	2
4	4	1	1	1	1	4	4	3	3
4	4	1	1	1	1	4	4	2	2
4	4	4	4	1	1	4	4	3	3
2	2	1	1	1	1	4	4	2	2
2	2	1	1	4	4	4	4	3	3
4	4	1	1	1	1	4	4	3	3
2	2	4	4	1	1	4	4	2	2
4	4	4	4	1	1	4	4	2	2
2	2	1	1	4	4	4	4	3	3
2	2	1	1	1	1	1	1	3	3
4	4	1	1	4	4	1	1	3	3
4	4	4	4	1	1	4	4	2	2
2	2	1	1	1	1	4	4	2	2
2	2	4	4	1	1	4	4	2	2
4	4	1	1	4	4	4	4	3	3
2	2	1	1	4	4	4	4	3	3
4	4	1	1	1	1	4	4	3	3
4	4	1	1	1	1	4	4	2	2
4	4	1	1	1	1	4	4	3	3
4	4	1	1	1	1	4	4	3	3
4	4	1	1	4	4	4	4	3	3
2	2	1	1	1	1	4	4	2	2
4	4	1	1	1	1	4	4	2	2
4	4	1	1	1	1	4	4	3	3
2	2	1	1	4	4	4	4	3	3
2	2	4	4	1	1	4	4	3	3
4	4	1	1	1	1	4	4	3	3
4	4	1	1	1	1	4	4	2	2
2	2	1	1	1	1	4	4	3	3
4	4	1	1	1	1	4	4	2	2
4	4	1	1	1	1	1	1	2	2
4	4	1	1	1	1	4	4	2	2
2	2	1	1	1	1	4	4	2	2
4	4	1	1	1	1	4	4	3	3
4	4	1	1	1	1	4	4	2	2
4	4	1	1	1	1	4	4	2	2
2	2	1	1	1	1	4	4	2	2
4	4	4	4	1	1	4	4	2	2
2	2	1	1	1	1	4	4	2	2
4	4	1	1	1	1	4	4	3	3

For Review Only

4	4	1	1	1	1	4	4	2	2
2	2	1	1	4	4	4	4	3	3
2	2	4	4	1	1	4	4	2	2
2	2	1	1	1	1	4	4	2	2
4	4	1	1	1	1	4	4	2	2
2	2	1	1	1	1	4	4	2	2
4	4	1	1	4	4	4	4	3	3
4	4	1	1	1	1	4	4	3	3
2	2	1	1	4	4	4	4	3	3
2	2	4	4	1	1	4	4	2	2
4	4	4	4	1	1	4	4	3	3
4	4	1	1	1	1	4	4	3	3
4	4	4	4	1	1	4	4	2	2
4	4	1	1	1	1	4	4	2	2
2	2	1	1	1	1	4	4	3	3
2	2	1	1	4	4	4	4	3	3
4	4	1	1	1	1	4	4	3	3
4	4	1	1	1	1	4	4	2	2
4	4	4	4	1	1	4	4	2	2
2	2	4	4	1	1	4	4	2	2
4	4	4	4	1	1	4	4	3	3
4	4	1	1	1	1	4	4	3	3
2	2	1	1	4	4	4	4	3	3
4	4	1	1	1	1	4	4	2	2
2	2	1	1	1	1	4	4	3	3
4	4	1	1	1	1	4	4	2	2
2	2	4	4	1	1	4	4	2	2
4	4	1	1	1	1	4	4	2	2
4	4	1	1	4	4	4	4	3	3
4	4	1	1	1	1	4	4	2	2
4	4	1	1	1	1	4	4	2	2
4	4	4	4	1	1	4	4	2	2
2	2	4	4	1	1	1	1	2	2
2	2	1	1	1	1	4	4	3	3
4	4	4	4	1	1	1	1	2	2
4	4	1	1	1	1	4	4	2	2
4	4	1	1	4	4	4	4	3	3
4	4	4	4	1	1	4	4	2	2
2	2	1	1	1	1	4	4	2	2
2	2	1	1	1	1	4	4	2	2
4	4	4	4	1	1	4	4	2	2
4	4	1	1	1	1	4	4	2	2
2	2	1	1	4	4	4	4	3	3
4	4	4	4	1	1	4	4	2	2
2	2	1	1	1	1	4	4	2	2
2	2	4	4	4	4	4	4	3	3
2	2	1	1	4	4	4	4	3	3
2	2	1	1	4	4	4	4	3	3
4	4	1	1	1	1	4	4	2	2
2	2	1	1	1	1	4	4	2	2
4	4	4	4	4	4	4	4	3	3
4	4	1	1	4	4	4	4	3	3
2	2	1	1	4	4	1	1	3	3
4	4	4	4	1	1	4	4	2	2
2	2	1	1	4	4	4	4	3	3

For Review Only

4	4	1	1	1	1	4	4	2	2
2	2	1	1	1	1	4	4	3	3
4	4	4	4	1	1	4	4	2	2
2	2	1	1	4	4	4	4	3	3
2	2	1	1	4	4	4	4	3	3
4	4	4	4	1	1	4	4	2	2
4	4	1	1	4	4	4	4	3	3
2	2	1	1	4	4	4	4	3	3
2	2	4	4	1	1	4	4	2	2
2	2	1	1	4	4	4	4	3	3
4	4	1	1	1	1	4	4	2	2
2	2	1	1	4	4	4	4	3	3
2	2	1	1	1	1	4	4	2	2
4	4	1	1	1	1	4	4	2	2
4	4	4	4	1	1	4	4	2	2
2	2	4	4	1	1	4	4	2	2
2	2	1	1	4	4	4	4	3	3
4	4	1	1	1	1	4	4	3	3
2	2	1	1	1	1	4	4	2	2
4	4	1	1	1	1	4	4	2	2
4	4	1	1	1	1	4	4	2	2
4	4	1	1	1	1	4	4	2	2
2	2	4	4	1	1	4	4	2	2
4	4	1	1	4	4	4	4	3	3
2	2	1	1	4	4	4	4	3	3
4	4	1	1	1	1	4	4	2	2
2	2	1	1	4	4	4	4	3	3
2	2	4	4	1	1	4	4	2	2
2	2	1	1	1	1	4	4	3	3
4	4	4	4	1	1	4	4	2	2
2	2	1	1	4	4	4	4	3	3
4	4	1	1	1	1	4	4	2	2
4	4	1	1	1	1	4	4	2	2
2	2	1	1	1	1	4	4	3	3
2	2	1	1	4	4	1	1	3	3
2	2	1	1	4	4	4	4	3	3
4	4	1	1	1	1	4	4	2	2
4	4	1	1	1	1	4	4	2	2
4	4	4	4	1	1	4	4	2	2
2	2	1	1	4	4	4	4	3	3
2	2	4	4	1	1	1	1	2	2
4	4	1	1	1	1	1	1	2	2
4	4	1	1	4	4	4	4	3	3
4	4	1	1	1	1	4	4	2	2
2	2	1	1	4	4	4	4	3	3
2	2	1	1	1	1	4	4	2	2
2	2	1	1	1	1	4	4	2	2
2	2	1	1	4	4	4	4	3	3
2	2	4	4	1	1	4	4	2	2
2	2	1	1	1	1	4	4	3	3
4	4	1	1	1	1	4	4	2	2
2	2	1	1	4	4	4	4	3	3
4	4	4	4	1	1	4	4	2	2
2	2	4	4	1	1	4	4	2	2
2	2	1	1	4	4	4	4	3	3

For Review Only

2	2	4	4	1	1	4	4	2	2
2	2	1	1	1	1	4	4	2	2

For Review Only

SNP2813	SNP2813	SNP2814	SNP2814	SNP2815	SNP2815	SNP2816	SNP2816	SNP2817	SNP2817
2	2	3	3	1	1	1	1	3	3
3	3	3	3	1	1	1	1	3	3
2	2	3	3	1	1	2	2	3	3
2	2	2	2	1	1	1	1	3	3
2	2	2	2	1	1	1	1	3	3
2	2	2	2	1	1	1	1	3	3
2	2	2	2	1	1	1	1	3	3
3	3	3	3	1	1	1	1	3	3
3	3	2	2	1	1	1	1	3	3
2	2	3	3	1	1	1	1	3	3
3	3	2	2	1	1	1	1	3	3
2	2	3	3	1	1	1	1	3	3
3	3	3	3	1	1	1	1	3	3
3	3	2	2	1	1	1	1	3	3
3	3	3	3	1	1	1	1	3	3
2	2	2	2	1	1	1	1	3	3
2	2	3	3	1	1	1	1	3	3
2	2	3	3	1	1	1	1	3	3
2	2	2	2	1	1	1	1	3	3
2	2	3	3	1	1	1	1	3	3
3	3	2	2	1	1	1	1	3	3
2	2	2	2	1	1	1	1	3	3
3	3	3	3	2	2	1	1	3	3
3	3	2	2	1	1	1	1	3	3
2	2	2	2	1	1	1	1	3	3
2	2	2	2	1	1	1	1	3	3
2	2	2	2	1	1	1	1	3	3
3	3	3	3	1	1	1	1	3	3
3	3	2	2	1	1	1	1	3	3
3	3	3	3	1	1	1	1	3	3
3	3	2	2	1	1	1	1	3	3
2	2	2	2	1	1	1	1	3	3
2	2	2	2	1	1	1	1	3	3
2	2	2	2	1	1	1	1	3	3
2	2	3	3	1	1	1	1	3	3
2	2	3	3	1	1	1	1	3	3
2	2	2	2	1	1	1	1	3	3
2	2	2	2	1	1	1	1	3	3
3	3	3	3	1	1	1	1	3	3
2	2	3	3	1	1	1	1	3	3
3	3	3	3	1	1	1	1	3	3
3	3	2	2	1	1	1	1	3	3
2	2	3	3	1	1	1	1	3	3
2	2	3	3	1	1	1	1	3	3
2	2	2	2	1	1	1	1	3	3
3	3	3	3	1	1	1	1	3	3
3	3	2	2	1	1	1	1	2	2
3	3	2	2	1	1	1	1	3	3
2	2	3	3	1	1	1	1	3	3
3	3	2	2	1	1	1	1	3	3
2	2	2	2	1	1	1	1	3	3
2	2	2	2	1	1	1	1	3	3

For Review Only

2	2	2	2	1	1	1	1	3	3
2	2	2	2	1	1	1	1	3	3
3	3	2	2	1	1	1	1	3	3
3	3	3	3	1	1	1	1	3	3
2	2	2	2	1	1	1	1	3	3
3	3	2	2	1	1	1	1	3	3
2	2	2	2	1	1	1	1	3	3
3	3	3	3	1	1	1	1	3	3
3	3	3	3	1	1	1	1	3	3
3	3	3	3	1	1	1	1	3	3
3	3	2	2	1	1	1	1	2	2
3	3	2	2	1	1	1	1	2	2
2	2	2	2	1	1	1	1	3	3
3	3	2	2	1	1	1	1	3	3
2	2	3	3	1	1	1	1	3	3
2	2	3	3	1	1	1	1	3	3
2	2	2	2	1	1	2	2	3	3
3	3	2	2	1	1	1	1	3	3
3	3	2	2	1	1	1	1	3	3
2	2	2	2	1	1	1	1	3	3
3	3	2	2	1	1	1	1	2	2
3	3	3	3	1	1	2	2	3	3
3	3	2	2	1	1	1	1	3	3
2	2	2	2	1	1	1	1	3	3
2	2	2	2	1	1	1	1	3	3
2	2	2	2	1	1	1	1	3	3
2	2	3	3	1	1	1	1	3	3
3	3	3	3	1	1	1	1	3	3
2	2	2	2	1	1	1	1	3	3
2	2	2	2	1	1	1	1	3	3
3	3	2	2	1	1	1	1	3	3
3	3	3	3	1	1	1	1	3	3
3	3	3	3	1	1	1	1	3	3
2	2	2	2	1	1	1	1	3	3
2	2	3	3	1	1	1	1	3	3
2	2	2	2	1	1	1	1	3	3
3	3	2	2	1	1	1	1	2	2
3	3	3	3	1	1	1	1	3	3
2	2	2	2	1	1	1	1	3	3
2	2	2	2	1	1	1	1	3	3
2	2	2	2	1	1	1	1	3	3
2	2	2	2	1	1	1	1	3	3
2	2	3	3	1	1	1	1	2	2
3	3	2	2	1	1	1	1	2	2
3	3	2	2	1	1	1	1	3	3
2	2	2	2	1	1	1	1	3	3
2	2	2	2	1	1	1	1	3	3
2	2	2	2	1	1	1	1	3	3
2	2	2	2	1	1	1	1	3	3
2	2	2	2	1	1	1	1	3	3
2	2	2	2	1	1	1	1	3	3
3	3	3	3	1	1	1	1	3	3
3	3	3	3	1	1	1	1	3	3
3	3	3	3	1	1	1	1	3	3
2	2	2	2	1	1	1	1	3	3

For Review Only

2	2	2	2	1	1	1	1	3	3
2	2	2	2	1	1	1	1	3	3
2	2	2	2	1	1	1	1	3	3
3	3	2	2	1	1	1	1	3	3
2	2	2	2	1	1	1	1	3	3
2	2	2	2	1	1	1	1	3	3
2	2	2	2	1	1	1	1	3	3
3	3	2	2	1	1	1	1	3	3
2	2	2	2	1	1	1	1	3	3
3	3	2	2	1	1	1	1	3	3
3	3	3	3	1	1	1	1	3	3
2	2	3	3	1	1	1	1	3	3
2	2	2	2	1	1	1	1	2	2
2	2	2	2	1	1	1	1	3	3
2	2	2	2	1	1	1	1	3	3
3	3	3	3	1	1	1	1	3	3
2	2	3	3	1	1	1	1	3	3
3	3	2	2	1	1	1	1	3	3
2	2	2	2	1	1	1	1	3	3
2	2	3	3	1	1	1	1	3	3
3	3	3	3	1	1	1	1	3	3
2	2	2	2	1	1	1	1	3	3
2	2	2	2	1	1	2	2	3	3
3	3	3	3	1	1	1	1	3	3
3	3	3	3	2	2	1	1	3	3
2	2	3	3	1	1	2	2	3	3
3	3	2	2	2	2	1	1	3	3
2	2	2	2	1	1	1	1	3	3
2	2	2	2	1	1	1	1	2	2
2	2	3	3	1	1	1	1	3	3
3	3	3	3	1	1	1	1	3	3
3	3	3	3	1	1	1	1	3	3
2	2	2	2	1	1	1	1	3	3
3	3	3	3	1	1	1	1	3	3
2	2	2	2	1	1	1	1	3	3
3	3	3	3	1	1	1	1	3	3
3	3	2	2	1	1	1	1	3	3
2	2	2	2	1	1	1	1	3	3
3	3	2	2	1	1	1	1	3	3
3	3	3	3	1	1	1	1	3	3
3	3	3	3	1	1	1	1	3	3
3	3	2	2	1	1	1	1	3	3
3	3	2	2	1	1	1	1	3	3
3	3	3	3	1	1	1	1	3	3
2	2	3	3	1	1	1	1	3	3
2	2	2	2	1	1	2	2	3	3
2	2	2	2	1	1	1	1	3	3
2	2	2	2	1	1	1	1	3	3
3	3	3	3	1	1	1	1	3	3
2	2	2	2	1	1	1	1	3	3
2	2	2	2	1	1	1	1	3	3
3	3	2	2	1	1	1	1	3	3
2	2	2	2	1	1	1	1	3	3
3	3	2	2	1	1	1	1	3	3
3	3	3	3	1	1	1	1	3	3

For Review Only

2	2	2	2	1	1	1	1	3	3
2	2	3	3	1	1	1	1	2	2
2	2	2	2	1	1	1	1	3	3
3	3	2	2	2	2	1	1	3	3
2	2	2	2	1	1	1	1	3	3
2	2	2	2	1	1	1	1	3	3
3	3	3	3	1	1	1	1	3	3
3	3	3	3	1	1	1	1	3	3
2	2	3	3	1	1	1	1	3	3
2	2	2	2	1	1	1	1	3	3
2	2	2	2	1	1	1	1	3	3
2	2	3	3	1	1	1	1	3	3
2	2	2	2	1	1	1	1	3	3
2	2	2	2	1	1	1	1	3	3
3	3	2	2	1	1	1	1	3	3
3	3	3	3	1	1	1	1	3	3
3	3	3	3	1	1	1	1	3	3
2	2	2	2	1	1	1	1	3	3
2	2	2	2	1	1	1	1	3	3
2	2	2	2	1	1	1	1	3	3
3	3	3	3	1	1	1	1	3	3
2	2	3	3	1	1	1	1	3	3
3	3	3	3	1	1	1	1	3	3
3	3	2	2	1	1	1	1	2	2
3	3	2	2	1	1	1	1	3	3
2	2	2	2	1	1	1	1	3	3
3	3	2	2	1	1	1	1	3	3
2	2	2	2	1	1	1	1	3	3
2	2	3	3	1	1	1	1	3	3
3	3	2	2	1	1	1	1	3	3
2	2	2	2	1	1	1	1	3	3
2	2	2	2	1	1	1	1	3	3
2	2	2	2	2	2	2	2	3	3
3	3	2	2	1	1	1	1	3	3
2	2	2	2	1	1	1	1	3	3
3	3	2	2	1	1	1	1	2	2
2	2	3	3	1	1	1	1	3	3
2	2	2	2	1	1	1	1	3	3
2	2	2	2	1	1	1	1	3	3
2	2	2	2	1	1	1	1	3	3
2	2	3	3	1	1	2	2	3	3
2	2	2	2	1	1	1	1	3	3
3	3	3	3	1	1	1	1	2	2
2	2	2	2	1	1	1	1	3	3
3	3	3	3	1	1	1	1	3	3
2	2	3	3	1	1	1	1	3	3
3	3	3	3	1	1	1	1	3	3
3	3	3	3	1	1	1	1	3	3
2	2	2	2	1	1	1	1	2	2
2	2	2	2	2	2	1	1	3	3
2	2	3	3	1	1	1	1	3	3
2	2	3	3	1	1	1	1	3	3
3	3	3	3	2	2	2	2	3	3
3	3	2	2	1	1	1	1	2	2
3	3	3	3	1	1	1	1	3	3

For Review Only

3	3	2	2	1	1	1	1	3	3
2	2	2	2	1	1	1	1	3	3
2	2	3	3	1	1	2	2	3	3
3	3	3	3	2	2	1	1	3	3
2	2	3	3	1	1	1	1	3	3
2	2	2	2	1	1	1	1	3	3
2	2	3	3	1	1	1	1	3	3
3	3	3	3	1	1	1	1	2	2
3	3	2	2	1	1	1	1	3	3
3	3	3	3	1	1	1	1	2	2
3	3	3	3	1	1	1	1	3	3
3	3	3	3	1	1	1	1	3	3
2	2	2	2	2	2	1	1	3	3
2	2	2	2	1	1	1	1	3	3
2	2	3	3	1	1	2	2	3	3
2	2	2	2	1	1	1	1	3	3
3	3	3	3	1	1	1	1	3	3
2	2	3	3	1	1	1	1	3	3
3	3	2	2	1	1	1	1	3	3
2	2	2	2	1	1	1	1	3	3
2	2	2	2	1	1	1	1	3	3
2	2	2	2	1	1	1	1	3	3
2	2	2	2	1	1	1	1	3	3
2	2	3	3	1	1	1	1	3	3
3	3	3	3	1	1	1	1	3	3
2	2	2	2	1	1	1	1	3	3
3	3	3	3	1	1	1	1	2	2
2	2	2	2	1	1	1	1	3	3
2	2	3	3	1	1	1	1	3	3
2	2	2	2	1	1	1	1	2	2
3	3	3	3	1	1	1	1	3	3
3	3	3	3	1	1	1	1	3	3
2	2	2	2	1	1	1	1	3	3
2	2	2	2	1	1	1	1	3	3
3	3	3	3	2	2	2	2	3	3
3	3	3	3	1	1	1	1	3	3
3	3	2	2	1	1	1	1	3	3
3	3	2	2	1	1	1	1	2	2
2	2	3	3	1	1	1	1	3	3
3	3	3	3	1	1	1	1	3	3
2	2	2	2	1	1	1	1	3	3
2	2	2	2	1	1	2	2	3	3
2	2	3	3	1	1	1	1	3	3
2	2	2	2	1	1	1	1	3	3
2	2	3	3	1	1	1	1	3	3
2	2	2	2	1	1	1	1	3	3
2	2	2	2	1	1	1	1	3	3
2	2	3	3	1	1	1	1	3	3
3	3	2	2	1	1	1	1	3	3
3	3	3	3	1	1	1	1	2	2
2	2	2	2	1	1	1	1	3	3
3	3	3	3	1	1	1	1	3	3
2	2	3	3	1	1	2	2	3	3
2	2	2	2	1	1	1	1	3	3
2	2	3	3	1	1	1	1	3	3

For Review Only

2	2	2	2	1	1	1	1	3	3
2	2	3	3	1	1	1	1	2	2

For Review Only

SNP2818	SNP2818	SNP2819	SNP2819	SNP282	SNP282	SNP2820	SNP2820	SNP2821	SNP2821
3	3	4	4	4	4	3	3	4	4
3	3	4	4	4	4	3	3	4	4
2	2	1	1	4	4	3	3	4	4
3	3	4	4	4	4	3	3	4	4
3	3	4	4	4	4	3	3	4	4
3	3	4	4	4	4	3	3	4	4
3	3	4	4	4	4	3	3	4	4
3	3	4	4	4	4	3	3	4	4
3	3	4	4	4	4	3	3	4	4
3	3	4	4	4	4	3	3	4	4
3	3	4	4	4	4	1	1	2	2
3	3	4	4	4	4	3	3	4	4
3	3	4	4	4	4	3	3	4	4
3	3	4	4	4	4	3	3	4	4
3	3	4	4	4	4	3	3	4	4
3	3	4	4	4	4	3	3	4	4
3	3	4	4	4	4	3	3	4	4
3	3	1	1	4	4	3	3	4	4
3	3	4	4	4	4	3	3	4	4
3	3	4	4	1	1	3	3	4	4
3	3	4	4	4	4	3	3	4	4
3	3	4	4	4	4	3	3	4	4
3	3	4	4	4	4	1	1	2	2
3	3	4	4	1	1	3	3	4	4
3	3	4	4	4	4	3	3	4	4
3	3	4	4	4	4	3	3	4	4
3	3	4	4	4	4	3	3	4	4
3	3	4	4	4	4	3	3	4	4
3	3	4	4	4	4	3	3	4	4
3	3	4	4	1	1	3	3	4	4
3	3	4	4	4	4	3	3	4	4
3	3	4	4	4	4	3	3	4	4
3	3	4	4	4	4	3	3	4	4
3	3	4	4	4	4	3	3	4	4
3	3	4	4	4	4	1	1	4	4
3	3	4	4	4	4	3	3	4	4
3	3	1	1	4	4	3	3	4	4
3	3	4	4	4	4	3	3	4	4
3	3	4	4	4	4	1	1	2	2
3	3	4	4	4	4	1	1	2	2
3	3	4	4	1	1	3	3	4	4
3	3	4	4	1	1	3	3	4	4
3	3	4	4	1	1	3	3	4	4
3	3	1	1	4	4	3	3	4	4
3	3	4	4	4	4	3	3	4	4
3	3	4	4	4	4	3	3	4	4
2	2	1	1	4	4	3	3	4	4
3	3	4	4	4	4	3	3	4	4
3	3	4	4	4	4	3	3	4	4
3	3	4	4	4	4	3	3	4	4
3	3	4	4	4	4	3	3	4	4
3	3	4	4	4	4	1	1	2	2

For Review Only

3	3	4	4	4	4	3	3	4	4
3	3	4	4	4	4	3	3	4	4
3	3	4	4	4	4	3	3	4	4
3	3	4	4	4	4	3	3	4	4
3	3	4	4	4	4	3	3	4	4
3	3	4	4	4	4	3	3	4	4
3	3	4	4	4	4	3	3	4	4
3	3	4	4	4	4	3	3	4	4
3	3	1	1	4	4	3	3	4	4
3	3	4	4	4	4	3	3	4	4
2	2	1	1	4	4	3	3	4	4
2	2	1	1	4	4	3	3	4	4
3	3	4	4	4	4	3	3	4	4
3	3	4	4	4	4	3	3	4	4
3	3	4	4	4	4	3	3	4	4
3	3	4	4	4	4	3	3	4	4
2	2	4	4	4	4	3	3	4	4
3	3	4	4	4	4	3	3	4	4
3	3	4	4	4	4	3	3	4	4
3	3	4	4	4	4	3	3	4	4
2	2	1	1	4	4	3	3	4	4
2	2	1	1	4	4	3	3	4	4
3	3	4	4	4	4	3	3	4	4
3	3	4	4	4	4	3	3	4	4
3	3	4	4	4	4	3	3	4	4
3	3	4	4	4	4	3	3	4	4
3	3	4	4	1	1	3	3	4	4
3	3	4	4	4	4	1	1	2	2
3	3	4	4	4	4	3	3	4	4
3	3	4	4	4	4	3	3	4	4
3	3	4	4	1	1	3	3	4	4
3	3	4	4	4	4	3	3	4	4
3	3	4	4	4	4	1	1	2	2
3	3	4	4	4	4	3	3	4	4
3	3	4	4	4	4	3	3	4	4
3	3	4	4	4	4	3	3	4	4
3	3	1	1	1	1	3	3	4	4
3	3	4	4	4	4	3	3	4	4
3	3	4	4	4	4	3	3	4	4
3	3	4	4	4	4	3	3	4	4
3	3	4	4	4	4	3	3	4	4
3	3	4	4	4	4	3	3	4	4
3	3	1	1	4	4	3	3	4	4
2	2	1	1	4	4	3	3	4	4
3	3	4	4	4	4	3	3	4	4
3	3	4	4	4	4	3	3	4	4
3	3	4	4	4	4	3	3	4	4
3	3	4	4	4	4	3	3	4	4
3	3	4	4	1	1	3	3	4	4
3	3	4	4	4	4	3	3	4	4
3	3	4	4	4	4	3	3	4	4
3	3	4	4	4	4	1	1	2	2
3	3	4	4	4	4	3	3	4	4
3	3	4	4	4	4	3	3	4	4
3	3	4	4	4	4	3	3	4	4

For Review Only

3	3	4	4	4	4	3	3	2	2
3	3	1	1	4	4	3	3	4	4
3	3	4	4	4	4	3	3	4	4
3	3	1	1	4	4	3	3	4	4
3	3	4	4	4	4	3	3	4	4
3	3	4	4	4	4	3	3	4	4
3	3	4	4	4	4	3	3	4	4
3	3	4	4	4	4	3	3	4	4
3	3	4	4	4	4	3	3	4	4
3	3	4	4	4	4	3	3	4	4
3	3	4	4	4	4	3	3	4	4
3	3	4	4	4	4	3	3	4	4
3	3	1	1	4	4	3	3	4	4
3	3	4	4	4	4	3	3	4	4
3	3	4	4	4	4	3	3	4	4
3	3	4	4	4	4	3	3	4	4
3	3	4	4	4	4	3	3	4	4
3	3	4	4	4	4	3	3	4	4
3	3	1	1	4	4	3	3	4	4
3	3	4	4	4	4	3	3	4	4
3	3	4	4	4	4	3	3	4	4
3	3	4	4	4	4	3	3	4	4
2	2	1	1	4	4	3	3	4	4
3	3	4	4	4	4	3	3	4	4
3	3	4	4	4	4	3	3	4	4
2	2	1	1	4	4	3	3	4	4
3	3	4	4	4	4	3	3	4	4
3	3	4	4	4	4	3	3	4	4
2	2	1	1	4	4	3	3	4	4
3	3	4	4	1	1	3	3	4	4
3	3	1	1	4	4	1	1	4	4
3	3	4	4	4	4	3	3	4	4
3	3	4	4	4	4	3	3	4	4
3	3	4	4	4	4	3	3	4	4
3	3	4	4	4	4	3	3	4	4
3	3	1	1	4	4	3	3	4	4
3	3	1	1	4	4	3	3	4	4
3	3	4	4	4	4	3	3	4	4
3	3	1	1	4	4	1	1	2	2
3	3	4	4	4	4	1	1	2	2
3	3	4	4	4	4	1	1	2	2
3	3	1	1	1	1	3	3	4	4
3	3	4	4	4	4	3	3	4	4
3	3	4	4	4	4	3	3	4	4
3	3	4	4	4	4	3	3	4	4
2	2	1	1	4	4	3	3	4	4
3	3	4	4	4	4	3	3	4	4
3	3	1	1	4	4	3	3	4	4
3	3	4	4	4	4	3	3	4	4
3	3	4	4	4	4	3	3	4	4
3	3	4	4	4	4	3	3	4	4
3	3	4	4	4	4	3	3	4	4
3	3	4	4	4	4	3	3	4	4
3	3	4	4	4	4	3	3	4	4
3	3	4	4	4	4	3	3	4	4

For Review Only

3	3	4	4	4	4	3	3	4	4
3	3	1	1	4	4	3	3	4	4
3	3	4	4	4	4	3	3	4	4
3	3	4	4	4	4	3	3	4	4
3	3	4	4	1	1	3	3	4	4
3	3	4	4	4	4	3	3	4	4
3	3	4	4	4	4	3	3	4	4
3	3	4	4	4	4	3	3	4	4
3	3	4	4	4	4	3	3	4	4
3	3	4	4	4	4	3	3	4	4
3	3	4	4	4	4	3	3	4	4
3	3	4	4	4	4	3	3	4	4
3	3	4	4	1	1	3	3	4	4
3	3	4	4	4	4	3	3	4	4
3	3	1	1	4	4	3	3	4	4
3	3	4	4	4	4	1	1	2	2
3	3	4	4	4	4	3	3	4	4
3	3	4	4	4	4	3	3	4	4
3	3	4	4	4	4	3	3	4	4
3	3	4	4	4	4	3	3	4	4
3	3	4	4	4	4	3	3	4	4
3	3	4	4	4	4	3	3	4	4
3	3	4	4	4	4	3	3	4	4
2	2	1	1	4	4	3	3	4	4
3	3	4	4	4	4	3	3	4	4
3	3	4	4	4	4	3	3	4	4
3	3	4	4	4	4	3	3	4	4
3	3	4	4	4	4	3	3	4	4
3	3	4	4	4	4	3	3	4	4
3	3	4	4	4	4	3	3	4	4
3	3	4	4	1	1	3	3	4	4
3	3	4	4	4	4	3	3	4	4
2	2	1	1	4	4	3	3	4	4
3	3	1	1	4	4	3	3	4	4
3	3	4	4	1	1	3	3	4	4
2	2	1	1	4	4	3	3	4	4
2	2	4	4	4	4	3	3	4	4
3	3	1	1	4	4	3	3	4	4
3	3	1	1	4	4	3	3	4	4
3	3	1	1	1	1	3	3	4	4
2	2	1	1	4	4	3	3	4	4
3	3	4	4	4	4	3	3	4	4
3	3	1	1	4	4	3	3	4	4
3	3	4	4	4	4	3	3	4	4
3	3	4	4	4	4	3	3	4	4
3	3	4	4	4	4	3	3	4	4
3	3	4	4	4	4	1	1	2	2
3	3	1	1	4	4	1	1	2	2
3	3	1	1	4	4	3	3	4	4
3	3	4	4	4	4	3	3	4	4
3	3	1	1	4	4	3	3	4	4
3	3	4	4	4	4	3	3	4	4
2	2	1	1	1	1	3	3	4	4
2	2	1	1	4	4	3	3	4	4
3	3	4	4	1	1	1	1	2	2

For Review Only

3	3	4	4	4	4	3	3	4	4
3	3	4	4	1	1	3	3	4	4
2	2	1	1	4	4	3	3	4	4
3	3	4	4	4	4	3	3	4	4
3	3	1	1	4	4	3	3	4	4
3	3	4	4	4	4	3	3	4	4
3	3	4	4	4	4	3	3	4	4
2	2	1	1	1	1	3	3	4	4
3	3	4	4	1	1	3	3	4	4
3	3	1	1	4	4	1	1	2	2
3	3	4	4	4	4	3	3	4	4
3	3	4	4	4	4	3	3	4	4
3	3	4	4	4	4	3	3	4	4
3	3	4	4	4	4	3	3	4	4
2	2	1	1	1	1	3	3	4	4
3	3	4	4	4	4	1	1	2	2
3	3	1	1	4	4	3	3	4	4
3	3	4	4	4	4	3	3	4	4
3	3	1	1	4	4	3	3	4	4
3	3	4	4	4	4	3	3	4	4
3	3	4	4	1	1	3	3	4	4
3	3	4	4	4	4	3	3	4	4
3	3	4	4	4	4	3	3	4	4
3	3	4	4	4	4	3	3	4	4
3	3	4	4	4	4	3	3	4	4
3	3	4	4	4	4	3	3	4	4
3	3	1	1	4	4	1	1	2	2
3	3	4	4	4	4	3	3	4	4
3	3	4	4	4	4	3	3	4	4
2	2	1	1	4	4	3	3	4	4
3	3	1	1	4	4	1	1	2	2
3	3	4	4	4	4	3	3	4	4
3	3	4	4	4	4	3	3	4	4
3	3	4	4	4	4	3	3	4	4
2	2	1	1	4	4	3	3	4	4
3	3	1	1	4	4	1	1	2	2
3	3	4	4	4	4	3	3	4	4
2	2	1	1	4	4	3	3	4	4
3	3	4	4	4	4	3	3	4	4
3	3	4	4	4	4	1	1	2	2
3	3	4	4	1	1	3	3	4	4
2	2	1	1	4	4	3	3	4	4
3	3	4	4	4	4	3	3	4	4
3	3	4	4	4	4	3	3	4	4
3	3	4	4	4	4	3	3	4	4
3	3	4	4	4	4	3	3	4	4
3	3	1	1	4	4	3	3	4	4
3	3	4	4	4	4	3	3	4	4
3	3	4	4	4	4	3	3	4	4
3	3	1	1	4	4	1	1	2	2
3	3	4	4	4	4	3	3	4	4
3	3	4	4	4	4	1	1	2	2
2	2	1	1	4	4	3	3	4	4
3	3	4	4	1	1	3	3	4	4
3	3	4	4	4	4	1	1	2	2

For Review Only

3	3	4	4	4	4	3	3	4	4
2	2	1	1	4	4	3	3	4	4

For Review Only

SNP2822	SNP2822	SNP2823	SNP2823	SNP2824	SNP2824	SNP2825	SNP2825	SNP2826	SNP2826
4	4	2	2	2	2	1	1	1	1
4	4	1	1	2	2	1	1	1	1
4	4	2	2	2	2	1	1	1	1
4	4	1	1	2	2	1	1	1	1
4	4	2	2	2	2	1	1	1	1
4	4	2	2	2	2	4	4	1	1
4	4	2	2	2	2	4	4	1	1
4	4	2	2	2	2	1	1	1	1
4	4	2	2	2	2	4	4	2	2
4	4	2	2	2	2	1	1	1	1
4	4	1	1	2	2	1	1	1	1
4	4	1	1	2	2	1	1	1	1
4	4	2	2	2	2	4	4	2	2
4	4	2	2	2	2	1	1	1	1
4	4	2	2	2	2	4	4	1	1
4	4	1	1	2	2	1	1	1	1
4	4	1	1	2	2	1	1	1	1
4	4	1	1	2	2	4	4	1	1
1	1	1	1	2	2	1	1	1	1
4	4	1	1	2	2	1	1	1	1
4	4	1	1	1	1	1	1	1	1
4	4	2	2	2	2	1	1	1	1
4	4	2	2	1	1	1	1	1	1
1	1	1	1	2	2	1	1	1	1
4	4	2	2	2	2	1	1	1	1
4	4	2	2	2	2	1	1	1	1
4	4	1	1	2	2	1	1	1	1
4	4	2	2	2	2	1	1	1	1
4	4	2	2	2	2	4	4	2	2
4	4	2	2	2	2	1	1	1	1
4	4	1	1	2	2	4	4	2	2
4	4	2	2	2	2	1	1	1	1
4	4	2	2	2	2	4	4	2	2
4	4	2	2	2	2	4	4	2	2
4	4	2	2	2	2	1	1	1	1
1	1	1	1	2	2	4	4	2	2
4	4	2	2	2	2	1	1	1	1
4	4	2	2	2	2	1	1	1	1
4	4	2	2	1	1	1	1	1	1
4	4	2	2	2	2	1	1	1	1
4	4	2	2	2	2	1	1	1	1
4	4	2	2	2	2	1	1	1	1
4	4	2	2	2	2	1	1	1	1
4	4	2	2	2	2	1	1	1	1
4	4	2	2	2	2	1	1	1	1
4	4	1	1	2	2	1	1	1	1
4	4	1	1	2	2	1	1	1	1
1	1	1	1	2	2	4	4	1	1
4	4	2	2	2	2	1	1	1	1
4	4	2	2	2	2	4	4	1	1
1	1	2	2	2	2	1	1	1	1
1	1	2	2	2	2	1	1	1	1

For Review Only

4	4	2	2	2	2	4	4	2	2
4	4	2	2	2	2	4	4	2	2
4	4	1	1	2	2	1	1	1	1
4	4	1	1	2	2	4	4	2	2
1	1	1	1	2	2	1	1	1	1
1	1	2	2	2	2	1	1	1	1
1	1	2	2	2	2	1	1	1	1
4	4	2	2	1	1	1	1	1	1
4	4	2	2	2	2	1	1	1	1
4	4	2	2	2	2	1	1	1	1
4	4	1	1	2	2	1	1	1	1
4	4	1	1	2	2	1	1	1	1
4	4	2	2	2	2	4	4	2	2
4	4	2	2	2	2	4	4	2	2
4	4	2	2	2	2	4	4	1	1
4	4	2	2	2	2	1	1	1	1
4	4	2	2	2	2	1	1	1	1
1	1	2	2	2	2	1	1	1	1
4	4	2	2	2	2	4	4	2	2
4	4	2	2	2	2	1	1	1	1
4	4	2	2	2	2	1	1	1	1
4	4	2	2	2	2	1	1	1	1
4	4	2	2	2	2	4	4	2	2
4	4	2	2	2	2	1	1	1	1
4	4	2	2	2	2	1	1	1	1
4	4	2	2	2	2	4	4	1	1
4	4	2	2	1	1	1	1	1	1
4	4	1	1	1	1	1	1	1	1
1	1	2	2	2	2	1	1	1	1
1	1	1	1	2	2	4	4	2	2
4	4	2	2	2	2	4	4	2	2
1	1	2	2	2	2	1	1	1	1
4	4	1	1	1	1	1	1	1	1
4	4	2	2	2	2	4	4	2	2
4	4	2	2	2	2	1	1	1	1
4	4	2	2	2	2	1	1	1	1
4	4	1	1	2	2	1	1	1	1
4	4	1	1	2	2	4	4	2	2
4	4	1	1	2	2	1	1	1	1
4	4	2	2	2	2	1	1	1	1
4	4	2	2	2	2	4	4	2	2
4	4	1	1	2	2	4	4	2	2
1	1	1	1	2	2	1	1	1	1
4	4	1	1	2	2	1	1	1	1
1	1	1	1	2	2	1	1	1	1
4	4	2	2	2	2	1	1	1	1
4	4	2	2	2	2	1	1	1	1
1	1	2	2	2	2	1	1	1	1
1	1	2	2	2	2	1	1	1	1
4	4	2	2	2	2	1	1	1	1
4	4	2	2	2	2	1	1	1	1
4	4	2	2	1	1	1	1	1	1
4	4	2	2	1	1	1	1	1	1
4	4	2	2	2	2	1	1	1	1
1	1	2	2	2	2	1	1	1	1

For Review Only

1	1	2	2	2	2	1	1	1	1
4	4	2	2	2	2	1	1	1	1
1	1	1	1	2	2	4	4	2	2
4	4	2	2	2	2	1	1	1	1
4	4	1	1	2	2	1	1	1	1
4	4	1	1	2	2	4	4	1	1
4	4	1	1	2	2	1	1	1	1
1	1	2	2	2	2	4	4	1	1
1	1	1	1	1	1	1	1	1	1
4	4	2	2	2	2	4	4	1	1
4	4	2	2	2	2	1	1	1	1
4	4	2	2	2	2	1	1	1	1
1	1	1	1	2	2	1	1	1	1
4	4	2	2	2	2	1	1	1	1
4	4	2	2	2	2	4	4	2	2
4	4	2	2	2	2	1	1	1	1
4	4	2	2	2	2	1	1	1	1
4	4	2	2	2	2	4	4	1	1
4	4	2	2	2	2	1	1	1	1
4	4	2	2	1	1	1	1	1	1
4	4	2	2	2	2	1	1	1	1
1	1	1	1	2	2	1	1	1	1
4	4	2	2	2	2	4	4	2	2
1	1	2	2	1	1	4	4	2	2
4	4	2	2	2	2	1	1	1	1
4	4	2	2	2	2	1	1	1	1
4	4	1	1	2	2	1	1	1	1
1	1	2	2	2	2	1	1	1	1
4	4	2	2	2	2	1	1	1	1
1	1	1	1	2	2	1	1	1	1
4	4	2	2	2	2	4	4	2	2
4	4	2	2	2	2	1	1	1	1
4	4	2	2	2	2	4	4	2	2
4	4	1	1	2	2	1	1	1	1
4	4	2	2	2	2	4	4	2	2
4	4	2	2	2	2	4	4	1	1
4	4	2	2	2	2	1	1	1	1
4	4	2	2	2	2	4	4	2	2
4	4	2	2	2	2	4	4	2	2
4	4	2	2	1	1	1	1	1	1
4	4	2	2	2	2	1	1	1	1
4	4	2	2	2	2	1	1	1	1
1	1	2	2	2	2	1	1	1	1
4	4	2	2	2	2	1	1	1	1
1	1	2	2	2	2	4	4	2	2
4	4	2	2	2	2	1	1	1	1
1	1	2	2	2	2	1	1	1	1
4	4	2	2	2	2	1	1	1	1
4	4	2	2	1	1	1	1	1	1
1	1	1	1	2	2	1	1	1	1
4	4	2	2	2	2	1	1	1	1
4	4	1	1	1	1	1	1	1	1
1	1	2	2	2	2	1	1	1	1
4	4	2	2	2	2	1	1	1	1
4	4	2	2	2	2	1	1	1	1

For Review Only

1	1	2	2	2	2	1	1	1	1
1	1	2	2	2	2	1	1	1	1
4	4	2	2	2	2	1	1	1	1
4	4	2	2	2	2	1	1	1	1
4	4	2	2	2	2	1	1	1	1
4	4	2	2	2	2	4	4	1	1
4	4	2	2	2	2	4	4	1	1
4	4	2	2	2	2	1	1	1	1
4	4	2	2	2	2	1	1	1	1
1	1	1	1	2	2	4	4	2	2
4	4	1	1	2	2	1	1	1	1
1	1	2	2	2	2	1	1	1	1
4	4	2	2	2	2	1	1	1	1
4	4	2	2	2	2	1	1	1	1
1	1	2	2	2	2	4	4	2	2
4	4	1	1	1	1	1	1	1	1
4	4	2	2	2	2	1	1	1	1
4	4	1	1	2	2	1	1	1	1
4	4	2	2	2	2	1	1	1	1
4	4	1	1	2	2	1	1	1	1
4	4	2	2	2	2	1	1	1	1
1	1	2	2	2	2	4	4	2	2
4	4	2	2	2	2	1	1	1	1
4	4	1	1	2	2	1	1	1	1
4	4	2	2	2	2	1	1	2	2
4	4	2	2	2	2	4	4	2	2
4	4	1	1	2	2	1	1	1	1
4	4	2	2	2	2	4	4	1	1
4	4	2	2	2	2	1	1	1	1
1	1	1	1	2	2	4	4	2	2
4	4	2	2	2	2	4	4	2	2
1	1	2	2	2	2	4	4	2	2
4	4	2	2	2	2	1	1	1	1
1	1	2	2	2	2	1	1	1	1
4	4	1	1	1	1	1	1	1	1
4	4	1	1	2	2	1	1	1	1
4	4	2	2	2	2	1	1	1	1
4	4	2	2	2	2	1	1	1	1
4	4	2	2	2	2	1	1	1	1
4	4	2	2	2	2	1	1	1	1
4	4	2	2	2	2	1	1	1	1
1	1	2	2	2	2	1	1	1	1
4	4	2	2	1	1	1	1	1	1
4	4	2	2	2	2	1	1	1	1
1	1	1	1	2	2	1	1	1	1
1	1	2	2	2	2	4	4	1	1
4	4	1	1	1	1	1	1	1	1
4	4	2	2	1	1	4	4	2	2
1	1	1	1	2	2	1	1	1	1
4	4	1	1	2	2	4	4	2	2
4	4	2	2	2	2	1	1	1	1
4	4	2	2	1	1	1	1	1	1
4	4	2	2	2	2	1	1	1	1
4	4	1	1	2	2	1	1	1	1
4	4	2	2	1	1	1	1	1	1

For Review Only

4	4	2	2	2	2	1	1	1	1
4	4	2	2	2	2	1	1	1	1
1	1	2	2	2	2	1	1	1	1
4	4	2	2	2	2	1	1	1	1
4	4	2	2	2	2	1	1	1	1
4	4	2	2	2	2	1	1	1	1
4	4	1	1	2	2	1	1	1	1
4	4	1	1	2	2	1	1	1	1
1	1	2	2	2	2	1	1	1	1
4	4	2	2	1	1	1	1	1	1
4	4	2	2	2	2	1	1	1	1
4	4	2	2	2	2	4	4	2	2
4	4	1	1	2	2	1	1	1	1
1	1	2	2	2	2	1	1	1	1
4	4	2	2	2	2	1	1	1	1
1	1	1	1	2	2	1	1	1	1
4	4	2	2	2	2	1	1	1	1
4	4	2	2	1	1	1	1	1	1
4	4	2	2	2	2	1	1	1	1
4	4	2	2	2	2	1	1	1	1
4	4	2	2	2	2	1	1	1	1
1	1	2	2	2	2	1	1	1	1
1	1	1	1	2	2	1	1	1	1
1	1	2	2	2	2	1	1	1	1
4	4	2	2	1	1	1	1	1	1
4	4	2	2	2	2	1	1	1	1
4	4	2	2	1	1	1	1	1	1
4	4	1	1	2	2	1	1	1	1
4	4	2	2	2	2	4	4	1	1
4	4	2	2	2	2	1	1	1	1
4	4	2	2	1	1	1	1	1	1
1	1	2	2	2	2	4	4	1	1
1	1	2	2	1	1	1	1	1	1
4	4	2	2	2	2	1	1	1	1
4	4	2	2	2	2	1	1	1	1
4	4	2	2	1	1	4	4	2	2
1	1	2	2	2	2	1	1	1	1
4	4	1	1	2	2	1	1	1	1
1	1	1	1	2	2	4	4	2	2
4	4	2	2	1	1	1	1	1	1
4	4	1	1	2	2	1	1	1	1
4	4	2	2	2	2	1	1	1	1
1	1	2	2	2	2	1	1	1	1
4	4	1	1	2	2	4	4	2	2
4	4	2	2	1	1	4	4	1	1
4	4	1	1	2	2	1	1	1	1
4	4	2	2	1	1	1	1	1	1
4	4	2	2	1	1	1	1	1	1
4	4	2	2	1	1	4	4	2	2
4	4	2	2	1	1	1	1	1	1
1	1	2	2	2	2	1	1	1	1
4	4	2	2	1	1	1	1	1	1
4	4	2	2	2	2	1	1	1	1
4	4	1	1	2	2	1	1	1	1
4	4	2	2	2	2	1	1	1	1

For Review Only

1	1	1	1	2	2	1	1	1	1
4	4	2	2	2	2	4	4	2	2

For Review Only

SNP2827	SNP2827	SNP2828	SNP2828	SNP2829	SNP2829	SNP283	SNP283	SNP2830	SNP2830
1	1	1	1	4	4	1	1	4	4
1	1	3	3	4	4	1	1	4	4
3	3	3	3	1	1	1	1	1	1
1	1	1	1	4	4	1	1	1	1
1	1	3	3	4	4	4	4	1	1
1	1	3	3	4	4	1	1	1	1
1	1	1	1	4	4	1	1	4	4
1	1	1	1	4	4	1	1	1	1
1	1	1	1	4	4	1	1	4	4
1	1	3	3	4	4	4	4	4	4
1	1	3	3	4	4	1	1	4	4
3	3	3	3	4	4	1	1	1	1
1	1	3	3	4	4	4	4	1	1
1	1	1	1	4	4	1	1	4	4
1	1	3	3	1	1	1	1	1	1
1	1	3	3	4	4	4	4	1	1
1	1	3	3	4	4	4	4	1	1
3	3	3	3	1	1	1	1	1	1
1	1	3	3	1	1	1	1	1	1
1	1	1	1	4	4	4	4	4	4
1	1	3	3	4	4	4	4	1	1
1	1	3	3	4	4	4	4	1	1
1	1	3	3	4	4	1	1	4	4
1	1	3	3	4	4	1	1	1	1
3	3	3	3	1	1	1	1	1	1
1	1	3	3	4	4	1	1	4	4
1	1	1	1	4	4	1	1	1	1
1	1	3	3	4	4	1	1	1	1
1	1	3	3	4	4	4	4	4	4
1	1	3	3	4	4	4	4	4	4
3	3	3	3	1	1	1	1	1	1
1	1	3	3	4	4	1	1	1	1
1	1	3	3	4	4	1	1	1	1
1	1	1	1	4	4	4	4	4	4
1	1	3	3	4	4	1	1	4	4
1	1	3	3	4	4	1	1	4	4
1	1	1	1	4	4	1	1	4	4
1	1	3	3	4	4	1	1	1	1
1	1	3	3	4	4	4	4	4	4
1	1	3	3	4	4	1	1	4	4
1	1	3	3	4	4	4	4	4	4
1	1	3	3	4	4	4	4	4	4
1	1	1	1	4	4	4	4	1	1
1	1	3	3	4	4	1	1	1	1
1	1	1	1	4	4	1	1	1	1
1	1	1	1	4	4	1	1	1	1
3	3	3	3	1	1	1	1	1	1
1	1	3	3	4	4	1	1	4	4
1	1	3	3	4	4	4	4	4	4
1	1	1	1	4	4	1	1	1	1
1	1	1	1	4	4	1	1	1	1
1	1	3	3	4	4	4	4	4	4

For Review Only

1	1	3	3	4	4	1	1	1	1
1	1	3	3	4	4	1	1	1	1
3	3	3	3	1	1	1	1	1	1
1	1	3	3	4	4	1	1	1	1
1	1	3	3	1	1	1	1	1	1
1	1	1	1	4	4	1	1	1	1
1	1	1	1	4	4	1	1	1	1
1	1	3	3	4	4	4	4	4	4
1	1	3	3	4	4	1	1	4	4
1	1	3	3	4	4	1	1	1	1
3	3	3	3	1	1	1	1	1	1
3	3	3	3	1	1	1	1	1	1
1	1	3	3	4	4	1	1	1	1
1	1	3	3	4	4	4	4	4	4
1	1	3	3	4	4	1	1	1	1
1	1	3	3	4	4	1	1	1	1
1	1	1	1	4	4	1	1	4	4
3	3	3	3	1	1	1	1	1	1
3	3	3	3	1	1	1	1	1	1
1	1	1	1	4	4	1	1	4	4
1	1	3	3	1	1	1	1	1	1
1	1	3	3	4	4	4	4	4	4
1	1	3	3	4	4	4	4	4	4
3	3	3	3	1	1	4	4	1	1
1	1	1	1	4	4	1	1	1	1
3	3	3	3	1	1	1	1	1	1
1	1	3	3	4	4	1	1	4	4
1	1	3	3	4	4	4	4	4	4
1	1	3	3	4	4	1	1	4	4
1	1	3	3	4	4	1	1	1	1
1	1	3	3	4	4	1	1	1	1
1	1	3	3	4	4	4	4	4	4
1	1	3	3	4	4	4	4	4	4
1	1	3	3	4	4	1	1	1	1
1	1	3	3	4	4	1	1	1	1
1	1	1	1	4	4	1	1	1	1
1	1	1	1	4	4	1	1	4	4
1	1	3	3	4	4	4	4	4	4
1	1	1	1	4	4	1	1	1	1
1	1	1	1	4	4	1	1	1	1
1	1	3	3	4	4	1	1	1	1
1	1	3	3	4	4	1	1	1	1
1	1	3	3	4	4	1	1	4	4
3	3	3	3	1	1	1	1	1	1
1	1	1	1	4	4	1	1	4	4
1	1	1	1	4	4	1	1	1	1
1	1	1	1	4	4	4	4	4	4
1	1	3	3	4	4	1	1	4	4
1	1	3	3	4	4	1	1	4	4
1	1	3	3	4	4	1	1	4	4
1	1	3	3	4	4	1	1	1	1
1	1	3	3	4	4	4	4	4	4
1	1	3	3	4	4	4	4	4	4
1	1	3	3	4	4	1	1	1	1
3	3	3	3	1	1	1	1	1	1

For Review Only

1	1	3	3	4	4	1	1	1	1
1	1	1	1	4	4	1	1	1	1
1	1	3	3	4	4	1	1	4	4
1	1	1	1	4	4	4	4	4	4
1	1	1	1	4	4	1	1	1	1
1	1	1	1	4	4	1	1	1	1
3	3	3	3	1	1	1	1	1	1
1	1	3	3	1	1	1	1	1	1
1	1	3	3	4	4	4	4	4	4
1	1	3	3	4	4	4	4	1	1
1	1	3	3	4	4	1	1	1	1
1	1	3	3	4	4	1	1	1	1
1	1	3	3	4	4	1	1	4	4
3	3	3	3	1	1	1	1	1	1
1	1	3	3	4	4	1	1	1	1
1	1	1	1	4	4	1	1	1	1
1	1	3	3	4	4	1	1	4	4
1	1	3	3	4	4	1	1	1	1
1	1	1	1	4	4	4	4	4	4
1	1	1	1	4	4	1	1	1	1
1	1	3	3	4	4	1	1	1	1
3	3	3	3	4	4	1	1	1	1
3	3	3	3	1	1	1	1	1	1
1	1	3	3	4	4	4	4	1	1
1	1	3	3	4	4	4	4	4	4
1	1	3	3	4	4	1	1	1	1
1	1	3	3	4	4	1	1	1	1
1	1	3	3	4	4	1	1	4	4
1	1	3	3	1	1	1	1	1	1
3	3	3	3	1	1	1	1	4	4
1	1	3	3	4	4	4	4	4	4
1	1	3	3	4	4	1	1	1	1
1	1	3	3	4	4	1	1	1	1
1	1	1	1	4	4	1	1	1	1
1	1	1	1	4	4	4	4	4	4
1	1	3	3	4	4	1	1	4	4
1	1	1	1	4	4	4	4	4	4
1	1	1	1	4	4	1	1	1	1
1	1	3	3	4	4	1	1	4	4
1	1	3	3	4	4	4	4	4	4
1	1	3	3	4	4	1	1	4	4
1	1	1	1	4	4	4	4	4	4
3	3	3	3	1	1	1	1	1	1
1	1	3	3	4	4	1	1	1	1
1	1	3	3	4	4	1	1	4	4
1	1	1	1	4	4	1	1	4	4
1	1	1	1	4	4	1	1	1	1
1	1	1	1	4	4	1	1	1	1
1	1	1	1	4	4	4	4	1	1
1	1	3	3	4	4	1	1	1	1
1	1	3	3	4	4	1	1	1	1
1	1	3	3	4	4	4	4	4	4
1	1	1	1	4	4	1	1	1	1
1	1	3	3	4	4	1	1	1	1
1	1	3	3	4	4	1	1	1	1

For Review Only

1	1	1	1	4	4	1	1	1	1
1	1	3	3	4	4	1	1	4	4
1	1	3	3	4	4	4	4	1	1
1	1	3	3	4	4	4	4	4	4
1	1	1	1	4	4	1	1	1	1
1	1	3	3	4	4	4	4	1	1
1	1	3	3	4	4	4	4	4	4
1	1	3	3	4	4	1	1	1	1
1	1	3	3	4	4	1	1	1	1
1	1	1	1	4	4	1	1	1	1
1	1	3	3	4	4	1	1	1	1
1	1	3	3	4	4	1	1	1	1
1	1	3	3	4	4	1	1	1	1
1	1	1	1	4	4	1	1	4	4
1	1	1	1	4	4	4	4	4	4
1	1	3	3	4	4	1	1	4	4
1	1	1	1	4	4	1	1	1	1
3	3	3	3	1	1	1	1	1	1
1	1	3	3	4	4	1	1	1	1
3	3	3	3	1	1	1	1	1	1
1	1	3	3	4	4	1	1	1	1
1	1	3	3	4	4	4	4	4	4
1	1	3	3	4	4	4	4	4	4
3	3	3	3	1	1	1	1	1	1
1	1	1	1	4	4	1	1	1	1
1	1	1	1	4	4	4	4	4	4
1	1	1	1	4	4	1	1	1	1
1	1	3	3	4	4	1	1	4	4
1	1	3	3	4	4	1	1	4	4
3	3	3	3	1	1	1	1	1	1
3	3	3	3	1	1	1	1	1	1
3	3	3	3	1	1	1	1	1	1
1	1	3	3	4	4	1	1	1	1
1	1	1	1	4	4	4	4	4	4
1	1	3	3	4	4	1	1	4	4
3	3	3	3	1	1	1	1	1	1
1	1	3	3	4	4	1	1	1	1
1	1	3	3	4	4	1	1	4	4
1	1	1	1	4	4	1	1	1	1
1	1	3	3	4	4	1	1	4	4
3	3	3	3	1	1	1	1	1	1
1	1	1	1	4	4	1	1	1	1
1	1	3	3	4	4	4	4	4	4
1	1	3	3	4	4	1	1	4	4
1	1	3	3	4	4	1	1	1	1
1	1	3	3	4	4	4	4	1	1
1	1	3	3	4	4	1	1	4	4
1	1	3	3	4	4	4	4	4	4
1	1	1	1	4	4	4	4	4	4
1	1	1	1	4	4	1	1	1	1
1	1	3	3	1	1	1	1	1	1
1	1	1	1	4	4	4	4	4	4
1	1	3	3	4	4	4	4	4	4
3	3	3	3	1	1	1	1	1	1
1	1	3	3	4	4	4	4	4	4

For Review Only

3	3	3	3	1	1	1	1	1	1
1	1	3	3	4	4	1	1	4	4
3	3	3	3	1	1	1	1	1	1
1	1	3	3	4	4	1	1	1	1
1	1	1	1	4	4	1	1	1	1
1	1	3	3	4	4	1	1	4	4
1	1	1	1	4	4	4	4	1	1
1	1	1	1	4	4	4	4	1	1
1	1	3	3	4	4	1	1	4	4
1	1	3	3	4	4	4	4	4	4
1	1	3	3	4	4	1	1	1	1
1	1	3	3	4	4	1	1	4	4
1	1	1	1	4	4	1	1	1	1
3	3	3	3	1	1	1	1	1	1
3	3	3	3	1	1	1	1	1	1
1	1	3	3	4	4	1	1	4	4
1	1	3	3	4	4	4	4	1	1
1	1	3	3	4	4	1	1	4	4
1	1	3	3	4	4	4	4	4	4
1	1	1	1	4	4	1	1	1	1
1	1	3	3	4	4	4	4	4	4
1	1	3	3	4	4	1	1	4	4
1	1	1	1	4	4	1	1	1	1
1	1	3	3	4	4	1	1	4	4
1	1	3	3	4	4	4	4	4	4
1	1	3	3	4	4	1	1	1	1
1	1	3	3	4	4	4	4	4	4
1	1	1	1	4	4	1	1	1	1
1	1	3	3	4	4	1	1	1	1
3	3	3	3	1	1	1	1	1	1
1	1	3	3	4	4	1	1	4	4
1	1	1	1	4	4	1	1	1	1
1	1	1	1	4	4	1	1	4	4
1	1	1	1	4	4	1	1	1	1
1	1	3	3	4	4	4	4	4	4
1	1	3	3	4	4	4	4	4	4
3	3	3	3	1	1	1	1	1	1
3	3	3	3	1	1	1	1	1	1
1	1	3	3	4	4	1	1	4	4
1	1	3	3	4	4	4	4	4	4
1	1	3	3	4	4	1	1	4	4
1	1	3	3	4	4	4	4	4	4
1	1	3	3	4	4	1	1	4	4
1	1	3	3	4	4	1	1	1	1
1	1	3	3	4	4	1	1	1	1
1	1	1	1	4	4	1	1	1	1
1	1	1	1	4	4	1	1	1	1
1	1	3	3	4	4	1	1	4	4
3	3	3	3	1	1	1	1	1	1
1	1	3	3	4	4	4	4	4	4
1	1	3	3	4	4	1	1	1	1
1	1	3	3	4	4	4	4	4	4
3	3	3	3	1	1	1	1	1	1
1	1	1	1	4	4	1	1	1	1
1	1	3	3	4	4	1	1	4	4

For Review Only

CSSA 5585 Guilford Rd., Madison, WI 53711

1	1	3	3	4	4	1	1	1	1
3	3	3	3	1	1	1	1	1	1

For Review Only

SNP2831	SNP2831	SNP2832	SNP2832	SNP2833	SNP2833	SNP2834	SNP2834	SNP2835	SNP2835
4	4	4	4	1	1	1	1	3	3
4	4	4	4	2	2	1	1	3	3
1	1	3	3	2	2	4	4	4	4
4	4	3	3	2	2	1	1	3	3
4	4	3	3	2	2	4	4	4	4
4	4	4	4	2	2	4	4	3	3
4	4	4	4	2	2	1	1	3	3
4	4	4	4	2	2	1	1	3	3
4	4	4	4	2	2	4	4	3	3
4	4	4	4	2	2	1	1	3	3
4	4	4	4	2	2	1	1	3	3
1	1	3	3	2	2	4	4	4	4
1	1	3	3	2	2	4	4	4	4
4	4	4	4	1	1	1	1	3	3
1	1	3	3	2	2	4	4	4	4
4	4	3	3	2	2	4	4	3	3
1	1	3	3	2	2	4	4	4	4
1	1	3	3	1	1	1	1	3	3
1	1	3	3	2	2	4	4	4	4
4	4	4	4	2	2	1	1	3	3
1	1	3	3	2	2	4	4	4	4
1	1	3	3	2	2	4	4	4	4
4	4	4	4	2	2	1	1	3	3
4	4	4	4	2	2	1	1	3	3
1	1	3	3	2	2	4	4	4	4
4	4	4	4	1	1	1	1	3	3
4	4	4	4	2	2	1	1	3	3
4	4	3	3	2	2	1	1	4	4
4	4	4	4	2	2	1	1	3	3
4	4	4	4	2	2	1	1	3	3
1	1	3	3	2	2	4	4	4	4
1	1	3	3	2	2	1	1	3	3
1	1	3	3	2	2	1	1	3	3
4	4	4	4	1	1	1	1	3	3
4	4	3	3	2	2	4	4	3	3
4	4	4	4	1	1	1	1	4	4
4	4	4	4	1	1	1	1	3	3
4	4	4	4	2	2	1	1	3	3
4	4	4	4	2	2	1	1	3	3
4	4	4	4	2	2	1	1	3	3
4	4	4	4	2	2	1	1	3	3
4	4	4	4	2	2	1	1	3	3
4	4	4	4	2	2	1	1	3	3
4	4	3	3	2	2	4	4	4	4
4	4	4	4	2	2	1	1	3	3
4	4	4	4	2	2	1	1	3	3
4	4	3	3	2	2	4	4	4	4
4	4	4	4	1	1	1	1	3	3
4	4	4	4	2	2	1	1	3	3
4	4	3	3	2	2	4	4	4	4
4	4	4	4	2	2	1	1	3	3
4	4	4	4	2	2	1	1	3	3

For Review Only

1	1	3	3	2	2	4	4	4	4
1	1	3	3	2	2	1	1	3	3
4	4	4	4	2	2	1	1	3	3
1	1	3	3	2	2	4	4	4	4
1	1	3	3	2	2	4	4	4	4
4	4	4	4	2	2	1	1	3	3
4	4	4	4	2	2	1	1	3	3
4	4	4	4	2	2	1	1	3	3
4	4	4	4	1	1	1	1	3	3
4	4	3	3	2	2	4	4	4	4
1	1	3	3	2	2	4	4	4	4
1	1	3	3	2	2	4	4	4	4
1	1	3	3	2	2	4	4	4	4
4	4	4	4	2	2	1	1	3	3
4	4	4	4	2	2	1	1	3	3
4	4	4	4	2	2	4	4	3	3
4	4	4	4	1	1	1	1	3	3
1	1	3	3	1	1	1	1	3	3
1	1	3	3	2	2	4	4	4	4
4	4	4	4	1	1	4	4	3	3
1	1	3	3	2	2	4	4	4	4
4	4	4	4	2	2	1	1	3	3
4	4	4	4	2	2	1	1	3	3
1	1	3	3	1	1	1	1	3	3
4	4	4	4	2	2	1	1	3	3
1	1	3	3	2	2	4	4	4	4
4	4	4	4	2	2	1	1	3	3
4	4	4	4	2	2	1	1	3	3
4	4	4	4	1	1	1	1	3	3
1	1	3	3	2	2	4	4	4	4
1	1	3	3	2	2	4	4	4	4
4	4	4	4	1	1	1	1	3	3
4	4	4	4	2	2	1	1	3	3
1	1	3	3	2	2	4	4	4	4
4	4	4	4	2	2	1	1	3	3
4	4	4	4	2	2	1	1	3	3
4	4	4	4	2	2	1	1	3	3
4	4	4	4	1	1	1	1	3	3
4	4	4	4	2	2	1	1	3	3
4	4	4	4	2	2	1	1	3	3
1	1	3	3	2	2	4	4	4	4
1	1	3	3	2	2	4	4	4	4
4	4	4	4	2	2	1	1	4	4
1	1	3	3	2	2	4	4	4	4
4	4	4	4	1	1	4	4	3	3
4	4	4	4	1	1	1	1	3	3
4	4	4	4	1	1	1	1	3	3
4	4	4	4	1	1	1	1	3	3
4	4	4	4	1	1	1	1	3	3
4	4	4	4	2	2	1	1	3	3
4	4	4	4	1	1	1	1	3	3
4	4	4	4	2	2	1	1	3	3
4	4	4	4	2	2	1	1	3	3
4	4	4	4	2	2	4	4	4	4
1	1	3	3	2	2	4	4	4	4

For Review Only

4	4	4	4	2	2	1	1	3	3
4	4	4	4	2	2	1	1	3	3
4	4	4	4	1	1	1	1	3	3
4	4	4	4	1	1	1	1	3	3
4	4	4	4	1	1	1	1	3	3
4	4	4	4	2	2	4	4	4	4
1	1	3	3	2	2	4	4	4	4
1	1	3	3	2	2	4	4	4	4
4	4	4	4	2	2	1	1	3	3
4	4	4	4	2	2	1	1	3	3
4	4	4	4	2	2	4	4	4	4
4	4	4	4	2	2	1	1	3	3
4	4	4	4	2	2	1	1	3	3
4	4	3	3	2	2	4	4	4	4
4	4	4	4	1	1	4	4	4	4
4	4	4	4	2	2	1	1	3	3
4	4	4	4	1	1	1	1	3	3
4	4	3	3	2	2	4	4	4	4
4	4	4	4	1	1	1	1	3	3
1	1	4	4	2	2	4	4	4	4
4	4	4	4	2	2	4	4	4	4
1	1	3	3	1	1	1	1	4	4
1	1	3	3	2	2	4	4	4	4
4	4	4	4	2	2	1	1	3	3
4	4	4	4	2	2	1	1	3	3
1	1	3	3	1	1	1	1	3	3
4	4	4	4	2	2	4	4	4	4
4	4	4	4	1	1	1	1	3	3
1	1	3	3	2	2	4	4	4	4
4	4	4	4	1	1	1	1	3	3
4	4	4	4	2	2	1	1	3	3
4	4	3	3	2	2	1	1	3	3
1	1	3	3	2	2	4	4	4	4
4	4	3	3	2	2	4	4	4	4
4	4	4	4	1	1	1	1	3	3
4	4	3	3	2	2	4	4	4	4
4	4	4	4	1	1	1	1	3	3
4	4	4	4	2	2	4	4	4	4
4	4	4	4	2	2	1	1	3	3
4	4	4	4	2	2	1	1	3	3
4	4	4	4	2	2	1	1	3	3
4	4	4	4	1	1	1	1	3	3
1	1	3	3	2	2	4	4	4	4
4	4	4	4	2	2	4	4	3	3
4	4	4	4	2	2	1	1	3	3
4	4	4	4	1	1	4	4	3	3
4	4	4	4	2	2	1	1	3	3
4	4	4	4	2	2	4	4	3	3
4	4	4	4	2	2	1	1	3	3
1	1	3	3	2	2	4	4	4	4
4	4	4	4	2	2	1	1	3	3
4	4	4	4	2	2	1	1	3	3
4	4	4	4	1	1	1	1	3	3
4	4	4	4	1	1	1	1	3	3
4	4	4	4	2	2	4	4	3	3

For Review Only

4	4	4	4	2	2	1	1	3	3
4	4	4	4	1	1	1	1	3	3
1	1	3	3	2	2	4	4	4	4
4	4	4	4	1	1	1	1	3	3
4	4	4	4	1	1	1	1	3	3
4	4	4	4	2	2	1	1	3	3
4	4	4	4	2	2	1	1	3	3
4	4	4	4	2	2	4	4	4	4
4	4	3	3	2	2	1	1	4	4
1	1	3	3	2	2	4	4	4	4
4	4	3	3	2	2	4	4	4	4
4	4	4	4	1	1	1	1	3	3
1	1	3	3	2	2	4	4	4	4
4	4	4	4	1	1	4	4	3	3
4	4	4	4	1	1	1	1	3	3
4	4	4	4	2	2	1	1	3	3
4	4	4	4	2	2	1	1	3	3
1	1	3	3	2	2	4	4	4	4
1	1	3	3	2	2	4	4	4	4
1	1	3	3	2	2	4	4	4	4
4	4	4	4	2	2	4	4	4	4
4	4	4	4	2	2	1	1	3	3
4	4	4	4	2	2	1	1	3	3
1	1	3	3	2	2	4	4	4	4
1	1	3	3	2	2	4	4	4	4
4	4	4	4	1	1	1	1	3	3
4	4	4	4	2	2	1	1	3	3
4	4	4	4	1	1	1	1	3	3
4	4	4	4	1	1	1	1	3	3
1	1	3	3	1	1	1	1	3	3
1	1	3	3	2	2	4	4	4	4
1	1	3	3	2	2	4	4	4	4
1	1	3	3	2	2	4	4	4	4
4	4	4	4	1	1	1	1	3	3
4	4	4	4	2	2	4	4	4	4
4	4	3	3	2	2	4	4	4	4
4	4	3	3	2	2	4	4	4	4
4	4	4	4	1	1	1	1	3	3
4	4	4	4	2	2	4	4	4	4
4	4	4	4	2	2	1	1	3	3
1	1	3	3	2	2	4	4	4	4
4	4	4	4	2	2	1	1	3	3
4	4	4	4	2	2	1	1	3	3
4	4	4	4	2	2	1	1	3	3
4	4	4	4	1	1	1	1	4	4
4	4	4	4	2	2	4	4	4	4
4	4	4	4	2	2	1	1	3	3
4	4	4	4	2	2	1	1	3	3
4	4	4	4	1	1	1	1	3	3
4	4	4	4	2	2	1	1	3	3
4	4	3	3	2	2	4	4	4	4
4	4	4	4	1	1	1	1	3	3
4	4	4	4	2	2	1	1	3	3
1	1	3	3	2	2	4	4	4	4
4	4	4	4	2	2	1	1	3	3

For Review Only

1	1	3	3	1	1	1	1	3	3
4	4	4	4	2	2	1	1	3	3
1	1	3	3	2	2	4	4	4	4
4	4	4	4	2	2	4	4	4	4
4	4	4	4	2	2	1	1	3	3
4	4	4	4	1	1	1	1	3	3
4	4	4	4	2	2	1	1	3	3
4	4	4	4	2	2	1	1	3	3
4	4	4	4	2	2	1	1	3	3
4	4	4	4	2	2	1	1	3	3
4	4	3	3	2	2	4	4	4	4
4	4	4	4	2	2	1	1	3	3
4	4	4	4	2	2	1	1	3	3
4	4	4	4	1	1	1	1	3	3
1	1	3	3	2	2	4	4	4	4
4	4	4	4	2	2	1	1	3	3
1	1	3	3	1	1	1	1	3	3
4	4	4	4	2	2	1	1	3	3
4	4	4	4	2	2	1	1	3	3
4	4	4	4	2	2	1	1	3	3
4	4	4	4	1	1	1	1	3	3
4	4	4	4	1	1	1	1	3	3
4	4	4	4	2	2	4	4	4	4
4	4	4	4	1	1	1	1	3	3
4	4	4	4	2	2	1	1	3	3
1	1	3	3	2	2	4	4	4	4
4	4	4	4	2	2	1	1	3	3
4	4	4	4	2	2	1	1	3	3
1	1	3	3	2	2	4	4	4	4
1	1	3	3	2	2	4	4	4	4
4	4	4	4	2	2	1	1	3	3
4	4	4	4	2	2	1	1	3	3
4	4	4	4	1	1	1	1	3	3
4	4	4	4	2	2	1	1	3	3
4	4	4	4	2	2	1	1	3	3
4	4	4	4	2	2	1	1	3	3
1	1	3	3	1	1	1	1	3	3
1	1	3	3	2	2	4	4	4	4
4	4	4	4	1	1	1	1	3	3
4	4	4	4	2	2	1	1	3	3
4	4	4	4	2	2	4	4	4	4
4	4	4	4	1	1	4	4	4	4
4	4	4	4	1	1	1	1	3	3
1	1	3	3	2	2	4	4	4	4
4	4	4	4	2	2	1	1	3	3
4	4	4	4	2	2	1	1	3	3
4	4	4	4	2	2	4	4	4	4
4	4	4	4	2	2	1	1	3	3
1	1	3	3	2	2	4	4	4	4
4	4	4	4	2	2	1	1	3	3
1	1	3	3	2	2	4	4	4	4
4	4	4	4	2	2	1	1	3	3
1	1	3	3	2	2	4	4	4	4
4	4	4	4	2	2	1	1	3	3
4	4	4	4	2	2	1	1	3	3

For Review Only

1	1	3	3	2	2	4	4	4	4
1	1	3	3	2	2	4	4	4	4

For Review Only

SNP2836	SNP2836	SNP2837	SNP2837	SNP2838	SNP2838	SNP2839	SNP2839	SNP284	SNP284
3	3	3	3	1	1	4	4	2	2
3	3	3	3	1	1	3	3	2	2
2	2	3	3	2	2	4	4	2	2
3	3	4	4	2	2	3	3	2	2
2	2	3	3	1	1	4	4	3	3
2	2	4	4	2	2	4	4	2	2
3	3	3	3	1	1	4	4	2	2
3	3	3	3	1	1	3	3	2	2
2	2	3	3	1	1	4	4	2	2
3	3	3	3	1	1	4	4	3	3
3	3	3	3	1	1	4	4	2	2
2	2	3	3	2	2	3	3	2	2
2	2	3	3	2	2	4	4	2	2
3	3	3	3	1	1	3	3	2	2
2	2	3	3	2	2	4	4	2	2
3	3	3	3	2	2	4	4	3	3
2	2	4	4	2	2	4	4	3	3
3	3	4	4	2	2	4	4	2	2
2	2	4	4	2	2	3	3	2	2
3	3	3	3	1	1	3	3	3	3
2	2	4	4	2	2	3	3	3	3
2	2	4	4	2	2	4	4	3	3
3	3	3	3	1	1	3	3	2	2
3	3	3	3	1	1	4	4	2	2
2	2	4	4	2	2	3	3	2	2
3	3	3	3	1	1	4	4	2	2
3	3	4	4	2	2	3	3	2	2
2	2	3	3	1	1	3	3	2	2
3	3	4	4	2	2	4	4	3	3
3	3	3	3	1	1	4	4	3	3
2	2	3	3	2	2	3	3	2	2
3	3	4	4	2	2	4	4	2	2
3	3	4	4	2	2	4	4	2	2
3	3	3	3	1	1	3	3	3	3
2	2	3	3	1	1	4	4	2	2
3	3	3	3	1	1	3	3	2	2
3	3	3	3	1	1	4	4	2	2
3	3	4	4	1	1	4	4	2	2
3	3	3	3	1	1	3	3	3	3
3	3	3	3	1	1	4	4	2	2
3	3	3	3	1	1	3	3	3	3
3	3	4	4	2	2	4	4	3	3
3	3	3	3	1	1	4	4	3	3
2	2	4	4	2	2	4	4	2	2
3	3	3	3	1	1	4	4	2	2
3	3	4	4	2	2	4	4	2	2
2	2	3	3	2	2	4	4	2	2
3	3	3	3	1	1	4	4	2	2
3	3	3	3	1	1	4	4	3	3
2	2	4	4	2	2	4	4	2	2
3	3	4	4	2	2	4	4	2	2
3	3	3	3	1	1	3	3	2	2

2	2	4	4	2	2	4	4	2	2
3	3	4	4	2	2	4	4	2	2
3	3	4	4	2	2	4	4	2	2
2	2	4	4	2	2	4	4	2	2
2	2	4	4	2	2	3	3	2	2
3	3	4	4	2	2	3	3	2	2
3	3	4	4	2	2	4	4	2	2
3	3	3	3	1	1	3	3	3	3
3	3	3	3	1	1	4	4	2	2
2	2	3	3	1	1	3	3	2	2
2	2	3	3	2	2	3	3	2	2
2	2	3	3	2	2	3	3	2	2
2	2	4	4	2	2	4	4	2	2
3	3	4	4	2	2	4	4	3	3
2	2	4	4	1	1	3	3	2	2
3	3	3	3	1	1	4	4	2	2
2	2	3	3	1	1	3	3	2	2
3	3	4	4	2	2	4	4	2	2
2	2	3	3	2	2	4	4	2	2
3	3	3	3	1	1	4	4	2	2
2	2	3	3	2	2	3	3	2	2
3	3	3	3	1	1	4	4	3	3
3	3	4	4	2	2	4	4	3	3
3	3	4	4	2	2	3	3	3	3
3	3	3	3	1	1	4	4	2	2
2	2	3	3	1	1	3	3	2	2
3	3	3	3	1	1	4	4	2	2
3	3	3	3	1	1	3	3	3	3
3	3	3	3	1	1	4	4	2	2
2	2	4	4	2	2	3	3	2	2
2	2	4	4	2	2	4	4	2	2
3	3	3	3	1	1	3	3	3	3
3	3	3	3	1	1	3	3	3	3
2	2	4	4	2	2	4	4	2	2
2	2	4	4	2	2	4	4	2	2
3	3	3	3	1	1	4	4	2	2
3	3	3	3	1	1	4	4	2	2
3	3	3	3	1	1	4	4	3	3
3	3	4	4	2	2	4	4	2	2
3	3	3	3	1	1	4	4	2	2
2	2	4	4	2	2	4	4	2	2
2	2	4	4	2	2	3	3	2	2
3	3	4	4	2	2	4	4	2	2
2	2	3	3	2	2	3	3	2	2
3	3	3	3	1	1	3	3	2	2
3	3	4	4	2	2	3	3	2	2
3	3	3	3	1	1	4	4	3	3
3	3	3	3	1	1	3	3	2	2
3	3	4	4	2	2	4	4	2	2
3	3	3	3	1	1	3	3	2	2
3	3	3	3	1	1	3	3	2	2
3	3	3	3	1	1	4	4	3	3
3	3	3	3	1	1	3	3	3	3
3	3	4	4	1	1	3	3	2	2
2	2	3	3	2	2	3	3	2	2

For Review Only

3	3	3	3	2	2	4	4	2	2
3	3	4	4	2	2	3	3	2	2
3	3	3	3	1	1	4	4	2	2
3	3	3	3	1	1	4	4	3	3
3	3	4	4	2	2	4	4	2	2
2	2	3	3	1	1	4	4	2	2
2	2	3	3	2	2	4	4	2	2
2	2	4	4	2	2	3	3	2	2
3	3	3	3	1	1	3	3	3	3
3	3	3	3	1	1	3	3	3	3
2	2	4	4	2	2	3	3	2	2
3	3	4	4	1	1	4	4	2	2
3	3	3	3	1	1	4	4	2	2
2	2	3	3	1	1	3	3	2	2
3	3	4	4	2	2	3	3	2	2
2	2	3	3	2	2	4	4	2	2
3	3	3	3	1	1	3	3	2	2
2	2	3	3	1	1	3	3	2	2
3	3	3	3	1	1	4	4	3	3
2	2	3	3	2	2	4	4	2	2
2	2	3	3	1	1	3	3	2	2
2	2	4	4	2	2	4	4	2	2
2	2	3	3	2	2	3	3	2	2
3	3	3	3	1	1	3	3	3	3
3	3	3	3	1	1	4	4	2	2
3	3	4	4	2	2	4	4	2	2
2	2	3	3	2	2	4	4	2	2
2	2	3	3	1	1	3	3	2	2
2	2	4	4	2	2	3	3	2	2
3	3	4	4	2	2	3	3	2	2
3	3	3	3	1	1	3	3	3	3
2	2	3	3	1	1	3	3	2	2
2	2	4	4	2	2	4	4	2	2
3	3	4	4	2	2	4	4	2	2
3	3	3	3	1	1	3	3	3	3
2	2	3	3	2	2	3	3	2	2
3	3	3	3	1	1	4	4	3	3
2	2	3	3	2	2	3	3	2	2
3	3	3	3	1	1	3	3	2	2
3	3	3	3	1	1	4	4	3	3
3	3	3	3	1	1	4	4	2	2
3	3	3	3	1	1	4	4	3	3
2	2	3	3	2	2	3	3	2	2
3	3	3	3	1	1	3	3	2	2
3	3	3	3	1	1	3	3	2	2
3	3	3	3	1	1	4	4	2	2
3	3	4	4	2	2	4	4	2	2
2	2	4	4	2	2	3	3	2	2
3	3	3	3	1	1	3	3	2	2
2	2	4	4	2	2	4	4	2	2
3	3	3	3	1	1	4	4	2	2
3	3	3	3	1	1	4	4	3	3
3	3	3	3	1	1	4	4	2	2
3	3	3	3	1	1	3	3	2	2
3	3	3	3	1	1	3	3	2	2

For Review Only

3	3	4	4	2	2	4	4	2	2
3	3	3	3	1	1	4	4	2	2
2	2	3	3	2	2	4	4	3	3
3	3	3	3	1	1	4	4	3	3
3	3	4	4	2	2	3	3	2	2
3	3	3	3	1	1	4	4	3	3
3	3	3	3	1	1	4	4	3	3
2	2	3	3	1	1	3	3	2	2
2	2	3	3	1	1	4	4	2	2
2	2	4	4	2	2	3	3	2	2
2	2	4	4	2	2	4	4	2	2
3	3	3	3	1	1	3	3	2	2
2	2	4	4	2	2	3	3	2	2
3	3	3	3	1	1	3	3	2	2
3	3	3	3	1	1	3	3	3	3
3	3	3	3	1	1	4	4	2	2
3	3	3	3	1	1	3	3	2	2
2	2	3	3	1	1	3	3	2	2
2	2	4	4	2	2	4	4	2	2
2	2	3	3	2	2	4	4	2	2
2	2	3	3	1	1	3	3	2	2
3	3	3	3	1	1	4	4	3	3
3	3	3	3	1	1	4	4	3	3
2	2	3	3	2	2	3	3	2	2
2	2	4	4	2	2	3	3	2	2
3	3	3	3	1	1	3	3	3	3
3	3	4	4	2	2	3	3	2	2
3	3	3	3	1	1	3	3	2	2
3	3	3	3	1	1	4	4	2	2
3	3	4	4	2	2	4	4	2	2
2	2	3	3	1	1	4	4	2	2
2	2	3	3	2	2	3	3	2	2
2	2	3	3	2	2	4	4	2	2
3	3	3	3	1	1	3	3	3	3
2	2	3	3	1	1	4	4	2	2
2	2	3	3	2	2	3	3	2	2
2	2	3	3	2	2	4	4	2	2
3	3	4	4	2	2	4	4	2	2
2	2	4	4	2	2	3	3	2	2
3	3	3	3	1	1	3	3	2	2
2	2	3	3	2	2	4	4	2	2
3	3	4	4	2	2	4	4	2	2
3	3	3	3	1	1	4	4	3	3
3	3	3	3	1	1	4	4	2	2
3	3	3	3	1	1	3	3	2	2
2	2	4	4	2	2	3	3	3	3
3	3	3	3	1	1	4	4	2	2
3	3	3	3	1	1	4	4	3	3
3	3	3	3	1	1	3	3	3	3
3	3	4	4	2	2	4	4	2	2
2	2	4	4	2	2	3	3	2	2
3	3	3	3	1	1	4	4	3	3
3	3	3	3	1	1	4	4	3	3
2	2	3	3	2	2	3	3	2	2
3	3	3	3	1	1	4	4	3	3

For Review Only

3	3	4	4	2	2	4	4	2	2
3	3	3	3	1	1	3	3	2	2
2	2	3	3	2	2	4	4	2	2
2	2	3	3	1	1	4	4	2	2
3	3	3	3	1	1	4	4	2	2
3	3	3	3	1	1	4	4	2	2
3	3	4	4	2	2	4	4	3	3
3	3	3	3	1	1	4	4	3	3
3	3	3	3	1	1	3	3	2	2
3	3	3	3	1	1	3	3	3	3
2	2	3	3	1	1	3	3	2	2
3	3	3	3	1	1	3	3	2	2
3	3	4	4	2	2	4	4	2	2
3	3	4	4	2	2	4	4	2	2
2	2	3	3	2	2	4	4	2	2
3	3	3	3	1	1	4	4	2	2
3	3	4	4	2	2	4	4	3	3
3	3	3	3	1	1	4	4	2	2
3	3	3	3	1	1	4	4	3	3
3	3	4	4	2	2	4	4	2	2
3	3	3	3	1	1	4	4	3	3
3	3	3	3	1	1	4	4	2	2
2	2	4	4	2	2	4	4	2	2
3	3	3	3	1	1	4	4	2	2
3	3	3	3	1	1	3	3	3	3
2	2	3	3	1	1	3	3	2	2
3	3	3	3	1	1	3	3	3	3
3	3	4	4	2	2	3	3	2	2
2	2	4	4	2	2	4	4	2	2
2	2	4	4	2	2	3	3	2	2
3	3	3	3	1	1	4	4	2	2
3	3	4	4	2	2	3	3	2	2
3	3	3	3	1	1	3	3	2	2
2	2	4	4	1	1	3	3	2	2
3	3	3	3	1	1	4	4	3	3
3	3	3	3	1	1	3	3	3	3
2	2	4	4	2	2	4	4	2	2
2	2	3	3	2	2	3	3	2	2
3	3	3	3	1	1	3	3	2	2
3	3	3	3	1	1	4	4	3	3
2	2	4	4	2	2	4	4	2	2
2	2	4	4	2	2	3	3	3	3
3	3	3	3	1	1	4	4	2	2
2	2	4	4	2	2	4	4	2	2
2	2	4	4	2	2	4	4	2	2
3	3	4	4	2	2	4	4	2	2
2	2	4	4	2	2	4	4	2	2
3	3	3	3	1	1	3	3	2	2
2	2	3	3	2	2	3	3	2	2
3	3	3	3	1	1	3	3	3	3
2	2	4	4	2	2	3	3	2	2
3	3	3	3	1	1	4	4	3	3
2	2	3	3	2	2	4	4	2	2
3	3	4	4	2	2	3	3	2	2
3	3	3	3	1	1	4	4	2	2

For Review Only

2	2	4	4	2	2	4	4	2	2
2	2	4	4	2	2	3	3	2	2

For Review Only

SNP2840	SNP2840	SNP2841	SNP2841	SNP2842	SNP2842	SNP2843	SNP2843	SNP2844	SNP2844
4	4	4	4	2	2	2	2	3	3
1	1	2	2	2	2	2	2	3	3
4	4	4	4	4	4	2	2	3	3
1	1	2	2	2	2	3	3	3	3
4	4	4	4	2	2	2	2	3	3
1	1	2	2	2	2	3	3	3	3
4	4	4	4	2	2	2	2	2	2
1	1	2	2	2	2	2	2	3	3
4	4	4	4	2	2	2	2	3	3
4	4	4	4	2	2	2	2	3	3
4	4	4	4	2	2	2	2	3	3
4	4	4	4	4	4	2	2	3	3
4	4	4	4	2	2	3	3	3	3
1	1	2	2	2	2	2	2	2	2
4	4	4	4	2	2	2	2	2	2
4	4	4	4	2	2	2	2	3	3
4	4	4	4	2	2	3	3	3	3
4	4	4	4	2	2	3	3	3	3
1	1	2	2	2	2	2	2	3	3
4	4	4	4	2	2	2	2	3	3
4	4	4	4	2	2	3	3	3	3
4	4	4	4	2	2	3	3	3	3
1	1	2	2	2	2	2	2	3	3
4	4	4	4	2	2	2	2	2	2
1	1	2	2	2	2	3	3	3	3
4	4	4	4	2	2	2	2	2	2
1	1	2	2	2	2	3	3	3	3
1	1	4	4	2	2	2	2	3	3
4	4	4	4	2	2	2	2	3	3
1	1	2	2	2	2	2	2	3	3
1	1	2	2	2	2	3	3	3	3
4	4	4	4	2	2	3	3	3	3
4	4	4	4	2	2	3	3	3	3
1	1	2	2	2	2	3	3	2	2
4	4	4	4	2	2	2	2	3	3
1	1	2	2	2	2	2	2	3	3
4	4	4	4	2	2	2	2	3	3
4	4	4	4	2	2	3	3	3	3
4	4	4	4	2	2	2	2	3	3
4	4	4	4	2	2	2	2	3	3
1	1	2	2	2	2	2	2	3	3
4	4	4	4	2	2	2	2	3	3
4	4	4	4	2	2	2	2	3	3
4	4	4	4	2	2	3	3	3	3
4	4	4	4	2	2	2	2	3	3
4	4	4	4	2	2	3	3	3	3
4	4	4	4	2	2	2	2	3	3
4	4	4	4	2	2	2	2	2	2
4	4	4	4	2	2	2	2	3	3
4	4	4	4	4	4	3	3	3	3
4	4	4	4	2	2	3	3	2	2
1	1	2	2	2	2	2	2	3	3

For Review Only

4	4	4	4	2	2	3	3	3	3
4	4	4	4	2	2	3	3	3	3
1	1	4	4	2	2	3	3	3	3
4	4	4	4	2	2	3	3	3	3
1	1	2	2	2	2	2	2	3	3
1	1	2	2	2	2	3	3	3	3
4	4	4	4	2	2	3	3	3	3
1	1	2	2	2	2	2	2	3	3
4	4	4	4	2	2	3	3	3	3
1	1	4	4	2	2	2	2	3	3
1	1	2	2	2	2	2	2	3	3
1	1	2	2	2	2	2	2	3	3
4	4	4	4	2	2	3	3	3	3
4	4	4	4	2	2	2	2	3	3
1	1	4	4	2	2	2	2	3	3
4	4	4	4	2	2	2	2	2	2
1	1	2	2	4	4	2	2	3	3
4	4	4	4	2	2	3	3	3	3
4	4	4	4	2	2	2	2	3	3
4	4	4	4	2	2	2	2	3	3
1	1	2	2	2	2	2	2	3	3
4	4	4	4	2	2	2	2	3	3
4	4	4	4	2	2	2	2	3	3
1	1	2	2	2	2	3	3	3	3
4	4	4	4	2	2	2	2	3	3
1	1	2	2	2	2	2	2	3	3
4	4	4	4	2	2	2	2	3	3
4	4	4	4	2	2	2	2	3	3
4	4	4	4	2	2	2	2	2	2
1	1	2	2	2	2	3	3	3	3
4	4	4	4	2	2	3	3	3	3
1	1	2	2	2	2	2	2	2	2
1	1	2	2	2	2	2	2	3	3
4	4	4	4	2	2	3	3	3	3
4	4	4	4	2	2	3	3	3	3
4	4	4	4	2	2	2	2	3	3
4	4	4	4	2	2	2	2	3	3
4	4	4	4	2	2	3	3	3	3
4	4	4	4	2	2	3	3	3	3
4	4	4	4	2	2	2	2	3	3
4	4	4	4	4	4	3	3	3	3
1	1	2	2	2	2	3	3	3	3
4	4	4	4	2	2	3	3	2	2
1	1	2	2	2	2	2	2	3	3
1	1	4	4	2	2	2	2	3	3
1	1	2	2	2	2	2	2	3	3
4	4	4	4	2	2	2	2	2	2
1	1	2	2	2	2	2	2	3	3
4	4	4	4	2	2	3	3	2	2
1	1	4	4	2	2	2	2	3	3
1	1	2	2	2	2	2	2	3	3
4	4	4	4	2	2	2	2	3	3
1	1	2	2	2	2	2	2	3	3
4	4	4	4	2	2	2	2	3	3
1	1	2	2	2	2	2	2	2	2

For Review Only

4	4	4	4	2	2	2	2	2	2
1	1	2	2	2	2	3	3	3	3
4	4	4	4	2	2	3	3	3	3
4	4	4	4	2	2	2	2	2	2
4	4	4	4	2	2	2	2	3	3
4	4	4	4	2	2	2	2	3	3
4	4	4	4	2	2	2	2	3	3
1	1	2	2	2	2	2	2	3	3
4	4	4	4	2	2	2	2	3	3
1	1	4	4	2	2	2	2	3	3
4	4	2	2	2	2	3	3	3	3
4	4	4	4	2	2	2	2	3	3
4	4	4	4	2	2	2	2	3	3
1	1	2	2	2	2	2	2	3	3
1	1	2	2	4	4	3	3	2	2
4	4	4	4	2	2	3	3	2	2
1	1	4	4	2	2	3	3	3	3
4	4	4	4	2	2	2	2	2	2
4	4	4	4	2	2	2	2	2	2
4	4	4	4	2	2	3	3	3	3
1	1	4	4	2	2	2	2	2	2
4	4	4	4	2	2	3	3	3	3
1	1	2	2	4	4	2	2	3	3
4	4	4	4	2	2	2	2	2	2
4	4	4	4	2	2	2	2	3	3
4	4	4	4	2	2	3	3	3	3
4	4	4	4	2	2	2	2	3	3
1	1	2	2	2	2	2	2	3	3
1	1	2	2	2	2	3	3	3	3
1	1	2	2	2	2	3	3	3	3
1	1	2	2	2	2	2	2	3	3
1	1	4	4	2	2	2	2	3	3
4	4	4	4	4	4	3	3	3	3
4	4	4	4	2	2	3	3	2	2
1	1	2	2	2	2	3	3	2	2
4	4	4	4	2	2	2	2	3	3
4	4	4	4	2	2	2	2	2	2
1	1	2	2	2	2	3	3	3	3
4	4	4	4	2	2	2	2	3	3
1	1	4	4	2	2	2	2	3	3
4	4	4	4	2	2	2	2	3	3
4	4	4	4	2	2	2	2	2	2
1	1	2	2	2	2	2	2	2	2
1	1	4	4	2	2	2	2	3	3
1	1	4	4	2	2	2	2	3	3
4	4	4	4	2	2	2	2	3	3
4	4	4	4	2	2	3	3	2	2
1	1	2	2	2	2	3	3	2	2
4	4	4	4	2	2	2	2	3	3
4	4	4	4	2	2	3	3	2	2
4	4	4	4	2	2	2	2	2	2
4	4	4	4	2	2	2	2	3	3
4	4	4	4	2	2	2	2	2	2
1	1	2	2	2	2	2	2	3	3
1	1	4	4	2	2	2	2	3	3

For Review Only

4	4	4	4	2	2	3	3	3	3
4	4	4	4	2	2	3	3	3	3
4	4	4	4	2	2	2	2	3	3
4	4	4	4	2	2	3	3	2	2
1	1	2	2	2	2	2	2	3	3
4	4	4	4	2	2	2	2	3	3
4	4	4	4	2	2	2	2	2	2
4	4	4	4	2	2	2	2	2	2
4	4	4	4	2	2	2	2	3	3
1	1	2	2	2	2	3	3	3	3
4	4	4	4	2	2	3	3	2	2
4	4	4	4	2	2	2	2	2	2
1	1	2	2	2	2	3	3	2	2
1	1	4	4	2	2	2	2	3	3
1	1	2	2	2	2	2	2	2	2
4	4	4	4	2	2	2	2	3	3
4	4	4	4	2	2	2	2	2	2
1	1	2	2	2	2	2	2	3	3
4	4	4	4	2	2	3	3	3	3
4	4	4	4	2	2	2	2	3	3
4	4	4	4	2	2	2	2	3	3
4	4	4	4	2	2	2	2	3	3
4	4	4	4	2	2	2	2	3	3
1	1	2	2	2	2	2	2	3	3
1	1	4	4	2	2	3	3	3	3
1	1	2	2	2	2	2	2	2	2
1	1	2	2	2	2	3	3	2	2
1	1	2	2	2	2	2	2	2	2
4	4	4	4	2	2	3	3	3	3
4	4	4	4	2	2	3	3	3	3
4	4	4	4	2	2	2	2	3	3
1	1	2	2	2	2	3	3	3	3
4	4	4	4	2	2	2	2	3	3
1	1	2	2	2	2	2	2	2	2
4	4	4	4	4	4	2	2	3	3
1	1	2	2	2	2	2	2	3	3
4	4	4	4	2	2	3	3	3	3
4	4	4	4	2	2	3	3	3	3
1	1	2	2	2	2	3	3	2	2
1	1	2	2	2	2	2	2	2	2
4	4	4	4	4	4	2	2	3	3
4	4	4	4	2	2	3	3	2	2
4	4	4	4	2	2	2	2	3	3
4	4	4	4	2	2	2	2	3	3
1	1	4	4	2	2	2	2	3	3
1	1	4	4	2	2	3	3	3	3
4	4	4	4	2	2	2	2	3	3
4	4	4	4	2	2	2	2	3	3
1	1	2	2	2	2	2	2	2	2
4	4	4	4	2	2	3	3	3	3
1	1	2	2	2	2	3	3	3	3
4	4	4	4	2	2	2	2	3	3
4	4	4	4	2	2	2	2	3	3
1	1	2	2	2	2	2	2	3	3
4	4	4	4	2	2	2	2	3	3

For Review Only

4	4	4	4	2	2	3	3	2	2
1	1	2	2	2	2	2	2	3	3
4	4	4	4	4	4	2	2	3	3
4	4	4	4	2	2	2	2	3	3
4	4	4	4	2	2	2	2	3	3
4	4	4	4	2	2	2	2	2	2
4	4	4	4	2	2	3	3	3	3
4	4	4	4	2	2	2	2	3	3
1	1	4	4	2	2	2	2	2	2
1	1	2	2	2	2	2	2	3	3
4	4	4	4	2	2	2	2	3	3
1	1	2	2	2	2	2	2	3	3
4	4	4	4	2	2	3	3	3	3
4	4	4	4	2	2	3	3	2	2
4	4	4	4	4	4	2	2	3	3
4	4	4	4	2	2	2	2	3	3
4	4	4	4	2	2	3	3	3	3
4	4	4	4	2	2	2	2	3	3
4	4	4	4	2	2	2	2	3	3
4	4	4	4	2	2	3	3	3	3
4	4	4	4	2	2	2	2	3	3
4	4	4	4	2	2	2	2	2	2
4	4	4	4	2	2	3	3	3	3
4	4	4	4	2	2	3	3	2	2
1	1	2	2	2	2	2	2	3	3
4	4	4	4	2	2	2	2	3	3
1	1	2	2	2	2	2	2	3	3
1	1	2	2	2	2	3	3	3	3
4	4	4	4	2	2	3	3	3	3
1	1	2	2	2	2	3	3	3	3
4	4	4	4	2	2	2	2	3	3
1	1	4	4	2	2	3	3	2	2
1	1	2	2	2	2	2	2	3	3
1	1	2	2	2	2	3	3	2	2
4	4	4	4	2	2	2	2	3	3
4	4	4	4	2	2	2	2	3	3
4	4	4	4	2	2	3	3	3	3
1	1	2	2	2	2	2	2	3	3
1	1	2	2	2	2	2	2	3	3
4	4	4	4	2	2	2	2	3	3
4	4	4	4	4	4	3	3	3	3
1	1	2	2	4	4	3	3	3	3
4	4	4	4	2	2	3	3	2	2
4	4	4	4	2	2	3	3	3	3
4	4	4	4	4	4	3	3	3	3
4	4	4	4	2	2	3	3	3	3
4	4	4	4	2	2	3	3	3	3
1	1	2	2	2	2	2	2	3	3
1	1	4	4	2	2	2	2	3	3
1	1	2	2	2	2	2	2	3	3
1	1	2	2	4	4	3	3	3	3
4	4	4	4	2	2	2	2	3	3
4	4	4	4	4	4	2	2	3	3
1	1	2	2	2	2	3	3	2	2
4	4	4	4	2	2	2	2	3	3

For Review Only

4	4	4	4	2	2	3	3	3	3
1	1	2	2	2	2	3	3	3	3

For Review Only

SNP2845	SNP2845	SNP2846	SNP2846	SNP2847	SNP2847	SNP2848	SNP2848	SNP2849	SNP2849
3	3	2	2	3	3	1	1	1	1
3	3	2	2	2	2	1	1	1	1
2	2	3	3	3	3	4	4	1	1
2	2	3	3	3	3	4	4	1	1
2	2	3	3	3	3	4	4	1	1
2	2	3	3	3	3	4	4	4	4
3	3	2	2	3	3	1	1	1	1
3	3	2	2	3	3	1	1	1	1
3	3	2	2	3	3	1	1	1	1
3	3	2	2	3	3	1	1	1	1
3	3	2	2	2	2	1	1	1	1
2	2	3	3	3	3	4	4	1	1
2	2	3	3	3	3	4	4	4	4
3	3	2	2	2	2	1	1	1	1
2	2	3	3	3	3	4	4	1	1
2	2	3	3	3	3	4	4	1	1
2	2	3	3	3	3	4	4	4	4
2	2	3	3	3	3	4	4	4	4
3	3	2	2	2	2	1	1	1	1
3	3	2	2	2	2	1	1	1	1
2	2	3	3	3	3	4	4	4	4
2	2	3	3	3	3	4	4	4	4
3	3	2	2	3	3	1	1	1	1
3	3	2	2	3	3	1	1	1	1
2	2	3	3	2	2	4	4	4	4
3	3	2	2	3	3	1	1	1	1
2	2	3	3	2	2	4	4	4	4
2	2	3	3	3	3	4	4	1	1
3	3	2	2	3	3	1	1	1	1
3	3	2	2	2	2	1	1	1	1
2	2	3	3	2	2	4	4	4	4
2	2	3	3	3	3	4	4	4	4
2	2	3	3	3	3	4	4	4	4
3	3	2	2	2	2	1	1	1	1
2	2	2	2	3	3	4	4	1	1
3	3	2	2	2	2	1	1	1	1
3	3	2	2	3	3	1	1	1	1
2	2	3	3	3	3	4	4	1	1
3	3	2	2	2	2	1	1	1	1
3	3	2	2	3	3	1	1	1	1
3	3	2	2	2	2	1	1	1	1
3	3	2	2	3	3	1	1	1	1
3	3	2	2	2	2	1	1	1	1
2	2	3	3	3	3	4	4	4	4
3	3	2	2	2	2	1	1	1	1
2	2	3	3	3	3	4	4	4	4
2	2	3	3	3	3	4	4	1	1
3	3	2	2	3	3	1	1	1	1
3	3	3	3	3	3	1	1	1	1
2	2	3	3	3	3	4	4	4	4
2	2	3	3	3	3	4	4	4	4
3	3	2	2	3	3	1	1	1	1

For Review Only

2	2	3	3	3	3	4	4	4	4
2	2	3	3	3	3	4	4	4	4
2	2	3	3	3	3	4	4	4	4
2	2	3	3	3	3	4	4	4	4
3	3	2	2	2	2	1	1	1	1
2	2	3	3	2	2	4	4	4	4
2	2	3	3	3	3	4	4	4	4
3	3	2	2	2	2	1	1	1	1
2	2	3	3	3	3	4	4	4	4
2	2	3	3	3	3	4	4	1	1
2	2	3	3	2	2	4	4	1	1
2	2	3	3	2	2	4	4	1	1
2	2	3	3	3	3	4	4	4	4
3	3	2	2	3	3	1	1	1	1
2	2	3	3	3	3	4	4	1	1
2	2	2	2	3	3	1	1	1	1
2	2	2	2	3	3	4	4	1	1
2	2	3	3	3	3	4	4	4	4
2	2	3	3	3	3	4	4	1	1
3	3	2	2	3	3	1	1	1	1
2	2	3	3	2	2	4	4	1	1
3	3	2	2	3	3	1	1	1	1
3	3	3	3	3	3	1	1	1	1
2	2	3	3	2	2	4	4	4	4
3	3	2	2	2	2	1	1	1	1
2	2	3	3	2	2	4	4	1	1
3	3	2	2	3	3	1	1	1	1
3	3	2	2	3	3	1	1	1	1
3	3	2	2	3	3	1	1	1	1
2	2	3	3	3	3	4	4	4	4
2	2	3	3	3	3	4	4	4	4
3	3	2	2	2	2	1	1	1	1
3	3	2	2	2	2	1	1	1	1
2	2	3	3	3	3	4	4	4	4
2	2	3	3	3	3	4	4	4	4
3	3	2	2	2	2	1	1	1	1
3	3	2	2	2	2	1	1	1	1
2	2	3	3	3	3	4	4	4	4
2	2	3	3	3	3	4	4	4	4
3	3	2	2	2	2	1	1	1	1
2	2	3	3	3	3	4	4	4	4
2	2	3	3	2	2	4	4	4	4
2	2	3	3	3	3	4	4	4	4
2	2	3	3	2	2	4	4	1	1
3	3	2	2	3	3	1	1	1	1
3	3	2	2	2	2	1	1	1	1
3	3	2	2	3	3	1	1	1	1
3	3	2	2	2	2	1	1	1	1
2	2	3	3	3	3	4	4	4	4
3	3	2	2	3	3	1	1	1	1
3	3	3	3	2	2	1	1	1	1
3	3	2	2	3	3	1	1	1	1
3	3	2	2	2	2	1	1	1	1
3	3	3	3	2	2	4	4	1	1
2	2	3	3	2	2	4	4	1	1

For Review Only

2	2	3	3	3	3	4	4	1	1
2	2	3	3	2	2	4	4	1	1
2	2	3	3	3	3	4	4	4	4
3	3	2	2	3	3	1	1	1	1
3	3	2	2	3	3	1	1	1	1
3	3	2	2	3	3	1	1	1	1
2	2	3	3	3	3	4	4	1	1
2	2	3	3	2	2	4	4	4	4
3	3	2	2	3	3	1	1	1	1
3	3	2	2	2	2	1	1	1	1
2	2	3	3	2	2	4	4	4	4
2	2	3	3	3	3	4	4	1	1
3	3	2	2	3	3	1	1	1	1
2	2	3	3	3	3	4	4	1	1
2	2	3	3	2	2	1	1	1	1
2	2	3	3	3	3	4	4	4	4
2	2	3	3	2	2	4	4	4	4
2	2	3	3	3	3	4	4	1	1
3	3	2	2	2	2	1	1	1	1
2	2	3	3	3	3	4	4	4	4
2	2	3	3	3	3	4	4	1	1
2	2	3	3	2	2	4	4	4	4
2	2	3	3	2	2	4	4	1	1
3	3	2	2	3	3	1	1	1	1
3	3	2	2	3	3	1	1	1	1
3	3	2	2	3	3	1	1	1	1
2	2	3	3	3	3	4	4	1	1
3	3	2	2	2	2	1	1	1	1
2	2	3	3	3	3	4	4	4	4
2	2	3	3	3	3	4	4	4	4
3	3	2	2	3	3	1	1	1	1
2	2	3	3	3	3	4	4	1	1
2	2	3	3	3	3	4	4	4	4
2	2	3	3	3	3	4	4	1	1
3	3	2	2	2	2	1	1	1	1
2	2	3	3	3	3	4	4	1	1
3	3	2	2	3	3	1	1	1	1
3	3	2	2	3	3	1	1	1	1
3	3	2	2	3	3	1	1	1	1
3	3	2	2	2	2	1	1	1	1
3	3	2	2	3	3	1	1	1	1
3	3	2	2	3	3	1	1	1	1
2	2	3	3	2	2	4	4	1	1
3	3	3	3	2	2	1	1	1	1
3	3	2	2	2	2	1	1	1	1
3	3	2	2	3	3	1	1	1	1
2	2	3	3	3	3	4	4	4	4
2	2	3	3	2	2	4	4	4	4
2	2	3	3	3	3	1	1	1	1
2	2	3	3	3	3	4	4	4	4
2	2	3	3	3	3	4	4	1	1
3	3	2	2	3	3	1	1	1	1
3	3	2	2	3	3	4	4	1	1
3	3	2	2	2	2	1	1	1	1
2	2	3	3	3	3	4	4	1	1

For Review Only

2	2	3	3	3	3	4	4	4	4
2	2	3	3	3	3	4	4	4	4
2	2	3	3	3	3	4	4	1	1
2	2	3	3	3	3	4	4	4	4
3	3	2	2	2	2	1	1	1	1
2	2	3	3	3	3	4	4	1	1
2	2	3	3	3	3	4	4	1	1
2	2	3	3	3	3	4	4	1	1
2	2	3	3	3	3	4	4	1	1
2	2	3	3	3	3	4	4	4	4
2	2	3	3	3	3	4	4	1	1
3	3	2	2	3	3	4	4	1	1
2	2	3	3	3	3	4	4	4	4
3	3	2	2	3	3	1	1	1	1
3	3	2	2	2	2	1	1	1	1
3	3	2	2	2	2	1	1	1	1
3	3	2	2	3	3	1	1	1	1
2	2	3	3	2	2	4	4	1	1
2	2	3	3	3	3	4	4	4	4
2	2	3	3	3	3	4	4	1	1
2	2	3	3	3	3	1	1	1	1
3	3	2	2	2	2	1	1	1	1
3	3	2	2	3	3	1	1	1	1
2	2	3	3	2	2	4	4	1	1
2	2	3	3	2	2	4	4	4	4
3	3	2	2	2	2	1	1	1	1
2	2	3	3	3	3	4	4	4	4
2	2	3	3	2	2	4	4	1	1
2	2	3	3	3	3	4	4	4	4
2	2	3	3	3	3	4	4	4	4
3	3	2	2	3	3	1	1	1	1
2	2	3	3	3	3	4	4	1	1
2	2	3	3	3	3	4	4	1	1
3	3	2	2	2	2	1	1	1	1
2	2	3	3	3	3	4	4	1	1
2	2	3	3	2	2	4	4	1	1
2	2	3	3	3	3	4	4	1	1
2	2	3	3	3	3	4	4	4	4
2	2	3	3	2	2	4	4	4	4
3	3	2	2	2	2	1	1	1	1
2	2	3	3	3	3	4	4	1	1
2	2	3	3	3	3	4	4	4	4
3	3	2	2	3	3	1	1	1	1
3	3	2	2	3	3	1	1	1	1
3	3	2	2	3	3	1	1	1	1
2	2	3	3	3	3	4	4	4	4
3	3	2	2	2	2	1	1	1	1
3	3	2	2	3	3	1	1	1	1
3	3	2	2	2	2	1	1	1	1
2	2	3	3	3	3	4	4	4	4
2	2	3	3	2	2	4	4	4	4
3	3	2	2	3	3	1	1	1	1
3	3	2	2	3	3	1	1	1	1
2	2	3	3	2	2	4	4	1	1
3	3	2	2	3	3	1	1	1	1

For Review Only

2	2	3	3	3	3	4	4	4	4
3	3	2	2	2	2	1	1	1	1
2	2	3	3	3	3	4	4	1	1
2	2	3	3	3	3	4	4	1	1
3	3	2	2	3	3	1	1	1	1
2	2	3	3	3	3	4	4	4	4
2	2	3	3	3	3	4	4	4	4
3	3	2	2	3	3	1	1	1	1
3	3	2	2	3	3	1	1	1	1
3	3	2	2	2	2	1	1	1	1
2	2	3	3	3	3	4	4	1	1
3	3	2	2	2	2	1	1	1	1
2	2	3	3	3	3	4	4	4	4
2	2	3	3	3	3	4	4	4	4
2	2	3	3	3	3	4	4	1	1
3	3	2	2	2	2	4	4	4	4
3	3	2	2	3	3	1	1	1	1
3	3	2	2	3	3	1	1	1	1
3	3	2	2	3	3	1	1	1	1
2	2	3	3	3	3	4	4	4	4
2	2	3	3	3	3	4	4	4	4
3	3	2	2	3	3	1	1	1	1
2	2	3	3	3	3	4	4	4	4
2	2	3	3	3	3	4	4	4	4
3	3	2	2	2	2	1	1	1	1
2	2	3	3	3	3	4	4	1	1
3	3	2	2	2	2	1	1	1	1
2	2	3	3	3	3	4	4	4	4
2	2	3	3	3	3	4	4	4	4
2	2	3	3	2	2	4	4	4	4
3	3	2	2	3	3	1	1	1	1
2	2	3	3	3	3	4	4	4	4
3	3	2	2	3	3	1	1	1	1
2	2	3	3	2	2	4	4	4	4
3	3	2	2	3	3	1	1	1	1
3	3	2	2	3	3	1	1	1	1
2	2	3	3	3	3	4	4	4	4
2	2	3	3	2	2	4	4	1	1
3	3	2	2	2	2	1	1	1	1
3	3	2	2	3	3	1	1	1	1
2	2	3	3	3	3	4	4	4	4
2	2	3	3	2	2	4	4	4	4
2	2	3	3	3	3	4	4	4	4
2	2	3	3	3	3	4	4	4	4
2	2	3	3	3	3	4	4	4	4
2	2	3	3	3	3	4	4	4	4
2	2	3	3	3	3	4	4	4	4
3	3	2	2	2	2	1	1	1	1
2	2	3	3	3	3	4	4	1	1
3	3	2	2	2	2	1	1	1	1
2	2	3	3	3	3	4	4	4	4
3	3	2	2	3	3	1	1	1	1
2	2	3	3	3	3	4	4	1	1
2	2	3	3	2	2	4	4	4	4
3	3	2	2	3	3	1	1	1	1

For Review Only

2	2	3	3	2	2	4	4	4	4
2	2	3	3	2	2	4	4	4	4

For Review Only

SNP285	SNP285	SNP2850	SNP2850	SNP2851	SNP2851	SNP2852	SNP2852	SNP2853	SNP2853
2	2	2	2	2	2	4	4	1	1
2	2	2	2	2	2	4	4	1	1
2	2	3	3	4	4	4	4	4	4
2	2	2	2	2	2	4	4	1	1
2	2	2	2	2	2	4	4	1	1
2	2	2	2	2	2	4	4	4	4
1	1	2	2	2	2	4	4	1	1
2	2	2	2	2	2	4	4	1	1
1	1	2	2	2	2	4	4	1	1
2	2	2	2	2	2	4	4	1	1
1	1	2	2	2	2	1	1	1	1
2	2	3	3	4	4	4	4	4	4
1	1	2	2	2	2	4	4	1	1
2	2	2	2	2	2	4	4	1	1
1	1	2	2	4	4	4	4	1	1
2	2	3	3	2	2	4	4	1	1
1	1	2	2	2	2	4	4	1	1
2	2	2	2	2	2	4	4	1	1
2	2	2	2	2	2	4	4	1	1
2	2	2	2	2	2	4	4	1	1
1	1	2	2	2	2	4	4	1	1
1	1	2	2	2	2	4	4	1	1
2	2	2	2	2	2	1	1	1	1
2	2	2	2	2	2	4	4	1	1
1	1	2	2	2	2	4	4	1	1
1	1	2	2	2	2	4	4	1	1
2	2	2	2	2	2	4	4	4	4
1	1	2	2	2	2	4	4	1	1
1	1	2	2	2	2	4	4	1	1
2	2	2	2	2	2	4	4	1	1
2	2	2	2	2	2	4	4	1	1
2	2	2	2	2	2	4	4	1	1
1	1	2	2	2	2	4	4	1	1
2	2	2	2	2	2	4	4	1	1
2	2	2	2	4	4	4	4	1	1
2	2	2	2	2	2	4	4	4	4
2	2	2	2	2	2	4	4	1	1
2	2	2	2	2	2	4	4	4	4
2	2	2	2	2	2	1	1	1	1
2	2	2	2	2	2	1	1	1	1
2	2	2	2	2	2	4	4	1	1
2	2	2	2	2	2	4	4	1	1
2	2	2	2	2	2	4	4	1	1
1	1	2	2	2	2	4	4	1	1
2	2	2	2	2	2	4	4	1	1
2	2	2	2	2	2	4	4	4	4
1	1	3	3	4	4	4	4	1	1
1	1	2	2	2	2	4	4	1	1
2	2	2	2	2	2	4	4	1	1
2	2	2	2	2	2	4	4	1	1
1	1	2	2	2	2	4	4	1	1
1	1	2	2	2	2	1	1	4	4

For Review Only

1	1	2	2	2	2	4	4	1	1
1	1	2	2	2	2	4	4	1	1
2	2	2	2	2	2	4	4	4	4
1	1	2	2	2	2	4	4	1	1
2	2	2	2	2	2	4	4	1	1
2	2	2	2	2	2	4	4	1	1
2	2	2	2	2	2	4	4	4	4
2	2	2	2	2	2	4	4	1	1
2	2	2	2	2	2	4	4	1	1
2	2	2	2	2	2	4	4	1	1
2	2	3	3	4	4	4	4	1	1
2	2	3	3	4	4	4	4	1	1
1	1	2	2	2	2	4	4	1	1
1	1	2	2	2	2	4	4	1	1
2	2	2	2	2	2	4	4	1	1
2	2	2	2	2	2	4	4	1	1
1	1	2	2	2	2	4	4	1	1
2	2	2	2	2	2	4	4	1	1
2	2	3	3	4	4	4	4	1	1
2	2	2	2	2	2	4	4	1	1
2	2	3	3	4	4	4	4	1	1
2	2	2	2	2	2	1	1	1	1
1	1	2	2	2	2	4	4	1	1
2	2	2	2	2	2	4	4	1	1
2	2	2	2	2	2	4	4	1	1
2	2	3	3	4	4	4	4	1	1
1	1	2	2	2	2	4	4	1	1
2	2	2	2	2	2	1	1	1	1
1	1	2	2	2	2	4	4	1	1
2	2	2	2	2	2	4	4	1	1
1	1	2	2	2	2	4	4	1	1
2	2	2	2	2	2	4	4	1	1
2	2	2	2	2	2	1	1	1	1
1	1	2	2	2	2	4	4	1	1
2	2	2	2	2	2	4	4	1	1
2	2	2	2	2	2	4	4	1	1
2	2	2	2	2	2	4	4	1	1
1	1	2	2	2	2	4	4	1	1
2	2	2	2	2	2	4	4	1	1
2	2	2	2	2	2	4	4	1	1
2	2	2	2	2	2	4	4	1	1
2	2	2	2	2	2	4	4	1	1
1	1	2	2	2	2	4	4	1	1
2	2	3	3	4	4	4	4	1	1
1	1	2	2	2	2	4	4	1	1
2	2	2	2	2	2	4	4	1	1
2	2	2	2	2	2	4	4	1	1
1	1	2	2	2	2	4	4	4	4
1	1	2	2	2	2	4	4	1	1
2	2	2	2	2	2	1	1	1	1
2	2	2	2	2	2	4	4	1	1
2	2	2	2	2	2	1	1	1	1
2	2	2	2	2	2	4	4	1	1
2	2	2	2	2	2	4	4	1	1
1	1	3	3	4	4	4	4	1	1

For Review Only

2	2	2	2	2	2	4	4	1	1
2	2	2	2	2	2	4	4	1	1
2	2	2	2	2	2	4	4	1	1
2	2	2	2	2	2	4	4	1	1
2	2	2	2	2	2	4	4	1	1
1	1	2	2	2	2	4	4	1	1
2	2	3	3	4	4	4	4	1	1
2	2	2	2	2	2	4	4	1	1
1	1	2	2	2	2	4	4	1	1
2	2	2	2	2	2	4	4	1	1
2	2	2	2	2	2	4	4	1	1
1	1	2	2	2	2	4	4	1	1
1	1	2	2	2	2	4	4	4	4
2	2	2	2	4	4	4	4	1	1
2	2	2	2	2	2	4	4	1	1
1	1	2	2	2	2	4	4	1	1
1	1	2	2	2	2	4	4	1	1
1	1	3	3	4	4	4	4	1	1
2	2	2	2	2	2	4	4	1	1
2	2	2	2	2	2	4	4	1	1
1	1	2	2	2	2	4	4	1	1
2	2	2	2	2	2	4	4	1	1
1	1	2	2	4	4	4	4	4	4
2	2	2	2	2	2	4	4	1	1
2	2	2	2	2	2	4	4	1	1
2	2	2	2	2	2	4	4	1	1
2	2	2	2	2	2	4	4	1	1
2	2	2	2	2	2	4	4	1	1
2	2	2	2	2	2	4	4	4	4
2	2	2	2	2	2	4	4	1	1
2	2	2	2	2	2	1	1	1	1
1	1	2	2	2	2	4	4	1	1
2	2	2	2	2	2	4	4	1	1
1	1	2	2	2	2	4	4	1	1
2	2	2	2	2	2	4	4	1	1
2	2	2	2	2	2	4	4	1	1
2	2	2	2	2	2	4	4	1	1
2	2	2	2	2	2	4	4	4	4
1	1	2	2	2	2	1	1	1	1
2	2	2	2	2	2	1	1	1	1
2	2	2	2	2	2	1	1	1	1
2	2	2	2	2	2	4	4	1	1
1	1	3	3	4	4	4	4	1	1
2	2	2	2	2	2	4	4	1	1
1	1	2	2	2	2	4	4	1	1
1	1	2	2	2	2	4	4	1	1
1	1	2	2	2	2	4	4	1	1
1	1	2	2	2	2	4	4	1	1
2	2	2	2	2	2	4	4	1	1
1	1	2	2	2	2	4	4	1	1
1	1	2	2	2	2	4	4	1	1
2	2	2	2	2	2	4	4	1	1
2	2	2	2	2	2	4	4	1	1
1	1	2	2	2	2	4	4	1	1
2	2	2	2	2	2	4	4	1	1

For Review Only

1	1	2	2	2	2	4	4	1	1
2	2	2	2	2	2	4	4	1	1
1	1	3	3	4	4	4	4	1	1
2	2	2	2	2	2	4	4	1	1
1	1	2	2	2	2	4	4	1	1
2	2	2	2	2	2	4	4	1	1
1	1	2	2	2	2	4	4	1	1
2	2	2	2	2	2	4	4	1	1
1	1	2	2	2	2	4	4	1	1
2	2	2	2	2	2	4	4	1	1
1	1	2	2	2	2	4	4	1	1
2	2	2	2	2	2	4	4	1	1
1	1	2	2	2	2	4	4	1	1
2	2	2	2	2	2	4	4	1	1
2	2	2	2	2	2	4	4	1	1
1	1	2	2	2	2	1	1	1	1
2	2	2	2	2	2	4	4	1	1
2	2	3	3	4	4	4	4	4	4
1	1	2	2	2	2	4	4	1	1
2	2	3	3	4	4	4	4	1	1
2	2	2	2	2	2	4	4	1	1
2	2	2	2	2	2	4	4	1	1
2	2	2	2	2	2	1	1	1	1
2	2	3	3	4	4	4	4	1	1
2	2	2	2	2	2	4	4	1	1
2	2	2	2	2	2	4	4	1	1
1	1	2	2	2	2	4	4	1	1
1	1	2	2	2	2	4	4	1	1
2	2	2	2	2	2	4	4	1	1
2	2	2	2	2	2	4	4	1	1
1	1	2	2	2	2	4	4	1	1
1	1	3	3	4	4	4	4	1	1
2	2	3	3	4	4	4	4	4	4
2	2	2	2	2	2	4	4	1	1
1	1	2	2	2	2	4	4	1	1
2	2	3	3	4	4	4	4	1	1
2	2	2	2	4	4	4	4	1	1
2	2	2	2	2	2	4	4	1	1
1	1	2	2	2	2	4	4	1	1
1	1	2	2	2	2	4	4	1	1
2	2	3	3	4	4	4	4	4	4
1	1	2	2	2	2	4	4	1	1
2	2	2	2	2	2	1	1	1	1
2	2	2	2	2	2	1	1	4	4
2	2	2	2	2	2	4	4	1	1
2	2	2	2	2	2	4	4	1	1
1	1	2	2	2	2	1	1	1	1
1	1	2	2	2	2	1	1	1	1
2	2	2	2	2	2	4	4	1	1
2	2	2	2	2	2	4	4	4	4
1	1	2	2	2	2	4	4	1	1
2	2	2	2	2	2	4	4	1	1
2	2	2	2	2	2	1	1	1	1
2	2	3	3	4	4	4	4	1	1
2	2	2	2	2	2	1	1	1	1

For Review Only

2	2	2	2	2	2	4	4	1	1
2	2	2	2	2	2	4	4	1	1
1	1	3	3	4	4	4	4	4	4
1	1	2	2	2	2	4	4	1	1
2	2	2	2	2	2	4	4	1	1
1	1	2	2	2	2	4	4	1	1
1	1	2	2	2	2	4	4	1	1
2	2	2	2	2	2	4	4	1	1
1	1	2	2	2	2	4	4	1	1
2	2	2	2	2	2	1	1	1	1
1	1	2	2	2	2	4	4	1	1
1	1	2	2	2	2	4	4	1	1
2	2	2	2	2	2	4	4	1	1
1	1	2	2	2	2	4	4	1	1
1	1	3	3	4	4	4	4	4	4
2	2	2	2	2	2	4	4	1	1
2	2	2	2	2	2	4	4	1	1
2	2	2	2	2	2	4	4	1	1
1	1	2	2	2	2	1	1	1	1
2	2	2	2	2	2	4	4	4	4
2	2	2	2	2	2	4	4	1	1
1	1	2	2	2	2	4	4	1	1
2	2	2	2	2	2	4	4	1	1
2	2	2	2	2	2	4	4	1	1
2	2	2	2	2	2	4	4	1	1
2	2	3	3	4	4	4	4	1	1
2	2	2	2	2	2	4	4	1	1
2	2	2	2	2	2	4	4	4	4
2	2	2	2	2	2	4	4	1	1
1	1	2	2	2	2	4	4	4	4
1	1	2	2	2	2	1	1	1	1
1	1	2	2	2	2	4	4	1	1
2	2	2	2	2	2	4	4	1	1
1	1	2	2	2	2	4	4	1	1
2	2	2	2	2	2	1	1	1	1
2	2	2	2	2	2	1	1	1	1
2	2	2	2	2	2	4	4	1	1
2	2	3	3	4	4	4	4	1	1
2	2	2	2	2	2	4	4	4	4
2	2	2	2	2	2	1	1	1	1
1	1	2	2	2	2	4	4	4	4
1	1	2	2	4	4	4	4	1	1
2	2	2	2	2	2	4	4	1	1
1	1	2	2	2	2	4	4	1	1
2	2	2	2	2	2	4	4	1	1
2	2	2	2	2	2	4	4	1	1
1	1	2	2	2	2	4	4	1	1
1	1	2	2	2	2	4	4	4	4
2	2	3	3	4	4	4	4	1	1
2	2	2	2	2	2	4	4	1	1
2	2	2	2	2	2	4	4	1	1
2	2	2	2	2	2	1	1	1	1
2	2	3	3	4	4	4	4	4	4
1	1	2	2	2	2	4	4	1	1
2	2	2	2	2	2	1	1	4	4

For Review Only

1	1	2	2	2	2	4	4	1	1
2	2	2	2	2	2	4	4	4	4

For Review Only

SNP2854	SNP2854	SNP2855	SNP2855	SNP2856	SNP2856	SNP2857	SNP2857	SNP2858	SNP2858
3	3	2	2	1	1	3	3	2	2
3	3	2	2	1	1	3	3	2	2
3	3	2	2	1	1	3	3	2	2
3	3	2	2	1	1	3	3	2	2
3	3	2	2	1	1	3	3	2	2
3	3	2	2	1	1	3	3	2	2
3	3	2	2	1	1	3	3	2	2
3	3	2	2	1	1	3	3	2	2
3	3	2	2	1	1	3	3	2	2
2	2	3	3	1	1	3	3	4	4
3	3	2	2	1	1	3	3	2	2
3	3	2	2	1	1	3	3	2	2
3	3	2	2	1	1	3	3	2	2
3	3	2	2	1	1	3	3	2	2
3	3	2	2	1	1	3	3	2	2
3	3	2	2	1	1	3	3	2	2
2	2	3	3	1	1	3	3	2	2
3	3	2	2	1	1	3	3	2	2
3	3	2	2	1	1	3	3	2	2
2	2	3	3	1	1	3	3	2	2
3	3	2	2	1	1	3	3	2	2
3	3	2	2	1	1	3	3	4	4
3	3	2	2	1	1	3	3	2	2
3	3	2	2	1	1	3	3	4	4
3	3	2	2	1	1	3	3	2	2
3	3	2	2	1	1	3	3	2	2
3	3	2	2	1	1	3	3	2	2
3	3	2	2	1	1	3	3	2	2
3	3	2	2	1	1	3	3	2	2
2	2	3	3	1	1	3	3	4	4
3	3	2	2	1	1	3	3	2	2
3	3	2	2	1	1	3	3	2	2
3	3	2	2	1	1	3	3	2	2
3	3	2	2	1	1	3	3	2	2
3	3	2	2	1	1	3	3	2	2
3	3	2	2	1	1	1	1	2	2
3	3	2	2	1	1	3	3	2	2
3	3	2	2	1	1	3	3	2	2
3	3	2	2	4	4	3	3	2	2
3	3	2	2	4	4	3	3	2	2
2	2	3	3	1	1	3	3	4	4
3	3	2	2	1	1	3	3	2	2
2	2	3	3	1	1	3	3	2	2
3	3	2	2	1	1	3	3	2	2
3	3	3	3	1	1	3	3	2	2
3	3	2	2	1	1	1	1	2	2
3	3	2	2	1	1	1	1	2	2
3	3	2	2	1	1	3	3	2	2
2	2	3	3	1	1	3	3	2	2
3	3	2	2	1	1	3	3	2	2
3	3	2	2	1	1	3	3	2	2
3	3	2	2	1	1	3	3	2	2

For Review Only

3	3	2	2	1	1	3	3	2	2
3	3	2	2	1	1	3	3	2	2
3	3	2	2	1	1	3	3	2	2
2	2	3	3	1	1	3	3	2	2
3	3	2	2	1	1	3	3	2	2
3	3	2	2	1	1	3	3	2	2
3	3	2	2	1	1	3	3	2	2
3	3	2	2	1	1	1	1	4	4
3	3	2	2	1	1	3	3	4	4
3	3	2	2	1	1	3	3	2	2
3	3	2	2	1	1	1	1	2	2
3	3	2	2	1	1	1	1	2	2
3	3	2	2	1	1	3	3	2	2
3	3	2	2	1	1	3	3	2	2
3	3	2	2	1	1	3	3	2	2
3	3	2	2	1	1	3	3	2	2
3	3	2	2	1	1	3	3	2	2
3	3	2	2	1	1	3	3	2	2
3	3	2	2	1	1	3	3	2	2
3	3	2	2	1	1	3	3	2	2
3	3	2	2	1	1	1	1	2	2
3	3	2	2	1	1	3	3	2	2
3	3	2	2	1	1	3	3	2	2
3	3	2	2	1	1	3	3	2	2
3	3	3	3	1	1	3	3	2	2
3	3	2	2	1	1	3	3	2	2
3	3	2	2	1	1	3	3	4	4
3	3	2	2	1	1	3	3	2	2
3	3	2	2	1	1	3	3	2	2
3	3	2	2	1	1	3	3	2	2
3	3	2	2	1	1	3	3	2	2
3	3	2	2	1	1	3	3	2	2
3	3	2	2	1	1	3	3	4	4
3	3	2	2	1	1	3	3	2	2
3	3	2	2	1	1	3	3	2	2
3	3	3	3	1	1	3	3	2	2
3	3	3	3	1	1	3	3	4	4
2	2	3	3	1	1	3	3	2	2
3	3	2	2	1	1	3	3	2	2
3	3	3	3	1	1	3	3	2	2
3	3	2	2	1	1	1	1	2	2
3	3	2	2	1	1	3	3	2	2
3	3	2	2	1	1	3	3	2	2
3	3	2	2	1	1	1	1	2	2
3	3	2	2	1	1	3	3	2	2
3	3	2	2	1	1	3	3	4	4
3	3	2	2	1	1	3	3	2	2
3	3	2	2	1	1	3	3	2	2
3	3	2	2	1	1	3	3	2	2
3	3	2	2	1	1	3	3	2	2
3	3	2	2	1	1	1	1	2	2
3	3	2	2	1	1	3	3	2	2
3	3	2	2	1	1	3	3	4	4
3	3	2	2	1	1	3	3	2	2
3	3	2	2	1	1	3	3	2	2

For Review Only

3	3	2	2	1	1	3	3	2	2
3	3	2	2	1	1	3	3	2	2
3	3	2	2	1	1	1	1	2	2
3	3	2	2	1	1	3	3	2	2
3	3	2	2	1	1	3	3	2	2
3	3	2	2	1	1	3	3	2	2
3	3	2	2	1	1	3	3	2	2
3	3	3	3	1	1	3	3	2	2
3	3	2	2	1	1	3	3	2	2
3	3	2	2	1	1	3	3	2	2
3	3	2	2	1	1	3	3	2	2
3	3	2	2	1	1	3	3	2	2
3	3	2	2	1	1	3	3	2	2
3	3	2	2	1	1	3	3	2	2
3	3	2	2	1	1	3	3	2	2
3	3	2	2	4	4	3	3	2	2
3	3	2	2	1	1	3	3	2	2
3	3	2	2	1	1	3	3	2	2
3	3	2	2	1	1	3	3	2	2
3	3	2	2	1	1	3	3	2	2
3	3	2	2	1	1	3	3	2	2
3	3	2	2	1	1	3	3	2	2
3	3	2	2	1	1	3	3	2	2
3	3	2	2	1	1	3	3	2	2
3	3	2	2	1	1	3	3	2	2
3	3	2	2	1	1	3	3	2	2
3	3	2	2	1	1	3	3	2	2
3	3	2	2	1	1	3	3	2	2
3	3	2	2	1	1	1	1	2	2
3	3	2	2	1	1	3	3	2	2
3	3	2	2	1	1	3	3	2	2
3	3	2	2	1	1	3	3	2	2
3	3	2	2	1	1	1	1	2	2
3	3	2	2	1	1	3	3	2	2
3	3	2	2	1	1	3	3	2	2
3	3	2	2	1	1	3	3	2	2
3	3	2	2	1	1	3	3	2	2
3	3	3	3	1	1	1	1	2	2
3	3	2	2	1	1	3	3	2	2
3	3	2	2	1	1	3	3	2	2
3	3	2	2	1	1	3	3	2	2
3	3	2	2	1	1	3	3	2	2
3	3	2	2	1	1	3	3	2	2
3	3	2	2	1	1	3	3	2	2
3	3	2	2	1	1	3	3	2	2
3	3	2	2	1	1	3	3	2	2
3	3	2	2	1	1	3	3	2	2
3	3	3	3	1	1	3	3	2	2
3	3	2	2	1	1	3	3	2	2
3	3	2	2	1	1	3	3	2	2
3	3	2	2	1	1	3	3	2	2
3	3	2	2	1	1	3	3	4	4
3	3	2	2	1	1	3	3	2	2
3	3	2	2	1	1	3	3	2	2
3	3	2	2	1	1	3	3	2	2

For Review Only

CSSA 5585 Guilford Rd., Madison, WI 53711

3	3	2	2	1	1	1	1	2	2
3	3	2	2	1	1	3	3	2	2
3	3	3	3	1	1	3	3	2	2
3	3	2	2	1	1	3	3	4	4
3	3	2	2	1	1	3	3	2	2
3	3	2	2	1	1	3	3	2	2
3	3	2	2	1	1	3	3	2	2
3	3	2	2	1	1	3	3	2	2
3	3	2	2	1	1	3	3	2	2
3	3	2	2	1	1	3	3	2	2
3	3	2	2	1	1	3	3	2	2
3	3	2	2	1	1	3	3	2	2
3	3	2	2	1	1	3	3	2	2
3	3	2	2	1	1	3	3	2	2
3	3	2	2	1	1	3	3	2	2
3	3	2	2	1	1	3	3	2	2
3	3	2	2	1	1	3	3	2	2
3	3	2	2	1	1	3	3	2	2
3	3	2	2	1	1	3	3	2	2
3	3	2	2	1	1	3	3	2	2
3	3	2	2	1	1	3	3	2	2
3	3	2	2	1	1	3	3	4	4
3	3	2	2	1	1	3	3	2	2
3	3	2	2	1	1	1	1	2	2
3	3	2	2	1	1	3	3	2	2
3	3	2	2	1	1	3	3	2	2
3	3	2	2	1	1	3	3	2	2
3	3	2	2	1	1	3	3	2	2
3	3	2	2	1	1	3	3	2	2
3	3	2	2	1	1	3	3	2	2
3	3	2	2	1	1	3	3	2	2
3	3	2	2	1	1	3	3	2	2
3	3	2	2	1	1	3	3	2	2
3	3	2	2	1	1	3	3	2	2
3	3	2	2	1	1	3	3	2	2
3	3	2	2	1	1	1	1	2	2
3	3	2	2	1	1	3	3	2	2
3	3	2	2	1	1	3	3	2	2
3	3	3	3	1	1	3	3	2	2
3	3	2	2	1	1	3	3	2	2
3	3	2	2	1	1	3	3	2	2
3	3	2	2	1	1	3	3	2	2
3	3	2	2	1	1	3	3	4	4
3	3	2	2	1	1	3	3	2	2
3	3	2	2	1	1	3	3	2	2
3	3	2	2	1	1	3	3	2	2
3	3	2	2	1	1	3	3	2	2
3	3	2	2	1	1	3	3	4	4
3	3	2	2	1	1	3	3	2	2
2	2	2	2	1	1	3	3	2	2
3	3	2	2	1	1	1	1	4	4
3	3	2	2	1	1	3	3	2	2
3	3	2	2	1	1	3	3	2	2
3	3	2	2	1	1	1	1	2	2
3	3	2	2	1	1	3	3	4	4

For Review Only

3	3	2	2	1	1	3	3	2	2
3	3	3	3	1	1	3	3	2	2
3	3	2	2	1	1	3	3	2	2
3	3	2	2	1	1	3	3	2	2
3	3	2	2	1	1	3	3	2	2
3	3	2	2	1	1	3	3	2	2
3	3	2	2	1	1	3	3	2	2
3	3	2	2	1	1	3	3	2	2
3	3	2	2	1	1	3	3	2	2
3	3	2	2	1	1	3	3	2	2
3	3	2	2	1	1	3	3	2	2
3	3	2	2	1	1	3	3	2	2
3	3	2	2	1	1	3	3	2	2
3	3	2	2	1	1	3	3	2	2
3	3	2	2	1	1	3	3	2	2
3	3	2	2	1	1	3	3	2	2
3	3	2	2	1	1	3	3	4	4
3	3	2	2	1	1	3	3	2	2
3	3	2	2	1	1	3	3	4	4
3	3	2	2	1	1	3	3	2	2
3	3	2	2	4	4	3	3	2	2
3	3	2	2	1	1	3	3	2	2
3	3	2	2	1	1	3	3	2	2
3	3	2	2	4	4	3	3	2	2
3	3	2	2	1	1	1	1	4	4
3	3	2	2	1	1	3	3	2	2
3	3	2	2	1	1	3	3	4	4
3	3	2	2	1	1	1	1	2	2
3	3	2	2	1	1	3	3	2	2
3	3	2	2	1	1	1	1	2	2
3	3	2	2	1	1	3	3	2	2
3	3	2	2	1	1	3	3	2	2
3	3	2	2	1	1	3	3	2	2
3	3	2	2	1	1	3	3	2	2
3	3	2	2	1	1	3	3	2	2
3	3	2	2	1	1	3	3	2	2
3	3	2	2	1	1	3	3	2	2
3	3	2	2	1	1	1	1	2	2
3	3	2	2	1	1	1	1	2	2
3	3	2	2	4	4	3	3	2	2
3	3	2	2	1	1	3	3	4	4
3	3	2	2	1	1	3	3	2	2
3	3	2	2	4	4	3	3	2	2
3	3	2	2	1	1	3	3	2	2
3	3	2	2	1	1	3	3	2	2
3	3	2	2	1	1	3	3	2	2
3	3	3	3	1	1	3	3	2	2
3	3	2	2	1	1	3	3	2	2
3	3	2	2	1	1	3	3	2	2
3	3	2	2	1	1	3	3	4	4
3	3	2	2	1	1	3	3	2	2
3	3	2	2	1	1	3	3	2	2
3	3	2	2	1	1	3	3	2	2
3	3	2	2	1	1	1	1	2	2
3	3	2	2	4	4	3	3	2	2

For Review Only

3	3	2	2	1	1	3	3	2	2
3	3	2	2	1	1	3	3	2	2

For Review Only

SNP2859	SNP2859	SNP286	SNP286	SNP2860	SNP2860	SNP2861	SNP2861	SNP2862	SNP2862
2	2	3	3	2	2	2	2	2	2
2	2	1	1	2	2	2	2	2	2
2	2	3	3	2	2	2	2	2	2
2	2	3	3	2	2	2	2	2	2
2	2	1	1	2	2	2	2	2	2
2	2	1	1	2	2	3	3	2	2
4	4	3	3	2	2	2	2	2	2
4	4	1	1	3	3	2	2	2	2
2	2	3	3	2	2	2	2	2	2
4	4	1	1	2	2	2	2	2	2
4	4	1	1	2	2	2	2	2	2
2	2	3	3	2	2	2	2	2	2
2	2	1	1	2	2	2	2	2	2
4	4	1	1	3	3	2	2	2	2
2	2	1	1	2	2	2	2	2	2
2	2	3	3	2	2	2	2	2	2
2	2	3	3	2	2	2	2	2	2
2	2	1	1	2	2	2	2	2	2
2	2	3	3	2	2	2	2	2	2
2	2	1	1	3	3	3	3	3	3
2	2	1	1	2	2	2	2	2	2
4	4	3	3	2	2	2	2	2	2
4	4	1	1	3	3	2	2	2	2
4	4	1	1	2	2	2	2	2	2
2	2	1	1	2	2	2	2	2	2
2	2	1	1	2	2	2	2	2	2
2	2	1	1	2	2	2	2	2	2
2	2	1	1	2	2	2	2	2	2
2	2	1	1	2	2	2	2	2	2
4	4	1	1	2	2	2	2	2	2
4	4	1	1	2	2	2	2	2	2
4	4	3	3	2	2	2	2	2	2
2	2	1	1	2	2	2	2	2	2
2	2	1	1	2	2	2	2	2	2
2	2	1	1	2	2	2	2	2	2
2	2	1	1	2	2	2	2	2	2
2	2	1	1	2	2	2	2	2	2
2	2	1	1	2	2	2	2	2	2
4	4	1	1	2	2	2	2	2	2
4	4	1	1	2	2	2	2	2	2
4	4	1	1	2	2	2	2	2	2
2	2	1	1	2	2	2	2	2	2
4	4	1	1	2	2	2	2	2	2
2	2	3	3	2	2	2	2	2	2
4	4	1	1	2	2	2	2	2	2
2	2	3	3	2	2	2	2	2	2
2	2	1	1	2	2	2	2	2	2
4	4	1	1	2	2	2	2	2	2
4	4	3	3	2	2	2	2	2	2
2	2	1	1	2	2	2	2	2	2
2	2	1	1	2	2	2	2	2	2
2	2	1	1	2	2	2	2	2	2

For Review Only

2	2	1	1	2	2	2	2	2	2
2	2	1	1	2	2	2	2	2	2
4	4	3	3	2	2	2	2	2	2
2	2	3	3	2	2	2	2	2	2
2	2	3	3	2	2	2	2	2	2
2	2	1	1	2	2	2	2	2	2
2	2	1	1	2	2	2	2	2	2
4	4	1	1	2	2	2	2	2	2
4	4	3	3	2	2	2	2	2	2
2	2	1	1	2	2	2	2	2	2
2	2	3	3	2	2	2	2	2	2
2	2	1	1	2	2	2	2	2	2
2	2	1	1	2	2	2	2	2	2
2	2	1	1	2	2	2	2	2	2
2	2	1	1	2	2	2	2	2	2
2	2	1	1	2	2	2	2	2	2
2	2	1	1	2	2	2	2	2	2
2	2	1	1	2	2	2	2	2	2
2	2	3	3	2	2	2	2	2	2
2	2	3	3	2	2	2	2	2	2
2	2	1	1	2	2	2	2	2	2
2	2	1	1	2	2	2	2	2	2
2	2	1	1	2	2	2	2	2	2
2	2	1	1	3	3	2	2	2	2
4	4	1	1	2	2	2	2	2	2
2	2	1	1	2	2	2	2	2	2
4	4	1	1	2	2	2	2	2	2
4	4	1	1	2	2	2	2	2	2
2	2	1	1	2	2	2	2	2	2
2	2	3	3	2	2	3	3	3	3
2	2	1	1	2	2	2	2	2	2
2	2	1	1	2	2	2	2	2	2
4	4	1	1	3	3	2	2	2	2
2	2	1	1	2	2	2	2	2	2
2	2	1	1	2	2	2	2	2	2
4	4	1	1	2	2	2	2	2	2
4	4	1	1	3	3	3	3	3	3
2	2	1	1	2	2	2	2	2	2
2	2	3	3	2	2	2	2	2	2
4	4	1	1	2	2	2	2	2	2
4	4	3	3	2	2	2	2	2	2
2	2	3	3	2	2	2	2	2	2
4	4	3	3	2	2	2	2	2	2
2	2	3	3	2	2	2	2	2	2
4	4	3	3	2	2	2	2	2	2
4	4	3	3	2	2	2	2	2	2
2	2	1	1	2	2	2	2	2	2
4	4	1	1	2	2	2	2	2	2
2	2	1	1	2	2	2	2	2	2
2	2	1	1	2	2	2	2	2	2
2	2	1	1	2	2	2	2	2	2
4	4	1	1	3	3	2	2	2	2
4	4	3	3	2	2	2	2	2	2
2	2	1	1	2	2	2	2	2	2
2	2	1	1	2	2	2	2	2	2

For Review Only

2	2	1	1	2	2	2	2	2	2
2	2	1	1	2	2	2	2	2	2
2	2	1	1	2	2	2	2	2	2
2	2	1	1	2	2	2	2	2	2
2	2	3	3	2	2	2	2	2	2
2	2	1	1	2	2	2	2	2	2
2	2	3	3	2	2	2	2	2	2
2	2	1	1	2	2	2	2	2	2
2	2	1	1	2	2	2	2	2	2
2	2	1	1	2	2	2	2	2	2
2	2	1	1	3	3	2	2	2	2
2	2	1	1	2	2	2	2	2	2
4	4	3	3	2	2	2	2	2	2
2	2	1	1	2	2	2	2	2	2
2	2	1	1	2	2	2	2	2	2
2	2	1	1	2	2	2	2	2	2
4	4	1	1	2	2	2	2	2	2
2	2	1	1	2	2	2	2	2	2
2	2	1	1	2	2	2	2	2	2
2	2	3	3	2	2	2	2	2	2
2	2	1	1	2	2	2	2	2	2
2	2	1	1	2	2	2	2	2	2
2	2	1	1	2	2	2	2	2	2
4	4	1	1	3	3	2	2	2	2
2	2	1	1	2	2	2	2	2	2
2	2	1	1	2	2	2	2	2	2
2	2	3	3	2	2	2	2	2	2
2	2	1	1	2	2	2	2	2	2
2	2	3	3	2	2	2	2	2	2
2	2	1	1	2	2	2	2	2	2
2	2	1	1	2	2	2	2	2	2
2	2	1	1	2	2	2	2	2	2
4	4	3	3	2	2	2	2	2	2
2	2	1	1	2	2	2	2	2	2
2	2	1	1	2	2	2	2	2	2
2	2	1	1	2	2	2	2	2	2
2	2	1	1	2	2	2	2	2	2
4	4	1	1	2	2	3	3	3	3
2	2	1	1	2	2	2	2	2	2
4	4	1	1	2	2	2	2	2	2
4	4	1	1	2	2	2	2	2	2
2	2	1	1	2	2	2	2	2	2
2	2	1	1	2	2	2	2	2	2
4	4	1	1	2	2	2	2	2	2
2	2	1	1	2	2	2	2	2	2
2	2	1	1	2	2	2	2	2	2
2	2	1	1	2	2	2	2	2	2
4	4	1	1	2	2	3	3	3	3
4	4	1	1	2	2	2	2	2	2
2	2	1	1	2	2	2	2	2	2
2	2	1	1	2	2	2	2	2	2
4	4	1	1	2	2	2	2	2	2
2	2	1	1	2	2	2	2	2	2
4	4	1	1	2	2	2	2	2	2
4	4	1	1	2	2	2	2	2	2

For Review Only

4	4	1	1	2	2	2	2	2	2
4	4	1	1	2	2	2	2	2	2
4	4	1	1	2	2	2	2	2	2
4	4	1	1	2	2	2	2	2	2
2	2	1	1	2	2	2	2	2	2
2	2	3	3	2	2	2	2	2	2
4	4	1	1	2	2	2	2	2	2
2	2	1	1	2	2	2	2	2	2
2	2	1	1	2	2	2	2	2	2
2	2	3	3	2	2	3	3	3	3
2	2	1	1	2	2	2	2	2	2
4	4	1	1	2	2	2	2	2	2
2	2	3	3	2	2	2	2	2	2
2	2	3	3	2	2	2	2	2	2
2	2	1	1	2	2	2	2	2	2
4	4	1	1	2	2	2	2	2	2
2	2	1	1	3	3	2	2	2	2
2	2	3	3	2	2	2	2	2	2
2	2	1	1	2	2	2	2	2	2
2	2	3	3	2	2	2	2	2	2
2	2	1	1	2	2	2	2	2	2
2	2	1	1	3	3	2	2	2	2
2	2	1	1	2	2	2	2	2	2
2	2	1	1	2	2	2	2	2	2
2	2	1	1	2	2	3	3	2	2
2	2	1	1	2	2	2	2	2	2
2	2	1	1	2	2	2	2	2	2
2	2	1	1	2	2	2	2	2	2
4	4	3	3	2	2	2	2	2	2
4	4	3	3	2	2	2	2	2	2
2	2	1	1	2	2	2	2	2	2
2	2	3	3	2	2	3	3	3	3
4	4	3	3	2	2	2	2	2	2
2	2	1	1	2	2	2	2	2	2
2	2	3	3	2	2	2	2	2	2
2	2	1	1	2	2	2	2	2	2
2	2	1	1	2	2	2	2	2	2
2	2	3	3	2	2	2	2	2	2
4	4	1	1	2	2	3	3	3	3
4	4	1	1	2	2	3	3	3	3
2	2	3	3	2	2	2	2	2	2
2	2	1	1	2	2	2	2	2	2
4	4	1	1	3	3	2	2	2	2
2	2	1	1	2	2	2	2	2	2
2	2	3	3	2	2	2	2	2	2
2	2	3	3	2	2	2	2	2	2
4	4	1	1	2	2	2	2	2	2
4	4	1	1	2	2	2	2	2	2
2	2	1	1	3	3	2	2	2	2
4	4	3	3	2	2	2	2	2	2
4	4	3	3	2	2	2	2	2	2
2	2	1	1	2	2	2	2	2	2
2	2	1	1	2	2	2	2	2	2
2	2	3	3	2	2	2	2	2	2
4	4	1	1	2	2	2	2	2	2

For Review Only

2	2	1	1	3	3	2	2	2	2
2	2	1	1	3	3	2	2	2	2
2	2	3	3	2	2	3	3	2	2
2	2	1	1	2	2	2	2	2	2
4	4	1	1	2	2	2	2	2	2
2	2	3	3	2	2	2	2	2	2
2	2	1	1	2	2	2	2	2	2
4	4	1	1	2	2	3	3	3	3
4	4	1	1	2	2	2	2	2	2
4	4	1	1	3	3	2	2	2	2
2	2	1	1	2	2	2	2	2	2
2	2	1	1	2	2	2	2	2	2
2	2	3	3	2	2	2	2	2	2
4	4	1	1	2	2	2	2	2	2
2	2	1	1	2	2	2	2	2	2
2	2	1	1	2	2	2	2	2	2
4	4	1	1	2	2	2	2	2	2
2	2	1	1	2	2	2	2	2	2
4	4	1	1	2	2	2	2	2	2
2	2	1	1	2	2	2	2	2	2
4	4	1	1	2	2	2	2	2	2
2	2	1	1	2	2	2	2	2	2
2	2	1	1	2	2	2	2	2	2
4	4	1	1	2	2	2	2	2	2
4	4	1	1	2	2	2	2	2	2
2	2	3	3	2	2	3	3	2	2
4	4	1	1	3	3	2	2	2	2
2	2	3	3	2	2	2	2	2	2
2	2	1	1	2	2	2	2	2	2
2	2	3	3	2	2	2	2	2	2
4	4	1	1	2	2	2	2	2	2
4	4	1	1	2	2	2	2	2	2
2	2	3	3	3	3	2	2	2	2
4	4	1	1	2	2	3	3	3	3
2	2	1	1	2	2	2	2	2	2
4	4	1	1	2	2	2	2	2	2
2	2	3	3	2	2	2	2	2	2
2	2	1	1	2	2	2	2	2	2
2	2	1	1	2	2	2	2	2	2
4	4	1	1	2	2	2	2	2	2
4	4	3	3	2	2	2	2	2	2
2	2	1	1	2	2	2	2	2	2
4	4	1	1	2	2	2	2	2	2
2	2	3	3	2	2	2	2	2	2
2	2	1	1	2	2	2	2	2	2
2	2	1	1	2	2	2	2	2	2
4	4	1	1	2	2	2	2	2	2
2	2	3	3	2	2	2	2	2	2
4	4	1	1	2	2	2	2	2	2
4	4	1	1	3	3	2	2	2	2
2	2	3	3	2	2	2	2	2	2
4	4	1	1	2	2	2	2	2	2
2	2	3	3	2	2	2	2	2	2
2	2	3	3	2	2	3	3	3	3
4	4	1	1	2	2	2	2	2	2

For Review Only

2	2	1	1	2	2	2	2	2	2
4	4	1	1	2	2	2	2	2	2

For Review Only

SNP2863	SNP2863	SNP2864	SNP2864	SNP2865	SNP2865	SNP2866	SNP2866	SNP2867	SNP2867
1	1	1	1	2	2	2	2	3	3
1	1	1	1	1	1	2	2	2	2
1	1	1	1	1	1	2	2	2	2
1	1	1	1	1	1	2	2	2	2
1	1	1	1	2	2	2	2	3	3
1	1	1	1	1	1	2	2	2	2
1	1	1	1	2	2	2	2	3	3
1	1	1	1	1	1	2	2	2	2
1	1	1	1	2	2	2	2	3	3
1	1	4	4	2	2	2	2	2	2
1	1	1	1	1	1	2	2	2	2
1	1	1	1	1	1	2	2	2	2
1	1	4	4	1	1	2	2	2	2
1	1	1	1	1	1	2	2	2	2
1	1	1	1	1	1	2	2	3	3
1	1	1	1	1	1	2	2	2	2
1	1	4	4	2	2	2	2	3	3
1	1	1	1	1	1	2	2	2	2
1	1	1	1	1	1	2	2	2	2
4	4	4	4	1	1	3	3	2	2
1	1	1	1	2	2	2	2	3	3
1	1	1	1	2	2	2	2	3	3
1	1	1	1	1	1	2	2	2	2
1	1	1	1	1	1	2	2	3	3
1	1	1	1	2	2	2	2	3	3
1	1	1	1	1	1	2	2	2	2
1	1	1	1	1	1	2	2	2	2
1	1	1	1	1	1	2	2	2	2
1	1	1	1	2	2	2	2	3	3
1	1	4	4	1	1	2	2	2	2
1	1	1	1	1	1	2	2	2	2
1	1	1	1	2	2	2	2	2	2
1	1	1	1	1	1	2	2	2	2
1	1	1	1	2	2	2	2	3	3
1	1	1	1	1	1	2	2	2	2
1	1	1	1	1	1	2	2	2	2
1	1	1	1	1	1	2	2	2	2
1	1	1	1	1	1	2	2	2	2
1	1	1	1	1	1	2	2	2	2
1	1	1	1	1	1	2	2	2	2
1	1	4	4	2	2	2	2	3	3
1	1	1	1	2	2	2	2	3	3
1	1	4	4	1	1	2	2	2	2
1	1	1	1	2	2	2	2	3	3
1	1	1	1	1	1	2	2	2	2
1	1	1	1	1	1	2	2	2	2
1	1	1	1	1	1	2	2	2	2
1	1	1	1	1	1	2	2	3	3
1	1	4	4	2	2	2	2	3	3
1	1	1	1	1	1	2	2	2	2
1	1	1	1	1	1	2	2	2	2
1	1	1	1	2	2	2	2	3	3

For Review Only

1	1	1	1	2	2	2	2	3	3
1	1	1	1	1	1	2	2	2	2
1	1	1	1	1	1	2	2	2	2
1	1	4	4	2	2	2	2	3	3
1	1	1	1	1	1	2	2	2	2
1	1	1	1	1	1	2	2	2	2
1	1	1	1	1	1	2	2	2	2
1	1	1	1	2	2	2	2	3	3
1	1	1	1	2	2	2	2	3	3
1	1	1	1	1	1	2	2	2	2
1	1	1	1	1	1	2	2	2	2
1	1	1	1	1	1	2	2	2	2
1	1	1	1	2	2	2	2	3	3
1	1	1	1	2	2	2	2	3	3
1	1	1	1	1	1	2	2	2	2
1	1	1	1	1	1	2	2	2	2
1	1	1	1	1	1	2	2	2	2
1	1	1	1	1	1	2	2	2	2
1	1	1	1	1	1	2	2	3	3
1	1	1	1	2	2	2	2	3	3
1	1	1	1	1	1	2	2	2	2
1	1	1	1	1	1	2	2	2	2
1	1	1	1	2	2	2	2	2	2
1	1	1	1	1	1	2	2	2	2
1	1	1	1	1	1	2	2	2	2
1	1	1	1	1	1	2	2	2	2
1	1	1	1	1	1	2	2	2	2
1	1	1	1	1	1	2	2	2	2
1	1	1	1	1	1	2	2	2	2
4	4	1	1	1	1	3	3	2	2
1	1	1	1	1	1	2	2	3	3
1	1	1	1	2	2	2	2	3	3
1	1	1	1	1	1	2	2	2	2
1	1	1	1	2	2	2	2	3	3
1	1	1	1	2	2	2	2	3	3
1	1	1	1	1	1	2	2	2	2
4	4	1	1	1	1	2	2	2	2
1	1	4	4	2	2	2	2	3	3
1	1	1	1	1	1	2	2	2	2
1	1	1	1	1	1	2	2	2	2
1	1	1	1	2	2	2	2	3	3
1	1	1	1	1	1	2	2	2	2
1	1	1	1	1	1	2	2	2	2
1	1	1	1	1	1	2	2	2	2
1	1	1	1	2	2	2	2	3	3
1	1	1	1	1	1	2	2	2	2
1	1	1	1	2	2	2	2	3	3
1	1	1	1	1	1	2	2	2	2
1	1	1	1	1	1	2	2	2	2
1	1	1	1	1	1	2	2	2	2
1	1	1	1	1	1	2	2	3	3
1	1	1	1	1	1	2	2	2	2
1	1	1	1	1	1	2	2	3	3
1	1	1	1	2	2	2	2	2	2
1	1	1	1	1	1	2	2	3	3

For Review Only

1	1	1	1	1	1	2	2	2	2
1	1	1	1	1	1	2	2	2	2
1	1	1	1	2	2	2	2	3	3
1	1	1	1	2	2	2	2	3	3
1	1	1	1	1	1	2	2	2	2
1	1	1	1	1	1	2	2	2	2
1	1	1	1	1	1	2	2	2	2
1	1	1	1	1	1	2	2	2	2
1	1	1	1	1	1	2	2	2	2
1	1	1	1	1	1	2	2	2	2
1	1	1	1	1	1	2	2	2	2
1	1	1	1	1	1	2	2	2	2
1	1	1	1	1	1	2	2	2	2
1	1	1	1	1	1	2	2	2	2
1	1	1	1	1	1	2	2	2	2
1	1	1	1	1	1	2	2	2	2
1	1	1	1	2	2	2	2	3	3
1	1	1	1	1	1	2	2	2	2
1	1	1	1	2	2	2	2	2	2
1	1	1	1	2	2	2	2	3	3
1	1	1	1	1	1	2	2	2	2
4	4	1	1	2	2	2	2	2	2
1	1	1	1	1	1	2	2	2	2
1	1	1	1	1	1	2	2	2	2
1	1	1	1	1	1	2	2	2	2
1	1	1	1	1	1	2	2	2	2
1	1	1	1	2	2	2	2	3	3
1	1	1	1	2	2	2	2	3	3
1	1	1	1	1	1	2	2	2	2
1	1	4	4	2	2	2	2	3	3
1	1	1	1	2	2	2	2	3	3
1	1	1	1	1	1	2	2	2	2
1	1	1	1	2	2	2	2	3	3
1	1	1	1	1	1	2	2	2	2
1	1	1	1	2	2	2	2	3	3
1	1	1	1	1	1	2	2	2	2
1	1	1	1	2	2	2	2	3	3
4	4	1	1	1	1	3	3	2	2
1	1	1	1	2	2	2	2	3	3
1	1	1	1	1	1	2	2	2	2
1	1	1	1	1	1	2	2	2	2
1	1	1	1	2	2	2	2	3	3
1	1	1	1	1	1	2	2	3	3
1	1	4	4	1	1	2	2	2	2
1	1	1	1	1	1	2	2	2	2
1	1	1	1	2	2	2	2	3	3
1	1	1	1	1	1	2	2	2	2
4	4	1	1	1	1	3	3	2	2
1	1	1	1	1	1	2	2	2	2
1	1	1	1	1	1	2	2	2	2
1	1	1	1	1	1	2	2	2	2
1	1	1	1	1	1	2	2	2	2
1	1	1	1	1	1	2	2	2	2
1	1	1	1	1	1	2	2	2	2
1	1	1	1	2	2	2	2	2	2

For Review Only

1	1	1	1	2	2	2	2	3	3
1	1	1	1	1	1	2	2	2	2
1	1	1	1	2	2	2	2	3	3
1	1	1	1	1	1	2	2	2	2
1	1	1	1	1	1	2	2	2	2
1	1	1	1	1	1	2	2	2	2
1	1	1	1	1	1	2	2	2	2
1	1	1	1	1	1	2	2	2	2
1	1	1	1	1	1	2	2	2	2
4	4	1	1	1	1	3	3	2	2
1	1	1	1	1	1	2	2	2	2
1	1	1	1	1	1	2	2	3	3
1	1	1	1	1	1	2	2	2	2
1	1	1	1	1	1	2	2	2	2
1	1	1	1	2	2	2	2	3	3
1	1	1	1	1	1	2	2	2	2
1	1	1	1	1	1	2	2	2	2
1	1	1	1	1	1	2	2	2	2
1	1	1	1	1	1	2	2	2	2
1	1	1	1	1	1	2	2	2	2
1	1	1	1	1	1	2	2	2	2
1	1	1	1	1	1	2	2	2	2
1	1	1	1	1	1	2	2	2	2
1	1	1	1	1	1	2	2	2	2
4	4	1	1	1	1	2	2	2	2
1	1	1	1	2	2	2	2	3	3
1	1	1	1	1	1	2	2	3	3
1	1	1	1	1	1	2	2	2	2
1	1	1	1	1	1	2	2	2	2
1	1	1	1	2	2	2	2	3	3
1	1	1	1	1	1	2	2	2	2
4	4	1	1	1	1	3	3	2	2
1	1	1	1	2	2	2	2	3	3
1	1	1	1	2	2	2	2	3	3
1	1	1	1	1	1	2	2	2	2
1	1	1	1	1	1	2	2	2	2
1	1	1	1	1	1	2	2	2	2
1	1	1	1	1	1	2	2	2	2
4	4	1	1	1	1	3	3	2	2
4	4	1	1	1	1	3	3	2	2
1	1	1	1	1	1	2	2	2	2
1	1	1	1	1	1	2	2	2	2
1	1	1	1	1	1	2	2	2	2
1	1	1	1	1	1	2	2	2	2
1	1	1	1	1	1	2	2	3	3
1	1	1	1	1	1	2	2	2	2
1	1	1	1	1	1	2	2	2	2
1	1	1	1	2	2	2	2	3	3
1	1	1	1	1	1	2	2	2	2
1	1	1	1	2	2	2	2	3	3
1	1	1	1	1	1	2	2	2	2
1	1	1	1	2	2	2	2	3	3
1	1	1	1	1	1	2	2	2	2
1	1	1	1	1	1	2	2	2	2
1	1	1	1	1	1	2	2	2	2

For Review Only

1	1	1	1	1	1	2	2	2	2
1	1	1	1	1	1	2	2	2	2
4	4	1	1	1	1	2	2	2	2
1	1	1	1	1	1	2	2	2	2
1	1	1	1	2	2	2	2	3	3
1	1	1	1	1	1	2	2	2	2
1	1	1	1	1	1	2	2	2	2
4	4	1	1	1	1	3	3	2	2
1	1	1	1	2	2	2	2	3	3
1	1	1	1	1	1	2	2	2	2
1	1	1	1	1	1	2	2	2	2
1	1	1	1	1	1	2	2	2	2
1	1	1	1	1	1	2	2	2	2
1	1	1	1	1	1	2	2	2	2
1	1	1	1	1	1	2	2	2	2
1	1	1	1	2	2	2	2	3	3
1	1	1	1	1	1	2	2	2	2
1	1	1	1	2	2	2	2	2	2
1	1	1	1	1	1	2	2	2	2
1	1	1	1	1	1	2	2	2	2
1	1	1	1	1	1	2	2	2	2
1	1	1	1	1	1	2	2	2	2
1	1	1	1	1	1	2	2	2	2
1	1	1	1	1	1	2	2	2	2
1	1	1	1	2	2	2	2	3	3
4	4	1	1	1	1	3	3	2	2
1	1	1	1	1	1	2	2	2	2
1	1	1	1	1	1	2	2	2	2
1	1	1	1	2	2	2	2	3	3
1	1	1	1	1	1	2	2	2	2
1	1	1	1	1	1	2	2	2	2
1	1	1	1	1	1	2	2	2	2
1	1	1	1	1	1	2	2	2	2
4	4	1	1	1	1	3	3	2	2
1	1	1	1	1	1	2	2	2	2
1	1	1	1	2	2	2	2	2	2
1	1	1	1	1	1	2	2	2	2
1	1	1	1	1	1	2	2	2	2
1	1	1	1	1	1	2	2	2	2
1	1	1	1	1	1	2	2	2	2
1	1	1	1	1	1	2	2	2	2
1	1	1	1	1	1	2	2	2	2
1	1	1	1	1	1	2	2	2	2
1	1	1	1	2	2	2	2	3	3
1	1	1	1	1	1	2	2	2	2
1	1	1	1	1	1	2	2	2	2
1	1	1	1	1	1	2	2	2	2
1	1	1	1	1	1	2	2	3	3
1	1	1	1	1	1	2	2	2	2
1	1	1	1	1	1	2	2	2	2
1	1	1	1	1	1	2	2	2	2
1	1	1	1	1	1	2	2	2	2
1	1	1	1	1	1	2	2	2	2
4	4	1	1	1	1	3	3	2	2
1	1	1	1	1	1	2	2	2	2

1	1	1	1	2	2	2	2	3	3
1	1	1	1	1	1	2	2	2	2

For Review Only

SNP2868	SNP2868	SNP2869	SNP2869	SNP287	SNP287	SNP2870	SNP2870	SNP2871	SNP2871
1	1	2	2	4	4	4	4	3	3
1	1	2	2	4	4	4	4	3	3
1	1	2	2	4	4	4	4	3	3
1	1	2	2	1	1	4	4	3	3
1	1	2	2	4	4	4	4	3	3
1	1	2	2	4	4	4	4	3	3
1	1	2	2	4	4	4	4	3	3
1	1	2	2	1	1	4	4	3	3
1	1	2	2	4	4	4	4	3	3
1	1	2	2	1	1	4	4	3	3
4	4	2	2	1	1	4	4	3	3
1	1	2	2	4	4	4	4	3	3
1	1	2	2	4	4	4	4	3	3
1	1	2	2	1	1	4	4	3	3
1	1	2	2	4	4	4	4	3	3
1	1	2	2	4	4	4	4	3	3
1	1	2	2	4	4	4	4	3	3
1	1	2	2	1	1	4	4	3	3
1	1	2	2	4	4	4	4	3	3
1	1	2	2	4	4	4	4	3	3
1	1	2	2	4	4	4	4	3	3
4	4	2	2	4	4	4	4	3	3
1	1	4	4	1	1	3	3	3	3
1	1	2	2	1	1	4	4	2	2
1	1	2	2	4	4	4	4	3	3
1	1	2	2	4	4	4	4	3	3
1	1	2	2	4	4	4	4	2	2
1	1	2	2	4	4	4	4	3	3
1	1	2	2	4	4	4	4	3	3
1	1	2	2	1	1	4	4	3	3
1	1	2	2	4	4	4	4	3	3
1	1	2	2	4	4	4	4	3	3
1	1	2	2	4	4	4	4	3	3
1	1	2	2	1	1	4	4	3	3
1	1	2	2	1	1	4	4	3	3
1	1	2	2	1	1	4	4	3	3
1	1	2	2	4	4	4	4	3	3
1	1	2	2	1	1	4	4	3	3
4	4	4	4	4	4	3	3	3	3
4	4	2	2	1	1	4	4	3	3
1	1	2	2	4	4	4	4	3	3
1	1	2	2	4	4	4	4	3	3
1	1	2	2	4	4	4	4	3	3
1	1	2	2	1	1	4	4	3	3
1	1	2	2	4	4	4	4	3	3
1	1	2	2	4	4	4	4	3	3
1	1	2	2	4	4	4	4	3	3
4	4	2	2	4	4	4	4	2	2
4	4	2	2	1	1	4	4	3	3
1	1	2	2	4	4	4	4	3	3
1	1	2	2	4	4	4	4	3	3
1	1	2	2	1	1	4	4	3	3

For Review Only

1	1	2	2	4	4	4	4	3	3
1	1	2	2	4	4	4	4	3	3
1	1	2	2	4	4	4	4	3	3
1	1	2	2	4	4	4	4	3	3
1	1	2	2	4	4	4	4	3	3
1	1	2	2	4	4	4	4	3	3
1	1	2	2	4	4	4	4	3	3
1	1	2	2	1	1	4	4	3	3
1	1	2	2	4	4	4	4	3	3
1	1	2	2	4	4	4	4	3	3
1	1	2	2	4	4	4	4	3	3
1	1	2	2	4	4	4	4	3	3
1	1	2	2	4	4	4	4	3	3
1	1	2	2	4	4	4	4	3	3
1	1	2	2	4	4	4	4	3	3
1	1	2	2	4	4	4	4	3	3
1	1	2	2	4	4	4	4	3	3
1	1	2	2	4	4	4	4	3	3
1	1	2	2	4	4	4	4	3	3
1	1	2	2	4	4	4	4	3	3
1	1	2	2	4	4	4	4	3	3
4	4	2	2	4	4	4	4	3	3
1	1	2	2	4	4	4	4	3	3
1	1	2	2	4	4	4	4	3	3
1	1	2	2	4	4	4	4	3	3
1	1	2	2	1	1	4	4	3	3
1	1	2	2	4	4	4	4	3	3
1	1	4	4	1	1	3	3	3	3
1	1	2	2	4	4	4	4	3	3
1	1	2	2	4	4	4	4	3	3
1	1	2	2	4	4	4	4	3	3
1	1	2	2	1	1	4	4	2	2
1	1	2	2	1	1	4	4	3	3
1	1	2	2	4	4	4	4	3	3
1	1	2	2	4	4	4	4	3	3
1	1	2	2	4	4	4	4	3	3
1	1	2	2	4	4	4	4	3	3
1	1	2	2	4	4	4	4	3	3
1	1	2	2	1	1	4	4	3	3
1	1	2	2	4	4	4	4	3	3
4	4	2	2	4	4	4	4	3	3
1	1	2	2	4	4	4	4	3	3
1	1	2	2	4	4	4	4	3	3
1	1	2	2	4	4	4	4	3	3
1	1	2	2	1	1	4	4	3	3
1	1	2	2	4	4	4	4	3	3
1	1	2	2	1	1	4	4	3	3
1	1	2	2	4	4	4	4	2	2
1	1	2	2	4	4	4	4	2	2
1	1	2	2	1	1	4	4	3	3
1	1	2	2	4	4	4	4	3	3
4	4	4	4	1	1	3	3	3	3
1	1	2	2	1	1	4	4	3	3
1	1	2	2	4	4	4	4	3	3
1	1	2	2	1	1	4	4	3	3

For Review Only

1	1	2	2	4	4	4	4	3	3
1	1	2	2	1	1	4	4	3	3
1	1	2	2	4	4	4	4	3	3
1	1	2	2	1	1	4	4	3	3
1	1	2	2	4	4	4	4	3	3
1	1	2	2	4	4	4	4	3	3
1	1	2	2	4	4	4	4	3	3
1	1	2	2	4	4	4	4	2	2
1	1	4	4	4	4	3	3	2	2
1	1	2	2	4	4	4	4	3	3
1	1	2	2	4	4	4	4	3	3
1	1	2	2	4	4	4	4	3	3
1	1	2	2	4	4	4	4	3	3
1	1	2	2	1	1	4	4	3	3
1	1	2	2	4	4	4	4	2	2
1	1	2	2	4	4	4	4	3	3
1	1	2	2	4	4	4	4	3	3
1	1	2	2	4	4	4	4	3	3
1	1	2	2	1	1	4	4	3	3
1	1	4	4	4	4	4	4	3	3
1	1	2	2	4	4	4	4	3	3
1	1	2	2	4	4	4	4	3	3
1	1	2	2	4	4	4	4	3	3
1	1	2	2	1	1	4	4	3	3
1	1	2	2	4	4	4	4	3	3
4	4	2	2	1	1	4	4	3	3
1	1	2	2	4	4	4	4	3	3
1	1	2	2	4	4	4	4	3	3
1	1	2	2	4	4	4	4	3	3
1	1	2	2	1	1	4	4	3	3
1	1	4	4	1	1	4	4	3	3
1	1	2	2	4	4	4	4	3	3
4	4	2	2	4	4	4	4	3	3
1	1	2	2	4	4	4	4	3	3
1	1	2	2	1	1	4	4	3	3
1	1	2	2	4	4	4	4	3	3
1	1	2	2	1	1	4	4	3	3
1	1	2	2	4	4	4	4	3	3
1	1	2	2	4	4	4	4	3	3
1	1	4	4	1	1	3	3	3	3
4	4	2	2	4	4	4	4	3	3
1	1	2	2	1	1	4	4	3	3
1	1	2	2	1	1	4	4	3	3
1	1	2	2	4	4	4	4	3	3
1	1	4	4	4	4	3	3	3	3
1	1	2	2	1	1	4	4	3	3
1	1	2	2	4	4	4	4	3	3
1	1	2	2	4	4	4	4	3	3
1	1	2	2	4	4	4	4	3	3
1	1	2	2	4	4	4	4	3	3
1	1	2	2	4	4	4	4	3	3
1	1	2	2	1	1	4	4	3	3
1	1	2	2	1	1	4	4	3	3
1	1	2	2	4	4	4	4	2	2
1	1	2	2	4	4	4	4	3	3

For Review Only

1	1	2	2	4	4	4	4	3	3
1	1	2	2	4	4	4	4	3	3
1	1	2	2	4	4	4	4	3	3
1	1	2	2	4	4	4	4	3	3
1	1	2	2	4	4	4	4	3	3
1	1	2	2	1	1	4	4	3	3
1	1	2	2	1	1	4	4	3	3
1	1	2	2	4	4	4	4	3	3
1	1	2	2	1	1	4	4	3	3
1	1	2	2	1	1	4	4	3	3
1	1	2	2	1	1	4	4	3	3
4	4	2	2	1	1	4	4	2	2
1	1	2	2	4	4	4	4	3	3
1	1	2	2	1	1	4	4	3	3
1	1	2	2	1	1	4	4	3	3
4	4	2	2	1	1	4	4	3	3
1	1	2	2	4	4	4	4	3	3
1	1	2	2	4	4	4	4	3	3
1	1	2	2	4	4	4	4	2	2
1	1	2	2	4	4	4	4	3	3
1	1	2	2	4	4	4	4	3	3
1	1	2	2	1	1	4	4	2	2
1	1	2	2	4	4	4	4	3	3
1	1	2	2	4	4	4	4	3	3
1	1	2	2	1	1	4	4	3	3
1	1	2	2	1	1	4	4	3	3
1	1	2	2	4	4	4	4	3	3
1	1	2	2	4	4	4	4	3	3
1	1	2	2	4	4	4	4	3	3
1	1	2	2	4	4	4	4	3	3
1	1	2	2	4	4	4	4	3	3
1	1	2	2	4	4	4	4	3	3
1	1	2	2	4	4	4	4	3	3
1	1	2	2	1	1	4	4	3	3
1	1	2	2	4	4	4	4	3	3
1	1	2	2	4	4	4	4	3	3
1	1	2	2	4	4	4	4	3	3
1	1	2	2	1	1	4	4	3	3
1	1	2	2	4	4	4	4	3	3
1	1	2	2	4	4	4	4	2	2
1	1	2	2	4	4	4	4	3	3
1	1	2	2	4	4	4	4	3	3
1	1	2	2	1	1	4	4	3	3
1	1	2	2	4	4	4	4	3	3
1	1	2	2	1	1	4	4	3	3
1	1	2	2	4	4	4	4	3	3
4	4	4	4	1	1	3	3	3	3
1	1	4	4	1	1	3	3	3	3
1	1	2	2	1	1	4	4	3	3
1	1	2	2	4	4	4	4	3	3
1	1	2	2	4	4	4	4	3	3
1	1	2	2	4	4	4	4	3	3
1	1	2	2	4	4	4	4	3	3
1	1	2	2	4	4	4	4	3	3
1	1	4	4	1	1	3	3	3	3

1	1	2	2	1	1	4	4	3	3
1	1	2	2	4	4	4	4	3	3
1	1	2	2	4	4	4	4	3	3
1	1	2	2	4	4	4	4	3	3
1	1	2	2	4	4	4	4	3	3
1	1	2	2	4	4	4	4	3	3
1	1	2	2	1	1	4	4	3	3
1	1	2	2	1	1	4	4	3	3
1	1	2	2	1	1	4	4	3	3
4	4	2	2	1	1	4	4	3	3
1	1	2	2	4	4	4	4	3	3
1	1	2	2	4	4	4	4	3	3
1	1	2	2	1	1	4	4	3	3
1	1	2	2	4	4	4	4	2	2
1	1	2	2	4	4	4	4	3	3
1	1	2	2	1	1	4	4	3	3
1	1	2	2	1	1	4	4	3	3
1	1	2	2	1	1	4	4	3	3
1	1	4	4	1	1	3	3	3	3
1	1	2	2	1	1	4	4	3	3
4	4	2	2	4	4	4	4	3	3
1	1	2	2	4	4	4	4	3	3
1	1	2	2	4	4	4	4	3	3
4	4	2	2	1	1	4	4	3	3
1	1	2	2	1	1	4	4	3	3
1	1	2	2	4	4	4	4	3	3
1	1	2	2	1	1	4	4	3	3
1	1	2	2	4	4	4	4	3	3
1	1	2	2	4	4	4	4	3	3
1	1	2	2	4	4	4	4	3	3
4	4	4	4	1	1	3	3	3	3
1	1	2	2	4	4	4	4	3	3
1	1	2	2	1	1	4	4	3	3
1	1	2	2	4	4	4	4	2	2
4	4	2	2	4	4	4	4	3	3
1	1	2	2	1	1	4	4	3	3
1	1	2	2	1	1	4	4	3	3
1	1	2	2	4	4	4	4	3	3
1	1	2	2	1	1	4	4	3	3
4	4	4	4	1	1	3	3	3	3
1	1	2	2	1	1	4	4	3	3
1	1	2	2	4	4	4	4	3	3
4	4	2	2	1	1	4	4	3	3
1	1	2	2	4	4	4	4	3	3
1	1	4	4	4	4	3	3	3	3
1	1	2	2	1	1	4	4	3	3
1	1	2	2	1	1	4	4	3	3
1	1	2	2	4	4	4	4	3	3
1	1	2	2	4	4	4	4	3	3
1	1	2	2	1	1	4	4	3	3
1	1	2	2	1	1	4	4	3	3
1	1	4	4	1	1	3	3	3	3
1	1	2	2	4	4	4	4	3	3
1	1	2	2	4	4	4	4	3	3
4	4	2	2	1	1	4	4	3	3

For Review Only

1	1	2	2	1	1	4	4	3	3
1	1	2	2	4	4	4	4	3	3

For Review Only

SNP2872	SNP2872	SNP2873	SNP2873	SNP2874	SNP2874	SNP2875	SNP2875	SNP2876	SNP2876
2	2	4	4	2	2	4	4	4	4
2	2	4	4	2	2	1	1	1	1
2	2	4	4	2	2	1	1	1	1
2	2	4	4	2	2	1	1	1	1
2	2	4	4	2	2	4	4	4	4
2	2	3	3	2	2	1	1	4	4
2	2	4	4	2	2	4	4	4	4
2	2	4	4	3	3	1	1	4	4
2	2	4	4	2	2	4	4	4	4
2	2	4	4	2	2	1	1	4	4
2	2	4	4	2	2	1	1	4	4
2	2	4	4	2	2	1	1	1	1
2	2	4	4	3	3	4	4	4	4
2	2	4	4	2	2	4	4	4	4
2	2	4	4	2	2	4	4	4	4
2	2	4	4	2	2	1	1	1	1
2	2	4	4	2	2	4	4	4	4
2	2	4	4	3	3	4	4	4	4
2	2	4	4	2	2	1	1	1	1
2	2	3	3	3	3	1	1	4	4
2	2	4	4	2	2	1	1	4	4
2	2	4	4	2	2	4	4	4	4
2	2	4	4	2	2	1	1	4	4
3	3	4	4	2	2	4	4	4	4
2	2	4	4	2	2	1	1	1	1
2	2	4	4	2	2	1	1	1	1
2	2	4	4	2	2	4	4	4	4
2	2	4	4	2	2	4	4	4	4
2	2	4	4	2	2	4	4	4	4
2	2	3	3	3	3	4	4	4	4
2	2	4	4	2	2	1	1	1	1
2	2	4	4	2	2	1	1	1	1
2	2	4	4	2	2	4	4	4	4
2	2	4	4	2	2	4	4	4	4
2	2	4	4	2	2	1	1	4	4
2	2	4	4	2	2	1	1	1	1
2	2	4	4	2	2	4	4	4	4
2	2	4	4	2	2	1	1	4	4
2	2	4	4	2	2	1	1	4	4
2	2	4	4	2	2	1	1	1	1
2	2	4	4	3	3	4	4	4	4
2	2	4	4	2	2	1	1	4	4
2	2	4	4	2	2	1	1	4	4
2	2	4	4	2	2	4	4	4	4
2	2	4	4	2	2	1	1	4	4
2	2	4	4	2	2	1	1	1	1
2	2	4	4	2	2	1	1	1	1
3	3	4	4	2	2	4	4	4	4
2	2	4	4	2	2	4	4	4	4
2	2	4	4	2	2	4	4	4	4
2	2	4	4	2	2	1	1	1	1
2	2	4	4	2	2	4	4	4	4

For Review Only

2	2	4	4	2	2	4	4	4	4
2	2	4	4	2	2	4	4	4	4
2	2	4	4	2	2	4	4	4	4
2	2	4	4	2	2	4	4	4	4
2	2	4	4	2	2	1	1	1	1
2	2	4	4	2	2	1	1	1	1
2	2	4	4	2	2	1	1	1	1
2	2	4	4	2	2	4	4	4	4
2	2	4	4	2	2	4	4	4	4
2	2	4	4	2	2	4	4	4	4
2	2	4	4	2	2	1	1	1	1
2	2	4	4	2	2	1	1	1	1
2	2	4	4	2	2	4	4	4	4
2	2	4	4	2	2	4	4	4	4
2	2	4	4	2	2	4	4	4	4
2	2	4	4	2	2	4	4	4	4
2	2	4	4	2	2	4	4	4	4
2	2	4	4	2	2	4	4	4	4
2	2	4	4	2	2	4	4	4	4
2	2	4	4	2	2	4	4	4	4
2	2	4	4	2	2	1	1	4	4
2	2	4	4	2	2	1	1	4	4
2	2	4	4	2	2	4	4	4	4
2	2	4	4	2	2	1	1	4	4
2	2	4	4	2	2	1	1	4	4
2	2	4	4	2	2	1	1	1	1
2	2	4	4	2	2	4	4	4	4
2	2	4	4	3	3	4	4	4	4
2	2	4	4	2	2	1	1	1	1
2	2	4	4	2	2	4	4	4	4
2	2	4	4	2	2	4	4	4	4
3	3	4	4	2	2	1	1	4	4
2	2	4	4	3	3	4	4	4	4
2	2	4	4	2	2	4	4	4	4
2	2	4	4	2	2	4	4	4	4
2	2	4	4	2	2	1	1	4	4
2	2	4	4	2	2	1	1	4	4
2	2	4	4	3	3	4	4	4	4
2	2	3	3	2	2	1	1	4	4
2	2	4	4	2	2	1	1	4	4
2	2	4	4	2	2	4	4	4	4
2	2	4	4	2	2	1	1	1	1
2	2	4	4	2	2	1	1	1	1
2	2	4	4	2	2	4	4	1	1
2	2	4	4	2	2	4	4	4	4
2	2	4	4	2	2	1	1	1	1
2	2	4	4	2	2	1	1	4	4
3	3	4	4	2	2	1	1	4	4
3	3	4	4	2	2	1	1	1	1
2	2	4	4	2	2	1	1	4	4
2	2	4	4	2	2	1	1	1	1
2	2	4	4	2	2	1	1	4	4
2	2	4	4	2	2	4	4	4	4
2	2	4	4	2	2	1	1	4	4
2	2	4	4	2	2	1	1	1	1

For Review Only

2	2	4	4	2	2	1	1	1	1
2	2	4	4	2	2	1	1	4	4
2	2	4	4	2	2	4	4	4	4
2	2	4	4	2	2	1	1	4	4
2	2	4	4	2	2	1	1	1	1
2	2	4	4	2	2	1	1	1	1
2	2	4	4	2	2	1	1	1	1
3	3	4	4	2	2	1	1	4	4
3	3	4	4	2	2	1	1	4	4
2	2	4	4	2	2	1	1	4	4
2	2	4	4	2	2	1	1	4	4
3	3	4	4	2	2	4	4	1	1
2	2	4	4	2	2	4	4	4	4
2	2	4	4	2	2	1	1	4	4
3	3	3	3	2	2	4	4	4	4
2	2	4	4	2	2	4	4	4	4
2	2	4	4	2	2	4	4	4	4
2	2	4	4	2	2	4	4	4	4
2	2	4	4	2	2	1	1	4	4
2	2	4	4	2	2	4	4	4	4
2	2	4	4	2	2	4	4	4	4
2	2	4	4	2	2	4	4	4	4
2	2	4	4	2	2	1	1	1	1
2	2	4	4	2	2	1	1	4	4
2	2	4	4	2	2	1	1	4	4
2	2	4	4	2	2	1	1	4	4
2	2	4	4	2	2	4	4	4	4
2	2	4	4	2	2	1	1	4	4
2	2	4	4	2	2	1	1	1	1
2	2	4	4	2	2	4	4	4	4
2	2	4	4	2	2	4	4	4	4
2	2	4	4	2	2	1	1	4	4
2	2	4	4	2	2	4	4	4	4
2	2	4	4	2	2	1	1	4	4
2	2	4	4	2	2	4	4	4	4
2	2	4	4	2	2	4	4	4	4
2	2	4	4	2	2	1	1	4	4
2	2	4	4	2	2	4	4	4	4
2	2	4	4	2	2	4	4	4	4
2	2	4	4	2	2	4	4	4	4
2	2	4	4	2	2	1	1	4	4
2	2	4	4	2	2	4	4	4	4
2	2	4	4	2	2	1	1	1	1
2	2	4	4	2	2	4	4	4	4
2	2	4	4	2	2	4	4	4	4
2	2	4	4	2	2	1	1	4	4
2	2	4	4	2	2	1	1	1	1
2	2	3	3	2	2	1	1	1	1
2	2	4	4	2	2	4	4	4	4
2	2	4	4	2	2	1	1	1	1
2	2	4	4	2	2	1	1	1	1
2	2	4	4	2	2	1	1	4	4
2	2	4	4	2	2	1	1	4	4
3	3	4	4	2	2	1	1	1	1
2	2	4	4	2	2	1	1	4	4

For Review Only

2	2	4	4	2	2	4	4	4	4
2	2	4	4	2	2	1	1	4	4
2	2	4	4	2	2	4	4	4	4
2	2	4	4	2	2	1	1	4	4
2	2	4	4	2	2	1	1	1	1
2	2	4	4	2	2	4	4	4	4
2	2	4	4	2	2	4	4	4	4
2	2	4	4	2	2	4	4	4	4
2	2	4	4	2	2	4	4	4	4
2	2	4	4	2	2	4	4	4	4
2	2	4	4	2	2	1	1	1	1
3	3	4	4	2	2	1	1	4	4
2	2	4	4	2	2	1	1	1	1
2	2	4	4	2	2	4	4	4	4
2	2	4	4	2	2	1	1	4	4
2	2	4	4	2	2	1	1	4	4
2	2	4	4	2	2	4	4	4	4
2	2	4	4	2	2	1	1	1	1
3	3	4	4	2	2	1	1	1	1
2	2	4	4	2	2	1	1	1	1
2	2	4	4	2	2	4	4	4	4
3	3	4	4	2	2	4	4	4	4
2	2	4	4	2	2	1	1	4	4
2	2	4	4	2	2	1	1	1	1
2	2	3	3	2	2	4	4	4	4
2	2	4	4	2	2	4	4	4	4
2	2	4	4	2	2	1	1	1	1
2	2	3	3	2	2	4	4	4	4
2	2	4	4	2	2	4	4	4	4
2	2	4	4	2	2	4	4	4	4
2	2	4	4	2	2	4	4	4	4
2	2	4	4	2	2	4	4	4	4
2	2	4	4	2	2	4	4	4	4
2	2	4	4	2	2	1	1	4	4
2	2	4	4	2	2	4	4	4	4
2	2	4	4	2	2	1	1	1	1
2	2	4	4	2	2	1	1	1	1
2	2	4	4	2	2	4	4	4	4
2	2	3	3	2	2	1	1	1	1
3	3	3	3	2	2	1	1	1	1
2	2	4	4	2	2	1	1	1	1
2	2	4	4	2	2	1	1	1	1
2	2	4	4	2	2	4	4	4	4
2	2	4	4	2	2	4	4	4	4
2	2	4	4	2	2	1	1	4	4
2	2	4	4	2	2	4	4	4	4
2	2	4	4	2	2	1	1	4	4
2	2	4	4	3	3	4	4	4	4
2	2	4	4	2	2	1	1	4	4
2	2	4	4	2	2	4	4	4	4
2	2	4	4	2	2	1	1	1	1
2	2	4	4	2	2	4	4	4	4
2	2	4	4	2	2	1	1	4	4
2	2	4	4	2	2	1	1	1	1
2	2	4	4	2	2	1	1	4	4

For Review Only

2	2	4	4	2	2	4	4	4	4
2	2	4	4	2	2	1	1	4	4
2	2	3	3	2	2	1	1	1	1
2	2	4	4	2	2	4	4	4	4
2	2	4	4	3	3	4	4	4	4
2	2	4	4	2	2	4	4	4	4
2	2	4	4	2	2	1	1	4	4
2	2	3	3	3	3	1	1	4	4
2	2	4	4	2	2	1	1	4	4
2	2	4	4	2	2	4	4	4	4
2	2	4	4	2	2	4	4	4	4
2	2	4	4	2	2	1	1	4	4
2	2	4	4	2	2	1	1	4	4
3	3	4	4	2	2	1	1	1	1
2	2	4	4	2	2	1	1	1	1
2	2	4	4	2	2	4	4	4	4
2	2	4	4	2	2	1	1	4	4
2	2	4	4	2	2	4	4	4	4
2	2	4	4	2	2	1	1	4	4
2	2	4	4	2	2	1	1	1	1
2	2	4	4	2	2	1	1	4	4
2	2	4	4	2	2	1	1	1	1
2	2	4	4	2	2	4	4	4	4
2	2	4	4	2	2	1	1	4	4
2	2	4	4	2	2	4	4	4	4
3	3	3	3	2	2	1	1	4	4
2	2	4	4	3	3	4	4	4	4
2	2	4	4	2	2	1	1	1	1
2	2	4	4	2	2	4	4	4	4
2	2	4	4	2	2	1	1	1	1
2	2	4	4	2	2	4	4	4	4
2	2	4	4	2	2	1	1	1	1
2	2	4	4	2	2	4	4	4	4
3	3	3	3	2	2	1	1	1	1
2	2	4	4	2	2	1	1	4	4
2	2	4	4	2	2	4	4	4	4
2	2	4	4	2	2	4	4	4	4
2	2	4	4	2	2	1	1	1	1
2	2	4	4	2	2	1	1	1	1
2	2	4	4	2	2	1	1	4	4
2	2	4	4	2	2	4	4	4	4
2	2	4	4	2	2	4	4	4	4
2	2	4	4	2	2	1	1	4	4
2	2	4	4	2	2	4	4	4	4
2	2	4	4	2	2	4	4	4	4
2	2	4	4	2	2	1	1	4	4
2	2	3	3	2	2	1	1	1	1
2	2	4	4	2	2	1	1	1	1
2	2	4	4	2	2	1	1	4	4
2	2	4	4	3	3	4	4	4	4
2	2	4	4	2	2	4	4	4	4
2	2	4	4	2	2	1	1	4	4
2	2	4	4	2	2	1	1	1	1
2	2	3	3	2	2	1	1	1	1
2	2	4	4	2	2	1	1	1	1

For Review Only

2	2	4	4	2	2	4	4	4	4
2	2	4	4	2	2	1	1	1	1

For Review Only

SNP2877	SNP2877	SNP2878	SNP2878	SNP2879	SNP2879	SNP288	SNP288	SNP2880	SNP2880
3	3	4	4	3	3	1	1	1	1
2	2	2	2	2	2	1	1	1	1
2	2	2	2	2	2	1	1	1	1
3	3	4	4	2	2	1	1	1	1
2	2	2	2	2	2	1	1	2	2
2	2	2	2	2	2	1	1	1	1
2	2	2	2	2	2	1	1	1	1
2	2	2	2	3	3	1	1	1	1
2	2	2	2	3	3	1	1	1	1
2	2	2	2	2	2	1	1	1	1
2	2	2	2	2	2	1	1	1	1
2	2	2	2	2	2	1	1	1	1
2	2	2	2	2	2	1	1	1	1
3	3	4	4	3	3	1	1	2	2
2	2	2	2	2	2	1	1	1	1
2	2	2	2	2	2	1	1	1	1
2	2	2	2	3	3	1	1	1	1
2	2	2	2	3	3	1	1	1	1
2	2	2	2	2	2	1	1	1	1
2	2	2	2	2	2	1	1	1	1
2	2	2	2	2	2	1	1	2	2
2	2	2	2	2	2	1	1	1	1
2	2	2	2	3	3	1	1	1	1
2	2	2	2	2	2	1	1	1	1
2	2	2	2	2	2	1	1	1	1
2	2	2	2	2	2	1	1	1	1
2	2	2	2	2	2	1	1	1	1
2	2	2	2	2	2	1	1	1	1
2	2	2	2	2	2	1	1	1	1
2	2	2	2	2	2	4	4	2	2
2	2	2	2	2	2	1	1	1	1
2	2	2	2	2	2	1	1	1	1
2	2	2	2	3	3	1	1	1	1
3	3	4	4	2	2	1	1	2	2
2	2	2	2	2	2	1	1	1	1
2	2	2	2	2	2	1	1	1	1
3	3	4	4	2	2	1	1	1	1
3	3	4	4	3	3	4	4	1	1
2	2	2	2	3	3	1	1	2	2
2	2	2	2	2	2	4	4	1	1
2	2	2	2	2	2	1	1	1	1
2	2	2	2	3	3	1	1	1	1
2	2	2	2	2	2	1	1	1	1
2	2	2	2	3	3	1	1	1	1
2	2	2	2	2	2	4	4	1	1
2	2	2	2	2	2	1	1	1	1
2	2	2	2	2	2	1	1	1	1
2	2	2	2	2	2	1	1	1	1
2	2	2	2	2	2	1	1	1	1
2	2	2	2	2	2	1	1	1	1
2	2	2	2	2	2	1	1	1	1
2	2	2	2	2	2	1	1	1	1

For Review Only

2	2	2	2	2	2	1	1	1	1
2	2	2	2	3	3	1	1	1	1
2	2	2	2	2	2	1	1	1	1
2	2	2	2	3	3	1	1	2	2
2	2	2	2	2	2	1	1	1	1
2	2	2	2	2	2	1	1	1	1
2	2	2	2	2	2	1	1	1	1
2	2	2	2	2	2	1	1	1	1
2	2	2	2	3	3	1	1	1	1
2	2	2	2	2	2	1	1	1	1
2	2	2	2	2	2	1	1	1	1
2	2	2	2	2	2	1	1	1	1
2	2	2	2	2	2	1	1	1	1
2	2	2	2	2	2	1	1	1	1
2	2	2	2	2	2	1	1	1	1
2	2	2	2	2	2	1	1	1	1
3	3	4	4	2	2	1	1	1	1
2	2	2	2	2	2	1	1	1	1
2	2	2	2	2	2	1	1	1	1
2	2	2	2	3	3	1	1	1	1
2	2	2	2	2	2	1	1	1	1
2	2	2	2	2	2	4	4	1	1
2	2	2	2	3	3	1	1	1	1
2	2	2	2	2	2	1	1	1	1
2	2	2	2	2	2	4	4	1	1
3	3	4	4	2	2	1	1	1	1
2	2	2	2	2	2	4	4	1	1
2	2	2	2	2	2	1	1	1	1
2	2	2	2	2	2	1	1	1	1
3	3	4	4	2	2	1	1	1	1
2	2	2	2	3	3	1	1	1	1
2	2	2	2	2	2	1	1	1	1
2	2	2	2	2	2	1	1	2	2
2	2	2	2	2	2	1	1	1	1
2	2	2	2	2	2	1	1	1	1
2	2	2	2	2	2	4	4	1	1
2	2	2	2	3	3	1	1	1	1
2	2	2	2	2	2	1	1	1	1
2	2	2	2	2	2	1	1	1	1
2	2	2	2	2	2	1	1	1	1
2	2	2	2	2	2	1	1	1	1
3	3	4	4	2	2	1	1	1	1
2	2	2	2	3	3	1	1	1	1
2	2	2	2	2	2	1	1	1	1
2	2	2	2	2	2	1	1	1	1
2	2	2	2	2	2	1	1	1	1
3	3	4	4	2	2	1	1	2	2
2	2	2	2	2	2	1	1	1	1
2	2	2	2	2	2	1	1	1	1
2	2	2	2	3	3	1	1	1	1
3	3	2	2	2	2	1	1	1	1
2	2	2	2	3	3	1	1	1	1
2	2	2	2	3	3	1	1	1	1
2	2	2	2	2	2	1	1	1	1
2	2	2	2	3	3	1	1	1	1

2	2	2	2	2	2	1	1	1	1
2	2	2	2	2	2	4	4	1	1
2	2	2	2	2	2	1	1	1	1
2	2	2	2	2	2	1	1	1	1
2	2	2	2	2	2	1	1	1	1
2	2	2	2	2	2	1	1	1	1
2	2	2	2	2	2	1	1	1	1
2	2	2	2	2	2	1	1	1	1
2	2	2	2	2	2	1	1	2	2
2	2	2	2	2	2	1	1	1	1
3	3	2	2	2	2	1	1	1	1
2	2	2	2	2	2	1	1	1	1
2	2	2	2	2	2	1	1	1	1
2	2	2	2	2	2	1	1	1	1
2	2	2	2	2	2	1	1	1	1
2	2	2	2	2	2	1	1	1	1
2	2	2	2	2	2	1	1	1	1
2	2	2	2	2	2	1	1	1	1
2	2	2	2	2	2	1	1	1	1
2	2	2	2	3	3	1	1	1	1
2	2	2	2	2	2	1	1	1	1
2	2	2	2	2	2	1	1	1	1
2	2	2	2	2	2	1	1	1	1
2	2	2	2	2	2	1	1	1	1
2	2	2	2	2	2	1	1	1	1
2	2	2	2	2	2	1	1	1	1
2	2	2	2	2	2	1	1	1	1
3	3	4	4	2	2	1	1	1	1
2	2	2	2	2	2	1	1	1	1
2	2	2	2	2	2	1	1	1	1
2	2	2	2	2	2	1	1	2	2
2	2	2	2	2	2	1	1	1	1
2	2	2	2	2	2	1	1	1	1
2	2	2	2	2	2	1	1	1	1
3	3	4	4	2	2	1	1	2	2
2	2	2	2	3	3	1	1	1	1
2	2	2	2	2	2	1	1	1	1
2	2	2	2	2	2	1	1	1	1
2	2	2	2	2	2	1	1	1	1
2	2	2	2	2	2	1	1	1	1
2	2	2	2	2	2	1	1	1	1
3	3	4	4	2	2	1	1	2	2
3	3	4	4	2	2	1	1	1	1
2	2	2	2	2	2	1	1	1	1
2	2	2	2	2	2	1	1	1	1
2	2	2	2	2	2	1	1	1	1
2	2	2	2	2	2	1	1	1	1
2	2	2	2	2	2	1	1	1	1
2	2	4	4	2	2	1	1	1	1
2	2	2	2	2	2	1	1	1	1
2	2	2	2	2	2	1	1	1	1
2	2	2	2	2	2	1	1	2	2
2	2	2	2	2	2	1	1	1	1
2	2	2	2	2	2	1	1	1	1
2	2	2	2	2	2	1	1	1	1

For Review Only

2	2	2	2	2	2	1	1	1	1
2	2	2	2	3	3	1	1	1	1
2	2	2	2	2	2	1	1	1	1
2	2	2	2	2	2	1	1	1	1
3	3	4	4	2	2	1	1	1	1
3	3	4	4	3	3	1	1	1	1
2	2	2	2	2	2	1	1	1	1
2	2	2	2	2	2	1	1	1	1
2	2	2	2	3	3	1	1	1	1
2	2	2	2	2	2	1	1	1	1
2	2	2	2	2	2	1	1	1	1
3	3	2	2	2	2	1	1	1	1
2	2	2	2	2	2	1	1	1	1
2	2	2	2	2	2	1	1	1	1
3	3	4	4	2	2	1	1	2	2
2	2	2	2	3	3	1	1	1	1
2	2	2	2	2	2	1	1	1	1
2	2	2	2	2	2	1	1	1	1
2	2	2	2	2	2	1	1	1	1
2	2	2	2	2	2	1	1	1	1
2	2	2	2	2	2	1	1	1	1
2	2	2	2	2	2	1	1	1	1
2	2	2	2	2	2	4	4	1	1
2	2	2	2	2	2	1	1	1	1
2	2	2	2	2	2	1	1	1	1
3	3	4	4	2	2	1	1	2	2
2	2	2	2	2	2	1	1	1	1
2	2	2	2	2	2	1	1	1	1
2	2	2	2	2	2	1	1	1	1
2	2	2	2	2	2	1	1	1	1
2	2	2	2	2	2	1	1	1	1
2	2	2	2	2	2	1	1	1	1
2	2	2	2	2	2	1	1	1	1
3	3	4	4	2	2	1	1	2	2
3	3	4	4	2	2	1	1	1	1
2	2	2	2	2	2	1	1	1	1
2	2	2	2	2	2	4	4	1	1
3	3	4	4	3	3	1	1	1	1
2	2	2	2	2	2	1	1	1	1
2	2	2	2	2	2	1	1	1	1
2	2	2	2	2	2	1	1	1	1
2	2	2	2	2	2	1	1	1	1
2	2	2	2	3	3	1	1	2	2
2	2	2	2	2	2	1	1	1	1
2	2	2	2	2	2	1	1	1	1
2	2	2	2	3	3	1	1	1	1
2	2	2	2	3	3	1	1	1	1
2	2	2	2	2	2	1	1	1	1
2	2	2	2	3	3	1	1	1	1
2	2	2	2	2	2	1	1	1	1
2	2	2	2	3	3	1	1	1	1
3	3	4	4	2	2	1	1	1	1
2	2	2	2	2	2	4	4	1	1
2	2	2	2	2	2	1	1	1	1
2	2	2	2	3	3	1	1	1	1

For Review Only

3	3	4	4	2	2	1	1	1	1
2	2	2	2	2	2	1	1	1	1
2	2	2	2	2	2	1	1	1	1
2	2	2	2	2	2	1	1	1	1
2	2	2	2	2	2	1	1	1	1
2	2	2	2	2	2	1	1	1	1
2	2	2	2	2	2	1	1	1	1
2	2	2	2	2	2	1	1	1	1
2	2	2	2	2	2	1	1	1	1
2	2	2	2	2	2	1	1	1	1
2	2	2	2	2	2	1	1	1	1
2	2	2	2	3	3	1	1	1	1
2	2	2	2	2	2	1	1	1	1
2	2	2	2	2	2	1	1	1	1
2	2	2	2	2	2	1	1	1	1
2	2	2	2	2	2	1	1	1	1
2	2	2	2	3	3	1	1	1	1
2	2	2	2	2	2	1	1	2	2
2	2	2	2	2	2	4	4	1	1
3	3	4	4	3	3	4	4	1	1
2	2	2	2	2	2	4	4	1	1
2	2	2	2	2	2	1	1	1	1
2	2	2	2	2	2	1	1	1	1
3	3	4	4	3	3	1	1	1	1
2	2	2	2	2	2	1	1	1	1
2	2	2	2	2	2	1	1	1	1
2	2	2	2	2	2	1	1	2	2
2	2	2	2	2	2	1	1	1	1
2	2	2	2	2	2	1	1	1	1
2	2	2	2	2	2	1	1	1	1
2	2	2	2	3	3	1	1	2	2
2	2	2	2	2	2	1	1	1	1
2	2	2	2	3	3	1	1	1	1
2	2	2	2	2	2	1	1	1	1
2	2	2	2	2	2	4	4	1	1
2	2	2	2	2	2	1	1	2	2
2	2	2	2	2	2	1	1	1	1
2	2	2	2	2	2	1	1	1	1
2	2	2	2	2	2	1	1	1	1
2	2	2	2	3	3	1	1	1	1
3	3	4	4	2	2	1	1	1	1
2	2	2	2	2	2	1	1	1	1
3	3	4	4	3	3	1	1	1	1
2	2	2	2	2	2	1	1	1	1
2	2	2	2	2	2	1	1	1	1
3	3	4	4	2	2	1	1	1	1
2	2	2	2	2	2	1	1	1	1
2	2	2	2	3	3	1	1	1	1
2	2	2	2	2	2	1	1	1	1
2	2	2	2	2	2	1	1	2	2
3	3	4	4	2	2	1	1	1	1
2	2	2	2	3	3	1	1	1	1
2	2	2	2	2	2	1	1	1	1
2	2	2	2	2	2	1	1	1	1
3	3	4	4	3	3	1	1	1	1

For Review Only

2	2	2	2	2	2	1	1	1	1
2	2	2	2	2	2	1	1	1	1

For Review Only

SNP2881	SNP2881	SNP2882	SNP2882	SNP2883	SNP2883	SNP2884	SNP2884	SNP2885	SNP2885
2	2	2	2	3	3	3	3	2	2
3	3	3	3	4	4	1	1	4	4
3	3	3	3	4	4	1	1	4	4
3	3	2	2	3	3	1	1	4	4
2	2	3	3	4	4	3	3	2	2
2	2	3	3	4	4	1	1	2	2
2	2	3	3	4	4	3	3	2	2
2	2	3	3	4	4	3	3	4	4
2	2	2	2	3	3	3	3	2	2
2	2	3	3	4	4	3	3	2	2
2	2	3	3	4	4	1	1	2	2
3	3	3	3	4	4	1	1	4	4
2	2	3	3	4	4	3	3	2	2
2	2	3	3	3	3	3	3	2	2
2	2	2	2	3	3	3	3	2	2
3	3	2	2	3	3	1	1	4	4
2	2	2	2	3	3	3	3	2	2
2	2	3	3	4	4	3	3	2	2
3	3	3	3	4	4	1	1	2	2
2	2	3	3	4	4	3	3	2	2
2	2	3	3	4	4	3	3	4	4
2	2	2	2	3	3	3	3	2	2
2	2	3	3	4	4	3	3	2	2
2	2	3	3	4	4	3	3	2	2
2	2	2	2	3	3	3	3	2	2
3	3	3	3	4	4	3	3	4	4
2	2	3	3	4	4	1	1	2	2
2	2	3	3	3	3	1	1	2	2
2	2	2	2	3	3	3	3	2	2
2	2	2	2	3	3	3	3	4	4
3	3	3	3	4	4	1	1	4	4
3	3	3	3	4	4	1	1	2	2
2	2	2	2	3	3	3	3	2	2
2	2	2	2	3	3	3	3	2	2
2	2	3	3	4	4	1	1	2	2
3	3	3	3	4	4	1	1	4	4
2	2	2	2	3	3	1	1	2	2
3	3	3	3	4	4	3	3	2	2
2	2	3	3	4	4	3	3	2	2
3	3	3	3	4	4	3	3	4	4
2	2	3	3	4	4	3	3	2	2
2	2	2	2	3	3	3	3	4	4
2	2	3	3	4	4	3	3	2	2
2	2	2	2	3	3	3	3	2	2
2	2	3	3	4	4	3	3	2	2
3	3	3	3	4	4	1	1	4	4
3	3	3	3	4	4	1	1	2	2
2	2	3	3	4	4	3	3	2	2
2	2	2	2	3	3	3	3	2	2
2	2	3	3	4	4	1	1	2	2
3	3	3	3	4	4	1	1	4	4
2	2	3	3	4	4	1	1	2	2

For Review Only

2	2	2	2	3	3	3	3	2	2
2	2	2	2	3	3	3	3	2	2
2	2	3	3	4	4	1	1	2	2
2	2	2	2	3	3	3	3	2	2
3	3	3	3	4	4	1	1	2	2
3	3	3	3	4	4	1	1	4	4
3	3	3	3	4	4	1	1	4	4
2	2	3	3	4	4	3	3	2	2
2	2	3	3	4	4	3	3	2	2
2	2	3	3	4	4	1	1	2	2
3	3	3	3	4	4	1	1	4	4
3	3	3	3	4	4	1	1	4	4
2	2	2	2	3	3	3	3	2	2
2	2	2	2	3	3	3	3	2	2
2	2	3	3	3	3	1	1	2	2
2	2	3	3	4	4	1	1	2	2
2	2	3	3	4	4	1	1	2	2
2	2	3	3	4	4	1	1	2	2
2	2	2	2	3	3	3	3	2	2
2	2	3	3	4	4	3	3	2	2
3	3	3	3	4	4	1	1	4	4
2	2	3	3	4	4	1	1	2	2
2	2	2	2	4	4	3	3	2	2
2	2	3	3	4	4	1	1	2	2
2	2	3	3	4	4	3	3	2	2
3	3	3	3	4	4	1	1	4	4
2	2	3	3	3	3	3	3	2	2
2	2	3	3	4	4	3	3	2	2
3	3	3	3	4	4	1	1	4	4
2	2	2	2	3	3	1	1	2	2
2	2	2	2	3	3	3	3	2	2
2	2	3	3	4	4	1	1	2	2
2	2	3	3	4	4	3	3	2	2
2	2	2	2	3	3	3	3	2	2
2	2	3	3	4	4	1	1	2	2
2	2	3	3	4	4	3	3	2	2
2	2	3	3	4	4	3	3	2	2
2	2	2	2	3	3	3	3	2	2
2	2	3	3	4	4	1	1	2	2
2	2	3	3	4	4	3	3	2	2
2	2	3	3	4	4	3	3	2	2
3	3	2	2	3	3	1	1	4	4
3	3	3	3	4	4	3	3	4	4
3	3	3	3	4	4	1	1	4	4
2	2	2	2	3	3	1	1	2	2
3	3	3	3	4	4	1	1	4	4
2	2	2	2	3	3	3	3	2	2
2	2	3	3	4	4	1	1	2	2
3	3	3	3	4	4	3	3	2	2
2	2	3	3	4	4	3	3	2	2
3	3	3	3	4	4	1	1	2	2
2	2	3	3	4	4	3	3	2	2
2	2	3	3	4	4	3	3	2	2
2	2	3	3	4	4	1	1	2	2
3	3	3	3	4	4	3	3	2	2

For Review Only

3	3	3	3	4	4	1	1	4	4
2	2	3	3	4	4	3	3	2	2
2	2	3	3	4	4	1	1	2	2
2	2	3	3	4	4	3	3	2	2
3	3	3	3	4	4	1	1	4	4
3	3	3	3	4	4	1	1	4	4
3	3	2	2	3	3	1	1	4	4
2	2	3	3	4	4	3	3	2	2
2	2	3	3	4	4	3	3	2	2
2	2	3	3	4	4	1	1	2	2
2	2	3	3	4	4	1	1	2	2
2	2	3	3	4	4	1	1	2	2
2	2	3	3	4	4	3	3	2	2
2	2	3	3	4	4	1	1	2	2
2	2	2	2	4	4	1	1	4	4
3	3	3	3	4	4	1	1	2	2
2	2	3	3	4	4	3	3	2	2
2	2	3	3	4	4	1	1	4	4
2	2	3	3	4	4	3	3	2	2
2	2	3	3	4	4	3	3	2	2
2	2	3	3	4	4	1	1	2	2
2	2	3	3	4	4	1	1	2	2
3	3	3	3	4	4	1	1	4	4
2	2	3	3	4	4	3	3	2	2
2	2	3	3	4	4	1	1	2	2
2	2	3	3	4	4	1	1	2	2
2	2	3	3	4	4	3	3	2	2
2	2	2	2	3	3	3	3	2	2
3	3	3	3	4	4	1	1	4	4
2	2	3	3	4	4	1	1	2	2
2	2	3	3	4	4	3	3	2	2
2	2	3	3	4	4	1	1	2	2
2	2	3	3	4	4	3	3	2	2
3	3	3	3	4	4	1	1	2	2
2	2	2	2	3	3	3	3	2	2
2	2	3	3	4	4	3	3	2	2
2	2	3	3	4	4	3	3	2	2
2	2	3	3	4	4	1	1	2	2
2	2	3	3	4	4	1	1	2	2
2	2	3	3	4	4	3	3	2	2
2	2	3	3	4	4	1	1	2	2
2	2	2	2	3	3	3	3	2	2
3	3	3	3	4	4	1	1	2	2
2	2	2	2	4	4	3	3	2	2
2	2	3	3	4	4	3	3	2	2
2	2	2	2	3	3	1	1	2	2
3	3	3	3	4	4	1	1	4	4
3	3	3	3	4	4	3	3	4	4
2	2	3	3	3	3	1	1	2	2
3	3	3	3	4	4	1	1	2	2
3	3	3	3	4	4	1	1	4	4
2	2	3	3	4	4	1	1	2	2
2	2	3	3	4	4	1	1	2	2
3	3	3	3	4	4	1	1	4	4
2	2	3	3	4	4	1	1	2	2

For Review Only

2	2	3	3	4	4	1	1	2	2
2	2	3	3	4	4	3	3	2	2
2	2	3	3	4	4	3	3	2	2
2	2	3	3	4	4	1	1	2	2
3	3	2	2	3	3	1	1	4	4
2	2	3	3	4	4	1	1	2	2
2	2	3	3	4	4	3	3	2	2
2	2	3	3	4	4	1	1	2	2
2	2	2	2	4	4	3	3	2	2
2	2	2	2	3	3	1	1	2	2
3	3	2	2	4	4	1	1	4	4
2	2	3	3	4	4	3	3	2	2
3	3	3	3	3	3	1	1	2	2
2	2	3	3	4	4	1	1	2	2
2	2	3	3	4	4	3	3	2	2
2	2	3	3	4	4	3	3	2	2
2	2	3	3	4	4	1	1	2	2
3	3	3	3	4	4	3	3	4	4
3	3	3	3	4	4	3	3	4	4
3	3	2	2	3	3	1	1	4	4
2	2	3	3	4	4	1	1	2	2
2	2	3	3	4	4	3	3	2	2
2	2	3	3	4	4	1	1	2	2
3	3	3	3	4	4	1	1	4	4
2	2	2	2	3	3	3	3	2	2
2	2	2	2	3	3	1	1	2	2
3	3	3	3	4	4	3	3	2	2
2	2	3	3	4	4	3	3	2	2
2	2	3	3	4	4	1	1	2	2
2	2	2	2	3	3	1	1	2	2
2	2	3	3	4	4	1	1	2	2
2	2	2	2	3	3	1	1	2	2
2	2	3	3	4	4	3	3	2	2
2	2	2	2	3	3	3	3	2	2
2	2	2	2	3	3	1	1	2	2
3	3	3	3	4	4	1	1	4	4
3	3	3	3	4	4	1	1	4	4
2	2	2	2	3	3	3	3	2	2
3	3	3	3	4	4	3	3	4	4
3	3	2	2	3	3	3	3	4	4
3	3	3	3	4	4	1	1	4	4
3	3	3	3	4	4	1	1	4	4
2	2	3	3	4	4	3	3	2	2
2	2	3	3	4	4	1	1	2	2
2	2	3	3	4	4	1	1	2	2
2	2	3	3	4	4	3	3	2	2
2	2	3	3	4	4	3	3	2	2
2	2	3	3	4	4	3	3	2	2
2	2	3	3	4	4	3	3	2	2
2	2	2	2	3	3	3	3	2	2
3	3	3	3	4	4	3	3	4	4
2	2	3	3	4	4	1	1	2	2
2	2	3	3	4	4	1	1	2	2
3	3	3	3	4	4	1	1	4	4
2	2	3	3	4	4	3	3	2	2

For Review Only

2	2	3	3	4	4	1	1	2	2
2	2	3	3	4	4	3	3	2	2
3	3	3	3	4	4	1	1	4	4
2	2	3	3	4	4	1	1	2	2
2	2	3	3	4	4	3	3	2	2
2	2	3	3	4	4	3	3	2	2
2	2	3	3	4	4	3	3	2	2
3	3	3	3	4	4	3	3	2	2
2	2	3	3	4	4	3	3	2	2
2	2	3	3	4	4	3	3	2	2
2	2	3	3	4	4	1	1	2	2
2	2	3	3	4	4	3	3	2	2
2	2	3	3	4	4	1	1	2	2
3	3	3	3	4	4	1	1	4	4
3	3	3	3	4	4	1	1	4	4
2	2	3	3	4	4	1	1	2	2
2	2	3	3	4	4	3	3	2	2
2	2	3	3	4	4	1	1	2	2
2	2	3	3	4	4	1	1	2	2
3	3	3	3	4	4	3	3	2	2
2	2	3	3	4	4	1	1	2	2
3	3	3	3	4	4	1	1	4	4
2	2	3	3	4	4	1	1	2	2
2	2	3	3	4	4	3	3	2	2
2	2	3	3	4	4	3	3	2	2
2	2	3	3	3	3	3	3	2	2
2	2	3	3	4	4	3	3	2	2
3	3	2	2	3	3	3	3	4	4
2	2	3	3	4	4	3	3	2	2
3	3	3	3	4	4	1	1	4	4
2	2	3	3	4	4	3	3	2	2
3	3	3	3	4	4	1	1	4	4
2	2	3	3	4	4	3	3	2	2
3	3	3	3	4	4	3	3	4	4
2	2	3	3	4	4	1	1	2	2
2	2	3	3	4	4	3	3	2	2
2	2	3	3	4	4	3	3	2	2
3	3	3	3	4	4	1	1	4	4
3	3	3	3	4	4	1	1	4	4
2	2	3	3	4	4	3	3	2	2
2	2	3	3	4	4	3	3	2	2
2	2	3	3	4	4	1	1	2	2
2	2	3	3	4	4	3	3	2	2
2	2	2	2	3	3	3	3	2	2
2	2	3	3	4	4	3	3	2	2
2	2	3	3	4	4	1	1	2	2
3	3	3	3	4	4	3	3	4	4
3	3	3	3	4	4	3	3	2	2
2	2	3	3	4	4	1	1	2	2
2	2	3	3	4	4	3	3	2	2
2	2	2	2	3	3	1	1	2	2
2	2	3	3	4	4	3	3	2	2
3	3	3	3	4	4	1	1	4	4
3	3	3	3	4	4	3	3	4	4
3	3	3	3	4	4	3	3	4	4

For Review Only

2	2	3	3	4	4	1	1	2	2
3	3	3	3	3	3	1	1	4	4

For Review Only

SNP2886	SNP2886	SNP2887	SNP2887	SNP2888	SNP2888	SNP2889	SNP2889	SNP289	SNP289
1	1	4	4	2	2	2	2	2	2
1	1	1	1	2	2	2	2	2	2
1	1	1	1	2	2	2	2	2	2
4	4	4	4	2	2	2	2	2	2
1	1	1	1	2	2	2	2	2	2
1	1	1	1	2	2	2	2	2	2
1	1	1	1	2	2	2	2	2	2
4	4	4	4	2	2	2	2	2	2
4	4	4	4	2	2	2	2	2	2
4	4	4	4	2	2	2	2	2	2
1	1	1	1	2	2	2	2	2	2
1	1	4	4	2	2	2	2	2	2
4	4	4	4	2	2	2	2	2	2
1	1	1	1	2	2	2	2	2	2
1	1	4	4	2	2	2	2	2	2
1	1	1	1	2	2	2	2	2	2
4	4	4	4	2	2	2	2	2	2
4	4	4	4	2	2	2	2	2	2
1	1	1	1	2	2	2	2	2	2
1	1	1	1	2	2	2	2	2	2
1	1	1	1	2	2	2	2	2	2
4	4	4	4	2	2	2	2	2	2
1	1	4	4	2	2	3	3	2	2
1	1	1	1	2	2	2	2	2	2
4	4	4	4	2	2	2	2	2	2
1	1	1	1	2	2	2	2	2	2
1	1	1	1	2	2	2	2	2	2
1	1	4	4	2	2	2	2	2	2
1	1	4	4	2	2	3	3	2	2
1	1	4	4	2	2	2	2	3	3
1	1	1	1	2	2	2	2	2	2
4	4	4	4	2	2	2	2	2	2
4	4	4	4	2	2	2	2	2	2
1	1	1	1	2	2	2	2	2	2
1	1	4	4	2	2	2	2	2	2
1	1	1	1	2	2	2	2	2	2
4	4	4	4	2	2	2	2	2	2
1	1	1	1	2	2	2	2	2	2
1	1	4	4	3	3	2	2	2	2
1	1	4	4	2	2	3	3	3	3
1	1	4	4	2	2	3	3	2	2
1	1	4	4	2	2	3	3	2	2
4	4	4	4	2	2	2	2	2	2
1	1	4	4	2	2	3	3	2	2
4	4	4	4	2	2	2	2	3	3
1	1	1	1	2	2	2	2	2	2
1	1	1	1	2	2	2	2	2	2
1	1	4	4	2	2	2	2	2	2
4	4	4	4	3	3	2	2	2	2
1	1	1	1	2	2	3	3	2	2
1	1	1	1	2	2	2	2	2	2
1	1	1	1	2	2	2	2	2	2

For Review Only

4	4	4	4	2	2	2	2	2	2
4	4	4	4	2	2	2	2	2	2
4	4	4	4	2	2	2	2	2	2
1	1	4	4	2	2	2	2	2	2
1	1	1	1	2	2	2	2	2	2
1	1	1	1	2	2	2	2	2	2
1	1	1	1	2	2	2	2	2	2
1	1	4	4	2	2	3	3	2	2
1	1	4	4	2	2	3	3	2	2
1	1	1	1	2	2	2	2	2	2
1	1	1	1	2	2	2	2	2	2
1	1	1	1	2	2	2	2	2	2
4	4	4	4	2	2	2	2	2	2
1	1	4	4	2	2	3	3	2	2
1	1	4	4	2	2	2	2	2	2
4	4	4	4	2	2	2	2	2	2
1	1	1	1	2	2	2	2	2	2
4	4	4	4	2	2	2	2	2	2
4	4	4	4	2	2	2	2	2	2
1	1	4	4	2	2	2	2	2	2
1	1	1	1	2	2	2	2	2	2
1	1	4	4	2	2	2	2	3	3
4	4	4	4	2	2	2	2	2	2
1	1	1	1	2	2	2	2	2	2
4	4	4	4	2	2	2	2	2	2
1	1	1	1	2	2	2	2	2	2
4	4	4	4	2	2	2	2	3	3
1	1	4	4	3	3	2	2	2	2
1	1	1	1	2	2	2	2	2	2
1	1	1	1	2	2	2	2	2	2
4	4	4	4	2	2	2	2	2	2
1	1	4	4	2	2	2	2	2	2
1	1	4	4	2	2	2	2	2	2
4	4	4	4	2	2	2	2	2	2
1	1	1	1	2	2	2	2	2	2
4	4	4	4	2	2	2	2	3	3
1	1	4	4	2	2	3	3	2	2
4	4	4	4	2	2	2	2	2	2
1	1	4	4	2	2	3	3	2	2
4	4	4	4	2	2	2	2	2	2
1	1	1	1	2	2	2	2	2	2
1	1	1	1	2	2	2	2	2	2
1	1	1	1	2	2	2	2	2	2
1	1	1	1	2	2	2	2	2	2
1	1	1	1	2	2	2	2	2	2
1	1	1	1	2	2	2	2	2	2
1	1	1	1	2	2	2	2	2	2
1	1	1	1	2	2	2	2	2	2
1	1	4	4	2	2	3	3	2	2
1	1	4	4	2	2	2	2	2	2
1	1	1	1	2	2	2	2	2	2
1	1	4	4	2	2	2	2	2	2
1	1	4	4	2	2	3	3	2	2
1	1	4	4	2	2	2	2	2	2
1	1	4	4	2	2	3	3	2	2

1	1	1	1	2	2	2	2	2	2
4	4	4	4	2	2	2	2	3	3
1	1	1	1	2	2	2	2	2	2
1	1	1	1	2	2	2	2	2	2
1	1	1	1	2	2	2	2	2	2
1	1	1	1	2	2	2	2	2	2
1	1	1	1	2	2	2	2	2	2
1	1	4	4	2	2	3	3	2	2
1	1	4	4	2	2	2	2	2	2
1	1	1	1	2	2	2	2	2	2
1	1	1	1	2	2	2	2	2	2
1	1	4	4	2	2	2	2	2	2
1	1	1	1	2	2	2	2	2	2
1	1	1	1	2	2	2	2	2	2
1	1	4	4	2	2	2	2	2	2
1	1	4	4	2	2	2	2	2	2
4	4	4	4	2	2	2	2	2	2
1	1	1	1	2	2	2	2	2	2
1	1	1	1	2	2	2	2	2	2
4	4	4	4	2	2	2	2	2	2
1	1	4	4	2	2	2	2	2	2
1	1	1	1	2	2	2	2	2	2
1	1	1	1	2	2	2	2	2	2
1	1	1	1	2	2	2	2	2	2
1	1	4	4	2	2	2	2	2	2
4	4	4	4	2	2	2	2	2	2
1	1	4	4	2	2	2	2	2	2
4	4	4	4	2	2	2	2	2	2
1	1	1	1	2	2	2	2	2	2
1	1	1	1	2	2	2	2	2	2
1	1	4	4	2	2	2	2	2	2
1	1	4	4	2	2	2	2	2	2
1	1	1	1	2	2	2	2	2	2
1	1	4	4	2	2	2	2	2	2
1	1	1	1	2	2	2	2	2	2
1	1	4	4	2	2	2	2	2	2
1	1	1	1	2	2	2	2	2	2
1	1	1	1	2	2	2	2	2	2
1	1	4	4	2	2	2	2	2	2
1	1	4	4	3	3	2	2	2	2
4	4	4	4	2	2	2	2	2	2
1	1	1	1	2	2	2	2	2	2
1	1	4	4	2	2	3	3	2	2
1	1	4	4	2	2	2	2	2	2
1	1	1	1	2	2	2	2	2	2
4	4	4	4	2	2	2	2	2	2
1	1	1	1	2	2	2	2	2	2
1	1	1	1	2	2	2	2	2	2
1	1	4	4	2	2	2	2	2	2
1	1	1	1	2	2	2	2	2	2
1	1	1	1	2	2	2	2	2	2
1	1	4	4	2	2	2	2	2	2
4	4	4	4	2	2	2	2	2	2
1	1	1	1	2	2	2	2	2	2
1	1	1	1	2	2	2	2	2	2

For Review Only

1	1	1	1	2	2	2	2	2	2
1	1	4	4	2	2	3	3	2	2
4	4	4	4	2	2	2	2	2	2
1	1	4	4	2	2	2	2	2	2
4	4	4	4	2	2	2	2	2	2
1	1	1	1	2	2	2	2	2	2
1	1	4	4	3	3	2	2	2	2
1	1	4	4	2	2	2	2	2	2
1	1	4	4	2	2	2	2	2	2
1	1	1	1	2	2	2	2	2	2
1	1	4	4	2	2	3	3	2	2
1	1	4	4	2	2	3	3	2	2
1	1	4	4	2	2	2	2	2	2
1	1	4	4	2	2	2	2	2	2
1	1	1	1	2	2	2	2	2	2
1	1	4	4	3	3	2	2	2	2
1	1	1	1	2	2	2	2	2	2
1	1	1	1	2	2	2	2	2	2
1	1	1	1	2	2	2	2	2	2
1	1	1	1	2	2	2	2	2	2
1	1	4	4	2	2	2	2	2	2
1	1	1	1	2	2	2	2	2	2
1	1	4	4	2	2	2	2	3	3
1	1	4	4	2	2	2	2	2	2
1	1	4	4	2	2	3	3	2	2
1	1	1	1	2	2	2	2	2	2
1	1	1	1	2	2	2	2	2	2
1	1	4	4	2	2	2	2	2	2
1	1	4	4	2	2	3	3	2	2
4	4	4	4	2	2	2	2	2	2
1	1	4	4	2	2	3	3	2	2
1	1	1	1	2	2	2	2	2	2
1	1	1	1	2	2	2	2	2	2
1	1	1	1	2	2	2	2	2	2
4	4	4	4	2	2	2	2	2	2
1	1	1	1	2	2	2	2	2	2
1	1	1	1	2	2	2	2	3	3
4	4	4	4	2	2	2	2	2	2
1	1	1	1	2	2	2	2	2	2
4	4	4	4	2	2	2	2	2	2
1	1	1	1	2	2	2	2	2	2
1	1	1	1	2	2	2	2	2	2
1	1	1	1	2	2	2	2	2	2
1	1	1	1	2	2	2	2	2	2
1	1	1	1	3	3	2	2	2	2
1	1	1	1	3	3	2	2	2	2
1	1	4	4	3	3	2	2	2	2
1	1	4	4	3	3	2	2	2	2
1	1	1	1	2	2	2	2	2	2
4	4	4	4	2	2	2	2	2	2
1	1	1	1	2	2	2	2	2	2
1	1	4	4	2	2	2	2	2	2
1	1	4	4	2	2	2	2	3	3
1	1	1	1	2	2	2	2	2	2
1	1	4	4	3	3	2	2	2	2

For Review Only

1	1	1	1	2	2	2	2	2	2
1	1	4	4	2	2	2	2	2	2
1	1	1	1	2	2	2	2	2	2
1	1	4	4	2	2	2	2	2	2
4	4	4	4	2	2	2	2	2	2
1	1	1	1	2	2	2	2	2	2
1	1	4	4	2	2	3	3	2	2
4	4	4	4	2	2	2	2	2	2
1	1	4	4	2	2	2	2	2	2
1	1	4	4	3	3	2	2	2	2
1	1	4	4	2	2	2	2	2	2
1	1	1	1	2	2	2	2	2	2
1	1	4	4	2	2	3	3	2	2
1	1	1	1	2	2	2	2	2	2
1	1	1	1	2	2	2	2	2	2
1	1	4	4	2	2	3	3	2	2
4	4	4	4	2	2	2	2	2	2
1	1	4	4	2	2	2	2	2	2
4	4	4	4	3	3	2	2	3	3
1	1	1	1	2	2	2	2	3	3
4	4	4	4	2	2	2	2	3	3
1	1	1	1	2	2	2	2	2	2
1	1	1	1	2	2	2	2	2	2
1	1	4	4	2	2	3	3	2	2
1	1	4	4	2	2	3	3	2	2
1	1	4	4	2	2	2	2	2	2
1	1	4	4	2	2	2	2	2	2
4	4	4	4	2	2	2	2	2	2
4	4	4	4	2	2	2	2	2	2
1	1	1	1	2	2	2	2	2	2
1	1	4	4	2	2	2	2	2	2
1	1	1	1	2	2	2	2	2	2
1	1	4	4	2	2	3	3	2	2
1	1	1	1	2	2	2	2	2	2
1	1	4	4	2	2	2	2	3	3
1	1	4	4	2	2	2	2	2	2
1	1	1	1	2	2	2	2	2	2
1	1	1	1	2	2	2	2	2	2
1	1	1	1	2	2	2	2	2	2
1	1	4	4	3	3	2	2	2	2
4	4	4	4	2	2	2	2	2	2
1	1	1	1	2	2	2	2	2	2
1	1	4	4	2	2	3	3	2	2
4	4	4	4	2	2	2	2	2	2
1	1	4	4	3	3	2	2	2	2
1	1	1	1	2	2	2	2	2	2
1	1	4	4	2	2	2	2	2	2
1	1	1	1	2	2	2	2	2	2
4	4	4	4	2	2	2	2	2	2
1	1	4	4	2	2	2	2	2	2
4	4	4	4	2	2	2	2	2	2
1	1	4	4	2	2	2	2	2	2
1	1	1	1	2	2	2	2	2	2
1	1	1	1	2	2	2	2	2	2
1	1	1	1	2	2	2	2	2	2

For Review Only

1	1	1	1	2	2	2	2	2	2
4	4	4	4	2	2	2	2	2	2

For Review Only

SNP2890	SNP2890	SNP2891	SNP2891	SNP2892	SNP2892	SNP2893	SNP2893	SNP2894	SNP2894
2	2	2	2	3	3	4	4	1	1
2	2	3	3	2	2	4	4	1	1
2	2	3	3	2	2	4	4	1	1
2	2	3	3	2	2	4	4	1	1
2	2	2	2	3	3	4	4	1	1
2	2	3	3	2	2	4	4	1	1
2	2	2	2	3	3	4	4	1	1
2	2	2	2	3	3	4	4	1	1
2	2	2	2	3	3	4	4	1	1
2	2	2	2	3	3	4	4	1	1
2	2	2	2	3	3	4	4	1	1
2	2	3	3	2	2	4	4	1	1
2	2	2	2	3	3	4	4	1	1
2	2	2	2	3	3	4	4	1	1
2	2	2	2	3	3	4	4	1	1
2	2	3	3	2	2	4	4	1	1
2	2	2	2	3	3	4	4	1	1
2	2	2	2	3	3	4	4	1	1
2	2	2	2	3	3	1	1	4	4
2	2	2	2	3	3	4	4	1	1
2	2	2	2	3	3	4	4	1	1
2	2	2	2	3	3	4	4	1	1
3	3	2	2	3	3	4	4	1	1
2	2	2	2	3	3	4	4	1	1
2	2	2	2	3	3	4	4	1	1
2	2	3	3	2	2	4	4	1	1
2	2	2	2	3	3	4	4	1	1
2	2	3	3	3	3	4	4	1	1
3	3	2	2	3	3	4	4	1	1
2	2	2	2	3	3	4	4	1	1
2	2	3	3	2	2	4	4	1	1
2	2	2	2	3	3	4	4	1	1
2	2	2	2	3	3	4	4	1	1
2	2	2	2	3	3	4	4	1	1
2	2	2	2	3	3	4	4	1	1
2	2	3	3	2	2	4	4	1	1
2	2	2	2	3	3	4	4	1	1
2	2	2	2	3	3	4	4	1	1
2	2	2	2	3	3	4	4	1	1
3	3	3	3	2	2	4	4	1	1
3	3	2	2	3	3	4	4	1	1
2	2	2	2	3	3	4	4	1	1
2	2	2	2	3	3	4	4	1	1
3	3	2	2	2	2	4	4	1	1
2	2	2	2	3	3	4	4	1	1
2	2	3	3	2	2	4	4	1	1
2	2	2	2	2	2	4	4	1	1
2	2	2	2	3	3	4	4	1	1
2	2	2	2	3	3	4	4	1	1
2	2	2	2	3	3	4	4	1	1
2	2	3	3	2	2	4	4	1	1
2	2	2	2	3	3	4	4	1	1

2	2	2	2	3	3	4	4	1	1
2	2	2	2	3	3	4	4	1	1
2	2	2	2	3	3	4	4	1	1
2	2	2	2	3	3	4	4	1	1
2	2	2	2	3	3	1	1	4	4
2	2	3	3	2	2	4	4	1	1
2	2	3	3	2	2	4	4	1	1
3	3	2	2	3	3	4	4	1	1
3	3	2	2	3	3	4	4	1	1
2	2	3	3	3	3	4	4	1	1
2	2	3	3	2	2	4	4	1	1
2	2	3	3	2	2	4	4	1	1
2	2	2	2	3	3	4	4	1	1
3	3	2	2	3	3	4	4	1	1
2	2	3	3	3	3	4	4	1	1
2	2	2	2	3	3	4	4	1	1
2	2	2	2	3	3	4	4	1	1
2	2	2	2	3	3	4	4	1	1
2	2	2	2	3	3	4	4	1	1
2	2	2	2	3	3	4	4	1	1
2	2	3	3	2	2	4	4	1	1
2	2	2	2	3	3	4	4	1	1
2	2	2	2	3	3	4	4	1	1
2	2	2	2	3	3	4	4	1	1
2	2	2	2	3	3	4	4	1	1
2	2	2	2	2	2	4	4	1	1
2	2	2	2	3	3	4	4	1	1
2	2	2	2	3	3	1	1	4	4
2	2	3	3	2	2	4	4	1	1
2	2	2	2	3	3	4	4	1	1
2	2	2	2	3	3	4	4	4	4
2	2	2	2	3	3	4	4	1	1
2	2	2	2	3	3	1	1	4	4
2	2	2	2	3	3	4	4	1	1
2	2	2	2	3	3	4	4	1	1
2	2	2	2	3	3	4	4	1	1
3	3	2	2	3	3	4	4	1	1
2	2	2	2	3	3	4	4	1	1
3	3	2	2	3	3	4	4	1	1
2	2	2	2	3	3	4	4	1	1
2	2	2	2	3	3	4	4	1	1
2	2	3	3	2	2	4	4	1	1
2	2	3	3	2	2	4	4	1	1
2	2	3	3	2	2	4	4	1	1
2	2	2	2	3	3	4	4	1	1
2	2	3	3	2	2	4	4	1	1
2	2	2	2	3	3	4	4	1	1
2	2	2	2	3	3	4	4	4	4
3	3	3	3	2	2	4	4	1	1
2	2	2	2	3	3	4	4	1	1
2	2	2	2	3	3	4	4	1	1
2	2	2	2	3	3	4	4	1	1
3	3	2	2	3	3	4	4	1	1
2	2	2	2	3	3	4	4	1	1
3	3	2	2	3	3	4	4	1	1

For Review Only

2	2	3	3	2	2	4	4	1	1
2	2	3	3	3	3	4	4	1	1
2	2	2	2	3	3	4	4	1	1
2	2	2	2	3	3	4	4	1	1
2	2	3	3	2	2	4	4	1	1
2	2	3	3	2	2	4	4	1	1
2	2	3	3	2	2	4	4	1	1
2	2	2	2	3	3	4	4	1	1
2	2	3	3	3	3	4	4	1	1
2	2	3	3	2	2	4	4	1	1
2	2	2	2	3	3	4	4	1	1
2	2	3	3	3	3	4	4	1	1
2	2	2	2	3	3	4	4	1	1
2	2	2	2	2	2	4	4	1	1
2	2	3	3	3	3	1	1	1	1
2	2	3	3	2	2	4	4	1	1
2	2	2	2	3	3	4	4	1	1
2	2	3	3	3	3	4	4	1	1
2	2	2	2	3	3	4	4	1	1
2	2	2	2	3	3	4	4	1	1
2	2	3	3	3	3	4	4	1	1
2	2	2	2	3	3	4	4	1	1
2	2	3	3	3	3	4	4	1	1
2	2	2	2	3	3	4	4	1	1
2	2	3	3	3	3	4	4	1	1
2	2	2	2	3	3	4	4	1	1
2	2	2	2	3	3	4	4	1	1
2	2	2	2	3	3	4	4	1	1
2	2	3	3	2	2	4	4	1	1
2	2	2	2	3	3	1	1	4	4
2	2	2	2	3	3	4	4	1	1
2	2	2	2	3	3	4	4	1	1
2	2	2	2	3	3	4	4	1	1
2	2	3	3	2	2	4	4	1	1
2	2	2	2	3	3	4	4	1	1
2	2	2	2	3	3	4	4	1	1
2	2	2	2	3	3	4	4	1	1
2	2	2	2	3	3	4	4	1	1
2	2	2	2	3	3	4	4	1	1
2	2	2	2	3	3	4	4	1	1
2	2	2	2	3	3	4	4	1	1
2	2	2	2	3	3	4	4	1	1
3	3	2	2	3	3	4	4	1	1
2	2	2	2	3	3	4	4	1	1
2	2	2	2	3	3	4	4	1	1
2	2	2	2	3	3	4	4	1	1
2	2	3	3	2	2	4	4	1	1
2	2	3	3	2	2	4	4	1	1
2	2	2	2	3	3	4	4	1	1
2	2	3	3	2	2	4	4	1	1
2	2	3	3	2	2	4	4	1	1
2	2	2	2	3	3	4	4	1	1
2	2	2	2	3	3	4	4	1	1
2	2	3	3	2	2	4	4	1	1
3	3	2	2	3	3	4	4	1	1

For Review Only

2	2	2	2	3	3	4	4	1	1
3	3	2	2	3	3	4	4	1	1
2	2	2	2	3	3	4	4	1	1
2	2	2	2	3	3	4	4	1	1
2	2	3	3	2	2	4	4	1	1
2	2	2	2	3	3	1	1	4	4
2	2	2	2	3	3	1	1	4	4
2	2	3	3	3	3	4	4	1	1
2	2	2	2	3	3	4	4	1	1
2	2	2	2	3	3	4	4	1	1
3	3	3	3	2	2	4	4	1	1
3	3	2	2	3	3	4	4	1	1
2	2	3	3	2	2	4	4	1	1
2	2	2	2	3	3	4	4	1	1
2	2	2	2	3	3	4	4	1	1
2	2	2	2	3	3	4	4	1	1
2	2	2	2	3	3	4	4	1	1
2	2	3	3	2	2	4	4	1	1
2	2	3	3	2	2	4	4	1	1
2	2	3	3	2	2	4	4	1	1
2	2	3	3	2	2	4	4	1	1
2	2	2	2	3	3	4	4	4	4
2	2	2	2	3	3	4	4	1	1
2	2	3	3	2	2	4	4	1	1
3	3	2	2	3	3	4	4	1	1
2	2	2	2	3	3	4	4	1	1
2	2	2	2	3	3	4	4	1	1
2	2	2	2	3	3	4	4	1	1
3	3	2	2	3	3	4	4	1	1
2	2	2	2	3	3	4	4	1	1
3	3	2	2	3	3	4	4	1	1
2	2	2	2	3	3	4	4	1	1
2	2	2	2	3	3	4	4	1	1
2	2	2	2	3	3	4	4	1	1
2	2	2	2	3	3	4	4	1	1
2	2	3	3	2	2	4	4	1	1
2	2	3	3	2	2	4	4	1	1
2	2	2	2	3	3	4	4	1	1
2	2	3	3	2	2	4	4	1	1
2	2	3	3	2	2	4	4	1	1
2	2	3	3	2	2	4	4	1	1
2	2	3	3	2	2	4	4	1	1
2	2	2	2	3	3	4	4	1	1
2	2	3	3	2	2	4	4	1	1
2	2	2	2	3	3	4	4	1	1
2	2	3	3	3	3	4	4	1	1
2	2	2	2	3	3	4	4	1	1
2	2	2	2	3	3	1	1	4	4
2	2	2	2	3	3	4	4	1	1
2	2	2	2	3	3	4	4	1	1
2	2	3	3	2	2	4	4	1	1
2	2	2	2	3	3	4	4	1	1
2	2	2	2	3	3	4	4	1	1
2	2	3	3	2	2	4	4	1	1
2	2	2	2	3	3	4	4	1	1

For Review Only

2	2	2	2	3	3	4	4	1	1
2	2	2	2	3	3	4	4	1	1
2	2	3	3	2	2	4	4	1	1
2	2	2	2	3	3	4	4	1	1
2	2	2	2	3	3	4	4	1	1
2	2	2	2	3	3	4	4	1	1
3	3	2	2	3	3	4	4	1	1
2	2	2	2	3	3	4	4	1	1
2	2	3	3	3	3	4	4	1	1
2	2	2	2	3	3	4	4	1	1
2	2	2	2	3	3	4	4	1	1
2	2	2	2	3	3	4	4	1	1
3	3	2	2	3	3	4	4	1	1
2	2	3	3	2	2	4	4	1	1
2	2	3	3	2	2	4	4	1	1
3	3	2	2	3	3	4	4	1	1
2	2	2	2	3	3	4	4	1	1
2	2	2	2	3	3	1	1	4	4
2	2	2	2	3	3	4	4	1	1
2	2	2	2	3	3	4	4	1	1
2	2	2	2	3	3	4	4	1	1
2	2	3	3	2	2	4	4	1	1
2	2	2	2	3	3	4	4	1	1
3	3	2	2	3	3	4	4	1	1
3	3	2	2	3	3	4	4	1	1
2	2	2	2	3	3	4	4	1	1
2	2	2	2	3	3	1	1	4	4
2	2	3	3	2	2	4	4	1	1
2	2	2	2	3	3	4	4	1	1
2	2	3	3	2	2	4	4	1	1
2	2	2	2	3	3	4	4	1	1
2	2	3	3	2	2	4	4	1	1
3	3	2	2	3	3	4	4	1	1
2	2	3	3	2	2	4	4	1	1
2	2	2	2	3	3	4	4	1	1
2	2	2	2	3	3	4	4	1	1
2	2	2	2	3	3	4	4	1	1
2	2	3	3	2	2	4	4	1	1
2	2	3	3	2	2	4	4	1	1
2	2	2	2	3	3	1	1	1	1
2	2	2	2	3	3	4	4	1	1
2	2	2	2	3	3	1	1	1	1
2	2	2	2	3	3	4	4	1	1
2	2	2	2	3	3	4	4	1	1
2	2	3	3	3	3	4	4	4	4
2	2	2	2	3	3	4	4	1	1
2	2	3	3	2	2	4	4	1	1
2	2	3	3	2	2	4	4	1	1
2	2	2	2	3	3	4	4	1	1
2	2	2	2	3	3	1	1	4	4
2	2	2	2	3	3	1	1	4	4
2	2	2	2	3	3	4	4	1	1
2	2	3	3	2	2	4	4	1	1
2	2	3	3	2	2	4	4	1	1
2	2	3	3	2	2	4	4	1	1

For Review Only

2	2	2	2	3	3	4	4	1	1
2	2	3	3	2	2	4	4	1	1

For Review Only

SNP2895	SNP2895	SNP2896	SNP2896	SNP2897	SNP2897	SNP2898	SNP2898	SNP2899	SNP2899
3	3	2	2	2	2	1	1	3	3
3	3	2	2	2	2	4	4	4	4
2	2	2	2	2	2	1	1	3	3
3	3	2	2	2	2	4	4	4	4
3	3	2	2	2	2	1	1	3	3
3	3	2	2	2	2	4	4	3	3
3	3	2	2	2	2	1	1	3	3
3	3	2	2	2	2	1	1	4	4
3	3	2	2	2	2	1	1	3	3
3	3	2	2	2	2	1	1	3	3
3	3	2	2	2	2	1	1	3	3
3	3	2	2	2	2	1	1	3	3
3	3	2	2	2	2	1	1	3	3
3	3	2	2	2	2	1	1	3	3
3	3	2	2	2	2	1	1	3	3
3	3	2	2	2	2	1	1	3	3
3	3	3	3	3	3	1	1	3	3
3	3	2	2	2	2	1	1	3	3
3	3	2	2	2	2	1	1	3	3
3	3	2	2	2	2	1	1	3	3
3	3	2	2	3	3	4	4	3	3
3	3	2	2	2	2	1	1	3	3
3	3	2	2	3	3	1	1	3	3
3	3	2	2	2	2	1	1	3	3
3	3	2	2	2	2	1	1	3	3
3	3	2	2	2	2	1	1	3	3
2	2	2	2	2	2	1	1	3	3
3	3	2	2	2	2	1	1	3	3
3	3	2	2	2	2	1	1	3	3
3	3	2	2	2	2	4	4	3	3
3	3	2	2	2	2	1	1	3	3
3	3	2	2	2	2	1	1	3	3
3	3	3	3	3	3	1	1	3	3
3	3	2	2	2	2	1	1	3	3
3	3	2	2	2	2	1	1	3	3
3	3	2	2	2	2	4	4	4	4
3	3	2	2	2	2	1	1	3	3
3	3	2	2	2	2	1	1	3	3
3	3	2	2	3	3	1	1	3	3
3	3	2	2	2	2	1	1	3	3
3	3	2	2	2	2	1	1	3	3
3	3	2	2	2	2	4	4	3	3
3	3	2	2	2	2	1	1	4	4
3	3	3	3	3	3	1	1	3	3
3	3	2	2	2	2	1	1	3	3
2	2	2	2	2	2	1	1	3	3
3	3	2	2	2	2	1	1	4	4
3	3	2	2	2	2	1	1	3	3
3	3	2	2	2	2	1	1	3	3
3	3	2	2	2	2	1	1	3	3
3	3	2	2	2	2	1	1	3	3
3	3	2	2	2	2	1	1	3	3

3	3	2	2	2	2	1	1	3	3
3	3	3	3	3	3	1	1	3	3
3	3	2	2	2	2	1	1	3	3
3	3	3	3	3	3	1	1	3	3
3	3	2	2	2	2	1	1	3	3
3	3	2	2	2	2	4	4	4	4
3	3	2	2	2	2	1	1	3	3
3	3	2	2	2	2	1	1	3	3
3	3	2	2	2	2	1	1	3	3
3	3	2	2	2	2	1	1	3	3
3	3	2	2	2	2	4	4	4	4
3	3	2	2	2	2	4	4	4	4
3	3	2	2	2	2	1	1	3	3
3	3	2	2	2	2	1	1	3	3
3	3	2	2	2	2	1	1	3	3
3	3	2	2	2	2	1	1	3	3
3	3	2	2	2	2	1	1	3	3
3	3	2	2	2	2	1	1	3	3
3	3	2	2	2	2	1	1	3	3
3	3	2	2	2	2	1	1	3	3
3	3	2	2	2	2	1	1	4	4
3	3	2	2	2	2	1	1	3	3
3	3	2	2	2	2	1	1	3	3
3	3	2	2	2	2	1	1	3	3
3	3	2	2	2	2	1	1	3	3
3	3	2	2	2	2	1	1	3	3
3	3	2	2	2	2	1	1	3	3
3	3	2	2	3	3	1	1	3	3
3	3	2	2	2	2	1	1	3	3
3	3	2	2	2	2	1	1	3	3
3	3	2	2	2	2	1	1	3	3
3	3	2	2	2	2	1	1	3	3
3	3	2	2	2	2	1	1	3	3
3	3	2	2	2	2	1	1	3	3
3	3	2	2	2	2	1	1	3	3
3	3	2	2	2	2	1	1	3	3
3	3	2	2	2	2	1	1	4	4
3	3	2	2	3	3	1	1	3	3
3	3	2	2	2	2	1	1	3	3
3	3	2	2	2	2	1	1	4	4
3	3	2	2	2	2	1	1	3	3
3	3	2	2	2	2	4	4	4	4
3	3	2	2	2	2	1	1	3	3
3	3	2	2	2	2	4	4	4	4
3	3	2	2	2	2	1	1	3	3
3	3	2	2	2	2	4	4	4	4
3	3	3	3	3	3	1	1	3	3
3	3	2	2	2	2	1	1	3	3
3	3	2	2	2	2	1	1	3	3
3	3	2	2	2	2	1	1	3	3
3	3	2	2	2	2	1	1	3	3
3	3	2	2	2	2	1	1	3	3
3	3	3	3	3	3	1	1	3	3
3	3	2	2	3	3	1	1	3	3
3	3	2	2	2	2	1	1	3	3

For Review Only

3	3	2	2	2	2	1	1	3	3
3	3	2	2	2	2	1	1	3	3
3	3	2	2	2	2	1	1	3	3
3	3	3	3	2	2	1	1	3	3
3	3	2	2	2	2	1	1	3	3
3	3	2	2	2	2	1	1	3	3
3	3	2	2	2	2	1	1	3	3
3	3	2	2	2	2	1	1	3	3
3	3	2	2	2	2	1	1	3	3
3	3	2	2	2	2	1	1	3	3
3	3	2	2	2	2	1	1	3	3
3	3	2	2	2	2	1	1	3	3
2	2	2	2	2	2	1	1	3	3
3	3	2	2	2	2	1	1	3	3
3	3	2	2	2	2	1	1	3	3
3	3	2	2	2	2	1	1	3	3
3	3	2	2	2	2	1	1	3	3
3	3	2	2	2	2	1	1	3	3
3	3	3	3	2	2	1	1	3	3
3	3	2	2	2	2	1	1	3	3
3	3	2	2	2	2	1	1	3	3
3	3	2	2	2	2	1	1	3	3
3	3	2	2	2	2	4	4	4	4
3	3	2	2	3	3	1	1	3	3
3	3	2	2	2	2	1	1	3	3
3	3	2	2	2	2	1	1	3	3
3	3	2	2	2	2	1	1	3	3
3	3	2	2	2	2	1	1	3	3
3	3	2	2	2	2	4	4	4	4
3	3	2	2	2	2	1	1	3	3
3	3	2	2	2	2	1	1	3	3
3	3	2	2	2	2	1	1	3	3
3	3	2	2	2	2	1	1	3	3
3	3	2	2	2	2	1	1	3	3
3	3	2	2	2	2	1	1	3	3
3	3	2	2	2	2	1	1	3	3
3	3	2	2	2	2	1	1	3	3
3	3	2	2	2	2	1	1	3	3
3	3	2	2	2	2	1	1	3	3
3	3	2	2	3	3	1	1	3	3
3	3	2	2	2	2	1	1	3	3
3	3	2	2	2	2	1	1	3	3
3	3	2	2	2	2	1	1	3	3
3	3	2	2	2	2	1	1	3	3
3	3	2	2	2	2	1	1	3	3
3	3	2	2	2	2	1	1	3	3
3	3	2	2	2	2	1	1	3	3
3	3	2	2	2	2	4	4	4	4
3	3	2	2	2	2	1	1	3	3
3	3	2	2	2	2	1	1	3	3
3	3	2	2	2	2	1	1	3	3
3	3	2	2	2	2	1	1	3	3
3	3	2	2	2	2	1	1	3	3
3	3	2	2	2	2	4	4	4	4
3	3	2	2	2	2	1	1	3	3

For Review Only

3	3	2	2	2	2	1	1	3	3
3	3	2	2	2	2	1	1	3	3
3	3	2	2	2	2	1	1	3	3
3	3	2	2	2	2	1	1	3	3
3	3	2	2	2	2	4	4	4	4
3	3	2	2	2	2	1	1	3	3
3	3	2	2	2	2	1	1	3	3
3	3	2	2	2	2	1	1	3	3
3	3	2	2	2	2	1	1	3	3
3	3	2	2	2	2	1	1	3	3
3	3	2	2	2	2	1	1	3	3
3	3	2	2	2	2	1	1	3	3
3	3	2	2	2	2	1	1	3	3
3	3	2	2	2	2	1	1	3	3
3	3	3	3	3	3	1	1	3	3
3	3	2	2	3	3	1	1	3	3
3	3	2	2	2	2	1	1	3	3
3	3	2	2	2	2	1	1	4	4
3	3	2	2	2	2	1	1	3	3
3	3	2	2	2	2	1	1	3	3
3	3	2	2	2	2	1	1	3	3
3	3	2	2	2	2	1	1	3	3
3	3	2	2	2	2	1	1	3	3
3	3	2	2	2	2	4	4	4	4
3	3	2	2	2	2	1	1	3	3
3	3	2	2	2	2	1	1	3	3
3	3	2	2	2	2	1	1	3	3
3	3	2	2	2	2	1	1	3	3
3	3	2	2	2	2	1	1	3	3
3	3	2	2	2	2	1	1	3	3
3	3	2	2	2	2	1	1	3	3
3	3	2	2	2	2	1	1	3	3
3	3	2	2	2	2	1	1	3	3
3	3	3	3	3	3	1	1	3	3
3	3	2	2	2	2	1	1	3	3
3	3	2	2	2	2	4	4	4	4
3	3	2	2	2	2	1	1	3	3
3	3	2	2	2	2	1	1	3	3
3	3	2	2	2	2	4	4	4	4
3	3	2	2	2	2	4	4	4	4
2	2	2	2	2	2	1	1	3	3
3	3	2	2	2	2	1	1	3	3
3	3	2	2	3	3	1	1	3	3
3	3	2	2	2	2	1	1	3	3
3	3	2	2	2	2	1	1	3	3
3	3	2	2	3	3	1	1	3	3
3	3	2	2	3	3	1	1	3	3
3	3	2	2	2	2	1	1	3	3
3	3	3	3	3	3	1	1	3	3
3	3	2	2	2	2	1	1	3	3
3	3	2	2	3	3	4	4	4	4
3	3	2	2	2	2	1	1	3	3
3	3	2	2	2	2	1	1	3	3
3	3	2	2	2	2	4	4	4	4
3	3	2	2	2	2	1	1	3	3

3	3	2	2	2	2	1	1	3	3
3	3	2	2	2	2	1	1	3	3
3	3	2	2	2	2	1	1	3	3
3	3	2	2	2	2	1	1	3	3
3	3	2	2	2	2	1	1	3	3
3	3	2	2	2	2	1	1	3	3
3	3	2	2	2	2	1	1	3	3
3	3	2	2	2	2	1	1	3	3
3	3	2	2	2	2	1	1	3	3
3	3	2	2	2	2	1	1	3	3
3	3	2	2	2	2	1	1	3	3
3	3	2	2	2	2	1	1	3	3
3	3	2	2	2	2	1	1	3	3
3	3	2	2	2	2	1	1	3	3
2	2	2	2	2	2	1	1	3	3
3	3	2	2	2	2	1	1	3	3
3	3	2	2	3	3	1	1	3	3
3	3	2	2	2	2	1	1	3	3
3	3	2	2	2	2	1	1	3	3
3	3	2	2	3	3	1	1	3	3
3	3	2	2	2	2	1	1	3	3
3	3	2	2	2	2	1	1	3	3
3	3	2	2	2	2	1	1	3	3
3	3	2	2	3	3	1	1	3	3
3	3	2	2	2	2	1	1	3	3
3	3	2	2	2	2	1	1	3	3
3	3	2	2	2	2	1	1	3	3
3	3	2	2	2	2	4	4	4	4
3	3	2	2	2	2	1	1	3	3
3	3	2	2	2	2	4	4	4	4
3	3	2	2	3	3	1	1	3	3
3	3	2	2	2	2	1	1	3	3
3	3	2	2	2	2	1	1	3	3
3	3	2	2	2	2	1	1	4	4
3	3	2	2	2	2	1	1	3	3
3	3	2	2	3	3	1	1	3	3
3	3	2	2	2	2	1	1	3	3
3	3	2	2	2	2	4	4	4	4
3	3	2	2	2	2	4	4	4	4
3	3	2	2	3	3	1	1	3	3
3	3	2	2	2	2	1	1	3	3
3	3	2	2	2	2	1	1	3	3
3	3	2	2	3	3	1	1	3	3
3	3	2	2	2	2	1	1	3	3
3	3	2	2	2	2	1	1	3	3
3	3	2	2	2	2	1	1	3	3
3	3	2	2	2	2	1	1	3	3
2	2	2	2	2	2	1	1	3	3
3	3	2	2	2	2	1	1	4	4
3	3	2	2	2	2	1	1	3	3
3	3	2	2	2	2	1	1	3	3
3	3	2	2	3	3	1	1	3	3
2	2	2	2	2	2	1	1	3	3
2	2	2	2	2	2	4	4	4	4
3	3	2	2	3	3	1	1	3	3

For Review Only

3	3	2	2	2	2	1	1	3	3
3	3	2	2	2	2	4	4	4	4

For Review Only

SNP290	SNP290	SNP2900	SNP2900	SNP2901	SNP2901	SNP2902	SNP2902	SNP2903	SNP2903
3	3	1	1	2	2	3	3	4	4
3	3	1	1	2	2	3	3	4	4
3	3	1	1	3	3	3	3	4	4
3	3	1	1	2	2	3	3	4	4
3	3	1	1	3	3	3	3	4	4
3	3	1	1	3	3	3	3	4	4
3	3	1	1	3	3	3	3	4	4
3	3	4	4	2	2	3	3	4	4
3	3	1	1	2	2	3	3	4	4
3	3	1	1	2	2	1	1	1	1
3	3	1	1	3	3	3	3	4	4
3	3	1	1	3	3	3	3	4	4
3	3	1	1	2	2	3	3	4	4
3	3	1	1	2	2	3	3	4	4
3	3	1	1	2	2	3	3	4	4
3	3	1	1	2	2	3	3	4	4
3	3	1	1	2	2	3	3	4	4
3	3	1	1	2	2	1	1	1	1
3	3	1	1	2	2	3	3	4	4
3	3	4	4	2	2	3	3	1	1
3	3	1	1	2	2	3	3	4	4
3	3	1	1	3	3	3	3	4	4
3	3	1	1	2	2	3	3	4	4
3	3	1	1	2	2	3	3	4	4
3	3	1	1	3	3	3	3	4	4
3	3	1	1	3	3	3	3	4	4
3	3	1	1	2	2	3	3	4	4
3	3	1	1	2	2	3	3	4	4
3	3	1	1	3	3	3	3	4	4
1	1	1	1	2	2	1	1	1	1
3	3	1	1	3	3	3	3	4	4
3	3	1	1	2	2	3	3	4	4
3	3	1	1	2	2	3	3	4	4
3	3	1	1	2	2	3	3	4	4
3	3	1	1	2	2	3	3	4	4
3	3	4	4	2	2	3	3	4	4
3	3	1	1	2	2	3	3	4	4
1	1	1	1	2	2	3	3	4	4
3	3	1	1	2	2	3	3	4	4
1	1	1	1	2	2	3	3	4	4
3	3	1	1	2	2	3	3	4	4
3	3	1	1	2	2	1	1	4	4
3	3	1	1	2	2	3	3	1	1
3	3	1	1	2	2	3	3	4	4
1	1	1	1	2	2	1	1	1	1
3	3	1	1	3	3	3	3	4	4
3	3	4	4	3	3	3	3	4	4
3	3	1	1	2	2	3	3	4	4
3	3	1	1	2	2	3	3	4	4
3	3	1	1	3	3	3	3	4	4
3	3	1	1	3	3	3	3	4	4
3	3	1	1	3	3	3	3	4	4

For Review Only

3	3	1	1	3	3	3	3	4	4
3	3	1	1	2	2	3	3	4	4
3	3	1	1	3	3	3	3	4	4
3	3	1	1	3	3	3	3	4	4
3	3	1	1	2	2	3	3	4	4
3	3	4	4	2	2	3	3	4	4
3	3	1	1	2	2	3	3	4	4
3	3	1	1	2	2	3	3	4	4
3	3	1	1	3	3	3	3	4	4
3	3	1	1	2	2	3	3	4	4
3	3	4	4	2	2	3	3	4	4
3	3	4	4	2	2	3	3	4	4
3	3	1	1	3	3	3	3	4	4
3	3	1	1	3	3	3	3	4	4
3	3	1	1	2	2	3	3	4	4
3	3	1	1	2	2	3	3	4	4
3	3	1	1	3	3	3	3	4	4
3	3	1	1	3	3	3	3	4	4
3	3	1	1	3	3	3	3	4	4
3	3	1	1	2	2	3	3	4	4
3	3	1	1	2	2	3	3	4	4
1	1	1	1	2	2	3	3	4	4
3	3	1	1	2	2	3	3	4	4
3	3	1	1	3	3	3	3	4	4
1	1	1	1	2	2	1	1	1	1
3	3	1	1	3	3	3	3	4	4
1	1	1	1	2	2	3	3	4	4
3	3	1	1	2	2	3	3	4	4
3	3	1	1	3	3	3	3	4	4
3	3	1	1	2	2	3	3	4	4
3	3	1	1	3	3	3	3	4	4
3	3	1	1	3	3	3	3	4	4
3	3	1	1	2	2	3	3	4	4
3	3	1	1	3	3	3	3	4	4
3	3	1	1	3	3	3	3	4	4
1	1	1	1	2	2	1	1	1	1
3	3	4	4	2	2	3	3	4	4
3	3	1	1	2	2	3	3	4	4
3	3	1	1	2	2	3	3	4	4
3	3	4	4	2	2	1	1	1	1
3	3	1	1	3	3	3	3	4	4
3	3	4	4	2	2	3	3	4	4
3	3	1	1	2	2	1	1	1	1
3	3	4	4	2	2	3	3	4	4
3	3	1	1	3	3	3	3	4	4
3	3	1	1	3	3	3	3	4	4
3	3	1	1	2	2	3	3	4	4
3	3	1	1	2	2	3	3	4	4
3	3	1	1	2	2	3	3	4	4
3	3	1	1	2	2	3	3	4	4
3	3	1	1	3	3	3	3	4	4
3	3	1	1	2	2	3	3	4	4
3	3	1	1	2	2	3	3	4	4
3	3	1	1	2	2	3	3	4	4
3	3	1	1	2	2	3	3	4	4

For Review Only

3	3	1	1	2	2	3	3	4	4
1	1	4	4	2	2	1	1	1	1
3	3	1	1	3	3	3	3	4	4
3	3	1	1	2	2	3	3	4	4
3	3	1	1	2	2	3	3	4	4
3	3	1	1	3	3	3	3	4	4
3	3	1	1	2	2	3	3	4	4
3	3	1	1	2	2	3	3	4	4
3	3	1	1	2	2	3	3	4	4
3	3	1	1	2	2	3	3	4	4
3	3	1	1	2	2	3	3	4	4
3	3	1	1	2	2	3	3	4	4
3	3	1	1	3	3	3	3	4	4
3	3	1	1	3	3	3	3	4	4
3	3	1	1	2	2	3	3	4	4
3	3	1	1	2	2	3	3	4	4
3	3	1	1	3	3	3	3	4	4
3	3	1	1	2	2	3	3	4	4
3	3	1	1	2	2	3	3	4	4
3	3	1	1	3	3	3	3	4	4
3	3	1	1	2	2	3	3	4	4
3	3	1	1	2	2	3	3	4	4
3	3	1	1	3	3	3	3	4	4
3	3	1	1	2	2	3	3	4	4
3	3	1	1	2	2	3	3	4	4
3	3	1	1	2	2	3	3	4	4
3	3	1	1	3	3	3	3	4	4
3	3	1	1	2	2	3	3	4	4
3	3	1	1	2	2	3	3	4	4
3	3	1	1	2	2	3	3	4	4
3	3	1	1	2	2	3	3	4	4
3	3	1	1	2	2	3	3	4	4
3	3	1	1	3	3	3	3	4	4
3	3	1	1	2	2	3	3	4	4
3	3	1	1	2	2	3	3	4	4
3	3	1	1	2	2	3	3	4	4
3	3	1	1	2	2	3	3	4	4
3	3	1	1	3	3	3	3	4	4
3	3	1	1	2	2	3	3	4	4
3	3	1	1	2	2	3	3	4	4
3	3	1	1	2	2	3	3	4	4
3	3	1	1	2	2	3	3	4	4
3	3	1	1	3	3	3	3	4	4
3	3	1	1	2	2	3	3	4	4
3	3	1	1	2	2	3	3	4	4
3	3	1	1	3	3	3	3	4	4
3	3	1	1	3	3	3	3	4	4
3	3	4	4	2	2	3	3	4	4
3	3	1	1	2	2	3	3	4	4
3	3	1	1	3	3	3	3	4	4
3	3	1	1	2	2	3	3	4	4
3	3	1	1	3	3	3	3	4	4
3	3	1	1	3	3	3	3	4	4
3	3	4	4	2	2	3	3	4	4
3	3	1	1	2	2	3	3	4	4

For Review Only

3	3	1	1	3	3	3	3	4	4
3	3	1	1	2	2	1	1	1	1
3	3	1	1	2	2	1	1	1	1
3	3	1	1	2	2	3	3	4	4
3	3	1	1	3	3	3	3	4	4
3	3	1	1	2	2	3	3	4	4
3	3	1	1	2	2	3	3	4	4
3	3	1	1	2	2	3	3	4	4
3	3	1	1	2	2	3	3	4	4
3	3	1	1	2	2	3	3	4	4
3	3	1	1	3	3	3	3	4	4
3	3	1	1	2	2	3	3	4	4
3	3	1	1	2	2	3	3	4	4
3	3	1	1	2	2	3	3	4	4
3	3	1	1	2	2	3	3	4	4
3	3	1	1	2	2	3	3	4	4
3	3	1	1	2	2	3	3	4	4
3	3	1	1	2	2	3	3	4	4
3	3	1	1	2	2	3	3	4	4
3	3	1	1	2	2	3	3	4	4
3	3	1	1	2	2	3	3	4	4
3	3	1	1	2	2	3	3	4	4
1	1	1	1	2	2	3	3	4	4
3	3	4	4	2	2	3	3	4	4
3	3	1	1	2	2	1	1	1	1
3	3	1	1	3	3	3	3	4	4
3	3	1	1	2	2	3	3	4	4
3	3	1	1	2	2	3	3	4	4
3	3	1	1	2	2	1	1	1	1
3	3	1	1	2	2	1	1	1	1
3	3	1	1	3	3	3	3	4	4
3	3	1	1	2	2	3	3	4	4
3	3	1	1	3	3	3	3	4	4
3	3	1	1	2	2	3	3	4	4
3	3	1	1	3	3	3	3	4	4
3	3	4	4	2	2	3	3	4	4
3	3	1	1	2	2	3	3	4	4
3	3	1	1	2	2	3	3	4	4
3	3	4	4	2	2	3	3	4	4
3	3	4	4	2	2	3	3	4	4
3	3	1	1	3	3	3	3	4	4
3	3	1	1	3	3	3	3	4	4
3	3	1	1	2	2	3	3	4	4
3	3	1	1	2	2	3	3	4	4
3	3	1	1	2	2	3	3	4	4
3	3	1	1	2	2	3	3	4	4
3	3	1	1	2	2	3	3	4	4
3	3	1	1	2	2	3	3	4	4
3	3	1	1	2	2	3	3	4	4
3	3	1	1	2	2	1	1	1	1
3	3	4	4	2	2	3	3	4	4
3	3	1	1	3	3	3	3	4	4
1	1	1	1	2	2	3	3	4	4
3	3	4	4	2	2	3	3	4	4
3	3	1	1	2	2	3	3	4	4

For Review Only

3	3	1	1	3	3	3	3	4	4
3	3	1	1	3	3	3	3	4	4
3	3	1	1	3	3	3	3	4	4
3	3	1	1	2	2	3	3	4	4
3	3	1	1	2	2	3	3	4	4
3	3	1	1	3	3	3	3	4	4
3	3	1	1	2	2	3	3	4	4
3	3	1	1	2	2	1	1	1	1
3	3	1	1	2	2	3	3	4	4
3	3	1	1	2	2	3	3	4	4
3	3	1	1	2	2	3	3	4	4
3	3	1	1	3	3	3	3	4	4
3	3	1	1	2	2	3	3	4	4
3	3	1	1	3	3	3	3	4	4
3	3	1	1	2	2	1	1	1	1
3	3	1	1	2	2	3	3	4	4
3	3	1	1	2	2	3	3	4	4
3	3	1	1	3	3	3	3	4	4
1	1	1	1	2	2	3	3	4	4
1	1	1	1	2	2	3	3	4	4
1	1	1	1	3	3	3	3	4	4
3	3	1	1	3	3	3	3	4	4
3	3	1	1	3	3	3	3	4	4
3	3	1	1	2	2	3	3	4	4
3	3	1	1	2	2	3	3	4	4
3	3	1	1	3	3	3	3	4	4
3	3	1	1	2	2	3	3	4	4
3	3	1	1	2	2	3	3	4	4
3	3	1	1	2	2	3	3	4	4
3	3	4	4	2	2	3	3	4	4
3	3	1	1	2	2	3	3	4	4
3	3	1	1	3	3	3	3	4	4
3	3	1	1	2	2	1	1	1	1
3	3	1	1	2	2	3	3	4	4
1	1	1	1	2	2	3	3	4	4
3	3	1	1	2	2	3	3	4	4
3	3	1	1	3	3	3	3	4	4
3	3	4	4	2	2	3	3	4	4
3	3	4	4	2	2	3	3	4	4
3	3	1	1	2	2	3	3	4	4
3	3	1	1	2	2	3	3	4	4
3	3	1	1	2	2	3	3	4	4
3	3	1	1	2	2	3	3	4	4
3	3	1	1	3	3	3	3	4	4
3	3	1	1	2	2	3	3	4	4
3	3	1	1	2	2	3	3	4	4
3	3	1	1	2	2	3	3	4	4
3	3	1	1	2	2	1	1	1	1
3	3	4	4	3	3	3	3	4	4
3	3	1	1	2	2	3	3	4	4
3	3	1	1	2	2	3	3	4	4
3	3	1	1	2	2	3	3	4	4
3	3	1	1	3	3	3	3	4	4
3	3	1	1	2	2	3	3	4	4
3	3	1	1	2	2	3	3	4	4

For Review Only

3	3	1	1	3	3	3	3	4	4
3	3	1	1	2	2	3	3	4	4

For Review Only

SNP2904	SNP2904	SNP2905	SNP2905	SNP2906	SNP2906	SNP2907	SNP2907	SNP2908	SNP2908
4	4	4	4	1	1	3	3	1	1
4	4	4	4	1	1	3	3	1	1
4	4	4	4	1	1	3	3	1	1
4	4	4	4	1	1	3	3	1	1
4	4	1	1	1	1	3	3	1	1
4	4	1	1	1	1	3	3	1	1
4	4	1	1	1	1	3	3	1	1
4	4	4	4	1	1	3	3	1	1
4	4	4	4	1	1	3	3	1	1
4	4	4	4	1	1	3	3	1	1
4	4	1	1	4	4	3	3	4	4
4	4	4	4	1	1	3	3	1	1
4	4	1	1	1	1	3	3	1	1
4	4	4	4	1	1	3	3	1	1
4	4	4	4	1	1	3	3	1	1
4	4	1	1	1	1	3	3	1	1
4	4	4	4	1	1	3	3	1	1
1	1	4	4	1	1	3	3	1	1
4	4	1	1	1	1	3	3	1	1
1	1	4	4	1	1	3	3	1	1
4	4	4	4	1	1	3	3	1	1
4	4	1	1	1	1	3	3	1	1
4	4	4	4	4	4	3	3	4	4
4	4	4	4	1	1	3	3	1	1
4	4	1	1	1	1	3	3	1	1
4	4	4	4	1	1	3	3	1	1
4	4	1	1	1	1	3	3	1	1
4	4	4	4	1	1	3	3	1	1
4	4	1	1	1	1	3	3	1	1
1	1	4	4	1	1	3	3	4	4
4	4	1	1	1	1	3	3	1	1
4	4	1	1	1	1	3	3	1	1
4	4	4	4	1	1	3	3	1	1
4	4	4	4	1	1	3	3	1	1
4	4	4	4	1	1	3	3	1	1
4	4	4	4	1	1	3	3	1	1
4	4	4	4	1	1	3	3	1	1
4	4	4	4	4	4	2	2	1	1
4	4	4	4	1	1	3	3	1	1
4	4	4	4	1	1	3	3	1	1
4	4	4	4	1	1	3	3	4	4
1	1	4	4	1	1	3	3	1	1
1	1	4	4	1	1	3	3	1	1
4	4	4	4	1	1	3	3	1	1
1	1	4	4	1	1	3	3	1	1
4	4	4	4	1	1	3	3	1	1
4	4	4	4	1	1	3	3	1	1
4	4	4	4	1	1	3	3	1	1
4	4	4	4	1	1	3	3	1	1
4	4	4	4	1	1	3	3	1	1
4	4	4	4	1	1	3	3	1	1
4	4	1	1	1	1	3	3	1	1

For Review Only

4	4	1	1	1	1	3	3	1	1
4	4	4	4	1	1	3	3	1	1
4	4	1	1	1	1	3	3	1	1
4	4	1	1	1	1	3	3	1	1
4	4	1	1	1	1	3	3	1	1
4	4	4	4	1	1	3	3	1	1
4	4	1	1	1	1	3	3	1	1
4	4	4	4	1	1	3	3	1	1
4	4	4	4	1	1	3	3	1	1
4	4	4	4	1	1	3	3	1	1
4	4	4	4	1	1	3	3	1	1
4	4	4	4	1	1	3	3	1	1
4	4	1	1	1	1	3	3	1	1
4	4	1	1	1	1	3	3	1	1
4	4	4	4	1	1	3	3	1	1
4	4	4	4	1	1	3	3	1	1
4	4	4	4	1	1	3	3	1	1
4	4	1	1	1	1	3	3	1	1
4	4	1	1	1	1	3	3	1	1
4	4	4	4	1	1	3	3	1	1
4	4	4	4	1	1	3	3	1	1
4	4	4	4	4	4	2	2	4	4
4	4	4	4	1	1	3	3	1	1
4	4	1	1	1	1	3	3	1	1
1	1	4	4	1	1	3	3	1	1
4	4	1	1	1	1	3	3	1	1
4	4	4	4	1	1	3	3	1	1
4	4	4	4	1	1	3	3	1	1
4	4	4	4	1	1	3	3	1	1
4	4	1	1	1	1	3	3	1	1
4	4	1	1	1	1	3	3	1	1
4	4	4	4	1	1	3	3	1	1
4	4	4	4	4	4	3	3	4	4
4	4	1	1	1	1	3	3	1	1
4	4	1	1	1	1	3	3	1	1
1	1	4	4	1	1	3	3	1	1
4	4	4	4	1	1	3	3	4	4
4	4	4	4	1	1	3	3	1	1
4	4	4	4	1	1	2	2	1	1
1	1	4	4	1	1	3	3	1	1
4	4	1	1	1	1	3	3	1	1
4	4	1	1	1	1	3	3	1	1
4	4	4	4	1	1	3	3	1	1
4	4	4	4	1	1	3	3	1	1
4	4	4	4	1	1	3	3	1	1
4	4	4	4	1	1	3	3	1	1
4	4	4	4	1	1	3	3	1	1
4	4	1	1	1	1	3	3	1	1
4	4	4	4	1	1	3	3	1	1
4	4	4	4	1	1	3	3	1	1
4	4	1	1	1	1	3	3	1	1
4	4	4	4	1	1	3	3	1	1
4	4	4	4	1	1	3	3	1	1
4	4	4	4	1	1	3	3	1	1
4	4	4	4	1	1	3	3	1	1

For Review Only

4	4	4	4	1	1	2	2	1	1
1	1	4	4	1	1	3	3	1	1
4	4	4	4	1	1	3	3	1	1
4	4	4	4	1	1	3	3	1	1
4	4	1	1	1	1	3	3	4	4
4	4	4	4	1	1	3	3	1	1
4	4	1	1	1	1	3	3	1	1
4	4	1	1	1	1	3	3	1	1
4	4	4	4	1	1	3	3	1	1
4	4	4	4	1	1	3	3	1	1
4	4	1	1	4	4	3	3	4	4
4	4	4	4	1	1	3	3	1	1
4	4	4	4	1	1	3	3	1	1
4	4	1	1	1	1	3	3	1	1
4	4	4	4	1	1	3	3	1	1
4	4	4	4	1	1	3	3	1	1
4	4	4	4	1	1	3	3	1	1
4	4	4	4	1	1	3	3	1	1
4	4	1	1	1	1	3	3	1	1
4	4	1	1	1	1	3	3	1	1
4	4	4	4	1	1	3	3	1	1
4	4	1	1	1	1	3	3	1	1
4	4	4	4	1	1	3	3	1	1
4	4	4	4	4	4	3	3	4	4
4	4	4	4	1	1	3	3	1	1
4	4	4	4	4	4	2	2	4	4
4	4	1	1	1	1	3	3	1	1
4	4	4	4	1	1	3	3	1	1
4	4	1	1	1	1	3	3	1	1
4	4	1	1	1	1	3	3	1	1
4	4	4	4	1	1	3	3	1	1
4	4	4	4	1	1	3	3	1	1
4	4	1	1	1	1	3	3	1	1
4	4	4	4	1	1	3	3	1	1
4	4	4	4	1	1	3	3	1	1
4	4	1	1	1	1	3	3	1	1
4	4	4	4	1	1	3	3	1	1
4	4	4	4	1	1	3	3	1	1
4	4	1	1	1	1	3	3	1	1
4	4	4	4	4	4	3	3	1	1
4	4	4	4	4	4	3	3	4	4
4	4	4	4	1	1	3	3	1	1
4	4	1	1	1	1	3	3	1	1
4	4	4	4	1	1	3	3	4	4
4	4	4	4	4	4	3	3	4	4
4	4	4	4	1	1	3	3	1	1
4	4	4	4	1	1	3	3	1	1
4	4	1	1	1	1	3	3	1	1
4	4	4	4	1	1	3	3	1	1
4	4	4	4	1	1	3	3	1	1
4	4	4	4	1	1	3	3	1	1
4	4	4	4	1	1	3	3	1	1
4	4	1	1	1	1	3	3	1	1
4	4	4	4	1	1	3	3	1	1
4	4	4	4	1	1	3	3	1	1

For Review Only

4	4	4	4	1	1	3	3	1	1
4	4	4	4	1	1	2	2	4	4
1	1	4	4	1	1	3	3	4	4
4	4	4	4	4	4	2	2	4	4
4	4	4	4	1	1	3	3	1	1
4	4	1	1	1	1	3	3	1	1
4	4	4	4	1	1	2	2	1	1
4	4	4	4	1	1	3	3	1	1
4	4	4	4	1	1	3	3	1	1
4	4	1	1	1	1	3	3	1	1
4	4	4	4	1	1	3	3	1	1
4	4	4	4	1	1	3	3	1	1
4	4	1	1	1	1	3	3	1	1
4	4	4	4	1	1	3	3	1	1
4	4	4	4	1	1	3	3	1	1
4	4	4	4	4	4	3	3	4	4
4	4	4	4	1	1	2	2	1	1
4	4	1	1	1	1	3	3	1	1
4	4	4	4	1	1	3	3	1	1
4	4	1	1	1	1	3	3	1	1
4	4	4	4	1	1	3	3	1	1
4	4	4	4	4	4	3	3	4	4
4	4	4	4	4	4	2	2	4	4
4	4	4	4	1	1	3	3	1	1
1	1	4	4	1	1	3	3	1	1
4	4	1	1	1	1	3	3	1	1
4	4	4	4	1	1	3	3	1	1
4	4	4	4	1	1	3	3	1	1
1	1	4	4	1	1	3	3	1	1
1	1	4	4	1	1	3	3	1	1
4	4	4	4	4	4	3	3	4	4
4	4	1	1	1	1	3	3	1	1
4	4	1	1	1	1	3	3	4	4
4	4	4	4	1	1	3	3	1	1
4	4	4	4	1	1	3	3	1	1
4	4	4	4	1	1	3	3	1	1
4	4	4	4	1	1	2	2	1	1
4	4	4	4	1	1	3	3	1	1
4	4	1	1	1	1	3	3	1	1
4	4	4	4	1	1	3	3	1	1
4	4	4	4	1	1	3	3	1	1
4	4	4	4	1	1	3	3	1	1
4	4	4	4	1	1	3	3	4	4
4	4	4	4	1	1	2	2	1	1
4	4	4	4	1	1	3	3	1	1
4	4	4	4	1	1	3	3	1	1
4	4	4	4	4	4	3	3	4	4
4	4	4	4	1	1	3	3	1	1
4	4	4	4	1	1	3	3	1	1
1	1	4	4	1	1	3	3	1	1
4	4	4	4	1	1	2	2	1	1
4	4	4	4	1	1	3	3	1	1
4	4	4	4	1	1	2	2	1	1
4	4	4	4	1	1	3	3	1	1
4	4	4	4	1	1	3	3	4	4

For Review Only

4	4	1	1	1	1	3	3	1	1
4	4	4	4	1	1	3	3	1	1
4	4	4	4	1	1	3	3	1	1
4	4	4	4	1	1	2	2	1	1
4	4	4	4	1	1	3	3	1	1
4	4	4	4	1	1	3	3	1	1
4	4	4	4	1	1	3	3	1	1
1	1	4	4	1	1	3	3	1	1
4	4	4	4	1	1	3	3	1	1
4	4	4	4	1	1	3	3	1	1
4	4	4	4	1	1	3	3	1	1
4	4	4	4	4	4	3	3	4	4
4	4	4	4	1	1	2	2	1	1
4	4	1	1	1	1	3	3	1	1
1	1	4	4	1	1	3	3	1	1
4	4	4	4	1	1	2	2	1	1
4	4	4	4	1	1	3	3	1	1
4	4	4	4	1	1	3	3	1	1
4	4	4	4	1	1	2	2	1	1
4	4	4	4	4	4	2	2	4	4
4	4	4	4	1	1	3	3	1	1
4	4	4	4	1	1	3	3	1	1
4	4	4	4	1	1	3	3	1	1
4	4	4	4	1	1	2	2	1	1
4	4	4	4	1	1	3	3	1	1
4	4	1	1	1	1	3	3	1	1
4	4	4	4	4	4	3	3	4	4
4	4	1	1	1	1	3	3	1	1
4	4	4	4	1	1	3	3	1	1
4	4	1	1	1	1	3	3	1	1
4	4	4	4	4	4	3	3	4	4
4	4	1	1	1	1	3	3	1	1
1	1	4	4	1	1	3	3	1	1
4	4	1	1	1	1	3	3	1	1
4	4	4	4	4	4	2	2	4	4
4	4	4	4	4	4	3	3	4	4
4	4	1	1	1	1	3	3	1	1
4	4	4	4	1	1	3	3	1	1
4	4	4	4	1	1	3	3	1	1
4	4	4	4	1	1	3	3	1	1
4	4	4	4	1	1	3	3	1	1
4	4	1	1	1	1	3	3	1	1
4	4	4	4	4	4	2	2	4	4
4	4	1	1	1	1	3	3	1	1
4	4	4	4	1	1	3	3	1	1
4	4	1	1	1	1	2	2	1	1
4	4	1	1	1	1	3	3	1	1
1	1	4	4	1	1	3	3	1	1
4	4	4	4	1	1	3	3	1	1
4	4	4	4	4	4	3	3	4	4
4	4	1	1	1	1	3	3	1	1
4	4	4	4	1	1	3	3	1	1
4	4	4	4	1	1	3	3	1	1
4	4	1	1	1	1	3	3	1	1
4	4	4	4	1	1	2	2	1	1

4	4	1	1	1	1	3	3	1	1
4	4	1	1	1	1	3	3	1	1

For Review Only

SNP2909	SNP2909	SNP291	SNP291	SNP2910	SNP2910	SNP2911	SNP2911	SNP2912	SNP2912
4	4	4	4	3	3	1	1	4	4
4	4	4	4	3	3	1	1	4	4
4	4	4	4	3	3	2	2	2	2
4	4	4	4	2	2	1	1	2	2
4	4	4	4	3	3	2	2	2	2
4	4	4	4	3	3	2	2	2	2
4	4	4	4	2	2	1	1	4	4
4	4	4	4	2	2	1	1	4	4
4	4	4	4	2	2	1	1	4	4
4	4	4	4	2	2	1	1	4	4
4	4	4	4	3	3	2	2	2	2
4	4	4	4	3	3	1	1	2	2
4	4	4	4	3	3	1	1	2	2
4	4	4	4	2	2	1	1	4	4
4	4	4	4	3	3	2	2	2	2
4	4	4	4	3	3	2	2	2	2
4	4	4	4	2	2	1	1	4	4
4	4	4	4	2	2	1	1	4	4
4	4	4	4	3	3	2	2	2	2
4	4	4	4	2	2	1	1	4	4
4	4	4	4	2	2	1	1	4	4
4	4	4	4	3	3	2	2	2	2
2	2	4	4	2	2	1	1	4	4
4	4	4	4	2	2	1	1	4	4
4	4	4	4	3	3	2	2	2	2
4	4	4	4	2	2	1	1	2	2
4	4	4	4	2	2	1	1	2	2
4	4	4	4	3	3	1	1	2	2
4	4	4	4	3	3	2	2	2	2
4	4	4	4	2	2	1	1	4	4
4	4	4	4	3	3	2	2	2	2
4	4	4	4	2	2	1	1	2	2
4	4	4	4	2	2	1	1	4	4
4	4	4	4	2	2	1	1	4	4
2	2	4	4	3	3	1	1	2	2
4	4	4	4	3	3	2	2	2	2
4	4	4	4	2	2	1	1	4	4
4	4	4	4	2	2	1	1	4	4
4	4	4	4	2	2	1	1	4	4
4	4	4	4	2	2	1	1	4	4
4	4	4	4	2	2	1	1	4	4
4	4	4	4	2	2	1	1	4	4
4	4	4	4	2	2	1	1	4	4
4	4	4	4	2	2	1	1	4	4
4	4	4	4	2	2	1	1	4	4
4	4	4	4	3	3	2	2	2	2
4	4	4	4	3	3	2	2	2	2
4	4	4	4	2	2	1	1	4	4
4	4	4	4	3	3	1	1	4	4
4	4	4	4	3	3	1	1	2	2
4	4	4	4	3	3	2	2	2	2
4	4	4	4	3	3	2	2	2	2

For Review Only

4	4	4	4	3	3	2	2	2	2
4	4	4	4	2	2	1	1	4	4
4	4	4	4	2	2	1	1	2	2
4	4	4	4	2	2	1	1	4	4
4	4	4	4	3	3	2	2	2	2
4	4	4	4	3	3	2	2	2	2
4	4	4	4	2	2	1	1	2	2
4	4	3	3	2	2	1	1	4	4
4	4	4	4	2	2	1	1	4	4
4	4	4	4	3	3	1	1	2	2
4	4	4	4	3	3	2	2	2	2
4	4	4	4	3	3	2	2	2	2
4	4	4	4	3	3	2	2	2	2
4	4	4	4	3	3	2	2	2	2
4	4	4	4	3	3	1	1	2	2
4	4	4	4	2	2	1	1	4	4
4	4	4	4	3	3	2	2	2	2
4	4	4	4	3	3	2	2	2	2
4	4	4	4	3	3	2	2	2	2
4	4	4	4	2	2	1	1	4	4
4	4	4	4	3	3	1	1	2	2
4	4	4	4	2	2	1	1	4	4
4	4	4	4	3	3	1	1	4	4
4	4	4	4	2	2	1	1	4	4
4	4	4	4	2	2	1	1	4	4
4	4	4	4	3	3	2	2	2	2
4	4	4	4	2	2	1	1	4	4
2	2	4	4	2	2	1	1	4	4
4	4	4	4	2	2	1	1	2	2
4	4	4	4	2	2	1	1	2	2
4	4	4	4	3	3	2	2	2	2
4	4	4	4	2	2	1	1	4	4
2	2	4	4	2	2	1	1	4	4
4	4	4	4	3	3	2	2	2	2
4	4	4	4	2	2	1	1	4	4
4	4	4	4	2	2	1	1	4	4
4	4	4	4	2	2	1	1	4	4
4	4	4	4	3	3	1	1	2	2
4	4	4	4	2	2	1	1	4	4
4	4	4	4	2	2	1	1	4	4
4	4	4	4	3	3	2	2	2	2
4	4	4	4	2	2	1	1	2	2
4	4	4	4	2	2	1	1	4	4
4	4	4	4	3	3	2	2	2	2
4	4	4	4	3	3	2	2	2	2
4	4	4	4	3	3	2	2	2	2
4	4	4	4	2	2	1	1	4	4
4	4	4	4	2	2	1	1	2	2
4	4	4	4	2	2	1	1	4	4
4	4	4	4	2	2	1	1	4	4
4	4	4	4	2	2	1	1	4	4
2	2	4	4	2	2	1	1	4	4
4	4	3	3	2	2	1	1	4	4
4	4	4	4	2	2	1	1	4	4
4	4	4	4	2	2	1	1	4	4

For Review Only

4	4	4	4	3	3	1	1	2	2
4	4	4	4	2	2	1	1	4	4
4	4	4	4	3	3	2	2	2	2
4	4	4	4	2	2	1	1	4	4
4	4	4	4	2	2	1	1	2	2
4	4	4	4	3	3	2	2	2	2
4	4	4	4	3	3	2	2	2	2
4	4	4	4	3	3	1	1	2	2
4	4	4	4	2	2	1	1	4	4
4	4	4	4	2	2	1	1	2	2
4	4	4	4	2	2	1	1	4	4
4	4	4	4	3	3	1	1	2	2
4	4	4	4	2	2	1	1	2	2
4	4	4	4	3	3	1	1	2	2
4	4	4	4	2	2	1	1	2	2
4	4	4	4	3	3	1	1	2	2
4	4	4	4	2	2	1	1	2	2
4	4	4	4	3	3	1	1	2	2
4	4	4	4	2	2	1	1	4	4
4	4	4	4	3	3	2	2	2	2
4	4	4	4	3	3	1	1	2	2
4	4	4	4	2	2	2	2	2	2
4	4	4	4	3	3	2	2	2	2
2	2	4	4	2	2	1	1	4	4
4	4	4	4	3	3	1	1	2	2
4	4	4	4	2	2	1	1	4	4
4	4	4	4	3	3	2	2	2	2
4	4	4	4	2	2	1	1	4	4
4	4	4	4	2	2	1	1	2	2
4	4	4	4	3	3	2	2	4	4
2	2	3	3	2	2	1	1	4	4
4	4	4	4	3	3	1	1	2	2
4	4	4	4	3	3	2	2	2	2
4	4	4	4	3	3	1	1	2	2
4	4	4	4	2	2	1	1	4	4
4	4	4	4	3	3	1	1	4	4
4	4	4	4	2	2	1	1	4	4
4	4	4	4	3	3	2	2	2	2
4	4	4	4	3	3	1	1	4	4
2	2	4	4	2	2	1	1	4	4
4	4	4	4	2	2	1	1	2	2
4	4	4	4	2	2	1	1	4	4
4	4	4	4	3	3	2	2	2	2
4	4	4	4	2	2	1	1	4	4
4	4	4	4	2	2	1	1	4	4
4	4	3	3	3	3	2	2	2	2
4	4	4	4	3	3	2	2	2	2
4	4	4	4	3	3	2	2	2	2
4	4	4	4	2	2	1	1	4	4
4	4	4	4	3	3	1	1	2	2
4	4	4	4	3	3	2	2	2	2
4	4	4	4	2	2	1	1	4	4
4	4	3	3	3	3	1	1	2	2
4	4	4	4	3	3	2	2	2	2
4	4	4	4	3	3	1	1	4	4

4	4	4	4	3	3	2	2	2	2
4	4	4	4	2	2	1	1	4	4
4	4	4	4	2	2	1	1	4	4
4	4	4	4	3	3	1	1	2	2
4	4	4	4	3	3	2	2	2	2
4	4	4	4	2	2	1	1	4	4
2	2	4	4	2	2	1	1	4	4
4	4	4	4	3	3	1	1	2	2
2	2	4	4	2	2	1	1	4	4
4	4	4	4	2	2	1	1	4	4
4	4	4	4	3	3	2	2	2	2
4	4	4	4	2	2	1	1	4	4
4	4	4	4	2	2	2	2	2	2
4	4	4	4	3	3	1	1	2	2
4	4	3	3	2	2	1	1	4	4
4	4	4	4	2	2	1	1	4	4
4	4	3	3	3	3	1	1	2	2
4	4	4	4	2	2	1	1	4	4
4	4	4	4	2	2	1	1	4	4
4	4	4	4	3	3	2	2	2	2
4	4	4	4	3	3	1	1	2	2
4	4	4	4	2	2	1	1	4	4
4	4	4	4	2	2	1	1	4	4
4	4	4	4	3	3	2	2	2	2
4	4	4	4	2	2	1	1	4	4
4	4	4	4	3	3	2	2	2	2
4	4	4	4	2	2	1	1	2	2
4	4	4	4	2	2	1	1	2	2
4	4	4	4	2	2	1	1	4	4
4	4	4	4	2	2	1	1	4	4
4	4	4	4	2	2	1	1	4	4
4	4	4	4	2	2	1	1	2	2
4	4	4	4	2	2	1	1	4	4
4	4	3	3	2	2	1	1	4	4
4	4	4	4	2	2	1	1	4	4
4	4	4	4	3	3	2	2	2	2
4	4	4	4	2	2	1	1	4	4
4	4	4	4	2	2	1	1	4	4
4	4	4	4	3	3	2	2	2	2
4	4	4	4	2	2	1	1	4	4
4	4	4	4	3	3	2	2	2	2
4	4	4	4	3	3	2	2	2	2
4	4	4	4	2	2	1	1	4	4
4	4	4	4	3	3	1	1	2	2
4	4	4	4	3	3	1	1	4	4
4	4	4	4	3	3	1	1	2	2
4	4	4	4	2	2	1	1	4	4
2	2	4	4	2	2	1	1	4	4
4	4	4	4	2	2	1	1	4	4
4	4	4	4	2	2	1	1	4	4
4	4	4	4	2	2	1	1	4	4
4	4	3	3	2	2	1	1	4	4
4	4	4	4	2	2	1	1	4	4
4	4	4	4	3	3	2	2	2	2
2	2	4	4	2	2	1	1	4	4

For Review Only

4	4	3	3	3	3	2	2	2	2
4	4	4	4	2	2	1	1	4	4
4	4	4	4	3	3	2	2	2	2
4	4	4	4	2	2	1	1	4	4
4	4	4	4	2	2	1	1	4	4
4	4	4	4	2	2	1	1	2	2
2	2	4	4	2	2	1	1	4	4
4	4	4	4	2	2	1	1	4	4
4	4	4	4	3	3	1	1	4	4
2	2	4	4	2	2	1	1	4	4
4	4	4	4	3	3	1	1	2	2
4	4	4	4	2	2	1	1	4	4
4	4	4	4	2	2	1	1	4	4
4	4	4	4	3	3	2	2	2	2
4	4	4	4	2	2	1	1	4	4
4	4	4	4	2	2	1	1	4	4
2	2	4	4	2	2	1	1	4	4
2	2	4	4	2	2	1	1	4	4
4	4	3	3	2	2	1	1	4	4
4	4	4	4	2	2	1	1	4	4
4	4	4	4	3	3	2	2	2	2
4	4	4	4	2	2	1	1	2	2
4	4	4	4	2	2	1	1	4	4
4	4	4	4	2	2	1	1	4	4
4	4	3	3	2	2	1	1	4	4
4	4	4	4	3	3	1	1	2	2
2	2	4	4	2	2	1	1	4	4
4	4	4	4	2	2	1	1	2	2
4	4	4	4	3	3	1	1	2	2
4	4	4	4	2	2	1	1	2	2
2	2	4	4	2	2	1	1	4	4
4	4	4	4	3	3	2	2	2	2
4	4	4	4	2	2	1	1	4	4
4	4	4	4	3	3	2	2	2	2
4	4	4	4	2	2	1	1	4	4
2	2	4	4	2	2	1	1	4	4
4	4	4	4	3	3	2	2	2	2
4	4	4	4	3	3	2	2	2	2
4	4	4	4	3	3	2	2	2	2
4	4	4	4	2	2	1	1	4	4
4	4	4	4	2	2	1	1	4	4
4	4	4	4	2	2	1	1	4	4
4	4	4	4	2	2	1	1	4	4
4	4	4	4	3	3	2	2	2	2
4	4	4	4	3	3	1	1	2	2
4	4	4	4	2	2	1	1	4	4
4	4	4	4	3	3	2	2	2	2
4	4	4	4	2	2	1	1	4	4
4	4	4	4	3	3	1	1	2	2
2	2	4	4	2	2	1	1	4	4
4	4	4	4	3	3	2	2	4	4
4	4	4	4	2	2	1	1	4	4
4	4	4	4	3	3	2	2	2	2
4	4	4	4	2	2	1	1	2	2
4	4	4	4	2	2	1	1	4	4

For Review Only

4	4	4	4	3	3	2	2	2	2
4	4	4	4	3	3	2	2	2	2

For Review Only

SNP2913	SNP2913	SNP2914	SNP2914	SNP2915	SNP2915	SNP2916	SNP2916	SNP2917	SNP2917
4	4	4	4	3	3	1	1	3	3
4	4	4	4	3	3	1	1	3	3
4	4	1	1	2	2	1	1	3	3
4	4	1	1	2	2	1	1	3	3
4	4	1	1	2	2	1	1	3	3
4	4	1	1	2	2	1	1	3	3
4	4	4	4	3	3	1	1	3	3
4	4	4	4	3	3	1	1	3	3
4	4	1	1	2	2	1	1	3	3
4	4	4	4	3	3	1	1	3	3
1	1	1	1	2	2	1	1	3	3
4	4	1	1	2	2	1	1	3	3
4	4	1	1	2	2	1	1	3	3
4	4	4	4	3	3	1	1	3	3
4	4	4	4	2	2	1	1	3	3
4	4	1	1	2	2	1	1	3	3
4	4	4	4	3	3	1	1	3	3
4	4	4	4	3	3	1	1	3	3
4	4	1	1	2	2	1	1	3	3
4	4	4	4	3	3	1	1	3	3
4	4	4	4	3	3	1	1	3	3
4	4	1	1	2	2	1	1	3	3
1	1	4	4	3	3	3	3	2	2
4	4	4	4	3	3	1	1	3	3
4	4	1	1	2	2	1	1	3	3
4	4	1	1	2	2	1	1	3	3
4	4	1	1	2	2	1	1	3	3
4	4	4	4	2	2	1	1	3	3
4	4	1	1	2	2	1	1	3	3
1	1	4	4	3	3	1	1	3	3
4	4	1	1	2	2	1	1	3	3
4	4	1	1	2	2	1	1	3	3
4	4	4	4	3	3	1	1	3	3
4	4	4	4	2	2	1	1	3	3
4	4	4	4	2	2	1	1	3	3
4	4	4	4	3	3	1	1	3	3
4	4	4	4	3	3	1	1	3	3
1	1	4	4	3	3	3	3	2	2
4	4	4	4	3	3	1	1	3	3
4	4	4	4	3	3	1	1	3	3
1	1	4	4	3	3	1	1	3	3
4	4	4	4	3	3	1	1	3	3
4	4	4	4	3	3	1	1	3	3
4	4	4	4	3	3	1	1	3	3
4	4	4	4	3	3	1	1	3	3
4	4	1	1	2	2	1	1	3	3
4	4	1	1	2	2	1	1	3	3
4	4	4	4	3	3	1	1	3	3
4	4	4	4	3	3	1	1	3	3
4	4	4	4	2	2	1	1	3	3
4	4	1	1	2	2	1	1	3	3
4	4	1	1	2	2	1	1	3	3

For Review Only

4	4	1	1	2	2	1	1	3	3
4	4	4	4	3	3	1	1	3	3
4	4	1	1	2	2	1	1	3	3
4	4	4	4	3	3	1	1	3	3
4	4	1	1	2	2	1	1	3	3
4	4	1	1	2	2	1	1	3	3
4	4	1	1	2	2	1	1	3	3
4	4	4	4	3	3	1	1	3	3
4	4	4	4	3	3	1	1	3	3
4	4	4	4	2	2	1	1	3	3
4	4	1	1	2	2	1	1	3	3
4	4	1	1	2	2	1	1	3	3
4	4	1	1	2	2	1	1	3	3
4	4	1	1	2	2	1	1	3	3
4	4	4	4	2	2	1	1	3	3
4	4	4	4	3	3	1	1	3	3
4	4	1	1	2	2	1	1	3	3
4	4	1	1	2	2	1	1	3	3
4	4	1	1	2	2	1	1	3	3
4	4	4	4	3	3	1	1	3	3
4	4	1	1	2	2	1	1	3	3
1	1	4	4	3	3	1	1	3	3
4	4	4	4	3	3	3	3	3	3
4	4	4	4	3	3	1	1	3	3
4	4	4	4	3	3	1	1	3	3
4	4	1	1	2	2	1	1	3	3
4	4	4	4	3	3	1	1	3	3
1	1	4	4	3	3	3	3	2	2
4	4	1	1	2	2	1	1	3	3
4	4	1	1	2	2	1	1	3	3
4	4	1	1	2	2	1	1	3	3
4	4	4	4	3	3	1	1	3	3
1	1	4	4	3	3	3	3	2	2
4	4	1	1	2	2	1	1	3	3
4	4	4	4	3	3	1	1	3	3
4	4	4	4	3	3	1	1	3	3
1	1	4	4	3	3	1	1	3	3
4	4	4	4	2	2	1	1	3	3
4	4	4	4	3	3	3	3	2	2
4	4	4	4	3	3	1	1	3	3
4	4	4	4	2	2	1	1	3	3
4	4	1	1	2	2	1	1	3	3
4	4	4	4	3	3	1	1	3	3
4	4	1	1	2	2	1	1	3	3
4	4	1	1	2	2	1	1	3	3
4	4	1	1	2	2	1	1	3	3
4	4	4	4	3	3	1	1	3	3
4	4	1	1	2	2	1	1	3	3
4	4	4	4	3	3	1	1	3	3
4	4	4	4	3	3	1	1	3	3
4	4	4	4	2	2	1	1	3	3
1	1	4	4	3	3	1	1	3	3
4	4	4	4	3	3	1	1	3	3
4	4	4	4	2	2	1	1	3	3
4	4	4	4	3	3	1	1	3	3

For Review Only

4	4	4	4	2	2	3	3	3	3
4	4	4	4	3	3	1	1	3	3
4	4	1	1	2	2	1	1	3	3
4	4	4	4	3	3	1	1	3	3
4	4	1	1	2	2	1	1	3	3
4	4	1	1	2	2	1	1	3	3
4	4	1	1	2	2	1	1	3	3
4	4	4	4	2	2	3	3	3	3
4	4	4	4	3	3	1	1	3	3
4	4	4	4	3	3	1	1	3	3
1	1	4	4	3	3	1	1	3	3
4	4	4	4	2	2	1	1	3	3
4	4	1	1	2	2	1	1	3	3
4	4	1	1	2	2	1	1	3	3
4	4	4	4	2	2	1	1	3	3
4	4	4	4	2	2	1	1	3	3
4	4	1	1	2	2	1	1	3	3
4	4	1	1	2	2	1	1	3	3
4	4	4	4	3	3	1	1	3	3
4	4	1	1	2	2	1	1	3	3
4	4	4	4	2	2	1	1	3	3
4	4	1	1	2	2	1	1	3	3
4	4	1	1	2	2	1	1	3	3
1	1	4	4	3	3	3	3	2	2
1	1	4	4	2	2	1	1	3	3
1	1	4	4	3	3	3	3	2	2
4	4	1	1	2	2	1	1	3	3
4	4	4	4	3	3	1	1	3	3
4	4	1	1	2	2	1	1	3	3
4	4	4	4	3	3	1	1	3	3
4	4	4	4	3	3	3	3	3	3
4	4	4	4	2	2	1	1	3	3
4	4	1	1	2	2	1	1	3	3
4	4	4	4	2	2	1	1	3	3
4	4	4	4	2	2	1	1	3	3
4	4	4	4	2	2	1	1	2	2
4	4	4	4	3	3	1	1	3	3
4	4	1	1	2	2	1	1	3	3
4	4	1	1	2	2	1	1	3	3
4	4	4	4	3	3	3	3	2	2
1	1	1	1	2	2	1	1	3	3
4	4	4	4	3	3	1	1	3	3
4	4	1	1	2	2	1	1	3	3
4	4	4	4	3	3	1	1	3	3
1	1	4	4	3	3	1	1	3	3
4	4	1	1	2	2	1	1	3	3
4	4	1	1	2	2	1	1	3	3
4	4	1	1	2	2	1	1	3	3
4	4	4	4	3	3	1	1	3	3
4	4	1	1	2	2	1	1	3	3
4	4	4	4	2	2	1	1	3	3
4	4	4	4	3	3	1	1	3	3
4	4	1	1	2	2	1	1	3	3
4	4	1	1	2	2	1	1	3	3
4	4	4	4	2	2	1	1	3	3

For Review Only

4	4	1	1	2	2	1	1	3	3
1	1	4	4	3	3	3	3	2	2
4	4	4	4	3	3	1	1	3	3
1	1	4	4	2	2	3	3	3	3
4	4	1	1	2	2	1	1	3	3
4	4	4	4	3	3	1	1	3	3
4	4	4	4	3	3	3	3	2	2
4	4	4	4	2	2	1	1	3	3
4	4	4	4	3	3	3	3	3	3
4	4	4	4	3	3	1	1	3	3
4	4	1	1	2	2	1	1	3	3
4	4	4	4	3	3	1	1	3	3
4	4	4	4	2	2	1	1	3	3
4	4	4	4	2	2	1	1	3	3
4	4	4	4	3	3	1	1	3	3
1	1	4	4	3	3	1	1	3	3
4	4	4	4	2	2	1	1	3	3
4	4	4	4	3	3	1	1	3	3
4	4	4	4	3	3	1	1	3	3
4	4	1	1	2	2	1	1	3	3
4	4	4	4	2	2	1	1	3	3
1	1	4	4	3	3	1	1	3	3
1	1	4	4	3	3	1	1	3	3
4	4	1	1	2	2	1	1	3	3
4	4	4	4	3	3	1	1	3	3
4	4	1	1	2	2	1	1	3	3
4	4	4	4	2	2	1	1	3	3
4	4	4	4	2	2	1	1	3	3
4	4	4	4	3	3	1	1	3	3
4	4	4	4	3	3	1	1	3	3
1	1	4	4	3	3	1	1	3	3
4	4	1	1	2	2	1	1	3	3
4	4	4	4	3	3	1	1	3	3
4	4	4	4	3	3	1	1	3	3
4	4	4	4	3	3	1	1	3	3
4	4	1	1	2	2	1	1	3	3
4	4	4	4	3	3	3	3	2	2
4	4	4	4	3	3	1	1	3	3
4	4	1	1	2	2	1	1	3	3
4	4	4	4	3	3	1	1	3	3
4	4	1	1	2	2	1	1	3	3
4	4	1	1	2	2	1	1	3	3
1	1	4	4	3	3	3	3	2	2
4	4	4	4	2	2	1	1	3	3
4	4	4	4	2	2	1	1	3	3
4	4	4	4	2	2	3	3	3	3
1	1	4	4	3	3	1	1	3	3
4	4	4	4	3	3	3	3	2	2
4	4	4	4	3	3	1	1	3	3
4	4	4	4	3	3	1	1	3	3
4	4	4	4	3	3	1	1	3	3
4	4	4	4	3	3	1	1	3	3
4	4	4	4	3	3	1	1	3	3
4	4	1	1	2	2	1	1	3	3
1	1	4	4	3	3	1	1	3	3

For Review Only

4	4	1	1	2	2	1	1	3	3
4	4	4	4	3	3	1	1	3	3
4	4	1	1	2	2	1	1	3	3
4	4	4	4	3	3	1	1	3	3
4	4	4	4	3	3	1	1	3	3
4	4	1	1	2	2	1	1	3	3
4	4	4	4	3	3	3	3	2	2
4	4	4	4	3	3	1	1	3	3
4	4	4	4	3	3	1	1	3	3
1	1	4	4	3	3	3	3	2	2
4	4	4	4	2	2	1	1	3	3
1	1	4	4	3	3	1	1	3	3
4	4	4	4	3	3	3	3	2	2
4	4	1	1	2	2	1	1	3	3
4	4	4	4	3	3	1	1	3	3
4	4	4	4	3	3	3	3	2	2
4	4	4	4	3	3	3	3	2	2
4	4	4	4	3	3	3	3	3	3
4	4	4	4	3	3	1	1	3	3
1	1	4	4	3	3	3	3	2	2
4	4	1	1	2	2	1	1	3	3
4	4	1	1	2	2	1	1	3	3
4	4	4	4	3	3	1	1	3	3
4	4	4	4	3	3	3	3	2	2
4	4	4	4	3	3	1	1	3	3
4	4	4	4	2	2	1	1	3	3
1	1	4	4	3	3	3	3	2	2
4	4	4	4	2	2	1	1	3	3
4	4	4	4	3	3	1	1	3	3
4	4	1	1	2	2	1	1	3	3
1	1	4	4	3	3	1	1	3	3
4	4	1	1	2	2	1	1	3	3
4	4	4	4	3	3	1	1	3	3
4	4	1	1	2	2	1	1	3	3
1	1	4	4	3	3	3	3	2	2
1	1	4	4	3	3	3	3	2	2
4	4	1	1	2	2	1	1	3	3
4	4	1	1	2	2	1	1	3	3
4	4	4	4	3	3	1	1	3	3
4	4	4	4	3	3	1	1	3	3
4	4	4	4	3	3	1	1	3	3
4	4	4	4	3	3	1	1	3	3
1	1	4	4	3	3	3	3	2	2
4	4	1	1	2	2	1	1	3	3
4	4	4	4	2	2	1	1	3	3
4	4	4	4	3	3	3	3	2	2
4	4	1	1	2	2	1	1	3	3
4	4	4	4	3	3	1	1	3	3
4	4	4	4	2	2	1	1	3	3
1	1	4	4	3	3	3	3	2	2
4	4	4	4	3	3	1	1	3	3
4	4	4	4	3	3	1	1	3	3
4	4	1	1	2	2	1	1	3	3
4	4	1	1	2	2	1	1	3	3
4	4	4	4	3	3	3	3	2	2

For Review Only

CSSA 5585 Guilford Rd., Madison, WI 53711

4	4	1	1	2	2	1	1	3	3
4	4	1	1	2	2	1	1	3	3

For Review Only

SNP2918	SNP2918	SNP2919	SNP2919	SNP292	SNP292	SNP2920	SNP2920	SNP2921	SNP2921
3	3	2	2	4	4	2	2	2	2
3	3	2	2	4	4	2	2	2	2
2	2	2	2	4	4	3	3	2	2
3	3	2	2	4	4	2	2	3	3
3	3	2	2	1	1	2	2	2	2
3	3	2	2	1	1	2	2	2	2
3	3	2	2	1	1	2	2	2	2
3	3	2	2	4	4	2	2	2	2
3	3	2	2	1	1	2	2	2	2
3	3	2	2	4	4	2	2	2	2
3	3	3	3	4	4	2	2	2	2
3	3	2	2	4	4	3	3	2	2
3	3	2	2	1	1	2	2	2	2
3	3	2	2	4	4	2	2	2	2
3	3	2	2	1	1	2	2	2	2
2	2	2	2	4	4	3	3	3	3
3	3	2	2	4	4	2	2	2	2
3	3	2	2	1	1	2	2	2	2
3	3	2	2	4	4	3	3	3	3
3	3	2	2	4	4	2	2	2	2
3	3	2	2	1	1	2	2	2	2
3	3	2	2	1	1	2	2	2	2
3	3	3	3	4	4	2	2	2	2
3	3	2	2	4	4	2	2	2	2
3	3	2	2	1	1	2	2	2	2
3	3	2	2	1	1	2	2	2	2
3	3	2	2	4	4	2	2	2	2
3	3	2	2	4	4	2	2	2	2
3	3	2	2	1	1	2	2	2	2
3	3	2	2	4	4	2	2	2	2
3	3	2	2	4	4	2	2	2	2
3	3	2	2	4	4	3	3	3	3
3	3	2	2	1	1	2	2	2	2
3	3	2	2	1	1	2	2	2	2
3	3	2	2	4	4	2	2	2	2
3	3	2	2	4	4	2	2	2	2
3	3	2	2	4	4	2	2	2	2
3	3	2	2	1	1	2	2	2	2
3	3	2	2	4	4	2	2	2	2
3	3	2	2	4	4	2	2	2	2
3	3	2	2	4	4	2	2	2	2
3	3	2	2	4	4	2	2	2	2
3	3	2	2	4	4	2	2	2	2
3	3	2	2	4	4	2	2	2	2
3	3	2	2	4	4	2	2	2	2
2	2	2	2	4	4	3	3	2	2
2	2	2	2	4	4	3	3	2	2
3	3	2	2	1	1	2	2	2	2
3	3	2	2	4	4	2	2	2	2
3	3	2	2	1	1	2	2	2	2
2	2	2	2	4	4	3	3	2	2
2	2	2	2	4	4	3	3	3	3

For Review Only

3	3	2	2	1	1	2	2	2	2
3	3	2	2	1	1	2	2	2	2
2	2	2	2	4	4	2	2	3	3
3	3	2	2	1	1	2	2	2	2
3	3	2	2	4	4	3	3	3	3
3	3	2	2	4	4	2	2	2	2
3	3	2	2	4	4	3	3	3	3
3	3	2	2	4	4	2	2	2	2
3	3	2	2	1	1	2	2	2	2
3	3	2	2	1	1	2	2	2	2
3	3	2	2	4	4	2	2	2	2
3	3	2	2	4	4	2	2	2	2
3	3	2	2	1	1	2	2	2	2
3	3	2	2	1	1	2	2	2	2
3	3	2	2	1	1	2	2	2	2
3	3	2	2	1	1	2	2	2	2
3	3	2	2	4	4	3	3	2	2
3	3	2	2	1	1	3	3	3	3
3	3	2	2	4	4	2	2	2	2
3	3	2	2	1	1	2	2	2	2
3	3	2	2	4	4	2	2	2	2
3	3	3	3	4	4	2	2	2	2
3	3	2	2	1	1	2	2	2	2
3	3	2	2	1	1	2	2	2	2
3	3	2	2	4	4	2	2	2	2
2	2	2	2	4	4	3	3	3	3
3	3	2	2	4	4	2	2	2	2
3	3	2	2	4	4	2	2	2	2
3	3	2	2	4	4	2	2	2	2
3	3	2	2	1	1	3	3	3	3
3	3	2	2	1	1	2	2	2	2
3	3	2	2	1	1	2	2	2	2
3	3	2	2	4	4	2	2	2	2
3	3	2	2	1	1	2	2	2	2
3	3	2	2	1	1	2	2	2	2
3	3	2	2	4	4	2	2	2	2
3	3	2	2	4	4	2	2	2	2
3	3	2	2	1	1	2	2	2	2
3	3	2	2	1	1	2	2	2	2
3	3	2	2	4	4	2	2	2	2
3	3	2	2	1	1	2	2	2	2
3	3	2	2	4	4	3	3	3	3
3	3	2	2	4	4	2	2	2	2
3	3	2	2	4	4	2	2	2	2
3	3	2	2	1	1	2	2	2	2
2	2	2	2	1	1	3	3	2	2
3	3	2	2	4	4	2	2	2	2
3	3	2	2	4	4	2	2	2	2
3	3	2	2	4	4	2	2	2	2
3	3	2	2	4	4	2	2	2	2
3	3	2	2	4	4	2	2	2	2
3	3	3	3	4	4	2	2	2	2
3	3	2	2	4	4	2	2	2	2
3	3	2	2	4	4	3	3	2	2
3	3	2	2	4	4	2	2	2	2

For Review Only

3	3	2	2	4	4	3	3	2	2
3	3	2	2	1	1	2	2	2	2
2	2	2	2	1	1	3	3	2	2
3	3	2	2	4	4	2	2	2	2
3	3	2	2	4	4	2	2	2	2
2	2	2	2	1	1	3	3	2	2
2	2	2	2	4	4	3	3	3	3
3	3	2	2	4	4	2	2	2	2
3	3	2	2	4	4	2	2	2	2
3	3	2	2	4	4	2	2	2	2
3	3	2	2	1	1	2	2	2	2
3	3	2	2	1	1	2	2	2	2
3	3	2	2	1	1	2	2	2	2
3	3	2	2	4	4	2	2	2	2
2	2	2	2	4	4	2	2	2	2
3	3	2	2	4	4	2	2	2	2
3	3	2	2	1	1	2	2	2	2
3	3	2	2	4	4	3	3	2	2
3	3	2	2	4	4	2	2	2	2
3	3	2	2	4	4	2	2	2	2
3	3	2	2	1	1	2	2	2	2
3	3	2	2	1	1	2	2	2	2
2	2	2	2	4	4	3	3	2	2
3	3	2	2	4	4	2	2	2	2
3	3	2	2	1	1	3	3	2	2
3	3	3	3	4	4	2	2	2	2
3	3	2	2	4	4	2	2	2	2
3	3	2	2	4	4	2	2	2	2
3	3	2	2	1	1	2	2	2	2
3	3	2	2	4	4	2	2	2	2
3	3	2	2	4	4	2	2	2	2
3	3	2	2	1	1	2	2	2	2
3	3	2	2	1	1	2	2	2	2
3	3	2	2	4	4	2	2	2	2
3	3	2	2	1	1	2	2	2	2
3	3	2	2	1	1	2	2	2	2
3	3	2	2	1	1	2	2	2	2
3	3	2	2	4	4	2	2	2	2
3	3	2	2	4	4	2	2	2	2
3	3	3	3	4	4	2	2	2	2
3	3	3	3	4	4	2	2	2	2
3	3	2	2	4	4	2	2	2	2
3	3	2	2	4	4	3	3	3	3
3	3	2	2	4	4	2	2	2	2
3	3	2	2	1	1	2	2	2	2
3	3	2	2	4	4	3	3	2	2
2	2	2	2	4	4	3	3	2	2
3	3	2	2	4	4	2	2	2	2
3	3	2	2	4	4	2	2	2	2
3	3	2	2	4	4	2	2	2	2
3	3	2	2	1	1	2	2	2	2
3	3	2	2	4	4	2	2	2	2
3	3	2	2	1	1	2	2	3	3
3	3	2	2	4	4	2	2	2	2
3	3	2	2	4	4	2	2	2	2

For Review Only

2	2	2	2	4	4	3	3	2	2
3	3	3	3	4	4	2	2	2	2
3	3	2	2	4	4	2	2	2	2
3	3	2	2	1	1	2	2	2	2
2	2	2	2	4	4	3	3	2	2
3	3	2	2	4	4	2	2	2	2
3	3	2	2	4	4	2	2	2	2
3	3	2	2	1	1	2	2	2	2
3	3	3	3	4	4	2	2	2	2
3	3	2	2	1	1	2	2	2	2
3	3	2	2	4	4	2	2	2	2
3	3	2	2	4	4	2	2	2	2
2	2	2	2	4	4	3	3	2	2
3	3	2	2	4	4	3	3	2	2
3	3	2	2	4	4	2	2	2	2
3	3	3	3	4	4	2	2	2	2
3	3	2	2	1	1	2	2	2	2
3	3	2	2	4	4	2	2	2	2
3	3	2	2	4	4	2	2	2	2
2	2	2	2	4	4	3	3	2	2
3	3	2	2	1	1	2	2	2	2
3	3	2	2	4	4	2	2	2	2
3	3	3	3	4	4	2	2	2	2
3	3	2	2	4	4	2	2	2	2
3	3	2	2	1	1	2	2	2	2
3	3	2	2	4	4	2	2	2	2
3	3	2	2	1	1	2	2	2	2
3	3	2	2	1	1	2	2	2	2
3	3	2	2	1	1	2	2	2	2
3	3	2	2	1	1	2	2	2	2
3	3	2	2	4	4	2	2	2	2
3	3	2	2	1	1	3	3	3	3
3	3	2	2	4	4	2	2	2	2
3	3	2	2	4	4	2	2	2	2
3	3	2	2	4	4	2	2	2	2
3	3	2	2	4	4	2	2	2	2
3	3	2	2	4	4	2	2	2	2
3	3	2	2	4	4	2	2	2	2
3	3	2	2	4	4	2	2	2	2
3	3	2	2	1	1	2	2	2	2
2	2	2	2	4	4	3	3	2	2
2	2	2	2	4	4	3	3	2	2
3	3	2	2	4	4	2	2	2	2
3	3	2	2	1	1	2	2	2	2
2	2	2	2	4	4	3	3	2	2
3	3	2	2	4	4	2	2	2	2
3	3	3	3	1	1	2	2	2	2
3	3	2	2	4	4	2	2	2	2
3	3	2	2	4	4	2	2	2	2
3	3	2	2	1	1	2	2	2	2
3	3	2	2	1	1	2	2	2	2
3	3	2	2	1	1	2	2	2	2
3	3	2	2	4	4	2	2	2	2
3	3	2	2	4	4	2	2	2	2
3	3	3	3	4	4	2	2	2	2

For Review Only

3	3	2	2	1	1	3	3	3	3
3	3	2	2	4	4	2	2	2	2
2	2	2	2	1	1	3	3	2	2
3	3	2	2	4	4	2	2	2	2
3	3	2	2	4	4	2	2	2	2
3	3	2	2	1	1	2	2	2	2
3	3	2	2	4	4	2	2	2	2
3	3	2	2	4	4	2	2	2	2
3	3	2	2	4	4	2	2	2	2
3	3	2	2	4	4	2	2	2	2
3	3	2	2	1	1	2	2	2	2
3	3	2	2	1	1	2	2	2	2
3	3	2	2	1	1	2	2	2	2
2	2	2	2	1	1	3	3	3	3
3	3	2	2	1	1	2	2	2	2
3	3	2	2	4	4	2	2	2	2
3	3	2	2	4	4	2	2	2	2
3	3	2	2	4	4	2	2	2	2
3	3	2	2	4	4	2	2	2	2
3	3	3	3	1	1	2	2	2	2
3	3	2	2	4	4	2	2	2	2
3	3	2	2	4	4	2	2	2	2
3	3	2	2	4	4	2	2	2	2
3	3	2	2	4	4	2	2	2	2
3	3	2	2	4	4	2	2	2	2
3	3	2	2	4	4	2	2	2	2
3	3	2	2	4	4	2	2	2	2
3	3	2	2	4	4	3	3	3	3
3	3	2	2	1	1	2	2	2	2
3	3	2	2	1	1	2	2	2	2
3	3	3	3	4	4	2	2	2	2
3	3	2	2	4	4	2	2	2	2
3	3	2	2	4	4	2	2	2	2
3	3	2	2	1	1	2	2	2	2
3	3	3	3	4	4	2	2	2	2
3	3	2	2	4	4	2	2	2	2
3	3	2	2	1	1	2	2	2	2
3	3	2	2	4	4	2	2	2	2
3	3	2	2	4	4	2	2	2	2
3	3	2	2	4	4	2	2	2	2
3	3	2	2	4	4	2	2	2	2
3	3	2	2	4	4	2	2	2	2
3	3	3	3	4	4	2	2	2	2
3	3	2	2	1	1	2	2	2	2
3	3	2	2	4	4	2	2	2	2
3	3	2	2	1	1	2	2	2	2
3	3	2	2	4	4	2	2	2	2
3	3	2	2	4	4	2	2	2	2
3	3	2	2	4	4	2	2	2	2
3	3	2	2	4	4	2	2	2	2
3	3	2	2	4	4	2	2	2	2
3	3	2	2	4	4	2	2	2	2
2	2	2	2	4	4	3	3	2	2
3	3	2	2	1	1	3	3	3	3
3	3	2	2	4	4	2	2	2	2

3	3	2	2	4	4	2	2	2	2
2	2	2	2	1	1	3	3	3	3

For Review Only

SNP2922	SNP2922	SNP2923	SNP2923	SNP2924	SNP2924	SNP2925	SNP2925	SNP2926	SNP2926
4	4	1	1	4	4	4	4	3	3
4	4	1	1	4	4	3	3	3	3
4	4	1	1	4	4	4	4	3	3
1	1	1	1	4	4	4	4	3	3
4	4	1	1	4	4	4	4	3	3
4	4	1	1	4	4	4	4	2	2
4	4	1	1	4	4	3	3	3	3
4	4	1	1	1	1	4	4	3	3
4	4	1	1	4	4	4	4	3	3
4	4	1	1	1	1	4	4	3	3
4	4	1	1	4	4	4	4	3	3
1	1	1	1	4	4	4	4	3	3
4	4	1	1	4	4	4	4	3	3
4	4	1	1	4	4	3	3	3	3
4	4	1	1	4	4	4	4	3	3
1	1	4	4	4	4	4	4	3	3
4	4	1	1	1	1	4	4	3	3
4	4	1	1	4	4	4	4	3	3
1	1	4	4	4	4	4	4	3	3
4	4	1	1	4	4	4	4	3	3
4	4	1	1	4	4	4	4	3	3
4	4	1	1	4	4	4	4	3	3
4	4	1	1	1	1	3	3	3	3
4	4	1	1	4	4	4	4	3	3
4	4	1	1	4	4	4	4	3	3
4	4	1	1	4	4	4	4	2	2
4	4	1	1	4	4	4	4	3	3
4	4	1	1	4	4	4	4	3	3
4	4	1	1	4	4	4	4	3	3
4	4	1	1	4	4	4	4	3	3
4	4	4	4	4	4	4	4	3	3
1	1	4	4	4	4	3	3	3	3
4	4	1	1	4	4	3	3	3	3
4	4	1	1	4	4	4	4	3	3
4	4	1	1	4	4	4	4	3	3
4	4	1	1	4	4	4	4	3	3
4	4	1	1	4	4	4	4	3	3
4	4	1	1	4	4	3	3	3	3
4	4	1	1	4	4	3	3	3	3
4	4	1	1	4	4	4	4	3	3
4	4	1	1	1	1	4	4	3	3
4	4	1	1	4	4	4	4	3	3
4	4	1	1	4	4	4	4	3	3
4	4	1	1	4	4	4	4	3	3
4	4	1	1	4	4	4	4	3	3
1	1	1	1	4	4	4	4	3	3
4	4	1	1	4	4	4	4	3	3
4	4	1	1	4	4	4	4	3	3
4	4	1	1	4	4	4	4	3	3
4	4	1	1	4	4	4	4	3	3
4	4	1	1	4	4	4	4	2	2
1	1	4	4	4	4	4	4	3	3

For Review Only

4	4	1	1	4	4	4	4	3	3
4	4	1	1	4	4	3	3	3	3
1	1	4	4	4	4	4	4	3	3
4	4	1	1	4	4	4	4	3	3
1	1	4	4	4	4	4	4	3	3
4	4	4	4	4	4	4	4	3	3
1	1	4	4	4	4	4	4	3	3
4	4	1	1	4	4	4	4	3	3
4	4	1	1	4	4	4	4	3	3
4	4	1	1	4	4	4	4	3	3
4	4	4	4	4	4	4	4	3	3
4	4	4	4	4	4	4	4	3	3
4	4	1	1	4	4	4	4	3	3
4	4	1	1	4	4	4	4	3	3
4	4	1	1	4	4	4	4	2	2
4	4	1	1	4	4	3	3	3	3
4	4	1	1	4	4	4	4	3	3
1	1	4	4	1	1	4	4	3	3
4	4	1	1	4	4	4	4	3	3
4	4	1	1	4	4	4	4	3	3
4	4	4	4	4	4	4	4	3	3
4	4	1	1	4	4	4	4	3	3
4	4	1	1	4	4	4	4	3	3
4	4	1	1	1	1	4	4	2	2
4	4	1	1	4	4	4	4	3	3
1	1	4	4	4	4	4	4	3	3
4	4	1	1	4	4	4	4	3	3
4	4	1	1	1	1	3	3	3	3
4	4	1	1	4	4	4	4	2	2
1	1	4	4	4	4	4	4	3	3
4	4	1	1	4	4	4	4	3	3
4	4	1	1	4	4	4	4	3	3
4	4	1	1	1	1	3	3	3	3
4	4	1	1	4	4	4	4	3	3
4	4	1	1	4	4	4	4	3	3
4	4	1	1	4	4	4	4	3	3
4	4	1	1	4	4	4	4	3	3
4	4	1	1	4	4	4	4	3	3
4	4	1	1	4	4	3	3	3	3
4	4	1	1	4	4	4	4	3	3
4	4	1	1	4	4	4	4	3	3
1	1	4	4	4	4	4	4	3	3
4	4	1	1	4	4	3	3	2	2
4	4	4	4	4	4	4	4	3	3
4	4	4	4	4	4	4	4	3	3
4	4	1	1	4	4	4	4	3	3
4	4	1	1	4	4	4	4	3	3
4	4	1	1	4	4	4	4	3	3
4	4	1	1	4	4	4	4	3	3
4	4	1	1	4	4	3	3	3	3
4	4	4	4	4	4	4	4	3	3
4	4	1	1	4	4	3	3	3	3
4	4	1	1	1	1	4	4	3	3
4	4	1	1	4	4	4	4	3	3
4	4	1	1	4	4	3	3	3	3

For Review Only

4	4	1	1	4	4	4	4	3	3
4	4	1	1	4	4	4	4	3	3
4	4	1	1	4	4	4	4	3	3
4	4	1	1	4	4	4	4	3	3
4	4	1	1	4	4	4	4	3	3
4	4	1	1	4	4	4	4	3	3
1	1	4	4	4	4	4	4	3	3
4	4	1	1	4	4	4	4	3	3
4	4	1	1	4	4	4	4	3	3
4	4	1	1	4	4	4	4	3	3
4	4	1	1	4	4	4	4	3	3
4	4	1	1	4	4	4	4	3	3
4	4	1	1	1	1	4	4	3	3
1	1	1	1	4	4	4	4	3	3
4	4	1	1	4	4	4	4	2	2
4	4	1	1	4	4	4	4	3	3
4	4	1	1	4	4	4	4	2	2
4	4	1	1	4	4	4	4	3	3
4	4	1	1	4	4	4	4	3	3
4	4	1	1	4	4	4	4	3	3
4	4	1	1	4	4	4	4	3	3
1	1	1	1	4	4	4	4	3	3
4	4	1	1	4	4	4	4	3	3
4	4	1	1	1	1	3	3	3	3
4	4	1	1	4	4	4	4	3	3
4	4	1	1	4	4	3	3	3	3
4	4	1	1	4	4	4	4	2	2
4	4	1	1	4	4	4	4	3	3
4	4	1	1	4	4	4	4	3	3
4	4	1	1	4	4	4	4	3	3
4	4	1	1	4	4	4	4	3	3
4	4	1	1	4	4	4	4	3	3
4	4	1	1	4	4	4	4	3	3
4	4	1	1	4	4	4	4	3	3
4	4	1	1	4	4	4	4	3	3
4	4	1	1	4	4	4	4	3	3
4	4	1	1	4	4	4	4	3	3
4	4	1	1	4	4	4	4	3	3
4	4	1	1	4	4	4	4	3	3
4	4	1	1	4	4	3	3	3	3
4	4	1	1	4	4	4	4	2	2
4	4	1	1	4	4	4	4	3	3
1	1	4	4	4	4	4	4	3	3
4	4	1	1	4	4	4	4	3	3
4	4	1	1	4	4	4	4	2	2
4	4	1	1	4	4	4	4	2	2
4	4	1	1	4	4	4	4	3	3
4	4	1	1	4	4	4	4	3	3
4	4	1	1	4	4	3	3	3	3
4	4	1	1	4	4	4	4	3	3
4	4	1	1	4	4	4	4	3	3
4	4	1	1	4	4	3	3	3	3
1	1	4	4	4	4	4	4	3	3
4	4	4	4	4	4	4	4	3	3
4	4	1	1	4	4	4	4	3	3

For Review Only

4	4	1	1	4	4	4	4	3	3
4	4	1	1	4	4	3	3	3	3
4	4	1	1	4	4	4	4	3	3
1	1	1	1	4	4	4	4	3	3
4	4	1	1	4	4	4	4	3	3
4	4	1	1	4	4	4	4	3	3
4	4	1	1	4	4	4	4	3	3
4	4	1	1	4	4	4	4	3	3
4	4	1	1	4	4	3	3	3	3
4	4	1	1	4	4	4	4	3	3
4	4	1	1	4	4	4	4	3	3
4	4	1	1	4	4	4	4	3	3
4	4	1	1	4	4	4	4	3	3
4	4	1	1	4	4	4	4	3	3
4	4	1	1	4	4	4	4	3	3
4	4	1	1	4	4	4	4	3	3
1	1	1	1	4	4	4	4	3	3
4	4	1	1	4	4	4	4	3	3
4	4	1	1	4	4	4	4	3	3
1	1	1	1	4	4	4	4	3	3
4	4	1	1	4	4	4	4	3	3
4	4	1	1	4	4	4	4	3	3
4	4	1	1	4	4	4	4	3	3
4	4	4	4	4	4	4	4	3	3
4	4	1	1	4	4	4	4	3	3
4	4	1	1	4	4	4	4	3	3
1	1	1	1	4	4	4	4	3	3
4	4	1	1	4	4	4	4	2	2
4	4	1	1	1	1	4	4	2	2
4	4	1	1	4	4	4	4	3	3
4	4	1	1	1	1	4	4	3	3
1	1	4	4	1	1	4	4	3	3
4	4	1	1	4	4	4	4	3	3
4	4	1	1	4	4	4	4	3	3
4	4	1	1	4	4	3	3	3	3
4	4	4	4	4	4	4	4	3	3
4	4	1	1	4	4	3	3	3	3
4	4	1	1	4	4	3	3	3	3
4	4	1	1	4	4	4	4	3	3
4	4	1	1	4	4	3	3	3	3
4	4	1	1	4	4	4	4	3	3
4	4	1	1	4	4	4	4	3	3
4	4	1	1	4	4	3	3	3	3
4	4	1	1	4	4	4	4	3	3
4	4	1	1	4	4	4	4	3	3
4	4	1	1	4	4	4	4	3	3
4	4	1	1	4	4	4	4	3	3
4	4	1	1	4	4	4	4	3	3
4	4	1	1	4	4	4	4	3	3
4	4	1	1	4	4	4	4	3	3
4	4	1	1	4	4	4	4	3	3
4	4	1	1	1	1	4	4	3	3
4	4	1	1	4	4	3	3	3	3
4	4	4	4	4	4	4	4	3	3
4	4	1	1	4	4	3	3	3	3

For Review Only

1	1	4	4	4	4	4	4	3	3
4	4	1	1	4	4	4	4	3	3
4	4	1	1	4	4	4	4	3	3
4	4	1	1	4	4	4	4	3	3
4	4	1	1	1	1	4	4	3	3
4	4	1	1	4	4	4	4	2	2
4	4	1	1	4	4	3	3	3	3
4	4	1	1	1	1	4	4	3	3
4	4	1	1	4	4	4	4	3	3
4	4	1	1	1	1	3	3	3	3
4	4	1	1	4	4	4	4	3	3
4	4	1	1	1	1	4	4	3	3
4	4	1	1	4	4	3	3	3	3
1	1	4	4	4	4	4	4	3	3
4	4	1	1	4	4	3	3	3	3
4	4	1	1	4	4	3	3	3	3
4	4	1	1	4	4	3	3	3	3
4	4	1	1	4	4	4	4	3	3
4	4	1	1	4	4	3	3	3	3
4	4	1	1	4	4	3	3	3	3
4	4	1	1	4	4	4	4	2	2
4	4	1	1	4	4	4	4	2	2
4	4	1	1	4	4	4	4	3	3
4	4	1	1	4	4	3	3	3	3
4	4	1	1	4	4	4	4	3	3
4	4	1	1	4	4	4	4	3	3
4	4	1	1	1	1	4	4	3	3
1	1	4	4	4	4	4	4	3	3
4	4	1	1	4	4	4	4	3	3
4	4	1	1	4	4	4	4	3	3
4	4	1	1	1	1	3	3	3	3
4	4	1	1	4	4	4	4	3	3
4	4	1	1	4	4	4	4	3	3
4	4	1	1	4	4	4	4	3	3
4	4	1	1	4	4	3	3	3	3
4	4	1	1	1	1	3	3	3	3
4	4	1	1	4	4	4	4	3	3
4	4	4	4	4	4	4	4	3	3
4	4	1	1	4	4	4	4	3	3
4	4	1	1	4	4	3	3	3	3
4	4	1	1	4	4	4	4	3	3
4	4	1	1	4	4	4	4	3	3
4	4	1	1	4	4	3	3	3	3
4	4	1	1	4	4	4	4	3	3
4	4	1	1	4	4	3	3	3	3
4	4	1	1	4	4	3	3	3	3
4	4	1	1	4	4	4	4	3	3
4	4	1	1	4	4	3	3	2	2
4	4	1	1	4	4	4	4	3	3
4	4	1	1	1	1	4	4	3	3
4	4	1	1	4	4	4	4	3	3
4	4	1	1	4	4	3	3	3	3
4	4	1	1	4	4	4	4	3	3
1	1	4	4	4	4	4	4	3	3
4	4	1	1	4	4	3	3	3	3

For Review Only

4	4	1	1	4	4	4	4	3	3
1	1	4	4	4	4	4	4	2	2

For Review Only

SNP2927	SNP2927	SNP2928	SNP2928	SNP2929	SNP2929	SNP293	SNP293	SNP2930	SNP2930
1	1	3	3	3	3	4	4	4	4
1	1	3	3	3	3	4	4	4	4
1	1	3	3	3	3	3	3	4	4
1	1	3	3	3	3	4	4	4	4
1	1	3	3	3	3	4	4	4	4
2	2	3	3	1	1	3	3	1	1
1	1	3	3	3	3	4	4	4	4
1	1	1	1	3	3	3	3	4	4
1	1	3	3	3	3	4	4	4	4
1	1	1	1	3	3	3	3	4	4
1	1	3	3	3	3	4	4	4	4
1	1	3	3	3	3	3	3	4	4
1	1	3	3	3	3	4	4	4	4
1	1	3	3	3	3	4	4	4	4
1	1	3	3	3	3	4	4	4	4
1	1	3	3	3	3	3	3	4	4
1	1	1	1	3	3	4	4	4	4
1	1	1	1	3	3	3	3	4	4
1	1	3	3	3	3	3	3	4	4
1	1	3	3	3	3	4	4	4	4
1	1	3	3	3	3	4	4	4	4
1	1	3	3	3	3	4	4	4	4
1	1	3	3	3	3	4	4	4	4
1	1	3	3	3	3	3	3	4	4
1	1	3	3	3	3	4	4	4	4
2	2	3	3	1	1	3	3	1	1
1	1	3	3	3	3	3	3	4	4
1	1	3	3	3	3	4	4	4	4
1	1	3	3	3	3	3	3	4	4
1	1	1	1	3	3	4	4	4	4
1	1	3	3	3	3	3	3	4	4
1	1	3	3	3	3	3	3	4	4
1	1	3	3	3	3	3	3	4	4
1	1	3	3	3	3	4	4	4	4
1	1	3	3	3	3	4	4	4	4
1	1	3	3	3	3	3	3	4	4
1	1	3	3	3	3	4	4	4	4
1	1	3	3	3	3	4	4	4	4
1	1	3	3	3	3	4	4	4	4
1	1	3	3	3	3	4	4	4	4
1	1	3	3	3	3	4	4	4	4
1	1	3	3	3	3	3	3	4	4
1	1	3	3	3	3	3	3	4	4
1	1	1	1	3	3	4	4	4	4
1	1	1	1	3	3	3	3	4	4
1	1	3	3	3	3	3	3	4	4
1	1	3	3	3	3	3	3	4	4
1	1	3	3	3	3	4	4	4	4
1	1	1	1	3	3	4	4	4	4
1	1	3	3	3	3	4	4	4	4
1	1	3	3	3	3	3	3	4	4
1	1	3	3	3	3	4	4	4	4

For Review Only

1	1	3	3	3	3	4	4	4	4
1	1	3	3	3	3	3	3	4	4
1	1	3	3	3	3	3	3	4	4
1	1	1	1	3	3	4	4	4	4
1	1	3	3	3	3	3	3	4	4
1	1	3	3	3	3	3	3	4	4
1	1	3	3	3	3	3	3	4	4
1	1	3	3	3	3	4	4	4	4
1	1	3	3	3	3	4	4	4	4
1	1	3	3	3	3	4	4	4	4
1	1	3	3	3	3	3	3	4	4
1	1	3	3	3	3	3	3	4	4
1	1	3	3	3	3	4	4	4	4
1	1	3	3	3	3	3	3	4	4
1	1	3	3	1	1	4	4	1	1
1	1	3	3	3	3	4	4	4	4
1	1	3	3	3	3	4	4	4	4
1	1	3	3	3	3	3	3	4	4
1	1	3	3	3	3	3	3	4	4
1	1	3	3	3	3	4	4	4	4
1	1	3	3	3	3	4	4	4	4
1	1	3	3	3	3	4	4	4	4
1	1	3	3	3	3	4	4	1	1
2	2	3	3	1	1	4	4	1	1
1	1	1	1	3	3	3	3	4	4
1	1	3	3	3	3	3	3	4	4
1	1	1	1	3	3	3	3	4	4
1	1	3	3	3	3	4	4	4	4
2	2	3	3	1	1	3	3	1	1
1	1	3	3	3	3	3	3	4	4
1	1	3	3	3	3	4	4	4	4
1	1	3	3	3	3	4	4	4	4
1	1	3	3	3	3	4	4	4	4
1	1	3	3	3	3	4	4	4	4
1	1	3	3	3	3	4	4	4	4
1	1	1	1	3	3	3	3	4	4
1	1	1	1	3	3	4	4	4	4
1	1	3	3	3	3	4	4	4	4
1	1	3	3	3	3	4	4	4	4
1	1	1	1	3	3	4	4	4	4
1	1	3	3	3	3	3	3	4	4
1	1	3	3	3	3	3	3	4	4
1	1	3	3	3	3	4	4	4	4
1	1	3	3	3	3	3	3	4	4
1	1	3	3	3	3	4	4	4	4
1	1	3	3	3	3	4	4	4	4
1	1	3	3	3	3	4	4	4	4
1	1	3	3	3	3	4	4	4	4
1	1	3	3	3	3	3	3	4	4
1	1	3	3	3	3	4	4	4	4
1	1	3	3	3	3	3	3	4	4
1	1	3	3	3	3	4	4	4	4
1	1	3	3	3	3	4	4	4	4
1	1	3	3	3	3	4	4	4	4
1	1	3	3	3	3	4	4	4	4

For Review Only

1	1	3	3	3	3	3	3	4	4
1	1	3	3	3	3	4	4	4	4
1	1	3	3	3	3	3	3	4	4
1	1	3	3	3	3	4	4	4	4
1	1	3	3	3	3	3	3	4	4
1	1	3	3	3	3	3	3	4	4
1	1	3	3	3	3	3	3	4	4
1	1	3	3	3	3	3	3	4	4
1	1	3	3	3	3	3	3	4	4
1	1	3	3	3	3	4	4	4	4
1	1	3	3	3	3	4	4	4	4
1	1	3	3	3	3	4	4	1	1
1	1	3	3	3	3	4	4	4	4
1	1	3	3	3	3	4	4	4	4
2	2	3	3	3	3	3	3	1	1
1	1	3	3	3	3	4	4	4	4
2	2	3	3	1	1	4	4	1	1
1	1	3	3	3	3	4	4	4	4
1	1	3	3	3	3	4	4	4	4
1	1	3	3	3	3	3	3	4	4
1	1	3	3	3	3	4	4	4	4
1	1	3	3	3	3	3	3	4	4
1	1	3	3	3	3	3	3	4	4
1	1	3	3	3	3	4	4	4	4
1	1	3	3	3	3	3	3	4	4
1	1	3	3	3	3	4	4	4	4
1	1	3	3	3	3	4	4	4	4
1	1	3	3	3	3	3	3	4	4
1	1	3	3	3	3	3	3	4	4
1	1	3	3	3	3	3	3	4	4
1	1	3	3	3	3	4	4	4	4
1	1	3	3	3	3	4	4	4	4
1	1	3	3	3	3	3	3	4	4
1	1	3	3	3	3	4	4	4	4
1	1	3	3	3	3	4	4	4	4
1	1	3	3	3	3	4	4	4	4
1	1	3	3	3	3	4	4	4	4
1	1	3	3	3	3	4	4	4	4
1	1	3	3	3	3	4	4	4	4
1	1	3	3	3	3	4	4	4	4
2	2	3	3	1	1	4	4	1	1
1	1	3	3	3	3	4	4	4	4
1	1	3	3	3	3	3	3	4	4
1	1	1	1	3	3	4	4	4	4
1	1	3	3	3	3	4	4	1	1
1	1	3	3	3	3	4	4	1	1
1	1	3	3	3	3	3	3	4	4
1	1	3	3	3	3	3	3	4	4
1	1	3	3	3	3	4	4	4	4
1	1	3	3	3	3	3	3	4	4
1	1	3	3	3	3	3	3	4	4
1	1	3	3	3	3	4	4	4	4
1	1	3	3	3	3	3	3	4	4
1	1	3	3	3	3	3	3	4	4
1	1	3	3	3	3	4	4	4	4

For Review Only

1	1	3	3	3	3	3	3	4	4
1	1	3	3	3	3	4	4	4	4
1	1	1	1	3	3	4	4	4	4
1	1	3	3	3	3	4	4	4	4
1	1	3	3	3	3	4	4	4	4
1	1	3	3	3	3	4	4	4	4
1	1	3	3	3	3	4	4	4	4
1	1	3	3	3	3	4	4	4	4
1	1	3	3	3	3	4	4	4	4
1	1	3	3	3	3	3	3	4	4
1	1	3	3	3	3	4	4	4	4
1	1	3	3	3	3	3	3	4	4
1	1	3	3	3	3	4	4	4	4
1	1	3	3	3	3	3	3	4	4
1	1	3	3	3	3	4	4	4	4
1	1	3	3	3	3	4	4	4	4
1	1	3	3	3	3	4	4	4	4
1	1	3	3	3	3	4	4	4	4
1	1	3	3	3	3	3	3	4	4
1	1	3	3	3	3	3	3	4	4
1	1	3	3	3	3	4	4	4	4
1	1	3	3	3	3	4	4	4	4
1	1	3	3	3	3	4	4	4	4
1	1	3	3	3	3	3	3	4	4
1	1	3	3	3	3	4	4	4	4
1	1	3	3	3	3	4	4	4	4
1	1	3	3	3	3	4	4	4	4
1	1	3	3	3	3	4	4	1	1
2	2	3	3	1	1	3	3	1	1
1	1	3	3	3	3	4	4	4	4
1	1	3	3	3	3	4	4	4	4
1	1	3	3	3	3	3	3	4	4
1	1	3	3	3	3	4	4	4	4
1	1	3	3	3	3	4	4	4	4
1	1	3	3	3	3	4	4	4	4
1	1	3	3	3	3	3	3	4	4
1	1	3	3	3	3	3	3	4	4
1	1	3	3	3	3	4	4	4	4
1	1	3	3	3	3	3	3	4	4
1	1	3	3	3	3	3	3	4	4
1	1	3	3	3	3	3	3	4	4
1	1	3	3	3	3	3	3	4	4
1	1	3	3	3	3	4	4	4	4
1	1	3	3	3	3	4	4	4	4
1	1	3	3	3	3	4	4	4	4
1	1	3	3	3	3	4	4	4	4
1	1	3	3	3	3	4	4	4	4
1	1	3	3	3	3	4	4	4	4
1	1	3	3	3	3	4	4	4	4
1	1	3	3	3	3	4	4	4	4
1	1	3	3	3	3	4	4	4	4
1	1	3	3	3	3	4	4	4	4
1	1	3	3	3	3	4	4	4	4
1	1	3	3	3	3	3	3	4	4
1	1	3	3	3	3	4	4	4	4

For Review Only

1	1	3	3	3	3	3	3	4	4
1	1	3	3	3	3	4	4	4	4
1	1	3	3	3	3	3	3	4	4
1	1	3	3	3	3	4	4	4	4
1	1	3	3	3	3	4	4	4	4
2	2	3	3	1	1	3	3	1	1
1	1	3	3	3	3	4	4	4	4
1	1	3	3	3	3	4	4	4	4
1	1	3	3	3	3	4	4	4	4
1	1	3	3	3	3	4	4	4	4
1	1	3	3	3	3	4	4	4	4
1	1	3	3	3	3	4	4	4	4
1	1	3	3	3	3	3	3	4	4
1	1	3	3	3	3	3	3	4	4
1	1	3	3	3	3	3	3	4	4
1	1	3	3	3	3	4	4	4	4
1	1	3	3	3	3	4	4	4	4
1	1	3	3	3	3	4	4	4	4
1	1	3	3	3	3	3	3	4	4
1	1	3	3	3	3	4	4	4	4
2	2	3	3	1	1	4	4	1	1
2	2	3	3	1	1	3	3	1	1
1	1	3	3	3	3	4	4	4	4
1	1	3	3	3	3	4	4	4	4
1	1	3	3	3	3	4	4	4	4
1	1	3	3	3	3	4	4	4	4
1	1	3	3	3	3	4	4	4	4
1	1	3	3	3	3	3	3	4	4
1	1	3	3	3	3	4	4	4	4
1	1	3	3	3	3	3	3	4	4
1	1	3	3	3	3	4	4	4	4
1	1	3	3	3	3	3	3	4	4
1	1	1	1	3	3	3	3	4	4
1	1	3	3	3	3	4	4	4	4
1	1	3	3	3	3	4	4	4	4
1	1	3	3	3	3	4	4	4	4
1	1	3	3	3	3	4	4	4	4
1	1	3	3	3	3	3	3	4	4
1	1	3	3	3	3	3	3	4	4
1	1	3	3	3	3	4	4	4	4
1	1	3	3	3	3	3	3	4	4
1	1	3	3	3	3	4	4	4	4
1	1	3	3	3	3	4	4	4	4
1	1	3	3	3	3	4	4	4	4
1	1	3	3	3	3	4	4	4	4
1	1	3	3	3	3	3	3	4	4
1	1	3	3	3	3	4	4	4	4
1	1	3	3	3	3	3	3	4	4
1	1	3	3	3	3	3	3	4	4
1	1	3	3	3	3	4	4	4	4
1	1	3	3	3	3	4	4	4	4
1	1	3	3	3	3	4	4	4	4
1	1	3	3	3	3	3	3	4	4
1	1	3	3	3	3	4	4	4	4
1	1	3	3	3	3	4	4	4	4

For Review Only

1	1	3	3	3	3	4	4	4	4
2	2	3	3	1	1	3	3	1	1

For Review Only

SNP2931	SNP2931	SNP2932	SNP2932	SNP2933	SNP2933	SNP2934	SNP2934	SNP2935	SNP2935
1	1	4	4	4	4	3	3	4	4
1	1	4	4	4	4	3	3	4	4
1	1	4	4	4	4	3	3	4	4
1	1	4	4	4	4	3	3	4	4
1	1	4	4	4	4	3	3	4	4
2	2	1	1	2	2	2	2	2	2
1	1	4	4	4	4	3	3	4	4
1	1	4	4	4	4	3	3	4	4
1	1	4	4	4	4	3	3	4	4
1	1	4	4	4	4	3	3	4	4
1	1	4	4	4	4	3	3	4	4
1	1	4	4	4	4	3	3	4	4
1	1	4	4	4	4	3	3	4	4
1	1	4	4	4	4	3	3	4	4
1	1	4	4	4	4	3	3	4	4
1	1	4	4	4	4	3	3	4	4
1	1	4	4	4	4	3	3	4	4
1	1	4	4	4	4	3	3	4	4
1	1	4	4	4	4	3	3	4	4
1	1	4	4	4	4	3	3	4	4
1	1	4	4	4	4	3	3	4	4
1	1	4	4	4	4	3	3	4	4
1	1	4	4	4	4	3	3	4	4
1	1	4	4	4	4	3	3	4	4
1	1	4	4	4	4	3	3	4	4
2	2	1	1	2	2	2	2	2	2
1	1	4	4	4	4	3	3	4	4
1	1	4	4	4	4	3	3	4	4
1	1	4	4	4	4	3	3	4	4
1	1	4	4	4	4	3	3	4	4
1	1	4	4	4	4	3	3	4	4
1	1	4	4	4	4	3	3	4	4
1	1	4	4	4	4	3	3	4	4
1	1	4	4	4	4	3	3	4	4
1	1	4	4	4	4	3	3	4	4
1	1	4	4	4	4	3	3	4	4
1	1	4	4	4	4	3	3	4	4
1	1	4	4	4	4	3	3	4	4
1	1	4	4	4	4	3	3	4	4
1	1	4	4	4	4	3	3	4	4
1	1	4	4	4	4	3	3	4	4
1	1	4	4	4	4	3	3	4	4
1	1	4	4	4	4	3	3	4	4
1	1	4	4	4	4	3	3	4	4
1	1	4	4	4	4	3	3	4	4
1	1	4	4	4	4	3	3	4	4
1	1	4	4	4	4	3	3	4	4
2	2	4	4	4	4	3	3	4	4
1	1	4	4	4	4	3	3	4	4
1	1	4	4	4	4	3	3	4	4
1	1	4	4	4	4	3	3	4	4
1	1	4	4	4	4	3	3	4	4

For Review Only

1	1	4	4	4	4	3	3	4	4
1	1	4	4	4	4	3	3	4	4
1	1	4	4	4	4	3	3	4	4
1	1	4	4	4	4	3	3	4	4
1	1	4	4	4	4	3	3	4	4
1	1	4	4	4	4	3	3	4	4
1	1	4	4	4	4	3	3	4	4
1	1	4	4	4	4	3	3	4	4
1	1	4	4	4	4	3	3	4	4
1	1	4	4	4	4	3	3	4	4
1	1	4	4	4	4	3	3	4	4
1	1	4	4	4	4	3	3	4	4
1	1	4	4	4	4	3	3	4	4
1	1	4	4	4	4	3	3	4	4
2	2	4	4	4	4	3	3	2	2
1	1	4	4	4	4	3	3	4	4
1	1	4	4	4	4	3	3	4	4
1	1	4	4	4	4	3	3	4	4
1	1	4	4	4	4	3	3	4	4
1	1	4	4	4	4	3	3	4	4
1	1	4	4	4	4	3	3	4	4
1	1	4	4	4	4	3	3	4	4
1	1	4	4	4	4	3	3	4	4
2	2	1	1	2	2	2	2	2	2
1	1	4	4	4	4	3	3	4	4
1	1	4	4	4	4	3	3	4	4
1	1	4	4	4	4	3	3	4	4
1	1	4	4	4	4	3	3	4	4
2	2	1	1	2	2	2	2	2	2
1	1	4	4	4	4	3	3	4	4
1	1	4	4	4	4	3	3	4	4
1	1	4	4	4	4	3	3	4	4
1	1	4	4	4	4	3	3	4	4
1	1	4	4	4	4	3	3	4	4
1	1	4	4	4	4	3	3	4	4
1	1	4	4	4	4	3	3	4	4
1	1	4	4	4	4	3	3	4	4
1	1	4	4	4	4	3	3	4	4
1	1	4	4	4	4	3	3	4	4
1	1	4	4	4	4	3	3	4	4
1	1	4	4	4	4	3	3	4	4
1	1	4	4	4	4	3	3	4	4
1	1	4	4	4	4	3	3	4	4
1	1	4	4	4	4	3	3	4	4
1	1	4	4	4	4	3	3	4	4
1	1	4	4	4	4	3	3	4	4
1	1	4	4	4	4	3	3	4	4
1	1	4	4	4	4	3	3	4	4
1	1	4	4	4	4	3	3	4	4
1	1	4	4	4	4	3	3	4	4
1	1	4	4	4	4	3	3	4	4
1	1	4	4	4	4	3	3	4	4
1	1	4	4	4	4	3	3	4	4
1	1	4	4	4	4	3	3	4	4
1	1	4	4	4	4	3	3	4	4

1	1	4	4	4	4	3	3	4	4
1	1	4	4	4	4	3	3	4	4
1	1	4	4	4	4	3	3	4	4
1	1	4	4	4	4	3	3	4	4
1	1	4	4	4	4	3	3	4	4
1	1	4	4	4	4	3	3	4	4
1	1	4	4	4	4	3	3	4	4
1	1	4	4	4	4	3	3	4	4
1	1	4	4	4	4	3	3	4	4
1	1	4	4	4	4	3	3	4	4
1	1	4	4	4	4	3	3	4	4
1	1	4	4	4	4	3	3	4	4
1	1	4	4	4	4	3	3	4	4
1	1	4	4	4	4	3	3	4	4
2	2	4	4	4	4	3	3	4	4
1	1	4	4	4	4	3	3	4	4
2	2	1	1	2	2	2	2	2	2
1	1	4	4	4	4	3	3	4	4
1	1	4	4	4	4	3	3	4	4
1	1	4	4	4	4	3	3	4	4
1	1	4	4	4	4	3	3	4	4
1	1	4	4	4	4	3	3	4	4
1	1	4	4	4	4	3	3	4	4
1	1	4	4	4	4	3	3	4	4
1	1	4	4	4	4	3	3	4	4
1	1	4	4	4	4	3	3	4	4
2	2	4	4	4	4	3	3	4	4
1	1	4	4	4	4	3	3	4	4
1	1	4	4	4	4	3	3	4	4
1	1	4	4	4	4	3	3	4	4
1	1	4	4	4	4	3	3	4	4
1	1	4	4	4	4	3	3	4	4
1	1	4	4	4	4	3	3	4	4
1	1	4	4	4	4	3	3	4	4
1	1	4	4	4	4	3	3	4	4
1	1	4	4	4	4	3	3	4	4
1	1	4	4	4	4	3	3	4	4
1	1	4	4	4	4	3	3	4	4
2	2	4	4	4	4	3	3	4	4
1	1	4	4	4	4	3	3	4	4
2	2	1	1	2	2	2	2	2	2
1	1	4	4	4	4	3	3	4	4
1	1	4	4	4	4	3	3	4	4
1	1	4	4	4	4	3	3	4	4
2	2	4	4	4	4	3	3	4	4
1	1	4	4	4	4	3	3	4	4
1	1	4	4	4	4	3	3	4	4
1	1	4	4	4	4	3	3	4	4
1	1	4	4	4	4	3	3	4	4
1	1	4	4	4	4	3	3	4	4
1	1	4	4	4	4	3	3	4	4
1	1	4	4	4	4	3	3	4	4
1	1	4	4	4	4	3	3	4	4
1	1	4	4	4	4	3	3	4	4
1	1	4	4	4	4	3	3	4	4

For Review Only

1	1	4	4	4	4	3	3	4	4
1	1	4	4	4	4	3	3	4	4
1	1	4	4	4	4	3	3	4	4
1	1	4	4	4	4	3	3	4	4
1	1	4	4	4	4	3	3	4	4
1	1	4	4	4	4	3	3	4	4
1	1	4	4	4	4	3	3	4	4
1	1	4	4	4	4	3	3	4	4
1	1	4	4	4	4	3	3	4	4
1	1	4	4	4	4	3	3	4	4
1	1	4	4	4	4	3	3	4	4
1	1	4	4	4	4	3	3	4	4
1	1	4	4	4	4	3	3	4	4
1	1	4	4	4	4	3	3	4	4
1	1	4	4	4	4	3	3	4	4
1	1	4	4	4	4	3	3	4	4
1	1	4	4	4	4	3	3	4	4
1	1	4	4	4	4	3	3	4	4
1	1	4	4	4	4	3	3	4	4
1	1	4	4	4	4	3	3	4	4
1	1	4	4	4	4	3	3	4	4
1	1	4	4	4	4	3	3	4	4
1	1	4	4	4	4	3	3	4	4
1	1	4	4	4	4	3	3	4	4
1	1	4	4	4	4	3	3	4	4
1	1	4	4	4	4	3	3	4	4
1	1	4	4	4	4	3	3	4	4
2	2	4	4	4	4	3	3	4	4
2	2	1	1	2	2	2	2	2	2
1	1	4	4	4	4	3	3	4	4
1	1	4	4	4	4	3	3	4	4
1	1	4	4	4	4	3	3	4	4
1	1	4	4	4	4	3	3	4	4
1	1	4	4	4	4	3	3	4	4
1	1	4	4	4	4	3	3	4	4
1	1	4	4	4	4	3	3	4	4
1	1	4	4	4	4	3	3	4	4
1	1	4	4	4	4	3	3	4	4
1	1	4	4	4	4	3	3	4	4
1	1	4	4	4	4	3	3	4	4
1	1	4	4	4	4	3	3	4	4
1	1	4	4	4	4	3	3	4	4
1	1	4	4	4	4	3	3	4	4
1	1	4	4	4	4	3	3	4	4
1	1	4	4	4	4	3	3	4	4
1	1	4	4	4	4	3	3	4	4
1	1	4	4	4	4	3	3	4	4
1	1	4	4	4	4	3	3	4	4
1	1	4	4	4	4	3	3	4	4
1	1	4	4	4	4	3	3	4	4
1	1	4	4	4	4	3	3	4	4
1	1	4	4	4	4	3	3	4	4
1	1	4	4	4	4	3	3	4	4
1	1	4	4	4	4	3	3	4	4
1	1	4	4	4	4	3	3	4	4

For Review Only

1	1	4	4	4	4	3	3	4	4
1	1	4	4	4	4	3	3	4	4
1	1	4	4	4	4	3	3	4	4
1	1	4	4	4	4	3	3	4	4
1	1	4	4	4	4	3	3	4	4
2	2	1	1	2	2	2	2	2	2
1	1	4	4	4	4	3	3	4	4
1	1	4	4	4	4	3	3	4	4
1	1	4	4	4	4	3	3	4	4
1	1	4	4	4	4	3	3	4	4
1	1	4	4	4	4	3	3	4	4
1	1	4	4	4	4	3	3	4	4
1	1	4	4	4	4	3	3	4	4
1	1	4	4	4	4	3	3	4	4
1	1	4	4	4	4	3	3	4	4
1	1	4	4	4	4	3	3	4	4
1	1	4	4	4	4	3	3	4	4
1	1	4	4	4	4	3	3	4	4
1	1	4	4	4	4	3	3	4	4
1	1	4	4	4	4	3	3	4	4
2	2	1	1	2	2	2	2	2	2
2	2	1	1	2	2	2	2	2	2
1	1	4	4	4	4	3	3	4	4
1	1	4	4	4	4	3	3	4	4
1	1	4	4	4	4	3	3	4	4
1	1	4	4	4	4	3	3	4	4
1	1	4	4	4	4	3	3	4	4
1	1	4	4	4	4	3	3	4	4
1	1	4	4	4	4	3	3	4	4
1	1	4	4	4	4	3	3	4	4
1	1	4	4	4	4	3	3	4	4
1	1	4	4	4	4	3	3	4	4
1	1	4	4	4	4	3	3	4	4
1	1	4	4	4	4	3	3	4	4
1	1	4	4	4	4	3	3	4	4
1	1	4	4	4	4	3	3	4	4
1	1	4	4	4	4	3	3	4	4
1	1	4	4	4	4	3	3	4	4
1	1	4	4	4	4	3	3	4	4
1	1	4	4	4	4	3	3	4	4
1	1	4	4	4	4	3	3	4	4
1	1	4	4	4	4	3	3	4	4
1	1	4	4	4	4	3	3	4	4
1	1	4	4	4	4	3	3	4	4
1	1	4	4	4	4	3	3	4	4
1	1	4	4	4	4	3	3	4	4
1	1	4	4	4	4	3	3	4	4
1	1	4	4	4	4	3	3	4	4
1	1	4	4	4	4	3	3	4	4
1	1	4	4	4	4	3	3	4	4
1	1	4	4	4	4	3	3	4	4
1	1	4	4	4	4	3	3	4	4
1	1	4	4	4	4	3	3	4	4
1	1	4	4	4	4	3	3	4	4
1	1	4	4	4	4	3	3	4	4

For Review Only

1	1	4	4	4	4	3	3	4	4
2	2	1	1	2	2	2	2	2	2

For Review Only

SNP2936	SNP2936	SNP2937	SNP2937	SNP2938	SNP2938	SNP2939	SNP2939	SNP294	SNP294
1	1	4	4	1	1	3	3	2	2
1	1	4	4	1	1	3	3	2	2
1	1	4	4	1	1	3	3	3	3
1	1	4	4	1	1	3	3	2	2
1	1	4	4	1	1	3	3	2	2
4	4	1	1	1	1	3	3	2	2
1	1	4	4	1	1	2	2	2	2
1	1	4	4	1	1	3	3	2	2
1	1	4	4	1	1	2	2	2	2
1	1	4	4	1	1	3	3	2	2
1	1	4	4	3	3	2	2	3	3
1	1	4	4	1	1	3	3	2	2
1	1	4	4	1	1	3	3	2	2
1	1	4	4	1	1	3	3	2	2
1	1	4	4	1	1	3	3	2	2
1	1	4	4	1	1	2	2	3	3
1	1	4	4	3	3	3	3	2	2
1	1	4	4	1	1	3	3	2	2
1	1	4	4	1	1	2	2	3	3
1	1	4	4	1	1	3	3	3	3
1	1	4	4	1	1	3	3	2	2
1	1	4	4	1	1	3	3	2	2
1	1	4	4	3	3	3	3	2	2
1	1	4	4	3	3	3	3	2	2
1	1	4	4	1	1	3	3	2	2
4	4	1	1	1	1	2	2	2	2
1	1	4	4	1	1	3	3	3	3
1	1	4	4	1	1	3	3	2	2
1	1	4	4	1	1	3	3	2	2
1	1	4	4	1	1	3	3	3	3
1	1	4	4	1	1	3	3	2	2
1	1	4	4	1	1	3	3	2	2
1	1	4	4	1	1	2	2	2	2
1	1	4	4	3	3	3	3	2	2
1	1	4	4	1	1	3	3	3	3
1	1	4	4	1	1	2	2	2	2
1	1	4	4	1	1	3	3	2	2
1	1	4	4	3	3	3	3	2	2
1	1	4	4	3	3	3	3	3	3
1	1	4	4	3	3	3	3	2	2
1	1	4	4	1	1	3	3	3	3
1	1	4	4	1	1	3	3	2	2
1	1	4	4	1	1	3	3	3	3
1	1	4	4	1	1	3	3	2	2
1	1	4	4	1	1	3	3	2	2
1	1	4	4	1	1	3	3	2	2
1	1	4	4	1	1	3	3	3	3
1	1	4	4	1	1	3	3	2	2
1	1	4	4	1	1	3	3	2	2
1	1	4	4	1	1	3	3	2	2
1	1	4	4	1	1	3	3	3	3
1	1	4	4	1	1	3	3	3	3

For Review Only

1	1	4	4	1	1	3	3	2	2
1	1	4	4	1	1	2	2	2	2
1	1	4	4	1	1	2	2	3	3
1	1	4	4	1	1	3	3	2	2
1	1	4	4	1	1	2	2	3	3
1	1	4	4	1	1	3	3	2	2
1	1	4	4	1	1	3	3	2	2
1	1	4	4	1	1	3	3	2	2
1	1	4	4	1	1	3	3	2	2
1	1	4	4	1	1	3	3	2	2
1	1	4	4	1	1	3	3	2	2
1	1	4	4	1	1	3	3	2	2
1	1	4	4	1	1	3	3	2	2
1	1	4	4	1	1	3	3	2	2
1	1	1	1	1	1	3	3	2	2
1	1	4	4	1	1	3	3	2	2
1	1	4	4	1	1	3	3	3	3
1	1	4	4	1	1	3	3	2	2
1	1	4	4	1	1	3	3	2	2
1	1	4	4	1	1	3	3	2	2
1	1	4	4	1	1	3	3	2	2
1	1	4	4	3	3	3	3	2	2
1	1	4	4	1	1	2	2	2	2
1	1	1	1	1	1	2	2	2	2
1	1	4	4	1	1	3	3	2	2
1	1	4	4	1	1	3	3	3	3
1	1	4	4	1	1	3	3	3	3
1	1	4	4	1	1	3	3	3	3
4	4	1	1	1	1	2	2	3	3
1	1	4	4	1	1	2	2	2	2
1	1	4	4	1	1	3	3	2	2
1	1	4	4	1	1	3	3	2	2
1	1	4	4	3	3	3	3	3	3
1	1	4	4	1	1	3	3	2	2
1	1	4	4	1	1	3	3	2	2
1	1	4	4	1	1	3	3	2	2
1	1	4	4	1	1	3	3	2	2
1	1	4	4	1	1	3	3	2	2
1	1	4	4	3	3	3	3	2	2
1	1	4	4	1	1	3	3	2	2
1	1	4	4	1	1	3	3	2	2
1	1	4	4	1	1	2	2	2	2
1	1	4	4	1	1	2	2	2	2
1	1	4	4	1	1	3	3	2	2
1	1	4	4	1	1	3	3	2	2
1	1	4	4	1	1	3	3	2	2
1	1	4	4	3	3	3	3	2	2
1	1	4	4	1	1	3	3	2	2
1	1	4	4	1	1	2	2	2	2
1	1	4	4	1	1	3	3	3	3
1	1	4	4	1	1	3	3	2	2
1	1	4	4	3	3	3	3	2	2
1	1	4	4	3	3	3	3	2	2
1	1	4	4	1	1	3	3	2	2
1	1	4	4	1	1	3	3	3	3

For Review Only

1	1	4	4	1	1	3	3	3	3
1	1	4	4	1	1	3	3	2	2
1	1	4	4	1	1	3	3	2	2
1	1	4	4	3	3	3	3	2	2
1	1	4	4	1	1	3	3	3	3
1	1	4	4	1	1	3	3	2	2
1	1	4	4	1	1	3	3	3	3
1	1	4	4	1	1	3	3	2	2
1	1	4	4	1	1	2	2	2	2
1	1	4	4	1	1	3	3	2	2
1	1	4	4	1	1	3	3	2	2
1	1	4	4	1	1	3	3	2	2
1	1	4	4	1	1	3	3	2	2
1	1	4	4	1	1	3	3	2	2
1	1	4	4	1	1	3	3	3	3
1	1	4	4	1	1	3	3	2	2
1	1	1	1	1	1	2	2	2	2
1	1	4	4	1	1	3	3	3	3
1	1	4	4	3	3	3	3	2	2
1	1	4	4	1	1	3	3	2	2
1	1	4	4	1	1	3	3	3	3
1	1	4	4	1	1	3	3	2	2
1	1	4	4	1	1	3	3	2	2
1	1	4	4	1	1	3	3	3	3
1	1	4	4	3	3	3	3	2	2
1	1	4	4	3	3	3	3	2	2
1	1	1	1	1	1	3	3	2	2
1	1	4	4	1	1	3	3	2	2
1	1	4	4	1	1	3	3	2	2
1	1	4	4	1	1	2	2	3	3
1	1	4	4	3	3	3	3	2	2
1	1	4	4	1	1	3	3	3	3
1	1	4	4	1	1	3	3	2	2
1	1	4	4	1	1	3	3	2	2
1	1	4	4	3	3	3	3	2	2
1	1	4	4	1	1	3	3	2	2
1	1	4	4	3	3	3	3	2	2
1	1	4	4	1	1	3	3	2	2
1	1	1	1	1	1	2	2	2	2
1	1	4	4	1	1	3	3	2	2
4	4	1	1	3	3	2	2	2	2
1	1	4	4	3	3	3	3	2	2
1	1	4	4	1	1	3	3	3	3
1	1	4	4	1	1	3	3	2	2
1	1	1	1	1	1	3	3	2	2
1	1	4	4	3	3	3	3	2	2
1	1	4	4	1	1	3	3	3	3
1	1	4	4	1	1	3	3	2	2
1	1	4	4	1	1	3	3	2	2
1	1	4	4	1	1	3	3	3	3
1	1	4	4	1	1	3	3	2	2
1	1	4	4	3	3	2	2	3	3
1	1	4	4	1	1	3	3	2	2
1	1	4	4	1	1	3	3	3	3
1	1	4	4	1	1	3	3	2	2

For Review Only

1	1	4	4	1	1	3	3	3	3
1	1	4	4	3	3	3	3	3	3
1	1	4	4	1	1	3	3	2	2
1	1	4	4	3	3	3	3	2	2
1	1	4	4	1	1	3	3	3	3
1	1	4	4	1	1	3	3	2	2
1	1	4	4	1	1	3	3	3	3
1	1	4	4	1	1	3	3	2	2
1	1	4	4	1	1	3	3	3	3
1	1	4	4	1	1	2	2	2	2
1	1	4	4	1	1	3	3	2	2
1	1	4	4	1	1	3	3	2	2
1	1	4	4	1	1	3	3	2	2
1	1	4	4	1	1	3	3	2	2
1	1	4	4	3	3	3	3	2	2
1	1	4	4	3	3	2	2	2	2
1	1	4	4	1	1	3	3	2	2
1	1	4	4	1	1	3	3	3	3
1	1	4	4	1	1	3	3	3	3
1	1	4	4	1	1	3	3	2	2
1	1	4	4	1	1	3	3	2	2
1	1	4	4	1	1	3	3	3	3
1	1	4	4	3	3	3	3	2	2
1	1	4	4	1	1	3	3	2	2
1	1	4	4	1	1	3	3	2	2
1	1	4	4	3	3	2	2	2	2
1	1	4	4	1	1	3	3	2	2
1	1	1	1	1	1	3	3	2	2
4	4	1	1	1	1	2	2	2	2
1	1	4	4	1	1	3	3	2	2
1	1	4	4	1	1	3	3	2	2
1	1	4	4	1	1	2	2	2	2
1	1	4	4	1	1	3	3	2	2
1	1	4	4	3	3	3	3	2	2
1	1	4	4	1	1	3	3	2	2
1	1	4	4	1	1	3	3	2	2
1	1	4	4	1	1	3	3	3	3
1	1	4	4	1	1	3	3	2	2
1	1	4	4	1	1	3	3	2	2
1	1	4	4	1	1	2	2	2	2
1	1	4	4	1	1	3	3	3	3
1	1	4	4	1	1	3	3	3	3
1	1	4	4	1	1	3	3	2	2
1	1	4	4	1	1	3	3	2	2
1	1	4	4	1	1	3	3	2	2
1	1	4	4	1	1	3	3	2	2
1	1	4	4	3	3	2	2	2	2
1	1	4	4	1	1	3	3	3	3
1	1	4	4	1	1	3	3	2	2
1	1	4	4	1	1	3	3	2	2
1	1	4	4	1	1	3	3	2	2
1	1	4	4	3	3	3	3	2	2
1	1	4	4	3	3	3	3	3	3
1	1	4	4	1	1	3	3	2	2
1	1	4	4	3	3	3	3	2	2

For Review Only

1	1	4	4	1	1	3	3	2	2
1	1	4	4	1	1	3	3	2	2
1	1	4	4	1	1	3	3	2	2
1	1	4	4	1	1	3	3	2	2
1	1	4	4	3	3	3	3	3	3
4	4	1	1	1	1	2	2	2	2
1	1	4	4	3	3	3	3	3	3
1	1	4	4	1	1	3	3	3	3
1	1	4	4	1	1	3	3	2	2
1	1	4	4	1	1	3	3	2	2
1	1	4	4	1	1	3	3	2	2
1	1	4	4	1	1	3	3	2	2
1	1	4	4	3	3	3	3	2	2
1	1	4	4	1	1	3	3	3	3
1	1	4	4	1	1	3	3	2	2
1	1	4	4	1	1	3	3	2	2
1	1	4	4	3	3	3	3	3	3
1	1	4	4	1	1	3	3	2	2
1	1	4	4	3	3	3	3	3	3
1	1	4	4	3	3	2	2	2	2
4	4	1	1	1	1	2	2	2	2
4	4	1	1	1	1	2	2	3	3
1	1	4	4	1	1	3	3	3	3
1	1	4	4	1	1	3	3	3	3
1	1	4	4	1	1	3	3	2	2
1	1	4	4	1	1	3	3	2	2
1	1	4	4	1	1	3	3	2	2
1	1	4	4	1	1	3	3	2	2
1	1	4	4	1	1	3	3	2	2
1	1	4	4	1	1	3	3	2	2
1	1	4	4	3	3	3	3	3	3
1	1	4	4	1	1	2	2	3	3
1	1	4	4	1	1	3	3	2	2
1	1	4	4	1	1	3	3	2	2
1	1	4	4	3	3	3	3	2	2
1	1	4	4	1	1	3	3	3	3
1	1	4	4	1	1	3	3	2	2
1	1	4	4	1	1	3	3	2	2
1	1	4	4	1	1	2	2	2	2
1	1	4	4	3	3	3	3	3	3
1	1	4	4	1	1	3	3	2	2
1	1	4	4	1	1	3	3	3	3
1	1	4	4	3	3	3	3	3	3
1	1	4	4	1	1	3	3	2	2
1	1	4	4	1	1	3	3	2	2
1	1	4	4	3	3	3	3	2	2
1	1	4	4	1	1	3	3	2	2
1	1	4	4	1	1	3	3	2	2
1	1	4	4	1	1	3	3	3	3
1	1	4	4	3	3	3	3	3	3
1	1	4	4	1	1	2	2	3	3
1	1	4	4	3	3	3	3	3	3
1	1	4	4	1	1	3	3	3	3
1	1	4	4	1	1	3	3	2	2
1	1	4	4	3	3	3	3	2	2

For Review Only

1	1	4	4	1	1	2	2	2	2
4	4	1	1	1	1	2	2	2	2

For Review Only

SNP2940	SNP2940	SNP2941	SNP2941	SNP2942	SNP2942	SNP2943	SNP2943	SNP2944	SNP2944
4	4	4	4	1	1	2	2	3	3
4	4	1	1	1	1	2	2	3	3
4	4	4	4	1	1	2	2	3	3
4	4	4	4	1	1	2	2	3	3
4	4	4	4	1	1	1	1	3	3
4	4	4	4	4	4	2	2	3	3
4	4	4	4	1	1	2	2	3	3
4	4	4	4	1	1	2	2	3	3
4	4	4	4	1	1	2	2	3	3
4	4	4	4	1	1	2	2	3	3
1	1	4	4	4	4	1	1	2	2
4	4	4	4	1	1	2	2	3	3
4	4	4	4	1	1	2	2	2	2
4	4	4	4	1	1	2	2	3	3
4	4	4	4	1	1	2	2	3	3
4	4	4	4	4	4	2	2	3	3
4	4	4	4	1	1	2	2	3	3
4	4	4	4	1	1	2	2	3	3
4	4	4	4	4	4	2	2	3	3
4	4	4	4	1	1	2	2	3	3
4	4	4	4	1	1	2	2	3	3
4	4	4	4	1	1	1	1	3	3
4	4	4	4	1	1	2	2	2	2
4	4	1	1	1	1	2	2	3	3
4	4	4	4	1	1	1	1	3	3
4	4	4	4	4	4	1	1	3	3
4	4	4	4	1	1	2	2	3	3
4	4	4	4	1	1	2	2	3	3
4	4	4	4	1	1	2	2	3	3
4	4	1	1	1	1	2	2	3	3
4	4	4	4	1	1	2	2	3	3
4	4	4	4	1	1	2	2	3	3
4	4	4	4	1	1	2	2	3	3
1	1	1	1	4	4	2	2	3	3
4	4	4	4	1	1	2	2	2	2
4	4	4	4	1	1	2	2	3	3
4	4	4	4	1	1	2	2	3	3
1	1	4	4	1	1	2	2	2	2
1	1	4	4	1	1	2	2	2	2
1	1	4	4	1	1	2	2	2	2
4	4	4	4	1	1	2	2	3	3
4	4	4	4	1	1	2	2	3	3
4	4	4	4	1	1	2	2	3	3
4	4	1	1	1	1	2	2	3	3
4	4	4	4	1	1	2	2	3	3
4	4	4	4	1	1	2	2	3	3
4	4	4	4	1	1	2	2	3	3
4	4	4	4	1	1	2	2	3	3
4	4	1	1	1	1	2	2	3	3
4	4	4	4	1	1	1	1	3	3
4	4	4	4	1	1	2	2	3	3
4	4	4	4	4	4	2	2	3	3

For Review Only

4	4	4	4	1	1	1	1	3	3
4	4	4	4	1	1	2	2	3	3
4	4	4	4	4	4	2	2	3	3
4	4	4	4	1	1	2	2	3	3
4	4	4	4	4	4	2	2	3	3
4	4	4	4	1	1	2	2	3	3
4	4	4	4	4	4	1	1	3	3
4	4	4	4	1	1	2	2	3	3
4	4	4	4	1	1	2	2	3	3
4	4	4	4	1	1	2	2	3	3
4	4	4	4	1	1	2	2	3	3
4	4	4	4	1	1	2	2	3	3
4	4	4	4	1	1	1	1	3	3
4	4	4	4	1	1	2	2	3	3
4	4	4	4	1	1	2	2	3	3
4	4	4	4	1	1	2	2	3	3
4	4	4	4	1	1	2	2	3	3
1	1	4	4	1	1	2	2	3	3
4	4	4	4	1	1	1	1	3	3
4	4	4	4	1	1	2	2	3	3
4	4	4	4	1	1	2	2	3	3
1	1	4	4	1	1	2	2	2	2
4	4	4	4	1	1	2	2	3	3
1	1	4	4	4	4	1	1	3	3
4	4	4	4	1	1	2	2	3	3
4	4	4	4	4	4	2	2	3	3
4	4	4	4	1	1	2	2	3	3
1	1	4	4	1	1	2	2	2	2
4	4	4	4	4	4	1	1	3	3
4	4	4	4	4	4	1	1	3	3
4	4	4	4	1	1	1	1	3	3
4	4	4	4	1	1	2	2	3	3
1	1	4	4	1	1	2	2	2	2
4	4	4	4	1	1	1	1	3	3
4	4	4	4	4	4	1	1	3	3
4	4	4	4	1	1	2	2	3	3
4	4	4	4	1	1	2	2	3	3
4	4	4	4	1	1	2	2	3	3
1	1	4	4	1	1	2	2	2	2
4	4	4	4	1	1	2	2	3	3
4	4	4	4	1	1	1	1	3	3
4	4	4	4	4	4	1	1	3	3
4	4	4	4	4	4	1	1	3	3
4	4	4	4	1	1	2	2	3	3
4	4	4	4	1	1	2	2	3	3
4	4	4	4	1	1	2	2	3	3
4	4	4	4	1	1	2	2	2	2
4	4	4	4	1	1	2	2	3	3
4	4	4	4	1	1	2	2	3	3
4	4	4	4	1	1	2	2	2	2
4	4	4	4	1	1	2	2	3	3
1	1	1	1	1	1	2	2	2	2
4	4	4	4	1	1	2	2	3	3
1	1	4	4	1	1	2	2	2	2
4	4	4	4	1	1	2	2	3	3

For Review Only

4	4	4	4	1	1	2	2	3	3
4	4	4	4	1	1	2	2	3	3
4	4	4	4	4	4	2	2	3	3
4	4	1	1	1	1	2	2	2	2
4	4	4	4	1	1	2	2	3	3
4	4	4	4	4	4	2	2	3	3
4	4	4	4	4	4	2	2	3	3
4	4	4	4	4	4	2	2	3	3
4	4	4	4	1	1	2	2	3	3
4	4	4	4	1	1	2	2	3	3
4	4	4	4	4	4	1	1	2	2
4	4	4	4	1	1	2	2	2	2
1	1	4	4	1	1	2	2	3	3
4	4	4	4	1	1	2	2	3	3
4	4	4	4	1	1	2	2	3	3
4	4	4	4	1	1	2	2	3	3
4	4	4	4	4	4	1	1	3	3
4	4	4	4	1	1	2	2	2	2
4	4	1	1	1	1	2	2	2	2
4	4	4	4	1	1	1	1	3	3
4	4	4	4	1	1	2	2	3	3
4	4	4	4	1	1	2	2	3	3
4	4	4	4	1	1	2	2	3	3
1	1	1	1	1	1	2	2	2	2
4	4	4	4	1	1	2	2	3	3
1	1	4	4	1	1	2	2	2	2
4	4	4	4	4	4	1	1	3	3
4	4	4	4	1	1	2	2	3	3
4	4	4	4	1	1	2	2	3	3
4	4	4	4	4	4	1	1	3	3
4	4	4	4	1	1	2	2	2	2
4	4	4	4	1	1	2	2	2	2
4	4	4	4	1	1	1	1	3	3
4	4	4	4	1	1	2	2	2	2
1	1	1	1	4	4	2	2	3	3
4	4	4	4	1	1	2	2	3	3
4	4	1	1	1	1	2	2	2	2
4	4	4	4	4	4	2	2	3	3
4	4	4	4	4	4	1	1	2	2
4	4	1	1	1	1	2	2	2	2
1	1	4	4	4	4	1	1	2	2
4	4	1	1	1	1	2	2	2	2
4	4	4	4	1	1	2	2	3	3
4	4	4	4	1	1	2	2	3	3
4	4	4	4	1	1	1	1	3	3
4	4	4	4	1	1	2	2	2	2
4	4	4	4	1	1	2	2	2	2
4	4	4	4	1	1	2	2	3	3
4	4	4	4	1	1	2	2	2	2
4	4	4	4	1	1	2	2	3	3
4	4	4	4	1	1	2	2	3	3
1	1	4	4	1	1	2	2	2	2
4	4	4	4	1	1	2	2	3	3
4	4	4	4	1	1	2	2	3	3
4	4	4	4	1	1	2	2	2	2

For Review Only

4	4	4	4	1	1	2	2	3	3
1	1	4	4	1	1	2	2	2	2
4	4	4	4	1	1	2	2	3	3
1	1	4	4	1	1	2	2	2	2
4	4	4	4	1	1	2	2	3	3
4	4	1	1	1	1	2	2	3	3
4	4	4	4	1	1	2	2	2	2
4	4	4	4	1	1	2	2	3	3
4	4	4	4	1	1	2	2	2	2
4	4	4	4	4	4	1	1	3	3
4	4	4	4	1	1	2	2	2	2
4	4	4	4	1	1	2	2	2	2
4	4	4	4	1	1	2	2	2	2
4	4	4	4	1	1	2	2	2	2
4	4	4	4	1	1	2	2	2	2
1	1	4	4	4	4	1	1	2	2
4	4	4	4	1	1	2	2	3	3
4	4	4	4	1	1	2	2	3	3
4	4	4	4	1	1	2	2	3	3
4	4	4	4	4	4	2	2	3	3
4	4	4	4	1	1	2	2	2	2
4	4	4	4	1	1	2	2	2	2
1	1	4	4	1	1	2	2	2	2
4	4	4	4	1	1	2	2	3	3
4	4	4	4	1	1	2	2	3	3
1	1	4	4	4	4	1	1	3	3
4	4	4	4	1	1	2	2	3	3
4	4	4	4	4	4	2	2	3	3
1	1	4	4	4	4	1	1	3	3
4	4	4	4	1	1	1	1	3	3
4	4	4	4	1	1	2	2	3	3
1	1	4	4	4	4	1	1	3	3
4	4	4	4	1	1	2	2	3	3
4	4	4	4	1	1	2	2	2	2
4	4	4	4	1	1	2	2	3	3
4	4	4	4	1	1	2	2	3	3
4	4	4	4	1	1	2	2	3	3
4	4	4	4	1	1	2	2	3	3
4	4	4	4	1	1	2	2	3	3
4	4	4	4	4	4	1	1	3	3
4	4	4	4	1	1	2	2	3	3
4	4	4	4	1	1	2	2	3	3
1	1	4	4	1	1	2	2	2	2
4	4	4	4	1	1	2	2	3	3
4	4	4	4	1	1	2	2	3	3
4	4	4	4	1	1	2	2	3	3
1	1	4	4	4	4	1	1	2	2
4	4	4	4	1	1	2	2	3	3
4	4	4	4	1	1	2	2	3	3
4	4	4	4	1	1	2	2	3	3
4	4	4	4	1	1	2	2	3	3
4	4	4	4	1	1	2	2	3	3
1	1	4	4	1	1	2	2	2	2
4	4	4	4	1	1	2	2	3	3
1	1	4	4	1	1	2	2	2	2

For Review Only

4	4	4	4	1	1	2	2	3	3
4	4	4	4	1	1	2	2	3	3
4	4	4	4	1	1	2	2	3	3
4	4	4	4	1	1	2	2	2	2
4	4	4	4	1	1	2	2	3	3
4	4	4	4	1	1	1	1	3	3
1	1	4	4	1	1	2	2	2	2
1	1	4	4	1	1	2	2	3	3
4	4	1	1	1	1	2	2	2	2
1	1	4	4	1	1	2	2	2	2
4	4	4	4	1	1	2	2	3	3
4	4	4	4	1	1	2	2	3	3
1	1	4	4	1	1	2	2	2	2
4	4	4	4	1	1	2	2	3	3
4	4	4	4	1	1	2	2	3	3
4	4	4	4	1	1	2	2	3	3
1	1	4	4	1	1	2	2	2	2
4	4	4	4	1	1	2	2	2	2
1	1	4	4	1	1	2	2	2	2
1	1	4	4	1	1	2	2	2	2
4	4	4	4	4	4	2	2	3	3
4	4	4	4	4	4	1	1	3	3
4	4	4	4	1	1	2	2	3	3
4	4	4	4	1	1	2	2	3	3
4	4	4	4	1	1	2	2	3	3
4	4	4	4	1	1	1	1	3	3
1	1	4	4	1	1	2	2	2	2
4	4	4	4	4	4	1	1	3	3
4	4	4	4	1	1	2	2	3	3
4	4	4	4	1	1	2	2	3	3
1	1	1	1	1	1	2	2	2	2
4	4	4	4	4	4	1	1	3	3
4	4	4	4	1	1	2	2	3	3
4	4	4	4	1	1	2	2	3	3
1	1	4	4	1	1	2	2	2	2
1	1	4	4	1	1	2	2	2	2
4	4	4	4	1	1	1	1	3	3
4	4	4	4	1	1	2	2	3	3
4	4	4	4	1	1	2	2	3	3
1	1	4	4	1	1	2	2	2	2
4	4	4	4	1	1	2	2	3	3
4	4	4	4	1	1	2	2	3	3
1	1	4	4	1	1	2	2	2	2
4	4	4	4	1	1	1	1	3	3
4	4	4	4	1	1	2	2	3	3
1	1	4	4	1	1	2	2	2	2
4	4	4	4	1	1	2	2	2	2
4	4	4	4	1	1	2	2	3	3
4	4	4	4	1	1	2	2	3	3
4	4	4	4	1	1	2	2	2	2
4	4	4	4	4	4	1	1	3	3
1	1	4	4	1	1	2	2	2	2
4	4	4	4	1	1	2	2	3	3
4	4	4	4	4	4	1	1	3	3
1	1	1	1	1	1	2	2	2	2

For Review Only

4	4	4	4	4	4	1	1	3	3
4	4	4	4	4	4	2	2	3	3

For Review Only

SNP2945	SNP2945	SNP2946	SNP2946	SNP2947	SNP2947	SNP2948	SNP2948	SNP2949	SNP2949
1	1	3	3	1	1	2	2	3	3
4	4	3	3	2	2	2	2	3	3
1	1	3	3	1	1	2	2	4	4
1	1	3	3	1	1	3	3	3	3
4	4	4	4	1	1	2	2	4	4
4	4	4	4	1	1	3	3	4	4
4	4	3	3	1	1	2	2	3	3
4	4	3	3	2	2	2	2	3	3
4	4	3	3	1	1	2	2	3	3
1	1	3	3	1	1	2	2	3	3
4	4	4	4	1	1	3	3	3	3
1	1	3	3	1	1	2	2	4	4
4	4	3	3	1	1	2	2	4	4
1	1	3	3	1	1	2	2	4	4
1	1	4	4	1	1	2	2	4	4
4	4	4	4	1	1	2	2	4	4
1	1	3	3	1	1	2	2	3	3
1	1	3	3	1	1	2	2	3	3
4	4	4	4	1	1	2	2	4	4
1	1	3	3	2	2	2	2	3	3
4	4	3	3	2	2	2	2	3	3
4	4	3	3	1	1	2	2	4	4
1	1	3	3	1	1	2	2	3	3
1	1	3	3	1	1	2	2	3	3
4	4	4	4	1	1	2	2	4	4
4	4	4	4	1	1	3	3	3	3
1	1	3	3	1	1	2	2	4	4
1	1	3	3	1	1	2	2	4	4
4	4	3	3	2	2	2	2	3	3
4	4	3	3	1	1	2	2	4	4
1	1	3	3	1	1	2	2	4	4
1	1	3	3	1	1	2	2	3	3
4	4	3	3	1	1	2	2	3	3
1	1	3	3	1	1	2	2	3	3
1	1	3	3	1	1	2	2	4	4
4	4	3	3	1	1	2	2	3	3
4	4	3	3	2	2	2	2	3	3
4	4	3	3	1	1	2	2	4	4
1	1	3	3	1	1	2	2	3	3
4	4	3	3	1	1	2	2	3	3
4	4	3	3	2	2	2	2	3	3
4	4	3	3	2	2	2	2	3	3
4	4	3	3	2	2	2	2	3	3
4	4	3	3	1	1	2	2	4	4
4	4	3	3	1	1	2	2	3	3
1	1	3	3	1	1	2	2	4	4
1	1	3	3	1	1	2	2	4	4
4	4	3	3	1	1	2	2	3	3
4	4	3	3	1	1	2	2	4	4
4	4	4	4	1	1	3	3	3	3
1	1	3	3	1	1	2	2	4	4
4	4	4	4	1	1	2	2	4	4

For Review Only

4	4	4	4	1	1	2	2	4	4
4	4	3	3	1	1	2	2	3	3
4	4	4	4	1	1	2	2	4	4
1	1	3	3	1	1	2	2	4	4
4	4	4	4	1	1	2	2	4	4
1	1	3	3	1	1	2	2	4	4
4	4	4	4	1	1	3	3	3	3
4	4	3	3	2	2	2	2	3	3
1	1	3	3	1	1	2	2	3	3
1	1	3	3	1	1	2	2	4	4
1	1	3	3	1	1	2	2	4	4
1	1	3	3	1	1	2	2	4	4
4	4	4	4	1	1	2	2	4	4
4	4	3	3	2	2	2	2	3	3
1	1	3	3	1	1	3	3	4	4
4	4	3	3	2	2	2	2	3	3
1	1	3	3	1	1	2	2	4	4
1	1	3	3	1	1	2	2	4	4
4	4	4	4	1	1	2	2	4	4
1	1	3	3	1	1	2	2	3	3
1	1	3	3	1	1	2	2	4	4
1	1	3	3	1	1	2	2	4	4
4	4	3	3	1	1	2	2	3	3
4	4	4	4	1	1	3	3	3	3
4	4	3	3	1	1	2	2	3	3
4	4	4	4	1	1	2	2	4	4
4	4	3	3	1	1	2	2	4	4
1	1	3	3	1	1	2	2	3	3
4	4	4	4	1	1	3	3	3	3
4	4	4	4	1	1	2	2	3	3
4	4	4	4	1	1	2	2	4	4
4	4	3	3	1	1	2	2	3	3
4	4	3	3	1	1	2	2	3	3
4	4	4	4	1	1	2	2	4	4
4	4	4	4	1	1	3	3	3	3
4	4	3	3	1	1	2	2	3	3
4	4	3	3	2	2	2	2	3	3
4	4	3	3	1	1	2	2	4	4
1	1	3	3	1	1	2	2	3	3
4	4	3	3	1	1	2	2	3	3
4	4	4	4	1	1	2	2	4	4
4	4	4	4	1	1	3	3	3	3
4	4	4	4	1	1	3	3	3	3
1	1	3	3	1	1	2	2	4	4
1	1	3	3	1	1	2	2	4	4
1	1	3	3	1	1	2	2	4	4
1	1	3	3	1	1	2	2	3	3
1	1	3	3	1	1	2	2	4	4
4	4	3	3	1	1	2	2	3	3
1	1	3	3	1	1	2	2	4	4
1	1	3	3	1	1	2	2	4	4
4	4	3	3	1	1	2	2	3	3
1	1	3	3	1	1	2	2	3	3
1	1	3	3	1	1	2	2	3	3
1	1	3	3	1	1	2	2	4	4

For Review Only

1	1	3	3	1	1	2	2	4	4
1	1	3	3	1	1	2	2	4	4
4	4	4	4	1	1	2	2	4	4
4	4	3	3	1	1	2	2	3	3
1	1	3	3	1	1	2	2	4	4
4	4	4	4	1	1	2	2	4	4
4	4	4	4	1	1	2	2	4	4
1	1	4	4	1	1	2	2	4	4
4	4	3	3	1	1	2	2	3	3
1	1	3	3	1	1	2	2	3	3
4	4	3	3	1	1	2	2	4	4
4	4	3	3	1	1	3	3	4	4
4	4	3	3	2	2	2	2	3	3
4	4	4	4	1	1	3	3	4	4
1	1	3	3	1	1	2	2	4	4
1	1	3	3	1	1	2	2	4	4
4	4	4	4	1	1	3	3	3	3
1	1	3	3	1	1	2	2	4	4
4	4	4	4	1	1	2	2	3	3
4	4	4	4	1	1	2	2	4	4
1	1	3	3	1	1	2	2	4	4
1	1	3	3	1	1	2	2	4	4
1	1	3	3	1	1	2	2	4	4
1	1	3	3	1	1	2	2	3	3
1	1	3	3	1	1	2	2	4	4
1	1	3	3	1	1	2	2	3	3
4	4	4	4	1	1	2	2	4	4
1	1	3	3	1	1	2	2	3	3
1	1	3	3	1	1	2	2	4	4
4	4	4	4	1	1	3	3	3	3
4	4	3	3	1	1	2	2	3	3
1	1	3	3	1	1	2	2	4	4
4	4	4	4	1	1	2	2	4	4
1	1	3	3	1	1	2	2	4	4
1	1	3	3	1	1	2	2	3	3
4	4	3	3	1	1	2	2	4	4
1	1	3	3	1	1	2	2	3	3
4	4	4	4	1	1	2	2	3	3
4	4	4	4	1	1	3	3	3	3
4	4	3	3	1	1	2	2	3	3
4	4	4	4	1	1	3	3	3	3
4	4	4	4	1	1	2	2	3	3
1	1	3	3	1	1	2	2	4	4
1	1	3	3	1	1	2	2	3	3
4	4	3	3	2	2	3	3	3	3
1	1	3	3	1	1	2	2	4	4
1	1	3	3	1	1	2	2	4	4
1	1	3	3	1	1	2	2	4	4
4	4	3	3	1	1	2	2	3	3
1	1	3	3	1	1	2	2	4	4
1	1	3	3	1	1	2	2	4	4
4	4	3	3	1	1	2	2	3	3
1	1	3	3	1	1	2	2	4	4
1	1	3	3	1	1	2	2	4	4
1	1	3	3	1	1	2	2	4	4

For Review Only

1	1	3	3	1	1	2	2	4	4
1	1	3	3	1	1	2	2	3	3
4	4	3	3	1	1	2	2	3	3
1	1	3	3	1	1	2	2	4	4
1	1	3	3	1	1	2	2	4	4
4	4	4	4	1	1	2	2	3	3
1	1	3	3	1	1	2	2	3	3
1	1	3	3	1	1	2	2	4	4
4	4	3	3	1	1	2	2	4	4
4	4	4	4	1	1	2	2	3	3
1	1	3	3	1	1	2	2	4	4
4	4	3	3	1	1	2	2	3	3
1	1	3	3	1	1	2	2	4	4
1	1	3	3	1	1	2	2	4	4
1	1	3	3	1	1	2	2	3	3
4	4	4	4	1	1	3	3	3	3
1	1	4	4	1	1	2	2	3	3
1	1	3	3	1	1	3	3	3	3
4	4	3	3	1	1	2	2	3	3
4	4	4	4	1	1	2	2	4	4
1	1	3	3	1	1	2	2	4	4
4	4	3	3	1	1	2	2	3	3
1	1	3	3	1	1	2	2	4	4
1	1	3	3	1	1	2	2	4	4
1	1	3	3	1	1	2	2	3	3
4	4	4	4	1	1	3	3	3	3
1	1	3	3	1	1	3	3	4	4
4	4	4	4	1	1	3	3	3	3
4	4	4	4	1	1	3	3	3	3
4	4	4	4	1	1	2	2	4	4
1	1	3	3	1	1	2	2	3	3
4	4	4	4	1	1	2	2	3	3
1	1	3	3	1	1	2	2	3	3
1	1	3	3	1	1	2	2	3	3
4	4	3	3	2	2	2	2	3	3
1	1	3	3	1	1	2	2	4	4
1	1	3	3	1	1	2	2	4	4
4	4	3	3	2	2	2	2	3	3
1	1	3	3	1	1	2	2	4	4
4	4	4	4	1	1	3	3	3	3
1	1	3	3	1	1	2	2	4	4
1	1	3	3	1	1	2	2	4	4
4	4	3	3	1	1	2	2	3	3
1	1	3	3	1	1	2	2	4	4
1	1	3	3	1	1	2	2	4	4
1	1	3	3	1	1	2	2	4	4
4	4	4	4	1	1	3	3	3	3
4	4	3	3	1	1	2	2	3	3
1	1	3	3	1	1	2	2	3	3
4	4	3	3	2	2	2	2	3	3
1	1	3	3	1	1	2	2	4	4
4	4	3	3	2	2	2	2	3	3
1	1	3	3	1	1	2	2	3	3
1	1	3	3	1	1	2	2	4	4
1	1	3	3	1	1	2	2	3	3

For Review Only

1	1	3	3	1	1	2	2	4	4
1	1	3	3	1	1	2	2	3	3
1	1	3	3	1	1	2	2	4	4
1	1	3	3	1	1	2	2	4	4
4	4	3	3	2	2	2	2	3	3
4	4	4	4	1	1	3	3	3	3
4	4	3	3	1	1	2	2	3	3
4	4	3	3	2	2	2	2	3	3
1	1	3	3	1	1	2	2	4	4
1	1	3	3	1	1	2	2	3	3
1	1	3	3	1	1	2	2	4	4
4	4	3	3	2	2	2	2	3	3
1	1	3	3	1	1	2	2	3	3
1	1	3	3	1	1	2	2	4	4
1	1	3	3	1	1	2	2	4	4
1	1	3	3	1	1	2	2	3	3
1	1	3	3	1	1	2	2	3	3
1	1	3	3	1	1	2	2	3	3
1	1	3	3	1	1	2	2	3	3
4	4	3	3	1	1	2	2	3	3
4	4	4	4	1	1	3	3	3	3
4	4	4	4	1	1	3	3	3	3
1	1	3	3	1	1	2	2	3	3
1	1	3	3	1	1	2	2	3	3
4	4	3	3	2	2	2	2	3	3
4	4	4	4	1	1	2	2	4	4
4	4	3	3	2	2	2	2	3	3
4	4	4	4	1	1	3	3	3	3
4	4	3	3	1	1	2	2	4	4
1	1	3	3	1	1	2	2	4	4
4	4	3	3	1	1	2	2	3	3
4	4	4	4	1	1	3	3	3	3
4	4	3	3	1	1	2	2	3	3
1	1	3	3	1	1	2	2	4	4
1	1	3	3	1	1	2	2	3	3
4	4	3	3	1	1	2	2	3	3
4	4	4	4	1	1	2	2	4	4
1	1	3	3	1	1	2	2	4	4
4	4	3	3	1	1	2	2	3	3
1	1	3	3	1	1	2	2	3	3
1	1	3	3	1	1	2	2	3	3
1	1	3	3	1	1	2	2	3	3
1	1	3	3	1	1	2	2	3	3
4	4	4	4	1	1	2	2	4	4
1	1	3	3	1	1	2	2	4	4
1	1	3	3	1	1	2	2	3	3
1	1	3	3	1	1	2	2	4	4
1	1	3	3	1	1	2	2	4	4
4	4	3	3	1	1	2	2	3	3
4	4	3	3	2	2	2	2	3	3
4	4	4	4	1	1	3	3	3	3
1	1	3	3	1	1	2	2	3	3
1	1	3	3	1	1	2	2	4	4
4	4	4	4	1	1	3	3	3	3
4	4	3	3	1	1	2	2	3	3

For Review Only

1	1	4	4	1	1	3	3	3	3
4	4	4	4	1	1	2	2	4	4

For Review Only

SNP295	SNP295	SNP2950	SNP2950	SNP2951	SNP2951	SNP2952	SNP2952	SNP2953	SNP2953
3	3	3	3	4	4	2	2	4	4
2	2	3	3	4	4	2	2	4	4
2	2	3	3	4	4	2	2	4	4
2	2	3	3	4	4	2	2	4	4
2	2	3	3	1	1	2	2	3	3
3	3	3	3	4	4	2	2	4	4
2	2	3	3	4	4	2	2	3	3
2	2	3	3	4	4	3	3	4	4
2	2	3	3	4	4	2	2	4	4
2	2	3	3	4	4	2	2	4	4
2	2	1	1	4	4	2	2	4	4
2	2	3	3	4	4	2	2	4	4
2	2	3	3	4	4	2	2	3	3
2	2	3	3	4	4	2	2	4	4
2	2	3	3	1	1	2	2	4	4
2	2	3	3	4	4	2	2	4	4
2	2	3	3	4	4	3	3	4	4
3	3	3	3	4	4	2	2	4	4
2	2	3	3	4	4	2	2	4	4
2	2	3	3	4	4	2	2	4	4
2	2	3	3	4	4	2	2	4	4
2	2	3	3	1	1	2	2	3	3
2	2	3	3	4	4	2	2	4	4
2	2	3	3	4	4	2	2	4	4
2	2	3	3	1	1	2	2	4	4
2	2	1	1	4	4	3	3	4	4
2	2	3	3	4	4	2	2	4	4
2	2	3	3	4	4	2	2	4	4
2	2	3	3	4	4	2	2	4	4
2	2	3	3	4	4	2	2	4	4
2	2	3	3	4	4	2	2	4	4
2	2	3	3	4	4	2	2	4	4
2	2	3	3	4	4	2	2	3	3
3	3	3	3	4	4	2	2	4	4
2	2	3	3	4	4	2	2	4	4
2	2	3	3	4	4	2	2	4	4
2	2	3	3	4	4	2	2	4	4
2	2	3	3	4	4	2	2	4	4
2	2	3	3	4	4	2	2	4	4
2	2	3	3	4	4	2	2	4	4
2	2	3	3	4	4	2	2	4	4
2	2	3	3	4	4	2	2	4	4
2	2	3	3	4	4	2	2	4	4
2	2	3	3	4	4	2	2	3	3
2	2	3	3	4	4	2	2	4	4
2	2	3	3	4	4	2	2	4	4
2	2	3	3	4	4	2	2	4	4
2	2	3	3	4	4	2	2	4	4
2	2	3	3	4	4	2	2	3	3
2	2	3	3	4	4	2	2	4	4
2	2	3	3	4	4	2	2	4	4
2	2	3	3	4	4	2	2	4	4

For Review Only

2	2	3	3	1	1	2	2	4	4
2	2	3	3	4	4	2	2	3	3
2	2	3	3	4	4	2	2	4	4
2	2	3	3	1	1	2	2	3	3
2	2	3	3	4	4	2	2	4	4
2	2	3	3	4	4	2	2	4	4
2	2	1	1	4	4	2	2	4	4
2	2	3	3	4	4	2	2	4	4
2	2	3	3	4	4	2	2	3	3
2	2	3	3	4	4	2	2	4	4
2	2	3	3	4	4	2	2	4	4
2	2	3	3	4	4	2	2	4	4
2	2	3	3	1	1	2	2	4	4
2	2	3	3	4	4	2	2	4	4
3	3	3	3	4	4	2	2	4	4
2	2	3	3	4	4	2	2	4	4
2	2	3	3	4	4	2	2	4	4
2	2	3	3	4	4	2	2	4	4
2	2	3	3	1	1	2	2	3	3
2	2	3	3	4	4	2	2	3	3
2	2	3	3	4	4	2	2	4	4
2	2	3	3	4	4	2	2	4	4
2	2	3	3	4	4	2	2	4	4
3	3	1	1	4	4	2	2	4	4
2	2	3	3	4	4	2	2	4	4
2	2	3	3	4	4	2	2	4	4
2	2	3	3	4	4	2	2	4	4
2	2	3	3	4	4	2	2	4	4
2	2	1	1	4	4	3	3	4	4
3	3	3	3	4	4	2	2	3	3
2	2	3	3	1	1	2	2	4	4
3	3	3	3	4	4	2	2	4	4
2	2	3	3	4	4	2	2	4	4
2	2	3	3	1	1	2	2	4	4
2	2	1	1	4	4	3	3	4	4
2	2	3	3	4	4	2	2	4	4
2	2	3	3	4	4	2	2	4	4
2	2	3	3	4	4	2	2	3	3
2	2	3	3	4	4	2	2	4	4
2	2	3	3	4	4	2	2	4	4
2	2	3	3	4	4	2	2	3	3
2	2	3	3	4	4	2	2	4	4
2	2	1	1	4	4	2	2	4	4
2	2	3	3	4	4	2	2	4	4
3	3	3	3	4	4	2	2	3	3
2	2	3	3	4	4	2	2	4	4
2	2	3	3	4	4	3	3	4	4
2	2	3	3	4	4	2	2	4	4
2	2	3	3	4	4	2	2	4	4
2	2	3	3	4	4	2	2	4	4
2	2	3	3	4	4	2	2	4	4
2	2	3	3	4	4	2	2	4	4
2	2	3	3	4	4	2	2	4	4
2	2	3	3	4	4	2	2	4	4
2	2	3	3	4	4	2	2	4	4

For Review Only

2	2	3	3	4	4	2	2	4	4
2	2	3	3	4	4	2	2	4	4
2	2	3	3	4	4	2	2	3	3
2	2	3	3	4	4	3	3	4	4
2	2	3	3	4	4	2	2	4	4
3	3	3	3	4	4	2	2	4	4
2	2	3	3	4	4	2	2	4	4
2	2	3	3	4	4	2	2	4	4
2	2	3	3	4	4	2	2	4	4
2	2	3	3	4	4	2	2	4	4
2	2	3	3	4	4	2	2	4	4
2	2	3	3	4	4	2	2	4	4
2	2	3	3	4	4	2	2	4	4
2	2	3	3	4	4	2	2	4	4
2	2	3	3	4	4	2	2	4	4
2	2	3	3	4	4	2	2	4	4
2	2	1	1	4	4	3	3	3	3
2	2	3	3	4	4	2	2	4	4
2	2	3	3	4	4	3	3	4	4
2	2	3	3	1	1	2	2	3	3
2	2	3	3	4	4	2	2	4	4
2	2	3	3	4	4	2	2	3	3
2	2	3	3	4	4	2	2	4	4
2	2	3	3	4	4	2	2	4	4
2	2	3	3	4	4	2	2	4	4
2	2	3	3	4	4	2	2	4	4
2	2	3	3	4	4	2	2	4	4
2	2	3	3	4	4	2	2	4	4
3	3	3	3	4	4	2	2	4	4
2	2	3	3	4	4	2	2	4	4
2	2	3	3	4	4	2	2	4	4
2	2	3	3	4	4	2	2	4	4
2	2	3	3	1	1	2	2	3	3
2	2	3	3	4	4	2	2	4	4
3	3	3	3	4	4	2	2	4	4
2	2	3	3	1	1	2	2	4	4
2	2	3	3	4	4	2	2	3	3
2	2	3	3	4	4	2	2	4	4
2	2	3	3	4	4	2	2	4	4
2	2	3	3	4	4	2	2	4	4
2	2	1	1	4	4	3	3	4	4
2	2	3	3	4	4	2	2	4	4
2	2	3	3	4	4	2	2	4	4
2	2	3	3	4	4	2	2	4	4
2	2	3	3	4	4	2	2	4	4
2	2	3	3	4	4	2	2	4	4
2	2	3	3	4	4	2	2	4	4
2	2	3	3	4	4	2	2	4	4
2	2	3	3	4	4	2	2	4	4
2	2	3	3	4	4	2	2	4	4
2	2	3	3	4	4	2	2	4	4
2	2	3	3	4	4	2	2	4	4
3	3	3	3	4	4	2	2	4	4
2	2	3	3	4	4	2	2	4	4
2	2	3	3	4	4	2	2	4	4

For Review Only

2	2	3	3	4	4	2	2	4	4
2	2	3	3	4	4	2	2	4	4
2	2	3	3	4	4	2	2	4	4
2	2	3	3	4	4	2	2	4	4
2	2	3	3	4	4	2	2	4	4
2	2	3	3	4	4	3	3	4	4
2	2	3	3	4	4	2	2	4	4
2	2	3	3	4	4	2	2	4	4
2	2	3	3	4	4	2	2	4	4
3	3	3	3	4	4	2	2	4	4
2	2	3	3	4	4	2	2	4	4
2	2	3	3	4	4	2	2	4	4
2	2	3	3	4	4	2	2	3	3
2	2	3	3	4	4	2	2	4	4
2	2	3	3	4	4	3	3	3	3
2	2	1	1	4	4	2	2	4	4
2	2	3	3	4	4	2	2	4	4
2	2	3	3	4	4	2	2	4	4
2	2	3	3	4	4	2	2	4	4
2	2	3	3	4	4	2	2	4	4
2	2	3	3	4	4	2	2	4	4
2	2	3	3	4	4	2	2	4	4
2	2	3	3	4	4	2	2	4	4
2	2	3	3	4	4	2	2	4	4
2	2	3	3	4	4	2	2	3	3
2	2	1	1	4	4	2	2	4	4
2	2	3	3	4	4	2	2	4	4
2	2	3	3	4	4	3	3	4	4
2	2	1	1	4	4	3	3	4	4
3	3	3	3	1	1	2	2	3	3
2	2	3	3	4	4	3	3	4	4
3	3	3	3	4	4	2	2	4	4
2	2	3	3	4	4	2	2	4	4
2	2	3	3	4	4	2	2	4	4
2	2	3	3	4	4	2	2	4	4
2	2	3	3	4	4	2	2	4	4
2	2	3	3	4	4	2	2	4	4
2	2	3	3	4	4	2	2	4	4
2	2	3	3	4	4	2	2	4	4
2	2	1	1	4	4	2	2	4	4
2	2	3	3	4	4	2	2	4	4
2	2	3	3	4	4	2	2	4	4
2	2	3	3	4	4	3	3	4	4
2	2	3	3	4	4	2	2	4	4
2	2	3	3	4	4	2	2	4	4
2	2	3	3	4	4	2	2	4	4
2	2	1	1	4	4	2	2	4	4
2	2	3	3	4	4	2	2	3	3
2	2	3	3	4	4	2	2	4	4
2	2	3	3	4	4	2	2	3	3
3	3	3	3	4	4	2	2	4	4
2	2	3	3	4	4	2	2	4	4
2	2	3	3	4	4	2	2	4	4
2	2	3	3	4	4	2	2	4	4
2	2	3	3	4	4	2	2	4	4

For Review Only

3	3	3	3	4	4	2	2	4	4
2	2	3	3	4	4	2	2	4	4
2	2	3	3	4	4	2	2	4	4
2	2	3	3	4	4	2	2	4	4
2	2	3	3	4	4	2	2	4	4
2	2	1	1	4	4	3	3	4	4
2	2	3	3	4	4	3	3	4	4
2	2	3	3	4	4	2	2	4	4
2	2	3	3	4	4	2	2	4	4
2	2	3	3	4	4	2	2	4	4
2	2	3	3	4	4	2	2	4	4
2	2	3	3	4	4	2	2	4	4
2	2	3	3	4	4	2	2	4	4
2	2	3	3	4	4	2	2	4	4
2	2	3	3	4	4	2	2	4	4
2	2	3	3	4	4	2	2	4	4
2	2	3	3	4	4	2	2	4	4
2	2	3	3	4	4	2	2	4	4
2	2	3	3	4	4	2	2	4	4
2	2	3	3	4	4	2	2	4	4
2	2	3	3	4	4	2	2	4	4
2	2	1	1	4	4	3	3	4	4
2	2	3	3	4	4	2	2	4	4
2	2	3	3	4	4	2	2	4	4
2	2	3	3	4	4	2	2	4	4
2	2	3	3	4	4	2	2	4	4
2	2	3	3	4	4	2	2	4	4
2	2	1	1	4	4	2	2	4	4
2	2	3	3	4	4	2	2	4	4
3	3	3	3	4	4	2	2	4	4
2	2	3	3	4	4	3	3	4	4
2	2	1	1	4	4	2	2	4	4
2	2	3	3	4	4	2	2	4	4
2	2	3	3	4	4	2	2	4	4
2	2	3	3	4	4	2	2	4	4
2	2	3	3	4	4	2	2	3	3
3	3	3	3	4	4	2	2	4	4
2	2	3	3	4	4	2	2	4	4
2	2	3	3	4	4	2	2	4	4
2	2	3	3	4	4	3	3	4	4
2	2	3	3	4	4	2	2	4	4
2	2	3	3	4	4	2	2	4	4
2	2	3	3	4	4	2	2	4	4
2	2	3	3	1	1	2	2	3	3
2	2	3	3	4	4	2	2	4	4
2	2	3	3	4	4	2	2	4	4
2	2	3	3	4	4	2	2	4	4
2	2	3	3	4	4	2	2	4	4
2	2	3	3	4	4	2	2	4	4
2	2	3	3	4	4	2	2	4	4
2	2	1	1	4	4	3	3	4	4
2	2	3	3	4	4	3	3	4	4
2	2	3	3	4	4	2	2	4	4
2	2	1	1	4	4	2	2	4	4
2	2	3	3	4	4	2	2	4	4

For Review Only

2	2	1	1	4	4	2	2	4	4
2	2	3	3	4	4	2	2	4	4

For Review Only

SNP2954	SNP2954	SNP2955	SNP2955	SNP2956	SNP2956	SNP2957	SNP2957	SNP2958	SNP2958
2	2	1	1	4	4	1	1	1	1
2	2	2	2	4	4	1	1	1	1
4	4	2	2	4	4	1	1	1	1
2	2	2	2	4	4	1	1	1	1
2	2	2	2	4	4	1	1	4	4
2	2	2	2	4	4	1	1	4	4
2	2	2	2	4	4	1	1	1	1
2	2	1	1	4	4	1	1	4	4
2	2	2	2	4	4	1	1	1	1
2	2	2	2	4	4	1	1	1	1
2	2	2	2	4	4	1	1	1	1
4	4	2	2	4	4	1	1	1	1
2	2	2	2	4	4	1	1	4	4
2	2	2	2	4	4	1	1	1	1
2	2	2	2	4	4	1	1	4	4
2	2	2	2	4	4	1	1	4	4
4	4	2	2	4	4	1	1	4	4
2	2	2	2	4	4	1	1	4	4
2	2	2	2	4	4	1	1	4	4
2	2	2	2	4	4	1	1	4	4
2	2	2	2	4	4	1	1	1	1
2	2	2	2	4	4	1	1	4	4
4	4	2	2	4	4	1	1	1	1
2	2	2	2	4	4	1	1	4	4
2	2	2	2	4	4	1	1	4	4
2	2	1	1	4	4	1	1	4	4
2	2	2	2	4	4	1	1	4	4
2	2	2	2	4	4	1	1	1	1
2	2	2	2	4	4	1	1	1	1
4	4	2	2	4	4	1	1	4	4
4	4	2	2	1	1	2	2	4	4
2	2	2	2	4	4	1	1	1	1
2	2	2	2	4	4	1	1	1	1
2	2	2	2	4	4	1	1	1	1
2	2	2	2	4	4	1	1	1	1
2	2	2	2	4	4	1	1	4	4
2	2	2	2	4	4	1	1	1	1
2	2	2	2	4	4	1	1	1	1
2	2	2	2	4	4	1	1	1	1
2	2	2	2	4	4	1	1	4	4
2	2	2	2	4	4	1	1	1	1
2	2	2	2	4	4	1	1	1	1
2	2	2	2	4	4	1	1	1	1
4	4	2	2	4	4	1	1	4	4
2	2	2	2	4	4	1	1	1	1
4	4	2	2	4	4	1	1	1	1
4	4	2	2	4	4	1	1	1	1
2	2	2	2	4	4	1	1	4	4
4	4	2	2	4	4	1	1	4	4
2	2	2	2	4	4	1	1	4	4
4	4	2	2	4	4	1	1	1	1
2	2	2	2	4	4	1	1	1	1

For Review Only

2	2	2	2	4	4	1	1	4	4
2	2	2	2	4	4	1	1	1	1
2	2	2	2	4	4	2	2	4	4
2	2	2	2	4	4	1	1	4	4
2	2	2	2	4	4	1	1	4	4
2	2	2	2	4	4	1	1	1	1
4	4	2	2	4	4	1	1	1	1
2	2	2	2	4	4	1	1	1	1
2	2	2	2	4	4	1	1	1	1
2	2	2	2	4	4	1	1	1	1
2	2	2	2	4	4	1	1	1	1
2	2	2	2	4	4	1	1	1	1
2	2	2	2	4	4	1	1	4	4
2	2	2	2	4	4	1	1	1	1
2	2	2	2	4	4	1	1	1	1
2	2	2	2	4	4	1	1	1	1
4	4	2	2	4	4	1	1	1	1
4	4	2	2	1	1	2	2	4	4
2	2	2	2	4	4	1	1	4	4
2	2	2	2	4	4	1	1	1	1
2	2	2	2	4	4	1	1	1	1
4	4	2	2	1	1	2	2	4	4
2	2	2	2	4	4	1	1	1	1
2	2	2	2	4	4	1	1	1	1
2	2	2	2	4	4	1	1	1	1
2	2	2	2	4	4	1	1	1	1
2	2	2	2	4	4	1	1	4	4
2	2	2	2	4	4	1	1	1	1
2	2	1	1	4	4	1	1	4	4
2	2	1	1	4	4	1	1	4	4
2	2	2	2	4	4	1	1	4	4
2	2	2	2	4	4	1	1	4	4
2	2	2	2	4	4	1	1	4	4
2	2	2	2	4	4	1	1	4	4
2	2	1	1	4	4	1	1	4	4
2	2	2	2	4	4	1	1	1	1
2	2	2	2	4	4	1	1	1	1
2	2	2	2	4	4	1	1	1	1
4	4	2	2	4	4	1	1	1	1
2	2	2	2	4	4	1	1	1	1
2	2	2	2	4	4	1	1	4	4
2	2	1	1	4	4	1	1	4	4
2	2	2	2	4	4	1	1	4	4
2	2	2	2	4	4	1	1	1	1
2	2	2	2	4	4	1	1	1	1
4	4	2	2	4	4	1	1	1	1
2	2	2	2	4	4	1	1	4	4
4	4	2	2	1	1	2	2	4	4
2	2	2	2	4	4	1	1	4	4
2	2	2	2	4	4	1	1	1	1
2	2	2	2	4	4	1	1	1	1
2	2	2	2	4	4	1	1	4	4
2	2	2	2	4	4	1	1	4	4
2	2	2	2	4	4	1	1	1	1
2	2	2	2	4	4	1	1	1	1

For Review Only

4	4	2	2	4	4	1	1	1	1
2	2	2	2	4	4	1	1	4	4
4	4	2	2	1	1	2	2	4	4
2	2	2	2	4	4	1	1	4	4
4	4	2	2	4	4	1	1	1	1
4	4	2	2	1	1	2	2	4	4
2	2	2	2	4	4	1	1	4	4
2	2	2	2	4	4	1	1	1	1
2	2	2	2	4	4	1	1	4	4
2	2	2	2	4	4	1	1	4	4
2	2	2	2	4	4	1	1	1	1
2	2	2	2	4	4	1	1	1	1
2	2	2	2	4	4	1	1	1	1
2	2	2	2	4	4	1	1	1	1
2	2	2	2	4	4	1	1	1	1
2	2	2	2	4	4	1	1	1	1
2	2	1	1	4	4	1	1	4	4
2	2	2	2	4	4	1	1	1	1
2	2	2	2	4	4	1	1	4	4
2	2	2	2	4	4	1	1	4	4
2	2	2	2	4	4	1	1	1	1
4	4	2	2	4	4	1	1	4	4
4	4	2	2	4	4	2	2	1	1
2	2	2	2	4	4	1	1	1	1
4	4	2	2	4	4	2	2	1	1
4	4	2	2	4	4	1	1	1	1
2	2	2	2	4	4	1	1	1	1
2	2	2	2	4	4	1	1	4	4
4	4	2	2	1	1	2	2	4	4
2	2	2	2	4	4	1	1	4	4
2	2	2	2	4	4	1	1	4	4
2	2	2	2	4	4	1	1	1	1
2	2	2	2	4	4	1	1	4	4
2	2	2	2	4	4	1	1	1	1
2	2	2	2	4	4	1	1	1	1
2	2	2	2	4	4	1	1	1	1
2	2	2	2	4	4	1	1	4	4
2	2	2	2	4	4	1	1	1	1
2	2	2	2	4	4	1	1	1	1
2	2	2	2	4	4	1	1	4	4
2	2	1	1	4	4	1	1	4	4
2	2	2	2	4	4	1	1	4	4
4	4	2	2	1	1	2	2	4	4
2	2	2	2	4	4	1	1	4	4
2	2	1	1	4	4	1	1	1	1
4	4	2	2	4	4	2	2	1	1
4	4	2	2	4	4	1	1	1	1
2	2	1	1	4	4	1	1	4	4
2	2	2	2	4	4	1	1	1	1
4	4	2	2	4	4	2	2	1	1
2	2	2	2	4	4	1	1	1	1
2	2	2	2	4	4	1	1	4	4
2	2	2	2	4	4	2	2	1	1
2	2	2	2	4	4	1	1	1	1
2	2	2	2	4	4	1	1	1	1

For Review Only

4	4	2	2	4	4	1	1	1	1
4	4	2	2	4	4	1	1	1	1
2	2	2	2	4	4	1	1	1	1
4	4	2	2	4	4	2	2	1	1
4	4	2	2	4	4	1	1	1	1
2	2	2	2	4	4	1	1	4	4
2	2	2	2	4	4	1	1	1	1
2	2	2	2	4	4	1	1	1	1
2	2	2	2	4	4	1	1	1	1
2	2	1	1	4	4	1	1	4	4
4	4	2	2	4	4	1	1	1	1
2	2	2	2	4	4	1	1	4	4
4	4	2	2	4	4	1	1	1	1
4	4	2	2	4	4	2	2	1	1
2	2	2	2	4	4	1	1	4	4
2	2	2	2	4	4	1	1	1	1
2	2	2	2	4	4	2	2	1	1
2	2	2	2	4	4	1	1	4	4
2	2	2	2	4	4	1	1	4	4
2	2	2	2	4	4	1	1	4	4
2	2	2	2	4	4	1	1	1	1
2	2	2	2	4	4	1	1	4	4
4	4	2	2	1	1	2	2	4	4
2	2	2	2	4	4	1	1	1	1
2	2	1	1	4	4	1	1	4	4
2	2	2	2	4	4	1	1	1	1
2	2	2	2	4	4	1	1	1	1
2	2	2	2	4	4	1	1	4	4
2	2	1	1	4	4	1	1	1	1
2	2	2	2	4	4	1	1	4	4
2	2	1	1	4	4	1	1	4	4
2	2	1	1	4	4	1	1	4	4
2	2	2	2	4	4	1	1	1	1
2	2	2	2	4	4	1	1	4	4
2	2	2	2	4	4	1	1	1	1
2	2	2	2	4	4	1	1	1	1
2	2	2	2	4	4	1	1	4	4
2	2	2	2	4	4	1	1	1	1
2	2	1	1	4	4	1	1	4	4
2	2	2	2	4	4	1	1	4	4
4	4	2	2	4	4	1	1	1	1
4	4	2	2	4	4	1	1	1	1
2	2	2	2	4	4	1	1	1	1
2	2	2	2	4	4	2	2	4	4
2	2	2	2	4	4	1	1	1	1
2	2	2	2	4	4	1	1	1	1
2	2	2	2	4	4	1	1	1	1
2	2	2	2	4	4	1	1	4	4
2	2	2	2	4	4	1	1	4	4
2	2	2	2	4	4	1	1	1	1
4	4	2	2	1	1	2	2	4	4
2	2	2	2	4	4	1	1	1	1
4	4	2	2	4	4	1	1	1	1
2	2	2	2	4	4	1	1	1	1
2	2	2	2	4	4	1	1	4	4

For Review Only

4	4	2	2	1	1	2	2	4	4
2	2	2	2	4	4	1	1	4	4
4	4	2	2	4	4	1	1	1	1
2	2	2	2	4	4	2	2	4	4
2	2	2	2	4	4	1	1	1	1
2	2	1	1	4	4	1	1	4	4
2	2	2	2	4	4	1	1	4	4
4	4	2	2	4	4	1	1	4	4
4	4	2	2	4	4	1	1	4	4
2	2	2	2	4	4	1	1	4	4
2	2	2	2	4	4	1	1	1	1
2	2	2	2	4	4	1	1	1	1
4	4	2	2	4	4	1	1	1	1
4	4	2	2	1	1	2	2	4	4
4	4	2	2	4	4	1	1	1	1
4	4	2	2	4	4	1	1	1	1
4	4	2	2	4	4	1	1	1	1
2	2	2	2	4	4	1	1	4	4
2	2	2	2	4	4	1	1	1	1
2	2	2	2	4	4	1	1	1	1
2	2	2	2	4	4	1	1	1	1
2	2	1	1	4	4	1	1	4	4
2	2	2	2	4	4	1	1	4	4
4	4	2	2	4	4	1	1	1	1
2	2	2	2	4	4	1	1	1	1
2	2	2	2	4	4	1	1	4	4
2	2	2	2	4	4	1	1	4	4
4	4	2	2	4	4	1	1	1	1
2	2	2	2	4	4	1	1	4	4
4	4	2	2	1	1	2	2	4	4
4	4	2	2	4	4	1	1	1	1
2	2	2	2	4	4	1	1	4	4
2	2	2	2	4	4	1	1	1	1
2	2	1	1	4	4	1	1	4	4
4	4	2	2	4	4	1	1	1	1
2	2	2	2	4	4	1	1	4	4
4	4	2	2	1	1	2	2	4	4
2	2	2	2	4	4	1	1	1	1
2	2	2	2	4	4	1	1	4	4
2	2	2	2	4	4	1	1	4	4
2	2	2	2	4	4	1	1	4	4
2	2	2	2	4	4	1	1	1	1
4	4	2	2	4	4	1	1	1	1
2	2	2	2	4	4	1	1	4	4
2	2	2	2	4	4	1	1	1	1
4	4	2	2	4	4	1	1	1	1
2	2	1	1	4	4	1	1	4	4
2	2	2	2	4	4	1	1	1	1
2	2	2	2	4	4	1	1	1	1
2	2	2	2	4	4	1	1	4	4
2	2	2	2	4	4	1	1	4	4
2	2	2	2	4	4	1	1	4	4
4	4	2	2	4	4	1	1	1	1
4	4	2	2	4	4	1	1	1	1
2	2	2	2	4	4	1	1	4	4

For Review Only

2	2	2	2	4	4	1	1	1	1
2	2	2	2	4	4	1	1	1	1

For Review Only

SNP2959	SNP2959	SNP296	SNP296	SNP2960	SNP2960	SNP2961	SNP2961	SNP2962	SNP2962
1	1	2	2	2	2	4	4	2	2
1	1	3	3	2	2	4	4	1	1
1	1	3	3	2	2	4	4	2	2
1	1	3	3	2	2	4	4	2	2
1	1	2	2	2	2	4	4	2	2
1	1	2	2	2	2	4	4	2	2
1	1	2	2	2	2	4	4	2	2
1	1	3	3	2	2	4	4	2	2
1	1	2	2	2	2	4	4	2	2
1	1	3	3	2	2	4	4	2	2
1	1	2	2	2	2	4	4	1	1
1	1	3	3	2	2	4	4	2	2
4	4	2	2	2	2	4	4	2	2
1	1	3	3	2	2	4	4	2	2
1	1	3	3	2	2	4	4	2	2
1	1	3	3	2	2	4	4	2	2
1	1	3	3	3	3	1	1	2	2
1	1	2	2	2	2	4	4	2	2
1	1	3	3	2	2	4	4	2	2
1	1	2	2	2	2	4	4	2	2
1	1	2	2	2	2	4	4	1	1
1	1	2	2	2	2	4	4	2	2
1	1	3	3	2	2	4	4	1	1
1	1	3	3	2	2	4	4	2	2
1	1	2	2	2	2	4	4	2	2
1	1	3	3	2	2	4	4	2	2
1	1	3	3	2	2	4	4	2	2
1	1	3	3	2	2	4	4	1	1
1	1	2	2	2	2	4	4	2	2
1	1	2	2	3	3	1	1	2	2
4	4	3	3	3	3	1	1	2	2
1	1	3	3	2	2	4	4	2	2
1	1	2	2	2	2	4	4	2	2
1	1	2	2	3	3	1	1	2	2
1	1	3	3	2	2	4	4	2	2
1	1	3	3	2	2	4	4	2	2
1	1	3	3	2	2	4	4	2	2
1	1	2	2	2	2	4	4	1	1
1	1	2	2	2	2	4	4	1	1
1	1	3	3	2	2	4	4	1	1
1	1	2	2	2	2	4	4	1	1
1	1	2	2	2	2	4	4	1	1
1	1	2	2	2	2	4	4	2	2
1	1	3	3	3	3	1	1	2	2
1	1	3	3	2	2	4	4	2	2
1	1	3	3	2	2	4	4	2	2
1	1	3	3	2	2	4	4	2	2
1	1	3	3	2	2	4	4	2	2
1	1	2	2	3	3	1	1	2	2
1	1	3	3	2	2	4	4	2	2
1	1	3	3	2	2	4	4	2	2
1	1	3	3	2	2	4	4	2	2

For Review Only

1	1	2	2	2	2	4	4	2	2
1	1	2	2	2	2	4	4	2	2
1	1	3	3	2	2	4	4	2	2
1	1	2	2	2	2	4	4	2	2
1	1	3	3	2	2	4	4	2	2
1	1	3	3	2	2	4	4	2	2
1	1	3	3	3	3	1	1	2	2
1	1	3	3	2	2	4	4	2	2
1	1	2	2	2	2	4	4	2	2
1	1	3	3	2	2	4	4	1	1
1	1	3	3	2	2	4	4	2	2
1	1	3	3	2	2	4	4	2	2
1	1	2	2	2	2	4	4	2	2
1	1	2	2	2	2	4	4	2	2
1	1	3	3	2	2	4	4	2	2
1	1	2	2	2	2	4	4	2	2
1	1	2	2	2	2	4	4	2	2
4	4	3	3	3	3	1	1	2	2
1	1	3	3	2	2	4	4	2	2
1	1	2	2	2	2	4	4	2	2
1	1	3	3	2	2	4	4	2	2
4	4	3	3	2	2	4	4	1	1
1	1	2	2	2	2	4	4	2	2
1	1	2	2	2	2	4	4	2	2
1	1	3	3	2	2	4	4	2	2
1	1	3	3	2	2	4	4	1	1
1	1	2	2	2	2	4	4	2	2
1	1	2	2	2	2	4	4	2	2
1	1	3	3	2	2	4	4	2	2
1	1	2	2	2	2	4	4	1	1
1	1	3	3	2	2	4	4	2	2
1	1	2	2	2	2	4	4	2	2
1	1	2	2	2	2	4	4	2	2
1	1	2	2	2	2	4	4	2	2
1	1	2	2	2	2	4	4	2	2
1	1	3	3	2	2	4	4	2	2
1	1	3	3	2	2	4	4	1	1
1	1	2	2	2	2	4	4	2	2
1	1	2	2	2	2	4	4	1	1
1	1	3	3	2	2	4	4	2	2
1	1	2	2	2	2	4	4	2	2
1	1	2	2	2	2	4	4	2	2
1	1	3	3	2	2	4	4	2	2
1	1	3	3	2	2	4	4	2	2
1	1	2	2	2	2	4	4	2	2
1	1	3	3	3	3	1	1	1	1
1	1	3	3	2	2	4	4	2	2
4	4	3	3	2	2	4	4	2	2
1	1	3	3	2	2	4	4	2	2
1	1	3	3	2	2	4	4	1	1
1	1	3	3	2	2	4	4	2	2
1	1	2	2	2	2	4	4	1	1
1	1	3	3	2	2	4	4	2	2
1	1	3	3	2	2	4	4	2	2
1	1	3	3	2	2	4	4	2	2

For Review Only

1	1	2	2	2	2	4	4	2	2
1	1	3	3	3	3	1	1	2	2
4	4	3	3	3	3	1	1	2	2
1	1	3	3	2	2	4	4	1	1
1	1	3	3	2	2	4	4	1	1
4	4	2	2	3	3	1	1	2	2
1	1	3	3	2	2	4	4	1	1
1	1	3	3	2	2	4	4	1	1
1	1	3	3	2	2	4	4	1	1
1	1	3	3	2	2	4	4	1	1
1	1	2	2	2	2	4	4	2	2
1	1	3	3	2	2	4	4	1	1
1	1	3	3	2	2	4	4	2	2
1	1	3	3	2	2	4	4	1	1
1	1	3	3	2	2	4	4	2	2
1	1	3	3	2	2	4	4	2	2
1	1	3	3	2	2	4	4	2	2
1	1	3	3	2	2	4	4	1	1
1	1	3	3	2	2	4	4	2	2
1	1	3	3	2	2	4	4	1	1
1	1	3	3	2	2	4	4	1	1
4	4	2	2	2	2	4	4	2	2
4	4	3	3	3	3	1	1	2	2
1	1	2	2	2	2	4	4	1	1
1	1	3	3	2	2	4	4	1	1
1	1	3	3	2	2	4	4	1	1
1	1	3	3	2	2	4	4	2	2
1	1	3	3	2	2	4	4	2	2
4	4	2	2	2	2	4	4	2	2
1	1	2	2	2	2	4	4	2	2
1	1	3	3	2	2	4	4	1	1
1	1	3	3	2	2	4	4	2	2
1	1	2	2	2	2	4	4	2	2
1	1	3	3	2	2	4	4	2	2
1	1	2	2	3	3	1	1	2	2
1	1	2	2	2	2	4	4	2	2
1	1	3	3	2	2	4	4	1	1
1	1	3	3	2	2	4	4	2	2
1	1	3	3	2	2	4	4	2	2
1	1	3	3	2	2	4	4	1	1
1	1	2	2	2	2	4	4	1	1
1	1	3	3	2	2	4	4	1	1
4	4	3	3	3	3	1	1	2	2
1	1	3	3	2	2	4	4	2	2
1	1	3	3	2	2	4	4	2	2
4	4	3	3	3	3	1	1	2	2
1	1	3	3	2	2	4	4	2	2
1	1	3	3	2	2	4	4	1	1
1	1	3	3	2	2	4	4	2	2
4	4	3	3	2	2	4	4	2	2
1	1	3	3	2	2	4	4	2	2
1	1	2	2	2	2	4	4	2	2
4	4	2	2	3	3	4	4	2	2
1	1	3	3	2	2	4	4	2	2
1	1	3	3	2	2	4	4	1	1

1	1	3	3	2	2	4	4	2	2
1	1	2	2	2	2	4	4	1	1
1	1	3	3	2	2	4	4	2	2
4	4	3	3	2	2	4	4	1	1
1	1	2	2	3	3	1	1	1	1
1	1	3	3	2	2	4	4	2	2
1	1	2	2	2	2	4	4	1	1
1	1	3	3	2	2	4	4	1	1
1	1	2	2	2	2	4	4	1	1
1	1	2	2	2	2	4	4	2	2
1	1	3	3	2	2	4	4	1	1
1	1	3	3	2	2	4	4	1	1
1	1	3	3	2	2	4	4	1	1
4	4	3	3	3	3	1	1	2	2
1	1	3	3	2	2	4	4	1	1
1	1	3	3	2	2	4	4	1	1
4	4	2	2	3	3	4	4	2	2
1	1	3	3	2	2	4	4	1	1
4	4	3	3	2	2	4	4	2	2
1	1	3	3	2	2	4	4	1	1
1	1	3	3	3	3	4	4	2	2
1	1	2	2	2	2	4	4	2	2
4	4	3	3	2	2	4	4	1	1
1	1	3	3	2	2	4	4	2	2
1	1	3	3	2	2	4	4	2	2
1	1	2	2	2	2	4	4	2	2
1	1	3	3	2	2	4	4	1	1
1	1	3	3	2	2	4	4	2	2
1	1	3	3	2	2	4	4	2	2
1	1	2	2	2	2	4	4	2	2
1	1	3	3	2	2	4	4	2	2
1	1	2	2	2	2	4	4	2	2
1	1	3	3	2	2	4	4	2	2
1	1	3	3	2	2	4	4	1	1
1	1	3	3	2	2	4	4	2	2
1	1	3	3	2	2	4	4	2	2
1	1	3	3	2	2	4	4	2	2
1	1	2	2	2	2	4	4	2	2
1	1	3	3	2	2	4	4	1	1
1	1	3	3	2	2	4	4	2	2
1	1	3	3	2	2	4	4	2	2
1	1	3	3	2	2	4	4	2	2
1	1	3	3	2	2	4	4	1	1
4	4	3	3	2	2	4	4	1	1
1	1	3	3	2	2	4	4	2	2
1	1	3	3	2	2	4	4	2	2
1	1	3	3	2	2	4	4	1	1
1	1	2	2	2	2	4	4	2	2
1	1	3	3	2	2	4	4	2	2
1	1	3	3	2	2	4	4	2	2
4	4	2	2	2	2	4	4	2	2
1	1	3	3	2	2	4	4	2	2
1	1	2	2	2	2	4	4	1	1
1	1	3	3	2	2	4	4	2	2
1	1	3	3	2	2	4	4	1	1

For Review Only

4	4	2	2	3	3	1	1	2	2
1	1	3	3	3	3	4	4	2	2
1	1	2	2	2	2	4	4	1	1
4	4	3	3	2	2	4	4	1	1
1	1	2	2	2	2	4	4	2	2
1	1	3	3	2	2	4	4	2	2
1	1	3	3	2	2	4	4	2	2
1	1	2	2	3	3	1	1	2	2
1	1	3	3	3	3	1	1	2	2
1	1	2	2	2	2	4	4	1	1
1	1	3	3	2	2	4	4	1	1
1	1	3	3	2	2	4	4	2	2
1	1	2	2	2	2	4	4	1	1
4	4	2	2	2	2	4	4	2	2
1	1	3	3	2	2	4	4	2	2
1	1	3	3	2	2	4	4	2	2
1	1	2	2	2	2	4	4	1	1
1	1	3	3	2	2	4	4	2	2
1	1	2	2	2	2	4	4	2	2
4	4	2	2	2	2	4	4	1	1
1	1	3	3	2	2	4	4	1	1
1	1	3	3	2	2	4	4	2	2
1	1	2	2	2	2	4	4	2	2
1	1	2	2	2	2	4	4	1	1
1	1	3	3	2	2	4	4	2	2
1	1	3	3	2	2	4	4	2	2
1	1	3	3	2	2	4	4	2	2
1	1	3	3	3	3	1	1	1	1
1	1	2	2	2	2	4	4	2	2
4	4	2	2	2	2	4	4	2	2
1	1	2	2	2	2	4	4	1	1
1	1	3	3	2	2	4	4	2	2
1	1	3	3	2	2	4	4	2	2
1	1	3	3	2	2	4	4	2	2
1	1	3	3	2	2	4	4	1	1
1	1	2	2	2	2	4	4	2	2
4	4	2	2	3	3	1	1	2	2
1	1	3	3	2	2	4	4	2	2
1	1	3	3	2	2	4	4	2	2
1	1	2	2	2	2	4	4	2	2
1	1	3	3	2	2	4	4	2	2
1	1	2	2	2	2	4	4	2	2
1	1	3	3	2	2	4	4	1	1
1	1	2	2	2	2	4	4	2	2
1	1	3	3	2	2	4	4	2	2
1	1	2	2	2	2	4	4	1	1
1	1	3	3	2	2	4	4	1	1
1	1	3	3	2	2	4	4	2	2
1	1	3	3	2	2	4	4	2	2
1	1	2	2	2	2	4	4	2	2
1	1	3	3	2	2	4	4	1	1
1	1	2	2	2	2	4	4	1	1
1	1	3	3	2	2	4	4	2	2
1	1	3	3	3	3	1	1	2	2
1	1	3	3	2	2	4	4	1	1

For Review Only

1	1	3	3	2	2	4	4	2	2
1	1	2	2	2	2	4	4	1	1

For Review Only

SNP2963	SNP2963	SNP2964	SNP2964	SNP2965	SNP2965	SNP2966	SNP2966	SNP2967	SNP2967
4	4	1	1	1	1	4	4	3	3
3	3	1	1	1	1	4	4	3	3
4	4	1	1	1	1	4	4	3	3
4	4	1	1	1	1	4	4	3	3
4	4	1	1	1	1	4	4	3	3
4	4	1	1	1	1	4	4	3	3
4	4	1	1	1	1	4	4	3	3
4	4	1	1	1	1	4	4	4	4
4	4	1	1	1	1	4	4	3	3
4	4	4	4	1	1	4	4	3	3
4	4	4	4	4	4	4	4	3	3
4	4	1	1	1	1	4	4	3	3
4	4	1	1	1	1	4	4	3	3
4	4	1	1	1	1	4	4	3	3
4	4	4	4	1	1	4	4	3	3
4	4	1	1	1	1	4	4	3	3
4	4	4	4	1	1	1	1	3	3
4	4	1	1	1	1	4	4	3	3
4	4	1	1	1	1	4	4	3	3
4	4	1	1	1	1	4	4	3	3
3	3	1	1	1	1	4	4	3	3
4	4	1	1	1	1	4	4	3	3
4	4	4	4	4	4	4	4	3	3
3	3	1	1	1	1	4	4	3	3
4	4	1	1	1	1	4	4	3	3
4	4	1	1	1	1	4	4	4	4
4	4	1	1	1	1	4	4	3	3
4	4	4	4	1	1	4	4	3	3
4	4	1	1	1	1	4	4	3	3
4	4	4	4	1	1	1	1	3	3
4	4	1	1	1	1	4	4	3	3
4	4	1	1	1	1	4	4	3	3
4	4	1	1	1	1	4	4	3	3
4	4	1	1	1	1	1	1	3	3
4	4	4	4	1	1	4	4	3	3
4	4	1	1	1	1	1	1	3	3
4	4	4	4	1	1	4	4	3	3
4	4	4	4	1	1	4	4	3	3
4	4	4	4	4	4	1	1	3	3
4	4	4	4	1	1	4	4	3	3
4	4	4	4	1	1	4	4	3	3
3	3	1	1	1	1	4	4	3	3
4	4	1	1	1	1	4	4	3	3
4	4	1	1	1	1	1	1	3	3
4	4	1	1	1	1	4	4	3	3
4	4	1	1	1	1	4	4	3	3
4	4	1	1	1	1	4	4	3	3
4	4	4	4	1	1	4	4	3	3
4	4	1	1	1	1	1	1	3	3
4	4	4	4	1	1	4	4	3	3
4	4	1	1	1	1	4	4	3	3
3	3	1	1	1	1	4	4	3	3

For Review Only

4	4	1	1	1	1	4	4	3	3
4	4	1	1	1	1	4	4	3	3
4	4	1	1	1	1	4	4	3	3
4	4	1	1	1	1	4	4	3	3
4	4	1	1	1	1	4	4	3	3
4	4	1	1	1	1	4	4	3	3
4	4	1	1	1	1	4	4	3	3
4	4	4	4	1	1	4	4	3	3
4	4	1	1	1	1	4	4	3	3
4	4	4	4	1	1	4	4	3	3
4	4	1	1	1	1	4	4	3	3
4	4	1	1	1	1	4	4	3	3
4	4	1	1	1	1	4	4	3	3
4	4	1	1	1	1	4	4	3	3
4	4	4	4	1	1	4	4	3	3
4	4	4	4	1	1	4	4	3	3
4	4	4	4	1	1	4	4	3	3
4	4	4	4	1	1	4	4	3	3
4	4	1	1	1	1	4	4	3	3
4	4	1	1	1	1	4	4	3	3
4	4	4	4	1	1	4	4	3	3
4	4	4	4	1	1	4	4	3	3
4	4	1	1	1	1	4	4	3	3
4	4	1	1	1	1	4	4	3	3
4	4	1	1	1	1	4	4	3	3
3	3	1	1	1	1	4	4	3	3
4	4	4	4	1	1	1	1	3	3
4	4	4	4	1	1	4	4	3	3
4	4	1	1	1	1	4	4	4	4
3	3	4	4	1	1	4	4	3	3
4	4	1	1	1	1	4	4	3	3
4	4	4	4	1	1	4	4	3	3
4	4	4	4	1	1	4	4	3	3
4	4	1	1	1	1	4	4	3	3
4	4	1	1	1	1	4	4	3	3
4	4	1	1	1	1	4	4	3	3
4	4	4	4	1	1	4	4	3	3
4	4	4	4	1	1	1	1	3	3
4	4	4	4	1	1	4	4	3	3
4	4	1	1	1	1	4	4	3	3
4	4	1	1	1	1	4	4	4	4
4	4	1	1	1	1	4	4	3	3
4	4	1	1	1	1	4	4	3	3
4	4	1	1	1	1	4	4	3	3
4	4	4	4	1	1	4	4	3	3
3	3	1	1	1	1	4	4	3	3
4	4	1	1	1	1	4	4	3	3
4	4	1	1	1	1	4	4	3	3
4	4	1	1	1	1	1	1	3	3
4	4	4	4	1	1	4	4	3	3
4	4	1	1	1	1	4	4	3	3
4	4	4	4	4	4	4	4	3	3
4	4	4	4	1	1	4	4	3	3
4	4	4	4	1	1	4	4	3	3
4	4	4	4	1	1	4	4	3	3

4	4	4	4	1	1	4	4	3	3
4	4	4	4	1	1	1	1	3	3
4	4	1	1	1	1	4	4	3	3
4	4	1	1	1	1	4	4	3	3
4	4	1	1	1	1	4	4	3	3
4	4	1	1	1	1	4	4	3	3
4	4	1	1	1	1	4	4	3	3
4	4	1	1	1	1	4	4	4	4
4	4	1	1	1	1	1	1	3	3
4	4	4	4	1	1	4	4	3	3
4	4	4	4	1	1	4	4	3	3
4	4	4	4	1	1	4	4	3	3
4	4	4	4	1	1	4	4	3	3
4	4	4	4	1	1	4	4	3	3
4	4	4	4	1	1	4	4	3	3
4	4	4	4	1	1	4	4	3	3
4	4	4	4	1	1	4	4	4	4
4	4	4	4	1	1	4	4	3	3
4	4	1	1	1	1	4	4	3	3
4	4	4	4	1	1	4	4	3	3
4	4	4	4	1	1	4	4	3	3
4	4	4	4	1	1	4	4	3	3
4	4	1	1	1	1	4	4	3	3
4	4	4	4	1	1	4	4	3	3
4	4	4	4	1	1	4	4	3	3
4	4	4	4	1	1	4	4	3	3
4	4	1	1	1	1	4	4	3	3
4	4	4	4	1	1	4	4	3	3
4	4	1	1	1	1	4	4	3	3
4	4	4	4	1	1	4	4	3	3
3	3	1	1	1	1	4	4	3	3
4	4	4	4	1	1	4	4	3	3
4	4	1	1	1	1	4	4	4	4
4	4	4	4	1	1	4	4	3	3
4	4	1	1	1	1	1	1	3	3
4	4	1	1	1	1	4	4	3	3
4	4	1	1	1	1	4	4	3	3
4	4	4	4	1	1	4	4	3	3
4	4	4	4	4	4	4	4	3	3
4	4	4	4	4	4	4	4	3	3
4	4	4	4	1	1	4	4	4	4
3	3	1	1	1	1	4	4	3	3
4	4	4	4	1	1	4	4	3	3
4	4	4	4	1	1	4	4	3	3
4	4	4	4	1	1	4	4	3	3
4	4	4	4	1	1	4	4	3	3
4	4	4	4	1	1	4	4	3	3
4	4	1	1	1	1	4	4	4	4
4	4	4	4	1	1	4	4	3	3
4	4	1	1	1	1	4	4	3	3
4	4	4	4	1	1	4	4	3	3
4	4	4	4	1	1	4	4	3	3
4	4	1	1	1	1	4	4	4	4
4	4	1	1	1	1	4	4	3	3
4	4	4	4	1	1	4	4	3	3

For Review Only

4	4	1	1	1	1	4	4	3	3
4	4	4	4	1	1	4	4	3	3
4	4	1	1	1	1	4	4	3	3
4	4	4	4	4	4	4	4	3	3
3	3	1	1	1	1	4	4	3	3
4	4	1	1	1	1	4	4	3	3
4	4	1	1	1	1	4	4	3	3
4	4	4	4	1	1	4	4	3	3
4	4	4	4	1	1	1	1	3	3
4	4	1	1	1	1	4	4	3	3
4	4	4	4	1	1	4	4	3	3
4	4	4	4	1	1	4	4	3	3
4	4	4	4	1	1	4	4	3	3
4	4	4	4	1	1	4	4	3	3
4	4	1	1	1	1	4	4	3	3
4	4	4	4	4	4	4	4	3	3
4	4	1	1	1	1	4	4	3	3
4	4	1	1	1	1	4	4	3	3
4	4	1	1	1	1	4	4	3	3
4	4	1	1	1	1	4	4	3	3
4	4	4	4	1	1	4	4	3	3
4	4	4	4	1	1	4	4	3	3
4	4	4	4	1	1	4	4	3	3
4	4	1	1	1	1	4	4	3	3
4	4	1	1	1	1	4	4	4	4
4	4	1	1	1	1	4	4	3	3
4	4	4	4	1	1	4	4	3	3
4	4	4	4	1	1	4	4	3	3
4	4	4	4	1	1	4	4	4	4
4	4	1	1	1	1	4	4	3	3
4	4	1	1	1	1	4	4	3	3
4	4	4	4	1	1	4	4	3	3
4	4	1	1	1	1	4	4	3	3
4	4	1	1	1	1	4	4	3	3
4	4	1	1	1	1	4	4	3	3
4	4	1	1	1	1	4	4	3	3
4	4	4	4	1	1	4	4	3	3
4	4	4	4	1	1	4	4	3	3
4	4	1	1	1	1	4	4	4	4
4	4	4	4	1	1	4	4	3	3
4	4	1	1	1	1	4	4	3	3
4	4	4	4	1	1	4	4	3	3
4	4	4	4	4	4	4	4	3	3
4	4	1	1	1	1	4	4	3	3
4	4	1	1	1	1	4	4	3	3
4	4	1	1	1	1	4	4	3	3
4	4	4	4	4	4	4	4	3	3
4	4	1	1	1	1	4	4	3	3
4	4	1	1	1	1	4	4	3	3
4	4	1	1	1	1	4	4	3	3
4	4	1	1	1	1	4	4	3	3
4	4	4	4	1	1	4	4	3	3
4	4	4	4	1	1	4	4	3	3
4	4	1	1	1	1	4	4	3	3
4	4	4	4	1	1	4	4	3	3

For Review Only

4	4	1	1	1	1	4	4	3	3
4	4	4	4	1	1	4	4	3	3
4	4	4	4	1	1	4	4	3	3
4	4	4	4	1	1	4	4	3	3
4	4	1	1	1	1	4	4	3	3
4	4	1	1	1	1	4	4	3	3
4	4	4	4	1	1	4	4	3	3
4	4	1	1	1	1	1	1	3	3
4	4	1	1	1	1	4	4	3	3
4	4	4	4	4	4	4	4	3	3
4	4	4	4	1	1	4	4	3	3
4	4	1	1	1	1	4	4	3	3
4	4	4	4	1	1	4	4	3	3
4	4	4	4	1	1	4	4	3	3
4	4	1	1	1	1	4	4	3	3
4	4	1	1	1	1	4	4	3	3
4	4	4	4	4	4	4	4	3	3
4	4	4	4	1	1	1	1	3	3
4	4	4	4	1	1	4	4	3	3
4	4	4	4	1	1	4	4	3	3
4	4	1	1	1	1	4	4	3	3
4	4	1	1	1	1	4	4	4	4
4	4	1	1	1	1	1	1	3	3
4	4	4	4	1	1	4	4	3	3
4	4	4	4	1	1	4	4	3	3
4	4	1	1	1	1	4	4	3	3
4	4	4	4	1	1	4	4	3	3
4	4	1	1	1	1	4	4	3	3
4	4	1	1	1	1	4	4	3	3
4	4	1	1	1	1	4	4	3	3
4	4	4	4	4	4	4	4	3	3
4	4	4	4	1	1	1	1	3	3
4	4	1	1	1	1	4	4	3	3
4	4	1	1	1	1	4	4	4	4
4	4	4	4	1	1	4	4	3	3
3	3	1	1	1	1	4	4	3	3
4	4	4	4	1	1	4	4	3	3
4	4	1	1	1	1	4	4	3	3
4	4	1	1	1	1	1	1	3	3
4	4	4	4	1	1	1	1	3	3
4	4	1	1	1	1	4	4	3	3
4	4	1	1	1	1	4	4	3	3
4	4	4	4	1	1	4	4	3	3
4	4	1	1	1	1	4	4	3	3
4	4	1	1	1	1	4	4	3	3
4	4	4	4	1	1	4	4	3	3
4	4	4	4	4	4	4	4	4	4
4	4	4	4	1	1	4	4	3	3
4	4	1	1	1	1	4	4	3	3
4	4	4	4	1	1	4	4	3	3
3	3	1	1	1	1	4	4	3	3
4	4	4	4	4	4	1	1	3	3
4	4	1	1	1	1	4	4	3	3
4	4	1	1	1	1	4	4	3	3
4	4	4	4	1	1	4	4	3	3

For Review Only

4	4	1	1	1	1	4	4	3	3
4	4	1	1	1	1	4	4	3	3

For Review Only

SNP2968	SNP2968	SNP2969	SNP2969	SNP297	SNP297	SNP2970	SNP2970	SNP2971	SNP2971
1	1	1	1	4	4	3	3	4	4
1	1	1	1	4	4	3	3	4	4
1	1	1	1	1	1	3	3	4	4
1	1	1	1	1	1	3	3	4	4
4	4	4	4	4	4	2	2	1	1
1	1	1	1	4	4	3	3	4	4
1	1	1	1	4	4	3	3	4	4
1	1	1	1	4	4	3	3	4	4
1	1	1	1	4	4	3	3	4	4
1	1	1	1	4	4	3	3	4	4
1	1	4	4	4	4	2	2	1	1
1	1	1	1	1	1	3	3	4	4
1	1	1	1	4	4	3	3	4	4
1	1	1	1	4	4	3	3	4	4
1	1	4	4	4	4	3	3	4	4
1	1	1	1	1	1	3	3	4	4
1	1	1	1	4	4	3	3	4	4
1	1	1	1	4	4	3	3	4	4
1	1	1	1	1	1	3	3	4	4
1	1	1	1	4	4	3	3	4	4
1	1	1	1	4	4	3	3	4	4
4	4	4	4	4	4	2	2	1	1
1	1	1	1	4	4	3	3	4	4
1	1	1	1	4	4	3	3	4	4
1	1	1	1	4	4	3	3	4	4
1	1	1	1	4	4	3	3	4	4
1	1	1	1	4	4	3	3	4	4
1	1	1	1	1	1	3	3	4	4
1	1	1	1	4	4	3	3	4	4
1	1	1	1	4	4	3	3	4	4
1	1	1	1	4	4	3	3	4	4
1	1	1	1	1	1	3	3	4	4
1	1	1	1	4	4	3	3	4	4
1	1	1	1	4	4	3	3	4	4
1	1	1	1	4	4	3	3	4	4
1	1	1	1	1	1	3	3	4	4
1	1	1	1	4	4	3	3	4	4
1	1	1	1	4	4	3	3	4	4
1	1	1	1	4	4	3	3	4	4
1	1	1	1	4	4	3	3	4	4
1	1	1	1	4	4	3	3	4	4
1	1	1	1	4	4	3	3	4	4
1	1	1	1	4	4	3	3	4	4
1	1	1	1	4	4	3	3	4	4
1	1	1	1	4	4	2	2	4	4
1	1	1	1	1	1	3	3	4	4
1	1	1	1	4	4	3	3	4	4
1	1	1	1	4	4	3	3	4	4
1	1	1	1	4	4	3	3	4	4
1	1	4	4	4	4	2	2	1	1
1	1	1	1	4	4	3	3	4	4
1	1	1	1	4	4	3	3	4	4

For Review Only

1	1	1	1	4	4	3	3	4	4
1	1	1	1	4	4	3	3	4	4
1	1	1	1	1	1	3	3	4	4
4	4	4	4	4	4	2	2	1	1
1	1	1	1	1	1	3	3	4	4
1	1	1	1	4	4	3	3	4	4
1	1	1	1	4	4	3	3	4	4
1	1	1	1	4	4	3	3	4	4
1	1	1	1	1	1	3	3	4	4
1	1	1	1	4	4	3	3	4	4
1	1	1	1	1	1	3	3	4	4
1	1	1	1	4	4	3	3	4	4
1	1	1	1	4	4	3	3	4	4
1	1	1	1	4	4	3	3	4	4
1	1	1	1	4	4	3	3	4	4
1	1	1	1	4	4	3	3	4	4
1	1	1	1	4	4	3	3	4	4
1	1	1	1	4	4	3	3	4	4
4	4	4	4	1	1	2	2	1	1
1	1	1	1	4	4	3	3	4	4
1	1	1	1	4	4	3	3	4	4
1	1	1	1	4	4	3	3	4	4
1	1	1	1	4	4	3	3	4	4
1	1	1	1	4	4	3	3	4	4
1	1	1	1	4	4	2	2	4	4
1	1	1	1	4	4	3	3	4	4
1	1	1	1	4	4	3	3	4	4
1	1	1	1	4	4	3	3	4	4
1	1	1	1	4	4	3	3	4	4
4	4	4	4	1	1	2	2	1	1
4	4	4	4	4	4	2	2	1	1
1	1	1	1	4	4	3	3	4	4
1	1	1	1	4	4	2	2	4	4
1	1	1	1	4	4	3	3	4	4
1	1	1	1	4	4	3	3	4	4
1	1	1	1	4	4	2	2	4	4
1	1	1	1	4	4	3	3	4	4
1	1	1	1	4	4	3	3	4	4
1	1	1	1	1	1	3	3	4	4
1	1	1	1	4	4	3	3	4	4
1	1	1	1	4	4	3	3	4	4
4	4	4	4	1	1	3	3	1	1
1	1	1	1	1	1	3	3	4	4
1	1	1	1	4	4	3	3	4	4
1	1	1	1	4	4	2	2	4	4
1	1	1	1	4	4	3	3	4	4
1	1	1	1	4	4	3	3	4	4
1	1	1	1	4	4	3	3	4	4
1	1	1	1	4	4	3	3	4	4
1	1	1	1	4	4	3	3	4	4
1	1	1	1	4	4	3	3	4	4
1	1	1	1	4	4	3	3	4	4
1	1	1	1	1	1	3	3	4	4
1	1	1	1	1	1	3	3	4	4
1	1	1	1	4	4	3	3	4	4

For Review Only

1	1	1	1	4	4	3	3	4	4
1	1	1	1	4	4	3	3	4	4
1	1	1	1	4	4	3	3	4	4
1	1	1	1	4	4	3	3	4	4
1	1	1	1	1	1	3	3	4	4
1	1	1	1	4	4	3	3	4	4
1	1	1	1	1	1	3	3	4	4
1	1	1	1	4	4	3	3	4	4
1	1	1	1	4	4	3	3	4	4
1	1	1	1	4	4	3	3	4	4
1	1	1	1	4	4	3	3	4	4
1	1	1	1	4	4	3	3	4	4
1	1	1	1	4	4	3	3	4	4
1	1	1	1	4	4	3	3	4	4
1	1	1	1	4	4	3	3	4	4
1	1	1	1	4	4	3	3	4	4
1	1	1	1	4	4	3	3	4	4
1	1	1	1	4	4	3	3	4	4
1	1	1	1	4	4	3	3	4	4
4	4	4	4	1	1	2	2	1	1
1	1	1	1	4	4	3	3	4	4
1	1	1	1	4	4	3	3	4	4
1	1	1	1	4	4	3	3	4	4
1	1	1	1	4	4	3	3	4	4
1	1	1	1	4	4	3	3	4	4
1	1	1	1	4	4	3	3	4	4
1	1	1	1	1	1	3	3	4	4
1	1	1	1	4	4	3	3	4	4
1	1	1	1	1	1	3	3	4	4
1	1	1	1	4	4	3	3	4	4
1	1	1	1	4	4	3	3	4	4
1	1	1	1	4	4	3	3	4	4
1	1	1	1	4	4	3	3	4	4
1	1	1	1	4	4	3	3	4	4
1	1	1	1	4	4	3	3	4	4
1	1	1	1	4	4	2	2	4	4
1	1	1	1	4	4	3	3	4	4
4	4	4	4	4	4	2	2	1	1
1	1	1	1	4	4	3	3	4	4
1	1	1	1	4	4	2	2	4	4
1	1	1	1	4	4	3	3	4	4
1	1	1	1	4	4	3	3	4	4
1	1	1	1	4	4	3	3	4	4
1	1	1	1	4	4	3	3	4	4
1	1	1	1	4	4	3	3	4	4
1	1	1	1	4	4	3	3	4	4
1	1	1	1	4	4	3	3	4	4
1	1	1	1	4	4	3	3	4	4
1	1	1	1	4	4	3	3	4	4
1	1	1	1	4	4	3	3	4	4
1	1	1	1	4	4	3	3	4	4
1	1	1	1	4	4	3	3	4	4
1	1	1	1	4	4	3	3	4	4
1	1	1	1	4	4	3	3	4	4
1	1	1	1	4	4	3	3	4	4

For Review Only

1	1	1	1	4	4	3	3	4	4
1	1	1	1	4	4	3	3	4	4
1	1	1	1	4	4	2	2	4	4
1	1	1	1	4	4	3	3	4	4
1	1	1	1	4	4	3	3	4	4
1	1	1	1	1	1	3	3	4	4
4	4	1	1	4	4	3	3	4	4
1	1	1	1	4	4	3	3	4	4
1	1	1	1	4	4	3	3	4	4
1	1	1	1	1	1	3	3	4	4
1	1	1	1	4	4	3	3	4	4
1	1	1	1	4	4	3	3	4	4
1	1	1	1	4	4	3	3	4	4
1	1	1	1	4	4	3	3	4	4
1	1	1	1	4	4	3	3	4	4
1	1	4	4	4	4	2	2	1	1
1	1	1	1	4	4	3	3	4	4
1	1	1	1	4	4	3	3	4	4
1	1	1	1	4	4	3	3	4	4
1	1	1	1	1	1	3	3	4	4
1	1	1	1	4	4	3	3	4	4
1	1	1	1	4	4	3	3	4	4
1	1	1	1	4	4	3	3	4	4
1	1	1	1	4	4	3	3	4	4
1	1	1	1	1	1	3	3	4	4
1	1	4	4	4	4	2	2	1	1
1	1	1	1	4	4	3	3	4	4
1	1	1	1	4	4	3	3	4	4
1	1	1	1	1	1	3	3	4	4
4	4	4	4	4	4	2	2	1	1
1	1	1	1	4	4	3	3	4	4
4	4	4	4	1	1	2	2	1	1
1	1	1	1	1	1	3	3	4	4
1	1	1	1	4	4	3	3	4	4
1	1	1	1	1	1	3	3	4	4
1	1	1	1	4	4	3	3	4	4
1	1	1	1	4	4	3	3	4	4
1	1	1	1	1	1	3	3	4	4
1	1	1	1	4	4	3	3	4	4
1	1	1	1	4	4	3	3	4	4
1	1	1	1	1	1	3	3	4	4
1	1	1	1	4	4	3	3	4	4
1	1	1	1	4	4	3	3	4	4
1	1	1	1	4	4	3	3	4	4
1	1	1	1	4	4	3	3	4	4
1	1	1	1	1	1	3	3	4	4
1	1	4	4	4	4	2	2	1	1
1	1	1	1	4	4	2	2	1	1
1	1	1	1	4	4	3	3	4	4
1	1	1	1	1	1	3	3	4	4
1	1	1	1	1	1	3	3	4	4
1	1	1	1	4	4	3	3	4	4
1	1	1	1	4	4	3	3	4	4
1	1	1	1	1	1	3	3	4	4
1	1	1	1	4	4	3	3	4	4

For Review Only

1	1	1	1	4	4	3	3	4	4
1	1	1	1	4	4	3	3	4	4
1	1	1	1	1	1	3	3	4	4
1	1	1	1	4	4	3	3	4	4
1	1	1	1	4	4	3	3	4	4
1	1	1	1	1	1	3	3	4	4
1	1	1	1	4	4	3	3	4	4
1	1	1	1	4	4	3	3	4	4
1	1	1	1	4	4	3	3	4	4
1	1	1	1	4	4	3	3	4	4
1	1	1	1	4	4	3	3	4	4
1	1	1	1	4	4	3	3	4	4
1	1	1	1	1	1	3	3	4	4
1	1	1	1	4	4	3	3	4	4
1	1	1	1	4	4	3	3	4	4
1	1	1	1	1	1	3	3	4	4
1	1	1	1	4	4	3	3	4	4
1	1	1	1	4	4	3	3	4	4
4	4	4	4	4	4	2	2	1	1
1	1	1	1	4	4	3	3	4	4
1	1	1	1	4	4	3	3	4	4
1	1	1	1	4	4	3	3	4	4
1	1	1	1	1	1	3	3	4	4
1	1	1	1	4	4	3	3	4	4
1	1	1	1	4	4	3	3	4	4
1	1	1	1	1	1	3	3	4	4
1	1	1	1	4	4	3	3	4	4
1	1	1	1	1	1	3	3	4	4
1	1	1	1	4	4	3	3	4	4
1	1	1	1	1	1	3	3	4	4
1	1	1	1	4	4	3	3	4	4
1	1	1	1	4	4	3	3	4	4
1	1	1	1	1	1	3	3	4	4
1	1	1	1	4	4	3	3	4	4
1	1	1	1	4	4	3	3	4	4
1	1	1	1	4	4	3	3	4	4
1	1	1	1	4	4	3	3	4	4
1	1	1	1	4	4	3	3	4	4
1	1	1	1	1	1	3	3	4	4
1	1	1	1	4	4	3	3	4	4
4	4	4	4	1	1	2	2	1	1
1	1	1	1	4	4	3	3	4	4
1	1	1	1	4	4	3	3	4	4
4	4	4	4	4	4	2	2	1	1
1	1	1	1	4	4	3	3	4	4
1	1	1	1	4	4	3	3	4	4
1	1	1	1	4	4	3	3	4	4
1	1	1	1	1	1	3	3	4	4
1	1	1	1	1	1	3	3	4	4
1	1	1	1	4	4	3	3	4	4
1	1	1	1	1	1	3	3	4	4
1	1	1	1	4	4	3	3	4	4
1	1	1	1	1	1	3	3	4	4
1	1	1	1	1	1	3	3	4	4
1	1	1	1	4	4	3	3	4	4

For Review Only

1	1	4	4	1	1	2	2	1	1
1	1	1	1	4	4	3	3	4	4

For Review Only

SNP2972	SNP2972	SNP2973	SNP2973	SNP2974	SNP2974	SNP2975	SNP2975	SNP2976	SNP2976
3	3	2	2	3	3	2	2	2	2
3	3	2	2	4	4	2	2	3	3
2	2	2	2	3	3	2	2	3	3
3	3	2	2	3	3	2	2	2	2
2	2	2	2	4	4	1	1	2	2
3	3	2	2	3	3	2	2	2	2
2	2	2	2	4	4	1	1	3	3
2	2	2	2	4	4	1	1	2	2
2	2	2	2	4	4	1	1	2	2
2	2	2	2	4	4	1	1	3	3
2	2	2	2	4	4	1	1	2	2
2	2	2	2	3	3	2	2	2	2
2	2	2	2	4	4	1	1	2	2
2	2	4	4	4	4	1	1	3	3
2	2	2	2	4	4	1	1	2	2
2	2	2	2	4	4	1	1	2	2
2	2	2	2	4	4	1	1	3	3
3	3	2	2	3	3	2	2	3	3
3	3	2	2	3	3	2	2	2	2
2	2	2	2	4	4	1	1	3	3
2	2	2	2	4	4	1	1	3	3
2	2	2	2	4	4	1	1	2	2
2	2	2	2	4	4	1	1	3	3
2	2	2	2	4	4	1	1	3	3
2	2	2	2	4	4	1	1	3	3
3	3	2	2	3	3	2	2	2	2
2	2	2	2	4	4	1	1	2	2
2	2	2	2	4	4	1	1	2	2
3	3	2	2	3	3	2	2	2	2
3	3	2	2	4	4	1	1	3	3
3	3	2	2	3	3	2	2	2	2
3	3	2	2	3	3	2	2	3	3
2	2	2	2	4	4	1	1	3	3
2	2	2	2	4	4	1	1	3	3
2	2	2	2	4	4	1	1	3	3
3	3	2	2	3	3	2	2	2	2
2	2	2	2	4	4	1	1	3	3
2	2	2	2	4	4	1	1	3	3
2	2	2	2	4	4	1	1	3	3
2	2	2	2	4	4	1	1	3	3
2	2	2	2	4	4	1	1	3	3
2	2	2	2	4	4	1	1	2	2
2	2	2	2	4	4	1	1	3	3
2	2	2	2	4	4	1	1	3	3
2	2	2	2	4	4	1	1	3	3
2	2	2	2	3	3	2	2	3	3
2	2	2	2	4	4	1	1	3	3
3	3	2	2	4	4	1	1	3	3
3	3	2	2	4	4	1	1	3	3
2	2	2	2	4	4	1	1	2	2
3	3	2	2	3	3	2	2	3	3
3	3	4	4	3	3	2	2	3	3

For Review Only

3	3	2	2	3	3	2	2	3	3
2	2	2	2	4	4	1	1	3	3
2	2	2	2	3	3	2	2	3	3
2	2	2	2	4	4	1	1	2	2
3	3	2	2	3	3	2	2	2	2
2	2	2	2	4	4	1	1	2	2
3	3	2	2	3	3	2	2	2	2
2	2	2	2	4	4	1	1	3	3
2	2	2	2	3	3	1	1	2	2
2	2	2	2	4	4	1	1	2	2
2	2	2	2	4	4	1	1	2	2
2	2	2	2	4	4	1	1	2	2
3	3	2	2	3	3	2	2	3	3
3	3	2	2	3	3	2	2	2	2
3	3	2	2	3	3	2	2	2	2
2	2	2	2	3	3	2	2	3	3
2	2	2	2	4	4	1	1	3	3
2	2	2	2	4	4	1	1	2	2
2	2	2	2	3	3	2	2	2	2
2	2	2	2	4	4	1	1	3	3
2	2	2	2	4	4	1	1	2	2
2	2	2	2	4	4	1	1	2	2
2	2	2	2	3	3	2	2	2	2
2	2	2	2	4	4	1	1	2	2
2	2	2	2	4	4	1	1	3	3
3	3	2	2	4	4	1	1	2	2
2	2	2	2	4	4	1	1	3	3
2	2	4	4	4	4	1	1	3	3
3	3	2	2	3	3	2	2	2	2
3	3	2	2	3	3	2	2	2	2
2	2	2	2	4	4	1	1	2	2
2	2	2	2	4	4	1	1	3	3
2	2	4	4	4	4	1	1	3	3
3	3	2	2	3	3	2	2	3	3
3	3	2	2	3	3	2	2	2	2
2	2	2	2	4	4	1	1	3	3
3	3	2	2	4	4	1	1	3	3
2	2	2	2	4	4	1	1	3	3
2	2	2	2	4	4	1	1	2	2
2	2	2	2	4	4	1	1	3	3
2	2	2	2	4	4	1	1	2	2
3	3	2	2	3	3	2	2	2	2
3	3	2	2	3	3	2	2	3	3
2	2	2	2	4	4	1	1	2	2
3	3	2	2	3	3	2	2	3	3
3	3	2	2	4	4	2	2	2	2
2	2	4	4	4	4	1	1	3	3
3	3	2	2	3	3	2	2	3	3
3	3	2	2	3	3	2	2	3	3
2	2	2	2	3	3	1	1	2	2
2	2	2	2	4	4	1	1	2	2
2	2	2	2	4	4	1	1	2	2
2	2	4	4	4	4	1	1	3	3
2	2	2	2	4	4	1	1	2	2
2	2	4	4	4	4	1	1	3	3

3	3	2	2	3	3	2	2	3	3
2	2	2	2	4	4	1	1	3	3
2	2	2	2	4	4	1	1	2	2
2	2	4	4	4	4	1	1	3	3
2	2	2	2	3	3	2	2	2	2
3	3	2	2	3	3	2	2	2	2
2	2	2	2	4	4	1	1	2	2
2	2	2	2	4	4	1	1	2	2
3	3	2	2	3	3	2	2	3	3
3	3	2	2	3	3	2	2	2	2
3	3	2	2	3	3	2	2	2	2
3	3	2	2	3	3	1	1	2	2
2	2	2	2	4	4	1	1	3	3
2	2	2	2	4	4	1	1	2	2
3	3	2	2	3	3	1	1	2	2
2	2	2	2	4	4	1	1	3	3
2	2	2	2	4	4	1	1	2	2
2	2	2	2	4	4	1	1	2	2
2	2	4	4	4	4	1	1	3	3
2	2	2	2	4	4	1	1	2	2
2	2	2	2	4	4	1	1	2	2
3	3	2	2	3	3	2	2	2	2
3	3	2	2	3	3	2	2	2	2
2	2	4	4	4	4	1	1	3	3
2	2	2	2	3	3	1	1	2	2
2	2	2	2	4	4	1	1	3	3
2	2	2	2	4	4	1	1	2	2
2	2	2	2	4	4	1	1	2	2
3	3	2	2	3	3	2	2	2	2
2	2	2	2	3	3	1	1	2	2
2	2	4	4	4	4	1	1	3	3
2	2	2	2	4	4	1	1	2	2
2	2	2	2	4	4	1	1	2	2
2	2	2	2	4	4	1	1	3	3
2	2	2	2	4	4	1	1	3	3
2	2	2	2	4	4	1	1	2	2
2	2	4	4	4	4	1	1	3	3
2	2	2	2	4	4	1	1	2	2
2	2	2	2	4	4	1	1	2	2
2	2	4	4	4	4	1	1	3	3
2	2	2	2	4	4	1	1	2	2
2	2	2	2	4	4	1	1	3	3
2	2	4	4	4	4	1	1	3	3
2	2	2	2	4	4	1	1	3	3
2	2	2	2	4	4	1	1	2	2
2	2	2	2	4	4	1	1	2	2
3	3	2	2	3	3	2	2	3	3
2	2	2	2	4	4	1	1	2	2
2	2	2	2	3	3	1	1	3	3
2	2	2	2	3	3	1	1	2	2
3	3	2	2	3	3	2	2	2	2
2	2	2	2	4	4	1	1	2	2
2	2	2	2	4	4	1	1	2	2
2	2	2	2	4	4	1	1	2	2
2	2	2	2	4	4	1	1	2	2

2	2	2	2	3	3	2	2	3	3
2	2	2	2	4	4	1	1	3	3
3	3	2	2	4	4	2	2	3	3
2	2	2	2	4	4	1	1	2	2
3	3	2	2	3	3	2	2	2	2
3	3	2	2	3	3	2	2	3	3
2	2	2	2	4	4	1	1	2	2
2	2	2	2	4	4	1	1	2	2
2	2	2	2	4	4	1	1	2	2
3	3	2	2	3	3	2	2	2	2
2	2	2	2	4	4	1	1	2	2
2	2	2	2	4	4	1	1	3	3
3	3	2	2	3	3	2	2	2	2
2	2	2	2	4	4	1	1	2	2
2	2	4	4	4	4	1	1	3	3
2	2	2	2	4	4	1	1	2	2
2	2	2	2	4	4	1	1	2	2
2	2	2	2	4	4	2	2	2	2
3	3	2	2	3	3	2	2	3	3
2	2	2	2	4	4	1	1	2	2
2	2	2	2	3	3	1	1	2	2
2	2	2	2	4	4	1	1	2	2
2	2	2	2	4	4	1	1	2	2
2	2	2	2	4	4	1	1	2	2
2	2	2	2	4	4	1	1	2	2
2	2	2	2	4	4	1	1	2	2
2	2	2	2	4	4	1	1	2	2
2	2	2	2	4	4	1	1	2	2
2	2	2	2	4	4	1	1	2	2
2	2	2	2	3	3	2	2	2	2
2	2	2	2	4	4	1	1	2	2
2	2	2	2	4	4	1	1	2	2
3	3	2	2	4	4	2	2	2	2
2	2	4	4	4	4	1	1	3	3
3	3	2	2	3	3	2	2	2	2
2	2	2	2	4	4	1	1	2	2
3	3	2	2	3	3	2	2	2	2
3	3	2	2	3	3	2	2	3	3
2	2	2	2	4	4	1	1	2	2
2	2	2	2	4	4	1	1	3	3
2	2	2	2	3	3	2	2	3	3
2	2	2	2	4	4	1	1	3	3
2	2	2	2	4	4	1	1	3	3
3	3	2	2	3	3	2	2	2	2
2	2	2	2	4	4	1	1	3	3
2	2	2	2	3	3	1	1	2	2
2	2	2	2	4	4	1	1	2	2
2	2	2	2	4	4	1	1	2	2
2	2	2	2	4	4	1	1	3	3
2	2	2	2	4	4	1	1	3	3
3	3	2	2	3	3	2	2	2	2
2	2	2	2	4	4	1	1	3	3
2	2	2	2	4	4	1	1	3	3
2	2	2	2	4	4	1	1	2	2
2	2	2	2	4	4	1	1	2	2

2	2	2	2	4	4	1	1	2	2
2	2	2	2	4	4	1	1	2	2
2	2	2	2	3	3	1	1	3	3
2	2	2	2	4	4	1	1	2	2
2	2	2	2	4	4	1	1	3	3
2	2	2	2	3	3	2	2	2	2
2	2	2	2	4	4	1	1	3	3
2	2	2	2	4	4	1	1	3	3
2	2	2	2	4	4	1	1	3	3
2	2	4	4	4	4	1	1	3	3
2	2	2	2	3	3	1	1	2	2
2	2	2	2	4	4	1	1	3	3
2	2	2	2	4	4	1	1	2	2
3	3	2	2	3	3	2	2	2	2
2	2	2	2	3	3	2	2	2	2
3	3	2	2	3	3	2	2	3	3
2	2	2	2	4	4	1	1	3	3
2	2	2	2	4	4	1	1	2	2
2	2	2	2	4	4	1	1	2	2
2	2	2	2	4	4	1	1	3	3
2	2	2	2	4	4	1	1	2	2
3	3	2	2	3	3	2	2	2	2
2	2	2	2	4	4	1	1	2	2
2	2	2	2	4	4	1	1	3	3
2	2	2	2	4	4	1	1	3	3
3	3	2	2	3	3	2	2	2	2
2	2	4	4	4	4	1	1	3	3
3	3	2	2	3	3	2	2	2	2
2	2	2	2	4	4	1	1	3	3
3	3	2	2	3	3	2	2	2	2
2	2	2	2	4	4	1	1	2	2
3	3	2	2	3	3	2	2	2	2
2	2	2	2	4	4	1	1	3	3
2	2	2	2	4	4	1	1	2	2
2	2	2	2	4	4	1	1	2	2
2	2	4	4	4	4	1	1	3	3
2	2	2	2	4	4	1	1	2	2
2	2	2	2	4	4	1	1	2	2
3	3	2	2	3	3	2	2	2	2
2	2	2	2	4	4	1	1	3	3
3	3	2	2	3	3	2	2	2	2
2	2	2	2	4	4	1	1	2	2
2	2	2	2	4	4	1	1	3	3
2	2	2	2	4	4	1	1	2	2
3	3	2	2	3	3	2	2	2	2
2	2	2	2	4	4	1	1	3	3
2	2	2	2	4	4	1	1	2	2
2	2	2	2	4	4	1	1	3	3
2	2	2	2	4	4	1	1	3	3
2	2	4	4	4	4	1	1	3	3
2	2	2	2	4	4	1	1	2	2
2	2	2	2	4	4	1	1	3	3
2	2	2	2	3	3	2	2	3	3
3	3	2	2	3	3	2	2	2	2
2	2	2	2	4	4	1	1	3	3

For Review Only

3	3	2	2	3	3	2	2	2	2
3	3	2	2	3	3	2	2	2	2

For Review Only

SNP2977	SNP2977	SNP2978	SNP2978	SNP2979	SNP2979	SNP298	SNP298	SNP2980	SNP2980
1	1	1	1	3	3	2	2	3	3
1	1	1	1	4	4	3	3	2	2
1	1	4	4	3	3	2	2	2	2
1	1	1	1	4	4	2	2	2	2
2	2	4	4	3	3	3	3	2	2
1	1	4	4	3	3	2	2	2	2
2	2	4	4	3	3	2	2	2	2
2	2	4	4	3	3	2	2	2	2
2	2	4	4	3	3	2	2	2	2
2	2	4	4	3	3	3	3	3	3
2	2	4	4	3	3	3	3	2	2
1	1	4	4	3	3	2	2	2	2
2	2	4	4	3	3	2	2	2	2
1	1	4	4	3	3	3	3	2	2
2	2	4	4	3	3	2	2	2	2
2	2	4	4	3	3	3	3	2	2
2	2	4	4	3	3	2	2	2	2
1	1	4	4	3	3	3	3	2	2
1	1	1	1	4	4	3	3	2	2
1	1	4	4	3	3	3	3	2	2
2	2	4	4	3	3	3	3	2	2
2	2	4	4	3	3	2	2	2	2
1	1	4	4	3	3	2	2	2	2
2	2	4	4	3	3	3	3	2	2
2	2	4	4	3	3	2	2	2	2
1	1	4	4	3	3	2	2	2	2
2	2	4	4	3	3	2	2	2	2
2	2	4	4	3	3	2	2	2	2
1	1	1	1	4	4	3	3	3	3
2	2	4	4	3	3	3	3	2	2
1	1	1	1	4	4	2	2	2	2
1	1	1	1	4	4	2	2	2	2
2	2	4	4	3	3	2	2	2	2
1	1	4	4	3	3	3	3	2	2
2	2	4	4	3	3	2	2	2	2
1	1	1	1	4	4	3	3	2	2
1	1	4	4	3	3	3	3	2	2
2	2	4	4	3	3	2	2	2	2
2	2	4	4	3	3	2	2	2	2
2	2	4	4	3	3	2	2	2	2
2	2	4	4	3	3	3	3	2	2
2	2	4	4	3	3	3	3	2	2
2	2	4	4	3	3	3	3	2	2
2	2	4	4	3	3	3	3	2	2
2	2	4	4	3	3	3	3	2	2
1	1	4	4	3	3	2	2	2	2
2	2	4	4	3	3	2	2	2	2
2	2	4	4	3	3	2	2	2	2
2	2	4	4	3	3	3	3	2	2
1	1	4	4	3	3	2	2	2	2
1	1	1	1	4	4	2	2	2	2
1	1	1	1	4	4	2	2	2	2

For Review Only

1	1	1	1	4	4	2	2	2	2
2	2	4	4	3	3	2	2	2	2
2	2	4	4	3	3	3	3	3	3
2	2	4	4	3	3	2	2	2	2
1	1	1	1	4	4	2	2	2	2
2	2	4	4	3	3	2	2	2	2
1	1	4	4	3	3	2	2	2	2
2	2	4	4	3	3	3	3	2	2
2	2	4	4	3	3	3	3	2	2
2	2	4	4	3	3	2	2	2	2
2	2	4	4	3	3	2	2	2	2
2	2	4	4	3	3	2	2	2	2
1	1	1	1	4	4	2	2	2	2
1	1	1	1	4	4	3	3	3	3
2	2	4	4	3	3	2	2	2	2
1	1	4	4	3	3	3	3	2	2
1	1	4	4	3	3	3	3	2	2
1	1	4	4	3	3	2	2	2	2
2	2	4	4	3	3	2	2	2	2
2	2	4	4	3	3	2	2	2	2
2	2	4	4	3	3	2	2	2	2
2	2	4	4	3	3	2	2	2	2
1	1	1	1	4	4	3	3	3	3
2	2	4	4	3	3	3	3	2	2
2	2	4	4	3	3	3	3	2	2
2	2	4	4	3	3	3	3	2	2
1	1	4	4	3	3	2	2	2	2
2	2	4	4	3	3	2	2	2	2
1	1	4	4	3	3	2	2	2	2
2	2	4	4	3	3	3	3	2	2
2	2	4	4	3	3	2	2	2	2
2	2	4	4	3	3	3	3	2	2
2	2	4	4	3	3	3	3	2	2
1	1	1	1	4	4	2	2	2	2
1	1	4	4	3	3	3	3	2	2
2	2	4	4	3	3	3	3	2	2
2	2	4	4	3	3	2	2	2	2
2	2	4	4	3	3	2	2	2	2
2	2	4	4	3	3	3	3	2	2
2	2	4	4	3	3	3	3	2	2
2	2	4	4	3	3	3	3	2	2
1	1	1	1	4	4	3	3	2	2
1	1	1	1	4	4	3	3	2	2
2	2	4	4	3	3	2	2	2	2
2	2	4	4	3	3	3	3	3	3
1	1	1	1	4	4	2	2	2	2
1	1	4	4	3	3	2	2	2	2
1	1	4	4	3	3	3	3	2	2
1	1	4	4	3	3	2	2	2	2
2	2	4	4	3	3	2	2	2	2
2	2	4	4	3	3	3	3	2	2
2	2	4	4	3	3	2	2	2	2
2	2	4	4	3	3	3	3	2	2
2	2	4	4	3	3	2	2	2	2
2	2	4	4	3	3	3	3	2	2

For Review Only

1	1	1	1	4	4	2	2	2	2
2	2	4	4	3	3	2	2	2	2
2	2	4	4	3	3	3	3	2	2
1	1	4	4	3	3	3	3	2	2
2	2	1	1	3	3	2	2	2	2
1	1	4	4	3	3	2	2	2	2
2	2	4	4	3	3	3	3	2	2
2	2	4	4	3	3	3	3	2	2
1	1	4	4	3	3	2	2	2	2
2	2	4	4	4	4	3	3	2	2
1	1	4	4	3	3	2	2	2	2
2	2	4	4	3	3	2	2	2	2
1	1	4	4	3	3	3	3	2	2
2	2	4	4	3	3	3	3	2	2
2	2	4	4	3	3	3	3	2	2
2	2	4	4	3	3	2	2	2	2
1	1	4	4	3	3	2	2	2	2
2	2	4	4	4	4	2	2	2	2
1	1	4	4	3	3	3	3	2	2
2	2	4	4	3	3	2	2	2	2
2	2	4	4	3	3	2	2	2	2
2	2	4	4	3	3	2	2	2	2
1	1	1	1	4	4	3	3	2	2
2	2	4	4	3	3	2	2	2	2
2	2	4	4	4	4	2	2	2	2
2	2	4	4	3	3	3	3	2	2
2	2	4	4	3	3	2	2	2	2
2	2	4	4	3	3	3	3	3	3
1	1	1	1	4	4	2	2	2	2
1	1	4	4	3	3	2	2	2	2
1	1	4	4	3	3	2	2	2	2
2	2	4	4	3	3	2	2	2	2
2	2	4	4	3	3	3	3	2	2
2	2	4	4	4	4	2	2	2	2
1	1	4	4	3	3	3	3	2	2
2	2	4	4	3	3	2	2	2	2
1	1	4	4	3	3	3	3	2	2
2	2	4	4	3	3	3	3	2	2
2	2	4	4	3	3	2	2	2	2
2	2	4	4	3	3	2	2	2	2
2	2	4	4	3	3	2	2	2	2
1	1	4	4	3	3	3	3	2	2
2	2	4	4	3	3	3	3	2	2
2	2	4	4	3	3	3	3	2	2
2	2	4	4	3	3	3	3	2	2
2	2	4	4	3	3	3	3	2	2
1	1	1	1	3	3	2	2	2	2
2	2	4	4	3	3	2	2	2	2
2	2	4	4	3	3	2	2	2	2
2	2	4	4	3	3	2	2	2	2
1	1	4	4	3	3	2	2	2	2
2	2	4	4	3	3	2	2	2	2
2	2	4	4	3	3	3	3	2	2
2	2	4	4	3	3	2	2	2	2
2	2	4	4	3	3	2	2	2	2

For Review Only

2	2	4	4	3	3	3	3	2	2
2	2	4	4	3	3	3	3	2	2
1	1	1	1	4	4	3	3	2	2
2	2	4	4	3	3	3	3	2	2
1	1	1	1	4	4	2	2	2	2
1	1	4	4	3	3	2	2	2	2
2	2	4	4	3	3	3	3	2	2
2	2	4	4	3	3	2	2	2	2
2	2	4	4	3	3	3	3	2	2
2	2	4	4	3	3	2	2	2	2
1	1	4	4	3	3	2	2	2	2
2	2	4	4	3	3	3	3	2	2
2	2	4	4	3	3	3	3	2	2
2	2	4	4	3	3	2	2	2	2
1	1	4	4	3	3	3	3	2	2
2	2	4	4	3	3	3	3	2	2
2	2	4	4	3	3	3	3	2	2
1	1	1	1	3	3	3	3	2	2
1	1	4	4	3	3	3	3	2	2
2	2	4	4	3	3	3	3	2	2
2	2	4	4	3	3	2	2	2	2
2	2	4	4	3	3	3	3	2	2
2	2	4	4	3	3	3	3	2	2
2	2	4	4	3	3	2	2	2	2
2	2	4	4	3	3	3	3	2	2
1	1	4	4	3	3	3	3	2	2
2	2	4	4	3	3	2	2	2	2
1	1	4	4	3	3	2	2	2	2
1	1	4	4	3	3	2	2	2	2
2	2	4	4	3	3	2	2	2	2
2	2	4	4	3	3	3	3	2	2
1	1	4	4	3	3	2	2	2	2
1	1	1	1	4	4	2	2	2	2
1	1	4	4	3	3	3	3	2	2
2	2	4	4	3	3	2	2	2	2
2	2	4	4	3	3	2	2	2	2
1	1	1	1	4	4	3	3	2	2
1	1	4	4	3	3	3	3	2	2
2	2	4	4	3	3	2	2	2	2
2	2	4	4	3	3	3	3	2	2
1	1	4	4	3	3	2	2	2	2
2	2	4	4	3	3	2	2	2	2
1	1	4	4	3	3	3	3	2	2
2	2	4	4	3	3	2	2	2	2
2	2	4	4	3	3	2	2	2	2
2	2	4	4	3	3	2	2	2	2
2	2	4	4	3	3	3	3	2	2
2	2	4	4	3	3	3	3	2	2
2	2	4	4	3	3	3	3	2	2
2	2	4	4	3	3	2	2	2	2
1	1	4	4	3	3	2	2	2	2
1	1	4	4	3	3	3	3	2	2
2	2	4	4	3	3	2	2	2	2
2	2	4	4	3	3	2	2	2	2
2	2	4	4	3	3	2	2	2	2

For Review Only

2	2	4	4	3	3	3	3	2	2
2	2	4	4	3	3	2	2	3	3
2	2	4	4	3	3	2	2	2	2
2	2	4	4	3	3	3	3	2	2
2	2	4	4	3	3	3	3	2	2
1	1	1	1	4	4	2	2	2	2
2	2	4	4	3	3	2	2	2	2
2	2	4	4	3	3	2	2	2	2
2	2	4	4	3	3	3	3	2	2
2	2	4	4	3	3	3	3	2	2
2	2	4	4	3	3	2	2	2	2
2	2	4	4	3	3	3	3	2	2
2	2	4	4	3	3	3	3	2	2
1	1	4	4	3	3	3	3	2	2
1	1	4	4	3	3	2	2	2	2
2	2	4	4	3	3	3	3	3	3
2	2	4	4	3	3	2	2	2	2
2	2	4	4	3	3	3	3	2	2
2	2	4	4	3	3	3	3	2	2
2	2	4	4	3	3	2	2	2	2
2	2	4	4	3	3	3	3	2	2
1	1	1	1	4	4	2	2	2	2
2	2	4	4	3	3	3	3	2	2
2	2	4	4	3	3	3	3	2	2
2	2	4	4	3	3	3	3	2	2
1	1	4	4	3	3	2	2	2	2
2	2	4	4	3	3	3	3	2	2
1	1	1	1	4	4	2	2	2	2
2	2	4	4	3	3	3	3	2	2
1	1	1	1	4	4	2	2	2	2
2	2	4	4	3	3	3	3	2	2
1	1	1	1	4	4	2	2	2	2
1	1	4	4	3	3	2	2	2	2
2	2	4	4	3	3	2	2	2	2
2	2	4	4	3	3	3	3	2	2
2	2	4	4	3	3	3	3	2	2
1	1	4	4	3	3	3	3	2	2
2	2	4	4	3	3	2	2	2	2
1	1	1	1	4	4	3	3	2	2
2	2	4	4	3	3	2	2	2	2
2	2	4	4	3	3	2	2	2	2
2	2	4	4	3	3	3	3	2	2
2	2	4	4	3	3	3	3	2	2
2	2	4	4	3	3	2	2	2	2
1	1	4	4	3	3	2	2	2	2
2	2	4	4	3	3	3	3	2	2
2	2	4	4	3	3	2	2	2	2
2	2	4	4	3	3	3	3	2	2
2	2	4	4	3	3	3	3	2	2
2	2	4	4	3	3	3	3	2	2
1	1	4	4	3	3	2	2	2	2
2	2	4	4	3	3	2	2	2	2
1	1	4	4	3	3	2	2	2	2
1	1	1	1	4	4	2	2	2	2
2	2	4	4	3	3	3	3	2	2

For Review Only

2	2	4	4	3	3	3	3	3	3
1	1	1	1	4	4	3	3	3	3

For Review Only

SNP2981	SNP2981	SNP2982	SNP2982	SNP2983	SNP2983	SNP2984	SNP2984	SNP2985	SNP2985
2	2	2	2	4	4	1	1	4	4
4	4	2	2	1	1	1	1	1	1
2	2	2	2	4	4	1	1	4	4
2	2	2	2	4	4	1	1	4	4
2	2	2	2	4	4	1	1	4	4
4	4	2	2	1	1	1	1	4	4
2	2	2	2	4	4	1	1	4	4
2	2	2	2	1	1	4	4	1	1
2	2	2	2	4	4	1	1	4	4
4	4	2	2	1	1	4	4	1	1
2	2	3	3	4	4	1	1	4	4
2	2	2	2	4	4	1	1	4	4
2	2	3	3	4	4	1	1	4	4
2	2	3	3	4	4	1	1	4	4
2	2	2	2	4	4	1	1	4	4
2	2	2	2	4	4	1	1	4	4
2	2	2	2	1	1	4	4	1	1
4	4	2	2	1	1	1	1	4	4
2	2	2	2	1	1	4	4	1	1
2	2	2	2	1	1	1	1	1	1
4	4	2	2	4	4	1	1	4	4
2	2	2	2	4	4	1	1	4	4
2	2	3	3	4	4	1	1	4	4
2	2	3	3	1	1	1	1	1	1
2	2	2	2	4	4	1	1	4	4
2	2	2	2	4	4	1	1	4	4
2	2	2	2	4	4	1	1	4	4
2	2	2	2	4	4	1	1	1	1
2	2	2	2	4	4	1	1	4	4
2	2	2	2	1	1	4	4	1	1
4	4	2	2	4	4	1	1	1	1
2	2	2	2	1	1	4	4	1	1
2	2	2	2	4	4	1	1	4	4
2	2	2	2	1	1	4	4	1	1
2	2	3	3	4	4	1	1	4	4
2	2	2	2	4	4	1	1	1	1
2	2	3	3	4	4	1	1	4	4
2	2	2	2	4	4	1	1	4	4
2	2	2	2	4	4	1	1	4	4
2	2	2	2	4	4	1	1	4	4
2	2	2	2	4	4	4	4	1	1
2	2	2	2	4	4	1	1	4	4
2	2	2	2	4	4	1	1	4	4
2	2	2	2	4	4	1	1	4	4
2	2	2	2	4	4	1	1	4	4
2	2	2	2	4	4	1	1	4	4
2	2	2	2	1	1	1	1	4	4
2	2	2	2	4	4	1	1	4	4
2	2	2	2	4	4	1	1	4	4
2	2	2	2	4	4	1	1	4	4
2	2	2	2	4	4	1	1	4	4
2	2	2	2	4	4	1	1	4	4

For Review Only

2	2	2	2	4	4	1	1	4	4
2	2	2	2	4	4	1	1	4	4
4	4	2	2	4	4	1	1	4	4
2	2	2	2	4	4	1	1	4	4
2	2	2	2	1	1	4	4	1	1
2	2	2	2	1	1	1	1	4	4
2	2	2	2	4	4	1	1	4	4
2	2	2	2	4	4	1	1	4	4
2	2	2	2	4	4	1	1	4	4
2	2	2	2	4	4	1	1	4	4
2	2	2	2	4	4	1	1	4	4
2	2	2	2	1	1	1	1	4	4
2	2	2	2	4	4	1	1	4	4
2	2	2	2	4	4	1	1	4	4
2	2	2	2	4	4	1	1	4	4
2	2	3	3	4	4	1	1	4	4
2	2	2	2	4	4	1	1	4	4
2	2	3	3	4	4	1	1	4	4
2	2	2	2	4	4	1	1	4	4
2	2	2	2	4	4	1	1	4	4
2	2	2	2	1	1	1	1	4	4
2	2	3	3	4	4	1	1	4	4
2	2	2	2	4	4	1	1	4	4
2	2	2	2	4	4	1	1	4	4
2	2	2	2	4	4	1	1	4	4
2	2	2	2	4	4	1	1	4	4
4	4	2	2	4	4	1	1	4	4
2	2	2	2	4	4	1	1	4	4
2	2	2	2	4	4	1	1	4	4
2	2	2	2	4	4	1	1	4	4
2	2	2	2	4	4	1	1	4	4
2	2	2	2	1	1	4	4	1	1
2	2	2	2	4	4	1	1	4	4
2	2	2	2	4	4	1	1	4	4
4	4	2	2	1	1	4	4	1	1
2	2	2	2	4	4	1	1	4	4
2	2	2	2	4	4	1	1	4	4
2	2	2	2	4	4	1	1	4	4
2	2	3	3	4	4	1	1	4	4
2	2	2	2	4	4	1	1	4	4
2	2	2	2	4	4	1	1	4	4
2	2	2	2	4	4	1	1	1	1
2	2	2	2	4	4	1	1	4	4
2	2	2	2	4	4	1	1	4	4
2	2	2	2	1	1	4	4	4	4
4	4	2	2	1	1	4	4	4	4
2	2	3	3	4	4	1	1	4	4
2	2	2	2	4	4	1	1	4	4
2	2	2	2	4	4	1	1	4	4
2	2	2	2	4	4	1	1	4	4
2	2	2	2	1	1	1	1	4	4
2	2	3	3	4	4	1	1	4	4
2	2	2	2	1	1	4	4	1	1
2	2	2	2	4	4	1	1	4	4
2	2	2	2	4	4	1	1	4	4

For Review Only

2	2	2	2	4	4	1	1	4	4
4	4	2	2	4	4	1	1	4	4
2	2	2	2	4	4	1	1	1	1
2	2	2	2	4	4	1	1	4	4
2	2	2	2	4	4	1	1	4	4
2	2	2	2	4	4	1	1	4	4
2	2	2	2	4	4	1	1	4	4
2	2	2	2	4	4	1	1	4	4
2	2	2	2	4	4	1	1	4	4
2	2	2	2	4	4	1	1	1	1
4	4	2	2	4	4	1	1	4	4
2	2	2	2	4	4	1	1	4	4
2	2	2	2	4	4	1	1	4	4
2	2	2	2	4	4	1	1	4	4
2	2	2	2	4	4	1	1	4	4
2	2	2	2	4	4	1	1	4	4
2	2	3	3	4	4	1	1	4	4
2	2	2	2	4	4	1	1	4	4
2	2	3	3	4	4	1	1	4	4
2	2	2	2	4	4	1	1	4	4
2	2	2	2	4	4	1	1	4	4
2	2	2	2	4	4	1	1	4	4
2	2	2	2	4	4	1	1	1	1
2	2	2	2	4	4	1	1	4	4
2	2	2	2	4	4	1	1	4	4
2	2	3	3	4	4	1	1	4	4
2	2	2	2	4	4	1	1	4	4
2	2	2	2	4	4	1	1	4	4
2	2	2	2	4	4	1	1	4	4
2	2	2	2	4	4	1	1	4	4
2	2	3	3	4	4	1	1	4	4
2	2	2	2	4	4	1	1	4	4
2	2	2	2	4	4	1	1	4	4
2	2	2	2	4	4	1	1	4	4
2	2	2	2	1	1	4	4	1	1
2	2	3	3	4	4	1	1	4	4
2	2	3	3	4	4	1	1	4	4
2	2	2	2	4	4	1	1	4	4
2	2	3	3	4	4	1	1	4	4
2	2	3	3	4	4	1	1	4	4
2	2	3	3	4	4	1	1	4	4
2	2	3	3	4	4	1	1	4	4
2	2	2	2	4	4	1	1	4	4
2	2	3	3	4	4	1	1	4	4
2	2	2	2	4	4	1	1	4	4
4	4	2	2	4	4	1	1	4	4
2	2	2	2	4	4	1	1	4	4
2	2	2	2	4	4	1	1	4	4
2	2	3	3	4	4	1	1	4	4
2	2	2	2	4	4	1	1	4	4
2	2	2	2	4	4	1	1	4	4
2	2	3	3	4	4	1	1	4	4
2	2	3	3	4	4	4	4	1	1
2	2	2	2	1	1	1	1	4	4
2	2	2	2	4	4	1	1	4	4

For Review Only

2	2	2	2	4	4	1	1	4	4
2	2	3	3	4	4	1	1	4	4
2	2	3	3	4	4	1	1	4	4
2	2	3	3	4	4	1	1	4	4
4	4	2	2	1	1	4	4	1	1
4	4	2	2	1	1	4	4	1	1
2	2	2	2	4	4	1	1	4	4
2	2	2	2	4	4	1	1	4	4
2	2	2	2	1	1	1	1	4	4
2	2	2	2	1	1	4	4	1	1
2	2	2	2	4	4	1	1	4	4
2	2	3	3	4	4	1	1	4	4
2	2	2	2	4	4	1	1	4	4
2	2	2	2	4	4	1	1	4	4
2	2	3	3	4	4	1	1	4	4
2	2	3	3	4	4	1	1	4	4
2	2	2	2	4	4	4	4	4	4
4	4	2	2	4	4	4	4	1	1
2	2	2	2	4	4	1	1	4	4
2	2	2	2	4	4	1	1	4	4
2	2	2	2	4	4	1	1	4	4
2	2	2	2	4	4	1	1	4	4
2	2	3	3	4	4	1	1	4	4
2	2	2	2	1	1	1	1	4	4
2	2	2	2	4	4	1	1	4	4
2	2	2	2	1	1	4	4	1	1
2	2	2	2	4	4	1	1	4	4
2	2	2	2	4	4	1	1	4	4
2	2	3	3	4	4	1	1	4	4
2	2	2	2	4	4	1	1	4	4
2	2	3	3	4	4	1	1	4	4
2	2	3	3	4	4	1	1	4	4
2	2	3	3	4	4	1	1	4	4
2	2	3	3	4	4	1	1	4	4
2	2	2	2	4	4	1	1	4	4
2	2	2	2	1	1	1	1	4	4
2	2	2	2	4	4	1	1	4	4
4	4	2	2	4	4	1	1	4	4
2	2	2	2	4	4	1	1	4	4
2	2	2	2	1	1	4	4	1	1
2	2	2	2	4	4	1	1	4	4
2	2	2	2	4	4	1	1	4	4
2	2	3	3	4	4	1	1	4	4
2	2	2	2	4	4	1	1	4	4
2	2	2	2	4	4	1	1	1	1
2	2	2	2	1	1	1	1	4	4
2	2	3	3	4	4	1	1	4	4
2	2	2	2	4	4	1	1	4	4
2	2	3	3	4	4	1	1	4	4
2	2	3	3	4	4	1	1	4	4
2	2	2	2	4	4	1	1	4	4
2	2	2	2	1	1	4	4	1	1
2	2	3	3	4	4	1	1	4	4
2	2	2	2	4	4	1	1	4	4
2	2	3	3	4	4	1	1	4	4

For Review Only

2	2	3	3	4	4	4	4	1	1
4	4	2	2	4	4	1	1	4	4
2	2	2	2	4	4	1	1	4	4
2	2	2	2	4	4	1	1	4	4
2	2	3	3	4	4	1	1	4	4
2	2	2	2	4	4	1	1	4	4
2	2	3	3	4	4	1	1	4	4
4	4	2	2	4	4	1	1	4	4
2	2	3	3	4	4	1	1	4	4
2	2	2	2	4	4	1	1	4	4
2	2	2	2	4	4	1	1	4	4
2	2	2	2	4	4	1	1	4	4
2	2	3	3	4	4	1	1	4	4
2	2	2	2	4	4	1	1	4	4
2	2	2	2	4	4	1	1	4	4
2	2	2	2	4	4	1	1	4	4
2	2	2	2	4	4	1	1	4	4
2	2	2	2	4	4	1	1	4	4
2	2	3	3	4	4	1	1	4	4
2	2	3	3	4	4	1	1	4	4
2	2	2	2	4	4	1	1	4	4
2	2	2	2	4	4	1	1	4	4
2	2	2	2	4	4	1	1	4	4
2	2	2	2	4	4	1	1	4	4
2	2	2	2	4	4	1	1	4	4
2	2	2	2	4	4	1	1	4	4
2	2	2	2	4	4	1	1	4	4
2	2	2	2	4	4	1	1	4	4
2	2	2	2	4	4	1	1	4	4
2	2	2	2	4	4	1	1	4	4
2	2	2	2	4	4	1	1	4	4
2	2	2	2	4	4	1	1	4	4
2	2	3	3	4	4	1	1	4	4
2	2	2	2	4	4	1	1	4	4
2	2	3	3	4	4	1	1	4	4
2	2	2	2	4	4	1	1	4	4
2	2	3	3	1	1	4	4	1	1
2	2	2	2	1	1	1	1	4	4
2	2	2	2	4	4	1	1	1	1
2	2	3	3	4	4	1	1	4	4
2	2	2	2	4	4	1	1	4	4
2	2	2	2	4	4	1	1	4	4
2	2	3	3	4	4	1	1	4	4
2	2	2	2	4	4	1	1	4	4
2	2	2	2	1	1	4	4	1	1
2	2	3	3	4	4	1	1	4	4
2	2	3	3	4	4	1	1	4	4
2	2	2	2	4	4	1	1	4	4
2	2	2	2	4	4	1	1	4	4
2	2	2	2	4	4	1	1	4	4
2	2	2	2	4	4	1	1	1	1
2	2	3	3	4	4	1	1	4	4
2	2	2	2	4	4	1	1	4	4
2	2	2	2	4	4	1	1	4	4
2	2	3	3	4	4	1	1	4	4

For Review Only

2	2	2	2	4	4	1	1	4	4
2	2	2	2	4	4	1	1	4	4

For Review Only

SNP2986	SNP2986	SNP2987	SNP2987	SNP2988	SNP2988	SNP2989	SNP2989	SNP299	SNP299
3	3	1	1	3	3	4	4	1	1
2	2	1	1	3	3	4	4	1	1
3	3	1	1	3	3	4	4	1	1
3	3	1	1	3	3	4	4	1	1
3	3	1	1	3	3	4	4	4	4
3	3	1	1	3	3	4	4	1	1
3	3	1	1	3	3	4	4	1	1
2	2	4	4	2	2	4	4	4	4
3	3	1	1	3	3	4	4	1	1
2	2	4	4	2	2	4	4	1	1
3	3	1	1	2	2	4	4	1	1
3	3	1	1	3	3	4	4	1	1
3	3	1	1	3	3	4	4	1	1
3	3	4	4	3	3	4	4	1	1
3	3	1	1	3	3	4	4	1	1
3	3	4	4	3	3	4	4	1	1
2	2	4	4	2	2	4	4	1	1
3	3	1	1	3	3	4	4	1	1
2	2	1	1	3	3	4	4	1	1
2	2	4	4	3	3	4	4	1	1
3	3	1	1	2	2	4	4	1	1
3	3	1	1	3	3	4	4	1	1
3	3	1	1	2	2	4	4	1	1
2	2	4	4	2	2	4	4	1	1
3	3	1	1	2	2	4	4	1	1
3	3	1	1	3	3	4	4	1	1
3	3	1	1	2	2	3	3	1	1
3	3	1	1	3	3	4	4	1	1
3	3	1	1	2	2	4	4	1	1
2	2	1	1	2	2	4	4	1	1
2	2	4	4	3	3	4	4	1	1
2	2	4	4	2	2	4	4	1	1
3	3	1	1	3	3	4	4	1	1
2	2	4	4	2	2	4	4	4	4
3	3	1	1	3	3	4	4	1	1
2	2	4	4	3	3	4	4	1	1
3	3	4	4	2	2	4	4	1	1
3	3	1	1	2	2	4	4	1	1
3	3	1	1	2	2	4	4	1	1
3	3	1	1	2	2	4	4	1	1
2	2	1	1	2	2	4	4	4	4
3	3	1	1	2	2	4	4	1	1
3	3	1	1	2	2	4	4	4	4
3	3	1	1	3	3	4	4	1	1
3	3	1	1	2	2	4	4	4	4
3	3	1	1	3	3	4	4	1	1
3	3	1	1	3	3	4	4	1	1
3	3	1	1	3	3	4	4	1	1
3	3	1	1	3	3	4	4	1	1
3	3	1	1	3	3	4	4	1	1
3	3	1	1	3	3	4	4	1	1
3	3	1	1	3	3	4	4	1	1

For Review Only

3	3	1	1	2	2	4	4	1	1
3	3	1	1	3	3	4	4	1	1
3	3	1	1	3	3	4	4	1	1
3	3	1	1	3	3	4	4	1	1
2	2	1	1	3	3	4	4	1	1
3	3	1	1	3	3	4	4	1	1
3	3	1	1	3	3	4	4	1	1
3	3	1	1	2	2	4	4	1	1
3	3	1	1	3	3	4	4	1	1
3	3	1	1	3	3	4	4	1	1
3	3	1	1	3	3	4	4	1	1
3	3	1	1	3	3	4	4	1	1
3	3	1	1	2	2	4	4	1	1
3	3	1	1	2	2	4	4	1	1
3	3	1	1	3	3	4	4	1	1
3	3	4	4	2	2	4	4	1	1
3	3	1	1	2	2	4	4	1	1
3	3	1	1	3	3	4	4	1	1
3	3	1	1	3	3	4	4	1	1
3	3	1	1	3	3	4	4	1	1
3	3	1	1	3	3	4	4	1	1
3	3	1	1	2	2	4	4	1	1
3	3	1	1	2	2	4	4	1	1
3	3	1	1	3	3	4	4	1	1
3	3	1	1	2	2	4	4	4	4
3	3	1	1	3	3	4	4	1	1
3	3	1	1	3	3	4	4	4	4
3	3	1	1	2	2	4	4	1	1
3	3	1	1	3	3	4	4	1	1
3	3	1	1	3	3	4	4	1	1
3	3	1	1	3	3	4	4	1	1
2	2	4	4	3	3	4	4	1	1
3	3	1	1	2	2	4	4	1	1
3	3	1	1	2	2	4	4	1	1
2	2	4	4	2	2	4	4	1	1
3	3	1	1	2	2	4	4	4	4
3	3	1	1	3	3	4	4	1	1
3	3	1	1	2	2	3	3	1	1
3	3	1	1	2	2	4	4	1	1
3	3	1	1	2	2	4	4	4	4
3	3	1	1	3	3	4	4	1	1
2	2	4	4	3	3	4	4	1	1
3	3	1	1	3	3	4	4	1	1
3	3	1	1	3	3	4	4	1	1
3	3	1	1	3	3	4	4	1	1
2	2	1	1	3	3	4	4	1	1
3	3	1	1	3	3	4	4	1	1
3	3	1	1	3	3	4	4	1	1
3	3	1	1	3	3	4	4	1	1
3	3	1	1	2	2	4	4	1	1
3	3	1	1	3	3	4	4	1	1
3	3	1	1	2	2	4	4	1	1
2	2	4	4	2	2	4	4	1	1
3	3	1	1	3	3	4	4	1	1
3	3	1	1	2	2	3	3	1	1

For Review Only

3	3	1	1	3	3	4	4	1	1
3	3	1	1	3	3	4	4	1	1
2	2	4	4	3	3	4	4	1	1
3	3	1	1	3	3	4	4	1	1
3	3	1	1	3	3	4	4	1	1
3	3	1	1	3	3	4	4	1	1
3	3	4	4	3	3	4	4	1	1
3	3	1	1	3	3	4	4	1	1
3	3	1	1	3	3	4	4	1	1
2	2	1	1	3	3	4	4	1	1
3	3	1	1	3	3	4	4	1	1
3	3	1	1	3	3	4	4	1	1
3	3	1	1	3	3	4	4	1	1
3	3	1	1	3	3	4	4	1	1
3	3	1	1	2	2	4	4	1	1
3	3	1	1	3	3	4	4	1	1
3	3	1	1	3	3	4	4	1	1
3	3	1	1	3	3	4	4	1	1
3	3	1	1	3	3	4	4	1	1
3	3	1	1	3	3	4	4	1	1
3	3	1	1	3	3	4	4	1	1
3	3	1	1	3	3	4	4	1	1
2	2	4	4	3	3	4	4	1	1
3	3	4	4	2	2	4	4	4	4
3	3	1	1	3	3	4	4	1	1
3	3	1	1	2	2	4	4	1	1
3	3	1	1	3	3	4	4	1	1
3	3	1	1	3	3	4	4	1	1
3	3	1	1	3	3	4	4	1	1
3	3	1	1	3	3	4	4	1	1
3	3	1	1	3	3	4	4	1	1
3	3	1	1	3	3	4	4	1	1
3	3	1	1	3	3	4	4	1	1
3	3	1	1	3	3	4	4	1	1
2	2	4	4	2	2	4	4	4	4
3	3	1	1	3	3	4	4	1	1
3	3	4	4	2	2	4	4	1	1
3	3	1	1	2	2	4	4	1	1
3	3	1	1	2	2	4	4	1	1
3	3	1	1	2	2	4	4	1	1
3	3	1	1	3	3	4	4	1	1
3	3	1	1	3	3	4	4	1	1
3	3	1	1	2	2	3	3	1	1
3	3	1	1	3	3	4	4	1	1
3	3	1	1	2	2	4	4	1	1
3	3	1	1	3	3	4	4	1	1
3	3	1	1	3	3	4	4	1	1
3	3	4	4	2	2	4	4	1	1
3	3	1	1	3	3	4	4	1	1
3	3	1	1	3	3	4	4	1	1
3	3	1	1	3	3	4	4	1	1
3	3	1	1	2	2	4	4	1	1
3	3	4	4	2	2	4	4	1	1
3	3	1	1	3	3	4	4	1	1
3	3	1	1	3	3	4	4	1	1

For Review Only

3	3	1	1	3	3	4	4	1	1
3	3	1	1	2	2	4	4	1	1
3	3	1	1	2	2	3	3	1	1
3	3	1	1	2	2	4	4	1	1
2	2	1	1	3	3	4	4	1	1
2	2	4	4	2	2	4	4	1	1
3	3	1	1	2	2	4	4	1	1
3	3	1	1	3	3	4	4	1	1
3	3	1	1	2	2	4	4	1	1
2	2	4	4	3	3	4	4	1	1
3	3	1	1	3	3	4	4	1	1
3	3	1	1	2	2	4	4	1	1
3	3	1	1	3	3	4	4	1	1
3	3	1	1	3	3	4	4	1	1
3	3	1	1	3	3	4	4	1	1
3	3	1	1	2	2	4	4	1	1
3	3	1	1	3	3	4	4	1	1
2	2	1	1	3	3	4	4	1	1
3	3	1	1	3	3	4	4	1	1
3	3	4	4	3	3	4	4	1	1
3	3	1	1	3	3	4	4	1	1
3	3	1	1	2	2	4	4	1	1
3	3	1	1	2	2	4	4	1	1
3	3	1	1	3	3	4	4	1	1
3	3	1	1	3	3	4	4	1	1
2	2	4	4	2	2	4	4	4	4
3	3	1	1	2	2	3	3	4	4
3	3	1	1	3	3	4	4	1	1
3	3	1	1	3	3	4	4	1	1
3	3	1	1	3	3	4	4	1	1
3	3	1	1	3	3	4	4	1	1
3	3	1	1	3	3	4	4	1	1
3	3	1	1	2	2	3	3	1	1
3	3	1	1	3	3	4	4	1	1
3	3	1	1	3	3	4	4	1	1
3	3	1	1	3	3	4	4	1	1
3	3	1	1	3	3	4	4	1	1
3	3	1	1	3	3	4	4	1	1
3	3	4	4	2	2	4	4	1	1
2	2	4	4	2	2	4	4	1	1
3	3	1	1	3	3	4	4	1	1
3	3	1	1	3	3	4	4	1	1
3	3	4	4	2	2	4	4	1	1
3	3	1	1	3	3	4	4	1	1
2	2	4	4	3	3	4	4	1	1
3	3	1	1	3	3	4	4	1	1
3	3	1	1	2	2	4	4	1	1
3	3	1	1	2	2	3	3	1	1
3	3	1	1	3	3	4	4	1	1
3	3	1	1	2	2	4	4	1	1
3	3	1	1	3	3	4	4	1	1
2	2	4	4	2	2	4	4	1	1
3	3	1	1	2	2	4	4	1	1
3	3	1	1	3	3	4	4	1	1
3	3	1	1	2	2	4	4	1	1

For Review Only

2	2	4	4	2	2	4	4	1	1
3	3	1	1	3	3	4	4	1	1
3	3	1	1	3	3	4	4	1	1
3	3	1	1	3	3	4	4	1	1
3	3	1	1	2	2	4	4	4	4
3	3	1	1	3	3	4	4	1	1
3	3	1	1	2	2	4	4	1	1
3	3	1	1	3	3	4	4	4	4
3	3	4	4	3	3	4	4	1	1
3	3	1	1	2	2	4	4	1	1
3	3	1	1	3	3	4	4	1	1
3	3	1	1	2	2	4	4	4	4
3	3	1	1	2	2	4	4	1	1
3	3	1	1	3	3	4	4	1	1
3	3	1	1	3	3	4	4	1	1
3	3	1	1	3	3	4	4	1	1
3	3	1	1	2	2	4	4	1	1
3	3	1	1	3	3	4	4	1	1
3	3	1	1	2	2	4	4	1	1
3	3	1	1	2	2	4	4	1	1
3	3	1	1	3	3	4	4	1	1
3	3	1	1	3	3	4	4	1	1
3	3	4	4	3	3	4	4	1	1
3	3	1	1	2	2	3	3	1	1
3	3	1	1	3	3	4	4	1	1
3	3	1	1	2	2	4	4	1	1
3	3	1	1	2	2	4	4	1	1
3	3	1	1	3	3	4	4	1	1
3	3	1	1	2	2	4	4	1	1
3	3	1	1	3	3	4	4	1	1
3	3	1	1	2	2	4	4	1	1
3	3	1	1	3	3	4	4	1	1
3	3	4	4	3	3	4	4	1	1
3	3	4	4	2	2	4	4	1	1
3	3	1	1	2	2	4	4	1	1
3	3	4	4	2	2	4	4	1	1
2	2	1	1	3	3	4	4	1	1
3	3	1	1	3	3	4	4	1	1
3	3	4	4	3	3	4	4	1	1
3	3	1	1	2	2	4	4	1	1
3	3	1	1	3	3	4	4	1	1
3	3	1	1	3	3	3	3	1	1
3	3	1	1	2	2	4	4	1	1
3	3	1	1	3	3	4	4	1	1
2	2	4	4	2	2	4	4	1	1
3	3	1	1	3	3	4	4	1	1
3	3	1	1	2	2	4	4	1	1
3	3	1	1	2	2	3	3	1	1
3	3	1	1	3	3	4	4	4	4
3	3	1	1	2	2	4	4	1	1
2	2	4	4	3	3	4	4	1	1
3	3	1	1	2	2	4	4	1	1
3	3	1	1	3	3	4	4	1	1
3	3	1	1	3	3	4	4	1	1
3	3	1	1	2	2	4	4	1	1

For Review Only

3	3	1	1	3	3	4	4	1	1
3	3	1	1	3	3	4	4	1	1

For Review Only

SNP2990	SNP2990	SNP2991	SNP2991	SNP2992	SNP2992	SNP2993	SNP2993	SNP2994	SNP2994
4	4	3	3	2	2	3	3	4	4
4	4	3	3	3	3	3	3	4	4
4	4	3	3	2	2	3	3	4	4
4	4	3	3	2	2	3	3	4	4
4	4	3	3	2	2	3	3	4	4
4	4	3	3	2	2	4	4	4	4
4	4	3	3	2	2	3	3	4	4
4	4	4	4	2	2	3	3	4	4
4	4	3	3	2	2	3	3	4	4
4	4	4	4	2	2	3	3	1	1
4	4	4	4	2	2	3	3	1	1
4	4	3	3	2	2	3	3	4	4
4	4	3	3	2	2	3	3	4	4
4	4	4	4	2	2	3	3	4	4
4	4	3	3	2	2	3	3	4	4
4	4	4	4	2	2	3	3	4	4
1	1	4	4	2	2	3	3	4	4
4	4	3	3	2	2	3	3	4	4
4	4	3	3	2	2	4	4	4	4
4	4	3	3	2	2	3	3	4	4
4	4	3	3	2	2	3	3	4	4
4	4	3	3	2	2	3	3	4	4
4	4	4	4	2	2	3	3	1	1
4	4	4	4	2	2	3	3	4	4
4	4	3	3	2	2	3	3	4	4
4	4	3	3	2	2	3	3	4	4
4	4	3	3	2	2	3	3	4	4
4	4	3	3	2	2	3	3	4	4
4	4	3	3	2	2	3	3	4	4
4	4	4	4	2	2	3	3	4	4
4	4	3	3	2	2	3	3	4	4
4	4	3	3	2	2	3	3	4	4
4	4	3	3	2	2	3	3	4	4
4	4	4	4	2	2	3	3	4	4
4	4	3	3	2	2	3	3	1	1
4	4	3	3	2	2	3	3	4	4
1	1	4	4	2	2	3	3	1	1
4	4	4	4	2	2	3	3	4	4
4	4	4	4	2	2	3	3	1	1
4	4	4	4	2	2	3	3	1	1
4	4	4	4	2	2	3	3	1	1
4	4	3	3	2	2	3	3	4	4
4	4	4	4	2	2	3	3	4	4
4	4	3	3	2	2	3	3	4	4
4	4	4	4	2	2	3	3	4	4
4	4	3	3	2	2	3	3	4	4
4	4	3	3	3	3	3	3	4	4
4	4	3	3	2	2	3	3	4	4
4	4	3	3	2	2	3	3	4	4
4	4	3	3	2	2	3	3	4	4
4	4	3	3	2	2	3	3	4	4
4	4	3	3	2	2	3	3	4	4

For Review Only

4	4	3	3	2	2	3	3	4	4
4	4	3	3	2	2	3	3	4	4
4	4	3	3	3	3	3	3	4	4
4	4	3	3	2	2	3	3	4	4
4	4	3	3	2	2	4	4	4	4
4	4	3	3	3	3	3	3	4	4
4	4	3	3	2	2	3	3	4	4
1	1	4	4	2	2	3	3	1	1
4	4	3	3	2	2	3	3	4	4
4	4	3	3	2	2	3	3	4	4
4	4	3	3	2	2	3	3	4	4
4	4	3	3	3	3	3	3	4	4
4	4	3	3	2	2	3	3	4	4
4	4	3	3	2	2	3	3	4	4
4	4	3	3	2	2	3	3	4	4
4	4	4	4	2	2	3	3	4	4
4	4	4	4	2	2	3	3	1	1
4	4	4	4	2	2	3	3	4	4
4	4	3	3	2	2	3	3	4	4
4	4	3	3	2	2	3	3	4	4
4	4	3	3	2	2	3	3	4	4
4	4	4	4	2	2	3	3	1	1
4	4	3	3	2	2	3	3	4	4
4	4	3	3	3	3	4	4	4	4
4	4	4	4	2	2	3	3	4	4
4	4	3	3	3	3	3	3	4	4
4	4	3	3	2	2	3	3	4	4
4	4	4	4	2	2	3	3	4	4
4	4	3	3	2	2	3	3	4	4
4	4	3	3	2	2	3	3	4	4
4	4	3	3	2	2	3	3	4	4
4	4	3	3	2	2	3	3	4	4
4	4	4	4	2	2	3	3	1	1
4	4	3	3	2	2	3	3	4	4
1	1	4	4	2	2	3	3	1	1
4	4	4	4	2	2	3	3	4	4
4	4	3	3	2	2	3	3	4	4
4	4	4	4	2	2	3	3	1	1
4	4	4	4	2	2	3	3	1	1
4	4	4	4	2	2	3	3	4	4
4	4	3	3	2	2	3	3	4	4
4	4	3	3	2	2	3	3	4	4
4	4	3	3	2	2	3	3	4	4
4	4	3	3	2	2	3	3	4	4
4	4	3	3	2	2	3	3	4	4
4	4	3	3	3	3	3	3	4	4
4	4	4	4	2	2	3	3	1	1
4	4	3	3	2	2	3	3	4	4
4	4	3	3	2	2	3	3	4	4
4	4	3	3	2	2	3	3	1	1
4	4	3	3	2	2	3	3	4	4
4	4	4	4	2	2	3	3	4	4
4	4	4	4	2	2	3	3	4	4
4	4	3	3	2	2	3	3	4	4
4	4	3	3	2	2	4	4	1	1

For Review Only

4	4	3	3	2	2	3	3	4	4
4	4	3	3	2	2	3	3	4	4
4	4	3	3	2	2	3	3	4	4
4	4	4	4	2	2	3	3	1	1
4	4	3	3	2	2	3	3	4	4
4	4	3	3	2	2	3	3	4	4
4	4	4	4	2	2	3	3	4	4
4	4	3	3	2	2	3	3	4	4
4	4	3	3	2	2	3	3	4	4
4	4	3	3	2	2	3	3	4	4
4	4	3	3	2	2	3	3	4	4
4	4	3	3	2	2	3	3	4	4
4	4	3	3	2	2	3	3	4	4
4	4	3	3	2	2	3	3	4	4
4	4	4	4	2	2	3	3	4	4
4	4	3	3	2	2	3	3	4	4
4	4	4	4	2	2	3	3	4	4
4	4	3	3	2	2	3	3	4	4
4	4	4	4	2	2	3	3	1	1
4	4	3	3	2	2	3	3	4	4
4	4	3	3	2	2	3	3	4	4
4	4	3	3	2	2	3	3	4	4
4	4	3	3	2	2	3	3	4	4
4	4	4	4	2	2	3	3	4	4
4	4	4	4	2	2	3	3	1	1
4	4	4	4	2	2	3	3	1	1
4	4	3	3	2	2	3	3	4	4
4	4	3	3	2	2	3	3	4	4
4	4	3	3	2	2	3	3	4	4
4	4	4	4	2	2	3	3	4	4
4	4	4	4	2	2	3	3	1	1
4	4	3	3	2	2	3	3	4	4
4	4	3	3	2	2	3	3	4	4
4	4	3	3	2	2	3	3	4	4
4	4	4	4	2	2	3	3	4	4
4	4	3	3	2	2	3	3	4	4
1	1	4	4	2	2	3	3	1	1
4	4	3	3	2	2	4	4	4	4
4	4	4	4	2	2	3	3	1	1
4	4	4	4	2	2	3	3	1	1
4	4	3	3	2	2	3	3	4	4
4	4	4	4	2	2	3	3	1	1
4	4	3	3	2	2	4	4	1	1
4	4	4	4	2	2	3	3	4	4
4	4	4	4	2	2	3	3	4	4
4	4	3	3	2	2	3	3	4	4
4	4	3	3	2	2	3	3	4	4
4	4	3	3	2	2	3	3	4	4
1	1	4	4	2	2	3	3	4	4
4	4	3	3	2	2	3	3	4	4
4	4	3	3	2	2	3	3	4	4
4	4	4	4	2	2	3	3	1	1
4	4	4	4	2	2	3	3	4	4
4	4	3	3	3	3	3	3	4	4
4	4	3	3	2	2	3	3	4	4

For Review Only

4	4	3	3	2	2	3	3	4	4
4	4	4	4	2	2	3	3	1	1
4	4	3	3	2	2	3	3	4	4
4	4	4	4	2	2	3	3	1	1
4	4	3	3	3	3	3	3	4	4
1	1	4	4	2	2	3	3	4	4
4	4	4	4	2	2	3	3	1	1
4	4	3	3	2	2	3	3	4	4
4	4	3	3	2	2	3	3	4	4
4	4	3	3	2	2	3	3	4	4
4	4	3	3	2	2	3	3	4	4
4	4	4	4	2	2	3	3	4	4
4	4	3	3	2	2	3	3	4	4
4	4	3	3	2	2	3	3	4	4
4	4	4	4	2	2	3	3	1	1
4	4	4	4	2	2	3	3	1	1
4	4	4	4	2	2	3	3	4	4
4	4	3	3	3	3	3	3	4	4
4	4	3	3	2	2	3	3	4	4
4	4	4	4	2	2	3	3	4	4
4	4	3	3	2	2	3	3	4	4
4	4	4	4	2	2	3	3	4	4
4	4	4	4	2	2	3	3	1	1
4	4	3	3	3	3	3	3	4	4
4	4	3	3	2	2	3	3	4	4
4	4	4	4	2	2	3	3	4	4
4	4	3	3	2	2	4	4	1	1
4	4	4	4	2	2	3	3	4	4
4	4	4	4	2	2	3	3	4	4
4	4	3	3	2	2	3	3	4	4
4	4	3	3	3	3	4	4	4	4
4	4	3	3	2	2	3	3	4	4
4	4	3	3	2	2	3	3	4	4
4	4	4	4	2	2	3	3	1	1
4	4	3	3	2	2	3	3	4	4
4	4	3	3	3	3	3	3	4	4
4	4	3	3	2	2	3	3	4	4
4	4	4	4	2	2	3	3	1	1
4	4	3	3	2	2	3	3	4	4
4	4	3	3	2	2	3	3	4	4
4	4	3	3	2	2	3	3	4	4
4	4	3	3	2	2	3	3	4	4
4	4	4	4	2	2	3	3	1	1
4	4	3	3	2	2	3	3	4	4
4	4	3	3	2	2	3	3	4	4
4	4	3	3	2	2	3	3	4	4
4	4	4	4	2	2	3	3	1	1
4	4	4	4	2	2	3	3	1	1
4	4	4	4	2	2	3	3	4	4
4	4	4	4	2	2	3	3	1	1
4	4	3	3	2	2	3	3	4	4
4	4	4	4	2	2	3	3	1	1
4	4	4	4	2	2	3	3	1	1
4	4	3	3	2	2	3	3	4	4
4	4	4	4	2	2	3	3	1	1

For Review Only

4	4	4	4	2	2	3	3	4	4
4	4	3	3	2	2	3	3	4	4
4	4	3	3	2	2	3	3	4	4
4	4	3	3	2	2	3	3	1	1
4	4	4	4	2	2	3	3	4	4
4	4	3	3	2	2	3	3	4	4
4	4	4	4	2	2	3	3	1	1
4	4	3	3	2	2	3	3	1	1
4	4	3	3	2	2	3	3	4	4
4	4	4	4	2	2	3	3	4	4
4	4	3	3	2	2	3	3	4	4
4	4	4	4	2	2	3	3	4	4
4	4	4	4	2	2	3	3	1	1
4	4	3	3	2	2	3	3	4	4
4	4	3	3	2	2	3	3	4	4
4	4	3	3	2	2	3	3	4	4
4	4	4	4	2	2	3	3	1	1
4	4	4	4	2	2	3	3	4	4
4	4	4	4	2	2	3	3	1	1
4	4	4	4	2	2	3	3	1	1
4	4	3	3	3	3	4	4	4	4
4	4	3	3	2	2	3	3	4	4
4	4	4	4	2	2	3	3	4	4
4	4	3	3	2	2	4	4	1	1
1	1	4	4	2	2	3	3	1	1
4	4	3	3	2	2	3	3	4	4
4	4	4	4	2	2	3	3	4	4
4	4	3	3	2	2	3	3	4	4
4	4	4	4	2	2	3	3	4	4
4	4	3	3	2	2	3	3	4	4
4	4	4	4	2	2	3	3	4	4
4	4	3	3	2	2	3	3	4	4
4	4	4	4	2	2	3	3	4	4
4	4	3	3	2	2	3	3	4	4
4	4	4	4	2	2	3	3	1	1
4	4	4	4	2	2	3	3	4	4
4	4	3	3	2	2	3	3	4	4
4	4	3	3	3	3	3	3	4	4
4	4	3	3	2	2	3	3	4	4
4	4	4	4	2	2	3	3	1	1
4	4	3	3	2	2	3	3	4	4
4	4	4	4	2	2	3	3	4	4
4	4	4	4	2	2	3	3	1	1
4	4	3	3	2	2	3	3	4	4
1	1	4	4	2	2	3	3	4	4
4	4	3	3	2	2	3	3	4	4
4	4	3	3	2	2	3	3	4	4
4	4	3	3	2	2	3	3	4	4
4	4	4	4	2	2	3	3	4	4
4	4	4	4	2	2	3	3	4	4
4	4	3	3	2	2	3	3	4	4
4	4	4	4	2	2	3	3	1	1
4	4	3	3	2	2	3	3	4	4
4	4	3	3	2	2	3	3	4	4
4	4	4	4	2	2	3	3	1	1

For Review Only

4	4	3	3	2	2	3	3	4	4
4	4	3	3	2	2	4	4	4	4

For Review Only

SNP2995	SNP2995	SNP2996	SNP2996	SNP2997	SNP2997	SNP2998	SNP2998	SNP2999	SNP2999
1	1	2	2	4	4	4	4	4	4
1	1	2	2	1	1	4	4	4	4
4	4	4	4	1	1	3	3	1	1
4	4	4	4	1	1	3	3	1	1
4	4	2	2	4	4	4	4	4	4
1	1	2	2	1	1	4	4	4	4
4	4	2	2	4	4	4	4	4	4
1	1	2	2	4	4	4	4	4	4
1	1	2	2	4	4	4	4	4	4
1	1	2	2	4	4	4	4	4	4
1	1	2	2	4	4	4	4	4	4
4	4	4	4	1	1	3	3	1	1
1	1	2	2	4	4	4	4	1	1
1	1	2	2	4	4	4	4	4	4
1	1	2	2	4	4	4	4	1	1
1	1	2	2	4	4	4	4	4	4
1	1	2	2	4	4	4	4	4	4
4	4	4	4	1	1	3	3	1	1
1	1	2	2	1	1	3	3	1	1
4	4	4	4	1	1	3	3	1	1
1	1	2	2	4	4	4	4	4	4
4	4	2	2	4	4	4	4	4	4
1	1	2	2	4	4	4	4	4	4
1	1	2	2	4	4	4	4	4	4
4	4	4	4	1	1	3	3	1	1
1	1	2	2	1	1	3	3	1	1
4	4	4	4	1	1	3	3	1	1
1	1	2	2	4	4	4	4	1	1
1	1	2	2	4	4	4	4	4	4
1	1	2	2	4	4	4	4	4	4
4	4	4	4	1	1	3	3	1	1
1	1	2	2	1	1	4	4	4	4
4	4	2	2	4	4	4	4	4	4
1	1	2	2	4	4	4	4	4	4
1	1	2	2	4	4	4	4	1	1
4	4	4	4	1	1	3	3	1	1
1	1	2	2	4	4	4	4	4	4
1	1	2	2	4	4	4	4	4	4
1	1	2	2	4	4	4	4	4	4
1	1	2	2	4	4	4	4	4	4
1	1	2	2	4	4	4	4	4	4
1	1	2	2	4	4	4	4	4	4
1	1	2	2	4	4	4	4	4	4
4	4	2	2	4	4	4	4	4	4
1	1	2	2	4	4	4	4	4	4
4	4	4	4	1	1	3	3	1	1
4	4	4	4	1	1	3	3	1	1
1	1	2	2	4	4	4	4	1	1
4	4	2	2	4	4	4	4	4	4
4	4	2	2	1	1	3	3	1	1
4	4	4	4	1	1	3	3	1	1
1	1	2	2	4	4	4	4	4	4

For Review Only

4	4	4	4	1	1	3	3	1	1
4	4	2	2	4	4	4	4	4	4
1	1	4	4	1	1	3	3	1	1
4	4	2	2	4	4	4	4	4	4
1	1	2	2	1	1	3	3	1	1
4	4	4	4	1	1	3	3	1	1
1	1	2	2	1	1	3	3	1	1
1	1	2	2	4	4	4	4	4	4
4	4	2	2	4	4	4	4	4	4
1	1	2	2	4	4	4	4	1	1
1	1	4	4	1	1	4	4	4	4
4	4	4	4	1	1	3	3	1	1
4	4	4	4	1	1	3	3	1	1
1	1	2	2	4	4	4	4	4	4
1	1	2	2	4	4	4	4	1	1
1	1	2	2	4	4	4	4	4	4
1	1	2	2	4	4	4	4	1	1
4	4	4	4	1	1	4	4	1	1
4	4	4	4	1	1	3	3	1	1
4	4	2	2	4	4	4	4	4	4
4	4	4	4	1	1	4	4	1	1
1	1	2	2	4	4	4	4	4	4
1	1	2	2	4	4	4	4	4	4
1	1	2	2	1	1	3	3	1	1
1	1	2	2	4	4	4	4	4	4
1	1	2	2	1	1	4	4	4	4
1	1	2	2	4	4	4	4	4	4
1	1	2	2	4	4	4	4	4	4
1	1	2	2	1	1	3	3	1	1
1	1	2	2	4	4	4	4	4	4
1	1	2	2	4	4	4	4	4	4
1	1	2	2	1	1	4	4	1	1
1	1	2	2	4	4	4	4	4	4
4	4	4	4	1	1	3	3	1	1
1	1	2	2	4	4	4	4	4	4
1	1	2	2	4	4	4	4	4	4
1	1	2	2	4	4	4	4	4	4
1	1	2	2	4	4	4	4	4	4
1	1	2	2	4	4	4	4	4	4
1	1	2	2	4	4	4	4	4	4
4	4	2	2	4	4	4	4	4	4
4	4	4	4	1	1	3	3	1	1
4	4	4	4	1	1	3	3	1	1
1	1	4	4	1	1	4	4	4	4
1	1	2	2	4	4	4	4	4	4
1	1	2	2	1	1	4	4	4	4
1	1	2	2	4	4	4	4	4	4
1	1	2	2	1	1	3	3	1	1
1	1	2	2	1	1	3	3	1	1
1	1	2	2	4	4	4	4	1	1
1	1	2	2	1	1	4	4	4	4
1	1	2	2	4	4	4	4	4	4
1	1	2	2	4	4	4	4	4	4
1	1	2	2	4	4	3	3	1	1
1	1	2	2	4	4	4	4	4	4

For Review Only

4	4	4	4	1	1	3	3	1	1
1	1	2	2	4	4	4	4	4	4
4	4	4	4	1	1	3	3	1	1
1	1	2	2	4	4	4	4	4	4
1	1	4	4	1	1	4	4	4	4
4	4	4	4	1	1	3	3	1	1
1	1	2	2	4	4	4	4	4	4
1	1	2	2	1	1	4	4	4	4
1	1	2	2	1	1	3	3	1	1
1	1	2	2	1	1	3	3	1	1
1	1	2	2	4	4	4	4	4	4
1	1	2	2	4	4	4	4	1	1
4	4	4	4	1	1	3	3	1	1
1	1	2	2	1	1	4	4	1	1
4	4	2	2	4	4	4	4	1	1
1	1	2	2	1	1	4	4	1	1
4	4	4	4	1	1	3	3	1	1
4	4	2	2	1	1	4	4	1	1
1	1	2	2	4	4	4	4	4	4
1	1	2	2	4	4	4	4	4	4
1	1	2	2	4	4	4	4	1	1
1	1	2	2	4	4	4	4	4	4
1	1	2	2	1	1	4	4	1	1
1	1	2	2	4	4	4	4	4	4
4	4	2	2	4	4	4	4	1	1
1	1	2	2	4	4	4	4	4	4
1	1	2	2	4	4	4	4	1	1
1	1	2	2	4	4	4	4	4	4
4	4	4	4	1	1	3	3	1	1
4	4	4	4	1	1	3	3	1	1
1	1	2	2	4	4	4	4	4	4
1	1	2	2	4	4	4	4	1	1
4	4	2	2	4	4	4	4	4	4
1	1	2	2	4	4	4	4	1	1
1	1	2	2	4	4	4	4	4	4
1	1	4	4	4	4	4	4	1	1
1	1	2	2	4	4	4	4	4	4
1	1	2	2	1	1	4	4	4	4
1	1	2	2	4	4	4	4	4	4
1	1	2	2	4	4	4	4	4	4
4	4	2	2	4	4	4	4	4	4
1	1	2	2	4	4	4	4	4	4
1	1	2	2	4	4	4	4	4	4
1	1	2	2	4	4	4	4	4	4
1	1	2	2	4	4	4	4	4	4
1	1	2	2	1	1	4	4	4	4
4	4	4	4	1	1	3	3	1	1
1	1	2	2	4	4	4	4	4	4
1	1	2	2	4	4	4	4	1	1
4	4	4	4	1	1	3	3	1	1
1	1	2	2	1	1	3	3	1	1
1	1	2	2	4	4	4	4	4	4
1	1	2	2	4	4	4	4	4	4
4	4	4	4	1	1	3	3	1	1
1	1	2	2	4	4	4	4	4	4

For Review Only

For Review Only

4	4	4	4	1	1	3	3	1	1
1	1	2	2	4	4	4	4	4	4
4	4	4	4	1	1	3	3	1	1
1	1	2	2	4	4	4	4	4	4
4	4	4	4	1	1	3	3	1	1
4	4	4	4	1	1	3	3	1	1
1	1	2	2	4	4	4	4	4	4
1	1	2	2	4	4	4	4	1	1
1	1	2	2	4	4	4	4	1	1
1	1	2	2	4	4	4	4	4	4
1	1	2	2	1	1	3	3	1	1
1	1	2	2	4	4	4	4	4	4
4	4	2	2	1	1	3	3	1	1
1	1	4	4	1	1	4	4	1	1
1	1	2	2	4	4	4	4	4	4
1	1	2	2	4	4	4	4	4	4
1	1	2	2	4	4	4	4	1	1
1	1	2	2	1	1	4	4	4	4
1	1	2	2	1	1	3	3	1	1
1	1	2	2	4	4	4	4	4	4
1	1	2	2	4	4	4	4	1	1
1	1	2	2	4	4	4	4	4	4
1	1	2	2	4	4	4	4	4	4
4	4	4	4	1	1	3	3	1	1
1	1	2	2	4	4	4	4	4	4
1	1	2	2	4	4	4	4	4	4
1	1	2	2	4	4	4	4	4	4
4	4	2	2	4	4	4	4	1	1
4	4	4	4	1	1	3	3	1	1
4	4	2	2	1	1	3	3	1	1
1	1	2	2	1	1	3	3	1	1
1	1	2	2	4	4	4	4	4	4
4	4	4	4	1	1	3	3	1	1
1	1	2	2	4	4	4	4	4	4
4	4	4	4	1	1	3	3	1	1
4	4	4	4	1	1	3	3	1	1
4	4	4	4	1	1	3	3	1	1
1	1	2	2	4	4	4	4	4	4
1	1	2	2	4	4	4	4	4	4
1	1	2	2	4	4	4	4	4	4
4	4	4	4	1	1	3	3	1	1
1	1	2	2	1	1	3	3	1	1
1	1	2	2	4	4	4	4	4	4
1	1	2	2	1	1	3	3	1	1
1	1	2	2	1	1	3	3	4	4
4	4	4	4	1	1	3	3	1	1
1	1	2	2	4	4	4	4	4	4
1	1	2	2	4	4	4	4	4	4
1	1	2	2	4	4	4	4	4	4
1	1	2	2	4	4	4	4	4	4
1	1	2	2	1	1	3	3	1	1
1	1	2	2	4	4	4	4	4	4
1	1	2	2	4	4	4	4	4	4
1	1	4	4	1	1	4	4	4	4
1	1	2	2	4	4	4	4	4	4

1	1	2	2	4	4	4	4	4	4
1	1	2	2	4	4	4	4	4	4
1	1	2	2	1	1	3	3	1	1
1	1	2	2	4	4	4	4	1	1
1	1	2	2	4	4	4	4	4	4
4	4	4	4	1	1	3	3	1	1
1	1	2	2	4	4	4	4	4	4
1	1	2	2	4	4	4	4	4	4
1	1	2	2	4	4	4	4	4	4
1	1	2	2	4	4	4	4	4	4
1	1	2	2	4	4	4	4	1	1
1	1	2	2	4	4	4	4	4	4
1	1	2	2	4	4	4	4	4	4
1	1	2	2	4	4	4	4	4	4
4	4	4	4	1	1	3	3	4	4
1	1	2	2	4	4	4	4	4	4
1	1	2	2	4	4	4	4	4	4
1	1	2	2	4	4	4	4	4	4
1	1	2	2	4	4	4	4	4	4
1	1	2	2	4	4	4	4	4	4
1	1	2	2	1	1	3	3	1	1
4	4	4	4	1	1	3	3	1	1
1	1	2	2	4	4	4	4	4	4
1	1	2	2	4	4	4	4	4	4
1	1	2	2	4	4	4	4	4	4
4	4	2	2	4	4	4	4	1	1
1	1	2	2	4	4	4	4	4	4
4	4	4	4	1	1	3	3	1	1
1	1	2	2	4	4	4	4	4	4
1	1	2	2	4	4	4	4	4	4
1	1	2	2	4	4	4	4	4	4
4	4	4	4	1	1	3	3	1	1
4	4	4	4	1	1	3	3	1	1
1	1	2	2	4	4	4	4	4	4
1	1	2	2	4	4	4	4	4	4
1	1	2	2	4	4	4	4	4	4
4	4	2	2	4	4	4	4	4	4
4	4	4	4	1	1	3	3	1	1
4	4	4	4	1	1	3	3	1	1
1	1	2	2	4	4	4	4	4	4
4	4	4	4	1	1	3	3	1	1
1	1	2	2	4	4	4	4	4	4
1	1	2	2	4	4	4	4	4	4
4	4	2	2	4	4	4	4	4	4
4	4	4	4	1	1	3	3	1	1
4	4	4	4	1	1	3	3	1	1
1	1	2	2	4	4	4	4	4	4
4	4	2	2	1	1	3	3	1	1
1	1	2	2	4	4	4	4	4	4
1	1	2	2	4	4	4	4	4	4
4	4	4	4	1	1	3	3	1	1
1	1	2	2	4	4	4	4	4	4
4	4	4	4	1	1	3	3	1	1
4	4	4	4	1	1	3	3	1	1
1	1	2	2	4	4	4	4	4	4

For Review Only

1	1	2	2	4	4	4	4	4	4
4	4	4	4	1	1	3	3	1	1

For Review Only

SNP300	SNP300	SNP3000	SNP3000	SNP3001	SNP3001	SNP3002	SNP3002	SNP3003	SNP3003
4	4	3	3	1	1	1	1	1	1
4	4	4	4	4	4	1	1	1	1
4	4	4	4	4	4	4	4	4	4
4	4	4	4	4	4	4	4	4	4
4	4	3	3	1	1	1	1	1	1
4	4	4	4	4	4	1	1	1	1
1	1	3	3	1	1	1	1	1	1
4	4	3	3	1	1	1	1	1	1
4	4	3	3	1	1	1	1	1	1
4	4	3	3	1	1	1	1	1	1
4	4	3	3	1	1	1	1	1	1
4	4	4	4	4	4	4	4	4	4
4	4	4	4	1	1	1	1	1	1
4	4	4	4	1	1	1	1	1	1
4	4	4	4	1	1	1	1	1	1
4	4	3	3	1	1	1	1	1	1
4	4	3	3	1	1	1	1	1	1
4	4	4	4	4	4	4	4	1	1
4	4	4	4	4	4	4	4	1	1
1	1	4	4	1	1	4	4	1	1
4	4	3	3	1	1	1	1	1	1
1	1	3	3	1	1	1	1	1	1
1	1	3	3	1	1	1	1	1	1
4	4	3	3	1	1	1	1	1	1
4	4	4	4	4	4	4	4	1	1
4	4	4	4	4	4	4	4	1	1
4	4	4	4	4	4	4	4	4	4
4	4	4	4	1	1	1	1	1	1
4	4	3	3	1	1	1	1	1	1
4	4	3	3	1	1	1	1	1	1
4	4	4	4	4	4	4	4	1	1
4	4	4	4	4	4	1	1	1	1
4	4	3	3	1	1	1	1	1	1
4	4	3	3	1	1	1	1	1	1
4	4	4	4	1	1	1	1	1	1
4	4	4	4	4	4	4	4	1	1
4	4	3	3	1	1	1	1	1	1
4	4	4	4	1	1	1	1	1	1
1	1	3	3	1	1	1	1	1	1
1	1	3	3	1	1	1	1	1	1
4	4	3	3	1	1	1	1	1	1
4	4	3	3	1	1	1	1	1	1
4	4	3	3	1	1	1	1	1	1
4	4	3	3	1	1	1	1	1	1
4	4	3	3	1	1	1	1	1	1
4	4	4	4	4	4	4	4	1	1
4	4	4	4	1	1	4	4	1	1
4	4	3	3	1	1	1	1	1	1
4	4	4	4	1	1	1	1	1	1
4	4	4	4	4	4	4	4	1	1
4	4	4	4	4	4	4	4	4	4
1	1	3	3	1	1	1	1	1	1

For Review Only

4	4	4	4	4	4	4	4	1	1
4	4	3	3	1	1	1	1	1	1
4	4	4	4	4	4	4	4	1	1
1	1	3	3	1	1	1	1	1	1
4	4	4	4	4	4	4	4	1	1
4	4	4	4	4	4	4	4	1	1
4	4	4	4	4	4	4	4	1	1
1	1	3	3	1	1	1	1	1	1
4	4	3	3	1	1	1	1	1	1
4	4	4	4	1	1	1	1	1	1
4	4	4	4	4	4	1	1	1	1
4	4	4	4	4	4	4	4	1	1
4	4	4	4	4	4	4	4	1	1
4	4	3	3	1	1	1	1	1	1
4	4	4	4	1	1	1	1	1	1
4	4	3	3	1	1	1	1	1	1
4	4	3	3	1	1	1	1	1	1
4	4	4	4	4	4	1	1	1	1
4	4	4	4	4	4	4	4	1	1
4	4	3	3	1	1	1	1	1	1
4	4	4	4	1	1	1	1	1	1
4	4	3	3	1	1	1	1	1	1
4	4	3	3	1	1	1	1	1	1
4	4	4	4	4	4	1	1	4	4
4	4	3	3	1	1	1	1	1	1
4	4	4	4	4	4	1	1	4	4
4	4	3	3	1	1	1	1	1	1
1	1	3	3	1	1	1	1	1	1
4	4	4	4	4	4	4	4	1	1
4	4	3	3	1	1	1	1	1	1
1	1	3	3	1	1	1	1	1	1
1	1	4	4	1	1	1	1	1	1
1	1	3	3	1	1	1	1	1	1
4	4	4	4	4	4	4	4	1	1
4	4	3	3	1	1	1	1	1	1
4	4	3	3	1	1	1	1	1	1
1	1	3	3	1	1	1	1	1	1
4	4	3	3	1	1	1	1	1	1
1	1	3	3	1	1	1	1	1	1
4	4	3	3	1	1	1	1	1	1
4	4	3	3	1	1	1	1	1	1
4	4	4	4	4	4	4	4	1	1
4	4	4	4	4	4	4	4	4	4
4	4	4	4	4	4	1	1	1	1
4	4	3	3	1	1	1	1	1	1
1	1	4	4	4	4	1	1	1	1
4	4	3	3	1	1	1	1	1	1
4	4	4	4	4	4	4	4	1	1
4	4	4	4	4	4	4	4	1	1
4	4	4	4	1	1	1	1	1	1
4	4	4	4	1	1	1	1	1	1
1	1	3	3	1	1	1	1	1	1
4	4	3	3	1	1	1	1	1	1
4	4	4	4	1	1	1	1	1	1
4	4	3	3	1	1	1	1	1	1

For Review Only

4	4	4	4	4	4	4	4	4	4
4	4	3	3	1	1	1	1	1	1
4	4	4	4	4	4	4	4	1	1
4	4	3	3	1	1	1	1	1	1
4	4	4	4	4	4	1	1	1	1
4	4	4	4	4	4	4	4	4	4
4	4	3	3	1	1	1	1	1	1
4	4	4	4	4	4	1	1	4	4
4	4	4	4	4	4	4	4	1	1
4	4	4	4	1	1	4	4	1	1
4	4	4	4	1	1	1	1	1	1
4	4	4	4	1	1	1	1	1	1
4	4	4	4	4	4	4	4	4	4
4	4	4	4	1	1	1	1	1	1
4	4	4	4	4	4	1	1	1	1
4	4	4	4	1	1	4	4	1	1
4	4	4	4	4	4	4	4	1	1
4	4	4	4	4	4	1	1	1	1
4	4	3	3	1	1	1	1	1	1
4	4	3	3	1	1	1	1	4	4
4	4	4	4	1	1	1	1	1	1
4	4	3	3	1	1	1	1	1	1
4	4	4	4	1	1	1	1	1	1
1	1	3	3	1	1	1	1	1	1
4	4	3	3	1	1	1	1	1	1
4	4	3	3	1	1	1	1	1	1
4	4	4	4	1	1	1	1	1	1
4	4	3	3	1	1	1	1	1	1
4	4	4	4	4	4	4	4	4	4
4	4	4	4	4	4	4	4	1	1
1	1	3	3	1	1	1	1	1	1
4	4	4	4	1	1	1	1	1	1
4	4	3	3	1	1	1	1	1	1
4	4	4	4	1	1	1	1	4	4
4	4	3	3	1	1	1	1	1	1
4	4	3	3	1	1	1	1	1	1
4	4	3	3	1	1	1	1	1	1
4	4	4	4	4	4	1	1	1	1
4	4	3	3	1	1	1	1	1	1
4	4	3	3	1	1	1	1	1	1
4	4	3	3	1	1	1	1	1	1
4	4	3	3	1	1	1	1	1	1
4	4	3	3	1	1	1	1	1	1
4	4	3	3	1	1	1	1	1	1
4	4	3	3	1	1	1	1	1	1
1	1	4	4	4	4	1	1	4	4
4	4	4	4	4	4	4	4	4	4
4	4	3	3	1	1	1	1	1	1
4	4	4	4	1	1	1	1	1	1
4	4	4	4	4	4	4	4	4	4
4	4	4	4	4	4	4	4	1	1
4	4	3	3	1	1	1	1	1	1
4	4	3	3	1	1	1	1	1	1
4	4	4	4	4	4	4	4	1	1
4	4	3	3	1	1	1	1	1	1

For Review Only

4	4	4	4	4	4	4	4	1	1
4	4	3	3	1	1	1	1	1	1
4	4	4	4	4	4	4	4	1	1
4	4	3	3	1	1	1	1	1	1
4	4	4	4	4	4	4	4	1	1
1	1	4	4	4	4	4	4	1	1
4	4	3	3	1	1	1	1	1	1
4	4	4	4	1	1	1	1	1	1
4	4	3	3	1	1	1	1	1	1
4	4	3	3	1	1	1	1	1	1
4	4	4	4	1	1	4	4	1	1
4	4	3	3	1	1	1	1	1	1
4	4	4	4	4	4	4	4	4	4
4	4	4	4	4	4	1	1	1	1
1	1	3	3	1	1	1	1	1	1
4	4	3	3	1	1	1	1	1	1
4	4	4	4	1	1	1	1	1	1
4	4	4	4	4	4	1	1	1	1
4	4	4	4	4	4	4	4	1	1
4	4	3	3	1	1	1	1	1	1
4	4	4	4	1	1	4	4	1	1
1	1	3	3	1	1	1	1	1	1
4	4	3	3	1	1	1	1	1	1
4	4	4	4	4	4	4	4	1	1
1	1	3	3	1	1	1	1	1	1
4	4	3	3	1	1	1	1	1	1
4	4	3	3	1	1	1	1	1	1
4	4	4	4	4	4	1	1	1	1
4	4	4	4	4	4	4	4	1	1
4	4	4	4	4	4	4	4	1	1
4	4	4	4	4	4	1	1	4	4
4	4	3	3	1	1	1	1	1	1
4	4	4	4	4	4	4	4	1	1
1	1	3	3	1	1	1	1	1	1
4	4	4	4	4	4	4	4	1	1
4	4	4	4	4	4	4	4	1	1
4	4	4	4	4	4	4	4	1	1
4	4	3	3	1	1	1	1	1	1
4	4	3	3	1	1	1	1	1	1
4	4	3	3	1	1	1	1	1	1
4	4	4	4	4	4	4	4	4	4
4	4	4	4	4	4	4	4	1	1
1	1	3	3	1	1	1	1	1	1
4	4	4	4	4	4	4	4	1	1
4	4	4	4	4	4	1	1	1	1
4	4	4	4	4	4	4	4	1	1
4	4	3	3	1	1	1	1	1	1
1	1	3	3	1	1	1	1	1	1
4	4	3	3	1	1	1	1	1	1
4	4	3	3	1	1	1	1	1	1
4	4	4	4	4	4	4	4	1	1
4	4	3	3	1	1	1	1	1	1
4	4	3	3	1	1	1	1	1	1
4	4	4	4	4	4	1	1	1	1
1	1	3	3	1	1	1	1	1	1

For Review Only

4	4	3	3	1	1	1	1	1	1
4	4	3	3	1	1	1	1	1	1
4	4	4	4	1	1	1	1	1	1
4	4	4	4	1	1	1	1	1	1
4	4	3	3	1	1	1	1	1	1
4	4	4	4	4	4	4	4	4	4
4	4	3	3	1	1	1	1	1	1
1	1	3	3	1	1	1	1	1	1
4	4	4	4	1	1	1	1	1	1
1	1	3	3	1	1	1	1	1	1
4	4	4	4	4	4	1	1	1	1
4	4	3	3	1	1	1	1	1	1
4	4	3	3	1	1	1	1	1	1
4	4	3	3	1	1	1	1	1	1
4	4	3	3	1	1	1	1	1	1
4	4	3	3	1	1	1	1	1	1
1	1	3	3	1	1	1	1	1	1
4	4	3	3	1	1	1	1	1	1
4	4	3	3	1	1	1	1	1	1
4	4	3	3	1	1	1	1	1	1
1	1	4	4	4	4	1	1	1	1
4	4	4	4	4	4	4	4	4	4
4	4	3	3	1	1	1	1	1	1
4	4	3	3	1	1	1	1	1	1
1	1	3	3	1	1	1	1	1	1
4	4	4	4	4	4	4	4	1	1
1	1	3	3	1	1	1	1	1	1
4	4	4	4	4	4	4	4	4	4
4	4	3	3	1	1	1	1	1	1
4	4	3	3	1	1	1	1	1	1
1	1	3	3	1	1	1	1	1	1
4	4	4	4	4	4	4	4	1	1
4	4	4	4	4	4	4	4	1	1
4	4	3	3	1	1	1	1	1	1
4	4	3	3	1	1	1	1	1	1
1	1	3	3	1	1	1	1	1	1
4	4	3	3	1	1	1	1	1	1
4	4	4	4	4	4	4	4	1	1
4	4	4	4	4	4	4	4	1	1
1	1	3	3	1	1	1	1	1	1
4	4	4	4	4	4	4	4	1	1
4	4	3	3	1	1	1	1	1	1
4	4	3	3	1	1	1	1	1	1
4	4	3	3	1	1	1	1	1	1
4	4	4	4	4	4	4	4	1	1
4	4	4	4	4	4	4	4	4	4
1	1	3	3	1	1	1	1	1	1
4	4	4	4	4	4	4	4	1	1
4	4	3	3	1	1	1	1	1	1
1	1	3	3	1	1	1	1	1	1
4	4	4	4	4	4	4	4	1	1
1	1	3	3	1	1	1	1	1	1
4	4	4	4	4	4	4	4	1	1
4	4	4	4	4	4	4	4	4	4
1	1	3	3	1	1	1	1	1	1

For Review Only

4	4	3	3	1	1	1	1	1	1
4	4	4	4	4	4	4	4	4	4

For Review Only

SNP3004	SNP3004	SNP3005	SNP3005	SNP3006	SNP3006	SNP3007	SNP3007	SNP3008	SNP3008
4	4	3	3	3	3	2	2	1	1
4	4	3	3	3	3	2	2	1	1
3	3	3	3	3	3	2	2	1	1
3	3	3	3	3	3	2	2	1	1
4	4	3	3	3	3	2	2	1	1
3	3	3	3	3	3	2	2	1	1
4	4	3	3	3	3	2	2	1	1
4	4	3	3	3	3	2	2	1	1
4	4	3	3	3	3	2	2	1	1
4	4	3	3	3	3	2	2	1	1
4	4	3	3	3	3	2	2	1	1
3	3	3	3	3	3	2	2	1	1
4	4	3	3	3	3	2	2	1	1
4	4	3	3	3	3	2	2	1	1
4	4	3	3	3	3	2	2	1	1
3	3	1	1	2	2	3	3	1	1
4	4	3	3	3	3	2	2	1	1
4	4	3	3	3	3	2	2	1	1
4	4	3	3	3	3	2	2	1	1
4	4	3	3	3	3	2	2	1	1
4	4	3	3	3	3	2	2	1	1
4	4	3	3	3	3	2	2	1	1
4	4	3	3	3	3	2	2	1	1
4	4	3	3	3	3	2	2	1	1
4	4	3	3	3	3	2	2	1	1
4	4	1	1	3	3	3	3	1	1
3	3	3	3	3	3	2	2	1	1
4	4	3	3	3	3	2	2	1	1
4	4	3	3	3	3	2	2	1	1
4	4	3	3	3	3	2	2	1	1
4	4	3	3	3	3	2	2	1	1
4	4	3	3	3	3	2	2	4	4
4	4	3	3	3	3	2	2	1	1
4	4	3	3	3	3	2	2	4	4
4	4	3	3	3	3	2	2	1	1
3	3	3	3	3	3	2	2	1	1
4	4	3	3	3	3	2	2	1	1
4	4	3	3	3	3	2	2	1	1
4	4	1	1	3	3	2	2	1	1
4	4	3	3	3	3	2	2	1	1
4	4	3	3	3	3	2	2	1	1
4	4	3	3	3	3	2	2	1	1
4	4	3	3	3	3	2	2	1	1
4	4	3	3	3	3	2	2	1	1
4	4	3	3	3	3	2	2	1	1
3	3	3	3	3	3	2	2	1	1
4	4	3	3	3	3	2	2	1	1
4	4	3	3	3	3	2	2	1	1
4	4	3	3	3	3	2	2	1	1
4	4	3	3	3	3	3	3	1	1
3	3	3	3	3	3	2	2	1	1
4	4	3	3	3	3	2	2	4	4

For Review Only

4	4	3	3	3	3	2	2	1	1
4	4	3	3	3	3	2	2	1	1
4	4	3	3	3	3	2	2	1	1
4	4	3	3	3	3	2	2	1	1
4	4	3	3	3	3	2	2	1	1
4	4	3	3	3	3	2	2	1	1
4	4	1	1	3	3	3	3	1	1
4	4	3	3	3	3	3	3	1	1
4	4	3	3	3	3	2	2	1	1
3	3	3	3	3	3	2	2	1	1
4	4	3	3	3	3	2	2	1	1
4	4	3	3	3	3	2	2	1	1
4	4	3	3	3	3	2	2	1	1
4	4	3	3	3	3	2	2	1	1
4	4	3	3	3	3	2	2	1	1
4	4	3	3	3	3	2	2	1	1
4	4	3	3	3	3	2	2	1	1
4	4	3	3	3	3	2	2	1	1
4	4	3	3	3	3	3	3	1	1
4	4	3	3	3	3	2	2	1	1
4	4	3	3	3	3	2	2	1	1
4	4	3	3	3	3	2	2	1	1
4	4	3	3	3	3	2	2	1	1
4	4	3	3	3	3	2	2	1	1
4	4	3	3	3	3	2	2	1	1
4	4	1	1	2	2	3	3	1	1
4	4	3	3	3	3	2	2	1	1
4	4	3	3	3	3	2	2	1	1
4	4	1	1	3	3	2	2	4	4
3	3	1	1	2	2	3	3	1	1
4	4	3	3	3	3	2	2	1	1
3	3	1	1	2	2	3	3	1	1
4	4	3	3	2	2	2	2	1	1
4	4	3	3	3	3	2	2	1	1
4	4	3	3	3	3	2	2	1	1
4	4	3	3	3	3	2	2	1	1
4	4	3	3	3	3	2	2	1	1
4	4	3	3	3	3	2	2	1	1
4	4	3	3	3	3	2	2	1	1
4	4	3	3	3	3	2	2	1	1
4	4	3	3	3	3	2	2	1	1
3	3	3	3	3	3	2	2	1	1
3	3	3	3	3	3	2	2	1	1
4	4	3	3	3	3	2	2	1	1
3	3	1	1	3	3	2	2	1	1
4	4	3	3	3	3	2	2	1	1
4	4	3	3	3	3	2	2	1	1
4	4	1	1	3	3	3	3	1	1
4	4	1	1	3	3	2	2	1	1
4	4	3	3	3	3	2	2	1	1
4	4	3	3	3	3	2	2	1	1
4	4	3	3	3	3	2	2	4	4
4	4	3	3	3	3	2	2	1	1
3	3	3	3	2	2	2	2	4	4
4	4	3	3	3	3	2	2	1	1

For Review Only

3	3	3	3	3	3	2	2	1	1
4	4	3	3	3	3	2	2	1	1
3	3	3	3	3	3	2	2	1	1
4	4	3	3	3	3	2	2	1	1
4	4	3	3	3	3	2	2	1	1
3	3	3	3	3	3	2	2	1	1
3	3	1	1	2	2	3	3	1	1
4	4	3	3	3	3	2	2	1	1
4	4	1	1	3	3	2	2	4	4
4	4	3	3	3	3	2	2	1	1
4	4	3	3	3	3	2	2	1	1
4	4	3	3	3	3	2	2	1	1
3	3	3	3	3	3	2	2	1	1
4	4	3	3	3	3	3	3	1	1
4	4	3	3	3	3	2	2	1	1
4	4	3	3	3	3	2	2	1	1
4	4	3	3	3	3	2	2	1	1
4	4	3	3	3	3	2	2	1	1
4	4	3	3	3	3	2	2	1	1
3	3	3	3	3	3	2	2	1	1
4	4	3	3	3	3	2	2	1	1
4	4	3	3	2	2	3	3	1	1
4	4	3	3	3	3	3	3	1	1
4	4	3	3	3	3	2	2	1	1
4	4	3	3	3	3	2	2	1	1
4	4	3	3	3	3	2	2	1	1
3	3	3	3	3	3	2	2	1	1
4	4	3	3	3	3	2	2	1	1
3	3	3	3	3	3	2	2	1	1
4	4	3	3	3	3	2	2	1	1
3	3	1	1	3	3	2	2	1	1
4	4	3	3	3	3	2	2	1	1
4	4	3	3	3	3	2	2	1	1
4	4	3	3	3	3	2	2	1	1
4	4	3	3	3	3	2	2	4	4
4	4	3	3	3	3	2	2	4	4
4	4	3	3	3	3	2	2	1	1
3	3	3	3	3	3	2	2	1	1
4	4	3	3	3	3	2	2	4	4
4	4	3	3	3	3	2	2	1	1
4	4	3	3	3	3	2	2	1	1
3	3	3	3	3	3	2	2	1	1
4	4	3	3	3	3	2	2	1	1
4	4	3	3	3	3	2	2	1	1
4	4	3	3	3	3	2	2	1	1
3	3	3	3	3	3	2	2	1	1
3	3	3	3	3	3	2	2	1	1
4	4	3	3	3	3	2	2	1	1
4	4	3	3	3	3	2	2	1	1
3	3	3	3	3	3	2	2	1	1
4	4	3	3	3	3	2	2	1	1
4	4	3	3	3	3	2	2	1	1
4	4	3	3	3	3	2	2	1	1
4	4	3	3	3	3	2	2	1	1
4	4	3	3	3	3	2	2	1	1

For Review Only

4	4	3	3	3	3	3	3	1	1
4	4	3	3	3	3	2	2	1	1
4	4	3	3	3	3	3	3	4	4
4	4	3	3	3	3	2	2	1	1
4	4	3	3	3	3	2	2	1	1
4	4	3	3	3	3	2	2	1	1
4	4	3	3	3	3	2	2	1	1
4	4	3	3	3	3	2	2	1	1
4	4	3	3	3	3	2	2	4	4
3	3	1	1	2	2	3	3	1	1
4	4	3	3	3	3	2	2	1	1
4	4	3	3	3	3	2	2	1	1
3	3	3	3	3	3	2	2	1	1
4	4	3	3	3	3	2	2	1	1
3	3	1	1	2	2	2	2	4	4
4	4	3	3	3	3	2	2	1	1
4	4	3	3	3	3	2	2	1	1
4	4	3	3	3	3	2	2	1	1
4	4	3	3	3	3	2	2	1	1
3	3	1	1	2	2	2	2	1	1
4	4	3	3	3	3	2	2	1	1
4	4	3	3	3	3	2	2	1	1
4	4	3	3	3	3	2	2	1	1
4	4	3	3	3	3	2	2	1	1
4	4	3	3	3	3	2	2	1	1
4	4	3	3	3	3	2	2	4	4
4	4	3	3	3	3	2	2	1	1
4	4	3	3	3	3	2	2	1	1
4	4	3	3	3	3	2	2	1	1
4	4	3	3	3	3	2	2	1	1
4	4	3	3	3	3	2	2	1	1
3	3	1	1	2	2	3	3	1	1
4	4	3	3	3	3	3	3	4	4
3	3	3	3	3	3	2	2	4	4
4	4	3	3	3	3	2	2	4	4
4	4	3	3	3	3	2	2	1	1
3	3	3	3	3	3	2	2	1	1
4	4	3	3	3	3	2	2	1	1
4	4	3	3	3	3	2	2	1	1
4	4	3	3	3	3	2	2	1	1
3	3	3	3	3	3	2	2	1	1
4	4	1	1	3	3	3	3	1	1
4	4	3	3	3	3	2	2	4	4
4	4	3	3	3	3	2	2	4	4
4	4	3	3	3	3	2	2	1	1
4	4	3	3	3	3	2	2	1	1
4	4	3	3	3	3	2	2	1	1
4	4	3	3	3	3	2	2	1	1
4	4	3	3	3	3	2	2	1	1
4	4	3	3	3	3	2	2	1	1
4	4	1	1	3	3	3	3	1	1
4	4	3	3	3	3	2	2	1	1
4	4	3	3	3	3	2	2	1	1
4	4	3	3	3	3	2	2	1	1
4	4	3	3	2	2	2	2	4	4

For Review Only

4	4	3	3	3	3	3	3	1	1
4	4	3	3	3	3	2	2	4	4
3	3	3	3	3	3	2	2	1	1
4	4	3	3	3	3	2	2	1	1
4	4	3	3	3	3	2	2	1	1
4	4	3	3	3	3	2	2	4	4
4	4	3	3	3	3	2	2	1	1
4	4	3	3	3	3	2	2	4	4
4	4	3	3	3	3	2	2	1	1
4	4	3	3	3	3	2	2	1	1
4	4	3	3	3	3	2	2	1	1
4	4	3	3	3	3	2	2	1	1
4	4	3	3	3	3	2	2	1	1
4	4	3	3	3	3	2	2	1	1
4	4	3	3	3	3	2	2	1	1
4	4	3	3	3	3	2	2	1	1
4	4	3	3	3	3	2	2	1	1
4	4	3	3	3	3	2	2	1	1
4	4	3	3	3	3	2	2	1	1
4	4	3	3	3	3	2	2	1	1
4	4	3	3	3	3	2	2	1	1
3	3	3	3	3	3	2	2	1	1
4	4	3	3	3	3	2	2	1	1
4	4	3	3	3	3	2	2	1	1
4	4	3	3	3	3	3	3	1	1
4	4	3	3	3	3	2	2	1	1
4	4	3	3	3	3	2	2	1	1
3	3	3	3	3	3	2	2	1	1
4	4	3	3	3	3	2	2	1	1
3	3	1	1	2	2	3	3	1	1
4	4	3	3	3	3	2	2	1	1
3	3	3	3	3	3	2	2	1	1
4	4	3	3	3	3	2	2	1	1
4	4	3	3	3	3	2	2	1	1
4	4	3	3	3	3	2	2	1	1
4	4	3	3	3	3	2	2	1	1
4	4	3	3	3	3	2	2	1	1
4	4	3	3	3	3	2	2	1	1
3	3	3	3	3	3	2	2	1	1
4	4	3	3	3	3	2	2	1	1
4	4	3	3	3	3	2	2	4	4
4	4	3	3	3	3	2	2	1	1
4	4	3	3	3	3	2	2	1	1
4	4	3	3	3	3	2	2	1	1
4	4	3	3	3	3	2	2	1	1
3	3	3	3	3	3	2	2	1	1
4	4	3	3	3	3	2	2	1	1
4	4	3	3	3	3	2	2	1	1
4	4	3	3	3	3	2	2	1	1
4	4	3	3	3	3	2	2	1	1
4	4	3	3	3	3	2	2	4	4
4	4	3	3	3	3	2	2	1	1
3	3	3	3	3	3	2	2	1	1
3	3	3	3	3	3	2	2	1	1
4	4	3	3	3	3	2	2	1	1

For Review Only

4	4	3	3	3	3	2	2	1	1
3	3	3	3	3	3	2	2	4	4

For Review Only

SNP3009	SNP3009	SNP301	SNP301	SNP3010	SNP3010	SNP3011	SNP3011	SNP3012	SNP3012
4	4	2	2	4	4	1	1	4	4
4	4	2	2	4	4	1	1	2	2
4	4	2	2	4	4	1	1	2	2
4	4	2	2	4	4	1	1	2	2
4	4	2	2	4	4	1	1	4	4
4	4	2	2	4	4	1	1	2	2
4	4	2	2	4	4	1	1	4	4
4	4	2	2	4	4	1	1	2	2
4	4	2	2	4	4	1	1	4	4
4	4	4	4	4	4	1	1	2	2
4	4	2	2	4	4	1	1	4	4
4	4	2	2	4	4	1	1	2	2
4	4	2	2	4	4	1	1	2	2
1	1	2	2	4	4	1	1	4	4
4	4	2	2	4	4	1	1	4	4
4	4	2	2	4	4	1	1	2	2
4	4	2	2	4	4	1	1	4	4
4	4	2	2	4	4	1	1	2	2
4	4	2	2	4	4	1	1	2	2
4	4	2	2	4	4	1	1	2	2
4	4	2	2	4	4	1	1	4	4
4	4	2	2	4	4	1	1	4	4
4	4	2	2	4	4	1	1	4	4
4	4	2	2	4	4	1	1	4	4
4	4	2	2	4	4	1	1	2	2
1	1	2	2	4	4	4	4	4	4
4	4	2	2	4	4	1	1	2	2
4	4	2	2	4	4	1	1	4	4
4	4	2	2	4	4	1	1	4	4
4	4	4	4	4	4	1	1	4	4
4	4	2	2	4	4	1	1	4	4
4	4	2	2	4	4	1	1	2	2
4	4	2	2	4	4	1	1	4	4
4	4	2	2	1	1	4	4	4	4
4	4	2	2	1	1	4	4	4	4
4	4	2	2	4	4	1	1	2	2
4	4	2	2	4	4	1	1	4	4
4	4	4	4	4	4	1	1	4	4
4	4	2	2	4	4	4	4	4	4
4	4	2	2	1	1	4	4	4	4
4	4	2	2	4	4	1	1	4	4
4	4	2	2	4	4	1	1	4	4
4	4	2	2	4	4	1	1	4	4
4	4	2	2	4	4	1	1	4	4
4	4	4	4	4	4	1	1	4	4
4	4	2	2	4	4	1	1	2	2
4	4	2	2	4	4	1	1	2	2
4	4	2	2	1	1	4	4	2	2
4	4	2	2	4	4	1	1	4	4
4	4	2	2	4	4	4	4	4	4
4	4	2	2	4	4	1	1	2	2
4	4	2	2	4	4	1	1	2	2

For Review Only

4	4	2	2	4	4	1	1	2	2
4	4	2	2	4	4	1	1	4	4
4	4	2	2	4	4	1	1	2	2
4	4	2	2	4	4	1	1	4	4
4	4	2	2	4	4	1	1	2	2
4	4	2	2	4	4	1	1	2	2
1	1	2	2	4	4	4	4	4	4
4	4	2	2	4	4	1	1	2	2
4	4	2	2	1	1	1	1	4	4
4	4	2	2	4	4	1	1	4	4
4	4	2	2	4	4	1	1	4	4
4	4	2	2	4	4	1	1	2	2
4	4	2	2	4	4	1	1	2	2
4	4	2	2	4	4	1	1	4	4
4	4	2	2	1	1	4	4	2	2
4	4	2	2	4	4	1	1	4	4
4	4	2	2	4	4	1	1	4	4
4	4	2	2	1	1	4	4	4	4
4	4	2	2	1	1	4	4	4	4
4	4	2	2	4	4	1	1	4	4
4	4	2	2	4	4	1	1	2	2
4	4	4	4	4	4	4	4	4	4
4	4	2	2	4	4	1	1	4	4
4	4	2	2	4	4	1	1	4	4
4	4	4	4	4	4	1	1	4	4
4	4	2	2	4	4	1	1	2	2
4	4	4	4	4	4	1	1	4	4
4	4	2	2	4	4	1	1	4	4
1	1	2	2	4	4	4	4	4	4
4	4	2	2	1	1	4	4	4	4
4	4	2	2	4	4	1	1	4	4
4	4	2	2	1	1	1	1	2	2
4	4	2	2	4	4	1	1	4	4
4	4	2	2	4	4	1	1	2	2
4	4	2	2	4	4	1	1	2	2
4	4	4	4	4	4	1	1	4	4
4	4	2	2	4	4	1	1	4	4
4	4	2	2	4	4	1	1	4	4
4	4	2	2	4	4	1	1	4	4
4	4	2	2	4	4	1	1	4	4
4	4	2	2	4	4	1	1	4	4
4	4	2	2	4	4	1	1	2	2
4	4	2	2	4	4	1	1	4	4
4	4	2	2	4	4	1	1	4	4
4	4	2	2	1	1	4	4	2	2
4	4	2	2	4	4	1	1	2	2
1	1	2	2	4	4	1	1	4	4
1	1	2	2	4	4	4	4	4	4
4	4	2	2	4	4	1	1	2	2
4	4	2	2	4	4	1	1	4	4
4	4	2	2	4	4	1	1	4	4
4	4	2	2	4	4	1	1	4	4
4	4	2	2	4	4	1	1	2	2
4	4	2	2	1	1	4	4	4	4
4	4	2	2	4	4	1	1	4	4

For Review Only

4	4	2	2	4	4	1	1	2	2
4	4	4	4	4	4	1	1	4	4
4	4	2	2	4	4	1	1	2	2
1	1	2	2	4	4	1	1	4	4
4	4	2	2	4	4	1	1	2	2
1	1	2	2	4	4	4	4	4	4
4	4	2	2	4	4	1	1	2	2
4	4	2	2	4	4	1	1	4	4
4	4	2	2	4	4	1	1	2	2
4	4	2	2	4	4	1	1	2	2
4	4	2	2	4	4	1	1	4	4
4	4	2	2	1	1	4	4	4	4
4	4	2	2	4	4	1	1	2	2
4	4	2	2	4	4	1	1	2	2
4	4	2	2	4	4	1	1	4	4
1	1	2	2	4	4	4	4	4	4
4	4	2	2	1	1	4	4	4	4
4	4	2	2	4	4	1	1	4	4
1	1	2	2	4	4	1	1	4	4
4	4	2	2	4	4	1	1	2	2
4	4	2	2	4	4	1	1	4	4
4	4	2	2	1	1	4	4	4	4
4	4	2	2	4	4	1	1	2	2
4	4	2	2	4	4	1	1	4	4
4	4	2	2	4	4	4	4	4	4
4	4	2	2	1	1	4	4	4	4
4	4	2	2	4	4	4	4	4	4
4	4	2	2	1	1	4	4	4	4
4	4	2	2	4	4	1	1	2	2
4	4	2	2	1	1	4	4	4	4
1	1	2	2	4	4	1	1	4	4
4	4	2	2	4	4	4	4	4	4
4	4	2	2	4	4	1	1	4	4
4	4	2	2	4	4	1	1	4	4
4	4	2	2	1	1	4	4	4	4
4	4	2	2	1	1	4	4	4	4
4	4	2	2	4	4	1	1	4	4
4	4	2	2	4	4	1	1	2	2
4	4	2	2	4	4	1	1	4	4
4	4	2	2	4	4	1	1	4	4
4	4	2	2	1	1	4	4	4	4
1	1	2	2	4	4	1	1	4	4
4	4	2	2	4	4	1	1	4	4
4	4	2	2	4	4	1	1	2	2
4	4	2	2	4	4	1	1	4	4
4	4	2	2	1	1	4	4	4	4
4	4	2	2	4	4	1	1	2	2
4	4	2	2	4	4	1	1	4	4
4	4	2	2	4	4	1	1	4	4
4	4	2	2	4	4	1	1	2	2
4	4	2	2	4	4	4	4	4	4
4	4	2	2	1	1	4	4	4	4
1	1	2	2	1	1	4	4	4	4
4	4	2	2	4	4	1	1	2	2
4	4	2	2	4	4	1	1	4	4

For Review Only

4	4	2	2	1	1	4	4	4	4
4	4	2	2	4	4	1	1	4	4
4	4	2	2	4	4	1	1	2	2
4	4	2	2	4	4	4	4	4	4
4	4	2	2	4	4	1	1	2	2
4	4	2	2	4	4	1	1	2	2
4	4	2	2	4	4	1	1	4	4
4	4	2	2	4	4	1	1	4	4
4	4	2	2	1	1	4	4	4	4
4	4	2	2	1	1	4	4	4	4
4	4	2	2	4	4	1	1	2	2
4	4	2	2	1	1	1	1	4	4
4	4	2	2	1	1	4	4	2	2
4	4	2	2	1	1	4	4	4	4
4	4	2	2	4	4	1	1	4	4
4	4	2	2	4	4	1	1	4	4
1	1	2	2	1	1	1	1	4	4
4	4	2	2	4	4	1	1	2	2
4	4	2	2	4	4	1	1	2	2
4	4	2	2	4	4	1	1	2	2
4	4	2	2	4	4	4	4	4	4
4	4	2	2	4	4	1	1	4	4
4	4	4	4	1	1	4	4	4	4
4	4	2	2	4	4	1	1	2	2
4	4	2	2	4	4	1	1	4	4
4	4	2	2	1	1	4	4	4	4
4	4	2	2	4	4	1	1	4	4
4	4	2	2	1	1	4	4	4	4
4	4	2	2	1	1	4	4	4	4
4	4	2	2	1	1	4	4	4	4
4	4	2	2	4	4	1	1	4	4
4	4	2	2	1	1	4	4	4	4
4	4	2	2	4	4	1	1	2	2
1	1	2	2	4	4	1	1	4	4
4	4	2	2	1	1	4	4	4	4
4	4	2	2	4	4	1	1	2	2
4	4	4	4	4	4	1	1	2	2
4	4	2	2	4	4	1	1	2	2
4	4	2	2	4	4	1	1	4	4
4	4	2	2	4	4	1	1	4	4
4	4	2	2	4	4	1	1	2	2
1	1	2	2	4	4	4	4	4	4
4	4	2	2	4	4	4	4	4	4
1	1	2	2	4	4	4	4	4	4
1	1	2	2	4	4	4	4	4	4
4	4	2	2	4	4	1	1	2	2
4	4	2	2	4	4	1	1	4	4
4	4	2	2	4	4	1	1	4	4
4	4	2	2	4	4	1	1	4	4
4	4	2	2	4	4	1	1	4	4
1	1	2	2	4	4	4	4	4	4
4	4	2	2	4	4	1	1	2	2
4	4	4	4	1	1	4	4	4	4
4	4	2	2	4	4	1	1	4	4
4	4	2	2	4	4	1	1	4	4

For Review Only

4	4	2	2	1	1	4	4	4	4
4	4	2	2	4	4	1	1	2	2
4	4	2	2	4	4	1	1	2	2
4	4	2	2	1	1	1	1	4	4
4	4	2	2	4	4	1	1	2	2
4	4	2	2	1	1	1	1	2	2
4	4	2	2	1	1	4	4	4	4
4	4	2	2	1	1	4	4	4	4
4	4	2	2	4	4	1	1	4	4
4	4	2	2	4	4	1	1	4	4
4	4	2	2	1	1	4	4	4	4
4	4	2	2	4	4	1	1	4	4
4	4	2	2	4	4	1	1	4	4
4	4	2	2	4	4	1	1	4	4
4	4	2	2	4	4	1	1	4	4
4	4	2	2	1	1	4	4	4	4
4	4	2	2	4	4	1	1	4	4
4	4	2	2	1	1	4	4	2	2
4	4	4	4	1	1	4	4	4	4
4	4	4	4	4	4	1	1	4	4
4	4	4	4	4	4	1	1	4	4
4	4	2	2	4	4	1	1	2	2
4	4	2	2	1	1	4	4	2	2
4	4	2	2	4	4	1	1	4	4
4	4	2	2	4	4	1	1	2	2
4	4	2	2	4	4	1	1	4	4
4	4	2	2	4	4	4	4	2	2
4	4	2	2	4	4	1	1	2	2
4	4	2	2	4	4	1	1	2	2
4	4	2	2	4	4	1	1	2	2
4	4	2	2	4	4	4	4	2	2
4	4	2	2	4	4	1	1	2	2
4	4	2	2	1	1	4	4	4	4
4	4	2	2	4	4	1	1	4	4
4	4	4	4	1	1	4	4	4	4
4	4	2	2	4	4	4	4	2	2
4	4	2	2	4	4	1	1	4	4
4	4	2	2	4	4	1	1	2	2
4	4	2	2	4	4	1	1	2	2
4	4	2	2	1	1	4	4	4	4
4	4	2	2	1	1	4	4	4	4
4	4	2	2	4	4	4	4	4	4
4	4	2	2	4	4	1	1	4	4
4	4	2	2	4	4	1	1	4	4
4	4	2	2	4	4	1	1	2	2
4	4	2	2	4	4	1	1	4	4
4	4	2	2	4	4	1	1	4	4
4	4	2	2	4	4	1	1	2	2
4	4	2	2	4	4	1	1	4	4
4	4	2	2	4	4	4	4	2	2
4	4	2	2	1	1	1	1	2	2
1	1	2	2	4	4	4	4	4	4
4	4	2	2	4	4	1	1	2	2
4	4	2	2	4	4	1	1	2	2
4	4	2	2	4	4	1	1	4	4

For Review Only

4	4	2	2	1	1	4	4	4	4
4	4	2	2	1	1	4	4	4	4

For Review Only

SNP3013	SNP3013	SNP3014	SNP3014	SNP3015	SNP3015	SNP3016	SNP3016	SNP3017	SNP3017
4	4	2	2	3	3	4	4	3	3
4	4	3	3	1	1	4	4	3	3
4	4	3	3	1	1	4	4	3	3
4	4	3	3	1	1	4	4	3	3
4	4	2	2	3	3	4	4	3	3
4	4	3	3	1	1	4	4	3	3
4	4	2	2	3	3	4	4	3	3
4	4	3	3	1	1	1	1	2	2
4	4	2	2	3	3	4	4	3	3
4	4	3	3	1	1	4	4	3	3
4	4	2	2	3	3	1	1	2	2
4	4	3	3	1	1	4	4	3	3
4	4	3	3	3	3	1	1	3	3
1	1	2	2	3	3	1	1	2	2
1	1	2	2	3	3	4	4	3	3
4	4	3	3	1	1	4	4	3	3
4	4	2	2	3	3	1	1	2	2
4	4	3	3	1	1	4	4	3	3
4	4	3	3	1	1	4	4	3	3
4	4	3	3	1	1	4	4	3	3
4	4	2	2	3	3	1	1	2	2
4	4	2	2	3	3	4	4	3	3
4	4	2	2	3	3	1	1	2	2
1	1	2	2	3	3	1	1	2	2
4	4	3	3	1	1	4	4	3	3
4	4	3	3	1	1	1	1	2	2
4	4	3	3	1	1	4	4	3	3
4	4	2	2	1	1	4	4	3	3
4	4	2	2	3	3	1	1	2	2
4	4	2	2	3	3	1	1	2	2
4	4	2	2	3	3	4	4	3	3
4	4	3	3	3	3	4	4	3	3
4	4	2	2	3	3	4	4	3	3
4	4	2	2	3	3	1	1	2	2
1	1	2	2	1	1	4	4	3	3
4	4	3	3	3	3	4	4	3	3
4	4	2	2	3	3	4	4	3	3
4	4	2	2	3	3	1	1	2	2
4	4	2	2	3	3	1	1	2	2
1	1	3	3	1	1	4	4	3	3
4	4	2	2	3	3	1	1	2	2
4	4	2	2	3	3	1	1	2	2
4	4	2	2	3	3	4	4	3	3
4	4	2	2	3	3	4	4	3	3
4	4	2	2	3	3	4	4	3	3
4	4	3	3	1	1	4	4	3	3
4	4	3	3	3	3	4	4	3	3
4	4	2	2	1	1	4	4	3	3
4	4	2	2	3	3	4	4	3	3
1	1	2	2	1	1	4	4	3	3
4	4	3	3	1	1	4	4	3	3
4	4	2	2	1	1	4	4	3	3

4	4	3	3	1	1	4	4	3	3
4	4	2	2	3	3	4	4	3	3
4	4	2	2	1	1	4	4	3	3
4	4	2	2	3	3	4	4	3	3
4	4	3	3	1	1	4	4	3	3
4	4	3	3	3	3	4	4	3	3
4	4	3	3	1	1	1	1	2	2
4	4	3	3	1	1	1	1	2	2
4	4	2	2	3	3	4	4	3	3
4	4	2	2	1	1	4	4	3	3
4	4	2	2	3	3	4	4	3	3
4	4	3	3	3	3	4	4	3	3
4	4	3	3	1	1	4	4	3	3
4	4	2	2	3	3	1	1	2	2
1	1	2	2	1	1	4	4	3	3
4	4	2	2	3	3	1	1	2	2
4	4	2	2	3	3	1	1	2	2
1	1	3	3	1	1	4	4	3	3
1	1	2	2	1	1	4	4	3	3
4	4	2	2	3	3	4	4	3	3
4	4	3	3	3	3	4	4	3	3
1	1	2	2	1	1	1	1	2	2
4	4	2	2	3	3	1	1	2	2
4	4	2	2	3	3	4	4	3	3
4	4	2	2	3	3	4	4	3	3
4	4	3	3	1	1	4	4	3	3
4	4	2	2	3	3	1	1	2	2
4	4	2	2	1	1	1	1	2	2
4	4	3	3	1	1	1	1	2	2
1	1	2	2	1	1	4	4	3	3
4	4	2	2	3	3	1	1	3	3
4	4	3	3	1	1	4	4	3	3
4	4	2	2	3	3	1	1	2	2
4	4	3	3	1	1	4	4	3	3
4	4	3	3	1	1	4	4	3	3
4	4	2	2	3	3	4	4	3	3
4	4	2	2	3	3	1	1	2	2
4	4	2	2	1	1	4	4	3	3
4	4	2	2	3	3	1	1	2	2
4	4	2	2	3	3	4	4	3	3
4	4	2	2	3	3	4	4	3	3
4	4	3	3	3	3	4	4	3	3
4	4	3	3	1	1	4	4	3	3
4	4	2	2	3	3	4	4	3	3
4	4	3	3	1	1	4	4	3	3
4	4	3	3	1	1	4	4	3	3
1	1	2	2	3	3	4	4	3	3
4	4	3	3	1	1	1	1	2	2
4	4	3	3	1	1	4	4	3	3
4	4	2	2	1	1	4	4	3	3
4	4	2	2	3	3	4	4	3	3
4	4	2	2	3	3	1	1	2	2
4	4	3	3	1	1	4	4	3	3
1	1	2	2	1	1	4	4	3	3
4	4	2	2	3	3	4	4	3	3

For Review Only

4	4	3	3	1	1	4	4	3	3
4	4	2	2	3	3	1	1	3	3
4	4	3	3	3	3	4	4	3	3
1	1	2	2	3	3	4	4	3	3
4	4	3	3	1	1	4	4	3	3
4	4	3	3	1	1	1	1	2	2
4	4	3	3	1	1	4	4	3	3
4	4	2	2	1	1	4	4	3	3
4	4	3	3	1	1	4	4	3	3
4	4	3	3	1	1	4	4	3	3
4	4	2	2	3	3	4	4	3	3
1	1	2	2	1	1	4	4	3	3
4	4	3	3	1	1	4	4	3	3
4	4	3	3	1	1	4	4	3	3
4	4	2	2	1	1	4	4	3	3
4	4	2	2	1	1	4	4	3	3
1	1	3	3	1	1	4	4	3	3
4	4	2	2	1	1	4	4	3	3
1	1	2	2	3	3	1	1	2	2
4	4	3	3	1	1	4	4	3	3
4	4	2	2	1	1	4	4	3	3
1	1	2	2	1	1	4	4	3	3
4	4	3	3	1	1	4	4	3	3
4	4	2	2	3	3	1	1	2	2
1	1	3	3	1	1	4	4	3	3
1	1	3	3	1	1	1	1	2	2
4	4	2	2	1	1	4	4	3	3
1	1	2	2	1	1	1	1	2	2
4	4	3	3	1	1	4	4	3	3
1	1	3	3	1	1	4	4	3	3
1	1	2	2	1	1	1	1	2	2
1	1	2	2	1	1	4	4	3	3
4	4	2	2	3	3	4	4	3	3
4	4	3	3	1	1	4	4	3	3
4	4	2	2	3	3	1	1	2	2
1	1	2	2	1	1	1	1	2	2
4	4	2	2	3	3	4	4	3	3
4	4	3	3	1	1	4	4	3	3
1	1	2	2	1	1	1	1	2	2
4	4	2	2	3	3	1	1	2	2
1	1	3	3	1	1	1	1	2	2
1	1	2	2	1	1	4	4	3	3
4	4	2	2	3	3	4	4	3	3
4	4	3	3	1	1	4	4	3	3
4	4	2	2	3	3	4	4	3	3
1	1	2	2	1	1	4	4	3	3
4	4	3	3	1	1	4	4	3	3
4	4	2	2	3	3	4	4	3	3
1	1	2	2	1	1	1	1	2	2
4	4	3	3	1	1	4	4	3	3
4	4	3	3	1	1	4	4	3	3
1	1	2	2	1	1	1	1	2	2
1	1	2	2	1	1	1	1	3	3
4	4	3	3	3	3	4	4	3	3
4	4	3	3	1	1	4	4	3	3

For Review Only

1	1	2	2	1	1	1	1	2	2
4	4	2	2	3	3	1	1	2	2
4	4	3	3	1	1	4	4	3	3
4	4	2	2	3	3	4	4	3	3
4	4	3	3	1	1	4	4	3	3
4	4	3	3	1	1	4	4	3	3
4	4	2	2	3	3	4	4	3	3
4	4	2	2	1	1	4	4	3	3
1	1	2	2	1	1	1	1	3	3
1	1	2	2	1	1	4	4	3	3
4	4	3	3	1	1	4	4	3	3
4	4	2	2	3	3	1	1	2	2
1	1	3	3	1	1	4	4	3	3
1	1	2	2	1	1	4	4	3	3
4	4	2	2	3	3	1	1	2	2
4	4	2	2	3	3	1	1	2	2
1	1	2	2	1	1	1	1	2	2
4	4	3	3	1	1	4	4	3	3
4	4	3	3	1	1	4	4	3	3
4	4	3	3	1	1	4	4	3	3
4	4	2	2	1	1	4	4	3	3
4	4	2	2	3	3	1	1	2	2
1	1	3	3	1	1	1	1	2	2
4	4	3	3	3	3	4	4	3	3
4	4	2	2	3	3	1	1	2	2
4	4	2	2	3	3	1	1	2	2
4	4	2	2	3	3	4	4	3	3
1	1	2	2	1	1	4	4	3	3
1	1	3	3	1	1	4	4	3	3
1	1	2	2	1	1	4	4	3	3
4	4	2	2	3	3	4	4	3	3
1	1	2	2	1	1	4	4	3	3
4	4	3	3	1	1	4	4	3	3
1	1	2	2	3	3	4	4	3	3
1	1	2	2	1	1	4	4	3	3
4	4	3	3	3	3	4	4	3	3
4	4	3	3	1	1	4	4	3	3
4	4	3	3	1	1	4	4	3	3
4	4	2	2	3	3	4	4	3	3
4	4	2	2	3	3	4	4	3	3
4	4	3	3	1	1	4	4	3	3
4	4	3	3	1	1	1	1	2	2
1	1	2	2	1	1	1	1	2	2
4	4	3	3	1	1	4	4	3	3
4	4	2	2	1	1	4	4	2	2
4	4	3	3	1	1	4	4	3	3
4	4	2	2	3	3	1	1	2	2
4	4	2	2	3	3	4	4	3	3
1	1	2	2	1	1	1	1	2	2
4	4	2	2	3	3	1	1	2	2
4	4	3	3	1	1	1	1	2	2
4	4	3	3	1	1	4	4	3	3
1	1	3	3	1	1	1	1	2	2
4	4	2	2	3	3	4	4	3	3
4	4	2	2	1	1	1	1	2	2

For Review Only

1	1	2	2	1	1	1	1	2	2
4	4	3	3	1	1	4	4	3	3
4	4	3	3	1	1	4	4	3	3
1	1	3	3	1	1	4	4	3	3
4	4	3	3	1	1	4	4	3	3
4	4	3	3	1	1	4	4	3	3
1	1	2	2	1	1	1	1	2	2
4	4	2	2	1	1	1	1	2	2
4	4	2	2	1	1	4	4	3	3
4	4	2	2	3	3	1	1	2	2
1	1	2	2	1	1	4	4	3	3
4	4	2	2	3	3	4	4	3	3
4	4	2	2	3	3	1	1	2	2
4	4	2	2	3	3	1	1	2	2
4	4	2	2	3	3	4	4	3	3
1	1	2	2	1	1	4	4	3	3
4	4	2	2	3	3	1	1	2	2
4	4	3	3	1	1	4	4	3	3
1	1	2	2	1	1	1	1	2	2
4	4	2	2	3	3	1	1	2	2
4	4	2	2	3	3	4	4	3	3
4	4	3	3	1	1	4	4	3	3
4	4	3	3	1	1	4	4	3	3
4	4	2	2	3	3	4	4	3	3
4	4	3	3	1	1	1	1	2	2
4	4	3	3	1	1	4	4	3	3
4	4	2	2	3	3	1	1	2	2
4	4	3	3	1	1	4	4	3	3
4	4	3	3	1	1	4	4	3	3
4	4	3	3	1	1	4	4	3	3
4	4	2	2	3	3	1	1	2	2
4	4	3	3	1	1	4	4	3	3
1	1	3	3	1	1	4	4	3	3
4	4	2	2	3	3	4	4	3	3
1	1	3	3	1	1	1	1	2	2
4	4	2	2	3	3	1	1	2	2
4	4	2	2	3	3	4	4	3	3
4	4	3	3	3	3	4	4	3	3
4	4	3	3	3	3	4	4	3	3
1	1	2	2	1	1	1	1	2	2
1	1	2	2	1	1	1	1	2	2
1	1	2	2	1	1	1	1	2	2
4	4	2	2	3	3	1	1	2	2
4	4	2	2	3	3	4	4	3	3
4	4	3	3	1	1	4	4	3	3
4	4	2	2	3	3	1	1	2	2
4	4	2	2	3	3	4	4	3	3
4	4	3	3	1	1	4	4	3	3
4	4	2	2	3	3	4	4	3	3
4	4	2	2	3	3	1	1	2	2
4	4	3	3	1	1	4	4	3	3
4	4	2	2	3	3	1	1	2	2
4	4	3	3	1	1	4	4	3	3
4	4	3	3	1	1	4	4	3	3
4	4	2	2	3	3	1	1	2	2

For Review Only

1	1	2	2	1	1	4	4	3	3
1	1	2	2	1	1	4	4	3	3

For Review Only

SNP3018	SNP3018	SNP3019	SNP3019	SNP302	SNP302	SNP3020	SNP3020	SNP3021	SNP3021
4	4	3	3	4	4	2	2	4	4
1	1	3	3	4	4	2	2	4	4
4	4	3	3	4	4	2	2	4	4
1	1	3	3	4	4	2	2	4	4
4	4	3	3	4	4	2	2	4	4
4	4	3	3	4	4	2	2	4	4
4	4	3	3	4	4	2	2	4	4
4	4	3	3	4	4	2	2	4	4
4	4	3	3	4	4	2	2	4	4
4	4	3	3	4	4	2	2	4	4
1	1	1	1	1	1	3	3	4	4
4	4	3	3	4	4	2	2	4	4
4	4	3	3	4	4	2	2	4	4
1	1	1	1	4	4	2	2	4	4
4	4	3	3	4	4	2	2	4	4
4	4	1	1	1	1	2	2	4	4
4	4	1	1	4	4	3	3	1	1
4	4	3	3	4	4	2	2	4	4
4	4	3	3	4	4	2	2	4	4
4	4	3	3	4	4	3	3	1	1
4	4	1	1	4	4	3	3	4	4
4	4	3	3	1	1	2	2	4	4
4	4	1	1	4	4	3	3	4	4
4	4	3	3	4	4	3	3	4	4
4	4	3	3	4	4	2	2	4	4
4	4	3	3	4	4	2	2	4	4
1	1	3	3	4	4	2	2	4	4
4	4	3	3	4	4	2	2	4	4
4	4	1	1	4	4	2	2	4	4
4	4	3	3	4	4	3	3	1	1
1	1	3	3	4	4	2	2	4	4
1	1	3	3	4	4	2	2	4	4
4	4	3	3	4	4	2	2	4	4
4	4	3	3	4	4	3	3	1	1
4	4	1	1	4	4	2	2	4	4
1	1	3	3	4	4	2	2	4	4
1	1	1	1	4	4	3	3	4	4
4	4	1	1	4	4	3	3	4	4
1	1	1	1	4	4	3	3	4	4
4	4	3	3	4	4	3	3	1	1
4	4	1	1	4	4	3	3	1	1
4	4	1	1	1	1	2	2	4	4
4	4	3	3	1	1	2	2	4	4
4	4	1	1	4	4	2	2	4	4
4	4	3	3	4	4	2	2	4	4
4	4	3	3	4	4	2	2	4	4
1	1	3	3	4	4	2	2	4	4
4	4	3	3	4	4	2	2	4	4
4	4	1	1	4	4	2	2	4	4
4	4	3	3	4	4	2	2	4	4
1	1	3	3	4	4	2	2	4	4
4	4	3	3	4	4	2	2	4	4

For Review Only

4	4	3	3	4	4	2	2	4	4
4	4	3	3	4	4	2	2	4	4
4	4	3	3	4	4	2	2	4	4
4	4	3	3	4	4	2	2	4	4
4	4	3	3	4	4	2	2	4	4
1	1	3	3	4	4	2	2	4	4
4	4	3	3	4	4	2	2	4	4
4	4	3	3	4	4	2	2	4	4
1	1	1	1	4	4	3	3	4	4
4	4	3	3	4	4	2	2	4	4
4	4	3	3	4	4	2	2	4	4
1	1	3	3	4	4	2	2	4	4
4	4	3	3	4	4	2	2	4	4
4	4	1	1	4	4	2	2	4	4
4	4	3	3	4	4	2	2	4	4
4	4	1	1	4	4	2	2	4	4
4	4	1	1	4	4	3	3	4	4
4	4	3	3	4	4	2	2	4	4
4	4	3	3	4	4	2	2	4	4
4	4	3	3	4	4	2	2	4	4
1	1	3	3	4	4	2	2	4	4
4	4	1	1	4	4	3	3	4	4
4	4	1	1	4	4	2	2	4	4
1	1	3	3	4	4	2	2	4	4
4	4	3	3	4	4	2	2	4	4
1	1	3	3	4	4	2	2	4	4
4	4	1	1	4	4	3	3	1	1
4	4	3	3	4	4	3	3	4	4
4	4	3	3	4	4	2	2	4	4
4	4	3	3	4	4	2	2	4	4
4	4	3	3	4	4	2	2	4	4
1	1	3	3	4	4	2	2	4	4
1	1	1	1	4	4	3	3	4	4
4	4	3	3	4	4	2	2	4	4
4	4	3	3	4	4	2	2	4	4
4	4	3	3	4	4	2	2	4	4
4	4	1	1	4	4	3	3	1	1
4	4	3	3	4	4	2	2	4	4
4	4	1	1	4	4	3	3	4	4
4	4	3	3	4	4	2	2	4	4
4	4	3	3	4	4	2	2	4	4
1	1	3	3	4	4	2	2	4	4
1	1	3	3	1	1	2	2	4	4
4	4	3	3	4	4	2	2	4	4
4	4	1	1	4	4	2	2	4	4
1	1	3	3	4	4	2	2	4	4
1	1	1	1	4	4	2	2	4	4
4	4	3	3	4	4	2	2	4	4
4	4	3	3	4	4	2	2	4	4
4	4	3	3	4	4	2	2	4	4
1	1	3	3	4	4	2	2	4	4
1	1	1	1	1	1	3	3	4	4
4	4	3	3	4	4	2	2	4	4
4	4	3	3	4	4	2	2	4	4
4	4	3	3	4	4	2	2	4	4

For Review Only

4	4	3	3	4	4	2	2	4	4
4	4	3	3	4	4	3	3	4	4
1	1	3	3	4	4	2	2	4	4
1	1	1	1	4	4	3	3	4	4
1	1	3	3	4	4	2	2	4	4
4	4	3	3	4	4	2	2	4	4
4	4	1	1	4	4	2	2	4	4
4	4	3	3	4	4	2	2	4	4
4	4	3	3	4	4	2	2	4	4
4	4	3	3	4	4	2	2	4	4
4	4	3	3	4	4	2	2	1	1
4	4	3	3	4	4	2	2	4	4
1	1	3	3	4	4	2	2	4	4
4	4	3	3	4	4	2	2	4	4
4	4	3	3	4	4	2	2	4	4
4	4	3	3	4	4	2	2	4	4
4	4	3	3	4	4	2	2	4	4
4	4	3	3	4	4	2	2	4	4
1	1	1	1	4	4	2	2	4	4
1	1	3	3	4	4	2	2	4	4
4	4	3	3	4	4	2	2	4	4
4	4	3	3	4	4	2	2	4	4
4	4	3	3	4	4	2	2	4	4
1	1	1	1	4	4	3	3	4	4
4	4	3	3	4	4	3	3	4	4
4	4	1	1	4	4	3	3	4	4
4	4	3	3	4	4	2	2	4	4
4	4	3	3	4	4	3	3	1	1
1	1	3	3	4	4	2	2	4	4
4	4	3	3	4	4	2	2	4	4
1	1	1	1	4	4	3	3	4	4
4	4	3	3	4	4	2	2	4	4
4	4	3	3	4	4	2	2	4	4
4	4	3	3	4	4	2	2	4	4
4	4	3	3	4	4	3	3	1	1
4	4	3	3	4	4	2	2	4	4
1	1	1	1	4	4	3	3	4	4
4	4	3	3	4	4	2	2	4	4
4	4	3	3	4	4	3	3	4	4
1	1	3	3	4	4	3	3	4	4
4	4	1	1	4	4	3	3	4	4
1	1	3	3	4	4	2	2	4	4
4	4	3	3	4	4	2	2	4	4
4	4	3	3	4	4	2	2	4	4
4	4	3	3	4	4	2	2	4	4
1	1	3	3	4	4	2	2	4	4
4	4	3	3	4	4	2	2	4	4
4	4	3	3	4	4	2	2	4	4
4	4	1	1	4	4	3	3	4	4
4	4	3	3	4	4	2	2	4	4
4	4	3	3	4	4	2	2	4	4
1	1	1	1	4	4	3	3	4	4
4	4	3	3	4	4	2	2	4	4
1	1	3	3	4	4	2	2	4	4
4	4	3	3	4	4	2	2	4	4

For Review Only

1	1	1	1	4	4	2	2	4	4
4	4	1	1	1	1	3	3	4	4
4	4	3	3	4	4	2	2	4	4
4	4	1	1	4	4	3	3	4	4
4	4	3	3	4	4	2	2	4	4
4	4	3	3	4	4	2	2	4	4
4	4	3	3	1	1	3	3	4	4
4	4	3	3	4	4	2	2	4	4
1	1	3	3	4	4	2	2	4	4
4	4	3	3	4	4	2	2	4	4
4	4	3	3	4	4	2	2	4	4
4	4	3	3	4	4	2	2	4	4
4	4	3	3	4	4	2	2	4	4
4	4	3	3	4	4	2	2	4	4
1	1	1	1	4	4	2	2	4	4
1	1	1	1	1	1	3	3	4	4
4	4	3	3	4	4	2	2	4	4
1	1	3	3	4	4	2	2	4	4
4	4	3	3	4	4	2	2	4	4
4	4	1	1	4	4	2	2	4	4
4	4	3	3	4	4	2	2	4	4
1	1	1	1	4	4	3	3	4	4
4	4	1	1	4	4	3	3	4	4
1	1	3	3	4	4	2	2	4	4
4	4	1	1	4	4	2	2	4	4
4	4	3	3	4	4	3	3	1	1
4	4	3	3	4	4	2	2	4	4
4	4	3	3	4	4	2	2	4	4
4	4	3	3	4	4	2	2	4	4
4	4	3	3	4	4	2	2	4	4
1	1	3	3	4	4	2	2	4	4
4	4	3	3	4	4	2	2	4	4
4	4	3	3	4	4	2	2	4	4
4	4	1	1	4	4	2	2	4	4
4	4	3	3	4	4	2	2	4	4
1	1	3	3	4	4	2	2	4	4
4	4	3	3	4	4	2	2	4	4
4	4	3	3	4	4	3	3	1	1
4	4	3	3	4	4	2	2	4	4
1	1	3	3	4	4	2	2	4	4
4	4	3	3	4	4	2	2	4	4
4	4	3	3	4	4	2	2	4	4
4	4	1	1	4	4	3	3	4	4
4	4	3	3	4	4	2	2	4	4
4	4	3	3	4	4	2	2	4	4
4	4	3	3	4	4	2	2	4	4
1	1	1	1	1	1	3	3	4	4
4	4	3	3	1	1	3	3	1	1
4	4	3	3	4	4	3	3	1	1
4	4	1	1	4	4	3	3	4	4
4	4	3	3	4	4	2	2	4	4
4	4	3	3	4	4	2	2	4	4
4	4	1	1	4	4	3	3	4	4
4	4	3	3	4	4	2	2	4	4
1	1	1	1	1	1	3	3	4	4

4	4	1	1	4	4	3	3	1	1
1	1	3	3	4	4	3	3	1	1
4	4	3	3	4	4	2	2	4	4
4	4	1	1	4	4	2	2	4	4
4	4	3	3	4	4	2	2	4	4
1	1	3	3	4	4	2	2	4	4
4	4	1	1	1	1	3	3	4	4
4	4	1	1	1	1	2	2	1	1
4	4	3	3	4	4	2	2	4	4
1	1	3	3	4	4	3	3	4	4
4	4	3	3	4	4	2	2	4	4
1	1	3	3	4	4	2	2	4	4
4	4	1	1	4	4	3	3	4	4
4	4	3	3	4	4	2	2	4	4
4	4	3	3	4	4	2	2	4	4
4	4	1	1	4	4	2	2	4	4
4	4	1	1	1	1	3	3	4	4
4	4	1	1	4	4	2	2	4	4
1	1	1	1	4	4	3	3	4	4
4	4	1	1	4	4	3	3	4	4
1	1	3	3	4	4	2	2	4	4
1	1	3	3	4	4	2	2	4	4
4	4	1	1	4	4	2	2	4	4
4	4	3	3	4	4	2	2	4	4
4	4	3	3	4	4	2	2	4	4
4	4	3	3	4	4	2	2	4	4
1	1	1	1	4	4	3	3	4	4
1	1	3	3	4	4	2	2	4	4
4	4	3	3	4	4	2	2	4	4
4	4	3	3	4	4	2	2	4	4
1	1	1	1	1	1	3	3	4	4
4	4	3	3	4	4	2	2	4	4
4	4	3	3	4	4	2	2	4	4
4	4	3	3	4	4	2	2	4	4
4	4	1	1	4	4	3	3	4	4
1	1	1	1	4	4	3	3	4	4
4	4	3	3	4	4	2	2	4	4
1	1	3	3	4	4	2	2	4	4
1	1	3	3	4	4	2	2	4	4
1	1	1	1	4	4	3	3	4	4
4	4	3	3	4	4	2	2	4	4
4	4	3	3	4	4	2	2	4	4
4	4	1	1	4	4	3	3	4	4
4	4	3	3	4	4	2	2	4	4
4	4	3	3	4	4	2	2	4	4
4	4	1	1	4	4	3	3	4	4
4	4	3	3	1	1	2	2	4	4
4	4	3	3	4	4	2	2	4	4
4	4	3	3	4	4	2	2	4	4
1	1	1	1	4	4	3	3	4	4
4	4	3	3	4	4	2	2	4	4
4	4	1	1	1	1	3	3	4	4
4	4	3	3	4	4	2	2	4	4
1	1	3	3	4	4	2	2	4	4
4	4	1	1	4	4	3	3	4	4

For Review Only

4	4	1	1	4	4	2	2	4	4
4	4	3	3	4	4	2	2	4	4

For Review Only

SNP3022	SNP3022	SNP3023	SNP3023	SNP3024	SNP3024	SNP3025	SNP3025	SNP3026	SNP3026
1	1	1	1	3	3	3	3	2	2
1	1	1	1	3	3	1	1	3	3
1	1	1	1	3	3	3	3	2	2
1	1	1	1	3	3	1	1	3	3
1	1	4	4	3	3	3	3	2	2
1	1	1	1	3	3	1	1	3	3
1	1	4	4	3	3	3	3	2	2
4	4	1	1	3	3	3	3	2	2
1	1	4	4	3	3	3	3	2	2
1	1	1	1	3	3	3	3	2	2
4	4	1	1	3	3	3	3	2	2
1	1	1	1	3	3	1	1	2	2
1	1	1	1	3	3	3	3	2	2
1	1	1	1	1	1	3	3	2	2
1	1	1	1	3	3	1	1	2	2
1	1	4	4	3	3	3	3	2	2
4	4	4	4	1	1	3	3	2	2
1	1	1	1	3	3	3	3	2	2
1	1	1	1	3	3	1	1	3	3
1	1	4	4	1	1	3	3	2	2
4	4	4	4	3	3	3	3	2	2
1	1	4	4	3	3	3	3	2	2
1	1	1	1	3	3	3	3	2	2
4	4	1	1	1	1	3	3	2	2
1	1	4	4	3	3	3	3	2	2
1	1	1	1	3	3	1	1	3	3
1	1	1	1	3	3	1	1	3	3
1	1	1	1	3	3	3	3	2	2
1	1	4	4	3	3	3	3	2	2
4	4	4	4	1	1	3	3	2	2
1	1	1	1	3	3	3	3	2	2
1	1	1	1	3	3	1	1	3	3
1	1	4	4	3	3	3	3	2	2
4	4	4	4	1	1	3	3	2	2
1	1	1	1	3	3	1	1	2	2
1	1	1	1	3	3	1	1	3	3
4	4	4	4	1	1	3	3	2	2
1	1	1	1	3	3	3	3	2	2
4	4	4	4	1	1	3	3	2	2
1	1	1	1	3	3	3	3	2	2
4	4	4	4	3	3	3	3	2	2
1	1	4	4	3	3	3	3	2	2
1	1	1	1	3	3	3	3	2	2
1	1	1	1	3	3	3	3	2	2
1	1	1	1	3	3	3	3	2	2
1	1	1	1	3	3	3	3	2	2
1	1	1	1	3	3	1	1	3	3
1	1	1	1	3	3	1	1	2	2
1	1	4	4	3	3	3	3	2	2
1	1	1	1	3	3	3	3	2	2
1	1	1	1	3	3	1	1	3	3
1	1	4	4	3	3	3	3	2	2

For Review Only

1	1	4	4	3	3	3	3	2	2
1	1	4	4	3	3	3	3	2	2
1	1	1	1	3	3	3	3	2	2
1	1	4	4	3	3	3	3	2	2
1	1	1	1	3	3	1	1	3	3
1	1	1	1	3	3	1	1	3	3
1	1	1	1	3	3	3	3	2	2
4	4	1	1	1	1	3	3	2	2
1	1	4	4	3	3	3	3	2	2
1	1	1	1	3	3	1	1	2	2
1	1	1	1	3	3	1	1	3	3
1	1	1	1	3	3	1	1	3	3
1	1	4	4	3	3	3	3	2	2
1	1	1	1	3	3	3	3	2	2
1	1	1	1	3	3	1	1	2	2
4	4	1	1	3	3	3	3	2	2
1	1	4	4	3	3	3	3	2	2
1	1	1	1	3	3	3	3	2	2
1	1	4	4	3	3	3	3	2	2
1	1	4	4	3	3	3	3	2	2
1	1	1	1	3	3	1	1	2	2
1	1	4	4	3	3	3	3	2	2
1	1	4	4	3	3	3	3	2	2
1	1	1	1	3	3	3	3	2	2
1	1	1	1	3	3	3	3	2	2
1	1	1	1	3	3	1	1	2	2
1	1	1	1	3	3	3	3	2	2
4	4	4	4	1	1	3	3	2	2
1	1	1	1	3	3	1	1	2	2
1	1	4	4	3	3	3	3	2	2
1	1	4	4	3	3	3	3	2	2
1	1	4	4	3	3	1	1	3	3
4	4	4	4	1	1	3	3	2	2
1	1	4	4	3	3	3	3	2	2
1	1	1	1	3	3	3	3	2	2
1	1	1	1	3	3	3	3	2	2
4	4	4	4	1	1	3	3	2	2
1	1	4	4	3	3	3	3	2	2
1	1	4	4	3	3	3	3	2	2
1	1	1	1	3	3	3	3	2	2
1	1	4	4	3	3	1	1	2	2
1	1	1	1	3	3	1	1	3	3
1	1	1	1	3	3	1	1	3	3
1	1	1	1	3	3	1	1	3	3
1	1	1	1	3	3	1	1	2	2
1	1	1	1	3	3	1	1	3	3
1	1	4	4	3	3	3	3	2	2
1	1	1	1	3	3	3	3	2	2
1	1	1	1	3	3	1	1	3	3
1	1	1	1	3	3	3	3	2	2
1	1	4	4	3	3	1	1	2	2
4	4	1	1	1	1	3	3	2	2
1	1	1	1	3	3	1	1	3	3
1	1	4	4	3	3	3	3	2	2
1	1	1	1	3	3	3	3	2	2

For Review Only

1	1	1	1	3	3	1	1	3	3
1	1	4	4	1	1	1	1	3	3
1	1	1	1	3	3	1	1	3	3
4	4	4	4	1	1	3	3	2	2
1	1	1	1	3	3	1	1	3	3
1	1	1	1	3	3	3	3	2	2
1	1	4	4	3	3	3	3	2	2
1	1	1	1	3	3	1	1	2	2
1	1	1	1	3	3	1	1	3	3
1	1	1	1	3	3	1	1	2	2
4	4	4	4	3	3	3	3	2	2
1	1	1	1	3	3	1	1	2	2
1	1	1	1	3	3	1	1	3	3
1	1	1	1	3	3	1	1	2	2
1	1	1	1	1	1	3	3	2	2
1	1	1	1	3	3	3	3	2	2
1	1	1	1	3	3	3	3	2	2
1	1	1	1	3	3	1	1	2	2
1	1	1	1	1	1	3	3	2	2
1	1	4	4	3	3	3	3	2	2
1	1	1	1	3	3	1	1	2	2
1	1	4	4	3	3	3	3	2	2
1	1	1	1	3	3	3	3	2	2
4	4	4	4	1	1	3	3	2	2
1	1	1	1	3	3	3	3	2	2
1	1	4	4	3	3	3	3	2	2
1	1	1	1	3	3	3	3	2	2
1	1	4	4	3	3	3	3	2	2
1	1	1	1	3	3	1	1	3	3
1	1	1	1	3	3	3	3	2	2
4	4	4	4	1	1	3	3	2	2
1	1	1	1	3	3	1	1	2	2
1	1	4	4	3	3	3	3	2	2
1	1	1	1	3	3	3	3	2	2
4	4	4	4	1	1	3	3	2	2
4	4	4	4	1	1	3	3	2	2
4	4	4	4	1	1	3	3	2	2
1	1	1	1	3	3	3	3	2	2
1	1	1	1	3	3	1	1	2	2
4	4	4	4	1	1	3	3	2	2
1	1	4	4	3	3	3	3	2	2
1	1	1	1	1	1	3	3	2	2
1	1	1	1	3	3	3	3	2	2
4	4	1	1	3	3	3	3	2	2
1	1	1	1	3	3	3	3	2	2
1	1	1	1	3	3	1	1	3	3
1	1	1	1	3	3	1	1	2	2
1	1	4	4	3	3	1	1	3	3
4	4	4	4	3	3	3	3	2	2
1	1	1	1	3	3	1	1	3	3
1	1	1	1	3	3	1	1	2	2
4	4	1	1	3	3	3	3	2	2
4	4	4	4	1	1	3	3	2	2
1	1	1	1	3	3	1	1	3	3
1	1	1	1	3	3	1	1	2	2

For Review Only

1	1	1	1	3	3	1	1	3	3
1	1	4	4	3	3	1	1	2	2
1	1	1	1	3	3	3	3	2	2
1	1	4	4	3	3	3	3	2	2
1	1	1	1	3	3	3	3	2	2
1	1	1	1	3	3	3	3	2	2
1	1	1	1	3	3	3	3	2	2
1	1	1	1	3	3	1	1	2	2
1	1	1	1	3	3	1	1	3	3
1	1	4	4	3	3	3	3	2	2
1	1	1	1	3	3	1	1	2	2
1	1	1	1	1	1	3	3	2	2
1	1	1	1	3	3	1	1	2	2
1	1	1	1	3	3	1	1	2	2
1	1	4	4	1	1	3	3	2	2
4	4	1	1	1	1	3	3	2	2
4	4	4	4	1	1	3	3	2	2
1	1	1	1	3	3	1	1	3	3
1	1	1	1	3	3	3	3	2	2
1	1	4	4	3	3	3	3	2	2
1	1	1	1	3	3	1	1	2	2
4	4	1	1	1	1	3	3	2	2
1	1	4	4	3	3	3	3	2	2
1	1	1	1	3	3	1	1	3	3
1	1	4	4	3	3	3	3	2	2
4	4	4	4	1	1	3	3	2	2
1	1	1	1	3	3	1	1	2	2
1	1	1	1	3	3	3	3	2	2
1	1	1	1	3	3	3	3	2	2
1	1	1	1	3	3	3	3	2	2
1	1	1	1	3	3	3	3	2	2
1	1	4	4	3	3	3	3	2	2
1	1	1	1	3	3	3	3	2	2
4	4	4	4	1	1	3	3	2	2
1	1	4	4	3	3	3	3	2	2
1	1	1	1	3	3	1	1	3	3
1	1	1	1	3	3	1	1	2	2
1	1	4	4	1	1	3	3	2	2
1	1	4	4	3	3	1	1	3	3
1	1	4	4	3	3	1	1	3	3
1	1	1	1	3	3	3	3	2	2
1	1	1	1	3	3	3	3	2	2
4	4	4	4	1	1	3	3	2	2
1	1	1	1	3	3	3	3	2	2
1	1	1	1	3	3	3	3	2	2
1	1	1	1	3	3	3	3	2	2
4	4	1	1	1	1	3	3	2	2
1	1	1	1	3	3	3	3	2	2
4	4	4	4	1	1	3	3	2	2
1	1	4	4	3	3	3	3	2	2
1	1	1	1	3	3	1	1	3	3
1	1	1	1	3	3	3	3	2	2
1	1	4	4	3	3	3	3	2	2
1	1	1	1	3	3	1	1	3	3
4	4	1	1	1	1	3	3	2	2

For Review Only

4	4	4	4	1	1	3	3	2	2
1	1	1	1	3	3	1	1	3	3
1	1	1	1	3	3	3	3	2	2
1	1	4	4	3	3	3	3	2	2
1	1	4	4	1	1	3	3	2	2
1	1	1	1	3	3	1	1	3	3
1	1	4	4	3	3	3	3	2	2
4	4	1	1	3	3	3	3	2	2
1	1	4	4	3	3	3	3	2	2
4	4	4	4	1	1	3	3	2	2
1	1	1	1	3	3	1	1	2	2
1	1	1	1	3	3	3	3	2	2
1	1	4	4	3	3	3	3	2	2
1	1	1	1	1	1	3	3	2	2
1	1	4	4	3	3	3	3	2	2
1	1	4	4	3	3	3	3	2	2
1	1	4	4	3	3	3	3	2	2
4	4	4	4	1	1	3	3	2	2
4	4	4	4	3	3	3	3	2	2
1	1	4	4	3	3	3	3	2	2
1	1	1	1	3	3	3	3	2	2
1	1	1	1	3	3	1	1	3	3
1	1	4	4	3	3	3	3	2	2
1	1	1	1	3	3	3	3	2	2
4	4	1	1	1	1	3	3	2	2
1	1	1	1	3	3	1	1	3	3
4	4	1	1	1	1	3	3	2	2
1	1	1	1	3	3	1	1	3	3
1	1	4	4	1	1	3	3	2	2
1	1	4	4	3	3	3	3	2	2
4	4	1	1	1	1	3	3	2	2
1	1	1	1	3	3	1	1	2	2
1	1	1	1	3	3	3	3	2	2
1	1	4	4	3	3	1	1	2	2
1	1	4	4	3	3	3	3	2	2
4	4	1	1	1	1	3	3	2	2
1	1	4	4	3	3	3	3	2	2
1	1	1	1	3	3	1	1	3	3
1	1	1	1	3	3	1	1	3	3
4	4	4	4	1	1	3	3	2	2
1	1	1	1	3	3	3	3	2	2
1	1	1	1	3	3	1	1	3	3
1	1	4	4	3	3	3	3	2	2
1	1	4	4	3	3	3	3	2	2
1	1	1	1	3	3	3	3	2	2
1	1	4	4	3	3	3	3	2	2
1	1	4	4	3	3	1	1	3	3
1	1	4	4	3	3	3	3	2	2
1	1	1	1	3	3	3	3	2	2
4	4	1	1	1	1	3	3	2	2
1	1	1	1	3	3	3	3	2	2
1	1	4	4	1	1	3	3	2	2
1	1	1	1	3	3	3	3	2	2
1	1	1	1	3	3	1	1	3	3
1	1	4	4	3	3	3	3	2	2

For Review Only

1	1	4	4	3	3	3	3	2	2
1	1	4	4	3	3	3	3	2	2

For Review Only

SNP3027	SNP3027	SNP3028	SNP3028	SNP3029	SNP3029	SNP303	SNP303	SNP3030	SNP3030
2	2	2	2	1	1	2	2	3	3
1	1	3	3	1	1	2	2	3	3
2	2	2	2	1	1	2	2	3	3
1	1	3	3	1	1	2	2	3	3
2	2	2	2	1	1	3	3	3	3
1	1	3	3	1	1	2	2	3	3
2	2	2	2	1	1	2	2	3	3
2	2	2	2	1	1	3	3	3	3
2	2	2	2	1	1	2	2	3	3
2	2	2	2	1	1	2	2	3	3
2	2	2	2	4	4	2	2	2	2
2	2	2	2	1	1	2	2	3	3
2	2	2	2	4	4	2	2	2	2
2	2	2	2	4	4	2	2	2	2
2	2	2	2	4	4	2	2	3	3
2	2	2	2	4	4	2	2	2	2
2	2	2	2	1	1	2	2	3	3
2	2	2	2	1	1	2	2	3	3
1	1	3	3	1	1	2	2	3	3
2	2	2	2	1	1	2	2	3	3
2	2	2	2	4	4	2	2	2	2
2	2	2	2	1	1	2	2	2	2
2	2	2	2	4	4	2	2	2	2
2	2	2	2	4	4	2	2	2	2
2	2	2	2	1	1	2	2	3	3
1	1	3	3	1	1	2	2	3	3
1	1	3	3	1	1	2	2	3	3
2	2	2	2	1	1	2	2	3	3
2	2	2	2	1	1	2	2	3	3
2	2	2	2	4	4	2	2	2	2
2	2	2	2	4	4	2	2	2	2
1	1	3	3	1	1	2	2	3	3
2	2	2	2	1	1	2	2	3	3
2	2	2	2	4	4	2	2	2	2
2	2	2	2	1	1	2	2	3	3
1	1	3	3	1	1	2	2	3	3
2	2	2	2	1	1	2	2	2	2
2	2	2	2	4	4	2	2	3	3
2	2	2	2	4	4	2	2	2	2
2	2	2	2	4	4	2	2	2	2
2	2	2	2	1	1	3	3	2	2
2	2	2	2	1	1	2	2	3	3
2	2	2	2	1	1	3	3	3	3
2	2	2	2	1	1	2	2	2	2
2	2	2	2	1	1	3	3	3	3
2	2	2	2	1	1	2	2	3	3
1	1	3	3	1	1	2	2	3	3
1	1	3	3	1	1	2	2	3	3
2	2	2	2	1	1	2	2	2	2
2	2	2	2	4	4	2	2	3	3
1	1	3	3	1	1	2	2	3	3
2	2	2	2	1	1	2	2	3	3

For Review Only

2	2	2	2	1	1	2	2	3	3
2	2	2	2	1	1	2	2	3	3
2	2	2	2	1	1	2	2	3	3
2	2	2	2	1	1	2	2	2	2
1	1	3	3	1	1	2	2	3	3
1	1	3	3	1	1	2	2	3	3
2	2	2	2	1	1	2	2	3	3
2	2	2	2	4	4	2	2	2	2
2	2	2	2	1	1	2	2	3	3
2	2	2	2	4	4	2	2	2	2
1	1	3	3	1	1	2	2	3	3
1	1	3	3	1	1	2	2	3	3
2	2	2	2	1	1	2	2	3	3
2	2	2	2	1	1	2	2	3	3
2	2	3	3	4	4	2	2	3	3
2	2	2	2	1	1	2	2	3	3
2	2	2	2	4	4	2	2	3	3
2	2	2	2	1	1	2	2	3	3
2	2	2	2	4	4	2	2	2	2
2	2	2	2	1	1	2	2	3	3
2	2	3	3	4	4	2	2	3	3
2	2	2	2	1	1	2	2	3	3
2	2	2	2	1	1	2	2	3	3
2	2	2	2	1	1	2	2	3	3
2	2	2	2	1	1	3	3	3	3
1	1	3	3	1	1	2	2	3	3
2	2	2	2	1	1	3	3	2	2
2	2	2	2	4	4	2	2	2	2
2	2	2	2	1	1	2	2	3	3
2	2	2	2	4	4	2	2	2	2
2	2	2	2	1	1	2	2	3	3
1	1	3	3	1	1	2	2	3	3
2	2	2	2	4	4	2	2	2	2
2	2	2	2	1	1	2	2	3	3
2	2	2	2	1	1	2	2	3	3
2	2	2	2	1	1	3	3	3	3
2	2	2	2	1	1	2	2	3	3
2	2	2	2	4	4	2	2	3	3
2	2	2	2	1	1	2	2	3	3
2	2	2	2	1	1	3	3	3	3
2	2	2	2	1	1	2	2	3	3
1	1	3	3	4	4	2	2	3	3
1	1	3	3	1	1	2	2	3	3
1	1	3	3	1	1	2	2	3	3
2	2	2	2	4	4	2	2	2	2
1	1	3	3	1	1	2	2	3	3
2	2	2	2	4	4	2	2	2	2
2	2	2	2	1	1	2	2	3	3
1	1	3	3	1	1	2	2	3	3
2	2	2	2	1	1	2	2	3	3
2	2	3	3	4	4	2	2	3	3
2	2	2	2	4	4	2	2	2	2
1	1	3	3	1	1	2	2	3	3
2	2	2	2	4	4	2	2	3	3
2	2	2	2	1	1	2	2	3	3

For Review Only

1	1	3	3	1	1	2	2	3	3
2	2	2	2	1	1	2	2	3	3
1	1	3	3	1	1	2	2	3	3
2	2	2	2	4	4	2	2	2	2
1	1	3	3	1	1	2	2	3	3
2	2	2	2	1	1	2	2	3	3
2	2	2	2	4	4	3	3	2	2
1	1	3	3	4	4	2	2	2	2
1	1	3	3	1	1	2	2	3	3
2	2	2	2	1	1	2	2	3	3
2	2	2	2	4	4	2	2	3	3
2	2	2	2	4	4	2	2	3	3
1	1	3	3	1	1	2	2	3	3
2	2	3	3	1	1	2	2	3	3
2	2	2	2	4	4	2	2	2	2
2	2	2	2	4	4	2	2	3	3
2	2	2	2	1	1	2	2	3	3
2	2	2	2	1	1	2	2	3	3
2	2	2	2	4	4	2	2	2	2
2	2	2	2	4	4	2	2	3	3
2	2	2	2	4	4	2	2	2	2
2	2	2	2	4	4	2	2	2	2
2	2	2	2	1	1	2	2	3	3
2	2	2	2	4	4	2	2	2	2
2	2	3	3	1	1	2	2	3	3
2	2	2	2	1	1	2	2	3	3
2	2	2	2	4	4	2	2	2	2
2	2	2	2	1	1	2	2	3	3
1	1	3	3	1	1	2	2	3	3
2	2	2	2	4	4	2	2	3	3
2	2	2	2	4	4	2	2	2	2
2	2	2	2	4	4	2	2	3	3
2	2	2	2	1	1	2	2	3	3
2	2	2	2	4	4	2	2	3	3
2	2	2	2	4	4	2	2	2	2
2	2	2	2	4	4	2	2	2	2
2	2	2	2	4	4	2	2	2	2
2	2	2	2	1	1	2	2	3	3
2	2	2	2	4	4	2	2	2	2
2	2	2	2	4	4	2	2	2	2
2	2	2	2	1	1	2	2	3	3
2	2	2	2	4	4	2	2	2	2
2	2	2	2	1	1	2	2	3	3
2	2	2	2	1	1	2	2	2	2
2	2	2	2	1	1	2	2	3	3
1	1	3	3	1	1	2	2	3	3
1	1	3	3	1	1	2	2	3	3
1	1	3	3	1	1	2	2	3	3
2	2	2	2	4	4	2	2	2	2
1	1	3	3	1	1	2	2	3	3
2	2	2	2	1	1	2	2	3	3
2	2	2	2	4	4	2	2	2	2
2	2	2	2	1	1	2	2	3	3
1	1	3	3	1	1	2	2	3	3
2	2	2	2	4	4	2	2	2	2

For Review Only

1	1	3	3	4	4	2	2	2	2
2	2	2	2	4	4	2	2	3	3
2	2	2	2	1	1	2	2	3	3
2	2	2	2	1	1	2	2	3	3
2	2	2	2	1	1	2	2	3	3
2	2	2	2	1	1	2	2	3	3
2	2	2	2	4	4	2	2	3	3
2	2	3	3	1	1	2	2	2	2
1	1	3	3	4	4	2	2	3	3
2	2	2	2	4	4	2	2	2	2
1	1	3	3	1	1	2	2	2	2
2	2	2	2	4	4	2	2	2	2
2	2	3	3	1	1	2	2	3	3
2	2	2	2	1	1	2	2	3	3
2	2	2	2	4	4	2	2	2	2
2	2	2	2	4	4	2	2	2	2
2	2	2	2	1	1	2	2	2	2
2	2	3	3	1	1	2	2	3	3
2	2	2	2	1	1	2	2	3	3
2	2	2	2	4	4	3	3	2	2
2	2	2	2	1	1	2	2	3	3
2	2	2	2	4	4	2	2	2	2
2	2	2	2	1	1	2	2	3	3
1	1	3	3	1	1	2	2	3	3
2	2	2	2	1	1	2	2	2	2
2	2	2	2	4	4	2	2	2	2
2	2	2	2	1	1	3	3	3	3
2	2	2	2	1	1	2	2	3	3
2	2	2	2	1	1	2	2	3	3
2	2	2	2	1	1	2	2	3	3
2	2	2	2	1	1	2	2	3	3
2	2	2	2	4	4	2	2	2	2
2	2	2	2	1	1	2	2	3	3
2	2	2	2	4	4	2	2	2	2
2	2	2	2	1	1	2	2	3	3
1	1	3	3	1	1	2	2	3	3
2	2	3	3	1	1	2	2	3	3
2	2	2	2	1	1	2	2	3	3
1	1	3	3	1	1	2	2	3	3
1	1	3	3	1	1	2	2	3	3
2	2	2	2	1	1	2	2	3	3
2	2	2	2	1	1	2	2	3	3
2	2	2	2	4	4	2	2	2	2
2	2	2	2	1	1	2	2	3	3
2	2	2	2	4	4	2	2	2	2
2	2	2	2	1	1	2	2	3	3
2	2	2	2	4	4	2	2	2	2
2	2	2	2	4	4	2	2	2	2
2	2	2	2	4	4	2	2	2	2
2	2	2	2	1	1	2	2	2	2
1	1	3	3	1	1	2	2	3	3
2	2	2	2	1	1	2	2	3	3
2	2	2	2	1	1	2	2	3	3
1	1	3	3	1	1	2	2	3	3
2	2	2	2	4	4	2	2	2	2

For Review Only

2	2	2	2	4	4	2	2	2	2
1	1	3	3	1	1	2	2	3	3
2	2	2	2	1	1	2	2	3	3
2	2	2	2	4	4	2	2	3	3
2	2	2	2	1	1	3	3	3	3
1	1	3	3	1	1	2	2	3	3
2	2	2	2	1	1	2	2	3	3
2	2	2	2	1	1	3	3	3	3
2	2	2	2	4	4	2	2	2	2
2	2	2	2	4	4	2	2	2	2
2	2	3	3	4	4	2	2	2	2
2	2	2	2	1	1	3	3	3	3
2	2	2	2	1	1	2	2	3	3
2	2	2	2	4	4	2	2	2	2
2	2	2	2	4	4	2	2	2	2
2	2	2	2	4	4	2	2	2	2
2	2	2	2	1	1	2	2	3	3
2	2	2	2	4	4	2	2	2	2
2	2	2	2	1	1	2	2	3	3
2	2	2	2	1	1	2	2	3	3
2	2	2	2	1	1	2	2	3	3
1	1	3	3	1	1	2	2	3	3
2	2	2	2	4	4	2	2	2	2
2	2	2	2	1	1	2	2	3	3
2	2	2	2	4	4	2	2	2	2
1	1	3	3	1	1	2	2	3	3
2	2	2	2	4	4	2	2	2	2
1	1	3	3	1	1	2	2	3	3
2	2	2	2	1	1	2	2	3	3
2	2	2	2	4	4	2	2	2	2
2	2	2	2	4	4	2	2	2	2
2	2	2	2	1	1	2	2	3	3
2	2	2	2	1	1	2	2	3	3
1	1	3	3	1	1	2	2	3	3
2	2	2	2	1	1	2	2	3	3
2	2	2	2	4	4	2	2	2	2
2	2	2	2	1	1	2	2	3	3
1	1	3	3	1	1	2	2	3	3
1	1	3	3	1	1	2	2	3	3
2	2	2	2	4	4	2	2	2	2
2	2	2	2	1	1	2	2	3	3
1	1	3	3	1	1	2	2	3	3
2	2	2	2	1	1	2	2	3	3
2	2	2	2	1	1	2	2	3	3
2	2	2	2	1	1	2	2	3	3
2	2	2	2	1	1	2	2	3	3
1	1	3	3	1	1	2	2	3	3
2	2	2	2	1	1	2	2	3	3
2	2	2	2	1	1	3	3	3	3
2	2	2	2	4	4	2	2	2	2
2	2	2	2	4	4	2	2	2	2
2	2	2	2	4	4	2	2	2	2
2	2	2	2	1	1	2	2	3	3
1	1	3	3	1	1	2	2	3	3
2	2	2	2	1	1	2	2	3	3

For Review Only

2	2	2	2	4	4	2	2	2	2
2	2	2	2	1	1	2	2	3	3

For Review Only

SNP3031	SNP3031	SNP3032	SNP3032	SNP3033	SNP3033	SNP3034	SNP3034	SNP3035	SNP3035
4	4	3	3	4	4	3	3	4	4
4	4	3	3	4	4	1	1	4	4
4	4	3	3	4	4	3	3	1	1
4	4	3	3	3	3	3	3	4	4
4	4	3	3	3	3	1	1	4	4
4	4	3	3	3	3	3	3	4	4
4	4	3	3	3	3	1	1	4	4
4	4	3	3	3	3	3	3	4	4
4	4	3	3	3	3	3	3	4	4
4	4	3	3	3	3	3	3	4	4
1	1	2	2	3	3	3	3	4	4
4	4	3	3	4	4	3	3	4	4
4	4	3	3	4	4	3	3	4	4
1	1	2	2	4	4	3	3	1	1
4	4	3	3	4	4	3	3	4	4
1	1	2	2	3	3	1	1	4	4
4	4	3	3	3	3	1	1	4	4
4	4	3	3	4	4	3	3	1	1
4	4	3	3	3	3	1	1	4	4
4	4	3	3	4	4	3	3	1	1
1	1	2	2	4	4	1	1	4	4
4	4	3	3	4	4	1	1	4	4
1	1	2	2	3	3	3	3	4	4
1	1	2	2	3	3	3	3	4	4
4	4	3	3	3	3	1	1	4	4
4	4	3	3	3	3	1	1	4	4
4	4	3	3	3	3	3	3	4	4
4	4	3	3	4	4	3	3	1	1
4	4	3	3	3	3	3	3	4	4
1	1	2	2	3	3	3	3	4	4
1	1	2	2	3	3	1	1	4	4
4	4	3	3	4	4	3	3	1	1
4	4	3	3	3	3	1	1	4	4
1	1	2	2	3	3	3	3	4	4
4	4	3	3	3	3	3	3	4	4
4	4	3	3	3	3	3	3	4	4
4	4	3	3	4	4	3	3	1	1
4	4	3	3	3	3	3	3	4	4
1	1	2	2	3	3	3	3	4	4
4	4	3	3	4	4	3	3	4	4
1	1	2	2	4	4	1	1	4	4
4	4	3	3	3	3	3	3	4	4
4	4	3	3	3	3	3	3	4	4
1	1	2	2	4	4	1	1	4	4
4	4	3	3	3	3	3	3	4	4
4	4	3	3	4	4	3	3	4	4
4	4	3	3	3	3	3	3	4	4
4	4	3	3	3	3	1	1	4	4
1	1	2	2	4	4	1	1	4	4
4	4	3	3	4	4	3	3	4	4
4	4	3	3	3	3	3	3	4	4
1	1	2	2	3	3	3	3	4	4

For Review Only

4	4	3	3	3	3	1	1	4	4
4	4	3	3	3	3	1	1	4	4
4	4	3	3	4	4	3	3	4	4
1	1	2	2	4	4	1	1	4	4
4	4	3	3	4	4	1	1	4	4
4	4	3	3	3	3	3	3	4	4
4	4	3	3	4	4	3	3	1	1
1	1	2	2	3	3	3	3	4	4
4	4	3	3	4	4	3	3	1	1
4	4	3	3	3	3	3	3	4	4
4	4	3	3	4	4	1	1	4	4
4	4	3	3	3	3	3	3	4	4
4	4	3	3	3	3	1	1	4	4
4	4	3	3	3	3	3	3	4	4
4	4	3	3	4	4	3	3	4	4
4	4	2	2	4	4	1	1	4	4
4	4	3	3	3	3	3	3	4	4
4	4	3	3	4	4	3	3	1	1
1	1	3	3	4	4	1	1	4	4
4	4	3	3	3	3	1	1	4	4
4	4	3	3	3	3	3	3	4	4
4	4	3	3	3	3	3	3	4	4
4	4	3	3	3	3	3	3	4	4
4	4	3	3	4	4	1	1	4	4
4	4	3	3	3	3	3	3	4	4
4	4	3	3	3	3	3	3	4	4
1	1	2	2	4	4	1	1	4	4
1	1	2	2	3	3	3	3	4	4
4	4	3	3	4	4	3	3	1	1
1	1	2	2	3	3	1	1	4	4
4	4	3	3	3	3	1	1	4	4
4	4	3	3	4	4	1	1	4	4
1	1	2	2	3	3	3	3	4	4
4	4	3	3	3	3	1	1	4	4
4	4	3	3	4	4	3	3	1	1
4	4	3	3	3	3	3	3	4	4
4	4	3	3	3	3	3	3	4	4
4	4	3	3	3	3	3	3	4	4
4	4	3	3	3	3	3	3	4	4
4	4	3	3	3	3	3	3	4	4
4	4	3	3	3	3	1	1	4	4
4	4	3	3	3	3	3	3	4	4
4	4	3	3	3	3	3	3	4	4
4	4	3	3	4	4	1	1	4	4
1	1	2	2	3	3	1	1	4	4
4	4	3	3	4	4	1	1	4	4
1	1	2	2	3	3	3	3	4	4
4	4	3	3	4	4	3	3	1	1
4	4	3	3	4	4	3	3	1	1
4	4	3	3	4	4	3	3	1	1
4	4	3	3	3	3	1	1	4	4
1	1	2	2	3	3	3	3	4	4
4	4	3	3	3	3	3	3	4	4
4	4	3	3	4	4	3	3	4	4
4	4	3	3	4	4	3	3	1	1

For Review Only

4	4	3	3	4	4	1	1	4	4
4	4	3	3	3	3	3	3	4	4
4	4	3	3	3	3	3	3	4	4
1	1	2	2	3	3	3	3	4	4
4	4	3	3	4	4	3	3	4	4
4	4	3	3	3	3	3	3	4	4
1	1	2	2	3	3	1	1	4	4
4	4	3	3	3	3	3	3	4	4
4	4	3	3	4	4	3	3	1	1
4	4	3	3	4	4	3	3	1	1
4	4	3	3	4	4	3	3	1	1
4	4	3	3	4	4	3	3	4	4
4	4	3	3	3	3	3	3	4	4
4	4	3	3	4	4	3	3	4	4
4	4	3	3	3	3	3	3	4	4
4	4	3	3	3	3	3	3	4	4
4	4	3	3	3	3	3	3	1	1
4	4	3	3	4	4	3	3	4	4
1	1	2	2	3	3	3	3	4	4
4	4	3	3	3	3	3	3	4	4
4	4	3	3	4	4	3	3	4	4
1	1	2	2	3	3	1	1	4	4
4	4	3	3	4	4	3	3	1	1
1	1	2	2	3	3	3	3	4	4
4	4	3	3	3	3	3	3	4	4
4	4	3	3	3	3	3	3	4	4
4	4	3	3	3	3	3	3	4	4
4	4	3	3	4	4	3	3	1	1
4	4	3	3	4	4	3	3	4	4
4	4	3	3	4	4	3	3	1	1
1	1	2	2	3	3	3	3	4	4
4	4	3	3	4	4	3	3	4	4
4	4	3	3	3	3	1	1	4	4
4	4	3	3	4	4	3	3	4	4
1	1	2	2	3	3	3	3	4	4
4	4	3	3	3	3	3	3	4	4
1	1	2	2	3	3	3	3	4	4
4	4	3	3	4	4	3	3	1	1
4	4	3	3	4	4	3	3	1	1
1	1	2	2	3	3	3	3	4	4
4	4	3	3	3	3	3	3	4	4
1	1	2	2	3	3	3	3	4	4
4	4	3	3	4	4	3	3	1	1
4	4	3	3	3	3	3	3	4	4
4	4	3	3	4	4	3	3	4	4
4	4	3	3	4	4	1	1	4	4
4	4	3	3	4	4	1	1	4	4
4	4	3	3	3	3	1	1	4	4
4	4	3	3	3	3	3	3	4	4
4	4	3	3	3	3	3	3	4	4
4	4	3	3	4	4	3	3	4	4
1	1	2	2	3	3	3	3	4	4
4	4	3	3	4	4	3	3	4	4
4	4	3	3	3	3	3	3	4	4
4	4	3	3	3	3	3	3	4	4

For Review Only

4	4	3	3	4	4	3	3	4	4
4	4	3	3	3	3	3	3	4	4
4	4	3	3	4	4	1	1	4	4
4	4	3	3	4	4	3	3	4	4
4	4	3	3	3	3	3	3	4	4
4	4	3	3	3	3	1	1	4	4
4	4	3	3	4	4	3	3	4	4
4	4	3	3	4	4	3	3	4	4
4	4	3	3	3	3	3	3	4	4
1	1	2	2	3	3	1	1	4	4
4	4	3	3	4	4	3	3	4	4
4	4	2	2	3	3	3	3	4	4
4	4	3	3	3	3	3	3	4	4
4	4	3	3	4	4	3	3	4	4
1	1	2	2	3	3	3	3	4	4
1	1	2	2	3	3	3	3	4	4
4	4	3	3	4	4	3	3	1	1
4	4	3	3	4	4	1	1	4	4
4	4	3	3	4	4	3	3	4	4
1	1	2	2	3	3	1	1	4	4
4	4	3	3	4	4	1	1	4	4
1	1	2	2	3	3	3	3	4	4
4	4	3	3	3	3	3	3	4	4
4	4	3	3	3	3	3	3	4	4
1	1	2	2	4	4	1	1	4	4
1	1	2	2	3	3	3	3	4	4
4	4	3	3	4	4	3	3	4	4
4	4	3	3	4	4	3	3	1	1
4	4	3	3	4	4	1	1	4	4
4	4	3	3	3	3	1	1	4	4
4	4	3	3	4	4	1	1	4	4
1	1	2	2	3	3	1	1	4	4
4	4	3	3	4	4	1	1	4	4
1	1	2	2	3	3	3	3	4	4
4	4	3	3	3	3	1	1	4	4
4	4	3	3	3	3	3	3	4	4
4	4	3	3	4	4	3	3	4	4
4	4	3	3	4	4	3	3	1	1
4	4	3	3	4	4	1	1	4	4
4	4	3	3	4	4	1	1	4	4
4	4	3	3	4	4	3	3	1	1
4	4	3	3	4	4	3	3	1	1
1	1	2	2	3	3	3	3	4	4
4	4	3	3	4	4	3	3	4	4
4	4	2	2	4	4	3	3	1	1
4	4	3	3	4	4	3	3	4	4
1	1	2	2	3	3	3	3	4	4
4	4	3	3	4	4	3	3	4	4
1	1	2	2	3	3	1	1	4	4
1	1	2	2	4	4	1	1	4	4
4	4	3	3	3	3	1	1	4	4
4	4	3	3	3	3	3	3	4	4
4	4	3	3	3	3	3	3	4	4
4	4	3	3	4	4	1	1	4	4
1	1	2	2	3	3	3	3	4	4

For Review Only

1	1	2	2	3	3	3	3	4	4
4	4	3	3	3	3	3	3	4	4
4	4	3	3	4	4	1	1	4	4
4	4	3	3	3	3	3	3	4	4
4	4	3	3	3	3	3	3	4	4
4	4	3	3	3	3	3	3	4	4
4	4	3	3	3	3	3	3	4	4
4	4	3	3	3	3	3	3	4	4
1	1	2	2	3	3	3	3	4	4
1	1	2	2	3	3	3	3	4	4
4	4	3	3	3	3	3	3	4	4
4	4	3	3	4	4	1	1	4	4
4	4	3	3	3	3	3	3	4	4
1	1	2	2	3	3	3	3	4	4
4	4	3	3	4	4	3	3	4	4
1	1	3	3	3	3	1	1	4	4
4	4	3	3	3	3	3	3	4	4
1	1	2	2	3	3	3	3	4	4
4	4	3	3	3	3	1	1	4	4
4	4	3	3	3	3	3	3	4	4
4	4	3	3	4	4	1	1	4	4
4	4	3	3	3	3	3	3	4	4
1	1	2	2	3	3	1	1	4	4
4	4	3	3	4	4	3	3	1	1
1	1	2	2	3	3	3	3	4	4
4	4	3	3	3	3	3	3	4	4
1	1	2	2	3	3	3	3	4	4
4	4	3	3	3	3	3	3	4	4
4	4	3	3	3	3	3	3	4	4
1	1	2	2	3	3	3	3	4	4
1	1	2	2	3	3	3	3	4	4
4	4	3	3	4	4	3	3	1	1
4	4	3	3	4	4	3	3	1	1
4	4	3	3	4	4	3	3	4	4
4	4	3	3	3	3	3	3	4	4
1	1	3	3	3	3	3	3	4	4
4	4	3	3	4	4	3	3	1	1
4	4	3	3	3	3	3	3	4	4
4	4	3	3	3	3	3	3	4	4
1	1	2	2	3	3	3	3	4	4
4	4	3	3	4	4	1	1	4	4
4	4	3	3	3	3	3	3	4	4
4	4	3	3	3	3	3	3	4	4
4	4	3	3	3	3	1	1	4	4
4	4	3	3	3	3	3	3	4	4
4	4	3	3	3	3	3	3	4	4
4	4	3	3	4	4	1	1	4	4
4	4	3	3	3	3	3	3	1	1
4	4	3	3	3	3	3	3	4	4
1	1	3	3	3	3	3	3	4	4
1	1	2	2	3	3	3	3	4	4
1	1	2	2	3	3	3	3	4	4
4	4	3	3	4	4	3	3	1	1
4	4	3	3	3	3	3	3	4	4
4	4	3	3	3	3	3	3	4	4

For Review Only

1	1	2	2	3	3	1	1	4	4
4	4	3	3	4	4	3	3	1	1

For Review Only

SNP3036	SNP3036	SNP3037	SNP3037	SNP3038	SNP3038	SNP3039	SNP3039	SNP304	SNP304
1	1	2	2	1	1	4	4	2	2
3	3	2	2	1	1	4	4	2	2
1	1	2	2	1	1	4	4	2	2
3	3	2	2	1	1	4	4	2	2
3	3	1	1	4	4	1	1	1	1
3	3	2	2	1	1	4	4	2	2
3	3	1	1	4	4	1	1	2	2
3	3	2	2	1	1	4	4	1	1
3	3	2	2	1	1	4	4	2	2
3	3	2	2	1	1	4	4	2	2
3	3	1	1	4	4	1	1	2	2
3	3	2	2	1	1	4	4	2	2
3	3	1	1	4	4	4	4	2	2
1	1	2	2	1	1	4	4	2	2
3	3	1	1	1	1	4	4	2	2
3	3	1	1	4	4	1	1	2	2
3	3	1	1	4	4	1	1	2	2
1	1	2	2	1	1	4	4	2	2
3	3	2	2	1	1	4	4	2	2
1	1	2	2	1	1	4	4	2	2
3	3	1	1	4	4	1	1	2	2
3	3	1	1	4	4	1	1	2	2
3	3	1	1	4	4	1	1	2	2
3	3	1	1	4	4	1	1	2	2
3	3	1	1	4	4	1	1	2	2
3	3	1	1	4	4	1	1	2	2
3	3	2	2	1	1	4	4	2	2
3	3	1	1	1	1	4	4	2	2
3	3	2	2	1	1	4	4	2	2
3	3	1	1	4	4	1	1	2	2
3	3	1	1	4	4	1	1	2	2
1	1	2	2	1	1	4	4	2	2
3	3	1	1	4	4	1	1	2	2
3	3	1	1	4	4	1	1	2	2
3	3	2	2	1	1	4	4	2	2
3	3	2	2	1	1	4	4	2	2
1	1	2	2	1	1	4	4	2	2
3	3	2	2	1	1	4	4	2	2
3	3	1	1	4	4	1	1	2	2
3	3	1	1	4	4	1	1	2	2
3	3	1	1	4	4	1	1	1	1
3	3	2	2	1	1	4	4	2	2
3	3	2	2	1	1	4	4	1	1
3	3	1	1	4	4	1	1	2	2
3	3	2	2	1	1	4	4	1	1
3	3	1	1	1	1	4	4	2	2
3	3	2	2	1	1	4	4	2	2
3	3	1	1	4	4	1	1	2	2
3	3	1	1	4	4	1	1	2	2
3	3	2	2	1	1	4	4	2	2
3	3	2	2	1	1	4	4	2	2
3	3	1	1	4	4	1	1	2	2

For Review Only

3	3	1	1	4	4	1	1	2	2
3	3	1	1	4	4	1	1	2	2
1	1	2	2	1	1	4	4	2	2
3	3	1	1	4	4	1	1	2	2
3	3	2	2	1	1	4	4	2	2
3	3	2	2	1	1	4	4	2	2
1	1	2	2	1	1	4	4	2	2
3	3	1	1	4	4	1	1	2	2
1	1	2	2	1	1	4	4	2	2
3	3	1	1	4	4	4	4	2	2
3	3	2	2	1	1	4	4	2	2
3	3	2	2	1	1	4	4	2	2
3	3	1	1	4	4	1	1	2	2
3	3	2	2	1	1	4	4	2	2
3	3	1	1	1	1	4	4	2	2
3	3	1	1	4	4	1	1	2	2
3	3	2	2	1	1	4	4	2	2
1	1	2	2	1	1	4	4	2	2
3	3	2	2	1	1	4	4	2	2
3	3	1	1	4	4	1	1	2	2
3	3	2	2	1	1	4	4	2	2
3	3	1	1	4	4	4	4	2	2
3	3	2	2	1	1	4	4	2	2
3	3	2	2	1	1	4	4	2	2
3	3	2	2	1	1	4	4	1	1
3	3	2	2	1	1	4	4	2	2
3	3	1	1	4	4	1	1	1	1
3	3	1	1	4	4	1	1	2	2
1	1	2	2	1	1	4	4	2	2
3	3	1	1	4	4	1	1	2	2
3	3	1	1	4	4	1	1	2	2
3	3	2	2	1	1	4	4	2	2
3	3	1	1	4	4	1	1	2	2
3	3	1	1	4	4	1	1	2	2
1	1	2	2	1	1	4	4	2	2
3	3	2	2	1	1	4	4	1	1
3	3	2	2	1	1	4	4	2	2
3	3	1	1	4	4	1	1	2	2
3	3	2	2	1	1	4	4	2	2
3	3	2	2	4	4	4	4	1	1
3	3	1	1	4	4	1	1	2	2
3	3	2	2	1	1	4	4	2	2
3	3	2	2	1	1	4	4	2	2
3	3	2	2	1	1	4	4	2	2
3	3	1	1	4	4	4	4	2	2
3	3	2	2	1	1	4	4	2	2
3	3	1	1	4	4	1	1	2	2
1	1	2	2	1	1	4	4	2	2
1	1	2	2	1	1	4	4	2	2
1	1	2	2	1	1	4	4	2	2
3	3	2	2	1	1	4	4	2	2
3	3	1	1	4	4	1	1	2	2
3	3	2	2	1	1	4	4	2	2
1	1	1	1	1	1	4	4	2	2
1	1	2	2	1	1	4	4	2	2

For Review Only

3	3	2	2	1	1	4	4	2	2
3	3	2	2	1	1	4	4	1	1
3	3	2	2	1	1	4	4	2	2
3	3	1	1	4	4	1	1	2	2
3	3	2	2	1	1	4	4	2	2
3	3	2	2	1	1	4	4	2	2
3	3	1	1	4	4	1	1	2	2
3	3	1	1	4	4	4	4	2	2
1	1	2	2	1	1	4	4	2	2
3	3	1	1	4	4	4	4	2	2
3	3	1	1	1	1	4	4	2	2
3	3	1	1	4	4	4	4	2	2
3	3	2	2	1	1	4	4	2	2
3	3	2	2	1	1	4	4	2	2
3	3	1	1	4	4	4	4	2	2
3	3	1	1	4	4	4	4	2	2
1	1	2	2	1	1	4	4	2	2
3	3	1	1	1	1	4	4	2	2
3	3	1	1	4	4	1	1	2	2
3	3	2	2	1	1	4	4	2	2
3	3	1	1	4	4	4	4	2	2
3	3	2	2	4	4	4	4	2	2
1	1	2	2	1	1	4	4	2	2
3	3	1	1	4	4	1	1	2	2
3	3	2	2	1	1	4	4	2	2
3	3	2	2	1	1	4	4	2	2
3	3	1	1	4	4	1	1	2	2
1	1	2	2	1	1	4	4	2	2
3	3	2	2	1	1	4	4	2	2
1	1	2	2	1	1	4	4	2	2
3	3	1	1	4	4	1	1	2	2
3	3	1	1	4	4	4	4	1	1
3	3	1	1	4	4	1	1	2	2
3	3	1	1	1	1	4	4	2	2
3	3	1	1	4	4	1	1	2	2
3	3	1	1	4	4	4	4	2	2
3	3	1	1	4	4	1	1	2	2
1	1	2	2	1	1	4	4	2	2
3	3	1	1	4	4	1	1	2	2
3	3	1	1	4	4	1	1	2	2
3	3	2	2	1	1	4	4	2	2
3	3	1	1	4	4	1	1	2	2
1	1	2	2	1	1	4	4	2	2
3	3	1	1	4	4	4	4	1	1
3	3	2	2	1	1	4	4	1	1
3	3	2	2	1	1	4	4	2	2
3	3	2	2	1	1	4	4	2	2
3	3	2	2	1	1	4	4	2	2
3	3	1	1	4	4	4	4	2	2
3	3	1	1	4	4	4	4	2	2
3	3	2	2	1	1	4	4	2	2
3	3	1	1	4	4	1	1	2	2
3	3	2	2	1	1	4	4	2	2
3	3	2	2	1	1	4	4	2	2
3	3	1	1	4	4	1	1	2	2

For Review Only

3	3	1	1	4	4	1	1	2	2
3	3	2	2	1	1	4	4	2	2
3	3	2	2	1	1	4	4	2	2
3	3	2	2	1	1	4	4	2	2
3	3	2	2	1	1	4	4	2	2
3	3	1	1	4	4	1	1	2	2
3	3	1	1	4	4	4	4	2	2
3	3	1	1	4	4	4	4	2	2
3	3	1	1	4	4	4	4	2	2
3	3	1	1	4	4	1	1	2	2
3	3	1	1	4	4	4	4	2	2
3	3	2	2	4	4	4	4	2	2
3	3	2	2	4	4	4	4	2	2
3	3	2	2	1	1	4	4	2	2
3	3	1	1	4	4	1	1	2	2
3	3	1	1	4	4	1	1	2	2
3	3	1	1	1	1	4	4	2	2
3	3	2	2	1	1	4	4	2	2
3	3	2	2	1	1	4	4	2	2
3	3	1	1	4	4	1	1	2	2
3	3	1	1	4	4	4	4	2	2
3	3	1	1	4	4	1	1	2	2
3	3	2	2	1	1	4	4	2	2
3	3	2	2	1	1	4	4	2	2
3	3	1	1	4	4	1	1	2	2
3	3	1	1	4	4	1	1	2	2
3	3	2	2	1	1	4	4	1	1
1	1	2	2	1	1	4	4	2	2
3	3	2	2	1	1	4	4	2	2
3	3	1	1	4	4	1	1	2	2
3	3	2	2	1	1	4	4	2	2
3	3	1	1	4	4	1	1	2	2
3	3	2	2	1	1	4	4	2	2
3	3	1	1	4	4	1	1	2	2
3	3	1	1	4	4	1	1	2	2
3	3	2	2	1	1	4	4	2	2
3	3	2	2	1	1	4	4	2	2
1	1	2	2	1	1	4	4	2	2
3	3	2	2	1	1	4	4	2	2
3	3	2	2	1	1	4	4	2	2
1	1	2	2	1	1	4	4	2	2
1	1	2	2	1	1	4	4	2	2
3	3	1	1	4	4	1	1	2	2
3	3	2	2	1	1	4	4	2	2
3	3	1	1	1	1	4	4	2	2
3	3	2	2	1	1	4	4	2	2
3	3	1	1	4	4	1	1	2	2
3	3	1	1	4	4	1	1	2	2
3	3	1	1	4	4	1	1	2	2
3	3	1	1	4	4	1	1	2	2
3	3	1	1	4	4	1	1	2	2
3	3	2	2	1	1	4	4	2	2
3	3	2	2	1	1	4	4	2	2
3	3	2	2	1	1	4	4	2	2
3	3	1	1	4	4	1	1	2	2

For Review Only

3	3	1	1	4	4	1	1	2	2
3	3	2	2	1	1	4	4	2	2
3	3	2	2	1	1	4	4	2	2
3	3	2	2	1	1	4	4	2	2
3	3	2	2	1	1	4	4	1	1
3	3	2	2	1	1	4	4	2	2
3	3	2	2	1	1	4	4	2	2
3	3	2	2	1	1	4	4	1	1
3	3	1	1	4	4	1	1	2	2
3	3	1	1	4	4	1	1	2	2
3	3	1	1	4	4	4	4	2	2
3	3	2	2	1	1	4	4	1	1
3	3	2	2	1	1	4	4	2	2
3	3	1	1	4	4	1	1	2	2
3	3	1	1	4	4	1	1	2	2
3	3	1	1	4	4	1	1	2	2
3	3	2	2	1	1	4	4	2	2
3	3	1	1	4	4	1	1	2	2
3	3	1	1	4	4	1	1	2	2
3	3	2	2	1	1	4	4	2	2
3	3	2	2	1	1	4	4	2	2
3	3	2	2	1	1	4	4	2	2
3	3	2	2	1	1	4	4	2	2
1	1	2	2	1	1	4	4	2	2
3	3	1	1	4	4	1	1	2	2
3	3	2	2	1	1	4	4	2	2
3	3	1	1	4	4	1	1	2	2
3	3	2	2	1	1	4	4	2	2
3	3	1	1	4	4	4	4	1	1
3	3	1	1	4	4	1	1	2	2
3	3	1	1	4	4	1	1	2	2
3	3	2	2	1	1	4	4	2	2
1	1	2	2	1	1	4	4	2	2
3	3	2	2	1	1	4	4	2	2
3	3	2	2	1	1	4	4	2	2
3	3	1	1	4	4	1	1	2	2
1	1	2	2	1	1	4	4	2	2
3	3	2	2	1	1	4	4	2	2
3	3	2	2	1	1	4	4	2	2
3	3	1	1	4	4	1	1	2	2
3	3	2	2	1	1	4	4	2	2
3	3	2	2	1	1	4	4	2	2
3	3	2	2	1	1	4	4	2	2
3	3	1	1	4	4	1	1	2	2
3	3	2	2	1	1	4	4	2	2
3	3	2	2	1	1	4	4	2	2
3	3	2	2	1	1	4	4	2	2
1	1	2	2	1	1	4	4	2	2
3	3	2	2	1	1	4	4	1	1
3	3	1	1	4	4	1	1	2	2
3	3	1	1	4	4	1	1	2	2
3	3	1	1	4	4	1	1	2	2
1	1	2	2	1	1	4	4	2	2
3	3	2	2	1	1	4	4	2	2
3	3	2	2	1	1	4	4	2	2

For Review Only

3	3	1	1	4	4	1	1	2	2
1	1	2	2	1	1	4	4	2	2

For Review Only

SNP3040	SNP3040	SNP3041	SNP3041	SNP3042	SNP3042	SNP3043	SNP3043	SNP3044	SNP3044
2	2	2	2	2	2	3	3	1	1
2	2	2	2	2	2	3	3	1	1
2	2	2	2	2	2	3	3	1	1
2	2	2	2	2	2	3	3	1	1
4	4	2	2	3	3	3	3	1	1
2	2	2	2	2	2	3	3	1	1
4	4	2	2	3	3	3	3	1	1
2	2	2	2	2	2	3	3	1	1
2	2	2	2	2	2	3	3	1	1
2	2	3	3	2	2	2	2	1	1
2	2	3	3	3	3	2	2	1	1
2	2	2	2	2	2	3	3	1	1
2	2	3	3	3	3	2	2	1	1
2	2	2	2	2	2	2	2	1	1
2	2	2	2	2	2	3	3	1	1
2	2	2	2	2	2	3	3	1	1
4	4	2	2	3	3	3	3	1	1
2	2	2	2	2	2	2	2	1	1
4	4	2	2	2	2	3	3	1	1
2	2	2	2	2	2	2	2	1	1
2	2	2	2	2	2	3	3	1	1
2	2	2	2	3	3	2	2	1	1
2	2	3	3	3	3	2	2	1	1
2	2	2	2	3	3	2	2	1	1
2	2	2	2	3	3	3	3	1	1
4	4	2	2	3	3	3	3	1	1
2	2	2	2	2	2	3	3	1	1
2	2	2	2	2	2	3	3	1	1
2	2	2	2	2	2	3	3	1	1
2	2	3	3	3	3	2	2	1	1
2	2	2	2	3	3	3	3	1	1
2	2	2	2	2	2	3	3	1	1
4	4	2	2	3	3	3	3	1	1
2	2	3	3	3	3	3	3	1	1
2	2	2	2	2	2	3	3	1	1
2	2	2	2	2	2	3	3	1	1
2	2	2	2	2	2	3	3	1	1
2	2	2	2	2	2	2	2	1	1
2	2	2	2	3	3	2	2	1	1
2	2	3	3	2	2	2	2	1	1
2	2	3	3	3	3	2	2	1	1
2	2	2	2	2	2	3	3	1	1
2	2	3	3	2	2	3	3	1	1
2	2	3	3	2	2	2	2	1	1
2	2	3	3	3	3	2	2	1	1
2	2	2	2	2	2	3	3	1	1
2	2	2	2	2	2	3	3	1	1
4	4	2	2	3	3	3	3	1	1
2	2	3	3	3	3	2	2	1	1
2	2	2	2	2	2	3	3	1	1
2	2	2	2	2	2	3	3	1	1
2	2	2	2	3	3	3	3	1	1

For Review Only

4	4	2	2	3	3	3	3	1	1
4	4	2	2	3	3	3	3	1	1
2	2	2	2	2	2	3	3	1	1
2	2	3	3	3	3	3	3	1	1
4	4	2	2	2	2	3	3	1	1
2	2	2	2	2	2	3	3	1	1
2	2	2	2	2	2	3	3	2	2
2	2	3	3	3	3	2	2	1	1
2	2	2	2	2	2	3	3	1	1
2	2	2	2	2	2	2	2	1	1
2	2	2	2	2	2	3	3	1	1
2	2	2	2	2	2	3	3	1	1
4	4	2	2	3	3	3	3	1	1
2	2	2	2	2	2	3	3	1	1
2	2	2	2	2	2	3	3	1	1
2	2	3	3	2	2	2	2	1	1
2	2	2	2	2	2	3	3	1	1
2	2	3	3	3	3	3	3	1	1
4	4	2	2	2	2	3	3	1	1
4	4	2	2	3	3	3	3	1	1
2	2	2	2	2	2	2	2	1	1
2	2	2	2	2	2	2	2	1	1
2	2	2	2	2	2	3	3	1	1
2	2	2	2	2	2	3	3	1	1
2	2	3	3	3	3	2	2	1	1
2	2	2	2	2	2	3	3	1	1
2	2	3	3	3	3	2	2	1	1
2	2	3	3	3	3	2	2	1	1
2	2	2	2	2	2	3	3	2	2
2	2	2	2	2	2	3	3	1	1
4	4	2	2	3	3	3	3	1	1
2	2	2	2	2	2	2	2	1	1
2	2	3	3	3	3	2	2	1	1
4	4	2	2	3	3	3	3	1	1
2	2	2	2	2	2	3	3	1	1
2	2	3	3	3	3	2	2	1	1
2	2	2	2	2	2	3	3	1	1
2	2	2	2	3	3	2	2	1	1
2	2	2	2	2	2	3	3	1	1
2	2	3	3	3	3	2	2	1	1
4	4	2	2	3	3	3	3	1	1
2	2	2	2	2	2	3	3	1	1
2	2	2	2	2	2	3	3	1	1
2	2	2	2	2	2	3	3	1	1
2	2	2	2	3	3	3	3	1	1
2	2	2	2	2	2	3	3	1	1
2	2	3	3	3	3	2	2	1	1
2	2	2	2	2	2	3	3	2	2
2	2	2	2	2	2	3	3	2	2
2	2	2	2	2	2	3	3	1	1
2	2	2	2	2	2	3	3	1	1
2	2	3	3	3	3	2	2	1	1
2	2	2	2	2	2	3	3	1	1
2	2	2	2	2	2	2	2	1	1
2	2	2	2	2	2	3	3	1	1

For Review Only

2	2	2	2	2	2	3	3	1	1
2	2	2	2	2	2	2	2	1	1
2	2	2	2	2	2	3	3	1	1
2	2	3	3	3	3	2	2	1	1
2	2	2	2	2	2	3	3	1	1
2	2	2	2	2	2	2	2	1	1
2	2	2	2	2	2	3	3	1	1
2	2	2	2	2	2	3	3	1	1
2	2	2	2	2	2	3	3	2	2
2	2	2	2	2	2	3	3	2	2
2	2	2	2	2	2	2	2	1	1
2	2	2	2	2	2	2	2	1	1
2	2	2	2	2	2	3	3	1	1
2	2	2	2	2	2	3	3	1	1
2	2	2	2	2	2	2	2	1	1
2	2	2	2	2	2	3	3	2	2
2	2	3	3	3	3	3	3	1	1
2	2	2	2	2	2	3	3	1	1
2	2	3	3	3	3	2	2	1	1
2	2	2	2	2	2	3	3	1	1
2	2	2	2	2	2	2	2	1	1
2	2	2	2	2	2	3	3	1	1
2	2	2	2	2	2	3	3	1	1
2	2	3	3	3	3	2	2	1	1
2	2	2	2	2	2	3	3	1	1
2	2	2	2	2	2	2	2	1	1
4	4	2	2	2	2	3	3	2	2
2	2	2	2	2	2	3	3	1	1
2	2	2	2	2	2	3	3	1	1
2	2	3	3	3	3	3	3	1	1
2	2	3	3	3	3	2	2	1	1
2	2	2	2	2	2	2	2	1	1
4	4	2	2	3	3	3	3	1	1
2	2	2	2	2	2	3	3	1	1
2	2	3	3	3	3	3	3	1	1
2	2	2	2	3	3	2	2	1	1
2	2	3	3	3	3	2	2	1	1
2	2	2	2	2	2	3	3	1	1
2	2	2	2	3	3	2	2	1	1
2	2	3	3	3	3	2	2	1	1
2	2	2	2	2	2	2	2	1	1
2	2	3	3	3	3	2	2	1	1
2	2	2	2	2	2	3	3	1	1
2	2	2	2	3	3	2	2	1	1
2	2	2	2	2	2	3	3	1	1
4	4	2	2	2	2	3	3	1	1
2	2	2	2	2	2	3	3	1	1
2	2	2	2	2	2	3	3	2	2
2	2	2	2	2	2	2	2	1	1
2	2	2	2	2	2	3	3	2	2
2	2	2	2	2	2	3	3	2	2
2	2	3	3	3	3	2	2	1	1
2	2	2	2	3	3	3	3	1	1
2	2	2	2	2	2	3	3	2	2
2	2	2	2	2	2	2	2	1	1

For Review Only

2	2	3	3	3	3	3	3	1	1
2	2	3	3	3	3	2	2	1	1
2	2	2	2	2	2	3	3	1	1
2	2	2	2	2	2	2	2	1	1
2	2	2	2	2	2	3	3	1	1
4	4	2	2	3	3	3	3	1	1
2	2	2	2	2	2	2	2	1	1
2	2	2	2	2	2	2	2	1	1
2	2	2	2	2	2	2	2	1	1
2	2	2	2	2	2	3	3	1	1
2	2	2	2	2	2	3	3	1	1
2	2	2	2	2	2	2	2	1	1
2	2	2	2	2	2	3	3	1	1
2	2	2	2	2	2	3	3	1	1
2	2	3	3	3	3	2	2	1	1
2	2	3	3	3	3	2	2	1	1
2	2	2	2	3	3	3	3	1	1
2	2	2	2	2	2	3	3	1	1
2	2	2	2	2	2	3	3	2	2
2	2	2	2	2	2	3	3	1	1
2	2	2	2	2	2	2	2	1	1
2	2	3	3	3	3	2	2	1	1
2	2	2	2	2	2	2	2	1	1
2	2	2	2	2	2	3	3	1	1
2	2	3	3	3	3	2	2	1	1
2	2	3	3	3	3	3	3	1	1
2	2	2	2	2	2	3	3	1	1
2	2	2	2	2	2	3	3	1	1
2	2	3	3	3	3	3	3	1	1
4	4	2	2	3	3	3	3	1	1
2	2	2	2	2	2	3	3	1	1
2	2	2	2	2	2	3	3	1	1
2	2	2	2	2	2	3	3	1	1
2	2	3	3	3	3	2	2	1	1
4	4	2	2	3	3	3	3	1	1
2	2	2	2	2	2	3	3	1	1
2	2	2	2	2	2	3	3	1	1
2	2	2	2	2	2	2	2	1	1
2	2	2	2	2	2	3	3	2	2
2	2	2	2	2	2	3	3	1	1
2	2	2	2	2	2	3	3	1	1
2	2	2	2	2	2	3	3	2	2
2	2	3	3	3	3	2	2	1	1
2	2	2	2	3	3	3	3	2	2
2	2	2	2	2	2	2	2	1	1
2	2	2	2	2	2	3	3	1	1
2	2	3	3	3	3	2	2	1	1
2	2	3	3	2	2	3	3	1	1
2	2	3	3	3	3	3	3	1	1
2	2	3	3	3	3	3	3	1	1
2	2	2	2	2	2	3	3	1	1
2	2	2	2	2	2	3	3	1	1
2	2	2	2	2	2	2	2	1	1
2	2	2	2	2	2	3	3	1	1
2	2	3	3	3	3	2	2	1	1

For Review Only

2	2	2	2	3	3	2	2	1	1
2	2	2	2	2	2	3	3	1	1
4	4	2	2	2	2	3	3	1	1
2	2	2	2	2	2	2	2	1	1
2	2	2	2	2	2	3	3	1	1
2	2	2	2	2	2	2	2	1	1
2	2	2	2	2	2	3	3	1	1
2	2	3	3	3	3	2	2	1	1
2	2	2	2	3	3	2	2	1	1
2	2	3	3	3	3	2	2	1	1
2	2	2	2	2	2	2	2	1	1
2	2	2	2	2	2	3	3	1	1
2	2	2	2	2	2	3	3	1	1
2	2	3	3	3	3	2	2	1	1
2	2	3	3	2	2	3	3	1	1
2	2	2	2	3	3	3	3	1	1
2	2	2	2	2	2	2	2	1	1
2	2	2	2	2	2	3	3	2	2
4	4	2	2	3	3	3	3	1	1
2	2	2	2	2	2	2	2	1	1
2	2	2	2	2	2	3	3	1	1
2	2	2	2	2	2	2	2	1	1
2	2	2	2	2	2	3	3	1	1
2	2	2	2	2	2	3	3	1	1
2	2	3	3	3	3	2	2	1	1
2	2	2	2	2	2	3	3	1	1
2	2	3	3	3	3	2	2	1	1
2	2	2	2	2	2	3	3	1	1
2	2	2	2	2	2	3	3	1	1
2	2	2	2	2	2	3	3	1	1
2	2	3	3	3	3	2	2	1	1
2	2	2	2	2	2	2	2	1	1
2	2	3	3	3	3	3	3	1	1
2	2	2	2	2	2	3	3	2	2
2	2	2	2	2	2	2	2	1	1
2	2	3	3	3	3	2	2	1	1
2	2	3	3	3	3	3	3	1	1
2	2	2	2	2	2	3	3	1	1
2	2	2	2	2	2	3	3	1	1
2	2	3	3	3	3	2	2	1	1
2	2	2	2	2	2	3	3	1	1
2	2	3	3	3	3	3	3	1	1
2	2	2	2	2	2	2	2	1	1
4	4	2	2	3	3	3	3	1	1
2	2	2	2	2	2	3	3	1	1
2	2	2	2	3	3	3	3	1	1
2	2	2	2	2	2	3	3	2	2
2	2	2	2	2	2	2	2	1	1
2	2	3	3	3	3	2	2	1	1
2	2	3	3	3	3	2	2	1	1
2	2	2	2	2	2	3	3	1	1
2	2	3	3	3	3	2	2	1	1
2	2	2	2	2	2	3	3	1	1
2	2	2	2	2	2	3	3	1	1
2	2	2	2	2	2	2	2	1	1

For Review Only

2	2	2	2	3	3	3	3	1	1
2	2	2	2	2	2	3	3	1	1

For Review Only

SNP3045	SNP3045	SNP3046	SNP3046	SNP3047	SNP3047	SNP3048	SNP3048	SNP3049	SNP3049
2	2	3	3	1	1	2	2	4	4
2	2	3	3	1	1	2	2	4	4
2	2	3	3	1	1	2	2	4	4
2	2	3	3	1	1	2	2	4	4
2	2	3	3	1	1	2	2	4	4
2	2	3	3	1	1	2	2	4	4
2	2	3	3	1	1	2	2	4	4
2	2	3	3	1	1	2	2	4	4
2	2	3	3	1	1	2	2	4	4
2	2	3	3	1	1	2	2	4	4
2	2	3	3	1	1	2	2	4	4
2	2	3	3	1	1	2	2	4	4
2	2	3	3	1	1	2	2	4	4
2	2	3	3	1	1	2	2	4	4
2	2	3	3	1	1	2	2	4	4
2	2	3	3	1	1	2	2	4	4
2	2	3	3	1	1	2	2	4	4
2	2	3	3	1	1	2	2	4	4
2	2	3	3	1	1	2	2	4	4
2	2	3	3	1	1	2	2	4	4
2	2	3	3	1	1	2	2	4	4
2	2	3	3	1	1	2	2	4	4
2	2	3	3	1	1	2	2	4	4
2	2	3	3	1	1	2	2	4	4
2	2	3	3	1	1	2	2	4	4
2	2	3	3	1	1	2	2	4	4
2	2	3	3	1	1	2	2	4	4
2	2	3	3	1	1	2	2	4	4
2	2	3	3	1	1	2	2	4	4
2	2	3	3	1	1	2	2	4	4
2	2	3	3	1	1	2	2	4	4
2	2	3	3	1	1	2	2	4	4
2	2	3	3	1	1	2	2	4	4
2	2	3	3	1	1	2	2	4	4
2	2	3	3	1	1	2	2	4	4
2	2	3	3	1	1	2	2	4	4
2	2	3	3	1	1	2	2	4	4
2	2	3	3	1	1	2	2	4	4
2	2	3	3	1	1	2	2	4	4
2	2	3	3	1	1	2	2	4	4
2	2	3	3	1	1	2	2	4	4
2	2	3	3	1	1	2	2	4	4
2	2	3	3	1	1	2	2	4	4
2	2	3	3	1	1	2	2	4	4
2	2	3	3	1	1	2	2	4	4
2	2	3	3	1	1	2	2	4	4
2	2	3	3	1	1	2	2	4	4
2	2	3	3	1	1	2	2	4	4
2	2	3	3	1	1	2	2	4	4
2	2	3	3	1	1	2	2	4	4
2	2	3	3	1	1	2	2	4	4
2	2	3	3	1	1	2	2	4	4

For Review Only

2	2	3	3	1	1	2	2	4	4
2	2	3	3	1	1	2	2	4	4
2	2	3	3	1	1	2	2	4	4
2	2	3	3	1	1	2	2	4	4
2	2	3	3	1	1	2	2	4	4
2	2	3	3	1	1	2	2	4	4
1	1	2	2	4	4	4	4	2	2
2	2	3	3	1	1	2	2	4	4
2	2	3	3	1	1	2	2	4	4
2	2	3	3	1	1	2	2	4	4
2	2	3	3	1	1	2	2	4	4
2	2	3	3	1	1	2	2	4	4
2	2	3	3	1	1	2	2	4	4
2	2	3	3	1	1	2	2	4	4
2	2	3	3	1	1	2	2	4	4
2	2	3	3	1	1	2	2	4	4
2	2	3	3	1	1	2	2	4	4
2	2	3	3	1	1	2	2	4	4
2	2	3	3	1	1	2	2	4	4
2	2	3	3	1	1	2	2	4	4
2	2	3	3	1	1	2	2	4	4
2	2	3	3	1	1	2	2	4	4
2	2	3	3	1	1	2	2	4	4
2	2	3	3	1	1	2	2	4	4
2	2	3	3	1	1	2	2	4	4
2	2	3	3	1	1	2	2	4	4
2	2	3	3	1	1	2	2	4	4
2	2	3	3	1	1	2	2	4	4
1	1	2	2	4	4	4	4	2	2
2	2	3	3	1	1	2	2	4	4
2	2	3	3	1	1	2	2	4	4
2	2	3	3	1	1	2	2	4	4
2	2	3	3	1	1	2	2	4	4
2	2	3	3	1	1	2	2	4	4
2	2	3	3	1	1	2	2	4	4
2	2	3	3	1	1	2	2	4	4
2	2	3	3	1	1	2	2	4	4
2	2	3	3	1	1	2	2	4	4
2	2	3	3	1	1	2	2	4	4
2	2	3	3	1	1	2	2	4	4
2	2	3	3	1	1	2	2	4	4
2	2	3	3	1	1	2	2	4	4
2	2	3	3	1	1	2	2	4	4
2	2	3	3	1	1	2	2	4	4
2	2	3	3	1	1	2	2	4	4
2	2	3	3	1	1	2	2	4	4
2	2	3	3	1	1	2	2	4	4
1	1	2	2	4	4	4	4	2	2
1	1	2	2	4	4	4	4	2	2
2	2	3	3	1	1	2	2	4	4
2	2	3	3	1	1	2	2	4	4
2	2	3	3	1	1	2	2	4	4
2	2	3	3	1	1	2	2	4	4
2	2	3	3	1	1	2	2	4	4
2	2	3	3	1	1	2	2	4	4

For Review Only

2	2	3	3	1	1	2	2	4	4
2	2	3	3	1	1	2	2	4	4
2	2	3	3	1	1	2	2	4	4
2	2	3	3	1	1	2	2	4	4
2	2	3	3	1	1	2	2	4	4
2	2	3	3	1	1	2	2	4	4
2	2	3	3	1	1	2	2	4	4
2	2	3	3	1	1	2	2	4	4
1	1	2	2	4	4	4	4	2	2
1	1	3	3	1	1	2	2	4	4
2	2	3	3	1	1	2	2	4	4
2	2	3	3	1	1	2	2	4	4
2	2	3	3	1	1	2	2	4	4
2	2	3	3	1	1	2	2	4	4
2	2	3	3	1	1	2	2	4	4
1	1	3	3	4	4	2	2	2	2
2	2	3	3	1	1	2	2	4	4
2	2	3	3	1	1	2	2	4	4
2	2	3	3	1	1	2	2	4	4
2	2	3	3	1	1	2	2	4	4
2	2	3	3	1	1	2	2	4	4
2	2	3	3	1	1	2	2	4	4
2	2	3	3	1	1	2	2	4	4
2	2	3	3	1	1	2	2	4	4
2	2	3	3	1	1	2	2	4	4
2	2	3	3	1	1	2	2	4	4
1	1	3	3	4	4	2	2	2	2
2	2	3	3	1	1	2	2	4	4
2	2	3	3	1	1	2	2	4	4
2	2	3	3	1	1	2	2	4	4
2	2	3	3	1	1	2	2	4	4
2	2	3	3	1	1	2	2	4	4
2	2	3	3	1	1	2	2	4	4
2	2	3	3	1	1	4	4	4	4
2	2	3	3	1	1	2	2	4	4
2	2	3	3	1	1	2	2	4	4
2	2	3	3	1	1	2	2	4	4
2	2	3	3	1	1	2	2	4	4
2	2	3	3	1	1	2	2	4	4
2	2	3	3	1	1	2	2	4	4
2	2	3	3	1	1	2	2	4	4
2	2	3	3	1	1	2	2	4	4
2	2	3	3	1	1	2	2	4	4
2	2	3	3	1	1	2	2	4	4
1	1	3	3	4	4	2	2	4	4
2	2	3	3	1	1	2	2	4	4
2	2	3	3	1	1	2	2	4	4
1	1	2	2	4	4	4	4	2	2
2	2	3	3	1	1	2	2	4	4
1	1	3	3	4	4	2	2	2	2
1	1	3	3	4	4	2	2	2	2
2	2	3	3	1	1	2	2	4	4
2	2	3	3	1	1	2	2	4	4
1	1	2	2	4	4	4	4	2	2
2	2	3	3	1	1	2	2	4	4

For Review Only

2	2	3	3	1	1	2	2	4	4
2	2	3	3	1	1	2	2	4	4
2	2	3	3	1	1	2	2	4	4
2	2	3	3	1	1	2	2	4	4
2	2	3	3	1	1	2	2	4	4
2	2	3	3	1	1	2	2	4	4
2	2	3	3	1	1	2	2	4	4
1	1	3	3	1	1	2	2	4	4
2	2	3	3	1	1	2	2	4	4
2	2	3	3	1	1	2	2	4	4
2	2	3	3	1	1	2	2	4	4
2	2	3	3	1	1	2	2	4	4
2	2	3	3	1	1	2	2	4	4
2	2	3	3	1	1	2	2	4	4
2	2	3	3	1	1	2	2	4	4
2	2	3	3	1	1	2	2	4	4
2	2	3	3	1	1	2	2	4	4
2	2	3	3	1	1	2	2	4	4
1	1	2	2	4	4	4	4	2	2
2	2	3	3	1	1	2	2	4	4
2	2	3	3	1	1	2	2	4	4
2	2	3	3	1	1	2	2	4	4
2	2	3	3	1	1	2	2	4	4
2	2	3	3	1	1	2	2	4	4
2	2	3	3	1	1	2	2	4	4
2	2	3	3	1	1	2	2	4	4
2	2	3	3	1	1	2	2	4	4
2	2	3	3	1	1	2	2	4	4
2	2	3	3	1	1	2	2	4	4
2	2	3	3	1	1	2	2	4	4
2	2	3	3	1	1	2	2	4	4
2	2	3	3	1	1	2	2	4	4
2	2	3	3	1	1	2	2	4	4
2	2	3	3	1	1	2	2	4	4
2	2	3	3	1	1	2	2	4	4
2	2	3	3	1	1	2	2	4	4
2	2	3	3	1	1	2	2	4	4
2	2	3	3	1	1	2	2	4	4
1	1	2	2	4	4	4	4	2	2
2	2	3	3	1	1	2	2	4	4
2	2	3	3	1	1	2	2	4	4
1	1	2	2	4	4	2	2	2	2
2	2	3	3	1	1	2	2	4	4
1	1	3	3	4	4	2	2	2	2
1	1	3	3	4	4	2	2	4	4
2	2	3	3	1	1	2	2	4	4
2	2	3	3	1	1	2	2	4	4
2	2	3	3	1	1	2	2	4	4
2	2	3	3	1	1	2	2	4	4
2	2	3	3	1	1	2	2	4	4
2	2	3	3	1	1	2	2	4	4
2	2	3	3	1	1	2	2	4	4
2	2	3	3	1	1	2	2	4	4
2	2	3	3	1	1	2	2	4	4
2	2	3	3	1	1	2	2	4	4

For Review Only

2	2	3	3	1	1	2	2	4	4
2	2	3	3	4	4	4	4	4	4
2	2	3	3	1	1	2	2	4	4
2	2	3	3	1	1	2	2	4	4
2	2	3	3	1	1	2	2	4	4
2	2	3	3	1	1	2	2	4	4
2	2	3	3	1	1	2	2	4	4
2	2	3	3	1	1	2	2	4	4
2	2	3	3	1	1	2	2	4	4
2	2	3	3	1	1	2	2	4	4
2	2	3	3	1	1	2	2	4	4
2	2	3	3	1	1	2	2	4	4
2	2	3	3	1	1	2	2	4	4
1	1	3	3	1	1	2	2	4	4
2	2	3	3	1	1	2	2	4	4
2	2	2	2	1	1	2	2	4	4
2	2	3	3	1	1	2	2	4	4
2	2	3	3	1	1	2	2	4	4
2	2	3	3	1	1	2	2	4	4
2	2	3	3	1	1	2	2	4	4
2	2	3	3	1	1	2	2	4	4
2	2	3	3	1	1	2	2	4	4
2	2	3	3	1	1	2	2	4	4
2	2	3	3	1	1	2	2	4	4
2	2	3	3	1	1	2	2	4	4
2	2	3	3	1	1	2	2	4	4
2	2	3	3	1	1	2	2	4	4
2	2	3	3	1	1	2	2	4	4
2	2	3	3	1	1	2	2	4	4
2	2	3	3	1	1	2	2	4	4
2	2	3	3	1	1	2	2	4	4
2	2	3	3	1	1	2	2	4	4
2	2	3	3	1	1	2	2	4	4
1	1	2	2	4	4	4	4	2	2
2	2	3	3	1	1	2	2	4	4
2	2	3	3	1	1	2	2	4	4
2	2	3	3	1	1	2	2	4	4
2	2	3	3	1	1	2	2	4	4
2	2	3	3	1	1	2	2	4	4
2	2	3	3	1	1	2	2	4	4
2	2	3	3	1	1	2	2	4	4
2	2	3	3	1	1	2	2	4	4
2	2	3	3	1	1	2	2	4	4
2	2	3	3	1	1	2	2	4	4
2	2	3	3	1	1	2	2	4	4
2	2	3	3	1	1	2	2	4	4
1	1	2	2	4	4	2	2	2	2
2	2	3	3	1	1	2	2	4	4
2	2	3	3	1	1	2	2	4	4
2	2	3	3	1	1	2	2	4	4
2	2	3	3	1	1	2	2	4	4
2	2	3	3	1	1	2	2	4	4
2	2	3	3	1	1	2	2	4	4
2	2	3	3	1	1	2	2	4	4
2	2	3	3	1	1	2	2	4	4

For Review Only

2	2	2	2	1	1	2	2	4	4
2	2	3	3	1	1	2	2	4	4

For Review Only

SNP305	SNP305	SNP3050	SNP3050	SNP3051	SNP3051	SNP3052	SNP3052	SNP3053	SNP3053
1	1	1	1	2	2	4	4	3	3
1	1	1	1	2	2	4	4	3	3
1	1	1	1	2	2	4	4	3	3
1	1	1	1	2	2	4	4	3	3
4	4	1	1	2	2	1	1	3	3
1	1	1	1	2	2	4	4	3	3
1	1	1	1	2	2	1	1	3	3
4	4	1	1	2	2	4	4	3	3
1	1	1	1	2	2	4	4	3	3
1	1	1	1	2	2	4	4	3	3
1	1	1	1	2	2	4	4	2	2
1	1	1	1	2	2	4	4	3	3
1	1	1	1	2	2	4	4	3	3
1	1	1	1	2	2	4	4	3	3
1	1	1	1	2	2	4	4	3	3
1	1	1	1	2	2	4	4	3	3
1	1	1	1	2	2	1	1	3	3
1	1	3	3	3	3	4	4	2	2
1	1	1	1	2	2	4	4	3	3
1	1	1	1	2	2	4	4	3	3
1	1	1	1	2	2	4	4	3	3
1	1	1	1	2	2	4	4	3	3
1	1	1	1	2	2	4	4	2	2
1	1	1	1	2	2	4	4	3	3
1	1	1	1	2	2	1	1	3	3
1	1	1	1	2	2	1	1	3	3
1	1	1	1	2	2	4	4	3	3
1	1	1	1	2	2	4	4	3	3
1	1	1	1	2	2	4	4	3	3
1	1	1	1	2	2	4	4	3	3
1	1	1	1	2	2	1	1	3	3
1	1	1	1	2	2	4	4	3	3
1	1	1	1	2	2	1	1	3	3
4	4	1	1	2	2	4	4	3	3
1	1	1	1	2	2	4	4	3	3
1	1	1	1	2	2	4	4	3	3
1	1	1	1	2	2	4	4	3	3
1	1	1	1	2	2	4	4	3	3
1	1	1	1	2	2	4	4	2	2
1	1	1	1	2	2	4	4	3	3
4	4	1	1	2	2	4	4	3	3
1	1	1	1	2	2	4	4	3	3
4	4	1	1	2	2	4	4	3	3
1	1	1	1	2	2	4	4	3	3
4	4	1	1	2	2	4	4	3	3
1	1	1	1	2	2	4	4	3	3
1	1	1	1	2	2	4	4	3	3
1	1	1	1	2	2	1	1	3	3
1	1	1	1	2	2	4	4	2	2
1	1	1	1	2	2	4	4	3	3
1	1	1	1	2	2	4	4	3	3
1	1	1	1	2	2	4	4	3	3

For Review Only

1	1	1	1	2	2	1	1	3	3
1	1	1	1	2	2	1	1	3	3
1	1	1	1	2	2	4	4	3	3
1	1	1	1	2	2	1	1	3	3
1	1	1	1	2	2	4	4	3	3
1	1	1	1	2	2	4	4	3	3
1	1	3	3	3	3	4	4	2	2
1	1	1	1	2	2	4	4	3	3
1	1	1	1	2	2	4	4	3	3
1	1	1	1	2	2	4	4	3	3
1	1	1	1	2	2	4	4	3	3
1	1	1	1	2	2	4	4	3	3
1	1	1	1	2	2	1	1	3	3
1	1	1	1	2	2	4	4	3	3
1	1	1	1	2	2	4	4	3	3
1	1	1	1	2	2	4	4	3	3
1	1	1	1	2	2	4	4	2	2
1	1	1	1	2	2	4	4	3	3
1	1	1	1	2	2	4	4	3	3
1	1	1	1	2	2	1	1	3	3
1	1	1	1	2	2	4	4	3	3
1	1	1	1	2	2	4	4	3	3
1	1	1	1	2	2	4	4	3	3
1	1	1	1	2	2	4	4	3	3
4	4	1	1	2	2	4	4	3	3
1	1	1	1	2	2	4	4	3	3
4	4	1	1	2	2	4	4	3	3
1	1	1	1	2	2	4	4	2	2
1	1	3	3	3	3	4	4	2	2
1	1	1	1	2	2	1	1	3	3
1	1	1	1	2	2	1	1	3	3
1	1	1	1	2	2	4	4	3	3
1	1	1	1	2	2	4	4	2	2
1	1	1	1	2	2	1	1	3	3
1	1	1	1	2	2	4	4	3	3
4	4	1	1	2	2	4	4	3	3
1	1	1	1	2	2	4	4	3	3
1	1	1	1	2	2	4	4	3	3
1	1	1	1	2	2	4	4	3	3
4	4	1	1	2	2	4	4	3	3
1	1	1	1	2	2	1	1	3	3
1	1	1	1	2	2	4	4	3	3
1	1	1	1	2	2	4	4	3	3
1	1	1	1	2	2	4	4	3	3
1	1	1	1	2	2	4	4	3	3
1	1	1	1	2	2	4	4	3	3
1	1	1	1	2	2	4	4	3	3
1	1	3	3	3	3	4	4	2	2
1	1	3	3	3	3	4	4	2	2
1	1	1	1	2	2	4	4	3	3
1	1	1	1	2	2	4	4	3	3
1	1	1	1	2	2	4	4	2	2
1	1	1	1	2	2	4	4	3	3
1	1	1	1	2	2	4	4	3	3
1	1	1	1	2	2	4	4	3	3

For Review Only

1	1	1	1	2	2	4	4	3	3
4	4	1	1	2	2	4	4	3	3
1	1	1	1	2	2	4	4	3	3
1	1	1	1	2	2	4	4	3	3
1	1	1	1	2	2	4	4	3	3
1	1	1	1	2	2	4	4	3	3
4	4	1	1	2	2	4	4	3	3
1	1	1	1	2	2	4	4	3	3
1	1	3	3	3	3	4	4	2	2
1	1	1	1	2	2	4	4	3	3
1	1	1	1	2	2	4	4	3	3
1	1	1	1	2	2	4	4	3	3
1	1	1	1	2	2	4	4	3	3
1	1	1	1	2	2	4	4	3	3
1	1	1	1	2	2	4	4	3	3
1	1	1	1	3	3	4	4	2	2
1	1	1	1	2	2	4	4	3	3
1	1	1	1	2	2	4	4	3	3
1	1	1	1	2	2	4	4	3	3
4	4	1	1	2	2	4	4	2	2
1	1	1	1	2	2	4	4	3	3
1	1	1	1	2	2	1	1	3	3
1	1	1	1	2	2	4	4	3	3
4	4	1	1	2	2	4	4	2	2
1	1	1	1	2	2	4	4	3	3
1	1	1	1	2	2	4	4	3	3
1	1	1	1	2	2	4	4	3	3
1	1	1	1	2	2	4	4	3	3
1	1	1	1	2	2	4	4	3	3
1	1	1	1	2	2	1	1	3	3
1	1	1	1	2	2	4	4	3	3
1	1	1	1	2	2	4	4	3	3
1	1	1	1	2	2	1	1	3	3
1	1	1	1	2	2	4	4	3	3
4	4	1	1	2	2	4	4	3	3
1	1	1	1	2	2	4	4	3	3
1	1	1	1	2	2	4	4	3	3
4	4	1	1	2	2	4	4	3	3
1	1	1	1	2	2	4	4	2	2
1	1	1	1	2	2	4	4	2	2
1	1	1	1	2	2	4	4	3	3
1	1	1	1	2	2	4	4	3	3
1	1	1	1	2	2	4	4	3	3
4	4	1	1	2	2	4	4	2	2
4	4	1	1	2	2	4	4	3	3
1	1	1	1	2	2	4	4	3	3
1	1	1	1	2	2	4	4	3	3
1	1	3	3	3	3	4	4	2	2
1	1	1	1	2	2	4	4	3	3
1	1	1	1	2	2	4	4	3	3
1	1	1	1	2	2	4	4	3	3
1	1	1	1	2	2	4	4	2	2
1	1	1	1	2	2	4	4	3	3
1	1	3	3	3	3	4	4	2	2
1	1	1	1	2	2	4	4	3	3

For Review Only

1	1	1	1	2	2	4	4	3	3
1	1	1	1	2	2	4	4	2	2
1	1	1	1	2	2	4	4	3	3
1	1	1	1	2	2	4	4	3	3
1	1	1	1	2	2	4	4	3	3
1	1	1	1	2	2	4	4	3	3
1	1	1	1	2	2	4	4	3	3
1	1	3	3	2	2	4	4	3	3
1	1	1	1	2	2	4	4	3	3
1	1	1	1	2	2	1	1	3	3
1	1	1	1	2	2	4	4	3	3
1	1	1	1	2	2	4	4	3	3
1	1	1	1	2	2	4	4	3	3
1	1	1	1	2	2	4	4	3	3
1	1	1	1	2	2	4	4	3	3
1	1	1	1	2	2	4	4	2	2
1	1	1	1	2	2	4	4	3	3
1	1	1	1	2	2	4	4	3	3
1	1	3	3	3	3	4	4	2	2
4	4	1	1	2	2	4	4	3	3
1	1	1	1	2	2	4	4	3	3
1	1	1	1	2	2	4	4	2	2
1	1	1	1	2	2	4	4	3	3
1	1	1	1	2	2	4	4	3	3
1	1	1	1	2	2	4	4	2	2
4	4	1	1	2	2	4	4	3	3
4	4	1	1	2	2	4	4	3	3
1	1	1	1	2	2	4	4	3	3
1	1	1	1	2	2	4	4	3	3
1	1	1	1	2	2	4	4	3	3
1	1	1	1	2	2	4	4	3	3
1	1	1	1	2	2	4	4	3	3
1	1	1	1	2	2	4	4	3	3
1	1	1	1	2	2	4	4	3	3
1	1	1	1	2	2	4	4	2	2
1	1	1	1	2	2	4	4	3	3
1	1	1	1	2	2	4	4	3	3
1	1	1	1	2	2	4	4	3	3
1	1	3	3	3	3	4	4	2	2
1	1	1	1	2	2	4	4	3	3
1	1	1	1	2	2	4	4	3	3
1	1	3	3	3	3	4	4	2	2
1	1	1	1	2	2	4	4	3	3
1	1	1	1	2	2	4	4	3	3
1	1	1	1	2	2	4	4	3	3
1	1	1	1	2	2	1	1	3	3
1	1	1	1	2	2	4	4	2	2
1	1	1	1	2	2	4	4	3	3
1	1	1	1	2	2	4	4	3	3
1	1	1	1	2	2	4	4	3	3
1	1	1	1	2	2	4	4	3	3
1	1	1	1	2	2	4	4	3	3
1	1	1	1	2	2	4	4	2	2
1	1	1	1	2	2	4	4	3	3
1	1	1	1	2	2	4	4	2	2

For Review Only

1	1	1	1	2	2	4	4	3	3
1	1	1	1	3	3	1	1	3	3
1	1	1	1	2	2	1	1	3	3
4	4	1	1	2	2	4	4	3	3
4	4	1	1	2	2	4	4	3	3
1	1	1	1	2	2	4	4	3	3
1	1	1	1	2	2	4	4	3	3
4	4	1	1	2	2	4	4	3	3
1	1	1	1	2	2	4	4	3	3
1	1	1	1	2	2	4	4	2	2
1	1	1	1	2	2	4	4	3	3
4	4	1	1	2	2	4	4	3	3
1	1	1	1	2	2	4	4	3	3
1	1	1	1	2	2	4	4	3	3
1	1	1	1	2	2	4	4	3	3
1	1	1	1	2	2	4	4	3	3
1	1	1	1	2	2	4	4	2	2
1	1	1	1	2	2	4	4	3	3
1	1	1	1	2	2	1	1	3	3
1	1	1	1	2	2	4	4	3	3
1	1	1	1	2	2	4	4	3	3
4	4	1	1	2	2	4	4	3	3
1	1	1	1	2	2	1	1	3	3
1	1	1	1	2	2	4	4	3	3
1	1	1	1	2	2	4	4	3	3
1	1	1	1	2	2	4	4	3	3
1	1	1	1	2	2	4	4	2	2
1	1	1	1	2	2	4	4	3	3
4	4	1	1	2	2	4	4	3	3
1	1	1	1	2	2	1	1	3	3
1	1	1	1	2	2	4	4	2	2
1	1	1	1	2	2	4	4	2	2
1	1	1	1	2	2	4	4	3	3
1	1	3	3	3	3	4	4	3	3
1	1	1	1	2	2	4	4	3	3
1	1	1	1	2	2	4	4	2	2
1	1	1	1	2	2	4	4	3	3
1	1	1	1	2	2	4	4	3	3
1	1	1	1	2	2	4	4	3	3
1	1	1	1	2	2	4	4	2	2
1	1	1	1	2	2	1	1	3	3
1	1	1	1	2	2	4	4	3	3
1	1	1	1	2	2	4	4	3	3
1	1	1	1	2	2	1	1	3	3
1	1	1	1	2	2	4	4	3	3
1	1	1	1	2	2	1	1	3	3
1	1	3	3	3	3	4	4	2	2
4	4	1	1	2	2	4	4	3	3
4	4	1	1	2	2	4	4	3	3
1	1	1	1	2	2	4	4	2	2
1	1	1	1	2	2	1	1	3	3
1	1	1	1	2	2	4	4	2	2
1	1	1	1	2	2	4	4	3	3
1	1	1	1	2	2	4	4	3	3
1	1	1	1	2	2	4	4	3	3

For Review Only

1	1	1	1	2	2	4	4	3	3
1	1	1	1	2	2	4	4	3	3

For Review Only

SNP3054	SNP3054	SNP3055	SNP3055	SNP3056	SNP3056	SNP3057	SNP3057	SNP3058	SNP3058
4	4	3	3	4	4	4	4	2	2
4	4	3	3	4	4	4	4	4	4
4	4	3	3	4	4	4	4	4	4
4	4	3	3	4	4	2	2	4	4
4	4	3	3	4	4	2	2	4	4
4	4	3	3	4	4	4	4	4	4
4	4	3	3	4	4	2	2	4	4
4	4	3	3	4	4	2	2	4	4
4	4	3	3	4	4	4	4	4	4
4	4	3	3	4	4	2	2	4	4
4	4	3	3	4	4	4	4	2	2
4	4	3	3	4	4	4	4	4	4
4	4	3	3	4	4	2	2	4	4
4	4	3	3	4	4	4	4	4	4
4	4	3	3	4	4	2	2	4	4
4	4	3	3	4	4	2	2	4	4
4	4	3	3	4	4	2	2	4	4
4	4	1	1	3	3	4	4	4	4
4	4	3	3	4	4	4	4	4	4
4	4	3	3	4	4	2	2	4	4
4	4	3	3	4	4	4	4	4	4
4	4	3	3	4	4	2	2	4	4
4	4	3	3	4	4	4	4	2	2
4	4	3	3	4	4	4	4	4	4
4	4	3	3	4	4	2	2	4	4
4	4	3	3	4	4	2	2	4	4
4	4	3	3	4	4	2	2	4	4
4	4	3	3	4	4	2	2	4	4
4	4	3	3	4	4	4	4	4	4
4	4	3	3	4	4	2	2	4	4
4	4	3	3	4	4	2	2	4	4
4	4	3	3	4	4	4	4	2	2
4	4	3	3	4	4	2	2	4	4
4	4	3	3	4	4	4	4	4	4
4	4	3	3	4	4	2	2	4	4
4	4	3	3	4	4	4	4	4	4
4	4	3	3	4	4	2	2	4	4
4	4	3	3	4	4	2	2	4	4
4	4	3	3	4	4	4	4	2	2
4	4	3	3	4	4	2	2	4	4
4	4	3	3	4	4	4	4	4	4
4	4	3	3	4	4	4	4	4	4
4	4	3	3	4	4	2	2	4	4
4	4	3	3	4	4	2	2	4	4
4	4	3	3	4	4	2	2	4	4
4	4	3	3	4	4	4	4	4	4
4	4	3	3	4	4	4	4	4	4
4	4	3	3	4	4	2	2	4	4
4	4	3	3	4	4	4	4	4	4
4	4	3	3	4	4	2	2	2	2
4	4	3	3	4	4	2	2	4	4
4	4	3	3	4	4	4	4	4	4

4	4	3	3	4	4	2	2	4	4
4	4	3	3	4	4	2	2	4	4
4	4	3	3	4	4	4	4	4	4
4	4	3	3	4	4	2	2	4	4
4	4	3	3	4	4	4	4	4	4
4	4	3	3	4	4	2	2	4	4
1	1	1	1	3	3	2	2	4	4
4	4	3	3	4	4	2	2	4	4
4	4	3	3	4	4	2	2	4	4
4	4	3	3	4	4	2	2	4	4
4	4	3	3	4	4	4	4	4	4
4	4	3	3	4	4	4	4	4	4
4	4	3	3	4	4	2	2	4	4
4	4	3	3	4	4	4	4	4	4
4	4	3	3	4	4	2	2	4	4
4	4	3	3	4	4	2	2	4	4
4	4	3	3	4	4	4	4	2	2
4	4	3	3	4	4	4	4	2	2
4	4	3	3	4	4	4	4	4	4
4	4	3	3	4	4	2	2	4	4
4	4	3	3	4	4	4	4	4	4
4	4	3	3	4	4	2	2	4	4
4	4	3	3	4	4	4	4	4	4
4	4	3	3	4	4	4	4	4	4
4	4	3	3	4	4	2	2	4	4
4	4	3	3	4	4	2	2	4	4
4	4	3	3	4	4	2	2	4	4
4	4	3	3	4	4	4	4	2	2
1	1	1	1	3	3	2	2	4	4
4	4	3	3	4	4	2	2	4	4
4	4	3	3	4	4	2	2	4	4
4	4	3	3	4	4	2	2	4	4
4	4	3	3	4	4	4	4	2	2
4	4	3	3	4	4	2	2	4	4
4	4	3	3	4	4	4	4	2	2
4	4	3	3	4	4	2	2	4	4
4	4	3	3	4	4	4	4	4	4
4	4	3	3	4	4	2	2	4	4
4	4	3	3	4	4	2	2	4	4
4	4	3	3	4	4	4	4	4	4
4	4	3	3	4	4	2	2	4	4
4	4	3	3	4	4	4	4	4	4
4	4	3	3	4	4	2	2	4	4
4	4	3	3	4	4	2	2	4	4
4	4	3	3	4	4	2	2	4	4
4	4	3	3	4	4	2	2	4	4
4	4	3	3	4	4	4	4	4	4
1	1	1	1	3	3	2	2	4	4
1	1	1	1	3	3	2	2	4	4
4	4	3	3	4	4	4	4	4	4
4	4	3	3	4	4	4	4	4	4
4	4	3	3	4	4	4	4	2	2
4	4	3	3	4	4	2	2	4	4
4	4	3	3	4	4	4	4	2	2
4	4	3	3	4	4	4	4	4	4

For Review Only

4	4	3	3	4	4	4	4	4	4
4	4	3	3	4	4	2	2	4	4
4	4	3	3	4	4	4	4	4	4
4	4	3	3	4	4	4	4	4	4
4	4	3	3	4	4	2	2	4	4
4	4	3	3	4	4	2	2	4	4
4	4	3	3	4	4	4	4	4	4
4	4	3	3	4	4	2	2	4	4
1	1	1	1	3	3	2	2	4	4
4	4	3	3	4	4	2	2	4	4
4	4	3	3	4	4	4	4	4	4
4	4	3	3	4	4	2	2	4	4
4	4	3	3	4	4	4	4	4	4
4	4	3	3	4	4	2	2	4	4
4	4	3	3	4	4	2	2	4	4
1	1	3	3	3	3	2	2	4	4
4	4	3	3	4	4	4	4	4	4
4	4	3	3	4	4	2	2	4	4
4	4	3	3	4	4	4	4	4	4
4	4	3	3	4	4	4	4	2	2
4	4	3	3	4	4	2	2	4	4
4	4	3	3	4	4	2	2	4	4
4	4	3	3	4	4	4	4	4	4
4	4	3	3	4	4	4	4	2	2
4	4	3	3	4	4	2	2	4	4
4	4	3	3	4	4	2	2	4	4
1	1	3	3	3	3	2	2	4	4
4	4	3	3	4	4	4	4	2	2
4	4	3	3	4	4	4	4	4	4
4	4	3	3	4	4	4	4	4	4
4	4	3	3	4	4	4	4	4	4
4	4	3	3	4	4	2	2	4	4
4	4	3	3	4	4	2	2	4	4
4	4	3	3	4	4	2	2	4	4
4	4	3	3	4	4	2	2	4	4
4	4	3	3	4	4	4	4	4	4
4	4	3	3	4	4	2	2	4	4
4	4	3	3	4	4	2	2	4	4
4	4	3	3	4	4	2	2	2	2
4	4	3	3	4	4	4	4	2	2
4	4	3	3	4	4	2	2	4	4
4	4	3	3	4	4	2	2	4	4
4	4	3	3	4	4	4	4	4	4
4	4	3	3	4	4	2	2	4	4
4	4	3	3	4	4	2	2	4	4
4	4	3	3	4	4	2	2	4	4
4	4	3	3	4	4	2	2	4	4
1	1	1	1	3	3	2	2	4	4
4	4	3	3	4	4	2	2	4	4
1	1	3	3	3	3	2	2	4	4
1	1	3	3	3	3	2	2	4	4
4	4	3	3	4	4	4	4	2	2
4	4	3	3	4	4	2	2	2	2
1	1	1	1	3	3	2	2	4	4
4	4	3	3	4	4	2	2	4	4

For Review Only

4	4	3	3	4	4	2	2	4	4
4	4	3	3	4	4	4	4	2	2
4	4	3	3	4	4	2	2	4	4
4	4	3	3	4	4	2	2	4	4
4	4	3	3	4	4	2	2	4	4
4	4	3	3	4	4	2	2	4	4
4	4	3	3	4	4	2	2	4	4
4	4	3	3	4	4	2	2	4	4
4	4	3	3	4	4	4	4	4	4
4	4	3	3	4	4	2	2	4	4
4	4	3	3	4	4	2	2	4	4
4	4	3	3	4	4	2	2	4	4
4	4	3	3	4	4	2	2	4	4
4	4	3	3	4	4	4	4	4	4
4	4	3	3	4	4	4	4	4	4
4	4	3	3	4	4	4	4	2	2
4	4	3	3	4	4	2	2	4	4
4	4	3	3	4	4	2	2	4	4
1	1	3	3	3	3	2	2	4	4
4	4	3	3	4	4	4	4	4	4
4	4	3	3	4	4	2	2	4	4
4	4	3	3	4	4	4	4	2	2
4	4	3	3	4	4	2	2	4	4
4	4	3	3	4	4	4	4	4	4
4	4	3	3	4	4	4	4	4	4
4	4	3	3	4	4	4	4	4	4
4	4	3	3	4	4	4	4	4	4
4	4	3	3	4	4	2	2	4	4
4	4	3	3	4	4	4	4	4	4
4	4	3	3	4	4	4	4	4	4
4	4	3	3	4	4	4	4	4	4
4	4	3	3	4	4	4	4	4	4
4	4	3	3	4	4	2	2	4	4
4	4	3	3	4	4	4	4	4	4
4	4	3	3	4	4	4	4	4	4
4	4	3	3	4	4	4	4	4	4
4	4	3	3	4	4	4	4	4	4
4	4	3	3	4	4	2	2	4	4
1	1	1	1	3	3	2	2	4	4
4	4	3	3	4	4	2	2	4	4
4	4	3	3	4	4	4	4	4	4
1	1	1	1	3	3	2	2	4	4
4	4	3	3	4	4	2	2	2	2
1	1	3	3	3	3	2	2	4	4
4	4	3	3	4	4	2	2	2	2
4	4	3	3	4	4	4	4	2	2
4	4	3	3	4	4	4	4	2	2
4	4	3	3	4	4	2	2	4	4
4	4	3	3	4	4	4	4	4	4
4	4	3	3	4	4	2	2	4	4
4	4	3	3	4	4	2	2	4	4
4	4	3	3	4	4	2	2	4	4
4	4	3	3	4	4	4	4	2	2
4	4	3	3	4	4	4	4	4	4
4	4	3	3	4	4	4	4	2	2

4	4	3	3	4	4	2	2	4	4
4	4	3	3	4	4	4	4	4	4
4	4	3	3	4	4	4	4	2	2
4	4	3	3	4	4	2	2	4	4
4	4	3	3	4	4	4	4	4	4
4	4	3	3	4	4	2	2	4	4
4	4	3	3	4	4	2	2	4	4
4	4	3	3	4	4	2	2	4	4
4	4	3	3	4	4	2	2	4	4
4	4	3	3	4	4	4	4	2	2
4	4	3	3	4	4	2	2	4	4
4	4	3	3	4	4	4	4	2	2
4	4	3	3	4	4	2	2	4	4
4	4	3	3	4	4	2	2	4	4
4	4	3	3	4	4	4	4	4	4
4	4	3	3	4	4	2	2	4	4
4	4	3	3	4	4	4	4	2	2
4	4	3	3	4	4	4	4	4	4
4	4	3	3	4	4	2	2	4	4
4	4	3	3	4	4	2	2	4	4
4	4	3	3	4	4	4	4	4	4
4	4	3	3	4	4	2	2	4	4
4	4	3	3	4	4	2	2	4	4
4	4	3	3	4	4	4	4	4	4
4	4	3	3	4	4	2	2	4	4
4	4	3	3	4	4	2	2	4	4
4	4	3	3	4	4	4	4	2	2
4	4	3	3	4	4	4	4	4	4
4	4	3	3	4	4	2	2	4	4
4	4	3	3	4	4	4	4	4	4
4	4	3	3	4	4	4	4	2	2
4	4	3	3	4	4	4	4	2	2
4	4	3	3	4	4	4	4	4	4
1	1	1	1	3	3	2	2	4	4
4	4	3	3	4	4	2	2	4	4
4	4	3	3	4	4	4	4	2	2
4	4	3	3	4	4	4	4	2	2
4	4	3	3	4	4	4	4	4	4
4	4	3	3	4	4	4	4	4	4
4	4	3	3	4	4	4	4	2	2
4	4	3	3	4	4	2	2	4	4
4	4	3	3	4	4	2	2	4	4
4	4	3	3	4	4	2	2	4	4
4	4	3	3	4	4	2	2	4	4
4	4	3	3	4	4	2	2	4	4
4	4	3	3	4	4	2	2	4	4
1	1	3	3	3	3	2	2	4	4
4	4	3	3	4	4	2	2	4	4
4	4	3	3	4	4	4	4	4	4
4	4	3	3	4	4	4	4	2	2
4	4	3	3	4	4	4	4	4	4
4	4	3	3	4	4	4	4	2	2
4	4	3	3	4	4	4	4	4	4
4	4	3	3	4	4	2	2	4	4
4	4	3	3	4	4	2	2	4	4

For Review Only

4	4	3	3	4	4	4	4	4	4
4	4	3	3	4	4	4	4	4	4

For Review Only

SNP3059	SNP3059	SNP306	SNP306	SNP3060	SNP3060	SNP3061	SNP3061	SNP3062	SNP3062
2	2	1	1	2	2	3	3	4	4
4	4	1	1	3	3	2	2	4	4
4	4	1	1	3	3	2	2	4	4
4	4	1	1	3	3	2	2	4	4
2	2	1	1	3	3	3	3	4	4
4	4	1	1	3	3	2	2	1	1
2	2	4	4	3	3	3	3	4	4
2	2	1	1	3	3	3	3	4	4
4	4	1	1	3	3	3	3	4	4
2	2	1	1	3	3	3	3	4	4
2	2	1	1	3	3	3	3	4	4
4	4	1	1	3	3	2	2	4	4
2	2	4	4	2	2	3	3	4	4
2	2	1	1	3	3	3	3	4	4
2	2	4	4	3	3	3	3	4	4
2	2	1	1	3	3	3	3	4	4
2	2	4	4	3	3	3	3	4	4
2	2	1	1	2	2	3	3	1	1
4	4	1	1	3	3	2	2	1	1
2	2	1	1	3	3	3	3	4	4
4	4	4	4	3	3	2	2	4	4
2	2	4	4	3	3	3	3	4	4
2	2	1	1	3	3	3	3	4	4
2	2	1	1	3	3	3	3	4	4
2	2	4	4	3	3	3	3	4	4
2	2	4	4	3	3	3	3	4	4
4	4	1	1	3	3	2	2	4	4
2	2	4	4	3	3	3	3	4	4
4	4	1	1	3	3	2	2	4	4
2	2	1	1	3	3	3	3	4	4
2	2	1	1	3	3	3	3	4	4
2	2	1	1	2	2	3	3	4	4
2	2	4	4	3	3	3	3	4	4
2	2	1	1	3	3	3	3	4	4
2	2	4	4	3	3	2	2	4	4
4	4	1	1	3	3	2	2	4	4
2	2	1	1	3	3	3	3	4	4
2	2	1	1	3	3	3	3	1	1
2	2	1	1	3	3	3	3	4	4
2	2	1	1	3	3	3	3	1	1
2	2	1	1	2	2	3	3	4	4
4	4	1	1	3	3	3	3	4	4
2	2	1	1	3	3	3	3	4	4
2	2	4	4	2	2	3	3	4	4
2	2	1	1	3	3	3	3	4	4
4	4	4	4	3	3	2	2	4	4
4	4	4	4	3	3	2	2	4	4
2	2	4	4	3	3	3	3	4	4
4	4	1	1	3	3	2	2	4	4
2	2	4	4	3	3	3	3	4	4
2	2	4	4	3	3	3	3	4	4
2	2	1	1	3	3	3	3	1	1

For Review Only

2	2	4	4	3	3	3	3	4	4
2	2	4	4	3	3	3	3	4	4
4	4	1	1	3	3	2	2	1	1
2	2	4	4	3	3	3	3	4	4
4	4	1	1	3	3	2	2	1	1
4	4	1	1	3	3	2	2	4	4
2	2	1	1	3	3	3	3	4	4
2	2	1	1	3	3	3	3	4	4
2	2	1	1	3	3	3	3	4	4
2	2	1	1	3	3	3	3	4	4
4	4	1	1	3	3	2	2	4	4
4	4	1	1	3	3	2	2	4	4
2	2	4	4	3	3	3	3	4	4
4	4	1	1	3	3	2	2	4	4
2	2	4	4	3	3	3	3	4	4
2	2	1	1	3	3	3	3	4	4
2	2	1	1	3	3	3	3	4	4
2	2	1	1	2	2	3	3	4	4
4	4	1	1	3	3	2	2	4	4
2	2	1	1	3	3	3	3	4	4
4	4	1	1	3	3	2	2	4	4
2	2	1	1	3	3	3	3	4	4
4	4	1	1	3	3	2	2	4	4
4	4	1	1	3	3	2	2	1	1
2	2	1	1	3	3	3	3	4	4
4	4	1	1	3	3	2	2	4	4
2	2	4	4	3	3	2	2	4	4
2	2	1	1	3	3	3	3	4	4
2	2	4	4	3	3	3	3	4	4
2	2	1	1	3	3	3	3	4	4
2	2	4	4	3	3	3	3	4	4
2	2	1	1	2	2	3	3	4	4
2	2	1	1	3	3	3	3	4	4
2	2	4	4	3	3	3	3	4	4
2	2	1	1	2	2	3	3	1	1
2	2	1	1	3	3	3	3	4	4
4	4	1	1	3	3	2	2	4	4
2	2	4	4	3	3	3	3	4	4
2	2	1	1	3	3	3	3	4	4
2	2	1	1	2	2	3	3	4	4
2	2	1	1	3	3	3	3	4	4
4	4	1	1	3	3	2	2	4	4
2	2	4	4	3	3	3	3	4	4
4	4	1	1	3	3	2	2	4	4
2	2	4	4	3	3	3	3	4	4
4	4	4	4	3	3	2	2	4	4
2	2	1	1	3	3	3	3	4	4
2	2	4	4	3	3	3	3	4	4
2	2	4	4	3	3	3	3	4	4
2	2	1	1	3	3	3	3	4	4
4	4	1	1	3	3	2	2	4	4
2	2	1	1	3	3	3	3	4	4
2	2	1	1	3	3	3	3	4	4
2	2	1	1	3	3	3	3	1	1
4	4	4	4	3	3	2	2	4	4

For Review Only

4	4	1	1	3	3	2	2	4	4
2	2	1	1	3	3	3	3	4	4
4	4	4	4	3	3	2	2	4	4
2	2	4	4	3	3	3	3	4	4
4	4	1	1	3	3	2	2	4	4
2	2	4	4	3	3	3	3	4	4
2	2	1	1	3	3	3	3	4	4
2	2	1	1	3	3	3	3	1	1
2	2	4	4	3	3	3	3	4	4
2	2	4	4	3	3	3	3	4	4
2	2	4	4	2	2	3	3	4	4
2	2	4	4	3	3	3	3	4	4
2	2	4	4	3	3	3	3	1	1
2	2	1	1	3	3	3	3	4	4
2	2	1	1	3	3	3	3	4	4
2	2	4	4	3	3	3	3	4	4
2	2	4	4	3	3	3	3	1	1
2	2	4	4	3	3	3	3	4	4
2	2	1	1	2	2	3	3	4	4
2	2	1	1	3	3	3	3	4	4
2	2	4	4	3	3	3	3	4	4
2	2	1	1	3	3	3	3	4	4
4	4	4	4	3	3	2	2	4	4
2	2	1	1	3	3	3	3	4	4
2	2	1	1	3	3	3	3	4	4
2	2	1	1	3	3	3	3	1	1
2	2	1	1	3	3	3	3	4	4
2	2	1	1	3	3	3	3	1	1
4	4	1	1	3	3	2	2	4	4
2	2	1	1	3	3	3	3	1	1
2	2	1	1	3	3	3	3	4	4
2	2	4	4	3	3	3	3	4	4
2	2	1	1	3	3	3	3	4	4
2	2	4	4	3	3	3	3	4	4
2	2	1	1	3	3	3	3	4	4
2	2	4	4	3	3	3	3	4	4
2	2	1	1	3	3	3	3	4	4
4	4	1	1	3	3	2	2	4	4
2	2	4	4	3	3	3	3	4	4
2	2	1	1	3	3	3	3	4	4
2	2	1	1	3	3	3	3	1	1
2	2	1	1	2	2	3	3	4	4
4	4	4	4	3	3	2	2	4	4
2	2	1	1	2	2	3	3	4	4
2	2	4	4	2	2	3	3	4	4
2	2	4	4	3	3	3	3	4	4
2	2	4	4	3	3	2	2	4	4
2	2	4	4	3	3	3	3	4	4
2	2	1	1	3	3	3	3	4	4
2	2	4	4	3	3	3	3	4	4
2	2	4	4	3	3	3	3	4	4
2	2	1	1	3	3	3	3	4	4
2	2	1	1	2	2	3	3	4	4
2	2	4	4	3	3	3	3	4	4
2	2	1	1	3	3	3	3	4	4

For Review Only

2	2	4	4	3	3	3	3	4	4
2	2	1	1	3	3	3	3	4	4
2	2	4	4	3	3	3	3	4	4
4	4	1	1	3	3	3	3	4	4
2	2	4	4	3	3	3	3	4	4
2	2	1	1	3	3	3	3	4	4
2	2	1	1	3	3	3	3	4	4
2	2	4	4	3	3	3	3	4	4
4	4	4	4	3	3	2	2	4	4
2	2	4	4	3	3	3	3	4	4
4	4	4	4	3	3	2	2	4	4
2	2	1	1	3	3	3	3	4	4
2	2	4	4	3	3	2	2	4	4
2	2	4	4	3	3	3	3	1	1
2	2	1	1	3	3	3	3	4	4
2	2	1	1	3	3	3	3	4	4
2	2	4	4	3	3	3	3	4	4
4	4	1	1	3	3	2	2	4	4
2	2	4	4	3	3	3	3	4	4
2	2	1	1	3	3	3	3	4	4
2	2	4	4	3	3	3	3	4	4
2	2	1	1	3	3	3	3	4	4
2	2	1	1	3	3	3	3	1	1
4	4	1	1	3	3	2	2	4	4
4	4	1	1	3	3	2	2	4	4
2	2	1	1	3	3	3	3	4	4
4	4	4	4	3	3	2	2	4	4
2	2	4	4	3	3	3	3	4	4
2	2	1	1	3	3	3	3	1	1
2	2	1	1	3	3	3	3	4	4
4	4	4	4	3	3	2	2	1	1
2	2	4	4	3	3	3	3	1	1
2	2	1	1	3	3	3	3	4	4
2	2	1	1	3	3	3	3	4	4
2	2	4	4	2	2	3	3	4	4
4	4	1	1	3	3	2	2	4	4
4	4	1	1	3	3	2	2	4	4
2	2	1	1	3	3	3	3	4	4
2	2	4	4	3	3	3	3	4	4
2	2	4	4	3	3	3	3	4	4
4	4	1	1	3	3	2	2	4	4
2	2	4	4	3	3	3	3	4	4
2	2	1	1	3	3	3	3	4	4
2	2	1	1	3	3	3	3	4	4
2	2	1	1	3	3	3	3	4	4
2	2	1	1	3	3	3	3	1	1
2	2	1	1	3	3	3	3	4	4
2	2	4	4	3	3	3	3	4	4
2	2	1	1	2	2	3	3	4	4
2	2	1	1	3	3	3	3	4	4
2	2	4	4	3	3	3	3	4	4
2	2	1	1	3	3	3	3	4	4
2	2	1	1	3	3	3	3	4	4
4	4	1	1	3	3	2	2	4	4
2	2	1	1	3	3	3	3	4	4

For Review Only

2	2	1	1	3	3	3	3	4	4
2	2	1	1	3	3	3	3	1	1
2	2	4	4	3	3	3	3	1	1
2	2	4	4	3	3	3	3	4	4
4	4	1	1	3	3	2	2	4	4
2	2	1	1	3	3	3	3	4	4
2	2	4	4	3	3	3	3	4	4
2	2	1	1	3	3	3	3	4	4
2	2	4	4	3	3	3	3	4	4
2	2	1	1	3	3	3	3	4	4
2	2	4	4	3	3	3	3	4	4
2	2	4	4	3	3	2	2	4	4
2	2	1	1	3	3	3	3	4	4
2	2	4	4	3	3	3	3	4	4
4	4	4	4	3	3	2	2	4	4
2	2	1	1	3	3	3	3	4	4
2	2	1	1	3	3	3	3	4	4
2	2	1	1	2	2	3	3	4	4
2	2	4	4	3	3	3	3	4	4
2	2	1	1	3	3	3	3	1	1
4	4	1	1	3	3	2	2	1	1
2	2	4	4	3	3	3	3	4	4
2	2	1	1	3	3	3	3	4	4
4	4	1	1	3	3	2	2	4	4
2	2	1	1	3	3	3	3	4	4
2	2	1	1	3	3	3	3	4	4
2	2	1	1	3	3	3	3	4	4
4	4	1	1	3	3	2	2	4	4
2	2	1	1	3	3	3	3	4	4
2	2	4	4	3	3	3	3	4	4
2	2	4	4	3	3	3	3	4	4
2	2	4	4	3	3	3	3	4	4
2	2	1	1	3	3	3	3	1	1
2	2	4	4	3	3	3	3	4	4
2	2	1	1	3	3	3	3	4	4
2	2	1	1	3	3	3	3	4	4
2	2	1	1	2	2	3	3	4	4
4	4	1	1	3	3	2	2	4	4
4	4	1	1	3	3	2	2	4	4
2	2	1	1	3	3	3	3	4	4
2	2	1	1	3	3	3	3	4	4
2	2	4	4	3	3	3	3	4	4
2	2	1	1	3	3	3	3	1	1
2	2	4	4	3	3	3	3	4	4
2	2	1	1	3	3	3	3	4	4
2	2	1	1	3	3	3	3	4	4
2	2	4	4	3	3	3	3	4	4
2	2	4	4	3	3	3	3	4	4
2	2	1	1	2	2	3	3	4	4
2	2	1	1	3	3	3	3	4	4
2	2	1	1	2	2	3	3	1	1
2	2	1	1	3	3	3	3	4	4
4	4	1	1	3	3	2	2	4	4
2	2	4	4	3	3	3	3	4	4
2	2	1	1	3	3	3	3	1	1

For Review Only

2	2	1	1	3	3	3	3	4	4
4	4	1	1	3	3	2	2	4	4

For Review Only

SNP3063	SNP3063	SNP3064	SNP3064	SNP3065	SNP3065	SNP3066	SNP3066	SNP3067	SNP3067
3	3	4	4	4	4	3	3	3	3
3	3	4	4	4	4	4	4	2	2
3	3	4	4	4	4	3	3	3	3
3	3	4	4	4	4	3	3	3	3
4	4	4	4	1	1	3	3	3	3
4	4	4	4	4	4	3	3	3	3
4	4	2	2	1	1	4	4	2	2
3	3	4	4	4	4	4	4	2	2
3	3	4	4	1	1	3	3	3	3
3	3	4	4	4	4	4	4	2	2
4	4	4	4	1	1	4	4	2	2
3	3	4	4	4	4	3	3	3	3
4	4	4	4	4	4	3	3	3	3
3	3	4	4	4	4	3	3	2	2
3	3	4	4	4	4	3	3	3	3
3	3	2	2	1	1	4	4	2	2
4	4	2	2	1	1	4	4	2	2
3	3	4	4	4	4	3	3	3	3
4	4	4	4	4	4	3	3	3	3
3	3	4	4	4	4	3	3	3	3
3	3	4	4	4	4	4	4	2	2
3	3	4	4	4	4	4	4	2	2
3	3	2	2	4	4	4	4	2	2
3	3	4	4	4	4	4	4	2	2
4	4	2	2	1	1	4	4	2	2
4	4	4	4	4	4	3	3	3	3
3	3	4	4	4	4	3	3	3	3
4	4	4	4	4	4	3	3	3	3
3	3	4	4	4	4	3	3	3	3
3	3	4	4	4	4	4	4	2	2
4	4	2	2	1	1	4	4	2	2
4	4	4	4	4	4	3	3	3	3
4	4	2	2	1	1	4	4	2	2
3	3	4	4	4	4	4	4	2	2
3	3	4	4	4	4	3	3	3	3
3	3	4	4	4	4	3	3	3	3
3	3	4	4	4	4	3	3	3	3
4	4	4	4	4	4	3	3	3	3
3	3	4	4	4	4	4	4	2	2
4	4	4	4	4	4	3	3	3	3
3	3	4	4	4	4	4	4	2	2
3	3	4	4	4	4	3	3	3	3
3	3	4	4	4	4	4	4	2	2
3	3	4	4	4	4	3	3	3	3
3	3	4	4	4	4	4	4	2	2
3	3	4	4	4	4	3	3	3	3
3	3	4	4	4	4	3	3	3	3
4	4	4	4	4	4	3	3	3	3
3	3	4	4	4	4	3	3	3	3
4	4	2	2	4	4	4	4	3	3
3	3	4	4	4	4	4	4	2	2
4	4	4	4	4	4	3	3	3	3

For Review Only

4	4	2	2	1	1	4	4	2	2
4	4	2	2	1	1	4	4	2	2
4	4	4	4	4	4	3	3	3	3
4	4	2	2	1	1	4	4	2	2
4	4	4	4	4	4	3	3	3	3
3	3	4	4	4	4	3	3	3	3
3	3	4	4	4	4	3	3	3	3
3	3	4	4	4	4	4	4	2	2
3	3	4	4	4	4	4	4	2	2
4	4	2	2	1	1	3	3	3	3
3	3	4	4	4	4	3	3	3	3
3	3	4	4	4	4	3	3	3	3
4	4	2	2	1	1	4	4	2	2
3	3	4	4	4	4	3	3	3	3
4	4	4	4	4	4	3	3	3	3
3	3	4	4	4	4	4	4	2	2
3	3	2	2	4	4	4	4	2	2
4	4	2	2	1	1	4	4	2	2
3	3	4	4	4	4	3	3	3	3
4	4	2	2	1	1	4	4	2	2
3	3	4	4	4	4	3	3	3	3
4	4	4	4	4	4	3	3	3	3
4	4	4	4	4	4	3	3	3	3
4	4	4	4	4	4	3	3	3	3
3	3	4	4	4	4	4	4	2	2
3	3	4	4	4	4	3	3	3	3
3	3	4	4	4	4	4	4	2	2
3	3	4	4	4	4	4	4	2	2
3	3	4	4	4	4	3	3	3	3
4	4	2	2	1	1	4	4	2	2
4	4	2	2	1	1	4	4	2	2
3	3	4	4	4	4	4	4	2	2
3	3	2	2	4	4	3	3	2	2
4	4	2	2	1	1	4	4	2	2
3	3	4	4	4	4	3	3	3	3
3	3	4	4	4	4	4	4	2	2
3	3	4	4	4	4	4	4	2	2
3	3	4	4	4	4	4	4	2	2
3	3	4	4	4	4	4	4	2	2
3	3	4	4	4	4	4	4	2	2
4	4	2	2	1	1	4	4	2	2
4	4	4	4	1	1	3	3	3	3
3	3	4	4	4	4	4	4	2	2
3	3	4	4	4	4	3	3	3	3
4	4	2	2	1	1	4	4	2	2
3	3	4	4	4	4	3	3	2	2
3	3	4	4	4	4	3	3	2	2
3	3	4	4	4	4	3	3	3	3
3	3	4	4	4	4	3	3	3	3
4	4	4	4	4	4	3	3	3	3
3	3	4	4	4	4	3	3	3	3
3	3	2	2	4	4	4	4	2	2
3	3	4	4	4	4	4	4	2	2
3	3	4	4	4	4	3	3	3	3
3	3	4	4	4	4	3	3	3	3

For Review Only

3	3	4	4	4	4	3	3	3	3
4	4	4	4	4	4	3	3	3	3
3	3	4	4	4	4	3	3	3	3
3	3	4	4	4	4	4	4	2	2
3	3	4	4	4	4	3	3	3	3
3	3	4	4	4	4	4	4	2	2
3	3	2	2	1	1	4	4	2	2
4	4	4	4	1	1	3	3	3	3
3	3	4	4	4	4	3	3	3	3
3	3	4	4	4	4	3	3	3	3
3	3	4	4	4	4	3	3	3	3
4	4	4	4	4	4	3	3	3	3
3	3	4	4	4	4	3	3	3	3
4	4	4	4	4	4	3	3	3	3
3	3	4	4	4	4	4	4	2	2
3	3	4	4	4	4	3	3	3	3
3	3	4	4	4	4	3	3	3	3
3	3	4	4	4	4	3	3	3	3
3	3	4	4	4	4	4	4	2	2
3	3	2	2	4	4	4	4	2	2
4	4	4	4	4	4	3	3	3	3
4	4	2	2	1	1	4	4	2	2
3	3	4	4	4	4	3	3	2	2
3	3	4	4	4	4	3	3	2	2
4	4	4	4	4	4	3	3	3	3
4	4	4	4	4	4	3	3	3	3
4	4	4	4	4	4	3	3	3	3
3	3	4	4	4	4	3	3	3	3
3	3	4	4	1	1	3	3	3	3
4	4	4	4	4	4	3	3	3	3
3	3	4	4	4	4	3	3	2	2
3	3	4	4	4	4	3	3	3	3
4	4	2	2	1	1	4	4	2	2
3	3	4	4	4	4	3	3	3	3
3	3	4	4	4	4	4	4	2	2
3	3	4	4	4	4	3	3	3	3
3	3	4	4	4	4	4	4	2	2
3	3	4	4	4	4	3	3	3	3
3	3	4	4	4	4	4	4	3	3
3	3	4	4	4	4	3	3	2	2
4	4	4	4	4	4	3	3	3	3
3	3	4	4	4	4	4	4	2	2
3	3	4	4	4	4	3	3	3	3
3	3	4	4	4	4	3	3	3	3
4	4	4	4	4	4	3	3	2	2
4	4	4	4	4	4	3	3	2	2
3	3	4	4	4	4	3	3	3	3
3	3	4	4	4	4	3	3	3	3
3	3	4	4	4	4	3	3	3	3
4	4	4	4	4	4	3	3	3	3
3	3	4	4	4	4	3	3	3	3
3	3	4	4	4	4	4	4	2	2
4	4	2	2	1	1	3	3	2	2
3	3	4	4	4	4	3	3	3	3
3	3	4	4	4	4	3	3	3	3

For Review Only

3	3	4	4	4	4	4	4	2	2
3	3	2	2	4	4	4	4	2	2
3	3	4	4	4	4	3	3	3	3
4	4	4	4	4	4	3	3	3	3
3	3	4	4	4	4	4	4	2	2
3	3	4	4	4	4	4	4	2	2
4	4	4	4	4	4	4	4	3	3
3	3	4	4	4	4	3	3	3	3
3	3	4	4	4	4	3	3	3	3
4	4	2	2	1	1	4	4	2	2
3	3	4	4	4	4	3	3	3	3
4	4	4	4	4	4	3	3	3	3
3	3	4	4	4	4	3	3	3	3
4	4	4	4	4	4	3	3	3	3
3	3	4	4	4	4	4	4	2	2
4	4	4	4	4	4	4	4	2	2
4	4	2	2	1	1	3	3	2	2
3	3	4	4	4	4	4	4	2	2
3	3	4	4	4	4	3	3	3	3
3	3	2	2	1	1	4	4	2	2
3	3	4	4	4	4	3	3	3	3
3	3	2	2	4	4	4	4	2	2
4	4	4	4	4	4	3	3	3	3
3	3	4	4	4	4	3	3	3	3
3	3	2	2	1	1	4	4	2	2
3	3	4	4	4	4	4	4	2	2
3	3	4	4	4	4	3	3	3	3
3	3	4	4	4	4	4	4	2	2
3	3	4	4	4	4	3	3	3	3
3	3	4	4	4	4	3	3	3	3
4	4	4	4	4	4	3	3	3	3
3	3	4	4	4	4	3	3	3	3
3	3	4	4	4	4	3	3	3	3
3	3	4	4	4	4	4	4	2	2
3	3	4	4	4	4	3	3	3	3
3	3	4	4	4	4	3	3	3	3
4	4	4	4	4	4	3	3	3	3
3	3	4	4	4	4	4	4	2	2
3	3	4	4	4	4	3	3	3	3
3	3	4	4	4	4	4	4	2	2
3	3	4	4	4	4	3	3	3	3
3	3	4	4	4	4	3	3	3	3
4	4	4	4	4	4	3	3	2	2
4	4	4	4	4	4	3	3	3	3
3	3	4	4	4	4	3	3	3	3
3	3	4	4	4	4	3	3	3	3
4	4	4	4	4	4	4	4	2	2
3	3	4	4	4	4	4	4	3	3
3	3	4	4	4	4	4	4	2	2
4	4	4	4	1	1	4	4	2	2
3	3	4	4	4	4	4	4	2	2
3	3	4	4	4	4	3	3	3	3
3	3	2	2	4	4	4	4	2	2
3	3	4	4	4	4	3	3	3	3
3	3	4	4	4	4	4	4	2	2

For Review Only

3	3	4	4	4	4	4	4	2	2
3	3	4	4	4	4	3	3	3	3
3	3	4	4	4	4	3	3	3	3
4	4	4	4	4	4	3	3	3	3
3	3	4	4	4	4	4	4	2	2
3	3	4	4	4	4	3	3	2	2
3	3	4	4	4	4	4	4	2	2
3	3	4	4	4	4	4	4	2	2
3	3	4	4	4	4	3	3	3	3
3	3	4	4	4	4	3	3	2	2
3	3	4	4	4	4	3	3	3	3
3	3	4	4	4	4	3	3	3	3
3	3	4	4	4	4	4	4	2	2
3	3	4	4	4	4	4	4	2	2
3	3	4	4	4	4	3	3	3	3
3	3	4	4	4	4	4	4	2	2
3	3	4	4	4	4	4	4	2	2
3	3	4	4	4	4	3	3	3	3
4	4	2	2	1	1	4	4	2	2
4	4	4	4	4	4	3	3	3	3
4	4	4	4	4	4	3	3	3	3
3	3	4	4	4	4	4	4	2	2
4	4	2	2	1	1	4	4	2	2
3	3	4	4	4	4	3	3	3	3
3	3	4	4	4	4	4	4	2	2
4	4	4	4	4	4	3	3	3	3
3	3	2	2	4	4	4	4	2	2
3	3	4	4	4	4	3	3	3	3
4	4	4	4	1	1	4	4	2	2
4	4	2	2	1	1	4	4	2	2
3	3	2	2	4	4	3	3	2	2
3	3	2	2	4	4	4	4	2	2
3	3	4	4	4	4	3	3	3	3
3	3	4	4	4	4	3	3	3	3
4	4	4	4	4	4	3	3	3	3
3	3	2	2	4	4	3	3	2	2
4	4	2	2	1	1	4	4	2	2
3	3	4	4	4	4	3	3	3	3
3	3	4	4	4	4	3	3	3	3
3	3	4	4	4	4	4	4	2	2
3	3	4	4	4	4	3	3	3	3
3	3	4	4	4	4	4	4	2	2
4	4	4	4	4	4	3	3	3	3
4	4	2	2	1	1	4	4	2	2
3	3	4	4	4	4	4	4	2	2
4	4	2	2	1	1	4	4	2	2
3	3	4	4	4	4	3	3	3	3
4	4	2	2	1	1	4	4	2	2
3	3	4	4	4	4	4	4	2	2
3	3	2	2	4	4	4	4	2	2
4	4	4	4	4	4	3	3	3	3
3	3	2	2	4	4	3	3	2	2
3	3	4	4	4	4	3	3	3	3
4	4	4	4	4	4	3	3	3	3
4	4	4	4	4	4	3	3	3	3

For Review Only

4	4	2	2	1	1	4	4	2	2
3	3	4	4	4	4	3	3	3	3

For Review Only

SNP3068	SNP3068	SNP3069	SNP3069	SNP307	SNP307	SNP3070	SNP3070	SNP3071	SNP3071
1	1	1	1	3	3	4	4	2	2
4	4	4	4	2	2	4	4	2	2
1	1	1	1	2	2	4	4	2	2
1	1	1	1	2	2	3	3	1	1
1	1	1	1	3	3	4	4	1	1
1	1	1	1	3	3	3	3	1	1
1	1	1	1	2	2	4	4	2	2
4	4	4	4	2	2	4	4	2	2
1	1	1	1	2	2	3	3	1	1
4	4	4	4	2	2	4	4	2	2
4	4	4	4	2	2	4	4	2	2
1	1	1	1	2	2	4	4	2	2
1	1	1	1	2	2	4	4	2	2
4	4	1	1	2	2	4	4	2	2
1	1	1	1	2	2	4	4	1	1
4	4	4	4	2	2	3	3	1	1
4	4	4	4	2	2	4	4	2	2
1	1	1	1	3	3	4	4	2	2
1	1	1	1	2	2	3	3	1	1
1	1	1	1	2	2	3	3	1	1
4	4	4	4	2	2	4	4	2	2
4	4	4	4	2	2	4	4	2	2
4	4	4	4	2	2	4	4	2	2
4	4	4	4	2	2	4	4	2	2
4	4	4	4	2	2	4	4	2	2
1	1	1	1	2	2	3	3	1	1
1	1	1	1	2	2	3	3	1	1
1	1	1	1	2	2	4	4	1	1
1	1	1	1	2	2	3	3	1	1
4	4	4	4	2	2	4	4	2	2
4	4	4	4	2	2	3	3	1	1
1	1	1	1	2	2	3	3	1	1
4	4	4	4	2	2	4	4	2	2
4	4	4	4	3	3	4	4	2	2
4	4	4	4	2	2	4	4	1	1
1	1	1	1	2	2	3	3	1	1
1	1	1	1	2	2	3	3	1	1
1	1	1	1	2	2	3	3	1	1
4	4	4	4	2	2	4	4	2	2
1	1	1	1	2	2	3	3	1	1
4	4	4	4	2	2	4	4	2	2
1	1	1	1	2	2	3	3	1	1
4	4	4	4	2	2	4	4	2	2
1	1	1	1	2	2	3	3	1	1
4	4	4	4	2	2	4	4	2	2
1	1	1	1	2	2	4	4	2	2
1	1	1	1	2	2	3	3	1	1
1	1	1	1	2	2	3	3	1	1
1	1	1	1	2	2	3	3	1	1
1	1	1	1	2	2	4	4	1	1
4	4	4	4	2	2	4	4	2	2
1	1	1	1	2	2	3	3	1	1

For Review Only

4	4	4	4	2	2	4	4	2	2
4	4	4	4	2	2	4	4	2	2
1	1	1	1	2	2	4	4	2	2
4	4	4	4	2	2	4	4	2	2
1	1	1	1	2	2	3	3	1	1
1	1	1	1	2	2	3	3	1	1
1	1	1	1	2	2	3	3	1	1
4	4	4	4	2	2	4	4	2	2
4	4	4	4	2	2	4	4	2	2
1	1	1	1	2	2	4	4	1	1
1	1	1	1	2	2	3	3	1	1
1	1	1	1	2	2	3	3	1	1
4	4	4	4	2	2	4	4	2	2
1	1	1	1	2	2	3	3	1	1
1	1	1	1	3	3	4	4	1	1
4	4	4	4	2	2	4	4	2	2
4	4	1	1	2	2	4	4	2	2
4	4	4	4	3	3	4	4	2	2
1	1	1	1	2	2	3	3	1	1
4	4	4	4	2	2	4	4	2	2
1	1	1	1	2	2	3	3	1	1
1	1	1	1	2	2	3	3	1	1
1	1	1	1	2	2	3	3	1	1
1	1	1	1	3	3	4	4	2	2
4	4	4	4	2	2	4	4	2	2
1	1	1	1	2	2	3	3	1	1
4	4	4	4	2	2	4	4	2	2
4	4	4	4	2	2	4	4	2	2
1	1	1	1	2	2	3	3	1	1
4	4	4	4	3	3	3	3	1	1
4	4	4	4	2	2	4	4	2	2
4	4	4	4	3	3	4	4	2	2
1	1	4	4	2	2	4	4	2	2
4	4	4	4	2	2	4	4	2	2
1	1	1	1	2	2	4	4	2	2
4	4	4	4	2	2	4	4	2	2
4	4	4	4	2	2	4	4	2	2
4	4	1	1	2	2	4	4	2	2
4	4	4	4	2	2	4	4	2	2
4	4	4	4	2	2	4	4	2	2
4	4	4	4	2	2	4	4	2	2
1	1	4	4	2	2	3	3	1	1
4	4	4	4	2	2	4	4	2	2
1	1	1	1	2	2	3	3	1	1
4	4	4	4	3	3	4	4	2	2
4	4	1	1	2	2	3	3	1	1
4	4	4	4	2	2	4	4	2	2
1	1	1	1	2	2	3	3	1	1
1	1	1	1	2	2	3	3	1	1
1	1	1	1	2	2	3	3	1	1
1	1	1	1	2	2	3	3	1	1
4	4	4	4	2	2	4	4	2	2
4	4	4	4	2	2	4	4	2	2
1	1	1	1	2	2	4	4	1	1
1	1	1	1	2	2	3	3	1	1

For Review Only

1	1	1	1	2	2	3	3	1	1
1	1	1	1	2	2	4	4	1	1
1	1	1	1	2	2	3	3	1	1
4	4	4	4	2	2	4	4	2	2
1	1	1	1	2	2	3	3	1	1
4	4	4	4	3	3	4	4	2	2
4	4	4	4	2	2	3	3	1	1
1	1	1	1	3	3	3	3	1	1
1	1	1	1	2	2	3	3	1	1
1	1	1	1	2	2	3	3	1	1
1	1	1	1	3	3	4	4	2	2
1	1	1	1	2	2	3	3	1	1
1	1	1	1	2	2	3	3	1	1
1	1	1	1	2	2	4	4	1	1
4	4	4	4	2	2	4	4	2	2
1	1	1	1	2	2	4	4	1	1
1	1	1	1	2	2	3	3	1	1
1	1	1	1	2	2	4	4	1	1
4	4	4	4	2	2	4	4	2	2
4	4	4	4	2	2	4	4	2	2
1	1	1	1	2	2	4	4	1	1
4	4	4	4	2	2	4	4	2	2
4	4	1	1	2	2	4	4	1	1
1	1	4	4	2	2	4	4	2	2
1	1	1	1	2	2	3	3	1	1
1	1	1	1	2	2	3	3	1	1
1	1	1	1	2	2	3	3	1	1
1	1	1	1	2	2	3	3	1	1
1	1	1	1	3	3	3	3	1	1
1	1	1	1	2	2	3	3	1	1
1	1	4	4	2	2	4	4	2	2
1	1	1	1	2	2	4	4	1	1
4	4	4	4	2	2	4	4	2	2
1	1	1	1	2	2	3	3	1	1
4	4	4	4	3	3	4	4	2	2
4	4	1	1	3	3	4	4	2	2
4	4	4	4	2	2	4	4	2	2
1	1	1	1	2	2	4	4	2	2
4	4	4	4	2	2	4	4	2	2
1	1	4	4	2	2	4	4	2	2
1	1	1	1	2	2	4	4	1	1
4	4	4	4	2	2	4	4	2	2
1	1	1	1	2	2	3	3	1	1
4	4	1	1	2	2	4	4	1	1
1	1	4	4	2	2	4	4	2	2
4	4	4	4	2	2	3	3	1	1
1	1	1	1	2	2	3	3	1	1
1	1	1	1	2	2	3	3	1	1
1	1	1	1	2	2	4	4	2	2
1	1	1	1	2	2	3	3	1	1
1	1	1	1	2	2	3	3	1	1
4	4	4	4	2	2	4	4	2	2
4	4	4	4	3	3	4	4	2	2
1	1	1	1	2	2	3	3	1	1
1	1	1	1	2	2	4	4	1	1

For Review Only

4	4	4	4	2	2	4	4	2	2
4	4	4	4	2	2	4	4	2	2
1	1	1	1	2	2	3	3	1	1
1	1	1	1	3	3	3	3	1	1
4	4	4	4	2	2	4	4	2	2
4	4	4	4	2	2	4	4	2	2
1	1	4	4	2	2	4	4	1	1
1	1	1	1	2	2	4	4	1	1
1	1	1	1	2	2	4	4	1	1
4	4	4	4	3	3	3	3	1	1
1	1	1	1	2	2	3	3	1	1
4	4	1	1	2	2	4	4	1	1
1	1	1	1	3	3	3	3	1	1
1	1	1	1	2	2	3	3	1	1
4	4	4	4	2	2	4	4	2	2
4	4	4	4	2	2	4	4	2	2
4	4	1	1	3	3	4	4	2	2
4	4	1	1	2	2	3	3	1	1
1	1	1	1	2	2	3	3	1	1
4	4	4	4	2	2	3	3	1	1
1	1	1	1	2	2	4	4	1	1
4	4	4	4	2	2	4	4	2	2
1	1	1	1	2	2	3	3	1	1
1	1	1	1	2	2	3	3	1	1
4	4	4	4	2	2	4	4	2	2
4	4	4	4	2	2	4	4	2	2
1	1	1	1	2	2	3	3	1	1
4	4	4	4	3	3	4	4	2	2
1	1	1	1	2	2	3	3	1	1
1	1	1	1	3	3	3	3	1	1
1	1	1	1	2	2	4	4	2	2
1	1	1	1	3	3	3	3	1	1
1	1	1	1	2	2	3	3	1	1
4	4	4	4	2	2	4	4	2	2
1	1	1	1	2	2	3	3	1	1
1	1	1	1	2	2	3	3	1	1
1	1	1	1	2	2	3	3	1	1
4	4	4	4	2	2	4	4	2	2
1	1	1	1	2	2	3	3	1	1
4	4	4	4	2	2	4	4	2	2
1	1	1	1	2	2	4	4	2	2
1	1	1	1	2	2	3	3	1	1
1	1	4	4	2	2	4	4	2	2
1	1	1	1	3	3	3	3	1	1
1	1	1	1	2	2	4	4	2	2
1	1	1	1	2	2	3	3	1	1
4	4	4	4	2	2	4	4	2	2
4	4	4	4	2	2	4	4	2	2
4	4	4	4	2	2	4	4	2	2
4	4	4	4	2	2	4	4	2	2
4	4	4	4	3	3	4	4	2	2
1	1	1	1	2	2	3	3	1	1
4	4	4	4	2	2	4	4	2	2
1	1	1	1	2	2	3	3	1	1
4	4	4	4	2	2	4	4	2	2

For Review Only

4	4	4	4	3	3	4	4	2	2
1	1	1	1	2	2	3	3	1	1
1	1	1	1	2	2	3	3	1	1
1	1	1	1	2	2	4	4	1	1
4	4	4	4	2	2	4	4	2	2
1	1	1	1	3	3	3	3	1	1
4	4	4	4	2	2	4	4	2	2
4	4	4	4	2	2	4	4	2	2
1	1	1	1	2	2	4	4	1	1
1	1	4	4	2	2	4	4	2	2
1	1	1	1	2	2	4	4	1	1
1	1	1	1	2	2	3	3	1	1
4	4	4	4	2	2	4	4	2	2
4	4	4	4	2	2	4	4	2	2
1	1	1	1	2	2	3	3	1	1
4	4	4	4	2	2	4	4	2	2
4	4	4	4	2	2	4	4	2	2
1	1	4	4	2	2	4	4	1	1
4	4	4	4	2	2	4	4	2	2
1	1	1	1	2	2	3	3	1	1
1	1	1	1	2	2	4	4	2	2
4	4	1	1	2	2	3	3	1	1
4	4	4	4	2	2	4	4	2	2
1	1	1	1	2	2	3	3	1	1
4	4	4	4	2	2	4	4	2	2
1	1	1	1	3	3	3	3	1	1
4	4	4	4	2	2	4	4	2	2
1	1	1	1	2	2	3	3	1	1
4	4	4	4	2	2	4	4	2	2
4	4	4	4	3	3	3	3	1	1
1	1	4	4	2	2	4	4	2	2
4	4	4	4	2	2	4	4	2	2
1	1	1	1	2	2	3	3	1	1
1	1	1	1	2	2	4	4	1	1
1	1	1	1	2	2	3	3	1	1
1	1	4	4	2	2	4	4	2	2
4	4	4	4	3	3	4	4	2	2
1	1	1	1	2	2	3	3	1	1
1	1	1	1	2	2	3	3	1	1
4	4	4	4	2	2	4	4	2	2
1	1	1	1	2	2	3	3	1	1
4	4	4	4	2	2	4	4	2	2
1	1	1	1	2	2	3	3	1	1
4	4	4	4	2	2	4	4	2	2
4	4	4	4	2	2	4	4	2	2
4	4	4	4	2	2	4	4	2	2
1	1	1	1	2	2	3	3	1	1
4	4	1	1	2	2	4	4	2	2
4	4	4	4	2	2	4	4	1	1
4	4	4	4	2	2	4	4	2	2
1	1	1	1	2	2	3	3	1	1
1	1	4	4	2	2	4	4	2	2
1	1	1	1	2	2	4	4	2	2
1	1	1	1	2	2	3	3	1	1
1	1	1	1	2	2	3	3	1	1

For Review Only

4	4	4	4	2	2	4	4	2	2
1	1	1	1	2	2	3	3	1	1

For Review Only

SNP3072	SNP3072	SNP3073	SNP3073	SNP3074	SNP3074	SNP3075	SNP3075	SNP3076	SNP3076
3	3	2	2	3	3	4	4	3	3
2	2	1	1	3	3	4	4	3	3
3	3	2	2	2	2	2	2	3	3
3	3	2	2	2	2	2	2	3	3
3	3	2	2	2	2	2	2	3	3
3	3	2	2	2	2	2	2	3	3
2	2	1	1	3	3	2	2	3	3
2	2	1	1	3	3	4	4	3	3
3	3	2	2	2	2	2	2	3	3
2	2	1	1	3	3	4	4	3	3
2	2	1	1	3	3	2	2	3	3
3	3	2	2	2	2	2	2	3	3
3	3	2	2	3	3	4	4	3	3
2	2	1	1	3	3	4	4	3	3
3	3	2	2	2	2	2	2	3	3
3	3	2	2	2	2	2	2	3	3
2	2	1	1	3	3	2	2	3	3
3	3	1	1	3	3	4	4	3	3
3	3	2	2	2	2	2	2	3	3
3	3	2	2	2	2	2	2	3	3
2	2	1	1	3	3	2	2	3	3
2	2	1	1	3	3	2	2	3	3
2	2	1	1	3	3	4	4	3	3
2	2	1	1	3	3	4	4	3	3
2	2	1	1	3	3	2	2	3	3
3	3	2	2	2	2	4	4	3	3
3	3	2	2	2	2	2	2	3	3
3	3	2	2	2	2	2	2	3	3
3	3	2	2	2	2	2	2	3	3
2	2	1	1	3	3	2	2	3	3
3	3	2	2	2	2	2	2	3	3
3	3	1	1	3	3	2	2	3	3
2	2	1	1	3	3	2	2	3	3
2	2	1	1	3	3	4	4	3	3
3	3	2	2	2	2	4	4	3	3
3	3	2	2	2	2	4	4	1	1
3	3	2	2	2	2	2	2	3	3
3	3	2	2	2	2	2	2	3	3
2	2	1	1	3	3	2	2	3	3
3	3	2	2	2	2	2	2	3	3
2	2	1	1	3	3	2	2	3	3
3	3	2	2	2	2	2	2	3	3
2	2	1	1	3	3	4	4	3	3
3	3	2	2	2	2	2	2	3	3
2	2	1	1	3	3	4	4	3	3
3	3	2	2	2	2	2	2	3	3
3	3	2	2	2	2	2	2	1	1
3	3	2	2	2	2	4	4	3	3
3	3	2	2	2	2	2	2	3	3
3	3	2	2	2	2	2	2	3	3
2	2	1	1	3	3	2	2	3	3
3	3	2	2	2	2	2	2	3	3

For Review Only

2	2	1	1	3	3	2	2	3	3
2	2	1	1	3	3	2	2	3	3
3	3	2	2	2	2	2	2	3	3
2	2	1	1	3	3	2	2	3	3
3	3	2	2	2	2	2	2	3	3
3	3	2	2	2	2	2	2	1	1
3	3	2	2	2	2	2	2	3	3
2	2	1	1	3	3	2	2	3	3
2	2	1	1	3	3	2	2	3	3
3	3	2	2	2	2	2	2	3	3
3	3	2	2	2	2	2	2	1	1
3	3	2	2	2	2	2	2	1	1
2	2	1	1	3	3	2	2	3	3
3	3	2	2	2	2	2	2	3	3
3	3	2	2	2	2	2	2	3	3
2	2	1	1	3	3	2	2	3	3
2	2	2	2	3	3	2	2	1	1
2	2	2	2	2	2	2	2	3	3
3	3	2	2	2	2	4	4	1	1
2	2	1	1	3	3	2	2	3	3
3	3	2	2	2	2	2	2	1	1
3	3	2	2	2	2	2	2	3	3
3	3	2	2	2	2	2	2	3	3
2	2	1	1	3	3	2	2	3	3
2	2	1	1	3	3	4	4	3	3
3	3	2	2	2	2	2	2	1	1
2	2	1	1	3	3	4	4	3	3
2	2	1	1	3	3	4	4	3	3
3	3	2	2	2	2	2	2	3	3
3	3	2	2	2	2	2	2	3	3
2	2	1	1	3	3	2	2	3	3
2	2	1	1	3	3	2	2	3	3
2	2	1	1	3	3	4	4	3	3
2	2	1	1	3	3	2	2	3	3
2	2	1	1	3	3	4	4	3	3
2	2	1	1	3	3	4	4	3	3
2	2	1	1	3	3	2	2	3	3
3	3	1	1	3	3	2	2	3	3
2	2	1	1	3	3	4	4	3	3
2	2	1	1	3	3	4	4	3	3
2	2	1	1	3	3	2	2	3	3
3	3	2	2	2	2	2	2	3	3
2	2	2	2	3	3	2	2	3	3
3	3	2	2	2	2	2	2	1	1
2	2	2	2	3	3	2	2	3	3
3	3	2	2	2	2	2	2	3	3
2	2	2	2	3	3	4	4	3	3
3	3	2	2	2	2	2	2	3	3
3	3	2	2	2	2	2	2	3	3
3	3	2	2	2	2	2	2	3	3
3	3	2	2	2	2	2	2	3	3
2	2	1	1	3	3	2	2	3	3
2	2	2	2	3	3	2	2	3	3
3	3	2	2	2	2	2	2	3	3
3	3	2	2	2	2	2	2	3	3

For Review Only

3	3	2	2	2	2	2	2	3	3
3	3	2	2	2	2	2	2	3	3
3	3	2	2	2	2	4	4	1	1
2	2	2	2	3	3	4	4	3	3
3	3	2	2	2	2	2	2	3	3
2	2	1	1	3	3	2	2	3	3
3	3	2	2	2	2	2	2	3	3
3	3	2	2	2	2	2	2	3	3
3	3	2	2	2	2	2	2	3	3
3	3	2	2	2	2	2	2	3	3
3	3	2	2	3	3	2	2	3	3
3	3	2	2	2	2	2	2	3	3
3	3	2	2	2	2	2	2	3	3
3	3	2	2	2	2	2	2	3	3
2	2	2	2	3	3	2	2	1	1
3	3	2	2	2	2	2	2	3	3
3	3	2	2	2	2	2	2	3	3
3	3	2	2	2	2	2	2	3	3
2	2	2	2	3	3	4	4	3	3
2	2	1	1	3	3	2	2	3	3
3	3	2	2	2	2	2	2	3	3
3	3	2	2	2	2	2	2	3	3
3	3	2	2	2	2	4	4	1	1
2	2	1	1	3	3	2	2	3	3
3	3	2	2	2	2	2	2	3	3
3	3	2	2	2	2	2	2	3	3
3	3	2	2	2	2	2	2	1	1
3	3	2	2	2	2	2	2	3	3
3	3	2	2	2	2	2	2	3	3
3	3	2	2	2	2	2	2	3	3
2	2	2	2	3	3	4	4	3	3
3	3	2	2	2	2	4	4	3	3
2	2	1	1	3	3	2	2	3	3
3	3	2	2	2	2	2	2	3	3
2	2	1	1	3	3	4	4	3	3
3	3	2	2	2	2	2	2	3	3
2	2	2	2	3	3	4	4	3	3
3	3	2	2	2	2	2	2	3	3
2	2	2	2	3	3	2	2	3	3
2	2	1	1	3	3	2	2	3	3
3	3	2	2	2	2	2	2	3	3
2	2	2	2	3	3	4	4	3	3
3	3	2	2	2	2	2	2	3	3
3	3	2	2	2	2	2	2	3	3
3	3	2	2	3	3	2	2	3	3
2	2	2	2	2	2	2	2	1	1
3	3	2	2	2	2	2	2	3	3
3	3	2	2	2	2	2	2	3	3
3	3	2	2	3	3	2	2	3	3
3	3	2	2	2	2	2	2	3	3
3	3	2	2	2	2	2	2	3	3
2	2	1	1	3	3	2	2	3	3
3	3	2	2	3	3	2	2	3	3
3	3	2	2	2	2	4	4	1	1
3	3	2	2	2	2	2	2	3	3

For Review Only

2	2	1	1	3	3	2	2	3	3
3	3	2	2	3	3	2	2	3	3
3	3	2	2	2	2	2	2	3	3
3	3	2	2	2	2	4	4	3	3
2	2	2	2	3	3	4	4	3	3
2	2	1	1	3	3	4	4	3	3
3	3	2	2	2	2	2	2	3	3
3	3	2	2	2	2	2	2	3	3
3	3	2	2	3	3	2	2	3	3
3	3	2	2	2	2	2	2	3	3
3	3	2	2	2	2	2	2	3	3
3	3	2	2	2	2	2	2	3	3
3	3	2	2	2	2	2	2	3	3
3	3	2	2	2	2	2	2	3	3
2	2	1	1	3	3	4	4	3	3
2	2	1	1	3	3	2	2	3	3
3	3	2	2	2	2	2	2	3	3
3	3	2	2	2	2	2	2	3	3
3	3	2	2	2	2	2	2	3	3
3	3	2	2	2	2	2	2	3	3
3	3	2	2	2	2	2	2	3	3
2	2	1	1	3	3	2	2	3	3
3	3	2	2	2	2	2	2	3	3
3	3	2	2	2	2	2	2	1	1
3	3	1	1	3	3	2	2	3	3
2	2	1	1	3	3	4	4	3	3
3	3	2	2	2	2	2	2	3	3
2	2	2	2	3	3	2	2	3	3
3	3	2	2	2	2	2	2	3	3
3	3	2	2	2	2	4	4	3	3
2	2	1	1	3	3	2	2	3	3
3	3	2	2	2	2	2	2	3	3
3	3	2	2	2	2	2	2	3	3
2	2	1	1	3	3	4	4	3	3
3	3	2	2	2	2	2	2	3	3
3	3	2	2	2	2	2	2	1	1
3	3	2	2	2	2	2	2	3	3
2	2	1	1	3	3	4	4	3	3
3	3	2	2	2	2	2	2	3	3
2	2	1	1	3	3	2	2	3	3
3	3	2	2	2	2	2	2	3	3
3	3	2	2	2	2	2	2	3	3
2	2	1	1	3	3	4	4	3	3
3	3	2	2	2	2	2	2	3	3
3	3	2	2	2	2	4	4	3	3
3	3	2	2	2	2	4	4	1	1
2	2	1	1	3	3	2	2	3	3
2	2	1	1	3	3	2	2	3	3
2	2	1	1	3	3	2	2	3	3
2	2	1	1	3	3	2	2	3	3
2	2	1	1	3	3	4	4	3	3
3	3	2	2	2	2	4	4	3	3
2	2	1	1	3	3	2	2	3	3
3	3	2	2	2	2	2	2	1	1
2	2	1	1	3	3	4	4	3	3

For Review Only

2	2	1	1	3	3	4	4	3	3
3	3	2	2	2	2	2	2	3	3
3	3	2	2	2	2	4	4	1	1
3	3	2	2	2	2	2	2	3	3
2	2	1	1	3	3	2	2	3	3
3	3	2	2	2	2	2	2	3	3
2	2	1	1	3	3	4	4	3	3
2	2	2	2	3	3	4	4	3	3
3	3	2	2	2	2	2	2	3	3
2	2	1	1	3	3	2	2	3	3
3	3	2	2	2	2	2	2	3	3
3	3	2	2	2	2	2	2	3	3
2	2	1	1	3	3	4	4	3	3
2	2	1	1	3	3	2	2	3	3
3	3	2	2	2	2	2	2	3	3
2	2	1	1	3	3	2	2	3	3
2	2	1	1	3	3	2	2	3	3
3	3	2	2	2	2	2	2	3	3
2	2	1	1	3	3	2	2	3	3
3	3	2	2	2	2	2	2	3	3
2	2	1	1	3	3	2	2	3	3
3	3	2	2	2	2	2	2	3	3
2	2	1	1	3	3	2	2	3	3
3	3	2	2	2	2	2	2	3	3
2	2	1	1	3	3	2	2	3	3
3	3	2	2	2	2	2	2	3	3
2	2	1	1	3	3	2	2	3	3
3	3	2	2	2	2	2	2	3	3
2	2	1	1	3	3	2	2	3	3
3	3	2	2	2	2	4	4	1	1
2	2	1	1	3	3	4	4	3	3
2	2	1	1	3	3	2	2	3	3
3	3	2	2	2	2	2	2	3	3
3	3	2	2	2	2	2	2	3	3
3	3	2	2	2	2	2	2	3	3
2	2	1	1	3	3	4	4	3	3
2	2	1	1	3	3	2	2	3	3
3	3	2	2	2	2	2	2	1	1
3	3	2	2	2	2	4	4	1	1
2	2	1	1	3	3	4	4	3	3
3	3	2	2	2	2	2	2	3	3
2	2	1	1	3	3	2	2	1	1
3	3	2	2	2	2	2	2	3	3
2	2	1	1	3	3	2	2	3	3
2	2	1	1	3	3	2	2	3	3
2	2	1	1	3	3	2	2	3	3
3	3	2	2	2	2	2	2	3	3
2	2	1	1	3	3	4	4	3	3
3	3	2	2	2	2	4	4	3	3
2	2	1	1	3	3	2	2	3	3
3	3	2	2	2	2	2	2	3	3
2	2	1	1	3	3	2	2	3	3
3	3	2	2	2	2	2	2	3	3
3	3	2	2	2	2	2	2	3	3
3	3	2	2	2	2	2	2	3	3

For Review Only

2	2	1	1	3	3	2	2	3	3
3	3	2	2	2	2	4	4	3	3

For Review Only

SNP3077	SNP3077	SNP3078	SNP3078	SNP3079	SNP3079	SNP308	SNP308	SNP3080	SNP3080
3	3	3	3	3	3	1	1	3	3
3	3	1	1	4	4	1	1	1	1
2	2	3	3	3	3	1	1	1	1
2	2	3	3	3	3	1	1	1	1
2	2	3	3	3	3	1	1	1	1
2	2	3	3	3	3	1	1	1	1
2	2	3	3	3	3	4	4	1	1
3	3	1	1	4	4	1	1	1	1
2	2	3	3	3	3	1	1	1	1
3	3	1	1	4	4	1	1	1	1
3	3	1	1	4	4	1	1	1	1
2	2	3	3	3	3	1	1	1	1
3	3	3	3	3	3	1	1	1	1
3	3	3	3	4	4	1	1	1	1
2	2	3	3	3	3	1	1	1	1
2	2	3	3	3	3	1	1	1	1
2	2	3	3	3	3	4	4	1	1
3	3	3	3	4	4	1	1	1	1
2	2	3	3	3	3	1	1	1	1
2	2	3	3	3	3	1	1	1	1
3	3	1	1	4	4	4	4	1	1
2	2	3	3	3	3	4	4	1	1
3	3	1	1	4	4	4	4	1	1
3	3	1	1	4	4	1	1	1	1
2	2	3	3	3	3	4	4	1	1
2	2	3	3	3	3	4	4	1	1
2	2	3	3	3	3	1	1	1	1
2	2	3	3	3	3	1	1	1	1
2	2	3	3	3	3	1	1	1	1
3	3	1	1	4	4	1	1	1	1
2	2	3	3	3	3	1	1	1	1
2	2	3	3	3	3	1	1	1	1
2	2	3	3	3	3	4	4	1	1
3	3	3	3	4	4	1	1	1	1
3	3	1	1	4	4	4	4	1	1
3	3	3	3	4	4	1	1	1	1
3	3	3	3	3	3	1	1	3	3
2	2	3	3	3	3	1	1	1	1
3	3	1	1	4	4	1	1	1	1
2	2	3	3	3	3	4	4	1	1
3	3	1	1	4	4	1	1	1	1
2	2	3	3	3	3	1	1	1	1
3	3	1	1	4	4	1	1	1	1
2	2	3	3	3	3	4	4	1	1
3	3	1	1	4	4	1	1	1	1
2	2	3	3	3	3	1	1	1	1
3	3	3	3	3	3	4	4	1	1
2	2	3	3	3	3	4	4	1	1
2	2	3	3	3	3	1	1	1	1
2	2	3	3	3	3	4	4	1	1
3	3	1	1	4	4	4	4	1	1
2	2	3	3	3	3	1	1	1	1

For Review Only

2	2	3	3	3	3	4	4	1	1
2	2	3	3	3	3	4	4	1	1
2	2	3	3	3	3	1	1	1	1
2	2	3	3	3	3	4	4	1	1
2	2	3	3	3	3	1	1	1	1
3	3	3	3	4	4	1	1	1	1
2	2	3	3	3	3	1	1	1	1
3	3	1	1	4	4	1	1	1	1
3	3	1	1	4	4	1	1	1	1
2	2	3	3	3	3	1	1	1	1
3	3	3	3	4	4	1	1	1	1
3	3	3	3	4	4	1	1	1	1
2	2	3	3	3	3	4	4	1	1
2	2	3	3	3	3	1	1	1	1
2	2	3	3	3	3	1	1	1	1
3	3	1	1	3	3	1	1	1	1
3	3	3	3	4	4	4	4	1	1
3	3	3	3	4	4	1	1	1	1
3	3	3	3	3	3	1	1	3	3
2	2	3	3	3	3	1	1	1	1
3	3	3	3	4	4	1	1	1	1
2	2	3	3	3	3	4	4	1	1
2	2	3	3	3	3	1	1	1	1
2	2	3	3	3	3	1	1	1	1
3	3	1	1	4	4	1	1	1	1
3	3	3	3	3	3	1	1	1	1
3	3	1	1	4	4	4	4	1	1
3	3	1	1	4	4	1	1	1	1
2	2	3	3	3	3	4	4	1	1
2	2	3	3	3	3	1	1	1	1
2	2	3	3	3	3	1	1	1	1
3	3	1	1	4	4	1	1	1	1
3	3	1	1	4	4	1	1	1	1
2	2	3	3	3	3	4	4	1	1
3	3	3	3	4	4	1	1	1	1
3	3	1	1	4	4	1	1	1	1
2	2	1	1	3	3	1	1	1	1
3	3	1	1	4	4	4	4	1	1
3	3	1	1	4	4	1	1	1	1
3	3	1	1	4	4	1	1	1	1
2	2	3	3	3	3	1	1	1	1
2	2	3	3	3	3	1	1	1	1
3	3	3	3	4	4	4	4	1	1
3	3	3	3	4	4	1	1	1	1
2	2	3	3	3	3	4	4	1	1
2	2	3	3	3	3	4	4	1	1
3	3	3	3	4	4	1	1	1	1
2	2	3	3	3	3	4	4	1	1
2	2	3	3	3	3	4	4	1	1
2	2	3	3	3	3	4	4	1	1
3	3	3	3	3	3	1	1	1	1
3	3	1	1	4	4	1	1	1	1
2	2	1	1	4	4	1	1	1	1
2	2	1	1	3	3	1	1	1	1
3	3	3	3	3	3	4	4	3	3

For Review Only

2	2	3	3	3	3	4	4	1	1
2	2	1	1	3	3	1	1	1	1
3	3	3	3	4	4	1	1	1	1
3	3	3	3	3	3	1	1	1	1
2	2	3	3	3	3	1	1	1	1
3	3	1	1	4	4	4	4	1	1
2	2	3	3	3	3	1	1	1	1
2	2	3	3	3	3	1	1	1	1
2	2	3	3	3	3	4	4	1	1
2	2	3	3	3	3	1	1	1	1
3	3	3	3	3	3	1	1	1	1
2	2	3	3	3	3	1	1	1	1
3	3	3	3	3	3	4	4	3	3
3	3	3	3	3	3	1	1	1	1
3	3	3	3	4	4	1	1	1	1
2	2	3	3	3	3	1	1	1	1
3	3	3	3	3	3	4	4	3	3
2	2	1	1	3	3	1	1	1	1
3	3	3	3	4	4	1	1	1	1
3	3	3	3	3	3	1	1	3	3
2	2	3	3	3	3	4	4	1	1
2	2	3	3	3	3	1	1	1	1
3	3	3	3	4	4	1	1	1	1
3	3	1	1	4	4	1	1	1	1
2	2	3	3	3	3	4	4	1	1
2	2	3	3	3	3	4	4	1	1
2	2	3	3	3	3	1	1	1	1
2	2	3	3	3	3	1	1	1	1
2	2	3	3	3	3	1	1	1	1
2	2	3	3	3	3	1	1	1	1
3	3	3	3	4	4	1	1	1	1
2	2	1	1	3	3	1	1	1	1
2	2	3	3	3	3	1	1	1	1
2	2	3	3	3	3	1	1	1	1
3	3	3	3	4	4	1	1	1	1
3	3	1	1	3	3	1	1	3	3
3	3	3	3	4	4	1	1	1	1
2	2	3	3	3	3	1	1	1	1
2	2	1	1	3	3	4	4	1	1
3	3	1	1	4	4	1	1	1	1
2	2	1	1	3	3	4	4	1	1
3	3	3	3	4	4	1	1	1	1
2	2	3	3	3	3	4	4	1	1
3	3	1	1	4	4	1	1	1	1
2	2	3	3	3	3	4	4	1	1
3	3	3	3	4	4	4	4	1	1
2	2	3	3	3	3	4	4	1	1
2	2	3	3	3	3	4	4	1	1
3	3	3	3	4	4	1	1	1	1
2	2	3	3	3	3	4	4	1	1
2	2	3	3	3	3	1	1	1	1
3	3	1	1	4	4	1	1	1	1
3	3	3	3	3	3	1	1	1	1
3	3	3	3	4	4	4	4	1	1
3	3	1	1	3	3	1	1	1	1

For Review Only

3	3	3	3	4	4	4	4	1	1
3	3	3	3	3	3	4	4	3	3
2	2	3	3	3	3	4	4	1	1
2	2	1	1	3	3	4	4	1	1
2	2	3	3	3	3	4	4	1	1
2	2	3	3	3	3	1	1	1	1
3	3	1	1	3	3	4	4	1	1
2	2	3	3	3	3	1	1	1	1
2	2	1	1	3	3	1	1	1	1
2	2	3	3	3	3	1	1	1	1
2	2	3	3	3	3	4	4	1	1
2	2	3	3	3	3	4	4	1	1
2	2	3	3	3	3	4	4	1	1
3	3	3	3	3	3	1	1	1	1
3	3	3	3	4	4	1	1	1	1
3	3	1	1	4	4	1	1	1	1
3	3	3	3	3	3	1	1	1	1
2	2	3	3	3	3	1	1	1	1
2	2	3	3	3	3	4	4	1	1
2	2	3	3	3	3	1	1	1	1
2	2	3	3	3	3	1	1	1	1
3	3	1	1	4	4	1	1	1	1
2	2	3	3	3	3	4	4	1	1
3	3	3	3	4	4	1	1	1	1
2	2	3	3	3	3	1	1	1	1
3	3	3	3	4	4	1	1	1	1
2	2	3	3	3	3	1	1	1	1
3	3	1	1	4	4	1	1	1	1
2	2	3	3	3	3	1	1	1	1
2	2	3	3	3	3	1	1	1	1
2	2	3	3	3	3	4	4	1	1
3	3	3	3	3	3	4	4	3	3
2	2	3	3	3	3	1	1	1	1
3	3	3	3	4	4	1	1	1	1
2	2	3	3	3	3	1	1	1	1
3	3	3	3	4	4	1	1	1	1
2	2	3	3	3	3	1	1	1	1
3	3	3	3	4	4	1	1	1	1
2	2	3	3	3	3	4	4	1	1
3	3	1	1	4	4	4	4	1	1
2	2	3	3	3	3	1	1	1	1
2	2	3	3	3	3	4	4	1	1
3	3	1	1	4	4	1	1	1	1
2	2	3	3	3	3	1	1	1	1
2	2	3	3	3	3	1	1	1	1
3	3	3	3	4	4	1	1	1	1
3	3	1	1	4	4	1	1	1	1
3	3	1	1	4	4	4	4	1	1
3	3	3	3	4	4	1	1	1	1
2	2	1	1	3	3	1	1	1	1
2	2	3	3	3	3	4	4	1	1
3	3	3	3	3	3	1	1	3	3
3	3	1	1	4	4	4	4	1	1
3	3	3	3	4	4	1	1	1	1
2	2	1	1	4	4	1	1	1	1

For Review Only

3	3	1	1	4	4	1	1	1	1
2	2	3	3	3	3	1	1	1	1
3	3	3	3	4	4	1	1	1	1
2	2	3	3	3	3	4	4	1	1
2	2	3	3	3	3	1	1	1	1
2	2	3	3	3	3	1	1	1	1
3	3	1	1	4	4	1	1	1	1
3	3	1	1	4	4	1	1	1	1
2	2	3	3	3	3	1	1	1	1
3	3	1	1	4	4	1	1	1	1
2	2	1	1	3	3	1	1	1	1
3	3	3	3	3	3	4	4	1	1
3	3	1	1	4	4	1	1	1	1
2	2	3	3	3	3	4	4	1	1
2	2	3	3	3	3	4	4	1	1
3	3	1	1	4	4	1	1	1	1
3	3	1	1	4	4	4	4	1	1
3	3	1	1	4	4	1	1	1	1
2	2	3	3	3	3	4	4	1	1
2	2	3	3	3	3	1	1	1	1
2	2	3	3	3	3	1	1	1	1
2	2	3	3	3	3	4	4	1	1
3	3	3	3	4	4	1	1	1	1
2	2	3	3	3	3	4	4	1	1
3	3	1	1	4	4	1	1	1	1
2	2	3	3	3	3	1	1	1	1
3	3	1	1	4	4	1	1	1	1
2	2	3	3	3	3	1	1	1	1
2	2	3	3	3	3	1	1	1	1
3	3	3	3	4	4	4	4	1	1
3	3	1	1	4	4	4	4	1	1
3	3	1	1	4	4	4	4	1	1
3	3	3	3	3	3	1	1	3	3
2	2	3	3	3	3	4	4	1	1
2	2	3	3	3	3	4	4	1	1
3	3	1	1	4	4	1	1	1	1
3	3	3	3	4	4	1	1	1	1
3	3	3	3	4	4	1	1	1	1
3	3	3	3	4	4	1	1	1	1
3	3	1	1	4	4	1	1	1	1
2	2	3	3	3	3	1	1	1	1
3	3	3	3	4	4	4	4	1	1
2	2	3	3	3	3	4	4	1	1
2	2	3	3	3	3	4	4	1	1
3	3	1	1	4	4	1	1	1	1
2	2	3	3	3	3	1	1	1	1
2	2	3	3	3	3	4	4	1	1
3	3	3	3	3	3	4	4	3	3
3	3	1	1	3	3	1	1	1	1
3	3	1	1	4	4	1	1	1	1
2	2	3	3	3	3	1	1	1	1
3	3	1	1	4	4	1	1	1	1
2	2	3	3	3	3	1	1	1	1
2	2	3	3	3	3	4	4	1	1
2	2	3	3	3	3	4	4	1	1

For Review Only

2	2	3	3	3	3	1	1	1	1
2	2	3	3	3	3	1	1	1	1

For Review Only

SNP3081	SNP3081	SNP3082	SNP3082	SNP3083	SNP3083	SNP3084	SNP3084	SNP3085	SNP3085
3	3	1	1	2	2	4	4	4	4
3	3	4	4	2	2	4	4	3	3
3	3	4	4	2	2	4	4	3	3
3	3	4	4	2	2	4	4	3	3
3	3	4	4	3	3	1	1	4	4
3	3	4	4	2	2	4	4	3	3
3	3	4	4	3	3	1	1	4	4
3	3	4	4	2	2	4	4	3	3
3	3	4	4	3	3	1	1	3	3
3	3	4	4	2	2	4	4	3	3
3	3	4	4	2	2	4	4	3	3
3	3	4	4	2	2	4	4	3	3
3	3	4	4	2	2	4	4	4	4
3	3	4	4	2	2	4	4	3	3
3	3	4	4	2	2	4	4	4	4
3	3	4	4	3	3	1	1	4	4
3	3	4	4	3	3	1	1	4	4
3	3	4	4	3	3	1	1	4	4
3	3	4	4	2	2	4	4	3	3
3	3	4	4	3	3	1	1	4	4
3	3	4	4	2	2	4	4	3	3
3	3	4	4	3	3	1	1	4	4
3	3	4	4	2	2	4	4	3	3
3	3	4	4	2	2	4	4	3	3
3	3	4	4	3	3	1	1	4	4
3	3	4	4	2	2	4	4	3	3
3	3	4	4	2	2	4	4	3	3
3	3	4	4	3	3	1	1	4	4
3	3	4	4	2	2	4	4	3	3
3	3	4	4	2	2	4	4	3	3
3	3	4	4	3	3	1	1	4	4
3	3	4	4	3	3	1	1	4	4
3	3	4	4	3	3	1	1	4	4
3	3	4	4	2	2	4	4	3	3
3	3	4	4	2	2	4	4	3	3
3	3	4	4	2	2	4	4	3	3
1	1	1	1	2	2	4	4	3	3
3	3	4	4	3	3	1	1	4	4
3	3	4	4	2	2	4	4	3	3
3	3	4	4	3	3	1	1	4	4
3	3	4	4	2	2	4	4	3	3
3	3	4	4	2	2	4	4	3	3
3	3	4	4	2	2	4	4	3	3
3	3	4	4	2	2	4	4	3	3
3	3	4	4	2	2	4	4	3	3
3	3	4	4	2	2	4	4	3	3
3	3	4	4	2	2	4	4	3	3
3	3	4	4	2	2	4	4	3	3
3	3	4	4	2	2	4	4	3	3
3	3	4	4	3	3	1	1	4	4
3	3	4	4	2	2	4	4	3	3
3	3	4	4	3	3	1	1	4	4

For Review Only

3	3	4	4	3	3	1	1	4	4
3	3	4	4	3	3	1	1	4	4
3	3	4	4	2	2	4	4	3	3
3	3	4	4	3	3	1	1	4	4
3	3	4	4	2	2	4	4	3	3
3	3	4	4	2	2	4	4	3	3
3	3	4	4	2	2	4	4	3	3
3	3	4	4	2	2	4	4	3	3
3	3	4	4	2	2	4	4	3	3
3	3	4	4	3	3	1	1	4	4
3	3	4	4	2	2	4	4	3	3
3	3	4	4	2	2	4	4	3	3
3	3	4	4	3	3	1	1	4	4
3	3	4	4	2	2	4	4	3	3
3	3	4	4	3	3	4	4	4	4
3	3	4	4	2	2	4	4	4	4
3	3	4	4	2	2	4	4	3	3
3	3	4	4	2	2	4	4	3	3
1	1	1	1	2	2	4	4	4	4
3	3	4	4	3	3	1	1	4	4
3	3	4	4	2	2	4	4	3	3
3	3	4	4	3	3	1	1	4	4
3	3	4	4	2	2	4	4	3	3
3	3	4	4	2	2	4	4	3	3
3	3	4	4	2	2	4	4	3	3
3	3	4	4	3	3	1	1	4	4
3	3	4	4	2	2	4	4	3	3
3	3	4	4	2	2	4	4	3	3
3	3	4	4	2	2	4	4	3	3
3	3	4	4	3	3	1	1	4	4
3	3	4	4	3	3	1	1	4	4
3	3	4	4	2	2	4	4	3	3
3	3	4	4	2	2	4	4	3	3
3	3	4	4	3	3	1	1	4	4
3	3	4	4	3	3	1	1	4	4
3	3	4	4	2	2	4	4	3	3
3	3	4	4	2	2	4	4	3	3
3	3	4	4	2	2	4	4	3	3
3	3	4	4	2	2	4	4	3	3
3	3	4	4	2	2	4	4	3	3
3	3	4	4	3	3	1	1	4	4
3	3	4	4	3	3	4	4	4	4
3	3	4	4	2	2	4	4	3	3
3	3	4	4	2	2	4	4	3	3
3	3	4	4	3	3	1	1	4	4
3	3	4	4	2	2	4	4	4	4
3	3	4	4	2	2	4	4	3	3
3	3	4	4	2	2	4	4	3	3
3	3	4	4	2	2	4	4	3	3
3	3	4	4	3	3	1	1	4	4
3	3	4	4	3	3	4	4	3	3
3	3	4	4	2	2	4	4	3	3
3	3	4	4	2	2	4	4	3	3
3	3	4	4	3	3	4	4	3	3
1	1	1	1	2	2	4	4	4	4

For Review Only

3	3	4	4	2	2	4	4	3	3
3	3	4	4	3	3	1	1	4	4
3	3	4	4	2	2	4	4	3	3
3	3	4	4	2	2	4	4	3	3
3	3	4	4	3	3	1	1	4	4
3	3	4	4	2	2	4	4	3	3
3	3	4	4	3	3	1	1	4	4
3	3	4	4	2	2	1	1	3	3
3	3	4	4	2	2	4	4	3	3
3	3	4	4	3	3	4	4	3	3
3	3	4	4	3	3	1	1	4	4
3	3	4	4	3	3	1	1	4	4
1	1	1	1	2	2	4	4	3	3
3	3	4	4	3	3	1	1	4	4
3	3	4	4	3	3	4	4	4	4
3	3	4	4	2	2	4	4	3	3
1	1	1	1	2	2	4	4	3	3
3	3	4	4	3	3	4	4	3	3
3	3	4	4	2	2	4	4	3	3
1	1	1	1	2	2	4	4	4	4
3	3	4	4	2	2	4	4	4	4
3	3	4	4	3	3	1	1	4	4
3	3	4	4	3	3	4	4	3	3
3	3	4	4	2	2	4	4	3	3
3	3	4	4	3	3	1	1	4	4
3	3	4	4	3	3	1	1	4	4
3	3	4	4	2	2	4	4	3	3
3	3	4	4	3	3	1	1	4	4
3	3	4	4	3	3	1	1	4	4
3	3	4	4	3	3	1	1	4	4
3	3	4	4	2	2	4	4	3	3
3	3	4	4	3	3	1	1	4	4
3	3	4	4	3	3	1	1	4	4
3	3	4	4	2	2	1	1	4	4
3	3	4	4	2	2	4	4	3	3
3	3	4	4	2	2	4	4	3	3
3	3	4	4	2	2	4	4	3	3
3	3	4	4	2	2	4	4	4	4
3	3	4	4	2	2	4	4	3	3
3	3	4	4	2	2	4	4	3	3
3	3	4	4	3	3	1	1	4	4
3	3	4	4	2	2	4	4	3	3
3	3	4	4	2	2	4	4	3	3
3	3	4	4	3	3	4	4	3	3
3	3	4	4	2	2	4	4	3	3
3	3	4	4	2	2	4	4	3	3
3	3	4	4	2	2	4	4	3	3
3	3	4	4	2	2	4	4	3	3
3	3	4	4	2	2	4	4	3	3
3	3	4	4	2	2	4	4	3	3
3	3	4	4	2	2	4	4	3	3
3	3	4	4	2	2	4	4	3	3
3	3	4	4	2	2	4	4	3	3
3	3	4	4	2	2	4	4	3	3
3	3	4	4	3	3	4	4	4	4

For Review Only

3	3	4	4	2	2	4	4	3	3
1	1	1	1	2	2	4	4	3	3
3	3	4	4	3	3	1	1	4	4
3	3	4	4	3	3	1	1	4	4
3	3	4	4	3	3	1	1	4	4
3	3	4	4	3	3	1	1	4	4
3	3	4	4	3	3	4	4	4	4
3	3	4	4	3	3	1	1	4	4
3	3	4	4	2	2	1	1	3	3
3	3	4	4	3	3	1	1	4	4
3	3	4	4	2	2	4	4	3	3
3	3	4	4	3	3	1	1	4	4
3	3	4	4	3	3	1	1	4	4
3	3	4	4	2	2	4	4	3	3
3	3	4	4	2	2	4	4	3	3
3	3	4	4	2	2	4	4	3	3
3	3	4	4	2	2	4	4	3	3
3	3	4	4	2	2	4	4	4	4
3	3	4	4	2	2	4	4	3	3
3	3	4	4	3	3	1	1	4	4
3	3	4	4	3	3	4	4	4	4
3	3	4	4	2	2	4	4	3	3
3	3	4	4	3	3	1	1	4	4
3	3	4	4	2	2	4	4	3	3
3	3	4	4	3	3	1	1	4	4
3	3	4	4	2	2	4	4	3	3
3	3	4	4	2	2	4	4	3	3
3	3	4	4	2	2	4	4	3	3
3	3	4	4	3	3	1	1	4	4
3	3	4	4	3	3	1	1	4	4
3	3	4	4	2	2	4	4	3	3
1	1	1	1	2	2	4	4	4	4
3	3	4	4	3	3	1	1	4	4
3	3	4	4	2	2	4	4	3	3
3	3	4	4	3	3	1	1	4	4
3	3	4	4	2	2	4	4	3	3
3	3	4	4	3	3	1	1	4	4
3	3	4	4	2	2	4	4	3	3
3	3	4	4	2	2	4	4	3	3
3	3	4	4	2	2	4	4	3	3
3	3	4	4	2	2	4	4	3	3
3	3	4	4	2	2	4	4	3	3
3	3	4	4	2	2	4	4	3	3
3	3	4	4	3	3	1	1	4	4
3	3	4	4	3	3	1	1	4	4
3	3	4	4	2	2	4	4	3	3
3	3	4	4	2	2	4	4	3	3
3	3	4	4	2	2	4	4	3	3
3	3	4	4	2	2	4	4	3	3
3	3	4	4	2	2	4	4	3	3
3	3	4	4	3	3	1	1	4	4
1	1	1	1	2	2	4	4	3	3
3	3	4	4	2	2	4	4	3	3
3	3	4	4	2	2	4	4	3	3
3	3	4	4	2	2	4	4	3	3

For Review Only

3	3	4	4	2	2	4	4	3	3
3	3	4	4	3	3	1	1	4	4
3	3	4	4	3	3	4	4	3	3
3	3	4	4	3	3	1	1	4	4
3	3	4	4	2	2	4	4	3	3
3	3	4	4	2	2	4	4	4	4
3	3	4	4	2	2	4	4	3	3
3	3	4	4	2	2	4	4	3	3
3	3	4	4	2	2	4	4	3	3
3	3	4	4	2	2	4	4	3	3
3	3	4	4	3	3	1	1	4	4
3	3	4	4	2	2	4	4	3	3
3	3	4	4	2	2	4	4	3	3
3	3	4	4	2	2	4	4	3	3
3	3	4	4	2	2	4	4	3	3
3	3	4	4	2	2	4	4	3	3
3	3	4	4	2	2	4	4	3	3
3	3	4	4	2	2	4	4	3	3
3	3	4	4	3	3	1	1	4	4
3	3	4	4	3	3	1	1	4	4
3	3	4	4	2	2	4	4	3	3
3	3	4	4	2	2	4	4	3	3
3	3	4	4	2	2	4	4	3	3
3	3	4	4	3	3	1	1	4	4
3	3	4	4	2	2	4	4	3	3
3	3	4	4	3	3	1	1	3	3
3	3	4	4	2	2	4	4	3	3
3	3	4	4	2	2	4	4	3	3
3	3	4	4	3	3	1	1	4	4
3	3	4	4	3	3	1	1	4	4
3	3	4	4	2	2	4	4	3	3
3	3	4	4	2	2	4	4	3	3
1	1	1	1	2	2	4	4	3	3
3	3	4	4	2	2	4	4	3	3
3	3	4	4	3	3	1	1	4	4
3	3	4	4	2	2	4	4	3	3
3	3	4	4	2	2	4	4	3	3
3	3	4	4	2	2	4	4	3	3
3	3	4	4	2	2	4	4	3	3
3	3	4	4	2	2	4	4	3	3
3	3	4	4	3	3	1	1	4	4
3	3	4	4	2	2	4	4	4	4
3	3	4	4	3	3	1	1	4	4
3	3	4	4	3	3	1	1	4	4
3	3	4	4	2	2	4	4	3	3
3	3	4	4	3	3	1	1	4	4
3	3	4	4	2	2	4	4	3	3
1	1	1	1	2	2	4	4	4	4
3	3	4	4	2	2	4	4	3	3
3	3	4	4	2	2	4	4	3	3
3	3	4	4	3	3	1	1	4	4
3	3	4	4	2	2	4	4	3	3
3	3	4	4	2	2	4	4	3	3
3	3	4	4	2	2	4	4	3	3
3	3	4	4	3	3	1	1	4	4

For Review Only

3	3	4	4	3	3	1	1	4	4
3	3	4	4	2	2	4	4	3	3

For Review Only

SNP3086	SNP3086	SNP3087	SNP3087	SNP3088	SNP3088	SNP3089	SNP3089	SNP309	SNP309
1	1	1	1	1	1	3	3	1	1
3	3	1	1	1	1	3	3	1	1
3	3	1	1	1	1	3	3	2	2
3	3	1	1	1	1	3	3	2	2
3	3	1	1	1	1	3	3	1	1
3	3	1	1	1	1	3	3	1	1
3	3	2	2	4	4	4	4	1	1
3	3	1	1	1	1	3	3	1	1
3	3	2	2	1	1	4	4	1	1
3	3	1	1	1	1	3	3	1	1
3	3	1	1	1	1	3	3	1	1
3	3	1	1	1	1	3	3	2	2
3	3	1	1	1	1	3	3	1	1
3	3	1	1	1	1	3	3	1	1
3	3	1	1	1	1	3	3	1	1
3	3	1	1	1	1	3	3	1	1
3	3	2	2	4	4	4	4	1	1
3	3	1	1	1	1	3	3	1	1
3	3	1	1	1	1	3	3	1	1
3	3	1	1	1	1	3	3	1	1
3	3	1	1	1	1	3	3	1	1
3	3	2	2	4	4	4	4	1	1
3	3	1	1	1	1	3	3	1	1
3	3	1	1	1	1	3	3	1	1
3	3	1	1	4	4	4	4	1	1
3	3	1	1	1	1	3	3	2	2
3	3	1	1	1	1	3	3	2	2
3	3	1	1	1	1	3	3	1	1
3	3	1	1	1	1	3	3	1	1
3	3	1	1	1	1	3	3	1	1
3	3	1	1	1	1	3	3	2	2
3	3	1	1	1	1	3	3	1	1
3	3	2	2	4	4	4	4	1	1
3	3	1	1	1	1	3	3	1	1
3	3	1	1	1	1	3	3	1	1
3	3	1	1	1	1	3	3	1	1
1	1	1	1	1	1	3	3	1	1
3	3	2	2	4	4	4	4	2	2
3	3	1	1	1	1	3	3	1	1
3	3	2	2	4	4	4	4	1	1
3	3	1	1	1	1	3	3	1	1
3	3	1	1	1	1	3	3	1	1
3	3	1	1	1	1	3	3	1	1
3	3	1	1	1	1	3	3	1	1
3	3	1	1	1	1	3	3	1	1
3	3	1	1	1	1	3	3	2	2
3	3	1	1	1	1	3	3	1	1
3	3	1	1	1	1	3	3	2	2
3	3	1	1	1	1	3	3	1	1
3	3	1	1	1	1	3	3	1	1
3	3	1	1	1	1	3	3	1	1
3	3	2	2	4	4	4	4	1	1

For Review Only

3	3	2	2	4	4	4	4	1	1
3	3	2	2	4	4	4	4	1	1
3	3	1	1	1	1	3	3	1	1
3	3	2	2	4	4	4	4	1	1
3	3	1	1	1	1	3	3	1	1
3	3	1	1	1	1	3	3	1	1
3	3	1	1	1	1	3	3	2	2
3	3	1	1	1	1	3	3	1	1
3	3	1	1	1	1	3	3	1	1
3	3	1	1	1	1	3	3	1	1
3	3	1	1	1	1	3	3	2	2
3	3	1	1	1	1	3	3	2	2
3	3	2	2	4	4	4	4	1	1
3	3	1	1	1	1	3	3	1	1
3	3	1	1	1	1	3	3	1	1
3	3	1	1	1	1	3	3	1	1
3	3	1	1	1	1	3	3	1	1
3	3	1	1	1	1	3	3	1	1
1	1	2	2	4	4	4	4	1	1
3	3	1	1	1	1	3	3	1	1
3	3	1	1	1	1	3	3	2	2
3	3	1	1	1	1	3	3	1	1
3	3	1	1	1	1	3	3	1	1
3	3	1	1	1	1	3	3	1	1
3	3	1	1	1	1	3	3	1	1
3	3	1	1	1	1	3	3	1	1
3	3	1	1	1	1	3	3	1	1
3	3	1	1	1	1	3	3	1	1
3	3	1	1	1	1	3	3	2	2
3	3	1	1	1	1	3	3	1	1
3	3	1	1	4	4	4	4	1	1
3	3	1	1	1	1	3	3	1	1
3	3	1	1	1	1	3	3	1	1
3	3	2	2	4	4	4	4	1	1
3	3	1	1	1	1	3	3	1	1
3	3	1	1	1	1	3	3	1	1
3	3	1	1	1	1	3	3	1	1
3	3	1	1	1	1	3	3	1	1
3	3	1	1	1	1	3	3	1	1
3	3	1	1	1	1	3	3	1	1
3	3	2	2	4	4	4	4	1	1
3	3	1	1	1	1	3	3	1	1
3	3	1	1	1	1	3	3	1	1
3	3	1	1	1	1	3	3	2	2
3	3	1	1	1	1	3	3	1	1
3	3	1	1	1	1	3	3	1	1
3	3	1	1	1	1	3	3	1	1
3	3	1	1	1	1	3	3	1	1
3	3	1	1	1	1	3	3	1	1
3	3	1	1	1	1	3	3	1	1
3	3	1	1	1	1	3	3	2	2
3	3	1	1	1	1	3	3	1	1
3	3	1	1	1	1	3	3	1	1
3	3	1	1	1	1	3	3	2	2
1	1	1	1	1	1	3	3	1	1

For Review Only

3	3	1	1	1	1	3	3	1	1
3	3	1	1	1	1	3	3	2	2
3	3	1	1	1	1	3	3	1	1
3	3	1	1	1	1	3	3	1	1
3	3	1	1	1	1	3	3	1	1
3	3	1	1	1	1	3	3	2	2
3	3	1	1	1	1	3	3	1	1
3	3	1	1	1	1	3	3	2	2
3	3	1	1	1	1	3	3	1	1
3	3	1	1	1	1	3	3	1	1
3	3	1	1	1	1	3	3	1	1
3	3	1	1	1	1	3	3	2	2
1	1	1	1	1	1	3	3	1	1
3	3	1	1	1	1	3	3	1	1
3	3	1	1	1	1	3	3	2	2
3	3	1	1	1	1	3	3	1	1
1	1	1	1	1	1	3	3	1	1
3	3	1	1	1	1	3	3	2	2
3	3	1	1	1	1	3	3	1	1
1	1	1	1	1	1	3	3	1	1
3	3	1	1	1	1	3	3	1	1
3	3	2	2	4	4	4	4	1	1
3	3	1	1	1	1	3	3	1	1
3	3	1	1	1	1	3	3	1	1
3	3	1	1	1	1	3	3	2	2
3	3	2	2	4	4	4	4	1	1
3	3	1	1	1	1	3	3	2	2
3	3	2	2	4	4	4	4	1	1
3	3	1	1	1	1	3	3	1	1
3	3	1	1	1	1	3	3	1	1
3	3	1	1	1	1	3	3	1	1
3	3	1	1	1	1	3	3	1	1
3	3	2	2	4	4	4	4	1	1
3	3	1	1	1	1	3	3	1	1
3	3	1	1	1	1	3	3	1	1
3	3	1	1	1	1	3	3	1	1
3	3	1	1	1	1	3	3	1	1
3	3	1	1	1	1	3	3	1	1
3	3	1	1	1	1	3	3	1	1
3	3	1	1	1	1	3	3	1	1
3	3	1	1	4	4	3	3	2	2
3	3	1	1	1	1	3	3	1	1
3	3	1	1	1	1	3	3	1	1
3	3	1	1	1	1	3	3	1	1
3	3	1	1	1	1	3	3	1	1
3	3	1	1	1	1	3	3	1	1
3	3	1	1	1	1	3	3	1	1
3	3	1	1	1	1	3	3	1	1
3	3	1	1	1	1	3	3	1	1
3	3	1	1	1	1	3	3	2	2
3	3	1	1	1	1	3	3	1	1
3	3	1	1	1	1	3	3	1	1
3	3	1	1	1	1	3	3	1	1
3	3	1	1	1	1	3	3	2	2
3	3	1	1	1	1	3	3	1	1

For Review Only

3	3	1	1	1	1	3	3	1	1
1	1	1	1	1	1	3	3	1	1
3	3	1	1	1	1	3	3	1	1
3	3	1	1	1	1	3	3	1	1
3	3	2	2	4	4	4	4	1	1
3	3	2	2	4	4	4	4	1	1
3	3	1	1	1	1	3	3	1	1
3	3	1	1	1	1	3	3	1	1
3	3	1	1	1	1	3	3	1	1
3	3	1	1	1	1	3	3	1	1
3	3	1	1	1	1	3	3	2	2
3	3	1	1	4	4	3	3	1	1
3	3	1	1	1	1	3	3	1	1
3	3	1	1	1	1	3	3	1	1
3	3	1	1	1	1	3	3	1	1
3	3	1	1	1	1	3	3	1	1
3	3	1	1	1	1	3	3	1	1
3	3	1	1	1	1	3	3	1	1
3	3	1	1	1	1	3	3	1	1
3	3	1	1	1	1	3	3	1	1
3	3	1	1	1	1	3	3	1	1
3	3	1	1	1	1	3	3	1	1
3	3	2	2	4	4	3	3	1	1
3	3	1	1	1	1	3	3	2	2
3	3	1	1	4	4	3	3	1	1
3	3	1	1	1	1	3	3	1	1
3	3	1	1	1	1	3	3	2	2
3	3	1	1	1	1	3	3	1	1
3	3	1	1	1	1	3	3	1	1
3	3	2	2	4	4	4	4	1	1
3	3	1	1	1	1	3	3	1	1
1	1	1	1	1	1	3	3	1	1
3	3	1	1	1	1	3	3	2	2
3	3	1	1	1	1	3	3	1	1
3	3	1	1	1	1	3	3	1	1
3	3	1	1	1	1	3	3	2	2
3	3	1	1	4	4	3	3	1	1
3	3	1	1	1	1	3	3	1	1
3	3	1	1	1	1	3	3	1	1
3	3	1	1	1	1	3	3	1	1
3	3	1	1	1	1	3	3	2	2
3	3	1	1	1	1	3	3	1	1
3	3	1	1	1	1	3	3	1	1
3	3	1	1	1	1	3	3	1	1
3	3	1	1	1	1	3	3	2	2
3	3	1	1	1	1	3	3	1	1
3	3	1	1	1	1	3	3	1	1
3	3	1	1	1	1	3	3	1	1
3	3	1	1	1	1	3	3	1	1
3	3	1	1	1	1	3	3	1	1
3	3	2	2	4	4	4	4	1	1
3	3	1	1	1	1	3	3	1	1
3	3	1	1	1	1	3	3	1	1
3	3	1	1	1	1	3	3	2	2
3	3	1	1	1	1	3	3	1	1

3	3	1	1	1	1	3	3	1	1
3	3	1	1	1	1	3	3	1	1
3	3	1	1	1	1	3	3	1	1
3	3	1	1	1	1	3	3	1	1
3	3	1	1	1	1	3	3	1	1
3	3	1	1	1	1	3	3	2	2
3	3	1	1	1	1	3	3	1	1
3	3	1	1	1	1	3	3	1	1
3	3	1	1	1	1	3	3	1	1
3	3	1	1	1	1	3	3	1	1
3	3	1	1	1	1	3	3	1	1
3	3	1	1	1	1	3	3	1	1
3	3	1	1	1	1	3	3	1	1
3	3	1	1	1	1	3	3	1	1
3	3	1	1	1	1	3	3	1	1
3	3	1	1	1	1	3	3	1	1
3	3	1	1	1	1	3	3	1	1
3	3	1	1	1	1	3	3	1	1
3	3	1	1	1	1	3	3	1	1
3	3	2	2	4	4	4	4	2	2
3	3	1	1	1	1	3	3	1	1
3	3	1	1	1	1	3	3	2	2
3	3	2	2	4	4	4	4	1	1
3	3	2	2	4	4	4	4	1	1
3	3	1	1	1	1	3	3	1	1
3	3	1	1	1	1	3	3	1	1
3	3	1	1	1	1	3	3	1	1
3	3	1	1	1	1	3	3	2	2
3	3	2	2	4	4	3	3	1	1
3	3	1	1	1	1	3	3	1	1
3	3	1	1	1	1	3	3	1	1
3	3	1	1	1	1	3	3	1	1
1	1	1	1	1	1	3	3	1	1
3	3	1	1	1	1	3	3	1	1
3	3	1	1	1	1	3	3	1	1
3	3	1	1	1	1	3	3	1	1
3	3	1	1	1	1	3	3	1	1
3	3	1	1	1	1	3	3	2	2
3	3	1	1	1	1	3	3	1	1
3	3	1	1	1	1	3	3	1	1
1	1	1	1	1	1	3	3	1	1
3	3	1	1	1	1	3	3	1	1
3	3	2	2	4	4	4	4	1	1
3	3	2	2	4	4	4	4	1	1
3	3	1	1	1	1	3	3	2	2
3	3	2	2	4	4	4	4	1	1
3	3	1	1	1	1	3	3	1	1
1	1	1	1	1	1	3	3	1	1
3	3	1	1	1	1	3	3	1	1
3	3	1	1	1	1	3	3	1	1
3	3	2	2	4	4	4	4	1	1
3	3	1	1	1	1	3	3	1	1
3	3	1	1	1	1	3	3	2	2
3	3	1	1	1	1	3	3	2	2
3	3	2	2	4	4	4	4	1	1

For Review Only

3	3	2	2	4	4	4	4	1	1
3	3	1	1	1	1	3	3	1	1

For Review Only

SNP3090	SNP3090	SNP3091	SNP3091	SNP3092	SNP3092	SNP3093	SNP3093	SNP3094	SNP3094
4	4	1	1	4	4	4	4	1	1
4	4	1	1	4	4	4	4	1	1
4	4	1	1	4	4	4	4	1	1
4	4	1	1	4	4	4	4	1	1
1	1	1	1	4	4	4	4	1	1
4	4	1	1	4	4	4	4	1	1
1	1	4	4	2	2	2	2	3	3
4	4	1	1	4	4	4	4	1	1
1	1	1	1	4	4	4	4	1	1
4	4	1	1	4	4	4	4	1	1
4	4	1	1	4	4	4	4	1	1
4	4	1	1	4	4	4	4	1	1
4	4	1	1	4	4	4	4	1	1
4	4	1	1	4	4	4	4	1	1
1	1	1	1	4	4	4	4	1	1
4	4	1	1	4	4	4	4	1	1
1	1	4	4	2	2	2	2	3	3
4	4	1	1	4	4	4	4	1	1
4	4	1	1	4	4	4	4	1	1
4	4	1	1	4	4	4	4	1	1
4	4	1	1	4	4	4	4	1	1
1	1	4	4	2	2	2	2	3	3
4	4	1	1	4	4	4	4	1	1
4	4	1	1	4	4	4	4	1	1
1	1	4	4	4	4	2	2	3	3
4	4	1	1	4	4	4	4	1	1
4	4	1	1	4	4	4	4	1	1
4	4	1	1	4	4	4	4	1	1
4	4	1	1	4	4	4	4	1	1
4	4	1	1	4	4	4	4	1	1
4	4	1	1	4	4	4	4	1	1
4	4	1	1	4	4	4	4	1	1
1	1	4	4	2	2	2	2	3	3
4	4	1	1	4	4	4	4	1	1
4	4	1	1	4	4	4	4	1	1
4	4	1	1	4	4	4	4	1	1
4	4	1	1	4	4	4	4	1	1
1	1	4	4	2	2	2	2	3	3
4	4	1	1	4	4	4	4	1	1
1	1	4	4	2	2	2	2	3	3
4	4	1	1	4	4	4	4	1	1
4	4	1	1	4	4	4	4	1	1
4	4	1	1	4	4	4	4	1	1
4	4	1	1	4	4	4	4	1	1
4	4	1	1	4	4	4	4	1	1
4	4	1	1	4	4	4	4	1	1
4	4	1	1	4	4	4	4	1	1
4	4	1	1	4	4	4	4	1	1
4	4	1	1	4	4	4	4	1	1
1	1	1	1	2	2	4	4	3	3
4	4	1	1	4	4	4	4	1	1
1	1	4	4	2	2	2	2	3	3

For Review Only

1	1	4	4	2	2	2	2	3	3
1	1	4	4	2	2	4	4	1	1
4	4	1	1	4	4	4	4	1	1
1	1	4	4	2	2	2	2	3	3
4	4	1	1	4	4	4	4	1	1
4	4	1	1	4	4	4	4	1	1
4	4	1	1	4	4	4	4	1	1
4	4	1	1	4	4	4	4	1	1
4	4	1	1	4	4	4	4	1	1
4	4	1	1	4	4	4	4	1	1
4	4	1	1	4	4	4	4	1	1
4	4	1	1	4	4	4	4	1	1
1	1	4	4	2	2	2	2	3	3
4	4	1	1	4	4	4	4	1	1
4	4	1	1	4	4	4	4	1	1
1	1	1	1	4	4	4	4	1	1
4	4	1	1	4	4	4	4	1	1
4	4	1	1	4	4	4	4	1	1
1	1	4	4	2	2	2	2	3	3
1	1	1	1	4	4	4	4	1	1
4	4	1	1	4	4	4	4	1	1
4	4	1	1	4	4	4	4	1	1
4	4	1	1	4	4	4	4	1	1
4	4	1	1	4	4	4	4	1	1
4	4	1	1	4	4	4	4	1	1
4	4	1	1	4	4	4	4	1	1
4	4	1	1	4	4	4	4	1	1
4	4	1	1	4	4	4	4	1	1
4	4	1	1	4	4	4	4	1	1
4	4	1	1	4	4	4	4	1	1
1	1	4	4	2	2	2	2	3	3
4	4	1	1	4	4	4	4	1	1
4	4	1	1	4	4	4	4	1	1
1	1	4	4	2	2	2	2	3	3
4	4	1	1	4	4	4	4	1	1
4	4	1	1	4	4	4	4	1	1
1	1	1	1	4	4	4	4	1	1
4	4	1	1	4	4	4	4	1	1
4	4	1	1	4	4	4	4	1	1
4	4	1	1	4	4	4	4	1	1
1	1	4	4	2	2	2	2	3	3
1	1	1	1	4	4	4	4	1	1
4	4	1	1	4	4	4	4	1	1
4	4	1	1	4	4	4	4	1	1
4	4	1	1	4	4	4	4	1	1
4	4	1	1	4	4	4	4	1	1
4	4	1	1	4	4	4	4	1	1
4	4	1	1	4	4	4	4	1	1
4	4	1	1	4	4	4	4	1	1
4	4	1	1	4	4	4	4	1	1
1	1	1	1	4	4	4	4	1	1
4	4	1	1	4	4	4	4	1	1
4	4	1	1	4	4	4	4	1	1
4	4	1	1	4	4	4	4	1	1
4	4	1	1	4	4	4	4	1	1

4	4	1	1	4	4	4	4	1	1
1	1	1	1	4	4	4	4	1	1
4	4	1	1	4	4	4	4	1	1
4	4	1	1	4	4	4	4	1	1
4	4	1	1	4	4	4	4	1	1
4	4	1	1	4	4	4	4	1	1
4	4	1	1	4	4	4	4	1	1
4	4	1	1	4	4	4	4	1	1
4	4	1	1	4	4	4	4	1	1
4	4	1	1	4	4	4	4	1	1
1	1	1	1	4	4	4	4	1	1
1	1	1	1	4	4	4	4	1	1
4	4	1	1	4	4	4	4	1	1
4	4	1	1	4	4	4	4	1	1
4	4	1	1	4	4	4	4	1	1
4	4	1	1	4	4	4	4	1	1
4	4	1	1	4	4	4	4	1	1
4	4	1	1	4	4	4	4	1	1
4	4	1	1	4	4	4	4	1	1
4	4	1	1	4	4	4	4	1	1
4	4	1	1	4	4	4	4	1	1
1	1	4	4	2	2	4	4	3	3
4	4	1	1	4	4	4	4	1	1
4	4	1	1	4	4	4	4	1	1
4	4	1	1	4	4	4	4	1	1
1	1	4	4	2	2	2	2	3	3
4	4	1	1	4	4	4	4	1	1
1	1	4	4	2	2	2	2	3	3
4	4	1	1	4	4	4	4	1	1
1	1	1	1	4	4	4	4	1	1
4	4	1	1	4	4	4	4	1	1
4	4	1	1	4	4	4	4	1	1
1	1	4	4	2	2	2	2	3	3
1	1	1	1	4	4	4	4	1	1
4	4	1	1	4	4	4	4	1	1
4	4	1	1	4	4	4	4	1	1
4	4	1	1	4	4	4	4	1	1
4	4	1	1	4	4	4	4	1	1
4	4	1	1	4	4	4	4	1	1
4	4	1	1	4	4	4	4	1	1
1	1	1	1	4	4	4	4	1	1
4	4	1	1	4	4	4	4	1	1
4	4	1	1	4	4	4	4	1	1
4	4	1	1	4	4	4	4	1	1
4	4	1	1	4	4	4	4	1	1
4	4	1	1	4	4	4	4	1	1
4	4	1	1	4	4	4	4	1	1
4	4	1	1	4	4	4	4	1	1
4	4	1	1	4	4	4	4	1	1
4	4	1	1	4	4	4	4	1	1
4	4	1	1	4	4	4	4	1	1
4	4	1	1	4	4	4	4	1	1
4	4	1	1	4	4	4	4	1	1
4	4	1	1	4	4	4	4	1	1
4	4	1	1	4	4	4	4	1	1

4	4	1	1	4	4	4	4	1	1
4	4	1	1	4	4	4	4	1	1
4	4	1	1	4	4	4	4	1	1
1	1	1	1	4	4	4	4	1	1
1	1	4	4	2	2	2	2	3	3
1	1	4	4	2	2	2	2	3	3
4	4	1	1	4	4	4	4	1	1
4	4	1	1	4	4	4	4	1	1
4	4	1	1	4	4	4	4	1	1
4	4	1	1	4	4	4	4	1	1
4	4	1	1	4	4	4	4	1	1
1	1	1	1	2	2	4	4	1	1
4	4	1	1	4	4	4	4	1	1
4	4	1	1	4	4	4	4	1	1
4	4	1	1	4	4	4	4	1	1
4	4	1	1	4	4	4	4	1	1
4	4	1	1	4	4	4	4	1	1
4	4	1	1	4	4	4	4	1	1
4	4	1	1	4	4	4	4	1	1
4	4	1	1	4	4	4	4	1	1
4	4	1	1	4	4	4	4	1	1
4	4	1	1	4	4	4	4	1	1
1	1	1	1	2	2	4	4	1	1
4	4	1	1	4	4	4	4	1	1
1	1	4	4	2	2	4	4	3	3
4	4	1	1	4	4	4	4	1	1
4	4	1	1	4	4	4	4	1	1
4	4	1	1	4	4	4	4	1	1
4	4	1	1	4	4	4	4	1	1
1	1	4	4	2	2	2	2	3	3
4	4	1	1	4	4	4	4	1	1
4	4	1	1	4	4	4	4	1	1
4	4	1	1	4	4	4	4	1	1
4	4	1	1	4	4	4	4	1	1
4	4	1	1	4	4	4	4	1	1
4	4	1	1	4	4	4	4	1	1
4	4	1	1	4	4	4	4	1	1
4	4	1	1	4	4	4	4	1	1
4	4	1	1	4	4	4	4	1	1
4	4	1	1	4	4	4	4	1	1
4	4	1	1	4	4	4	4	1	1
4	4	1	1	4	4	4	4	1	1
4	4	1	1	4	4	4	4	1	1
4	4	1	1	4	4	4	4	1	1
4	4	1	1	4	4	4	4	1	1
4	4	1	1	4	4	4	4	1	1
4	4	1	1	4	4	4	4	1	1
4	4	1	1	4	4	4	4	1	1
4	4	1	1	4	4	4	4	1	1
4	4	1	1	4	4	4	4	1	1
1	1	4	4	2	2	2	2	3	3
4	4	1	1	4	4	4	4	1	1
4	4	1	1	4	4	4	4	1	1
4	4	1	1	4	4	4	4	1	1
4	4	1	1	4	4	4	4	1	1

For Review Only

4	4	1	1	4	4	4	4	1	1
4	4	1	1	4	4	4	4	1	1
4	4	1	1	4	4	4	4	1	1
1	1	1	1	4	4	4	4	1	1
4	4	1	1	4	4	4	4	1	1
4	4	1	1	4	4	4	4	1	1
4	4	1	1	4	4	4	4	1	1
4	4	1	1	4	4	4	4	1	1
4	4	1	1	4	4	4	4	1	1
4	4	1	1	4	4	4	4	1	1
4	4	1	1	4	4	4	4	1	1
4	4	1	1	4	4	4	4	1	1
4	4	1	1	4	4	4	4	1	1
4	4	1	1	4	4	4	4	1	1
4	4	1	1	4	4	4	4	1	1
4	4	1	1	4	4	4	4	1	1
4	4	1	1	4	4	4	4	1	1
4	4	1	1	4	4	4	4	1	1
4	4	1	1	4	4	4	4	1	1
1	1	4	4	2	2	2	2	3	3
4	4	1	1	4	4	4	4	1	1
4	4	1	1	4	4	4	4	1	1
1	1	4	4	2	2	2	2	3	3
1	1	4	4	2	2	2	2	3	3
4	4	1	1	4	4	4	4	1	1
4	4	1	1	4	4	4	4	1	1
4	4	1	1	4	4	4	4	1	1
4	4	1	1	4	4	4	4	1	1
1	1	1	1	4	4	4	4	1	1
4	4	1	1	4	4	4	4	1	1
4	4	1	1	4	4	4	4	1	1
4	4	1	1	4	4	4	4	1	1
4	4	1	1	4	4	4	4	1	1
4	4	1	1	4	4	4	4	1	1
4	4	1	1	4	4	4	4	1	1
4	4	1	1	4	4	4	4	1	1
4	4	1	1	4	4	4	4	1	1
4	4	1	1	4	4	4	4	1	1
4	4	1	1	4	4	4	4	1	1
4	4	1	1	4	4	4	4	1	1
4	4	1	1	4	4	4	4	1	1
4	4	1	1	4	4	4	4	1	1
1	1	4	4	2	2	2	2	3	3
1	1	4	4	2	2	2	2	3	3
4	4	1	1	4	4	4	4	1	1
1	1	4	4	2	2	2	2	3	3
4	4	1	1	4	4	4	4	1	1
4	4	1	1	4	4	4	4	1	1
1	1	1	1	4	4	4	4	1	1
4	4	1	1	4	4	4	4	1	1
1	1	4	4	2	2	2	2	3	3
4	4	1	1	4	4	4	4	1	1
4	4	1	1	4	4	4	4	1	1
4	4	1	1	4	4	4	4	1	1
1	1	4	4	2	2	2	2	3	3

For Review Only

1	1	4	4	2	2	2	2	3	3
4	4	1	1	4	4	4	4	1	1

For Review Only

SNP3095	SNP3095	SNP3096	SNP3096	SNP3097	SNP3097	SNP3098	SNP3098	SNP3099	SNP3099
3	3	4	4	3	3	1	1	2	2
3	3	4	4	3	3	2	2	1	1
3	3	4	4	3	3	1	1	2	2
3	3	4	4	3	3	2	2	2	2
3	3	4	4	2	2	2	2	2	2
3	3	4	4	3	3	1	1	2	2
3	3	2	2	2	2	2	2	2	2
3	3	4	4	3	3	2	2	2	2
3	3	4	4	3	3	2	2	2	2
3	3	4	4	3	3	2	2	1	1
3	3	4	4	3	3	1	1	1	1
3	3	4	4	3	3	1	1	2	2
3	3	2	2	3	3	2	2	2	2
3	3	4	4	3	3	1	1	1	1
3	3	4	4	3	3	1	1	2	2
3	3	4	4	2	2	2	2	2	2
2	2	2	2	2	2	2	2	2	2
3	3	4	4	3	3	1	1	2	2
3	3	4	4	3	3	1	1	2	2
3	3	4	4	3	3	1	1	2	2
3	3	4	4	3	3	2	2	1	1
2	2	4	4	3	3	2	2	1	1
3	3	4	4	3	3	1	1	1	1
3	3	4	4	3	3	1	1	2	2
2	2	2	2	2	2	2	2	2	2
3	3	4	4	3	3	1	1	2	2
3	3	4	4	3	3	2	2	2	2
3	3	4	4	3	3	1	1	2	2
3	3	4	4	3	3	2	2	1	1
3	3	4	4	3	3	2	2	1	1
3	3	2	2	2	2	2	2	2	2
3	3	4	4	3	3	2	2	2	2
2	2	2	2	2	2	2	2	2	2
3	3	4	4	3	3	1	1	1	1
3	3	4	4	3	3	2	2	2	2
3	3	4	4	3	3	2	2	2	2
3	3	4	4	3	3	1	1	2	2
2	2	2	2	2	2	2	2	2	2
3	3	4	4	3	3	1	1	1	1
2	2	2	2	2	2	2	2	2	2
3	3	4	4	3	3	2	2	1	1
3	3	4	4	3	3	1	1	2	2
3	3	4	4	3	3	2	2	1	1
3	3	4	4	3	3	2	2	1	1
3	3	4	4	3	3	2	2	1	1
3	3	4	4	3	3	1	1	2	2
3	3	4	4	3	3	2	2	2	2
3	3	4	4	3	3	1	1	2	2
3	3	4	4	3	3	2	2	1	1
3	3	4	4	2	2	1	1	2	2
3	3	4	4	3	3	2	2	1	1
2	2	2	2	2	2	2	2	2	2

For Review Only

2	2	2	2	2	2	2	2	2	2
2	2	2	2	2	2	2	2	2	2
3	3	4	4	3	3	1	1	2	2
2	2	2	2	2	2	2	2	2	2
3	3	4	4	3	3	1	1	2	2
3	3	4	4	3	3	2	2	2	2
3	3	4	4	3	3	2	2	2	2
3	3	4	4	3	3	2	2	1	1
3	3	4	4	3	3	2	2	1	1
3	3	4	4	3	3	1	1	2	2
3	3	4	4	3	3	1	1	2	2
3	3	4	4	3	3	2	2	2	2
2	2	2	2	2	2	2	2	2	2
3	3	4	4	3	3	2	2	1	1
3	3	4	4	3	3	1	1	2	2
3	3	4	4	3	3	1	1	2	2
3	3	4	4	3	3	1	1	2	2
3	3	4	4	3	3	1	1	2	2
2	2	2	2	2	2	2	2	2	2
3	3	4	4	2	2	2	2	2	2
3	3	4	4	3	3	2	2	2	2
3	3	4	4	3	3	2	2	1	1
3	3	4	4	3	3	2	2	1	1
3	3	4	4	3	3	1	1	2	2
3	3	4	4	3	3	2	2	1	1
3	3	4	4	3	3	2	2	2	2
3	3	4	4	3	3	2	2	1	1
3	3	4	4	3	3	2	2	1	1
3	3	4	4	3	3	1	1	1	1
3	3	4	4	2	2	2	2	2	2
2	2	2	2	2	2	2	2	2	2
3	3	4	4	3	3	2	2	1	1
3	3	4	4	3	3	1	1	2	2
2	2	2	2	2	2	2	2	2	2
3	3	4	4	2	2	2	2	2	2
3	3	4	4	3	3	2	2	1	1
3	3	4	4	3	3	2	2	1	1
3	3	4	4	3	3	2	2	1	1
3	3	4	4	3	3	2	2	2	2
3	3	4	4	3	3	2	2	1	1
2	2	2	2	2	2	2	2	2	2
3	3	4	4	2	2	2	2	2	2
3	3	4	4	3	3	2	2	1	1
3	3	4	4	3	3	1	1	2	2
3	3	4	4	3	3	2	2	2	2
3	3	4	4	3	3	1	1	2	2
3	3	4	4	3	3	1	1	1	1
3	3	4	4	3	3	2	2	2	2
3	3	4	4	3	3	1	1	2	2
3	3	4	4	3	3	1	1	2	2
3	3	4	4	3	3	1	1	2	2
3	3	4	4	3	3	1	1	1	1
3	3	4	4	3	3	2	2	1	1
3	3	4	4	3	3	1	1	2	2
3	3	4	4	3	3	1	1	2	2

For Review Only

3	3	4	4	3	3	2	2	2	2
3	3	4	4	3	3	1	1	2	2
3	3	4	4	3	3	2	2	2	2
3	3	4	4	3	3	1	1	2	2
3	3	4	4	3	3	1	1	2	2
3	3	4	4	3	3	1	1	2	2
3	3	4	4	2	2	2	2	2	2
3	3	4	4	3	3	1	1	2	2
3	3	4	4	3	3	1	1	2	2
3	3	4	4	3	3	2	2	2	2
3	3	4	4	3	3	2	2	2	2
3	3	2	2	3	3	1	1	2	2
3	3	4	4	3	3	1	1	2	2
3	3	4	4	3	3	1	1	2	2
3	3	4	4	3	3	1	1	2	2
3	3	4	4	3	3	2	2	2	2
3	3	4	4	3	3	1	1	2	2
3	3	4	4	3	3	1	1	2	2
3	3	4	4	3	3	1	1	1	1
3	3	4	4	3	3	1	1	2	2
3	3	4	4	3	3	2	2	2	2
2	2	2	2	2	2	2	2	2	2
3	3	4	4	3	3	1	1	2	2
3	3	4	4	3	3	1	1	1	1
3	3	4	4	3	3	2	2	2	2
2	2	2	2	2	2	2	2	2	2
3	3	4	4	3	3	2	2	2	2
2	2	2	2	2	2	2	2	2	2
3	3	4	4	3	3	2	2	2	2
3	3	4	4	3	3	1	1	2	2
3	3	4	4	3	3	1	1	1	1
3	3	4	4	3	3	1	1	2	2
2	2	2	2	2	2	2	2	2	2
3	3	4	4	3	3	1	1	2	2
3	3	4	4	3	3	1	1	1	1
3	3	4	4	3	3	2	2	2	2
3	3	4	4	3	3	1	1	1	1
3	3	4	4	3	3	1	1	2	2
3	3	4	4	3	3	1	1	2	2
3	3	4	4	3	3	2	2	1	1
3	3	4	4	3	3	1	1	2	2
3	3	4	4	3	3	1	1	1	1
3	3	4	4	3	3	1	1	2	2
3	3	4	4	3	3	2	2	2	2
3	3	4	4	3	3	1	1	2	2
3	3	4	4	3	3	1	1	2	2
3	3	4	4	3	3	2	2	2	2
3	3	4	4	3	3	2	2	2	2
3	3	4	4	3	3	2	2	2	2
3	3	4	4	3	3	1	1	2	2
3	3	4	4	3	3	2	2	2	2
3	3	4	4	3	3	2	2	1	1
3	3	4	4	3	3	1	1	2	2
3	3	4	4	3	3	2	2	2	2
3	3	4	4	3	3	2	2	2	2

For Review Only

3	3	4	4	3	3	1	1	2	2
3	3	4	4	3	3	1	1	2	2
3	3	4	4	3	3	2	2	2	2
3	3	4	4	3	3	1	1	2	2
2	2	2	2	2	2	2	2	2	2
2	2	2	2	2	2	2	2	2	2
3	3	4	4	3	3	2	2	2	2
3	3	4	4	3	3	1	1	2	2
3	3	4	4	3	3	1	1	2	2
3	3	4	4	2	2	2	2	2	2
3	3	4	4	3	3	2	2	2	2
2	2	4	4	3	3	2	2	1	1
3	3	4	4	3	3	2	2	2	2
3	3	4	4	3	3	1	1	2	2
3	3	4	4	3	3	1	1	1	1
3	3	4	4	3	3	1	1	1	1
3	3	4	4	3	3	1	1	2	2
3	3	4	4	3	3	1	1	2	2
3	3	4	4	3	3	2	2	2	2
3	3	4	4	3	3	2	2	2	2
3	3	4	4	3	3	1	1	2	2
3	3	4	4	3	3	2	2	1	1
3	3	4	4	2	2	2	2	2	2
3	3	4	4	3	3	2	2	2	2
2	2	2	2	2	2	2	2	2	2
3	3	4	4	3	3	1	1	1	1
3	3	4	4	3	3	1	1	2	2
3	3	4	4	3	3	1	1	2	2
3	3	4	4	3	3	1	1	2	2
2	2	2	2	2	2	2	2	2	2
3	3	4	4	3	3	1	1	2	2
3	3	4	4	3	3	1	1	2	2
3	3	4	4	2	2	2	2	2	2
3	3	4	4	3	3	1	1	1	1
3	3	4	4	3	3	1	1	1	1
3	3	4	4	3	3	2	2	2	2
3	3	4	4	2	2	2	2	2	2
3	3	4	4	3	3	1	1	2	2
3	3	4	4	3	3	2	2	2	2
3	3	4	4	3	3	2	2	1	1
3	3	4	4	3	3	1	1	2	2
3	3	4	4	3	3	2	2	2	2
3	3	4	4	3	3	2	2	1	1
3	3	4	4	3	3	1	1	2	2
3	3	4	4	3	3	1	1	2	2
3	3	4	4	2	2	2	2	2	2
3	3	4	4	3	3	1	1	1	1
3	3	4	4	3	3	2	2	1	1
3	3	4	4	3	3	1	1	2	2
3	3	4	4	3	3	2	2	2	2
2	2	2	2	2	2	2	2	2	2
3	3	4	4	3	3	1	1	2	2
3	3	4	4	3	3	2	2	1	1
3	3	4	4	3	3	1	1	2	2
3	3	4	4	3	3	1	1	1	1

For Review Only

3	3	4	4	3	3	1	1	2	2
3	3	4	4	2	2	2	2	2	2
3	3	4	4	2	2	2	2	2	2
3	3	4	4	3	3	1	1	2	2
3	3	4	4	3	3	1	1	2	2
3	3	4	4	3	3	1	1	1	1
3	3	4	4	3	3	2	2	1	1
3	3	4	4	3	3	2	2	1	1
3	3	4	4	3	3	1	1	2	2
3	3	4	4	3	3	1	1	1	1
3	3	4	4	3	3	1	1	2	2
3	3	4	4	3	3	1	1	2	2
3	3	4	4	3	3	2	2	1	1
3	3	4	4	3	3	2	2	2	2
3	3	4	4	3	3	2	2	2	2
3	3	4	4	3	3	2	2	1	1
3	3	4	4	3	3	1	1	1	1
3	3	4	4	3	3	2	2	1	1
3	3	4	4	3	3	1	1	2	2
2	2	2	2	2	2	2	2	2	2
3	3	4	4	3	3	1	1	2	2
3	3	4	4	3	3	1	1	1	1
2	2	2	2	2	2	2	2	2	2
2	2	2	2	2	2	2	2	2	2
3	3	4	4	3	3	2	2	1	1
3	3	4	4	3	3	1	1	2	2
3	3	4	4	3	3	1	1	1	1
3	3	4	4	3	3	2	2	2	2
3	3	4	4	3	3	1	1	2	2
3	3	4	4	2	2	2	2	2	2
3	3	4	4	3	3	1	1	2	2
3	3	4	4	3	3	1	1	2	2
3	3	4	4	3	3	1	1	2	2
3	3	4	4	3	3	2	2	2	2
3	3	4	4	3	3	2	2	1	1
3	3	4	4	3	3	1	1	2	2
3	3	4	4	3	3	1	1	2	2
3	3	4	4	3	3	2	2	2	2
3	3	4	4	3	3	2	2	2	2
3	3	4	4	3	3	1	1	1	1
3	3	2	2	2	2	2	2	2	2
3	3	4	4	3	3	1	1	2	2
2	2	2	2	2	2	2	2	2	2
2	2	2	2	2	2	2	2	2	2
3	3	4	4	3	3	1	1	1	1
2	2	2	2	2	2	2	2	2	2
3	3	4	4	3	3	2	2	2	2
3	3	4	4	3	3	2	2	1	1
3	3	4	4	3	3	2	2	2	2
3	3	4	4	3	3	1	1	1	1
2	2	2	2	2	2	2	2	2	2
3	3	4	4	3	3	1	1	1	1
3	3	4	4	3	3	1	1	2	2
3	3	4	4	3	3	2	2	2	2
2	2	2	2	2	2	2	2	2	2

For Review Only

2	2	4	4	2	2	2	2	2	2
3	3	4	4	3	3	1	1	2	2

For Review Only

SNP310	SNP310	SNP3100	SNP3100	SNP3101	SNP3101	SNP3102	SNP3102	SNP3103	SNP3103
1	1	2	2	3	3	3	3	1	1
4	4	2	2	1	1	3	3	1	1
4	4	2	2	3	3	3	3	3	3
1	1	2	2	3	3	3	3	1	1
1	1	2	2	3	3	3	3	1	1
1	1	2	2	3	3	3	3	1	1
1	1	2	2	3	3	3	3	3	3
4	4	2	2	3	3	3	3	1	1
1	1	2	2	3	3	3	3	1	1
1	1	4	4	1	1	3	3	1	1
4	4	2	2	1	1	2	2	1	1
1	1	2	2	3	3	3	3	3	3
1	1	2	2	1	1	3	3	3	3
4	4	4	4	1	1	3	3	1	1
1	1	2	2	3	3	3	3	1	1
4	4	2	2	1	1	2	2	3	3
1	1	2	2	1	1	3	3	3	3
1	1	2	2	3	3	3	3	3	3
1	1	2	2	3	3	3	3	1	1
1	1	2	2	3	3	3	3	1	1
1	1	4	4	1	1	3	3	1	1
1	1	4	4	1	1	2	2	1	1
4	4	2	2	1	1	2	2	1	1
1	1	2	2	3	3	2	2	1	1
1	1	2	2	3	3	3	3	3	3
4	4	2	2	3	3	3	3	1	1
4	4	2	2	3	3	3	3	1	1
1	1	2	2	1	1	3	3	1	1
1	1	2	2	3	3	3	3	1	1
1	1	4	4	1	1	2	2	1	1
1	1	2	2	3	3	3	3	1	1
1	1	2	2	3	3	3	3	1	1
1	1	2	2	3	3	3	3	3	3
4	4	4	4	1	1	3	3	1	1
1	1	2	2	1	1	2	2	1	1
4	4	2	2	3	3	3	3	1	1
1	1	2	2	3	3	3	3	1	1
4	4	2	2	3	3	3	3	3	3
4	4	2	2	1	1	2	2	1	1
4	4	2	2	3	3	3	3	3	3
1	1	2	2	1	1	3	3	1	1
1	1	2	2	3	3	3	3	1	1
1	1	2	2	1	1	3	3	1	1
1	1	2	2	3	3	3	3	1	1
1	1	2	2	1	1	3	3	1	1
1	1	2	2	3	3	3	3	3	3
1	1	2	2	3	3	3	3	1	1
4	4	2	2	3	3	3	3	3	3
1	1	2	2	3	3	3	3	1	1
1	1	2	2	3	3	3	3	3	3
4	4	4	4	3	3	3	3	1	1
4	4	2	2	3	3	3	3	3	3

For Review Only

1	1	2	2	3	3	3	3	3	3
1	1	2	2	3	3	3	3	3	3
4	4	2	2	3	3	3	3	1	1
1	1	2	2	3	3	3	3	3	3
1	1	2	2	3	3	3	3	1	1
4	4	2	2	3	3	3	3	1	1
1	1	2	2	3	3	3	3	1	1
4	4	4	4	1	1	2	2	1	1
4	4	4	4	1	1	3	3	1	1
1	1	2	2	1	1	3	3	1	1
4	4	2	2	3	3	3	3	1	1
4	4	2	2	3	3	3	3	1	1
1	1	2	2	3	3	3	3	3	3
1	1	2	2	3	3	3	3	1	1
1	1	2	2	3	3	3	3	1	1
1	1	2	2	3	3	3	3	1	1
4	4	2	2	1	1	2	2	1	1
1	1	2	2	3	3	3	3	1	1
1	1	2	2	3	3	3	3	1	1
1	1	2	2	3	3	3	3	1	1
4	4	2	2	3	3	3	3	1	1
1	1	2	2	3	3	2	2	1	1
1	1	2	2	3	3	3	3	1	1
4	4	2	2	3	3	3	3	1	1
1	1	2	2	1	1	3	3	1	1
4	4	2	2	3	3	3	3	1	1
1	1	4	4	3	3	3	3	1	1
4	4	2	2	1	1	2	2	1	1
1	1	2	2	1	1	2	2	1	1
1	1	2	2	3	3	3	3	1	1
1	1	2	2	3	3	3	3	3	3
1	1	4	4	3	3	3	3	1	1
4	4	2	2	1	1	2	2	1	1
1	1	2	2	3	3	3	3	3	3
4	4	2	2	3	3	3	3	3	3
1	1	2	2	1	1	3	3	1	1
1	1	4	4	1	1	3	3	1	1
1	1	2	2	1	1	3	3	1	1
1	1	2	2	3	3	3	3	3	3
1	1	2	2	1	1	3	3	1	1
1	1	2	2	3	3	3	3	3	3
1	1	2	2	3	3	3	3	1	1
1	1	4	4	3	3	3	3	1	1
4	4	2	2	3	3	3	3	1	1
4	4	2	2	3	3	3	3	1	1
4	4	2	2	3	3	3	3	1	1
1	1	4	4	1	1	3	3	1	1
1	1	2	2	3	3	3	3	1	1
4	4	2	2	3	3	3	3	3	3
1	1	2	2	3	3	2	2	1	1
4	4	2	2	3	3	3	3	1	1
4	4	2	2	1	1	2	2	1	1
4	4	4	4	1	1	2	2	1	1
4	4	2	2	1	1	2	2	1	1
1	1	2	2	3	3	3	3	1	1

For Review Only

4	4	2	2	3	3	3	3	1	1
4	4	2	2	1	1	2	2	1	1
1	1	2	2	3	3	3	3	1	1
1	1	2	2	1	1	3	3	1	1
4	4	2	2	1	1	3	3	1	1
1	1	2	2	3	3	2	2	1	1
1	1	2	2	1	1	3	3	3	3
1	1	2	2	3	3	2	2	1	1
4	4	2	2	3	3	3	3	3	3
1	1	2	2	1	1	3	3	1	1
1	1	2	2	3	3	3	3	1	1
1	1	2	2	3	3	3	3	1	1
1	1	2	2	3	3	3	3	1	1
4	4	2	2	3	3	3	3	1	1
4	4	2	2	1	1	3	3	1	1
1	1	2	2	3	3	3	3	1	1
4	4	2	2	3	3	3	3	1	1
1	1	2	2	1	1	2	2	1	1
1	1	4	4	1	1	3	3	1	1
4	4	2	2	3	3	3	3	1	1
1	1	2	2	3	3	3	3	1	1
1	1	2	2	3	3	3	3	3	3
4	4	2	2	3	3	3	3	1	1
4	4	2	2	1	1	2	2	1	1
1	1	2	2	3	3	3	3	1	1
1	1	2	2	3	3	3	3	3	3
4	4	2	2	3	3	3	3	1	1
1	1	2	2	3	3	2	2	1	1
1	1	2	2	3	3	3	3	1	1
1	1	2	2	3	3	3	3	3	3
1	1	4	4	1	1	3	3	1	1
1	1	2	2	3	3	2	2	1	1
1	1	2	2	3	3	3	3	3	3
1	1	2	2	1	1	3	3	1	1
4	4	4	4	1	1	3	3	1	1
1	1	2	2	3	3	3	3	1	1
4	4	4	4	1	1	3	3	1	1
4	4	2	2	3	3	2	2	1	1
4	4	2	2	1	1	2	2	1	1
4	4	2	2	1	1	2	2	1	1
4	4	2	2	1	1	2	2	1	1
1	1	4	4	1	1	3	3	1	1
1	1	2	2	3	3	3	3	1	1
1	1	4	4	1	1	3	3	1	1
4	4	2	2	3	3	3	3	1	1
4	4	2	2	3	3	3	3	1	1
1	1	2	2	3	3	3	3	1	1
4	4	2	2	3	3	3	3	1	1
1	1	2	2	1	1	2	2	1	1
4	4	2	2	3	3	3	3	1	1
4	4	2	2	3	3	2	2	1	1
4	4	4	4	1	1	2	2	1	1
1	1	2	2	1	1	3	3	1	1
1	1	2	2	3	3	3	3	1	1
4	4	4	4	1	1	2	2	1	1

For Review Only

1	1	2	2	3	3	3	3	1	1
1	1	2	2	3	3	3	3	1	1
1	1	2	2	1	1	3	3	1	1
1	1	2	2	1	1	2	2	1	1
4	4	2	2	3	3	3	3	3	3
4	4	2	2	3	3	3	3	3	3
4	4	2	2	3	3	2	2	1	1
1	1	2	2	1	1	3	3	1	1
4	4	2	2	1	1	2	2	1	1
1	1	2	2	3	3	3	3	1	1
1	1	2	2	3	3	2	2	1	1
4	4	4	4	1	1	3	3	1	1
1	1	2	2	3	3	3	3	1	1
1	1	2	2	3	3	3	3	1	1
1	1	4	4	1	1	3	3	1	1
4	4	2	2	1	1	2	2	1	1
1	1	2	2	1	1	3	3	1	1
4	4	2	2	3	3	3	3	1	1
1	1	2	2	3	3	3	3	1	1
1	1	2	2	1	1	3	3	3	3
1	1	2	2	1	1	3	3	1	1
4	4	2	2	3	3	2	2	1	1
1	1	2	2	3	3	2	2	1	1
4	4	2	2	3	3	3	3	1	1
1	1	2	2	3	3	3	3	3	3
4	4	4	4	1	1	3	3	1	1
1	1	2	2	3	3	2	2	1	1
4	4	2	2	3	3	3	3	1	1
1	1	2	2	3	3	3	3	1	1
1	1	2	2	3	3	3	3	3	3
4	4	2	2	3	3	3	3	1	1
4	4	2	2	3	3	3	3	1	1
4	4	2	2	3	3	3	3	1	1
1	1	4	4	1	1	3	3	1	1
1	1	4	4	1	1	3	3	1	1
4	4	2	2	3	3	3	3	1	1
4	4	2	2	3	3	3	3	1	1
1	1	2	2	1	1	3	3	1	1
4	4	2	2	3	3	3	3	1	1
4	4	4	4	3	3	3	3	1	1
4	4	2	2	3	3	3	3	3	3
1	1	2	2	3	3	3	3	1	1
4	4	2	2	1	1	2	2	1	1
1	1	2	2	3	3	2	2	1	1
4	4	2	2	3	3	3	3	1	1
4	4	2	2	3	3	3	3	1	1
4	4	2	2	1	1	2	2	1	1
4	4	2	2	3	3	2	2	1	1
1	1	2	2	1	1	3	3	1	1
1	1	2	2	3	3	3	3	1	1
1	1	2	2	3	3	3	3	3	3
4	4	2	2	3	3	3	3	1	1
4	4	2	2	3	3	2	2	1	1
4	4	2	2	3	3	3	3	1	1
4	4	2	2	1	1	2	2	1	1

For Review Only

1	1	2	2	1	1	3	3	1	1
4	4	2	2	3	3	3	3	1	1
1	1	2	2	3	3	3	3	1	1
1	1	2	2	3	3	3	3	1	1
1	1	2	2	3	3	3	3	1	1
1	1	2	2	1	1	3	3	1	1
1	1	4	4	1	1	2	2	1	1
1	1	4	4	1	1	3	3	1	1
1	1	2	2	3	3	2	2	1	1
4	4	4	4	1	1	2	2	1	1
1	1	2	2	1	1	3	3	1	1
1	1	2	2	3	3	3	3	1	1
1	1	4	4	1	1	2	2	1	1
1	1	2	2	3	3	3	3	1	1
4	4	2	2	3	3	3	3	1	1
1	1	4	4	1	1	2	2	1	1
1	1	2	2	1	1	2	2	1	1
4	4	2	2	1	1	2	2	1	1
4	4	2	2	3	3	3	3	1	1
4	4	2	2	3	3	3	3	3	3
4	4	2	2	3	3	3	3	1	1
4	4	2	2	1	1	2	2	1	1
1	1	2	2	1	1	3	3	1	1
4	4	2	2	3	3	3	3	1	1
4	4	4	4	1	1	2	2	1	1
4	4	2	2	3	3	3	3	1	1
4	4	2	2	1	1	2	2	1	1
1	1	2	2	3	3	3	3	1	1
1	1	2	2	3	3	3	3	1	1
1	1	2	2	3	3	3	3	1	1
4	4	2	2	1	1	2	2	1	1
4	4	2	2	3	3	3	3	3	3
1	1	2	2	3	3	3	3	1	1
4	4	2	2	3	3	3	3	1	1
1	1	2	2	3	3	2	2	1	1
4	4	2	2	1	1	2	2	1	1
1	1	2	2	3	3	3	3	1	1
4	4	2	2	3	3	3	3	1	1
4	4	2	2	3	3	3	3	1	1
4	4	2	2	1	1	2	2	1	1
1	1	2	2	3	3	3	3	3	3
4	4	2	2	3	3	3	3	1	1
4	4	2	2	3	3	3	3	3	3
1	1	2	2	3	3	3	3	3	3
4	4	2	2	1	1	2	2	1	1
1	1	2	2	3	3	3	3	1	1
4	4	2	2	3	3	3	3	1	1
1	1	4	4	1	1	2	2	1	1
4	4	2	2	1	1	3	3	1	1
4	4	2	2	3	3	2	2	1	1
1	1	2	2	3	3	3	3	3	3
1	1	2	2	1	1	2	2	1	1
4	4	2	2	3	3	3	3	3	3
4	4	2	2	3	3	3	3	1	1
4	4	2	2	3	3	3	3	3	3

For Review Only

1	1	2	2	3	3	3	3	3	3
4	4	2	2	3	3	3	3	1	1

For Review Only

SNP3104	SNP3104	SNP3105	SNP3105	SNP3106	SNP3106	SNP3107	SNP3107	SNP3108	SNP3108
2	2	3	3	2	2	3	3	3	3
2	2	3	3	2	2	3	3	3	3
3	3	3	3	2	2	3	3	3	3
2	2	3	3	2	2	3	3	3	3
2	2	3	3	2	2	3	3	3	3
2	2	3	3	2	2	3	3	3	3
3	3	4	4	4	4	3	3	3	3
2	2	3	3	2	2	3	3	3	3
2	2	3	3	2	2	3	3	3	3
2	2	3	3	2	2	3	3	3	3
3	3	4	4	2	2	1	1	2	2
2	2	3	3	2	2	3	3	3	3
3	3	3	3	4	4	3	3	3	3
3	3	4	4	4	4	3	3	3	3
2	2	3	3	2	2	3	3	3	3
3	3	4	4	4	4	3	3	3	3
3	3	4	4	4	4	3	3	3	3
3	3	4	4	2	2	3	3	3	3
2	2	3	3	2	2	3	3	3	3
2	2	3	3	2	2	3	3	3	3
2	2	3	3	2	2	3	3	3	3
2	2	3	3	2	2	3	3	3	3
3	3	4	4	2	2	1	1	2	2
2	2	3	3	2	2	3	3	3	3
3	3	4	4	4	4	3	3	3	3
2	2	3	3	2	2	3	3	3	3
2	2	3	3	2	2	3	3	3	3
2	2	3	3	2	2	3	3	3	3
2	2	3	3	2	2	3	3	3	3
2	2	3	3	2	2	3	3	3	3
2	2	3	3	2	2	3	3	3	3
2	2	3	3	2	2	3	3	3	3
3	3	4	4	4	4	3	3	3	3
3	3	4	4	2	2	3	3	3	3
2	2	3	3	2	2	3	3	2	2
2	2	3	3	2	2	3	3	3	3
2	2	3	3	2	2	3	3	3	3
3	3	4	4	4	4	3	3	3	3
3	3	4	4	2	2	1	1	2	2
3	3	4	4	4	4	3	3	3	3
2	2	3	3	2	2	3	3	3	3
2	2	3	3	2	2	3	3	3	3
2	2	3	3	2	2	3	3	3	3
2	2	3	3	2	2	3	3	3	3
2	2	3	3	2	2	3	3	3	3
3	3	3	3	2	2	3	3	3	3
2	2	3	3	2	2	3	3	3	3
3	3	4	4	4	4	3	3	3	3
2	2	3	3	2	2	3	3	3	3
2	2	4	4	2	2	3	3	3	3
2	2	3	3	2	2	3	3	3	3
3	3	4	4	4	4	3	3	3	3

3	3	4	4	4	4	3	3	3	3
3	3	4	4	4	4	3	3	3	3
2	2	3	3	2	2	3	3	3	3
3	3	4	4	4	4	3	3	3	3
2	2	3	3	2	2	3	3	3	3
2	2	3	3	2	2	3	3	3	3
2	2	3	3	2	2	3	3	3	3
2	2	3	3	2	2	3	3	3	3
2	2	3	3	2	2	3	3	3	3
2	2	3	3	2	2	3	3	3	3
2	2	3	3	2	2	3	3	3	3
2	2	3	3	2	2	3	3	3	3
3	3	4	4	4	4	3	3	3	3
2	2	3	3	2	2	3	3	3	3
2	2	3	3	2	2	3	3	3	3
2	2	3	3	2	2	3	3	3	3
3	3	4	4	2	2	3	3	3	3
2	2	3	3	2	2	3	3	3	3
2	2	3	3	2	2	3	3	3	3
2	2	3	3	2	2	3	3	3	3
2	2	3	3	2	2	3	3	3	3
3	3	4	4	2	2	3	3	2	2
2	2	3	3	2	2	3	3	3	3
2	2	3	3	2	2	3	3	3	3
2	2	3	3	2	2	3	3	3	3
2	2	3	3	2	2	3	3	3	3
2	2	3	3	2	2	3	3	3	3
3	3	4	4	2	2	1	1	2	2
3	3	4	4	2	2	1	1	2	2
2	2	3	3	2	2	3	3	3	3
3	3	4	4	4	4	3	3	3	3
2	2	3	3	2	2	3	3	3	3
3	3	4	4	2	2	1	1	2	2
3	3	4	4	4	4	3	3	3	3
3	3	4	4	2	2	3	3	3	3
2	2	3	3	2	2	3	3	3	3
2	2	3	3	2	2	3	3	3	3
2	2	3	3	2	2	3	3	3	3
3	3	4	4	4	4	3	3	3	3
2	2	3	3	2	2	3	3	3	3
2	2	4	4	2	2	3	3	3	3
2	2	3	3	2	2	3	3	3	3
2	2	3	3	2	2	3	3	3	3
2	2	3	3	2	2	3	3	3	3
2	2	3	3	2	2	3	3	3	3
2	2	3	3	2	2	3	3	3	3
3	3	4	4	2	2	3	3	3	3
2	2	3	3	2	2	3	3	3	3
3	3	4	4	4	4	3	3	3	3
2	2	3	3	2	2	3	3	3	3
2	2	3	3	2	2	3	3	3	3
3	3	4	4	2	2	1	1	2	2
2	2	3	3	2	2	3	3	3	3
2	2	3	3	4	4	3	3	3	3
2	2	3	3	2	2	3	3	3	3

For Review Only

2	2	3	3	2	2	3	3	3	3
2	2	3	3	2	2	3	3	3	3
2	2	3	3	2	2	3	3	3	3
3	3	4	4	2	2	3	3	3	3
2	2	3	3	2	2	3	3	3	3
2	2	3	3	2	2	3	3	3	3
3	3	4	4	4	4	3	3	3	3
2	2	3	3	2	2	3	3	3	3
3	3	4	4	4	4	3	3	3	3
2	2	3	3	2	2	3	3	3	3
3	3	3	3	2	2	3	3	3	3
2	2	3	3	2	2	3	3	3	3
2	2	3	3	2	2	3	3	3	3
2	2	3	3	2	2	3	3	3	3
2	2	3	3	2	2	3	3	3	3
2	2	3	3	2	2	3	3	3	3
2	2	3	3	2	2	3	3	3	3
2	2	3	3	4	4	3	3	2	2
3	3	4	4	4	4	3	3	3	3
2	2	3	3	2	2	3	3	3	3
2	2	3	3	2	2	3	3	3	3
3	3	4	4	4	4	3	3	3	3
2	2	3	3	2	2	3	3	3	3
3	3	4	4	2	2	1	1	2	2
3	3	3	3	2	2	3	3	2	2
3	3	4	4	4	4	3	3	3	3
2	2	3	3	2	2	3	3	3	3
2	2	3	3	2	2	3	3	3	3
2	2	3	3	2	2	3	3	3	3
2	2	3	3	2	2	3	3	3	3
3	3	4	4	2	2	3	3	3	3
2	2	3	3	2	2	3	3	3	3
3	3	4	4	4	4	3	3	3	3
3	3	4	4	2	2	3	3	3	3
3	3	4	4	2	2	3	3	3	3
2	2	3	3	2	2	3	3	3	3
2	2	3	3	2	2	3	3	3	3
3	3	4	4	4	4	3	3	3	3
3	3	4	4	2	2	3	3	2	2
3	3	4	4	2	2	1	1	2	2
3	3	4	4	2	2	3	3	2	2
3	3	4	4	2	2	3	3	3	3
2	2	3	3	2	2	3	3	3	3
2	2	3	3	2	2	3	3	3	3
2	2	3	3	2	2	3	3	3	3
2	2	3	3	2	2	3	3	3	3
2	2	3	3	2	2	3	3	3	3
2	2	3	3	2	2	3	3	3	3
2	2	3	3	2	2	3	3	3	3
2	2	3	3	2	2	3	3	3	3
2	2	3	3	2	2	3	3	3	3
2	2	3	3	2	2	3	3	3	3
2	2	3	3	2	2	3	3	3	3
2	2	3	3	2	2	3	3	3	3
2	2	3	3	2	2	3	3	3	3

For Review Only

2	2	3	3	2	2	3	3	3	3
2	2	3	3	2	2	3	3	3	3
2	2	3	3	2	2	3	3	3	3
3	3	4	4	2	2	3	3	2	2
3	3	4	4	4	4	3	3	3	3
3	3	4	4	4	4	3	3	3	3
3	3	4	4	2	2	3	3	2	2
2	2	3	3	2	2	3	3	3	3
2	2	3	3	2	2	3	3	2	2
2	2	3	3	2	2	3	3	3	3
2	2	3	3	2	2	3	3	3	3
2	2	3	3	2	2	3	3	3	3
2	2	3	3	2	2	3	3	3	3
2	2	3	3	2	2	3	3	3	3
3	3	4	4	2	2	3	3	3	3
3	3	4	4	2	2	1	1	2	2
2	2	3	3	2	2	3	3	3	3
2	2	3	3	2	2	3	3	3	3
2	2	3	3	2	2	3	3	3	3
3	3	4	4	4	4	3	3	3	3
2	2	3	3	2	2	3	3	3	3
3	3	4	4	2	2	1	1	2	2
3	3	4	4	2	2	3	3	2	2
2	2	3	3	2	2	3	3	3	3
3	3	4	4	4	4	3	3	3	3
3	3	4	4	2	2	3	3	3	3
2	2	3	3	2	2	3	3	3	3
2	2	3	3	2	2	3	3	3	3
2	2	3	3	2	2	3	3	3	3
3	3	4	4	4	4	3	3	3	3
2	2	3	3	2	2	3	3	3	3
2	2	3	3	2	2	3	3	3	3
2	2	3	3	2	2	3	3	3	3
3	3	4	4	2	2	3	3	3	3
2	2	3	3	2	2	3	3	3	3
2	2	3	3	2	2	3	3	3	3
2	2	3	3	2	2	3	3	3	3
2	2	3	3	2	2	3	3	3	3
2	2	3	3	2	2	3	3	3	3
2	2	3	3	2	2	3	3	3	3
2	2	3	3	2	2	3	3	3	3
2	2	3	3	2	2	3	3	3	3
3	3	4	4	4	4	3	3	3	3
3	3	4	4	2	2	3	3	2	2
2	2	3	3	2	2	3	3	3	3
2	2	3	3	2	2	3	3	3	3
3	3	4	4	2	2	1	1	2	2
3	3	4	4	2	2	1	1	2	2
2	2	3	3	2	2	3	3	3	3
2	2	3	3	2	2	3	3	3	3
3	3	4	4	4	4	3	3	3	3
2	2	3	3	2	2	3	3	3	3
3	3	4	4	2	2	1	1	2	2
2	2	3	3	2	2	3	3	3	3
3	3	4	4	2	2	1	1	2	2

For Review Only

3	3	4	4	2	2	3	3	3	3
2	2	3	3	2	2	3	3	3	3
2	2	3	3	2	2	3	3	3	3
2	2	3	3	2	2	3	3	3	3
2	2	3	3	2	2	3	3	3	3
2	2	3	3	2	2	3	3	3	3
3	3	4	4	2	2	1	1	2	2
2	2	3	3	2	2	3	3	3	3
2	2	3	3	2	2	3	3	3	3
3	3	4	4	2	2	1	1	2	2
2	2	3	3	2	2	3	3	3	3
2	2	3	3	2	2	3	3	3	3
3	3	4	4	2	2	1	1	2	2
2	2	3	3	2	2	3	3	3	3
2	2	3	3	2	2	3	3	3	3
3	3	4	4	2	2	3	3	2	2
3	3	4	4	2	2	1	1	2	2
3	3	4	4	2	2	3	3	2	2
2	2	3	3	2	2	3	3	3	3
3	3	4	4	4	4	3	3	3	3
2	2	3	3	2	2	3	3	3	3
2	2	3	3	2	2	3	3	3	3
2	2	3	3	2	2	3	3	3	3
2	2	3	3	2	2	3	3	3	3
2	2	3	3	2	2	3	3	3	3
2	2	3	3	2	2	3	3	3	3
3	3	4	4	4	4	3	3	3	3
2	2	3	3	2	2	3	3	3	3
2	2	3	3	2	2	3	3	3	3
2	2	3	3	2	2	3	3	3	3
3	3	4	4	2	2	1	1	2	2
3	3	4	4	4	4	1	1	2	2
2	2	3	3	2	2	3	3	3	3
2	2	3	3	2	2	3	3	3	3
3	3	4	4	2	2	3	3	3	3
3	3	4	4	2	2	1	1	2	2
2	2	3	3	2	2	3	3	3	3
2	2	3	3	2	2	3	3	3	3
2	2	3	3	2	2	3	3	3	3
3	3	4	4	2	2	1	1	2	2
2	2	3	3	2	2	3	3	3	3
2	2	3	3	2	2	3	3	3	3
3	3	4	4	4	4	3	3	3	3
3	3	4	4	4	4	3	3	3	3
3	3	4	4	4	4	3	3	3	3
3	3	4	4	2	2	1	1	2	2
2	2	3	3	2	2	3	3	3	3
2	2	3	3	2	2	3	3	3	3
2	2	3	3	2	2	3	3	3	3
3	3	4	4	2	2	1	1	2	2
2	2	3	3	2	2	3	3	3	3
3	3	4	4	2	2	1	1	2	2
3	3	3	3	2	2	3	3	3	3
2	2	3	3	2	2	3	3	3	3
3	3	4	4	4	4	3	3	3	3

For Review Only

3	3	4	4	4	4	3	3	3	3
2	2	3	3	2	2	3	3	3	3

For Review Only

SNP3109	SNP3109	SNP311	SNP311	SNP3110	SNP3110	SNP3111	SNP3111	SNP3112	SNP3112
1	1	3	3	2	2	3	3	2	2
1	1	3	3	2	2	3	3	2	2
1	1	3	3	2	2	3	3	2	2
1	1	3	3	2	2	3	3	2	2
1	1	3	3	2	2	3	3	2	2
1	1	3	3	2	2	3	3	2	2
1	1	2	2	2	2	3	3	2	2
2	2	3	3	1	1	1	1	2	2
1	1	2	2	2	2	3	3	2	2
2	2	3	3	1	1	1	1	2	2
2	2	3	3	1	1	1	1	2	2
1	1	3	3	2	2	3	3	2	2
2	2	2	2	2	2	3	3	2	2
1	1	3	3	2	2	3	3	2	2
1	1	2	2	2	2	3	3	2	2
1	1	3	3	2	2	3	3	2	2
1	1	2	2	2	2	3	3	2	2
1	1	3	3	2	2	3	3	2	2
1	1	3	3	2	2	1	1	2	2
2	2	3	3	1	1	1	1	2	2
2	2	2	2	1	1	1	1	2	2
2	2	2	2	2	2	1	1	2	2
2	2	2	2	1	1	1	1	1	1
1	1	3	3	2	2	3	3	2	2
1	1	2	2	2	2	3	3	2	2
1	1	2	2	2	2	3	3	2	2
1	1	3	3	2	2	3	3	2	2
1	1	2	2	2	2	3	3	2	2
2	2	2	2	1	1	1	1	2	2
1	1	3	3	1	1	1	1	2	2
1	1	3	3	2	2	3	3	2	2
1	1	3	3	2	2	3	3	2	2
1	1	2	2	2	2	3	3	2	2
1	1	3	3	2	2	3	3	1	1
2	2	2	2	2	2	3	3	2	2
1	1	3	3	2	2	3	3	2	2
1	1	3	3	2	2	3	3	2	2
1	1	3	3	2	2	3	3	2	2
2	2	2	2	1	1	1	1	2	2
1	1	2	2	2	2	3	3	2	2
2	2	2	2	1	1	1	1	2	2
2	2	3	3	2	2	1	1	2	2
2	2	3	3	1	1	1	1	2	2
2	2	2	2	1	1	1	1	2	2
1	1	2	2	2	2	3	3	2	2
1	1	2	2	2	2	3	3	2	2
1	1	2	2	2	2	3	3	2	2
1	1	2	2	2	2	3	3	2	2
2	2	3	3	2	2	3	3	2	2
1	1	3	3	2	2	3	3	2	2
2	2	2	2	1	1	1	1	2	2
1	1	3	3	2	2	3	3	2	2

For Review Only

1	1	2	2	2	2	3	3	2	2
1	1	2	2	2	2	3	3	2	2
1	1	3	3	1	1	1	1	2	2
1	1	2	2	2	2	3	3	2	2
1	1	3	3	2	2	1	1	2	2
1	1	3	3	2	2	3	3	2	2
1	1	3	3	2	2	3	3	2	2
2	2	3	3	1	1	1	1	2	2
1	1	3	3	2	2	3	3	2	2
1	1	2	2	2	2	3	3	2	2
1	1	3	3	2	2	3	3	2	2
1	1	3	3	2	2	3	3	2	2
1	1	2	2	2	2	3	3	2	2
2	2	2	2	1	1	1	1	2	2
1	1	2	2	2	2	3	3	2	2
1	1	3	3	2	2	3	3	2	2
2	2	2	2	1	1	1	1	2	2
1	1	3	3	2	2	3	3	2	2
1	1	3	3	2	2	3	3	2	2
1	1	3	3	2	2	3	3	2	2
1	1	3	3	2	2	3	3	2	2
1	1	2	2	1	1	3	3	2	2
2	2	3	3	1	1	1	1	2	2
1	1	3	3	1	1	1	1	2	2
1	1	2	2	2	2	3	3	2	2
1	1	3	3	2	2	3	3	2	2
2	2	2	2	1	1	1	1	2	2
2	2	3	3	1	1	1	1	2	2
2	2	2	2	1	1	1	1	2	2
1	1	3	3	2	2	3	3	2	2
1	1	3	3	2	2	3	3	2	2
1	1	3	3	2	2	3	3	2	2
2	2	3	3	2	2	3	3	2	2
1	1	2	2	2	2	3	3	2	2
1	1	3	3	2	2	3	3	2	2
1	1	2	2	2	2	3	3	2	2
1	1	3	3	2	2	3	3	2	2
2	2	2	2	2	2	3	3	2	2
1	1	3	3	2	2	3	3	2	2
1	1	3	3	2	2	3	3	2	2
1	1	3	3	2	2	3	3	2	2
1	1	3	3	2	2	3	3	2	2
2	2	2	2	1	1	1	1	2	2
1	1	3	3	2	2	3	3	2	2
1	1	2	2	2	2	3	3	2	2
1	1	2	2	1	1	1	1	2	2
1	1	3	3	2	2	3	3	1	1
1	1	2	2	2	2	3	3	2	2
1	1	2	2	2	2	3	3	2	2
1	1	2	2	2	2	3	3	2	2
1	1	3	3	2	2	3	3	2	2
2	2	2	2	1	1	1	1	1	1
2	2	3	3	1	1	1	1	2	2
2	2	3	3	1	1	1	1	1	1
1	1	2	2	2	2	3	3	2	2

For Review Only

1	1	2	2	2	2	3	3	2	2
1	1	3	3	2	2	3	3	2	2
1	1	2	2	2	2	3	3	2	2
1	1	2	2	2	2	3	3	2	2
1	1	3	3	2	2	3	3	2	2
1	1	2	2	2	2	3	3	2	2
1	1	3	3	2	2	3	3	2	2
1	1	3	3	2	2	3	3	2	2
1	1	2	2	2	2	3	3	2	2
2	2	2	2	2	2	3	3	2	2
1	1	3	3	2	2	3	3	2	2
1	1	2	2	2	2	3	3	2	2
1	1	2	2	2	2	3	3	2	2
1	1	3	3	2	2	3	3	2	2
1	1	3	3	2	2	3	3	2	2
1	1	2	2	2	2	3	3	2	2
1	1	2	2	2	2	3	3	2	2
2	2	2	2	2	2	3	3	2	2
1	1	3	3	2	2	3	3	1	1
1	1	3	3	2	2	3	3	2	2
2	2	2	2	2	2	1	1	2	2
1	1	3	3	2	2	3	3	2	2
1	1	2	2	2	2	3	3	2	2
2	2	2	2	1	1	1	1	1	1
1	1	2	2	2	2	3	3	2	2
1	1	2	2	2	2	3	3	2	2
1	1	3	3	1	1	1	1	2	2
1	1	3	3	2	2	3	3	2	2
1	1	3	3	2	2	3	3	2	2
1	1	3	3	2	2	3	3	2	2
1	1	3	3	2	2	3	3	1	1
2	2	2	2	2	2	1	1	2	2
1	1	3	3	2	2	3	3	2	2
1	1	2	2	2	2	3	3	2	2
1	1	3	3	2	2	3	3	1	1
1	1	3	3	2	2	3	3	2	2
1	1	3	3	2	2	3	3	2	2
1	1	3	3	2	2	3	3	2	2
2	2	2	2	2	2	3	3	2	2
2	2	3	3	2	2	3	3	2	2
2	2	2	2	2	2	3	3	2	2
1	1	3	3	2	2	3	3	2	2
1	1	2	2	2	2	3	3	2	2
2	2	2	2	2	2	3	3	2	2
1	1	2	2	2	2	3	3	2	2
1	1	2	2	2	2	1	1	2	2
1	1	2	2	2	2	3	3	2	2
1	1	2	2	2	2	3	3	2	2
1	1	2	2	2	2	3	3	2	2
1	1	2	2	2	2	3	3	2	2
1	1	2	2	2	2	3	3	2	2
2	2	3	3	1	1	1	1	2	2
1	1	3	3	2	2	3	3	2	2
1	1	2	2	2	2	3	3	2	2
2	2	2	2	2	2	3	3	2	2

For Review Only

1	1	2	2	2	2	3	3	2	2
1	1	2	2	2	2	3	3	2	2
1	1	2	2	2	2	3	3	2	2
2	2	2	2	2	2	3	3	2	2
1	1	2	2	2	2	3	3	2	2
1	1	3	3	2	2	3	3	2	2
1	1	2	2	1	1	3	3	2	2
1	1	2	2	2	2	3	3	2	2
2	2	3	3	2	2	3	3	2	2
1	1	3	3	2	2	3	3	2	2
1	1	3	3	2	2	3	3	2	2
1	1	3	3	2	2	3	3	2	2
1	1	2	2	2	2	3	3	2	2
1	1	2	2	2	2	3	3	2	2
1	1	3	3	2	2	3	3	1	1
2	2	3	3	1	1	1	1	2	2
2	2	2	2	2	2	3	3	2	2
1	1	3	3	1	1	3	3	2	2
1	1	2	2	2	2	3	3	2	2
1	1	3	3	2	2	3	3	2	2
1	1	2	2	2	2	3	3	2	2
1	1	3	3	1	1	1	1	2	2
1	1	2	2	2	2	3	3	2	2
1	1	3	3	2	2	3	3	2	2
1	1	3	3	2	2	3	3	2	2
1	1	3	3	2	2	3	3	1	1
1	1	2	2	2	2	3	3	2	2
1	1	2	2	2	2	3	3	2	2
1	1	3	3	2	2	3	3	2	2
1	1	3	3	2	2	3	3	2	2
1	1	2	2	2	2	3	3	2	2
1	1	2	2	2	2	3	3	2	2
1	1	3	3	2	2	3	3	2	2
1	1	3	3	2	2	3	3	1	1
1	1	2	2	2	2	3	3	2	2
1	1	3	3	2	2	3	3	2	2
1	1	3	3	2	2	3	3	2	2
1	1	2	2	2	2	3	3	1	1
1	1	2	2	2	2	3	3	2	2
2	2	2	2	1	1	1	1	2	2
1	1	3	3	2	2	3	3	2	2
1	1	2	2	2	2	3	3	2	2
2	2	3	3	1	1	1	1	2	2
1	1	2	2	2	2	3	3	2	2
1	1	3	3	2	2	3	3	2	2
1	1	3	3	2	2	3	3	2	2
2	2	3	3	1	1	1	1	2	2
1	1	2	2	1	1	1	1	2	2
1	1	3	3	2	2	3	3	2	2
1	1	3	3	2	2	3	3	2	2
1	1	2	2	2	2	3	3	2	2
1	1	3	3	2	2	3	3	2	2
1	1	2	2	1	1	1	1	2	2
1	1	3	3	2	2	3	3	2	2
2	2	2	2	1	1	1	1	1	1

For Review Only

1	1	3	3	2	2	3	3	2	2
2	2	2	2	1	1	1	1	2	2
1	1	2	2	2	2	3	3	2	2
1	1	2	2	2	2	3	3	2	2
1	1	3	3	2	2	3	3	2	2
1	1	3	3	2	2	3	3	2	2
2	2	2	2	1	1	1	1	1	1
2	2	3	3	1	1	1	1	2	2
2	2	2	2	2	2	3	3	2	2
1	1	3	3	2	2	3	3	2	2
1	1	2	2	2	2	3	3	2	2
1	1	2	2	2	2	3	3	2	2
2	2	3	3	1	1	1	1	1	1
1	1	2	2	2	2	3	3	2	2
1	1	2	2	2	2	3	3	2	2
2	2	3	3	1	1	1	1	1	1
2	2	2	2	1	1	1	1	1	1
1	1	3	3	1	1	3	3	2	2
1	1	2	2	2	2	3	3	2	2
1	1	3	3	2	2	3	3	2	2
2	2	3	3	1	1	1	1	2	2
1	1	2	2	2	2	3	3	2	2
1	1	3	3	2	2	3	3	2	2
1	1	2	2	2	2	3	3	2	2
2	2	3	3	1	1	1	1	2	2
1	1	3	3	2	2	3	3	2	2
2	2	3	3	1	1	1	1	1	1
2	2	3	3	2	2	3	3	2	2
1	1	3	3	2	2	3	3	2	2
1	1	2	2	2	2	3	3	2	2
2	2	2	2	2	2	3	3	2	2
2	2	2	2	1	1	1	1	2	2
1	1	3	3	2	2	3	3	2	2
1	1	2	2	2	2	3	3	2	2
1	1	2	2	2	2	3	3	2	2
2	2	3	3	2	2	3	3	2	2
1	1	3	3	2	2	3	3	2	2
1	1	3	3	2	2	3	3	2	2
1	1	3	3	2	2	3	3	2	2
2	2	3	3	1	1	1	1	2	2
1	1	3	3	2	2	3	3	2	2
1	1	2	2	2	2	3	3	2	2
1	1	2	2	2	2	3	3	2	2
1	1	2	2	2	2	3	3	2	2
1	1	3	3	2	2	3	3	2	2
2	2	3	3	1	1	1	1	1	1
1	1	2	2	2	2	3	3	2	2
2	2	2	2	1	1	1	1	2	2
1	1	3	3	2	2	3	3	2	2
1	1	3	3	1	1	3	3	2	2
1	1	3	3	2	2	3	3	2	2
2	2	3	3	1	1	1	1	2	2
1	1	3	3	2	2	3	3	2	2
1	1	2	2	2	2	3	3	2	2
1	1	2	2	2	2	3	3	2	2

For Review Only

1	1	3	3	2	2	3	3	2	2
1	1	3	3	1	1	1	1	2	2

For Review Only

SNP3113	SNP3113	SNP3114	SNP3114	SNP3115	SNP3115	SNP3116	SNP3116	SNP3117	SNP3117
2	2	1	1	1	1	2	2	4	4
2	2	1	1	4	4	2	2	3	3
2	2	1	1	1	1	2	2	4	4
2	2	1	1	1	1	1	1	3	3
2	2	1	1	1	1	2	2	4	4
2	2	1	1	1	1	1	1	3	3
2	2	1	1	1	1	1	1	4	4
2	2	1	1	1	1	2	2	3	3
2	2	1	1	4	4	2	2	3	3
2	2	1	1	4	4	2	2	3	3
2	2	1	1	4	4	2	2	3	3
2	2	1	1	1	1	2	2	4	4
2	2	1	1	1	1	2	2	3	3
2	2	4	4	1	1	2	2	4	4
2	2	1	1	1	1	2	2	4	4
2	2	1	1	1	1	1	1	4	4
2	2	1	1	1	1	1	1	4	4
2	2	1	1	1	1	2	2	4	4
2	2	1	1	1	1	1	1	3	3
2	2	1	1	4	4	2	2	3	3
2	2	1	1	1	1	2	2	3	3
2	2	1	1	1	1	2	2	3	3
3	3	4	4	1	1	2	2	3	3
2	2	1	1	1	1	2	2	3	3
2	2	1	1	1	1	1	1	4	4
2	2	1	1	1	1	2	2	3	3
2	2	1	1	1	1	1	1	3	3
2	2	1	1	1	1	2	2	3	3
2	2	1	1	4	4	2	2	3	3
2	2	1	1	4	4	2	2	3	3
2	2	1	1	1	1	2	2	4	4
2	2	1	1	1	1	2	2	3	3
2	2	1	1	1	1	1	1	4	4
3	3	1	1	1	1	2	2	4	4
3	3	1	1	1	1	2	2	3	3
2	2	1	1	1	1	1	1	3	3
2	2	1	1	1	1	2	2	4	4
2	2	1	1	1	1	1	1	4	4
2	2	1	1	4	4	2	2	3	3
2	2	1	1	1	1	1	1	4	4
2	2	1	1	1	1	2	2	3	3
2	2	1	1	4	4	2	2	3	3
2	2	1	1	1	1	1	1	4	4
2	2	1	1	1	1	1	1	4	4
2	2	1	1	1	1	1	1	4	4
2	2	1	1	1	1	2	2	4	4
2	2	1	1	1	1	1	1	3	3
2	2	1	1	1	1	1	1	4	4
2	2	1	1	1	1	1	1	4	4
2	2	1	1	1	1	1	1	4	4
2	2	1	1	1	1	2	2	3	3
2	2	1	1	1	1	1	1	4	4

For Review Only

2	2	1	1	1	1	1	1	4	4
2	2	1	1	1	1	1	1	4	4
2	2	1	1	1	1	2	2	3	3
2	2	1	1	1	1	1	1	4	4
2	2	1	1	1	1	1	1	3	3
2	2	1	1	1	1	1	1	3	3
2	2	1	1	1	1	1	1	3	3
2	2	1	1	4	4	2	2	4	4
2	2	1	1	4	4	2	2	3	3
2	2	1	1	1	1	2	2	3	3
2	2	1	1	1	1	2	2	4	4
2	2	1	1	1	1	1	1	3	3
2	2	1	1	1	1	1	1	4	4
2	2	1	1	4	4	2	2	3	3
2	2	1	1	1	1	1	1	3	3
2	2	1	1	1	1	1	1	4	4
2	2	1	1	1	1	1	1	4	4
2	2	1	1	1	1	2	2	3	3
2	2	1	1	1	1	2	2	4	4
2	2	1	1	1	1	2	2	4	4
2	2	1	1	1	1	1	1	3	3
2	2	1	1	1	1	2	2	3	3
2	2	1	1	1	1	2	2	3	3
2	2	1	1	1	1	2	2	4	4
2	2	1	1	1	1	1	1	4	4
2	2	1	1	1	1	1	1	4	4
2	2	1	1	1	1	2	2	3	3
2	2	1	1	4	4	2	2	3	3
2	2	1	1	4	4	2	2	3	3
2	2	1	1	1	1	2	2	4	4
2	2	1	1	1	1	1	1	4	4
2	2	1	1	1	1	1	1	4	4
2	2	1	1	4	4	2	2	3	3
2	2	1	1	1	1	1	1	4	4
2	2	1	1	1	1	2	2	4	4
2	2	1	1	1	1	1	1	4	4
2	2	1	1	1	1	2	2	3	3
2	2	1	1	1	1	2	2	3	3
2	2	1	1	1	1	2	2	3	3
2	2	1	1	1	1	2	2	4	4
2	2	1	1	1	1	1	1	4	4
2	2	1	1	1	1	2	2	4	4
2	2	1	1	1	1	2	2	3	3
2	2	1	1	1	1	2	2	4	4
2	2	1	1	1	1	2	2	4	4
2	2	1	1	1	1	2	2	3	3
2	2	1	1	1	1	2	2	4	4
2	2	1	1	1	1	1	1	3	3
2	2	1	1	1	1	1	1	4	4
2	2	1	1	1	1	2	2	3	3
2	2	1	1	1	1	2	2	3	3
2	2	4	4	4	4	2	2	3	3
2	2	1	1	4	4	2	2	3	3
2	2	1	1	1	1	2	2	4	4
2	2	1	1	1	1	1	1	3	3

For Review Only

2	2	1	1	1	1	1	1	3	3
2	2	1	1	1	1	2	2	4	4
2	2	1	1	1	1	1	1	4	4
2	2	4	4	1	1	2	2	4	4
2	2	1	1	1	1	2	2	3	3
2	2	1	1	1	1	2	2	4	4
2	2	1	1	1	1	1	1	4	4
2	2	1	1	1	1	2	2	3	3
2	2	1	1	1	1	1	1	4	4
2	2	1	1	1	1	2	2	3	3
2	2	1	1	1	1	2	2	4	4
2	2	1	1	1	1	2	2	3	3
2	2	1	1	1	1	1	1	3	3
2	2	1	1	1	1	1	1	3	3
2	2	1	1	1	1	1	1	4	4
2	2	1	1	1	1	2	2	3	3
2	2	1	1	1	1	1	1	3	3
2	2	1	1	1	1	2	2	3	3
3	3	1	1	1	1	2	2	4	4
2	2	1	1	1	1	1	1	3	3
2	2	1	1	1	1	2	2	3	3
2	2	1	1	1	1	2	2	4	4
2	2	1	1	1	1	2	2	3	3
3	3	4	4	4	4	2	2	3	3
2	2	1	1	1	1	2	2	3	3
2	2	1	1	1	1	2	2	4	4
2	2	1	1	1	1	2	2	4	4
2	2	1	1	1	1	2	2	4	4
2	2	1	1	1	1	1	1	3	3
2	2	1	1	1	1	1	1	4	4
3	3	1	1	1	1	2	2	4	4
2	2	1	1	1	1	2	2	3	3
2	2	1	1	1	1	1	1	4	4
2	2	1	1	1	1	2	2	3	3
3	3	1	1	1	1	2	2	4	4
2	2	1	1	1	1	2	2	3	3
2	2	4	4	1	1	2	2	4	4
2	2	1	1	1	1	1	1	3	3
2	2	1	1	1	1	2	2	4	4
2	2	1	1	4	4	2	2	3	3
2	2	1	1	1	1	2	2	3	3
2	2	1	1	1	1	2	2	4	4
2	2	1	1	1	1	2	2	3	3
2	2	1	1	1	1	2	2	3	3
2	2	1	1	1	1	1	1	4	4
2	2	1	1	1	1	2	2	3	3
2	2	1	1	1	1	1	1	3	3
2	2	1	1	1	1	1	1	3	3
3	3	1	1	1	1	2	2	3	3
2	2	1	1	1	1	1	1	3	3
2	2	1	1	1	1	1	1	3	3
2	2	1	1	4	4	2	2	3	3
2	2	1	1	1	1	2	2	3	3
2	2	1	1	1	1	1	1	3	3
2	2	1	1	1	1	2	2	3	3

For Review Only

2	2	1	1	1	1	2	2	4	4
2	2	1	1	1	1	1	1	3	3
2	2	1	1	1	1	1	1	4	4
2	2	1	1	1	1	2	2	3	3
2	2	1	1	1	1	1	1	4	4
2	2	1	1	1	1	1	1	4	4
2	2	1	1	1	1	1	1	3	3
2	2	1	1	1	1	2	2	3	3
2	2	1	1	1	1	2	2	3	3
2	2	1	1	1	1	1	1	4	4
2	2	1	1	1	1	1	1	3	3
2	2	1	1	1	1	1	1	4	4
2	2	1	1	1	1	2	2	4	4
2	2	1	1	1	1	2	2	3	3
3	3	1	1	1	1	2	2	4	4
2	2	1	1	4	4	2	2	3	3
2	2	1	1	1	1	2	2	3	3
2	2	1	1	1	1	2	2	4	4
2	2	1	1	1	1	1	1	3	3
2	2	1	1	1	1	1	1	4	4
2	2	1	1	1	1	2	2	3	3
2	2	1	1	1	1	2	2	3	3
2	2	1	1	1	1	2	2	3	3
2	2	1	1	1	1	1	1	3	3
2	2	1	1	1	1	1	1	4	4
3	3	1	1	1	1	2	2	4	4
2	2	1	1	1	1	2	2	4	4
2	2	1	1	1	1	2	2	3	3
2	2	1	1	1	1	1	1	3	3
2	2	1	1	1	1	1	1	3	3
2	2	1	1	1	1	2	2	4	4
2	2	1	1	1	1	1	1	3	3
2	2	1	1	1	1	2	2	4	4
3	3	1	1	1	1	2	2	4	4
2	2	1	1	1	1	2	2	4	4
2	2	1	1	1	1	1	1	3	3
2	2	1	1	1	1	1	1	3	3
3	3	1	1	1	1	2	2	4	4
2	2	1	1	1	1	1	1	3	3
2	2	1	1	1	1	2	2	3	3
2	2	1	1	1	1	2	2	4	4
2	2	1	1	1	1	1	1	3	3
3	3	1	1	4	4	2	2	3	3
2	2	1	1	1	1	2	2	3	3
2	2	1	1	1	1	2	2	3	3
2	2	1	1	1	1	2	2	4	4
2	2	1	1	4	4	2	2	3	3
2	2	1	1	1	1	2	2	3	3
2	2	4	4	4	4	2	2	4	4
2	2	1	1	1	1	1	1	4	4
2	2	1	1	1	1	2	2	3	3
2	2	1	1	1	1	2	2	4	4
2	2	1	1	1	1	2	2	3	3
2	2	1	1	1	1	2	2	4	4
3	3	4	4	1	1	2	2	3	3

For Review Only

2	2	1	1	1	1	2	2	4	4
2	2	1	1	1	1	2	2	4	4
2	2	1	1	1	1	2	2	4	4
2	2	1	1	1	1	2	2	3	3
2	2	1	1	1	1	2	2	4	4
2	2	1	1	1	1	2	2	4	4
3	3	1	1	1	1	2	2	3	3
2	2	1	1	1	1	2	2	3	3
2	2	1	1	1	1	2	2	4	4
2	2	1	1	4	4	2	2	3	3
2	2	1	1	1	1	2	2	3	3
2	2	1	1	1	1	2	2	4	4
3	3	4	4	1	1	2	2	3	3
2	2	1	1	1	1	1	1	3	3
2	2	1	1	1	1	1	1	3	3
3	3	4	4	1	1	2	2	3	3
3	3	4	4	1	1	2	2	3	3
2	2	4	4	4	4	2	2	3	3
2	2	1	1	1	1	2	2	4	4
2	2	1	1	1	1	1	1	4	4
2	2	1	1	4	4	2	2	3	3
2	2	1	1	1	1	2	2	4	4
2	2	1	1	1	1	1	1	4	4
2	2	1	1	1	1	2	2	3	3
2	2	1	1	4	4	2	2	4	4
2	2	1	1	1	1	2	2	3	3
3	3	4	4	4	4	2	2	3	3
2	2	1	1	1	1	1	1	4	4
2	2	1	1	1	1	2	2	4	4
2	2	1	1	1	1	1	1	3	3
2	2	1	1	4	4	2	2	3	3
2	2	1	1	4	4	2	2	3	3
2	2	1	1	1	1	1	1	3	3
2	2	1	1	1	1	1	1	3	3
2	2	1	1	1	1	2	2	3	3
2	2	1	1	4	4	2	2	3	3
2	2	1	1	1	1	2	2	3	3
2	2	1	1	1	1	1	1	3	3
2	2	1	1	1	1	1	1	3	3
2	2	1	1	4	4	2	2	3	3
2	2	1	1	1	1	1	1	4	4
2	2	1	1	1	1	2	2	4	4
2	2	1	1	1	1	1	1	4	4
2	2	1	1	1	1	1	1	4	4
2	2	1	1	1	1	1	1	4	4
3	3	4	4	1	1	2	2	4	4
2	2	1	1	1	1	1	1	3	3
2	2	1	1	1	1	2	2	4	4
2	2	1	1	1	1	2	2	3	3
2	2	1	1	1	1	2	2	3	3
2	2	1	1	1	1	1	1	4	4
2	2	1	1	4	4	2	2	3	3
2	2	1	1	1	1	2	2	4	4
2	2	1	1	1	1	1	1	3	3
2	2	1	1	1	1	1	1	4	4

For Review Only

2	2	1	1	1	1	2	2	4	4
2	2	1	1	1	1	2	2	4	4

For Review Only

SNP3118	SNP3118	SNP3119	SNP3119	SNP312	SNP312	SNP3120	SNP3120	SNP3121	SNP3121
1	1	3	3	2	2	1	1	2	2
2	2	4	4	2	2	1	1	1	1
1	1	3	3	2	2	2	2	2	2
2	2	3	3	2	2	2	2	2	2
1	1	3	3	2	2	1	1	2	2
2	2	3	3	2	2	2	2	2	2
1	1	3	3	2	2	2	2	2	2
2	2	4	4	2	2	1	1	1	1
1	1	3	3	2	2	2	2	2	2
2	2	4	4	2	2	1	1	2	2
2	2	4	4	2	2	1	1	1	1
1	1	3	3	2	2	2	2	2	2
1	1	3	3	2	2	2	2	2	2
1	1	3	3	2	2	1	1	2	2
1	1	3	3	2	2	2	2	2	2
1	1	3	3	4	4	2	2	2	2
1	1	3	3	2	2	2	2	2	2
1	1	3	3	2	2	1	1	2	2
2	2	3	3	4	4	2	2	2	2
2	2	4	4	4	4	1	1	1	1
2	2	4	4	2	2	1	1	1	1
2	2	4	4	2	2	1	1	1	1
2	2	4	4	4	4	1	1	2	2
2	2	3	3	2	2	1	1	1	1
1	1	3	3	2	2	2	2	2	2
2	2	3	3	4	4	2	2	2	2
2	2	3	3	4	4	2	2	2	2
1	1	3	3	2	2	2	2	2	2
2	2	3	3	2	2	1	1	1	1
2	2	4	4	2	2	1	1	1	1
1	1	3	3	4	4	2	2	2	2
2	2	3	3	2	2	2	2	2	2
1	1	3	3	2	2	2	2	2	2
1	1	3	3	2	2	2	2	2	2
1	1	3	3	4	4	2	2	2	2
1	1	3	3	4	4	2	2	2	2
1	1	3	3	2	2	2	2	2	2
1	1	3	3	2	2	2	2	2	2
2	2	4	4	2	2	1	1	1	1
1	1	3	3	2	2	2	2	1	1
2	2	3	3	2	2	1	1	1	1
2	2	3	3	2	2	1	1	1	1
1	1	3	3	4	4	2	2	2	2
1	1	3	3	2	2	2	2	2	2
1	1	3	3	2	2	2	2	2	2
1	1	3	3	4	4	2	2	2	2
1	1	3	3	4	4	2	2	2	2
1	1	3	3	4	4	2	2	2	2
1	1	3	3	2	2	2	2	2	2
2	2	3	3	4	4	2	2	2	2
2	2	4	4	4	4	1	1	2	2
1	1	3	3	4	4	2	2	2	2

For Review Only

1	1	3	3	2	2	2	2	2	2
1	1	3	3	2	2	2	2	2	2
1	1	3	3	4	4	2	2	2	2
1	1	3	3	2	2	2	2	2	2
2	2	3	3	4	4	2	2	2	2
2	2	3	3	4	4	2	2	2	2
2	2	3	3	4	4	2	2	2	2
1	1	3	3	2	2	1	1	1	1
2	2	4	4	2	2	1	1	1	1
1	1	3	3	4	4	2	2	2	2
1	1	3	3	4	4	2	2	2	2
1	1	3	3	4	4	2	2	2	2
1	1	3	3	2	2	2	2	2	2
2	2	3	3	2	2	1	1	1	1
2	2	3	3	2	2	2	2	2	2
1	1	3	3	2	2	2	2	2	2
1	1	3	3	2	2	2	2	2	2
2	2	3	3	4	4	2	2	2	2
1	1	3	3	4	4	2	2	2	2
1	1	3	3	2	2	2	2	2	2
1	1	3	3	4	4	2	2	2	2
2	2	4	4	2	2	2	2	2	2
2	2	3	3	2	2	1	1	2	2
1	1	3	3	2	2	2	2	2	2
1	1	3	3	2	2	2	2	2	2
1	1	3	3	4	4	2	2	2	2
2	2	4	4	4	4	1	1	2	2
2	2	4	4	2	2	1	1	2	2
2	2	4	4	4	4	1	1	1	1
1	1	3	3	4	4	2	2	2	2
1	1	3	3	2	2	2	2	2	2
1	1	3	3	2	2	2	2	2	2
2	2	4	4	2	2	1	1	2	2
1	1	3	3	2	2	2	2	2	2
1	1	3	3	2	2	1	1	2	2
1	1	3	3	2	2	2	2	2	2
2	2	4	4	2	2	1	1	2	2
2	2	4	4	2	2	2	2	2	2
2	2	4	4	2	2	1	1	2	2
1	1	3	3	2	2	2	2	2	2
1	1	3	3	2	2	2	2	2	2
1	1	3	3	2	2	2	2	2	2
2	2	4	4	2	2	1	1	2	2
1	1	3	3	4	4	2	2	2	2
1	1	3	3	2	2	2	2	2	2
2	2	3	3	4	4	2	2	2	2
1	1	3	3	2	2	1	1	2	2
2	2	3	3	4	4	2	2	2	2
1	1	3	3	2	2	2	2	2	2
2	2	3	3	4	4	2	2	2	2
1	1	3	3	2	2	2	2	2	2
2	2	4	4	2	2	1	1	1	1
2	2	4	4	2	2	1	1	1	1
1	1	3	3	2	2	2	2	2	2
2	2	3	3	4	4	2	2	2	2

For Review Only

2	2	3	3	2	2	2	2	2	2
1	1	3	3	2	2	2	2	2	2
1	1	3	3	4	4	2	2	2	2
1	1	3	3	2	2	2	2	2	2
2	2	3	3	4	4	2	2	2	2
1	1	3	3	2	2	2	2	2	2
1	1	3	3	4	4	2	2	2	2
1	1	3	3	2	2	2	2	2	2
1	1	3	3	2	2	2	2	2	2
1	1	3	3	4	4	2	2	2	2
1	1	3	3	2	2	1	1	2	2
1	1	3	3	4	4	2	2	2	2
2	2	3	3	4	4	2	2	2	2
1	1	3	3	4	4	2	2	2	2
1	1	3	3	4	4	1	1	2	2
1	1	3	3	4	4	2	2	2	2
2	2	3	3	4	4	2	2	2	2
1	1	3	3	4	4	2	2	2	2
1	1	3	3	2	2	1	1	2	2
2	2	3	3	2	2	2	2	2	2
1	1	3	3	4	4	2	2	2	2
1	1	3	3	4	4	2	2	2	2
1	1	3	3	2	2	2	2	2	2
2	2	4	4	2	2	1	1	2	2
2	2	3	3	4	4	2	2	2	2
1	1	3	3	4	4	2	2	2	2
1	1	3	3	4	4	2	2	2	2
1	1	3	3	2	2	1	1	2	2
1	1	3	3	2	2	2	2	2	2
1	1	3	3	4	4	2	2	2	2
1	1	3	3	2	2	1	1	2	2
1	1	3	3	4	4	2	2	2	2
1	1	3	3	2	2	2	2	2	2
1	1	3	3	4	4	2	2	2	2
1	1	3	3	2	2	2	2	2	2
1	1	3	3	2	2	1	1	2	2
1	1	3	3	2	2	1	1	1	1
2	2	3	3	4	4	2	2	2	2
1	1	3	3	2	2	2	2	2	2
2	2	4	4	2	2	1	1	1	1
1	1	3	3	4	4	2	2	2	2
1	1	3	3	2	2	1	1	2	2
2	2	3	3	4	4	2	2	2	2
1	1	3	3	2	2	1	1	2	2
1	1	3	3	4	4	2	2	2	2
1	1	3	3	4	4	2	2	2	2
2	2	3	3	4	4	2	2	2	2
2	2	3	3	2	2	2	2	2	2
2	2	3	3	2	2	1	1	2	2
1	1	3	3	4	4	2	2	2	2
2	2	3	3	4	4	2	2	2	2
2	2	4	4	2	2	1	1	1	1
2	2	3	3	2	2	2	2	2	2
1	1	3	3	4	4	2	2	2	2
1	1	3	3	2	2	2	2	2	2

For Review Only

1	1	3	3	2	2	2	2	2	2
2	2	3	3	2	2	2	2	2	2
1	1	3	3	2	2	2	2	2	2
1	1	3	3	4	4	2	2	2	2
1	1	3	3	4	4	2	2	2	2
1	1	3	3	2	2	2	2	2	2
1	1	3	3	2	2	1	1	2	2
1	1	3	3	4	4	2	2	2	2
2	2	3	3	2	2	2	2	2	2
1	1	3	3	2	2	2	2	2	2
2	2	3	3	4	4	2	2	2	2
1	1	3	3	2	2	2	2	2	2
1	1	3	3	2	2	2	2	2	2
1	1	3	3	4	4	2	2	2	2
1	1	3	3	2	2	1	1	2	2
2	2	4	4	2	2	1	1	1	1
2	2	3	3	2	2	2	2	2	2
2	2	3	3	2	2	2	2	2	2
2	2	3	3	2	2	2	2	2	2
1	1	3	3	4	4	2	2	2	2
1	1	3	3	2	2	2	2	2	2
2	2	4	4	2	2	1	1	2	2
2	2	4	4	2	2	1	1	2	2
1	1	3	3	4	4	2	2	2	2
1	1	3	3	2	2	2	2	2	2
1	1	3	3	2	2	2	2	2	2
1	1	3	3	4	4	2	2	2	2
1	1	3	3	4	4	2	2	2	2
2	2	3	3	4	4	2	2	2	2
2	2	3	3	2	2	2	2	2	2
1	1	3	3	2	2	1	1	1	1
2	2	3	3	4	4	2	2	2	2
1	1	3	3	4	4	2	2	2	2
1	1	3	3	2	2	1	1	2	2
1	1	3	3	2	2	2	2	2	2
1	1	3	3	4	4	2	2	2	2
1	1	3	3	2	2	2	2	2	2
2	2	3	3	2	2	2	2	1	1
2	2	3	3	2	2	2	2	2	2
2	2	4	4	2	2	1	1	2	2
1	1	3	3	2	2	2	2	2	2
2	2	3	3	4	4	2	2	2	2
2	2	4	4	2	2	1	1	2	2
2	2	3	3	2	2	2	2	2	2
1	1	3	3	2	2	2	2	2	2
1	1	3	3	4	4	2	2	2	2
2	2	4	4	2	2	1	1	1	1
2	2	4	4	2	2	1	1	2	2
1	1	3	3	2	2	1	1	1	1
1	1	3	3	4	4	2	2	2	2
2	2	4	4	2	2	1	1	2	2
1	1	3	3	2	2	2	2	2	2
2	2	4	4	2	2	1	1	2	2
1	1	3	3	4	4	2	2	2	2
2	2	4	4	2	2	1	1	1	1

For Review Only

1	1	3	3	2	2	1	1	2	2
1	1	3	3	2	2	2	2	2	2
1	1	3	3	2	2	2	2	2	2
2	2	3	3	2	2	2	2	2	2
1	1	3	3	2	2	2	2	2	2
1	1	3	3	2	2	2	2	2	2
2	2	4	4	2	2	1	1	1	1
2	2	4	4	4	4	1	1	1	1
1	1	3	3	2	2	2	2	2	2
2	2	4	4	2	2	1	1	2	2
1	1	3	3	4	4	2	2	2	2
1	1	3	3	4	4	1	1	1	1
2	2	4	4	2	2	1	1	2	2
2	2	3	3	2	2	2	2	2	2
2	2	3	3	2	2	2	2	2	2
2	2	4	4	2	2	1	1	2	2
2	2	4	4	2	2	1	1	2	2
2	2	4	4	2	2	1	1	1	1
1	1	3	3	2	2	1	1	2	2
1	1	3	3	2	2	2	2	2	2
2	2	4	4	2	2	1	1	1	1
1	1	3	3	4	4	2	2	2	2
1	1	3	3	2	2	2	2	2	2
1	1	3	3	2	2	2	2	2	2
1	1	3	3	2	2	1	1	1	1
1	1	3	3	2	2	2	2	2	2
2	2	4	4	2	2	1	1	2	2
1	1	3	3	4	4	2	2	2	2
1	1	3	3	2	2	2	2	2	2
1	1	3	3	2	2	2	2	2	2
2	2	4	4	2	2	1	1	1	1
2	2	4	4	2	2	1	1	1	1
2	2	3	3	2	2	2	2	2	2
2	2	3	3	4	4	2	2	2	2
2	2	4	4	2	2	1	1	2	2
2	2	4	4	2	2	1	1	2	2
2	2	3	3	2	2	2	2	2	2
1	1	3	3	4	4	2	2	2	2
1	1	3	3	4	4	2	2	2	2
2	2	4	4	2	2	1	1	1	1
1	1	3	3	2	2	2	2	2	2
1	1	3	3	2	2	2	2	2	2
1	1	3	3	4	4	2	2	2	2
1	1	3	3	2	2	2	2	2	2
1	1	3	3	2	2	2	2	2	2
1	1	3	3	2	2	2	2	2	2
2	2	3	3	2	2	2	2	2	2
1	1	3	3	4	4	2	2	2	2
1	1	3	3	2	2	2	2	2	2
2	2	4	4	2	2	1	1	2	2
1	1	3	3	4	4	2	2	2	2
2	2	4	4	2	2	1	1	1	1
1	1	3	3	2	2	2	2	2	2
2	2	3	3	2	2	2	2	2	2
1	1	3	3	2	2	2	2	2	2

For Review Only

1	1	3	3	2	2	1	1	2	2
1	1	3	3	2	2	2	2	2	2

For Review Only

SNP3122	SNP3122	SNP3123	SNP3123	SNP3124	SNP3124	SNP3125	SNP3125	SNP3126	SNP3126
2	2	3	3	4	4	2	2	4	4
4	4	2	2	2	2	4	4	2	2
2	2	3	3	4	4	2	2	4	4
2	2	3	3	4	4	2	2	4	4
2	2	3	3	4	4	2	2	4	4
2	2	3	3	4	4	2	2	4	4
2	2	3	3	4	4	2	2	4	4
4	4	2	2	2	2	2	2	4	4
2	2	3	3	4	4	2	2	4	4
2	2	3	3	4	4	2	2	4	4
4	4	2	2	2	2	2	2	4	4
2	2	3	3	4	4	2	2	4	4
2	2	2	2	2	2	2	2	4	4
4	4	2	2	2	2	4	4	2	2
2	2	3	3	4	4	2	2	4	4
2	2	3	3	4	4	2	2	4	4
2	2	3	3	4	4	2	2	4	4
2	2	3	3	4	4	2	2	4	4
2	2	3	3	4	4	2	2	4	4
2	2	3	3	4	4	2	2	4	4
4	4	2	2	2	2	4	4	2	2
4	4	2	2	2	2	4	4	2	2
4	4	2	2	2	2	2	2	4	4
4	4	2	2	2	2	2	2	4	4
2	2	3	3	4	4	2	2	4	4
2	2	2	2	2	2	2	2	4	4
2	2	3	3	4	4	2	2	4	4
2	2	3	3	4	4	2	2	4	4
4	4	3	3	4	4	2	2	4	4
4	4	2	2	2	2	4	4	2	2
2	2	3	3	4	4	2	2	4	4
2	2	3	3	4	4	2	2	4	4
2	2	3	3	4	4	2	2	4	4
2	2	2	2	2	2	4	4	2	2
2	2	2	2	2	2	4	4	4	4
2	2	3	3	4	4	2	2	4	4
2	2	3	3	4	4	2	2	4	4
2	2	3	3	4	4	2	2	4	4
4	4	2	2	2	2	2	2	4	4
2	2	2	2	2	2	2	2	4	4
4	4	2	2	2	2	4	4	2	2
4	4	3	3	4	4	2	2	4	4
2	2	3	3	4	4	2	2	4	4
2	2	3	3	4	4	2	2	4	4
2	2	3	3	4	4	2	2	4	4
2	2	3	3	4	4	2	2	4	4
2	2	3	3	4	4	2	2	4	4
2	2	3	3	4	4	2	2	4	4
2	2	3	3	4	4	2	2	4	4
2	2	3	3	4	4	2	2	4	4
2	2	3	3	4	4	2	2	4	4
2	2	3	3	4	4	2	2	4	4

For Review Only

2	2	3	3	4	4	2	2	4	4
2	2	3	3	4	4	2	2	4	4
2	2	3	3	4	4	2	2	4	4
2	2	3	3	4	4	2	2	4	4
2	2	3	3	4	4	2	2	4	4
2	2	3	3	4	4	2	2	4	4
2	2	3	3	4	4	2	2	4	4
4	4	2	2	2	2	4	4	2	2
4	4	2	2	2	2	4	4	2	2
2	2	2	2	2	2	2	2	4	4
2	2	3	3	4	4	2	2	4	4
2	2	3	3	4	4	2	2	4	4
2	2	3	3	4	4	2	2	4	4
4	4	3	3	4	4	2	2	4	4
2	2	3	3	2	2	2	2	4	4
2	2	2	2	2	2	2	2	4	4
2	2	3	3	4	4	2	2	4	4
2	2	3	3	4	4	2	2	4	4
2	2	3	3	4	4	2	2	4	4
2	2	3	3	4	4	2	2	4	4
2	2	3	3	4	4	2	2	4	4
2	2	3	3	2	2	2	2	4	4
4	4	3	3	2	2	2	2	4	4
2	2	3	3	4	4	2	2	4	4
2	2	3	3	4	4	2	2	4	4
2	2	3	3	4	4	2	2	4	4
2	2	3	3	4	4	2	2	4	4
4	4	2	2	2	2	2	2	4	4
4	4	2	2	2	2	2	2	4	4
2	2	3	3	4	4	2	2	4	4
2	2	3	3	4	4	2	2	4	4
2	2	3	3	4	4	2	2	4	4
4	4	2	2	2	2	2	2	4	4
2	2	3	3	4	4	2	2	4	4
2	2	3	3	4	4	4	4	2	2
2	2	3	3	4	4	2	2	4	4
2	2	3	3	4	4	2	2	2	2
2	2	2	2	4	4	4	4	2	2
4	4	2	2	2	2	2	2	4	4
2	2	3	3	4	4	2	2	4	4
2	2	3	3	4	4	2	2	4	4
2	2	3	3	4	4	2	2	4	4
2	2	3	3	4	4	2	2	4	4
2	2	3	3	4	4	2	2	4	4
2	2	3	3	4	4	2	2	4	4
2	2	3	3	4	4	2	2	4	4
4	4	2	2	2	2	2	2	4	4
2	2	3	3	4	4	2	2	4	4
2	2	3	3	4	4	2	2	4	4
2	2	2	2	2	2	2	2	4	4
2	2	3	3	4	4	2	2	4	4
4	4	2	2	2	2	2	2	4	4
4	4	2	2	2	2	4	4	2	2
2	2	2	2	2	2	2	2	4	4
2	2	3	3	4	4	2	2	4	4

For Review Only

2	2	3	3	4	4	2	2	4	4
2	2	3	3	4	4	2	2	4	4
2	2	3	3	4	4	2	2	4	4
2	2	3	3	4	4	4	4	2	2
2	2	3	3	4	4	2	2	4	4
2	2	3	3	4	4	2	2	4	4
2	2	3	3	4	4	2	2	4	4
2	2	3	3	2	2	2	2	4	4
2	2	3	3	4	4	2	2	4	4
2	2	2	2	2	2	2	2	4	4
2	2	3	3	4	4	2	2	4	4
2	2	3	3	4	4	2	2	4	4
2	2	3	3	4	4	2	2	4	4
2	2	3	3	4	4	2	2	4	4
2	2	3	3	4	4	4	4	4	4
2	2	2	2	2	2	2	2	4	4
2	2	3	3	4	4	2	2	4	4
2	2	3	3	4	4	2	2	4	4
2	2	3	3	4	4	4	4	2	2
2	2	3	3	4	4	2	2	4	4
2	2	3	3	4	4	2	2	4	4
2	2	3	3	4	4	2	2	4	4
2	2	3	3	4	4	2	2	4	4
4	4	2	2	2	2	2	2	4	4
2	2	3	3	2	2	2	2	4	4
2	2	3	3	4	4	2	2	4	4
2	2	3	3	4	4	2	2	4	4
2	2	3	3	4	4	2	2	4	4
2	2	3	3	4	4	2	2	4	4
2	2	3	3	4	4	2	2	4	4
4	4	2	2	2	2	4	4	4	4
2	2	2	2	2	2	2	2	4	4
2	2	3	3	4	4	2	2	4	4
2	2	3	3	4	4	2	2	4	4
2	2	2	2	2	2	4	4	2	2
2	2	2	2	4	4	2	2	4	4
4	4	2	2	2	2	4	4	2	2
2	2	3	3	4	4	2	2	4	4
2	2	2	2	2	2	4	4	4	4
4	4	2	2	2	2	2	2	4	4
2	2	2	2	2	2	2	2	4	4
4	4	2	2	2	2	2	2	4	4
2	2	3	3	4	4	2	2	4	4
2	2	2	2	2	2	4	4	4	4
2	2	2	2	4	4	4	4	2	2
2	2	3	3	4	4	2	2	4	4
2	2	3	3	4	4	2	2	4	4
2	2	3	3	4	4	2	2	4	4
4	4	2	2	2	2	4	4	4	4
2	2	3	3	4	4	2	2	4	4
2	2	3	3	4	4	2	2	4	4
4	4	2	2	2	2	4	4	2	2
2	2	2	2	2	2	2	2	4	4
2	2	3	3	4	4	2	2	4	4
2	2	2	2	2	2	4	4	4	4

For Review Only

2	2	3	3	4	4	2	2	4	4
2	2	3	3	4	4	2	2	4	4
2	2	3	3	4	4	2	2	4	4
4	4	2	2	2	2	2	2	4	4
2	2	3	3	4	4	2	2	4	4
2	2	3	3	4	4	2	2	4	4
2	2	2	2	2	2	2	2	4	4
2	2	2	2	2	2	2	2	4	4
2	2	2	2	2	2	2	2	4	4
2	2	3	3	4	4	2	2	4	4
2	2	2	2	2	2	2	2	4	4
2	2	2	2	2	2	2	2	4	4
2	2	3	3	4	4	2	2	4	4
2	2	3	3	4	4	2	2	4	4
4	4	2	2	2	2	2	2	4	4
4	4	2	2	2	2	2	2	4	4
2	2	2	2	2	2	2	2	4	4
2	2	3	3	4	4	2	2	4	4
2	2	3	3	4	4	2	2	4	4
2	2	3	3	4	4	2	2	4	4
2	2	3	3	2	2	2	2	4	4
4	4	3	3	4	4	2	2	4	4
4	4	2	2	2	2	2	2	4	4
2	2	3	3	4	4	2	2	4	4
2	2	2	2	2	2	4	4	2	2
2	2	2	2	2	2	4	4	2	2
2	2	3	3	4	4	2	2	4	4
2	2	3	3	4	4	2	2	4	4
2	2	3	3	4	4	2	2	4	4
2	2	3	3	4	4	2	2	4	4
4	4	2	2	2	2	4	4	2	2
2	2	3	3	4	4	2	2	4	4
2	2	3	3	4	4	2	2	4	4
4	4	2	2	2	2	2	2	4	4
2	2	3	3	4	4	4	4	2	2
2	2	3	3	4	4	2	2	4	4
2	2	3	3	4	4	2	2	4	4
4	4	2	2	2	2	4	4	2	2
2	2	3	3	4	4	2	2	4	4
2	2	3	3	4	4	2	2	4	4
2	2	3	3	4	4	2	2	4	4
2	2	3	3	4	4	2	2	4	4
4	4	2	2	2	2	4	4	2	2
2	2	3	3	4	4	2	2	4	4
2	2	3	3	4	4	2	2	4	4
2	2	3	3	4	4	2	2	4	4
4	4	2	2	2	2	2	2	4	4
4	4	2	2	2	2	2	2	4	4
4	4	2	2	2	2	4	4	2	2
2	2	3	3	4	4	2	2	4	4
4	4	2	2	2	2	2	2	4	4
2	2	3	3	4	4	2	2	4	4
4	4	2	2	2	2	2	2	4	4
2	2	3	3	4	4	2	2	4	4
4	4	2	2	2	2	2	2	4	4

For Review Only

4	4	2	2	2	2	4	4	2	2
2	2	3	3	4	4	2	2	4	4
2	2	3	3	4	4	2	2	4	4
2	2	2	2	2	2	2	2	4	4
2	2	3	3	4	4	2	2	4	4
2	2	3	3	4	4	2	2	4	4
4	4	2	2	2	2	4	4	2	2
4	4	2	2	2	2	4	4	2	2
2	2	2	2	2	2	2	2	4	4
4	4	2	2	2	2	2	2	4	4
2	2	2	2	2	2	2	2	4	4
4	4	2	2	2	2	4	4	2	2
4	4	2	2	2	2	2	2	4	4
2	2	3	3	4	4	2	2	4	4
2	2	3	3	4	4	2	2	4	4
4	4	2	2	2	2	2	2	4	4
4	4	2	2	2	2	2	2	4	4
4	4	2	2	2	2	4	4	2	2
2	2	3	3	4	4	2	2	4	4
2	2	3	3	4	4	2	2	4	4
4	4	2	2	2	2	2	2	4	4
2	2	3	3	4	4	2	2	4	4
2	2	3	3	4	4	2	2	4	4
2	2	3	3	4	4	2	2	4	4
4	4	2	2	2	2	4	4	2	2
2	2	3	3	4	4	2	2	4	4
4	4	2	2	2	2	2	2	4	4
2	2	3	3	4	4	2	2	4	4
2	2	3	3	4	4	2	2	4	4
2	2	3	3	4	4	2	2	4	4
4	4	2	2	2	2	2	2	4	4
4	4	2	2	2	2	2	2	4	4
2	2	3	3	4	4	2	2	4	4
2	2	3	3	4	4	2	2	4	4
4	4	2	2	2	2	2	2	4	4
4	4	2	2	2	2	2	2	4	4
2	2	3	3	4	4	2	2	4	4
2	2	3	3	4	4	2	2	4	4
2	2	3	3	4	4	2	2	4	4
4	4	2	2	2	2	2	2	4	4
2	2	3	3	4	4	2	2	4	4
2	2	3	3	4	4	2	2	4	4
2	2	3	3	4	4	2	2	4	4
2	2	3	3	4	4	2	2	4	4
2	2	3	3	4	4	2	2	4	4
2	2	3	3	4	4	2	2	4	4
2	2	3	3	4	4	2	2	4	4
2	2	3	3	4	4	2	2	4	4
2	2	2	2	4	4	2	2	4	4
4	4	2	2	2	2	2	2	4	4
2	2	3	3	4	4	2	2	4	4
4	4	2	2	2	2	2	2	4	4
2	2	3	3	4	4	2	2	4	4
2	2	3	3	4	4	2	2	4	4
2	2	3	3	4	4	2	2	4	4

2	2	3	3	4	4	2	2	4	4
2	2	3	3	4	4	2	2	4	4

For Review Only

SNP3127	SNP3127	SNP313	SNP313	SNP314	SNP314	SNP315	SNP315	SNP316	SNP316
3	3	1	1	3	3	2	2	1	1
3	3	1	1	3	3	2	2	4	4
3	3	1	1	3	3	2	2	4	4
3	3	3	3	3	3	2	2	4	4
3	3	1	1	3	3	2	2	1	1
3	3	1	1	4	4	3	3	1	1
3	3	1	1	3	3	3	3	1	1
3	3	1	1	3	3	2	2	4	4
3	3	1	1	3	3	3	3	1	1
3	3	3	3	3	3	2	2	4	4
3	3	1	1	3	3	3	3	4	4
3	3	3	3	3	3	2	2	4	4
3	3	3	3	3	3	3	3	1	1
3	3	3	3	3	3	2	2	4	4
3	3	3	3	3	3	2	2	1	1
3	3	3	3	3	3	2	2	4	4
3	3	1	1	3	3	2	2	4	4
3	3	1	1	4	4	3	3	1	1
3	3	3	3	3	3	2	2	4	4
3	3	3	3	3	3	2	2	4	4
3	3	1	1	4	4	3	3	4	4
1	1	1	1	3	3	3	3	1	1
3	3	3	3	3	3	2	2	4	4
3	3	3	3	3	3	2	2	4	4
3	3	1	1	3	3	3	3	1	1
3	3	3	3	3	3	2	2	1	1
3	3	3	3	3	3	2	2	4	4
3	3	3	3	3	3	2	2	4	4
3	3	1	1	3	3	3	3	1	1
1	1	3	3	3	3	3	3	4	4
3	3	3	3	3	3	2	2	4	4
3	3	1	1	3	3	2	2	4	4
3	3	1	1	3	3	3	3	1	1
3	3	1	1	4	4	3	3	1	1
3	3	3	3	3	3	2	2	4	4
3	3	3	3	3	3	2	2	4	4
3	3	1	1	3	3	2	2	4	4
3	3	1	1	4	4	2	2	4	4
3	3	1	1	3	3	3	3	4	4
3	3	1	1	3	3	2	2	4	4
3	3	3	3	3	3	3	3	4	4
3	3	3	3	3	3	3	3	4	4
3	3	3	3	3	3	2	2	4	4
3	3	1	1	3	3	3	3	4	4
3	3	3	3	3	3	2	2	4	4
3	3	3	3	3	3	2	2	1	1
3	3	3	3	3	3	2	2	4	4
3	3	3	3	3	3	2	2	4	4
3	3	3	3	3	3	3	3	4	4
3	3	3	3	3	3	2	2	4	4
3	3	3	3	3	3	2	2	4	4
3	3	3	3	3	3	2	2	4	4

For Review Only

3	3	1	1	3	3	2	2	1	1
3	3	1	1	3	3	3	3	1	1
3	3	3	3	3	3	2	2	4	4
3	3	1	1	3	3	3	3	1	1
3	3	3	3	3	3	2	2	4	4
3	3	3	3	3	3	2	2	4	4
3	3	3	3	3	3	2	2	4	4
1	1	1	1	3	3	2	2	4	4
1	1	1	1	4	4	3	3	4	4
3	3	3	3	3	3	2	2	4	4
3	3	3	3	3	3	2	2	4	4
3	3	3	3	3	3	2	2	4	4
3	3	1	1	3	3	3	3	1	1
3	3	1	1	3	3	2	2	1	1
3	3	3	3	3	3	2	2	1	1
3	3	1	1	4	4	3	3	4	4
3	3	1	1	3	3	3	3	4	4
3	3	3	3	3	3	2	2	1	1
3	3	3	3	3	3	2	2	4	4
3	3	1	1	3	3	3	3	1	1
3	3	3	3	3	3	2	2	4	4
3	3	1	1	3	3	3	3	4	4
3	3	1	1	3	3	3	3	1	1
3	3	1	1	4	4	3	3	1	1
3	3	3	3	3	3	2	2	4	4
3	3	3	3	3	3	2	2	4	4
3	3	3	3	3	3	3	3	4	4
3	3	1	1	3	3	3	3	4	4
3	3	3	3	3	3	2	2	4	4
3	3	3	3	3	3	2	2	4	4
3	3	1	1	3	3	2	2	4	4
3	3	1	1	4	4	3	3	1	1
3	3	1	1	3	3	3	3	4	4
3	3	1	1	3	3	3	3	1	1
1	1	1	1	4	4	3	3	4	4
3	3	3	3	3	3	2	2	4	4
3	3	1	1	3	3	2	2	4	4
3	3	1	1	3	3	3	3	1	1
3	3	1	1	4	4	3	3	4	4
3	3	3	3	3	3	2	2	4	4
3	3	1	1	3	3	3	3	1	1
3	3	1	1	3	3	2	2	4	4
3	3	3	3	3	3	2	2	4	4
3	3	3	3	3	3	2	2	4	4
3	3	1	1	3	3	2	2	4	4
3	3	3	3	3	3	2	2	1	1
3	3	3	3	3	3	3	3	4	4
3	3	3	3	3	3	2	2	4	4
3	3	3	3	3	3	2	2	4	4
3	3	3	3	3	3	2	2	4	4
3	3	3	3	3	3	2	2	4	4
3	3	1	1	3	3	3	3	4	4
1	1	1	1	3	3	2	2	4	4
3	3	1	1	3	3	2	2	4	4
3	3	3	3	3	3	2	2	4	4

For Review Only

3	3	1	1	3	3	3	3	4	4
3	3	1	1	3	3	2	2	4	4
3	3	3	3	3	3	2	2	1	1
3	3	3	3	3	3	2	2	4	4
3	3	1	1	3	3	2	2	4	4
3	3	1	1	4	4	3	3	1	1
3	3	3	3	3	3	2	2	4	4
3	3	3	3	3	3	2	2	4	4
3	3	3	3	3	3	2	2	4	4
3	3	3	3	3	3	2	2	4	4
3	3	1	1	4	4	2	2	1	1
3	3	3	3	3	3	2	2	4	4
3	3	3	3	3	3	2	2	1	1
3	3	3	3	3	3	2	2	4	4
3	3	3	3	3	3	2	2	4	4
3	3	3	3	3	3	2	2	4	4
3	3	3	3	3	3	2	2	1	1
3	3	3	3	3	3	2	2	4	4
3	3	3	3	3	3	2	2	4	4
3	3	3	3	3	3	2	2	4	4
3	3	3	3	3	3	2	2	4	4
3	3	1	1	3	3	3	3	1	1
3	3	1	1	3	3	2	2	4	4
3	3	1	1	3	3	3	3	4	4
3	3	3	3	3	3	2	2	4	4
3	3	3	3	3	3	2	2	4	4
3	3	3	3	3	3	2	2	1	1
3	3	3	3	3	3	2	2	4	4
3	3	1	1	4	4	3	3	1	1
3	3	3	3	3	3	2	2	4	4
3	3	3	3	3	3	2	2	4	4
3	3	3	3	3	3	2	2	4	4
3	3	1	1	3	3	3	3	1	1
3	3	3	3	3	3	2	2	4	4
3	3	1	1	4	4	3	3	1	1
3	3	1	1	4	4	3	3	1	1
3	3	3	3	3	3	2	2	4	4
3	3	3	3	3	3	2	2	4	4
3	3	1	1	3	3	2	2	4	4
3	3	1	1	3	3	3	3	4	4
3	3	3	3	3	3	2	2	4	4
3	3	3	3	3	3	2	2	4	4
3	3	3	3	3	3	2	2	4	4
3	3	3	3	3	3	3	3	4	4
1	1	3	3	3	3	2	2	1	1
3	3	3	3	3	3	2	2	4	4
3	3	3	3	3	3	2	2	4	4
3	3	3	3	3	3	2	2	4	4
3	3	3	3	3	3	2	2	4	4
3	3	3	3	3	3	2	2	4	4
3	3	3	3	3	3	2	2	4	4
1	1	1	1	3	3	3	3	4	4
3	3	1	1	4	4	3	3	1	1
3	3	3	3	3	3	2	2	4	4
3	3	3	3	3	3	2	2	4	4

For Review Only

3	3	3	3	3	3	2	2	4	4
3	3	3	3	3	3	2	2	4	4
3	3	1	1	3	3	2	2	4	4
3	3	3	3	3	3	2	2	4	4
3	3	3	3	3	3	2	2	4	4
3	3	1	1	3	3	2	2	4	4
3	3	1	1	3	3	3	3	4	4
3	3	3	3	3	3	2	2	4	4
3	3	1	1	3	3	3	3	4	4
3	3	1	1	3	3	2	2	4	4
3	3	3	3	3	3	2	2	4	4
3	3	3	3	3	3	3	3	4	4
3	3	1	1	3	3	3	3	4	4
3	3	3	3	3	3	2	2	4	4
3	3	3	3	3	3	2	2	4	4
3	3	1	1	3	3	2	2	4	4
3	3	1	1	4	4	3	3	1	1
3	3	1	1	3	3	3	3	4	4
3	3	3	3	3	3	2	2	4	4
3	3	3	3	3	3	2	2	4	4
3	3	3	3	3	3	2	2	4	4
3	3	1	1	3	3	3	3	4	4
3	3	1	1	3	3	2	2	4	4
3	3	3	3	3	3	2	2	4	4
1	1	3	3	3	3	2	2	1	1
3	3	3	3	3	3	3	3	4	4
3	3	3	3	3	3	2	2	4	4
3	3	3	3	3	3	2	2	1	1
3	3	3	3	3	3	2	2	1	1
3	3	1	1	4	4	3	3	1	1
1	1	1	1	4	4	3	3	1	1
3	3	3	3	3	3	2	2	4	4
3	3	3	3	3	3	2	2	1	1
3	3	3	3	3	3	2	2	4	4
1	1	1	1	4	4	3	3	1	1
3	3	3	3	3	3	2	2	4	4
3	3	3	3	3	3	2	2	4	4
1	1	1	1	3	3	2	2	4	4
3	3	3	3	3	3	2	2	4	4
3	3	1	1	4	4	3	3	1	1
3	3	1	1	3	3	2	2	4	4
3	3	3	3	3	3	2	2	4	4
1	1	3	3	3	3	2	2	4	4
3	3	1	1	3	3	2	2	1	1
3	3	3	3	3	3	2	2	4	4
3	3	3	3	3	3	2	2	4	4
3	3	1	1	3	3	2	2	4	4
3	3	1	1	3	3	3	3	4	4
3	3	3	3	3	3	2	2	4	4
3	3	3	3	3	3	2	2	4	4
3	3	1	1	4	4	3	3	1	1
3	3	3	3	3	3	2	2	4	4
3	3	1	1	3	3	3	3	4	4
3	3	3	3	3	3	2	2	4	4
3	3	3	3	3	3	2	2	4	4

For Review Only

3	3	1	1	4	4	3	3	1	1
3	3	1	1	3	3	2	2	4	4
3	3	1	1	4	4	3	3	4	4
3	3	3	3	3	3	2	2	4	4
3	3	3	3	3	3	3	3	4	4
3	3	3	3	3	3	3	3	4	4
1	1	3	3	3	3	2	2	4	4
3	3	3	3	3	3	2	2	4	4
3	3	3	3	3	3	2	2	4	4
3	3	1	1	3	3	3	3	4	4
3	3	3	3	3	3	2	2	4	4
1	1	3	3	3	3	2	2	1	1
3	3	1	1	4	4	2	2	4	4
3	3	3	3	4	4	3	3	1	1
3	3	1	1	4	4	3	3	1	1
3	3	3	3	3	3	2	2	4	4
3	3	1	1	3	3	3	3	4	4
3	3	1	1	3	3	2	2	4	4
3	3	1	1	3	3	3	3	4	4
3	3	1	1	4	4	2	2	4	4
3	3	1	1	4	4	3	3	1	1
3	3	3	3	3	3	2	2	4	4
3	3	1	1	3	3	2	2	4	4
3	3	1	1	3	3	3	3	4	4
1	1	1	1	3	3	2	2	4	4
3	3	1	1	4	4	2	2	1	1
3	3	3	3	3	3	2	2	4	4
3	3	3	3	3	3	2	2	4	4
3	3	3	3	3	3	3	3	4	4
3	3	1	1	4	4	3	3	1	1
3	3	1	1	3	3	3	3	4	4
3	3	3	3	3	3	2	2	4	4
3	3	3	3	3	3	2	2	4	4
3	3	3	3	3	3	2	2	1	1
3	3	1	1	3	3	2	2	4	4
3	3	1	1	3	3	3	3	4	4
3	3	1	1	4	4	3	3	1	1
3	3	3	3	3	3	2	2	4	4
3	3	3	3	3	3	2	2	4	4
3	3	1	1	3	3	3	3	4	4
3	3	1	1	4	4	3	3	4	4
3	3	1	1	3	3	3	3	4	4
3	3	3	3	3	3	2	2	4	4
3	3	1	1	3	3	3	3	1	1
3	3	1	1	3	3	2	2	4	4
3	3	1	1	4	4	3	3	4	4
3	3	3	3	3	3	2	2	4	4
3	3	3	3	3	3	2	2	4	4
3	3	1	1	3	3	2	2	4	4
3	3	1	1	3	3	3	3	4	4
3	3	3	3	3	3	2	2	4	4
3	3	1	1	3	3	3	3	4	4
3	3	1	1	3	3	2	2	4	4
3	3	1	1	4	4	3	3	1	1
3	3	1	1	3	3	2	2	4	4

For Review Only

3	3	3	3	3	3	2	2	4	4
3	3	1	1	3	3	3	3	1	1

For Review Only

SNP317	SNP317	SNP318	SNP318	SNP319	SNP319	SNP320	SNP320	SNP321	SNP321
3	3	3	3	3	3	2	2	4	4
2	2	3	3	3	3	2	2	4	4
2	2	3	3	3	3	2	2	4	4
2	2	3	3	2	2	2	2	4	4
3	3	3	3	3	3	2	2	4	4
3	3	2	2	3	3	2	2	4	4
3	3	3	3	3	3	2	2	1	1
2	2	2	2	3	3	2	2	4	4
3	3	3	3	3	3	2	2	1	1
2	2	3	3	3	3	2	2	4	4
2	2	3	3	3	3	2	2	4	4
2	2	3	3	2	2	2	2	4	4
3	3	3	3	3	3	1	1	1	1
2	2	3	3	3	3	2	2	4	4
2	2	3	3	3	3	2	2	1	1
2	2	3	3	2	2	2	2	4	4
2	2	3	3	3	3	2	2	4	4
3	3	3	3	3	3	2	2	4	4
2	2	3	3	2	2	2	2	4	4
2	2	3	3	3	3	2	2	4	4
3	3	3	3	3	3	1	1	1	1
3	3	3	3	3	3	2	2	1	1
2	2	2	2	3	3	1	1	4	4
2	2	3	3	3	3	2	2	4	4
3	3	2	2	2	2	2	2	1	1
3	3	3	3	3	3	2	2	1	1
2	2	2	2	2	2	1	1	4	4
2	2	3	3	2	2	2	2	4	4
3	3	3	3	3	3	2	2	1	1
2	2	3	3	3	3	2	2	4	4
2	2	2	2	2	2	2	2	4	4
2	2	3	3	2	2	2	2	4	4
3	3	3	3	3	3	2	2	1	1
3	3	3	3	3	3	1	1	4	4
2	2	3	3	2	2	2	2	4	4
2	2	3	3	2	2	2	2	4	4
2	2	3	3	3	3	2	2	4	4
3	3	3	3	3	3	2	2	1	1
2	2	2	2	3	3	1	1	4	4
2	2	3	3	3	3	2	2	4	4
2	2	2	2	3	3	2	2	4	4
2	2	3	3	3	3	2	2	4	4
2	2	3	3	3	3	2	2	4	4
2	2	3	3	3	3	2	2	4	4
2	2	3	3	3	3	2	2	4	4
2	2	3	3	3	3	2	2	4	4
2	2	3	3	2	2	2	2	4	4
2	2	2	2	3	3	2	2	4	4
2	2	3	3	3	3	2	2	4	4
2	2	3	3	2	2	2	2	4	4
2	2	3	3	3	3	2	2	4	4
2	2	3	3	2	2	2	2	4	4

For Review Only

3	3	3	3	3	3	2	2	1	1
3	3	3	3	3	3	2	2	1	1
2	2	3	3	2	2	2	2	4	4
3	3	3	3	3	3	2	2	1	1
2	2	3	3	2	2	2	2	4	4
2	2	3	3	2	2	2	2	4	4
2	2	3	3	3	3	2	2	4	4
2	2	3	3	3	3	1	1	4	4
3	3	3	3	3	3	2	2	4	4
2	2	3	3	2	2	2	2	4	4
2	2	3	3	2	2	2	2	4	4
2	2	3	3	2	2	2	2	4	4
3	3	3	3	3	3	2	2	1	1
3	3	3	3	3	3	2	2	1	1
3	3	3	3	3	3	2	2	4	4
2	2	3	3	3	3	2	2	1	1
2	2	3	3	3	3	1	1	4	4
3	3	3	3	3	3	2	2	1	1
2	2	3	3	2	2	2	2	4	4
3	3	3	3	3	3	2	2	1	1
2	2	3	3	2	2	2	2	4	4
2	2	3	3	3	3	2	2	4	4
3	3	3	3	3	3	2	2	1	1
3	3	3	3	2	2	2	2	4	4
2	2	3	3	3	3	2	2	4	4
2	2	3	3	3	3	2	2	4	4
2	2	3	3	2	2	2	2	4	4
2	2	3	3	2	2	2	2	4	4
2	2	3	3	3	3	2	2	4	4
2	2	3	3	2	2	2	2	4	4
2	2	3	3	3	3	2	2	1	1
3	3	3	3	3	3	2	2	4	4
2	2	3	3	3	3	1	1	4	4
3	3	3	3	3	3	2	2	1	1
3	3	3	3	3	3	2	2	1	1
2	2	3	3	3	3	2	2	4	4
2	2	2	2	3	3	2	2	4	4
3	3	3	3	3	3	2	2	1	1
3	3	3	3	2	2	1	1	1	1
2	2	3	3	3	3	2	2	4	4
3	3	3	3	3	3	1	1	1	1
2	2	3	3	2	2	2	2	4	4
2	2	3	3	2	2	2	2	4	4
2	2	3	3	2	2	2	2	4	4
2	2	3	3	2	2	1	1	4	4
3	3	3	3	2	2	2	2	1	1
2	2	2	2	3	3	2	2	4	4
2	2	3	3	3	3	2	2	4	4
2	2	3	3	2	2	2	2	4	4
2	2	3	3	2	2	2	2	4	4
2	2	3	3	2	2	2	2	4	4
2	2	2	2	3	3	1	1	4	4
2	2	3	3	3	3	1	1	4	4
2	2	3	3	2	2	1	1	4	4
2	2	3	3	2	2	2	2	4	4

For Review Only

2	2	3	3	3	3	2	2	4	4
2	2	3	3	3	3	2	2	4	4
3	3	3	3	3	3	2	2	1	1
2	2	3	3	3	3	1	1	4	4
3	3	3	3	2	2	2	2	4	4
3	3	3	3	3	3	2	2	4	4
2	2	3	3	2	2	2	2	4	4
2	2	3	3	3	3	2	2	4	4
2	2	2	2	2	2	1	1	4	4
2	2	3	3	2	2	1	1	4	4
3	3	3	3	3	3	2	2	1	1
2	2	3	3	3	3	2	2	4	4
3	3	3	3	2	2	2	2	1	1
2	2	3	3	2	2	2	2	4	4
2	2	3	3	2	2	2	2	4	4
2	2	3	3	2	2	2	2	4	4
3	3	3	3	3	3	2	2	1	1
2	2	3	3	2	2	2	2	4	4
2	2	3	3	3	3	2	2	4	4
2	2	3	3	2	2	2	2	4	4
2	2	3	3	2	2	2	2	4	4
3	3	3	3	3	3	2	2	1	1
2	2	3	3	2	2	2	2	4	4
2	2	3	3	3	3	2	2	4	4
2	2	3	3	3	3	2	2	4	4
2	2	3	3	3	3	2	2	4	4
3	3	3	3	2	2	2	2	4	4
2	2	3	3	3	3	2	2	4	4
3	3	2	2	2	2	2	2	4	4
2	2	3	3	3	3	2	2	4	4
2	2	3	3	3	3	2	2	4	4
2	2	3	3	2	2	2	2	4	4
3	3	3	3	3	3	1	1	1	1
2	2	3	3	3	3	2	2	4	4
3	3	3	3	3	3	1	1	4	4
3	3	3	3	3	3	2	2	4	4
2	2	3	3	3	3	1	1	4	4
2	2	3	3	3	3	2	2	4	4
2	2	3	3	3	3	2	2	4	4
2	2	3	3	3	3	2	2	4	4
2	2	3	3	2	2	2	2	4	4
2	2	3	3	3	3	2	2	4	4
2	2	3	3	2	2	2	2	4	4
2	2	3	3	3	3	2	2	4	4
2	2	3	3	3	3	2	2	1	1
2	2	3	3	3	3	2	2	4	4
2	2	3	3	3	3	2	2	4	4
2	2	3	3	2	2	2	2	4	4
2	2	3	3	3	3	1	1	4	4
2	2	3	3	3	3	2	2	4	4
2	2	3	3	3	3	2	2	4	4
2	2	3	3	3	3	2	2	4	4
3	3	3	3	3	3	2	2	4	4
2	2	3	3	2	2	2	2	4	4
2	2	3	3	3	3	1	1	4	4

For Review Only

2	2	3	3	3	3	2	2	4	4
2	2	3	3	3	3	2	2	4	4
2	2	3	3	2	2	2	2	4	4
2	2	3	3	3	3	2	2	4	4
2	2	3	3	2	2	2	2	4	4
2	2	3	3	3	3	2	2	4	4
2	2	3	3	2	2	1	1	4	4
2	2	3	3	2	2	2	2	4	4
2	2	2	2	2	2	1	1	4	4
2	2	3	3	2	2	2	2	4	4
2	2	3	3	3	3	2	2	4	4
2	2	3	3	2	2	2	2	4	4
2	2	3	3	2	2	2	2	4	4
2	2	3	3	3	3	2	2	4	4
2	2	3	3	3	3	1	1	4	4
2	2	3	3	3	3	2	2	4	4
3	3	3	3	2	2	2	2	4	4
3	3	3	3	2	2	2	2	4	4
2	2	3	3	3	3	2	2	4	4
2	2	3	3	2	2	2	2	4	4
2	2	3	3	2	2	2	2	4	4
2	2	3	3	3	3	1	1	4	4
2	2	3	3	3	3	2	2	4	4
2	2	3	3	2	2	2	2	4	4
3	3	3	3	2	2	2	2	1	1
2	2	3	3	3	3	1	1	4	4
2	2	3	3	2	2	2	2	4	4
3	3	3	3	3	3	2	2	4	4
3	3	3	3	2	2	2	2	1	1
3	3	3	3	3	3	2	2	4	4
3	3	3	3	2	2	2	2	4	4
2	2	3	3	2	2	2	2	4	4
3	3	3	3	2	2	2	2	4	4
2	2	3	3	3	3	1	1	4	4
3	3	3	3	3	3	2	2	4	4
2	2	3	3	2	2	2	2	4	4
2	2	3	3	2	2	2	2	4	4
2	2	3	3	3	3	2	2	4	4
2	2	3	3	2	2	2	2	4	4
3	3	3	3	2	2	2	2	4	4
2	2	3	3	3	3	2	2	4	4
2	2	3	3	3	3	2	2	4	4
2	2	3	3	3	3	2	2	4	4
2	2	3	3	2	2	2	2	4	4
2	2	3	3	2	2	2	2	4	4
2	2	3	3	3	3	1	1	4	4
2	2	3	3	3	3	2	2	4	4
2	2	3	3	3	3	1	1	4	4
2	2	3	3	3	3	2	2	4	4
2	2	2	2	2	2	1	1	4	4
3	3	2	2	2	2	2	2	4	4
2	2	3	3	3	3	1	1	4	4
2	2	2	2	3	3	1	1	4	4
2	2	3	3	2	2	2	2	4	4
2	2	2	2	3	3	1	1	4	4

For Review Only

3	3	3	3	3	3	2	2	4	4
2	2	3	3	3	3	2	2	4	4
3	3	2	2	2	2	2	2	1	1
2	2	3	3	2	2	2	2	4	4
2	2	3	3	2	2	2	2	4	4
3	3	3	3	3	3	2	2	4	4
2	2	2	2	2	2	2	2	4	4
2	2	2	2	3	3	2	2	4	4
2	2	3	3	2	2	2	2	4	4
2	2	3	3	3	3	1	1	4	4
2	2	3	3	2	2	2	2	4	4
3	3	3	3	2	2	1	1	1	1
3	3	3	3	2	2	2	2	1	1
3	3	3	3	3	3	2	2	4	4
3	3	3	3	2	2	2	2	4	4
2	2	3	3	2	2	2	2	4	4
2	2	3	3	3	3	1	1	4	4
2	2	3	3	3	3	1	1	4	4
2	2	3	3	3	3	1	1	4	4
3	3	3	3	3	3	2	2	1	1
3	3	3	3	3	3	2	2	4	4
2	2	3	3	3	3	2	2	4	4
2	2	3	3	2	2	2	2	4	4
2	2	3	3	2	2	2	2	4	4
2	2	3	3	3	3	1	1	4	4
3	3	3	3	2	2	2	2	4	4
2	2	3	3	3	3	1	1	4	4
2	2	3	3	2	2	2	2	4	4
2	2	3	3	2	2	2	2	4	4
3	3	2	2	2	2	2	2	4	4
2	2	3	3	3	3	1	1	4	4
2	2	3	3	3	3	2	2	4	4
2	2	2	2	3	3	2	2	4	4
3	3	3	3	2	2	1	1	1	1
2	2	3	3	3	3	2	2	4	4
2	2	3	3	3	3	1	1	4	4
2	2	3	3	3	3	2	2	4	4
2	2	3	3	2	2	2	2	4	4
2	2	3	3	2	2	2	2	4	4
2	2	2	2	3	3	1	1	4	4
3	3	3	3	3	3	2	2	4	4
3	3	3	3	3	3	2	2	4	4
2	2	3	3	2	2	2	2	4	4
3	3	3	3	3	3	2	2	1	1
2	2	3	3	2	2	1	1	4	4
3	3	3	3	3	3	1	1	1	1
2	2	3	3	2	2	2	2	4	4
2	2	3	3	2	2	2	2	4	4
2	2	3	3	2	2	2	2	4	4
2	2	3	3	3	3	1	1	4	4
2	2	3	3	2	2	2	2	4	4
2	2	3	3	3	3	1	1	4	4
2	2	3	3	3	3	2	2	4	4
3	3	3	3	2	2	2	2	4	4
2	2	3	3	3	3	2	2	4	4

For Review Only

2	2	3	3	2	2	2	2	4	4
3	3	3	3	2	2	2	2	1	1

For Review Only

SNP322	SNP322	SNP323	SNP323	SNP324	SNP324	SNP325	SNP325	SNP326	SNP326
3	3	3	3	2	2	4	4	2	2
2	2	1	1	2	2	4	4	4	4
3	3	3	3	3	3	3	3	2	2
2	2	1	1	3	3	3	3	2	2
2	2	3	3	2	2	4	4	4	4
2	2	3	3	2	2	3	3	2	2
3	3	3	3	2	2	4	4	2	2
2	2	1	1	2	2	4	4	2	2
3	3	1	1	2	2	4	4	4	4
2	2	3	3	2	2	4	4	4	4
2	2	1	1	2	2	4	4	4	4
3	3	3	3	3	3	3	3	2	2
2	2	3	3	2	2	4	4	2	2
2	2	1	1	2	2	4	4	4	4
2	2	3	3	2	2	4	4	2	2
2	2	1	1	2	2	4	4	4	4
3	3	3	3	2	2	4	4	2	2
2	2	3	3	2	2	4	4	4	4
2	2	1	1	2	2	4	4	4	4
2	2	1	1	2	2	4	4	4	4
2	2	3	3	2	2	4	4	4	4
3	3	3	3	2	2	4	4	2	2
2	2	3	3	2	2	4	4	4	4
2	2	1	1	2	2	4	4	4	4
2	2	3	3	2	2	4	4	2	2
2	2	3	3	3	3	3	3	2	2
2	2	1	1	2	2	4	4	2	2
2	2	3	3	2	2	4	4	2	2
2	2	1	1	2	2	4	4	4	4
2	2	1	1	2	2	4	4	4	4
2	2	1	1	3	3	3	3	2	2
2	2	1	1	2	2	4	4	2	2
3	3	3	3	2	2	4	4	2	2
2	2	1	1	2	2	4	4	2	2
2	2	3	3	2	2	4	4	4	4
2	2	1	1	2	2	4	4	4	4
2	2	1	1	2	2	4	4	4	4
2	2	3	3	2	2	4	4	2	2
2	2	3	3	2	2	4	4	4	4
2	2	3	3	2	2	4	4	2	2
2	2	3	3	2	2	4	4	2	2
2	2	1	1	2	2	4	4	4	4
2	2	1	1	2	2	4	4	4	4
2	2	3	3	2	2	4	4	4	4
2	2	3	3	2	2	4	4	4	4
3	3	3	3	2	2	4	4	2	2
2	2	3	3	2	2	4	4	2	2
2	2	3	3	2	2	3	3	2	2
3	3	1	1	2	2	4	4	4	4
2	2	3	3	2	2	4	4	2	2
2	2	3	3	2	2	4	4	2	2
2	2	1	1	2	2	4	4	2	2

For Review Only

2	2	3	3	2	2	4	4	2	2
3	3	3	3	2	2	4	4	2	2
2	2	1	1	2	2	4	4	4	4
3	3	3	3	2	2	4	4	2	2
2	2	1	1	2	2	4	4	4	4
2	2	1	1	2	2	4	4	2	2
2	2	1	1	3	3	3	3	2	2
2	2	1	1	2	2	4	4	4	4
3	3	1	1	2	2	4	4	4	4
2	2	3	3	2	2	4	4	2	2
2	2	1	1	3	3	3	3	2	2
2	2	1	1	3	3	3	3	2	2
2	2	3	3	2	2	4	4	2	2
2	2	1	1	2	2	4	4	4	4
2	2	3	3	2	2	4	4	2	2
2	2	3	3	2	2	4	4	4	4
3	3	3	3	2	2	4	4	4	4
3	3	3	3	2	2	4	4	2	2
2	2	1	1	2	2	4	4	2	2
3	3	3	3	2	2	4	4	2	2
2	2	1	1	3	3	4	4	2	2
2	2	3	3	2	2	4	4	2	2
2	2	1	1	2	2	4	4	4	4
2	2	1	1	2	2	4	4	4	4
2	2	3	3	2	2	4	4	4	4
2	2	1	1	2	2	4	4	4	4
2	2	3	3	2	2	4	4	2	2
2	2	1	1	2	2	4	4	4	4
2	2	3	3	3	3	3	3	2	2
2	2	1	1	2	2	4	4	4	4
2	2	3	3	2	2	4	4	2	2
2	2	1	1	2	2	4	4	4	4
2	2	1	1	2	2	4	4	4	4
2	2	3	3	2	2	4	4	2	2
2	2	3	3	2	2	4	4	4	4
2	2	3	3	2	2	4	4	4	4
2	2	1	1	2	2	4	4	2	2
2	2	3	3	2	2	4	4	2	2
2	2	3	3	2	2	4	4	2	2
2	2	3	3	2	2	4	4	4	4
3	3	3	3	2	2	4	4	4	4
2	2	1	1	2	2	4	4	4	4
2	2	3	3	2	2	4	4	4	4
2	2	1	1	3	3	3	3	2	2
2	2	3	3	2	2	4	4	4	4
2	2	3	3	2	2	4	4	2	2
2	2	1	1	2	2	4	4	2	2
2	2	3	3	2	2	4	4	4	4
2	2	3	3	2	2	4	4	2	2
2	2	3	3	2	2	4	4	2	2
2	2	1	1	2	2	4	4	4	4
2	2	3	3	2	2	4	4	2	2
3	3	1	1	2	2	4	4	4	4
2	2	1	1	2	2	4	4	2	2
2	2	3	3	2	2	4	4	4	4

For Review Only

2	2	3	3	2	2	4	4	2	2
2	2	1	1	2	2	4	4	2	2
2	2	3	3	2	2	4	4	4	4
2	2	3	3	2	2	4	4	4	4
2	2	1	1	2	2	4	4	2	2
2	2	3	3	3	3	3	3	2	2
2	2	1	1	2	2	4	4	4	4
2	2	1	1	2	2	4	4	2	2
2	2	3	3	2	2	4	4	4	4
2	2	3	3	2	2	4	4	4	4
2	2	3	3	2	2	4	4	2	2
2	2	3	3	2	2	4	4	2	2
2	2	3	3	2	2	4	4	2	2
2	2	1	1	2	2	4	4	2	2
2	2	1	1	2	2	4	4	4	4
2	2	3	3	2	2	4	4	2	2
2	2	3	3	2	2	4	4	2	2
2	2	3	3	2	2	4	4	2	2
2	2	1	1	2	2	4	4	4	4
2	2	1	1	2	2	4	4	4	4
2	2	3	3	2	2	4	4	2	2
3	3	1	1	2	2	4	4	4	4
2	2	3	3	2	2	4	4	4	4
2	2	1	1	2	2	4	4	4	4
2	2	3	3	2	2	3	3	2	2
2	2	3	3	2	2	4	4	4	4
2	2	3	3	2	2	4	4	2	2
2	2	1	1	2	2	4	4	4	4
2	2	1	1	2	2	3	3	2	2
2	2	1	1	2	2	4	4	2	2
2	2	1	1	2	2	4	4	4	4
2	2	3	3	2	2	4	4	2	2
3	3	3	3	2	2	4	4	4	4
2	2	3	3	2	2	4	4	2	2
2	2	1	1	2	2	4	4	2	2
2	2	1	1	2	2	4	4	2	2
2	2	3	3	2	2	4	4	2	2
2	2	1	1	3	3	3	3	2	2
2	2	3	3	3	3	4	4	4	4
2	2	1	1	2	2	4	4	4	4
2	2	3	3	2	2	4	4	2	2
2	2	1	1	2	2	4	4	4	4
2	2	3	3	2	2	4	4	4	4
2	2	1	1	2	2	4	4	2	2
2	2	3	3	2	2	4	4	4	4
2	2	3	3	2	2	4	4	4	4
2	2	3	3	2	2	4	4	2	2
2	2	3	3	2	2	4	4	2	2
2	2	1	1	2	2	4	4	2	2
2	2	3	3	2	2	4	4	2	2
2	2	3	3	2	2	4	4	2	2
2	2	3	3	2	2	4	4	2	2
3	3	3	3	2	2	4	4	4	4
2	2	3	3	3	3	3	3	2	2
2	2	1	1	2	2	4	4	2	2

For Review Only

For Review Only

2	2	3	3	2	2	4	4	4	4
2	2	3	3	2	2	4	4	4	4
2	2	3	3	2	2	4	4	4	4
2	2	3	3	2	2	4	4	4	4
2	2	3	3	2	2	4	4	2	2
3	3	1	1	2	2	4	4	2	2
2	2	3	3	2	2	4	4	4	4
2	2	3	3	2	2	4	4	2	2
2	2	3	3	2	2	4	4	4	4
2	2	1	1	2	2	4	4	2	2
2	2	3	3	2	2	3	3	2	2
2	2	3	3	2	2	4	4	4	4
2	2	3	3	2	2	4	4	2	2
2	2	3	3	2	2	4	4	2	2
2	2	1	1	2	2	4	4	2	2
2	2	1	1	2	2	4	4	4	4
2	2	3	3	2	2	4	4	4	4
2	2	1	1	2	2	4	4	4	4
2	2	3	3	2	2	4	4	4	4
2	2	1	1	2	2	4	4	4	4
2	2	3	3	2	2	3	3	2	2
2	2	1	1	2	2	4	4	4	4
2	2	3	3	2	2	4	4	4	4
2	2	1	1	3	3	3	3	2	2
2	2	1	1	2	2	4	4	4	4
2	2	1	1	2	2	4	4	2	2
2	2	3	3	3	3	3	3	2	2
2	2	3	3	2	2	4	4	2	2
2	2	1	1	2	2	4	4	2	2
3	3	3	3	2	2	4	4	2	2
2	2	3	3	2	2	4	4	4	4
2	2	3	3	2	2	4	4	2	2
2	2	1	1	2	2	4	4	2	2
2	2	1	1	2	2	4	4	2	2
2	2	3	3	2	2	4	4	2	2
2	2	1	1	3	3	3	3	2	2
2	2	1	1	2	2	4	4	4	4
3	3	3	3	2	2	4	4	4	4
2	2	3	3	2	2	4	4	2	2
2	2	3	3	2	2	4	4	4	4
3	3	3	3	3	3	3	3	2	2
2	2	3	3	2	2	4	4	2	2
2	2	1	1	2	2	4	4	4	4
2	2	3	3	2	2	4	4	2	2
2	2	1	1	2	2	4	4	2	2
3	3	1	1	2	2	4	4	2	2
2	2	1	1	2	2	4	4	4	4
2	2	3	3	2	2	4	4	4	4
2	2	1	1	2	2	4	4	4	4
2	2	1	1	2	2	4	4	4	4
2	2	3	3	2	2	3	3	2	2
2	2	1	1	2	2	4	4	4	4
2	2	3	3	2	2	4	4	4	4
2	2	1	1	3	3	3	3	2	2
2	2	1	1	2	2	4	4	4	4

2	2	3	3	2	2	4	4	4	4
2	2	3	3	2	2	4	4	2	2
2	2	3	3	2	2	3	3	2	2
2	2	3	3	2	2	4	4	4	4
2	2	1	1	2	2	4	4	4	4
3	3	3	3	3	3	3	3	2	2
2	2	3	3	2	2	3	3	2	2
2	2	1	1	2	2	4	4	2	2
2	2	3	3	2	2	4	4	4	4
2	2	1	1	2	2	4	4	4	4
2	2	3	3	2	2	4	4	2	2
2	2	3	3	2	2	4	4	4	4
2	2	3	3	2	2	4	4	4	4
2	2	3	3	2	2	4	4	4	4
2	2	3	3	2	2	4	4	2	2
2	2	1	1	2	2	4	4	4	4
2	2	3	3	2	2	4	4	2	2
2	2	1	1	2	2	4	4	2	2
2	2	3	3	2	2	4	4	4	4
2	2	3	3	3	3	3	3	2	2
2	2	1	1	2	2	4	4	4	4
2	2	3	3	3	3	3	3	2	2
2	2	1	1	2	2	4	4	4	4
2	2	3	3	2	2	4	4	4	4
2	2	1	1	2	2	4	4	4	4
2	2	1	1	2	2	4	4	2	2
2	2	1	1	2	2	4	4	4	4
2	2	1	1	3	3	3	3	2	2
2	2	1	1	2	2	4	4	2	2
2	2	3	3	2	2	3	3	2	2
2	2	3	3	2	2	4	4	4	4
2	2	3	3	2	2	4	4	2	2
2	2	1	1	2	2	4	4	2	2
2	2	3	3	2	2	4	4	2	2
2	2	3	3	2	2	4	4	4	4
2	2	1	1	2	2	4	4	4	4
3	3	3	3	2	2	4	4	4	4
2	2	1	1	3	3	3	3	2	2
2	2	1	1	2	2	4	4	4	4
2	2	1	1	2	2	4	4	2	2
2	2	1	1	2	2	4	4	2	2
2	2	3	3	2	2	4	4	4	4
2	2	3	3	2	2	4	4	4	4
3	3	3	3	2	2	4	4	2	2
2	2	3	3	2	2	4	4	2	2
2	2	3	3	2	2	4	4	2	2
2	2	3	3	2	2	4	4	2	2
2	2	3	3	2	2	4	4	4	4
2	2	3	3	2	2	4	4	4	4
2	2	1	1	2	2	4	4	4	4
2	2	1	1	2	2	4	4	2	2
2	2	1	1	2	2	4	4	2	2
3	3	3	3	3	3	3	3	2	2
2	2	3	3	3	3	3	3	2	2
2	2	3	3	2	2	4	4	2	2

For Review Only

2	2	1	1	2	2	4	4	4	4
2	2	1	1	2	2	4	4	4	4

For Review Only

SNP327	SNP327	SNP328	SNP328	SNP329	SNP329	SNP330	SNP330	SNP331	SNP331
2	2	3	3	4	4	1	1	1	1
1	1	3	3	4	4	1	1	1	1
2	2	1	1	4	4	2	2	1	1
2	2	3	3	2	2	1	1	1	1
1	1	3	3	4	4	1	1	1	1
2	2	3	3	4	4	2	2	1	1
2	2	3	3	4	4	1	1	1	1
2	2	3	3	4	4	2	2	1	1
2	2	3	3	4	4	1	1	1	1
1	1	3	3	4	4	2	2	1	1
1	1	3	3	4	4	1	1	4	4
2	2	1	1	4	4	2	2	1	1
2	2	3	3	4	4	1	1	1	1
1	1	1	1	4	4	1	1	1	1
2	2	3	3	4	4	1	1	1	1
1	1	1	1	2	2	2	2	1	1
2	2	3	3	4	4	1	1	1	1
1	1	3	3	4	4	2	2	1	1
1	1	1	1	2	2	2	2	1	1
1	1	3	3	4	4	1	1	1	1
1	1	3	3	4	4	1	1	1	1
2	2	3	3	4	4	1	1	1	1
1	1	3	3	4	4	1	1	4	4
1	1	3	3	4	4	1	1	1	1
2	2	3	3	2	2	1	1	1	1
2	2	3	3	4	4	2	2	1	1
2	2	1	1	2	2	2	2	1	1
2	2	3	3	2	2	1	1	1	1
1	1	3	3	4	4	2	2	1	1
1	1	3	3	4	4	1	1	1	1
2	2	3	3	2	2	2	2	1	1
2	2	3	3	2	2	2	2	1	1
2	2	3	3	4	4	2	2	1	1
2	2	3	3	4	4	1	1	1	1
1	1	1	1	4	4	1	1	1	1
1	1	1	1	2	2	2	2	1	1
1	1	3	3	4	4	1	1	1	1
2	2	3	3	4	4	1	1	1	1
1	1	1	1	4	4	1	1	1	1
2	2	3	3	4	4	1	1	1	1
2	2	3	3	4	4	2	2	1	1
1	1	1	1	4	4	2	2	1	1
1	1	1	1	4	4	1	1	1	1
1	1	3	3	4	4	1	1	1	1
1	1	3	3	4	4	2	2	1	1
2	2	3	3	4	4	2	2	1	1
2	2	1	1	2	2	2	2	1	1
2	2	3	3	4	4	1	1	1	1
1	1	3	3	4	4	1	1	1	1
2	2	3	3	4	4	1	1	1	1
2	2	1	1	4	4	2	2	1	1
2	2	1	1	2	2	1	1	4	4

For Review Only

2	2	3	3	4	4	1	1	1	1
2	2	3	3	4	4	2	2	1	1
1	1	1	1	2	2	2	2	1	1
2	2	3	3	4	4	1	1	1	1
1	1	1	1	2	2	2	2	1	1
2	2	3	3	2	2	1	1	1	1
2	2	3	3	4	4	2	2	1	1
1	1	3	3	4	4	1	1	1	1
1	1	3	3	4	4	1	1	1	1
2	2	3	3	4	4	1	1	1	1
2	2	3	3	2	2	1	1	1	1
2	2	3	3	2	2	1	1	1	1
2	2	3	3	4	4	1	1	1	1
1	1	3	3	4	4	2	2	1	1
2	2	3	3	4	4	1	1	1	1
1	1	3	3	4	4	1	1	1	1
1	1	1	1	4	4	1	1	4	4
2	2	3	3	4	4	2	2	1	1
2	2	3	3	2	2	2	2	1	1
2	2	3	3	4	4	1	1	1	1
2	2	3	3	2	2	1	1	1	1
2	2	3	3	4	4	1	1	1	1
1	1	3	3	4	4	1	1	1	1
1	1	3	3	2	2	2	2	1	1
1	1	3	3	4	4	2	2	1	1
1	1	1	1	4	4	2	2	1	1
2	2	1	1	2	2	2	2	1	1
1	1	3	3	4	4	1	1	4	4
2	2	1	1	4	4	2	2	1	1
1	1	1	1	2	2	1	1	1	1
2	2	3	3	4	4	1	1	1	1
1	1	3	3	4	4	1	1	1	1
1	1	3	3	4	4	1	1	4	4
2	2	3	3	4	4	1	1	1	1
1	1	3	3	4	4	1	1	1	1
1	1	3	3	4	4	2	2	1	1
2	2	3	3	4	4	1	1	1	1
2	2	3	3	4	4	1	1	1	1
2	2	3	3	2	2	1	1	1	1
1	1	3	3	4	4	1	1	1	1
1	1	3	3	4	4	1	1	1	1
1	1	1	1	2	2	2	2	1	1
1	1	3	3	2	2	1	1	1	1
2	2	3	3	2	2	1	1	1	1
1	1	3	3	4	4	1	1	1	1
2	2	3	3	2	2	1	1	1	1
2	2	1	1	4	4	1	1	1	1
1	1	1	1	4	4	1	1	1	1
2	2	1	1	2	2	2	2	1	1
2	2	1	1	2	2	1	1	1	1
1	1	3	3	2	2	2	2	1	1
2	2	3	3	4	4	1	1	4	4
1	1	3	3	4	4	1	1	1	1
2	2	3	3	2	2	1	1	1	1
1	1	1	1	2	2	1	1	1	1

For Review Only

2	2	1	1	4	4	2	2	1	1
2	2	3	3	4	4	1	1	1	1
1	1	3	3	4	4	2	2	1	1
2	2	1	1	4	4	1	1	1	1
2	2	1	1	2	2	2	2	1	1
2	2	3	3	4	4	2	2	1	1
1	1	1	1	2	2	2	2	1	1
2	2	1	1	4	4	1	1	1	1
1	1	3	3	2	2	2	2	1	1
2	2	3	3	4	4	1	1	1	1
2	2	3	3	4	4	1	1	1	1
2	2	3	3	4	4	1	1	1	1
2	2	3	3	2	2	2	2	1	1
2	2	3	3	2	2	2	2	4	4
1	1	3	3	2	2	1	1	1	1
2	2	3	3	4	4	1	1	1	1
2	2	3	3	4	4	2	2	1	1
2	2	3	3	2	2	1	1	1	1
1	1	1	1	4	4	1	1	1	1
1	1	3	3	2	2	2	2	4	4
2	2	3	3	2	2	1	1	1	1
1	1	3	3	4	4	1	1	1	1
1	1	1	1	2	2	2	2	1	1
1	1	3	3	4	4	1	1	1	1
2	2	3	3	4	4	1	1	1	1
1	1	3	3	4	4	1	1	1	1
2	2	3	3	2	2	1	1	1	1
1	1	1	1	4	4	2	2	1	1
2	2	3	3	2	2	2	2	1	1
2	2	3	3	4	4	1	1	1	1
2	2	1	1	4	4	1	1	1	1
2	2	3	3	2	2	1	1	1	1
1	1	3	3	4	4	1	1	1	1
2	2	1	1	4	4	1	1	1	1
2	2	3	3	4	4	1	1	1	1
2	2	3	3	4	4	1	1	1	1
2	2	1	1	4	4	1	1	1	1
2	2	3	3	4	4	1	1	1	1
2	2	3	3	4	4	1	1	4	4
1	1	3	3	4	4	1	1	1	1
1	1	3	3	2	2	1	1	1	1
1	1	1	1	4	4	1	1	1	1
1	1	1	1	2	2	1	1	1	1
2	2	3	3	4	4	2	2	1	1
1	1	1	1	4	4	1	1	1	1
1	1	1	1	4	4	1	1	1	1
2	2	1	1	4	4	2	2	1	1
2	2	3	3	2	2	2	2	1	1
2	2	3	3	4	4	1	1	1	1
2	2	1	1	4	4	1	1	1	1
2	2	3	3	4	4	2	2	1	1
2	2	1	1	4	4	1	1	1	1
2	2	3	3	4	4	1	1	1	1
2	2	1	1	2	2	1	1	1	1
2	2	3	3	4	4	1	1	1	1

For Review Only

1	1	1	1	4	4	2	2	1	1
1	1	3	3	4	4	1	1	1	1
1	1	3	3	2	2	1	1	1	1
1	1	3	3	4	4	1	1	1	1
2	2	1	1	2	2	1	1	1	1
2	2	3	3	4	4	1	1	1	1
1	1	1	1	2	2	1	1	4	4
2	2	3	3	4	4	1	1	1	1
1	1	3	3	4	4	1	1	4	4
2	2	3	3	4	4	1	1	1	1
2	2	1	1	4	4	2	2	1	1
1	1	3	3	2	2	1	1	1	1
1	1	3	3	2	2	1	1	1	1
2	2	3	3	4	4	1	1	1	1
2	2	1	1	4	4	1	1	1	1
1	1	3	3	4	4	1	1	4	4
1	1	3	3	4	4	1	1	1	1
1	1	3	3	2	2	1	1	1	1
1	1	1	1	4	4	2	2	1	1
1	1	3	3	2	2	2	2	1	1
2	2	3	3	2	2	1	1	1	1
1	1	3	3	4	4	1	1	1	1
1	1	3	3	4	4	1	1	1	1
2	2	3	3	2	2	1	1	1	1
1	1	3	3	2	2	1	1	1	1
2	2	1	1	4	4	1	1	1	1
2	2	3	3	2	2	1	1	1	1
2	2	3	3	4	4	1	1	1	1
2	2	3	3	2	2	2	2	1	1
2	2	3	3	4	4	1	1	1	1
1	1	3	3	2	2	1	1	1	1
2	2	1	1	2	2	1	1	1	1
2	2	3	3	2	2	1	1	1	1
2	2	1	1	4	4	1	1	1	1
2	2	3	3	4	4	1	1	1	1
2	2	3	3	2	2	1	1	1	1
1	1	1	1	2	2	2	2	1	1
1	1	3	3	4	4	1	1	1	1
2	2	3	3	2	2	2	2	1	1
1	1	3	3	2	2	1	1	1	1
2	2	1	1	4	4	2	2	1	1
2	2	1	1	4	4	2	2	1	1
1	1	3	3	4	4	1	1	1	1
2	2	3	3	2	2	1	1	1	1
2	2	1	1	4	4	1	1	4	4
2	2	3	3	4	4	1	1	1	1
1	1	3	3	4	4	1	1	4	4
1	1	3	3	4	4	1	1	4	4
1	1	3	3	4	4	1	1	1	1
1	1	3	3	2	2	1	1	1	1
2	2	3	3	2	2	1	1	1	1
1	1	1	1	4	4	1	1	1	1
1	1	3	3	4	4	1	1	4	4
2	2	3	3	2	2	1	1	1	1
1	1	3	3	4	4	1	1	1	1

For Review Only

1	1	3	3	4	4	1	1	1	1
2	2	3	3	4	4	1	1	1	1
2	2	3	3	2	2	2	2	1	1
1	1	3	3	2	2	1	1	1	1
1	1	3	3	2	2	1	1	1	1
2	2	3	3	4	4	1	1	1	1
2	2	1	1	2	2	1	1	1	1
2	2	1	1	4	4	1	1	1	1
1	1	3	3	2	2	1	1	1	1
1	1	3	3	4	4	1	1	4	4
2	2	3	3	2	2	1	1	1	1
1	1	3	3	2	2	1	1	1	1
1	1	3	3	2	2	2	2	1	1
1	1	3	3	4	4	2	2	1	1
2	2	3	3	2	2	2	2	1	1
1	1	3	3	4	4	1	1	1	1
2	2	1	1	4	4	1	1	1	1
2	2	3	3	4	4	1	1	1	1
1	1	3	3	4	4	1	1	4	4
2	2	3	3	4	4	1	1	1	1
1	1	3	3	4	4	1	1	1	1
2	2	1	1	4	4	2	2	1	1
1	1	3	3	4	4	1	1	1	1
1	1	1	1	2	2	1	1	1	1
1	1	3	3	4	4	1	1	1	1
2	2	3	3	2	2	1	1	1	1
1	1	3	3	4	4	1	1	1	1
2	2	3	3	2	2	2	2	1	1
2	2	3	3	2	2	1	1	1	1
2	2	3	3	2	2	2	2	1	1
1	1	3	3	4	4	1	1	1	1
2	2	1	1	4	4	2	2	1	1
2	2	3	3	4	4	2	2	1	1
2	2	3	3	2	2	1	1	1	1
1	1	3	3	4	4	1	1	1	1
1	1	3	3	4	4	1	1	4	4
1	1	3	3	4	4	1	1	1	1
2	2	3	3	2	2	1	1	1	1
1	1	1	1	2	2	2	2	1	1
2	2	1	1	4	4	1	1	1	1
2	2	3	3	4	4	2	2	1	1
1	1	3	3	4	4	1	1	1	1
1	1	1	1	2	2	1	1	1	1
2	2	3	3	4	4	1	1	1	1
2	2	3	3	2	2	1	1	1	1
2	2	3	3	4	4	2	2	1	1
2	2	3	3	2	2	1	1	1	1
1	1	1	1	2	2	1	1	1	1
1	1	1	1	2	2	2	2	1	1
1	1	3	3	4	4	1	1	4	4
2	2	3	3	2	2	2	2	1	1
2	2	1	1	4	4	1	1	1	1
2	2	1	1	4	4	2	2	1	1
2	2	3	3	2	2	1	1	1	1
1	1	3	3	4	4	1	1	1	1

For Review Only

1	1	3	3	4	4	1	1	1	1
1	1	3	3	2	2	2	2	1	1

For Review Only

SNP332	SNP332	SNP333	SNP333	SNP334	SNP334	SNP335	SNP335	SNP336	SNP336
2	2	2	2	1	1	1	1	1	1
1	1	2	2	1	1	1	1	4	4
2	2	2	2	4	4	1	1	1	1
2	2	2	2	4	4	1	1	4	4
1	1	3	3	1	1	1	1	4	4
2	2	2	2	4	4	1	1	1	1
1	1	3	3	4	4	1	1	1	1
1	1	2	2	4	4	3	3	4	4
1	1	2	2	1	1	1	1	4	4
1	1	2	2	1	1	1	1	4	4
1	1	3	3	1	1	1	1	4	4
2	2	2	2	4	4	1	1	4	4
1	1	2	2	4	4	1	1	1	1
1	1	2	2	1	1	1	1	4	4
2	2	2	2	4	4	1	1	1	1
2	2	2	2	1	1	1	1	4	4
1	1	3	3	4	4	1	1	1	1
2	2	2	2	1	1	1	1	1	1
2	2	2	2	1	1	1	1	4	4
1	1	3	3	1	1	1	1	4	4
1	1	3	3	4	4	1	1	1	1
1	1	3	3	4	4	1	1	1	1
1	1	3	3	4	4	1	1	1	1
1	1	2	2	1	1	1	1	4	4
2	2	2	2	4	4	1	1	1	1
1	1	2	2	4	4	1	1	1	1
2	2	2	2	4	4	1	1	4	4
2	2	2	2	4	4	1	1	4	4
1	1	2	2	1	1	1	1	4	4
1	1	3	3	1	1	1	1	4	4
2	2	2	2	4	4	1	1	4	4
2	2	2	2	4	4	1	1	4	4
1	1	2	2	4	4	1	1	1	1
1	1	2	2	1	1	1	1	4	4
1	1	2	2	4	4	1	1	1	1
2	2	2	2	1	1	1	1	4	4
1	1	2	2	1	1	1	1	4	4
1	1	2	2	4	4	1	1	1	1
1	1	3	3	4	4	1	1	4	4
1	1	2	2	4	4	1	1	1	1
1	1	2	2	4	4	3	3	4	4
1	1	2	2	1	1	1	1	4	4
1	1	2	2	1	1	1	1	4	4
1	1	3	3	1	1	1	1	1	1
1	1	2	2	1	1	1	1	4	4
2	2	2	2	1	1	1	1	1	1
2	2	2	2	4	4	1	1	1	1
1	1	2	2	4	4	1	1	1	1
1	1	3	3	4	4	1	1	4	4
2	2	2	2	4	4	1	1	1	1
1	1	2	2	4	4	1	1	1	1
2	2	2	2	4	4	1	1	4	4

For Review Only

1	1	2	2	4	4	1	1	1	1
1	1	2	2	4	4	1	1	1	1
2	2	2	2	1	1	1	1	4	4
1	1	3	3	4	4	1	1	1	1
2	2	2	2	1	1	1	1	4	4
2	2	2	2	4	4	1	1	4	4
1	1	2	2	4	4	1	1	4	4
1	1	3	3	4	4	1	1	4	4
2	2	2	2	1	1	1	1	4	4
2	2	2	2	1	1	1	1	4	4
2	2	2	2	4	4	1	1	4	4
2	2	2	2	4	4	1	1	4	4
1	1	2	2	4	4	1	1	1	1
1	1	2	2	1	1	1	1	4	4
2	2	2	2	4	4	1	1	4	4
1	1	2	2	4	4	1	1	1	1
2	2	3	3	1	1	1	1	1	1
2	2	2	2	4	4	1	1	1	1
2	2	2	2	4	4	1	1	4	4
1	1	2	2	1	1	1	1	1	1
2	2	2	2	4	4	1	1	4	4
1	1	2	2	4	4	1	1	1	1
1	1	3	3	1	1	1	1	4	4
2	2	2	2	1	1	1	1	4	4
1	1	2	2	1	1	1	1	4	4
1	1	2	2	1	1	1	1	4	4
1	1	2	2	4	4	1	1	1	1
1	1	2	2	1	1	1	1	4	4
1	1	2	2	4	4	1	1	1	1
2	2	2	2	1	1	1	1	4	4
1	1	3	3	4	4	1	1	4	4
1	1	2	2	1	1	1	1	4	4
1	1	2	2	1	1	1	1	4	4
1	1	2	2	4	4	1	1	1	1
2	2	2	2	1	1	1	1	1	1
1	1	2	2	1	1	1	1	4	4
1	1	3	3	4	4	3	3	4	4
1	1	2	2	4	4	1	1	1	1
2	2	3	3	1	1	1	1	1	1
1	1	3	3	1	1	1	1	1	1
1	1	2	2	4	4	1	1	1	1
2	2	2	2	1	1	1	1	4	4
2	2	2	2	1	1	1	1	1	1
2	2	2	2	4	4	1	1	4	4
1	1	2	2	4	4	1	1	1	1
2	2	2	2	4	4	1	1	4	4
1	1	2	2	4	4	3	3	4	4
1	1	3	3	1	1	1	1	1	1
2	2	2	2	4	4	1	1	1	1
2	2	2	2	4	4	1	1	1	1
2	2	2	2	1	1	1	1	4	4
1	1	2	2	4	4	3	3	4	4
2	2	2	2	4	4	1	1	4	4
2	2	2	2	4	4	1	1	4	4
2	2	2	2	1	1	1	1	1	1

For Review Only

1	1	2	2	4	4	1	1	1	1
1	1	3	3	4	4	1	1	4	4
1	1	2	2	1	1	1	1	1	1
1	1	2	2	4	4	1	1	1	1
2	2	2	2	4	4	1	1	4	4
1	1	2	2	4	4	1	1	1	1
2	2	2	2	1	1	1	1	4	4
2	2	2	2	4	4	1	1	4	4
2	2	2	2	4	4	1	1	1	1
2	2	2	2	4	4	1	1	4	4
1	1	2	2	4	4	1	1	1	1
1	1	2	2	4	4	1	1	4	4
2	2	2	2	1	1	1	1	1	1
2	2	2	2	4	4	1	1	4	4
2	2	2	2	4	4	1	1	4	4
1	1	2	2	4	4	1	1	4	4
2	2	2	2	4	4	1	1	1	1
2	2	2	2	4	4	1	1	4	4
1	1	2	2	1	1	1	1	4	4
2	2	2	2	4	4	1	1	4	4
2	2	2	2	4	4	1	1	4	4
2	2	2	2	1	1	1	1	4	4
2	2	2	2	1	1	1	1	1	1
1	1	2	2	1	1	1	1	4	4
1	1	2	2	4	4	1	1	1	1
1	1	2	2	1	1	1	1	1	1
2	2	2	2	4	4	1	1	4	4
1	1	2	2	1	1	1	1	4	4
2	2	2	2	4	4	1	1	4	4
1	1	2	2	4	4	1	1	4	4
1	1	2	2	1	1	1	1	4	4
2	2	2	2	4	4	1	1	4	4
1	1	2	2	4	4	1	1	1	1
1	1	2	2	4	4	1	1	4	4
1	1	2	2	1	1	1	1	4	4
1	1	2	2	4	4	1	1	1	1
1	1	2	2	1	1	1	1	1	1
2	2	2	2	4	4	1	1	4	4
1	1	2	2	4	4	1	1	1	1
1	1	3	3	1	1	1	1	4	4
2	2	2	2	4	4	1	1	1	1
1	1	2	2	1	1	1	1	4	4
2	2	2	2	1	1	1	1	1	1
1	1	2	2	4	4	3	3	4	4
1	1	2	2	1	1	1	1	1	1
1	1	2	2	1	1	1	1	1	1
1	1	2	2	4	4	1	1	1	1
2	2	2	2	4	4	1	1	1	1
1	1	2	2	4	4	1	1	4	4
1	1	2	2	4	4	1	1	1	1
1	1	2	2	4	4	1	1	1	1
1	1	2	2	4	4	1	1	1	1
2	2	2	2	1	1	1	1	1	1
2	2	2	2	4	4	1	1	1	1
1	1	2	2	4	4	1	1	4	4

For Review Only

1	1	2	2	1	1	1	1	1	1
1	1	2	2	1	1	1	1	1	1
2	2	2	2	1	1	1	1	1	1
1	1	2	2	1	1	1	1	1	1
1	1	2	2	1	1	1	1	1	1
2	2	3	3	4	4	1	1	4	4
1	1	2	2	1	1	1	1	1	1
2	2	2	2	4	4	1	1	4	4
1	1	2	2	4	4	1	1	4	4
1	1	2	2	4	4	1	1	4	4
1	1	2	2	4	4	1	1	1	1
2	2	2	2	1	1	1	1	4	4
2	2	2	2	1	1	1	1	1	1
1	1	2	2	4	4	1	1	1	1
1	1	2	2	1	1	1	1	4	4
1	1	3	3	1	1	1	1	4	4
2	2	2	2	1	1	1	1	1	1
2	2	2	2	1	1	1	1	4	4
1	1	2	2	1	1	1	1	1	1
2	2	2	2	1	1	1	1	4	4
2	2	2	2	4	4	1	1	4	4
1	1	2	2	1	1	1	1	4	4
1	1	2	2	1	1	1	1	1	1
2	2	2	2	4	4	1	1	4	4
2	2	3	3	1	1	1	1	4	4
1	1	3	3	1	1	1	1	4	4
2	2	2	2	4	4	1	1	4	4
1	1	2	2	4	4	1	1	4	4
2	2	2	2	4	4	1	1	4	4
1	1	2	2	4	4	1	1	1	1
2	2	2	2	1	1	1	1	1	1
2	2	2	2	4	4	1	1	1	1
2	2	2	2	4	4	1	1	4	4
1	1	2	2	1	1	1	1	4	4
1	1	2	2	4	4	1	1	4	4
2	2	2	2	4	4	1	1	4	4
1	1	2	2	1	1	1	1	4	4
2	2	2	2	1	1	1	1	1	1
2	2	2	2	4	4	1	1	1	1
2	2	2	2	1	1	1	1	1	1
2	2	2	2	4	4	1	1	1	1
1	1	2	2	4	4	1	1	1	1
1	1	3	3	1	1	1	1	4	4
2	2	2	2	4	4	1	1	1	1
2	2	3	3	4	4	1	1	4	4
2	2	2	2	4	4	3	3	4	4
1	1	3	3	1	1	1	1	4	4
1	1	2	2	1	1	1	1	1	1
1	1	2	2	1	1	1	1	4	4
2	2	2	2	4	4	1	1	4	4
2	2	2	2	4	4	1	1	1	1
1	1	2	2	4	4	1	1	4	4
1	1	2	2	4	4	1	1	1	1
2	2	2	2	4	4	1	1	4	4
1	1	3	3	4	4	1	1	4	4

For Review Only

1	1	2	2	1	1	1	1	1	1
1	1	2	2	4	4	1	1	4	4
2	2	2	2	4	4	1	1	1	1
2	2	2	2	1	1	1	1	1	1
2	2	2	2	1	1	1	1	4	4
2	2	2	2	4	4	1	1	1	1
2	2	2	2	4	4	1	1	4	4
1	1	3	3	4	4	3	3	4	4
1	1	2	2	1	1	1	1	1	1
1	1	2	2	1	1	1	1	4	4
2	2	2	2	4	4	1	1	4	4
2	2	2	2	1	1	1	1	1	1
2	2	2	2	1	1	1	1	1	1
1	1	2	2	1	1	1	1	1	1
2	2	2	2	4	4	1	1	1	1
1	1	3	3	1	1	1	1	4	4
1	1	2	2	4	4	3	3	1	1
1	1	3	3	4	4	1	1	4	4
1	1	2	2	1	1	1	1	1	1
1	1	3	3	4	4	1	1	1	1
1	1	3	3	1	1	1	1	4	4
1	1	2	2	4	4	1	1	1	1
1	1	3	3	1	1	1	1	4	4
2	2	2	2	1	1	1	1	1	1
1	1	3	3	4	4	1	1	4	4
2	2	2	2	4	4	1	1	4	4
1	1	2	2	1	1	1	1	4	4
2	2	2	2	4	4	1	1	4	4
2	2	2	2	4	4	1	1	4	4
2	2	2	2	4	4	1	1	1	1
1	1	2	2	1	1	1	1	1	1
1	1	2	2	4	4	1	1	1	1
1	1	2	2	4	4	3	3	4	4
2	2	3	3	4	4	1	1	1	1
1	1	2	2	1	1	1	1	1	1
1	1	2	2	1	1	1	1	4	4
1	1	3	3	1	1	1	1	1	1
2	2	2	2	4	4	1	1	4	4
2	2	2	2	1	1	1	1	4	4
1	1	3	3	4	4	3	3	4	4
1	1	2	2	4	4	1	1	4	4
1	1	2	2	1	1	1	1	1	1
2	2	2	2	1	1	1	1	1	1
1	1	3	3	4	4	1	1	1	1
2	2	2	2	4	4	1	1	1	1
1	1	2	2	1	1	1	1	1	1
2	2	3	3	4	4	1	1	1	1
2	2	2	2	1	1	1	1	1	1
2	2	2	2	1	1	1	1	4	4
1	1	2	2	1	1	1	1	4	4
2	2	2	2	4	4	1	1	4	4
1	1	3	3	4	4	3	3	4	4
2	2	2	2	4	4	1	1	1	1
2	2	3	3	4	4	1	1	1	1
1	1	3	3	1	1	1	1	1	1

For Review Only

1	1	3	3	1	1	1	1	4	4
2	2	2	2	1	1	1	1	4	4

For Review Only

SNP337	SNP337	SNP338	SNP338	SNP339	SNP339	SNP340	SNP340	SNP341	SNP341
3	3	1	1	1	1	3	3	2	2
3	3	1	1	4	4	3	3	4	4
3	3	1	1	1	1	3	3	4	4
2	2	1	1	4	4	3	3	4	4
3	3	1	1	1	1	3	3	2	2
2	2	1	1	1	1	3	3	2	2
3	3	1	1	1	1	3	3	2	2
3	3	4	4	4	4	3	3	2	2
3	3	1	1	4	4	3	3	2	2
3	3	1	1	1	1	3	3	4	4
3	3	1	1	4	4	3	3	2	2
3	3	1	1	4	4	3	3	4	4
3	3	1	1	1	1	3	3	4	4
3	3	1	1	4	4	3	3	4	4
3	3	1	1	1	1	3	3	4	4
3	3	1	1	4	4	3	3	4	4
3	3	1	1	1	1	3	3	2	2
2	2	1	1	1	1	3	3	2	2
3	3	1	1	4	4	1	1	4	4
3	3	1	1	4	4	3	3	2	2
3	3	1	1	1	1	3	3	2	2
3	3	1	1	1	1	3	3	2	2
3	3	4	4	4	4	3	3	2	2
3	3	1	1	4	4	3	3	4	4
3	3	1	1	1	1	3	3	2	2
3	3	1	1	1	1	3	3	4	4
3	3	1	1	4	4	3	3	4	4
3	3	1	1	4	4	3	3	4	4
3	3	1	1	4	4	3	3	2	2
3	3	1	1	4	4	3	3	2	2
3	3	1	1	4	4	3	3	4	4
3	3	1	1	4	4	3	3	4	4
3	3	1	1	4	4	3	3	2	2
3	3	1	1	4	4	3	3	2	2
3	3	1	1	1	1	1	1	4	4
3	3	1	1	4	4	3	3	4	4
3	3	4	4	4	4	3	3	2	2
3	3	4	4	1	1	3	3	2	2
3	3	1	1	1	1	3	3	2	2
3	3	1	1	1	1	3	3	2	2
3	3	4	4	1	1	3	3	2	2
3	3	1	1	4	4	3	3	2	2
3	3	4	4	4	4	3	3	4	4
3	3	1	1	1	1	3	3	2	2
3	3	4	4	1	1	3	3	4	4
3	3	1	1	1	1	3	3	4	4
2	2	4	4	4	4	3	3	4	4
3	3	1	1	1	1	3	3	4	4
3	3	1	1	4	4	3	3	2	2
3	3	1	1	1	1	3	3	4	4
3	3	1	1	1	1	3	3	4	4
3	3	1	1	4	4	3	3	4	4

For Review Only

3	3	1	1	1	1	3	3	2	2
3	3	1	1	1	1	3	3	2	2
3	3	1	1	4	4	3	3	4	4
3	3	1	1	1	1	3	3	2	2
3	3	1	1	4	4	1	1	4	4
2	2	4	4	4	4	3	3	4	4
3	3	1	1	4	4	3	3	4	4
3	3	1	1	4	4	3	3	4	4
3	3	1	1	4	4	1	1	2	2
3	3	1	1	4	4	3	3	4	4
2	2	1	1	4	4	1	1	4	4
2	2	4	4	4	4	1	1	4	4
3	3	1	1	1	1	3	3	2	2
3	3	1	1	4	4	3	3	2	2
3	3	1	1	1	1	3	3	4	4
3	3	4	4	1	1	3	3	2	2
3	3	1	1	4	4	3	3	2	2
3	3	1	1	1	1	3	3	4	4
3	3	4	4	4	4	3	3	4	4
3	3	1	1	1	1	3	3	2	2
3	3	4	4	4	4	3	3	4	4
3	3	1	1	1	1	3	3	2	2
3	3	1	1	4	4	3	3	2	2
3	3	1	1	4	4	3	3	2	2
3	3	4	4	1	1	3	3	4	4
3	3	1	1	4	4	3	3	4	4
3	3	4	4	1	1	3	3	4	4
3	3	4	4	4	4	3	3	2	2
3	3	1	1	1	1	3	3	4	4
3	3	1	1	4	4	3	3	4	4
3	3	1	1	4	4	3	3	2	2
3	3	1	1	4	4	3	3	2	2
3	3	4	4	4	4	3	3	2	2
3	3	1	1	1	1	3	3	2	2
2	2	1	1	1	1	3	3	2	2
3	3	4	4	1	1	3	3	4	4
3	3	1	1	4	4	3	3	2	2
3	3	1	1	1	1	3	3	2	2
3	3	1	1	1	1	3	3	2	2
3	3	4	4	1	1	3	3	4	4
3	3	1	1	4	4	3	3	2	2
3	3	1	1	4	4	3	3	4	4
3	3	1	1	1	1	3	3	4	4
2	2	1	1	4	4	1	1	4	4
3	3	1	1	1	1	3	3	2	2
3	3	1	1	1	1	3	3	4	4
3	3	4	4	4	4	3	3	4	4
3	3	1	1	1	1	3	3	4	4
3	3	1	1	1	1	3	3	4	4
3	3	4	4	1	1	1	1	4	4
3	3	1	1	4	4	3	3	4	4
3	3	1	1	1	1	3	3	2	2
3	3	1	1	4	4	1	1	2	2
3	3	1	1	4	4	3	3	2	2
3	3	4	4	1	1	3	3	4	4

For Review Only

3	3	1	1	1	1	3	3	2	2
3	3	1	1	4	4	3	3	4	4
3	3	1	1	4	4	3	3	4	4
3	3	4	4	1	1	3	3	4	4
3	3	1	1	4	4	3	3	4	4
3	3	1	1	1	1	3	3	2	2
3	3	1	1	4	4	3	3	4	4
3	3	1	1	4	4	3	3	4	4
3	3	1	1	1	1	3	3	4	4
3	3	1	1	4	4	3	3	4	4
3	3	1	1	1	1	3	3	2	2
3	3	1	1	4	4	3	3	4	4
3	3	1	1	1	1	3	3	4	4
3	3	1	1	4	4	3	3	4	4
3	3	1	1	4	4	3	3	4	4
3	3	1	1	1	1	3	3	4	4
3	3	1	1	1	1	3	3	4	4
3	3	1	1	1	1	3	3	4	4
3	3	4	4	4	4	3	3	4	4
3	3	1	1	4	4	3	3	2	2
3	3	1	1	4	4	3	3	4	4
3	3	1	1	4	4	3	3	4	4
3	3	1	1	1	1	3	3	4	4
3	3	4	4	4	4	3	3	2	2
3	3	1	1	1	1	3	3	4	4
3	3	4	4	1	1	1	1	2	2
3	3	1	1	4	4	3	3	4	4
3	3	1	1	4	4	3	3	4	4
3	3	1	1	4	4	3	3	2	2
3	3	1	1	4	4	3	3	2	2
3	3	4	4	4	4	1	1	4	4
3	3	1	1	4	4	3	3	4	4
3	3	1	1	1	1	3	3	2	2
3	3	1	1	1	1	3	3	4	4
3	3	1	1	4	4	3	3	2	2
3	3	1	1	4	4	3	3	2	2
3	3	4	4	1	1	1	1	4	4
3	3	1	1	4	4	3	3	4	4
3	3	1	1	1	1	3	3	4	4
3	3	4	4	4	4	3	3	2	2
3	3	1	1	1	1	3	3	4	4
3	3	4	4	4	4	1	1	4	4
3	3	1	1	1	1	3	3	4	4
3	3	1	1	4	4	3	3	2	2
3	3	1	1	1	1	1	1	4	4
3	3	1	1	1	1	3	3	4	4
3	3	1	1	1	1	3	3	4	4
2	2	1	1	1	1	3	3	4	4
3	3	1	1	4	4	3	3	4	4
3	3	1	1	1	1	3	3	4	4
3	3	1	1	4	4	3	3	4	4
3	3	1	1	1	1	1	1	2	2
3	3	1	1	1	1	3	3	2	2
2	2	1	1	1	1	1	1	4	4
3	3	1	1	4	4	3	3	4	4

For Review Only

3	3	1	1	1	1	3	3	4	4
3	3	1	1	1	1	3	3	4	4
3	3	1	1	1	1	3	3	2	2
3	3	4	4	1	1	3	3	2	2
3	3	1	1	1	1	3	3	4	4
3	3	1	1	4	4	3	3	2	2
3	3	1	1	1	1	3	3	2	2
3	3	1	1	4	4	3	3	4	4
3	3	4	4	4	4	3	3	2	2
3	3	1	1	4	4	3	3	2	2
3	3	1	1	1	1	3	3	4	4
3	3	1	1	4	4	3	3	2	2
3	3	1	1	1	1	3	3	2	2
3	3	1	1	4	4	3	3	4	4
3	3	4	4	4	4	1	1	4	4
3	3	1	1	4	4	3	3	2	2
3	3	1	1	1	1	3	3	2	2
3	3	1	1	4	4	3	3	2	2
3	3	1	1	1	1	3	3	4	4
3	3	1	1	4	4	3	3	4	4
3	3	1	1	4	4	3	3	4	4
3	3	1	1	4	4	3	3	2	2
3	3	1	1	1	1	1	1	2	2
2	2	4	4	4	4	1	1	4	4
3	3	1	1	4	4	3	3	2	2
3	3	1	1	4	4	3	3	4	4
3	3	4	4	4	4	3	3	4	4
3	3	1	1	4	4	3	3	4	4
3	3	1	1	4	4	3	3	4	4
3	3	1	1	1	1	3	3	2	2
3	3	4	4	1	1	1	1	4	4
3	3	1	1	1	1	3	3	4	4
3	3	1	1	4	4	3	3	4	4
3	3	4	4	4	4	1	1	4	4
3	3	1	1	4	4	3	3	4	4
2	2	4	4	4	4	1	1	4	4
3	3	1	1	4	4	3	3	4	4
3	3	1	1	1	1	3	3	2	2
2	2	1	1	1	1	3	3	4	4
2	2	1	1	1	1	1	1	2	2
3	3	1	1	1	1	3	3	4	4
3	3	1	1	1	1	3	3	4	4
3	3	4	4	4	4	3	3	2	2
3	3	1	1	1	1	3	3	4	4
3	3	1	1	4	4	3	3	4	4
3	3	1	1	4	4	3	3	4	4
3	3	1	1	4	4	3	3	2	2
3	3	1	1	1	1	3	3	2	2
3	3	1	1	4	4	3	3	4	4
3	3	1	1	4	4	3	3	4	4
3	3	1	1	1	1	3	3	2	2
3	3	4	4	4	4	3	3	4	4
3	3	4	4	1	1	1	1	2	2
2	2	1	1	4	4	1	1	4	4
3	3	4	4	4	4	3	3	2	2

For Review Only

3	3	1	1	1	1	3	3	2	2
3	3	1	1	1	1	3	3	4	4
3	3	1	1	1	1	3	3	2	2
3	3	1	1	1	1	3	3	2	2
3	3	4	4	4	4	3	3	2	2
3	3	1	1	1	1	3	3	4	4
3	3	1	1	4	4	3	3	4	4
3	3	4	4	4	4	3	3	4	4
3	3	1	1	4	4	3	3	4	4
3	3	4	4	4	4	3	3	2	2
3	3	1	1	4	4	3	3	4	4
3	3	4	4	1	1	1	1	4	4
3	3	1	1	1	1	3	3	2	2
3	3	1	1	1	1	3	3	2	2
3	3	1	1	1	1	3	3	2	2
3	3	1	1	4	4	3	3	4	4
3	3	1	1	4	4	3	3	2	2
3	3	4	4	4	4	3	3	4	4
3	3	1	1	1	1	3	3	2	2
3	3	4	4	1	1	3	3	2	2
3	3	1	1	4	4	3	3	4	4
3	3	1	1	1	1	3	3	4	4
3	3	1	1	4	4	3	3	2	2
3	3	1	1	1	1	3	3	2	2
3	3	1	1	4	4	3	3	4	4
3	3	1	1	4	4	3	3	2	2
3	3	1	1	4	4	3	3	2	2
3	3	1	1	4	4	3	3	4	4
3	3	1	1	4	4	3	3	2	2
3	3	1	1	1	1	3	3	2	2
3	3	4	4	1	1	3	3	2	2
3	3	1	1	1	1	3	3	4	4
3	3	4	4	4	4	3	3	4	4
2	2	1	1	1	1	3	3	4	4
3	3	4	4	1	1	1	1	2	2
3	3	4	4	4	4	3	3	2	2
3	3	1	1	1	1	3	3	2	2
2	2	4	4	4	4	1	1	4	4
3	3	1	1	4	4	3	3	4	4
3	3	1	1	4	4	3	3	2	2
3	3	1	1	4	4	3	3	2	2
3	3	1	1	1	1	3	3	2	2
3	3	1	1	1	1	1	1	4	4
3	3	1	1	1	1	3	3	2	2
2	2	1	1	1	1	3	3	4	4
3	3	1	1	1	1	3	3	2	2
2	2	1	1	1	1	3	3	4	4
3	3	4	4	1	1	3	3	4	4
3	3	1	1	1	1	3	3	4	4
3	3	1	1	4	4	3	3	2	2
3	3	1	1	4	4	3	3	2	2
3	3	4	4	4	4	3	3	2	2
3	3	1	1	1	1	3	3	4	4
3	3	1	1	1	1	3	3	2	2
3	3	1	1	1	1	3	3	4	4

For Review Only

3	3	1	1	4	4	3	3	4	4
2	2	1	1	4	4	3	3	2	2

For Review Only

SNP342	SNP342	SNP343	SNP343	SNP344	SNP344	SNP345	SNP345	SNP346	SNP346
2	2	3	3	2	2	2	2	2	2
3	3	3	3	2	2	2	2	2	2
2	2	3	3	2	2	3	3	4	4
3	3	2	2	2	2	2	2	2	2
3	3	2	2	2	2	2	2	2	2
2	2	3	3	2	2	2	2	2	2
2	2	3	3	2	2	2	2	2	2
3	3	3	3	2	2	2	2	2	2
2	2	3	3	2	2	2	2	2	2
3	3	2	2	2	2	2	2	2	2
3	3	3	3	2	2	2	2	2	2
2	2	3	3	2	2	2	2	2	2
2	2	2	2	2	2	2	2	2	2
3	3	3	3	2	2	2	2	2	2
2	2	3	3	2	2	2	2	2	2
3	3	2	2	3	3	2	2	2	2
2	2	2	2	2	2	2	2	2	2
2	2	3	3	2	2	3	3	2	2
3	3	3	3	2	2	2	2	4	4
3	3	2	2	2	2	3	3	2	2
2	2	2	2	2	2	2	2	2	2
2	2	2	2	2	2	2	2	2	2
2	2	3	3	2	2	2	2	2	2
3	3	3	3	2	2	3	3	2	2
2	2	3	3	2	2	2	2	4	4
2	2	3	3	2	2	2	2	2	2
3	3	3	3	2	2	2	2	2	2
2	2	3	3	2	2	2	2	2	2
3	3	2	2	2	2	3	3	2	2
3	3	2	2	2	2	2	2	2	2
3	3	3	3	2	2	3	3	4	4
3	3	3	3	2	2	3	3	2	2
2	2	3	3	2	2	2	2	2	2
3	3	2	2	2	2	2	2	2	2
2	2	3	3	3	3	2	2	2	2
3	3	3	3	2	2	3	3	2	2
3	3	3	3	2	2	2	2	2	2
2	2	3	3	2	2	2	2	2	2
3	3	2	2	2	2	2	2	2	2
2	2	3	3	2	2	2	2	2	2
3	3	2	2	2	2	3	3	2	2
3	3	2	2	2	2	3	3	2	2
3	3	3	3	2	2	2	2	2	2
2	2	3	3	2	2	2	2	2	2
3	3	3	3	2	2	3	3	2	2
2	2	3	3	2	2	3	3	4	4
2	2	3	3	3	3	2	2	2	2
2	2	3	3	2	2	2	2	2	2
3	3	2	2	2	2	2	2	2	2
2	2	3	3	2	2	2	2	2	2
2	2	3	3	2	2	2	2	2	2
3	3	2	2	2	2	2	2	2	2

For Review Only

2	2	3	3	2	2	2	2	2	2
2	2	3	3	2	2	2	2	2	2
3	3	3	3	2	2	3	3	2	2
2	2	3	3	2	2	2	2	2	2
3	3	3	3	2	2	2	2	4	4
3	3	3	3	3	3	2	2	2	2
3	3	3	3	2	2	2	2	2	2
3	3	2	2	2	2	2	2	2	2
3	3	3	3	2	2	2	2	4	4
2	2	3	3	2	2	2	2	2	2
3	3	3	3	2	2	2	2	2	2
3	3	3	3	3	3	2	2	2	2
2	2	3	3	2	2	2	2	2	2
3	3	2	2	2	2	3	3	2	2
2	2	3	3	2	2	2	2	2	2
2	2	3	3	2	2	2	2	2	2
2	2	3	3	2	2	2	2	2	2
2	2	3	3	2	2	3	3	2	2
3	3	3	3	2	2	2	2	2	2
2	2	3	3	2	2	2	2	2	2
3	3	3	3	3	3	2	2	2	2
2	2	2	2	2	2	2	2	2	2
3	3	2	2	2	2	2	2	2	2
3	3	2	2	2	2	3	3	4	4
3	3	3	3	2	2	3	3	2	2
3	3	3	3	2	2	2	2	4	4
2	2	3	3	2	2	2	2	2	2
3	3	2	2	2	2	2	2	2	2
2	2	3	3	2	2	2	2	2	2
3	3	3	3	3	3	2	2	2	2
2	2	3	3	2	2	3	3	2	2
3	3	2	2	2	2	2	2	2	2
3	3	2	2	2	2	2	2	2	2
2	2	3	3	2	2	2	2	2	2
2	2	3	3	2	2	2	2	2	2
3	3	3	3	2	2	3	3	2	2
3	3	3	3	2	2	3	3	2	2
2	2	2	2	2	2	2	2	2	2
2	2	3	3	2	2	2	2	2	2
2	2	3	3	2	2	2	2	2	2
2	2	3	3	2	2	2	2	2	2
3	3	3	3	2	2	3	3	2	2
2	2	3	3	2	2	2	2	2	2
3	3	3	3	2	2	2	2	2	2
2	2	3	3	2	2	2	2	2	2
2	2	3	3	2	2	2	2	4	4
3	3	2	2	2	2	2	2	2	2
2	2	3	3	2	2	2	2	2	2
2	2	3	3	2	2	2	2	4	4
2	2	3	3	2	2	2	2	2	2
3	3	3	3	2	2	2	2	2	2
3	3	2	2	2	2	2	2	2	2
3	3	2	2	2	2	2	2	4	4
3	3	3	3	2	2	2	2	4	4
2	2	3	3	2	2	2	2	4	4

For Review Only

2	2	3	3	2	2	2	2	2	2
3	3	3	3	2	2	2	2	2	2
2	2	3	3	2	2	3	3	2	2
2	2	2	2	2	2	2	2	2	2
3	3	3	3	2	2	2	2	2	2
2	2	3	3	2	2	2	2	2	2
3	3	3	3	3	3	2	2	2	2
3	3	3	3	2	2	2	2	2	2
2	2	2	2	2	2	2	2	2	2
2	2	2	2	2	2	3	3	2	2
2	2	3	3	2	2	2	2	2	2
2	2	3	3	2	2	2	2	2	2
2	2	3	3	2	2	3	3	2	2
3	3	3	3	2	2	2	2	2	2
3	3	3	3	2	2	2	2	2	2
2	2	3	3	2	2	2	2	2	2
2	2	3	3	2	2	3	3	2	2
2	2	3	3	2	2	2	2	2	2
3	3	2	2	2	2	2	2	2	2
3	3	3	3	2	2	2	2	2	2
2	2	2	2	2	2	2	2	2	2
3	3	3	3	3	3	2	2	2	2
2	2	3	3	2	2	3	3	2	2
3	3	2	2	2	2	2	2	2	2
2	2	2	2	2	2	2	2	2	2
2	2	3	3	2	2	2	2	2	2
2	2	3	3	2	2	2	2	4	4
3	3	3	3	2	2	2	2	2	2
3	3	3	3	2	2	3	3	2	2
3	3	3	3	2	2	2	2	2	2
3	3	2	2	2	2	2	2	2	2
2	2	2	2	2	2	2	2	2	2
2	2	3	3	2	2	2	2	2	2
2	2	3	3	2	2	2	2	2	2
3	3	2	2	2	2	2	2	2	2
3	3	2	2	2	2	2	2	2	2
2	2	2	2	2	2	2	2	2	2
3	3	3	3	2	2	2	2	2	2
2	2	3	3	2	2	2	2	2	2
3	3	2	2	2	2	2	2	2	2
2	2	2	2	2	2	2	2	2	2
3	3	2	2	2	2	2	2	2	2
2	2	3	3	2	2	2	2	4	4
3	3	2	2	2	2	3	3	2	2
2	2	3	3	2	2	2	2	2	2
2	2	3	3	2	2	2	2	2	2
2	2	3	3	2	2	2	2	2	2
2	2	3	3	2	2	2	2	2	2
2	2	2	2	2	2	2	2	2	2
2	2	3	3	2	2	2	2	2	2
2	2	3	3	2	2	2	2	2	2
2	2	2	2	2	2	2	2	2	2
2	2	3	3	2	2	2	2	4	4
2	2	3	3	2	2	2	2	2	2
2	2	2	2	2	2	2	2	2	2

2	2	3	3	2	2	2	2	2	2
2	2	3	3	2	2	2	2	2	2
2	2	2	2	2	2	2	2	2	2
2	2	2	2	2	2	2	2	2	2
2	2	3	3	2	2	2	2	2	2
3	3	2	2	2	2	2	2	4	4
2	2	2	2	2	2	2	2	2	2
2	2	2	2	2	2	2	2	2	2
2	2	2	2	2	2	2	2	2	2
3	3	3	3	3	3	2	2	2	2
2	2	3	3	2	2	2	2	2	2
2	2	3	3	2	2	3	3	2	2
2	2	3	3	2	2	2	2	2	2
2	2	3	3	2	2	2	2	2	2
3	3	2	2	2	2	2	2	2	2
3	3	3	3	2	2	2	2	2	2
2	2	2	2	2	2	2	2	4	4
3	3	3	3	2	2	2	2	4	4
2	2	3	3	2	2	3	3	2	2
3	3	3	3	3	3	2	2	2	2
2	2	3	3	2	2	2	2	2	2
3	3	3	3	2	2	2	2	2	2
2	2	2	2	2	2	2	2	2	2
3	3	3	3	3	3	2	2	2	2
3	3	3	3	2	2	2	2	4	4
3	3	2	2	2	2	2	2	2	2
2	2	3	3	3	3	2	2	2	2
2	2	3	3	2	2	2	2	2	2
3	3	3	3	2	2	2	2	2	2
2	2	3	3	2	2	2	2	2	2
2	2	3	3	2	2	2	2	4	4
2	2	3	3	3	3	2	2	4	4
3	3	3	3	2	2	2	2	4	4
3	3	2	2	2	2	2	2	2	2
2	2	3	3	2	2	2	2	4	4
3	3	3	3	3	3	2	2	2	2
2	2	3	3	2	2	2	2	2	2
2	2	3	3	2	2	2	2	4	4
2	2	3	3	2	2	2	2	2	2
2	2	3	3	2	2	2	2	2	2
2	2	3	3	2	2	3	3	4	4
2	2	3	3	2	2	2	2	2	2
3	3	2	2	2	2	2	2	2	2
2	2	3	3	2	2	2	2	2	2
3	3	3	3	2	2	2	2	2	2
3	3	2	2	2	2	2	2	4	4
3	3	3	3	2	2	2	2	2	2
2	2	2	2	2	2	2	2	2	2
3	3	2	2	2	2	2	2	2	2
3	3	2	2	2	2	2	2	2	2
2	2	3	3	2	2	2	2	2	2
3	3	2	2	2	2	2	2	2	2
2	2	2	2	2	2	2	2	2	2
3	3	3	3	2	2	2	2	2	2
3	3	2	2	2	2	2	2	2	2

For Review Only

2	2	3	3	2	2	2	2	2	2
3	3	3	3	2	2	2	2	2	2
2	2	3	3	2	2	3	3	2	2
2	2	2	2	2	2	2	2	2	2
3	3	3	3	2	2	2	2	2	2
2	2	3	3	2	2	2	2	4	4
2	2	2	2	2	2	2	2	2	2
3	3	2	2	2	2	2	2	2	2
2	2	2	2	2	2	2	2	2	2
3	3	2	2	2	2	2	2	2	2
2	2	3	3	2	2	2	2	2	2
2	2	3	3	2	2	2	2	4	4
2	2	3	3	2	2	2	2	2	2
2	2	3	3	3	3	2	2	2	2
2	2	3	3	2	2	3	3	4	4
3	3	3	3	2	2	2	2	2	2
2	2	2	2	2	2	2	2	2	2
2	2	3	3	2	2	2	2	2	2
2	2	2	2	2	2	2	2	2	2
2	2	3	3	2	2	2	2	2	2
3	3	2	2	2	2	2	2	2	2
2	2	3	3	2	2	2	2	2	2
3	3	3	3	2	2	2	2	2	2
2	2	3	3	2	2	2	2	4	4
3	3	2	2	2	2	2	2	2	2
2	2	2	2	2	2	2	2	2	2
3	3	2	2	2	2	2	2	2	2
3	3	3	3	3	3	2	2	2	2
3	3	3	3	2	2	2	2	2	2
2	2	3	3	2	2	3	3	2	2
2	2	3	3	2	2	2	2	2	2
2	2	3	3	2	2	3	3	2	2
3	3	3	3	2	2	3	3	2	2
2	2	3	3	2	2	2	2	2	2
2	2	2	2	2	2	2	2	2	2
3	3	2	2	2	2	2	2	2	2
2	2	3	3	2	2	2	2	2	2
3	3	3	3	3	3	2	2	2	2
3	3	3	3	2	2	3	3	2	2
3	3	2	2	2	2	2	2	2	2
3	3	3	3	2	2	3	3	4	4
2	2	3	3	2	2	2	2	2	2
2	2	3	3	2	2	2	2	4	4
2	2	3	3	2	2	2	2	2	2
2	2	3	3	3	3	2	2	2	2
2	2	3	3	2	2	2	2	2	2
2	2	3	3	2	2	2	2	2	2
2	2	3	3	2	2	2	2	4	4
2	2	3	3	2	2	2	2	2	2
3	3	2	2	2	2	2	2	2	2
3	3	3	3	2	2	2	2	2	2
3	3	2	2	2	2	2	2	2	2
2	2	3	3	2	2	3	3	4	4
2	2	3	3	3	3	2	2	2	2
2	2	3	3	2	2	2	2	2	2

For Review Only

3	3	3	3	2	2	2	2	2	2
3	3	3	3	2	2	3	3	2	2

For Review Only

SNP347	SNP347	SNP348	SNP348	SNP349	SNP349	SNP350	SNP350	SNP351	SNP351
3	3	2	2	3	3	1	1	1	1
3	3	1	1	3	3	1	1	3	3
3	3	2	2	3	3	4	4	3	3
2	2	2	2	3	3	1	1	3	3
3	3	1	1	3	3	1	1	3	3
3	3	2	2	3	3	1	1	3	3
3	3	1	1	3	3	1	1	3	3
2	2	1	1	3	3	1	1	3	3
3	3	1	1	3	3	1	1	3	3
3	3	2	2	3	3	4	4	3	3
3	3	1	1	3	3	1	1	3	3
3	3	2	2	3	3	4	4	3	3
3	3	1	1	3	3	1	1	3	3
3	3	1	1	3	3	1	1	3	3
3	3	2	2	3	3	1	1	3	3
3	3	2	2	3	3	1	1	3	3
3	3	1	1	3	3	1	1	3	3
3	3	2	2	3	3	1	1	3	3
2	2	2	2	3	3	1	1	3	3
3	3	2	2	3	3	4	4	3	3
3	3	1	1	3	3	1	1	3	3
3	3	1	1	3	3	1	1	3	3
3	3	1	1	3	3	1	1	3	3
3	3	1	1	3	3	1	1	3	3
3	3	2	2	3	3	1	1	3	3
3	3	1	1	3	3	1	1	1	1
3	3	2	2	3	3	4	4	3	3
3	3	2	2	3	3	1	1	3	3
3	3	2	2	3	3	4	4	3	3
3	3	1	1	3	3	1	1	3	3
3	3	2	2	3	3	1	1	3	3
3	3	2	2	3	3	4	4	3	3
3	3	1	1	3	3	1	1	3	3
3	3	1	1	3	3	1	1	3	3
3	3	2	2	3	3	1	1	3	3
3	3	2	2	3	3	4	4	3	3
3	3	1	1	3	3	1	1	3	3
3	3	1	1	3	3	1	1	3	3
3	3	1	1	3	3	1	1	3	3
3	3	1	1	3	3	1	1	3	3
2	2	1	1	3	3	4	4	3	3
3	3	2	2	3	3	4	4	1	1
2	2	2	2	3	3	1	1	3	3
2	2	1	1	3	3	1	1	3	3
2	2	1	1	3	3	4	4	3	3
3	3	2	2	3	3	4	4	3	3
2	2	2	2	2	2	1	1	3	3
3	3	1	1	3	3	1	1	3	3
2	2	1	1	3	3	1	1	1	1
3	3	2	2	3	3	1	1	1	1
3	3	2	2	3	3	1	1	3	3
3	3	2	2	3	3	1	1	3	3

For Review Only

3	3	1	1	3	3	1	1	3	3
3	3	1	1	3	3	1	1	3	3
3	3	2	2	3	3	1	1	3	3
3	3	1	1	3	3	1	1	3	3
2	2	2	2	3	3	1	1	3	3
2	2	2	2	2	2	1	1	3	3
2	2	1	1	3	3	4	4	3	3
3	3	1	1	3	3	1	1	3	3
3	3	2	2	3	3	1	1	3	3
3	3	2	2	3	3	1	1	3	3
3	3	2	2	3	3	1	1	3	3
2	2	2	2	2	2	1	1	3	3
3	3	1	1	3	3	1	1	3	3
3	3	2	2	3	3	4	4	3	3
3	3	2	2	3	3	1	1	3	3
3	3	2	2	3	3	1	1	3	3
3	3	2	2	3	3	1	1	3	3
3	3	2	2	3	3	1	1	1	1
2	2	2	2	3	3	4	4	3	3
3	3	1	1	3	3	1	1	3	3
2	2	2	2	2	2	1	1	3	3
3	3	1	1	3	3	1	1	3	3
3	3	2	2	3	3	1	1	3	3
3	3	2	2	3	3	4	4	3	3
2	2	1	1	3	3	4	4	3	3
3	3	2	2	3	3	1	1	3	3
2	2	2	2	3	3	1	1	3	3
3	3	1	1	3	3	1	1	3	3
3	3	1	1	3	3	1	1	3	3
2	2	2	2	2	2	1	1	3	3
2	2	2	2	3	3	4	4	3	3
3	3	1	1	3	3	1	1	3	3
3	3	1	1	3	3	1	1	3	3
3	3	1	1	3	3	1	1	3	3
3	3	2	2	3	3	1	1	3	3
2	2	1	1	3	3	4	4	3	3
2	2	2	2	3	3	4	4	3	3
3	3	1	1	3	3	1	1	3	3
3	3	2	2	3	3	1	1	3	3
3	3	1	1	3	3	1	1	3	3
3	3	1	1	3	3	1	1	3	3
3	3	2	2	3	3	4	4	3	3
3	3	2	2	3	3	1	1	3	3
3	3	2	2	3	3	1	1	3	3
3	3	1	1	3	3	1	1	3	3
3	3	2	2	3	3	1	1	1	1
3	3	1	1	3	3	1	1	3	3
3	3	1	1	3	3	1	1	3	3
3	3	2	2	3	3	1	1	3	3
2	2	2	2	3	3	1	1	3	3
2	2	2	2	3	3	1	1	3	3
3	3	1	1	3	3	1	1	3	3
3	3	2	2	3	3	1	1	3	3
3	3	2	2	3	3	1	1	3	3
2	2	2	2	3	3	1	1	3	3

For Review Only

3	3	1	1	3	3	1	1	3	3
2	2	1	1	3	3	1	1	3	3
3	3	2	2	3	3	4	4	1	1
2	2	1	1	3	3	1	1	3	3
3	3	2	2	3	3	1	1	3	3
3	3	1	1	3	3	4	4	3	3
2	2	2	2	2	2	1	1	3	3
3	3	1	1	3	3	1	1	3	3
3	3	2	2	3	3	1	1	3	3
3	3	2	2	3	3	1	1	3	3
3	3	1	1	3	3	1	1	3	3
3	3	2	2	3	3	1	1	3	3
3	3	2	2	3	3	1	1	1	1
3	3	2	2	3	3	1	1	3	3
3	3	2	2	3	3	1	1	3	3
3	3	1	1	3	3	1	1	3	3
3	3	1	1	3	3	1	1	1	1
3	3	2	2	3	3	1	1	3	3
2	2	1	1	3	3	1	1	3	3
3	3	2	2	3	3	1	1	3	3
3	3	2	2	3	3	1	1	3	3
2	2	1	1	3	3	1	1	3	3
3	3	2	2	3	3	1	1	3	3
3	3	1	1	3	3	1	1	3	3
3	3	1	1	3	3	1	1	3	3
3	3	1	1	3	3	1	1	3	3
3	3	2	2	3	3	1	1	1	1
3	3	1	1	3	3	1	1	3	3
2	2	2	2	3	3	1	1	3	3
3	3	2	2	3	3	1	1	3	3
3	3	1	1	3	3	1	1	3	3
3	3	2	2	3	3	1	1	3	3
3	3	1	1	3	3	1	1	3	3
3	3	1	1	3	3	1	1	3	3
3	3	1	1	3	3	1	1	3	3
3	3	1	1	3	3	1	1	3	3
3	3	1	1	3	3	1	1	3	3
3	3	1	1	3	3	1	1	3	3
3	3	1	1	3	3	1	1	3	3
3	3	1	1	3	3	1	1	3	3
3	3	2	2	3	3	1	1	3	3
3	3	1	1	3	3	1	1	3	3
3	3	2	2	3	3	1	1	3	3
3	3	1	1	3	3	1	1	3	3
2	2	1	1	3	3	1	1	1	1
3	3	1	1	3	3	1	1	3	3
3	3	1	1	3	3	1	1	3	3
3	3	2	2	3	3	1	1	3	3
3	3	1	1	3	3	1	1	3	3
3	3	1	1	3	3	1	1	3	3
3	3	1	1	3	3	1	1	3	3
3	3	1	1	3	3	1	1	3	3
3	3	2	2	3	3	1	1	1	1
3	3	2	2	3	3	1	1	3	3
3	3	1	1	3	3	1	1	3	3

For Review Only

3	3	1	1	3	3	1	1	3	3
3	3	1	1	3	3	1	1	3	3
3	3	2	2	3	3	1	1	3	3
3	3	1	1	3	3	1	1	3	3
3	3	2	2	3	3	1	1	3	3
2	2	2	2	3	3	1	1	3	3
3	3	2	2	3	3	1	1	3	3
3	3	2	2	3	3	1	1	3	3
3	3	1	1	3	3	1	1	3	3
2	2	1	1	2	2	1	1	3	3
3	3	1	1	3	3	1	1	3	3
3	3	2	2	3	3	1	1	3	3
3	3	2	2	3	3	1	1	3	3
3	3	1	1	3	3	1	1	3	3
3	3	1	1	3	3	1	1	3	3
3	3	1	1	3	3	1	1	3	3
3	3	2	2	3	3	1	1	1	1
3	3	2	2	3	3	1	1	3	3
3	3	2	2	3	3	1	1	3	3
2	2	2	2	2	2	1	1	3	3
3	3	2	2	3	3	1	1	3	3
3	3	1	1	3	3	1	1	3	3
3	3	1	1	3	3	1	1	3	3
2	2	2	2	2	2	1	1	3	3
3	3	1	1	3	3	1	1	3	3
3	3	1	1	3	3	1	1	3	3
2	2	2	2	3	3	1	1	3	3
3	3	1	1	3	3	1	1	3	3
3	3	2	2	3	3	1	1	1	1
3	3	1	1	3	3	1	1	1	1
3	3	2	2	3	3	1	1	3	3
2	2	2	2	2	2	1	1	3	3
3	3	2	2	3	3	1	1	1	1
3	3	1	1	3	3	1	1	3	3
3	3	2	2	3	3	1	1	3	3
2	2	2	2	2	2	1	1	3	3
3	3	2	2	3	3	1	1	3	3
3	3	2	2	3	3	1	1	3	3
3	3	2	2	3	3	1	1	3	3
3	3	2	2	3	3	1	1	3	3
3	3	2	2	3	3	4	4	3	3
3	3	1	1	3	3	1	1	3	3
3	3	1	1	3	3	1	1	3	3
3	3	2	2	3	3	1	1	3	3
3	3	2	2	3	3	1	1	3	3
3	3	2	2	3	3	1	1	3	3
3	3	1	1	3	3	1	1	3	3
3	3	1	1	3	3	1	1	3	3
3	3	1	1	3	3	1	1	3	3
3	3	2	2	3	3	1	1	3	3
2	2	2	2	3	3	1	1	3	3
3	3	1	1	3	3	1	1	3	3
3	3	1	1	3	3	1	1	3	3
3	3	2	2	3	3	1	1	3	3
3	3	1	1	3	3	1	1	3	3

For Review Only

3	3	1	1	3	3	1	1	1	1
3	3	1	1	3	3	1	1	3	3
3	3	2	2	3	3	1	1	3	3
3	3	2	2	3	3	1	1	3	3
3	3	1	1	3	3	1	1	3	3
3	3	2	2	3	3	1	1	3	3
3	3	2	2	3	3	1	1	3	3
2	2	1	1	3	3	1	1	3	3
3	3	2	2	3	3	1	1	3	3
3	3	1	1	3	3	1	1	3	3
3	3	2	2	3	3	1	1	3	3
2	2	2	2	3	3	1	1	1	1
3	3	2	2	3	3	4	4	3	3
2	2	1	1	2	2	1	1	3	3
3	3	2	2	3	3	4	4	3	3
3	3	2	2	3	3	1	1	3	3
3	3	1	1	3	3	1	1	3	3
3	3	1	1	3	3	1	1	3	3
3	3	1	1	3	3	1	1	3	3
3	3	1	1	3	3	1	1	3	3
3	3	1	1	3	3	1	1	3	3
3	3	1	1	3	3	1	1	3	3
3	3	2	2	3	3	1	1	3	3
3	3	2	2	3	3	1	1	3	3
3	3	1	1	3	3	1	1	3	3
3	3	2	2	3	3	1	1	1	1
3	3	1	1	3	3	1	1	3	3
2	2	2	2	3	3	4	4	3	3
3	3	1	1	3	3	1	1	3	3
2	2	2	2	3	3	1	1	3	3
3	3	1	1	3	3	1	1	3	3
3	3	1	1	3	3	4	4	3	3
2	2	1	1	3	3	1	1	3	3
3	3	2	2	3	3	1	1	1	1
3	3	1	1	3	3	1	1	3	3
3	3	1	1	3	3	1	1	3	3
3	3	1	1	3	3	1	1	1	1
2	2	2	2	2	2	1	1	3	3
3	3	2	2	3	3	4	4	3	3
3	3	1	1	3	3	1	1	3	3
3	3	2	2	3	3	1	1	3	3
3	3	1	1	3	3	1	1	3	3
3	3	2	2	3	3	1	1	3	3
3	3	1	1	3	3	1	1	3	3
2	2	2	2	3	3	1	1	3	3
3	3	1	1	3	3	4	4	3	3
3	3	2	2	3	3	1	1	3	3
2	2	2	2	3	3	1	1	3	3
3	3	2	2	3	3	1	1	3	3
3	3	1	1	3	3	1	1	3	3
3	3	2	2	3	3	1	1	3	3
3	3	1	1	3	3	1	1	3	3
3	3	2	2	3	3	4	4	3	3
2	2	2	2	2	2	1	1	3	3
3	3	1	1	3	3	1	1	3	3

For Review Only

3	3	2	2	3	3	1	1	3	3
3	3	2	2	3	3	1	1	3	3

For Review Only

SNP352	SNP352	SNP353	SNP353	SNP354	SNP354	SNP355	SNP355	SNP356	SNP356
1	1	4	4	1	1	1	1	1	1
1	1	4	4	1	1	4	4	1	1
4	4	4	4	1	1	1	1	1	1
1	1	4	4	1	1	4	4	1	1
1	1	4	4	1	1	4	4	1	1
1	1	4	4	3	3	4	4	1	1
1	1	4	4	1	1	1	1	1	1
1	1	4	4	1	1	4	4	1	1
1	1	4	4	1	1	1	1	1	1
1	1	4	4	1	1	4	4	1	1
1	1	4	4	3	3	4	4	1	1
1	1	4	4	1	1	4	4	1	1
1	1	1	1	1	1	4	4	1	1
1	1	4	4	1	1	4	4	1	1
1	1	4	4	1	1	4	4	1	1
1	1	4	4	1	1	4	4	1	1
1	1	4	4	1	1	1	1	1	1
1	1	4	4	1	1	4	4	1	1
1	1	4	4	1	1	4	4	1	1
1	1	4	4	1	1	4	4	1	1
1	1	4	4	1	1	4	4	1	1
1	1	1	1	1	1	1	1	4	4
1	1	4	4	3	3	4	4	1	1
1	1	4	4	1	1	4	4	1	1
1	1	4	4	1	1	4	4	1	1
1	1	4	4	1	1	4	4	1	1
1	1	4	4	1	1	4	4	1	1
1	1	4	4	1	1	4	4	1	1
1	1	1	1	1	1	4	4	4	4
1	1	1	1	1	1	4	4	4	4
1	1	4	4	1	1	4	4	1	1
1	1	4	4	1	1	4	4	1	1
1	1	4	4	1	1	1	1	1	1
1	1	4	4	1	1	4	4	1	1
1	1	4	4	1	1	4	4	1	1
1	1	4	4	1	1	4	4	1	1
1	1	4	4	1	1	4	4	1	1
1	1	4	4	1	1	4	4	4	4
1	1	4	4	3	3	4	4	1	1
1	1	4	4	1	1	4	4	1	1
1	1	1	1	3	3	4	4	4	4
1	1	1	1	3	3	4	4	4	4
1	1	4	4	1	1	4	4	1	1
1	1	4	4	1	1	4	4	1	1
1	1	4	4	1	1	4	4	1	1
1	1	4	4	1	1	1	1	1	1
1	1	4	4	1	1	4	4	1	1
1	1	4	4	3	3	4	4	1	1
1	1	4	4	1	1	1	1	1	1
1	1	4	4	1	1	4	4	1	1
1	1	4	4	1	1	4	4	1	1
1	1	4	4	1	1	4	4	1	1

For Review Only

1	1	4	4	1	1	4	4	1	1
1	1	4	4	1	1	1	1	1	1
1	1	4	4	1	1	4	4	1	1
1	1	4	4	1	1	1	1	1	1
1	1	4	4	1	1	4	4	1	1
1	1	4	4	1	1	4	4	1	1
1	1	4	4	1	1	4	4	1	1
1	1	4	4	1	1	4	4	1	1
4	4	4	4	1	1	1	1	1	1
1	1	4	4	1	1	4	4	1	1
1	1	4	4	1	1	4	4	1	1
1	1	4	4	1	1	4	4	1	1
1	1	4	4	1	1	4	4	1	1
1	1	1	1	1	1	4	4	4	4
4	4	4	4	1	1	4	4	1	1
1	1	4	4	1	1	4	4	1	1
1	1	4	4	1	1	4	4	1	1
1	1	4	4	1	1	1	1	1	1
1	1	4	4	3	3	4	4	1	1
1	1	4	4	1	1	1	1	1	1
1	1	4	4	1	1	4	4	1	1
1	1	4	4	1	1	4	4	1	1
1	1	1	1	1	1	4	4	4	4
1	1	4	4	1	1	4	4	1	1
1	1	4	4	1	1	4	4	1	1
1	1	4	4	1	1	4	4	1	1
1	1	1	1	1	1	4	4	4	4
1	1	4	4	1	1	4	4	1	1
1	1	4	4	1	1	4	4	1	1
1	1	4	4	1	1	4	4	1	1
1	1	4	4	1	1	4	4	1	1
1	1	4	4	1	1	4	4	1	1
1	1	4	4	1	1	4	4	1	1
1	1	4	4	1	1	4	4	1	1
1	1	4	4	1	1	4	4	1	1
1	1	4	4	1	1	4	4	1	1
1	1	4	4	1	1	4	4	1	1
1	1	4	4	1	1	4	4	1	1
1	1	4	4	1	1	4	4	1	1
1	1	4	4	1	1	4	4	1	1
1	1	4	4	1	1	1	1	1	1
1	1	4	4	1	1	4	4	1	1
1	1	1	1	1	1	4	4	4	4
1	1	4	4	1	1	4	4	1	1
1	1	4	4	1	1	1	1	1	1
1	1	4	4	1	1	4	4	1	1
1	1	4	4	3	3	4	4	1	1
1	1	4	4	1	1	4	4	1	1
1	1	4	4	1	1	4	4	1	1
1	1	4	4	3	3	4	4	1	1
1	1	4	4	3	3	4	4	1	1
1	1	4	4	3	3	4	4	1	1
4	4	4	4	1	1	1	1	1	1
1	1	4	4	1	1	4	4	1	1
1	1	4	4	3	3	4	4	1	1

For Review Only

1	1	4	4	1	1	4	4	1	1
1	1	1	1	1	1	4	4	4	4
1	1	4	4	1	1	4	4	1	1
1	1	4	4	3	3	4	4	1	1
1	1	4	4	1	1	4	4	1	1
1	1	4	4	1	1	4	4	1	1
1	1	4	4	1	1	4	4	1	1
1	1	4	4	1	1	4	4	1	1
1	1	4	4	1	1	4	4	1	1
4	4	4	4	1	1	4	4	1	1
1	1	4	4	1	1	4	4	1	1
1	1	4	4	1	1	4	4	1	1
1	1	4	4	1	1	4	4	1	1
1	1	4	4	1	1	4	4	1	1
1	1	4	4	1	1	4	4	1	1
1	1	4	4	1	1	4	4	1	1
1	1	4	4	1	1	4	4	1	1
1	1	4	4	1	1	4	4	1	1
1	1	4	4	3	3	4	4	1	1
1	1	4	4	1	1	4	4	1	1
1	1	4	4	1	1	4	4	1	1
1	1	4	4	1	1	4	4	1	1
1	1	4	4	1	1	4	4	1	1
1	1	4	4	3	3	4	4	1	1
1	1	4	4	1	1	4	4	1	1
1	1	4	4	1	1	4	4	1	1
4	4	4	4	1	1	4	4	1	1
1	1	4	4	1	1	4	4	1	1
1	1	4	4	3	3	4	4	1	1
1	1	4	4	1	1	4	4	1	1
1	1	4	4	1	1	4	4	1	1
1	1	4	4	1	1	4	4	1	1
1	1	4	4	1	1	1	1	1	1
1	1	4	4	1	1	4	4	1	1
1	1	4	4	1	1	4	4	1	1
1	1	4	4	3	3	1	1	1	1
1	1	4	4	3	3	4	4	1	1
1	1	4	4	1	1	4	4	1	1
1	1	4	4	1	1	4	4	1	1
1	1	4	4	1	1	4	4	1	1
1	1	4	4	1	1	4	4	1	1
1	1	4	4	3	3	4	4	1	1
1	1	4	4	1	1	4	4	1	1
1	1	4	4	1	1	4	4	1	1
1	1	4	4	1	1	4	4	1	1
1	1	4	4	1	1	4	4	1	1
1	1	4	4	1	1	4	4	1	1
1	1	4	4	1	1	4	4	1	1
1	1	4	4	1	1	4	4	1	1
1	1	4	4	1	1	4	4	1	1
1	1	4	4	1	1	4	4	1	1
1	1	4	4	1	1	4	4	1	1
1	1	4	4	1	1	1	1	1	1
1	1	4	4	1	1	4	4	1	1
1	1	4	4	1	1	4	4	1	1

For Review Only

1	1	4	4	1	1	4	4	1	1
1	1	1	1	1	1	4	4	4	4
1	1	4	4	1	1	4	4	1	1
1	1	4	4	1	1	4	4	1	1
1	1	4	4	1	1	4	4	1	1
1	1	4	4	1	1	4	4	1	1
1	1	4	4	1	1	4	4	1	1
1	1	4	4	1	1	4	4	1	1
1	1	4	4	3	3	4	4	1	1
1	1	4	4	1	1	1	1	1	1
1	1	4	4	1	1	4	4	1	1
1	1	4	4	1	1	4	4	1	1
1	1	4	4	1	1	4	4	1	1
1	1	4	4	1	1	4	4	1	1
1	1	4	4	1	1	4	4	1	1
1	1	4	4	3	3	4	4	1	1
1	1	4	4	1	1	1	1	1	1
1	1	4	4	1	1	4	4	1	1
1	1	4	4	1	1	4	4	1	1
1	1	4	4	1	1	4	4	1	1
1	1	4	4	1	1	4	4	1	1
1	1	4	4	1	1	4	4	1	1
1	1	4	4	1	1	4	4	1	1
1	1	4	4	1	1	4	4	1	1
1	1	1	1	1	1	4	4	1	1
1	1	4	4	1	1	4	4	1	1
1	1	4	4	1	1	4	4	1	1
1	1	4	4	1	1	4	4	1	1
1	1	4	4	1	1	4	4	1	1
1	1	4	4	1	1	1	1	1	1
1	1	4	4	1	1	4	4	1	1
1	1	4	4	1	1	4	4	1	1
4	4	4	4	1	1	4	4	1	1
1	1	4	4	1	1	4	4	1	1
4	4	4	4	1	1	1	1	1	1
1	1	4	4	1	1	4	4	1	1
1	1	1	1	1	1	4	4	4	4
1	1	4	4	1	1	1	1	1	1
1	1	4	4	1	1	4	4	1	1
1	1	4	4	1	1	4	4	1	1
4	4	4	4	1	1	1	1	1	1
1	1	4	4	1	1	4	4	1	1
1	1	4	4	3	3	4	4	1	1
1	1	4	4	1	1	4	4	1	1
1	1	4	4	1	1	4	4	1	1
4	4	4	4	1	1	1	1	1	1
1	1	4	4	3	3	4	4	1	1
1	1	4	4	3	3	4	4	1	1
1	1	4	4	1	1	4	4	1	1
1	1	4	4	1	1	4	4	1	1
1	1	4	4	3	3	4	4	1	1
1	1	4	4	1	1	4	4	1	1
1	1	4	4	1	1	4	4	1	1
1	1	4	4	1	1	4	4	1	1
1	1	4	4	1	1	4	4	1	1

For Review Only

1	1	4	4	1	1	4	4	1	1
1	1	4	4	1	1	4	4	1	1
1	1	4	4	1	1	4	4	1	1
1	1	4	4	1	1	4	4	1	1
1	1	4	4	1	1	4	4	1	1
4	4	4	4	1	1	1	1	1	1
1	1	4	4	1	1	4	4	1	1
1	1	4	4	1	1	4	4	1	1
1	1	4	4	3	3	4	4	1	1
1	1	4	4	3	3	4	4	1	1
1	1	4	4	1	1	4	4	1	1
1	1	4	4	1	1	4	4	1	1
1	1	4	4	1	1	4	4	1	1
1	1	4	4	1	1	4	4	1	1
1	1	4	4	1	1	4	4	1	1
1	1	4	4	3	3	1	1	1	1
1	1	1	1	3	3	4	4	4	4
1	1	4	4	3	3	4	4	1	1
1	1	1	1	1	1	4	4	4	4
1	1	1	1	1	1	4	4	4	4
1	1	1	1	1	1	4	4	4	4
1	1	4	4	1	1	4	4	1	1
1	1	4	4	1	1	1	1	1	1
1	1	4	4	1	1	4	4	1	1
1	1	4	4	1	1	4	4	1	1
1	1	4	4	1	1	4	4	1	1
1	1	4	4	3	3	4	4	1	1
1	1	4	4	1	1	4	4	1	1
1	1	4	4	1	1	4	4	1	1
1	1	4	4	3	3	4	4	1	1
1	1	4	4	3	3	4	4	1	1
1	1	4	4	3	3	4	4	1	1
1	1	4	4	3	3	4	4	1	1
1	1	4	4	1	1	4	4	1	1
1	1	4	4	1	1	4	4	1	1
1	1	4	4	3	3	4	4	1	1
1	1	4	4	1	1	1	1	1	1
1	1	4	4	1	1	4	4	1	1
1	1	4	4	1	1	4	4	1	1
1	1	4	4	3	3	4	4	1	1
4	4	4	4	1	1	1	1	1	1
1	1	4	4	1	1	4	4	1	1
1	1	4	4	1	1	4	4	1	1
1	1	4	4	1	1	1	1	1	1
1	1	4	4	1	1	4	4	1	1
1	1	4	4	1	1	1	1	1	1
1	1	4	4	1	1	4	4	1	1
1	1	4	4	3	3	4	4	1	1
1	1	4	4	1	1	4	4	1	1
1	1	4	4	3	3	4	4	1	1
1	1	4	4	1	1	4	4	1	1
1	1	4	4	1	1	4	4	1	1
4	4	4	4	1	1	1	1	1	1
1	1	4	4	1	1	4	4	1	1
1	1	4	4	1	1	4	4	1	1

For Review Only

1	1	4	4	1	1	1	1	1	1
1	1	4	4	1	1	4	4	1	1

For Review Only

SNP357	SNP357	SNP358	SNP358	SNP359	SNP359	SNP360	SNP360	SNP361	SNP361
3	3	2	2	1	1	4	4	1	1
3	3	2	2	1	1	4	4	1	1
3	3	2	2	1	1	4	4	1	1
3	3	2	2	1	1	4	4	1	1
3	3	2	2	1	1	4	4	1	1
3	3	2	2	1	1	4	4	1	1
3	3	2	2	1	1	4	4	1	1
3	3	4	4	1	1	4	4	1	1
3	3	2	2	1	1	4	4	1	1
3	3	2	2	1	1	4	4	1	1
3	3	2	2	1	1	4	4	1	1
3	3	2	2	1	1	4	4	1	1
2	2	2	2	3	3	4	4	1	1
3	3	2	2	1	1	1	1	1	1
3	3	2	2	1	1	4	4	1	1
3	3	2	2	1	1	4	4	1	1
3	3	2	2	1	1	4	4	1	1
3	3	2	2	1	1	4	4	1	1
3	3	4	4	1	1	4	4	1	1
3	3	4	4	1	1	4	4	1	1
3	3	2	2	1	1	4	4	1	1
2	2	2	2	3	3	1	1	1	1
3	3	2	2	1	1	4	4	1	1
3	3	2	2	1	1	4	4	1	1
3	3	2	2	1	1	4	4	1	1
3	3	2	2	1	1	4	4	1	1
3	3	2	2	1	1	4	4	1	1
3	3	2	2	1	1	4	4	1	1
2	2	2	2	3	3	1	1	1	1
2	2	2	2	3	3	1	1	1	1
3	3	2	2	1	1	4	4	1	1
3	3	2	2	1	1	4	4	1	1
3	3	2	2	1	1	4	4	1	1
3	3	2	2	1	1	4	4	1	1
3	3	2	2	1	1	4	4	1	1
3	3	2	2	1	1	4	4	1	1
3	3	2	2	1	1	4	4	1	1
2	2	2	2	3	3	1	1	1	1
3	3	2	2	1	1	4	4	1	1
3	3	2	2	1	1	4	4	1	1
2	2	2	2	3	3	1	1	4	4
2	2	2	2	3	3	1	1	4	4
3	3	4	4	1	1	4	4	1	1
3	3	2	2	1	1	4	4	1	1
3	3	2	2	1	1	4	4	1	1
3	3	2	2	1	1	4	4	1	1
3	3	4	4	1	1	4	4	1	1
3	3	2	2	1	1	4	4	1	1
3	3	2	2	1	1	4	4	1	1
3	3	2	2	1	1	4	4	1	1
3	3	2	2	1	1	4	4	1	1
3	3	2	2	1	1	4	4	1	1

For Review Only

3	3	2	2	1	1	4	4	1	1
3	3	2	2	1	1	4	4	1	1
3	3	2	2	1	1	4	4	1	1
3	3	2	2	1	1	4	4	1	1
3	3	4	4	1	1	4	4	1	1
3	3	4	4	1	1	4	4	1	1
3	3	2	2	1	1	4	4	1	1
3	3	2	2	1	1	4	4	1	1
3	3	2	2	1	1	4	4	1	1
3	3	2	2	1	1	4	4	1	1
3	3	4	4	1	1	4	4	1	1
3	3	4	4	1	1	4	4	1	1
3	3	2	2	1	1	4	4	1	1
2	2	2	2	3	3	1	1	1	1
3	3	2	2	1	1	4	4	1	1
3	3	2	2	1	1	4	4	1	1
3	3	2	2	1	1	4	4	1	1
3	3	4	4	1	1	4	4	1	1
3	3	2	2	1	1	4	4	1	1
3	3	2	2	1	1	4	4	1	1
3	3	2	2	1	1	4	4	1	1
3	3	2	2	1	1	4	4	1	1
2	2	2	2	3	3	4	4	1	1
3	3	4	4	1	1	4	4	1	1
3	3	2	2	1	1	4	4	1	1
3	3	4	4	1	1	4	4	1	1
2	2	2	2	3	3	1	1	1	1
3	3	2	2	1	1	4	4	1	1
3	3	2	2	1	1	4	4	1	1
3	3	2	2	1	1	4	4	1	1
3	3	2	2	1	1	4	4	1	1
3	3	2	2	1	1	4	4	1	1
3	3	2	2	1	1	4	4	1	1
3	3	2	2	1	1	4	4	1	1
3	3	2	2	1	1	4	4	1	1
3	3	2	2	1	1	4	4	1	1
3	3	4	4	1	1	4	4	1	1
3	3	2	2	1	1	4	4	1	1
3	3	2	2	1	1	4	4	1	1
3	3	2	2	1	1	4	4	1	1
3	3	2	2	1	1	4	4	1	1
3	3	2	2	1	1	4	4	1	1
2	2	2	2	3	3	1	1	1	1
3	3	4	4	1	1	4	4	1	1
3	3	2	2	1	1	4	4	1	1
3	3	2	2	1	1	4	4	1	1
3	3	2	2	1	1	4	4	4	4
3	3	2	2	1	1	4	4	1	1
3	3	2	2	1	1	4	4	1	1
3	3	2	2	1	1	4	4	1	1
3	3	2	2	1	1	4	4	1	1
3	3	2	2	1	1	4	4	1	1
3	3	2	2	1	1	4	4	1	1
3	3	2	2	1	1	4	4	1	1
3	3	2	2	1	1	4	4	1	1

For Review Only

3	3	2	2	1	1	4	4	1	1
2	2	2	2	3	3	1	1	1	1
3	3	2	2	1	1	4	4	1	1
3	3	2	2	1	1	1	1	1	1
3	3	2	2	1	1	4	4	1	1
3	3	2	2	1	1	4	4	1	1
3	3	2	2	1	1	4	4	1	1
3	3	2	2	1	1	4	4	1	1
3	3	2	2	1	1	4	4	1	1
3	3	2	2	1	1	4	4	1	1
3	3	2	2	1	1	4	4	1	1
3	3	2	2	1	1	4	4	1	1
3	3	4	4	1	1	4	4	1	1
3	3	2	2	1	1	4	4	1	1
3	3	4	4	1	1	4	4	1	1
3	3	2	2	1	1	4	4	1	1
3	3	4	4	1	1	4	4	1	1
3	3	2	2	1	1	4	4	1	1
3	3	2	2	1	1	4	4	1	1
3	3	2	2	1	1	4	4	1	1
3	3	2	2	1	1	4	4	1	1
3	3	2	2	1	1	4	4	1	1
3	3	2	2	1	1	4	4	1	1
3	3	2	2	1	1	1	1	4	4
3	3	2	2	1	1	4	4	1	1
3	3	2	2	1	1	4	4	1	1
3	3	2	2	1	1	4	4	1	1
3	3	2	2	1	1	4	4	1	1
3	3	2	2	1	1	4	4	1	1
3	3	2	2	1	1	4	4	1	1
3	3	2	2	1	1	4	4	1	1
3	3	2	2	1	1	4	4	1	1
3	3	2	2	1	1	4	4	1	1
3	3	2	2	1	1	4	4	1	1
3	3	2	2	1	1	4	4	1	1
3	3	2	2	1	1	4	4	1	1
3	3	2	2	1	1	4	4	4	4
3	3	2	2	1	1	4	4	1	1
3	3	2	2	1	1	4	4	1	1
3	3	2	2	1	1	4	4	1	1
3	3	2	2	1	1	4	4	1	1
3	3	2	2	1	1	4	4	4	4
3	3	2	2	1	1	4	4	1	1
3	3	2	2	1	1	4	4	1	1
3	3	2	2	1	1	4	4	1	1
3	3	2	2	1	1	4	4	1	1
3	3	2	2	1	1	4	4	1	1
3	3	2	2	1	1	4	4	1	1
3	3	2	2	1	1	4	4	1	1
3	3	2	2	1	1	4	4	1	1
3	3	2	2	1	1	4	4	1	1
3	3	2	2	1	1	4	4	1	1
3	3	2	2	1	1	4	4	1	1
3	3	4	4	1	1	4	4	1	1
2	2	2	2	1	1	1	1	1	1

For Review Only

3	3	2	2	1	1	4	4	1	1
2	2	2	2	3	3	1	1	1	1
3	3	2	2	1	1	4	4	1	1
3	3	2	2	1	1	4	4	1	1
3	3	2	2	1	1	4	4	1	1
3	3	2	2	1	1	4	4	1	1
3	3	2	2	1	1	4	4	4	4
3	3	2	2	1	1	4	4	1	1
3	3	2	2	1	1	4	4	1	1
3	3	2	2	1	1	4	4	1	1
3	3	2	2	1	1	4	4	1	1
3	3	2	2	1	1	4	4	1	1
3	3	2	2	1	1	4	4	1	1
3	3	2	2	1	1	4	4	1	1
3	3	2	2	1	1	1	1	1	1
3	3	2	2	1	1	4	4	1	1
3	3	2	2	1	1	1	1	1	1
3	3	2	2	1	1	4	4	1	1
3	3	2	2	1	1	4	4	1	1
3	3	2	2	1	1	4	4	1	1
3	3	2	2	1	1	4	4	1	1
3	3	2	2	1	1	1	1	1	1
3	3	2	2	1	1	4	4	1	1
3	3	4	4	1	1	4	4	1	1
3	3	2	2	3	3	1	1	1	1
3	3	2	2	1	1	4	4	1	1
3	3	2	2	1	1	4	4	1	1
3	3	2	2	1	1	4	4	1	1
3	3	2	2	1	1	4	4	1	1
3	3	2	2	1	1	4	4	1	1
3	3	2	2	1	1	4	4	1	1
3	3	4	4	1	1	4	4	1	1
3	3	2	2	1	1	4	4	1	1
3	3	2	2	1	1	1	1	1	1
3	3	2	2	1	1	4	4	1	1
3	3	4	4	1	1	4	4	1	1
2	2	2	2	3	3	4	4	1	1
3	3	2	2	1	1	4	4	1	1
3	3	2	2	1	1	4	4	1	1
3	3	4	4	1	1	4	4	1	1
3	3	2	2	1	1	4	4	1	1
3	3	2	2	1	1	4	4	1	1
3	3	2	2	1	1	4	4	1	1
3	3	2	2	1	1	4	4	1	1
3	3	2	2	1	1	4	4	1	1
3	3	2	2	1	1	4	4	1	1
3	3	2	2	1	1	4	4	1	1
3	3	2	2	1	1	4	4	4	4
3	3	2	2	1	1	4	4	1	1
3	3	2	2	1	1	4	4	1	1
3	3	2	2	1	1	4	4	1	1
3	3	2	2	1	1	4	4	1	1
3	3	2	2	1	1	4	4	1	1
3	3	4	4	1	1	4	4	1	1
3	3	2	2	1	1	4	4	1	1

For Review Only

3	3	2	2	1	1	4	4	1	1
3	3	4	4	1	1	4	4	1	1
3	3	2	2	1	1	4	4	1	1
3	3	2	2	1	1	4	4	1	1
3	3	2	2	1	1	4	4	1	1
3	3	2	2	1	1	4	4	1	1
3	3	2	2	1	1	4	4	1	1
3	3	2	2	1	1	4	4	1	1
3	3	2	2	1	1	4	4	4	4
3	3	2	2	1	1	4	4	4	4
3	3	2	2	1	1	4	4	1	1
3	3	2	2	1	1	4	4	1	1
3	3	2	2	1	1	4	4	1	1
3	3	2	2	1	1	4	4	1	1
3	3	2	2	1	1	4	4	1	1
3	3	2	2	1	1	4	4	4	4
2	2	2	2	3	3	4	4	1	1
3	3	2	2	1	1	4	4	1	1
2	2	2	2	3	3	1	1	1	1
2	2	2	2	3	3	1	1	1	1
2	2	2	2	3	3	1	1	1	1
3	3	2	2	1	1	4	4	1	1
3	3	2	2	1	1	4	4	1	1
3	3	2	2	1	1	4	4	1	1
3	3	2	2	1	1	4	4	1	1
3	3	2	2	1	1	4	4	1	1
3	3	2	2	1	1	1	1	4	4
3	3	2	2	1	1	4	4	1	1
3	3	2	2	1	1	4	4	1	1
3	3	2	2	1	1	4	4	1	1
3	3	2	2	1	1	1	1	4	4
3	3	2	2	1	1	4	4	1	1
3	3	2	2	1	1	4	4	1	1
3	3	2	2	1	1	4	4	1	1
3	3	2	2	1	1	4	4	1	1
3	3	2	2	1	1	1	1	4	4
3	3	2	2	1	1	4	4	1	1
3	3	4	4	1	1	4	4	1	1
3	3	2	2	1	1	4	4	1	1
3	3	2	2	1	1	4	4	1	1
3	3	2	2	1	1	4	4	1	1
3	3	2	2	1	1	4	4	1	1
3	3	2	2	1	1	4	4	1	1
3	3	2	2	1	1	4	4	1	1
3	3	2	2	1	1	4	4	1	1
3	3	2	2	1	1	4	4	1	1
3	3	2	2	1	1	4	4	1	1
3	3	2	2	1	1	4	4	1	1
3	3	2	2	1	1	4	4	1	1
3	3	2	2	1	1	4	4	4	4
3	3	2	2	1	1	4	4	1	1
3	3	2	2	1	1	1	1	1	1
3	3	2	2	1	1	4	4	1	1
3	3	2	2	1	1	4	4	1	1
3	3	2	2	1	1	4	4	1	1

For Review Only

3	3	2	2	1	1	4	4	1	1
3	3	4	4	1	1	4	4	1	1

For Review Only

SNP362	SNP362	SNP363	SNP363	SNP364	SNP364	SNP365	SNP365	SNP366	SNP366
1	1	4	4	4	4	1	1	4	4
3	3	4	4	4	4	2	2	2	2
1	1	4	4	4	4	2	2	2	2
1	1	4	4	4	4	1	1	2	2
3	3	4	4	4	4	1	1	2	2
3	3	4	4	4	4	1	1	4	4
1	1	4	4	4	4	1	1	4	4
3	3	4	4	4	4	2	2	2	2
3	3	4	4	4	4	1	1	4	4
3	3	4	4	4	4	1	1	2	2
1	1	4	4	4	4	1	1	2	2
1	1	4	4	4	4	2	2	2	2
1	1	4	4	4	4	1	1	2	2
3	3	4	4	4	4	1	1	2	2
1	1	4	4	4	4	1	1	2	2
3	3	4	4	4	4	2	2	2	2
1	1	4	4	4	4	1	1	2	2
1	1	4	4	4	4	1	1	2	2
3	3	4	4	4	4	1	1	2	2
1	1	4	4	4	4	1	1	2	2
3	3	4	4	4	4	1	1	2	2
1	1	4	4	4	4	1	1	2	2
3	3	4	4	4	4	1	1	2	2
3	3	4	4	4	4	2	2	2	2
3	3	4	4	4	4	1	1	4	4
1	1	4	4	4	4	1	1	2	2
3	3	4	4	4	4	1	1	2	2
1	1	4	4	4	4	2	2	2	2
1	1	4	4	4	4	2	2	2	2
3	3	4	4	4	4	1	1	2	2
3	3	4	4	4	4	1	1	2	2
1	1	4	4	4	4	1	1	2	2
1	1	4	4	4	4	2	2	4	4
3	3	4	4	4	4	1	1	2	2
1	1	4	4	4	4	1	1	2	2
3	3	4	4	4	4	2	2	2	2
3	3	4	4	4	4	1	1	4	4
1	1	4	4	4	4	2	2	2	2
3	3	4	4	4	4	1	1	2	2
1	1	4	4	1	1	2	2	2	2
3	3	3	3	4	4	1	1	2	2
1	1	3	3	4	4	2	2	2	2
1	1	4	4	4	4	1	1	2	2
3	3	4	4	4	4	1	1	4	4
3	3	4	4	4	4	1	1	2	2
1	1	4	4	4	4	2	2	2	2
1	1	4	4	4	4	2	2	2	2
1	1	4	4	4	4	1	1	2	2
3	3	4	4	4	4	2	2	4	4
1	1	4	4	4	4	1	1	2	2
1	1	4	4	4	4	1	1	2	2
1	1	4	4	4	4	1	1	2	2

For Review Only

1	1	4	4	4	4	1	1	4	4
1	1	4	4	4	4	2	2	4	4
3	3	4	4	4	4	1	1	2	2
1	1	4	4	4	4	1	1	2	2
3	3	4	4	4	4	1	1	2	2
1	1	4	4	4	4	2	2	2	2
1	1	4	4	4	4	1	1	2	2
3	3	4	4	4	4	1	1	2	2
3	3	4	4	4	4	1	1	2	2
1	1	4	4	4	4	2	2	2	2
1	1	4	4	4	4	2	2	2	2
3	3	4	4	4	4	2	2	2	2
1	1	4	4	4	4	1	1	4	4
1	1	4	4	4	4	2	2	2	2
1	1	4	4	4	4	1	1	2	2
3	3	4	4	4	4	2	2	2	2
3	3	4	4	4	4	1	1	2	2
1	1	4	4	4	4	1	1	2	2
1	1	4	4	4	4	1	1	2	2
1	1	4	4	4	4	1	1	4	4
3	3	4	4	4	4	2	2	2	2
1	1	4	4	4	4	1	1	2	2
1	1	4	4	4	4	1	1	4	4
3	3	4	4	4	4	2	2	2	2
3	3	4	4	4	4	1	1	2	2
3	3	4	4	4	4	1	1	2	2
1	1	4	4	4	4	2	2	2	2
3	3	4	4	4	4	1	1	2	2
1	1	4	4	4	4	1	1	2	2
3	3	4	4	4	4	2	2	2	2
1	1	4	4	4	4	1	1	2	2
3	3	4	4	4	4	2	2	2	2
3	3	4	4	4	4	1	1	2	2
1	1	4	4	4	4	1	1	4	4
1	1	4	4	4	4	1	1	2	2
3	3	4	4	4	4	1	1	2	2
3	3	4	4	4	4	1	1	2	2
1	1	4	4	4	4	1	1	4	4
3	3	4	4	4	4	1	1	2	2
3	3	4	4	4	4	1	1	2	2
1	1	4	4	4	4	2	2	2	2
3	3	4	4	4	4	2	2	2	2
3	3	4	4	4	4	1	1	2	2
1	1	4	4	4	4	2	2	2	2
3	3	4	4	1	1	1	1	2	2
1	1	4	4	4	4	2	2	2	2
3	3	3	3	4	4	1	1	2	2
3	3	4	4	4	4	1	1	2	2
1	1	4	4	4	4	1	1	2	2
1	1	4	4	4	4	1	1	2	2
1	1	4	4	4	4	1	1	2	2
3	3	4	4	4	4	2	2	2	2
3	3	4	4	4	4	1	1	2	2
1	1	4	4	4	4	2	2	2	2
3	3	4	4	4	4	1	1	2	2

For Review Only

1	1	4	4	4	4	1	1	2	2
1	1	4	4	4	4	1	1	2	2
3	3	4	4	4	4	2	2	2	2
1	1	4	4	4	4	1	1	2	2
1	1	4	4	4	4	2	2	2	2
1	1	4	4	4	4	2	2	2	2
3	3	4	4	4	4	2	2	2	2
3	3	4	4	4	4	1	1	2	2
3	3	4	4	4	4	1	1	2	2
3	3	4	4	4	4	2	2	2	2
1	1	4	4	1	1	1	1	4	4
1	1	4	4	4	4	2	2	2	2
3	3	4	4	4	4	1	1	2	2
3	3	4	4	4	4	1	1	2	2
1	1	4	4	4	4	1	1	2	2
1	1	4	4	4	4	1	1	2	2
1	1	4	4	4	4	1	1	2	2
1	1	4	4	4	4	2	2	2	2
3	3	4	4	4	4	1	1	2	2
1	1	4	4	4	4	1	1	2	2
1	1	4	4	4	4	2	2	2	2
1	1	4	4	4	4	2	2	2	2
3	3	4	4	4	4	2	2	2	2
3	3	3	3	4	4	1	1	2	2
1	1	4	4	4	4	1	1	2	2
1	1	4	4	4	4	1	1	2	2
1	1	4	4	4	4	2	2	2	2
3	3	4	4	4	4	1	1	2	2
1	1	4	4	4	4	1	1	2	2
1	1	4	4	4	4	1	1	2	2
3	3	4	4	1	1	1	1	2	2
1	1	4	4	4	4	1	1	2	2
1	1	4	4	4	4	2	2	2	2
1	1	4	4	4	4	1	1	2	2
3	3	4	4	4	4	1	1	2	2
1	1	4	4	4	4	1	1	2	2
3	3	3	3	4	4	1	1	2	2
1	1	4	4	4	4	2	2	2	2
1	1	4	4	4	4	1	1	2	2
1	1	4	4	4	4	1	1	2	2
1	1	4	4	4	4	2	2	2	2
3	3	3	3	1	1	1	1	2	2
3	3	4	4	4	4	1	1	2	2
3	3	4	4	4	4	1	1	2	2
1	1	4	4	4	4	1	1	2	2
3	3	4	4	4	4	2	2	2	2
1	1	4	4	4	4	1	1	2	2
1	1	4	4	4	4	1	1	2	2
3	3	4	4	1	1	1	1	2	2
1	1	4	4	4	4	1	1	2	2
1	1	4	4	4	4	1	1	2	2
1	1	4	4	4	4	1	1	2	2
3	3	4	4	4	4	1	1	2	2
3	3	4	4	4	4	2	2	2	2
3	3	4	4	4	4	1	1	2	2

For Review Only

3	3	4	4	4	4	2	2	2	2
3	3	4	4	4	4	1	1	2	2
3	3	4	4	4	4	2	2	2	2
1	1	4	4	4	4	1	1	2	2
1	1	4	4	4	4	2	2	2	2
1	1	4	4	4	4	1	1	2	2
1	1	4	4	4	4	1	1	2	2
1	1	4	4	4	4	2	2	2	2
3	3	4	4	4	4	1	1	2	2
1	1	4	4	1	1	2	2	2	2
1	1	4	4	4	4	1	1	2	2
3	3	4	4	4	4	1	1	2	2
3	3	4	4	4	4	1	1	2	2
3	3	4	4	1	1	1	1	2	2
3	3	4	4	1	1	1	1	2	2
1	1	4	4	4	4	1	1	2	2
1	1	4	4	4	4	2	2	2	2
3	3	4	4	4	4	2	2	2	2
3	3	4	4	4	4	1	1	2	2
1	1	4	4	4	4	2	2	2	2
1	1	4	4	4	4	2	2	2	2
3	3	4	4	4	4	1	1	2	2
1	1	4	4	4	4	1	1	2	2
3	3	4	4	4	4	2	2	2	2
3	3	4	4	4	4	2	2	2	2
3	3	4	4	4	4	1	1	2	2
1	1	4	4	4	4	1	1	2	2
1	1	4	4	4	4	1	1	2	2
1	1	4	4	4	4	1	1	2	2
1	1	4	4	4	4	2	2	2	2
3	3	4	4	4	4	1	1	2	2
1	1	4	4	4	4	2	2	2	2
1	1	4	4	4	4	2	2	2	2
3	3	4	4	1	1	1	1	2	2
1	1	4	4	4	4	2	2	2	2
3	3	4	4	4	4	2	2	2	2
3	3	4	4	4	4	1	1	2	2
3	3	4	4	4	4	1	1	4	4
1	1	4	4	4	4	1	1	2	2
3	3	4	4	4	4	1	1	2	2
1	1	4	4	4	4	2	2	2	2
1	1	4	4	4	4	1	1	2	2
3	3	4	4	4	4	1	1	2	2
1	1	4	4	1	1	2	2	2	2
1	1	4	4	4	4	2	2	2	2
3	3	4	4	4	4	2	2	2	2
1	1	4	4	4	4	1	1	2	2
3	3	3	3	4	4	1	1	2	2
3	3	4	4	4	4	1	1	2	2
3	3	4	4	4	4	1	1	2	2
1	1	4	4	4	4	1	1	2	2
3	3	4	4	1	1	1	1	2	2
3	3	4	4	1	1	1	1	2	2
1	1	4	4	4	4	2	2	2	2
3	3	4	4	4	4	1	1	2	2

For Review Only

3	3	4	4	4	4	1	1	2	2
1	1	4	4	4	4	1	1	2	2
1	1	4	4	4	4	2	2	2	2
1	1	4	4	4	4	1	1	2	2
1	1	4	4	4	4	2	2	2	2
1	1	4	4	4	4	1	1	2	2
1	1	4	4	4	4	1	1	2	2
3	3	4	4	4	4	1	1	2	2
3	3	3	3	4	4	1	1	2	2
3	3	3	3	4	4	1	1	2	2
1	1	4	4	4	4	1	1	2	2
1	1	4	4	4	4	1	1	2	2
1	1	4	4	4	4	1	1	2	2
3	3	4	4	4	4	1	1	2	2
1	1	4	4	4	4	2	2	2	2
1	1	3	3	4	4	1	1	2	2
3	3	4	4	4	4	2	2	2	2
1	1	4	4	1	1	1	1	2	2
3	3	4	4	4	4	1	1	2	2
1	1	4	4	4	4	1	1	2	2
3	3	4	4	4	4	1	1	2	2
1	1	4	4	4	4	1	1	2	2
3	3	4	4	4	4	1	1	2	2
3	3	4	4	4	4	1	1	2	2
3	3	4	4	4	4	1	1	2	2
1	1	4	4	4	4	1	1	2	2
3	3	3	3	4	4	1	1	2	2
1	1	4	4	4	4	1	1	2	2
1	1	4	4	4	4	2	2	2	2
1	1	4	4	4	4	1	1	2	2
3	3	3	3	4	4	1	1	2	2
1	1	4	4	4	4	2	2	2	2
3	3	4	4	1	1	1	1	2	2
1	1	4	4	4	4	1	1	2	2
1	1	4	4	4	4	1	1	2	2
3	3	3	3	4	4	1	1	2	2
3	3	4	4	4	4	1	1	2	2
3	3	4	4	4	4	2	2	2	2
3	3	4	4	4	4	2	2	2	2
3	3	4	4	4	4	1	1	2	2
1	1	4	4	4	4	1	1	2	2
1	1	4	4	4	4	1	1	2	2
1	1	4	4	4	4	1	1	2	2
1	1	4	4	4	4	1	1	2	2
3	3	4	4	4	4	1	1	2	2
3	3	4	4	4	4	1	1	2	2
1	1	4	4	4	4	1	1	2	2
3	3	4	4	4	4	1	1	2	2
3	3	4	4	4	4	1	1	2	2
3	3	3	3	4	4	1	1	2	2
1	1	4	4	4	4	1	1	2	2
3	3	4	4	4	4	1	1	2	2
1	1	4	4	4	4	2	2	2	2
1	1	4	4	4	4	1	1	2	2
1	1	4	4	1	1	1	1	2	2

For Review Only

1	1	4	4	4	4	1	1	2	2
3	3	4	4	4	4	1	1	2	2

For Review Only

SNP367	SNP367	SNP368	SNP368	SNP369	SNP369	SNP370	SNP370	SNP371	SNP371
4	4	4	4	2	2	2	2	1	1
3	3	1	1	2	2	2	2	1	1
4	4	1	1	2	2	2	2	1	1
3	3	4	4	1	1	2	2	4	4
3	3	4	4	2	2	2	2	1	1
4	4	4	4	2	2	2	2	1	1
4	4	4	4	1	1	2	2	4	4
3	3	1	1	2	2	2	2	1	1
3	3	1	1	2	2	2	2	1	1
3	3	4	4	2	2	2	2	1	1
3	3	4	4	2	2	2	2	1	1
3	3	1	1	2	2	2	2	1	1
4	4	1	1	2	2	2	2	1	1
3	3	4	4	2	2	2	2	1	1
3	3	4	4	2	2	2	2	1	1
3	3	1	1	2	2	2	2	1	1
4	4	4	4	2	2	2	2	1	1
4	4	4	4	1	1	2	2	4	4
3	3	4	4	2	2	2	2	1	1
3	3	4	4	2	2	2	2	1	1
4	4	4	4	1	1	2	2	4	4
4	4	4	4	1	1	2	2	4	4
3	3	1	1	1	1	2	2	4	4
3	3	4	4	2	2	2	2	1	1
4	4	4	4	2	2	2	2	1	1
4	4	4	4	2	2	2	2	1	1
3	3	4	4	2	2	2	2	1	1
3	3	1	1	2	2	2	2	1	1
3	3	1	1	2	2	2	2	1	1
3	3	4	4	1	1	2	2	4	4
3	3	4	4	2	2	2	2	1	1
3	3	4	4	2	2	2	2	1	1
4	4	1	1	2	2	2	2	1	1
3	3	4	4	2	2	2	2	1	1
3	3	4	4	2	2	2	2	1	1
3	3	1	1	2	2	2	2	1	1
3	3	4	4	1	1	2	2	4	4
4	4	1	1	2	2	2	2	1	1
3	3	4	4	2	2	2	2	1	1
3	3	1	1	2	2	2	2	1	1
3	3	4	4	2	2	2	2	4	4
3	3	1	1	2	2	2	2	1	1
3	3	4	4	2	2	2	2	1	1
4	4	4	4	2	2	2	2	1	1
3	3	4	4	2	2	2	2	1	1
4	4	1	1	2	2	2	2	1	1
4	4	4	4	2	2	4	4	1	1
4	4	4	4	2	2	2	2	1	1
3	3	1	1	2	2	2	2	1	1
3	3	4	4	2	2	2	2	1	1
4	4	4	4	2	2	2	2	1	1
3	3	4	4	2	2	2	2	1	1

For Review Only

4	4	4	4	2	2	2	2	1	1
4	4	1	1	2	2	2	2	1	1
3	3	4	4	2	2	2	2	1	1
4	4	4	4	1	1	2	2	4	4
3	3	4	4	2	2	2	2	1	1
3	3	1	1	2	2	4	4	1	1
3	3	4	4	2	2	2	2	1	1
3	3	4	4	2	2	2	2	1	1
3	3	4	4	2	2	2	2	1	1
3	3	1	1	2	2	2	2	1	1
3	3	1	1	2	2	2	2	1	1
3	3	1	1	2	2	4	4	1	1
4	4	4	4	2	2	2	2	1	1
3	3	1	1	2	2	2	2	1	1
3	3	4	4	2	2	2	2	1	1
4	4	1	1	2	2	2	2	1	1
3	3	4	4	2	2	2	2	1	1
4	4	4	4	2	2	2	2	1	1
3	3	4	4	2	2	2	2	1	1
4	4	4	4	2	2	2	2	1	1
3	3	1	1	2	2	4	4	1	1
3	3	4	4	2	2	2	2	1	1
3	3	1	1	2	2	2	2	1	1
3	3	1	1	2	2	2	2	1	1
3	3	4	4	2	2	2	2	1	1
3	3	4	4	2	2	2	2	1	1
4	4	1	1	2	2	2	2	1	1
3	3	4	4	1	1	2	2	4	4
4	4	4	4	2	2	2	2	1	1
3	3	1	1	2	2	2	2	1	1
3	3	4	4	1	1	2	2	4	4
3	3	1	1	2	2	2	2	1	1
3	3	4	4	2	2	2	2	1	1
4	4	4	4	2	2	2	2	1	1
4	4	4	4	2	2	2	2	1	1
3	3	4	4	2	2	2	2	1	1
3	3	4	4	1	1	2	2	4	4
4	4	4	4	2	2	2	2	1	1
4	4	4	4	2	2	2	2	1	1
4	4	4	4	2	2	2	2	1	1
3	3	1	1	2	2	2	2	1	1
3	3	1	1	2	2	2	2	1	1
4	4	4	4	2	2	4	4	1	1
3	3	1	1	2	2	2	2	1	1
4	4	4	4	2	2	2	2	1	1
4	4	1	1	2	2	2	2	1	1
3	3	4	4	2	2	2	2	1	1
4	4	4	4	2	2	2	2	1	1
4	4	4	4	2	2	2	2	1	1
3	3	4	4	2	2	2	2	1	1
3	3	4	4	2	2	2	2	1	1
3	3	4	4	2	2	2	2	1	1
3	3	4	4	2	2	2	2	1	1
3	3	4	4	2	2	2	2	1	1
4	4	4	4	2	2	2	2	1	1

For Review Only

3	3	4	4	2	2	2	2	1	1
3	3	4	4	2	2	2	2	1	1
3	3	1	1	2	2	2	2	1	1
4	4	4	4	2	2	2	2	1	1
3	3	1	1	2	2	2	2	1	1
4	4	1	1	2	2	2	2	1	1
3	3	1	1	2	2	2	2	1	1
3	3	4	4	2	2	2	2	1	1
4	4	4	4	2	2	2	2	1	1
3	3	1	1	2	2	2	2	1	1
3	3	4	4	2	2	2	2	1	1
3	3	1	1	2	2	2	2	1	1
4	4	4	4	2	2	4	4	1	1
3	3	4	4	2	2	2	2	1	1
3	3	4	4	2	2	2	2	1	1
3	3	1	1	2	2	2	2	1	1
4	4	4	4	2	2	4	4	1	1
3	3	1	1	2	2	2	2	1	1
3	3	4	4	2	2	2	2	1	1
3	3	4	4	2	2	2	2	1	1
3	3	4	4	2	2	2	2	1	1
3	3	1	1	2	2	4	4	1	1
4	4	4	4	2	2	2	2	1	1
3	3	4	4	1	1	2	2	1	1
3	3	1	1	2	2	2	2	1	1
3	3	4	4	2	2	2	2	1	1
3	3	1	1	2	2	2	2	1	1
3	3	4	4	2	2	2	2	1	1
3	3	4	4	2	2	2	2	1	1
3	3	4	4	2	2	4	4	1	1
3	3	4	4	2	2	2	2	1	1
3	3	4	4	2	2	2	2	1	1
4	4	1	1	2	2	2	2	1	1
3	3	4	4	2	2	2	2	1	1
3	3	4	4	2	2	2	2	1	1
3	3	4	4	2	2	2	2	1	1
4	4	4	4	2	2	2	2	1	1
3	3	1	1	2	2	2	2	1	1
4	4	4	4	2	2	2	2	1	1
3	3	4	4	2	2	2	2	1	1
3	3	1	1	2	2	4	4	1	1
3	3	4	4	2	2	2	2	1	1
4	4	4	4	2	2	2	2	1	1
3	3	4	4	2	2	2	2	1	1
4	4	4	4	2	2	2	2	1	1
4	4	1	1	2	2	2	2	1	1
4	4	4	4	2	2	2	2	1	1
4	4	4	4	2	2	2	2	1	1
3	3	4	4	2	2	2	2	1	1
4	4	4	4	2	2	2	2	1	1
3	3	4	4	2	2	2	2	1	1
4	4	4	4	2	2	2	2	1	1
4	4	4	4	2	2	2	2	1	1
4	4	1	1	2	2	2	2	1	1
3	3	4	4	2	2	2	2	1	1

For Review Only

4	4	1	1	2	2	2	2	1	1
3	3	4	4	2	2	4	4	1	1
3	3	1	1	2	2	2	2	1	1
3	3	4	4	2	2	2	2	1	1
4	4	1	1	2	2	2	2	1	1
3	3	4	4	2	2	2	2	1	1
3	3	4	4	2	2	2	2	1	1
3	3	4	4	2	2	2	2	1	1
3	3	4	4	2	2	2	2	1	1
3	3	1	1	2	2	2	2	1	1
4	4	4	4	2	2	2	2	1	1
3	3	4	4	2	2	2	2	1	1
4	4	4	4	2	2	2	2	1	1
3	3	1	1	2	2	2	2	1	1
3	3	4	4	2	2	2	2	1	1
3	3	4	4	2	2	2	2	1	1
3	3	4	4	2	2	2	2	1	1
3	3	1	1	2	2	2	2	1	1
4	4	4	4	2	2	2	2	1	1
3	3	1	1	2	2	2	2	1	1
3	3	4	4	2	2	2	2	1	1
3	3	4	4	2	2	2	2	1	1
3	3	4	4	2	2	2	2	1	1
3	3	1	1	2	2	4	4	1	1
3	3	1	1	2	2	2	2	1	1
3	3	4	4	2	2	2	2	1	1
3	3	4	4	2	2	2	2	1	1
3	3	4	4	2	2	2	2	1	1
3	3	4	4	2	2	4	4	1	1
4	4	1	1	2	2	2	2	1	1
4	4	4	4	2	2	2	2	1	1
4	4	1	1	2	2	2	2	1	1
3	3	1	1	2	2	2	2	1	1
3	3	4	4	2	2	2	2	1	1
3	3	1	1	2	2	2	2	1	1
3	3	1	1	2	2	4	4	1	1
3	3	4	4	2	2	2	2	1	1
4	4	4	4	2	2	2	2	1	1
4	4	4	4	2	2	2	2	1	1
4	4	4	4	2	2	2	2	1	1
4	4	1	1	2	2	2	2	1	1
4	4	4	4	2	2	2	2	1	1
3	3	4	4	2	2	2	2	1	1
3	3	1	1	2	2	2	2	1	1
3	3	1	1	2	2	2	2	1	1
3	3	1	1	2	2	2	2	1	1
3	3	4	4	2	2	2	2	1	1
4	4	4	4	2	2	2	2	1	1
3	3	4	4	2	2	2	2	1	1
3	3	4	4	2	2	2	2	1	1
3	3	4	4	2	2	2	2	1	1
3	3	4	4	1	1	2	2	4	4
3	3	4	4	2	2	2	2	1	1
3	3	1	1	2	2	2	2	1	1
3	3	4	4	2	2	2	2	4	4

For Review Only

4	4	4	4	2	2	2	2	1	1
3	3	4	4	2	2	2	2	4	4
3	3	1	1	2	2	2	2	1	1
3	3	4	4	2	2	2	2	1	1
3	3	1	1	2	2	2	2	1	1
4	4	1	1	2	2	2	2	1	1
3	3	4	4	2	2	2	2	1	1
3	3	4	4	2	2	2	2	1	1
3	3	4	4	2	2	2	2	1	1
3	3	4	4	2	2	2	2	1	1
3	3	4	4	2	2	2	2	1	1
4	4	4	4	2	2	2	2	1	1
4	4	4	4	2	2	2	2	1	1
4	4	4	4	2	2	2	2	1	1
4	4	1	1	2	2	2	2	1	1
3	3	4	4	2	2	2	2	1	1
3	3	1	1	2	2	2	2	1	1
3	3	4	4	2	2	2	2	1	1
4	4	4	4	2	2	2	2	1	1
4	4	4	4	2	2	2	2	1	1
3	3	4	4	2	2	2	2	1	1
4	4	4	4	2	2	2	2	1	1
3	3	4	4	2	2	2	2	1	1
3	3	4	4	2	2	2	2	1	1
3	3	4	4	2	2	2	2	1	1
3	3	4	4	2	2	2	2	4	4
3	3	4	4	2	2	2	2	1	1
3	3	4	4	2	2	2	2	1	1
3	3	1	1	2	2	2	2	1	1
3	3	4	4	2	2	2	2	1	1
4	4	4	4	2	2	2	2	1	1
4	4	1	1	2	2	2	2	1	1
3	3	4	4	2	2	2	2	1	1
4	4	4	4	2	2	2	2	1	1
3	3	4	4	2	2	2	2	1	1
3	3	4	4	2	2	2	2	1	1
4	4	4	4	2	2	2	2	1	1
3	3	1	1	2	2	4	4	1	1
3	3	1	1	2	2	2	2	1	1
3	3	4	4	2	2	2	2	1	1
3	3	4	4	2	2	2	2	1	1
4	4	4	4	2	2	2	2	1	1
3	3	4	4	2	2	2	2	1	1
4	4	4	4	2	2	2	2	1	1
4	4	4	4	2	2	2	2	1	1
3	3	4	4	2	2	2	2	1	1
4	4	4	4	2	2	2	2	1	1
4	4	4	4	2	2	2	2	1	1
3	3	4	4	2	2	2	2	1	1
3	3	4	4	2	2	2	2	1	1
3	3	4	4	2	2	2	2	1	1
3	3	4	4	2	2	2	2	1	1
4	4	1	1	2	2	2	2	1	1
4	4	4	4	2	2	2	2	1	1
3	3	4	4	2	2	2	2	1	1

For Review Only

CSSA 5585 Guilford Rd., Madison, WI 53711

3	3	4	4	2	2	2	2	1	1
3	3	4	4	2	2	2	2	1	1

For Review Only

SNP372	SNP372	SNP373	SNP373	SNP374	SNP374	SNP375	SNP375	SNP376	SNP376
1	1	3	3	3	3	3	3	2	2
4	4	3	3	3	3	3	3	3	3
1	1	3	3	3	3	3	3	3	3
4	4	3	3	2	2	3	3	2	2
4	4	3	3	3	3	2	2	2	2
1	1	3	3	2	2	3	3	2	2
1	1	3	3	3	3	3	3	2	2
4	4	3	3	3	3	2	2	3	3
4	4	3	3	3	3	2	2	2	2
4	4	3	3	3	3	2	2	2	2
4	4	3	3	3	3	3	3	2	2
4	4	3	3	3	3	3	3	3	3
1	1	3	3	3	3	2	2	2	2
4	4	3	3	2	2	2	2	2	2
1	1	3	3	3	3	3	3	2	2
4	4	3	3	3	3	3	3	3	3
4	4	3	3	3	3	3	3	2	2
1	1	3	3	3	3	3	3	2	2
4	4	3	3	3	3	3	3	2	2
4	4	3	3	2	2	3	3	2	2
1	1	3	3	3	3	3	3	2	2
1	1	3	3	3	3	3	3	2	2
4	4	3	3	3	3	2	2	2	2
4	4	3	3	2	2	2	2	2	2
1	1	3	3	3	3	3	3	2	2
1	1	3	3	3	3	3	3	3	3
4	4	3	3	3	3	3	3	2	2
4	4	3	3	3	3	3	3	3	3
4	4	3	3	3	3	3	3	2	2
4	4	3	3	3	3	2	2	2	2
4	4	3	3	3	3	3	3	2	2
4	4	3	3	2	2	3	3	2	2
1	1	3	3	3	3	3	3	3	3
4	4	3	3	3	3	2	2	2	2
4	4	3	3	3	3	3	3	3	3
4	4	3	3	2	2	2	2	3	3
4	4	3	3	3	3	2	2	2	2
1	1	3	3	3	3	3	3	3	3
4	4	3	3	3	3	2	2	2	2
4	4	3	3	2	2	2	2	3	3
4	4	3	3	2	2	2	2	2	2
4	4	3	3	2	2	3	3	2	2
4	4	3	3	3	3	3	3	2	2
1	1	3	3	2	2	2	2	2	2
4	4	3	3	3	3	3	3	2	2
1	1	3	3	3	3	3	3	3	3
1	1	3	3	2	2	3	3	3	3
1	1	3	3	3	3	3	3	2	2
4	4	3	3	2	2	2	2	3	3
1	1	3	3	3	3	3	3	2	2
1	1	3	3	2	2	3	3	3	3
4	4	3	3	3	3	3	3	2	2

For Review Only

1	1	3	3	3	3	3	3	2	2
1	1	3	3	3	3	3	3	3	3
4	4	2	2	2	2	3	3	2	2
1	1	3	3	3	3	3	3	2	2
4	4	3	3	3	3	3	3	2	2
4	4	3	3	3	3	3	3	3	3
4	4	3	3	3	3	3	3	2	2
4	4	3	3	3	3	2	2	2	2
4	4	3	3	3	3	2	2	2	2
4	4	3	3	3	3	3	3	3	3
4	4	3	3	3	3	3	3	3	3
4	4	3	3	3	3	3	3	3	3
1	1	3	3	3	3	3	3	2	2
4	4	3	3	3	3	3	3	2	2
1	1	3	3	3	3	3	3	3	3
1	1	3	3	3	3	2	2	3	3
4	4	3	3	3	3	2	2	2	2
1	1	3	3	3	3	3	3	2	2
4	4	3	3	3	3	3	3	2	2
1	1	3	3	3	3	3	3	2	2
4	4	3	3	3	3	3	3	3	3
4	4	3	3	2	2	3	3	2	2
4	4	3	3	3	3	2	2	2	2
4	4	3	3	3	3	3	3	3	3
4	4	3	3	3	3	3	3	2	2
4	4	3	3	3	3	3	3	2	2
1	1	3	3	2	2	3	3	3	3
4	4	3	3	2	2	2	2	2	2
1	1	3	3	2	2	3	3	3	3
4	4	3	3	3	3	3	3	3	3
4	4	3	3	3	3	3	3	2	2
4	4	3	3	3	3	2	2	3	3
4	4	3	3	2	2	2	2	2	2
4	4	3	3	3	3	3	3	2	2
1	1	3	3	3	3	3	3	2	2
4	4	3	3	3	3	3	3	2	2
4	4	3	3	3	3	2	2	2	2
1	1	3	3	3	3	3	3	2	2
1	1	3	3	3	3	2	2	2	2
1	1	3	3	3	3	3	3	2	2
1	1	3	3	3	3	3	3	3	3
4	4	3	3	3	3	3	3	3	3
1	1	3	3	3	3	2	2	2	2
4	4	3	3	3	3	3	3	3	3
1	1	3	3	3	3	2	2	2	2
1	1	3	3	3	3	3	3	3	3
4	4	3	3	2	2	2	2	2	2
1	1	3	3	2	2	2	2	2	2
1	1	3	3	2	2	3	3	3	3
4	4	3	3	3	3	3	3	2	2
4	4	3	3	3	3	3	3	2	2
4	4	3	3	3	3	2	2	2	2
4	4	3	3	2	2	2	2	2	2
4	4	3	3	3	3	3	3	2	2
1	1	3	3	3	3	2	2	2	2

For Review Only

4	4	3	3	3	3	3	3	3	3
4	4	3	3	2	2	3	3	2	2
4	4	3	3	3	3	2	2	3	3
1	1	3	3	2	2	2	2	2	2
4	4	3	3	3	3	3	3	3	3
1	1	2	2	2	2	3	3	3	3
4	4	3	3	3	3	3	3	3	3
4	4	3	3	2	2	3	3	2	2
1	1	3	3	3	3	2	2	3	3
4	4	3	3	3	3	2	2	3	3
1	1	3	3	3	3	3	3	2	2
1	1	3	3	3	3	3	3	2	2
1	1	3	3	3	3	2	2	2	2
4	4	3	3	3	3	3	3	2	2
4	4	3	3	3	3	3	3	2	2
1	1	3	3	3	3	2	2	3	3
1	1	3	3	3	3	3	3	2	2
4	4	3	3	3	3	3	3	3	3
4	4	3	3	2	2	2	2	2	2
4	4	3	3	3	3	3	3	2	2
4	4	3	3	3	3	3	3	3	3
4	4	3	3	3	3	3	3	3	3
1	1	3	3	3	3	3	3	2	2
4	4	3	3	3	3	2	2	2	2
4	4	3	3	2	2	3	3	3	3
4	4	3	3	3	3	3	3	2	2
4	4	3	3	3	3	3	3	3	3
4	4	3	3	3	3	2	2	2	2
4	4	2	2	2	2	3	3	2	2
4	4	3	3	3	3	3	3	2	2
4	4	3	3	3	3	2	2	2	2
4	4	3	3	3	3	3	3	2	2
1	1	3	3	3	3	3	3	3	3
4	4	3	3	3	3	2	2	3	3
4	4	3	3	3	3	2	2	2	2
4	4	3	3	3	3	2	2	3	3
1	1	3	3	2	2	2	2	2	2
4	4	3	3	3	3	3	3	3	3
4	4	3	3	3	3	2	2	2	2
4	4	3	3	3	3	3	3	2	2
4	4	3	3	3	3	3	3	3	3
4	4	3	3	3	3	2	2	2	2
1	1	3	3	3	3	2	2	3	3
4	4	3	3	3	3	2	2	2	2
1	1	3	3	2	2	3	3	2	2
1	1	3	3	3	3	2	2	3	3
1	1	3	3	2	2	3	3	3	3
1	1	3	3	3	3	3	3	3	3
4	4	3	3	3	3	2	2	2	2
1	1	3	3	2	2	3	3	3	3
4	4	3	3	3	3	3	3	3	3
1	1	3	3	2	2	3	3	2	2
1	1	3	3	3	3	3	3	3	3
1	1	3	3	2	2	3	3	3	3
4	4	3	3	3	3	2	2	2	2

For Review Only

1	1	3	3	2	2	3	3	3	3
4	4	3	3	3	3	2	2	2	2
1	1	3	3	2	2	3	3	3	3
4	4	3	3	3	3	3	3	3	3
1	1	3	3	3	3	3	3	3	3
4	4	3	3	2	2	3	3	2	2
4	4	3	3	2	2	3	3	2	2
4	4	3	3	3	3	3	3	3	3
4	4	3	3	2	2	3	3	2	2
4	4	3	3	3	3	3	3	3	3
1	1	3	3	2	2	3	3	3	3
4	4	3	3	3	3	2	2	2	2
1	1	3	3	3	3	3	3	2	2
4	4	3	3	3	3	2	2	3	3
4	4	3	3	2	2	2	2	2	2
4	4	3	3	2	2	3	3	2	2
1	1	3	3	3	3	3	3	3	3
4	4	3	3	3	3	3	3	3	3
1	1	3	3	2	2	2	2	2	2
4	4	3	3	3	3	3	3	3	3
1	1	3	3	3	3	3	3	3	3
4	4	3	3	2	2	2	2	2	2
4	4	3	3	2	2	3	3	2	2
4	4	3	3	3	3	3	3	3	3
4	4	3	3	3	3	2	2	3	3
4	4	3	3	2	2	2	2	2	2
4	4	3	3	3	3	3	3	2	2
4	4	3	3	3	3	3	3	2	2
4	4	3	3	3	3	3	3	2	2
1	1	3	3	3	3	3	3	3	3
1	1	3	3	3	3	2	2	2	2
1	1	3	3	3	3	3	3	3	3
4	4	3	3	3	3	3	3	3	3
4	4	3	3	3	3	2	2	2	2
4	4	3	3	3	3	3	3	3	3
4	4	3	3	3	3	3	3	3	3
4	4	3	3	2	2	3	3	3	3
1	1	3	3	3	3	2	2	2	2
1	1	3	3	3	3	3	3	3	3
1	1	3	3	3	3	2	2	2	2
1	1	3	3	3	3	3	3	3	3
1	1	3	3	2	2	3	3	3	3
4	4	3	3	3	3	2	2	2	2
4	4	3	3	2	2	3	3	3	3
4	4	3	3	3	3	3	3	3	3
4	4	3	3	3	3	2	2	3	3
4	4	3	3	2	2	3	3	2	2
1	1	3	3	2	2	2	2	2	2
4	4	3	3	2	2	2	2	2	2
4	4	3	3	3	3	2	2	2	2
4	4	2	2	2	2	3	3	2	2
4	4	3	3	3	3	2	2	2	2
4	4	3	3	3	3	2	2	2	2
4	4	3	3	3	3	3	3	3	3
4	4	3	3	3	3	2	2	2	2

For Review Only

1	1	3	3	3	3	3	3	3	3
4	4	3	3	2	2	3	3	2	2
4	4	3	3	3	3	3	3	3	3
4	4	3	3	3	3	3	3	3	3
4	4	3	3	3	3	3	3	3	3
1	1	3	3	2	2	3	3	3	3
4	4	3	3	2	2	3	3	2	2
4	4	3	3	2	2	2	2	2	2
4	4	3	3	2	2	2	2	3	3
4	4	3	3	3	3	2	2	2	2
4	4	3	3	3	3	3	3	3	3
1	1	3	3	3	3	3	3	2	2
1	1	3	3	3	3	3	3	2	2
1	1	3	3	2	2	3	3	3	3
1	1	3	3	3	3	3	3	3	3
4	4	2	2	2	2	3	3	2	2
4	4	3	3	3	3	2	2	3	3
4	4	3	3	3	3	3	3	2	2
1	1	3	3	2	2	2	2	2	2
1	1	3	3	3	3	3	3	2	2
4	4	3	3	3	3	3	3	2	2
1	1	3	3	2	2	3	3	2	2
4	4	3	3	3	3	3	3	2	2
4	4	3	3	3	3	2	2	2	2
4	4	3	3	3	3	2	2	2	2
4	4	3	3	3	3	3	3	2	2
4	4	3	3	3	3	2	2	2	2
4	4	3	3	3	3	3	3	2	2
4	4	3	3	3	3	3	3	3	3
4	4	2	2	2	2	3	3	2	2
1	1	3	3	3	3	2	2	2	2
1	1	3	3	2	2	3	3	3	3
4	4	3	3	3	3	2	2	2	2
1	1	3	3	3	3	3	3	3	3
4	4	3	3	2	2	3	3	2	2
4	4	3	3	2	2	2	2	2	2
1	1	3	3	3	3	3	3	3	3
4	4	3	3	3	3	3	3	3	3
4	4	3	3	2	2	2	2	3	3
4	4	3	3	3	3	2	2	2	2
4	4	3	3	3	3	3	3	2	2
1	1	3	3	3	3	3	3	2	2
4	4	3	3	3	3	3	3	2	2
1	1	3	3	3	3	3	3	2	2
1	1	3	3	3	3	3	3	2	2
1	1	3	3	3	3	2	2	2	2
1	1	3	3	3	3	3	3	2	2
1	1	3	3	3	3	2	2	2	2
4	4	3	3	3	3	3	3	3	3
4	4	3	3	2	2	2	2	2	2
4	4	3	3	3	3	3	3	2	2
4	4	3	3	3	3	2	2	2	2
1	1	3	3	3	3	3	3	3	3
1	1	3	3	3	3	3	3	2	2
4	4	3	3	2	2	3	3	2	2

For Review Only

4	4	2	2	2	2	3	3	2	2
4	4	3	3	3	3	3	3	2	2

For Review Only

SNP377	SNP377	SNP378	SNP378	SNP379	SNP379	SNP380	SNP380	SNP381	SNP381
4	4	1	1	2	2	1	1	3	3
1	1	1	1	2	2	4	4	2	2
1	1	1	1	2	2	4	4	2	2
4	4	1	1	3	3	1	1	3	3
4	4	1	1	2	2	1	1	3	3
1	1	1	1	3	3	4	4	3	3
4	4	1	1	2	2	1	1	3	3
1	1	1	1	2	2	4	4	2	2
4	4	1	1	3	3	4	4	3	3
1	1	1	1	3	3	4	4	3	3
4	4	1	1	3	3	1	1	3	3
1	1	1	1	2	2	4	4	2	2
1	1	1	1	2	2	4	4	3	3
1	1	3	3	3	3	4	4	3	3
1	1	1	1	2	2	4	4	3	3
4	4	1	1	3	3	1	1	2	2
1	1	1	1	2	2	4	4	3	3
1	1	1	1	2	2	4	4	3	3
4	4	1	1	3	3	1	1	3	3
4	4	1	1	2	2	1	1	3	3
4	4	1	1	2	2	1	1	3	3
1	1	1	1	2	2	4	4	3	3
1	1	1	1	3	3	4	4	3	3
4	4	3	3	3	3	1	1	3	3
1	1	1	1	2	2	4	4	3	3
4	4	1	1	2	2	1	1	2	2
1	1	1	1	3	3	4	4	3	3
1	1	1	1	2	2	4	4	2	2
4	4	1	1	3	3	1	1	3	3
1	1	1	1	2	2	4	4	3	3
1	1	1	1	3	3	4	4	3	3
4	4	1	1	3	3	1	1	3	3
1	1	1	1	2	2	4	4	2	2
1	1	1	1	3	3	4	4	3	3
1	1	1	1	2	2	4	4	2	2
1	1	3	3	3	3	4	4	2	2
4	4	1	1	3	3	1	1	3	3
1	1	1	1	2	2	4	4	2	2
1	1	1	1	2	2	4	4	3	3
1	1	1	1	3	3	4	4	2	2
1	1	3	3	2	2	4	4	3	3
1	1	1	1	3	3	1	1	3	3
1	1	1	1	2	2	4	4	3	3
1	1	1	1	2	2	4	4	3	3
1	1	1	1	2	2	4	4	3	3
1	1	1	1	2	2	4	4	2	2
4	4	1	1	2	2	1	1	2	2
4	4	1	1	2	2	1	1	3	3
1	1	3	3	2	2	4	4	2	2
4	4	1	1	2	2	1	1	2	2
4	4	1	1	2	2	1	1	2	2
1	1	1	1	3	3	4	4	3	3

For Review Only

1	1	1	1	2	2	4	4	3	3
1	1	1	1	2	2	4	4	2	2
1	1	3	3	3	3	4	4	3	3
1	1	1	1	2	2	4	4	3	3
4	4	1	1	3	3	1	1	3	3
1	1	1	1	3	3	4	4	2	2
1	1	1	1	3	3	4	4	2	2
1	1	1	1	2	2	4	4	3	3
1	1	1	1	2	2	1	1	3	3
1	1	1	1	2	2	4	4	2	2
1	1	1	1	3	3	4	4	2	2
1	1	1	1	3	3	4	4	2	2
1	1	1	1	2	2	4	4	3	3
4	4	1	1	3	3	1	1	3	3
1	1	1	1	2	2	4	4	2	2
4	4	1	1	2	2	1	1	2	2
4	4	1	1	3	3	4	4	3	3
4	4	1	1	2	2	1	1	2	2
1	1	1	1	3	3	4	4	3	3
1	1	1	1	2	2	4	4	3	3
1	1	1	1	3	3	4	4	2	2
4	4	1	1	3	3	1	1	3	3
1	1	1	1	3	3	4	4	3	3
1	1	1	1	2	2	4	4	2	2
1	1	1	1	2	2	4	4	3	3
1	1	1	1	3	3	4	4	3	3
1	1	1	1	2	2	4	4	2	2
1	1	1	1	2	2	4	4	3	3
4	4	1	1	2	2	1	1	2	2
1	1	1	1	2	2	4	4	2	2
1	1	1	1	2	2	4	4	3	3
1	1	3	3	2	2	4	4	2	2
1	1	1	1	3	3	4	4	3	3
1	1	1	1	2	2	4	4	3	3
1	1	1	1	2	2	4	4	3	3
1	1	1	1	2	2	4	4	3	3
1	1	1	1	3	3	4	4	3	3
1	1	1	1	2	2	4	4	3	3
4	4	1	1	3	3	1	1	3	3
1	1	1	1	2	2	4	4	3	3
1	1	1	1	2	2	4	4	2	2
1	1	1	1	3	3	4	4	2	2
1	1	1	1	2	2	4	4	3	3
1	1	1	1	3	3	4	4	2	2
4	4	1	1	2	2	1	1	3	3
1	1	1	1	2	2	4	4	2	2
1	1	3	3	3	3	4	4	3	3
4	4	3	3	2	2	4	4	3	3
1	1	3	3	2	2	4	4	2	2
1	1	1	1	3	3	4	4	3	3
1	1	1	1	3	3	4	4	3	3
1	1	1	1	2	2	4	4	3	3
1	1	1	1	2	2	4	4	3	3
4	4	1	1	3	3	4	4	3	3
1	1	1	1	2	2	4	4	3	3

For Review Only

1	1	1	1	3	3	4	4	2	2
1	1	1	1	3	3	4	4	3	3
1	1	1	1	2	2	4	4	2	2
1	1	3	3	2	2	4	4	3	3
1	1	1	1	3	3	4	4	2	2
1	1	3	3	2	2	4	4	2	2
1	1	1	1	3	3	4	4	2	2
4	4	3	3	3	3	1	1	3	3
1	1	1	1	2	2	4	4	2	2
4	4	1	1	2	2	1	1	2	2
1	1	1	1	2	2	4	4	3	3
1	1	1	1	2	2	4	4	2	2
1	1	1	1	2	2	4	4	3	3
1	1	1	1	3	3	4	4	3	3
1	1	1	1	3	3	4	4	3	3
1	1	1	1	2	2	1	1	2	2
1	1	1	1	2	2	4	4	3	3
1	1	1	1	2	2	4	4	2	2
1	1	3	3	3	3	4	4	3	3
1	1	1	1	3	3	4	4	3	3
1	1	1	1	2	2	4	4	2	2
1	1	1	1	3	3	4	4	2	2
1	1	1	1	2	2	4	4	3	3
1	1	1	1	3	3	4	4	3	3
4	4	1	1	2	2	1	1	3	3
1	1	1	1	3	3	4	4	3	3
1	1	1	1	2	2	4	4	2	2
4	4	1	1	3	3	1	1	3	3
1	1	3	3	3	3	4	4	3	3
1	1	1	1	3	3	4	4	3	3
1	1	3	3	2	2	4	4	3	3
1	1	1	1	2	2	4	4	3	3
1	1	1	1	2	2	4	4	2	2
1	1	1	1	2	2	4	4	2	2
1	1	1	1	3	3	4	4	3	3
1	1	1	1	2	2	4	4	3	3
1	1	3	3	3	3	4	4	3	3
1	1	1	1	3	3	4	4	2	2
1	1	1	1	2	2	4	4	3	3
1	1	1	1	2	2	1	1	3	3
1	1	1	1	3	3	4	4	2	2
1	1	3	3	3	3	4	4	3	3
4	4	1	1	2	2	1	1	2	2
1	1	1	1	2	2	4	4	3	3
4	4	1	1	2	2	1	1	3	3
4	4	1	1	2	2	4	4	2	2
4	4	1	1	2	2	1	1	2	2
1	1	3	3	2	2	4	4	2	2
1	1	1	1	3	3	4	4	3	3
1	1	1	1	2	2	4	4	2	2
4	4	1	1	2	2	1	1	2	2
1	1	1	1	3	3	4	4	3	3
1	1	1	1	2	2	4	4	2	2
4	4	1	1	2	2	1	1	2	2
1	1	1	1	2	2	4	4	3	3

For Review Only

4	4	1	1	2	2	1	1	2	2
4	4	1	1	3	3	1	1	3	3
4	4	1	1	2	2	4	4	2	2
1	1	1	1	3	3	4	4	2	2
1	1	1	1	2	2	4	4	2	2
1	1	1	1	3	3	4	4	3	3
1	1	1	1	2	2	4	4	3	3
1	1	1	1	2	2	4	4	2	2
1	1	1	1	2	2	4	4	3	3
4	4	1	1	3	3	1	1	2	2
1	1	1	1	2	2	4	4	3	3
1	1	3	3	3	3	4	4	3	3
1	1	1	1	2	2	4	4	3	3
4	4	1	1	2	2	4	4	3	3
1	1	3	3	3	3	4	4	3	3
1	1	1	1	3	3	4	4	3	3
1	1	1	1	2	2	4	4	2	2
1	1	1	1	2	2	4	4	2	2
1	1	1	1	2	2	4	4	3	3
1	1	1	1	3	3	4	4	2	2
1	1	1	1	2	2	4	4	2	2
1	1	1	1	3	3	4	4	3	3
4	4	1	1	3	3	1	1	3	3
1	1	1	1	3	3	4	4	2	2
1	1	1	1	3	3	4	4	2	2
1	1	3	3	3	3	4	4	3	3
1	1	1	1	2	2	4	4	3	3
1	1	1	1	2	2	4	4	3	3
1	1	1	1	2	2	4	4	3	3
1	1	1	1	2	2	4	4	2	2
1	1	1	1	2	2	1	1	3	3
4	4	1	1	2	2	1	1	2	2
4	4	1	1	2	2	1	1	2	2
1	1	3	3	3	3	4	4	3	3
1	1	1	1	2	2	4	4	2	2
1	1	1	1	3	3	4	4	2	2
1	1	1	1	3	3	4	4	2	2
4	4	1	1	2	2	1	1	3	3
1	1	3	3	2	2	4	4	2	2
1	1	1	1	2	2	4	4	3	3
1	1	1	1	2	2	4	4	2	2
4	4	1	1	2	2	1	1	2	2
1	1	1	1	3	3	4	4	3	3
1	1	1	1	2	2	4	4	2	2
1	1	1	1	2	2	4	4	2	2
1	1	1	1	3	3	4	4	2	2
1	1	1	1	3	3	4	4	3	3
1	1	1	1	2	2	4	4	3	3
1	1	1	1	3	3	4	4	3	3
1	1	1	1	3	3	4	4	3	3
1	1	3	3	3	3	4	4	3	3
1	1	1	1	3	3	4	4	3	3
4	4	1	1	3	3	1	1	3	3
1	1	1	1	3	3	4	4	2	2
1	1	1	1	3	3	4	4	3	3

For Review Only

1	1	1	1	2	2	4	4	2	2
1	1	3	3	3	3	4	4	3	3
4	4	1	1	2	2	4	4	2	2
1	1	1	1	2	2	4	4	3	3
1	1	1	1	2	2	4	4	2	2
4	4	1	1	2	2	1	1	3	3
1	1	1	1	2	2	4	4	3	3
1	1	3	3	2	2	4	4	3	3
1	1	1	1	2	2	4	4	2	2
1	1	1	1	2	2	4	4	3	3
1	1	1	1	2	2	4	4	2	2
1	1	1	1	2	2	4	4	3	3
4	4	1	1	2	2	1	1	3	3
4	4	3	3	2	2	1	1	2	2
1	1	1	1	2	2	4	4	2	2
1	1	3	3	2	2	4	4	3	3
1	1	1	1	3	3	4	4	2	2
1	1	1	1	3	3	4	4	3	3
1	1	1	1	2	2	4	4	3	3
1	1	1	1	2	2	4	4	3	3
1	1	1	1	3	3	4	4	3	3
4	4	1	1	2	2	1	1	3	3
4	4	1	1	3	3	1	1	3	3
1	1	1	1	3	3	4	4	3	3
1	1	1	1	2	2	4	4	3	3
1	1	1	1	2	2	4	4	3	3
1	1	1	1	3	3	4	4	3	3
1	1	1	1	3	3	4	4	3	3
1	1	1	1	2	2	4	4	2	2
1	1	3	3	3	3	4	4	3	3
1	1	1	1	2	2	4	4	3	3
1	1	1	1	2	2	4	4	2	2
1	1	1	1	3	3	4	4	3	3
4	4	1	1	2	2	1	1	2	2
4	4	1	1	3	3	1	1	3	3
1	1	1	1	3	3	4	4	3	3
4	4	1	1	2	2	1	1	2	2
1	1	1	1	3	3	4	4	2	2
1	1	3	3	3	3	4	4	2	2
1	1	1	1	2	2	4	4	3	3
1	1	1	1	3	3	4	4	3	3
1	1	1	1	2	2	4	4	3	3
1	1	1	1	3	3	4	4	3	3
1	1	1	1	2	2	4	4	3	3
1	1	1	1	2	2	4	4	3	3
4	4	1	1	2	2	1	1	3	3
1	1	3	3	2	2	4	4	3	3
1	1	1	1	2	2	4	4	3	3
1	1	1	1	2	2	4	4	2	2
1	1	1	1	2	2	4	4	3	3
1	1	1	1	3	3	4	4	3	3
1	1	1	1	2	2	4	4	3	3
1	1	1	1	2	2	4	4	2	2
1	1	1	1	2	2	4	4	3	3
1	1	1	1	2	2	4	4	3	3

For Review Only

4	4	3	3	3	3	1	1	3	3
1	1	1	1	3	3	4	4	3	3

For Review Only

SNP382	SNP382	SNP383	SNP383	SNP384	SNP384	SNP385	SNP385	SNP386	SNP386
2	2	3	3	3	3	1	1	2	2
2	2	3	3	2	2	4	4	1	1
2	2	3	3	3	3	4	4	1	1
2	2	3	3	3	3	4	4	2	2
2	2	3	3	2	2	4	4	2	2
3	3	3	3	3	3	1	1	2	2
2	2	3	3	3	3	4	4	2	2
2	2	1	1	3	3	4	4	1	1
3	3	3	3	3	3	4	4	2	2
2	2	1	1	3	3	4	4	2	2
3	3	3	3	2	2	4	4	2	2
2	2	3	3	3	3	4	4	1	1
2	2	3	3	3	3	4	4	2	2
3	3	1	1	3	3	4	4	2	2
2	2	3	3	3	3	4	4	2	2
3	3	3	3	3	3	4	4	1	1
2	2	3	3	2	2	4	4	2	2
2	2	3	3	3	3	4	4	2	2
3	3	3	3	2	2	4	4	2	2
2	2	3	3	3	3	4	4	2	2
2	2	3	3	3	3	4	4	2	2
2	2	3	3	3	3	4	4	2	2
3	3	1	1	2	2	4	4	2	2
3	3	1	1	2	2	4	4	2	2
2	2	3	3	3	3	1	1	2	2
3	3	3	3	3	3	4	4	1	1
3	3	3	3	3	3	4	4	2	2
3	3	3	3	3	3	4	4	2	2
3	3	3	3	2	2	4	4	2	2
2	2	1	1	3	3	4	4	2	2
3	3	3	3	3	3	1	1	2	2
2	2	3	3	3	3	4	4	2	2
2	2	3	3	3	3	4	4	1	1
3	3	1	1	3	3	4	4	2	2
3	3	3	3	2	2	4	4	2	2
3	3	1	1	3	3	4	4	1	1
3	3	1	1	3	3	4	4	2	2
2	2	3	3	3	3	4	4	1	1
3	3	1	1	3	3	4	4	2	2
3	3	3	3	3	3	4	4	1	1
2	2	1	1	2	2	4	4	2	2
3	3	3	3	2	2	4	4	2	2
2	2	3	3	3	3	4	4	2	2
2	2	1	1	3	3	1	1	2	2
2	2	3	3	3	3	4	4	2	2
2	2	3	3	3	3	4	4	1	1
2	2	3	3	3	3	4	4	1	1
2	2	3	3	2	2	1	1	2	2
2	2	1	1	3	3	4	4	2	2
2	2	3	3	3	3	4	4	2	2
2	2	3	3	3	3	4	4	1	1
3	3	3	3	3	3	4	4	2	2

For Review Only

2	2	3	3	3	3	1	1	2	2
2	2	3	3	3	3	4	4	1	1
3	3	3	3	3	3	1	1	2	2
2	2	3	3	3	3	4	4	2	2
3	3	3	3	3	3	4	4	2	2
3	3	3	3	3	3	4	4	1	1
2	2	3	3	3	3	4	4	2	2
2	2	1	1	2	2	4	4	2	2
2	2	1	1	3	3	4	4	2	2
3	3	3	3	3	3	4	4	2	2
2	2	3	3	3	3	4	4	1	1
3	3	3	3	3	3	4	4	1	1
2	2	3	3	3	3	1	1	2	2
3	3	3	3	2	2	4	4	2	2
3	3	3	3	3	3	4	4	2	2
2	2	3	3	3	3	4	4	1	1
3	3	1	1	2	2	4	4	2	2
2	2	3	3	3	3	4	4	2	2
3	3	3	3	2	2	1	1	2	2
2	2	3	3	3	3	1	1	2	2
3	3	3	3	3	3	4	4	2	2
3	3	3	3	3	3	1	1	2	2
3	3	3	3	3	3	4	4	2	2
2	2	3	3	3	3	4	4	1	1
2	2	3	3	3	3	4	4	2	2
3	3	3	3	3	3	4	4	2	2
2	2	3	3	3	3	4	4	1	1
3	3	1	1	2	2	4	4	2	2
2	2	3	3	3	3	4	4	1	1
3	3	3	3	3	3	4	4	1	1
2	2	3	3	3	3	4	4	2	2
2	2	1	1	3	3	4	4	2	2
3	3	1	1	2	2	4	4	2	2
2	2	3	3	3	3	1	1	2	2
2	2	3	3	3	3	1	1	2	2
2	2	3	3	3	3	4	4	2	2
3	3	1	1	2	2	4	4	2	2
2	2	3	3	3	3	1	1	2	2
3	3	1	1	3	3	4	4	2	2
2	2	3	3	3	3	1	1	2	2
2	2	3	3	3	3	4	4	1	1
3	3	3	3	3	3	4	4	2	2
2	2	1	1	3	3	1	1	2	2
2	2	3	3	3	3	4	4	1	1
2	2	1	1	3	3	4	4	2	2
2	2	3	3	3	3	4	4	2	2
2	2	1	1	3	3	4	4	2	2
2	2	1	1	3	3	1	1	2	2
2	2	3	3	3	3	4	4	1	1
3	3	3	3	2	2	1	1	2	2
3	3	3	3	3	3	4	4	2	2
3	3	1	1	2	2	4	4	2	2
2	2	1	1	2	2	4	4	2	2
3	3	3	3	2	2	4	4	2	2
2	2	1	1	2	2	1	1	2	2

For Review Only

3	3	3	3	3	3	4	4	1	1
3	3	3	3	3	3	4	4	2	2
3	3	3	3	3	3	4	4	1	1
2	2	1	1	3	3	4	4	2	2
2	2	3	3	3	3	4	4	2	2
3	3	3	3	3	3	4	4	1	1
3	3	3	3	3	3	4	4	1	1
3	3	3	3	3	3	1	1	2	2
2	2	1	1	3	3	4	4	1	1
2	2	1	1	3	3	4	4	2	2
2	2	3	3	3	3	1	1	2	2
3	3	3	3	3	3	4	4	2	2
2	2	1	1	3	3	1	1	2	2
3	3	3	3	3	3	4	4	2	2
2	2	3	3	3	3	4	4	2	2
3	3	3	3	3	3	4	4	1	1
2	2	3	3	3	3	1	1	2	2
3	3	3	3	3	3	4	4	2	2
2	2	1	1	3	3	4	4	2	2
3	3	3	3	2	2	1	1	2	2
3	3	3	3	3	3	4	4	2	2
3	3	3	3	3	3	4	4	2	2
2	2	3	3	3	3	1	1	2	2
3	3	1	1	2	2	4	4	2	2
3	3	3	3	3	3	4	4	2	2
3	3	3	3	3	3	1	1	2	2
3	3	3	3	3	3	4	4	1	1
3	3	1	1	3	3	4	4	2	2
2	2	3	3	3	3	1	1	2	2
3	3	3	3	3	3	4	4	2	2
3	3	1	1	3	3	4	4	2	2
3	3	3	3	3	3	4	4	2	2
2	2	3	3	3	3	4	4	1	1
3	3	3	3	3	3	4	4	2	2
3	3	1	1	3	3	4	4	2	2
3	3	1	1	3	3	4	4	2	2
2	2	1	1	3	3	4	4	2	2
3	3	3	3	3	3	4	4	1	1
2	2	3	3	2	2	1	1	2	2
3	3	3	3	2	2	4	4	2	2
3	3	3	3	3	3	4	4	2	2
2	2	1	1	3	3	4	4	2	2
2	2	1	1	3	3	4	4	1	1
3	3	3	3	3	3	4	4	2	2
2	2	3	3	3	3	4	4	2	2
2	2	1	1	3	3	4	4	1	1
2	2	3	3	3	3	4	4	1	1
2	2	3	3	3	3	4	4	1	1
3	3	1	1	3	3	4	4	2	2
3	3	3	3	3	3	4	4	1	1
2	2	3	3	3	3	4	4	1	1
2	2	3	3	2	2	4	4	2	2
2	2	3	3	3	3	4	4	2	2
2	2	3	3	3	3	4	4	1	1
3	3	3	3	3	3	4	4	2	2

For Review Only

2	2	3	3	3	3	4	4	1	1
2	2	1	1	2	2	1	1	2	2
2	2	3	3	3	3	4	4	1	1
3	3	3	3	3	3	4	4	2	2
2	2	3	3	3	3	4	4	2	2
3	3	3	3	3	3	4	4	2	2
3	3	1	1	3	3	4	4	2	2
2	2	3	3	3	3	4	4	2	2
3	3	3	3	2	2	4	4	2	2
3	3	3	3	3	3	4	4	1	1
3	3	3	3	3	3	1	1	2	2
3	3	3	3	3	3	4	4	2	2
2	2	3	3	3	3	1	1	2	2
3	3	1	1	3	3	4	4	2	2
3	3	1	1	3	3	4	4	2	2
3	3	3	3	2	2	4	4	2	2
3	3	3	3	3	3	4	4	2	2
3	3	3	3	3	3	4	4	2	2
3	3	1	1	3	3	1	1	2	2
3	3	3	3	3	3	4	4	1	1
3	3	3	3	3	3	4	4	2	2
3	3	1	1	3	3	4	4	2	2
3	3	3	3	2	2	1	1	2	2
3	3	3	3	3	3	4	4	1	1
3	3	3	3	2	2	4	4	2	2
3	3	1	1	3	3	4	4	2	2
2	2	3	3	3	3	1	1	2	2
3	3	3	3	3	3	4	4	2	2
2	2	3	3	3	3	4	4	2	2
2	2	3	3	3	3	4	4	1	1
2	2	1	1	3	3	4	4	2	2
2	2	3	3	3	3	4	4	1	1
3	3	3	3	3	3	4	4	1	1
2	2	1	1	3	3	4	4	2	2
3	3	3	3	3	3	4	4	2	2
3	3	3	3	3	3	4	4	1	1
3	3	3	3	3	3	4	4	2	2
2	2	1	1	3	3	1	1	2	2
2	2	3	3	3	3	4	4	1	1
2	2	1	1	3	3	1	1	2	2
2	2	3	3	3	3	4	4	1	1
2	2	3	3	3	3	4	4	1	1
3	3	1	1	2	2	4	4	2	2
3	3	3	3	3	3	4	4	1	1
3	3	3	3	3	3	4	4	2	2
2	2	1	1	3	3	4	4	2	2
3	3	3	3	2	2	4	4	2	2
2	2	1	1	2	2	4	4	2	2
2	2	1	1	3	3	4	4	2	2
2	2	3	3	3	3	4	4	2	2
3	3	3	3	2	2	4	4	2	2
2	2	1	1	3	3	4	4	2	2
3	3	1	1	2	2	1	1	2	2
2	2	3	3	3	3	4	4	1	1
3	3	1	1	2	2	4	4	2	2

For Review Only

2	2	3	3	3	3	4	4	1	1
3	3	3	3	3	3	4	4	2	2
3	3	3	3	3	3	4	4	2	2
3	3	3	3	3	3	1	1	2	2
2	2	3	3	3	3	4	4	1	1
2	2	3	3	3	3	4	4	2	2
3	3	3	3	3	3	4	4	2	2
2	2	1	1	3	3	4	4	2	2
3	3	1	1	3	3	4	4	2	2
3	3	1	1	2	2	4	4	2	2
3	3	3	3	3	3	4	4	2	2
2	2	3	3	3	3	4	4	2	2
2	2	3	3	2	2	4	4	2	2
2	2	3	3	3	3	4	4	1	1
2	2	3	3	3	3	4	4	1	1
3	3	3	3	3	3	4	4	2	2
3	3	1	1	3	3	4	4	1	1
3	3	3	3	2	2	4	4	2	2
2	2	1	1	2	2	1	1	2	2
2	2	3	3	3	3	4	4	2	2
3	3	3	3	3	3	4	4	2	2
2	2	3	3	3	3	1	1	2	2
3	3	3	3	3	3	4	4	2	2
3	3	1	1	3	3	1	1	2	2
2	2	1	1	2	2	4	4	2	2
3	3	3	3	3	3	4	4	2	2
3	3	1	1	2	2	4	4	2	2
2	2	3	3	3	3	4	4	2	2
2	2	3	3	3	3	4	4	2	2
3	3	3	3	3	3	1	1	2	2
2	2	1	1	2	2	4	4	2	2
2	2	3	3	2	2	4	4	1	1
3	3	1	1	2	2	1	1	2	2
2	2	3	3	3	3	4	4	2	2
3	3	3	3	2	2	1	1	2	2
3	3	1	1	2	2	4	4	2	2
2	2	3	3	3	3	4	4	1	1
3	3	3	3	3	3	4	4	1	1
3	3	1	1	3	3	4	4	1	1
3	3	1	1	3	3	4	4	2	2
3	3	3	3	3	3	1	1	2	2
2	2	3	3	3	3	4	4	2	2
3	3	3	3	3	3	1	1	2	2
2	2	3	3	3	3	4	4	2	2
2	2	3	3	3	3	1	1	2	2
2	2	1	1	3	3	4	4	2	2
2	2	3	3	3	3	4	4	2	2
2	2	1	1	3	3	1	1	2	2
2	2	3	3	3	3	4	4	1	1
3	3	1	1	2	2	4	4	2	2
3	3	3	3	3	3	4	4	2	2
3	3	1	1	3	3	4	4	2	2
2	2	3	3	3	3	4	4	1	1
2	2	3	3	3	3	1	1	2	2
3	3	3	3	3	3	4	4	2	2

For Review Only

3	3	3	3	3	3	4	4	2	2
2	2	3	3	3	3	1	1	2	2

For Review Only

SNP387	SNP387	SNP388	SNP388	SNP389	SNP389	SNP390	SNP390	SNP391	SNP391
3	3	2	2	2	2	1	1	3	3
4	4	2	2	2	2	1	1	2	2
3	3	2	2	2	2	1	1	3	3
3	3	2	2	2	2	1	1	3	3
4	4	2	2	1	1	2	2	2	2
4	4	2	2	1	1	1	1	3	3
3	3	2	2	2	2	1	1	3	3
3	3	2	2	1	1	1	1	2	2
3	3	2	2	1	1	1	1	2	2
3	3	2	2	1	1	1	1	2	2
3	3	2	2	1	1	1	1	2	2
3	3	2	2	2	2	1	1	3	3
3	3	2	2	2	2	1	1	3	3
3	3	2	2	1	1	1	1	3	3
3	3	2	2	1	1	1	1	2	2
4	4	2	2	1	1	2	2	2	2
4	4	2	2	2	2	1	1	3	3
3	3	2	2	2	2	1	1	3	3
4	4	2	2	1	1	1	1	2	2
3	3	2	2	1	1	1	1	2	2
3	3	2	2	2	2	2	2	3	3
4	4	2	2	2	2	2	2	3	3
4	4	3	3	1	1	1	1	2	2
3	3	2	2	1	1	1	1	2	2
4	4	2	2	2	2	1	1	3	3
3	3	2	2	2	2	1	1	2	2
4	4	2	2	2	2	1	1	2	2
3	3	2	2	2	2	1	1	2	2
3	3	2	2	1	1	2	2	2	2
3	3	2	2	1	1	2	2	2	2
4	4	2	2	1	1	1	1	2	2
3	3	2	2	2	2	1	1	2	2
3	3	2	2	2	2	1	1	3	3
3	3	2	2	1	1	1	1	2	2
3	3	2	2	2	2	1	1	2	2
3	3	2	2	1	1	1	1	2	2
4	4	3	3	1	1	1	1	3	3
3	3	2	2	2	2	1	1	3	3
4	4	2	2	1	1	2	2	2	2
3	3	2	2	1	1	1	1	2	2
3	3	2	2	2	2	2	2	2	2
3	3	2	2	1	1	2	2	2	2
3	3	2	2	2	2	1	1	2	2
3	3	2	2	2	2	1	1	3	3
4	4	2	2	1	1	1	1	2	2
3	3	2	2	2	2	1	1	3	3
3	3	2	2	2	2	1	1	3	3
3	3	2	2	2	2	1	1	3	3
3	3	2	2	1	1	2	2	2	2
3	3	2	2	2	2	1	1	2	2
3	3	2	2	2	2	1	1	3	3
3	3	2	2	2	2	1	1	2	2

For Review Only

4	4	2	2	2	2	1	1	3	3
3	3	2	2	2	2	1	1	3	3
4	4	2	2	1	1	1	1	3	3
4	4	2	2	2	2	1	1	3	3
4	4	2	2	1	1	1	1	2	2
3	3	2	2	1	1	1	1	2	2
3	3	2	2	2	2	1	1	3	3
4	4	3	3	1	1	2	2	2	2
3	3	2	2	1	1	1	1	2	2
3	3	2	2	2	2	1	1	2	2
4	4	2	2	2	2	1	1	3	3
4	4	2	2	1	1	1	1	2	2
4	4	2	2	2	2	1	1	3	3
3	3	2	2	1	1	2	2	2	2
3	3	2	2	2	2	1	1	2	2
3	3	2	2	2	2	1	1	2	2
3	3	2	2	1	1	1	1	2	2
3	3	2	2	2	2	1	1	3	3
4	4	2	2	1	1	1	1	2	2
3	3	2	2	2	2	1	1	3	3
4	4	2	2	1	1	1	1	2	2
3	3	2	2	1	1	2	2	2	2
3	3	2	2	1	1	2	2	2	2
4	4	2	2	2	2	2	2	2	2
4	4	2	2	1	1	1	1	2	2
4	4	2	2	1	1	1	1	2	2
3	3	2	2	2	2	1	1	3	3
3	3	3	3	1	1	1	1	2	2
3	3	2	2	2	2	1	1	3	3
3	3	2	2	1	1	1	1	2	2
3	3	2	2	1	1	1	1	2	2
4	4	3	3	1	1	1	1	2	2
3	3	2	2	1	1	2	2	2	2
4	4	2	2	2	2	1	1	3	3
3	3	2	2	1	1	1	1	3	3
4	4	2	2	1	1	1	1	2	2
3	3	2	2	1	1	1	1	2	2
3	3	2	2	2	2	2	2	3	3
3	3	2	2	1	1	1	1	2	2
4	4	2	2	2	2	1	1	3	3
3	3	2	2	2	2	1	1	3	3
3	3	2	2	1	1	1	1	2	2
3	3	2	2	1	1	1	1	2	2
4	4	2	2	2	2	1	1	3	3
4	4	3	3	2	2	1	1	3	3
3	3	2	2	2	2	1	1	3	3
4	4	3	3	1	1	1	1	2	2
3	3	2	2	1	1	1	1	3	3
3	3	2	2	2	2	1	1	3	3
3	3	2	2	1	1	1	1	2	2
4	4	2	2	2	2	1	1	2	2
4	4	3	3	2	2	1	1	2	2
4	4	3	3	1	1	2	2	2	2
3	3	2	2	2	2	1	1	2	2
3	3	2	2	1	1	1	1	2	2

For Review Only

3	3	2	2	1	1	1	1	2	2
3	3	2	2	1	1	1	1	2	2
3	3	2	2	2	2	1	1	2	2
4	4	2	2	2	2	1	1	3	3
3	3	2	2	2	2	1	1	3	3
3	3	2	2	2	2	1	1	2	2
4	4	2	2	1	1	1	1	2	2
3	3	2	2	2	2	1	1	2	2
4	4	3	3	2	2	2	2	3	3
3	3	2	2	1	1	1	1	3	3
4	4	2	2	2	2	1	1	3	3
3	3	2	2	2	2	1	1	2	2
3	3	2	2	1	1	1	1	2	2
3	3	2	2	2	2	1	1	2	2
4	4	2	2	2	2	1	1	2	2
3	3	2	2	2	2	1	1	2	2
3	3	2	2	1	1	1	1	2	2
3	3	2	2	2	2	1	1	2	2
4	4	2	2	2	2	1	1	2	2
3	3	2	2	1	1	1	1	2	2
3	3	2	2	2	2	1	1	2	2
3	3	2	2	1	1	1	1	2	2
4	4	2	2	1	1	1	1	3	3
4	4	2	2	1	1	1	1	2	2
3	3	2	2	1	1	2	2	2	2
3	3	2	2	1	1	1	1	2	2
3	3	2	2	2	2	1	1	2	2
3	3	2	2	1	1	1	1	2	2
3	3	2	2	2	2	1	1	3	3
4	4	2	2	1	1	1	1	2	2
3	3	3	3	1	1	1	1	2	2
3	3	2	2	1	1	1	1	2	2
3	3	2	2	2	2	1	1	3	3
3	3	2	2	2	2	1	1	2	2
3	3	2	2	1	1	1	1	2	2
3	3	2	2	2	2	1	1	3	3
4	4	3	3	1	1	1	1	2	2
4	4	2	2	1	1	1	1	2	2
3	3	2	2	2	2	1	1	2	2
3	3	2	2	1	1	1	1	2	2
3	3	2	2	1	1	1	1	2	2
4	4	3	3	1	1	2	2	2	2
3	3	2	2	1	1	1	1	2	2
3	3	2	2	1	1	1	1	2	2
3	3	2	2	2	2	1	1	3	3
3	3	2	2	2	2	1	1	3	3
3	3	2	2	2	2	1	1	3	3
3	3	2	2	2	2	1	1	3	3
3	3	2	2	1	1	1	1	2	2
3	3	2	2	2	2	1	1	3	3
3	3	2	2	2	2	1	1	3	3
3	3	2	2	1	1	2	2	2	2
3	3	2	2	2	2	1	1	3	3
4	4	2	2	2	2	1	1	3	3
3	3	3	3	2	2	1	1	2	2

For Review Only

4	4	2	2	2	2	1	1	3	3
3	3	2	2	1	1	1	1	2	2
4	4	2	2	2	2	2	2	3	3
3	3	2	2	1	1	2	2	2	2
3	3	2	2	2	2	1	1	3	3
3	3	2	2	1	1	2	2	2	2
3	3	2	2	2	2	1	1	3	3
3	3	2	2	2	2	1	1	2	2
4	4	2	2	2	2	1	1	2	2
4	4	2	2	1	1	1	1	2	2
3	3	2	2	2	2	1	1	3	3
3	3	2	2	1	1	1	1	2	2
3	3	2	2	2	2	1	1	3	3
3	3	2	2	2	2	1	1	2	2
3	3	2	2	1	1	1	1	2	2
3	3	2	2	1	1	1	1	2	2
3	3	2	2	2	2	2	2	2	2
3	3	2	2	1	1	1	1	2	2
4	4	3	3	2	2	1	1	2	2
4	4	2	2	1	1	1	1	2	2
3	3	2	2	2	2	1	1	2	2
3	3	2	2	1	1	1	1	2	2
3	3	2	2	1	1	2	2	2	2
4	4	2	2	1	1	1	1	2	2
3	3	2	2	1	1	1	1	2	2
3	3	2	2	1	1	1	1	2	2
3	3	2	2	2	2	1	1	2	2
3	3	2	2	1	1	1	1	2	2
3	3	2	2	1	1	1	1	2	2
4	4	2	2	2	2	1	1	3	3
3	3	2	2	1	1	1	1	2	2
3	3	2	2	1	1	1	1	2	2
3	3	2	2	1	1	1	1	2	2
3	3	2	2	1	1	1	1	2	2
3	3	2	2	2	2	1	1	2	2
4	4	2	2	1	1	1	1	2	2
4	4	2	2	1	1	1	1	2	2
4	4	3	3	2	2	1	1	3	3
3	3	2	2	2	2	1	1	3	3
4	4	2	2	1	1	1	1	2	2
3	3	2	2	2	2	1	1	3	3
3	3	2	2	2	2	1	1	3	3
4	4	3	3	1	1	1	1	2	2
3	3	2	2	2	2	1	1	2	2
3	3	2	2	1	1	1	1	2	2
4	4	3	3	2	2	2	2	2	2
3	3	2	2	1	1	1	1	2	2
3	3	2	2	2	2	1	1	3	3
3	3	2	2	1	1	1	1	2	2
3	3	2	2	2	2	1	1	3	3
3	3	2	2	1	1	1	1	2	2
4	4	3	3	1	1	2	2	2	2
3	3	2	2	1	1	2	2	2	2
4	4	2	2	2	2	1	1	3	3
4	4	2	2	1	1	1	1	2	2

For Review Only

4	4	2	2	2	2	1	1	3	3
3	3	2	2	1	1	1	1	2	2
3	3	2	2	2	2	1	1	2	2
3	3	2	2	1	1	2	2	2	2
3	3	2	2	1	1	1	1	2	2
3	3	2	2	2	2	1	1	3	3
3	3	2	2	1	1	2	2	2	2
3	3	2	2	1	1	1	1	2	2
3	3	3	3	2	2	1	1	2	2
3	3	3	3	1	1	2	2	2	2
3	3	2	2	2	2	1	1	2	2
3	3	2	2	1	1	1	1	2	2
3	3	2	2	2	2	1	1	3	3
4	4	2	2	1	1	1	1	3	3
3	3	2	2	1	1	1	1	2	2
3	3	2	2	1	1	1	1	2	2
4	4	3	3	1	1	2	2	2	2
3	3	2	2	1	1	1	1	2	2
3	3	2	2	2	2	2	2	3	3
3	3	2	2	2	2	1	1	3	3
4	4	2	2	1	1	2	2	2	2
3	3	2	2	2	2	1	1	3	3
4	4	2	2	1	1	1	1	2	2
3	3	2	2	1	1	1	1	2	2
4	4	3	3	1	1	2	2	2	2
3	3	2	2	2	2	1	1	2	2
4	4	3	3	1	1	2	2	2	2
3	3	2	2	2	2	1	1	3	3
3	3	2	2	1	1	1	1	2	2
3	3	2	2	1	1	1	1	2	2
4	4	2	2	2	2	1	1	3	3
3	3	2	2	2	2	1	1	3	3
3	3	2	2	1	1	1	1	2	2
3	3	2	2	2	2	1	1	3	3
3	3	2	2	1	1	2	2	2	2
4	4	2	2	1	1	1	1	2	2
4	4	2	2	2	2	1	1	3	3
4	4	2	2	1	1	1	1	2	2
3	3	2	2	1	1	1	1	2	2
3	3	2	2	1	1	2	2	2	2
3	3	2	2	1	1	1	1	2	2
4	4	2	2	2	2	1	1	3	3
3	3	2	2	1	1	1	1	2	2
4	4	2	2	2	2	1	1	3	3
4	4	2	2	2	2	1	1	3	3
3	3	2	2	2	2	1	1	3	3
3	3	2	2	2	2	1	1	3	3
3	3	2	2	1	1	1	1	2	2
4	4	2	2	2	2	1	1	3	3
4	4	3	3	1	1	2	2	2	2
3	3	2	2	2	2	1	1	2	2
3	3	2	2	1	1	1	1	2	2
3	3	2	2	2	2	1	1	3	3
3	3	2	2	1	1	1	1	2	2
3	3	2	2	1	1	1	1	2	2

For Review Only

3	3	2	2	1	1	1	1	2	2
4	4	2	2	2	2	1	1	2	2

For Review Only

SNP392	SNP392	SNP393	SNP393	SNP394	SNP394	SNP395	SNP395	SNP396	SNP396
2	2	1	1	4	4	3	3	3	3
3	3	1	1	1	1	3	3	3	3
2	2	3	3	1	1	3	3	3	3
2	2	1	1	4	4	3	3	3	3
3	3	1	1	1	1	3	3	3	3
2	2	1	1	4	4	2	2	3	3
2	2	1	1	4	4	3	3	3	3
3	3	1	1	4	4	3	3	3	3
3	3	1	1	4	4	3	3	2	2
3	3	1	1	4	4	3	3	3	3
3	3	1	1	4	4	3	3	3	3
2	2	3	3	1	1	3	3	3	3
3	3	1	1	4	4	3	3	3	3
2	2	1	1	4	4	3	3	2	2
3	3	1	1	4	4	3	3	2	2
3	3	1	1	4	4	3	3	2	2
2	2	1	1	4	4	3	3	3	3
2	2	1	1	1	1	3	3	3	3
3	3	1	1	4	4	3	3	3	3
3	3	1	1	4	4	3	3	3	3
2	2	1	1	4	4	3	3	3	3
2	2	1	1	4	4	3	3	3	3
3	3	1	1	4	4	3	3	3	3
3	3	1	1	4	4	3	3	3	3
2	2	1	1	4	4	3	3	2	2
3	3	1	1	4	4	3	3	3	3
3	3	1	1	4	4	2	2	3	3
3	3	1	1	4	4	3	3	2	2
3	3	1	1	4	4	3	3	3	3
3	3	1	1	4	4	3	3	3	3
3	3	1	1	4	4	3	3	2	2
3	3	1	1	4	4	3	3	3	3
2	2	1	1	4	4	3	3	3	3
3	3	1	1	4	4	3	3	3	3
3	3	1	1	4	4	3	3	2	2
3	3	1	1	4	4	3	3	3	3
2	2	1	1	4	4	3	3	3	3
2	2	1	1	4	4	3	3	3	3
3	3	1	1	4	4	3	3	3	3
3	3	1	1	4	4	3	3	3	3
3	3	1	1	4	4	3	3	3	3
3	3	1	1	4	4	3	3	3	3
3	3	1	1	4	4	3	3	3	3
3	3	1	1	4	4	3	3	3	3
3	3	1	1	4	4	3	3	3	3
2	2	3	3	1	1	3	3	3	3
2	2	1	1	4	4	3	3	2	2
2	2	1	1	4	4	3	3	3	3
3	3	1	1	4	4	3	3	3	3
3	3	1	1	4	4	3	3	3	3
2	2	1	1	4	4	3	3	2	2
3	3	1	1	4	4	3	3	3	3

2	2	1	1	4	4	3	3	3	3
2	2	1	1	4	4	3	3	3	3
2	2	1	1	4	4	2	2	2	2
2	2	1	1	4	4	3	3	3	3
3	3	1	1	4	4	3	3	3	3
3	3	3	3	4	4	3	3	2	2
2	2	3	3	4	4	3	3	3	3
3	3	1	1	4	4	3	3	3	3
3	3	1	1	4	4	3	3	3	3
3	3	1	1	4	4	3	3	2	2
2	2	1	1	4	4	2	2	3	3
3	3	3	3	4	4	2	2	2	2
2	2	1	1	4	4	3	3	3	3
3	3	1	1	4	4	3	3	3	3
3	3	1	1	4	4	3	3	3	3
2	2	1	1	4	4	3	3	3	3
3	3	1	1	4	4	3	3	3	3
3	3	1	1	4	4	3	3	3	3
3	3	1	1	4	4	3	3	2	2
2	2	1	1	4	4	3	3	3	3
3	3	1	1	4	4	3	3	2	2
3	3	1	1	4	4	3	3	3	3
3	3	1	1	4	4	3	3	2	2
3	3	1	1	4	4	3	3	2	2
3	3	1	1	4	4	3	3	3	3
3	3	1	1	4	4	3	3	3	3
2	2	1	1	1	1	3	3	3	3
3	3	3	3	4	4	3	3	3	3
2	2	1	1	4	4	3	3	3	3
3	3	1	1	4	4	3	3	2	2
3	3	1	1	4	4	3	3	3	3
3	3	1	1	4	4	3	3	3	3
3	3	1	1	4	4	3	3	3	3
2	2	1	1	4	4	3	3	3	3
2	2	1	1	4	4	3	3	3	3
3	3	1	1	4	4	3	3	3	3
3	3	1	1	4	4	3	3	3	3
2	2	1	1	4	4	3	3	3	3
3	3	1	1	4	4	3	3	3	3
2	2	1	1	4	4	3	3	3	3
2	2	1	1	4	4	3	3	3	3
3	3	3	3	1	1	3	3	2	2
3	3	1	1	4	4	3	3	3	3
2	2	1	1	4	4	2	2	3	3
2	2	1	1	4	4	3	3	3	3
2	2	1	1	4	4	3	3	2	2
3	3	3	3	4	4	3	3	3	3
2	2	3	3	4	4	3	3	3	3
2	2	1	1	4	4	3	3	3	3
3	3	1	1	1	1	3	3	2	2
3	3	1	1	4	4	3	3	3	3
3	3	3	3	4	4	3	3	3	3
3	3	1	1	4	4	3	3	3	3
3	3	1	1	4	4	3	3	3	3
3	3	1	1	1	1	3	3	2	2

For Review Only

3	3	1	1	4	4	3	3	3	3
3	3	1	1	4	4	3	3	3	3
3	3	1	1	4	4	3	3	3	3
2	2	3	3	4	4	3	3	3	3
2	2	1	1	4	4	3	3	2	2
3	3	1	1	4	4	3	3	3	3
3	3	3	3	4	4	3	3	2	2
3	3	1	1	1	1	3	3	3	3
2	2	1	1	4	4	3	3	3	3
3	3	1	1	4	4	3	3	3	3
3	3	1	1	4	4	3	3	2	2
2	2	1	1	4	4	3	3	3	3
3	3	3	3	4	4	3	3	3	3
3	3	1	1	4	4	3	3	2	2
2	2	1	1	4	4	3	3	2	2
3	3	1	1	4	4	3	3	3	3
3	3	1	1	4	4	3	3	3	3
3	3	1	1	4	4	3	3	3	3
2	2	3	3	4	4	3	3	3	3
3	3	1	1	4	4	3	3	3	3
2	2	1	1	4	4	3	3	2	2
3	3	1	1	4	4	3	3	2	2
3	3	3	3	4	4	2	2	3	3
3	3	3	3	4	4	3	3	3	3
3	3	1	1	4	4	3	3	3	3
3	3	1	1	1	1	3	3	3	3
3	3	1	1	4	4	3	3	3	3
3	3	1	1	4	4	3	3	3	3
2	2	1	1	4	4	3	3	3	3
3	3	1	1	1	1	2	2	3	3
3	3	3	3	4	4	3	3	3	3
3	3	1	1	4	4	3	3	3	3
2	2	1	1	4	4	3	3	3	3
3	3	1	1	4	4	2	2	2	2
3	3	1	1	4	4	3	3	3	3
3	3	3	3	4	4	3	3	3	3
3	3	3	3	4	4	3	3	3	3
3	3	1	1	4	4	3	3	2	2
2	2	1	1	4	4	3	3	3	3
3	3	1	1	4	4	3	3	3	3
3	3	1	1	4	4	3	3	3	3
3	3	3	3	4	4	3	3	3	3
3	3	1	1	4	4	3	3	2	2
3	3	1	1	4	4	3	3	3	3
2	2	1	1	4	4	3	3	3	3
2	2	1	1	4	4	3	3	2	2
2	2	1	1	4	4	3	3	3	3
2	2	1	1	4	4	3	3	2	2
3	3	1	1	4	4	3	3	2	2
2	2	1	1	4	4	3	3	3	3
2	2	1	1	4	4	3	3	3	3
3	3	1	1	4	4	3	3	3	3
2	2	1	1	4	4	3	3	3	3
2	2	3	3	4	4	2	2	2	2
3	3	1	1	4	4	3	3	3	3

For Review Only

2	2	1	1	4	4	3	3	3	3
3	3	1	1	1	1	3	3	3	3
2	2	1	1	4	4	3	3	3	3
3	3	1	1	1	1	3	3	3	3
2	2	1	1	1	1	3	3	2	2
3	3	1	1	4	4	3	3	3	3
3	3	1	1	4	4	3	3	2	2
3	3	1	1	4	4	3	3	3	3
3	3	1	1	4	4	3	3	3	3
3	3	1	1	4	4	3	3	3	3
2	2	1	1	4	4	3	3	3	3
3	3	1	1	4	4	3	3	2	2
2	2	3	3	4	4	3	3	2	2
3	3	1	1	4	4	3	3	3	3
3	3	3	3	4	4	3	3	3	3
3	3	1	1	4	4	3	3	3	3
2	2	1	1	4	4	3	3	3	3
3	3	1	1	4	4	3	3	2	2
3	3	1	1	4	4	3	3	3	3
3	3	3	3	4	4	3	3	2	2
3	3	1	1	4	4	3	3	3	3
3	3	3	3	4	4	3	3	3	3
3	3	1	1	1	1	3	3	3	3
3	3	3	3	4	4	2	2	2	2
3	3	1	1	4	4	3	3	2	2
3	3	1	1	4	4	3	3	3	3
3	3	3	3	4	4	3	3	2	2
3	3	1	1	4	4	3	3	3	3
3	3	1	1	4	4	3	3	3	3
2	2	1	1	4	4	3	3	3	3
3	3	1	1	4	4	3	3	2	2
3	3	1	1	4	4	3	3	2	2
3	3	1	1	4	4	3	3	3	3
3	3	3	3	4	4	3	3	3	3
3	3	1	1	4	4	3	3	3	3
3	3	3	3	4	4	2	2	2	2
3	3	3	3	4	4	3	3	2	2
2	2	1	1	4	4	3	3	3	3
2	2	1	1	4	4	3	3	2	2
3	3	1	1	4	4	2	2	2	2
2	2	3	3	1	1	3	3	3	3
2	2	1	1	4	4	3	3	3	3
3	3	3	3	4	4	3	3	3	3
3	3	1	1	4	4	3	3	3	3
3	3	1	1	4	4	3	3	3	3
3	3	1	1	4	4	3	3	3	3
3	3	1	1	4	4	3	3	3	3
2	2	3	3	4	4	3	3	3	3
3	3	1	1	4	4	3	3	3	3
2	2	3	3	4	4	3	3	3	3
3	3	1	1	4	4	3	3	2	2
3	3	1	1	4	4	3	3	3	3
3	3	1	1	1	1	3	3	3	3
2	2	1	1	4	4	2	2	3	3
3	3	1	1	4	4	3	3	3	3

For Review Only

2	2	1	1	1	1	3	3	3	3
3	3	1	1	4	4	3	3	2	2
3	3	1	1	4	4	3	3	2	2
3	3	1	1	4	4	3	3	3	3
3	3	1	1	4	4	3	3	3	3
2	2	1	1	4	4	3	3	2	2
3	3	1	1	4	4	3	3	3	3
3	3	1	1	4	4	3	3	3	3
3	3	3	3	4	4	3	3	3	3
3	3	3	3	4	4	3	3	3	3
3	3	1	1	4	4	3	3	3	3
3	3	1	1	4	4	3	3	3	3
2	2	1	1	4	4	3	3	3	3
2	2	1	1	4	4	3	3	3	3
3	3	3	3	1	1	3	3	3	3
3	3	1	1	4	4	3	3	3	3
3	3	1	1	4	4	3	3	3	3
3	3	1	1	4	4	3	3	3	3
2	2	1	1	4	4	3	3	3	3
2	2	1	1	1	1	3	3	3	3
3	3	1	1	4	4	3	3	3	3
2	2	1	1	1	1	3	3	3	3
3	3	1	1	4	4	3	3	3	3
3	3	1	1	4	4	3	3	2	2
3	3	1	1	4	4	3	3	3	3
3	3	1	1	4	4	3	3	3	3
3	3	3	3	4	4	3	3	3	3
2	2	3	3	4	4	3	3	3	3
3	3	1	1	4	4	3	3	3	3
3	3	1	1	4	4	3	3	2	2
2	2	1	1	4	4	3	3	3	3
2	2	1	1	4	4	3	3	3	3
3	3	1	1	4	4	3	3	3	3
2	2	1	1	4	4	3	3	2	2
3	3	1	1	1	1	3	3	3	3
3	3	3	3	4	4	3	3	3	3
2	2	1	1	1	1	3	3	3	3
3	3	3	3	4	4	2	2	2	2
3	3	1	1	4	4	3	3	3	3
3	3	3	3	4	4	3	3	3	3
3	3	1	1	1	1	3	3	3	3
2	2	1	1	4	4	2	2	3	3
3	3	1	1	4	4	3	3	2	2
2	2	1	1	1	1	3	3	3	3
2	2	1	1	4	4	3	3	2	2
2	2	1	1	4	4	3	3	3	3
2	2	3	3	4	4	3	3	2	2
3	3	3	3	4	4	3	3	2	2
3	3	1	1	4	4	3	3	3	3
3	3	3	3	4	4	3	3	3	3
3	3	1	1	1	1	2	2	2	2
3	3	3	3	4	4	3	3	3	3
2	2	3	3	1	1	3	3	3	3
3	3	3	3	4	4	3	3	3	3
3	3	1	1	4	4	3	3	3	3

For Review Only

3	3	1	1	4	4	3	3	3	3
3	3	1	1	4	4	3	3	2	2

For Review Only

SNP397	SNP397	SNP398	SNP398	SNP399	SNP399	SNP400	SNP400	SNP401	SNP401
4	4	2	2	4	4	2	2	1	1
4	4	2	2	1	1	2	2	1	1
4	4	2	2	4	4	2	2	1	1
2	2	2	2	1	1	2	2	1	1
4	4	2	2	4	4	3	3	1	1
4	4	2	2	4	4	2	2	1	1
2	2	2	2	4	4	2	2	1	1
2	2	2	2	4	4	2	2	1	1
4	4	2	2	4	4	2	2	1	1
2	2	2	2	4	4	3	3	1	1
2	2	2	2	1	1	2	2	2	2
4	4	2	2	4	4	2	2	1	1
4	4	2	2	4	4	2	2	1	1
2	2	4	4	1	1	2	2	1	1
4	4	4	4	4	4	2	2	1	1
2	2	4	4	4	4	2	2	1	1
2	2	2	2	1	1	3	3	1	1
4	4	2	2	4	4	2	2	1	1
2	2	2	2	1	1	2	2	1	1
2	2	2	2	4	4	3	3	1	1
2	2	2	2	4	4	3	3	1	1
2	2	2	2	4	4	2	2	1	1
2	2	2	2	1	1	2	2	2	2
4	4	2	2	4	4	2	2	1	1
4	4	4	4	1	1	2	2	1	1
2	2	2	2	4	4	2	2	1	1
2	2	2	2	4	4	2	2	1	1
4	4	2	2	4	4	2	2	1	1
2	2	2	2	4	4	3	3	1	1
2	2	2	2	4	4	3	3	1	1
4	4	4	4	1	1	2	2	1	1
2	2	2	2	4	4	2	2	1	1
4	4	2	2	1	1	2	2	1	1
2	2	2	2	4	4	3	3	1	1
4	4	4	4	4	4	2	2	2	2
2	2	2	2	4	4	2	2	1	1
2	2	2	2	1	1	2	2	1	1
2	2	2	2	4	4	2	2	1	1
2	2	2	2	1	1	3	3	2	2
2	2	2	2	1	1	2	2	1	1
2	2	2	2	4	4	3	3	1	1
2	2	2	2	4	4	3	3	1	1
2	2	2	2	4	4	2	2	1	1
4	4	2	2	1	1	2	2	1	1
2	2	2	2	4	4	2	2	1	1
4	4	2	2	4	4	2	2	1	1
2	2	4	4	4	4	2	2	2	2
4	4	2	2	4	4	2	2	1	1
2	2	2	2	4	4	3	3	1	1
4	4	2	2	4	4	2	2	1	1
2	2	4	4	4	4	2	2	1	1
2	2	2	2	4	4	2	2	1	1

For Review Only

4	4	2	2	1	1	2	2	1	1
4	4	2	2	1	1	2	2	1	1
4	4	4	4	4	4	2	2	1	1
2	2	4	4	1	1	2	2	1	1
2	2	2	2	1	1	2	2	1	1
2	2	4	4	4	4	2	2	2	2
2	2	2	2	4	4	2	2	1	1
2	2	2	2	4	4	3	3	1	1
2	2	2	2	1	1	2	2	1	1
4	4	2	2	4	4	2	2	1	1
2	2	2	2	1	1	2	2	1	1
2	2	4	4	4	4	2	2	2	2
4	4	2	2	1	1	2	2	1	1
2	2	2	2	4	4	3	3	1	1
4	4	2	2	4	4	2	2	1	1
2	2	2	2	4	4	2	2	1	1
4	4	2	2	1	1	2	2	1	1
4	4	2	2	4	4	2	2	1	1
4	4	4	4	4	4	2	2	1	1
4	4	2	2	1	1	2	2	1	1
2	2	4	4	4	4	2	2	2	2
4	4	2	2	1	1	3	3	2	2
4	4	2	2	1	1	3	3	1	1
2	2	4	4	4	4	3	3	1	1
2	2	2	2	4	4	2	2	1	1
2	2	2	2	4	4	2	2	1	1
4	4	2	2	4	4	2	2	1	1
2	2	2	2	1	1	3	3	2	2
2	2	2	2	4	4	2	2	1	1
2	2	4	4	1	1	2	2	1	1
2	2	2	2	4	4	2	2	1	1
2	2	2	2	4	4	2	2	1	1
2	2	2	2	1	1	3	3	2	2
4	4	2	2	1	1	2	2	1	1
2	2	2	2	4	4	2	2	1	1
2	2	2	2	4	4	2	2	1	1
2	2	2	2	4	4	2	2	1	1
4	4	2	2	1	1	3	3	1	1
2	2	2	2	1	1	2	2	1	1
2	2	2	2	4	4	2	2	1	1
2	2	2	2	1	1	2	2	1	1
4	4	4	4	4	4	2	2	1	1
4	4	2	2	1	1	2	2	1	1
2	2	2	2	1	1	2	2	1	1
2	2	2	2	1	1	2	2	1	1
2	2	2	2	1	1	2	2	1	1
2	2	2	2	1	1	3	3	1	1
2	2	2	2	4	4	2	2	1	1
2	2	2	2	4	4	2	2	1	1
4	4	4	4	4	4	2	2	1	1
4	4	2	2	4	4	2	2	1	1
2	2	2	2	1	1	3	3	2	2
2	2	2	2	1	1	3	3	1	1
4	4	2	2	4	4	2	2	1	1
4	4	4	4	4	4	2	2	1	1

For Review Only

2	2	2	2	4	4	2	2	2	2
2	2	2	2	4	4	2	2	2	2
2	2	2	2	4	4	2	2	1	1
2	2	2	2	1	1	3	3	2	2
4	4	2	2	1	1	2	2	1	1
2	2	2	2	4	4	2	2	1	1
2	2	4	4	4	4	2	2	1	1
4	4	4	4	4	4	2	2	1	1
2	2	2	2	4	4	3	3	1	1
2	2	2	2	4	4	2	2	1	1
4	4	2	2	1	1	2	2	1	1
4	4	2	2	4	4	2	2	1	1
4	4	4	4	4	4	2	2	1	1
2	2	2	2	4	4	2	2	1	1
4	4	4	4	4	4	2	2	1	1
4	4	2	2	4	4	2	2	1	1
4	4	2	2	4	4	2	2	1	1
2	2	2	2	4	4	2	2	1	1
2	2	2	2	1	1	3	3	2	2
4	4	2	2	1	1	2	2	2	2
4	4	2	2	4	4	2	2	2	2
4	4	4	4	1	1	2	2	1	1
2	2	2	2	4	4	2	2	1	1
2	2	2	2	1	1	3	3	2	2
4	4	2	2	1	1	2	2	1	1
4	4	2	2	1	1	2	2	2	2
2	2	2	2	4	4	2	2	1	1
2	2	2	2	1	1	2	2	1	1
2	2	2	2	4	4	2	2	1	1
4	4	2	2	4	4	2	2	1	1
2	2	2	2	1	1	3	3	2	2
4	4	2	2	4	4	2	2	1	1
2	2	2	2	1	1	2	2	1	1
4	4	2	2	4	4	2	2	1	1
2	2	2	2	4	4	3	3	1	1
4	4	2	2	1	1	3	3	1	1
2	2	2	2	1	1	3	3	1	1
2	2	2	2	4	4	2	2	1	1
4	4	2	2	4	4	2	2	1	1
2	2	2	2	1	1	3	3	2	2
4	4	2	2	1	1	2	2	2	2
2	2	2	2	1	1	3	3	1	1
4	4	4	4	1	1	2	2	1	1
4	4	2	2	4	4	3	3	1	1
2	2	2	2	4	4	2	2	1	1
2	2	2	2	4	4	2	2	1	1
2	2	2	2	4	4	2	2	1	1
2	2	4	4	4	4	2	2	2	2
2	2	2	2	4	4	2	2	1	1
2	2	2	2	4	4	2	2	1	1
2	2	2	2	4	4	2	2	1	1
2	2	2	2	4	4	3	3	1	1
4	4	2	2	4	4	2	2	1	1
2	2	4	4	4	4	2	2	2	2
4	4	2	2	4	4	2	2	1	1

For Review Only

2	2	2	2	4	4	2	2	1	1
4	4	2	2	4	4	2	2	2	2
2	2	2	2	4	4	3	3	1	1
4	4	2	2	1	1	3	3	2	2
4	4	2	2	4	4	2	2	1	1
2	2	2	2	4	4	3	3	1	1
4	4	4	4	1	1	3	3	2	2
4	4	2	2	4	4	2	2	1	1
4	4	2	2	1	1	2	2	1	1
2	2	2	2	1	1	2	2	1	1
2	2	2	2	4	4	2	2	1	1
4	4	4	4	4	4	2	2	1	1
4	4	4	4	4	4	2	2	1	1
4	4	2	2	4	4	2	2	1	1
2	2	2	2	1	1	3	3	1	1
2	2	2	2	1	1	2	2	2	2
4	4	2	2	4	4	2	2	1	1
4	4	4	4	1	1	2	2	1	1
4	4	2	2	4	4	2	2	1	1
2	2	4	4	4	4	2	2	1	1
4	4	2	2	4	4	2	2	1	1
2	2	2	2	1	1	2	2	2	2
4	4	2	2	1	1	3	3	2	2
2	2	4	4	4	4	2	2	2	2
2	2	2	2	1	1	2	2	1	1
2	2	2	2	4	4	3	3	1	1
4	4	4	4	4	4	2	2	1	1
4	4	2	2	4	4	2	2	1	1
2	2	2	2	1	1	2	2	1	1
2	2	2	2	4	4	2	2	1	1
2	2	4	4	1	1	2	2	1	1
2	2	4	4	4	4	2	2	1	1
2	2	2	2	1	1	2	2	1	1
2	2	2	2	1	1	3	3	1	1
2	2	2	2	1	1	2	2	1	1
2	2	4	4	4	4	2	2	2	2
2	2	2	2	4	4	2	2	1	1
4	4	2	2	1	1	2	2	1	1
2	2	4	4	4	4	2	2	2	2
4	4	4	4	4	4	2	2	1	1
4	4	2	2	4	4	2	2	1	1
2	2	2	2	4	4	2	2	1	1
2	2	2	2	1	1	3	3	2	2
2	2	2	2	1	1	2	2	1	1
2	2	2	2	1	1	2	2	1	1
4	4	2	2	4	4	3	3	1	1
2	2	2	2	1	1	2	2	2	2
2	2	4	4	1	1	3	3	2	2
2	2	2	2	1	1	3	3	1	1
2	2	2	2	4	4	2	2	1	1
2	2	4	4	4	4	2	2	1	1
2	2	2	2	4	4	3	3	1	1
4	4	2	2	1	1	3	3	2	2
2	2	2	2	1	1	2	2	1	1
2	2	2	2	1	1	3	3	2	2

For Review Only

4	4	2	2	4	4	2	2	1	1
2	2	2	2	4	4	2	2	1	1
4	4	4	4	1	1	2	2	1	1
4	4	2	2	4	4	2	2	2	2
2	2	2	2	4	4	2	2	1	1
4	4	4	4	4	4	2	2	1	1
2	2	2	2	1	1	3	3	1	1
2	2	2	2	4	4	3	3	1	1
2	2	2	2	1	1	2	2	1	1
2	2	2	2	1	1	3	3	2	2
4	4	2	2	4	4	2	2	1	1
2	2	2	2	4	4	2	2	1	1
2	2	2	2	1	1	2	2	1	1
2	2	2	2	4	4	2	2	1	1
4	4	2	2	4	4	2	2	1	1
2	2	2	2	1	1	2	2	1	1
2	2	2	2	1	1	3	3	2	2
2	2	2	2	4	4	2	2	1	1
4	4	2	2	1	1	3	3	1	1
4	4	2	2	4	4	2	2	1	1
2	2	2	2	4	4	3	3	1	1
4	4	2	2	4	4	2	2	1	1
2	2	2	2	4	4	2	2	1	1
4	4	4	4	1	1	2	2	1	1
2	2	2	2	4	4	3	3	1	1
2	2	2	2	1	1	2	2	1	1
2	2	2	2	1	1	3	3	2	2
2	2	2	2	4	4	2	2	1	1
4	4	2	2	4	4	2	2	1	1
2	2	4	4	4	4	2	2	1	1
2	2	2	2	1	1	2	2	2	2
2	2	2	2	4	4	2	2	1	1
4	4	2	2	1	1	2	2	1	1
2	2	4	4	4	4	2	2	2	2
4	4	2	2	1	1	3	3	2	2
2	2	2	2	1	1	3	3	2	2
4	4	2	2	1	1	2	2	1	1
2	2	4	4	4	4	2	2	2	2
2	2	2	2	4	4	2	2	1	1
2	2	2	2	1	1	3	3	2	2
4	4	2	2	1	1	2	2	1	1
2	2	2	2	1	1	2	2	2	2
4	4	4	4	1	1	2	2	1	1
4	4	2	2	1	1	2	2	1	1
4	4	4	4	4	4	2	2	2	2
2	2	2	2	1	1	2	2	1	1
2	2	4	4	1	1	2	2	2	2
4	4	4	4	4	4	2	2	1	1
2	2	2	2	4	4	2	2	1	1
2	2	2	2	1	1	3	3	2	2
4	4	2	2	4	4	2	2	1	1
2	2	2	2	1	1	3	3	2	2
4	4	2	2	4	4	2	2	1	1
2	2	2	2	4	4	2	2	1	1
2	2	2	2	1	1	2	2	1	1

For Review Only

2	2	2	2	1	1	2	2	1	1
4	4	4	4	1	1	2	2	1	1

For Review Only

SNP402	SNP402	SNP403	SNP403	SNP404	SNP404	SNP405	SNP405	SNP406	SNP406
4	4	4	4	1	1	3	3	3	3
4	4	4	4	3	3	3	3	3	3
4	4	4	4	1	1	3	3	3	3
4	4	4	4	1	1	3	3	3	3
2	2	1	1	3	3	3	3	3	3
4	4	4	4	3	3	3	3	3	3
4	4	4	4	1	1	4	4	3	3
4	4	4	4	1	1	3	3	3	3
4	4	4	4	1	1	3	3	3	3
2	2	1	1	1	1	3	3	3	3
4	4	4	4	1	1	3	3	3	3
4	4	4	4	1	1	3	3	3	3
4	4	4	4	1	1	3	3	3	3
4	4	4	4	1	1	3	3	3	3
4	4	4	4	1	1	3	3	3	3
2	2	1	1	3	3	3	3	3	3
2	2	1	1	3	3	3	3	3	3
4	4	4	4	1	1	4	4	3	3
4	4	4	4	3	3	3	3	3	3
2	2	1	1	1	1	4	4	3	3
2	2	1	1	1	1	4	4	3	3
2	2	1	1	3	3	3	3	3	3
4	4	4	4	1	1	3	3	3	3
4	4	4	4	1	1	3	3	3	3
4	4	4	4	3	3	3	3	3	3
4	4	4	4	1	1	3	3	3	3
4	4	4	4	3	3	3	3	3	3
4	4	4	4	1	1	3	3	3	3
2	2	1	1	1	1	4	4	3	3
2	2	1	1	1	1	3	3	3	3
4	4	4	4	3	3	3	3	3	3
4	4	4	4	1	1	4	4	3	3
4	4	4	4	1	1	4	4	3	3
2	2	1	1	1	1	3	3	3	3
4	4	4	4	1	1	4	4	3	3
4	4	4	4	1	1	3	3	3	3
4	4	4	4	1	1	3	3	3	3
4	4	4	4	1	1	3	3	3	3
2	2	1	1	1	1	3	3	3	3
4	4	4	4	1	1	4	4	3	3
2	2	1	1	1	1	3	3	3	3
2	2	1	1	1	1	4	4	3	3
4	4	4	4	1	1	3	3	3	3
4	4	4	4	1	1	3	3	3	3
4	4	4	4	3	3	3	3	3	3
4	4	4	4	1	1	3	3	3	3
4	4	4	4	1	1	4	4	2	2
4	4	4	4	1	1	3	3	3	3
2	2	1	1	1	1	3	3	3	3
4	4	4	4	3	3	3	3	3	3
4	4	4	4	1	1	4	4	2	2
4	4	4	4	1	1	3	3	3	3

For Review Only

4	4	4	4	3	3	3	3	3	3
4	4	4	4	1	1	4	4	3	3
4	4	4	4	3	3	3	3	3	3
4	4	4	4	3	3	3	3	3	3
4	4	4	4	3	3	3	3	3	3
4	4	4	4	1	1	4	4	2	2
4	4	4	4	1	1	3	3	3	3
2	2	1	1	1	1	3	3	3	3
4	4	4	4	1	1	3	3	3	3
4	4	4	4	1	1	3	3	3	3
4	4	4	4	3	3	3	3	3	3
4	4	4	4	3	3	3	3	2	2
4	4	4	4	3	3	3	3	3	3
2	2	1	1	1	1	4	4	3	3
4	4	4	4	1	1	3	3	3	3
4	4	4	4	1	1	3	3	3	3
4	4	4	4	1	1	3	3	3	3
4	4	4	4	1	1	4	4	3	3
4	4	4	4	3	3	3	3	3	3
4	4	4	4	1	1	3	3	3	3
4	4	4	4	1	1	3	3	2	2
2	2	1	1	1	1	4	4	3	3
4	4	4	4	1	1	4	4	3	3
2	2	1	1	3	3	3	3	3	3
4	4	4	4	3	3	3	3	3	3
4	4	4	4	3	3	3	3	3	3
4	4	4	4	1	1	3	3	3	3
2	2	1	1	1	1	3	3	3	3
4	4	4	4	1	1	3	3	3	3
4	4	4	4	1	1	3	3	3	3
4	4	4	4	1	1	3	3	3	3
2	2	1	1	1	1	3	3	3	3
2	2	1	1	1	1	3	3	3	3
4	4	4	4	3	3	3	3	3	3
4	4	4	4	1	1	4	4	3	3
4	4	4	4	3	3	3	3	3	3
4	4	4	4	1	1	3	3	3	3
2	2	1	1	1	1	3	3	3	3
4	4	4	4	1	1	3	3	3	3
4	4	4	4	3	3	3	3	3	3
4	4	4	4	1	1	4	4	3	3
4	4	4	4	3	3	3	3	3	3
4	4	4	4	1	1	3	3	3	3
4	4	4	4	3	3	3	3	3	3
4	4	4	4	1	1	3	3	3	3
4	4	4	4	1	1	4	4	3	3
2	2	1	1	1	1	3	3	3	3
4	4	4	4	1	1	3	3	3	3
4	4	4	4	1	1	4	4	3	3
4	4	4	4	1	1	3	3	3	3
4	4	4	4	3	3	3	3	3	3
2	2	1	1	1	1	4	4	3	3
2	2	1	1	1	1	3	3	3	3
2	2	1	1	1	1	3	3	3	3
4	4	4	4	1	1	3	3	3	3

For Review Only

4	4	4	4	1	1	4	4	3	3
4	4	4	4	1	1	3	3	3	3
4	4	4	4	1	1	3	3	3	3
2	2	1	1	1	1	3	3	3	3
4	4	4	4	1	1	3	3	3	3
4	4	4	4	1	1	3	3	3	3
4	4	4	4	3	3	3	3	3	3
4	4	4	4	1	1	3	3	3	3
2	2	1	1	3	3	3	3	3	3
2	2	4	4	1	1	3	3	3	3
4	4	4	4	3	3	3	3	3	3
4	4	4	4	1	1	3	3	3	3
4	4	4	4	1	1	3	3	3	3
4	4	4	4	1	1	3	3	3	3
4	4	4	4	3	3	3	3	3	3
4	4	4	4	1	1	4	4	3	3
4	4	4	4	1	1	4	4	3	3
4	4	4	4	1	1	4	4	3	3
2	2	1	1	3	3	3	3	3	3
4	4	4	4	1	1	3	3	3	3
4	4	4	4	1	1	3	3	3	3
4	4	4	4	1	1	3	3	2	2
4	4	4	4	1	1	3	3	3	3
2	2	1	1	1	1	3	3	3	3
4	4	4	4	1	1	4	4	3	3
4	4	4	4	1	1	3	3	3	3
4	4	4	4	1	1	3	3	3	3
4	4	4	4	1	1	3	3	3	3
4	4	4	4	1	1	3	3	3	3
4	4	4	4	3	3	4	4	3	3
2	2	1	1	1	1	3	3	3	3
4	4	4	4	1	1	3	3	3	3
4	4	4	4	1	1	4	4	3	3
4	4	4	4	1	1	4	4	3	3
2	2	1	1	1	1	3	3	3	3
4	4	4	4	1	1	4	4	3	3
2	2	1	1	1	1	3	3	3	3
4	4	4	4	3	3	3	3	3	3
4	4	4	4	1	1	3	3	3	3
2	2	1	1	1	1	3	3	3	3
4	4	4	4	1	1	3	3	3	3
2	2	1	1	1	1	3	3	3	3
4	4	4	4	1	1	3	3	3	3
4	4	4	4	1	1	3	3	3	3
4	4	4	4	1	1	3	3	3	3
4	4	4	4	1	1	3	3	3	3
4	4	4	4	1	1	4	4	3	3
4	4	4	4	1	1	4	4	2	2
4	4	4	4	1	1	3	3	3	3
4	4	4	4	1	1	4	4	3	3
4	4	4	4	1	1	4	4	3	3
2	2	1	1	1	1	4	4	3	3
4	4	4	4	1	1	3	3	3	3
4	4	4	4	3	3	3	3	3	3
4	4	4	4	1	1	3	3	3	3

For Review Only

4	4	4	4	3	3	3	3	3	3
4	4	4	4	1	1	3	3	3	3
2	2	1	1	1	1	3	3	3	3
2	2	1	1	1	1	3	3	3	3
4	4	4	4	1	1	3	3	3	3
2	2	1	1	1	1	4	4	3	3
4	4	4	4	1	1	3	3	3	3
4	4	4	4	1	1	4	4	3	3
4	4	4	4	1	1	3	3	3	3
4	4	4	4	3	3	3	3	3	3
4	4	4	4	1	1	4	4	3	3
4	4	4	4	1	1	3	3	3	3
4	4	4	4	1	1	3	3	3	3
4	4	4	4	1	1	3	3	3	3
2	2	1	1	1	1	3	3	3	3
4	4	4	4	1	1	3	3	3	3
4	4	4	4	1	1	3	3	3	3
4	4	4	4	3	3	3	3	3	3
4	4	4	4	1	1	3	3	3	3
4	4	4	4	1	1	3	3	3	3
4	4	4	4	1	1	3	3	3	3
4	4	4	4	1	1	3	3	3	3
2	2	1	1	1	1	3	3	3	3
4	4	4	4	3	3	3	3	2	2
4	4	4	4	1	1	3	3	3	3
2	2	1	1	1	1	3	3	3	3
4	4	4	4	1	1	3	3	3	3
4	4	4	4	1	1	4	4	3	3
4	4	4	4	1	1	4	4	3	3
4	4	4	4	3	3	3	3	3	3
4	4	4	4	1	1	3	3	3	3
4	4	4	4	1	1	4	4	3	3
4	4	4	4	1	1	3	3	3	3
2	2	1	1	1	1	3	3	3	3
4	4	4	4	1	1	4	4	3	3
4	4	4	4	3	3	3	3	2	2
4	4	4	4	1	1	3	3	2	2
4	4	4	4	1	1	3	3	3	3
4	4	4	4	1	1	4	4	2	2
4	4	4	4	3	3	3	3	3	3
4	4	4	4	1	1	3	3	3	3
4	4	4	4	1	1	4	4	3	3
2	2	1	1	1	1	3	3	3	3
4	4	4	4	1	1	4	4	3	3
4	4	4	4	1	1	3	3	3	3
2	2	1	1	1	1	3	3	3	3
4	4	4	4	1	1	3	3	3	3
2	2	1	1	1	1	3	3	3	3
2	2	1	1	1	1	3	3	3	3
4	4	4	4	1	1	3	3	3	3
4	4	4	4	1	1	3	3	3	3
2	2	1	1	1	1	3	3	3	3
2	2	1	1	1	1	3	3	3	3
4	4	4	4	3	3	3	3	3	3
2	2	1	1	1	1	3	3	3	3

4	4	4	4	3	3	3	3	3	3
4	4	4	4	1	1	4	4	3	3
4	4	4	4	1	1	3	3	3	3
4	4	4	4	1	1	3	3	3	3
4	4	4	4	1	1	4	4	3	3
4	4	4	4	1	1	3	3	3	3
2	2	1	1	1	1	3	3	3	3
2	2	1	1	1	1	3	3	3	3
4	4	4	4	1	1	3	3	3	3
2	2	1	1	1	1	3	3	3	3
4	4	4	4	1	1	3	3	3	3
4	4	4	4	1	1	3	3	3	3
4	4	4	4	1	1	3	3	3	3
4	4	4	4	3	3	3	3	3	3
4	4	4	4	1	1	4	4	3	3
4	4	4	4	1	1	3	3	3	3
2	2	1	1	1	1	3	3	3	3
4	4	4	4	1	1	3	3	3	3
2	2	1	1	1	1	3	3	3	3
4	4	4	4	1	1	3	3	3	3
2	2	1	1	3	3	3	3	3	3
4	4	4	4	1	1	3	3	3	3
4	4	4	4	3	3	3	3	3	3
4	4	4	4	1	1	3	3	3	3
2	2	1	1	1	1	3	3	3	3
4	4	4	4	1	1	3	3	3	3
2	2	1	1	1	1	3	3	3	3
4	4	4	4	1	1	3	3	3	3
4	4	4	4	1	1	4	4	3	3
4	4	4	4	1	1	3	3	3	3
4	4	4	4	1	1	3	3	3	3
4	4	4	4	1	1	4	4	3	3
4	4	4	4	1	1	3	3	3	3
4	4	4	4	1	1	4	4	2	2
2	2	1	1	1	1	3	3	3	3
2	2	1	1	1	1	3	3	3	3
4	4	4	4	3	3	3	3	3	3
4	4	4	4	3	3	3	3	2	2
4	4	4	4	1	1	3	3	3	3
2	2	1	1	1	1	3	3	3	3
4	4	4	4	1	1	3	3	3	3
4	4	4	4	3	3	3	3	3	3
4	4	4	4	1	1	3	3	3	3
4	4	4	4	3	3	3	3	3	3
4	4	4	4	1	1	3	3	2	2
4	4	4	4	1	1	3	3	3	3
4	4	4	4	1	1	4	4	2	2
4	4	4	4	1	1	3	3	3	3
4	4	4	4	3	3	3	3	3	3
2	2	1	1	1	1	3	3	3	3
4	4	4	4	1	1	3	3	3	3
2	2	1	1	1	1	3	3	3	3
4	4	4	4	1	1	3	3	3	3
4	4	4	4	1	1	3	3	3	3
4	4	4	4	1	1	3	3	3	3

For Review Only

4	4	4	4	1	1	3	3	3	3
4	4	4	4	3	3	3	3	3	3

For Review Only

SNP407	SNP407	SNP408	SNP408	SNP409	SNP409	SNP410	SNP410	SNP411	SNP411
4	4	4	4	4	4	2	2	2	2
1	1	4	4	4	4	2	2	2	2
1	1	3	3	4	4	3	3	2	2
1	1	3	3	1	1	2	2	2	2
4	4	4	4	1	1	2	2	2	2
4	4	3	3	4	4	2	2	4	4
4	4	3	3	1	1	2	2	2	2
4	4	4	4	4	4	3	3	4	4
4	4	4	4	4	4	2	2	2	2
1	1	4	4	1	1	2	2	2	2
4	4	3	3	4	4	3	3	2	2
1	1	3	3	4	4	3	3	2	2
4	4	3	3	1	1	2	2	2	2
1	1	3	3	4	4	2	2	2	2
4	4	4	4	4	4	2	2	4	4
4	4	4	4	4	4	2	2	4	4
1	1	3	3	4	4	3	3	2	2
4	4	3	3	4	4	2	2	4	4
4	4	3	3	4	4	2	2	2	2
4	4	4	4	4	4	3	3	2	2
1	1	3	3	4	4	3	3	2	2
1	1	3	3	4	4	2	2	2	2
1	1	4	4	4	4	3	3	2	2
4	4	4	4	4	4	2	2	4	4
4	4	3	3	4	4	2	2	2	2
1	1	4	4	4	4	3	3	2	2
1	1	4	4	4	4	2	2	2	2
4	4	4	4	4	4	2	2	2	2
1	1	4	4	4	4	3	3	2	2
4	4	4	4	1	1	2	2	2	2
4	4	4	4	4	4	3	3	4	4
1	1	4	4	4	4	2	2	2	2
1	1	3	3	4	4	3	3	2	2
1	1	4	4	4	4	3	3	2	2
4	4	4	4	4	4	2	2	2	2
4	4	4	4	4	4	3	3	4	4
1	1	3	3	1	1	2	2	2	2
4	4	3	3	4	4	2	2	4	4
1	1	4	4	4	4	3	3	2	2
4	4	4	4	4	4	3	3	2	2
4	4	4	4	1	1	2	2	2	2
1	1	4	4	1	1	2	2	2	2
4	4	4	4	1	1	2	2	4	4
4	4	3	3	1	1	2	2	2	2
4	4	4	4	1	1	2	2	4	4
1	1	3	3	4	4	3	3	2	2
1	1	3	3	4	4	2	2	2	2
4	4	3	3	4	4	2	2	2	2
4	4	4	4	1	1	2	2	4	4
4	4	3	3	4	4	2	2	2	2
1	1	3	3	4	4	2	2	2	2
1	1	4	4	4	4	2	2	2	2

For Review Only

4	4	3	3	4	4	3	3	2	2
1	1	3	3	4	4	3	3	2	2
4	4	3	3	4	4	3	3	2	2
4	4	3	3	4	4	3	3	2	2
4	4	3	3	4	4	2	2	2	2
4	4	4	4	4	4	2	2	2	2
1	1	3	3	4	4	2	2	2	2
1	1	4	4	1	1	2	2	2	2
1	1	4	4	1	1	2	2	2	2
4	4	4	4	4	4	2	2	2	2
4	4	3	3	4	4	2	2	4	4
4	4	4	4	4	4	2	2	4	4
4	4	3	3	4	4	3	3	2	2
1	1	4	4	4	4	3	3	2	2
4	4	4	4	4	4	2	2	2	2
4	4	3	3	4	4	2	2	4	4
4	4	4	4	1	1	2	2	2	2
4	4	3	3	4	4	3	3	4	4
4	4	4	4	4	4	3	3	2	2
4	4	4	4	4	4	3	3	2	2
4	4	4	4	4	4	2	2	4	4
4	4	4	4	4	4	2	2	2	2
4	4	4	4	4	4	3	3	2	2
1	1	4	4	4	4	2	2	2	2
4	4	4	4	1	1	2	2	4	4
1	1	4	4	1	1	2	2	2	2
1	1	3	3	1	1	2	2	2	2
1	1	4	4	4	4	3	3	2	2
1	1	3	3	4	4	3	3	2	2
4	4	4	4	4	4	3	3	2	2
1	1	4	4	4	4	2	2	2	2
4	4	4	4	1	1	2	2	4	4
1	1	4	4	4	4	3	3	2	2
4	4	3	3	4	4	3	3	2	2
1	1	3	3	4	4	3	3	2	2
4	4	4	4	1	1	2	2	4	4
1	1	4	4	1	1	2	2	2	2
4	4	3	3	1	1	2	2	2	2
1	1	4	4	1	1	2	2	2	2
4	4	3	3	4	4	3	3	4	4
1	1	3	3	4	4	3	3	2	2
4	4	4	4	4	4	3	3	2	2
4	4	3	3	4	4	3	3	2	2
4	4	3	3	4	4	2	2	4	4
4	4	3	3	4	4	2	2	2	2
4	4	3	3	4	4	3	3	2	2
1	1	4	4	4	4	3	3	2	2
1	1	3	3	4	4	3	3	2	2
1	1	3	3	4	4	2	2	2	2
1	1	4	4	4	4	2	2	2	2
4	4	4	4	4	4	2	2	4	4
1	1	4	4	4	4	3	3	2	2
1	1	4	4	1	1	2	2	2	2
4	4	4	4	4	4	2	2	2	2
4	4	3	3	4	4	2	2	4	4

For Review Only

4	4	4	4	4	4	3	3	2	2
1	1	4	4	4	4	2	2	2	2
4	4	4	4	4	4	3	3	4	4
1	1	3	3	4	4	2	2	2	2
1	1	3	3	4	4	2	2	2	2
4	4	4	4	1	1	2	2	4	4
4	4	4	4	4	4	3	3	4	4
4	4	4	4	4	4	2	2	4	4
1	1	3	3	4	4	2	2	2	2
1	1	3	3	4	4	2	2	2	2
4	4	3	3	4	4	2	2	2	2
4	4	4	4	4	4	2	2	2	2
4	4	3	3	4	4	3	3	2	2
1	1	4	4	4	4	2	2	2	2
4	4	4	4	4	4	2	2	2	2
4	4	4	4	4	4	2	2	2	2
4	4	3	3	4	4	3	3	4	4
4	4	4	4	4	4	2	2	2	2
1	1	3	3	4	4	2	2	2	2
4	4	4	4	4	4	3	3	4	4
4	4	4	4	4	4	2	2	2	2
4	4	4	4	4	4	2	2	4	4
1	1	3	3	4	4	3	3	2	2
1	1	4	4	4	4	3	3	2	2
1	1	4	4	4	4	2	2	2	2
1	1	4	4	1	1	2	2	2	2
1	1	4	4	4	4	3	3	2	2
1	1	4	4	4	4	3	3	2	2
1	1	3	3	4	4	2	2	2	2
4	4	3	3	4	4	2	2	2	2
4	4	4	4	4	4	2	2	2	2
4	4	4	4	4	4	2	2	2	2
1	1	3	3	4	4	3	3	2	2
4	4	4	4	4	4	3	3	4	4
1	1	4	4	4	4	3	3	2	2
1	1	4	4	4	4	2	2	2	2
1	1	4	4	4	4	2	2	2	2
1	1	4	4	4	4	3	3	2	2
4	4	4	4	4	4	2	2	2	2
4	4	4	4	4	4	3	3	2	2
4	4	4	4	4	4	3	3	2	2
1	1	4	4	4	4	3	3	2	2
1	1	3	3	4	4	3	3	2	2
1	1	4	4	4	4	2	2	2	2
1	1	3	3	1	1	2	2	2	2
4	4	3	3	4	4	2	2	2	2
1	1	3	3	4	4	3	3	2	2
1	1	3	3	4	4	3	3	2	2
1	1	3	3	1	1	2	2	2	2
1	1	4	4	4	4	3	3	2	2
1	1	4	4	4	4	3	3	2	2
1	1	3	3	1	1	2	2	2	2
1	1	3	3	4	4	2	2	2	2
4	4	3	3	4	4	3	3	4	4
4	4	4	4	4	4	2	2	2	2

For Review Only

4	4	3	3	4	4	2	2	4	4
1	1	3	3	4	4	3	3	2	2
1	1	3	3	4	4	2	2	2	2
1	1	4	4	4	4	2	2	2	2
1	1	3	3	4	4	2	2	2	2
4	4	4	4	1	1	2	2	4	4
4	4	4	4	4	4	2	2	2	2
4	4	4	4	4	4	2	2	2	2
4	4	4	4	1	1	2	2	2	2
4	4	4	4	4	4	3	3	2	2
1	1	4	4	4	4	3	3	2	2
4	4	4	4	4	4	2	2	2	2
4	4	3	3	4	4	2	2	2	2
4	4	4	4	4	4	2	2	2	2
1	1	4	4	4	4	2	2	2	2
4	4	3	3	4	4	3	3	2	2
1	1	3	3	4	4	2	2	2	2
1	1	4	4	4	4	3	3	2	2
1	1	4	4	4	4	3	3	2	2
4	4	4	4	4	4	3	3	4	4
4	4	4	4	4	4	2	2	2	2
1	1	4	4	1	1	2	2	2	2
1	1	4	4	4	4	2	2	2	2
4	4	4	4	4	4	2	2	4	4
4	4	4	4	4	4	3	3	4	4
1	1	4	4	4	4	3	3	2	2
4	4	3	3	4	4	2	2	4	4
4	4	3	3	4	4	2	2	2	2
1	1	4	4	4	4	3	3	2	2
1	1	3	3	4	4	3	3	2	2
1	1	3	3	4	4	2	2	2	2
4	4	3	3	4	4	3	3	2	2
1	1	4	4	4	4	3	3	2	2
1	1	4	4	4	4	2	2	2	2
4	4	4	4	4	4	2	2	2	2
4	4	4	4	4	4	2	2	4	4
1	1	4	4	4	4	3	3	2	2
4	4	4	4	4	4	2	2	2	2
1	1	3	3	4	4	3	3	2	2
4	4	3	3	4	4	2	2	4	4
1	1	3	3	4	4	3	3	2	2
1	1	3	3	4	4	3	3	2	2
1	1	4	4	4	4	3	3	2	2
4	4	4	4	4	4	3	3	2	2
4	4	4	4	1	1	2	2	2	2
4	4	3	3	4	4	2	2	2	2
4	4	3	3	4	4	3	3	2	2
1	1	3	3	4	4	3	3	2	2
1	1	4	4	4	4	2	2	2	2
4	4	3	3	4	4	3	3	2	2
1	1	4	4	4	4	2	2	2	2
1	1	4	4	1	1	2	2	2	2
1	1	4	4	1	1	2	2	2	2
4	4	3	3	4	4	2	2	4	4
1	1	4	4	4	4	3	3	2	2

For Review Only

1	1	3	3	4	4	2	2	2	2
4	4	4	4	4	4	2	2	2	2
4	4	3	3	4	4	3	3	2	2
4	4	4	4	4	4	2	2	2	2
4	4	4	4	1	1	2	2	4	4
4	4	3	3	4	4	3	3	2	2
1	1	4	4	4	4	2	2	2	2
1	1	4	4	1	1	2	2	2	2
4	4	4	4	1	1	2	2	2	2
4	4	4	4	4	4	3	3	2	2
4	4	4	4	4	4	2	2	2	2
1	1	3	3	1	1	2	2	2	2
1	1	3	3	4	4	3	3	2	2
1	1	3	3	4	4	3	3	2	2
1	1	3	3	4	4	3	3	2	2
4	4	4	4	4	4	3	3	2	2
4	4	4	4	4	4	3	3	2	2
4	4	3	3	4	4	2	2	4	4
4	4	3	3	4	4	2	2	2	2
1	1	3	3	1	1	2	2	2	2
1	1	4	4	4	4	3	3	2	2
4	4	3	3	4	4	3	3	4	4
4	4	3	3	4	4	3	3	4	4
4	4	4	4	4	4	2	2	4	4
1	1	4	4	1	1	2	2	2	2
4	4	4	4	4	4	2	2	2	2
1	1	4	4	4	4	3	3	2	2
1	1	3	3	4	4	3	3	2	2
4	4	4	4	4	4	2	2	2	2
1	1	4	4	4	4	2	2	2	2
1	1	3	3	4	4	3	3	2	2
4	4	3	3	4	4	3	3	4	4
4	4	4	4	4	4	2	2	4	4
1	1	3	3	4	4	3	3	2	2
1	1	4	4	1	1	2	2	2	2
4	4	4	4	4	4	3	3	2	2
1	1	3	3	4	4	3	3	2	2
4	4	4	4	4	4	2	2	4	4
4	4	4	4	4	4	3	3	4	4
1	1	4	4	4	4	3	3	2	2
4	4	4	4	4	4	3	3	4	4
1	1	3	3	4	4	2	2	2	2
4	4	4	4	4	4	2	2	4	4
4	4	3	3	4	4	3	3	2	2
4	4	4	4	4	4	2	2	2	2
1	1	3	3	4	4	3	3	2	2
1	1	3	3	4	4	3	3	2	2
1	1	4	4	1	1	2	2	2	2
1	1	4	4	4	4	3	3	2	2
4	4	4	4	4	4	3	3	2	2
4	4	4	4	4	4	2	2	2	2
1	1	4	4	4	4	3	3	2	2
1	1	3	3	4	4	3	3	2	2
1	1	3	3	4	4	3	3	2	2
4	4	4	4	4	4	3	3	4	4

For Review Only

4	4	3	3	4	4	3	3	2	2
4	4	4	4	4	4	3	3	4	4

For Review Only

SNP412	SNP412	SNP413	SNP413	SNP414	SNP414	SNP415	SNP415	SNP416	SNP416
3	3	2	2	3	3	3	3	3	3
3	3	3	3	2	2	1	1	3	3
2	2	2	2	3	3	3	3	3	3
2	2	3	3	2	2	3	3	3	3
3	3	3	3	2	2	1	1	3	3
3	3	3	3	2	2	3	3	3	3
3	3	3	3	2	2	3	3	3	3
3	3	3	3	2	2	1	1	3	3
3	3	3	3	2	2	1	1	3	3
3	3	2	2	3	3	1	1	3	3
3	3	3	3	2	2	3	3	3	3
2	2	3	3	3	3	3	3	3	3
3	3	3	3	2	2	3	3	3	3
3	3	3	3	2	2	3	3	3	3
3	3	3	3	2	2	1	1	3	3
3	3	3	3	2	2	1	1	3	3
3	3	3	3	2	2	3	3	3	3
3	3	3	3	2	2	3	3	3	3
3	3	3	3	2	2	3	3	3	3
3	3	3	3	2	2	1	1	3	3
3	3	3	3	2	2	3	3	3	3
3	3	3	3	2	2	3	3	3	3
3	3	3	3	2	2	1	1	3	3
3	3	3	3	2	2	1	1	3	3
3	3	3	3	2	2	3	3	3	3
2	2	3	3	2	2	1	1	3	3
3	3	3	3	2	2	1	1	3	3
3	3	3	3	2	2	1	1	3	3
3	3	3	3	2	2	1	1	3	3
3	3	3	3	2	2	1	1	3	3
3	3	2	2	3	3	1	1	3	3
3	3	2	2	3	3	1	1	3	3
3	3	3	3	2	2	3	3	3	3
3	3	3	3	2	2	1	1	3	3
3	3	3	3	2	2	1	1	3	3
3	3	3	3	2	2	1	1	3	3
3	3	3	3	2	2	3	3	3	3
2	2	3	3	2	2	3	3	3	3
3	3	3	3	2	2	1	1	3	3
3	3	3	3	2	2	1	1	3	3
3	3	3	3	2	2	1	1	3	3
3	3	3	3	2	2	1	1	3	3
3	3	3	3	2	2	1	1	3	3
3	3	3	3	2	2	3	3	3	3
3	3	3	3	2	2	1	1	3	3
3	3	3	3	3	3	3	3	3	3
3	3	3	3	2	2	3	3	3	3
3	3	3	3	2	2	1	1	3	3
3	3	3	3	2	2	1	1	3	3
3	3	3	3	2	2	1	1	3	3
3	3	3	3	2	2	3	3	3	3
3	3	2	2	3	3	1	1	3	3

3	3	3	3	2	2	3	3	3	3
3	3	3	3	2	2	3	3	3	3
3	3	3	3	2	2	3	3	3	3
3	3	3	3	2	2	3	3	3	3
3	3	3	3	2	2	3	3	3	3
3	3	3	3	2	2	1	1	3	3
2	2	3	3	2	2	1	1	3	3
3	3	3	3	2	2	1	1	3	3
3	3	3	3	2	2	1	1	3	3
3	3	3	3	2	2	1	1	3	3
3	3	3	3	2	2	3	3	2	2
3	3	3	3	2	2	1	1	2	2
3	3	3	3	2	2	3	3	3	3
3	3	3	3	2	2	1	1	3	3
3	3	3	3	2	2	1	1	3	3
3	3	3	3	2	2	1	1	3	3
3	3	3	3	2	2	1	1	3	3
3	3	3	3	2	2	3	3	3	3
3	3	2	2	3	3	1	1	3	3
3	3	3	3	2	2	1	1	3	3
3	3	3	3	2	2	1	1	2	2
3	3	3	3	2	2	1	1	3	3
3	3	3	3	2	2	1	1	3	3
3	3	3	3	2	2	1	1	3	3
3	3	3	3	2	2	1	1	3	3
3	3	3	3	2	2	1	1	3	3
3	3	3	3	2	2	3	3	3	3
3	3	3	3	2	2	1	1	3	3
2	2	3	3	2	2	3	3	3	3
3	3	3	3	2	2	1	1	3	3
3	3	3	3	2	2	1	1	3	3
3	3	3	3	2	2	1	1	3	3
3	3	3	3	2	2	1	1	3	3
3	3	3	3	2	2	3	3	3	3
3	3	3	3	2	2	3	3	3	3
3	3	3	3	2	2	1	1	3	3
3	3	2	2	3	3	1	1	3	3
3	3	3	3	2	2	3	3	3	3
3	3	3	3	2	2	1	1	3	3
3	3	3	3	2	2	3	3	3	3
3	3	3	3	2	2	3	3	3	3
3	3	2	2	2	2	1	1	3	3
3	3	3	3	2	2	3	3	3	3
3	3	3	3	2	2	3	3	2	2
3	3	3	3	2	2	3	3	3	3
3	3	3	3	2	2	1	1	3	3
3	3	3	3	2	2	1	1	3	3
3	3	3	3	2	2	3	3	3	3
3	3	3	3	2	2	3	3	3	3
3	3	3	3	2	2	1	1	3	3
2	2	3	3	2	2	1	1	3	3
3	3	3	3	2	2	1	1	3	3
3	3	3	3	2	2	1	1	3	3
3	3	3	3	2	2	1	1	3	3
3	3	3	3	2	2	3	3	3	3

For Review Only

3	3	3	3	2	2	1	1	3	3
3	3	3	3	2	2	1	1	3	3
3	3	3	3	2	2	1	1	3	3
3	3	3	3	2	2	3	3	3	3
3	3	3	3	2	2	3	3	3	3
2	2	3	3	2	2	1	1	3	3
3	3	3	3	2	2	1	1	3	3
3	3	3	3	2	2	1	1	3	3
3	3	3	3	2	2	3	3	3	3
3	3	3	3	2	2	1	1	3	3
3	3	3	3	2	2	1	1	3	3
3	3	3	3	2	2	3	3	3	3
3	3	3	3	2	2	3	3	3	3
3	3	3	3	2	2	1	1	3	3
3	3	3	3	2	2	1	1	3	3
3	3	3	3	2	2	1	1	3	3
3	3	3	3	2	2	3	3	3	3
3	3	3	3	2	2	3	3	3	3
3	3	3	3	2	2	3	3	3	3
3	3	3	3	2	2	1	1	3	3
3	3	3	3	2	2	1	1	3	3
3	3	3	3	2	2	1	1	3	3
3	3	3	3	2	2	3	3	3	3
3	3	3	3	2	2	1	1	3	3
3	3	3	3	2	2	1	1	3	3
3	3	3	3	2	2	1	1	3	3
3	3	3	3	2	2	1	1	3	3
3	3	3	3	2	2	1	1	3	3
3	3	3	3	2	2	3	3	3	3
3	3	3	3	2	2	3	3	2	2
3	3	3	3	2	2	1	1	3	3
3	3	3	3	2	2	1	1	3	3
3	3	3	3	2	2	3	3	3	3
3	3	3	3	2	2	1	1	3	3
3	3	3	3	2	2	1	1	3	3
3	3	3	3	2	2	1	1	3	3
3	3	3	3	2	2	1	1	3	3
3	3	3	3	2	2	1	1	3	3
2	2	3	3	2	2	3	3	3	3
3	3	3	3	2	2	1	1	3	3
3	3	3	3	2	2	1	1	3	3
3	3	3	3	2	2	1	1	3	3
3	3	3	3	2	2	3	3	3	3
3	3	3	3	2	2	1	1	3	3
3	3	3	3	2	2	3	3	3	3
3	3	3	3	2	2	3	3	3	3
3	3	3	3	2	2	3	3	3	3
3	3	3	3	2	2	1	1	3	3
3	3	3	3	2	2	1	1	3	3
3	3	3	3	2	2	1	1	3	3
3	3	3	3	2	2	1	1	3	3
3	3	3	3	2	2	3	3	3	3
3	3	3	3	2	2	3	3	3	3
3	3	3	3	2	2	3	3	2	2
3	3	3	3	2	2	1	1	3	3

For Review Only

3	3	3	3	2	2	3	3	3	3
3	3	3	3	2	2	3	3	3	3
3	3	3	3	2	2	3	3	3	3
3	3	3	3	2	2	1	1	3	3
3	3	3	3	2	2	3	3	3	3
3	3	3	3	2	2	1	1	3	3
3	3	3	3	2	2	3	3	3	3
3	3	3	3	2	2	1	1	3	3
3	3	3	3	2	2	1	1	3	3
3	3	3	3	2	2	1	1	3	3
2	2	3	3	2	2	1	1	3	3
3	3	3	3	2	2	1	1	3	3
3	3	3	3	2	2	3	3	3	3
3	3	3	3	2	2	1	1	3	3
3	3	3	3	2	2	1	1	3	3
3	3	3	3	2	2	3	3	3	3
3	3	3	3	2	2	1	1	3	3
3	3	3	3	2	2	1	1	3	3
3	3	3	3	2	2	1	1	3	3
3	3	3	3	2	2	1	1	3	3
3	3	3	3	2	2	1	1	3	3
3	3	3	3	2	2	1	1	3	3
3	3	3	3	2	2	1	1	3	3
3	3	3	3	2	2	1	1	2	2
3	3	3	3	2	2	1	1	3	3
3	3	3	3	2	2	1	1	3	3
2	2	3	3	2	2	3	3	3	3
3	3	3	3	2	2	3	3	3	3
3	3	3	3	2	2	1	1	3	3
3	3	3	3	2	2	3	3	3	3
3	3	3	3	2	2	3	3	3	3
3	3	3	3	2	2	3	3	3	3
3	3	2	2	3	3	1	1	3	3
3	3	3	3	2	2	1	1	3	3
3	3	3	3	2	2	1	1	3	3
3	3	3	3	2	2	1	1	2	2
3	3	3	3	2	2	1	1	3	3
3	3	3	3	2	2	1	1	3	3
3	3	3	3	2	2	1	1	3	3
3	3	2	2	3	3	3	3	2	2
2	2	2	2	3	3	3	3	3	3
3	3	3	3	2	2	3	3	3	3
3	3	3	3	2	2	1	1	3	3
3	3	3	3	2	2	1	1	3	3
3	3	3	3	2	2	1	1	3	3
3	3	3	3	2	2	3	3	3	3
3	3	3	3	2	2	3	3	3	3
3	3	3	3	2	2	3	3	3	3
3	3	3	3	2	2	1	1	3	3
3	3	3	3	2	2	3	3	3	3
3	3	3	3	2	2	1	1	3	3
3	3	3	3	2	2	1	1	3	3
3	3	3	3	2	2	1	1	3	3
3	3	3	3	2	2	3	3	2	2
3	3	3	3	2	2	1	1	3	3

For Review Only

3	3	3	3	2	2	3	3	3	3
3	3	3	3	2	2	1	1	3	3
3	3	3	3	2	2	3	3	3	3
3	3	3	3	2	2	1	1	3	3
3	3	3	3	2	2	1	1	3	3
2	2	3	3	2	2	3	3	3	3
3	3	3	3	2	2	1	1	3	3
3	3	3	3	2	2	1	1	3	3
3	3	3	3	2	2	3	3	3	3
3	3	3	3	2	2	1	1	3	3
3	3	3	3	2	2	1	1	3	3
3	3	3	3	2	2	3	3	3	3
3	3	3	3	2	2	3	3	3	3
3	3	3	3	2	2	3	3	3	3
3	3	3	3	2	2	3	3	3	3
3	3	2	2	3	3	1	1	3	3
3	3	3	3	2	2	1	1	3	3
3	3	3	3	2	2	1	1	3	3
3	3	3	3	2	2	3	3	3	3
2	2	3	3	2	2	3	3	3	3
3	3	3	3	2	2	1	1	3	3
2	2	3	3	2	2	3	3	3	3
3	3	2	2	3	3	3	3	3	3
3	3	3	3	2	2	1	1	3	3
3	3	3	3	2	2	1	1	3	3
3	3	3	3	2	2	1	1	3	3
3	3	3	3	2	2	1	1	3	3
2	2	3	3	2	2	3	3	3	3
3	3	3	3	2	2	1	1	3	3
3	3	3	3	2	2	1	1	3	3
3	3	3	3	2	2	3	3	3	3
3	3	3	3	2	2	3	3	3	3
3	3	3	3	2	2	1	1	3	3
3	3	3	3	2	2	3	3	3	3
3	3	3	3	2	2	1	1	3	3
3	3	3	3	2	2	1	1	3	3
3	3	3	3	2	2	3	3	3	3
3	3	3	3	2	2	1	1	2	2
3	3	3	3	2	2	1	1	3	3
3	3	3	3	2	2	1	1	3	3
3	3	3	3	2	2	1	1	3	3
3	3	3	3	2	2	3	3	3	3
3	3	3	3	2	2	1	1	3	3
3	3	3	3	2	2	3	3	3	3
3	3	3	3	2	2	1	1	3	3
3	3	3	3	2	2	3	3	3	3
3	3	3	3	2	2	1	1	3	3
3	3	3	3	2	2	3	3	3	3
3	3	2	2	3	3	1	1	3	3
3	3	3	3	2	2	1	1	3	3
3	3	3	3	2	2	1	1	3	3
3	3	3	3	2	2	1	1	3	3
2	2	2	2	3	3	3	3	3	3
2	2	3	3	2	2	3	3	3	3
2	2	3	3	2	2	1	1	3	3

For Review Only

3	3	2	2	3	3	3	3	3	3
3	3	3	3	2	2	1	1	3	3

For Review Only

SNP417	SNP417	SNP418	SNP418	SNP419	SNP419	SNP420	SNP420	SNP421	SNP421
1	1	2	2	2	2	3	3	4	4
2	2	3	3	2	2	3	3	4	4
2	2	3	3	2	2	3	3	4	4
1	1	2	2	2	2	3	3	4	4
1	1	2	2	2	2	3	3	4	4
1	1	2	2	2	2	3	3	4	4
1	1	2	2	2	2	3	3	4	4
1	1	2	2	2	2	3	3	4	4
1	1	2	2	2	2	3	3	4	4
1	1	2	2	2	2	3	3	4	4
1	1	2	2	2	2	3	3	4	4
1	1	2	2	2	2	3	3	4	4
1	1	2	2	2	2	3	3	4	4
1	1	2	2	2	2	3	3	4	4
1	1	2	2	2	2	3	3	4	4
1	1	2	2	2	2	3	3	4	4
1	1	2	2	2	2	3	3	4	4
1	1	2	2	4	4	3	3	4	4
1	1	2	2	2	2	2	2	2	2
1	1	2	2	4	4	3	3	4	4
1	1	2	2	4	4	3	3	4	4
2	2	3	3	2	2	3	3	4	4
2	2	3	3	2	2	3	3	4	4
1	1	2	2	2	2	3	3	4	4
1	1	2	2	2	2	3	3	4	4
1	1	2	2	2	2	3	3	4	4
1	1	2	2	2	2	3	3	4	4
1	1	2	2	2	2	3	3	4	4
2	2	3	3	4	4	3	3	4	4
1	1	2	2	2	2	3	3	4	4
1	1	2	2	2	2	3	3	4	4
1	1	2	2	4	4	3	3	4	4
2	2	3	3	4	4	3	3	4	4
2	2	3	3	2	2	3	3	4	4
1	1	2	2	2	2	3	3	2	2
1	1	2	2	2	2	2	2	2	2
1	1	2	2	2	2	3	3	4	4
1	1	2	2	2	2	3	3	4	4
2	2	3	3	2	2	3	3	4	4
1	1	2	2	2	2	3	3	4	4
1	1	2	2	2	2	3	3	4	4
2	2	3	3	4	4	3	3	4	4
1	1	2	2	2	2	3	3	4	4
1	1	2	2	2	2	3	3	4	4
1	1	2	2	2	2	3	3	4	4
1	1	2	2	2	2	3	3	4	4
1	1	2	2	2	2	2	2	2	2
1	1	2	2	2	2	3	3	4	4
1	1	2	2	2	2	3	3	4	4
1	1	2	2	2	2	3	3	4	4
1	1	2	2	2	2	2	2	2	2
1	1	2	2	2	2	3	3	4	4

For Review Only

1	1	2	2	2	2	3	3	4	4
2	2	3	3	4	4	3	3	4	4
1	1	2	2	2	2	3	3	4	4
1	1	2	2	2	2	3	3	4	4
1	1	2	2	2	2	2	2	2	2
1	1	2	2	2	2	2	2	2	2
1	1	2	2	2	2	3	3	4	4
1	1	2	2	2	2	3	3	4	4
1	1	2	2	2	2	3	3	4	4
1	1	2	2	2	2	3	3	4	4
1	1	2	2	2	2	3	3	4	4
1	1	2	2	2	2	2	2	2	2
1	1	2	2	2	2	3	3	4	4
2	2	3	3	4	4	3	3	4	4
1	1	2	2	2	2	3	3	4	4
1	1	2	2	2	2	3	3	4	4
1	1	2	2	2	2	3	3	4	4
1	1	2	2	2	2	3	3	4	4
1	1	2	2	2	2	3	3	4	4
1	1	2	2	2	2	3	3	4	4
1	1	2	2	2	2	3	3	4	4
1	1	2	2	2	2	3	3	4	4
1	1	2	2	4	4	3	3	4	4
1	1	2	2	2	2	3	3	4	4
1	1	2	2	2	2	3	3	4	4
1	1	2	2	2	2	3	3	4	4
2	2	2	2	2	2	3	3	4	4
1	1	2	2	2	2	3	3	4	4
1	1	2	2	2	2	3	3	4	4
1	1	2	2	2	2	3	3	4	4
2	2	3	3	2	2	3	3	4	4
1	1	2	2	2	2	3	3	4	4
2	2	3	3	2	2	3	3	4	4
1	1	2	2	2	2	3	3	4	4
1	1	2	2	4	4	3	3	4	4
1	1	2	2	2	2	3	3	4	4
2	2	3	3	2	2	3	3	4	4
1	1	2	2	2	2	3	3	4	4
1	1	2	2	2	2	3	3	4	4
1	1	2	2	2	2	3	3	4	4
1	1	2	2	4	4	3	3	4	4
2	2	3	3	2	2	3	3	4	4
1	1	2	2	2	2	3	3	4	4
1	1	2	2	2	2	3	3	4	4
1	1	2	2	2	2	3	3	4	4
1	1	2	2	2	2	3	3	2	2
1	1	2	2	2	2	3	3	4	4
2	2	3	3	2	2	3	3	4	4
1	1	2	2	2	2	3	3	4	4
1	1	2	2	2	2	3	3	4	4
1	1	2	2	2	2	3	3	4	4
2	2	3	3	2	2	3	3	4	4
1	1	2	2	2	2	3	3	4	4
1	1	2	2	2	2	3	3	4	4
1	1	2	2	2	2	3	3	4	4

For Review Only

1	1	2	2	2	2	3	3	4	4
1	1	2	2	2	2	3	3	4	4
1	1	2	2	2	2	3	3	4	4
1	1	2	2	2	2	3	3	4	4
2	2	2	2	2	2	3	3	4	4
1	1	2	2	2	2	3	3	4	4
1	1	2	2	2	2	3	3	4	4
1	1	2	2	2	2	3	3	4	4
1	1	2	2	2	2	3	3	4	4
1	1	2	2	2	2	3	3	4	4
1	1	2	2	2	2	3	3	4	4
1	1	2	2	2	2	3	3	4	4
1	1	2	2	2	2	3	3	4	4
1	1	2	2	2	2	3	3	4	4
1	1	2	2	2	2	3	3	4	4
1	1	2	2	2	2	3	3	4	4
1	1	2	2	2	2	2	2	2	2
1	1	2	2	2	2	3	3	4	4
1	1	2	2	2	2	3	3	4	4
1	1	2	2	2	2	3	3	4	4
1	1	2	2	2	2	3	3	4	4
1	1	2	2	2	2	2	2	4	4
2	2	3	3	2	2	3	3	4	4
2	2	3	3	2	2	3	3	4	4
1	1	2	2	2	2	3	3	4	4
1	1	2	2	2	2	3	3	4	4
1	1	2	2	2	2	3	3	4	4
1	1	2	2	2	2	3	3	4	4
2	2	3	3	2	2	3	3	4	4
1	1	2	2	2	2	3	3	4	4
1	1	2	2	2	2	3	3	4	4
1	1	2	2	2	2	3	3	4	4
1	1	2	2	4	4	3	3	4	4
1	1	2	2	2	2	3	3	4	4
2	2	3	3	2	2	3	3	4	4
2	2	3	3	2	2	3	3	4	4
1	1	2	2	2	2	3	3	4	4
1	1	2	2	2	2	3	3	4	4
1	1	2	2	2	2	3	3	4	4
1	1	2	2	2	2	3	3	4	4
1	1	2	2	2	2	3	3	4	4
1	1	2	2	2	2	3	3	4	4
2	2	3	3	2	2	3	3	4	4
2	2	3	3	2	2	3	3	4	4
1	1	2	2	2	2	3	3	4	4
1	1	2	2	2	2	3	3	4	4
1	1	2	2	2	2	3	3	4	4
1	1	2	2	2	2	2	2	2	2
1	1	3	3	2	2	3	3	4	4
1	1	2	2	2	2	3	3	4	4
1	1	2	2	2	2	3	3	4	4
1	1	2	2	2	2	3	3	4	4
1	1	2	2	2	2	3	3	4	4
1	1	2	2	2	2	2	2	2	2
1	1	2	2	2	2	3	3	4	4

1	1	2	2	2	2	3	3	4	4
1	1	2	2	2	2	3	3	4	4
1	1	2	2	2	2	3	3	4	4
1	1	2	2	2	2	3	3	4	4
2	2	3	3	2	2	3	3	4	4
1	1	2	2	2	2	3	3	4	4
1	1	2	2	2	2	3	3	4	4
1	1	2	2	2	2	3	3	4	4
1	1	2	2	2	2	3	3	4	4
1	1	2	2	2	2	3	3	4	4
1	1	2	2	2	2	3	3	4	4
1	1	2	2	2	2	3	3	4	4
1	1	2	2	2	2	3	3	4	4
1	1	2	2	2	2	3	3	4	4
1	1	2	2	2	2	3	3	4	4
1	1	2	2	2	2	3	3	4	4
2	2	2	2	2	2	3	3	4	4
2	2	3	3	2	2	2	2	2	2
1	1	2	2	2	2	3	3	4	4
1	1	2	2	2	2	3	3	4	4
1	1	2	2	2	2	3	3	4	4
2	2	3	3	2	2	3	3	4	4
1	1	2	2	2	2	3	3	4	4
1	1	2	2	2	2	2	2	2	2
1	1	2	2	2	2	3	3	4	4
2	2	3	3	2	2	3	3	4	4
1	1	2	2	2	2	3	3	4	4
1	1	2	2	2	2	3	3	2	2
1	1	2	2	2	2	3	3	4	4
1	1	2	2	2	2	3	3	4	4
1	1	2	2	2	2	3	3	4	4
1	1	2	2	2	2	3	3	4	4
1	1	2	2	2	2	3	3	4	4
1	1	2	2	2	2	3	3	4	4
1	1	2	2	4	4	3	3	4	4
1	1	2	2	2	2	2	2	2	2
1	1	2	2	2	2	2	2	2	2
1	1	2	2	2	2	3	3	4	4
1	1	2	2	2	2	2	2	2	2
1	1	2	2	2	2	3	3	4	4
2	2	3	3	2	2	3	3	4	4
1	1	2	2	2	2	3	3	4	4
2	2	3	3	2	2	3	3	4	4
1	1	2	2	2	2	3	3	4	4
1	1	2	2	2	2	3	3	4	4
1	1	2	2	2	2	3	3	4	4
1	1	2	2	2	2	3	3	4	4
2	2	3	3	2	2	3	3	4	4
2	2	3	3	2	2	3	3	4	4
1	1	2	2	2	2	3	3	4	4
2	2	3	3	2	2	3	3	4	4
1	1	2	2	2	2	3	3	4	4
1	1	2	2	2	2	3	3	4	4
1	1	2	2	2	2	3	3	4	4
2	2	3	3	2	2	3	3	4	4

For Review Only

2	2	3	3	2	2	3	3	4	4
1	1	2	2	2	2	3	3	4	4
1	1	2	2	2	2	3	3	4	4
1	1	2	2	2	2	3	3	4	4
1	1	2	2	4	4	3	3	4	4
1	1	2	2	2	2	3	3	4	4
1	1	2	2	2	2	3	3	4	4
1	1	2	2	2	2	3	3	4	4
1	1	2	2	2	2	3	3	4	4
1	1	3	3	2	2	3	3	4	4
1	1	2	2	2	2	3	3	4	4
1	1	2	2	2	2	3	3	4	4
1	1	2	2	2	2	3	3	4	4
1	1	2	2	2	2	3	3	4	4
2	2	3	3	2	2	3	3	4	4
1	1	2	2	2	2	3	3	4	4
1	1	2	2	2	2	3	3	4	4
1	1	2	2	2	2	3	3	4	4
1	1	2	2	2	2	3	3	4	4
2	2	3	3	2	2	3	3	4	4
2	2	3	3	2	2	3	3	4	4
1	1	2	2	2	2	3	3	4	4
1	1	2	2	2	2	3	3	4	4
1	1	2	2	2	2	3	3	4	4
1	1	2	2	2	2	3	3	4	4
1	1	2	2	2	2	3	3	4	4
2	2	3	3	2	2	3	3	4	4
1	1	2	2	2	2	3	3	4	4
1	1	2	2	2	2	3	3	4	4
2	2	3	3	2	2	3	3	4	4
2	2	3	3	2	2	3	3	4	4
1	1	2	2	2	2	3	3	4	4
1	1	2	2	2	2	3	3	4	4
1	1	2	2	2	2	2	2	2	2
1	1	2	2	2	2	3	3	4	4
1	1	2	2	2	2	3	3	4	4
2	2	2	2	2	2	3	3	4	4
1	1	2	2	2	2	2	2	2	2
1	1	2	2	2	2	2	2	2	2
2	2	3	3	2	2	3	3	4	4
1	1	2	2	2	2	3	3	4	4
2	2	3	3	2	2	2	2	2	2
1	1	2	2	2	2	3	3	4	4
1	1	2	2	2	2	3	3	4	4
1	1	2	2	2	2	2	2	2	2
1	1	2	2	2	2	3	3	4	4
2	2	2	2	2	2	2	2	2	2
1	1	2	2	2	2	3	3	4	4
1	1	2	2	2	2	3	3	4	4
1	1	2	2	2	2	3	3	4	4
1	1	2	2	2	2	3	3	4	4
2	2	3	3	2	2	3	3	4	4
2	2	3	3	2	2	3	3	4	4
1	1	2	2	2	2	3	3	4	4
1	1	2	2	2	2	3	3	4	4

For Review Only

1	1	2	2	2	2	3	3	4	4
1	1	2	2	2	2	3	3	4	4

For Review Only

SNP422	SNP422	SNP423	SNP423	SNP424	SNP424	SNP425	SNP425	SNP426	SNP426
3	3	4	4	1	1	3	3	4	4
1	1	3	3	1	1	3	3	4	4
3	3	4	4	1	1	3	3	3	3
3	3	4	4	1	1	3	3	3	3
1	1	3	3	1	1	3	3	4	4
3	3	4	4	1	1	3	3	3	3
3	3	4	4	1	1	3	3	3	3
1	1	3	3	1	1	3	3	4	4
1	1	3	3	1	1	3	3	4	4
1	1	3	3	1	1	3	3	4	4
3	3	4	4	1	1	3	3	3	3
3	3	4	4	1	1	3	3	3	3
3	3	4	4	1	1	3	3	4	4
3	3	4	4	2	2	3	3	3	3
3	3	4	4	1	1	3	3	4	4
1	1	3	3	1	1	3	3	4	4
3	3	4	4	1	1	3	3	3	3
3	3	4	4	1	1	2	2	3	3
3	3	4	4	1	1	2	2	3	3
1	1	3	3	1	1	3	3	4	4
3	3	4	4	1	1	3	3	3	3
3	3	4	4	1	1	3	3	3	3
1	1	3	3	1	1	3	3	4	4
1	1	3	3	2	2	3	3	4	4
3	3	4	4	1	1	3	3	3	3
1	1	3	3	1	1	3	3	4	4
1	1	3	3	1	1	3	3	4	4
3	3	4	4	1	1	3	3	4	4
1	1	3	3	1	1	3	3	4	4
1	1	3	3	1	1	3	3	4	4
1	1	3	3	1	1	3	3	4	4
1	1	3	3	1	1	3	3	4	4
3	3	4	4	1	1	3	3	3	3
1	1	3	3	1	1	3	3	4	4
3	3	4	4	1	1	3	3	4	4
1	1	3	3	1	1	3	3	4	4
3	3	4	4	1	1	3	3	3	3
3	3	4	4	1	1	3	3	3	3
1	1	3	3	1	1	3	3	4	4
1	1	3	3	1	1	3	3	4	4
1	1	3	3	1	1	3	3	4	4
1	1	3	3	1	1	3	3	4	4
1	1	3	3	1	1	3	3	4	4
3	3	4	4	1	1	3	3	3	3
1	1	3	3	1	1	3	3	4	4
3	3	4	4	1	1	3	3	3	3
3	3	4	4	1	1	2	2	3	3
3	3	4	4	1	1	3	3	3	3
1	1	4	4	1	1	3	3	4	4
3	3	4	4	1	1	3	3	3	3
3	3	4	4	1	1	3	3	3	3
3	3	4	4	1	1	3	3	4	4

For Review Only

3	3	4	4	1	1	3	3	3	3
3	3	4	4	1	1	3	3	3	3
3	3	4	4	1	1	3	3	3	3
3	3	4	4	1	1	3	3	3	3
3	3	4	4	1	1	2	2	3	3
1	1	3	3	1	1	2	2	4	4
1	1	3	3	1	1	3	3	4	4
1	1	3	3	1	1	3	3	4	4
1	1	3	3	1	1	3	3	4	4
3	3	3	3	1	1	3	3	4	4
3	3	4	4	1	1	3	3	3	3
1	1	3	3	1	1	2	2	4	4
3	3	4	4	1	1	3	3	3	3
1	1	3	3	1	1	3	3	4	4
3	3	4	4	1	1	3	3	4	4
3	3	4	4	1	1	3	3	3	3
3	3	3	3	1	1	3	3	4	4
3	3	4	4	2	2	3	3	3	3
1	1	3	3	1	1	3	3	4	4
1	1	3	3	1	1	3	3	4	4
1	1	3	3	1	1	3	3	4	4
1	1	3	3	1	1	3	3	4	4
1	1	3	3	1	1	3	3	4	4
1	1	3	3	1	1	3	3	4	4
1	1	3	3	1	1	3	3	4	4
1	1	3	3	1	1	3	3	4	4
3	3	4	4	1	1	3	3	3	3
1	1	3	3	1	1	3	3	4	4
3	3	4	4	1	1	3	3	3	3
1	1	3	3	1	1	3	3	4	4
1	1	3	3	1	1	3	3	3	3
1	1	3	3	1	1	3	3	4	4
1	1	3	3	1	1	3	3	4	4
3	3	4	4	1	1	3	3	3	3
3	3	4	4	1	1	3	3	3	3
1	1	3	3	1	1	3	3	4	4
1	1	3	3	1	1	3	3	4	4
3	3	4	4	1	1	3	3	3	3
1	1	3	3	1	1	3	3	4	4
3	3	4	4	1	1	3	3	3	3
3	3	4	4	1	1	3	3	3	3
1	1	3	3	1	1	3	3	4	4
3	3	4	4	1	1	3	3	3	3
3	3	4	4	1	1	3	3	3	3
3	3	4	4	1	1	3	3	3	3
1	1	3	3	1	1	2	2	4	4
1	1	3	3	1	1	3	3	4	4
3	3	4	4	1	1	3	3	3	3
3	3	4	4	1	1	3	3	3	3
1	1	3	3	2	2	3	3	4	4
1	1	3	3	1	1	3	3	4	4
1	1	3	3	1	1	3	3	4	4
1	1	3	3	1	1	3	3	4	4
1	1	3	3	1	1	3	3	4	4
3	3	4	4	2	2	3	3	3	3

For Review Only

1	1	3	3	1	1	3	3	4	4
1	1	3	3	1	1	3	3	4	4
1	1	3	3	1	1	3	3	4	4
3	3	4	4	1	1	3	3	3	3
3	3	4	4	1	1	3	3	3	3
1	1	3	3	1	1	3	3	4	4
3	3	3	3	2	2	3	3	4	4
1	1	3	3	1	1	3	3	4	4
3	3	4	4	1	1	3	3	3	3
3	3	4	4	1	1	3	3	4	4
3	3	4	4	2	2	3	3	3	3
3	3	4	4	1	1	3	3	4	4
3	3	4	4	1	1	3	3	3	3
3	3	4	4	1	1	3	3	4	4
3	3	4	4	1	1	3	3	4	4
3	3	4	4	1	1	3	3	4	4
3	3	4	4	1	1	3	3	3	3
3	3	4	4	1	1	3	3	3	3
3	3	4	4	1	1	2	2	3	3
1	1	3	3	1	1	3	3	4	4
3	3	4	4	1	1	3	3	3	3
1	1	3	3	1	1	3	3	4	4
3	3	4	4	1	1	3	3	3	3
1	1	3	3	1	1	3	3	4	4
1	1	3	3	1	1	3	3	4	4
1	1	3	3	1	1	3	3	4	4
3	3	3	3	1	1	3	3	4	4
1	1	3	3	1	1	3	3	4	4
3	3	4	4	1	1	3	3	3	3
3	3	4	4	2	2	3	3	3	3
1	1	3	3	1	1	3	3	4	4
3	3	4	4	1	1	3	3	4	4
3	3	4	4	1	1	3	3	3	3
3	3	4	4	1	1	3	3	4	4
1	1	3	3	1	1	3	3	4	4
3	3	4	4	1	1	3	3	4	4
1	1	3	3	1	1	2	2	4	4
1	1	3	3	1	1	3	3	4	4
3	3	4	4	1	1	3	3	3	3
1	1	3	3	1	1	3	3	4	4
1	1	3	3	1	1	3	3	4	4
1	1	3	3	1	1	3	3	4	4
3	3	4	4	2	2	3	3	3	3
1	1	4	4	1	1	3	3	4	4
3	3	4	4	1	1	3	3	3	3
3	3	4	4	1	1	3	3	3	3
3	3	4	4	1	1	3	3	3	3
1	1	3	3	1	1	3	3	4	4
3	3	4	4	1	1	3	3	4	4
3	3	4	4	1	1	3	3	4	4
3	3	4	4	1	1	3	3	4	4
3	3	3	3	1	1	3	3	4	4
3	3	4	4	1	1	3	3	3	3
3	3	4	4	1	1	3	3	3	3
1	1	4	4	1	1	3	3	4	4

For Review Only

3	3	4	4	1	1	3	3	3	3
3	3	4	4	1	1	3	3	3	3
3	3	4	4	1	1	3	3	3	3
1	1	3	3	1	1	3	3	4	4
3	3	4	4	1	1	3	3	3	3
1	1	3	3	1	1	3	3	4	4
3	3	4	4	1	1	3	3	4	4
3	3	4	4	1	1	3	3	4	4
1	1	3	3	1	1	3	3	4	4
1	1	3	3	1	1	2	2	4	4
3	3	4	4	1	1	3	3	4	4
1	1	3	3	1	1	3	3	4	4
3	3	4	4	1	1	3	3	3	3
3	3	4	4	1	1	3	3	4	4
1	1	3	3	1	1	3	3	4	4
3	3	4	4	1	1	3	3	3	3
3	3	4	4	1	1	3	3	3	3
1	1	3	3	1	1	3	3	4	4
1	1	3	3	1	1	3	3	4	4
1	1	3	3	2	2	3	3	4	4
3	3	4	4	1	1	3	3	4	4
1	1	3	3	1	1	3	3	4	4
1	1	3	3	1	1	3	3	4	4
1	1	3	3	1	1	2	2	4	4
1	1	3	3	1	1	3	3	4	4
1	1	3	3	1	1	3	3	4	4
3	3	4	4	1	1	3	3	3	3
3	3	4	4	1	1	3	3	3	3
1	1	3	3	1	1	3	3	4	4
3	3	4	4	2	2	3	3	3	3
3	3	4	4	1	1	3	3	3	3
3	3	4	4	1	1	3	3	3	3
1	1	3	3	1	1	3	3	4	4
1	1	3	3	1	1	3	3	4	4
3	3	4	4	2	2	3	3	4	4
1	1	3	3	1	1	2	2	4	4
1	1	3	3	1	1	3	3	4	4
1	1	3	3	1	1	3	3	4	4
1	1	3	3	1	1	3	3	4	4
3	3	4	4	1	1	3	3	3	3
3	3	4	4	1	1	3	3	3	3
3	3	4	4	1	1	3	3	3	3
1	1	3	3	1	1	3	3	4	4
1	1	3	3	1	1	3	3	4	4
1	1	3	3	2	2	3	3	4	4
3	3	4	4	1	1	3	3	3	3
3	3	4	4	1	1	3	3	3	3
3	3	4	4	1	1	3	3	3	3
1	1	3	3	2	2	3	3	4	4
3	3	4	4	1	1	3	3	3	3
1	1	3	3	1	1	3	3	4	4
1	1	3	3	1	1	3	3	4	4
1	1	3	3	1	1	3	3	4	4
3	3	4	4	1	1	3	3	3	3
1	1	3	3	1	1	3	3	4	4

For Review Only

3	3	4	4	2	2	3	3	3	3
1	1	3	3	2	2	3	3	4	4
3	3	4	4	1	1	3	3	3	3
3	3	4	4	1	1	3	3	4	4
1	1	3	3	1	1	3	3	4	4
3	3	4	4	1	1	3	3	3	3
3	3	4	4	1	1	3	3	4	4
1	1	3	3	1	1	3	3	4	4
3	3	4	4	2	2	3	3	4	4
1	1	3	3	1	1	3	3	4	4
3	3	4	4	1	1	3	3	4	4
3	3	4	4	1	1	3	3	3	3
3	3	4	4	1	1	3	3	3	3
3	3	4	4	1	1	3	3	3	3
3	3	4	4	1	1	3	3	3	3
1	1	3	3	1	1	3	3	4	4
1	1	3	3	1	1	3	3	4	4
3	3	4	4	1	1	3	3	3	3
3	3	4	4	1	1	3	3	3	3
3	3	4	4	1	1	3	3	3	3
1	1	3	3	1	1	3	3	4	4
3	3	4	4	1	1	3	3	3	3
3	3	4	4	1	1	3	3	3	3
1	1	3	3	1	1	3	3	4	4
1	1	3	3	1	1	3	3	4	4
1	1	3	3	1	1	3	3	4	4
1	1	3	3	1	1	3	3	4	4
3	3	4	4	1	1	3	3	3	3
1	1	3	3	1	1	3	3	4	4
1	1	3	3	1	1	3	3	4	4
3	3	4	4	1	1	3	3	3	3
3	3	4	4	1	1	3	3	3	3
1	1	3	3	2	2	3	3	4	4
3	3	4	4	1	1	3	3	3	3
1	1	3	3	1	1	3	3	4	4
1	1	3	3	1	1	3	3	4	4
3	3	4	4	2	2	3	3	3	3
1	1	3	3	1	1	2	2	4	4
1	1	3	3	1	1	3	3	4	4
1	1	3	3	1	1	3	3	4	4
1	1	3	3	1	1	2	2	4	4
3	3	4	4	2	2	3	3	3	3
1	1	3	3	1	1	3	3	4	4
3	3	4	4	1	1	3	3	3	3
1	1	3	3	1	1	3	3	4	4
3	3	4	4	1	1	3	3	3	3
1	1	3	3	1	1	3	3	4	4
3	3	4	4	1	1	3	3	4	4
1	1	4	4	1	1	3	3	4	4
1	1	3	3	1	1	3	3	4	4
1	1	3	3	1	1	2	2	4	4
1	1	3	3	1	1	3	3	4	4
3	3	4	4	1	1	3	3	3	3
3	3	4	4	1	1	3	3	3	3
1	1	3	3	1	1	3	3	4	4

For Review Only

3	3	4	4	1	1	3	3	3	3
1	1	3	3	1	1	3	3	4	4

For Review Only

SNP427	SNP427	SNP428	SNP428	SNP429	SNP429	SNP430	SNP430	SNP431	SNP431
3	3	1	1	3	3	1	1	4	4
1	1	4	4	3	3	2	2	4	4
3	3	1	1	3	3	2	2	4	4
3	3	1	1	3	3	2	2	4	4
1	1	1	1	3	3	1	1	4	4
3	3	1	1	3	3	2	2	4	4
3	3	1	1	3	3	2	2	4	4
1	1	1	1	3	3	2	2	4	4
1	1	1	1	3	3	1	1	4	4
1	1	1	1	3	3	2	2	4	4
3	3	1	1	3	3	1	1	4	4
3	3	1	1	3	3	2	2	4	4
3	3	1	1	3	3	2	2	4	4
3	3	1	1	4	4	2	2	4	4
3	3	1	1	3	3	2	2	4	4
1	1	1	1	4	4	2	2	4	4
3	3	1	1	3	3	2	2	4	4
3	3	4	4	3	3	2	2	4	4
3	3	1	1	3	3	1	1	4	4
1	1	1	1	3	3	2	2	3	3
3	3	1	1	3	3	2	2	3	3
3	3	1	1	3	3	2	2	4	4
1	1	1	1	3	3	2	2	4	4
1	1	1	1	4	4	2	2	4	4
3	3	1	1	3	3	1	1	3	3
1	1	1	1	3	3	2	2	4	4
1	1	1	1	3	3	2	2	3	3
3	3	1	1	3	3	2	2	3	3
1	1	1	1	3	3	2	2	3	3
1	1	1	1	3	3	2	2	4	4
1	1	1	1	3	3	2	2	3	3
1	1	1	1	3	3	2	2	4	4
3	3	1	1	3	3	2	2	4	4
1	1	1	1	3	3	2	2	4	4
3	3	1	1	3	3	2	2	4	4
1	1	1	1	3	3	2	2	4	4
3	3	4	4	3	3	2	2	4	4
3	3	4	4	3	3	2	2	4	4
1	1	1	1	3	3	2	2	4	4
1	1	1	1	3	3	2	2	4	4
1	1	1	1	3	3	2	2	4	4
1	1	1	1	3	3	2	2	3	3
1	1	1	1	3	3	2	2	4	4
3	3	4	4	3	3	2	2	4	4
1	1	1	1	3	3	2	2	4	4
3	3	1	1	3	3	2	2	4	4
3	3	1	1	3	3	2	2	4	4
3	3	1	1	3	3	1	1	3	3
1	1	4	4	3	3	2	2	4	4
3	3	1	1	3	3	2	2	4	4
3	3	1	1	3	3	2	2	4	4
3	3	1	1	3	3	2	2	3	3

For Review Only

3	3	1	1	3	3	1	1	4	4
3	3	1	1	3	3	2	2	4	4
3	3	1	1	3	3	2	2	4	4
3	3	1	1	3	3	2	2	4	4
3	3	1	1	3	3	1	1	4	4
1	1	1	1	3	3	2	2	4	4
1	1	1	1	3	3	2	2	4	4
1	1	4	4	3	3	2	2	4	4
1	1	1	1	3	3	2	2	4	4
3	3	1	1	3	3	2	2	4	4
3	3	1	1	3	3	2	2	4	4
1	1	1	1	3	3	2	2	4	4
3	3	1	1	3	3	1	1	4	4
1	1	1	1	3	3	2	2	3	3
3	3	1	1	3	3	2	2	4	4
3	3	1	1	4	4	2	2	4	4
3	3	1	1	3	3	2	2	4	4
3	3	1	1	4	4	2	2	4	4
1	1	1	1	3	3	1	1	4	4
1	1	1	1	3	3	1	1	4	4
1	1	1	1	3	3	2	2	4	4
1	1	1	1	3	3	2	2	4	4
1	1	1	1	3	3	1	1	4	4
1	1	1	1	3	3	2	2	4	4
1	1	1	1	3	3	2	2	4	4
1	1	1	1	3	3	2	2	4	4
3	3	1	1	3	3	2	2	4	4
1	1	1	1	3	3	2	2	4	4
3	3	1	1	3	3	2	2	4	4
1	1	1	1	3	3	2	2	4	4
1	1	1	1	3	3	2	2	4	4
1	1	1	1	3	3	2	2	4	4
1	1	1	1	3	3	2	2	4	4
3	3	1	1	3	3	1	1	4	4
3	3	1	1	3	3	1	1	4	4
1	1	1	1	3	3	2	2	4	4
1	1	1	1	3	3	2	2	4	4
3	3	1	1	3	3	1	1	4	4
1	1	1	1	3	3	2	2	4	4
3	3	1	1	3	3	2	2	4	4
3	3	1	1	3	3	2	2	4	4
1	1	1	1	3	3	2	2	4	4
3	3	1	1	3	3	1	1	4	4
3	3	1	1	3	3	2	2	4	4
3	3	1	1	3	3	2	2	4	4
1	1	1	1	3	3	1	1	4	4
1	1	1	1	3	3	2	2	4	4
3	3	1	1	3	3	2	2	4	4
3	3	1	1	3	3	2	2	4	4
1	1	1	1	4	4	2	2	4	4
1	1	1	1	3	3	2	2	4	4
1	1	1	1	3	3	2	2	4	4
1	1	4	4	3	3	2	2	4	4
1	1	1	1	3	3	2	2	4	4
3	3	1	1	4	4	2	2	4	4

For Review Only

1	1	1	1	3	3	2	2	4	4
1	1	1	1	3	3	2	2	4	4
1	1	1	1	3	3	2	2	4	4
3	3	1	1	3	3	2	2	4	4
3	3	1	1	3	3	2	2	4	4
1	1	1	1	3	3	2	2	4	4
1	1	1	1	4	4	2	2	4	4
1	1	1	1	3	3	2	2	3	3
3	3	1	1	3	3	2	2	4	4
3	3	1	1	3	3	2	2	4	4
3	3	1	1	4	4	1	1	4	4
3	3	1	1	3	3	2	2	4	4
3	3	1	1	3	3	1	1	4	4
3	3	1	1	3	3	2	2	4	4
3	3	4	4	3	3	2	2	4	4
3	3	1	1	3	3	2	2	4	4
3	3	1	1	3	3	2	2	4	4
3	3	1	1	3	3	2	2	4	4
3	3	1	1	3	3	2	2	4	4
1	1	1	1	3	3	2	2	4	4
3	3	1	1	3	3	2	2	4	4
1	1	1	1	3	3	2	2	4	4
3	3	1	1	3	3	2	2	4	4
1	1	1	1	3	3	2	2	4	4
1	1	1	1	3	3	2	2	4	4
1	1	1	1	3	3	2	2	4	4
1	1	1	1	3	3	2	2	4	4
1	1	1	1	3	3	2	2	4	4
3	3	1	1	3	3	2	2	3	3
3	3	1	1	4	4	1	1	4	4
1	1	1	1	3	3	2	2	4	4
3	3	1	1	3	3	2	2	4	4
3	3	1	1	3	3	2	2	4	4
3	3	1	1	3	3	2	2	4	4
1	1	1	1	3	3	2	2	4	4
1	1	1	1	3	3	2	2	4	4
1	1	1	1	3	3	2	2	4	4
1	1	1	1	3	3	2	2	4	4
3	3	1	1	3	3	2	2	4	4
1	1	1	1	3	3	2	2	4	4
1	1	1	1	3	3	2	2	4	4
1	1	1	1	3	3	2	2	4	4
3	3	1	1	4	4	2	2	4	4
1	1	1	1	3	3	2	2	4	4
3	3	1	1	3	3	2	2	4	4
3	3	1	1	3	3	2	2	4	4
3	3	1	1	3	3	2	2	4	4
1	1	1	1	3	3	2	2	4	4
3	3	1	1	3	3	2	2	4	4
3	3	1	1	3	3	2	2	4	4
3	3	1	1	3	3	2	2	4	4
1	1	4	4	3	3	2	2	4	4
3	3	1	1	3	3	2	2	4	4
3	3	1	1	3	3	2	2	4	4
1	1	4	4	3	3	2	2	4	4

For Review Only

3	3	1	1	3	3	2	2	4	4
3	3	1	1	3	3	1	1	4	4
3	3	4	4	3	3	2	2	4	4
1	1	1	1	3	3	2	2	4	4
3	3	1	1	3	3	2	2	4	4
1	1	4	4	3	3	2	2	4	4
3	3	1	1	3	3	2	2	4	4
3	3	1	1	3	3	2	2	4	4
1	1	1	1	3	3	1	1	4	4
1	1	1	1	3	3	2	2	4	4
3	3	1	1	3	3	2	2	4	4
1	1	4	4	3	3	2	2	4	4
3	3	1	1	3	3	2	2	4	4
3	3	1	1	3	3	2	2	4	4
1	1	1	1	3	3	2	2	4	4
3	3	1	1	3	3	1	1	4	4
3	3	1	1	3	3	2	2	4	4
1	1	1	1	3	3	2	2	4	4
1	1	1	1	3	3	2	2	4	4
1	1	1	1	4	4	2	2	4	4
3	3	1	1	3	3	2	2	4	4
1	1	1	1	3	3	2	2	4	4
1	1	1	1	3	3	2	2	4	4
1	1	1	1	3	3	2	2	4	4
1	1	1	1	3	3	2	2	4	4
1	1	1	1	3	3	2	2	4	4
3	3	1	1	3	3	2	2	4	4
3	3	1	1	3	3	2	2	4	4
1	1	1	1	3	3	2	2	4	4
3	3	1	1	4	4	2	2	4	4
3	3	1	1	3	3	2	2	4	4
3	3	1	1	3	3	2	2	4	4
1	1	1	1	3	3	2	2	4	4
1	1	1	1	3	3	2	2	4	4
3	3	1	1	4	4	2	2	3	3
1	1	1	1	3	3	2	2	4	4
1	1	1	1	3	3	2	2	4	4
1	1	4	4	3	3	1	1	4	4
1	1	1	1	3	3	2	2	4	4
3	3	1	1	3	3	2	2	4	4
3	3	1	1	3	3	2	2	4	4
3	3	1	1	3	3	2	2	4	4
1	1	1	1	3	3	2	2	4	4
1	1	4	4	3	3	2	2	4	4
1	1	1	1	3	3	2	2	4	4
3	3	4	4	3	3	2	2	4	4
3	3	1	1	3	3	1	1	4	4
3	3	1	1	3	3	2	2	4	4
1	1	1	1	4	4	2	2	4	4
3	3	1	1	3	3	2	2	4	4
1	1	1	1	3	3	2	2	3	3
1	1	1	1	3	3	2	2	4	4
1	1	1	1	3	3	2	2	4	4
3	3	1	1	3	3	2	2	4	4
1	1	1	1	3	3	2	2	4	4

For Review Only

3	3	1	1	4	4	2	2	4	4
1	1	1	1	4	4	2	2	4	4
3	3	1	1	3	3	2	2	3	3
3	3	1	1	3	3	2	2	4	4
1	1	1	1	3	3	2	2	3	3
3	3	1	1	3	3	2	2	4	4
3	3	1	1	3	3	2	2	4	4
1	1	1	1	3	3	2	2	4	4
3	3	1	1	4	4	2	2	4	4
1	1	1	1	3	3	2	2	4	4
3	3	1	1	3	3	2	2	4	4
3	3	1	1	3	3	2	2	3	3
3	3	1	1	3	3	2	2	4	4
3	3	1	1	3	3	2	2	4	4
3	3	1	1	3	3	2	2	4	4
1	1	1	1	3	3	2	2	4	4
1	1	1	1	3	3	2	2	4	4
3	3	1	1	3	3	2	2	4	4
3	3	1	1	3	3	1	1	4	4
3	3	4	4	3	3	2	2	4	4
1	1	1	1	3	3	2	2	4	4
3	3	1	1	3	3	2	2	4	4
3	3	1	1	3	3	2	2	4	4
1	1	1	1	3	3	2	2	4	4
1	1	4	4	3	3	2	2	4	4
1	1	4	4	3	3	2	2	4	4
1	1	1	1	3	3	2	2	4	4
3	3	1	1	3	3	2	2	4	4
1	1	1	1	3	3	2	2	4	4
1	1	1	1	3	3	2	2	3	3
3	3	1	1	3	3	2	2	4	4
3	3	1	1	3	3	2	2	4	4
1	1	1	1	3	3	2	2	4	4
3	3	1	1	3	3	2	2	4	4
1	1	1	1	3	3	2	2	4	4
1	1	1	1	3	3	2	2	4	4
3	3	1	1	4	4	2	2	4	4
1	1	1	1	3	3	2	2	4	4
1	1	1	1	3	3	2	2	4	4
1	1	1	1	3	3	2	2	4	4
1	1	1	1	3	3	2	2	4	4
3	3	1	1	4	4	2	2	4	4
1	1	1	1	3	3	2	2	4	4
3	3	1	1	3	3	1	1	4	4
1	1	4	4	3	3	1	1	4	4
3	3	1	1	3	3	2	2	4	4
1	1	1	1	3	3	2	2	4	4
3	3	1	1	3	3	2	2	4	4
3	3	1	1	3	3	2	2	4	4
1	1	1	1	3	3	2	2	4	4
1	1	1	1	3	3	1	1	3	3
1	1	1	1	3	3	2	2	4	4
3	3	1	1	3	3	2	2	4	4
3	3	1	1	3	3	2	2	4	4
1	1	4	4	3	3	2	2	4	4

For Review Only

3	3	1	1	3	3	1	1	4	4
1	1	1	1	3	3	2	2	4	4

For Review Only

SNP432	SNP432	SNP433	SNP433	SNP434	SNP434	SNP435	SNP435	SNP436	SNP436
2	2	1	1	1	1	1	1	3	3
3	3	3	3	1	1	1	1	3	3
3	3	3	3	1	1	1	1	3	3
3	3	1	1	1	1	1	1	3	3
3	3	1	1	1	1	1	1	2	2
3	3	3	3	1	1	1	1	3	3
3	3	1	1	1	1	1	1	3	3
3	3	1	1	1	1	1	1	3	3
3	3	1	1	1	1	1	1	2	2
3	3	1	1	1	1	1	1	3	3
3	3	1	1	1	1	1	1	2	2
3	3	3	3	1	1	1	1	3	3
3	3	1	1	1	1	1	1	3	3
3	3	1	1	1	1	1	1	3	3
2	2	3	3	1	1	1	1	3	3
3	3	1	1	1	1	1	1	3	3
3	3	1	1	1	1	1	1	3	3
3	3	3	3	1	1	1	1	3	3
3	3	1	1	1	1	1	1	3	3
3	3	1	1	1	1	4	4	3	3
3	3	1	1	1	1	4	4	3	3
3	3	1	1	1	1	1	1	3	3
3	3	1	1	1	1	1	1	3	3
3	3	3	3	1	1	1	1	3	3
3	3	1	1	1	1	1	1	3	3
3	3	3	3	4	4	1	1	3	3
3	3	3	3	1	1	1	1	3	3
3	3	3	3	1	1	1	1	3	3
3	3	1	1	1	1	1	1	3	3
3	3	1	1	1	1	4	4	3	3
3	3	3	3	1	1	1	1	3	3
3	3	1	1	1	1	1	1	3	3
3	3	3	3	1	1	1	1	3	3
3	3	1	1	1	1	1	1	3	3
3	3	3	3	1	1	1	1	3	3
3	3	3	3	4	4	1	1	3	3
3	3	1	1	1	1	1	1	3	3
3	3	3	3	4	4	1	1	3	3
3	3	1	1	1	1	4	4	3	3
2	2	3	3	4	4	4	4	3	3
3	3	1	1	1	1	1	1	3	3
3	3	1	1	1	1	1	1	3	3
3	3	3	3	1	1	1	1	3	3
3	3	1	1	1	1	1	1	3	3
3	3	3	3	1	1	1	1	3	3
3	3	3	3	1	1	1	1	3	3
2	2	3	3	4	4	1	1	3	3
3	3	1	1	1	1	1	1	3	3
3	3	3	3	4	4	1	1	3	3
3	3	1	1	1	1	1	1	3	3
3	3	3	3	4	4	1	1	3	3
3	3	1	1	1	1	4	4	3	3

3	3	1	1	1	1	1	1	3	3
3	3	3	3	1	1	1	1	3	3
3	3	1	1	1	1	4	4	3	3
3	3	1	1	1	1	1	1	3	3
3	3	1	1	1	1	1	1	3	3
2	2	3	3	1	1	1	1	3	3
3	3	3	3	1	1	1	1	3	3
3	3	1	1	1	1	4	4	3	3
3	3	1	1	1	1	1	1	3	3
3	3	3	3	1	1	1	1	3	3
3	3	3	3	1	1	1	1	3	3
3	3	3	3	1	1	1	1	3	3
3	3	1	1	1	1	1	1	3	3
3	3	1	1	1	1	1	1	3	3
3	3	3	3	1	1	1	1	3	3
3	3	3	3	4	4	1	1	3	3
3	3	1	1	1	1	1	1	2	2
2	2	3	3	1	1	1	1	3	3
3	3	1	1	1	1	1	1	3	3
2	2	1	1	1	1	1	1	3	3
3	3	3	3	1	1	1	1	3	3
3	3	3	3	1	1	4	4	3	3
3	3	1	1	1	1	1	1	3	3
3	3	1	1	1	1	1	1	3	3
3	3	3	3	1	1	1	1	3	3
3	3	1	1	1	1	1	1	3	3
3	3	3	3	1	1	1	1	3	3
3	3	1	1	1	1	4	4	3	3
3	3	3	3	4	4	1	1	3	3
3	3	3	3	4	4	1	1	3	3
3	3	1	1	1	1	1	1	3	3
3	3	3	3	1	1	1	1	3	3
3	3	1	1	1	1	4	4	3	3
3	3	1	1	1	1	1	1	3	3
3	3	1	1	1	1	1	1	3	3
3	3	3	3	1	1	1	1	3	3
3	3	1	1	1	1	1	1	3	3
3	3	1	1	1	1	1	1	3	3
3	3	1	1	1	1	1	1	3	3
3	3	3	3	1	1	1	1	3	3
3	3	3	3	1	1	1	1	3	3
3	3	3	3	1	1	1	1	3	3
3	3	1	1	1	1	1	1	3	3
3	3	3	3	1	1	1	1	3	3
3	3	3	3	1	1	1	1	3	3
2	2	1	1	1	1	1	1	3	3
3	3	1	1	1	1	1	1	3	3
3	3	3	3	1	1	1	1	3	3
2	2	3	3	4	4	1	1	3	3
3	3	1	1	1	1	4	4	3	3
3	3	3	3	4	4	1	1	3	3
3	3	1	1	1	1	4	4	3	3
3	3	1	1	1	1	4	4	3	3
3	3	1	1	1	1	1	1	3	3
3	3	3	3	1	1	1	1	3	3

For Review Only

3	3	3	3	4	4	4	4	3	3
3	3	3	3	1	1	4	4	3	3
3	3	3	3	4	4	1	1	3	3
3	3	3	3	1	1	1	1	3	3
3	3	3	3	1	1	1	1	3	3
3	3	3	3	4	4	1	1	3	3
3	3	1	1	1	1	1	1	3	3
3	3	3	3	1	1	1	1	3	3
3	3	3	3	4	4	1	1	3	3
3	3	3	3	1	1	1	1	3	3
3	3	1	1	1	1	1	1	3	3
3	3	3	3	1	1	1	1	3	3
3	3	1	1	1	1	1	1	3	3
3	3	3	3	1	1	1	1	3	3
3	3	3	3	1	1	1	1	3	3
3	3	3	3	1	1	1	1	3	3
2	2	3	3	1	1	1	1	3	3
3	3	3	3	1	1	1	1	3	3
3	3	1	1	1	1	1	1	3	3
3	3	3	3	1	1	1	1	3	3
2	2	3	3	1	1	1	1	3	3
3	3	1	1	1	1	1	1	3	3
3	3	3	3	1	1	1	1	3	3
3	3	1	1	1	1	4	4	3	3
3	3	3	3	1	1	1	1	3	3
3	3	1	1	1	1	1	1	3	3
3	3	3	3	4	4	1	1	3	3
3	3	1	1	1	1	1	1	3	3
3	3	3	3	1	1	1	1	3	3
3	3	1	1	1	1	1	1	2	2
3	3	1	1	1	1	4	4	3	3
3	3	3	3	1	1	1	1	3	3
3	3	3	3	1	1	1	1	3	3
2	2	3	3	4	4	1	1	3	3
3	3	1	1	1	1	1	1	3	3
3	3	1	1	1	1	1	1	3	3
3	3	1	1	1	1	1	1	3	3
3	3	3	3	1	1	1	1	3	3
3	3	3	3	1	1	1	1	3	3
3	3	1	1	1	1	4	4	3	3
3	3	3	3	1	1	4	4	3	3
3	3	1	1	1	1	1	1	3	3
3	3	3	3	1	1	4	4	3	3
3	3	3	3	1	1	1	1	3	3
3	3	1	1	1	1	1	1	3	3
3	3	3	3	4	4	4	4	3	3
2	2	3	3	4	4	1	1	3	3
2	2	3	3	4	4	1	1	3	3
3	3	1	1	1	1	1	1	3	3
3	3	3	3	4	4	1	1	3	3
2	2	3	3	4	4	1	1	3	3
2	2	1	1	1	1	1	1	3	3
3	3	3	3	1	1	4	4	3	3
3	3	3	3	1	1	1	1	3	3
3	3	3	3	1	1	1	1	3	3

For Review Only

3	3	1	1	1	1	1	1	3	3
3	3	1	1	1	1	1	1	3	3
3	3	3	3	4	4	1	1	3	3
3	3	3	3	1	1	1	1	3	3
3	3	3	3	1	1	1	1	3	3
2	2	3	3	4	4	4	4	3	3
3	3	3	3	1	1	1	1	3	3
3	3	3	3	1	1	1	1	3	3
3	3	1	1	1	1	1	1	3	3
3	3	3	3	4	4	1	1	3	3
3	3	3	3	4	4	1	1	3	3
3	3	3	3	1	1	4	4	3	3
3	3	3	3	1	1	1	1	3	3
3	3	3	3	1	1	1	1	3	3
3	3	1	1	1	1	4	4	3	3
3	3	1	1	1	1	4	4	2	2
3	3	3	3	1	1	1	1	3	3
3	3	3	3	1	1	1	1	3	3
3	3	3	3	1	1	1	1	3	3
3	3	1	1	1	1	1	1	3	3
3	3	3	3	1	1	1	1	3	3
3	3	1	1	1	1	4	4	3	3
3	3	1	1	1	1	4	4	3	3
3	3	3	3	1	1	1	1	3	3
3	3	3	3	4	4	1	1	3	3
3	3	1	1	1	1	1	1	3	3
3	3	3	3	1	1	1	1	3	3
3	3	3	3	1	1	1	1	3	3
2	2	1	1	1	1	1	1	3	3
3	3	3	3	1	1	1	1	3	3
3	3	1	1	1	1	1	1	3	3
2	2	3	3	4	4	1	1	3	3
3	3	3	3	4	4	1	1	3	3
3	3	1	1	1	1	4	4	3	3
2	2	3	3	1	1	1	1	3	3
3	3	3	3	1	1	1	1	3	3
3	3	3	3	1	1	1	1	3	3
3	3	1	1	1	1	1	1	3	3
2	2	3	3	4	4	1	1	3	3
3	3	3	3	1	1	1	1	3	3
3	3	3	3	1	1	1	1	3	3
2	2	3	3	4	4	1	1	3	3
3	3	1	1	1	1	4	4	3	3
3	3	3	3	1	1	1	1	3	3
3	3	3	3	4	4	1	1	3	3
3	3	3	3	4	4	1	1	3	3
3	3	1	1	1	1	4	4	2	2
3	3	1	1	1	1	1	1	3	3
3	3	1	1	1	1	1	1	3	3
3	3	3	3	1	1	1	1	3	3
3	3	3	3	1	1	1	1	3	3
3	3	1	1	1	1	4	4	3	3
3	3	1	1	1	1	4	4	3	3
3	3	3	3	1	1	1	1	3	3
3	3	1	1	1	1	4	4	3	3

For Review Only

3	3	3	3	1	1	4	4	3	3
3	3	1	1	1	1	1	1	3	3
3	3	3	3	1	1	1	1	3	3
3	3	3	3	1	1	1	1	3	3
3	3	3	3	4	4	1	1	3	3
3	3	3	3	1	1	1	1	3	3
3	3	3	3	1	1	4	4	3	3
3	3	1	1	1	1	4	4	3	3
3	3	3	3	1	1	1	1	3	3
3	3	1	1	1	1	4	4	3	3
3	3	3	3	1	1	1	1	3	3
3	3	1	1	1	1	1	1	3	3
3	3	1	1	1	1	1	1	3	3
3	3	3	3	4	4	1	1	3	3
2	2	3	3	1	1	1	1	3	3
3	3	1	1	1	1	1	1	2	2
3	3	3	3	4	4	1	1	3	3
3	3	3	3	1	1	1	1	3	3
3	3	1	1	1	1	4	4	3	3
3	3	3	3	1	1	1	1	3	3
3	3	1	1	1	1	1	1	3	3
3	3	3	3	1	1	1	1	3	3
3	3	3	3	1	1	1	1	3	3
3	3	3	3	1	1	4	4	3	3
3	3	1	1	1	1	4	4	3	3
3	3	1	1	1	1	1	1	3	3
3	3	1	1	1	1	4	4	3	3
3	3	3	3	1	1	1	1	3	3
3	3	3	3	1	1	1	1	3	3
3	3	3	3	1	1	1	1	3	3
3	3	1	1	1	1	4	4	3	3
3	3	3	3	4	4	1	1	3	3
3	3	3	3	1	1	1	1	3	3
2	2	3	3	4	4	1	1	3	3
3	3	1	1	1	1	4	4	3	3
3	3	1	1	1	1	4	4	3	3
3	3	3	3	1	1	1	1	3	3
3	3	3	3	1	1	1	1	3	3
3	3	3	3	4	4	1	1	3	3
3	3	1	1	1	1	4	4	3	3
2	2	3	3	1	1	1	1	3	3
3	3	1	1	1	1	1	1	3	3
3	3	3	3	1	1	4	4	3	3
3	3	1	1	1	1	1	1	2	2
3	3	1	1	1	1	1	1	3	3
3	3	3	3	1	1	1	1	3	3
2	2	1	1	1	1	4	4	3	3
3	3	3	3	1	1	1	1	3	3
3	3	3	3	4	4	1	1	3	3
3	3	1	1	1	1	4	4	3	3
3	3	1	1	1	1	1	1	2	2
3	3	1	1	1	1	4	4	3	3
3	3	3	3	1	1	1	1	3	3
3	3	3	3	1	1	1	1	3	3
3	3	3	3	4	4	4	4	3	3

For Review Only

3	3	1	1	1	1	1	1	2	2
3	3	3	3	1	1	1	1	3	3

For Review Only

SNP437	SNP437	SNP438	SNP438	SNP439	SNP439	SNP440	SNP440	SNP441	SNP441
2	2	3	3	2	2	1	1	2	2
2	2	3	3	3	3	2	2	3	3
2	2	3	3	2	2	1	1	2	2
1	1	4	4	2	2	2	2	3	3
2	2	3	3	2	2	2	2	3	3
2	2	3	3	2	2	1	1	2	2
2	2	3	3	2	2	1	1	2	2
1	1	4	4	2	2	2	2	3	3
2	2	3	3	2	2	2	2	3	3
1	1	4	4	2	2	2	2	3	3
2	2	3	3	2	2	1	1	2	2
2	2	3	3	2	2	2	2	2	2
2	2	3	3	2	2	1	1	2	2
1	1	4	4	2	2	1	1	2	2
2	2	3	3	2	2	1	1	2	2
1	1	4	4	2	2	2	2	3	3
1	1	4	4	2	2	1	1	2	2
2	2	3	3	3	3	1	1	2	2
2	2	3	3	2	2	1	1	2	2
2	2	3	3	2	2	2	2	3	3
1	1	4	4	2	2	1	1	2	2
1	1	3	3	2	2	1	1	2	2
1	1	4	4	2	2	2	2	3	3
2	2	3	3	2	2	2	2	3	3
2	2	3	3	2	2	1	1	2	2
2	2	3	3	2	2	2	2	3	3
2	2	3	3	2	2	2	2	3	3
2	2	3	3	2	2	2	2	2	2
1	1	4	4	2	2	2	2	3	3
1	1	4	4	2	2	2	2	3	3
1	1	4	4	2	2	2	2	3	3
1	1	4	4	2	2	2	2	3	3
2	2	3	3	2	2	1	1	2	2
1	1	4	4	3	3	2	2	3	3
2	2	3	3	2	2	2	2	2	2
2	2	3	3	2	2	2	2	3	3
1	1	4	4	3	3	1	1	2	2
2	2	3	3	2	2	1	1	2	2
1	1	4	4	2	2	2	2	3	3
2	2	3	3	2	2	2	2	3	3
1	1	4	4	2	2	2	2	3	3
1	1	4	4	2	2	2	2	3	3
2	2	3	3	2	2	2	2	3	3
2	2	3	3	3	3	1	1	2	2
2	2	3	3	2	2	2	2	3	3
2	2	3	3	2	2	1	1	2	2
2	2	3	3	2	2	1	1	2	2
2	2	3	3	2	2	1	1	2	2
2	2	3	3	3	3	1	1	2	2
2	2	3	3	2	2	1	1	2	2
2	2	3	3	2	2	1	1	2	2
2	2	3	3	2	2	2	2	3	3

For Review Only

2	2	3	3	2	2	1	1	2	2
2	2	3	3	2	2	1	1	2	2
2	2	3	3	2	2	1	1	2	2
1	1	4	4	2	2	1	1	2	2
2	2	3	3	2	2	1	1	2	2
2	2	3	3	2	2	2	2	3	3
2	2	3	3	2	2	2	2	3	3
1	1	4	4	3	3	2	2	3	3
1	1	4	4	2	2	2	2	3	3
2	2	3	3	2	2	2	2	2	2
2	2	3	3	2	2	2	2	3	3
2	2	3	3	2	2	2	2	3	3
2	2	3	3	2	2	1	1	2	2
1	1	4	4	2	2	2	2	3	3
2	2	3	3	2	2	2	2	2	2
2	2	3	3	2	2	2	2	2	2
2	2	3	3	2	2	2	2	3	3
1	1	4	4	2	2	1	1	2	2
2	2	3	3	2	2	2	2	3	3
2	2	3	3	2	2	2	2	3	3
2	2	3	3	2	2	2	2	3	3
2	2	3	3	2	2	2	2	3	3
2	2	3	3	2	2	2	2	3	3
1	1	4	4	2	2	2	2	3	3
2	2	3	3	2	2	2	2	3	3
2	2	3	3	2	2	2	2	3	3
2	2	3	3	2	2	1	1	2	2
1	1	4	4	2	2	2	2	3	3
2	2	3	3	2	2	1	1	2	2
2	2	3	3	2	2	2	2	3	3
1	1	4	4	2	2	2	2	2	2
2	2	3	3	2	2	2	2	3	3
1	1	4	4	2	2	2	2	3	3
2	2	3	3	2	2	1	1	2	2
2	2	3	3	2	2	1	1	2	2
2	2	3	3	2	2	2	2	3	3
2	2	4	4	2	2	2	2	3	3
2	2	3	3	2	2	1	1	2	2
1	1	4	4	2	2	2	2	3	3
2	2	3	3	2	2	1	1	2	2
2	2	3	3	2	2	1	1	2	2
2	2	3	3	2	2	2	2	3	3
2	2	3	3	2	2	1	1	2	2
2	2	3	3	2	2	2	2	3	3
2	2	4	4	2	2	1	1	2	2
2	2	3	3	2	2	2	2	3	3
1	1	4	4	2	2	2	2	3	3
1	1	4	4	2	2	1	1	2	2
2	2	3	3	2	2	1	1	2	2
1	1	4	4	2	2	2	2	3	3
2	2	3	3	2	2	2	2	3	3
1	1	4	4	2	2	2	2	3	3
1	1	4	4	3	3	2	2	3	3
1	1	3	3	2	2	2	2	3	3
1	1	4	4	2	2	1	1	2	2

For Review Only

2	2	3	3	2	2	2	2	3	3
2	2	3	3	2	2	2	2	3	3
2	2	3	3	2	2	2	2	3	3
1	1	4	4	2	2	1	1	2	2
2	2	3	3	2	2	2	2	3	3
2	2	3	3	2	2	2	2	3	3
1	1	4	4	2	2	2	2	3	3
1	1	4	4	2	2	2	2	3	3
2	2	3	3	2	2	1	1	2	2
2	2	3	3	2	2	1	1	2	2
2	2	3	3	2	2	1	1	2	2
2	2	3	3	2	2	2	2	2	2
2	2	3	3	2	2	1	1	2	2
2	2	3	3	2	2	2	2	3	3
1	1	4	4	2	2	2	2	3	3
2	2	3	3	2	2	2	2	2	2
1	1	4	4	2	2	1	1	2	2
2	2	3	3	2	2	2	2	2	2
1	1	4	4	2	2	1	1	2	2
2	2	3	3	2	2	2	2	3	3
2	2	3	3	2	2	2	2	2	2
1	1	3	3	2	2	2	2	3	3
1	1	4	4	2	2	1	1	2	2
1	1	4	4	2	2	2	2	3	3
2	2	3	3	2	2	2	2	2	2
1	1	4	4	2	2	2	2	3	3
2	2	3	3	2	2	2	2	2	2
1	1	4	4	2	2	2	2	3	3
1	1	4	4	2	2	2	2	3	3
2	2	3	3	2	2	1	1	2	2
1	1	4	4	2	2	2	2	3	3
2	2	3	3	2	2	2	2	3	3
2	2	3	3	2	2	1	1	2	2
2	2	3	3	2	2	1	1	2	2
1	1	4	4	3	3	2	2	3	3
2	2	3	3	2	2	1	1	2	2
1	1	4	4	2	2	2	2	3	3
2	2	3	3	2	2	2	2	3	3
2	2	3	3	2	2	2	2	2	2
1	1	3	3	2	2	2	2	3	3
2	2	3	3	2	2	2	2	3	3
1	1	4	4	2	2	2	2	3	3
2	2	3	3	2	2	1	1	2	2
2	2	3	3	3	3	2	2	3	3
1	1	4	4	2	2	1	1	2	2
2	2	3	3	2	2	1	1	2	2
2	2	3	3	2	2	1	1	2	2
2	2	3	3	2	2	2	2	3	3
1	1	3	3	2	2	2	2	3	3
2	2	3	3	2	2	2	2	2	2
2	2	3	3	2	2	2	2	2	2
1	1	4	4	3	3	2	2	3	3
2	2	3	3	2	2	1	1	2	2
2	2	3	3	2	2	1	1	2	2
2	2	3	3	2	2	2	2	3	3

For Review Only

1	1	4	4	2	2	1	1	2	2
2	2	3	3	2	2	1	1	2	2
2	2	3	3	3	3	1	1	2	2
2	2	3	3	2	2	2	2	3	3
2	2	3	3	2	2	1	1	2	2
2	2	3	3	3	3	2	2	3	3
2	2	3	3	2	2	2	2	2	2
2	2	3	3	2	2	2	2	2	2
2	2	3	3	2	2	2	2	3	3
2	2	3	3	2	2	2	2	3	3
2	2	3	3	2	2	2	2	3	3
2	2	3	3	2	2	2	2	3	3
2	2	3	3	2	2	2	2	2	2
2	2	3	3	2	2	2	2	2	2
1	1	4	4	2	2	2	2	3	3
2	2	3	3	2	2	1	1	2	2
2	2	3	3	2	2	2	2	2	2
2	2	3	3	2	2	2	2	3	3
1	1	4	4	2	2	2	2	3	3
1	1	4	4	2	2	2	2	3	3
2	2	3	3	2	2	2	2	2	2
1	1	4	4	2	2	2	2	3	3
1	1	4	4	2	2	2	2	3	3
2	2	3	3	2	2	2	2	3	3
2	2	3	3	2	2	2	2	3	3
1	1	4	4	2	2	2	2	3	3
2	2	3	3	2	2	2	2	2	2
1	1	4	4	2	2	1	1	2	2
1	1	4	4	2	2	2	2	3	3
2	2	3	3	2	2	1	1	2	2
1	1	4	4	2	2	1	1	2	2
2	2	3	3	2	2	1	1	2	2
2	2	3	3	2	2	2	2	3	3
1	1	4	4	2	2	2	2	3	3
2	2	3	3	2	2	1	1	2	2
2	2	3	3	2	2	2	2	3	3
2	2	3	3	2	2	2	2	3	3
2	2	3	3	3	3	2	2	3	3
2	2	3	3	2	2	2	2	3	3
1	1	4	4	2	2	1	1	2	2
2	2	3	3	2	2	1	1	2	2
2	2	3	3	2	2	1	1	2	2
1	1	4	4	2	2	2	2	3	3
2	2	3	3	2	2	2	2	3	3
2	2	3	3	2	2	2	2	3	3
2	2	3	3	2	2	1	1	2	2
2	2	3	3	2	2	1	1	2	2
1	1	4	4	2	2	1	1	2	2
1	1	4	4	2	2	2	2	3	3
2	2	3	3	2	2	2	2	3	3
1	1	4	4	2	2	2	2	3	3
1	1	4	4	2	2	2	2	3	3
1	1	4	4	2	2	2	2	3	3
2	2	3	3	2	2	2	2	3	3
1	1	4	4	2	2	2	2	3	3

For Review Only

2	2	3	3	2	2	1	1	2	2
2	2	3	3	2	2	2	2	3	3
2	2	3	3	2	2	1	1	2	2
2	2	3	3	2	2	2	2	2	2
2	2	3	3	2	2	2	2	3	3
1	1	4	4	2	2	1	1	2	2
2	2	3	3	2	2	2	2	2	2
1	1	4	4	2	2	2	2	3	3
2	2	4	4	2	2	2	2	2	2
1	1	4	4	2	2	2	2	3	3
2	2	3	3	2	2	2	2	2	2
1	1	4	4	2	2	1	1	2	2
1	1	4	4	2	2	1	1	2	2
2	2	3	3	2	2	1	1	2	2
2	2	3	3	2	2	1	1	2	2
2	2	3	3	2	2	2	2	3	3
2	2	3	3	2	2	2	2	3	3
2	2	3	3	2	2	2	2	3	3
2	2	3	3	2	2	1	1	2	2
2	2	3	3	2	2	1	1	2	2
1	1	4	4	2	2	2	2	3	3
1	1	4	4	2	2	1	1	2	2
2	2	3	3	2	2	2	2	3	3
2	2	3	3	2	2	2	2	3	3
1	1	4	4	3	3	2	2	3	3
1	1	4	4	2	2	2	2	3	3
1	1	4	4	2	2	2	2	3	3
2	2	3	3	2	2	2	2	3	3
2	2	3	3	2	2	2	2	3	3
1	1	4	4	2	2	2	2	3	3
1	1	4	4	2	2	1	1	2	2
2	2	3	3	2	2	1	1	2	2
1	1	3	3	2	2	2	2	3	3
2	2	3	3	2	2	1	1	2	2
1	1	4	4	2	2	2	2	3	3
1	1	4	4	2	2	2	2	3	3
2	2	3	3	2	2	1	1	2	2
2	2	3	3	2	2	2	2	3	3
2	2	3	3	2	2	2	2	3	3
1	1	4	4	2	2	2	2	3	3
1	1	4	4	2	2	1	1	2	2
1	1	4	4	2	2	1	1	2	2
2	2	3	3	2	2	2	2	3	3
2	2	3	3	2	2	1	1	2	2
2	2	3	3	2	2	2	2	3	3
2	2	3	3	2	2	1	1	2	2
1	1	4	4	2	2	2	2	3	3
2	2	3	3	2	2	1	1	2	2
2	2	3	3	2	2	2	2	2	2
1	1	4	4	2	2	2	2	3	3
2	2	3	3	2	2	2	2	3	3
1	1	4	4	2	2	2	2	3	3
2	2	3	3	2	2	1	1	2	2
2	2	3	3	2	2	1	1	2	2
2	2	3	3	3	3	2	2	3	3

For Review Only

2	2	3	3	2	2	1	1	2	2
1	1	4	4	2	2	2	2	3	3

For Review Only

SNP442	SNP442	SNP443	SNP443	SNP444	SNP444	SNP445	SNP445	SNP446	SNP446
2	2	4	4	1	1	2	2	3	3
2	2	4	4	1	1	1	1	3	3
2	2	4	4	1	1	2	2	3	3
2	2	1	1	1	1	2	2	3	3
2	2	4	4	1	1	2	2	3	3
2	2	4	4	1	1	2	2	3	3
2	2	4	4	1	1	2	2	3	3
2	2	4	4	1	1	1	1	3	3
2	2	4	4	1	1	2	2	3	3
2	2	4	4	1	1	2	2	3	3
2	2	4	4	1	1	2	2	3	3
2	2	1	1	1	1	2	2	3	3
2	2	4	4	1	1	2	2	3	3
2	2	1	1	1	1	2	2	3	3
2	2	4	4	1	1	2	2	3	3
2	2	1	1	1	1	2	2	3	3
2	2	4	4	1	1	2	2	3	3
2	2	4	4	1	1	2	2	3	3
3	3	4	4	1	1	2	2	3	3
2	2	4	4	1	1	1	1	3	3
2	2	4	4	1	1	2	2	2	2
2	2	4	4	1	1	2	2	2	2
2	2	4	4	1	1	2	2	3	3
2	2	4	4	1	1	1	1	3	3
2	2	1	1	1	1	2	2	3	3
2	2	1	1	3	3	2	2	3	3
2	2	1	1	3	3	2	2	2	2
2	2	1	1	1	1	2	2	3	3
2	2	4	4	1	1	2	2	3	3
2	2	4	4	1	1	2	2	2	2
2	2	1	1	1	1	2	2	3	3
2	2	4	4	1	1	2	2	3	3
2	2	4	4	1	1	2	2	3	3
2	2	4	4	1	1	1	1	3	3
2	2	1	1	1	1	2	2	3	3
2	2	1	1	1	1	2	2	3	3
2	2	4	4	1	1	2	2	3	3
2	2	4	4	1	1	1	1	2	2
2	2	4	4	1	1	2	2	3	3
2	2	4	4	1	1	1	1	3	3
2	2	4	4	1	1	2	2	3	3
2	2	4	4	1	1	2	2	3	3
2	2	4	4	1	1	1	1	3	3
2	2	4	4	1	1	1	1	2	2
2	2	4	4	1	1	1	1	3	3
2	2	4	4	1	1	2	2	3	3
3	3	1	1	1	1	2	2	3	3
2	2	4	4	1	1	1	1	3	3
2	2	4	4	1	1	2	2	2	2
2	2	4	4	1	1	2	2	3	3
2	2	1	1	1	1	2	2	3	3
2	2	1	1	1	1	2	2	3	3

For Review Only

2	2	4	4	1	1	2	2	3	3
2	2	4	4	1	1	2	2	3	3
2	2	1	1	3	3	2	2	3	3
2	2	4	4	1	1	2	2	3	3
3	3	4	4	1	1	2	2	3	3
3	3	1	1	1	1	2	2	3	3
2	2	4	4	1	1	1	1	3	3
2	2	4	4	1	1	1	1	3	3
2	2	4	4	1	1	2	2	3	3
2	2	1	1	1	1	2	2	3	3
2	2	1	1	1	1	2	2	3	3
3	3	1	1	1	1	2	2	3	3
2	2	4	4	1	1	2	2	3	3
2	2	4	4	1	1	2	2	3	3
2	2	4	4	1	1	2	2	3	3
2	2	4	4	1	1	2	2	3	3
2	2	4	4	1	1	2	2	3	3
2	2	4	4	1	1	2	2	3	3
2	2	1	1	3	3	2	2	3	3
2	2	4	4	1	1	2	2	3	3
3	3	1	1	1	1	2	2	3	3
2	2	4	4	1	1	2	2	3	3
2	2	4	4	1	1	2	2	3	3
2	2	1	1	1	1	2	2	3	3
2	2	4	4	1	1	1	1	3	3
2	2	1	1	1	1	2	2	3	3
2	2	4	4	1	1	2	2	2	2
2	2	4	4	1	1	2	2	3	3
2	2	1	1	1	1	2	2	3	3
2	2	4	4	1	1	2	2	3	3
2	2	4	4	1	1	1	1	3	3
2	2	4	4	1	1	1	1	3	3
2	2	4	4	1	1	2	2	3	3
2	2	4	4	1	1	2	2	3	3
2	2	4	4	1	1	2	2	3	3
2	2	4	4	1	1	1	1	3	3
2	2	4	4	1	1	1	1	2	2
2	2	4	4	1	1	1	1	2	2
2	2	1	1	3	3	2	2	3	3
2	2	4	4	1	1	2	2	3	3
2	2	4	4	1	1	2	2	3	3
2	2	4	4	1	1	2	2	3	3
2	2	1	1	1	1	2	2	3	3
2	2	1	1	1	1	2	2	3	3
2	2	4	4	1	1	2	2	3	3
2	2	1	1	1	1	2	2	3	3
2	2	4	4	1	1	1	1	3	3
2	2	4	4	1	1	2	2	3	3
2	2	4	4	1	1	2	2	3	3
2	2	1	1	1	1	2	2	3	3
2	2	1	1	1	1	2	2	3	3
2	2	4	4	1	1	2	2	3	3
2	2	4	4	1	1	1	1	3	3
3	3	1	1	1	1	1	1	3	3
2	2	1	1	1	1	2	2	3	3

For Review Only

2	2	4	4	1	1	2	2	3	3
2	2	4	4	1	1	2	2	2	2
2	2	1	1	3	3	2	2	3	3
2	2	4	4	1	1	1	1	3	3
2	2	1	1	1	1	2	2	3	3
2	2	1	1	3	3	2	2	3	3
2	2	1	1	3	3	2	2	3	3
2	2	4	4	1	1	2	2	3	3
2	2	1	1	1	1	2	2	3	3
2	2	1	1	1	1	2	2	3	3
2	2	4	4	1	1	2	2	3	3
2	2	4	4	1	1	2	2	3	3
2	2	1	1	3	3	2	2	3	3
2	2	1	1	1	1	2	2	3	3
2	2	1	1	1	1	2	2	3	3
2	2	4	4	1	1	1	1	3	3
2	2	1	1	3	3	2	2	3	3
2	2	1	1	1	1	2	2	3	3
2	2	4	4	1	1	1	1	3	3
2	2	4	4	1	1	2	2	3	3
2	2	1	1	1	1	2	2	3	3
2	2	1	1	1	1	2	2	3	3
2	2	4	4	1	1	2	2	3	3
2	2	4	4	1	1	1	1	3	3
2	2	4	4	1	1	2	2	3	3
2	2	4	4	1	1	2	2	3	3
3	3	4	4	1	1	2	2	3	3
2	2	4	4	1	1	2	2	3	3
2	2	1	1	3	3	2	2	3	3
2	2	4	4	1	1	1	1	3	3
2	2	4	4	1	1	2	2	3	3
2	2	1	1	1	1	2	2	3	3
2	2	4	4	1	1	2	2	3	3
2	2	1	1	1	1	2	2	3	3
2	2	4	4	1	1	1	1	3	3
2	2	4	4	1	1	2	2	3	3
2	2	4	4	1	1	1	1	3	3
2	2	4	4	1	1	1	1	3	3
2	2	1	1	1	1	2	2	3	3
2	2	4	4	1	1	2	2	3	3
2	2	1	1	1	1	2	2	3	3
2	2	4	4	1	1	1	1	3	3
2	2	1	1	1	1	2	2	3	3
2	2	4	4	1	1	2	2	2	2
2	2	4	4	1	1	2	2	3	3
2	2	4	4	1	1	2	2	3	3
2	2	4	4	1	1	1	1	3	3
2	2	1	1	1	1	2	2	3	3
2	2	4	4	1	1	1	1	3	3
2	2	1	1	3	3	2	2	3	3
2	2	4	4	1	1	1	1	3	3
2	2	4	4	1	1	1	1	3	3
2	2	4	4	1	1	2	2	3	3
2	2	1	1	1	1	2	2	3	3
2	2	4	4	1	1	2	2	3	3

For Review Only

2	2	4	4	1	1	1	1	3	3
2	2	4	4	1	1	2	2	2	2
3	3	4	4	1	1	2	2	3	3
2	2	4	4	1	1	2	2	3	3
2	2	1	1	1	1	2	2	3	3
2	2	4	4	1	1	2	2	3	3
2	2	1	1	1	1	2	2	3	3
2	2	1	1	1	1	2	2	3	3
2	2	4	4	1	1	2	2	3	3
2	2	4	4	1	1	2	2	3	3
3	3	4	4	1	1	1	1	3	3
2	2	1	1	1	1	2	2	3	3
2	2	1	1	1	1	2	2	3	3
2	2	4	4	1	1	2	2	3	3
2	2	4	4	1	1	1	1	3	3
2	2	4	4	1	1	2	2	3	3
2	2	4	4	1	1	2	2	3	3
2	2	1	1	1	1	2	2	3	3
2	2	1	1	3	3	2	2	3	3
2	2	1	1	3	3	2	2	3	3
2	2	1	1	1	1	2	2	3	3
2	2	4	4	1	1	2	2	3	3
2	2	4	4	1	1	2	2	3	3
3	3	1	1	1	1	2	2	3	3
2	2	4	4	1	1	2	2	3	3
2	2	4	4	1	1	1	1	3	3
2	2	1	1	1	1	2	2	3	3
2	2	1	1	1	1	2	2	3	3
2	2	1	1	3	3	2	2	2	2
2	2	4	4	1	1	2	2	3	3
2	2	4	4	1	1	2	2	3	3
2	2	4	4	1	1	2	2	3	3
2	2	4	4	1	1	2	2	3	3
2	2	4	4	1	1	1	1	3	3
2	2	4	4	1	1	2	2	3	3
3	3	1	1	1	1	2	2	3	3
2	2	1	1	1	1	2	2	2	2
2	2	4	4	1	1	2	2	3	3
2	2	1	1	1	1	2	2	3	3
2	2	1	1	1	1	2	2	3	3
2	2	4	4	1	1	2	2	3	3
2	2	4	4	1	1	1	1	3	3
2	2	4	4	1	1	2	2	3	3
2	2	4	4	1	1	2	2	3	3
2	2	1	1	1	1	2	2	3	3
2	2	4	4	1	1	2	2	3	3
2	2	4	4	1	1	2	2	3	3
2	2	4	4	1	1	2	2	3	3
2	2	4	4	1	1	2	2	3	3
2	2	1	1	3	3	2	2	3	3
2	2	1	1	3	3	2	2	3	3
2	2	4	4	1	1	1	1	3	3
2	2	4	4	1	1	2	2	3	3
2	2	1	1	1	1	2	2	3	3
2	2	4	4	1	1	2	2	3	3

For Review Only

2	2	4	4	1	1	1	1	3	3
2	2	4	4	1	1	1	1	3	3
2	2	4	4	1	1	2	2	3	3
2	2	4	4	1	1	2	2	3	3
2	2	1	1	1	1	2	2	3	3
2	2	1	1	1	1	2	2	3	3
2	2	1	1	3	3	2	2	3	3
2	2	4	4	1	1	2	2	2	2
2	2	4	4	1	1	1	1	3	3
2	2	4	4	1	1	2	2	3	3
2	2	1	1	1	1	2	2	3	3
2	2	1	1	1	1	2	2	3	3
2	2	1	1	3	3	2	2	3	3
2	2	4	4	1	1	1	1	3	3
2	2	4	4	1	1	2	2	3	3
2	2	4	4	1	1	2	2	3	3
2	2	4	4	1	1	2	2	2	2
2	2	4	4	1	1	2	2	3	3
2	2	4	4	1	1	2	2	3	3
2	2	4	4	1	1	1	1	2	2
2	2	4	4	1	1	1	1	2	2
2	2	4	4	1	1	1	1	3	3
2	2	1	1	1	1	2	2	3	3
2	2	1	1	1	1	2	2	3	3
2	2	4	4	1	1	1	1	3	3
2	2	1	1	1	1	2	2	3	3
2	2	4	4	1	1	2	2	3	3
2	2	1	1	3	3	2	2	3	3
2	2	4	4	1	1	2	2	3	3
2	2	1	1	3	3	2	2	3	3
2	2	4	4	1	1	2	2	3	3
2	2	4	4	1	1	1	1	3	3
2	2	4	4	1	1	2	2	3	3
2	2	1	1	1	1	2	2	3	3
2	2	4	4	1	1	2	2	3	3
2	2	4	4	1	1	2	2	3	3
2	2	4	4	1	1	2	2	3	3
3	3	1	1	1	1	2	2	3	3
2	2	1	1	1	1	2	2	3	3
2	2	4	4	1	1	2	2	3	3
2	2	4	4	1	1	2	2	3	3
2	2	1	1	1	1	2	2	3	3
2	2	1	1	1	1	2	2	3	3
2	2	4	4	1	1	2	2	3	3
3	3	1	1	1	1	2	2	3	3
2	2	4	4	1	1	2	2	3	3
2	2	1	1	1	1	2	2	3	3
2	2	1	1	1	1	2	2	3	3
2	2	4	4	1	1	2	2	3	3
2	2	4	4	1	1	2	2	3	3
2	2	4	4	1	1	2	2	3	3
2	2	4	4	1	1	2	2	3	3
2	2	4	4	1	1	2	2	3	3
2	2	1	1	3	3	2	2	3	3
2	2	4	4	1	1	1	1	3	3

For Review Only

2	2	4	4	1	1	2	2	3	3
2	2	1	1	1	1	2	2	3	3

For Review Only

SNP447	SNP447	SNP448	SNP448	SNP449	SNP449	SNP450	SNP450	SNP451	SNP451
1	1	3	3	4	4	3	3	2	2
1	1	3	3	4	4	1	1	2	2
1	1	2	2	4	4	3	3	4	4
1	1	3	3	4	4	3	3	4	4
1	1	3	3	4	4	1	1	4	4
1	1	3	3	4	4	1	1	4	4
1	1	3	3	4	4	1	1	4	4
1	1	3	3	4	4	1	1	4	4
1	1	3	3	4	4	1	1	4	4
1	1	3	3	4	4	1	1	4	4
1	1	3	3	4	4	1	1	4	4
1	1	2	2	4	4	3	3	4	4
4	4	3	3	4	4	1	1	4	4
1	1	3	3	4	4	1	1	4	4
1	1	3	3	4	4	3	3	4	4
1	1	3	3	4	4	1	1	4	4
1	1	3	3	4	4	1	1	4	4
1	1	3	3	4	4	1	1	2	2
1	1	3	3	4	4	1	1	2	2
1	1	3	3	4	4	1	1	4	4
1	1	3	3	1	1	3	3	4	4
1	1	3	3	4	4	1	1	4	4
1	1	3	3	4	4	1	1	4	4
1	1	3	3	4	4	1	1	2	2
1	1	3	3	4	4	1	1	2	2
1	1	3	3	4	4	3	3	4	4
4	4	3	3	1	1	1	1	4	4
1	1	3	3	4	4	1	1	4	4
1	1	3	3	4	4	3	3	4	4
4	4	2	2	1	1	1	1	4	4
1	1	3	3	4	4	1	1	4	4
1	1	3	3	4	4	1	1	4	4
1	1	3	3	4	4	1	1	4	4
1	1	3	3	4	4	1	1	4	4
1	1	3	3	4	4	1	1	4	4
1	1	3	3	4	4	1	1	4	4
1	1	3	3	4	4	1	1	4	4
4	4	3	3	1	1	3	3	4	4
1	1	3	3	4	4	1	1	4	4
1	1	3	3	4	4	3	3	4	4
4	4	3	3	4	4	1	1	4	4
1	1	3	3	4	4	3	3	4	4
1	1	3	3	4	4	1	1	2	2
1	1	3	3	1	1	1	1	2	2
1	1	3	3	4	4	1	1	2	2
1	1	2	2	4	4	3	3	4	4
1	1	3	3	4	4	3	3	4	4
1	1	3	3	4	4	1	1	2	2
4	4	3	3	1	1	1	1	4	4
1	1	3	3	4	4	1	1	4	4
1	1	3	3	4	4	1	1	4	4
1	1	2	2	4	4	1	1	4	4

For Review Only

1	1	3	3	4	4	1	1	2	2
1	1	3	3	4	4	1	1	4	4
1	1	3	3	4	4	1	1	2	2
1	1	3	3	4	4	1	1	4	4
1	1	3	3	4	4	1	1	2	2
1	1	3	3	4	4	3	3	4	4
1	1	3	3	4	4	3	3	4	4
1	1	3	3	4	4	1	1	4	4
1	1	3	3	4	4	1	1	4	4
1	1	3	3	4	4	1	1	4	4
1	1	3	3	4	4	1	1	2	2
1	1	3	3	4	4	1	1	2	2
1	1	3	3	4	4	1	1	2	2
1	1	3	3	4	4	3	3	4	4
1	1	3	3	4	4	1	1	4	4
1	1	3	3	4	4	1	1	4	4
1	1	3	3	4	4	1	1	4	4
1	1	3	3	4	4	3	3	4	4
1	1	3	3	4	4	1	1	4	4
1	1	3	3	4	4	3	3	2	2
1	1	3	3	4	4	1	1	4	4
1	1	3	3	4	4	1	1	4	4
1	1	3	3	4	4	3	3	4	4
1	1	3	3	4	4	1	1	4	4
1	1	3	3	4	4	1	1	2	2
1	1	2	2	4	4	1	1	4	4
1	1	3	3	1	1	1	1	4	4
1	1	3	3	4	4	1	1	4	4
1	1	3	3	4	4	3	3	4	4
1	1	3	3	4	4	1	1	4	4
1	1	3	3	4	4	1	1	4	4
1	1	3	3	4	4	1	1	4	4
1	1	3	3	4	4	1	1	4	4
1	1	3	3	4	4	1	1	2	2
1	1	3	3	4	4	1	1	2	2
1	1	3	3	4	4	1	1	2	2
4	4	3	3	1	1	1	1	4	4
4	4	3	3	4	4	1	1	2	2
1	1	3	3	4	4	1	1	4	4
1	1	3	3	4	4	1	1	2	2
1	1	3	3	4	4	1	1	4	4
1	1	3	3	4	4	1	1	4	4
1	1	3	3	4	4	1	1	2	2
1	1	3	3	4	4	1	1	2	2
1	1	3	3	4	4	1	1	4	4
1	1	3	3	4	4	3	3	4	4
1	1	3	3	4	4	1	1	4	4
1	1	3	3	4	4	1	1	4	4
1	1	2	2	4	4	3	3	4	4
1	1	3	3	4	4	1	1	4	4
1	1	3	3	4	4	3	3	4	4
1	1	3	3	4	4	1	1	4	4
1	1	3	3	4	4	1	1	4	4
1	1	3	3	4	4	1	1	4	4
1	1	3	3	4	4	1	1	4	4

For Review Only

1	1	2	2	4	4	1	1	4	4
4	4	3	3	1	1	1	1	4	4
1	1	3	3	4	4	1	1	4	4
1	1	3	3	4	4	1	1	4	4
1	1	3	3	4	4	1	1	4	4
1	1	3	3	4	4	3	3	4	4
1	1	3	3	4	4	1	1	4	4
1	1	3	3	4	4	1	1	4	4
1	1	2	2	4	4	1	1	4	4
1	1	3	3	4	4	1	1	4	4
1	1	3	3	4	4	1	1	4	4
1	1	3	3	4	4	1	1	4	4
1	1	3	3	1	1	1	1	4	4
1	1	2	2	4	4	1	1	4	4
1	1	3	3	4	4	1	1	4	4
1	1	3	3	4	4	1	1	4	4
1	1	3	3	4	4	3	3	4	4
1	1	3	3	4	4	1	1	4	4
1	1	3	3	4	4	1	1	4	4
1	1	3	3	4	4	1	1	2	2
1	1	3	3	4	4	1	1	4	4
1	1	3	3	4	4	1	1	4	4
1	1	3	3	4	4	1	1	4	4
1	1	3	3	4	4	1	1	4	4
1	1	3	3	4	4	3	3	4	4
1	1	3	3	4	4	1	1	4	4
1	1	2	2	4	4	1	1	4	4
1	1	3	3	4	4	1	1	4	4
1	1	3	3	4	4	1	1	4	4
1	1	3	3	4	4	3	3	4	4
1	1	3	3	4	4	1	1	4	4
1	1	3	3	4	4	1	1	4	4
1	1	3	3	4	4	1	1	4	4
1	1	3	3	4	4	1	1	4	4
1	1	3	3	4	4	1	1	4	4
1	1	3	3	4	4	3	3	4	4
1	1	3	3	4	4	1	1	4	4
1	1	3	3	4	4	1	1	4	4
1	1	3	3	4	4	3	3	2	2
1	1	3	3	4	4	1	1	4	4
1	1	3	3	4	4	1	1	4	4
1	1	3	3	4	4	1	1	4	4
1	1	3	3	4	4	1	1	4	4
4	4	3	3	4	4	1	1	4	4
1	1	3	3	4	4	1	1	4	4
1	1	3	3	4	4	1	1	4	4
1	1	3	3	4	4	3	3	4	4
1	1	3	3	4	4	3	3	4	4
1	1	3	3	4	4	1	1	4	4
1	1	3	3	4	4	3	3	4	4
1	1	3	3	4	4	3	3	4	4
1	1	3	3	4	4	3	3	4	4
1	1	3	3	4	4	1	1	4	4
1	1	3	3	4	4	1	1	2	2
1	1	3	3	4	4	1	1	4	4

For Review Only

1	1	3	3	4	4	1	1	4	4
4	4	3	3	1	1	1	1	4	4
1	1	2	2	4	4	1	1	4	4
1	1	3	3	4	4	1	1	4	4
1	1	3	3	4	4	1	1	2	2
1	1	3	3	4	4	3	3	4	4
1	1	3	3	4	4	1	1	4	4
1	1	3	3	4	4	1	1	4	4
1	1	3	3	4	4	1	1	4	4
1	1	3	3	4	4	1	1	4	4
1	1	3	3	4	4	3	3	4	4
1	1	3	3	4	4	1	1	4	4
1	1	3	3	4	4	1	1	4	4
1	1	3	3	4	4	1	1	4	4
1	1	3	3	4	4	1	1	4	4
1	1	3	3	4	4	1	1	4	4
1	1	3	3	4	4	1	1	4	4
1	1	3	3	4	4	1	1	2	2
1	1	3	3	4	4	1	1	4	4
1	1	3	3	4	4	1	1	4	4
1	1	3	3	4	4	3	3	4	4
1	1	3	3	4	4	1	1	4	4
1	1	3	3	4	4	1	1	4	4
1	1	3	3	4	4	1	1	2	2
1	1	3	3	4	4	1	1	4	4
1	1	3	3	4	4	1	1	4	4
1	1	3	3	4	4	3	3	4	4
1	1	3	3	4	4	1	1	4	4
4	4	3	3	1	1	3	3	4	4
1	1	3	3	4	4	1	1	4	4
1	1	3	3	4	4	1	1	4	4
1	1	3	3	4	4	3	3	4	4
1	1	2	2	4	4	1	1	4	4
1	1	3	3	4	4	1	1	4	4
1	1	3	3	4	4	3	3	4	4
1	1	3	3	4	4	1	1	2	2
4	4	3	3	1	1	1	1	4	4
1	1	3	3	4	4	1	1	2	2
1	1	3	3	4	4	3	3	4	4
1	1	2	2	4	4	1	1	4	4
1	1	2	2	4	4	3	3	4	4
1	1	3	3	4	4	3	3	4	4
1	1	3	3	4	4	1	1	4	4
1	1	3	3	4	4	3	3	4	4
1	1	3	3	4	4	1	1	4	4
1	1	3	3	4	4	1	1	4	4
1	1	3	3	4	4	1	1	4	4
1	1	3	3	4	4	1	1	4	4
1	1	3	3	4	4	1	1	4	4
1	1	3	3	4	4	1	1	4	4
1	1	3	3	4	4	1	1	4	4
1	1	3	3	4	4	1	1	4	4
1	1	3	3	4	4	1	1	4	4
1	1	3	3	4	4	1	1	2	2
1	1	3	3	4	4	1	1	4	4

For Review Only

1	1	3	3	4	4	1	1	4	4
1	1	3	3	4	4	1	1	4	4
1	1	3	3	4	4	1	1	4	4
1	1	3	3	4	4	1	1	4	4
1	1	2	2	4	4	3	3	4	4
1	1	3	3	4	4	3	3	4	4
1	1	2	2	4	4	1	1	4	4
4	4	3	3	1	1	1	1	4	4
1	1	3	3	4	4	1	1	4	4
1	1	3	3	4	4	1	1	4	4
1	1	3	3	4	4	1	1	4	4
1	1	3	3	4	4	1	1	4	4
1	1	3	3	4	4	1	1	4	4
1	1	3	3	4	4	1	1	4	4
1	1	2	2	4	4	3	3	4	4
1	1	3	3	4	4	1	1	4	4
4	4	3	3	1	1	1	1	4	4
1	1	2	2	4	4	1	1	2	2
1	1	3	3	4	4	1	1	2	2
4	4	3	3	1	1	3	3	4	4
4	4	3	3	1	1	1	1	4	4
1	1	3	3	4	4	3	3	4	4
1	1	3	3	4	4	1	1	2	2
1	1	3	3	4	4	1	1	2	2
1	1	3	3	4	4	1	1	4	4
1	1	2	2	4	4	1	1	4	4
1	1	3	3	4	4	1	1	4	4
1	1	3	3	4	4	3	3	4	4
1	1	3	3	4	4	1	1	4	4
1	1	3	3	4	4	1	1	4	4
1	1	3	3	4	4	1	1	4	4
1	1	3	3	4	4	1	1	4	4
1	1	3	3	4	4	1	1	4	4
1	1	3	3	4	4	3	3	4	4
1	1	3	3	4	4	1	1	4	4
1	1	3	3	4	4	1	1	4	4
1	1	3	3	4	4	1	1	4	4
1	1	3	3	4	4	1	1	2	2
1	1	3	3	4	4	1	1	4	4
1	1	3	3	4	4	1	1	4	4
1	1	3	3	4	4	3	3	4	4
1	1	3	3	4	4	1	1	4	4
1	1	3	3	4	4	1	1	2	2
1	1	3	3	4	4	1	1	4	4
1	1	3	3	4	4	1	1	2	2
1	1	3	3	4	4	1	1	4	4
1	1	3	3	4	4	3	3	4	4
1	1	3	3	4	4	1	1	4	4
1	1	3	3	4	4	1	1	4	4
1	1	3	3	4	4	1	1	4	4
1	1	3	3	4	4	1	1	2	2
1	1	3	3	4	4	1	1	4	4
1	1	2	2	4	4	3	3	4	4
1	1	3	3	4	4	3	3	4	4
1	1	3	3	4	4	3	3	4	4

For Review Only

1	1	3	3	4	4	1	1	4	4
1	1	2	2	4	4	1	1	4	4

For Review Only

SNP452	SNP452	SNP453	SNP453	SNP454	SNP454	SNP455	SNP455	SNP456	SNP456
4	4	2	2	3	3	2	2	1	1
1	1	2	2	3	3	2	2	1	1
1	1	2	2	3	3	1	1	3	3
1	1	2	2	3	3	2	2	1	1
1	1	1	1	3	3	1	1	1	1
1	1	2	2	3	3	1	1	3	3
1	1	2	2	3	3	1	1	3	3
1	1	2	2	3	3	2	2	1	1
1	1	2	2	3	3	1	1	3	3
1	1	2	2	3	3	2	2	1	1
1	1	1	1	3	3	2	2	1	1
1	1	2	2	3	3	1	1	1	1
1	1	2	2	3	3	1	1	1	1
1	1	2	2	3	3	2	2	1	1
1	1	2	2	3	3	1	1	3	3
1	1	2	2	3	3	1	1	3	3
1	1	2	2	3	3	2	2	1	1
1	1	2	2	3	3	1	1	1	1
1	1	2	2	3	3	2	2	1	1
4	4	2	2	3	3	2	2	1	1
4	4	2	2	3	3	2	2	1	1
4	4	1	1	3	3	1	1	3	3
1	1	2	2	3	3	2	2	1	1
1	1	2	2	3	3	2	2	1	1
1	1	2	2	3	3	1	1	3	3
1	1	2	2	1	1	1	1	3	3
1	1	2	2	3	3	2	2	1	1
1	1	2	2	3	3	1	1	1	1
4	4	1	1	3	3	1	1	3	3
1	1	2	2	3	3	2	2	1	1
1	1	2	2	3	3	1	1	3	3
1	1	2	2	3	3	1	1	3	3
1	1	2	2	3	3	1	1	3	3
1	1	1	1	3	3	1	1	1	1
1	1	2	2	3	3	1	1	1	1
1	1	2	2	3	3	2	2	1	1
1	1	2	2	3	3	1	1	1	1
1	1	2	2	3	3	2	2	1	1
1	1	2	2	3	3	2	2	1	1
4	4	2	2	3	3	2	2	1	1
1	1	2	2	3	3	2	2	1	1
4	4	1	1	3	3	1	1	1	1
1	1	2	2	3	3	2	2	1	1
1	1	2	2	3	3	2	2	1	1
1	1	2	2	3	3	2	2	1	1
1	1	2	2	3	3	1	1	3	3
4	4	2	2	1	1	1	1	3	3
1	1	1	1	3	3	1	1	3	3
1	1	2	2	3	3	1	1	1	1
4	4	2	2	3	3	1	1	1	1
1	1	2	2	1	1	1	1	3	3
1	1	2	2	3	3	1	1	3	3

For Review Only

1	1	2	2	3	3	1	1	3	3
1	1	2	2	3	3	1	1	3	3
1	1	2	2	3	3	2	2	1	1
1	1	2	2	3	3	1	1	3	3
1	1	2	2	3	3	2	2	1	1
4	4	2	2	1	1	2	2	1	1
1	1	2	2	3	3	2	2	1	1
1	1	2	2	3	3	2	2	1	1
1	1	1	1	3	3	1	1	3	3
1	1	2	2	3	3	1	1	1	1
1	1	2	2	3	3	2	2	1	1
1	1	2	2	1	1	2	2	1	1
1	1	2	2	3	3	1	1	3	3
4	4	1	1	3	3	1	1	3	3
1	1	2	2	3	3	1	1	1	1
1	1	2	2	3	3	2	2	1	1
1	1	2	2	3	3	2	2	1	1
4	4	2	2	3	3	1	1	3	3
1	1	2	2	3	3	1	1	3	3
4	4	2	2	3	3	1	1	3	3
1	1	2	2	3	3	2	2	1	1
4	4	2	2	3	3	2	2	1	1
4	4	2	2	3	3	1	1	3	3
1	1	2	2	3	3	2	2	1	1
1	1	2	2	3	3	2	2	1	1
1	1	2	2	3	3	2	2	1	1
1	1	2	2	3	3	2	2	1	1
1	1	1	1	3	3	2	2	1	1
1	1	2	2	1	1	1	1	3	3
1	1	2	2	3	3	1	1	3	3
1	1	2	2	3	3	1	1	3	3
1	1	2	2	3	3	2	2	1	1
1	1	2	2	3	3	2	2	1	1
1	1	2	2	3	3	1	1	3	3
1	1	1	1	3	3	1	1	3	3
1	1	2	2	3	3	2	2	1	1
1	1	2	2	3	3	1	1	3	3
1	1	2	2	3	3	1	1	3	3
1	1	2	2	3	3	2	2	1	1
1	1	1	1	3	3	2	2	1	1
1	1	2	2	3	3	1	1	3	3
1	1	2	2	3	3	1	1	3	3
1	1	2	2	3	3	2	2	1	1
1	1	2	2	3	3	2	2	1	1
1	1	2	2	3	3	2	2	1	1
4	4	2	2	3	3	2	2	1	1
1	1	1	1	3	3	2	2	1	1
1	1	2	2	3	3	2	2	1	1
4	4	2	2	3	3	2	2	1	1
1	1	2	2	3	3	2	2	1	1
1	1	2	2	3	3	2	2	1	1
1	1	1	1	3	3	2	2	1	1
1	1	2	2	3	3	2	2	1	1
1	1	2	2	1	1	2	2	1	1
1	1	2	2	3	3	2	2	1	1

For Review Only

4	4	2	2	1	1	2	2	1	1
1	1	2	2	3	3	2	2	1	1
1	1	2	2	3	3	1	1	3	3
1	1	1	1	3	3	2	2	1	1
1	1	2	2	3	3	1	1	1	1
1	1	2	2	3	3	1	1	3	3
1	1	2	2	3	3	1	1	3	3
1	1	2	2	3	3	2	2	1	1
1	1	2	2	3	3	2	2	1	1
1	1	2	2	3	3	1	1	1	1
1	1	2	2	3	3	2	2	1	1
1	1	2	2	3	3	1	1	1	1
1	1	2	2	3	3	1	1	3	3
1	1	2	2	3	3	1	1	1	1
1	1	2	2	3	3	2	2	1	1
4	4	2	2	3	3	1	1	1	1
4	4	2	2	3	3	1	1	3	3
1	1	2	2	3	3	1	1	1	1
1	1	2	2	3	3	2	2	1	1
1	1	2	2	3	3	2	2	1	1
1	1	2	2	3	3	1	1	1	1
1	1	2	2	3	3	1	1	3	3
1	1	2	2	3	3	1	1	3	3
1	1	1	1	3	3	2	2	1	1
4	4	2	2	3	3	2	2	1	1
1	1	2	2	3	3	2	2	1	1
1	1	2	2	1	1	1	1	1	1
1	1	2	2	3	3	1	1	1	1
1	1	2	2	3	3	1	1	3	3
4	4	2	2	3	3	2	2	1	1
1	1	1	1	3	3	2	2	1	1
1	1	2	2	3	3	1	1	1	1
1	1	2	2	3	3	1	1	3	3
4	4	2	2	1	1	1	1	3	3
1	1	1	1	3	3	1	1	1	1
4	4	2	2	3	3	1	1	3	3
1	1	1	1	3	3	2	2	1	1
1	1	2	2	3	3	2	2	1	1
1	1	1	1	3	3	2	2	1	1
1	1	1	1	3	3	2	2	1	1
1	1	2	2	3	3	2	2	1	1
1	1	1	1	3	3	1	1	1	1
1	1	2	2	3	3	2	2	1	1
1	1	2	2	3	3	2	2	1	1
1	1	2	2	3	3	1	1	3	3
1	1	2	2	3	3	2	2	1	1
4	4	2	2	1	1	1	1	3	3
4	4	2	2	1	1	2	2	1	1
1	1	2	2	3	3	1	1	1	1
4	4	2	2	1	1	1	1	3	3
4	4	2	2	3	3	1	1	3	3
4	4	2	2	3	3	2	2	1	1
1	1	2	2	3	3	1	1	1	1
1	1	2	2	1	1	1	1	3	3
1	1	2	2	3	3	2	2	1	1

For Review Only

1	1	2	2	3	3	1	1	3	3
1	1	2	2	3	3	2	2	1	1
1	1	2	2	3	3	2	2	1	1
1	1	2	2	3	3	1	1	3	3
4	4	2	2	3	3	2	2	1	1
4	4	2	2	3	3	2	2	1	1
1	1	2	2	3	3	2	2	1	1
1	1	2	2	3	3	1	1	1	1
1	1	1	1	3	3	2	2	1	1
1	1	2	2	1	1	2	2	1	1
1	1	2	2	1	1	1	1	1	1
1	1	2	2	3	3	2	2	1	1
1	1	2	2	3	3	2	2	1	1
1	1	2	2	3	3	2	2	1	1
1	1	1	1	3	3	2	2	1	1
1	1	1	1	3	3	2	2	1	1
1	1	2	2	3	3	1	1	1	1
1	1	2	2	3	3	2	2	1	1
1	1	2	2	1	1	1	1	3	3
1	1	2	2	3	3	1	1	3	3
1	1	2	2	3	3	1	1	1	1
1	1	2	2	3	3	2	2	1	1
1	1	2	2	3	3	2	2	1	1
1	1	2	2	1	1	2	2	1	1
1	1	2	2	3	3	1	1	1	1
1	1	1	1	3	3	2	2	1	1
1	1	2	2	3	3	1	1	1	1
4	4	2	2	3	3	1	1	3	3
4	4	2	2	3	3	1	1	3	3
1	1	2	2	3	3	1	1	3	3
1	1	2	2	3	3	2	2	1	1
4	4	2	2	3	3	1	1	3	3
1	1	2	2	3	3	1	1	3	3
1	1	1	1	3	3	2	2	1	1
4	4	2	2	3	3	2	2	1	1
1	1	2	2	1	1	2	2	1	1
1	1	2	2	1	1	1	1	3	3
1	1	2	2	3	3	1	1	1	1
4	4	2	2	1	1	2	2	1	1
1	1	2	2	3	3	2	2	1	1
1	1	2	2	3	3	1	1	3	3
4	4	2	2	1	1	1	1	3	3
1	1	1	1	3	3	2	2	1	1
4	4	2	2	3	3	1	1	1	1
1	1	2	2	3	3	1	1	1	1
1	1	2	2	3	3	2	2	1	1
1	1	1	1	3	3	2	2	1	1
1	1	1	1	3	3	2	2	1	1
1	1	2	2	3	3	2	2	1	1
1	1	2	2	3	3	2	2	1	1
1	1	2	2	3	3	1	1	3	3
1	1	2	2	3	3	2	2	1	1
1	1	1	1	3	3	2	2	1	1
1	1	2	2	3	3	2	2	1	1
1	1	1	1	3	3	2	2	1	1

For Review Only

1	1	2	2	3	3	1	1	3	3
4	4	2	2	3	3	2	2	1	1
1	1	2	2	3	3	1	1	3	3
1	1	2	2	3	3	2	2	1	1
4	4	2	2	3	3	1	1	1	1
1	1	2	2	3	3	1	1	3	3
1	1	2	2	3	3	1	1	3	3
1	1	1	1	3	3	2	2	1	1
1	1	2	2	3	3	1	1	1	1
1	1	1	1	3	3	2	2	1	1
1	1	2	2	3	3	1	1	1	1
1	1	2	2	3	3	1	1	3	3
1	1	2	2	3	3	2	2	1	1
1	1	2	2	3	3	1	1	3	3
4	4	2	2	3	3	2	2	1	1
1	1	2	2	3	3	2	2	1	1
1	1	2	2	3	3	2	2	1	1
1	1	2	2	3	3	2	2	1	1
1	1	2	2	3	3	2	2	1	1
1	1	2	2	3	3	2	2	1	1
1	1	2	2	3	3	2	2	1	1
1	1	2	2	3	3	1	1	3	3
1	1	2	2	3	3	1	1	3	3
1	1	2	2	3	3	2	2	1	1
1	1	2	2	3	3	2	2	1	1
1	1	2	2	3	3	1	1	1	1
1	1	2	2	3	3	2	2	1	1
1	1	2	2	3	3	2	2	1	1
4	4	2	2	3	3	1	1	1	1
1	1	2	2	3	3	1	1	3	3
1	1	1	1	3	3	2	2	1	1
4	4	1	1	3	3	1	1	3	3
1	1	2	2	3	3	2	2	1	1
4	4	2	2	3	3	2	2	1	1
1	1	2	2	3	3	2	2	1	1
1	1	1	1	3	3	2	2	1	1
1	1	2	2	3	3	1	1	3	3
1	1	2	2	1	1	2	2	1	1
1	1	2	2	3	3	2	2	1	1
1	1	2	2	3	3	2	2	1	1
4	4	2	2	1	1	1	1	3	3
1	1	2	2	3	3	2	2	1	1
1	1	2	2	3	3	2	2	1	1
1	1	2	2	3	3	1	1	3	3
1	1	2	2	3	3	1	1	1	1
1	1	2	2	3	3	2	2	1	1
4	4	2	2	3	3	2	2	1	1
1	1	2	2	3	3	1	1	3	3
1	1	2	2	3	3	1	1	3	3
1	1	2	2	3	3	2	2	1	1
1	1	2	2	3	3	1	1	3	3
1	1	1	1	3	3	2	2	1	1
1	1	2	2	3	3	1	1	3	3
1	1	2	2	3	3	2	2	1	1
1	1	2	2	3	3	2	2	1	1

For Review Only

1	1	2	2	3	3	2	2	1	1
1	1	2	2	3	3	1	1	3	3

For Review Only

SNP457	SNP457	SNP458	SNP458	SNP459	SNP459	SNP460	SNP460	SNP461	SNP461
2	2	1	1	3	3	4	4	1	1
2	2	1	1	3	3	4	4	1	1
2	2	1	1	3	3	4	4	1	1
4	4	1	1	3	3	4	4	1	1
2	2	1	1	3	3	4	4	1	1
2	2	1	1	1	1	1	1	1	1
2	2	1	1	1	1	1	1	1	1
2	2	1	1	3	3	4	4	1	1
2	2	1	1	3	3	1	1	1	1
2	2	1	1	3	3	4	4	1	1
2	2	1	1	3	3	4	4	1	1
2	2	1	1	3	3	4	4	1	1
2	2	1	1	3	3	4	4	1	1
4	4	1	1	3	3	4	4	1	1
2	2	1	1	3	3	4	4	1	1
2	2	1	1	1	1	1	1	1	1
2	2	1	1	3	3	4	4	1	1
2	2	1	1	3	3	4	4	1	1
2	2	1	1	3	3	4	4	1	1
2	2	4	4	3	3	4	4	1	1
2	2	4	4	3	3	4	4	1	1
2	2	1	1	1	1	4	4	1	1
2	2	1	1	3	3	4	4	1	1
2	2	1	1	3	3	4	4	1	1
4	4	1	1	1	1	1	1	1	1
2	2	1	1	1	1	1	1	4	4
2	2	1	1	3	3	4	4	1	1
2	2	1	1	3	3	4	4	1	1
2	2	1	1	1	1	1	1	1	1
2	2	4	4	3	3	4	4	1	1
4	4	1	1	1	1	1	1	1	1
2	2	1	1	3	3	4	4	1	1
2	2	1	1	1	1	1	1	1	1
2	2	1	1	3	3	4	4	1	1
2	2	1	1	3	3	4	4	1	1
4	4	1	1	3	3	4	4	1	1
2	2	1	1	3	3	4	4	1	1
2	2	1	1	3	3	4	4	1	1
2	2	4	4	3	3	4	4	1	1
2	2	4	4	3	3	4	4	1	1
2	2	1	1	3	3	4	4	1	1
2	2	1	1	3	3	4	4	1	1
2	2	1	1	3	3	4	4	1	1
2	2	1	1	3	3	4	4	1	1
2	2	1	1	3	3	4	4	1	1
2	2	1	1	3	3	4	4	1	1
4	4	1	1	3	3	4	4	4	4
2	2	1	1	1	1	1	1	1	1
2	2	1	1	3	3	4	4	1	1
2	2	1	1	1	1	1	1	1	1
4	4	1	1	3	3	4	4	4	4
2	2	4	4	1	1	1	1	1	1

For Review Only

2	2	1	1	1	1	1	1	1	1
2	2	1	1	1	1	1	1	1	1
2	2	1	1	3	3	4	4	1	1
2	2	1	1	1	1	1	1	1	1
2	2	1	1	3	3	4	4	1	1
4	4	1	1	3	3	4	4	1	1
2	2	1	1	3	3	4	4	1	1
2	2	4	4	3	3	4	4	1	1
2	2	1	1	1	1	1	1	1	1
2	2	1	1	3	3	4	4	1	1
4	4	1	1	3	3	4	4	1	1
4	4	1	1	3	3	4	4	1	1
2	2	1	1	1	1	1	1	1	1
2	2	1	1	1	1	1	1	1	1
2	2	1	1	3	3	4	4	1	1
2	2	1	1	3	3	4	4	1	1
2	2	1	1	3	3	4	4	1	1
2	2	1	1	1	1	1	1	1	1
2	2	1	1	1	1	1	1	1	1
2	2	1	1	1	1	1	1	1	1
4	4	1	1	3	3	4	4	1	1
2	2	4	4	3	3	4	4	1	1
2	2	1	1	1	1	1	1	1	1
4	4	1	1	3	3	4	4	1	1
2	2	1	1	3	3	4	4	1	1
2	2	1	1	3	3	4	4	1	1
2	2	1	1	3	3	4	4	1	1
2	2	4	4	3	3	4	4	1	1
2	2	1	1	3	3	4	4	4	4
2	2	1	1	3	3	4	4	1	1
2	2	1	1	1	1	1	1	1	1
2	2	1	1	3	3	4	4	1	1
2	2	4	4	3	3	4	4	1	1
2	2	1	1	1	1	1	1	1	1
2	2	1	1	1	1	1	1	1	1
2	2	1	1	3	3	4	4	1	1
2	2	1	1	1	1	1	1	1	1
2	2	1	1	1	1	1	1	1	1
2	2	1	1	3	3	4	4	1	1
2	2	1	1	3	3	4	4	1	1
2	2	1	1	1	1	1	1	1	1
2	2	1	1	3	3	4	4	1	1
2	2	1	1	3	3	4	4	1	1
4	4	1	1	3	3	4	4	1	1
2	2	1	1	3	3	4	4	1	1
4	4	1	1	1	1	1	1	1	1
2	2	1	1	3	3	4	4	1	1
2	2	1	1	3	3	4	4	1	1
2	2	1	1	3	3	4	4	4	4
2	2	4	4	3	3	4	4	1	1
4	4	1	1	3	3	4	4	1	1
2	2	4	4	3	3	4	4	1	1
2	2	4	4	3	3	4	4	1	1
2	2	1	1	3	3	4	4	1	1
4	4	1	1	3	3	4	4	1	1

For Review Only

2	2	4	4	3	3	4	4	4	4
2	2	1	1	3	3	4	4	1	1
2	2	1	1	1	1	1	1	4	4
2	2	1	1	3	3	4	4	1	1
2	2	1	1	3	3	4	4	1	1
2	2	1	1	1	1	1	1	1	1
2	2	1	1	1	1	1	1	1	1
2	2	1	1	3	3	4	4	1	1
2	2	1	1	3	3	4	4	4	4
2	2	1	1	3	3	1	1	4	4
2	2	1	1	3	3	4	4	1	1
2	2	1	1	3	3	4	4	1	1
2	2	1	1	1	1	1	1	1	1
2	2	1	1	3	3	4	4	1	1
4	4	1	1	3	3	4	4	1	1
2	2	1	1	3	3	4	4	4	4
2	2	1	1	3	3	1	1	1	1
2	2	1	1	3	3	4	4	1	1
2	2	1	1	3	3	4	4	1	1
2	2	1	1	3	3	4	4	1	1
2	2	1	1	3	3	4	4	1	1
4	4	1	1	1	1	1	1	1	1
2	2	1	1	3	3	4	4	1	1
2	2	4	4	3	3	4	4	1	1
2	2	1	1	3	3	4	4	1	1
2	2	1	1	3	3	4	4	1	1
2	2	1	1	3	3	4	4	1	1
2	2	1	1	3	3	4	4	1	1
2	2	1	1	3	3	1	1	1	1
2	2	1	1	3	3	4	4	1	1
2	2	4	4	3	3	4	4	1	1
2	2	1	1	3	3	4	4	1	1
2	2	1	1	1	1	4	4	1	1
2	2	1	1	3	3	4	4	1	1
2	2	1	1	3	3	4	4	1	1
2	2	1	1	3	3	4	4	1	1
2	2	1	1	3	3	4	4	1	1
2	2	1	1	3	3	4	4	1	1
2	2	1	1	3	3	4	4	1	1
2	2	4	4	3	3	4	4	1	1
2	2	4	4	3	3	4	4	1	1
2	2	1	1	3	3	4	4	1	1
4	4	4	4	3	3	4	4	1	1
2	2	1	1	3	3	4	4	1	1
2	2	1	1	3	3	4	4	1	1
2	2	4	4	3	3	4	4	1	1
2	2	1	1	3	3	4	4	4	4
4	4	1	1	3	3	4	4	4	4
2	2	4	4	3	3	4	4	1	1
2	2	1	1	3	3	4	4	4	4
2	2	1	1	3	3	4	4	4	4
2	2	1	1	3	3	4	4	1	1
2	2	4	4	3	3	1	1	1	1
4	4	1	1	3	3	4	4	1	1
2	2	1	1	3	3	4	4	1	1

For Review Only

2	2	1	1	3	3	4	4	1	1
2	2	4	4	3	3	4	4	1	1
2	2	1	1	3	3	4	4	1	1
2	2	1	1	1	1	1	1	1	1
2	2	1	1	3	3	4	4	1	1
2	2	4	4	3	3	4	4	1	1
4	4	1	1	3	3	4	4	1	1
2	2	1	1	3	3	4	4	1	1
2	2	1	1	3	3	4	4	1	1
2	2	1	1	3	3	4	4	1	1
2	2	1	1	3	3	4	4	1	1
2	2	4	4	3	3	4	4	1	1
2	2	1	1	3	3	4	4	1	1
2	2	1	1	3	3	4	4	1	1
2	2	4	4	3	3	4	4	1	1
2	2	4	4	3	3	4	4	1	1
2	2	4	4	3	3	1	1	1	1
2	2	1	1	3	3	4	4	1	1
2	2	1	1	3	3	4	4	1	1
2	2	1	1	3	3	1	1	1	1
2	2	1	1	3	3	4	4	1	1
2	2	4	4	3	3	4	4	1	1
2	2	4	4	3	3	4	4	1	1
4	4	1	1	3	3	4	4	1	1
2	2	1	1	3	3	4	4	1	1
2	2	1	1	3	3	4	4	1	1
2	2	1	1	3	3	4	4	1	1
2	2	1	1	3	3	4	4	1	1
2	2	1	1	1	1	1	1	1	1
2	2	1	1	1	1	1	1	1	1
2	2	1	1	3	3	4	4	1	1
2	2	1	1	3	3	4	4	1	1
2	2	1	1	1	1	1	1	1	1
2	2	4	4	3	3	4	4	1	1
2	2	1	1	3	3	4	4	1	1
4	4	1	1	3	3	4	4	1	1
4	4	1	1	3	3	4	4	1	1
2	2	1	1	3	3	4	4	1	1
4	4	1	1	3	3	4	4	4	4
2	2	1	1	3	3	4	4	1	1
2	2	1	1	3	3	4	4	1	1
2	2	1	1	3	3	4	4	4	4
2	2	4	4	3	3	4	4	1	1
2	2	1	1	3	3	4	4	1	1
2	2	4	4	3	3	4	4	1	1
2	2	1	1	3	3	4	4	1	1
2	2	4	4	3	3	4	4	1	1
2	2	1	1	3	3	4	4	1	1
2	2	1	1	3	3	4	4	1	1
2	2	1	1	3	3	4	4	1	1
2	2	1	1	3	3	1	1	1	1
2	2	4	4	3	3	4	4	1	1
2	2	4	4	3	3	4	4	1	1
4	4	1	1	3	3	4	4	1	1
2	2	4	4	3	3	4	4	1	1

For Review Only

2	2	4	4	1	1	1	1	1	1
2	2	1	1	3	3	4	4	1	1
2	2	1	1	1	1	1	1	1	1
2	2	1	1	3	3	4	4	1	1
2	2	1	1	3	3	4	4	1	1
4	4	1	1	3	3	4	4	1	1
2	2	4	4	3	3	4	4	1	1
2	2	4	4	3	3	4	4	1	1
2	2	1	1	3	3	4	4	1	1
2	2	4	4	3	3	4	4	1	1
2	2	1	1	3	3	4	4	1	1
2	2	1	1	1	1	1	1	1	1
2	2	1	1	3	3	4	4	1	1
2	2	1	1	1	1	1	1	4	4
2	2	1	1	3	3	4	4	1	1
2	2	1	1	3	3	4	4	1	1
2	2	4	4	3	3	4	4	1	1
2	2	1	1	3	3	4	4	1	1
2	2	4	4	3	3	4	4	1	1
2	2	1	1	3	3	4	4	1	1
2	2	1	1	3	3	4	4	1	1
2	2	1	1	3	3	4	4	1	1
2	2	1	1	3	3	4	4	1	1
4	4	4	4	3	3	4	4	1	1
2	2	4	4	3	3	4	4	1	1
2	2	1	1	3	3	4	4	1	1
2	2	4	4	3	3	4	4	1	1
2	2	1	1	3	3	4	4	1	1
2	2	1	1	3	3	4	4	1	1
2	2	1	1	3	3	1	1	1	1
2	2	4	4	3	3	4	4	1	1
2	2	1	1	3	3	4	4	1	1
2	2	1	1	3	3	4	4	1	1
4	4	1	1	3	3	4	4	4	4
2	2	4	4	3	3	4	4	1	1
2	2	4	4	3	3	4	4	1	1
2	2	1	1	1	1	1	1	1	1
4	4	1	1	3	3	4	4	1	1
4	4	1	1	3	3	4	4	1	1
2	2	4	4	3	3	4	4	1	1
2	2	1	1	1	1	1	1	1	1
2	2	1	1	3	3	4	4	1	1
4	4	4	4	3	3	4	4	1	1
2	2	1	1	1	1	1	1	1	1
4	4	1	1	3	3	4	4	1	1
2	2	1	1	3	3	4	4	1	1
4	4	4	4	3	3	4	4	1	1
4	4	1	1	1	1	1	1	1	1
2	2	1	1	3	3	4	4	4	4
2	2	4	4	3	3	4	4	1	1
2	2	1	1	1	1	1	1	1	1
2	2	4	4	3	3	4	4	1	1
2	2	1	1	3	3	4	4	1	1
2	2	1	1	3	3	4	4	1	1
2	2	4	4	3	3	4	4	1	1

For Review Only

2	2	1	1	3	3	4	4	1	1
2	2	1	1	3	3	4	4	1	1

For Review Only

SNP462	SNP462	SNP463	SNP463	SNP464	SNP464	SNP465	SNP465	SNP466	SNP466
1	1	3	3	4	4	3	3	2	2
1	1	2	2	4	4	3	3	2	2
3	3	3	3	2	2	3	3	2	2
3	3	2	2	4	4	3	3	2	2
3	3	2	2	4	4	3	3	2	2
1	1	3	3	2	2	3	3	2	2
1	1	3	3	2	2	3	3	2	2
1	1	2	2	4	4	3	3	2	2
3	3	2	2	4	4	3	3	2	2
3	3	2	2	4	4	3	3	2	2
3	3	3	3	2	2	3	3	2	2
3	3	2	2	4	4	3	3	2	2
1	1	3	3	2	2	3	3	2	2
3	3	3	3	2	2	3	3	2	2
1	1	3	3	2	2	3	3	2	2
1	1	2	2	4	4	3	3	2	2
3	3	3	3	2	2	3	3	2	2
1	1	3	3	2	2	3	3	2	2
3	3	3	3	2	2	3	3	2	2
1	1	2	2	4	4	3	3	2	2
1	1	3	3	2	2	3	3	2	2
1	1	3	3	2	2	3	3	2	2
3	3	3	3	2	2	3	3	2	2
3	3	2	2	4	4	3	3	2	2
1	1	3	3	4	4	2	2	2	2
1	1	2	2	4	4	3	3	1	1
3	3	2	2	4	4	3	3	2	2
3	3	2	2	4	4	3	3	2	2
1	1	2	2	4	4	3	3	2	2
1	1	2	2	4	4	3	3	2	2
1	1	2	2	4	4	3	3	2	2
3	3	2	2	4	4	3	3	2	2
1	1	3	3	2	2	3	3	2	2
3	3	2	2	4	4	3	3	2	2
1	1	2	2	4	4	3	3	2	2
3	3	2	2	4	4	3	3	2	2
1	1	3	3	4	4	3	3	2	2
3	3	3	3	2	2	3	3	2	2
1	1	2	2	4	4	3	3	2	2
3	3	3	3	2	2	3	3	2	2
1	1	2	2	4	4	3	3	2	2
1	1	2	2	4	4	3	3	2	2
3	3	2	2	4	4	3	3	2	2
3	3	3	3	2	2	3	3	2	2
3	3	2	2	4	4	3	3	2	2
3	3	2	2	4	4	3	3	2	2
1	1	3	3	2	2	3	3	2	2
1	1	3	3	2	2	3	3	2	2
1	1	2	2	4	4	3	3	2	2
3	3	3	3	2	2	3	3	2	2
1	1	3	3	2	2	3	3	1	1
1	1	2	2	4	4	2	2	2	2

For Review Only

1	1	3	3	2	2	3	3	2	2
1	1	3	3	2	2	3	3	2	2
3	3	3	3	2	2	3	3	2	2
1	1	3	3	2	2	3	3	2	2
3	3	3	3	2	2	3	3	2	2
3	3	2	2	4	4	2	2	2	2
3	3	2	2	4	4	3	3	2	2
3	3	2	2	4	4	3	3	2	2
1	1	2	2	4	4	3	3	2	2
3	3	2	2	4	4	3	3	2	2
3	3	2	2	4	4	3	3	2	2
3	3	2	2	4	4	2	2	2	2
1	1	3	3	2	2	3	3	2	2
1	1	2	2	4	4	3	3	2	2
3	3	2	2	4	4	3	3	2	2
1	1	3	3	2	2	3	3	2	2
3	3	3	3	2	2	3	3	2	2
1	1	3	3	2	2	3	3	2	2
1	1	2	2	4	4	3	3	2	2
1	1	2	2	4	4	3	3	2	2
3	3	2	2	4	4	2	2	2	2
3	3	2	2	4	4	3	3	2	2
1	1	2	2	4	4	3	3	2	2
1	1	2	2	4	4	3	3	2	2
3	3	2	2	4	4	3	3	2	2
1	1	2	2	4	4	2	2	2	2
1	1	3	3	2	2	3	3	2	2
3	3	2	2	4	4	3	3	2	2
1	1	3	3	2	2	3	3	1	1
3	3	2	2	4	4	3	3	2	2
1	1	2	2	2	2	3	3	2	2
1	1	2	2	4	4	3	3	2	2
3	3	2	2	4	4	3	3	2	2
1	1	3	3	2	2	3	3	2	2
1	1	3	3	2	2	3	3	2	2
3	3	2	2	4	4	3	3	2	2
1	1	2	2	4	4	3	3	2	2
1	1	3	3	2	2	3	3	2	2
1	1	3	3	2	2	3	3	2	2
3	3	3	3	2	2	3	3	2	2
1	1	3	3	2	2	3	3	2	2
3	3	2	2	4	4	3	3	2	2
3	3	3	3	2	2	3	3	2	2
3	3	2	2	4	4	3	3	2	2
3	3	3	3	2	2	3	3	2	2
1	1	2	2	4	4	3	3	2	2
3	3	2	2	4	4	3	3	2	2
1	1	3	3	2	2	3	3	2	2
1	1	3	3	2	2	3	3	2	2
3	3	3	3	2	2	3	3	2	2
3	3	2	2	4	4	3	3	2	2
3	3	2	2	4	4	3	3	2	2
3	3	2	2	4	4	3	3	2	2
3	3	2	2	4	4	3	3	2	2
3	3	3	3	2	2	3	3	2	2

For Review Only

1	1	3	3	2	2	3	3	1	1
3	3	2	2	4	4	3	3	2	2
1	1	2	2	4	4	3	3	2	2
3	3	3	3	2	2	3	3	2	2
3	3	2	2	4	4	3	3	2	2
1	1	2	2	4	4	3	3	2	2
1	1	2	2	4	4	3	3	2	2
3	3	2	2	4	4	2	2	2	2
3	3	3	3	2	2	3	3	2	2
1	1	3	3	2	2	3	3	2	2
3	3	3	3	2	2	3	3	2	2
3	3	2	2	2	2	3	3	2	2
1	1	3	3	2	2	2	2	2	2
3	3	2	2	4	4	3	3	2	2
3	3	2	2	4	4	3	3	2	2
3	3	2	2	2	2	3	3	2	2
1	1	3	3	2	2	3	3	2	2
3	3	3	3	2	2	3	3	2	2
3	3	3	3	2	2	3	3	2	2
3	3	2	2	4	4	3	3	2	2
3	3	2	2	4	4	3	3	2	2
3	3	2	2	4	4	3	3	2	2
3	3	3	3	2	2	3	3	2	2
3	3	2	2	4	4	3	3	2	2
3	3	3	3	2	2	3	3	1	1
1	1	3	3	2	2	3	3	2	2
3	3	2	2	2	2	3	3	2	2
3	3	2	2	4	4	3	3	2	2
1	1	2	2	4	4	2	2	2	2
1	1	3	3	2	2	3	3	2	2
3	3	2	2	4	4	3	3	2	2
3	3	2	2	4	4	3	3	2	2
1	1	3	3	2	2	3	3	2	2
3	3	3	3	2	2	3	3	2	2
3	3	2	2	4	4	3	3	2	2
1	1	2	2	2	2	3	3	2	2
3	3	2	2	4	4	3	3	2	2
3	3	2	2	4	4	2	2	2	2
3	3	3	3	2	2	3	3	2	2
3	3	2	2	4	4	3	3	2	2
3	3	2	2	4	4	3	3	2	2
3	3	2	2	4	4	3	3	2	2
3	3	3	3	2	2	3	3	2	2
3	3	2	2	4	4	3	3	2	2
1	1	3	3	2	2	3	3	1	1
3	3	3	3	2	2	3	3	2	2
1	1	3	3	2	2	3	3	1	1
3	3	2	2	4	4	3	3	1	1
3	3	2	2	4	4	3	3	2	2
1	1	2	2	4	4	3	3	1	1
1	1	2	2	4	4	3	3	2	2
3	3	2	2	4	4	3	3	2	2
1	1	3	3	2	2	3	3	2	2
1	1	3	3	2	2	2	2	1	1
3	3	2	2	4	4	3	3	2	2

For Review Only

1	1	3	3	2	2	3	3	2	2
3	3	3	3	2	2	3	3	2	2
3	3	3	3	2	2	3	3	2	2
1	1	2	2	4	4	3	3	2	2
3	3	3	3	2	2	3	3	2	2
3	3	2	2	4	4	3	3	2	2
3	3	2	2	4	4	3	3	2	2
3	3	2	2	4	4	3	3	2	2
3	3	2	2	4	4	3	3	2	2
3	3	2	2	4	4	3	3	2	2
1	1	2	2	4	4	3	3	1	1
3	3	2	2	4	4	3	3	2	2
3	3	3	3	4	4	3	3	2	2
3	3	3	3	4	4	3	3	2	2
3	3	2	2	4	4	3	3	2	2
3	3	3	3	2	2	3	3	2	2
3	3	3	3	2	2	3	3	2	2
3	3	2	2	4	4	3	3	2	2
1	1	2	2	4	4	3	3	1	1
1	1	2	2	4	4	3	3	2	2
3	3	2	2	4	4	3	3	2	2
3	3	2	2	4	4	3	3	2	2
3	3	2	2	4	4	3	3	2	2
3	3	2	2	4	4	2	2	2	2
3	3	2	2	4	4	3	3	2	2
3	3	2	2	4	4	3	3	2	2
3	3	2	2	4	4	3	3	2	2
3	3	3	3	2	2	3	3	2	2
1	1	2	2	4	4	3	3	2	2
1	1	3	3	2	2	3	3	2	2
1	1	2	2	4	4	3	3	2	2
3	3	3	3	2	2	3	3	2	2
1	1	2	2	4	4	3	3	2	2
3	3	2	2	4	4	3	3	2	2
1	1	2	2	4	4	3	3	2	2
3	3	2	2	4	4	2	2	2	2
3	3	2	2	4	4	3	3	2	2
1	1	2	2	4	4	3	3	2	2
3	3	2	2	4	4	3	3	1	1
1	1	3	3	2	2	2	2	2	2
3	3	3	3	2	2	3	3	2	2
1	1	3	3	2	2	3	3	1	1
3	3	2	2	4	4	3	3	2	2
3	3	2	2	4	4	3	3	2	2
3	3	2	2	4	4	3	3	2	2
3	3	3	3	2	2	3	3	2	2
3	3	3	3	2	2	3	3	2	2
3	3	2	2	2	2	3	3	2	2
3	3	2	2	4	4	3	3	2	2
3	3	2	2	4	4	3	3	2	2
1	1	2	2	4	4	2	2	2	2
1	1	2	2	4	4	3	3	2	2
3	3	3	3	2	2	3	3	2	2
3	3	2	2	4	4	3	3	2	2
3	3	2	2	4	4	3	3	2	2

For Review Only

1	1	3	3	2	2	3	3	2	2
1	1	2	2	4	4	3	3	2	2
1	1	3	3	2	2	3	3	2	2
3	3	3	3	2	2	3	3	2	2
1	1	2	2	4	4	3	3	2	2
1	1	3	3	2	2	3	3	2	2
1	1	2	2	4	4	3	3	2	2
3	3	2	2	4	4	3	3	2	2
3	3	2	2	4	4	3	3	2	2
3	3	2	2	4	4	3	3	2	2
3	3	2	2	4	4	3	3	2	2
1	1	3	3	2	2	3	3	2	2
1	1	3	3	2	2	3	3	2	2
1	1	3	3	2	2	3	3	2	2
3	3	3	3	2	2	3	3	2	2
3	3	2	2	4	4	3	3	2	2
1	1	3	3	2	2	3	3	2	2
3	3	3	3	2	2	3	3	2	2
3	3	3	3	2	2	3	3	2	2
1	1	3	3	2	2	3	3	2	2
1	1	2	2	4	4	3	3	2	2
1	1	3	3	2	2	3	3	2	2
3	3	3	3	2	2	3	3	2	2
1	1	2	2	4	4	3	3	2	2
3	3	2	2	4	4	3	3	2	2
3	3	2	2	4	4	3	3	2	2
3	3	2	2	4	4	3	3	2	2
3	3	2	2	4	4	3	3	2	2
1	1	2	2	4	4	3	3	2	2
1	1	2	2	4	4	2	2	2	2
3	3	3	3	2	2	3	3	2	2
1	1	3	3	2	2	3	3	2	2
3	3	2	2	4	4	3	3	2	2
3	3	3	3	2	2	3	3	2	2
3	3	3	3	2	2	3	3	2	2
3	3	2	2	4	4	3	3	2	2
1	1	3	3	2	2	3	3	2	2
3	3	2	2	4	4	2	2	2	2
3	3	2	2	4	4	3	3	2	2
1	1	2	2	4	4	3	3	2	2
1	1	2	2	4	4	3	3	2	2
3	3	3	3	2	2	3	3	2	2
3	3	2	2	4	4	3	3	2	2
1	1	3	3	2	2	3	3	2	2
3	3	2	2	4	4	3	3	2	2
1	1	3	3	2	2	3	3	2	2
3	3	2	2	4	4	3	3	1	1
1	1	3	3	2	2	2	2	2	2
3	3	3	3	2	2	3	3	2	2
3	3	2	2	4	4	3	3	2	2
1	1	2	2	4	4	3	3	2	2
3	3	2	2	4	4	3	3	2	2
3	3	3	3	2	2	3	3	2	2
3	3	3	3	2	2	3	3	2	2
3	3	2	2	4	4	3	3	2	2

For Review Only

3	3	3	3	2	2	3	3	2	2
3	3	2	2	4	4	3	3	2	2

For Review Only

SNP467	SNP467	SNP468	SNP468	SNP469	SNP469	SNP470	SNP470	SNP471	SNP471
3	3	2	2	4	4	1	1	2	2
3	3	2	2	4	4	1	1	2	2
1	1	2	2	4	4	1	1	2	2
1	1	2	2	4	4	1	1	2	2
1	1	2	2	4	4	1	1	2	2
3	3	2	2	4	4	1	1	2	2
1	1	2	2	4	4	1	1	2	2
1	1	2	2	4	4	1	1	2	2
3	3	2	2	4	4	1	1	2	2
3	3	2	2	4	4	1	1	2	2
3	3	2	2	4	4	1	1	2	2
3	3	2	2	4	4	1	1	2	2
1	1	2	2	4	4	1	1	2	2
1	1	2	2	4	4	1	1	2	2
3	3	2	2	4	4	1	1	2	2
3	3	2	2	4	4	1	1	2	2
1	1	2	2	4	4	1	1	2	2
3	3	2	2	4	4	1	1	4	4
1	1	2	2	4	4	1	1	2	2
3	3	4	4	4	4	1	1	2	2
3	3	4	4	4	4	1	1	2	2
1	1	4	4	4	4	1	1	2	2
3	3	2	2	3	3	3	3	2	2
3	3	2	2	4	4	1	1	2	2
1	1	2	2	4	4	1	1	2	2
1	1	2	2	4	4	1	1	2	2
1	1	4	4	4	4	1	1	2	2
3	3	2	2	4	4	1	1	2	2
3	3	2	2	4	4	1	1	2	2
1	1	4	4	4	4	1	1	4	4
1	1	2	2	4	4	1	1	2	2
3	3	2	2	4	4	1	1	2	2
1	1	2	2	4	4	1	1	2	2
3	3	2	2	4	4	1	1	2	2
3	3	2	2	4	4	1	1	2	2
1	1	2	2	4	4	1	1	2	2
3	3	2	2	4	4	1	1	4	4
3	3	2	2	4	4	1	1	2	2
3	3	2	2	4	4	1	1	2	2
3	3	2	2	4	4	1	1	4	4
1	1	2	2	4	4	1	1	2	2
3	3	2	2	4	4	1	1	2	2
3	3	2	2	4	4	1	1	2	2
3	3	2	2	4	4	1	1	2	2
3	3	2	2	4	4	1	1	2	2
3	3	2	2	4	4	1	1	2	2
1	1	2	2	4	4	1	1	2	2
3	3	2	2	4	4	1	1	2	2
3	3	4	4	4	4	1	1	2	2
3	3	2	2	4	4	1	1	2	2
1	1	2	2	4	4	1	1	2	2
3	3	2	2	4	4	1	1	2	2

For Review Only

3	3	2	2	4	4	1	1	2	2
1	1	2	2	4	4	1	1	2	2
3	3	2	2	4	4	1	1	2	2
1	1	2	2	4	4	1	1	2	2
1	1	2	2	4	4	1	1	2	2
1	1	2	2	4	4	1	1	2	2
3	3	2	2	4	4	1	1	2	2
3	3	2	2	4	4	1	1	2	2
1	1	2	2	4	4	1	1	2	2
3	3	2	2	4	4	1	1	2	2
1	1	2	2	4	4	1	1	2	2
1	1	2	2	4	4	1	1	2	2
3	3	2	2	4	4	1	1	2	2
3	3	2	2	4	4	1	1	2	2
3	3	2	2	4	4	1	1	2	2
3	3	2	2	4	4	1	1	2	2
3	3	2	2	4	4	1	1	2	2
3	3	2	2	4	4	1	1	2	2
3	3	2	2	4	4	1	1	2	2
1	1	2	2	4	4	1	1	2	2
3	3	2	2	4	4	1	1	2	2
3	3	2	2	3	3	3	3	2	2
3	3	2	2	4	4	1	1	2	2
1	1	2	2	4	4	1	1	2	2
3	3	2	2	4	4	1	1	2	2
1	1	2	2	4	4	1	1	2	2
3	3	2	2	4	4	1	1	2	2
3	3	4	4	4	4	1	1	4	4
1	1	2	2	4	4	1	1	2	2
3	3	2	2	3	3	3	3	2	2
3	3	2	2	4	4	1	1	2	2
3	3	2	2	4	4	1	1	2	2
3	3	4	4	4	4	1	1	2	2
3	3	2	2	4	4	1	1	2	2
3	3	2	2	4	4	1	1	2	2
3	3	2	2	4	4	1	1	2	2
3	3	2	2	4	4	1	1	2	2
1	1	4	4	4	4	1	1	2	2
1	1	2	2	3	3	3	3	2	2
1	1	2	2	4	4	1	1	2	2
1	1	2	2	4	4	1	1	2	2
3	3	2	2	3	3	1	1	2	2
1	1	2	2	4	4	1	1	2	2
1	1	2	2	4	4	1	1	2	2
3	3	2	2	3	3	3	3	2	2
1	1	2	2	4	4	1	1	2	2
3	3	2	2	4	4	1	1	2	2
3	3	2	2	4	4	1	1	2	2
1	1	2	2	4	4	1	1	2	2
3	3	2	2	4	4	1	1	2	2
1	1	2	2	4	4	1	1	2	2
3	3	2	2	4	4	1	1	2	2
3	3	2	2	4	4	1	1	4	4
3	3	2	2	4	4	1	1	2	2
1	1	2	2	4	4	1	1	2	2

For Review Only

3	3	2	2	4	4	1	1	2	2
3	3	2	2	4	4	1	1	2	2
1	1	2	2	4	4	1	1	2	2
3	3	2	2	4	4	1	1	2	2
1	1	2	2	4	4	1	1	2	2
1	1	2	2	4	4	1	1	2	2
3	3	2	2	4	4	1	1	2	2
3	3	2	2	4	4	1	1	2	2
1	1	2	2	4	4	1	1	2	2
1	1	2	2	4	4	1	1	2	2
3	3	2	2	4	4	1	1	2	2
3	3	2	2	4	4	1	1	2	2
1	1	2	2	4	4	1	1	2	2
3	3	2	2	4	4	1	1	2	2
1	1	2	2	4	4	1	1	2	2
3	3	2	2	4	4	1	1	2	2
1	1	2	2	4	4	1	1	2	2
3	3	2	2	4	4	1	1	2	2
3	3	2	2	4	4	1	1	2	2
3	3	2	2	4	4	1	1	2	2
3	3	2	2	4	4	1	1	2	2
3	3	2	2	4	4	1	1	2	2
3	3	2	2	4	4	1	1	2	2
3	3	2	2	4	4	1	1	2	2
3	3	2	2	3	3	1	1	2	2
3	3	2	2	4	4	1	1	2	2
1	1	2	2	4	4	1	1	2	2
3	3	2	2	4	4	1	1	2	2
3	3	2	2	4	4	1	1	2	2
3	3	2	2	4	4	1	1	2	2
3	3	4	4	4	4	1	1	2	2
3	3	2	2	4	4	1	1	2	2
1	1	2	2	4	4	1	1	2	2
3	3	2	2	4	4	1	1	2	2
3	3	2	2	4	4	1	1	2	2
3	3	2	2	4	4	1	1	2	2
3	3	2	2	4	4	1	1	2	2
3	3	2	2	4	4	1	1	2	2
3	3	2	2	4	4	1	1	2	2
3	3	2	2	4	4	1	1	2	2
3	3	2	2	3	3	3	3	2	2
3	3	2	2	4	4	1	1	2	2
1	1	2	2	4	4	1	1	2	2
3	3	2	2	4	4	1	1	2	2
3	3	2	2	4	4	1	1	2	2
3	3	2	2	4	4	1	1	2	2
3	3	2	2	4	4	1	1	2	2
1	1	2	2	4	4	1	1	2	2
3	3	2	2	4	4	1	1	2	2
1	1	2	2	4	4	1	1	2	2
3	3	2	2	4	4	1	1	2	2
3	3	2	2	4	4	1	1	4	4
3	3	2	2	4	4	1	1	2	2
1	1	2	2	4	4	1	1	2	2
3	3	2	2	4	4	1	1	2	2

For Review Only

3	3	2	2	4	4	1	1	2	2
3	3	4	4	4	4	1	1	2	2
3	3	2	2	4	4	1	1	4	4
3	3	2	2	4	4	1	1	2	2
3	3	2	2	4	4	1	1	2	2
3	3	2	2	4	4	1	1	4	4
3	3	2	2	4	4	1	1	2	2
3	3	2	2	4	4	1	1	2	2
3	3	2	2	4	4	1	1	2	2
3	3	2	2	3	3	3	3	2	2
3	3	2	2	4	4	1	1	2	2
3	3	2	2	4	4	1	1	2	2
3	3	2	2	4	4	1	1	2	2
3	3	2	2	4	4	1	1	2	2
3	3	4	4	4	4	1	1	2	2
3	3	2	2	4	4	1	1	2	2
3	3	2	2	4	4	1	1	2	2
1	1	2	2	4	4	1	1	2	2
1	1	2	2	4	4	1	1	2	2
3	3	2	2	4	4	1	1	2	2
3	3	2	2	4	4	1	1	2	2
3	3	2	2	4	4	1	1	4	4
3	3	2	2	3	3	3	3	2	2
1	1	2	2	4	4	1	1	2	2
3	3	2	2	4	4	1	1	2	2
3	3	2	2	4	4	1	1	2	2
3	3	2	2	4	4	1	1	2	2
3	3	2	2	4	4	1	1	2	2
1	1	2	2	4	4	1	1	2	2
1	1	2	2	4	4	1	1	2	2
3	3	2	2	4	4	1	1	2	2
3	3	2	2	3	3	3	3	2	2
1	1	2	2	4	4	1	1	2	2
3	3	4	4	4	4	1	1	2	2
3	3	2	2	4	4	1	1	2	2
1	1	2	2	4	4	1	1	2	2
1	1	2	2	4	4	1	1	2	2
3	3	2	2	4	4	1	1	4	4
1	1	2	2	4	4	1	1	2	2
1	1	2	2	4	4	1	1	2	2
1	1	2	2	4	4	1	1	2	2
3	3	2	2	4	4	1	1	2	2
3	3	2	2	4	4	1	1	2	2
3	3	2	2	4	4	1	1	2	2
3	3	2	2	4	4	1	1	2	2
3	3	2	2	4	4	1	1	2	2
3	3	2	2	4	4	1	1	2	2
3	3	2	2	4	4	1	1	4	4
3	3	2	2	4	4	1	1	2	2
1	1	2	2	4	4	1	1	2	2
3	3	2	2	4	4	1	1	2	2
3	3	2	2	4	4	1	1	2	2
3	3	2	2	3	3	3	3	4	4
1	1	2	2	4	4	1	1	2	2
3	3	2	2	4	4	1	1	4	4

For Review Only

3	3	2	2	4	4	1	1	2	2
3	3	2	2	4	4	1	1	2	2
3	3	2	2	3	3	3	3	2	2
3	3	2	2	3	3	3	3	2	2
3	3	2	2	4	4	1	1	2	2
1	1	2	2	4	4	1	1	2	2
3	3	2	2	4	4	1	1	2	2
1	1	2	2	4	4	1	1	2	2
3	3	2	2	4	4	1	1	2	2
3	3	4	4	4	4	1	1	4	4
3	3	2	2	4	4	1	1	2	2
1	1	2	2	4	4	1	1	2	2
1	1	2	2	3	3	3	3	2	2
1	1	2	2	4	4	1	1	2	2
1	1	2	2	4	4	1	1	2	2
3	3	2	2	3	3	3	3	2	2
3	3	2	2	4	4	1	1	2	2
3	3	2	2	3	3	3	3	2	2
1	1	2	2	4	4	1	1	4	4
3	3	2	2	4	4	1	1	2	2
3	3	2	2	4	4	1	1	2	2
3	3	2	2	4	4	1	1	2	2
3	3	2	2	3	3	3	3	2	2
1	1	2	2	4	4	1	1	2	2
3	3	2	2	4	4	1	1	2	2
1	1	2	2	4	4	1	1	2	2
3	3	4	4	4	4	1	1	4	4
3	3	2	2	4	4	1	1	2	2
3	3	2	2	4	4	1	1	2	2
3	3	2	2	4	4	1	1	2	2
3	3	2	2	4	4	1	1	2	2
3	3	2	2	4	4	1	1	2	2
3	3	2	2	4	4	1	1	2	2
1	1	2	2	4	4	1	1	2	2
3	3	2	2	3	3	3	3	2	2
3	3	4	4	4	4	1	1	4	4
1	1	2	2	4	4	1	1	2	2
1	1	2	2	4	4	1	1	2	2
1	1	2	2	4	4	1	1	2	2
3	3	2	2	4	4	1	1	2	2
3	3	2	2	3	3	3	3	2	2
3	3	2	2	4	4	1	1	2	2
1	1	2	2	4	4	1	1	2	2
1	1	2	2	4	4	1	1	2	2
1	1	2	2	4	4	1	1	2	2
1	1	2	2	3	3	3	3	2	2
1	1	2	2	4	4	1	1	2	2
1	1	2	2	4	4	1	1	2	2
3	3	2	2	4	4	1	1	2	2
3	3	4	4	4	4	1	1	4	4
1	1	2	2	3	3	3	3	2	2
3	3	2	2	4	4	1	1	2	2
1	1	2	2	4	4	1	1	2	2
3	3	2	2	4	4	1	1	2	2
3	3	2	2	4	4	1	1	4	4

For Review Only

3	3	2	2	3	3	3	3	2	2
1	1	2	2	4	4	1	1	2	2

For Review Only

SNP472	SNP472	SNP473	SNP473	SNP474	SNP474	SNP475	SNP475	SNP476	SNP476
2	2	4	4	4	4	2	2	1	1
2	2	4	4	4	4	2	2	1	1
2	2	4	4	4	4	3	3	1	1
2	2	4	4	4	4	2	2	1	1
2	2	4	4	4	4	2	2	1	1
2	2	4	4	4	4	2	2	1	1
2	2	4	4	4	4	3	3	1	1
2	2	4	4	4	4	2	2	1	1
2	2	4	4	4	4	3	3	1	1
2	2	4	4	4	4	2	2	1	1
2	2	4	4	4	4	2	2	1	1
2	2	4	4	4	4	2	2	1	1
2	2	4	4	4	4	3	3	1	1
2	2	4	4	4	4	2	2	1	1
2	2	4	4	4	4	3	3	1	1
2	2	4	4	4	4	2	2	1	1
2	2	4	4	4	4	3	3	1	1
2	2	4	4	4	4	2	2	1	1
2	2	4	4	4	4	3	3	1	1
2	2	4	4	4	4	2	2	1	1
2	2	4	4	4	4	2	2	1	1
2	2	4	4	4	4	3	3	1	1
4	4	3	3	1	1	2	2	1	1
2	2	4	4	4	4	2	2	1	1
2	2	4	4	4	4	2	2	1	1
2	2	4	4	4	4	3	3	4	4
2	2	4	4	4	4	3	3	4	4
2	2	4	4	4	4	2	2	1	1
2	2	4	4	4	4	2	2	1	1
2	2	4	4	4	4	2	2	1	1
2	2	4	4	4	4	2	2	1	1
2	2	4	4	4	4	2	2	1	1
2	2	4	4	4	4	3	3	1	1
2	2	4	4	4	4	2	2	1	1
2	2	4	4	4	4	2	2	1	1
2	2	4	4	4	4	2	2	1	1
2	2	4	4	4	4	2	2	1	1
2	2	4	4	4	4	2	2	1	1
2	2	4	4	4	4	2	2	1	1
4	4	3	3	1	1	2	2	1	1
2	2	4	4	4	4	2	2	1	1
2	2	4	4	4	4	2	2	1	1
2	2	4	4	4	4	2	2	1	1
2	2	4	4	4	4	2	2	1	1
2	2	4	4	4	4	2	2	1	1
2	2	4	4	4	4	2	2	1	1
2	2	4	4	4	4	2	2	1	1
2	2	4	4	4	4	2	2	1	1
2	2	4	4	4	4	2	2	1	1
2	2	4	4	4	4	2	2	1	1
2	2	4	4	4	4	2	2	1	1
2	2	4	4	4	4	2	2	1	1

For Review Only

2	2	4	4	4	4	2	2	1	1
2	2	4	4	4	4	3	3	1	1
2	2	4	4	4	4	2	2	1	1
2	2	4	4	4	4	3	3	1	1
2	2	4	4	4	4	3	3	1	1
2	2	4	4	4	4	2	2	1	1
2	2	4	4	4	4	2	2	1	1
2	2	4	4	4	4	2	2	1	1
2	2	4	4	4	4	3	3	1	1
2	2	4	4	4	4	2	2	1	1
2	2	4	4	4	4	2	2	1	1
2	2	4	4	4	4	2	2	1	1
2	2	4	4	4	4	2	2	1	1
2	2	4	4	4	4	2	2	1	1
2	2	4	4	4	4	2	2	1	1
2	2	4	4	4	4	2	2	1	1
2	2	4	4	4	4	2	2	1	1
2	2	4	4	4	4	2	2	1	1
2	2	4	4	4	4	2	2	1	1
2	2	4	4	4	4	3	3	1	1
2	2	4	4	4	4	2	2	1	1
4	4	3	3	1	1	2	2	1	1
2	2	4	4	4	4	2	2	1	1
2	2	4	4	4	4	2	2	1	1
2	2	4	4	4	4	2	2	1	1
2	2	4	4	4	4	3	3	4	4
2	2	4	4	4	4	2	2	1	1
2	2	4	4	4	4	2	2	1	1
2	2	4	4	4	4	2	2	1	1
4	4	3	3	1	1	2	2	1	1
2	2	4	4	4	4	2	2	1	1
2	2	4	4	4	4	2	2	1	1
2	2	4	4	4	4	2	2	1	1
2	2	4	4	4	4	2	2	1	1
2	2	4	4	4	4	2	2	1	1
2	2	4	4	4	4	2	2	1	1
2	2	4	4	4	4	2	2	1	1
2	2	4	4	4	4	2	2	1	1
4	4	3	3	1	1	3	3	4	4
2	2	4	4	4	4	2	2	1	1
2	2	4	4	4	4	3	3	1	1
4	4	3	3	1	1	2	2	1	1
2	2	4	4	4	4	3	3	4	4
2	2	4	4	4	4	2	2	1	1
4	4	4	4	1	1	2	2	1	1
2	2	4	4	4	4	2	2	1	1
2	2	4	4	4	4	2	2	1	1
2	2	4	4	4	4	2	2	1	1
2	2	4	4	4	4	3	3	4	4
2	2	4	4	4	4	2	2	1	1
2	2	4	4	4	4	2	2	1	1
2	2	4	4	4	4	2	2	1	1
2	2	4	4	4	4	2	2	1	1
2	2	4	4	4	4	2	2	1	1
2	2	4	4	4	4	2	2	1	1

For Review Only

2	2	4	4	4	4	2	2	1	1
2	2	4	4	4	4	2	2	1	1
2	2	4	4	4	4	3	3	4	4
2	2	4	4	4	4	2	2	1	1
2	2	4	4	4	4	2	2	1	1
2	2	4	4	4	4	3	3	4	4
2	2	4	4	4	4	2	2	1	1
2	2	4	4	4	4	2	2	1	1
2	2	4	4	4	4	3	3	4	4
2	2	4	4	4	4	2	2	1	1
2	2	4	4	4	4	2	2	1	1
2	2	4	4	4	4	2	2	1	1
2	2	4	4	4	4	3	3	4	4
2	2	4	4	4	4	3	3	4	4
2	2	4	4	4	4	2	2	1	1
2	2	4	4	4	4	2	2	1	1
2	2	4	4	4	4	3	3	4	4
2	2	4	4	4	4	2	2	1	1
2	2	4	4	4	4	2	2	1	1
2	2	4	4	4	4	2	2	1	1
2	2	4	4	4	4	2	2	1	1
4	4	3	3	1	1	2	2	1	1
2	2	4	4	4	4	2	2	1	1
2	2	4	4	4	4	2	2	1	1
2	2	4	4	4	4	2	2	1	1
2	2	4	4	4	4	2	2	1	1
2	2	4	4	4	4	3	3	1	1
2	2	4	4	4	4	2	2	1	1
2	2	4	4	4	4	2	2	1	1
2	2	4	4	4	4	2	2	1	1
2	2	4	4	4	4	2	2	1	1
2	2	4	4	4	4	2	2	1	1
2	2	4	4	4	4	3	3	1	1
2	2	4	4	4	4	2	2	1	1
2	2	4	4	4	4	2	2	1	1
2	2	4	4	4	4	2	2	1	1
2	2	4	4	4	4	2	2	1	1
2	2	4	4	4	4	2	2	1	1
2	2	4	4	4	4	2	2	4	4
2	2	4	4	4	4	2	2	1	1
4	4	3	3	1	1	2	2	1	1
2	2	4	4	4	4	2	2	1	1
2	2	4	4	4	4	2	2	1	1
2	2	4	4	4	4	2	2	1	1
2	2	4	4	4	4	2	2	1	1
2	2	4	4	4	4	2	2	1	1
2	2	4	4	4	4	2	2	1	1
2	2	4	4	4	4	2	2	1	1
2	2	4	4	4	4	2	2	1	1
2	2	4	4	4	4	3	3	4	4
2	2	4	4	4	4	2	2	1	1
2	2	4	4	4	4	2	2	1	1
2	2	4	4	4	4	2	2	1	1
2	2	4	4	4	4	2	2	1	1
2	2	4	4	4	4	2	2	1	1

For Review Only

2	2	4	4	4	4	2	2	1	1
2	2	4	4	4	4	2	2	1	1
2	2	4	4	4	4	2	2	1	1
2	2	4	4	4	4	2	2	1	1
2	2	4	4	4	4	2	2	1	1
2	2	4	4	4	4	2	2	1	1
2	2	4	4	4	4	2	2	1	1
2	2	4	4	4	4	2	2	1	1
2	2	4	4	4	4	2	2	1	1
4	4	3	3	1	1	2	2	1	1
2	2	4	4	4	4	2	2	1	1
2	2	4	4	4	4	2	2	1	1
2	2	4	4	4	4	2	2	1	1
2	2	4	4	4	4	2	2	1	1
2	2	4	4	4	4	2	2	1	1
2	2	4	4	4	4	2	2	1	1
2	2	4	4	4	4	2	2	1	1
2	2	4	4	4	4	2	2	1	1
2	2	4	4	4	4	3	3	4	4
2	2	4	4	4	4	2	2	1	1
2	2	4	4	4	4	2	2	1	1
2	2	4	4	4	4	2	2	1	1
4	4	3	3	1	1	2	2	1	1
2	2	4	4	4	4	2	2	1	1
2	2	4	4	4	4	2	2	1	1
2	2	4	4	4	4	2	2	1	1
2	2	4	4	4	4	2	2	1	1
2	2	4	4	4	4	2	2	1	1
2	2	4	4	4	4	3	3	1	1
2	2	4	4	4	4	3	3	1	1
2	2	4	4	4	4	2	2	1	1
4	4	3	3	1	1	2	2	1	1
2	2	4	4	4	4	3	3	1	1
2	2	4	4	4	4	2	2	1	1
2	2	4	4	4	4	2	2	1	1
2	2	4	4	4	4	2	2	1	1
2	2	4	4	4	4	2	2	1	1
2	2	4	4	4	4	2	2	1	1
2	2	4	4	4	4	2	2	1	1
2	2	4	4	4	4	3	3	4	4
2	2	4	4	4	4	3	3	1	1
2	2	4	4	4	4	2	2	1	1
2	2	4	4	4	4	2	2	1	1
2	2	4	4	4	4	2	2	1	1
2	2	4	4	4	4	2	2	4	4
2	2	4	4	4	4	2	2	1	1
2	2	4	4	4	4	2	2	1	1
2	2	4	4	4	4	2	2	1	1
2	2	4	4	4	4	2	2	1	1
2	2	4	4	4	4	3	3	4	4
2	2	4	4	4	4	2	2	1	1
2	2	4	4	4	4	2	2	1	1
4	4	3	3	1	1	2	2	1	1
2	2	4	4	4	4	2	2	1	1
2	2	4	4	4	4	2	2	1	1

For Review Only

2	2	4	4	4	4	2	2	1	1
2	2	4	4	4	4	2	2	1	1
4	4	3	3	1	1	2	2	1	1
4	4	3	3	1	1	2	2	1	1
2	2	4	4	4	4	2	2	1	1
2	2	4	4	4	4	2	2	1	1
2	2	4	4	4	4	2	2	1	1
2	2	4	4	4	4	2	2	1	1
2	2	4	4	4	4	2	2	1	1
2	2	4	4	4	4	2	2	1	1
2	2	4	4	4	4	2	2	1	1
2	2	4	4	4	4	3	3	4	4
4	4	3	3	1	1	3	3	4	4
2	2	4	4	4	4	3	3	4	4
2	2	4	4	4	4	3	3	1	1
4	4	3	3	1	1	2	2	1	1
2	2	4	4	4	4	2	2	1	1
4	4	3	3	1	1	2	2	1	1
2	2	4	4	4	4	3	3	1	1
2	2	4	4	4	4	2	2	1	1
2	2	4	4	4	4	2	2	1	1
2	2	4	4	4	4	2	2	1	1
4	4	3	3	1	1	3	3	1	1
2	2	4	4	4	4	2	2	1	1
2	2	4	4	4	4	2	2	1	1
2	2	4	4	4	4	3	3	4	4
2	2	4	4	4	4	2	2	1	1
2	2	4	4	4	4	2	2	1	1
2	2	4	4	4	4	2	2	1	1
2	2	4	4	4	4	2	2	1	1
2	2	4	4	4	4	2	2	1	1
2	2	4	4	4	4	2	2	1	1
2	2	4	4	4	4	2	2	1	1
2	2	4	4	4	4	2	2	1	1
4	4	3	3	1	1	2	2	1	1
2	2	4	4	4	4	2	2	1	1
2	2	4	4	4	4	3	3	1	1
2	2	4	4	4	4	2	2	1	1
2	2	4	4	4	4	2	2	1	1
2	2	4	4	4	4	2	2	1	1
4	4	3	3	1	1	2	2	1	1
2	2	4	4	4	4	2	2	1	1
2	2	4	4	4	4	2	2	1	1
2	2	4	4	4	4	3	3	1	1
2	2	4	4	4	4	2	2	1	1
4	4	3	3	1	1	3	3	1	1
2	2	4	4	4	4	2	2	1	1
2	2	4	4	4	4	2	2	1	1
2	2	4	4	4	4	2	2	1	1
2	2	4	4	4	4	2	2	1	1
4	4	3	3	1	1	2	2	1	1
2	2	4	4	4	4	2	2	1	1
2	2	4	4	4	4	3	3	1	1
2	2	4	4	4	4	2	2	1	1
2	2	4	4	4	4	2	2	1	1

For Review Only

4	4	3	3	1	1	2	2	1	1
2	2	4	4	4	4	3	3	4	4

For Review Only

SNP477	SNP477	SNP478	SNP478	SNP479	SNP479	SNP480	SNP480	SNP481	SNP481
2	2	4	4	1	1	1	1	4	4
4	4	4	4	1	1	1	1	4	4
2	2	4	4	1	1	1	1	4	4
2	2	4	4	1	1	1	1	4	4
2	2	4	4	1	1	1	1	4	4
4	4	4	4	1	1	1	1	4	4
2	2	4	4	1	1	1	1	4	4
2	2	4	4	1	1	1	1	4	4
2	2	4	4	1	1	1	1	4	4
2	2	4	4	1	1	1	1	4	4
4	4	4	4	1	1	1	1	4	4
2	2	4	4	1	1	1	1	4	4
2	2	4	4	1	1	1	1	4	4
4	4	4	4	1	1	1	1	4	4
2	2	4	4	1	1	1	1	4	4
2	2	4	4	1	1	1	1	1	1
2	2	4	4	1	1	1	1	4	4
2	2	4	4	1	1	1	1	4	4
2	2	4	4	1	1	1	1	4	4
2	2	4	4	1	1	1	1	4	4
2	2	4	4	1	1	1	1	4	4
2	2	4	4	1	1	1	1	4	4
2	2	1	1	4	4	3	3	4	4
2	2	4	4	1	1	1	1	4	4
2	2	4	4	1	1	1	1	4	4
2	2	4	4	1	1	1	1	4	4
4	4	4	4	1	1	1	1	4	4
2	2	4	4	1	1	1	1	4	4
2	2	4	4	1	1	1	1	4	4
4	4	4	4	1	1	1	1	4	4
2	2	4	4	1	1	1	1	4	4
2	2	4	4	1	1	1	1	4	4
2	2	4	4	1	1	1	1	4	4
4	4	4	4	1	1	1	1	4	4
2	2	4	4	1	1	1	1	4	4
2	2	4	4	1	1	1	1	4	4
2	2	4	4	1	1	1	1	4	4
2	2	4	4	1	1	1	1	4	4
2	2	4	4	1	1	1	1	1	1
4	4	1	1	4	4	3	3	4	4
2	2	4	4	1	1	1	1	4	4
2	2	4	4	1	1	1	1	4	4
2	2	4	4	1	1	1	1	4	4
2	2	4	4	1	1	1	1	4	4
4	4	4	4	1	1	1	1	4	4
2	2	4	4	1	1	1	1	4	4
2	2	4	4	1	1	1	1	4	4
2	2	4	4	1	1	1	1	4	4
2	2	4	4	1	1	1	1	4	4
2	2	1	1	1	1	3	3	4	4
2	2	4	4	1	1	1	1	4	4
2	2	4	4	1	1	1	1	4	4

For Review Only

2	2	4	4	1	1	1	1	4	4
2	2	4	4	1	1	1	1	4	4
2	2	4	4	1	1	1	1	4	4
2	2	4	4	1	1	1	1	4	4
2	2	4	4	1	1	1	1	4	4
2	2	4	4	1	1	1	1	4	4
2	2	4	4	1	1	1	1	4	4
4	4	4	4	1	1	1	1	4	4
2	2	4	4	1	1	1	1	4	4
2	2	4	4	1	1	1	1	4	4
4	4	4	4	1	1	1	1	4	4
2	2	4	4	1	1	1	1	4	4
2	2	4	4	1	1	1	1	4	4
2	2	4	4	1	1	1	1	4	4
2	2	4	4	1	1	1	1	4	4
2	2	4	4	1	1	1	1	4	4
2	2	1	1	1	1	1	1	4	4
2	2	4	4	1	1	1	1	4	4
2	2	4	4	1	1	1	1	4	4
2	2	4	4	1	1	1	1	4	4
2	2	4	4	1	1	1	1	4	4
2	2	1	1	4	4	3	3	1	1
2	2	4	4	1	1	1	1	4	4
4	4	4	4	1	1	1	1	4	4
4	4	4	4	1	1	1	1	4	4
2	2	4	4	1	1	1	1	4	4
2	2	4	4	1	1	1	1	4	4
2	2	4	4	1	1	1	1	1	1
2	2	4	4	1	1	1	1	4	4
2	2	1	1	4	4	3	3	4	4
4	4	4	4	1	1	1	1	4	4
2	2	4	4	1	1	1	1	4	4
4	4	4	4	1	1	1	1	1	1
2	2	4	4	1	1	1	1	4	4
2	2	4	4	1	1	1	1	4	4
4	4	4	4	1	1	1	1	4	4
2	2	4	4	1	1	1	1	4	4
2	2	4	4	1	1	1	1	4	4
2	2	1	1	4	4	3	3	4	4
2	2	4	4	1	1	1	1	4	4
2	2	4	4	1	1	1	1	4	4
2	2	1	1	4	4	3	3	4	4
2	2	4	4	1	1	1	1	4	4
4	4	4	4	1	1	1	1	4	4
2	2	1	1	4	4	1	1	4	4
4	4	4	4	1	1	1	1	4	4
2	2	4	4	1	1	1	1	4	4
2	2	4	4	1	1	1	1	4	4
2	2	4	4	1	1	1	1	4	4
2	2	1	1	4	4	3	3	4	4
4	4	4	4	1	1	1	1	4	4
2	2	4	4	1	1	1	1	1	1
4	4	4	4	1	1	1	1	4	4
2	2	4	4	1	1	1	1	4	4
2	2	4	4	1	1	1	1	4	4

For Review Only

2	2	4	4	1	1	1	1	4	4
2	2	4	4	1	1	1	1	4	4
2	2	4	4	1	1	1	1	4	4
2	2	4	4	1	1	1	1	1	1
4	4	4	4	1	1	1	1	4	4
2	2	4	4	1	1	1	1	4	4
2	2	4	4	1	1	1	1	4	4
2	2	4	4	1	1	1	1	4	4
4	4	4	4	1	1	1	1	1	1
2	2	4	4	1	1	1	1	4	4
2	2	4	4	1	1	1	1	4	4
2	2	4	4	1	1	1	1	4	4
2	2	4	4	1	1	1	1	4	4
2	2	4	4	1	1	1	1	4	4
4	4	4	4	1	1	1	1	4	4
2	2	4	4	1	1	1	1	4	4
4	4	4	4	1	1	1	1	4	4
2	2	4	4	1	1	1	1	4	4
2	2	4	4	1	1	1	1	1	1
2	2	1	1	1	1	1	1	4	4
2	2	4	4	1	1	1	1	4	4
2	2	1	1	4	4	1	1	4	4
2	2	4	4	1	1	1	1	4	4
2	2	4	4	1	1	1	1	1	1
2	2	1	1	1	1	1	1	4	4
2	2	4	4	1	1	1	1	4	4
2	2	4	4	1	1	1	1	4	4
2	2	4	4	1	1	1	1	4	4
2	2	4	4	1	1	1	1	4	4
2	2	4	4	4	4	1	1	4	4
2	2	4	4	1	1	1	1	1	1
2	2	4	4	1	1	1	1	4	4
2	2	4	4	1	1	1	1	4	4
2	2	4	4	1	1	1	1	4	4
4	4	4	4	1	1	1	1	4	4
2	2	4	4	1	1	1	1	4	4
2	2	4	4	1	1	1	1	1	1
2	2	4	4	1	1	1	1	4	4
4	4	4	4	1	1	1	1	4	4
2	2	4	4	1	1	1	1	1	1
2	2	1	1	4	4	3	3	4	4
2	2	4	4	1	1	1	1	1	1
4	4	4	4	1	1	1	1	4	4
2	2	4	4	1	1	1	1	4	4
2	2	4	4	1	1	1	1	4	4
4	4	4	4	1	1	1	1	4	4
2	2	4	4	1	1	1	1	4	4
4	4	4	4	1	1	1	1	4	4
2	2	4	4	1	1	1	1	4	4
2	2	4	4	1	1	1	1	4	4
2	2	4	4	1	1	1	1	4	4
4	4	4	4	1	1	1	1	4	4
2	2	4	4	1	1	1	1	4	4
2	2	4	4	1	1	1	1	4	4
2	2	4	4	1	1	1	1	4	4

For Review Only

2	2	4	4	1	1	1	1	4	4
2	2	1	1	1	1	1	1	4	4
2	2	4	4	1	1	1	1	4	4
2	2	4	4	1	1	1	1	4	4
4	4	4	4	1	1	1	1	4	4
4	4	4	4	1	1	1	1	4	4
4	4	4	4	1	1	1	1	4	4
2	2	4	4	1	1	1	1	4	4
2	2	4	4	4	4	1	1	4	4
4	4	1	1	4	4	3	3	4	4
2	2	4	4	1	1	1	1	4	4
4	4	4	4	1	1	1	1	4	4
2	2	4	4	1	1	1	1	4	4
4	4	4	4	1	1	1	1	4	4
2	2	4	4	1	1	1	1	4	4
4	4	4	4	1	1	1	1	4	4
2	2	4	4	1	1	1	1	4	4
4	4	4	4	1	1	1	1	4	4
2	2	4	4	1	1	1	1	4	4
2	2	4	4	4	4	1	1	4	4
2	2	4	4	1	1	1	1	4	4
4	4	4	4	1	1	1	1	4	4
2	2	1	1	4	4	3	3	1	1
2	2	4	4	1	1	1	1	4	4
2	2	4	4	1	1	1	1	4	4
4	4	4	4	1	1	1	1	4	4
2	2	4	4	1	1	1	1	4	4
4	4	4	4	1	1	1	1	4	4
2	2	4	4	1	1	1	1	4	4
2	2	4	4	1	1	1	1	4	4
2	2	4	4	1	1	1	1	4	4
2	2	1	1	4	4	3	3	4	4
2	2	4	4	1	1	1	1	4	4
2	2	4	4	1	1	1	1	4	4
2	2	4	4	1	1	1	1	4	4
2	2	4	4	1	1	1	1	4	4
2	2	4	4	1	1	1	1	4	4
2	2	4	4	1	1	1	1	4	4
4	4	4	4	1	1	1	1	4	4
2	2	4	4	1	1	1	1	4	4
2	2	4	4	1	1	1	1	4	4
2	2	4	4	1	1	1	1	4	4
4	4	4	4	1	1	1	1	1	1
2	2	4	4	1	1	1	1	4	4
2	2	4	4	1	1	1	1	4	4
2	2	4	4	1	1	1	1	4	4
4	4	4	4	1	1	1	1	4	4
4	4	4	4	1	1	1	1	1	1
4	4	4	4	1	1	1	1	4	4
2	2	4	4	1	1	1	1	4	4
2	2	4	4	1	1	1	1	4	4
4	4	4	4	1	1	1	1	4	4
2	2	1	1	4	4	3	3	1	1
4	4	4	4	1	1	1	1	4	4
2	2	4	4	1	1	1	1	1	1

For Review Only

2	2	4	4	1	1	1	1	4	4
2	2	4	4	1	1	1	1	4	4
2	2	1	1	4	4	3	3	4	4
2	2	1	1	4	4	3	3	4	4
2	2	4	4	1	1	1	1	4	4
2	2	4	4	1	1	1	1	4	4
4	4	4	4	1	1	1	1	4	4
4	4	4	4	1	1	1	1	4	4
2	2	4	4	1	1	1	1	4	4
4	4	4	4	1	1	1	1	1	1
2	2	4	4	1	1	1	1	4	4
4	4	4	4	1	1	1	1	4	4
2	2	1	1	4	4	3	3	4	4
2	2	4	4	1	1	1	1	4	4
2	2	4	4	1	1	1	1	4	4
4	4	1	1	4	4	3	3	4	4
4	4	4	4	1	1	1	1	1	1
4	4	1	1	4	4	3	3	4	4
4	4	4	4	1	1	1	1	1	1
2	2	4	4	1	1	1	1	4	4
4	4	4	4	1	1	1	1	1	1
2	2	4	4	1	1	1	1	4	4
2	2	1	1	4	4	3	3	4	4
4	4	4	4	1	1	1	1	4	4
4	4	4	4	1	1	1	1	4	4
2	2	4	4	1	1	1	1	4	4
2	2	4	4	1	1	1	1	1	1
2	2	4	4	1	1	1	1	4	4
2	2	4	4	1	1	1	1	4	4
2	2	4	4	1	1	1	1	4	4
4	4	4	4	1	1	1	1	4	4
2	2	4	4	1	1	1	1	4	4
2	2	4	4	1	1	1	1	4	4
4	4	4	4	1	1	1	1	4	4
2	2	1	1	4	4	3	3	1	1
2	2	4	4	1	1	1	1	1	1
4	4	4	4	1	1	1	1	4	4
2	2	4	4	1	1	1	1	4	4
2	2	4	4	1	1	1	1	4	4
4	4	4	4	1	1	1	1	1	1
2	2	1	1	4	4	3	3	4	4
4	4	4	4	1	1	1	1	4	4
4	4	4	4	1	1	1	1	4	4
2	2	4	4	1	1	1	1	4	4
2	2	4	4	1	1	1	1	4	4
2	2	1	1	4	4	3	3	4	4
2	2	4	4	1	1	1	1	4	4
2	2	4	4	1	1	1	1	4	4
2	2	4	4	1	1	1	1	4	4
2	2	4	4	1	1	1	1	1	1
4	4	1	1	4	4	3	3	4	4
4	4	4	4	1	1	1	1	1	1
2	2	4	4	1	1	1	1	4	4
2	2	4	4	1	1	1	1	4	4
4	4	4	4	1	1	1	1	4	4

For Review Only

4	4	1	1	4	4	3	3	4	4
4	4	4	4	1	1	1	1	4	4

For Review Only

SNP482	SNP482	SNP483	SNP483	SNP484	SNP484	SNP485	SNP485	SNP486	SNP486
4	4	2	2	1	1	3	3	4	4
1	1	3	3	1	1	3	3	4	4
1	1	3	3	1	1	2	2	4	4
4	4	3	3	1	1	2	2	4	4
4	4	3	3	1	1	3	3	1	1
1	1	2	2	1	1	3	3	4	4
4	4	3	3	1	1	3	3	1	1
4	4	3	3	1	1	3	3	4	4
4	4	3	3	1	1	3	3	1	1
4	4	3	3	1	1	3	3	1	1
1	1	3	3	4	4	3	3	4	4
1	1	3	3	1	1	2	2	4	4
4	4	3	3	1	1	3	3	4	4
1	1	3	3	1	1	3	3	1	1
4	4	3	3	1	1	3	3	4	4
4	4	3	3	1	1	3	3	4	4
4	4	3	3	1	1	3	3	4	4
4	4	3	3	4	4	3	3	4	4
4	4	2	2	1	1	3	3	4	4
4	4	3	3	1	1	3	3	1	1
4	4	3	3	1	1	3	3	4	4
1	1	3	3	1	1	3	3	1	1
4	4	3	3	1	1	3	3	4	4
4	4	3	3	1	1	3	3	4	4
4	4	2	2	1	1	3	3	1	1
4	4	3	3	1	1	2	2	4	4
1	1	2	2	1	1	3	3	4	4
4	4	3	3	1	1	3	3	4	4
4	4	3	3	1	1	3	3	1	1
1	1	3	3	1	1	3	3	4	4
4	4	2	2	1	1	3	3	1	1
4	4	3	3	1	1	3	3	4	4
4	4	3	3	1	1	3	3	1	1
1	1	3	3	1	1	3	3	1	1
4	4	3	3	1	1	3	3	4	4
4	4	2	2	1	1	3	3	4	4
4	4	2	2	1	1	3	3	4	4
1	1	2	2	1	1	2	2	4	4
4	4	3	3	1	1	3	3	4	4
4	4	2	2	1	1	3	3	4	4
1	1	3	3	1	1	3	3	4	4
4	4	3	3	1	1	3	3	4	4
4	4	2	2	1	1	3	3	4	4
4	4	2	2	1	1	3	3	1	1
1	1	2	2	1	1	3	3	4	4
1	1	3	3	1	1	2	2	4	4
4	4	3	3	1	1	3	3	4	4
4	4	2	2	1	1	3	3	4	4
1	1	2	2	1	1	3	3	4	4
4	4	2	2	1	1	3	3	4	4
4	4	3	3	1	1	3	3	4	4
1	1	3	3	1	1	3	3	4	4

For Review Only

4	4	2	2	1	1	3	3	1	1
4	4	3	3	1	1	3	3	1	1
4	4	2	2	1	1	3	3	4	4
4	4	3	3	1	1	3	3	1	1
4	4	2	2	1	1	3	3	4	4
1	1	3	3	1	1	3	3	4	4
1	1	2	2	1	1	2	2	4	4
1	1	2	2	1	1	3	3	4	4
4	4	3	3	1	1	3	3	4	4
4	4	3	3	1	1	3	3	4	4
1	1	3	3	1	1	3	3	4	4
1	1	3	3	1	1	3	3	4	4
4	4	2	2	1	1	3	3	1	1
4	4	3	3	1	1	3	3	1	1
4	4	3	3	1	1	3	3	4	4
4	4	3	3	1	1	3	3	4	4
4	4	3	3	1	1	3	3	4	4
4	4	3	3	1	1	3	3	4	4
4	4	3	3	1	1	3	3	4	4
4	4	2	2	1	1	3	3	1	1
4	4	3	3	1	1	3	3	4	4
4	4	3	3	1	1	3	3	4	4
4	4	3	3	1	1	3	3	1	1
1	1	3	3	1	1	3	3	1	1
1	1	2	2	1	1	3	3	4	4
1	1	3	3	1	1	3	3	4	4
4	4	3	3	1	1	3	3	1	1
1	1	3	3	1	1	3	3	4	4
4	4	3	3	1	1	2	2	4	4
4	4	3	3	4	4	3	3	1	1
1	1	3	3	1	1	3	3	4	4
4	4	3	3	1	1	3	3	4	4
1	1	3	3	1	1	3	3	4	4
4	4	3	3	1	1	3	3	1	1
4	4	2	2	1	1	3	3	4	4
1	1	2	2	1	1	3	3	4	4
1	1	3	3	1	1	3	3	1	1
4	4	2	2	1	1	3	3	1	1
4	4	2	2	1	1	3	3	4	4
4	4	2	2	1	1	3	3	4	4
4	4	3	3	1	1	3	3	1	1
1	1	3	3	1	1	2	2	4	4
4	4	2	2	1	1	3	3	4	4
1	1	3	3	1	1	3	3	4	4
4	4	3	3	1	1	3	3	4	4
1	1	2	2	4	4	3	3	4	4
1	1	3	3	1	1	3	3	1	1
1	1	3	3	1	1	3	3	4	4
4	4	3	3	4	4	3	3	4	4
4	4	2	2	1	1	3	3	4	4
1	1	2	2	1	1	2	2	4	4
1	1	3	3	1	1	3	3	4	4
1	1	3	3	1	1	3	3	4	4
4	4	3	3	1	1	3	3	1	1
1	1	3	3	1	1	3	3	4	4

For Review Only

4	4	3	3	1	1	3	3	4	4
4	4	3	3	1	1	3	3	4	4
4	4	3	3	1	1	3	3	4	4
1	1	3	3	1	1	3	3	1	1
1	1	2	2	1	1	3	3	4	4
4	4	3	3	1	1	2	2	4	4
1	1	3	3	1	1	3	3	4	4
4	4	2	2	4	4	3	3	4	4
1	1	3	3	4	4	3	3	4	4
4	4	3	3	1	1	3	3	4	4
4	4	2	2	1	1	3	3	1	1
4	4	3	3	1	1	3	3	4	4
4	4	3	3	1	1	3	3	4	4
1	1	3	3	1	1	3	3	4	4
1	1	3	3	1	1	3	3	4	4
4	4	3	3	1	1	3	3	4	4
4	4	3	3	1	1	3	3	4	4
4	4	3	3	1	1	3	3	4	4
1	1	3	3	1	1	3	3	1	1
4	4	3	3	1	1	3	3	4	4
4	4	3	3	1	1	3	3	4	4
4	4	3	3	1	1	3	3	4	4
1	1	3	3	1	1	3	3	4	4
1	1	3	3	1	1	3	3	4	4
4	4	3	3	1	1	3	3	4	4
4	4	3	3	1	1	3	3	4	4
4	4	2	2	1	1	3	3	4	4
4	4	2	2	4	4	3	3	1	1
4	4	3	3	1	1	3	3	4	4
4	4	3	3	1	1	3	3	4	4
1	1	2	2	1	1	3	3	1	1
4	4	3	3	1	1	3	3	4	4
4	4	3	3	1	1	3	3	1	1
4	4	3	3	1	1	3	3	4	4
1	1	3	3	1	1	3	3	1	1
4	4	3	3	1	1	3	3	4	4
1	1	3	3	1	1	3	3	1	1
4	4	2	2	1	1	3	3	4	4
4	4	3	3	1	1	3	3	4	4
1	1	3	3	1	1	3	3	4	4
4	4	3	3	4	4	3	3	4	4
1	1	3	3	1	1	3	3	1	1
1	1	3	3	1	1	3	3	4	4
1	1	3	3	1	1	3	3	4	4
4	4	2	2	1	1	3	3	4	4
1	1	2	2	1	1	3	3	4	4
4	4	3	3	1	1	3	3	4	4
1	1	3	3	1	1	3	3	4	4
4	4	2	2	1	1	3	3	4	4
4	4	3	3	1	1	3	3	4	4
4	4	3	3	1	1	3	3	4	4
1	1	3	3	1	1	3	3	4	4
1	1	3	3	1	1	3	3	4	4
1	1	3	3	1	1	3	3	4	4
4	4	3	3	1	1	3	3	4	4

For Review Only

4	4	3	3	1	1	3	3	4	4
4	4	3	3	1	1	3	3	4	4
4	4	2	2	4	4	3	3	4	4
4	4	3	3	1	1	3	3	4	4
1	1	3	3	1	1	3	3	4	4
1	1	2	2	1	1	3	3	4	4
1	1	3	3	1	1	3	3	4	4
4	4	3	3	1	1	3	3	4	4
1	1	3	3	1	1	3	3	4	4
1	1	3	3	1	1	3	3	4	4
4	4	2	2	1	1	3	3	4	4
4	4	3	3	1	1	3	3	4	4
1	1	2	2	1	1	3	3	4	4
1	1	3	3	1	1	3	3	4	4
1	1	2	2	1	1	3	3	1	1
1	1	3	3	4	4	3	3	4	4
4	4	3	3	1	1	3	3	4	4
1	1	3	3	1	1	3	3	4	4
4	4	3	3	1	1	3	3	4	4
1	1	3	3	1	1	3	3	4	4
4	4	3	3	1	1	3	3	4	4
1	1	3	3	1	1	3	3	1	1
4	4	3	3	1	1	3	3	4	4
1	1	3	3	1	1	3	3	4	4
4	4	2	2	1	1	3	3	1	1
1	1	3	3	1	1	3	3	1	1
1	1	2	2	1	1	2	2	4	4
1	1	3	3	1	1	3	3	4	4
4	4	2	2	4	4	3	3	4	4
4	4	3	3	1	1	3	3	4	4
4	4	2	2	1	1	3	3	1	1
4	4	3	3	4	4	3	3	1	1
4	4	2	2	4	4	3	3	4	4
1	1	2	2	1	1	3	3	1	1
4	4	3	3	1	1	3	3	1	1
1	1	3	3	1	1	3	3	4	4
1	1	3	3	1	1	2	2	4	4
4	4	2	2	1	1	3	3	4	4
1	1	3	3	1	1	3	3	4	4
4	4	3	3	1	1	3	3	1	1
1	1	3	3	1	1	2	2	4	4
4	4	3	3	1	1	3	3	4	4
1	1	3	3	1	1	3	3	1	1
4	4	3	3	1	1	3	3	4	4
1	1	3	3	1	1	3	3	1	1
4	4	3	3	1	1	3	3	4	4
1	1	3	3	4	4	3	3	4	4
1	1	3	3	1	1	3	3	1	1
1	1	2	2	1	1	3	3	4	4
1	1	3	3	1	1	3	3	4	4
4	4	3	3	1	1	3	3	4	4
1	1	2	2	1	1	3	3	1	1
4	4	3	3	1	1	3	3	4	4
1	1	3	3	1	1	3	3	4	4
1	1	3	3	1	1	3	3	4	4

For Review Only

1	1	3	3	1	1	3	3	4	4
1	1	2	2	4	4	3	3	1	1
4	4	3	3	1	1	3	3	4	4
4	4	3	3	1	1	3	3	4	4
4	4	2	2	1	1	3	3	4	4
4	4	3	3	1	1	2	2	4	4
4	4	3	3	1	1	3	3	4	4
1	1	3	3	1	1	3	3	4	4
1	1	3	3	1	1	3	3	4	4
1	1	3	3	1	1	3	3	4	4
4	4	3	3	1	1	3	3	4	4
1	1	2	2	1	1	3	3	4	4
4	4	2	2	1	1	3	3	4	4
4	4	3	3	1	1	3	3	4	4
1	1	3	3	1	1	3	3	4	4
4	4	3	3	1	1	3	3	4	4
1	1	2	2	1	1	3	3	4	4
1	1	3	3	1	1	3	3	4	4
4	4	2	2	1	1	3	3	1	1
1	1	3	3	1	1	2	2	4	4
1	1	3	3	1	1	3	3	1	1
4	4	3	3	1	1	2	2	4	4
4	4	3	3	1	1	3	3	4	4
4	4	3	3	1	1	3	3	4	4
1	1	2	2	1	1	3	3	4	4
4	4	2	2	1	1	3	3	1	1
1	1	3	3	1	1	3	3	1	1
1	1	2	2	4	4	2	2	4	4
4	4	2	2	1	1	3	3	4	4
4	4	3	3	1	1	3	3	4	4
4	4	3	3	1	1	3	3	4	4
4	4	2	2	1	1	3	3	4	4
1	1	3	3	1	1	3	3	1	1
1	1	3	3	1	1	3	3	4	4
4	4	3	3	1	1	3	3	4	4
1	1	2	2	1	1	3	3	1	1
1	1	3	3	1	1	3	3	4	4
1	1	3	3	1	1	3	3	4	4
4	4	2	2	1	1	3	3	4	4
4	4	3	3	1	1	3	3	4	4
4	4	3	3	1	1	3	3	4	4
1	1	2	2	1	1	3	3	1	1
4	4	3	3	1	1	3	3	4	4
4	4	3	3	1	1	3	3	1	1
1	1	2	2	1	1	3	3	4	4
4	4	2	2	1	1	3	3	4	4
1	1	3	3	1	1	3	3	4	4
1	1	3	3	1	1	3	3	4	4
4	4	3	3	1	1	3	3	4	4
1	1	3	3	1	1	3	3	1	1
1	1	3	3	1	1	3	3	4	4
4	4	3	3	1	1	3	3	4	4
1	1	3	3	1	1	2	2	4	4
1	1	2	2	4	4	2	2	4	4
4	4	2	2	1	1	2	2	4	4

For Review Only

1	1	3	3	4	4	3	3	4	4
1	1	3	3	4	4	3	3	4	4

For Review Only

SNP487	SNP487	SNP488	SNP488	SNP489	SNP489	SNP490	SNP490	SNP491	SNP491
3	3	2	2	2	2	3	3	3	3
3	3	2	2	2	2	3	3	3	3
3	3	2	2	2	2	3	3	3	3
3	3	2	2	2	2	3	3	3	3
3	3	4	4	2	2	3	3	3	3
3	3	2	2	2	2	3	3	3	3
3	3	2	2	2	2	3	3	3	3
3	3	4	4	2	2	3	3	3	3
3	3	2	2	2	2	3	3	3	3
3	3	2	2	2	2	3	3	3	3
3	3	4	4	2	2	3	3	3	3
3	3	2	2	2	2	3	3	3	3
3	3	2	2	2	2	3	3	3	3
3	3	2	2	2	2	3	3	3	3
3	3	2	2	2	2	3	3	3	3
3	3	2	2	1	1	4	4	3	3
3	3	2	2	2	2	3	3	3	3
3	3	2	2	2	2	3	3	3	3
3	3	2	2	2	2	3	3	3	3
3	3	4	4	2	2	3	3	3	3
3	3	4	4	2	2	3	3	3	3
3	3	2	2	2	2	3	3	3	3
3	3	4	4	2	2	3	3	3	3
3	3	4	4	2	2	3	3	3	3
3	3	2	2	2	2	3	3	3	3
4	4	2	2	2	2	3	3	1	1
4	4	2	2	2	2	3	3	3	3
3	3	4	4	2	2	3	3	3	3
3	3	2	2	2	2	3	3	3	3
3	3	2	2	2	2	3	3	3	3
3	3	2	2	2	2	3	3	3	3
3	3	2	2	2	2	3	3	3	3
3	3	2	2	2	2	3	3	3	3
3	3	2	2	2	2	3	3	3	3
3	3	4	4	2	2	3	3	1	1
3	3	2	2	1	1	3	3	3	3
3	3	2	2	2	2	3	3	1	1
3	3	4	4	2	2	3	3	3	3
3	3	4	4	1	1	4	4	3	3
3	3	4	4	1	1	3	3	3	3
3	3	4	4	2	2	3	3	3	3
3	3	2	2	2	2	3	3	3	3
3	3	4	4	2	2	3	3	3	3
3	3	4	4	2	2	3	3	3	3
3	3	4	4	2	2	3	3	3	3
3	3	2	2	2	2	3	3	3	3
3	3	2	2	2	2	3	3	1	1
3	3	2	2	2	2	3	3	1	1
3	3	2	2	2	2	3	3	3	3
3	3	2	2	2	2	3	3	3	3
3	3	2	2	2	2	3	3	1	1
3	3	4	4	1	1	3	3	3	3

For Review Only

3	3	2	2	2	2	3	3	3	3
3	3	2	2	2	2	3	3	3	3
3	3	2	2	2	2	3	3	3	3
3	3	2	2	2	2	3	3	3	3
3	3	2	2	2	2	3	3	3	3
3	3	2	2	2	2	3	3	3	3
3	3	2	2	2	2	3	3	3	3
3	3	4	4	1	1	3	3	3	3
3	3	2	2	2	2	3	3	1	1
3	3	2	2	2	2	3	3	3	3
3	3	2	2	2	2	3	3	3	3
3	3	2	2	2	2	3	3	3	3
3	3	2	2	2	2	3	3	3	3
3	3	2	2	2	2	3	3	3	3
3	3	4	4	2	2	3	3	3	3
3	3	4	4	2	2	3	3	3	3
3	3	2	2	2	2	3	3	3	3
3	3	2	2	2	2	3	3	1	1
3	3	2	2	2	2	3	3	3	3
3	3	2	2	2	2	3	3	3	3
3	3	2	2	2	2	3	3	3	3
3	3	4	4	1	1	4	4	3	3
3	3	2	2	2	2	3	3	3	3
3	3	2	2	2	2	3	3	3	3
3	3	4	4	2	2	3	3	3	3
4	4	2	2	2	2	3	3	3	3
3	3	4	4	2	2	3	3	3	3
3	3	2	2	1	1	4	4	3	3
3	3	2	2	2	2	3	3	1	1
3	3	2	2	2	2	3	3	3	3
3	3	4	4	2	2	3	3	3	3
3	3	2	2	2	2	3	3	3	3
3	3	4	4	1	1	4	4	3	3
3	3	2	2	2	2	3	3	3	3
3	3	2	2	2	2	3	3	3	3
3	3	4	4	2	2	3	3	3	3
3	3	4	4	2	2	3	3	3	3
3	3	4	4	2	2	3	3	3	3
4	4	2	2	2	2	3	3	3	3
3	3	4	4	2	2	3	3	3	3
3	3	2	2	2	2	3	3	3	3
3	3	2	2	1	1	3	3	3	3
4	4	2	2	2	2	3	3	1	1
3	3	2	2	2	2	3	3	3	3
3	3	2	2	2	2	3	3	3	3
3	3	2	2	2	2	3	3	3	3
3	3	2	2	2	2	3	3	3	3
3	3	2	2	2	2	3	3	3	3
3	3	2	2	2	2	3	3	3	3
3	3	4	4	2	2	3	3	3	3
3	3	2	2	2	2	3	3	3	3
3	3	4	4	1	1	4	4	3	3
3	3	4	4	1	1	3	3	3	3
3	3	2	2	2	2	3	3	3	3
3	3	2	2	2	2	3	3	3	3

For Review Only

3	3	2	2	1	1	3	3	3	3
3	3	4	4	2	2	3	3	3	3
4	4	2	2	1	1	3	3	3	3
3	3	2	2	2	2	4	4	3	3
3	3	2	2	2	2	3	3	3	3
4	4	2	2	2	2	3	3	3	3
3	3	2	2	2	2	3	3	3	3
3	3	4	4	2	2	3	3	3	3
3	3	2	2	2	2	4	4	3	3
3	3	2	2	2	2	3	3	3	3
3	3	4	4	2	2	3	3	3	3
3	3	4	4	2	2	3	3	3	3
4	4	2	2	2	2	3	3	3	3
3	3	2	2	2	2	3	3	3	3
3	3	2	2	2	2	3	3	3	3
3	3	4	4	2	2	3	3	1	1
3	3	2	2	2	2	3	3	3	3
3	3	2	2	2	2	3	3	3	3
3	3	2	2	2	2	4	4	3	3
3	3	4	4	2	2	3	3	3	3
3	3	4	4	2	2	3	3	3	3
3	3	2	2	2	2	3	3	3	3
3	3	2	2	2	2	3	3	3	3
3	3	2	2	1	1	4	4	3	3
3	3	2	2	2	2	3	3	3	3
3	3	4	4	2	2	3	3	3	3
3	3	2	2	2	2	3	3	3	3
3	3	4	4	2	2	3	3	3	3
3	3	2	2	2	2	3	3	3	3
3	3	4	4	2	2	3	3	3	3
3	3	2	2	1	1	4	4	3	3
3	3	4	4	2	2	3	3	3	3
3	3	2	2	2	2	3	3	3	3
3	3	4	4	2	2	3	3	3	3
3	3	2	2	2	2	3	3	3	3
3	3	2	2	2	2	3	3	3	3
3	3	2	2	2	2	4	4	3	3
3	3	4	4	2	2	3	3	3	3
4	4	2	2	2	2	3	3	3	3
3	3	4	4	1	1	4	4	3	3
3	3	4	4	1	1	3	3	3	3
3	3	2	2	2	2	3	3	3	3
3	3	2	2	1	1	3	3	3	3
3	3	4	4	2	2	3	3	3	3
3	3	2	2	1	1	3	3	1	1
3	3	2	2	1	1	3	3	3	3
3	3	2	2	2	2	3	3	1	1
3	3	4	4	2	2	3	3	3	3
3	3	4	4	1	1	3	3	1	1
4	4	2	2	2	2	3	3	3	3
3	3	2	2	2	2	3	3	1	1
3	3	4	4	2	2	3	3	3	3
3	3	2	2	1	1	3	3	3	3
3	3	2	2	2	2	3	3	3	3
3	3	4	4	2	2	3	3	3	3

For Review Only

3	3	2	2	2	2	3	3	1	1
3	3	4	4	1	1	3	3	1	1
3	3	4	4	2	2	3	3	3	3
3	3	4	4	2	2	3	3	3	3
3	3	2	2	2	2	3	3	3	3
3	3	2	2	1	1	3	3	3	3
3	3	4	4	2	2	3	3	3	3
3	3	2	2	2	2	3	3	3	3
3	3	4	4	2	2	3	3	3	3
3	3	2	2	2	2	3	3	3	3
3	3	2	2	2	2	3	3	1	1
3	3	2	2	1	1	3	3	3	3
3	3	2	2	2	2	3	3	3	3
3	3	2	2	2	2	3	3	3	3
3	3	2	2	1	1	3	3	3	3
3	3	4	4	1	1	3	3	3	3
3	3	2	2	2	2	3	3	3	3
3	3	2	2	2	2	3	3	3	3
4	4	2	2	2	2	3	3	3	3
3	3	2	2	2	2	3	3	3	3
3	3	2	2	2	2	3	3	3	3
3	3	4	4	1	1	3	3	3	3
3	3	4	4	1	1	4	4	3	3
3	3	2	2	2	2	3	3	3	3
3	3	2	2	2	2	3	3	3	3
3	3	2	2	2	2	3	3	3	3
3	3	2	2	2	2	3	3	3	3
3	3	2	2	2	2	3	3	3	3
4	4	2	2	2	2	3	3	3	3
3	3	2	2	2	2	3	3	3	3
3	3	2	2	2	2	3	3	1	1
3	3	2	2	2	2	3	3	1	1
3	3	2	2	2	2	3	3	1	1
3	3	2	2	1	1	3	3	3	3
3	3	2	2	2	2	3	3	3	3
3	3	2	2	2	2	3	3	3	3
3	3	2	2	1	1	3	3	3	3
3	3	2	2	2	2	3	3	3	3
3	3	4	4	2	2	3	3	3	3
3	3	2	2	2	2	3	3	3	3
3	3	2	2	2	2	3	3	3	3
3	3	2	2	2	2	3	3	1	1
3	3	4	4	1	1	4	4	3	3
3	3	4	4	2	2	3	3	3	3
3	3	2	2	2	2	4	4	3	3
3	3	2	2	2	2	3	3	3	3
3	3	4	4	1	1	3	3	3	3
3	3	4	4	2	2	4	4	3	3
3	3	4	4	2	2	3	3	3	3
4	4	2	2	2	2	3	3	3	3
3	3	2	2	2	2	3	3	3	3
3	3	4	4	1	1	3	3	3	3
3	3	4	4	1	1	4	4	3	3
3	3	2	2	2	2	3	3	3	3
3	3	4	4	1	1	4	4	3	3

For Review Only

3	3	2	2	1	1	3	3	3	3
3	3	4	4	2	2	3	3	3	3
3	3	2	2	2	2	3	3	1	1
3	3	4	4	2	2	3	3	3	3
3	3	2	2	2	2	3	3	3	3
3	3	2	2	2	2	3	3	1	1
3	3	2	2	1	1	3	3	3	3
3	3	4	4	2	2	3	3	3	3
3	3	2	2	2	2	3	3	3	3
3	3	2	2	1	1	4	4	3	3
3	3	2	2	2	2	3	3	3	3
4	4	2	2	2	2	3	3	3	3
4	4	2	2	2	2	3	3	3	3
4	4	2	2	2	2	3	3	1	1
3	3	2	2	2	2	3	3	3	3
3	3	2	2	2	2	3	3	3	3
3	3	4	4	1	1	4	4	3	3
3	3	2	2	2	2	3	3	3	3
3	3	2	2	1	1	4	4	3	3
3	3	4	4	2	2	3	3	3	3
3	3	4	4	2	2	4	4	3	3
3	3	2	2	2	2	3	3	1	1
3	3	2	2	2	2	3	3	1	1
3	3	2	2	1	1	3	3	3	3
3	3	4	4	1	1	3	3	3	3
3	3	2	2	2	2	3	3	3	3
3	3	4	4	1	1	4	4	3	3
3	3	2	2	2	2	3	3	3	3
3	3	2	2	2	2	3	3	3	3
3	3	2	2	2	2	3	3	3	3
3	3	4	4	1	1	3	3	3	3
3	3	2	2	2	2	3	3	1	1
3	3	2	2	2	2	3	3	3	3
3	3	4	4	2	2	3	3	3	3
3	3	4	4	1	1	4	4	3	3
3	3	4	4	1	1	4	4	3	3
3	3	2	2	2	2	3	3	3	3
3	3	2	2	2	2	3	3	3	3
3	3	2	2	1	1	3	3	3	3
3	3	4	4	1	1	4	4	3	3
3	3	2	2	2	2	3	3	3	3
3	3	2	2	2	2	3	3	3	3
3	3	2	2	1	1	3	3	3	3
3	3	2	2	2	2	3	3	3	3
3	3	2	2	2	2	3	3	3	3
3	3	2	2	2	2	3	3	3	3
3	3	4	4	1	1	3	3	3	3
3	3	2	2	2	2	3	3	3	3
3	3	2	2	2	2	3	3	3	3
3	3	4	4	1	1	4	4	3	3
3	3	2	2	2	2	3	3	3	3
3	3	4	4	1	1	4	4	3	3
3	3	2	2	2	2	3	3	3	3
3	3	2	2	2	2	3	3	3	3
3	3	4	4	1	1	3	3	3	3

For Review Only

CSSA 5585 Guilford Rd., Madison, WI 53711

3	3	2	2	2	2	3	3	3	3
3	3	2	2	2	2	3	3	3	3

For Review Only

SNP492	SNP492	SNP493	SNP493	SNP494	SNP494	SNP495	SNP495	SNP496	SNP496
4	4	2	2	3	3	3	3	2	2
4	4	2	2	3	3	3	3	2	2
4	4	2	2	3	3	3	3	2	2
4	4	2	2	3	3	3	3	2	2
4	4	2	2	3	3	3	3	3	3
4	4	1	1	3	3	3	3	2	2
4	4	2	2	3	3	3	3	2	2
4	4	2	2	3	3	3	3	3	3
4	4	2	2	3	3	2	2	2	2
4	4	2	2	3	3	3	3	3	3
4	4	2	2	3	3	3	3	3	3
4	4	2	2	3	3	3	3	2	2
4	4	2	2	3	3	3	3	2	2
1	1	2	2	3	3	2	2	2	2
4	4	1	1	3	3	3	3	2	2
4	4	2	2	3	3	3	3	2	2
4	4	2	2	3	3	3	3	2	2
4	4	2	2	3	3	2	2	2	2
4	4	2	2	3	3	3	3	2	2
4	4	2	2	3	3	3	3	3	3
4	4	2	2	3	3	3	3	2	2
4	4	2	2	3	3	3	3	2	2
1	1	2	2	3	3	3	3	3	3
4	4	2	2	3	3	2	2	3	3
4	4	1	1	3	3	3	3	2	2
4	4	1	1	3	3	3	3	2	2
4	4	2	2	3	3	3	3	2	2
4	4	2	2	3	3	3	3	3	3
4	4	2	2	3	3	3	3	2	2
4	4	2	2	3	3	2	2	2	2
4	4	1	1	3	3	3	3	2	2
4	4	2	2	3	3	3	3	2	2
4	4	2	2	3	3	3	3	2	2
1	1	2	2	3	3	2	2	2	2
1	1	2	2	3	3	3	3	3	3
4	4	2	2	3	3	2	2	2	2
4	4	1	1	3	3	2	2	2	2
4	4	2	2	3	3	3	3	3	3
4	4	2	2	3	3	3	3	3	3
1	1	2	2	3	3	3	3	3	3
4	4	2	2	3	3	3	3	3	3
4	4	2	2	3	3	3	3	2	2
4	4	2	2	3	3	3	3	3	3
4	4	2	2	3	3	2	2	3	3
4	4	2	2	3	3	2	2	3	3
4	4	2	2	3	3	3	3	2	2
4	4	1	1	3	3	3	3	2	2
4	4	1	1	3	3	3	3	2	2
4	4	2	2	3	3	2	2	2	2
4	4	2	2	3	3	3	3	2	2
4	4	1	1	3	3	2	2	2	2
4	4	2	2	3	3	3	3	3	3

For Review Only

4	4	2	2	3	3	3	3	2	2
4	4	2	2	3	3	3	3	2	2
4	4	1	1	3	3	3	3	2	2
4	4	2	2	3	3	3	3	2	2
4	4	2	2	3	3	3	3	2	2
4	4	1	1	3	3	3	3	2	2
4	4	2	2	3	3	3	3	2	2
4	4	2	2	3	3	2	2	3	3
4	4	1	1	3	3	2	2	2	2
4	4	2	2	3	3	3	3	2	2
4	4	1	1	3	3	2	2	2	2
4	4	1	1	3	3	2	2	2	2
4	4	2	2	3	3	3	3	2	2
4	4	2	2	3	3	3	3	2	2
4	4	1	1	3	3	3	3	3	3
4	4	2	2	3	3	2	2	2	2
1	1	2	2	3	3	2	2	3	3
4	4	1	1	3	3	3	3	2	2
4	4	2	2	3	3	3	3	2	2
4	4	2	2	3	3	3	3	2	2
4	4	1	1	3	3	2	2	2	2
4	4	2	2	3	3	3	3	3	3
4	4	2	2	3	3	3	3	3	3
4	4	1	1	3	3	3	3	2	2
4	4	2	2	3	3	2	2	3	3
4	4	2	2	3	3	2	2	2	2
4	4	2	2	3	3	3	3	3	3
1	1	2	2	3	3	2	2	2	2
4	4	1	1	3	3	3	3	2	2
4	4	2	2	3	3	3	3	2	2
4	4	2	2	3	3	3	3	3	3
4	4	2	2	3	3	2	2	2	2
1	1	2	2	3	3	2	2	3	3
4	4	2	2	3	3	3	3	2	2
4	4	2	2	3	3	2	2	2	2
4	4	2	2	3	3	2	2	3	3
4	4	2	2	3	3	3	3	3	3
4	4	2	2	3	3	3	3	3	3
4	4	2	2	3	3	2	2	2	2
4	4	2	2	3	3	2	2	3	3
4	4	2	2	3	3	3	3	2	2
4	4	2	2	3	3	3	3	2	2
4	4	1	1	3	3	2	2	2	2
4	4	1	1	3	3	2	2	2	2
4	4	2	2	3	3	2	2	2	2
4	4	1	1	3	3	3	3	2	2
1	1	2	2	2	2	2	2	2	2
4	4	2	2	3	3	2	2	2	2
4	4	2	2	3	3	3	3	2	2
4	4	2	2	3	3	3	3	3	3
4	4	1	1	3	3	3	3	2	2
4	4	2	2	3	3	2	2	3	3
4	4	2	2	3	3	2	2	3	3
4	4	2	2	3	3	2	2	3	3
4	4	2	2	3	3	2	2	2	2

For Review Only

4	4	1	1	3	3	3	3	2	2
4	4	2	2	3	3	3	3	3	3
4	4	2	2	3	3	2	2	2	2
1	1	2	2	3	3	2	2	2	2
4	4	1	1	3	3	3	3	2	2
4	4	2	2	3	3	3	3	2	2
4	4	2	2	3	3	3	3	2	2
4	4	1	1	3	3	3	3	2	2
4	4	2	2	3	3	2	2	2	2
4	4	2	2	3	3	2	2	2	2
4	4	2	2	3	3	3	3	2	2
4	4	2	2	3	3	3	3	3	3
4	4	1	1	3	3	2	2	2	2
4	4	2	2	3	3	2	2	2	2
4	4	1	1	3	3	3	3	2	2
4	4	1	1	3	3	3	3	2	2
4	4	1	1	3	3	3	3	2	2
4	4	1	1	3	3	3	3	2	2
1	1	2	2	2	2	2	2	2	2
4	4	2	2	3	3	3	3	3	3
4	4	1	1	3	3	3	3	3	3
4	4	2	2	3	3	3	3	2	2
4	4	2	2	3	3	2	2	2	2
4	4	2	2	3	3	2	2	2	2
4	4	1	1	3	3	3	3	3	3
4	4	2	2	2	2	3	3	3	3
4	4	1	1	3	3	3	3	2	2
4	4	2	2	3	3	2	2	2	2
4	4	1	1	3	3	3	3	2	2
4	4	1	1	3	3	3	3	3	3
4	4	2	2	3	3	2	2	2	2
4	4	2	2	3	3	2	2	3	3
4	4	2	2	3	3	3	3	2	2
4	4	1	1	3	3	3	3	2	2
1	1	2	2	3	3	2	2	2	2
4	4	2	2	3	3	3	3	2	2
1	1	2	2	2	2	2	2	2	2
4	4	1	1	3	3	3	3	2	2
4	4	2	2	3	3	3	3	2	2
1	1	2	2	3	3	3	3	3	3
4	4	2	2	3	3	3	3	3	3
1	1	2	2	3	3	2	2	2	2
4	4	2	2	3	3	2	2	2	2
4	4	2	2	2	2	3	3	3	3
4	4	1	1	3	3	3	3	2	2
4	4	2	2	3	3	2	2	2	2
4	4	1	1	3	3	3	3	2	2
4	4	2	2	3	3	3	3	3	3
4	4	2	2	3	3	2	2	3	3
4	4	1	1	3	3	3	3	2	2
4	4	1	1	3	3	3	3	2	2
4	4	2	2	2	2	3	3	3	3
1	1	2	2	3	3	3	3	2	2
4	4	1	1	3	3	2	2	2	2
4	4	2	2	3	3	3	3	3	3

For Review Only

4	4	1	1	3	3	3	3	2	2
4	4	1	1	3	3	2	2	3	3
4	4	1	1	3	3	2	2	3	3
4	4	2	2	3	3	3	3	3	3
4	4	1	1	3	3	3	3	2	2
4	4	2	2	3	3	3	3	2	2
1	1	2	2	3	3	3	3	3	3
4	4	2	2	3	3	3	3	2	2
4	4	2	2	3	3	2	2	3	3
4	4	2	2	3	3	3	3	2	2
4	4	1	1	3	3	3	3	2	2
4	4	2	2	3	3	3	3	2	2
4	4	2	2	3	3	3	3	2	2
4	4	2	2	3	3	2	2	2	2
1	1	2	2	3	3	2	2	2	2
1	1	2	2	3	3	3	3	3	3
1	1	2	2	3	3	3	3	2	2
4	4	1	1	3	3	3	3	2	2
4	4	2	2	3	3	2	2	2	2
4	4	2	2	3	3	3	3	2	2
4	4	1	1	3	3	3	3	2	2
1	1	2	2	3	3	3	3	3	3
4	4	2	2	2	2	3	3	3	3
4	4	1	1	3	3	2	2	2	2
4	4	2	2	3	3	2	2	2	2
1	1	2	2	3	3	2	2	2	2
4	4	2	2	3	3	3	3	2	2
4	4	2	2	3	3	3	3	2	2
4	4	2	2	3	3	3	3	2	2
4	4	2	2	3	3	3	3	2	2
4	4	1	1	3	3	2	2	2	2
4	4	1	1	3	3	3	3	2	2
4	4	1	1	3	3	2	2	2	2
4	4	2	2	3	3	2	2	2	2
4	4	2	2	3	3	3	3	2	2
4	4	1	1	3	3	2	2	2	2
4	4	2	2	3	3	3	3	2	2
4	4	2	2	3	3	2	2	2	2
4	4	2	2	3	3	3	3	3	3
4	4	1	1	3	3	3	3	2	2
4	4	2	2	3	3	3	3	2	2
4	4	1	1	3	3	3	3	2	2
1	1	2	2	3	3	2	2	3	3
4	4	2	2	3	3	3	3	3	3
4	4	2	2	3	3	2	2	2	2
4	4	1	1	3	3	2	2	2	2
1	1	2	2	3	3	3	3	3	3
1	1	2	2	3	3	2	2	3	3
4	4	2	2	3	3	2	2	3	3
4	4	2	2	3	3	3	3	2	2
4	4	1	1	3	3	3	3	2	2
4	4	2	2	3	3	2	2	3	3
4	4	2	2	2	2	2	2	3	3
4	4	1	1	3	3	2	2	2	2
1	1	2	2	3	3	2	2	3	3

For Review Only

1	1	2	2	3	3	3	3	2	2
4	4	1	1	3	3	3	3	2	2
4	4	1	1	3	3	3	3	2	2
4	4	2	2	3	3	3	3	3	3
4	4	2	2	3	3	3	3	2	2
4	4	1	1	3	3	3	3	2	2
1	1	2	2	3	3	2	2	2	2
4	4	2	2	3	3	2	2	3	3
1	1	2	2	3	3	2	2	2	2
1	1	2	2	3	3	2	2	2	2
4	4	2	2	3	3	3	3	2	2
4	4	1	1	3	3	3	3	2	2
4	4	2	2	3	3	3	3	2	2
4	4	1	1	3	3	3	3	2	2
4	4	2	2	3	3	3	3	2	2
1	1	2	2	3	3	3	3	2	2
1	1	2	2	3	3	2	2	3	3
4	4	2	2	3	3	3	3	2	2
1	1	2	2	3	3	2	2	2	2
4	4	2	2	3	3	3	3	3	3
4	4	1	1	3	3	3	3	3	3
4	4	1	1	3	3	3	3	2	2
4	4	1	1	3	3	3	3	2	2
1	1	2	2	3	3	2	2	2	2
4	4	2	2	3	3	2	2	3	3
4	4	2	2	3	3	3	3	2	2
4	4	2	2	3	3	2	2	3	3
4	4	2	2	3	3	3	3	2	2
4	4	2	2	3	3	3	3	2	2
4	4	1	1	3	3	3	3	2	2
1	1	2	2	3	3	2	2	3	3
1	1	1	1	3	3	3	3	2	2
1	1	2	2	3	3	2	2	2	2
4	4	2	2	3	3	3	3	3	3
4	4	2	2	2	2	3	3	3	3
1	1	2	2	3	3	2	2	3	3
4	4	2	2	3	3	3	3	2	2
4	4	1	1	3	3	2	2	2	2
4	4	2	2	3	3	2	2	2	2
1	1	2	2	3	3	2	2	3	3
4	4	2	2	3	3	3	3	2	2
4	4	2	2	3	3	2	2	2	2
1	1	2	2	3	3	3	3	2	2
4	4	2	2	3	3	3	3	2	2
4	4	2	2	3	3	2	2	2	2
4	4	2	2	3	3	2	2	2	2
4	4	2	2	3	3	3	3	3	3
4	4	2	2	3	3	2	2	2	2
4	4	1	1	3	3	2	2	2	2
4	4	2	2	3	3	2	2	3	3
4	4	2	2	3	3	3	3	2	2
1	1	2	2	3	3	2	2	3	3
4	4	2	2	3	3	3	3	2	2
4	4	2	2	3	3	3	3	2	2
1	1	2	2	3	3	3	3	3	3

For Review Only

4	4	2	2	3	3	3	3	2	2
4	4	1	1	3	3	3	3	2	2

For Review Only

SNP497	SNP497	SNP498	SNP498	SNP499	SNP499	SNP500	SNP500	SNP501	SNP501
4	4	3	3	3	3	4	4	3	3
4	4	3	3	3	3	1	1	3	3
4	4	3	3	3	3	1	1	3	3
4	4	3	3	3	3	1	1	3	3
4	4	3	3	3	3	4	4	3	3
4	4	2	2	3	3	1	1	2	2
4	4	3	3	3	3	4	4	3	3
4	4	3	3	3	3	1	1	3	3
4	4	3	3	3	3	4	4	3	3
4	4	3	3	3	3	4	4	3	3
4	4	3	3	3	3	4	4	3	3
4	4	3	3	3	3	1	1	3	3
4	4	3	3	3	3	4	4	3	3
4	4	3	3	3	3	4	4	3	3
4	4	3	3	3	3	1	1	3	3
4	4	3	3	3	3	4	4	3	3
4	4	3	3	3	3	1	1	3	3
4	4	3	3	3	3	1	1	3	3
4	4	3	3	3	3	1	1	3	3
4	4	3	3	3	3	4	4	3	3
4	4	3	3	3	3	4	4	3	3
4	4	3	3	3	3	4	4	3	3
1	1	3	3	3	3	4	4	3	3
4	4	3	3	3	3	1	1	3	3
4	4	2	2	3	3	4	4	2	2
4	4	3	3	2	2	1	1	3	3
4	4	3	3	3	3	1	1	3	3
4	4	3	3	3	3	4	4	3	3
4	4	3	3	3	3	4	4	3	3
4	4	3	3	3	3	1	1	3	3
4	4	2	2	3	3	4	4	2	2
4	4	3	3	3	3	1	1	3	3
4	4	3	3	3	3	4	4	3	3
4	4	3	3	3	3	4	4	3	3
4	4	3	3	3	3	1	1	3	3
4	4	3	3	3	3	4	4	3	3
4	4	3	3	3	3	1	1	3	3
4	4	3	3	3	3	4	4	3	3
4	4	3	3	3	3	1	1	3	3
1	1	3	3	3	3	1	1	3	3
4	4	3	3	3	3	1	1	3	3
4	4	3	3	3	3	1	1	3	3
4	4	3	3	3	3	1	1	3	3
4	4	3	3	3	3	4	4	3	3
4	4	3	3	3	3	1	1	3	3
4	4	3	3	3	3	1	1	3	3
4	4	3	3	2	2	1	1	3	3
4	4	3	3	2	2	1	1	3	3
4	4	3	3	3	3	1	1	3	3
4	4	3	3	3	3	1	1	3	3
4	4	3	3	2	2	1	1	3	3
4	4	3	3	3	3	4	4	3	3

For Review Only

4	4	3	3	3	3	4	4	3	3
4	4	3	3	3	3	4	4	3	3
4	4	3	3	3	3	1	1	3	3
4	4	3	3	3	3	4	4	3	3
4	4	3	3	3	3	1	1	3	3
4	4	2	2	3	3	1	1	2	2
4	4	3	3	3	3	1	1	3	3
4	4	3	3	3	3	1	1	3	3
4	4	3	3	3	3	4	4	3	3
4	4	3	3	3	3	4	4	3	3
4	4	2	2	3	3	1	1	2	2
4	4	2	2	3	3	1	1	2	2
4	4	3	3	3	3	4	4	3	3
4	4	3	3	3	3	4	4	3	3
4	4	3	3	3	3	4	4	2	2
4	4	3	3	3	3	4	4	3	3
4	4	3	3	3	3	4	4	3	3
4	4	3	3	2	2	1	1	3	3
4	4	3	3	3	3	1	1	3	3
4	4	3	3	3	3	4	4	3	3
4	4	2	2	3	3	1	1	3	3
1	1	3	3	3	3	4	4	3	3
4	4	3	3	3	3	4	4	3	3
4	4	2	2	3	3	4	4	2	2
4	4	3	3	3	3	1	1	3	3
4	4	3	3	3	3	4	4	3	3
4	4	3	3	3	3	4	4	3	3
4	4	3	3	3	3	4	4	3	3
4	4	3	3	2	2	1	1	3	3
1	1	3	3	3	3	4	4	3	3
4	4	3	3	3	3	4	4	3	3
4	4	3	3	3	3	4	4	3	3
4	4	3	3	3	3	1	1	3	3
4	4	3	3	3	3	4	4	3	3
4	4	3	3	3	3	4	4	3	3
4	4	3	3	3	3	1	1	3	3
4	4	3	3	3	3	4	4	3	3
4	4	3	3	3	3	4	4	3	3
1	1	3	3	3	3	4	4	3	3
4	4	3	3	3	3	1	1	3	3
4	4	3	3	3	3	4	4	3	3
1	1	3	3	3	3	1	1	3	3
4	4	3	3	2	2	1	1	3	3
4	4	2	2	3	3	1	1	2	2
1	1	3	3	3	3	1	1	3	3
4	4	2	2	3	3	4	4	2	2
4	4	3	3	3	3	4	4	3	3
4	4	3	3	3	3	4	4	3	3
4	4	3	3	3	3	4	4	3	3
4	4	3	3	3	3	1	1	3	3
4	4	3	3	3	3	1	1	2	2
4	4	3	3	3	3	4	4	3	3
4	4	3	3	3	3	1	1	3	3
4	4	3	3	3	3	4	4	3	3
4	4	3	3	3	3	1	1	3	3

For Review Only

4	4	3	3	3	3	1	1	3	3
4	4	3	3	3	3	1	1	3	3
4	4	3	3	3	3	4	4	3	3
4	4	3	3	3	3	4	4	3	3
4	4	2	2	3	3	1	1	2	2
4	4	3	3	3	3	1	1	3	3
4	4	3	3	3	3	1	1	3	3
4	4	3	3	3	3	4	4	2	2
4	4	3	3	3	3	1	1	3	3
4	4	3	3	2	2	4	4	3	3
4	4	3	3	3	3	4	4	3	3
4	4	3	3	3	3	4	4	3	3
4	4	2	2	3	3	1	1	2	2
4	4	3	3	3	3	4	4	3	3
4	4	3	3	3	3	1	1	3	3
4	4	3	3	3	3	1	1	3	3
4	4	3	3	3	3	1	1	3	3
4	4	3	3	3	3	4	4	3	3
4	4	3	3	3	3	4	4	3	3
4	4	3	3	3	3	1	1	3	3
4	4	3	3	3	3	4	4	3	3
1	1	3	3	3	3	4	4	3	3
4	4	3	3	3	3	1	1	3	3
4	4	3	3	3	3	4	4	3	3
4	4	3	3	3	3	1	1	3	3
4	4	3	3	3	3	1	1	3	3
4	4	3	3	3	3	4	4	2	2
4	4	3	3	3	3	4	4	3	3
4	4	2	2	3	3	1	1	2	2
4	4	3	3	3	3	4	4	3	3
4	4	3	3	3	3	4	4	3	3
4	4	3	3	3	3	4	4	3	3
4	4	3	3	3	3	4	4	3	3
4	4	2	2	3	3	4	4	2	2
4	4	3	3	3	3	4	4	3	3
4	4	3	3	3	3	4	4	3	3
4	4	3	3	3	3	4	4	3	3
4	4	2	2	3	3	1	1	2	2
4	4	3	3	3	3	1	1	3	3
4	4	3	3	3	3	1	1	3	3
1	1	3	3	3	3	1	1	3	3
4	4	3	3	3	3	4	4	3	3
4	4	3	3	3	3	1	1	3	3
4	4	3	3	3	3	1	1	3	3
4	4	3	3	3	3	4	4	3	3
4	4	3	3	3	3	1	1	3	3
4	4	3	3	2	2	1	1	3	3
4	4	3	3	3	3	1	1	3	3
4	4	3	3	3	3	4	4	3	3
4	4	2	2	3	3	1	1	2	2
4	4	3	3	2	2	1	1	3	3
4	4	3	3	3	3	1	1	3	3
4	4	3	3	3	3	1	1	3	3
4	4	2	2	3	3	1	1	2	2
4	4	3	3	3	3	4	4	3	3

For Review Only

4	4	3	3	2	2	1	1	3	3
4	4	3	3	2	2	1	1	3	3
4	4	3	3	3	3	1	1	3	3
4	4	3	3	3	3	4	4	3	3
4	4	3	3	3	3	1	1	3	3
4	4	3	3	3	3	1	1	3	3
4	4	3	3	3	3	4	4	3	3
4	4	3	3	3	3	1	1	3	3
4	4	3	3	3	3	1	1	3	3
1	1	3	3	3	3	1	1	3	3
4	4	3	3	2	2	1	1	3	3
4	4	3	3	3	3	4	4	3	3
4	4	3	3	3	3	1	1	2	2
4	4	3	3	3	3	4	4	3	3
4	4	3	3	3	3	4	4	3	3
4	4	3	3	3	3	1	1	3	3
4	4	3	3	3	3	4	4	3	3
4	4	2	2	3	3	4	4	2	2
4	4	3	3	3	3	1	1	3	3
4	4	3	3	3	3	1	1	3	3
4	4	3	3	3	3	4	4	3	3
4	4	3	3	3	3	4	4	3	3
1	1	3	3	3	3	4	4	3	3
4	4	2	2	3	3	1	1	2	2
4	4	3	3	3	3	4	4	3	3
4	4	3	3	3	3	4	4	3	3
4	4	3	3	3	3	1	1	3	3
4	4	3	3	3	3	4	4	3	3
4	4	3	3	3	3	1	1	3	3
4	4	3	3	3	3	1	1	3	3
4	4	3	3	3	3	4	4	3	3
1	1	3	3	2	2	4	4	3	3
4	4	3	3	3	3	4	4	3	3
4	4	3	3	3	3	4	4	3	3
4	4	3	3	3	3	4	4	3	3
4	4	2	2	3	3	1	1	2	2
4	4	3	3	3	3	1	1	3	3
4	4	3	3	3	3	1	1	3	3
4	4	3	3	3	3	1	1	3	3
4	4	2	2	3	3	4	4	2	2
4	4	3	3	3	3	1	1	3	3
4	4	3	3	2	2	1	1	3	3
4	4	3	3	3	3	4	4	3	3
4	4	3	3	3	3	4	4	3	3
4	4	3	3	3	3	1	1	3	3
4	4	3	3	3	3	1	1	3	3
4	4	3	3	3	3	1	1	3	3
4	4	3	3	3	3	4	4	3	3
4	4	3	3	3	3	1	1	3	3
4	4	3	3	3	3	1	1	3	3
4	4	2	2	3	3	1	1	2	2
4	4	3	3	3	3	4	4	3	3
1	1	3	3	3	3	4	4	3	3
4	4	2	2	3	3	1	1	2	2
4	4	3	3	3	3	4	4	3	3

For Review Only

4	4	3	3	3	3	1	1	3	3
4	4	2	2	3	3	4	4	2	2
1	1	3	3	2	2	1	1	3	3
1	1	3	3	3	3	1	1	3	3
4	4	3	3	3	3	1	1	3	3
4	4	3	3	2	2	4	4	3	3
4	4	3	3	3	3	1	1	3	3
4	4	3	3	3	3	1	1	3	3
4	4	3	3	3	3	1	1	3	3
4	4	3	3	3	3	4	4	3	3
4	4	3	3	3	3	4	4	3	3
4	4	3	3	3	3	1	1	3	3
1	1	3	3	3	3	4	4	3	3
4	4	3	3	2	2	1	1	3	3
4	4	3	3	3	3	1	1	3	3
1	1	3	3	3	3	4	4	3	3
4	4	3	3	3	3	1	1	3	3
1	1	3	3	3	3	4	4	3	3
4	4	3	3	3	3	4	4	3	3
4	4	3	3	3	3	4	4	3	3
4	4	2	2	3	3	4	4	2	2
4	4	3	3	2	2	1	1	3	3
1	1	3	3	2	2	4	4	3	3
4	4	3	3	3	3	1	1	3	3
4	4	3	3	3	3	1	1	3	3
4	4	3	3	3	3	4	4	3	3
4	4	3	3	3	3	4	4	3	3
4	4	3	3	3	3	1	1	3	3
4	4	3	3	3	3	4	4	3	3
4	4	2	2	3	3	1	1	2	2
4	4	3	3	3	3	1	1	3	3
4	4	3	3	3	3	1	1	3	3
4	4	3	3	3	3	4	4	3	3
4	4	3	3	3	3	1	1	3	3
1	1	3	3	3	3	1	1	3	3
4	4	3	3	3	3	4	4	3	3
4	4	3	3	3	3	1	1	3	3
4	4	2	2	3	3	1	1	2	2
4	4	3	3	3	3	4	4	3	3
4	4	3	3	3	3	1	1	3	3
1	1	3	3	3	3	4	4	3	3
4	4	3	3	3	3	4	4	3	3
4	4	3	3	3	3	1	1	3	3
4	4	3	3	3	3	4	4	3	3
4	4	3	3	3	3	1	1	3	3
1	1	3	3	3	3	4	4	3	3
4	4	3	3	3	3	1	1	3	3
4	4	2	2	3	3	4	4	2	2
4	4	3	3	3	3	1	1	3	3
4	4	3	3	3	3	4	4	3	3
1	1	3	3	3	3	4	4	3	3
4	4	3	3	3	3	1	1	3	3
4	4	3	3	3	3	1	1	3	3
4	4	3	3	3	3	1	1	3	3
4	4	3	3	3	3	1	1	3	3

For Review Only

1	1	3	3	3	3	4	4	3	3
4	4	2	2	3	3	1	1	2	2

For Review Only

SNP502	SNP502	SNP503	SNP503	SNP504	SNP504	SNP505	SNP505	SNP506	SNP506
1	1	1	1	1	1	2	2	1	1
4	4	1	1	2	2	2	2	3	3
1	1	1	1	1	1	2	2	3	3
1	1	1	1	1	1	2	2	3	3
1	1	1	1	1	1	2	2	3	3
1	1	1	1	2	2	2	2	1	1
1	1	1	1	1	1	2	2	3	3
1	1	1	1	1	1	2	2	1	1
1	1	1	1	1	1	2	2	1	1
4	4	1	1	1	1	2	2	1	1
4	4	1	1	2	2	2	2	3	3
1	1	1	1	1	1	2	2	3	3
4	4	1	1	2	2	2	2	3	3
1	1	1	1	2	2	2	2	1	1
1	1	1	1	2	2	2	2	3	3
1	1	1	1	1	1	2	2	3	3
4	4	1	1	2	2	2	2	1	1
4	4	1	1	1	1	2	2	3	3
1	1	1	1	1	1	2	2	1	1
4	4	1	1	2	2	2	2	3	3
4	4	1	1	1	1	2	2	1	1
4	4	1	1	2	2	2	2	3	3
4	4	4	4	2	2	2	2	3	3
1	1	1	1	1	1	2	2	1	1
1	1	1	1	2	2	2	2	1	1
1	1	1	1	2	2	3	3	3	3
1	1	1	1	2	2	2	2	1	1
4	4	1	1	2	2	2	2	3	3
4	4	1	1	1	1	2	2	1	1
4	4	1	1	2	2	2	2	3	3
1	1	1	1	2	2	2	2	1	1
1	1	1	1	1	1	2	2	1	1
1	1	1	1	2	2	2	2	1	1
4	4	1	1	2	2	2	2	1	1
4	4	1	1	2	2	3	3	3	3
1	1	1	1	1	1	2	2	1	1
1	1	1	1	1	1	2	2	3	3
1	1	1	1	2	2	2	2	3	3
4	4	1	1	2	2	2	2	3	3
1	1	4	4	2	2	2	2	3	3
4	4	1	1	2	2	2	2	3	3
4	4	1	1	2	2	2	2	3	3
1	1	1	1	2	2	2	2	1	1
1	1	1	1	2	2	2	2	3	3
1	1	1	1	2	2	2	2	1	1
1	1	1	1	1	1	2	2	3	3
1	1	1	1	2	2	3	3	3	3
4	4	1	1	2	2	3	3	3	3
1	1	1	1	2	2	2	2	3	3
4	4	4	4	1	1	3	3	3	3
1	1	1	1	2	2	3	3	3	3
4	4	1	1	1	1	2	2	3	3

For Review Only

1	1	1	1	2	2	2	2	1	1
1	1	1	1	2	2	2	2	1	1
1	1	1	1	1	1	2	2	1	1
4	4	1	1	2	2	2	2	1	1
1	1	1	1	1	1	2	2	1	1
1	1	1	1	2	2	2	2	3	3
1	1	1	1	1	1	2	2	3	3
4	4	1	1	2	2	2	2	3	3
4	4	1	1	1	1	2	2	3	3
1	1	1	1	2	2	2	2	3	3
1	1	1	1	2	2	2	2	3	3
1	1	1	1	2	2	2	2	3	3
1	1	1	1	2	2	2	2	1	1
4	4	1	1	1	1	2	2	1	1
4	4	1	1	2	2	2	2	3	3
4	4	1	1	1	1	2	2	3	3
4	4	1	1	2	2	2	2	1	1
4	4	1	1	1	1	3	3	3	3
1	1	1	1	1	1	2	2	1	1
1	1	1	1	2	2	2	2	1	1
1	1	1	1	2	2	2	2	3	3
4	4	4	4	1	1	2	2	3	3
1	1	1	1	2	2	2	2	1	1
1	1	1	1	2	2	2	2	1	1
1	1	1	1	2	2	2	2	1	1
1	1	1	1	1	1	2	2	3	3
4	4	1	1	2	2	2	2	3	3
4	4	1	1	2	2	2	2	3	3
1	1	1	1	2	2	3	3	3	3
1	1	4	4	1	1	2	2	3	3
1	1	1	1	1	1	2	2	3	3
1	1	1	1	2	2	2	2	3	3
4	4	1	1	2	2	2	2	3	3
1	1	1	1	2	2	2	2	1	1
4	4	1	1	1	1	2	2	3	3
1	1	1	1	2	2	2	2	1	1
1	1	1	1	1	1	2	2	3	3
4	4	1	1	2	2	2	2	3	3
4	4	4	4	1	1	2	2	3	3
1	1	1	1	2	2	2	2	1	1
4	4	1	1	2	2	2	2	1	1
1	1	4	4	1	1	2	2	3	3
1	1	1	1	2	2	3	3	3	3
1	1	1	1	2	2	2	2	3	3
1	1	4	4	2	2	2	2	3	3
1	1	1	1	2	2	2	2	3	3
1	1	1	1	2	2	2	2	3	3
1	1	1	1	1	1	2	2	1	1
1	1	1	1	1	1	2	2	3	3
1	1	1	1	2	2	2	2	3	3
1	1	1	1	2	2	2	2	1	1
4	4	1	1	2	2	2	2	3	3
4	4	1	1	2	2	2	2	3	3
4	4	1	1	1	1	2	2	3	3
1	1	1	1	2	2	2	2	3	3

For Review Only

4	4	1	1	2	2	3	3	3	3
4	4	1	1	2	2	2	2	3	3
1	1	1	1	1	1	3	3	1	1
1	1	1	1	2	2	2	2	3	3
1	1	1	1	2	2	2	2	3	3
1	1	1	1	1	1	2	2	3	3
1	1	1	1	1	1	2	2	3	3
1	1	1	1	2	2	2	2	1	1
1	1	1	1	2	2	2	2	3	3
4	4	1	1	1	1	2	2	3	3
1	1	1	1	1	1	2	2	3	3
4	4	1	1	2	2	2	2	3	3
1	1	1	1	2	2	2	2	1	1
1	1	1	1	1	1	2	2	3	3
4	4	1	1	2	2	2	2	3	3
4	4	1	1	2	2	3	3	3	3
4	4	1	1	2	2	3	3	1	1
1	1	1	1	2	2	2	2	3	3
1	1	1	1	2	2	2	2	3	3
4	4	1	1	2	2	2	2	1	1
4	4	1	1	2	2	2	2	3	3
1	1	4	4	1	1	2	2	1	1
1	1	1	1	1	1	2	2	3	3
4	4	1	1	2	2	2	2	3	3
4	4	1	1	1	1	3	3	3	3
1	1	1	1	1	1	2	2	3	3
4	4	1	1	2	2	2	2	1	1
1	1	1	1	1	1	2	2	3	3
1	1	1	1	2	2	2	2	3	3
4	4	1	1	2	2	3	3	3	3
1	1	1	1	2	2	2	2	3	3
4	4	1	1	2	2	2	2	3	3
1	1	1	1	2	2	2	2	1	1
4	4	1	1	2	2	3	3	3	3
4	4	1	1	2	2	2	2	1	1
4	4	1	1	2	2	2	2	3	3
1	1	1	1	2	2	2	2	3	3
1	1	1	1	2	2	2	2	1	1
4	4	1	1	2	2	2	2	3	3
4	4	1	1	2	2	2	2	3	3
4	4	4	4	1	1	2	2	3	3
1	1	1	1	2	2	2	2	3	3
4	4	1	1	1	1	2	2	3	3
4	4	1	1	1	1	2	2	3	3
1	1	1	1	1	1	3	3	3	3
1	1	1	1	2	2	2	2	3	3
1	1	1	1	2	2	3	3	3	3
1	1	1	1	1	1	2	2	3	3
4	4	1	1	2	2	2	2	3	3
1	1	1	1	2	2	2	2	3	3
1	1	1	1	2	2	3	3	1	1
4	4	1	1	2	2	2	2	3	3
4	4	1	1	2	2	2	2	3	3
1	1	1	1	2	2	2	2	3	3
4	4	1	1	2	2	2	2	3	3

For Review Only

1	1	1	1	2	2	3	3	3	3
4	4	1	1	2	2	3	3	3	3
1	1	1	1	2	2	2	2	3	3
1	1	1	1	2	2	2	2	3	3
1	1	1	1	2	2	2	2	3	3
1	1	1	1	2	2	2	2	3	3
4	4	1	1	2	2	2	2	3	3
4	4	1	1	2	2	2	2	3	3
4	4	1	1	2	2	2	2	3	3
1	1	4	4	2	2	2	2	1	1
1	1	1	1	2	2	3	3	3	3
1	1	1	1	2	2	2	2	3	3
1	1	1	1	2	2	2	2	3	3
1	1	1	1	2	2	2	2	3	3
1	1	1	1	2	2	2	2	3	3
4	4	1	1	2	2	2	2	3	3
4	4	1	1	2	2	2	2	3	3
1	1	1	1	2	2	2	2	3	3
1	1	1	1	1	1	2	2	3	3
1	1	1	1	1	1	2	2	3	3
4	4	1	1	2	2	2	2	3	3
1	1	1	1	2	2	2	2	3	3
4	4	4	4	1	1	2	2	3	3
1	1	1	1	2	2	2	2	3	3
4	4	1	1	2	2	2	2	3	3
4	4	1	1	2	2	2	2	1	1
1	1	1	1	1	1	2	2	3	3
4	4	1	1	2	2	2	2	1	1
4	4	1	1	2	2	2	2	3	3
1	1	1	1	1	1	2	2	3	3
1	1	1	1	1	1	2	2	3	3
4	4	4	4	1	1	3	3	3	3
1	1	1	1	2	2	2	2	3	3
1	1	1	1	2	2	2	2	3	3
1	1	1	1	2	2	2	2	3	3
1	1	1	1	2	2	2	2	3	3
1	1	1	1	1	1	2	2	3	3
1	1	1	1	1	1	2	2	1	1
1	1	1	1	1	1	2	2	3	3
1	1	1	1	2	2	2	2	1	1
1	1	1	1	1	1	2	2	3	3
1	1	1	1	2	2	3	3	3	3
1	1	1	1	2	2	2	2	1	1
4	4	1	1	2	2	2	2	3	3
1	1	1	1	2	2	2	2	3	3
1	1	1	1	2	2	2	2	1	1
4	4	1	1	2	2	2	2	3	3
4	4	1	1	2	2	2	2	3	3
1	1	1	1	2	2	2	2	1	1
4	4	1	1	1	1	2	2	3	3
4	4	1	1	2	2	2	2	3	3
1	1	1	1	2	2	2	2	3	3
4	4	4	4	1	1	2	2	3	3
1	1	1	1	2	2	2	2	3	3
4	4	1	1	2	2	2	2	3	3

For Review Only

4	4	1	1	2	2	2	2	3	3
4	4	1	1	2	2	2	2	3	3
1	1	4	4	2	2	3	3	3	3
4	4	4	4	2	2	2	2	3	3
1	1	1	1	1	1	3	3	1	1
1	1	1	1	1	1	3	3	3	3
4	4	1	1	2	2	2	2	3	3
4	4	1	1	2	2	2	2	3	3
1	1	1	1	2	2	2	2	3	3
4	4	1	1	2	2	2	2	3	3
4	4	1	1	2	2	2	2	3	3
1	1	1	1	2	2	2	2	3	3
4	4	4	4	1	1	2	2	1	1
1	1	1	1	2	2	3	3	1	1
1	1	1	1	1	1	2	2	3	3
1	1	4	4	2	2	2	2	3	3
1	1	1	1	2	2	2	2	3	3
4	4	4	4	2	2	2	2	3	3
4	4	1	1	2	2	2	2	3	3
1	1	1	1	2	2	2	2	3	3
1	1	1	1	2	2	2	2	3	3
1	1	1	1	2	2	3	3	3	3
1	1	4	4	2	2	3	3	1	1
1	1	1	1	2	2	2	2	3	3
4	4	1	1	2	2	2	2	3	3
1	1	1	1	2	2	2	2	1	1
4	4	1	1	2	2	2	2	3	3
1	1	1	1	1	1	2	2	3	3
1	1	1	1	2	2	2	2	1	1
1	1	1	1	2	2	2	2	3	3
4	4	1	1	2	2	2	2	3	3
1	1	1	1	2	2	3	3	3	3
1	1	1	1	2	2	2	2	3	3
1	1	1	1	1	1	2	2	3	3
4	4	4	4	1	1	2	2	3	3
4	4	1	1	2	2	2	2	3	3
1	1	1	1	1	1	2	2	1	1
1	1	1	1	2	2	2	2	3	3
1	1	1	1	1	1	2	2	1	1
4	4	1	1	2	2	2	2	3	3
1	1	4	4	1	1	2	2	1	1
1	1	1	1	2	2	2	2	3	3
4	4	1	1	2	2	2	2	3	3
1	1	1	1	2	2	2	2	1	1
1	1	1	1	2	2	2	2	3	3
1	1	4	4	1	1	2	2	1	1
1	1	1	1	2	2	2	2	3	3
1	1	1	1	2	2	2	2	3	3
1	1	1	1	2	2	2	2	3	3
4	4	1	1	2	2	2	2	3	3
1	1	4	4	2	2	2	2	3	3
4	4	1	1	2	2	2	2	3	3
1	1	1	1	1	1	2	2	3	3
1	1	1	1	1	1	2	2	3	3
1	1	1	1	2	2	2	2	3	3

For Review Only

1	1	4	4	1	1	2	2	3	3
4	4	1	1	2	2	2	2	3	3

For Review Only

SNP507	SNP507	SNP508	SNP508	SNP509	SNP509	SNP510	SNP510	SNP511	SNP511
3	3	3	3	4	4	1	1	4	4
1	1	3	3	4	4	3	3	4	4
1	1	3	3	4	4	1	1	4	4
1	1	3	3	1	1	3	3	4	4
1	1	3	3	4	4	3	3	4	4
3	3	3	3	4	4	1	1	4	4
1	1	3	3	4	4	1	1	4	4
1	1	2	2	4	4	3	3	4	4
1	1	3	3	4	4	3	3	4	4
1	1	3	3	1	1	3	3	4	4
1	1	3	3	4	4	3	3	4	4
1	1	3	3	4	4	1	1	4	4
1	1	3	3	4	4	1	1	4	4
1	1	3	3	4	4	1	1	4	4
1	1	3	3	4	4	1	1	4	4
1	1	3	3	4	4	3	3	4	4
1	1	3	3	1	1	1	1	4	4
3	3	3	3	1	1	1	1	4	4
1	1	3	3	4	4	1	1	4	4
1	1	3	3	4	4	3	3	4	4
1	1	3	3	1	1	1	1	4	4
1	1	3	3	4	4	1	1	4	4
1	1	3	3	4	4	3	3	2	2
1	1	3	3	1	1	3	3	4	4
1	1	3	3	4	4	1	1	4	4
1	1	3	3	4	4	3	3	4	4
1	1	3	3	4	4	3	3	4	4
1	1	3	3	4	4	1	1	4	4
1	1	3	3	1	1	3	3	4	4
1	1	2	2	4	4	3	3	4	4
1	1	3	3	4	4	3	3	4	4
1	1	3	3	4	4	3	3	4	4
1	1	3	3	4	4	1	1	4	4
1	1	3	3	4	4	3	3	4	4
1	1	3	3	4	4	1	1	4	4
1	1	3	3	4	4	3	3	4	4
1	1	3	3	4	4	1	1	4	4
1	1	3	3	4	4	1	1	4	4
1	1	3	3	4	4	3	3	4	4
1	1	3	3	4	4	3	3	2	2
1	1	3	3	4	4	3	3	4	4
1	1	3	3	4	4	3	3	4	4
1	1	3	3	4	4	3	3	4	4
1	1	3	3	4	4	1	1	4	4
1	1	2	2	4	4	3	3	4	4
1	1	3	3	4	4	1	1	4	4
1	1	3	3	4	4	1	1	4	4
1	1	3	3	1	1	3	3	4	4
1	1	2	2	4	4	3	3	4	4
1	1	3	3	1	1	1	1	4	4
1	1	3	3	4	4	1	1	4	4
1	1	3	3	4	4	3	3	4	4

For Review Only

1	1	3	3	4	4	1	1	4	4
1	1	3	3	4	4	1	1	4	4
1	1	3	3	4	4	1	1	4	4
1	1	3	3	4	4	1	1	4	4
1	1	3	3	4	4	1	1	4	4
1	1	3	3	4	4	3	3	4	4
1	1	3	3	4	4	3	3	4	4
1	1	3	3	1	1	3	3	4	4
1	1	3	3	1	1	3	3	4	4
1	1	3	3	4	4	1	1	4	4
1	1	3	3	4	4	3	3	4	4
1	1	3	3	4	4	3	3	4	4
1	1	3	3	4	4	1	1	4	4
1	1	3	3	1	1	3	3	4	4
1	1	3	3	4	4	1	1	4	4
3	3	3	3	1	1	1	1	4	4
1	1	3	3	4	4	3	3	4	4
1	1	3	3	1	1	1	1	4	4
1	1	3	3	1	1	3	3	4	4
1	1	3	3	4	4	3	3	4	4
1	1	3	3	4	4	3	3	4	4
1	1	3	3	4	4	3	3	2	2
1	1	3	3	4	4	3	3	4	4
1	1	3	3	4	4	1	1	4	4
1	1	2	2	4	4	3	3	4	4
1	1	3	3	4	4	3	3	4	4
1	1	3	3	1	1	1	1	4	4
1	1	3	3	4	4	3	3	4	4
1	1	3	3	4	4	1	1	4	4
1	1	3	3	4	4	3	3	2	2
1	1	3	3	4	4	1	1	4	4
1	1	3	3	4	4	3	3	4	4
1	1	3	3	4	4	3	3	4	4
1	1	3	3	4	4	1	1	4	4
3	3	3	3	1	1	1	1	4	4
1	1	2	2	4	4	3	3	4	4
1	1	3	3	1	1	3	3	4	4
1	1	2	2	4	4	3	3	4	4
1	1	3	3	4	4	3	3	2	2
1	1	3	3	1	1	1	1	4	4
1	1	3	3	4	4	1	1	4	4
1	1	3	3	4	4	3	3	2	2
1	1	3	3	4	4	1	1	4	4
1	1	3	3	4	4	3	3	4	4
3	3	3	3	4	4	3	3	2	2
1	1	3	3	4	4	3	3	4	4
1	1	3	3	4	4	3	3	4	4
1	1	3	3	1	1	1	1	4	4
1	1	3	3	4	4	1	1	4	4
1	1	3	3	4	4	3	3	4	4
1	1	3	3	4	4	3	3	4	4
1	1	3	3	4	4	3	3	4	4
1	1	3	3	1	1	3	3	4	4
1	1	3	3	4	4	3	3	4	4
1	1	3	3	1	1	1	1	4	4

For Review Only

1	1	3	3	4	4	3	3	4	4
1	1	3	3	4	4	3	3	4	4
1	1	3	3	4	4	3	3	4	4
1	1	3	3	4	4	1	1	4	4
1	1	3	3	4	4	3	3	4	4
1	1	3	3	4	4	3	3	4	4
1	1	3	3	4	4	3	3	4	4
1	1	3	3	1	1	3	3	4	4
1	1	3	3	4	4	1	1	4	4
1	1	3	3	4	4	1	1	4	4
3	3	3	3	4	4	1	1	4	4
1	1	3	3	4	4	1	1	4	4
1	1	3	3	4	4	1	1	4	4
1	1	3	3	4	4	3	3	4	4
1	1	3	3	4	4	3	3	4	4
1	1	3	3	4	4	1	1	4	4
1	1	3	3	1	1	1	1	4	4
1	1	3	3	4	4	1	1	4	4
1	1	3	3	4	4	1	1	4	4
1	1	3	3	1	1	3	3	4	4
1	1	3	3	4	4	1	1	4	4
1	1	3	3	4	4	3	3	2	2
1	1	3	3	4	4	1	1	4	4
1	1	3	3	4	4	3	3	4	4
1	1	3	3	4	4	3	3	2	2
1	1	3	3	4	4	3	3	4	4
1	1	3	3	4	4	1	1	4	4
1	1	3	3	4	4	3	3	4	4
1	1	3	3	4	4	3	3	4	4
1	1	3	3	1	1	1	1	4	4
1	1	3	3	4	4	3	3	4	4
1	1	3	3	4	4	1	1	4	4
1	1	3	3	4	4	1	1	4	4
1	1	3	3	4	4	1	1	4	4
1	1	3	3	4	4	3	3	4	4
1	1	3	3	4	4	1	1	4	4
1	1	3	3	4	4	3	3	4	4
1	1	3	3	4	4	3	3	4	4
1	1	3	3	1	1	3	3	4	4
1	1	3	3	4	4	3	3	4	4
1	1	3	3	1	1	3	3	2	2
1	1	3	3	4	4	3	3	4	4
1	1	3	3	1	1	1	1	4	4
1	1	2	2	4	4	3	3	4	4
1	1	3	3	4	4	1	1	4	4
3	3	3	3	4	4	1	1	4	4
1	1	3	3	4	4	1	1	4	4
1	1	3	3	4	4	3	3	4	4
1	1	3	3	4	4	1	1	4	4
1	1	3	3	4	4	1	1	4	4
1	1	3	3	4	4	1	1	4	4
1	1	3	3	1	1	3	3	4	4
1	1	3	3	1	1	1	1	4	4
1	1	3	3	4	4	1	1	4	4
1	1	3	3	4	4	3	3	4	4

For Review Only

1	1	3	3	4	4	1	1	4	4
1	1	3	3	4	4	1	1	4	4
1	1	3	3	4	4	1	1	4	4
1	1	3	3	4	4	1	1	4	4
3	3	3	3	4	4	1	1	4	4
1	1	3	3	4	4	3	3	4	4
1	1	3	3	4	4	1	1	4	4
1	1	3	3	4	4	1	1	4	4
1	1	3	3	4	4	3	3	4	4
1	1	3	3	4	4	3	3	2	2
1	1	3	3	4	4	1	1	4	4
1	1	3	3	4	4	3	3	4	4
1	1	3	3	4	4	1	1	4	4
3	3	3	3	4	4	1	1	4	4
1	1	3	3	4	4	3	3	4	4
1	1	3	3	4	4	3	3	4	4
1	1	3	3	4	4	1	1	4	4
1	1	3	3	4	4	3	3	4	4
1	1	3	3	4	4	3	3	4	4
1	1	3	3	4	4	3	3	4	4
1	1	3	3	4	4	1	1	4	4
1	1	3	3	4	4	3	3	4	4
1	1	3	3	4	4	3	3	2	2
1	1	3	3	4	4	3	3	4	4
1	1	3	3	1	1	3	3	4	4
1	1	3	3	4	4	3	3	4	4
1	1	3	3	1	1	1	1	4	4
1	1	3	3	1	1	1	1	4	4
1	1	3	3	1	1	3	3	4	4
1	1	3	3	4	4	1	1	4	4
3	3	3	3	4	4	1	1	4	4
1	1	3	3	1	1	1	1	2	2
1	1	3	3	4	4	3	3	4	4
1	1	3	3	4	4	3	3	4	4
1	1	3	3	4	4	1	1	4	4
1	1	3	3	4	4	3	3	4	4
1	1	3	3	4	4	3	3	4	4
1	1	3	3	4	4	3	3	4	4
1	1	3	3	4	4	3	3	4	4
1	1	3	3	4	4	1	1	4	4
1	1	3	3	4	4	1	1	4	4
1	1	3	3	4	4	1	1	4	4
1	1	3	3	1	1	3	3	4	4
1	1	3	3	4	4	3	3	4	4
1	1	3	3	4	4	3	3	4	4
1	1	3	3	4	4	1	1	4	4
1	1	3	3	4	4	3	3	4	4
1	1	3	3	4	4	1	1	4	4
1	1	3	3	4	4	3	3	4	4
1	1	3	3	4	4	3	3	4	4
1	1	3	3	4	4	3	3	4	4
1	1	3	3	4	4	3	3	4	4
1	1	3	3	4	4	3	3	2	2
1	1	3	3	4	4	3	3	4	4
1	1	3	3	4	4	3	3	4	4

For Review Only

1	1	3	3	1	1	1	1	4	4
1	1	2	2	4	4	3	3	4	4
1	1	3	3	4	4	1	1	2	2
1	1	3	3	4	4	1	1	2	2
3	3	3	3	4	4	3	3	4	4
1	1	3	3	4	4	1	1	4	4
1	1	3	3	4	4	3	3	4	4
1	1	3	3	4	4	3	3	4	4
1	1	3	3	4	4	1	1	4	4
1	1	3	3	4	4	3	3	4	4
1	1	3	3	4	4	1	1	4	4
1	1	3	3	4	4	1	1	4	4
1	1	3	3	4	4	1	1	2	2
1	1	3	3	4	4	1	1	4	4
1	1	3	3	4	4	1	1	4	4
3	3	3	3	4	4	3	3	2	2
1	1	3	3	4	4	3	3	4	4
1	1	3	3	4	4	1	1	2	2
1	1	3	3	4	4	1	1	4	4
1	1	3	3	4	4	1	1	4	4
1	1	3	3	4	4	1	1	4	4
1	1	3	3	4	4	1	1	4	4
3	3	3	3	4	4	1	1	2	2
1	1	3	3	4	4	3	3	4	4
1	1	3	3	1	1	3	3	4	4
1	1	3	3	4	4	3	3	4	4
1	1	3	3	4	4	3	3	4	4
1	1	3	3	1	1	3	3	4	4
1	1	3	3	4	4	3	3	4	4
1	1	3	3	4	4	3	3	4	4
1	1	3	3	1	1	1	1	4	4
1	1	3	3	4	4	1	1	4	4
3	3	3	3	1	1	3	3	4	4
1	1	3	3	4	4	1	1	4	4
1	1	3	3	4	4	3	3	2	2
1	1	3	3	4	4	3	3	4	4
1	1	3	3	4	4	1	1	4	4
1	1	3	3	4	4	3	3	4	4
1	1	3	3	4	4	3	3	4	4
1	1	3	3	4	4	3	3	4	4
1	1	3	3	4	4	3	3	4	4
1	1	3	3	4	4	1	1	4	4
1	1	3	3	4	4	3	3	4	4
1	1	3	3	4	4	1	1	4	4
1	1	3	3	4	4	1	1	4	4
1	1	3	3	4	4	1	1	2	2
1	1	3	3	4	4	3	3	4	4
1	1	3	3	1	1	1	1	4	4
1	1	3	3	4	4	1	1	4	4
3	3	3	3	4	4	3	3	4	4
1	1	3	3	4	4	3	3	4	4
1	1	3	3	4	4	3	3	4	4
1	1	3	3	4	4	1	1	4	4
1	1	3	3	1	1	1	1	4	4
1	1	3	3	4	4	3	3	4	4

For Review Only

3	3	3	3	4	4	3	3	2	2
1	1	3	3	1	1	3	3	4	4

For Review Only

SNP512	SNP512	SNP513	SNP513	SNP514	SNP514	SNP515	SNP515	SNP516	SNP516
4	4	3	3	1	1	3	3	3	3
4	4	3	3	4	4	3	3	2	2
4	4	3	3	4	4	3	3	3	3
4	4	3	3	4	4	3	3	3	3
4	4	3	3	4	4	3	3	3	3
4	4	3	3	1	1	3	3	2	2
4	4	3	3	4	4	3	3	3	3
4	4	3	3	4	4	3	3	3	3
4	4	3	3	4	4	3	3	3	3
4	4	3	3	4	4	3	3	2	2
4	4	3	3	4	4	2	2	2	2
4	4	3	3	4	4	3	3	3	3
4	4	3	3	4	4	3	3	3	3
4	4	3	3	4	4	3	3	3	3
4	4	3	3	4	4	3	3	3	3
4	4	3	3	4	4	3	3	3	3
4	4	3	3	4	4	3	3	2	2
4	4	3	3	4	4	3	3	2	2
4	4	3	3	1	1	3	3	3	3
4	4	3	3	4	4	3	3	3	3
4	4	3	3	4	4	3	3	3	3
4	4	3	3	4	4	3	3	3	3
1	1	2	2	4	4	3	3	2	2
4	4	3	3	4	4	3	3	2	2
4	4	3	3	1	1	3	3	3	3
4	4	3	3	4	4	3	3	3	3
4	4	2	2	1	1	3	3	3	3
4	4	3	3	4	4	3	3	3	3
4	4	3	3	4	4	3	3	2	2
4	4	3	3	4	4	3	3	3	3
4	4	3	3	1	1	3	3	3	3
4	4	3	3	4	4	3	3	3	3
4	4	3	3	4	4	3	3	3	3
4	4	3	3	1	1	3	3	2	2
4	4	3	3	4	4	3	3	3	3
4	4	3	3	4	4	3	3	3	3
4	4	3	3	4	4	3	3	3	3
4	4	3	3	4	4	3	3	3	3
4	4	3	3	4	4	3	3	2	2
1	1	2	2	4	4	3	3	2	2
4	4	3	3	4	4	3	3	3	3
4	4	3	3	4	4	3	3	2	2
4	4	3	3	4	4	3	3	2	2
4	4	3	3	4	4	3	3	3	3
4	4	3	3	4	4	3	3	2	2
4	4	3	3	4	4	3	3	3	3
4	4	3	3	4	4	3	3	3	3
4	4	3	3	1	1	3	3	2	2
4	4	3	3	4	4	3	3	3	3
4	4	3	3	4	4	3	3	3	3
4	4	3	3	4	4	3	3	3	3
4	4	3	3	1	1	3	3	3	3

For Review Only

4	4	3	3	1	1	3	3	3	3
4	4	3	3	4	4	3	3	3	3
4	4	3	3	4	4	3	3	2	2
4	4	3	3	4	4	3	3	3	3
4	4	3	3	1	1	3	3	3	3
4	4	3	3	4	4	3	3	3	3
4	4	3	3	1	1	3	3	3	3
4	4	3	3	4	4	3	3	2	2
4	4	3	3	4	4	3	3	2	2
4	4	3	3	4	4	3	3	3	3
4	4	3	3	4	4	3	3	3	3
4	4	3	3	4	4	3	3	3	3
4	4	3	3	1	1	3	3	3	3
4	4	3	3	4	4	3	3	2	2
4	4	3	3	4	4	3	3	3	3
4	4	3	3	4	4	3	3	2	2
4	4	3	3	4	4	3	3	2	2
4	4	3	3	4	4	3	3	3	3
4	4	3	3	4	4	3	3	2	2
4	4	3	3	1	1	3	3	2	2
4	4	3	3	4	4	3	3	3	3
1	1	2	2	4	4	3	3	2	2
1	1	2	2	4	4	3	3	3	3
4	4	3	3	4	4	3	3	3	3
4	4	3	3	4	4	3	3	2	2
4	4	3	3	1	1	3	3	3	3
4	4	3	3	4	4	3	3	3	3
4	4	3	3	4	4	2	2	2	2
4	4	3	3	4	4	3	3	3	3
1	1	2	2	4	4	3	3	3	3
4	4	3	3	1	1	3	3	3	3
4	4	3	3	4	4	3	3	2	2
4	4	3	3	1	1	2	2	2	2
4	4	3	3	1	1	3	3	3	3
4	4	3	3	4	4	3	3	2	2
4	4	3	3	4	4	3	3	2	2
4	4	3	3	1	1	3	3	2	2
4	4	3	3	1	1	3	3	3	3
1	1	2	2	1	1	3	3	2	2
4	4	3	3	4	4	3	3	2	2
4	4	3	3	4	4	3	3	3	3
1	1	2	2	4	4	3	3	3	3
4	4	3	3	1	1	3	3	3	3
4	4	3	3	4	4	3	3	3	3
1	1	2	2	4	4	3	3	3	3
4	4	3	3	4	4	3	3	3	3
4	4	3	3	4	4	2	2	3	3
4	4	3	3	4	4	3	3	3	3
4	4	3	3	4	4	3	3	3	3
1	1	2	2	4	4	3	3	3	3
4	4	3	3	1	1	3	3	3	3
4	4	3	3	4	4	2	2	2	2
4	4	3	3	1	1	3	3	2	2
4	4	3	3	4	4	3	3	3	3
4	4	3	3	4	4	3	3	2	2

For Review Only

4	4	3	3	4	4	3	3	3	3
4	4	3	3	4	4	3	3	2	2
4	4	3	3	4	4	3	3	3	3
4	4	3	3	4	4	2	2	3	3
4	4	3	3	4	4	3	3	3	3
4	4	3	3	1	1	3	3	3	3
4	4	3	3	4	4	3	3	3	3
4	4	3	3	4	4	3	3	3	3
4	4	3	3	4	4	3	3	3	3
4	4	3	3	4	4	3	3	3	3
4	4	3	3	1	1	3	3	3	3
4	4	3	3	4	4	3	3	3	3
4	4	3	3	4	4	3	3	3	3
4	4	3	3	4	4	3	3	3	3
4	4	3	3	4	4	3	3	3	3
4	4	3	3	4	4	3	3	3	3
4	4	3	3	4	4	3	3	3	3
4	4	3	3	4	4	3	3	3	3
4	4	3	3	4	4	2	2	3	3
1	1	2	2	4	4	3	3	2	2
4	4	3	3	4	4	3	3	3	3
1	1	2	2	4	4	3	3	3	3
4	4	3	3	4	4	3	3	3	3
4	4	3	3	4	4	2	2	3	3
4	4	2	2	4	4	2	2	3	3
4	4	3	3	4	4	3	3	2	2
4	4	3	3	4	4	3	3	3	3
4	4	3	3	4	4	3	3	3	3
4	4	3	3	4	4	3	3	3	3
4	4	3	3	4	4	3	3	3	3
4	4	3	3	4	4	3	3	2	2
4	4	3	3	4	4	3	3	3	3
4	4	3	3	4	4	3	3	3	3
4	4	3	3	4	4	3	3	3	3
4	4	3	3	1	1	3	3	2	2
4	4	3	3	4	4	3	3	3	3
4	4	3	3	4	4	2	2	3	3
4	4	3	3	4	4	3	3	3	3
4	4	3	3	4	4	3	3	2	2
4	4	3	3	4	4	2	2	2	2
1	1	2	2	4	4	3	3	3	3
4	4	3	3	4	4	2	2	2	2
4	4	3	3	4	4	3	3	3	3
4	4	3	3	4	4	3	3	3	3
4	4	3	3	4	4	3	3	3	3
4	4	3	3	4	4	3	3	3	3
4	4	3	3	4	4	3	3	3	3
4	4	3	3	4	4	3	3	3	3
4	4	3	3	4	4	3	3	3	3
4	4	3	3	4	4	3	3	3	3
4	4	3	3	4	4	3	3	3	3
4	4	3	3	4	4	3	3	2	2
4	4	3	3	4	4	3	3	3	3
4	4	3	3	4	4	3	3	3	3
4	4	3	3	4	4	3	3	3	3

For Review Only

4	4	3	3	1	1	3	3	3	3
4	4	3	3	4	4	3	3	2	2
4	4	3	3	4	4	3	3	3	3
4	4	3	3	4	4	3	3	2	2
4	4	3	3	4	4	3	3	3	3
4	4	3	3	4	4	3	3	2	2
4	4	3	3	4	4	3	3	2	2
4	4	3	3	4	4	3	3	3	3
1	1	2	2	4	4	2	2	2	2
1	1	2	2	4	4	3	3	3	3
4	4	3	3	4	4	3	3	3	3
4	4	3	3	4	4	3	3	3	3
4	4	3	3	1	1	3	3	3	3
4	4	3	3	4	4	3	3	3	3
4	4	3	3	4	4	3	3	3	3
4	4	3	3	4	4	2	2	2	2
4	4	3	3	4	4	3	3	3	3
4	4	3	3	4	4	3	3	3	3
4	4	3	3	4	4	3	3	2	2
4	4	3	3	4	4	3	3	3	3
4	4	3	3	4	4	3	3	3	3
4	4	3	3	1	1	3	3	3	3
1	1	2	2	4	4	3	3	2	2
4	4	3	3	4	4	3	3	3	3
4	4	3	3	4	4	3	3	2	2
4	4	3	3	1	1	2	2	2	2
4	4	3	3	1	1	3	3	3	3
4	4	3	3	4	4	3	3	3	3
4	4	3	3	4	4	3	3	3	3
4	4	3	3	4	4	3	3	3	3
4	4	3	3	4	4	3	3	3	3
1	1	2	2	4	4	3	3	3	3
4	4	3	3	4	4	3	3	3	3
4	4	3	3	4	4	3	3	3	3
4	4	3	3	4	4	3	3	3	3
4	4	3	3	4	4	3	3	3	3
4	4	3	3	4	4	3	3	3	3
4	4	3	3	1	1	3	3	2	2
4	4	3	3	4	4	3	3	3	3
4	4	3	3	4	4	3	3	3	3
4	4	3	3	4	4	3	3	3	3
4	4	3	3	4	4	3	3	3	3
4	4	3	3	4	4	3	3	3	3
4	4	3	3	4	4	3	3	3	3
4	4	2	2	4	4	3	3	3	3
4	4	3	3	4	4	3	3	3	3
4	4	3	3	4	4	2	2	2	2
4	4	3	3	1	1	3	3	2	2
4	4	3	3	1	1	3	3	2	2
4	4	3	3	4	4	3	3	3	3
4	4	3	3	4	4	3	3	3	3
4	4	3	3	4	4	3	3	3	3
1	1	2	2	4	4	2	2	2	2
4	4	3	3	4	4	3	3	3	3
4	4	3	3	4	4	2	2	2	2

For Review Only

4	4	3	3	4	4	3	3	2	2
4	4	3	3	4	4	3	3	2	2
1	1	2	2	4	4	3	3	3	3
1	1	2	2	4	4	3	3	3	3
4	4	3	3	4	4	3	3	3	3
4	4	3	3	4	4	3	3	3	3
4	4	3	3	4	4	3	3	2	2
4	4	3	3	4	4	3	3	2	2
4	4	3	3	4	4	3	3	3	3
4	4	3	3	4	4	2	2	2	2
4	4	3	3	4	4	3	3	3	3
4	4	3	3	4	4	3	3	3	3
1	1	2	2	1	1	3	3	2	2
4	4	3	3	4	4	3	3	3	3
4	4	3	3	4	4	3	3	3	3
1	1	2	2	4	4	3	3	3	3
4	4	3	3	4	4	3	3	2	2
1	1	2	2	4	4	2	2	2	2
4	4	3	3	1	1	3	3	2	2
4	4	3	3	4	4	3	3	2	2
4	4	3	3	1	1	3	3	3	3
4	4	3	3	4	4	3	3	3	3
1	1	2	2	4	4	3	3	3	3
4	4	3	3	4	4	3	3	3	3
4	4	3	3	4	4	3	3	2	2
4	4	3	3	4	4	3	3	3	3
4	4	3	3	4	4	3	3	2	2
4	4	3	3	1	1	3	3	3	3
4	4	3	3	4	4	3	3	3	3
4	4	3	3	4	4	3	3	3	3
4	4	3	3	4	4	3	3	2	2
4	4	3	3	4	4	3	3	3	3
4	4	3	3	4	4	3	3	2	2
4	4	3	3	4	4	3	3	3	3
1	1	2	2	4	4	2	2	2	2
4	4	3	3	4	4	3	3	2	2
4	4	3	3	4	4	3	3	3	3
4	4	3	3	4	4	3	3	3	3
4	4	3	3	4	4	3	3	3	3
4	4	3	3	4	4	3	3	2	2
4	4	3	3	4	4	3	3	3	3
4	4	3	3	4	4	3	3	3	3
4	4	3	3	4	4	3	3	3	3
4	4	3	3	4	4	3	3	3	3
4	4	3	3	4	4	3	3	3	3
1	1	2	2	1	1	2	2	3	3
4	4	3	3	1	1	3	3	3	3
4	4	3	3	4	4	3	3	2	2
4	4	3	3	4	4	3	3	3	3
4	4	3	3	1	1	3	3	2	2
4	4	3	3	4	4	3	3	3	3
4	4	3	3	4	4	2	2	2	2
4	4	3	3	4	4	3	3	3	3
4	4	3	3	1	1	3	3	3	3
4	4	3	3	4	4	3	3	2	2

For Review Only

1	1	2	2	4	4	3	3	3	3
4	4	3	3	4	4	3	3	3	3

For Review Only

SNP517	SNP517	SNP518	SNP518	SNP519	SNP519	SNP520	SNP520	SNP521	SNP521
3	3	4	4	2	2	1	1	4	4
2	2	4	4	2	2	1	1	2	2
2	2	4	4	3	3	2	2	2	2
3	3	4	4	2	2	1	1	2	2
3	3	4	4	2	2	1	1	4	4
3	3	1	1	2	2	1	1	2	2
3	3	4	4	2	2	1	1	4	4
3	3	4	4	2	2	1	1	4	4
3	3	4	4	2	2	1	1	2	2
3	3	1	1	2	2	1	1	2	2
3	3	4	4	2	2	1	1	2	2
3	3	4	4	3	3	1	1	2	2
3	3	4	4	2	2	1	1	2	2
3	3	4	4	2	2	1	1	4	4
3	3	4	4	2	2	1	1	4	4
3	3	4	4	2	2	1	1	2	2
3	3	1	1	2	2	1	1	4	4
2	2	1	1	2	2	1	1	4	4
3	3	4	4	2	2	1	1	2	2
3	3	4	4	2	2	1	1	4	4
3	3	1	1	2	2	1	1	2	2
3	3	1	1	2	2	1	1	2	2
3	3	4	4	2	2	1	1	2	2
3	3	1	1	2	2	1	1	2	2
2	2	4	4	2	2	1	1	2	2
3	3	4	4	3	3	2	2	4	4
2	2	4	4	2	2	2	2	2	2
3	3	4	4	2	2	1	1	2	2
3	3	1	1	2	2	1	1	2	2
3	3	1	1	2	2	2	2	2	2
3	3	4	4	2	2	1	1	2	2
3	3	4	4	2	2	1	1	2	2
3	3	4	4	2	2	1	1	4	4
3	3	1	1	2	2	1	1	4	4
3	3	1	1	2	2	1	1	2	2
3	3	1	1	2	2	2	2	2	2
3	3	4	4	2	2	1	1	2	2
3	3	4	4	2	2	1	1	2	2
3	3	1	1	2	2	1	1	2	2
3	3	4	4	2	2	1	1	2	2
3	3	1	1	2	2	1	1	2	2
3	3	1	1	2	2	1	1	2	2
3	3	1	1	2	2	1	1	2	2
3	3	4	4	2	2	1	1	4	4
3	3	1	1	2	2	1	1	2	2
2	2	4	4	3	3	2	2	2	2
2	2	4	4	2	2	1	1	4	4
3	3	1	1	2	2	1	1	4	4
3	3	4	4	2	2	1	1	2	2
3	3	4	4	2	2	1	1	2	2
3	3	1	1	3	3	1	1	4	4
3	3	4	4	2	2	2	2	2	2

For Review Only

3	3	4	4	2	2	1	1	4	4
3	3	4	4	2	2	1	1	4	4
3	3	1	1	2	2	1	1	2	2
3	3	4	4	2	2	1	1	4	4
3	3	4	4	2	2	1	1	2	2
2	2	1	1	2	2	1	1	2	2
3	3	4	4	2	2	1	1	2	2
3	3	1	1	2	2	1	1	2	2
3	3	1	1	2	2	1	1	2	2
3	3	4	4	2	2	1	1	2	2
2	2	1	1	2	2	1	1	2	2
2	2	1	1	2	2	1	1	2	2
3	3	4	4	2	2	1	1	4	4
3	3	1	1	2	2	1	1	2	2
3	3	4	4	2	2	1	1	2	2
2	2	1	1	2	2	1	1	4	4
2	2	4	4	2	2	1	1	2	2
2	2	4	4	2	2	1	1	2	2
3	3	1	1	3	3	1	1	2	2
3	3	1	1	2	2	1	1	4	4
3	3	1	1	2	2	1	1	2	2
3	3	4	4	2	2	1	1	2	2
3	3	4	4	2	2	1	1	2	2
3	3	4	4	2	2	1	1	2	2
3	3	1	1	2	2	1	1	2	2
3	3	4	4	2	2	2	2	2	2
3	3	1	1	2	2	1	1	2	2
3	3	1	1	2	2	1	1	2	2
3	3	4	4	3	3	1	1	4	4
3	3	4	4	2	2	1	1	4	4
3	3	4	4	2	2	1	1	4	4
3	3	1	1	2	2	1	1	2	2
3	3	4	4	2	2	2	2	2	2
3	3	4	4	2	2	1	1	4	4
3	3	1	1	2	2	1	1	4	4
3	3	1	1	2	2	1	1	2	2
3	3	4	4	2	2	1	1	4	4
3	3	1	1	2	2	1	1	4	4
3	3	1	1	2	2	2	2	2	2
3	3	1	1	2	2	1	1	2	2
3	3	4	4	2	2	1	1	4	4
2	2	4	4	2	2	1	1	2	2
3	3	4	4	2	2	2	2	4	4
2	2	1	1	2	2	1	1	2	2
2	2	4	4	2	2	1	1	4	4
2	2	4	4	2	2	1	1	2	2
3	3	1	1	2	2	1	1	2	2
3	3	4	4	2	2	1	1	2	2
3	3	4	4	2	2	1	1	2	2
2	2	4	4	2	2	1	1	2	2
3	3	1	1	2	2	2	2	2	2
3	3	1	1	2	2	1	1	2	2
3	3	1	1	2	2	1	1	2	2
3	3	4	4	2	2	1	1	2	2
2	2	1	1	2	2	1	1	2	2

For Review Only

3	3	1	1	3	3	1	1	4	4
3	3	4	4	2	2	1	1	2	2
3	3	4	4	2	2	2	2	2	2
3	3	1	1	2	2	1	1	2	2
2	2	4	4	3	3	2	2	2	2
3	3	4	4	3	3	2	2	2	2
2	2	4	4	2	2	1	1	2	2
2	2	4	4	2	2	1	1	2	2
3	3	4	4	3	3	1	1	2	2
3	3	4	4	3	3	1	1	2	2
2	2	4	4	2	2	1	1	4	4
3	3	4	4	2	2	1	1	2	2
3	3	4	4	2	2	2	2	2	2
3	3	4	4	2	2	2	2	2	2
3	3	4	4	2	2	1	1	2	2
3	3	4	4	2	2	1	1	2	2
3	3	4	4	2	2	1	1	2	2
3	3	4	4	2	2	1	1	2	2
3	3	1	1	2	2	1	1	4	4
3	3	1	1	2	2	1	1	4	4
3	3	4	4	2	2	1	1	2	2
3	3	4	4	2	2	1	1	4	4
2	2	4	4	2	2	1	1	2	2
3	3	1	1	2	2	1	1	2	2
3	3	4	4	2	2	1	1	2	2
3	3	4	4	2	2	1	1	2	2
3	3	4	4	2	2	1	1	2	2
2	2	1	1	2	2	1	1	2	2
2	2	4	4	2	2	1	1	2	2
2	2	4	4	2	2	1	1	2	2
3	3	1	1	2	2	1	1	2	2
3	3	4	4	2	2	1	1	2	2
3	3	4	4	2	2	1	1	4	4
3	3	4	4	3	3	1	1	2	2
3	3	1	1	2	2	1	1	4	4
3	3	1	1	3	3	1	1	2	2
3	3	1	1	2	2	1	1	4	4
3	3	4	4	2	2	1	1	2	2
3	3	4	4	3	3	1	1	4	4
3	3	4	4	2	2	1	1	2	2
3	3	4	4	2	2	1	1	2	2
3	3	1	1	2	2	1	1	4	4
2	2	4	4	2	2	1	1	2	2
3	3	4	4	2	2	1	1	2	2
3	3	4	4	2	2	1	1	2	2
2	2	1	1	2	2	1	1	2	2
3	3	4	4	3	3	1	1	4	4
2	2	4	4	2	2	1	1	2	2
3	3	4	4	2	2	1	1	2	2
3	3	4	4	3	3	2	2	2	2
3	3	4	4	3	3	1	1	4	4
3	3	1	1	2	2	1	1	2	2
3	3	4	4	2	2	1	1	2	2
3	3	1	1	2	2	1	1	2	2
3	3	4	4	2	2	1	1	2	2

For Review Only

3	3	4	4	3	3	1	1	4	4
3	3	4	4	2	2	1	1	4	4
2	2	4	4	2	2	1	1	2	2
3	3	4	4	2	2	1	1	2	2
3	3	4	4	2	2	1	1	2	2
3	3	4	4	2	2	1	1	2	2
3	3	4	4	2	2	1	1	2	2
3	3	4	4	2	2	1	1	4	4
3	3	4	4	2	2	1	1	2	2
2	2	1	1	2	2	1	1	2	2
3	3	4	4	3	3	1	1	4	4
3	3	4	4	2	2	1	1	2	2
2	2	4	4	2	2	1	1	2	2
2	2	1	1	2	2	1	1	2	2
3	3	1	1	2	2	1	1	2	2
3	3	4	4	2	2	1	1	2	2
3	3	4	4	2	2	1	1	2	2
3	3	4	4	2	2	1	1	2	2
3	3	1	1	2	2	2	2	2	2
2	2	4	4	2	2	1	1	2	2
3	3	4	4	2	2	1	1	2	2
3	3	1	1	2	2	2	2	2	2
3	3	4	4	2	2	1	1	2	2
2	2	1	1	2	2	1	1	2	2
3	3	4	4	2	2	1	1	4	4
3	3	1	1	2	2	1	1	4	4
3	3	4	4	2	2	1	1	2	2
3	3	4	4	2	2	1	1	4	4
2	2	4	4	2	2	2	2	2	2
2	2	4	4	2	2	1	1	2	2
2	2	4	4	2	2	1	1	2	2
3	3	4	4	2	2	1	1	4	4
2	2	4	4	2	2	1	1	2	2
3	3	1	1	2	2	1	1	4	4
3	3	4	4	2	2	1	1	4	4
2	2	1	1	2	2	1	1	2	2
3	3	1	1	2	2	2	2	2	2
3	3	1	1	2	2	1	1	2	2
2	2	4	4	2	2	1	1	2	2
3	3	1	1	2	2	1	1	2	2
2	2	4	4	2	2	2	2	2	2
3	3	4	4	3	3	1	1	4	4
3	3	1	1	2	2	1	1	4	4
2	2	4	4	2	2	1	1	2	2
3	3	4	4	2	2	1	1	2	2
3	3	4	4	2	2	1	1	2	2
3	3	4	4	2	2	1	1	2	2
3	3	4	4	2	2	2	2	2	2
3	3	1	1	2	2	1	1	4	4
3	3	4	4	2	2	2	2	2	2
3	3	4	4	3	3	1	1	2	2
3	3	1	1	2	2	1	1	2	2
3	3	1	1	2	2	1	1	2	2
2	2	1	1	2	2	1	1	2	2
3	3	1	1	2	2	1	1	2	2

2	2	4	4	2	2	1	1	4	4
3	3	4	4	2	2	2	2	2	2
2	2	4	4	2	2	1	1	2	2
3	3	4	4	2	2	1	1	2	2
3	3	4	4	2	2	2	2	2	2
3	3	1	1	3	3	2	2	4	4
3	3	1	1	2	2	1	1	2	2
3	3	1	1	2	2	1	1	2	2
3	3	4	4	2	2	1	1	4	4
3	3	4	4	2	2	1	1	2	2
3	3	4	4	2	2	1	1	2	2
3	3	4	4	3	3	1	1	2	2
3	3	1	1	2	2	2	2	2	2
3	3	4	4	2	2	2	2	4	4
2	2	4	4	3	3	2	2	2	2
3	3	4	4	2	2	1	1	2	2
3	3	4	4	2	2	1	1	2	2
3	3	4	4	2	2	1	1	4	4
3	3	1	1	3	3	1	1	2	2
3	3	1	1	2	2	1	1	2	2
3	3	4	4	2	2	1	1	2	2
2	2	4	4	3	3	1	1	4	4
3	3	4	4	2	2	1	1	4	4
2	2	4	4	2	2	1	1	2	2
3	3	1	1	2	2	1	1	2	2
3	3	4	4	3	3	2	2	4	4
3	3	4	4	2	2	2	2	4	4
3	3	4	4	2	2	1	1	2	2
3	3	4	4	2	2	1	1	4	4
2	2	4	4	2	2	1	1	2	2
3	3	1	1	2	2	1	1	2	2
3	3	1	1	3	3	1	1	2	2
2	2	1	1	2	2	1	1	2	2
3	3	1	1	2	2	1	1	2	2
3	3	4	4	2	2	1	1	2	2
3	3	4	4	2	2	1	1	2	2
3	3	4	4	2	2	1	1	2	2
2	2	1	1	2	2	1	1	2	2
3	3	1	1	2	2	2	2	2	2
3	3	1	1	2	2	1	1	2	2
3	3	4	4	2	2	1	1	2	2
3	3	4	4	2	2	1	1	2	2
2	2	4	4	2	2	1	1	2	2
3	3	4	4	2	2	1	1	4	4
3	3	1	1	3	3	1	1	2	2
3	3	4	4	2	2	1	1	2	2
2	2	4	4	2	2	1	1	2	2
3	3	1	1	2	2	1	1	2	2
3	3	4	4	2	2	1	1	2	2
3	3	4	4	2	2	1	1	4	4
3	3	4	4	2	2	1	1	2	2
3	3	1	1	2	2	1	1	2	2
2	2	4	4	3	3	2	2	2	2
3	3	4	4	2	2	1	1	2	2
3	3	4	4	2	2	1	1	2	2

For Review Only

3	3	4	4	2	2	1	1	2	2
3	3	4	4	2	2	2	2	2	2

For Review Only

SNP522	SNP522	SNP523	SNP523	SNP524	SNP524	SNP525	SNP525	SNP526	SNP526
4	4	4	4	1	1	1	1	4	4
4	4	4	4	1	1	1	1	4	4
4	4	4	4	1	1	1	1	4	4
4	4	4	4	1	1	1	1	3	3
4	4	4	4	1	1	1	1	4	4
4	4	4	4	1	1	1	1	4	4
4	4	4	4	1	1	1	1	4	4
4	4	4	4	1	1	1	1	4	4
4	4	4	4	1	1	1	1	4	4
4	4	4	4	4	4	4	4	4	4
3	3	1	1	1	1	1	1	4	4
4	4	4	4	1	1	1	1	4	4
4	4	4	4	1	1	1	1	4	4
4	4	4	4	1	1	1	1	4	4
4	4	4	4	1	1	1	1	4	4
4	4	4	4	1	1	1	1	4	4
4	4	4	4	1	1	1	1	4	4
4	4	4	4	1	1	1	1	4	4
4	4	4	4	1	1	1	1	4	4
4	4	4	4	1	1	1	1	4	4
4	4	4	4	1	1	1	1	4	4
4	4	4	4	1	1	1	1	4	4
4	4	4	4	1	1	1	1	4	4
4	4	4	4	1	1	1	1	4	4
4	4	4	4	1	1	1	1	4	4
4	4	4	4	1	1	1	1	4	4
4	4	4	4	1	1	1	1	3	3
4	4	4	4	1	1	1	1	4	4
4	4	4	4	1	1	1	1	4	4
4	4	4	4	1	1	1	1	4	4
4	4	4	4	4	4	4	4	4	4
4	4	4	4	1	1	1	1	4	4
4	4	4	4	1	1	1	1	4	4
4	4	4	4	1	1	1	1	4	4
4	4	4	4	1	1	1	1	4	4
4	4	4	4	1	1	1	1	4	4
4	4	4	4	1	1	1	1	3	3
4	4	4	4	1	1	1	1	4	4
4	4	4	4	1	1	1	1	4	4
4	4	4	4	1	1	1	1	4	4
4	4	4	4	1	1	1	1	4	4
4	4	4	4	1	1	1	1	4	4
4	4	4	4	1	1	1	1	4	4
4	4	4	4	4	4	4	4	4	4
4	4	4	4	1	1	1	1	4	4
4	4	4	4	1	1	1	1	4	4
4	4	4	4	1	1	1	1	4	4
4	4	4	4	1	1	1	1	4	4
4	4	4	4	1	1	1	1	4	4
4	4	4	4	1	1	1	1	3	3
4	4	4	4	1	1	1	1	4	4
4	4	4	4	1	1	1	1	4	4

For Review Only

4	4	4	4	1	1	1	1	4	4
4	4	4	4	1	1	1	1	4	4
4	4	4	4	1	1	1	1	3	3
4	4	4	4	1	1	1	1	4	4
4	4	4	4	1	1	1	1	4	4
4	4	4	4	1	1	1	1	4	4
4	4	4	4	1	1	1	1	4	4
4	4	4	4	1	1	1	1	4	4
4	4	4	4	1	1	1	1	3	3
4	4	4	4	1	1	1	1	4	4
4	4	4	4	1	1	1	1	4	4
4	4	4	4	1	1	1	1	4	4
4	4	4	4	1	1	1	1	4	4
4	4	4	4	1	1	1	1	4	4
4	4	4	4	1	1	1	1	4	4
4	4	4	4	1	1	1	1	4	4
4	4	4	4	1	1	1	1	4	4
4	4	4	4	1	1	1	1	3	3
4	4	4	4	1	1	1	1	4	4
4	4	4	4	1	1	1	1	4	4
4	4	4	4	1	1	1	1	4	4
4	4	4	4	1	1	1	1	3	3
4	4	4	4	1	1	1	1	4	4
4	4	4	4	4	4	4	4	4	4
4	4	4	4	1	1	1	1	4	4
4	4	4	4	1	1	1	1	4	4
4	4	4	4	4	4	4	4	4	4
4	4	4	4	1	1	1	1	4	4
4	4	4	4	1	1	1	1	4	4
3	3	1	1	1	1	1	1	4	4
4	4	4	4	4	4	1	1	4	4
4	4	4	4	1	1	1	1	4	4
4	4	4	4	1	1	1	1	4	4
4	4	4	4	1	1	1	1	4	4
4	4	4	4	1	1	1	1	4	4
4	4	4	4	1	1	1	1	4	4
4	4	4	4	4	4	4	4	4	4
4	4	4	4	1	1	1	1	4	4
4	4	4	4	1	1	1	1	3	3
4	4	4	4	1	1	1	1	4	4
4	4	4	4	1	1	1	1	4	4
3	3	1	1	1	1	1	1	4	4
4	4	4	4	1	1	1	1	4	4
4	4	4	4	1	1	1	1	4	4
3	3	1	1	1	1	1	1	3	3
4	4	4	4	1	1	1	1	4	4
4	4	4	4	1	1	1	1	4	4
4	4	4	4	1	1	1	1	4	4
4	4	4	4	1	1	1	1	4	4
4	4	4	4	1	1	1	1	3	3
4	4	4	4	1	1	1	1	4	4
4	4	4	4	1	1	1	1	4	4
4	4	4	4	1	1	1	1	4	4
4	4	4	4	1	1	1	1	4	4
4	4	4	4	1	1	1	1	4	4

For Review Only

4	4	4	4	1	1	1	1	4	4
4	4	4	4	1	1	1	1	3	3
4	4	4	4	1	1	1	1	4	4
4	4	4	4	1	1	1	1	4	4
4	4	4	4	1	1	1	1	4	4
4	4	4	4	1	1	1	1	4	4
4	4	4	4	1	1	1	1	4	4
4	4	4	4	1	1	1	1	4	4
4	4	4	4	1	1	1	1	4	4
4	4	4	4	1	1	1	1	4	4
4	4	4	4	1	1	1	1	4	4
4	4	4	4	1	1	1	1	4	4
4	4	4	4	1	1	1	1	4	4
4	4	4	4	1	1	1	1	4	4
4	4	4	4	1	1	1	1	4	4
4	4	4	4	1	1	1	1	4	4
4	4	4	4	1	1	1	1	4	4
4	4	4	4	1	1	1	1	4	4
4	4	4	4	1	1	1	1	4	4
4	4	4	4	1	1	1	1	4	4
4	4	4	4	1	1	1	1	4	4
4	4	4	4	1	1	1	1	4	4
4	4	4	4	1	1	1	1	4	4
4	4	4	4	1	1	1	1	4	4
4	4	4	4	1	1	1	1	3	3
4	4	4	4	1	1	1	1	3	3
4	4	4	4	1	1	1	1	3	3
4	4	4	4	1	1	1	1	4	4
4	4	4	4	1	1	1	1	4	4
4	4	4	4	1	1	1	1	4	4
4	4	4	4	1	1	1	1	4	4
4	4	4	4	1	1	1	1	4	4
4	4	4	4	1	1	1	1	4	4
4	4	4	4	1	1	1	1	4	4
4	4	4	4	1	1	1	1	4	4
4	4	4	4	1	1	1	1	4	4
4	4	4	4	1	1	1	1	4	4
4	4	4	4	1	1	1	1	4	4
4	4	4	4	1	1	1	1	4	4
3	3	4	4	1	1	1	1	4	4
4	4	1	1	1	1	1	1	3	3
4	4	4	4	1	1	1	1	4	4
4	4	4	4	1	1	1	1	3	3
4	4	4	4	1	1	1	1	4	4
4	4	4	4	1	1	1	1	3	3
4	4	4	4	1	1	1	1	4	4
4	4	4	4	1	1	1	1	4	4
4	4	4	4	1	1	1	1	4	4
4	4	4	4	1	1	1	1	3	3
4	4	4	4	1	1	1	1	4	4
4	4	4	4	1	1	1	1	4	4
4	4	4	4	1	1	1	1	4	4
4	4	4	4	1	1	1	1	3	3
4	4	4	4	1	1	1	1	4	4
4	4	4	4	1	1	1	1	4	4

For Review Only

4	4	4	4	1	1	1	1	4	4
4	4	4	4	1	1	1	1	4	4
4	4	4	4	1	1	1	1	4	4
4	4	4	4	1	1	1	1	4	4
4	4	4	4	1	1	1	1	4	4
4	4	4	4	1	1	1	1	4	4
4	4	4	4	1	1	1	1	4	4
4	4	4	4	1	1	1	1	4	4
4	4	4	4	1	1	1	1	3	3
3	3	1	1	1	1	1	1	4	4
4	4	4	4	1	1	1	1	4	4
4	4	4	4	1	1	1	1	4	4
4	4	4	4	1	1	1	1	4	4
4	4	4	4	1	1	1	1	3	3
4	4	4	4	1	1	1	1	4	4
3	3	1	1	1	1	1	1	4	4
4	4	4	4	1	1	1	1	3	3
4	4	4	4	4	4	4	4	4	4
4	4	4	4	1	1	1	1	4	4
4	4	4	4	1	1	1	1	4	4
4	4	4	4	1	1	1	1	4	4
4	4	4	4	1	1	1	1	4	4
4	4	4	4	1	1	1	1	3	3
4	4	4	4	1	1	1	1	4	4
4	4	4	4	1	1	1	1	4	4
4	4	4	4	1	1	1	1	4	4
4	4	4	4	1	1	1	1	4	4
4	4	4	4	1	1	1	1	4	4
4	4	4	4	1	1	1	1	4	4
4	4	4	4	1	1	1	1	4	4
4	4	4	4	4	4	4	4	3	3
3	3	1	1	1	1	1	1	4	4
4	4	4	4	4	4	4	4	4	4
4	4	4	4	1	1	1	1	4	4
4	4	4	4	4	4	4	4	4	4
4	4	4	4	1	1	1	1	4	4
4	4	4	4	1	1	1	1	4	4
4	4	4	4	1	1	1	1	4	4
4	4	4	4	1	1	1	1	4	4
4	4	4	4	1	1	1	1	4	4
4	4	4	4	1	1	1	1	4	4
4	4	4	4	1	1	1	1	4	4
4	4	4	4	1	1	1	1	4	4
4	4	4	4	1	1	1	1	4	4
4	4	4	4	1	1	1	1	4	4
4	4	4	4	1	1	1	1	4	4
3	3	1	1	1	1	1	1	4	4
4	4	4	4	1	1	1	1	4	4
4	4	4	4	1	1	1	1	4	4
4	4	4	4	1	1	1	1	4	4
4	4	4	4	1	1	1	1	4	4
4	4	4	4	1	1	1	1	4	4
4	4	4	4	1	1	1	1	3	3
4	4	4	4	1	1	1	1	4	4
4	4	4	4	1	1	1	1	4	4

For Review Only

4	4	4	4	1	1	1	1	4	4
4	4	4	4	1	1	1	1	4	4
4	4	4	4	1	1	1	1	3	3
4	4	4	4	1	1	1	1	3	3
4	4	4	4	1	1	1	1	4	4
4	4	4	4	1	1	1	1	4	4
4	4	4	4	1	1	1	1	4	4
4	4	4	4	1	1	1	1	4	4
4	4	4	4	1	1	1	1	4	4
4	4	4	4	1	1	1	1	4	4
4	4	4	4	1	1	1	1	4	4
4	4	4	4	1	1	1	1	4	4
4	4	4	4	1	1	1	1	3	3
4	4	4	4	1	1	1	1	4	4
4	4	4	4	1	1	1	1	4	4
3	3	1	1	1	1	1	1	4	4
4	4	4	4	1	1	1	1	4	4
4	4	4	4	1	1	1	1	4	4
4	4	4	4	1	1	1	1	4	4
4	4	4	4	1	1	1	1	4	4
4	4	4	4	4	4	4	4	4	4
4	4	4	4	1	1	1	1	4	4
4	4	4	4	1	1	1	1	3	3
4	4	4	4	1	1	1	1	4	4
4	4	4	4	1	1	1	1	4	4
4	4	4	4	1	1	1	1	4	4
4	4	4	4	1	1	1	1	4	4
4	4	4	4	1	1	1	1	4	4
4	4	4	4	1	1	1	1	4	4
4	4	4	4	1	1	1	1	4	4
4	4	4	4	1	1	1	1	4	4
4	4	4	4	1	1	1	1	4	4
4	4	4	4	1	1	1	1	4	4
4	4	4	4	1	1	1	1	4	4
4	4	4	4	1	1	1	1	3	3
4	4	4	4	1	1	1	1	4	4
4	4	4	4	1	1	1	1	3	3
4	4	4	4	1	1	1	1	4	4
4	4	4	4	1	1	1	1	4	4
4	4	4	4	1	1	1	1	4	4
4	4	4	4	1	1	1	1	3	3
4	4	4	4	1	1	1	1	3	3
4	4	4	4	1	1	1	1	4	4
4	4	4	4	1	1	1	1	4	4
4	4	4	4	1	1	1	1	4	4
4	4	4	4	1	1	1	1	3	3
4	4	4	4	1	1	1	1	4	4
4	4	4	4	1	1	1	1	4	4
4	4	4	4	1	1	1	1	4	4
4	4	4	4	1	1	1	1	4	4
3	3	1	1	4	4	4	4	4	4
4	4	4	4	1	1	1	1	4	4
4	4	4	4	1	1	1	1	4	4
4	4	4	4	1	1	1	1	4	4
4	4	4	4	1	1	1	1	4	4

For Review Only

3	3	1	1	1	1	1	1	4	4
4	4	4	4	1	1	1	1	4	4

For Review Only

SNP527	SNP527	SNP528	SNP528	SNP529	SNP529	SNP530	SNP530	SNP531	SNP531
2	2	3	3	3	3	3	3	4	4
2	2	3	3	3	3	3	3	4	4
2	2	3	3	3	3	3	3	4	4
2	2	3	3	3	3	3	3	4	4
2	2	3	3	3	3	3	3	4	4
2	2	3	3	3	3	3	3	4	4
2	2	3	3	3	3	3	3	4	4
2	2	3	3	3	3	3	3	4	4
2	2	3	3	2	2	3	3	4	4
2	2	3	3	3	3	2	2	4	4
2	2	1	1	3	3	3	3	1	1
2	2	3	3	3	3	3	3	4	4
2	2	3	3	3	3	3	3	1	1
2	2	3	3	2	2	3	3	4	4
2	2	3	3	3	3	3	3	4	4
2	2	3	3	3	3	3	3	4	4
2	2	3	3	3	3	3	3	4	4
2	2	3	3	3	3	3	3	4	4
2	2	3	3	3	3	2	2	4	4
3	3	3	3	3	3	3	3	4	4
2	2	3	3	3	3	3	3	4	4
2	2	3	3	3	3	3	3	4	4
3	3	3	3	3	3	3	3	4	4
2	2	3	3	3	3	3	3	4	4
2	2	3	3	3	3	3	3	4	4
2	2	3	3	3	3	2	2	4	4
2	2	3	3	3	3	3	3	1	1
2	2	1	1	3	3	3	3	4	4
2	2	3	3	3	3	3	3	4	4
3	3	3	3	3	3	3	3	4	4
2	2	3	3	3	3	2	2	4	4
2	2	3	3	3	3	3	3	4	4
2	2	3	3	3	3	3	3	4	4
2	2	3	3	2	2	3	3	4	4
2	2	3	3	3	3	2	2	4	4
2	2	3	3	3	3	3	3	4	4
2	2	3	3	3	3	3	3	4	4
2	2	3	3	3	3	3	3	4	4
2	2	3	3	3	3	3	3	4	4
3	3	3	3	3	3	3	3	4	4
3	3	3	3	3	3	3	3	4	4
2	2	3	3	3	3	3	3	4	4
2	2	3	3	3	3	3	3	4	4
2	2	3	3	2	2	2	2	4	4
3	3	3	3	3	3	2	2	4	4
2	2	3	3	3	3	3	3	4	4
2	2	3	3	3	3	2	2	4	4
2	2	3	3	3	3	2	2	4	4
3	3	3	3	2	2	2	2	4	4
2	2	3	3	3	3	3	3	1	1
2	2	3	3	3	3	2	2	4	4
2	2	3	3	3	3	2	2	4	4

For Review Only

2	2	3	3	3	3	3	3	4	4
2	2	3	3	3	3	3	3	4	4
2	2	3	3	3	3	3	3	4	4
2	2	3	3	3	3	3	3	4	4
2	2	3	3	3	3	3	3	4	4
2	2	3	3	3	3	3	3	4	4
2	2	3	3	3	3	3	3	4	4
2	2	3	3	3	3	2	2	4	4
2	2	3	3	2	2	3	3	4	4
2	2	3	3	3	3	3	3	4	4
2	2	3	3	3	3	3	3	4	4
2	2	3	3	3	3	3	3	4	4
2	2	3	3	3	3	3	3	4	4
2	2	3	3	3	3	3	3	4	4
2	2	3	3	3	3	2	2	4	4
2	2	3	3	2	2	3	3	4	4
2	2	3	3	3	3	2	2	4	4
2	2	3	3	3	3	3	3	4	4
2	2	3	3	3	3	3	3	4	4
2	2	3	3	3	3	3	3	4	4
2	2	3	3	3	3	3	3	4	4
3	3	3	3	3	3	3	3	4	4
2	2	3	3	3	3	3	3	4	4
2	2	3	3	3	3	2	2	4	4
3	3	3	3	3	3	2	2	4	4
2	2	3	3	3	3	3	3	4	4
3	3	3	3	3	3	2	2	4	4
2	2	3	3	2	2	3	3	4	4
2	2	3	3	3	3	2	2	4	4
2	2	1	1	3	3	3	3	1	1
2	2	1	1	3	3	2	2	4	4
2	2	3	3	3	3	3	3	4	4
2	2	3	3	3	3	3	3	4	4
2	2	3	3	3	3	3	3	4	4
2	2	3	3	3	3	3	3	4	4
3	3	3	3	3	3	2	2	4	4
2	2	1	1	3	3	2	2	4	4
2	2	3	3	3	3	3	3	4	4
3	3	3	3	3	3	3	3	4	4
2	2	3	3	3	3	2	2	4	4
2	2	3	3	3	3	3	3	4	4
2	2	1	1	3	3	3	3	1	1
2	2	3	3	3	3	2	2	4	4
2	2	3	3	3	3	3	3	4	4
2	2	1	1	2	2	3	3	1	1
2	2	3	3	3	3	3	3	4	4
2	2	3	3	3	3	3	3	4	4
2	2	3	3	3	3	3	3	4	4
2	2	3	3	3	3	3	3	4	4
2	2	3	3	3	3	3	3	4	4
2	2	3	3	3	3	3	3	4	4
2	2	3	3	3	3	3	3	4	4
2	2	3	3	2	2	3	3	4	4
2	2	3	3	3	3	3	3	4	4
2	2	3	3	2	2	3	3	4	4

For Review Only

2	2	3	3	3	3	3	3	4	4
2	2	3	3	3	3	3	3	4	4
2	2	3	3	3	3	3	3	4	4
2	2	3	3	3	3	3	3	4	4
2	2	3	3	3	3	2	2	4	4
2	2	3	3	3	3	3	3	4	4
2	2	3	3	3	3	3	3	4	4
2	2	3	3	3	3	3	3	4	4
2	2	3	3	3	3	2	2	4	4
2	2	3	3	3	3	3	3	4	4
2	2	3	3	3	3	3	3	4	4
2	2	3	3	3	3	2	2	4	4
2	2	3	3	3	3	3	3	4	4
2	2	3	3	3	3	3	3	4	4
2	2	3	3	3	3	3	3	1	1
2	2	3	3	3	3	2	2	4	4
2	2	3	3	3	3	3	3	4	4
2	2	3	3	3	3	3	3	4	4
2	2	3	3	3	3	3	3	4	4
2	2	3	3	3	3	3	3	4	4
2	2	3	3	3	3	2	2	4	4
2	2	3	3	3	3	3	3	1	1
2	2	3	3	3	3	3	3	1	1
2	2	3	3	2	2	3	3	4	4
3	3	3	3	3	3	2	2	4	4
2	2	3	3	3	3	3	3	4	4
2	2	3	3	3	3	3	3	4	4
2	2	3	3	2	2	3	3	4	4
2	2	3	3	3	3	3	3	4	4
2	2	3	3	3	3	3	3	4	4
2	2	3	3	3	3	3	3	4	4
2	2	3	3	3	3	3	3	4	4
2	2	3	3	3	3	3	3	4	4
2	2	3	3	3	3	2	2	4	4
2	2	3	3	2	2	3	3	4	4
2	2	3	3	3	3	3	3	4	4
2	2	3	3	2	2	3	3	4	4
2	2	3	3	3	3	3	3	4	4
2	2	3	3	3	3	3	3	4	4
2	2	3	3	3	3	3	3	1	1
3	3	3	3	3	3	3	3	1	1
2	2	3	3	2	2	3	3	4	4
2	2	3	3	2	2	3	3	4	4
2	2	3	3	3	3	3	3	4	4
2	2	3	3	3	3	2	2	4	4
2	2	3	3	3	3	3	3	4	4
2	2	3	3	3	3	2	2	4	4
2	2	3	3	3	3	3	3	4	4
2	2	3	3	3	3	3	3	4	4
2	2	3	3	3	3	2	2	4	4
2	2	3	3	3	3	2	2	4	4
2	2	3	3	3	3	3	3	4	4
2	2	3	3	3	3	3	3	1	1
2	2	3	3	3	3	2	2	4	4
2	2	3	3	3	3	3	3	4	4

For Review Only

2	2	3	3	3	3	2	2	4	4
2	2	3	3	3	3	2	2	4	4
2	2	3	3	3	3	3	3	4	4
2	2	3	3	3	3	3	3	4	4
2	2	3	3	3	3	3	3	4	4
2	2	3	3	3	3	3	3	4	4
2	2	3	3	3	3	3	3	1	1
2	2	3	3	3	3	3	3	4	4
2	2	3	3	3	3	3	3	4	4
2	2	1	1	3	3	3	3	1	1
2	2	3	3	3	3	2	2	4	4
2	2	3	3	3	3	3	3	4	4
2	2	3	3	3	3	2	2	4	4
2	2	3	3	3	3	3	3	4	4
2	2	3	3	3	3	3	3	4	4
2	2	1	1	3	3	3	3	1	1
2	2	3	3	3	3	3	3	4	4
2	2	3	3	3	3	2	2	4	4
2	2	3	3	3	3	2	2	4	4
2	2	3	3	3	3	3	3	4	4
2	2	3	3	3	3	3	3	4	4
2	2	3	3	3	3	3	3	4	4
3	3	3	3	3	3	3	3	4	4
2	2	3	3	3	3	3	3	4	4
2	2	3	3	2	2	3	3	4	4
2	2	3	3	2	2	3	3	4	4
2	2	3	3	3	3	3	3	4	4
2	2	3	3	3	3	3	3	4	4
3	3	3	3	3	3	3	3	4	4
2	2	3	3	3	3	3	3	4	4
2	2	3	3	2	2	2	2	4	4
2	2	1	1	3	3	3	3	1	1
2	2	3	3	3	3	2	2	4	4
2	2	3	3	3	3	3	3	4	4
2	2	3	3	3	3	2	2	4	4
2	2	3	3	3	3	3	3	4	4
2	2	3	3	3	3	3	3	4	4
2	2	3	3	3	3	3	3	4	4
2	2	3	3	3	3	3	3	4	4
2	2	3	3	3	3	3	3	4	4
2	2	3	3	3	3	3	3	4	4
2	2	3	3	3	3	2	2	4	4
2	2	3	3	3	3	3	3	4	4
2	2	3	3	3	3	3	3	4	4
2	2	3	3	3	3	3	3	4	4
2	2	3	3	3	3	3	3	1	1
2	2	1	1	3	3	3	3	1	1
2	2	3	3	3	3	3	3	1	1
2	2	3	3	3	3	3	3	4	4
2	2	3	3	3	3	3	3	4	4
2	2	3	3	3	3	3	3	4	4
2	2	3	3	3	3	3	3	4	4
3	3	3	3	3	3	3	3	4	4
2	2	3	3	3	3	3	3	4	4
2	2	3	3	2	2	3	3	4	4

2	2	3	3	3	3	3	3	4	4
3	3	3	3	3	3	3	3	4	4
2	2	3	3	3	3	3	3	1	1
3	3	3	3	3	3	3	3	4	4
2	2	3	3	3	3	3	3	4	4
2	2	3	3	3	3	2	2	4	4
2	2	3	3	3	3	2	2	4	4
2	2	3	3	2	2	3	3	4	4
2	2	3	3	3	3	3	3	4	4
2	2	3	3	2	2	3	3	4	4
2	2	3	3	3	3	3	3	4	4
2	2	3	3	3	3	3	3	4	4
3	3	3	3	3	3	3	3	4	4
2	2	3	3	3	3	3	3	4	4
2	2	3	3	3	3	2	2	4	4
3	3	1	1	3	3	3	3	1	1
2	2	3	3	3	3	3	3	4	4
2	2	3	3	3	3	3	3	4	4
2	2	3	3	3	3	3	3	4	4
2	2	3	3	3	3	3	3	4	4
3	3	3	3	3	3	2	2	4	4
2	2	3	3	3	3	2	2	4	4
2	2	3	3	3	3	3	3	4	4
2	2	3	3	2	2	3	3	4	4
2	2	3	3	3	3	3	3	4	4
2	2	3	3	3	3	3	3	4	4
2	2	3	3	3	3	3	3	4	4
2	2	3	3	3	3	3	3	4	4
2	2	3	3	3	3	3	3	4	4
2	2	3	3	3	3	3	3	4	4
2	2	3	3	3	3	3	3	4	4
2	2	3	3	3	3	2	2	4	4
2	2	3	3	3	3	3	3	4	4
2	2	3	3	3	3	3	3	4	4
3	3	3	3	3	3	3	3	4	4
2	2	3	3	3	3	3	3	4	4
2	2	3	3	3	3	3	3	1	1
2	2	3	3	3	3	3	3	4	4
2	2	3	3	3	3	3	3	4	4
2	2	3	3	3	3	3	3	4	4
2	2	3	3	3	3	2	2	4	4
2	2	3	3	2	2	3	3	4	4
2	2	3	3	3	3	3	3	4	4
2	2	3	3	3	3	3	3	4	4
2	2	3	3	3	3	3	3	4	4
3	3	3	3	3	3	3	3	4	4
2	2	3	3	3	3	3	3	4	4
2	2	3	3	3	3	3	3	4	4
2	2	3	3	3	3	3	3	4	4
2	2	3	3	3	3	3	3	4	4
2	2	1	1	3	3	2	2	1	1
2	2	3	3	3	3	3	3	4	4
2	2	3	3	3	3	3	3	4	4
2	2	3	3	3	3	3	3	4	4
2	2	3	3	3	3	3	3	4	4

For Review Only

2	2	1	1	3	3	3	3	1	1
2	2	3	3	3	3	3	3	4	4

For Review Only

SNP532	SNP532	SNP533	SNP533	SNP534	SNP534	SNP535	SNP535	SNP536	SNP536
1	1	2	2	1	1	3	3	3	3
1	1	2	2	1	1	3	3	3	3
1	1	2	2	1	1	3	3	3	3
1	1	2	2	1	1	3	3	3	3
1	1	2	2	1	1	3	3	2	2
1	1	2	2	1	1	3	3	3	3
1	1	2	2	1	1	3	3	2	2
1	1	3	3	1	1	3	3	2	2
1	1	2	2	1	1	3	3	3	3
1	1	3	3	1	1	3	3	3	3
2	2	2	2	1	1	3	3	3	3
1	1	2	2	1	1	3	3	3	3
1	1	2	2	1	1	3	3	3	3
1	1	2	2	1	1	3	3	3	3
1	1	2	2	1	1	3	3	3	3
1	1	2	2	1	1	3	3	2	2
1	1	2	2	1	1	3	3	3	3
1	1	2	2	1	1	3	3	3	3
1	1	2	2	1	1	3	3	3	3
1	1	3	3	1	1	3	3	2	2
1	1	3	3	1	1	3	3	3	3
1	1	2	2	1	1	3	3	2	2
1	1	2	2	1	1	3	3	2	2
1	1	2	2	1	1	3	3	3	3
1	1	2	2	1	1	3	3	2	2
1	1	2	2	1	1	3	3	3	3
1	1	2	2	1	1	3	3	3	3
1	1	2	2	1	1	3	3	3	3
1	1	2	2	1	1	3	3	3	3
1	1	3	3	1	1	3	3	2	2
1	1	2	2	1	1	3	3	3	3
1	1	3	3	1	1	3	3	2	2
1	1	3	3	1	1	3	3	2	2
1	1	2	2	2	2	3	3	2	2
1	1	2	2	1	1	3	3	3	3
1	1	2	2	1	1	3	3	2	2
1	1	2	2	1	1	3	3	3	3
1	1	2	2	1	1	3	3	3	3
1	1	2	2	1	1	3	3	2	2
1	1	2	2	1	1	3	3	2	2
1	1	3	3	1	1	3	3	3	3
1	1	2	2	1	1	3	3	3	3
1	1	2	2	1	1	2	2	3	3
1	1	2	2	1	1	3	3	3	3
1	1	2	2	1	1	2	2	2	2
1	1	2	2	1	1	3	3	2	2
1	1	2	2	1	1	3	3	3	3
1	1	2	2	1	1	3	3	2	2
1	1	2	2	1	1	3	3	2	2
1	1	2	2	1	1	3	3	2	2
1	1	2	2	1	1	3	3	3	3
1	1	2	2	1	1	3	3	3	3

For Review Only

1	1	2	2	1	1	3	3	2	2
1	1	3	3	1	1	3	3	2	2
1	1	2	2	1	1	2	2	3	3
1	1	2	2	1	1	3	3	2	2
1	1	2	2	1	1	3	3	3	3
1	1	2	2	1	1	3	3	3	3
1	1	2	2	2	2	3	3	3	3
1	1	2	2	1	1	3	3	3	3
1	1	2	2	1	1	3	3	2	2
1	1	2	2	1	1	3	3	3	3
1	1	2	2	1	1	2	2	3	3
1	1	2	2	1	1	2	2	3	3
1	1	2	2	1	1	3	3	2	2
1	1	2	2	1	1	3	3	3	3
1	1	2	2	1	1	3	3	3	3
1	1	2	2	1	1	3	3	2	2
1	1	2	2	1	1	3	3	3	3
1	1	2	2	1	1	3	3	2	2
1	1	3	3	1	1	3	3	3	3
1	1	2	2	1	1	3	3	2	2
1	1	2	2	1	1	2	2	3	3
1	1	2	2	1	1	3	3	2	2
1	1	2	2	1	1	3	3	3	3
1	1	2	2	1	1	2	2	2	2
1	1	2	2	1	1	2	2	2	2
1	1	2	2	1	1	2	2	3	3
1	1	2	2	1	1	3	3	2	2
1	1	2	2	1	1	3	3	2	2
1	1	2	2	1	1	3	3	3	3
2	2	2	2	1	1	3	3	3	3
1	1	2	2	1	1	3	3	2	2
1	1	2	2	1	1	3	3	3	3
1	1	2	2	1	1	3	3	2	2
1	1	2	2	1	1	3	3	2	2
1	1	2	2	1	1	3	3	2	2
1	1	2	2	1	1	2	2	2	2
1	1	2	2	1	1	3	3	3	3
1	1	3	3	1	1	3	3	2	2
1	1	2	2	1	1	3	3	2	2
1	1	3	3	1	1	2	2	2	2
1	1	2	2	1	1	3	3	2	2
2	2	2	2	1	1	3	3	3	3
1	1	2	2	1	1	3	3	2	2
1	1	2	2	1	1	2	2	3	3
2	2	2	2	1	1	3	3	2	2
1	1	2	2	2	2	3	3	3	3
1	1	2	2	1	1	3	3	3	3
1	1	2	2	1	1	3	3	3	3
1	1	2	2	2	2	3	3	2	2
1	1	2	2	2	2	3	3	2	2
1	1	2	2	1	1	3	3	3	3
1	1	2	2	1	1	3	3	3	3
1	1	2	2	1	1	3	3	3	3
1	1	2	2	1	1	3	3	3	3
1	1	3	3	2	2	3	3	2	2

For Review Only

1	1	2	2	1	1	3	3	3	3
1	1	2	2	1	1	3	3	3	3
1	1	2	2	1	1	3	3	3	3
1	1	2	2	1	1	3	3	3	3
1	1	2	2	1	1	3	3	3	3
1	1	2	2	1	1	3	3	3	3
1	1	2	2	1	1	3	3	3	3
1	1	2	2	1	1	3	3	2	2
1	1	2	2	2	2	3	3	3	3
1	1	2	2	1	1	3	3	2	2
1	1	2	2	1	1	3	3	2	2
1	1	2	2	1	1	3	3	3	3
1	1	2	2	1	1	3	3	3	3
1	1	2	2	1	1	2	2	3	3
1	1	2	2	1	1	2	2	3	3
1	1	2	2	1	1	3	3	3	3
1	1	2	2	1	1	3	3	3	3
1	1	2	2	1	1	3	3	3	3
1	1	2	2	1	1	3	3	3	3
1	1	2	2	1	1	3	3	2	2
1	1	2	2	1	1	3	3	3	3
1	1	2	2	1	1	3	3	3	3
1	1	2	2	1	1	2	2	2	2
1	1	2	2	2	2	3	3	2	2
1	1	2	2	1	1	3	3	2	2
1	1	2	2	1	1	3	3	2	2
1	1	2	2	1	1	3	3	2	2
1	1	2	2	1	1	3	3	2	2
1	1	2	2	1	1	3	3	2	2
1	1	2	2	2	2	3	3	2	2
1	1	2	2	1	1	3	3	3	3
1	1	2	2	1	1	3	3	3	3
1	1	2	2	1	1	3	3	2	2
1	1	2	2	1	1	3	3	3	3
1	1	2	2	2	2	3	3	2	2
1	1	2	2	1	1	3	3	2	2
1	1	2	2	1	1	3	3	3	3
1	1	2	2	1	1	3	3	3	3
1	1	2	2	2	2	3	3	3	3
1	1	2	2	1	1	3	3	3	3
1	1	2	2	1	1	3	3	2	2
1	1	2	2	1	1	3	3	2	2
1	1	2	2	2	2	3	3	2	2
1	1	2	2	1	1	3	3	3	3
1	1	2	2	1	1	3	3	2	2
1	1	2	2	1	1	3	3	2	2
1	1	2	2	1	1	3	3	3	3
1	1	2	2	1	1	3	3	3	3
1	1	2	2	1	1	2	2	3	3
1	1	2	2	1	1	3	3	3	3
1	1	2	2	1	1	3	3	3	3
1	1	3	3	1	1	3	3	2	2
1	1	2	2	1	1	3	3	3	3
1	1	2	2	1	1	2	2	3	3
1	1	2	2	1	1	3	3	3	3

For Review Only

1	1	2	2	2	2	3	3	3	3
1	1	2	2	1	1	3	3	3	3
1	1	2	2	1	1	2	2	2	2
1	1	2	2	1	1	3	3	3	3
1	1	2	2	1	1	3	3	3	3
1	1	2	2	1	1	3	3	2	2
1	1	2	2	1	1	3	3	3	3
1	1	2	2	1	1	3	3	3	3
1	1	2	2	1	1	2	2	2	2
2	2	2	2	2	2	3	3	3	3
1	1	2	2	1	1	3	3	3	3
1	1	2	2	1	1	3	3	3	3
1	1	2	2	1	1	3	3	3	3
1	1	2	2	1	1	3	3	3	3
1	1	2	2	1	1	3	3	3	3
2	2	2	2	1	1	3	3	3	3
1	1	2	2	1	1	3	3	2	2
1	1	2	2	1	1	3	3	3	3
1	1	2	2	1	1	3	3	2	2
1	1	2	2	1	1	3	3	3	3
1	1	2	2	1	1	3	3	3	3
1	1	2	2	1	1	2	2	2	2
1	1	2	2	1	1	3	3	2	2
1	1	2	2	1	1	2	2	3	3
1	1	2	2	1	1	3	3	3	3
1	1	2	2	2	2	3	3	2	2
1	1	2	2	1	1	3	3	3	3
1	1	2	2	1	1	3	3	2	2
1	1	2	2	1	1	3	3	2	2
1	1	2	2	2	2	3	3	2	2
1	1	2	2	2	2	3	3	3	3
2	2	2	2	1	1	3	3	3	3
1	1	2	2	2	2	3	3	2	2
1	1	2	2	1	1	3	3	3	3
1	1	2	2	1	1	3	3	2	2
1	1	2	2	1	1	2	2	3	3
1	1	2	2	1	1	3	3	3	3
1	1	2	2	1	1	3	3	2	2
1	1	2	2	1	1	3	3	3	3
1	1	2	2	1	1	3	3	2	2
1	1	2	2	1	1	3	3	3	3
1	1	2	2	1	1	3	3	3	3
1	1	2	2	1	1	3	3	2	2
1	1	2	2	1	1	3	3	3	3
1	1	2	2	1	1	3	3	2	2
2	2	2	2	1	1	3	3	3	3
2	2	2	2	1	1	3	3	3	3
2	2	2	2	1	1	3	3	3	3
1	1	2	2	1	1	3	3	3	3
1	1	2	2	1	1	3	3	3	3
1	1	2	2	1	1	3	3	3	3
1	1	2	2	1	1	3	3	3	3
1	1	2	2	1	1	3	3	2	2
1	1	2	2	1	1	2	2	3	3
1	1	2	2	1	1	3	3	2	2

1	1	2	2	2	2	3	3	2	2
1	1	2	2	1	1	3	3	2	2
2	2	2	2	1	1	3	3	3	3
1	1	2	2	1	1	3	3	2	2
1	1	2	2	1	1	3	3	2	2
1	1	2	2	1	1	3	3	3	3
1	1	2	2	1	1	3	3	2	2
1	1	3	3	1	1	3	3	2	2
1	1	2	2	1	1	3	3	3	3
1	1	2	2	1	1	3	3	2	2
1	1	2	2	1	1	3	3	3	3
1	1	2	2	2	2	3	3	3	3
1	1	2	2	1	1	3	3	2	2
1	1	2	2	2	2	2	2	2	2
1	1	2	2	1	1	3	3	3	3
2	2	2	2	1	1	3	3	2	2
1	1	2	2	1	1	3	3	3	3
1	1	2	2	1	1	3	3	2	2
1	1	2	2	1	1	3	3	3	3
1	1	2	2	1	1	3	3	3	3
1	1	2	2	1	1	2	2	2	2
1	1	2	2	1	1	3	3	3	3
1	1	2	2	1	1	3	3	2	2
1	1	2	2	1	1	3	3	3	3
1	1	2	2	1	1	3	3	3	3
1	1	2	2	1	1	3	3	3	3
1	1	2	2	1	1	3	3	2	2
1	1	2	2	1	1	3	3	3	3
1	1	2	2	1	1	3	3	2	2
1	1	2	2	1	1	3	3	2	2
1	1	2	2	1	1	3	3	2	2
1	1	2	2	1	1	3	3	3	3
1	1	2	2	2	2	3	3	3	3
1	1	2	2	1	1	3	3	3	3
1	1	2	2	1	1	3	3	2	2
1	1	2	2	1	1	3	3	2	2
1	1	2	2	1	1	3	3	2	2
1	1	2	2	1	1	2	2	3	3
1	1	2	2	1	1	3	3	2	2
1	1	2	2	1	1	3	3	2	2
1	1	2	2	1	1	3	3	3	3
1	1	2	2	1	1	3	3	3	3
1	1	2	2	1	1	3	3	2	2
1	1	2	2	1	1	3	3	2	2
1	1	2	2	1	1	2	2	3	3
1	1	2	2	1	1	3	3	2	2
1	1	2	2	1	1	3	3	3	3
1	1	2	2	1	1	3	3	2	2
1	1	2	2	1	1	2	2	3	3
1	1	2	2	1	1	3	3	2	2
2	2	2	2	1	1	3	3	3	3
1	1	2	2	1	1	3	3	2	2
1	1	2	2	1	1	3	3	3	3
1	1	2	2	1	1	3	3	2	2
1	1	2	2	1	1	3	3	3	3

For Review Only

2	2	2	2	1	1	3	3	2	2
1	1	2	2	1	1	2	2	3	3

For Review Only

SNP537	SNP537	SNP538	SNP538	SNP539	SNP539	SNP540	SNP540	SNP541	SNP541
1	1	4	4	4	4	3	3	3	3
1	1	4	4	4	4	3	3	3	3
1	1	4	4	4	4	3	3	4	4
1	1	4	4	4	4	3	3	4	4
1	1	4	4	1	1	2	2	4	4
1	1	4	4	4	4	3	3	3	3
1	1	4	4	4	4	3	3	3	3
1	1	4	4	4	4	3	3	4	4
1	1	4	4	4	4	3	3	4	4
1	1	4	4	4	4	3	3	4	4
1	1	4	4	1	1	2	2	3	3
1	1	4	4	4	4	3	3	4	4
1	1	4	4	4	4	3	3	4	4
1	1	4	4	1	1	3	3	4	4
1	1	4	4	4	4	3	3	4	4
1	1	4	4	4	4	3	3	4	4
1	1	4	4	4	4	3	3	3	3
1	1	4	4	4	4	3	3	3	3
1	1	4	4	4	4	3	3	4	4
1	1	4	4	1	1	2	2	4	4
1	1	4	4	1	1	2	2	3	3
1	1	4	4	4	4	3	3	3	3
1	1	1	1	4	4	3	3	4	4
1	1	4	4	4	4	3	3	4	4
1	1	4	4	4	4	3	3	4	4
1	1	4	4	1	1	3	3	3	3
1	1	4	4	4	4	3	3	4	4
1	1	4	4	4	4	3	3	4	4
1	1	4	4	4	4	3	3	3	3
1	1	4	4	1	1	2	2	4	4
2	2	4	4	4	4	3	3	3	3
1	1	4	4	4	4	3	3	3	3
1	1	4	4	4	4	3	3	3	3
1	1	4	4	1	1	2	2	4	4
1	1	1	1	1	1	3	3	4	4
1	1	4	4	4	4	3	3	4	4
1	1	4	4	4	4	3	3	4	4
1	1	4	4	4	4	3	3	4	4
1	1	1	1	4	4	3	3	4	4
1	1	1	1	4	4	3	3	4	4
1	1	4	4	1	1	2	2	4	4
1	1	4	4	4	4	3	3	4	4
1	1	4	4	4	4	3	3	4	4
2	2	1	1	4	4	3	3	3	3
2	2	4	4	4	4	3	3	4	4
1	1	4	4	4	4	3	3	4	4
1	1	4	4	4	4	3	3	3	3
1	1	4	4	4	4	3	3	4	4
1	1	4	4	4	4	3	3	3	3
1	1	4	4	4	4	3	3	4	4
2	2	4	4	4	4	3	3	3	3
1	1	4	4	4	4	3	3	3	3

For Review Only

1	1	4	4	4	4	3	3	3	3
1	1	4	4	4	4	3	3	3	3
1	1	4	4	4	4	3	3	3	3
1	1	4	4	4	4	3	3	3	3
1	1	4	4	4	4	3	3	4	4
1	1	4	4	4	4	3	3	3	3
1	1	4	4	1	1	2	2	3	3
1	1	1	1	4	4	3	3	4	4
1	1	4	4	4	4	3	3	4	4
1	1	4	4	4	4	3	3	4	4
1	1	4	4	4	4	3	3	3	3
1	1	4	4	4	4	3	3	3	3
1	1	4	4	4	4	3	3	3	3
1	1	4	4	4	4	3	3	3	3
1	1	4	4	4	4	3	3	4	4
1	1	4	4	4	4	3	3	4	4
2	2	4	4	4	4	3	3	4	4
1	1	4	4	4	4	3	3	4	4
1	1	4	4	4	4	3	3	3	3
1	1	4	4	4	4	3	3	3	3
1	1	4	4	4	4	3	3	4	4
1	1	1	1	4	4	3	3	4	4
1	1	4	4	4	4	3	3	3	3
2	2	4	4	4	4	3	3	3	3
2	2	4	4	4	4	3	3	4	4
1	1	4	4	4	4	3	3	4	4
2	2	4	4	4	4	3	3	4	4
1	1	1	1	4	4	3	3	4	4
1	1	4	4	1	1	2	2	3	3
1	1	4	4	4	4	3	3	3	3
1	1	4	4	4	4	3	3	4	4
1	1	4	4	4	4	3	3	4	4
1	1	4	4	4	4	3	3	4	4
1	1	4	4	4	4	3	3	3	3
1	1	4	4	4	4	3	3	3	3
2	2	4	4	4	4	3	3	4	4
2	2	4	4	4	4	3	3	4	4
1	1	4	4	1	1	2	2	4	4
1	1	4	4	4	4	3	3	4	4
2	2	4	4	4	4	3	3	4	4
1	1	4	4	4	4	3	3	3	3
1	1	4	4	4	4	3	3	4	4
2	2	4	4	4	4	3	3	4	4
1	1	4	4	4	4	3	3	3	3
1	1	4	4	4	4	3	3	3	3
1	1	4	4	4	4	3	3	4	4
1	1	4	4	1	1	2	2	4	4
1	1	4	4	1	1	3	3	4	4
1	1	4	4	1	1	2	2	4	4
1	1	4	4	4	4	3	3	4	4
1	1	4	4	4	4	3	3	3	3
1	1	4	4	4	4	3	3	4	4
1	1	1	1	4	4	3	3	3	3
1	1	4	4	4	4	3	3	4	4
1	1	4	4	4	4	3	3	4	4

1	1	1	1	4	4	3	3	3	3
1	1	1	1	4	4	3	3	4	4
1	1	4	4	4	4	3	3	4	4
1	1	4	4	1	1	2	2	4	4
1	1	4	4	4	4	3	3	3	3
1	1	4	4	4	4	3	3	4	4
1	1	4	4	4	4	3	3	3	3
1	1	4	4	4	4	3	3	4	4
2	2	4	4	1	1	3	3	4	4
1	1	4	4	4	4	3	3	4	4
1	1	4	4	4	4	3	3	4	4
1	1	4	4	4	4	3	3	4	4
1	1	4	4	4	4	3	3	4	4
1	1	4	4	4	4	3	3	4	4
1	1	4	4	4	4	3	3	4	4
1	1	4	4	1	1	3	3	4	4
1	1	4	4	4	4	3	3	4	4
1	1	4	4	1	1	3	3	4	4
1	1	4	4	1	1	2	2	4	4
1	1	1	1	4	4	3	3	3	3
1	1	4	4	4	4	3	3	4	4
1	1	4	4	4	4	3	3	4	4
1	1	4	4	4	4	3	3	4	4
1	1	1	1	4	4	3	3	4	4
1	1	4	4	1	1	2	2	3	3
1	1	1	1	4	4	3	3	4	4
1	1	4	4	4	4	3	3	4	4
1	1	4	4	4	4	3	3	4	4
1	1	4	4	4	4	3	3	3	3
1	1	4	4	4	4	3	3	4	4
1	1	4	4	4	4	3	3	4	4
1	1	4	4	4	4	3	3	4	4
1	1	4	4	4	4	3	3	3	3
1	1	4	4	4	4	3	3	4	4
1	1	4	4	1	1	2	2	4	4
1	1	4	4	4	4	3	3	4	4
1	1	4	4	1	1	2	2	4	4
1	1	4	4	4	4	3	3	4	4
1	1	4	4	1	1	3	3	4	4
1	1	4	4	1	1	3	3	4	4
1	1	4	4	4	4	3	3	3	3
1	1	4	4	1	1	2	2	4	4
1	1	4	4	4	4	3	3	4	4
1	1	1	1	1	1	3	3	4	4
1	1	4	4	4	4	3	3	4	4
1	1	4	4	4	4	3	3	3	3
1	1	4	4	1	1	2	2	3	3
2	2	4	4	4	4	3	3	4	4
1	1	4	4	4	4	3	3	4	4
1	1	4	4	4	4	3	3	3	3
1	1	4	4	1	1	3	3	4	4
1	1	1	1	4	4	3	3	4	4
1	1	4	4	4	4	3	3	4	4
1	1	4	4	4	4	3	3	3	3
1	1	4	4	4	4	3	3	4	4

For Review Only

1	1	4	4	1	1	2	2	4	4
2	2	1	1	4	4	3	3	4	4
1	1	4	4	4	4	3	3	4	4
1	1	1	1	4	4	3	3	4	4
1	1	4	4	4	4	3	3	4	4
1	1	4	4	4	4	3	3	4	4
1	1	4	4	4	4	3	3	4	4
1	1	4	4	4	4	3	3	4	4
1	1	4	4	1	1	3	3	4	4
1	1	4	4	4	4	3	3	4	4
1	1	4	4	1	1	3	3	3	3
1	1	4	4	4	4	3	3	4	4
1	1	4	4	1	1	3	3	4	4
1	1	4	4	4	4	3	3	4	4
1	1	4	4	4	4	3	3	4	4
1	1	4	4	1	1	2	2	3	3
1	1	4	4	4	4	3	3	4	4
2	2	4	4	4	4	3	3	3	3
1	1	4	4	4	4	3	3	3	3
1	1	4	4	4	4	3	3	4	4
1	1	4	4	4	4	3	3	4	4
1	1	4	4	4	4	3	3	4	4
1	1	1	1	4	4	3	3	4	4
1	1	4	4	4	4	3	3	3	3
1	1	4	4	4	4	3	3	3	3
1	1	4	4	1	1	2	2	4	4
1	1	4	4	4	4	3	3	4	4
1	1	4	4	4	4	3	3	4	4
1	1	4	4	4	4	3	3	4	4
1	1	4	4	4	4	3	3	4	4
2	2	4	4	4	4	3	3	4	4
1	1	4	4	4	4	3	3	3	3
2	2	4	4	4	4	3	3	3	3
1	1	4	4	4	4	3	3	4	4
2	2	4	4	4	4	3	3	4	4
1	1	4	4	4	4	3	3	3	3
1	1	4	4	4	4	3	3	4	4
1	1	4	4	4	4	3	3	3	3
2	2	4	4	4	4	3	3	4	4
1	1	4	4	4	4	3	3	4	4
1	1	4	4	4	4	3	3	4	4
1	1	4	4	1	1	2	2	3	3
1	1	4	4	1	1	2	2	4	4
1	1	4	4	4	4	3	3	4	4
1	1	4	4	4	4	3	3	4	4
1	1	4	4	4	4	3	3	4	4
1	1	4	4	1	1	2	2	3	3
1	1	4	4	4	4	3	3	4	4
1	1	4	4	4	4	3	3	4	4
1	1	4	4	4	4	3	3	4	4
1	1	4	4	4	4	3	3	3	3
1	1	1	1	4	4	3	3	4	4
1	1	1	1	4	4	3	3	4	4
1	1	4	4	4	4	3	3	3	3
1	1	1	1	4	4	3	3	4	4

For Review Only

1	1	4	4	4	4	3	3	4	4
1	1	4	4	4	4	2	2	4	4
1	1	4	4	4	4	3	3	4	4
1	1	1	1	4	4	3	3	4	4
1	1	4	4	4	4	3	3	4	4
1	1	4	4	1	1	3	3	4	4
1	1	4	4	1	1	3	3	4	4
1	1	4	4	4	4	3	3	4	4
1	1	4	4	4	4	3	3	4	4
1	1	1	1	4	4	3	3	4	4
1	1	4	4	4	4	3	3	4	4
1	1	4	4	4	4	3	3	4	4
1	1	4	4	4	4	3	3	3	3
1	1	4	4	4	4	3	3	4	4
1	1	4	4	4	4	3	3	4	4
1	1	4	4	4	4	3	3	4	4
1	1	1	1	4	4	3	3	4	4
1	1	1	1	4	4	3	3	4	4
1	1	4	4	4	4	3	3	4	4
1	1	1	1	4	4	3	3	4	4
2	2	1	1	4	4	3	3	4	4
1	1	4	4	1	1	2	2	3	3
1	1	4	4	4	4	3	3	4	4
1	1	4	4	4	4	3	3	3	3
1	1	1	1	4	4	3	3	4	4
1	1	4	4	4	4	3	3	3	3
1	1	4	4	4	4	3	3	4	4
1	1	4	4	4	4	3	3	3	3
1	1	4	4	4	4	3	3	4	4
1	1	4	4	4	4	3	3	3	3
1	1	1	1	4	4	3	3	4	4
1	1	4	4	1	1	2	2	4	4
1	1	4	4	4	4	3	3	4	4
2	2	4	4	4	4	3	3	4	4
1	1	1	1	4	4	3	3	4	4
1	1	1	1	4	4	3	3	4	4
1	1	4	4	4	4	3	3	3	3
1	1	4	4	4	4	3	3	3	3
1	1	4	4	4	4	3	3	4	4
1	1	1	1	4	4	3	3	4	4
2	2	4	4	4	4	3	3	3	3
1	1	4	4	4	4	3	3	4	4
1	1	4	4	4	4	3	3	3	3
1	1	4	4	4	4	3	3	3	3
1	1	4	4	4	4	3	3	4	4
1	1	4	4	4	4	3	3	3	3
2	2	4	4	4	4	3	3	4	4
1	1	4	4	4	4	3	3	4	4
1	1	4	4	4	4	3	3	4	4
1	1	1	1	4	4	3	3	4	4
1	1	4	4	4	4	3	3	3	3
1	1	4	4	1	1	2	2	4	4
1	1	4	4	4	4	3	3	4	4
1	1	4	4	4	4	3	3	3	3
1	1	1	1	4	4	3	3	4	4

For Review Only

1	1	4	4	4	4	3	3	3	3
1	1	4	4	4	4	3	3	4	4

For Review Only

SNP542	SNP542	SNP543	SNP543	SNP544	SNP544	SNP545	SNP545	SNP546	SNP546
1	1	2	2	2	2	2	2	4	4
1	1	3	3	3	3	2	2	4	4
4	4	2	2	2	2	2	2	4	4
1	1	2	2	2	2	2	2	4	4
1	1	2	2	2	2	2	2	4	4
1	1	2	2	2	2	2	2	4	4
1	1	2	2	2	2	2	2	4	4
1	1	3	3	3	3	2	2	4	4
1	1	2	2	2	2	2	2	4	4
1	1	3	3	3	3	2	2	4	4
1	1	3	3	3	3	2	2	4	4
4	4	2	2	2	2	2	2	4	4
1	1	2	2	2	2	2	2	4	4
1	1	2	2	2	2	2	2	4	4
1	1	2	2	2	2	2	2	4	4
4	4	2	2	2	2	2	2	4	4
1	1	3	3	3	3	2	2	4	4
1	1	3	3	3	3	2	2	4	4
4	4	2	2	2	2	2	2	4	4
1	1	2	2	2	2	2	2	4	4
1	1	3	3	3	3	2	2	4	4
1	1	3	3	3	3	2	2	4	4
1	1	3	3	3	3	4	4	1	1
4	4	3	3	3	3	2	2	4	4
1	1	2	2	2	2	2	2	4	4
1	1	2	2	2	2	2	2	4	4
1	1	2	2	2	2	2	2	4	4
1	1	2	2	3	3	2	2	4	4
1	1	3	3	3	3	2	2	4	4
1	1	3	3	3	3	2	2	4	4
4	4	2	2	2	2	2	2	4	4
4	4	2	2	2	2	2	2	4	4
1	1	2	2	2	2	2	2	4	4
1	1	3	3	3	3	2	2	4	4
1	1	2	2	3	3	2	2	4	4
1	1	2	2	2	2	2	2	4	4
1	1	2	2	2	2	2	2	4	4
1	1	2	2	2	2	2	2	4	4
1	1	3	3	3	3	2	2	4	4
1	1	3	3	3	3	2	2	1	1
1	1	3	3	3	3	2	2	4	4
1	1	3	3	3	3	2	2	4	4
1	1	3	3	3	3	2	2	4	4
4	4	2	2	2	2	2	2	4	4
1	1	3	3	3	3	4	4	1	1
4	4	2	2	2	2	2	2	4	4
4	4	2	2	2	2	2	2	4	4
1	1	3	3	3	3	2	2	4	4
1	1	2	2	2	2	2	2	4	4
1	1	3	3	2	2	2	2	4	4
1	1	2	2	2	2	2	2	4	4
1	1	2	2	2	2	2	2	4	4

For Review Only

1	1	2	2	2	2	2	2	4	4
1	1	2	2	2	2	2	2	4	4
1	1	2	2	2	2	2	2	4	4
1	1	2	2	2	2	2	2	4	4
4	4	2	2	2	2	2	2	4	4
4	4	2	2	2	2	2	2	4	4
4	4	2	2	2	2	2	2	4	4
1	1	3	3	3	3	2	2	4	4
1	1	3	3	3	3	2	2	4	4
1	1	2	2	2	2	2	2	4	4
4	4	2	2	2	2	2	2	4	4
4	4	2	2	2	2	2	2	4	4
1	1	2	2	2	2	2	2	4	4
1	1	3	3	3	3	2	2	4	4
1	1	2	2	2	2	2	2	4	4
1	1	3	3	3	3	2	2	4	4
1	1	3	3	3	3	2	2	4	4
4	4	3	3	3	3	2	2	4	4
4	4	3	3	3	3	2	2	4	4
1	1	2	2	2	2	2	2	4	4
4	4	2	2	2	2	2	2	4	4
1	1	3	3	3	3	2	2	4	4
1	1	2	2	2	2	2	2	4	4
1	1	2	2	2	2	2	2	4	4
1	1	3	3	3	3	4	4	1	1
1	1	2	2	2	2	2	2	4	4
4	4	3	3	3	3	4	4	1	1
1	1	3	3	3	3	2	2	4	4
1	1	2	2	2	2	2	2	4	4
4	4	2	2	2	2	2	2	4	4
4	4	2	2	2	2	2	2	4	4
4	4	3	3	3	3	2	2	4	4
1	1	3	3	3	3	2	2	4	4
1	1	2	2	2	2	2	2	4	4
1	1	3	3	3	3	2	2	4	4
1	1	3	3	3	3	4	4	1	1
4	4	3	3	3	3	2	2	4	4
1	1	3	3	2	2	4	4	1	1
1	1	3	3	3	3	4	4	1	1
1	1	2	2	2	2	2	2	4	4
1	1	2	2	2	2	2	2	4	4
4	4	2	2	2	2	2	2	4	4
1	1	2	2	2	2	2	2	4	4
4	4	2	2	2	2	2	2	4	4
1	1	2	2	2	2	2	2	4	4
1	1	2	2	2	2	2	2	4	4
1	1	3	3	3	3	2	2	4	4
4	4	3	3	3	3	2	2	4	4
4	4	2	2	2	2	2	2	4	4
1	1	2	2	2	2	4	4	4	4
1	1	3	3	3	3	2	2	4	4
1	1	3	3	3	3	2	2	4	4
1	1	3	3	3	3	4	4	4	4
1	1	3	3	3	3	2	2	4	4
1	1	3	3	3	3	4	4	4	4

For Review Only

1	1	2	2	3	3	2	2	4	4
1	1	2	2	2	2	2	2	4	4
1	1	2	2	2	2	2	2	4	4
1	1	3	3	3	3	2	2	4	4
1	1	2	2	2	2	2	2	4	4
1	1	2	2	2	2	2	2	4	4
4	4	2	2	2	2	2	2	4	4
4	4	2	2	2	2	2	2	4	4
1	1	2	2	2	2	2	2	4	4
1	1	3	3	3	3	2	2	4	4
1	1	2	2	2	2	2	2	4	4
1	1	2	2	2	2	2	2	4	4
1	1	3	3	3	3	2	2	4	4
1	1	2	2	2	2	2	2	4	4
1	1	2	2	2	2	2	2	4	4
1	1	2	2	2	2	2	2	4	4
1	1	3	3	3	3	2	2	4	4
1	1	2	2	2	2	2	2	4	4
1	1	3	3	3	3	2	2	4	4
1	1	3	3	3	3	2	2	4	4
1	1	2	2	2	2	2	2	4	4
4	4	2	2	2	2	2	2	4	4
4	4	3	3	3	3	2	2	4	4
1	1	3	3	3	3	2	2	4	4
1	1	2	2	2	2	2	2	4	4
1	1	3	3	3	3	2	2	4	4
4	4	2	2	2	2	2	2	4	4
1	1	2	2	2	2	2	2	4	4
1	1	2	2	2	2	2	2	4	4
1	1	3	3	3	3	4	4	4	4
1	1	3	3	3	3	2	2	4	4
1	1	2	2	2	2	2	2	4	4
1	1	2	2	2	2	2	2	4	4
1	1	2	2	2	2	2	2	4	4
1	1	3	3	3	3	2	2	4	4
1	1	3	3	3	3	2	2	4	4
1	1	3	3	3	3	2	2	4	4
4	4	2	2	2	2	2	2	4	4
1	1	3	3	3	3	2	2	4	4
1	1	3	3	3	3	2	2	4	4
1	1	3	3	3	3	4	4	1	1
1	1	3	3	3	3	4	4	4	4
1	1	3	3	3	3	2	2	4	4
1	1	3	3	3	3	2	2	4	4
1	1	3	3	3	3	2	2	4	4
1	1	2	2	2	2	2	2	4	4
1	1	2	2	2	2	2	2	4	4
4	4	2	2	2	2	2	2	4	4
1	1	3	3	3	3	2	2	4	4
1	1	2	2	2	2	2	2	4	4
1	1	2	2	2	2	2	2	4	4
1	1	3	3	3	3	4	4	4	4
4	4	3	3	3	3	2	2	4	4
1	1	2	2	2	2	2	2	4	4
1	1	3	3	3	3	2	2	4	4

For Review Only

1	1	2	2	2	2	2	2	4	4
1	1	3	3	3	3	2	2	4	4
1	1	3	3	3	3	2	2	4	4
1	1	3	3	3	3	2	2	4	4
1	1	3	3	3	3	2	2	4	4
1	1	3	3	3	3	4	4	4	4
1	1	3	3	3	3	2	2	4	4
1	1	2	2	2	2	2	2	4	4
1	1	3	3	3	3	2	2	4	4
1	1	2	2	2	2	2	2	4	4
1	1	3	3	2	2	2	2	4	4
4	4	2	2	2	2	2	2	4	4
1	1	2	2	2	2	2	2	4	4
1	1	2	2	2	2	2	2	4	4
1	1	3	3	3	3	2	2	4	4
1	1	3	3	3	3	2	2	4	4
4	4	3	3	3	3	2	2	4	4
1	1	2	2	2	2	2	2	4	4
1	1	3	3	3	3	2	2	4	4
4	4	2	2	2	2	2	2	4	4
1	1	2	2	2	2	2	2	4	4
1	1	3	3	3	3	2	2	4	4
1	1	3	3	3	3	2	2	1	1
4	4	2	2	2	2	2	2	4	4
1	1	3	3	2	2	2	2	4	4
1	1	3	3	3	3	2	2	4	4
1	1	3	3	3	3	2	2	4	4
1	1	3	3	3	3	2	2	4	4
1	1	3	3	3	3	2	2	1	1
1	1	2	2	2	2	2	2	4	4
1	1	2	2	2	2	2	2	4	4
4	4	3	3	3	3	2	2	4	4
1	1	2	2	2	2	2	2	4	4
1	1	3	3	3	3	2	2	4	4
1	1	2	2	2	2	2	2	4	4
4	4	2	2	2	2	2	2	4	4
1	1	2	2	2	2	2	2	4	4
1	1	2	2	2	2	2	2	4	4
4	4	2	2	2	2	2	2	4	4
1	1	2	2	2	2	2	2	4	4
4	4	2	2	2	2	2	2	4	4
1	1	2	2	2	2	2	2	4	4
4	4	3	3	3	3	2	2	4	4
1	1	3	3	3	3	2	2	4	4
1	1	2	2	2	2	2	2	4	4
1	1	2	2	2	2	2	2	4	4
1	1	3	3	3	3	2	2	4	4
1	1	3	3	3	3	2	2	4	4
1	1	3	3	3	3	2	2	4	4
4	4	2	2	2	2	2	2	4	4
1	1	2	2	2	2	2	2	4	4
1	1	3	3	3	3	2	2	4	4
1	1	3	3	3	3	2	2	1	1
4	4	2	2	2	2	2	2	4	4
1	1	3	3	3	3	2	2	4	4

For Review Only

4	4	3	3	3	3	2	2	4	4
4	4	3	3	3	3	2	2	4	4
1	1	2	2	2	2	2	2	4	4
1	1	2	2	2	2	2	2	1	1
1	1	2	2	2	2	2	2	4	4
1	1	2	2	2	2	2	2	4	4
1	1	3	3	3	3	2	2	4	4
1	1	3	3	3	3	2	2	4	4
1	1	3	3	2	2	2	2	4	4
1	1	3	3	3	3	2	2	4	4
1	1	2	2	2	2	2	2	4	4
1	1	3	3	3	3	2	2	4	4
1	1	3	3	3	3	4	4	1	1
4	4	2	2	2	2	2	2	4	4
1	1	2	2	2	2	2	2	4	4
1	1	2	2	2	2	2	2	1	1
1	1	3	3	3	3	2	2	4	4
1	1	3	3	3	3	2	2	4	4
1	1	3	3	3	3	2	2	4	4
1	1	2	2	2	2	2	2	4	4
1	1	2	2	2	2	2	2	1	1
1	1	2	2	2	2	2	2	4	4
1	1	2	2	2	2	2	2	4	4
1	1	2	2	2	2	2	2	4	4
1	1	3	3	3	3	2	2	4	4
1	1	2	2	2	2	2	2	4	4
1	1	3	3	3	3	2	2	4	4
4	4	3	3	3	3	2	2	4	4
1	1	2	2	2	2	2	2	4	4
1	1	2	2	2	2	2	2	4	4
1	1	3	3	3	3	2	2	4	4
1	1	2	2	2	2	2	2	4	4
1	1	3	3	3	3	2	2	4	4
4	4	2	2	2	2	2	2	4	4
1	1	3	3	3	3	2	2	1	1
1	1	3	3	3	3	2	2	4	4
4	4	2	2	2	2	2	2	4	4
4	4	2	2	2	2	2	2	4	4
1	1	2	2	2	2	2	2	4	4
1	1	3	3	3	3	2	2	4	4
1	1	2	2	2	2	2	2	4	4
1	1	3	3	3	3	2	2	4	4
1	1	2	2	2	2	2	2	4	4
1	1	2	2	2	2	2	2	4	4
1	1	2	2	2	2	2	2	4	4
1	1	2	2	2	2	4	4	1	1
4	4	2	2	2	2	2	2	4	4
1	1	3	3	3	3	2	2	4	4
4	4	3	3	3	3	2	2	4	4
1	1	3	3	3	3	2	2	4	4
1	1	3	3	3	3	2	2	4	4
1	1	3	3	3	3	2	2	4	4
4	4	2	2	2	2	2	2	4	4
4	4	3	3	3	3	2	2	4	4
1	1	3	3	3	3	2	2	4	4

For Review Only

1	1	2	2	2	2	2	2	4	4
4	4	3	3	3	3	2	2	4	4

For Review Only

SNP547	SNP547	SNP548	SNP548	SNP549	SNP549	SNP550	SNP550	SNP551	SNP551
3	3	4	4	4	4	4	4	1	1
1	1	2	2	4	4	4	4	1	1
3	3	2	2	4	4	2	2	2	2
1	1	2	2	4	4	4	4	2	2
3	3	2	2	2	2	2	2	2	2
3	3	2	2	4	4	4	4	1	1
3	3	2	2	4	4	4	4	1	1
1	1	2	2	4	4	2	2	2	2
3	3	2	2	2	2	4	4	1	1
1	1	2	2	4	4	2	2	2	2
1	1	2	2	4	4	4	4	1	1
1	1	2	2	4	4	4	4	2	2
3	3	2	2	4	4	4	4	2	2
3	3	2	2	4	4	2	2	2	2
1	1	2	2	2	2	4	4	2	2
3	3	2	2	4	4	4	4	2	2
1	1	2	2	2	2	4	4	1	1
1	1	2	2	4	4	4	4	1	1
3	3	2	2	4	4	2	2	2	2
1	1	2	2	4	4	2	2	2	2
1	1	2	2	4	4	4	4	1	1
1	1	2	2	2	2	4	4	1	1
1	1	2	2	2	2	2	2	2	2
1	1	2	2	4	4	2	2	2	2
3	3	4	4	4	4	2	2	2	2
3	3	4	4	4	4	4	4	1	1
3	3	2	2	4	4	4	4	1	1
1	1	2	2	4	4	4	4	2	2
1	1	2	2	4	4	4	4	1	1
1	1	2	2	4	4	2	2	2	2
3	3	2	2	4	4	4	4	1	1
3	3	2	2	4	4	4	4	1	1
3	3	2	2	4	4	4	4	1	1
1	1	2	2	4	4	2	2	2	2
1	1	2	2	4	4	2	2	2	2
3	3	2	2	4	4	2	2	2	2
3	3	2	2	2	2	2	2	2	2
3	3	2	2	4	4	2	2	2	2
1	1	2	2	4	4	2	2	2	2
1	1	2	2	2	2	2	2	2	2
1	1	2	2	4	4	2	2	2	2
1	1	2	2	4	4	2	2	2	2
1	1	2	2	4	4	2	2	2	2
3	3	2	2	4	4	4	4	1	1
1	1	2	2	2	2	2	2	2	2
1	1	2	2	4	4	4	4	2	2
3	3	4	4	4	4	4	4	1	1
1	1	4	4	4	4	2	2	2	2
3	3	2	2	4	4	4	4	1	1
1	1	2	2	4	4	2	2	2	2
3	3	4	4	4	4	4	4	1	1
1	1	4	4	4	4	4	4	1	1

For Review Only

3	3	4	4	2	2	4	4	1	1
3	3	2	2	4	4	4	4	1	1
3	3	2	2	4	4	4	4	1	1
3	3	2	2	2	2	4	4	1	1
3	3	2	2	4	4	2	2	2	2
3	3	2	2	4	4	4	4	1	1
3	3	2	2	4	4	4	4	1	1
1	1	2	2	4	4	2	2	2	2
1	1	2	2	2	2	2	2	2	2
1	1	2	2	4	4	4	4	2	2
1	1	2	2	4	4	4	4	1	1
3	3	2	2	4	4	4	4	1	1
3	3	4	4	2	2	4	4	1	1
1	1	2	2	4	4	4	4	1	1
1	1	2	2	4	4	4	4	2	2
1	1	2	2	2	2	2	2	2	2
1	1	2	2	4	4	4	4	1	1
1	1	2	2	4	4	2	2	2	2
1	1	2	2	4	4	4	4	1	1
3	3	4	4	2	2	4	4	1	1
3	3	2	2	4	4	4	4	1	1
1	1	2	2	4	4	2	2	2	2
3	3	2	2	4	4	4	4	1	1
3	3	2	2	4	4	4	4	1	1
1	1	2	2	2	2	2	2	2	2
3	3	4	4	4	4	2	2	2	2
1	1	2	2	2	2	2	2	2	2
1	1	2	2	4	4	2	2	2	2
3	3	4	4	4	4	4	4	1	1
3	3	2	2	4	4	4	4	1	1
3	3	2	2	2	2	2	2	2	2
1	1	2	2	4	4	2	2	2	2
1	1	2	2	4	4	2	2	2	2
3	3	4	4	2	2	4	4	1	1
1	1	2	2	4	4	4	4	1	1
1	1	2	2	2	2	2	2	2	2
1	1	2	2	4	4	2	2	2	2
1	1	4	4	2	2	2	2	2	2
1	1	2	2	2	2	2	2	2	2
1	1	2	2	4	4	2	2	2	2
3	3	2	2	2	2	4	4	1	1
3	3	2	2	4	4	2	2	2	2
3	3	2	2	2	2	2	2	2	2
3	3	2	2	4	4	4	4	1	1
1	1	2	2	2	2	4	4	1	1
3	3	2	2	4	4	2	2	2	2
1	1	2	2	4	4	2	2	2	2
1	1	2	2	4	4	2	2	2	2
3	3	4	4	4	4	4	4	2	2
3	3	2	2	2	2	2	2	2	2
1	1	2	2	4	4	4	4	1	1
1	1	2	2	4	4	2	2	2	2
1	1	2	2	4	4	4	4	1	1
1	1	2	2	4	4	4	4	2	2
1	1	2	2	2	2	2	2	2	2

For Review Only

1	1	4	4	4	4	4	4	1	1
1	1	2	2	4	4	2	2	2	2
3	3	4	4	4	4	2	2	2	2
1	1	2	2	4	4	2	2	2	2
3	3	2	2	4	4	4	4	1	1
3	3	4	4	4	4	2	2	2	2
3	3	2	2	4	4	4	4	1	1
3	3	2	2	4	4	2	2	2	2
3	3	4	4	4	4	4	4	2	2
1	1	4	4	4	4	4	4	2	2
3	3	2	2	2	2	4	4	1	1
1	1	2	2	4	4	2	2	2	2
1	1	2	2	4	4	2	2	2	2
3	3	2	2	4	4	4	4	2	2
1	1	2	2	4	4	4	4	2	2
3	3	2	2	4	4	4	4	2	2
1	1	2	2	4	4	2	2	2	2
1	1	2	2	4	4	4	4	2	2
1	1	2	2	4	4	2	2	2	2
1	1	2	2	4	4	4	4	1	1
1	1	2	2	4	4	4	4	2	2
1	1	2	2	4	4	2	2	2	2
1	1	2	2	2	2	2	2	2	2
1	1	2	2	4	4	2	2	2	2
1	1	2	2	4	4	4	4	1	1
1	1	2	2	2	2	2	2	2	2
3	3	2	2	4	4	4	4	1	1
3	3	2	2	2	2	4	4	2	2
1	1	2	2	4	4	4	4	1	1
1	1	2	2	2	2	2	2	2	2
1	1	2	2	4	4	2	2	2	2
1	1	2	2	4	4	4	4	2	2
3	3	2	2	2	2	4	4	1	1
1	1	2	2	4	4	4	4	1	1
1	1	2	2	4	4	2	2	2	2
1	1	2	2	4	4	2	2	2	2
1	1	2	2	4	4	2	2	2	2
3	3	2	2	4	4	2	2	2	2
1	1	4	4	4	4	4	4	2	2
1	1	2	2	4	4	2	2	2	2
1	1	2	2	2	2	4	4	1	1
1	1	2	2	2	2	2	2	2	2
1	1	2	2	2	2	2	2	2	2
1	1	2	2	4	4	2	2	2	2
1	1	2	2	4	4	4	4	2	2
3	3	2	2	2	2	4	4	1	1
3	3	4	4	4	4	4	4	1	1
3	3	4	4	4	4	2	2	2	2
1	1	2	2	4	4	2	2	2	2
3	3	4	4	4	4	4	4	1	1
3	3	4	4	4	4	4	4	2	2
1	1	2	2	2	2	2	2	2	2
1	1	2	2	4	4	2	2	2	2
3	3	2	2	4	4	4	4	1	1
1	1	2	2	4	4	4	4	2	2

For Review Only

3	3	2	2	4	4	2	2	2	2
1	1	2	2	4	4	2	2	2	2
1	1	2	2	4	4	2	2	2	2
1	1	2	2	4	4	2	2	2	2
1	1	2	2	4	4	4	4	1	1
1	1	2	2	2	2	2	2	2	2
1	1	2	2	2	2	4	4	2	2
1	1	2	2	4	4	4	4	2	2
1	1	2	2	4	4	2	2	2	2
3	3	2	2	4	4	2	2	2	2
3	3	2	2	4	4	4	4	1	1
1	1	2	2	4	4	2	2	2	2
3	3	2	2	4	4	4	4	2	2
1	1	2	2	2	2	4	4	2	2
1	1	2	2	4	4	2	2	2	2
1	1	2	2	4	4	4	4	1	1
1	1	2	2	4	4	2	2	2	2
3	3	2	2	4	4	4	4	2	2
1	1	2	2	4	4	4	4	1	1
3	3	2	2	4	4	4	4	2	2
1	1	2	2	4	4	4	4	2	2
1	1	2	2	4	4	2	2	2	2
1	1	2	2	2	2	2	2	2	2
3	3	2	2	4	4	4	4	1	1
1	1	2	2	4	4	4	4	1	1
1	1	2	2	4	4	2	2	2	2
1	1	2	2	4	4	4	4	2	2
1	1	2	2	4	4	2	2	2	2
1	1	2	2	2	2	2	2	2	2
3	3	2	2	4	4	2	2	2	2
3	3	2	2	4	4	2	2	2	2
1	1	2	2	4	4	4	4	1	1
3	3	2	2	4	4	4	4	1	1
1	1	2	2	4	4	2	2	2	2
3	3	2	2	2	2	4	4	2	2
3	3	2	2	4	4	4	4	1	1
3	3	2	2	4	4	4	4	1	1
1	1	4	4	2	2	4	4	1	1
3	3	4	4	4	4	2	2	2	2
3	3	2	2	4	4	2	2	2	2
3	3	2	2	4	4	2	2	2	2
3	3	4	4	4	4	4	4	1	1
1	1	4	4	4	4	2	2	2	2
1	1	2	2	4	4	4	4	2	2
3	3	2	2	4	4	2	2	2	2
1	1	2	2	4	4	2	2	2	2
1	1	2	2	4	4	4	4	1	1
1	1	2	2	4	4	2	2	2	2
1	1	2	2	4	4	2	2	2	2
1	1	2	2	4	4	2	2	2	2
1	1	2	2	4	4	4	4	1	1
1	1	2	2	4	4	2	2	2	2
1	1	2	2	2	2	2	2	2	2
3	3	2	2	4	4	4	4	1	1
1	1	2	2	4	4	2	2	2	2

For Review Only

1	1	2	2	4	4	2	2	2	2
1	1	2	2	4	4	2	2	2	2
3	3	2	2	4	4	2	2	2	2
1	1	2	2	2	2	4	4	2	2
3	3	2	2	4	4	2	2	2	2
3	3	2	2	4	4	2	2	2	2
1	1	2	2	4	4	4	4	2	2
1	1	2	2	4	4	2	2	2	2
1	1	2	2	4	4	2	2	2	2
1	1	2	2	4	4	2	2	2	2
1	1	2	2	4	4	4	4	2	2
1	1	2	2	4	4	2	2	2	2
1	1	2	2	2	2	4	4	1	1
3	3	4	4	4	4	4	4	2	2
3	3	2	2	4	4	4	4	1	1
3	3	2	2	2	2	2	2	2	2
1	1	2	2	4	4	2	2	2	2
1	1	2	2	2	2	2	2	2	2
1	1	4	4	4	4	2	2	2	2
3	3	4	4	4	4	2	2	2	2
3	3	2	2	2	2	2	2	2	2
3	3	2	2	4	4	4	4	1	1
3	3	2	2	2	2	2	2	2	2
3	3	2	2	4	4	4	4	1	1
1	1	2	2	4	4	2	2	2	2
3	3	4	4	4	4	4	4	2	2
1	1	2	2	4	4	2	2	2	2
1	1	2	2	4	4	4	4	1	1
3	3	2	2	4	4	2	2	2	2
3	3	2	2	4	4	4	4	1	1
1	1	2	2	4	4	2	2	2	2
3	3	2	2	4	4	2	2	2	2
1	1	2	2	4	4	2	2	2	2
1	1	2	2	4	4	4	4	1	1
1	1	2	2	2	2	2	2	2	2
1	1	2	2	4	4	2	2	2	2
3	3	2	2	4	4	4	4	1	1
3	3	2	2	4	4	4	4	1	1
3	3	2	2	4	4	2	2	2	2
1	1	2	2	4	4	2	2	2	2
3	3	2	2	4	4	4	4	1	1
1	1	2	2	4	4	2	2	2	2
3	3	2	2	2	2	4	4	1	1
3	3	2	2	2	2	4	4	1	1
1	1	2	2	4	4	4	4	2	2
3	3	2	2	2	2	4	4	1	1
3	3	2	2	4	4	2	2	2	2
1	1	2	2	4	4	2	2	2	2
1	1	4	4	4	4	2	2	2	2
1	1	2	2	4	4	2	2	2	2
1	1	2	2	2	2	4	4	1	1
1	1	2	2	4	4	2	2	2	2
3	3	2	2	4	4	2	2	2	2
1	1	4	4	4	4	4	4	1	1
1	1	2	2	4	4	2	2	2	2

For Review Only

3	3	2	2	2	2	4	4	1	1
1	1	4	4	4	4	2	2	2	2

For Review Only

SNP552	SNP552	SNP553	SNP553	SNP554	SNP554	SNP555	SNP555	SNP556	SNP556
3	3	1	1	4	4	3	3	1	1
3	3	1	1	4	4	3	3	1	1
3	3	4	4	1	1	3	3	1	1
3	3	4	4	1	1	1	1	1	1
3	3	1	1	1	1	1	1	1	1
3	3	4	4	1	1	3	3	1	1
3	3	1	1	1	1	3	3	1	1
2	2	1	1	1	1	1	1	1	1
3	3	1	1	1	1	3	3	1	1
3	3	1	1	1	1	1	1	1	1
3	3	1	1	1	1	3	3	1	1
3	3	4	4	1	1	3	3	1	1
2	2	1	1	4	4	1	1	1	1
3	3	1	1	1	1	1	1	1	1
2	2	1	1	4	4	1	1	1	1
3	3	4	4	1	1	1	1	1	1
3	3	4	4	1	1	3	3	1	1
3	3	4	4	1	1	3	3	1	1
3	3	4	4	1	1	1	1	1	1
2	2	1	1	1	1	1	1	1	1
3	3	1	1	1	1	3	3	1	1
3	3	1	1	4	4	3	3	4	4
2	2	1	1	4	4	1	1	4	4
3	3	4	4	1	1	1	1	1	1
2	2	1	1	1	1	1	1	1	1
3	3	1	1	4	4	3	3	1	1
3	3	4	4	1	1	1	1	1	1
2	2	4	4	4	4	1	1	1	1
3	3	1	1	1	1	3	3	1	1
2	2	4	4	4	4	1	1	4	4
3	3	4	4	1	1	3	3	1	1
3	3	1	1	1	1	3	3	1	1
3	3	1	1	1	1	3	3	1	1
2	2	1	1	1	1	1	1	1	1
2	2	4	4	4	4	1	1	4	4
3	3	1	1	1	1	1	1	1	1
3	3	4	4	1	1	1	1	1	1
3	3	1	1	4	4	1	1	1	1
3	3	1	1	1	1	1	1	1	1
2	2	4	4	4	4	1	1	4	4
3	3	1	1	4	4	1	1	1	1
3	3	4	4	1	1	1	1	1	1
2	2	1	1	4	4	1	1	1	1
3	3	4	4	1	1	3	3	1	1
2	2	1	1	1	1	1	1	1	1
3	3	4	4	1	1	3	3	1	1
3	3	4	4	4	4	3	3	1	1
3	3	1	1	4	4	1	1	1	1
3	3	1	1	4	4	3	3	4	4
3	3	1	1	4	4	1	1	1	1
3	3	1	1	4	4	3	3	1	1
3	3	1	1	1	1	3	3	1	1

For Review Only

3	3	1	1	1	1	3	3	1	1
3	3	1	1	1	1	3	3	1	1
3	3	4	4	1	1	1	1	1	1
3	3	1	1	1	1	3	3	1	1
3	3	4	4	1	1	1	1	1	1
3	3	4	4	1	1	3	3	1	1
3	3	4	4	1	1	3	3	1	1
3	3	4	4	4	4	1	1	1	1
3	3	1	1	1	1	1	1	1	1
2	2	4	4	4	4	3	3	1	1
3	3	4	4	1	1	3	3	1	1
3	3	4	4	1	1	3	3	1	1
3	3	1	1	1	1	3	3	1	1
3	3	1	1	1	1	3	3	1	1
3	3	4	4	1	1	1	1	1	1
2	2	1	1	1	1	1	1	1	1
3	3	1	1	1	1	1	1	1	1
3	3	4	4	4	4	1	1	1	1
3	3	4	4	1	1	3	3	1	1
3	3	1	1	1	1	3	3	1	1
3	3	4	4	1	1	1	1	1	1
2	2	1	1	4	4	1	1	1	1
2	2	1	1	1	1	3	3	1	1
3	3	4	4	1	1	3	3	1	1
2	2	1	1	1	1	1	1	1	1
3	3	4	4	1	1	1	1	1	1
3	3	1	1	4	4	1	1	4	4
2	2	1	1	1	1	1	1	1	1
3	3	1	1	4	4	3	3	1	1
3	3	4	4	1	1	3	3	1	1
3	3	4	4	1	1	1	1	1	1
3	3	4	4	1	1	1	1	1	1
2	2	1	1	4	4	1	1	1	1
3	3	1	1	1	1	3	3	1	1
3	3	1	1	1	1	3	3	1	1
2	2	1	1	1	1	1	1	1	1
3	3	4	4	1	1	1	1	1	1
2	2	1	1	4	4	1	1	4	4
2	2	1	1	4	4	1	1	4	4
2	2	1	1	4	4	1	1	1	1
3	3	1	1	1	1	3	3	1	1
3	3	4	4	1	1	3	3	1	1
3	3	1	1	4	4	1	1	1	1
3	3	4	4	1	1	3	3	1	1
3	3	1	1	1	1	1	1	1	1
3	3	4	4	1	1	1	1	1	1
2	2	1	1	1	1	1	1	1	1
3	3	4	4	1	1	1	1	1	1
3	3	4	4	1	1	1	1	1	1
2	2	4	4	4	4	1	1	1	1
3	3	1	1	1	1	3	3	1	1
3	3	1	1	1	1	1	1	1	1
3	3	4	4	4	4	3	3	1	1
2	2	4	4	4	4	1	1	1	1
2	2	4	4	4	4	1	1	1	1

For Review Only

3	3	1	1	4	4	3	3	1	1
3	3	4	4	4	4	1	1	1	1
3	3	1	1	4	4	1	1	1	1
3	3	1	1	1	1	1	1	1	1
3	3	4	4	1	1	3	3	1	1
3	3	1	1	1	1	1	1	1	1
3	3	4	4	1	1	3	3	1	1
2	2	4	4	1	1	1	1	1	1
3	3	1	1	1	1	1	1	1	1
3	3	1	1	4	4	1	1	1	1
2	2	1	1	1	1	3	3	1	1
3	3	1	1	4	4	3	3	1	1
3	3	1	1	1	1	1	1	1	1
3	3	4	4	1	1	3	3	1	1
3	3	1	1	1	1	1	1	1	1
2	2	1	1	4	4	1	1	1	1
3	3	4	4	1	1	1	1	1	1
3	3	4	4	4	4	1	1	1	1
3	3	1	1	1	1	1	1	1	1
3	3	1	1	4	4	3	3	1	1
2	2	4	4	4	4	1	1	1	1
3	3	4	4	1	1	1	1	1	1
3	3	4	4	1	1	3	3	1	1
2	2	1	1	1	1	1	1	1	1
2	2	1	1	4	4	3	3	1	1
2	2	1	1	4	4	1	1	1	1
3	3	4	4	4	4	1	1	1	1
3	3	1	1	1	1	1	1	1	1
3	3	1	1	1	1	3	3	1	1
3	3	4	4	4	4	1	1	4	4
2	2	1	1	4	4	1	1	1	1
3	3	1	1	4	4	1	1	1	1
3	3	1	1	1	1	3	3	1	1
3	3	4	4	4	4	3	3	1	1
2	2	1	1	1	1	1	1	1	1
2	2	1	1	1	1	1	1	1	1
2	2	1	1	1	1	1	1	1	1
3	3	4	4	1	1	1	1	1	1
2	2	1	1	4	4	1	1	1	1
2	2	1	1	1	1	1	1	1	1
3	3	4	4	4	4	3	3	4	4
3	3	1	1	1	1	1	1	1	1
3	3	4	4	4	4	1	1	1	1
2	2	1	1	4	4	1	1	1	1
3	3	4	4	4	4	1	1	1	1
3	3	4	4	4	4	1	1	1	1
3	3	1	1	4	4	3	3	1	1
2	2	4	4	1	1	1	1	1	1
2	2	1	1	4	4	1	1	1	1
3	3	1	1	1	1	3	3	1	1
3	3	1	1	4	4	1	1	1	1
3	3	4	4	4	4	1	1	1	1
2	2	4	4	1	1	1	1	1	1
3	3	1	1	1	1	3	3	1	1
3	3	4	4	4	4	1	1	1	1

For Review Only

CSSA 5585 Guilford Rd., Madison, WI 53711

3	3	1	1	4	4	1	1	1	1
2	2	1	1	4	4	1	1	1	1
2	2	4	4	4	4	1	1	1	1
3	3	4	4	1	1	1	1	1	1
3	3	1	1	1	1	1	1	1	1
3	3	4	4	4	4	1	1	1	1
2	2	1	1	4	4	1	1	1	1
2	2	4	4	4	4	1	1	1	1
3	3	1	1	1	1	1	1	1	1
3	3	4	4	1	1	1	1	1	1
3	3	1	1	4	4	3	3	1	1
2	2	4	4	1	1	1	1	1	1
3	3	1	1	1	1	1	1	1	1
3	3	4	4	4	4	1	1	1	1
2	2	1	1	1	1	1	1	1	1
3	3	1	1	1	1	3	3	1	1
2	2	4	4	1	1	1	1	1	1
3	3	1	1	1	1	3	3	1	1
3	3	1	1	1	1	3	3	1	1
3	3	4	4	1	1	1	1	1	1
3	3	4	4	4	4	1	1	1	1
3	3	1	1	4	4	1	1	1	1
2	2	1	1	4	4	1	1	4	4
3	3	4	4	1	1	3	3	1	1
3	3	1	1	1	1	3	3	1	1
2	2	1	1	1	1	1	1	1	1
3	3	4	4	1	1	1	1	1	1
2	2	4	4	1	1	1	1	1	1
3	3	1	1	1	1	1	1	4	4
3	3	4	4	1	1	1	1	1	1
3	3	4	4	1	1	1	1	1	1
3	3	4	4	4	4	3	3	1	1
3	3	1	1	4	4	3	3	1	1
2	2	1	1	1	1	1	1	1	1
3	3	4	4	1	1	1	1	1	1
3	3	4	4	1	1	3	3	1	1
3	3	1	1	1	1	3	3	1	1
3	3	1	1	4	4	3	3	1	1
2	2	4	4	1	1	1	1	1	1
2	2	1	1	1	1	1	1	1	1
3	3	4	4	1	1	3	3	1	1
3	3	1	1	4	4	3	3	1	1
2	2	1	1	1	1	1	1	1	1
2	2	4	4	1	1	1	1	1	1
2	2	4	4	1	1	1	1	1	1
2	2	4	4	4	4	1	1	1	1
3	3	1	1	1	1	3	3	1	1
2	2	1	1	4	4	1	1	1	1
3	3	1	1	1	1	1	1	1	1
2	2	4	4	1	1	1	1	1	1
3	3	1	1	1	1	3	3	1	1
3	3	1	1	1	1	1	1	1	1
2	2	1	1	4	4	1	1	4	4
3	3	4	4	1	1	3	3	1	1
2	2	1	1	1	1	1	1	1	1

For Review Only

2	2	4	4	1	1	1	1	1	1
2	2	4	4	4	4	1	1	1	1
3	3	1	1	4	4	3	3	1	1
2	2	1	1	4	4	1	1	4	4
3	3	4	4	1	1	1	1	1	1
2	2	4	4	4	4	3	3	1	1
2	2	1	1	1	1	1	1	1	1
3	3	1	1	4	4	1	1	1	1
3	3	1	1	1	1	1	1	1	1
2	2	1	1	4	4	1	1	1	1
3	3	4	4	1	1	1	1	1	1
2	2	4	4	4	4	1	1	1	1
3	3	1	1	4	4	3	3	4	4
3	3	4	4	4	4	1	1	1	1
3	3	1	1	1	1	1	1	1	1
2	2	1	1	1	1	1	1	4	4
3	3	1	1	1	1	1	1	1	1
3	3	1	1	1	1	1	1	1	1
3	3	1	1	4	4	1	1	1	1
3	3	1	1	4	4	1	1	1	1
2	2	4	4	1	1	1	1	4	4
3	3	1	1	4	4	3	3	1	1
3	3	1	1	4	4	1	1	1	1
3	3	1	1	1	1	3	3	1	1
3	3	4	4	4	4	1	1	1	1
3	3	4	4	1	1	3	3	1	1
2	2	1	1	1	1	1	1	1	1
3	3	4	4	1	1	3	3	1	1
3	3	4	4	1	1	1	1	1	1
3	3	1	1	1	1	3	3	1	1
2	2	1	1	1	1	1	1	1	1
3	3	1	1	4	4	1	1	1	1
2	2	1	1	1	1	1	1	1	1
2	2	4	4	1	1	1	1	1	1
2	2	1	1	4	4	1	1	4	4
2	2	1	1	1	1	1	1	1	1
3	3	4	4	1	1	3	3	1	1
3	3	4	4	1	1	3	3	1	1
3	3	1	1	1	1	1	1	1	1
2	2	1	1	1	1	1	1	1	1
3	3	1	1	4	4	3	3	1	1
2	2	1	1	1	1	1	1	1	1
3	3	4	4	1	1	3	3	1	1
3	3	1	1	1	1	3	3	1	1
3	3	4	4	4	4	1	1	1	1
3	3	1	1	4	4	3	3	4	4
2	2	4	4	1	1	1	1	1	1
3	3	1	1	1	1	1	1	1	1
3	3	4	4	4	4	1	1	1	1
2	2	1	1	1	1	1	1	1	1
3	3	1	1	1	1	3	3	1	1
3	3	1	1	1	1	1	1	1	1
3	3	4	4	1	1	3	3	1	1
3	3	4	4	1	1	3	3	1	1
2	2	4	4	1	1	1	1	1	1

For Review Only

3	3	1	1	1	1	3	3	1	1
2	2	4	4	1	1	1	1	1	1

For Review Only

SNP557	SNP557	SNP558	SNP558	SNP559	SNP559	SNP560	SNP560	SNP561	SNP561
3	3	3	3	4	4	2	2	3	3
4	4	3	3	3	3	2	2	3	3
3	3	1	1	4	4	4	4	3	3
3	3	1	1	4	4	4	4	3	3
3	3	1	1	4	4	4	4	3	3
3	3	3	3	4	4	2	2	3	3
3	3	3	3	4	4	2	2	3	3
4	4	1	1	4	4	4	4	3	3
3	3	1	1	4	4	4	4	3	3
4	4	3	3	4	4	2	2	3	3
4	4	1	1	4	4	4	4	3	3
3	3	1	1	4	4	4	4	3	3
4	4	3	3	4	4	2	2	3	3
3	3	3	3	4	4	4	4	3	3
4	4	1	1	4	4	4	4	3	3
3	3	1	1	4	4	4	4	3	3
4	4	3	3	4	4	2	2	3	3
4	4	3	3	4	4	2	2	3	3
3	3	3	3	4	4	2	2	3	3
4	4	3	3	4	4	2	2	3	3
4	4	3	3	4	4	2	2	3	3
4	4	3	3	4	4	2	2	3	3
4	4	3	3	4	4	2	2	3	3
4	4	3	3	4	4	2	2	3	3
3	3	3	3	4	4	2	2	3	3
3	3	3	3	4	4	2	2	3	3
3	3	3	3	4	4	2	2	3	3
4	4	3	3	4	4	2	2	3	3
4	4	3	3	4	4	2	2	3	3
4	4	3	3	4	4	2	2	3	3
3	3	3	3	4	4	2	2	3	3
3	3	3	3	4	4	2	2	3	3
3	3	1	1	4	4	4	4	3	3
4	4	3	3	4	4	2	2	3	3
4	4	1	1	4	4	4	4	3	3
3	3	3	3	4	4	2	2	3	3
3	3	3	3	4	4	2	2	2	2
3	3	3	3	4	4	2	2	3	3
4	4	3	3	4	4	2	2	3	3
4	4	3	3	4	4	2	2	3	3
4	4	3	3	4	4	2	2	3	3
4	4	3	3	4	4	2	2	3	3
4	4	3	3	3	3	2	2	3	3
3	3	1	1	4	4	4	4	3	3
4	4	3	3	3	3	2	2	3	3
3	3	1	1	4	4	4	4	3	3
3	3	3	3	4	4	2	2	3	3
4	4	3	3	4	4	2	2	3	3
3	3	3	3	4	4	2	2	3	3
4	4	3	3	4	4	4	4	3	3
3	3	3	3	4	4	2	2	3	3
4	4	3	3	4	4	2	2	3	3

For Review Only

3	3	3	3	4	4	2	2	3	3
3	3	1	1	4	4	4	4	3	3
3	3	3	3	4	4	2	2	3	3
3	3	3	3	4	4	2	2	3	3
3	3	3	3	4	4	2	2	3	3
3	3	3	3	4	4	2	2	3	3
3	3	3	3	4	4	2	2	3	3
4	4	3	3	4	4	2	2	3	3
4	4	3	3	4	4	2	2	3	3
4	4	1	1	4	4	4	4	3	3
3	3	3	3	3	3	2	2	3	3
3	3	3	3	3	3	2	2	3	3
3	3	3	3	4	4	2	2	3	3
4	4	3	3	4	4	2	2	3	3
4	4	1	1	4	4	2	2	3	3
4	4	3	3	4	4	2	2	3	3
4	4	1	1	4	4	4	4	3	3
4	4	3	3	4	4	2	2	3	3
4	4	1	1	4	4	4	4	3	3
3	3	3	3	4	4	2	2	3	3
3	3	3	3	3	3	2	2	3	3
4	4	3	3	4	4	2	2	3	3
3	3	1	1	4	4	4	4	3	3
3	3	3	3	4	4	2	2	3	3
4	4	3	3	3	3	2	2	3	3
3	3	3	3	3	3	2	2	3	3
4	4	1	1	4	4	4	4	3	3
4	4	3	3	4	4	2	2	3	3
3	3	3	3	4	4	2	2	3	3
4	4	1	1	4	4	4	4	3	3
3	3	3	3	4	4	2	2	3	3
4	4	3	3	4	4	2	2	3	3
4	4	3	3	4	4	2	2	3	3
3	3	3	3	4	4	2	2	3	3
3	3	1	1	4	4	4	4	3	3
4	4	3	3	3	3	2	2	3	3
4	4	3	3	4	4	2	2	3	3
4	4	3	3	4	4	2	2	3	3
4	4	3	3	4	4	2	2	3	3
4	4	3	3	3	3	2	2	3	3
3	3	1	1	4	4	4	4	3	3
3	3	1	1	4	4	4	4	3	3
3	3	3	3	4	4	2	2	3	3
3	3	3	3	3	3	2	2	3	3
3	3	1	1	4	4	4	4	3	3
3	3	3	3	4	4	2	2	3	3
4	4	3	3	4	4	2	2	3	3
3	3	3	3	4	4	2	2	3	3
3	3	3	3	4	4	2	2	3	3
3	3	1	1	4	4	4	4	3	3
4	4	3	3	4	4	2	2	3	3
4	4	3	3	4	4	2	2	3	3
4	4	3	3	4	4	2	2	3	3
4	4	3	3	4	4	2	2	3	3
4	4	3	3	4	4	2	2	3	3

For Review Only

4	4	3	3	4	4	2	2	3	3
3	3	1	1	4	4	4	4	3	3
3	3	3	3	4	4	2	2	3	3
4	4	3	3	4	4	2	2	3	3
3	3	3	3	3	3	2	2	3	3
3	3	3	3	4	4	2	2	3	3
3	3	1	1	4	4	4	4	3	3
3	3	3	3	4	4	2	2	3	3
3	3	3	3	4	4	2	2	3	3
4	4	3	3	4	4	2	2	3	3
3	3	3	3	4	4	2	2	3	3
4	4	3	3	4	4	2	2	3	3
4	4	3	3	4	4	2	2	3	3
3	3	3	3	3	3	2	2	3	3
3	3	3	3	3	3	2	2	3	3
3	3	3	3	4	4	2	2	3	3
4	4	3	3	4	4	2	2	3	3
4	4	3	3	4	4	4	4	3	3
4	4	3	3	4	4	2	2	3	3
4	4	3	3	4	4	2	2	3	3
4	4	3	3	4	4	2	2	3	3
3	3	1	1	4	4	4	4	3	3
4	4	3	3	3	3	2	2	3	3
4	4	3	3	4	4	2	2	3	3
3	3	3	3	4	4	2	2	3	3
4	4	3	3	4	4	2	2	3	3
3	3	3	3	4	4	2	2	3	3
3	3	3	3	4	4	2	2	3	3
3	3	3	3	4	4	2	2	3	3
4	4	1	1	4	4	4	4	3	3
4	4	3	3	4	4	2	2	3	3
4	4	3	3	4	4	2	2	3	3
3	3	1	1	4	4	4	4	3	3
3	3	3	3	4	4	2	2	3	3
4	4	3	3	4	4	2	2	3	3
4	4	1	1	4	4	2	2	3	3
4	4	3	3	4	4	2	2	3	3
3	3	3	3	4	4	2	2	3	3
4	4	3	3	4	4	2	2	3	3
4	4	1	1	4	4	4	4	3	3
4	4	1	1	4	4	4	4	3	3
4	4	3	3	4	4	2	2	3	3
4	4	1	1	4	4	4	4	2	2
4	4	1	1	4	4	2	2	3	3
4	4	1	1	4	4	4	4	3	3
3	3	3	3	4	4	2	2	3	3
3	3	3	3	4	4	2	2	3	3
3	3	3	3	4	4	2	2	3	3
4	4	3	3	4	4	2	2	3	3
3	3	3	3	4	4	2	2	3	3
3	3	3	3	4	4	2	2	3	3
4	4	3	3	4	4	2	2	3	3
4	4	1	1	4	4	4	4	2	2
3	3	3	3	3	3	2	2	3	3
4	4	3	3	4	4	2	2	3	3

For Review Only

4	4	3	3	4	4	2	2	3	3
4	4	3	3	4	4	2	2	3	3
4	4	3	3	3	3	2	2	3	3
4	4	1	1	4	4	2	2	3	3
3	3	3	3	4	4	2	2	3	3
4	4	3	3	4	4	2	2	3	3
4	4	1	1	4	4	4	4	3	3
4	4	3	3	4	4	4	4	3	3
4	4	1	1	4	4	4	4	2	2
3	3	1	1	4	4	4	4	3	3
3	3	3	3	4	4	2	2	3	3
4	4	3	3	4	4	2	2	3	3
3	3	3	3	4	4	2	2	3	3
4	4	1	1	4	4	4	4	3	3
4	4	3	3	4	4	2	2	3	3
4	4	1	1	4	4	4	4	3	3
4	4	1	1	4	4	4	4	3	3
3	3	1	1	3	3	2	2	3	3
4	4	3	3	4	4	2	2	3	3
3	3	1	1	4	4	4	4	3	3
4	4	3	3	4	4	2	2	3	3
4	4	3	3	4	4	2	2	3	3
4	4	3	3	4	4	2	2	3	3
3	3	3	3	3	3	2	2	3	3
4	4	3	3	4	4	2	2	3	3
4	4	3	3	4	4	2	2	3	3
3	3	3	3	4	4	2	2	3	3
4	4	3	3	4	4	2	2	3	3
4	4	3	3	4	4	2	2	2	2
3	3	3	3	4	4	2	2	3	3
4	4	1	1	4	4	4	4	2	2
4	4	1	1	4	4	4	4	3	3
3	3	3	3	4	4	2	2	3	3
3	4	3	3	4	4	2	2	3	3
3	3	3	3	4	4	2	2	3	3
3	3	3	3	3	3	2	2	3	3
3	3	3	3	4	4	2	2	3	3
3	3	3	3	4	4	2	2	3	3
3	3	3	3	4	4	2	2	3	3
3	3	3	3	3	3	2	2	3	3
3	3	1	1	4	4	4	4	3	3
3	3	3	3	4	4	2	2	3	3
4	4	3	3	4	4	2	2	3	3
4	4	3	3	4	4	2	2	3	3
3	3	3	3	4	4	2	2	3	3
4	4	1	1	4	4	4	4	3	3
4	4	1	1	4	4	4	4	3	3
4	4	1	1	4	4	4	4	3	3
4	4	3	3	4	4	2	2	3	3
4	4	3	3	4	4	2	2	3	3
4	4	3	3	4	4	2	2	3	3
4	4	3	3	4	4	2	2	3	3
4	4	3	3	4	4	2	2	3	3
3	3	3	3	3	3	2	2	3	3
4	4	3	3	4	4	2	2	3	3

4	4	1	1	4	4	4	4	3	3
3	3	3	3	4	4	2	2	3	3
3	3	1	1	4	4	4	4	3	3
4	4	3	3	4	4	2	2	3	3
3	3	3	3	4	4	2	2	3	3
3	3	3	3	4	4	2	2	3	3
4	4	3	3	4	4	2	2	3	3
4	4	3	3	4	4	2	2	3	3
3	3	3	3	4	4	2	2	3	3
4	4	3	3	4	4	2	2	3	3
4	4	3	3	4	4	2	2	3	3
4	4	1	1	4	4	4	4	3	3
4	4	3	3	4	4	2	2	2	2
3	3	3	3	3	3	2	2	3	3
3	3	1	1	4	4	4	4	3	3
3	3	1	1	4	4	4	4	3	3
4	4	3	3	4	4	2	2	3	3
4	4	1	1	4	4	2	2	3	3
4	4	3	3	4	4	2	2	3	3
4	4	3	3	4	4	2	2	3	3
3	3	3	3	4	4	2	2	3	3
3	3	3	3	4	4	2	2	3	3
3	3	1	1	4	4	2	2	3	3
3	3	3	3	4	4	2	2	3	3
4	4	3	3	4	4	2	2	3	3
4	4	1	1	4	4	2	2	3	3
4	4	3	3	4	4	2	2	3	3
3	3	3	3	4	4	2	2	3	3
3	3	3	3	4	4	2	2	3	3
3	3	3	3	4	4	2	2	3	3
4	4	3	3	4	4	2	2	3	3
3	3	3	3	4	4	2	2	3	3
4	4	1	1	4	4	4	4	3	3
3	3	3	3	4	4	2	2	3	3
4	4	3	3	4	4	2	2	3	3
4	4	3	3	4	4	2	2	3	3
3	3	1	1	4	4	4	4	2	2
3	3	3	3	3	3	2	2	3	3
3	3	3	3	4	4	2	2	3	3
4	4	3	3	4	4	2	2	3	3
3	3	3	3	4	4	2	2	2	2
3	3	3	3	4	4	2	2	3	3
3	3	3	3	4	4	2	2	3	3
3	3	1	1	4	4	4	4	3	3
4	4	3	3	4	4	2	2	3	3
3	3	3	3	4	4	2	2	2	2
3	3	3	3	4	4	2	2	3	3
4	4	3	3	4	4	2	2	3	3
4	4	3	3	3	3	2	2	3	3
4	4	3	3	4	4	2	2	3	3
3	3	1	1	4	4	4	4	2	2
4	4	3	3	4	4	2	2	3	3
3	3	1	1	4	4	4	4	3	3
3	3	3	3	4	4	2	2	3	3
4	4	3	3	4	4	2	2	3	3

For Review Only

3	3	1	1	4	4	4	4	3	3
3	3	3	3	3	3	2	2	3	3

For Review Only

SNP562	SNP562	SNP563	SNP563	SNP564	SNP564	SNP565	SNP565	SNP566	SNP566
1	1	1	1	1	1	4	4	2	2
1	1	1	1	1	1	4	4	2	2
1	1	4	4	1	1	1	1	2	2
1	1	1	1	1	1	4	4	2	2
1	1	1	1	1	1	4	4	4	4
1	1	4	4	1	1	1	1	2	2
1	1	1	1	1	1	4	4	2	2
1	1	1	1	1	1	4	4	2	2
1	1	1	1	1	1	4	4	4	4
1	1	1	1	1	1	4	4	2	2
1	1	4	4	1	1	1	1	2	2
1	1	4	4	1	1	1	1	2	2
1	1	1	1	1	1	4	4	2	2
1	1	1	1	1	1	4	4	2	2
1	1	1	1	1	1	4	4	2	2
1	1	1	1	1	1	4	4	2	2
1	1	4	4	1	1	1	1	2	2
1	1	1	1	1	1	4	4	2	2
1	1	4	4	1	1	1	1	2	2
1	1	4	4	1	1	1	1	2	2
1	1	1	1	1	1	4	4	2	2
1	1	1	1	1	1	4	4	2	2
1	1	1	1	1	1	4	4	2	2
1	1	4	4	1	1	1	1	2	2
1	1	4	4	1	1	1	1	2	2
1	1	1	1	1	1	4	4	2	2
1	1	4	4	1	1	1	1	2	2
1	1	1	1	1	1	4	4	2	2
1	1	1	1	1	1	4	4	2	2
1	1	1	1	1	1	4	4	2	2
1	1	4	4	1	1	1	1	2	2
1	1	4	4	1	1	1	1	2	2
1	1	4	4	1	1	1	1	2	2
1	1	1	1	1	1	4	4	2	2
1	1	1	1	1	1	4	4	2	2
1	1	1	1	1	1	4	4	2	2
3	3	4	4	1	1	4	4	2	2
1	1	1	1	1	1	4	4	2	2
1	1	1	1	1	1	4	4	2	2
1	1	1	1	1	1	4	4	2	2
1	1	1	1	1	1	4	4	2	2
1	1	1	1	1	1	4	4	2	2
1	1	1	1	1	1	4	4	2	2
1	1	4	4	1	1	1	1	4	4
1	1	1	1	1	1	4	4	2	2
1	1	4	4	1	1	1	1	2	2
1	1	1	1	1	1	4	4	2	2
1	1	1	1	1	1	4	4	2	2
1	1	1	1	1	1	4	4	2	2
3	3	1	1	4	4	4	4	2	2
1	1	1	1	1	1	4	4	2	2
1	1	4	4	1	1	1	1	2	2

For Review Only

1	1	4	4	1	1	1	1	4	4
1	1	4	4	1	1	1	1	2	2
1	1	4	4	1	1	1	1	2	2
1	1	4	4	1	1	1	1	4	4
1	1	4	4	1	1	1	1	2	2
1	1	4	4	1	1	1	1	2	2
1	1	4	4	1	1	1	1	2	2
1	1	1	1	1	1	4	4	2	2
3	3	4	4	1	1	4	4	4	4
1	1	1	1	1	1	4	4	2	2
1	1	4	4	1	1	1	1	2	2
1	1	4	4	1	1	1	1	2	2
1	1	4	4	1	1	1	1	4	4
1	1	1	1	1	1	4	4	2	2
1	1	4	4	1	1	4	4	2	2
1	1	1	1	1	1	4	4	2	2
1	1	1	1	1	1	4	4	2	2
3	3	1	1	4	4	4	4	2	2
1	1	4	4	1	1	1	1	2	2
1	1	4	4	1	1	1	1	4	4
1	1	4	4	1	1	1	1	2	2
1	1	1	1	1	1	4	4	2	2
1	1	1	1	1	1	1	1	2	2
1	1	4	4	1	1	1	1	2	2
1	1	1	1	1	1	4	4	2	2
1	1	4	4	1	1	1	1	2	2
1	1	1	1	1	1	4	4	2	2
1	1	1	1	1	1	4	4	2	2
1	1	1	1	1	1	4	4	2	2
1	1	4	4	1	1	1	1	2	2
1	1	1	1	1	1	4	4	4	4
1	1	1	1	1	1	4	4	2	2
1	1	1	1	1	1	4	4	2	2
1	1	4	4	1	1	1	1	4	4
1	1	1	1	1	1	4	4	2	2
1	1	1	1	1	1	4	4	2	2
1	1	1	1	1	1	4	4	2	2
1	1	4	4	1	1	1	1	2	2
1	1	1	1	1	1	4	4	2	2
1	1	1	1	1	1	4	4	2	2
1	1	4	4	1	1	1	1	4	4
1	1	4	4	1	1	1	1	2	2
1	1	1	1	1	1	4	4	4	4
1	1	4	4	1	1	1	1	2	2
1	1	1	1	1	1	4	4	2	2
1	1	4	4	1	1	1	1	2	2
1	1	1	1	1	1	4	4	2	2
1	1	4	4	1	1	1	1	2	2
1	1	4	4	1	1	1	1	2	2
1	1	1	1	1	1	4	4	2	2
1	1	4	4	1	1	1	1	2	2
1	1	1	1	1	1	4	4	2	2
1	1	1	1	1	1	4	4	2	2
1	1	1	1	1	1	4	4	2	2
1	1	1	1	1	1	4	4	2	2

For Review Only

1	1	1	1	1	1	4	4	2	2
1	1	1	1	1	1	4	4	2	2
1	1	1	1	1	1	4	4	2	2
1	1	1	1	1	1	4	4	2	2
1	1	4	4	1	1	1	1	2	2
1	1	1	1	1	1	4	4	2	2
1	1	4	4	1	1	1	1	2	2
1	1	4	4	1	1	1	1	2	2
1	1	1	1	1	1	4	4	2	2
1	1	1	1	1	1	4	4	2	2
1	1	1	1	1	1	4	4	2	2
1	1	1	1	1	1	4	4	2	2
1	1	4	4	1	1	1	1	2	2
1	1	4	4	1	1	1	1	2	2
1	1	1	1	1	1	4	4	2	2
1	1	1	1	1	1	4	4	2	2
1	1	1	1	1	1	4	4	2	2
1	1	1	1	1	1	4	4	2	2
1	1	1	1	1	1	4	4	2	2
1	1	1	1	1	1	4	4	2	2
1	1	1	1	1	1	4	4	2	2
1	1	4	4	1	1	1	1	2	2
1	1	4	4	1	1	1	1	4	4
1	1	1	1	1	1	4	4	2	2
1	1	1	1	1	1	4	4	2	2
1	1	1	1	1	1	4	4	4	4
1	1	1	1	1	1	4	4	4	4
1	1	1	1	1	1	4	4	4	4
1	1	4	4	1	1	1	1	2	2
1	1	1	1	1	1	4	4	2	2
1	1	1	1	1	1	4	4	2	2
1	1	1	1	1	1	4	4	2	2
1	1	4	4	1	1	1	1	4	4
1	1	1	1	1	1	4	4	2	2
1	1	1	1	1	1	4	4	2	2
1	1	1	1	1	1	4	4	2	2
1	1	1	1	1	1	4	4	2	2
1	1	4	4	1	1	1	1	2	2
1	1	1	1	1	1	4	4	4	4
1	1	1	1	1	1	4	4	2	2
1	1	1	1	1	1	4	4	2	2
1	1	1	1	1	1	4	4	2	2
3	3	1	1	4	4	4	4	4	4
1	1	1	1	1	1	4	4	2	2
1	1	1	1	1	1	4	4	2	2
1	1	1	1	1	1	4	4	4	4
1	1	1	1	1	1	4	4	2	2
1	1	4	4	1	1	1	1	2	2
3	3	1	1	1	1	4	4	2	2
1	1	4	4	1	1	1	1	2	2
1	1	1	1	1	1	4	4	2	2
1	1	1	1	1	1	4	4	2	2
3	3	1	1	4	4	4	4	2	2
1	1	4	4	1	1	1	1	2	2
1	1	1	1	1	1	4	4	2	2

For Review Only

1	1	1	1	1	1	4	4	2	2
1	1	1	1	1	1	4	4	2	2
1	1	1	1	1	1	4	4	2	2
1	1	1	1	1	1	4	4	2	2
1	1	4	4	1	1	1	1	2	2
1	1	1	1	1	1	4	4	2	2
1	1	1	1	1	1	4	4	2	2
1	1	1	1	1	1	4	4	2	2
1	1	1	1	1	1	4	4	2	2
1	1	4	4	1	1	1	1	2	2
1	1	1	1	1	1	4	4	2	2
1	1	1	1	1	1	4	4	2	2
1	1	4	4	1	1	1	1	2	2
1	1	1	1	1	1	4	4	2	2
1	1	1	1	1	1	4	4	2	2
1	1	1	1	1	1	4	4	2	2
3	3	1	1	1	1	4	4	2	2
1	1	4	4	1	1	1	1	2	2
1	1	4	4	1	1	1	1	2	2
1	1	1	1	1	1	1	1	2	2
1	1	1	1	1	1	4	4	2	2
1	1	1	1	1	1	4	4	2	2
1	1	1	1	1	1	4	4	2	2
1	1	4	4	1	1	1	1	2	2
1	1	1	1	1	1	1	1	2	2
1	1	1	1	1	1	4	4	2	2
1	1	4	4	1	1	1	1	2	2
1	1	1	1	1	1	4	4	2	2
3	3	1	1	4	4	4	4	2	2
1	1	4	4	1	1	1	1	2	2
3	3	4	4	1	1	4	4	2	2
1	1	1	1	1	1	4	4	2	2
1	1	1	1	1	1	4	4	2	2
1	1	1	1	1	1	4	4	2	2
1	1	4	4	1	1	1	1	4	4
1	1	4	4	1	1	1	1	2	2
1	1	4	4	1	1	4	4	2	2
1	1	1	1	1	1	4	4	4	4
1	1	4	4	1	1	1	1	2	2
1	1	4	4	1	1	1	1	2	2
1	1	4	4	1	1	1	1	2	2
1	1	1	1	1	1	4	4	2	2
1	1	1	1	1	1	4	4	2	2
1	1	1	1	1	1	4	4	2	2
1	1	1	1	1	1	4	4	2	2
1	1	1	1	1	1	4	4	2	2
1	1	1	1	1	1	4	4	2	2
1	1	1	1	1	1	4	4	2	2
1	1	1	1	1	1	4	4	2	2
1	1	4	4	1	1	1	1	2	2
1	1	4	4	1	1	1	1	2	2
1	1	1	1	1	1	4	4	2	2
1	1	1	1	1	1	4	4	2	2
1	1	4	4	1	1	1	1	2	2
1	1	1	1	1	1	4	4	2	2

For Review Only

1	1	1	1	1	1	4	4	2	2
1	1	1	1	1	1	4	4	2	2
1	1	1	1	1	1	4	4	2	2
1	1	1	1	1	1	4	4	2	2
1	1	1	1	1	1	4	4	2	2
1	1	1	1	1	1	4	4	2	2
1	1	1	1	1	1	4	4	2	2
1	1	1	1	1	1	4	4	2	2
1	1	1	1	1	1	4	4	2	2
1	1	1	1	1	1	4	4	2	2
1	1	1	1	1	1	4	4	2	2
1	1	1	1	1	1	4	4	2	2
3	3	1	1	4	4	4	4	2	2
1	1	1	1	1	1	4	4	2	2
1	1	4	4	1	1	1	1	2	2
1	1	1	1	1	1	4	4	2	2
1	1	1	1	1	1	4	4	2	2
1	1	1	1	1	1	4	4	2	2
1	1	1	1	1	1	4	4	2	2
1	1	1	1	1	1	4	4	2	2
1	1	4	4	1	1	1	1	2	2
1	1	1	1	1	1	4	4	2	2
1	1	1	1	1	1	4	4	4	4
1	1	1	1	1	1	4	4	2	2
1	1	1	1	1	1	4	4	2	2
1	1	1	1	1	1	4	4	2	2
1	1	1	1	1	1	4	4	2	2
1	1	4	4	1	1	1	1	2	2
1	1	4	4	1	1	1	1	2	2
1	1	4	4	1	1	1	1	2	2
1	1	1	1	1	1	4	4	2	2
1	1	1	1	1	1	4	4	2	2
1	1	1	1	1	1	4	4	2	2
1	1	4	4	1	1	1	1	2	2
1	1	1	1	1	1	4	4	2	2
1	1	1	1	1	1	4	4	2	2
3	3	1	1	4	4	4	4	2	2
1	1	4	4	1	1	1	1	2	2
1	1	1	1	1	1	4	4	2	2
1	1	1	1	1	1	4	4	2	2
3	3	1	1	4	4	4	4	2	2
1	1	1	1	1	1	4	4	2	2
1	1	1	1	1	1	4	4	4	4
1	1	4	4	1	1	1	1	4	4
1	1	1	1	1	1	4	4	2	2
3	3	1	1	4	4	4	4	2	2
1	1	1	1	1	1	4	4	2	2
1	1	4	4	1	1	1	1	2	2
1	1	1	1	1	1	4	4	2	2
1	1	1	1	1	1	4	4	2	2
3	3	1	1	4	4	4	4	4	4
1	1	1	1	1	1	4	4	2	2
1	1	4	4	1	1	1	1	2	2
1	1	4	4	1	1	1	1	2	2
1	1	1	1	1	1	4	4	2	2

For Review Only

1	1	4	4	1	1	1	1	2	2
1	1	4	4	1	1	1	1	2	2

For Review Only

SNP567	SNP567	SNP568	SNP568	SNP569	SNP569	SNP570	SNP570	SNP571	SNP571
4	4	4	4	4	4	2	2	3	3
4	4	1	1	4	4	2	2	3	3
3	3	1	1	3	3	2	2	2	2
3	3	1	1	4	4	4	4	2	2
3	3	4	4	4	4	4	4	2	2
4	4	4	4	4	4	4	4	3	3
4	4	4	4	4	4	2	2	3	3
3	3	1	1	4	4	4	4	2	2
3	3	1	1	4	4	2	2	2	2
4	4	1	1	4	4	4	4	3	3
3	3	4	4	4	4	2	2	2	2
3	3	1	1	3	3	2	2	2	2
4	4	4	4	4	4	2	2	3	3
4	4	1	1	3	3	2	2	3	3
4	4	4	4	4	4	4	4	2	2
3	3	1	1	3	3	2	2	2	2
4	4	4	4	4	4	4	4	3	3
4	4	4	4	4	4	4	4	3	3
4	4	4	4	3	3	2	2	3	3
4	4	4	4	4	4	4	4	3	3
4	4	4	4	4	4	4	4	3	3
4	4	4	4	4	4	4	4	3	3
4	4	1	1	4	4	2	2	2	2
4	4	1	1	4	4	4	4	3	3
4	4	4	4	3	3	2	2	3	3
4	4	1	1	3	3	2	2	3	3
4	4	4	4	3	3	2	2	3	3
4	4	4	4	3	3	2	2	3	3
4	4	4	4	4	4	4	4	3	3
4	4	1	1	4	4	4	4	3	3
4	4	4	4	3	3	2	2	3	3
4	4	4	4	4	4	4	4	3	3
3	3	4	4	4	4	2	2	2	2
4	4	1	1	4	4	4	4	3	3
3	3	4	4	3	3	4	4	2	2
4	4	1	1	3	3	2	2	3	3
4	4	1	1	4	4	2	2	3	3
4	4	1	1	3	3	4	4	3	3
4	4	1	1	4	4	2	2	3	3
4	4	4	4	4	4	4	4	3	3
4	4	1	1	4	4	4	4	3	3
3	3	4	4	4	4	4	4	3	3
4	4	1	1	4	4	4	4	3	3
3	3	1	1	4	4	4	4	2	2
4	4	1	1	4	4	4	4	3	3
3	3	1	1	3	3	2	2	2	2
4	4	4	4	3	3	2	2	3	3
4	4	4	4	4	4	4	4	3	3
4	4	1	1	4	4	4	4	3	3
4	4	4	4	4	4	4	4	3	3
4	4	1	1	3	3	2	2	3	3
4	4	1	1	3	3	2	2	3	3

For Review Only

4	4	4	4	4	4	4	4	3	3
3	3	4	4	4	4	2	2	2	2
4	4	4	4	3	3	2	2	3	3
4	4	4	4	4	4	4	4	3	3
4	4	4	4	3	3	2	2	3	3
4	4	4	4	3	3	2	2	3	3
4	4	4	4	4	4	4	4	3	3
4	4	1	1	4	4	4	4	3	3
4	4	1	1	4	4	4	4	3	3
3	3	1	1	3	3	2	2	2	2
4	4	1	1	3	3	2	2	3	3
4	4	1	1	3	3	2	2	3	3
4	4	4	4	4	4	4	4	3	3
4	4	4	4	4	4	4	4	3	3
4	4	4	4	4	4	4	4	3	3
4	4	1	1	4	4	2	2	3	3
3	3	1	1	4	4	2	2	2	2
4	4	4	4	4	4	4	4	2	2
3	3	4	4	4	4	2	2	2	2
4	4	4	4	4	4	2	2	3	3
4	4	1	1	3	3	2	2	3	3
4	4	4	4	4	4	4	4	3	3
3	3	4	4	4	4	4	4	2	2
4	4	4	4	3	3	2	2	3	3
4	4	1	1	4	4	4	4	3	3
4	4	1	1	3	3	2	2	3	3
3	3	4	4	4	4	4	4	2	2
4	4	1	1	4	4	2	2	3	3
4	4	1	1	4	4	4	4	3	3
3	3	1	1	3	3	2	2	2	2
4	4	4	4	4	4	4	4	3	3
4	4	1	1	4	4	4	4	3	3
4	4	1	1	4	4	4	4	3	3
4	4	4	4	4	4	4	4	3	3
3	3	1	1	4	4	4	4	2	2
4	4	1	1	4	4	4	4	3	3
4	4	4	4	4	4	4	4	3	3
4	4	4	4	4	4	4	4	3	3
4	4	1	1	3	3	2	2	3	3
4	4	1	1	4	4	4	4	3	3
3	3	4	4	4	4	4	4	2	2
3	3	1	1	3	3	2	2	2	2
4	4	1	1	3	3	2	2	3	3
4	4	1	1	3	3	2	2	3	3
3	3	1	1	3	3	2	2	2	2
4	4	4	4	3	3	2	2	3	3
4	4	1	1	4	4	4	4	3	3
4	4	1	1	4	4	4	4	3	3
4	4	4	4	4	4	4	4	3	3
3	3	4	4	3	3	2	2	2	2
4	4	1	1	3	3	2	2	3	3
4	4	1	1	4	4	4	4	3	3
4	4	1	1	4	4	4	4	3	3
3	3	4	4	3	3	2	2	2	2
4	4	1	1	3	3	2	2	2	2

For Review Only

4	4	4	4	4	4	4	4	3	3
3	3	4	4	4	4	4	4	2	2
4	4	1	1	3	3	2	2	3	3
3	3	4	4	4	4	4	4	3	3
4	4	4	4	3	3	2	2	3	3
4	4	1	1	3	3	2	2	3	3
3	3	1	1	3	3	2	2	2	2
4	4	4	4	4	4	4	4	3	3
4	4	1	1	3	3	2	2	3	3
4	4	1	1	3	3	4	4	3	3
4	4	4	4	4	4	2	2	3	3
4	4	4	4	3	3	4	4	3	3
4	4	1	1	3	3	2	2	3	3
4	4	1	1	3	3	2	2	3	3
4	4	1	1	3	3	2	2	3	3
4	4	4	4	4	4	4	4	3	3
4	4	4	4	3	3	2	2	2	2
3	3	4	4	3	3	2	2	3	3
3	3	4	4	4	4	4	4	3	3
4	4	4	4	4	4	2	2	3	3
4	4	4	4	3	3	2	2	3	3
3	3	4	4	3	3	2	2	2	2
4	4	1	1	3	3	2	2	3	3
4	4	1	1	4	4	4	4	3	3
4	4	4	4	4	4	4	4	3	3
4	4	4	4	4	4	4	4	3	3
4	4	4	4	4	4	4	4	3	3
4	4	1	1	4	4	4	4	3	3
4	4	4	4	3	3	2	2	3	3
3	3	4	4	4	4	4	4	2	2
4	4	1	1	4	4	4	4	3	3
4	4	4	4	3	3	2	2	3	3
3	3	4	4	4	4	4	4	2	2
4	4	4	4	4	4	4	4	3	3
4	4	1	1	4	4	4	4	3	3
3	3	4	4	4	4	4	4	2	2
4	4	1	1	4	4	4	4	3	3
4	4	1	1	4	4	4	4	3	3
4	4	4	4	4	4	4	4	3	3
3	3	4	4	4	4	2	2	2	2
3	3	4	4	3	3	2	2	3	3
4	4	1	1	4	4	4	4	3	3
3	3	1	1	3	3	2	2	2	2
4	4	1	1	4	4	4	4	3	3
3	3	1	1	4	4	4	4	2	2
4	4	1	1	4	4	2	2	3	3
4	4	4	4	3	3	4	4	3	3
4	4	4	4	4	4	4	4	3	3
4	4	1	1	4	4	2	2	3	3
4	4	1	1	3	3	2	2	3	3
4	4	4	4	3	3	2	2	3	3
4	4	4	4	4	4	4	4	3	3
3	3	1	1	4	4	4	4	2	2
4	4	1	1	3	3	2	2	3	3
4	4	4	4	4	4	2	2	3	3

4	4	4	4	4	4	4	4	3	3
4	4	1	1	4	4	4	4	3	3
4	4	1	1	4	4	4	4	3	3
3	3	4	4	4	4	4	4	2	2
4	4	4	4	3	3	2	2	3	3
4	4	4	4	4	4	4	4	3	3
3	3	4	4	3	3	2	2	2	2
4	4	4	4	3	3	2	2	3	3
3	3	1	1	4	4	2	2	2	2
3	3	4	4	4	4	2	2	2	2
4	4	1	1	3	3	4	4	3	3
4	4	1	1	3	3	2	2	3	3
4	4	1	1	3	3	2	2	3	3
3	3	1	1	4	4	2	2	2	2
4	4	1	1	4	4	2	2	3	3
3	3	4	4	4	4	2	2	2	2
3	3	1	1	4	4	2	2	2	2
4	4	1	1	3	3	2	2	3	3
4	4	1	1	3	3	2	2	3	3
3	3	1	1	3	3	2	2	2	2
4	4	4	4	3	3	2	2	3	3
4	4	1	1	4	4	4	4	3	3
4	4	4	4	4	4	4	4	3	3
4	4	1	1	3	3	2	2	3	3
4	4	1	1	4	4	4	4	3	3
4	4	1	1	4	4	4	4	3	3
4	4	1	1	3	3	2	2	3	3
4	4	4	4	3	3	2	2	2	2
4	4	4	4	3	3	2	2	3	3
3	3	4	4	4	4	4	4	3	3
3	3	1	1	4	4	2	2	2	2
3	3	4	4	3	3	2	2	2	2
4	4	4	4	4	4	4	4	3	3
4	4	1	1	4	4	2	2	3	3
4	4	4	4	4	4	2	2	3	3
4	4	1	1	3	3	2	2	3	3
4	4	1	1	3	3	2	2	3	3
4	4	1	1	4	4	2	2	3	3
4	4	4	4	4	4	4	4	3	3
4	4	1	1	4	4	4	4	3	3
3	3	1	1	3	3	2	2	2	2
4	4	4	4	4	4	4	4	3	3
3	3	1	1	4	4	4	4	3	3
4	4	4	4	4	4	4	4	3	3
4	4	4	4	3	3	2	2	3	3
3	3	1	1	4	4	2	2	2	2
3	3	4	4	4	4	2	2	2	2
3	3	1	1	4	4	4	4	2	2
4	4	1	1	4	4	4	4	3	3
4	4	4	4	3	3	2	2	3	3
4	4	4	4	4	4	4	4	3	3
4	4	4	4	4	4	4	4	3	3
4	4	1	1	4	4	4	4	3	3
4	4	1	1	3	3	2	2	3	3
4	4	1	1	4	4	2	2	3	3

For Review Only

3	3	1	1	4	4	4	4	2	2
4	4	4	4	4	4	4	4	3	3
3	3	4	4	3	3	2	2	2	2
4	4	4	4	4	4	2	2	3	3
4	4	4	4	4	4	4	4	3	3
4	4	1	1	3	3	2	2	3	3
4	4	4	4	4	4	2	2	3	3
4	4	1	1	4	4	4	4	3	3
4	4	1	1	4	4	2	2	3	3
4	4	1	1	4	4	4	4	3	3
4	4	4	4	3	3	2	2	3	3
3	3	4	4	4	4	2	2	2	2
4	4	4	4	3	3	2	2	3	3
4	4	4	4	3	3	2	2	3	3
3	3	4	4	3	3	2	2	2	2
3	3	4	4	4	4	4	4	2	2
4	4	1	1	4	4	2	2	3	3
4	4	4	4	4	4	4	4	3	3
4	4	1	1	4	4	4	4	3	3
4	4	1	1	4	4	4	4	3	3
4	4	4	4	4	4	4	4	3	3
4	4	1	1	4	4	4	4	3	3
4	4	4	4	4	4	2	2	3	3
4	4	1	1	3	3	2	2	3	3
4	4	1	1	4	4	4	4	3	3
3	3	4	4	3	3	2	2	2	2
4	4	1	1	4	4	4	4	3	3
4	4	1	1	4	4	2	2	3	3
4	4	4	4	4	4	4	4	3	3
4	4	4	4	4	4	2	2	3	3
4	4	1	1	4	4	2	2	3	3
3	3	4	4	4	4	4	4	2	2
3	3	1	1	4	4	2	2	2	2
4	4	4	4	4	4	4	4	3	3
4	4	4	4	4	4	4	4	3	3
4	4	1	1	4	4	4	4	3	3
3	3	1	1	4	4	4	4	2	2
4	4	1	1	3	3	2	2	3	3
4	4	1	1	3	3	2	2	3	3
4	4	1	1	4	4	4	4	3	3
4	4	4	4	4	4	2	2	3	3
4	4	1	1	4	4	4	4	3	3
4	4	4	4	3	3	2	2	3	3
3	3	4	4	4	4	4	4	2	2
4	4	1	1	3	3	2	2	3	3
4	4	1	1	4	4	4	4	3	3
4	4	4	4	4	4	4	4	3	3
4	4	1	1	3	3	2	2	2	2
4	4	1	1	3	3	2	2	3	3
4	4	4	4	4	4	4	4	3	3
3	3	4	4	4	4	4	4	2	2
4	4	1	1	4	4	4	4	3	3
3	3	1	1	3	3	2	2	2	2
4	4	1	1	3	3	2	2	3	3
4	4	1	1	4	4	4	4	3	3

For Review Only

3	3	4	4	4	4	4	4	2	2
4	4	1	1	3	3	2	2	3	3

For Review Only

SNP572	SNP572	SNP573	SNP573	SNP574	SNP574	SNP575	SNP575	SNP576	SNP576
4	4	2	2	1	1	1	1	1	1
4	4	2	2	1	1	1	1	1	1
1	1	2	2	4	4	3	3	1	1
1	1	2	2	4	4	1	1	1	1
1	1	4	4	4	4	1	1	1	1
4	4	2	2	1	1	1	1	1	1
4	4	4	4	1	1	1	1	1	1
1	1	2	2	4	4	1	1	4	4
1	1	4	4	4	4	1	1	4	4
4	4	2	2	1	1	1	1	4	4
1	1	2	2	4	4	1	1	1	1
1	1	2	2	4	4	3	3	1	1
4	4	2	2	1	1	1	1	1	1
4	4	2	2	1	1	3	3	4	4
1	1	4	4	4	4	1	1	1	1
1	1	2	2	4	4	1	1	1	1
4	4	4	4	1	1	1	1	1	1
4	4	2	2	1	1	1	1	1	1
4	4	4	4	1	1	3	3	1	1
4	4	2	2	1	1	1	1	1	1
4	4	2	2	1	1	1	1	1	1
4	4	2	2	4	4	1	1	1	1
1	1	2	2	1	1	1	1	1	1
4	4	2	2	1	1	1	1	1	1
4	4	4	4	1	1	3	3	1	1
4	4	2	2	1	1	3	3	1	1
4	4	2	2	1	1	3	3	1	1
1	1	4	4	4	4	1	1	1	1
4	4	2	2	1	1	1	1	1	1
4	4	2	2	1	1	1	1	4	4
4	4	4	4	1	1	3	3	1	1
4	4	2	2	1	1	1	1	1	1
1	1	2	2	4	4	1	1	1	1
1	1	2	2	1	1	1	1	4	4
1	1	2	2	4	4	1	1	1	1
4	4	2	2	1	1	3	3	1	1
4	4	2	2	1	1	1	1	4	4
4	4	2	2	1	1	1	1	1	1
1	1	2	2	1	1	1	1	4	4
4	4	2	2	1	1	1	1	1	1
4	4	2	2	1	1	1	1	4	4
4	4	2	2	4	4	1	1	1	1
4	4	2	2	1	1	3	3	1	1
1	1	2	2	4	4	1	1	4	4
4	4	2	2	1	1	3	3	1	1
1	1	2	2	4	4	3	3	1	1
4	4	2	2	1	1	3	3	1	1
4	4	2	2	1	1	1	1	1	1
4	4	2	2	1	1	1	1	4	4
1	1	4	4	4	4	1	1	1	1
4	4	2	2	1	1	3	3	4	4
4	4	4	4	1	1	1	1	4	4

For Review Only

4	4	4	4	1	1	1	1	1	1
1	1	2	2	4	4	1	1	1	1
4	4	2	2	1	1	3	3	1	1
4	4	4	4	1	1	1	1	1	1
4	4	4	4	1	1	3	3	1	1
4	4	2	2	1	1	3	3	1	1
4	4	2	2	1	1	1	1	1	1
4	4	2	2	1	1	1	1	4	4
4	4	4	4	1	1	1	1	4	4
1	1	4	4	4	4	1	1	1	1
4	4	2	2	1	1	3	3	1	1
4	4	2	2	1	1	3	3	1	1
4	4	4	4	1	1	1	1	1	1
4	4	2	2	1	1	1	1	1	1
1	1	2	2	1	1	1	1	1	1
4	4	4	4	1	1	1	1	4	4
1	1	2	2	4	4	1	1	1	1
1	1	2	2	4	4	1	1	1	1
1	1	2	2	4	4	1	1	1	1
4	4	4	4	1	1	1	1	1	1
4	4	2	2	1	1	1	1	1	1
4	4	2	2	1	1	1	1	1	1
1	1	2	2	4	4	1	1	1	1
4	4	2	2	1	1	3	3	1	1
4	4	2	2	1	1	3	3	1	1
4	4	2	2	1	1	3	3	1	1
1	1	2	2	4	4	1	1	1	1
1	1	2	2	1	1	1	1	4	4
4	4	2	2	1	1	1	1	1	1
1	1	4	4	4	4	1	1	4	4
4	4	4	4	1	1	1	1	1	1
4	4	2	2	1	1	1	1	4	4
4	4	2	2	1	1	1	1	4	4
4	4	4	4	1	1	1	1	1	1
1	1	2	2	4	4	1	1	4	4
4	4	2	2	1	1	3	3	1	1
4	4	2	2	1	1	1	1	4	4
4	4	2	2	1	1	1	1	1	1
4	4	2	2	1	1	3	3	4	4
4	4	2	2	1	1	3	3	1	1
1	1	4	4	4	4	1	1	1	1
1	1	4	4	4	4	1	1	1	1
4	4	4	4	1	1	3	3	4	4
4	4	2	2	1	1	3	3	1	1
1	1	4	4	4	4	1	1	4	4
4	4	2	2	1	1	3	3	1	1
4	4	2	2	1	1	1	1	4	4
4	4	4	4	1	1	1	1	1	1
4	4	2	2	1	1	1	1	1	1
1	1	2	2	4	4	1	1	1	1
4	4	2	2	1	1	3	3	1	1
4	4	2	2	1	1	1	1	4	4
4	4	2	2	1	1	1	1	4	4
1	1	4	4	4	4	1	1	1	1
1	1	2	2	4	4	3	3	4	4

For Review Only

4	4	2	2	1	1	1	1	1	1
1	1	2	2	4	4	1	1	1	1
4	4	2	2	1	1	3	3	1	1
4	4	2	2	1	1	1	1	1	1
4	4	2	2	1	1	3	3	1	1
4	4	2	2	1	1	3	3	1	1
1	1	4	4	4	4	1	1	1	1
4	4	4	4	1	1	1	1	1	1
4	4	2	2	1	1	1	1	4	4
4	4	2	2	1	1	1	1	4	4
4	4	2	2	1	1	1	1	1	1
4	4	4	4	1	1	1	1	1	1
4	4	2	2	1	1	3	3	4	4
4	4	2	2	1	1	3	3	1	1
4	4	4	4	1	1	3	3	1	1
4	4	2	2	1	1	1	1	1	1
1	1	2	2	4	4	3	3	1	1
1	1	2	2	4	4	1	1	1	1
4	4	2	2	1	1	1	1	1	1
4	4	4	4	1	1	1	1	1	1
1	1	2	2	1	1	1	1	1	1
1	1	4	4	4	4	1	1	1	1
4	4	4	4	1	1	1	1	1	1
1	1	2	2	1	1	1	1	4	4
4	4	2	2	1	1	1	1	1	1
4	4	4	4	1	1	1	1	1	1
4	4	4	4	1	1	1	1	1	1
1	1	4	4	1	1	1	1	4	4
4	4	2	2	1	1	3	3	1	1
1	1	2	2	4	4	1	1	1	1
4	4	2	2	1	1	1	1	4	4
4	4	4	4	1	1	1	1	1	1
1	1	4	4	4	4	1	1	1	1
4	4	4	4	1	1	1	1	1	1
1	1	2	2	1	1	1	1	4	4
1	1	2	2	4	4	1	1	1	1
4	4	2	2	1	1	1	1	4	4
4	4	4	4	1	1	1	1	1	1
4	4	4	4	1	1	1	1	1	1
1	1	2	2	4	4	1	1	1	1
4	4	2	2	1	1	1	1	1	1
4	4	2	2	1	1	1	1	4	4
1	1	4	4	4	4	3	3	4	4
4	4	2	2	1	1	1	1	4	4
1	1	2	2	4	4	1	1	4	4
4	4	4	4	1	1	1	1	4	4
4	4	2	2	1	1	1	1	1	1
4	4	2	2	1	1	1	1	1	1
4	4	2	2	1	1	1	1	4	4
4	4	2	2	1	1	3	3	1	1
4	4	2	2	1	1	1	1	1	1
4	4	2	2	1	1	1	1	1	1
1	1	4	4	4	4	1	1	1	1
4	4	2	2	1	1	3	3	1	1
4	4	2	2	1	1	1	1	1	1

For Review Only

4	4	2	2	1	1	1	1	1	1
4	4	2	2	1	1	1	1	4	4
4	4	2	2	1	1	1	1	1	1
1	1	2	2	4	4	1	1	1	1
4	4	2	2	1	1	1	1	1	1
4	4	2	2	1	1	1	1	1	1
1	1	2	2	4	4	1	1	1	1
1	1	2	2	4	4	1	1	1	1
1	1	2	2	4	4	1	1	1	1
1	1	4	4	4	4	1	1	1	1
4	4	2	2	1	1	1	1	1	1
4	4	2	2	1	1	1	1	4	4
4	4	2	2	1	1	1	1	4	4
1	1	4	4	4	4	1	1	4	4
4	4	2	2	1	1	1	1	1	1
1	1	2	2	4	4	1	1	1	1
1	1	2	2	4	4	1	1	1	1
4	4	2	2	1	1	3	3	1	1
4	4	2	2	1	1	3	3	4	4
1	1	4	4	4	4	1	1	1	1
4	4	4	4	1	1	3	3	1	1
4	4	2	2	1	1	1	1	4	4
4	4	2	2	1	1	1	1	1	1
4	4	2	2	1	1	3	3	1	1
4	4	2	2	4	4	1	1	4	4
1	1	2	2	1	1	1	1	4	4
4	4	2	2	1	1	3	3	1	1
1	1	2	2	4	4	1	1	1	1
4	4	2	2	1	1	1	1	1	1
1	1	2	2	1	1	1	1	1	1
1	1	2	2	4	4	1	1	4	4
1	1	4	4	4	4	1	1	1	1
4	4	2	2	1	1	1	1	1	1
4	4	2	2	1	1	1	1	1	1
4	4	4	4	1	1	1	1	1	1
4	4	2	2	1	1	3	3	1	1
4	4	2	2	1	1	3	3	1	1
4	4	4	4	1	1	1	1	4	4
4	4	2	2	1	1	1	1	1	1
4	4	4	4	1	1	1	1	1	1
1	1	2	2	4	4	3	3	1	1
4	4	2	2	1	1	1	1	1	1
1	1	2	2	1	1	1	1	4	4
4	4	2	2	1	1	1	1	1	1
4	4	2	2	1	1	3	3	1	1
1	1	2	2	4	4	1	1	4	4
1	1	2	2	4	4	1	1	1	1
1	1	2	2	4	4	1	1	4	4
4	4	2	2	1	1	1	1	4	4
4	4	2	2	1	1	3	3	1	1
4	4	2	2	1	1	1	1	1	1
4	4	2	2	1	1	1	1	4	4
4	4	2	2	1	1	1	1	4	4
4	4	2	2	1	1	3	3	1	1
4	4	2	2	1	1	1	1	4	4

For Review Only

1	1	2	2	4	4	1	1	1	1
4	4	2	2	1	1	1	1	1	1
1	1	4	4	4	4	1	1	1	1
4	4	2	2	1	1	1	1	1	1
4	4	2	2	1	1	3	3	1	1
4	4	2	2	1	1	3	3	1	1
4	4	2	2	1	1	1	1	1	1
4	4	2	2	1	1	1	1	4	4
4	4	2	2	1	1	1	1	4	4
1	1	2	2	1	1	1	1	1	1
1	1	2	2	1	1	1	1	1	1
1	1	2	2	4	4	1	1	1	1
4	4	2	2	1	1	3	3	1	1
4	4	4	4	1	1	3	3	1	1
1	1	2	2	4	4	3	3	1	1
1	1	2	2	4	4	1	1	1	1
4	4	2	2	1	1	1	1	4	4
4	4	4	4	1	1	1	1	1	1
4	4	2	2	1	1	1	1	4	4
4	4	2	2	1	1	1	1	1	1
4	4	2	2	1	1	1	1	1	1
4	4	2	2	1	1	1	1	1	1
4	4	4	4	1	1	1	1	1	1
4	4	2	2	1	1	3	3	4	4
4	4	2	2	1	1	1	1	4	4
1	1	2	2	4	4	3	3	1	1
4	4	2	2	1	1	1	1	4	4
4	4	2	2	1	1	1	1	1	1
4	4	4	4	1	1	1	1	1	1
4	4	2	2	1	1	1	1	1	1
4	4	2	2	1	1	1	1	4	4
1	1	2	2	1	1	1	1	1	1
1	1	2	2	4	4	1	1	4	4
4	4	2	2	1	1	1	1	1	1
4	4	2	2	1	1	1	1	1	1
4	4	2	2	1	1	1	1	4	4
1	1	2	2	4	4	1	1	1	1
4	4	2	2	1	1	3	3	1	1
4	4	2	2	1	1	3	3	1	1
4	4	2	2	1	1	1	1	4	4
4	4	4	4	1	1	1	1	1	1
4	4	2	2	1	1	1	1	1	1
4	4	4	4	1	1	3	3	1	1
1	1	4	4	4	4	1	1	1	1
4	4	2	2	1	1	3	3	1	1
4	4	2	2	1	1	1	1	4	4
4	4	2	2	1	1	1	1	1	1
1	1	2	2	4	4	3	3	4	4
4	4	2	2	1	1	3	3	1	1
4	4	2	2	1	1	1	1	4	4
1	1	4	4	4	4	1	1	4	4
4	4	2	2	1	1	1	1	4	4
1	1	2	2	4	4	3	3	1	1
4	4	2	2	1	1	3	3	1	1
4	4	2	2	1	1	1	1	1	1

For Review Only

1	1	4	4	4	4	1	1	1	1
4	4	4	4	1	1	3	3	1	1

For Review Only

SNP577	SNP577	SNP578	SNP578	SNP579	SNP579	SNP580	SNP580	SNP581	SNP581
1	1	4	4	2	2	1	1	2	2
1	1	4	4	2	2	1	1	2	2
1	1	4	4	2	2	1	1	2	2
2	2	4	4	2	2	1	1	2	2
2	2	4	4	2	2	1	1	2	2
1	1	4	4	2	2	1	1	2	2
1	1	4	4	3	3	1	1	2	2
2	2	4	4	2	2	1	1	3	3
1	1	4	4	3	3	1	1	2	2
2	2	4	4	3	3	1	1	2	2
1	1	4	4	2	2	1	1	2	2
1	1	4	4	2	2	1	1	2	2
1	1	4	4	3	3	1	1	2	2
2	2	4	4	3	3	1	1	2	2
1	1	4	4	2	2	1	1	2	2
2	2	4	4	2	2	1	1	2	2
1	1	4	4	3	3	1	1	2	2
1	1	4	4	3	3	1	1	2	2
1	1	4	4	2	2	1	1	2	2
2	2	4	4	3	3	1	1	2	2
1	1	4	4	3	3	1	1	2	2
1	1	4	4	3	3	1	1	2	2
2	2	4	4	3	3	1	1	2	2
2	2	1	1	3	3	1	1	3	3
2	2	4	4	2	2	1	1	2	2
1	1	4	4	2	2	1	1	2	2
1	1	4	4	2	2	1	1	2	2
1	1	4	4	3	3	1	1	2	2
1	1	4	4	3	3	1	1	3	3
2	2	4	4	3	3	1	1	2	2
1	1	4	4	2	2	1	1	2	2
1	1	4	4	3	3	1	1	2	2
1	1	4	4	3	3	1	1	2	2
2	2	4	4	3	3	1	1	2	2
1	1	4	4	2	2	1	1	2	2
2	2	4	4	3	3	4	4	2	2
2	2	4	4	3	3	1	1	2	2
2	2	4	4	2	2	1	1	2	2
2	2	4	4	3	3	1	1	2	2
1	1	4	4	2	2	1	1	2	2
2	2	4	4	3	3	1	1	2	2
2	2	4	4	3	3	1	1	2	2
2	2	4	4	3	3	1	1	2	2
1	1	4	4	2	2	1	1	3	3
2	2	4	4	3	3	1	1	2	2
1	1	4	4	2	2	1	1	2	2
1	1	4	4	2	2	1	1	2	2
1	1	4	4	2	2	1	1	3	3
1	1	4	4	2	2	1	1	2	2
1	1	4	4	3	3	1	1	2	2
1	1	4	4	2	2	1	1	2	2
1	1	4	4	2	2	1	1	2	2

For Review Only

1	1	4	4	2	2	1	1	2	2
1	1	4	4	3	3	1	1	2	2
1	1	4	4	2	2	1	1	2	2
1	1	4	4	3	3	1	1	2	2
1	1	4	4	2	2	1	1	2	2
1	1	4	4	3	3	1	1	2	2
1	1	4	4	2	2	1	1	2	2
2	2	4	4	3	3	1	1	2	2
1	1	4	4	3	3	1	1	2	2
1	1	4	4	3	3	1	1	2	2
1	1	4	4	2	2	1	1	2	2
1	1	4	4	3	3	4	4	2	2
1	1	4	4	2	2	1	1	2	2
1	1	4	4	3	3	1	1	3	3
1	1	4	4	2	2	1	1	2	2
2	2	4	4	3	3	1	1	2	2
1	1	4	4	2	2	1	1	2	2
1	1	4	4	2	2	1	1	2	2
1	1	4	4	3	3	1	1	2	2
1	1	4	4	2	2	1	1	2	2
1	1	4	4	3	3	1	1	2	2
2	2	4	4	3	3	1	1	2	2
1	1	4	4	3	3	1	1	3	3
1	1	4	4	3	3	1	1	2	2
2	2	4	4	3	3	1	1	2	2
2	2	4	4	2	2	1	1	2	2
2	2	4	4	3	3	1	1	2	2
2	2	4	4	3	3	1	1	2	2
1	1	4	4	2	2	1	1	2	2
1	1	4	4	2	2	1	1	2	2
2	2	4	4	3	3	1	1	3	3
2	2	1	1	2	2	1	1	3	3
2	2	4	4	3	3	1	1	2	2
1	1	4	4	2	2	1	1	2	2
1	1	4	4	2	2	1	1	2	2
2	2	4	4	3	3	1	1	2	2
2	2	4	4	3	3	1	1	3	3
2	2	4	4	2	2	1	1	2	2
2	2	4	4	2	2	1	1	2	2
2	2	4	4	3	3	1	1	2	2
1	1	4	4	2	2	1	1	2	2
1	1	4	4	2	2	1	1	2	2
1	1	4	4	3	3	1	1	2	2
1	1	4	4	2	2	1	1	2	2
1	1	4	4	2	2	1	1	2	2
2	2	4	4	2	2	1	1	2	2
2	2	1	1	3	3	1	1	3	3
2	2	1	1	2	2	1	1	2	2
1	1	1	1	2	2	1	1	2	2
2	2	4	4	2	2	1	1	2	2
1	1	4	4	2	2	1	1	2	2
2	2	4	4	3	3	1	1	2	2
1	1	4	4	3	3	1	1	2	2
1	1	4	4	3	3	1	1	2	2
2	2	4	4	2	2	1	1	2	2

For Review Only

1	1	4	4	2	2	1	1	3	3
2	2	4	4	2	2	1	1	2	2
2	2	4	4	2	2	1	1	2	2
2	2	4	4	3	3	1	1	3	3
1	1	4	4	2	2	1	1	2	2
2	2	4	4	2	2	1	1	2	2
2	2	4	4	2	2	1	1	2	2
2	2	4	4	3	3	1	1	3	3
1	1	4	4	2	2	1	1	2	2
1	1	4	4	3	3	4	4	2	2
1	1	1	1	3	3	1	1	2	2
1	1	4	4	3	3	1	1	2	2
1	1	4	4	3	3	1	1	2	2
1	1	4	4	2	2	1	1	2	2
1	1	4	4	2	2	1	1	2	2
1	1	4	4	3	3	1	1	2	2
1	1	4	4	2	2	1	1	2	2
1	1	4	4	3	3	1	1	2	2
2	2	1	1	3	3	1	1	3	3
1	1	4	4	2	2	1	1	2	2
1	1	4	4	3	3	1	1	2	2
1	1	4	4	2	2	1	1	2	2
1	1	4	4	2	2	1	1	2	2
2	2	4	4	3	3	1	1	2	2
1	1	4	4	3	3	1	1	2	2
2	2	4	4	3	3	1	1	2	2
2	2	4	4	2	2	1	1	3	3
2	2	4	4	3	3	4	4	3	3
1	1	4	4	2	2	1	1	2	2
2	2	4	4	2	2	1	1	2	2
2	2	1	1	3	3	1	1	2	2
1	1	4	4	3	3	1	1	2	2
1	1	4	4	2	2	1	1	2	2
1	1	4	4	3	3	1	1	2	2
2	2	4	4	3	3	1	1	2	2
2	2	4	4	3	3	1	1	2	2
2	2	4	4	3	3	1	1	3	3
1	1	4	4	2	2	1	1	3	3
1	1	4	4	2	2	1	1	2	2
2	2	4	4	3	3	1	1	2	2
1	1	4	4	3	3	1	1	2	2
2	2	4	4	3	3	1	1	2	2
2	2	4	4	3	3	1	1	2	2
2	2	4	4	3	3	1	1	2	2
1	1	4	4	2	2	1	1	2	2
2	2	4	4	2	2	1	1	2	2
1	1	4	4	2	2	1	1	2	2
2	2	4	4	2	2	1	1	3	3
1	1	4	4	3	3	1	1	2	2
1	1	4	4	2	2	1	1	2	2
1	1	4	4	2	2	1	1	2	2
2	2	4	4	3	3	1	1	2	2
2	2	4	4	3	3	1	1	2	2
1	1	4	4	3	3	4	4	2	2
2	2	4	4	3	3	1	1	2	2

For Review Only

1	1	4	4	2	2	1	1	2	2
2	2	4	4	3	3	1	1	2	2
2	2	4	4	2	2	1	1	2	2
2	2	4	4	3	3	1	1	2	2
1	1	4	4	2	2	1	1	2	2
2	2	4	4	2	2	1	1	3	3
1	1	4	4	2	2	1	1	2	2
1	1	4	4	3	3	1	1	2	2
2	2	4	4	2	2	1	1	2	2
2	2	4	4	2	2	1	1	2	2
1	1	4	4	3	3	1	1	2	2
2	2	4	4	3	3	1	1	2	2
1	1	4	4	3	3	1	1	2	2
2	2	4	4	2	2	1	1	2	2
2	2	1	1	3	3	1	1	2	2
1	1	4	4	2	2	1	1	2	2
1	1	4	4	3	3	1	1	2	2
1	1	4	4	2	2	1	1	2	2
1	1	4	4	3	3	1	1	2	2
2	2	4	4	2	2	1	1	2	2
1	1	4	4	3	3	1	1	2	2
2	2	1	1	3	3	1	1	2	2
2	2	4	4	3	3	1	1	3	3
1	1	4	4	3	3	4	4	2	2
1	1	4	4	2	2	1	1	2	2
2	2	1	1	3	3	1	1	2	2
1	1	4	4	2	2	1	1	2	2
1	1	4	4	3	3	1	1	2	2
2	2	4	4	3	3	1	1	2	2
2	2	4	4	3	3	4	4	2	2
1	1	4	4	2	2	1	1	2	2
1	1	4	4	2	2	1	1	2	2
1	1	4	4	2	2	1	1	3	3
2	2	1	1	3	3	1	1	2	2
1	1	4	4	2	2	1	1	3	3
1	1	4	4	3	3	4	4	2	2
1	1	4	4	2	2	1	1	2	2
1	1	4	4	2	2	1	1	2	2
2	2	4	4	2	2	1	1	3	3
2	2	4	4	3	3	1	1	3	3
1	1	4	4	2	2	1	1	2	2
1	1	4	4	2	2	1	1	2	2
2	2	1	1	3	3	1	1	2	2
1	1	4	4	2	2	1	1	2	2
2	2	4	4	2	2	1	1	2	2
2	2	4	4	3	3	1	1	2	2
1	1	4	4	2	2	1	1	2	2
2	2	4	4	2	2	1	1	2	2
2	2	4	4	3	3	1	1	3	3
2	2	4	4	3	3	1	1	2	2
1	1	4	4	2	2	1	1	3	3
2	2	1	1	3	3	1	1	2	2
2	2	4	4	3	3	1	1	3	3
1	1	4	4	2	2	1	1	2	2
2	2	4	4	3	3	1	1	2	2

For Review Only

2	2	4	4	3	3	1	1	3	3
2	2	4	4	2	2	1	1	2	2
1	1	4	4	2	2	1	1	2	2
1	1	4	4	3	3	1	1	2	2
2	2	1	1	3	3	1	1	2	2
2	2	4	4	3	3	1	1	2	2
1	1	4	4	3	3	1	1	2	2
2	2	4	4	3	3	1	1	2	2
2	2	4	4	3	3	1	1	3	3
2	2	4	4	3	3	1	1	2	2
1	1	4	4	3	3	1	1	2	2
2	2	4	4	2	2	1	1	2	2
2	2	4	4	2	2	1	1	2	2
1	1	1	1	2	2	1	1	2	2
1	1	4	4	2	2	1	1	2	2
2	2	4	4	2	2	1	1	2	2
2	2	4	4	2	2	1	1	2	2
1	1	4	4	2	2	4	4	3	3
2	2	1	1	2	2	1	1	2	2
2	2	4	4	2	2	1	1	2	2
2	2	4	4	3	3	1	1	2	2
1	1	4	4	3	3	1	1	2	2
1	1	4	4	2	2	4	4	2	2
1	1	4	4	2	2	1	1	2	2
2	2	4	4	3	3	1	1	2	2
1	1	4	4	3	3	1	1	2	2
2	2	1	1	3	3	1	1	2	2
1	1	4	4	2	2	1	1	2	2
2	2	4	4	3	3	1	1	2	2
1	1	4	4	2	2	1	1	2	2
2	2	4	4	3	3	1	1	2	2
2	2	4	4	3	3	4	4	2	2
2	2	4	4	2	2	1	1	2	2
1	1	4	4	2	2	1	1	3	3
2	2	4	4	3	3	1	1	3	3
2	2	4	4	3	3	1	1	2	2
1	1	4	4	3	3	1	1	2	2
1	1	4	4	3	3	4	4	2	2
2	2	4	4	3	3	4	4	2	2
2	2	4	4	3	3	1	1	2	2
1	1	4	4	2	2	1	1	3	3
2	2	4	4	3	3	1	1	3	3
1	1	4	4	2	2	1	1	2	2
1	1	4	4	2	2	1	1	2	2
1	1	4	4	3	3	1	1	2	2
1	1	4	4	2	2	1	1	2	2
2	2	4	4	3	3	1	1	3	3
2	2	4	4	2	2	1	1	2	2
1	1	4	4	2	2	1	1	2	2
2	2	1	1	3	3	1	1	2	2
1	1	4	4	2	2	1	1	3	3
2	2	4	4	3	3	1	1	2	2
1	1	4	4	2	2	1	1	2	2
1	1	4	4	2	2	1	1	2	2
2	2	4	4	2	2	1	1	2	2

For Review Only

1	1	4	4	2	2	1	1	2	2
2	2	4	4	3	3	4	4	2	2

For Review Only

SNP582	SNP582	SNP583	SNP583	SNP584	SNP584	SNP585	SNP585	SNP586	SNP586
4	4	1	1	4	4	1	1	3	3
2	2	1	1	4	4	1	1	2	2
2	2	1	1	4	4	1	1	2	2
2	2	1	1	4	4	1	1	2	2
2	2	1	1	4	4	1	1	2	2
4	4	1	1	1	1	3	3	3	3
4	4	1	1	4	4	1	1	3	3
4	4	1	1	1	1	3	3	3	3
2	2	1	1	4	4	1	1	2	2
4	4	1	1	1	1	3	3	3	3
4	4	4	4	4	4	1	1	3	3
2	2	1	1	4	4	1	1	2	2
2	2	1	1	4	4	1	1	3	3
4	4	1	1	1	1	3	3	2	2
2	2	1	1	4	4	1	1	2	2
4	4	4	4	4	4	1	1	3	3
2	2	1	1	4	4	1	1	2	2
4	4	1	1	1	1	3	3	3	3
2	2	1	1	4	4	1	1	2	2
4	4	1	1	1	1	3	3	3	3
4	4	1	1	1	1	3	3	3	3
4	4	1	1	4	4	3	3	3	3
4	4	4	4	4	4	1	1	3	3
4	4	4	4	4	4	1	1	3	3
2	2	1	1	4	4	1	1	2	2
2	2	1	1	4	4	1	1	2	2
2	2	1	1	4	4	1	1	2	2
2	2	1	1	4	4	1	1	2	2
4	4	1	1	1	1	3	3	3	3
4	4	1	1	1	1	3	3	3	3
2	2	1	1	4	4	1	1	2	2
4	4	4	4	4	4	1	1	3	3
4	4	4	4	4	4	1	1	3	3
4	4	1	1	1	1	3	3	3	3
2	2	1	1	4	4	1	1	2	2
4	4	1	1	1	1	3	3	3	3
4	4	1	1	4	4	3	3	3	3
4	4	1	1	4	4	1	1	3	3
4	4	4	4	4	4	1	1	3	3
4	4	1	1	4	4	1	1	2	2
4	4	1	1	1	1	3	3	3	3
4	4	1	1	1	1	3	3	3	3
4	4	1	1	1	1	3	3	3	3
4	4	1	1	1	1	3	3	3	3
4	4	1	1	1	1	3	3	3	3
4	4	4	4	4	4	1	1	2	2
2	2	1	1	4	4	1	1	2	2
4	4	1	1	1	1	3	3	3	3
4	4	1	1	1	1	3	3	3	3
2	2	1	1	4	4	1	1	2	2
2	2	1	1	4	4	1	1	2	2
2	2	1	1	4	4	1	1	2	2

For Review Only

2	2	1	1	4	4	1	1	2	2
4	4	4	4	4	4	1	1	3	3
4	4	1	1	1	1	3	3	3	3
2	2	1	1	4	4	1	1	2	2
2	2	1	1	4	4	1	1	2	2
2	2	1	1	4	4	1	1	2	2
2	2	1	1	4	4	1	1	2	2
4	4	4	4	4	4	1	1	3	3
2	2	1	1	4	4	1	1	2	2
2	2	1	1	4	4	1	1	2	2
2	2	1	1	4	4	1	1	2	2
2	2	1	1	4	4	1	1	2	2
2	2	1	1	4	4	1	1	2	2
4	4	1	1	1	1	3	3	3	3
2	2	1	1	4	4	3	3	2	2
2	2	1	1	4	4	1	1	2	2
2	2	1	1	4	4	1	1	2	2
2	2	1	1	4	4	1	1	2	2
4	4	1	1	1	1	3	3	3	3
2	2	1	1	4	4	1	1	2	2
2	2	1	1	4	4	1	1	2	2
2	2	1	1	4	4	1	1	2	2
4	4	1	1	1	1	3	3	3	3
4	4	4	4	4	4	1	1	3	3
4	4	1	1	1	1	3	3	3	3
2	2	1	1	4	4	1	1	2	2
4	4	4	4	4	4	1	1	3	3
4	4	1	1	1	1	3	3	3	3
2	2	1	1	4	4	1	1	2	2
2	2	1	1	4	4	1	1	2	2
2	2	1	1	4	4	1	1	2	2
4	4	4	4	4	4	1	1	3	3
4	4	4	4	4	4	1	1	3	3
2	2	1	1	4	4	1	1	2	2
4	4	1	1	1	1	3	3	3	3
4	4	1	1	1	1	3	3	3	3
4	4	1	1	1	1	3	3	3	3
4	4	1	1	1	1	3	3	3	3
4	4	4	4	4	4	1	1	3	3
4	4	1	1	4	4	1	1	3	3
2	2	1	1	4	4	1	1	2	2
2	2	1	1	4	4	1	1	2	2
2	2	1	1	4	4	1	1	2	2
2	2	1	1	4	4	1	1	2	2
2	2	1	1	4	4	1	1	2	2
2	2	1	1	4	4	1	1	2	2
4	4	1	1	1	1	3	3	3	3
2	2	1	1	4	4	1	1	2	2
4	4	4	4	4	4	1	1	3	3
4	4	1	1	4	4	1	1	3	3
2	2	1	1	4	4	1	1	2	2
4	4	4	4	4	4	1	1	3	3
4	4	4	4	4	4	1	1	3	3
2	2	1	1	4	4	1	1	2	2
4	4	1	1	1	1	3	3	3	3

For Review Only

2	2	1	1	4	4	1	1	2	2
4	4	4	4	4	4	1	1	3	3
2	2	1	1	1	1	1	1	2	2
4	4	1	1	1	1	3	3	3	3
2	2	1	1	4	4	1	1	2	2
2	2	1	1	4	4	1	1	2	2
2	2	1	1	4	4	1	1	2	2
4	4	1	1	4	4	1	1	2	2
2	2	1	1	4	4	1	1	2	2
4	4	1	1	4	4	3	3	2	2
2	2	1	1	4	4	1	1	3	3
2	2	1	1	4	4	1	1	2	2
4	4	1	1	4	4	3	3	3	3
2	2	1	1	4	4	1	1	2	2
2	2	1	1	1	1	1	1	3	3
2	2	1	1	4	4	1	1	2	2
2	2	1	1	4	4	1	1	2	2
2	2	1	1	4	4	1	1	2	2
4	4	1	1	1	1	3	3	3	3
2	2	1	1	4	4	1	1	2	2
2	2	1	1	4	4	1	1	2	2
2	2	1	1	4	4	1	1	2	2
2	2	1	1	4	4	1	1	2	2
4	4	1	1	1	1	3	3	3	3
4	4	1	1	4	4	1	1	2	2
2	2	1	1	4	4	1	1	2	2
2	2	1	1	4	4	1	1	2	2
2	2	1	1	4	4	1	1	2	2
2	2	1	1	4	4	1	1	2	2
4	4	4	4	4	4	1	1	3	3
4	4	1	1	4	4	3	3	3	3
2	2	1	1	4	4	1	1	2	2
2	2	1	1	4	4	1	1	2	2
2	2	1	1	4	4	1	1	2	2
4	4	1	1	1	1	3	3	3	3
2	2	1	1	1	1	1	1	2	2
4	4	1	1	1	1	3	3	3	3
2	2	1	1	4	4	1	1	2	2
2	2	1	1	4	4	1	1	2	2
4	4	4	4	4	4	1	1	3	3
4	4	4	4	4	4	1	1	3	3
4	4	1	1	4	4	3	3	3	3
2	2	1	1	4	4	1	1	2	2
2	2	1	1	4	4	1	1	3	3
2	2	1	1	1	1	3	3	2	2
2	2	1	1	4	4	1	1	2	2
2	2	1	1	4	4	1	1	2	2
2	2	1	1	4	4	1	1	2	2
2	2	1	1	4	4	3	3	2	2
2	2	1	1	4	4	1	1	2	2
2	2	1	1	4	4	1	1	2	2
4	4	4	4	4	4	1	1	3	3
2	2	1	1	4	4	1	1	2	2
2	2	1	1	4	4	1	1	2	2
2	2	1	1	4	4	1	1	2	2

For Review Only

2	2	1	1	4	4	1	1	2	2
4	4	1	1	1	1	3	3	2	2
4	4	1	1	1	1	3	3	3	3
2	2	1	1	4	4	1	1	3	3
2	2	1	1	4	4	1	1	2	2
4	4	4	4	4	4	1	1	3	3
2	2	1	1	4	4	1	1	2	2
2	2	1	1	4	4	1	1	2	2
2	2	1	1	4	4	1	1	2	2
2	2	1	1	4	4	1	1	2	2
2	2	1	1	4	4	1	1	2	2
4	4	1	1	4	4	1	1	3	3
2	2	1	1	4	4	1	1	2	2
2	2	1	1	4	4	1	1	2	2
4	4	1	1	1	1	3	3	3	3
4	4	4	4	4	4	1	1	3	3
2	2	1	1	1	1	1	1	2	2
2	2	1	1	4	4	1	1	2	2
2	2	1	1	4	4	1	1	2	2
2	2	1	1	4	4	1	1	2	2
2	2	1	1	4	4	1	1	2	2
4	4	1	1	1	1	3	3	3	3
4	4	4	4	4	4	1	1	3	3
2	2	1	1	4	4	1	1	2	2
4	4	1	1	4	4	3	3	3	3
4	4	1	1	1	1	3	3	3	3
2	2	1	1	4	4	1	1	2	2
2	2	1	1	4	4	1	1	2	2
4	4	4	4	4	4	1	1	3	3
2	2	1	1	4	4	1	1	2	2
2	2	1	1	4	4	1	1	2	2
2	2	1	1	4	4	1	1	2	2
4	4	1	1	1	1	3	3	3	3
4	4	1	1	1	1	3	3	3	3
2	2	1	1	4	4	1	1	2	2
2	2	1	1	4	4	1	1	2	2
4	4	4	4	4	4	1	1	2	2
2	2	1	1	4	4	1	1	2	2
2	2	1	1	4	4	1	1	2	2
2	2	1	1	4	4	1	1	2	2
2	2	1	1	4	4	1	1	2	2
2	2	1	1	4	4	1	1	2	2
4	4	4	4	4	4	1	1	3	3
4	4	1	1	4	4	1	1	3	3
4	4	1	1	1	1	3	3	2	2
2	2	1	1	4	4	1	1	2	2
4	4	4	4	4	4	1	1	3	3
4	4	4	4	4	4	1	1	3	3
4	4	1	1	1	1	3	3	3	3
2	2	1	1	4	4	1	1	2	2
2	2	1	1	4	4	1	1	2	2
4	4	1	1	1	1	3	3	3	3
4	4	4	4	4	4	1	1	3	3
2	2	1	1	4	4	1	1	2	2
4	4	4	4	4	4	1	1	3	3

For Review Only

2	2	1	1	4	4	1	1	2	2
4	4	4	4	4	4	1	1	3	3
2	2	1	1	4	4	1	1	2	2
4	4	4	4	4	4	1	1	3	3
4	4	1	1	1	1	3	3	3	3
2	2	1	1	4	4	1	1	2	2
4	4	1	1	1	1	3	3	3	3
4	4	1	1	1	1	3	3	3	3
4	4	4	4	4	4	1	1	3	3
4	4	1	1	1	1	3	3	3	3
2	2	1	1	4	4	1	1	2	2
2	2	1	1	4	4	1	1	2	2
4	4	4	4	4	4	1	1	3	3
2	2	1	1	4	4	1	1	2	2
2	2	1	1	4	4	1	1	2	2
4	4	4	4	4	4	1	1	3	3
4	4	4	4	4	4	1	1	3	3
2	2	1	1	4	4	1	1	3	3
2	2	1	1	4	4	1	1	2	2
4	4	4	4	4	4	1	1	3	3
4	4	4	4	4	4	1	1	3	3
2	2	1	1	4	4	1	1	2	2
2	2	1	1	4	4	1	1	2	2
4	4	4	4	4	4	1	1	3	3
4	4	4	4	4	4	1	1	3	3
2	2	1	1	4	4	1	1	2	2
4	4	4	4	4	4	1	1	3	3
2	2	1	1	4	4	1	1	2	2
2	2	1	1	4	4	1	1	2	2
2	2	1	1	4	4	1	1	2	2
4	4	4	4	4	4	1	1	3	3
2	2	1	1	4	4	1	1	2	2
4	4	1	1	1	1	3	3	3	3
2	2	1	1	4	4	1	1	2	2
4	4	4	4	4	4	1	1	3	3
4	4	1	1	1	1	3	3	3	3
2	2	1	1	4	4	1	1	2	2
2	2	1	1	4	4	1	1	2	2
4	4	1	1	1	1	3	3	3	3
4	4	4	4	4	4	1	1	3	3
2	2	1	1	4	4	1	1	2	2
4	4	1	1	1	1	3	3	3	3
2	2	1	1	4	4	1	1	2	2
2	2	1	1	4	4	1	1	2	2
2	2	1	1	4	4	1	1	2	2
4	4	4	4	4	4	1	1	3	3
4	4	4	4	4	4	1	1	3	3
2	2	1	1	4	4	1	1	2	2
2	2	1	1	1	1	1	1	2	2
4	4	4	4	4	4	1	1	3	3
2	2	1	1	4	4	1	1	2	2
4	4	4	4	4	4	1	1	3	3
2	2	1	1	4	4	1	1	2	2
4	4	1	1	1	1	3	3	3	3
4	4	1	1	1	1	3	3	3	3

For Review Only

2	2	1	1	4	4	1	1	2	2
2	2	1	1	4	4	1	1	2	2

For Review Only

SNP587	SNP587	SNP588	SNP588	SNP589	SNP589	SNP590	SNP590	SNP591	SNP591
4	4	3	3	1	1	2	2	4	4
4	4	2	2	4	4	2	2	4	4
4	4	2	2	4	4	2	2	4	4
4	4	3	3	4	4	3	3	4	4
4	4	3	3	4	4	3	3	4	4
4	4	3	3	1	1	2	2	1	1
4	4	3	3	1	1	2	2	4	4
4	4	2	2	1	1	3	3	4	4
4	4	3	3	4	4	2	2	4	4
3	3	3	3	1	1	3	3	4	4
4	4	3	3	1	1	2	2	4	4
4	4	2	2	4	4	2	2	4	4
4	4	3	3	4	4	2	2	4	4
4	4	2	2	1	1	3	3	1	1
4	4	3	3	4	4	2	2	4	4
4	4	3	3	1	1	3	3	1	1
4	4	2	2	4	4	2	2	4	4
4	4	2	2	1	1	3	3	4	4
4	4	3	3	4	4	2	2	4	4
4	4	2	2	1	1	3	3	4	4
4	4	2	2	1	1	2	2	4	4
3	3	3	3	1	1	2	2	4	4
4	4	2	2	1	1	3	3	4	4
4	4	3	3	1	1	3	3	4	4
4	4	3	3	4	4	3	3	4	4
4	4	2	2	4	4	2	2	4	4
4	4	2	2	4	4	2	2	4	4
4	4	2	2	4	4	2	2	4	4
4	4	3	3	1	1	2	2	4	4
4	4	2	2	1	1	3	3	4	4
4	4	2	2	4	4	2	2	4	4
4	4	3	3	1	1	2	2	1	1
4	4	3	3	1	1	2	2	4	4
4	4	3	3	1	1	3	3	4	4
4	4	3	3	4	4	3	3	4	4
4	4	3	3	1	1	3	3	4	4
4	4	2	2	1	1	3	3	4	4
3	3	2	2	1	1	3	3	4	4
4	4	3	3	4	4	3	3	4	4
4	4	2	2	1	1	2	2	4	4
3	3	2	2	1	1	3	3	4	4
4	4	2	2	4	4	3	3	4	4
3	3	2	2	1	1	3	3	4	4
3	3	2	2	1	1	2	2	4	4
4	4	2	2	1	1	3	3	4	4
4	4	2	2	4	4	2	2	4	4
4	4	2	2	4	4	2	2	4	4
4	4	3	3	4	4	3	3	4	4
3	3	2	2	4	4	2	2	4	4
4	4	3	3	4	4	2	2	4	4
4	4	2	2	4	4	2	2	4	4
4	4	3	3	4	4	2	2	4	4

For Review Only

4	4	3	3	4	4	2	2	4	4
4	4	3	3	1	1	2	2	4	4
4	4	3	3	1	1	3	3	1	1
4	4	2	2	4	4	2	2	4	4
4	4	3	3	4	4	2	2	4	4
4	4	2	2	4	4	2	2	4	4
4	4	3	3	4	4	2	2	4	4
4	4	3	3	1	1	3	3	4	4
4	4	3	3	4	4	2	2	4	4
4	4	3	3	4	4	2	2	4	4
4	4	2	2	4	4	2	2	4	4
4	4	2	2	4	4	2	2	4	4
4	4	3	3	4	4	2	2	4	4
4	4	3	3	1	1	2	2	4	4
4	4	3	3	4	4	2	2	4	4
4	4	3	3	4	4	3	3	4	4
4	4	2	2	4	4	2	2	4	4
4	4	2	2	4	4	2	2	4	4
4	4	2	2	1	1	2	2	4	4
4	4	3	3	4	4	2	2	4	4
4	4	3	3	4	4	2	2	4	4
4	4	3	3	4	4	3	3	4	4
4	4	3	3	1	1	2	2	4	4
4	4	3	3	1	1	2	2	1	1
4	4	2	2	1	1	3	3	4	4
4	4	2	2	4	4	3	3	4	4
4	4	3	3	1	1	3	3	4	4
4	4	3	3	1	1	3	3	4	4
4	4	2	2	4	4	2	2	4	4
4	4	3	3	4	4	3	3	4	4
4	4	3	3	4	4	3	3	4	4
4	4	3	3	1	1	3	3	4	4
4	4	3	3	1	1	3	3	4	4
4	4	3	3	4	4	2	2	4	4
4	4	3	3	1	1	3	3	4	4
4	4	2	2	1	1	3	3	4	4
4	4	3	3	1	1	3	3	4	4
4	4	3	3	1	1	3	3	4	4
4	4	3	3	1	1	3	3	4	4
4	4	2	2	1	1	3	3	4	4
4	4	3	3	4	4	2	2	4	4
4	4	3	3	4	4	2	2	1	1
4	4	2	2	4	4	2	2	4	4
4	4	2	2	4	4	2	2	4	4
4	4	3	3	4	4	2	2	4	4
4	4	3	3	4	4	2	2	1	1
4	4	3	3	1	1	3	3	4	4
4	4	3	3	4	4	3	3	4	4
4	4	3	3	1	1	2	2	4	4
4	4	2	2	1	1	3	3	4	4
4	4	3	3	4	4	2	2	1	1
4	4	3	3	1	1	3	3	4	4
4	4	2	2	1	1	2	2	4	4
4	4	3	3	4	4	3	3	4	4
3	3	2	2	1	1	3	3	4	4

For Review Only

4	4	3	3	4	4	2	2	4	4
3	3	2	2	4	4	3	3	4	4
4	4	3	3	4	4	3	3	4	4
4	4	3	3	1	1	3	3	4	4
4	4	3	3	4	4	2	2	4	4
4	4	2	2	4	4	3	3	4	4
4	4	3	3	4	4	3	3	1	1
4	4	2	2	1	1	3	3	4	4
3	3	2	2	4	4	2	2	4	4
4	4	3	3	4	4	2	2	4	4
4	4	3	3	4	4	3	3	4	4
4	4	2	2	4	4	2	2	4	4
4	4	3	3	1	1	2	2	4	4
4	4	2	2	4	4	2	2	4	4
4	4	3	3	4	4	2	2	4	4
4	4	3	3	4	4	2	2	4	4
4	4	3	3	4	4	2	2	4	4
4	4	3	3	4	4	2	2	4	4
4	4	3	3	1	1	3	3	4	4
4	4	3	3	4	4	2	2	4	4
4	4	3	3	4	4	2	2	4	4
4	4	3	3	4	4	2	2	1	1
4	4	3	3	4	4	2	2	4	4
4	4	3	3	1	1	3	3	4	4
4	4	3	3	4	4	2	2	4	4
4	4	3	3	4	4	3	3	4	4
4	4	3	3	4	4	3	3	4	4
4	4	2	2	4	4	3	3	4	4
4	4	2	2	4	4	2	2	4	4
4	4	3	3	1	1	2	2	4	4
4	4	3	3	1	1	3	3	4	4
4	4	3	3	4	4	2	2	4	4
4	4	3	3	4	4	2	2	4	4
4	4	2	2	4	4	2	2	4	4
4	4	3	3	1	1	3	3	4	4
4	4	3	3	4	4	3	3	4	4
4	4	3	3	1	1	3	3	4	4
4	4	2	2	4	4	2	2	1	1
4	4	3	3	4	4	2	2	4	4
4	4	3	3	1	1	3	3	4	4
4	4	3	3	1	1	2	2	4	4
4	4	3	3	4	4	3	3	4	4
4	4	2	2	4	4	2	2	4	4
4	4	3	3	4	4	3	3	4	4
4	4	3	3	1	1	2	2	4	4
4	4	2	2	4	4	2	2	1	1
4	4	2	2	4	4	2	2	4	4
4	4	3	3	4	4	3	3	4	4
4	4	3	3	1	1	3	3	4	4
4	4	2	2	4	4	2	2	4	4
4	4	2	2	4	4	2	2	4	4
4	4	2	2	1	1	3	3	4	4
4	4	3	3	4	4	3	3	4	4
4	4	2	2	4	4	2	2	4	4
4	4	2	2	4	4	3	3	4	4

For Review Only

4	4	2	2	4	4	2	2	4	4
4	4	2	2	1	1	3	3	4	4
4	4	3	3	1	1	3	3	4	4
4	4	3	3	4	4	2	2	4	4
4	4	3	3	4	4	2	2	4	4
4	4	2	2	1	1	3	3	4	4
4	4	3	3	4	4	2	2	4	4
4	4	3	3	4	4	2	2	4	4
4	4	3	3	4	4	3	3	4	4
4	4	3	3	4	4	2	2	4	4
4	4	2	2	4	4	2	2	4	4
4	4	3	3	1	1	3	3	4	4
4	4	2	2	4	4	2	2	4	4
4	4	3	3	4	4	3	3	4	4
4	4	3	3	1	1	3	3	4	4
4	4	3	3	1	1	2	2	4	4
4	4	2	2	4	4	3	3	4	4
4	4	3	3	4	4	2	2	4	4
4	4	2	2	4	4	2	2	4	4
4	4	3	3	4	4	3	3	1	1
4	4	3	3	4	4	2	2	4	4
4	4	3	3	1	1	3	3	4	4
4	4	3	3	1	1	3	3	4	4
4	4	2	2	4	4	2	2	4	4
4	4	3	3	1	1	2	2	4	4
4	4	3	3	1	1	3	3	4	4
4	4	3	3	4	4	2	2	4	4
4	4	3	3	4	4	2	2	4	4
4	4	3	3	1	1	2	2	4	4
4	4	3	3	4	4	2	2	4	4
4	4	3	3	4	4	2	2	1	1
4	4	3	3	4	4	3	3	4	4
4	4	3	3	1	1	3	3	4	4
4	4	3	3	1	1	3	3	4	4
4	4	3	3	4	4	2	2	4	4
4	4	2	2	4	4	2	2	4	4
4	4	2	2	1	1	2	2	4	4
4	4	3	3	4	4	2	2	4	4
4	4	3	3	4	4	3	3	4	4
4	4	3	3	4	4	3	3	4	4
4	4	2	2	4	4	2	2	4	4
4	4	2	2	4	4	2	2	4	4
4	4	3	3	1	1	3	3	4	4
4	4	3	3	1	1	2	2	4	4
4	4	3	3	1	1	3	3	4	4
4	4	3	3	4	4	3	3	1	1
4	4	3	3	1	1	2	2	4	4
4	4	3	3	1	1	3	3	4	4
4	4	3	3	1	1	3	3	4	4
4	4	3	3	4	4	3	3	4	4
4	4	2	2	4	4	2	2	4	4
4	4	3	3	1	1	3	3	4	4
4	4	3	3	1	1	3	3	4	4
4	4	2	2	4	4	2	2	4	4
4	4	3	3	1	1	3	3	4	4

For Review Only

4	4	2	2	4	4	3	3	4	4
3	3	3	3	1	1	2	2	4	4
4	4	2	2	4	4	2	2	4	4
4	4	3	3	1	1	3	3	4	4
4	4	3	3	1	1	3	3	1	1
4	4	3	3	4	4	3	3	4	4
4	4	2	2	1	1	2	2	4	4
3	3	2	2	1	1	3	3	4	4
4	4	3	3	1	1	3	3	4	4
4	4	3	3	1	1	3	3	4	4
4	4	3	3	4	4	2	2	4	4
4	4	3	3	4	4	3	3	4	4
4	4	3	3	1	1	3	3	4	4
4	4	3	3	4	4	2	2	4	4
4	4	2	2	4	4	2	2	4	4
4	4	3	3	1	1	3	3	4	4
4	4	2	2	1	1	3	3	4	4
4	4	2	2	1	1	2	2	4	4
4	4	3	3	4	4	3	3	4	4
3	3	2	2	1	1	3	3	4	4
4	4	3	3	1	1	3	3	1	1
4	4	2	2	4	4	2	2	4	4
4	4	3	3	4	4	2	2	1	1
4	4	3	3	1	1	2	2	4	4
4	4	3	3	1	1	3	3	4	4
4	4	3	3	4	4	2	2	4	4
4	4	3	3	1	1	3	3	4	4
4	4	3	3	4	4	2	2	4	4
4	4	3	3	4	4	3	3	4	4
4	4	2	2	4	4	2	2	1	1
4	4	2	2	1	1	3	3	4	4
4	4	2	2	4	4	3	3	4	4
4	4	3	3	1	1	3	3	4	4
4	4	3	3	4	4	2	2	4	4
4	4	3	3	1	1	3	3	4	4
4	4	3	3	1	1	3	3	4	4
4	4	2	2	4	4	2	2	4	4
4	4	2	2	4	4	2	2	4	4
4	4	3	3	1	1	3	3	4	4
4	4	3	3	1	1	3	3	4	4
4	4	3	3	4	4	2	2	4	4
4	4	2	2	1	1	3	3	4	4
4	4	2	2	4	4	2	2	1	1
4	4	3	3	4	4	2	2	4	4
4	4	2	2	4	4	2	2	4	4
4	4	3	3	1	1	2	2	4	4
4	4	3	3	1	1	3	3	4	4
4	4	3	3	4	4	3	3	4	4
3	3	3	3	4	4	2	2	4	4
4	4	3	3	1	1	3	3	4	4
4	4	3	3	4	4	2	2	4	4
4	4	3	3	1	1	3	3	4	4
4	4	2	2	4	4	2	2	4	4
4	4	3	3	1	1	2	2	4	4
4	4	2	2	1	1	3	3	4	4

For Review Only

4	4	3	3	4	4	2	2	4	4
4	4	2	2	4	4	3	3	4	4

For Review Only

SNP592	SNP592	SNP593	SNP593	SNP594	SNP594	SNP595	SNP595	SNP596	SNP596
4	4	1	1	2	2	3	3	3	3
2	2	1	1	2	2	3	3	3	3
2	2	1	1	2	2	3	3	3	3
4	4	1	1	2	2	3	3	3	3
4	4	4	4	2	2	3	3	3	3
2	2	1	1	2	2	3	3	3	3
4	4	1	1	2	2	3	3	3	3
4	4	4	4	2	2	3	3	3	3
4	4	1	1	2	2	3	3	3	3
4	4	4	4	1	1	2	2	3	3
2	2	1	1	2	2	3	3	3	3
2	2	1	1	2	2	3	3	3	3
4	4	1	1	2	2	3	3	3	3
4	4	4	4	2	2	3	3	3	3
2	2	1	1	2	2	3	3	3	3
4	4	1	1	2	2	3	3	3	3
4	4	1	1	1	1	2	2	3	3
4	4	1	1	1	1	3	3	3	3
2	2	1	1	2	2	3	3	3	3
4	4	4	4	2	2	3	3	3	3
4	4	1	1	2	2	3	3	3	3
4	4	1	1	2	2	3	3	3	3
4	4	4	4	2	2	3	3	3	3
4	4	4	4	1	1	2	2	3	3
2	2	1	1	2	2	3	3	3	3
2	2	1	1	2	2	3	3	3	3
4	4	1	1	2	2	3	3	3	3
4	4	1	1	2	2	3	3	3	3
4	4	1	1	2	2	3	3	3	3
4	4	1	1	2	2	3	3	3	3
2	2	1	1	2	2	3	3	3	3
2	2	1	1	2	2	3	3	3	3
2	2	1	1	2	2	3	3	3	3
4	4	4	4	1	1	2	2	3	3
4	4	1	1	2	2	3	3	3	3
4	4	4	4	2	2	3	3	3	3
4	4	4	4	2	2	3	3	3	3
4	4	4	4	2	2	3	3	3	3
4	4	4	4	2	2	3	3	2	2
2	2	1	1	2	2	3	3	3	3
4	4	4	4	2	2	3	3	3	3
4	4	4	4	2	2	3	3	3	3
4	4	4	4	2	2	3	3	3	3
4	4	1	1	2	2	2	2	3	3
4	4	4	4	2	2	3	3	3	3
2	2	1	1	2	2	3	3	3	3
2	2	1	1	2	2	3	3	3	3
4	4	4	4	2	2	3	3	3	3
4	4	1	1	2	2	3	3	3	3
2	2	1	1	2	2	3	3	3	3
2	2	1	1	2	2	3	3	3	3
2	2	1	1	2	2	3	3	3	3

For Review Only

4	4	1	1	2	2	3	3	3	3
2	2	1	1	2	2	3	3	3	3
4	4	1	1	2	2	3	3	3	3
4	4	1	1	2	2	3	3	3	3
2	2	1	1	2	2	3	3	3	3
2	2	1	1	2	2	3	3	3	3
2	2	1	1	2	2	3	3	3	3
4	4	4	4	2	2	3	3	3	3
4	4	1	1	2	2	3	3	3	3
4	4	1	1	2	2	3	3	3	3
2	2	1	1	2	2	3	3	3	3
2	2	1	1	2	2	3	3	3	3
4	4	1	1	2	2	3	3	3	3
4	4	1	1	2	2	3	3	3	3
4	4	1	1	2	2	3	3	3	3
4	4	1	1	2	2	3	3	3	3
4	4	1	1	2	2	3	3	3	3
2	2	1	1	2	2	3	3	3	3
4	4	1	1	2	2	3	3	3	3
4	4	1	1	2	2	3	3	3	3
2	2	1	1	2	2	3	3	3	3
4	4	4	4	2	2	3	3	3	3
4	4	1	1	2	2	3	3	3	3
2	2	1	1	2	2	3	3	3	3
4	4	4	4	2	2	3	3	3	3
4	4	4	4	2	2	3	3	3	3
4	4	4	4	2	2	3	3	3	3
4	4	4	4	2	2	3	3	2	2
2	2	1	1	2	2	3	3	3	3
4	4	1	1	2	2	3	3	3	3
4	4	4	4	1	1	2	2	3	3
4	4	4	4	1	1	3	3	3	3
4	4	4	4	2	2	3	3	3	3
4	4	1	1	2	2	3	3	3	3
4	4	1	1	2	2	3	3	3	3
4	4	4	4	2	2	3	3	3	3
4	4	4	4	1	1	2	2	3	3
4	4	4	4	2	2	3	3	3	3
4	4	4	4	2	2	3	3	3	3
4	4	4	4	2	2	3	3	3	3
4	4	1	1	2	2	3	3	3	3
2	2	1	1	2	2	3	3	3	3
2	2	1	1	2	2	3	3	3	3
2	2	1	1	2	2	3	3	3	3
2	2	1	1	2	2	3	3	3	3
2	2	1	1	2	2	3	3	3	3
4	4	4	4	2	2	3	3	3	3
4	4	4	4	1	1	2	2	3	3
2	2	1	1	2	2	3	3	3	3
4	4	4	4	2	2	3	3	3	3
2	2	1	1	2	2	3	3	3	3
4	4	4	4	2	2	3	3	3	3
4	4	1	1	2	2	3	3	3	3
4	4	1	1	2	2	3	3	3	3
4	4	4	4	2	2	3	3	3	3

For Review Only

2	2	1	1	2	2	3	3	3	3
4	4	4	4	2	2	3	3	3	3
4	4	4	4	2	2	3	3	3	3
4	4	4	4	1	1	2	2	3	3
2	2	1	1	2	2	3	3	3	3
4	4	4	4	2	2	3	3	3	3
2	2	1	1	2	2	3	3	3	3
4	4	4	4	2	2	3	3	3	3
2	2	1	1	2	2	3	3	3	3
4	4	1	1	2	2	3	3	3	3
4	4	1	1	2	2	3	3	3	3
4	4	1	1	2	2	3	3	3	3
2	2	1	1	2	2	3	3	3	3
2	2	1	1	2	2	3	3	3	3
4	4	1	1	2	2	3	3	3	3
2	2	1	1	2	2	3	3	3	3
2	2	1	1	2	2	3	3	3	3
2	2	1	1	2	2	3	3	3	3
4	4	4	4	1	1	2	2	3	3
4	4	1	1	2	2	3	3	3	3
4	4	1	1	2	2	3	3	3	3
2	2	1	1	2	2	3	3	3	3
2	2	1	1	2	2	3	3	3	3
4	4	4	4	2	2	3	3	3	3
4	4	1	1	2	2	3	3	3	3
4	4	4	4	2	2	3	3	3	3
4	4	4	4	2	2	3	3	3	3
2	2	1	1	2	2	3	3	3	3
2	2	1	1	2	2	3	3	3	3
2	2	1	1	2	2	3	3	3	3
4	4	4	4	2	2	3	3	3	3
4	4	1	1	2	2	3	3	3	3
4	4	1	1	2	2	3	3	3	3
4	4	1	1	2	2	3	3	3	3
4	4	4	4	1	1	2	2	3	3
4	4	4	4	2	2	3	3	3	3
4	4	4	4	2	2	3	3	3	3
2	2	1	1	2	2	3	3	3	3
4	4	1	1	2	2	3	3	3	3
4	4	4	4	2	2	3	3	2	2
2	2	1	1	2	2	3	3	3	3
4	4	4	4	1	1	2	2	3	3
2	2	1	1	2	2	3	3	3	3
4	4	1	1	1	1	3	3	3	3
4	4	1	1	2	2	3	3	3	3
2	2	1	1	2	2	3	3	3	3
2	2	1	1	2	2	3	3	3	3
4	4	4	4	2	2	3	3	3	3
4	4	1	1	2	2	3	3	3	3
2	2	1	1	2	2	3	3	3	3
4	4	1	1	2	2	3	3	3	3
4	4	4	4	2	2	3	3	3	3
4	4	4	4	2	2	3	3	3	3
2	2	1	1	2	2	3	3	3	3
4	4	1	1	2	2	3	3	3	3

2	2	1	1	2	2	3	3	3	3
4	4	4	4	2	2	3	3	3	3
2	2	1	1	2	2	3	3	3	3
4	4	1	1	2	2	3	3	3	3
4	4	1	1	2	2	3	3	3	3
4	4	4	4	2	2	3	3	3	3
4	4	1	1	2	2	3	3	3	3
4	4	1	1	2	2	3	3	3	3
4	4	4	4	1	1	2	2	2	2
2	2	1	1	2	2	3	3	3	3
4	4	1	1	2	2	3	3	3	3
4	4	4	4	1	1	2	2	3	3
4	4	1	1	2	2	3	3	3	3
4	4	1	1	2	2	3	3	3	3
4	4	4	4	2	2	3	3	3	3
2	2	1	1	2	2	3	3	3	3
4	4	1	1	2	2	3	3	3	3
2	2	1	1	2	2	3	3	3	3
2	2	1	1	2	2	3	3	3	3
2	2	1	1	2	2	3	3	3	3
4	4	1	1	2	2	3	3	3	3
4	4	4	4	2	2	3	3	3	3
4	4	4	4	2	2	3	3	3	3
2	2	1	1	2	2	3	3	3	3
4	4	1	1	2	2	3	3	3	3
4	4	4	4	2	2	3	3	3	3
4	4	1	1	2	2	3	3	3	3
4	4	1	1	2	2	3	3	3	3
2	2	1	1	2	2	3	3	3	3
2	2	1	1	2	2	3	3	3	3
4	4	1	1	2	2	3	3	3	3
4	4	1	1	2	2	3	3	3	3
4	4	4	4	2	2	3	3	3	3
4	4	4	4	2	2	3	3	3	3
4	4	1	1	2	2	3	3	3	3
2	2	1	1	2	2	3	3	3	3
2	2	1	1	2	2	3	3	3	3
4	4	1	1	2	2	3	3	3	3
4	4	4	4	2	2	3	3	3	3
4	4	4	4	2	2	3	3	3	3
2	2	1	1	2	2	3	3	3	3
2	2	1	1	2	2	3	3	3	3
4	4	4	4	2	2	3	3	2	2
4	4	1	1	2	2	3	3	3	3
4	4	4	4	1	1	2	2	3	3
2	2	1	1	2	2	3	3	3	3
2	2	1	1	2	2	3	3	3	3
4	4	4	4	2	2	3	3	3	3
4	4	4	4	2	2	3	3	3	3
4	4	4	4	2	2	3	3	3	3
2	2	1	1	2	2	3	3	3	3
4	4	4	4	2	2	3	3	3	3
4	4	4	4	2	2	3	3	3	3
2	2	1	1	2	2	3	3	3	3
4	4	4	4	2	2	3	3	2	2

For Review Only

4	4	4	4	2	2	3	3	3	3
4	4	1	1	2	2	3	3	3	3
2	2	1	1	2	2	3	3	3	3
4	4	1	1	2	2	3	3	3	3
4	4	4	4	2	2	3	3	3	3
2	2	1	1	2	2	3	3	3	3
4	4	4	4	2	2	3	3	2	2
4	4	4	4	2	2	3	3	3	3
4	4	4	4	2	2	3	3	3	3
4	4	4	4	2	2	3	3	3	3
4	4	1	1	2	2	3	3	3	3
4	4	4	4	2	2	3	3	3	3
4	4	4	4	2	2	3	3	3	3
2	2	1	1	2	2	3	3	3	3
4	4	1	1	2	2	3	3	3	3
4	4	4	4	2	2	3	3	3	3
4	4	4	4	2	2	3	3	2	2
4	4	1	1	2	2	3	3	3	3
4	4	4	4	2	2	3	3	3	3
4	4	4	4	2	2	3	3	3	3
4	4	4	4	2	2	3	3	2	2
2	2	1	1	2	2	3	3	3	3
2	2	1	1	2	2	3	3	3	3
2	2	1	1	2	2	3	3	3	3
4	4	4	4	2	2	3	3	3	3
4	4	1	1	2	2	2	2	3	3
4	4	4	4	2	2	3	3	3	3
2	2	1	1	2	2	3	3	3	3
4	4	4	4	2	2	3	3	3	3
2	2	1	1	2	2	3	3	3	3
4	4	4	4	2	2	3	3	3	3
4	4	4	4	2	2	3	3	3	3
4	4	4	4	2	2	3	3	3	3
2	2	1	1	2	2	3	3	3	3
4	4	4	4	2	2	3	3	2	2
4	4	4	4	2	2	3	3	3	3
4	4	1	1	2	2	3	3	3	3
2	2	1	1	2	2	3	3	3	3
4	4	4	4	2	2	3	3	3	3
4	4	4	4	2	2	3	3	2	2
2	2	1	1	2	2	3	3	3	3
4	4	4	4	2	2	3	3	3	3
2	2	1	1	2	2	3	3	3	3
4	4	1	1	2	2	3	3	3	3
4	4	4	4	2	2	3	3	3	3
4	4	1	1	2	2	3	3	3	3
4	4	4	4	2	2	3	3	3	3
4	4	4	4	2	2	3	3	3	3
2	2	1	1	2	2	3	3	3	3
4	4	4	4	2	2	3	3	3	3
2	2	1	1	2	2	3	3	3	3
4	4	4	4	2	2	3	3	3	3
2	2	1	1	2	2	3	3	3	3
2	2	1	1	2	2	3	3	3	3
4	4	4	4	2	2	3	3	2	2

For Review Only

2	2	1	1	2	2	3	3	3	3
4	4	4	4	2	2	3	3	3	3

For Review Only

SNP597	SNP597	SNP598	SNP598	SNP599	SNP599	SNP600	SNP600	SNP601	SNP601
3	3	2	2	3	3	1	1	4	4
3	3	2	2	3	3	4	4	3	3
3	3	2	2	3	3	4	4	4	4
3	3	3	3	3	3	4	4	3	3
2	2	3	3	2	2	1	1	4	4
3	3	2	2	3	3	4	4	4	4
3	3	2	2	3	3	1	1	4	4
2	2	3	3	2	2	4	4	3	3
3	3	2	2	3	3	1	1	4	4
2	2	3	3	2	2	4	4	4	4
3	3	2	2	3	3	4	4	3	3
3	3	2	2	3	3	4	4	3	3
3	3	2	2	3	3	4	4	3	3
2	2	3	3	2	2	4	4	4	4
3	3	2	2	3	3	4	4	3	3
3	3	3	3	3	3	4	4	4	4
3	3	2	2	3	3	4	4	3	3
3	3	3	3	3	3	4	4	3	3
3	3	2	2	3	3	4	4	3	3
2	2	3	3	2	2	4	4	4	4
3	3	2	2	3	3	1	1	3	3
3	3	2	2	3	3	4	4	3	3
2	2	3	3	2	2	4	4	3	3
2	2	3	3	2	2	4	4	3	3
3	3	2	2	3	3	4	4	4	4
3	3	2	2	3	3	4	4	4	4
3	3	3	3	2	2	4	4	4	4
3	3	2	2	3	3	4	4	3	3
3	3	2	2	3	3	1	1	3	3
2	2	3	3	2	2	4	4	3	3
3	3	2	2	3	3	4	4	4	4
3	3	2	2	3	3	4	4	4	4
3	3	2	2	3	3	4	4	4	4
2	2	3	3	2	2	4	4	3	3
3	3	2	2	2	2	4	4	3	3
2	2	3	3	2	2	4	4	3	3
2	2	3	3	2	2	4	4	4	4
2	2	2	2	3	3	4	4	4	4
3	3	2	2	3	3	4	4	3	3
3	3	2	2	3	3	4	4	3	3
2	2	3	3	2	2	4	4	3	3
3	3	3	3	2	2	1	1	3	3
2	2	3	3	2	2	4	4	3	3
3	3	2	2	3	3	4	4	4	4
2	2	3	3	2	2	4	4	3	3
3	3	2	2	3	3	4	4	3	3
3	3	2	2	3	3	4	4	4	4
2	2	3	3	2	2	4	4	3	3
3	3	2	2	3	3	4	4	4	4
3	3	2	2	3	3	4	4	3	3
3	3	2	2	3	3	4	4	3	3
3	3	2	2	3	3	4	4	3	3

For Review Only

3	3	2	2	3	3	4	4	4	4
3	3	2	2	3	3	4	4	4	4
3	3	3	3	2	2	4	4	4	4
3	3	2	2	3	3	4	4	4	4
3	3	2	2	3	3	4	4	3	3
3	3	2	2	3	3	4	4	3	3
3	3	2	2	3	3	4	4	4	4
2	2	3	3	2	2	4	4	3	3
3	3	2	2	3	3	4	4	3	3
3	3	2	2	3	3	4	4	3	3
3	3	2	2	3	3	4	4	3	3
2	2	2	2	3	3	4	4	3	3
3	3	2	2	3	3	4	4	4	4
3	3	2	2	3	3	1	1	3	3
3	3	2	2	3	3	4	4	3	3
2	2	2	2	3	3	4	4	3	3
3	3	3	3	3	3	4	4	3	3
3	3	2	2	3	3	4	4	3	3
3	3	2	2	3	3	4	4	3	3
3	3	2	2	3	3	4	4	4	4
3	3	2	2	3	3	4	4	3	3
2	2	3	3	2	2	4	4	3	3
3	3	2	2	3	3	4	4	4	4
3	3	2	2	3	3	4	4	4	4
2	2	3	3	2	2	4	4	3	3
2	2	3	3	2	2	4	4	3	3
2	2	3	3	2	2	4	4	3	3
3	3	2	2	3	3	4	4	3	3
3	3	2	2	3	3	4	4	4	4
3	3	3	3	3	3	4	4	3	3
2	2	3	3	2	2	4	4	4	4
2	2	3	3	2	2	4	4	3	3
2	2	3	3	2	2	4	4	3	3
3	3	2	2	3	3	4	4	4	4
3	3	3	3	3	3	1	1	3	3
2	2	3	3	2	2	4	4	3	3
2	2	3	3	2	2	4	4	3	3
2	2	3	3	2	2	4	4	3	3
2	2	3	3	2	2	4	4	3	3
2	2	3	3	2	2	4	4	3	3
3	3	2	2	3	3	4	4	4	4
3	3	2	2	3	3	4	4	4	4
3	3	2	2	3	3	4	4	4	4
3	3	2	2	3	3	4	4	3	3
3	3	2	2	3	3	4	4	4	4
3	3	2	2	3	3	4	4	4	4
2	2	3	3	2	2	4	4	3	3
2	2	3	3	2	2	4	4	3	3
3	3	2	2	3	3	4	4	4	4
2	2	3	3	2	2	4	4	4	4
3	3	2	2	3	3	4	4	4	4
3	3	2	2	3	3	4	4	3	3
3	3	2	2	3	3	4	4	3	3
3	3	2	2	3	3	4	4	3	3
2	2	3	3	2	2	4	4	3	3

For Review Only

3	3	2	2	3	3	4	4	3	3
2	2	3	3	2	2	4	4	3	3
2	2	3	3	2	2	4	4	4	4
2	2	2	2	3	3	4	4	3	3
3	3	2	2	3	3	4	4	4	4
2	2	3	3	2	2	4	4	4	4
3	3	3	3	3	3	4	4	4	4
2	2	3	3	2	2	4	4	4	4
3	3	2	2	3	3	4	4	4	4
3	3	2	2	2	2	4	4	3	3
3	3	2	2	3	3	1	1	4	4
3	3	2	2	3	3	4	4	3	3
3	3	2	2	3	3	4	4	3	3
3	3	2	2	3	3	4	4	3	3
3	3	2	2	2	2	4	4	4	4
3	3	2	2	3	3	4	4	3	3
3	3	2	2	3	3	4	4	3	3
3	3	2	2	3	3	4	4	3	3
2	2	3	3	2	2	4	4	3	3
3	3	2	2	3	3	4	4	3	3
3	3	2	2	3	3	4	4	3	3
3	3	2	2	3	3	4	4	4	4
3	3	2	2	3	3	4	4	3	3
2	2	3	3	2	2	4	4	3	3
2	2	2	2	2	2	4	4	3	3
2	2	3	3	2	2	4	4	3	3
3	3	2	2	3	3	4	4	4	4
3	3	2	2	3	3	4	4	4	4
3	3	2	2	3	3	4	4	3	3
3	3	2	2	3	3	4	4	3	3
3	3	2	2	3	3	4	4	3	3
3	3	2	2	3	3	4	4	3	3
3	3	2	2	3	3	4	4	4	4
3	3	2	2	3	3	4	4	4	4
2	2	3	3	2	2	4	4	3	3
2	2	3	3	2	2	4	4	3	3
2	2	2	2	2	2	4	4	3	3
3	3	2	2	3	3	4	4	4	4
3	3	2	2	3	3	4	4	3	3
3	3	2	2	3	3	4	4	3	3
3	3	2	2	3	3	4	4	3	3
2	2	3	3	2	2	4	4	3	3
3	3	2	2	3	3	4	4	3	3
3	3	3	3	2	2	4	4	3	3
3	3	2	2	3	3	4	4	4	4
3	3	2	2	3	3	4	4	4	4
3	3	2	2	3	3	4	4	4	4
2	2	3	3	2	2	4	4	3	3
2	2	2	2	3	3	4	4	3	3
3	3	2	2	3	3	4	4	4	4
3	3	2	2	3	3	4	4	4	4
2	2	3	3	2	2	4	4	3	3
2	2	3	3	2	2	4	4	3	3
3	3	2	2	3	3	4	4	3	3
2	2	2	2	3	3	4	4	3	3

For Review Only

3	3	2	2	3	3	4	4	4	4
2	2	3	3	2	2	4	4	3	3
3	3	2	2	3	3	4	4	3	3
3	3	2	2	3	3	4	4	3	3
3	3	3	3	3	3	4	4	4	4
2	2	3	3	2	2	4	4	3	3
3	3	2	2	3	3	4	4	3	3
2	2	2	2	3	3	4	4	3	3
2	2	2	2	2	2	4	4	3	3
3	3	2	2	3	3	4	4	4	4
3	3	2	2	3	3	4	4	4	4
2	2	3	3	2	2	4	4	3	3
3	3	3	3	3	3	4	4	4	4
3	3	2	2	3	3	4	4	3	3
2	2	3	3	2	2	4	4	3	3
3	3	2	2	3	3	4	4	3	3
2	2	3	3	2	2	4	4	3	3
3	3	2	2	3	3	4	4	3	3
3	3	2	2	3	3	4	4	3	3
3	3	3	3	2	2	4	4	4	4
3	3	2	2	3	3	4	4	3	3
2	2	3	3	2	2	4	4	3	3
2	2	3	3	2	2	4	4	3	3
3	3	2	2	3	3	4	4	3	3
3	3	2	2	3	3	4	4	3	3
2	2	3	3	2	2	4	4	3	3
3	3	2	2	3	3	4	4	3	3
3	3	2	2	3	3	4	4	3	3
3	3	2	2	3	3	4	4	3	3
3	3	2	2	3	3	4	4	4	4
3	3	3	3	3	3	4	4	4	4
3	3	3	3	3	3	4	4	3	3
3	3	2	2	3	3	1	1	3	3
2	2	3	3	2	2	4	4	3	3
2	2	3	3	2	2	4	4	3	3
3	3	2	2	3	3	4	4	3	3
3	3	2	2	3	3	4	4	3	3
3	3	2	2	3	3	4	4	4	4
2	2	3	3	2	2	4	4	4	4
2	2	3	3	2	2	4	4	3	3
3	3	2	2	3	3	4	4	3	3
3	3	2	2	3	3	4	4	4	4
3	3	2	2	2	2	4	4	3	3
3	3	2	2	3	3	4	4	3	3
2	2	3	3	2	2	4	4	3	3
3	3	2	2	3	3	4	4	3	3
3	3	2	2	3	3	4	4	3	3
2	2	3	3	2	2	4	4	3	3
2	2	3	3	2	2	4	4	3	3
2	2	3	3	2	2	4	4	3	3
3	3	2	2	3	3	4	4	3	3
2	2	3	3	2	2	1	1	3	3
2	2	3	3	2	2	4	4	3	3
3	3	2	2	3	3	4	4	3	3
3	3	2	2	2	2	4	4	3	3

For Review Only

2	2	3	3	2	2	4	4	3	3
3	3	2	2	3	3	4	4	3	3
3	3	2	2	3	3	4	4	4	4
3	3	2	2	2	2	4	4	3	3
2	2	3	3	2	2	4	4	4	4
3	3	2	2	3	3	4	4	4	4
3	3	2	2	3	3	4	4	3	3
2	2	3	3	3	3	4	4	3	3
2	2	2	2	3	3	4	4	3	3
2	2	3	3	2	2	4	4	3	3
3	3	2	2	3	3	4	4	3	3
2	2	3	3	2	2	4	4	3	3
2	2	3	3	2	2	4	4	3	3
3	3	2	2	3	3	4	4	4	4
3	3	3	3	3	3	4	4	4	4
2	2	3	3	2	2	4	4	4	4
3	3	2	2	3	3	4	4	3	3
3	3	2	2	3	3	4	4	3	3
2	2	3	3	2	2	4	4	3	3
2	2	3	3	2	2	4	4	3	3
3	3	2	2	3	3	4	4	4	4
3	3	2	2	3	3	4	4	4	4
3	3	2	2	3	3	4	4	4	4
3	3	2	2	3	3	4	4	3	3
2	2	3	3	2	2	4	4	3	3
3	3	2	2	3	3	4	4	4	4
2	2	3	3	2	2	4	4	3	3
3	3	2	2	3	3	4	4	3	3
2	2	2	2	3	3	4	4	4	4
3	3	2	2	3	3	4	4	4	4
2	2	3	3	2	2	4	4	3	3
2	2	3	3	2	2	4	4	3	3
2	2	3	3	2	2	4	4	3	3
3	3	2	2	3	3	4	4	3	3
2	2	2	2	2	2	4	4	3	3
2	2	2	2	2	2	4	4	3	3
3	3	2	2	3	3	4	4	4	4
3	3	2	2	3	3	4	4	3	3
2	2	3	3	2	2	4	4	3	3
3	3	2	2	3	3	4	4	3	3
3	3	2	2	3	3	4	4	3	3
2	2	3	3	2	2	4	4	4	4
3	3	2	2	3	3	4	4	4	4
3	3	2	2	3	3	4	4	4	4
3	3	3	3	3	3	4	4	3	3
3	3	2	2	3	3	4	4	4	4
3	3	3	3	2	2	4	4	4	4
2	2	3	3	2	2	4	4	3	3
3	3	2	2	3	3	4	4	3	3
2	2	3	3	2	2	4	4	3	3
3	3	2	2	3	3	4	4	4	4
2	2	3	3	2	2	4	4	3	3
3	3	2	2	3	3	4	4	4	4
3	3	2	2	3	3	4	4	3	3
3	3	2	2	3	3	4	4	3	3

For Review Only

3	3	2	2	3	3	4	4	4	4
2	2	3	3	2	2	4	4	4	4

For Review Only

SNP602	SNP602	SNP603	SNP603	SNP604	SNP604	SNP605	SNP605	SNP606	SNP606
2	2	4	4	3	3	1	1	2	2
2	2	4	4	3	3	4	4	2	2
2	2	4	4	3	3	1	1	2	2
2	2	4	4	3	3	1	1	2	2
4	4	1	1	3	3	1	1	4	4
2	2	4	4	3	3	4	4	2	2
4	4	4	4	2	2	1	1	2	2
2	2	1	1	2	2	4	4	2	2
2	2	4	4	2	2	1	1	2	2
2	2	1	1	2	2	1	1	4	4
2	2	4	4	3	3	4	4	2	2
2	2	4	4	3	3	1	1	2	2
2	2	4	4	2	2	1	1	2	2
2	2	1	1	2	2	4	4	2	2
2	2	4	4	3	3	1	1	2	2
2	2	4	4	3	3	1	1	4	4
2	2	4	4	3	3	4	4	4	4
2	2	4	4	2	2	4	4	2	2
2	2	4	4	3	3	4	4	2	2
2	2	1	1	2	2	1	1	2	2
4	4	4	4	2	2	4	4	4	4
2	2	4	4	3	3	4	4	4	4
2	2	1	1	2	2	4	4	2	2
2	2	1	1	2	2	4	4	2	2
2	2	4	4	2	2	1	1	2	2
2	2	4	4	3	3	1	1	2	2
2	2	4	4	3	3	1	1	2	2
2	2	4	4	2	2	1	1	2	2
4	4	4	4	2	2	4	4	4	4
2	2	1	1	2	2	4	4	4	4
2	2	4	4	2	2	1	1	2	2
2	2	4	4	2	2	1	1	2	2
2	2	4	4	2	2	1	1	2	2
2	2	1	1	2	2	4	4	4	4
2	2	4	4	3	3	4	4	2	2
2	2	1	1	2	2	1	1	2	2
2	2	1	1	3	3	1	1	2	2
2	2	4	4	3	3	1	1	2	2
2	2	4	4	2	2	4	4	4	4
2	2	4	4	3	3	4	4	2	2
2	2	1	1	2	2	4	4	2	2
4	4	4	4	2	2	4	4	4	4
2	2	1	1	2	2	4	4	2	2
2	2	4	4	2	2	1	1	2	2
2	2	1	1	2	2	4	4	2	2
2	2	4	4	3	3	1	1	2	2
2	2	4	4	3	3	1	1	2	2
2	2	1	1	3	3	4	4	2	2
2	2	4	4	2	2	1	1	4	4
2	2	4	4	3	3	1	1	2	2
2	2	4	4	2	2	1	1	2	2
2	2	4	4	2	2	1	1	2	2

For Review Only

2	2	4	4	3	3	1	1	2	2
2	2	4	4	2	2	1	1	2	2
2	2	4	4	3	3	4	4	2	2
2	2	4	4	3	3	1	1	2	2
2	2	4	4	3	3	4	4	2	2
2	2	4	4	3	3	4	4	2	2
2	2	4	4	3	3	1	1	2	2
2	2	1	1	2	2	4	4	4	4
2	2	4	4	2	2	4	4	2	2
2	2	4	4	2	2	1	1	2	2
2	2	4	4	2	2	4	4	2	2
2	2	4	4	2	2	4	4	2	2
2	2	4	4	3	3	1	1	2	2
4	4	4	4	2	2	4	4	4	4
2	2	4	4	3	3	1	1	2	2
2	2	4	4	2	2	4	4	2	2
2	2	4	4	3	3	4	4	2	2
2	2	4	4	3	3	1	1	2	2
2	2	4	4	3	3	4	4	2	2
2	2	4	4	2	2	1	1	2	2
2	2	4	4	2	2	4	4	2	2
2	2	1	1	3	3	4	4	4	4
4	4	4	4	2	2	1	1	4	4
2	2	4	4	2	2	1	1	2	2
2	2	1	1	2	2	4	4	2	2
2	2	1	1	2	2	1	1	2	2
2	2	1	1	2	2	4	4	2	2
2	2	4	4	2	2	4	4	4	4
2	2	4	4	3	3	1	1	2	2
2	2	4	4	2	2	1	1	2	2
2	2	1	1	3	3	1	1	2	2
2	2	1	1	2	2	4	4	2	2
2	2	1	1	2	2	4	4	4	4
2	2	4	4	3	3	1	1	2	2
4	4	4	4	2	2	1	1	2	2
2	2	1	1	2	2	4	4	2	2
2	2	1	1	2	2	4	4	2	2
2	2	1	1	3	3	1	1	4	4
2	2	1	1	2	2	4	4	2	2
2	2	1	1	2	2	4	4	2	2
2	2	4	4	3	3	1	1	2	2
2	2	4	4	3	3	1	1	2	2
2	2	4	4	2	2	1	1	2	2
2	2	4	4	2	2	4	4	2	2
2	2	4	4	2	2	1	1	2	2
2	2	4	4	3	3	1	1	2	2
2	2	1	1	2	2	4	4	4	4
2	2	1	1	2	2	4	4	2	2
2	2	4	4	3	3	1	1	2	2
2	2	1	1	2	2	1	1	2	2
2	2	4	4	3	3	1	1	2	2
2	2	4	4	2	2	4	4	4	4
2	2	4	4	2	2	4	4	4	4
2	2	4	4	2	2	1	1	2	2
2	2	1	1	2	2	4	4	2	2

For Review Only

2	2	4	4	3	3	1	1	2	2
2	2	1	1	3	3	1	1	2	2
2	2	1	1	2	2	1	1	2	2
2	2	4	4	2	2	4	4	4	4
2	2	4	4	2	2	1	1	2	2
2	2	1	1	3	3	1	1	2	2
2	2	4	4	3	3	1	1	2	2
2	2	1	1	2	2	1	1	2	2
2	2	4	4	2	2	1	1	4	4
2	2	1	1	2	2	4	4	2	2
4	4	4	4	3	3	1	1	2	2
2	2	4	4	2	2	1	1	2	2
2	2	4	4	2	2	4	4	2	2
2	2	4	4	2	2	1	1	2	2
2	2	4	4	2	2	1	1	2	2
2	2	4	4	3	3	1	1	2	2
2	2	4	4	3	3	4	4	2	2
2	2	4	4	2	2	1	1	2	2
2	2	1	1	2	2	4	4	4	4
2	2	4	4	2	2	4	4	2	2
2	2	4	4	2	2	1	1	2	2
2	2	4	4	3	3	1	1	2	2
2	2	4	4	2	2	4	4	2	2
2	2	1	1	2	2	4	4	4	4
2	2	4	4	3	3	1	1	2	2
2	2	1	1	3	3	4	4	2	2
2	2	4	4	3	3	1	1	2	2
2	2	4	4	2	2	1	1	2	2
2	2	4	4	3	3	1	1	2	2
2	2	4	4	3	3	4	4	2	2
2	2	1	1	2	2	4	4	4	4
2	2	1	1	2	2	1	1	2	2
2	2	4	4	3	3	1	1	2	2
2	2	4	4	3	3	1	1	2	2
2	2	1	1	2	2	4	4	4	4
2	2	1	1	2	2	4	4	2	2
2	2	1	1	2	2	4	4	4	4
2	2	4	4	2	2	4	4	2	2
4	4	4	4	3	3	4	4	2	2
2	2	4	4	2	2	4	4	4	4
2	2	4	4	3	3	1	1	2	2
2	2	1	1	2	2	4	4	4	4
2	2	4	4	2	2	1	1	2	2
2	2	4	4	2	2	4	4	2	2
2	2	4	4	2	2	1	1	2	2
2	2	4	4	2	2	1	1	2	2
2	2	4	4	3	3	1	1	2	2
2	2	1	1	3	3	1	1	2	2
2	2	1	1	2	2	4	4	2	2
2	2	4	4	3	3	1	1	2	2
2	2	1	1	3	3	1	1	2	2
2	2	1	1	3	3	4	4	4	4
2	2	1	1	2	2	1	1	2	2
2	2	4	4	2	2	4	4	2	2
2	2	4	4	2	2	4	4	2	2

For Review Only

2	2	4	4	3	3	1	1	2	2
2	2	1	1	2	2	4	4	2	2
2	2	4	4	2	2	4	4	2	2
2	2	4	4	3	3	4	4	4	4
2	2	4	4	3	3	4	4	2	2
2	2	1	1	3	3	4	4	4	4
2	2	4	4	2	2	4	4	2	2
2	2	1	1	2	2	1	1	2	2
2	2	1	1	3	3	4	4	2	2
2	2	4	4	3	3	1	1	2	2
2	2	4	4	2	2	1	1	2	2
2	2	1	1	3	3	4	4	2	2
2	2	4	4	2	2	1	1	2	2
2	2	4	4	2	2	1	1	2	2
2	2	1	1	2	2	4	4	4	4
2	2	4	4	3	3	4	4	2	2
2	2	4	4	2	2	4	4	2	2
2	2	4	4	3	3	1	1	2	2
2	2	4	4	2	2	4	4	2	2
2	2	4	4	3	3	1	1	2	2
2	2	4	4	2	2	1	1	2	2
2	2	1	1	2	2	4	4	2	2
2	2	1	1	3	3	4	4	4	4
2	2	4	4	2	2	4	4	2	2
2	2	4	4	2	2	1	1	2	2
2	2	1	1	2	2	4	4	4	4
2	2	4	4	3	3	4	4	2	2
2	2	4	4	2	2	4	4	2	2
2	2	4	4	3	3	1	1	2	2
2	2	4	4	3	3	1	1	2	2
2	2	4	4	2	2	1	1	2	2
2	2	4	4	3	3	1	1	2	2
4	4	4	4	3	3	1	1	2	2
2	2	1	1	2	2	4	4	4	4
2	2	1	1	2	2	4	4	2	2
2	2	4	4	2	2	4	4	2	2
2	2	4	4	3	3	1	1	2	2
2	2	4	4	3	3	1	1	2	2
2	2	1	1	3	3	1	1	2	2
2	2	1	1	2	2	1	1	2	2
2	2	4	4	3	3	1	1	2	2
2	2	4	4	3	3	1	1	2	2
2	2	4	4	2	2	4	4	4	4
2	2	1	1	3	3	4	4	2	2
2	2	1	1	2	2	4	4	2	2
2	2	4	4	2	2	1	1	4	4
2	2	4	4	3	3	4	4	2	2
2	2	1	1	2	2	4	4	4	4
2	2	1	1	2	2	4	4	4	4
2	2	1	1	3	3	1	1	2	2
2	2	4	4	3	3	1	1	2	2
4	4	1	1	2	2	4	4	4	4
2	2	1	1	2	2	4	4	4	4
2	2	4	4	2	2	4	4	2	2
2	2	4	4	2	2	4	4	4	4

For Review Only

2	2	1	1	3	3	1	1	2	2
2	2	4	4	2	2	4	4	2	2
2	2	4	4	3	3	1	1	2	2
2	2	4	4	2	2	4	4	2	2
2	2	1	1	2	2	1	1	2	2
2	2	4	4	3	3	1	1	2	2
2	2	4	4	2	2	4	4	4	4
2	2	1	1	2	2	4	4	2	2
2	2	1	1	2	2	1	1	2	2
2	2	1	1	2	2	4	4	4	4
2	2	4	4	2	2	1	1	2	2
2	2	1	1	3	3	4	4	2	2
2	2	1	1	3	3	4	4	2	2
2	2	4	4	3	3	1	1	2	2
2	2	4	4	3	3	1	1	2	2
2	2	1	1	3	3	1	1	2	2
2	2	4	4	2	2	4	4	4	4
2	2	1	1	2	2	4	4	2	2
2	2	1	1	2	2	4	4	4	4
2	2	1	1	3	3	4	4	2	2
2	2	4	4	2	2	1	1	4	4
2	2	4	4	3	3	1	1	2	2
2	2	4	4	3	3	1	1	2	2
2	2	4	4	2	2	4	4	2	2
2	2	1	1	2	2	4	4	4	4
2	2	4	4	3	3	1	1	2	2
2	2	1	1	2	2	4	4	4	4
2	2	4	4	3	3	4	4	2	2
2	2	1	1	2	2	1	1	2	2
2	2	4	4	3	3	1	1	2	2
2	2	1	1	2	2	4	4	2	2
2	2	1	1	2	2	1	1	2	2
2	2	1	1	2	2	4	4	2	2
2	2	4	4	3	3	1	1	2	2
2	2	1	1	3	3	4	4	4	4
2	2	1	1	2	2	4	4	4	4
2	2	4	4	3	3	1	1	2	2
2	2	4	4	2	2	4	4	2	2
2	2	1	1	2	2	1	1	2	2
2	2	4	4	2	2	4	4	4	4
2	2	4	4	2	2	4	4	2	2
2	2	1	1	2	2	4	4	2	2
2	2	4	4	3	3	4	4	2	2
2	2	4	4	3	3	1	1	2	2
2	2	4	4	2	2	1	1	2	2
2	2	4	4	2	2	1	1	2	2
2	2	1	1	3	3	1	1	2	2
2	2	1	1	2	2	4	4	2	2
2	2	4	4	2	2	4	4	2	2
2	2	1	1	2	2	4	4	4	4
2	2	4	4	2	2	4	4	2	2
2	2	1	1	2	2	4	4	4	4
2	2	4	4	3	3	1	1	2	2
2	2	4	4	3	3	4	4	2	2
2	2	4	4	3	3	4	4	2	2

For Review Only

2	2	4	4	3	3	1	1	2	2
2	2	1	1	3	3	1	1	2	2

For Review Only

SNP607	SNP607	SNP608	SNP608	SNP609	SNP609	SNP610	SNP610	SNP611	SNP611
2	2	1	1	2	2	4	4	2	2
2	2	1	1	2	2	1	1	1	1
2	2	1	1	2	2	4	4	2	2
2	2	1	1	3	3	4	4	2	2
2	2	1	1	3	3	4	4	2	2
2	2	1	1	2	2	4	4	2	2
2	2	1	1	2	2	4	4	2	2
2	2	1	1	2	2	1	1	1	1
2	2	1	1	2	2	4	4	2	2
1	1	2	2	3	3	4	4	2	2
2	2	1	1	2	2	1	1	1	1
2	2	1	1	2	2	4	4	2	2
2	2	1	1	3	3	4	4	2	2
1	1	2	2	2	2	4	4	2	2
2	2	1	1	2	2	4	4	2	2
2	2	1	1	3	3	4	4	2	2
1	1	2	2	3	3	1	1	1	1
2	2	2	2	2	2	1	1	1	1
2	2	1	1	2	2	4	4	2	2
1	1	2	2	2	2	4	4	2	2
1	1	2	2	3	3	1	1	1	1
1	1	2	2	3	3	1	1	1	1
1	1	2	2	2	2	1	1	1	1
1	1	2	2	2	2	1	1	1	1
2	2	1	1	2	2	4	4	2	2
2	2	1	1	2	2	4	4	2	2
2	2	1	1	2	2	4	4	2	2
2	2	1	1	2	2	4	4	2	2
2	2	2	2	3	3	4	4	1	1
1	1	2	2	3	3	1	1	1	1
2	2	1	1	2	2	4	4	2	2
1	1	2	2	2	2	4	4	2	2
2	2	1	1	2	2	4	4	2	2
1	1	2	2	3	3	1	1	1	1
2	2	1	1	2	2	1	1	2	2
2	2	2	2	2	2	4	4	2	2
1	1	2	2	2	2	4	4	2	2
2	2	1	1	2	2	4	4	2	2
1	1	2	2	3	3	1	1	1	1
2	2	1	1	2	2	1	1	1	1
1	1	2	2	3	3	1	1	1	1
2	2	1	1	3	3	4	4	2	2
1	1	2	2	2	2	1	1	1	1
2	2	1	1	2	2	4	4	2	2
1	1	2	2	2	2	1	1	1	1
2	2	1	1	2	2	4	4	2	2
2	2	1	1	2	2	4	4	2	2
2	2	1	1	2	2	1	1	1	1
2	2	1	1	3	3	4	4	2	2
2	2	1	1	2	2	4	4	2	2
2	2	1	1	2	2	4	4	2	2
2	2	1	1	2	2	4	4	2	2

For Review Only

2	2	1	1	2	2	4	4	2	2
2	2	1	1	2	2	4	4	2	2
2	2	1	1	2	2	4	4	2	2
1	1	2	2	2	2	4	4	2	2
2	2	1	1	2	2	4	4	2	2
2	2	1	1	2	2	4	4	2	2
2	2	1	1	2	2	4	4	2	2
1	1	2	2	3	3	1	1	1	1
1	1	1	1	2	2	1	1	1	1
2	2	1	1	2	2	4	4	2	2
2	2	1	1	2	2	4	4	2	2
2	2	2	2	2	2	4	4	2	2
2	2	1	1	2	2	4	4	2	2
2	2	2	2	3	3	4	4	1	1
2	2	1	1	2	2	4	4	2	2
2	2	2	2	2	2	1	1	1	1
2	2	1	1	2	2	1	1	1	1
2	2	1	1	2	2	4	4	2	2
2	2	1	1	2	2	1	1	1	1
2	2	1	1	2	2	4	4	2	2
2	2	2	2	3	3	4	4	2	2
1	1	2	2	3	3	1	1	1	1
2	2	1	1	3	3	4	4	2	2
1	1	2	2	2	2	4	4	2	2
1	1	2	2	2	2	1	1	1	1
2	2	1	1	2	2	4	4	2	2
2	2	1	1	2	2	1	1	1	1
1	1	2	2	3	3	1	1	1	1
2	2	1	1	2	2	4	4	2	2
2	2	1	1	2	2	4	4	2	2
2	2	1	1	2	2	4	4	2	2
2	2	1	1	2	2	1	1	1	1
1	1	2	2	3	3	1	1	1	1
2	2	1	1	2	2	4	4	2	2
2	2	1	1	2	2	4	4	2	2
1	1	2	2	2	2	1	1	1	1
2	2	1	1	2	2	1	1	1	1
2	2	1	1	3	3	4	4	2	2
2	2	1	1	2	2	1	1	1	1
1	1	2	2	2	2	1	1	1	1
2	2	1	1	2	2	4	4	2	2
2	2	1	1	2	2	4	4	2	2
1	1	2	2	2	2	4	4	2	2
2	2	1	1	2	2	4	4	2	2
2	2	1	1	2	2	4	4	2	2
2	2	1	1	2	2	4	4	2	2
1	1	2	2	3	3	1	1	1	1
2	2	1	1	2	2	1	1	1	1
2	2	1	1	2	2	4	4	2	2
2	2	1	1	2	2	4	4	2	2
2	2	1	1	2	2	4	4	2	2
1	1	2	2	3	3	1	1	1	1
1	1	2	2	3	3	1	1	1	1
1	1	1	1	2	2	1	1	2	2
2	2	1	1	2	2	1	1	1	1

For Review Only

2	2	1	1	2	2	4	4	2	2
2	2	1	1	2	2	4	4	2	2
2	2	1	1	2	2	4	4	2	2
1	1	2	2	3	3	1	1	1	1
2	2	1	1	2	2	4	4	2	2
2	2	1	1	2	2	4	4	2	2
2	2	1	1	2	2	4	4	2	2
2	2	1	1	2	2	4	4	2	2
2	2	1	1	3	3	4	4	2	2
2	2	1	1	3	3	1	1	1	1
1	1	2	2	2	2	4	4	2	2
2	2	1	1	2	2	4	4	2	2
2	2	2	2	2	2	1	1	2	2
2	2	1	1	2	2	4	4	2	2
2	2	1	1	2	2	4	4	2	2
2	2	1	1	2	2	4	4	2	2
2	2	1	1	2	2	1	1	2	2
2	2	1	1	2	2	4	4	2	2
1	1	2	2	3	3	1	1	1	1
2	2	1	1	2	2	1	1	1	1
2	2	1	1	2	2	4	4	2	2
2	2	1	1	2	2	4	4	2	2
2	2	1	1	2	2	1	1	1	1
1	1	2	2	3	3	1	1	1	1
2	2	1	1	2	2	4	4	2	2
1	1	2	2	2	2	1	1	1	1
2	2	1	1	2	2	4	4	2	2
2	2	2	2	2	2	4	4	2	2
2	2	1	1	2	2	4	4	2	2
2	2	1	1	2	2	4	4	2	2
1	1	2	2	3	3	1	1	1	1
2	2	1	1	2	2	4	4	2	2
2	2	1	1	2	2	4	4	2	2
2	2	1	1	2	2	4	4	1	1
1	1	2	2	3	3	1	1	1	1
2	2	2	2	2	2	4	4	2	2
1	1	2	2	3	3	1	1	1	1
2	2	1	1	2	2	4	4	2	2
2	2	1	1	2	2	1	1	1	1
2	2	1	1	3	3	1	1	1	1
1	1	2	2	2	2	4	4	2	2
1	1	2	2	3	3	1	1	1	1
2	2	1	1	2	2	4	4	2	2
2	2	1	1	3	3	1	1	1	1
2	2	1	1	2	2	4	4	2	2
2	2	1	1	2	2	4	4	2	2
2	2	1	1	2	2	4	4	2	2
2	2	1	1	2	2	4	4	2	2
2	2	2	2	2	2	1	1	2	2
2	2	1	1	2	2	4	4	2	2
2	2	1	1	2	2	4	4	2	2
1	1	2	2	3	3	1	1	1	1
2	2	1	1	2	2	4	4	2	2
2	2	2	2	2	2	4	4	2	2
2	2	2	2	2	2	1	1	1	1

For Review Only

2	2	1	1	2	2	4	4	2	2
1	1	2	2	2	2	1	1	1	1
2	2	1	1	2	2	4	4	2	2
2	2	1	1	3	3	1	1	1	1
2	2	1	1	2	2	4	4	2	2
2	2	1	1	3	3	1	1	1	1
2	2	1	1	2	2	1	1	1	1
2	2	1	1	2	2	4	4	2	2
2	2	1	1	2	2	1	1	1	1
2	2	1	1	2	2	4	4	2	2
2	2	1	1	2	2	4	4	2	2
2	2	1	1	2	2	4	4	2	2
2	2	1	1	2	2	4	4	2	2
2	2	1	1	2	2	4	4	2	2
1	1	2	2	3	3	1	1	1	1
2	2	1	1	2	2	1	1	1	1
2	2	1	1	2	2	4	4	1	1
2	2	1	1	2	2	4	4	2	2
2	2	2	2	2	2	1	1	1	1
2	2	1	1	2	2	4	4	2	2
2	2	1	1	2	2	4	4	2	2
1	1	2	2	2	2	1	1	1	1
1	1	2	2	3	3	1	1	1	1
2	2	2	2	2	2	4	4	2	2
2	2	1	1	2	2	4	4	1	1
1	1	2	2	3	3	1	1	1	1
2	2	1	1	2	2	1	1	1	1
2	2	1	1	2	2	1	1	1	1
1	1	2	2	2	2	4	4	2	2
2	2	2	2	2	2	4	4	2	2
2	2	1	1	2	2	4	4	2	2
2	2	1	1	2	2	4	4	2	2
2	2	1	1	2	2	4	4	2	2
1	1	2	2	3	3	1	1	1	1
2	2	1	1	2	2	4	4	2	2
2	2	2	2	2	2	4	4	2	2
2	2	1	1	2	2	4	4	2	2
2	2	1	1	2	2	4	4	2	2
2	2	1	1	2	2	4	4	2	2
2	2	1	1	2	2	4	4	2	2
2	2	1	1	2	2	4	4	2	2
2	2	1	1	2	2	4	4	2	2
1	1	2	2	3	3	1	1	1	1
2	2	1	1	2	2	4	4	2	2
2	2	1	1	3	3	4	4	2	2
2	2	1	1	3	3	4	4	2	2
2	2	1	1	2	2	1	1	1	1
2	2	1	1	3	3	1	1	1	1
1	1	2	2	3	3	1	1	1	1
1	1	2	2	2	2	4	4	2	2
2	2	1	1	2	2	4	4	2	2
1	1	2	2	3	3	1	1	1	1
1	1	2	2	3	3	1	1	1	1
2	2	1	1	2	2	4	4	2	2
1	1	2	2	3	3	1	1	1	1

For Review Only

2	2	1	1	2	2	4	4	2	2
2	2	1	1	2	2	1	1	1	1
2	2	1	1	2	2	4	4	2	2
2	2	1	1	2	2	1	1	1	1
1	1	2	2	2	2	4	4	2	2
2	2	1	1	2	2	4	4	2	2
2	2	1	1	3	3	1	1	1	1
2	2	2	2	2	2	1	1	1	1
2	2	1	1	2	2	4	4	2	2
1	1	2	2	3	3	1	1	1	1
2	2	1	1	2	2	4	4	2	2
2	2	1	1	2	2	1	1	1	1
2	2	1	1	2	2	1	1	1	1
2	2	1	1	2	2	4	4	2	2
2	2	1	1	2	2	4	4	2	2
2	2	1	1	2	2	4	4	2	2
2	2	1	1	3	3	1	1	1	1
2	2	1	1	2	2	1	1	1	1
2	2	1	1	3	3	1	1	1	1
2	2	1	1	2	2	1	1	1	1
2	2	1	1	3	3	4	4	2	2
2	2	1	1	2	2	4	4	2	2
2	2	1	1	2	2	4	4	2	2
2	2	1	1	2	2	1	1	1	1
1	1	2	2	3	3	1	1	1	1
2	2	1	1	2	2	4	4	2	2
1	1	2	2	3	3	1	1	1	1
2	2	1	1	2	2	1	1	1	1
2	2	1	1	2	2	4	4	2	2
2	2	1	1	2	2	4	4	2	2
1	1	2	2	2	2	1	1	1	1
2	2	2	2	2	2	4	4	2	2
2	2	1	1	2	2	1	1	1	1
2	2	1	1	2	2	4	4	2	2
1	1	2	2	3	3	1	1	1	1
1	1	2	2	3	3	1	1	1	1
2	2	1	1	2	2	4	4	2	2
2	2	2	2	2	2	4	4	2	2
2	2	2	2	2	2	4	4	2	2
1	1	2	2	3	3	1	1	1	1
2	2	1	1	2	2	4	4	2	2
1	1	2	2	2	2	4	4	2	2
2	2	1	1	2	2	4	4	2	2
2	2	1	1	2	2	4	4	2	2
1	1	2	2	2	2	4	4	2	2
2	2	1	1	2	2	4	4	2	2
2	2	2	2	2	2	4	4	2	2
2	2	1	1	2	2	1	1	1	1
2	2	1	1	2	2	1	1	1	1
1	1	1	1	3	3	1	1	1	1
2	2	1	1	2	2	4	4	2	2
1	1	2	2	3	3	1	1	1	1
2	2	1	1	2	2	4	4	2	2
2	2	1	1	2	2	1	1	1	1
2	2	1	1	2	2	1	1	1	1

For Review Only

2	2	1	1	2	2	4	4	2	2
1	1	2	2	2	2	4	4	2	2

For Review Only

SNP612	SNP612	SNP613	SNP613	SNP614	SNP614	SNP615	SNP615	SNP616	SNP616
2	2	2	2	3	3	3	3	2	2
2	2	2	2	2	2	2	2	2	2
2	2	2	2	2	2	2	2	2	2
1	1	3	3	2	2	2	2	2	2
1	1	3	3	3	3	3	3	2	2
2	2	2	2	3	3	3	3	2	2
2	2	2	2	3	3	3	3	2	2
1	1	2	2	3	3	3	3	2	2
2	2	2	2	3	3	3	3	2	2
1	1	3	3	3	3	3	3	4	4
2	2	2	2	3	3	3	3	2	2
2	2	2	2	2	2	2	2	2	2
2	2	2	2	3	3	3	3	2	2
1	1	2	2	3	3	3	3	4	4
2	2	2	2	3	3	3	3	2	2
1	1	3	3	3	3	3	3	2	2
2	2	3	3	3	3	3	3	4	4
1	1	2	2	3	3	3	3	2	2
2	2	2	2	3	3	3	3	2	2
1	1	2	2	3	3	3	3	4	4
2	2	3	3	3	3	3	3	2	2
2	2	3	3	3	3	3	3	2	2
1	1	2	2	3	3	3	3	4	4
1	1	2	2	3	3	3	3	2	2
1	1	2	2	3	3	3	3	2	2
2	2	2	2	2	2	2	2	2	2
2	2	2	2	2	2	2	2	2	2
2	2	2	2	3	3	2	2	2	2
2	2	3	3	3	3	3	3	2	2
1	1	3	3	3	3	3	3	4	4
2	2	2	2	3	3	3	3	2	2
2	2	2	2	3	3	3	3	4	4
2	2	2	2	3	3	3	3	2	2
1	1	3	3	3	3	3	3	4	4
2	2	2	2	3	3	2	2	2	2
1	1	2	2	3	3	3	3	2	2
1	1	2	2	3	3	3	3	2	2
1	1	2	2	3	3	3	3	2	2
1	1	3	3	3	3	3	3	4	4
2	2	2	2	3	3	3	3	2	2
1	1	3	3	3	3	3	3	2	2
1	1	3	3	3	3	3	3	2	2
1	1	2	2	3	3	3	3	2	2
1	1	2	2	3	3	3	3	2	2
1	1	2	2	3	3	3	3	2	2
2	2	2	2	3	3	3	3	2	2
2	2	2	2	2	2	2	2	2	2
1	1	2	2	3	3	3	3	2	2
1	1	3	3	3	3	3	3	2	2
2	2	2	2	3	3	3	3	2	2
2	2	2	2	2	2	2	2	2	2
2	2	2	2	3	3	3	3	2	2

For Review Only

2	2	2	2	3	3	3	3	2	2
2	2	2	2	3	3	3	3	2	2
2	2	2	2	3	3	3	3	2	2
2	2	2	2	3	3	3	3	4	4
2	2	2	2	3	3	3	3	2	2
2	2	2	2	2	2	2	2	2	2
2	2	2	2	2	2	2	2	2	2
1	1	3	3	3	3	3	3	2	2
2	2	2	2	3	3	3	3	2	2
2	2	2	2	3	3	3	3	2	2
2	2	2	2	2	2	2	2	2	2
2	2	2	2	2	2	2	2	2	2
2	2	2	2	3	3	3	3	2	2
2	2	3	3	3	3	3	3	2	2
2	2	2	2	3	3	3	3	2	2
1	1	2	2	3	3	3	3	2	2
2	2	2	2	2	2	2	2	2	2
2	2	2	2	3	3	3	3	2	2
2	2	2	2	3	3	3	3	2	2
2	2	2	2	3	3	3	3	2	2
2	2	2	2	3	3	2	2	2	2
2	2	3	3	3	3	2	2	4	4
2	2	3	3	3	3	3	3	2	2
2	2	2	2	3	3	3	3	4	4
1	1	2	2	3	3	3	3	2	2
1	1	2	2	2	2	2	2	2	2
1	1	2	2	3	3	3	3	2	2
1	1	3	3	3	3	3	3	4	4
2	2	2	2	2	2	2	2	2	2
1	1	2	2	3	3	3	3	2	2
1	1	2	2	3	3	3	3	2	2
1	1	2	2	3	3	3	3	2	2
1	1	3	3	3	3	3	3	4	4
2	2	2	2	3	3	3	3	2	2
1	1	2	2	3	3	3	3	2	2
1	1	2	2	3	3	3	3	2	2
1	1	2	2	3	3	3	3	2	2
1	1	3	3	3	3	3	3	2	2
1	1	2	2	3	3	3	3	2	2
1	1	2	2	3	3	3	3	4	4
2	2	2	2	3	3	3	3	2	2
2	2	2	2	3	3	3	3	2	2
2	2	2	2	3	3	3	3	2	2
2	2	2	2	2	2	2	2	2	2
2	2	2	2	3	3	3	3	2	2
2	2	2	2	2	2	2	2	2	2
1	1	3	3	3	3	3	3	4	4
1	1	2	2	3	3	2	2	2	2
2	2	2	2	3	3	3	3	2	2
1	1	2	2	3	3	3	3	2	2
2	2	2	2	2	2	2	2	2	2
1	1	3	3	3	3	3	3	4	4
2	2	3	3	3	3	3	3	2	2
2	2	2	2	2	2	2	2	2	2
1	1	2	2	3	3	3	3	2	2

For Review Only

2	2	2	2	2	2	2	2	2	2
1	1	2	2	3	3	3	3	2	2
1	1	2	2	3	3	2	2	2	2
1	1	3	3	3	3	3	3	4	4
2	2	2	2	2	2	2	2	2	2
1	1	2	2	2	2	2	2	2	2
1	1	2	2	2	2	3	3	2	2
1	1	2	2	3	3	3	3	2	2
2	2	3	3	2	2	2	2	2	2
2	2	3	3	2	2	2	2	2	2
2	2	2	2	3	3	3	3	2	2
2	2	2	2	3	3	2	2	2	2
2	2	2	2	3	3	3	3	4	4
2	2	2	2	2	2	2	2	2	2
2	2	2	2	3	3	2	2	2	2
2	2	2	2	3	3	2	2	2	2
2	2	2	2	3	3	3	3	2	2
2	2	2	2	3	3	2	2	2	2
1	1	3	3	3	3	3	3	4	4
2	2	2	2	3	3	3	3	2	2
2	2	2	2	3	3	2	2	2	2
2	2	2	2	3	3	3	3	2	2
2	2	2	2	3	3	3	3	2	2
1	1	3	3	3	3	3	3	2	2
1	1	2	2	2	2	2	2	2	2
1	1	2	2	3	3	3	3	4	4
2	2	2	2	3	3	3	3	2	2
2	2	2	2	3	3	3	3	2	2
2	2	2	2	3	3	3	3	2	2
2	2	2	2	3	3	3	3	2	2
1	1	3	3	3	3	3	3	4	4
2	2	2	2	3	3	3	3	4	4
2	2	2	2	3	3	3	3	2	2
2	2	2	2	3	3	2	2	2	2
1	1	2	2	3	3	3	3	4	4
1	1	2	2	3	3	3	3	4	4
1	1	3	3	3	3	3	3	4	4
2	2	2	2	3	3	3	3	2	2
2	2	2	2	2	2	2	2	2	2
1	1	3	3	3	3	3	3	2	2
2	2	2	2	3	3	3	3	4	4
1	1	3	3	3	3	3	3	4	4
2	2	2	2	3	3	3	3	2	2
1	1	3	3	3	3	3	3	2	2
1	1	2	2	3	3	3	3	2	2
2	2	2	2	3	3	3	3	2	2
2	2	2	2	2	2	2	2	2	2
1	1	2	2	2	2	2	2	2	2
1	1	2	2	3	3	3	3	2	2
2	2	2	2	2	2	2	2	2	2
2	2	2	2	2	2	2	2	2	2
1	1	3	3	3	3	3	3	4	4
1	1	2	2	3	3	3	3	2	2
2	2	2	2	2	2	2	2	2	2
1	1	3	3	3	3	3	3	2	2

For Review Only

2	2	2	2	2	2	2	2	2	2
1	1	2	2	3	3	3	3	4	4
2	2	2	2	3	3	3	3	2	2
2	2	3	3	3	3	3	3	2	2
2	2	2	2	2	2	2	2	2	2
1	1	3	3	3	3	3	3	2	2
1	1	2	2	3	3	3	3	2	2
2	2	2	2	3	3	2	2	2	2
1	1	2	2	3	3	3	3	2	2
2	2	2	2	3	3	3	3	2	2
2	2	2	2	2	2	2	2	2	2
1	1	2	2	3	3	3	3	2	2
2	2	2	2	2	2	2	2	2	2
2	2	2	2	3	3	3	3	2	2
1	1	3	3	3	3	3	3	4	4
2	2	2	2	3	3	3	3	2	2
2	2	2	2	3	3	3	3	2	2
2	2	2	2	2	2	2	2	2	2
2	2	2	2	2	2	2	2	2	2
1	1	2	2	2	2	3	3	2	2
2	2	2	2	3	3	3	3	2	2
1	1	2	2	3	3	3	3	4	4
1	1	3	3	3	3	3	3	4	4
2	2	2	2	2	2	2	2	2	2
2	2	2	2	3	3	3	3	2	2
1	1	3	3	3	3	3	3	4	4
2	2	2	2	3	3	2	2	2	2
2	2	2	2	3	3	3	3	2	2
2	2	2	2	3	3	3	3	4	4
2	2	2	2	3	3	3	3	2	2
1	1	2	2	2	2	2	2	2	2
1	1	2	2	3	3	3	3	2	2
2	2	2	2	3	3	3	3	2	2
1	1	3	3	3	3	3	3	4	4
1	1	2	2	3	3	3	3	2	2
2	2	2	2	2	2	2	2	2	2
2	2	2	2	3	3	3	3	2	2
2	2	2	2	3	3	3	3	2	2
1	1	2	2	2	2	2	2	2	2
1	1	2	2	3	3	3	3	2	2
2	2	2	2	2	2	2	2	2	2
2	2	2	2	2	2	2	2	2	2
1	1	3	3	3	3	3	3	4	4
2	2	2	2	3	3	3	3	2	2
1	1	2	2	3	3	3	3	2	2
2	2	3	3	3	3	2	2	2	2
2	2	2	2	3	3	3	3	2	2
1	1	3	3	3	3	3	3	2	2
1	1	3	3	3	3	3	3	4	4
1	1	2	2	2	2	2	2	4	4
2	2	2	2	2	2	2	2	2	2
1	1	3	3	3	3	3	3	4	4
1	1	3	3	3	3	3	3	4	4
2	2	2	2	2	2	2	2	2	2
1	1	3	3	3	3	3	3	4	4

For Review Only

1	1	2	2	3	3	3	3	2	2
2	2	2	2	3	3	3	3	2	2
2	2	2	2	3	3	3	3	2	2
2	2	2	2	3	3	3	3	2	2
1	1	2	2	3	3	3	3	4	4
2	2	2	2	2	2	2	2	2	2
2	2	3	3	3	3	3	3	2	2
1	1	2	2	3	3	3	3	2	2
1	1	2	2	3	3	3	3	2	2
1	1	3	3	3	3	3	3	4	4
2	2	2	2	3	3	2	2	2	2
1	1	2	2	2	2	2	2	2	2
1	1	2	2	3	3	3	3	2	2
1	1	2	2	2	2	2	2	2	2
2	2	2	2	2	2	2	2	2	2
1	1	2	2	3	3	3	3	2	2
1	1	3	3	3	3	3	3	2	2
2	2	2	2	3	3	3	3	2	2
1	1	3	3	2	2	2	2	2	2
1	1	2	2	3	3	3	3	2	2
1	1	3	3	3	3	3	3	2	2
2	2	2	2	2	2	2	2	2	2
2	2	2	2	3	3	3	3	2	2
2	2	2	2	3	3	3	3	2	2
1	1	3	3	3	3	3	3	2	2
2	2	2	2	3	3	2	2	2	2
1	1	3	3	3	3	3	3	4	4
2	2	2	2	2	2	2	2	2	2
1	1	2	2	3	3	3	3	2	2
2	2	2	2	3	3	3	3	2	2
1	1	2	2	3	3	3	3	4	4
1	1	2	2	2	2	2	2	2	2
1	1	2	2	3	3	3	3	2	2
2	2	2	2	2	2	2	2	2	2
1	1	3	3	3	3	3	3	4	4
1	1	3	3	3	3	3	3	4	4
2	2	2	2	3	3	3	3	2	2
2	2	2	2	2	2	2	2	2	2
1	1	2	2	3	3	3	3	2	2
1	1	3	3	3	3	3	3	4	4
1	1	2	2	3	3	3	3	2	2
1	1	2	2	3	3	3	3	2	2
2	2	2	2	3	3	3	3	2	2
2	2	2	2	3	3	3	3	2	2
2	2	2	2	2	2	2	2	2	2
2	2	2	2	3	3	3	3	2	2
1	1	2	2	3	3	3	3	4	4
1	1	2	2	3	3	3	3	2	2
2	2	2	2	3	3	2	2	2	2
1	1	3	3	3	3	3	3	4	4
2	2	2	2	3	3	3	3	2	2
1	1	3	3	3	3	3	3	4	4
2	2	2	2	2	2	2	2	2	2
2	2	2	2	3	3	3	3	2	2
1	1	2	2	3	3	3	3	2	2

For Review Only

2	2	2	2	3	3	3	3	2	2
1	1	2	2	2	2	2	2	2	2

For Review Only

SNP617	SNP617	SNP618	SNP618	SNP619	SNP619	SNP620	SNP620	SNP621	SNP621
1	1	4	4	4	4	3	3	4	4
4	4	4	4	4	4	1	1	4	4
1	1	4	4	1	1	3	3	4	4
1	1	4	4	1	1	3	3	4	4
1	1	4	4	1	1	3	3	2	2
1	1	4	4	1	1	3	3	4	4
1	1	4	4	1	1	3	3	4	4
4	4	4	4	1	1	1	1	4	4
1	1	4	4	1	1	3	3	4	4
4	4	4	4	1	1	1	1	2	2
1	1	4	4	1	1	3	3	4	4
1	1	4	4	4	4	3	3	4	4
4	4	4	4	1	1	1	1	4	4
4	4	4	4	1	1	1	1	4	4
1	1	4	4	1	1	3	3	4	4
1	1	4	4	1	1	1	1	4	4
1	1	4	4	4	4	3	3	2	2
1	1	4	4	1	1	3	3	4	4
1	1	4	4	1	1	3	3	4	4
1	1	4	4	1	1	3	3	4	4
1	1	4	4	1	1	3	3	2	2
4	4	4	4	4	4	1	1	4	4
4	4	4	4	1	1	1	1	2	2
4	4	4	4	1	1	1	1	4	4
1	1	4	4	1	1	3	3	4	4
1	1	4	4	4	4	3	3	4	4
1	1	4	4	4	4	3	3	4	4
1	1	4	4	1	1	3	3	4	4
1	1	4	4	1	1	3	3	2	2
4	4	4	4	1	1	1	1	2	2
1	1	4	4	1	1	3	3	4	4
1	1	4	4	4	4	3	3	4	4
1	1	4	4	1	1	3	3	4	4
1	1	4	4	1	1	3	3	2	2
1	1	2	2	4	4	1	1	4	4
1	1	4	4	1	1	3	3	4	4
1	1	4	4	4	4	3	3	4	4
4	4	4	4	1	1	3	3	4	4
4	4	2	2	1	1	1	1	4	4
4	4	4	4	1	1	1	1	4	4
4	4	4	4	1	1	1	1	2	2
1	1	4	4	1	1	3	3	2	2
4	4	4	4	1	1	3	3	4	4
4	4	4	4	1	1	1	1	4	4
1	1	4	4	1	1	3	3	4	4
1	1	4	4	4	4	3	3	4	4
1	1	4	4	4	4	3	3	4	4
4	4	4	4	1	1	3	3	4	4
4	4	4	4	4	4	1	1	4	4
1	1	4	4	1	1	3	3	4	4
1	1	4	4	4	4	3	3	4	4
1	1	4	4	1	1	3	3	4	4

For Review Only

1	1	4	4	4	4	3	3	4	4
1	1	4	4	1	1	3	3	4	4
1	1	4	4	1	1	3	3	4	4
1	1	4	4	4	4	3	3	4	4
1	1	4	4	1	1	3	3	4	4
1	1	4	4	4	4	3	3	4	4
1	1	4	4	1	1	3	3	4	4
1	1	4	4	1	1	3	3	2	2
1	1	4	4	4	4	3	3	4	4
1	1	4	4	4	4	3	3	4	4
1	1	4	4	1	1	3	3	4	4
1	1	4	4	4	4	3	3	4	4
1	1	4	4	4	4	3	3	4	4
1	1	4	4	1	1	3	3	2	2
1	1	4	4	1	1	3	3	4	4
1	1	4	4	4	4	3	3	4	4
1	1	2	2	4	4	3	3	4	4
1	1	4	4	1	1	3	3	4	4
1	1	4	4	1	1	3	3	4	4
1	1	4	4	1	1	3	3	4	4
1	1	4	4	1	1	3	3	4	4
1	1	4	4	1	1	3	3	4	4
1	1	4	4	1	1	3	3	4	4
1	1	4	4	1	1	3	3	2	2
1	1	4	4	1	1	3	3	4	4
1	1	4	4	4	4	3	3	4	4
1	1	4	4	4	4	3	3	4	4
4	4	2	2	1	1	1	1	4	4
1	1	4	4	1	1	3	3	4	4
1	1	4	4	1	1	3	3	4	4
1	1	4	4	1	1	3	3	4	4
1	1	4	4	1	1	3	3	4	4
4	4	4	4	1	1	1	1	2	2
1	1	4	4	4	4	3	3	4	4
1	1	4	4	1	1	3	3	4	4
1	1	4	4	1	1	3	3	4	4
1	1	4	4	1	1	3	3	4	4
1	1	4	4	4	4	3	3	2	2
1	1	4	4	4	4	3	3	4	4
1	1	4	4	1	1	3	3	4	4
1	1	4	4	4	4	3	3	4	4
1	1	4	4	1	1	3	3	4	4
1	1	4	4	4	4	3	3	4	4
1	1	4	4	1	1	3	3	4	4
1	1	4	4	4	4	3	3	4	4
1	1	4	4	1	1	3	3	4	4
1	1	4	4	1	1	3	3	4	4
1	1	4	4	1	1	3	3	4	4
1	1	4	4	1	1	3	3	4	4
1	1	4	4	1	1	3	3	4	4
1	1	4	4	1	1	3	3	4	4
4	4	4	4	1	1	1	1	4	4
1	1	4	4	1	1	3	3	2	2
1	1	4	4	1	1	3	3	4	4
4	4	4	4	1	1	1	1	4	4

For Review Only

1	1	4	4	1	1	3	3	4	4
4	4	2	2	1	1	1	1	4	4
1	1	4	4	4	4	3	3	4	4
4	4	4	4	1	1	1	1	4	4
1	1	4	4	1	1	3	3	4	4
1	1	4	4	4	4	3	3	4	4
1	1	4	4	1	1	3	3	4	4
1	1	4	4	1	1	3	3	4	4
4	4	4	4	4	4	1	1	2	2
1	1	4	4	1	1	3	3	4	4
1	1	4	4	1	1	3	3	4	4
1	1	4	4	1	1	1	1	4	4
1	1	4	4	4	4	3	3	4	4
1	1	4	4	1	1	3	3	4	4
1	1	4	4	1	1	3	3	4	4
1	1	4	4	1	1	3	3	4	4
1	1	4	4	1	1	3	3	4	4
1	1	4	4	1	1	3	3	4	4
1	1	4	4	1	1	3	3	4	4
1	1	4	4	1	1	3	3	4	4
1	1	4	4	1	1	3	3	4	4
1	1	4	4	1	1	3	3	4	4
1	1	4	4	4	4	3	3	4	4
4	4	2	2	1	1	1	1	4	4
1	1	4	4	1	1	3	3	4	4
1	1	2	2	1	1	3	3	4	4
1	1	4	4	1	1	3	3	2	2
1	1	4	4	1	1	3	3	4	4
1	1	4	4	4	4	3	3	4	4
1	1	4	4	1	1	3	3	2	2
4	4	4	4	1	1	1	1	2	2
1	1	4	4	1	1	3	3	4	4
1	1	4	4	1	1	3	3	4	4
1	1	4	4	1	1	3	3	4	4
1	1	4	4	1	1	3	3	2	2
1	1	4	4	1	1	1	1	4	4
4	4	4	4	1	1	1	1	2	2
1	1	4	4	1	1	3	3	4	4
4	4	4	4	1	1	1	1	4	4
4	4	4	4	1	1	1	1	2	2
1	1	4	4	1	1	3	3	4	4
4	4	4	4	1	1	1	1	2	2
1	1	4	4	1	1	3	3	4	4
1	1	4	4	4	4	1	1	4	4
1	1	4	4	1	1	3	3	4	4
1	1	4	4	1	1	3	3	4	4
1	1	4	4	1	1	3	3	4	4
1	1	4	4	1	1	3	3	4	4
1	1	4	4	4	4	3	3	4	4
1	1	4	4	4	4	3	3	4	4
1	1	4	4	1	1	3	3	4	4
1	1	4	4	1	1	3	3	2	2
1	1	4	4	1	1	3	3	4	4
1	1	4	4	4	4	3	3	4	4
1	1	4	4	4	4	3	3	4	4

For Review Only

1	1	4	4	1	1	3	3	4	4
1	1	2	2	4	4	3	3	4	4
1	1	4	4	1	1	3	3	2	2
1	1	2	2	1	1	3	3	4	4
1	1	4	4	1	1	3	3	4	4
1	1	4	4	1	1	3	3	4	4
4	4	4	4	1	1	1	1	4	4
1	1	4	4	1	1	3	3	4	4
4	4	4	4	4	4	1	1	4	4
1	1	4	4	4	4	3	3	4	4
1	1	4	4	1	1	3	3	4	4
4	4	4	4	1	1	1	1	4	4
1	1	4	4	1	1	3	3	4	4
1	1	4	4	4	4	3	3	4	4
1	1	4	4	1	1	3	3	4	4
4	4	4	4	1	1	1	1	4	4
1	1	4	4	1	1	3	3	4	4
1	1	4	4	1	1	3	3	4	4
4	4	4	4	4	4	1	1	4	4
1	1	4	4	1	1	3	3	4	4
1	1	4	4	1	1	3	3	4	4
4	4	4	4	1	1	1	1	4	4
1	1	4	4	1	1	3	3	2	2
1	1	4	4	1	1	3	3	4	4
4	4	4	4	1	1	3	3	4	4
1	1	4	4	1	1	3	3	2	2
1	1	4	4	4	4	3	3	4	4
1	1	4	4	1	1	3	3	4	4
1	1	4	4	4	4	3	3	4	4
1	1	4	4	4	4	3	3	4	4
1	1	4	4	1	1	3	3	4	4
1	1	4	4	1	1	3	3	4	4
1	1	4	4	1	1	3	3	2	2
1	1	4	4	4	4	3	3	4	4
1	1	4	4	1	1	3	3	2	2
1	1	4	4	4	4	3	3	4	4
1	1	4	4	1	1	3	3	4	4
1	1	4	4	4	4	3	3	4	4
1	1	4	4	1	1	3	3	4	4
1	1	4	4	1	1	3	3	4	4
1	1	4	4	4	4	3	3	4	4
1	1	4	4	1	1	3	3	4	4
4	4	4	4	1	1	1	1	4	4
1	1	4	4	4	4	3	3	4	4
1	1	4	4	4	4	3	3	2	2
1	1	4	4	4	4	3	3	2	2
4	4	4	4	1	1	1	1	4	4
4	4	4	4	1	1	1	1	4	4
1	1	4	4	1	1	3	3	2	2
1	1	4	4	4	4	3	3	4	4
1	1	4	4	1	1	3	3	4	4
1	1	4	4	1	1	3	3	2	2
1	1	4	4	1	1	3	3	2	2
1	1	4	4	1	1	3	3	4	4
4	4	4	4	1	1	1	1	2	2

For Review Only

1	1	4	4	1	1	3	3	4	4
4	4	4	4	4	4	1	1	2	2
1	1	4	4	1	1	3	3	4	4
1	1	4	4	1	1	3	3	4	4
1	1	4	4	1	1	3	3	4	4
1	1	4	4	4	4	3	3	4	4
4	4	4	4	1	1	1	1	2	2
4	4	4	4	1	1	1	1	4	4
4	4	4	4	1	1	1	1	4	4
4	4	4	4	1	1	1	1	2	2
1	1	4	4	1	1	3	3	4	4
1	1	4	4	1	1	3	3	4	4
1	1	4	4	4	4	3	3	4	4
1	1	4	4	4	4	3	3	4	4
1	1	4	4	4	4	3	3	4	4
4	4	4	4	4	4	1	1	4	4
4	4	4	4	1	1	1	1	2	2
1	1	4	4	1	1	3	3	4	4
4	4	4	4	4	4	1	1	2	2
4	4	4	4	1	1	1	1	4	4
1	1	4	4	1	1	3	3	2	2
1	1	4	4	1	1	3	3	4	4
1	1	4	4	4	4	3	3	4	4
4	4	4	4	1	1	1	1	4	4
1	1	4	4	1	1	3	3	2	2
1	1	4	4	4	4	3	3	4	4
4	4	4	4	1	1	1	1	2	2
1	1	4	4	4	4	3	3	4	4
1	1	4	4	4	4	3	3	4	4
1	1	4	4	4	4	3	3	4	4
4	4	4	4	1	1	1	1	4	4
1	1	4	4	1	1	3	3	4	4
1	1	4	4	4	4	3	3	4	4
1	1	4	4	1	1	3	3	4	4
1	1	4	4	1	1	3	3	2	2
4	4	2	2	1	1	1	1	2	2
1	1	4	4	4	4	3	3	4	4
1	1	4	4	4	4	3	3	4	4
1	1	4	4	1	1	3	3	4	4
4	4	2	2	1	1	1	1	4	4
1	1	4	4	1	1	3	3	2	2
1	1	4	4	1	1	3	3	2	2
4	4	4	4	1	1	1	1	4	4
1	1	4	4	4	4	3	3	4	4
1	1	4	4	4	4	3	3	4	4
1	1	4	4	4	4	3	3	4	4
4	4	4	4	1	1	1	1	4	4
1	1	4	4	1	1	3	3	4	4
1	1	4	4	4	4	3	3	4	4
4	4	4	4	1	1	1	1	2	2
1	1	4	4	4	4	3	3	4	4
4	4	4	4	1	1	1	1	4	4
1	1	4	4	4	4	3	3	4	4
1	1	4	4	4	4	3	3	4	4
4	4	4	4	1	1	1	1	4	4

For Review Only

1	1	4	4	4	4	3	3	4	4
1	1	4	4	1	1	3	3	4	4

For Review Only

SNP622	SNP622	SNP623	SNP623	SNP624	SNP624	SNP625	SNP625	SNP626	SNP626
1	1	4	4	1	1	2	2	4	4
1	1	3	3	4	4	2	2	4	4
1	1	3	3	1	1	2	2	4	4
3	3	4	4	1	1	2	2	4	4
3	3	4	4	1	1	2	2	4	4
1	1	3	3	1	1	2	2	4	4
1	1	3	3	1	1	2	2	1	1
3	3	4	4	4	4	2	2	4	4
1	1	3	3	1	1	2	2	1	1
3	3	4	4	4	4	2	2	4	4
1	1	3	3	1	1	2	2	4	4
1	1	3	3	1	1	2	2	4	4
1	1	4	4	1	1	2	2	4	4
3	3	4	4	4	4	3	3	4	4
1	1	4	4	1	1	2	2	4	4
3	3	4	4	1	1	2	2	4	4
1	1	3	3	1	1	2	2	4	4
3	3	4	4	1	1	2	2	4	4
1	1	3	3	1	1	2	2	4	4
3	3	4	4	1	1	2	2	4	4
1	1	3	3	1	1	2	2	4	4
1	1	3	3	4	4	2	2	1	1
3	3	4	4	4	4	2	2	4	4
3	3	4	4	4	4	3	3	4	4
3	3	4	4	1	1	2	2	1	1
1	1	3	3	1	1	2	2	4	4
1	1	3	3	1	1	2	2	4	4
1	1	4	4	1	1	2	2	4	4
1	1	3	3	1	1	2	2	1	1
3	3	4	4	4	4	2	2	4	4
1	1	3	3	1	1	2	2	4	4
1	1	3	3	1	1	2	2	4	4
1	1	3	3	1	1	2	2	1	1
3	3	4	4	1	1	3	3	4	4
1	1	4	4	1	1	2	2	4	4
3	3	4	4	1	1	3	3	4	4
3	3	4	4	1	1	3	3	4	4
3	3	4	4	4	4	3	3	4	4
3	3	4	4	4	4	2	2	4	4
1	1	4	4	4	4	2	2	4	4
3	3	4	4	4	4	2	2	4	4
3	3	4	4	1	1	2	2	4	4
3	3	4	4	4	4	2	2	4	4
3	3	4	4	4	4	3	3	4	4
3	3	4	4	1	1	2	2	4	4
1	1	3	3	1	1	2	2	4	4
1	1	3	3	1	1	2	2	4	4
3	3	4	4	1	1	3	3	4	4
1	1	4	4	4	4	3	3	4	4
1	1	3	3	1	1	2	2	4	4
1	1	3	3	1	1	2	2	4	4
1	1	3	3	1	1	2	2	4	4

For Review Only

1	1	3	3	1	1	2	2	1	1
1	1	3	3	1	1	2	2	1	1
3	3	4	4	4	4	3	3	4	4
1	1	3	3	1	1	2	2	4	4
1	1	3	3	1	1	2	2	4	4
1	1	3	3	1	1	2	2	4	4
1	1	3	3	1	1	2	2	4	4
3	3	4	4	1	1	3	3	4	4
1	1	3	3	1	1	2	2	4	4
3	3	4	4	1	1	3	3	4	4
1	1	3	3	1	1	2	2	4	4
1	1	3	3	1	1	2	2	4	4
1	1	3	3	1	1	2	2	1	1
1	1	3	3	1	1	2	2	1	1
1	1	4	4	1	1	2	2	4	4
3	3	4	4	1	1	3	3	4	4
1	1	3	3	1	1	2	2	4	4
1	1	3	3	1	1	2	2	4	4
1	1	3	3	1	1	2	2	4	4
1	1	3	3	1	1	2	2	4	4
1	1	4	4	1	1	2	2	4	4
1	1	4	4	1	1	2	2	4	4
1	1	3	3	1	1	2	2	1	1
1	1	3	3	1	1	2	2	4	4
3	3	4	4	1	1	2	2	4	4
3	3	4	4	1	1	3	3	4	4
3	3	4	4	1	1	2	2	1	1
3	3	4	4	4	4	3	3	4	4
1	1	3	3	1	1	2	2	4	4
3	3	4	4	1	1	3	3	4	4
3	3	4	4	1	1	3	3	4	4
3	3	4	4	1	1	2	2	4	4
3	3	4	4	4	4	2	2	4	4
1	1	3	3	1	1	2	2	1	1
3	3	4	4	1	1	2	2	4	4
3	3	4	4	1	1	2	2	4	4
3	3	4	4	1	1	3	3	4	4
3	3	4	4	1	1	2	2	1	1
3	3	4	4	1	1	2	2	4	4
3	3	4	4	1	1	2	2	4	4
1	1	3	3	1	1	2	2	1	1
1	1	3	3	1	1	2	2	4	4
1	1	3	3	4	4	2	2	4	4
1	1	3	3	1	1	2	2	4	4
1	1	3	3	1	1	2	2	4	4
1	1	3	3	1	1	2	2	4	4
3	3	4	4	1	1	3	3	4	4
3	3	4	4	1	1	3	3	4	4
3	3	4	4	1	1	3	3	4	4
3	3	4	4	1	1	2	2	4	4
1	1	3	3	1	1	2	2	4	4
3	3	4	4	4	4	3	3	4	4
1	1	3	3	1	1	2	2	4	4
1	1	4	4	1	1	2	2	4	4
3	3	4	4	4	4	3	3	4	4

For Review Only

1	1	3	3	1	1	2	2	4	4
1	1	4	4	4	4	2	2	4	4
3	3	4	4	1	1	3	3	4	4
3	3	4	4	4	4	3	3	4	4
1	1	3	3	1	1	2	2	4	4
3	3	4	4	1	1	2	2	4	4
3	3	4	4	1	1	2	2	4	4
3	3	4	4	1	1	3	3	4	4
3	3	4	4	4	4	3	3	4	4
1	1	4	4	1	1	2	2	4	4
1	1	3	3	1	1	2	2	4	4
1	1	4	4	1	1	2	2	4	4
1	1	3	3	1	1	2	2	4	4
1	1	3	3	1	1	2	2	4	4
3	3	4	4	1	1	2	2	4	4
1	1	3	3	1	1	2	2	4	4
1	1	3	3	1	1	2	2	4	4
1	1	4	4	1	1	2	2	4	4
3	3	4	4	1	1	3	3	4	4
1	1	3	3	1	1	2	2	4	4
1	1	4	4	1	1	2	2	4	4
1	1	3	3	1	1	2	2	4	4
1	1	3	3	1	1	2	2	4	4
3	3	4	4	4	4	3	3	4	4
1	1	4	4	1	1	2	2	4	4
3	3	4	4	1	1	2	2	4	4
1	1	3	3	1	1	2	2	4	4
1	1	3	3	1	1	2	2	4	4
1	1	3	3	1	1	2	2	4	4
1	1	3	3	1	1	2	2	4	4
3	3	4	4	4	4	2	2	4	4
1	1	4	4	1	1	2	2	4	4
1	1	3	3	1	1	2	2	1	1
1	1	4	4	1	1	2	2	4	4
3	3	4	4	1	1	3	3	4	4
3	3	4	4	1	1	2	2	4	4
3	3	4	4	4	4	3	3	4	4
1	1	3	3	1	1	2	2	4	4
1	1	4	4	4	4	3	3	4	4
3	3	4	4	4	4	2	2	4	4
1	1	3	3	1	1	2	2	4	4
3	3	4	4	4	4	3	3	4	4
1	1	3	3	1	1	2	2	4	4
3	3	4	4	4	4	2	2	4	4
1	1	4	4	1	1	2	2	4	4
1	1	3	3	1	1	2	2	4	4
1	1	3	3	1	1	2	2	4	4
3	3	4	4	1	1	3	3	4	4
3	3	4	4	1	1	3	3	4	4
1	1	3	3	1	1	2	2	4	4
1	1	4	4	1	1	2	2	4	4
3	3	4	4	1	1	3	3	4	4
3	3	4	4	1	1	3	3	4	4
1	1	3	3	1	1	2	2	4	4
3	3	4	4	1	1	3	3	4	4

For Review Only

1	1	3	3	1	1	2	2	4	4
3	3	4	4	4	4	2	2	4	4
1	1	3	3	1	1	2	2	4	4
1	1	4	4	1	1	2	2	4	4
1	1	4	4	1	1	2	2	4	4
3	3	4	4	1	1	3	3	4	4
1	1	4	4	4	4	2	2	4	4
1	1	4	4	1	1	3	3	4	4
3	3	4	4	4	4	3	3	4	4
1	1	3	3	1	1	2	2	4	4
1	1	3	3	1	1	2	2	4	4
3	3	4	4	4	4	2	2	4	4
1	1	3	3	1	1	2	2	4	4
1	1	4	4	1	1	2	2	4	4
3	3	4	4	1	1	3	3	4	4
1	1	3	3	4	4	2	2	4	4
3	3	4	4	1	1	2	2	4	4
1	1	3	3	1	1	2	2	4	4
1	1	3	3	4	4	2	2	4	4
3	3	4	4	1	1	2	2	4	4
1	1	4	4	1	1	2	2	4	4
3	3	4	4	4	4	2	2	4	4
3	3	4	4	1	1	2	2	4	4
1	1	3	3	1	1	2	2	4	4
1	1	3	3	1	1	2	2	4	4
3	3	4	4	1	1	3	3	4	4
1	1	4	4	1	1	2	2	4	4
1	1	4	4	1	1	2	2	4	4
1	1	3	3	4	4	2	2	4	4
3	3	3	3	1	1	2	2	4	4
3	3	4	4	1	1	3	3	4	4
3	3	4	4	1	1	3	3	4	4
1	1	3	3	1	1	2	2	4	4
3	3	4	4	1	1	3	3	4	4
1	1	4	4	1	1	2	2	4	4
1	1	3	3	1	1	2	2	4	4
1	1	3	3	1	1	2	2	4	4
1	1	3	3	4	4	2	2	4	4
3	3	4	4	1	1	3	3	4	4
3	3	4	4	1	1	3	3	4	4
1	1	3	3	1	1	2	2	4	4
1	1	3	3	1	1	2	2	4	4
3	3	4	4	4	4	2	2	4	4
1	1	4	4	1	1	2	2	4	4
3	3	4	4	1	1	2	2	4	4
1	1	3	3	1	1	2	2	4	4
1	1	3	3	4	4	2	2	4	4
3	3	4	4	4	4	2	2	4	4
3	3	4	4	1	1	3	3	4	4
3	3	4	4	1	1	2	2	4	4
1	1	3	3	1	1	2	2	4	4
3	3	4	4	1	1	3	3	4	4
3	3	4	4	1	1	2	2	4	4
1	1	3	3	1	1	2	2	4	4
3	3	4	4	4	4	3	3	4	4

For Review Only

3	3	4	4	1	1	3	3	4	4
1	1	4	4	4	4	2	2	4	4
1	1	3	3	1	1	2	2	4	4
1	1	4	4	1	1	2	2	4	4
3	3	4	4	1	1	2	2	4	4
1	1	3	3	1	1	2	2	4	4
1	1	4	4	4	4	2	2	4	4
3	3	4	4	4	4	3	3	4	4
3	3	4	4	4	4	3	3	4	4
1	1	4	4	4	4	3	3	4	4
1	1	4	4	1	1	2	2	4	4
3	3	4	4	1	1	2	2	1	1
3	3	4	4	1	1	2	2	4	4
3	3	4	4	1	1	3	3	4	4
1	1	3	3	1	1	2	2	4	4
3	3	4	4	4	4	2	2	1	1
3	3	4	4	4	4	3	3	4	4
1	1	4	4	1	1	2	2	4	4
3	3	4	4	4	4	2	2	4	4
3	3	4	4	4	4	3	3	4	4
3	3	4	4	1	1	3	3	4	4
1	1	3	3	1	1	2	2	4	4
1	1	3	3	1	1	2	2	4	4
1	1	3	3	4	4	2	2	4	4
3	3	4	4	1	1	3	3	4	4
1	1	4	4	1	1	2	2	4	4
3	3	4	4	4	4	2	2	4	4
1	1	3	3	1	1	2	2	4	4
3	3	4	4	1	1	2	2	4	4
1	1	3	3	1	1	2	2	4	4
3	3	4	4	4	4	2	2	4	4
3	3	4	4	1	1	3	3	4	4
3	3	4	4	1	1	3	3	4	4
1	1	3	3	1	1	2	2	4	4
3	3	4	4	1	1	3	3	4	4
3	3	4	4	4	4	2	2	4	4
1	1	3	3	1	1	2	2	4	4
1	1	3	3	1	1	2	2	4	4
3	3	4	4	1	1	3	3	4	4
3	3	4	4	4	4	3	3	4	4
1	1	3	3	1	1	2	2	4	4
3	3	4	4	1	1	3	3	4	4
1	1	3	3	4	4	2	2	4	4
1	1	3	3	1	1	2	2	1	1
1	1	4	4	1	1	2	2	4	4
1	1	3	3	1	1	2	2	4	4
3	3	4	4	4	4	3	3	4	4
3	3	4	4	1	1	3	3	4	4
1	1	3	3	1	1	2	2	4	4
3	3	4	4	4	4	2	2	4	4
1	1	3	3	1	1	2	2	4	4
3	3	4	4	4	4	3	3	4	4
1	1	3	3	1	1	2	2	4	4
1	1	3	3	1	1	2	2	4	4
3	3	4	4	4	4	3	3	4	4

For Review Only

1	1	3	3	1	1	2	2	1	1
3	3	4	4	1	1	3	3	4	4

For Review Only

SNP627	SNP627	SNP628	SNP628	SNP629	SNP629	SNP630	SNP630	SNP631	SNP631
4	4	2	2	4	4	2	2	2	2
4	4	2	2	4	4	2	2	3	3
4	4	2	2	4	4	3	3	3	3
1	1	2	2	3	3	3	3	2	2
1	1	3	3	3	3	2	2	2	2
1	1	2	2	3	3	3	3	2	2
1	1	2	2	3	3	3	3	2	2
1	1	2	2	3	3	2	2	2	2
1	1	2	2	3	3	2	2	2	2
1	1	2	2	3	3	2	2	2	2
1	1	2	2	3	3	2	2	2	2
4	4	2	2	4	4	3	3	2	2
1	1	2	2	3	3	2	2	2	2
1	1	2	2	3	3	2	2	2	2
4	4	2	2	3	3	2	2	2	2
1	1	2	2	3	3	3	3	2	2
4	4	2	2	4	4	2	2	3	3
1	1	3	3	3	3	2	2	2	2
1	1	2	2	3	3	2	2	2	2
1	1	3	3	3	3	2	2	2	2
1	1	3	3	3	3	2	2	2	2
4	4	2	2	4	4	2	2	3	3
4	4	2	2	3	3	2	2	3	3
1	1	2	2	3	3	2	2	2	2
1	1	3	3	3	3	2	2	2	2
4	4	2	2	4	4	2	2	3	3
4	4	2	2	4	4	2	2	3	3
4	4	2	2	3	3	2	2	2	2
1	1	2	2	3	3	2	2	2	2
1	1	2	2	3	3	2	2	2	2
1	1	2	2	3	3	2	2	2	2
4	4	2	2	4	4	3	3	3	3
1	1	2	2	3	3	2	2	2	2
1	1	2	2	3	3	2	2	2	2
4	4	2	2	3	3	2	2	2	2
4	4	2	2	3	3	2	2	2	2
4	4	2	2	4	4	2	2	3	3
1	1	2	2	3	3	2	2	2	2
1	1	2	2	3	3	2	2	2	2
1	1	2	2	3	3	2	2	2	2
1	1	2	2	3	3	2	2	2	2
1	1	2	2	3	3	2	2	2	2
1	1	2	2	3	3	2	2	2	2
1	1	2	2	3	3	2	2	2	2
1	1	2	2	3	3	2	2	2	2
4	4	2	2	4	4	3	3	3	3
4	4	2	2	4	4	2	2	3	3
1	1	2	2	3	3	2	2	2	2
4	4	2	2	4	4	2	2	3	3
4	4	2	2	3	3	2	2	3	3
4	4	2	2	4	4	2	2	3	3
1	1	2	2	3	3	3	3	2	2

For Review Only

4	4	3	3	4	4	2	2	3	3
1	1	2	2	3	3	2	2	2	2
1	1	2	2	3	3	2	2	2	2
4	4	3	3	4	4	2	2	3	3
1	1	2	2	3	3	2	2	2	2
4	4	2	2	4	4	3	3	3	3
1	1	2	2	3	3	3	3	2	2
1	1	2	2	3	3	2	2	2	2
4	4	2	2	4	4	2	2	3	3
4	4	2	2	3	3	2	2	2	2
1	1	2	2	3	3	2	2	2	2
4	4	2	2	4	4	3	3	3	3
4	4	3	3	4	4	2	2	3	3
4	4	2	2	3	3	2	2	2	2
4	4	2	2	3	3	2	2	2	2
1	1	2	2	3	3	2	2	2	2
1	1	2	2	3	3	2	2	2	2
1	1	3	3	3	3	2	2	2	2
1	1	2	2	3	3	2	2	2	2
1	1	2	2	3	3	2	2	2	2
4	4	2	2	3	3	3	3	2	2
1	1	2	2	3	3	2	2	2	2
1	1	2	2	3	3	2	2	2	2
1	1	2	2	3	3	3	3	2	2
1	1	2	2	3	3	2	2	2	2
4	4	2	2	4	4	3	3	3	3
4	4	2	2	3	3	2	2	2	2
1	1	2	2	3	3	2	2	2	2
1	1	2	2	3	3	2	2	2	2
1	1	2	2	3	3	2	2	2	2
1	1	3	3	3	3	2	2	2	2
1	1	2	2	3	3	2	2	2	2
1	1	2	2	3	3	2	2	2	2
4	4	3	3	4	4	2	2	3	3
1	1	3	3	3	3	2	2	2	2
1	1	2	2	3	3	2	2	2	2
1	1	2	2	3	3	2	2	2	2
4	4	2	2	4	4	2	2	3	3
4	4	2	2	4	4	2	2	3	3
1	1	2	2	3	3	2	2	2	2
4	4	2	2	4	4	2	2	3	3
1	1	2	2	3	3	3	3	2	2
4	4	2	2	4	4	2	2	3	3
1	1	2	2	3	3	2	2	2	2
4	4	2	2	4	4	2	2	3	3
1	1	2	2	3	3	2	2	2	2
1	1	2	2	3	3	2	2	2	2
1	1	2	2	3	3	3	3	2	2
1	1	2	2	3	3	3	3	2	2
1	1	3	3	3	3	2	2	2	2
1	1	2	2	3	3	2	2	2	2
1	1	2	2	3	3	2	2	2	2
1	1	2	2	3	3	2	2	2	2
1	1	2	2	3	3	2	2	2	2
1	1	2	2	3	3	2	2	2	2

For Review Only

1	1	3	3	3	3	2	2	2	2
1	1	2	2	3	3	2	2	2	2
4	4	2	2	4	4	2	2	3	3
1	1	2	2	3	3	2	2	2	2
4	4	2	2	3	3	2	2	3	3
4	4	2	2	4	4	2	2	3	3
1	1	2	2	3	3	3	3	2	2
1	1	2	2	3	3	3	3	2	2
4	4	2	2	3	3	2	2	2	2
4	4	2	2	3	3	2	2	2	2
1	1	2	2	3	3	2	2	2	2
1	1	2	2	3	3	2	2	2	2
4	4	2	2	4	4	3	3	3	3
4	4	2	2	3	3	3	3	2	2
1	1	2	2	3	3	3	3	2	2
4	4	2	2	3	3	2	2	2	2
1	1	2	2	3	3	2	2	2	2
4	4	2	2	3	3	3	3	2	2
1	1	2	2	3	3	2	2	2	2
4	4	2	2	3	3	2	2	3	3
4	4	2	2	3	3	2	2	2	2
1	1	2	2	3	3	2	2	2	2
4	4	2	2	4	4	3	3	2	2
1	1	2	2	3	3	2	2	2	2
1	1	2	2	3	3	2	2	2	2
4	4	2	2	3	3	2	2	2	2
1	1	3	3	3	3	3	3	2	2
1	1	2	2	3	3	2	2	2	2
4	4	2	2	4	4	3	3	3	3
1	1	2	2	3	3	2	2	2	2
4	4	2	2	3	3	2	2	2	2
4	4	2	2	3	3	2	2	2	2
4	4	2	2	4	4	2	2	3	3
1	1	2	2	3	3	2	2	2	2
1	1	2	2	3	3	2	2	2	2
4	4	2	2	3	3	2	2	3	3
1	1	2	2	3	3	2	2	2	2
1	1	2	2	3	3	3	3	2	2
1	1	2	2	3	3	2	2	2	2
1	1	2	2	3	3	2	2	2	2
1	1	2	2	3	3	2	2	2	2
1	1	2	2	3	3	2	2	2	2
1	1	3	3	3	3	2	2	2	2
1	1	2	2	3	3	2	2	2	2
1	1	2	2	3	3	2	2	2	2
1	1	2	2	3	3	2	2	2	2
1	1	3	3	3	3	2	2	2	2
1	1	2	2	3	3	2	2	2	2
1	1	2	2	3	3	2	2	2	2
4	4	2	2	4	4	3	3	3	3
1	1	3	3	3	3	2	2	2	2
1	1	2	2	3	3	2	2	2	2
1	1	2	2	3	3	2	2	2	2
4	4	2	2	4	4	3	3	3	3
1	1	2	2	3	3	2	2	2	2

For Review Only

1	1	2	2	3	3	2	2	2	2
1	1	2	2	3	3	2	2	2	2
1	1	2	2	3	3	2	2	2	2
1	1	2	2	3	3	2	2	2	2
1	1	3	3	3	3	2	2	2	2
1	1	2	2	3	3	2	2	2	2
1	1	2	2	3	3	2	2	2	2
4	4	2	2	3	3	2	2	2	2
4	4	2	2	3	3	2	2	3	3
4	4	2	2	4	4	2	2	3	3
1	1	2	2	3	3	2	2	2	2
1	1	2	2	3	3	2	2	2	2
1	1	3	3	3	3	3	3	2	2
1	1	2	2	3	3	2	2	2	2
4	4	2	2	3	3	2	2	2	2
1	1	2	2	3	3	2	2	2	2
1	1	2	2	3	3	2	2	2	2
1	1	2	2	3	3	2	2	2	2
4	4	2	2	4	4	2	2	3	3
1	1	2	2	3	3	2	2	2	2
4	4	2	2	3	3	2	2	2	2
1	1	2	2	3	3	2	2	2	2
1	1	2	2	3	3	2	2	2	2
4	4	2	2	4	4	3	3	3	3
1	1	2	2	3	3	2	2	2	2
1	1	2	2	3	3	2	2	2	2
4	4	2	2	3	3	3	3	2	2
1	1	2	2	3	3	2	2	2	2
4	4	2	2	4	4	2	2	3	3
4	4	3	3	4	4	2	2	3	3
1	1	2	2	3	3	2	2	2	2
1	1	2	2	3	3	2	2	2	2
1	1	3	3	3	3	2	2	2	2
4	4	2	2	3	3	2	2	2	2
1	1	2	2	3	3	2	2	2	2
4	4	2	2	4	4	3	3	3	3
1	1	2	2	3	3	3	3	2	2
4	4	2	2	4	4	2	2	3	3
1	1	2	2	3	3	2	2	2	2
1	1	2	2	3	3	3	3	2	2
4	4	2	2	4	4	3	3	3	3
1	1	3	3	3	3	2	2	2	2
1	1	2	2	3	3	2	2	2	2
4	4	2	2	3	3	2	2	2	2
4	4	2	2	4	4	2	2	2	2
4	4	2	2	3	3	3	3	2	2
1	1	2	2	3	3	2	2	2	2
1	1	2	2	3	3	2	2	2	2
1	1	2	2	3	3	2	2	2	2
4	4	2	2	4	4	3	3	3	3
1	1	2	2	3	3	3	3	2	2
1	1	2	2	3	3	2	2	2	2
1	1	2	2	3	3	2	2	2	2
1	1	2	2	3	3	2	2	2	2
4	4	2	2	3	3	2	2	3	3

For Review Only

1	1	3	3	3	3	2	2	2	2
1	1	2	2	3	3	2	2	2	2
1	1	2	2	3	3	2	2	2	2
4	4	2	2	3	3	2	2	2	2
1	1	2	2	3	3	2	2	2	2
4	4	2	2	4	4	2	2	3	3
4	4	2	2	3	3	2	2	2	2
1	1	2	2	3	3	2	2	2	2
1	1	2	2	3	3	3	3	2	2
4	4	2	2	3	3	2	2	3	3
4	4	2	2	3	3	2	2	2	2
1	1	2	2	3	3	2	2	2	2
4	4	2	2	4	4	2	2	3	3
4	4	2	2	4	4	2	2	3	3
4	4	3	3	4	4	3	3	3	3
4	4	2	2	4	4	2	2	3	3
4	4	2	2	3	3	2	2	2	2
1	1	3	3	3	3	2	2	2	2
4	4	2	2	4	4	2	2	3	3
1	1	2	2	3	3	2	2	2	2
1	1	2	2	3	3	2	2	2	2
1	1	2	2	3	3	2	2	2	2
4	4	2	2	4	4	2	2	3	3
1	1	2	2	3	3	2	2	2	2
1	1	2	2	3	3	2	2	2	2
4	4	3	3	3	3	3	3	3	3
1	1	2	2	3	3	2	2	2	2
4	4	2	2	3	3	3	3	3	3
4	4	3	3	4	4	2	2	3	3
4	4	2	2	4	4	3	3	3	3
4	4	2	2	3	3	2	2	3	3
1	1	2	2	3	3	2	2	2	2
4	4	2	2	4	4	2	2	3	3
1	1	2	2	3	3	3	3	2	2
1	1	2	2	3	3	2	2	2	2
1	1	2	2	3	3	2	2	2	2
4	4	3	3	4	4	2	2	3	3
4	4	2	2	4	4	3	3	3	3
1	1	2	2	3	3	2	2	2	2
1	1	2	2	3	3	2	2	2	2
1	1	2	2	3	3	2	2	2	2
1	1	3	3	3	3	2	2	2	2
1	1	2	2	3	3	2	2	2	2
4	4	3	3	4	4	2	2	3	3
4	4	2	2	4	4	3	3	3	3
4	4	2	2	4	4	2	2	3	3
1	1	2	2	3	3	2	2	2	2
1	1	2	2	3	3	2	2	2	2
4	4	2	2	4	4	2	2	3	3
1	1	2	2	3	3	2	2	2	2
4	4	2	2	4	4	2	2	2	2
1	1	2	2	3	3	2	2	2	2
4	4	2	2	4	4	3	3	3	3
4	4	2	2	4	4	3	3	3	3
1	1	2	2	3	3	2	2	2	2

For Review Only

4	4	2	2	4	4	2	2	3	3
1	1	2	2	3	3	3	3	2	2

For Review Only

SNP632	SNP632	SNP633	SNP633	SNP634	SNP634	SNP635	SNP635	SNP636	SNP636
4	4	2	2	4	4	3	3	3	3
4	4	2	2	2	2	3	3	3	3
4	4	4	4	2	2	3	3	3	3
4	4	2	2	4	4	3	3	3	3
3	3	2	2	4	4	1	1	3	3
4	4	4	4	2	2	3	3	3	3
4	4	2	2	4	4	3	3	3	3
3	3	2	2	2	2	3	3	3	3
4	4	2	2	2	2	3	3	3	3
3	3	2	2	2	2	1	1	1	1
4	4	2	2	2	2	3	3	1	1
4	4	4	4	4	4	3	3	3	3
4	4	2	2	4	4	3	3	3	3
4	4	2	2	2	2	3	3	3	3
4	4	2	2	4	4	3	3	3	3
4	4	2	2	2	2	1	1	3	3
4	4	2	2	2	2	1	1	3	3
4	4	2	2	4	4	3	3	3	3
4	4	4	4	4	4	3	3	3	3
3	3	2	2	4	4	3	3	3	3
3	3	2	2	4	4	1	1	3	3
4	4	2	2	2	2	1	1	3	3
4	4	2	2	2	2	1	1	1	1
4	4	2	2	2	2	3	3	3	3
4	4	4	4	4	4	3	3	3	3
4	4	2	2	2	2	1	1	3	3
4	4	2	2	2	2	3	3	3	3
4	4	2	2	2	2	3	3	3	3
3	3	2	2	2	2	1	1	3	3
3	3	2	2	2	2	1	1	3	3
4	4	4	4	2	2	3	3	3	3
4	4	2	2	2	2	3	3	3	3
4	4	2	2	2	2	3	3	3	3
4	4	2	2	2	2	1	1	3	3
4	4	2	2	2	2	3	3	1	1
3	3	2	2	4	4	3	3	3	3
4	4	2	2	2	2	3	3	3	3
4	4	2	2	4	4	3	3	3	3
4	4	2	2	2	2	1	1	1	1
4	4	2	2	2	2	3	3	1	1
3	3	2	2	2	2	1	1	3	3
3	3	2	2	2	2	1	1	3	3
4	4	2	2	2	2	3	3	3	3
4	4	2	2	2	2	3	3	3	3
4	4	4	4	2	2	3	3	1	1
4	4	4	4	4	4	3	3	3	3
4	4	2	2	2	2	1	1	3	3
4	4	2	2	2	2	1	1	1	1
4	4	2	2	2	2	1	1	3	3
4	4	2	2	4	4	3	3	3	3
4	4	2	2	4	4	1	1	3	3
4	4	4	4	4	4	3	3	3	3

For Review Only

4	4	2	2	4	4	3	3	3	3
4	4	2	2	2	2	3	3	3	3
4	4	2	2	2	2	3	3	3	3
4	4	2	2	4	4	3	3	3	3
4	4	4	4	4	4	3	3	3	3
4	4	4	4	2	2	1	1	3	3
3	3	4	4	2	2	3	3	3	3
4	4	2	2	2	2	1	1	3	3
4	4	2	2	2	2	3	3	3	3
4	4	2	2	2	2	3	3	3	3
4	4	4	4	2	2	3	3	3	3
4	4	4	4	2	2	3	3	3	3
4	4	2	2	4	4	3	3	3	3
3	3	2	2	2	2	1	1	3	3
4	4	2	2	4	4	3	3	3	3
4	4	2	2	2	2	3	3	3	3
4	4	2	2	2	2	3	3	1	1
4	4	2	2	4	4	3	3	3	3
4	4	4	4	2	2	3	3	3	3
4	4	2	2	2	2	3	3	3	3
4	4	2	2	2	2	3	3	3	3
4	4	2	2	2	2	1	1	1	1
3	3	2	2	2	2	1	1	3	3
4	4	4	4	2	2	1	1	3	3
4	4	4	4	2	2	3	3	1	1
4	4	4	4	4	4	3	3	3	3
4	4	2	2	2	2	3	3	3	3
4	4	2	2	2	2	1	1	1	1
3	3	2	2	2	2	1	1	3	3
4	4	2	2	4	4	3	3	3	3
3	3	2	2	4	4	3	3	3	3
4	4	2	2	2	2	3	3	3	3
4	4	2	2	2	2	1	1	1	1
4	4	2	2	4	4	3	3	3	3
4	4	2	2	4	4	1	1	3	3
4	4	4	4	2	2	3	3	1	1
3	3	2	2	2	2	3	3	3	3
4	4	2	2	2	2	1	1	3	3
4	4	2	2	2	2	3	3	1	1
4	4	2	2	2	2	3	3	3	3
4	4	2	2	4	4	3	3	3	3
4	4	4	4	2	2	3	3	3	3
4	4	2	2	4	4	3	3	3	3
4	4	4	4	2	2	3	3	3	3
4	4	4	4	4	4	3	3	3	3
4	4	2	2	2	2	3	3	3	3
4	4	2	2	2	2	1	1	3	3
3	3	4	4	2	2	3	3	3	3
3	3	2	2	4	4	1	1	3	3
4	4	2	2	4	4	3	3	3	3
3	3	2	2	2	2	3	3	3	3
4	4	2	2	2	2	1	1	1	1
4	4	2	2	2	2	1	1	3	3
4	4	2	2	2	2	3	3	3	3
4	4	2	2	2	2	3	3	3	3

For Review Only

4	4	2	2	4	4	1	1	3	3
4	4	2	2	4	4	3	3	3	3
4	4	2	2	2	2	3	3	3	3
4	4	2	2	2	2	1	1	3	3
4	4	2	2	2	2	3	3	3	3
4	4	2	2	2	2	1	1	3	3
4	4	2	2	2	2	3	3	3	3
4	4	4	4	4	4	3	3	3	3
4	4	2	2	2	2	1	1	3	3
4	4	2	2	4	4	3	3	1	1
4	4	2	2	4	4	3	3	3	3
4	4	2	2	2	2	3	3	3	3
4	4	2	2	2	2	3	3	1	1
4	4	4	4	4	4	3	3	3	3
4	4	2	2	2	2	3	3	3	3
4	4	2	2	4	4	3	3	3	3
4	4	2	2	2	2	3	3	3	3
4	4	2	2	4	4	1	1	3	3
4	4	2	2	2	2	1	1	3	3
4	4	2	2	2	2	3	3	1	1
4	4	2	2	4	4	3	3	3	3
4	4	2	2	2	2	3	3	3	3
4	4	4	4	2	2	3	3	3	3
4	4	2	2	2	2	1	1	1	1
4	4	2	2	2	2	1	1	3	3
4	4	2	2	2	2	3	3	1	1
4	4	2	2	4	4	3	3	3	3
4	4	2	2	2	2	3	3	3	3
4	4	4	4	4	4	3	3	3	3
3	3	2	2	4	4	1	1	3	3
4	4	2	2	2	2	1	1	3	3
4	4	2	2	4	4	3	3	3	3
4	4	2	2	4	4	3	3	3	3
4	4	2	2	4	4	3	3	3	3
4	4	2	2	2	2	1	1	3	3
4	4	2	2	4	4	3	3	3	3
4	4	2	2	2	2	1	1	3	3
4	4	4	4	2	2	3	3	3	3
4	4	2	2	2	2	3	3	1	1
4	4	2	2	2	2	1	1	1	1
4	4	2	2	2	2	3	3	3	3
4	4	2	2	2	2	1	1	3	3
4	4	4	4	4	4	3	3	3	3
4	4	2	2	2	2	3	3	1	1
3	3	2	2	2	2	3	3	3	3
4	4	2	2	2	2	3	3	3	3
4	4	2	2	4	4	1	1	3	3
4	4	4	4	2	2	3	3	3	3
4	4	2	2	2	2	3	3	3	3
4	4	2	2	2	2	1	1	3	3
3	3	2	2	4	4	3	3	3	3
4	4	2	2	2	2	1	1	3	3
4	4	2	2	4	4	3	3	3	3
4	4	4	4	2	2	3	3	3	3
4	4	2	2	2	2	3	3	3	3

For Review Only

3	3	2	2	2	2	3	3	3	3
4	4	2	2	2	2	3	3	1	1
4	4	2	2	4	4	1	1	3	3
4	4	2	2	2	2	1	1	3	3
4	4	2	2	4	4	3	3	3	3
4	4	2	2	2	2	1	1	1	1
4	4	2	2	2	2	3	3	1	1
4	4	2	2	4	4	3	3	3	3
4	4	2	2	2	2	3	3	1	1
4	4	4	4	2	2	3	3	3	3
4	4	2	2	4	4	3	3	3	3
4	4	2	2	2	2	3	3	3	3
4	4	4	4	4	4	3	3	3	3
4	4	2	2	2	2	3	3	3	3
4	4	2	2	2	2	1	1	3	3
4	4	2	2	2	2	3	3	1	1
4	4	2	2	4	4	1	1	3	3
4	4	2	2	2	2	3	3	3	3
4	4	2	2	2	2	3	3	3	3
4	4	2	2	2	2	3	3	3	3
4	4	2	2	2	2	3	3	3	3
4	4	2	2	2	2	3	3	3	3
4	4	2	2	2	2	1	1	1	1
4	4	4	4	2	2	3	3	3	3
3	3	2	2	2	2	3	3	3	3
4	4	2	2	2	2	1	1	3	3
4	4	4	4	4	4	3	3	3	3
4	4	2	2	2	2	3	3	3	3
4	4	2	2	4	4	3	3	3	3
4	4	4	4	4	4	3	3	3	3
4	4	2	2	2	2	3	3	3	3
4	4	2	2	4	4	3	3	3	3
4	4	2	2	4	4	1	1	3	3
4	4	2	2	2	2	1	1	3	3
4	4	2	2	2	2	1	1	3	3
4	4	4	4	2	2	3	3	3	3
4	4	2	2	4	4	3	3	3	3
4	4	2	2	2	2	3	3	3	3
4	4	4	4	2	2	3	3	3	3
4	4	4	4	4	4	3	3	3	3
4	4	4	4	2	2	3	3	3	3
3	3	2	2	4	4	1	1	3	3
4	4	2	2	2	2	1	1	1	1
4	4	2	2	2	2	3	3	3	3
4	4	2	2	4	4	1	1	3	3
4	4	2	2	4	4	1	1	3	3
4	4	2	2	2	2	3	3	1	1
4	4	2	2	2	2	1	1	1	1
4	4	2	2	2	2	1	1	3	3
4	4	4	4	4	4	3	3	3	3
4	4	4	4	2	2	3	3	3	3
4	4	2	2	2	2	1	1	3	3
4	4	2	2	2	2	1	1	1	1
4	4	4	4	2	2	3	3	3	3
4	4	2	2	2	2	1	1	1	1

For Review Only

4	4	2	2	4	4	3	3	3	3
3	3	2	2	2	2	1	1	3	3
4	4	2	2	2	2	3	3	3	3
4	4	2	2	2	2	3	3	1	1
4	4	2	2	2	2	3	3	3	3
4	4	2	2	2	2	1	1	3	3
4	4	2	2	2	2	1	1	1	1
3	3	2	2	2	2	3	3	3	3
4	4	2	2	2	2	1	1	3	3
4	4	2	2	2	2	1	1	1	1
4	4	2	2	4	4	3	3	3	3
4	4	2	2	2	2	3	3	1	1
4	4	2	2	2	2	3	3	1	1
4	4	4	4	2	2	1	1	3	3
4	4	4	4	4	4	3	3	3	3
4	4	2	2	4	4	3	3	3	3
4	4	2	2	2	2	1	1	1	1
4	4	2	2	2	2	3	3	1	1
4	4	2	2	2	2	1	1	1	1
4	4	2	2	2	2	3	3	3	3
4	4	4	4	2	2	3	3	3	3
3	3	2	2	2	2	1	1	3	3
4	4	2	2	2	2	3	3	3	3
4	4	2	2	2	2	3	3	1	1
4	4	2	2	2	2	1	1	3	3
4	4	4	4	4	4	3	3	3	3
4	4	2	2	2	2	1	1	1	1
4	4	4	4	4	4	3	3	3	3
4	4	2	2	4	4	3	3	3	3
4	4	4	4	2	2	3	3	3	3
4	4	2	2	2	2	3	3	1	1
3	3	2	2	2	2	3	3	1	1
4	4	2	2	2	2	3	3	1	1
4	4	4	4	2	2	1	1	3	3
4	4	2	2	2	2	1	1	1	1
4	4	2	2	2	2	1	1	1	1
4	4	2	2	4	4	3	3	3	3
4	4	4	4	2	2	3	3	3	3
3	3	2	2	4	4	3	3	3	3
4	4	2	2	2	2	1	1	1	1
4	4	2	2	2	2	1	1	3	3
4	4	2	2	4	4	1	1	3	3
4	4	2	2	2	2	3	3	3	3
4	4	2	2	4	4	3	3	3	3
4	4	2	2	2	2	3	3	3	3
4	4	2	2	2	2	3	3	3	3
4	4	2	2	2	2	3	3	3	3
4	4	2	2	2	2	3	3	1	1
4	4	2	2	2	2	3	3	3	3
4	4	2	2	2	2	1	1	1	1
4	4	2	2	4	4	3	3	3	3
4	4	2	2	2	2	1	1	1	1
4	4	4	4	2	2	3	3	3	3
4	4	4	4	4	4	3	3	3	3
4	4	2	2	2	2	3	3	1	1

For Review Only

4	4	2	2	4	4	3	3	3	3
4	4	2	2	2	2	3	3	3	3

For Review Only

SNP637	SNP637	SNP638	SNP638	SNP639	SNP639	SNP640	SNP640	SNP641	SNP641
1	1	3	3	4	4	4	4	4	4
1	1	3	3	4	4	4	4	4	4
1	1	3	3	4	4	4	4	4	4
1	1	3	3	4	4	4	4	4	4
4	4	4	4	3	3	2	2	1	1
1	1	3	3	4	4	4	4	4	4
1	1	3	3	4	4	4	4	1	1
1	1	3	3	4	4	4	4	4	4
1	1	3	3	4	4	4	4	4	4
4	4	4	4	4	4	4	4	4	4
1	1	3	3	4	4	4	4	1	1
1	1	3	3	4	4	4	4	4	4
1	1	4	4	4	4	4	4	1	1
1	1	3	3	3	3	2	2	4	4
1	1	3	3	4	4	4	4	1	1
1	1	4	4	4	4	4	4	1	1
4	4	4	4	4	4	4	4	1	1
1	1	3	3	4	4	4	4	1	1
1	1	3	3	3	3	2	2	1	1
1	1	3	3	3	3	2	2	1	1
4	4	4	4	3	3	2	2	1	1
4	4	4	4	4	4	4	4	1	1
4	4	4	4	4	4	4	4	1	1
1	1	3	3	4	4	4	4	1	1
1	1	3	3	3	3	2	2	4	4
4	4	4	4	4	4	4	4	4	4
1	1	3	3	4	4	4	4	4	4
1	1	3	3	4	4	4	4	1	1
4	4	4	4	4	4	4	4	1	1
4	4	4	4	4	4	4	4	1	1
1	1	3	3	4	4	4	4	4	4
1	1	3	3	4	4	4	4	1	1
1	1	3	3	4	4	4	4	1	1
4	4	4	4	4	4	4	4	1	1
1	1	4	4	4	4	4	4	1	1
1	1	3	3	3	3	2	2	1	1
1	1	3	3	4	4	4	4	1	1
1	1	3	3	4	4	4	4	4	4
4	4	4	4	4	4	4	4	1	1
1	1	3	3	4	4	4	4	1	1
4	4	4	4	4	4	4	4	4	4
1	1	4	4	4	4	4	4	1	1
1	1	3	3	4	4	4	4	4	4
1	1	3	3	4	4	4	4	4	4
1	1	3	3	4	4	4	4	4	4
1	1	3	3	4	4	4	4	4	4
4	4	4	4	4	4	4	4	1	1
4	4	4	4	4	4	4	4	4	4
4	4	4	4	4	4	4	4	4	4
1	1	3	3	4	4	4	4	1	1
4	4	4	4	3	3	2	2	1	1
1	1	3	3	4	4	4	4	1	1

For Review Only

1	1	3	3	3	3	2	2	1	1
1	1	3	3	4	4	4	4	1	1
1	1	3	3	3	3	2	2	4	4
1	1	3	3	3	3	2	2	1	1
1	1	3	3	3	3	2	2	1	1
4	4	4	4	3	3	2	2	4	4
1	1	3	3	4	4	4	4	1	1
4	4	4	4	4	4	4	4	1	1
1	1	3	3	4	4	4	4	1	1
1	1	3	3	4	4	4	4	1	1
1	1	3	3	3	3	2	2	4	4
1	1	3	3	3	3	2	2	4	4
1	1	3	3	3	3	2	2	1	1
4	4	4	4	4	4	4	4	1	1
1	1	3	3	4	4	4	4	4	4
1	1	3	3	4	4	4	4	4	4
1	1	3	3	4	4	4	4	1	1
1	1	3	3	3	3	2	2	1	1
1	1	3	3	4	4	4	4	1	1
1	1	3	3	4	4	4	4	4	4
1	1	3	3	4	4	2	2	4	4
4	4	4	4	4	4	4	4	1	1
1	1	4	4	4	4	4	4	1	1
1	1	4	4	4	4	4	4	4	4
1	1	3	3	4	4	4	4	4	4
1	1	3	3	3	3	2	2	4	4
1	1	3	3	4	4	4	4	1	1
4	4	4	4	4	4	4	4	1	1
4	4	4	4	4	4	4	4	4	4
1	1	3	3	3	3	2	2	4	4
1	1	3	3	3	3	4	4	1	1
1	1	3	3	4	4	4	4	1	1
4	4	4	4	4	4	4	4	1	1
1	1	3	3	3	3	2	2	1	1
4	4	4	4	4	4	4	4	4	4
1	1	3	3	4	4	4	4	4	4
1	1	3	3	4	4	4	4	4	4
4	4	4	4	4	4	4	4	1	1
1	1	3	3	4	4	4	4	1	1
1	1	3	3	4	4	4	4	4	4
1	1	3	3	4	4	4	4	1	1
1	1	3	3	4	4	4	4	4	4
1	1	3	3	3	3	4	4	1	1
1	1	3	3	3	3	2	2	4	4
1	1	3	3	4	4	4	4	1	1
1	1	3	3	4	4	4	4	1	1
4	4	4	4	4	4	4	4	1	1
1	1	3	3	4	4	4	4	1	1
1	1	4	4	4	4	4	4	1	1
1	1	3	3	3	3	2	2	4	4
1	1	3	3	4	4	4	4	1	1
4	4	4	4	4	4	4	4	1	1
4	4	4	4	4	4	4	4	1	1
1	1	3	3	4	4	4	4	1	1
1	1	3	3	4	4	4	4	4	4

For Review Only

4	4	4	4	4	4	4	4	1	1
1	1	3	3	4	4	4	4	1	1
1	1	3	3	4	4	4	4	1	1
4	4	4	4	4	4	4	4	1	1
1	1	3	3	4	4	4	4	1	1
4	4	4	4	4	4	4	4	4	4
1	1	3	3	4	4	4	4	4	4
1	1	3	3	4	4	4	4	1	1
4	4	4	4	4	4	4	4	1	1
1	1	3	3	4	4	4	4	1	1
1	1	3	3	4	4	4	4	1	1
1	1	3	3	4	4	4	4	1	1
1	1	3	3	4	4	4	4	1	1
1	1	3	3	4	4	2	2	1	1
1	1	3	3	4	4	4	4	1	1
1	1	3	3	4	4	4	4	1	1
1	1	3	3	4	4	4	4	1	1
1	1	3	3	4	4	4	4	4	4
4	4	4	4	4	4	4	4	1	1
1	1	3	3	4	4	4	4	1	1
1	1	3	3	4	4	4	4	1	1
1	1	3	3	4	4	4	4	1	1
1	1	3	3	4	4	4	4	4	4
4	4	4	4	4	4	4	4	1	1
4	4	4	4	4	4	4	4	1	1
1	1	3	3	4	4	4	4	1	1
1	1	4	4	3	3	2	2	1	1
1	1	3	3	4	4	4	4	1	1
1	1	3	3	4	4	4	4	1	1
1	1	4	4	3	3	2	2	4	4
4	4	4	4	4	4	4	4	1	1
1	1	3	3	4	4	4	4	1	1
1	1	3	3	4	4	4	4	1	1
1	1	4	4	4	4	4	4	1	1
4	4	4	4	4	4	4	4	1	1
1	1	3	3	3	3	4	4	1	1
4	4	4	4	4	4	4	4	1	1
1	1	3	3	4	4	2	2	4	4
1	1	3	3	4	4	4	4	1	1
4	4	4	4	4	4	4	4	1	1
1	1	3	3	4	4	4	4	1	1
4	4	4	4	4	4	4	4	1	1
1	1	3	3	3	3	2	2	4	4
1	1	3	3	4	4	4	4	1	1
1	1	3	3	4	4	4	4	1	1
1	1	3	3	4	4	2	2	1	1
4	4	4	4	3	3	2	2	4	4
1	1	3	3	3	3	2	2	1	1
1	1	3	3	4	4	4	4	1	1
4	4	4	4	4	4	4	4	4	4
1	1	3	3	3	3	2	2	4	4
4	4	4	4	4	4	4	4	1	1
1	1	3	3	4	4	4	4	4	4
1	1	3	3	3	3	2	2	4	4
1	1	4	4	4	4	4	4	1	1

For Review Only

1	1	3	3	4	4	4	4	1	1
1	1	3	3	4	4	4	4	1	1
1	1	4	4	4	4	4	4	4	4
4	4	4	4	4	4	4	4	1	1
1	1	3	3	3	3	2	2	4	4
4	4	4	4	4	4	4	4	1	1
1	1	3	3	4	4	4	4	1	1
1	1	3	3	4	4	4	4	1	1
1	1	3	3	4	4	4	4	1	1
1	1	3	3	4	4	4	4	1	1
1	1	4	4	4	4	4	4	4	4
1	1	3	3	4	4	4	4	1	1
1	1	3	3	3	3	2	2	1	1
1	1	3	3	4	4	4	4	1	1
4	4	4	4	4	4	4	4	1	1
1	1	3	3	4	4	4	4	1	1
1	1	3	3	4	4	4	4	4	4
1	1	3	3	3	3	2	2	4	4
1	1	3	3	4	4	4	4	1	1
1	1	3	3	4	4	4	4	1	1
1	1	3	3	4	4	4	4	1	1
1	1	3	3	4	4	4	4	1	1
4	4	4	4	4	4	4	4	1	1
1	1	3	3	3	3	2	2	4	4
1	1	3	3	4	4	4	4	1	1
4	4	4	4	4	4	4	4	1	1
1	1	3	3	3	3	2	2	4	4
1	1	3	3	4	4	4	4	1	1
1	1	3	3	4	4	4	4	1	1
1	1	3	3	3	3	2	2	1	1
1	1	3	3	3	3	2	2	4	4
1	1	3	3	4	4	4	4	1	1
4	4	4	4	4	4	4	4	1	1
4	4	4	4	4	4	4	4	1	1
1	1	4	4	3	3	2	2	1	1
1	1	3	3	3	3	2	2	4	4
1	1	3	3	3	3	2	2	4	4
1	1	3	3	4	4	4	4	1	1
1	1	3	3	4	4	4	4	1	1
1	1	3	3	3	3	2	2	4	4
1	1	3	3	4	4	4	4	4	4
4	4	4	4	3	3	2	2	4	4
4	4	4	4	4	4	4	4	1	1
1	1	3	3	4	4	4	4	1	1
1	1	4	4	4	4	4	4	1	1
4	4	4	4	4	4	4	4	1	1
1	1	3	3	4	4	4	4	1	1
1	1	4	4	4	4	4	4	1	1
4	4	4	4	4	4	4	4	1	1
1	1	3	3	4	4	4	4	1	1
1	1	3	3	4	4	4	4	1	1
4	4	4	4	4	4	4	4	1	1
4	4	4	4	4	4	4	4	1	1
1	1	3	3	3	3	2	2	4	4
4	4	4	4	4	4	4	4	1	1

For Review Only

1	1	3	3	4	4	4	4	4	4
1	1	4	4	4	4	4	4	1	1
1	1	3	3	4	4	4	4	1	1
1	1	3	3	4	4	4	4	1	1
1	1	3	3	4	4	4	4	1	1
1	1	4	4	4	4	4	4	1	1
4	4	4	4	4	4	4	4	1	1
1	1	3	3	4	4	4	4	1	1
4	4	4	4	4	4	4	4	1	1
4	4	4	4	4	4	4	4	1	1
1	1	3	3	4	4	4	4	1	1
1	1	3	3	4	4	4	4	1	1
1	1	3	3	4	4	4	4	1	1
4	4	4	4	4	4	4	4	1	1
1	1	3	3	3	3	2	2	4	4
1	1	3	3	3	3	2	2	1	1
4	4	4	4	4	4	4	4	1	1
1	1	3	3	4	4	4	4	1	1
4	4	4	4	4	4	4	4	1	1
1	1	3	3	4	4	4	4	4	4
1	1	3	3	4	4	4	4	4	4
4	4	4	4	4	4	4	4	4	4
1	1	3	3	4	4	4	4	1	1
1	1	3	3	4	4	4	4	4	4
4	4	4	4	4	4	4	4	1	1
1	1	3	3	4	4	4	4	1	1
4	4	4	4	4	4	4	4	1	1
1	1	3	3	3	3	2	2	4	4
1	1	3	3	4	4	4	4	1	1
1	1	3	3	4	4	4	4	1	1
1	1	3	3	4	4	4	4	1	1
1	1	3	3	4	4	4	4	1	1
1	1	3	3	4	4	4	4	1	1
1	1	4	4	3	3	2	2	1	1
4	4	4	4	4	4	4	4	1	1
4	4	4	4	4	4	4	4	1	1
1	1	3	3	3	3	2	2	4	4
1	1	3	3	3	3	2	2	4	4
1	1	3	3	3	3	2	2	1	1
4	4	4	4	4	4	4	4	1	1
1	1	4	4	3	3	2	2	1	1
1	1	4	4	3	3	2	2	4	4
1	1	3	3	3	3	2	2	1	1
1	1	3	3	3	3	2	2	1	1
1	1	3	3	3	3	2	2	1	1
1	1	3	3	4	4	4	4	1	1
1	1	3	3	4	4	4	4	1	1
1	1	3	3	4	4	4	4	1	1
1	1	3	3	4	4	4	4	4	4
4	4	4	4	4	4	4	4	1	1
1	1	3	3	4	4	4	4	1	1
4	4	4	4	4	4	4	4	1	1
1	1	3	3	4	4	4	4	4	4
1	1	3	3	3	3	2	2	4	4
1	1	3	3	4	4	4	4	4	4

For Review Only

1	1	3	3	3	3	2	2	1	1
1	1	3	3	4	4	4	4	1	1

For Review Only

SNP642	SNP642	SNP643	SNP643	SNP644	SNP644	SNP645	SNP645	SNP646	SNP646
1	1	1	1	2	2	2	2	3	3
1	1	1	1	2	2	2	2	3	3
1	1	1	1	2	2	2	2	3	3
1	1	1	1	2	2	2	2	3	3
1	1	1	1	2	2	2	2	2	2
1	1	1	1	2	2	2	2	3	3
1	1	1	1	2	2	2	2	2	2
1	1	1	1	1	1	2	2	2	2
1	1	1	1	2	2	2	2	2	2
1	1	1	1	2	2	2	2	2	2
4	4	4	4	2	2	2	2	2	2
1	1	1	1	2	2	2	2	3	3
1	1	1	1	2	2	2	2	2	2
1	1	1	1	2	2	2	2	2	2
1	1	1	1	2	2	2	2	2	2
1	1	1	1	2	2	2	2	3	3
1	1	1	1	2	2	2	2	2	2
1	1	1	1	2	2	2	2	2	2
1	1	1	1	2	2	3	3	2	2
1	1	1	1	2	2	2	2	2	2
1	1	1	1	2	2	2	2	2	2
1	1	1	1	2	2	2	2	2	2
4	4	4	4	2	2	2	2	2	2
1	1	1	1	1	1	2	2	2	2
1	1	1	1	2	2	2	2	3	3
1	1	1	1	2	2	2	2	3	3
1	1	1	1	2	2	2	2	3	3
1	1	4	4	2	2	2	2	2	2
1	1	1	1	2	2	2	2	2	2
1	1	1	1	1	1	2	2	2	2
1	1	1	1	2	2	2	2	3	3
1	1	1	1	2	2	2	2	2	2
1	1	1	1	2	2	2	2	2	2
1	1	1	1	1	1	2	2	2	2
1	1	4	4	2	2	2	2	2	2
1	1	1	1	2	2	3	3	3	3
1	1	1	1	2	2	2	2	2	2
1	1	1	1	2	2	2	2	3	3
4	4	4	4	2	2	2	2	2	2
4	4	4	4	2	2	2	2	2	2
1	1	4	4	2	2	2	2	2	2
1	1	1	1	2	2	2	2	2	2
1	1	1	1	2	2	2	2	2	2
1	1	1	1	2	2	2	2	2	2
1	1	1	1	2	2	2	2	3	3
1	1	1	1	2	2	2	2	3	3
1	1	1	1	2	2	2	2	2	2
4	4	4	4	2	2	2	2	2	2
1	1	1	1	2	2	2	2	2	2
1	1	1	1	2	2	2	2	2	2
1	1	1	1	1	1	2	2	2	2
1	1	4	4	2	2	2	2	3	3

For Review Only

1	1	1	1	2	2	2	2	2	2
1	1	1	1	2	2	2	2	2	2
1	1	1	1	2	2	2	2	2	2
1	1	1	1	2	2	2	2	2	2
1	1	1	1	2	2	3	3	2	2
1	1	1	1	2	2	2	2	3	3
1	1	1	1	2	2	2	2	2	2
1	1	1	1	1	1	2	2	2	2
1	1	1	1	2	2	2	2	2	2
1	1	1	1	2	2	2	2	2	2
1	1	1	1	2	2	2	2	3	3
1	1	1	1	2	2	2	2	3	3
1	1	1	1	2	2	2	2	2	2
1	1	1	1	2	2	2	2	2	2
1	1	1	1	2	2	2	2	2	2
1	1	4	4	2	2	2	2	2	2
4	4	4	4	2	2	2	2	2	2
1	1	1	1	2	2	2	2	2	2
1	1	1	1	2	2	2	2	2	2
1	1	1	1	2	2	2	2	3	3
1	1	1	1	2	2	2	2	2	2
4	4	4	4	2	2	2	2	2	2
1	1	1	1	2	2	2	2	2	2
1	1	1	1	2	2	2	2	3	3
1	1	1	1	2	2	2	2	3	3
1	1	1	1	1	1	2	2	2	2
1	1	4	4	2	2	2	2	2	2
1	1	1	1	2	2	2	2	3	3
1	1	1	1	2	2	2	2	3	3
1	1	1	1	2	2	2	2	3	3
1	1	1	1	2	2	2	2	2	2
1	1	1	1	1	1	2	2	2	2
4	4	4	4	2	2	2	2	2	2
1	1	1	1	2	2	2	2	2	2
1	1	1	1	2	2	2	2	2	2
1	1	1	1	2	2	2	2	3	3
1	1	1	1	2	2	2	2	2	2
1	1	1	1	2	2	2	2	2	2
1	1	1	1	1	1	2	2	2	2
1	1	1	1	2	2	2	2	3	3
1	1	1	1	2	2	2	2	2	2
1	1	1	1	2	2	2	2	3	3
1	1	1	1	1	1	2	2	2	2
1	1	1	1	2	2	2	2	3	3
1	1	1	1	1	1	2	2	2	2
1	1	1	1	2	2	2	2	2	2
1	1	1	1	2	2	2	2	2	2
1	1	1	1	2	2	2	2	2	2
1	1	1	1	2	2	2	2	2	2
1	1	1	1	2	2	2	2	2	2
1	1	1	1	2	2	2	2	3	3
4	4	4	4	2	2	2	2	2	2
1	1	1	1	1	1	2	2	2	2
1	1	1	1	2	2	2	2	2	2
1	1	1	1	2	2	2	2	2	2

For Review Only

1	1	1	1	2	2	2	2	2	2
1	1	1	1	2	2	2	2	2	2
1	1	1	1	2	2	2	2	3	3
1	1	1	1	2	2	2	2	2	2
1	1	1	1	2	2	2	2	2	2
1	1	1	1	2	2	2	2	3	3
1	1	1	1	2	2	2	2	3	3
1	1	1	1	2	2	2	2	3	3
1	1	1	1	2	2	2	2	2	2
1	1	4	4	1	1	3	3	2	2
1	1	1	1	2	2	2	2	2	2
1	1	1	1	2	2	2	2	2	2
1	1	1	1	1	1	2	2	2	2
1	1	1	1	1	1	2	2	2	2
1	1	1	1	2	2	2	2	2	2
1	1	4	4	2	2	2	2	2	2
1	1	1	1	2	2	2	2	2	2
1	1	1	1	2	2	2	2	2	2
1	1	1	1	2	2	2	2	2	2
4	4	4	4	2	2	2	2	2	2
1	1	1	1	2	2	2	2	2	2
1	1	1	1	2	2	2	2	2	2
1	1	1	1	2	2	2	2	2	2
4	4	4	4	2	2	2	2	2	2
1	1	4	4	2	2	2	2	3	3
4	4	4	4	2	2	2	2	2	2
1	1	1	1	2	2	2	2	2	2
1	1	1	1	1	1	3	3	2	2
1	1	1	1	2	2	2	2	2	2
1	1	1	1	2	2	2	2	3	3
1	1	4	4	2	2	2	2	2	2
1	1	1	1	2	2	2	2	2	2
1	1	1	1	2	2	2	2	2	2
1	1	1	1	2	2	2	2	3	3
1	1	1	1	1	1	2	2	2	2
1	1	1	1	2	2	2	2	2	2
1	1	1	1	1	1	2	2	2	2
1	1	1	1	2	2	2	2	3	3
4	4	4	4	2	2	2	2	2	2
1	1	4	4	2	2	2	2	2	2
1	1	1	1	2	2	2	2	2	2
1	1	1	1	1	1	2	2	2	2
1	1	1	1	2	2	2	2	2	2
1	1	1	1	1	1	2	2	2	2
1	1	1	1	1	1	3	3	2	2
1	1	1	1	2	2	2	2	2	2
1	1	1	1	2	2	2	2	3	3
1	1	1	1	2	2	2	2	2	2
1	1	1	1	2	2	2	2	2	2
1	1	1	1	2	2	2	2	3	3
1	1	1	1	2	2	2	2	3	3
1	1	1	1	2	2	2	2	2	2
1	1	4	4	2	2	2	2	3	3
1	1	1	1	2	2	2	2	3	3
1	1	4	4	2	2	2	2	2	2

For Review Only

1	1	1	1	2	2	2	2	2	2
4	4	4	4	1	1	2	2	2	2
1	1	1	1	2	2	2	2	3	3
1	1	1	1	2	2	2	2	2	2
1	1	1	1	2	2	2	2	3	3
4	4	4	4	2	2	2	2	2	2
4	4	4	4	2	2	2	2	2	2
1	1	1	1	2	2	2	2	2	2
4	4	4	4	2	2	2	2	2	2
1	1	1	1	2	2	2	2	2	2
1	1	1	1	2	2	2	2	3	3
1	1	1	1	2	2	2	2	2	2
1	1	1	1	2	2	2	2	2	2
1	1	4	4	2	2	2	2	2	2
1	1	1	1	2	2	2	2	3	3
4	4	4	4	2	2	2	2	2	2
1	1	4	4	2	2	2	2	2	2
1	1	1	1	2	2	2	2	3	3
1	1	1	1	1	1	3	3	2	2
1	1	1	1	2	2	2	2	3	3
1	1	1	1	2	2	2	2	2	2
1	1	1	1	2	2	2	2	2	2
4	4	4	4	2	2	2	2	2	2
1	1	1	1	2	2	2	2	3	3
1	1	1	1	1	1	2	2	2	2
1	1	1	1	1	1	2	2	2	2
1	1	1	1	2	2	2	2	3	3
1	1	1	1	2	2	2	2	2	2
1	1	1	1	2	2	2	2	2	2
1	1	1	1	2	2	2	2	2	2
1	1	1	1	2	2	3	3	2	2
1	1	1	1	2	2	2	2	2	2
1	1	1	1	2	2	2	2	2	2
1	1	1	1	2	2	2	2	3	3
1	1	1	1	2	2	2	2	2	2
1	1	1	1	2	2	2	2	3	3
1	1	1	1	2	2	2	2	2	2
1	1	1	1	2	2	2	2	2	2
1	1	1	1	2	2	2	2	2	2
1	1	1	1	2	2	2	2	3	3
1	1	1	1	2	2	2	2	3	3
1	1	1	1	2	2	2	2	3	3
4	4	4	4	1	1	2	2	2	2
1	1	4	4	2	2	2	2	2	2
1	1	1	1	2	2	2	2	2	2
1	1	1	1	2	2	2	2	2	2
4	4	4	4	2	2	2	2	2	2
4	4	4	4	2	2	2	2	2	2
1	1	1	1	1	1	2	2	2	2
1	1	1	1	2	2	2	2	2	2
1	1	1	1	2	2	2	2	2	2
1	1	1	1	1	1	2	2	2	2
4	4	4	4	2	2	2	2	2	2
1	1	1	1	2	2	2	2	3	3
4	4	4	4	1	1	2	2	2	2

For Review Only

1	1	1	1	2	2	2	2	3	3
1	1	1	1	2	2	2	2	2	2
1	1	1	1	2	2	2	2	2	2
4	4	4	4	2	2	2	2	2	2
1	1	1	1	2	2	2	2	2	2
1	1	1	1	2	2	2	2	3	3
1	1	1	1	2	2	2	2	3	3
1	1	1	1	1	1	2	2	2	2
1	1	1	1	1	1	2	2	2	2
1	1	1	1	2	2	2	2	3	3
1	1	1	1	2	2	2	2	2	2
4	4	4	4	2	2	3	3	2	2
1	1	1	1	2	2	2	2	2	2
1	1	1	1	2	2	2	2	2	2
1	1	1	1	2	2	2	2	3	3
1	1	1	1	2	2	2	2	2	2
4	4	4	4	2	2	2	2	2	2
1	1	1	1	2	2	2	2	2	2
4	4	4	4	2	2	2	2	2	2
1	1	1	1	2	2	2	2	3	3
1	1	1	1	2	2	2	2	3	3
1	1	1	1	2	2	2	2	3	3
1	1	1	1	2	2	2	2	2	2
4	4	4	4	2	2	2	2	2	2
1	1	1	1	1	1	2	2	2	2
1	1	1	1	2	2	2	2	2	2
4	4	4	4	2	2	2	2	2	2
1	1	1	1	2	2	2	2	3	3
1	1	1	1	2	2	2	2	2	2
1	1	1	1	2	2	2	2	2	2
4	4	4	4	1	1	2	2	2	2
4	4	4	4	2	2	3	3	3	3
4	4	4	4	2	2	2	2	3	3
1	1	1	1	2	2	2	2	2	2
4	4	4	4	2	2	2	2	2	2
4	4	4	4	1	1	2	2	2	2
1	1	1	1	2	2	2	2	3	3
1	1	1	1	2	2	2	2	3	3
1	1	1	1	2	2	3	3	3	3
4	4	4	4	1	1	2	2	2	2
1	1	1	1	2	2	2	2	2	2
1	1	1	1	2	2	2	2	2	2
1	1	1	1	2	2	2	2	2	2
1	1	1	1	2	2	2	2	2	2
1	1	1	1	2	2	2	2	2	2
1	1	1	1	1	1	2	2	2	2
1	1	1	1	2	2	2	2	2	2
1	1	1	1	1	1	2	2	2	2
1	1	1	1	2	2	2	2	3	3
4	4	4	4	2	2	2	2	2	2
1	1	1	1	2	2	2	2	3	3
4	4	4	4	2	2	2	2	2	2
1	1	1	1	2	2	2	2	3	3
1	1	1	1	2	2	2	2	3	3
4	4	4	4	2	2	2	2	3	3

For Review Only

1	1	1	1	2	2	2	2	2	2
1	1	1	1	2	2	3	3	2	2

For Review Only

SNP647	SNP647	SNP648	SNP648	SNP649	SNP649	SNP650	SNP650	SNP651	SNP651
4	4	1	1	4	4	3	3	3	3
1	1	1	1	4	4	3	3	3	3
1	1	1	1	4	4	3	3	3	3
4	4	1	1	4	4	3	3	3	3
1	1	1	1	4	4	2	2	3	3
4	4	4	4	4	4	3	3	3	3
4	4	4	4	4	4	3	3	3	3
1	1	1	1	4	4	3	3	3	3
4	4	1	1	4	4	3	3	3	3
1	1	1	1	4	4	2	2	3	3
1	1	1	1	4	4	3	3	3	3
1	1	1	1	4	4	3	3	3	3
1	1	1	1	4	4	3	3	3	3
1	1	1	1	4	4	3	3	3	3
1	1	1	1	4	4	3	3	3	3
1	1	1	1	4	4	2	2	3	3
1	1	1	1	4	4	2	2	3	3
1	1	1	1	4	4	3	3	3	3
1	1	1	1	4	4	3	3	2	2
1	1	1	1	4	4	3	3	3	3
1	1	1	1	4	4	3	3	3	3
1	1	1	1	4	4	2	2	3	3
1	1	1	1	4	4	3	3	3	3
1	1	1	1	4	4	3	3	3	3
1	1	1	1	4	4	3	3	3	3
1	1	1	1	4	4	2	2	3	3
1	1	1	1	4	4	3	3	3	3
1	1	1	1	4	4	3	3	3	3
1	1	1	1	4	4	2	2	2	2
1	1	1	1	4	4	2	2	3	3
1	1	1	1	4	4	3	3	3	3
1	1	1	1	4	4	3	3	2	2
1	1	1	1	4	4	3	3	3	3
1	1	1	1	4	4	2	2	3	3
1	1	1	1	4	4	3	3	3	3
1	1	1	1	4	4	3	3	3	3
4	4	4	4	1	1	3	3	3	3
1	1	4	4	4	4	3	3	3	3
1	1	1	1	4	4	2	2	3	3
1	1	1	1	4	4	3	3	3	3
1	1	1	1	4	4	2	2	3	3
1	1	1	1	4	4	3	3	3	3
1	1	1	1	4	4	3	3	3	3
1	1	1	1	1	1	3	3	3	3
1	1	1	1	4	4	3	3	3	3
1	1	1	1	4	4	3	3	3	3
1	1	1	1	4	4	2	2	3	3
1	1	1	1	4	4	2	2	3	3
1	1	1	1	4	4	3	3	3	3
4	4	1	1	4	4	3	3	3	3
1	1	1	1	4	4	2	2	3	3
1	1	1	1	4	4	3	3	3	3

For Review Only

For Review Only

1	1	1	1	4	4	3	3	3	3
1	1	1	1	4	4	3	3	3	3
1	1	1	1	4	4	3	3	3	3
1	1	1	1	4	4	3	3	3	3
1	1	1	1	4	4	3	3	2	2
1	1	1	1	4	4	2	2	3	3
1	1	1	1	4	4	3	3	3	3
1	1	1	1	4	4	2	2	3	3
1	1	1	1	1	1	3	3	3	3
1	1	1	1	4	4	3	3	3	3
1	1	1	1	4	4	3	3	3	3
1	1	1	1	4	4	3	3	3	3
1	1	1	1	4	4	3	3	3	3
1	1	1	1	4	4	2	2	2	2
4	4	1	1	4	4	3	3	3	3
4	4	1	1	4	4	3	3	3	3
1	1	1	1	4	4	3	3	3	3
1	1	1	1	4	4	3	3	3	3
1	1	1	1	4	4	3	3	3	3
4	4	4	4	4	4	3	3	3	3
1	1	1	1	4	4	3	3	3	3
1	1	1	1	4	4	2	2	3	3
1	1	1	1	4	4	2	2	2	2
1	1	1	1	4	4	3	3	3	3
1	1	1	1	4	4	3	3	3	3
1	1	1	1	4	4	3	3	3	3
1	1	1	1	4	4	3	3	3	3
1	1	1	1	4	4	2	2	3	3
1	1	1	1	4	4	2	2	3	3
4	4	1	1	4	4	3	3	3	3
1	1	1	1	4	4	3	3	3	3
1	1	1	1	4	4	3	3	3	3
1	1	1	1	4	4	2	2	3	3
1	1	1	1	4	4	3	3	3	3
1	1	1	1	4	4	2	2	3	3
1	1	1	1	4	4	3	3	3	3
1	1	1	1	4	4	3	3	3	3
1	1	1	1	4	4	2	2	3	3
1	1	1	1	4	4	3	3	3	3
1	1	1	1	4	4	3	3	3	3
1	1	1	1	4	4	3	3	3	3
1	1	1	1	4	4	3	3	3	3
1	1	1	1	4	4	3	3	3	3
1	1	1	1	4	4	3	3	3	3
1	1	1	1	4	4	3	3	3	3
4	4	4	4	4	4	3	3	2	2
1	1	1	1	4	4	2	2	3	3
1	1	1	1	4	4	3	3	3	3
1	1	1	1	4	4	2	2	3	3
1	1	1	1	4	4	3	3	3	3
4	4	4	4	4	4	3	3	3	3
1	1	1	1	4	4	2	2	3	3
1	1	1	1	1	1	2	2	3	3
1	1	1	1	4	4	3	3	3	3
1	1	1	1	4	4	3	3	3	3

1	1	1	1	4	4	2	2	3	3
1	1	1	1	4	4	3	3	3	3
1	1	1	1	4	4	3	3	3	3
1	1	1	1	4	4	2	2	3	3
1	1	1	1	4	4	3	3	3	3
1	1	1	1	4	4	2	2	3	3
1	1	1	1	4	4	3	3	3	3
1	1	1	1	4	4	3	3	3	3
1	1	1	1	4	4	2	2	3	3
1	1	1	1	4	4	2	2	3	3
1	1	1	1	1	1	3	3	3	3
1	1	1	1	4	4	3	3	3	3
1	1	1	1	4	4	3	3	3	3
1	1	1	1	4	4	3	3	3	3
4	4	4	4	4	4	3	3	3	3
1	1	1	1	4	4	2	2	3	3
1	1	1	1	4	4	3	3	3	3
1	1	1	1	4	4	2	2	3	3
1	1	1	1	1	1	2	2	3	3
1	1	1	1	4	4	3	3	3	3
1	1	1	1	4	4	3	3	3	3
1	1	1	1	4	4	3	3	3	3
1	1	1	1	4	4	3	3	3	3
1	1	1	1	4	4	2	2	3	3
1	1	1	1	4	4	2	2	3	3
1	1	1	1	4	4	3	3	3	3
1	1	1	1	4	4	3	3	3	3
4	4	4	4	4	4	3	3	3	3
1	1	1	1	4	4	3	3	2	2
1	1	1	1	4	4	3	3	3	3
1	1	1	1	4	4	2	2	3	3
1	1	1	1	4	4	3	3	3	3
1	1	1	1	4	4	3	3	3	3
1	1	1	1	4	4	3	3	3	3
1	1	1	1	4	4	2	2	3	3
1	1	1	1	4	4	3	3	3	3
1	1	1	1	1	1	2	2	3	3
1	1	1	1	4	4	3	3	3	3
1	1	1	1	4	4	3	3	3	3
1	1	1	1	4	4	2	2	3	3
4	4	4	4	4	4	3	3	3	3
1	1	1	1	1	1	2	2	3	3
1	1	1	1	4	4	3	3	3	3
1	1	1	1	4	4	2	2	3	3
1	1	1	1	4	4	3	3	3	3
1	1	1	1	4	4	3	3	3	3
1	1	1	1	4	4	2	2	3	3
1	1	1	1	4	4	3	3	3	3
1	1	1	1	4	4	3	3	3	3
1	1	1	1	4	4	2	2	3	3
1	1	1	1	4	4	3	3	3	3
1	1	1	1	4	4	2	2	3	3
1	1	1	1	4	4	3	3	3	3
1	1	1	1	4	4	3	3	3	3
1	1	1	1	4	4	3	3	3	3

For Review Only

1	1	1	1	4	4	3	3	3	3
1	1	1	1	4	4	3	3	3	3
1	1	1	1	4	4	3	3	3	3
1	1	1	1	4	4	2	2	3	3
1	1	1	1	4	4	3	3	3	3
1	1	1	1	4	4	2	2	3	3
1	1	1	1	4	4	3	3	3	3
1	1	1	1	4	4	3	3	3	3
1	1	1	1	4	4	3	3	3	3
1	1	1	1	4	4	3	3	3	3
1	1	1	1	4	4	2	2	3	3
1	1	1	1	4	4	3	3	3	3
1	1	1	1	4	4	3	3	3	3
1	1	1	1	4	4	3	3	3	3
1	1	1	1	1	1	2	2	3	3
1	1	1	1	4	4	3	3	3	3
1	1	1	1	4	4	3	3	3	3
1	1	1	1	4	4	3	3	2	2
1	1	1	1	4	4	3	3	3	3
1	1	1	1	4	4	3	3	3	3
1	1	1	1	4	4	3	3	3	3
1	1	1	1	4	4	3	3	3	3
1	1	1	1	4	4	2	2	3	3
1	1	1	1	4	4	3	3	3	3
1	1	1	1	4	4	3	3	3	3
1	1	1	1	4	4	2	2	3	3
1	1	1	1	4	4	3	3	3	3
1	1	1	1	4	4	3	3	3	3
4	4	4	4	4	4	3	3	3	3
1	1	1	1	4	4	3	3	3	3
1	1	1	1	4	4	3	3	3	3
4	4	4	4	4	4	3	3	3	3
4	4	1	1	4	4	3	3	3	3
1	1	1	1	1	1	2	2	3	3
1	1	1	1	4	4	3	3	2	2
1	1	1	1	4	4	3	3	3	3
1	1	1	1	4	4	3	3	3	3
4	4	4	4	4	4	3	3	3	3
1	1	1	1	4	4	3	3	3	3
1	1	1	1	4	4	3	3	3	3
1	1	1	1	4	4	3	3	3	3
1	1	1	1	4	4	2	2	3	3
1	1	1	1	4	4	2	2	3	3
1	1	1	1	4	4	3	3	3	3
1	1	1	1	4	4	3	3	3	3
1	1	1	1	4	4	2	2	3	3
1	1	1	1	4	4	3	3	3	3
1	1	1	1	4	4	2	2	3	3
1	1	1	1	1	1	2	2	3	3
1	1	1	1	4	4	3	3	3	3
4	4	1	1	4	4	3	3	3	3
1	1	1	1	1	1	2	2	3	3
1	1	1	1	4	4	2	2	3	3
1	1	1	1	4	4	3	3	3	3
1	1	1	1	4	4	2	2	3	3

For Review Only

1	1	1	1	4	4	3	3	3	3
1	1	1	1	4	4	3	3	3	3
1	1	1	1	4	4	3	3	3	3
1	1	1	1	4	4	3	3	3	3
1	1	1	1	4	4	3	3	3	3
4	4	4	4	4	4	2	2	2	2
1	1	1	1	4	4	2	2	3	3
1	1	1	1	4	4	3	3	3	3
1	1	1	1	4	4	2	2	3	3
1	1	1	1	4	4	2	2	3	3
1	1	1	1	4	4	3	3	3	3
1	1	1	1	4	4	3	3	3	3
1	1	1	1	4	4	3	3	3	3
4	4	4	4	4	4	2	2	3	3
1	1	1	1	4	4	3	3	3	3
1	1	1	1	4	4	3	3	3	3
1	1	1	1	4	4	2	2	3	3
1	1	1	1	4	4	3	3	3	3
1	1	1	1	4	4	2	2	3	3
1	1	1	1	4	4	3	3	3	3
1	1	1	1	4	4	3	3	3	3
1	1	1	1	4	4	2	2	3	3
1	1	1	1	4	4	3	3	3	3
1	1	1	1	4	4	3	3	3	3
1	1	1	1	4	4	2	2	3	3
1	1	1	1	4	4	3	3	3	3
1	1	1	1	4	4	2	2	3	3
1	1	1	1	4	4	3	3	2	2
1	1	1	1	4	4	3	3	3	3
1	1	1	1	4	4	3	3	3	3
1	1	1	1	4	4	3	3	3	3
1	1	1	1	4	4	3	3	3	3
1	1	1	1	4	4	3	3	3	3
1	1	1	1	4	4	2	2	3	3
1	1	1	1	4	4	2	2	3	3
1	1	1	1	4	4	2	2	3	3
1	1	1	1	4	4	3	3	3	3
1	1	1	1	4	4	3	3	3	3
1	1	1	1	4	4	3	3	3	3
1	1	1	1	4	4	2	2	3	3
1	1	1	1	4	4	3	3	2	2
1	1	1	1	4	4	3	3	3	3
1	1	1	1	4	4	3	3	3	3
1	1	1	1	4	4	3	3	3	3
1	1	1	1	4	4	3	3	3	3
1	1	1	1	4	4	3	3	3	3
1	1	1	1	4	4	3	3	3	3
1	1	1	1	4	4	3	3	3	3
1	1	1	1	4	4	3	3	3	3
1	1	1	1	4	4	2	2	3	3
4	4	4	4	4	4	3	3	3	3
1	1	1	1	4	4	2	2	3	3
1	1	1	1	4	4	3	3	3	3
1	1	1	1	4	4	3	3	2	2
1	1	1	1	1	1	3	3	3	3

For Review Only

1	1	1	1	4	4	3	3	3	3
4	4	4	4	4	4	3	3	3	3

For Review Only

SNP652	SNP652	SNP653	SNP653	SNP654	SNP654	SNP655	SNP655	SNP656	SNP656
1	1	2	2	4	4	3	3	2	2
1	1	2	2	4	4	3	3	1	1
1	1	2	2	1	1	3	3	1	1
1	1	2	2	4	4	3	3	1	1
1	1	2	2	4	4	2	2	1	1
1	1	2	2	4	4	3	3	1	1
1	1	2	2	1	1	3	3	1	1
1	1	2	2	4	4	3	3	1	1
1	1	2	2	4	4	3	3	1	1
1	1	2	2	4	4	2	2	1	1
1	1	2	2	1	1	3	3	1	1
1	1	2	2	1	1	3	3	1	1
1	1	2	2	4	4	3	3	1	1
1	1	2	2	4	4	3	3	2	2
1	1	2	2	4	4	3	3	1	1
1	1	2	2	1	1	2	2	1	1
1	1	2	2	4	4	2	2	1	1
1	1	2	2	4	4	3	3	2	2
2	2	1	1	4	4	3	3	1	1
1	1	2	2	4	4	3	3	2	2
1	1	2	2	4	4	2	2	1	1
1	1	2	2	4	4	2	2	1	1
1	1	2	2	1	1	3	3	1	1
1	1	2	2	4	4	3	3	1	1
1	1	2	2	4	4	3	3	1	1
1	1	2	2	4	4	2	2	1	1
1	1	2	2	1	1	3	3	1	1
1	1	2	2	4	4	3	3	1	1
2	2	1	1	4	4	2	2	1	1
1	1	2	2	4	4	2	2	1	1
1	1	2	2	4	4	3	3	2	2
2	2	1	1	1	1	3	3	1	1
1	1	2	2	1	1	3	3	2	2
1	1	2	2	4	4	2	2	1	1
1	1	2	2	1	1	3	3	1	1
1	1	2	2	4	4	3	3	1	1
1	1	2	2	1	1	3	3	1	1
1	1	2	2	1	1	3	3	1	1
1	1	2	2	1	1	2	2	1	1
1	1	2	2	4	4	3	3	1	1
1	1	2	2	4	4	2	2	1	1
2	2	1	1	4	4	2	2	1	1
1	1	2	2	4	4	3	3	1	1
1	1	2	2	1	1	3	3	2	2
1	1	2	2	4	4	3	3	1	1
1	1	2	2	4	4	3	3	1	1
1	1	2	2	4	4	2	2	1	1
1	1	2	2	4	4	2	2	1	1
1	1	2	2	4	4	2	2	2	2
1	1	2	2	4	4	3	3	1	1
1	1	2	2	1	1	2	2	1	1
1	1	2	2	4	4	3	3	1	1

For Review Only

1	1	2	2	4	4	3	3	1	1
1	1	2	2	1	1	3	3	2	2
1	1	2	2	4	4	3	3	2	2
1	1	2	2	4	4	3	3	1	1
2	2	1	1	4	4	3	3	1	1
1	1	2	2	1	1	2	2	2	2
1	1	2	2	4	4	3	3	1	1
1	1	2	2	4	4	2	2	1	1
1	1	2	2	1	1	3	3	1	1
1	1	2	2	4	4	3	3	1	1
1	1	2	2	4	4	3	3	2	2
1	1	2	2	1	1	3	3	2	2
1	1	2	2	4	4	3	3	1	1
2	2	1	1	4	4	2	2	1	1
1	1	2	2	4	4	3	3	1	1
1	1	2	2	4	4	3	3	1	1
1	1	2	2	1	1	3	3	1	1
1	1	2	2	4	4	3	3	2	2
1	1	2	2	4	4	3	3	1	1
1	1	2	2	4	4	3	3	1	1
1	1	2	2	1	1	3	3	1	1
1	1	2	2	4	4	2	2	1	1
2	2	1	1	4	4	2	2	1	1
1	1	2	2	1	1	3	3	2	2
1	1	2	2	4	4	3	3	1	1
1	1	2	2	4	4	3	3	2	2
1	1	2	2	4	4	3	3	2	2
1	1	2	2	4	4	2	2	1	1
1	1	2	2	4	4	2	2	1	1
1	1	2	2	4	4	2	2	1	1
1	1	2	2	4	4	3	3	1	1
1	1	2	2	4	4	3	3	1	1
1	1	2	2	4	4	2	2	1	1
1	1	2	2	4	4	3	3	1	1
1	1	2	2	1	1	2	2	2	2
1	1	2	2	4	4	3	3	1	1
1	1	2	2	4	4	3	3	1	1
1	1	2	2	4	4	2	2	1	1
1	1	2	2	4	4	3	3	1	1
1	1	2	2	4	4	3	3	1	1
1	1	2	2	4	4	3	3	2	2
1	1	2	2	4	4	3	3	1	1
1	1	2	2	4	4	3	3	2	2
1	1	2	2	4	4	3	3	2	2
1	1	2	2	4	4	3	3	1	1
2	2	1	1	4	4	3	3	1	1
1	1	2	2	1	1	2	2	1	1
1	1	2	2	4	4	3	3	1	1
1	1	2	2	4	4	2	2	1	1
1	1	2	2	4	4	3	3	1	1
1	1	2	2	4	4	3	3	1	1
1	1	2	2	1	1	2	2	1	1
1	1	2	2	1	1	2	2	1	1
1	1	2	2	4	4	2	2	1	1
1	1	2	2	4	4	3	3	1	1

For Review Only

1	1	2	2	1	1	2	2	1	1
1	1	2	2	1	1	3	3	1	1
1	1	2	2	4	4	3	3	1	1
1	1	2	2	1	1	2	2	1	1
2	2	1	1	4	4	3	3	1	1
1	1	2	2	4	4	2	2	1	1
1	1	2	2	4	4	3	3	1	1
1	1	2	2	1	1	3	3	1	1
1	1	2	2	1	1	2	2	1	1
1	1	2	2	4	4	2	2	1	1
1	1	2	2	1	1	3	3	1	1
1	1	2	2	4	4	3	3	1	1
1	1	2	2	4	4	3	3	1	1
1	1	2	2	4	4	3	3	1	1
1	1	2	2	4	4	3	3	1	1
1	1	2	2	4	4	3	3	1	1
1	1	2	2	4	4	3	3	1	1
1	1	2	2	4	4	3	3	1	1
1	1	2	2	1	1	2	2	1	1
1	1	2	2	4	4	3	3	1	1
1	1	2	2	4	4	3	3	1	1
1	1	2	2	4	4	3	3	1	1
1	1	2	2	4	4	3	3	1	1
1	1	2	2	4	4	2	2	1	1
1	1	2	2	4	4	2	2	1	1
1	1	2	2	4	4	3	3	1	1
1	1	2	2	4	4	3	3	1	1
1	1	2	2	4	4	3	3	1	1
2	2	1	1	4	4	3	3	1	1
1	1	2	2	1	1	3	3	2	2
1	1	2	2	4	4	2	2	1	1
1	1	2	2	4	4	3	3	1	1
1	1	2	2	4	4	3	3	2	2
1	1	2	2	4	4	2	2	1	1
1	1	2	2	4	4	2	2	1	1
1	1	2	2	4	4	3	3	1	1
1	1	2	2	1	1	2	2	1	1
1	1	2	2	4	4	3	3	2	2
1	1	2	2	4	4	3	3	1	1
1	1	2	2	1	1	2	2	1	1
1	1	2	2	1	1	3	3	1	1
1	1	2	2	4	4	2	2	1	1
1	1	2	2	4	4	3	3	2	2
1	1	2	2	4	4	2	2	1	1
1	1	2	2	1	1	3	3	1	1
1	1	2	2	4	4	3	3	2	2
1	1	2	2	1	1	2	2	1	1
1	1	2	2	1	1	3	3	1	1
1	1	2	2	4	4	2	2	1	1
1	1	2	2	4	4	2	2	1	1
1	1	2	2	4	4	3	3	1	1
1	1	2	2	4	4	2	2	1	1
1	1	2	2	4	4	3	3	1	1
1	1	2	2	4	4	3	3	2	2
1	1	2	2	4	4	2	2	1	1

For Review Only

1	1	2	2	1	1	3	3	1	1
1	1	2	2	4	4	3	3	1	1
1	1	2	2	4	4	3	3	1	1
1	1	2	2	1	1	2	2	1	1
1	1	1	1	4	4	3	3	2	2
1	1	2	2	1	1	2	2	1	1
1	1	2	2	4	4	3	3	1	1
1	1	2	2	4	4	3	3	1	1
1	1	2	2	1	1	3	3	1	1
1	1	2	2	4	4	3	3	1	1
1	1	2	2	4	4	2	2	1	1
1	1	2	2	4	4	3	3	1	1
1	1	2	2	4	4	2	2	1	1
1	1	2	2	4	4	3	3	1	1
1	1	2	2	1	1	2	2	1	1
1	1	2	2	1	1	3	3	1	1
1	1	2	2	4	4	3	3	1	1
2	2	1	1	4	4	3	3	2	2
1	1	2	2	4	4	3	3	1	1
1	1	2	2	4	4	3	3	1	1
1	1	2	2	4	4	3	3	1	1
1	1	2	2	4	4	3	3	1	1
1	1	2	2	4	4	2	2	1	1
1	1	2	2	1	1	3	3	2	2
1	1	2	2	4	4	3	3	1	1
1	1	2	2	4	4	3	3	1	1
1	1	2	2	4	4	3	3	1	1
1	1	2	2	4	4	3	3	1	1
1	1	2	2	4	4	3	3	1	1
1	1	2	2	4	4	3	3	1	1
1	1	2	2	4	4	3	3	1	1
1	1	2	2	4	4	2	2	1	1
1	1	2	2	1	1	3	3	1	1
1	1	2	2	1	1	2	2	1	1
2	2	1	1	4	4	3	3	2	2
1	1	2	2	1	1	3	3	2	2
1	1	2	2	4	4	3	3	1	1
1	1	2	2	4	4	3	3	1	1
1	1	2	2	1	1	3	3	1	1
1	1	2	2	4	4	3	3	1	1
1	1	2	2	1	1	3	3	1	1
1	1	2	2	1	1	2	2	1	1
1	1	2	2	1	1	2	2	1	1
1	1	2	2	1	1	3	3	2	2
1	1	2	2	4	4	3	3	1	1
1	1	2	2	1	1	2	2	2	2
1	1	2	2	1	1	3	3	1	1
1	1	2	2	1	1	2	2	1	1
1	1	2	2	4	4	2	2	1	1
1	1	2	2	4	4	3	3	1	1
1	1	2	2	4	4	3	3	1	1
1	1	2	2	1	1	2	2	1	1
1	1	2	2	4	4	2	2	1	1
1	1	2	2	4	4	3	3	2	2
1	1	2	2	1	1	2	2	1	1

For Review Only

1	1	2	2	4	4	3	3	1	1
1	1	2	2	4	4	3	3	1	1
1	1	2	2	4	4	3	3	1	1
1	1	2	2	4	4	3	3	1	1
1	1	2	2	4	4	3	3	1	1
2	2	1	1	4	4	2	2	1	1
1	1	2	2	1	1	2	2	1	1
1	1	2	2	4	4	3	3	1	1
1	1	2	2	4	4	2	2	1	1
1	1	2	2	1	1	2	2	1	1
1	1	2	2	4	4	2	2	1	1
1	1	2	2	1	1	3	3	1	1
1	1	2	2	4	4	3	3	1	1
1	1	2	2	4	4	2	2	1	1
1	1	2	2	1	1	3	3	1	1
1	1	2	2	4	4	3	3	1	1
1	1	2	2	1	1	2	2	2	2
1	1	2	2	1	1	3	3	1	1
1	1	2	2	4	4	2	2	1	1
1	1	2	2	1	1	3	3	1	1
1	1	2	2	4	4	3	3	2	2
1	1	2	2	4	4	2	2	1	1
1	1	2	2	4	4	3	3	1	1
1	1	2	2	1	1	3	3	1	1
1	1	2	2	4	4	2	2	1	1
1	1	2	2	4	4	3	3	1	1
1	1	2	2	4	4	2	2	1	1
1	1	2	2	1	1	3	3	1	1
1	1	2	2	4	4	3	3	1	1
1	1	2	2	4	4	3	3	1	1
1	1	2	2	1	1	3	3	1	1
1	1	2	2	1	1	3	3	1	1
1	1	2	2	1	1	3	3	1	1
1	1	2	2	1	1	2	2	1	1
1	1	2	2	4	4	2	2	1	1
1	1	2	2	1	1	2	2	1	1
1	1	2	2	4	4	3	3	1	1
1	1	2	2	1	1	3	3	2	2
1	1	2	2	4	4	3	3	1	1
1	1	2	2	1	1	2	2	1	1
2	2	1	1	4	4	3	3	2	2
1	1	2	2	4	4	3	3	2	2
1	1	2	2	4	4	3	3	2	2
1	1	2	2	4	4	3	3	1	1
1	1	2	2	1	1	3	3	2	2
1	1	2	2	4	4	3	3	1	1
1	1	2	2	1	1	3	3	1	1
1	1	2	2	4	4	3	3	2	2
1	1	2	2	4	4	3	3	1	1
1	1	2	2	4	4	2	2	1	1
1	1	2	2	4	4	3	3	1	1
1	1	2	2	1	1	2	2	1	1
1	1	2	2	1	1	3	3	1	1
2	2	1	1	4	4	3	3	1	1
1	1	2	2	1	1	3	3	1	1

1	1	2	2	4	4	3	3	1	1
1	1	2	2	4	4	3	3	1	1

For Review Only

SNP657	SNP657	SNP658	SNP658	SNP659	SNP659	SNP660	SNP660	SNP661	SNP661
3	3	2	2	3	3	2	2	1	1
2	2	4	4	3	3	2	2	1	1
3	3	4	4	3	3	1	1	4	4
3	3	4	4	3	3	2	2	1	1
3	3	4	4	3	3	2	2	1	1
2	2	4	4	3	3	2	2	1	1
3	3	4	4	3	3	1	1	4	4
2	2	4	4	3	3	2	2	1	1
3	3	4	4	3	3	2	2	1	1
2	2	4	4	3	3	2	2	1	1
2	2	4	4	3	3	1	1	4	4
3	3	4	4	3	3	1	1	4	4
3	3	2	2	3	3	2	2	1	1
3	3	2	2	3	3	1	1	4	4
3	3	4	4	3	3	2	2	1	1
2	2	4	4	3	3	1	1	1	1
2	2	4	4	3	3	2	2	1	1
3	3	2	2	3	3	2	2	1	1
3	3	4	4	3	3	2	2	1	1
3	3	4	4	4	4	2	2	1	1
3	3	4	4	4	4	2	2	1	1
2	2	4	4	3	3	2	2	1	1
2	2	4	4	4	4	1	1	4	4
3	3	4	4	3	3	2	2	1	1
3	3	4	4	3	3	2	2	1	1
3	3	4	4	3	3	2	2	1	1
3	3	4	4	3	3	1	1	4	4
2	2	4	4	3	3	2	2	1	1
2	2	4	4	3	3	2	2	1	1
2	2	4	4	4	4	2	2	1	1
3	3	4	4	3	3	2	2	1	1
2	2	4	4	3	3	1	1	4	4
3	3	2	2	3	3	1	1	4	4
2	2	4	4	3	3	2	2	1	1
2	2	4	4	3	3	2	2	4	4
3	3	4	4	3	3	2	2	1	1
2	2	4	4	3	3	1	1	4	4
3	3	4	4	3	3	1	1	4	4
2	2	4	4	3	3	1	1	4	4
2	2	4	4	3	3	2	2	1	1
2	2	4	4	3	3	2	2	1	1
2	2	4	4	3	3	2	2	1	1
2	2	4	4	3	3	2	2	1	1
3	3	2	2	3	3	2	2	1	1
2	2	4	4	3	3	2	2	1	1
2	2	4	4	3	3	2	2	1	1
2	2	4	4	3	3	2	2	4	4
2	2	4	4	3	3	2	2	1	1
3	3	2	2	3	3	2	2	1	1
2	2	2	2	3	3	2	2	1	1
3	3	4	4	3	3	1	1	4	4
3	3	4	4	3	3	2	2	1	1

For Review Only

3	3	4	4	3	3	2	2	1	1
3	3	2	2	3	3	1	1	4	4
3	3	2	2	3	3	2	2	1	1
3	3	4	4	3	3	2	2	1	1
3	3	4	4	3	3	2	2	1	1
3	3	2	2	3	3	1	1	4	4
3	3	4	4	3	3	2	2	1	1
2	2	4	4	4	4	2	2	1	1
2	2	4	4	3	3	1	1	4	4
3	3	4	4	3	3	2	2	1	1
3	3	2	2	3	3	2	2	1	1
3	3	2	2	3	3	1	1	4	4
3	3	4	4	3	3	2	2	1	1
2	2	4	4	3	3	2	2	1	1
2	2	4	4	3	3	1	1	1	1
2	2	4	4	3	3	2	2	1	1
2	2	4	4	4	4	1	1	4	4
3	3	2	2	3	3	2	2	1	1
2	2	4	4	3	3	2	2	1	1
3	3	4	4	3	3	2	2	1	1
3	3	4	4	3	3	1	1	4	4
2	2	4	4	3	3	2	2	1	1
2	2	4	4	3	3	2	2	1	1
3	3	2	2	3	3	1	1	4	4
2	2	4	4	3	3	2	2	1	1
3	3	2	2	3	3	2	2	1	1
3	3	4	4	3	3	2	2	1	1
2	2	4	4	3	3	1	1	4	4
3	3	4	4	3	3	2	2	1	1
3	3	4	4	3	3	2	2	1	1
3	3	4	4	3	3	2	2	1	1
2	2	4	4	3	3	2	2	1	1
2	2	4	4	3	3	2	2	1	1
3	3	4	4	3	3	2	2	1	1
3	3	2	2	3	3	1	1	4	4
2	2	4	4	3	3	2	2	1	1
2	2	4	4	3	3	2	2	1	1
3	3	2	2	3	3	2	2	1	1
3	3	4	4	3	3	2	2	1	1
2	2	4	4	3	3	2	2	1	1
3	3	2	2	3	3	2	2	1	1
2	2	4	4	3	3	2	2	1	1
3	3	2	2	3	3	2	2	1	1
3	3	2	2	3	3	2	2	1	1
3	3	4	4	3	3	2	2	1	1
2	2	4	4	3	3	2	2	1	1
2	2	4	4	4	4	1	1	4	4
3	3	4	4	3	3	2	2	1	1
2	2	4	4	3	3	2	2	1	1
3	3	4	4	3	3	2	2	1	1
2	2	4	4	3	3	1	1	4	4
2	2	4	4	3	3	1	1	4	4
2	2	4	4	4	4	1	1	4	4
3	3	4	4	3	3	2	2	1	1
2	2	4	4	3	3	2	2	1	1

For Review Only

3	3	4	4	3	3	1	1	4	4
3	3	4	4	4	4	1	1	4	4
3	3	4	4	3	3	2	2	1	1
2	2	4	4	3	3	1	1	4	4
2	2	4	4	3	3	1	1	4	4
3	3	4	4	3	3	2	2	1	1
2	2	4	4	3	3	2	2	1	1
3	3	4	4	3	3	1	1	4	4
2	2	4	4	3	3	1	1	4	4
2	2	4	4	3	3	2	2	1	1
2	2	4	4	3	3	1	1	4	4
3	3	4	4	3	3	2	2	1	1
2	2	4	4	3	3	2	2	1	1
3	3	2	2	3	3	2	2	1	1
3	3	4	4	3	3	2	2	1	1
3	3	4	4	3	3	2	2	1	1
2	2	4	4	3	3	2	2	1	1
3	3	4	4	3	3	2	2	1	1
2	2	4	4	3	3	1	1	4	4
2	2	4	4	3	3	2	2	1	1
3	3	4	4	3	3	2	2	1	1
2	2	4	4	3	3	2	2	1	1
2	2	4	4	3	3	2	2	1	1
2	2	4	4	3	3	2	2	1	1
3	3	4	4	3	3	1	1	1	1
2	2	4	4	4	4	2	2	1	1
3	3	4	4	3	3	2	2	1	1
3	3	4	4	3	3	2	2	1	1
2	2	4	4	3	3	2	2	1	1
3	3	2	2	4	4	1	1	4	4
2	2	4	4	3	3	2	2	1	1
3	3	4	4	3	3	2	2	1	1
3	3	2	2	3	3	2	2	1	1
3	3	4	4	3	3	1	1	1	1
2	2	4	4	3	3	2	2	1	1
3	3	4	4	3	3	2	2	1	1
2	2	4	4	3	3	1	1	4	4
3	3	2	2	3	3	2	2	1	1
2	2	4	4	3	3	2	2	1	1
2	2	4	4	3	3	2	2	1	1
3	3	4	4	3	3	1	1	4	4
2	2	4	4	3	3	2	2	1	1
3	3	2	2	3	3	2	2	1	1
2	2	4	4	4	4	2	2	1	1
3	3	4	4	3	3	2	2	1	1
3	3	2	2	3	3	2	2	1	1
3	3	4	4	3	3	1	1	4	4
3	3	4	4	3	3	1	1	4	4
2	2	4	4	3	3	1	1	1	1
3	3	4	4	3	3	2	2	1	1
3	3	4	4	3	3	2	2	1	1
2	2	4	4	4	4	2	2	1	1
2	2	4	4	3	3	2	2	1	1
3	3	2	2	3	3	2	2	4	4
3	3	4	4	4	4	2	2	1	1

For Review Only

For Review Only

3	3	4	4	3	3	1	1	4	4
2	2	4	4	4	4	2	2	1	1
3	3	4	4	3	3	2	2	1	1
2	2	4	4	4	4	2	2	1	1
3	3	2	2	3	3	2	2	1	1
2	2	4	4	3	3	1	1	4	4
2	2	4	4	3	3	2	2	1	1
3	3	4	4	3	3	2	2	1	1
2	2	4	4	3	3	2	2	1	1
3	3	4	4	3	3	2	2	1	1
3	3	4	4	3	3	2	2	1	1
3	3	4	4	3	3	1	1	1	1
3	3	4	4	3	3	2	2	1	1
3	3	4	4	3	3	2	2	1	1
2	2	4	4	3	3	1	1	4	4
2	2	4	4	3	3	1	1	4	4
2	2	4	4	3	3	2	2	1	1
3	3	4	4	3	3	2	2	1	1
2	2	4	4	3	3	2	2	1	1
2	2	4	4	3	3	2	2	1	1
3	3	4	4	3	3	2	2	1	1
2	2	4	4	3	3	2	2	1	1
2	2	4	4	3	3	2	2	1	1
3	3	2	2	3	3	1	1	4	4
2	2	4	4	3	3	2	2	1	1
2	2	4	4	3	3	2	2	1	1
3	3	4	4	3	3	2	2	1	1
2	2	4	4	3	3	2	2	1	1
2	2	4	4	3	3	2	2	1	1
3	3	4	4	3	3	2	2	1	1
3	3	2	2	3	3	2	2	1	1
3	3	4	4	3	3	2	2	1	1
3	3	4	4	3	3	1	1	4	4
2	2	4	4	3	3	1	1	4	4
3	3	2	2	3	3	2	2	1	1
3	3	2	2	3	3	1	1	4	4
3	3	4	4	3	3	2	2	1	1
3	3	4	4	3	3	2	2	1	1
3	3	4	4	3	3	1	1	4	4
3	3	4	4	3	3	2	2	1	1
3	3	4	4	3	3	1	1	4	4
3	3	4	4	3	3	1	1	4	4
2	2	4	4	4	4	1	1	4	4
3	3	4	4	3	3	1	1	4	4
3	3	4	4	3	3	2	2	1	1
3	3	2	2	3	3	1	1	4	4
2	2	4	4	3	3	1	1	4	4
2	2	4	4	3	3	1	1	1	1
2	2	4	4	3	3	2	2	1	1
3	3	4	4	3	3	2	2	1	1
3	3	4	4	3	3	2	2	1	1
2	2	4	4	4	4	1	1	4	4
2	2	4	4	3	3	2	2	1	1
3	3	2	2	3	3	2	2	1	1
2	2	4	4	3	3	1	1	4	4

2	2	4	4	4	4	2	2	1	1
2	2	4	4	3	3	2	2	1	1
3	3	4	4	3	3	2	2	1	1
2	2	4	4	3	3	2	2	1	1
2	2	4	4	3	3	2	2	1	1
3	3	4	4	3	3	2	2	1	1
3	3	4	4	3	3	1	1	4	4
2	2	4	4	4	4	2	2	1	1
2	2	4	4	3	3	2	2	1	1
2	2	4	4	3	3	1	1	4	4
3	3	4	4	3	3	2	2	1	1
2	2	4	4	3	3	1	1	4	4
3	3	4	4	3	3	2	2	1	1
3	3	4	4	3	3	2	2	1	1
3	3	4	4	3	3	1	1	4	4
3	3	4	4	3	3	2	2	1	1
3	3	2	2	4	4	1	1	4	4
2	2	4	4	3	3	1	1	4	4
2	2	4	4	3	3	2	2	1	1
2	2	4	4	4	4	1	1	4	4
3	3	2	2	3	3	2	2	1	1
2	2	4	4	3	3	2	2	1	1
2	2	4	4	3	3	2	2	1	1
2	2	4	4	3	3	1	1	4	4
2	2	4	4	4	4	2	2	1	1
3	3	4	4	3	3	2	2	1	1
2	2	4	4	3	3	2	2	1	1
2	2	4	4	3	3	2	2	1	1
3	3	4	4	3	3	2	2	1	1
2	2	4	4	3	3	2	2	1	1
2	2	4	4	4	4	1	1	4	4
2	2	4	4	3	3	1	1	4	4
2	2	4	4	3	3	1	1	4	4
3	3	4	4	3	3	1	1	4	4
2	2	4	4	3	3	2	2	1	1
2	2	4	4	3	3	1	1	4	4
3	3	4	4	3	3	2	2	1	1
3	3	2	2	3	3	1	1	4	4
3	3	4	4	3	3	2	2	1	1
2	2	4	4	3	3	1	1	4	4
3	3	2	2	3	3	2	2	1	1
3	3	2	2	3	3	2	2	1	1
3	3	2	2	3	3	2	2	1	1
3	3	4	4	3	3	2	2	1	1
3	3	2	2	3	3	1	1	4	4
3	3	4	4	3	3	2	2	1	1
3	3	4	4	3	3	1	1	4	4
3	3	4	4	3	3	2	2	1	1
2	2	4	4	3	3	2	2	1	1
2	2	4	4	3	3	2	2	1	1
3	3	4	4	3	3	2	2	1	1
2	2	4	4	3	3	1	1	4	4
3	3	4	4	3	3	1	1	4	4
2	2	4	4	3	3	2	2	1	1
2	2	4	4	3	3	1	1	4	4

For Review Only

3	3	4	4	3	3	2	2	1	1
3	3	4	4	3	3	2	2	1	1

For Review Only

SNP662	SNP662	SNP663	SNP663	SNP664	SNP664	SNP665	SNP665	SNP666	SNP666
4	4	4	4	1	1	2	2	2	2
3	3	1	1	1	1	2	2	2	2
4	4	4	4	2	2	2	2	2	2
3	3	4	4	1	1	2	2	2	2
4	4	4	4	1	1	2	2	2	2
3	3	4	4	1	1	2	2	2	2
4	4	4	4	1	1	2	2	2	2
3	3	4	4	2	2	2	2	2	2
3	3	4	4	1	1	2	2	2	2
4	4	4	4	1	1	2	2	2	2
3	3	4	4	2	2	2	2	2	2
3	3	4	4	2	2	2	2	2	2
4	4	4	4	1	1	2	2	2	2
4	4	4	4	1	1	2	2	2	2
3	3	4	4	1	1	2	2	2	2
3	3	4	4	2	2	2	2	2	2
3	3	4	4	1	1	2	2	2	2
4	4	4	4	1	1	2	2	2	2
3	3	4	4	1	1	2	2	2	2
4	4	4	4	1	1	2	2	2	2
3	3	4	4	1	1	2	2	2	2
3	3	4	4	1	1	2	2	2	2
3	3	4	4	1	1	2	2	2	2
3	3	1	1	1	1	3	3	2	2
4	4	4	4	1	1	2	2	2	2
4	4	4	4	1	1	2	2	3	3
4	4	4	4	1	1	2	2	2	2
3	3	4	4	1	1	2	2	2	2
3	3	4	4	1	1	2	2	2	2
3	3	1	1	1	1	2	2	2	2
4	4	4	4	1	1	2	2	2	2
3	3	4	4	2	2	2	2	2	2
4	4	4	4	2	2	2	2	2	2
3	3	1	1	1	1	2	2	2	2
3	3	4	4	1	1	2	2	2	2
4	4	4	4	1	1	2	2	2	2
3	3	4	4	1	1	2	2	2	2
4	4	4	4	2	2	2	2	2	2
3	3	4	4	1	1	2	2	2	2
3	3	4	4	1	1	2	2	2	2
3	3	4	4	1	1	2	2	2	2
3	3	4	4	1	1	2	2	2	2
3	3	4	4	1	1	2	2	2	2
4	4	4	4	1	1	2	2	2	2
3	3	4	4	1	1	2	2	2	2
4	4	4	4	1	1	2	2	2	2
3	3	4	4	1	1	2	2	3	3
3	3	4	4	1	1	3	3	2	2
4	4	4	4	2	2	2	2	2	2
3	3	4	4	1	1	2	2	2	2
4	4	1	1	1	1	2	2	3	3
3	3	4	4	1	1	2	2	3	3

For Review Only

4	4	4	4	1	1	2	2	2	2
4	4	4	4	2	2	2	2	2	2
4	4	4	4	2	2	2	2	2	2
4	4	4	4	1	1	2	2	2	2
3	3	4	4	1	1	2	2	2	2
4	4	4	4	1	1	2	2	3	3
4	4	4	4	1	1	3	3	3	3
3	3	1	1	1	1	2	2	2	2
3	3	4	4	1	1	2	2	2	2
3	3	4	4	1	1	2	2	2	2
4	4	4	4	1	1	2	2	2	2
4	4	4	4	1	1	2	2	2	2
4	4	4	4	1	1	2	2	2	2
3	3	4	4	1	1	2	2	2	2
3	3	4	4	1	1	2	2	2	2
3	3	4	4	1	1	2	2	2	2
3	3	4	4	1	1	2	2	2	2
4	4	4	4	2	2	2	2	2	2
3	3	4	4	1	1	2	2	2	2
4	4	4	4	1	1	2	2	2	2
4	4	4	4	1	1	2	2	2	2
3	3	4	4	1	1	2	2	2	2
3	3	4	4	1	1	2	2	2	2
4	4	4	4	1	1	2	2	2	2
3	3	4	4	1	1	2	2	2	2
4	4	4	4	1	1	2	2	2	2
4	4	4	4	2	2	2	2	2	2
3	3	4	4	1	1	2	2	2	2
4	4	4	4	1	1	2	2	3	3
4	4	4	4	1	1	2	2	2	2
4	4	4	4	1	1	2	2	2	2
3	3	1	1	1	1	2	2	3	3
3	3	4	4	1	1	2	2	2	2
4	4	4	4	1	1	2	2	2	2
4	4	4	4	2	2	2	2	2	2
3	3	4	4	1	1	2	2	2	2
3	3	4	4	1	1	2	2	2	2
3	3	4	4	1	1	2	2	2	2
4	4	1	1	1	1	2	2	2	2
3	3	4	4	1	1	2	2	2	2
4	4	4	4	2	2	2	2	2	2
3	3	4	4	1	1	2	2	2	2
4	4	1	1	1	1	2	2	2	2
4	4	4	4	1	1	2	2	2	2
4	4	1	1	1	1	2	2	2	2
3	3	4	4	1	1	2	2	2	2
3	3	1	1	1	1	2	2	2	2
4	4	4	4	1	1	3	3	3	3
3	3	4	4	1	1	3	3	3	3
4	4	4	4	1	1	2	2	2	2
3	3	4	4	1	1	2	2	3	3
3	3	4	4	1	1	2	2	2	2
3	3	1	1	1	1	2	2	2	2
3	3	4	4	1	1	2	2	2	2
3	3	4	4	2	2	2	2	2	2

For Review Only

3	3	4	4	1	1	2	2	3	3
3	3	4	4	1	1	2	2	2	2
4	4	4	4	1	1	2	2	3	3
3	3	4	4	1	1	2	2	2	2
3	3	4	4	1	1	2	2	2	2
4	4	4	4	1	1	2	2	2	2
3	3	4	4	2	2	2	2	2	2
3	3	4	4	1	1	2	2	2	2
3	3	4	4	1	1	2	2	3	3
3	3	4	4	1	1	2	2	2	2
4	4	4	4	1	1	2	2	2	2
3	3	4	4	1	1	2	2	2	2
3	3	1	1	1	1	2	2	2	2
3	3	4	4	1	1	2	2	2	2
3	3	4	4	1	1	2	2	2	2
3	3	4	4	1	1	2	2	2	2
3	3	4	4	2	2	2	2	2	2
3	3	4	4	1	1	2	2	2	2
3	3	4	4	1	1	2	2	2	2
3	3	4	4	1	1	2	2	2	2
3	3	4	4	1	1	2	2	2	2
3	3	4	4	2	2	2	2	2	2
3	3	4	4	1	1	2	2	2	2
3	3	1	1	1	1	2	2	2	2
3	3	4	4	1	1	3	3	2	2
3	3	4	4	1	1	2	2	2	2
4	4	4	4	1	1	2	2	2	2
4	4	1	1	1	1	2	2	2	2
3	3	4	4	1	1	2	2	3	3
4	4	4	4	1	1	2	2	2	2
3	3	1	1	1	1	2	2	2	2
3	3	4	4	1	1	2	2	2	2
4	4	4	4	2	2	2	2	2	2
3	3	4	4	1	1	3	3	2	2
3	3	1	1	1	1	2	2	2	2
3	3	4	4	1	1	2	2	2	2
3	3	1	1	1	1	2	2	2	2
4	4	4	4	1	1	3	3	2	2
3	3	4	4	1	1	2	2	2	2
3	3	4	4	1	1	2	2	2	2
4	4	4	4	1	1	2	2	2	2
3	3	1	1	1	1	2	2	2	2
4	4	4	4	1	1	2	2	3	3
3	3	1	1	1	1	2	2	2	2
3	3	1	1	1	1	3	3	2	2
4	4	1	1	1	1	2	2	2	2
4	4	4	4	1	1	3	3	3	3
4	4	4	4	1	1	2	2	3	3
4	4	4	4	1	1	2	2	2	2
4	4	4	4	1	1	2	2	3	3
4	4	4	4	1	1	2	2	3	3
3	3	4	4	1	1	2	2	2	2
3	3	4	4	1	1	2	2	2	2
4	4	4	4	1	1	2	2	2	2
3	3	4	4	1	1	2	2	2	2

For Review Only

4	4	4	4	1	1	3	3	2	2
3	3	1	1	1	1	2	2	2	2
3	3	4	4	1	1	2	2	2	2
3	3	4	4	1	1	2	2	2	2
4	4	4	4	1	1	2	2	2	2
3	3	4	4	2	2	2	2	2	2
3	3	4	4	1	1	2	2	2	2
4	4	4	4	1	1	2	2	2	2
3	3	4	4	1	1	2	2	2	2
4	4	4	4	1	1	2	2	2	2
4	4	4	4	1	1	2	2	2	2
3	3	4	4	1	1	2	2	2	2
4	4	4	4	1	1	2	2	2	2
3	3	4	4	1	1	2	2	2	2
3	3	4	4	1	1	2	2	2	2
3	3	4	4	2	2	2	2	2	2
3	3	4	4	1	1	2	2	2	2
4	4	1	1	1	1	2	2	2	2
3	3	1	1	1	1	2	2	3	3
3	3	4	4	2	2	2	2	2	2
3	3	4	4	1	1	2	2	2	2
3	3	4	4	1	1	2	2	2	2
3	3	4	4	1	1	2	2	2	2
4	4	4	4	1	1	2	2	2	2
3	3	1	1	1	1	2	2	2	2
3	3	1	1	1	1	2	2	2	2
3	3	4	4	1	1	2	2	2	2
3	3	4	4	1	1	2	2	2	2
3	3	4	4	1	1	2	2	2	2
4	4	4	4	1	1	2	2	2	2
4	4	4	4	2	2	2	2	2	2
4	4	4	4	1	1	2	2	2	2
4	4	4	4	1	1	2	2	2	2
3	3	4	4	1	1	2	2	2	2
4	4	4	4	1	1	2	2	2	2
4	4	4	4	1	1	2	2	2	2
4	4	4	4	1	1	2	2	3	3
4	4	4	4	1	1	2	2	2	2
4	4	4	4	1	1	2	2	3	3
4	4	4	4	1	1	2	2	3	3
4	4	4	4	2	2	2	2	2	2
4	4	4	4	1	1	3	3	3	3
3	3	1	1	1	1	2	2	2	2
3	3	4	4	1	1	2	2	2	2
4	4	4	4	1	1	2	2	2	2
4	4	4	4	1	1	2	2	2	2
3	3	4	4	2	2	2	2	2	2
3	3	4	4	2	2	2	2	2	2
3	3	1	1	1	1	2	2	2	2
3	3	4	4	1	1	2	2	2	2
3	3	4	4	1	1	2	2	3	3
3	3	1	1	1	1	2	2	2	2
3	3	4	4	1	1	2	2	2	2
4	4	4	4	1	1	2	2	2	2
3	3	1	1	1	1	2	2	2	2

For Review Only

3	3	4	4	1	1	2	2	3	3
3	3	4	4	1	1	2	2	2	2
4	4	4	4	1	1	2	2	2	2
3	3	4	4	1	1	2	2	2	2
3	3	4	4	1	1	2	2	2	2
4	4	4	4	1	1	2	2	2	2
4	4	4	4	1	1	2	2	2	2
3	3	1	1	1	1	2	2	2	2
3	3	1	1	1	1	2	2	2	2
3	3	4	4	1	1	2	2	2	2
3	3	4	4	1	1	2	2	2	2
3	3	4	4	2	2	2	2	2	2
4	4	4	4	1	1	2	2	2	2
4	4	4	4	1	1	2	2	3	3
4	4	4	4	2	2	2	2	2	2
4	4	4	4	2	2	2	2	2	2
4	4	4	4	1	1	2	2	2	2
3	3	4	4	1	1	2	2	2	2
3	3	4	4	1	1	2	2	2	2
3	3	4	4	2	2	2	2	2	2
4	4	4	4	1	1	2	2	2	2
3	3	4	4	1	1	3	3	2	2
3	3	4	4	1	1	2	2	2	2
3	3	4	4	1	1	2	2	2	2
3	3	1	1	1	1	2	2	2	2
4	4	4	4	1	1	2	2	2	2
3	3	1	1	1	1	2	2	2	2
3	3	4	4	1	1	2	2	2	2
3	3	4	4	1	1	2	2	2	2
3	3	4	4	1	1	2	2	3	3
3	3	1	1	1	1	2	2	2	2
3	3	4	4	1	1	3	3	2	2
3	3	4	4	1	1	2	2	2	2
4	4	4	4	1	1	2	2	3	3
3	3	4	4	1	1	2	2	2	2
3	3	1	1	1	1	2	2	2	2
4	4	4	4	1	1	2	2	3	3
4	4	4	4	1	1	2	2	2	2
4	4	4	4	1	1	2	2	2	2
3	3	1	1	1	1	2	2	2	2
4	4	4	4	1	1	2	2	2	2
4	4	4	4	1	1	2	2	3	3
4	4	4	4	1	1	2	2	2	2
4	4	4	4	1	1	2	2	2	2
4	4	4	4	1	1	2	2	2	2
4	4	1	1	1	1	2	2	2	2
4	4	4	4	1	1	2	2	3	3
4	4	1	1	2	2	2	2	2	2
3	3	4	4	1	1	2	2	3	3
3	3	1	1	1	1	2	2	2	2
4	4	4	4	2	2	2	2	2	2
3	3	1	1	1	1	2	2	2	2
4	4	4	4	2	2	2	2	2	2
3	3	4	4	1	1	2	2	3	3
3	3	4	4	2	2	2	2	2	2

For Review Only

4	4	4	4	2	2	2	2	2	2
4	4	4	4	1	1	2	2	3	3

For Review Only

SNP667	SNP667	SNP668	SNP668	SNP669	SNP669	SNP670	SNP670	SNP671	SNP671
1	1	2	2	3	3	2	2	4	4
1	1	4	4	3	3	2	2	4	4
1	1	2	2	3	3	2	2	1	1
1	1	2	2	3	3	2	2	4	4
2	2	2	2	3	3	2	2	4	4
1	1	2	2	3	3	2	2	4	4
1	1	2	2	3	3	2	2	1	1
1	1	2	2	3	3	2	2	4	4
1	1	2	2	2	2	2	2	4	4
1	1	2	2	3	3	2	2	4	4
1	1	4	4	2	2	2	2	1	1
1	1	2	2	3	3	2	2	4	4
1	1	4	4	3	3	2	2	4	4
1	1	2	2	2	2	2	2	4	4
1	1	4	4	3	3	2	2	4	4
1	1	4	4	3	3	2	2	1	1
1	1	2	2	3	3	2	2	1	1
1	1	2	2	3	3	2	2	1	1
1	1	4	4	3	3	2	2	1	1
1	1	2	2	3	3	2	2	4	4
2	2	2	2	3	3	2	2	4	4
1	1	2	2	3	3	2	2	4	4
1	1	2	2	2	2	2	2	4	4
1	1	2	2	3	3	2	2	4	4
2	2	4	4	2	2	2	2	4	4
1	1	2	2	3	3	2	2	4	4
1	1	2	2	3	3	2	2	4	4
1	1	4	4	3	3	2	2	4	4
1	1	2	2	3	3	2	2	1	1
1	1	2	2	3	3	2	2	4	4
1	1	4	4	2	2	2	2	4	4
1	1	4	4	3	3	2	2	1	1
1	1	2	2	3	3	2	2	1	1
1	1	2	2	3	3	2	2	4	4
1	1	2	2	2	2	2	2	4	4
1	1	2	2	3	3	2	2	4	4
1	1	2	2	2	2	2	2	4	4
1	1	2	2	2	2	2	2	1	1
1	1	2	2	2	2	2	2	4	4
1	1	2	2	2	2	2	2	1	1
1	1	2	2	3	3	2	2	4	4
1	1	2	2	3	3	2	2	1	1
1	1	2	2	3	3	2	2	4	4
1	1	2	2	2	2	2	2	4	4
1	1	2	2	3	3	2	2	4	4
1	1	2	2	3	3	2	2	1	1
1	1	2	2	3	3	2	2	4	4
1	1	2	2	3	3	2	2	4	4
1	1	2	2	3	3	2	2	1	1
1	1	4	4	3	3	2	2	4	4
1	1	2	2	3	3	2	2	4	4
1	1	4	4	3	3	2	2	4	4

For Review Only

2	2	2	2	3	3	2	2	4	4
1	1	2	2	3	3	2	2	1	1
1	1	4	4	2	2	2	2	1	1
2	2	2	2	3	3	2	2	4	4
1	1	4	4	3	3	2	2	1	1
1	1	2	2	3	3	2	2	4	4
1	1	2	2	3	3	2	2	4	4
1	1	4	4	3	3	3	3	4	4
1	1	2	2	2	2	2	2	4	4
1	1	4	4	3	3	2	2	1	1
1	1	2	2	3	3	2	2	4	4
1	1	2	2	3	3	2	2	4	4
2	2	2	2	3	3	2	2	4	4
1	1	2	2	3	3	2	2	1	1
1	1	2	2	2	2	2	2	4	4
1	1	2	2	3	3	2	2	4	4
1	1	2	2	3	3	2	2	4	4
1	1	4	4	2	2	2	2	1	1
1	1	4	4	3	3	2	2	4	4
1	1	4	4	3	3	2	2	4	4
1	1	2	2	3	3	2	2	4	4
1	1	4	4	2	2	2	2	4	4
1	1	2	2	3	3	2	2	4	4
1	1	2	2	3	3	2	2	4	4
1	1	2	2	3	3	2	2	4	4
1	1	2	2	3	3	2	2	1	1
1	1	2	2	3	3	3	3	1	1
1	1	2	2	2	2	2	2	4	4
1	1	2	2	3	3	2	2	4	4
1	1	4	4	3	3	2	2	4	4
2	2	2	2	3	3	2	2	1	1
1	1	4	4	3	3	2	2	4	4
1	1	2	2	3	3	2	2	4	4
2	2	2	2	3	3	2	2	4	4
1	1	2	2	3	3	2	2	1	1
1	1	2	2	3	3	2	2	4	4
1	1	2	2	3	3	3	3	1	1
1	1	2	2	3	3	2	2	4	4
1	1	2	2	3	3	2	2	4	4
1	1	2	2	3	3	3	3	4	4
1	1	2	2	3	3	2	2	1	1
1	1	4	4	3	3	2	2	4	4
1	1	4	4	3	3	3	3	4	4
1	1	2	2	3	3	2	2	4	4
1	1	4	4	2	2	2	2	1	1
1	1	2	2	3	3	2	2	4	4
1	1	4	4	2	2	2	2	4	4
1	1	4	4	3	3	2	2	4	4
1	1	2	2	3	3	2	2	4	4
1	1	2	2	2	2	2	2	4	4
1	1	2	2	3	3	2	2	4	4
1	1	2	2	2	2	2	2	4	4
1	1	4	4	2	2	2	2	4	4
1	1	4	4	3	3	2	2	4	4
1	1	4	4	3	3	3	3	1	1

For Review Only

2	2	2	2	3	3	2	2	4	4
1	1	2	2	2	2	2	2	4	4
1	1	2	2	3	3	2	2	4	4
1	1	2	2	2	2	2	2	4	4
1	1	4	4	3	3	2	2	1	1
1	1	2	2	3	3	2	2	4	4
1	1	4	4	3	3	2	2	1	1
1	1	2	2	2	2	2	2	4	4
1	1	2	2	3	3	2	2	4	4
1	1	4	4	3	3	2	2	4	4
1	1	2	2	2	2	2	2	4	4
1	1	4	4	2	2	2	2	4	4
1	1	4	4	3	3	3	3	4	4
1	1	4	4	3	3	2	2	4	4
1	1	4	4	3	3	2	2	4	4
1	1	2	2	3	3	2	2	4	4
1	1	4	4	3	3	2	2	1	1
1	1	2	2	3	3	2	2	4	4
1	1	2	2	2	2	2	2	4	4
1	1	4	4	2	2	2	2	4	4
1	1	4	4	2	2	2	2	4	4
1	1	4	4	3	3	2	2	1	1
1	1	4	4	3	3	2	2	4	4
1	1	2	2	3	3	2	2	4	4
1	1	2	2	3	3	2	2	4	4
1	1	2	2	3	3	2	2	4	4
1	1	4	4	3	3	2	2	4	4
1	1	2	2	3	3	2	2	4	4
1	1	2	2	3	3	2	2	4	4
1	1	4	4	3	3	2	2	4	4
1	1	4	4	3	3	2	2	4	4
1	1	4	4	2	2	2	2	4	4
1	1	2	2	3	3	2	2	1	1
1	1	4	4	3	3	2	2	4	4
1	1	2	2	3	3	2	2	4	4
1	1	2	2	2	2	2	2	4	4
1	1	4	4	2	2	2	2	4	4
1	1	4	4	3	3	2	2	4	4
1	1	2	2	2	2	2	2	4	4
1	1	2	2	2	2	2	2	4	4
1	1	4	4	3	3	2	2	4	4
1	1	4	4	3	3	3	3	4	4
1	1	4	4	2	2	2	2	4	4
1	1	2	2	3	3	2	2	4	4
1	1	2	2	3	3	2	2	4	4
1	1	2	2	3	3	2	2	1	1
2	2	2	2	3	3	2	2	4	4
1	1	2	2	3	3	2	2	4	4
1	1	4	4	2	2	2	2	4	4
1	1	2	2	3	3	2	2	4	4
2	2	2	2	3	3	2	2	4	4
1	1	2	2	3	3	3	3	4	4
1	1	4	4	2	2	2	2	4	4
1	1	2	2	3	3	2	2	4	4
1	1	4	4	2	2	2	2	4	4

For Review Only

2	2	2	2	3	3	2	2	4	4
1	1	4	4	3	3	3	3	4	4
1	1	2	2	2	2	2	2	1	1
1	1	4	4	2	2	2	2	4	4
1	1	2	2	3	3	2	2	4	4
1	1	4	4	2	2	2	2	1	1
1	1	2	2	2	2	2	2	4	4
1	1	2	2	2	2	2	2	4	4
1	1	2	2	2	2	2	2	4	4
2	2	4	4	3	3	2	2	4	4
1	1	2	2	3	3	2	2	4	4
1	1	2	2	2	2	2	2	4	4
1	1	2	2	3	3	2	2	1	1
1	1	2	2	3	3	2	2	4	4
1	1	4	4	2	2	2	2	4	4
1	1	4	4	2	2	2	2	1	1
1	1	4	4	2	2	2	2	4	4
1	1	4	4	3	3	2	2	4	4
1	1	2	2	3	3	2	2	4	4
1	1	4	4	3	3	2	2	1	1
1	1	4	4	3	3	2	2	4	4
1	1	2	2	3	3	2	2	4	4
1	1	4	4	2	2	2	2	4	4
1	1	2	2	3	3	2	2	4	4
1	1	2	2	3	3	2	2	1	1
1	1	2	2	3	3	2	2	4	4
1	1	4	4	3	3	2	2	4	4
1	1	4	4	3	3	2	2	4	4
1	1	2	2	3	3	2	2	4	4
2	2	4	4	3	3	2	2	4	4
1	1	2	2	3	3	2	2	1	1
1	1	4	4	3	3	2	2	4	4
1	1	4	4	2	2	2	2	1	1
1	1	2	2	2	2	2	2	4	4
1	1	2	2	3	3	2	2	4	4
1	1	2	2	3	3	2	2	4	4
1	1	2	2	2	2	2	2	4	4
1	1	4	4	3	3	2	2	4	4
1	1	2	2	3	3	2	2	4	4
1	1	4	4	3	3	2	2	4	4
1	1	2	2	3	3	2	2	1	1
2	2	2	2	3	3	2	2	4	4
1	1	2	2	2	2	2	2	4	4
1	1	4	4	2	2	2	2	4	4
1	1	2	2	2	2	2	2	1	1
1	1	4	4	2	2	2	2	4	4
1	1	4	4	2	2	2	2	1	1
1	1	2	2	2	2	2	2	1	1
1	1	2	2	3	3	3	3	4	4
1	1	2	2	3	3	2	2	4	4
1	1	4	4	3	3	2	2	4	4
1	1	4	4	2	2	2	2	1	1
1	1	2	2	2	2	2	2	4	4
1	1	2	2	3	3	2	2	4	4
1	1	2	2	2	2	2	2	4	4

For Review Only

1	1	4	4	3	3	2	2	4	4
1	1	2	2	3	3	2	2	4	4
1	1	2	2	3	3	2	2	4	4
1	1	4	4	2	2	2	2	4	4
1	1	2	2	3	3	2	2	4	4
1	1	4	4	3	3	2	2	4	4
1	1	2	2	2	2	2	2	4	4
1	1	2	2	3	3	3	3	4	4
1	1	2	2	2	2	2	2	4	4
1	1	2	2	2	2	2	2	4	4
1	1	4	4	2	2	2	2	4	4
1	1	4	4	3	3	2	2	1	1
1	1	2	2	3	3	2	2	4	4
1	1	4	4	3	3	2	2	4	4
1	1	2	2	3	3	2	2	1	1
1	1	2	2	3	3	2	2	1	1
1	1	2	2	2	2	2	2	4	4
1	1	4	4	2	2	2	2	4	4
1	1	2	2	3	3	2	2	4	4
1	1	2	2	2	2	2	2	1	1
1	1	2	2	3	3	2	2	4	4
1	1	2	2	3	3	2	2	4	4
1	1	4	4	3	3	2	2	4	4
1	1	2	2	2	2	2	2	4	4
1	1	4	4	3	3	2	2	4	4
1	1	4	4	2	2	2	2	4	4
1	1	2	2	3	3	2	2	4	4
1	1	2	2	3	3	2	2	4	4
1	1	2	2	3	3	2	2	4	4
1	1	4	4	3	3	2	2	4	4
1	1	2	2	2	2	2	2	4	4
1	1	2	2	3	3	2	2	4	4
1	1	2	2	3	3	2	2	4	4
1	1	4	4	3	3	2	2	4	4
1	1	2	2	2	2	2	2	4	4
1	1	2	2	2	2	2	2	4	4
2	2	4	4	3	3	2	2	4	4
1	1	2	2	3	3	2	2	4	4
1	1	2	2	3	3	2	2	4	4
1	1	2	2	2	2	2	2	4	4
1	1	2	2	3	3	2	2	4	4
1	1	2	2	3	3	2	2	4	4
1	1	2	2	2	2	2	2	4	4
2	2	2	2	3	3	2	2	4	4
1	1	2	2	3	3	2	2	4	4
1	1	2	2	3	3	2	2	4	4
1	1	2	2	2	2	2	2	4	4
1	1	4	4	3	3	2	2	1	1
1	1	2	2	3	3	2	2	4	4
1	1	2	2	3	3	2	2	4	4
1	1	2	2	3	3	2	2	1	1
1	1	2	2	2	2	2	2	4	4
1	1	2	2	3	3	2	2	1	1
1	1	4	4	3	3	2	2	4	4
1	1	2	2	2	2	2	2	1	1

For Review Only

1	1	4	4	3	3	2	2	1	1
1	1	4	4	3	3	2	2	4	4

For Review Only

SNP672	SNP672	SNP673	SNP673	SNP674	SNP674	SNP675	SNP675	SNP676	SNP676
4	4	1	1	3	3	4	4	1	1
4	4	1	1	4	4	4	4	1	1
4	4	4	4	4	4	3	3	1	1
4	4	1	1	3	3	4	4	1	1
4	4	1	1	3	3	4	4	1	1
4	4	1	1	3	3	4	4	4	4
4	4	1	1	3	3	4	4	1	1
4	4	4	4	4	4	3	3	1	1
4	4	1	1	3	3	4	4	1	1
4	4	1	1	3	3	4	4	1	1
1	1	4	4	4	4	3	3	1	1
4	4	4	4	4	4	3	3	1	1
4	4	1	1	3	3	4	4	1	1
4	4	1	1	3	3	4	4	1	1
4	4	1	1	3	3	4	4	1	1
4	4	4	4	4	4	3	3	1	1
4	4	1	1	3	3	4	4	1	1
1	1	1	1	3	3	4	4	1	1
4	4	1	1	3	3	4	4	1	1
4	4	1	1	3	3	4	4	4	4
4	4	1	1	3	3	4	4	1	1
4	4	1	1	3	3	4	4	1	1
1	1	1	1	3	3	4	4	1	1
4	4	1	1	3	3	4	4	4	4
4	4	1	1	3	3	4	4	1	1
4	4	1	1	3	3	4	4	1	1
4	4	1	1	3	3	4	4	1	1
4	4	4	4	3	3	4	4	1	1
4	4	1	1	3	3	4	4	1	1
1	1	1	1	3	3	4	4	1	1
4	4	1	1	3	3	4	4	1	1
4	4	4	4	4	4	3	3	1	1
4	4	4	4	4	4	3	3	1	1
4	4	1	1	3	3	4	4	1	1
4	4	1	1	3	3	4	4	1	1
4	4	1	1	3	3	4	4	1	1
4	4	1	1	3	3	4	4	1	1
1	1	4	4	4	4	3	3	1	1
1	1	1	1	3	3	4	4	1	1
1	1	4	4	4	4	4	4	1	1
4	4	1	1	3	3	4	4	4	4
4	4	1	1	3	3	4	4	1	1
4	4	1	1	3	3	4	4	1	1
4	4	1	1	3	3	4	4	1	1
4	4	1	1	3	3	4	4	1	1
4	4	4	4	4	4	4	4	1	1
4	4	1	1	3	3	4	4	1	1
4	4	1	1	3	3	4	4	1	1
4	4	4	4	4	4	4	4	1	1
4	4	1	1	3	3	4	4	1	1
4	4	1	1	3	3	4	4	1	1
4	4	1	1	3	3	4	4	1	1

For Review Only

4	4	1	1	3	3	4	4	1	1
4	4	4	4	4	4	3	3	1	1
4	4	1	1	3	3	4	4	1	1
4	4	1	1	3	3	4	4	1	1
4	4	1	1	3	3	4	4	1	1
4	4	1	1	3	3	4	4	1	1
4	4	1	1	3	3	4	4	1	1
1	1	1	1	3	3	4	4	1	1
4	4	1	1	3	3	4	4	1	1
4	4	4	4	4	4	4	4	1	1
4	4	1	1	3	3	4	4	1	1
4	4	1	1	3	3	4	4	1	1
4	4	1	1	3	3	4	4	1	1
4	4	1	1	3	3	4	4	1	1
4	4	1	1	3	3	4	4	1	1
4	4	1	1	3	3	4	4	1	1
4	4	1	1	3	3	4	4	1	1
4	4	4	4	3	3	4	4	1	1
4	4	1	1	3	3	4	4	1	1
4	4	1	1	3	3	4	4	1	1
4	4	1	1	3	3	4	4	1	1
4	4	1	1	3	3	4	4	1	1
4	4	1	1	3	3	4	4	1	1
4	4	1	1	3	3	4	4	1	1
4	4	1	1	3	3	4	4	1	1
4	4	1	1	3	3	4	4	1	1
4	4	4	4	4	4	3	3	1	1
4	4	1	1	3	3	4	4	1	1
4	4	1	1	3	3	4	4	1	1
4	4	1	1	3	3	4	4	1	1
4	4	1	1	3	3	4	4	1	1
4	4	1	1	3	3	4	4	4	4
4	4	1	1	3	3	4	4	1	1
4	4	1	1	3	3	4	4	1	1
4	4	4	4	4	4	3	3	1	1
4	4	1	1	3	3	4	4	1	1
4	4	1	1	3	3	4	4	1	1
4	4	1	1	3	3	4	4	1	1
4	4	1	1	3	3	4	4	1	1
4	4	1	1	3	3	4	4	1	1
4	4	4	4	4	4	3	3	1	1
4	4	1	1	3	3	4	4	1	1
4	4	1	1	3	3	4	4	1	1
4	4	1	1	3	3	4	4	1	1
4	4	4	4	4	4	3	3	1	1
4	4	4	4	3	3	4	4	1	1
4	4	1	1	3	3	4	4	1	1
4	4	1	1	3	3	4	4	1	1
4	4	1	1	3	3	4	4	1	1
4	4	1	1	3	3	4	4	1	1
4	4	1	1	3	3	4	4	1	1
1	1	1	1	3	3	4	4	1	1
1	1	1	1	3	3	4	4	1	1
4	4	4	4	3	3	4	4	1	1
4	4	4	4	4	4	4	4	1	1

For Review Only

4	4	4	4	3	3	4	4	1	1
1	1	1	1	3	3	4	4	1	1
4	4	1	1	3	3	4	4	1	1
4	4	1	1	3	3	4	4	1	1
4	4	4	4	3	3	4	4	1	1
4	4	1	1	3	3	4	4	1	1
4	4	4	4	4	4	3	3	1	1
4	4	1	1	3	3	4	4	1	1
4	4	1	1	3	3	4	4	1	1
4	4	1	1	3	3	4	4	1	1
4	4	4	4	3	3	4	4	1	1
4	4	4	4	3	3	4	4	1	1
4	4	1	1	3	3	4	4	1	1
4	4	1	1	3	3	4	4	1	1
4	4	1	1	4	4	4	4	1	1
4	4	1	1	3	3	4	4	1	1
4	4	4	4	3	3	4	4	1	1
4	4	4	4	3	3	4	4	1	1
4	4	1	1	3	3	4	4	1	1
4	4	4	4	3	3	4	4	1	1
4	4	1	1	3	3	4	4	1	1
4	4	4	4	4	4	3	3	1	1
4	4	1	1	3	3	4	4	4	4
4	4	1	1	3	3	4	4	1	1
4	4	1	1	3	3	4	4	1	1
1	1	1	1	3	3	4	4	1	1
4	4	4	4	3	3	4	4	1	1
4	4	1	1	3	3	4	4	1	1
4	4	1	1	3	3	4	4	1	1
4	4	1	1	3	3	4	4	1	1
4	4	1	1	3	3	4	4	1	1
4	4	1	1	3	3	4	4	1	1
4	4	4	4	4	4	3	3	1	1
4	4	1	1	3	3	4	4	1	1
4	4	1	1	3	3	4	4	1	1
4	4	1	1	3	3	4	4	1	1
4	4	1	1	3	3	4	4	1	1
4	4	1	1	3	3	4	4	1	1
4	4	4	4	3	3	4	4	1	1
1	1	4	4	4	4	3	3	1	1
4	4	1	1	3	3	4	4	1	1
4	4	1	1	3	3	4	4	1	1
4	4	1	1	3	3	4	4	1	1
4	4	1	1	3	3	4	4	1	1
4	4	1	1	3	3	4	4	1	1
4	4	4	4	4	4	4	4	1	1
4	4	1	1	3	3	4	4	1	1
4	4	1	1	3	3	4	4	1	1
4	4	1	1	3	3	4	4	1	1
4	4	1	1	3	3	4	4	1	1
4	4	1	1	3	3	4	4	1	1
1	1	1	1	3	3	4	4	1	1
4	4	1	1	3	3	4	4	1	1
4	4	1	1	3	3	4	4	1	1
4	4	1	1	3	3	4	4	1	1

For Review Only

4	4	1	1	3	3	4	4	1	1
1	1	1	1	3	3	4	4	1	1
4	4	4	4	4	4	4	4	1	1
1	1	1	1	3	3	4	4	1	1
4	4	1	1	4	4	4	4	1	1
4	4	4	4	4	4	3	3	1	1
4	4	1	1	3	3	4	4	1	1
4	4	1	1	3	3	4	4	1	1
4	4	1	1	3	3	4	4	1	1
4	4	1	1	3	3	4	4	1	1
4	4	1	1	3	3	4	4	1	1
4	4	1	1	3	3	4	4	4	4
4	4	1	1	3	3	4	4	1	1
4	4	1	1	3	3	4	4	1	1
4	4	1	1	3	3	4	4	1	1
1	1	4	4	4	4	3	3	1	1
4	4	1	1	3	3	4	4	1	1
4	4	1	1	4	4	4	4	1	1
4	4	1	1	3	3	4	4	4	4
4	4	4	4	4	4	3	3	1	1
4	4	1	1	3	3	4	4	1	1
4	4	1	1	3	3	4	4	4	4
4	4	1	1	3	3	4	4	1	1
4	4	1	1	3	3	4	4	1	1
1	1	1	1	3	3	4	4	1	1
4	4	1	1	3	3	4	4	1	1
4	4	1	1	3	3	4	4	1	1
4	4	4	4	3	3	4	4	1	1
4	4	1	1	3	3	4	4	1	1
4	4	1	1	3	3	4	4	1	1
4	4	1	1	3	3	4	4	1	1
4	4	1	1	3	3	4	4	1	1
4	4	4	4	3	3	4	4	1	1
4	4	1	1	3	3	4	4	1	1
4	4	4	4	3	3	4	4	1	1
4	4	1	1	3	3	4	4	1	1
4	4	1	1	3	3	4	4	1	1
4	4	1	1	3	3	4	4	1	1
4	4	1	1	3	3	4	4	1	1
4	4	1	1	3	3	4	4	1	1
4	4	4	4	4	4	3	3	1	1
4	4	1	1	3	3	4	4	1	1
1	1	1	1	3	3	4	4	1	1
4	4	1	1	3	3	4	4	1	1
4	4	4	4	3	3	4	4	1	1
4	4	1	1	3	3	4	4	1	1
1	1	4	4	4	4	3	3	1	1
4	4	4	4	4	4	3	3	1	1
4	4	1	1	3	3	4	4	4	4
4	4	1	1	3	3	4	4	1	1
4	4	1	1	3	3	4	4	1	1
1	1	4	4	3	3	4	4	1	1
4	4	1	1	3	3	4	4	1	1
4	4	1	1	3	3	4	4	1	1
4	4	1	1	3	3	4	4	1	1

For Review Only

4	4	1	1	3	3	4	4	1	1
4	4	1	1	3	3	4	4	1	1
4	4	4	4	4	4	4	4	1	1
4	4	1	1	3	3	4	4	1	1
4	4	1	1	3	3	4	4	1	1
4	4	1	1	3	3	4	4	1	1
4	4	1	1	3	3	4	4	1	1
4	4	1	1	3	3	4	4	4	4
4	4	1	1	3	3	4	4	1	1
4	4	1	1	3	3	4	4	1	1
4	4	4	4	3	3	4	4	1	1
4	4	1	1	3	3	4	4	1	1
4	4	1	1	3	3	4	4	1	1
4	4	1	1	3	3	4	4	1	1
4	4	4	4	4	4	3	3	1	1
4	4	4	4	4	4	3	3	1	1
1	1	1	1	3	3	4	4	1	1
4	4	1	1	3	3	4	4	1	1
4	4	1	1	3	3	4	4	1	1
1	1	4	4	3	3	4	4	1	1
4	4	1	1	3	3	4	4	1	1
4	4	1	1	3	3	4	4	1	1
4	4	1	1	3	3	4	4	1	1
4	4	1	1	3	3	4	4	1	1
1	1	1	1	3	3	4	4	1	1
4	4	1	1	3	3	4	4	1	1
4	4	1	1	3	3	4	4	1	1
4	4	1	1	3	3	4	4	1	1
4	4	1	1	3	3	4	4	1	1
4	4	1	1	3	3	4	4	1	1
1	1	1	1	3	3	4	4	1	1
4	4	1	1	3	3	4	4	1	1
4	4	1	1	3	3	4	4	1	1
4	4	1	1	3	3	4	4	1	1
4	4	1	1	3	3	4	4	1	1
4	4	1	1	3	3	4	4	1	1
4	4	1	1	3	3	4	4	1	1
4	4	1	1	3	3	4	4	1	1
4	4	1	1	3	3	4	4	1	1
1	1	1	1	3	3	4	4	1	1
4	4	1	1	3	3	4	4	1	1
4	4	4	4	4	4	4	4	1	1
4	4	1	1	3	3	4	4	1	1
4	4	1	1	3	3	4	4	1	1
4	4	1	1	3	3	4	4	1	1
4	4	1	1	3	3	4	4	1	1
4	4	1	1	3	3	4	4	1	1
4	4	4	4	4	4	4	4	1	1
4	4	1	1	3	3	4	4	4	4
4	4	1	1	3	3	4	4	1	1
4	4	4	4	4	4	3	3	1	1
1	1	1	1	3	3	4	4	1	1
4	4	4	4	4	4	3	3	1	1
4	4	1	1	3	3	4	4	1	1
1	1	4	4	4	4	3	3	1	1

For Review Only

4	4	4	4	4	4	3	3	1	1
4	4	4	4	3	3	4	4	1	1

For Review Only

SNP677	SNP677	SNP678	SNP678	SNP679	SNP679	SNP680	SNP680	SNP681	SNP681
4	4	1	1	2	2	3	3	4	4
2	2	1	1	1	1	3	3	1	1
2	2	1	1	2	2	3	3	1	1
2	2	1	1	2	2	3	3	4	4
4	4	1	1	2	2	3	3	4	4
2	2	3	3	2	2	3	3	4	4
4	4	1	1	2	2	3	3	4	4
2	2	1	1	1	1	3	3	1	1
4	4	1	1	2	2	3	3	4	4
2	2	1	1	1	1	3	3	4	4
2	2	1	1	2	2	3	3	1	1
2	2	1	1	2	2	3	3	1	1
4	4	3	3	2	2	3	3	4	4
4	4	1	1	2	2	3	3	4	4
4	4	1	1	2	2	3	3	4	4
4	4	1	1	1	1	3	3	1	1
2	2	1	1	2	2	3	3	4	4
2	2	1	1	2	2	3	3	4	4
4	4	1	1	2	2	3	3	4	4
2	2	3	3	2	2	3	3	4	4
2	2	1	1	2	2	3	3	4	4
4	4	3	3	2	2	3	3	4	4
2	2	1	1	2	2	3	3	4	4
4	4	3	3	2	2	3	3	4	4
4	4	1	1	2	2	3	3	4	4
2	2	1	1	2	2	3	3	4	4
2	2	1	1	2	2	3	3	4	4
2	2	1	1	2	2	3	3	4	4
4	4	1	1	2	2	3	3	4	4
2	2	1	1	2	2	3	3	4	4
2	2	1	1	2	2	3	3	4	4
2	2	1	1	2	2	3	3	1	1
4	4	1	1	2	2	3	3	1	1
4	4	1	1	2	2	3	3	4	4
4	4	1	1	2	2	3	3	4	4
4	4	3	3	2	2	3	3	4	4
2	2	1	1	2	2	3	3	4	4
2	2	1	1	1	1	3	3	1	1
4	4	1	1	2	2	3	3	4	4
2	2	3	3	2	2	3	3	1	1
2	2	3	3	2	2	3	3	4	4
4	4	1	1	2	2	1	1	4	4
2	2	1	1	2	2	3	3	4	4
2	2	1	1	1	1	1	1	4	4
4	4	1	1	2	2	1	1	4	4
2	2	1	1	2	2	3	3	1	1
2	2	1	1	2	2	3	3	4	4
2	2	1	1	2	2	3	3	4	4
2	2	1	1	1	1	3	3	1	1
4	4	1	1	2	2	3	3	4	4
4	4	3	3	2	2	3	3	4	4
4	4	1	1	2	2	3	3	4	4

For Review Only

4	4	1	1	2	2	3	3	4	4
4	4	1	1	2	2	3	3	1	1
2	2	1	1	2	2	3	3	4	4
2	2	1	1	2	2	3	3	4	4
4	4	1	1	2	2	3	3	4	4
2	2	1	1	2	2	3	3	4	4
2	2	1	1	2	2	3	3	4	4
2	2	1	1	2	2	3	3	4	4
4	4	1	1	2	2	3	3	4	4
4	4	1	1	2	2	3	3	4	4
2	2	1	1	2	2	3	3	4	4
2	2	1	1	2	2	3	3	4	4
4	4	1	1	2	2	3	3	4	4
4	4	1	1	2	2	3	3	4	4
2	2	3	3	2	2	3	3	4	4
4	4	1	1	2	2	3	3	4	4
4	4	1	1	2	2	1	1	4	4
4	4	3	3	2	2	3	3	4	4
2	2	1	1	2	2	3	3	4	4
4	4	1	1	2	2	3	3	4	4
2	2	1	1	2	2	3	3	4	4
4	4	1	1	2	2	3	3	4	4
4	4	1	1	2	2	3	3	4	4
2	2	1	1	2	2	3	3	4	4
4	4	1	1	2	2	1	1	4	4
2	2	1	1	2	2	3	3	4	4
2	2	1	1	2	2	3	3	1	1
2	2	1	1	2	2	3	3	4	4
2	2	1	1	2	2	3	3	4	4
4	4	1	1	1	1	3	3	4	4
2	2	1	1	2	2	3	3	4	4
4	4	3	3	2	2	3	3	4	4
4	4	1	1	2	2	3	3	4	4
4	4	1	1	2	2	3	3	4	4
2	2	1	1	2	2	3	3	1	1
4	4	1	1	2	2	1	1	4	4
2	2	1	1	2	2	3	3	4	4
4	4	3	3	2	2	3	3	4	4
4	4	1	1	2	2	3	3	4	4
4	4	3	3	2	2	1	1	4	4
4	4	1	1	2	2	3	3	1	1
2	2	1	1	2	2	3	3	4	4
4	4	1	1	2	2	3	3	4	4
2	2	1	1	2	2	3	3	4	4
4	4	3	3	2	2	3	3	1	1
2	2	1	1	2	2	3	3	4	4
2	2	1	1	2	2	1	1	4	4
4	4	1	1	2	2	3	3	4	4
2	2	1	1	2	2	3	3	4	4
4	4	1	1	2	2	3	3	4	4
2	2	1	1	2	2	3	3	4	4
2	2	1	1	2	2	3	3	4	4
2	2	3	3	2	2	3	3	4	4
4	4	1	1	2	2	3	3	4	4
4	4	1	1	1	1	3	3	4	4

For Review Only

4	4	1	1	2	2	3	3	4	4
4	4	3	3	2	2	3	3	4	4
4	4	1	1	2	2	3	3	4	4
2	2	1	1	2	2	1	1	4	4
2	2	1	1	2	2	3	3	1	1
2	2	1	1	2	2	3	3	4	4
4	4	1	1	1	1	3	3	1	1
2	2	1	1	2	2	3	3	4	4
2	2	1	1	2	2	3	3	4	4
4	4	1	1	2	2	3	3	4	4
4	4	1	1	2	2	3	3	4	4
4	4	1	1	2	2	3	3	4	4
4	4	1	1	2	2	3	3	4	4
4	4	1	1	2	2	3	3	4	4
4	4	1	1	2	2	3	3	1	1
2	2	1	1	2	2	3	3	4	4
4	4	1	1	2	2	3	3	4	4
4	4	1	1	2	2	3	3	4	4
2	2	1	1	2	2	1	1	4	4
4	4	1	1	2	2	3	3	4	4
4	4	1	1	2	2	3	3	4	4
4	4	1	1	1	1	3	3	1	1
2	2	3	3	2	2	3	3	4	4
2	2	1	1	2	2	3	3	4	4
4	4	1	1	2	2	3	3	4	4
4	4	1	1	2	2	3	3	4	4
4	4	1	1	2	2	3	3	4	4
2	2	1	1	2	2	3	3	4	4
2	2	1	1	2	2	3	3	4	4
2	2	3	3	2	2	3	3	4	4
2	2	1	1	2	2	3	3	4	4
4	4	3	3	2	2	3	3	4	4
4	4	1	1	2	2	3	3	1	1
4	4	1	1	2	2	3	3	4	4
4	4	1	1	2	2	3	3	4	4
4	4	1	1	2	2	3	3	4	4
4	4	1	1	2	2	3	3	4	4
2	2	1	1	2	2	3	3	4	4
4	4	1	1	2	2	3	3	4	4
4	4	1	1	2	2	3	3	1	1
4	4	3	3	2	2	3	3	4	4
2	2	1	1	2	2	1	1	4	4
4	4	3	3	2	2	3	3	4	4
4	4	3	3	2	2	3	3	4	4
4	4	1	1	2	2	3	3	4	4
4	4	1	1	2	2	3	3	4	4
4	4	1	1	2	2	3	3	4	4
2	2	3	3	2	2	3	3	4	4
4	4	1	1	2	2	3	3	4	4
2	2	1	1	2	2	3	3	4	4
4	4	1	1	2	2	3	3	4	4
2	2	1	1	1	1	3	3	4	4
2	2	3	3	2	2	3	3	4	4
2	2	1	1	2	2	3	3	4	4
2	2	1	1	2	2	3	3	4	4

For Review Only

CSSA 5585 Guilford Rd., Madison, WI 53711

2	2	1	1	2	2	3	3	4	4
4	4	1	1	2	2	1	1	4	4
4	4	3	3	2	2	3	3	4	4
4	4	1	1	2	2	3	3	4	4
4	4	3	3	2	2	3	3	1	1
2	2	1	1	2	2	3	3	1	1
4	4	1	1	2	2	3	3	1	1
4	4	1	1	2	2	3	3	4	4
2	2	1	1	2	2	3	3	4	4
4	4	3	3	2	2	3	3	4	4
4	4	1	1	2	2	3	3	4	4
4	4	3	3	2	2	3	3	4	4
2	2	1	1	2	2	3	3	4	4
4	4	1	1	2	2	3	3	4	4
2	2	1	1	2	2	1	1	4	4
2	2	1	1	2	2	3	3	1	1
2	2	3	3	2	2	3	3	4	4
4	4	1	1	2	2	3	3	1	1
2	2	3	3	2	2	3	3	4	4
4	4	1	1	1	1	3	3	1	1
4	4	1	1	2	2	3	3	4	4
2	2	3	3	2	2	3	3	4	4
4	4	1	1	2	2	3	3	4	4
2	2	1	1	2	2	3	3	4	4
4	4	1	1	2	2	3	3	4	4
4	4	1	1	2	2	3	3	4	4
2	2	1	1	2	2	3	3	4	4
4	4	1	1	2	2	3	3	4	4
4	4	1	1	2	2	3	3	4	4
2	2	1	1	2	2	3	3	4	4
4	4	1	1	2	2	3	3	4	4
4	4	3	3	2	2	3	3	4	4
4	4	1	1	2	2	3	3	4	4
2	2	1	1	2	2	1	1	4	4
2	2	1	1	2	2	3	3	4	4
2	2	1	1	2	2	3	3	4	4
4	4	3	3	2	2	3	3	4	4
4	4	1	1	2	2	3	3	4	4
2	2	1	1	2	2	3	3	4	4
2	2	3	3	2	2	3	3	4	4
2	2	1	1	2	2	3	3	1	1
4	4	1	1	2	2	3	3	4	4
4	4	1	1	2	2	3	3	4	4
2	2	1	1	2	2	3	3	4	4
2	4	1	1	2	2	3	3	4	4
2	2	1	1	2	2	3	3	4	4
2	2	1	1	2	2	3	3	1	1
2	2	1	1	2	2	3	3	1	1
4	4	3	3	2	2	1	1	4	4
2	2	3	3	2	2	3	3	4	4
2	2	1	1	2	2	3	3	4	4
2	2	1	1	2	2	1	1	4	4
4	4	1	1	2	2	3	3	4	4
2	2	1	1	2	2	3	3	4	4
2	2	1	1	2	2	3	3	4	4

For Review Only

2	2	1	1	2	2	3	3	4	4
2	2	1	1	2	2	3	3	4	4
2	2	1	1	2	2	3	3	1	1
4	4	1	1	2	2	3	3	4	4
4	4	1	1	2	2	3	3	4	4
2	2	1	1	2	2	3	3	4	4
4	4	1	1	2	2	3	3	4	4
2	2	3	3	2	2	3	3	4	4
4	4	3	3	2	2	3	3	4	4
4	4	1	1	2	2	3	3	4	4
4	4	1	1	2	2	3	3	4	4
4	4	1	1	1	1	3	3	4	4
4	4	1	1	2	2	3	3	4	4
4	4	1	1	2	2	3	3	4	4
2	2	3	3	2	2	3	3	1	1
4	4	3	3	2	2	3	3	1	1
2	2	1	1	2	2	3	3	1	1
4	4	1	1	2	2	3	3	4	4
2	2	1	1	2	2	3	3	4	4
2	2	1	1	1	1	3	3	4	4
2	2	1	1	2	2	3	3	4	4
2	2	1	1	2	2	3	3	4	4
4	4	1	1	2	2	3	3	4	4
4	4	1	1	2	2	3	3	4	4
2	2	1	1	2	2	3	3	4	4
2	2	1	1	2	2	3	3	4	4
2	2	1	1	2	2	3	3	4	4
2	2	1	1	2	2	3	3	4	4
4	4	1	1	2	2	3	3	4	4
2	2	1	1	2	2	3	3	4	4
2	2	1	1	2	2	3	3	1	1
4	4	1	1	2	2	3	3	4	4
4	4	1	1	2	2	3	3	4	4
2	2	3	3	2	2	3	3	4	4
4	4	1	1	2	2	3	3	4	4
4	4	1	1	2	2	1	1	4	4
2	2	1	1	2	2	3	3	4	4
2	2	1	1	2	2	3	3	4	4
4	4	3	3	2	2	3	3	4	4
4	4	1	1	2	2	3	3	4	4
4	4	1	1	2	2	3	3	4	4
2	2	3	3	2	2	3	3	1	1
4	4	1	1	2	2	3	3	4	4
4	4	1	1	2	2	3	3	4	4
2	2	1	1	2	2	3	3	4	4
2	2	1	1	2	2	3	3	4	4
2	2	1	1	2	2	3	3	4	4
4	4	1	1	2	2	3	3	1	1
2	2	3	3	2	2	3	3	4	4
2	2	1	1	2	2	3	3	4	4
4	4	1	1	2	2	3	3	1	1
2	2	1	1	2	2	3	3	4	4
2	2	1	1	2	2	3	3	1	1
2	2	1	1	2	2	3	3	4	4
2	2	1	1	2	2	3	3	1	1

For Review Only

4	4	3	3	2	2	3	3	1	1
2	2	1	1	2	2	3	3	4	4

For Review Only

SNP682	SNP682	SNP683	SNP683	SNP684	SNP684	SNP685	SNP685	SNP686	SNP686
1	1	1	1	2	2	3	3	3	3
1	1	1	1	2	2	3	3	3	3
1	1	3	3	2	2	3	3	3	3
1	1	1	1	2	2	3	3	3	3
1	1	1	1	2	2	3	3	3	3
1	1	1	1	2	2	3	3	3	3
1	1	1	1	2	2	3	3	3	3
1	1	1	1	2	2	3	3	3	3
1	1	1	1	2	2	3	3	3	3
1	1	1	1	2	2	3	3	3	3
1	1	1	1	3	3	2	2	3	3
1	1	3	3	2	2	3	3	3	3
1	1	1	1	2	2	3	3	3	3
1	1	1	1	2	2	3	3	3	3
1	1	1	1	2	2	3	3	3	3
1	1	1	1	2	2	3	3	3	3
1	1	1	1	2	2	3	3	3	3
1	1	1	1	3	3	2	2	3	3
1	1	3	3	2	2	3	3	3	3
1	1	1	1	2	2	3	3	3	3
1	1	1	1	2	2	3	3	3	3
1	1	1	1	2	2	3	3	3	3
1	1	1	1	3	3	2	2	3	3
4	4	1	1	2	2	3	3	1	1
1	1	1	1	2	2	3	3	3	3
1	1	3	3	2	2	3	3	3	3
1	1	1	1	2	2	3	3	3	3
1	1	1	1	2	2	3	3	3	3
1	1	1	1	2	2	3	3	3	3
1	1	1	1	3	3	2	2	3	3
1	1	1	1	2	2	3	3	3	3
1	1	3	3	2	2	3	3	3	3
1	1	3	3	2	2	3	3	3	3
4	4	1	1	2	2	3	3	1	1
1	1	3	3	2	2	3	3	3	3
1	1	1	1	2	2	3	3	3	3
1	1	1	1	2	2	3	3	3	3
1	1	1	1	2	2	2	2	3	3
1	1	1	1	3	3	2	2	3	3
1	1	1	1	3	3	2	2	3	3
1	1	1	1	2	2	3	3	3	3
4	4	1	1	2	2	3	3	1	1
1	1	1	1	2	2	3	3	1	1
4	4	1	1	2	2	3	3	1	1
4	4	1	1	2	2	3	3	1	1
1	1	3	3	2	2	3	3	3	3
4	4	3	3	2	2	3	3	3	3
1	1	1	1	2	2	3	3	3	3
1	1	1	1	2	2	3	3	3	3
1	1	1	1	2	2	3	3	3	3
1	1	3	3	2	2	3	3	3	3
1	1	1	1	2	2	3	3	3	3

For Review Only

1	1	1	1	2	2	3	3	3	3
1	1	3	3	2	2	3	3	3	3
1	1	3	3	2	2	3	3	3	3
1	1	1	1	2	2	3	3	3	3
1	1	3	3	2	2	3	3	3	3
1	1	3	3	2	2	3	3	3	3
1	1	3	3	2	2	3	3	3	3
1	1	1	1	3	3	2	2	3	3
1	1	1	1	2	2	3	3	3	3
1	1	3	3	2	2	3	3	3	3
1	1	3	3	2	2	3	3	3	3
1	1	3	3	2	2	3	3	3	3
1	1	1	1	2	2	3	3	3	3
1	1	1	1	2	2	3	3	3	3
1	1	1	1	2	2	3	3	3	3
1	1	1	1	2	2	3	3	3	3
4	4	3	3	2	2	3	3	1	1
1	1	1	1	2	2	3	3	3	3
1	1	1	1	2	2	3	3	3	3
1	1	1	1	2	2	3	3	3	3
1	1	3	3	2	2	3	3	3	3
1	1	1	1	2	2	3	3	3	3
1	1	1	1	2	2	3	3	3	3
1	1	3	3	2	2	3	3	3	3
4	4	1	1	2	2	3	3	1	1
1	1	3	3	2	2	3	3	3	3
1	1	3	3	2	2	3	3	3	3
1	1	1	1	2	2	3	3	3	3
1	1	3	3	2	2	3	3	3	3
1	1	1	1	2	2	3	3	3	3
1	1	1	1	2	2	3	3	3	3
4	4	1	1	2	2	3	3	1	1
1	1	1	1	2	2	3	3	3	3
1	1	1	1	2	2	3	3	3	3
4	4	1	1	2	2	3	3	1	1
4	4	1	1	2	2	3	3	1	1
1	1	1	1	2	2	3	3	3	3
1	1	3	3	2	2	3	3	3	3
1	1	3	3	2	2	3	3	3	3
4	4	1	1	2	2	3	3	1	1
1	1	1	1	2	2	3	3	3	3
1	1	1	1	2	2	3	3	3	3
1	1	1	1	2	2	3	3	3	3
1	1	3	3	2	2	3	3	3	3
1	1	1	1	2	2	3	3	3	3
1	1	1	1	2	2	3	3	3	3
4	4	3	3	2	2	3	3	1	1
4	4	3	3	2	2	3	3	1	1
1	1	1	1	2	2	3	3	3	3
1	1	1	1	2	2	3	3	3	3
1	1	1	1	2	2	3	3	3	3
1	1	1	1	3	3	2	2	3	3
1	1	1	1	3	3	2	2	3	3
1	1	3	3	2	2	3	3	3	3
1	1	1	1	2	2	3	3	3	3

For Review Only

1	1	1	1	2	2	2	2	3	3
1	1	1	1	3	3	2	2	3	3
1	1	1	1	2	2	3	3	3	3
4	4	3	3	2	2	3	3	1	1
1	1	3	3	2	2	3	3	3	3
1	1	3	3	2	2	3	3	3	3
1	1	1	1	2	2	3	3	3	3
4	4	1	1	2	2	3	3	1	1
4	4	1	1	2	2	3	3	1	1
1	1	3	3	2	2	3	3	3	3
1	1	1	1	2	2	3	3	3	3
1	1	3	3	2	2	3	3	3	3
1	1	1	1	2	2	3	3	3	3
1	1	3	3	2	2	3	3	3	3
4	4	1	1	2	2	3	3	3	3
1	1	3	3	2	2	3	3	3	3
1	1	1	1	2	2	3	3	3	3
1	1	3	3	2	2	3	3	3	3
4	4	3	3	2	2	3	3	1	1
1	1	1	1	2	2	2	2	3	3
1	1	3	3	2	2	3	3	3	3
1	1	1	1	2	2	3	3	3	3
1	1	1	1	2	2	3	3	3	3
1	1	1	1	2	2	3	3	3	3
1	1	3	3	2	2	3	3	3	3
1	1	3	3	3	3	2	2	3	3
1	1	3	3	2	2	3	3	3	3
1	1	1	1	2	2	3	3	3	3
1	1	3	3	2	2	3	3	3	3
1	1	1	1	2	2	3	3	3	3
4	4	1	1	2	2	3	3	1	1
1	1	3	3	2	2	3	3	3	3
1	1	1	1	2	2	3	3	3	3
1	1	1	1	2	2	3	3	3	3
4	4	1	1	2	2	3	3	1	1
4	4	1	1	2	2	3	3	3	3
4	4	3	3	2	2	3	3	1	1
1	1	3	3	2	2	3	3	3	3
1	1	3	3	2	2	3	3	3	3
1	1	1	1	3	3	3	3	3	3
1	1	1	1	2	2	3	3	3	3
4	4	1	1	2	2	3	3	1	1
1	1	1	1	2	2	3	3	3	3
4	4	1	1	2	2	3	3	1	1
1	1	1	1	2	2	3	3	3	3
1	1	1	1	2	2	3	3	3	3
1	1	3	3	2	2	3	3	3	3
1	1	3	3	2	2	3	3	3	3
1	1	1	1	2	2	3	3	3	3
1	1	3	3	2	2	3	3	3	3
1	1	3	3	2	2	3	3	3	3
1	1	1	1	3	3	2	2	3	3
4	4	3	3	2	2	3	3	3	3
1	1	3	3	2	2	3	3	3	3
1	1	1	1	2	2	2	2	3	3

For Review Only

1	1	3	3	2	2	3	3	3	3
4	4	1	1	3	3	2	2	1	1
4	4	3	3	2	2	3	3	1	1
1	1	1	1	3	3	2	2	3	3
4	4	3	3	2	2	3	3	3	3
1	1	1	1	2	2	3	3	3	3
1	1	1	1	2	2	3	3	3	3
1	1	3	3	2	2	3	3	3	3
1	1	3	3	2	2	3	3	3	3
1	1	1	1	2	2	3	3	3	3
4	4	3	3	2	2	3	3	3	3
4	4	1	1	2	2	3	3	3	3
1	1	1	1	2	2	3	3	3	3
1	1	1	1	2	2	3	3	3	3
4	4	1	1	2	2	3	3	1	1
1	1	1	1	3	3	2	2	3	3
1	1	3	3	2	2	3	3	3	3
1	1	3	3	2	2	3	3	3	3
1	1	1	1	2	2	3	3	3	3
1	1	1	1	2	2	3	3	3	3
1	1	3	3	2	2	3	3	3	3
1	1	1	1	2	2	3	3	3	3
1	1	1	1	2	2	3	3	3	3
1	1	3	3	2	2	3	3	3	3
1	1	1	1	3	3	2	2	3	3
4	4	1	1	2	2	3	3	1	1
1	1	3	3	2	2	3	3	3	3
4	4	1	1	2	2	3	3	1	1
1	1	1	1	2	2	3	3	3	3
1	1	1	1	2	2	3	3	3	3
1	1	3	3	2	2	3	3	3	3
1	1	1	1	2	2	3	3	3	3
1	1	3	3	2	2	3	3	3	3
4	4	1	1	2	2	3	3	1	1
1	1	1	1	2	2	3	3	3	3
1	1	3	3	2	2	3	3	3	3
1	1	1	1	2	2	3	3	3	3
1	1	1	1	2	2	3	3	3	3
1	1	3	3	2	2	3	3	3	3
1	1	3	3	2	2	3	3	3	3
1	1	3	3	2	2	3	3	3	3
1	1	3	3	2	2	3	3	3	3
1	1	1	1	3	3	2	2	3	3
1	1	3	3	2	2	3	3	3	3
4	4	3	3	2	2	3	3	3	3
4	4	1	1	2	2	3	3	1	1
1	1	1	1	3	3	2	2	3	3
1	1	1	1	2	2	3	3	3	3
4	4	1	1	2	2	3	3	1	1
1	1	1	1	2	2	3	3	3	3
1	1	3	3	2	2	3	3	3	3
4	4	1	1	3	3	2	2	1	1
1	1	1	1	2	2	3	3	3	3
1	1	3	3	2	2	3	3	3	3
1	1	1	1	2	2	3	3	3	3

For Review Only

1	1	3	3	2	2	3	3	3	3
1	1	1	1	2	2	3	3	3	3
1	1	3	3	2	2	3	3	3	3
1	1	1	1	2	2	3	3	3	3
1	1	1	1	2	2	3	3	3	3
1	1	1	1	2	2	3	3	3	3
1	1	1	1	2	2	3	3	3	3
1	1	3	3	2	2	3	3	3	3
4	4	1	1	2	2	3	3	1	1
1	1	1	1	2	2	3	3	3	3
1	1	3	3	2	2	3	3	3	3
1	1	3	3	2	2	3	3	3	3
1	1	3	3	2	2	3	3	3	3
4	4	3	3	2	2	3	3	1	1
1	1	3	3	2	2	3	3	3	3
1	1	1	1	2	2	3	3	3	3
1	1	1	1	3	3	2	2	3	3
1	1	1	1	2	2	2	2	3	3
1	1	3	3	2	2	3	3	3	3
1	1	1	1	3	3	2	2	3	3
1	1	3	3	2	2	3	3	3	3
1	1	3	3	2	2	3	3	3	3
1	1	1	1	2	2	3	3	3	3
1	1	1	1	2	2	3	3	3	3
1	1	1	1	3	3	2	2	3	3
4	4	1	1	2	2	3	3	3	3
1	1	1	1	2	2	3	3	3	3
1	1	3	3	2	2	3	3	3	3
1	1	1	1	2	2	3	3	3	3
1	1	3	3	2	2	3	3	3	3
1	1	1	1	3	3	2	2	3	3
1	1	3	3	2	2	3	3	3	3
1	1	1	1	2	2	3	3	3	3
1	1	3	3	2	2	3	3	3	3
1	1	1	1	2	2	3	3	3	3
1	1	1	1	2	2	3	3	3	3
1	1	1	1	2	2	3	3	3	3
1	1	3	3	2	2	3	3	3	3
1	1	1	1	2	2	3	3	3	3
1	1	1	1	3	3	2	2	3	3
1	1	1	1	2	2	3	3	3	3
1	1	1	1	2	2	3	3	3	3
1	1	1	1	2	2	3	3	3	3
1	1	1	1	2	2	3	3	3	3
1	1	3	3	2	2	3	3	3	3
1	1	1	1	2	2	3	3	3	3
1	1	1	1	2	2	3	3	3	3
1	1	1	1	2	2	3	3	3	3
4	4	1	1	2	2	3	3	1	1
1	1	1	1	2	2	3	3	3	3
1	1	1	1	2	2	3	3	3	3
1	1	1	1	3	3	2	2	3	3
1	1	3	3	2	2	3	3	3	3
1	1	3	3	2	2	3	3	3	3
1	1	1	1	3	3	2	2	3	3

For Review Only

1	1	1	1	2	2	3	3	3	3
1	1	3	3	2	2	3	3	3	3

For Review Only

SNP687	SNP687	SNP688	SNP688	SNP689	SNP689	SNP690	SNP690	SNP691	SNP691
2	2	4	4	4	4	2	2	3	3
3	3	4	4	4	4	4	4	1	1
2	2	4	4	4	4	4	4	1	1
2	2	4	4	4	4	2	2	1	1
2	2	4	4	4	4	4	4	1	1
2	2	4	4	4	4	4	4	1	1
2	2	4	4	4	4	2	2	3	3
2	2	4	4	4	4	2	2	1	1
2	2	4	4	4	4	2	2	3	3
2	2	4	4	4	4	2	2	3	3
3	3	1	1	1	1	4	4	3	3
2	2	4	4	4	4	2	2	1	1
2	2	4	4	4	4	2	2	3	3
2	2	1	1	1	1	2	2	1	1
2	2	4	4	4	4	2	2	1	1
2	2	4	4	4	4	2	2	1	1
3	3	4	4	4	4	4	4	1	1
2	2	4	4	4	4	4	4	3	3
2	2	4	4	4	4	2	2	1	1
2	2	4	4	4	4	4	4	1	1
2	2	1	1	4	4	4	4	1	1
2	2	4	4	4	4	4	4	1	1
3	3	1	1	1	1	4	4	3	3
2	2	4	4	4	4	4	4	3	3
2	2	4	4	4	4	4	4	1	1
2	2	4	4	4	4	2	2	1	1
2	2	4	4	4	4	2	2	1	1
2	2	1	1	4	4	2	2	3	3
2	2	1	1	4	4	2	2	1	1
3	3	4	4	4	4	4	4	3	3
2	2	4	4	4	4	4	4	1	1
2	2	4	4	4	4	4	4	1	1
2	2	4	4	4	4	2	2	3	3
3	3	4	4	4	4	4	4	1	1
3	3	1	1	1	1	4	4	1	1
2	2	4	4	4	4	2	2	1	1
3	3	1	1	1	1	4	4	1	1
2	2	1	1	1	1	2	2	3	3
3	3	1	1	1	1	4	4	3	3
3	3	1	1	1	1	4	4	3	3
2	2	4	4	4	4	4	4	1	1
2	2	1	1	4	4	2	2	1	1
2	2	1	1	4	4	2	2	3	3
2	2	1	1	1	1	2	2	1	1
2	2	1	1	4	4	2	2	3	3
2	2	4	4	4	4	2	2	1	1
2	2	4	4	4	4	4	4	1	1
3	3	4	4	4	4	4	4	3	3
2	2	4	4	4	4	2	2	1	1
2	2	1	1	4	4	2	2	3	3
2	2	4	4	4	4	2	2	1	1
2	2	4	4	4	4	2	2	3	3

For Review Only

2	2	4	4	4	4	4	4	3	3
2	2	4	4	4	4	2	2	3	3
2	2	4	4	4	4	2	2	1	1
2	2	4	4	4	4	4	4	1	1
2	2	4	4	4	4	2	2	1	1
2	2	4	4	4	4	2	2	1	1
2	2	4	4	4	4	2	2	3	3
3	3	1	1	1	1	4	4	3	3
3	3	1	1	1	1	4	4	1	1
2	2	1	1	4	4	2	2	3	3
2	2	4	4	4	4	2	2	1	1
2	2	4	4	4	4	2	2	1	1
2	2	4	4	4	4	4	4	3	3
2	2	1	1	4	4	2	2	1	1
2	2	1	1	4	4	4	4	3	3
3	3	1	1	1	1	4	4	3	3
3	3	4	4	4	4	4	4	3	3
2	2	4	4	4	4	2	2	1	1
2	2	4	4	4	4	4	4	1	1
2	2	4	4	4	4	2	2	3	3
2	2	4	4	4	4	2	2	3	3
3	3	1	1	4	4	4	4	1	1
2	2	4	4	4	4	2	2	3	3
2	2	4	4	4	4	2	2	1	1
2	2	1	1	4	4	2	2	3	3
2	2	4	4	4	4	2	2	1	1
2	2	1	1	1	1	2	2	1	1
3	3	4	4	4	4	4	4	3	3
2	2	4	4	4	4	2	2	3	3
2	2	4	4	4	4	4	4	1	1
2	2	4	4	4	4	4	4	1	1
2	2	4	4	4	4	4	4	3	3
3	3	4	4	4	4	4	4	1	1
2	2	4	4	4	4	4	4	3	3
2	2	4	4	4	4	2	2	1	1
2	2	1	1	4	4	2	2	3	3
2	2	1	1	1	1	2	2	1	1
2	2	4	4	4	4	2	2	1	1
2	2	4	4	4	4	4	4	1	1
2	2	1	1	1	1	2	2	3	3
2	2	4	4	4	4	2	2	1	1
2	2	4	4	4	4	4	4	1	1
2	2	1	1	1	1	2	2	1	1
2	2	4	4	4	4	2	2	1	1
2	2	4	4	4	4	2	2	1	1
2	2	4	4	4	4	2	2	1	1
3	3	4	4	4	4	4	4	3	3
2	2	4	4	4	4	2	2	3	3
2	2	4	4	4	4	2	2	1	1
2	2	1	1	1	1	4	4	1	1
2	2	4	4	4	4	2	2	1	1
2	2	1	1	1	1	4	4	3	3
2	2	1	1	1	1	4	4	3	3
2	2	4	4	4	4	4	4	3	3
2	2	1	1	1	1	4	4	1	1

For Review Only

2	2	4	4	4	4	2	2	1	1
2	2	1	1	1	1	2	2	3	3
2	2	4	4	4	4	2	2	1	1
3	3	4	4	4	4	4	4	3	3
2	2	4	4	4	4	2	2	1	1
2	2	4	4	4	4	2	2	3	3
2	2	4	4	4	4	2	2	1	1
2	2	4	4	4	4	2	2	3	3
2	2	4	4	4	4	2	2	1	1
3	3	4	4	4	4	2	2	3	3
2	2	1	1	4	4	4	4	3	3
2	2	1	1	4	4	2	2	3	3
3	3	1	1	1	1	4	4	1	1
2	2	4	4	4	4	2	2	1	1
2	2	1	1	4	4	2	2	1	1
2	2	1	1	4	4	2	2	3	3
3	3	4	4	4	4	4	4	1	1
2	2	4	4	4	4	2	2	3	3
3	3	1	1	1	1	4	4	3	3
3	3	1	1	4	4	4	4	3	3
2	2	4	4	4	4	2	2	3	3
2	2	4	4	4	4	4	4	3	3
2	2	4	4	4	4	4	4	1	1
3	3	4	4	4	4	4	4	3	3
3	3	1	1	4	4	2	2	1	1
3	3	4	4	4	4	4	4	3	3
2	2	4	4	4	4	2	2	1	1
2	2	4	4	4	4	4	4	3	3
2	2	4	4	4	4	2	2	1	1
2	2	4	4	4	4	2	2	1	1
2	2	4	4	4	4	4	4	3	3
2	2	1	1	4	4	2	2	3	3
2	2	4	4	4	4	2	2	1	1
2	2	4	4	4	4	2	2	3	3
3	3	4	4	4	4	4	4	1	1
2	2	4	4	4	4	4	4	3	3
3	3	1	1	1	1	4	4	3	3
2	2	4	4	4	4	2	2	3	3
2	2	4	4	4	4	2	2	3	3
3	3	1	1	4	4	4	4	3	3
2	2	1	1	4	4	2	2	3	3
3	3	1	1	1	1	4	4	3	3
2	2	4	4	4	4	2	2	1	1
2	2	1	1	4	4	4	4	3	3
2	2	4	4	4	4	2	2	3	3
2	2	4	4	4	4	2	2	3	3
2	2	4	4	4	4	2	2	3	3
2	2	4	4	4	4	2	2	3	3
2	2	1	1	1	1	2	2	3	3
2	2	4	4	4	4	2	2	1	1
2	2	4	4	4	4	2	2	3	3
3	3	1	1	1	1	4	4	3	3
3	3	4	4	4	4	4	4	1	1
2	2	4	4	4	4	2	2	1	1
2	2	1	1	4	4	2	2	3	3

For Review Only

2	2	4	4	4	4	4	4	3	3
3	3	1	1	1	1	4	4	3	3
2	2	4	4	4	4	2	2	1	1
2	2	1	1	4	4	4	4	3	3
2	2	4	4	4	4	2	2	1	1
3	3	4	4	4	4	4	4	3	3
3	3	4	4	4	4	4	4	3	3
2	2	4	4	4	4	2	2	1	1
3	3	1	1	4	4	4	4	1	1
2	2	4	4	4	4	2	2	1	1
2	2	4	4	4	4	2	2	1	1
2	2	1	1	1	1	2	2	1	1
2	2	4	4	4	4	2	2	1	1
2	2	4	4	4	4	2	2	3	3
3	3	1	1	1	1	4	4	3	3
3	3	1	1	1	1	4	4	3	3
2	2	4	4	4	4	2	2	1	1
2	2	4	4	4	4	2	2	1	1
2	2	4	4	4	4	4	4	1	1
2	2	4	4	4	4	2	2	3	3
2	2	1	1	4	4	2	2	3	3
2	2	4	4	4	4	4	4	3	3
3	3	1	1	1	1	4	4	1	1
2	2	4	4	4	4	2	2	1	1
2	2	1	1	4	4	2	2	3	3
3	3	4	4	4	4	4	4	1	1
2	2	4	4	4	4	2	2	1	1
3	3	4	4	4	4	4	4	3	3
2	2	4	4	4	4	4	4	1	1
2	2	4	4	4	4	4	4	1	1
2	2	4	4	4	4	2	2	3	3
2	2	4	4	4	4	2	2	1	1
2	2	4	4	4	4	2	2	1	1
3	3	1	1	1	1	4	4	1	1
2	2	1	1	1	1	2	2	1	1
2	2	4	4	4	4	2	2	1	1
2	2	1	1	1	1	2	2	3	3
2	2	1	1	1	1	4	4	3	3
2	2	4	4	4	4	2	2	3	3
2	2	4	4	4	4	2	2	1	1
2	2	4	4	4	4	4	4	1	1
2	2	4	4	4	4	2	2	3	3
3	3	4	4	4	4	4	4	3	3
2	2	1	1	1	1	2	2	1	1
2	2	1	1	1	1	2	2	1	1
2	2	4	4	4	4	4	4	3	3
3	3	1	1	1	1	4	4	3	3
3	3	4	4	4	4	4	4	3	3
2	2	1	1	1	1	4	4	3	3
2	2	4	4	4	4	2	2	1	1
2	2	4	4	4	4	4	4	3	3
3	3	1	1	1	1	4	4	3	3
3	3	1	1	1	1	4	4	1	1
2	2	4	4	4	4	2	2	1	1
3	3	1	1	1	1	4	4	3	3

For Review Only

3	3	4	4	4	4	4	4	3	3
2	2	4	4	4	4	2	2	1	1
2	2	4	4	4	4	4	4	1	1
3	3	1	1	1	1	4	4	3	3
2	2	4	4	4	4	2	2	3	3
2	2	4	4	4	4	4	4	1	1
2	2	1	1	1	1	2	2	1	1
2	2	1	1	1	1	4	4	3	3
2	2	1	1	1	1	2	2	1	1
2	2	4	4	4	4	4	4	3	3
2	2	1	1	4	4	2	2	3	3
3	3	4	4	4	4	4	4	3	3
2	2	4	4	4	4	4	4	1	1
2	2	4	4	4	4	4	4	1	1
2	2	4	4	4	4	2	2	1	1
2	2	4	4	4	4	2	2	1	1
2	2	1	1	1	1	2	2	3	3
3	3	1	1	1	1	4	4	3	3
3	3	4	4	4	4	4	4	1	1
3	3	1	1	1	1	4	4	3	3
2	2	4	4	4	4	2	2	1	1
2	2	4	4	4	4	4	4	3	3
2	2	4	4	4	4	4	4	1	1
3	3	1	1	1	1	4	4	1	1
3	3	1	1	1	1	4	4	3	3
2	2	4	4	4	4	4	4	1	1
3	3	4	4	4	4	4	4	1	1
2	2	4	4	4	4	2	2	1	1
2	2	4	4	4	4	2	2	3	3
2	2	4	4	4	4	4	4	1	1
3	3	1	1	1	1	4	4	3	3
3	3	4	4	4	4	4	4	3	3
3	3	4	4	4	4	4	4	1	1
2	2	4	4	4	4	2	2	3	3
3	3	1	1	1	1	4	4	1	1
3	3	1	1	1	1	4	4	3	3
2	2	4	4	4	4	4	4	1	1
2	2	4	4	4	4	2	2	1	1
2	2	4	4	4	4	2	2	1	1
3	3	1	1	1	1	4	4	3	3
2	2	4	4	4	4	2	2	1	1
2	2	4	4	4	4	2	2	3	3
2	2	4	4	4	4	2	2	3	3
2	2	4	4	4	4	4	4	1	1
2	2	4	4	4	4	2	2	1	1
2	2	4	4	4	4	2	2	1	1
2	2	1	1	1	1	2	2	3	3
2	2	4	4	4	4	2	2	1	1
2	2	1	1	4	4	4	4	1	1
3	3	4	4	4	4	4	4	3	3
2	2	1	1	1	1	2	2	1	1
3	3	4	4	4	4	4	4	3	3
2	2	4	4	4	4	4	4	3	3
2	2	4	4	4	4	2	2	1	1
3	3	1	1	1	1	4	4	3	3

For Review Only

2	2	4	4	4	4	2	2	1	1
2	2	1	1	1	1	2	2	1	1

For Review Only

SNP692	SNP692	SNP693	SNP693	SNP694	SNP694	SNP695	SNP695	SNP696	SNP696
3	3	1	1	1	1	3	3	2	2
4	4	4	4	4	4	3	3	4	4
3	3	4	4	4	4	3	3	2	2
3	3	4	4	4	4	3	3	2	2
3	3	1	1	1	1	3	3	2	2
3	3	4	4	4	4	3	3	2	2
3	3	1	1	1	1	3	3	2	2
3	3	4	4	4	4	4	4	2	2
3	3	1	1	1	1	3	3	2	2
3	3	4	4	4	4	3	3	2	2
4	4	4	4	4	4	3	3	2	2
3	3	4	4	4	4	3	3	2	2
3	3	4	4	4	4	3	3	2	2
3	3	1	1	1	1	4	4	2	2
3	3	1	1	4	4	3	3	2	2
3	3	4	4	4	4	4	4	2	2
4	4	4	4	1	1	3	3	2	2
3	3	1	1	1	1	3	3	2	2
3	3	4	4	4	4	3	3	2	2
3	3	1	1	1	1	3	3	2	2
3	3	1	1	1	1	3	3	2	2
3	3	1	1	1	1	3	3	2	2
4	4	4	4	4	4	3	3	2	2
3	3	4	4	4	4	3	3	2	2
3	3	4	4	4	4	3	3	2	2
3	3	4	4	4	4	4	4	2	2
3	3	4	4	4	4	4	4	2	2
3	3	4	4	4	4	3	3	2	2
3	3	1	1	1	1	3	3	2	2
4	4	4	4	4	4	3	3	2	2
3	3	4	4	4	4	3	3	2	2
3	3	1	1	1	1	3	3	2	2
3	3	1	1	1	1	3	3	2	2
3	3	4	4	4	4	3	3	2	2
4	4	4	4	4	4	3	3	2	2
3	3	4	4	4	4	4	4	2	2
4	4	1	1	1	1	3	3	2	2
4	4	4	4	4	4	4	4	4	4
4	4	4	4	4	4	3	3	2	2
4	4	4	4	4	4	3	3	4	4
4	4	4	4	4	4	3	3	2	2
3	3	1	1	1	1	3	3	2	2
3	3	4	4	4	4	3	3	2	2
3	3	1	1	1	1	3	3	2	2
3	3	4	4	4	4	3	3	2	2
3	3	4	4	4	4	3	3	2	2
3	3	1	1	1	1	3	3	2	2
4	4	4	4	4	4	3	3	2	2
3	3	1	1	1	1	3	3	4	4
3	3	1	1	4	4	3	3	2	2
3	3	4	4	4	4	4	4	2	2
4	4	4	4	4	4	4	4	2	2

For Review Only

3	3	1	1	1	1	3	3	2	2
3	3	1	1	1	1	3	3	2	2
3	3	4	4	4	4	4	4	2	2
3	3	1	1	1	1	3	3	2	2
3	3	4	4	4	4	3	3	2	2
3	3	1	1	4	4	4	4	2	2
3	3	1	1	1	1	4	4	2	2
4	4	4	4	4	4	3	3	2	2
4	4	4	4	4	4	3	3	2	2
4	4	4	4	4	4	4	4	2	2
3	3	4	4	4	4	4	4	2	2
3	3	4	4	4	4	4	4	2	2
3	3	1	1	1	1	3	3	2	2
3	3	1	1	1	1	3	3	2	2
3	3	4	4	4	4	3	3	2	2
4	4	4	4	4	4	3	3	2	2
4	4	1	1	1	1	3	3	2	2
3	3	1	1	1	1	4	4	4	4
3	3	1	1	1	1	3	3	2	2
3	3	1	1	1	1	3	3	2	2
3	3	4	4	4	4	4	4	2	2
4	4	4	4	4	4	3	3	2	2
3	3	1	1	1	1	3	3	2	2
3	3	4	4	4	4	3	3	2	2
3	3	4	4	4	4	3	3	2	2
3	3	4	4	4	4	4	4	2	2
3	3	4	4	4	4	3	3	2	2
4	4	4	4	4	4	3	3	2	2
3	3	4	4	4	4	4	4	2	2
3	3	4	4	4	4	3	3	2	2
3	3	4	4	4	4	3	3	2	2
3	3	4	4	4	4	3	3	2	2
4	4	4	4	4	4	3	3	2	2
3	3	1	1	1	1	3	3	2	2
3	3	4	4	4	4	3	3	2	2
3	3	4	4	4	4	3	3	2	2
3	3	4	4	4	4	3	3	2	2
3	3	1	1	4	4	3	3	2	2
3	3	4	4	4	4	3	3	2	2
3	3	4	4	4	4	3	3	2	2
3	3	1	1	1	1	3	3	2	2
3	3	4	4	4	4	3	3	2	2
3	3	4	4	4	4	4	4	2	2
3	3	4	4	4	4	4	4	2	2
3	3	4	4	4	4	3	3	2	2
3	3	1	1	1	1	3	3	4	4
4	4	4	4	4	4	3	3	2	2
3	3	1	1	1	1	4	4	2	2
3	3	1	1	1	1	4	4	2	2
3	3	4	4	4	4	3	3	2	2
3	3	4	4	4	4	3	3	2	2
4	4	4	4	4	4	3	3	2	2
3	3	4	4	4	4	3	3	2	2
3	3	4	4	4	4	3	3	2	2
3	3	1	1	1	1	3	3	4	4

For Review Only

4	4	4	4	4	4	4	4	2	2
3	3	4	4	4	4	4	4	2	2
3	3	4	4	4	4	4	4	2	2
3	3	1	1	1	1	3	3	2	2
3	3	4	4	4	4	3	3	4	4
3	3	4	4	4	4	4	4	2	2
3	3	4	4	4	4	4	4	2	2
3	3	4	4	4	4	3	3	2	2
4	4	4	4	4	4	4	4	2	2
4	4	4	4	4	4	3	3	2	2
3	3	1	1	1	1	3	3	2	2
4	4	4	4	4	4	3	3	4	4
3	3	4	4	4	4	3	3	2	2
3	3	4	4	4	4	4	4	2	2
3	3	4	4	4	4	4	4	4	4
4	4	4	4	4	4	3	3	2	2
4	4	1	1	1	1	3	3	4	4
3	3	4	4	4	4	4	4	2	2
3	3	1	1	1	1	3	3	2	2
4	4	4	4	4	4	3	3	2	2
3	3	4	4	4	4	4	4	2	2
3	3	4	4	4	4	3	3	2	2
3	3	4	4	4	4	3	3	2	2
4	4	4	4	4	4	3	3	2	2
4	4	4	4	4	4	4	4	2	2
4	4	4	4	4	4	3	3	2	2
3	3	1	1	1	1	3	3	4	4
3	3	4	4	4	4	3	3	2	2
3	3	1	1	1	1	3	3	2	2
3	3	1	1	4	4	4	4	2	2
4	4	4	4	4	4	3	3	2	2
3	3	4	4	4	4	4	4	2	2
3	3	1	1	1	1	3	3	2	2
3	3	4	4	4	4	4	4	2	2
3	3	4	4	4	4	3	3	2	2
4	4	1	1	1	1	4	4	2	2
4	4	4	4	4	4	3	3	2	2
3	3	4	4	4	4	4	4	2	2
4	4	4	4	4	4	3	3	2	2
4	4	4	4	4	4	3	3	2	2
3	3	4	4	4	4	4	4	2	2
4	4	4	4	4	4	3	3	2	2
3	3	1	1	1	1	4	4	2	2
4	4	4	4	4	4	3	3	2	2
3	3	4	4	4	4	4	4	2	2
3	3	4	4	4	4	4	4	4	4
3	3	1	1	4	4	4	4	2	2
3	3	1	1	1	1	4	4	2	2
3	3	4	4	4	4	3	3	2	2
3	3	4	4	4	4	4	4	2	2
3	3	1	1	4	4	4	4	2	2
4	4	1	1	1	1	3	3	2	2
4	4	4	4	4	4	3	3	2	2
3	3	4	4	4	4	4	4	2	2
4	4	4	4	4	4	3	3	2	2

For Review Only

3	3	1	1	1	1	3	3	2	2
4	4	4	4	4	4	3	3	2	2
3	3	4	4	4	4	4	4	4	4
4	4	4	4	4	4	3	3	2	2
3	3	4	4	4	4	4	4	4	4
4	4	1	1	1	1	3	3	4	4
4	4	4	4	4	4	3	3	2	2
3	3	4	4	1	1	4	4	2	2
4	4	4	4	4	4	3	3	2	2
3	3	1	1	1	1	3	3	2	2
3	3	4	4	4	4	3	3	2	2
4	4	4	4	1	1	3	3	2	2
3	3	4	4	4	4	4	4	2	2
4	4	4	4	4	4	3	3	4	4
4	4	4	4	4	4	3	3	2	2
4	4	4	4	4	4	3	3	2	2
4	4	4	4	4	4	3	3	2	2
3	3	4	4	4	4	4	4	4	4
3	3	4	4	4	4	3	3	2	2
3	3	4	4	4	4	4	4	2	2
4	4	1	1	4	4	3	3	2	2
3	3	4	4	4	4	3	3	2	2
4	4	4	4	4	4	3	3	2	2
3	3	4	4	4	4	4	4	2	2
3	3	4	4	4	4	3	3	2	2
3	3	4	4	4	4	3	3	2	2
3	3	4	4	4	4	4	4	2	2
4	4	4	4	4	4	3	3	4	4
3	3	1	1	1	1	3	3	2	2
3	3	1	1	1	1	3	3	2	2
3	3	4	4	4	4	3	3	2	2
3	3	1	1	1	1	4	4	2	2
3	3	1	1	1	1	3	3	4	4
4	4	4	4	4	4	3	3	2	2
3	3	1	1	1	1	4	4	4	4
3	3	4	4	4	4	4	4	2	2
3	3	4	4	4	4	4	4	2	2
3	3	1	1	1	1	3	3	2	2
3	3	1	1	1	1	4	4	2	2
3	3	4	4	4	4	4	4	2	2
3	3	4	4	4	4	3	3	2	2
3	3	1	1	4	4	4	4	2	2
4	4	4	4	4	4	3	3	2	2
3	3	1	1	1	1	4	4	2	2
3	3	1	1	4	4	4	4	4	4
3	3	4	4	4	4	3	3	2	2
4	4	4	4	4	4	3	3	2	2
4	4	4	4	4	4	3	3	2	2
3	3	4	4	4	4	3	3	2	2
3	3	4	4	4	4	4	4	2	2
3	3	1	1	1	1	3	3	2	2
3	3	4	4	4	4	3	3	4	4
4	4	4	4	4	4	3	3	2	2
3	3	4	4	4	4	4	4	2	2
4	4	4	4	4	4	3	3	4	4

For Review Only

4	4	4	4	4	4	3	3	2	2
3	3	1	1	1	1	3	3	2	2
3	3	1	1	1	1	3	3	2	2
4	4	4	4	4	4	3	3	2	2
3	3	1	1	1	1	3	3	2	2
3	3	4	4	4	4	3	3	2	2
3	3	4	4	4	4	3	3	4	4
4	4	4	4	4	4	3	3	2	2
4	4	4	4	4	4	4	4	2	2
4	4	4	4	4	4	3	3	2	2
4	4	4	4	4	4	3	3	2	2
4	4	1	1	1	1	3	3	2	2
3	3	4	4	4	4	3	3	2	2
3	3	1	1	1	1	3	3	2	2
3	3	4	4	4	4	4	4	2	2
3	3	4	4	4	4	3	3	2	2
3	3	4	4	4	4	3	3	2	2
4	4	4	4	1	1	3	3	2	2
4	4	4	4	4	4	3	3	2	2
4	4	4	4	4	4	3	3	4	4
3	3	4	4	4	4	3	3	2	2
3	3	4	4	4	4	3	3	2	2
3	3	1	1	4	4	3	3	2	2
4	4	1	1	1	1	3	3	2	2
4	4	4	4	4	4	3	3	2	2
3	3	4	4	4	4	3	3	2	2
4	4	4	4	4	4	3	3	2	2
3	3	4	4	4	4	3	3	2	2
3	3	1	1	1	1	3	3	2	2
3	3	1	1	1	1	3	3	2	2
4	4	4	4	4	4	3	3	2	2
4	4	4	4	4	4	3	3	2	2
4	4	4	4	4	4	3	3	2	2
3	3	4	4	1	1	4	4	2	2
4	4	4	4	4	4	3	3	2	2
4	4	4	4	4	4	3	3	2	2
3	3	4	4	4	4	3	3	2	2
3	3	4	4	4	4	4	4	2	2
3	3	4	4	4	4	4	4	2	2
4	4	4	4	4	4	3	3	2	2
3	3	1	1	1	1	4	4	2	2
3	3	1	1	1	1	4	4	4	4
3	3	4	4	4	4	4	4	2	2
3	3	1	1	1	1	3	3	2	2
3	3	4	4	4	4	4	4	2	2
3	3	4	4	4	4	4	4	2	2
3	3	1	1	1	1	4	4	2	2
3	3	4	4	4	4	4	4	4	4
3	3	4	4	4	4	3	3	2	2
4	4	4	4	4	4	3	3	2	2
3	3	1	1	1	1	3	3	2	2
4	4	4	4	4	4	3	3	2	2
3	3	4	4	4	4	3	3	2	2
3	3	4	4	4	4	3	3	2	2
4	4	4	4	4	4	3	3	4	4

For Review Only

3	3	4	4	4	4	3	3	2	2
3	3	1	1	1	1	4	4	4	4

For Review Only

SNP697	SNP697	SNP698	SNP698	SNP699	SNP699	SNP700	SNP700	SNP701	SNP701
4	4	1	1	2	2	3	3	4	4
2	2	4	4	2	2	3	3	4	4
2	2	1	1	2	2	3	3	4	4
4	4	1	1	2	2	3	3	4	4
2	2	1	1	2	2	3	3	4	4
2	2	1	1	2	2	3	3	4	4
4	4	1	1	2	2	3	3	4	4
4	4	4	4	2	2	3	3	4	4
4	4	1	1	2	2	3	3	4	4
2	2	4	4	2	2	3	3	4	4
2	2	4	4	2	2	3	3	4	4
2	2	1	1	2	2	3	3	4	4
4	4	4	4	2	2	3	3	4	4
4	4	1	1	2	2	3	3	4	4
4	4	4	4	2	2	3	3	4	4
4	4	1	1	2	2	3	3	4	4
2	2	1	1	2	2	3	3	4	4
2	2	1	1	2	2	3	3	4	4
4	4	4	4	2	2	3	3	4	4
2	2	1	1	2	2	3	3	4	4
2	2	1	1	2	2	3	3	4	4
2	2	1	1	2	2	3	3	4	4
2	2	4	4	2	2	3	3	4	4
2	2	4	4	2	2	3	3	4	4
4	4	4	4	3	3	1	1	4	4
4	4	1	1	2	2	3	3	4	4
4	4	4	4	3	3	1	1	4	4
4	4	4	4	2	2	3	3	4	4
2	2	1	1	2	2	3	3	4	4
2	2	4	4	2	2	3	3	3	3
4	4	4	4	3	3	1	1	4	4
2	2	1	1	2	2	3	3	4	4
4	4	1	1	2	2	3	3	4	4
2	2	4	4	2	2	3	3	4	4
2	2	4	4	2	2	3	3	4	4
4	4	1	1	2	2	3	3	4	4
2	2	1	1	2	2	3	3	4	4
4	4	1	1	2	2	3	3	4	4
2	2	4	4	2	2	3	3	4	4
2	2	1	1	2	2	3	3	4	4
2	2	4	4	2	2	3	3	4	4
2	2	1	1	2	2	3	3	4	4
2	2	4	4	2	2	3	3	4	4
4	4	1	1	2	2	3	3	4	4
2	2	4	4	2	2	3	3	4	4
2	2	1	1	2	2	3	3	4	4
2	2	1	1	2	2	3	3	4	4
2	2	4	4	2	2	3	3	4	4
4	4	1	1	2	2	3	3	4	4
2	2	1	1	2	2	3	3	4	4
4	4	4	4	2	2	3	3	4	4
4	4	4	4	2	2	3	3	4	4

For Review Only

2	2	1	1	2	2	3	3	4	4
4	4	1	1	2	2	3	3	4	4
4	4	4	4	3	3	1	1	4	4
2	2	1	1	2	2	3	3	4	4
4	4	4	4	2	2	3	3	4	4
4	4	1	1	2	2	3	3	4	4
4	4	1	1	2	2	3	3	4	4
2	2	4	4	2	2	3	3	4	4
2	2	1	1	2	2	3	3	4	4
4	4	4	4	2	2	3	3	4	4
4	4	4	4	3	3	1	1	4	4
4	4	4	4	3	3	1	1	4	4
2	2	1	1	2	2	3	3	4	4
2	2	1	1	2	2	3	3	4	4
4	4	4	4	2	2	3	3	4	4
4	4	1	1	2	2	3	3	4	4
2	2	1	1	2	2	3	3	4	4
4	4	1	1	2	2	3	3	4	4
2	2	1	1	2	2	3	3	4	4
4	4	1	1	2	2	3	3	4	4
4	4	4	4	2	2	1	1	4	4
2	2	4	4	2	2	3	3	4	4
2	2	1	1	2	2	3	3	4	4
2	2	4	4	3	3	1	1	4	4
2	2	4	4	2	2	3	3	4	4
4	4	4	4	2	2	3	3	4	4
4	4	4	4	2	2	3	3	4	4
2	2	1	1	2	2	3	3	4	4
4	4	1	1	2	2	3	3	4	4
2	2	4	4	2	2	3	3	4	4
2	2	1	1	2	2	3	3	4	4
2	2	1	1	2	2	3	3	4	4
2	2	4	4	2	2	3	3	4	4
2	2	1	1	2	2	3	3	4	4
4	4	1	1	2	2	3	3	4	4
2	2	4	4	2	2	3	3	4	4
2	2	4	4	2	2	3	3	4	4
4	4	1	1	2	2	3	3	4	4
2	2	4	4	2	2	3	3	3	3
4	4	4	4	2	2	3	3	4	4
4	4	1	1	2	2	3	3	4	4
2	2	1	1	2	2	3	3	4	4
4	4	4	4	2	2	3	3	4	4
4	4	4	4	3	3	1	1	4	4
4	4	4	4	2	2	3	3	4	4
2	2	1	1	2	2	3	3	4	4
2	2	4	4	2	2	3	3	4	4
4	4	1	1	2	2	3	3	4	4
4	4	1	1	2	2	3	3	4	4
2	2	4	4	3	3	3	3	4	4
4	4	1	1	2	2	3	3	4	4
2	2	4	4	2	2	3	3	4	4
2	2	4	4	2	2	3	3	4	4
2	2	4	4	2	2	3	3	4	4
2	2	1	1	2	2	3	3	4	4

For Review Only

4	4	4	4	2	2	3	3	4	4
4	4	4	4	2	2	3	3	4	4
4	4	1	1	2	2	3	3	4	4
2	2	4	4	2	2	3	3	4	4
2	2	4	4	2	2	3	3	4	4
4	4	1	1	2	2	3	3	4	4
4	4	1	1	2	2	3	3	4	4
2	2	4	4	2	2	1	1	4	4
4	4	4	4	2	2	3	3	4	4
2	2	4	4	2	2	3	3	4	4
4	4	1	1	2	2	3	3	4	4
4	4	4	4	2	2	3	3	4	4
2	2	4	4	2	2	3	3	4	4
4	4	4	4	2	2	3	3	4	4
4	4	4	4	2	2	3	3	4	4
2	2	4	4	2	2	3	3	4	4
2	2	1	1	2	2	3	3	4	4
4	4	4	4	2	2	3	3	4	4
2	2	1	1	2	2	3	3	4	4
2	2	4	4	2	2	3	3	4	4
4	4	4	4	2	2	3	3	4	4
2	2	4	4	2	2	3	3	4	4
2	2	4	4	3	3	1	1	4	4
2	2	4	4	2	2	3	3	4	4
2	2	1	1	2	2	3	3	4	4
2	2	4	4	2	2	3	3	3	3
4	4	1	1	2	2	3	3	4	4
2	2	4	4	2	2	3	3	4	4
4	4	1	1	2	2	3	3	4	4
4	4	1	1	2	2	3	3	4	4
2	2	4	4	2	2	3	3	4	4
4	4	4	4	2	2	3	3	4	4
4	4	1	1	2	2	3	3	4	4
4	4	1	1	2	2	3	3	4	4
2	2	4	4	2	2	3	3	4	4
2	2	1	1	2	2	3	3	4	4
2	2	4	4	2	2	3	3	4	4
4	4	4	4	2	2	3	3	4	4
2	2	1	1	2	2	3	3	4	4
2	2	4	4	2	2	3	3	4	4
4	4	4	4	2	2	3	3	4	4
2	2	4	4	2	2	3	3	4	4
4	4	1	1	2	2	3	3	4	4
2	2	4	4	2	2	3	3	4	4
4	4	4	4	2	2	3	3	4	4
4	4	4	4	2	2	3	3	4	4
4	4	1	1	2	2	3	3	4	4
4	4	1	1	2	2	3	3	4	4
2	2	4	4	2	2	3	3	4	4
4	4	1	1	2	2	3	3	4	4
4	4	1	1	2	2	3	3	4	4
2	2	1	1	2	2	3	3	4	4
4	4	4	4	2	2	3	3	4	4
4	4	4	4	3	3	1	1	4	4
2	2	4	4	2	2	3	3	4	4

For Review Only

2	2	1	1	2	2	3	3	4	4
2	2	4	4	2	2	3	3	3	3
4	4	4	4	2	2	3	3	4	4
2	2	4	4	2	2	3	3	4	4
4	4	4	4	2	2	3	3	4	4
2	2	1	1	2	2	3	3	4	4
2	2	4	4	2	2	3	3	4	4
4	4	4	4	2	2	3	3	4	4
2	2	4	4	2	2	3	3	4	4
2	2	1	1	2	2	3	3	4	4
4	4	4	4	2	2	3	3	4	4
4	4	1	1	2	2	3	3	4	4
4	4	4	4	2	2	3	3	4	4
2	2	4	4	2	2	3	3	4	4
2	2	4	4	2	2	3	3	4	4
2	2	4	4	2	2	3	3	4	4
4	4	4	4	2	2	3	3	4	4
4	4	4	4	2	2	3	3	4	4
2	2	1	1	2	2	3	3	4	4
4	4	1	1	2	2	3	3	4	4
4	4	4	4	2	2	3	3	4	4
2	2	4	4	2	2	3	3	4	4
2	2	4	4	2	2	3	3	3	3
4	4	4	4	3	3	1	1	4	4
2	2	4	4	2	2	3	3	4	4
2	2	4	4	2	2	3	3	4	4
4	4	1	1	2	2	3	3	4	4
2	2	4	4	2	2	3	3	4	4
2	2	1	1	2	2	3	3	4	4
2	2	1	1	2	2	3	3	4	4
2	2	1	1	2	2	3	3	4	4
4	4	1	1	2	2	3	3	4	4
4	4	1	1	2	2	3	3	4	4
2	2	1	1	2	2	3	3	4	4
4	4	1	1	2	2	3	3	4	4
4	4	4	4	3	3	1	1	4	4
4	4	1	1	2	2	3	3	4	4
2	2	1	1	2	2	3	3	4	4
4	4	1	1	2	2	3	3	4	4
4	4	4	4	3	3	1	1	4	4
2	2	1	1	2	2	3	3	4	4
4	4	1	1	2	2	3	3	4	4
2	2	4	4	2	2	3	3	4	4
4	4	1	1	2	2	3	3	4	4
4	4	1	1	2	2	3	3	4	4
2	2	4	4	2	2	3	3	4	4
2	2	4	4	2	2	3	3	4	4
2	2	4	4	3	3	1	1	4	4
2	2	4	4	2	2	3	3	4	4
4	4	4	4	2	2	3	3	3	3
2	2	1	1	2	2	3	3	4	4
2	2	4	4	2	2	3	3	4	4
2	2	4	4	2	2	3	3	3	3
4	4	4	4	3	3	1	1	4	4
2	2	4	4	2	2	3	3	4	4

For Review Only

2	2	4	4	2	2	3	3	4	4
2	2	1	1	2	2	3	3	4	4
2	2	1	1	2	2	3	3	4	4
2	2	4	4	2	2	3	3	4	4
2	2	1	1	2	2	3	3	4	4
2	2	1	1	2	2	3	3	4	4
2	2	1	1	2	2	3	3	4	4
2	2	4	4	2	2	3	3	4	4
4	4	4	4	2	2	3	3	4	4
2	2	1	1	2	2	3	3	4	4
4	4	4	4	2	2	3	3	4	4
2	2	1	1	2	2	3	3	4	4
2	2	4	4	2	2	3	3	3	3
2	2	1	1	2	2	3	3	4	4
4	4	1	1	2	2	3	3	4	4
4	4	4	4	2	2	3	3	3	3
4	4	4	4	2	2	3	3	4	4
2	2	4	4	2	2	3	3	4	4
2	2	4	4	2	2	3	3	4	4
2	2	1	1	2	2	3	3	4	4
2	2	4	4	3	3	1	1	4	4
2	2	1	1	2	2	3	3	4	4
2	2	1	1	2	2	3	3	4	4
2	2	1	1	2	2	3	3	4	4
2	2	4	4	2	2	3	3	4	4
2	2	1	1	2	2	3	3	4	4
2	2	4	4	3	3	1	1	4	4
4	4	1	1	2	2	3	3	4	4
2	2	1	1	2	2	3	3	4	4
2	2	1	1	2	2	3	3	4	4
2	2	4	4	2	2	3	3	4	4
2	2	1	1	2	2	3	3	4	4
2	2	1	1	2	2	3	3	4	4
4	4	4	4	2	2	3	3	4	4
2	2	4	4	2	2	3	3	4	4
2	2	4	4	2	2	3	3	4	4
2	2	4	4	2	2	3	3	4	4
4	4	4	4	3	3	1	1	4	4
4	4	1	1	2	2	3	3	4	4
2	2	4	4	2	2	3	3	4	4
4	4	1	1	2	2	3	3	4	4
4	4	1	1	2	2	3	3	4	4
4	4	4	4	2	2	3	3	4	4
2	2	1	1	2	2	3	3	4	4
4	4	4	4	2	2	3	3	4	4
4	4	4	4	2	2	3	3	4	4
4	4	1	1	2	2	3	3	4	4
4	4	4	4	2	2	3	3	4	4
2	2	4	4	2	2	3	3	4	4
2	2	4	4	3	3	1	1	4	4
2	2	1	1	2	2	3	3	4	4
2	2	4	4	2	2	3	3	4	4
2	2	1	1	2	2	3	3	4	4
4	4	1	1	2	2	3	3	4	4
2	2	1	1	2	2	3	3	4	4

For Review Only

4	4	4	4	2	2	3	3	4	4
4	4	1	1	2	2	3	3	4	4

For Review Only

SNP702	SNP702	SNP703	SNP703	SNP704	SNP704	SNP705	SNP705	SNP706	SNP706
2	2	4	4	2	2	4	4	3	3
2	2	4	4	1	1	1	1	3	3
2	2	4	4	1	1	1	1	3	3
2	2	4	4	2	2	1	1	3	3
2	2	4	4	2	2	1	1	3	3
2	2	4	4	2	2	1	1	3	3
2	2	4	4	2	2	4	4	3	3
2	2	4	4	1	1	4	4	3	3
2	2	4	4	2	2	4	4	3	3
2	2	4	4	2	2	1	1	3	3
2	2	4	4	2	2	1	1	2	2
2	2	4	4	2	2	4	4	3	3
2	2	4	4	2	2	4	4	2	2
2	2	4	4	2	2	4	4	3	3
2	2	4	4	2	2	4	4	3	3
2	2	4	4	2	2	4	4	2	2
2	2	4	4	2	2	4	4	3	3
2	2	4	4	2	2	1	1	3	3
2	2	4	4	2	2	4	4	3	3
2	2	4	4	2	2	1	1	3	3
2	2	4	4	2	2	1	1	3	3
2	2	4	4	2	2	1	1	3	3
2	2	4	4	2	2	1	1	2	2
2	2	4	4	2	2	4	4	2	2
2	2	4	4	2	2	1	1	3	3
2	2	4	4	2	2	4	4	3	3
2	2	4	4	2	2	4	4	3	3
2	2	4	4	2	2	4	4	3	3
2	2	4	4	2	2	1	1	3	3
1	1	2	2	2	2	4	4	3	3
2	2	4	4	2	2	1	1	2	2
2	2	4	4	1	1	1	1	3	3
2	2	4	4	1	1	4	4	3	3
2	2	4	4	2	2	4	4	2	2
2	2	4	4	2	2	1	1	2	2
2	2	4	4	2	2	4	4	2	2
2	2	4	4	2	2	1	1	3	3
2	2	4	4	2	2	4	4	3	3
2	2	4	4	2	2	1	1	3	3
2	2	4	4	1	1	1	1	3	3
2	2	4	4	2	2	1	1	3	3
1	1	2	2	2	2	1	1	3	3
2	2	4	4	2	2	4	4	3	3
2	2	4	4	2	2	4	4	3	3
2	2	4	4	2	2	1	1	3	3
2	2	4	4	2	2	4	4	3	3
2	2	4	4	2	2	1	1	3	3
2	2	4	4	2	2	4	4	3	3
2	2	4	4	1	1	4	4	3	3
2	2	4	4	2	2	1	1	2	2
2	2	4	4	2	2	4	4	3	3
2	2	4	4	2	2	1	1	3	3

For Review Only

2	2	4	4	2	2	1	1	3	3
2	2	4	4	1	1	4	4	3	3
2	2	4	4	2	2	4	4	3	3
2	2	4	4	2	2	1	1	3	3
2	2	4	4	2	2	4	4	3	3
2	2	4	4	2	2	4	4	3	3
2	2	4	4	2	2	4	4	3	3
2	2	4	4	2	2	4	4	3	3
2	2	4	4	2	2	4	4	2	2
2	2	4	4	2	2	4	4	2	2
2	2	4	4	2	2	4	4	3	3
2	2	4	4	2	2	4	4	2	2
2	2	4	4	2	2	1	1	3	3
2	2	4	4	2	2	1	1	3	3
2	2	4	4	2	2	4	4	3	3
2	2	4	4	2	2	4	4	2	2
2	2	4	4	2	2	1	1	3	3
2	2	4	4	1	1	4	4	3	3
2	2	4	4	2	2	1	1	2	2
2	2	4	4	2	2	4	4	3	3
2	2	4	4	2	2	4	4	3	3
2	2	2	2	2	2	1	1	3	3
2	2	4	4	2	2	4	4	3	3
2	2	4	4	2	2	4	4	2	2
2	2	4	4	2	2	1	1	3	3
2	2	4	4	2	2	1	1	3	3
2	2	4	4	1	1	4	4	3	3
2	2	4	4	2	2	4	4	3	3
2	2	4	4	2	2	4	4	3	3
2	2	4	4	2	2	4	4	2	2
2	2	4	4	2	2	1	1	3	3
2	2	4	4	2	2	4	4	2	2
2	2	4	4	2	2	1	1	3	3
2	2	4	4	2	2	1	1	2	2
2	2	4	4	1	1	4	4	2	2
2	2	4	4	2	2	1	1	3	3
2	2	4	4	2	2	1	1	3	3
2	2	4	4	2	2	4	4	3	3
1	1	2	2	2	2	1	1	2	2
2	2	4	4	2	2	4	4	3	3
2	2	4	4	2	2	4	4	3	3
2	2	4	4	2	2	1	1	2	2
2	2	4	4	2	2	4	4	3	3
2	2	4	4	2	2	4	4	3	3
2	2	4	4	2	2	4	4	2	2
2	2	4	4	1	1	1	1	3	3
2	2	4	4	2	2	4	4	2	2
2	2	4	4	2	2	4	4	3	3
2	2	4	4	2	2	4	4	3	3
2	2	4	4	2	2	1	1	3	3
2	2	4	4	2	2	4	4	3	3
2	2	4	4	2	2	1	1	2	2
2	2	4	4	2	2	4	4	3	3
2	2	4	4	1	1	1	1	2	2
2	2	4	4	1	1	1	1	3	3

For Review Only

1	1	2	2	2	2	4	4	3	3
2	2	4	4	2	2	4	4	2	2
2	2	4	4	2	2	4	4	2	2
2	2	4	4	2	2	4	4	2	2
2	2	4	4	1	1	4	4	3	3
2	2	4	4	2	2	4	4	2	2
2	2	4	4	2	2	4	4	2	2
2	2	4	4	2	2	4	4	2	2
2	2	4	4	2	2	4	4	3	3
2	2	4	4	2	2	4	4	3	3
2	2	4	4	2	2	4	4	2	2
2	2	4	4	2	2	4	4	3	3
2	2	4	4	2	2	4	4	3	3
2	2	4	4	2	2	1	1	3	3
2	2	4	4	1	1	1	1	2	2
2	2	4	4	2	2	4	4	3	3
2	2	4	4	1	1	4	4	3	3
2	2	4	4	2	2	4	4	3	3
2	2	4	4	2	2	4	4	2	2
2	2	4	4	2	2	1	1	3	3
2	2	4	4	2	2	4	4	3	3
2	2	4	4	2	2	4	4	2	2
2	2	4	4	2	2	4	4	2	2
2	2	4	4	2	2	1	1	3	3
1	1	4	4	2	2	4	4	3	3
1	1	2	2	2	2	1	1	3	3
2	2	4	4	1	1	4	4	3	3
2	2	4	4	2	2	1	1	2	2
2	2	4	4	2	2	4	4	2	2
2	2	4	4	2	2	4	4	3	3
2	2	4	4	2	2	4	4	2	2
2	2	4	4	2	2	4	4	3	3
2	2	4	4	1	1	4	4	3	3
2	2	4	4	2	2	4	4	2	2
2	2	4	4	2	2	4	4	2	2
2	2	4	4	2	2	4	4	2	2
2	2	4	4	2	2	1	1	2	2
2	2	4	4	2	2	4	4	3	3
2	2	4	4	2	2	4	4	3	3
2	2	4	4	2	2	1	1	2	2
2	2	4	4	2	2	4	4	3	3
2	2	4	4	2	2	4	4	2	2
2	2	4	4	2	2	4	4	3	3
2	2	4	4	2	2	4	4	3	3
2	2	4	4	2	2	4	4	3	3
2	2	4	4	1	1	4	4	3	3
2	2	4	4	2	2	4	4	3	3
2	2	4	4	2	2	4	4	3	3
2	2	4	4	2	2	1	1	2	2
2	2	4	4	2	2	4	4	3	3
2	2	4	4	2	2	4	4	3	3
2	2	4	4	2	2	1	1	3	3
2	2	4	4	2	2	4	4	2	2
2	2	4	4	2	2	4	4	3	3
2	2	2	2	2	2	4	4	2	2

For Review Only

2	2	4	4	2	2	1	1	3	3
1	1	2	2	2	2	1	1	3	3
2	2	4	4	1	1	4	4	3	3
1	1	2	2	2	2	1	1	2	2
2	2	4	4	1	1	4	4	2	2
2	2	4	4	1	1	1	1	3	3
2	2	4	4	2	2	1	1	2	2
2	2	4	4	2	2	4	4	2	2
2	2	4	4	2	2	1	1	2	2
2	2	4	4	2	2	4	4	2	2
2	2	4	4	2	2	4	4	3	3
2	2	4	4	2	2	4	4	2	2
2	2	4	4	2	2	4	4	3	3
2	2	4	4	2	2	4	4	3	3
2	2	4	4	2	2	4	4	2	2
2	2	4	4	2	2	1	1	2	2
2	2	4	4	2	2	4	4	2	2
2	2	4	4	1	1	1	1	3	3
2	2	4	4	2	2	4	4	3	3
2	2	4	4	2	2	4	4	2	2
2	2	4	4	2	2	4	4	2	2
2	2	4	4	2	2	4	4	3	3
1	1	2	2	2	2	1	1	3	3
2	2	4	4	2	2	4	4	3	3
2	2	4	4	2	2	1	1	2	2
2	2	4	4	2	2	4	4	3	3
2	2	4	4	2	2	4	4	3	3
2	2	4	4	2	2	4	4	2	2
2	2	4	4	2	2	1	1	3	3
2	2	4	4	2	2	1	1	2	2
2	2	4	4	2	2	4	4	2	2
2	2	4	4	2	2	4	4	2	2
2	2	4	4	1	1	4	4	3	3
2	2	4	4	2	2	1	1	2	2
2	2	4	4	1	1	4	4	2	2
2	2	4	4	2	2	4	4	3	3
2	2	4	4	2	2	4	4	3	3
2	2	4	4	2	2	1	1	3	3
2	2	4	4	2	2	4	4	3	3
2	2	4	4	2	2	4	4	3	3
2	2	4	4	1	1	1	1	3	3
2	2	4	4	2	2	4	4	3	3
2	2	4	4	2	2	4	4	2	2
2	2	4	4	2	2	4	4	3	3
1	1	2	2	2	2	4	4	2	2
2	2	4	4	2	2	1	1	3	3
2	2	4	4	2	2	1	1	2	2
2	2	4	4	2	2	1	1	3	3
2	2	4	4	2	2	4	4	2	2
1	1	2	2	2	2	4	4	3	3
2	2	4	4	2	2	1	1	2	2
2	2	4	4	1	1	4	4	2	2
1	1	2	2	2	2	1	1	3	3
2	2	4	4	2	2	4	4	3	3
2	2	4	4	2	2	1	1	2	2

For Review Only

2	2	4	4	2	2	4	4	2	2
2	2	4	4	2	2	1	1	3	3
2	2	4	4	2	2	1	1	2	2
1	1	2	2	2	2	1	1	3	3
2	2	4	4	2	2	1	1	3	3
2	2	4	4	2	2	1	1	3	3
2	2	4	4	2	2	1	1	3	3
2	2	4	4	2	2	1	1	3	3
2	2	4	4	2	2	1	1	3	3
2	2	4	4	2	2	1	1	2	2
2	2	4	4	2	2	4	4	2	2
2	2	4	4	2	2	4	4	3	3
1	1	2	2	2	2	1	1	2	2
2	2	4	4	2	2	1	1	3	3
2	2	4	4	1	1	4	4	3	3
1	1	2	2	2	2	4	4	2	2
2	2	4	4	2	2	4	4	3	3
1	1	2	2	2	2	4	4	3	3
2	2	4	4	2	2	4	4	3	3
2	2	4	4	1	1	4	4	3	3
2	2	4	4	2	2	4	4	2	2
2	2	4	4	2	2	1	1	3	3
2	2	4	4	2	2	1	1	3	3
2	2	4	4	2	2	1	1	3	3
2	2	4	4	2	2	4	4	3	3
2	2	4	4	2	2	4	4	2	2
2	2	4	4	2	2	1	1	3	3
2	2	4	4	2	2	4	4	3	3
2	2	4	4	2	2	1	1	3	3
2	2	4	4	2	2	1	1	2	2
2	2	4	4	2	2	1	1	3	3
2	2	4	4	2	2	1	1	3	3
2	2	4	4	2	2	1	1	3	3
2	2	4	4	2	2	4	4	3	3
2	2	4	4	2	2	1	1	3	3
2	2	4	4	2	2	1	1	2	2
2	2	4	4	2	2	1	1	2	2
2	2	4	4	2	2	4	4	3	3
2	2	4	4	2	2	4	4	2	2
2	2	4	4	2	2	1	1	3	3
2	2	4	4	2	2	4	4	3	3
2	2	4	4	1	1	4	4	2	2
2	2	4	4	2	2	4	4	3	3
2	2	4	4	2	2	1	1	3	3
2	2	4	4	2	2	4	4	3	3
2	2	4	4	2	2	4	4	2	2
2	2	4	4	2	2	4	4	3	3
2	2	4	4	1	1	4	4	2	2
2	2	4	4	2	2	4	4	3	3
2	2	4	4	2	2	1	1	3	3
2	2	4	4	2	2	1	1	3	3
2	2	4	4	2	2	1	1	2	2
2	2	4	4	1	1	1	1	3	3
2	2	4	4	2	2	4	4	3	3
2	2	4	4	1	1	1	1	3	3

For Review Only

2	2	4	4	2	2	4	4	2	2
2	2	4	4	1	1	1	1	3	3

For Review Only

SNP707	SNP707	SNP708	SNP708	SNP709	SNP709	SNP710	SNP710	SNP711	SNP711
2	2	1	1	3	3	2	2	2	2
2	2	1	1	3	3	3	3	2	2
2	2	1	1	3	3	3	3	2	2
2	2	1	1	3	3	3	3	2	2
2	2	1	1	3	3	3	3	2	2
2	2	1	1	3	3	3	3	2	2
2	2	1	1	3	3	2	2	2	2
2	2	1	1	3	3	2	2	2	2
2	2	1	1	3	3	2	2	2	2
2	2	1	1	2	2	3	3	2	2
2	2	4	4	3	3	3	3	2	2
2	2	1	1	3	3	3	3	2	2
2	2	1	1	3	3	3	3	2	2
2	2	1	1	3	3	2	2	2	2
2	2	1	1	3	3	3	3	2	2
2	2	1	1	3	3	3	3	2	2
2	2	1	1	3	3	2	2	2	2
2	2	1	1	3	3	3	3	2	2
2	2	1	1	3	3	3	3	2	2
2	2	1	1	2	2	3	3	2	2
2	2	1	1	3	3	3	3	2	2
2	2	1	1	3	3	3	3	2	2
2	2	4	4	3	3	3	3	2	2
2	2	4	4	3	3	2	2	2	2
2	2	1	1	3	3	3	3	2	2
2	2	1	1	3	3	2	2	2	2
2	2	1	1	3	3	2	2	2	2
2	2	1	1	3	3	3	3	2	2
2	2	1	1	3	3	3	3	2	2
2	2	1	1	2	2	2	2	2	2
2	2	4	4	3	3	3	3	2	2
2	2	1	1	3	3	3	3	2	2
2	2	1	1	3	3	2	2	2	2
2	2	4	4	3	3	2	2	4	4
2	2	1	1	3	3	3	3	2	2
2	2	1	1	3	3	3	3	2	2
2	2	1	1	3	3	3	3	2	2
2	2	1	1	3	3	3	3	2	2
2	2	1	1	3	3	3	3	2	2
2	2	1	1	3	3	3	3	2	2
2	2	1	1	3	3	3	3	2	2
2	2	1	1	3	3	3	3	2	2
2	2	1	1	2	2	3	3	2	2
2	2	1	1	3	3	2	2	2	2
2	2	1	1	2	2	3	3	2	2
2	2	1	1	3	3	3	3	2	2
2	2	1	1	3	3	3	3	2	2
2	2	1	1	3	3	3	3	2	2
2	2	1	1	3	3	3	3	2	2
2	2	1	1	3	3	3	3	2	2
2	2	1	1	3	3	3	3	2	2
2	2	1	1	3	3	3	3	2	2

For Review Only

For Review Only

2	2	1	1	3	3	3	3	2	2
2	2	1	1	3	3	2	2	2	2
2	2	1	1	3	3	2	2	2	2
2	2	1	1	3	3	3	3	2	2
2	2	1	1	3	3	3	3	2	2
2	2	1	1	3	3	3	3	2	2
2	2	1	1	3	3	2	2	2	2
2	2	1	1	3	3	2	2	2	2
2	2	1	1	3	3	2	2	2	2
2	2	1	1	3	3	3	3	2	2
2	2	1	1	3	3	3	3	2	2
2	2	1	1	3	3	3	3	2	2
2	2	1	1	3	3	3	3	2	2
2	2	1	1	3	3	3	3	2	2
2	2	1	1	3	3	3	3	2	2
2	2	1	1	3	3	2	2	2	2
2	2	1	1	3	3	3	3	2	2
2	2	1	1	3	3	2	2	2	2
2	2	1	1	3	3	3	3	2	2
2	2	1	1	3	3	2	2	2	2
2	2	1	1	3	3	3	3	2	2
2	2	1	1	3	3	3	3	2	2
2	2	1	1	3	3	3	3	2	2
2	2	1	1	3	3	2	2	2	2
2	2	1	1	2	2	3	3	2	2
2	2	1	1	3	3	3	3	2	2
2	2	1	1	3	3	2	2	2	2
2	2	1	1	3	3	3	3	2	2
2	2	1	1	3	3	2	2	2	2
2	2	1	1	3	3	2	2	2	2
2	2	1	1	3	3	3	3	2	2
2	2	1	1	3	3	2	2	2	2
2	2	1	1	3	3	3	3	2	2
2	2	1	1	3	3	3	3	2	2
2	2	1	1	3	3	2	2	2	2
2	2	1	1	2	2	3	3	2	2
2	2	1	1	3	3	3	3	2	2
2	2	1	1	3	3	3	3	2	2
2	2	1	1	3	3	3	3	2	2
2	2	1	1	3	3	3	3	2	2
2	2	1	1	3	3	2	2	2	2
2	2	1	1	3	3	3	3	2	2
2	2	1	1	3	3	2	2	2	2
2	2	1	1	3	3	3	3	2	2
2	2	1	1	3	3	2	2	2	2
2	2	1	1	3	3	3	3	2	2
2	2	1	1	3	3	2	2	4	4
2	2	1	1	3	3	2	2	2	2
2	2	1	1	3	3	2	2	2	2
2	2	1	1	3	3	3	3	2	2
2	2	1	1	3	3	3	3	2	2
2	2	4	4	3	3	3	3	2	2
2	2	1	1	3	3	3	3	2	2
2	2	1	1	3	3	3	3	2	2
2	2	1	1	3	3	3	3	2	2

2	2	1	1	3	3	3	3	2	2
2	2	1	1	3	3	3	3	2	2
2	2	1	1	3	3	2	2	2	2
2	2	1	1	3	3	2	2	4	4
2	2	1	1	3	3	3	3	2	2
2	2	1	1	3	3	2	2	2	2
2	2	1	1	3	3	3	3	2	2
2	2	1	1	3	3	3	3	2	2
2	2	1	1	3	3	3	3	2	2
3	3	1	1	3	3	3	3	2	2
2	2	1	1	3	3	2	2	2	2
2	2	1	1	3	3	3	3	2	2
2	2	1	1	3	3	2	2	2	2
2	2	1	1	3	3	3	3	2	2
2	2	1	1	3	3	3	3	2	2
2	2	1	1	3	3	3	3	2	2
2	2	1	1	3	3	2	2	2	2
2	2	1	1	3	3	3	3	2	2
2	2	4	4	3	3	2	2	4	4
2	2	1	1	3	3	3	3	2	2
2	2	1	1	3	3	3	3	2	2
2	2	1	1	3	3	3	3	2	2
2	2	1	1	3	3	3	3	2	2
2	2	1	1	3	3	3	3	2	2
2	2	1	1	3	3	3	3	2	2
2	2	1	1	3	3	3	3	2	2
2	2	1	1	3	3	3	3	2	2
2	2	1	1	3	3	3	3	2	2
2	2	1	1	3	3	3	3	2	2
2	2	1	1	3	3	3	3	2	2
2	2	4	4	3	3	3	3	4	4
2	2	1	1	3	3	3	3	2	2
2	2	1	1	3	3	2	2	2	2
2	2	1	1	3	3	3	3	4	4
2	2	4	4	3	3	2	2	4	4
2	2	1	1	3	3	3	3	2	2
3	3	1	1	3	3	3	3	2	2
2	2	1	1	3	3	2	2	2	2
2	2	1	1	3	3	3	3	2	2
2	2	4	4	3	3	3	3	4	4
2	2	1	1	3	3	3	3	2	2
2	2	4	4	3	3	3	3	4	4
2	2	1	1	3	3	2	2	2	2
2	2	1	1	2	2	3	3	2	2
2	2	1	1	3	3	2	2	2	2
2	2	1	1	3	3	2	2	2	2
2	2	1	1	3	3	2	2	2	2
2	2	1	1	3	3	3	3	2	2
2	2	1	1	3	3	3	3	2	2
2	2	1	1	3	3	2	2	2	2
2	2	1	1	3	3	2	2	2	2
2	2	1	1	3	3	3	3	2	2
2	2	1	1	3	3	3	3	2	2
2	2	1	1	3	3	3	3	2	2
2	2	1	1	3	3	2	2	2	2

For Review Only

2	2	1	1	3	3	3	3	2	2
2	2	1	1	3	3	3	3	2	2
2	2	1	1	3	3	3	3	2	2
2	2	1	1	3	3	3	3	2	2
2	2	1	1	3	3	3	3	2	2
2	2	1	1	3	3	3	3	2	2
2	2	1	1	3	3	3	3	2	2
2	2	1	1	3	3	3	3	2	2
2	2	1	1	3	3	3	3	2	2
2	2	1	1	3	3	3	3	2	2
2	2	1	1	3	3	2	2	2	2
2	2	1	1	3	3	3	3	2	2
2	2	1	1	3	3	2	2	2	2
2	2	1	1	3	3	3	3	2	2
2	2	4	4	3	3	3	3	4	4
2	2	4	4	3	3	3	3	4	4
2	2	1	1	3	3	3	3	2	2
2	2	1	1	3	3	3	3	2	2
2	2	1	1	3	3	2	2	2	2
2	2	1	1	3	3	3	3	2	2
2	2	1	1	3	3	3	3	2	2
2	2	1	1	3	3	2	2	2	2
2	2	1	1	3	3	3	3	2	2
2	2	1	1	3	3	3	3	2	2
2	2	1	1	3	3	3	3	2	2
2	2	1	1	3	3	2	2	4	4
2	2	1	1	3	3	2	2	2	2
2	2	1	1	3	3	3	3	2	2
2	2	1	1	3	3	3	3	2	2
2	2	1	1	3	3	3	3	2	2
2	2	1	1	3	3	2	2	2	2
2	2	1	1	3	3	3	3	2	2
2	2	1	1	3	3	2	2	2	2
2	2	4	4	3	3	3	3	4	4
2	2	1	1	3	3	3	3	2	2
2	2	1	1	3	3	3	3	2	2
2	2	1	1	3	3	3	3	2	2
2	2	1	1	3	3	3	3	2	2
2	2	1	1	3	3	2	2	2	2
2	2	1	1	3	3	3	3	2	2
2	2	1	1	3	3	3	3	2	2
2	2	1	1	3	3	2	2	2	2
3	3	4	4	3	3	3	3	2	2
2	2	1	1	3	3	3	3	2	2
2	2	4	4	3	3	3	3	4	4
2	2	1	1	3	3	3	3	2	2
2	2	4	4	3	3	3	3	4	4
2	2	1	1	3	3	3	3	2	2
2	2	4	4	3	3	3	3	4	4
2	2	1	1	3	3	3	3	2	2
2	2	1	1	3	3	3	3	2	2
2	2	1	1	3	3	2	2	2	2
2	2	1	1	3	3	3	3	2	2
2	2	1	1	3	3	3	3	2	2
2	2	4	4	3	3	3	3	2	2

For Review Only

2	2	1	1	3	3	3	3	2	2
2	2	1	1	2	2	3	3	2	2
2	2	1	1	3	3	3	3	2	2
2	2	1	1	3	3	3	3	2	2
2	2	1	1	3	3	3	3	2	2
2	2	1	1	3	3	3	3	2	2
2	2	1	1	3	3	3	3	2	2
2	2	1	1	2	2	3	3	2	2
2	2	1	1	3	3	3	3	2	2
2	2	4	4	3	3	3	3	4	4
2	2	1	1	3	3	3	3	2	2
2	2	1	1	3	3	3	3	2	2
2	2	1	1	3	3	3	3	2	2
2	2	1	1	3	3	3	3	2	2
2	2	1	1	3	3	3	3	2	2
2	2	1	1	3	3	2	2	2	2
2	2	1	1	3	3	2	2	2	2
2	2	1	1	3	3	3	3	2	2
2	2	1	1	3	3	3	3	2	2
2	2	1	1	3	3	2	2	2	2
2	2	1	1	3	3	2	2	2	2
2	2	1	1	3	3	3	3	2	2
2	2	1	1	3	3	3	3	2	2
2	2	1	1	3	3	3	3	2	2
2	2	1	1	3	3	2	2	2	2
2	2	1	1	3	3	3	3	2	2
2	2	1	1	3	3	3	3	2	2
2	2	1	1	3	3	2	2	2	2
2	2	1	1	3	3	3	3	2	2
2	2	1	1	3	3	3	3	2	2
2	2	1	1	3	3	3	3	2	2
2	2	1	1	3	3	3	3	2	2
2	2	1	1	3	3	3	3	2	2
2	2	1	1	3	3	3	3	2	2
2	2	1	1	3	3	3	3	2	2
2	2	1	1	3	3	3	3	4	4
2	2	1	1	3	3	3	3	2	2
2	2	1	1	3	3	3	3	2	2
2	2	1	1	3	3	3	3	2	2
2	2	1	1	3	3	3	3	2	2
2	2	1	1	3	3	3	3	2	2
2	2	1	1	3	3	2	2	2	2
2	2	1	1	3	3	2	2	2	2
2	2	1	1	3	3	3	3	2	2
2	2	1	1	3	3	3	3	2	2
2	2	4	4	3	3	2	2	2	2
2	2	1	1	3	3	2	2	2	2
2	2	1	1	3	3	2	2	2	2
2	2	1	1	2	2	3	3	2	2
2	2	1	1	3	3	3	3	2	2
2	2	1	1	3	3	3	3	2	2
2	2	4	4	3	3	3	3	2	2
2	2	1	1	3	3	3	3	2	2
2	2	1	1	3	3	2	2	2	2
2	2	1	1	3	3	3	3	2	2

For Review Only

2	2	1	1	3	3	2	2	2	2
2	2	1	1	3	3	3	3	2	2

For Review Only

SNP712	SNP712	SNP713	SNP713	SNP714	SNP714	SNP715	SNP715	SNP716	SNP716
3	3	3	3	3	3	2	2	4	4
3	3	3	3	3	3	2	2	4	4
3	3	3	3	3	3	2	2	4	4
3	3	3	3	3	3	2	2	2	2
3	3	3	3	3	3	2	2	4	4
3	3	3	3	3	3	2	2	4	4
3	3	3	3	3	3	2	2	4	4
2	2	4	4	3	3	4	4	4	4
3	3	3	3	3	3	2	2	4	4
3	3	3	3	3	3	2	2	4	4
3	3	3	3	3	3	4	4	4	4
3	3	3	3	3	3	2	2	2	2
3	3	3	3	3	3	2	2	4	4
3	3	4	4	3	3	2	2	2	2
3	3	3	3	3	3	2	2	4	4
3	3	3	3	3	3	2	2	4	4
3	3	3	3	3	3	2	2	4	4
3	3	3	3	3	3	2	2	4	4
3	3	3	3	3	3	2	2	4	4
3	3	3	3	3	3	2	2	4	4
3	3	3	3	3	3	2	2	4	4
3	3	3	3	3	3	2	2	4	4
3	3	3	3	2	2	4	4	4	4
3	3	3	3	3	3	2	2	4	4
3	3	3	3	3	3	2	2	4	4
2	2	4	4	3	3	2	2	2	2
2	2	4	4	3	3	2	2	2	2
3	3	3	3	3	3	2	2	4	4
3	3	3	3	3	3	2	2	4	4
3	3	3	3	3	3	4	4	4	4
3	3	3	3	3	3	2	2	4	4
3	3	3	3	2	2	2	2	4	4
3	3	3	3	3	3	2	2	4	4
3	3	3	3	3	3	4	4	4	4
3	3	3	3	2	2	2	2	4	4
3	3	3	3	3	3	2	2	4	4
3	3	3	3	3	3	2	2	4	4
3	3	3	3	3	3	4	4	4	4
3	3	3	3	3	3	2	2	4	4
3	3	3	3	3	3	2	2	4	4
3	3	3	3	3	3	4	4	4	4
3	3	3	3	3	3	2	2	4	4
3	3	3	3	3	3	2	2	4	4
3	3	3	3	2	2	2	2	4	4
3	3	3	3	3	3	2	2	4	4
3	3	4	4	3	3	2	2	2	2
3	3	3	3	3	3	2	2	4	4
3	3	3	3	3	3	2	2	4	4
3	3	3	3	3	3	4	4	4	4
3	3	4	4	3	3	2	2	4	4
3	3	3	3	3	3	2	2	4	4
3	3	3	3	3	3	2	2	4	4

3	3	3	3	3	3	2	2	4	4
3	3	3	3	3	3	2	2	4	4
3	3	4	4	3	3	2	2	2	2
3	3	3	3	3	3	2	2	4	4
3	3	3	3	3	3	2	2	4	4
3	3	3	3	3	3	2	2	4	4
2	2	4	4	3	3	2	2	2	2
3	3	3	3	3	3	4	4	4	4
3	3	3	3	3	3	2	2	4	4
3	3	3	3	3	3	2	2	4	4
3	3	3	3	3	3	2	2	4	4
3	3	3	3	3	3	2	2	4	4
3	3	3	3	3	3	2	2	4	4
3	3	3	3	3	3	2	2	4	4
3	3	3	3	3	3	2	2	4	4
3	3	3	3	2	2	2	2	4	4
3	3	3	3	3	3	2	2	4	4
2	2	4	4	3	3	2	2	4	4
3	3	3	3	3	3	2	2	4	4
3	3	3	3	3	3	2	2	4	4
3	3	3	3	3	3	2	2	4	4
3	3	3	3	3	3	2	2	4	4
3	3	3	3	3	3	2	2	4	4
2	2	4	4	2	2	2	2	2	2
3	3	3	3	3	3	2	2	4	4
3	3	3	3	3	3	2	2	4	4
3	3	3	3	2	2	2	2	4	4
3	3	3	3	3	3	4	4	4	4
2	2	4	4	3	3	2	2	2	2
2	2	4	4	3	3	2	2	2	2
3	3	3	3	3	3	2	2	4	4
3	3	3	3	3	3	2	2	4	4
3	3	3	3	3	3	4	4	4	4
3	3	3	3	3	3	2	2	4	4
3	3	3	3	3	3	2	2	4	4
3	3	3	3	3	3	2	2	4	4
3	3	3	3	3	3	4	4	4	4
3	3	3	3	3	3	4	4	4	4
3	3	3	3	3	3	2	2	4	4
3	3	3	3	3	3	4	4	4	4
2	2	4	4	3	3	2	2	2	2
3	3	3	3	3	3	2	2	4	4
3	3	4	4	3	3	4	4	2	2
3	3	3	3	3	3	2	2	4	4
3	3	3	3	3	3	2	2	4	4
3	3	3	3	2	2	2	2	2	2
3	3	3	3	3	3	2	2	4	4
2	2	4	4	3	3	2	2	2	2
2	2	4	4	3	3	2	2	2	2
3	3	3	3	3	3	2	2	4	4
3	3	4	4	3	3	2	2	2	2
3	3	3	3	3	3	4	4	4	4
3	3	3	3	3	3	2	2	4	4
3	3	3	3	3	3	2	2	4	4
3	3	3	3	3	3	4	4	4	4

For Review Only

3	3	4	4	3	3	2	2	4	4
3	3	3	3	3	3	4	4	4	4
2	2	4	4	3	3	2	2	2	2
3	3	3	3	3	3	2	2	4	4
3	3	3	3	3	3	2	2	2	2
2	2	4	4	3	3	2	2	2	2
3	3	3	3	3	3	2	2	4	4
3	3	3	3	3	3	2	2	4	4
3	3	3	3	3	3	2	2	4	4
3	3	3	3	3	3	2	2	4	4
3	3	3	3	3	3	2	2	4	4
3	3	3	3	3	3	2	2	4	4
3	3	3	3	3	3	4	4	4	4
3	3	3	3	3	3	2	2	4	4
3	3	3	3	2	2	2	2	4	4
3	3	3	3	3	3	2	2	4	4
3	3	3	3	3	3	2	2	4	4
3	3	3	3	3	3	2	2	4	4
3	3	3	3	3	3	4	4	4	4
3	3	3	3	3	3	2	2	4	4
3	3	3	3	3	3	2	2	4	4
3	3	3	3	3	3	2	2	4	4
3	3	3	3	2	2	2	2	4	4
3	3	3	3	3	3	4	4	4	4
3	3	3	3	3	3	2	2	4	4
3	3	3	3	3	3	2	2	4	4
3	3	3	3	2	2	2	2	4	4
3	3	3	3	3	3	2	2	4	4
3	3	3	3	3	3	2	2	4	4
3	3	3	3	2	2	2	2	4	4
3	3	3	3	3	3	2	2	4	4
3	3	3	3	3	3	2	2	4	4
2	2	4	4	3	3	2	2	2	2
3	3	3	3	3	3	2	2	4	4
3	3	3	3	3	3	4	4	4	4
3	3	4	4	3	3	2	2	4	4
3	3	3	3	3	3	4	4	4	4
3	3	4	4	3	3	2	2	2	2
3	3	3	3	3	3	2	2	4	4
3	3	3	3	3	3	4	4	4	4
3	3	3	3	3	3	2	2	4	4
3	3	3	3	3	3	2	2	4	4
2	2	4	4	3	3	2	2	4	4
3	3	3	3	3	3	2	2	4	4
3	3	4	4	3	3	2	2	2	2
2	2	4	4	3	3	2	2	2	2
2	2	4	4	3	3	2	2	2	2
3	3	3	3	3	3	2	2	4	4
3	3	3	3	3	3	2	2	4	4
2	2	4	4	3	3	2	2	2	2
2	2	4	4	3	3	2	2	2	2
3	3	3	3	3	3	4	4	4	4
3	3	3	3	3	3	2	2	4	4
3	3	3	3	3	3	2	2	4	4
3	3	3	3	3	3	2	2	4	4

For Review Only

3	3	3	3	3	3	2	2	4	4
3	3	3	3	3	3	4	4	4	4
3	3	3	3	2	2	2	2	4	4
3	3	3	3	3	3	2	2	4	4
3	3	3	3	3	3	2	2	2	2
3	3	3	3	3	3	4	4	4	4
3	3	3	3	3	3	4	4	4	4
3	3	3	3	3	3	2	2	4	4
3	3	3	3	3	3	2	2	4	4
3	3	3	3	3	3	2	2	4	4
2	2	4	4	3	3	2	2	2	2
3	3	3	3	3	3	2	2	4	4
2	2	4	4	3	3	2	2	2	2
3	3	3	3	3	3	2	2	4	4
3	3	3	3	3	3	2	2	4	4
3	3	3	3	3	3	4	4	4	4
3	3	3	3	3	3	2	2	4	4
3	3	3	3	3	3	2	2	4	4
3	3	3	3	3	3	2	2	4	4
3	3	3	3	3	3	2	2	4	4
3	3	3	3	3	3	2	2	4	4
3	3	3	3	3	3	2	2	4	4
3	3	3	3	3	3	2	2	4	4
3	3	3	3	3	3	2	2	4	4
3	3	3	3	2	2	2	2	4	4
3	3	3	3	3	3	4	4	4	4
3	3	4	4	3	3	2	2	2	2
3	3	3	3	3	3	2	2	4	4
3	3	3	3	3	3	2	2	4	4
3	3	3	3	3	3	2	2	4	4
2	2	4	4	3	3	2	2	2	2
3	3	3	3	3	3	2	2	4	4
2	2	4	4	2	2	2	2	2	2
3	3	3	3	3	3	2	2	4	4
3	3	3	3	2	2	2	2	4	4
3	3	3	3	3	3	2	2	4	4
3	3	3	3	3	3	2	2	4	4
3	3	3	3	3	3	2	2	4	4
2	2	4	4	3	3	2	2	2	2
3	3	3	3	3	3	2	2	4	4
3	3	3	3	3	3	2	2	4	4
2	2	4	4	3	3	2	2	2	2
3	3	3	3	3	3	2	2	4	4
3	3	3	3	2	2	2	2	4	4
3	3	3	3	3	3	2	2	4	4
3	3	3	3	3	3	2	2	4	4
3	3	3	3	3	3	4	4	4	4
3	3	3	3	3	3	4	4	4	4
3	3	3	3	3	3	2	2	4	4
3	3	3	3	3	3	2	2	4	4
3	3	3	3	3	3	2	2	4	4
3	3	3	3	3	3	2	2	4	4
3	3	3	3	3	3	2	2	4	4
3	3	3	3	3	3	2	2	4	4
3	3	3	3	3	3	2	2	4	4

For Review Only

3	3	3	3	3	3	2	2	4	4
3	3	3	3	2	2	2	2	4	4
3	3	3	3	3	3	2	2	4	4
3	3	3	3	3	3	2	2	4	4
3	3	3	3	3	3	4	4	4	4
3	3	3	3	3	3	2	2	4	4
3	3	3	3	3	3	2	2	4	4
3	3	3	3	3	3	4	4	4	4
3	3	3	3	3	3	2	2	4	4
3	3	3	3	3	3	4	4	4	4
3	3	3	3	3	3	2	2	4	4
3	3	3	3	3	3	2	2	4	4
3	3	3	3	3	3	2	2	4	4
3	3	3	3	3	3	2	2	4	4
3	3	3	3	3	3	2	2	4	4
2	2	4	4	3	3	2	2	2	2
2	2	4	4	3	3	4	4	2	2
3	3	3	3	3	3	2	2	4	4
3	3	3	3	3	3	2	2	4	4
3	3	3	3	3	3	4	4	4	4
2	2	4	4	3	3	2	2	2	2
3	3	3	3	3	3	2	2	4	4
3	3	3	3	3	3	2	2	4	4
3	3	3	3	3	3	4	4	4	4
3	3	3	3	3	3	4	4	4	4
3	3	3	3	3	3	2	2	4	4
3	3	3	3	3	3	4	4	4	4
2	2	4	4	3	3	2	2	2	2
3	3	3	3	3	3	2	2	4	4
3	3	3	3	3	3	2	2	4	4
3	3	3	3	3	3	4	4	4	4
3	3	3	3	3	3	2	2	4	4
3	3	3	3	3	3	2	2	4	4
3	3	3	3	3	3	2	2	4	4
3	3	3	3	3	3	2	2	4	4
3	3	3	3	3	3	4	4	4	4
3	3	3	3	3	3	2	2	4	4
3	3	3	3	3	3	2	2	4	4
3	3	3	3	3	3	2	2	4	4
3	3	3	3	3	3	4	4	4	4
3	3	3	3	2	2	2	2	4	4
2	2	4	4	2	2	2	2	2	2
3	3	4	4	3	3	2	2	2	2
3	3	3	3	3	3	2	2	4	4
3	3	3	3	3	3	2	2	4	4
2	2	4	4	3	3	2	2	2	2
2	2	4	4	3	3	4	4	2	2
3	3	4	4	3	3	2	2	2	2
3	3	3	3	3	3	2	2	4	4
3	3	3	3	3	3	4	4	4	4
3	3	4	4	3	3	2	2	2	2
3	3	3	3	3	3	4	4	4	4
3	3	3	3	3	3	2	2	4	4
2	2	4	4	3	3	4	4	2	2
3	3	3	3	2	2	2	2	4	4

For Review Only

2	2	4	4	3	3	2	2	2	2
3	3	3	3	3	3	2	2	4	4

For Review Only

SNP717	SNP717	SNP718	SNP718	SNP719	SNP719	SNP720	SNP720	SNP721	SNP721
1	1	1	1	1	1	3	3	3	3
1	1	1	1	3	3	3	3	3	3
1	1	1	1	1	1	2	2	3	3
1	1	1	1	1	1	3	3	3	3
1	1	1	1	1	1	3	3	3	3
4	4	1	1	1	1	3	3	3	3
1	1	1	1	1	1	3	3	3	3
1	1	1	1	1	1	2	2	4	4
1	1	1	1	1	1	3	3	3	3
1	1	1	1	1	1	2	2	3	3
4	4	4	4	1	1	3	3	4	4
1	1	1	1	1	1	2	2	3	3
1	1	1	1	1	1	2	2	3	3
1	1	1	1	1	1	3	3	4	4
1	1	1	1	1	1	2	2	3	3
1	1	1	1	1	1	2	2	3	3
1	1	1	1	3	3	3	3	3	3
1	1	1	1	1	1	3	3	3	3
1	1	1	1	1	1	2	2	3	3
1	1	1	1	1	1	2	2	3	3
1	1	1	1	1	1	3	3	3	3
1	1	1	1	1	1	3	3	4	4
1	1	1	1	1	1	3	3	4	4
1	1	1	1	1	1	3	3	3	3
1	1	1	1	1	1	3	3	3	3
1	1	1	1	1	1	2	2	3	3
1	1	1	1	1	1	3	3	3	3
1	1	1	1	1	1	2	2	3	3
1	1	1	1	1	1	3	3	3	3
1	1	1	1	1	1	2	2	4	4
1	1	1	1	1	1	3	3	3	3
1	1	1	1	1	1	2	2	3	3
1	1	1	1	1	1	2	2	3	3
4	4	4	4	3	3	3	3	4	4
1	1	1	1	1	1	2	2	4	4
1	1	1	1	1	1	2	2	3	3
1	1	1	1	1	1	3	3	4	4
1	1	1	1	1	1	3	3	4	4
1	1	1	1	1	1	2	2	4	4
1	1	1	1	1	1	2	2	4	4
1	1	1	1	1	1	2	2	4	4
1	1	1	1	1	1	2	2	3	3
1	1	1	1	1	1	3	3	3	3
1	1	1	1	1	1	3	3	4	4
1	1	1	1	1	1	3	3	4	4
1	1	1	1	1	1	2	2	3	3
1	1	1	1	1	1	2	2	3	3
1	1	1	1	1	1	2	2	3	3
1	1	1	1	1	1	3	3	4	4
1	1	1	1	1	1	3	3	3	3
1	1	1	1	1	1	2	2	3	3
1	1	1	1	1	1	2	2	3	3

For Review Only

1	1	1	1	1	1	3	3	3	3
1	1	1	1	1	1	2	2	3	3
1	1	1	1	1	1	2	2	3	3
1	1	1	1	1	1	3	3	3	3
1	1	1	1	1	1	2	2	3	3
1	1	1	1	1	1	2	2	3	3
1	1	1	1	1	1	2	2	3	3
1	1	1	1	3	3	3	3	4	4
1	1	1	1	3	3	3	3	4	4
1	1	1	1	1	1	2	2	3	3
1	1	1	1	1	1	2	2	3	3
1	1	1	1	1	1	2	2	3	3
1	1	1	1	1	1	3	3	3	3
1	1	1	1	1	1	3	3	3	3
4	4	1	1	1	1	2	2	3	3
1	1	1	1	1	1	3	3	4	4
1	1	1	1	1	1	2	2	3	3
1	1	1	1	1	1	3	3	3	3
1	1	1	1	1	1	3	3	3	3
1	1	1	1	1	1	3	3	3	3
1	1	1	1	1	1	2	2	3	3
1	1	1	1	1	1	2	2	4	4
1	1	1	1	1	1	3	3	3	3
1	1	1	1	1	1	2	2	3	3
1	1	1	1	1	1	3	3	4	4
4	4	1	1	1	1	2	2	3	3
1	1	1	1	1	1	2	2	3	3
1	1	1	1	3	3	2	2	3	3
1	1	1	1	1	1	2	2	3	3
1	1	1	1	1	1	3	3	3	3
1	1	1	1	1	1	2	2	3	3
1	1	1	1	1	1	3	3	3	3
1	1	1	1	3	3	2	2	3	3
1	1	1	1	1	1	3	3	3	3
1	1	1	1	1	1	3	3	3	3
1	1	1	1	1	1	3	3	4	4
1	1	1	1	1	1	3	3	3	3
1	1	1	1	1	1	3	3	4	4
1	1	1	1	1	1	2	2	3	3
1	1	1	1	1	1	2	2	4	4
1	1	1	1	1	1	3	3	3	3
1	1	1	1	1	1	2	2	3	3
1	1	1	1	1	1	3	3	3	3
1	1	1	1	1	1	2	2	3	3
1	1	1	1	1	1	3	3	3	3
1	1	1	1	1	1	3	3	3	3
1	1	1	1	1	1	3	3	3	3
1	1	1	1	1	1	2	2	3	3
1	1	1	1	1	1	3	3	3	3
1	1	1	1	1	1	2	2	3	3
1	1	1	1	1	1	3	3	3	3
4	4	4	4	1	1	2	2	4	4
1	1	1	1	1	1	2	2	4	4
1	1	1	1	1	1	2	2	3	3
1	1	1	1	1	1	2	2	3	3

For Review Only

1	1	1	1	1	1	2	2	3	3
1	1	1	1	1	1	2	2	4	4
1	1	1	1	1	1	2	2	3	3
1	1	1	1	3	3	3	3	3	3
1	1	1	1	1	1	2	2	3	3
1	1	1	1	1	1	2	2	3	3
1	1	1	1	1	1	3	3	3	3
1	1	1	1	1	1	3	3	3	3
1	1	1	1	1	1	3	3	4	4
1	1	1	1	1	1	2	2	3	3
1	1	1	1	1	1	3	3	4	4
1	1	1	1	1	1	2	2	3	3
1	1	1	1	3	3	3	3	3	3
4	4	1	1	1	1	2	2	3	3
1	1	1	1	1	1	3	3	3	3
1	1	1	1	1	1	2	2	3	3
1	1	1	1	3	3	3	3	3	3
1	1	1	1	1	1	2	2	3	3
1	1	1	1	3	3	3	3	4	4
1	1	1	1	1	1	3	3	3	3
1	1	1	1	1	1	2	2	3	3
1	1	1	1	1	1	3	3	3	3
1	1	1	1	1	1	3	3	3	3
1	1	1	1	3	3	2	2	4	4
1	1	1	1	1	1	2	2	3	3
1	1	1	1	1	1	3	3	4	4
1	1	1	1	1	1	2	2	3	3
1	1	1	1	1	1	3	3	3	3
1	1	1	1	1	1	2	2	3	3
1	1	1	1	1	1	3	3	3	3
1	1	4	4	1	1	2	2	4	4
1	1	1	1	1	1	2	2	3	3
1	1	1	1	1	1	3	3	3	3
1	1	1	1	1	1	2	2	3	3
4	4	4	4	3	3	3	3	4	4
1	1	1	1	1	1	3	3	3	3
1	1	1	1	1	1	3	3	4	4
1	1	1	1	1	1	2	2	3	3
1	1	1	1	1	1	2	2	3	3
1	1	1	1	1	1	2	2	4	4
1	1	1	1	1	1	2	2	3	3
1	1	1	1	1	1	3	3	3	3
1	1	1	1	1	1	2	2	3	3
1	1	1	1	1	1	2	2	3	3
1	1	1	1	1	1	2	2	3	3
1	1	1	1	1	1	3	3	3	3
1	1	1	1	1	1	2	2	3	3
1	1	1	1	1	1	2	2	3	3
1	1	1	1	1	1	2	2	4	4
1	1	1	1	1	1	2	2	3	3
1	1	1	1	1	1	2	2	3	3
1	1	1	1	1	1	2	2	4	4
1	1	1	1	1	1	3	3	3	3
1	1	1	1	1	1	2	2	3	3
1	1	1	1	1	1	3	3	4	4

For Review Only

1	1	1	1	1	1	2	2	3	3
1	1	1	1	1	1	2	2	3	3
1	1	1	1	1	1	3	3	4	4
1	1	1	1	1	1	3	3	4	4
1	1	1	1	1	1	2	2	3	3
1	1	1	1	1	1	2	2	4	4
1	1	1	1	1	1	2	2	3	3
1	1	1	1	1	1	2	2	3	3
1	1	1	1	1	1	3	3	3	3
1	1	1	1	1	1	2	2	3	3
1	1	1	1	1	1	2	2	3	3
1	1	1	1	1	1	2	2	4	4
1	1	1	1	1	1	3	3	3	3
1	1	1	1	1	1	3	3	3	3
1	1	1	1	1	1	2	2	3	3
1	1	1	1	1	1	2	2	4	4
1	1	1	1	1	1	3	3	3	3
1	1	1	1	1	1	2	2	3	3
1	1	1	1	1	1	3	3	3	3
1	1	1	1	1	1	3	3	3	3
1	1	1	1	1	1	2	2	3	3
1	1	1	1	1	1	2	2	3	3
1	1	1	1	3	3	2	2	4	4
1	1	1	1	1	1	2	2	3	3
1	1	1	1	1	1	2	2	3	3
1	1	1	1	3	3	3	3	4	4
1	1	1	1	1	1	2	2	3	3
1	1	1	1	1	1	3	3	3	3
1	1	1	1	1	1	3	3	3	3
1	1	1	1	1	1	3	3	3	3
1	1	1	1	1	1	2	2	3	3
1	1	1	1	1	1	3	3	3	3
1	1	1	1	1	1	3	3	4	4
1	1	1	1	1	1	2	2	3	3
1	1	1	1	1	1	3	3	4	4
1	1	1	1	1	1	2	2	3	3
1	1	1	1	1	1	2	2	4	4
1	1	1	1	1	1	3	3	4	4
1	1	1	1	1	1	2	2	3	3
1	1	1	1	1	1	2	2	3	3
1	1	1	1	1	1	2	2	3	3
1	1	1	1	1	1	2	2	3	3
1	1	1	1	1	1	2	2	3	3
1	1	1	1	1	1	2	2	4	4
1	1	1	1	1	1	2	2	3	3
1	1	1	1	1	1	3	3	3	3
1	1	1	1	1	1	2	2	4	4
1	1	1	1	1	1	3	3	3	3
4	4	4	4	3	3	3	3	3	3
1	1	1	1	1	1	3	3	3	3
1	1	1	1	1	1	2	2	3	3
1	1	1	1	3	3	2	2	4	4
4	4	4	4	1	1	2	2	4	4
1	1	1	1	1	1	2	2	3	3
1	1	1	1	1	1	2	2	4	4

For Review Only

1	1	1	1	1	1	3	3	3	3
1	1	1	1	1	1	3	3	3	3
1	1	1	1	1	1	2	2	3	3
1	1	1	1	1	1	2	2	4	4
1	1	1	1	1	1	3	3	3	3
1	1	1	1	1	1	3	3	3	3
1	1	1	1	1	1	2	2	4	4
1	1	1	1	1	1	2	2	4	4
1	1	1	1	1	1	2	2	3	3
4	4	4	4	3	3	2	2	3	3
1	1	1	1	1	1	2	2	3	3
1	1	1	1	3	3	2	2	3	3
1	1	1	1	1	1	2	2	3	3
1	1	1	1	1	1	2	2	3	3
1	1	1	1	1	1	2	2	3	3
1	1	1	1	1	1	3	3	3	3
1	1	1	1	1	1	2	2	4	4
1	1	1	1	3	3	2	2	3	3
1	1	1	1	1	1	3	3	3	3
1	1	1	1	1	1	3	3	4	4
1	1	1	1	1	1	2	2	3	3
1	1	1	1	1	1	2	2	3	3
1	1	1	1	1	1	3	3	3	3
1	1	1	1	1	1	2	2	4	4
1	1	1	1	3	3	3	3	4	4
1	1	1	1	1	1	2	2	3	3
1	1	1	1	1	1	2	2	3	3
1	1	1	1	1	1	2	2	3	3
1	1	1	1	1	1	3	3	3	3
1	1	1	1	1	1	2	2	3	3
1	1	1	1	1	1	2	2	3	3
1	1	1	1	1	1	2	2	3	3
1	1	1	1	1	1	3	3	3	3
1	1	1	1	1	1	2	2	3	3
1	1	1	1	3	3	2	2	4	4
4	4	4	4	3	3	2	2	3	3
1	1	1	1	1	1	3	3	3	3
1	1	1	1	1	1	2	2	3	3
1	1	1	1	1	1	2	2	3	3
1	1	1	1	1	1	2	2	4	4
1	1	1	1	1	1	3	3	3	3
1	1	1	1	1	1	3	3	3	3
1	1	1	1	1	1	2	2	3	3
1	1	1	1	1	1	3	3	3	3
1	1	1	1	1	1	2	2	3	3
4	4	4	4	1	1	3	3	3	3
1	1	1	1	1	1	2	2	4	4
1	1	1	1	1	1	3	3	3	3
1	1	1	1	1	1	3	3	3	3
4	4	4	4	1	1	2	2	3	3
1	1	1	1	1	1	3	3	3	3
1	1	1	1	1	1	2	2	4	4
1	1	1	1	1	1	2	2	3	3
1	1	1	1	1	1	2	2	4	4
1	1	1	1	1	1	2	2	4	4

For Review Only

1	1	1	1	1	1	3	3	3	3
1	1	1	1	1	1	2	2	3	3

For Review Only

SNP722	SNP722	SNP723	SNP723	SNP724	SNP724	SNP725	SNP725	SNP726	SNP726
4	4	1	1	4	4	4	4	1	1
4	4	1	1	4	4	1	1	1	1
4	4	1	1	4	4	1	1	3	3
4	4	1	1	4	4	1	1	1	1
4	4	1	1	4	4	4	4	1	1
4	4	1	1	4	4	4	4	1	1
4	4	1	1	4	4	4	4	1	1
4	4	1	1	2	2	4	4	1	1
4	4	1	1	4	4	4	4	1	1
4	4	1	1	4	4	1	1	1	1
1	1	2	2	2	2	4	4	1	1
4	4	1	1	4	4	1	1	3	3
4	4	1	1	4	4	4	4	1	1
4	4	2	2	4	4	1	1	1	1
4	4	1	1	4	4	4	4	3	3
4	4	1	1	4	4	4	4	1	1
4	4	1	1	4	4	4	4	1	1
4	4	1	1	4	4	1	1	1	1
4	4	1	1	4	4	4	4	3	3
4	4	1	1	2	2	4	4	1	1
4	4	1	1	4	4	1	1	1	1
4	4	1	1	4	4	4	4	1	1
4	4	1	1	2	2	4	4	1	1
1	1	2	2	4	4	4	4	1	1
4	4	1	1	4	4	1	1	1	1
4	4	1	1	4	4	4	4	3	3
4	4	1	1	4	4	1	1	1	1
4	4	1	1	4	4	4	4	3	3
4	4	1	1	4	4	1	1	1	1
4	4	1	1	2	2	4	4	1	1
4	4	1	1	4	4	1	1	1	1
4	4	1	1	4	4	4	4	3	3
4	4	1	1	4	4	4	4	3	3
1	1	2	2	4	4	1	1	1	1
4	4	1	1	2	2	4	4	3	3
1	1	2	2	4	4	4	4	3	3
4	4	1	1	4	4	1	1	1	1
4	4	1	1	2	2	4	4	1	1
4	4	1	1	4	4	1	1	3	3
4	4	1	1	4	4	1	1	3	3
4	4	1	1	2	2	4	4	1	1
4	4	1	1	4	4	1	1	1	1
4	4	1	1	4	4	4	4	1	1
4	4	1	1	4	4	4	4	1	1
4	4	1	1	4	4	1	1	1	1
4	4	1	1	4	4	4	4	3	3
4	4	1	1	4	4	4	4	3	3
4	4	1	1	4	4	4	4	3	3
4	4	1	1	2	2	4	4	1	1
4	4	1	1	4	4	4	4	1	1
4	4	1	1	4	4	1	1	3	3
4	4	1	1	4	4	4	4	3	3

For Review Only

4	4	1	1	4	4	4	4	1	1
4	4	1	1	4	4	4	4	3	3
4	4	1	1	4	4	4	4	3	3
4	4	1	1	4	4	4	4	1	1
4	4	1	1	4	4	4	4	3	3
4	4	1	1	4	4	4	4	3	3
4	4	1	1	4	4	1	1	3	3
4	4	1	1	4	4	1	1	1	1
4	4	1	1	4	4	1	1	1	1
4	4	1	1	4	4	4	4	3	3
4	4	1	1	4	4	4	4	3	3
4	4	1	1	4	4	4	4	3	3
4	4	1	1	4	4	4	4	1	1
4	4	1	1	4	4	1	1	1	1
4	4	1	1	4	4	4	4	3	3
4	4	1	1	4	4	1	1	1	1
4	4	1	1	4	4	4	4	3	3
4	4	1	1	4	4	4	4	1	1
4	4	1	1	4	4	4	4	1	1
4	4	1	1	4	4	4	4	3	3
4	4	1	1	4	4	4	4	3	3
4	4	1	1	4	4	4	4	3	3
4	4	1	1	4	4	4	4	1	1
4	4	1	1	4	4	4	4	3	3
4	4	1	1	4	4	1	1	1	1
4	4	1	1	4	4	4	4	3	3
4	4	1	1	4	4	4	4	3	3
4	4	1	1	2	2	4	4	3	3
4	4	1	1	4	4	4	4	3	3
4	4	1	1	4	4	4	4	1	1
1	1	2	2	4	4	4	4	1	1
4	4	1	1	4	4	4	4	1	1
4	4	1	1	2	2	4	4	3	3
4	4	1	1	4	4	4	4	1	1
4	4	1	1	4	4	4	4	1	1
4	4	1	1	4	4	1	1	1	1
1	1	2	2	2	2	4	4	1	1
4	4	1	1	2	2	4	4	1	1
4	4	1	1	4	4	4	4	3	3
4	4	1	1	4	4	4	4	1	1
4	4	1	1	4	4	4	4	1	1
4	4	1	1	4	4	4	4	3	3
4	4	1	1	2	2	4	4	1	1
4	4	1	1	4	4	4	4	3	3
4	4	1	1	4	4	4	4	1	1
4	4	1	1	4	4	1	1	1	1
4	4	2	2	4	4	1	1	1	1
1	1	2	2	4	4	1	1	3	3
4	4	1	1	4	4	4	4	1	1
4	4	1	1	2	2	4	4	3	3
4	4	1	1	4	4	4	4	3	3
1	1	2	2	4	4	4	4	3	3
4	4	1	1	4	4	1	1	1	1
4	4	1	1	4	4	4	4	3	3
4	4	1	1	2	2	4	4	1	1

For Review Only

4	4	1	1	4	4	1	1	3	3
4	4	1	1	2	2	4	4	3	3
1	1	2	2	4	4	4	4	3	3
4	4	2	2	4	4	4	4	1	1
4	4	1	1	4	4	1	1	3	3
4	4	1	1	4	4	4	4	3	3
4	4	1	1	4	4	1	1	1	1
1	1	2	2	4	4	4	4	1	1
4	4	1	1	2	2	4	4	1	1
4	4	1	1	4	4	4	4	3	3
4	4	1	1	4	4	1	1	1	1
4	4	1	1	4	4	4	4	3	3
4	4	1	1	2	2	4	4	1	1
4	4	1	1	4	4	4	4	3	3
4	4	1	1	4	4	1	1	1	1
4	4	1	1	4	4	4	4	3	3
4	4	1	1	4	4	4	4	1	1
4	4	1	1	4	4	4	4	3	3
4	4	2	2	4	4	1	1	1	1
4	4	1	1	2	2	4	4	1	1
4	4	1	1	4	4	4	4	3	3
4	4	1	1	4	4	4	4	1	1
4	4	1	1	4	4	4	4	1	1
4	4	1	1	4	4	4	4	3	3
4	4	1	1	4	4	1	1	3	3
4	4	1	1	2	2	4	4	1	1
4	4	1	1	4	4	4	4	3	3
1	1	2	2	4	4	1	1	1	1
4	4	1	1	4	4	1	1	3	3
4	4	1	1	4	4	4	4	1	1
1	1	2	2	2	2	4	4	3	3
4	4	1	1	4	4	4	4	3	3
4	4	1	1	4	4	4	4	1	1
4	4	1	1	4	4	4	4	1	1
1	1	2	2	4	4	1	1	1	1
4	4	1	1	4	4	4	4	3	3
4	4	2	2	4	4	1	1	1	1
4	4	1	1	4	4	4	4	1	1
4	4	1	1	4	4	4	4	1	1
4	4	1	1	2	2	4	4	3	3
4	4	1	1	4	4	4	4	3	3
4	4	2	2	4	4	1	1	1	1
4	4	1	1	4	4	4	4	3	3
4	4	1	1	4	4	4	4	1	1
4	4	1	1	4	4	4	4	3	3
4	4	1	1	4	4	4	4	1	1
4	4	1	1	4	4	1	1	3	3
4	4	1	1	4	4	4	4	3	3
4	4	1	1	4	4	4	4	3	3
4	4	1	1	4	4	4	4	3	3
4	4	1	1	4	4	4	4	3	3
4	4	1	1	4	4	4	4	1	1
4	4	1	1	4	4	4	4	1	1
4	4	1	1	4	4	4	4	3	3
4	4	1	1	4	4	4	4	1	1

For Review Only

4	4	1	1	4	4	4	4	3	3
4	4	1	1	2	2	4	4	1	1
4	4	1	1	4	4	1	1	1	1
4	4	1	1	2	2	4	4	1	1
4	4	1	1	4	4	1	1	3	3
4	4	1	1	4	4	1	1	3	3
4	4	1	1	4	4	4	4	3	3
4	4	1	1	4	4	4	4	3	3
4	4	1	1	4	4	4	4	1	1
4	4	1	1	4	4	4	4	1	1
4	4	1	1	4	4	4	4	3	3
4	4	2	2	2	2	4	4	3	3
4	4	1	1	4	4	4	4	1	1
4	4	1	1	4	4	4	4	1	1
4	4	2	2	4	4	1	1	3	3
4	4	1	1	2	2	4	4	3	3
4	4	1	1	4	4	4	4	3	3
4	4	1	1	4	4	1	1	3	3
4	4	1	1	4	4	4	4	1	1
4	4	1	1	4	4	1	1	1	1
4	4	1	1	4	4	4	4	3	3
4	4	1	1	4	4	4	4	3	3
4	4	1	1	2	2	4	4	3	3
4	4	1	1	4	4	4	4	3	3
4	4	1	1	4	4	1	1	1	1
4	4	2	2	4	4	1	1	1	1
4	4	1	1	4	4	4	4	3	3
4	4	1	1	4	4	4	4	1	1
4	4	1	1	4	4	4	4	1	1
4	4	1	1	4	4	4	4	1	1
4	4	1	1	4	4	4	4	3	3
4	4	1	1	4	4	4	4	1	1
4	4	1	1	4	4	1	1	1	1
4	4	2	2	4	4	1	1	3	3
4	4	1	1	4	4	1	1	1	1
4	4	1	1	4	4	4	4	3	3
4	4	1	1	2	2	4	4	3	3
4	4	1	1	4	4	1	1	1	1
4	4	1	1	4	4	4	4	3	3
4	4	1	1	4	4	4	4	3	3
4	4	1	1	4	4	1	1	3	3
4	4	1	1	4	4	1	1	3	3
4	4	1	1	4	4	1	1	3	3
4	4	1	1	4	4	4	4	3	3
1	1	1	1	4	4	4	4	1	1
4	4	1	1	4	4	1	1	1	1
4	4	1	1	2	2	4	4	3	3
4	4	1	1	4	4	4	4	1	1
1	1	2	2	4	4	4	4	1	1
4	4	1	1	4	4	1	1	1	1
4	4	1	1	4	4	4	4	3	3
4	4	2	2	4	4	1	1	1	1
1	1	2	2	2	2	4	4	3	3
4	4	1	1	4	4	4	4	3	3
1	1	2	2	2	2	4	4	3	3

For Review Only

4	4	1	1	4	4	4	4	1	1
4	4	1	1	4	4	1	1	1	1
4	4	1	1	4	4	4	4	3	3
4	4	1	1	2	2	4	4	3	3
4	4	1	1	4	4	1	1	1	1
4	4	1	1	4	4	4	4	1	1
4	4	1	1	2	2	4	4	3	3
4	4	1	1	2	2	4	4	1	1
4	4	1	1	4	4	4	4	3	3
1	1	2	2	2	2	4	4	3	3
4	4	1	1	4	4	4	4	3	3
4	4	1	1	4	4	1	1	3	3
4	4	1	1	4	4	4	4	3	3
1	1	2	2	4	4	4	4	3	3
4	4	1	1	4	4	1	1	3	3
4	4	1	1	4	4	1	1	1	1
4	4	1	1	2	2	4	4	3	3
4	4	1	1	4	4	1	1	3	3
4	4	1	1	4	4	4	4	1	1
4	4	1	1	2	2	4	4	1	1
4	4	1	1	4	4	4	4	3	3
4	4	1	1	4	4	4	4	3	3
4	4	1	1	4	4	4	4	1	1
4	4	1	1	2	2	4	4	3	3
4	4	1	1	4	4	1	1	1	1
4	4	1	1	4	4	4	4	1	1
4	4	1	1	2	2	4	4	3	3
4	4	1	1	4	4	1	1	3	3
4	4	1	1	4	4	4	4	1	1
4	4	1	1	4	4	1	1	3	3
4	4	1	1	4	4	4	4	3	3
4	4	1	1	4	4	1	1	3	3
4	4	2	2	4	4	1	1	1	1
4	4	1	1	4	4	4	4	3	3
4	4	1	1	2	2	4	4	3	3
1	1	1	1	4	4	4	4	3	3
4	4	1	1	4	4	4	4	1	1
4	4	1	1	4	4	4	4	3	3
1	1	2	2	4	4	4	4	3	3
4	4	1	1	4	4	1	1	3	3
4	4	1	1	4	4	4	4	1	1
4	4	1	1	4	4	4	4	1	1
4	4	1	1	4	4	4	4	3	3
4	4	1	1	4	4	4	4	1	1
4	4	1	1	4	4	4	4	3	3
1	1	2	2	4	4	4	4	1	1
4	4	1	1	2	2	4	4	3	3
4	4	1	1	4	4	4	4	1	1
4	4	1	1	2	2	4	4	1	1
1	1	1	1	2	2	4	4	3	3
4	4	1	1	4	4	4	4	1	1
4	4	1	1	2	2	4	4	3	3
4	4	1	1	4	4	1	1	3	3
4	4	1	1	4	4	1	1	3	3
4	4	1	1	4	4	1	1	3	3

For Review Only

4	4	1	1	4	4	4	4	1	1
4	4	1	1	4	4	1	1	3	3

For Review Only

SNP727	SNP727	SNP728	SNP728	SNP729	SNP729	SNP730	SNP730	SNP731	SNP731
3	3	1	1	1	1	2	2	1	1
2	2	3	3	1	1	3	3	1	1
3	3	1	1	1	1	2	2	1	1
3	3	1	1	1	1	2	2	1	1
3	3	1	1	1	1	2	2	1	1
3	3	1	1	1	1	2	2	1	1
3	3	1	1	1	1	2	2	1	1
3	3	1	1	1	1	2	2	1	1
3	3	1	1	1	1	2	2	4	4
3	3	1	1	1	1	2	2	1	1
2	2	1	1	4	4	2	2	1	1
3	3	1	1	1	1	2	2	1	1
3	3	1	1	1	1	2	2	1	1
3	3	1	1	1	1	2	2	1	1
3	3	1	1	1	1	2	2	1	1
3	3	1	1	1	1	2	2	4	4
2	2	3	3	1	1	3	3	1	1
3	3	1	1	1	1	2	2	1	1
3	3	1	1	1	1	2	2	1	1
3	3	3	3	4	4	2	2	4	4
3	3	3	3	1	1	3	3	1	1
3	3	3	3	1	1	3	3	1	1
2	2	1	1	1	1	2	2	1	1
3	3	1	1	1	1	2	2	1	1
3	3	1	1	1	1	2	2	1	1
3	3	1	1	1	1	2	2	1	1
3	3	1	1	1	1	2	2	1	1
3	3	1	1	1	1	2	2	1	1
3	3	1	1	1	1	2	2	4	4
2	2	3	3	4	4	3	3	4	4
3	3	1	1	1	1	2	2	1	1
3	3	1	1	1	1	2	2	1	1
3	3	1	1	1	1	2	2	1	1
3	3	1	1	1	1	2	2	1	1
3	3	1	1	1	1	2	2	4	4
3	3	1	1	1	1	2	2	1	1
3	3	1	1	1	1	2	2	1	1
3	3	1	1	1	1	2	2	1	1
2	2	1	1	1	1	2	2	1	1
2	2	1	1	1	1	2	2	1	1
3	3	3	3	4	4	3	3	1	1
3	3	1	1	4	4	2	2	1	1
3	3	1	1	1	1	2	2	4	4
3	3	1	1	4	4	2	2	1	1
3	3	1	1	1	1	2	2	4	4
3	3	1	1	1	1	2	2	1	1
3	3	1	1	1	1	2	2	1	1
2	2	1	1	1	1	2	2	1	1
3	3	1	1	1	1	2	2	1	1
3	3	1	1	1	1	2	2	1	1
3	3	1	1	1	1	2	2	1	1
3	3	1	1	1	1	2	2	1	1

For Review Only

3	3	1	1	1	1	2	2	1	1
3	3	1	1	1	1	2	2	1	1
3	3	1	1	1	1	2	2	1	1
3	3	1	1	4	4	2	2	1	1
3	3	1	1	1	1	2	2	1	1
3	3	1	1	1	1	2	2	1	1
3	3	1	1	1	1	2	2	1	1
2	2	3	3	1	1	3	3	1	1
2	2	3	3	1	1	3	3	1	1
3	3	1	1	1	1	2	2	1	1
3	3	1	1	1	1	2	2	1	1
3	3	1	1	1	1	2	2	1	1
3	3	1	1	1	1	2	2	1	1
3	3	1	1	1	1	2	2	4	4
3	3	1	1	1	1	2	2	1	1
2	2	1	1	1	1	3	3	4	4
2	2	1	1	1	1	2	2	1	1
3	3	1	1	1	1	2	2	1	1
3	3	1	1	1	1	2	2	1	1
3	3	1	1	1	1	2	2	1	1
3	3	1	1	1	1	2	2	1	1
3	3	1	1	1	1	2	2	1	1
3	3	1	1	1	1	2	2	4	4
3	3	1	1	1	1	2	2	1	1
3	3	1	1	1	1	2	2	4	4
3	3	1	1	1	1	2	2	4	4
3	3	1	1	1	1	2	2	1	1
3	3	1	1	1	1	2	2	1	1
3	3	1	1	1	1	2	2	1	1
3	3	1	1	1	1	2	2	1	1
3	3	1	1	1	1	2	2	1	1
3	3	1	1	1	1	2	2	1	1
3	3	1	1	1	1	3	3	1	1
3	3	1	1	1	1	2	2	1	1
3	3	1	1	1	1	2	2	1	1
3	3	1	1	1	1	2	2	4	4
3	3	3	3	1	1	3	3	4	4
3	3	1	1	4	4	2	2	1	1
3	3	1	1	1	1	2	2	1	1
3	3	1	1	1	1	2	2	1	1
2	2	3	3	1	1	3	3	1	1
3	3	1	1	1	1	2	2	1	1
3	3	1	1	1	1	2	2	1	1
3	3	1	1	1	1	2	2	1	1
3	3	1	1	1	1	2	2	1	1
3	3	1	1	1	1	2	2	1	1
3	3	1	1	1	1	3	3	1	1
3	3	1	1	1	1	2	2	1	1
3	3	1	1	1	1	2	2	1	1
3	3	1	1	1	1	2	2	1	1
3	3	1	1	1	1	2	2	1	1
3	3	1	1	4	4	2	2	1	1
3	3	1	1	1	1	2	2	1	1
3	3	1	1	1	1	2	2	1	1
3	3	1	1	1	1	2	2	1	1

For Review Only

3	3	1	1	1	1	2	2	1	1
3	3	1	1	1	1	2	2	1	1
3	3	1	1	1	1	2	2	1	1
3	3	1	1	1	1	3	3	1	1
3	3	1	1	1	1	2	2	1	1
3	3	1	1	1	1	2	2	1	1
3	3	1	1	1	1	2	2	1	1
3	3	1	1	1	1	2	2	1	1
3	3	1	1	1	1	2	2	1	1
3	3	3	3	1	1	2	2	1	1
3	3	1	1	1	1	2	2	1	1
3	3	1	1	1	1	2	2	1	1
2	2	1	1	1	1	3	3	1	1
3	3	1	1	1	1	2	2	1	1
3	3	1	1	1	1	2	2	1	1
3	3	1	1	1	1	2	2	1	1
3	3	3	3	1	1	3	3	1	1
3	3	1	1	1	1	2	2	1	1
3	3	1	1	1	1	3	3	1	1
2	2	1	1	1	1	2	2	1	1
3	3	1	1	1	1	2	2	1	1
3	3	1	1	1	1	2	2	1	1
3	3	1	1	1	1	2	2	1	1
3	3	1	1	1	1	3	3	1	1
3	3	1	1	1	1	2	2	1	1
2	2	1	1	1	1	2	2	1	1
3	3	1	1	1	1	2	2	1	1
3	3	1	1	1	1	2	2	1	1
3	3	1	1	1	1	2	2	1	1
3	3	1	1	1	1	2	2	1	1
3	3	1	1	1	1	2	2	1	1
3	3	1	1	1	1	2	2	1	1
2	2	3	3	1	1	3	3	1	1
3	3	1	1	1	1	2	2	1	1
3	3	1	1	1	1	2	2	1	1
3	3	1	1	1	1	2	2	1	1
3	3	1	1	1	1	2	2	1	1
3	3	1	1	1	1	2	2	1	1
2	2	1	1	1	1	2	2	1	1
3	3	1	1	1	1	2	2	1	1
2	2	1	1	1	1	2	2	1	1
3	3	1	1	1	1	3	3	1	1
3	3	1	1	1	1	2	2	1	1
3	3	1	1	1	1	2	2	1	1
3	3	1	1	1	1	2	2	1	1
3	3	1	1	1	1	2	2	1	1
3	3	1	1	1	1	2	2	1	1
3	3	1	1	1	1	2	2	1	1
3	3	1	1	1	1	2	2	1	1
3	3	1	1	1	1	2	2	1	1
3	3	1	1	1	1	2	2	1	1
3	3	1	1	1	1	2	2	1	1
3	3	1	1	1	1	2	2	1	1
3	3	1	1	1	1	2	2	1	1
3	3	1	1	1	1	2	2	1	1

For Review Only

3	3	1	1	1	1	2	2	1	1
3	3	1	1	1	1	2	2	1	1
3	3	1	1	1	1	2	2	1	1
3	3	1	1	1	1	2	2	1	1
3	3	1	1	1	1	2	2	1	1
2	2	1	1	1	1	2	2	1	1
2	2	1	1	1	1	2	2	1	1
3	3	1	1	1	1	2	2	1	1
3	3	1	1	1	1	2	2	1	1
3	3	1	1	1	1	2	2	1	1
3	3	1	1	1	1	2	2	1	1
3	3	1	1	1	1	2	2	1	1
3	3	1	1	1	1	2	2	1	1
2	2	1	1	1	1	2	2	1	1
3	3	1	1	1	1	3	3	1	1
2	2	1	1	1	1	2	2	1	1
3	3	1	1	1	1	2	2	1	1
3	3	1	1	1	1	2	2	1	1
3	3	1	1	1	1	2	2	1	1
3	3	1	1	1	1	2	2	1	1
3	3	1	1	1	1	2	2	1	1
3	3	1	1	1	1	2	2	1	1
3	3	1	1	1	1	2	2	1	1
3	3	1	1	1	1	2	2	1	1
3	3	1	1	4	4	2	2	4	4
3	3	1	1	1	1	2	2	1	1
3	3	1	1	1	1	2	2	1	1
3	3	1	1	1	1	2	2	1	1
3	3	1	1	1	1	2	2	1	1
3	3	1	1	1	1	2	2	1	1
3	3	1	1	1	1	2	2	1	1
3	3	1	1	1	1	2	2	1	1
3	3	1	1	1	1	2	2	1	1
3	3	1	1	1	1	2	2	1	1
3	3	1	1	1	1	2	2	1	1
3	3	1	1	1	1	2	2	1	1
3	3	1	1	1	1	2	2	1	1
3	3	1	1	1	1	2	2	1	1
3	3	1	1	1	1	2	2	1	1
3	3	1	1	1	1	2	2	1	1
3	3	1	1	1	1	2	2	1	1
3	3	1	1	1	1	2	2	1	1
3	3	1	1	1	1	3	3	1	1
3	3	1	1	1	1	2	2	1	1
3	3	1	1	1	1	2	2	1	1
3	3	1	1	1	1	2	2	1	1
2	2	1	1	1	1	2	2	1	1
3	3	1	1	1	1	2	2	1	1
3	3	1	1	1	1	2	2	1	1
3	3	1	1	1	1	2	2	1	1
3	3	1	1	1	1	2	2	1	1
2	2	1	1	1	1	3	3	1	1
2	2	1	1	1	1	2	2	1	1
3	3	1	1	1	1	2	2	1	1
3	3	1	1	1	1	2	2	4	4

For Review Only

3	3	1	1	1	1	2	2	1	1
3	3	1	1	1	1	2	2	4	4
3	3	1	1	1	1	2	2	1	1
3	3	1	1	1	1	2	2	1	1
3	3	1	1	1	1	2	2	1	1
3	3	1	1	1	1	2	2	1	1
3	3	1	1	1	1	2	2	1	1
3	3	3	3	1	1	3	3	4	4
3	3	1	1	1	1	2	2	1	1
3	3	1	1	1	1	2	2	1	1
3	3	1	1	1	1	2	2	1	1
3	3	1	1	1	1	3	3	1	1
3	3	1	1	1	1	2	2	1	1
3	3	1	1	1	1	2	2	1	1
3	3	1	1	1	1	2	2	1	1
3	3	1	1	1	1	2	2	1	1
3	3	1	1	1	1	2	2	1	1
3	3	3	3	1	1	3	3	1	1
3	3	1	1	1	1	3	3	1	1
3	3	1	1	1	1	2	2	1	1
3	3	1	1	1	1	2	2	1	1
3	3	1	1	1	1	3	3	1	1
3	3	1	1	1	1	2	2	1	1
2	2	1	1	1	1	2	2	4	4
2	2	3	3	1	1	3	3	1	1
3	3	1	1	4	4	2	2	4	4
3	3	1	1	1	1	2	2	1	1
3	3	1	1	1	1	2	2	1	1
3	3	1	1	1	1	2	2	1	1
3	3	1	1	1	1	2	2	1	1
3	3	1	1	1	1	3	3	1	1
2	2	1	1	1	1	2	2	1	1
2	2	1	1	1	1	2	2	1	1
3	3	1	1	1	1	2	2	1	1
3	3	1	1	1	1	2	2	1	1
3	3	1	1	1	1	3	3	1	1
3	3	1	1	1	1	2	2	1	1
3	3	1	1	1	1	2	2	1	1
3	3	1	1	1	1	2	2	1	1
2	2	1	1	1	1	2	2	1	1
3	3	1	1	1	1	2	2	1	1
3	3	1	1	1	1	2	2	1	1
3	3	1	1	1	1	2	2	4	4
3	3	1	1	1	1	2	2	1	1
3	3	1	1	1	1	2	2	1	1
3	3	1	1	4	4	2	2	1	1
3	3	1	1	1	1	2	2	1	1
3	3	1	1	1	1	2	2	1	1
3	3	1	1	1	1	2	2	1	1
3	3	1	1	1	1	2	2	1	1
3	3	1	1	1	1	2	2	1	1
2	2	1	1	1	1	2	2	1	1
3	3	1	1	1	1	2	2	1	1
3	3	1	1	1	1	2	2	1	1
2	2	1	1	1	1	2	2	4	4

For Review Only

3	3	1	1	1	1	2	2	1	1
3	3	1	1	1	1	2	2	1	1

For Review Only

SNP732	SNP732	SNP733	SNP733	SNP734	SNP734	SNP735	SNP735	SNP736	SNP736
1	1	3	3	3	3	4	4	4	4
1	1	3	3	2	2	4	4	2	2
1	1	3	3	2	2	4	4	2	2
1	1	3	3	3	3	4	4	2	2
4	4	3	3	3	3	4	4	4	4
4	4	3	3	2	2	4	4	2	2
4	4	3	3	2	2	4	4	2	2
1	1	3	3	3	3	4	4	2	2
4	4	3	3	3	3	4	4	4	4
1	1	3	3	2	2	4	4	2	2
1	1	4	4	2	2	4	4	4	4
4	4	3	3	2	2	4	4	2	2
4	4	3	3	2	2	4	4	2	2
1	1	3	3	3	3	4	4	4	4
4	4	3	3	2	2	4	4	2	2
1	1	3	3	3	3	4	4	2	2
4	4	3	3	3	3	4	4	2	2
1	1	3	3	2	2	4	4	2	2
1	1	3	3	2	2	4	4	2	2
4	4	4	4	3	3	4	4	4	4
4	4	3	3	3	3	4	4	2	2
4	4	3	3	3	3	4	4	2	2
1	1	4	4	3	3	4	4	2	2
1	1	3	3	3	3	4	4	4	4
1	1	3	3	3	3	4	4	2	2
1	1	3	3	3	3	4	4	4	4
1	1	3	3	2	2	4	4	2	2
4	4	3	3	3	3	4	4	2	2
4	4	3	3	3	3	4	4	4	4
1	1	4	4	2	2	4	4	2	2
1	1	3	3	2	2	4	4	2	2
4	4	3	3	2	2	2	2	2	2
4	4	3	3	3	3	2	2	4	4
1	1	3	3	3	3	4	4	4	4
1	1	3	3	3	3	4	4	2	2
1	1	3	3	2	2	4	4	2	2
1	1	3	3	3	3	4	4	4	4
1	1	3	3	3	3	4	4	2	2
1	1	4	4	3	3	4	4	4	4
4	4	3	3	2	2	4	4	2	2
1	1	4	4	2	2	4	4	2	2
1	1	4	4	3	3	4	4	4	4
1	1	3	3	3	3	4	4	2	2
1	1	4	4	3	3	4	4	2	2
1	1	3	3	3	3	4	4	2	2
4	4	3	3	3	3	4	4	2	2
1	1	3	3	3	3	4	4	4	4
1	1	3	3	3	3	4	4	2	2
1	1	3	3	2	2	4	4	2	2
1	1	3	3	3	3	4	4	2	2
1	1	3	3	3	3	4	4	4	4
1	1	3	3	2	2	4	4	2	2

For Review Only

4	4	3	3	3	3	4	4	2	2
4	4	3	3	3	3	2	2	4	4
1	1	3	3	3	3	4	4	4	4
4	4	4	4	3	3	4	4	4	4
1	1	3	3	2	2	4	4	2	2
1	1	3	3	2	2	4	4	2	2
4	4	3	3	3	3	4	4	4	4
1	1	3	3	3	3	4	4	2	2
4	4	4	4	3	3	4	4	2	2
1	1	3	3	3	3	4	4	2	2
1	1	3	3	3	3	4	4	2	2
1	1	3	3	2	2	4	4	2	2
4	4	3	3	3	3	4	4	2	2
4	4	3	3	3	3	4	4	4	4
4	4	3	3	3	3	4	4	2	2
1	1	3	3	3	3	4	4	2	2
1	1	3	3	3	3	4	4	4	4
1	1	3	3	2	2	4	4	2	2
4	4	3	3	2	2	4	4	2	2
4	4	3	3	3	3	4	4	2	2
1	1	3	3	2	2	4	4	2	2
1	1	4	4	2	2	4	4	2	2
4	4	3	3	3	3	4	4	4	4
4	4	3	3	2	2	2	2	2	2
1	1	3	3	3	3	4	4	2	2
1	1	3	3	2	2	4	4	2	2
1	1	3	3	3	3	4	4	4	4
4	4	4	4	3	3	4	4	4	4
4	4	3	3	3	3	4	4	4	4
4	4	3	3	3	3	2	2	2	2
1	1	3	3	2	2	4	4	2	2
1	1	3	3	2	2	4	4	2	2
4	4	4	4	2	2	4	4	2	2
4	4	3	3	3	3	4	4	2	2
1	1	4	4	3	3	4	4	2	2
1	1	3	3	3	3	4	4	2	2
1	1	3	3	2	2	4	4	2	2
4	4	4	4	3	3	4	4	2	2
1	1	3	3	3	3	4	4	4	4
1	1	3	3	3	3	4	4	2	2
4	4	3	3	3	3	4	4	4	4
4	4	3	3	2	2	2	2	2	2
1	1	3	3	3	3	4	4	2	2
1	1	3	3	3	3	4	4	2	2
1	1	3	3	2	2	4	4	2	2
1	1	3	3	3	3	4	4	2	2
1	1	3	3	2	2	4	4	2	2
1	1	3	3	2	2	4	4	2	2
1	1	3	3	2	2	4	4	2	2
1	1	3	3	3	3	4	4	2	2
1	1	3	3	3	3	4	4	2	2
1	1	4	4	3	3	4	4	4	4
4	4	3	3	3	3	4	4	2	2
1	1	3	3	3	3	4	4	4	4
1	1	3	3	3	3	4	4	2	2

For Review Only

4	4	3	3	3	3	4	4	2	2
1	1	3	3	2	2	4	4	2	2
1	1	3	3	3	3	4	4	2	2
1	1	3	3	2	2	4	4	2	2
1	1	3	3	3	3	4	4	2	2
1	1	3	3	2	2	4	4	2	2
1	1	3	3	3	3	4	4	2	2
1	1	3	3	2	2	4	4	2	2
1	1	3	3	3	3	4	4	2	2
4	4	3	3	3	3	4	4	2	2
4	4	3	3	3	3	2	2	2	2
4	4	3	3	3	3	4	4	2	2
1	1	3	3	3	3	4	4	2	2
1	1	3	3	2	2	4	4	2	2
4	4	3	3	3	3	4	4	2	2
4	4	3	3	3	3	4	4	4	4
1	1	3	3	3	3	4	4	2	2
4	4	3	3	2	2	4	4	2	2
1	1	3	3	2	2	4	4	2	2
4	4	4	4	3	3	4	4	2	2
4	4	3	3	3	3	4	4	2	2
4	4	3	3	3	3	2	2	2	2
1	1	3	3	2	2	2	2	2	2
1	1	3	3	2	2	4	4	2	2
1	1	3	3	3	3	4	4	4	4
1	1	4	4	3	3	4	4	4	4
4	4	3	3	3	3	4	4	4	4
1	1	3	3	3	3	4	4	2	2
4	4	3	3	2	2	4	4	2	2
4	4	3	3	3	3	4	4	2	2
1	1	3	3	3	3	4	4	4	4
4	4	3	3	3	3	4	4	2	2
4	4	3	3	3	3	4	4	4	4
4	4	3	3	3	3	4	4	2	2
1	1	3	3	3	3	4	4	4	4
1	1	3	3	3	3	4	4	4	4
1	1	3	3	2	2	4	4	2	2
1	1	3	3	2	2	4	4	2	2
1	1	3	3	2	2	4	4	2	2
1	1	3	3	3	3	4	4	2	2
4	4	4	4	3	3	4	4	2	2
1	1	3	3	2	2	4	4	2	2
1	1	3	3	2	2	4	4	2	2
1	1	3	3	2	2	4	4	2	2
4	4	3	3	3	3	4	4	2	2
4	4	3	3	3	3	2	2	2	2
4	4	3	3	3	3	4	4	4	4
1	1	3	3	3	3	4	4	4	4
1	1	3	3	2	2	4	4	2	2
4	4	3	3	3	3	4	4	2	2
4	4	3	3	3	3	4	4	4	4
1	1	3	3	3	3	4	4	2	2
1	1	3	3	2	2	4	4	2	2
4	4	3	3	2	2	4	4	2	2
1	1	3	3	3	3	4	4	2	2

For Review Only

4	4	3	3	3	3	4	4	2	2
1	1	3	3	3	3	4	4	2	2
1	1	3	3	3	3	4	4	2	2
1	1	3	3	3	3	4	4	4	4
1	1	3	3	3	3	4	4	2	2
1	1	3	3	3	3	4	4	4	4
1	1	4	4	2	2	4	4	2	2
1	1	3	3	3	3	4	4	2	2
1	1	4	4	3	3	4	4	2	2
4	4	3	3	2	2	2	2	2	2
4	4	3	3	3	3	4	4	4	4
1	1	3	3	2	2	4	4	2	2
4	4	3	3	2	2	4	4	2	2
4	4	3	3	3	3	2	2	2	2
1	1	3	3	2	2	4	4	2	2
1	1	4	4	3	3	4	4	4	4
1	1	3	3	3	3	4	4	2	2
1	1	4	4	3	3	4	4	2	2
4	4	3	3	2	2	4	4	2	2
1	1	3	3	3	3	4	4	2	2
1	1	3	3	3	3	4	4	2	2
1	1	4	4	2	2	4	4	2	2
1	1	4	4	2	2	4	4	2	2
1	1	3	3	2	2	4	4	2	2
4	4	3	3	2	2	4	4	2	2
1	1	3	3	3	3	4	4	4	4
4	4	3	3	2	2	4	4	2	2
1	1	3	3	3	3	4	4	2	2
1	1	3	3	3	3	4	4	2	2
1	1	3	3	2	2	4	4	2	2
1	1	3	3	2	2	4	4	2	2
4	4	3	3	3	3	2	2	2	2
4	4	4	4	3	3	4	4	4	4
1	1	3	3	2	2	4	4	2	2
4	4	3	3	3	3	4	4	2	2
1	1	3	3	2	2	4	4	2	2
4	4	3	3	2	2	4	4	2	2
1	1	3	3	3	3	4	4	2	2
1	1	3	3	3	3	4	4	4	4
1	1	3	3	2	2	4	4	2	2
1	1	3	3	2	2	4	4	2	2
4	4	3	3	3	3	4	4	4	4
1	1	3	3	3	3	4	4	4	4
1	1	3	3	3	3	4	4	2	2
1	1	3	3	3	3	4	4	4	4
1	1	3	3	3	3	4	4	2	2
1	1	4	4	3	3	4	4	4	4
1	1	4	4	2	2	4	4	2	2
1	1	3	3	3	3	4	4	4	4
1	1	3	3	3	3	4	4	2	2
1	1	3	3	3	3	4	4	2	2
1	1	3	3	3	3	4	4	2	2
1	1	4	4	2	2	4	4	2	2
1	1	3	3	3	3	4	4	2	2
1	1	4	4	3	3	4	4	4	4

For Review Only

1	1	3	3	3	3	4	4	4	4
1	1	3	3	2	2	4	4	2	2
1	1	4	4	3	3	4	4	4	4
4	4	4	4	3	3	4	4	2	2
1	1	3	3	2	2	4	4	2	2
1	1	3	3	3	3	4	4	4	4
1	1	4	4	2	2	4	4	2	2
1	1	3	3	3	3	4	4	2	2
1	1	3	3	3	3	4	4	2	2
1	1	4	4	3	3	4	4	4	4
4	4	3	3	3	3	4	4	2	2
1	1	3	3	3	3	4	4	2	2
1	1	3	3	3	3	4	4	4	4
1	1	3	3	2	2	4	4	2	2
1	1	3	3	2	2	4	4	2	2
1	1	3	3	2	2	4	4	2	2
1	1	3	3	3	3	4	4	4	4
4	4	3	3	2	2	4	4	2	2
1	1	4	4	2	2	4	4	2	2
1	1	3	3	3	3	4	4	2	2
1	1	3	3	3	3	4	4	4	4
1	1	3	3	3	3	4	4	4	4
4	4	3	3	3	3	4	4	4	4
1	1	4	4	2	2	4	4	2	2
1	1	3	3	3	3	4	4	2	2
1	1	4	4	3	3	4	4	2	2
1	1	4	4	2	2	4	4	2	2
4	4	3	3	2	2	4	4	2	2
1	1	3	3	3	3	4	4	2	2
1	1	3	3	2	2	4	4	2	2
1	1	4	4	3	3	4	4	4	4
1	1	3	3	3	3	4	4	2	2
1	1	3	3	3	3	4	4	4	4
4	4	3	3	3	3	4	4	4	4
1	1	4	4	2	2	4	4	2	2
1	1	4	4	2	2	4	4	2	2
4	4	3	3	2	2	4	4	2	2
1	1	3	3	2	2	4	4	2	2
1	1	3	3	2	2	4	4	2	2
1	1	4	4	2	2	4	4	2	2
4	4	4	4	3	3	4	4	2	2
1	1	3	3	3	3	4	4	2	2
1	1	4	4	2	2	4	4	2	2
4	4	3	3	3	3	4	4	4	4
1	1	3	3	3	3	4	4	2	2
4	4	4	4	3	3	4	4	4	4
1	1	3	3	3	3	4	4	4	4
1	1	4	4	3	3	4	4	2	2
1	1	3	3	3	3	4	4	2	2
1	1	3	3	2	2	4	4	2	2
1	1	3	3	3	3	4	4	2	2
1	1	4	4	3	3	4	4	4	4
1	1	3	3	2	2	4	4	2	2
1	1	3	3	2	2	4	4	2	2
1	1	3	3	2	2	4	4	2	2

For Review Only

4	4	3	3	2	2	4	4	4	4
1	1	4	4	2	2	4	4	2	2

For Review Only

SNP737	SNP737	SNP738	SNP738	SNP739	SNP739	SNP740	SNP740	SNP741	SNP741
1	1	3	3	3	3	3	3	3	3
1	1	3	3	3	3	3	3	3	3
2	2	4	4	3	3	1	1	3	3
1	1	3	3	3	3	3	3	3	3
2	2	3	3	3	3	3	3	3	3
1	1	3	3	3	3	3	3	3	3
1	1	3	3	3	3	3	3	3	3
1	1	3	3	3	3	3	3	3	3
1	1	3	3	3	3	3	3	3	3
1	1	3	3	3	3	1	1	1	1
1	1	3	3	3	3	3	3	3	3
1	1	3	3	3	3	3	3	3	3
1	1	3	3	3	3	3	3	3	3
1	1	3	3	3	3	3	3	3	3
2	2	3	3	3	3	3	3	3	3
1	1	3	3	3	3	3	3	3	3
1	1	3	3	3	3	3	3	3	3
1	1	3	3	3	3	3	3	3	3
1	1	3	3	3	3	3	3	3	3
1	1	3	3	3	3	1	1	1	1
1	1	3	3	3	3	3	3	3	3
1	1	3	3	3	3	3	3	3	3
1	1	3	3	3	3	1	1	1	1
1	1	3	3	2	2	1	1	1	1
2	2	3	3	3	3	3	3	3	3
1	1	3	3	3	3	3	3	3	3
1	1	3	3	3	3	3	3	3	3
1	1	3	3	3	3	3	3	3	3
1	1	3	3	3	3	1	1	1	1
1	1	3	3	3	3	1	1	1	1
1	1	3	3	3	3	3	3	3	3
1	1	4	4	3	3	1	1	3	3
1	1	4	4	3	3	1	1	3	3
1	1	3	3	2	2	3	3	3	3
1	1	3	3	3	3	3	3	3	3
1	1	3	3	2	2	3	3	3	3
1	1	3	3	3	3	1	1	1	1
1	1	3	3	3	3	3	3	3	3
1	1	3	3	3	3	3	3	3	3
1	1	3	3	3	3	3	3	3	3
1	1	3	3	3	3	1	1	1	1
1	1	3	3	3	3	1	1	1	1
1	1	3	3	3	3	1	1	1	1
1	1	3	3	3	3	3	3	3	3
1	1	3	3	3	3	1	1	1	1
2	2	3	3	3	3	3	3	3	3
1	1	3	3	3	3	3	3	3	3
1	1	3	3	3	3	3	3	3	3
1	1	3	3	3	3	3	3	3	3
1	1	3	3	3	3	3	3	1	1
1	1	3	3	3	3	3	3	3	3
1	1	3	3	3	3	3	3	3	3

For Review Only

2	2	3	3	3	3	3	3	3	3
1	1	4	4	3	3	1	1	3	3
1	1	3	3	3	3	3	3	3	3
2	2	3	3	3	3	1	1	1	1
1	1	3	3	3	3	3	3	3	3
1	1	3	3	3	3	3	3	3	3
1	1	3	3	3	3	3	3	3	3
1	1	3	3	3	3	1	1	1	1
1	1	3	3	3	3	1	1	1	1
1	1	3	3	3	3	3	3	3	3
1	1	3	3	3	3	3	3	3	3
1	1	3	3	3	3	3	3	3	3
2	2	3	3	3	3	3	3	3	3
1	1	3	3	3	3	1	1	1	1
1	1	3	3	3	3	3	3	3	3
1	1	3	3	3	3	1	1	1	1
1	1	3	3	3	3	3	3	3	3
1	1	3	3	3	3	3	3	3	3
1	1	3	3	3	3	3	3	3	3
1	1	3	3	3	3	3	3	3	3
1	1	3	3	3	3	3	3	3	3
1	1	3	3	3	3	1	1	1	1
1	1	3	3	3	3	3	3	3	3
1	1	3	3	3	3	1	1	1	1
1	1	3	3	3	3	1	1	1	1
1	1	3	3	3	3	3	3	3	3
1	1	4	4	3	3	3	3	3	3
1	1	3	3	3	3	3	3	3	3
1	1	3	3	3	3	3	3	3	3
1	1	3	3	3	3	3	3	3	3
2	2	3	3	3	3	3	3	3	3
1	1	3	3	2	2	3	3	3	3
1	1	3	3	2	2	3	3	3	3
2	2	3	3	3	3	3	3	3	3
1	1	4	4	3	3	1	1	3	3
1	1	3	3	3	3	1	1	1	1
1	1	3	3	3	3	3	3	3	3
1	1	3	3	3	3	3	3	3	3
1	1	3	3	3	3	3	3	3	3
1	1	3	3	3	3	1	1	1	1
1	1	4	4	3	3	1	1	3	3
1	1	3	3	3	3	3	3	3	3
1	1	3	3	3	3	3	3	3	3
1	1	3	3	3	3	3	3	3	3
1	1	3	3	3	3	3	3	3	3
1	1	3	3	3	3	3	3	3	3
1	1	3	3	2	2	3	3	3	3
1	1	3	3	3	3	3	3	3	3
1	1	3	3	3	3	3	3	3	3
2	2	3	3	3	3	1	1	1	1
1	1	3	3	3	3	3	3	3	3
1	1	3	3	3	3	1	1	1	1
1	1	3	3	3	3	1	1	1	1
1	1	3	3	3	3	3	3	3	3
1	1	3	3	3	3	3	3	3	3

For Review Only

1	1	3	3	3	3	3	3	3	3
1	1	3	3	3	3	1	1	1	1
1	1	3	3	3	3	3	3	3	3
1	1	3	3	3	3	3	3	3	3
1	1	3	3	3	3	3	3	3	3
1	1	3	3	3	3	3	3	3	3
1	1	3	3	3	3	3	3	3	3
1	1	3	3	3	3	3	3	3	3
1	1	3	3	3	3	3	3	3	3
1	1	3	3	2	2	3	3	3	3
1	1	3	3	3	3	3	3	3	3
1	1	3	3	3	3	3	3	3	3
1	1	3	3	3	3	1	1	1	1
1	1	3	3	3	3	3	3	3	3
1	1	3	3	3	3	3	3	3	3
1	1	3	3	3	3	3	3	3	3
1	1	3	3	3	3	3	3	3	3
1	1	3	3	3	3	3	3	3	3
1	1	3	3	3	3	3	3	3	3
1	1	3	3	3	3	3	3	3	3
1	1	3	3	3	3	3	3	3	3
1	1	3	3	3	3	3	3	3	3
1	1	3	3	3	3	3	3	3	3
1	1	3	3	3	3	3	3	3	3
1	1	3	3	3	3	3	3	3	3
1	1	3	3	3	3	1	1	1	1
2	2	3	3	3	3	3	3	3	3
1	1	3	3	3	3	3	3	3	3
1	1	3	3	3	3	3	3	3	3
1	1	3	3	3	3	3	3	3	3
1	1	3	3	3	3	3	3	3	3
1	1	3	3	3	3	3	3	3	3
1	1	4	4	3	3	1	1	3	3
1	1	3	3	3	3	3	3	3	3
1	1	3	3	2	2	3	3	3	3
1	1	3	3	3	3	3	3	3	3
1	1	3	3	3	3	3	3	3	3
1	1	3	3	3	3	3	3	3	3
1	1	3	3	3	3	3	3	3	3
1	1	3	3	3	3	3	3	3	3
1	1	3	3	3	3	1	1	1	1
1	1	3	3	3	3	3	3	3	3
1	1	3	3	3	3	3	3	3	3
1	1	3	3	3	3	3	3	3	3
1	1	3	3	3	3	3	3	3	3
1	1	3	3	3	3	3	3	3	3
1	1	3	3	3	3	3	3	3	3
1	1	3	3	3	3	3	3	3	3
1	1	3	3	3	3	3	3	3	3
1	1	3	3	3	3	3	3	3	3
1	1	3	3	3	3	3	3	3	3
1	1	3	3	3	3	1	1	1	1
1	1	3	3	3	3	3	3	3	3
1	1	3	3	3	3	3	3	3	3
1	1	3	3	3	3	3	3	3	3

1	1	3	3	3	3	3	3	3	3
1	1	3	3	3	3	1	1	1	1
1	1	3	3	3	3	3	3	3	3
1	1	3	3	3	3	3	3	3	3
1	1	3	3	3	3	3	3	3	3
1	1	3	3	3	3	1	1	3	3
1	1	3	3	3	3	3	3	3	3
1	1	3	3	3	3	3	3	3	3
1	1	3	3	3	3	3	3	3	3
2	2	3	3	3	3	3	3	3	3
1	1	3	3	3	3	3	3	3	3
1	1	3	3	3	3	3	3	3	3
1	1	3	3	3	3	3	3	3	3
1	1	3	3	3	3	3	3	3	3
1	1	3	3	2	2	3	3	3	3
1	1	3	3	3	3	3	3	3	3
1	1	3	3	3	3	3	3	3	3
1	1	3	3	3	3	3	3	3	3
1	1	3	3	3	3	3	3	3	3
1	1	3	3	3	3	3	3	3	3
1	1	3	3	3	3	3	3	3	3
1	1	3	3	2	2	3	3	3	3
1	1	3	3	3	3	1	1	1	1
1	1	3	3	3	3	3	3	3	3
1	1	3	3	3	3	1	1	1	1
1	1	3	3	2	2	3	3	3	3
1	1	3	3	3	3	3	3	3	3
1	1	3	3	3	3	3	3	3	3
1	1	3	3	3	3	3	3	1	1
2	2	3	3	3	3	1	1	1	1
1	1	3	3	3	3	3	3	3	3
1	1	3	3	3	3	3	3	3	3
1	1	3	3	3	3	3	3	3	3
1	1	3	3	2	2	3	3	3	3
1	1	3	3	3	3	3	3	3	3
1	1	3	3	3	3	3	3	3	3
1	1	3	3	3	3	3	3	3	3
1	1	3	3	3	3	3	3	3	3
1	1	3	3	3	3	3	3	3	3
1	1	3	3	3	3	3	3	3	3
2	2	4	4	3	3	1	1	3	3
1	1	3	3	3	3	3	3	3	3
1	1	3	3	3	3	3	3	3	3
1	1	3	3	3	3	3	3	3	3
1	1	3	3	3	3	3	3	3	3
1	1	3	3	3	3	3	3	3	3
1	1	3	3	3	3	3	3	3	3
1	1	3	3	3	3	3	3	3	3
1	1	3	3	3	3	3	3	3	3
1	1	3	3	3	3	1	1	1	1
1	1	3	3	3	3	3	3	3	3
1	1	3	3	3	3	3	3	3	3
1	1	3	3	3	3	1	1	1	1
1	1	3	3	3	3	3	3	3	3
1	1	3	3	3	3	3	3	3	3

For Review Only

1	1	3	3	3	3	3	3	3	3
1	1	3	3	3	3	1	1	1	1
2	2	3	3	3	3	3	3	3	3
1	1	3	3	3	3	1	1	1	1
1	1	3	3	3	3	1	1	1	1
1	1	3	3	3	3	3	3	3	3
1	1	3	3	3	3	3	3	3	3
1	1	3	3	3	3	1	1	1	1
1	1	3	3	3	3	3	3	3	3
1	1	3	3	2	2	3	3	3	3
1	1	3	3	3	3	3	3	3	3
1	1	3	3	3	3	3	3	3	3
1	1	3	3	3	3	3	3	3	3
1	1	3	3	3	3	3	3	3	3
1	1	4	4	3	3	1	1	3	3
1	1	3	3	3	3	3	3	3	3
1	1	3	3	3	3	3	3	3	3
1	1	3	3	2	2	3	3	3	3
1	1	3	3	3	3	3	3	3	3
1	1	3	3	3	3	3	3	3	3
1	1	3	3	3	3	3	3	1	1
1	1	3	3	3	3	3	3	3	3
1	1	3	3	3	3	3	3	3	3
1	1	3	3	3	3	3	3	3	3
1	1	3	3	3	3	1	1	1	1
1	1	3	3	3	3	3	3	3	3
1	1	3	3	2	2	3	3	3	3
1	1	3	3	3	3	3	3	3	3
1	1	3	3	3	3	3	3	3	3
1	1	3	3	3	3	3	3	3	3
1	1	3	3	3	3	3	3	3	3
1	1	3	3	3	3	3	3	3	3
1	1	3	3	3	3	3	3	3	3
1	1	3	3	3	3	3	3	3	3
1	1	3	3	3	3	1	1	1	1
1	1	3	3	3	3	3	3	3	3
2	2	3	3	3	3	3	3	3	3
1	1	3	3	3	3	3	3	3	3
1	1	3	3	2	2	3	3	3	3
1	1	3	3	3	3	3	3	3	3
1	1	3	3	3	3	3	3	3	3
1	1	3	3	3	3	3	3	3	3
1	1	3	3	3	3	3	3	3	3
2	2	3	3	3	3	3	3	3	3
1	1	3	3	3	3	3	3	3	3
1	1	3	3	3	3	3	3	3	3
1	1	3	3	3	3	3	3	3	3
1	1	3	3	3	3	3	3	3	3
1	1	3	3	3	3	3	3	3	3
1	1	3	3	2	2	3	3	3	3
1	1	3	3	3	3	3	3	3	3
1	1	3	3	3	3	3	3	3	3
2	2	4	4	3	3	1	1	3	3
1	1	3	3	3	3	3	3	3	3
1	1	3	3	3	3	1	1	3	3

For Review Only

1	1	3	3	3	3	3	3	3	3
1	1	3	3	3	3	3	3	3	3

For Review Only

SNP742	SNP742	SNP743	SNP743	SNP744	SNP744	SNP745	SNP745	SNP746	SNP746
2	2	1	1	1	1	1	1	2	2
1	1	2	2	1	1	4	4	2	2
1	1	1	1	1	1	4	4	3	3
2	2	2	2	1	1	1	1	2	2
2	2	2	2	1	1	1	1	2	2
2	2	2	2	1	1	1	1	2	2
2	2	2	2	1	1	1	1	2	2
1	1	1	1	2	2	4	4	2	2
2	2	2	2	1	1	1	1	2	2
1	1	2	2	1	1	4	4	2	2
1	1	2	2	1	1	4	4	2	2
1	1	2	2	1	1	4	4	3	3
2	2	2	2	1	1	1	1	2	2
1	1	2	2	1	1	4	4	2	2
2	2	2	2	1	1	1	1	2	2
1	1	2	2	1	1	4	4	2	2
1	1	2	2	1	1	4	4	2	2
1	1	2	2	1	1	4	4	2	2
2	2	2	2	1	1	1	1	3	3
2	2	2	2	1	1	1	1	2	2
1	1	1	1	1	1	4	4	2	2
2	2	2	2	1	1	1	1	2	2
1	1	2	2	1	1	4	4	2	2
1	1	1	1	1	1	4	4	2	2
2	2	2	2	1	1	1	1	2	2
1	1	1	1	1	1	4	4	3	3
2	2	1	1	1	1	1	1	2	2
2	2	2	2	1	1	4	4	2	2
2	2	2	2	1	1	1	1	2	2
1	1	2	2	1	1	1	1	2	2
2	2	2	2	1	1	1	1	2	2
2	2	2	2	1	1	1	1	2	2
2	2	2	2	1	1	1	1	2	2
1	1	2	2	1	1	1	1	2	2
2	2	2	2	1	1	1	1	2	2
1	1	1	1	1	1	4	4	2	2
1	1	2	2	1	1	4	4	2	2
1	1	2	2	2	2	4	4	2	2
1	1	2	2	1	1	4	4	2	2
1	1	2	2	2	2	4	4	2	2
1	1	2	2	1	1	1	1	2	2
2	2	2	2	1	1	1	1	2	2
1	1	2	2	1	1	4	4	2	2
2	2	2	2	1	1	1	1	2	2
1	1	2	2	1	1	4	4	2	2
1	1	2	2	1	1	4	4	3	3
2	2	2	2	1	1	1	1	3	3
2	2	1	1	1	1	1	1	2	2
1	1	2	2	1	1	4	4	2	2
2	2	2	2	1	1	1	1	2	2
1	1	2	2	1	1	4	4	3	3
2	2	2	2	1	1	1	1	2	2

For Review Only

2	2	2	2	1	1	1	1	2	2
2	2	2	2	1	1	1	1	2	2
2	2	2	2	1	1	1	1	2	2
1	1	2	2	1	1	1	1	2	2
2	2	2	2	1	1	1	1	3	3
2	2	2	2	1	1	1	1	3	3
1	1	1	1	1	1	4	4	3	3
1	1	2	2	1	1	4	4	2	2
2	2	2	2	1	1	1	1	2	2
2	2	2	2	1	1	4	4	2	2
2	2	2	2	1	1	1	1	3	3
2	2	2	2	1	1	1	1	3	3
2	2	2	2	1	1	1	1	2	2
2	2	2	2	1	1	1	1	2	2
2	2	2	2	1	1	4	4	2	2
1	1	2	2	1	1	4	4	2	2
1	1	1	1	1	1	4	4	3	3
2	2	2	2	1	1	1	1	2	2
2	2	2	2	1	1	1	1	2	2
2	2	2	2	1	1	1	1	3	3
2	2	2	2	1	1	4	4	2	2
1	1	2	2	1	1	4	4	2	2
2	2	2	2	1	1	1	1	2	2
2	2	2	2	1	1	1	1	3	3
1	1	2	2	1	1	4	4	2	2
1	1	2	2	1	1	4	4	3	3
1	1	2	2	1	1	4	4	3	3
1	1	2	2	1	1	4	4	2	2
1	1	1	1	1	1	4	4	3	3
1	1	2	2	1	1	4	4	2	2
2	2	2	2	1	1	1	1	2	2
1	1	2	2	1	1	4	4	2	2
1	1	2	2	1	1	4	4	2	2
2	2	2	2	1	1	1	1	2	2
2	2	2	2	1	1	1	1	2	2
1	1	2	2	1	1	4	4	2	2
2	2	2	2	1	1	1	1	2	2
2	2	2	2	1	1	1	1	2	2
1	1	2	2	1	1	4	4	3	3
1	1	2	2	1	1	4	4	2	2
2	2	2	2	1	1	1	1	2	2
2	2	2	2	1	1	1	1	2	2
2	2	1	1	1	1	1	1	2	2
2	2	2	2	1	1	1	1	3	3
2	2	2	2	1	1	1	1	2	2
2	2	2	2	2	2	1	1	2	2
1	1	2	2	1	1	4	4	2	2
1	1	1	1	1	1	4	4	3	3
1	1	1	1	1	1	4	4	2	2
2	2	2	2	1	1	4	4	2	2
2	2	2	2	1	1	1	1	2	2
1	1	2	2	1	1	4	4	2	2
1	1	2	2	1	1	4	4	2	2
1	1	2	2	1	1	4	4	2	2
2	2	2	2	2	2	1	1	2	2

For Review Only

1	1	2	2	1	1	4	4	2	2
1	1	2	2	1	1	4	4	2	2
1	1	1	1	1	1	4	4	2	2
1	1	2	2	1	1	4	4	2	2
1	1	2	2	2	2	4	4	3	3
2	2	2	2	1	1	1	1	3	3
1	1	2	2	1	1	4	4	2	2
1	1	1	1	1	1	4	4	2	2
1	1	1	1	1	1	4	4	2	2
2	2	2	2	1	1	4	4	2	2
1	1	2	2	1	1	4	4	2	2
2	2	2	2	1	1	4	4	2	2
2	2	1	1	1	1	1	1	2	2
2	2	2	2	1	1	4	4	3	3
2	2	1	1	2	2	4	4	2	2
2	2	2	2	1	1	4	4	2	2
2	2	1	1	2	2	4	4	2	2
2	2	2	2	1	1	4	4	2	2
1	1	2	2	1	1	4	4	2	2
1	1	2	2	1	1	4	4	2	2
2	2	2	2	1	1	4	4	2	2
1	1	2	2	1	1	4	4	2	2
2	2	1	1	1	1	1	1	2	2
1	1	2	2	1	1	4	4	2	2
1	1	2	2	1	1	4	4	2	2
1	1	2	2	1	1	4	4	2	2
2	2	2	2	2	2	4	4	2	2
1	1	2	2	1	1	4	4	2	2
2	2	2	2	1	1	1	1	3	3
1	1	2	2	1	1	4	4	2	2
1	1	2	2	1	1	4	4	2	2
2	2	2	2	1	1	4	4	2	2
2	2	2	2	1	1	1	1	2	2
2	2	2	2	1	1	4	4	2	2
1	1	2	2	1	1	1	1	2	2
1	1	1	1	1	1	1	1	3	3
1	1	2	2	1	1	4	4	2	2
2	2	2	2	1	1	1	1	2	2
1	1	2	2	1	1	4	4	2	2
1	1	2	2	1	1	4	4	2	2
1	1	2	2	1	1	4	4	2	2
1	1	2	2	1	1	4	4	2	2
2	2	2	2	1	1	1	1	2	2
1	1	2	2	1	1	4	4	2	2
2	2	2	2	1	1	4	4	2	2
2	2	2	2	2	2	1	1	2	2
1	1	2	2	1	1	4	4	3	3
1	1	1	1	1	1	4	4	3	3
2	2	2	2	1	1	4	4	2	2
1	1	2	2	1	1	4	4	3	3
1	1	1	1	1	1	4	4	2	2
1	1	2	2	1	1	4	4	2	2
2	2	2	2	1	1	4	4	2	2
1	1	2	2	1	1	4	4	3	3
1	1	2	2	1	1	4	4	2	2

For Review Only

1	1	2	2	1	1	4	4	3	3
1	1	2	2	1	1	4	4	2	2
2	2	2	2	2	2	4	4	2	2
2	2	2	2	1	1	4	4	2	2
2	2	2	2	1	1	1	1	2	2
1	1	2	2	1	1	4	4	2	2
1	1	1	1	1	1	4	4	2	2
2	2	2	2	1	1	4	4	2	2
1	1	1	1	1	1	4	4	2	2
2	2	2	2	1	1	1	1	2	2
1	1	2	2	1	1	4	4	3	3
1	1	2	2	1	1	4	4	2	2
2	2	2	2	1	1	4	4	2	2
2	2	2	2	2	2	4	4	2	2
1	1	2	2	1	1	4	4	2	2
1	1	2	2	1	1	4	4	2	2
2	2	2	2	1	1	1	1	2	2
2	2	1	1	1	1	1	1	3	3
2	2	2	2	1	1	1	1	2	2
1	1	2	2	1	1	4	4	2	2
2	2	2	2	1	1	4	4	2	2
1	1	1	1	1	1	4	4	2	2
1	1	2	2	1	1	4	4	2	2
2	2	2	2	1	1	1	1	3	3
2	2	2	2	1	1	1	1	2	2
1	1	2	2	1	1	4	4	2	2
1	1	2	2	1	1	4	4	2	2
2	2	2	2	2	2	1	1	2	2
2	2	2	2	1	1	1	1	2	2
2	2	2	2	1	1	1	1	2	2
2	2	1	1	1	1	1	1	3	3
1	1	2	2	1	1	4	4	2	2
2	2	2	2	2	2	1	1	2	2
1	1	2	2	1	1	4	4	2	2
2	2	2	2	2	2	1	1	2	2
2	2	2	2	1	1	1	1	3	3
2	2	2	2	1	1	4	4	2	2
2	2	1	1	1	1	4	4	2	2
1	1	2	2	1	1	4	4	3	3
1	1	2	2	1	1	4	4	3	3
1	1	1	1	1	1	4	4	3	3
1	1	2	2	1	1	4	4	3	3
1	1	1	1	1	1	4	4	2	2
2	2	2	2	1	1	4	4	2	2
2	2	2	2	1	1	4	4	2	2
2	2	2	2	1	1	4	4	2	2
1	1	2	2	1	1	4	4	2	2
1	1	1	1	1	1	4	4	2	2
1	1	2	2	1	1	4	4	2	2
1	1	2	2	1	1	4	4	2	2
2	2	2	2	1	1	4	4	3	3
1	1	2	2	2	2	4	4	2	2
1	1	2	2	1	1	4	4	2	2
2	2	2	2	1	1	1	1	3	3
1	1	2	2	1	1	4	4	2	2

For Review Only

2	2	2	2	1	1	1	1	2	2
2	2	2	2	1	1	1	1	2	2
2	2	2	2	1	1	4	4	3	3
1	1	2	2	1	1	4	4	2	2
2	2	2	2	1	1	1	1	2	2
2	2	2	2	1	1	1	1	2	2
2	2	2	2	2	2	1	1	2	2
1	1	2	2	1	1	4	4	2	2
2	2	2	2	1	1	1	1	2	2
1	1	2	2	1	1	4	4	2	2
2	2	2	2	1	1	4	4	2	2
2	2	1	1	1	1	1	1	2	2
1	1	2	2	1	1	4	4	3	3
1	1	1	1	1	1	4	4	3	3
1	1	1	1	1	1	4	4	3	3
2	2	2	2	1	1	1	1	2	2
1	1	2	2	1	1	4	4	2	2
1	1	2	2	1	1	4	4	2	2
1	1	2	2	1	1	4	4	2	2
1	1	2	2	2	2	4	4	2	2
2	2	2	2	2	2	1	1	3	3
1	1	2	2	1	1	4	4	3	3
1	1	2	2	1	1	4	4	2	2
1	1	2	2	1	1	4	4	2	2
1	1	2	2	1	1	4	4	2	2
2	2	2	2	2	2	1	1	2	2
1	1	2	2	1	1	4	4	2	2
1	1	2	2	1	1	4	4	3	3
2	2	2	2	1	1	1	1	2	2
2	2	2	2	1	1	1	1	3	3
1	1	2	2	1	1	4	4	2	2
1	1	2	2	1	1	4	4	3	3
1	1	2	2	1	1	4	4	2	2
1	1	2	2	1	1	4	4	3	3
1	1	2	2	1	1	4	4	2	2
1	1	2	2	1	1	4	4	2	2
2	2	2	2	1	1	1	1	2	2
2	2	2	2	1	1	1	1	3	3
1	1	1	1	1	1	4	4	2	2
1	1	2	2	1	1	4	4	2	2
2	2	2	2	1	1	1	1	2	2
2	2	2	2	2	2	1	1	2	2
1	1	2	2	1	1	4	4	2	2
2	2	2	2	1	1	1	1	2	2
1	1	2	2	1	1	4	4	3	3
1	1	2	2	1	1	4	4	2	2
1	1	2	2	1	1	4	4	2	2
2	2	2	2	2	2	1	1	2	2
2	2	1	1	1	1	4	4	2	2
1	1	1	1	1	1	4	4	2	2
2	2	2	2	2	2	1	1	2	2
1	1	2	2	1	1	4	4	2	2
1	1	1	1	1	1	4	4	3	3
1	1	2	2	1	1	4	4	3	3
1	1	2	2	1	1	4	4	2	2

For Review Only

2	2	2	2	1	1	1	1	2	2
2	2	2	2	1	1	1	1	3	3

For Review Only

SNP747	SNP747	SNP748	SNP748	SNP749	SNP749	SNP750	SNP750	SNP751	SNP751
2	2	3	3	2	2	1	1	3	3
4	4	3	3	2	2	1	1	1	1
4	4	3	3	2	2	1	1	1	1
4	4	3	3	2	2	4	4	3	3
2	2	3	3	2	2	4	4	3	3
4	4	3	3	2	2	4	4	3	3
2	2	3	3	2	2	4	4	3	3
4	4	3	3	3	3	1	1	1	1
2	2	3	3	3	3	4	4	3	3
4	4	3	3	2	2	1	1	1	1
2	2	2	2	3	3	1	1	1	1
4	4	3	3	2	2	1	1	1	1
4	4	3	3	3	3	1	1	3	3
2	2	3	3	2	2	1	1	1	1
2	2	3	3	3	3	4	4	3	3
4	4	3	3	2	2	1	1	1	1
2	2	3	3	3	3	1	1	1	1
2	2	3	3	2	2	1	1	3	3
2	2	3	3	2	2	4	4	3	3
2	2	3	3	2	2	1	1	1	1
2	2	3	3	3	3	1	1	1	1
2	2	2	2	3	3	4	4	3	3
4	4	2	2	3	3	1	1	1	1
4	4	3	3	3	3	1	1	1	1
4	4	3	3	2	2	4	4	3	3
4	4	3	3	3	3	1	1	1	1
2	2	3	3	2	2	4	4	3	3
4	4	3	3	3	3	1	1	3	3
2	2	3	3	3	3	4	4	3	3
4	4	3	3	3	3	1	1	3	3
4	4	3	3	2	2	4	4	3	3
2	2	3	3	2	2	4	4	3	3
2	2	3	3	2	2	4	4	3	3
4	4	3	3	2	2	1	1	1	1
2	2	3	3	3	3	1	1	3	3
4	4	3	3	3	3	1	1	1	1
2	2	3	3	3	3	1	1	1	1
4	4	3	3	3	3	1	1	1	1
4	4	2	2	3	3	1	1	1	1
4	4	2	2	3	3	1	1	1	1
4	4	2	2	3	3	1	1	1	1
2	2	3	3	3	3	1	1	3	3
4	4	3	3	3	3	1	1	1	1
2	2	3	3	2	2	1	1	1	1
4	4	3	3	3	3	1	1	1	1
4	4	3	3	3	3	1	1	1	1
2	2	3	3	2	2	1	1	1	1
2	2	2	2	3	3	1	1	1	1
2	2	3	3	3	3	1	1	3	3
2	2	3	3	2	2	4	4	3	3
4	4	3	3	2	2	1	1	1	1
4	4	3	3	2	2	4	4	3	3

2	2	3	3	2	2	4	4	3	3
2	2	3	3	2	2	4	4	3	3
4	4	3	3	2	2	4	4	3	3
2	2	3	3	2	2	1	1	1	1
2	2	3	3	2	2	4	4	3	3
2	2	3	3	2	2	1	1	1	1
2	2	3	3	2	2	1	1	1	1
4	4	3	3	3	3	1	1	1	1
2	2	3	3	2	2	4	4	3	3
4	4	3	3	3	3	1	1	3	3
2	2	3	3	2	2	1	1	1	1
2	2	3	3	2	2	1	1	1	1
2	2	3	3	2	2	4	4	3	3
2	2	3	3	3	3	4	4	3	3
4	4	3	3	3	3	1	1	3	3
2	2	2	2	3	3	1	1	1	1
2	2	2	2	2	2	1	1	1	1
2	2	3	3	2	2	4	4	3	3
2	2	3	3	2	2	4	4	3	3
2	2	3	3	2	2	4	4	3	3
2	2	3	3	2	2	1	1	1	1
2	2	2	2	3	3	1	1	1	1
2	2	3	3	3	3	4	4	3	3
4	4	3	3	2	2	1	1	3	3
4	4	3	3	3	3	1	1	1	1
4	4	3	3	2	2	4	4	3	3
4	4	3	3	3	3	1	1	1	1
4	4	2	2	3	3	1	1	1	1
4	4	3	3	3	3	1	1	1	1
2	2	3	3	2	2	1	1	1	1
2	2	3	3	2	2	4	4	3	3
4	4	3	3	3	3	1	1	1	1
4	4	2	2	3	3	1	1	1	1
2	2	3	3	2	2	4	4	3	3
2	2	3	3	2	2	4	4	3	3
4	4	3	3	3	3	1	1	1	1
2	2	3	3	2	2	4	4	3	3
4	4	3	3	3	3	1	1	1	1
4	4	3	3	2	2	1	1	1	1
4	4	3	3	3	3	1	1	1	1
2	2	3	3	2	2	4	4	3	3
4	4	3	3	2	2	4	4	3	3
4	4	3	3	3	3	4	4	3	3
2	2	3	3	2	2	1	1	1	1
4	4	3	3	2	2	1	1	3	3
2	2	3	3	2	2	1	1	1	1
4	4	2	2	2	2	1	1	1	1
2	2	3	3	2	2	1	1	1	1
2	2	3	3	3	3	1	1	1	1
4	4	3	3	3	3	1	1	3	3
4	4	3	3	2	2	1	1	1	1
4	4	2	2	3	3	1	1	1	1
4	4	3	3	3	3	1	1	1	1
4	4	3	3	3	3	1	1	3	3
2	2	3	3	3	3	4	4	3	3

For Review Only

2	2	3	3	2	2	1	1	1	1
4	4	3	3	3	3	1	1	1	1
4	4	3	3	3	3	1	1	1	1
2	2	2	2	3	3	1	1	1	1
4	4	3	3	2	2	1	1	3	3
4	4	3	3	3	3	4	4	3	3
4	4	3	3	2	2	1	1	1	1
4	4	3	3	3	3	1	1	1	1
2	2	3	3	3	3	1	1	1	1
4	4	3	3	3	3	1	1	3	3
2	2	3	3	3	3	1	1	1	1
4	4	3	3	3	3	1	1	1	1
4	4	2	2	3	3	1	1	3	3
4	4	3	3	3	3	4	4	3	3
4	4	3	3	3	3	1	1	3	3
4	4	3	3	3	3	1	1	1	1
2	2	3	3	2	2	1	1	3	3
4	4	3	3	3	3	1	1	3	3
2	2	2	2	2	2	1	1	1	1
4	4	2	2	3	3	1	1	1	1
4	4	3	3	3	3	1	1	3	3
2	2	3	3	2	2	1	1	1	1
4	4	3	3	2	2	4	4	3	3
4	4	2	2	2	2	1	1	1	1
4	4	3	3	3	3	1	1	1	1
2	2	2	2	3	3	1	1	1	1
2	2	3	3	3	3	4	4	3	3
4	4	3	3	2	2	1	1	1	1
2	2	3	3	3	3	4	4	3	3
4	4	3	3	2	2	1	1	1	1
4	4	3	3	2	2	1	1	1	1
4	4	3	3	3	3	1	1	1	1
2	2	3	3	2	2	4	4	3	3
4	4	3	3	3	3	1	1	1	1
4	4	3	3	2	2	1	1	1	1
4	4	3	3	2	2	1	1	3	3
2	2	2	2	2	2	1	1	1	1
2	2	3	3	2	2	4	4	3	3
4	4	2	2	3	3	1	1	1	1
4	4	2	2	3	3	1	1	1	1
4	4	2	2	3	3	1	1	1	1
4	4	2	2	3	3	1	1	1	1
2	2	3	3	2	2	4	4	3	3
4	4	3	3	3	3	1	1	1	1
4	4	3	3	2	2	1	1	3	3
4	4	3	3	2	2	4	4	3	3
4	4	3	3	2	2	1	1	1	1
2	2	3	3	2	2	1	1	1	1
4	4	3	3	3	3	4	4	3	3
4	4	3	3	3	3	1	1	1	1
2	2	3	3	3	3	1	1	1	1
2	2	3	3	3	3	1	1	1	1
2	2	2	2	2	2	4	4	3	3
2	2	3	3	3	3	1	1	1	1
4	4	2	2	3	3	1	1	1	1

For Review Only

2	2	3	3	2	2	1	1	1	1
4	4	2	2	3	3	1	1	1	1
4	4	3	3	3	3	1	1	1	1
2	2	3	3	3	3	1	1	3	3
4	4	3	3	2	2	1	1	3	3
2	2	2	2	2	2	1	1	1	1
4	4	3	3	2	2	1	1	1	1
4	4	3	3	3	3	1	1	3	3
4	4	2	2	3	3	1	1	1	1
2	2	3	3	2	2	4	4	3	3
4	4	3	3	3	3	1	1	1	1
2	2	3	3	2	2	1	1	1	1
4	4	3	3	2	2	1	1	1	1
4	4	3	3	2	2	4	4	3	3
4	4	3	3	2	2	1	1	1	1
2	2	2	2	3	3	1	1	1	1
4	4	3	3	2	2	4	4	3	3
4	4	3	3	2	2	4	4	3	3
4	4	3	3	3	3	4	4	3	3
4	4	3	3	2	2	1	1	1	1
4	4	3	3	3	3	1	1	3	3
4	4	3	3	3	3	1	1	1	1
2	2	2	2	3	3	1	1	1	1
2	2	3	3	2	2	1	1	1	1
4	4	3	3	3	3	4	4	3	3
4	4	3	3	2	2	1	1	1	1
4	4	3	3	2	2	1	1	1	1
4	4	3	3	2	2	1	1	3	3
2	2	3	3	2	2	1	1	3	3
2	2	3	3	2	2	4	4	3	3
2	2	3	3	2	2	4	4	3	3
2	2	3	3	2	2	1	1	1	1
2	2	3	3	2	2	4	4	3	3
4	4	2	2	2	2	1	1	1	1
2	2	3	3	3	3	4	4	3	3
2	2	3	3	2	2	1	1	1	1
4	4	3	3	3	3	1	1	1	1
2	2	3	3	3	3	1	1	3	3
2	2	3	3	2	2	1	1	1	1
2	2	3	3	2	2	1	1	1	1
4	4	3	3	2	2	1	1	1	1
2	2	3	3	2	2	1	1	1	1
4	4	3	3	2	2	1	1	1	1
2	2	3	3	2	2	1	1	3	3
2	2	3	3	3	3	1	1	3	3
2	2	3	3	2	2	1	1	3	3
2	2	2	2	3	3	1	1	1	1
4	4	3	3	2	2	1	1	1	1
4	4	3	3	2	2	1	1	1	1
2	2	3	3	2	2	1	1	1	1
2	2	3	3	3	3	1	1	3	3
4	4	2	2	3	3	1	1	1	1
2	2	2	2	3	3	1	1	1	1
2	2	3	3	2	2	1	1	1	1
4	4	2	2	3	3	1	1	1	1

For Review Only

2	2	2	2	2	2	4	4	3	3
4	4	3	3	2	2	4	4	3	3
2	2	3	3	2	2	1	1	3	3
4	4	2	2	3	3	1	1	1	1
2	2	3	3	2	2	4	4	3	3
4	4	3	3	2	2	4	4	3	3
2	2	3	3	3	3	4	4	3	3
4	4	3	3	3	3	1	1	1	1
4	4	3	3	3	3	1	1	3	3
4	4	2	2	3	3	1	1	1	1
4	4	3	3	3	3	1	1	3	3
2	2	2	2	2	2	4	4	3	3
2	2	3	3	2	2	1	1	1	1
4	4	3	3	3	3	1	1	1	1
4	4	3	3	2	2	1	1	1	1
2	2	3	3	2	2	1	1	1	1
2	2	3	3	3	3	1	1	1	1
4	4	3	3	3	3	1	1	1	1
2	2	2	2	2	2	1	1	1	1
4	4	3	3	3	3	1	1	1	1
4	4	3	3	2	2	4	4	3	3
4	4	2	2	3	3	1	1	1	1
2	2	3	3	2	2	1	1	1	1
2	2	2	2	3	3	1	1	1	1
4	4	3	3	3	3	1	1	1	1
2	2	3	3	2	2	1	1	3	3
4	4	2	2	3	3	1	1	1	1
4	4	3	3	2	2	1	1	1	1
2	2	3	3	2	2	4	4	3	3
2	2	3	3	3	3	4	4	3	3
4	4	2	2	3	3	1	1	1	1
4	4	3	3	2	2	1	1	1	1
4	4	2	2	2	2	1	1	1	1
4	4	3	3	3	3	1	1	1	1
2	2	2	2	3	3	1	1	1	1
4	4	2	2	3	3	1	1	1	1
2	2	3	3	2	2	4	4	3	3
2	2	3	3	2	2	1	1	1	1
4	4	3	3	3	3	1	1	1	1
4	4	2	2	3	3	1	1	1	1
2	2	3	3	2	2	4	4	3	3
4	4	3	3	2	2	4	4	3	3
2	2	3	3	2	2	1	1	1	1
2	2	3	3	2	2	4	4	3	3
2	2	3	3	2	2	1	1	1	1
4	4	3	3	2	2	1	1	1	1
2	2	3	3	3	3	1	1	1	1
4	4	3	3	2	2	4	4	3	3
4	4	3	3	3	3	1	1	3	3
4	4	2	2	3	3	1	1	1	1
2	2	3	3	2	2	4	4	3	3
4	4	2	2	3	3	1	1	1	1
4	4	3	3	2	2	1	1	1	1
4	4	3	3	3	3	1	1	1	1
4	4	2	2	3	3	1	1	1	1

For Review Only

2	2	3	3	2	2	1	1	3	3
2	2	3	3	2	2	1	1	1	1

For Review Only

SNP752	SNP752	SNP753	SNP753	SNP754	SNP754	SNP755	SNP755	SNP756	SNP756
1	1	3	3	3	3	4	4	1	1
1	1	3	3	4	4	4	4	1	1
1	1	3	3	3	3	4	4	4	4
1	1	3	3	3	3	4	4	4	4
1	1	3	3	3	3	4	4	4	4
1	1	3	3	3	3	4	4	4	4
1	1	1	1	3	3	4	4	4	4
4	4	1	1	4	4	4	4	1	1
1	1	1	1	3	3	4	4	4	4
1	1	1	1	3	3	4	4	1	1
4	4	3	3	4	4	4	4	1	1
1	1	3	3	3	3	4	4	4	4
1	1	3	3	3	3	4	4	1	1
1	1	1	1	4	4	4	4	1	1
1	1	3	3	3	3	4	4	1	1
1	1	3	3	3	3	4	4	4	4
4	4	1	1	3	3	4	4	1	1
1	1	1	1	4	4	4	4	1	1
1	1	3	3	3	3	4	4	4	4
1	1	1	1	4	4	4	4	1	1
4	4	3	3	4	4	4	4	1	1
1	1	3	3	3	3	4	4	4	4
4	4	1	1	4	4	4	4	1	1
4	4	1	1	4	4	4	4	1	1
1	1	3	3	3	3	4	4	4	4
4	4	3	3	3	3	4	4	4	4
1	1	3	3	4	4	1	1	1	1
1	1	3	3	3	3	4	4	1	1
4	4	1	1	3	3	4	4	4	4
4	4	1	1	4	4	4	4	1	1
1	1	3	3	3	3	4	4	4	4
1	1	3	3	3	3	4	4	4	4
1	1	3	3	3	3	4	4	4	4
1	1	1	1	3	3	4	4	1	1
1	1	3	3	3	3	4	4	4	4
4	4	1	1	4	4	1	1	1	1
4	4	1	1	4	4	4	4	1	1
4	4	3	3	3	3	1	1	1	1
4	4	1	1	4	4	4	4	1	1
4	4	1	1	4	4	4	4	1	1
4	4	1	1	4	4	4	4	1	1
4	4	1	1	4	4	4	4	1	1
4	4	1	1	3	3	4	4	1	1
1	1	3	3	4	4	4	4	1	1
4	4	1	1	3	3	4	4	1	1
4	4	3	3	3	3	4	4	4	4
1	1	3	3	4	4	4	4	1	1
4	4	3	3	3	3	4	4	1	1
1	1	3	3	4	4	4	4	1	1
1	1	1	1	3	3	4	4	4	4
1	1	3	3	3	3	4	4	4	4
1	1	3	3	3	3	4	4	4	4

For Review Only

1	1	3	3	3	3	4	4	4	4
1	1	3	3	3	3	4	4	4	4
1	1	3	3	3	3	4	4	4	4
1	1	1	1	4	4	4	4	1	1
1	1	3	3	3	3	4	4	4	4
1	1	3	3	4	4	4	4	1	1
1	1	3	3	3	3	4	4	4	4
4	4	1	1	3	3	4	4	1	1
1	1	1	1	3	3	4	4	4	4
1	1	3	3	3	3	4	4	1	1
1	1	3	3	4	4	4	4	1	1
1	1	1	1	4	4	4	4	1	1
1	1	3	3	3	3	4	4	4	4
4	4	1	1	3	3	4	4	4	4
1	1	3	3	3	3	4	4	1	1
4	4	1	1	4	4	4	4	1	1
1	1	3	3	3	3	4	4	1	1
1	1	3	3	3	3	4	4	4	4
1	1	3	3	3	3	4	4	4	4
1	1	3	3	3	3	4	4	1	1
1	1	1	1	4	4	4	4	1	1
1	1	1	1	4	4	4	4	1	1
4	4	3	3	3	3	4	4	4	4
1	1	1	1	3	3	4	4	4	4
4	4	1	1	3	3	4	4	1	1
1	1	1	1	3	3	4	4	4	4
4	4	3	3	3	3	4	4	1	1
4	4	1	1	4	4	4	4	1	1
4	4	3	3	3	3	4	4	4	4
1	1	3	3	3	3	4	4	4	4
1	1	1	1	3	3	4	4	4	4
4	4	3	3	4	4	4	4	1	1
4	4	1	1	4	4	4	4	1	1
1	1	3	3	3	3	4	4	4	4
1	1	3	3	3	3	4	4	4	4
4	4	1	1	3	3	4	4	1	1
1	1	1	1	3	3	4	4	4	4
4	4	3	3	4	4	4	4	1	1
1	1	3	3	3	3	4	4	4	4
4	4	1	1	3	3	4	4	1	1
1	1	3	3	3	3	4	4	4	4
1	1	3	3	3	3	1	1	4	4
4	4	3	3	3	3	4	4	1	1
1	1	3	3	4	4	4	4	1	1
1	1	3	3	3	3	4	4	4	4
1	1	1	1	4	4	4	4	1	1
1	1	1	1	4	4	4	4	1	1
1	1	1	1	4	4	4	4	1	1
4	4	3	3	3	3	4	4	4	4
4	4	1	1	4	4	4	4	1	1
1	1	3	3	4	4	4	4	1	1
4	4	1	1	4	4	4	4	1	1
4	4	1	1	3	3	4	4	1	1
4	4	3	3	3	3	4	4	1	1
4	4	3	3	4	4	4	4	1	1

For Review Only

1	1	3	3	3	3	4	4	1	1
4	4	1	1	4	4	4	4	1	1
4	4	3	3	4	4	1	1	1	1
4	4	1	1	4	4	4	4	1	1
1	1	3	3	3	3	4	4	4	4
4	4	3	3	3	3	4	4	4	4
1	1	3	3	3	3	4	4	4	4
4	4	3	3	4	4	4	4	1	1
4	4	3	3	3	3	4	4	1	1
4	4	3	3	4	4	4	4	4	4
1	1	3	3	4	4	4	4	1	1
1	1	3	3	3	3	4	4	1	1
4	4	1	1	3	3	4	4	1	1
1	1	3	3	3	3	4	4	4	4
4	4	3	3	4	4	4	4	1	1
1	1	3	3	3	3	4	4	1	1
1	1	3	3	3	3	4	4	1	1
1	1	3	3	3	3	4	4	1	1
1	1	1	1	4	4	4	4	1	1
4	4	3	3	3	3	4	4	1	1
1	1	3	3	3	3	4	4	1	1
1	1	3	3	3	3	4	4	4	4
1	1	3	3	3	3	1	1	4	4
1	1	1	1	3	3	4	4	1	1
1	1	3	3	4	4	4	4	1	1
4	4	1	1	4	4	4	4	1	1
4	4	3	3	3	3	4	4	1	1
1	1	1	1	4	4	4	4	1	1
1	1	3	3	3	3	4	4	4	4
1	1	3	3	3	3	4	4	1	1
4	4	1	1	4	4	4	4	1	1
1	1	3	3	3	3	4	4	1	1
1	1	3	3	3	3	4	4	4	4
1	1	3	3	3	3	4	4	1	1
1	1	1	1	3	3	4	4	1	1
1	1	3	3	3	3	4	4	1	1
1	1	1	1	4	4	4	4	1	1
1	1	3	3	3	3	4	4	4	4
4	4	3	3	3	3	4	4	1	1
4	4	3	3	4	4	4	4	1	1
1	1	1	1	4	4	4	4	1	1
4	4	1	1	4	4	4	4	1	1
1	1	3	3	4	4	4	4	1	1
1	1	1	1	3	3	4	4	1	1
1	1	1	1	3	3	1	1	4	4
1	1	1	1	3	3	4	4	1	1
1	1	3	3	3	3	4	4	4	4
1	1	3	3	4	4	4	4	1	1
1	1	3	3	4	4	4	4	1	1
4	4	3	3	3	3	4	4	4	4
1	1	3	3	3	3	4	4	4	4
4	4	1	1	3	3	4	4	1	1
1	1	3	3	3	3	4	4	1	1
4	4	1	1	3	3	4	4	4	4
4	4	3	3	3	3	4	4	1	1

For Review Only

1	1	3	3	3	3	4	4	4	4
4	4	1	1	3	3	4	4	1	1
1	1	3	3	3	3	4	4	1	1
4	4	3	3	3	3	4	4	4	4
1	1	3	3	3	3	4	4	1	1
1	1	3	3	3	3	4	4	1	1
1	1	3	3	3	3	4	4	1	1
1	1	3	3	3	3	4	4	1	1
1	1	3	3	3	3	4	4	1	1
1	1	3	3	3	3	4	4	4	4
4	4	3	3	3	3	1	1	4	4
1	1	3	3	4	4	4	4	1	1
1	1	3	3	3	3	4	4	4	4
1	1	3	3	3	3	4	4	1	1
1	1	1	1	4	4	4	4	1	1
4	4	3	3	4	4	4	4	1	1
1	1	3	3	3	3	4	4	1	1
1	1	3	3	3	3	4	4	4	4
4	4	3	3	3	3	4	4	4	4
1	1	3	3	3	3	4	4	4	4
1	1	3	3	3	3	4	4	1	1
4	4	1	1	4	4	4	4	1	1
4	4	1	1	4	4	4	4	1	1
1	1	1	1	4	4	4	4	1	1
4	4	1	1	3	3	4	4	4	4
1	1	1	1	4	4	4	4	1	1
1	1	1	1	3	3	4	4	4	4
1	1	1	1	3	3	4	4	1	1
1	1	1	1	3	3	4	4	1	1
1	1	1	1	3	3	4	4	4	4
1	1	3	3	3	3	4	4	4	4
1	1	3	3	3	3	4	4	4	4
1	1	3	3	3	3	4	4	4	4
1	1	1	1	4	4	4	4	1	1
4	4	3	3	3	3	4	4	4	4
1	1	1	1	4	4	4	4	1	1
4	4	1	1	3	3	1	1	1	1
4	4	3	3	3	3	4	4	1	1
1	1	3	3	4	4	4	4	1	1
1	1	1	1	4	4	4	4	1	1
1	1	3	3	3	3	4	4	4	4
1	1	3	3	3	3	4	4	4	4
1	1	1	1	4	4	4	4	1	1
1	1	3	3	3	3	4	4	1	1
4	4	3	3	3	3	1	1	1	1
1	1	3	3	3	3	4	4	1	1
4	4	3	3	4	4	4	4	1	1
1	1	3	3	4	4	4	4	1	1
1	1	1	1	4	4	4	4	1	1
1	1	1	1	3	3	4	4	4	4
4	4	3	3	3	3	4	4	1	1
4	4	3	3	4	4	4	4	1	1
4	4	1	1	4	4	4	4	1	1
1	1	3	3	4	4	4	4	1	1
4	4	1	1	4	4	4	4	1	1

For Review Only

1	1	3	3	3	3	4	4	4	4
1	1	1	1	3	3	4	4	4	4
1	1	3	3	3	3	4	4	4	4
4	4	1	1	4	4	4	4	1	1
1	1	1	1	3	3	4	4	4	4
1	1	3	3	3	3	4	4	1	1
4	4	3	3	3	3	4	4	4	4
4	4	1	1	4	4	4	4	1	1
4	4	3	3	3	3	4	4	1	1
4	4	1	1	4	4	4	4	1	1
1	1	3	3	3	3	4	4	1	1
1	1	3	3	3	3	4	4	4	4
1	1	3	3	3	3	4	4	4	4
4	4	3	3	4	4	4	4	1	1
1	1	3	3	3	3	4	4	4	4
1	1	3	3	3	3	4	4	1	1
4	4	1	1	4	4	4	4	1	1
4	4	3	3	4	4	4	4	1	1
1	1	3	3	4	4	4	4	1	1
4	4	3	3	3	3	4	4	1	1
1	1	1	1	3	3	4	4	4	4
4	4	3	3	3	3	4	4	4	4
1	1	3	3	3	3	4	4	4	4
4	4	1	1	4	4	4	4	1	1
4	4	1	1	3	3	4	4	1	1
1	1	3	3	4	4	4	4	1	1
4	4	1	1	4	4	4	4	1	1
1	1	3	3	3	3	4	4	4	4
1	1	3	3	3	3	4	4	4	4
1	1	3	3	3	3	4	4	4	4
4	4	1	1	4	4	4	4	1	1
1	1	1	1	3	3	4	4	4	4
1	1	3	3	4	4	4	4	1	1
4	4	3	3	4	4	4	4	1	1
4	4	1	1	4	4	4	4	1	1
4	4	1	1	4	4	4	4	1	1
1	1	3	3	3	3	4	4	4	4
1	1	1	1	4	4	4	4	1	1
4	4	1	1	4	4	1	1	1	1
4	4	1	1	4	4	4	4	1	1
1	1	3	3	3	3	4	4	4	4
1	1	1	1	4	4	4	4	1	1
1	1	1	1	4	4	4	4	1	1
1	1	3	3	3	3	4	4	4	4
1	1	1	1	3	3	4	4	1	1
1	1	3	3	4	4	1	1	1	1
4	4	3	3	4	4	4	4	1	1
1	1	3	3	3	3	4	4	4	4
1	1	3	3	3	3	4	4	1	1
1	1	1	1	4	4	4	4	1	1
1	1	3	3	3	3	4	4	4	4
4	4	1	1	4	4	4	4	1	1
1	1	3	3	3	3	4	4	4	4
4	4	1	1	4	4	4	4	1	1
4	4	3	3	4	4	4	4	1	1

For Review Only

1	1	3	3	3	3	4	4	1	1
1	1	1	1	4	4	4	4	1	1

For Review Only

SNP757	SNP757	SNP758	SNP758	SNP759	SNP759	SNP760	SNP760	SNP761	SNP761
3	3	1	1	4	4	1	1	2	2
2	2	1	1	4	4	1	1	1	1
3	3	1	1	4	4	1	1	2	2
3	3	1	1	4	4	1	1	2	2
3	3	1	1	4	4	1	1	2	2
3	3	1	1	4	4	1	1	2	2
3	3	1	1	4	4	4	4	2	2
2	2	1	1	4	4	4	4	1	1
3	3	1	1	4	4	4	4	2	2
2	2	3	3	3	3	4	4	1	1
2	2	1	1	4	4	1	1	1	1
3	3	1	1	4	4	1	1	2	2
3	3	1	1	4	4	1	1	2	2
2	2	1	1	4	4	4	4	1	1
3	3	1	1	4	4	1	1	2	2
3	3	1	1	4	4	1	1	2	2
2	2	1	1	4	4	4	4	1	1
3	3	1	1	3	3	4	4	1	1
3	3	1	1	4	4	1	1	2	2
2	2	1	1	3	3	4	4	1	1
2	2	1	1	3	3	1	1	1	1
3	3	1	1	4	4	1	1	2	2
2	2	3	3	3	3	4	4	1	1
2	2	1	1	4	4	4	4	1	1
3	3	1	1	4	4	1	1	2	2
3	3	1	1	4	4	1	1	2	2
2	2	1	1	4	4	1	1	1	1
3	3	1	1	4	4	1	1	2	2
3	3	1	1	3	3	4	4	2	2
2	2	1	1	4	4	4	4	1	1
3	3	1	1	4	4	1	1	1	1
3	3	1	1	3	3	1	1	2	2
3	3	1	1	3	3	1	1	2	2
2	2	3	3	3	3	4	4	1	1
3	3	1	1	4	4	1	1	2	2
2	2	1	1	4	4	4	4	1	1
2	2	1	1	4	4	4	4	1	1
2	2	1	1	4	4	1	1	1	1
2	2	3	3	3	3	4	4	2	2
2	2	1	1	4	4	1	1	1	1
2	2	1	1	4	4	4	4	1	1
2	2	1	1	3	3	4	4	1	1
2	2	1	1	4	4	1	1	1	1
2	2	1	1	4	4	1	1	1	1
2	2	1	1	4	4	1	1	1	1
3	3	1	1	4	4	1	1	2	2
2	2	1	1	3	3	1	1	1	1
2	2	1	1	4	4	1	1	1	1
2	2	1	1	4	4	1	1	1	1
3	3	1	1	4	4	1	1	2	2
3	3	1	1	4	4	1	1	2	2
3	3	1	1	4	4	1	1	2	2

For Review Only

3	3	1	1	4	4	1	1	2	2
3	3	1	1	3	3	1	1	2	2
3	3	1	1	4	4	1	1	2	2
2	2	1	1	4	4	4	4	1	1
3	3	1	1	4	4	1	1	2	2
2	2	1	1	3	3	1	1	1	1
3	3	1	1	3	3	1	1	2	2
2	2	1	1	4	4	4	4	1	1
3	3	1	1	3	3	4	4	2	2
3	3	1	1	4	4	1	1	2	2
2	2	1	1	4	4	1	1	1	1
2	2	1	1	4	4	4	4	1	1
3	3	1	1	4	4	1	1	2	2
3	3	1	1	3	3	4	4	2	2
3	3	1	1	4	4	1	1	2	2
2	2	1	1	4	4	4	4	1	1
2	2	1	1	3	3	1	1	1	1
3	3	1	1	3	3	1	1	1	1
3	3	1	1	4	4	1	1	2	2
3	3	1	1	4	4	1	1	2	2
2	2	1	1	4	4	4	4	1	1
2	2	1	1	4	4	4	4	2	2
3	3	1	1	4	4	1	1	2	2
3	3	1	1	4	4	4	4	1	1
2	2	1	1	4	4	1	1	1	1
3	3	1	1	4	4	4	4	2	2
2	2	1	1	4	4	1	1	1	1
2	2	3	3	3	3	4	4	1	1
3	3	1	1	4	4	1	1	2	2
3	3	1	1	4	4	1	1	2	2
3	3	1	1	4	4	4	4	2	2
2	2	1	1	4	4	1	1	1	1
2	2	3	3	3	3	4	4	1	1
3	3	1	1	4	4	1	1	2	2
3	3	1	1	3	3	1	1	1	1
2	2	1	1	4	4	1	1	1	1
3	3	1	1	4	4	4	4	2	2
2	2	1	1	4	4	1	1	1	1
3	3	3	3	3	3	1	1	2	2
2	2	1	1	4	4	4	4	1	1
3	3	1	1	4	4	1	1	2	2
3	3	1	1	4	4	1	1	2	2
3	3	1	1	4	4	1	1	2	2
2	2	1	1	4	4	1	1	1	1
3	3	1	1	4	4	1	1	2	2
2	2	1	1	3	3	1	1	1	1
2	2	3	3	3	3	4	4	1	1
2	2	1	1	4	4	4	4	1	1
3	3	1	1	3	3	1	1	2	2
2	2	1	1	4	4	4	4	2	2
2	2	1	1	4	4	1	1	1	1
2	2	1	1	4	4	4	4	1	1
2	2	1	1	4	4	4	4	1	1
3	3	1	1	4	4	1	1	1	1
2	2	1	1	4	4	1	1	1	1

For Review Only

3	3	1	1	4	4	1	1	2	2
2	2	1	1	4	4	4	4	1	1
2	2	1	1	4	4	1	1	1	1
2	2	1	1	3	3	4	4	1	1
3	3	1	1	4	4	1	1	2	2
3	3	1	1	4	4	1	1	2	2
3	3	1	1	4	4	1	1	2	2
2	2	1	1	4	4	1	1	1	1
3	3	1	1	4	4	1	1	2	2
3	3	1	1	4	4	1	1	1	1
2	2	1	1	4	4	1	1	1	1
3	3	1	1	4	4	1	1	2	2
3	3	1	1	4	4	4	4	2	2
3	3	1	1	4	4	1	1	2	2
2	2	1	1	4	4	1	1	1	1
3	3	1	1	4	4	1	1	2	2
3	3	1	1	3	3	1	1	1	1
3	3	1	1	4	4	1	1	2	2
2	2	1	1	3	3	4	4	1	1
2	2	1	1	4	4	1	1	1	1
3	3	1	1	4	4	1	1	2	2
3	3	1	1	4	4	1	1	2	2
3	3	1	1	4	4	1	1	2	2
2	2	3	3	3	3	4	4	1	1
3	3	1	1	4	4	1	1	2	2
2	2	1	1	4	4	4	4	1	1
3	3	1	1	3	3	1	1	2	2
2	2	1	1	4	4	4	4	1	1
3	3	1	1	4	4	1	1	2	2
2	2	1	1	4	4	1	1	1	1
2	2	3	3	3	3	4	4	1	1
3	3	1	1	4	4	4	4	2	2
3	3	1	1	4	4	1	1	2	2
3	3	1	1	4	4	1	1	2	2
2	2	3	3	3	3	4	4	1	1
2	2	1	1	3	3	1	1	1	1
2	2	1	1	3	3	4	4	1	1
3	3	1	1	4	4	1	1	2	2
3	3	1	1	4	4	1	1	2	2
2	2	1	1	4	4	1	1	2	2
2	2	1	1	4	4	4	4	1	1
2	2	3	3	3	3	4	4	1	1
2	2	1	1	4	4	1	1	1	1
2	2	1	1	4	4	4	4	1	1
3	3	1	1	4	4	4	4	2	2
3	3	3	3	3	3	1	1	2	2
3	3	1	1	3	3	1	1	2	2
2	2	1	1	3	3	1	1	1	1
2	2	1	1	3	3	1	1	1	1
3	3	1	1	4	4	1	1	2	2
3	3	1	1	3	3	1	1	2	2
2	2	1	1	3	3	4	4	1	1
3	3	1	1	4	4	1	1	2	2
3	3	1	1	4	4	4	4	2	2
2	2	1	1	4	4	1	1	1	1

For Review Only

3	3	1	1	3	3	1	1	2	2
2	2	1	1	4	4	4	4	1	1
2	2	1	1	4	4	1	1	1	1
3	3	1	1	4	4	1	1	2	2
3	3	1	1	4	4	1	1	2	2
2	2	1	1	3	3	1	1	1	1
3	3	1	1	4	4	1	1	1	1
3	3	1	1	4	4	1	1	2	2
3	3	1	1	4	4	1	1	2	2
3	3	1	1	4	4	1	1	2	2
3	3	1	1	4	4	1	1	2	2
2	2	1	1	4	4	1	1	1	1
3	3	1	1	4	4	1	1	2	2
3	3	3	3	3	3	1	1	2	2
2	2	3	3	3	3	4	4	1	1
2	2	1	1	4	4	1	1	2	2
3	3	1	1	4	4	1	1	2	2
3	3	1	1	4	4	1	1	2	2
3	3	1	1	4	4	1	1	1	1
3	3	1	1	4	4	1	1	2	2
3	3	1	1	4	4	1	1	2	2
2	2	3	3	4	4	4	4	1	1
2	2	1	1	4	4	4	4	2	2
2	2	1	1	4	4	4	4	1	1
3	3	1	1	4	4	4	4	2	2
2	2	3	3	3	3	4	4	1	1
3	3	1	1	4	4	4	4	2	2
3	3	1	1	3	3	1	1	1	1
3	3	1	1	3	3	4	4	2	2
3	3	1	1	4	4	4	4	2	2
3	3	1	1	4	4	1	1	1	1
3	3	1	1	3	3	1	1	2	2
3	3	1	1	3	3	1	1	2	2
2	2	3	3	3	3	4	4	1	1
3	3	1	1	4	4	1	1	1	1
2	2	1	1	4	4	4	4	1	1
3	3	1	1	4	4	4	4	2	2
3	3	1	1	4	4	1	1	2	2
2	2	1	1	3	3	1	1	1	1
2	2	1	1	4	4	4	4	1	1
3	3	1	1	4	4	1	1	2	2
3	3	1	1	3	3	1	1	2	2
2	2	3	3	3	3	4	4	1	1
3	3	1	1	3	3	1	1	2	2
3	3	1	1	3	3	1	1	2	2
3	3	1	1	4	4	1	1	2	2
2	2	1	1	4	4	1	1	2	2
2	2	3	3	3	3	1	1	1	1
2	2	1	1	4	4	4	4	1	1
3	3	1	1	4	4	4	4	2	2
3	3	1	1	4	4	1	1	2	2
2	2	1	1	4	4	1	1	1	1
2	2	1	1	4	4	4	4	1	1
2	2	1	1	4	4	1	1	1	1
2	2	1	1	4	4	4	4	1	1

For Review Only

3	3	1	1	4	4	1	1	2	2
3	3	1	1	3	3	4	4	2	2
3	3	1	1	4	4	1	1	2	2
2	2	1	1	4	4	4	4	2	2
3	3	1	1	3	3	4	4	2	2
3	3	1	1	4	4	1	1	2	2
3	3	1	1	3	3	1	1	2	2
2	2	1	1	4	4	4	4	1	1
3	3	3	3	3	3	1	1	2	2
2	2	3	3	3	3	4	4	1	1
3	3	1	1	4	4	1	1	2	2
3	3	1	1	4	4	1	1	1	1
3	3	1	1	4	4	1	1	2	2
2	2	1	1	4	4	1	1	1	1
3	3	1	1	4	4	1	1	2	2
3	3	1	1	4	4	1	1	2	2
2	2	1	1	4	4	1	1	1	1
2	2	1	1	4	4	1	1	1	1
2	2	1	1	4	4	1	1	2	2
2	2	1	1	4	4	1	1	1	1
3	3	1	1	4	4	4	4	1	1
3	3	1	1	4	4	1	1	2	2
3	3	1	1	4	4	1	1	2	2
2	2	1	1	4	4	4	4	1	1
2	2	1	1	4	4	4	4	1	1
2	2	1	1	4	4	1	1	1	1
2	2	3	3	3	3	4	4	1	1
3	3	1	1	4	4	1	1	2	2
3	3	1	1	4	4	1	1	2	2
3	3	1	1	4	4	1	1	2	2
2	2	3	3	3	3	1	1	1	1
3	3	1	1	4	4	4	4	2	2
2	2	3	3	3	3	1	1	1	1
3	3	1	1	4	4	1	1	2	2
2	2	1	1	4	4	4	4	2	2
2	2	3	3	3	3	4	4	1	1
3	3	1	1	4	4	1	1	2	2
2	2	1	1	4	4	4	4	1	1
2	2	1	1	4	4	4	4	1	1
2	2	3	3	3	3	4	4	1	1
3	3	1	1	4	4	1	1	2	2
2	2	1	1	4	4	1	1	1	1
2	2	1	1	4	4	4	4	1	1
3	3	1	1	4	4	1	1	2	2
3	3	1	1	4	4	4	4	2	2
2	2	3	3	3	3	1	1	1	1
2	2	1	1	3	3	1	1	1	1
3	3	1	1	4	4	1	1	1	1
3	3	1	1	4	4	1	1	2	2
2	2	3	3	3	3	4	4	1	1
3	3	1	1	4	4	1	1	2	2
2	2	3	3	3	3	4	4	1	1
3	3	1	1	4	4	1	1	2	2
2	2	1	1	4	4	4	4	1	1
2	2	1	1	4	4	1	1	1	1

For Review Only

3	3	1	1	4	4	1	1	2	2
2	2	1	1	4	4	4	4	1	1

For Review Only

SNP762	SNP762	SNP763	SNP763	SNP764	SNP764	SNP765	SNP765	SNP766	SNP766
4	4	1	1	4	4	3	3	2	2
4	4	1	1	4	4	3	3	1	1
4	4	1	1	4	4	3	3	2	2
4	4	4	4	4	4	3	3	2	2
4	4	1	1	4	4	3	3	2	2
4	4	1	1	4	4	3	3	1	1
4	4	4	4	2	2	3	3	2	2
2	2	1	1	2	2	3	3	1	1
4	4	4	4	2	2	3	3	2	2
2	2	1	1	2	2	3	3	1	1
4	4	4	4	4	4	3	3	1	1
4	4	1	1	4	4	3	3	2	2
4	4	4	4	4	4	3	3	1	1
4	4	4	4	2	2	3	3	2	2
4	4	4	4	4	4	3	3	2	2
4	4	4	4	4	4	3	3	1	1
2	2	4	4	2	2	3	3	1	1
4	4	4	4	2	2	3	3	1	1
4	4	1	1	4	4	3	3	2	2
4	4	4	4	2	2	3	3	1	1
4	4	4	4	4	4	3	3	1	1
4	4	1	1	4	4	3	3	2	2
4	4	4	4	2	2	3	3	1	1
4	4	1	1	2	2	2	2	2	2
4	4	4	4	4	4	3	3	2	2
4	4	1	1	4	4	3	3	2	2
4	4	4	4	4	4	3	3	2	2
4	4	4	4	2	2	3	3	1	1
4	4	1	1	2	2	3	3	2	2
4	4	1	1	2	2	3	3	1	1
4	4	4	4	4	4	3	3	2	2
4	4	1	1	4	4	3	3	1	1
4	4	4	4	4	4	3	3	2	2
4	4	4	4	2	2	3	3	2	2
4	4	4	4	2	2	3	3	2	2
4	4	1	1	2	2	2	2	2	2
4	4	1	1	2	2	3	3	2	2
2	2	1	1	4	4	3	3	1	1
4	4	4	4	2	2	3	3	1	1
4	4	4	4	4	4	3	3	1	1
2	2	4	4	2	2	3	3	1	1
4	4	1	1	2	2	3	3	1	1
4	4	1	1	2	2	3	3	1	1
4	4	1	1	4	4	3	3	1	1
2	2	1	1	2	2	3	3	1	1
4	4	1	1	4	4	3	3	2	2
4	4	4	4	4	4	3	3	1	1
4	4	4	4	4	4	3	3	1	1
2	2	1	1	4	4	3	3	1	1
4	4	1	1	2	2	3	3	1	1
4	4	1	1	4	4	3	3	2	2
4	4	1	1	4	4	3	3	1	1

For Review Only

4	4	4	4	4	4	3	3	2	2
4	4	4	4	4	4	3	3	2	2
4	4	4	4	4	4	3	3	1	1
4	4	4	4	2	2	3	3	1	1
4	4	1	1	4	4	3	3	2	2
4	4	1	1	4	4	3	3	1	1
4	4	1	1	4	4	3	3	2	2
2	2	1	1	2	2	3	3	1	1
4	4	1	1	2	2	3	3	1	1
4	4	4	4	4	4	3	3	1	1
4	4	1	1	4	4	3	3	1	1
4	4	1	1	2	2	3	3	1	1
4	4	4	4	4	4	3	3	2	2
4	4	1	1	2	2	3	3	2	2
4	4	4	4	4	4	3	3	1	1
4	4	1	1	2	2	3	3	1	1
2	2	4	4	4	4	3	3	1	1
4	4	1	1	4	4	3	3	2	2
4	4	1	1	4	4	3	3	2	2
4	4	4	4	4	4	3	3	2	2
4	4	1	1	2	2	3	3	1	1
4	4	4	4	2	2	3	3	1	1
4	4	4	4	4	4	3	3	2	2
4	4	1	1	2	2	3	3	2	2
2	2	1	1	2	2	3	3	1	1
4	4	1	1	2	2	3	3	2	2
2	2	4	4	4	4	3	3	1	1
4	4	4	4	2	2	3	3	1	1
4	4	1	1	4	4	3	3	2	2
4	4	1	1	4	4	3	3	1	1
4	4	1	1	2	2	3	3	1	1
4	4	1	1	2	2	2	2	2	2
4	4	1	1	2	2	3	3	1	1
4	4	4	4	4	4	3	3	2	2
4	4	1	1	4	4	3	3	2	2
2	2	1	1	2	2	3	3	1	1
4	4	1	1	2	2	3	3	1	1
4	4	4	4	4	4	3	3	1	1
4	4	1	1	4	4	3	3	1	1
2	2	4	4	2	2	3	3	1	1
4	4	4	4	4	4	3	3	2	2
4	4	1	1	4	4	3	3	2	2
2	2	1	1	4	4	3	3	2	2
4	4	1	1	4	4	3	3	1	1
4	4	1	1	4	4	3	3	2	2
4	4	1	1	4	4	3	3	1	1
4	4	1	1	2	2	3	3	1	1
4	4	1	1	2	2	3	3	2	2
4	4	1	1	4	4	3	3	2	2
4	4	4	4	2	2	3	3	1	1
4	4	1	1	4	4	3	3	1	1
4	4	4	4	2	2	3	3	1	1
2	2	1	1	2	2	3	3	1	1
4	4	4	4	4	4	3	3	1	1
4	4	1	1	4	4	3	3	2	2

For Review Only

4	4	4	4	4	4	3	3	1	1
4	4	4	4	2	2	3	3	1	1
4	4	1	1	4	4	2	2	2	2
4	4	1	1	2	2	3	3	1	1
4	4	1	1	4	4	3	3	2	2
4	4	1	1	4	4	3	3	2	2
4	4	1	1	4	4	3	3	1	1
4	4	4	4	4	4	3	3	1	1
2	2	1	1	4	4	3	3	1	1
4	4	4	4	2	2	3	3	1	1
4	4	1	1	4	4	3	3	2	2
4	4	4	4	4	4	3	3	1	1
4	4	1	1	2	2	3	3	2	2
4	4	1	1	4	4	3	3	2	2
4	4	1	1	4	4	3	3	1	1
4	4	4	4	4	4	3	3	1	1
2	2	1	1	4	4	3	3	1	1
4	4	1	1	4	4	3	3	1	1
4	4	1	1	2	2	3	3	1	1
2	2	4	4	4	4	3	3	1	1
4	4	4	4	2	2	3	3	1	1
4	4	1	1	4	4	3	3	1	1
4	4	1	1	4	4	3	3	1	1
4	4	4	4	2	2	3	3	2	2
4	4	4	4	2	2	3	3	1	1
4	4	1	1	2	2	3	3	1	1
4	4	1	1	4	4	3	3	1	1
4	4	1	1	2	2	2	2	2	2
4	4	1	1	4	4	3	3	2	2
2	2	4	4	4	4	3	3	1	1
4	4	1	1	2	2	3	3	1	1
4	4	4	4	2	2	3	3	1	1
4	4	4	4	4	4	3	3	2	2
4	4	4	4	4	4	3	3	2	2
4	4	4	4	2	2	3	3	2	2
4	4	4	4	2	2	3	3	2	2
4	4	1	1	2	2	3	3	1	1
4	4	1	1	4	4	3	3	2	2
4	4	4	4	4	4	3	3	2	2
4	4	4	4	2	2	3	3	1	1
4	4	4	4	2	2	3	3	1	1
4	4	1	1	2	2	3	3	1	1
4	4	1	1	4	4	3	3	2	2
2	2	4	4	2	2	3	3	1	1
4	4	1	1	4	4	3	3	2	2
4	4	1	1	4	4	3	3	2	2
4	4	1	1	4	4	3	3	2	2
4	4	1	1	4	4	3	3	2	2
4	4	4	4	2	2	3	3	2	2
4	4	1	1	4	4	3	3	2	2
4	4	1	1	4	4	3	3	2	2
2	2	1	1	2	2	3	3	1	1
4	4	1	1	4	4	3	3	2	2
4	4	1	1	2	2	3	3	2	2
2	2	4	4	2	2	3	3	1	1

For Review Only

4	4	1	1	4	4	3	3	2	2
2	2	1	1	2	2	3	3	1	1
2	2	1	1	4	4	3	3	1	1
4	4	4	4	4	4	3	3	1	1
4	4	1	1	4	4	3	3	1	1
2	2	4	4	4	4	3	3	1	1
4	4	4	4	4	4	3	3	1	1
4	4	4	4	2	2	3	3	1	1
4	4	4	4	4	4	3	3	1	1
4	4	4	4	4	4	3	3	2	2
4	4	4	4	4	4	3	3	2	2
4	4	4	4	2	2	3	3	1	1
4	4	1	1	4	4	3	3	1	1
4	4	4	4	4	4	3	3	2	2
4	4	1	1	2	2	2	2	2	2
4	4	4	4	2	2	3	3	1	1
4	4	1	1	2	2	3	3	1	1
4	4	1	1	4	4	3	3	1	1
4	4	1	1	4	4	3	3	2	2
4	4	1	1	4	4	3	3	1	1
4	4	4	4	2	2	3	3	1	1
4	4	1	1	2	2	2	2	1	1
4	4	4	4	2	2	3	3	1	1
4	4	1	1	2	2	3	3	1	1
4	4	4	4	2	2	3	3	2	2
4	4	4	4	2	2	3	3	2	2
4	4	1	1	2	2	3	3	2	2
4	4	4	4	2	2	3	3	2	2
4	4	1	1	2	2	3	3	2	2
4	4	1	1	2	2	3	3	2	2
4	4	4	4	4	4	3	3	2	2
4	4	1	1	4	4	3	3	1	1
4	4	1	1	4	4	3	3	1	1
4	4	1	1	2	2	2	2	2	2
4	4	4	4	4	4	3	3	2	2
4	4	1	1	2	2	3	3	1	1
4	4	1	1	4	4	3	3	1	1
4	4	1	1	4	4	3	3	2	2
4	4	1	1	4	4	3	3	2	2
4	4	1	1	4	4	3	3	2	2
4	4	1	1	4	4	3	3	2	2
4	4	1	1	4	4	3	3	2	2
4	4	1	1	2	2	3	3	1	1
4	4	4	4	4	4	3	3	1	1
4	4	4	4	2	2	2	2	1	1
2	2	4	4	2	2	3	3	1	1
4	4	4	4	4	4	3	3	1	1
4	4	1	1	4	4	3	3	1	1
4	4	1	1	2	2	2	2	2	2
4	4	4	4	2	2	3	3	2	2
2	2	1	1	4	4	3	3	1	1
4	4	1	1	4	4	3	3	2	2
4	4	4	4	2	2	3	3	1	1
4	4	1	1	4	4	3	3	1	1
4	4	4	4	2	2	3	3	1	1

For Review Only

4	4	4	4	4	4	3	3	2	2
4	4	4	4	2	2	3	3	1	1
4	4	1	1	4	4	3	3	1	1
4	4	4	4	2	2	3	3	1	1
4	4	4	4	2	2	3	3	1	1
4	4	1	1	4	4	3	3	1	1
4	4	4	4	2	2	3	3	1	1
2	2	1	1	2	2	3	3	1	1
4	4	4	4	2	2	3	3	2	2
4	4	1	1	2	2	3	3	1	1
4	4	4	4	2	2	3	3	1	1
4	4	1	1	4	4	3	3	2	2
4	4	1	1	4	4	3	3	1	1
4	4	1	1	4	4	3	3	2	2
4	4	1	1	4	4	3	3	2	2
4	4	4	4	4	4	3	3	2	2
4	4	4	4	2	2	3	3	1	1
4	4	4	4	4	4	3	3	1	1
4	4	4	4	4	4	3	3	1	1
2	2	1	1	4	4	3	3	1	1
4	4	4	4	4	4	3	3	2	2
4	4	1	1	4	4	3	3	2	2
4	4	1	1	4	4	3	3	1	1
4	4	4	4	2	2	3	3	1	1
2	2	1	1	2	2	3	3	1	1
2	2	1	1	4	4	3	3	1	1
4	4	4	4	2	2	3	3	1	1
4	4	1	1	4	4	3	3	2	2
4	4	4	4	2	2	3	3	1	1
4	4	1	1	4	4	3	3	2	2
4	4	4	4	2	2	3	3	1	1
4	4	4	4	2	2	3	3	2	2
4	4	4	4	4	4	2	2	2	2
4	4	1	1	4	4	3	3	2	2
4	4	4	4	2	2	3	3	1	1
4	4	4	4	2	2	3	3	1	1
4	4	1	1	4	4	3	3	2	2
4	4	1	1	2	2	3	3	1	1
4	4	1	1	2	2	2	2	2	2
4	4	4	4	2	2	3	3	1	1
4	4	1	1	4	4	3	3	1	1
4	4	4	4	4	4	3	3	1	1
4	4	4	4	2	2	3	3	1	1
4	4	4	4	4	4	3	3	2	2
2	2	4	4	2	2	3	3	1	1
4	4	1	1	4	4	3	3	1	1
4	4	4	4	4	4	3	3	2	2
4	4	4	4	4	4	3	3	2	2
2	2	1	1	4	4	3	3	1	1
4	4	4	4	2	2	3	3	1	1
4	4	4	4	4	4	3	3	2	2
4	4	4	4	2	2	3	3	1	1
4	4	1	1	4	4	3	3	2	2
4	4	1	1	4	4	3	3	2	2
4	4	4	4	4	4	3	3	1	1

For Review Only

4	4	1	1	4	4	3	3	2	2
4	4	1	1	4	4	3	3	2	2

For Review Only

SNP767	SNP767	SNP768	SNP768	SNP769	SNP769	SNP770	SNP770	SNP771	SNP771
4	4	4	4	2	2	2	2	1	1
3	3	4	4	2	2	2	2	1	1
4	4	1	1	2	2	2	2	4	4
4	4	1	1	2	2	2	2	1	1
4	4	1	1	2	2	2	2	1	1
3	3	4	4	3	3	2	2	1	1
4	4	1	1	2	2	2	2	1	1
3	3	4	4	2	2	2	2	4	4
4	4	1	1	2	2	2	2	1	1
3	3	1	1	2	2	2	2	4	4
3	3	4	4	2	2	3	3	1	1
4	4	1	1	2	2	2	2	4	4
4	4	4	4	2	2	2	2	1	1
4	4	1	1	2	2	2	2	1	1
4	4	1	1	2	2	2	2	1	1
3	3	1	1	2	2	2	2	1	1
3	3	1	1	2	2	2	2	1	1
3	3	4	4	3	3	2	2	1	1
4	4	1	1	2	2	2	2	4	4
3	3	4	4	2	2	2	2	4	4
3	3	4	4	2	2	2	2	4	4
4	4	1	1	2	2	2	2	1	1
3	3	4	4	2	2	2	2	1	1
4	4	1	1	2	2	2	2	1	1
4	4	1	1	2	2	2	2	1	1
4	4	1	1	2	2	2	2	4	4
4	4	1	1	2	2	2	2	1	1
3	3	4	4	2	2	2	2	1	1
4	4	1	1	2	2	2	2	1	1
3	3	4	4	2	2	2	2	4	4
4	4	1	1	2	2	2	2	1	1
3	3	4	4	3	3	2	2	1	1
4	4	1	1	2	2	2	2	1	1
4	4	4	4	2	2	2	2	1	1
4	4	4	4	2	2	2	2	4	4
4	4	1	1	2	2	2	2	4	4
4	4	1	1	2	2	2	2	1	1
3	3	1	1	2	2	2	2	1	1
3	3	4	4	3	3	3	3	4	4
3	3	4	4	2	2	2	2	4	4
3	3	4	4	2	2	2	2	4	4
3	3	4	4	3	3	2	2	4	4
3	3	4	4	2	2	2	2	1	1
3	3	4	4	3	3	3	3	1	1
3	3	4	4	3	3	2	2	1	1
4	4	1	1	2	2	2	2	4	4
3	3	4	4	3	3	2	2	4	4
3	3	4	4	3	3	2	2	4	4
3	3	1	1	2	2	2	2	1	1
4	4	1	1	2	2	2	2	1	1
4	4	1	1	2	2	2	2	4	4
3	3	4	4	3	3	2	2	4	4

For Review Only

4	4	1	1	2	2	2	2	1	1
4	4	1	1	2	2	2	2	1	1
3	3	4	4	2	2	2	2	4	4
3	3	4	4	2	2	2	2	1	1
4	4	1	1	2	2	2	2	4	4
3	3	4	4	3	3	2	2	4	4
4	4	1	1	2	2	2	2	4	4
3	3	1	1	2	2	2	2	1	1
3	3	4	4	3	3	2	2	1	1
3	3	1	1	2	2	2	2	1	1
3	3	4	4	3	3	2	2	4	4
3	3	4	4	3	3	2	2	4	4
4	4	4	4	2	2	2	2	1	1
4	4	1	1	2	2	2	2	1	1
3	3	4	4	2	2	2	2	1	1
4	4	1	1	2	2	2	2	1	1
3	3	4	4	2	2	2	2	4	4
4	4	1	1	2	2	2	2	1	1
4	4	1	1	2	2	2	2	4	4
4	4	1	1	2	2	2	2	1	1
3	3	4	4	2	2	2	2	4	4
3	3	4	4	3	3	2	2	4	4
4	4	1	1	2	2	2	2	1	1
4	4	1	1	2	2	2	2	4	4
3	3	4	4	3	3	2	2	1	1
4	4	1	1	2	2	2	2	4	4
3	3	1	1	2	2	2	2	4	4
3	3	4	4	2	2	3	3	4	4
4	4	1	1	2	2	2	2	4	4
3	3	1	1	2	2	2	2	1	1
3	3	4	4	3	3	2	2	1	1
4	4	1	1	2	2	2	2	1	1
3	3	4	4	2	2	3	3	4	4
4	4	1	1	2	2	2	2	1	1
4	4	1	1	2	2	3	3	1	1
3	3	4	4	3	3	2	2	1	1
3	3	4	4	2	2	2	2	1	1
3	3	4	4	2	2	2	2	1	1
3	3	4	4	3	3	3	3	4	4
3	3	4	4	3	3	2	2	4	4
4	4	1	1	2	2	2	2	1	1
4	4	1	1	2	2	2	2	4	4
4	4	1	1	2	2	2	2	1	1
3	3	4	4	3	3	2	2	4	4
4	4	4	4	3	3	2	2	1	1
3	3	4	4	3	3	2	2	1	1
3	3	1	1	2	2	2	2	1	1
4	4	1	1	2	2	2	2	4	4
4	4	1	1	2	2	2	2	1	1
3	3	4	4	2	2	2	2	4	4
3	3	4	4	3	3	2	2	1	1
3	3	4	4	2	2	3	3	4	4
3	3	1	1	2	2	2	2	1	1
3	3	4	4	2	2	2	2	4	4
4	4	1	1	2	2	2	2	4	4

For Review Only

3	3	4	4	2	2	2	2	1	1
3	3	4	4	3	3	2	2	1	1
4	4	1	1	2	2	2	2	4	4
3	3	1	1	2	2	2	2	1	1
4	4	1	1	2	2	2	2	4	4
4	4	1	1	2	2	2	2	4	4
3	3	1	1	2	2	2	2	1	1
4	4	1	1	2	2	2	2	1	1
3	3	4	4	2	2	2	2	1	1
3	3	4	4	2	2	2	2	4	4
4	4	4	4	2	2	2	2	1	1
3	3	4	4	2	2	2	2	1	1
4	4	1	1	2	2	2	2	1	1
4	4	4	4	2	2	2	2	4	4
3	3	4	4	3	3	2	2	1	1
3	3	4	4	2	2	2	2	4	4
3	3	1	1	2	2	2	2	1	1
3	3	4	4	2	2	2	2	1	1
3	3	1	1	2	2	2	2	1	1
3	3	4	4	2	2	2	2	1	1
3	3	4	4	2	2	2	2	4	4
3	3	1	1	2	2	2	2	1	1
3	3	4	4	3	3	2	2	1	1
3	3	1	1	2	2	3	3	4	4
3	3	4	4	2	2	2	2	4	4
3	3	4	4	2	2	3	3	1	1
3	3	4	4	2	2	2	2	1	1
4	4	1	1	2	2	2	2	1	1
4	4	1	1	2	2	2	2	4	4
3	3	1	1	2	2	2	2	1	1
3	3	4	4	2	2	3	3	4	4
3	3	4	4	2	2	2	2	4	4
4	4	1	1	2	2	2	2	1	1
4	4	1	1	2	2	2	2	1	1
4	4	4	4	2	2	2	2	1	1
4	4	1	1	2	2	2	2	1	1
3	3	1	1	2	2	2	2	1	1
4	4	1	1	2	2	2	2	4	4
4	4	1	1	2	2	3	3	4	4
3	3	4	4	2	2	3	3	4	4
3	3	4	4	2	2	2	2	4	4
3	3	1	1	2	2	2	2	1	1
4	4	1	1	2	2	2	2	4	4
3	3	4	4	2	2	2	2	1	1
4	4	1	1	2	2	2	2	4	4
4	4	1	1	2	2	2	2	1	1
4	4	1	1	2	2	2	2	4	4
4	4	1	1	2	2	2	2	4	4
3	3	1	1	2	2	2	2	1	1
4	4	1	1	2	2	2	2	4	4
4	4	1	1	2	2	2	2	1	1
3	3	1	1	2	2	2	2	4	4
4	4	1	1	2	2	2	2	1	1
4	4	1	1	2	2	2	2	4	4
3	3	4	4	2	2	2	2	1	1

For Review Only

4	4	1	1	2	2	2	2	4	4
3	3	4	4	3	3	3	3	4	4
3	3	4	4	2	2	2	2	1	1
3	3	4	4	2	2	2	2	1	1
3	3	4	4	2	2	2	2	1	1
3	3	1	1	2	2	2	2	4	4
3	3	4	4	3	3	2	2	1	1
4	4	4	4	2	2	2	2	1	1
3	3	1	1	2	2	2	2	1	1
4	4	4	4	3	3	2	2	1	1
4	4	1	1	2	2	2	2	4	4
3	3	4	4	2	2	2	2	4	4
4	4	1	1	2	2	2	2	1	1
4	4	1	1	2	2	2	2	1	1
4	4	1	1	2	2	2	2	4	4
3	3	4	4	3	3	3	3	4	4
4	4	4	4	2	2	2	2	1	1
3	3	4	4	3	3	2	2	4	4
4	4	1	1	2	2	2	2	1	1
3	3	1	1	2	2	2	2	1	1
3	3	4	4	2	2	2	2	1	1
3	3	4	4	2	2	2	2	4	4
3	3	4	4	3	3	3	3	4	4
3	3	4	4	3	3	2	2	4	4
4	4	1	1	2	2	2	2	4	4
4	4	1	1	2	2	2	2	1	1
4	4	1	1	2	2	2	2	1	1
4	4	4	4	2	2	2	2	1	1
4	4	1	1	2	2	2	2	1	1
4	4	1	1	2	2	2	2	1	1
4	4	1	1	2	2	2	2	4	4
3	3	1	1	2	2	2	2	1	1
3	3	4	4	3	3	2	2	1	1
4	4	1	1	2	2	2	2	4	4
4	4	1	1	2	2	2	2	1	1
3	3	4	4	3	3	2	2	4	4
3	3	4	4	2	2	2	2	4	4
4	4	1	1	2	2	2	2	1	1
4	4	1	1	2	2	2	2	4	4
4	4	1	1	2	2	2	2	4	4
4	4	1	1	2	2	2	2	4	4
4	4	1	1	2	2	2	2	4	4
3	3	4	4	2	2	2	2	4	4
3	3	4	4	2	2	2	2	1	1
3	3	4	4	2	2	2	2	1	1
3	3	4	4	2	2	2	2	1	1
3	3	4	4	3	3	3	3	4	4
3	3	4	4	2	2	3	3	1	1
4	4	1	1	2	2	2	2	1	1
4	4	1	1	2	2	2	2	1	1
3	3	1	1	2	2	2	2	4	4
4	4	1	1	2	2	2	2	1	1
3	3	4	4	2	2	3	3	4	4
3	3	4	4	3	3	2	2	4	4
3	3	4	4	2	2	3	3	4	4

For Review Only

4	4	1	1	2	2	2	2	1	1
3	3	4	4	3	3	2	2	1	1
3	3	4	4	2	2	2	2	4	4
3	3	4	4	2	2	2	2	1	1
3	3	4	4	3	3	2	2	1	1
3	3	1	1	2	2	2	2	1	1
3	3	4	4	3	3	3	3	4	4
3	3	4	4	2	2	2	2	4	4
3	3	4	4	2	2	2	2	1	1
3	3	4	4	2	2	3	3	4	4
3	3	4	4	2	2	2	2	1	1
4	4	1	1	2	2	3	3	4	4
3	3	4	4	3	3	3	3	4	4
4	4	1	1	2	2	2	2	4	4
4	4	1	1	2	2	2	2	4	4
4	4	4	4	3	3	3	3	1	1
3	3	4	4	2	2	3	3	4	4
3	3	4	4	2	2	2	2	1	1
3	3	4	4	2	2	2	2	1	1
3	3	1	1	2	2	2	2	1	1
4	4	1	1	2	2	2	2	4	4
4	4	1	1	2	2	2	2	4	4
3	3	1	1	2	2	2	2	1	1
3	3	4	4	2	2	2	2	4	4
3	3	1	1	2	2	2	2	1	1
3	3	4	4	2	2	2	2	1	1
3	3	4	4	2	2	3	3	4	4
4	4	1	1	2	2	2	2	4	4
3	3	4	4	2	2	2	2	1	1
4	4	1	1	2	2	2	2	4	4
3	3	4	4	2	2	2	2	4	4
4	4	1	1	2	2	3	3	4	4
4	4	1	1	2	2	2	2	1	1
4	4	1	1	2	2	2	2	4	4
3	3	4	4	3	3	2	2	4	4
3	3	4	4	2	2	3	3	4	4
4	4	1	1	2	2	2	2	1	1
3	3	4	4	3	3	2	2	4	4
4	4	1	1	2	2	2	2	4	4
3	3	4	4	2	2	3	3	4	4
3	3	4	4	3	3	2	2	1	1
3	3	4	4	3	3	2	2	1	1
3	3	4	4	2	2	2	2	4	4
4	4	1	1	2	2	2	2	1	1
3	3	1	1	2	2	2	2	4	4
3	3	4	4	2	2	2	2	1	1
4	4	1	1	2	2	2	2	4	4
4	4	4	4	2	2	2	2	1	1
3	3	4	4	3	3	2	2	1	1
3	3	4	4	2	2	3	3	4	4
4	4	4	4	3	3	2	2	1	1
3	3	4	4	2	2	2	2	4	4
4	4	1	1	2	2	2	2	4	4
4	4	1	1	2	2	2	2	4	4
3	3	4	4	2	2	2	2	4	4

For Review Only

4	4	4	4	3	3	2	2	1	1
4	4	1	1	2	2	2	2	4	4

For Review Only

SNP772	SNP772	SNP773	SNP773	SNP774	SNP774	SNP775	SNP775	SNP776	SNP776
4	4	3	3	4	4	4	4	1	1
4	4	3	3	4	4	4	4	1	1
4	4	3	3	4	4	4	4	1	1
4	4	3	3	1	1	4	4	1	1
4	4	3	3	4	4	4	4	1	1
4	4	3	3	4	4	4	4	2	2
4	4	3	3	4	4	4	4	1	1
4	4	3	3	4	4	4	4	1	1
4	4	3	3	4	4	4	4	1	1
4	4	3	3	4	4	4	4	1	1
4	4	3	3	4	4	4	4	1	1
4	4	3	3	1	1	4	4	1	1
4	4	3	3	4	4	4	4	1	1
4	4	3	3	4	4	4	4	1	1
4	4	3	3	4	4	4	4	1	1
4	4	3	3	4	4	4	4	1	1
4	4	3	3	4	4	4	4	1	1
4	4	3	3	4	4	4	4	1	1
1	1	4	4	1	1	4	4	1	1
4	4	3	3	1	1	4	4	1	1
4	4	3	3	4	4	4	4	1	1
4	4	3	3	4	4	4	4	1	1
4	4	3	3	4	4	4	4	1	1
4	4	3	3	4	4	4	4	1	1
4	4	3	3	4	4	4	4	1	1
4	4	3	3	4	4	4	4	1	1
4	4	3	3	4	4	4	4	1	1
4	4	3	3	4	4	4	4	1	1
4	4	3	3	4	4	4	4	1	1
4	4	3	3	1	1	4	4	1	1
1	1	4	4	1	1	4	4	1	1
1	1	4	4	1	1	4	4	1	1
4	4	3	3	4	4	4	4	1	1
4	4	3	3	4	4	4	4	1	1
4	4	3	3	4	4	1	1	1	1
1	1	4	4	1	1	1	1	1	1
4	4	3	3	4	4	4	4	1	1
4	4	3	3	4	4	4	4	1	1
4	4	3	3	4	4	4	4	1	1
4	4	3	3	4	4	4	4	1	1
4	4	3	3	4	4	4	4	1	1
4	4	3	3	4	4	4	4	1	1
4	4	3	3	4	4	4	4	1	1
4	4	3	3	4	4	4	4	1	1
4	4	3	3	4	4	4	4	2	2
4	4	3	3	4	4	4	4	1	1
1	1	4	4	4	4	1	1	1	1
4	4	3	3	4	4	4	4	1	1
4	4	3	3	4	4	4	4	1	1
4	4	3	3	4	4	4	4	1	1
4	4	3	3	4	4	4	4	1	1
4	4	3	3	4	4	4	4	1	1

For Review Only

4	4	3	3	4	4	4	4	1	1
4	4	3	3	4	4	4	4	1	1
4	4	3	3	4	4	4	4	2	2
4	4	3	3	4	4	4	4	1	1
1	1	4	4	1	1	4	4	1	1
1	1	4	4	4	4	1	1	1	1
4	4	3	3	4	4	4	4	1	1
4	4	3	3	4	4	4	4	1	1
4	4	3	3	4	4	4	4	2	2
4	4	3	3	4	4	4	4	1	1
1	1	4	4	4	4	1	1	1	1
1	1	4	4	4	4	1	1	1	1
4	4	3	3	4	4	4	4	1	1
4	4	3	3	4	4	4	4	1	1
4	4	3	3	4	4	4	4	1	1
4	4	3	3	4	4	4	4	1	1
4	4	3	3	4	4	4	4	2	2
4	4	3	3	4	4	4	4	1	1
4	4	3	3	4	4	4	4	1	1
4	4	3	3	4	4	4	4	1	1
4	4	4	4	4	4	1	1	1	1
4	4	3	3	4	4	4	4	1	1
4	4	3	3	4	4	4	4	1	1
4	4	3	3	4	4	4	4	1	1
4	4	3	3	4	4	4	4	2	2
4	4	3	3	4	4	4	4	1	1
4	4	3	3	4	4	4	4	1	1
4	4	3	3	4	4	4	4	1	1
4	4	3	3	4	4	4	4	1	1
1	1	4	4	1	1	4	4	1	1
4	4	3	3	4	4	4	4	2	2
4	4	3	3	4	4	4	4	1	1
4	4	3	3	4	4	4	4	1	1
4	4	3	3	4	4	4	4	1	1
4	4	3	3	4	4	4	4	1	1
4	4	3	3	4	4	4	4	2	2
4	4	3	3	4	4	1	1	2	2
4	4	3	3	4	4	4	4	1	1
4	4	3	3	4	4	4	4	1	1
4	4	3	3	4	4	4	4	2	2
4	4	3	3	4	4	4	4	1	1
1	1	4	4	4	4	1	1	1	1
4	4	3	3	4	4	4	4	1	1
1	1	4	4	4	4	1	1	1	1
4	4	3	3	4	4	4	4	1	1
1	1	4	4	1	1	4	4	1	1
4	4	3	3	4	4	4	4	1	1
4	4	3	3	4	4	4	4	1	1
4	4	3	3	4	4	4	4	1	1
4	4	3	3	4	4	1	1	1	1
4	4	3	3	4	4	1	1	1	1
4	4	3	3	4	4	4	4	1	1
4	4	3	3	4	4	4	4	1	1
4	4	3	3	4	4	4	4	1	1
4	4	3	3	4	4	4	4	1	1

For Review Only

4	4	3	3	4	4	4	4	1	1
4	4	3	3	4	4	1	1	1	1
4	4	3	3	4	4	4	4	1	1
4	4	3	3	4	4	4	4	1	1
1	1	4	4	4	4	4	4	1	1
4	4	3	3	4	4	4	4	1	1
4	4	3	3	4	4	4	4	1	1
4	4	3	3	4	4	4	4	1	1
4	4	3	3	4	4	4	4	1	1
4	4	3	3	4	4	4	4	1	1
4	4	3	3	4	4	4	4	1	1
4	4	3	3	4	4	4	4	1	1
4	4	3	3	4	4	4	4	1	1
4	4	3	3	4	4	4	4	1	1
4	4	3	3	4	4	4	4	1	1
4	4	3	3	4	4	4	4	1	1
4	4	3	3	4	4	4	4	1	1
4	4	3	3	4	4	4	4	1	1
4	4	3	3	4	4	4	4	1	1
4	4	3	3	4	4	4	4	1	1
4	4	3	3	4	4	4	4	1	1
4	4	3	3	1	1	4	4	1	1
1	1	4	4	4	4	4	4	1	1
4	4	3	3	4	4	4	4	1	1
4	4	3	3	4	4	4	4	1	1
4	4	3	3	4	4	4	4	1	1
4	4	3	3	4	4	4	4	1	1
4	4	3	3	4	4	4	4	1	1
4	4	3	3	1	1	4	4	1	1
4	4	3	3	4	4	1	1	1	1
4	4	3	3	4	4	4	4	1	1
4	4	3	3	4	4	4	4	1	1
4	4	3	3	4	4	4	4	1	1
4	4	3	3	4	4	4	4	1	1
4	4	3	3	4	4	4	4	1	1
4	4	3	3	1	1	4	4	1	1
4	4	3	3	4	4	4	4	1	1
4	4	3	3	4	4	4	4	1	1
4	4	3	3	4	4	4	4	1	1
4	4	3	3	4	4	4	4	1	1
4	4	3	3	4	4	4	4	1	1
4	4	3	3	4	4	4	4	1	1
4	4	3	3	4	4	4	4	1	1
4	4	3	3	4	4	4	4	1	1
4	4	3	3	4	4	4	4	2	2
4	4	3	3	4	4	4	4	1	1
4	4	3	3	4	4	4	4	1	1
4	4	3	3	4	4	1	1	1	1
4	4	3	3	4	4	4	4	1	1
4	4	3	3	4	4	4	4	1	1
4	4	3	3	4	4	4	4	1	1
4	4	3	3	4	4	4	4	1	1
4	4	3	3	4	4	4	4	1	1
1	1	4	4	4	4	1	1	1	1
4	4	3	3	4	4	4	4	1	1

For Review Only

4	4	3	3	4	4	1	1	1	1
4	4	3	3	4	4	4	4	2	2
4	4	3	3	1	1	4	4	1	1
4	4	3	3	4	4	4	4	1	1
4	4	3	3	1	1	4	4	1	1
4	4	3	3	4	4	4	4	1	1
4	4	3	3	4	4	4	4	1	1
4	4	3	3	4	4	4	4	1	1
4	4	3	3	4	4	4	4	1	1
4	4	3	3	4	4	4	4	1	1
4	4	3	3	4	4	4	4	1	1
4	4	3	3	4	4	4	4	1	1
4	4	3	3	4	4	4	4	1	1
4	4	3	3	4	4	4	4	1	1
4	4	3	3	4	4	4	4	1	1
4	4	3	3	4	4	4	4	1	1
4	4	3	3	4	4	4	4	1	1
4	4	3	3	4	4	4	4	1	1
4	4	3	3	4	4	4	4	1	1
4	4	3	3	4	4	4	4	1	1
4	4	3	3	4	4	4	4	1	1
4	4	3	3	4	4	4	4	1	1
4	4	3	3	4	4	4	4	1	1
1	1	4	4	4	4	1	1	1	1
4	4	3	3	4	4	4	4	1	1
4	4	3	3	4	4	4	4	1	1
4	4	3	3	1	1	4	4	1	1
4	4	3	3	4	4	4	4	1	1
4	4	3	3	4	4	4	4	1	1
4	4	3	3	4	4	4	4	1	1
4	4	3	3	4	4	4	4	1	1
1	1	4	4	1	1	4	4	1	1
4	4	3	3	4	4	4	4	1	1
4	4	3	3	4	4	4	4	1	1
1	1	4	4	1	1	4	4	1	1
1	1	4	4	4	4	1	1	1	1
1	1	4	4	4	4	1	1	1	1
4	4	3	3	4	4	4	4	1	1
4	4	3	3	4	4	1	1	1	1
4	4	3	3	4	4	4	4	1	1
4	4	3	3	4	4	4	4	1	1
4	4	3	3	4	4	4	4	1	1
4	4	3	3	4	4	4	4	1	1
4	4	3	3	4	4	4	4	1	1
4	4	3	3	4	4	1	1	1	1
4	4	3	3	1	1	4	4	1	1
4	4	3	3	4	4	4	4	1	1
4	4	3	3	4	4	4	4	1	1
4	4	3	3	4	4	4	4	1	1
4	4	3	3	1	1	4	4	1	1
4	4	3	3	4	4	4	4	1	1
4	4	3	3	4	4	4	4	1	1
4	4	3	3	4	4	4	4	1	1
1	1	3	3	4	4	1	1	1	1
4	4	3	3	4	4	4	4	1	1

For Review Only

4	4	3	3	4	4	4	4	1	1
4	4	3	3	4	4	1	1	1	1
4	4	3	3	4	4	4	4	1	1
4	4	3	3	4	4	4	4	1	1
4	4	3	3	4	4	1	1	2	2
4	4	3	3	4	4	4	4	1	1
4	4	3	3	4	4	4	4	1	1
4	4	3	3	4	4	4	4	1	1
4	4	3	3	4	4	4	4	1	1
4	4	3	3	4	4	4	4	1	1
4	4	3	3	4	4	4	4	1	1
4	4	3	3	4	4	4	4	2	2
4	4	3	3	4	4	4	4	1	1
4	4	3	3	4	4	4	4	1	1
1	1	4	4	4	4	1	1	1	1
1	1	4	4	1	1	4	4	1	1
4	4	3	3	4	4	4	4	1	1
4	4	3	3	4	4	4	4	1	1
4	4	3	3	4	4	4	4	1	1
4	4	3	3	4	4	4	4	1	1
4	4	3	3	4	4	4	4	1	1
4	4	3	3	4	4	4	4	1	1
4	4	3	3	4	4	1	1	1	1
4	4	3	3	4	4	4	4	1	1
4	4	3	3	4	4	4	4	1	1
4	4	3	3	4	4	4	4	1	1
4	4	3	3	4	4	4	4	1	1
1	1	4	4	1	1	4	4	1	1
4	4	3	3	4	4	1	1	1	1
4	4	3	3	4	4	4	4	1	1
4	4	3	3	4	4	4	4	1	1
4	4	3	3	4	4	4	4	1	1
4	4	3	3	4	4	4	4	1	1
4	4	3	3	4	4	1	1	1	1
4	4	3	3	4	4	4	4	1	1
4	4	3	3	4	4	4	4	1	1
4	4	3	3	4	4	4	4	1	1
1	1	4	4	4	4	1	1	1	1
1	1	4	4	4	4	1	1	1	1
4	4	3	3	4	4	4	4	1	1
1	1	4	4	1	1	4	4	1	1
4	4	3	3	4	4	4	4	2	2
4	4	3	3	4	4	4	4	1	1
4	4	3	3	4	4	4	4	1	1
4	4	3	3	4	4	1	1	1	1
4	4	3	3	4	4	4	4	1	1
4	4	3	3	4	4	1	1	1	1
4	4	3	3	4	4	4	4	1	1
4	4	3	3	4	4	4	4	1	1
4	4	3	3	4	4	4	4	1	1
1	1	4	4	1	1	4	4	2	2
4	4	3	3	4	4	4	4	1	1
4	4	3	3	4	4	4	4	1	1
1	1	4	4	1	1	4	4	1	1
4	4	3	3	4	4	4	4	1	1

For Review Only

1	1	4	4	1	1	4	4	1	1
4	4	3	3	4	4	4	4	1	1

For Review Only

SNP777	SNP777	SNP778	SNP778	SNP779	SNP779	SNP780	SNP780	SNP781	SNP781
2	2	1	1	3	3	2	2	2	2
2	2	1	1	2	2	3	3	2	2
2	2	4	4	3	3	3	3	2	2
3	3	1	1	2	2	2	2	2	2
2	2	1	1	3	3	2	2	2	2
2	2	4	4	3	3	2	2	2	2
2	2	4	4	3	3	2	2	3	3
2	2	1	1	3	3	2	2	2	2
2	2	1	1	3	3	2	2	2	2
2	2	1	1	3	3	3	3	2	2
2	2	1	1	2	2	2	2	3	3
2	2	4	4	3	3	3	3	2	2
2	2	1	1	2	2	2	2	2	2
2	2	1	1	2	2	2	2	2	2
2	2	1	1	2	2	2	2	2	2
2	2	1	1	2	2	3	3	2	2
2	2	1	1	2	2	2	2	2	2
2	2	1	1	2	2	3	3	2	2
2	2	1	1	2	2	2	2	2	2
2	2	1	1	2	2	3	3	2	2
2	2	1	1	2	2	3	3	2	2
2	2	1	1	3	3	2	2	2	2
2	2	1	1	2	2	2	2	3	3
2	2	1	1	2	2	2	2	2	2
2	2	1	1	2	2	2	2	2	2
2	2	1	1	2	2	3	3	2	2
2	2	1	1	2	2	3	3	2	2
2	2	1	1	2	2	2	2	2	2
2	2	1	1	3	3	3	3	2	2
2	2	1	1	3	3	2	2	2	2
2	2	1	1	2	2	3	3	2	2
2	2	1	1	3	3	3	3	3	3
2	2	1	1	2	2	3	3	3	3
2	2	1	1	2	2	2	2	2	2
2	2	1	1	2	2	2	2	2	2
2	2	1	1	2	2	2	2	2	2
2	2	1	1	2	2	2	2	2	2
2	2	1	1	2	2	3	3	3	3
2	2	1	1	2	2	2	2	3	3
2	2	1	1	2	2	2	2	2	2
2	2	1	1	3	3	2	2	2	2
2	2	1	1	2	2	3	3	2	2
2	2	1	1	3	3	2	2	2	2
2	2	1	1	3	3	2	2	2	2
2	2	1	1	3	3	2	2	2	2
2	2	1	1	2	2	3	3	2	2
2	2	1	1	2	2	3	3	2	2
2	2	1	1	2	2	2	2	2	2
2	2	1	1	3	3	2	2	2	2
2	2	1	1	2	2	3	3	2	2
2	2	1	1	2	2	3	3	2	2
2	2	4	4	3	3	2	2	2	2

For Review Only

2	2	1	1	2	2	2	2	2	2
2	2	1	1	2	2	3	3	3	3
2	2	1	1	2	2	2	2	2	2
2	2	1	1	2	2	2	2	2	2
2	2	1	1	2	2	2	2	2	2
2	2	4	4	3	3	3	3	2	2
2	2	1	1	2	2	3	3	2	2
2	2	1	1	3	3	2	2	2	2
2	2	1	1	3	3	2	2	2	2
2	2	1	1	2	2	2	2	2	2
2	2	1	1	2	2	3	3	2	2
2	2	4	4	3	3	3	3	2	2
2	2	1	1	2	2	2	2	2	2
2	2	1	1	3	3	3	3	2	2
2	2	1	1	2	2	2	2	2	2
2	2	1	1	2	2	2	2	2	2
2	2	1	1	2	2	3	3	2	2
2	2	1	1	2	2	3	3	2	2
2	2	1	1	2	2	2	2	2	2
2	2	1	1	2	2	2	2	2	2
2	2	4	4	2	2	2	2	2	2
2	2	1	1	2	2	2	2	2	2
2	2	1	1	2	2	3	3	2	2
2	2	4	4	3	3	3	3	3	3
2	2	1	1	3	3	2	2	2	2
2	2	4	4	3	3	2	2	2	2
2	2	1	1	2	2	2	2	3	3
2	2	1	1	3	3	2	2	2	2
2	2	1	1	2	2	3	3	2	2
2	2	1	1	2	2	2	2	2	2
2	2	1	1	3	3	2	2	2	2
2	2	1	1	2	2	2	2	2	2
2	2	1	1	2	2	2	2	3	3
2	2	1	1	2	2	2	2	2	2
2	2	1	1	2	2	2	2	2	2
2	2	1	1	3	3	2	2	2	2
2	2	1	1	3	3	2	2	2	2
2	2	1	1	2	2	2	2	2	2
2	2	1	1	2	2	2	2	2	2
2	2	1	1	3	3	2	2	3	3
2	2	1	1	2	2	2	2	2	2
2	2	4	4	2	2	2	2	2	2
2	2	1	1	3	3	2	2	3	3
2	2	1	1	2	2	3	3	2	2
2	2	1	1	2	2	2	2	2	2
2	2	1	1	2	2	3	3	2	2
2	2	1	1	2	2	2	2	2	2
2	2	4	4	3	3	2	2	2	2
2	2	4	4	3	3	3	3	2	2
2	2	1	1	2	2	2	2	2	2
2	2	1	1	2	2	3	3	2	2
2	2	1	1	2	2	2	2	3	3
2	2	1	1	3	3	2	2	2	2
2	2	1	1	2	2	2	2	2	2
2	2	1	1	3	3	2	2	3	3

For Review Only

2	2	1	1	2	2	2	2	2	2
2	2	1	1	2	2	2	2	3	3
2	2	1	1	2	2	2	2	2	2
2	2	1	1	2	2	3	3	2	2
2	2	1	1	2	2	3	3	2	2
2	2	1	1	2	2	3	3	2	2
2	2	1	1	2	2	3	3	2	2
2	2	1	1	2	2	2	2	3	3
2	2	1	1	2	2	2	2	3	3
2	2	1	1	2	2	2	2	2	2
2	2	1	1	2	2	2	2	2	2
2	2	1	1	2	2	2	2	2	2
2	2	1	1	3	3	2	2	3	3
2	2	1	1	3	3	2	2	2	2
2	2	1	1	2	2	2	2	2	2
2	2	1	1	2	2	2	2	2	2
2	2	1	1	3	3	2	2	2	2
2	2	1	1	3	3	2	2	2	2
2	2	1	1	2	2	3	3	2	2
2	2	1	1	3	3	2	2	2	2
2	2	1	1	2	2	2	2	2	2
2	2	1	1	2	2	2	2	2	2
2	2	4	4	2	2	2	2	2	2
2	2	1	1	2	2	2	2	3	3
2	2	1	1	2	2	2	2	2	2
2	2	1	1	2	2	2	2	3	3
2	2	1	1	2	2	2	2	3	3
2	2	1	1	2	2	2	2	2	2
2	2	4	4	3	3	2	2	2	2
2	2	1	1	2	2	3	3	3	3
2	2	1	1	2	2	2	2	2	2
2	2	1	1	2	2	2	2	2	2
2	2	1	1	2	2	2	2	2	2
2	2	1	1	2	2	2	2	2	2
2	2	1	1	2	2	2	2	2	2
2	2	1	1	2	2	2	2	2	2
2	2	1	1	2	2	3	3	2	2
2	2	4	4	3	3	2	2	2	2
3	3	1	1	3	3	2	2	3	3
2	2	1	1	2	2	2	2	2	2
2	2	1	1	2	2	2	2	2	2
2	2	1	1	2	2	2	2	2	2
2	2	1	1	2	2	2	2	2	2
2	2	1	1	2	2	2	2	2	2
2	2	1	1	2	2	2	2	2	2
2	2	1	1	3	3	2	2	2	2
2	2	1	1	2	2	3	3	2	2
2	2	1	1	2	2	3	3	2	2
2	2	1	1	2	2	2	2	2	2
2	2	1	1	2	2	3	3	2	2
2	2	1	1	2	2	3	3	2	2
2	2	1	1	3	3	3	3	2	2
2	2	1	1	2	2	2	2	2	2
2	2	4	4	3	3	3	3	2	2
3	3	1	1	2	2	2	2	2	2

For Review Only

2	2	1	1	2	2	3	3	2	2
2	2	1	1	3	3	2	2	3	3
2	2	1	1	2	2	2	2	3	3
2	2	1	1	2	2	2	2	3	3
2	2	1	1	2	2	2	2	2	2
2	2	1	1	2	2	3	3	2	2
2	2	1	1	2	2	2	2	2	2
2	2	1	1	2	2	2	2	2	2
2	2	1	1	2	2	2	2	2	2
2	2	1	1	2	2	2	2	3	3
2	2	1	1	2	2	3	3	2	2
2	2	1	1	2	2	2	2	2	2
2	2	1	1	3	3	2	2	2	2
2	2	1	1	2	2	2	2	2	2
2	2	1	1	2	2	2	2	2	2
2	2	1	1	2	2	2	2	3	3
2	2	1	1	2	2	2	2	2	2
2	2	1	1	2	2	2	2	2	2
2	2	1	1	2	2	2	2	2	2
2	2	1	1	2	2	3	3	2	2
2	2	1	1	2	2	2	2	2	2
2	2	1	1	2	2	2	2	2	2
2	2	1	1	2	2	2	2	2	2
2	2	4	4	3	3	2	2	2	2
2	2	1	1	2	2	2	2	2	2
2	2	1	1	2	2	2	2	2	2
2	2	4	4	3	3	3	3	2	2
2	2	1	1	2	2	2	2	2	2
2	2	1	1	3	3	3	3	2	2
2	2	1	1	2	2	2	2	2	2
2	2	1	1	2	2	2	2	2	2
2	2	1	1	2	2	3	3	2	2
2	2	1	1	2	2	3	3	3	3
2	2	1	1	2	2	2	2	2	2
2	2	1	1	2	2	2	2	2	2
2	2	4	4	3	3	3	3	2	2
2	2	4	4	2	2	3	3	2	2
2	2	1	1	3	3	2	2	2	2
2	2	1	1	2	2	3	3	2	2
2	2	4	4	3	3	3	3	2	2
2	2	4	4	3	3	3	3	2	2
2	2	1	1	2	2	3	3	2	2
2	2	1	1	2	2	2	2	2	2
2	2	1	1	2	2	3	3	2	2
2	2	1	1	2	2	3	3	2	2
2	2	1	1	2	2	2	2	2	2
2	2	1	1	2	2	2	2	3	3
2	2	1	1	2	2	2	2	3	3
2	2	1	1	2	2	2	2	2	2
2	2	1	1	2	2	2	2	2	2
2	2	1	1	3	3	2	2	2	2
2	2	1	1	3	3	2	2	2	2
2	2	1	1	2	2	2	2	2	2
2	2	1	1	2	2	3	3	2	2
2	2	1	1	2	2	2	2	3	3

For Review Only

2	2	1	1	2	2	2	2	2	2
2	2	1	1	2	2	2	2	2	2
2	2	1	1	2	2	2	2	2	2
2	2	1	1	2	2	2	2	2	2
2	2	1	1	3	3	3	3	2	2
2	2	1	1	2	2	2	2	2	2
2	2	1	1	2	2	3	3	3	3
2	2	1	1	3	3	2	2	3	3
2	2	1	1	2	2	2	2	2	2
2	2	1	1	2	2	2	2	3	3
2	2	1	1	2	2	2	2	2	2
2	2	1	1	2	2	2	2	2	2
2	2	1	1	2	2	2	2	2	2
2	2	1	1	2	2	2	2	2	2
2	2	4	4	3	3	3	3	2	2
2	2	1	1	2	2	2	2	2	2
2	2	1	1	2	2	2	2	3	3
2	2	1	1	2	2	2	2	2	2
2	2	1	1	2	2	2	2	2	2
2	2	1	1	3	3	3	3	3	3
2	2	1	1	2	2	3	3	2	2
2	2	1	1	2	2	3	3	2	2
2	2	1	1	2	2	2	2	2	2
2	2	1	1	2	2	2	2	3	3
2	2	1	1	3	3	2	2	2	2
2	2	1	1	2	2	2	2	2	2
2	2	1	1	2	2	2	2	3	3
2	2	4	4	3	3	3	3	2	2
2	2	1	1	2	2	2	2	2	2
2	2	4	4	3	3	2	2	2	2
2	2	1	1	2	2	2	2	3	3
2	2	1	1	2	2	2	2	2	2
2	2	1	1	2	2	2	2	2	2
2	2	1	1	2	2	2	2	2	2
2	2	1	1	2	2	2	2	2	2
2	2	1	1	2	2	2	2	3	3
2	2	1	1	2	2	2	2	2	2
2	2	4	4	3	3	3	3	2	2
2	2	1	1	2	2	2	2	2	2
2	2	1	1	2	2	2	2	3	3
2	2	1	1	2	2	2	2	2	2
2	2	1	1	2	2	3	3	2	2
2	2	1	1	2	2	2	2	2	2
2	2	1	1	2	2	2	2	2	2
2	2	1	1	2	2	2	2	2	2
2	2	1	1	2	2	2	2	2	2
2	2	1	1	2	2	3	3	3	3
2	2	1	1	2	2	2	2	2	2
2	2	1	1	2	2	2	2	2	2
2	2	1	1	2	2	2	2	3	3
2	2	1	1	2	2	2	2	3	3
2	2	1	1	2	2	2	2	3	3
2	2	4	4	3	3	3	3	2	2
2	2	4	4	3	3	3	3	2	2
2	2	1	1	2	2	3	3	2	2

For Review Only

2	2	1	1	2	2	2	2	3	3
2	2	4	4	3	3	2	2	2	2

For Review Only

SNP782	SNP782	SNP783	SNP783	SNP784	SNP784	SNP785	SNP785	SNP786	SNP786
1	1	1	1	3	3	3	3	3	3
3	3	1	1	2	2	3	3	4	4
1	1	1	1	3	3	2	2	4	4
1	1	1	1	3	3	3	3	4	4
1	1	1	1	3	3	3	3	3	3
1	1	1	1	3	3	2	2	3	3
1	1	1	1	3	3	3	3	4	4
3	3	1	1	2	2	2	2	3	3
1	1	1	1	3	3	3	3	3	3
3	3	1	1	2	2	3	3	3	3
3	3	1	1	3	3	3	3	3	3
1	1	1	1	3	3	2	2	4	4
1	1	1	1	3	3	2	2	3	3
1	1	1	1	3	3	2	2	3	3
1	1	1	1	3	3	2	2	3	3
1	1	1	1	3	3	3	3	4	4
3	3	1	1	2	2	3	3	4	4
1	1	1	1	3	3	2	2	4	4
1	1	1	1	3	3	3	3	3	3
3	3	1	1	3	3	3	3	4	4
3	3	1	1	2	2	3	3	4	4
1	1	2	2	3	3	3	3	3	3
3	3	2	2	2	2	3	3	3	3
1	1	1	1	3	3	3	3	3	3
1	1	1	1	3	3	3	3	4	4
1	1	1	1	3	3	2	2	4	4
1	1	1	1	3	3	2	2	4	4
1	1	1	1	3	3	2	2	4	4
1	1	2	2	3	3	3	3	4	4
3	3	1	1	2	2	3	3	3	3
1	1	1	1	3	3	2	2	4	4
1	1	1	1	3	3	3	3	4	4
1	1	1	1	3	3	2	2	4	4
1	1	1	1	3	3	2	2	3	3
1	1	1	1	3	3	2	2	4	4
1	1	1	1	3	3	2	2	3	3
1	1	1	1	3	3	2	2	3	3
3	3	1	1	2	2	3	3	4	4
3	3	1	1	2	2	3	3	3	3
1	1	1	1	3	3	2	2	4	4
3	3	2	2	2	2	3	3	3	3
3	3	2	2	3	3	2	2	4	4
1	1	1	1	3	3	2	2	3	3
3	3	1	1	3	3	2	2	3	3
1	1	1	1	3	3	3	3	3	3
1	1	1	1	3	3	2	2	4	4
1	1	1	1	3	3	2	2	4	4
1	1	1	1	3	3	3	3	4	4
3	3	1	1	2	2	3	3	3	3
1	1	1	1	3	3	3	3	4	4
1	1	1	1	3	3	2	2	4	4
1	1	1	1	3	3	2	2	3	3

For Review Only

1	1	1	1	3	3	3	3	3	3
1	1	1	1	3	3	2	2	4	4
1	1	1	1	3	3	3	3	3	3
3	3	1	1	2	2	3	3	3	3
1	1	1	1	3	3	3	3	3	3
1	1	1	1	3	3	2	2	4	4
1	1	1	1	3	3	2	2	4	4
3	3	1	1	2	2	3	3	3	3
1	1	1	1	3	3	3	3	3	3
1	1	1	1	3	3	2	2	4	4
1	1	1	1	3	3	2	2	4	4
1	1	1	1	3	3	2	2	4	4
1	1	1	1	3	3	3	3	3	3
1	1	2	2	3	3	3	3	4	4
1	1	1	1	3	3	2	2	3	3
1	1	2	2	3	3	2	2	3	3
3	3	1	1	3	3	3	3	4	4
1	1	1	1	3	3	2	2	4	4
1	1	1	1	3	3	3	3	3	3
1	1	1	1	3	3	3	3	3	3
1	1	1	1	3	3	2	2	4	4
3	3	1	1	3	3	3	3	4	4
1	1	2	2	3	3	2	2	4	4
1	1	1	1	3	3	2	2	4	4
1	1	1	1	3	3	3	3	3	3
1	1	1	1	3	3	3	3	3	3
3	3	1	1	2	2	3	3	4	4
3	3	1	1	2	2	3	3	3	3
1	1	1	1	3	3	2	2	4	4
1	1	1	1	3	3	3	3	4	4
1	1	1	1	3	3	3	3	3	3
1	1	1	1	3	3	2	2	3	3
3	3	2	2	3	3	3	3	3	3
1	1	1	1	3	3	3	3	3	3
1	1	1	1	3	3	2	2	3	3
1	1	1	1	3	3	3	3	3	3
1	1	1	1	3	3	3	3	3	3
3	3	1	1	2	2	3	3	3	3
1	1	1	1	3	3	3	3	3	3
1	1	1	1	3	3	3	3	3	3
1	1	1	1	3	3	2	2	3	3
1	1	1	1	3	3	3	3	4	4
1	1	1	1	3	3	3	3	3	3
1	1	1	1	3	3	2	2	4	4
1	1	1	1	3	3	3	3	3	3
1	1	1	1	3	3	2	2	4	4
1	1	1	1	3	3	2	2	3	3
1	1	1	1	3	3	2	2	4	4
1	1	1	1	3	3	2	2	4	4
1	1	1	1	3	3	2	2	3	3
1	1	1	1	3	3	2	2	4	4
3	3	1	1	3	3	3	3	3	3
3	3	1	1	2	2	3	3	3	3
1	1	1	1	3	3	2	2	3	3
1	1	1	1	3	3	2	2	3	3

For Review Only

1	1	1	1	3	3	2	2	4	4
1	1	1	1	3	3	3	3	4	4
1	1	1	1	3	3	2	2	3	3
1	1	1	1	3	3	2	2	4	4
1	1	1	1	3	3	2	2	4	4
1	1	1	1	3	3	2	2	4	4
1	1	1	1	3	3	3	3	4	4
1	1	1	1	3	3	3	3	4	4
1	1	1	1	3	3	2	2	3	3
1	1	1	1	3	3	2	2	4	4
1	1	1	1	3	3	2	2	3	3
1	1	1	1	3	3	2	2	4	4
1	1	1	1	3	3	3	3	3	3
1	1	1	1	3	3	2	2	3	3
1	1	1	1	3	3	2	2	3	3
1	1	1	1	3	3	2	2	4	4
1	1	1	1	3	3	2	2	4	4
1	1	1	1	3	3	2	2	4	4
1	1	1	1	3	3	2	2	4	4
1	1	2	2	3	3	2	2	3	3
1	1	1	1	3	3	2	2	4	4
1	1	1	1	3	3	3	3	4	4
1	1	1	1	3	3	3	3	4	4
3	3	2	2	2	2	3	3	3	3
3	3	1	1	3	3	2	2	4	4
1	1	2	2	3	3	2	2	3	3
1	1	1	1	3	3	2	2	4	4
1	1	1	1	3	3	2	2	3	3
1	1	1	1	3	3	3	3	3	3
3	3	1	1	2	2	3	3	4	4
3	3	2	2	2	2	3	3	3	3
1	1	1	1	3	3	3	3	3	3
1	1	1	1	3	3	2	2	3	3
1	1	1	1	3	3	2	2	4	4
1	1	1	1	3	3	2	2	3	3
1	1	1	1	3	3	2	2	4	4
1	1	1	1	3	3	2	2	4	4
1	1	1	1	3	3	3	3	3	3
1	1	1	1	3	3	2	2	4	4
3	3	1	1	2	2	3	3	3	3
1	1	2	2	3	3	2	2	4	4
1	1	1	1	3	3	2	2	3	3
1	1	1	1	3	3	2	2	3	3
1	1	1	1	2	2	3	3	3	3
1	1	1	1	3	3	2	2	3	3
1	1	1	1	3	3	3	3	3	3
1	1	1	1	3	3	2	2	4	4
1	1	1	1	3	3	2	2	4	4
1	1	1	1	3	3	2	2	3	3
1	1	1	1	3	3	2	2	4	4
1	1	1	1	3	3	2	2	4	4
3	3	1	1	2	2	3	3	4	4
1	1	1	1	3	3	2	2	3	3
1	1	1	1	3	3	2	2	4	4
1	1	1	1	3	3	2	2	4	4

For Review Only

1	1	1	1	3	3	2	2	4	4
1	1	2	2	3	3	3	3	3	3
1	1	1	1	3	3	2	2	3	3
1	1	1	1	3	3	3	3	3	3
1	1	1	1	3	3	2	2	4	4
3	3	1	1	2	2	3	3	4	4
1	1	1	1	3	3	2	2	3	3
1	1	1	1	3	3	2	2	4	4
1	1	1	1	3	3	3	3	3	3
1	1	1	1	3	3	3	3	3	3
1	1	1	1	3	3	2	2	4	4
1	1	1	1	3	3	2	2	3	3
1	1	1	1	3	3	2	2	4	4
1	1	1	1	2	2	2	2	3	3
1	1	1	1	3	3	3	3	3	3
3	3	1	1	2	2	3	3	3	3
1	1	1	1	3	3	2	2	3	3
1	1	1	1	3	3	3	3	4	4
1	1	1	1	3	3	2	2	3	3
1	1	1	1	3	3	3	3	4	4
1	1	1	1	3	3	2	2	4	4
1	1	1	1	3	3	3	3	3	3
3	3	1	1	3	3	3	3	3	3
1	1	1	1	3	3	2	2	4	4
1	1	2	2	3	3	3	3	3	3
1	1	1	1	3	3	2	2	3	3
1	1	1	1	3	3	2	2	4	4
1	1	1	1	3	3	2	2	4	4
1	1	1	1	3	3	3	3	4	4
1	1	1	1	3	3	3	3	3	3
1	1	1	1	3	3	2	2	3	3
1	1	1	1	3	3	3	3	4	4
1	1	1	1	3	3	3	3	4	4
1	1	1	1	3	3	3	3	3	3
1	1	1	1	3	3	2	2	4	4
1	1	1	1	3	3	2	2	4	4
1	1	1	1	3	3	2	2	4	4
1	1	1	1	3	3	3	3	3	3
1	1	1	1	3	3	2	2	4	4
1	1	1	1	3	3	3	3	4	4
1	1	1	1	3	3	2	2	4	4
1	1	1	1	3	3	2	2	4	4
3	3	1	1	2	2	3	3	3	3
1	1	1	1	3	3	2	2	4	4
1	1	1	1	3	3	2	2	4	4
1	1	1	1	3	3	3	3	3	3
3	3	1	1	2	2	3	3	3	3
1	1	1	1	3	3	3	3	3	3
1	1	1	1	3	3	2	2	3	3
1	1	1	1	3	3	2	2	3	3
1	1	1	1	3	3	3	3	3	3
1	1	2	2	3	3	2	2	3	3
3	3	2	2	3	3	3	3	3	3
1	1	1	1	3	3	2	2	4	4
3	3	1	1	2	2	2	2	3	3

For Review Only

1	1	1	1	3	3	3	3	3	3
1	1	1	1	3	3	3	3	4	4
1	1	1	1	3	3	2	2	3	3
1	1	1	1	3	3	2	2	3	3
1	1	1	1	3	3	3	3	4	4
1	1	1	1	3	3	3	3	4	4
1	1	1	1	3	3	2	2	4	4
3	3	1	1	2	2	3	3	3	3
1	1	1	1	2	2	3	3	3	3
3	3	1	1	3	3	3	3	3	3
1	1	1	1	3	3	2	2	4	4
1	1	1	1	3	3	3	3	3	3
1	1	1	1	3	3	3	3	3	3
1	1	1	1	3	3	2	2	3	3
1	1	1	1	3	3	3	3	4	4
1	1	1	1	3	3	2	2	3	3
3	3	1	1	2	2	3	3	3	3
3	3	1	1	3	3	3	3	3	3
1	1	1	1	3	3	3	3	3	3
3	3	1	1	2	2	3	3	4	4
1	1	1	1	3	3	2	2	4	4
1	1	1	1	3	3	3	3	4	4
1	1	1	1	3	3	3	3	3	3
1	1	1	1	3	3	2	2	3	3
3	3	1	1	2	2	3	3	3	3
3	3	1	1	3	3	2	2	3	3
3	3	2	2	2	2	3	3	3	3
1	1	1	1	3	3	2	2	4	4
1	1	1	1	3	3	3	3	4	4
1	1	1	1	3	3	3	3	3	3
1	1	2	2	3	3	2	2	3	3
1	1	1	1	3	3	2	2	4	4
3	3	2	2	2	2	2	2	3	3
1	1	1	1	3	3	2	2	3	3
3	3	1	1	3	3	3	3	3	3
1	1	1	1	3	3	3	3	3	3
1	1	1	1	3	3	3	3	3	3
1	1	1	1	3	3	2	2	4	4
1	1	1	1	3	3	2	2	3	3
1	1	1	1	3	3	2	2	3	3
1	1	1	1	3	3	3	3	3	3
1	1	1	1	3	3	2	2	4	4
1	1	1	1	3	3	2	2	3	3
1	1	1	1	3	3	3	3	3	3
1	1	1	1	3	3	2	2	4	4
3	3	1	1	3	3	3	3	3	3
1	1	1	1	3	3	2	2	4	4
1	1	1	1	3	3	2	2	3	3
1	1	1	1	3	3	2	2	3	3
1	1	2	2	3	3	3	3	3	3
1	1	1	1	3	3	3	3	3	3
3	3	1	1	2	2	3	3	3	3
1	1	1	1	3	3	2	2	4	4
1	1	1	1	3	3	3	3	4	4
1	1	1	1	3	3	2	2	4	4

For Review Only

1	1	1	1	3	3	2	2	3	3
1	1	1	1	3	3	3	3	3	3

For Review Only

SNP787	SNP787	SNP788	SNP788	SNP789	SNP789	SNP790	SNP790	SNP791	SNP791
4	4	3	3	1	1	4	4	4	4
1	1	3	3	1	1	2	2	2	2
1	1	3	3	1	1	4	4	4	4
1	1	3	3	1	1	4	4	4	4
4	4	3	3	1	1	4	4	4	4
4	4	3	3	1	1	4	4	4	4
1	1	3	3	1	1	2	2	4	4
4	4	3	3	1	1	4	4	2	2
4	4	3	3	1	1	4	4	2	2
4	4	3	3	1	1	4	4	4	4
4	4	3	3	4	4	4	4	4	4
1	1	3	3	1	1	4	4	4	4
1	1	3	3	1	1	4	4	4	4
4	4	3	3	1	1	2	2	4	4
1	1	3	3	1	1	4	4	4	4
1	1	3	3	1	1	4	4	4	4
1	1	3	3	1	1	4	4	4	4
1	1	3	3	4	4	4	4	2	2
4	4	3	3	1	1	4	4	4	4
1	1	3	3	1	1	4	4	4	4
1	1	3	3	1	1	4	4	4	4
4	4	3	3	1	1	4	4	4	4
4	4	3	3	4	4	2	2	4	4
4	4	3	3	1	1	4	4	4	4
1	1	3	3	1	1	4	4	4	4
1	1	3	3	1	1	2	2	4	4
1	1	3	3	1	1	4	4	4	4
1	1	3	3	1	1	4	4	4	4
1	1	3	3	1	1	4	4	2	2
4	4	3	3	1	1	4	4	4	4
1	1	3	3	1	1	4	4	4	4
1	1	3	3	1	1	2	2	4	4
1	1	3	3	1	1	2	2	4	4
4	4	3	3	4	4	4	4	4	4
1	1	3	3	1	1	2	2	2	2
4	4	4	4	1	1	2	2	4	4
4	4	3	3	1	1	2	2	4	4
1	1	3	3	4	4	4	4	2	2
4	4	4	4	4	4	4	4	2	2
1	1	4	4	4	4	2	2	2	2
4	4	3	3	1	1	4	4	4	4
1	1	3	3	1	1	4	4	2	2
4	4	3	3	1	1	4	4	4	4
4	4	3	3	1	1	2	2	4	4
4	4	3	3	1	1	4	4	2	2
1	1	3	3	1	1	2	2	4	4
1	1	3	3	1	1	4	4	4	4
1	1	4	4	1	1	4	4	4	4
4	4	3	3	1	1	4	4	4	4
4	4	3	3	1	1	2	2	4	4
1	1	3	3	1	1	4	4	4	4
4	4	4	4	1	1	4	4	4	4

For Review Only

4	4	3	3	1	1	4	4	4	4
1	1	3	3	1	1	2	2	4	4
4	4	3	3	1	1	4	4	2	2
4	4	3	3	1	1	4	4	4	4
4	4	3	3	1	1	4	4	4	4
1	1	3	3	1	1	4	4	4	4
1	1	3	3	1	1	4	4	4	4
4	4	4	4	4	4	2	2	4	4
4	4	3	3	1	1	2	2	4	4
1	1	3	3	1	1	4	4	4	4
1	1	3	3	1	1	4	4	4	4
1	1	3	3	1	1	4	4	4	4
4	4	3	3	1	1	4	4	4	4
1	1	3	3	1	1	4	4	2	2
1	1	3	3	1	1	4	4	2	2
4	4	3	3	1	1	4	4	4	4
1	1	3	3	1	1	4	4	4	4
1	1	3	3	1	1	4	4	4	4
4	4	3	3	1	1	4	4	4	4
4	4	3	3	1	1	4	4	4	4
1	1	3	3	1	1	4	4	4	4
1	1	4	4	1	1	2	2	2	2
1	1	3	3	1	1	4	4	2	2
1	1	3	3	1	1	2	2	4	4
4	4	3	3	1	1	4	4	2	2
4	4	3	3	1	1	2	2	4	4
1	1	3	3	1	1	4	4	2	2
4	4	4	4	4	4	2	2	4	4
1	1	3	3	1	1	2	2	4	4
1	1	3	3	1	1	2	2	4	4
4	4	3	3	4	4	4	4	2	2
4	4	3	3	1	1	2	2	4	4
4	4	4	4	4	4	2	2	2	2
4	4	3	3	1	1	4	4	4	4
4	4	3	3	1	1	2	2	4	4
4	4	3	3	1	1	4	4	2	2
4	4	3	3	4	4	2	2	2	2
4	4	3	3	1	1	4	4	4	4
4	4	3	3	1	1	4	4	4	4
4	4	3	3	1	1	4	4	2	2
4	4	3	3	1	1	4	4	4	4
1	1	4	4	1	1	2	2	4	4
4	4	3	3	1	1	4	4	4	4
1	1	3	3	1	1	4	4	4	4
4	4	3	3	1	1	4	4	4	4
1	1	3	3	1	1	2	2	4	4
4	4	3	3	4	4	2	2	4	4
1	1	3	3	1	1	4	4	4	4
1	1	3	3	1	1	2	2	4	4
4	4	3	3	1	1	4	4	2	2
1	1	3	3	1	1	4	4	4	4
4	4	4	4	4	4	4	4	2	2
4	4	4	4	4	4	2	2	2	2
1	1	3	3	1	1	4	4	4	4
4	4	3	3	1	1	4	4	2	2

For Review Only

1	1	4	4	1	1	4	4	4	4
1	1	3	3	4	4	4	4	4	4
4	4	4	4	1	1	2	2	4	4
1	1	3	3	1	1	2	2	2	2
1	1	3	3	1	1	4	4	4	4
1	1	3	3	1	1	2	2	4	4
1	1	3	3	1	1	4	4	4	4
1	1	3	3	1	1	4	4	4	4
4	4	3	3	1	1	4	4	4	4
1	1	3	3	1	1	4	4	4	4
4	4	3	3	4	4	4	4	4	4
1	1	3	3	1	1	4	4	4	4
4	4	3	3	1	1	4	4	2	2
4	4	3	3	1	1	4	4	4	4
4	4	3	3	1	1	2	2	4	4
1	1	3	3	1	1	4	4	4	4
1	1	3	3	1	1	4	4	4	4
1	1	3	3	1	1	4	4	4	4
1	1	3	3	1	1	2	2	4	4
4	4	3	3	4	4	2	2	4	4
1	1	3	3	1	1	4	4	2	2
1	1	3	3	1	1	4	4	4	4
1	1	3	3	1	1	4	4	4	4
4	4	4	4	4	4	2	2	4	4
1	1	3	3	1	1	4	4	2	2
4	4	3	3	4	4	4	4	4	4
1	1	3	3	1	1	2	2	4	4
4	4	3	3	1	1	2	2	4	4
4	4	3	3	1	1	4	4	4	4
1	1	3	3	4	4	2	2	4	4
4	4	4	4	4	4	2	2	4	4
1	1	3	3	1	1	4	4	2	2
4	4	3	3	1	1	4	4	4	4
1	1	3	3	1	1	4	4	4	4
4	4	3	3	4	4	4	4	4	4
1	1	3	3	1	1	4	4	2	2
1	1	3	3	4	4	2	2	4	4
4	4	3	3	1	1	4	4	4	4
1	1	3	3	1	1	2	2	4	4
4	4	4	4	4	4	4	4	4	4
1	1	4	4	1	1	4	4	2	2
4	4	3	3	4	4	2	2	4	4
4	4	4	4	1	1	4	4	4	4
4	4	3	3	1	1	4	4	4	4
4	4	4	4	1	1	2	2	4	4
4	4	4	4	1	1	4	4	4	4
1	1	3	3	1	1	4	4	4	4
1	1	3	3	1	1	4	4	4	4
4	4	3	3	1	1	4	4	4	4
1	1	3	3	1	1	2	2	4	4
1	1	3	3	1	1	4	4	4	4
1	1	3	3	4	4	2	2	2	2
4	4	3	3	1	1	4	4	4	4
1	1	3	3	1	1	2	2	4	4
1	1	3	3	1	1	2	2	4	4

For Review Only

1	1	3	3	1	1	4	4	4	4
4	4	4	4	4	4	4	4	4	4
4	4	3	3	1	1	4	4	2	2
4	4	3	3	4	4	4	4	4	4
1	1	3	3	1	1	4	4	4	4
1	1	4	4	1	1	2	2	4	4
4	4	3	3	4	4	4	4	2	2
1	1	3	3	1	1	4	4	4	4
4	4	3	3	1	1	2	2	4	4
4	4	3	3	1	1	2	2	4	4
1	1	3	3	1	1	4	4	4	4
4	4	3	3	1	1	4	4	4	4
1	1	3	3	1	1	4	4	4	4
4	4	3	3	1	1	2	2	4	4
4	4	4	4	1	1	2	2	4	4
4	4	4	4	4	4	4	4	2	2
4	4	3	3	1	1	4	4	4	4
1	1	3	3	1	1	4	4	4	4
4	4	3	3	1	1	2	2	4	4
1	1	3	3	1	1	4	4	4	4
1	1	3	3	1	1	4	4	4	4
4	4	4	4	4	4	2	2	2	2
4	4	4	4	1	1	2	2	2	2
1	1	3	3	1	1	4	4	4	4
4	4	3	3	1	1	4	4	4	4
4	4	3	3	4	4	4	4	4	4
1	1	3	3	1	1	4	4	4	4
1	1	3	3	4	4	4	4	4	4
1	1	3	3	1	1	4	4	4	4
4	4	3	3	1	1	4	4	4	4
4	4	3	3	1	1	4	4	4	4
1	1	3	3	1	1	2	2	4	4
1	1	3	3	1	1	2	2	4	4
4	4	4	4	1	1	2	2	4	4
1	1	3	3	1	1	4	4	4	4
1	1	3	3	1	1	4	4	4	4
1	1	4	4	1	1	2	2	4	4
4	4	3	3	1	1	2	2	4	4
1	1	3	3	1	1	4	4	4	4
1	1	3	3	1	1	4	4	4	4
1	1	3	3	1	1	4	4	4	4
1	1	3	3	1	1	4	4	4	4
4	4	4	4	4	4	2	2	4	4
1	1	3	3	1	1	2	2	2	2
1	1	4	4	1	1	2	2	4	4
4	4	3	3	1	1	4	4	4	4
4	4	4	4	4	4	4	4	2	2
4	4	3	3	4	4	2	2	2	2
4	4	3	3	1	1	2	2	2	2
4	4	3	3	1	1	4	4	4	4
4	4	3	3	1	1	4	4	2	2
4	4	4	4	4	4	2	2	4	4
4	4	4	4	1	1	2	2	2	2
1	1	3	3	1	1	4	4	4	4
4	4	4	4	4	4	4	4	2	2

For Review Only

4	4	3	3	1	1	4	4	2	2
1	1	3	3	4	4	4	4	4	4
4	4	3	3	1	1	2	2	4	4
4	4	3	3	1	1	4	4	2	2
1	1	3	3	1	1	4	4	4	4
1	1	3	3	1	1	4	4	4	4
1	1	4	4	4	4	4	4	2	2
4	4	3	3	4	4	2	2	2	2
4	4	3	3	4	4	2	2	4	4
4	4	4	4	4	4	2	2	4	4
1	1	3	3	1	1	4	4	2	2
4	4	3	3	1	1	4	4	4	4
4	4	3	3	1	1	4	4	4	4
4	4	3	3	1	1	2	2	4	4
1	1	3	3	1	1	4	4	4	4
4	4	3	3	1	1	4	4	2	2
4	4	4	4	4	4	2	2	2	2
4	4	3	3	1	1	4	4	4	4
4	4	3	3	1	1	2	2	2	2
1	1	3	3	4	4	4	4	2	2
1	1	3	3	4	4	4	4	2	2
1	1	3	3	1	1	2	2	4	4
4	4	3	3	1	1	4	4	4	4
4	4	4	4	4	4	4	4	2	2
4	4	4	4	4	4	2	2	4	4
4	4	3	3	1	1	4	4	4	4
4	4	4	4	4	4	2	2	4	4
1	1	3	3	1	1	4	4	4	4
1	1	3	3	1	1	4	4	4	4
4	4	3	3	1	1	4	4	2	2
4	4	4	4	4	4	2	2	2	2
1	1	3	3	1	1	4	4	4	4
4	4	3	3	4	4	2	2	4	4
4	4	3	3	1	1	2	2	4	4
4	4	4	4	1	1	2	2	2	2
4	4	4	4	4	4	2	2	2	2
4	4	3	3	1	1	4	4	4	4
1	1	3	3	1	1	4	4	4	4
4	4	4	4	1	1	2	2	4	4
4	4	4	4	4	4	4	4	2	2
4	4	3	3	1	1	2	2	4	4
1	1	3	3	1	1	4	4	4	4
4	4	4	4	4	4	4	4	2	2
4	4	3	3	1	1	4	4	4	4
1	1	3	3	1	1	4	4	4	4
4	4	3	3	1	1	4	4	4	4
1	1	4	4	1	1	4	4	2	2
4	4	3	3	1	1	4	4	2	2
4	4	3	3	1	1	4	4	2	2
4	4	4	4	4	4	2	2	4	4
4	4	3	3	1	1	4	4	4	4
4	4	4	4	4	4	4	4	2	2
1	1	3	3	1	1	4	4	4	4
1	1	3	3	1	1	4	4	4	4
1	1	4	4	4	4	2	2	2	2

For Review Only

4	4	3	3	1	1	2	2	4	4
4	4	3	3	1	1	2	2	4	4

For Review Only

SNP792	SNP792	SNP793	SNP793	SNP794	SNP794	SNP795	SNP795	SNP796	SNP796
1	1	4	4	4	4	1	1	3	3
1	1	4	4	4	4	1	1	2	2
4	4	4	4	4	4	4	4	3	3
4	4	4	4	4	4	1	1	3	3
1	1	4	4	4	4	1	1	3	3
4	4	4	4	4	4	4	4	2	2
4	4	4	4	4	4	1	1	3	3
1	1	4	4	4	4	1	1	3	3
1	1	3	3	4	4	1	1	3	3
1	1	4	4	4	4	1	1	2	2
4	4	4	4	4	4	1	1	2	2
4	4	4	4	4	4	4	4	3	3
4	4	4	4	4	4	1	1	3	3
1	1	4	4	4	4	1	1	2	2
4	4	4	4	4	4	1	1	3	3
4	4	3	3	4	4	1	1	3	3
4	4	4	4	4	4	1	1	2	2
1	1	4	4	4	4	1	1	2	2
4	4	4	4	4	4	1	1	3	3
4	4	3	3	1	1	1	1	2	2
4	4	4	4	4	4	1	1	2	2
4	4	4	4	4	4	1	1	3	3
4	4	4	4	4	4	1	1	2	2
4	4	4	4	4	4	1	1	3	3
4	4	4	4	4	4	1	1	3	3
4	4	4	4	4	4	1	1	3	3
4	4	4	4	4	4	1	1	3	3
4	4	4	4	4	4	1	1	3	3
1	1	3	3	1	1	1	1	3	3
1	1	3	3	1	1	1	1	2	2
4	4	4	4	4	4	1	1	3	3
4	4	4	4	4	4	1	1	3	3
4	4	4	4	4	4	1	1	3	3
4	4	4	4	4	4	1	1	3	3
1	1	4	4	4	4	1	1	3	3
4	4	4	4	4	4	1	1	3	3
4	4	4	4	4	4	1	1	3	3
1	1	4	4	4	4	1	1	2	2
4	4	4	4	4	4	1	1	2	2
1	1	4	4	4	4	1	1	2	2
1	1	4	4	4	4	1	1	2	2
4	4	3	3	1	1	1	1	3	3
4	4	3	3	1	1	1	1	3	3
1	1	4	4	4	4	1	1	3	3
1	1	3	3	1	1	1	1	2	2
4	4	4	4	4	4	1	1	3	3
4	4	4	4	4	4	1	1	3	3
1	1	4	4	4	4	1	1	3	3
1	1	4	4	4	4	1	1	2	2
4	4	4	4	4	4	1	1	3	3
4	4	4	4	4	4	1	1	3	3
4	4	4	4	4	4	1	1	3	3

For Review Only

4	4	4	4	4	4	1	1	3	3
4	4	4	4	4	4	1	1	3	3
1	1	4	4	4	4	1	1	3	3
4	4	4	4	4	4	1	1	2	2
4	4	4	4	4	4	1	1	3	3
4	4	4	4	4	4	4	4	3	3
4	4	4	4	4	4	1	1	3	3
1	1	4	4	4	4	1	1	2	2
1	1	4	4	4	4	1	1	3	3
4	4	4	4	4	4	1	1	3	3
4	4	4	4	4	4	4	4	3	3
4	4	4	4	4	4	4	4	3	3
4	4	4	4	4	4	1	1	3	3
1	1	3	3	1	1	1	1	3	3
4	4	4	4	4	4	1	1	2	2
4	4	4	4	1	1	1	1	3	3
1	1	4	4	4	4	1	1	2	2
4	4	4	4	4	4	1	1	3	3
4	4	4	4	4	4	1	1	3	3
4	4	4	4	4	4	1	1	3	3
4	4	4	4	4	4	1	1	3	3
1	1	4	4	4	4	1	1	2	2
4	4	3	3	1	1	1	1	3	3
4	4	4	4	4	4	4	4	3	3
1	1	3	3	1	1	1	1	2	2
4	4	3	3	1	1	4	4	3	3
4	4	4	4	4	4	1	1	2	2
1	1	4	4	4	4	1	1	2	2
4	4	4	4	4	4	1	1	3	3
4	4	4	4	4	4	1	1	3	3
1	1	4	4	4	4	1	1	3	3
4	4	4	4	4	4	1	1	3	3
1	1	4	4	4	4	1	1	2	2
4	4	4	4	4	4	1	1	3	3
4	4	4	4	4	4	1	1	3	3
1	1	3	3	1	1	1	1	2	2
1	1	4	4	4	4	1	1	3	3
4	4	4	4	4	4	1	1	2	2
4	4	4	4	4	4	1	1	3	3
1	1	4	4	4	4	1	1	2	2
4	4	4	4	4	4	1	1	3	3
4	4	4	4	4	4	4	4	3	3
1	1	4	4	4	4	1	1	3	3
4	4	4	4	4	4	4	4	3	3
4	4	4	4	4	4	1	1	3	3
4	4	4	4	4	4	1	1	3	3
4	4	4	4	4	4	1	1	3	3
4	4	4	4	4	4	4	4	3	3
4	4	4	4	4	4	4	4	3	3
1	1	4	4	4	4	1	1	3	3
4	4	4	4	4	4	1	1	3	3
4	4	4	4	4	4	1	1	2	2
1	1	4	4	4	4	1	1	2	2
4	4	4	4	4	4	1	1	3	3
1	1	4	4	4	4	1	1	3	3

For Review Only

4	4	4	4	4	4	1	1	3	3
4	4	4	4	4	4	1	1	3	3
4	4	4	4	4	4	1	1	3	3
1	1	4	4	4	4	1	1	2	2
4	4	4	4	4	4	4	4	3	3
4	4	4	4	4	4	1	1	3	3
4	4	4	4	4	4	1	1	3	3
4	4	4	4	4	4	1	1	3	3
1	1	4	4	4	4	1	1	3	3
4	4	4	4	4	4	1	1	3	3
1	1	4	4	4	4	1	1	3	3
4	4	4	4	4	4	1	1	3	3
1	1	4	4	4	4	1	1	3	3
4	4	4	4	4	4	4	4	2	2
4	4	4	4	4	4	1	1	3	3
4	4	4	4	4	4	1	1	3	3
4	4	4	4	4	4	1	1	2	2
4	4	4	4	4	4	1	1	3	3
1	1	4	4	4	4	1	1	3	3
1	1	4	4	4	4	1	1	2	2
4	4	4	4	4	4	1	1	3	3
4	4	4	4	4	4	1	1	3	3
4	4	4	4	4	4	1	1	3	3
1	1	4	4	4	4	1	1	3	3
1	1	4	4	4	4	1	1	3	3
4	4	4	4	4	4	1	1	2	2
4	4	4	4	4	4	1	1	3	3
4	4	4	4	4	4	1	1	3	3
4	4	4	4	4	4	4	4	3	3
4	4	4	4	4	4	1	1	3	3
1	1	4	4	4	4	1	1	2	2
4	4	4	4	4	4	1	1	3	3
4	4	4	4	4	4	1	1	3	3
4	4	4	4	4	4	1	1	3	3
4	4	4	4	4	4	1	1	3	3
4	4	4	4	4	4	1	1	3	3
1	1	4	4	4	4	1	1	3	3
4	4	4	4	4	4	4	4	3	3
1	1	4	4	4	4	1	1	3	3
4	4	4	4	4	4	1	1	2	2
4	4	4	4	4	4	1	1	2	2
4	4	4	4	4	4	1	1	3	3
4	4	4	4	4	4	1	1	3	3
1	1	4	4	4	4	1	1	3	3
4	4	4	4	4	4	1	1	3	3
1	1	4	4	4	4	1	1	3	3
4	4	4	4	4	4	1	1	3	3
4	4	4	4	4	4	1	1	3	3
4	4	4	4	4	4	1	1	3	3
4	4	4	4	4	4	4	4	3	3
4	4	4	4	4	4	1	1	3	3
1	1	4	4	4	4	1	1	2	2
4	4	4	4	4	4	1	1	2	2
4	4	4	4	4	4	4	4	3	3
4	4	4	4	4	4	1	1	3	3

For Review Only

4	4	4	4	4	4	1	1	3	3
1	1	4	4	4	4	1	1	2	2
4	4	4	4	4	4	1	1	3	3
4	4	4	4	4	4	1	1	3	3
4	4	4	4	4	4	1	1	3	3
1	1	4	4	4	4	1	1	3	3
1	1	4	4	4	4	1	1	2	2
4	4	4	4	4	4	1	1	3	3
4	4	4	4	4	4	1	1	3	3
4	4	4	4	4	4	1	1	3	3
4	4	4	4	4	4	1	1	3	3
1	1	4	4	4	4	1	1	3	3
4	4	4	4	4	4	1	1	3	3
1	1	4	4	4	4	1	1	3	3
1	1	4	4	4	4	1	1	3	3
4	4	4	4	4	4	1	1	2	2
4	4	4	4	4	4	1	1	2	2
4	4	4	4	4	4	1	1	3	3
4	4	4	4	4	4	1	1	3	3
4	4	4	4	4	4	1	1	3	3
4	4	4	4	4	4	1	1	3	3
4	4	4	4	4	4	1	1	2	2
1	1	4	4	4	4	1	1	2	2
4	4	4	4	4	4	4	4	3	3
4	4	3	3	1	1	1	1	3	3
4	4	4	4	4	4	1	1	2	2
4	4	4	4	4	4	4	4	3	3
4	4	4	4	4	4	1	1	3	3
1	1	4	4	4	4	1	1	3	3
4	4	4	4	4	4	1	1	3	3
4	4	4	4	4	4	1	1	3	3
4	4	4	4	4	4	1	1	3	3
4	4	4	4	4	4	1	1	3	3
1	1	4	4	4	4	1	1	2	2
4	4	4	4	4	4	1	1	3	3
4	4	4	4	4	4	4	4	3	3
4	4	4	4	4	4	4	4	3	3
1	1	4	4	4	4	1	1	3	3
4	4	4	4	4	4	1	1	3	3
4	4	4	4	4	4	4	4	3	3
4	4	4	4	4	4	4	4	3	3
4	4	4	4	4	4	1	1	3	3
1	1	4	4	4	4	1	1	2	2
4	4	4	4	4	4	1	1	3	3
4	4	4	4	4	4	1	1	3	3
4	4	4	4	4	4	1	1	3	3
4	4	4	4	4	4	1	1	2	2
1	1	4	4	4	4	1	1	2	2
1	1	4	4	4	4	1	1	3	3
4	4	4	4	4	4	1	1	3	3
4	4	4	4	4	4	1	1	3	3
1	1	4	4	4	4	1	1	3	3
1	1	4	4	4	4	1	1	2	2
4	4	4	4	4	4	4	4	3	3
4	4	3	3	4	4	1	1	2	2

For Review Only

1	1	4	4	4	4	1	1	3	3
4	4	4	4	1	1	1	1	3	3
4	4	4	4	4	4	1	1	3	3
1	1	4	4	4	4	1	1	2	2
1	1	4	4	4	4	1	1	3	3
4	4	4	4	4	4	1	1	3	3
1	1	4	4	4	4	1	1	3	3
1	1	3	3	1	1	1	1	2	2
4	4	4	4	4	4	1	1	3	3
1	1	4	4	4	4	1	1	2	2
4	4	4	4	4	4	1	1	3	3
4	4	4	4	4	4	1	1	2	2
4	4	4	4	4	4	1	1	3	3
4	4	4	4	4	4	1	1	3	3
4	4	4	4	4	4	4	4	2	2
1	1	4	4	4	4	1	1	3	3
4	4	4	4	4	4	1	1	2	2
1	1	4	4	4	4	1	1	2	2
1	1	4	4	4	4	1	1	2	2
1	1	4	4	4	4	1	1	2	2
1	1	4	4	4	4	1	1	3	3
4	4	4	4	4	4	1	1	3	3
4	4	4	4	4	4	1	1	3	3
1	1	3	3	4	4	1	1	2	2
1	1	4	4	4	4	1	1	2	2
4	4	3	3	4	4	1	1	3	3
1	1	4	4	4	4	1	1	2	2
4	4	4	4	4	4	4	4	3	3
4	4	4	4	4	4	1	1	3	3
4	4	4	4	4	4	4	4	3	3
4	4	4	4	4	4	1	1	2	2
4	4	4	4	4	4	1	1	3	3
1	1	4	4	4	4	1	1	3	3
4	4	4	4	4	4	1	1	3	3
1	1	4	4	4	4	1	1	2	2
1	1	4	4	4	4	1	1	2	2
4	4	4	4	4	4	1	1	3	3
4	4	4	4	4	4	4	4	3	3
4	4	4	4	4	4	1	1	3	3
1	1	4	4	4	4	1	1	2	2
4	4	4	4	4	4	1	1	3	3
4	4	4	4	4	4	1	1	3	3
1	1	3	3	4	4	1	1	2	2
4	4	4	4	4	4	1	1	3	3
4	4	4	4	4	4	1	1	3	3
4	4	4	4	4	4	1	1	3	3
4	4	4	4	4	4	1	1	3	3
1	1	4	4	4	4	1	1	3	3
4	4	4	4	4	4	1	1	3	3
1	1	4	4	4	4	1	1	2	2
4	4	4	4	4	4	1	1	3	3
1	1	4	4	4	4	1	1	2	2
4	4	4	4	4	4	4	4	3	3
4	4	4	4	4	4	4	4	2	2
1	1	3	3	4	4	1	1	2	2

For Review Only

4	4	4	4	4	4	1	1	3	3
4	4	4	4	4	4	4	4	3	3

For Review Only

SNP797	SNP797	SNP798	SNP798	SNP799	SNP799	SNP800	SNP800	SNP801	SNP801
1	1	1	1	4	4	4	4	2	2
1	1	1	1	3	3	3	3	1	1
1	1	1	1	3	3	3	3	1	1
1	1	1	1	3	3	3	3	1	1
1	1	1	1	3	3	3	3	2	2
1	1	1	1	3	3	3	3	1	1
1	1	1	1	4	4	4	4	2	2
1	1	1	1	3	3	3	3	1	1
1	1	1	1	3	3	3	3	2	2
1	1	1	1	3	3	3	3	1	1
1	1	3	3	3	3	3	3	1	1
1	1	1	1	3	3	3	3	1	1
1	1	1	1	3	3	3	3	1	1
3	3	1	1	3	3	3	3	1	1
1	1	1	1	3	3	3	3	1	1
1	1	1	1	3	3	3	3	1	1
1	1	3	3	3	3	3	3	1	1
1	1	1	1	3	3	3	3	1	1
3	3	1	1	3	3	3	3	1	1
1	1	1	1	3	3	3	3	1	1
1	1	3	3	3	3	3	3	1	1
1	1	1	1	3	3	3	3	1	1
1	1	3	3	3	3	3	3	1	1
1	1	3	3	3	3	3	3	1	1
1	1	3	3	3	3	3	3	1	1
3	3	1	1	3	3	3	3	1	1
3	3	1	1	3	3	3	3	1	1
1	1	1	1	3	3	3	3	1	1
1	1	1	1	3	3	3	3	1	1
1	1	1	1	4	4	3	3	1	1
3	3	1	1	3	3	3	3	1	1
1	1	1	1	3	3	3	3	1	1
1	1	1	1	4	4	4	4	2	2
3	3	1	1	3	3	3	3	1	1
1	1	1	1	3	3	3	3	1	1
1	1	3	3	3	3	3	3	1	1
1	1	3	3	3	3	3	3	1	1
1	1	1	1	3	3	3	3	1	1
1	1	1	1	3	3	3	3	1	1
1	1	3	3	3	3	3	3	1	1
1	1	1	1	3	3	3	3	1	1
1	1	1	1	3	3	3	3	1	1
1	1	1	1	3	3	3	3	1	1
1	1	1	1	4	4	4	4	2	2
1	1	1	1	3	3	3	3	1	1
1	1	1	1	3	3	3	3	1	1
3	3	1	1	3	3	3	3	1	1
1	1	1	1	3	3	3	3	1	1
1	1	1	1	3	3	4	4	2	2
1	1	3	3	3	3	3	3	1	1
3	3	1	1	3	3	3	3	1	1
1	1	1	1	3	3	3	3	1	1

For Review Only

1	1	1	1	3	3	3	3	1	1
1	1	1	1	4	4	4	4	2	2
1	1	1	1	3	3	3	3	1	1
1	1	3	3	3	3	3	3	2	2
3	3	1	1	3	3	3	3	1	1
1	1	1	1	3	3	3	3	1	1
3	3	1	1	3	3	3	3	1	1
1	1	1	1	3	3	3	3	1	1
1	1	1	1	3	3	3	3	1	1
1	1	1	1	3	3	3	3	1	1
1	1	1	1	3	3	3	3	1	1
1	1	1	1	3	3	3	3	1	1
1	1	1	1	3	3	3	3	1	1
1	1	1	1	3	3	3	3	1	1
1	1	1	1	3	3	3	3	1	1
1	1	1	1	3	3	3	3	1	1
3	3	1	1	3	3	3	3	1	1
1	1	3	3	3	3	3	3	1	1
1	1	3	3	3	3	3	3	1	1
1	1	1	1	4	4	4	4	2	2
1	1	1	1	3	3	3	3	1	1
1	1	1	1	3	3	3	3	1	1
1	1	1	1	3	3	3	3	1	1
1	1	1	1	3	3	3	3	1	1
1	1	1	1	3	3	3	3	1	1
1	1	1	1	3	3	3	3	1	1
1	1	1	1	4	4	4	4	2	2
1	1	1	1	3	3	3	3	1	1
3	3	1	1	3	3	3	3	1	1
1	1	3	3	3	3	3	3	1	1
1	1	1	1	3	3	3	3	1	1
1	1	3	3	3	3	3	3	1	1
1	1	1	1	3	3	3	3	1	1
1	1	1	1	3	3	3	3	1	1
1	1	1	1	3	3	3	3	1	1
1	1	1	1	3	3	3	3	1	1
1	1	1	1	3	3	3	3	1	1
1	1	3	3	3	3	3	3	2	2
1	1	3	3	3	3	3	3	1	1
1	1	1	1	4	4	4	4	2	2
1	1	3	3	4	4	4	4	2	2
1	1	1	1	3	3	3	3	1	1
1	1	1	1	3	3	3	3	1	1
1	1	1	1	3	3	3	3	1	1
1	1	3	3	4	4	4	4	2	2
3	3	1	1	3	3	3	3	1	1
1	1	1	1	3	3	3	3	1	1
1	1	1	1	3	3	3	3	1	1
1	1	1	1	3	3	3	3	1	1
1	1	1	1	4	4	4	4	2	2
1	1	1	1	3	3	3	3	1	1
1	1	1	1	3	3	3	3	1	1
1	1	1	1	3	3	3	3	1	1
1	1	1	1	3	3	3	3	1	1
1	1	1	1	3	3	3	3	1	1

For Review Only

1	1	1	1	3	3	3	3	1	1
1	1	3	3	4	4	3	3	1	1
1	1	1	1	3	3	3	3	1	1
1	1	1	1	3	3	3	3	1	1
1	1	1	1	3	3	3	3	1	1
1	1	3	3	3	3	3	3	1	1
1	1	3	3	3	3	3	3	1	1
1	1	1	1	3	3	3	3	1	1
1	1	1	1	3	3	3	3	1	1
1	1	3	3	3	3	3	3	1	1
1	1	1	1	3	3	3	3	1	1
1	1	1	1	3	3	3	3	1	1
1	1	1	1	3	3	3	3	1	1
1	1	1	1	3	3	3	3	1	1
1	1	1	1	3	3	3	3	1	1
1	1	1	1	3	3	3	3	1	1
1	1	1	1	3	3	3	3	1	1
1	1	1	1	3	3	3	3	1	1
1	1	1	1	3	3	3	3	1	1
1	1	1	1	3	3	3	3	1	1
1	1	1	1	3	3	3	3	1	1
1	1	3	3	3	3	3	3	1	1
1	1	1	1	3	3	3	3	1	1
1	1	1	1	3	3	3	3	1	1
3	3	1	1	3	3	3	3	1	1
1	1	3	3	3	3	3	3	1	1
1	1	3	3	3	3	3	3	1	1
1	1	3	3	3	3	3	3	1	1
1	1	1	1	3	3	3	3	1	1
1	1	1	1	3	3	3	3	1	1
1	1	1	1	3	3	3	3	1	1
1	1	1	1	3	3	3	3	1	1
1	1	3	3	4	4	4	4	2	2
1	1	1	1	3	3	3	3	1	1
3	3	1	1	3	3	3	3	1	1
1	1	3	3	3	3	3	3	1	1
1	1	1	1	3	3	3	3	1	1
1	1	1	1	3	3	3	3	1	1
1	1	1	1	3	3	3	3	1	1
1	1	1	1	3	3	3	3	1	1
1	1	1	1	3	3	3	3	1	1
3	3	1	1	3	3	3	3	1	1
1	1	3	3	3	3	3	3	1	1
1	1	1	1	3	3	3	3	1	1
1	1	1	1	4	4	4	4	1	1
1	1	1	1	3	3	3	3	1	1
3	3	1	1	3	3	3	3	1	1
3	3	1	1	3	3	3	3	1	1
1	1	1	1	3	3	3	3	1	1
1	1	1	1	3	3	3	3	1	1
3	3	1	1	3	3	3	3	1	1
1	1	1	1	3	3	3	3	1	1
1	1	1	1	3	3	3	3	1	1
1	1	1	1	3	3	3	3	1	1
1	1	1	1	3	3	3	3	1	1

For Review Only

3	3	1	1	3	3	3	3	1	1
1	1	1	1	3	3	3	3	1	1
1	1	1	1	3	3	3	3	1	1
1	1	3	3	3	3	3	3	1	1
1	1	1	1	3	3	3	3	1	1
3	3	1	1	3	3	3	3	1	1
1	1	1	1	3	3	3	3	1	1
1	1	1	1	3	3	3	3	1	1
1	1	3	3	3	3	3	3	1	1
1	1	3	3	3	3	3	3	1	1
1	1	1	1	3	3	3	3	1	1
1	1	3	3	3	3	3	3	1	1
1	1	1	1	3	3	3	3	1	1
1	1	1	1	3	3	3	3	1	1
1	1	1	1	3	3	3	3	1	1
1	1	1	1	3	3	3	3	1	1
1	1	1	1	3	3	3	3	1	1
1	1	1	1	3	3	3	3	1	1
1	1	3	3	3	3	3	3	1	1
1	1	3	3	3	3	3	3	1	1
1	1	1	1	3	3	3	3	1	1
1	1	1	1	3	3	3	3	1	1
1	1	1	1	3	3	3	3	1	1
1	1	1	1	3	3	3	3	1	1
1	1	1	1	3	3	3	3	1	1
3	3	1	1	3	3	3	3	1	1
1	1	1	1	3	3	3	3	1	1
1	1	1	1	3	3	3	3	1	1
1	1	1	1	3	3	3	3	1	1
1	1	3	3	3	3	3	3	1	1
3	3	1	1	3	3	3	3	1	1
3	3	1	1	3	3	3	3	1	1
1	1	3	3	3	3	3	3	1	1
1	1	1	1	3	3	3	3	1	1
3	3	1	1	3	3	3	3	1	1
1	1	1	1	3	3	3	3	1	1
1	1	1	1	3	3	3	3	1	1
1	1	1	1	3	3	3	3	1	1
3	3	1	1	3	3	3	3	1	1
1	1	1	1	3	3	3	3	1	1
1	1	1	1	3	3	3	3	1	1
3	3	1	1	3	3	3	3	1	1
1	1	1	1	3	3	3	3	1	1
1	1	3	3	3	3	3	3	1	1
3	3	3	3	3	3	3	3	1	1
1	1	1	1	3	3	3	3	1	1
1	1	1	1	3	3	3	3	1	1
1	1	1	1	3	3	3	3	1	1
1	1	3	3	4	4	4	4	2	2
1	1	1	1	3	3	3	3	1	1
1	1	1	1	3	3	3	3	1	1
1	1	1	1	3	3	3	3	1	1
1	1	1	1	3	3	3	3	1	1
1	1	1	1	3	3	3	3	1	1
1	1	1	1	3	3	3	3	1	1

For Review Only

1	1	1	1	3	3	3	3	1	1
1	1	3	3	4	4	3	3	1	1
1	1	3	3	3	3	3	3	1	1
1	1	1	1	3	3	3	3	1	1
1	1	1	1	3	3	3	3	1	1
1	1	1	1	3	3	3	3	1	1
1	1	3	3	3	3	3	3	1	1
1	1	1	1	3	3	3	3	1	1
1	1	1	1	3	3	4	4	2	2
1	1	1	1	3	3	3	3	1	1
1	1	1	1	3	3	3	3	1	1
1	1	3	3	3	3	3	3	1	1
1	1	3	3	3	3	3	3	1	1
1	1	1	1	3	3	3	3	1	1
1	1	1	1	3	3	3	3	1	1
1	1	1	1	3	3	3	3	1	1
1	1	1	1	3	3	3	3	1	1
1	1	1	1	3	3	4	4	2	2
1	1	1	1	3	3	3	3	1	1
1	1	1	1	3	3	3	3	1	1
1	1	3	3	3	3	3	3	1	1
3	3	1	1	3	3	3	3	1	1
1	1	1	1	3	3	3	3	1	1
1	1	3	3	3	3	3	3	1	1
1	1	1	1	3	3	3	3	1	1
1	1	1	1	3	3	3	3	1	1
1	1	1	1	3	3	3	3	1	1
1	1	1	1	3	3	3	3	1	1
1	1	1	1	3	3	3	3	1	1
1	1	1	1	3	3	3	3	1	1
1	1	1	1	3	3	3	3	1	1
1	1	1	1	3	3	3	3	1	1
3	3	1	1	3	3	3	3	1	1
1	1	1	1	3	3	3	3	1	1
1	1	1	1	3	3	3	3	1	1
1	1	1	1	3	3	3	3	1	1
1	1	3	3	3	3	3	3	1	1
1	1	1	1	3	3	3	3	1	1
1	1	3	3	3	3	3	3	1	1
1	1	1	1	3	3	3	3	1	1
1	1	3	3	3	3	3	3	1	1
1	1	3	3	3	3	3	3	1	1
1	1	3	3	3	3	3	3	1	1
1	1	3	3	3	3	3	3	2	2
1	1	1	1	3	3	3	3	1	1
1	1	3	3	3	3	3	3	1	1
1	1	1	1	3	3	3	3	1	1
1	1	1	1	3	3	3	3	1	1
3	3	1	1	3	3	3	3	1	1
1	1	1	1	3	3	3	3	1	1
1	1	3	3	3	3	3	3	1	1
1	1	1	1	3	3	3	3	1	1
1	1	1	1	3	3	3	3	1	1
1	1	1	1	3	3	3	3	1	1
1	1	3	3	3	3	3	3	1	1

For Review Only

1	1	3	3	3	3	3	3	1	1
1	1	1	1	3	3	3	3	1	1

For Review Only

SNP802	SNP802	SNP803	SNP803	SNP804	SNP804	SNP805	SNP805	SNP806	SNP806
1	1	3	3	2	2	2	2	2	2
1	1	3	3	2	2	1	1	2	2
3	3	3	3	2	2	1	1	3	3
1	1	3	3	2	2	1	1	2	2
3	3	3	3	2	2	1	1	2	2
1	1	3	3	2	2	1	1	2	2
1	1	2	2	2	2	1	1	2	2
1	1	3	3	2	2	1	1	2	2
1	1	3	3	2	2	1	1	2	2
1	1	3	3	2	2	1	1	2	2
1	1	3	3	1	1	1	1	2	2
3	3	3	3	2	2	1	1	3	3
1	1	3	3	2	2	1	1	2	2
1	1	3	3	2	2	1	1	2	2
1	1	3	3	2	2	1	1	2	2
1	1	3	3	2	2	1	1	2	2
1	1	3	3	1	1	1	1	2	2
1	1	3	3	2	2	1	1	2	2
1	1	3	3	2	2	1	1	3	3
1	1	3	3	2	2	1	1	2	2
1	1	3	3	1	1	1	1	2	2
1	1	3	3	2	2	1	1	2	2
1	1	3	3	1	1	1	1	2	2
1	1	3	3	1	1	1	1	2	2
3	3	3	3	1	1	1	1	2	2
1	1	3	3	2	2	1	1	3	3
1	1	3	3	2	2	1	1	2	2
1	1	3	3	2	2	1	1	2	2
1	1	3	3	2	2	1	1	2	2
1	1	3	3	2	2	1	1	2	2
1	1	3	3	2	2	1	1	2	2
1	1	3	3	2	2	1	1	2	2
1	1	2	2	2	2	2	2	2	2
1	1	3	3	2	2	1	1	2	2
1	1	3	3	2	2	1	1	2	2
1	1	3	3	1	1	1	1	2	2
1	1	3	3	1	1	1	1	2	2
1	1	3	3	2	2	1	1	2	2
1	1	3	3	2	2	1	1	2	2
1	1	3	3	1	1	1	1	2	2
1	1	3	3	2	2	1	1	2	2
1	1	3	3	2	2	1	1	2	2
1	1	3	3	2	2	1	1	2	2
1	1	3	3	2	2	2	2	2	2
1	1	3	3	2	2	1	1	2	2
3	3	3	3	1	1	1	1	3	3
1	1	3	3	2	2	1	1	3	3
1	1	3	3	2	2	1	1	2	2
1	1	2	2	2	2	2	2	2	2
1	1	3	3	2	2	1	1	2	2
1	1	3	3	2	2	1	1	3	3
1	1	3	3	2	2	1	1	2	2

For Review Only

3	3	3	3	2	2	1	1	2	2
1	1	2	2	2	2	2	2	2	2
1	1	3	3	2	2	1	1	3	3
3	3	3	3	1	1	1	1	2	2
1	1	3	3	2	2	1	1	3	3
1	1	3	3	2	2	1	1	3	3
1	1	3	3	2	2	1	1	3	3
1	1	3	3	2	2	1	1	2	2
1	1	3	3	2	2	1	1	2	2
1	1	3	3	2	2	1	1	2	2
1	1	3	3	2	2	1	1	3	3
1	1	3	3	2	2	1	1	3	3
3	3	3	3	2	2	1	1	2	2
1	1	3	3	2	2	1	1	2	2
1	1	3	3	2	2	1	1	2	2
1	1	3	3	2	2	1	1	2	2
1	1	3	3	2	2	1	1	3	3
1	1	3	3	1	1	1	1	2	2
1	1	3	3	1	1	1	1	2	2
1	1	2	2	2	2	2	2	2	2
1	1	3	3	2	2	1	1	3	3
1	1	3	3	2	2	1	1	2	2
1	1	3	3	2	2	1	1	2	2
1	1	3	3	2	2	1	1	3	3
1	1	3	3	2	2	1	1	2	2
1	1	3	3	2	2	1	1	3	3
1	1	2	2	2	2	2	2	3	3
1	1	3	3	2	2	1	1	2	2
1	1	3	3	2	2	1	1	3	3
1	1	3	3	1	1	1	1	2	2
3	3	3	3	2	2	1	1	2	2
1	1	3	3	1	1	1	1	2	2
1	1	3	3	2	2	1	1	2	2
3	3	3	3	2	2	1	1	2	2
1	1	3	3	2	2	1	1	2	2
1	1	3	3	2	2	1	1	2	2
1	1	3	3	2	2	1	1	2	2
3	3	3	3	1	1	1	1	2	2
1	1	3	3	1	1	1	1	3	3
1	1	3	3	2	2	1	1	2	2
1	1	2	2	1	1	2	2	2	2
1	1	3	3	2	2	1	1	2	2
1	1	3	3	2	2	1	1	2	2
1	1	3	3	2	2	1	1	3	3
1	1	2	2	1	1	2	2	2	2
1	1	3	3	2	2	1	1	2	2
1	1	3	3	2	2	1	1	2	2
1	1	3	3	2	2	1	1	3	3
1	1	3	3	2	2	1	1	2	2
1	1	2	2	2	2	2	2	2	2
1	1	3	3	2	2	1	1	2	2
1	1	3	3	2	2	1	1	2	2
1	1	3	3	2	2	1	1	2	2
1	1	3	3	2	2	1	1	3	3
1	1	3	3	2	2	1	1	2	2

For Review Only

1	1	3	3	2	2	1	1	2	2
1	1	3	3	1	1	1	1	2	2
1	1	3	3	2	2	1	1	2	2
1	1	3	3	2	2	1	1	2	2
1	1	3	3	2	2	1	1	3	3
1	1	3	3	2	2	1	1	3	3
1	1	3	3	1	1	1	1	2	2
1	1	3	3	2	2	1	1	2	2
1	1	3	3	2	2	1	1	2	2
1	1	3	3	2	2	1	1	3	3
1	1	3	3	2	2	1	1	2	2
1	1	2	2	2	2	1	1	2	2
1	1	3	3	2	2	1	1	2	2
1	1	3	3	2	2	1	1	3	3
1	1	3	3	2	2	1	1	2	2
1	1	3	3	2	2	1	1	3	3
1	1	3	3	2	2	1	1	2	2
1	1	3	3	2	2	1	1	2	2
1	1	3	3	2	2	1	1	2	2
1	1	3	3	2	2	1	1	2	2
1	1	3	3	2	2	1	1	2	2
1	1	3	3	1	1	1	1	2	2
1	1	3	3	2	2	1	1	2	2
1	1	3	3	2	2	1	1	2	2
1	1	3	3	2	2	1	1	2	2
1	1	3	3	1	1	1	1	2	2
1	1	3	3	1	1	1	1	2	2
1	1	3	3	1	1	1	1	2	2
1	1	3	3	2	2	1	1	3	3
1	1	3	3	2	2	1	1	2	2
1	1	3	3	2	2	1	1	2	2
1	1	3	3	2	2	1	1	2	2
1	1	2	2	1	1	2	2	2	2
1	1	3	3	2	2	1	1	2	2
1	1	3	3	2	2	1	1	2	2
1	1	3	3	2	2	1	1	2	2
1	1	3	3	2	2	1	1	2	2
1	1	3	3	2	2	1	1	3	3
1	1	3	3	2	2	1	1	2	2
1	1	3	3	2	2	1	1	2	2
1	1	3	3	2	2	1	1	2	2
1	1	3	3	2	2	1	1	2	2
1	1	3	3	1	1	1	1	2	2
1	1	3	3	2	2	1	1	2	2
1	1	3	3	2	2	2	2	2	2
1	1	3	3	2	2	1	1	2	2
1	1	3	3	2	2	1	1	3	3
1	1	3	3	2	2	1	1	3	3
1	1	3	3	2	2	1	1	2	2
1	1	3	3	2	2	1	1	3	3
1	1	3	3	2	2	1	1	2	2
1	1	3	3	2	2	1	1	2	2
1	1	3	3	1	1	1	1	2	2
1	1	3	3	2	2	1	1	3	3
1	1	3	3	2	2	1	1	2	2

For Review Only

1	1	3	3	2	2	1	1	3	3
1	1	3	3	2	2	1	1	2	2
1	1	3	3	2	2	1	1	2	2
1	1	3	3	1	1	1	1	2	2
1	1	3	3	2	2	1	1	2	2
1	1	3	3	2	2	1	1	2	2
1	1	3	3	2	2	1	1	2	2
1	1	3	3	2	2	1	1	2	2
1	1	3	3	1	1	1	1	2	2
1	1	3	3	1	1	1	1	2	2
1	1	3	3	2	2	1	1	3	3
1	1	3	3	2	2	1	1	2	2
1	1	3	3	2	2	1	1	2	2
1	1	3	3	2	2	1	1	2	2
1	1	3	3	2	2	1	1	2	2
1	1	3	3	2	2	1	1	2	2
1	1	3	3	1	1	1	1	2	2
1	1	3	3	2	2	1	1	3	3
1	1	3	3	1	1	1	1	2	2
1	1	3	3	1	1	1	1	2	2
1	1	3	3	2	2	1	1	2	2
1	1	3	3	2	2	1	1	2	2
1	1	3	3	2	2	1	1	2	2
1	1	3	3	2	2	1	1	3	3
1	1	3	3	2	2	1	1	2	2
1	1	3	3	2	2	1	1	2	2
1	1	3	3	2	2	1	1	2	2
1	1	3	3	2	2	1	1	2	2
1	1	3	3	2	2	1	1	2	2
1	1	3	3	1	1	1	1	2	2
1	1	3	3	2	2	1	1	3	3
1	1	3	3	2	2	1	1	2	2
1	1	3	3	1	1	1	1	2	2
1	1	3	3	2	2	1	1	2	2
1	1	3	3	2	2	1	1	2	2
1	1	3	3	2	2	1	1	3	3
1	1	3	3	2	2	1	1	2	2
1	1	3	3	2	2	1	1	2	2
1	1	3	3	2	2	1	1	3	3
1	1	3	3	2	2	1	1	3	3
3	3	3	3	2	2	1	1	3	3
1	1	3	3	2	2	1	1	3	3
1	1	3	3	2	2	1	1	2	2
1	1	3	3	1	1	1	1	2	2
1	1	3	3	2	2	1	1	2	2
1	1	3	3	2	2	1	1	2	2
1	1	3	3	2	2	1	1	2	2
1	1	3	3	2	2	1	1	2	2
1	1	2	2	1	1	2	2	2	2
1	1	3	3	2	2	1	1	2	2
1	1	3	3	2	2	1	1	3	3
1	1	3	3	2	2	1	1	2	2
1	1	3	3	2	2	1	1	2	2
1	1	3	3	2	2	1	1	3	3
1	1	3	3	2	2	1	1	2	2

For Review Only

1	1	3	3	2	2	1	1	2	2
1	1	3	3	2	2	1	1	2	2
1	1	3	3	1	1	1	1	3	3
1	1	3	3	2	2	1	1	2	2
1	1	3	3	2	2	1	1	2	2
1	1	3	3	2	2	1	1	2	2
1	1	3	3	1	1	1	1	2	2
1	1	3	3	2	2	1	1	2	2
1	1	3	3	2	2	1	1	2	2
1	1	3	3	2	2	1	1	2	2
1	1	3	3	2	2	1	1	2	2
1	1	3	3	2	2	1	1	3	3
1	1	3	3	1	1	1	1	3	3
1	1	3	3	2	2	1	1	3	3
1	1	3	3	2	2	1	1	3	3
1	1	3	3	2	2	1	1	2	2
1	1	3	3	2	2	1	1	2	2
1	1	2	2	2	2	1	1	2	2
1	1	3	3	2	2	1	1	2	2
1	1	3	3	2	2	1	1	2	2
1	1	3	3	1	1	1	1	3	3
1	1	3	3	2	2	1	1	3	3
1	1	3	3	2	2	1	1	2	2
1	1	3	3	1	1	1	1	2	2
1	1	3	3	2	2	1	1	2	2
1	1	3	3	2	2	1	1	2	2
1	1	3	3	2	2	1	1	2	2
1	1	3	3	2	2	1	1	3	3
1	1	3	3	2	2	1	1	2	2
1	1	3	3	2	2	1	1	3	3
1	1	3	3	2	2	1	1	2	2
1	1	3	3	2	2	1	1	3	3
1	1	3	3	2	2	1	1	2	2
1	1	3	3	2	2	1	1	3	3
1	1	3	3	2	2	1	1	2	2
1	1	3	3	2	2	1	1	2	2
3	3	3	3	1	1	1	1	2	2
1	1	3	3	2	2	1	1	3	3
1	1	3	3	1	1	1	1	2	2
1	1	3	3	2	2	1	1	2	2
1	1	3	3	1	1	1	1	2	2
1	1	3	3	1	1	1	1	2	2
1	1	3	3	1	1	1	1	2	2
3	3	3	3	1	1	1	1	2	2
1	1	3	3	2	2	1	1	3	3
1	1	3	3	1	1	1	1	2	2
1	1	3	3	2	2	1	1	2	2
1	1	3	3	2	2	1	1	2	2
1	1	3	3	2	2	1	1	2	2
1	1	3	3	2	2	1	1	2	2
1	1	3	3	1	1	1	1	2	2
1	1	3	3	2	2	1	1	2	2
3	3	3	3	2	2	1	1	3	3
1	1	3	3	2	2	1	1	3	3
1	1	3	3	1	1	1	1	2	2

For Review Only

1	1	3	3	1	1	1	1	2	2
1	1	3	3	2	2	1	1	3	3

For Review Only

SNP807	SNP807	SNP808	SNP808	SNP809	SNP809	SNP810	SNP810	SNP811	SNP811
4	4	1	1	4	4	3	3	3	3
3	3	2	2	4	4	3	3	3	3
4	4	2	2	2	2	3	3	3	3
3	3	2	2	4	4	3	3	3	3
3	3	2	2	4	4	3	3	3	3
3	3	1	1	4	4	3	3	3	3
3	3	1	1	4	4	3	3	3	3
3	3	2	2	2	2	3	3	3	3
3	3	2	2	4	4	3	3	3	3
3	3	2	2	2	2	3	3	3	3
3	3	2	2	4	4	2	2	3	3
4	4	2	2	4	4	3	3	3	3
3	3	2	2	4	4	3	3	3	3
4	4	2	2	4	4	3	3	3	3
3	3	2	2	4	4	3	3	3	3
4	4	2	2	4	4	3	3	3	3
3	3	2	2	4	4	3	3	3	3
3	3	2	2	4	4	2	2	3	3
3	3	2	2	2	2	3	3	3	3
3	3	2	2	4	4	3	3	3	3
3	3	2	2	4	4	3	3	3	3
3	3	2	2	4	4	3	3	3	3
3	3	2	2	4	4	2	2	2	2
3	3	2	2	4	4	2	2	3	3
3	3	2	2	4	4	3	3	3	3
4	4	2	2	4	4	3	3	3	3
3	3	2	2	4	4	3	3	3	3
4	4	2	2	4	4	3	3	3	3
3	3	2	2	4	4	3	3	3	3
3	3	2	2	4	4	2	2	2	2
3	3	2	2	4	4	3	3	3	3
4	4	2	2	2	2	2	2	3	3
3	3	1	1	4	4	3	3	3	3
4	4	2	2	4	4	2	2	2	2
4	4	1	1	4	4	3	3	3	3
4	4	1	1	4	4	3	3	3	3
3	3	2	2	4	4	2	2	3	3
3	3	2	2	4	4	3	3	3	3
3	3	2	2	4	4	3	3	2	2
3	3	2	2	4	4	3	3	3	3
3	3	2	2	4	4	3	3	3	3
3	3	2	2	4	4	3	3	3	3
3	3	2	2	2	2	3	3	3	3
3	3	1	1	4	4	3	3	3	3
3	3	2	2	2	2	3	3	3	3
4	4	2	2	2	2	3	3	3	3
4	4	2	2	4	4	3	3	3	3
4	4	2	2	4	4	2	2	3	3
3	3	1	1	4	4	3	3	3	3
4	4	2	2	4	4	3	3	3	3
3	3	2	2	4	4	3	3	3	3
4	4	2	2	4	4	3	3	3	3

For Review Only

3	3	2	2	4	4	3	3	3	3
3	3	1	1	4	4	3	3	3	3
4	4	2	2	4	4	3	3	3	3
3	3	2	2	4	4	3	3	3	3
3	3	2	2	2	2	3	3	3	3
4	4	2	2	4	4	3	3	3	3
4	4	2	2	4	4	3	3	3	3
3	3	2	2	4	4	3	3	3	3
3	3	2	2	4	4	2	2	3	3
4	4	2	2	4	4	3	3	3	3
4	4	2	2	2	2	3	3	3	3
4	4	2	2	4	4	3	3	3	3
3	3	2	2	4	4	3	3	3	3
3	3	2	2	4	4	3	3	3	3
4	4	2	2	4	4	3	3	3	3
3	3	1	1	4	4	3	3	3	3
3	3	2	2	4	4	3	3	3	3
3	3	2	2	4	4	3	3	3	3
3	3	2	2	4	4	3	3	3	3
4	4	1	1	4	4	3	3	3	3
4	4	2	2	4	4	3	3	3	3
3	3	2	2	4	4	3	3	3	3
3	3	2	2	4	4	3	3	3	3
3	3	2	2	4	4	3	3	3	3
3	3	2	2	2	2	3	3	3	3
3	3	2	2	4	4	3	3	3	3
3	3	1	1	4	4	3	3	3	3
3	3	2	2	4	4	2	2	2	2
4	4	2	2	4	4	3	3	3	3
4	4	2	2	4	4	3	3	3	3
3	3	2	2	4	4	3	3	3	3
4	4	2	2	4	4	2	2	2	2
3	3	2	2	4	4	2	2	2	2
3	3	2	2	4	4	3	3	3	3
3	3	2	2	4	4	2	2	3	3
3	3	2	2	2	2	3	3	3	3
3	3	2	2	4	4	3	3	2	2
3	3	2	2	4	4	3	3	3	3
3	3	1	1	4	4	3	3	2	2
3	3	1	1	2	2	3	3	3	3
3	3	1	1	4	4	3	3	3	3
4	4	1	1	4	4	3	3	3	3
3	3	2	2	4	4	3	3	3	3
4	4	2	2	2	2	3	3	3	3
4	4	1	1	4	4	3	3	3	3
3	3	2	2	4	4	3	3	3	3
4	4	2	2	4	4	2	2	2	2
4	4	2	2	4	4	3	3	3	3
4	4	2	2	4	4	3	3	3	3
3	3	1	1	4	4	3	3	3	3
3	3	2	2	4	4	3	3	3	3
3	3	2	2	4	4	3	3	2	2
3	3	2	2	4	4	2	2	3	3
4	4	2	2	4	4	3	3	3	3
3	3	2	2	4	4	3	3	3	3

For Review Only

4	4	2	2	4	4	3	3	3	3
4	4	2	2	4	4	3	3	3	3
4	4	2	2	4	4	3	3	3	3
3	3	2	2	4	4	2	2	3	3
4	4	2	2	4	4	3	3	3	3
4	4	2	2	4	4	3	3	3	3
4	4	2	2	4	4	3	3	3	3
4	4	2	2	4	4	3	3	3	3
4	4	2	2	4	4	3	3	3	3
4	4	2	2	4	4	3	3	3	3
3	3	1	1	4	4	3	3	3	3
3	3	2	2	4	4	3	3	3	3
4	4	2	2	4	4	3	3	3	3
3	3	2	2	4	4	3	3	3	3
4	4	2	2	4	4	3	3	3	3
3	3	2	2	4	4	3	3	3	3
3	3	2	2	4	4	3	3	3	3
3	3	2	2	4	4	3	3	3	3
4	4	2	2	4	4	2	2	3	3
3	3	2	2	4	4	2	2	3	3
4	4	2	2	4	4	3	3	3	3
4	4	2	2	4	4	3	3	3	3
3	3	1	1	4	4	3	3	3	3
3	3	2	2	4	4	3	3	2	2
4	4	2	2	4	4	3	3	3	3
3	3	2	2	4	4	3	3	3	3
3	3	2	2	4	4	3	3	2	2
4	4	2	2	4	4	2	2	2	2
4	4	2	2	4	4	3	3	3	3
3	3	2	2	4	4	3	3	3	3
3	3	2	2	4	4	2	2	2	2
4	4	2	2	4	4	3	3	3	3
3	3	1	1	4	4	3	3	3	3
4	4	2	2	4	4	3	3	3	3
4	4	2	2	4	4	2	2	2	2
4	4	2	2	4	4	3	3	3	3
4	4	2	2	4	4	2	2	3	3
3	3	2	2	4	4	3	3	3	3
4	4	2	2	4	4	2	2	3	3
3	3	2	2	4	4	2	2	3	3
3	3	2	2	4	4	3	3	3	3
3	3	2	2	4	4	3	3	2	2
4	4	2	2	4	4	3	3	3	3
3	3	2	2	4	4	3	3	2	2
4	4	1	1	4	4	3	3	3	3
3	3	2	2	4	4	2	2	2	2
4	4	2	2	4	4	3	3	3	3
4	4	2	2	4	4	3	3	3	3
3	3	2	2	4	4	3	3	2	2
4	4	2	2	4	4	3	3	3	3
3	3	2	2	4	4	3	3	3	3
3	3	2	2	4	4	2	2	3	3
4	4	2	2	4	4	2	2	3	3
3	3	2	2	4	4	3	3	3	3
3	3	2	2	4	4	3	3	3	3

For Review Only

3	3	2	2	4	4	3	3	3	3
3	3	2	2	4	4	3	3	3	3
4	4	2	2	4	4	3	3	3	3
4	4	2	2	4	4	3	3	3	3
3	3	2	2	4	4	3	3	3	3
3	3	2	2	4	4	3	3	3	3
3	3	2	2	4	4	3	3	3	3
4	4	2	2	4	4	3	3	3	3
4	4	2	2	4	4	3	3	3	3
4	4	2	2	4	4	3	3	3	3
3	3	2	2	2	2	3	3	3	3
3	3	2	2	4	4	3	3	3	3
4	4	2	2	4	4	3	3	3	3
4	4	2	2	4	4	2	2	2	2
3	3	2	2	4	4	2	2	2	2
3	3	2	2	4	4	2	2	3	3
4	4	2	2	4	4	3	3	3	3
3	3	2	2	4	4	3	3	3	3
3	3	2	2	4	4	3	3	2	2
4	4	2	2	4	4	3	3	3	3
4	4	2	2	4	4	3	3	3	3
3	3	2	2	4	4	2	2	3	3
3	3	2	2	4	4	2	2	3	3
4	4	2	2	4	4	3	3	3	3
3	3	2	2	4	4	3	3	3	3
4	4	2	2	4	4	2	2	2	2
3	3	2	2	4	4	3	3	3	3
4	4	2	2	4	4	3	3	3	3
4	4	2	2	4	4	3	3	3	3
4	4	2	2	4	4	3	3	3	3
3	3	2	2	4	4	3	3	3	3
4	4	2	2	4	4	2	2	3	3
3	3	2	2	4	4	2	2	2	2
4	4	2	2	4	4	2	2	2	2
3	3	2	2	4	4	3	3	3	3
4	4	2	2	4	4	3	3	3	3
3	3	2	2	4	4	3	3	3	3
4	4	2	2	4	4	2	2	3	3
4	4	2	2	4	4	3	3	3	3
3	3	2	2	4	4	3	3	3	3
4	4	2	2	2	2	3	3	3	3
3	3	2	2	4	4	3	3	3	3
3	3	2	2	4	4	2	2	2	2
4	4	2	2	4	4	3	3	3	3
4	4	1	1	4	4	3	3	3	3
3	3	2	2	4	4	3	3	2	2
3	3	2	2	4	4	2	2	3	3
3	3	2	2	4	4	2	2	2	2
4	4	1	1	4	4	3	3	2	2
3	3	2	2	4	4	3	3	3	3
4	4	2	2	4	4	3	3	3	3
3	3	1	1	4	4	3	3	3	3
3	3	2	2	4	4	3	3	2	2
4	4	2	2	2	2	3	3	3	3
3	3	1	1	4	4	3	3	2	2

For Review Only

4	4	2	2	4	4	2	2	3	3
4	4	2	2	4	4	2	2	3	3
4	4	2	2	4	4	2	2	3	3
3	3	2	2	4	4	2	2	3	3
3	3	2	2	4	4	3	3	3	3
4	4	2	2	4	4	3	3	3	3
3	3	1	1	4	4	3	3	3	3
3	3	2	2	4	4	3	3	2	2
3	3	2	2	4	4	3	3	2	2
3	3	2	2	4	4	2	2	2	2
4	4	2	2	4	4	3	3	3	3
3	3	2	2	4	4	2	2	3	3
3	3	2	2	4	4	2	2	3	3
4	4	2	2	4	4	2	2	2	2
3	3	2	2	2	2	3	3	3	3
3	3	2	2	4	4	3	3	3	3
3	3	2	2	4	4	3	3	2	2
3	3	2	2	4	4	3	3	3	3
3	3	2	2	4	4	3	3	3	3
3	3	2	2	4	4	3	3	3	3
3	3	2	2	4	4	3	3	3	3
4	4	2	2	4	4	3	3	3	3
4	4	2	2	4	4	3	3	3	3
3	3	2	2	4	4	3	3	3	3
3	3	2	2	4	4	3	3	3	3
3	3	2	2	4	4	3	3	3	3
3	3	2	2	4	4	2	2	2	2
3	3	2	2	4	4	3	3	3	3
3	3	2	2	4	4	3	3	3	3
4	4	2	2	4	4	3	3	3	3
3	3	2	2	4	4	2	2	2	2
3	3	1	1	4	4	3	3	3	3
4	4	2	2	4	4	2	2	2	2
4	4	2	2	4	4	3	3	3	3
3	3	2	2	4	4	2	2	3	3
3	3	2	2	4	4	2	2	2	2
4	4	2	2	4	4	3	3	3	3
4	4	2	2	4	4	3	3	3	3
4	4	1	1	4	4	3	3	3	3
3	3	2	2	4	4	3	3	2	2
4	4	2	2	4	4	3	3	3	3
4	4	2	2	4	4	2	2	3	3
3	3	2	2	4	4	3	3	3	3
3	3	2	2	4	4	3	3	3	3
4	4	2	2	4	4	3	3	3	3
3	3	2	2	4	4	2	2	2	2
4	4	2	2	4	4	2	2	3	3
3	3	2	2	4	4	3	3	3	3
3	3	2	2	4	4	2	2	3	3
3	3	2	2	4	4	2	2	2	2
4	4	2	2	4	4	2	2	3	3
3	3	2	2	4	4	3	3	2	2
4	4	2	2	2	2	3	3	3	3
3	3	2	2	4	4	3	3	3	3
3	3	2	2	4	4	3	3	3	3

For Review Only

3	3	2	2	4	4	3	3	3	3
3	3	2	2	4	4	3	3	3	3

For Review Only

SNP812	SNP812	SNP813	SNP813	SNP814	SNP814	SNP815	SNP815	SNP816	SNP816
1	1	1	1	1	1	3	3	2	2
2	2	4	4	1	1	3	3	2	2
2	2	4	4	1	1	3	3	2	2
1	1	1	1	1	1	3	3	2	2
2	2	4	4	1	1	3	3	2	2
1	1	4	4	1	1	3	3	2	2
1	1	1	1	4	4	3	3	2	2
2	2	4	4	1	1	3	3	2	2
1	1	1	1	1	1	3	3	2	2
2	2	4	4	1	1	3	3	2	2
1	1	4	4	1	1	3	3	2	2
2	2	4	4	1	1	3	3	2	2
2	2	1	1	1	1	3	3	2	2
1	1	4	4	1	1	2	2	2	2
1	1	1	1	1	1	3	3	2	2
2	2	4	4	1	1	3	3	2	2
2	2	4	4	1	1	3	3	2	2
1	1	1	1	4	4	3	3	2	2
1	1	1	1	4	4	3	3	2	2
2	2	4	4	1	1	3	3	2	2
2	2	4	4	1	1	3	3	2	2
2	2	4	4	1	1	3	3	2	2
1	1	4	4	1	1	3	3	2	2
2	2	4	4	1	1	2	2	2	2
2	2	4	4	1	1	3	3	2	2
2	2	4	4	1	1	3	3	2	2
1	1	1	1	4	4	3	3	2	2
1	1	1	1	1	1	3	3	2	2
2	2	4	4	1	1	3	3	2	2
1	1	4	4	1	1	3	3	2	2
2	2	4	4	1	1	3	3	2	2
1	1	1	1	4	4	3	3	2	2
1	1	1	1	4	4	3	3	2	2
1	1	4	4	1	1	3	3	2	2
2	2	1	1	1	1	3	3	2	2
2	2	4	4	1	1	2	2	2	2
1	1	1	1	4	4	2	2	2	2
1	1	1	1	4	4	3	3	2	2
1	1	4	4	1	1	3	3	2	2
1	1	4	4	1	1	3	3	2	2
2	2	4	4	1	1	3	3	2	2
2	2	1	1	1	1	3	3	2	2
2	2	4	4	1	1	3	3	2	2
1	1	1	1	4	4	3	3	2	2
2	2	4	4	1	1	3	3	2	2
2	2	4	4	1	1	3	3	2	2
2	2	4	4	1	1	3	3	1	1
1	1	4	4	1	1	3	3	2	2
1	1	1	1	4	4	3	3	2	2
2	2	1	1	1	1	3	3	2	2
2	2	4	4	1	1	3	3	2	2
1	1	4	4	1	1	3	3	2	2

For Review Only

2	2	4	4	1	1	3	3	2	2
1	1	1	1	4	4	3	3	2	2
1	1	1	1	4	4	3	3	2	2
2	2	4	4	1	1	3	3	2	2
1	1	1	1	4	4	3	3	2	2
2	2	4	4	1	1	3	3	2	2
2	2	4	4	1	1	3	3	2	2
2	2	4	4	1	1	3	3	2	2
1	1	1	1	4	4	3	3	2	2
1	1	4	4	1	1	3	3	2	2
2	2	4	4	1	1	3	3	2	2
2	2	4	4	1	1	3	3	2	2
2	2	4	4	1	1	3	3	2	2
2	2	4	4	1	1	3	3	2	2
1	1	4	4	1	1	3	3	2	2
1	1	4	4	1	1	3	3	2	2
1	1	4	4	1	1	3	3	2	2
2	2	4	4	1	1	3	3	2	2
2	2	4	4	1	1	3	3	2	2
1	1	1	1	4	4	3	3	2	2
1	1	4	4	1	1	3	3	2	2
1	1	4	4	1	1	3	3	2	2
2	2	4	4	1	1	3	3	2	2
2	2	4	4	1	1	3	3	2	2
2	2	4	4	1	1	3	3	2	2
2	2	4	4	1	1	3	3	2	2
1	1	1	1	4	4	3	3	2	2
1	1	4	4	1	1	3	3	2	2
2	2	4	4	1	1	3	3	2	2
1	1	1	1	4	4	3	3	2	2
2	2	4	4	1	1	3	3	2	2
2	2	4	4	1	1	2	2	2	2
1	1	4	4	1	1	3	3	2	2
2	2	4	4	1	1	3	3	2	2
1	1	1	1	4	4	3	3	2	2
2	2	4	4	1	1	3	3	2	2
2	2	4	4	1	1	3	3	2	2
2	2	4	4	1	1	3	3	2	2
2	2	1	1	4	4	3	3	2	2
1	1	1	1	1	1	3	3	2	2
1	1	1	1	4	4	3	3	2	2
2	2	4	4	1	1	3	3	2	2
1	1	1	1	1	1	3	3	2	2
2	2	4	4	1	1	3	3	1	1
1	1	1	1	4	4	3	3	2	2
1	1	4	4	1	1	3	3	2	2
1	1	4	4	1	1	2	2	2	2
2	2	4	4	1	1	3	3	2	2
2	2	4	4	1	1	3	3	2	2
1	1	1	1	4	4	3	3	2	2
2	2	4	4	1	1	3	3	2	2
1	1	4	4	1	1	3	3	2	2
2	2	4	4	1	1	2	2	2	2
1	1	4	4	1	1	3	3	1	1
1	1	1	1	4	4	3	3	2	2

For Review Only

1	1	4	4	1	1	3	3	2	2
1	1	1	1	1	1	3	3	2	2
2	2	4	4	1	1	3	3	2	2
1	1	4	4	1	1	2	2	2	2
2	2	4	4	1	1	3	3	2	2
2	2	4	4	1	1	3	3	2	2
2	2	4	4	1	1	3	3	2	2
1	1	1	1	4	4	3	3	2	2
1	1	4	4	1	1	3	3	2	2
2	2	4	4	1	1	3	3	2	2
1	1	4	4	1	1	2	2	2	2
1	1	1	1	1	1	3	3	2	2
2	2	4	4	1	1	3	3	2	2
1	1	4	4	1	1	3	3	2	2
1	1	1	1	1	1	3	3	2	2
1	1	1	1	1	1	3	3	2	2
1	1	4	4	1	1	3	3	2	2
2	2	1	1	1	1	3	3	2	2
1	1	4	4	1	1	2	2	2	2
1	1	4	4	1	1	3	3	2	2
1	1	1	1	1	1	3	3	2	2
1	1	1	1	4	4	3	3	2	2
2	2	4	4	1	1	3	3	2	2
1	1	4	4	1	1	3	3	2	2
1	1	4	4	1	1	3	3	2	2
1	1	4	4	1	1	3	3	2	2
2	2	4	4	1	1	3	3	2	2
2	2	1	1	4	4	3	3	2	2
1	1	1	1	1	1	3	3	2	2
2	2	4	4	1	1	3	3	2	2
1	1	4	4	1	1	3	3	2	2
1	1	4	4	1	1	3	3	2	2
1	1	1	1	4	4	3	3	2	2
1	1	1	1	1	1	3	3	2	2
1	1	4	4	1	1	3	3	2	2
1	1	4	4	1	1	3	3	2	2
1	1	4	4	1	1	2	2	2	2
1	1	1	1	4	4	3	3	2	2
1	1	4	4	1	1	3	3	2	2
1	1	4	4	1	1	3	3	2	2
1	1	4	4	1	1	3	3	2	2
1	1	4	4	1	1	2	2	2	2
2	2	4	4	1	1	3	3	2	2
1	1	4	4	1	1	3	3	2	2
2	2	4	4	1	1	3	3	2	2
2	2	1	1	4	4	3	3	2	2
2	2	4	4	1	1	3	3	2	2
2	2	4	4	1	1	3	3	2	2
1	1	1	1	1	1	3	3	2	2
2	2	4	4	1	1	3	3	2	2
2	2	4	4	1	1	3	3	2	2
1	1	1	1	4	4	2	2	2	2
1	1	4	4	1	1	3	3	2	2
2	2	4	4	1	1	3	3	2	2
2	2	4	4	1	1	3	3	2	2

For Review Only

1	1	1	1	4	4	3	3	2	2
1	1	4	4	1	1	3	3	2	2
1	1	1	1	1	1	3	3	2	2
1	1	1	1	4	4	3	3	2	2
1	1	1	1	1	1	3	3	2	2
1	1	4	4	1	1	3	3	2	2
1	1	4	4	1	1	3	3	2	2
2	2	1	1	1	1	3	3	2	2
1	1	4	4	1	1	3	3	2	2
1	1	1	1	4	4	3	3	2	2
2	2	4	4	1	1	3	3	2	2
1	1	1	1	1	1	3	3	2	2
1	1	4	4	1	1	3	3	2	2
2	2	1	1	1	1	3	3	2	2
1	1	4	4	1	1	2	2	2	2
1	1	4	4	1	1	3	3	2	2
1	1	4	4	1	1	3	3	2	2
2	2	4	4	1	1	3	3	2	2
2	2	4	4	1	1	3	3	2	2
2	2	4	4	1	1	3	3	2	2
1	1	4	4	1	1	3	3	2	2
2	2	4	4	1	1	2	2	2	2
1	1	4	4	1	1	3	3	2	2
2	2	4	4	1	1	3	3	2	2
2	2	4	4	1	1	3	3	2	2
1	1	4	4	1	1	3	3	2	2
1	1	1	1	1	1	3	3	2	2
1	1	4	4	1	1	3	3	2	2
1	1	1	1	4	4	3	3	2	2
2	2	4	4	1	1	3	3	2	2
2	2	4	4	1	1	3	3	2	2
1	1	1	1	4	4	3	3	2	2
2	2	4	4	1	1	2	2	2	2
1	1	4	4	1	1	2	2	2	2
1	1	1	1	4	4	3	3	2	2
2	2	4	4	1	1	3	3	2	2
2	2	4	4	1	1	3	3	2	2
1	1	1	1	4	4	3	3	2	2
2	2	4	4	1	1	3	3	2	2
2	2	4	4	1	1	3	3	2	2
2	2	4	4	1	1	3	3	2	2
2	2	4	4	1	1	3	3	2	2
1	1	4	4	1	1	3	3	2	2
1	1	1	1	1	1	3	3	2	2
1	1	1	1	1	1	2	2	2	2
2	2	1	1	1	1	3	3	2	2
1	1	4	4	1	1	3	3	2	2
2	2	4	4	1	1	3	3	2	2
2	2	4	4	1	1	2	2	2	2
1	1	4	4	1	1	3	3	2	2
1	1	1	1	4	4	3	3	2	2
1	1	4	4	1	1	2	2	2	2
1	1	4	4	1	1	3	3	2	2
2	2	4	4	1	1	3	3	2	2
1	1	4	4	1	1	2	2	2	2

For Review Only

1	1	4	4	1	1	3	3	2	2
1	1	4	4	1	1	3	3	2	2
2	2	1	1	4	4	3	3	2	2
1	1	4	4	1	1	3	3	2	2
2	2	4	4	1	1	3	3	2	2
2	2	1	1	4	4	3	3	2	2
1	1	4	4	1	1	2	2	2	2
2	2	4	4	1	1	3	3	2	2
2	2	4	4	1	1	3	3	2	2
1	1	4	4	1	1	2	2	2	2
1	1	4	4	1	1	3	3	2	2
1	1	4	4	1	1	3	3	2	2
2	2	1	1	4	4	3	3	2	2
2	2	1	1	4	4	3	3	2	2
2	2	4	4	1	1	3	3	2	2
1	1	1	1	4	4	3	3	2	2
2	2	1	1	4	4	3	3	2	2
1	1	4	4	1	1	2	2	2	2
1	1	4	4	1	1	3	3	2	2
2	2	4	4	1	1	3	3	2	2
2	2	4	4	1	1	3	3	2	2
1	1	1	1	4	4	3	3	2	2
2	2	4	4	1	1	3	3	2	2
1	1	4	4	1	1	3	3	2	2
2	2	4	4	1	1	3	3	2	2
2	2	1	1	1	1	3	3	2	2
1	1	4	4	1	1	3	3	2	2
2	2	4	4	1	1	3	3	2	2
2	2	4	4	1	1	3	3	2	2
1	1	1	1	4	4	3	3	2	2
1	1	4	4	1	1	2	2	2	2
2	2	4	4	1	1	3	3	2	2
1	1	4	4	1	1	2	2	2	2
2	2	4	4	1	1	3	3	2	2
1	1	4	4	1	1	3	3	2	2
1	1	4	4	1	1	3	3	2	2
2	2	4	4	1	1	3	3	2	2
2	2	4	4	1	1	3	3	2	2
2	2	4	4	1	1	2	2	2	2
1	1	4	4	1	1	3	3	2	2
1	1	1	1	4	4	3	3	2	2
1	1	1	1	4	4	3	3	2	2
1	1	1	1	4	4	3	3	2	2
2	2	4	4	1	1	3	3	2	2
2	2	4	4	1	1	3	3	2	2
2	2	1	1	4	4	3	3	2	2
1	1	4	4	1	1	3	3	2	2
1	1	1	1	4	4	3	3	2	2
2	2	1	1	4	4	3	3	2	2
1	1	4	4	1	1	3	3	2	2
1	1	1	1	4	4	3	3	2	2
1	1	4	4	1	1	3	3	2	2
2	2	4	4	1	1	3	3	2	2
2	2	4	4	1	1	3	3	2	2
1	1	4	4	1	1	2	2	2	2

For Review Only

1	1	1	1	4	4	3	3	2	2
1	1	1	1	4	4	3	3	2	2

For Review Only

SNP817	SNP817	SNP818	SNP818	SNP819	SNP819	SNP820	SNP820	SNP821	SNP821
4	4	4	4	3	3	4	4	2	2
4	4	4	4	3	3	4	4	2	2
4	4	4	4	3	3	4	4	2	2
4	4	4	4	3	3	4	4	2	2
4	4	4	4	3	3	4	4	2	2
4	4	4	4	3	3	4	4	2	2
2	2	4	4	2	2	4	4	2	2
2	2	4	4	2	2	1	1	2	2
4	4	1	1	3	3	4	4	2	2
2	2	4	4	2	2	1	1	2	2
2	2	4	4	3	3	4	4	2	2
4	4	4	4	3	3	4	4	2	2
4	4	4	4	3	3	4	4	2	2
4	4	4	4	2	2	4	4	2	2
4	4	4	4	3	3	4	4	2	2
4	4	1	1	3	3	1	1	4	4
4	4	4	4	2	2	4	4	2	2
4	4	4	4	2	2	4	4	2	2
2	2	4	4	3	3	4	4	2	2
4	4	1	1	2	2	4	4	4	4
4	4	4	4	3	3	4	4	2	2
4	4	4	4	3	3	4	4	2	2
2	2	4	4	2	2	4	4	2	2
2	2	4	4	2	2	4	4	2	2
4	4	4	4	3	3	4	4	2	2
4	4	4	4	3	3	4	4	2	2
4	4	4	4	3	3	4	4	2	2
4	4	4	4	3	3	4	4	2	2
4	4	1	1	2	2	4	4	4	4
2	2	1	1	2	2	4	4	4	4
4	4	4	4	3	3	4	4	2	2
2	2	4	4	3	3	4	4	2	2
2	2	4	4	3	3	4	4	2	2
2	2	4	4	2	2	4	4	2	2
4	4	1	1	3	3	4	4	2	2
2	2	4	4	2	2	4	4	2	2
4	4	4	4	2	2	4	4	2	2
4	4	4	4	3	3	4	4	2	2
2	2	4	4	2	2	4	4	2	2
4	4	4	4	3	3	4	4	2	2
2	2	4	4	2	2	4	4	2	2
4	4	1	1	2	2	4	4	4	4
4	4	1	1	2	2	1	1	4	4
4	4	4	4	3	3	4	4	2	2
4	4	1	1	2	2	1	1	4	4
4	4	4	4	3	3	4	4	2	2
4	4	4	4	3	3	4	4	2	2
4	4	4	4	3	3	4	4	2	2
4	4	4	4	3	3	4	4	2	2
4	4	4	4	3	3	4	4	2	2
4	4	4	4	3	3	4	4	2	2
4	4	4	4	3	3	1	1	2	2

For Review Only

4	4	4	4	3	3	4	4	2	2
2	2	4	4	3	3	4	4	2	2
4	4	4	4	3	3	4	4	2	2
4	4	4	4	2	2	4	4	2	2
2	2	4	4	3	3	4	4	2	2
4	4	4	4	3	3	4	4	2	2
4	4	4	4	3	3	4	4	2	2
2	2	4	4	2	2	4	4	2	2
2	2	4	4	2	2	4	4	2	2
4	4	4	4	3	3	4	4	2	2
4	4	4	4	3	3	4	4	2	2
4	4	4	4	2	2	4	4	2	2
4	4	4	4	3	3	4	4	2	2
4	4	1	1	2	2	4	4	4	4
4	4	4	4	3	3	4	4	2	2
2	2	1	1	2	2	4	4	4	4
4	4	4	4	3	3	4	4	2	2
4	4	4	4	3	3	4	4	2	2
4	4	4	4	3	3	4	4	2	2
4	4	4	4	3	3	4	4	2	2
4	4	4	4	2	2	4	4	2	2
4	4	4	4	2	2	4	4	2	2
4	4	1	1	3	3	4	4	2	2
4	4	4	4	2	2	4	4	2	2
4	4	1	1	2	2	1	1	4	4
2	2	1	1	2	2	1	1	4	4
2	2	4	4	3	3	4	4	2	2
2	2	4	4	2	2	4	4	2	2
4	4	4	4	3	3	4	4	2	2
4	4	4	4	3	3	4	4	2	2
4	4	1	1	2	2	4	4	2	2
2	2	4	4	3	3	4	4	2	2
2	2	4	4	2	2	4	4	4	4
4	4	4	4	3	3	4	4	2	2
2	2	4	4	3	3	4	4	2	2
4	4	1	1	2	2	1	1	4	4
2	2	1	1	2	2	4	4	4	4
4	4	4	4	3	3	4	4	2	2
2	2	4	4	3	3	4	4	2	2
4	4	4	4	2	2	1	1	2	2
4	4	4	4	3	3	4	4	2	2
4	4	4	4	3	3	4	4	2	2
2	2	4	4	3	3	4	4	2	2
4	4	4	4	3	3	4	4	2	2
2	2	4	4	3	3	4	4	2	2
4	4	4	4	3	3	4	4	2	2
2	2	4	4	2	2	4	4	2	2
4	4	4	4	2	2	4	4	2	2
4	4	4	4	3	3	4	4	2	2
4	4	4	4	2	2	4	4	2	2
4	4	4	4	3	3	4	4	2	2
2	2	4	4	2	2	4	4	2	2
2	2	4	4	2	2	4	4	2	2
4	4	4	4	3	3	4	4	2	2
4	4	4	4	3	3	4	4	2	2

For Review Only

4	4	4	4	3	3	4	4	2	2
4	4	4	4	2	2	1	1	2	2
2	2	4	4	3	3	4	4	2	2
4	4	4	4	2	2	4	4	2	2
4	4	4	4	3	3	4	4	2	2
4	4	4	4	3	3	4	4	2	2
4	4	4	4	3	3	4	4	2	2
4	4	4	4	3	3	1	1	2	2
2	2	4	4	3	3	4	4	2	2
4	4	4	4	3	3	4	4	2	2
4	4	4	4	3	3	4	4	2	2
4	4	4	4	3	3	4	4	2	2
2	2	4	4	2	2	4	4	2	2
4	4	4	4	3	3	1	1	2	2
4	4	4	4	3	3	4	4	2	2
2	2	4	4	3	3	4	4	2	2
4	4	4	4	3	3	4	4	2	2
4	4	4	4	3	3	4	4	2	2
4	4	4	4	2	2	4	4	2	2
4	4	4	4	3	3	4	4	2	2
4	4	4	4	3	3	4	4	2	2
4	4	4	4	3	3	4	4	2	2
4	4	4	4	3	3	4	4	2	2
2	2	4	4	2	2	4	4	2	2
4	4	4	4	3	3	4	4	2	2
2	2	4	4	2	2	1	1	2	2
2	2	4	4	2	2	4	4	2	2
2	2	4	4	3	3	4	4	2	2
4	4	4	4	3	3	4	4	2	2
2	2	4	4	3	3	4	4	2	2
2	2	4	4	2	2	4	4	2	2
4	4	4	4	3	3	4	4	2	2
4	4	4	4	3	3	4	4	2	2
4	4	4	4	3	3	4	4	2	2
2	2	4	4	2	2	4	4	2	2
4	4	4	4	3	3	4	4	2	2
4	4	4	4	2	2	4	4	2	2
2	2	4	4	3	3	4	4	2	2
4	4	4	4	3	3	4	4	2	2
2	2	4	4	3	3	4	4	2	2
2	2	4	4	2	2	1	1	2	2
2	2	4	4	2	2	4	4	2	2
4	4	4	4	3	3	4	4	2	2
2	2	4	4	2	2	4	4	2	2
2	2	4	4	3	3	4	4	2	2
2	2	4	4	3	3	4	4	2	2
4	4	4	4	3	3	4	4	2	2
4	4	4	4	3	3	4	4	2	2
4	4	4	4	3	3	4	4	2	2
4	4	4	4	3	3	4	4	2	2
4	4	4	4	3	3	4	4	2	2
4	4	4	4	2	2	4	4	2	2
2	2	4	4	3	3	4	4	2	2
4	4	4	4	2	2	4	4	2	2
4	4	4	4	3	3	4	4	2	2

For Review Only

4	4	4	4	3	3	4	4	2	2
2	2	4	4	2	2	1	1	2	2
4	4	4	4	2	2	4	4	2	2
2	2	4	4	3	3	1	1	2	2
4	4	4	4	3	3	4	4	2	2
4	4	4	4	3	3	4	4	2	2
2	2	4	4	3	3	4	4	2	2
4	4	4	4	3	3	4	4	2	2
2	2	4	4	3	3	1	1	2	2
4	4	4	4	3	3	4	4	2	2
4	4	4	4	3	3	4	4	2	2
4	4	1	1	3	3	4	4	2	2
4	4	4	4	3	3	4	4	2	2
2	2	4	4	3	3	4	4	2	2
2	2	4	4	2	2	4	4	2	2
2	2	4	4	3	3	4	4	2	2
4	4	4	4	3	3	4	4	2	2
4	4	4	4	3	3	4	4	2	2
2	2	4	4	3	3	4	4	2	2
4	4	4	4	3	3	4	4	2	2
2	2	4	4	3	3	4	4	2	2
2	2	4	4	2	2	4	4	2	2
2	2	4	4	2	2	1	1	2	2
4	4	4	4	2	2	4	4	2	2
2	2	4	4	2	2	4	4	4	4
2	2	4	4	2	2	4	4	2	2
4	4	4	4	2	2	4	4	2	2
4	4	4	4	3	3	4	4	2	2
4	4	4	4	3	3	4	4	2	2
4	4	4	4	3	3	4	4	2	2
2	2	4	4	3	3	4	4	2	2
4	4	4	4	3	3	4	4	2	2
2	2	4	4	2	2	4	4	2	2
2	2	4	4	2	2	4	4	2	2
4	4	4	4	3	3	4	4	2	2
4	4	4	4	2	2	4	4	2	2
4	4	4	4	3	3	4	4	2	2
4	4	4	4	3	3	4	4	2	2
4	4	4	4	3	3	4	4	2	2
4	4	4	4	3	3	4	4	2	2
4	4	4	4	3	3	4	4	2	2
4	4	4	4	3	3	4	4	2	2
2	2	4	4	2	2	4	4	2	2
4	4	4	4	3	3	4	4	2	2
4	4	4	4	2	2	4	4	4	4
2	2	4	4	3	3	4	4	2	2
2	2	4	4	3	3	4	4	2	2
2	2	4	4	3	3	4	4	2	2
2	2	4	4	2	2	4	4	2	2
2	2	4	4	2	2	4	4	2	2
4	4	4	4	3	3	4	4	2	2
2	2	4	4	3	3	4	4	2	2
2	2	4	4	2	2	4	4	2	2
4	4	4	4	3	3	4	4	2	2
2	2	1	1	2	2	4	4	4	4

For Review Only

2	2	4	4	3	3	4	4	2	2
2	2	1	1	2	2	4	4	4	4
4	4	4	4	3	3	4	4	2	2
2	2	4	4	2	2	4	4	2	2
4	4	4	4	2	2	4	4	2	2
4	4	4	4	3	3	4	4	2	2
4	4	1	1	3	3	1	1	2	2
2	2	1	1	2	2	4	4	4	4
2	2	4	4	3	3	4	4	2	2
2	2	4	4	2	2	4	4	2	2
4	4	4	4	3	3	4	4	2	2
4	4	4	4	3	3	4	4	2	2
2	2	4	4	3	3	4	4	2	2
2	2	4	4	3	3	4	4	2	2
4	4	4	4	3	3	4	4	2	2
2	2	4	4	3	3	4	4	2	2
2	2	4	4	3	3	4	4	2	2
2	2	4	4	3	3	4	4	2	2
2	2	4	4	3	3	4	4	2	2
4	4	4	4	3	3	4	4	2	2
4	4	4	4	3	3	4	4	2	2
4	4	4	4	3	3	4	4	2	2
2	2	4	4	3	3	4	4	2	2
4	4	1	1	2	2	4	4	4	4
2	2	4	4	2	2	4	4	2	2
2	2	4	4	3	3	1	1	2	2
2	2	4	4	2	2	4	4	2	2
4	4	4	4	3	3	4	4	2	2
4	4	4	4	3	3	4	4	2	2
4	4	4	4	3	3	4	4	2	2
2	2	4	4	3	3	4	4	2	2
4	4	4	4	2	2	4	4	2	2
2	2	4	4	3	3	4	4	2	2
2	2	4	4	3	3	4	4	2	2
2	2	4	4	2	2	4	4	2	2
2	2	4	4	2	2	4	4	2	2
2	2	4	4	3	3	4	4	2	2
4	4	4	4	2	2	4	4	2	2
2	2	4	4	2	2	4	4	2	2
2	2	4	4	2	2	4	4	2	2
4	4	4	4	3	3	4	4	2	2
4	4	4	4	3	3	4	4	2	2
2	2	1	1	2	2	4	4	4	4
4	4	4	4	3	3	4	4	2	2
4	4	4	4	3	3	4	4	2	2
2	2	4	4	3	3	4	4	2	2
4	4	4	4	3	3	4	4	2	2
2	2	4	4	3	3	4	4	2	2
4	4	4	4	3	3	4	4	2	2
2	2	4	4	2	2	4	4	2	2
4	4	4	4	3	3	4	4	2	2
2	2	4	4	2	2	4	4	2	2
4	4	4	4	3	3	4	4	2	2
4	4	4	4	3	3	4	4	2	2
4	4	1	1	3	3	4	4	4	4

For Review Only

2	2	4	4	3	3	4	4	2	2
4	4	4	4	3	3	4	4	2	2

For Review Only

SNP822	SNP822	SNP823	SNP823	SNP824	SNP824	SNP825	SNP825	SNP826	SNP826
1	1	2	2	3	3	2	2	2	2
1	1	2	2	3	3	2	2	2	2
4	4	1	1	3	3	2	2	2	2
1	1	2	2	3	3	2	2	2	2
1	1	2	2	3	3	2	2	2	2
1	1	2	2	3	3	2	2	2	2
1	1	2	2	2	2	1	1	3	3
1	1	2	2	2	2	2	2	2	2
1	1	2	2	2	2	2	2	2	2
1	1	2	2	2	2	2	2	2	2
1	1	2	2	3	3	2	2	2	2
4	4	1	1	3	3	2	2	2	2
1	1	2	2	3	3	2	2	2	2
1	1	2	2	2	2	2	2	2	2
1	1	2	2	3	3	2	2	2	2
1	1	2	2	3	3	2	2	2	2
1	1	2	2	2	2	2	2	2	2
1	1	2	2	2	2	2	2	2	2
4	4	1	1	3	3	2	2	2	2
1	1	2	2	2	2	2	2	2	2
1	1	2	2	3	3	2	2	2	2
1	1	2	2	3	3	2	2	2	2
1	1	2	2	2	2	2	2	2	2
1	1	2	2	2	2	2	2	2	2
1	1	2	2	3	3	2	2	2	2
4	4	1	1	3	3	2	2	2	2
1	1	2	2	3	3	2	2	2	2
1	1	2	2	3	3	2	2	2	2
1	1	2	2	2	2	2	2	2	2
1	1	2	2	2	2	2	2	2	2
1	1	2	2	3	3	2	2	2	2
4	4	2	2	3	3	2	2	2	2
4	4	2	2	3	3	2	2	2	2
1	1	2	2	2	2	2	2	2	2
1	1	2	2	3	3	2	2	2	2
1	1	2	2	2	2	2	2	2	2
1	1	2	2	2	2	2	2	2	2
1	1	2	2	3	3	2	2	2	2
1	1	2	2	2	2	2	2	2	2
1	1	2	2	3	3	2	2	2	2
1	1	2	2	2	2	2	2	2	2
1	1	2	2	2	2	2	2	2	2
1	1	2	2	2	2	2	2	2	2
1	1	2	2	3	3	2	2	2	2
1	1	2	2	2	2	2	2	2	2
4	4	1	1	3	3	2	2	2	2
4	4	1	1	3	3	2	2	2	2
1	1	2	2	3	3	2	2	2	2
1	1	2	2	3	3	2	2	2	2
1	1	2	2	3	3	2	2	2	2
4	4	1	1	3	3	2	2	2	2
1	1	2	2	3	3	1	1	3	3

For Review Only

1	1	2	2	3	3	2	2	2	2
4	4	2	2	3	3	2	2	2	2
1	1	1	1	3	3	2	2	2	2
1	1	2	2	2	2	2	2	2	2
4	4	1	1	3	3	2	2	2	2
4	4	1	1	3	3	1	1	3	3
4	4	1	1	3	3	2	2	2	2
1	1	2	2	2	2	2	2	2	2
1	1	2	2	2	2	2	2	2	2
1	1	2	2	3	3	2	2	2	2
4	4	1	1	3	3	1	1	3	3
4	4	1	1	2	2	1	1	3	3
1	1	2	2	3	3	2	2	2	2
1	1	2	2	2	2	2	2	2	2
1	1	2	2	3	3	2	2	2	2
1	1	2	2	2	2	2	2	2	2
4	4	2	2	3	3	2	2	2	2
1	1	2	2	3	3	2	2	2	2
1	1	2	2	3	3	2	2	2	2
1	1	2	2	3	3	2	2	2	2
1	1	2	2	3	3	1	1	3	3
1	1	2	2	2	2	2	2	2	2
1	1	2	2	3	3	2	2	2	2
4	4	1	1	2	2	1	1	3	3
1	1	2	2	2	2	2	2	2	2
4	4	1	1	2	2	1	1	3	3
4	4	1	1	3	3	2	2	2	2
1	1	2	2	3	3	2	2	2	2
4	4	1	1	3	3	2	2	2	2
1	1	2	2	3	3	2	2	2	2
1	1	2	2	2	2	2	2	2	2
1	1	2	2	3	3	2	2	2	2
1	1	2	2	2	2	2	2	2	2
1	1	2	2	3	3	2	2	2	2
1	1	2	2	3	3	2	2	2	2
1	1	2	2	2	2	2	2	2	2
1	1	2	2	2	2	2	2	2	2
1	1	2	2	3	3	2	2	2	2
4	4	1	1	3	3	2	2	2	2
1	1	2	2	2	2	2	2	2	2
1	1	2	2	3	3	2	2	2	2
1	1	2	2	3	3	2	2	2	2
1	1	2	2	3	3	2	2	2	2
4	4	1	1	3	3	1	1	3	3
1	1	2	2	3	3	2	2	2	2
1	1	2	2	3	3	2	2	2	2
1	1	2	2	2	2	2	2	2	2
4	4	1	1	2	2	2	2	2	2
1	1	2	2	3	3	1	1	3	3
1	1	2	2	2	2	2	2	2	2
1	1	2	2	3	3	1	1	2	2
1	1	2	2	2	2	2	2	2	2
1	1	2	2	2	2	2	2	2	2
1	1	2	2	3	3	2	2	2	2
1	1	2	2	3	3	2	2	2	2

For Review Only

1	1	2	2	3	3	2	2	2	2
1	1	2	2	2	2	2	2	2	2
1	1	2	2	3	3	2	2	2	2
1	1	2	2	2	2	2	2	2	2
4	4	2	2	3	3	1	1	2	2
4	4	1	1	3	3	2	2	2	2
1	1	2	2	3	3	2	2	2	2
1	1	2	2	3	3	1	1	2	2
1	1	2	2	3	3	2	2	2	2
4	4	2	2	3	3	2	2	2	2
1	1	2	2	3	3	2	2	2	2
1	1	2	2	3	3	2	2	2	2
1	1	2	2	2	2	2	2	2	2
4	4	2	2	3	3	1	1	2	2
1	1	2	2	3	3	2	2	2	2
4	4	2	2	3	3	2	2	2	2
1	1	2	2	3	3	2	2	2	2
4	4	2	2	3	3	2	2	2	2
1	1	2	2	2	2	2	2	2	2
1	1	2	2	3	3	2	2	2	2
1	1	2	2	3	3	2	2	2	2
1	1	2	2	3	3	2	2	2	2
1	1	2	2	3	3	2	2	2	2
1	1	2	2	2	2	2	2	2	2
4	4	1	1	3	3	2	2	2	2
1	1	2	2	2	2	2	2	2	2
1	1	2	2	2	2	2	2	3	3
1	1	2	2	3	3	2	2	2	2
4	4	1	1	3	3	2	2	2	2
1	1	2	2	3	3	2	2	2	2
1	1	2	2	2	2	2	2	2	2
1	1	2	2	2	2	2	2	2	2
1	1	2	2	3	3	2	2	2	2
1	1	2	2	3	3	1	1	2	2
1	1	2	2	2	2	2	2	2	2
1	1	2	2	3	3	2	2	2	2
1	1	2	2	2	2	2	2	2	2
1	1	2	2	3	3	1	1	3	3
1	1	2	2	3	3	2	2	2	2
1	1	2	2	3	3	2	2	2	2
1	1	2	2	2	2	2	2	2	2
1	1	2	2	2	2	2	2	2	2
1	1	2	2	3	3	2	2	2	2
1	1	2	2	2	2	2	2	2	2
1	1	2	2	3	3	2	2	2	2
1	1	2	2	3	3	2	2	2	2
4	4	1	1	3	3	2	2	2	2
4	4	1	1	3	3	2	2	2	2
1	1	2	2	3	3	2	2	2	2
4	4	1	1	3	3	1	1	3	3
1	1	2	2	3	3	2	2	2	2
1	1	2	2	2	2	2	2	2	2
1	1	2	2	3	3	2	2	2	2
4	4	1	1	2	2	1	1	3	3
1	1	2	2	3	3	2	2	2	2

For Review Only

CSSA 5585 Guilford Rd., Madison, WI 53711

4	4	1	1	3	3	2	2	2	2
1	1	2	2	2	2	2	2	2	2
1	1	2	2	2	2	2	2	2	2
1	1	2	2	3	3	2	2	2	2
4	4	2	2	3	3	2	2	2	2
1	1	2	2	3	3	2	2	2	2
1	1	2	2	3	3	2	2	2	2
1	1	2	2	3	3	2	2	2	2
1	1	2	2	3	3	2	2	2	2
1	1	2	2	3	3	2	2	2	2
4	4	1	1	3	3	2	2	2	2
1	1	2	2	3	3	2	2	2	2
1	1	2	2	3	3	2	2	2	2
1	1	2	2	3	3	2	2	2	2
1	1	2	2	2	2	2	2	2	2
1	1	2	2	3	3	2	2	2	2
1	1	2	2	3	3	2	2	2	2
4	4	2	2	3	3	1	1	2	2
1	1	2	2	3	3	2	2	2	2
1	1	2	2	3	3	2	2	2	2
1	1	2	2	3	3	2	2	2	2
1	1	2	2	2	2	2	2	2	2
1	1	2	2	2	2	2	2	2	2
4	4	1	1	2	2	1	1	3	3
1	1	2	2	2	2	2	2	2	2
1	1	2	2	2	2	2	2	2	2
1	1	2	2	2	2	2	2	2	2
1	1	2	2	3	3	2	2	2	2
1	1	2	2	3	3	2	2	2	2
1	1	2	2	3	3	2	2	2	2
4	4	1	1	3	3	2	2	2	2
1	1	2	2	3	3	2	2	2	2
1	1	2	2	2	2	2	2	2	2
1	1	2	2	2	2	2	2	2	2
1	1	2	2	3	3	2	2	2	2
4	4	1	1	2	2	1	1	3	3
1	1	2	2	3	3	2	2	2	2
1	1	2	2	3	3	2	2	2	2
4	4	1	1	3	3	2	2	2	2
4	4	1	1	3	3	1	1	3	3
4	4	1	1	3	3	2	2	2	2
4	4	1	1	3	3	2	2	2	2
1	1	2	2	2	2	2	2	2	2
1	1	2	2	3	3	2	2	2	2
1	1	2	2	2	2	2	2	2	2
1	1	2	2	3	3	2	2	2	2
1	1	2	2	3	3	2	2	2	2
1	1	2	2	3	3	2	2	2	2
1	1	2	2	2	2	2	2	2	2
1	1	2	2	2	2	2	2	2	2
4	4	1	1	3	3	2	2	3	3
1	1	2	2	3	3	2	2	2	2
1	1	2	2	2	2	2	2	2	2
4	4	1	1	3	3	1	1	3	3
1	1	2	2	2	2	2	2	2	2

For Review Only

1	1	2	2	3	3	2	2	2	2
1	1	2	2	2	2	2	2	2	2
4	4	1	1	3	3	2	2	2	2
1	1	2	2	2	2	2	2	2	2
1	1	2	2	2	2	2	2	2	2
1	1	2	2	3	3	2	2	2	2
1	1	2	2	3	3	2	2	2	2
1	1	2	2	2	2	2	2	2	2
1	1	2	2	3	3	2	2	2	2
1	1	2	2	2	2	2	2	2	2
1	1	2	2	3	3	2	2	2	2
4	4	2	2	3	3	2	2	2	2
4	4	1	1	3	3	2	2	2	2
4	4	1	1	3	3	2	2	2	2
4	4	1	1	3	3	2	2	2	2
1	1	2	2	3	3	2	2	2	2
1	1	2	2	3	3	2	2	2	2
1	1	2	2	3	3	2	2	2	2
1	1	2	2	3	3	2	2	2	2
1	1	2	2	3	3	2	2	2	2
4	4	1	1	3	3	2	2	2	2
4	4	1	1	3	3	2	2	2	2
1	1	2	2	3	3	2	2	2	2
1	1	2	2	2	2	2	2	2	2
1	1	2	2	2	2	2	2	2	2
1	1	2	2	3	3	2	2	2	2
1	1	2	2	2	2	2	2	2	2
4	4	1	1	3	3	2	2	2	2
1	1	2	2	3	3	2	2	2	2
4	4	1	1	3	3	1	1	3	3
1	1	2	2	2	2	2	2	2	2
4	4	1	1	2	2	2	2	2	2
1	1	2	2	3	3	2	2	2	2
4	4	1	1	3	3	2	2	2	2
1	1	2	2	2	2	2	2	2	2
1	1	2	2	2	2	2	2	2	2
1	1	2	2	3	3	2	2	2	2
4	4	1	1	2	2	1	1	3	3
1	1	2	2	2	2	2	2	2	2
1	1	2	2	2	2	2	2	2	2
1	1	2	2	3	3	2	2	2	2
1	1	2	2	3	3	2	2	2	2
1	1	2	2	2	2	2	2	2	2
1	1	2	2	3	3	2	2	2	2
4	4	1	1	3	3	2	2	2	2
1	1	2	2	3	3	2	2	2	2
1	1	2	2	3	3	2	2	2	2
1	1	2	2	3	3	2	2	2	2
1	1	2	2	3	3	2	2	2	2
1	1	2	2	2	2	2	2	2	2
1	1	2	2	3	3	2	2	2	2
1	1	2	2	2	2	2	2	2	2
4	4	1	1	3	3	2	2	2	2
4	4	1	1	3	3	2	2	2	2
1	1	2	2	3	3	2	2	2	2

For Review Only

1	1	2	2	3	3	2	2	2	2
4	4	1	1	3	3	1	1	3	3

For Review Only

SNP827	SNP827	SNP828	SNP828	SNP829	SNP829	SNP830	SNP830	SNP831	SNP831
4	4	4	4	4	4	4	4	3	3
1	1	3	3	4	4	4	4	1	1
1	1	4	4	2	2	4	4	3	3
1	1	4	4	4	4	4	4	3	3
1	1	4	4	4	4	4	4	3	3
1	1	3	3	4	4	4	4	3	3
1	1	4	4	2	2	4	4	3	3
4	4	3	3	4	4	4	4	1	1
1	1	3	3	4	4	4	4	3	3
1	1	3	3	4	4	4	4	1	1
4	4	3	3	4	4	1	1	1	1
1	1	4	4	4	4	4	4	3	3
4	4	3	3	4	4	4	4	3	3
4	4	4	4	4	4	1	1	1	1
1	1	4	4	2	2	4	4	3	3
4	4	4	4	4	4	4	4	3	3
1	1	3	3	4	4	4	4	1	1
4	4	3	3	4	4	4	4	3	3
1	1	4	4	4	4	4	4	3	3
1	1	3	3	4	4	1	1	1	1
4	4	3	3	4	4	4	4	1	1
1	1	4	4	4	4	4	4	3	3
4	4	3	3	4	4	4	4	1	1
1	1	4	4	4	4	1	1	1	1
1	1	4	4	4	4	4	4	3	3
4	4	4	4	4	4	4	4	3	3
1	1	4	4	2	2	4	4	3	3
1	1	3	3	4	4	4	4	3	3
1	1	4	4	4	4	4	4	1	1
4	4	3	3	4	4	1	1	1	1
1	1	4	4	2	2	4	4	3	3
1	1	4	4	4	4	4	4	3	3
1	1	4	4	4	4	4	4	3	3
4	4	4	4	4	4	4	4	1	1
4	4	4	4	4	4	4	4	3	3
4	4	4	4	4	4	1	1	3	3
4	4	4	4	4	4	4	4	3	3
4	4	3	3	4	4	4	4	3	3
4	4	3	3	4	4	4	4	1	1
1	1	4	4	4	4	1	1	3	3
4	4	3	3	4	4	4	4	1	1
4	4	3	3	4	4	4	4	1	1
4	4	4	4	4	4	4	4	1	1
4	4	3	3	4	4	4	4	3	3
4	4	3	3	4	4	4	4	1	1
1	1	4	4	4	4	4	4	3	3
4	4	4	4	4	4	4	4	3	3
1	1	4	4	4	4	4	4	3	3
1	1	3	3	4	4	1	1	3	3
1	1	4	4	4	4	4	4	3	3
1	1	4	4	4	4	4	4	1	1
1	1	4	4	2	2	4	4	1	1

For Review Only

1	1	4	4	4	4	4	4	3	3
1	1	4	4	4	4	4	4	3	3
1	1	4	4	4	4	4	4	3	3
1	1	3	3	4	4	4	4	3	3
1	1	4	4	4	4	4	4	3	3
4	4	4	4	2	2	4	4	3	3
4	4	4	4	4	4	4	4	3	3
4	4	3	3	4	4	4	4	1	1
1	1	4	4	4	4	4	4	3	3
1	1	4	4	4	4	4	4	3	3
1	1	4	4	2	2	4	4	3	3
4	4	4	4	2	2	4	4	3	3
1	1	4	4	4	4	4	4	3	3
1	1	4	4	4	4	4	4	1	1
1	1	3	3	4	4	4	4	3	3
4	4	4	4	4	4	4	4	1	1
1	1	3	3	4	4	4	4	3	3
1	1	4	4	4	4	4	4	3	3
1	1	4	4	4	4	4	4	3	3
1	1	4	4	4	4	4	4	3	3
1	1	4	4	2	2	4	4	3	3
1	1	3	3	4	4	4	4	1	1
1	1	4	4	4	4	4	4	3	3
1	1	3	3	2	2	4	4	1	1
4	4	3	3	4	4	4	4	1	1
4	4	4	4	2	2	4	4	3	3
1	1	3	3	4	4	4	4	3	3
4	4	3	3	4	4	4	4	1	1
4	4	4	4	4	4	4	4	3	3
1	1	4	4	4	4	4	4	3	3
4	4	4	4	4	4	4	4	3	3
4	4	4	4	4	4	1	1	3	3
4	4	3	3	4	4	4	4	1	1
1	1	4	4	4	4	4	4	3	3
4	4	4	4	4	4	4	4	3	3
4	4	3	3	4	4	4	4	1	1
4	4	4	4	4	4	4	4	1	1
1	1	3	3	4	4	1	1	3	3
4	4	4	4	4	4	4	4	3	3
4	4	3	3	4	4	4	4	3	3
1	1	4	4	4	4	4	4	3	3
1	1	4	4	2	2	4	4	3	3
1	1	4	4	4	4	4	4	3	3
1	1	4	4	2	2	4	4	3	3
4	4	4	4	4	4	4	4	3	3
1	1	3	3	4	4	4	4	3	3
4	4	4	4	4	4	1	1	1	1
1	1	4	4	4	4	4	4	3	3
4	4	4	4	2	2	4	4	3	3
4	4	3	3	4	4	4	4	3	3
1	1	4	4	2	2	4	4	3	3
4	4	3	3	4	4	4	4	1	1
1	1	4	4	4	4	1	1	1	1
4	4	4	4	4	4	4	4	3	3
4	4	4	4	4	4	4	4	3	3

For Review Only

4	4	3	3	4	4	4	4	3	3
1	1	3	3	4	4	4	4	3	3
4	4	4	4	4	4	4	4	3	3
1	1	3	3	4	4	1	1	1	1
1	1	4	4	2	2	4	4	3	3
4	4	4	4	4	4	4	4	3	3
1	1	4	4	4	4	4	4	3	3
4	4	4	4	2	2	4	4	3	3
4	4	3	3	4	4	4	4	3	3
1	1	4	4	4	4	1	1	3	3
1	1	4	4	4	4	1	1	1	1
1	1	3	3	4	4	4	4	3	3
1	1	4	4	4	4	4	4	3	3
1	1	4	4	2	2	4	4	3	3
1	1	3	3	4	4	4	4	3	3
4	4	4	4	4	4	4	4	3	3
1	1	3	3	4	4	4	4	3	3
1	1	4	4	2	2	4	4	3	3
4	4	4	4	4	4	1	1	1	1
1	1	3	3	4	4	4	4	1	1
1	1	3	3	4	4	4	4	3	3
1	1	4	4	4	4	4	4	3	3
1	1	4	4	2	2	4	4	3	3
4	4	3	3	4	4	4	4	1	1
4	4	3	3	4	4	4	4	3	3
4	4	4	4	4	4	4	4	1	1
4	4	3	3	4	4	4	4	3	3
1	1	4	4	4	4	4	4	3	3
1	1	4	4	4	4	4	4	3	3
1	1	3	3	4	4	4	4	3	3
4	4	3	3	4	4	4	4	3	3
1	1	3	3	4	4	4	4	3	3
1	1	4	4	4	4	4	4	3	3
1	1	4	4	4	4	4	4	3	3
4	4	4	4	4	4	4	4	1	1
1	1	4	4	4	4	4	4	3	3
4	4	4	4	4	4	1	1	1	1
1	1	4	4	2	2	4	4	1	1
4	4	4	4	4	4	4	4	3	3
4	4	3	3	4	4	4	4	1	1
1	1	3	3	4	4	4	4	1	1
1	1	4	4	4	4	1	1	1	1
1	1	4	4	4	4	4	4	3	3
4	4	3	3	4	4	4	4	1	1
4	4	3	3	4	4	4	4	1	1
4	4	4	4	4	4	4	4	3	3
4	4	4	4	4	4	4	4	3	3
1	1	4	4	4	4	4	4	3	3
4	4	4	4	4	4	1	1	3	3
4	4	4	4	2	2	4	4	3	3
4	4	4	4	4	4	4	4	3	3
4	4	3	3	4	4	1	1	3	3
4	4	4	4	4	4	4	4	3	3
4	4	4	4	2	2	4	4	3	3
4	4	3	3	4	4	4	4	3	3

For Review Only

1	1	4	4	4	4	4	4	3	3
4	4	3	3	4	4	4	4	3	3
4	4	3	3	4	4	1	1	3	3
1	1	4	4	4	4	4	4	3	3
1	1	3	3	4	4	4	4	3	3
4	4	4	4	4	4	4	4	1	1
4	4	3	3	4	4	4	4	3	3
1	1	4	4	4	4	4	4	3	3
1	1	4	4	4	4	4	4	1	1
1	1	4	4	4	4	4	4	3	3
4	4	4	4	4	4	4	4	3	3
1	1	3	3	4	4	4	4	3	3
1	1	4	4	4	4	4	4	3	3
4	4	4	4	4	4	4	4	3	3
4	4	4	4	4	4	1	1	1	1
4	4	3	3	4	4	1	1	1	1
1	1	4	4	4	4	4	4	1	1
1	1	4	4	2	2	4	4	3	3
4	4	4	4	4	4	4	4	3	3
1	1	4	4	4	4	4	4	3	3
1	1	3	3	4	4	4	4	3	3
1	1	3	3	4	4	1	1	3	3
4	4	3	3	4	4	4	4	1	1
4	4	4	4	2	2	4	4	3	3
1	1	4	4	4	4	4	4	1	1
4	4	4	4	4	4	4	4	1	1
1	1	4	4	4	4	4	4	3	3
1	1	4	4	4	4	4	4	1	1
4	4	4	4	4	4	4	4	3	3
1	1	4	4	4	4	4	4	3	3
1	1	3	3	4	4	4	4	3	3
1	1	4	4	4	4	4	4	3	3
4	4	4	4	4	4	1	1	3	3
4	4	4	4	4	4	1	1	1	1
1	1	3	3	4	4	4	4	3	3
4	4	4	4	2	2	4	4	3	3
4	4	4	4	2	2	4	4	3	3
4	4	4	4	4	4	4	4	3	3
1	1	4	4	4	4	4	4	3	3
1	1	4	4	2	2	4	4	3	3
1	1	4	4	2	2	4	4	3	3
4	4	4	4	4	4	4	4	3	3
4	4	3	3	4	4	4	4	1	1
1	1	4	4	4	4	4	4	3	3
1	1	4	4	4	4	1	1	3	3
1	1	4	4	4	4	4	4	3	3
4	4	3	3	4	4	1	1	1	1
4	4	3	3	4	4	4	4	3	3
4	4	4	4	4	4	1	1	1	1
4	4	4	4	4	4	4	4	1	1
1	1	4	4	4	4	4	4	3	3
4	4	4	4	4	4	1	1	1	1
4	4	3	3	4	4	4	4	1	1
1	1	4	4	2	2	4	4	3	3
4	4	4	4	4	4	1	1	1	1

For Review Only

4	4	4	4	4	4	4	4	1	1
1	1	4	4	4	4	4	4	1	1
1	1	4	4	4	4	4	4	3	3
1	1	3	3	4	4	4	4	1	1
4	4	4	4	4	4	4	4	3	3
1	1	4	4	4	4	4	4	3	3
4	4	4	4	4	4	1	1	1	1
4	4	3	3	4	4	4	4	1	1
4	4	3	3	4	4	4	4	3	3
4	4	3	3	4	4	1	1	1	1
1	1	3	3	4	4	4	4	3	3
4	4	3	3	4	4	4	4	1	1
1	1	4	4	4	4	4	4	3	3
4	4	4	4	4	4	4	4	3	3
1	1	3	3	2	2	4	4	3	3
4	4	4	4	4	4	4	4	3	3
4	4	3	3	4	4	4	4	3	3
4	4	4	4	4	4	1	1	1	1
4	4	3	3	4	4	4	4	1	1
4	4	3	3	4	4	4	4	1	1
4	4	3	3	4	4	4	4	1	1
4	4	4	4	4	4	4	4	3	3
1	1	4	4	4	4	4	4	3	3
4	4	4	4	4	4	4	4	1	1
4	4	3	3	4	4	4	4	1	1
1	1	3	3	4	4	4	4	3	3
4	4	3	3	4	4	4	4	1	1
1	1	4	4	4	4	4	4	3	3
4	4	4	4	4	4	4	4	3	3
1	1	4	4	2	2	4	4	3	3
4	4	3	3	4	4	1	1	1	1
4	4	4	4	4	4	4	4	3	3
4	4	4	4	4	4	1	1	1	1
4	4	4	4	4	4	4	4	3	3
4	4	3	3	4	4	4	4	1	1
4	4	3	3	4	4	4	4	1	1
1	1	4	4	4	4	4	4	3	3
4	4	4	4	2	2	4	4	3	3
4	4	4	4	4	4	1	1	3	3
4	4	4	4	4	4	4	4	1	1
1	1	4	4	4	4	4	4	3	3
4	4	3	3	4	4	4	4	3	3
4	4	4	4	4	4	4	4	1	1
1	1	4	4	4	4	4	4	3	3
1	1	4	4	4	4	4	4	3	3
1	1	4	4	4	4	4	4	3	3
1	1	4	4	4	4	1	1	1	1
1	1	4	4	4	4	4	4	3	3
1	1	3	3	4	4	4	4	3	3
4	4	4	4	4	4	4	4	1	1
4	4	4	4	4	4	4	4	3	3
4	4	4	4	4	4	4	4	1	1
1	1	4	4	2	2	4	4	3	3
1	1	3	3	4	4	4	4	3	3
4	4	4	4	4	4	1	1	1	1

For Review Only

1	1	4	4	4	4	4	4	3	3
1	1	4	4	2	2	4	4	3	3

For Review Only

SNP832	SNP832	SNP833	SNP833	SNP834	SNP834	SNP835	SNP835	SNP836	SNP836
3	3	4	4	2	2	4	4	1	1
3	3	4	4	2	2	1	1	1	1
3	3	4	4	2	2	1	1	1	1
3	3	4	4	2	2	4	4	4	4
3	3	4	4	2	2	4	4	4	4
3	3	4	4	3	3	4	4	4	4
3	3	4	4	2	2	4	4	4	4
3	3	4	4	3	3	1	1	1	1
3	3	4	4	2	2	4	4	4	4
3	3	4	4	3	3	1	1	1	1
2	2	3	3	2	2	1	1	1	1
3	3	4	4	2	2	1	1	1	1
3	3	4	4	2	2	4	4	4	4
2	2	3	3	3	3	1	1	1	1
3	3	4	4	2	2	4	4	1	1
3	3	4	4	2	2	4	4	4	4
3	3	4	4	3	3	1	1	1	1
3	3	4	4	2	2	4	4	1	1
3	3	4	4	2	2	1	1	1	1
3	3	4	4	3	3	1	1	1	1
3	3	4	4	3	3	1	1	1	1
3	3	4	4	2	2	4	4	4	4
3	3	4	4	3	3	1	1	1	1
3	3	4	4	3	3	4	4	1	1
3	3	4	4	2	2	4	4	4	4
3	3	4	4	2	2	1	1	1	1
3	3	4	4	2	2	4	4	1	1
3	3	4	4	2	2	4	4	1	1
3	3	4	4	3	3	4	4	4	4
3	3	4	4	3	3	1	1	1	1
3	3	4	4	2	2	4	4	4	4
3	3	4	4	2	2	4	4	4	4
3	3	4	4	2	2	4	4	4	4
3	3	4	4	3	3	4	4	4	4
3	3	4	4	2	2	4	4	4	4
3	3	4	4	2	2	4	4	1	1
2	2	4	4	2	2	1	1	1	1
3	3	4	4	2	2	1	1	1	1
3	3	4	4	2	2	1	1	1	1
3	3	3	3	2	2	1	1	1	1
3	3	4	4	2	2	1	1	1	1
3	3	4	4	3	3	1	1	1	1
3	3	4	4	3	3	4	4	4	4
3	3	4	4	2	2	1	1	1	1
3	3	4	4	3	3	1	1	1	1
3	3	4	4	2	2	1	1	1	1
3	3	4	4	2	2	4	4	4	4
3	3	4	4	2	2	4	4	4	4
3	3	4	4	2	2	1	1	1	1
3	3	4	4	2	2	4	4	4	4
3	3	4	4	3	3	4	4	4	4
3	3	4	4	2	2	4	4	4	4

For Review Only

3	3	4	4	2	2	4	4	4	4
3	3	4	4	2	2	4	4	4	4
3	3	4	4	2	2	4	4	1	1
3	3	4	4	2	2	1	1	1	1
3	3	4	4	2	2	1	1	1	1
3	3	4	4	2	2	4	4	4	4
3	3	4	4	2	2	1	1	1	1
3	3	4	4	3	3	1	1	1	1
3	3	4	4	2	2	4	4	4	4
3	3	4	4	2	2	4	4	4	4
3	3	4	4	2	2	4	4	4	4
3	3	4	4	2	2	4	4	4	4
3	3	4	4	2	2	4	4	4	4
3	3	4	4	3	3	4	4	4	4
3	3	4	4	2	2	4	4	4	4
3	3	4	4	3	3	1	1	4	4
3	3	4	4	2	2	1	1	1	1
3	3	4	4	2	2	4	4	4	4
3	3	4	4	2	2	4	4	4	4
3	3	4	4	2	2	4	4	1	1
3	3	4	4	2	2	4	4	4	4
3	3	4	4	2	2	1	1	1	1
3	3	4	4	3	3	4	4	4	4
3	3	4	4	3	3	1	1	1	1
3	3	4	4	3	3	1	1	1	1
3	3	4	4	2	2	1	1	1	1
3	3	4	4	2	2	1	1	1	1
3	3	4	4	2	2	1	1	1	1
3	3	4	4	2	2	1	1	1	1
3	3	4	4	2	2	4	4	4	4
3	3	4	4	2	2	1	1	1	1
3	3	4	4	3	3	4	4	4	4
3	3	4	4	2	2	1	1	1	1
3	3	4	4	2	2	4	4	4	4
3	3	4	4	2	2	4	4	4	4
3	3	4	4	3	3	1	1	1	1
3	3	4	4	2	2	1	1	1	1
3	3	4	4	2	2	1	1	1	1
3	3	4	4	2	2	4	4	4	4
3	3	4	4	2	2	1	1	1	1
3	3	4	4	2	2	4	4	4	4
3	3	4	4	2	2	1	1	1	1
3	3	4	4	3	3	4	4	4	4
3	3	4	4	2	2	4	4	4	4
3	3	4	4	2	2	4	4	4	4
3	3	4	4	2	2	4	4	4	4
2	2	3	3	3	3	1	1	1	1
3	3	4	4	2	2	4	4	4	4
3	3	4	4	2	2	1	1	1	1
3	3	4	4	2	2	1	1	1	1
3	3	4	4	2	2	4	4	4	4
2	2	4	4	2	2	1	1	1	1
2	2	3	3	3	3	1	1	1	1
3	3	4	4	3	3	1	1	4	4
3	3	4	4	2	2	1	1	1	1

For Review Only

3	3	4	4	2	2	1	1	1	1
3	3	4	4	2	2	4	4	4	4
3	3	4	4	2	2	4	4	1	1
3	3	3	3	3	3	1	1	1	1
3	3	4	4	2	2	4	4	4	4
3	3	4	4	2	2	4	4	1	1
3	3	4	4	2	2	4	4	4	4
3	3	4	4	2	2	4	4	1	1
3	3	4	4	2	2	1	1	1	1
3	3	4	4	2	2	4	4	1	1
3	3	3	3	3	3	4	4	4	4
3	3	4	4	2	2	4	4	4	4
3	3	4	4	3	3	4	4	1	1
3	3	4	4	2	2	4	4	1	1
3	3	4	4	2	2	1	1	1	1
3	3	4	4	2	2	4	4	4	4
3	3	4	4	3	3	4	4	4	4
3	3	4	4	2	2	4	4	4	4
2	2	3	3	3	3	1	1	1	1
3	3	4	4	3	3	1	1	1	1
3	3	4	4	2	2	4	4	4	4
3	3	4	4	2	2	4	4	4	4
3	3	4	4	2	2	1	1	1	1
3	3	4	4	2	2	1	1	1	1
3	3	4	4	2	2	1	1	1	1
3	3	4	4	2	2	1	1	1	1
3	3	4	4	2	2	4	4	4	4
3	3	4	4	2	2	4	4	1	1
3	3	4	4	2	2	4	4	4	4
3	3	4	4	2	2	1	1	1	1
3	3	4	4	2	2	1	1	1	1
3	3	4	4	2	2	4	4	4	4
3	3	4	4	2	2	4	4	4	4
3	3	4	4	2	2	4	4	4	4
3	3	4	4	3	3	4	4	4	4
3	3	4	4	2	2	4	4	1	1
2	2	3	3	3	3	1	1	1	1
3	3	4	4	3	3	4	4	1	1
3	3	4	4	2	2	1	1	1	1
3	3	4	4	2	2	1	1	1	1
3	3	4	4	2	2	1	1	1	1
2	2	3	3	3	3	1	1	1	1
3	3	4	4	2	2	4	4	4	4
3	3	4	4	2	2	1	1	1	1
3	3	4	4	3	3	1	1	1	1
3	3	4	4	2	2	4	4	4	4
3	3	4	4	2	2	1	1	1	1
3	3	4	4	2	2	4	4	1	1
3	3	4	4	2	2	4	4	4	4
3	3	4	4	2	2	1	1	1	1
3	3	4	4	2	2	4	4	4	4
3	3	3	3	2	2	1	1	1	1
3	3	4	4	3	3	4	4	1	1
3	3	4	4	2	2	4	4	4	4
3	3	4	4	2	2	4	4	4	4

For Review Only

3	3	4	4	2	2	4	4	4	4
3	3	4	4	2	2	1	1	1	1
3	3	4	4	2	2	1	1	1	1
3	3	4	4	2	2	4	4	4	4
3	3	4	4	2	2	4	4	4	4
3	3	4	4	2	2	4	4	4	4
3	3	4	4	2	2	1	1	1	1
3	3	4	4	2	2	4	4	1	1
3	3	4	4	2	2	4	4	4	4
3	3	4	4	2	2	4	4	4	4
3	3	4	4	2	2	1	1	1	1
3	3	4	4	3	3	1	1	1	1
3	3	4	4	2	2	4	4	4	4
3	3	4	4	2	2	4	4	4	4
2	2	3	3	3	3	1	1	1	1
3	3	3	3	2	2	1	1	1	1
2	2	4	4	2	2	4	4	4	4
3	3	4	4	2	2	4	4	4	4
3	3	4	4	2	2	4	4	4	4
3	3	4	4	2	2	4	4	4	4
3	3	4	4	2	2	4	4	4	4
3	3	4	4	3	3	1	1	1	1
3	3	4	4	2	2	1	1	1	1
3	3	4	4	2	2	4	4	4	4
3	3	4	4	3	3	4	4	4	4
2	2	4	4	3	3	1	1	1	1
3	3	4	4	2	2	1	1	1	1
3	3	4	4	3	3	4	4	4	4
3	3	4	4	2	2	1	1	1	1
3	3	4	4	2	2	4	4	4	4
3	3	4	4	2	2	1	1	1	1
3	3	4	4	2	2	4	4	4	4
3	3	3	3	2	2	4	4	4	4
2	2	3	3	3	3	1	1	1	1
3	3	4	4	2	2	4	4	1	1
3	3	4	4	2	2	4	4	4	4
3	3	4	4	2	2	4	4	4	4
3	3	4	4	2	2	1	1	1	1
3	3	4	4	2	2	4	4	1	1
3	3	4	4	2	2	4	4	1	1
3	3	4	4	2	2	1	1	1	1
3	3	4	4	2	2	4	4	4	4
3	3	4	4	3	3	1	1	1	1
3	3	4	4	2	2	1	1	1	1
2	2	3	3	2	2	4	4	1	1
3	3	4	4	2	2	4	4	4	4
3	3	3	3	2	2	1	1	1	1
3	3	4	4	2	2	1	1	1	1
2	2	4	4	3	3	1	1	1	1
3	3	4	4	2	2	1	1	1	1
3	3	4	4	2	2	4	4	4	4
2	2	3	3	3	3	1	1	1	1
3	3	4	4	2	2	1	1	1	1
3	3	4	4	2	2	4	4	4	4
3	3	3	3	2	2	1	1	1	1

For Review Only

3	3	4	4	3	3	4	4	4	4
3	3	4	4	3	3	4	4	4	4
3	3	4	4	2	2	1	1	1	1
3	3	4	4	2	2	1	1	1	1
3	3	4	4	3	3	1	1	1	1
3	3	4	4	2	2	4	4	1	1
3	3	3	3	2	2	4	4	4	4
3	3	4	4	3	3	1	1	1	1
3	3	4	4	2	2	1	1	1	1
3	3	3	3	2	2	1	1	1	1
3	3	4	4	2	2	4	4	4	4
3	3	4	4	2	2	1	1	1	1
3	3	4	4	2	2	4	4	4	4
3	3	4	4	2	2	4	4	4	4
3	3	4	4	2	2	1	1	1	1
3	3	4	4	2	2	1	1	1	1
3	3	4	4	2	2	1	1	1	1
3	3	3	3	2	2	1	1	1	1
3	3	4	4	3	3	1	1	1	1
3	3	4	4	2	2	1	1	1	1
3	3	4	4	3	3	1	1	1	1
3	3	4	4	2	2	1	1	1	1
3	3	4	4	2	2	4	4	4	4
3	3	4	4	2	2	1	1	4	4
3	3	4	4	3	3	1	1	1	1
3	3	4	4	2	2	1	1	1	1
3	3	4	4	2	2	1	1	1	1
3	3	4	4	2	2	1	1	1	1
3	3	4	4	2	2	4	4	4	4
3	3	4	4	2	2	4	4	4	4
3	3	3	3	2	2	1	1	1	1
3	3	4	4	2	2	4	4	4	4
2	2	3	3	3	3	1	1	1	1
3	3	4	4	2	2	4	4	1	1
3	3	4	4	2	2	1	1	1	1
3	3	4	4	3	3	1	1	1	1
3	3	4	4	2	2	4	4	4	4
3	3	4	4	2	2	4	4	4	4
3	3	4	4	2	2	4	4	1	1
3	3	4	4	2	2	1	1	1	1
3	3	4	4	2	2	4	4	4	4
3	3	4	4	2	2	1	1	1	1
3	3	4	4	3	3	1	1	1	1
3	3	4	4	2	2	4	4	4	4
3	3	4	4	2	2	4	4	4	4
3	3	4	4	2	2	4	4	4	4
3	3	3	3	2	2	4	4	1	1
3	3	4	4	3	3	4	4	4	4
3	3	4	4	2	2	1	1	1	1
3	3	4	4	2	2	1	1	1	1
3	3	4	4	2	2	4	4	4	4
3	3	4	4	2	2	1	1	1	1
3	3	4	4	2	2	1	1	1	1
3	3	4	4	2	2	1	1	1	1
3	3	3	3	2	2	1	1	1	1

For Review Only

3	3	4	4	2	2	1	1	1	1
3	3	4	4	2	2	1	1	1	1

For Review Only

SNP837	SNP837	SNP838	SNP838	SNP839	SNP839	SNP840	SNP840	SNP841	SNP841
2	2	2	2	1	1	3	3	1	1
3	3	3	3	1	1	4	4	3	3
3	3	2	2	4	4	4	4	1	1
2	2	2	2	1	1	3	3	1	1
2	2	2	2	1	1	3	3	1	1
2	2	3	3	1	1	4	4	3	3
2	2	2	2	1	1	3	3	1	1
3	3	3	3	4	4	3	3	3	3
2	2	3	3	1	1	4	4	3	3
3	3	3	3	1	1	3	3	3	3
3	3	3	3	1	1	3	3	3	3
3	3	2	2	4	4	4	4	1	1
2	2	2	2	1	1	3	3	1	1
3	3	3	3	1	1	3	3	3	3
2	2	2	2	1	1	3	3	3	3
2	2	2	2	1	1	3	3	3	3
3	3	3	3	1	1	4	4	3	3
2	2	2	2	1	1	3	3	1	1
3	3	2	2	4	4	4	4	1	1
3	3	3	3	4	4	4	4	3	3
3	3	3	3	4	4	3	3	3	3
2	2	2	2	1	1	4	4	1	1
3	3	3	3	1	1	3	3	3	3
3	3	3	3	1	1	3	3	3	3
2	2	2	2	1	1	4	4	1	1
3	3	2	2	4	4	3	3	1	1
3	3	2	2	1	1	4	4	1	1
2	2	2	2	4	4	3	3	3	3
2	2	3	3	1	1	4	4	3	3
3	3	3	3	1	1	3	3	3	3
3	3	2	2	1	1	4	4	1	1
2	2	2	2	4	4	3	3	1	1
2	2	2	2	4	4	3	3	1	1
2	2	3	3	1	1	3	3	3	3
2	2	2	2	4	4	3	3	1	1
3	3	2	2	1	1	3	3	1	1
3	3	2	2	1	1	3	3	1	1
3	3	2	2	1	1	3	3	1	1
3	3	3	3	4	4	3	3	3	3
3	3	3	3	1	1	3	3	3	3
3	3	3	3	4	4	3	3	3	3
2	2	3	3	4	4	3	3	3	3
2	2	3	3	1	1	3	3	3	3
2	2	2	2	1	1	3	3	1	1
2	2	3	3	1	1	3	3	3	3
3	3	2	2	4	4	3	3	1	1
2	2	2	2	4	4	3	3	1	1
2	2	3	3	4	4	3	3	3	3
2	2	2	2	1	1	4	4	1	1
2	2	2	2	1	1	3	3	1	1
2	2	3	3	4	4	4	4	3	3
2	2	2	2	4	4	3	3	3	3

For Review Only

2	2	2	2	1	1	4	4	1	1
2	2	2	2	4	4	3	3	1	1
2	2	2	2	4	4	4	4	1	1
3	3	2	2	1	1	4	4	1	1
3	3	2	2	4	4	4	4	1	1
2	2	2	2	4	4	3	3	1	1
3	3	2	2	4	4	3	3	1	1
3	3	3	3	4	4	3	3	3	3
2	2	2	2	1	1	4	4	1	1
2	2	2	2	1	1	3	3	3	3
2	2	2	2	4	4	4	4	1	1
2	2	2	2	4	4	3	3	1	1
2	2	2	2	1	1	4	4	1	1
2	2	3	3	1	1	4	4	3	3
2	2	2	2	1	1	3	3	3	3
2	2	3	3	1	1	3	3	3	3
2	2	2	2	4	4	3	3	3	3
2	2	2	2	1	1	3	3	1	1
2	2	2	2	4	4	4	4	1	1
3	3	2	2	1	1	4	4	1	1
2	2	2	2	4	4	3	3	1	1
3	3	3	3	4	4	3	3	3	3
2	2	3	3	1	1	4	4	3	3
3	3	3	3	4	4	3	3	3	3
2	2	3	3	1	1	3	3	3	3
3	3	2	2	4	4	3	3	1	1
3	3	2	2	4	4	4	4	1	1
3	3	3	3	1	1	3	3	3	3
3	3	2	2	4	4	3	3	1	1
2	2	2	2	1	1	4	4	1	1
3	3	2	2	1	1	3	3	1	1
3	3	3	3	4	4	3	3	3	3
3	3	3	3	1	1	3	3	3	3
2	2	2	2	1	1	4	4	1	1
2	2	2	2	1	1	3	3	1	1
2	2	3	3	1	1	3	3	3	3
3	3	3	3	1	1	3	3	3	3
3	3	2	2	1	1	4	4	1	1
2	2	2	2	4	4	3	3	1	1
2	2	2	2	1	1	3	3	3	3
2	2	2	2	1	1	4	4	1	1
3	3	2	2	1	1	4	4	1	1
2	2	3	3	1	1	3	3	3	3
2	2	2	2	4	4	4	4	1	1
2	2	2	2	1	1	3	3	1	1
2	2	2	2	1	1	4	4	1	1
3	3	3	3	1	1	3	3	3	3
2	2	2	2	4	4	4	4	1	1
3	3	2	2	1	1	3	3	1	1
3	3	2	2	1	1	3	3	1	1
2	2	3	3	1	1	3	3	3	3
3	3	3	3	4	4	3	3	3	3
3	3	3	3	1	1	4	4	3	3
3	3	2	2	4	4	3	3	3	3
3	3	2	2	4	4	3	3	1	1

For Review Only

3	3	2	2	4	4	3	3	3	3
2	2	2	2	4	4	3	3	1	1
3	3	2	2	1	1	3	3	1	1
3	3	3	3	1	1	4	4	3	3
2	2	2	2	4	4	4	4	1	1
3	3	2	2	4	4	3	3	1	1
2	2	2	2	1	1	4	4	3	3
3	3	2	2	1	1	3	3	3	3
2	2	2	2	1	1	3	3	3	3
3	3	2	2	4	4	3	3	3	3
2	2	3	3	1	1	3	3	1	1
2	2	2	2	4	4	3	3	3	3
3	3	3	3	1	1	3	3	3	3
2	2	2	2	4	4	3	3	1	1
3	3	2	2	1	1	3	3	1	1
2	2	2	2	4	4	3	3	3	3
2	2	3	3	1	1	3	3	3	3
2	2	2	2	4	4	3	3	3	3
3	3	3	3	1	1	3	3	3	3
3	3	3	3	1	1	4	4	3	3
2	2	2	2	4	4	3	3	3	3
2	2	2	2	1	1	4	4	1	1
3	3	2	2	1	1	4	4	1	1
3	3	3	3	1	1	3	3	3	3
3	3	3	3	4	4	3	3	3	3
3	3	3	3	1	1	3	3	3	3
2	2	2	2	1	1	3	3	3	3
3	3	2	2	1	1	4	4	1	1
2	2	2	2	4	4	4	4	3	3
2	2	2	2	1	1	3	3	1	1
3	3	3	3	4	4	3	3	3	3
2	2	2	2	4	4	3	3	3	3
2	2	2	2	1	1	4	4	1	1
2	2	2	2	1	1	3	3	3	3
2	2	3	3	1	1	3	3	3	3
2	2	2	2	1	1	3	3	3	3
3	3	3	3	1	1	3	3	3	3
3	3	3	3	4	4	4	4	3	3
3	3	3	3	4	4	3	3	3	3
3	3	3	3	4	4	3	3	3	3
3	3	3	3	4	4	4	4	3	3
3	3	3	3	1	1	4	4	3	3
2	2	2	2	4	4	3	3	1	1
2	2	3	3	1	1	3	3	3	3
2	2	3	3	1	1	3	3	3	3
2	2	2	2	1	1	3	3	1	1
3	3	2	2	4	4	3	3	1	1
3	3	2	2	4	4	4	4	1	1
2	2	2	2	4	4	3	3	1	1
3	3	2	2	4	4	3	3	3	3
2	2	2	2	4	4	3	3	1	1
3	3	2	2	4	4	3	3	1	1
2	2	2	2	1	1	3	3	3	3
2	2	2	2	4	4	3	3	1	1
2	2	2	2	1	1	3	3	3	3

For Review Only

2	2	2	2	4	4	3	3	1	1
2	2	3	3	4	4	3	3	3	3
2	2	2	2	1	1	3	3	3	3
2	2	2	2	1	1	3	3	1	1
2	2	2	2	4	4	4	4	1	1
2	2	3	3	4	4	3	3	3	3
3	3	2	2	4	4	3	3	3	3
2	2	2	2	4	4	3	3	3	3
2	2	3	3	1	1	3	3	3	3
2	2	2	2	1	1	3	3	1	1
3	3	2	2	4	4	3	3	1	1
3	3	3	3	4	4	3	3	3	3
3	3	2	2	1	1	3	3	1	1
2	2	2	2	1	1	3	3	3	3
3	3	3	3	4	4	3	3	3	3
3	3	3	3	4	4	3	3	3	3
2	2	2	2	1	1	3	3	1	1
2	2	2	2	4	4	4	4	1	1
2	2	2	2	1	1	3	3	1	1
2	2	2	2	1	1	4	4	3	3
2	2	2	2	4	4	3	3	3	3
3	3	3	3	4	4	4	4	3	3
3	3	3	3	1	1	3	3	3	3
2	2	2	2	4	4	3	3	1	1
2	2	3	3	4	4	4	4	3	3
3	3	3	3	1	1	3	3	3	3
3	3	2	2	4	4	4	4	3	3
2	2	3	3	1	1	3	3	3	3
2	2	2	2	1	1	3	3	1	1
2	2	2	2	1	1	4	4	1	1
3	3	2	2	4	4	4	4	1	1
2	2	2	2	1	1	4	4	1	1
2	2	2	2	1	1	3	3	1	1
3	3	3	3	4	4	3	3	3	3
3	3	2	2	1	1	3	3	1	1
2	2	2	2	4	4	3	3	1	1
2	2	2	2	1	1	3	3	1	1
3	3	2	2	1	1	3	3	1	1
3	3	2	2	4	4	4	4	1	1
3	3	2	2	4	4	3	3	1	1
3	3	2	2	4	4	4	4	1	1
2	2	2	2	4	4	3	3	1	1
3	3	3	3	1	1	3	3	3	3
3	3	2	2	4	4	4	4	3	3
3	3	2	2	1	1	3	3	1	1
2	2	2	2	1	1	4	4	1	1
3	3	3	3	4	4	3	3	3	3
3	3	2	2	1	1	3	3	1	1
3	3	3	3	1	1	3	3	3	3
3	3	3	3	1	1	3	3	3	3
2	2	2	2	4	4	3	3	1	1
3	3	3	3	1	1	3	3	3	3
3	3	3	3	4	4	3	3	3	3
2	2	2	2	4	4	4	4	1	1
3	3	3	3	1	1	3	3	3	3

For Review Only

2	2	3	3	1	1	3	3	3	3
2	2	3	3	1	1	3	3	3	3
3	3	2	2	4	4	3	3	1	1
3	3	3	3	1	1	3	3	3	3
3	3	3	3	1	1	3	3	3	3
2	2	2	2	1	1	3	3	1	1
2	2	3	3	4	4	3	3	3	3
3	3	3	3	4	4	3	3	3	3
3	3	2	2	4	4	3	3	3	3
3	3	3	3	1	1	3	3	3	3
2	2	2	2	4	4	3	3	3	3
2	2	3	3	4	4	3	3	3	3
2	2	2	2	4	4	3	3	1	1
2	2	2	2	4	4	3	3	1	1
2	2	2	2	4	4	4	4	1	1
3	3	2	2	1	1	3	3	1	1
3	3	2	2	1	1	3	3	1	1
3	3	3	3	1	1	3	3	3	3
3	3	3	3	1	1	3	3	3	3
3	3	3	3	1	1	3	3	3	3
3	3	3	3	4	4	3	3	3	3
3	3	2	2	4	4	3	3	1	1
2	2	2	2	1	1	3	3	1	1
3	3	3	3	4	4	3	3	3	3
3	3	3	3	4	4	3	3	3	3
2	2	2	2	1	1	3	3	1	1
3	3	3	3	1	1	4	4	3	3
3	3	2	2	4	4	4	4	3	3
2	2	2	2	1	1	3	3	3	3
2	2	2	2	4	4	3	3	1	1
3	3	3	3	4	4	3	3	3	3
2	2	2	2	4	4	3	3	1	1
3	3	3	3	1	1	3	3	3	3
3	3	2	2	4	4	3	3	1	1
3	3	3	3	4	4	3	3	3	3
3	3	3	3	1	1	3	3	3	3
2	2	2	2	1	1	3	3	1	1
2	2	2	2	4	4	3	3	1	1
3	3	2	2	1	1	3	3	1	1
3	3	3	3	4	4	3	3	3	3
2	2	2	2	1	1	3	3	1	1
3	3	2	2	1	1	3	3	1	1
3	3	3	3	4	4	3	3	3	3
2	2	2	2	1	1	4	4	1	1
2	2	2	2	4	4	3	3	1	1
2	2	3	3	1	1	4	4	3	3
3	3	3	3	4	4	3	3	3	3
2	2	3	3	1	1	4	4	3	3
3	3	2	2	1	1	4	4	1	1
3	3	3	3	1	1	3	3	3	3
2	2	2	2	1	1	3	3	1	1
2	2	3	3	1	1	3	3	3	3
3	3	2	2	4	4	4	4	1	1
2	2	2	2	4	4	4	4	3	3
3	3	3	3	4	4	3	3	3	3

For Review Only

3	3	2	2	1	1	4	4	1	1
3	3	2	2	4	4	4	4	1	1

For Review Only

SNP842	SNP842	SNP843	SNP843	SNP844	SNP844	SNP845	SNP845	SNP846	SNP846
2	2	4	4	3	3	2	2	4	4
1	1	1	1	4	4	1	1	4	4
2	2	4	4	3	3	2	2	1	1
2	2	4	4	3	3	2	2	4	4
2	2	4	4	3	3	2	2	4	4
2	2	1	1	4	4	2	2	4	4
2	2	4	4	3	3	2	2	4	4
2	2	1	1	4	4	2	2	4	4
1	1	4	4	4	4	1	1	4	4
2	2	1	1	4	4	2	2	4	4
1	1	1	1	4	4	1	1	4	4
2	2	4	4	3	3	2	2	4	4
2	2	4	4	3	3	2	2	4	4
2	2	1	1	4	4	2	2	4	4
2	2	1	1	4	4	1	1	4	4
2	2	1	1	4	4	2	2	4	4
2	2	1	1	4	4	2	2	4	4
2	2	4	4	3	3	2	2	4	4
2	2	4	4	3	3	2	2	4	4
2	2	1	1	4	4	2	2	4	4
2	2	1	1	4	4	2	2	4	4
2	2	4	4	3	3	2	2	4	4
1	1	1	1	4	4	1	1	4	4
2	2	1	1	4	4	2	2	4	4
2	2	4	4	3	3	2	2	4	4
2	2	4	4	3	3	2	2	4	4
2	2	4	4	3	3	2	2	4	4
2	2	4	4	4	4	1	1	4	4
1	1	1	1	4	4	1	1	4	4
1	1	1	1	4	4	1	1	4	4
2	2	4	4	3	3	2	2	1	1
2	2	1	1	4	4	1	1	4	4
2	2	4	4	3	3	2	2	4	4
2	2	1	1	4	4	2	2	4	4
2	2	4	4	4	4	2	2	4	4
2	2	4	4	3	3	2	2	4	4
2	2	4	4	3	3	2	2	4	4
2	2	4	4	4	4	2	2	4	4
2	2	1	1	4	4	2	2	4	4
2	2	1	1	4	4	2	2	4	4
1	1	1	1	4	4	1	1	4	4
1	1	1	1	4	4	1	1	4	4
2	2	1	1	4	4	2	2	4	4
2	2	4	4	3	3	2	2	4	4
2	2	1	1	4	4	2	2	4	4
2	2	4	4	3	3	2	2	1	1
2	2	4	4	3	3	2	2	4	4
1	1	1	1	4	4	2	2	4	4
2	2	4	4	3	3	2	2	4	4
2	2	4	4	4	4	2	2	4	4
2	2	1	1	4	4	2	2	4	4
2	2	4	4	4	4	2	2	1	1

For Review Only

2	2	4	4	3	3	2	2	4	4
2	2	4	4	3	3	2	2	4	4
2	2	4	4	3	3	2	2	4	4
2	2	4	4	3	3	2	2	4	4
2	2	4	4	3	3	2	2	4	4
2	2	4	4	3	3	2	2	4	4
2	2	4	4	3	3	2	2	4	4
1	1	1	1	4	4	1	1	4	4
2	2	4	4	3	3	2	2	4	4
2	2	4	4	4	4	2	2	4	4
2	2	4	4	3	3	2	2	1	1
2	2	4	4	3	3	2	2	4	4
2	2	4	4	3	3	2	2	4	4
1	1	1	1	4	4	1	1	4	4
2	2	4	4	4	4	2	2	4	4
2	2	1	1	4	4	2	2	4	4
2	2	4	4	4	4	2	2	4	4
2	2	4	4	4	4	1	1	4	4
2	2	4	4	3	3	2	2	4	4
2	2	4	4	3	3	2	2	4	4
2	2	4	4	3	3	2	2	4	4
2	2	1	1	4	4	2	2	4	4
1	1	1	1	4	4	1	1	4	4
2	2	1	1	4	4	2	2	4	4
2	2	1	1	4	4	2	2	4	4
2	2	4	4	3	3	2	2	1	1
2	2	4	4	3	3	2	2	4	4
2	2	1	1	4	4	2	2	4	4
2	2	4	4	3	3	2	2	4	4
2	2	4	4	3	3	2	2	4	4
2	2	4	4	3	3	2	2	4	4
2	2	1	1	4	4	2	2	4	4
1	1	1	1	4	4	1	1	4	4
2	2	4	4	3	3	2	2	4	4
2	2	4	4	3	3	2	2	4	4
2	2	1	1	4	4	2	2	4	4
2	2	1	1	4	4	2	2	4	4
2	2	4	4	3	3	2	2	4	4
2	2	4	4	3	3	2	2	4	4
2	2	4	4	4	4	1	1	4	4
2	2	4	4	3	3	2	2	4	4
2	2	4	4	3	3	2	2	1	1
2	2	1	1	4	4	2	2	4	4
2	2	4	4	3	3	2	2	1	1
2	2	4	4	3	3	2	2	4	4
2	2	4	4	3	3	2	2	4	4
1	1	1	1	4	4	1	1	4	4
2	2	4	4	3	3	2	2	4	4
2	2	4	4	3	3	2	2	4	4
2	2	4	4	3	3	2	2	4	4
2	2	1	1	4	4	2	2	1	1
1	1	1	1	4	4	1	1	4	4
2	2	1	1	4	4	2	2	4	4
2	2	4	4	3	3	1	1	4	4
2	2	4	4	3	3	2	2	4	4

For Review Only

1	1	4	4	4	4	2	2	4	4
2	2	4	4	4	4	1	1	4	4
2	2	4	4	3	3	2	2	4	4
1	1	1	1	4	4	1	1	4	4
2	2	4	4	3	3	2	2	1	1
2	2	4	4	3	3	2	2	4	4
2	2	1	1	4	4	2	2	4	4
2	2	1	1	4	4	2	2	4	4
2	2	4	4	4	4	1	1	4	4
2	2	4	4	3	3	1	1	4	4
2	2	4	4	4	4	2	2	4	4
2	2	4	4	4	4	2	2	4	4
2	2	1	1	4	4	2	2	4	4
2	2	4	4	3	3	2	2	1	1
2	2	4	4	3	3	2	2	4	4
2	2	4	4	4	4	2	2	4	4
1	1	1	1	4	4	1	1	4	4
2	2	4	4	4	4	2	2	4	4
2	2	1	1	4	4	2	2	4	4
2	2	1	1	4	4	1	1	4	4
2	2	4	4	4	4	2	2	4	4
2	2	1	1	4	4	1	1	4	4
2	2	4	4	3	3	2	2	1	1
1	1	1	1	4	4	1	1	4	4
2	2	4	4	4	4	2	2	4	4
1	1	1	1	4	4	2	2	4	4
2	2	4	4	4	4	2	2	4	4
2	2	4	4	3	3	2	2	4	4
2	2	1	1	4	4	1	1	4	4
2	2	4	4	4	4	2	2	4	4
1	1	1	1	4	4	1	1	4	4
2	2	4	4	4	4	2	2	4	4
2	2	4	4	3	3	2	2	4	4
2	2	4	4	4	4	2	2	4	4
2	2	1	1	4	4	2	2	4	4
2	2	1	1	4	4	2	2	4	4
1	1	1	1	4	4	1	1	4	4
2	2	1	1	4	4	2	2	4	4
1	1	1	1	4	4	1	1	4	4
1	1	1	1	4	4	1	1	4	4
1	1	1	1	4	4	2	2	4	4
1	1	1	1	4	4	1	1	4	4
2	2	4	4	4	4	2	2	4	4
1	1	1	1	4	4	1	1	4	4
2	2	1	1	4	4	2	2	4	4
2	2	4	4	3	3	2	2	4	4
2	2	4	4	3	3	2	2	4	4
2	2	4	4	3	3	2	2	4	4
2	2	4	4	3	3	2	2	4	4
2	2	4	4	4	4	2	2	1	1
2	2	4	4	3	3	2	2	4	4
2	2	4	4	3	3	2	2	4	4
2	2	1	1	4	4	2	2	4	4
2	2	4	4	3	3	2	2	4	4
1	1	4	4	4	4	1	1	4	4

For Review Only

2	2	1	1	4	4	1	1	4	4
1	1	1	1	4	4	2	2	4	4
2	2	4	4	4	4	2	2	4	4
2	2	4	4	4	4	1	1	4	4
2	2	4	4	3	3	2	2	4	4
2	2	1	1	4	4	2	2	4	4
2	2	4	4	4	4	2	2	4	4
2	2	4	4	4	4	2	2	4	4
1	1	1	1	4	4	1	1	4	4
2	2	1	1	4	4	1	1	4	4
2	2	4	4	3	3	2	2	4	4
2	2	1	1	4	4	2	2	4	4
2	2	4	4	3	3	2	2	4	4
2	2	4	4	4	4	2	2	4	4
1	1	1	1	4	4	1	1	4	4
1	1	1	1	4	4	1	1	4	4
2	2	4	4	4	4	2	2	4	4
2	2	4	4	3	3	2	2	1	1
2	2	4	4	3	3	2	2	4	4
2	2	1	1	4	4	2	2	4	4
2	2	4	4	4	4	2	2	4	4
2	2	1	1	4	4	2	2	4	4
1	1	1	1	4	4	2	2	4	4
2	2	4	4	3	3	2	2	4	4
2	2	1	1	4	4	1	1	4	4
2	2	1	1	4	4	2	2	4	4
2	2	1	1	4	4	2	2	4	4
2	2	1	1	4	4	1	1	4	4
2	2	4	4	3	3	2	2	4	4
2	2	4	4	4	4	2	2	4	4
2	2	4	4	3	3	2	2	4	4
2	2	4	4	4	4	1	1	4	4
2	2	4	4	3	3	2	2	4	4
1	1	1	1	4	4	1	1	4	4
2	2	4	4	3	3	2	2	4	4
2	2	4	4	3	3	2	2	4	4
2	2	4	4	3	3	2	2	4	4
2	2	4	4	4	4	2	2	4	4
2	2	4	4	3	3	2	2	4	4
2	2	4	4	3	3	2	2	4	4
2	2	4	4	3	3	2	2	1	1
2	2	4	4	3	3	2	2	4	4
1	1	1	1	4	4	1	1	4	4
2	2	4	4	4	4	2	2	4	4
2	2	4	4	3	3	2	2	4	4
2	2	1	1	4	4	1	1	4	4
1	1	1	1	4	4	1	1	4	4
2	2	4	4	3	3	2	2	4	4
2	2	1	1	4	4	2	2	4	4
2	2	1	1	4	4	2	2	4	4
2	2	1	1	4	4	1	1	4	4
2	2	1	1	4	4	2	2	4	4
1	1	1	1	4	4	2	2	4	4
2	2	4	4	3	3	2	2	1	1
1	1	1	1	4	4	1	1	4	4

For Review Only

2	2	1	1	4	4	2	2	4	4
1	1	1	1	4	4	1	1	4	4
2	2	1	1	4	4	1	1	4	4
1	1	1	1	4	4	1	1	4	4
2	2	1	1	4	4	2	2	4	4
2	2	4	4	3	3	2	2	4	4
1	1	1	1	4	4	1	1	4	4
1	1	1	1	4	4	1	1	4	4
2	2	4	4	4	4	1	1	4	4
1	1	1	1	4	4	1	1	4	4
2	2	4	4	4	4	2	2	4	4
1	1	1	1	4	4	2	2	4	4
2	2	4	4	3	3	2	2	4	4
2	2	4	4	3	3	2	2	4	4
2	2	4	4	3	3	2	2	1	1
2	2	4	4	4	4	2	2	4	4
2	2	4	4	3	3	2	2	4	4
2	2	1	1	4	4	2	2	4	4
1	1	1	1	4	4	1	1	4	4
1	1	1	1	4	4	2	2	4	4
1	1	1	1	4	4	1	1	4	4
2	2	4	4	3	3	2	2	4	4
2	2	4	4	3	3	2	2	4	4
2	2	1	1	4	4	2	2	4	4
1	1	1	1	4	4	1	1	4	4
2	2	4	4	3	3	2	2	4	4
1	1	1	1	4	4	1	1	4	4
2	2	4	4	4	4	2	2	4	4
2	2	4	4	3	3	2	2	4	4
2	2	1	1	4	4	1	1	1	1
1	1	1	1	4	4	1	1	4	4
2	2	4	4	3	3	2	2	4	4
2	2	1	1	4	4	2	2	4	4
2	2	4	4	3	3	2	2	4	4
1	1	1	1	4	4	2	2	4	4
1	1	1	1	4	4	2	2	4	4
2	2	4	4	3	3	2	2	4	4
2	2	4	4	3	3	2	2	4	4
2	2	4	4	3	3	2	2	4	4
2	2	1	1	4	4	2	2	4	4
2	2	4	4	3	3	2	2	4	4
2	2	4	4	3	3	2	2	4	4
2	2	1	1	4	4	2	2	4	4
2	2	4	4	3	3	2	2	4	4
2	2	4	4	4	4	2	2	4	4
2	2	1	1	4	4	2	2	4	4
1	1	1	1	4	4	1	1	4	4
2	2	1	1	4	4	2	2	4	4
2	2	4	4	3	3	2	2	4	4
1	1	1	1	4	4	2	2	4	4
2	2	4	4	3	3	2	2	4	4
1	1	1	1	4	4	1	1	4	4
2	2	4	4	3	3	2	2	1	1
2	2	1	1	4	4	2	2	4	4
2	2	1	1	4	4	2	2	4	4

For Review Only

2	2	4	4	4	4	1	1	4	4
2	2	4	4	3	3	2	2	4	4

For Review Only

SNP847	SNP847	SNP848	SNP848	SNP849	SNP849	SNP850	SNP850	SNP851	SNP851
3	3	4	4	3	3	1	1	3	3
1	1	4	4	3	3	1	1	3	3
3	3	4	4	2	2	1	1	2	2
3	3	4	4	3	3	1	1	2	2
3	3	4	4	3	3	1	1	3	3
3	3	4	4	3	3	1	1	2	2
3	3	4	4	3	3	1	1	2	2
3	3	4	4	2	2	1	1	3	3
3	3	4	4	3	3	1	1	2	2
3	3	4	4	2	2	1	1	3	3
1	1	2	2	3	3	3	3	2	2
3	3	4	4	2	2	1	1	2	2
3	3	4	4	3	3	1	1	2	2
3	3	4	4	2	2	1	1	2	2
3	3	4	4	2	2	1	1	2	2
3	3	4	4	3	3	1	1	2	2
3	3	4	4	3	3	1	1	2	2
3	3	2	2	3	3	1	1	3	3
3	3	4	4	2	2	1	1	2	2
3	3	4	4	2	2	1	1	3	3
3	3	4	4	3	3	1	1	2	2
3	3	4	4	3	3	1	1	3	3
1	1	4	4	3	3	3	3	2	2
3	3	4	4	3	3	1	1	3	3
3	3	4	4	3	3	1	1	2	2
3	3	4	4	2	2	1	1	2	2
3	3	4	4	3	3	1	1	3	3
3	3	4	4	2	2	1	1	2	2
3	3	4	4	3	3	1	1	2	2
1	1	4	4	2	2	1	1	3	3
3	3	4	4	3	3	1	1	2	2
3	3	4	4	2	2	1	1	2	2
3	3	4	4	2	2	1	1	3	3
3	3	4	4	3	3	1	1	2	2
3	3	4	4	2	2	1	1	2	2
3	3	4	4	3	3	1	1	2	2
3	3	4	4	3	3	1	1	2	2
3	3	4	4	3	3	1	1	3	3
1	1	4	4	3	3	1	1	2	2
3	3	2	2	3	3	1	1	2	2
1	1	4	4	2	2	1	1	3	3
3	3	2	2	2	2	1	1	2	2
3	3	4	4	3	3	1	1	3	3
3	3	2	2	3	3	1	1	3	3
3	3	2	2	3	3	1	1	3	3
3	3	4	4	2	2	1	1	2	2
3	3	4	4	2	2	1	1	2	2
1	1	4	4	3	3	3	3	2	2
3	3	4	4	3	3	1	1	3	3
3	3	4	4	3	3	1	1	3	3
3	3	4	4	2	2	1	1	2	2
3	3	4	4	3	3	1	1	2	2

For Review Only

3	3	4	4	3	3	1	1	3	3
3	3	4	4	2	2	1	1	3	3
3	3	4	4	2	2	1	1	3	3
1	1	4	4	3	3	1	1	2	2
3	3	4	4	2	2	1	1	2	2
3	3	4	4	2	2	1	1	2	2
3	3	4	4	2	2	1	1	2	2
1	1	4	4	3	3	1	1	3	3
3	3	4	4	3	3	1	1	3	3
3	3	4	4	3	3	1	1	2	2
3	3	4	4	2	2	1	1	2	2
3	3	4	4	2	2	1	1	2	2
3	3	4	4	3	3	1	1	3	3
3	3	4	4	3	3	1	1	2	2
3	3	4	4	2	2	1	1	2	2
3	3	4	4	2	2	1	1	2	2
3	3	4	4	2	2	1	1	2	2
3	3	4	4	3	3	1	1	2	2
3	3	4	4	3	3	1	1	2	2
3	3	4	4	3	3	1	1	3	3
3	3	4	4	2	2	1	1	2	2
3	3	4	4	3	3	1	1	2	2
3	3	4	4	3	3	1	1	2	2
3	3	4	4	2	2	1	1	2	2
3	3	2	2	3	3	1	1	3	3
3	3	4	4	2	2	1	1	2	2
3	3	4	4	2	2	1	1	3	3
3	3	4	4	3	3	1	1	2	2
3	3	4	4	2	2	1	1	2	2
3	3	4	4	3	3	1	1	2	2
3	3	4	4	3	3	1	1	2	2
3	3	4	4	3	3	1	1	2	2
1	1	4	4	3	3	3	3	2	2
3	3	4	4	3	3	1	1	3	3
3	3	4	4	3	3	1	1	2	2
3	3	2	2	3	3	1	1	3	3
3	3	4	4	3	3	1	1	2	2
3	3	4	4	3	3	1	1	2	2
3	3	4	4	2	2	1	1	2	2
1	1	2	2	3	3	1	1	3	3
3	3	4	4	3	3	1	1	2	2
3	3	4	4	3	3	1	1	2	2
3	3	4	4	2	2	1	1	3	3
3	3	4	4	2	2	1	1	2	2
3	3	4	4	3	3	1	1	2	2
3	3	4	4	3	3	1	1	2	2
1	1	4	4	3	3	1	1	3	3
3	3	4	4	2	2	1	1	2	2
3	3	4	4	2	2	1	1	2	2
3	3	4	4	3	3	1	1	2	2
3	3	4	4	3	3	1	1	2	2
1	1	2	2	3	3	1	1	2	2
3	3	4	4	3	3	1	1	2	2
3	3	4	4	3	3	1	1	2	2
3	3	4	4	3	3	1	1	3	3

For Review Only

3	3	4	4	3	3	1	1	2	2
3	3	4	4	3	3	1	1	3	3
3	3	4	4	3	3	1	1	2	2
1	1	4	4	3	3	1	1	3	3
3	3	4	4	2	2	1	1	2	2
3	3	4	4	2	2	1	1	2	2
3	3	4	4	3	3	1	1	2	2
3	3	4	4	3	3	1	1	2	2
1	1	4	4	2	2	1	1	3	3
1	1	4	4	2	2	1	1	2	2
3	3	4	4	3	3	1	1	3	3
3	3	4	4	3	3	1	1	2	2
3	3	4	4	3	3	1	1	3	3
3	3	4	4	2	2	1	1	2	2
3	3	4	4	3	3	1	1	2	2
3	3	4	4	2	2	1	1	2	2
1	1	4	4	3	3	1	1	3	3
3	3	4	4	2	2	1	1	2	2
3	3	4	4	3	3	1	1	3	3
1	1	4	4	3	3	3	3	3	3
3	3	2	2	2	2	1	1	2	2
3	3	4	4	3	3	1	1	2	2
3	3	4	4	3	3	1	1	2	2
1	1	4	4	2	2	1	1	2	2
3	3	4	4	2	2	1	1	2	2
3	3	4	4	3	3	1	1	2	2
3	3	4	4	3	3	1	1	2	2
3	3	4	4	3	3	1	1	2	2
3	3	4	4	2	2	1	1	2	2
3	3	4	4	3	3	1	1	2	2
1	1	2	2	3	3	1	1	2	2
3	3	4	4	2	2	1	1	2	2
3	3	4	4	3	3	1	1	2	2
3	3	4	4	2	2	1	1	2	2
3	3	4	4	3	3	1	1	2	2
3	3	2	2	3	3	1	1	2	2
1	1	4	4	2	2	1	1	3	3
3	3	4	4	2	2	1	1	2	2
1	1	4	4	2	2	3	3	2	2
1	1	4	4	3	3	3	3	2	2
3	3	4	4	3	3	1	1	2	2
1	1	4	4	3	3	1	1	2	2
3	3	4	4	2	2	1	1	2	2
1	1	4	4	2	2	1	1	2	2
3	3	2	2	2	2	1	1	2	2
3	3	4	4	3	3	1	1	3	3
3	3	4	4	2	2	1	1	2	2
3	3	4	4	2	2	1	1	2	2
1	1	4	4	3	3	1	1	2	2
3	3	4	4	2	2	1	1	2	2
3	3	4	4	2	2	1	1	2	2
3	3	4	4	2	2	1	1	3	3
3	3	2	2	3	3	1	1	2	2
3	3	4	4	2	2	1	1	2	2
1	1	4	4	3	3	1	1	3	3

For Review Only

3	3	4	4	2	2	1	1	2	2
3	3	2	2	3	3	1	1	3	3
3	3	4	4	3	3	1	1	2	2
1	1	4	4	3	3	1	1	2	2
3	3	4	4	2	2	1	1	2	2
3	3	4	4	3	3	1	1	2	2
3	3	4	4	3	3	1	1	2	2
3	3	4	4	2	2	1	1	2	2
1	1	4	4	3	3	1	1	2	2
3	3	4	4	3	3	1	1	2	2
3	3	4	4	2	2	1	1	2	2
3	3	4	4	3	3	1	1	2	2
3	3	4	4	3	3	1	1	2	2
3	3	4	4	3	3	1	1	2	2
1	1	4	4	3	3	1	1	3	3
1	1	4	4	3	3	3	3	2	2
3	3	2	2	3	3	1	1	2	2
3	3	4	4	2	2	1	1	2	2
3	3	4	4	3	3	1	1	2	2
3	3	4	4	3	3	1	1	2	2
3	3	4	4	2	2	1	1	2	2
3	3	4	4	3	3	1	1	2	2
3	3	4	4	3	3	1	1	2	2
3	3	4	4	2	2	1	1	2	2
3	3	4	4	2	2	1	1	3	3
3	3	4	4	3	3	1	1	2	2
3	3	4	4	2	2	1	1	2	2
1	1	4	4	3	3	1	1	2	2
3	3	4	4	3	3	1	1	3	3
3	3	4	4	3	3	1	1	2	2
3	3	4	4	2	2	1	1	2	2
3	3	4	4	3	3	1	1	2	2
3	3	4	4	3	3	1	1	2	2
1	1	4	4	3	3	1	1	3	3
3	3	4	4	3	3	1	1	2	2
3	3	4	4	2	2	1	1	2	2
3	3	4	4	3	3	1	1	2	2
3	3	4	4	3	3	1	1	3	3
3	3	4	4	2	2	1	1	2	2
3	3	4	4	2	2	1	1	2	2
3	3	4	4	2	2	1	1	2	2
3	3	4	4	2	2	1	1	2	2
1	1	4	4	3	3	1	1	2	2
3	3	4	4	2	2	1	1	2	2
3	3	4	4	3	3	1	1	2	2
3	3	4	4	3	3	1	1	2	2
1	1	4	4	3	3	3	3	2	2
3	3	4	4	3	3	1	1	2	2
3	3	2	2	3	3	1	1	2	2
3	3	4	4	3	3	1	1	2	2
3	3	4	4	2	2	1	1	2	2
3	3	4	4	3	3	1	1	3	3
3	3	4	4	3	3	1	1	2	2
3	3	4	4	2	2	1	1	2	2
1	1	4	4	3	3	3	3	2	2

For Review Only

3	3	4	4	2	2	1	1	2	2
1	1	4	4	3	3	1	1	3	3
3	3	4	4	2	2	1	1	2	2
1	1	4	4	3	3	1	1	2	2
3	3	4	4	3	3	1	1	3	3
3	3	4	4	3	3	1	1	2	2
1	1	4	4	3	3	3	3	2	2
1	1	4	4	2	2	1	1	3	3
3	3	4	4	2	2	1	1	2	2
1	1	4	4	3	3	1	1	2	2
3	3	4	4	2	2	1	1	2	2
3	3	4	4	2	2	1	1	2	2
3	3	4	4	2	2	1	1	2	2
3	3	4	4	2	2	1	1	2	2
3	3	2	2	2	2	1	1	2	2
3	3	4	4	3	3	1	1	2	2
3	3	4	4	2	2	1	1	2	2
3	3	2	2	3	3	1	1	3	3
1	1	4	4	2	2	1	1	2	2
3	3	4	4	3	3	1	1	3	3
1	1	4	4	2	2	1	1	2	2
3	3	4	4	2	2	1	1	2	2
3	3	4	4	3	3	1	1	2	2
3	3	2	2	2	2	1	1	2	2
1	1	4	4	3	3	1	1	3	3
3	3	2	2	3	3	1	1	2	2
1	1	4	4	3	3	3	3	2	2
3	3	4	4	2	2	1	1	2	2
3	3	4	4	3	3	1	1	3	3
3	3	4	4	2	2	1	1	2	2
1	1	4	4	3	3	3	3	2	2
3	3	4	4	2	2	1	1	2	2
3	3	2	2	3	3	1	1	2	2
3	3	4	4	2	2	1	1	2	2
3	3	4	4	3	3	1	1	2	2
3	3	4	4	2	2	1	1	2	2
3	3	4	4	3	3	1	1	2	2
3	3	4	4	2	2	1	1	2	2
3	3	4	4	3	3	1	1	2	2
3	3	2	2	3	3	1	1	2	2
3	3	4	4	3	3	1	1	2	2
3	3	4	4	3	3	1	1	2	2
3	3	2	2	3	3	1	1	2	2
3	3	4	4	3	3	1	1	2	2
3	3	4	4	2	2	1	1	2	2
3	3	4	4	3	3	1	1	2	2
1	1	4	4	3	3	3	3	2	2
3	3	4	4	3	3	1	1	3	3
3	3	4	4	3	3	1	1	3	3
3	3	4	4	2	2	1	1	2	2
3	3	4	4	3	3	1	1	2	2
1	1	4	4	3	3	1	1	2	2
3	3	4	4	2	2	1	1	2	2
3	3	2	2	2	2	1	1	2	2
3	3	2	2	3	3	1	1	2	2

For Review Only

3	3	4	4	3	3	1	1	2	2
3	3	4	4	2	2	1	1	2	2

For Review Only

SNP852	SNP852	SNP853	SNP853	SNP854	SNP854	SNP855	SNP855	SNP856	SNP856
2	2	1	1	1	1	1	1	3	3
2	2	1	1	4	4	1	1	3	3
1	1	1	1	1	1	1	1	3	3
1	1	1	1	1	1	1	1	3	3
2	2	1	1	1	1	4	4	1	1
1	1	1	1	4	4	1	1	3	3
1	1	3	3	1	1	4	4	3	3
2	2	3	3	4	4	4	4	1	1
1	1	1	1	4	4	1	1	1	1
2	2	1	1	4	4	4	4	1	1
1	1	1	1	4	4	4	4	1	1
1	1	1	1	1	1	1	1	3	3
1	1	1	1	1	1	4	4	1	1
1	1	3	3	4	4	4	4	1	1
1	1	1	1	4	4	1	1	3	3
1	1	1	1	1	1	1	1	3	3
1	1	3	3	4	4	1	1	3	3
2	2	3	3	1	1	1	1	3	3
1	1	1	1	1	1	4	4	1	1
2	2	3	3	4	4	1	1	3	3
1	1	1	1	4	4	1	1	3	3
2	2	1	1	1	1	4	4	1	1
1	1	1	1	4	4	4	4	1	1
2	2	1	1	4	4	4	4	1	1
1	1	1	1	1	1	1	1	3	3
1	1	1	1	1	1	4	4	1	1
2	2	1	1	1	1	1	1	3	3
1	1	1	1	1	1	1	1	3	3
1	1	1	1	4	4	1	1	3	3
2	2	1	1	4	4	4	4	1	1
1	1	1	1	1	1	1	1	3	3
1	1	1	1	1	1	4	4	1	1
2	2	1	1	1	1	4	4	1	1
1	1	3	3	4	4	4	4	1	1
1	1	1	1	1	1	1	1	3	3
1	1	3	3	1	1	4	4	1	1
1	1	1	1	1	1	1	1	3	3
2	2	1	1	1	1	1	1	3	3
1	1	1	1	4	4	4	4	1	1
1	1	1	1	4	4	1	1	3	3
2	2	1	1	4	4	4	4	1	1
1	1	1	1	4	4	1	1	3	3
2	2	1	1	4	4	4	4	1	1
2	2	1	1	1	1	4	4	1	1
2	2	1	1	4	4	1	1	3	3
1	1	1	1	1	1	1	1	3	3
1	1	1	1	1	1	1	1	3	3
1	1	1	1	4	4	1	1	3	3
2	2	1	1	1	1	4	4	1	1
1	1	3	3	1	1	4	4	3	3
1	1	1	1	4	4	4	4	1	1
1	1	1	1	1	1	1	1	3	3

For Review Only

2	2	1	1	1	1	4	4	1	1
2	2	1	1	1	1	4	4	1	1
2	2	1	1	1	1	1	1	3	3
1	1	1	1	1	1	4	4	1	1
1	1	1	1	1	1	4	4	1	1
1	1	1	1	1	1	1	1	3	3
1	1	1	1	1	1	1	1	3	3
2	2	1	1	4	4	4	4	1	1
2	2	3	3	1	1	4	4	1	1
1	1	1	1	1	1	1	1	3	3
1	1	1	1	1	1	1	1	3	3
1	1	1	1	1	1	1	1	3	3
2	2	1	1	1	1	4	4	1	1
1	1	1	1	4	4	1	1	3	3
1	1	1	1	1	1	1	1	3	3
1	1	3	3	4	4	1	1	3	3
1	1	1	1	1	1	1	1	3	3
1	1	1	1	1	1	1	1	3	3
1	1	1	1	1	1	4	4	1	1
2	2	1	1	1	1	1	1	3	3
1	1	1	1	1	1	1	1	3	3
1	1	1	1	4	4	1	1	3	3
1	1	1	1	4	4	1	1	3	3
1	1	1	1	4	4	1	1	3	3
2	2	1	1	4	4	1	1	3	3
1	1	3	3	1	1	4	4	1	1
2	2	1	1	1	1	4	4	1	1
1	1	1	1	4	4	4	4	1	1
1	1	1	1	1	1	4	4	1	1
1	1	1	1	1	1	1	1	3	3
1	1	1	1	1	1	4	4	1	1
1	1	1	1	4	4	4	4	1	1
1	1	1	1	4	4	4	4	1	1
2	2	1	1	1	1	4	4	1	1
1	1	1	1	1	1	4	4	1	1
2	2	1	1	4	4	1	1	3	3
1	1	3	3	4	4	4	4	1	1
1	1	1	1	1	1	4	4	1	1
1	1	1	1	1	1	4	4	1	1
2	2	1	1	1	1	1	1	3	3
1	1	1	1	1	1	4	4	1	1
1	1	1	1	1	1	1	1	3	3
2	2	1	1	4	4	4	4	1	1
1	1	1	1	1	1	1	1	3	3
1	1	3	3	1	1	4	4	1	1
1	1	1	1	1	1	1	1	3	3
2	2	3	3	4	4	4	4	1	1
1	1	3	3	1	1	1	1	3	3
1	1	1	1	1	1	1	1	3	3
1	1	1	1	1	1	4	4	1	1
1	1	1	1	1	1	1	1	3	3
1	1	1	1	4	4	4	4	1	1
1	1	3	3	4	4	4	4	1	1
2	2	1	1	1	1	1	1	1	1
2	2	1	1	1	1	4	4	1	1

For Review Only

1	1	1	1	1	1	1	1	3	3
2	2	1	1	1	1	1	1	3	3
1	1	1	1	1	1	4	4	1	1
2	2	3	3	4	4	1	1	3	3
1	1	1	1	1	1	1	1	3	3
1	1	1	1	1	1	4	4	1	1
1	1	1	1	1	1	1	1	3	3
1	1	1	1	4	4	1	1	3	3
2	2	1	1	1	1	1	1	1	1
1	1	1	1	1	1	4	4	1	1
2	2	1	1	1	1	4	4	1	1
1	1	1	1	4	4	1	1	3	3
2	2	1	1	4	4	4	4	1	1
1	1	1	1	1	1	4	4	1	1
1	1	1	1	1	1	1	1	3	3
1	1	1	1	1	1	1	1	3	3
1	1	1	1	4	4	1	1	3	3
1	1	1	1	1	1	4	4	3	3
2	2	3	3	4	4	1	1	3	3
2	2	1	1	4	4	4	4	1	1
1	1	1	1	1	1	1	1	3	3
1	1	1	1	1	1	1	1	3	3
1	1	1	1	1	1	1	1	3	3
1	1	1	1	4	4	4	4	1	1
1	1	1	1	1	1	1	1	3	3
1	1	1	1	4	4	4	4	1	1
1	1	1	1	1	1	4	4	1	1
1	1	1	1	1	1	4	4	1	1
1	1	1	1	1	1	1	1	3	3
1	1	1	1	1	1	1	1	3	3
1	1	3	3	4	4	1	1	1	1
1	1	1	1	1	1	1	1	3	3
1	1	1	1	1	1	4	4	1	1
1	1	1	1	1	1	1	1	3	3
1	1	3	3	4	4	4	4	1	1
1	1	1	1	1	1	4	4	1	1
2	2	3	3	4	4	1	1	3	3
1	1	1	1	4	4	4	4	1	1
1	1	1	1	1	1	1	1	3	3
1	1	1	1	4	4	4	4	1	1
1	1	1	1	4	4	4	4	1	1
1	1	3	3	4	4	4	4	1	1
1	1	1	1	1	1	4	4	1	1
1	1	1	1	4	4	1	1	3	3
1	1	1	1	1	1	4	4	1	1
2	2	1	1	1	1	4	4	1	1
1	1	1	1	1	1	1	1	3	3
1	1	1	1	1	1	1	1	3	3
1	1	1	1	1	1	1	1	1	1
1	1	1	1	1	1	4	4	1	1
1	1	1	1	1	1	1	1	3	3
2	2	1	1	1	1	1	1	3	3
1	1	1	1	4	4	4	4	1	1
1	1	1	1	1	1	1	1	3	3
1	1	1	1	1	1	1	1	3	3

1	1	1	1	1	1	1	1	3	3
2	2	1	1	4	4	4	4	1	1
1	1	1	1	1	1	1	1	3	3
1	1	1	1	1	1	4	4	1	1
1	1	1	1	1	1	1	1	3	3
1	1	1	1	4	4	1	1	3	3
1	1	1	1	4	4	4	4	1	1
2	2	1	1	1	1	1	1	3	3
1	1	1	1	4	4	4	4	1	1
1	1	1	1	1	1	1	1	3	3
1	1	1	1	1	1	1	1	3	3
1	1	3	3	4	4	1	1	3	3
1	1	1	1	1	1	1	1	3	3
1	1	1	1	1	1	4	4	1	1
2	2	3	3	4	4	4	4	1	1
1	1	1	1	4	4	4	4	1	1
1	1	1	1	1	1	4	4	1	1
1	1	1	1	1	1	1	1	3	3
1	1	1	1	1	1	4	4	1	1
1	1	1	1	1	1	1	1	3	3
1	1	1	1	1	1	1	1	3	3
1	1	1	1	4	4	4	4	1	1
1	1	1	1	4	4	4	4	1	1
1	1	1	1	1	1	1	1	3	3
1	1	1	1	4	4	4	4	1	1
1	1	3	3	4	4	4	4	1	1
1	1	3	3	1	1	1	1	3	3
1	1	1	1	4	4	1	1	3	3
2	2	1	1	1	1	1	1	3	3
1	1	1	1	1	1	4	4	1	1
1	1	1	1	1	1	4	4	1	1
1	1	1	1	1	1	1	1	3	3
1	1	1	1	1	1	4	4	1	1
2	2	3	3	4	4	4	4	1	1
1	1	1	1	1	1	1	1	3	3
1	1	1	1	1	1	1	1	3	3
1	1	1	1	1	1	1	1	3	3
2	2	1	1	1	1	1	1	3	3
1	1	1	1	1	1	1	1	3	3
1	1	1	1	1	1	4	4	1	1
1	1	1	1	1	1	1	1	3	3
1	1	1	1	1	1	1	1	3	3
1	1	1	1	4	4	4	4	1	1
1	1	1	1	1	1	1	1	3	3
1	1	1	1	1	1	1	1	3	3
1	1	1	1	1	1	4	4	1	1
1	1	1	1	4	4	4	4	1	1
1	1	1	1	1	1	4	4	1	1
1	1	3	3	4	4	4	4	1	1
1	1	1	1	4	4	4	4	1	1
1	1	1	1	1	1	1	1	3	3
2	2	1	1	4	4	4	4	1	1
1	1	1	1	4	4	4	4	1	1
1	1	1	1	1	1	1	1	3	3
1	1	1	1	4	4	4	4	1	1

For Review Only

1	1	1	1	4	4	4	4	1	1
2	2	1	1	4	4	1	1	3	3
1	1	1	1	1	1	1	1	3	3
1	1	1	1	4	4	4	4	1	1
2	2	3	3	4	4	1	1	3	3
1	1	1	1	1	1	4	4	1	1
1	1	1	1	4	4	1	1	3	3
2	2	1	1	4	4	4	4	1	1
1	1	1	1	4	4	4	4	1	1
1	1	1	1	4	4	4	4	1	1
1	1	1	1	1	1	1	1	3	3
1	1	1	1	1	1	1	1	3	3
1	1	1	1	1	1	4	4	1	1
1	1	1	1	1	1	4	4	1	1
1	1	1	1	1	1	1	1	3	3
1	1	1	1	1	1	4	4	1	1
1	1	1	1	1	1	4	4	1	1
2	2	1	1	4	4	4	4	1	1
1	1	1	1	4	4	4	4	1	1
2	2	1	1	4	4	1	1	3	3
1	1	1	1	4	4	1	1	3	3
1	1	1	1	1	1	4	4	1	1
1	1	1	1	1	1	4	4	1	1
1	1	3	3	4	4	4	4	1	1
2	2	1	1	4	4	4	4	1	1
1	1	1	1	1	1	4	4	1	1
1	1	1	1	4	4	4	4	1	1
1	1	1	1	1	1	1	1	3	3
2	2	1	1	1	1	1	1	3	3
1	1	1	1	1	1	1	1	3	3
1	1	1	1	4	4	4	4	1	1
1	1	3	3	1	1	1	1	3	3
1	1	1	1	4	4	4	4	1	1
1	1	1	1	1	1	4	4	1	1
1	1	1	1	4	4	4	4	1	1
1	1	1	1	4	4	4	4	1	1
1	1	1	1	1	1	4	4	1	1
1	1	1	1	1	1	1	1	3	3
1	1	3	3	1	1	4	4	1	1
1	1	1	1	4	4	4	4	1	1
1	1	1	1	1	1	1	1	3	3
1	1	1	1	1	1	1	1	3	3
1	1	3	3	4	4	4	4	1	1
1	1	1	1	1	1	4	4	1	1
1	1	1	1	1	1	1	1	3	3
1	1	1	1	4	4	4	4	1	1
1	1	1	1	4	4	1	1	3	3
2	2	1	1	4	4	4	4	1	1
1	1	1	1	1	1	1	1	3	3
1	1	1	1	4	4	4	4	1	1
1	1	1	1	1	1	1	1	3	3
1	1	1	1	4	4	4	4	1	1
1	1	1	1	1	1	1	1	3	3
1	1	1	1	1	1	1	1	3	3
1	1	1	1	4	4	1	1	3	3

For Review Only

1	1	1	1	1	1	4	4	1	1
1	1	1	1	1	1	1	1	3	3

For Review Only

SNP857	SNP857	SNP858	SNP858	SNP859	SNP859	SNP860	SNP860	SNP861	SNP861
2	2	2	2	2	2	3	3	1	1
2	2	1	1	2	2	3	3	4	4
2	2	2	2	2	2	2	2	1	1
2	2	2	2	4	4	3	3	1	1
3	3	2	2	2	2	3	3	1	1
2	2	1	1	2	2	3	3	4	4
2	2	2	2	2	2	3	3	1	1
3	3	1	1	2	2	2	2	4	4
3	3	2	2	2	2	3	3	1	1
3	3	1	1	2	2	2	2	4	4
3	3	1	1	2	2	3	3	4	4
2	2	2	2	2	2	2	2	1	1
2	2	2	2	2	2	3	3	4	4
3	3	2	2	2	2	3	3	4	4
2	2	1	1	2	2	3	3	4	4
2	2	2	2	2	2	3	3	1	1
2	2	1	1	2	2	3	3	4	4
2	2	2	2	2	2	3	3	1	1
3	3	2	2	2	2	2	2	1	1
2	2	1	1	2	2	2	2	4	4
2	2	1	1	2	2	2	2	4	4
3	3	2	2	2	2	3	3	1	1
3	3	1	1	2	2	3	3	4	4
3	3	1	1	2	2	3	3	4	4
2	2	2	2	2	2	3	3	1	1
3	3	2	2	2	2	2	2	1	1
2	2	2	2	2	2	3	3	1	1
2	2	2	2	2	2	3	3	4	4
2	2	1	1	2	2	3	3	4	4
3	3	1	1	2	2	2	2	4	4
2	2	2	2	2	2	3	3	1	1
3	3	2	2	4	4	2	2	1	1
3	3	2	2	4	4	2	2	1	1
3	3	2	2	2	2	3	3	4	4
2	2	1	1	2	2	3	3	4	4
3	3	2	2	2	2	3	3	1	1
2	2	2	2	2	2	3	3	1	1
2	2	2	2	2	2	3	3	1	1
3	3	1	1	2	2	3	3	4	4
2	2	1	1	4	4	3	3	4	4
3	3	1	1	2	2	2	2	4	4
2	2	1	1	2	2	2	2	4	4
2	2	1	1	2	2	3	3	4	4
3	3	2	2	2	2	3	3	4	4
2	2	1	1	2	2	3	3	4	4
2	2	2	2	2	2	2	2	1	1
2	2	1	1	2	2	2	2	4	4
2	2	1	1	2	2	3	3	4	4
3	3	2	2	2	2	3	3	1	1
2	2	2	2	2	2	3	3	1	1
3	3	1	1	4	4	2	2	4	4
3	3	2	2	2	2	3	3	1	1

For Review Only

3	3	2	2	2	2	3	3	1	1
3	3	2	2	4	4	2	2	1	1
2	2	1	1	2	2	2	2	4	4
3	3	2	2	2	2	3	3	1	1
3	3	2	2	2	2	2	2	1	1
2	2	1	1	2	2	2	2	4	4
2	2	2	2	2	2	2	2	1	1
3	3	1	1	2	2	3	3	4	4
3	3	2	2	4	4	3	3	1	1
2	2	2	2	2	2	3	3	4	4
2	2	1	1	2	2	2	2	4	4
2	2	1	1	2	2	2	2	4	4
3	3	2	2	2	2	3	3	1	1
2	2	1	1	2	2	3	3	4	4
2	2	2	2	2	2	3	3	4	4
3	3	1	1	4	4	3	3	4	4
2	2	2	2	2	2	2	2	4	4
2	2	2	2	4	4	3	3	4	4
3	3	2	2	2	2	2	2	1	1
2	2	2	2	2	2	3	3	1	1
2	2	1	1	2	2	3	3	4	4
2	2	1	1	2	2	3	3	4	4
2	2	1	1	2	2	3	3	4	4
2	2	1	1	2	2	2	2	4	4
2	2	1	1	2	2	3	3	4	4
3	3	2	2	2	2	2	2	1	1
3	3	2	2	2	2	2	2	1	1
3	3	1	1	2	2	3	3	4	4
3	3	2	2	2	2	2	2	1	1
2	2	2	2	2	2	3	3	1	1
3	3	2	2	2	2	3	3	4	4
3	3	1	1	2	2	3	3	4	4
3	3	1	1	2	2	3	3	4	4
3	3	2	2	2	2	3	3	1	1
3	3	2	2	4	4	3	3	1	1
2	2	1	1	2	2	3	3	4	4
3	3	1	1	2	2	3	3	4	4
3	3	2	2	2	2	3	3	1	1
3	3	2	2	2	2	2	2	1	1
2	2	2	2	2	2	2	2	4	4
3	3	2	2	2	2	3	3	1	1
2	2	2	2	4	4	3	3	1	1
3	3	1	1	2	2	3	3	4	4
2	2	1	1	2	2	2	2	4	4
3	3	2	2	2	2	3	3	1	1
2	2	2	2	2	2	3	3	1	1
2	2	1	1	2	2	3	3	4	4
2	2	2	2	2	2	2	2	1	1
2	2	2	2	2	2	3	3	1	1
3	3	2	2	2	2	3	3	4	4
2	2	1	1	2	2	3	3	4	4
3	3	1	1	2	2	3	3	4	4
3	3	1	1	4	4	3	3	4	4
3	3	1	1	2	2	3	3	4	4
3	3	2	2	2	2	2	2	1	1

For Review Only

3	3	2	2	2	2	3	3	1	1
2	2	2	2	2	2	3	3	4	4
3	3	2	2	2	2	3	3	1	1
2	2	1	1	2	2	3	3	4	4
2	2	2	2	2	2	3	3	1	1
3	3	2	2	2	2	2	2	1	1
2	2	2	2	2	2	3	3	1	1
2	2	1	1	2	2	3	3	4	4
2	2	2	2	2	2	3	3	4	4
3	3	1	1	2	2	2	2	1	1
3	3	1	1	2	2	3	3	4	4
2	2	1	1	2	2	3	3	1	1
3	3	1	1	2	2	3	3	4	4
2	2	2	2	4	4	3	3	4	4
2	2	2	2	2	2	3	3	1	1
2	2	2	2	2	2	3	3	4	4
2	2	1	1	2	2	3	3	4	4
2	2	2	2	2	2	3	3	4	4
2	2	1	1	2	2	3	3	4	4
3	3	1	1	2	2	3	3	4	4
2	2	2	2	2	2	3	3	4	4
3	3	2	2	2	2	3	3	1	1
2	2	2	2	4	4	3	3	1	1
3	3	1	1	2	2	3	3	4	4
2	2	2	2	2	2	2	2	1	1
3	3	1	1	2	2	3	3	4	4
3	3	2	2	2	2	3	3	1	1
3	3	2	2	2	2	3	3	1	1
2	2	2	2	2	2	2	2	1	1
2	2	2	2	2	2	3	3	1	1
3	3	1	1	2	2	3	3	4	4
2	2	2	2	2	2	3	3	4	4
3	3	2	2	2	2	3	3	1	1
2	2	2	2	2	2	3	3	4	4
3	3	2	2	2	2	3	3	4	4
2	2	1	1	4	4	3	3	4	4
2	2	1	1	2	2	3	3	4	4
3	3	1	1	2	2	2	2	4	4
3	3	1	1	2	2	3	3	4	4
3	3	1	1	2	2	3	3	4	4
3	3	1	1	2	2	3	3	4	4
3	3	1	1	2	2	3	3	4	4
3	3	2	2	2	2	3	3	4	4
3	3	2	2	2	2	3	3	4	4
3	3	1	1	2	2	3	3	4	4
3	3	2	2	2	2	3	3	1	1
2	2	2	2	2	2	2	2	1	1
2	2	2	2	2	2	2	2	4	4
2	2	2	2	2	2	3	3	4	4
3	3	2	2	2	2	2	2	4	4
2	2	2	2	4	4	3	3	1	1
2	2	2	2	2	2	2	2	1	1
3	3	2	2	2	2	3	3	4	4
2	2	1	1	4	4	2	2	4	4
2	2	2	2	2	2	3	3	4	4

For Review Only

2	2	2	2	4	4	2	2	1	1
3	3	1	1	2	2	3	3	4	4
2	2	2	2	2	2	3	3	4	4
3	3	2	2	2	2	3	3	4	4
2	2	2	2	2	2	3	3	1	1
2	2	1	1	4	4	3	3	4	4
3	3	2	2	2	2	3	3	4	4
2	2	2	2	2	2	3	3	4	4
3	3	1	1	2	2	3	3	4	4
2	2	2	2	2	2	3	3	1	1
2	2	2	2	2	2	2	2	4	4
3	3	1	1	2	2	3	3	4	4
2	2	2	2	2	2	3	3	4	4
3	3	2	2	2	2	3	3	4	4
3	3	1	1	2	2	3	3	4	4
3	3	1	1	2	2	3	3	4	4
3	3	1	1	2	2	3	3	4	4
2	2	2	2	4	4	2	2	1	1
3	3	2	2	4	4	3	3	1	1
2	2	2	2	2	2	3	3	1	1
2	2	2	2	2	2	3	3	4	4
3	3	1	1	2	2	3	3	4	4
3	3	1	1	2	2	3	3	4	4
2	2	1	1	2	2	2	2	4	4
3	3	1	1	2	2	2	2	4	4
3	3	2	2	2	2	3	3	4	4
2	2	2	2	2	2	3	3	4	4
2	2	1	1	2	2	3	3	4	4
2	2	2	2	2	2	3	3	1	1
3	3	2	2	2	2	3	3	4	4
3	3	2	2	2	2	2	2	1	1
2	2	2	2	2	2	3	3	1	1
3	3	2	2	4	4	3	3	1	1
3	3	1	1	2	2	3	3	4	4
2	2	2	2	2	2	3	3	1	1
2	2	1	1	2	2	2	2	4	4
2	2	2	2	4	4	3	3	1	1
2	2	2	2	2	2	3	3	1	1
2	2	2	2	2	2	2	2	4	4
3	3	2	2	4	4	2	2	4	4
2	2	2	2	2	2	2	2	1	1
2	2	2	2	4	4	2	2	1	1
3	3	1	1	2	2	3	3	4	4
2	2	2	2	2	2	3	3	4	4
2	2	2	2	2	2	3	3	1	1
3	3	2	2	4	4	3	3	1	1
3	3	1	1	2	2	3	3	4	4
3	3	2	2	2	2	3	3	1	1
3	3	1	1	2	2	3	3	4	4
3	3	1	1	2	2	3	3	4	4
2	2	2	2	2	2	2	2	1	1
3	3	1	1	2	2	3	3	4	4
3	3	1	1	2	2	3	3	4	4
2	2	1	1	2	2	2	2	4	4
3	3	1	1	2	2	3	3	4	4

For Review Only

3	3	1	1	2	2	3	3	4	4
2	2	2	2	4	4	3	3	4	4
2	2	2	2	2	2	2	2	1	1
3	3	1	1	2	2	3	3	4	4
2	2	1	1	2	2	3	3	4	4
3	3	2	2	2	2	3	3	1	1
2	2	1	1	2	2	3	3	4	4
3	3	1	1	2	2	3	3	4	4
3	3	2	2	2	2	3	3	4	4
3	3	1	1	2	2	3	3	4	4
2	2	2	2	2	2	3	3	4	4
2	2	1	1	2	2	3	3	4	4
3	3	2	2	2	2	2	2	1	1
3	3	2	2	2	2	2	2	1	1
2	2	2	2	2	2	2	2	4	4
3	3	2	2	4	4	3	3	1	1
3	3	2	2	2	2	3	3	1	1
3	3	1	1	2	2	3	3	4	4
3	3	1	1	2	2	3	3	4	4
2	2	1	1	2	2	3	3	4	4
2	2	1	1	2	2	2	2	4	4
3	3	2	2	2	2	2	2	1	1
3	3	2	2	2	2	3	3	1	1
3	3	1	1	4	4	3	3	4	4
3	3	1	1	2	2	3	3	4	4
3	3	2	2	2	2	3	3	1	1
3	3	1	1	2	2	3	3	4	4
2	2	2	2	2	2	2	2	1	1
2	2	2	2	2	2	3	3	4	4
2	2	2	2	2	2	2	2	1	1
3	3	1	1	2	2	3	3	4	4
2	2	2	2	2	2	2	2	1	1
3	3	1	1	2	2	3	3	4	4
3	3	2	2	2	2	2	2	4	4
3	3	1	1	2	2	3	3	4	4
3	3	1	1	2	2	3	3	4	4
3	3	2	2	2	2	3	3	1	1
2	2	1	1	2	2	2	2	4	4
3	3	2	2	2	2	3	3	1	1
3	3	1	1	4	4	3	3	4	4
2	2	2	2	4	4	3	3	1	1
2	2	2	2	2	2	3	3	1	1
3	3	1	1	4	4	3	3	4	4
3	3	2	2	2	2	3	3	1	1
2	2	2	2	2	2	2	2	1	1
3	3	1	1	2	2	3	3	4	4
2	2	1	1	2	2	3	3	4	4
3	3	1	1	4	4	3	3	4	4
2	2	2	2	2	2	3	3	4	4
3	3	1	1	2	2	3	3	4	4
2	2	2	2	4	4	3	3	1	1
3	3	1	1	2	2	3	3	4	4
2	2	2	2	2	2	2	2	1	1
2	2	2	2	2	2	2	2	1	1
2	2	1	1	4	4	3	3	4	4

For Review Only

3	3	2	2	4	4	3	3	1	1
2	2	2	2	2	2	2	2	1	1

For Review Only

SNP862	SNP862	SNP863	SNP863	SNP864	SNP864	SNP865	SNP865	SNP866	SNP866
2	2	2	2	4	4	2	2	1	1
2	2	2	2	4	4	3	3	4	4
3	3	2	2	4	4	2	2	1	1
3	3	2	2	4	4	2	2	1	1
3	3	2	2	1	1	2	2	1	1
3	3	3	3	4	4	3	3	4	4
2	2	2	2	4	4	2	2	1	1
3	3	3	3	4	4	3	3	4	4
2	2	3	3	4	4	2	2	4	4
3	3	3	3	1	1	3	3	4	4
3	3	3	3	4	4	3	3	4	4
3	3	2	2	4	4	2	2	1	1
2	2	2	2	1	1	2	2	1	1
3	3	3	3	4	4	3	3	4	4
3	3	2	2	4	4	3	3	4	4
3	3	2	2	1	1	2	2	1	1
3	3	3	3	1	1	3	3	4	4
3	3	2	2	4	4	2	2	1	1
3	3	2	2	4	4	2	2	1	1
3	3	3	3	1	1	2	2	4	4
3	3	3	3	1	1	3	3	4	4
3	3	2	2	1	1	2	2	1	1
3	3	3	3	4	4	3	3	4	4
3	3	3	3	1	1	3	3	4	4
3	3	2	2	4	4	2	2	1	1
3	3	2	2	4	4	2	2	1	1
3	3	2	2	4	4	2	2	1	1
3	3	2	2	4	4	2	2	1	1
3	3	3	3	1	1	3	3	4	4
3	3	3	3	1	1	3	3	4	4
3	3	2	2	4	4	2	2	1	1
3	3	2	2	4	4	2	2	1	1
2	2	2	2	4	4	2	2	1	1
3	3	3	3	1	1	3	3	4	4
3	3	3	3	4	4	3	3	4	4
3	3	2	2	4	4	2	2	1	1
2	2	2	2	4	4	2	2	1	1
3	3	2	2	4	4	2	2	1	1
3	3	3	3	1	1	3	3	4	4
3	3	3	3	4	4	3	3	4	4
3	3	3	3	1	1	3	3	4	4
3	3	3	3	4	4	3	3	4	4
3	3	3	3	1	1	3	3	4	4
3	3	2	2	4	4	2	2	1	1
3	3	3	3	4	4	3	3	4	4
3	3	2	2	4	4	2	2	1	1
3	3	3	3	4	4	3	3	4	4
3	3	3	3	4	4	3	3	4	4
3	3	2	2	1	1	2	2	1	1
3	3	2	2	4	4	2	2	1	1
3	3	3	3	4	4	3	3	4	4
3	3	2	2	4	4	2	2	1	1

For Review Only

2	2	2	2	4	4	2	2	1	1
2	2	2	2	4	4	2	2	1	1
2	2	3	3	4	4	3	3	4	4
3	3	2	2	4	4	2	2	1	1
3	3	2	2	4	4	2	2	1	1
3	3	3	3	4	4	3	3	4	4
3	3	2	2	4	4	2	2	1	1
3	3	3	3	1	1	3	3	4	4
2	2	2	2	4	4	2	2	1	1
3	3	2	2	4	4	2	2	1	1
3	3	3	3	4	4	3	3	4	4
3	3	3	3	4	4	3	3	4	4
2	2	2	2	4	4	2	2	1	1
3	3	3	3	1	1	3	3	4	4
3	3	2	2	4	4	2	2	1	1
2	2	3	3	4	4	3	3	4	4
3	3	2	2	4	4	2	2	1	1
3	3	3	3	4	4	3	3	4	4
2	2	2	2	4	4	2	2	1	1
2	2	2	2	4	4	2	2	1	1
3	3	2	2	4	4	3	3	4	4
2	2	3	3	1	1	3	3	4	4
3	3	3	3	1	1	3	3	4	4
3	3	3	3	1	1	3	3	4	4
3	3	3	3	4	4	3	3	4	4
3	3	2	2	4	4	2	2	1	1
3	3	2	2	4	4	2	2	1	1
3	3	3	3	1	1	3	3	4	4
3	3	2	2	4	4	2	2	1	1
2	2	2	2	4	4	2	2	1	1
3	3	2	2	4	4	2	2	1	1
3	3	3	3	1	1	3	3	4	4
3	3	3	3	1	1	3	3	4	4
2	2	2	2	4	4	2	2	1	1
3	3	2	2	4	4	2	2	1	1
3	3	3	3	4	4	3	3	4	4
3	3	3	3	4	4	3	3	4	4
3	3	2	2	1	1	2	2	1	1
3	3	2	2	4	4	2	2	1	1
3	3	2	2	4	4	2	2	1	1
3	3	2	2	4	4	2	2	1	1
2	2	2	2	4	4	2	2	1	1
3	3	3	3	4	4	3	3	4	4
3	3	3	3	4	4	3	3	4	4
3	3	2	2	4	4	2	2	1	1
3	3	2	2	4	4	3	3	4	4
3	3	3	3	1	1	3	3	4	4
3	3	2	2	4	4	2	2	1	1
2	2	2	2	4	4	2	2	1	1
3	3	3	3	4	4	3	3	4	4
3	3	3	3	4	4	3	3	4	4
3	3	3	3	1	1	3	3	4	4
3	3	3	3	1	1	3	3	4	4
3	3	2	2	4	4	2	2	1	1
3	3	2	2	4	4	2	2	1	1

For Review Only

3	3	2	2	4	4	2	2	1	1
3	3	2	2	4	4	3	3	1	1
3	3	2	2	4	4	2	2	1	1
3	3	3	3	1	1	3	3	4	4
3	3	2	2	4	4	3	3	4	4
3	3	2	2	4	4	2	2	1	1
2	2	2	2	4	4	2	2	1	1
3	3	3	3	4	4	3	3	4	4
3	3	2	2	1	1	2	2	1	1
3	3	2	2	4	4	2	2	1	1
3	3	3	3	4	4	3	3	4	4
3	3	2	2	4	4	2	2	1	1
3	3	3	3	4	4	3	3	4	4
3	3	2	2	4	4	2	2	1	1
3	3	2	2	4	4	3	3	1	1
3	3	2	2	4	4	2	2	1	1
3	3	3	3	4	4	3	3	4	4
3	3	2	2	4	4	2	2	1	1
3	3	3	3	1	1	3	3	4	4
3	3	2	2	4	4	3	3	4	4
3	3	2	2	4	4	2	2	1	1
2	2	2	2	4	4	2	2	1	1
3	3	2	2	4	4	2	2	1	1
3	3	3	3	1	1	3	3	4	4
3	3	2	2	4	4	2	2	1	1
3	3	3	3	4	4	3	3	4	4
2	2	2	2	4	4	2	2	1	1
3	3	2	2	4	4	2	2	1	1
3	3	2	2	4	4	2	2	1	1
3	3	2	2	4	4	2	2	1	1
3	3	3	3	1	1	3	3	4	4
3	3	2	2	4	4	2	2	1	1
3	3	2	2	4	4	2	2	1	1
3	3	2	2	4	4	2	2	1	1
3	3	3	3	1	1	3	3	4	4
3	3	3	3	1	1	3	3	4	4
3	3	3	3	1	1	2	2	4	4
3	3	3	3	4	4	3	3	4	4
3	3	2	2	4	4	3	3	4	4
3	3	3	3	1	1	3	3	4	4
3	3	3	3	1	1	3	3	4	4
3	3	3	3	1	1	3	3	4	4
3	3	3	3	4	4	3	3	4	4
3	3	2	2	4	4	3	3	4	4
3	3	3	3	4	4	3	3	4	4
2	2	2	2	4	4	2	2	1	1
3	3	2	2	4	4	2	2	1	1
3	3	3	3	4	4	3	3	4	4
3	3	2	2	4	4	2	2	1	1
3	3	2	2	4	4	2	2	1	1
3	3	2	2	4	4	2	2	1	1
3	3	2	2	1	1	2	2	1	1
3	3	2	2	4	4	3	3	4	4
3	3	3	3	4	4	3	3	4	4
3	3	2	2	4	4	2	2	1	1

For Review Only

3	3	2	2	4	4	2	2	1	1
3	3	3	3	4	4	3	3	4	4
3	3	2	2	1	1	2	2	1	1
3	3	2	2	1	1	2	2	1	1
3	3	2	2	4	4	2	2	1	1
3	3	3	3	1	1	3	3	4	4
3	3	2	2	4	4	2	2	1	1
3	3	2	2	4	4	2	2	1	1
3	3	3	3	4	4	3	3	4	4
2	2	2	2	4	4	2	2	1	1
3	3	2	2	4	4	3	3	1	1
3	3	3	3	4	4	3	3	4	4
3	3	2	2	4	4	2	2	1	1
3	3	2	2	4	4	2	2	1	1
3	3	3	3	1	1	3	3	4	4
3	3	3	3	4	4	3	3	4	4
3	3	2	2	4	4	3	3	4	4
3	3	2	2	4	4	3	3	4	4
3	3	2	2	4	4	2	2	1	1
2	2	2	2	4	4	2	2	1	1
3	3	2	2	4	4	2	2	1	1
3	3	3	3	1	1	3	3	4	4
3	3	3	3	1	1	3	3	4	4
3	3	3	3	4	4	3	3	4	4
3	3	3	3	4	4	3	3	4	4
3	3	3	3	1	1	3	3	4	4
3	3	2	2	4	4	2	2	1	1
3	3	3	3	4	4	3	3	4	4
3	3	2	2	4	4	2	2	1	1
3	3	3	3	4	4	3	3	4	4
2	2	2	2	4	4	2	2	1	1
2	2	2	2	4	4	2	2	1	1
2	2	2	2	4	4	2	2	1	1
3	3	3	3	1	1	3	3	4	4
2	2	2	2	4	4	2	2	1	1
3	3	3	3	4	4	3	3	4	4
3	3	2	2	4	4	2	2	1	1
2	2	2	2	4	4	2	2	1	1
3	3	3	3	4	4	3	3	4	4
3	3	3	3	4	4	3	3	4	4
3	3	2	2	4	4	2	2	1	1
3	3	2	2	4	4	2	2	1	1
3	3	3	3	1	1	3	3	4	4
3	3	2	2	4	4	2	2	1	1
2	2	2	2	4	4	2	2	1	1
3	3	2	2	1	1	2	2	1	1
3	3	3	3	4	4	3	3	4	4
3	3	2	2	1	1	2	2	1	1
3	3	3	3	1	1	3	3	4	4
3	3	3	3	1	1	3	3	4	4
3	3	2	2	4	4	2	2	1	1
3	3	3	3	1	1	3	3	4	4
2	2	3	3	1	1	3	3	4	4
3	3	3	3	4	4	3	3	4	4
3	3	3	3	1	1	3	3	4	4

For Review Only

3	3	3	3	4	4	3	3	4	4
3	3	3	3	4	4	3	3	4	4
2	2	2	2	4	4	2	2	1	1
3	3	3	3	4	4	3	3	4	4
3	3	3	3	4	4	3	3	4	4
2	2	2	2	4	4	2	2	1	1
3	3	3	3	1	1	3	3	4	4
3	3	3	3	1	1	3	3	4	4
3	3	3	3	4	4	2	2	1	1
3	3	3	3	1	1	3	3	4	4
3	3	2	2	4	4	2	2	1	1
2	2	3	3	4	4	3	3	4	4
3	3	2	2	4	4	2	2	1	1
3	3	2	2	4	4	2	2	1	1
3	3	3	3	4	4	3	3	4	4
2	2	2	2	4	4	2	2	1	1
3	3	2	2	1	1	2	2	1	1
3	3	3	3	4	4	3	3	4	4
3	3	3	3	1	1	3	3	4	4
3	3	3	3	4	4	3	3	4	4
3	3	3	3	1	1	3	3	4	4
3	3	2	2	4	4	2	2	1	1
2	2	2	2	4	4	2	2	1	1
3	3	3	3	4	4	3	3	4	4
3	3	3	3	1	1	3	3	4	4
3	3	2	2	4	4	2	2	1	1
3	3	3	3	1	1	3	3	4	4
3	3	2	2	4	4	2	2	1	1
3	3	2	2	4	4	2	2	1	1
2	2	2	2	4	4	2	2	1	1
3	3	3	3	4	4	3	3	4	4
3	3	2	2	4	4	2	2	1	1
3	3	3	3	4	4	3	3	4	4
3	3	3	3	4	4	3	3	4	4
2	2	3	3	1	1	3	3	4	4
3	3	3	3	1	1	3	3	4	4
3	3	2	2	4	4	2	2	1	1
3	3	3	3	4	4	3	3	4	4
3	3	2	2	4	4	2	2	1	1
3	3	3	3	1	1	3	3	4	4
2	2	2	2	4	4	2	2	1	1
3	3	2	2	1	1	2	2	1	1
3	3	3	3	4	4	3	3	4	4
3	3	2	2	4	4	2	2	1	1
3	3	2	2	4	4	2	2	1	1
3	3	3	3	4	4	3	3	4	4
3	3	3	3	4	4	3	3	4	4
3	3	3	3	4	4	3	3	4	4
3	3	2	2	4	4	2	2	1	1
3	3	3	3	1	1	3	3	4	4
2	2	2	2	4	4	2	2	1	1
3	3	3	3	1	1	3	3	4	4
3	3	2	2	4	4	2	2	1	1
3	3	2	2	4	4	2	2	1	1
3	3	3	3	4	4	3	3	4	4

For Review Only

2	2	2	2	4	4	2	2	1	1
3	3	2	2	4	4	2	2	1	1

For Review Only

SNP867	SNP867	SNP868	SNP868	SNP869	SNP869	SNP870	SNP870	SNP871	SNP871
1	1	3	3	4	4	3	3	3	3
4	4	3	3	4	4	3	3	3	3
4	4	3	3	1	1	3	3	4	4
4	4	3	3	4	4	2	2	3	3
4	4	3	3	1	1	3	3	3	3
1	1	2	2	1	1	3	3	3	3
4	4	3	3	4	4	3	3	3	3
4	4	3	3	1	1	3	3	3	3
4	4	3	3	4	4	3	3	3	3
4	4	3	3	1	1	2	2	3	3
1	1	2	2	1	1	2	2	3	3
4	4	3	3	1	1	3	3	4	4
4	4	3	3	4	4	3	3	3	3
4	4	2	2	1	1	3	3	3	3
4	4	3	3	1	1	3	3	3	3
4	4	3	3	4	4	3	3	3	3
4	4	3	3	1	1	3	3	3	3
4	4	3	3	1	1	3	3	3	3
1	1	3	3	1	1	3	3	4	4
1	1	3	3	1	1	3	3	4	4
1	1	2	2	1	1	3	3	3	3
4	4	3	3	1	1	3	3	3	3
1	1	2	2	1	1	3	3	3	3
4	4	2	2	1	1	3	3	3	3
1	1	3	3	1	1	3	3	3	3
1	1	3	3	1	1	3	3	4	4
4	4	3	3	1	1	3	3	3	3
4	4	3	3	1	1	3	3	3	3
1	1	2	2	1	1	3	3	3	3
1	1	2	2	1	1	3	3	3	3
4	4	3	3	1	1	3	3	3	3
4	4	3	3	1	1	3	3	3	3
4	4	3	3	4	4	2	2	3	3
4	4	2	2	1	1	3	3	3	3
1	1	2	2	1	1	3	3	3	3
4	4	3	3	1	1	3	3	3	3
1	1	3	3	4	4	3	3	3	3
1	1	3	3	1	1	3	3	3	3
1	1	2	2	1	1	3	3	3	3
1	1	2	2	1	1	3	3	3	3
1	1	3	3	1	1	3	3	3	3
1	1	2	2	1	1	2	2	3	3
4	4	3	3	1	1	3	3	3	3
1	1	3	3	1	1	2	2	3	3
4	4	3	3	1	1	2	2	3	3
1	1	3	3	1	1	3	3	4	4
1	1	2	2	1	1	3	3	4	4
1	1	2	2	1	1	2	2	3	3
1	1	3	3	1	1	3	3	3	3
4	4	3	3	4	4	3	3	3	3
4	4	2	2	1	1	2	2	4	4
4	4	3	3	1	1	2	2	3	3

For Review Only

4	4	3	3	4	4	3	3	3	3
4	4	3	3	4	4	2	2	3	3
4	4	2	2	4	4	3	3	3	3
4	4	3	3	1	1	3	3	3	3
1	1	3	3	1	1	3	3	4	4
4	4	2	2	1	1	3	3	4	4
4	4	3	3	1	1	3	3	4	4
1	1	2	2	1	1	3	3	3	3
1	1	3	3	4	4	2	2	3	3
4	4	3	3	4	4	3	3	3	3
1	1	2	2	1	1	3	3	4	4
1	1	2	2	1	1	3	3	4	4
4	4	3	3	4	4	3	3	3	3
1	1	2	2	1	1	3	3	3	3
4	4	3	3	4	4	3	3	3	3
1	1	2	2	4	4	2	2	3	3
4	4	3	3	1	1	3	3	4	4
1	1	2	2	1	1	2	2	3	3
4	4	3	3	4	4	3	3	3	3
4	4	3	3	4	4	3	3	3	3
4	4	3	3	1	1	3	3	3	3
4	4	3	3	4	4	3	3	3	3
4	4	3	3	4	4	3	3	3	3
4	4	2	2	1	1	3	3	4	4
4	4	3	3	1	1	2	2	3	3
1	1	3	3	1	1	2	2	4	4
1	1	3	3	1	1	3	3	4	4
1	1	2	2	1	1	3	3	3	3
1	1	3	3	1	1	3	3	4	4
4	4	3	3	4	4	3	3	3	3
4	4	3	3	1	1	3	3	3	3
4	4	2	2	1	1	3	3	3	3
1	1	2	2	1	1	3	3	3	3
4	4	3	3	4	4	3	3	3	3
1	1	3	3	1	1	2	2	3	3
4	4	3	3	1	1	2	2	3	3
1	1	2	2	1	1	3	3	3	3
1	1	3	3	4	4	3	3	3	3
4	4	3	3	1	1	3	3	4	4
1	1	3	3	1	1	2	2	3	3
4	4	3	3	1	1	3	3	3	3
4	4	3	3	4	4	3	3	3	3
4	4	2	2	1	1	3	3	3	3
1	1	2	2	1	1	3	3	4	4
4	4	3	3	1	1	3	3	3	3
4	4	2	2	1	1	3	3	3	3
4	4	2	2	1	1	3	3	3	3
4	4	3	3	1	1	3	3	4	4
1	1	3	3	4	4	3	3	3	3
4	4	2	2	4	4	3	3	3	3
4	4	2	2	1	1	3	3	3	3
1	1	2	2	1	1	2	2	3	3
4	4	2	2	1	1	2	2	3	3
4	4	3	3	4	4	3	3	4	4
4	4	3	3	1	1	3	3	3	3

For Review Only

4	4	3	3	1	1	3	3	3	3
1	1	3	3	1	1	3	3	3	3
4	4	3	3	1	1	3	3	3	3
4	4	2	2	1	1	3	3	3	3
4	4	2	2	1	1	3	3	4	4
1	1	3	3	1	1	3	3	4	4
4	4	3	3	4	4	3	3	3	3
1	1	2	2	1	1	3	3	3	3
1	1	3	3	4	4	3	3	3	3
4	4	3	3	1	1	3	3	4	4
1	1	2	2	4	4	2	2	3	3
4	4	3	3	1	1	3	3	3	3
4	4	3	3	1	1	3	3	3	3
4	4	3	3	1	1	2	2	4	4
4	4	3	3	1	1	3	3	3	3
4	4	3	3	1	1	2	2	4	4
1	1	3	3	1	1	3	3	3	3
1	1	3	3	1	1	3	3	3	3
4	4	2	2	1	1	3	3	3	3
4	4	2	2	1	1	3	3	3	3
4	4	3	3	1	1	3	3	3	3
4	4	3	3	4	4	3	3	3	3
1	1	3	3	1	1	3	3	3	3
1	1	3	3	1	1	3	3	3	3
4	4	3	3	4	4	3	3	3	3
1	1	2	2	1	1	3	3	3	3
4	4	3	3	4	4	3	3	3	3
1	1	3	3	1	1	3	3	3	3
1	1	3	3	1	1	3	3	4	4
1	1	3	3	1	1	3	3	3	3
1	1	2	2	1	1	3	3	3	3
4	4	3	3	1	1	3	3	3	3
4	4	3	3	1	1	3	3	3	3
4	4	3	3	4	4	3	3	3	3
4	4	2	2	1	1	3	3	3	3
4	4	2	2	1	1	3	3	3	3
4	4	2	2	1	1	3	3	3	3
4	4	2	2	1	1	3	3	4	4
4	4	2	2	1	1	3	3	3	3
1	1	2	2	1	1	3	3	3	3
4	4	2	2	4	4	3	3	3	3
4	4	2	2	1	1	3	3	3	3
1	1	2	2	1	1	3	3	3	3
4	4	3	3	1	1	3	3	3	3
1	1	2	2	1	1	3	3	3	3
4	4	3	3	4	4	3	3	3	3
4	4	3	3	1	1	3	3	4	4
4	4	3	3	1	1	3	3	4	4
4	4	3	3	4	4	2	2	3	3
1	1	3	3	1	1	3	3	4	4
4	4	3	3	1	1	2	2	3	3
4	4	3	3	1	1	3	3	3	3
4	4	2	2	1	1	3	3	3	3
4	4	2	2	1	1	2	2	4	4
1	1	3	3	1	1	3	3	3	3

For Review Only

4	4	3	3	1	1	2	2	4	4
4	4	2	2	1	1	2	2	3	3
1	1	3	3	1	1	3	3	4	4
1	1	3	3	1	1	3	3	3	3
1	1	3	3	1	1	3	3	3	3
1	1	2	2	1	1	2	2	3	3
1	1	3	3	1	1	3	3	3	3
4	4	3	3	1	1	3	3	3	3
4	4	2	2	4	4	3	3	3	3
4	4	3	3	4	4	3	3	3	3
1	1	3	3	4	4	2	2	4	4
4	4	2	2	1	1	3	3	3	3
4	4	3	3	4	4	3	3	3	3
4	4	3	3	4	4	3	3	3	3
4	4	2	2	1	1	3	3	3	3
1	1	2	2	1	1	3	3	3	3
4	4	2	2	1	1	3	3	3	3
4	4	3	3	1	1	2	2	4	4
1	1	3	3	1	1	2	2	3	3
4	4	3	3	4	4	3	3	3	3
4	4	3	3	1	1	3	3	3	3
4	4	2	2	1	1	3	3	3	3
1	1	3	3	4	4	3	3	3	3
1	1	2	2	1	1	3	3	4	4
1	1	2	2	1	1	3	3	3	3
4	4	2	2	1	1	3	3	3	3
4	4	3	3	1	1	3	3	3	3
1	1	3	3	1	1	3	3	3	3
1	1	3	3	1	1	3	3	3	3
4	4	2	2	1	1	3	3	3	3
1	1	3	3	4	4	3	3	4	4
4	4	3	3	4	4	3	3	3	3
1	1	3	3	4	4	2	2	3	3
4	4	2	2	1	1	3	3	3	3
1	1	3	3	4	4	3	3	3	3
1	1	2	2	1	1	3	3	4	4
1	1	3	3	1	1	3	3	3	3
1	1	3	3	4	4	3	3	3	3
4	4	3	3	1	1	3	3	4	4
1	1	3	3	1	1	2	2	4	4
4	4	3	3	1	1	3	3	4	4
4	4	3	3	1	1	2	2	4	4
1	1	2	2	1	1	3	3	3	3
1	1	3	3	4	4	3	3	3	3
4	4	3	3	4	4	3	3	3	3
1	1	3	3	1	1	2	2	3	3
1	1	2	2	1	1	3	3	3	3
1	1	3	3	1	1	3	3	3	3
4	4	2	2	1	1	3	3	3	3
4	4	2	2	1	1	3	3	3	3
1	1	3	3	1	1	3	3	4	4
4	4	2	2	1	1	3	3	3	3
1	1	2	2	4	4	3	3	3	3
1	1	2	2	1	1	3	3	4	4
1	1	2	2	1	1	3	3	3	3

For Review Only

4	4	2	2	1	1	3	3	3	3
1	1	2	2	4	4	2	2	3	3
4	4	3	3	4	4	3	3	4	4
4	4	3	3	4	4	3	3	3	3
4	4	3	3	1	1	3	3	3	3
1	1	3	3	4	4	3	3	3	3
1	1	2	2	1	1	3	3	3	3
1	1	2	2	1	1	3	3	4	4
4	4	3	3	1	1	3	3	3	3
1	1	2	2	1	1	3	3	3	3
4	4	3	3	1	1	3	3	3	3
1	1	3	3	4	4	2	2	4	4
4	4	3	3	1	1	3	3	4	4
1	1	3	3	1	1	3	3	4	4
1	1	3	3	1	1	2	2	4	4
4	4	3	3	4	4	2	2	3	3
1	1	3	3	1	1	3	3	3	3
4	4	2	2	4	4	3	3	3	3
4	4	3	3	1	1	3	3	3	3
1	1	2	2	1	1	3	3	3	3
1	1	2	2	1	1	3	3	4	4
1	1	3	3	1	1	3	3	4	4
4	4	3	3	4	4	3	3	3	3
1	1	2	2	1	1	3	3	3	3
1	1	2	2	1	1	3	3	3	3
1	1	3	3	1	1	2	2	3	3
1	1	2	2	1	1	3	3	3	3
4	4	3	3	1	1	3	3	3	3
4	4	3	3	4	4	3	3	3	3
1	1	3	3	4	4	3	3	4	4
1	1	2	2	1	1	3	3	3	3
4	4	3	3	1	1	3	3	4	4
4	4	2	2	1	1	3	3	3	3
1	1	3	3	1	1	3	3	4	4
1	1	3	3	4	4	3	3	3	3
1	1	2	2	1	1	3	3	3	3
4	4	3	3	1	1	3	3	3	3
1	1	2	2	1	1	3	3	4	4
4	4	3	3	1	1	3	3	3	3
1	1	2	2	1	1	3	3	3	3
1	1	3	3	4	4	2	2	3	3
1	1	3	3	1	1	3	3	3	3
1	1	2	2	1	1	3	3	3	3
4	4	3	3	1	1	3	3	3	3
4	4	3	3	1	1	3	3	4	4
4	4	2	2	1	1	3	3	3	3
1	1	2	2	1	1	3	3	3	3
1	1	2	2	1	1	2	2	3	3
1	1	3	3	1	1	3	3	3	3
1	1	3	3	1	1	3	3	3	3
1	1	3	3	4	4	3	3	3	3
1	1	2	2	1	1	3	3	3	3
4	4	3	3	1	1	3	3	4	4
1	1	3	3	4	4	2	2	4	4
1	1	2	2	1	1	3	3	3	3

For Review Only

4	4	3	3	4	4	2	2	3	3
4	4	3	3	1	1	3	3	4	4

For Review Only

SNP872	SNP872	SNP873	SNP873	SNP874	SNP874	SNP875	SNP875	SNP876	SNP876
2	2	4	4	1	1	3	3	2	2
2	2	4	4	4	4	2	2	3	3
2	2	4	4	1	1	3	3	2	2
2	2	4	4	1	1	3	3	2	2
1	1	1	1	1	1	2	2	3	3
2	2	4	4	1	1	2	2	3	3
2	2	4	4	1	1	2	2	3	3
2	2	4	4	4	4	2	2	3	3
2	2	4	4	1	1	2	2	3	3
2	2	4	4	1	1	2	2	3	3
2	2	4	4	1	1	2	2	3	3
2	2	4	4	1	1	3	3	2	2
2	2	4	4	1	1	3	3	3	3
1	1	1	1	1	1	2	2	3	3
2	2	4	4	1	1	2	2	3	3
2	2	4	4	1	1	2	2	3	3
1	1	4	4	4	4	2	2	3	3
2	2	4	4	1	1	2	2	3	3
1	1	1	1	1	1	2	2	3	3
2	2	4	4	1	1	2	2	3	3
2	2	4	4	4	4	2	2	3	3
2	2	4	4	1	1	2	2	3	3
2	2	4	4	1	1	2	2	3	3
1	1	1	1	1	1	2	2	2	2
2	2	4	4	1	1	2	2	3	3
2	2	4	4	1	1	3	3	2	2
2	2	4	4	1	1	2	2	3	3
2	2	4	4	1	1	2	2	3	3
2	2	4	4	1	1	2	2	3	3
2	2	4	4	4	4	2	2	3	3
2	2	4	4	1	1	2	2	3	3
2	2	4	4	1	1	2	2	3	3
2	2	4	4	1	1	3	3	2	2
1	1	1	1	1	1	2	2	3	3
2	2	4	4	1	1	2	2	3	3
1	1	1	1	1	1	2	2	3	3
2	2	4	4	1	1	2	2	3	3
2	2	4	4	4	4	2	2	3	3
2	2	4	4	1	1	2	2	3	3
2	2	4	4	1	1	2	2	3	3
2	2	4	4	4	4	2	2	3	3
2	2	4	4	1	1	2	2	3	3
2	2	4	4	1	1	2	2	3	3
2	2	4	4	1	1	2	2	3	3
2	2	4	4	1	1	2	2	3	3
2	2	4	4	1	1	3	3	2	2
2	2	4	4	1	1	3	3	2	2
2	2	4	4	1	1	3	3	2	2
2	2	4	4	4	4	2	2	3	3
2	2	4	4	1	1	3	3	2	2
2	2	4	4	1	1	3	3	2	2
1	1	1	1	1	1	3	3	2	2

For Review Only

1	1	1	1	1	1	2	2	3	3
2	2	4	4	1	1	3	3	2	2
2	2	4	4	1	1	2	2	3	3
1	1	1	1	4	4	2	2	3	3
1	1	1	1	1	1	2	2	3	3
2	2	4	4	1	1	3	3	2	2
2	2	4	4	1	1	3	3	2	2
2	2	4	4	4	4	2	2	3	3
2	2	4	4	1	1	2	2	3	3
2	2	4	4	1	1	2	2	3	3
2	2	4	4	1	1	3	3	2	2
2	2	4	4	1	1	3	3	2	2
1	1	1	1	1	1	2	2	3	3
2	2	4	4	1	1	2	2	3	3
2	2	4	4	1	1	2	2	3	3
2	2	4	4	1	1	3	3	2	2
2	2	4	4	1	1	2	2	3	3
2	2	4	4	1	1	3	3	2	2
2	2	4	4	1	1	2	2	3	3
1	1	1	1	1	1	3	3	2	2
2	2	4	4	1	1	2	2	3	3
2	2	4	4	1	1	2	2	3	3
2	2	4	4	1	1	3	3	2	2
2	2	4	4	1	1	3	3	2	2
2	2	4	4	1	1	2	2	3	3
2	2	4	4	1	1	2	2	3	3
2	2	4	4	4	4	2	2	3	3
2	2	4	4	1	1	2	2	3	3
2	2	4	4	1	1	3	3	2	2
1	1	1	1	1	1	2	2	3	3
2	2	4	4	1	1	2	2	3	3
1	1	1	1	1	1	2	2	3	3
2	2	4	4	1	1	2	2	3	3
1	1	1	1	1	1	2	2	3	3
2	2	4	4	1	1	3	3	2	2
2	2	4	4	1	1	2	2	3	3
2	2	4	4	1	1	2	2	3	3
2	2	4	4	4	4	2	2	3	3
1	1	1	1	1	1	2	2	3	3
2	2	4	4	1	1	2	2	3	3
1	1	1	1	1	1	2	2	3	3
1	1	1	1	1	1	2	2	3	3
2	2	4	4	1	1	2	2	3	3
2	2	4	4	1	1	3	3	2	2
2	2	4	4	1	1	3	3	2	2
2	2	4	4	1	1	2	2	3	3
1	1	1	1	1	1	2	2	3	3
2	2	4	4	1	1	2	2	3	3
2	2	4	4	1	1	3	3	2	2
2	2	4	4	1	1	2	2	3	3
2	2	4	4	1	1	2	2	3	3
2	2	4	4	1	1	2	2	3	3
1	1	1	1	1	1	3	3	2	2
2	2	1	1	1	1	2	2	3	3
2	2	4	4	1	1	2	2	3	3

For Review Only

2	2	4	4	1	1	2	2	3	3
2	2	4	4	1	1	2	2	3	3
2	2	4	4	1	1	2	2	3	3
1	1	1	1	1	1	2	2	3	3
2	2	4	4	1	1	3	3	3	3
2	2	4	4	1	1	3	3	3	3
2	2	4	4	1	1	2	2	3	3
1	1	1	1	1	1	2	2	3	3
2	2	4	4	1	1	3	3	2	2
2	2	4	4	1	1	2	2	3	3
2	2	4	4	1	1	3	3	2	2
2	2	4	4	1	1	3	3	3	3
2	2	4	4	1	1	3	3	2	2
1	1	1	1	1	1	2	2	3	3
2	2	4	4	1	1	2	2	3	3
2	2	4	4	1	1	2	2	3	3
2	2	4	4	1	1	2	2	2	2
2	2	4	4	1	1	2	2	3	3
1	1	1	1	1	1	2	2	3	3
2	2	4	4	1	1	2	2	3	3
2	2	4	4	1	1	2	2	3	3
1	1	1	1	1	1	2	2	3	3
2	2	4	4	1	1	2	2	3	3
2	2	4	4	4	4	2	2	3	3
2	2	4	4	1	1	2	2	2	2
2	2	4	4	1	1	2	2	3	3
1	1	1	1	1	1	2	2	3	3
1	1	1	1	1	1	2	2	3	3
2	2	4	4	1	1	2	2	3	3
2	2	4	4	1	1	2	2	3	3
2	2	4	4	1	1	2	2	3	3
2	2	4	4	1	1	2	2	3	3
1	1	1	1	1	1	2	2	3	3
2	2	4	4	1	1	2	2	3	3
1	1	1	1	1	1	2	2	3	3
2	2	4	4	1	1	2	2	2	2
1	1	1	1	1	1	2	2	3	3
2	2	4	4	1	1	2	2	3	3
2	2	4	4	1	1	3	3	3	3
2	2	4	4	1	1	2	2	3	3
2	2	4	4	1	1	2	2	3	3
1	1	1	1	1	1	2	2	3	3
2	2	4	4	1	1	3	3	2	2
2	2	4	4	1	1	2	2	3	3
2	2	4	4	1	1	3	3	2	2
1	1	1	1	1	1	2	2	3	3
2	2	4	4	1	1	3	3	2	2
2	2	4	4	1	1	2	2	3	3
2	2	4	4	1	1	3	3	3	3
2	2	4	4	1	1	3	3	2	2
2	2	4	4	1	1	3	3	2	2
2	2	4	4	4	4	2	2	3	3
2	2	4	4	1	1	3	3	3	3
2	2	4	4	1	1	3	3	2	2
2	2	4	4	1	1	2	2	3	3

For Review Only

2	2	4	4	1	1	3	3	2	2
2	2	4	4	1	1	2	2	3	3
2	2	4	4	1	1	2	2	3	3
2	2	4	4	1	1	2	2	3	3
1	1	1	1	1	1	2	2	3	3
2	2	4	4	1	1	3	3	2	2
2	2	4	4	1	1	2	2	3	3
2	2	4	4	1	1	2	2	3	3
2	2	4	4	1	1	2	2	3	3
1	1	1	1	1	1	3	3	2	2
2	2	4	4	1	1	3	3	2	2
2	2	4	4	1	1	2	2	3	3
2	2	4	4	1	1	3	3	2	2
1	1	1	1	1	1	2	2	3	3
1	1	1	1	1	1	2	2	3	3
2	2	4	4	1	1	2	2	3	3
2	2	4	4	1	1	2	2	3	3
2	2	4	4	1	1	3	3	2	2
2	2	4	4	1	1	3	3	2	2
2	2	4	4	1	1	2	2	3	3
2	2	4	4	1	1	2	2	3	3
1	1	1	1	1	1	2	2	3	3
2	2	4	4	1	1	2	2	3	3
2	2	4	4	1	1	3	3	2	2
2	2	4	4	1	1	2	2	3	3
1	1	1	1	1	1	2	2	3	3
2	2	4	4	1	1	3	3	2	2
2	2	4	4	1	1	2	2	3	3
2	2	4	4	1	1	3	3	2	2
2	2	4	4	1	1	3	3	2	2
2	2	4	4	1	1	3	3	2	2
1	1	1	1	1	1	2	2	3	3
2	2	4	4	1	1	3	3	2	2
1	1	1	1	1	1	2	2	3	3
2	2	4	4	1	1	3	3	2	2
2	2	4	4	1	1	3	3	2	2
2	2	4	4	1	1	2	2	3	3
2	2	4	4	1	1	3	3	2	2
2	2	4	4	1	1	2	2	3	3
2	2	4	4	1	1	3	3	2	2
2	2	4	4	1	1	3	3	2	2
2	2	4	4	1	1	3	3	2	2
2	2	4	4	1	1	2	2	3	3
2	2	4	4	1	1	2	2	3	3
2	2	4	4	4	4	2	2	3	3
2	2	4	4	1	1	3	3	2	2
2	2	4	4	1	1	2	2	3	3
2	2	4	4	1	1	2	2	3	3
1	1	1	1	1	1	2	2	3	3
2	2	4	4	1	1	3	3	2	2
2	2	4	4	1	1	2	2	3	3
1	1	1	1	1	1	2	2	3	3
2	2	4	4	1	1	2	2	3	3
2	2	4	4	1	1	3	3	2	2
2	2	4	4	1	1	2	2	3	3

For Review Only

2	2	4	4	1	1	3	3	2	2
2	2	4	4	1	1	3	3	2	2
2	2	4	4	1	1	3	3	2	2
2	2	4	4	1	1	2	2	3	3
2	2	4	4	1	1	2	2	3	3
2	2	4	4	1	1	3	3	2	2
2	2	4	4	1	1	2	2	3	3
2	2	4	4	1	1	2	2	3	3
2	2	4	4	1	1	2	2	3	3
2	2	4	4	1	1	2	2	3	3
2	2	4	4	1	1	2	2	3	3
2	2	4	4	1	1	2	2	3	3
1	1	1	1	1	1	2	2	3	3
2	2	4	4	1	1	2	2	3	3
2	2	4	4	1	1	3	3	2	2
2	2	4	4	1	1	3	3	2	2
2	2	4	4	1	1	2	2	3	3
1	1	1	1	1	1	2	2	3	3
2	2	4	4	1	1	2	2	3	3
2	2	4	4	4	4	2	2	3	3
2	2	4	4	1	1	3	3	2	2
2	2	4	4	1	1	3	3	2	2
1	1	1	1	1	1	2	2	3	3
2	2	4	4	1	1	2	2	3	3
2	2	4	4	4	4	2	2	3	3
2	2	4	4	1	1	2	2	3	3
2	2	4	4	1	1	2	2	3	3
2	2	4	4	1	1	3	3	2	2
2	2	4	4	1	1	2	2	3	3
2	2	4	4	1	1	2	2	3	3
2	2	4	4	1	1	2	2	3	3
2	2	4	4	1	1	3	3	3	3
1	1	1	1	1	1	2	2	3	3
2	2	4	4	1	1	2	2	3	3
2	2	4	4	1	1	2	2	3	3
2	2	4	4	1	1	2	2	3	3
2	2	4	4	1	1	3	3	2	2
2	2	4	4	1	1	3	3	2	2
1	1	1	1	1	1	2	2	3	3
2	2	4	4	1	1	2	2	3	3
2	2	4	4	1	1	3	3	2	2
1	1	1	1	1	1	2	2	3	3
2	2	4	4	1	1	2	2	3	3
1	1	1	1	1	1	2	2	3	3
2	2	4	4	1	1	3	3	2	2
1	1	1	1	1	1	3	3	2	2
2	2	4	4	1	1	2	2	3	3
2	2	4	4	1	1	3	3	2	2
2	2	4	4	1	1	2	2	3	3
2	2	4	4	1	1	2	2	3	3
2	2	4	4	1	1	3	3	2	2
2	2	4	4	1	1	2	2	3	3
2	2	4	4	1	1	3	3	2	2
2	2	4	4	1	1	2	2	2	2
2	2	4	4	1	1	2	2	3	3

For Review Only

1	1	1	1	1	1	3	3	2	2
2	2	4	4	1	1	2	2	3	3

For Review Only

SNP877	SNP877	SNP878	SNP878	SNP879	SNP879	SNP880	SNP880	SNP881	SNP881
3	3	4	4	3	3	4	4	3	3
3	3	4	4	3	3	4	4	3	3
3	3	4	4	3	3	2	2	1	1
3	3	4	4	3	3	4	4	3	3
2	2	1	1	4	4	4	4	3	3
3	3	4	4	3	3	4	4	3	3
3	3	4	4	3	3	4	4	3	3
3	3	4	4	3	3	4	4	3	3
3	3	1	1	4	4	4	4	3	3
3	3	4	4	3	3	4	4	3	3
3	3	4	4	3	3	4	4	3	3
3	3	4	4	3	3	2	2	1	1
3	3	1	1	3	3	4	4	3	3
2	2	1	1	4	4	4	4	3	3
3	3	4	4	3	3	4	4	3	3
3	3	4	4	3	3	4	4	3	3
2	2	1	1	4	4	4	4	3	3
3	3	4	4	3	3	4	4	3	3
2	2	1	1	4	4	2	2	1	1
3	3	4	4	3	3	4	4	3	3
3	3	4	4	3	3	4	4	3	3
3	3	4	4	4	4	4	4	3	3
3	3	4	4	3	3	4	4	3	3
2	2	1	1	4	4	4	4	3	3
3	3	4	4	3	3	4	4	3	3
3	3	4	4	3	3	2	2	1	1
3	3	4	4	3	3	4	4	3	3
3	3	4	4	3	3	4	4	3	3
3	3	4	4	3	3	4	4	3	3
3	3	4	4	3	3	4	4	3	3
3	3	4	4	3	3	4	4	3	3
3	3	4	4	3	3	2	2	3	3
3	3	4	4	3	3	2	2	3	3
3	3	4	4	3	3	4	4	3	3
3	3	4	4	3	3	4	4	3	3
2	2	1	1	4	4	4	4	3	3
3	3	4	4	3	3	4	4	3	3
3	3	4	4	3	3	4	4	3	3
3	3	4	4	3	3	4	4	3	3
3	3	4	4	3	3	4	4	3	3
3	3	4	4	3	3	4	4	3	3
3	3	4	4	3	3	4	4	3	3
3	3	4	4	3	3	4	4	3	3
3	3	4	4	3	3	4	4	3	3
3	3	4	4	3	3	4	4	3	3
3	3	4	4	3	3	2	2	1	1
3	3	4	4	3	3	4	4	1	1
3	3	4	4	3	3	4	4	3	3
3	3	4	4	3	3	4	4	3	3
3	3	1	1	3	3	4	4	3	3
3	3	4	4	3	3	2	2	1	1
2	2	1	1	4	4	4	4	3	3

For Review Only

2	2	1	1	4	4	4	4	3	3
3	3	4	4	3	3	2	2	3	3
3	3	4	4	3	3	2	2	3	3
2	2	1	1	4	4	4	4	3	3
2	2	1	1	4	4	2	2	1	1
3	3	4	4	3	3	2	2	1	1
3	3	4	4	3	3	2	2	1	1
3	3	4	4	3	3	4	4	3	3
3	3	4	4	3	3	4	4	3	3
3	3	4	4	3	3	4	4	3	3
3	3	4	4	3	3	2	2	1	1
3	3	4	4	3	3	2	2	1	1
2	2	1	1	4	4	4	4	3	3
3	3	4	4	3	3	4	4	3	3
3	3	4	4	3	3	4	4	3	3
3	3	4	4	3	3	4	4	3	3
3	3	4	4	3	3	4	4	3	3
3	3	4	4	3	3	4	4	3	3
3	3	4	4	3	3	4	4	3	3
2	2	1	1	4	4	4	4	3	3
3	3	4	4	3	3	4	4	1	1
3	3	4	4	3	3	4	4	3	3
3	3	1	1	3	3	4	4	3	3
3	3	4	4	3	3	2	2	1	1
3	3	4	4	3	3	4	4	3	3
3	3	4	4	3	3	2	2	1	1
3	3	4	4	3	3	2	2	1	1
3	3	4	4	3	3	4	4	3	3
3	3	4	4	3	3	2	2	1	1
2	2	1	1	4	4	4	4	3	3
3	3	1	1	3	3	4	4	3	3
2	2	1	1	4	4	4	4	3	3
3	3	4	4	3	3	4	4	3	3
2	2	1	1	4	4	4	4	3	3
3	3	4	4	3	3	4	4	3	3
3	3	4	4	3	3	4	4	3	3
3	3	4	4	3	3	4	4	3	3
3	3	4	4	3	3	4	4	3	3
2	2	1	1	4	4	2	2	1	1
3	3	4	4	3	3	4	4	3	3
2	2	1	1	4	4	4	4	3	3
2	2	1	1	4	4	4	4	3	3
3	3	4	4	3	3	4	4	3	3
3	3	4	4	3	3	2	2	1	1
3	3	1	1	4	4	4	4	3	3
3	3	4	4	3	3	4	4	3	3
2	2	1	1	4	4	4	4	3	3
3	3	4	4	3	3	2	2	1	1
3	3	4	4	3	3	4	4	3	3
3	3	4	4	3	3	4	4	3	3
3	3	4	4	3	3	4	4	3	3
3	3	4	4	3	3	4	4	3	3
2	2	1	1	4	4	4	4	3	3
3	3	1	1	4	4	4	4	3	3
3	3	4	4	3	3	4	4	3	3

For Review Only

3	3	4	4	3	3	4	4	3	3
3	3	4	4	3	3	4	4	3	3
3	3	1	1	4	4	4	4	3	3
2	2	1	1	4	4	4	4	3	3
3	3	4	4	3	3	2	2	1	1
3	3	4	4	3	3	2	2	1	1
3	3	4	4	3	3	4	4	3	3
2	2	1	1	4	4	4	4	3	3
3	3	4	4	3	3	4	4	3	3
3	3	1	1	3	3	4	4	1	1
3	3	4	4	3	3	4	4	3	3
3	3	1	1	3	3	4	4	3	3
3	3	4	4	3	3	4	4	3	3
3	3	1	1	3	3	4	4	1	1
3	3	1	1	4	4	4	4	3	3
3	3	4	4	3	3	2	2	1	1
3	3	4	4	3	3	4	4	3	3
3	3	4	4	3	3	4	4	1	1
2	2	1	1	4	4	4	4	3	3
3	3	4	4	3	3	4	4	3	3
3	3	4	4	3	3	4	4	3	3
2	2	1	1	4	4	4	4	3	3
3	3	4	4	3	3	4	4	3	3
3	3	4	4	3	3	4	4	3	3
3	3	4	4	3	3	2	2	3	3
3	3	4	4	3	3	4	4	3	3
3	3	4	4	3	3	4	4	3	3
2	2	1	1	4	4	4	4	3	3
3	3	4	4	3	3	2	2	1	1
3	3	4	4	3	3	4	4	3	3
3	3	4	4	4	4	4	4	3	3
3	3	1	1	3	3	4	4	3	3
2	2	1	1	4	4	4	4	3	3
3	3	1	1	3	3	4	4	3	3
3	3	4	4	3	3	4	4	3	3
3	3	4	4	3	3	4	4	3	3
2	2	1	1	4	4	4	4	3	3
3	3	4	4	3	3	4	4	3	3
3	3	4	4	3	3	4	4	3	3
3	3	4	4	3	3	4	4	3	3
3	3	4	4	3	3	4	4	3	3
2	2	1	1	4	4	4	4	3	3
3	3	4	4	3	3	4	4	3	3
3	3	4	4	3	3	4	4	3	3
3	3	4	4	3	3	4	4	3	3
2	2	1	1	4	4	4	4	3	3
3	3	4	4	3	3	2	2	1	1
3	3	4	4	3	3	2	2	1	1
3	3	1	1	4	4	4	4	3	3
3	3	4	4	3	3	2	2	1	1
3	3	1	1	4	4	4	4	3	3
2	2	1	1	4	4	4	4	3	3
3	3	4	4	3	3	4	4	3	3
3	3	4	4	3	3	2	2	1	1
3	3	4	4	3	3	4	4	3	3

For Review Only

3	3	4	4	3	3	2	2	1	1
3	3	4	4	3	3	4	4	3	3
3	3	4	4	3	3	4	4	3	3
3	3	4	4	3	3	4	4	3	3
2	2	1	1	4	4	4	4	3	3
3	3	4	4	3	3	4	4	3	3
3	3	4	4	3	3	4	4	3	3
3	3	1	1	4	4	4	4	3	3
3	3	1	1	4	4	4	4	3	3
2	2	1	1	4	4	4	4	3	3
3	3	4	4	3	3	4	4	1	1
3	3	4	4	3	3	4	4	3	3
3	3	4	4	3	3	4	4	3	3
2	2	1	1	4	4	4	4	3	3
2	2	1	1	4	4	4	4	3	3
3	3	4	4	3	3	4	4	3	3
3	3	4	4	3	3	4	4	3	3
3	3	4	4	3	3	2	2	1	1
3	3	4	4	3	3	4	4	3	3
3	3	4	4	3	3	4	4	3	3
3	3	4	4	3	3	4	4	3	3
2	2	1	1	4	4	4	4	3	3
3	3	4	4	3	3	4	4	3	3
3	3	4	4	3	3	2	2	3	3
3	3	4	4	3	3	4	4	3	3
3	3	4	4	3	3	4	4	3	3
3	3	4	4	3	3	4	4	3	3
3	3	4	4	3	3	4	4	3	3
3	3	4	4	3	3	4	4	3	3
3	3	4	4	3	3	4	4	3	3
3	3	4	4	3	3	2	2	1	1
2	2	1	1	4	4	4	4	3	3
3	3	4	4	3	3	4	4	3	3
2	2	1	1	4	4	4	4	3	3
3	3	4	4	3	3	4	4	3	3
3	3	4	4	3	3	2	2	1	1
3	3	4	4	3	3	4	4	3	3
3	3	4	4	3	3	4	4	3	3
3	3	4	4	3	3	2	2	1	1
3	3	4	4	3	3	2	2	1	1
3	3	4	4	3	3	2	2	1	1
3	3	4	4	3	3	2	2	1	1
3	3	4	4	3	3	4	4	3	3
3	3	4	4	3	3	4	4	3	3
3	3	4	4	4	4	4	4	3	3
3	3	4	4	3	3	4	4	3	3
3	3	4	4	3	3	4	4	3	3
3	3	4	4	3	3	4	4	3	3
3	3	4	4	3	3	4	4	3	3
3	3	4	4	3	3	4	4	3	3
3	3	4	4	3	3	2	2	1	1
2	2	1	1	4	4	4	4	3	3
3	3	4	4	3	3	4	4	3	3
3	3	4	4	3	3	2	2	1	1
3	3	4	4	3	3	4	4	3	3

For Review Only

3	3	4	4	3	3	4	4	3	3
3	3	4	4	3	3	4	4	3	3
3	3	1	1	3	3	2	2	1	1
3	3	4	4	3	3	4	4	3	3
3	3	4	4	3	3	4	4	3	3
3	3	4	4	3	3	4	4	3	3
3	3	4	4	3	3	4	4	3	3
3	3	4	4	3	3	4	4	3	3
3	3	1	1	4	4	4	4	3	3
3	3	4	4	3	3	4	4	3	3
3	3	4	4	3	3	4	4	3	3
3	3	4	4	3	3	2	2	1	1
2	2	1	1	4	4	2	2	1	1
3	3	4	4	3	3	2	2	1	1
3	3	4	4	3	3	2	2	1	1
3	3	4	4	3	3	4	4	3	3
3	3	4	4	3	3	4	4	3	3
2	2	1	1	4	4	4	4	3	3
3	3	4	4	3	3	4	4	3	3
3	3	4	4	3	3	4	4	3	3
3	3	4	4	3	3	2	2	1	1
3	3	4	4	3	3	2	2	1	1
2	2	1	1	4	4	4	4	3	3
3	3	4	4	3	3	4	4	3	3
3	3	4	4	3	3	4	4	3	3
3	3	4	4	3	3	4	4	3	3
3	3	4	4	3	3	4	4	3	3
3	3	4	4	3	3	2	2	1	1
3	3	1	1	4	4	4	4	3	3
3	3	4	4	3	3	2	2	1	1
3	3	4	4	3	3	4	4	3	3
3	3	4	4	3	3	2	2	1	1
2	2	1	1	4	4	4	4	3	3
3	3	4	4	3	3	2	2	1	1
3	3	4	4	3	3	4	4	3	3
3	3	4	4	3	3	4	4	3	3
3	3	4	4	3	3	4	4	3	3
3	3	4	4	3	3	2	2	1	1
2	2	1	1	4	4	4	4	3	3
3	3	4	4	3	3	4	4	3	3
3	3	4	4	3	3	4	4	3	3
2	2	1	1	4	4	4	4	3	3
3	3	4	4	3	3	4	4	3	3
2	2	1	1	4	4	4	4	3	3
3	3	4	4	3	3	2	2	1	1
2	2	1	1	4	4	4	4	3	3
3	3	4	4	3	3	4	4	3	3
3	3	4	4	3	3	4	4	3	3
3	3	4	4	3	3	4	4	3	3
3	3	4	4	3	3	4	4	3	3
3	3	4	4	3	3	4	4	3	3
3	3	4	4	3	3	4	4	3	3
3	3	4	4	3	3	2	2	1	1
3	3	4	4	3	3	2	2	1	1
3	3	4	4	3	3	4	4	3	3

For Review Only

2	2	1	1	4	4	4	4	3	3
3	3	4	4	3	3	2	2	1	1

For Review Only

SNP882	SNP882	SNP883	SNP883	SNP884	SNP884	SNP885	SNP885	SNP886	SNP886
3	3	2	2	1	1	1	1	2	2
3	3	2	2	1	1	1	1	2	2
3	3	2	2	1	1	1	1	2	2
3	3	2	2	4	4	4	4	2	2
3	3	2	2	1	1	4	4	2	2
3	3	2	2	4	4	4	4	2	2
4	4	2	2	1	1	4	4	2	2
3	3	2	2	1	1	4	4	2	2
4	4	2	2	1	1	1	1	2	2
3	3	2	2	1	1	4	4	2	2
3	3	3	3	4	4	1	1	2	2
3	3	2	2	1	1	4	4	2	2
3	3	2	2	1	1	1	1	2	2
3	3	2	2	4	4	1	1	2	2
3	3	2	2	1	1	1	1	2	2
3	3	2	2	4	4	4	4	2	2
4	4	2	2	4	4	4	4	2	2
3	3	2	2	1	1	1	1	2	2
3	3	2	2	1	1	4	4	2	2
3	3	2	2	1	1	4	4	2	2
3	3	2	2	4	4	1	1	2	2
4	4	2	2	1	1	4	4	2	2
3	3	3	3	4	4	1	1	2	2
3	3	2	2	4	4	1	1	1	1
3	3	2	2	4	4	4	4	2	2
3	3	2	2	1	1	4	4	2	2
3	3	2	2	1	1	4	4	2	2
3	3	2	2	1	1	1	1	2	2
4	4	2	2	4	4	1	1	2	2
3	3	2	2	1	1	1	1	2	2
3	3	2	2	1	1	4	4	2	2
3	3	2	2	4	4	4	4	2	2
3	3	2	2	1	1	4	4	2	2
3	3	2	2	4	4	1	1	1	1
3	3	2	2	1	1	1	1	2	2
3	3	2	2	4	4	1	1	1	1
3	3	2	2	4	4	4	4	2	2
3	3	2	2	1	1	1	1	2	2
3	3	3	3	4	4	1	1	2	2
3	3	3	3	4	4	1	1	2	2
3	3	2	2	1	1	1	1	2	2
3	3	2	2	4	4	1	1	2	2
3	3	2	2	1	1	4	4	2	2
3	3	2	2	1	1	4	4	2	2
3	3	2	2	1	1	4	4	2	2
3	3	2	2	1	1	4	4	2	2
3	3	2	2	1	1	4	4	2	2
3	3	3	3	1	1	1	1	2	2
4	4	2	2	1	1	4	4	2	2
3	3	2	2	4	4	4	4	2	2
3	3	2	2	1	1	1	1	2	2
3	3	2	2	1	1	1	1	2	2

For Review Only

4	4	2	2	1	1	4	4	2	2
3	3	2	2	1	1	4	4	2	2
3	3	2	2	4	4	1	1	2	2
4	4	2	2	4	4	4	4	2	2
3	3	2	2	1	1	4	4	2	2
3	3	2	2	1	1	4	4	2	2
3	3	2	2	1	1	4	4	2	2
3	3	2	2	1	1	1	1	2	2
4	4	2	2	1	1	4	4	2	2
3	3	2	2	1	1	1	1	2	2
3	3	2	2	1	1	4	4	2	2
3	3	2	2	1	1	4	4	2	2
4	4	2	2	1	1	4	4	2	2
4	4	2	2	4	4	1	1	2	2
3	3	2	2	1	1	1	1	2	2
3	3	2	2	4	4	1	1	2	2
3	3	2	2	4	4	1	1	2	2
3	3	2	2	4	4	1	1	2	2
4	4	2	2	4	4	4	4	2	2
4	4	2	2	1	1	4	4	2	2
3	3	2	2	1	1	4	4	2	2
3	3	2	2	4	4	1	1	2	2
4	4	2	2	1	1	1	1	2	2
3	3	2	2	1	1	1	1	2	2
3	3	2	2	1	1	4	4	2	2
3	3	2	2	1	1	4	4	2	2
4	4	2	2	1	1	4	4	2	2
3	3	3	3	4	4	1	1	2	2
3	3	2	2	1	1	4	4	2	2
3	3	2	2	4	4	4	4	2	2
3	3	2	2	4	4	4	4	2	2
3	3	2	2	4	4	1	1	1	1
3	3	3	3	4	4	1	1	2	2
4	4	2	2	1	1	4	4	2	2
3	3	2	2	4	4	4	4	2	2
3	3	2	2	1	1	4	4	2	2
3	3	2	2	4	4	4	4	2	2
4	4	2	2	4	4	4	4	2	2
3	3	2	2	4	4	1	1	2	2
3	3	2	2	1	1	1	1	2	2
3	3	2	2	4	4	4	4	2	2
3	3	2	2	1	1	1	1	2	2
3	3	2	2	1	1	1	1	2	2
3	3	2	2	1	1	4	4	2	2
3	3	2	2	4	4	4	4	2	2
3	3	2	2	4	4	4	4	2	2
3	3	2	2	4	4	1	1	1	1
3	3	2	2	4	4	4	4	2	2
3	3	2	2	1	1	4	4	2	2
3	3	2	2	1	1	1	1	2	2
3	3	2	2	1	1	1	1	2	2
3	3	3	3	4	4	1	1	2	2
4	4	2	2	4	4	4	4	2	2
3	3	2	2	1	1	1	1	2	2
3	3	2	2	1	1	4	4	2	2

For Review Only

3	3	2	2	1	1	1	1	2	2
3	3	2	2	1	1	1	1	2	2
3	3	2	2	1	1	4	4	2	2
3	3	2	2	1	1	1	1	1	1
3	3	2	2	1	1	1	1	2	2
3	3	2	2	4	4	4	4	2	2
3	3	2	2	4	4	4	4	2	2
3	3	2	2	1	1	1	1	2	2
3	3	2	2	1	1	1	1	2	2
3	3	2	2	1	1	1	1	2	2
3	3	2	2	1	1	4	4	2	2
3	3	2	2	1	1	1	1	2	2
3	3	2	2	1	1	4	4	2	2
3	3	2	2	1	1	1	1	2	2
3	3	2	2	4	4	4	4	2	2
3	3	2	2	1	1	1	1	2	2
3	3	2	2	1	1	1	1	2	2
3	3	2	2	1	1	1	1	2	2
3	3	2	2	1	1	1	1	2	2
4	4	3	3	1	1	1	1	2	2
3	3	2	2	1	1	1	1	2	2
4	4	2	2	4	4	4	4	2	2
3	3	2	2	1	1	1	1	2	2
3	3	2	2	4	4	1	1	2	2
3	3	2	2	1	1	1	1	2	2
3	3	3	3	4	4	1	1	2	2
3	3	2	2	1	1	4	4	1	1
3	3	2	2	1	1	4	4	2	2
3	3	2	2	1	1	4	4	2	2
3	3	2	2	1	1	4	4	2	2
3	3	2	2	4	4	1	1	2	2
3	3	2	2	1	1	1	1	2	2
4	4	2	2	4	4	4	4	2	2
3	3	2	2	1	1	1	1	2	2
3	3	2	2	4	4	1	1	1	1
3	3	2	2	4	4	1	1	2	2
3	3	2	2	1	1	1	1	2	2
3	3	2	2	1	1	1	1	2	2
3	3	3	3	4	4	1	1	2	2
3	3	3	3	4	4	1	1	2	2
3	3	3	3	4	4	1	1	2	2
3	3	2	2	4	4	1	1	2	2
3	3	2	2	4	4	1	1	2	2
3	3	2	2	1	1	1	1	2	2
3	3	2	2	1	1	1	1	2	2
3	3	2	2	1	1	4	4	2	2
3	3	2	2	1	1	4	4	2	2
3	3	2	2	1	1	4	4	2	2
3	3	2	2	4	4	4	4	2	2
3	3	2	2	1	1	1	1	2	2
3	3	2	2	1	1	4	4	2	2
3	3	2	2	1	1	1	1	1	1
3	3	2	2	4	4	1	1	2	2
3	3	2	2	1	1	4	4	2	2
3	3	2	2	1	1	1	1	2	2

For Review Only

3	3	2	2	1	1	4	4	2	2
3	3	3	3	1	1	1	1	2	2
3	3	2	2	1	1	4	4	2	2
3	3	2	2	4	4	1	1	2	2
3	3	2	2	1	1	4	4	2	2
3	3	3	3	4	4	1	1	2	2
3	3	2	2	4	4	1	1	2	2
3	3	2	2	1	1	1	1	2	2
3	3	3	3	4	4	1	1	2	2
4	4	2	2	4	4	4	4	2	2
3	3	2	2	1	1	4	4	2	2
3	3	2	2	4	4	1	1	2	2
3	3	2	2	4	4	1	1	2	2
3	3	2	2	1	1	1	1	2	2
3	3	2	2	4	4	1	1	2	2
3	3	3	3	4	4	1	1	2	2
3	3	2	2	4	4	1	1	2	2
3	3	2	2	1	1	4	4	2	2
3	3	2	2	4	4	4	4	2	2
3	3	2	2	4	4	4	4	2	2
3	3	2	2	4	4	1	1	2	2
3	3	2	2	4	4	1	1	1	1
3	3	2	2	4	4	1	1	2	2
3	3	2	2	1	1	4	4	2	2
4	4	2	2	1	1	1	1	2	2
3	3	2	2	4	4	1	1	1	1
3	3	2	2	4	4	1	1	2	2
3	3	2	2	1	1	1	1	2	2
3	3	2	2	4	4	4	4	2	2
4	4	2	2	4	4	1	1	2	2
3	3	2	2	4	4	4	4	2	2
3	3	2	2	1	1	4	4	2	2
3	3	2	2	4	4	4	4	1	1
3	3	2	2	4	4	1	1	2	2
3	3	2	2	4	4	1	1	2	2
3	3	2	2	1	1	4	4	2	2
3	3	2	2	1	1	4	4	2	2
3	3	2	2	4	4	4	4	2	2
3	3	2	2	1	1	4	4	2	2
3	3	2	2	1	1	4	4	2	2
3	3	2	2	1	1	4	4	2	2
3	3	2	2	1	1	4	4	2	2
3	3	3	3	4	4	1	1	2	2
3	3	2	2	4	4	1	1	2	2
3	3	2	2	4	4	1	1	2	2
3	3	2	2	1	1	4	4	2	2
3	3	3	3	4	4	1	1	2	2
3	3	2	2	4	4	4	4	2	2
3	3	2	2	4	4	1	1	1	1
3	3	2	2	4	4	1	1	2	2
3	3	2	2	1	1	4	4	2	2
3	3	2	2	1	1	1	1	2	2
3	3	3	3	4	4	1	1	2	2
3	3	2	2	1	1	4	4	2	2
3	3	3	3	4	4	1	1	2	2

For Review Only

3	3	2	2	1	1	1	1	2	2
3	3	2	2	1	1	1	1	2	2
3	3	2	2	1	1	4	4	2	2
3	3	2	2	1	1	1	1	2	2
3	3	2	2	1	1	4	4	2	2
3	3	2	2	4	4	4	4	2	2
3	3	3	3	4	4	1	1	2	2
3	3	2	2	1	1	1	1	2	2
3	3	2	2	1	1	1	1	2	2
3	3	2	2	4	4	1	1	2	2
3	3	2	2	1	1	1	1	2	2
3	3	2	2	4	4	1	1	2	2
3	3	2	2	4	4	1	1	2	2
3	3	2	2	4	4	4	4	2	2
3	3	2	2	1	1	4	4	2	2
3	3	2	2	4	4	4	4	2	2
3	3	2	2	4	4	4	4	2	2
3	3	2	2	1	1	1	1	2	2
3	3	3	3	4	4	1	1	2	2
3	3	3	3	1	1	1	1	2	2
3	3	3	3	4	4	1	1	2	2
3	3	2	2	1	1	4	4	2	2
3	3	2	2	4	4	4	4	2	2
3	3	3	3	4	4	1	1	2	2
3	3	2	2	1	1	1	1	2	2
3	3	2	2	4	4	4	4	2	2
3	3	3	3	4	4	1	1	2	2
3	3	2	2	4	4	4	4	2	2
3	3	2	2	1	1	4	4	2	2
3	3	2	2	1	1	4	4	2	2
3	3	2	2	4	4	1	1	2	2
3	3	2	2	1	1	1	1	2	2
3	3	2	2	4	4	1	1	2	2
3	3	2	2	1	1	4	4	2	2
3	3	2	2	4	4	1	1	2	2
3	3	3	3	4	4	1	1	2	2
3	3	2	2	4	4	4	4	2	2
3	3	2	2	1	1	4	4	2	2
3	3	2	2	4	4	1	1	1	1
3	3	3	3	4	4	1	1	2	2
3	3	2	2	4	4	1	1	2	2
3	3	2	2	1	1	4	4	2	2
3	3	2	2	4	4	1	1	2	2
4	4	2	2	4	4	4	4	2	2
3	3	2	2	1	1	4	4	2	2
4	4	2	2	4	4	1	1	2	2
3	3	3	3	4	4	1	1	2	2
3	3	2	2	1	1	1	1	2	2
3	3	2	2	1	1	4	4	2	2
3	3	2	2	4	4	1	1	2	2
3	3	2	2	4	4	4	4	2	2
3	3	3	3	4	4	1	1	2	2
3	3	2	2	1	1	4	4	2	2
3	3	2	2	4	4	4	4	2	2
3	3	3	3	4	4	1	1	2	2

For Review Only

4	4	2	2	4	4	4	4	2	2
3	3	2	2	1	1	4	4	2	2

For Review Only

SNP887	SNP887	SNP888	SNP888	SNP889	SNP889	SNP890	SNP890	SNP891	SNP891
2	2	1	1	2	2	4	4	3	3
2	2	1	1	2	2	3	3	3	3
2	2	1	1	2	2	3	3	1	1
2	2	1	1	2	2	3	3	3	3
2	2	1	1	2	2	4	4	3	3
2	2	1	1	2	2	3	3	3	3
2	2	1	1	2	2	4	4	3	3
2	2	1	1	2	2	3	3	3	3
3	3	1	1	2	2	4	4	3	3
3	3	1	1	2	2	3	3	3	3
2	2	4	4	2	2	3	3	3	3
2	2	1	1	2	2	3	3	3	3
2	2	1	1	2	2	3	3	3	3
2	2	1	1	2	2	3	3	3	3
2	2	1	1	2	2	4	4	3	3
2	2	1	1	2	2	3	3	3	3
2	2	1	1	2	2	4	4	3	3
2	2	1	1	2	2	3	3	3	3
2	2	1	1	2	2	3	3	3	3
2	2	1	1	2	2	3	3	3	3
2	2	1	1	2	2	3	3	3	3
2	2	1	1	2	2	4	4	1	1
2	2	1	1	2	2	3	3	3	3
3	3	1	1	3	3	3	3	3	3
2	2	1	1	2	2	3	3	3	3
2	2	1	1	2	2	3	3	3	3
2	2	1	1	2	2	3	3	3	3
2	2	1	1	2	2	3	3	3	3
3	3	1	1	2	2	4	4	3	3
3	3	1	1	2	2	3	3	3	3
2	2	1	1	2	2	3	3	3	3
2	2	1	1	2	2	3	3	3	3
2	2	1	1	2	2	3	3	3	3
2	2	1	1	3	3	3	3	3	3
2	2	1	1	2	2	3	3	3	3
2	2	1	1	3	3	3	3	3	3
3	3	1	1	2	2	3	3	3	3
2	2	1	1	2	2	3	3	1	1
2	2	1	1	2	2	3	3	3	3
2	2	1	1	2	2	3	3	3	3
3	3	1	1	2	2	3	3	3	3
3	3	4	4	2	2	3	3	3	3
3	3	1	1	2	2	3	3	3	3
2	2	4	4	2	2	3	3	3	3
3	3	4	4	2	2	3	3	3	3
2	2	1	1	2	2	3	3	1	1
2	2	1	1	2	2	3	3	3	3
2	2	1	1	2	2	3	3	3	3
2	2	1	1	2	2	4	4	3	3
2	2	1	1	2	2	3	3	3	3
2	2	1	1	2	2	3	3	3	3
2	2	1	1	2	2	3	3	3	3

For Review Only

2	2	1	1	2	2	4	4	3	3
2	2	1	1	2	2	3	3	3	3
2	2	1	1	2	2	3	3	3	3
3	3	1	1	2	2	4	4	1	1
2	2	1	1	2	2	3	3	3	3
2	2	1	1	2	2	3	3	3	3
2	2	1	1	2	2	3	3	3	3
3	3	1	1	2	2	3	3	3	3
3	3	1	1	2	2	4	4	3	3
2	2	1	1	2	2	3	3	3	3
2	2	1	1	2	2	3	3	3	3
3	3	1	1	2	2	3	3	3	3
2	2	1	1	2	2	4	4	3	3
3	3	1	1	2	2	4	4	3	3
2	2	1	1	2	2	3	3	3	3
2	2	1	1	2	2	3	3	3	3
2	2	1	1	2	2	3	3	3	3
2	2	1	1	2	2	3	3	3	3
2	2	1	1	2	2	4	4	3	3
2	2	1	1	2	2	4	4	3	3
2	2	1	1	2	2	3	3	3	3
3	3	1	1	2	2	3	3	3	3
2	2	1	1	2	2	4	4	3	3
3	3	1	1	2	2	3	3	3	3
3	3	4	4	2	2	3	3	3	3
2	2	1	1	2	2	3	3	3	3
2	2	1	1	2	2	4	4	3	3
2	2	1	1	2	2	3	3	3	3
2	2	1	1	2	2	3	3	3	3
2	2	1	1	2	2	3	3	3	3
2	2	1	1	2	2	3	3	1	1
2	2	1	1	3	3	4	4	3	3
2	2	1	1	2	2	3	3	3	3
2	2	1	1	2	2	4	4	3	3
2	2	1	1	2	2	3	3	3	3
3	3	4	4	2	2	3	3	3	3
2	2	1	1	2	2	3	3	3	3
2	2	1	1	2	2	4	4	1	1
2	2	1	1	2	2	3	3	3	3
3	3	4	4	2	2	3	3	3	3
2	2	1	1	2	2	4	4	3	3
2	2	1	1	2	2	3	3	3	3
2	2	1	1	2	2	3	3	3	3
2	2	1	1	2	2	3	3	3	3
2	2	1	1	2	2	3	3	3	3
2	2	1	1	2	2	3	3	3	3
2	2	1	1	3	3	3	3	3	3
2	2	1	1	2	2	3	3	3	3
2	2	1	1	2	2	4	4	3	3
3	3	1	1	2	2	3	3	3	3
2	2	1	1	2	2	3	3	3	3
2	2	4	4	2	2	3	3	3	3
3	3	1	1	2	2	4	4	3	3
2	2	1	1	2	2	3	3	3	3
2	2	1	1	2	2	3	3	3	3

For Review Only

2	2	1	1	2	2	3	3	3	3
2	2	1	1	2	2	3	3	3	3
2	2	1	1	2	2	3	3	3	3
2	2	1	1	2	2	3	3	3	3
2	2	1	1	2	2	3	3	3	3
2	2	1	1	2	2	3	3	3	3
2	2	1	1	2	2	3	3	3	3
2	2	1	1	2	2	3	3	3	3
2	2	1	1	2	2	3	3	3	3
2	2	1	1	2	2	3	3	3	3
2	2	1	1	2	2	3	3	3	3
2	2	1	1	2	2	3	3	3	3
2	2	1	1	2	2	3	3	3	3
2	2	1	1	2	2	3	3	3	3
2	2	1	1	2	2	4	4	3	3
2	2	1	1	2	2	3	3	3	3
2	2	1	1	2	2	3	3	3	3
2	2	1	1	2	2	3	3	3	3
2	2	1	1	2	2	3	3	3	3
2	2	1	1	2	2	4	4	3	3
2	2	1	1	2	2	3	3	3	3
2	2	1	1	2	2	4	4	3	3
2	2	1	1	2	2	3	3	3	3
3	3	4	4	2	2	3	3	3	3
2	2	1	1	2	2	3	3	3	3
3	3	1	1	2	2	3	3	3	3
2	2	1	1	3	3	3	3	1	1
2	2	1	1	2	2	3	3	3	3
2	2	1	1	2	2	3	3	3	3
2	2	1	1	2	2	3	3	3	3
2	2	1	1	2	2	3	3	3	3
2	2	1	1	3	3	3	3	3	3
2	2	1	1	2	2	4	4	3	3
2	2	1	1	2	2	3	3	3	3
2	2	1	1	3	3	3	3	3	3
2	2	1	1	2	2	3	3	3	3
2	2	1	1	2	2	3	3	3	3
2	2	1	1	2	2	3	3	3	3
2	2	1	1	2	2	3	3	3	3
2	2	1	1	2	2	3	3	3	3
3	3	1	1	2	2	3	3	3	3
2	2	1	1	2	2	3	3	3	3
2	2	1	1	2	2	3	3	3	3
2	2	1	1	2	2	3	3	3	3
2	2	1	1	2	2	3	3	3	3
2	2	1	1	2	2	3	3	1	1
2	2	1	1	2	2	3	3	3	3
2	2	1	1	2	2	3	3	3	3
3	3	1	1	2	2	3	3	3	3
2	2	1	1	2	2	3	3	3	3
2	2	1	1	2	2	3	3	3	3
3	3	1	1	3	3	3	3	3	3
2	2	1	1	2	2	3	3	3	3
3	3	1	1	2	2	3	3	3	3
2	2	1	1	2	2	3	3	3	3

For Review Only

2	2	1	1	2	2	3	3	3	3
3	3	4	4	2	2	3	3	3	3
2	2	1	1	2	2	3	3	3	3
2	2	1	1	2	2	3	3	3	3
2	2	1	1	2	2	4	4	3	3
2	2	1	1	2	2	3	3	3	3
2	2	1	1	2	2	3	3	3	3
2	2	1	1	2	2	3	3	3	3
2	2	1	1	2	2	3	3	3	3
2	2	1	1	2	2	4	4	3	3
2	2	1	1	2	2	3	3	3	3
2	2	1	1	2	2	3	3	3	3
2	2	1	1	2	2	3	3	3	3
2	2	1	1	2	2	3	3	3	3
2	2	1	1	2	2	3	3	3	3
2	2	1	1	2	2	3	3	3	3
2	2	1	1	2	2	3	3	3	3
2	2	1	1	2	2	3	3	3	3
2	2	1	1	2	2	3	3	3	3
2	2	1	1	2	2	3	3	3	3
2	2	1	1	2	2	3	3	3	3
2	2	1	1	3	3	3	3	3	3
3	3	1	1	2	2	3	3	3	3
3	3	1	1	2	2	3	3	3	3
3	3	1	1	2	2	4	4	3	3
2	2	1	1	3	3	3	3	3	3
2	2	1	1	2	2	3	3	3	3
2	2	1	1	2	2	3	3	3	3
2	2	1	1	2	2	3	3	3	3
2	2	1	1	2	2	4	4	3	3
2	2	1	1	2	2	3	3	3	3
2	2	1	1	2	2	3	3	3	3
2	2	1	1	3	3	3	3	1	1
2	2	1	1	2	2	3	3	3	3
2	2	1	1	2	2	3	3	3	3
3	3	1	1	2	2	3	3	3	3
2	2	1	1	2	2	3	3	3	3
2	2	1	1	2	2	3	3	3	3
2	2	1	1	2	2	3	3	3	3
2	2	1	1	2	2	3	3	3	3
2	2	1	1	2	2	3	3	3	3
2	2	1	1	2	2	3	3	3	3
3	3	1	1	2	2	3	3	3	3
2	2	1	1	2	2	3	3	3	3
2	2	1	1	2	2	3	3	3	3
3	3	1	1	2	2	3	3	3	3
2	2	1	1	2	2	3	3	3	3
2	2	1	1	2	2	3	3	1	1
2	2	1	1	3	3	3	3	3	3
3	3	1	1	2	2	3	3	3	3
2	2	1	1	2	2	3	3	3	3
2	2	1	1	2	2	3	3	3	3
3	3	1	1	2	2	3	3	3	3
2	2	1	1	2	2	3	3	3	3
2	2	1	1	2	2	3	3	3	3

For Review Only

2	2	1	1	2	2	3	3	3	3
3	3	1	1	2	2	3	3	3	3
2	2	1	1	2	2	3	3	3	3
3	3	1	1	2	2	4	4	3	3
2	2	1	1	2	2	3	3	3	3
2	2	1	1	2	2	3	3	3	3
2	2	1	1	2	2	3	3	3	3
3	3	1	1	2	2	3	3	3	3
2	2	1	1	2	2	3	3	3	3
3	3	1	1	2	2	3	3	3	3
2	2	1	1	2	2	3	3	3	3
2	2	4	4	2	2	3	3	3	3
2	2	1	1	2	2	3	3	3	3
2	2	1	1	2	2	3	3	3	3
2	2	1	1	2	2	3	3	3	3
2	2	1	1	2	2	3	3	1	1
2	2	1	1	2	2	3	3	1	1
2	2	1	1	2	2	3	3	3	3
2	2	1	1	2	2	3	3	3	3
2	2	1	1	2	2	3	3	3	3
2	2	1	1	2	2	3	3	3	3
2	2	1	1	2	2	3	3	3	3
2	2	1	1	2	2	3	3	1	1
2	2	1	1	2	2	3	3	3	3
3	3	1	1	2	2	3	3	3	3
2	2	4	4	2	2	3	3	3	3
2	2	1	1	2	2	3	3	3	3
2	2	1	1	2	2	3	3	3	3
2	2	1	1	2	2	3	3	3	3
2	2	1	1	2	2	3	3	3	3
2	2	1	1	2	2	3	3	3	3
2	2	1	1	2	2	3	3	3	3
2	2	1	1	2	2	3	3	3	3
2	2	1	1	2	2	3	3	3	3
3	3	1	1	2	2	3	3	3	3
2	2	1	1	2	2	3	3	3	3
2	2	1	1	2	2	3	3	1	1
3	3	1	1	2	2	3	3	3	3
2	2	1	1	3	3	3	3	3	3
2	2	1	1	2	2	3	3	3	3
2	2	1	1	2	2	3	3	3	3
2	2	1	1	2	2	3	3	1	1
2	2	1	1	2	2	3	3	3	3
2	2	1	1	2	2	4	4	1	1
2	2	1	1	2	2	3	3	3	3
2	2	1	1	2	2	4	4	3	3
2	2	1	1	2	2	3	3	3	3
2	2	1	1	2	2	3	3	3	3
2	2	1	1	2	2	3	3	3	3
2	2	1	1	2	2	3	3	3	3
2	2	1	1	2	2	3	3	3	3
2	2	1	1	2	2	3	3	3	3
2	2	1	1	2	2	3	3	1	1
2	2	4	4	2	2	3	3	3	3
2	2	1	1	2	2	3	3	3	3

For Review Only

2	2	1	1	2	2	4	4	1	1
2	2	1	1	2	2	3	3	3	3

For Review Only

SNP892	SNP892	SNP893	SNP893	SNP894	SNP894	SNP895	SNP895	SNP896	SNP896
2	2	4	4	1	1	1	1	1	1
2	2	4	4	1	1	1	1	1	1
3	3	4	4	1	1	3	3	1	1
2	2	4	4	1	1	1	1	1	1
3	3	4	4	1	1	1	1	1	1
3	3	4	4	1	1	1	1	1	1
3	3	4	4	4	4	1	1	1	1
3	3	4	4	1	1	1	1	2	2
3	3	4	4	1	1	1	1	1	1
3	3	4	4	1	1	1	1	2	2
2	2	4	4	1	1	1	1	1	1
2	2	4	4	1	1	3	3	1	1
2	2	4	4	1	1	1	1	1	1
2	2	4	4	1	1	1	1	2	2
3	3	4	4	1	1	1	1	1	1
2	2	4	4	1	1	1	1	1	1
3	3	4	4	1	1	1	1	1	1
3	3	4	4	1	1	1	1	1	1
2	2	4	4	1	1	3	3	1	1
3	3	4	4	1	1	1	1	1	1
2	2	4	4	1	1	1	1	1	1
3	3	4	4	1	1	1	1	1	1
3	3	4	4	1	1	1	1	1	1
3	3	4	4	1	1	1	1	1	1
3	3	4	4	1	1	1	1	2	2
2	2	4	4	1	1	3	3	1	1
3	3	4	4	1	1	1	1	1	1
3	3	4	4	1	1	1	1	1	1
3	3	4	4	1	1	1	1	1	1
3	3	4	4	1	1	1	1	1	1
3	3	4	4	1	1	1	1	2	2
2	2	4	4	1	1	1	1	1	1
2	2	4	4	1	1	1	1	1	1
2	2	4	4	1	1	1	1	1	1
2	2	4	4	1	1	1	1	1	1
2	2	4	4	1	1	1	1	2	2
2	2	4	4	1	1	1	1	1	1
3	3	4	4	1	1	1	1	1	1
2	2	4	4	1	1	1	1	1	1
3	3	4	4	1	1	1	1	1	1
2	2	4	4	1	1	1	1	1	1
2	2	4	4	1	1	1	1	1	1
3	3	4	4	1	1	1	1	2	2
3	3	4	4	1	1	1	1	2	2
3	3	4	4	1	1	1	1	2	2
2	2	4	4	1	1	3	3	1	1
3	3	4	4	1	1	3	3	2	2
3	3	4	4	1	1	1	1	1	1
3	3	4	4	1	1	1	1	1	1
2	2	4	4	1	1	1	1	1	1
2	2	4	4	1	1	3	3	2	2
2	2	4	4	1	1	1	1	1	1

For Review Only

3	3	4	4	1	1	1	1	1	1
2	2	4	4	1	1	1	1	1	1
3	3	4	4	1	1	1	1	1	1
3	3	4	4	1	1	1	1	1	1
2	2	4	4	1	1	3	3	1	1
3	3	3	3	4	4	3	3	2	2
2	2	4	4	1	1	3	3	1	1
3	3	4	4	1	1	1	1	1	1
3	3	4	4	1	1	1	1	1	1
2	2	4	4	1	1	1	1	1	1
3	3	4	4	1	1	3	3	1	1
3	3	3	3	4	4	3	3	2	2
3	3	4	4	1	1	1	1	1	1
3	3	4	4	1	1	1	1	1	1
2	2	4	4	1	1	1	1	1	1
2	2	4	4	1	1	1	1	1	1
2	2	4	4	1	1	3	3	2	2
3	3	4	4	1	1	1	1	1	1
3	3	4	4	1	1	1	1	1	1
3	3	4	4	1	1	1	1	1	1
3	3	4	4	1	1	1	1	1	1
2	2	4	4	1	1	1	1	1	1
3	3	4	4	1	1	1	1	1	1
2	2	3	3	4	4	3	3	2	2
3	3	4	4	1	1	1	1	2	2
3	3	4	4	1	1	3	3	1	1
3	3	4	4	1	1	3	3	1	1
2	2	4	4	1	1	1	1	1	1
2	2	4	4	1	1	3	3	1	1
2	2	4	4	1	1	1	1	1	1
3	3	4	4	1	1	1	1	1	1
3	3	4	4	1	1	1	1	1	1
2	2	4	4	1	1	1	1	1	1
3	3	4	4	1	1	1	1	1	1
2	2	4	4	1	1	1	1	1	1
3	3	4	4	1	1	1	1	2	2
3	3	4	4	1	1	1	1	1	1
3	3	4	4	1	1	1	1	1	1
2	2	4	4	1	1	3	3	1	1
3	3	4	4	1	1	1	1	2	2
3	3	4	4	1	1	1	1	1	1
3	3	4	4	1	1	1	1	1	1
3	3	4	4	1	1	1	1	1	1
3	3	4	4	1	1	3	3	1	1
3	3	4	4	1	1	1	1	1	1
2	2	4	4	1	1	1	1	1	1
3	3	4	4	1	1	1	1	1	1
3	3	4	4	1	1	3	3	1	1
2	2	3	3	4	4	1	1	1	1
3	3	4	4	1	1	1	1	2	2
2	2	4	4	1	1	1	1	2	2
2	2	4	4	1	1	1	1	1	1
3	3	4	4	1	1	1	1	1	1
2	2	4	4	1	1	1	1	1	1
3	3	4	4	1	1	1	1	2	2

For Review Only

2	2	4	4	1	1	1	1	1	1
2	2	4	4	1	1	1	1	1	1
2	2	4	4	1	1	1	1	1	1
3	3	4	4	1	1	1	1	1	1
2	2	4	4	1	1	3	3	1	1
3	3	4	4	1	1	3	3	1	1
2	2	4	4	1	1	1	1	1	1
2	2	4	4	1	1	1	1	2	2
2	2	4	4	1	1	1	1	1	1
2	2	4	4	1	1	1	1	2	2
2	2	4	4	1	1	1	1	1	1
2	2	4	4	1	1	1	1	1	1
3	3	4	4	1	1	1	1	1	1
2	2	4	4	1	1	3	3	1	1
2	2	4	4	1	1	1	1	1	1
2	2	4	4	1	1	3	3	1	1
3	3	4	4	1	1	1	1	1	1
2	2	4	4	1	1	3	3	1	1
3	3	4	4	1	1	1	1	1	1
3	3	4	4	1	1	1	1	1	1
2	2	4	4	1	1	3	3	1	1
2	2	4	4	1	1	1	1	1	1
3	3	4	4	1	1	1	1	2	2
2	2	4	4	1	1	1	1	1	1
2	2	4	4	1	1	1	1	1	1
2	2	4	4	1	1	1	1	1	1
2	2	4	4	1	1	1	1	1	1
3	3	4	4	1	1	1	1	1	1
2	2	4	4	1	1	3	3	1	1
3	3	4	4	1	1	1	1	1	1
2	2	4	4	1	1	1	1	1	1
2	2	4	4	1	1	1	1	1	1
3	3	4	4	1	1	1	1	1	1
2	2	4	4	1	1	1	1	1	1
2	2	4	4	1	1	1	1	1	1
2	2	4	4	1	1	1	1	1	1
3	3	4	4	1	1	1	1	1	1
2	2	3	3	4	4	1	1	2	2
2	2	4	4	1	1	1	1	1	1
2	2	4	4	1	1	1	1	1	1
2	2	4	4	1	1	1	1	1	1
3	3	4	4	1	1	1	1	1	1
2	2	4	4	1	1	1	1	2	2
2	2	4	4	1	1	1	1	1	1
2	2	4	4	1	1	1	1	1	1
2	2	4	4	1	1	1	1	1	1
2	2	4	4	1	1	3	3	1	1
3	3	4	4	1	1	3	3	1	1
3	3	4	4	1	1	1	1	1	1
2	2	4	4	1	1	3	3	1	1
2	2	4	4	1	1	1	1	1	1
2	2	4	4	1	1	1	1	1	1
2	2	4	4	1	1	1	1	1	1
2	2	3	3	4	4	3	3	1	1
2	2	4	4	1	1	1	1	1	1

For Review Only

2	2	4	4	1	1	3	3	1	1
2	2	4	4	1	1	1	1	1	1
3	3	4	4	1	1	1	1	1	1
2	2	4	4	1	1	1	1	1	1
2	2	4	4	1	1	1	1	1	1
3	3	4	4	1	1	1	1	1	1
2	2	4	4	1	1	1	1	1	1
2	2	4	4	1	1	1	1	1	1
2	2	4	4	1	1	1	1	1	1
2	2	4	4	1	1	1	1	1	1
2	2	4	4	1	1	3	3	1	1
2	2	4	4	1	1	1	1	2	2
2	2	4	4	1	1	1	1	1	1
2	2	4	4	1	1	1	1	1	1
3	3	4	4	1	1	1	1	1	1
2	2	4	4	1	1	1	1	1	1
2	2	4	4	1	1	1	1	1	1
2	2	4	4	1	1	3	3	1	1
2	2	4	4	1	1	1	1	1	1
2	2	4	4	1	1	1	1	1	1
2	2	4	4	1	1	1	1	1	1
3	3	4	4	1	1	1	1	1	1
2	2	4	4	1	1	1	1	1	1
3	3	3	3	4	4	3	3	2	2
3	3	4	4	1	1	1	1	1	1
2	2	4	4	1	1	1	1	1	1
2	2	4	4	1	1	1	1	2	2
3	3	4	4	1	1	1	1	1	1
3	3	4	4	1	1	1	1	2	2
3	3	4	4	1	1	1	1	1	1
3	3	4	4	1	1	3	3	1	1
2	2	4	4	1	1	1	1	1	1
2	2	4	4	1	1	1	1	1	1
3	3	4	4	1	1	1	1	1	1
3	3	4	4	1	1	1	1	1	1
3	3	3	3	1	1	3	3	2	2
2	2	4	4	1	1	1	1	1	1
2	2	4	4	1	1	1	1	1	1
3	3	4	4	1	1	3	3	1	1
3	3	3	3	4	4	3	3	2	2
3	3	4	4	1	1	3	3	1	1
2	2	4	4	1	1	3	3	1	1
2	2	4	4	1	1	1	1	1	1
2	2	4	4	1	1	3	3	1	1
2	2	4	4	1	1	1	1	1	1
3	3	4	4	1	1	1	1	1	1
2	2	4	4	1	1	1	1	1	1
2	2	4	4	1	1	1	1	1	1
3	3	4	4	1	1	1	1	1	1
3	3	4	4	1	1	1	1	1	1
3	3	4	4	1	1	3	3	1	1
3	3	4	4	1	1	1	1	1	1
2	2	4	4	1	1	1	1	1	1
3	3	4	4	1	1	3	3	1	1
2	2	4	4	1	1	1	1	1	1

For Review Only

3	3	4	4	1	1	1	1	1	1
2	2	4	4	1	1	1	1	1	1
3	3	4	4	1	1	3	3	1	1
2	2	4	4	1	1	1	1	1	1
3	3	4	4	1	1	1	1	1	1
3	3	4	4	1	1	1	1	1	1
2	2	4	4	1	1	1	1	1	1
3	3	4	4	1	1	1	1	1	1
3	3	4	4	1	1	1	1	1	1
2	2	4	4	1	1	1	1	1	1
2	2	4	4	1	1	1	1	1	1
2	2	4	4	1	1	3	3	1	1
2	2	4	4	1	1	3	3	1	1
2	2	4	4	1	1	3	3	1	1
3	3	4	4	1	1	3	3	1	1
2	2	4	4	1	1	1	1	1	1
2	2	4	4	1	1	1	1	1	1
2	2	4	4	1	1	1	1	1	1
2	2	4	4	1	1	1	1	1	1
3	3	4	4	1	1	1	1	1	1
2	2	4	4	1	1	3	3	1	1
2	2	4	4	1	1	3	3	1	1
2	2	4	4	1	1	1	1	1	1
3	3	4	4	1	1	1	1	1	1
3	3	4	4	1	1	1	1	1	1
2	2	4	4	1	1	1	1	1	1
2	2	4	4	1	1	1	1	1	1
2	2	4	4	1	1	3	3	1	1
3	3	4	4	1	1	1	1	1	1
3	3	4	4	1	1	3	3	1	1
2	2	4	4	1	1	1	1	1	1
2	2	4	4	1	1	3	3	1	1
3	3	4	4	1	1	1	1	1	1
2	2	4	4	1	1	3	3	1	1
2	2	4	4	1	1	1	1	1	1
2	2	4	4	1	1	1	1	1	1
2	2	4	4	1	1	1	1	1	1
3	3	3	3	4	4	3	3	2	2
2	2	4	4	1	1	1	1	2	2
2	2	4	4	1	1	1	1	1	1
3	3	4	4	1	1	1	1	1	1
3	3	4	4	1	1	1	1	2	2
3	3	4	4	1	1	1	1	1	1
3	3	4	4	1	1	1	1	1	1
3	3	4	4	1	1	3	3	2	2
3	3	4	4	1	1	1	1	1	1
2	2	4	4	1	1	1	1	1	1
3	3	4	4	1	1	1	1	2	2
3	3	4	4	1	1	1	1	2	2
2	2	4	4	1	1	1	1	1	1
2	2	4	4	1	1	1	1	1	1
2	2	4	4	1	1	1	1	1	1
3	3	4	4	1	1	3	3	1	1
2	2	4	4	1	1	3	3	2	2
2	2	4	4	1	1	1	1	1	1

For Review Only

2	2	4	4	1	1	1	1	1	1
2	2	3	3	4	4	3	3	1	1

For Review Only

SNP897	SNP897	SNP898	SNP898	SNP899	SNP899	SNP900	SNP900	SNP901	SNP901
2	2	2	2	2	2	1	1	3	3
3	3	2	2	2	2	3	3	3	3
3	3	2	2	2	2	1	1	3	3
3	3	2	2	2	2	1	1	2	2
3	3	2	2	2	2	1	1	3	3
3	3	2	2	2	2	1	1	2	2
2	2	2	2	2	2	1	1	3	3
3	3	2	2	2	2	1	1	3	3
3	3	2	2	2	2	1	1	3	3
3	3	1	1	2	2	1	1	3	3
3	3	2	2	3	3	3	3	3	3
3	3	2	2	2	2	1	1	3	3
3	3	2	2	2	2	1	1	3	3
3	3	2	2	2	2	3	3	2	2
3	3	2	2	2	2	1	1	3	3
2	2	2	2	2	2	1	1	3	3
3	3	2	2	2	2	1	1	3	3
3	3	2	2	2	2	1	1	3	3
3	3	2	2	2	2	1	1	3	3
3	3	2	2	2	2	1	1	3	3
3	3	2	2	2	2	3	3	2	2
3	3	2	2	2	2	1	1	3	3
3	3	1	1	3	3	3	3	3	3
3	3	2	2	2	2	1	1	3	3
3	3	2	2	2	2	1	1	3	3
3	3	2	2	2	2	1	1	3	3
3	3	2	2	2	2	1	1	3	3
3	3	2	2	2	2	1	1	3	3
3	3	2	2	2	2	3	3	2	2
3	3	1	1	2	2	3	3	3	3
3	3	2	2	2	2	1	1	3	3
3	3	2	2	2	2	1	1	2	2
3	3	2	2	2	2	1	1	3	3
3	3	1	1	2	2	3	3	3	3
3	3	2	2	2	2	1	1	3	3
3	3	2	2	2	2	1	1	3	3
3	3	2	2	2	2	1	1	3	3
3	3	2	2	2	2	1	1	3	3
3	3	1	1	3	3	3	3	3	3
3	3	2	2	3	3	3	3	3	3
3	3	1	1	2	2	3	3	3	3
3	3	2	2	2	2	3	3	2	2
3	3	2	2	2	2	1	1	3	3
3	3	2	2	2	2	3	3	3	3
3	3	2	2	2	2	1	1	3	3
3	3	2	2	2	2	1	1	3	3
3	3	2	2	2	2	1	1	3	3
3	3	2	2	2	2	1	1	3	3
3	3	2	2	2	2	3	3	3	3
3	3	2	2	2	2	1	1	3	3
3	3	2	2	2	2	1	1	3	3
2	2	2	2	2	2	1	1	3	3

For Review Only

3	3	2	2	2	2	1	1	3	3
3	3	2	2	2	2	1	1	3	3
3	3	2	2	2	2	1	1	3	3
3	3	2	2	2	2	1	1	3	3
3	3	2	2	2	2	1	1	3	3
3	3	2	2	2	2	1	1	3	3
3	3	2	2	2	2	1	1	3	3
3	3	2	2	2	2	3	3	3	3
3	3	1	1	2	2	1	1	3	3
3	3	2	2	2	2	1	1	3	3
2	2	2	2	2	2	1	1	3	3
3	3	2	2	2	2	1	1	3	3
3	3	2	2	2	2	1	1	3	3
3	3	2	2	2	2	3	3	2	2
3	3	2	2	2	2	1	1	3	3
3	3	2	2	2	2	3	3	2	2
3	3	2	2	2	2	3	3	2	2
3	3	2	2	2	2	1	1	2	2
3	3	2	2	2	2	1	1	2	2
3	3	2	2	2	2	1	1	3	3
3	3	2	2	2	2	1	1	3	3
2	2	2	2	2	2	3	3	2	2
3	3	2	2	2	2	3	3	2	2
3	3	2	2	2	2	3	3	3	3
3	3	2	2	2	2	1	1	3	3
2	2	2	2	2	2	1	1	3	3
3	3	2	2	2	2	1	1	3	3
3	3	1	1	2	2	3	3	3	3
3	3	2	2	2	2	1	1	3	3
3	3	2	2	2	2	1	1	2	2
3	3	2	2	2	2	1	1	2	2
3	3	2	2	2	2	3	3	3	3
3	3	1	1	2	2	3	3	3	3
3	3	2	2	2	2	1	1	3	3
3	3	2	2	2	2	1	1	2	2
3	3	2	2	2	2	1	1	3	3
3	3	2	2	2	2	1	1	2	2
3	3	2	2	2	2	1	1	3	3
3	3	1	1	2	2	1	1	3	3
3	3	2	2	2	2	1	1	3	3
3	3	2	2	2	2	1	1	3	3
3	3	2	2	2	2	1	1	3	3
3	3	2	2	2	2	1	1	2	2
2	2	2	2	2	2	1	1	3	3
3	3	2	2	2	2	1	1	3	3
2	2	2	2	2	2	1	1	3	3
3	3	1	1	2	2	3	3	3	3
3	3	2	2	2	2	1	1	2	2
2	2	2	2	2	2	1	1	3	3
2	2	2	2	2	2	1	1	3	3
3	3	2	2	2	2	1	1	3	3
3	3	2	2	2	2	3	3	3	3
3	3	2	2	2	2	1	1	3	3
3	3	2	2	2	2	1	1	3	3
3	3	2	2	2	2	1	1	3	3

For Review Only

3	3	2	2	2	2	1	1	3	3
3	3	2	2	3	3	3	3	3	3
3	3	2	2	2	2	1	1	3	3
3	3	2	2	2	2	3	3	3	3
2	2	2	2	2	2	1	1	3	3
3	3	2	2	2	2	1	1	3	3
2	2	2	2	2	2	1	1	3	3
3	3	2	2	2	2	1	1	3	3
2	2	2	2	2	2	3	3	3	3
3	3	2	2	2	2	1	1	3	3
3	3	2	2	2	2	1	1	3	3
3	3	2	2	2	2	1	1	3	3
3	3	2	2	2	2	1	1	3	3
2	2	2	2	2	2	1	1	3	3
3	3	2	2	2	2	1	1	3	3
3	3	2	2	2	2	1	1	3	3
3	3	2	2	2	2	3	3	3	3
3	3	2	2	2	2	1	1	3	3
3	3	2	2	2	2	3	3	3	3
3	3	2	2	2	2	1	1	3	3
3	3	2	2	2	2	1	1	3	3
3	3	2	2	2	2	1	1	2	2
3	3	2	2	2	2	1	1	3	3
3	3	1	1	2	2	3	3	3	3
2	2	2	2	2	2	3	3	3	3
3	3	2	2	3	3	3	3	3	3
2	2	2	2	2	2	1	1	3	3
3	3	2	2	2	2	1	1	3	3
2	2	2	2	2	2	1	1	3	3
3	3	2	2	2	2	1	1	2	2
3	3	1	1	2	2	3	3	3	3
3	3	2	2	2	2	1	1	3	3
3	3	2	2	2	2	1	1	3	3
3	3	2	2	2	2	1	1	3	3
3	3	1	1	2	2	3	3	3	3
3	3	2	2	2	2	1	1	3	3
3	3	2	2	2	2	3	3	3	3
3	3	2	2	2	2	1	1	3	3
3	3	2	2	2	2	1	1	3	3
3	3	2	2	2	2	3	3	3	3
3	3	2	2	2	2	3	3	3	3
3	3	1	1	2	2	3	3	3	3
3	3	2	2	2	2	1	1	2	2
3	3	2	2	2	2	1	1	3	3
3	3	2	2	2	2	3	3	3	3
3	3	2	2	2	2	1	1	3	3
3	3	2	2	2	2	1	1	3	3
3	3	2	2	2	2	1	1	3	3
3	3	2	2	2	2	1	1	3	3
3	3	2	2	2	2	1	1	3	3
3	3	2	2	2	2	1	1	3	3
3	3	2	2	2	2	1	1	3	3
3	3	2	2	2	2	1	1	3	3
3	3	2	2	2	2	1	1	3	3
3	3	2	2	2	2	1	1	3	3

For Review Only

3	3	2	2	2	2	1	1	3	3
3	3	2	2	3	3	3	3	2	2
3	3	2	2	2	2	1	1	3	3
3	3	2	2	3	3	1	1	3	3
2	2	2	2	2	2	1	1	3	3
3	3	2	2	2	2	3	3	2	2
3	3	2	2	2	2	1	1	3	3
3	3	2	2	2	2	1	1	3	3
2	2	2	2	2	2	3	3	3	3
3	3	2	2	2	2	1	1	3	3
2	2	2	2	2	2	1	1	3	3
3	3	2	2	2	2	3	3	3	3
3	3	2	2	2	2	1	1	3	3
3	3	2	2	2	2	1	1	2	2
3	3	2	2	2	2	3	3	3	3
3	3	2	2	3	3	3	3	3	3
3	3	2	2	2	2	1	1	3	3
2	2	2	2	2	2	1	1	3	3
3	3	2	2	2	2	1	1	3	3
2	2	2	2	2	2	1	1	3	3
3	3	2	2	2	2	1	1	3	3
3	3	2	2	2	2	3	3	3	3
2	2	2	2	2	2	3	3	2	2
3	3	2	2	2	2	1	1	3	3
3	3	2	2	2	2	1	1	3	3
3	3	1	1	2	2	3	3	3	3
2	2	2	2	2	2	1	1	2	2
3	3	2	2	2	2	3	3	3	3
3	3	2	2	2	2	1	1	3	3
3	3	2	2	2	2	1	1	2	2
3	3	2	2	2	2	1	1	2	2
3	3	2	2	2	2	1	1	3	3
2	2	2	2	2	2	1	1	2	2
3	3	2	2	2	2	3	3	3	3
3	3	2	2	2	2	1	1	2	2
3	3	2	2	2	2	1	1	3	3
3	3	2	2	2	2	1	1	3	3
3	3	2	2	2	2	1	1	2	2
3	3	2	2	2	2	1	1	3	3
3	3	2	2	2	2	1	1	3	3
3	3	2	2	2	2	1	1	3	3
3	3	2	2	2	2	1	1	3	3
3	3	1	1	2	2	3	3	3	3
2	2	2	2	2	2	1	1	3	3
3	3	2	2	2	2	1	1	3	3
3	3	1	1	2	2	1	1	3	3
3	3	2	2	3	3	3	3	3	3
3	3	2	2	2	2	1	1	3	3
3	3	2	2	2	2	3	3	3	3
3	3	2	2	2	2	1	1	3	3
3	3	2	2	2	2	1	1	3	3
3	3	2	2	2	2	3	3	3	3
2	2	1	1	2	2	3	3	2	2
2	2	2	2	2	2	1	1	3	3
3	3	1	1	3	3	3	3	3	3

For Review Only

3	3	2	2	2	2	3	3	3	3
3	3	2	2	2	2	3	3	3	3
3	3	2	2	2	2	1	1	3	3
2	2	2	2	2	2	3	3	3	3
3	3	2	2	2	2	3	3	3	3
3	3	2	2	2	2	1	1	3	3
3	3	2	2	2	2	3	3	3	3
3	3	1	1	2	2	3	3	3	3
3	3	2	2	2	2	1	1	3	3
3	3	1	1	2	2	3	3	3	3
3	3	2	2	2	2	1	1	3	3
3	3	1	1	2	2	3	3	3	3
3	3	2	2	2	2	1	1	3	3
3	3	2	2	2	2	1	1	3	3
3	3	2	2	2	2	1	1	3	3
2	2	2	2	2	2	1	1	3	3
3	3	1	1	3	3	1	1	3	3
3	3	2	2	2	2	3	3	3	3
3	3	2	2	2	2	3	3	3	3
3	3	2	2	2	2	3	3	3	3
3	3	2	2	3	3	3	3	3	3
3	3	2	2	2	2	1	1	3	3
3	3	2	2	2	2	1	1	2	2
3	3	2	2	3	3	3	3	3	3
3	3	2	2	2	2	3	3	3	3
2	2	2	2	2	2	1	1	3	3
3	3	1	1	2	2	3	3	3	3
2	2	2	2	2	2	1	1	2	2
3	3	2	2	2	2	1	1	3	3
3	3	2	2	2	2	1	1	3	3
3	3	1	1	3	3	3	3	3	3
3	3	2	2	2	2	1	1	3	3
3	3	1	1	2	2	3	3	2	2
3	3	2	2	2	2	1	1	3	3
2	2	2	2	2	2	3	3	2	2
3	3	1	1	3	3	3	3	3	3
3	3	2	2	2	2	1	1	2	2
3	3	2	2	2	2	1	1	3	3
3	3	2	2	2	2	1	1	3	3
3	3	1	1	3	3	3	3	3	3
3	3	2	2	2	2	1	1	2	2
3	3	2	2	2	2	1	1	3	3
3	3	2	2	3	3	1	1	3	3
3	3	2	2	2	2	1	1	3	3
3	3	2	2	2	2	1	1	3	3
3	3	1	1	2	2	3	3	3	3
3	3	2	2	2	2	3	3	3	3
3	3	2	2	2	2	1	1	3	3
3	3	2	2	2	2	1	1	2	2
3	3	1	1	2	2	3	3	3	3
2	2	2	2	2	2	1	1	2	2
3	3	1	1	2	2	3	3	3	3
3	3	2	2	2	2	1	1	3	3
2	2	2	2	2	2	1	1	2	2
3	3	2	2	3	3	3	3	3	3

For Review Only

2	2	2	2	2	2	1	1	3	3
3	3	2	2	2	2	1	1	3	3

For Review Only

SNP902	SNP902	SNP903	SNP903	SNP904	SNP904	SNP905	SNP905	SNP906	SNP906
4	4	1	1	3	3	3	3	4	4
4	4	1	1	3	3	3	3	1	1
4	4	4	4	2	2	3	3	4	4
4	4	1	1	3	3	3	3	4	4
4	4	1	1	3	3	3	3	4	4
4	4	1	1	3	3	3	3	4	4
2	2	1	1	3	3	3	3	4	4
4	4	1	1	3	3	3	3	4	4
4	4	1	1	3	3	3	3	4	4
4	4	1	1	3	3	3	3	4	4
4	4	1	1	3	3	3	3	1	1
4	4	4	4	2	2	3	3	4	4
4	4	1	1	3	3	3	3	4	4
4	4	1	1	3	3	1	1	1	1
4	4	1	1	3	3	3	3	4	4
4	4	1	1	3	3	3	3	4	4
4	4	1	1	3	3	3	3	4	4
4	4	1	1	3	3	3	3	4	4
4	4	4	4	2	2	3	3	4	4
4	4	1	1	3	3	3	3	4	4
4	4	1	1	3	3	3	3	1	1
4	4	1	1	3	3	3	3	4	4
4	4	1	1	3	3	3	3	1	1
4	4	1	1	3	3	3	3	1	1
4	4	1	1	3	3	3	3	4	4
4	4	4	4	2	2	3	3	4	4
4	4	1	1	3	3	3	3	4	4
4	4	1	1	3	3	3	3	4	4
4	4	1	1	3	3	1	1	1	1
4	4	1	1	3	3	1	1	4	4
4	4	1	1	3	3	3	3	4	4
4	4	1	1	3	3	3	3	4	4
4	4	1	1	3	3	3	3	4	4
4	4	1	1	3	3	1	1	1	1
4	4	1	1	3	3	1	1	4	4
4	4	1	1	3	3	3	3	4	4
4	4	1	1	3	3	3	3	4	4
4	4	1	1	3	3	3	3	4	4
4	4	1	1	3	3	3	3	1	1
4	4	1	1	3	3	3	3	1	1
4	4	1	1	3	3	1	1	1	1
4	4	1	1	3	3	1	1	1	1
4	4	1	1	3	3	3	3	4	4
2	2	1	1	3	3	1	1	4	4
4	4	1	1	3	3	3	3	4	4
4	4	4	4	2	2	3	3	4	4
4	4	4	4	2	2	3	3	4	4
4	4	1	1	3	3	3	3	1	1
4	4	1	1	3	3	1	1	4	4
4	4	1	1	3	3	3	3	4	4
4	4	4	4	2	2	3	3	4	4
4	4	1	1	3	3	3	3	4	4

For Review Only

4	4	1	1	3	3	3	3	4	4
4	4	1	1	3	3	3	3	4	4
4	4	1	1	3	3	3	3	4	4
4	4	1	1	3	3	3	3	4	4
4	4	4	4	2	2	3	3	4	4
4	4	4	4	2	2	3	3	4	4
4	4	4	4	2	2	3	3	4	4
4	4	1	1	3	3	3	3	1	1
4	4	1	1	3	3	3	3	4	4
4	4	1	1	3	3	3	3	4	4
4	4	4	4	2	2	3	3	4	4
4	4	4	4	2	2	3	3	4	4
4	4	1	1	3	3	3	3	4	4
4	4	1	1	3	3	1	1	1	1
4	4	1	1	3	3	3	3	4	4
4	4	1	1	3	3	3	3	1	1
4	4	4	4	2	2	3	3	1	1
4	4	1	1	3	3	3	3	4	4
4	4	1	1	3	3	3	3	4	4
4	4	1	1	3	3	3	3	4	4
4	4	4	4	2	2	3	3	4	4
2	2	1	1	3	3	3	3	1	1
4	4	1	1	3	3	3	3	1	1
4	4	4	4	2	2	3	3	1	1
4	4	1	1	3	3	3	3	4	4
4	4	4	4	2	2	3	3	4	4
4	4	4	4	2	2	3	3	4	4
4	4	1	1	3	3	3	3	1	1
4	4	4	4	2	2	3	3	4	4
4	4	1	1	3	3	3	3	4	4
4	4	1	1	3	3	3	3	4	4
4	4	1	1	3	3	3	3	4	4
4	4	1	1	3	3	3	3	1	1
4	4	1	1	3	3	3	3	4	4
4	4	1	1	3	3	3	3	4	4
4	4	1	1	3	3	3	3	4	4
4	4	1	1	3	3	3	3	4	4
4	4	1	1	3	3	3	3	4	4
4	4	4	4	2	2	3	3	4	4
4	4	1	1	3	3	3	3	4	4
4	4	1	1	3	3	3	3	4	4
4	4	1	1	3	3	3	3	4	4
4	4	1	1	3	3	3	3	4	4
4	4	4	4	2	2	3	3	4	4
4	4	1	1	3	3	3	3	4	4
4	4	1	1	3	3	3	3	4	4
4	4	1	1	3	3	1	1	1	1
4	4	4	4	2	2	3	3	4	4
2	2	1	1	3	3	3	3	4	4
4	4	1	1	3	3	3	3	4	4
4	4	1	1	3	3	3	3	4	4
4	4	1	1	3	3	1	1	1	1
4	4	1	1	3	3	3	3	4	4
4	4	1	1	3	3	3	3	4	4
4	4	1	1	3	3	3	3	4	4

4	4	1	1	3	3	3	3	4	4
4	4	1	1	3	3	3	3	4	4
4	4	1	1	3	3	3	3	4	4
4	4	1	1	3	3	1	1	1	1
4	4	4	4	2	2	3	3	4	4
4	4	4	4	2	2	3	3	4	4
2	2	1	1	3	3	3	3	4	4
4	4	1	1	3	3	3	3	4	4
2	2	1	1	3	3	3	3	4	4
4	4	4	4	2	2	3	3	4	4
4	4	1	1	3	3	3	3	4	4
4	4	1	1	3	3	3	3	4	4
4	4	1	1	3	3	3	3	4	4
4	4	4	4	2	2	3	3	4	4
4	4	1	1	3	3	3	3	4	4
4	4	4	4	2	2	3	3	4	4
4	4	1	1	3	3	3	3	4	4
4	4	1	1	3	3	3	3	4	4
4	4	1	1	3	3	1	1	1	1
4	4	1	1	3	3	3	3	1	1
4	4	1	1	3	3	3	3	4	4
2	2	1	1	3	3	3	3	4	4
4	4	1	1	3	3	3	3	4	4
4	4	1	1	3	3	3	3	1	1
4	4	1	1	3	3	3	3	4	4
4	4	1	1	3	3	3	3	1	1
4	4	1	1	3	3	3	3	4	4
4	4	1	1	3	3	3	3	4	4
4	4	4	4	2	2	3	3	4	4
4	4	1	1	3	3	3	3	4	4
4	4	1	1	3	3	1	1	1	1
4	4	1	1	3	3	3	3	4	4
4	4	1	1	3	3	3	3	4	4
4	4	1	1	3	3	3	3	4	4
4	4	1	1	3	3	1	1	1	1
4	4	1	1	3	3	3	3	4	4
4	4	1	1	3	3	1	1	1	1
4	4	4	4	2	2	3	3	4	4
4	4	1	1	3	3	3	3	1	1
4	4	1	1	3	3	3	3	1	1
4	4	1	1	3	3	3	3	1	1
4	4	1	1	3	3	1	1	1	1
4	4	1	1	3	3	3	3	4	4
4	4	1	1	3	3	3	3	1	1
4	4	1	1	3	3	1	1	1	1
4	4	1	1	3	3	3	3	4	4
4	4	4	4	2	2	3	3	4	4
4	4	4	4	2	2	3	3	4	4
4	4	1	1	3	3	3	3	4	4
4	4	4	4	2	2	3	3	4	4
4	4	1	1	3	3	3	3	4	4
4	4	1	1	3	3	3	3	4	4
4	4	1	1	3	3	3	3	1	1
4	4	4	4	2	2	3	3	4	4
4	4	1	1	3	3	3	3	4	4

For Review Only

4	4	4	4	2	2	3	3	4	4
4	4	1	1	3	3	3	3	1	1
4	4	1	1	3	3	3	3	4	4
4	4	1	1	3	3	3	3	4	4
4	4	1	1	3	3	3	3	4	4
4	4	1	1	3	3	3	3	1	1
4	4	1	1	3	3	3	3	4	4
4	4	1	1	2	2	3	3	4	4
4	4	1	1	3	3	3	3	1	1
4	4	1	1	3	3	3	3	4	4
2	2	4	4	2	2	3	3	4	4
4	4	1	1	3	3	3	3	1	1
4	4	1	1	3	3	3	3	4	4
4	4	1	1	3	3	3	3	4	4
4	4	1	1	3	3	1	1	1	1
4	4	1	1	3	3	3	3	1	1
4	4	1	1	3	3	1	1	4	4
4	4	1	1	2	2	3	3	4	4
4	4	1	1	3	3	3	3	4	4
2	2	1	1	3	3	3	3	4	4
4	4	1	1	3	3	3	3	4	4
4	4	1	1	3	3	1	1	1	1
4	4	1	1	3	3	3	3	1	1
4	4	4	4	2	2	3	3	4	4
4	4	1	1	3	3	1	1	1	1
4	4	1	1	3	3	1	1	1	1
4	4	1	1	3	3	3	3	4	4
4	4	1	1	3	3	3	3	1	1
4	4	1	1	3	3	3	3	4	4
4	4	1	1	3	3	3	3	4	4
4	4	4	4	2	2	3	3	4	4
4	4	1	1	3	3	3	3	4	4
2	2	1	1	3	3	3	3	4	4
4	4	1	1	3	3	1	1	1	1
4	4	1	1	3	3	3	3	4	4
4	4	4	4	2	2	3	3	4	4
4	4	1	1	3	3	3	3	4	4
4	4	1	1	3	3	3	3	4	4
4	4	4	4	2	2	3	3	4	4
4	4	4	4	2	2	3	3	4	4
4	4	4	4	2	2	3	3	4	4
4	4	4	4	2	2	3	3	4	4
4	4	1	1	3	3	3	3	1	1
4	4	4	4	2	2	3	3	4	4
4	4	1	1	3	3	3	3	4	4
4	4	1	1	3	3	3	3	4	4
4	4	1	1	3	3	3	3	1	1
4	4	1	1	3	3	3	3	4	4
4	4	1	1	3	3	1	1	1	1
4	4	1	1	3	3	3	3	4	4
4	4	4	4	2	2	3	3	4	4
4	4	1	1	3	3	1	1	1	1
2	2	1	1	3	3	3	3	1	1
4	4	4	4	2	2	3	3	4	4
4	4	1	1	3	3	3	3	1	1

For Review Only

4	4	1	1	3	3	1	1	1	1
4	4	1	1	3	3	1	1	1	1
4	4	4	4	2	2	3	3	4	4
4	4	1	1	3	3	3	3	1	1
4	4	1	1	3	3	3	3	1	1
4	4	1	1	3	3	3	3	4	4
4	4	1	1	3	3	3	3	1	1
4	4	1	1	3	3	1	1	1	1
4	4	1	1	3	3	3	3	4	4
4	4	1	1	3	3	1	1	1	1
4	4	1	1	3	3	3	3	4	4
4	4	4	4	2	2	3	3	1	1
4	4	4	4	2	2	3	3	4	4
4	4	4	4	2	2	3	3	4	4
4	4	4	4	2	2	3	3	4	4
2	2	1	1	3	3	3	3	4	4
4	4	1	1	3	3	3	3	4	4
4	4	1	1	3	3	1	1	1	1
4	4	1	1	3	3	3	3	1	1
4	4	1	1	3	3	3	3	1	1
4	4	4	4	2	2	3	3	1	1
4	4	4	4	2	2	3	3	4	4
4	4	1	1	3	3	3	3	4	4
4	4	1	1	3	3	3	3	1	1
4	4	1	1	3	3	3	3	1	1
4	4	1	1	3	3	3	3	4	4
4	4	1	1	3	3	3	3	1	1
4	4	4	4	2	2	3	3	4	4
4	4	1	1	3	3	3	3	4	4
4	4	4	4	2	2	3	3	4	4
4	4	1	1	3	3	3	3	1	1
4	4	4	4	2	2	3	3	4	4
4	4	1	1	3	3	1	1	1	1
4	4	4	4	2	2	3	3	4	4
2	2	1	1	3	3	3	3	1	1
4	4	1	1	3	3	1	1	1	1
4	4	1	1	3	3	3	3	4	4
4	4	4	4	2	2	3	3	4	4
4	4	1	1	3	3	3	3	4	4
4	4	1	1	3	3	3	3	1	1
4	4	1	1	3	3	3	3	4	4
4	4	1	1	3	3	3	3	4	4
4	4	1	1	3	3	3	3	4	4
4	4	1	1	3	3	3	3	4	4
4	4	4	4	2	2	3	3	4	4
4	4	1	1	3	3	1	1	1	1
4	4	1	1	3	3	3	3	1	1
4	4	1	1	3	3	3	3	4	4
4	4	1	1	3	3	3	3	4	4
4	4	1	1	3	3	3	3	1	1
2	2	1	1	3	3	3	3	4	4
4	4	1	1	3	3	1	1	1	1
4	4	4	4	2	2	3	3	4	4
4	4	4	4	2	2	3	3	4	4
4	4	1	1	3	3	3	3	1	1

For Review Only

2	2	1	1	3	3	3	3	4	4
4	4	4	4	2	2	3	3	4	4

For Review Only

SNP907	SNP907	SNP908	SNP908	SNP909	SNP909	SNP910	SNP910	SNP911	SNP911
2	2	4	4	4	4	2	2	2	2
2	2	4	4	4	4	3	3	2	2
2	2	4	4	4	4	2	2	2	2
2	2	4	4	1	1	2	2	2	2
2	2	4	4	1	1	2	2	2	2
2	2	4	4	4	4	2	2	2	2
2	2	4	4	4	4	2	2	2	2
2	2	4	4	4	4	2	2	2	2
2	2	4	4	4	4	3	3	2	2
2	2	4	4	4	4	2	2	2	2
2	2	2	2	1	1	3	3	2	2
2	2	4	4	4	4	2	2	3	3
2	2	4	4	4	4	2	2	2	2
2	2	4	4	1	1	3	3	2	2
2	2	4	4	4	4	2	2	2	2
2	2	4	4	1	1	2	2	2	2
2	2	4	4	4	4	2	2	2	2
2	2	4	4	1	1	2	2	2	2
2	2	4	4	4	4	2	2	2	2
2	2	4	4	1	1	2	2	2	2
2	2	4	4	4	4	3	3	2	2
2	2	4	4	4	4	2	2	2	2
2	2	4	4	1	1	3	3	2	2
2	2	4	4	1	1	3	3	2	2
2	2	4	4	4	4	2	2	2	2
2	2	4	4	1	1	2	2	2	2
2	2	4	4	4	4	2	2	2	2
2	2	4	4	1	1	2	2	2	2
2	2	4	4	4	4	3	3	2	2
2	2	4	4	1	1	3	3	2	2
2	2	4	4	4	4	2	2	2	2
1	1	4	4	4	4	2	2	2	2
1	1	4	4	4	4	2	2	2	2
2	2	4	4	1	1	3	3	2	2
2	2	4	4	1	1	3	3	2	2
2	2	4	4	1	1	2	2	2	2
2	2	4	4	4	4	2	2	2	2
2	2	4	4	1	1	2	2	3	3
2	2	4	4	1	1	3	3	2	2
2	2	4	4	4	4	3	3	2	2
2	2	4	4	1	1	3	3	2	2
2	2	2	2	1	1	3	3	2	2
2	2	4	4	1	1	2	2	2	2
2	2	2	2	1	1	2	2	2	2
2	2	2	2	1	1	2	2	2	2
2	2	4	4	1	1	2	2	2	2
2	2	4	4	4	4	2	2	2	2
2	2	4	4	1	1	2	2	2	2
2	2	4	4	4	4	2	2	3	3
2	2	4	4	4	4	2	2	2	2
2	2	4	4	4	4	2	2	2	2
2	2	4	4	4	4	2	2	2	2

For Review Only

2	2	4	4	4	4	2	2	2	2
1	1	4	4	4	4	2	2	2	2
2	2	4	4	4	4	2	2	2	2
2	2	4	4	4	4	2	2	2	2
2	2	4	4	4	4	2	2	2	2
2	2	4	4	4	4	2	2	2	2
2	2	4	4	1	1	2	2	2	2
2	2	4	4	1	1	3	3	2	2
2	2	4	4	4	4	2	2	2	2
2	2	4	4	1	1	2	2	2	2
2	2	4	4	4	4	2	2	2	2
2	2	4	4	4	4	2	2	2	2
2	2	4	4	4	4	2	2	2	2
2	2	4	4	4	4	3	3	2	2
2	2	4	4	4	4	2	2	2	2
2	2	4	4	4	4	3	3	2	2
2	2	4	4	1	1	3	3	2	2
1	1	4	4	4	4	2	2	3	3
2	2	4	4	4	4	2	2	2	2
2	2	4	4	4	4	2	2	2	2
2	2	4	4	4	4	2	2	2	2
2	2	4	4	1	1	3	3	2	2
2	2	4	4	4	4	3	3	2	2
1	1	4	4	4	4	3	3	2	2
2	2	2	2	1	1	2	2	2	2
2	2	4	4	1	1	2	2	2	2
2	2	4	4	4	4	2	2	2	2
2	2	4	4	1	1	3	3	2	2
2	2	4	4	1	1	2	2	2	2
2	2	4	4	4	4	2	2	2	2
2	2	4	4	1	1	2	2	2	2
2	2	4	4	4	4	2	2	2	2
2	2	4	4	1	1	3	3	2	2
2	2	4	4	4	4	2	2	2	2
2	2	4	4	1	1	2	2	2	2
2	2	2	2	1	1	2	2	2	2
2	2	4	4	1	1	2	2	2	2
2	2	4	4	4	4	2	2	2	2
2	2	2	2	1	1	2	2	2	2
2	2	2	2	1	1	2	2	2	2
2	2	4	4	4	4	2	2	2	2
2	2	4	4	4	4	2	2	2	2
1	1	4	4	4	4	2	2	2	2
2	2	4	4	4	4	2	2	2	2
2	2	4	4	4	4	2	2	2	2
2	2	4	4	4	4	2	2	2	2
2	2	4	4	1	1	3	3	2	2
2	2	4	4	1	1	2	2	2	2
2	2	4	4	1	1	2	2	2	2
2	2	4	4	1	1	2	2	2	2
2	2	4	4	4	4	2	2	2	2
2	2	4	4	1	1	3	3	2	2
2	2	4	4	4	4	2	2	2	2
2	2	4	4	4	4	2	2	2	2
2	2	4	4	1	1	2	2	2	2

For Review Only

2	2	4	4	1	1	2	2	2	2
2	2	4	4	4	4	2	2	2	2
2	2	4	4	1	1	2	2	2	2
2	2	4	4	1	1	3	3	2	2
2	2	4	4	4	4	2	2	2	2
2	2	4	4	4	4	2	2	2	2
2	2	4	4	4	4	2	2	2	2
2	2	2	2	4	4	2	2	2	2
2	2	4	4	1	1	2	2	2	2
2	2	4	4	4	4	2	2	2	2
2	2	4	4	4	4	2	2	2	2
2	2	4	4	1	1	2	2	2	2
1	1	4	4	4	4	2	2	2	2
2	2	4	4	4	4	2	2	2	2
2	2	4	4	4	4	2	2	2	2
2	2	4	4	1	1	2	2	2	2
1	1	4	4	4	4	3	3	2	2
2	2	4	4	1	1	2	2	2	2
2	2	4	4	1	1	3	3	2	2
2	2	2	2	4	4	3	3	2	2
2	2	4	4	1	1	2	2	2	2
2	2	4	4	4	4	2	2	2	2
2	2	4	4	4	4	2	2	2	2
2	2	4	4	1	1	3	3	2	2
2	2	4	4	1	1	3	3	2	2
2	2	4	4	1	1	3	3	2	2
2	2	4	4	4	4	2	2	2	2
2	2	4	4	4	4	2	2	2	2
2	2	4	4	4	4	2	2	2	2
1	1	4	4	4	4	2	2	2	2
2	2	4	4	1	1	3	3	2	2
2	2	4	4	4	4	2	2	2	2
2	2	4	4	4	4	2	2	2	2
2	2	4	4	1	1	2	2	2	2
2	2	4	4	1	1	3	3	2	2
2	2	4	4	1	1	3	3	2	2
2	2	4	4	1	1	3	3	2	2
2	2	4	4	4	4	2	2	2	2
2	2	4	4	1	1	3	3	2	2
2	2	4	4	1	1	3	3	2	2
2	2	4	4	4	4	3	3	2	2
2	2	4	4	1	1	3	3	2	2
2	2	4	4	4	4	2	2	2	2
2	2	4	4	1	1	3	3	2	2
2	2	4	4	1	1	3	3	2	2
2	2	4	4	4	4	2	2	2	2
2	2	4	4	1	1	2	2	2	2
2	2	4	4	4	4	2	2	2	2
2	2	4	4	1	1	2	2	2	2
2	2	4	4	1	1	2	2	2	2
2	2	4	4	1	1	2	2	2	2
2	2	4	4	1	1	2	2	2	2
2	2	4	4	1	1	3	3	2	2
2	2	4	4	4	4	2	2	2	2
2	2	4	4	1	1	2	2	2	2

For Review Only

2	2	4	4	4	4	2	2	2	2
2	2	2	2	1	1	3	3	2	2
2	2	4	4	1	1	2	2	2	2
2	2	4	4	1	1	3	3	2	2
2	2	4	4	4	4	2	2	2	2
2	2	4	4	1	1	3	3	2	2
2	2	4	4	1	1	2	2	2	2
2	2	4	4	1	1	2	2	2	2
2	2	4	4	1	1	3	3	2	2
2	2	4	4	4	4	2	2	2	2
2	2	4	4	1	1	2	2	2	2
2	2	4	4	1	1	3	3	2	2
2	2	4	4	4	4	2	2	2	2
2	2	4	4	1	1	2	2	2	2
2	2	4	4	1	1	3	3	2	2
2	2	4	4	1	1	3	3	2	2
2	2	4	4	1	1	2	2	2	2
2	2	4	4	4	4	2	2	2	2
2	2	4	4	1	1	2	2	2	2
2	2	4	4	4	4	2	2	2	2
2	2	4	4	1	1	2	2	2	2
2	2	4	4	1	1	3	3	2	2
2	2	4	4	1	1	3	3	2	2
2	2	4	4	4	4	2	2	2	2
2	2	4	4	4	4	3	3	2	2
2	2	4	4	1	1	3	3	2	2
2	2	4	4	4	4	2	2	2	2
2	2	4	4	4	4	3	3	2	2
2	2	4	4	1	1	2	2	2	2
2	2	4	4	4	4	2	2	2	2
2	2	4	4	4	4	2	2	2	2
2	2	4	4	4	4	2	2	2	2
2	2	4	4	4	4	2	2	2	2
2	2	4	4	1	1	2	2	2	2
2	2	4	4	1	1	2	2	2	2
2	2	4	4	4	4	2	2	2	2
2	2	4	4	1	1	2	2	2	2
2	2	4	4	4	4	2	2	2	2
2	2	4	4	4	4	2	2	2	2
1	1	4	4	4	4	2	2	2	2
2	2	4	4	4	4	2	2	2	2
2	2	4	4	1	1	2	2	2	2
2	2	4	4	1	1	3	3	2	2
2	2	4	4	1	1	2	2	2	2
2	2	4	4	1	1	2	2	2	2
2	2	4	4	4	4	2	2	2	2
2	2	4	4	1	1	3	3	2	2
2	2	4	4	1	1	2	2	2	2
2	2	4	4	1	1	3	3	2	2
2	2	4	4	1	1	2	2	2	2
1	1	4	4	4	4	2	2	2	2
2	2	4	4	1	1	3	3	2	2
2	2	4	4	1	1	3	3	2	2
2	2	4	4	4	4	2	2	2	2
2	2	4	4	1	1	3	3	2	2

For Review Only

2	2	4	4	1	1	3	3	2	2
2	2	4	4	1	1	3	3	2	2
2	2	4	4	4	4	2	2	2	2
2	2	2	2	1	1	3	3	2	2
2	2	4	4	1	1	3	3	2	2
1	1	4	4	4	4	2	2	2	2
2	2	2	2	1	1	3	3	2	2
2	2	4	4	1	1	3	3	2	2
2	2	4	4	1	1	3	3	2	2
2	2	4	4	1	1	3	3	2	2
2	2	4	4	1	1	2	2	2	2
2	2	2	2	1	1	3	3	2	2
2	2	4	4	1	1	2	2	2	2
2	2	4	4	1	1	2	2	2	2
2	2	4	4	4	4	2	2	2	2
2	2	4	4	1	1	2	2	2	2
2	2	4	4	1	1	2	2	2	2
2	2	4	4	4	4	3	3	2	2
2	2	4	4	1	1	3	3	2	2
2	2	4	4	1	1	3	3	2	2
2	2	4	4	1	1	3	3	2	2
2	2	4	4	1	1	2	2	2	2
2	2	4	4	4	4	2	2	2	2
2	2	4	4	4	4	3	3	2	2
2	2	4	4	1	1	3	3	2	2
2	2	2	2	4	4	2	2	2	2
2	2	4	4	1	1	3	3	2	2
2	2	4	4	4	4	2	2	2	2
2	2	4	4	1	1	2	2	2	2
1	1	4	4	4	4	2	2	2	2
2	2	4	4	1	1	3	3	2	2
2	2	4	4	1	1	2	2	2	2
2	2	4	4	1	1	3	3	2	2
2	2	4	4	1	1	2	2	2	2
2	2	4	4	1	1	3	3	2	2
2	2	4	4	1	1	3	3	2	2
2	2	4	4	4	4	2	2	2	2
2	2	4	4	4	4	2	2	2	2
2	2	4	4	1	1	2	2	2	2
2	2	4	4	1	1	3	3	2	2
2	2	4	4	4	4	2	2	2	2
2	2	4	4	4	4	2	2	3	3
2	2	4	4	1	1	2	2	2	2
2	2	4	4	4	4	2	2	2	2
2	2	4	4	1	1	2	2	2	2
2	2	4	4	4	4	3	3	2	2
2	2	4	4	1	1	3	3	2	2
2	2	4	4	4	4	2	2	2	2
2	2	2	2	4	4	2	2	2	2
2	2	4	4	1	1	3	3	2	2
2	2	4	4	4	4	2	2	2	2
2	2	4	4	1	1	3	3	2	2
2	2	4	4	4	4	2	2	2	2
2	2	2	2	4	4	2	2	2	2
2	2	4	4	1	1	3	3	2	2

For Review Only

2	2	4	4	4	4	2	2	2	2
2	2	4	4	4	4	2	2	2	2

For Review Only

SNP912	SNP912	SNP913	SNP913	SNP914	SNP914	SNP915	SNP915	SNP916	SNP916
2	2	2	2	4	4	2	2	4	4
2	2	2	2	4	4	2	2	4	4
2	2	2	2	4	4	2	2	1	1
2	2	2	2	4	4	2	2	1	1
2	2	2	2	4	4	2	2	4	4
2	2	2	2	4	4	3	3	1	1
2	2	2	2	4	4	2	2	1	1
2	2	2	2	4	4	2	2	4	4
2	2	2	2	4	4	3	3	4	4
2	2	2	2	4	4	2	2	4	4
2	2	2	2	4	4	3	3	1	1
2	2	2	2	4	4	2	2	1	1
2	2	2	2	4	4	2	2	4	4
2	2	2	2	4	4	2	2	4	4
2	2	2	2	4	4	2	2	4	4
2	2	2	2	4	4	3	3	4	4
2	2	2	2	4	4	3	3	1	1
2	2	2	2	4	4	2	2	4	4
2	2	2	2	4	4	2	2	4	4
2	2	2	2	4	4	2	2	4	4
2	2	2	2	4	4	3	3	4	4
2	2	2	2	4	4	3	3	1	1
2	2	2	2	4	4	3	3	1	1
2	2	2	2	1	1	3	3	1	1
2	2	2	2	4	4	3	3	1	1
2	2	2	2	4	4	2	2	1	1
2	2	2	2	4	4	3	3	1	1
2	2	2	2	4	4	3	3	1	1
2	2	2	2	4	4	3	3	4	4
2	2	2	2	4	4	2	2	4	4
2	2	2	2	4	4	2	2	4	4
2	2	2	2	4	4	2	2	1	1
2	2	2	2	4	4	2	2	4	4
2	2	2	2	1	1	2	2	1	1
2	2	2	2	4	4	3	3	1	1
2	2	2	2	1	1	2	2	1	1
2	2	2	2	4	4	3	3	1	1
2	2	2	2	4	4	2	2	4	4
2	2	2	2	4	4	3	3	1	1
2	2	2	2	4	4	3	3	1	1
2	2	2	2	4	4	2	2	4	4
4	4	3	3	4	4	3	3	4	4
2	2	2	2	4	4	2	2	4	4
4	4	3	3	4	4	2	2	1	1
4	4	3	3	4	4	2	2	4	4
2	2	2	2	4	4	3	3	1	1
2	2	2	2	4	4	2	2	1	1
2	2	2	2	4	4	3	3	1	1
2	2	2	2	4	4	2	2	4	4
2	2	2	2	4	4	2	2	4	4
2	2	2	2	4	4	2	2	1	1
2	2	2	2	4	4	2	2	4	4

For Review Only

2	2	2	2	4	4	2	2	4	4
2	2	2	2	4	4	2	2	4	4
2	2	2	2	4	4	3	3	1	1
2	2	2	2	4	4	3	3	1	1
2	2	2	2	4	4	2	2	4	4
2	2	2	2	4	4	2	2	1	1
2	2	2	2	4	4	2	2	1	1
2	2	2	2	4	4	2	2	4	4
2	2	2	2	4	4	2	2	4	4
2	2	2	2	4	4	3	3	4	4
2	2	2	2	4	4	2	2	4	4
2	2	2	2	4	4	2	2	1	1
2	2	2	2	4	4	2	2	4	4
2	2	2	2	4	4	3	3	4	4
2	2	2	2	4	4	3	3	1	1
2	2	2	2	4	4	2	2	1	1
2	2	2	2	4	4	2	2	1	1
2	2	2	2	4	4	2	2	4	4
2	2	2	2	4	4	2	2	4	4
2	2	2	2	4	4	2	2	4	4
2	2	2	2	4	4	2	2	1	1
2	2	2	2	4	4	3	3	1	1
2	2	2	2	4	4	3	3	4	4
2	2	2	2	4	4	2	2	1	1
4	4	3	3	4	4	2	2	4	4
2	2	2	2	4	4	2	2	4	4
2	2	2	2	4	4	2	2	4	4
2	2	2	2	4	4	3	3	1	1
2	2	2	2	4	4	2	2	1	1
2	2	2	2	4	4	2	2	4	4
2	2	2	2	4	4	3	3	1	1
2	2	2	2	1	1	2	2	1	1
2	2	2	2	4	4	3	3	1	1
2	2	2	2	4	4	2	2	4	4
2	2	2	2	4	4	2	2	4	4
4	4	3	3	4	4	2	2	4	4
2	2	2	2	4	4	3	3	1	1
2	2	2	2	4	4	3	3	1	1
4	4	3	3	4	4	2	2	4	4
4	4	3	3	4	4	3	3	1	1
2	2	2	2	4	4	3	3	1	1
2	2	2	2	4	4	2	2	1	1
2	2	2	2	4	4	2	2	4	4
2	2	2	2	4	4	2	2	1	1
2	2	2	2	4	4	3	3	1	1
2	2	2	2	4	4	3	3	1	1
2	2	2	2	1	1	2	2	1	1
2	2	2	2	4	4	2	2	4	4
2	2	2	2	4	4	2	2	1	1
2	2	2	2	4	4	3	3	4	4
2	2	2	2	4	4	2	2	4	4
2	2	2	2	4	4	3	3	1	1
2	2	2	2	4	4	3	3	1	1
2	2	2	2	4	4	2	2	4	4
2	2	2	2	4	4	2	2	4	4

For Review Only

2	2	2	2	4	4	3	3	1	1
2	2	2	2	4	4	2	2	1	1
2	2	2	2	4	4	2	2	1	1
2	2	2	2	4	4	2	2	4	4
2	2	2	2	4	4	2	2	4	4
2	2	2	2	4	4	2	2	1	1
2	2	2	2	4	4	3	3	1	1
2	2	2	2	4	4	2	2	1	1
2	2	2	2	4	4	2	2	4	4
2	2	2	2	4	4	2	2	4	4
2	2	2	2	4	4	2	2	4	4
2	2	2	2	4	4	3	3	4	4
4	4	3	3	4	4	2	2	4	4
2	2	2	2	4	4	2	2	1	1
2	2	2	2	4	4	2	2	1	1
2	2	2	2	4	4	2	2	1	1
2	2	2	2	4	4	2	2	4	4
2	2	2	2	4	4	2	2	4	4
2	2	2	2	4	4	2	2	1	1
4	4	3	3	4	4	2	2	4	4
2	2	2	2	4	4	3	3	1	1
2	2	2	2	4	4	2	2	4	4
2	2	2	2	4	4	2	2	1	1
2	2	2	2	4	4	3	3	1	1
2	2	2	2	4	4	3	3	1	1
2	2	2	2	4	4	2	2	4	4
2	2	2	2	4	4	3	3	1	1
2	2	2	2	4	4	2	2	1	1
2	2	2	2	4	4	2	2	4	4
2	2	2	2	4	4	3	3	4	4
2	2	2	2	4	4	3	3	1	1
2	2	2	2	4	4	3	3	4	4
2	2	2	2	4	4	3	3	1	1
2	2	2	2	4	4	2	2	4	4
2	2	2	2	1	1	2	2	1	1
2	2	2	2	4	4	3	3	4	4
2	2	2	2	4	4	2	2	4	4
2	2	2	2	4	4	2	2	1	1
2	2	2	2	4	4	3	3	1	1
2	2	2	2	4	4	3	3	1	1
2	2	2	2	4	4	3	3	4	4
2	2	2	2	1	1	3	3	1	1
2	2	2	2	4	4	2	2	4	4
2	2	2	2	4	4	3	3	4	4
2	2	2	2	4	4	3	3	1	1
2	2	2	2	4	4	2	2	4	4
2	2	2	2	4	4	2	2	1	1
2	2	2	2	4	4	2	2	1	1
2	2	2	2	4	4	2	2	1	1
2	2	2	2	4	4	2	2	4	4
2	2	2	2	4	4	2	2	1	1
2	2	2	2	1	1	2	2	4	4
2	2	2	2	4	4	2	2	1	1
2	2	2	2	4	4	2	2	1	1
2	2	2	2	4	4	2	2	4	4

For Review Only

2	2	2	2	4	4	2	2	1	1
4	4	3	3	4	4	2	2	4	4
2	2	2	2	4	4	3	3	4	4
2	2	2	2	4	4	3	3	1	1
2	2	2	2	4	4	2	2	4	4
2	2	2	2	4	4	2	2	4	4
2	2	2	2	4	4	3	3	1	1
2	2	2	2	4	4	2	2	1	1
2	2	2	2	4	4	3	3	1	1
2	2	2	2	4	4	3	3	1	1
2	2	2	2	4	4	2	2	1	1
2	2	2	2	4	4	3	3	1	1
2	2	2	2	4	4	3	3	4	4
2	2	2	2	4	4	2	2	4	4
2	2	2	2	4	4	2	2	4	4
2	2	2	2	4	4	3	3	1	1
2	2	2	2	4	4	3	3	4	4
2	2	2	2	4	4	2	2	4	4
2	2	2	2	4	4	2	2	1	1
2	2	2	2	4	4	3	3	4	4
2	2	2	2	4	4	3	3	1	1
2	2	2	2	1	1	3	3	1	1
2	2	2	2	4	4	3	3	1	1
2	2	2	2	4	4	2	2	1	1
2	2	2	2	4	4	2	2	4	4
2	2	2	2	1	1	2	2	1	1
2	2	2	2	4	4	2	2	4	4
2	2	2	2	4	4	2	2	4	4
2	2	2	2	4	4	2	2	4	4
2	2	2	2	4	4	2	2	4	4
2	2	2	2	4	4	2	2	1	1
2	2	2	2	4	4	2	2	1	1
2	2	2	2	4	4	3	3	1	1
2	2	2	2	4	4	2	2	1	1
2	2	2	2	4	4	3	3	1	1
2	2	2	2	4	4	2	2	1	1
2	2	2	2	4	4	2	2	1	1
2	2	2	2	4	4	2	2	4	4
2	2	2	2	4	4	2	2	1	1
2	2	2	2	4	4	2	2	1	1
2	2	2	2	4	4	2	2	1	1
2	2	2	2	4	4	2	2	1	1
2	2	2	2	4	4	3	3	1	1
2	2	2	2	4	4	3	3	1	1
2	2	2	2	4	4	3	3	4	4
2	2	2	2	4	4	2	2	4	4
2	2	2	2	4	4	3	3	1	1
2	2	2	2	4	4	3	3	1	1
2	2	2	2	1	1	3	3	1	1
2	2	2	2	4	4	3	3	4	4
2	2	2	2	4	4	2	2	4	4
2	2	2	2	4	4	2	2	4	4
2	2	2	2	4	4	3	3	1	1
2	2	2	2	4	4	2	2	4	4
2	2	2	2	4	4	3	3	1	1

For Review Only

2	2	2	2	4	4	3	3	1	1
2	2	2	2	4	4	2	2	4	4
2	2	2	2	4	4	2	2	1	1
2	2	2	2	4	4	3	3	4	4
2	2	2	2	4	4	2	2	4	4
2	2	2	2	4	4	2	2	1	1
4	4	3	3	4	4	3	3	1	1
2	2	2	2	4	4	2	2	4	4
2	2	2	2	4	4	3	3	1	1
2	2	2	2	4	4	3	3	1	1
2	2	2	2	4	4	3	3	4	4
4	4	2	2	4	4	3	3	1	1
2	2	2	2	4	4	3	3	1	1
2	2	2	2	4	4	2	2	1	1
2	2	2	2	4	4	2	2	1	1
2	2	2	2	4	4	3	3	4	4
2	2	2	2	4	4	3	3	1	1
2	2	2	2	1	1	3	3	1	1
2	2	2	2	4	4	3	3	1	1
2	2	2	2	4	4	2	2	4	4
2	2	2	2	4	4	3	3	1	1
2	2	2	2	4	4	2	2	1	1
2	2	2	2	4	4	2	2	4	4
2	2	2	2	4	4	3	3	1	1
2	2	2	2	4	4	2	2	4	4
2	2	2	2	4	4	3	3	1	1
2	2	2	2	4	4	3	3	1	1
2	2	2	2	4	4	2	2	4	4
2	2	2	2	4	4	2	2	4	4
2	2	2	2	4	4	2	2	4	4
2	2	2	2	4	4	3	3	1	1
2	2	2	2	4	4	3	3	1	1
2	2	2	2	4	4	2	2	4	4
2	2	2	2	4	4	2	2	1	1
2	2	2	2	4	4	3	3	1	1
2	2	2	2	4	4	3	3	1	1
2	2	2	2	4	4	2	2	4	4
2	2	2	2	4	4	2	2	1	1
2	2	2	2	1	1	2	2	1	1
2	2	2	2	4	4	3	3	1	1
2	2	2	2	4	4	2	2	4	4
2	2	2	2	4	4	2	2	1	1
2	2	2	2	4	4	3	3	1	1
2	2	2	2	4	4	3	3	1	1
2	2	2	2	4	4	2	2	4	4
2	2	2	2	4	4	3	3	1	1
2	2	2	2	4	4	3	3	1	1
2	2	2	2	4	4	2	2	4	4
4	4	2	2	4	4	3	3	1	1
2	2	2	2	4	4	3	3	1	1
2	2	2	2	4	4	3	3	1	1
2	2	2	2	4	4	3	3	1	1
2	2	2	2	4	4	2	2	1	1
4	4	3	3	4	4	2	2	4	4
2	2	2	2	4	4	3	3	1	1

For Review Only

2	2	2	2	4	4	3	3	1	1
2	2	2	2	4	4	2	2	1	1

For Review Only

SNP917	SNP917	SNP918	SNP918	SNP919	SNP919	SNP920	SNP920	SNP921	SNP921
2	2	2	2	3	3	2	2	4	4
3	3	1	1	3	3	1	1	1	1
2	2	2	2	2	2	1	1	4	4
2	2	1	1	3	3	1	1	4	4
2	2	2	2	2	2	2	2	4	4
2	2	2	2	2	2	1	1	4	4
2	2	2	2	2	2	2	2	4	4
2	2	2	2	2	2	2	2	4	4
3	3	1	1	3	3	2	2	4	4
2	2	2	2	2	2	1	1	4	4
3	3	1	1	3	3	2	2	1	1
2	2	2	2	2	2	1	1	4	4
2	2	1	1	3	3	1	1	4	4
3	3	1	1	3	3	2	2	1	1
2	2	2	2	3	3	1	1	4	4
2	2	2	2	2	2	1	1	4	4
2	2	2	2	2	2	2	2	4	4
3	3	1	1	3	3	1	1	1	1
2	2	2	2	2	2	1	1	4	4
2	2	2	2	2	2	1	1	4	4
3	3	1	1	3	3	2	2	1	1
2	2	2	2	2	2	2	2	4	4
3	3	1	1	3	3	2	2	1	1
3	3	1	1	3	3	2	2	1	1
2	2	2	2	2	2	1	1	4	4
2	2	2	2	2	2	2	2	4	4
2	2	2	2	3	3	1	1	1	1
2	2	1	1	3	3	1	1	4	4
3	3	1	1	3	3	2	2	1	1
3	3	1	1	3	3	2	2	1	1
2	2	2	2	2	2	2	2	4	4
2	2	2	2	2	2	2	2	4	4
2	2	2	2	2	2	2	2	4	4
3	3	1	1	3	3	1	1	1	1
3	3	1	1	3	3	1	1	4	4
2	2	2	2	2	2	2	2	4	4
2	2	2	2	2	2	1	1	4	4
2	2	2	2	3	3	1	1	4	4
3	3	1	1	3	3	2	2	1	1
3	3	1	1	3	3	1	1	1	1
3	3	1	1	3	3	2	2	1	1
3	3	1	1	3	3	2	2	1	1
2	2	2	2	2	2	2	2	4	4
3	3	1	1	3	3	2	2	1	1
2	2	2	2	2	2	1	1	4	4
2	2	2	2	2	2	1	1	4	4
2	2	2	2	2	2	1	1	4	4
3	3	1	1	3	3	1	1	1	1
3	3	1	1	3	3	2	2	1	1
2	2	2	2	2	2	1	1	4	4
2	2	2	2	2	2	2	2	4	4
2	2	1	1	3	3	1	1	4	4

For Review Only

2	2	2	2	2	2	2	2	4	4
2	2	2	2	2	2	2	2	4	4
3	3	1	1	2	2	1	1	4	4
2	2	2	2	2	2	2	2	4	4
2	2	2	2	2	2	1	1	4	4
2	2	2	2	2	2	1	1	4	4
2	2	2	2	2	2	1	1	4	4
3	3	1	1	3	3	2	2	1	1
2	2	2	2	2	2	1	1	4	4
2	2	1	1	3	3	1	1	4	4
2	2	2	2	2	2	2	2	4	4
2	2	2	2	2	2	2	2	4	4
2	2	2	2	2	2	2	2	4	4
3	3	1	1	3	3	2	2	1	1
2	2	1	1	3	3	1	1	4	4
2	2	1	1	3	3	2	2	1	1
2	2	1	1	3	3	1	1	1	1
2	2	1	1	3	3	1	1	4	4
2	2	2	2	2	2	2	2	4	4
2	2	2	2	2	2	1	1	4	4
2	2	2	2	3	3	1	1	4	4
3	3	1	1	3	3	1	1	1	1
3	3	1	1	3	3	2	2	1	1
3	3	1	1	3	3	1	1	1	1
2	2	2	2	2	2	1	1	4	4
2	2	2	2	2	2	1	1	4	4
2	2	2	2	2	2	2	2	4	4
3	3	1	1	3	3	2	2	1	1
2	2	2	2	2	2	2	2	4	4
3	3	1	1	3	3	1	1	1	1
2	2	2	2	2	2	2	2	4	4
2	2	2	2	2	2	1	1	4	4
3	3	1	1	3	3	2	2	1	1
2	2	2	2	3	3	2	2	4	4
2	2	2	2	2	2	1	1	4	4
2	2	2	2	2	2	1	1	4	4
2	2	2	2	2	2	2	2	4	4
2	2	2	2	2	2	2	2	4	4
2	2	1	1	3	3	2	2	1	1
2	2	1	1	3	3	1	1	4	4
2	2	2	2	2	2	2	2	4	4
2	2	2	2	2	2	1	1	4	4
2	2	2	2	2	2	2	2	4	4
2	2	2	2	2	2	2	2	4	4
2	2	2	2	2	2	2	2	4	4
2	2	2	2	2	2	1	1	4	4
3	3	1	1	3	3	1	1	1	1
2	2	2	2	2	2	1	1	4	4
2	2	2	2	2	2	1	1	4	4
2	2	2	2	2	2	2	2	4	4
2	2	2	2	2	2	1	1	4	4
3	3	1	1	3	3	2	2	1	1
2	2	2	2	2	2	2	2	4	4
3	3	1	1	3	3	1	1	4	4
2	2	2	2	2	2	2	2	4	4

For Review Only

2	2	1	1	3	3	1	1	4	4
2	2	1	1	2	2	1	1	4	4
2	2	2	2	2	2	2	2	4	4
3	3	1	1	3	3	1	1	1	1
2	2	2	2	2	2	1	1	4	4
2	2	2	2	2	2	2	2	4	4
2	2	2	2	2	2	1	1	4	4
2	2	2	2	2	2	1	1	4	4
2	2	2	2	2	2	1	1	1	1
2	2	1	1	3	3	2	2	4	4
2	2	2	2	3	3	2	2	1	1
2	2	1	1	3	3	1	1	4	4
2	2	2	2	2	2	2	2	4	4
2	2	2	2	3	3	1	1	4	4
2	2	2	2	2	2	1	1	4	4
2	2	1	1	3	3	1	1	4	4
3	3	1	1	3	3	1	1	1	1
2	2	2	2	2	2	2	2	4	4
3	3	1	1	3	3	1	1	1	1
2	2	1	1	3	3	2	2	1	1
2	2	1	1	3	3	1	1	4	4
2	2	1	1	3	3	2	2	4	4
2	2	2	2	2	2	2	2	4	4
3	3	1	1	3	3	2	2	1	1
2	2	1	1	3	3	1	1	1	1
3	3	1	1	3	3	2	2	1	1
2	2	2	2	2	2	1	1	4	4
2	2	2	2	2	2	2	2	4	4
2	2	2	2	2	2	1	1	4	4
2	2	2	2	2	2	1	1	4	4
3	3	1	1	3	3	1	1	1	1
2	2	1	1	3	3	1	1	4	4
2	2	2	2	2	2	2	2	4	4
2	2	1	1	3	3	1	1	4	4
3	3	1	1	3	3	1	1	1	1
2	2	1	1	3	3	2	2	4	4
3	3	1	1	3	3	1	1	1	1
2	2	2	2	2	2	1	1	4	4
2	2	1	1	3	3	1	1	1	1
3	3	1	1	3	3	2	2	1	1
3	3	1	1	3	3	2	2	1	1
3	3	1	1	3	3	1	1	1	1
2	2	1	1	3	3	2	2	4	4
2	2	1	1	3	3	2	2	1	1
3	3	1	1	3	3	2	2	1	1
2	2	2	2	2	2	2	2	4	4
2	2	2	2	2	2	1	1	4	4
2	2	2	2	2	2	1	1	4	4
2	2	1	1	3	3	2	2	4	4
2	2	1	1	3	3	2	2	4	4
2	2	2	2	2	2	1	1	4	4
3	3	1	1	3	3	1	1	1	1
2	2	1	1	3	3	2	2	1	1
2	2	2	2	2	2	2	2	4	4
2	2	1	1	3	3	2	2	4	4

For Review Only

2	2	2	2	2	2	1	1	4	4
3	3	1	1	3	3	2	2	1	1
2	2	2	2	2	2	1	1	4	4
2	2	1	1	3	3	2	2	1	1
2	2	2	2	2	2	1	1	4	4
2	2	2	2	2	2	1	1	4	4
2	2	1	1	3	3	2	2	4	4
2	2	1	1	3	3	1	1	4	4
3	3	1	1	3	3	1	1	1	1
2	2	2	2	2	2	2	2	4	4
2	2	2	2	3	3	1	1	4	4
3	3	1	1	3	3	1	1	1	1
2	2	2	2	3	3	1	1	4	4
2	2	1	1	3	3	2	2	4	4
3	3	1	1	3	3	1	1	1	1
3	3	1	1	3	3	2	2	1	1
2	2	1	1	3	3	2	2	4	4
2	2	2	2	2	2	1	1	4	4
2	2	2	2	2	2	1	1	4	4
2	2	2	2	2	2	1	1	4	4
2	2	1	1	3	3	2	2	4	4
3	3	1	1	3	3	2	2	1	1
3	3	1	1	3	3	2	2	1	1
2	2	2	2	2	2	2	2	4	4
3	3	1	1	3	3	2	2	1	1
3	3	1	1	3	3	1	1	1	1
2	2	2	2	2	2	1	1	4	4
3	3	1	1	3	3	1	1	1	1
2	2	2	2	2	2	1	1	4	4
2	2	1	1	3	3	2	2	4	4
2	2	2	2	2	2	2	2	4	4
2	2	2	2	2	2	1	1	4	4
3	3	1	1	3	3	1	1	1	1
3	3	1	1	3	3	1	1	1	1
2	2	1	1	3	3	1	1	1	1
2	2	2	2	2	2	2	2	4	4
2	2	2	2	2	2	1	1	4	4
2	2	2	2	2	2	1	1	4	4
2	2	2	2	2	2	1	1	4	4
2	2	2	2	2	2	2	2	4	4
2	2	2	2	2	2	1	1	4	4
2	2	2	2	2	2	1	1	4	4
3	3	1	1	3	3	2	2	1	1
2	2	1	1	3	3	1	1	4	4
2	2	2	2	2	2	1	1	4	4
2	2	2	2	2	2	1	1	4	4
2	2	1	1	3	3	2	2	1	1
2	2	2	2	2	2	2	2	4	4
3	3	1	1	3	3	1	1	1	1
2	2	1	1	3	3	2	2	4	4
2	2	2	2	2	2	1	1	4	4
3	3	1	1	3	3	2	2	1	1
3	3	1	1	3	3	2	2	1	1
2	2	2	2	2	2	2	2	4	4
3	3	1	1	3	3	2	2	1	1

For Review Only

3	3	1	1	3	3	2	2	1	1
3	3	1	1	3	3	1	1	4	4
2	2	2	2	2	2	1	1	4	4
3	3	1	1	3	3	2	2	1	1
3	3	1	1	3	3	1	1	1	1
2	2	2	2	3	3	2	2	1	1
3	3	1	1	3	3	1	1	1	1
3	3	1	1	3	3	1	1	1	1
2	2	1	1	3	3	1	1	4	4
3	3	1	1	3	3	2	2	1	1
2	2	1	1	3	3	1	1	4	4
3	3	1	1	3	3	2	2	1	1
2	2	1	1	3	3	2	2	1	1
2	2	2	2	2	2	2	2	4	4
2	2	2	2	2	2	1	1	4	4
2	2	2	2	2	2	2	2	4	4
2	2	2	2	2	2	1	1	4	4
3	3	1	1	3	3	2	2	1	1
3	3	1	1	3	3	2	2	1	1
3	3	1	1	3	3	1	1	1	1
3	3	1	1	3	3	1	1	1	1
2	2	2	2	2	2	2	2	4	4
3	3	1	1	3	3	2	2	1	1
3	3	1	1	3	3	2	2	1	1
3	3	1	1	3	3	2	2	1	1
2	2	2	2	3	3	2	2	4	4
3	3	1	1	3	3	2	2	1	1
2	2	2	2	2	2	1	1	4	4
2	2	1	1	3	3	1	1	4	4
2	2	2	2	2	2	1	1	4	4
3	3	1	1	3	3	2	2	1	1
2	2	2	2	2	2	1	1	4	4
3	3	1	1	3	3	1	1	1	1
2	2	2	2	2	2	2	2	4	4
3	3	1	1	3	3	2	2	1	1
3	3	1	1	3	3	2	2	1	1
2	2	2	2	2	2	2	2	4	4
2	2	2	2	2	2	2	2	4	4
2	2	2	2	2	2	2	2	4	4
3	3	1	1	3	3	1	1	1	1
2	2	1	1	3	3	1	1	1	1
2	2	2	2	2	2	1	1	4	4
2	2	2	2	2	2	2	2	4	4
2	2	2	2	2	2	2	2	4	4
2	2	2	2	2	2	2	2	4	4
3	3	1	1	3	3	1	1	1	1
2	2	1	1	3	3	1	1	1	1
2	2	2	2	2	2	2	2	4	4
2	2	2	2	2	2	1	1	4	4
3	3	1	1	3	3	2	2	1	1
2	2	2	2	3	3	2	2	4	4
3	3	1	1	3	3	1	1	1	1
2	2	2	2	2	2	1	1	4	4
2	2	2	2	2	2	1	1	4	4
3	3	1	1	3	3	1	1	1	1

For Review Only

2	2	2	2	2	2	2	2	4	4
2	2	2	2	2	2	1	1	4	4

For Review Only

SNP922	SNP922	SNP923	SNP923	SNP924	SNP924	SNP925	SNP925	SNP926	SNP926
3	3	4	4	3	3	2	2	4	4
3	3	4	4	3	3	2	2	4	4
3	3	4	4	3	3	2	2	1	1
3	3	4	4	3	3	2	2	4	4
3	3	4	4	3	3	2	2	4	4
3	3	4	4	3	3	2	2	4	4
3	3	4	4	3	3	2	2	4	4
3	3	4	4	3	3	2	2	4	4
3	3	4	4	3	3	2	2	4	4
3	3	4	4	3	3	2	2	4	4
3	3	4	4	3	3	2	2	4	4
3	3	4	4	3	3	2	2	1	1
3	3	4	4	3	3	2	2	4	4
3	3	4	4	3	3	2	2	4	4
3	3	4	4	3	3	2	2	4	4
3	3	4	4	3	3	2	2	4	4
3	3	4	4	3	3	2	2	4	4
3	3	4	4	3	3	2	2	4	4
3	3	4	4	3	3	2	2	1	1
3	3	4	4	3	3	2	2	1	1
3	3	4	4	3	3	2	2	4	4
3	3	4	4	3	3	2	2	4	4
3	3	4	4	3	3	2	2	4	4
3	3	4	4	3	3	2	2	4	4
3	3	4	4	3	3	2	2	4	4
3	3	4	4	3	3	2	2	1	1
3	3	4	4	3	3	2	2	4	4
3	3	4	4	3	3	2	2	4	4
3	3	4	4	3	3	2	2	4	4
3	3	4	4	3	3	2	2	4	4
3	3	4	4	3	3	2	2	4	4
3	3	4	4	1	1	2	2	4	4
3	3	4	4	1	1	2	2	4	4
3	3	4	4	3	3	2	2	4	4
3	3	4	4	3	3	2	2	4	4
3	3	4	4	3	3	2	2	4	4
3	3	4	4	3	3	2	2	4	4
3	3	4	4	3	3	2	2	4	4
3	3	4	4	3	3	2	2	4	4
3	3	4	4	3	3	3	3	4	4
3	3	4	4	3	3	2	2	4	4
3	3	1	1	3	3	2	2	4	4
3	3	4	4	3	3	2	2	4	4
3	3	1	1	1	1	2	2	4	4
3	3	1	1	3	3	2	2	4	4
3	3	4	4	3	3	2	2	1	1
3	3	4	4	3	3	2	2	1	1
3	3	4	4	3	3	2	2	4	4
3	3	4	4	3	3	2	2	4	4
3	3	4	4	3	3	2	2	4	4
3	3	4	4	3	3	2	2	1	1
2	2	4	4	3	3	2	2	4	4

For Review Only

3	3	4	4	3	3	2	2	4	4
3	3	4	4	1	1	2	2	4	4
3	3	4	4	3	3	2	2	4	4
3	3	4	4	3	3	2	2	4	4
3	3	4	4	3	3	2	2	1	1
3	3	4	4	3	3	2	2	1	1
3	3	4	4	3	3	2	2	1	1
3	3	4	4	3	3	2	2	4	4
3	3	4	4	3	3	2	2	4	4
3	3	4	4	3	3	2	2	4	4
2	2	4	4	3	3	2	2	1	1
3	3	4	4	3	3	2	2	1	1
3	3	4	4	3	3	2	2	4	4
3	3	4	4	3	3	2	2	4	4
3	3	4	4	3	3	2	2	4	4
3	3	4	4	3	3	2	2	4	4
3	3	4	4	3	3	2	2	1	1
3	3	4	4	3	3	2	2	4	4
3	3	4	4	3	3	2	2	4	4
3	3	4	4	3	3	2	2	4	4
3	3	4	4	3	3	2	2	4	4
3	3	4	4	3	3	2	2	4	4
3	3	4	4	3	3	2	2	4	4
3	3	4	4	3	3	2	2	1	1
3	3	1	1	3	3	2	2	4	4
2	2	4	4	3	3	2	2	1	1
3	3	4	4	3	3	2	2	1	1
3	3	4	4	3	3	2	2	4	4
3	3	4	4	3	3	2	2	1	1
3	3	4	4	3	3	2	2	4	4
3	3	4	4	3	3	2	2	4	4
3	3	4	4	3	3	2	2	4	4
3	3	4	4	3	3	3	3	4	4
3	3	4	4	3	3	2	2	4	4
3	3	4	4	3	3	2	2	4	4
3	3	1	1	3	3	2	2	4	4
3	3	4	4	3	3	2	2	4	4
3	3	4	4	3	3	2	2	4	4
3	3	1	1	3	3	2	2	1	1
3	3	1	1	3	3	2	2	4	4
3	3	4	4	3	3	2	2	4	4
3	3	4	4	3	3	2	2	4	4
3	3	4	4	1	1	2	2	4	4
2	2	4	4	3	3	2	2	1	1
3	3	4	4	3	3	2	2	4	4
2	2	4	4	3	3	2	2	4	4
3	3	4	4	3	3	3	3	4	4
3	3	4	4	3	3	2	2	1	1
3	3	4	4	3	3	2	2	4	4
3	3	4	4	3	3	2	2	4	4
3	3	4	4	3	3	2	2	4	4
3	3	4	4	3	3	2	2	4	4
3	3	4	4	3	3	2	2	4	4
3	3	4	4	3	3	2	2	1	1
3	3	4	4	3	3	2	2	4	4

For Review Only

3	3	4	4	3	3	3	3	4	4
3	3	4	4	3	3	2	2	4	4
3	3	4	4	3	3	2	2	4	4
3	3	4	4	3	3	3	3	4	4
3	3	4	4	3	3	2	2	1	1
3	3	4	4	3	3	2	2	1	1
3	3	4	4	3	3	2	2	4	4
3	3	4	4	3	3	2	2	4	4
3	3	4	4	1	1	2	2	4	4
3	3	4	4	3	3	2	2	1	1
3	3	4	4	3	3	2	2	4	4
3	3	4	4	3	3	2	2	4	4
3	3	1	1	1	1	2	2	4	4
3	3	4	4	3	3	2	2	1	1
3	3	4	4	3	3	2	2	4	4
3	3	4	4	3	3	2	2	4	4
3	3	4	4	3	3	2	2	4	4
3	3	4	4	3	3	2	2	1	1
3	3	4	4	3	3	3	3	4	4
3	3	1	1	1	1	2	2	4	4
3	3	4	4	3	3	2	2	4	4
3	3	4	4	3	3	2	2	4	4
3	3	4	4	3	3	2	2	4	4
3	3	4	4	3	3	2	2	4	4
3	3	4	4	3	3	2	2	4	4
3	3	4	4	3	3	2	2	4	4
3	3	4	4	3	3	2	2	4	4
3	3	4	4	3	3	2	2	4	4
2	2	4	4	3	3	2	2	1	1
3	3	4	4	1	1	2	2	4	4
3	3	4	4	3	3	2	2	4	4
3	3	4	4	3	3	2	2	4	4
3	3	4	4	3	3	2	2	4	4
3	3	4	4	3	3	2	2	4	4
3	3	4	4	3	3	2	2	4	4
3	3	4	4	3	3	2	2	4	4
3	3	4	4	3	3	3	3	4	4
3	3	4	4	3	3	2	2	4	4
3	3	4	4	3	3	2	2	4	4
3	3	4	4	3	3	2	2	4	4
3	3	4	4	3	3	2	2	4	4
3	3	4	4	3	3	2	2	4	4
3	3	4	4	3	3	2	2	4	4
3	3	4	4	3	3	2	2	4	4
3	3	1	1	3	3	3	3	4	4
3	3	4	4	3	3	2	2	4	4
3	3	4	4	3	3	2	2	1	1
3	3	4	4	3	3	2	2	1	1
3	3	4	4	3	3	2	2	4	4
3	3	4	4	3	3	2	2	1	1
3	3	4	4	3	3	2	2	4	4
3	3	4	4	3	3	2	2	4	4
3	3	4	4	3	3	2	2	4	4
3	3	4	4	3	3	2	2	1	1
3	3	4	4	3	3	2	2	4	4

For Review Only

3	3	4	4	3	3	2	2	1	1
3	3	1	1	3	3	2	2	4	4
3	3	4	4	3	3	2	2	4	4
3	3	4	4	3	3	2	2	4	4
2	2	4	4	3	3	2	2	4	4
3	3	4	4	3	3	2	2	4	4
3	3	4	4	3	3	2	2	4	4
3	3	4	4	3	3	2	2	4	4
3	3	4	4	3	3	2	2	4	4
3	3	4	4	3	3	2	2	4	4
3	3	4	4	3	3	2	2	4	4
3	3	4	4	3	3	2	2	4	4
3	3	4	4	3	3	2	2	4	4
3	3	4	4	3	3	2	2	4	4
3	3	4	4	3	3	2	2	4	4
3	3	4	4	3	3	2	2	4	4
3	3	4	4	3	3	2	2	4	4
2	2	4	4	3	3	2	2	4	4
3	3	4	4	3	3	2	2	4	4
3	3	4	4	3	3	2	2	4	4
3	3	4	4	3	3	2	2	4	4
3	3	4	4	3	3	2	2	4	4
3	3	4	4	3	3	3	3	4	4
3	3	4	4	3	3	2	2	1	1
3	3	4	4	3	3	2	2	4	4
3	3	4	4	3	3	2	2	4	4
3	3	4	4	3	3	2	2	4	4
3	3	4	4	3	3	2	2	4	4
3	3	4	4	3	3	2	2	4	4
3	3	4	4	3	3	2	2	4	4
3	3	4	4	3	3	2	2	1	1
3	3	4	4	3	3	2	2	4	4
3	3	4	4	3	3	2	2	4	4
3	3	4	4	3	3	2	2	4	4
3	3	4	4	3	3	2	2	4	4
3	3	4	4	3	3	2	2	1	1
3	3	4	4	3	3	2	2	4	4
3	3	4	4	3	3	2	2	4	4
3	3	4	4	3	3	2	2	1	1
3	3	4	4	3	3	2	2	1	1
3	3	4	4	3	3	2	2	1	1
3	3	4	4	3	3	2	2	1	1
3	3	4	4	3	3	2	2	4	4
3	3	4	4	3	3	2	2	4	4
3	3	4	4	3	3	2	2	4	4
3	3	4	4	3	3	2	2	4	4
3	3	4	4	3	3	2	2	4	4
3	3	4	4	3	3	2	2	4	4
3	3	4	4	3	3	2	2	4	4
3	3	4	4	3	3	2	2	4	4
3	3	4	4	3	3	2	2	1	1
3	3	4	4	3	3	2	2	4	4
3	3	4	4	3	3	2	2	4	4
2	2	4	4	3	3	2	2	1	1
3	3	4	4	3	3	2	2	4	4

For Review Only

For Review Only

3	3	4	4	3	3	2	2	4	4
3	3	4	4	3	3	2	2	4	4
3	3	4	4	3	3	2	2	1	1
3	3	4	4	1	1	3	3	4	4
3	3	4	4	3	3	2	2	4	4
3	3	4	4	3	3	2	2	4	4
3	3	1	1	3	3	2	2	4	4
3	3	4	4	3	3	2	2	4	4
3	3	4	4	3	3	2	2	4	4
3	3	4	4	3	3	2	2	4	4
3	3	4	4	3	3	2	2	4	4
3	3	1	1	3	3	2	2	1	1
3	3	4	4	3	3	2	2	1	1
3	3	4	4	3	3	2	2	1	1
3	3	4	4	3	3	2	2	1	1
3	3	4	4	3	3	2	2	4	4
3	3	4	4	3	3	2	2	4	4
3	3	4	4	3	3	2	2	4	4
3	3	4	4	3	3	2	2	4	4
3	3	4	4	3	3	2	2	4	4
3	3	4	4	3	3	2	2	1	1
3	3	4	4	3	3	2	2	1	1
3	3	4	4	3	3	2	2	4	4
3	3	4	4	3	3	2	2	4	4
3	3	4	4	3	3	2	2	4	4
3	3	4	4	3	3	2	2	4	4
3	3	4	4	3	3	2	2	4	4
3	3	4	4	3	3	2	2	1	1
3	3	4	4	3	3	2	2	4	4
3	3	4	4	3	3	2	2	1	1
3	3	4	4	3	3	2	2	4	4
3	3	4	4	3	3	2	2	1	1
3	3	4	4	3	3	2	2	4	4
3	3	4	4	3	3	2	2	1	1
3	3	4	4	3	3	2	2	4	4
3	3	4	4	3	3	2	2	4	4
3	3	4	4	3	3	2	2	4	4
3	3	4	4	3	3	2	2	1	1
3	3	4	4	3	3	2	2	4	4
3	3	4	4	3	3	2	2	4	4
3	3	4	4	3	3	2	2	4	4
3	3	4	4	3	3	2	2	4	4
3	3	4	4	3	3	2	2	4	4
3	3	4	4	3	3	2	2	4	4
3	3	4	4	3	3	2	2	1	1
3	3	4	4	3	3	2	2	4	4
3	3	4	4	3	3	2	2	4	4
3	3	4	4	1	1	2	2	4	4
3	3	4	4	3	3	2	2	4	4
3	3	4	4	3	3	3	3	4	4
3	3	4	4	1	1	2	2	4	4
3	3	4	4	3	3	2	2	4	4
3	3	4	4	3	3	2	2	1	1
3	3	1	1	3	3	2	2	1	1
3	3	4	4	3	3	2	2	4	4

3	3	4	4	3	3	2	2	4	4
3	3	4	4	3	3	2	2	1	1

For Review Only

SNP927	SNP927	SNP928	SNP928	SNP929	SNP929	SNP930	SNP930	SNP931	SNP931
4	4	4	4	3	3	3	3	2	2
4	4	4	4	3	3	3	3	2	2
4	4	1	1	2	2	2	2	4	4
4	4	4	4	2	2	2	2	2	2
4	4	4	4	3	3	3	3	2	2
4	4	4	4	3	3	3	3	2	2
4	4	4	4	3	3	3	3	2	2
4	4	4	4	2	2	2	2	2	2
2	2	4	4	3	3	3	3	2	2
2	2	4	4	2	2	2	2	2	2
4	4	4	4	3	3	3	3	2	2
4	4	1	1	2	2	2	2	2	2
2	2	4	4	3	3	3	3	2	2
2	2	4	4	3	3	3	3	2	2
4	4	4	4	2	2	2	2	2	2
4	4	4	4	3	3	3	3	2	2
4	4	4	4	2	2	2	2	2	2
2	2	4	4	2	2	2	2	2	2
4	4	4	4	2	2	2	2	2	2
4	4	4	4	3	3	2	2	2	2
4	4	4	4	3	3	3	3	2	2
4	4	4	4	3	3	3	3	2	2
2	2	4	4	3	3	3	3	2	2
2	2	4	4	3	3	2	2	2	2
4	4	4	4	2	2	2	2	2	2
4	4	1	1	3	3	3	3	4	4
4	4	4	4	2	2	2	2	2	2
2	2	4	4	3	3	2	2	2	2
2	2	4	4	3	3	3	3	2	2
2	2	4	4	3	3	3	3	2	2
4	4	4	4	2	2	2	2	4	4
4	4	4	4	2	2	2	2	2	2
4	4	4	4	3	3	3	3	2	2
2	2	4	4	3	3	3	3	2	2
4	4	4	4	3	3	3	3	2	2
2	2	4	4	2	2	2	2	2	2
2	2	4	4	3	3	3	3	2	2
4	4	4	4	3	3	2	2	2	2
2	2	4	4	3	3	3	3	2	2
4	4	4	4	3	3	3	3	2	2
2	2	4	4	3	3	3	3	2	2
2	2	4	4	3	3	3	3	2	2
2	2	4	4	2	2	2	2	2	2
4	4	4	4	3	3	3	3	2	2
2	2	4	4	2	2	2	2	2	2
4	4	1	1	3	3	2	2	2	2
4	4	4	4	3	3	3	3	4	4
4	4	4	4	3	3	3	3	2	2
4	4	4	4	3	3	3	3	2	2
4	4	4	4	3	3	3	3	2	2
4	4	1	1	2	2	2	2	4	4
4	4	4	4	3	3	2	2	2	2

For Review Only

4	4	4	4	3	3	3	3	2	2
4	4	4	4	3	3	3	3	2	2
4	4	4	4	2	2	2	2	2	2
2	2	4	4	3	3	3	3	2	2
4	4	4	4	2	2	2	2	2	2
4	4	1	1	2	2	2	2	2	2
4	4	1	1	3	3	3	3	4	4
2	2	4	4	3	3	3	3	2	2
4	4	4	4	3	3	3	3	2	2
2	2	4	4	3	3	2	2	2	2
4	4	1	1	2	2	2	2	2	2
4	4	1	1	2	2	2	2	2	2
4	4	4	4	3	3	3	3	2	2
2	2	4	4	3	3	3	3	2	2
4	4	4	4	3	3	2	2	2	2
4	4	4	4	3	3	3	3	2	2
2	2	1	1	3	3	3	3	2	2
4	4	4	4	3	3	3	3	4	4
4	4	4	4	2	2	2	2	2	2
4	4	4	4	3	3	3	3	2	2
4	4	4	4	3	3	2	2	2	2
2	2	4	4	3	3	3	3	2	2
4	4	4	4	3	3	3	3	2	2
2	2	1	1	3	3	3	3	2	2
2	2	4	4	2	2	2	2	2	2
4	4	1	1	2	2	2	2	2	2
4	4	4	4	3	3	3	3	2	2
2	2	4	4	3	3	3	3	2	2
4	4	1	1	3	3	3	3	4	4
4	4	4	4	3	3	3	3	2	2
4	4	4	4	3	3	3	3	2	2
4	4	4	4	2	2	2	2	2	2
2	2	4	4	3	3	3	3	2	2
4	4	4	4	3	3	3	3	2	2
4	4	4	4	3	3	3	3	2	2
2	2	4	4	2	2	2	2	2	2
4	4	4	4	2	2	2	2	2	2
4	4	4	4	3	3	3	3	2	2
4	4	1	1	3	3	3	3	2	2
2	2	4	4	3	3	3	3	2	2
4	4	4	4	3	3	3	3	2	2
4	4	4	4	2	2	2	2	4	4
4	4	4	4	2	2	2	2	2	2
4	4	1	1	2	2	2	2	2	2
4	4	4	4	3	3	2	2	2	2
4	4	4	4	2	2	2	2	2	2
2	2	4	4	3	3	3	3	2	2
2	2	1	1	3	3	3	3	2	2
4	4	4	4	3	3	3	3	2	2
2	2	4	4	3	3	3	3	2	2
4	4	4	4	2	2	2	2	2	2
2	2	4	4	3	3	3	3	2	2
4	4	4	4	2	2	2	2	2	2
2	2	1	1	3	3	3	3	2	2
4	4	4	4	3	3	3	3	2	2

For Review Only

CSSA 5585 Guilford Rd., Madison, WI 53711

4	4	4	4	3	3	3	3	2	2
2	2	4	4	3	3	2	2	2	2
4	4	4	4	2	2	2	2	4	4
2	2	4	4	3	3	3	3	2	2
4	4	1	1	2	2	2	2	2	2
4	4	1	1	3	3	3	3	2	2
4	4	4	4	3	3	3	3	2	2
2	2	4	4	3	3	2	2	2	2
2	2	4	4	3	3	3	3	2	2
2	2	1	1	3	3	3	3	2	2
2	2	4	4	2	2	2	2	2	2
2	2	4	4	3	3	3	3	2	2
4	4	4	4	2	2	2	2	2	2
4	4	1	1	3	3	2	2	2	2
4	4	4	4	3	3	2	2	2	2
4	4	1	1	3	3	3	3	2	2
4	4	4	4	3	3	3	3	4	4
4	4	4	4	3	3	2	2	2	2
2	2	4	4	3	3	3	3	2	2
4	4	4	4	3	3	3	3	2	2
2	2	4	4	3	3	2	2	2	2
4	4	4	4	2	2	2	2	2	2
4	4	4	4	2	2	2	2	4	4
4	4	4	4	3	3	3	3	2	2
2	2	1	1	3	3	3	3	2	2
2	2	4	4	3	3	3	3	2	2
2	2	4	4	3	3	3	3	2	2
4	4	4	4	3	3	3	3	2	2
2	2	1	1	3	3	2	2	2	2
4	4	4	4	3	3	3	3	2	2
2	2	4	4	3	3	3	3	2	2
2	2	4	4	2	2	2	2	2	2
4	4	4	4	3	3	3	3	2	2
4	4	4	4	3	3	3	3	2	2
2	2	4	4	3	3	3	3	2	2
4	4	4	4	3	3	3	3	2	2
2	2	4	4	3	3	3	3	2	2
4	4	4	4	2	2	2	2	2	2
4	4	1	1	3	3	3	3	2	2
4	4	4	4	3	3	3	3	2	2
2	2	4	4	3	3	3	3	2	2
2	2	4	4	3	3	3	3	2	2
4	4	4	4	3	3	3	3	4	4
2	2	4	4	3	3	3	3	2	2
4	4	4	4	3	3	3	3	2	2
4	4	4	4	3	3	3	3	2	2
4	4	1	1	3	3	3	3	4	4
4	4	1	1	2	2	2	2	4	4
2	2	4	4	3	3	3	3	2	2
4	4	1	1	3	3	2	2	2	2
4	4	4	4	3	3	3	3	4	4
2	2	4	4	2	2	2	2	2	2
4	4	4	4	3	3	3	3	4	4
4	4	1	1	2	2	2	2	2	2
2	2	4	4	3	3	3	3	2	2

For Review Only

CSSA 5585 Guilford Rd., Madison, WI 53711

4	4	1	1	2	2	2	2	4	4
2	2	4	4	3	3	3	3	2	2
4	4	4	4	2	2	2	2	2	2
2	2	4	4	3	3	3	3	2	2
4	4	4	4	2	2	2	2	2	2
4	4	4	4	2	2	2	2	2	2
4	4	4	4	3	3	3	3	2	2
4	4	4	4	3	3	2	2	2	2
4	4	4	4	3	3	3	3	2	2
4	4	4	4	2	2	2	2	2	2
4	4	1	1	3	3	3	3	4	4
2	2	4	4	3	3	3	3	2	2
4	4	4	4	3	3	2	2	2	2
4	4	4	4	3	3	3	3	2	2
2	2	4	4	3	3	3	3	2	2
4	4	4	4	3	3	3	3	2	2
4	4	4	4	3	3	3	3	2	2
4	4	1	1	2	2	2	2	2	2
4	4	4	4	2	2	2	2	2	2
4	4	4	4	3	3	3	3	2	2
2	2	4	4	3	3	2	2	2	2
2	2	4	4	3	3	3	3	2	2
2	2	4	4	3	3	3	3	2	2
4	4	1	1	2	2	2	2	2	2
2	2	4	4	3	3	3	3	2	2
2	2	4	4	3	3	3	3	2	2
4	4	4	4	3	3	2	2	2	2
2	2	4	4	3	3	3	3	2	2
4	4	4	4	3	3	3	3	2	2
4	4	4	4	3	3	3	3	2	2
4	4	1	1	2	2	2	2	2	2
4	4	4	4	2	2	2	2	4	4
4	4	4	4	2	2	2	2	2	2
2	2	4	4	3	3	3	3	2	2
4	4	4	4	3	3	3	3	2	2
4	4	1	1	2	2	2	2	2	2
4	4	4	4	3	3	3	3	4	4
4	4	4	4	3	3	3	3	2	2
4	4	1	1	2	2	2	2	4	4
4	4	1	1	2	2	2	2	2	2
4	4	1	1	2	2	2	2	4	4
4	4	1	1	3	3	3	3	4	4
4	4	4	4	3	3	3	3	2	2
4	4	4	4	3	3	3	3	2	2
2	2	4	4	2	2	2	2	2	2
2	2	4	4	3	3	2	2	2	2
4	4	4	4	3	3	3	3	2	2
4	4	4	4	3	3	3	3	2	2
2	2	4	4	3	3	3	3	2	2
2	2	4	4	3	3	3	3	2	2
2	2	1	1	2	2	2	2	2	2
4	4	4	4	3	3	3	3	2	2
2	2	4	4	3	3	3	3	2	2
4	4	1	1	2	2	2	2	2	2
2	2	4	4	3	3	3	3	2	2

For Review Only

4	4	4	4	3	3	3	3	2	2
2	2	4	4	3	3	3	3	2	2
2	2	1	1	2	2	2	2	2	2
2	2	4	4	3	3	3	3	2	2
4	4	4	4	3	3	3	3	2	2
4	4	4	4	3	3	3	3	4	4
2	2	4	4	3	3	3	3	2	2
2	2	4	4	3	3	3	3	2	2
2	2	4	4	3	3	3	3	2	2
2	2	4	4	3	3	3	3	2	2
2	2	4	4	3	3	2	2	2	2
4	4	1	1	3	3	3	3	2	2
4	4	1	1	3	3	3	3	2	2
4	4	1	1	3	3	3	3	2	2
4	4	1	1	2	2	2	2	4	4
4	4	4	4	2	2	2	2	2	2
2	2	4	4	3	3	3	3	2	2
4	4	4	4	3	3	3	3	2	2
4	4	4	4	3	3	3	3	2	2
4	4	4	4	3	3	3	3	2	2
4	4	1	1	3	3	3	3	2	2
4	4	1	1	3	3	3	3	4	4
4	4	4	4	3	3	3	3	2	2
4	4	4	4	3	3	3	3	2	2
2	2	4	4	3	3	3	3	2	2
4	4	4	4	3	3	2	2	2	2
2	2	4	4	3	3	3	3	2	2
4	4	1	1	3	3	2	2	2	2
4	4	4	4	3	3	3	3	2	2
2	2	1	1	2	2	2	2	2	2
2	2	4	4	3	3	3	3	2	2
4	4	1	1	2	2	2	2	2	2
4	4	4	4	3	3	3	3	2	2
4	4	1	1	2	2	2	2	4	4
2	2	4	4	3	3	3	3	2	2
2	2	4	4	3	3	3	3	2	2
4	4	4	4	3	3	3	3	4	4
4	4	1	1	2	2	2	2	2	2
2	2	4	4	2	2	2	2	2	2
2	2	4	4	3	3	3	3	2	2
4	4	4	4	3	3	3	3	2	2
4	4	4	4	2	2	2	2	2	2
4	4	4	4	2	2	2	2	2	2
4	4	4	4	3	3	3	3	2	2
4	4	1	1	2	2	2	2	2	2
4	4	4	4	3	3	3	3	2	2
4	4	4	4	3	3	3	3	2	2
4	4	4	4	2	2	2	2	2	2
4	4	4	4	2	2	2	2	2	2
2	2	4	4	3	3	3	3	2	2
4	4	4	4	3	3	3	3	2	2
2	2	4	4	3	3	3	3	2	2
4	4	1	1	2	2	2	2	4	4
4	4	1	1	3	3	3	3	2	2
4	4	4	4	3	3	3	3	2	2

For Review Only

4	4	4	4	2	2	2	2	2	2
4	4	1	1	3	3	3	3	2	2

For Review Only

SNP932	SNP932	SNP933	SNP933	SNP934	SNP934	SNP935	SNP935	SNP936	SNP936
2	2	2	2	3	3	3	3	2	2
2	2	3	3	3	3	3	3	2	2
2	2	2	2	3	3	2	2	3	3
2	2	2	2	3	3	3	3	2	2
2	2	2	2	3	3	3	3	2	2
2	2	2	2	3	3	3	3	2	2
2	2	2	2	3	3	3	3	2	2
2	2	2	2	4	4	3	3	2	2
2	2	2	2	3	3	3	3	2	2
2	2	2	2	4	4	3	3	2	2
2	2	2	2	3	3	3	3	2	2
2	2	2	2	3	3	2	2	2	2
2	2	2	2	4	4	3	3	2	2
2	2	3	3	3	3	3	3	3	3
2	2	2	2	3	3	3	3	3	3
2	2	3	3	3	3	3	3	3	3
2	2	2	2	3	3	3	3	2	2
2	2	2	2	3	3	3	3	2	2
2	2	2	2	3	3	2	2	2	2
2	2	2	2	3	3	3	3	2	2
2	2	2	2	4	4	3	3	2	2
2	2	2	2	3	3	3	3	2	2
2	2	3	3	4	4	3	3	3	3
2	2	2	2	4	4	3	3	2	2
2	2	2	2	3	3	3	3	2	2
2	2	2	2	4	4	2	2	2	2
2	2	2	2	3	3	3	3	2	2
2	2	2	2	3	3	3	3	2	2
2	2	2	2	4	4	3	3	2	2
2	2	2	2	4	4	3	3	2	2
2	2	2	2	4	4	3	3	3	3
2	2	2	2	3	3	3	3	3	3
2	2	2	2	3	3	3	3	2	2
2	2	2	2	4	4	3	3	2	2
2	2	2	2	3	3	3	3	2	2
2	2	3	3	4	4	3	3	2	2
2	2	2	2	3	3	3	3	2	2
2	2	2	2	3	3	3	3	2	2
2	2	2	2	4	4	3	3	3	3
2	2	3	3	3	3	3	3	3	3
2	2	2	2	4	4	3	3	2	2
3	3	2	2	3	3	3	3	2	2
2	2	2	2	4	4	3	3	2	2
3	3	3	3	3	3	3	3	2	2
3	3	2	2	3	3	3	3	2	2
2	2	2	2	3	3	2	2	2	2
2	2	2	2	3	3	2	2	2	2
2	2	2	2	3	3	3	3	2	2
2	2	2	2	3	3	3	3	2	2
2	2	2	2	3	3	3	3	2	2
2	2	2	2	4	4	2	2	2	2
2	2	2	2	3	3	3	3	2	2

For Review Only

2	2	2	2	3	3	3	3	2	2
2	2	2	2	3	3	3	3	2	2
2	2	3	3	3	3	3	3	3	3
2	2	2	2	3	3	3	3	2	2
2	2	2	2	3	3	2	2	2	2
2	2	2	2	3	3	2	2	3	3
2	2	2	2	3	3	2	2	2	2
2	2	3	3	4	4	3	3	2	2
2	2	2	2	4	4	3	3	2	2
2	2	2	2	3	3	3	3	2	2
2	2	2	2	3	3	2	2	2	2
2	2	2	2	3	3	2	2	3	3
2	2	2	2	3	3	3	3	2	2
2	2	2	2	4	4	3	3	2	2
2	2	2	2	3	3	3	3	2	2
2	2	2	2	4	4	3	3	2	2
2	2	2	2	3	3	2	2	2	2
2	2	2	2	3	3	3	3	2	2
2	2	2	2	3	3	3	3	2	2
2	2	2	2	3	3	3	3	2	2
2	2	2	2	3	3	3	3	3	3
2	2	2	2	3	3	3	3	2	2
2	2	2	2	4	4	3	3	2	2
2	2	2	2	3	3	2	2	2	2
3	3	2	2	3	3	3	3	2	2
2	2	2	2	3	3	2	2	2	2
2	2	2	2	3	3	2	2	2	2
2	2	3	3	4	4	3	3	3	3
2	2	2	2	4	4	2	2	2	2
2	2	2	2	3	3	3	3	2	2
2	2	2	2	3	3	3	3	2	2
2	2	3	3	3	3	3	3	2	2
2	2	3	3	4	4	3	3	3	3
2	2	2	2	3	3	3	3	2	2
2	2	2	2	3	3	3	3	2	2
3	3	2	2	3	3	3	3	2	2
2	2	2	2	4	4	3	3	2	2
2	2	2	2	3	3	3	3	2	2
3	3	2	2	4	4	3	3	2	2
3	3	2	2	3	3	3	3	2	2
2	2	2	2	3	3	3	3	2	2
2	2	2	2	3	3	3	3	3	3
2	2	2	2	4	4	3	3	2	2
2	2	2	2	3	3	2	2	2	2
2	2	2	2	4	4	3	3	2	2
2	2	2	2	3	3	3	3	2	2
2	2	2	2	4	4	3	3	2	2
2	2	2	2	3	3	2	2	2	2
2	2	2	2	3	3	3	3	3	3
2	2	2	2	3	3	3	3	2	2
2	2	2	2	3	3	3	3	2	2
2	2	3	3	4	4	3	3	3	3
2	2	3	3	4	4	3	3	3	3
2	2	2	2	3	3	3	3	2	2
2	2	2	2	3	3	3	3	2	2

For Review Only

2	2	2	2	3	3	3	3	2	2
2	2	2	2	3	3	3	3	2	2
2	2	2	2	4	4	3	3	2	2
2	2	2	2	3	3	3	3	2	2
2	2	2	2	3	3	2	2	2	2
2	2	3	3	4	4	2	2	2	2
2	2	2	2	3	3	3	3	2	2
2	2	2	2	3	3	3	3	2	2
2	2	2	2	4	4	3	3	2	2
2	2	2	2	4	4	3	3	2	2
2	2	2	2	3	3	3	3	2	2
2	2	2	2	3	3	3	3	2	2
3	3	2	2	4	4	3	3	2	2
2	2	2	2	3	3	2	2	2	2
2	2	2	2	3	3	3	3	2	2
2	2	2	2	3	3	3	3	2	2
2	2	2	2	3	3	3	3	2	2
2	2	2	2	3	3	3	3	2	2
2	2	2	2	3	3	3	3	2	2
3	3	2	2	3	3	3	3	2	2
2	2	2	2	3	3	3	3	2	2
2	2	2	2	3	3	3	3	2	2
2	2	2	2	4	4	3	3	3	3
2	2	3	3	4	4	3	3	3	3
2	2	2	2	3	3	3	3	2	2
2	2	2	2	3	3	3	3	2	2
2	2	2	2	3	3	3	3	2	2
2	2	3	3	4	4	3	3	2	2
2	2	2	2	3	3	2	2	2	2
2	2	2	2	3	3	3	3	2	2
2	2	3	3	4	4	3	3	3	3
2	2	2	2	3	3	3	3	2	2
2	2	2	2	3	3	3	3	2	2
2	2	2	2	3	3	3	3	2	2
2	2	2	2	4	4	3	3	2	2
2	2	3	3	4	4	3	3	2	2
2	2	2	2	3	3	3	3	2	2
2	2	2	2	3	3	3	3	3	3
2	2	3	3	3	3	3	3	3	3
2	2	3	3	3	3	3	3	3	3
2	2	2	2	3	3	3	3	2	2
2	2	3	3	4	4	3	3	3	3
2	2	2	2	4	4	3	3	2	2
2	2	2	2	4	4	3	3	2	2
2	2	2	2	4	4	3	3	2	2
2	2	2	2	4	4	3	3	2	2
2	2	2	2	3	3	2	2	2	2
2	2	2	2	3	3	2	2	2	2
2	2	2	2	4	4	3	3	2	2
2	2	2	2	4	4	2	2	2	2
2	2	2	2	3	3	3	3	2	2
2	2	3	3	3	3	3	3	2	2
2	2	2	2	4	4	3	3	2	2
2	2	2	2	3	3	2	2	3	3
2	2	2	2	3	3	3	3	2	2

For Review Only

2	2	2	2	3	3	2	2	2	2
3	3	2	2	4	4	3	3	2	2
2	2	2	2	3	3	3	3	2	2
2	2	2	2	3	3	3	3	2	2
2	2	2	2	3	3	3	3	2	2
2	2	2	2	3	3	3	3	2	2
2	2	3	3	3	3	3	3	3	3
2	2	2	2	3	3	3	3	2	2
2	2	2	2	3	3	3	3	2	2
2	2	2	2	3	3	3	3	2	2
2	2	2	2	3	3	2	2	2	2
2	2	2	2	3	3	3	3	2	2
2	2	2	2	3	3	3	3	2	2
2	2	2	2	4	4	3	3	2	2
2	2	2	2	4	4	3	3	2	2
2	2	3	3	3	3	3	3	3	3
2	2	2	2	4	4	3	3	2	2
2	2	2	2	3	3	2	2	2	2
2	2	3	3	3	3	3	3	2	2
2	2	2	2	3	3	3	3	2	2
2	2	2	2	3	3	3	3	2	2
2	2	3	3	4	4	3	3	3	3
2	2	2	2	3	3	3	3	2	2
2	2	2	2	3	3	2	2	3	3
2	2	2	2	4	4	3	3	2	2
2	2	2	2	4	4	3	3	2	2
2	2	2	2	3	3	3	3	2	2
2	2	2	2	3	3	3	3	2	2
2	2	2	2	3	3	3	3	2	2
2	2	2	2	3	3	3	3	2	2
2	2	2	2	4	4	2	2	2	2
2	2	2	2	3	3	3	3	2	2
2	2	2	2	3	3	3	3	2	2
2	2	2	2	4	4	3	3	2	2
2	2	2	2	3	3	3	3	2	2
2	2	2	2	3	3	2	2	3	3
2	2	2	2	3	3	3	3	3	3
2	2	2	2	3	3	3	3	2	2
2	2	2	2	3	3	2	2	2	2
2	2	2	2	4	4	2	2	3	3
2	2	2	2	3	3	2	2	3	3
2	2	2	2	3	3	2	2	2	2
2	2	3	3	4	4	3	3	3	3
2	2	2	2	3	3	3	3	2	2
2	2	2	2	3	3	3	3	2	2
2	2	2	2	4	4	3	3	2	2
2	2	3	3	3	3	3	3	3	3
2	2	3	3	4	4	3	3	3	3
2	2	3	3	4	4	3	3	3	3
2	2	2	2	3	3	3	3	2	2
2	2	2	2	3	3	2	2	2	2
2	2	2	2	4	4	3	3	2	2
2	2	2	2	4	4	3	3	2	2
2	2	2	2	3	3	2	2	2	2
2	2	3	3	4	4	3	3	3	3

For Review Only

2	2	3	3	4	4	3	3	3	3
2	2	2	2	3	3	3	3	2	2
2	2	3	3	3	3	2	2	2	2
2	2	2	2	3	3	3	3	2	2
2	2	2	2	3	3	3	3	2	2
2	2	2	2	4	4	3	3	2	2
3	3	3	3	3	3	3	3	3	3
2	2	2	2	4	4	3	3	2	2
2	2	2	2	4	4	3	3	3	3
2	2	3	3	4	4	3	3	3	3
2	2	2	2	3	3	3	3	2	2
3	3	2	2	4	4	3	3	2	2
2	2	2	2	3	3	3	3	2	2
2	2	2	2	4	4	2	2	3	3
2	2	2	2	3	3	2	2	3	3
2	2	3	3	3	3	3	3	3	3
2	2	3	3	4	4	3	3	3	3
2	2	2	2	4	4	3	3	2	2
2	2	3	3	4	4	3	3	3	3
2	2	2	2	3	3	3	3	2	2
2	2	3	3	3	3	2	2	3	3
2	2	2	2	4	4	2	2	2	2
2	2	2	2	4	4	3	3	2	2
2	2	3	3	3	3	3	3	3	3
2	2	3	3	4	4	3	3	2	2
3	3	2	2	4	4	3	3	2	2
2	2	3	3	4	4	3	3	3	3
2	2	2	2	3	3	2	2	2	2
2	2	2	2	3	3	3	3	2	2
2	2	2	2	3	3	2	2	2	2
2	2	3	3	4	4	3	3	3	3
2	2	2	2	3	3	2	2	2	2
2	2	2	2	4	4	3	3	2	2
2	2	2	2	4	4	3	3	2	2
2	2	2	2	3	3	3	3	2	2
2	2	3	3	4	4	3	3	3	3
2	2	2	2	4	4	3	3	2	2
2	2	2	2	3	3	2	2	3	3
2	2	3	3	4	4	3	3	2	2
2	2	3	3	4	4	3	3	3	3
2	2	2	2	3	3	3	3	2	2
2	2	2	2	3	3	3	3	2	2
2	2	3	3	3	3	3	3	3	3
2	2	2	2	3	3	3	3	2	2
2	2	2	2	3	3	2	2	2	2
2	2	2	2	4	4	3	3	2	2
2	2	3	3	3	3	3	3	3	3
2	2	2	2	4	4	3	3	2	2
2	2	2	2	3	3	3	3	2	2
2	2	3	3	4	4	3	3	3	3
2	2	2	2	3	3	3	3	2	2
2	2	3	3	4	4	3	3	3	3
2	2	2	2	3	3	2	2	3	3
3	3	2	2	3	3	2	2	2	2
2	2	3	3	3	3	3	3	3	3

For Review Only

2	2	2	2	3	3	3	3	2	2
2	2	2	2	3	3	2	2	3	3

For Review Only

SNP937	SNP937	SNP938	SNP938	SNP939	SNP939	SNP940	SNP940	SNP941	SNP941
3	3	3	3	1	1	2	2	2	2
3	3	3	3	1	1	2	2	2	2
4	4	3	3	4	4	2	2	3	3
3	3	3	3	1	1	2	2	2	2
4	4	3	3	1	1	2	2	2	2
3	3	3	3	1	1	2	2	3	3
4	4	3	3	1	1	2	2	2	2
3	3	2	2	4	4	2	2	3	3
4	4	3	3	1	1	2	2	2	2
3	3	2	2	4	4	2	2	3	3
3	3	3	3	1	1	2	2	2	2
4	4	3	3	4	4	2	2	3	3
4	4	3	3	1	1	2	2	2	2
3	3	3	3	1	1	2	2	2	2
4	4	3	3	1	1	2	2	2	2
3	3	3	3	1	1	2	2	2	2
4	4	2	2	1	1	2	2	2	2
3	3	3	3	1	1	2	2	2	2
4	4	3	3	4	4	2	2	3	3
4	4	3	3	4	4	2	2	3	3
4	4	3	3	4	4	2	2	3	3
4	4	2	2	1	1	2	2	2	2
3	3	3	3	1	1	2	2	2	2
4	4	3	3	4	4	2	2	2	2
3	3	3	3	1	1	2	2	2	2
4	4	3	3	4	4	2	2	3	3
4	4	3	3	1	1	2	2	2	2
4	4	3	3	4	4	2	2	2	2
4	4	3	3	1	1	2	2	2	2
4	4	3	3	4	4	2	2	3	3
3	3	3	3	1	1	2	2	2	2
3	3	3	3	1	1	2	2	2	2
3	3	3	3	1	1	2	2	2	2
3	3	3	3	1	1	2	2	2	2
3	3	3	3	4	4	2	2	3	3
3	3	3	3	1	1	2	2	2	2
4	4	3	3	1	1	2	2	2	2
4	4	3	3	1	1	2	2	2	2
3	3	3	3	1	1	2	2	2	2
4	4	3	3	4	4	2	2	2	2
4	4	3	3	4	4	2	2	3	3
4	4	3	3	4	4	2	2	3	3
3	3	3	3	4	4	3	3	3	3
4	4	2	2	1	1	2	2	2	2
3	3	2	2	1	1	3	3	2	2
4	4	3	3	4	4	2	2	3	3
3	3	3	3	4	4	2	2	3	3
4	4	3	3	1	1	2	2	2	2
4	4	2	2	1	1	2	2	2	2
4	4	3	3	1	1	2	2	2	2
4	4	3	3	4	4	2	2	3	3
3	3	3	3	4	4	2	2	3	3

For Review Only

4	4	3	3	1	1	2	2	2	2
3	3	3	3	1	1	2	2	2	2
4	4	3	3	1	1	2	2	3	3
4	4	2	2	1	1	2	2	2	2
4	4	3	3	4	4	2	2	3	3
4	4	3	3	4	4	2	2	3	3
3	3	3	3	4	4	2	2	3	3
4	4	3	3	1	1	2	2	2	2
4	4	3	3	1	1	2	2	2	2
4	4	3	3	4	4	2	2	2	2
3	3	3	3	4	4	2	2	3	3
3	3	3	3	4	4	2	2	3	3
4	4	3	3	1	1	2	2	2	2
4	4	3	3	1	1	2	2	2	2
4	4	3	3	1	1	2	2	2	2
4	4	3	3	1	1	2	2	2	2
4	4	3	3	4	4	2	2	3	3
4	4	3	3	1	1	2	2	2	2
4	4	3	3	4	4	2	2	3	3
4	4	3	3	1	1	2	2	2	2
3	3	3	3	4	4	2	2	2	2
4	4	3	3	4	4	2	2	3	3
4	4	3	3	4	4	2	2	2	2
3	3	3	3	4	4	2	2	3	3
3	3	2	2	1	1	3	3	2	2
4	4	3	3	4	4	2	2	3	3
4	4	3	3	4	4	2	2	3	3
4	4	3	3	1	1	2	2	2	2
4	4	3	3	4	4	2	2	3	3
4	4	3	3	1	1	2	2	2	2
4	4	2	2	1	1	2	2	2	2
3	3	3	3	1	1	2	2	2	2
4	4	3	3	4	4	2	2	3	3
4	4	3	3	1	1	2	2	2	2
4	4	3	3	1	1	2	2	2	2
3	3	2	2	1	1	3	3	2	2
4	4	3	3	4	4	2	2	3	3
4	4	2	2	4	4	2	2	2	2
4	4	3	3	1	1	3	3	3	3
4	4	3	3	4	4	3	3	3	3
4	4	3	3	1	1	2	2	2	2
4	4	3	3	1	1	2	2	2	2
3	3	3	3	4	4	2	2	3	3
3	3	3	3	4	4	2	2	3	3
3	3	3	3	1	1	2	2	2	2
3	3	3	3	1	1	2	2	2	2
4	4	3	3	1	1	2	2	2	2
3	3	3	3	4	4	2	2	3	3
4	4	3	3	1	1	2	2	2	2
3	3	3	3	4	4	2	2	3	3
3	3	3	3	4	4	2	2	2	2
3	3	3	3	1	1	2	2	2	2
3	3	3	3	1	1	2	2	2	2
4	4	3	3	4	4	2	2	3	3
3	3	3	3	4	4	2	2	3	3

For Review Only

3	3	3	3	4	4	2	2	2	2
4	4	3	3	1	1	2	2	2	2
4	4	3	3	1	1	2	2	2	2
4	4	3	3	1	1	2	2	2	2
4	4	3	3	4	4	2	2	3	3
4	4	3	3	4	4	2	2	3	3
4	4	3	3	1	1	2	2	2	2
4	4	3	3	1	1	2	2	2	2
4	4	3	3	4	4	2	2	2	2
3	3	3	3	4	4	2	2	3	3
4	4	3	3	1	1	2	2	2	2
4	4	3	3	4	4	2	2	2	2
3	3	2	2	4	4	3	3	3	3
4	4	3	3	4	4	2	2	3	3
4	4	3	3	1	1	2	2	2	2
4	4	3	3	4	4	2	2	3	3
4	4	3	3	4	4	2	2	3	3
4	4	3	3	4	4	2	2	2	2
3	3	3	3	1	1	2	2	2	2
3	3	3	3	4	4	3	3	3	3
4	4	3	3	4	4	2	2	2	2
4	4	3	3	1	1	2	2	2	2
4	4	3	3	1	1	2	2	2	2
4	4	3	3	1	1	2	2	2	2
4	4	3	3	4	4	2	2	3	3
4	4	3	3	1	1	3	3	2	2
3	3	3	3	1	1	2	2	2	2
3	3	3	3	1	1	2	2	2	2
4	4	3	3	4	4	2	2	3	3
3	3	3	3	4	4	2	2	3	3
4	4	3	3	1	1	2	2	2	2
4	4	3	3	4	4	2	2	2	2
4	4	3	3	1	1	2	2	2	2
4	4	3	3	1	1	2	2	2	2
3	3	3	3	1	1	2	2	2	2
4	4	3	3	1	1	2	2	2	2
3	3	3	3	1	1	2	2	2	2
4	4	3	3	1	1	2	2	2	2
3	3	3	3	4	4	2	2	2	2
4	4	3	3	1	1	2	2	2	2
4	4	3	3	1	1	2	2	2	2
4	4	3	3	1	1	2	2	2	2
3	3	3	3	1	1	2	2	2	2
4	4	3	3	4	4	2	2	2	2
4	4	3	3	4	4	2	2	3	3
4	4	3	3	1	1	2	2	2	2
4	4	3	3	4	4	2	2	3	3
3	3	3	3	4	4	2	2	3	3
4	4	3	3	1	1	2	2	2	2
4	4	3	3	4	4	2	2	3	3
4	4	3	3	4	4	2	2	2	2
3	3	2	2	1	1	2	2	2	2
4	4	3	3	1	1	2	2	2	2
3	3	3	3	4	4	2	2	3	3
4	4	3	3	4	4	2	2	2	2

For Review Only

3	3	3	3	4	4	2	2	3	3
3	3	3	3	1	1	3	3	2	2
4	4	3	3	4	4	2	2	2	2
4	4	3	3	1	1	2	2	2	2
3	3	3	3	4	4	2	2	2	2
4	4	3	3	4	4	2	2	3	3
4	4	3	3	4	4	2	2	2	2
4	4	3	3	4	4	2	2	2	2
4	4	3	3	1	1	2	2	2	2
4	4	3	3	1	1	2	2	2	2
4	4	3	3	4	4	2	2	3	3
3	3	3	3	4	4	2	2	3	3
4	4	3	3	4	4	2	2	2	2
4	4	3	3	1	1	2	2	2	2
3	3	3	3	1	1	2	2	2	2
3	3	3	3	1	1	2	2	2	2
4	4	3	3	1	1	2	2	2	2
3	3	3	3	4	4	2	2	3	3
4	4	3	3	1	1	2	2	2	2
4	4	3	3	1	1	2	2	2	2
4	4	3	3	1	1	2	2	2	2
3	3	3	3	1	1	2	2	2	2
3	3	3	3	4	4	2	2	3	3
3	3	3	3	4	4	2	2	3	3
4	4	3	3	4	4	2	2	3	3
3	3	3	3	1	1	2	2	2	2
4	4	3	3	4	4	2	2	2	2
4	4	3	3	1	1	2	2	2	2
4	4	3	3	1	1	2	2	2	2
4	4	3	3	1	1	2	2	2	2
4	4	3	3	4	4	2	2	3	3
4	4	3	3	1	1	2	2	2	2
3	3	3	3	1	1	2	2	2	2
3	3	3	3	1	1	2	2	2	2
4	4	3	3	1	1	2	2	2	2
3	3	3	3	4	4	2	2	3	3
3	3	3	3	1	1	2	2	2	2
4	4	3	3	1	1	2	2	2	2
3	3	3	3	4	4	2	2	3	3
4	4	3	3	4	4	2	2	3	3
4	4	3	3	4	4	2	2	2	2
4	4	3	3	4	4	2	2	3	3
4	4	3	3	4	4	2	2	3	3
3	3	3	3	1	1	2	2	3	3
3	3	3	3	1	1	2	2	2	2
4	4	3	3	1	1	2	2	2	2
3	3	3	3	1	1	2	2	2	2
4	4	3	3	1	1	2	2	2	2
3	3	3	3	1	1	2	2	2	2
3	3	3	3	1	1	2	2	2	2
4	4	3	3	4	4	2	2	3	3
4	4	3	3	1	1	2	2	2	2
3	3	3	3	1	1	2	2	2	2
3	3	3	3	4	4	2	2	3	3
4	4	3	3	1	1	2	2	2	2

For Review Only

4	4	3	3	1	1	2	2	2	2
4	4	3	3	1	1	2	2	2	2
4	4	3	3	4	4	2	2	3	3
4	4	3	3	4	4	3	3	3	3
3	3	3	3	1	1	2	2	2	2
4	4	3	3	1	1	2	2	2	2
4	4	3	3	1	1	3	3	2	2
4	4	3	3	4	4	2	2	3	3
4	4	3	3	1	1	2	2	2	2
4	4	3	3	1	1	2	2	2	2
4	4	3	3	4	4	2	2	2	2
4	4	3	3	4	4	3	3	3	3
4	4	3	3	1	1	2	2	2	2
4	4	3	3	4	4	2	2	3	3
4	4	3	3	4	4	2	2	3	3
3	3	3	3	1	1	2	2	2	2
4	4	2	2	1	1	2	2	2	2
4	4	3	3	4	4	2	2	2	2
3	3	3	3	1	1	2	2	2	2
4	4	3	3	1	1	2	2	2	2
4	4	3	3	4	4	2	2	3	3
4	4	3	3	4	4	2	2	3	3
4	4	3	3	1	1	2	2	2	2
4	4	3	3	1	1	2	2	2	2
4	4	3	3	1	1	2	2	2	2
3	3	3	3	1	1	2	2	2	2
4	4	3	3	1	1	2	2	2	2
4	4	3	3	4	4	2	2	3	3
4	4	3	3	1	1	2	2	2	2
4	4	3	3	4	4	2	2	3	3
4	4	3	3	1	1	2	2	2	2
4	4	3	3	4	4	2	2	3	3
3	3	3	3	1	1	2	2	2	2
4	4	3	3	4	4	2	2	3	3
3	3	3	3	1	1	2	2	2	2
4	4	3	3	1	1	2	2	2	2
4	4	2	2	1	1	2	2	2	2
3	3	3	3	4	4	2	2	3	3
3	3	3	3	1	1	2	2	2	2
3	3	3	3	1	1	2	2	2	2
4	4	3	3	1	1	2	2	2	2
3	3	3	3	1	1	2	2	2	2
3	3	3	3	4	4	2	2	3	3
4	4	2	2	1	1	2	2	2	2
4	4	3	3	4	4	2	2	3	3
4	4	3	3	1	1	2	2	2	2
3	3	3	3	1	1	2	2	2	2
4	4	3	3	4	4	2	2	3	3
4	4	3	3	4	4	3	3	2	2
4	4	3	3	4	4	2	2	3	3
3	3	3	3	4	4	2	2	3	3
3	3	3	3	1	1	3	3	2	2
4	4	3	3	4	4	2	2	3	3
3	3	3	3	4	4	2	2	3	3
4	4	3	3	4	4	2	2	3	3

For Review Only

4	4	3	3	1	1	2	2	2	2
4	4	3	3	4	4	2	2	3	3

For Review Only

SNP942	SNP942	SNP943	SNP943	SNP944	SNP944	SNP945	SNP945	SNP946	SNP946
1	1	4	4	1	1	3	3	2	2
1	1	4	4	1	1	3	3	2	2
1	1	4	4	1	1	3	3	2	2
4	4	4	4	1	1	1	1	2	2
4	4	4	4	1	1	1	1	2	2
1	1	4	4	1	1	3	3	2	2
1	1	4	4	1	1	3	3	2	2
1	1	4	4	1	1	3	3	2	2
1	1	4	4	1	1	1	1	2	2
1	1	4	4	1	1	3	3	2	2
4	4	2	2	1	1	1	1	2	2
1	1	4	4	1	1	3	3	2	2
4	4	4	4	1	1	1	1	3	3
1	1	4	4	1	1	3	3	2	2
1	1	4	4	1	1	3	3	3	3
4	4	4	4	1	1	1	1	2	2
1	1	4	4	1	1	3	3	3	3
4	4	2	2	1	1	1	1	2	2
1	1	4	4	1	1	3	3	3	3
4	4	2	2	1	1	1	1	2	2
1	1	4	4	1	1	3	3	2	2
1	1	4	4	1	1	3	3	3	3
4	4	2	2	1	1	1	1	2	2
4	4	2	2	1	1	1	1	2	2
1	1	4	4	1	1	3	3	2	2
1	1	4	4	1	1	3	3	2	2
1	1	4	4	1	1	3	3	2	2
4	4	4	4	1	1	1	1	2	2
1	1	4	4	1	1	3	3	2	2
4	4	2	2	1	1	1	1	2	2
1	1	4	4	1	1	3	3	2	2
1	1	4	4	1	1	3	3	3	3
1	1	4	4	1	1	3	3	2	2
1	1	4	4	1	1	3	3	2	2
4	4	4	4	1	1	1	1	2	2
1	1	4	4	1	1	3	3	2	2
1	1	4	4	1	1	3	3	2	2
1	1	4	4	1	1	3	3	2	2
4	4	2	2	1	1	1	1	2	2
4	4	4	4	1	1	3	3	2	2
4	4	2	2	1	1	1	1	2	2
4	4	2	2	1	1	1	1	2	2
4	4	2	2	1	1	1	1	2	2
4	4	2	2	1	1	1	1	2	2
4	4	2	2	1	1	1	1	2	2
1	1	4	4	1	1	3	3	2	2
1	1	4	4	1	1	3	3	2	2
4	4	2	2	1	1	1	1	2	2
1	1	4	4	1	1	1	1	2	2
1	1	4	4	1	1	3	3	2	2
1	1	4	4	1	1	3	3	2	2
1	1	4	4	1	1	3	3	2	2

For Review Only

1	1	4	4	1	1	3	3	2	2
1	1	4	4	1	1	3	3	2	2
1	1	4	4	1	1	3	3	2	2
1	1	4	4	1	1	3	3	2	2
1	1	4	4	1	1	3	3	3	3
1	1	4	4	1	1	3	3	2	2
1	1	4	4	1	1	3	3	2	2
4	4	2	2	1	1	1	1	2	2
1	1	4	4	1	1	3	3	2	2
1	1	4	4	1	1	3	3	2	2
1	1	4	4	1	1	3	3	2	2
1	1	4	4	1	1	3	3	2	2
1	1	4	4	1	1	3	3	2	2
1	1	4	4	1	1	3	3	2	2
4	4	4	4	1	1	1	1	2	2
1	1	4	4	1	1	3	3	2	2
4	4	4	4	1	1	1	1	2	2
1	1	4	4	1	1	3	3	2	2
1	1	4	4	1	1	3	3	2	2
1	1	4	4	1	1	3	3	2	2
1	1	4	4	1	1	3	3	2	2
4	4	2	2	1	1	1	1	2	2
4	4	4	4	1	1	3	3	2	2
1	1	4	4	1	1	3	3	2	2
4	4	2	2	1	1	1	1	2	2
1	1	4	4	1	1	3	3	2	2
1	1	4	4	1	1	3	3	2	2
4	4	2	2	1	1	1	1	2	2
1	1	4	4	1	1	3	3	2	2
1	1	4	4	1	1	3	3	2	2
4	4	2	2	1	1	1	1	2	2
1	1	4	4	1	1	3	3	2	2
4	4	2	2	1	1	1	1	2	2
1	1	4	4	1	1	3	3	2	2
4	4	2	2	1	1	1	1	2	2
4	4	2	2	1	1	1	1	2	2
4	4	2	2	1	1	1	1	2	2
1	1	4	4	1	1	3	3	3	3
4	4	2	2	1	1	1	1	2	2
4	4	2	2	1	1	1	1	2	2
1	1	4	4	1	1	3	3	3	3
1	1	4	4	1	1	3	3	2	2
1	1	4	4	1	1	3	3	2	2
1	1	4	4	1	1	3	3	2	2
1	1	4	4	1	1	3	3	2	2
1	1	4	4	4	4	3	3	2	2
1	1	4	4	1	1	3	3	2	2
4	4	4	4	1	1	1	1	2	2
1	1	4	4	1	1	3	3	2	2
1	1	4	4	1	1	3	3	2	2
1	1	4	4	1	1	3	3	2	2
4	4	2	2	1	1	1	1	2	2
1	1	4	4	1	1	3	3	2	2
1	1	4	4	1	1	1	1	2	2
1	1	4	4	1	1	3	3	2	2

For Review Only

4	4	2	2	1	1	1	1	2	2
4	4	4	4	1	1	1	1	2	2
1	1	4	4	1	1	3	3	2	2
1	1	4	4	1	1	3	3	2	2
1	1	4	4	4	4	3	3	2	2
1	1	4	4	1	1	3	3	2	2
1	1	4	4	1	1	3	3	2	2
4	4	2	2	1	1	1	1	2	2
1	1	4	4	1	1	3	3	2	2
4	4	4	4	1	1	3	3	2	2
1	1	4	4	1	1	3	3	2	2
4	4	4	4	1	1	3	3	2	2
1	1	4	4	1	1	3	3	2	2
1	1	4	4	1	1	3	3	2	2
1	1	4	4	1	1	3	3	2	2
4	4	4	4	1	1	1	1	2	2
1	1	4	4	1	1	3	3	2	2
4	4	2	2	1	1	1	1	2	2
1	1	4	4	1	1	3	3	2	2
1	1	4	4	1	1	3	3	2	2
4	4	4	4	1	1	3	3	2	2
1	1	4	4	1	1	3	3	2	2
1	1	4	4	1	1	3	3	2	2
4	4	2	2	1	1	1	1	2	2
4	4	4	4	1	1	1	1	2	2
4	4	2	2	1	1	1	1	2	2
1	1	4	4	1	1	3	3	2	2
1	1	4	4	1	1	3	3	2	2
1	1	4	4	1	1	3	3	2	2
1	1	4	4	1	1	3	3	2	2
4	4	2	2	1	1	1	1	2	2
4	4	4	4	1	1	1	1	2	2
1	1	4	4	1	1	3	3	3	3
4	4	4	4	1	1	1	1	2	2
1	1	4	4	1	1	3	3	2	2
1	1	4	4	1	1	1	1	2	2
1	1	4	4	1	1	3	3	2	2
1	1	4	4	1	1	3	3	2	2
1	1	4	4	1	1	1	1	2	2
4	4	2	2	1	1	1	1	2	2
1	1	4	4	1	1	3	3	2	2
1	1	4	4	1	1	3	3	2	2
1	1	4	4	1	1	3	3	2	2
4	4	4	4	1	1	1	1	2	2
4	4	4	4	1	1	1	1	2	2
1	1	4	4	1	1	3	3	2	2
4	4	2	2	1	1	1	1	2	2
1	1	4	4	1	1	3	3	2	2
4	4	4	4	1	1	1	1	2	2
1	1	4	4	1	1	3	3	2	2
1	1	4	4	1	1	3	3	2	2
4	4	2	2	1	1	1	1	2	2
1	1	4	4	1	1	3	3	2	2
1	1	4	4	1	1	3	3	2	2
4	4	4	4	1	1	1	1	2	2

For Review Only

1	1	4	4	1	1	3	3	2	2
4	4	2	2	1	1	1	1	2	2
4	4	2	2	1	1	1	1	2	2
4	4	4	4	1	1	1	1	2	2
1	1	4	4	1	1	3	3	2	2
4	4	2	2	1	1	1	1	3	3
4	4	2	2	1	1	1	1	2	2
4	4	4	4	1	1	3	3	2	2
4	4	4	4	1	1	1	1	2	2
1	1	4	4	1	1	3	3	2	2
4	4	2	2	1	1	1	1	2	2
4	4	2	2	1	1	1	1	2	2
4	4	4	4	1	1	1	1	2	2
1	1	4	4	1	1	3	3	2	2
1	1	4	4	1	1	3	3	2	2
4	4	2	2	1	1	1	1	2	2
1	1	4	4	1	1	3	3	2	2
1	1	4	4	4	4	3	3	2	2
1	1	4	4	1	1	3	3	2	2
1	1	4	4	1	1	3	3	2	2
4	4	4	4	1	1	3	3	2	2
4	4	2	2	1	1	1	1	2	2
4	4	2	2	1	1	1	1	2	2
1	1	4	4	1	1	3	3	2	2
1	1	4	4	1	1	1	1	3	3
1	1	4	4	1	1	3	3	2	2
1	1	4	4	1	1	3	3	2	2
1	1	4	4	1	1	3	3	2	2
1	1	4	4	1	1	3	3	2	2
1	1	4	4	1	1	3	3	3	3
1	1	4	4	1	1	3	3	2	2
1	1	4	4	1	1	3	3	2	2
1	1	4	4	1	1	3	3	2	2
1	1	4	4	1	1	3	3	2	2
1	1	4	4	1	1	3	3	2	2
1	1	4	4	1	1	3	3	2	2
4	4	4	4	1	1	1	1	2	2
1	1	4	4	1	1	3	3	2	2
1	1	4	4	1	1	3	3	2	2
1	1	4	4	1	1	3	3	2	2
1	1	4	4	1	1	3	3	2	2
4	4	2	2	1	1	1	1	2	2
4	4	2	2	1	1	1	1	2	2
4	4	4	4	1	1	1	1	2	2
1	1	4	4	4	4	3	3	2	2
1	1	4	4	1	1	3	3	2	2
4	4	2	2	1	1	1	1	2	2
4	4	2	2	1	1	1	1	2	2
1	1	4	4	1	1	3	3	2	2
4	4	2	2	1	1	1	1	2	2
1	1	4	4	1	1	3	3	2	2
1	1	4	4	1	1	3	3	2	2
4	4	2	2	1	1	1	1	2	2
1	1	4	4	1	1	3	3	2	2
4	4	2	2	1	1	1	1	2	2

For Review Only

1	1	4	4	1	1	3	3	2	2
4	4	2	2	1	1	1	1	2	2
1	1	4	4	1	1	3	3	2	2
4	4	4	4	1	1	1	1	2	2
4	4	2	2	1	1	1	1	2	2
1	1	4	4	1	1	3	3	2	2
4	4	2	2	1	1	1	1	2	2
4	4	2	2	1	1	1	1	2	2
1	1	4	4	1	1	3	3	2	2
4	4	2	2	1	1	1	1	2	2
4	4	4	4	1	1	3	3	2	2
4	4	2	2	1	1	1	1	2	2
4	4	2	2	1	1	1	1	2	2
1	1	4	4	1	1	3	3	2	2
1	1	4	4	1	1	3	3	2	2
4	4	2	2	1	1	1	1	2	2
4	4	2	2	1	1	1	1	2	2
1	1	4	4	1	1	3	3	2	2
4	4	2	2	1	1	1	1	3	3
1	1	4	4	1	1	3	3	2	2
4	4	4	4	1	1	1	1	2	2
1	1	4	4	1	1	3	3	2	2
1	1	4	4	1	1	1	1	2	2
1	1	4	4	1	1	3	3	2	2
4	4	2	2	1	1	1	1	2	2
1	1	4	4	1	1	3	3	2	2
4	4	2	2	1	1	1	1	2	2
1	1	4	4	1	1	3	3	2	2
4	4	2	2	1	1	1	1	2	2
1	1	4	4	1	1	3	3	2	2
4	4	2	2	1	1	1	1	2	2
4	4	2	2	1	1	1	1	2	2
1	1	4	4	1	1	3	3	2	2
1	1	4	4	1	1	3	3	2	2
4	4	2	2	1	1	1	1	2	2
4	4	2	2	1	1	1	1	2	2
1	1	4	4	1	1	3	3	3	3
1	1	4	4	1	1	3	3	2	2
1	1	4	4	1	1	3	3	2	2
4	4	2	2	1	1	1	1	2	2
1	1	4	4	1	1	3	3	2	2
1	1	4	4	1	1	3	3	2	2
4	4	2	2	1	1	1	1	2	2
1	1	4	4	1	1	3	3	3	3
4	4	4	4	1	1	1	1	2	2
1	1	4	4	1	1	3	3	3	3
4	4	2	2	1	1	1	1	2	2
1	1	4	4	1	1	3	3	2	2
1	1	4	4	1	1	3	3	2	2
4	4	2	2	1	1	1	1	2	2
1	1	4	4	1	1	3	3	2	2
4	4	2	2	1	1	1	1	2	2
1	1	4	4	1	1	3	3	2	2
1	1	4	4	1	1	3	3	2	2
4	4	2	2	1	1	1	1	2	2

For Review Only

1	1	4	4	1	1	3	3	2	2
1	1	4	4	1	1	3	3	2	2

For Review Only

SNP947	SNP947	SNP948	SNP948	SNP949	SNP949	SNP950	SNP950	SNP951	SNP951
2	2	4	4	3	3	3	3	4	4
2	2	4	4	3	3	3	3	4	4
4	4	4	4	3	3	3	3	4	4
4	4	2	2	3	3	3	3	4	4
4	4	4	4	3	3	3	3	4	4
4	4	4	4	3	3	3	3	4	4
4	4	4	4	3	3	3	3	4	4
4	4	4	4	3	3	3	3	4	4
4	4	4	4	3	3	3	3	4	4
4	4	4	4	3	3	3	3	4	4
4	4	4	4	3	3	2	2	4	4
4	4	4	4	3	3	3	3	4	4
4	4	4	4	3	3	3	3	4	4
4	4	4	4	3	3	3	3	4	4
4	4	4	4	3	3	3	3	4	4
4	4	2	2	3	3	3	3	4	4
4	4	4	4	3	3	3	3	4	4
4	4	4	4	3	3	3	3	4	4
4	4	4	4	3	3	3	3	4	4
4	4	4	4	3	3	2	2	4	4
4	4	4	4	3	3	3	3	4	4
4	4	4	4	3	3	3	3	4	4
4	4	4	4	3	3	2	2	4	4
4	4	4	4	3	3	3	3	4	4
4	4	4	4	3	3	3	3	4	4
4	4	4	4	3	3	3	3	4	4
4	4	4	4	3	3	3	3	4	4
4	4	4	4	3	3	3	3	4	4
4	4	4	4	3	3	3	3	4	4
4	4	4	4	3	3	2	2	4	4
4	4	4	4	3	3	3	3	4	4
4	4	4	4	3	3	3	3	4	4
2	2	4	4	3	3	3	3	4	4
4	4	4	4	3	3	3	3	1	1
4	4	4	4	3	3	2	2	4	4
4	4	4	4	3	3	3	3	4	4
2	2	4	4	3	3	3	3	4	4
4	4	4	4	3	3	3	3	4	4
4	4	4	4	3	3	2	2	4	4
4	4	4	4	3	3	3	3	4	4
4	4	4	4	3	3	2	2	4	4
4	4	4	4	3	3	2	2	4	4
4	4	4	4	2	2	3	3	4	4
4	4	4	4	3	3	3	3	4	4
4	4	4	4	2	2	3	3	4	4
4	4	4	4	3	3	3	3	4	4
4	4	4	4	3	3	3	3	4	4
4	4	4	4	3	3	3	3	4	4
4	4	4	4	3	3	3	3	4	4
4	4	4	4	3	3	3	3	4	4
4	4	2	2	3	3	3	3	4	4
4	4	4	4	3	3	3	3	4	4

For Review Only

2	2	4	4	3	3	3	3	4	4
2	2	4	4	3	3	3	3	4	4
4	4	4	4	3	3	3	3	4	4
4	4	4	4	3	3	3	3	4	4
4	4	4	4	3	3	3	3	4	4
4	4	4	4	3	3	3	3	4	4
4	4	4	4	3	3	3	3	4	4
4	4	4	4	3	3	3	3	1	1
2	2	4	4	3	3	3	3	4	4
4	4	4	4	3	3	3	3	4	4
4	4	4	4	3	3	3	3	4	4
4	4	4	4	3	3	3	3	4	4
2	2	4	4	3	3	3	3	4	4
4	4	4	4	3	3	3	3	4	4
4	4	4	4	3	3	3	3	4	4
2	2	4	4	3	3	3	3	4	4
4	4	4	4	3	3	3	3	4	4
4	4	4	4	3	3	3	3	4	4
4	4	4	4	3	3	3	3	4	4
2	2	4	4	3	3	3	3	4	4
4	4	4	4	3	3	3	3	4	4
4	4	4	4	3	3	2	2	4	4
2	2	4	4	3	3	3	3	4	4
4	4	4	4	3	3	3	3	4	4
4	4	4	4	2	2	3	3	4	4
4	4	4	4	3	3	3	3	4	4
4	4	4	4	3	3	3	3	4	4
4	4	4	4	2	2	2	2	4	4
4	4	4	4	3	3	3	3	4	4
4	4	4	4	3	3	3	3	4	4
4	4	4	4	3	3	3	3	4	4
4	4	4	4	2	2	3	3	4	4
4	4	4	4	3	3	2	2	4	4
2	2	4	4	3	3	3	3	4	4
4	4	4	4	3	3	2	2	1	1
4	4	4	4	2	2	3	3	4	4
4	4	4	4	3	3	3	3	4	4
4	4	4	4	3	3	3	3	4	4
4	4	4	4	2	2	3	3	4	4
4	4	4	4	2	2	3	3	4	4
4	4	4	4	3	3	3	3	4	4
4	4	4	4	3	3	3	3	4	4
4	4	2	2	3	3	3	3	4	4
4	4	4	4	3	3	3	3	4	4
4	4	4	4	3	3	3	3	4	4
4	4	4	4	3	3	3	3	4	4
4	4	4	4	3	3	3	3	4	4
4	4	4	4	3	3	3	3	4	4
4	4	4	4	3	3	3	3	4	4
4	4	4	4	3	3	3	3	4	4
4	4	2	2	3	3	3	3	4	4
4	4	4	4	3	3	2	2	4	4
4	4	4	4	3	3	3	3	4	4
4	4	4	4	3	3	3	3	4	4
4	4	4	4	3	3	3	3	4	4

For Review Only

4	4	4	4	3	3	2	2	4	4
4	4	4	4	3	3	3	3	4	4
4	4	4	4	3	3	3	3	4	4
4	4	4	4	3	3	3	3	4	4
4	4	4	4	3	3	3	3	4	4
4	4	4	4	2	2	3	3	4	4
4	4	2	2	3	3	3	3	4	4
4	4	4	4	3	3	3	3	4	4
4	4	4	4	3	3	3	3	4	4
4	4	4	4	3	3	3	3	4	4
4	4	4	4	3	3	3	3	4	4
4	4	4	4	3	3	3	3	4	4
4	4	4	4	2	2	3	3	4	4
4	4	4	4	3	3	3	3	4	4
4	4	4	4	3	3	3	3	4	4
4	4	4	4	3	3	3	3	4	4
4	4	4	4	3	3	3	3	4	4
4	4	4	4	3	3	3	3	4	4
4	4	4	4	3	3	3	3	1	1
4	4	4	4	3	3	3	3	4	4
4	4	4	4	3	3	3	3	4	4
4	4	4	4	3	3	3	3	4	4
4	4	4	4	3	3	3	3	4	4
4	4	4	4	3	3	2	2	4	4
4	4	4	4	3	3	3	3	4	4
4	4	4	4	3	3	2	2	4	4
4	4	4	4	3	3	3	3	4	4
4	4	4	4	3	3	3	3	4	4
4	4	4	4	3	3	3	3	4	4
4	4	4	4	3	3	3	3	4	4
4	4	4	4	3	3	2	2	4	4
4	4	4	4	3	3	3	3	4	4
4	4	4	4	3	3	3	3	4	4
4	4	4	4	3	3	3	3	4	4
4	4	4	4	3	3	3	3	1	1
4	4	4	4	3	3	3	3	4	4
4	4	4	4	3	3	3	3	1	1
4	4	2	2	3	3	3	3	4	4
4	4	4	4	3	3	3	3	4	4
4	4	4	4	3	3	2	2	4	4
4	4	4	4	3	3	3	3	4	4
4	4	4	4	3	3	3	3	1	1
4	4	4	4	3	3	3	3	4	4
4	4	4	4	3	3	3	3	4	4
4	4	4	4	3	3	3	3	4	4
4	4	4	4	3	3	3	3	4	4
4	4	4	4	3	3	2	2	4	4
4	4	2	2	3	3	3	3	4	4
4	4	4	4	3	3	3	3	1	1
4	4	4	4	3	3	3	3	4	4
4	4	4	4	3	3	3	3	4	4
4	4	4	4	3	3	2	2	4	4
4	4	4	4	3	3	3	3	4	4
4	4	4	4	3	3	3	3	4	4
4	4	4	4	3	3	3	3	4	4

For Review Only

4	4	4	4	3	3	3	3	4	4
4	4	4	4	2	2	2	2	4	4
4	4	4	4	3	3	3	3	4	4
4	4	4	4	3	3	3	3	4	4
4	4	4	4	3	3	3	3	4	4
4	4	4	4	3	3	3	3	4	4
4	4	4	4	3	3	2	2	4	4
4	4	4	4	3	3	3	3	4	4
4	4	4	4	3	3	3	3	4	4
4	4	4	4	3	3	3	3	4	4
4	4	4	4	3	3	2	2	4	4
4	4	4	4	3	3	3	3	4	4
4	4	4	4	3	3	3	3	4	4
4	4	4	4	3	3	3	3	4	4
4	4	4	4	2	2	3	3	1	1
4	4	4	4	3	3	2	2	4	4
4	4	4	4	3	3	3	3	4	4
4	4	4	4	3	3	3	3	4	4
4	4	4	4	3	3	3	3	4	4
4	4	2	2	3	3	3	3	4	4
4	4	4	4	3	3	3	3	4	4
4	4	4	4	3	3	3	3	4	4
4	4	4	4	3	3	2	2	4	4
4	4	4	4	3	3	3	3	4	4
4	4	4	4	3	3	3	3	4	4
4	4	4	4	3	3	3	3	1	1
4	4	2	2	3	3	3	3	4	4
4	4	4	4	3	3	3	3	4	4
4	4	4	4	3	3	3	3	4	4
4	4	4	4	3	3	3	3	4	4
2	2	4	4	3	3	3	3	4	4
4	4	4	4	3	3	3	3	4	4
4	4	4	4	3	3	3	3	4	4
4	4	4	4	3	3	3	3	1	1
4	4	4	4	3	3	3	3	4	4
4	4	4	4	3	3	3	3	4	4
4	4	4	4	3	3	3	3	4	4
2	2	4	4	3	3	3	3	4	4
4	4	2	2	3	3	3	3	4	4
4	4	2	2	3	3	3	3	4	4
4	4	4	4	3	3	3	3	4	4
4	4	4	4	3	3	2	2	4	4
4	4	4	4	3	3	3	3	1	1
4	4	4	4	3	3	3	3	4	4
4	4	4	4	3	3	3	3	1	1
4	4	4	4	3	3	3	3	4	4
4	4	4	4	3	3	2	2	4	4
4	4	4	4	3	3	2	2	4	4
4	4	4	4	3	3	3	3	4	4
4	4	4	4	3	3	3	3	4	4
4	4	4	4	3	3	3	3	4	4
4	4	4	4	3	3	3	3	4	4
4	4	4	4	3	3	2	2	4	4
4	4	4	4	3	3	3	3	4	4
4	4	4	4	3	3	2	2	4	4

For Review Only

4	4	4	4	3	3	3	3	4	4
4	4	4	4	3	3	2	2	4	4
4	4	4	4	3	3	3	3	4	4
4	4	4	4	3	3	3	3	4	4
4	4	4	4	3	3	2	2	4	4
4	4	4	4	3	3	3	3	4	4
4	4	4	4	2	2	2	2	4	4
4	4	4	4	3	3	3	3	4	4
4	4	4	4	3	3	3	3	4	4
4	4	4	4	3	3	2	2	4	4
4	4	4	4	3	3	3	3	4	4
2	2	4	4	3	3	2	2	4	4
4	4	4	4	3	3	3	3	4	4
4	4	4	4	3	3	3	3	4	4
4	4	2	2	3	3	3	3	4	4
4	4	4	4	3	3	2	2	4	4
4	4	4	4	3	3	2	2	4	4
4	4	4	4	3	3	3	3	1	1
4	4	4	4	3	3	2	2	4	4
4	4	4	4	3	3	3	3	4	4
4	4	4	4	3	3	3	3	4	4
4	4	4	4	3	3	3	3	4	4
4	4	4	4	3	3	3	3	4	4
4	4	4	4	3	3	3	3	4	4
4	4	4	4	3	3	3	3	1	1
4	4	4	4	3	3	3	3	4	4
4	4	4	4	3	3	2	2	4	4
4	4	2	2	3	3	3	3	4	4
2	2	4	4	3	3	2	2	4	4
4	4	4	4	3	3	3	3	4	4
4	4	4	4	3	3	2	2	4	4
4	4	4	4	3	3	2	2	4	4
4	4	4	4	3	3	3	3	4	4
4	4	2	2	3	3	3	3	4	4
4	4	4	4	3	3	2	2	4	4
4	4	4	4	3	3	2	2	4	4
4	4	4	4	3	3	3	3	4	4
4	4	4	4	3	3	3	3	4	4
4	4	4	4	3	3	3	3	4	4
4	4	4	4	3	3	2	2	4	4
4	4	4	4	3	3	3	3	4	4
4	4	4	4	3	3	3	3	4	4
4	4	2	2	3	3	2	2	4	4
4	4	4	4	3	3	3	3	4	4
4	4	4	4	3	3	3	3	4	4
4	4	4	4	3	3	3	3	4	4
4	4	4	4	3	3	2	2	4	4
4	4	2	2	3	3	3	3	4	4
4	4	4	4	3	3	3	3	4	4
4	4	4	4	3	3	2	2	4	4
4	4	4	4	3	3	3	3	4	4
4	4	4	4	3	3	2	2	4	4
4	4	4	4	3	3	3	3	4	4
4	4	2	2	3	3	3	3	4	4
4	4	4	4	3	3	2	2	4	4

For Review Only

4	4	4	4	3	3	3	3	4	4
4	4	4	4	3	3	3	3	4	4

For Review Only

SNP952	SNP952	SNP953	SNP953	SNP954	SNP954	SNP955	SNP955	SNP956	SNP956
4	4	1	1	2	2	4	4	3	3
4	4	1	1	2	2	4	4	3	3
4	4	2	2	3	3	4	4	3	3
4	4	1	1	2	2	4	4	3	3
4	4	1	1	2	2	4	4	3	3
4	4	1	1	2	2	4	4	3	3
4	4	1	1	2	2	4	4	3	3
4	4	2	2	3	3	4	4	3	3
4	4	1	1	2	2	4	4	3	3
4	4	2	2	3	3	4	4	3	3
4	4	1	1	2	2	4	4	3	3
4	4	2	2	3	3	4	4	3	3
4	4	1	1	2	2	4	4	3	3
4	4	1	1	2	2	4	4	3	3
4	4	1	1	2	2	4	4	3	3
4	4	1	1	2	2	4	4	3	3
4	4	1	1	2	2	4	4	3	3
4	4	1	1	2	2	4	4	3	3
4	4	2	2	3	3	4	4	3	3
4	4	2	2	3	3	4	4	3	3
4	4	1	1	2	2	4	4	3	3
4	4	1	1	2	2	4	4	3	3
1	1	1	1	2	2	4	4	3	3
4	4	1	1	2	2	4	4	3	3
4	4	1	1	2	2	4	4	3	3
4	4	2	2	3	3	4	4	3	3
4	4	1	1	2	2	4	4	3	3
4	4	1	1	2	2	4	4	3	3
4	4	1	1	2	2	4	4	3	3
1	1	1	1	2	2	4	4	3	3
4	4	1	1	2	2	4	4	3	3
4	4	1	1	2	2	4	4	3	3
4	4	1	1	2	2	4	4	3	3
1	1	1	1	2	2	3	3	4	4
4	4	1	1	2	2	4	4	3	3
4	4	1	1	2	2	4	4	3	3
4	4	1	1	2	2	4	4	3	3
4	4	1	1	2	2	4	4	3	3
1	1	1	1	2	2	4	4	3	3
4	4	1	1	2	2	4	4	3	3
4	4	2	2	3	3	4	4	3	3
4	4	2	2	3	3	4	4	3	3
4	4	2	2	3	3	4	4	3	3
4	4	1	1	2	2	4	4	3	3
4	4	1	1	2	2	4	4	3	3
4	4	2	2	3	3	4	4	3	3
4	4	2	2	3	3	4	4	3	3
4	4	1	1	2	2	4	4	3	3
4	4	1	1	2	2	4	4	3	3
4	4	1	1	2	2	4	4	3	3
4	4	2	2	3	3	4	4	3	3
4	4	1	1	2	2	4	4	3	3

4	4	1	1	2	2	4	4	3	3
4	4	1	1	2	2	4	4	3	3
4	4	2	2	3	3	4	4	3	3
4	4	1	1	2	2	4	4	3	3
4	4	2	2	3	3	4	4	3	3
4	4	2	2	3	3	4	4	3	3
4	4	2	2	3	3	4	4	3	3
4	4	1	1	2	2	3	3	4	4
4	4	1	1	2	2	4	4	3	3
4	4	1	1	2	2	4	4	3	3
4	4	2	2	3	3	4	4	3	3
4	4	2	2	3	3	4	4	3	3
4	4	1	1	2	2	4	4	3	3
4	4	1	1	2	2	4	4	3	3
4	4	1	1	2	2	4	4	3	3
4	4	1	1	2	2	4	4	3	3
4	4	2	2	3	3	4	4	3	3
4	4	1	1	2	2	4	4	3	3
4	4	2	2	3	3	4	4	3	3
4	4	1	1	2	2	4	4	3	3
4	4	2	2	3	3	4	4	3	3
4	4	1	1	2	2	4	4	3	3
4	4	1	1	2	2	4	4	3	3
4	4	2	2	3	3	4	4	3	3
4	4	1	1	2	2	4	4	3	3
4	4	2	2	3	3	4	4	3	3
4	4	2	2	3	3	4	4	3	3
1	1	1	1	2	2	4	4	3	3
4	4	2	2	3	3	4	4	3	3
4	4	1	1	2	2	4	4	3	3
4	4	1	1	2	2	4	4	3	3
4	4	1	1	2	2	4	4	3	3
1	1	1	1	2	2	4	4	3	3
4	4	1	1	2	2	4	4	3	3
4	4	1	1	2	2	3	3	4	4
4	4	1	1	2	2	4	4	3	3
4	4	2	2	3	3	4	4	3	3
4	4	1	1	2	2	4	4	3	3
1	1	1	1	2	2	4	4	3	3
4	4	2	2	3	3	4	4	3	3
4	4	1	1	2	2	4	4	3	3
4	4	1	1	2	2	4	4	3	3
4	4	2	2	3	3	4	4	3	3
4	4	2	2	3	3	4	4	3	3
4	4	1	1	2	2	4	4	3	3
4	4	1	1	2	2	4	4	3	3
1	1	1	1	2	2	4	4	3	3
4	4	2	2	3	3	4	4	3	3
4	4	1	1	2	2	4	4	3	3
4	4	1	1	2	2	4	4	3	3
4	4	1	1	2	2	4	4	3	3
4	4	1	1	2	2	4	4	3	3
4	4	1	1	2	2	4	4	3	3
4	4	1	1	2	2	4	4	3	3
4	4	2	2	3	3	4	4	3	3

For Review Only

4	4	1	1	2	2	4	4	3	3
4	4	1	1	2	2	4	4	3	3
4	4	1	1	2	2	4	4	3	3
4	4	1	1	2	2	3	3	4	4
4	4	2	2	3	3	4	4	3	3
4	4	2	2	3	3	4	4	3	3
4	4	1	1	2	2	4	4	3	3
4	4	1	1	2	2	4	4	3	3
1	1	1	1	2	2	4	4	3	3
4	4	2	2	3	3	4	4	3	3
4	4	1	1	2	2	4	4	3	3
4	4	1	1	2	2	4	4	3	3
4	4	2	2	3	3	4	4	3	3
4	4	2	2	3	3	4	4	3	3
4	4	1	1	2	2	4	4	3	3
4	4	2	2	3	3	4	4	3	3
4	4	2	2	3	3	4	4	3	3
4	4	2	2	2	2	4	4	3	3
4	4	1	1	2	2	3	3	4	4
4	4	2	2	3	3	4	4	3	3
4	4	1	1	2	2	4	4	3	3
4	4	1	1	2	2	4	4	3	3
4	4	1	1	2	2	4	4	3	3
4	4	1	1	2	2	4	4	3	3
4	4	1	1	2	2	4	4	3	3
4	4	1	1	2	2	4	4	3	3
4	4	2	2	2	2	4	4	3	3
4	4	1	1	2	2	4	4	3	3
4	4	2	2	3	3	4	4	3	3
4	4	1	1	2	2	4	4	3	3
1	1	1	1	2	2	4	4	3	3
4	4	1	1	3	3	4	4	3	3
4	4	1	1	2	2	4	4	3	3
4	4	1	1	2	2	4	4	3	3
1	1	1	1	2	2	3	3	4	4
4	4	1	1	2	2	4	4	3	3
4	4	1	1	2	2	3	3	4	4
4	4	2	2	3	3	4	4	3	3
4	4	1	1	3	3	4	4	3	3
4	4	1	1	2	2	4	4	3	3
4	4	1	1	2	2	4	4	3	3
1	1	1	1	2	2	3	3	4	4
4	4	1	1	2	2	4	4	3	3
4	4	1	1	2	2	4	4	3	3
4	4	1	1	2	2	4	4	3	3
4	4	1	1	2	2	4	4	3	3
4	4	2	2	3	3	4	4	3	3
4	4	2	2	3	3	4	4	3	3
4	4	1	1	2	2	4	4	3	3
4	4	2	2	3	3	4	4	3	3
4	4	1	1	2	2	4	4	3	3
4	4	2	2	3	3	4	4	3	3
4	4	1	1	2	2	4	4	3	3
4	4	2	2	3	3	4	4	3	3
4	4	1	1	2	2	4	4	3	3

For Review Only

4	4	2	2	3	3	4	4	3	3
4	4	1	1	2	2	4	4	3	3
4	4	1	1	2	2	4	4	3	3
4	4	1	1	2	2	4	4	3	3
4	4	1	1	2	2	4	4	3	3
4	4	1	1	2	2	4	4	3	3
4	4	1	1	2	2	4	4	3	3
4	4	1	1	2	2	4	4	3	3
4	4	1	1	2	2	4	4	3	3
4	4	1	1	2	2	4	4	3	3
4	4	2	2	3	3	4	4	3	3
4	4	1	1	2	2	4	4	3	3
4	4	2	2	3	3	4	4	3	3
4	4	1	1	2	2	4	4	3	3
1	1	1	1	2	2	3	3	4	4
4	4	1	1	2	2	4	4	3	3
4	4	1	1	2	2	4	4	3	3
4	4	2	2	3	3	4	4	3	3
4	4	1	1	2	2	4	4	3	3
4	4	1	1	2	2	4	4	3	3
4	4	1	1	2	2	4	4	3	3
4	4	1	1	2	2	4	4	3	3
4	4	1	1	2	2	4	4	3	3
4	4	2	2	3	3	4	4	3	3
4	4	2	2	3	3	4	4	3	3
1	1	1	1	2	2	3	3	4	4
4	4	1	1	2	2	4	4	3	3
4	4	1	1	2	2	4	4	3	3
4	4	1	1	2	2	4	4	3	3
4	4	1	1	2	2	4	4	3	3
4	4	2	2	3	3	4	4	3	3
4	4	1	1	2	2	4	4	3	3
4	4	1	1	2	2	4	4	3	3
1	1	1	1	2	2	3	3	4	4
4	4	1	1	2	2	4	4	3	3
4	4	2	2	3	3	4	4	3	3
4	4	1	1	2	2	4	4	3	3
4	4	1	1	2	2	4	4	3	3
4	4	2	2	3	3	4	4	3	3
4	4	2	2	3	3	4	4	3	3
4	4	2	2	3	3	4	4	3	3
4	4	2	2	3	3	4	4	3	3
4	4	1	1	2	2	4	4	3	3
4	4	2	2	3	3	4	4	3	3
4	4	1	1	2	2	4	4	3	3
1	1	1	1	2	2	4	4	3	3
4	4	1	1	2	2	4	4	3	3
1	1	1	1	2	2	4	4	3	3
1	1	1	1	2	2	4	4	3	3
4	4	1	1	2	2	4	4	3	3
4	4	2	2	3	3	4	4	3	3
4	4	1	1	2	2	4	4	3	3
4	4	1	1	2	2	4	4	3	3
4	4	2	2	3	3	4	4	3	3
4	4	1	1	2	2	4	4	3	3

For Review Only

4	4	1	1	2	2	4	4	3	3
4	4	1	1	2	2	4	4	3	3
4	4	2	2	3	3	4	4	3	3
4	4	1	1	2	2	4	4	3	3
4	4	1	1	2	2	4	4	3	3
4	4	1	1	2	2	4	4	3	3
4	4	1	1	2	2	4	4	3	3
4	4	2	2	3	3	4	4	3	3
4	4	1	1	2	2	4	4	3	3
1	1	1	1	2	2	4	4	3	3
4	4	1	1	2	2	4	4	3	3
4	4	2	2	3	3	4	4	3	3
4	4	1	1	2	2	4	4	3	3
4	4	2	2	3	3	4	4	3	3
4	4	2	2	3	3	4	4	3	3
4	4	1	1	2	2	4	4	3	3
1	1	1	1	2	2	4	4	3	3
4	4	1	1	2	2	3	3	4	4
4	4	1	1	2	2	3	3	3	3
4	4	1	1	2	2	4	4	3	3
4	4	2	2	3	3	4	4	3	3
4	4	2	2	3	3	4	4	3	3
4	4	1	1	2	2	4	4	3	3
4	4	1	1	2	2	4	4	3	3
4	4	1	1	2	2	3	3	4	4
4	4	1	1	2	2	4	4	3	3
1	1	1	1	2	2	4	4	3	3
4	4	2	2	3	3	4	4	3	3
4	4	1	1	2	2	4	4	3	3
4	4	2	2	3	3	4	4	3	3
4	4	1	1	2	2	4	4	3	3
4	4	2	2	3	3	4	4	3	3
1	1	1	1	2	2	4	4	3	3
4	4	2	2	3	3	4	4	3	3
4	4	1	1	2	2	4	4	3	3
4	4	1	1	2	2	4	4	3	3
4	4	1	1	2	2	4	4	3	3
4	4	2	2	3	3	4	4	3	3
4	4	1	1	2	2	4	4	3	3
4	4	1	1	2	2	4	4	3	3
4	4	1	1	2	2	4	4	3	3
4	4	1	1	2	2	4	4	3	3
4	4	1	1	2	2	4	4	3	3
4	4	1	1	2	2	4	4	3	3
4	4	2	2	3	3	4	4	3	3
1	1	1	1	2	2	4	4	3	3
4	4	1	1	2	2	4	4	3	3
4	4	1	1	3	3	4	4	3	3
4	4	1	1	2	2	4	4	3	3
1	1	1	1	2	2	4	4	3	3
4	4	1	1	2	2	4	4	3	3
1	1	1	1	2	2	4	4	3	3
4	4	2	2	3	3	4	4	3	3
4	4	2	2	3	3	4	4	3	3
4	4	1	1	2	2	4	4	3	3

For Review Only

4	4	1	1	2	2	4	4	3	3
4	4	2	2	3	3	4	4	3	3

For Review Only

SNP957	SNP957	SNP958	SNP958	SNP959	SNP959	SNP960	SNP960	SNP961	SNP961
3	3	4	4	1	1	2	2	2	2
3	3	3	3	1	1	2	2	2	2
3	3	4	4	1	1	2	2	4	4
3	3	4	4	1	1	3	3	2	2
3	3	4	4	1	1	2	2	2	2
3	3	4	4	1	1	2	2	2	2
3	3	4	4	1	1	2	2	2	2
3	3	4	4	1	1	2	2	2	2
3	3	4	4	1	1	2	2	2	2
3	3	4	4	1	1	2	2	2	2
3	3	4	4	1	1	2	2	2	2
3	3	4	4	1	1	2	2	2	2
3	3	4	4	1	1	2	2	2	2
3	3	4	4	1	1	2	2	2	2
1	1	4	4	1	1	2	2	2	2
3	3	3	3	1	1	3	3	2	2
3	3	4	4	1	1	2	2	2	2
3	3	4	4	1	1	2	2	2	2
1	1	4	4	1	1	2	2	2	2
3	3	4	4	1	1	2	2	2	2
3	3	4	4	1	1	2	2	2	2
1	1	4	4	1	1	2	2	2	2
3	3	3	3	1	1	2	2	2	2
3	3	4	4	1	1	2	2	2	2
1	1	4	4	1	1	2	2	2	2
3	3	4	4	1	1	2	2	2	2
3	3	3	3	1	1	2	2	2	2
3	3	4	4	1	1	2	2	2	2
3	3	4	4	1	1	2	2	2	2
3	3	4	4	1	1	2	2	2	2
1	1	4	4	1	1	2	2	2	2
1	1	4	4	1	1	2	2	4	4
3	3	4	4	1	1	2	2	2	2
3	3	4	4	1	1	2	2	2	2
3	3	4	4	1	1	2	2	2	2
3	3	4	4	1	1	2	2	2	2
3	3	4	4	1	1	2	2	2	2
1	1	4	4	1	1	2	2	2	2
3	3	4	4	1	1	2	2	2	2
3	3	3	3	1	1	2	2	2	2
3	3	4	4	1	1	2	2	2	2
3	3	4	4	1	1	2	2	2	2
3	3	4	4	1	1	2	2	2	2
1	1	4	4	1	1	2	2	4	4
3	3	4	4	1	1	2	2	2	2
3	3	4	4	1	1	2	2	4	4
3	3	4	4	1	1	2	2	2	2
3	3	4	4	1	1	2	2	2	2
1	1	4	4	1	1	2	2	2	2
3	3	4	4	1	1	2	2	4	4
3	3	4	4	1	1	3	3	2	2
3	3	4	4	1	1	2	2	2	2

For Review Only

3	3	4	4	1	1	2	2	2	2
3	3	4	4	1	1	2	2	2	2
1	1	3	3	1	1	2	2	2	2
1	1	4	4	1	1	2	2	2	2
1	1	4	4	1	1	2	2	2	2
1	1	4	4	1	1	2	2	2	2
3	3	4	4	1	1	2	2	2	2
3	3	3	3	1	1	2	2	2	2
3	3	3	3	1	1	2	2	2	2
3	3	4	4	1	1	2	2	2	2
1	1	3	3	1	1	2	2	2	2
1	1	4	4	1	1	2	2	2	2
3	3	4	4	1	1	2	2	2	2
3	3	4	4	1	1	2	2	2	2
1	1	4	4	1	1	2	2	2	2
3	3	3	3	1	1	2	2	2	2
3	3	4	4	1	1	2	2	4	4
3	3	4	4	1	1	2	2	2	2
3	3	4	4	1	1	2	2	2	2
3	3	4	4	1	1	2	2	2	2
1	1	4	4	1	1	2	2	2	2
3	3	4	4	1	1	2	2	2	2
3	3	4	4	1	1	2	2	2	2
1	1	4	4	1	1	2	2	2	2
3	3	4	4	1	1	2	2	2	2
3	3	4	4	1	1	2	2	2	2
3	3	4	4	1	1	2	2	4	4
3	3	3	3	1	1	2	2	2	2
3	3	4	4	1	1	2	2	2	2
3	3	4	4	1	1	2	2	4	4
1	1	4	4	1	1	2	2	2	2
1	1	4	4	1	1	2	2	2	2
3	3	3	3	1	1	2	2	2	2
3	3	4	4	1	1	2	2	2	2
3	3	4	4	1	1	2	2	2	2
3	3	4	4	1	1	2	2	2	2
3	3	4	4	1	1	2	2	2	2
1	1	4	4	1	1	2	2	2	2
3	3	3	3	1	1	2	2	2	2
3	3	4	4	1	1	2	2	2	2
3	3	4	4	1	1	2	2	2	2
3	3	4	4	1	1	2	2	2	2
3	3	4	4	1	1	3	3	2	2
1	1	3	3	1	1	2	2	2	2
3	3	4	4	1	1	2	2	2	2
1	1	4	4	1	1	2	2	2	2
3	3	4	4	4	4	2	2	2	2
3	3	4	4	1	1	2	2	2	2
3	3	4	4	1	1	2	2	2	2
1	1	4	4	4	4	2	2	2	2
1	1	3	3	1	1	2	2	2	2
3	3	3	3	1	1	2	2	2	2
3	3	4	4	1	1	2	2	2	2
3	3	4	4	1	1	2	2	2	2
3	3	4	4	4	4	2	2	2	2

For Review Only

3	3	4	4	1	1	2	2	2	2
3	3	4	4	1	1	2	2	2	2
3	3	4	4	1	1	2	2	2	2
3	3	4	4	4	4	2	2	2	2
1	1	3	3	1	1	2	2	2	2
3	3	4	4	1	1	2	2	2	2
3	3	4	4	1	1	3	3	2	2
1	1	4	4	1	1	2	2	2	2
3	3	3	3	1	1	2	2	2	2
3	3	4	4	1	1	2	2	2	2
3	3	4	4	1	1	2	2	2	2
3	3	4	4	1	1	2	2	2	2
3	3	4	4	1	1	2	2	2	2
3	3	4	4	1	1	2	2	2	2
1	1	4	4	1	1	2	2	2	2
3	3	4	4	1	1	2	2	2	2
3	3	4	4	1	1	2	2	2	2
3	3	4	4	1	1	2	2	2	2
3	3	4	4	4	4	2	2	2	2
3	3	4	4	1	1	2	2	2	2
3	3	4	4	1	1	2	2	2	2
3	3	4	4	1	1	2	2	2	2
1	1	4	4	1	1	2	2	4	4
3	3	3	3	1	1	2	2	2	2
3	3	4	4	1	1	2	2	2	2
3	3	3	3	1	1	2	2	2	2
1	1	4	4	1	1	2	2	2	2
3	3	4	4	1	1	2	2	2	2
3	3	4	4	1	1	2	2	2	2
3	3	4	4	1	1	2	2	2	2
3	3	3	3	1	1	2	2	2	2
3	3	3	3	1	1	2	2	2	2
3	3	4	4	1	1	2	2	2	2
3	3	4	4	1	1	2	2	2	2
3	3	4	4	1	1	2	2	2	2
3	3	4	4	1	1	2	2	2	2
3	3	4	4	4	4	2	2	2	2
3	3	4	4	1	1	3	3	2	2
3	3	3	3	1	1	2	2	2	2
3	3	3	3	1	1	2	2	2	2
3	3	4	4	1	1	2	2	2	2
3	3	4	4	1	1	2	2	2	2
3	3	4	4	1	1	2	2	2	2
3	3	3	3	1	1	2	2	2	2
3	3	4	4	1	1	2	2	2	2
1	1	3	3	1	1	2	2	2	2
3	3	4	4	1	1	2	2	2	2
1	1	4	4	1	1	2	2	2	2
3	3	4	4	1	1	2	2	2	2
3	3	4	4	1	1	2	2	2	2
3	3	4	4	1	1	2	2	2	2
3	3	3	3	1	1	2	2	2	2
3	3	4	4	1	1	2	2	2	2
1	1	4	4	1	1	2	2	2	2
3	3	3	3	1	1	2	2	2	2

For Review Only

3	3	4	4	1	1	2	2	2	2
3	3	4	4	1	1	2	2	2	2
3	3	4	4	1	1	2	2	2	2
3	3	4	4	1	1	2	2	2	2
1	1	4	4	1	1	2	2	2	2
3	3	4	4	1	1	2	2	2	2
1	1	3	3	1	1	2	2	2	2
3	3	4	4	1	1	2	2	2	2
3	3	4	4	1	1	2	2	2	2
3	3	4	4	1	1	2	2	2	2
3	3	4	4	1	1	2	2	2	2
3	3	3	3	1	1	2	2	2	2
3	3	4	4	1	1	2	2	4	4
1	1	4	4	1	1	2	2	2	2
3	3	4	4	1	1	2	2	2	2
3	3	3	3	1	1	2	2	2	2
3	3	4	4	1	1	2	2	2	2
1	1	3	3	1	1	2	2	2	2
3	3	4	4	1	1	2	2	2	2
3	3	4	4	1	1	3	3	2	2
3	3	4	4	1	1	2	2	2	2
3	3	3	3	1	1	2	2	2	2
3	3	4	4	1	1	2	2	2	2
1	1	4	4	1	1	2	2	2	2
3	3	4	4	1	1	2	2	4	4
3	3	4	4	1	1	2	2	2	2
3	3	4	4	1	1	3	3	2	2
3	3	4	4	1	1	2	2	2	2
3	3	3	3	1	1	2	2	2	2
3	3	4	4	1	1	2	2	4	4
1	1	4	4	1	1	2	2	2	2
3	3	4	4	1	1	2	2	4	4
3	3	4	4	1	1	2	2	2	2
3	3	4	4	1	1	2	2	2	2
1	1	4	4	1	1	2	2	2	2
1	1	4	4	1	1	2	2	2	2
3	3	4	4	1	1	2	2	2	2
3	3	4	4	1	1	2	2	2	2
1	1	4	4	1	1	2	2	2	2
1	1	4	4	1	1	2	2	2	2
3	3	4	4	1	1	2	2	4	4
3	3	4	4	1	1	2	2	2	2
3	3	3	3	1	1	2	2	2	2
1	1	4	4	1	1	2	2	2	2
3	3	4	4	1	1	2	2	2	2
1	1	4	4	1	1	2	2	2	2
3	3	3	3	1	1	2	2	2	2
1	1	3	3	1	1	2	2	2	2
3	3	3	3	1	1	2	2	2	2
3	3	4	4	1	1	2	2	2	2
3	3	4	4	1	1	2	2	2	2
3	3	4	4	4	4	2	2	2	2
3	3	4	4	1	1	2	2	2	2
1	1	3	3	1	1	2	2	2	2
3	3	3	3	1	1	2	2	2	2

For Review Only

3	3	3	3	1	1	2	2	2	2
3	3	4	4	1	1	2	2	2	2
3	3	4	4	1	1	2	2	2	2
3	3	4	4	1	1	2	2	2	2
3	3	4	4	1	1	2	2	2	2
1	1	4	4	1	1	2	2	2	2
3	3	3	3	1	1	2	2	2	2
3	3	4	4	1	1	2	2	2	2
3	3	4	4	1	1	2	2	2	2
3	3	3	3	1	1	2	2	2	2
3	3	4	4	1	1	2	2	2	2
3	3	4	4	1	1	2	2	2	2
3	3	3	3	1	1	2	2	2	2
3	3	4	4	1	1	2	2	2	2
1	1	4	4	1	1	2	2	4	4
3	3	3	3	1	1	2	2	2	2
1	1	3	3	1	1	2	2	2	2
3	3	4	4	1	1	2	2	2	2
3	3	3	3	1	1	2	2	2	2
3	3	4	4	4	4	2	2	2	2
3	3	3	3	1	1	2	2	2	2
3	3	4	4	1	1	2	2	2	2
3	3	4	4	1	1	3	3	2	2
3	3	3	3	1	1	2	2	2	2
3	3	3	3	1	1	2	2	2	2
3	3	4	4	1	1	2	2	2	2
3	3	3	3	1	1	2	2	2	2
3	3	4	4	1	1	3	3	2	2
3	3	4	4	1	1	2	2	2	2
3	3	3	3	1	1	2	2	2	2
3	3	3	3	1	1	2	2	2	2
3	3	4	4	1	1	2	2	2	2
3	3	4	4	4	4	2	2	2	2
1	1	4	4	1	1	2	2	2	2
3	3	4	4	1	1	2	2	4	4
3	3	3	3	1	1	2	2	2	2
1	1	4	4	1	1	2	2	2	2
1	1	4	4	1	1	2	2	2	2
3	3	4	4	1	1	2	2	2	2
3	3	3	3	1	1	2	2	2	2
3	3	4	4	1	1	2	2	2	2
1	1	4	4	1	1	2	2	2	2
3	3	3	3	1	1	3	3	2	2
1	1	4	4	1	1	2	2	2	2
3	3	4	4	1	1	2	2	2	2
3	3	4	4	1	1	2	2	2	2
1	1	3	3	1	1	2	2	2	2
3	3	4	4	1	1	3	3	2	2
3	3	4	4	1	1	2	2	2	2
3	3	3	3	1	1	2	2	2	2
3	3	4	4	1	1	2	2	4	4
3	3	3	3	1	1	2	2	2	2
3	3	4	4	1	1	2	2	4	4
3	3	4	4	1	1	3	3	2	2
3	3	3	3	1	1	2	2	2	2

For Review Only

3	3	4	4	1	1	2	2	2	2
3	3	4	4	1	1	2	2	2	2

For Review Only

SNP962	SNP962	SNP963	SNP963	SNP964	SNP964	SNP965	SNP965	SNP966	SNP966
2	2	4	4	4	4	4	4	2	2
2	2	3	3	3	3	4	4	3	3
2	2	4	4	4	4	4	4	2	2
3	3	4	4	4	4	4	4	3	3
2	2	4	4	4	4	4	4	2	2
2	2	4	4	4	4	4	4	3	3
2	2	4	4	4	4	4	4	2	2
2	2	4	4	4	4	4	4	2	2
2	2	4	4	4	4	4	4	3	3
2	2	4	4	4	4	4	4	2	2
2	2	3	3	3	3	1	1	3	3
2	2	4	4	4	4	4	4	2	2
2	2	4	4	4	4	4	4	3	3
2	2	3	3	3	3	4	4	3	3
2	2	4	4	4	4	4	4	3	3
3	3	4	4	4	4	4	4	3	3
2	2	4	4	4	4	4	4	2	2
2	2	4	4	4	4	4	4	2	2
2	2	4	4	4	4	4	4	3	3
2	2	4	4	3	3	4	4	3	3
2	2	3	3	3	3	1	1	3	3
2	2	4	4	4	4	4	4	3	3
2	2	3	3	3	3	1	1	3	3
2	2	3	3	3	3	4	4	3	3
2	2	4	4	4	4	4	4	3	3
2	2	4	4	4	4	4	4	2	2
2	2	4	4	4	4	4	4	2	2
2	2	4	4	4	4	4	4	3	3
2	2	3	3	3	3	1	1	3	3
2	2	3	3	3	3	1	1	3	3
2	2	4	4	4	4	4	4	2	2
2	2	4	4	4	4	4	4	3	3
2	2	4	4	4	4	4	4	2	2
2	2	3	3	3	3	4	4	3	3
2	2	4	4	4	4	4	4	2	2
2	2	4	4	4	4	4	4	3	3
2	2	4	4	4	4	4	4	2	2
2	2	4	4	4	4	4	4	2	2
2	2	3	3	3	3	1	1	3	3
2	2	3	3	3	3	1	1	3	3
2	2	3	3	3	3	1	1	3	3
2	2	3	3	3	3	1	1	3	3
2	2	4	4	4	4	4	4	2	2
2	2	3	3	3	3	1	1	3	3
2	2	4	4	4	4	4	4	2	2
2	2	4	4	4	4	4	4	2	2
2	2	4	4	4	4	4	4	2	2
2	2	3	3	4	4	4	4	3	3
2	2	4	4	4	4	4	4	3	3
2	2	4	4	4	4	4	4	2	2
3	3	4	4	4	4	4	4	2	2
2	2	4	4	4	4	4	4	3	3

For Review Only

2	2	4	4	4	4	4	4	2	2
2	2	4	4	4	4	4	4	2	2
2	2	4	4	4	4	4	4	3	3
2	2	4	4	4	4	4	4	3	3
2	2	4	4	4	4	4	4	3	3
2	2	4	4	4	4	4	4	3	3
2	2	4	4	4	4	4	4	2	2
2	2	3	3	3	3	1	1	3	3
2	2	4	4	4	4	4	4	2	2
2	2	4	4	4	4	4	4	3	3
2	2	4	4	4	4	4	4	3	3
2	2	4	4	4	4	4	4	3	3
2	2	4	4	4	4	4	4	2	2
2	2	3	3	3	3	1	1	3	3
2	2	4	4	4	4	4	4	3	3
2	2	3	3	4	4	4	4	3	3
2	2	3	3	4	4	4	4	2	2
2	2	4	4	4	4	4	4	2	2
2	2	4	4	4	4	4	4	2	2
2	2	4	4	4	4	4	4	2	2
2	2	4	4	4	4	4	4	3	3
2	2	3	3	3	3	1	1	3	3
2	2	3	3	3	3	1	1	3	3
2	2	4	4	4	4	4	4	3	3
2	2	4	4	4	4	4	4	2	2
2	2	4	4	4	4	4	4	3	3
2	2	3	3	3	3	4	4	3	3
2	2	3	3	3	3	1	1	3	3
2	2	4	4	4	4	4	4	2	2
2	2	4	4	4	4	4	4	3	3
2	2	4	4	4	4	4	4	3	3
2	2	4	4	4	4	4	4	3	3
2	2	3	3	3	3	1	1	3	3
2	2	4	4	4	4	4	4	2	2
2	2	4	4	4	4	4	4	2	2
2	2	4	4	4	4	4	4	2	2
2	2	3	3	3	3	1	1	3	3
2	2	4	4	4	4	4	4	3	3
2	2	4	4	4	4	4	4	2	2
2	2	4	4	4	4	4	4	3	3
2	2	4	4	4	4	4	4	2	2
2	2	4	4	4	4	4	4	3	3
3	3	4	4	4	4	4	4	2	2
2	2	4	4	4	4	4	4	3	3
2	2	4	4	4	4	4	4	3	3
2	2	4	4	4	4	4	4	3	3
2	2	3	3	3	3	4	4	3	3
2	2	4	4	4	4	4	4	2	2
2	2	4	4	4	4	4	4	2	2
2	2	4	4	4	4	4	4	3	3
2	2	4	4	4	4	4	4	3	3
2	2	3	3	3	3	1	1	3	3
2	2	3	3	3	3	4	4	2	2
2	2	4	4	4	4	4	4	3	3
2	2	4	4	4	4	4	4	2	2

For Review Only

2	2	4	4	4	4	4	4	3	3
2	2	4	4	4	4	4	4	3	3
2	2	4	4	4	4	4	4	3	3
2	2	3	3	3	3	4	4	3	3
2	2	4	4	4	4	4	4	3	3
2	2	4	4	4	4	4	4	3	3
3	3	4	4	4	4	4	4	3	3
2	2	4	4	4	4	4	4	3	3
2	2	4	4	4	4	4	4	3	3
3	3	4	4	3	3	4	4	3	3
2	2	4	4	4	4	4	4	2	2
2	2	3	3	4	4	4	4	3	3
2	2	4	4	4	4	4	4	2	2
2	2	4	4	4	4	4	4	3	3
2	2	4	4	4	4	4	4	3	3
2	2	4	4	4	4	4	4	3	3
2	2	3	3	3	3	1	1	3	3
2	2	4	4	4	4	4	4	3	3
2	2	3	3	3	3	4	4	3	3
2	2	3	3	4	4	1	1	3	3
2	2	4	4	4	4	4	4	3	3
2	2	4	4	4	4	4	4	2	2
2	2	4	4	4	4	4	4	3	3
2	2	3	3	3	3	4	4	3	3
2	2	4	4	4	4	4	4	3	3
2	2	3	3	3	3	1	1	3	3
2	2	4	4	4	4	4	4	3	3
2	2	4	4	4	4	4	4	2	2
3	3	4	4	4	4	4	4	3	3
2	2	4	4	4	4	4	4	3	3
2	2	3	3	3	3	4	4	3	3
2	2	4	4	4	4	4	4	3	3
2	2	4	4	4	4	4	4	2	2
2	2	4	4	4	4	4	4	3	3
2	2	3	3	3	3	4	4	3	3
2	2	4	4	3	3	4	4	2	2
2	2	3	3	3	3	4	4	3	3
3	3	4	4	4	4	4	4	2	2
2	2	3	3	3	3	1	1	3	3
2	2	3	3	3	3	1	1	3	3
2	2	3	3	3	3	1	1	3	3
2	2	3	3	3	3	4	4	3	3
2	2	4	4	4	4	4	4	2	2
2	2	3	3	3	3	4	4	3	3
2	2	3	3	3	3	4	4	3	3
2	2	4	4	4	4	4	4	3	3
2	2	4	4	4	4	4	4	2	2
2	2	4	4	4	4	4	4	3	3
2	2	4	4	4	4	4	4	3	3
2	2	4	4	4	4	4	4	3	3
2	2	4	4	4	4	4	4	2	2
2	2	4	4	4	4	4	4	2	2
2	2	3	3	4	4	4	4	3	3
2	2	4	4	4	4	4	4	3	3
2	2	4	4	4	4	4	4	3	3

For Review Only

CSSA 5585 Guilford Rd., Madison, WI 53711

2	2	4	4	4	4	4	4	2	2
2	2	3	3	3	3	1	1	3	3
2	2	4	4	4	4	4	4	2	2
2	2	3	3	3	3	1	1	3	3
2	2	4	4	4	4	4	4	3	3
2	2	4	4	4	4	4	4	2	2
2	2	4	4	4	4	4	4	3	3
2	2	4	4	4	4	4	4	3	3
2	2	3	3	3	3	4	4	3	3
2	2	4	4	4	4	4	4	2	2
2	2	4	4	4	4	4	4	3	3
2	2	3	3	3	3	4	4	3	3
2	2	4	4	4	4	4	4	2	2
2	2	4	4	4	4	4	4	3	3
2	2	3	3	3	3	4	4	3	3
2	2	3	3	3	3	1	1	3	3
2	2	4	4	4	4	4	4	3	3
2	2	4	4	4	4	4	4	3	3
2	2	4	4	4	4	4	4	3	3
3	3	4	4	4	4	4	4	3	3
2	2	4	4	4	4	4	4	3	3
2	2	3	3	3	3	4	4	3	3
2	2	3	3	3	3	1	1	3	3
2	2	4	4	4	4	4	4	3	3
2	2	3	3	3	3	4	4	3	3
2	2	3	3	3	3	4	4	3	3
3	3	4	4	4	4	4	4	3	3
2	2	3	3	3	3	1	1	3	3
2	2	4	4	4	4	4	4	2	2
2	2	4	4	4	4	4	4	2	2
2	2	4	4	4	4	4	4	3	3
2	2	4	4	4	4	4	4	2	2
2	2	4	4	4	4	4	4	2	2
2	2	3	3	3	3	1	1	3	3
2	2	4	4	4	4	4	4	3	3
2	2	4	4	4	4	4	4	3	3
2	2	4	4	4	4	4	4	2	2
2	2	4	4	4	4	4	4	2	2
2	2	4	4	4	4	4	4	3	3
2	2	4	4	4	4	4	4	3	3
2	2	4	4	4	4	4	4	2	2
2	2	4	4	4	4	4	4	2	2
2	2	3	3	3	3	1	1	3	3
2	2	4	4	4	4	4	4	3	3
2	2	4	4	4	4	4	4	3	3
2	2	4	4	4	4	4	4	3	3
2	2	3	3	3	3	1	1	3	3
2	2	4	4	4	4	4	4	3	3
2	2	3	3	3	3	4	4	3	3
2	2	4	4	4	4	4	4	3	3
2	2	4	4	4	4	4	4	2	2
2	2	3	3	3	3	4	4	3	3
2	2	3	3	3	3	1	1	3	3
2	2	4	4	4	4	4	4	3	3
2	2	3	3	3	3	1	1	3	3

For Review Only

2	2	3	3	3	3	1	1	3	3
2	2	3	3	3	3	1	1	3	3
2	2	4	4	4	4	4	4	2	2
2	2	3	3	3	3	1	1	3	3
2	2	3	3	3	3	1	1	3	3
2	2	4	4	4	4	4	4	2	2
2	2	4	4	3	3	4	4	3	3
2	2	3	3	3	3	1	1	3	3
2	2	4	4	4	4	4	4	3	3
2	2	3	3	3	3	1	1	3	3
2	2	4	4	4	4	4	4	3	3
2	2	3	3	3	3	1	1	3	3
2	2	4	4	4	4	4	4	2	2
2	2	4	4	4	4	4	4	2	2
2	2	4	4	4	4	4	4	3	3
2	2	4	4	4	4	4	4	3	3
2	2	4	4	4	4	4	4	3	3
2	2	3	3	3	3	4	4	3	3
2	2	3	3	3	3	1	1	3	3
2	2	4	4	4	4	4	4	2	2
2	2	3	3	3	3	1	1	3	3
2	2	4	4	4	4	4	4	2	2
3	3	4	4	4	4	4	4	3	3
2	2	3	3	3	3	4	4	3	3
2	2	3	3	3	3	1	1	3	3
3	3	4	4	4	4	4	4	3	3
2	2	3	3	3	3	1	1	3	3
3	3	4	4	4	4	4	4	3	3
2	2	4	4	4	4	4	4	3	3
2	2	4	4	4	4	4	4	2	2
2	2	3	3	3	3	1	1	3	3
2	2	4	4	4	4	4	4	3	3
2	2	3	3	3	3	4	4	3	3
2	2	4	4	4	4	4	4	3	3
2	2	3	3	3	3	4	4	3	3
2	2	3	3	3	3	4	4	3	3
2	2	4	4	4	4	4	4	3	3
2	2	4	4	4	4	4	4	3	3
2	2	4	4	4	4	4	4	3	3
2	2	3	3	3	3	1	1	3	3
2	2	4	4	4	4	4	4	2	2
2	2	4	4	4	4	4	4	2	2
3	3	4	4	4	4	4	4	2	2
2	2	4	4	4	4	4	4	3	3
2	2	4	4	4	4	4	4	2	2
2	2	3	3	3	3	4	4	3	3
2	2	3	3	3	3	1	1	3	3
3	3	4	4	4	4	4	4	2	2
2	2	4	4	4	4	4	4	3	3
2	2	3	3	3	3	1	1	3	3
2	2	4	4	4	4	4	4	3	3
2	2	3	3	3	3	1	1	3	3
2	2	4	4	4	4	4	4	2	2
3	3	4	4	4	4	4	4	3	3
2	2	3	3	3	3	1	1	3	3

For Review Only

2	2	4	4	4	4	4	4	3	3
2	2	4	4	4	4	4	4	3	3

For Review Only

SNP967	SNP967	SNP968	SNP968	SNP969	SNP969	SNP970	SNP970	SNP971	SNP971
1	1	3	3	3	3	1	1	2	2
1	1	2	2	4	4	1	1	3	3
4	4	3	3	3	3	1	1	3	3
4	4	3	3	3	3	1	1	3	3
4	4	3	3	3	3	1	1	2	2
1	1	2	2	4	4	1	1	2	2
4	4	3	3	3	3	1	1	2	2
1	1	2	2	4	4	1	1	2	2
4	4	3	3	3	3	1	1	2	2
1	1	2	2	4	4	1	1	2	2
1	1	2	2	4	4	1	1	3	3
4	4	3	3	3	3	1	1	3	3
1	1	3	3	3	3	1	1	2	2
1	1	2	2	4	4	4	4	3	3
1	1	3	3	3	3	1	1	2	2
4	4	3	3	3	3	1	1	3	3
1	1	2	2	4	4	1	1	2	2
1	1	2	2	4	4	1	1	2	2
1	1	2	2	4	4	1	1	3	3
1	1	2	2	4	4	1	1	2	2
1	1	2	2	4	4	1	1	3	3
4	4	3	3	3	3	4	4	3	3
1	1	2	2	4	4	1	1	3	3
4	4	3	3	3	3	1	1	3	3
4	4	3	3	3	3	1	1	2	2
4	4	3	3	3	3	1	1	2	2
4	4	3	3	3	3	1	1	2	2
1	1	3	3	3	3	1	1	2	2
4	4	3	3	3	3	1	1	3	3
1	1	2	2	4	4	1	1	3	3
4	4	3	3	3	3	1	1	2	2
1	1	2	2	4	4	4	4	3	3
1	1	2	2	4	4	1	1	2	2
1	1	2	2	4	4	4	4	3	3
1	1	2	2	4	4	1	1	2	2
1	1	2	2	4	4	1	1	3	3
1	1	2	2	4	4	1	1	2	2
1	1	2	2	4	4	1	1	3	3
1	1	2	2	4	4	1	1	3	3
1	1	2	2	4	4	1	1	3	3
1	1	2	2	4	4	1	1	3	3
1	1	2	2	4	4	1	1	3	3
1	1	2	2	4	4	1	1	2	2
1	1	2	2	4	4	1	1	3	3
1	1	2	2	4	4	1	1	2	2
4	4	3	3	3	3	1	1	3	3
4	4	3	3	3	3	1	1	2	2
4	4	3	3	3	3	1	1	3	3
1	1	2	2	4	4	1	1	3	3
1	1	3	3	3	3	1	1	2	2
4	4	3	3	3	3	1	1	2	2
1	1	2	2	4	4	1	1	3	3

For Review Only

4	4	3	3	3	3	1	1	2	2
1	1	2	2	4	4	1	1	2	2
1	1	2	2	4	4	1	1	3	3
1	1	2	2	4	4	4	4	2	2
1	1	2	2	4	4	1	1	3	3
4	4	3	3	3	3	1	1	3	3
4	4	3	3	3	3	1	1	2	2
1	1	2	2	4	4	1	1	3	3
1	1	2	2	4	4	1	1	2	2
1	1	3	3	3	3	1	1	2	2
4	4	3	3	3	3	1	1	3	3
4	4	3	3	3	3	1	1	3	3
4	4	3	3	3	3	1	1	2	2
4	4	3	3	3	3	1	1	3	3
1	1	3	3	3	3	1	1	2	2
1	1	2	2	4	4	1	1	2	2
1	1	2	2	4	4	1	1	3	3
4	4	3	3	3	3	1	1	2	2
1	1	2	2	4	4	1	1	2	2
4	4	3	3	3	3	1	1	2	2
4	4	3	3	3	3	1	1	3	3
1	1	2	2	4	4	1	1	3	3
1	1	2	2	3	3	1	1	3	3
4	4	3	3	3	3	1	1	3	3
1	1	2	2	4	4	1	1	2	2
4	4	3	3	3	3	1	1	3	3
1	1	2	2	4	4	4	4	3	3
1	1	2	2	4	4	1	1	3	3
4	4	3	3	3	3	1	1	2	2
4	4	3	3	3	3	4	4	3	3
1	1	2	2	4	4	4	4	3	3
1	1	2	2	4	4	1	1	3	3
1	1	2	2	4	4	1	1	3	3
4	4	3	3	3	3	1	1	2	2
4	4	3	3	3	3	1	1	2	2
1	1	2	2	4	4	1	1	2	2
1	1	2	2	4	4	1	1	3	3
1	1	2	2	4	4	1	1	3	3
1	1	2	2	4	4	1	1	2	2
1	1	2	2	4	4	1	1	2	2
4	4	3	3	3	3	1	1	2	2
4	4	3	3	3	3	1	1	3	3
1	1	3	3	3	3	1	1	2	2
4	4	3	3	3	3	1	1	3	3
4	4	3	3	3	3	1	1	3	3
4	4	3	3	3	3	1	1	3	3
1	1	2	2	4	4	4	4	3	3
4	4	3	3	3	3	1	1	2	2
4	4	3	3	3	3	1	1	2	2
1	1	3	3	3	3	1	1	3	3
4	4	3	3	3	3	1	1	3	3
1	1	2	2	4	4	1	1	3	3
4	4	3	3	3	3	1	1	2	2
1	1	3	3	3	3	1	1	3	3
4	4	3	3	3	3	1	1	2	2

4	4	3	3	3	3	1	1	3	3
1	1	2	2	4	4	1	1	3	3
1	1	2	2	4	4	1	1	3	3
1	1	2	2	4	4	4	4	3	3
4	4	3	3	3	3	1	1	2	2
1	1	2	2	4	4	1	1	3	3
4	4	3	3	3	3	1	1	3	3
4	4	3	3	3	3	1	1	2	2
4	4	3	3	3	3	1	1	2	2
1	1	3	3	3	3	1	1	2	2
1	1	2	2	4	4	1	1	2	2
1	1	3	3	3	3	4	4	2	2
1	1	2	2	4	4	1	1	2	2
4	4	3	3	3	3	1	1	2	2
1	1	3	3	3	3	1	1	3	3
1	1	3	3	3	3	1	1	2	2
1	1	3	3	3	3	1	1	3	3
1	1	3	3	3	3	1	1	2	2
1	1	2	2	4	4	4	4	3	3
1	1	2	2	4	4	1	1	3	3
1	1	3	3	3	3	1	1	2	2
4	4	3	3	3	3	1	1	2	2
1	1	3	3	3	3	1	1	3	3
1	1	2	2	4	4	1	1	3	3
1	1	3	3	4	4	1	1	3	3
1	1	2	2	4	4	1	1	3	3
1	1	3	3	3	3	1	1	3	3
1	1	2	2	4	4	1	1	2	2
4	4	3	3	3	3	1	1	2	2
1	1	2	2	4	4	4	4	3	3
1	1	2	2	4	4	1	1	3	3
1	1	3	3	3	3	1	1	2	2
4	4	3	3	3	3	1	1	2	2
1	1	3	3	3	3	1	1	2	2
1	1	2	2	4	4	4	4	3	3
1	1	3	3	4	4	1	1	2	2
1	1	2	2	4	4	4	4	3	3
4	4	3	3	3	3	1	1	2	2
1	1	3	3	3	3	1	1	3	3
1	1	2	2	4	4	1	1	3	3
1	1	3	3	3	3	1	1	3	3
1	1	2	2	4	4	4	4	3	3
4	4	3	3	3	3	1	1	2	2
1	1	2	2	4	4	1	1	3	3
4	4	2	2	3	3	4	4	3	3
4	4	3	3	3	3	4	4	3	3
4	4	3	3	3	3	1	1	2	2
4	4	3	3	3	3	1	1	2	2
1	1	3	3	3	3	1	1	2	2
4	4	3	3	3	3	1	1	2	2
4	4	3	3	3	3	1	1	2	2
1	1	2	2	4	4	1	1	2	2
1	1	3	3	3	3	1	1	3	3
4	4	3	3	3	3	1	1	3	3
1	1	3	3	3	3	1	1	3	3

For Review Only

4	4	3	3	3	3	1	1	2	2
1	1	2	2	4	4	1	1	3	3
1	1	3	3	3	3	1	1	2	2
4	4	3	3	3	3	1	1	3	3
4	4	3	3	3	3	1	1	2	2
1	1	2	2	4	4	1	1	2	2
1	1	2	2	4	4	1	1	2	2
1	1	3	3	3	3	1	1	2	2
4	4	3	3	3	3	1	1	3	3
4	4	3	3	3	3	1	1	2	2
4	4	3	3	3	3	1	1	2	2
1	1	2	2	4	4	1	1	3	3
4	4	3	3	3	3	1	1	2	2
4	4	3	3	3	3	4	4	3	3
1	1	2	2	4	4	1	1	3	3
1	1	2	2	4	4	1	1	3	3
1	1	3	3	4	4	1	1	3	3
4	4	3	3	3	3	1	1	3	3
4	4	3	3	3	3	1	1	3	3
4	4	3	3	3	3	1	1	2	2
1	1	3	3	3	3	1	1	2	2
1	1	2	2	4	4	1	1	3	3
1	1	2	2	4	4	1	1	3	3
4	4	3	3	3	3	1	1	3	3
1	1	2	2	4	4	1	1	3	3
1	1	2	2	4	4	4	4	3	3
4	4	3	3	3	3	1	1	2	2
1	1	3	3	3	3	1	1	3	3
4	4	3	3	3	3	1	1	2	2
4	4	3	3	3	3	1	1	2	2
4	4	3	3	3	3	1	1	3	3
4	4	3	3	3	3	1	1	2	2
4	4	3	3	3	3	1	1	2	2
4	4	2	2	4	4	1	1	3	3
4	4	3	3	3	3	1	1	3	3
4	4	3	3	3	3	1	1	3	3
4	4	3	3	3	3	1	1	2	2
4	4	3	3	3	3	1	1	2	2
4	4	3	3	3	3	1	1	2	2
4	4	3	3	3	3	1	1	3	3
4	4	3	3	3	3	1	1	3	3
4	4	3	3	3	3	1	1	2	2
1	1	2	2	4	4	1	1	3	3
1	1	3	3	3	3	1	1	3	3
1	1	3	3	3	3	1	1	3	3
4	4	3	3	3	3	1	1	2	2
1	1	2	2	4	4	1	1	3	3
1	1	2	2	4	4	4	4	3	3
1	1	2	2	4	4	4	4	3	3
4	4	3	3	3	3	1	1	2	2
4	4	3	3	3	3	1	1	2	2
1	1	2	2	4	4	4	4	3	3
1	1	2	2	4	4	1	1	3	3
4	4	3	3	3	3	1	1	3	3
1	1	2	2	4	4	1	1	3	3

For Review Only

4	4	3	3	3	3	4	4	3	3
4	4	3	3	3	3	1	1	3	3
4	4	3	3	3	3	1	1	2	2
1	1	2	2	4	4	1	1	3	3
1	1	2	2	4	4	1	1	3	3
1	1	2	2	4	4	1	1	2	2
1	1	2	2	4	4	1	1	2	2
1	1	2	2	4	4	1	1	3	3
1	1	3	3	3	3	1	1	2	2
1	1	2	2	4	4	1	1	3	3
1	1	3	3	3	3	1	1	2	2
1	1	2	2	4	4	1	1	3	3
4	4	3	3	3	3	1	1	2	2
4	4	3	3	3	3	1	1	2	2
4	4	3	3	3	3	1	1	3	3
4	4	3	3	3	3	4	4	3	3
1	1	2	2	4	4	4	4	3	3
1	1	2	2	4	4	1	1	3	3
1	1	2	2	4	4	1	1	3	3
1	1	2	2	4	4	1	1	3	3
4	4	3	3	3	3	1	1	3	3
4	4	3	3	3	3	1	1	2	2
4	4	3	3	3	3	4	4	3	3
1	1	2	2	4	4	1	1	3	3
1	1	2	2	4	4	1	1	3	3
1	1	2	2	4	4	1	1	3	3
1	1	2	2	4	4	1	1	3	3
4	4	3	3	3	3	1	1	3	3
4	4	3	3	3	3	1	1	2	2
4	4	3	3	3	3	1	1	2	2
1	1	2	2	4	4	1	1	3	3
1	1	2	2	4	4	1	1	2	2
1	1	2	2	4	4	4	4	3	3
4	4	3	3	3	3	1	1	3	3
1	1	2	2	4	4	1	1	3	3
1	1	2	2	4	4	1	1	3	3
4	4	3	3	3	3	4	4	3	3
4	4	3	3	3	3	1	1	3	3
1	1	2	2	4	4	1	1	3	3
1	1	2	2	4	4	1	1	3	3
4	4	3	3	3	3	1	1	2	2
4	4	3	3	3	3	1	1	2	2
1	1	2	2	4	4	1	1	2	2
4	4	3	3	3	3	4	4	3	3
4	4	3	3	3	3	1	1	2	2
4	4	3	3	3	3	4	4	3	3
4	4	3	3	3	3	1	1	3	3
4	4	3	3	3	3	1	1	2	2
1	1	2	2	4	4	1	1	3	3
1	1	2	2	4	4	1	1	3	3
1	1	2	2	4	4	4	4	3	3
1	1	2	2	4	4	1	1	3	3
4	4	3	3	3	3	1	1	3	3
4	4	3	3	3	3	1	1	3	3
1	1	2	2	4	4	1	1	3	3

For Review Only

4	4	3	3	3	3	4	4	3	3
4	4	3	3	3	3	1	1	3	3

For Review Only

SNP972	SNP972	SNP973	SNP973	SNP974	SNP974	SNP975	SNP975	SNP976	SNP976
3	3	4	4	3	3	2	2	2	2
2	2	3	3	3	3	2	2	2	2
3	3	4	4	1	1	2	2	3	3
2	2	3	3	3	3	2	2	2	2
3	3	4	4	3	3	2	2	2	2
2	2	3	3	1	1	2	2	2	2
3	3	4	4	3	3	2	2	2	2
2	2	3	3	1	1	3	3	2	2
2	2	3	3	3	3	2	2	2	2
2	2	3	3	1	1	3	3	2	2
2	2	3	3	3	3	2	2	3	3
3	3	4	4	1	1	2	2	3	3
2	2	3	3	3	3	2	2	2	2
2	2	3	3	3	3	2	2	2	2
3	3	3	3	3	3	2	2	2	2
3	3	4	4	3	3	2	2	3	3
2	2	3	3	3	3	2	2	2	2
2	2	3	3	3	3	2	2	2	2
2	2	3	3	1	1	2	2	2	2
2	2	3	3	3	3	2	2	2	2
2	2	3	3	1	1	3	3	2	2
3	3	4	4	3	3	2	2	2	2
2	2	3	3	3	3	2	2	2	2
3	3	4	4	3	3	3	3	2	2
3	3	4	4	3	3	2	2	2	2
3	3	4	4	3	3	2	2	3	3
3	3	4	4	3	3	2	2	3	3
2	2	3	3	3	3	2	2	2	2
3	3	4	4	3	3	3	3	2	2
2	2	3	3	3	3	2	2	2	2
3	3	4	4	3	3	3	3	3	3
2	2	3	3	3	3	2	2	2	2
2	2	3	3	3	3	2	2	2	2
2	2	3	3	3	3	2	2	2	2
2	2	3	3	3	3	2	2	3	3
3	3	4	4	3	3	3	3	3	3
2	2	3	3	3	3	2	2	3	3
2	2	3	3	3	3	2	2	2	2
2	2	3	3	3	3	2	2	2	2
2	2	3	3	3	3	2	2	2	2
2	2	3	3	1	1	2	2	2	2
2	2	3	3	1	1	3	3	2	2
2	2	3	3	1	1	3	3	3	3
2	2	3	3	3	3	2	2	2	2
2	2	3	3	3	3	2	2	3	3
3	3	4	4	1	1	2	2	3	3
3	3	4	4	3	3	2	2	3	3
3	3	4	4	3	3	2	2	3	3
2	2	3	3	3	3	2	2	2	2
2	2	3	3	3	3	2	2	2	2
3	3	4	4	3	3	3	3	3	3
2	2	3	3	3	3	2	2	2	2

For Review Only

3	3	4	4	3	3	2	2	2	2
2	2	3	3	3	3	2	2	2	2
2	2	3	3	1	1	2	2	2	2
2	2	3	3	3	3	2	2	2	2
2	2	3	3	1	1	2	2	2	2
3	3	4	4	3	3	2	2	3	3
3	3	4	4	1	1	2	2	3	3
2	2	3	3	3	3	3	3	2	2
2	2	3	3	3	3	2	2	3	3
2	2	3	3	3	3	2	2	2	2
3	3	4	4	3	3	2	2	3	3
3	3	4	4	3	3	2	2	3	3
3	3	4	4	3	3	2	2	2	2
3	3	4	4	3	3	3	3	2	2
2	2	3	3	3	3	2	2	2	2
2	2	3	3	3	3	2	2	2	2
2	2	3	3	1	1	2	2	2	2
3	3	4	4	3	3	3	3	2	2
2	2	3	3	3	3	2	2	2	2
3	3	4	4	3	3	2	2	2	2
3	3	4	4	3	3	2	2	3	3
2	2	3	3	3	3	2	2	2	2
2	2	3	3	3	3	3	3	2	2
3	3	4	4	1	1	2	2	3	3
2	2	3	3	3	3	2	2	3	3
3	3	4	4	1	1	2	2	3	3
2	2	3	3	1	1	2	2	2	2
2	2	3	3	3	3	3	3	3	3
3	3	4	4	3	3	2	2	3	3
3	3	4	4	3	3	2	2	3	3
2	2	3	3	3	3	2	2	3	3
2	2	3	3	3	3	2	2	2	2
2	2	3	3	3	3	2	2	3	3
3	3	4	4	3	3	2	2	2	2
2	2	3	3	3	3	2	2	2	2
2	2	3	3	3	3	2	2	3	3
2	2	3	3	1	1	3	3	2	2
2	2	3	3	3	3	2	2	2	2
2	2	3	3	3	3	2	2	3	3
2	2	3	3	1	1	2	2	3	3
3	3	4	4	3	3	2	2	2	2
3	3	4	4	3	3	2	2	3	3
3	3	3	3	3	3	3	3	2	2
3	3	4	4	3	3	2	2	3	3
3	3	4	4	3	3	2	2	2	2
3	3	3	3	3	3	2	2	3	3
2	2	3	3	3	3	2	2	3	3
3	3	4	4	1	1	2	2	3	3
3	3	4	4	3	3	2	2	3	3
2	2	3	3	3	3	2	2	2	2
3	3	4	4	3	3	2	2	3	3
2	2	3	3	3	3	3	3	3	3
3	3	4	4	3	3	3	3	2	2
2	2	3	3	3	3	2	2	2	2
3	3	4	4	3	3	2	2	3	3

For Review Only

2	2	3	3	3	3	2	2	2	2
2	2	3	3	3	3	2	2	2	2
3	3	4	4	3	3	3	3	3	3
2	2	3	3	3	3	2	2	3	3
3	3	4	4	1	1	2	2	3	3
2	2	3	3	3	3	2	2	2	2
3	3	4	4	3	3	2	2	2	2
3	3	3	3	3	3	2	2	2	2
3	3	4	4	3	3	2	2	3	3
2	2	3	3	3	3	2	2	3	3
2	2	3	3	3	3	2	2	2	2
2	2	3	3	3	3	2	2	2	2
2	2	3	3	3	3	3	3	3	3
2	2	3	3	3	3	2	2	3	3
2	2	3	3	3	3	2	2	2	2
3	3	3	3	3	3	2	2	2	2
2	2	3	3	1	1	2	2	2	2
2	2	3	3	3	3	2	2	3	3
2	2	3	3	3	3	3	3	3	3
2	2	3	3	3	3	2	2	3	3
2	2	3	3	3	3	2	2	2	2
3	3	4	4	3	3	2	2	3	3
3	3	3	3	1	1	3	3	3	3
2	2	3	3	3	3	3	3	2	2
2	2	4	4	3	3	2	2	2	2
2	2	3	3	3	3	2	2	2	2
3	3	4	4	3	3	2	2	3	3
3	3	4	4	3	3	2	2	3	3
3	3	4	4	1	1	2	2	3	3
2	2	3	3	3	3	2	2	3	3
2	2	3	3	3	3	2	2	2	2
2	2	3	3	3	3	2	2	2	2
3	3	4	4	3	3	2	2	2	2
2	2	3	3	3	3	2	2	2	2
2	2	3	3	3	3	2	2	2	2
2	2	4	4	3	3	3	3	2	2
2	2	3	3	3	3	3	3	3	3
3	3	4	4	3	3	2	2	2	2
2	2	4	4	3	3	2	2	3	3
2	2	3	3	3	3	2	2	2	2
2	2	4	4	3	3	2	2	2	2
2	2	3	3	3	3	2	2	3	3
3	3	4	4	3	3	3	3	2	2
2	2	3	3	3	3	2	2	2	2
2	2	4	4	3	3	3	3	3	3
3	3	4	4	3	3	2	2	2	2
3	3	4	4	3	3	2	2	3	3
3	3	4	4	1	1	2	2	3	3
2	2	3	3	3	3	3	3	2	2
3	3	4	4	3	3	2	2	3	3
3	3	4	4	3	3	2	2	3	3
2	2	3	3	1	1	2	2	2	2
2	2	3	3	3	3	2	2	2	2
3	3	4	4	3	3	2	2	3	3
2	2	3	3	3	3	2	2	2	2

For Review Only

3	3	4	4	3	3	2	2	2	2
2	2	3	3	3	3	3	3	3	3
2	2	3	3	3	3	2	2	3	3
2	2	3	3	3	3	2	2	2	2
3	3	4	4	3	3	2	2	3	3
2	2	3	3	3	3	2	2	2	2
2	2	3	3	3	3	2	2	2	2
2	2	3	3	3	3	2	2	2	2
3	3	4	4	3	3	2	2	2	2
3	3	4	4	3	3	2	2	2	2
3	3	4	4	3	3	2	2	3	3
2	2	3	3	3	3	2	2	2	2
3	3	3	3	3	3	2	2	2	2
3	3	4	4	3	3	2	2	2	2
2	2	3	3	3	3	2	2	2	2
2	2	3	3	3	3	2	2	3	3
2	2	3	3	3	3	2	2	2	2
3	3	3	3	3	3	2	2	3	3
3	3	4	4	3	3	2	2	3	3
3	3	4	4	3	3	2	2	2	2
2	2	3	3	3	3	2	2	2	2
2	2	3	3	3	3	2	2	2	2
2	2	3	3	3	3	2	2	2	2
3	3	4	4	3	3	2	2	3	3
2	2	3	3	1	1	3	3	2	2
2	2	3	3	3	3	2	2	2	2
3	3	4	4	3	3	2	2	3	3
2	2	3	3	3	3	2	2	2	2
3	3	4	4	3	3	2	2	2	2
3	3	4	4	3	3	2	2	2	2
3	3	4	4	1	1	2	2	3	3
3	3	4	4	3	3	2	2	3	3
3	3	4	4	3	3	2	2	3	3
2	2	3	3	3	3	2	2	2	2
3	3	4	4	3	3	2	2	3	3
3	3	4	4	3	3	2	2	3	3
2	2	3	3	3	3	2	2	2	2
3	3	4	4	3	3	2	2	2	2
3	3	4	4	3	3	2	2	3	3
3	3	4	4	1	1	2	2	2	2
3	3	4	4	1	1	2	2	3	3
3	3	4	4	3	3	2	2	3	3
2	2	3	3	3	3	3	3	2	2
2	2	3	3	3	3	2	2	2	2
2	2	3	3	3	3	2	2	2	2
2	2	3	3	3	3	2	2	3	3
2	2	3	3	3	3	2	2	3	3
2	2	3	3	3	3	2	2	2	2
2	2	3	3	3	3	2	2	2	2
3	3	4	4	3	3	2	2	3	3
2	2	3	3	3	3	2	2	2	2
2	2	3	3	3	3	3	3	3	3
2	2	3	3	3	3	3	3	2	2
3	3	4	4	3	3	2	2	3	3
2	2	3	3	3	3	3	3	3	3

For Review Only

3	3	4	4	3	3	3	3	2	2
3	3	4	4	3	3	2	2	2	2
3	3	4	4	1	1	2	2	3	3
2	2	3	3	3	3	2	2	2	2
2	2	3	3	3	3	2	2	2	2
2	2	3	3	3	3	2	2	2	2
2	2	3	3	3	3	2	2	3	3
2	2	3	3	1	1	3	3	2	2
2	2	3	3	3	3	2	2	2	2
2	2	3	3	3	3	2	2	2	2
2	2	3	3	3	3	2	2	2	2
2	2	3	3	1	1	3	3	3	3
3	3	4	4	3	3	2	2	3	3
3	3	4	4	3	3	2	2	3	3
3	3	4	4	1	1	2	2	3	3
3	3	4	4	3	3	2	2	2	2
2	2	3	3	3	3	2	2	2	2
2	2	3	3	3	3	2	2	2	2
2	2	3	3	3	3	2	2	2	2
2	2	3	3	3	3	2	2	2	2
3	3	4	4	1	1	2	2	3	3
3	3	4	4	3	3	2	2	3	3
3	3	4	4	3	3	3	3	3	3
2	2	3	3	3	3	2	2	2	2
2	2	3	3	3	3	3	3	2	2
2	2	3	3	3	3	2	2	2	2
2	2	3	3	3	3	2	2	3	3
3	3	4	4	1	1	2	2	3	3
2	2	3	3	3	3	2	2	2	2
3	3	4	4	1	1	2	2	3	3
2	2	3	3	3	3	3	3	3	3
3	3	4	4	3	3	2	2	3	3
2	2	3	3	3	3	2	2	2	2
3	3	4	4	3	3	2	2	3	3
2	2	3	3	3	3	2	2	2	2
2	2	3	3	3	3	3	3	3	3
3	3	4	4	3	3	3	3	2	2
3	3	4	4	3	3	2	2	3	3
3	3	4	4	3	3	2	2	3	3
2	2	3	3	3	3	3	3	2	2
3	3	4	4	3	3	2	2	3	3
3	3	4	4	3	3	2	2	2	2
2	2	3	3	3	3	2	2	2	2
3	3	4	4	3	3	2	2	2	2
3	3	4	4	1	1	2	2	3	3
3	3	4	4	3	3	2	2	3	3
3	3	4	4	3	3	2	2	3	3
3	3	4	4	3	3	3	3	2	2
2	2	3	3	1	1	2	2	3	3
2	2	3	3	3	3	2	2	3	3
3	3	4	4	3	3	2	2	2	2
2	2	3	3	3	3	2	2	2	2
3	3	4	4	1	1	2	2	3	3
3	3	4	4	1	1	2	2	3	3
2	2	3	3	3	3	2	2	2	2

For Review Only

3	3	4	4	3	3	2	2	2	2
3	3	4	4	1	1	2	2	2	2

For Review Only

SNP977	SNP977	SNP978	SNP978	SNP979	SNP979	SNP980	SNP980	SNP981	SNP981
1	1	3	3	1	1	1	1	1	1
1	1	3	3	1	1	1	1	4	4
1	1	3	3	1	1	1	1	4	4
3	3	3	3	1	1	1	1	4	4
1	1	3	3	1	1	1	1	1	1
1	1	3	3	1	1	1	1	1	1
1	1	3	3	1	1	1	1	1	1
1	1	3	3	3	3	4	4	1	1
1	1	3	3	1	1	1	1	4	4
1	1	3	3	3	3	4	4	1	1
3	3	3	3	1	1	1	1	4	4
1	1	3	3	1	1	1	1	4	4
1	1	3	3	1	1	1	1	4	4
1	1	3	3	1	1	1	1	4	4
1	1	3	3	1	1	4	4	4	4
1	1	3	3	3	3	4	4	4	4
1	1	3	3	1	1	1	1	1	1
1	1	3	3	3	3	4	4	1	1
1	1	3	3	1	1	1	1	4	4
3	3	3	3	1	1	1	1	4	4
3	3	3	3	1	1	4	4	4	4
1	1	3	3	1	1	1	1	4	4
1	1	3	3	1	1	1	1	4	4
1	1	3	3	3	3	4	4	4	4
1	1	3	3	3	3	4	4	4	4
1	1	3	3	3	3	4	4	4	4
1	1	3	3	1	1	1	1	4	4
1	1	3	3	1	1	4	4	4	4
3	3	3	3	1	1	1	1	4	4
3	3	3	3	1	1	1	1	4	4
3	3	3	3	1	1	1	1	4	4
1	1	3	3	1	1	1	1	4	4
1	1	3	3	1	1	1	1	1	1
3	3	3	3	1	1	1	1	4	4
1	1	3	3	1	1	1	1	1	1
1	1	3	3	3	3	4	4	4	4
1	1	3	3	1	1	1	1	1	1
1	1	3	3	1	1	4	4	4	4
1	1	3	3	1	1	1	1	4	4
1	1	3	3	1	1	1	1	4	4
3	3	3	3	1	1	1	1	4	4
3	3	3	3	1	1	1	1	4	4
1	1	1	1	3	3	4	4	1	1
3	3	3	3	1	1	1	1	4	4
1	1	1	1	3	3	4	4	1	1
1	1	3	3	3	3	4	4	4	4
1	1	3	3	1	1	4	4	1	1
1	1	3	3	3	3	4	4	4	4
1	1	3	3	1	1	1	1	4	4
3	3	3	3	1	1	1	1	1	1
1	1	3	3	1	1	1	1	4	4
1	1	3	3	1	1	1	1	4	4

For Review Only

1	1	3	3	1	1	1	1	1	1
1	1	3	3	1	1	1	1	1	1
1	1	3	3	3	3	4	4	4	4
1	1	3	3	1	1	1	1	4	4
1	1	3	3	1	1	1	1	4	4
1	1	3	3	1	1	1	1	4	4
3	3	3	3	1	1	1	1	4	4
3	3	3	3	1	1	1	1	4	4
1	1	3	3	1	1	1	1	1	1
1	1	3	3	1	1	4	4	4	4
1	1	3	3	3	3	4	4	4	4
1	1	3	3	3	3	4	4	4	4
1	1	3	3	1	1	1	1	1	1
3	3	3	3	1	1	1	1	4	4
1	1	3	3	1	1	4	4	4	4
1	1	3	3	1	1	1	1	4	4
1	1	3	3	1	1	1	1	4	4
1	1	3	3	3	3	4	4	1	1
3	3	3	3	1	1	1	1	1	1
1	1	3	3	1	1	1	1	1	1
1	1	3	3	3	3	4	4	4	4
1	1	3	3	1	1	1	1	4	4
3	3	3	3	1	1	1	1	4	4
1	1	3	3	3	3	4	4	4	4
1	1	1	1	3	3	4	4	1	1
1	1	3	3	1	1	1	1	4	4
3	3	3	3	1	1	1	1	1	1
1	1	3	3	1	1	1	1	4	4
1	1	3	3	1	1	4	4	4	4
1	1	3	3	1	1	1	1	4	4
1	1	3	3	1	1	1	1	4	4
1	1	3	3	3	3	4	4	4	4
3	3	3	3	1	1	4	4	4	4
1	1	3	3	1	1	1	1	1	1
1	1	3	3	1	1	1	1	1	1
1	1	1	1	3	3	4	4	1	1
3	3	3	3	1	1	1	1	4	4
3	3	3	3	1	1	1	1	4	4
1	1	1	1	1	1	1	1	1	1
3	3	1	1	1	1	1	1	4	4
1	1	3	3	1	1	1	1	1	1
1	1	3	3	1	1	1	1	4	4
1	1	3	3	3	3	4	4	4	4
1	1	3	3	3	3	4	4	4	4
1	1	3	3	3	3	4	4	4	4
1	1	3	3	1	1	1	1	4	4
1	1	3	3	1	1	1	1	4	4
3	3	3	3	1	1	1	1	4	4
3	3	3	3	1	1	1	1	4	4
1	1	3	3	1	1	4	4	4	4
1	1	3	3	1	1	4	4	4	4
3	3	3	3	1	1	1	1	4	4
1	1	3	3	1	1	1	1	1	1
1	1	3	3	1	1	1	1	4	4
1	1	3	3	3	3	4	4	1	1

For Review Only

1	1	3	3	1	1	1	1	4	4
1	1	3	3	3	3	4	4	4	4
1	1	3	3	3	3	4	4	4	4
1	1	3	3	1	1	1	1	4	4
1	1	3	3	1	1	1	1	4	4
1	1	3	3	3	3	4	4	4	4
1	1	3	3	1	1	1	1	4	4
1	1	3	3	1	1	4	4	4	4
1	1	3	3	1	1	1	1	4	4
1	1	3	3	3	3	4	4	4	4
1	1	3	3	1	1	1	1	1	1
1	1	3	3	1	1	4	4	4	4
1	1	1	1	3	3	4	4	4	4
1	1	3	3	3	3	4	4	4	4
1	1	3	3	1	1	4	4	4	4
1	1	3	3	1	1	4	4	4	4
1	1	3	3	3	3	4	4	4	4
1	1	3	3	3	3	4	4	4	4
1	1	3	3	1	1	1	1	4	4
1	1	1	1	1	1	4	4	4	4
1	1	3	3	1	1	4	4	4	4
1	1	3	3	1	1	1	1	1	1
1	1	3	3	3	3	4	4	4	4
1	1	3	3	1	1	1	1	4	4
1	1	3	3	1	1	1	1	4	4
1	1	3	3	3	3	4	4	4	4
1	1	3	3	3	3	1	1	4	4
1	1	3	3	3	3	1	1	1	1
1	1	3	3	1	1	1	1	1	1
1	1	1	1	1	1	1	1	4	4
1	1	3	3	1	1	1	1	4	4
1	1	3	3	1	1	4	4	4	4
1	1	3	3	1	1	1	1	1	1
1	1	3	3	1	1	4	4	4	4
3	3	3	3	1	1	1	1	4	4
3	3	3	3	1	1	1	1	4	4
1	1	3	3	1	1	1	1	4	4
1	1	3	3	3	3	4	4	4	4
1	1	3	3	1	1	4	4	4	4
3	3	3	3	1	1	1	1	4	4
1	1	3	3	1	1	4	4	4	4
1	1	3	3	1	1	1	1	4	4
1	1	3	3	3	3	4	4	1	1
1	1	3	3	1	1	1	1	4	4
1	1	3	3	3	3	4	4	4	4
1	1	3	3	1	1	1	1	4	4
1	1	3	3	3	3	4	4	4	4
1	1	3	3	3	3	4	4	4	4
1	1	3	3	1	1	1	1	4	4
1	1	3	3	3	3	4	4	4	4
1	1	3	3	1	1	4	4	4	4
1	1	3	3	1	1	1	1	1	1
3	3	3	3	1	1	1	1	4	4
1	1	3	3	3	3	4	4	4	4
1	1	3	3	1	1	1	1	4	4

For Review Only

3	3	3	3	1	1	1	1	1	1
1	1	1	1	3	3	4	4	4	4
3	3	3	3	1	1	1	1	1	1
1	1	3	3	3	3	4	4	4	4
1	1	3	3	1	1	1	1	1	1
1	1	3	3	1	1	1	1	1	1
1	1	3	3	3	3	4	4	4	4
1	1	3	3	1	1	4	4	4	4
3	3	3	3	1	1	1	1	4	4
1	1	3	3	1	1	1	1	1	1
1	1	3	3	3	3	4	4	4	4
1	1	3	3	3	3	4	4	4	4
1	1	3	3	1	1	1	1	4	4
1	1	3	3	1	1	4	4	4	4
1	1	3	3	1	1	1	1	4	4
3	3	3	3	1	1	1	1	4	4
1	1	3	3	1	1	1	1	4	4
1	1	3	3	1	1	1	1	4	4
1	1	3	3	3	3	4	4	4	4
1	1	3	3	1	1	1	1	4	4
1	1	3	3	1	1	4	4	4	4
1	1	3	3	3	3	4	4	4	4
1	1	3	3	1	1	1	1	4	4
1	1	3	3	3	3	4	4	4	4
3	3	3	3	1	1	4	4	4	4
3	3	3	3	1	1	1	1	4	4
1	1	3	3	3	3	1	1	4	4
1	1	3	3	3	3	4	4	4	4
1	1	3	3	3	3	4	4	1	1
3	3	3	3	1	1	1	1	1	1
1	1	3	3	1	1	4	4	4	4
1	1	3	3	1	1	1	1	1	1
1	1	3	3	3	3	1	1	1	1
1	1	3	3	1	1	1	1	4	4
1	1	3	3	1	1	1	1	4	4
1	1	3	3	3	3	4	4	4	4
1	1	3	3	1	1	1	1	1	1
1	1	3	3	1	1	1	1	1	1
1	1	3	3	3	3	4	4	4	4
1	1	3	3	1	1	1	1	4	4
1	1	3	3	1	1	1	1	4	4
1	1	3	3	1	1	1	1	4	4
1	1	3	3	1	1	1	1	4	4
1	1	3	3	1	1	4	4	4	4
1	1	3	3	1	1	1	1	4	4
1	1	3	3	1	1	1	1	4	4
3	3	3	3	1	1	1	1	4	4
3	3	3	3	1	1	1	1	4	4
1	1	3	3	1	1	1	1	4	4
3	3	3	3	1	1	1	1	4	4
1	1	3	3	1	1	4	4	1	1
1	1	3	3	1	1	1	1	4	4
1	1	3	3	1	1	1	1	4	4
1	1	3	3	3	3	4	4	4	4
3	3	3	3	1	1	1	1	4	4

For Review Only

3	3	3	3	1	1	1	1	4	4
1	1	3	3	3	3	4	4	4	4
1	1	3	3	1	1	1	1	1	1
1	1	3	3	1	1	1	1	4	4
1	1	3	3	3	3	4	4	1	1
1	1	3	3	3	3	4	4	1	1
1	1	1	1	1	1	1	1	4	4
3	3	3	3	1	1	1	1	4	4
3	3	3	3	1	1	4	4	4	4
1	1	3	3	1	1	1	1	4	4
1	1	3	3	1	1	4	4	4	4
1	1	1	1	3	3	4	4	4	4
1	1	3	3	1	1	1	1	1	1
1	1	3	3	1	1	1	1	1	1
1	1	3	3	1	1	1	1	4	4
1	1	3	3	3	3	4	4	1	1
3	3	3	3	1	1	1	1	4	4
1	1	3	3	1	1	1	1	4	4
3	3	3	3	1	1	1	1	4	4
1	1	3	3	3	3	4	4	4	4
1	1	3	3	3	3	4	4	4	4
1	1	3	3	3	3	4	4	1	1
1	1	3	3	1	1	1	1	4	4
1	1	3	3	3	3	4	4	4	4
3	3	3	3	1	1	1	1	4	4
1	1	3	3	1	1	1	1	4	4
3	3	3	3	1	1	1	1	4	4
1	1	3	3	1	1	1	1	4	4
1	1	3	3	1	1	4	4	1	1
1	1	3	3	1	1	1	1	1	1
3	3	3	3	1	1	1	1	4	4
1	1	3	3	1	1	1	1	4	4
1	1	3	3	3	3	4	4	4	4
1	1	3	3	3	3	4	4	4	4
1	1	3	3	1	1	1	1	4	4
3	3	3	3	1	1	1	1	4	4
3	3	3	3	1	1	1	1	4	4
1	1	3	3	3	3	4	4	4	4
1	1	3	3	3	3	4	4	4	4
3	3	3	3	1	1	1	1	4	4
1	1	3	3	1	1	1	1	1	1
1	1	3	3	1	1	1	1	1	1
1	1	3	3	3	3	4	4	4	4
1	1	3	3	1	1	1	1	4	4
1	1	3	3	1	1	1	1	4	4
1	1	3	3	1	1	1	1	4	4
3	3	3	3	1	1	1	1	4	4
1	1	3	3	3	3	4	4	4	4
1	1	3	3	1	1	1	1	4	4
3	3	3	3	1	1	1	1	4	4
1	1	3	3	1	1	1	1	1	1
3	3	3	3	1	1	1	1	4	4
1	1	3	3	1	1	1	1	4	4
1	1	3	3	1	1	1	1	4	4
1	1	3	3	1	1	1	1	4	4

For Review Only

1	1	3	3	3	3	4	4	1	1
1	1	3	3	1	1	1	1	4	4

For Review Only

SNP982	SNP982	SNP983	SNP983	SNP984	SNP984	SNP985	SNP985	SNP986	SNP986
3	3	1	1	1	1	2	2	3	3
3	3	1	1	1	1	2	2	3	3
3	3	1	1	1	1	2	2	3	3
3	3	1	1	1	1	2	2	3	3
3	3	1	1	1	1	2	2	3	3
3	3	1	1	1	1	2	2	2	2
2	2	4	4	4	4	2	2	3	3
2	2	4	4	4	4	2	2	3	3
3	3	1	1	1	1	2	2	3	3
2	2	4	4	4	4	2	2	3	3
3	3	1	1	1	1	2	2	3	3
3	3	1	1	1	1	2	2	3	3
3	3	1	1	1	1	2	2	3	3
3	3	1	1	1	1	2	2	3	3
3	3	1	1	1	1	2	2	3	3
2	2	4	4	4	4	2	2	3	3
3	3	1	1	1	1	2	2	3	3
2	2	4	4	4	4	2	2	3	3
3	3	1	1	1	1	2	2	3	3
3	3	1	1	1	1	2	2	3	3
3	3	1	1	1	1	2	2	3	3
3	3	1	1	1	1	2	2	3	3
3	3	1	1	1	1	2	2	3	3
2	2	4	4	4	4	2	2	3	3
2	2	4	4	4	4	2	2	3	3
2	2	4	4	4	4	2	2	2	2
3	3	1	1	1	1	2	2	3	3
3	3	1	1	1	1	2	2	3	3
3	3	1	1	1	1	2	2	3	3
3	3	1	1	1	1	2	2	3	3
3	3	1	1	1	1	2	2	3	3
2	2	4	4	4	4	2	2	3	3
2	2	4	4	4	4	2	2	3	3
3	3	1	1	1	1	2	2	3	3
3	3	1	1	1	1	2	2	3	3
2	2	4	4	4	4	2	2	3	3
3	3	1	1	1	1	2	2	3	3
2	2	4	4	4	4	2	2	3	3
3	3	1	1	1	1	2	2	3	3
3	3	1	1	1	1	2	2	3	3
3	3	1	1	1	1	2	2	3	3
3	3	1	1	1	1	2	2	3	3
2	2	4	4	4	4	2	2	3	3
3	3	1	1	1	1	2	2	3	3
2	2	4	4	4	4	2	2	3	3
2	2	4	4	4	4	2	2	3	3
2	2	4	4	4	4	2	2	3	3
2	2	4	4	4	4	2	2	3	3
3	3	1	1	1	1	2	2	3	3
2	2	1	1	1	1	2	2	3	3
3	3	1	1	1	1	2	2	3	3
2	2	4	4	4	4	2	2	3	3

For Review Only

3	3	1	1	1	1	2	2	3	3
2	2	4	4	4	4	2	2	3	3
2	2	4	4	4	4	2	2	3	3
3	3	1	1	1	1	2	2	3	3
3	3	1	1	1	1	2	2	3	3
3	3	1	1	1	1	2	2	2	2
3	3	1	1	1	1	2	2	3	3
3	3	1	1	1	1	2	2	3	3
3	3	1	1	1	1	2	2	3	3
3	3	1	1	1	1	2	2	3	3
2	2	4	4	4	4	2	2	3	3
2	2	4	4	4	4	2	2	3	3
3	3	1	1	1	1	2	2	3	3
3	3	1	1	1	1	2	2	3	3
3	3	1	1	1	1	2	2	2	2
3	3	1	1	1	1	2	2	3	3
3	3	1	1	1	1	2	2	3	3
2	2	4	4	4	4	2	2	3	3
3	3	1	1	1	1	2	2	3	3
3	3	1	1	1	1	2	2	3	3
3	3	4	4	1	1	2	2	3	3
3	3	1	1	1	1	2	2	3	3
3	3	1	1	1	1	2	2	3	3
2	2	4	4	4	4	2	2	3	3
2	2	4	4	4	4	2	2	3	3
3	3	1	1	1	1	2	2	3	3
3	3	1	1	1	1	2	2	3	3
3	3	1	1	1	1	2	2	3	3
2	2	4	4	4	4	2	2	2	2
3	3	1	1	1	1	2	2	3	3
3	3	1	1	1	1	2	2	3	3
3	3	1	1	4	4	2	2	3	3
3	3	1	1	1	1	2	2	3	3
3	3	1	1	1	1	2	2	3	3
2	2	4	4	4	4	2	2	3	3
2	2	4	4	4	4	2	2	3	3
3	3	1	1	1	1	2	2	3	3
3	3	1	1	1	1	2	2	3	3
3	3	1	1	1	1	2	2	3	3
3	3	1	1	1	1	2	2	3	3
3	3	1	1	1	1	2	2	3	3
3	3	1	1	1	1	2	2	2	2
2	2	4	4	4	4	2	2	3	3
2	2	4	4	4	4	2	2	3	3
3	3	4	4	4	4	2	2	3	3
3	3	1	1	1	1	2	2	3	3
3	3	1	1	1	1	2	2	3	3
3	3	1	1	1	1	2	2	3	3
3	3	1	1	1	1	2	2	3	3
3	3	1	1	1	1	2	2	3	3
2	2	4	4	4	4	2	2	2	2
3	3	1	1	1	1	2	2	3	3
3	3	1	1	1	1	2	2	3	3
3	3	1	1	1	1	2	2	3	3
2	2	4	4	4	4	2	2	3	3

For Review Only

3	3	1	1	1	1	2	2	3	3
2	2	4	4	4	4	2	2	3	3
2	2	4	4	4	4	2	2	3	3
3	3	1	1	1	1	2	2	3	3
3	3	1	1	1	1	2	2	3	3
2	2	4	4	4	4	2	2	2	2
3	3	1	1	1	1	2	2	3	3
3	3	1	1	1	1	2	2	3	3
3	3	1	1	1	1	2	2	3	3
2	2	4	4	4	4	2	2	3	3
3	3	1	1	1	1	2	2	3	3
3	3	4	4	1	1	2	2	3	3
2	2	4	4	4	4	2	2	3	3
2	2	1	1	1	1	2	2	3	3
3	3	1	1	1	1	2	2	3	3
3	3	1	1	1	1	2	2	3	3
2	2	4	4	4	4	2	2	3	3
3	3	4	4	1	1	2	2	3	3
3	3	1	1	1	1	2	2	3	3
2	2	4	4	4	4	2	2	3	3
3	3	1	1	1	1	2	2	3	3
3	3	1	1	1	1	2	2	3	3
2	2	4	4	4	4	2	2	3	3
3	3	1	1	1	1	2	2	3	3
3	3	1	1	1	1	2	2	3	3
2	2	4	4	4	4	2	2	3	3
3	3	4	4	4	4	2	2	3	3
3	3	4	4	4	4	2	2	3	3
3	3	1	1	1	1	2	2	3	3
3	3	1	1	1	1	2	2	2	2
3	3	1	1	1	1	2	2	3	3
3	3	1	1	1	1	2	2	3	3
3	3	1	1	1	1	2	2	3	3
2	2	1	1	1	1	2	2	2	2
3	3	1	1	1	1	2	2	3	3
3	3	1	1	1	1	2	2	3	3
3	3	1	1	1	1	2	2	3	3
2	2	4	4	4	4	2	2	3	3
3	3	1	1	1	1	2	2	3	3
3	3	1	1	1	1	2	2	3	3
3	3	1	1	1	1	2	2	3	3
3	3	1	1	1	1	2	2	3	3
2	2	4	4	4	4	2	2	3	3
3	3	1	1	1	1	2	2	3	3
2	2	4	4	4	4	2	2	3	3
3	3	1	1	1	1	2	2	3	3
2	2	4	4	1	1	2	2	2	2
2	2	4	4	4	4	2	2	3	3
3	3	1	1	1	1	2	2	3	3
2	2	4	4	4	4	2	2	2	2
3	3	1	1	1	1	2	2	2	2
3	3	1	1	1	1	2	2	3	3
3	3	1	1	1	1	2	2	3	3
2	2	4	4	4	4	2	2	3	3
3	3	1	1	1	1	2	2	3	3

For Review Only

3	3	1	1	1	1	2	2	3	3
2	2	4	4	4	4	2	2	3	3
3	3	1	1	1	1	2	2	3	3
3	3	1	1	1	1	2	2	3	3
2	2	4	4	4	4	2	2	3	3
3	3	1	1	1	1	2	2	3	3
2	2	4	4	1	1	3	3	3	3
3	3	1	1	1	1	2	2	3	3
3	3	1	1	1	1	2	2	3	3
3	3	1	1	1	1	2	2	3	3
3	3	4	4	4	4	2	2	3	3
2	2	4	4	4	4	3	3	3	3
3	3	1	1	1	1	2	2	3	3
3	3	1	1	1	1	2	2	3	3
3	3	1	1	1	1	2	2	3	3
3	3	1	1	1	1	2	2	3	3
3	3	1	1	1	1	2	2	3	3
3	3	1	1	1	1	2	2	3	3
2	2	4	4	4	4	2	2	3	3
3	3	1	1	1	1	2	2	3	3
3	3	1	1	1	1	2	2	3	3
2	2	4	4	4	4	2	2	3	3
3	3	1	1	1	1	2	2	3	3
2	2	4	4	4	4	2	2	3	3
3	3	1	1	1	1	2	2	3	3
3	3	1	1	1	1	2	2	3	3
3	3	1	1	1	1	2	2	3	3
2	2	4	4	4	4	2	2	3	3
2	2	4	4	4	4	2	2	3	3
3	3	1	1	1	1	2	2	3	3
3	3	1	1	1	1	2	2	3	3
3	3	1	1	1	1	2	2	3	3
3	3	4	4	4	4	2	2	3	3
3	3	1	1	1	1	2	2	3	3
3	3	1	1	1	1	2	2	3	3
2	2	4	4	4	4	2	2	3	3
3	3	1	1	1	1	2	2	2	2
3	3	1	1	1	1	2	2	3	3
2	2	4	4	4	4	2	2	3	3
3	3	1	1	1	1	2	2	3	3
3	3	1	1	1	1	2	2	3	3
3	3	1	1	1	1	2	2	2	2
3	3	1	1	1	1	2	2	3	3
3	3	1	1	1	1	2	2	3	3
3	3	4	4	4	4	2	2	3	3
3	3	1	1	1	1	2	2	3	3
3	3	1	1	1	1	2	2	3	3
3	3	1	1	1	1	2	2	3	3
3	3	1	1	1	1	2	2	3	3
3	3	1	1	1	1	2	2	3	3
3	3	1	1	1	1	2	2	3	3
3	3	1	1	1	1	2	2	3	3
3	3	1	1	1	1	2	2	3	3
2	2	4	4	4	4	2	2	3	3
3	3	1	1	1	1	2	2	3	3

For Review Only

3	3	1	1	1	1	2	2	3	3
2	2	4	4	4	4	2	2	3	3
3	3	1	1	1	1	2	2	3	3
3	3	1	1	1	1	2	2	3	3
2	2	4	4	4	4	2	2	3	3
2	2	4	4	4	4	2	2	2	2
3	3	1	1	1	1	3	3	3	3
3	3	1	1	1	1	2	2	3	3
3	3	1	1	1	1	2	2	3	3
3	3	1	1	1	1	2	2	3	3
3	3	1	1	1	1	2	2	2	2
3	3	4	4	4	4	2	2	3	3
3	3	1	1	1	1	2	2	3	3
3	3	1	1	1	1	2	2	3	3
3	3	1	1	1	1	2	2	3	3
2	2	4	4	4	4	2	2	3	3
3	3	1	1	1	1	2	2	3	3
3	3	1	1	1	1	2	2	3	3
3	3	1	1	1	1	2	2	3	3
2	2	4	4	4	4	2	2	3	3
2	2	4	4	4	4	2	2	3	3
2	2	4	4	4	4	2	2	2	2
3	3	1	1	1	1	2	2	3	3
2	2	4	4	4	4	2	2	3	3
3	3	1	1	1	1	2	2	3	3
3	3	1	1	1	1	2	2	3	3
3	3	1	1	1	1	2	2	3	3
3	3	1	1	1	1	2	2	3	3
3	3	1	1	1	1	2	2	3	3
3	3	1	1	1	1	2	2	3	3
3	3	1	1	1	1	2	2	3	3
3	3	1	1	1	1	2	2	3	3
2	2	4	4	4	4	2	2	3	3
2	2	4	4	4	4	2	2	3	3
3	3	1	1	1	1	2	2	3	3
3	3	1	1	1	1	2	2	3	3
3	3	1	1	1	1	2	2	3	3
2	2	4	4	4	4	2	2	3	3
2	2	4	4	4	4	2	2	3	3
3	3	1	1	1	1	2	2	3	3
3	3	1	1	1	1	2	2	3	3
2	2	4	4	4	4	2	2	3	3
2	2	4	4	4	4	2	2	3	3
3	3	1	1	1	1	2	2	3	3
3	3	1	1	1	1	2	2	3	3
3	3	1	1	1	1	2	2	3	3
3	3	1	1	1	1	2	2	3	3
2	2	4	4	4	4	2	2	3	3
3	3	1	1	1	1	2	2	3	3
3	3	1	1	1	1	2	2	3	3
2	2	4	4	4	4	2	2	3	3
3	3	1	1	1	1	2	2	3	3
3	3	1	1	1	1	2	2	3	3
3	3	1	1	1	1	2	2	3	3
3	3	1	1	1	1	2	2	3	3

For Review Only

2	2	4	4	4	4	2	2	3	3
3	3	1	1	1	1	2	2	3	3

For Review Only

SNP987	SNP987	SNP988	SNP988	SNP989	SNP989	SNP990	SNP990	SNP991	SNP991
1	1	2	2	2	2	3	3	4	4
1	1	2	2	2	2	3	3	4	4
1	1	2	2	2	2	3	3	4	4
1	1	2	2	2	2	3	3	4	4
1	1	2	2	2	2	3	3	4	4
1	1	2	2	2	2	2	2	1	1
1	1	2	2	2	2	3	3	4	4
1	1	2	2	2	2	3	3	4	4
1	1	2	2	2	2	3	3	4	4
1	1	2	2	2	2	2	2	1	1
1	1	2	2	2	2	3	3	4	4
1	1	2	2	2	2	3	3	4	4
1	1	2	2	2	2	3	3	4	4
1	1	2	2	2	2	3	3	4	4
1	1	2	2	2	2	3	3	4	4
1	1	2	2	2	2	3	3	4	4
1	1	2	2	2	2	3	3	4	4
1	1	2	2	2	2	3	3	4	4
1	1	2	2	2	2	3	3	4	4
1	1	2	2	2	2	3	3	4	4
1	1	2	2	2	2	3	3	4	4
1	1	2	2	2	2	3	3	4	4
1	1	2	2	2	2	3	3	4	4
2	2	3	3	2	2	3	3	4	4
1	1	2	2	2	2	3	3	4	4
1	1	2	2	2	2	2	2	1	1
2	2	3	3	2	2	3	3	4	4
1	1	2	2	2	2	3	3	4	4
1	1	2	2	2	2	3	3	4	4
1	1	2	2	2	2	3	3	4	4
1	1	2	2	2	2	3	3	4	4
1	1	2	2	2	2	3	3	4	4
1	1	2	2	2	2	3	3	4	4
1	1	2	2	2	2	3	3	4	4
1	1	2	2	2	2	3	3	4	4
1	1	2	2	2	2	3	3	4	4
1	1	2	2	2	2	3	3	4	4
1	1	2	2	2	2	3	3	4	4
1	1	2	2	2	2	3	3	4	4
1	1	2	2	2	2	3	3	4	4
1	1	2	2	2	2	2	2	1	1
1	1	2	2	2	2	3	3	4	4
1	1	2	2	2	2	3	3	4	4
1	1	2	2	2	2	3	3	4	4
1	1	2	2	2	2	3	3	4	4
1	1	2	2	2	2	3	3	4	4
2	2	3	3	2	2	3	3	4	4
1	1	2	2	2	2	3	3	4	4
1	1	2	2	2	2	3	3	4	4
1	1	2	2	2	2	3	3	4	4
1	1	2	2	2	2	3	3	4	4
1	1	2	2	2	2	3	3	4	4

For Review Only

1	1	2	2	2	2	3	3	4	4
1	1	2	2	2	2	3	3	4	4
1	1	2	2	2	2	3	3	4	4
1	1	2	2	2	2	3	3	4	4
1	1	2	2	2	2	3	3	4	4
2	2	3	3	2	2	2	2	1	1
1	1	2	2	2	2	3	3	4	4
1	1	2	2	2	2	3	3	4	4
1	1	2	2	2	2	3	3	4	4
1	1	2	2	2	2	3	3	4	4
2	2	3	3	2	2	3	3	4	4
2	2	3	3	2	2	3	3	4	4
1	1	2	2	2	2	3	3	4	4
1	1	2	2	2	2	3	3	4	4
1	1	2	2	2	2	3	3	4	4
1	1	2	2	2	2	3	3	4	4
1	1	2	2	2	2	3	3	4	4
1	1	2	2	2	2	3	3	4	4
1	1	2	2	2	2	3	3	4	4
1	1	2	2	2	2	3	3	4	4
2	2	3	3	2	2	3	3	4	4
1	1	2	2	2	2	3	3	4	4
1	1	2	2	2	2	3	3	4	4
1	1	2	2	2	2	3	3	4	4
1	1	2	2	2	2	3	3	4	4
1	1	2	2	3	3	3	3	4	4
1	1	2	2	2	2	3	3	4	4
1	1	2	2	2	2	3	3	4	4
1	1	2	2	2	2	2	2	1	1
2	2	3	3	2	2	3	3	4	4
2	2	3	3	2	2	3	3	4	4
1	1	2	2	2	2	3	3	4	4
1	1	2	2	2	2	3	3	4	4
1	1	2	2	2	2	3	3	4	4
1	1	2	2	2	2	3	3	4	4
1	1	2	2	2	2	3	3	4	4
1	1	2	2	2	2	3	3	4	4
1	1	2	2	2	2	3	3	4	4
1	1	2	2	3	3	3	3	4	4
1	1	2	2	2	2	3	3	4	4
1	1	2	2	2	2	3	3	4	4
2	2	3	3	2	2	2	2	1	1
1	1	2	2	2	2	3	3	4	4
2	2	3	3	2	2	3	3	4	4
2	2	3	3	2	2	3	3	4	4
1	1	2	2	2	2	3	3	4	4
1	1	2	2	2	2	3	3	4	4
2	2	3	3	2	2	3	3	4	4
1	1	2	2	2	2	3	3	4	4
1	1	2	2	2	2	3	3	4	4
2	2	3	3	3	3	2	2	1	1
1	1	2	2	2	2	3	3	4	4
1	1	2	2	2	2	3	3	4	4
1	1	2	2	2	2	3	3	4	4
2	2	3	3	2	2	3	3	4	4

For Review Only

1	1	2	2	2	2	3	3	1	1
1	1	2	2	2	2	3	3	4	4
1	1	2	2	2	2	3	3	4	4
1	1	2	2	2	2	3	3	4	4
1	1	2	2	2	2	3	3	4	4
1	1	2	2	2	2	2	2	1	1
1	1	2	2	2	2	3	3	4	4
1	1	3	3	2	2	3	3	4	4
1	1	2	2	2	2	3	3	4	4
1	1	2	2	2	2	3	3	4	4
1	1	2	2	2	2	3	3	4	4
1	1	2	2	2	2	3	3	4	4
1	1	2	2	2	2	3	3	4	4
1	1	3	3	2	2	3	3	4	4
1	1	2	2	2	2	3	3	4	4
1	1	2	2	2	2	3	3	4	4
1	1	2	2	2	2	3	3	4	4
1	1	2	2	2	2	3	3	4	4
1	1	2	2	2	2	3	3	4	4
1	1	2	2	2	2	3	3	4	4
1	1	2	2	2	2	3	3	4	4
2	2	3	3	2	2	3	3	4	4
1	1	2	2	2	2	3	3	4	4
1	1	2	2	2	2	3	3	4	4
1	1	2	2	2	2	3	3	4	4
1	1	2	2	2	2	3	3	4	4
1	1	2	2	2	2	3	3	4	4
2	2	3	3	2	2	3	3	4	4
2	2	3	3	2	2	3	3	4	4
1	1	2	2	2	2	2	2	1	1
1	1	2	2	2	2	3	3	4	4
1	1	2	2	2	2	3	3	4	4
1	1	2	2	2	2	3	3	4	4
1	1	2	2	2	2	2	2	4	4
1	1	2	2	2	2	3	3	4	4
1	1	2	2	2	2	3	3	4	4
1	1	2	2	2	2	3	3	4	4
1	1	2	2	2	2	3	3	4	4
1	1	2	2	2	2	3	3	4	4
1	1	2	2	2	2	3	3	4	4
1	1	2	2	2	2	3	3	4	4
1	1	2	2	2	2	3	3	4	4
1	1	2	2	2	2	3	3	4	4
1	1	2	2	2	2	3	3	4	4
1	1	2	2	2	2	3	3	4	4
1	1	2	2	2	2	3	3	4	4
1	1	2	2	2	2	2	2	1	1
1	1	2	2	3	3	3	3	4	4
1	1	2	2	2	2	3	3	4	4
1	1	2	2	2	2	3	3	1	1
1	1	2	2	2	2	2	2	1	1
1	1	2	2	2	2	3	3	4	4
1	1	2	2	2	2	3	3	4	4
1	1	2	2	2	2	3	3	4	4
1	1	2	2	2	2	3	3	4	4

For Review Only

1	1	2	2	2	2	3	3	4	4
1	1	2	2	2	2	3	3	4	4
1	1	2	2	2	2	3	3	4	4
1	1	2	2	2	2	3	3	4	4
1	1	2	2	2	2	3	3	4	4
1	1	2	2	2	2	3	3	4	4
1	1	2	2	2	2	3	3	4	4
1	1	2	2	2	2	3	3	4	4
1	1	2	2	2	2	3	3	1	1
1	1	2	2	2	2	3	3	4	4
1	1	2	2	2	2	3	3	4	4
1	1	2	2	2	2	3	3	4	4
1	1	2	2	2	2	3	3	4	4
1	1	2	2	2	2	3	3	4	4
1	1	2	2	2	2	3	3	4	4
1	1	2	2	2	2	3	3	4	4
1	1	2	2	2	2	3	3	4	4
2	2	3	3	2	2	3	3	4	4
1	1	2	2	2	2	3	3	4	4
1	1	2	2	2	2	3	3	4	4
1	1	2	2	2	2	3	3	4	4
1	1	2	2	2	2	3	3	4	4
1	1	2	2	2	2	3	3	4	4
2	2	3	3	2	2	3	3	4	4
1	1	2	2	2	2	3	3	4	4
1	1	2	2	2	2	3	3	4	4
1	1	2	2	2	2	3	3	4	4
1	1	2	2	2	2	3	3	4	4
1	1	2	2	2	2	3	3	4	4
1	1	2	2	2	2	3	3	4	4
1	1	2	2	2	2	3	3	4	4
2	2	3	3	2	2	3	3	4	4
2	2	3	3	2	2	3	3	4	4
1	1	2	2	2	2	3	3	4	4
1	1	2	2	2	2	3	3	4	4
2	2	3	3	2	2	3	3	4	4
1	1	2	2	2	2	3	3	1	1
1	1	2	2	2	2	3	3	4	4
2	2	3	3	2	2	3	3	4	4
1	1	2	2	3	3	3	3	4	4
1	1	2	2	2	2	3	3	4	4
1	1	2	2	2	2	2	2	1	1
1	1	2	2	2	2	3	3	4	4
1	1	2	2	2	2	3	3	4	4
1	1	2	2	2	2	3	3	4	4
1	1	2	2	2	2	3	3	4	4
1	1	2	2	2	2	3	3	4	4
1	1	2	2	2	2	3	3	4	4
1	1	2	2	2	2	3	3	4	4
1	1	2	2	2	2	3	3	4	4
1	1	2	2	2	2	3	3	4	4
1	1	2	2	2	2	3	3	4	4
1	1	2	2	2	2	3	3	4	4
1	1	3	3	2	2	3	3	4	4
1	1	2	2	2	2	3	3	4	4

For Review Only

1	1	2	2	2	2	3	3	4	4
1	1	2	2	2	2	3	3	4	4
1	1	2	2	2	2	3	3	4	4
1	1	2	2	2	2	3	3	4	4
1	1	2	2	2	2	3	3	4	4
1	1	2	2	2	2	2	2	4	4
1	1	2	2	2	2	3	3	4	4
1	1	2	2	2	2	3	3	4	4
1	1	2	2	2	2	3	3	4	4
1	1	2	2	2	2	3	3	4	4
1	1	2	2	2	2	3	3	4	4
1	1	2	2	2	2	3	3	4	4
1	1	2	2	3	3	3	3	4	4
2	2	3	3	2	2	3	3	4	4
1	1	2	2	2	2	3	3	4	4
1	1	2	2	2	2	3	3	4	4
1	1	2	2	2	2	3	3	4	4
1	1	2	2	2	2	3	3	4	4
1	1	2	2	2	2	3	3	4	4
1	1	2	2	2	2	3	3	4	4
1	1	2	2	2	2	3	3	4	4
1	1	2	2	2	2	2	2	1	1
2	2	3	3	2	2	3	3	4	4
1	1	2	2	2	2	3	3	4	4
1	1	2	2	2	2	3	3	4	4
1	1	2	2	2	2	3	3	4	4
1	1	2	2	2	2	3	3	4	4
1	1	2	2	2	2	3	3	4	4
1	1	2	2	2	2	3	3	4	4
2	2	3	3	2	2	3	3	4	4
1	1	2	2	2	2	3	3	4	4
1	1	2	2	2	2	3	3	1	1
1	1	2	2	2	2	3	3	4	4
2	2	2	2	2	2	3	3	4	4
1	1	2	2	2	2	3	3	4	4
1	1	2	2	2	2	3	3	4	4
1	1	2	2	2	2	3	3	4	4
2	2	3	3	2	2	3	3	4	4
1	1	2	2	2	2	3	3	4	4
1	1	2	2	2	2	3	3	4	4
2	2	3	3	2	2	3	3	4	4
1	1	2	2	2	2	3	3	4	4
1	1	2	2	2	2	3	3	4	4
1	1	2	2	2	2	3	3	4	4
1	1	2	2	2	2	3	3	4	4
1	1	2	2	2	2	3	3	4	4
2	2	3	3	2	2	3	3	4	4
1	1	2	2	2	2	3	3	4	4
1	1	2	2	2	2	3	3	4	4
1	1	2	2	2	2	3	3	4	4
1	1	2	2	2	2	3	3	4	4
1	1	2	2	2	2	3	3	4	4
1	1	2	2	2	2	3	3	4	4
1	1	2	2	3	3	3	3	4	4
1	1	2	2	2	2	3	3	4	4

For Review Only

1	1	2	2	2	2	3	3	4	4
1	1	2	2	3	3	3	3	4	4

For Review Only

SNP992	SNP992	SNP993	SNP993	SNP994	SNP994	SNP995	SNP995	SNP996	SNP996
1	1	4	4	1	1	2	2	2	2
1	1	4	4	1	1	2	2	1	1
1	1	1	1	3	3	2	2	1	1
1	1	4	4	3	3	2	2	1	1
1	1	4	4	1	1	2	2	2	2
1	1	4	4	3	3	2	2	2	2
1	1	4	4	1	1	2	2	2	2
1	1	4	4	1	1	3	3	2	2
1	1	4	4	1	1	2	2	2	2
1	1	4	4	3	3	3	3	2	2
1	1	4	4	1	1	2	2	1	1
1	1	4	4	3	3	2	2	1	1
1	1	4	4	1	1	2	2	1	1
1	1	4	4	1	1	2	2	2	2
1	1	4	4	1	1	3	3	2	2
1	1	4	4	3	3	3	3	1	1
1	1	4	4	1	1	2	2	2	2
1	1	4	4	1	1	3	3	1	1
1	1	4	4	1	1	2	2	2	2
1	1	4	4	3	3	2	2	1	1
1	1	4	4	1	1	2	2	2	2
1	1	4	4	1	1	2	2	2	2
1	1	4	4	1	1	2	2	2	2
1	1	4	4	1	1	3	3	2	2
1	1	4	4	1	1	3	3	1	1
4	4	4	4	3	3	3	3	2	2
4	4	4	4	1	1	2	2	1	1
1	1	4	4	1	1	3	3	1	1
1	1	4	4	1	1	2	2	2	2
1	1	4	4	1	1	2	2	2	2
1	1	4	4	1	1	2	2	2	2
1	1	1	1	1	1	2	2	2	2
1	1	1	1	1	1	2	2	2	2
1	1	4	4	1	1	2	2	2	2
1	1	4	4	1	1	3	3	1	1
1	1	4	4	1	1	2	2	2	2
1	1	4	4	1	1	2	2	2	2
1	1	1	1	3	3	3	3	1	1
1	1	4	4	1	1	2	2	2	2
1	1	4	4	1	1	2	2	1	1
1	1	4	4	3	3	2	2	2	2
1	1	4	4	1	1	2	2	2	2
1	1	4	4	1	1	3	3	2	2
1	1	4	4	1	1	2	2	2	2
1	1	4	4	1	1	3	3	1	1
1	1	4	4	3	3	3	3	1	1
4	4	4	4	3	3	3	3	1	1
1	1	4	4	1	1	3	3	2	2
1	1	1	1	1	1	2	2	2	2
1	1	4	4	1	1	2	2	1	1
4	4	4	4	1	1	2	2	2	2
1	1	4	4	3	3	2	2	1	1

For Review Only

1	1	4	4	1	1	2	2	2	2
1	1	1	1	1	1	2	2	2	2
1	1	4	4	3	3	3	3	1	1
1	1	4	4	1	1	2	2	2	2
1	1	4	4	1	1	2	2	2	2
4	4	4	4	3	3	2	2	2	2
4	4	4	4	3	3	2	2	1	1
1	1	4	4	1	1	2	2	2	2
1	1	4	4	1	1	2	2	2	2
1	1	4	4	1	1	3	3	1	1
1	1	4	4	1	1	3	3	2	2
1	1	4	4	1	1	3	3	2	2
1	1	4	4	1	1	2	2	2	2
1	1	4	4	1	1	2	2	2	2
1	1	4	4	3	3	3	3	1	1
1	1	4	4	3	3	2	2	2	2
1	1	4	4	3	3	3	3	1	1
1	1	1	1	3	3	3	3	1	1
1	1	4	4	1	1	2	2	2	2
1	1	4	4	1	1	2	2	2	2
1	1	4	4	1	1	3	3	2	2
1	1	4	4	1	1	2	2	1	1
1	1	4	4	1	1	2	2	2	2
1	1	4	4	1	1	3	3	1	1
1	1	4	4	1	1	3	3	1	1
1	1	4	4	3	3	3	3	2	2
1	1	1	1	1	1	2	2	2	2
1	1	4	4	1	1	2	2	2	2
4	4	4	4	3	3	3	3	2	2
1	1	4	4	1	1	2	2	2	2
1	1	4	4	1	1	2	2	2	2
1	1	4	4	1	1	3	3	2	2
1	1	4	4	1	1	2	2	2	2
1	1	4	4	1	1	2	2	2	2
1	1	1	1	1	1	2	2	1	1
1	1	4	4	1	1	3	3	1	1
1	1	4	4	1	1	2	2	2	2
1	1	4	4	1	1	2	2	2	2
1	1	4	4	1	1	2	2	2	2
1	1	4	4	1	1	2	2	1	1
1	1	1	1	1	1	2	2	2	2
1	1	4	4	3	3	2	2	2	2
1	1	4	4	1	1	3	3	2	2
1	1	4	4	1	1	3	3	2	2
1	1	1	1	1	1	3	3	2	2
1	1	4	4	3	3	2	2	1	1
1	1	4	4	1	1	2	2	2	2
1	1	4	4	1	1	2	2	1	1
4	4	4	4	3	3	2	2	1	1
1	1	4	4	1	1	3	3	2	2
1	1	4	4	3	3	3	3	2	2
1	1	4	4	1	1	2	2	2	2
1	1	4	4	1	1	2	2	2	2
1	1	4	4	1	1	2	2	1	1
1	1	1	1	1	1	3	3	2	2

For Review Only

4	4	4	4	1	1	2	2	1	1
1	1	4	4	1	1	3	3	1	1
4	4	4	4	1	1	3	3	2	2
1	1	4	4	1	1	2	2	2	2
1	1	4	4	1	1	2	2	1	1
1	1	4	4	3	3	3	3	2	2
1	1	4	4	3	3	2	2	1	1
1	1	4	4	1	1	2	2	1	1
1	1	4	4	1	1	2	2	2	2
1	1	4	4	1	1	3	3	2	2
1	1	1	1	1	1	2	2	1	1
1	1	4	4	1	1	3	3	1	1
1	1	4	4	1	1	3	3	2	2
1	1	4	4	3	3	3	3	2	2
1	1	4	4	1	1	2	2	2	2
4	4	4	4	1	1	3	3	1	1
1	1	1	1	3	3	3	3	1	1
1	1	4	4	1	1	3	3	2	2
1	1	4	4	1	1	2	2	2	2
1	1	4	4	1	1	3	3	2	2
1	1	4	4	1	1	3	3	1	1
1	1	4	4	1	1	2	2	2	2
1	1	4	4	1	1	3	3	2	2
1	1	4	4	1	1	2	2	2	2
1	1	4	4	3	3	2	2	2	2
1	1	4	4	1	1	3	3	1	1
1	1	1	1	1	1	2	2	1	1
1	1	4	4	1	1	2	2	2	2
1	1	1	1	3	3	2	2	1	1
1	1	4	4	3	3	2	2	2	2
1	1	4	4	1	1	2	2	2	2
1	1	4	4	1	1	3	3	1	1
1	1	1	1	1	1	2	2	2	2
1	1	4	4	1	1	3	3	1	1
1	1	4	4	1	1	2	2	1	1
1	1	4	4	1	1	2	2	2	2
1	1	4	4	1	1	2	2	2	2
1	1	4	4	1	1	3	3	2	2
1	1	4	4	3	3	3	3	1	1
1	1	4	4	1	1	2	2	1	1
1	1	4	4	1	1	3	3	1	1
1	1	4	4	1	1	2	2	2	2
1	1	4	4	1	1	3	3	2	2
1	1	4	4	1	1	2	2	1	1
1	1	4	4	1	1	3	3	2	2
1	1	1	1	1	1	2	2	2	2
4	4	4	4	3	3	3	3	2	2
4	4	4	4	3	3	3	3	1	1
1	1	4	4	1	1	2	2	1	1
4	4	4	4	3	3	3	3	2	2
4	4	4	4	3	3	2	2	2	2
1	1	4	4	3	3	2	2	1	1
1	1	4	4	1	1	2	2	2	2
1	1	4	4	1	1	3	3	2	2
1	1	4	4	1	1	2	2	1	1

For Review Only

1	1	1	1	3	3	2	2	1	1
1	1	4	4	1	1	3	3	2	2
1	1	1	1	1	1	2	2	1	1
1	1	4	4	1	1	3	3	1	1
1	1	4	4	1	1	2	2	1	1
1	1	1	1	3	3	2	2	1	1
1	1	4	4	1	1	2	2	1	1
1	1	4	4	1	1	3	3	1	1
1	1	4	4	1	1	2	2	2	2
1	1	4	4	3	3	2	2	1	1
4	4	4	4	1	1	3	3	1	1
1	1	4	4	1	1	3	3	1	1
1	1	4	4	3	3	3	3	1	1
1	1	4	4	1	1	3	3	2	2
1	1	4	4	1	1	2	2	1	1
1	1	4	4	1	1	2	2	1	1
1	1	4	4	1	1	3	3	2	2
1	1	4	4	1	1	2	2	1	1
4	4	4	4	1	1	3	3	2	2
1	1	4	4	3	3	2	2	1	1
1	1	4	4	1	1	3	3	1	1
1	1	4	4	1	1	2	2	2	2
1	1	4	4	1	1	2	2	1	1
1	1	4	4	1	1	3	3	2	2
1	1	4	4	1	1	3	3	2	2
1	1	4	4	1	1	2	2	2	2
1	1	4	4	3	3	3	3	1	1
1	1	4	4	1	1	3	3	1	1
1	1	4	4	3	3	3	3	1	1
1	1	1	1	1	1	2	2	2	2
1	1	1	1	3	3	3	3	2	2
1	1	4	4	3	3	2	2	1	1
1	1	1	1	1	1	2	2	1	1
1	1	4	4	1	1	2	2	1	1
1	1	1	1	1	1	2	2	1	1
1	1	4	4	1	1	3	3	2	2
1	1	4	4	3	3	2	2	2	2
1	1	4	4	1	1	2	2	2	2
4	4	4	4	3	3	3	3	1	1
1	1	4	4	1	1	3	3	1	1
1	1	1	1	3	3	2	2	1	1
4	4	4	4	3	3	2	2	2	2
1	1	4	4	1	1	2	2	2	2
1	1	4	4	3	3	3	3	1	1
1	1	4	4	1	1	2	2	1	1
1	1	4	4	1	1	3	3	2	2
1	1	4	4	1	1	2	2	1	1
1	1	1	1	1	1	2	2	2	2
1	1	4	4	1	1	2	2	2	2
1	1	4	4	1	1	2	2	1	1
1	1	1	1	3	3	3	3	1	1
1	1	4	4	1	1	2	2	2	2
1	1	4	4	1	1	2	2	2	2
1	1	4	4	1	1	3	3	2	2
1	1	4	4	1	1	2	2	2	2

For Review Only

1	1	4	4	1	1	2	2	2	2
1	1	4	4	3	3	3	3	1	1
1	1	1	1	3	3	2	2	1	1
1	1	4	4	1	1	2	2	1	1
1	1	4	4	3	3	3	3	1	1
4	4	4	4	3	3	3	3	2	2
1	1	4	4	1	1	2	2	1	1
1	1	4	4	1	1	2	2	2	2
1	1	4	4	1	1	2	2	2	2
1	1	4	4	1	1	2	2	2	2
1	1	4	4	1	1	3	3	1	1
1	1	1	1	1	1	3	3	2	2
1	1	4	4	1	1	2	2	1	1
4	4	4	4	1	1	3	3	1	1
1	1	1	1	3	3	3	3	1	1
1	1	4	4	1	1	3	3	1	1
1	1	4	4	1	1	2	2	2	2
1	1	4	4	1	1	2	2	2	2
1	1	4	4	3	3	2	2	2	2
1	1	1	1	3	3	3	3	1	1
1	1	4	4	1	1	3	3	1	1
4	4	4	4	3	3	3	3	2	2
1	1	4	4	1	1	2	2	2	2
1	1	4	4	1	1	3	3	2	2
1	1	4	4	1	1	2	2	2	2
1	1	4	4	1	1	2	2	2	2
1	1	4	4	1	1	2	2	2	2
1	1	4	4	3	3	2	2	1	1
1	1	4	4	1	1	3	3	1	1
1	1	1	1	3	3	2	2	1	1
1	1	4	4	1	1	2	2	2	2
1	1	4	4	1	1	2	2	2	2
1	1	4	4	1	1	3	3	2	2
4	4	4	4	1	1	3	3	1	1
1	1	4	4	1	1	2	2	1	1
1	1	4	4	1	1	2	2	2	2
1	1	4	4	1	1	2	2	2	2
1	1	4	4	1	1	3	3	2	2
1	1	4	4	1	1	2	2	2	2
1	1	4	4	1	1	2	2	2	2
1	1	1	1	1	1	2	2	2	2
1	1	1	1	1	1	2	2	1	1
1	1	4	4	1	1	3	3	1	1
1	1	4	4	1	1	2	2	2	2
1	1	4	4	1	1	2	2	2	2
1	1	4	4	1	1	2	2	2	2
4	4	4	4	3	3	2	2	1	1
1	1	1	1	1	1	3	3	2	2
4	4	4	4	1	1	3	3	1	1
1	1	4	4	1	1	2	2	2	2
1	1	4	4	3	3	2	2	1	1
1	1	4	4	1	1	2	2	2	2
1	1	1	1	3	3	2	2	1	1
1	1	4	4	3	3	2	2	1	1
1	1	1	1	1	1	2	2	1	1

For Review Only

1	1	4	4	1	1	3	3	1	1
1	1	4	4	1	1	2	2	2	2

For Review Only

SNP997	SNP997	SNP998	SNP998	SNP999	SNP999
2	2	2	2	2	2
2	2	2	2	2	2
2	2	2	2	2	2
2	2	2	2	2	2
2	2	2	2	2	2
2	2	2	2	3	3
2	2	2	2	2	2
2	2	2	2	2	2
2	2	2	2	2	2
2	2	2	2	2	2
2	2	2	2	2	2
2	2	2	2	2	2
2	2	2	2	2	2
2	2	2	2	2	2
2	2	2	2	2	2
2	2	2	2	2	2
2	2	2	2	2	2
2	2	2	2	2	2
2	2	2	2	3	3
2	2	2	2	2	2
2	2	2	2	2	2
2	2	2	2	2	2
2	2	2	2	2	2
2	2	2	2	3	3
2	2	2	2	2	2
1	1	3	3	2	2
2	2	2	2	2	2
2	2	2	2	2	2
2	2	2	2	2	2
2	2	2	2	2	2
2	2	2	2	3	3
2	2	2	2	2	2
2	2	2	2	2	2
2	2	2	2	2	2
1	1	3	3	2	2
2	2	2	2	2	2
2	2	2	2	2	2
2	2	2	2	2	2
2	2	2	2	2	2
2	2	2	2	3	3
2	2	2	2	2	2
2	2	2	2	2	2
2	2	2	2	2	2
2	2	2	2	2	2
2	2	2	2	2	2
2	2	2	2	2	2
1	1	3	3	2	2
2	2	2	2	2	2
2	2	2	2	2	2
2	2	2	2	2	2
2	2	2	2	2	2
2	2	2	2	2	2

2	2	2	2	2	2
2	2	2	2	2	2
2	2	2	2	2	2
2	2	2	2	2	2
2	2	2	2	3	3
2	2	2	2	2	2
1	1	3	3	2	2
2	2	2	2	2	2
2	2	2	2	2	2
2	2	2	2	2	2
2	2	2	2	2	2
2	2	2	2	2	2
2	2	2	2	2	2
2	2	2	2	2	2
2	2	2	2	3	3
2	2	2	2	2	2
2	2	2	2	2	2
2	2	2	2	2	2
2	2	2	2	2	2
2	2	2	2	2	2
2	2	2	2	2	2
2	2	2	2	3	3
2	2	2	2	2	2
2	2	2	2	3	3
2	2	2	2	2	2
2	2	2	2	3	3
2	2	2	2	2	2
2	2	2	2	2	2
1	1	3	3	2	2
2	2	2	2	2	2
2	2	2	2	2	2
2	2	2	2	3	3
2	2	2	2	3	3
2	2	2	2	2	2
2	2	2	2	2	2
2	2	2	2	2	2
2	2	2	2	2	2
2	2	2	2	2	2
1	1	3	3	2	2
2	2	2	2	2	2
2	2	2	2	2	2
2	2	2	2	2	2
2	2	2	2	2	2
2	2	2	2	2	2
2	2	2	2	2	2
2	2	2	2	2	2
2	2	2	2	2	2
2	2	2	2	2	2
1	1	3	3	2	2
2	2	2	2	2	2
2	2	2	2	2	2
2	2	2	2	2	2
2	2	3	3	2	2
2	2	2	2	2	2
2	2	2	2	2	2

2	2	2	2	2	2
2	2	2	2	2	2
2	2	2	2	2	2
2	2	2	2	2	2
2	2	2	2	2	2
2	2	2	2	2	2
2	2	2	2	2	2
2	2	2	2	2	2
2	2	2	2	2	2
2	2	2	2	2	2
2	2	2	2	2	2
2	2	2	2	2	2
2	2	2	2	2	2
2	2	2	2	3	3
2	2	2	2	2	2
2	2	2	2	2	2
2	2	2	2	2	2
2	2	2	2	2	2
2	2	2	2	2	2
2	2	2	2	2	2
2	2	2	2	2	2
2	2	2	2	2	2
2	2	2	2	2	2
2	2	2	2	3	3
2	2	2	2	3	3
2	2	2	2	3	3
2	2	2	2	2	2
2	2	2	2	3	3
2	2	2	2	2	2
2	2	2	2	2	2
2	2	2	2	2	2
2	2	2	2	2	2
2	2	2	2	2	2
2	2	2	2	2	2
2	2	2	2	2	2
2	2	2	2	2	2
2	2	2	2	2	2
2	2	2	2	2	2
2	2	2	2	2	2
2	2	2	2	2	2
2	2	2	2	2	2
2	2	2	2	2	2
1	1	3	3	2	2
2	2	2	2	2	2
2	2	2	2	2	2
2	2	2	2	2	2
1	1	3	3	2	2
2	2	2	2	2	2
2	2	2	2	2	2
2	2	2	2	2	2
1	1	3	3	2	2
2	2	2	2	2	2
2	2	2	2	2	2
2	2	2	2	2	2
2	2	2	2	2	2

2	2	2	2	2	2
2	2	2	2	2	2
2	2	2	2	2	2
2	2	2	2	2	2
2	2	2	2	2	2
2	2	2	2	3	3
2	2	2	2	3	3
2	2	2	2	2	2
2	2	2	2	2	2
2	2	2	2	2	2
2	2	2	2	2	2
2	2	2	2	3	3
1	1	3	3	2	2
2	2	2	2	2	2
2	2	2	2	2	2
2	2	2	2	2	2
2	2	2	2	2	2
2	2	2	2	2	2
2	2	2	2	2	2
2	2	2	2	2	2
2	2	2	2	2	2
2	2	2	2	3	3
2	2	2	2	3	3
2	2	2	2	2	2
2	2	2	2	2	2
2	2	2	2	2	2
2	2	2	2	2	2
2	2	2	2	2	2
2	2	2	2	2	2
1	1	3	3	2	2
2	2	2	2	2	2
2	2	2	2	2	2
1	1	3	3	2	2
2	2	2	2	2	2
2	2	2	2	3	3
2	2	2	2	2	2
1	1	3	3	2	2
2	2	2	2	2	2
2	2	2	2	3	3
2	2	2	2	2	2
2	2	2	2	2	2
1	1	3	3	2	2
2	2	2	2	3	3
2	2	2	2	2	2
2	2	2	2	3	3
2	2	2	2	2	2
2	2	2	2	2	2
2	2	2	2	3	3
2	2	2	2	2	2
2	2	2	2	2	2
2	2	2	2	2	2
2	2	2	2	2	2
2	2	2	2	3	3
2	2	2	2	2	2
2	2	2	2	2	2

For Review Only

2	2	2	2	2	2
2	2	2	2	2	2
2	2	2	2	2	2
2	2	2	2	2	2
2	2	2	2	2	2
2	2	2	2	2	2
1	1	2	2	2	2
2	2	2	2	2	2
2	2	2	2	2	2
2	2	2	2	2	2
2	2	2	2	2	2
2	2	2	2	2	2
1	1	3	3	2	2
2	2	2	2	2	2
2	2	2	2	2	2
2	2	2	2	2	2
2	2	2	2	2	2
2	2	2	2	2	2
2	2	2	2	3	3
2	2	2	2	2	2
2	2	2	2	3	3
1	1	3	3	2	2
2	2	2	2	2	2
2	2	2	2	3	3
2	2	2	2	2	2
2	2	2	2	2	2
2	2	2	2	3	3
2	2	2	2	2	2
2	2	2	2	2	2
2	2	2	2	2	2
2	2	2	2	2	2
2	2	2	2	2	2
2	2	2	2	2	2
2	2	2	2	3	3
2	2	2	2	3	3
2	2	2	2	2	2
2	2	2	2	2	2
2	2	2	2	2	2
2	2	2	2	2	2
2	2	2	2	3	3
1	1	3	3	2	2
1	1	3	3	2	2
2	2	2	2	3	3
2	2	2	2	2	2
2	2	2	2	2	2
2	2	2	2	2	2
2	2	2	2	3	3
2	2	2	2	2	2
2	2	2	2	2	2
2	2	2	2	3	3
2	2	2	2	2	2
2	2	2	2	3	3
2	2	2	2	2	2
2	2	2	2	2	2
2	2	2	2	3	3

For Review Only

2	2	2	2	2	2
2	2	2	2	2	2

For Review Only

Table S5 : Membership of 274 cowpea accessions as indicated by discriminant a

**Country**	**Number of clusters**	**Number of accessions**
Benin	3	42
Burkina-Faso	1	5
Côte d'Ivoire	2	3
Ghana	3	13
Liberia	2	2
Mali	2	2
Niger	1	2
Nigeria	3	40
Senegal	2	2
Central Africa Republic	2	2
Total Accessions		113
Kenya	2	3
Malawi	2	3
Mozambique	2	5
Uganda	3	76
Botswana	2	3
Lesotho	1	1
Swaziland	1	1
South Africa	1	1
Total Accessions		93
Egypt	2	2
Mauritania	1	1
Total Accessions		3
India	3	49
Iran	1	1
Pakistan	1	1
Siri Lanka	1	1
Yemen	1	1
Total Accessions		53
Brazil	2	2
Colombia	1	1
Guatemala	1	1
Honduras	1	1
Nicaragua	1	1
Puerto Rico	1	1
US	1	2
Total Accessions		9
Australia	1	3

For Review Only

For Review Only

For Review Only

For Review Only

For Review Only

For Review Only

analysis of principal components (DAPC)

Regions	Clusters/subpopulations 1	2	3
West and Central Africa	46	50	17
East and southern Africa	14	39	40
North Africa	0	2	1
Asia	14	23	16
America	4	3	2
Oceania	0	0	3

Accessions	Membership probabilities Cluster1	Cluster2	Cluster3
1	0.00	1.00	0.00
2	0.00	1.00	0.00
3	0.00	0.00	1.00
4	0.00	0.00	1.00
5	0.00	1.00	0.00
6	0.00	1.00	0.00
7	0.00	1.00	0.00
8	1.00	0.00	0.00
9	0.00	1.00	0.00
10	1.00	0.00	0.00
11	1.00	0.00	0.00
12	0.00	0.00	1.00
13	0.00	1.00	0.00
14	1.00	0.00	0.00
15	0.00	1.00	0.00
16	0.00	0.00	1.00
17	0.00	1.00	0.00
18	0.00	1.00	0.00
19	0.00	0.00	1.00
20	1.00	0.00	0.00
21	0.00	1.00	0.00
22	0.00	1.00	0.00
23	1.00	0.00	0.00
24	1.00	0.00	0.00
25	0.00	1.00	0.00
26	0.00	0.00	1.00
27	0.00	0.00	1.00
28	0.00	1.00	0.00
29	0.00	1.00	0.00
30	1.00	0.00	0.00
31	0.00	0.00	1.00
32	0.00	1.00	0.00
33	0.00	1.00	0.00
34	1.00	0.00	0.00
35	0.00	1.00	0.00
36	0.00	0.00	1.00
37	0.00	1.00	0.00
38	0.00	1.00	0.00
39	1.00	0.00	0.00
40	1.00	0.00	0.00

For Review Only

41	1.00	0.00	0.00
42	1.00	0.00	0.00
43	1.00	0.00	0.00
44	0.00	1.00	0.00
45	1.00	0.00	0.00
46	0.00	0.00	1.00
47	0.00	0.00	1.00
48	0.00	1.00	0.00
49	0.00	1.00	0.00
50	0.00	1.00	0.00
51	0.00	0.00	1.00
52	0.00	1.00	0.00
53	0.00	1.00	0.00
54	0.00	1.00	0.00
55	0.00	0.00	1.00
56	0.00	1.00	0.00
57	0.00	0.00	1.00
58	0.00	0.00	1.00
59	0.00	0.00	1.00
60	1.00	0.00	0.00
61	0.00	1.00	0.00
62	0.00	1.00	0.00
63	0.00	0.00	1.00
64	0.00	0.00	1.00
65	0.00	1.00	0.00
66	0.00	1.00	0.00
67	0.00	1.00	0.00
68	0.00	1.00	0.00
69	0.00	1.00	0.00
70	0.00	1.00	0.00
71	0.00	1.00	0.00
72	0.00	1.00	0.00
73	0.00	0.00	1.00
74	1.00	0.00	0.00
75	0.00	1.00	0.00
76	0.00	1.00	0.00
77	1.00	0.00	0.00
78	0.00	0.00	1.00
79	0.00	1.00	0.00
80	1.00	0.00	0.00
81	0.00	0.00	1.00
82	0.00	1.00	0.00
83	0.00	1.00	0.00
84	1.00	0.00	0.00
85	1.00	0.00	0.00
86	0.00	1.00	0.00
87	0.00	1.00	0.00
88	1.00	0.00	0.00
89	1.00	0.00	0.00
90	0.00	1.00	0.00
91	0.00	1.00	0.00
92	1.00	0.00	0.00
93	0.00	1.00	0.00
94	0.00	0.00	1.00
95	0.00	1.00	0.00

For Review Only

96	0.00	0.00	1.00
97	0.00	1.00	0.00
98	0.00	0.00	1.00
99	1.00	0.00	0.00
100	0.00	0.00	1.00
101	0.00	0.00	1.00
102	0.00	1.00	0.00
103	0.00	0.00	1.00
104	1.00	0.00	0.00
105	1.00	0.00	0.00
106	0.00	1.00	0.00
107	0.00	1.00	0.00
108	0.00	0.00	1.00
109	0.00	1.00	0.00
110	0.00	0.00	1.00
111	1.00	0.00	0.00
112	0.00	0.00	1.00
113	0.00	0.00	1.00
114	0.00	0.00	1.00
115	0.00	1.00	0.00
116	0.00	1.00	0.00
117	0.00	1.00	0.00
118	0.00	1.00	0.00
119	0.00	1.00	0.00
120	0.00	1.00	0.00
121	0.00	0.00	1.00
122	0.00	1.00	0.00
123	0.00	0.00	1.00
124	0.00	1.00	0.00
125	0.00	0.00	1.00
126	1.00	0.00	0.00
127	0.00	1.00	0.00
128	0.00	1.00	0.00
129	0.00	1.00	0.00
130	0.00	0.00	1.00
131	1.00	0.00	0.00
132	0.00	0.00	1.00
133	1.00	0.00	0.00
134	0.00	1.00	0.00
135	0.00	1.00	0.00
136	0.00	0.00	1.00
137	0.00	1.00	0.00
138	1.00	0.00	0.00
139	0.00	1.00	0.00
140	0.00	1.00	0.00
141	0.00	0.00	1.00
142	1.00	0.00	0.00
143	0.00	1.00	0.00
144	1.00	0.00	0.00
145	0.00	0.00	1.00
146	0.00	1.00	0.00
147	1.00	0.00	0.00
148	0.00	1.00	0.00
149	1.00	0.00	0.00
150	0.00	1.00	0.00

For Review Only

151	1.00	0.00	0.00
152	0.00	1.00	0.00
153	0.00	1.00	0.00
154	0.00	0.00	1.00
155	0.00	0.00	1.00
156	1.00	0.00	0.00
157	0.00	0.00	1.00
158	0.00	0.00	1.00
159	1.00	0.00	0.00
160	0.00	1.00	0.00
161	0.00	0.00	1.00
162	1.00	0.00	0.00
163	0.00	0.00	1.00
164	1.00	0.00	0.00
165	0.00	1.00	0.00
166	1.00	0.00	0.00
167	0.00	0.00	1.00
168	0.00	1.00	0.00
169	1.00	0.00	0.00
170	0.00	1.00	0.00
171	1.00	0.00	0.00
172	0.00	1.00	0.00
173	0.00	0.00	1.00
174	1.00	0.00	0.00
175	0.00	0.00	1.00
176	0.00	1.00	0.00
177	1.00	0.00	0.00
178	1.00	0.00	0.00
179	0.00	1.00	0.00
180	0.00	0.00	1.00
181	0.00	0.00	1.00
182	0.00	0.00	1.00
183	0.00	1.00	0.00
184	1.00	0.00	0.00
185	1.00	0.00	0.00
186	0.00	0.00	1.00
187	0.00	1.00	0.00
188	1.00	0.00	0.00
189	0.00	0.00	1.00
190	0.00	1.00	0.00
191	0.00	1.00	0.00
192	0.00	1.00	0.00
193	0.00	1.00	0.00
194	0.00	1.00	0.00
195	0.00	1.00	0.00
196	1.00	0.00	0.00
197	0.00	1.00	0.00
198	0.00	0.00	1.00
199	0.00	0.00	1.00
200	0.00	1.00	0.00
201	0.00	0.00	1.00
202	0.00	0.00	1.00
203	0.00	0.00	1.00
204	0.00	0.00	1.00
205	1.00	0.00	0.00

For Review Only

206	0.00	1.00	0.00
207	0.00	1.00	0.00
208	0.00	1.00	0.00
209	1.00	0.00	0.00
210	1.00	0.00	0.00
211	1.00	0.00	0.00
212	0.00	1.00	0.00
213	0.00	0.00	1.00
214	1.00	0.00	0.00
215	1.00	0.00	0.00
216	0.00	0.00	1.00
217	1.00	0.00	0.00
218	0.00	1.00	0.00
219	0.00	1.00	0.00
220	0.00	0.00	1.00
221	0.00	1.00	0.00
222	0.00	1.00	0.00
223	0.00	0.00	1.00
224	1.00	0.00	0.00
225	1.00	0.00	0.00
226	1.00	0.00	0.00
227	1.00	0.00	0.00
228	0.00	1.00	0.00
229	0.00	1.00	0.00
230	0.00	1.00	0.00
231	0.00	0.00	1.00
232	0.00	0.00	1.00
233	0.00	1.00	0.00
234	1.00	0.00	0.00
235	1.00	0.00	0.00
236	1.00	0.00	0.00
237	0.00	1.00	0.00
238	1.00	0.00	0.00
239	0.00	0.00	1.00
240	0.00	1.00	0.00
241	1.00	0.00	0.00
242	1.00	0.00	0.00
243	0.00	1.00	0.00
244	1.00	0.00	0.00
245	0.00	0.00	1.00
246	0.00	1.00	0.00
247	0.00	0.00	1.00
248	1.00	0.00	0.00
249	0.00	0.00	1.00
250	1.00	0.00	0.00
251	0.00	0.00	1.00
252	1.00	0.00	0.00
253	1.00	0.00	0.00
254	0.00	1.00	0.00
255	0.00	0.00	1.00
256	0.00	0.00	1.00
257	1.00	0.00	0.00
258	0.00	1.00	0.00
259	0.00	1.00	0.00
260	1.00	0.00	0.00

For Review Only

261	0.00	1.00	0.00
262	0.00	0.00	1.00
263	0.00	1.00	0.00
264	0.00	0.00	1.00
265	0.00	1.00	0.00
266	0.00	0.00	1.00
267	1.00	0.00	0.00
268	0.00	1.00	0.00
269	1.00	0.00	0.00
270	0.00	0.00	1.00
271	0.00	0.00	1.00
272	1.00	0.00	0.00
273	0.00	1.00	0.00
274	0.00	0.00	1.00

For Review Only

Table S6: Membership of 274 cowpea accessions as indicated by the Neighbour Joinin

Country	Number of clusters	Number of accessions	Regions
Benin	3	42	West and Central Africa
Burkina-Faso	2	5	
Côte d'Ivoire	2	3	
Ghana	3	13	
Liberia	1	2	
Mali	2	2	
Niger	1	2	
Nigeria	3	40	
Senegal	1	2	
Central Africa Republ	2	2	
Total Accessions		113	
Kenya	2	3	East and Southern Africa
Malawi	1	3	
Mozambique	3	5	
Uganda	3	76	
Botswana	2	3	
Lesotho	1	1	
Swaziland	1	1	
South Africa	1	1	
Total Accessions		93	
Egypt	2	2	North Africa
Mauritania	1	1	
Total Accessions		3	
India	3	49	Asia
Iran	1	1	
Pakistan	1	1	
Siri Lanka	1	1	
Yemen	1	1	
Total Accessions		53	
Brazil	2	2	America
Colombia	1	1	
Guatemala	1	1	
Honduras	1	1	
Nicaragua	1	1	
Puerto Rico	1	1	
US	2	2	
Total Accessions		9	
Australia	1	3	Oceania

For Review Only

ıg clustering method

Clusters/Subpopulations		
1	2	3
44	59	10
61	18	14
2	0	1
29	18	6
4	5	0
0	0	3

For Review Only

For Review Only

Table S7. Summary statistic of LD analsysis between pairs of SNPs across 11 c

	LG1			LG2		
**Distance**	**Mean (r^2)**	**SD (r^2)**	**N**	**Mean (r^2)**	**SD (r^2)**	**N**
0-1kb	0.854	0.262	26	0.505	0.377	19
1-20kb	0.516	0.305	31	0.325	0.284	59
20-40kb	0.482	0.325	37	0.378	0.302	36
40-60kb	0.529	0.383	44	0.391	0.263	37
60-80kb	0.428	0.287	40	0.302	0.250	40
80-100kb	0.386	0.265	45	0.295	0.228	51
0.1-0.5Mb	0.281	0.258	656	0.179	0.176	704
0.5-1Mb	0.223	0.240	540	0.140	0.144	586
1-5Mb	0.115	0.114	2642	0.123	0.130	2142
5-10Mb	0.109	0.092	434	0.182	0.219	190
10-20Mb	0.080	0.068	354	0.133	0.097	496
>20Mb	0.054	0.021	15	_	_	_
Total/Average	0.338		4864	0.268		4360

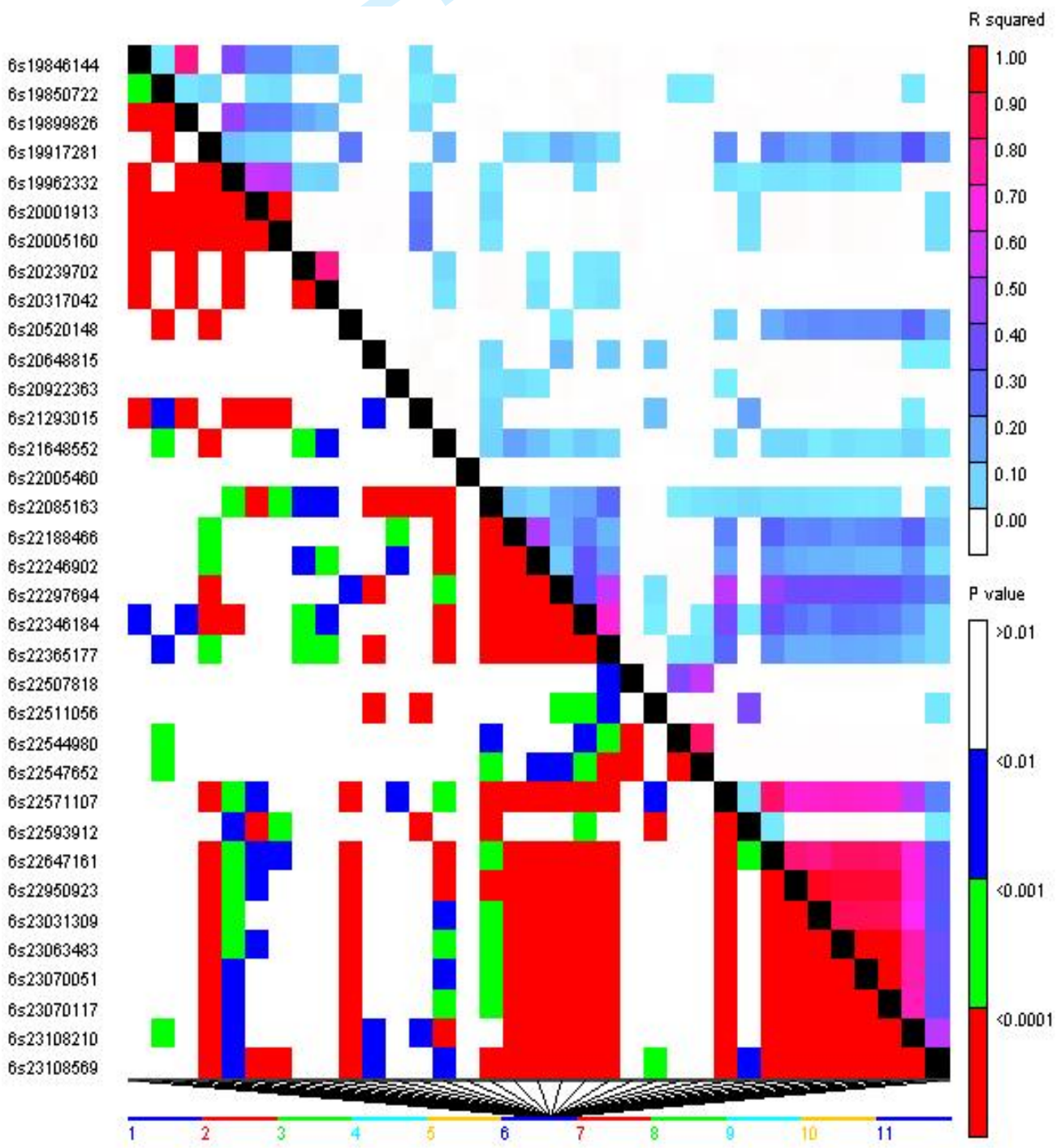

owpea chromosomes

LG3			LG4			LG5			
Mean (r²)	SD (r²)	N	Mean (r²)	SD (r²)	N	Mean (r²)	SD (r²)	N	Mean (r²)
0.554	0.409	13	0.401	0.328	24	0.768	0.324	26	0.638
0.471	0.354	49	0.325	0.300	61	0.580	0.357	67	0.576
0.409	0.304	46	0.300	0.180	25	0.321	0.321	62	0.493
0.269	0.264	51	0.333	0.235	38	0.334	0.289	33	0.463
0.340	0.293	60	0.337	0.323	45	0.325	0.296	30	0.357
0.381	0.309	41	0.339	0.282	33	0.244	0.269	29	0.292
0.220	0.210	641	0.182	0.179	564	0.174	0.166	713	0.235
0.170	0.161	682	0.119	0.113	520	0.128	0.127	633	0.146
0.122	0.114	3879	0.107	0.107	2211	0.112	0.107	1871	0.123
0.101	0.081	1257	0.114	0.112	827	0.097	0.082	646	0.102
0.097	0.076	490	0.093	0.075	571	0.123	0.123	237	0.210
0.135	0.114	10	0.066	0.016	2	0.106	0.079	257	_
0.273		7219	0.226		4921	0.276		4604	0.331

For Review Only

LG6			LG7			LG8			LG9
SD (r²)	N	Mean (r²)	SD (r²)	N	Mean (r²)	SD (r²)	N	Mean (r²)	SD (r²)
0.342	11.000	0.683	0.263	20	0.503	0.320	22	0.865	0.252
0.326	34.000	0.420	0.361	60	0.399	0.304	64	0.745	0.328
0.284	59.000	0.380	0.328	65	0.342	0.268	61	0.591	0.323
0.286	49.000	0.255	0.272	46	0.257	0.220	48	0.368	0.322
0.279	48.000	0.284	0.276	66	0.360	0.278	34	0.519	0.383
0.267	42.000	0.275	0.245	43	0.305	0.239	44	0.662	0.305
0.206	785.000	0.216	0.223	933	0.186	0.173	638	0.298	0.302
0.131	722.000	0.164	0.158	992	0.135	0.123	655	0.152	0.125
0.112	3270.000	0.120	0.102	4488	0.112	0.096	2566	0.124	0.107
0.095	494.000	0.116	0.088	682	0.121	0.102	201	0.109	0.093
0.154	99.000	0.088	0.047	371	0.129	0.107	305	0.069	0.046
_	_	_	_		0.107	0.081	143	_	_
	5613	0.273		7766	0.246		4781	0.409	

For Review Only

		LG10			LG11	
**N**	**Mean (r²)**	**SD (r²)**	**N**	**Mean (r²)**	**SD (r²)**	**N**
20	0.711	0.328	13	0.700	0.305	11
46	0.373	0.341	37	0.454	0.320	31
32	0.495	0.313	36	0.328	0.298	23
43	0.257	0.268	29	0.356	0.300	43
30	0.182	0.193	27	0.317	0.264	34
35	0.227	0.212	42	0.154	0.155	23
480	0.201	0.189	531	0.172	0.168	434
425	0.154	0.134	496	0.126	0.125	453
2059	0.129	0.114	2488	0.103	0.098	2040
768	0.120	0.089	1183	0.122	0.112	660
376	0.111	0.082	467	0.131	0.122	407
_	_	_	_	0.040		1
4314	0.269		5349	0.250		4160

For Review Only

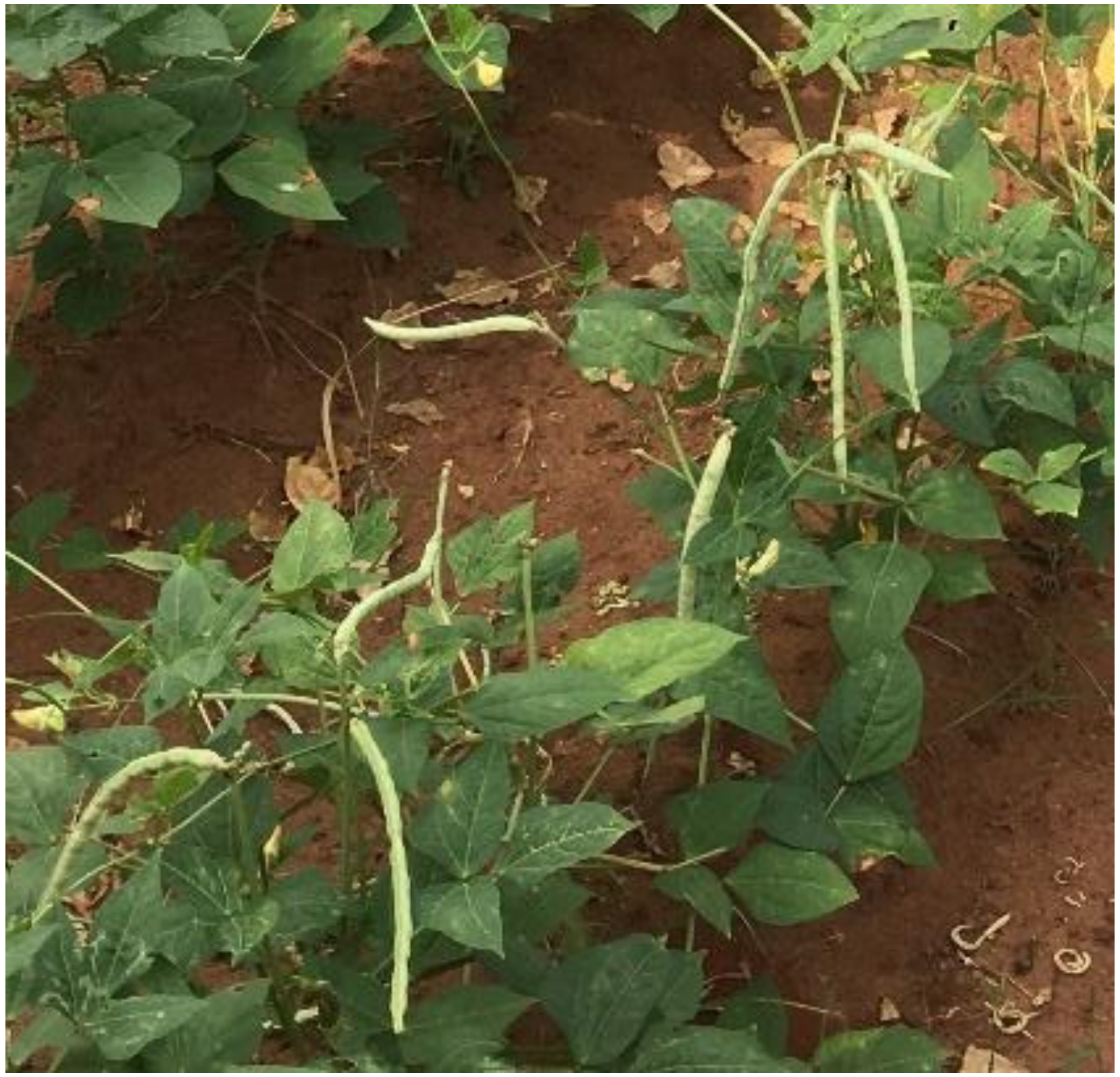

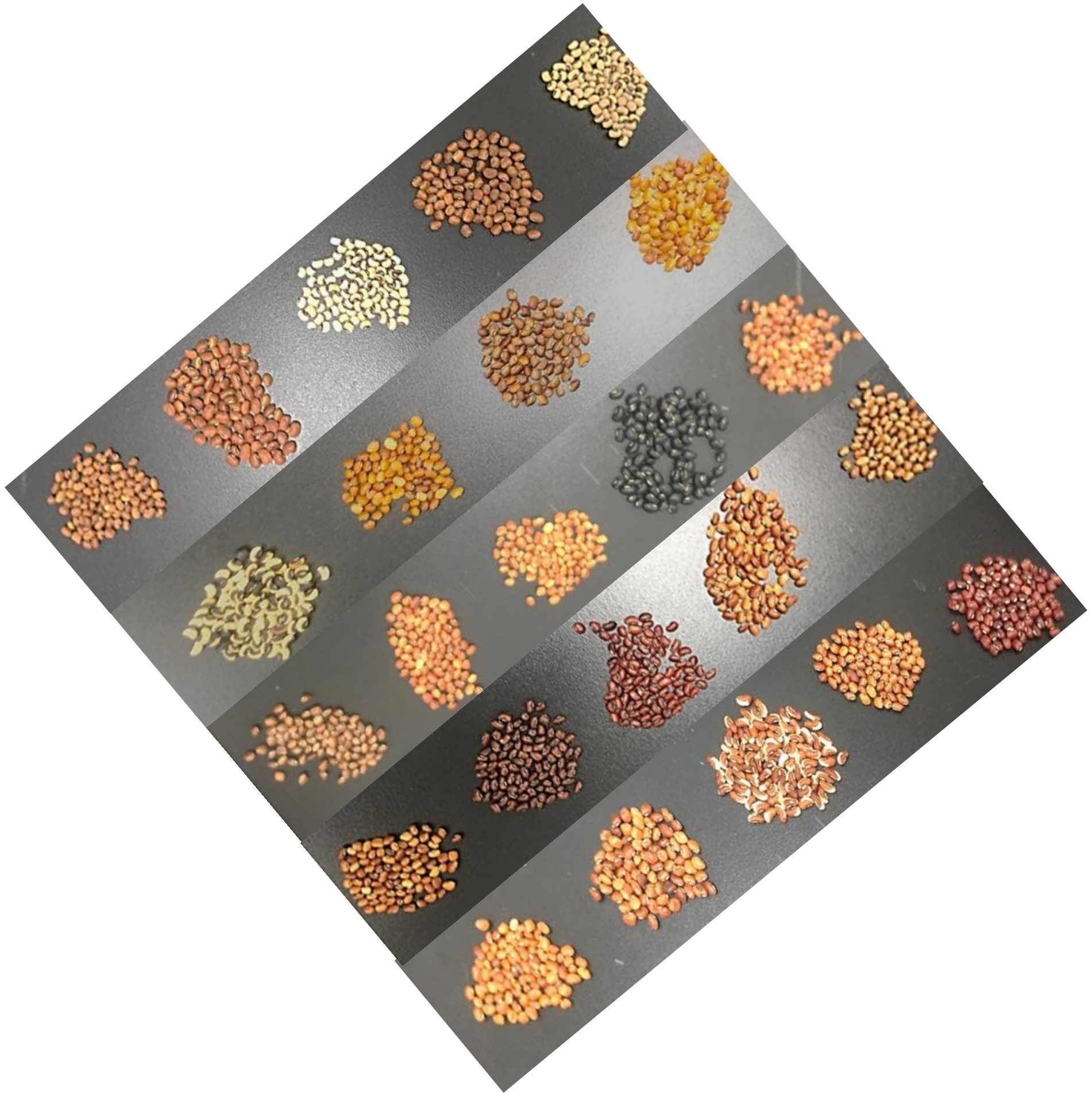

Supplement: Supplementary file 1 [file TPG2-14-e20113-s001.pdf]
